# Burden of disease scenarios for 204 countries and territories, 2022–2050: a forecasting analysis for the Global Burden of Disease Study 2021

**DOI:** 10.1016/S0140-6736(24)00685-8

**Published:** 2024-05-18

**Authors:** GBD 2021 Forecasting Collaborators, Stein Emil Vollset, Hazim S Ababneh, Yohannes Habtegiorgis Abate, Cristiana Abbafati, Rouzbeh Abbasgholizadeh, Mohammadreza Abbasian, Hedayat Abbastabar, Abdallah H A Abd Al Magied, Samar Abd ElHafeez, Atef Abdelkader, Michael Abdelmasseh, Sherief Abd-Elsalam, Parsa Abdi, Mohammad Abdollahi, Meriem Abdoun, Auwal Abdullahi, Mesfin Abebe, Olumide Abiodun, Richard Gyan Aboagye, Hassan Abolhassani, Mohamed Abouzid, Girma Beressa Aboye, Lucas Guimarães Abreu, Abdorrahim Absalan, Hasan Abualruz, Bilyaminu Abubakar, Hana Jihad Jihad Abukhadijah, Giovanni Addolorato, Victor Adekanmbi, Charles Oluwaseun Adetunji, Juliana Bunmi Adetunji, Temitayo Esther Adeyeoluwa, Rishan Adha, Ripon Kumar Adhikary, Qorinah Estiningtyas Sakilah Adnani, Leticia Akua Adzigbli, Fatemeh Afrashteh, Muhammad Sohail Afzal, Saira Afzal, Faith Agbozo, Antonella Agodi, Anurag Agrawal, Williams Agyemang-Duah, Bright Opoku Ahinkorah, Austin J Ahlstrom, Aqeel Ahmad, Firdos Ahmad, Muayyad M Ahmad, Sajjad Ahmad, Shahzaib Ahmad, Anisuddin Ahmed, Ayman Ahmed, Haroon Ahmed, Safoora Ahmed, Syed Anees Ahmed, Karolina Akinosoglou, Mohammed Ahmed Akkaif, Ashley E Akrami, Ema Akter, Salah Al Awaidy, Syed Mahfuz Al Hasan, Amjad S Al Mosa, Omar Al Ta'ani, Omar Ali Mohammed Al Zaabi, Fares Alahdab, Muaaz M Alajlani, Yazan Al-Ajlouni, Samer O Alalalmeh, Ziyad Al-Aly, Khurshid Alam, Noore Alam, Tahiya Alam, Zufishan Alam, Rasmieh Mustafa Al-amer, Fahad Mashhour Alanezi, Turki M Alanzi, Almaza Albakri, Wafa A Aldhaleei, Robert W Aldridge, Seyedeh Yasaman Alemohammad, Yihun Mulugeta Alemu, Adel Ali Saeed Al-Gheethi, Mohammed Khaled Al-Hanawi, Abid Ali, Amjad Ali, Iman Ali, Mohammed Usman Ali, Rafat Ali, Syed Shujait Shujait Ali, Victor Ekoche Ali, Waad Ali, Akram Al-Ibraheem, Gianfranco Alicandro, Sheikh Mohammad Alif, Syed Mohamed Aljunid, François Alla, Joseph Uy Almazan, Hesham M Al-Mekhlafi, Ahmed Yaseen Alqutaibi, Ahmad Alrawashdeh, Sahel Majed Alrousan, Salman Khalifah Al-Sabah, Mohammed A Alsabri, Zaid Altaany, Ala'a B. Al-Tammemi, Jaffar A Al-Tawfiq, Khalid A Altirkawi, Deborah Oyine Aluh, Nelson Alvis-Guzman, Mohammad Sami Al-Wardat, Yaser Mohammed Al-Worafi, Hany Aly, Mohammad Sharif Alyahya, Karem H Alzoubi, Walid Al-Zyoud, Reza Amani, Edward Kwabena Ameyaw, Tarek Tawfik Amin, Alireza Amindarolzarbi, Sohrab Amiri, Mohammad Hosein Amirzade-Iranaq, Hubert Amu, Dickson A Amugsi, Robert Ancuceanu, Deanna Anderlini, David B Anderson, Pedro Prata Andrade, Catalina Liliana Andrei, Tudorel Andrei, Erick Adrian Andrews, Abhishek Anil, Sneha Anil, Amir Anoushiravani, Catherine M Antony, Ernoiz Antriyandarti, Boluwatife Stephen Anuoluwa, Saeid Anvari, Anayochukwu Edward Anyasodor, Francis Appiah, Michele Aquilano, Juan Pablo Arab, Jalal Arabloo, Elshaimaa A Arafa, Mosab Arafat, Aleksandr Y Aravkin, Ali Ardekani, Demelash Areda, Brhane Berhe Aregawi, Abdulfatai Aremu, Hany Ariffin, Mesay Arkew, Keivan Armani, Anton A Artamonov, Ashokan Arumugam, Mohammad Asghari-Jafarabadi, Charlie Ashbaugh, Thomas Astell-Burt, Seyyed Shamsadin Athari, Prince Atorkey, Maha Moh'd Wahbi Atout, Avinash Aujayeb, Marcel Ausloos, Hamzeh Awad, Adedapo Wasiu Awotidebe, Haleh Ayatollahi, Jose L Ayuso-Mateos, Sina Azadnajafabad, Fahad Khan Azeez, Rui M S Azevedo, Muhammad Badar, Soroush Baghdadi, Mahboube Bagheri, Nasser Bagheri, Ruhai Bai, Jennifer L Baker, Abdulaziz T Bako, Senthilkumar Balakrishnan, Wondu Feyisa Balcha, Ovidiu Constantin Baltatu, Martina Barchitta, Erfan Bardideh, Suzanne Lyn Barker-Collo, Till Winfried Bärnighausen, Hiba Jawdat Barqawi, Sandra Barteit, Afisu Basiru, João Diogo Basso, Mohammad-Mahdi Bastan, Sanjay Basu, Matteo Bauckneht, Bernhard T Baune, Mohsen Bayati, Nebiyou Simegnew Bayileyegn, Amir Hossein Behnoush, Payam Behzadi, Maryam Beiranvand, Olorunjuwon Omolaja Bello, Luis Belo, Apostolos Beloukas, Maryam Bemanalizadeh, Isabela M Bensenor, Habib Benzian, Azizullah Beran, Zombor Berezvai, Robert S Bernstein, Paulo J G Bettencourt, Kebede A Beyene, Melak Gedamu Beyene, Devidas S Bhagat, Akshaya Srikanth Bhagavathula, Neeraj Bhala, Dinesh Bhandari, Ravi Bharadwaj, Nikha Bhardwaj, Pankaj Bhardwaj, Ashish Bhargava, Sonu Bhaskar, Vivek Bhat, Natalia V Bhattacharjee, Gurjit Kaur Bhatti, Jasvinder Singh Bhatti, Manpreet S Bhatti, Mohiuddin Ahmed Bhuiyan, Catherine Bisignano, Bijit Biswas, Tone Bjørge, Virginia Bodolica, Aadam Olalekan Bodunrin, Milad Bonakdar Hashemi, Berrak Bora Basara, Hamed Borhany, Samuel Adolf Bosoka, Alejandro Botero Carvajal, Souad Bouaoud, Soufiane Boufous, Christopher Boxe, Edward J Boyko, Oliver J Brady, Dejana Braithwaite, Michael Brauer, Javier Brazo-Sayavera, Hermann Brenner, Colin Stewart Brown, Annie J Browne, Traolach Brugha, Dana Bryazka, Norma B Bulamu, Danilo Buonsenso, Katrin Burkart, Richard A Burns, Reinhard Busse, Yasser Bustanji, Zahid A Butt, Florentino Luciano Caetano dos Santos, Mehtap Çakmak Barsbay, Daniela Calina, Luciana Aparecida Campos, Shujin Cao, Angelo Capodici, Rosario Cárdenas, Giulia Carreras, Andrea Carugno, Márcia Carvalho, Joao Mauricio Castaldelli-Maia, Giulio Castelpietra, Maria Sofia Cattaruzza, Arthur Caye, Luca Cegolon, Francieli Cembranel, Edina Cenko, Ester Cerin, Steven J Chadban, Joshua Chadwick, Chiranjib Chakraborty, Sandip Chakraborty, Julian Chalek, Jeffrey Shi Kai Chan, Rama Mohan Chandika, Sara Chandy, Jaykaran Charan, Anis Ahmad Chaudhary, Akhilanand Chaurasia, An-Tian Chen, Haowei Chen, Meng Xuan Chen, Simiao Chen, Nicolas Cherbuin, Gerald Chi, Fatemeh Chichagi, Odgerel Chimed-Ochir, Ritesh Chimoriya, Patrick R Ching, Jesus Lorenzo Chirinos-Caceres, Abdulaal Chitheer, Daniel Youngwhan Cho, William C S Cho, Dong-Woo Choi, Bryan Chong, Chean Lin Chong, Hitesh Chopra, Dinh-Toi Chu, Eric Chung, Muhammad Chutiyami, Justin T Clayton, Rebecca M Cogen, Aaron J Cohen, Alyssa Columbus, Haley Comfort, Joao Conde, Jon T Connolly, Ezra E K Cooper, Samuele Cortese, Natália Cruz-Martins, Alanna Gomes da Silva, Omid Dadras, Xiaochen Dai, Zhaoli Dai, Bronte E Dalton, Giovanni Damiani, Lalit Dandona, Rakhi Dandona, Jai K Das, Saswati Das, Subasish Das, Nihar Ranjan Dash, Kairat Davletov, Fernando Pio De la Hoz, Diego De Leo, Shayom Debopadhaya, Ivan Delgado-Enciso, Edgar Denova-Gutiérrez, Nikolaos Dervenis, Hardik Dineshbhai Desai, Vinoth Gnana Chellaiyan Devanbu, Syed Masudur Rahman Dewan, Kuldeep Dhama, Amol S Dhane, Sameer Dhingra, Diana Dias da Silva, Daniel Diaz, Luis Antonio Diaz, Michael J Diaz, Adriana Dima, Delaney D Ding, Thao Huynh Phuong Do, Camila Bruneli do Prado, Masoud Dodangeh, Milad Dodangeh, Phidelia Theresa Doegah, Sushil Dohare, Wanyue Dong, Mario D'Oria, Rajkumar Doshi, Robert Kokou Dowou, Haneil Larson Dsouza, Viola Dsouza, John Dube, Samuel C Dumith, Bruce B Duncan, Andre Rodrigues Duraes, Senbagam Duraisamy, Oyewole Christopher Durojaiye, Anar Dushpanova, Sulagna Dutta, Paulina Agnieszka Dzianach, Arkadiusz Marian Dziedzic, Ejemai Eboreime, Alireza Ebrahimi, Mohammad Ebrahimi Kalan, Hisham Atan Edinur, Ferry Efendi, Terje Andreas Eikemo, Ebrahim Eini, Temitope Cyrus Ekundayo, Rabie Adel El Arab, Iman El Sayed, Osman Elamin, Noha Mousaad Elemam, Ghada Metwally Tawfik ElGohary, Muhammed Elhadi, Omar Abdelsadek Abdou Elmeligy, Adel B Elmoselhi, Mohammed Elshaer, Ibrahim Elsohaby, Mohd. Elmagzoub Eltahir, Theophilus I Emeto, Babak Eshrati, Majid Eslami, Zahra Esmaeili, Natalia Fabin, Adeniyi Francis Fagbamigbe, Omotayo Francis Fagbule, Luca Falzone, Mohammad Fareed, Carla Sofia e Sá Farinha, MoezAlIslam Ezzat Mahmoud Faris, Andre Faro, Kiana Fasihi, Ali Fatehizadeh, Nelsensius Klau Fauk, Timur Fazylov, Valery L Feigin, Ginenus Fekadu, Xiaoqi Feng, Seyed-Mohammad Fereshtehnejad, Pietro Ferrara, Nuno Ferreira, Belete Sewasew Firew, Florian Fischer, Ida Fitriana, Joanne Flavel, Luisa S Flor, Morenike Oluwatoyin Folayan, Kristen Marie Foley, Marco Fonzo, Lisa M Force, Matteo Foschi, Alberto Freitas, Ni Kadek Yuni Fridayani, Kai Glenn Fukutaki, João M Furtado, Blima Fux, Peter Andras Gaal, Muktar A Gadanya, Silvano Gallus, Balasankar Ganesan, Mohammad Arfat Ganiyani, Rupesh K Gautam, Tilaye Gebru Gebi, Miglas W Gebregergis, Mesfin Gebrehiwot, Lemma Getacher, Genanew K A Getahun, Peter W Gething, Delaram J Ghadimi, Fataneh Ghadirian, Sadegh Ghafarian, Khalid Yaser Ghailan, MohammadReza Ghasemi, Ghazal Ghasempour Dabaghi, Ramy Mohamed Ghazy, Sama Ghoba, Ehsan Gholami, Ali Gholamrezanezhad, Nasim Gholizadeh, Mahsa Ghorbani, Pooyan Ghorbani Vajargah, Elena Ghotbi, Artyom Urievich Gil, Tiffany K Gill, Alem Girmay, James C Glasbey, Ekaterina Vladimirovna Glushkova, Elena V Gnedovskaya, Laszlo Göbölös, Mohamad Goldust, Pouya Goleij, Davide Golinelli, Sameer Vali Gopalani, Alessandra C Goulart, Mahdi Gouravani, Anmol Goyal, Michal Grivna, Giuseppe Grosso, Giovanni Guarducci, Mohammed Ibrahim Mohialdeen Gubari, Stefano Guicciardi, Rafael Alves Guimarães, Snigdha Gulati, David Gulisashvili, Damitha Asanga Gunawardane, Cui Guo, Anish Kumar Gupta, Rahul Gupta, Rajeev Gupta, Renu Gupta, Sapna Gupta, Vijai Kumar Gupta, Annie Haakenstad, Najah R Hadi, Nils Haep, Abdul Hafiz, Dariush Haghmorad, Demewoz Haile, Adel Hajj Ali, Ali Hajj Ali, Arvin Haj-Mirzaian, Esam S Halboub, Sebastian Haller, Rabih Halwani, Kanaan Hamagharib Abdullah, Nadia M Hamdy, Rifat Hamoudi, Nasrin Hanifi, Graeme J Hankey, Zaim Anan Haq, Md Rabiul Haque, Harapan Harapan, Arief Hargono, Josep Maria Haro, Ahmed I Hasaballah, S. M. Mahmudul Hasan, Mohammad Hasanian, Md Saquib Hasnain, Amr Hassan, Johannes Haubold, Simon I Hay, Jeffrey J Hebert, Omar E Hegazi, Mohammad Heidari, Mehdi Hemmati, Claire A Henson, Brenda Yuliana Herrera-Serna, Claudiu Herteliu, Majid Heydari, Kamal Hezam, Irma Hidayana, Yuta Hiraike, Nguyen Quoc Hoan, Ramesh Holla, Praveen Hoogar, Nobuyuki Horita, Md Mahbub Hossain, Hassan Hosseinzadeh, Mehdi Hosseinzadeh, Mihaela Hostiuc, Sorin Hostiuc, Chengxi Hu, Junjie Huang, Michael Hultström, Tsegaye Gebreyes Hundie, Aliza J Hunt, Kiavash Hushmandi, Javid Hussain, M. Azhar Hussain, Nawfal R Hussein, Hong-Han Huynh, Bing-Fang Hwang, Segun Emmanuel Ibitoye, Pulwasha Maria Iftikhar, Adalia I Ikiroma, Paul Chukwudi Ikwegbue, Irena M Ilic, Milena D Ilic, Mustapha Immurana, Mustafa Alhaji Isa, Md. Rabiul Islam, Sheikh Mohammed Shariful Islam, Faisal Ismail, Nahlah Elkudssiah Ismail, Gaetano Isola, Masao Iwagami, Ihoghosa Osamuyi Iyamu, Louis Jacob, Kathryn H Jacobsen, Morteza Jafarinia, Kasra Jahankhani, Nader Jahanmehr, Nityanand Jain, Ammar Abdulrahman Jairoun, Dr Ruchi Jakhmola Mani, Safayet Jamil, Roland Dominic G Jamora, Abubakar Ibrahim Jatau, Sabzali Javadov, Tahereh Javaheri, Shubha Jayaram, Sun Ha Jee, Jayakumar Jeganathan, Heng Jiang, Mohammad Jokar, Jost B Jonas, Nitin Joseph, Charity Ehimwenma Joshua, Mikk Jürisson, Vaishali K, Ali Kabir, Zubair Kabir, Vidya Kadashetti, Laleh R Kalankesh, Sanjay Kalra, Ashwin Kamath, Rajesh Kamath, Arun Kamireddy, Mona Kanaan, Tanuj Kanchan, Edmund Wedam Kanmiki, Kehinde Kazeem Kanmodi, Sushil Kumar Kansal, Asima Karim, Samad Karkhah, Faizan Zaffar Kashoo, Hengameh Kasraei, Molly B Kassel, Srinivasa Vittal Katikireddi, Joonas H Kauppila, Harkiran Kaur, Gbenga A Kayode, Foad Kazemi, Sina Kazemian, Fassikaw Kebede, Evie Shoshannah Kendal, Emmanuelle Kesse-Guyot, Shahram Khademvatan, Himanshu Khajuria, Amirmohammad Khalaji, Asaad Khalid, Nauman Khalid, Alireza Khalilian, Faham Khamesipour, Fayaz Khan, Mohammad Jobair Khan, Moien AB Khan, Shaghayegh Khanmohammadi, Khaled Khatab, Haitham Khatatbeh, Moawiah Mohammad Khatatbeh, Mahalaqua Nazli Khatib, Hamid Reza Khayat Kashani, Khalid A Kheirallah, Manoj Khokhar, Moein Khormali, Zahra Khorrami, Atulya Aman Khosla, Majid Khosravi, Mahmood Khosrowjerdi, Jagdish Khubchandani, Zemene Demelash Kifle, Grace Kim, Julie Sojin Kim, Min Seo Kim, Yun Jin Kim, Ruth W Kimokoti, Adnan Kisa, Sezer Kisa, Luke D Knibbs, Ann Kristin Skrindo Knudsen, Sonali Kochhar, Ali-Asghar Kolahi, Farzad Kompani, Gerbrand Koren, Oleksii Korzh, Kewal Krishan, Varun Krishna, Vijay Krishnamoorthy, Burcu Kucuk Bicer, Md Abdul Kuddus, Mohammed Kuddus, Ilari Kuitunen, Omar Kujan, Mukhtar Kulimbet, Vishnutheertha Kulkarni, G Anil Kumar, Harish Kumar, Nithin Kumar, Rakesh Kumar, Vijay Kumar, Amartya Kundu, Dian Kusuma, Frank Kyei-Arthur, Ville Kytö, Hmwe Hmwe Kyu, Carlo La Vecchia, Ben Lacey, Muhammad Awwal Ladan, Lucie Laflamme, Chandrakant Lahariya, Daphne Teck Ching Lai, Ratilal Lalloo, Tea Lallukka, Judit Lám, Qing Lan, Tuo Lan, Iván Landires, Francesco Lanfranchi, Berthold Langguth, Van Charles Lansingh, Ariane Laplante-Lévesque, Bagher Larijani, Anders O Larsson, Savita Lasrado, Paolo Lauriola, Hilary R Lawlor, Huu-Hoai Le, Long Khanh Dao Le, Nhi Huu Hanh Le, Thao Thi Thu Le, Trang Diep Thanh Le, Janet L Leasher, Doo Woong Lee, Munjae Lee, Paul H Lee, Sang-woong Lee, Seung Won Lee, Shaun Wen Huey Lee, Yo Han Lee, James Leigh, Elvynna Leong, Ming-Chieh Li, Massimo Libra, Virendra S Ligade, Lee-Ling Lim, Stephen S Lim, Liknaw Workie Limenh, Daniel Lindholm, Paulina A Lindstedt, Stefan Listl, Gang Liu, Shiwei Liu, Shuke Liu, Xiaofeng Liu, Xuefeng Liu, Erand Llanaj, Rubén López-Bueno, José Francisco López-Gil, Arianna Maever Loreche, Paulo A Lotufo, Rafael Lozano, Jailos Lubinda, Giancarlo Lucchetti, Lisha Luo, Jay B Lusk, Lei Lv, Hawraz Ibrahim M Amin, Zheng Feei Ma, Kelsey Lynn Maass, Nikolaos Machairas, Monika Machoy, Áurea M Madureira-Carvalho, Hassan Magdy Abd El Razek, Azzam A Maghazachi, D.R. Mahadeshwara Prasad, Mehrdad Mahalleh, Phetole Walter Mahasha, Mansour Adam Mahmoud, Elham Mahmoudi, Golnaz Mahmoudvand, Maureen Makama, Elaheh Malakan Rad, Kashish Malhotra, Ahmad Azam Malik, Deborah Carvalho Malta, Yosef Manla, Ali Mansour, Mohammad Hadi Mansouri, Pejman Mansouri, Vahid Mansouri, Marjan Mansourian, Mohammad Ali Mansournia, Bishnu P Marasini, Hamid Reza Marateb, Joemer C Maravilla, Parham Mardi, Abdoljalal Marjani, Hamed Markazi Moghadam, Carlos Alberto Marrugo Arnedo, Gabriel Martinez, Ramon Martinez-Piedra, Francisco Rogerlândio Martins-Melo, Miquel Martorell, Wolfgang Marx, Roy Rillera Marzo, Sahar Masoudi, Yasith Mathangasinghe, Alexander G Mathioudakis, Medha Mathur, Navgeet Mathur, Neeta Mathur, Fernanda Penido Matozinhos, Jishanth Mattumpuram, Richard James Maude, Andrea Maugeri, Mahsa Mayeli, Mohsen Mazidi, Antonio Mazzotti, John J McGrath, Martin McKee, Anna Laura W McKowen, Michael A McPhail, Steven M McPhail, Asim Mehmood, Kamran Mehrabani-Zeinabad, Sepideh Mehravar, Tesfahun Mekene Meto, Endalkachew Belayneh Melese, Max Alberto Mendez Mendez-Lopez, Walter Mendoza, Ritesh G Menezes, George A Mensah, Laverne G Mensah, Alexios-Fotios A Mentis, Sultan Ayoub Meo, Atte Meretoja, Tuomo J Meretoja, Abera M Mersha, Tomislav Mestrovic, Kukulege Chamila Dinushi Mettananda, Sachith Mettananda, Adquate Mhlanga, Laurette Mhlanga, Tomasz Miazgowski, Irmina Maria Michalek, Ana Carolina Micheletti Gomide Nogueira de Sá, Ted R Miller, Le Huu Nhat Minh, Alireza Mirahmadi, Antonio Mirijello, Erkin M Mirrakhimov, Roya Mirzaei, Philip B Mitchell, Chaitanya Mittal, Madeline E Moberg, Atousa Moghadam Fard, Seyedehfatemeh Mohajelin, Ashraf Mohamadkhani, Ahmed Ismail Mohamed, Jama Mohamed, Mouhand F H Mohamed, Nouh Saad Mohamed, Ameen Mosa Mohammad, Soheil Mohammadi, Hussen Mohammed, Mustapha Mohammed, Shafiu Mohammed, Ali H Mokdad, Mariam Molokhia, Shaher Mohammad Momani, Sara Momtazmanesh, Lorenzo Monasta, Stefania Mondello, Mohammad Ali Moni, Fateme Montazeri, AmirAli Moodi Ghalibaf, Maryam Moradi, Yousef Moradi, Paula Moraga, Lidia Morawska, Rafael Silveira Moreira, Negar Morovatdar, Shane Douglas Morrison, Abbas Mosapour, Jonathan F Mosser, Elias Mossialos, Rohith Motappa, Vincent Mougin, Parsa Mousavi, Matías Mrejen, Sumaira Mubarik, Ulrich Otto Mueller, Francesk Mulita, Kavita Munjal, Efrén Murillo-Zamora, Khaled M Musallam, Ana-Maria Musina, Ghulam Mustafa, Woojae Myung, Ayoub Nafei, Ahamarshan Jayaraman Nagarajan, Pirouz Naghavi, Ganesh R Naik, Gurudatta Naik, Firzan Nainu, Soroush Najdaghi, Noureddin Nakhostin Ansari, Vinay Nangia, Sreenivas Narasimha Swamy, Shumaila Nargus, Delaram Narimani Davani, Bruno Ramos Nascimento, Gustavo G Nascimento, Abdallah Y Naser, Abdulqadir J Nashwan, Zuhair S Natto, Javaid Nauman, Samidi N K Navaratna, Muhammad Naveed, Nawsherwan, Biswa Prakash Nayak, Vinod C Nayak, Hadush Negash, Ionut Negoi, Ruxandra Irina Negoi, Seyed Aria Nejadghaderi, Chakib Nejjari, Soroush Nematollahi, Henok Biresaw Netsere, Marie Ng, Georges Nguefack-Tsague, Josephine W Ngunjiri, Anh Hoang Nguyen, Dang H Nguyen, Duc Hoang Nguyen, Hau Thi Hien Nguyen, Nhan Nguyen, Nhien Ngoc Y Nguyen, Phat Tuan Nguyen, QuynhAnh P Nguyen, Van Thanh Nguyen, Duc Nguyen Tran Minh, Robina Khan Niazi, Yeshambel T Nigatu, Mahdieh Niknam, Ali Nikoobar, Amin Reza Nikpoor, Nasrin Nikravangolsefid, Efaq Ali Noman, Shuhei Nomura, Syed Toukir Ahmed Noor, Nafise Noroozi, Mehran Nouri, Majid Nozari, Chisom Adaobi Nri-Ezedi, George Ntaios, Mengistu H Nunemo, Dieta Nurrika, Jerry John Nutor, Chimezie Igwegbe Nzoputam, Ogochukwu Janet Nzoputam, Bogdan Oancea, Kehinde O Obamiro, Ismail A Odetokun, Michael Safo Oduro, Oluwaseun Adeolu Ogundijo, Adesola Adenike Ogunfowokan, Abiola Ogunkoya, Ayodipupo Sikiru Oguntade, In-Hwan Oh, Tolulope R Ojo-Akosile, Hassan Okati-Aliabad, Akinkunmi Paul Okekunle, Osaretin Christabel Okonji, Andrew T Olagunju, Matthew Idowu Olatubi, Gláucia Maria Moraes Oliveira, Bolajoko Olubukunola Olusanya, Jacob Olusegun Olusanya, Yinka Doris Oluwafemi, Hany A Omar, Goran Latif Omer, Sokking Ong, Sandersan Onie, Obinna E Onwujekwe, Abdulahi Opejin Opejin, Michal Ordak, Verner N Orish, Alberto Ortiz, Esteban Ortiz-Prado, Wael M S Osman, Sergej M Ostojic, Samuel M Ostroff, Uchechukwu Levi Osuagwu, Adrian Otoiu, Stanislav S Otstavnov, Amel Ouyahia, Mayowa O Owolabi, Oyetunde T Oyeyemi, Ahmad Ozair, Mahesh Padukudru P A, Alicia Padron-Monedero, Jagadish Rao Padubidri, Pramod Kumar Pal, Tamás Palicz, Feng Pan, Hai-Feng Pan, Songhomitra Panda-Jonas, Anamika Pandey, Victoria Pando-Robles, Helena Ullyartha Pangaribuan, Georgios D Panos, Leonidas D Panos, Ioannis Pantazopoulos, Anca Mihaela Pantea Stoian, Romil R Parikh, Eun-Kee Park, Seoyeon Park, Sungchul Park, Nicholas Parsons, Ashwaghosha Parthasarathi, Maja Pasovic, Roberto Passera, Jay Patel, Aslam Ramjan Pathan, Shankargouda Patil, Dimitrios Patoulias, Shrikant Pawar, Hamidreza Pazoki Toroudi, Spencer A Pease, Amy E Peden, Paolo Pedersini, Umberto Pensato, Veincent Christian Filipino Pepito, Prince Peprah, Marcos Pereira, Maria Odete Pereira, Arokiasamy Perianayagam, Norberto Perico, Simone Perna, Konrad Pesudovs, Fanny Emily Petermann-Rocha, Hoang Tran Pham, Anil K Philip, Michael R Phillips, Manon Pigeolet, Michael A Piradov, Enrico Pisoni, Evgenii Plotnikov, Dimitri Poddighe, Roman V Polibin, Ramesh Poluru, Ville T Ponkilainen, Djordje S Popovic, Maarten J Postma, Ahmad Pour-Rashidi, Disha Prabhu, Sergio I Prada, Jalandhar Pradhan, Pranil Man Singh Pradhan, Akila Prashant, Elton Junio Sady Prates, Tina Priscilla, Hery Purnobasuki, Bharathi M Purohit, Jagadeesh Puvvula, Nameer Hashim Qasim, Ibrahim Qattea, Asma Saleem Qazi, Gangzhen Qian, Mehrdad Rabiee Rad, Venkatraman Radhakrishnan, Hadi Raeisi Shahraki, Quinn Rafferty, Alberto Raggi, Cat Raggi, Nasiru Raheem, Fakher Rahim, Md Jillur Rahim, Sarvenaz Rahimibarghani, Md Mijanur Mijanur Rahman Rahman, Mosiur Rahman, Muhammad Aziz Rahman, Tafhimur Rahman, Amir Masoud Rahmani, Mohammad Rahmanian, Nazanin Rahmanian, Rahem Rahmati, Setyaningrum Rahmawaty, Diego Raimondo, Adarsh Raja, Prashant Rajput, Majed Ramadan, Shakthi Kumaran Ramasamy, Sheena Ramazanu, Pramod W Ramteke, Kritika Rana, Rishabh Kumar Rana, Chhabi Lal Ranabhat, Amey Rane, Chythra R Rao, Mithun Rao, Davide Rasella, Vahid Rashedi, Ahmed Mustafa Rashid, Ashkan Rasouli-Saravani, Prateek Rastogi, Azad Rasul, Devarajan Rathish, Giridhara Rathnaiah Babu, Santosh Kumar Rauniyar, Ramin Ravangard, David Laith Rawaf, Salman Rawaf, Rabail Zehra Raza, Elrashdy Moustafa Mohamed Redwan, Lennart Reifels, Marissa B Reitsma, Giuseppe Remuzzi, Kannan RR Rengasamy, Bhageerathy Reshmi, Serge Resnikoff, Stefano Restaino, Luis Felipe Reyes, Nazila Rezaei, Negar Rezaei, Zahra Sadat Rezaei, Mohsen Rezaeian, Taeho Gregory Rhee, Jennifer Rickard, Toshana Robalik, Hannah Elizabeth Robinson-Oden, Hermano Alexandre Lima Rocha, Mónica Rodrigues, Jefferson Antonio Buendia Rodriguez, Leonardo Roever, Debby Syahru Romadlon, Luca Ronfani, Moustaq karim khan Rony, Gholamreza Roshandel, Kunle Rotimi, Himanshu Sekhar Rout, Bedanta Roy, Enrico Rubagotti, Guilherme de Andrade Ruela, Susan Fred Rumisha, Tilleye Runghien, Michele Russo, Aly M A Saad, Korosh Saber, Maha Mohamed Saber-Ayad, Cameron John Sabet, Siamak Sabour, Perminder S Sachdev, Adam Saddler, Bashdar Abuzed Sadee, Masoumeh Sadeghi, Mohammad Reza Saeb, Umar Saeed, Sher Zaman Safi, Rajesh Sagar, Alireza Saghafi, Dominic Sagoe, Amirhossein Sahebkar, Pragyan Monalisa Sahoo, Mirza Rizwan Sajid, Nasir Salam, Payman Salamati, Afeez Abolarinwa Salami, Mohamed A Saleh, Leili Salehi, Marwa Rashad Salem, Aanuoluwa James Salemcity, Sohrab Salimi, Hossein Samadi Kafil, Saad Samargandy, Yoseph Leonardo Samodra, Abdallah M Samy, Juan Sanabria, Francesca Sanna, Milena M Santric-Milicevic, Bruno Piassi Sao Jose, Sivan Yegnanarayana Iyer Saraswathy, Aswini Saravanan, Rodrigo Sarmiento-Suárez, Gargi Sachin Sarode, Sachin C Sarode, Benn Sartorius, Maheswar Satpathy, Abu Sayeed, Nikolaos Scarmeas, Benedikt Michael Schaarschmidt, Christophe Schinckus, Art Schuermans, Austin E Schumacher, Aletta Elisabeth Schutte, David C Schwebel, Falk Schwendicke, Siddharthan Selvaraj, Mohammad H Semreen, Sabyasachi Senapati, Pallav Sengupta, Subramanian Senthilkumaran, Dragos Serban, Yashendra Sethi, Allen Seylani, Mahan Shafie, Pritik A Shah, Ataollah Shahbandi, Samiah Shahid, Wajeehah Shahid, Hamid R Shahsavari, Moyad Jamal Shahwan, Masood Ali Shaikh, Ali S Shalash, Ali Shamekh, Muhammad Aaqib Shamim, Mohd Shanawaz, Abhishek Shankar, Mohammed Shannawaz, Medha Sharath, Sadaf Sharfaei, Amin Sharifan, Javad Sharifi-Rad, Anupam Sharma, Manoj Sharma, Saurab Sharma, Vishal Sharma, Rajesh P Shastry, Maryam Shayan, Shashank Shekhar, Rekha R Shenoy, Mahabalesh Shetty, Pavanchand H Shetty, Premalatha K Shetty, Peilin Shi, Amir Shiani, Mika Shigematsu, Tariku Shimels, Rahman Shiri, Aminu Shittu, Ivy Shiue, K M Shivakumar, Sina Shool, Seyed Afshin Shorofi, Sunil Shrestha, Kerem Shuval, Yafei Si, Emmanuel Edwar Siddig, Jaspreet Kaur Sidhu, João Pedro Silva, Luís Manuel Lopes Rodrigues Silva, Soraia Silva, Thales Philipe R Silva, Colin R Simpson, Kyle E Simpson, Abhinav Singh, Balbir Bagicha Singh, Baljinder Singh, Harmanjit Singh, Jasbir Singh, Paramdeep Singh, Puneetpal Singh, Søren T Skou, Georgia Smith, Farrukh Sobia, Bogdan Socea, Shipra Solanki, Hamidreza Soleimani, Sameh S M Soliman, Yi Song, Ireneous N Soyiri, Michael Spartalis, Sandra Spearman, Chandrashekhar T Sreeramareddy, Jeffrey D Stanaway, Muhammad Haroon Stanikzai, Antonina V Starodubova, Dan J Stein, Caitlyn Steiner, Paschalis Steiropoulos, Leo Stockfelt, Mark A Stokes, Kurt Straif, Narayan Subedi, Rizwan Suliankatchi Abdulkader, Abida Sultana, Jing Sun, Johan Sundström, Chandan Kumar Swain, Lukasz Szarpak, Mindy D Szeto, Payam Tabaee Damavandi, Rafael Tabarés-Seisdedos, Ozra Tabatabaei Malazy, Seyed-Amir Tabatabaeizadeh, Shima Tabatabai, Karen M Tabb, Celine Tabche, Mohammad Tabish, Yasaman Taheri Abkenar, Moslem Taheri Soodejani, Jabeen Taiba, Iman M Talaat, Jacques Lukenze Tamuzi, Ker-Kan Tan, Haosu Tang, Nathan Y Tat, Razieh Tavakoli Oliaee, Seyed Mohammad Tavangar, Nuno Taveira, Abdelghani Tbakhi, Hadi Tehrani, Mohamad-Hani Temsah, Masayuki Teramoto, Behailu Terefe Tesfaye, Enoch Teye-Kwadjo, Pugazhenthan Thangaraju, Kavumpurathu Raman Thankappan, Rekha Thapar, Rasiah Thayakaran, Sathish Thirunavukkarasu, Nihal Thomas, Lau Caspar Thygesen, Jansje Henny Vera Ticoalu, Dinesh Timalsena, Tenaw Yimer Tiruye, Krishna Tiwari, Sojit Tomo, Marcello Tonelli, Roman Topor-Madry, Mathilde Touvier, Marcos Roberto Tovani-Palone, An Thien Tran, Jasmine T Tran, Nghia Minh Tran, Thang Huu Tran, Domenico Trico, Samuel Joseph Tromans, Thien Tan Tri Tai Truyen, Aristidis Tsatsakis, Evangelia Eirini Tsermpini, Munkhtuya Tumurkhuu, Steven T Turnock, Arit Udoh, Atta Ullah, Saeed Ullah, Sana Ullah, Srikanth Umakanthan, Muhammad Umar, Shehu Salihu Umar, Brigid Unim, Bhaskaran Unnikrishnan, Era Upadhyay, Jibrin Sammani Usman, Sanaz Vahdati, Asokan Govindaraj Vaithinathan, Omid Vakili, Rohollah Valizadeh, Jef Van den Eynde, Priya Vart, Shoban Babu Varthya, Tommi Juhani Vasankari, Milena Vasic, Narayanaswamy Venketasubramanian, Massimiliano Veroux, Georgios-Ioannis Verras, Dominique Vervoort, Mathavaswami Vijayageetha, Jorge Hugo Villafañe, Manish Vinayak, Francesco S Violante, Sergey Konstantinovitch Vladimirov, Vasily Vlassov, Bay Vo, Karn Vohra, Theo Vos, Abdul Wadood Wadood, Yasir Waheed, Fang Wang, Shaopan Wang, Shu Wang, Yanqing Wang, Yanzhong Wang, Yuan-Pang Wang, Mary Njeri Wanjau, Muhammad Waqas, Paul Ward, Abdul Waris, Emebet Gashaw Wassie, Stefanie Watson, Marcia R Weaver, Kosala Gayan Weerakoon, Robert G Weintraub, Haftom Legese Legese Weldetinsaa, Katherine M Wells, Yi Feng Wen, Ronny Westerman, Taweewat Wiangkham, Dakshitha Praneeth Wickramasinghe, Evi Widowati, Marcin W Wojewodzic, Dawit Habte Woldeyes, Axel Walter Wolf, Charles D A Wolfe, Chenkai Wu, Dongze Wu, Felicia Wu, Jiayuan Wu, Zenghong Wu, Sarah Wulf Hanson, Hong Xiao, Suowen Xu, Rakesh Yadav, Kazumasa Yamagishi, Danting Yang, Yuichiro Yano, Amir Yarahmadi, Iman Yazdani Nia, Pengpeng Ye, Renjulal Yesodharan, Subah Abderehim Yesuf, Saber Yezli, Arzu Yiğit, Vahit Yiğit, Zeamanuel Anteneh Yigzaw, Dehui Yin, Paul Yip, Naohiro Yonemoto, Yuyi You, Mustafa Z Younis, Chuanhua Yu, Elaine A Yu, Yong Yu, Chun-Wei Yuan, Hadiza Yusuf, Uzma Zafar, Nima Zafari, Mondal Hasan Zahid, Fathiah Zakham, Nazar Zaki, Taddese Alemu Zerfu, Haijun Zhang, Jingya Zhang, Liqun Zhang, Yunquan Zhang, Zhiqiang Zhang, Xiu-Ju George Zhao, Yang Zhao, Zhongyi Zhao, Chenwen Zhong, Bolun Zhou, Juexiao Zhou, Shangcheng Zhou, Bin Zhu, Abzal Zhumagaliuly, Magdalena Zielińska, Ghazal Zoghi, Alimuddin Zumla, Sa'ed H Zyoud, Samer H Zyoud, Amanda E Smith, Christopher J L Murray

## Abstract

**Background:**

Future trends in disease burden and drivers of health are of great interest to policy makers and the public at large. This information can be used for policy and long-term health investment, planning, and prioritisation. We have expanded and improved upon previous forecasts produced as part of the Global Burden of Diseases, Injuries, and Risk Factors Study (GBD) and provide a reference forecast (the most likely future), and alternative scenarios assessing disease burden trajectories if selected sets of risk factors were eliminated from current levels by 2050.

**Methods:**

Using forecasts of major drivers of health such as the Socio-demographic Index (SDI; a composite measure of lag-distributed income per capita, mean years of education, and total fertility under 25 years of age) and the full set of risk factor exposures captured by GBD, we provide cause-specific forecasts of mortality, years of life lost (YLLs), years lived with disability (YLDs), and disability-adjusted life-years (DALYs) by age and sex from 2022 to 2050 for 204 countries and territories, 21 GBD regions, seven super-regions, and the world. All analyses were done at the cause-specific level so that only risk factors deemed causal by the GBD comparative risk assessment influenced future trajectories of mortality for each disease. Cause-specific mortality was modelled using mixed-effects models with SDI and time as the main covariates, and the combined impact of causal risk factors as an offset in the model. At the all-cause mortality level, we captured unexplained variation by modelling residuals with an autoregressive integrated moving average model with drift attenuation. These all-cause forecasts constrained the cause-specific forecasts at successively deeper levels of the GBD cause hierarchy using cascading mortality models, thus ensuring a robust estimate of cause-specific mortality. For non-fatal measures (eg, low back pain), incidence and prevalence were forecasted from mixed-effects models with SDI as the main covariate, and YLDs were computed from the resulting prevalence forecasts and average disability weights from GBD. Alternative future scenarios were constructed by replacing appropriate reference trajectories for risk factors with hypothetical trajectories of gradual elimination of risk factor exposure from current levels to 2050. The scenarios were constructed from various sets of risk factors: environmental risks (Safer Environment scenario), risks associated with communicable, maternal, neonatal, and nutritional diseases (CMNNs; Improved Childhood Nutrition and Vaccination scenario), risks associated with major non-communicable diseases (NCDs; Improved Behavioural and Metabolic Risks scenario), and the combined effects of these three scenarios. Using the Shared Socioeconomic Pathways climate scenarios SSP2-4.5 as reference and SSP1-1.9 as an optimistic alternative in the Safer Environment scenario, we accounted for climate change impact on health by using the most recent Intergovernmental Panel on Climate Change temperature forecasts and published trajectories of ambient air pollution for the same two scenarios. Life expectancy and healthy life expectancy were computed using standard methods. The forecasting framework includes computing the age-sex-specific future population for each location and separately for each scenario. 95% uncertainty intervals (UIs) for each individual future estimate were derived from the 2·5th and 97·5th percentiles of distributions generated from propagating 500 draws through the multistage computational pipeline.

**Findings:**

In the reference scenario forecast, global and super-regional life expectancy increased from 2022 to 2050, but improvement was at a slower pace than in the three decades preceding the COVID-19 pandemic (beginning in 2020). Gains in future life expectancy were forecasted to be greatest in super-regions with comparatively low life expectancies (such as sub-Saharan Africa) compared with super-regions with higher life expectancies (such as the high-income super-region), leading to a trend towards convergence in life expectancy across locations between now and 2050. At the super-region level, forecasted healthy life expectancy patterns were similar to those of life expectancies. Forecasts for the reference scenario found that health will improve in the coming decades, with all-cause age-standardised DALY rates decreasing in every GBD super-region. The total DALY burden measured in counts, however, will increase in every super-region, largely a function of population ageing and growth. We also forecasted that both DALY counts and age-standardised DALY rates will continue to shift from CMNNs to NCDs, with the most pronounced shifts occurring in sub-Saharan Africa (60·1% [95% UI 56·8–63·1] of DALYs were from CMNNs in 2022 compared with 35·8% [31·0–45·0] in 2050) and south Asia (31·7% [29·2–34·1] to 15·5% [13·7–17·5]). This shift is reflected in the leading global causes of DALYs, with the top four causes in 2050 being ischaemic heart disease, stroke, diabetes, and chronic obstructive pulmonary disease, compared with 2022, with ischaemic heart disease, neonatal disorders, stroke, and lower respiratory infections at the top. The global proportion of DALYs due to YLDs likewise increased from 33·8% (27·4–40·3) to 41·1% (33·9–48·1) from 2022 to 2050, demonstrating an important shift in overall disease burden towards morbidity and away from premature death. The largest shift of this kind was forecasted for sub-Saharan Africa, from 20·1% (15·6–25·3) of DALYs due to YLDs in 2022 to 35·6% (26·5–43·0) in 2050. In the assessment of alternative future scenarios, the combined effects of the scenarios (Safer Environment, Improved Childhood Nutrition and Vaccination, and Improved Behavioural and Metabolic Risks scenarios) demonstrated an important decrease in the global burden of DALYs in 2050 of 15·4% (13·5–17·5) compared with the reference scenario, with decreases across super-regions ranging from 10·4% (9·7–11·3) in the high-income super-region to 23·9% (20·7–27·3) in north Africa and the Middle East. The Safer Environment scenario had its largest decrease in sub-Saharan Africa (5·2% [3·5–6·8]), the Improved Behavioural and Metabolic Risks scenario in north Africa and the Middle East (23·2% [20·2–26·5]), and the Improved Nutrition and Vaccination scenario in sub-Saharan Africa (2·0% [–0·6 to 3·6]).

**Interpretation:**

Globally, life expectancy and age-standardised disease burden were forecasted to improve between 2022 and 2050, with the majority of the burden continuing to shift from CMNNs to NCDs. That said, continued progress on reducing the CMNN disease burden will be dependent on maintaining investment in and policy emphasis on CMNN disease prevention and treatment. Mostly due to growth and ageing of populations, the number of deaths and DALYs due to all causes combined will generally increase. By constructing alternative future scenarios wherein certain risk exposures are eliminated by 2050, we have shown that opportunities exist to substantially improve health outcomes in the future through concerted efforts to prevent exposure to well established risk factors and to expand access to key health interventions.

**Funding:**

Bill & Melinda Gates Foundation.

## Introduction

Comprehensive forecasts and alternative future scenarios of disease burden are in high demand from policy makers and planners of health-care systems and are also of great interest to the general public. A key feature for health forecasts to be useful is that changes in population size and age composition of future populations are built in. Future population estimates are needed to enumerate effects of, for example, ageing populations on disease burden, so that health system capacity can be planned accordingly. Likewise, widespread reductions in fertility that have been observed in past decades have consequences for future school and education systems, the size of the workforce, and capacity of pension systems to support a growing older population.^1^, ^2^

Over the past several decades, a series of forecasting studies have been built on historical estimates of disease and injury burden from the Global Burden of Diseases, Injuries, and Risk Factors Study (GBD). GBD forecasts of mortality and disability-adjusted life-years (DALYs) were initially produced at the regional level for 1990–2020^3^ and were updated a decade later to include projections to 2030.^4^ In 2011, regional forecasts were published for communicable and non-communicable disease burden through 2060 that integrated GBD estimates with methods from the International Futures economic modelling framework,^5^ and more recently, forecasts of mortality and independent drivers of health to 2040 were published at the country level.^6^ A GBD-based analysis also produced detailed forecasts of population size, age structure, fertility, migration, and all-cause mortality for 195 countries and territories to 2100.^1^

In this study, we extended country-level GBD forecasts of disease burden to include non-fatal disease burden and present estimates of years lived with disability (YLDs), DALYs, and healthy life expectancy (HALE) for 204 countries and territories to 2050, in addition to measures of fatal disease burden—mortality, years of life lost (YLLs), and life expectancy.

## Methods

### Overview

We projected 359 causes of fatal and non-fatal disease burden from 2022 to 2050 for 204 countries and territories using GBD 2021 estimates.^7^, ^8^ Our modelling framework is multi-staged, using forecasts of independent drivers of health to inform forecasts of cause-specific health outcomes. In our framework, we forecasted drivers of disease, such as age-specific fertility rates by location, age-specific educational attainment by location, and projections of risk factor exposure—such as smoking—by location,^9^ in order to obtain forecasts of cause-specific and all-cause mortality and YLLs. Cause-specific forecasts for mortality–incidence ratios (MIRs) and mortality–prevalence ratios (MPRs) are combined with forecasts of mortality to produce estimates of non-fatal disease burden (YLDs) by age, sex, location, and cause (appendix 1 figure A). Forecasts of YLLs and YLDs are combined to produce forecasts of DALYs, and forecasts of mortality are used along with forecasts of fertility and migration to forecast population, which allows for all of the cause-specific burden measures to be aggregated to produce global estimates.

**Research in context**

**Evidence before this study**

The first forecasts of global disease and injury burden were published a quarter of a century ago. They were part of the first Global Burden of Diseases, Injuries, and Risk Factors Study (GBD) and provided baseline forecasts and optimistic and pessimistic scenarios globally and for eight regions, by sex, in 14 age groups, for nine disease clusters and their child causes from 1990 to 2020. Independent drivers of health in the forecasting regression models were income per capita, years of education, smoking intensity, and time. A 2006 update based on the same GBD framework extending these forecasts to 2030 was based on WHO Global Burden of Disease estimates from 2002. Hughes and colleagues—published in 2011—integrated GBD data with the International Futures modelling system to produce forecasts and scenarios to 2060 for three major cause groups (communicable diseases, non-communicable diseases, and injuries). This study used explicit forecasts of a wider set of drivers including childhood underweight, BMI, unsafe water, poor sanitation and hygiene, ambient and household air pollution, climate change, and motor vehicle ownership. Climate change was captured as mediated through agricultural yield on childhood mortality. In 2018, the most detailed GBD forecasts of mortality, risk factor exposure, and risk factor burden to 2040 were published for 195 countries and territories for 250 causes of death using 79 drivers of health. These health drivers included all GBD 2016 risk factors; selected vaccines; and the Socio-demographic Index (SDI), a composite measure of development that includes lag-distributed income per capita, mean years of educational attainment, and total fertility rate under 25 years. In addition to a reference forecast, optimistic (better for health) and pessimistic (worse for health) scenarios were also produced. These alternative scenarios were constructed from past rates of change for all drivers of health across locations by setting all in an either optimistic or pessimistic position.

**Added value of this study**

This study updates previous mortality forecasts and aligns with the GBD 2021 estimates. For the first time, country-level forecasts include a full set of burden measures: deaths, years of life lost, years lived with disability, disability-adjusted life-years (DALYs), life expectancy, and healthy life expectancy. Considering non-fatal diseases, this expands the number of diseases and injuries GBD forecasts from 250 to 359. The current study includes several methodological refinements. First, ambient mean temperature was added as a risk factor, allowing for forecasts of effects of temperature changes on cause-specific mortality as well as scenarios of different trajectories of mean global surface temperatures and associated effects on health. Second, we untangle the co-dependence between risk drivers via the modelling of intrinsic summary exposure values through the application of mediation factors (see appendix 1 section 2.1.6.2). This allows us to make independent predictions of the component risk trends and combine their future effects without over-counting. Third, we forecast risk factor exposures using an ensemble of models rather than a single rate of change model. The ensemble includes earlier rate of change models and adds a set of meta-regression—Bayesian, regularised, trimmed (MR-BRT) spline regression models that also allow for covariates (such as SDI), with predictive validity informing the sub-model selection. Fourth, in combination with GBD 2021 estimates, we present the first set of estimates and forecasts of fatal and non-fatal burden due to COVID-19 for the first 4 years of the pandemic (2020–23). This allows us to put the burden of COVID-19 in the context of total disease burden. Fifth, we evaluated the skill metric of our cause-specific forecasts for deaths and DALYs. Sixth, we also developed new, target-based scenarios rather than relying only on past rates of change applied across all drivers of health. The first alternative scenario forecasts the effects of eliminating exposure to unsafe water, sanitation, and hygiene and exposure to household air pollution by 2050, as well as the effects of an optimistic global temperature and ambient air pollution scenario on future location-specific, cause-specific disease burden. The second scenario demonstrates the effects on disease burden from eliminating smoking, diet, and metabolic risk factors including high BMI, high LDL cholesterol, high systolic blood pressure, and high fasting plasma glucose by 2050. The third scenario forecasts the effects of eliminating childhood malnutrition risks including child stunting and wasting, vitamin A deficiency, and non-optimal breastfeeding; as well as increasing vaccination rates for a range of common vaccines to 100% by 2050.

**Implications of all the available evidence**

Our reference scenario suggests that global age-standardised disease burden and life expectancy will continue to improve steadily to 2050, although at slower rates than in the several decades preceding the COVID-19 pandemic (beginning in 2020). The greatest health gains are projected to occur in locations with highest rates of deaths and DALYs as of 2022, resulting in a convergence of disease burden across regions. Across locations, the burden of disease will continue to shift from communicable, maternal, neonatal, and nutritional diseases to non-communicable diseases. Policy makers can use our location-specific reference and alternative scenarios—which forecast potential improvements in health outcomes if exposure to key groups of risk factors were eliminated by 2050—to plan for the future and set policy priorities that target the areas of health and risk exposure with the greatest room for improvement. While our framework does not account for every possible future threat to human health, our alternative future scenarios suggest that there are considerable opportunities to achieve better than expected health outcomes over the next several decades—particularly in locations with the highest rates of disease burden—if concerted efforts are made to reduce exposure to known risk factors.

This analysis complies with the Guidelines on Accurate and Transparent Health Estimates Reporting (appendix 1 table S2).^10^ Analyses were completed with Python version 3.10. All code used in the analysis can be found online. Summaries of the modelling methods used in the forecasts of independent drivers, risk factors, mortality, fertility, population, and non-fatal burden are presented in this report, with additional details available in appendix 1 (section 2). Details on the mortality and population forecasting frameworks were also reported previously.^1^, ^6^ We estimated 95% uncertainty intervals (UIs) by combining draw-level data from GBD with draws from the forecast-generating models, when sampling or posterior distributions were available. This allows the propagation of uncertainty through the entire modelling framework. We display the mean, 2·5th, and 97·5th percentiles of the draws for all scenarios in tables and only for the reference scenario in figures. We also produced alternative scenarios of disease burden to reflect potential impacts of health policies.

### Modelling

#### Independent drivers—demographic drivers

We modelled contraceptive met need and educational attainment using the weighted annualised rate of change described by Foreman and colleagues (2018)^6^ with cross-validation to select the parameters. For age-specific fertility rate (ASFR) forecasts, we forecasted completed cohort fertility by age 50 years by location and derived the implied ASFR using cohort-age-specific models, as detailed by the GBD 2021 Fertility and Forecasting Collaborators.^2^ We projected lag-distributed income (LDI) per capita, a moving average transformation of gross domestic product (GDP), by applying a decay function forward in time to estimate future GDP for each country and territory.^11^ Short-term GDP estimates included estimates of the economic impacts of COVID-19 from 2021 to 2026 (appendix 1 section 2.1.3). Finally, we standardised education—which included the estimated effects of disruptions in schooling due to COVID-19 on educational attainment (appendix 1 section 2.1.2)—LDI, and ASFR, and took the geometric mean to obtain location-specific forecasts of the Socio-demographic Index (SDI), used as a driver for mortality and non-fatal models to prevent issues with collinearity between the three component drivers. We forecasted coverage of key vaccines as described by Foreman and colleagues (2018)^6^ using a combination of linear mixed effects models for the following vaccines: third-dose diphtheria, tetanus, and pertussis (DTP3) vaccine; measles conjugate vaccine doses 1 and 2 (MCV1 and MCV2); *Haemophilus influenzae* type B (HiB) vaccine; pneumococcal conjugate (PCV3) vaccine; and rotavirus vaccine (Rota). Estimates of disruption in vaccine coverage during the COVID-19 pandemic were incorporated into the vaccine coverage forecasts (appendix 1 section 2.1.5).

#### Independent drivers—risk factors

We forecasted location-specific GBD summary exposure values (SEVs) for 68 risk factors using an ensemble model comprising six annualised rate of change models and six meta-regression—Bayesian, regularised, trimmed (MR-BRT) spline models driven by SDI. Within each model branch, we varied recency-weighting schemes among the six sub-models, from treating all years equally (recency weight=0) to exponentially weighting the most recent trends more heavily (see appendix 1 section 2.1.6). MR-BRT includes a range of statistical models—primarily non-linear and linear mixed effects models—and fitting procedures.^12^ Forecasts of population attributable fractions (PAFs) by cause and risk were computed using GBD past SEVs and relative risks.^9^, ^13^ Finally, we used novel risk factor mediation methodology that allowed for the impacts of changes to diet, BMI, fasting plasma glucose, smoking, and physical activity to be mediated through systolic blood pressure, LDL cholesterol, and plasma glucose levels. In this way, changes in diet, smoking, and BMI risk factors have secondary impacts on these mediator risk factors as well (see appendix 1 sections 2.1.6.2–2.1.6.3 for details).

Additional details on the methodology to incorporate risk exposure into the mortality modelling framework are described by Foreman and colleagues^6^ and in appendix 1 (section 2.1.6). Figures for select SEVs globally and by super-region for all scenarios can be found in appendix 2 (figure S1).

#### Independent drivers—temperature and particulate matter pollution

To forecast health impacts due to temperature and ambient particulate matter pollution, we employed a modelling approach distinct from the one described above. Using Coupled Model Intercomparison Project Phase 6 (CMIP6) forecasted gridded global temperatures for a reference and optimistic scenario with GBD estimates of relative risks for the association between temperature and cause-specific mortality—estimated using the methods for evaluating the association between cause-specific mortality and daily temperature published by Burkart and colleagues^14^—we calculated PAFs for each cause due to non-optimal temperature (high and low separately), using the methodology from GBD 2021.^9^ See appendix 1 (section 2.1.6.7) for more details. For ambient particulate matter pollution, we used data from a study by Turnock and colleagues,^15^ which translates the same CMIP6 scenarios into gridded ambient air particulate matter concentrations. We employed a similar GBD risk association strategy to calculate PAFs for relevant causes due to air pollution with GBD 2021 methods,^9^ as detailed in appendix 1 (section 2.1.6.8).

#### Mortality and YLLs

Our mortality framework used the methods of Foreman and colleagues^6^ and Vollset and colleagues^1^ with a few minor changes. In short, we forecasted 220 mutually exclusive, collectively exhaustive causes independently using a three-component model comprising (1) the underlying mortality modelled as a function of SDI, time, and cause-specific covariates, (2) a risk factor scalar capturing cause-specific combined risk factor effects, and (3) a random walk with attenuated drift for unexplained residual mortality. We used the model

$$\ln(m_{ilast}^{T})=\alpha_{ilas}+\beta_{is}{\text{SDI}}_{lt}+\theta_{ias}t+\ln(S_{ilast})+\varepsilon_{ilast}$$

where the first three terms of the right-hand side of the equation constitute the logarithm of the underlying (risk-deleted) mortality,

$$\ln(m_{ilast}^{U})$$

S_ilast_ is the risk factor scalar, and for the *i*th cause and *s*th sex,

$$\alpha_{ilas}\sim \text{N}(\beta_{\alpha ,is,}\tau_{\alpha ,is}^{2})$$

is a location-age-specific random intercept, β_is_ is a global fixed slope on SDI, and

$$\theta_{ilas}\sim \text{N}(\beta_{\theta ,is,}\tau_{\alpha ,is}^{2})$$

is an age-specific random slope on the secular time trend. Covariates also included risk factors for which the PAF for the risk and the cause is 1, such as type 2 diabetes and high fasting plasma glucose, because we could not compute a risk factor scalar from a PAF of 1. To forecast the residual trends not captured by risk factors, SDI, and global secular trends in the basic model, we used an autoregressive integrated moving average (ARIMA; 0,1,0) with attenuated drift (exponential decay parameter=0·1) on all-cause mortality, and for all other causes we used an ARIMA (0,1,0) without drift. This allowed the model to capture accelerating trends in all-cause mortality that would not be captured at the detailed cause level. We then cascaded the all-cause mortality envelope down to the most detailed levels in the hierarchy by scaling the cause-specific forecasts. Additional details are provided in appendix 1 (section 2.2). For five out of 284 causes (HIV, exposure to forces of nature, conflict and terrorism, executions, and COVID-19), we used alternative approaches for past events that are stochastic in nature or that are more suitable for capturing the unique transmission and intervention dynamics of the HIV epidemic. For COVID-19, due to severe data limitations after 2021 and high uncertainty around potential longer-term patterns in COVID-19 mortality, we chose to assume that COVID-19 deaths and DALYs will decline linearly to zero between 2023 and 2030. Detailed methods for these custom models are described in appendix 1 (section 2.2.3). We used the resulting estimates to produce forecasted life tables and as inputs to the population cohort-component model and the non-fatal burden estimates, described below and in detail in appendix 1 (sections 2.3–2.5).

Forecasted YLLs were computed as the product of the forecasted cause-specific death rates and standard life expectancy at each age (using the GBD 2021 reference life table).^7^, ^16^

#### Population

We used the methods of Vollset and colleagues^1^ to obtain forecasts of migration and population. Briefly, we used past migration estimates from the UN's Population Division of the Department of Economic and Social Affairs World Population Prospects 2022 report^17^, ^18^ to forecast net migration rates for each country or territory as a function of natural population increase and mortality from war and disaster. We then balanced migration rates to achieve zero net migration globally. We used these estimates in combination with age-sex-location-year-specific all-cause mortality rates and age-location-year-specific total fertility rates to calculate projections of population.^2^ Further information on the population and life table calculations are described in appendix 1 (sections 2.4, 2.5). Forecasts of population were used to produce counts for disease burden metrics and location, age, and sex aggregations (such as global, all-age, and all-sex rates).

#### Non-fatal burden

For the first time, we forecasted non-fatal disease burden (YLDs) by individually modelling 290 causes independently. In order to compute future YLDs, we first forecasted incidence and prevalence for all causes. For causes considered acute, we forecasted incidence using a mixed-effect model of the MIR and forecasted mortality, while for non-acute causes, we used a mixed-effect model to obtain prevalence either via the MPR or directly from GBD estimates of prevalence (such as in cases where mortality is not present or constitutes a very small portion of the disease burden for a given cause, such as chronic low back pain). Where applicable, we then converted these primary metrics into the secondary metric using the prevalence–incidence ratio (PIR); for instance, we obtained incidence for an acute cause, and then converted the incidence to prevalence using the forecasted PIR. For the MIRs, MPRs, and PIRs, we used the model:

$$\log(R_{a,s,l,y})=\beta_{0}+\beta_{1}{\text{SDI}}_{l,y}+\pi_{0:a,s,l}+\pi_{1:a,s,l}{\text{SDI}}_{l,y}+\varepsilon_{a,s,l,y}$$

where *R*_a,s,l,y_ is the age-sex-location-year-specific ratio for a given cause, with the covariate SDI_l,y_ being the location-year-specific SDI. π_0:a,s,l_ is the age-sex-location-specific random intercept, π_1:a,s,l_ is the age-sex-location-specific slope on SDI, and ε_a,s,l,y_ is the residual term. The direct prevalence model was similar, but in logit space without the random slope term. Causes that were exceptions to this general system included congenital causes where incidence only occurs at birth, and composite cause groups (other causes) for which prevalence and incidence do not have a good definition. For the former, we modelled both incidence and prevalence directly without using the PIR; for the latter, we modelled the YLD–YLL ratio and used forecasted YLLs to obtain YLD estimates. We then computed average disability weights across sequelae for each cause from the GBD 2019 estimates and multiplied these by the forecasts of prevalence to compute forecasts of YLDs. We added the YLDs to YLLs to obtain DALYs. The division of causes into different modelling strategies, causes that are exceptions to the standard strategies, and model details can be found in appendix 1 (section 2.3).

### Scenarios

In addition to a reference scenario, we produced the following alternative health scenarios to illustrate the potential health gains from improvements to risk exposure over the course of the next several decades. These scenarios are designed to illustrate the potential achievable benefit to better understand what is possible if exposure to a set of key modifiable risk factors were eliminated. The scenarios do not factor in cost or feasibility of eliminating exposure, or analyse specific policies that could be implemented; they simply forecast potential disease burden in the coming decades if risk exposure targets were realised. Target scenarios were applied to future years for all locations at the most granular age and sex level. In the rare instances that the reference scenario was more optimistic that the target scenario, the reference scenario values were used for those location-year-age-sex subgroups.

#### Safer environment

This scenario assumes that exposure to unsafe water, unsafe sanitation, unsafe hygiene, and household air pollution will be eliminated linearly by 2050 in all locations. In addition, forecasts of particulate matter air pollution and non-optimal temperature reflect carbon emissions trends from the Shared Socioeconomic Pathways SSP1-1.9 scenario as published in the CMIP6 climate projections, representing an aggressive decrease in emissions and reaching net 0 carbon dioxide emissions by 2050 for the alternative scenario.^19^, ^20^ The SSP2-4.5 scenario represents a “middle of the road” path for reducing emissions and stays truer to historical trends for the reference scenario. The resulting global temperature anomalies reported for 2100 were 2·7°C for the reference scenario and 1·9°C for the alternative scenario.^21^ We used the PM2·5 air pollution values reported for the SSP2-4.5 and SSP1-1.9 scenarios by Turnock and colleagues.^15^

#### Improved behavioural and metabolic risks

This scenario assumes exposure to high adult BMI, high systolic blood pressure, high LDL cholesterol, and high fasting plasma glucose are linearly eliminated by 2050 in all locations. It further assumes that exposure to non-optimal diet for all GBD diet-related risk factors is likewise eliminated by 2050; ie, all dietary components included in GBD will be consumed at the level that minimises health risk for that dietary component. In addition, we assumed a linear reduction of current tobacco smokers to zero by 2050 as well as no new smokers after 2022 in all locations.

#### Improved childhood nutrition and vaccination

This scenario assumes exposure to child growth failure (stunting, wasting, underweight), vitamin A and iron deficiency, and suboptimal breastfeeding (discontinued or non-exclusive) linearly decreases to zero by 2050 and assumes a linear increase in vaccine coverage to 100% in all locations by 2050 for the following vaccines: DTP3, MCV1, MCV2, Hib, PCV3, and Rota.

#### Combined

This scenario combines all the target-based trends from the Safer Environment, Improved Behavioural and Metabolic Risks, and Improved Childhood Nutrition and Vaccination scenarios.

### Model performance

To evaluate our model performance, we used the following skill metric,^22^ designed to evaluate forecasting accuracy for the validation period 2010–19:

$$\text{skill}=1-\frac{\text{RMSE}(\text{Model})}{\text{RMSE}(\text{Baseline}\;\text{Model})}$$

where Model is the Institute for Health Metrics and Evaluation (IHME) forecasting model and Baseline Model is a simple model where the value in the year 2009 was held constant over 2010–19. For each model, we calculated squared errors between observed and predicted values for each cause-sex-location-year and winsorised the errors at the 95% level. To calculate root mean square error values, we took the square root of the average of the winsorised squared errors across location-year. This skill metric was reported for both mortality and DALY estimates for males and females of all ages combined for level 0 (all causes combined), 1 (aggregates of non-communicable diseases [NCDs]; injuries; and a category combining communicable, maternal, neonatal, and nutritional diseases [CMNNs]), and 2 (disease and injury aggregate groupings within the Level 1 cause groups such as cardiovascular diseases and nutritional deficiencies) causes in the GBD cause hierarchy (appendix 1 tables E, F). A positive skill value indicates that the model being evaluated performs better than the baseline model (here, a simple model holding 2009 values constant in the future), and a negative value indicates that the evaluated model performs worse than baseline. A negative skill value thus suggests that a different modelling approach might be needed for that particular sex-cause.

We used a 10-year hold-out period to assess skill in order to strike a balance between maximising the available time series of data (beginning in 1990) and producing a hold-out forecast long enough to assess our annual projections sufficiently. We believe that the structural nature of our modelling approach (utilising risks and drivers with known relationships to disease outcomes) will result in more robust forecasts over long time horizons (20 or 30 years). Due to the limited time series of data we have, we were not able to assess the long-term predictability of our models and thus our 10-year hold outs might be more sensitive to noise in the data than they are of long term trends, leading to higher skill values over time.

### Presentation of estimates

Disease and injury burden estimates for 1990, 2022, and 2050 are given in death and DALY counts as well as all-age and age-standardised rates per 100 000 population. For changes over time, we present percentage changes over the time period 2022–50. All final estimates were computed using the mean estimate across 500 draws, and 95% UIs are given as the 2·5th and 97·5th ranked values across all 500 draws. UIs were computed for forecasted alternative scenarios but are only reported in the text and tables. For readability, figures only include UIs for the past and the future reference scenario.

### Role of the funding source

The funders of this study had no role in study design, data collection, data analysis, data interpretation, or the writing of the report.

## Results

We forecasted the complete set of burden estimates (deaths, YLLs, YLDs, DALYs, life expectancy, and HALE) by age and sex for 204 countries and territories from 2022 to 2050. Forecasts have been produced for a reference scenario (the most likely future in our framework, assuming the continuation of past trends and relationships) and for four alternative scenarios. Due to space limitations, YLL and YLD forecasts are available primarily in appendix 2 and on the GBD Foresight visualisation tool.

### Life expectancy and HALE, reference scenario

#### Global and super-regional trends

**Figure 1:**
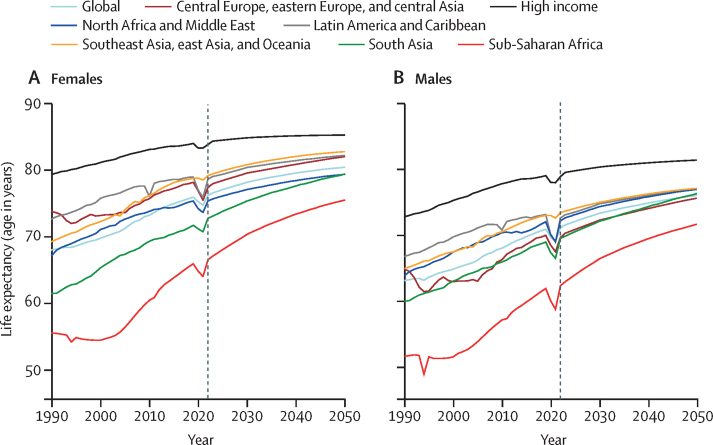
Global and super-regional life expectancy, 1990–2050 for females (A) and males (B) Forecasts are based on the reference scenario. The black vertical line indicates the year 2022 (the first forecast year).

In the reference scenario, we forecasted continued increases in global and super-regional life expectancy between 2022 (global life expectancy of 73·6 years [95% UI 72·7–74·4]) and 2050 (global 78·2 years [75·2–80·3]), but at slower growth rates than in the three decades preceding the COVID-19 pandemic in 2020–21^16^ (table 1, figure 1, appendix 2 table S1). Globally, life expectancy increased by an average of 0·27 years (0·25–0·29) annually between 1990 and 2019 compared with a 0·16-year (0·07–0·23) annual increase between 2022 and 2050, a statistically significant difference in growth rate (appendix 2 table S1). Life expectancy growth rates were significantly slower in the forecasted period than the past period for the high-income super-region; north Africa and the Middle East; and southeast Asia, east Asia, and Oceania. Overall and for both males and females, global life expectancy will continue to increase in all seven GBD super-regions. From 2022 to 2023, we estimate that life expectancies returned to or exceeded 2019 levels. By 2050, we forecast the global life expectancy for males to be just above 75 years and for females just above 80 years.

**Table 1:** Life expectancy and HALE at birth in 2022 and 2050 (reference scenario) by location for males and females

		**Reference scenario life expectancy at birth (years)**						**Reference scenario HALE at birth (years)**					
		Females			Males			Females			Males		
		2022	2030	2050	2022	2030	2050	2022	2030	2050	2022	2030	2050
**Global**		**76·2 (75·4–77·0)**	**78·2 (76·9–79·3)**	**80·5 (77·4–82·6)**	**71·1 (70·1–72·0)**	**73·3 (72·0–74·4)**	**76·0 (73·2–77·9)**	**64·7 (61·4–67·7)**	**66·3 (62·8–69·5)**	**67·5 (63·3–71·6)**	**62·6 (60·1–64·9)**	**64·3 (61·6–66·7)**	**66·0 (62·6–69·3)**
**Central Europe, eastern Europe, and central Asia**		**77·4 (76·7–78·0)**	**79·6 (78·5–80·6)**	**82·0 (79·7–84·1)**	**69·6 (68·8–70·3)**	**72·2 (71·2–73·2)**	**75·5 (73·3–77·4)**	**66·3 (62·9–69·1)**	**68·2 (64·8–71·2)**	**69·9 (66·0–73·5)**	**61·5 (59·1–63·6)**	**63·7 (61·2–66·0)**	**66·3 (63·1–69·3)**
Central Asia		75·3 (74·2–76·3)	78·3 (76·7–79·8)	81·4 (77·3–84·8)	68·9 (67·7–70·0)	72·3 (70·8–73·8)	75·8 (71·8–78·9)	64·9 (61·8–67·5)	67·5 (64·2–70·6)	69·7 (65·1–74·2)	61·5 (59·3–63·5)	64·4 (61·8–66·7)	67·1 (63·0–70·9)
	Armenia	79·5 (76·9–80·7)	82·2 (81·1–83·2)	84·8 (82·8–86·7)	72·7 (69·6–74·4)	76·2 (74·8–77·7)	79·7 (77·6–81·7)	68·5 (64·7–71·3)	70·9 (67·6–73·6)	72·8 (69·0–76·2)	64·7 (61·3–67·2)	67·6 (65·0–69·9)	70·4 (67·2–73·3)
	Azerbaijan	75·3 (74·1–76·5)	78·2 (76·5–79·8)	81·6 (77·5–85·3)	69·7 (68·3–71·2)	73·1 (71·4–74·8)	76·6 (72·9–79·7)	65·1 (62·2–67·7)	67·7 (64·5–70·6)	70·1 (65·5–74·7)	62·5 (60·2–64·6)	65·4 (62·9–67·8)	68·2 (64·5–71·7)
	Georgia	78·6 (77·4–79·7)	81·5 (80·3–82·8)	84·2 (81·7–86·4)	69·3 (68·2–70·4)	71·5 (70·4–72·6)	73·6 (71·5–75·3)	67·6 (64·3–70·5)	70·3 (67·1–73·3)	72·5 (68·5–76·4)	61·6 (59·2–63·6)	63·5 (61·1–65·6)	65·3 (62·5–68·0)
	Kazakhstan	74·0 (72·0–75·6)	78·5 (77·3–79·8)	81·6 (78·9–83·8)	66·0 (63·9–67·6)	71·0 (69·6–72·3)	75·0 (72·1–77·1)	63·8 (60·7–66·7)	67·5 (64·2–70·3)	69·6 (65·7–73·3)	58·9 (56·6–61·1)	63·0 (60·6–65·4)	66·1 (62·9–69·3)
	Kyrgyzstan	74·9 (72·8–76·6)	79·2 (77·4–81·0)	81·5 (78·0–85·0)	67·4 (65·1–69·3)	72·2 (70·1–74·3)	74·9 (71·0–78·3)	64·9 (61·7–68·0)	68·4 (64·9–71·4)	69·9 (65·6–74·4)	60·5 (57·8–62·8)	64·4 (61·6–67·0)	66·4 (62·4–70·1)
	Mongolia	76·0 (74·9–77·2)	77·7 (76·2–79·2)	80·4 (76·8–83·4)	67·1 (65·7–68·8)	68·6 (66·6–70·7)	70·3 (66·2–74·0)	65·4 (62·2–68·2)	67·1 (63·9–70·2)	69·0 (64·7–73·3)	59·4 (56·9–61·8)	60·7 (58·1–63·3)	62·0 (58·1–65·8)
	Tajikistan	74·8 (72·6–76·4)	77·2 (74·8–79·3)	80·1 (75·0–84·5)	71·3 (68·6–73·4)	74·4 (72·2–76·7)	77·7 (73·6–81·6)	64·6 (61·4–67·4)	66·9 (63·6–70·0)	69·0 (64·2–73·8)	63·4 (60·3–66·0)	66·2 (63·0–68·8)	68·8 (64·6–72·9)
	Turkmenistan	73·5 (70·6–75·9)	76·2 (72·8–79·2)	80·7 (74·1–86·3)	66·7 (63·6–69·4)	69·5 (66·0–72·5)	73·8 (67·6–79·1)	63·7 (60·1–67·0)	66·2 (62·2–70·0)	69·6 (63·4–75·8)	59·8 (56·7–62·7)	62·3 (58·9–65·4)	65·7 (60·2–70·9)
	Uzbekistan	75·8 (74·4–77·2)	77·8 (75·6–79·7)	81·2 (76·1–85·5)	70·9 (69·3–72·3)	73·0 (70·8–75·1)	76·4 (71·2–80·4)	65·1 (61·9–68·0)	66·9 (63·4–70·0)	69·3 (64·1–74·4)	63·2 (60·8–65·6)	65·0 (62·2–67·8)	67·7 (63·0–72·0)
Central Europe		79·9 (79·3–80·5)	81·9 (81·3–82·6)	83·6 (82·4–84·6)	73·2 (72·5–74·0)	75·6 (74·8–76·3)	77·6 (76·5–78·5)	68·5 (65·1–71·4)	70·4 (67·1–73·3)	71·4 (67·8–74·6)	64·6 (61·9–66·9)	66·6 (64·0–69·0)	68·2 (65·3–70·7)
	Albania	79·7 (78·5–80·9)	81·6 (80·3–83·0)	83·1 (81·1–85·0)	75·4 (73·7–76·9)	78·0 (76·5–79·8)	79·7 (77·5–81·7)	68·9 (65·4–71·8)	70·5 (66·9–73·6)	71·5 (67·6–74·9)	66·7 (63·8–69·3)	68·9 (66·0–71·5)	70·2 (66·8–73·1)
	Bosnia and Herzegovina	79·8 (78·1–81·6)	82·1 (80·4–84·1)	84·4 (81·5–87·1)	74·8 (72·3–77·4)	77·5 (75·5–80·2)	79·9 (77·4–82·8)	68·2 (64·5–71·5)	70·2 (66·4–73·5)	71·7 (67·4–75·9)	65·6 (62·5–68·6)	67·9 (64·6–71·2)	69·7 (65·6–73·2)
	Bulgaria	76·0 (74·9–77·2)	78·3 (77·1–79·5)	80·0 (78·2–81·8)	68·9 (67·6–70·5)	71·5 (70·1–73·0)	73·7 (71·7–75·4)	65·7 (62·5–68·5)	67·8 (64·5–70·5)	69·0 (65·6–72·0)	61·3 (58·8–63·5)	63·5 (61·0–65·9)	65·2 (62·5–67·9)
	Croatia	80·7 (80·0–81·6)	82·8 (82·0–83·7)	84·0 (82·9–85·0)	74·8 (73·8–75·9)	77·5 (76·6–78·6)	79·3 (78·3–80·4)	69·1 (65·7–72·1)	71·0 (67·5–73·9)	71·7 (68·1–74·8)	66·0 (63·3–68·3)	68·2 (65·4–70·6)	69·6 (66·7–72·0)
	Czechia	82·4 (81·8–83·3)	83·9 (83·1–84·8)	85·3 (84·0–86·6)	76·7 (75·7–77·7)	78·4 (77·4–79·4)	80·3 (79·0–81·5)	70·0 (66·4–73·2)	71·5 (67·9–74·6)	72·4 (68·6–75·8)	67·1 (64·2–69·6)	68·6 (65·7–71·3)	70·0 (67·1–72·7)
	Hungary	79·7 (78·8–80·6)	81·3 (80·3–82·3)	82·8 (81·2–84·2)	73·2 (72·1–74·4)	75·5 (74·4–76·8)	77·9 (76·4–79·2)	68·1 (64·6–71·2)	69·6 (66·2–72·7)	70·5 (66·8–73·8)	64·6 (61·9–67·1)	66·6 (63·8–69·2)	68·3 (65·3–71·1)
	Montenegro	77·8 (77·0–78·5)	79·5 (78·7–80·3)	81·0 (79·7–82·2)	72·3 (71·2–73·5)	74·5 (73·4–75·7)	76·6 (75·2–77·9)	66·9 (63·6–69·8)	68·7 (65·5–71·4)	69·6 (66·1–72·6)	64·1 (61·6–66·4)	66·2 (63·5–68·5)	67·8 (65·0–70·3)
	North Macedonia	74·8 (72·6–76·3)	78·2 (77·1–79·3)	79·9 (78·2–81·3)	71·0 (68·4–72·9)	75·4 (73·9–76·8)	77·2 (75·4–78·8)	64·7 (61·4–67·7)	67·6 (64·3–70·4)	68·7 (65·4–71·7)	63·2 (60·5–65·8)	66·8 (64·2–69·2)	68·2 (65·5–70·8)
	Poland	81·1 (80·2–82·0)	83·3 (82·5–84·2)	84·7 (83·8–85·8)	73·5 (72·5–74·6)	75·9 (74·8–77·0)	77·6 (76·5–78·8)	69·4 (66·0–72·3)	71·3 (67·9–74·5)	72·3 (68·6–75·7)	64·6 (62·0–66·9)	66·6 (63·9–69·0)	68·0 (65·1–70·5)
	Romania	79·0 (78·0–80·0)	80·8 (79·8–82·1)	82·9 (80·8–84·8)	71·7 (70·6–72·9)	73·7 (72·4–75·0)	76·0 (74·1–77·7)	68·2 (64·9–71·2)	69·9 (66·6–73·0)	71·3 (67·6–74·8)	63·6 (61·1–65·9)	65·3 (62·9–67·7)	67·2 (64·3–69·9)
	Serbia	77·8 (76·8–78·8)	79·7 (78·6–80·7)	81·4 (79·9–82·8)	73·0 (71·8–74·4)	75·3 (73·9–76·6)	77·2 (75·6–78·7)	67·1 (64·0–70·0)	68·8 (65·6–71·6)	69·6 (66·1–72·8)	64·8 (62·2–67·0)	66·6 (63·7–69·0)	67·9 (64·8–70·5)
	Slovakia	80·3 (79·3–81·3)	82·1 (81·0–83·2)	83·8 (82·0–85·3)	73·7 (72·7–74·9)	76·0 (74·8–77·3)	78·1 (76·7–79·5)	68·8 (65·2–71·7)	70·3 (66·9–73·4)	71·5 (67·6–74·7)	65·0 (62·3–67·5)	66·9 (64·1–69·5)	68·5 (65·5–71·3)
	Slovenia	84·8 (83·8–85·7)	86·4 (85·5–87·4)	87·4 (86·2–88·6)	78·5 (77·6–79·5)	80·6 (79·7–81·5)	82·1 (81·1–83·1)	71·8 (68·0–74·9)	73·5 (69·8–76·7)	74·1 (70·2–77·4)	68·6 (65·5–71·1)	70·5 (67·4–73·0)	71·6 (68·5–74·2)
Eastern Europe		77·2 (76·2–78·2)	79·2 (77·9–80·5)	81·9 (79·4–84·0)	68·3 (67·1–69·5)	70·7 (69·3–72·2)	74·7 (72·4–76·9)	65·9 (62·5–68·8)	67·7 (64·3–70·6)	69·5 (65·6–73·0)	60·3 (58·0–62·5)	62·4 (59·8–65·0)	65·4 (62·2–68·5)
	Belarus	76·7 (75·0–78·4)	80·3 (78·6–82·2)	83·0 (80·0–85·7)	67·1 (65·3–69·0)	70·6 (68·6–72·7)	73·6 (70·4–76·2)	66·2 (62·9–69·2)	69·0 (65·9–72·0)	70·9 (66·8–74·6)	59·9 (57·7–62·3)	62·8 (60·3–65·2)	65·1 (61·8–68·1)
	Estonia	81·8 (80·8–82·7)	84·3 (83·4–85·4)	85·8 (84·1–87·3)	73·6 (72·6–74·8)	76·8 (75·7–78·0)	79·2 (77·7–80·6)	69·7 (66·1–72·8)	72·0 (68·3–75·1)	73·2 (69·2–76·5)	64·8 (62·2–67·1)	67·6 (64·8–69·9)	69·4 (66·5–72·0)
	Latvia	78·5 (77·1–79·8)	81·7 (80·7–82·7)	83·7 (82·1–85·2)	69·6 (68·0–71·0)	73·1 (72·0–74·5)	75·7 (74·1–77·3)	67·4 (64·3–70·3)	70·1 (66·9–73·2)	71·5 (67·9–74·8)	61·7 (59·2–64·0)	64·7 (62·1–66·9)	66·6 (63·9–69·3)
	Lithuania	79·7 (78·6–80·8)	82·1 (81·1–83·1)	83·5 (81·9–85·0)	70·2 (69·1–71·5)	72·8 (71·7–74·1)	74·9 (73·4–76·4)	68·1 (64·6–70·9)	70·1 (66·6–73·1)	71·1 (67·3–74·5)	62·1 (59·7–64·3)	64·2 (61·8–66·6)	65·8 (63·0–68·4)
	Moldova	78·8 (77·6–79·9)	81·3 (79·5–83·0)	84·7 (80·3–88·1)	70·5 (69·0–71·8)	72·9 (71·1–74·6)	76·4 (72·5–79·4)	67·6 (64·3–70·8)	69·9 (66·1–73·5)	72·4 (67·1–77·7)	62·7 (60·2–65·1)	64·8 (62·1–67·5)	67·5 (63·5–71·4)
	Russia	77·0 (76·1–78·0)	79·2 (78·1–80·4)	82·0 (79·9–84·0)	68·2 (67·2–69·6)	71·0 (69·6–72·5)	75·1 (73·1–77·2)	65·7 (62·6–68·7)	67·7 (64·4–70·7)	69·5 (65·6–73·1)	60·2 (57·7–62·4)	62·6 (59·9–64·9)	65·7 (62·6–68·6)
	Ukraine	77·5 (73·9–80·7)	78·4 (74·7–81·8)	81·0 (76·5–85·3)	68·5 (64·4–72·6)	69·7 (65·4–74·3)	73·0 (68·0–78·2)	66·2 (62·4–69·7)	67·1 (63·4–71·1)	68·8 (64·3–73·8)	60·3 (56·9–64·5)	61·4 (57·8–65·7)	64·0 (59·6–69·0)
**High income**		**83·9 (83·7–84·0)**	**84·9 (84·7–85·1)**	**85·3 (84·8–85·7)**	**78·8 (78·7–79·0)**	**80·3 (80·1–80·4)**	**81·3 (81·0–81·6)**	**70·3 (66·3–73·6)**	**71·2 (67·3–74·6)**	**71·4 (67·3–74·7)**	**68·4 (65·4–70·9)**	**69·5 (66·6–72·1)**	**70·2 (67·1–72·8)**
Australasia		85·2 (85·1–85·3)	86·1 (85·9–86·3)	86·5 (86·1–86·9)	81·1 (81·0–81·2)	82·4 (82·2–82·6)	83·3 (83·0–83·6)	71·2 (67·1–74·6)	72·1 (68·0–75·5)	72·3 (68·3–75·8)	69·9 (66·7–72·7)	71·0 (67·8–73·8)	71·5 (68·1–74·4)
	Australia	85·5 (85·4–85·6)	86·4 (86·2–86·6)	86·8 (86·3–87·1)	81·2 (81·1–81·3)	82·5 (82·3–82·7)	83·4 (83·1–83·7)	71·4 (67·3–74·8)	72·3 (68·2–75·8)	72·5 (68·5–76·0)	70·0 (66·8–72·8)	71·1 (67·8–73·9)	71·5 (68·1–74·4)
	New Zealand	83·8 (83·7–84·1)	84·8 (84·5–85·0)	85·3 (84·7–85·8)	80·4 (80·2–80·6)	81·8 (81·6–82·1)	82·9 (82·4–83·3)	70·1 (66·2–73·5)	71·0 (67·1–74·4)	71·4 (67·3–74·7)	69·6 (66·5–72·2)	70·8 (67·6–73·5)	71·4 (68·2–74·2)
High-income Asia Pacific		87·7 (87·5–87·8)	88·7 (88·5–88·8)	89·1 (88·8–89·5)	81·9 (81·7–82·0)	83·1 (82·9–83·3)	84·4 (84·0–84·7)	75·1 (71·4–78·3)	75·9 (72·2–79·1)	76·1 (72·3–79·3)	71·9 (69·1–74·4)	72·9 (70·1–75·5)	73·8 (70·8–76·4)
	Brunei	78·3 (77·1–79·3)	79·4 (78·1–80·4)	80·5 (78·9–81·9)	75·0 (73·8–76·1)	76·4 (75·2–77·5)	78·0 (76·3–79·3)	67·7 (64·1–70·7)	68·5 (65·0–71·5)	68·9 (65·3–72·3)	66·0 (63·3–68·3)	67·1 (64·4–69·7)	68·1 (65·1–70·9)
	Japan	88·1 (87·9–88·2)	88·8 (88·6–88·9)	89·2 (88·9–89·5)	82·2 (82·0–82·3)	83·1 (82·9–83·3)	84·2 (83·8–84·5)	75·5 (71·6–78·6)	76·1 (72·3–79·3)	76·3 (72·4–79·5)	72·3 (69·5–74·8)	73·0 (70·2–75·5)	73·7 (70·8–76·3)
	Singapore	88·0 (87·7–88·2)	89·0 (88·7–89·3)	89·7 (89·0–90·2)	84·1 (83·8–84·3)	85·5 (85·1–85·9)	86·9 (86·2–87·5)	76·1 (72·4–79·1)	77·0 (73·3–80·0)	76·9 (73·0–80·1)	74·2 (71·3–76·5)	75·4 (72·4–77·8)	75·9 (72·7–78·6)
	South Korea	86·0 (85·3–86·3)	87·8 (87·5–88·0)	88·9 (88·3–89·4)	80·4 (79·6–80·6)	82·6 (82·3–82·8)	84·4 (83·9–84·9)	73·6 (70·0–76·7)	75·1 (71·5–78·2)	75·7 (71·9–79·0)	70·4 (67·7–72·9)	72·3 (69·5–74·8)	73·6 (70·6–76·3)
High-income North America		81·3 (81·1–81·4)	82·4 (82·1–82·6)	82·8 (82·2–83·4)	76·2 (75·9–76·4)	77·8 (77·5–78·0)	78·9 (78·3–79·4)	67·0 (62·9–70·4)	68·0 (63·9–71·4)	68·2 (64·1–71·7)	64·9 (61·7–67·6)	66·1 (63·0–68·8)	66·8 (63·6–69·6)
	Canada	84·6 (84·4–84·8)	85·5 (85·2–85·7)	85·8 (85·2–86·2)	80·5 (80·3–80·7)	81·7 (81·5–82·0)	82·6 (82·2–83·0)	71·3 (67·3–74·6)	72·0 (68·2–75·4)	72·0 (68·0–75·4)	69·8 (66·7–72·4)	70·7 (67·6–73·3)	71·1 (67·8–73·8)
	Greenland	77·1 (75·7–78·1)	79·8 (78·4–80·9)	81·7 (80·1–82·9)	71·5 (69·8–72·9)	74·0 (72·1–75·4)	75·9 (74·2–77·4)	65·3 (61·6–68·5)	67·5 (63·7–70·9)	69·0 (65·2–72·5)	62·8 (60·1–65·2)	64·7 (61·9–67·3)	66·1 (63·2–69·0)
	USA	80·9 (80·7–81·1)	82·0 (81·7–82·3)	82·4 (81·8–83·1)	75·7 (75·5–75·9)	77·3 (77·0–77·6)	78·4 (77·8–79·0)	66·5 (62·4–70·0)	67·5 (63·4–70·9)	67·8 (63·6–71·2)	64·3 (61·2–67·0)	65·6 (62·4–68·3)	66·3 (63·0–69·1)
Southern Latin America		81·0 (80·8–81·3)	82·3 (81·9–82·7)	83·2 (82·5–83·8)	75·6 (75·3–76·0)	77·4 (77·0–77·8)	79·0 (78·3–79·7)	68·6 (65·0–71·7)	69·7 (66·0–72·8)	70·1 (66·1–73·2)	66·5 (63·8–68·7)	67·9 (65·1–70·2)	68·8 (65·8–71·3)
	Argentina	80·3 (80·0–80·6)	81·3 (81·0–81·7)	82·2 (81·4–82·9)	74·9 (74·5–75·2)	76·3 (75·9–76·8)	77·8 (77·1–78·5)	68·3 (64·8–71·3)	69·2 (65·6–72·2)	69·5 (65·7–72·6)	66·0 (63·4–68·2)	67·1 (64·4–69·4)	68·0 (65·1–70·4)
	Chile	82·9 (82·7–83·2)	84·7 (84·4–85·0)	85·5 (84·9–86·0)	78·0 (77·7–78·3)	80·5 (80·2–80·9)	82·0 (81·4–82·7)	69·4 (65·5–72·8)	71·0 (67·0–74·4)	71·3 (67·1–74·8)	68·0 (65·1–70·4)	70·0 (67·0–72·6)	70·8 (67·6–73·5)
	Uruguay	80·4 (80·0–80·7)	81·7 (81·3–82·0)	82·7 (82·0–83·2)	73·4 (72·9–73·8)	75·0 (74·5–75·5)	76·6 (76·0–77·1)	68·2 (64·7–71·3)	69·4 (65·8–72·3)	70·0 (66·2–73·0)	64·8 (62·3–67·0)	66·1 (63·5–68·3)	67·1 (64·3–69·5)
Western Europe		84·6 (84·5–84·7)	85·6 (85·5–85·7)	86·1 (85·8–86·4)	80·1 (80·0–80·2)	81·5 (81·4–81·7)	82·6 (82·4–82·8)	71·1 (67·1–74·4)	72·2 (68·2–75·5)	72·5 (68·4–75·8)	70·0 (67·0–72·5)	71·3 (68·3–73·8)	72·0 (69·0–74·6)
	Andorra	86·2 (84·1–88·5)	87·2 (85·0–89·5)	87·6 (85·3–89·9)	81·6 (79·0–84·7)	83·0 (80·2–86·2)	83·8 (81·2–86·8)	72·1 (68·1–76·0)	73·2 (69·3–77·1)	73·6 (69·5–77·5)	71·4 (68·2–74·7)	72·7 (69·4–76·1)	73·4 (70·1–76·7)
	Austria	84·7 (84·5–84·9)	86·0 (85·7–86·2)	86·8 (86·2–87·3)	80·3 (80·1–80·5)	82·0 (81·7–82·3)	83·4 (82·8–83·8)	71·5 (67·7–74·8)	72·9 (69·2–76·1)	73·6 (69·8–76·8)	70·3 (67·3–72·7)	71·9 (69·0–74·3)	72·9 (70·0–75·4)
	Belgium	84·5 (84·2–84·7)	85·4 (85·1–85·7)	85·8 (85·3–86·3)	79·9 (79·6–80·1)	81·2 (81·0–81·5)	82·4 (82·0–82·8)	70·8 (66·6–74·3)	71·9 (67·8–75·3)	72·1 (68·0–75·5)	69·7 (66·7–72·3)	70·9 (68·0–73·4)	71·6 (68·6–74·3)
	Cyprus	83·5 (82·9–84·0)	84·7 (84·1–85·3)	85·5 (84·7–86·2)	79·6 (78·6–80·5)	81·1 (80·1–82·1)	82·5 (81·4–83·5)	71·0 (67·3–74·1)	72·2 (68·5–75·2)	72·6 (68·9–75·8)	70·2 (67·5–72·6)	71·5 (68·8–73·9)	72·4 (69·6–74·9)
	Denmark	83·2 (82·9–83·5)	84·4 (84·2–84·7)	84·8 (84·4–85·2)	79·3 (78·9–79·6)	81·1 (80·8–81·4)	82·2 (81·9–82·6)	70·6 (66·8–73·7)	71·8 (68·1–74·9)	72·1 (68·3–75·2)	69·6 (66·8–71·9)	71·1 (68·2–73·6)	71·9 (68·9–74·5)
	Finland	83·7 (83·1–84·1)	85·9 (85·6–86·2)	86·5 (86·1–87·0)	78·1 (77·4–78·6)	81·2 (80·8–81·5)	82·6 (82·1–83·0)	70·7 (66·9–73·9)	72·5 (68·6–75·8)	72·8 (68·9–76·2)	68·2 (65·3–70·6)	70·6 (67·5–73·2)	71·5 (68·3–74·2)
	France	85·9 (85·7–86·0)	86·9 (86·8–87·1)	87·2 (86·9–87·5)	80·3 (80·0–80·5)	81·7 (81·6–81·9)	82·6 (82·3–82·8)	71·9 (67·6–75·5)	73·0 (68·9–76·5)	73·1 (68·8–76·5)	70·1 (67·0–72·6)	71·4 (68·3–74·0)	71·8 (68·7–74·5)
	Germany	83·8 (83·6–83·9)	84·5 (84·4–84·7)	85·0 (84·7–85·3)	79·2 (79·1–79·4)	80·5 (80·3–80·6)	81·6 (81·2–81·9)	70·2 (66·1–73·5)	71·0 (67·1–74·3)	71·3 (67·3–74·7)	69·3 (66·4–71·8)	70·4 (67·6–72·9)	71·2 (68·3–73·7)
	Greece	82·9 (82·7–83·2)	84·4 (84·1–84·7)	85·0 (84·6–85·4)	77·7 (77·3–78·0)	79·7 (79·4–80·0)	81·1 (80·6–81·5)	70·1 (66·2–73·3)	71·4 (67·4–74·6)	71·6 (67·5–74·8)	68·1 (65·4–70·5)	69·7 (66·9–72·1)	70·6 (67·5–73·2)
	Iceland	84·7 (84·1–85·4)	86·2 (85·5–86·8)	87·0 (86·2–87·8)	81·9 (81·1–82·6)	83·3 (82·5–84·0)	84·3 (83·4–85·0)	71·8 (67·9–75·0)	73·2 (69·5–76·3)	73·9 (70·1–77·2)	71·7 (68·8–74·2)	73·0 (70·1–75·5)	73·7 (70·7–76·4)
	Ireland	85·0 (84·7–85·3)	86·2 (85·9–86·6)	87·0 (86·6–87·4)	81·6 (81·2–81·9)	83·4 (83·1–83·7)	84·9 (84·5–85·3)	71·4 (67·4–74·8)	72·7 (68·9–76·0)	73·3 (69·3–76·6)	71·1 (68·1–73·6)	72·8 (69·7–75·3)	73·8 (70·7–76·4)
	Israel	85·4 (84·9–85·6)	86·7 (86·2–87·0)	87·1 (86·4–87·6)	81·7 (80·8–82·2)	83·4 (82·4–83·9)	84·3 (83·2–84·9)	72·3 (68·5–75·6)	73·6 (69·7–76·8)	73·8 (69·8–77·1)	71·8 (68·9–74·3)	73·2 (70·3–75·8)	73·7 (70·7–76·3)
	Italy	85·1 (84·9–85·3)	86·3 (86·1–86·4)	86·8 (86·4–87·1)	80·8 (80·5–81·0)	82·3 (82·2–82·5)	83·4 (83·1–83·6)	71·8 (67·8–75·1)	72·9 (68·9–76·2)	73·1 (69·0–76·5)	70·6 (67·7–73·2)	71·9 (68·9–74·5)	72·6 (69·6–75·3)
	Luxembourg	85·2 (84·6–85·8)	86·0 (85·3–86·6)	86·2 (85·5–87·0)	81·0 (80·2–81·8)	82·1 (81·3–82·9)	82·9 (82·2–83·7)	72·0 (68·1–75·3)	72·8 (69·0–76·0)	73·0 (69·1–76·1)	71·0 (68·0–73·4)	72·0 (69·0–74·5)	72·6 (69·6–75·1)
	Malta	83·9 (83·1–84·7)	85·8 (85·0–86·5)	86·6 (85·7–87·5)	80·7 (79·8–81·6)	83·2 (82·3–83·9)	84·5 (83·6–85·3)	71·1 (67·2–74·3)	72·6 (68·5–75·8)	72·9 (68·9–76·4)	70·9 (67·8–73·4)	72·7 (69·7–75·3)	73·4 (70·2–76·3)
	Monaco	82·3 (80·8–83·9)	82·9 (81·4–84·7)	83·4 (81·8–85·2)	77·7 (76·4–78·8)	78·9 (77·5–79·9)	79·9 (78·5–81·1)	70·0 (66·0–73·3)	70·6 (66·8–74·0)	71·0 (67·2–74·4)	68·6 (65·6–71·1)	69·6 (66·6–72·1)	70·4 (67·4–72·9)
	Netherlands	83·8 (83·7–84·0)	84·3 (84·1–84·5)	84·6 (84·3–84·9)	80·7 (80·5–80·9)	81·6 (81·4–81·9)	82·6 (82·3–82·9)	70·9 (67·0–74·0)	71·5 (67·8–74·7)	71·7 (67·9–74·8)	70·9 (68·0–73·3)	71·8 (68·9–74·3)	72·4 (69·5–75·0)
	Norway	84·6 (83·9–84·9)	85·5 (85·3–85·7)	85·9 (85·6–86·2)	81·4 (80·5–81·7)	82·8 (82·6–83·0)	83·7 (83·4–84·0)	71·2 (67·2–74·6)	72·2 (68·4–75·5)	72·6 (68·7–75·8)	71·0 (68·1–73·6)	72·4 (69·4–74·9)	73·1 (70·2–75·7)
	Portugal	84·8 (84·5–85·0)	86·4 (86·2–86·7)	87·4 (86·9–87·9)	79·1 (78·8–79·4)	81·1 (80·8–81·3)	82·5 (82·1–82·8)	71·4 (67·3–74·6)	73·0 (69·1–76·2)	73·7 (69·6–77·0)	69·2 (66·3–71·6)	71·0 (68·0–73·5)	71·9 (68·9–74·5)
	San Marino	88·2 (85·4–90·9)	89·6 (86·6–92·8)	90·0 (86·9–93·1)	84·8 (81·9–87·3)	86·9 (83·9–89·8)	87·8 (85·0–90·5)	73·9 (69·6–78·0)	75·2 (70·8–79·6)	75·3 (70·8–80·0)	73·6 (70·1–77·0)	75·3 (71·7–79·0)	75·8 (72·1–79·6)
	Spain	86·0 (85·8–86·2)	87·1 (86·9–87·3)	87·5 (87·0–87·9)	80·5 (80·3–80·7)	82·1 (81·9–82·2)	83·3 (83·0–83·6)	72·3 (68·1–75·7)	73·5 (69·4–76·8)	73·6 (69·4–77·1)	70·7 (67·7–73·1)	72·1 (69·1–74·6)	72·9 (69·7–75·4)
	Sweden	85·2 (84·3–86·0)	86·0 (85·1–86·9)	86·5 (85·5–87·3)	82·2 (81·2–83·3)	83·5 (82·5–84·6)	84·3 (83·3–85·4)	71·9 (68·0–75·2)	72·7 (69·0–76·0)	73·0 (69·2–76·4)	71·9 (68·8–74·6)	73·0 (69·9–75·7)	73·6 (70·4–76·3)
	Switzerland	86·7 (86·5–86·9)	87·4 (87·1–87·6)	87·7 (87·3–88·0)	83·1 (82·9–83·4)	84·3 (84·0–84·5)	85·1 (84·7–85·4)	72·5 (68·4–76·1)	73·3 (69·4–76·8)	73·6 (69·6–77·0)	72·2 (69·1–74·9)	73·2 (70·2–75·9)	73·8 (70·7–76·5)
	UK	83·5 (83·3–83·6)	84·3 (84·1–84·5)	85·0 (84·6–85·3)	79·9 (79·8–80·1)	81·1 (80·9–81·3)	82·1 (81·8–82·5)	70·2 (66·2–73·5)	71·1 (67·3–74·4)	71·6 (67·7–74·9)	69·6 (66·6–72·1)	70·6 (67·6–73·3)	71·3 (68·3–74·0)
**Latin America and Caribbean**		**78·7 (77·6–79·5)**	**80·4 (78·8–81·7)**	**82·2 (79·5–84·4)**	**72·5 (71·4–73·5)**	**74·7 (73·1–75·9)**	**77·0 (74·6–78·8)**	**66·4 (62·6–69·4)**	**67·8 (64·0–71·1)**	**68·6 (64·2–72·7)**	**63·4 (60·5–65·9)**	**65·1 (62·1–67·8)**	**66·4 (62·9–69·7)**
Andean Latin America		78·0 (76·2–79·7)	80·8 (79·0–82·5)	82·3 (79·8–84·5)	73·9 (72·0–75·9)	77·6 (75·6–79·7)	79·3 (76·7–81·7)	67·0 (63·6–70·1)	69·3 (65·7–72·5)	70·1 (66·1–73·7)	65·4 (62·5–68·3)	68·4 (65·2–71·2)	69·3 (66·2–72·4)
	Bolivia	70·5 (67·7–72·9)	75·2 (72·1–77·5)	77·1 (73·7–79·9)	67·0 (64·0–69·9)	73·7 (70·4–76·3)	75·8 (72·2–78·8)	60·8 (57·3–64·0)	64·6 (60·9–68·0)	65·7 (61·7–69·5)	59·7 (56·6–62·8)	65·0 (61·5–68·3)	66·2 (62·5–69·7)
	Ecuador	79·6 (77·7–81·3)	81·1 (79·1–83·1)	82·4 (80·0–84·7)	74·8 (72·2–77·1)	76·9 (74·3–79·3)	78·4 (75·7–81·2)	68·1 (64·3–71·3)	69·4 (65·8–72·7)	69·8 (65·8–73·6)	65·8 (62·6–68·9)	67·4 (64·1–70·6)	67·8 (64·3–71·6)
	Peru	80·2 (78·0–82·4)	82·6 (80·2–84·7)	84·0 (81·3–86·7)	76·2 (73·8–78·6)	79·2 (76·5–81·8)	81·0 (78·0–83·8)	68·8 (65·4–72·0)	70·9 (67·3–74·2)	71·8 (67·8–75·6)	67·5 (64·3–70·5)	70·0 (66·6–73·2)	71·1 (67·7–74·4)
Caribbean		74·7 (64·4–77·0)	76·5 (72·3–78·7)	78·6 (67·9–82·0)	69·8 (56·5–72·3)	71·9 (68·1–74·2)	74·3 (59·6–77·5)	63·4 (54·9–67·2)	64·9 (59·4–68·7)	66·0 (57·0–70·6)	61·3 (49·5–64·7)	63·0 (58·8–66·3)	64·5 (51·7–68·5)
	Antigua and Barbuda	78·0 (77·5–78·4)	78·7 (77·9–79·4)	79·9 (77·9–81·4)	74·6 (74·0–75·0)	76·0 (75·3–76·6)	77·6 (75·7–78·8)	66·8 (63·3–69·6)	67·3 (63·8–70·1)	67·6 (63·7–71·0)	65·5 (62·7–67·9)	66·6 (63·7–69·1)	67·3 (64·0–70·0)
	The Bahamas	77·1 (74·5–79·4)	78·4 (75·8–80·8)	79·7 (76·7–82·5)	70·5 (67·3–73·2)	72·1 (68·9–75·2)	73·9 (70·8–76·7)	66·0 (62·5–69·6)	67·0 (63·3–70·8)	67·6 (63·6–71·7)	62·3 (59·3–65·6)	63·6 (60·4–66·9)	64·5 (61·2–68·2)
	Barbados	77·2 (75·0–79·3)	78·8 (76·5–80·9)	79·7 (77·1–82·0)	73·9 (71·3–76·3)	76·8 (74·1–79·5)	78·3 (75·3–81·0)	66·1 (62·3–69·6)	67·4 (63·4–70·8)	67·6 (63·6–71·5)	65·2 (61·9–68·3)	67·5 (64·0–70·8)	68·1 (64·5–71·5)
	Belize	77·0 (74·6–78·3)	79·1 (77·5–80·6)	81·1 (78·0–83·8)	71·9 (68·6–73·9)	74·2 (72·4–76·2)	75·9 (72·8–78·5)	65·8 (62·2–68·8)	67·6 (63·9–70·9)	68·7 (64·1–72·9)	63·4 (59·9–66·2)	65·2 (62·2–68·0)	66·0 (62·2–69·5)
	Bermuda	85·0 (83·0–86·4)	87·1 (85·1–88·5)	88·3 (86·2–89·7)	77·5 (75·6–78·9)	79·6 (77·7–81·0)	81·2 (79·4–82·7)	72·7 (68·8–76·0)	74·5 (70·5–77·8)	74·9 (70·8–78·5)	68·4 (65·6–71·0)	70·1 (67·1–72·8)	70·8 (67·7–73·7)
	Cuba	81·2 (80·0–82·3)	82·0 (80·8–83·2)	83·6 (81·7–85·2)	76·1 (74·7–77·6)	77·0 (75·5–78·5)	78·4 (76·5–80·1)	69·5 (66·0–72·6)	70·2 (66·8–73·2)	71·0 (67·2–74·3)	67·3 (64·7–69·8)	67·9 (65·3–70·5)	68·5 (65·5–71·3)
	Dominica	73·9 (71·6–75·7)	75·8 (73·4–77·5)	77·5 (74·3–79·8)	68·6 (66·1–70·7)	71·0 (68·3–73·2)	72·9 (69·8–75·4)	63·2 (59·5–66·3)	64·8 (61·0–68·1)	65·6 (61·4–69·6)	60·6 (57·6–63·3)	62·5 (59·3–65·5)	63·6 (59·9–66·9)
	Dominican Republic	78·6 (76·6–80·4)	80·0 (77·6–82·0)	82·3 (78·7–85·2)	72·5 (69·9–75·0)	74·1 (71·3–76·5)	76·7 (73·0–79·8)	66·9 (63·1–70·4)	68·0 (64·2–71·6)	69·0 (64·8–73·2)	63·6 (60·5–67·0)	64·9 (61·8–68·2)	66·3 (62·8–70·2)
	Grenada	75·1 (73·9–76·3)	76·6 (75·2–78·1)	78·2 (75·7–80·3)	70·3 (68·9–71·7)	72·0 (70·4–73·6)	73·8 (71·2–75·7)	64·3 (61·0–67·2)	65·4 (62·1–68·3)	66·1 (62·1–69·9)	62·0 (59·4–64·4)	63·3 (60·7–65·9)	64·2 (61·0–67·3)
	Guyana	70·9 (66·9–73·8)	74·7 (71·5–77·7)	77·8 (74·1–81·3)	64·1 (60·1–67·5)	67·7 (64·1–71·4)	70·8 (66·5–75·0)	60·0 (55·8–64·1)	62·8 (58·5–66·8)	64·6 (59·9–69·2)	55·9 (52·0–59·5)	58·5 (54·6–62·3)	60·6 (56·2–64·8)
	Haiti	63·4 (42·8–67·6)	66·2 (57·9–70·4)	70·9 (50·2–77·0)	61·8 (34·5–66·4)	65·1 (56·6–69·7)	69·4 (40·5–75·0)	53·7 (37·1–58·2)	56·0 (48·0–60·7)	59·3 (42·7–65·5)	54·4 (30·9–59·0)	57·1 (49·7–61·6)	60·4 (36·6–65·8)
	Jamaica	77·6 (74·7–80·4)	79·3 (76·2–82·4)	81·0 (77·3–84·6)	73·9 (70·7–77·0)	76·0 (72·5–79·3)	77·6 (74·1–80·8)	66·5 (62·7–70·0)	67·9 (64·0–71·6)	68·9 (64·4–73·2)	65·4 (62·1–68·9)	67·0 (63·5–70·5)	68·0 (64·1–71·6)
	Puerto Rico	84·4 (82·8–86·0)	86·3 (84·6–87·9)	87·2 (85·0–89·1)	76·7 (74·7–78·9)	79·3 (77·2–81·5)	81·2 (78·8–83·4)	71·5 (67·3–75·1)	73·3 (69·2–76·9)	73·8 (69·4–77·6)	66·9 (63·7–70·1)	69·1 (65·9–72·4)	70·3 (66·6–73·7)
	Saint Kitts and Nevis	76·5 (74·7–78·1)	78·3 (76·6–80·0)	80·3 (77·8–82·6)	69·9 (67·8–71·7)	72·1 (70·3–73·9)	74·7 (72·5–76·8)	65·6 (62·1–68·6)	67·0 (63·5–70·1)	68·1 (64·0–71·8)	61·6 (58·7–64·2)	63·2 (60·3–66·0)	64·9 (61·6–68·1)
	Saint Lucia	77·9 (75·6–80·1)	80·4 (78·2–82·4)	82·3 (79·2–84·9)	71·6 (68·9–74·3)	74·2 (71·5–76·7)	76·2 (73·1–78·9)	66·2 (62·1–69·5)	68·1 (64·1–71·7)	68·9 (64·3–73·0)	62·9 (59·5–66·0)	64·8 (61·6–67·9)	65·9 (62·1–69·4)
	Saint Vincent and the Grenadines	75·4 (73·7–76·8)	77·2 (75·5–78·9)	79·1 (76·4–81·3)	70·1 (68·4–71·6)	72·0 (70·3–73·6)	73·6 (71·3–75·6)	64·5 (61·1–67·6)	65·9 (62·4–69·1)	66·7 (62·6–70·5)	61·7 (59·0–64·3)	63·1 (60·1–65·8)	63·9 (60·7–67·1)
	Suriname	76·0 (73·4–78·6)	78·3 (75·5–81·0)	80·6 (76·4–84·2)	69·9 (66·5–73·4)	72·6 (69·1–76·5)	75·4 (70·6–80·0)	64·4 (60·6–68·0)	66·2 (62·1–70·2)	67·5 (62·5–72·1)	61·2 (57·8–64·8)	63·3 (59·4–67·3)	65·1 (60·5–69·7)
	Trinidad and Tobago	76·3 (73·4–78·8)	79·9 (77·2–82·8)	82·6 (79·3–85·8)	69·4 (66·1–72·4)	73·2 (69·9–76·6)	75·8 (72·4–79·2)	64·9 (61·0–68·6)	67·8 (63·6–71·7)	69·2 (64·7–73·7)	60·9 (57·6–64·1)	63·8 (60·3–67·4)	65·2 (61·3–69·1)
	Virgin Islands	81·5 (78·5–83·8)	84·7 (82·9–86·4)	85·9 (83·9–87·8)	70·9 (67·3–74·0)	74·7 (72·1–77·1)	77·0 (74·4–79·5)	69·1 (65·0–72·7)	71·6 (67·6–75·2)	71·9 (67·6–75·9)	62·1 (58·5–65·5)	65·2 (61·9–68·5)	66·7 (63·2–70·2)
Central Latin America		78·6 (77·2–79·8)	80·1 (77·8–82·0)	81·9 (78·6–84·6)	72·1 (70·4–73·5)	74·1 (71·9–76·0)	76·6 (73·5–79·0)	66·8 (63·3–69·9)	68·0 (64·3–71·5)	68·7 (64·1–72·9)	63·2 (60·5–65·9)	64·8 (61·7–67·8)	66·1 (62·4–69·7)
	Colombia	82·9 (81·1–84·6)	84·9 (83·2–86·7)	86·7 (84·0–89·0)	76·7 (74·6–78·9)	79·4 (77·2–81·5)	82·0 (79·3–84·3)	70·6 (67·1–73·8)	72·2 (68·4–75·6)	72·9 (68·5–76·9)	67·2 (64·3–70·2)	69·2 (66·0–72·4)	70·8 (67·2–74·2)
	Costa Rica	82·9 (81·9–84·0)	84·5 (83·4–85·7)	86·0 (84·2–87·7)	76·6 (75·2–77·9)	78·3 (77·0–79·7)	80·2 (78·3–82·0)	70·1 (66·3–73·4)	71·4 (67·5–74·8)	72·1 (67·9–76·1)	66·8 (63·8–69·8)	68·0 (64·9–71·0)	69·1 (65·9–72·4)
	El Salvador	79·0 (76·9–80·9)	80·7 (78·4–83·0)	81·9 (78·6–84·8)	70·1 (67·5–72·6)	72·5 (69·7–75·2)	74·7 (71·5–77·6)	67·4 (63·5–70·9)	68·8 (64·8–72·3)	69·2 (64·8–73·4)	61·2 (57·9–64·4)	63·0 (59·5–66·3)	64·2 (60·5–67·9)
	Guatemala	75·9 (74·4–77·4)	78·0 (76·3–79·7)	80·9 (78·0–83·4)	70·9 (68·9–72·9)	73·3 (71·3–75·3)	76·4 (73·6–78·7)	64·1 (60·5–67·4)	65·9 (62·3–69·5)	67·3 (63·2–71·7)	61·4 (58·3–64·5)	63·3 (60·2–66·5)	65·0 (61·5–68·7)
	Honduras	72·8 (69·6–74·8)	74·7 (71·5–77·0)	77·0 (73·6–79·9)	69·9 (65·4–71·9)	72·3 (67·3–74·2)	74·8 (70·3–77·2)	62·4 (58·6–65·7)	64·1 (60·2–67·5)	65·5 (61·2–69·5)	61·6 (57·5–64·5)	63·5 (59·3–66·4)	65·0 (60·8–68·5)
	Mexico	77·6 (76·3–79·0)	79·5 (77·9–81·0)	81·1 (78·7–83·3)	71·2 (69·4–73·1)	73·6 (71·7–75·8)	75·6 (73·0–78·2)	65·8 (62·2–68·9)	67·2 (63·6–70·4)	67·7 (63·3–71·5)	62·5 (59·7–65·6)	64·3 (61·2–67·6)	65·1 (61·8–68·9)
	Nicaragua	82·0 (79·9–83·6)	82·5 (80·5–84·3)	83·5 (80·5–86·2)	77·0 (74·1–78·9)	78·0 (75·0–80·0)	79·3 (75·7–82·3)	69·4 (65·4–73·0)	69·6 (65·8–73·4)	69·8 (65·6–74·3)	66·8 (63·4–69·9)	67·3 (63·9–70·6)	67·7 (63·7–71·9)
	Panama	82·3 (80·4–84·3)	84·1 (82·1–86·1)	85·7 (83·3–88·2)	76·8 (74·5–79·3)	79·2 (76·7–81·7)	81·3 (78·6–84·1)	69·7 (65·9–73·1)	71·0 (66·8–74·4)	71·5 (67·0–75·5)	67·1 (64·0–70·3)	68·7 (65·5–72·3)	69·8 (66·1–73·7)
	Venezuela	76·7 (72·7–79·6)	76·1 (66·5–82·5)	77·8 (65·3–85·8)	67·7 (62·9–71·4)	67·3 (58·4–73·3)	70·0 (57·9–77·7)	65·8 (61·4–69·6)	65·7 (57·1–72·2)	66·7 (55·8–74·7)	60·0 (55·6–63·7)	59·9 (52·2–65·4)	61·9 (52·0–68·9)
Tropical Latin America		79·8 (79·4–80·2)	81·5 (80·6–82·2)	83·2 (80·9–84·8)	73·3 (72·9–73·8)	75·2 (74·5–75·8)	77·4 (75·3–78·5)	66·5 (62·5–69·7)	67·8 (63·9–71·1)	68·7 (64·3–72·6)	63·6 (60·7–66·1)	65·0 (62·0–67·5)	66·2 (62·8–69·2)
	Brazil	79·8 (79·5–80·2)	81·5 (80·6–82·2)	83·2 (81·0–84·8)	73·3 (72·9–73·8)	75·2 (74·5–75·8)	77·4 (75·3–78·6)	66·5 (62·5–69·7)	67·8 (63·9–71·0)	68·7 (64·3–72·6)	63·6 (60·6–66·1)	65·0 (62·0–67·5)	66·2 (62·8–69·2)
	Paraguay	78·5 (76·1–80·7)	80·2 (77·5–82·7)	82·3 (78·7–85·3)	72·4 (69·3–75·1)	73·9 (70·6–76·7)	75·8 (71·9–78·9)	66·4 (62·4–69·9)	67·7 (63·6–71·4)	68·9 (64·1–73·0)	63·5 (60·3–66·7)	64·6 (61·3–68·0)	65·7 (61·8–69·2)
**North Africa and Middle East**		**75·4 (74·2–76·7)**	**77·1 (75·5–78·6)**	**79·4 (76·4–82·0)**	**72·1 (70·7–73·5)**	**74·3 (72·5–75·8)**	**76·9 (73·8–79·5)**	**63·5 (59·9–66·7)**	**64·8 (61·3–68·3)**	**65·8 (61·3–70·0)**	**63·3 (60·6–65·9)**	**64·9 (62·1–67·7)**	**66·0 (62·3–69·6)**
	Afghanistan	63·2 (60·6–65·9)	66·1 (63·0–69·1)	71·9 (67·1–76·6)	62·7 (59·8–65·7)	66·8 (63·9–69·9)	72·4 (67·9–77·0)	53·2 (49·7–56·6)	55·7 (51·9–59·2)	59·8 (54·5–64·8)	54·1 (50·8–57·5)	57·3 (53·7–60·7)	61·0 (56·3–65·8)
	Algeria	77·8 (76·6–78·9)	79·0 (77·5–80·3)	81·4 (78·8–83·7)	76·9 (75·3–78·4)	78·5 (76·6–80·3)	81·2 (78·2–84·0)	65·8 (62·1–68·8)	66·5 (62·9–69·9)	67·5 (63·1–71·6)	67·5 (64·6–70·2)	68·6 (65·5–71·5)	69·7 (65·8–73·4)
	Bahrain	76·9 (75·9–77·9)	78·3 (77·0–79·4)	80·0 (77·6–81·9)	75·4 (74·2–76·6)	77·1 (75·9–78·3)	79·0 (76·7–80·6)	64·5 (60·8–67·6)	65·6 (61·9–68·8)	66·2 (62·2–70·1)	66·1 (63·3–68·5)	67·4 (64·6–70·0)	68·1 (64·9–71·2)
	Egypt	71·4 (69·9–72·7)	72·7 (71·1–74·3)	75·1 (72·4–77·5)	69·7 (67·7–71·8)	71·2 (69·0–73·4)	73·2 (69·6–76·4)	61·2 (58·0–64·0)	62·3 (58·8–65·4)	63·2 (59·4–67·2)	62·0 (59·3–64·6)	63·0 (60·0–66·0)	63·5 (60·0–67·4)
	Iran	80·4 (79·5–81·0)	82·0 (80·9–83·0)	84·2 (81·4–86·5)	77·4 (75·9–78·1)	79·4 (77·9–80·4)	81·8 (78·6–84·1)	67·1 (63·0–70·4)	68·3 (64·2–71·7)	69·1 (64·4–73·5)	67·2 (64·2–69·8)	68·7 (65·7–71·5)	69·7 (65·6–73·5)
	Iraq	74·7 (72·0–77·1)	76·9 (73·9–79·2)	80·7 (76·3–84·0)	69·1 (65·7–72·5)	71·2 (67·8–74·5)	74·8 (70·3–78·8)	62·7 (58·9–66·2)	64·5 (60·6–68·2)	66·8 (61·7–71·3)	60·2 (56·6–63·7)	61·9 (58·3–65·7)	64·1 (59·7–68·3)
	Jordan	80·0 (78·4–81·4)	82·3 (80·5–83·9)	84·7 (82·1–87·2)	78·9 (76·6–80·9)	81·7 (79·4–83·9)	84·0 (80·8–86·8)	66·9 (63·1–70·3)	68·7 (64·7–72·2)	69·7 (65·3–74·0)	68·8 (65·8–71·7)	70·8 (67·5–74·0)	71·7 (68·0–75·5)
	Kuwait	87·0 (86·0–87·9)	87·8 (86·5–89·0)	88·9 (86·2–90·8)	80·1 (78·2–81·9)	80·9 (79·1–82·8)	82·4 (79·8–84·6)	71·9 (67·5–75·7)	72·6 (68·1–76·3)	72·6 (67·8–77·1)	69·5 (66·4–72·4)	69·9 (66·5–72·9)	70·2 (66·5–73·7)
	Lebanon	81·0 (79·5–82·2)	83·1 (81·7–84·5)	84·3 (82·1–86·3)	75·7 (73·9–77·3)	78·3 (76·6–80·0)	79·8 (77·3–81·7)	67·5 (63·5–71·1)	69·2 (65·5–73·0)	69·5 (65·1–73·8)	65·8 (62·8–68·6)	67·7 (64·7–70·7)	68·0 (64·4–71·4)
	Libya	74·0 (71·2–76·3)	76·1 (72·8–78·9)	77·6 (73·1–81·5)	69·7 (66·4–72·5)	72·4 (68·4–75·8)	73·8 (69·1–77·8)	62·3 (58·7–66·0)	63·9 (59·9–67·8)	64·3 (59·4–69·2)	61·2 (57·5–64·6)	63·1 (59·1–66·7)	63·4 (59·1–67·8)
	Morocco	74·5 (72·3–76·4)	76·5 (74·2–79·0)	79·4 (75·1–83·5)	72·1 (69·9–74·4)	74·6 (72·6–77·5)	77·3 (73·6–81·0)	62·6 (58·9–66·3)	64·2 (60·4–68·1)	65·3 (60·4–70·3)	63·3 (60·3–66·4)	65·2 (62·1–68·4)	66·3 (62·3–70·6)
	Oman	79·8 (78·1–81·5)	80·5 (78·4–82·7)	82·6 (78·5–86·4)	74·7 (72·9–76·3)	75·6 (73·8–77·6)	77·6 (74·2–80·5)	67·2 (63·6–70·8)	67·6 (63·7–71·5)	68·1 (63·1–73·2)	65·6 (63·0–68·6)	66·3 (63·3–69·4)	66·9 (63·0–70·8)
	Palestine	76·4 (74·2–77·9)	78·5 (76·3–80·0)	80·2 (77·2–82·6)	72·2 (68·1–74·1)	74·9 (70·6–77·1)	76·9 (72·3–79·5)	64·5 (61·1–67·9)	66·2 (62·3–69·6)	66·9 (62·8–71·2)	63·2 (59·4–66·2)	65·3 (60·8–68·4)	66·4 (62·5–70·1)
	Qatar	80·1 (78·4–81·6)	80·5 (78·7–82·1)	81·4 (78·9–83·3)	77·8 (75·7–79·7)	79·2 (77·2–81·1)	80·9 (78·6–83·1)	66·6 (62·8–70·1)	66·9 (63·1–70·3)	67·0 (62·9–70·9)	67·8 (64·8–70·6)	69·0 (65·9–72·0)	69·8 (66·6–73·1)
	Saudi Arabia	75·9 (73·6–77·9)	76·6 (74·2–78·8)	78·3 (74·5–81·3)	73·0 (71·1–74·7)	73·7 (71·4–75·6)	75·2 (71·1–78·3)	64·0 (60·7–67·1)	64·5 (60·9–67·8)	65·0 (60·5–69·3)	63·7 (61·0–66·3)	64·0 (61·2–66·8)	64·4 (60·5–68·3)
	Sudan	71·1 (68·0–73·7)	73·8 (70·2–77·2)	76·5 (67·3–82·4)	68·5 (65·1–71·2)	71·7 (67·9–75·0)	74·1 (66·5–79·6)	60·4 (56·7–64·2)	62·5 (58·4–66·8)	63·9 (56·2–70·4)	60·5 (56·8–63·7)	63·0 (59·1–66·8)	64·1 (57·2–69·6)
	Syria	73·5 (68·4–77·4)	74·6 (69·3–78·6)	76·5 (70·4–81·2)	67·9 (58·5–74·7)	69·3 (60·3–76·0)	71·7 (62·2–79·2)	62·1 (56·7–66·9)	62·8 (57·7–67·8)	62·3 (55·4–68·1)	59·6 (51·2–66·3)	60·4 (52·9–67·1)	60·2 (51·5–67·7)
	Tunisia	79·3 (77·0–81·6)	81·8 (79·3–84·3)	83·9 (80·5–87·0)	74·0 (71·3–76·6)	77·0 (74·0–79·6)	79·2 (75·7–82·4)	66·6 (62·7–70·2)	68·4 (64·2–72·2)	69·1 (64·3–73·7)	65·0 (61·8–68·1)	67·2 (63·9–70·6)	68·2 (64·3–72·1)
	Türkiye	80·1 (78·7–81·5)	81·8 (80·3–83·2)	83·4 (81·3–85·3)	75·0 (73·2–77·0)	77·2 (75·3–79·2)	79·5 (77·5–81·6)	67·6 (64·0–70·9)	68·9 (65·3–72·3)	69·6 (65·5–73·4)	66·4 (63·8–69·1)	68·0 (65·2–70·8)	69·1 (66·1–72·3)
	United Arab Emirates	71·9 (71·2–72·7)	72·4 (71·6–73·3)	73·2 (71·8–74·4)	79·0 (77·4–80·7)	80·3 (78·6–82·1)	81·6 (79·1–83·9)	61·2 (57·9–64·0)	61·9 (58·8–64·6)	62·1 (58·8–65·1)	68·4 (65·1–71·3)	69·5 (66·4–72·3)	70·0 (66·5–73·6)
	Yemen	70·2 (65·9–73·4)	72·7 (68·7–76·1)	77·0 (71·3–82·2)	66·4 (62·1–70·1)	69·0 (64·7–72·5)	72·8 (67·4–77·7)	58·5 (52·8–62·8)	60·6 (55·7–65·1)	63·6 (58·1–69·4)	58·5 (53·8–62·6)	60·5 (56·3–64·4)	63·0 (57·9–68·0)
**South Asia**		**72·8 (71·7–73·9)**	**75·4 (73·7–77·1)**	**79·4 (75·4–82·8)**	**69·5 (68·2–70·7)**	**72·1 (70·2–73·7)**	**76·3 (72·2–79·8)**	**61·1 (57·8–64·1)**	**63·1 (59·6–66·6)**	**65·6 (61·1–70·4)**	**60·8 (58·2–63·4)**	**62·8 (60·1–65·6)**	**65·6 (61·5–69·7)**
	Bangladesh	75·6 (73·1–77·7)	78·0 (75·3–80·4)	81·5 (77·1–85·2)	73·1 (70·1–75·6)	75·6 (72·5–78·2)	79·2 (74·8–82·4)	63·9 (60·5–67·0)	65·6 (62·0–69·0)	67·2 (62·5–71·9)	64·3 (61·1–67·3)	66·2 (62·9–69·3)	68·5 (64·4–72·3)
	Bhutan	74·1 (71·7–76·4)	77·2 (74·6–79·5)	80·8 (76·8–84·2)	71·5 (68·9–73·9)	74·9 (72·1–77·4)	78·4 (74·2–81·7)	63·3 (60·2–66·3)	65·4 (62·0–68·6)	67·6 (63·1–72·2)	63·1 (60·4–65·9)	65·6 (62·6–68·4)	67·7 (63·6–71·7)
	India	73·3 (72·1–74·6)	75·7 (74·0–77·5)	79·8 (75·9–83·3)	69·6 (68·2–71·0)	72·0 (70·1–73·8)	76·2 (72·1–79·7)	61·4 (58·0–64·5)	63·3 (59·6–66·8)	65·9 (61·5–70·8)	60·8 (58·2–63·4)	62·7 (60·0–65·6)	65·5 (61·5–69·7)
	Nepal	73·5 (71·2–75·5)	76·5 (74·0–78·6)	80·8 (76·8–84·2)	70·1 (67·6–72·3)	72·9 (70·4–75·2)	77·1 (73·0–80·4)	62·2 (59·0–65·1)	64·6 (61·1–67·9)	67·3 (62·8–71·8)	61·3 (58·8–63·9)	63·6 (60·7–66·5)	66·3 (62·3–70·3)
	Pakistan	68·5 (65·6–71·2)	71·4 (68·5–74·2)	75·7 (70·8–79·6)	67·3 (64·2–70·1)	70·5 (67·2–73·6)	75·0 (70·2–79·3)	58·0 (54·5–61·5)	60·2 (56·6–63·9)	62·7 (57·6–67·8)	59·0 (55·9–62·3)	61·5 (58·1–65·1)	64·4 (59·7–69·2)
**Southeast Asia, east Asia, and Oceania**		**79·2 (77·8–80·7)**	**80·8 (79·4–82·4)**	**82·8 (80·6–84·8)**	**73·3 (71·6–74·9)**	**74·9 (73·2–76·5)**	**77·0 (74·7–78·8)**	**68·7 (65·4–71·5)**	**69·9 (66·3–72·8)**	**70·7 (66·8–74·3)**	**65·6 (63·4–67·9)**	**66·8 (64·4–69·0)**	**68·0 (65·0–70·7)**
East Asia		81·0 (79·1–82·8)	82·7 (80·8–84·6)	84·6 (82·3–86·8)	75·2 (73·0–77·2)	76·6 (74·4–78·6)	78·6 (76·1–80·8)	70·5 (66·9–73·2)	71·6 (67·9–74·6)	72·4 (68·4–75·9)	67·4 (65·0–70·0)	68·4 (65·8–70·9)	69·4 (66·4–72·4)
	China	81·1 (79·1–82·9)	82·7 (80·7–84·7)	84·7 (82·3–86·9)	75·2 (73·0–77·3)	76·7 (74·4–78·7)	78·7 (76·0–80·9)	70·5 (66·9–73·3)	71·6 (67·9–74·6)	72·4 (68·5–76·0)	67·4 (65·0–70·1)	68·4 (65·8–71·0)	69·5 (66·5–72·4)
	North Korea	76·2 (73·7–78·4)	77·4 (74·6–79·7)	79·5 (75·4–82·6)	70·3 (68·0–72·5)	71·8 (69·2–74·0)	74·7 (70·8–77·6)	66·9 (63·9–69·8)	67·8 (64·6–70·9)	69·2 (65·1–73·0)	63·7 (61·3–66·2)	64·9 (62·3–67·5)	67·2 (63·6–70·3)
	Taiwan (Province of China)	84·6 (84·0–85·2)	85·6 (85·1–86·2)	86·2 (85·5–87·0)	78·0 (77·2–78·7)	79·0 (78·5–79·8)	80·2 (79·4–81·1)	72·5 (69·0–75·6)	73·3 (69·7–76·3)	73·1 (69·2–76·2)	69·2 (66·5–71·4)	69·9 (67·2–72·2)	70·4 (67·6–72·8)
Oceania		67·8 (65·1–70·0)	69·9 (67·0–72·2)	73·8 (68·6–77·9)	64·9 (62·2–67·2)	67·3 (64·5–69·7)	71·4 (67·5–74·9)	58·6 (55·5–61·4)	60·4 (57·0–63·4)	63·1 (58·5–67·6)	57·5 (54·6–60·0)	59·6 (56·7–62·3)	62·6 (58·8–66·2)
	American Samoa	72·9 (70·6–74·8)	73·2 (70·9–75·6)	75·0 (71·6–77·7)	69·4 (67·1–71·2)	70·1 (67·8–72·2)	72·4 (69·3–74·8)	62·0 (58·7–65·2)	62·6 (59·0–65·9)	63·4 (59·3–67·4)	60·9 (58·0–63·5)	61·7 (58·6–64·5)	63·0 (59·6–66·2)
	Cook Islands	79·9 (77·7–81·7)	80·9 (78·8–83·0)	82·7 (79·9–85·3)	73·0 (71·0–74·9)	74·0 (71·9–75·9)	75·3 (72·4–77·9)	68·0 (64·3–71·2)	68·7 (64·9–72·1)	69·2 (65·0–73·2)	64·4 (61·4–67·1)	65·0 (61·9–67·9)	65·1 (61·7–68·6)
	Federated States of Micronesia	69·8 (66·6–72·6)	70·5 (67·4–73·4)	71·9 (67·6–75·6)	64·7 (61·0–67·7)	65·5 (62·0–68·5)	67·4 (62·9–71·2)	60·6 (57·1–64·0)	60·9 (57·4–64·6)	61·4 (57·1–65·6)	57·9 (54·6–61·1)	58·5 (55·0–61·8)	59·4 (55·2–63·4)
	Fiji	70·4 (67·4–73·1)	72·0 (68·7–74·9)	75·1 (70·7–78·9)	66·0 (63·0–69·1)	67·7 (64·4–70·8)	70·7 (66·6–74·5)	60·8 (57·3–64·1)	62·0 (58·2–65·7)	63·8 (59·1–68·1)	58·7 (55·5–61·8)	60·1 (56·7–63·4)	62·0 (57·9–65·9)
	Guam	83·6 (81·4–85·1)	87·3 (85·7–88·8)	89·3 (86·1–91·8)	74·1 (71·5–76·0)	77·9 (76·0–79·7)	80·3 (77·4–82·9)	71·4 (67·5–75·1)	74·7 (70·5–78·6)	75·9 (71·0–80·7)	65·6 (62·4–68·5)	68·6 (65·6–71·4)	70·2 (66·5–73·8)
	Kiribati	67·1 (64·0–69·7)	68·7 (65·8–71·4)	71·9 (67·5–75·7)	61·2 (57·8–64·2)	63·1 (59·8–66·1)	66·8 (62·4–70·6)	58·0 (54·7–61·4)	59·1 (55·6–62·4)	60·9 (56·3–65·4)	54·7 (51·5–57·7)	56·2 (53·0–59·3)	58·6 (54·6–62·8)
	Marshall Islands	66·9 (63·6–69·7)	67·8 (64·3–71·1)	70·1 (64·7–74·5)	63·6 (60·0–66·7)	64·8 (61·0–68·1)	67·4 (62·3–72·0)	57·9 (54·4–61·3)	58·3 (54·4–62·0)	59·6 (54·3–64·4)	56·6 (53·0–59·8)	57·3 (53·5–60·8)	58·9 (54·2–63·4)
	Nauru	65·9 (62·3–68·8)	67·1 (63·4–70·3)	70·0 (64·3–74·5)	59·4 (55·8–62·7)	60·3 (56·5–64·1)	62·9 (57·1–67·7)	57·3 (53·7–60·6)	58·1 (54·4–61·8)	59·9 (54·6–64·8)	53·4 (50·1–56·6)	54·0 (50·4–57·5)	55·7 (50·6–60·4)
	Niue	70·1 (68·2–71·9)	71·2 (69·0–73·5)	73·8 (70·4–76·8)	65·7 (63·3–67·4)	66·9 (64·5–69·0)	69·7 (66·5–72·2)	60·5 (57·4–63·3)	61·1 (57·7–64·1)	62·4 (58·1–66·4)	58·5 (55·9–61·1)	59·3 (56·5–61·9)	61·0 (57·5–64·3)
	Northern Mariana Islands	76·3 (75·5–78·0)	77·2 (76·1–78·7)	78·2 (75·6–80·4)	71·1 (70·7–72·6)	71·7 (70·8–73·2)	73·1 (70·8–74·9)	66·0 (63·1–68·8)	66·8 (63·7–69·5)	67·1 (63·5–70·5)	63·4 (61·1–65·5)	63·8 (61·4–65·9)	64·5 (61·5–67·3)
	Palau	70·5 (68·3–72·6)	71·1 (68·7–73·2)	72·5 (69·2–75·0)	67·7 (65·0–70·4)	68·7 (65·8–71·4)	71·0 (66·9–74·1)	61·0 (58·2–63·7)	61·3 (58·3–64·2)	61·8 (58·2–65·2)	60·0 (57·2–62·8)	60·6 (57·7–63·4)	62·0 (58·3–65·3)
	Papua New Guinea	67·0 (64·0–69·7)	69·3 (66·2–72·1)	73·5 (68·1–78·0)	64·9 (61·6–67·6)	67·4 (64·1–70·2)	71·7 (67·5–75·3)	57·9 (54·6–60·9)	60·0 (56·7–63·1)	63·0 (58·2–67·6)	57·4 (54·2–60·2)	59·6 (56·5–62·6)	62·8 (58·9–66·6)
	Samoa	71·0 (68·3–73·2)	71·8 (68·7–74·2)	73·3 (69·2–76·6)	68·8 (65·9–70·8)	70·2 (67·2–72·2)	72·5 (68·8–75·7)	61·4 (57·9–64·8)	61·8 (58·3–65·3)	62·4 (58·4–66·7)	61·1 (58·2–63·8)	62·1 (58·7–64·9)	63·3 (59·7–67·1)
	Solomon Islands	68·3 (64·5–71·2)	68·9 (65·0–71·9)	71·3 (66·5–75·5)	63·7 (59·7–66·7)	64·4 (60·4–67·5)	67·3 (62·5–71·5)	59·3 (55·8–62·8)	59·7 (55·7–63·3)	61·1 (56·5–65·8)	57·3 (53·9–60·4)	57·8 (53·9–61·0)	59·7 (55·3–64·0)
	Tokelau	69·1 (66·8–71·2)	70·9 (68·3–73·3)	74·5 (70·4–78·0)	68·2 (66·1–70·1)	69·8 (67·7–71·9)	73·1 (69·6–75·9)	59·6 (56·5–62·6)	60·8 (57·2–64·1)	62·8 (58·0–67·1)	60·5 (57·8–63·0)	61·5 (58·6–64·4)	63·2 (59·3–66·9)
	Tonga	75·7 (72·9–78·1)	76·7 (73·9–79·3)	78·6 (75·2–81·6)	70·7 (68·1–73·0)	71·9 (69·3–74·3)	74·2 (70·9–76·9)	65·2 (61·4–68·7)	65·7 (61·8–69·3)	66·4 (62·0–70·6)	63·2 (60·1–66·2)	63·9 (60·8–67·0)	65·0 (61·2–68·7)
	Tuvalu	70·8 (68·0–73·2)	72·0 (69·2–74·5)	74·2 (69·9–77·7)	65·9 (62·8–68·7)	67·2 (63·9–69·9)	69·6 (64·9–73·0)	61·6 (58·3–64·7)	62·3 (58·9–65·6)	63·2 (58·8–67·5)	59·2 (56·4–62·1)	60·1 (57·2–63·0)	61·4 (57·5–65·0)
	Vanuatu	68·9 (66·3–71·0)	71·9 (69·7–74·1)	74·5 (70·7–78·0)	62·2 (59·4–64·5)	65·9 (63·5–68·2)	69·4 (65·4–73·0)	60·0 (57·0–62·9)	62·3 (59·2–65·6)	63·8 (59·7–68·2)	56·1 (53·4–58·7)	59·0 (56·4–61·9)	61·4 (57·6–65·3)
Southeast Asia		75·8 (74·4–77·0)	77·6 (76·0–78·9)	80·2 (77·7–82·3)	70·1 (68·6–71·5)	72·1 (70·5–73·5)	75·0 (72·7–77·0)	65·6 (62·4–68·3)	67·0 (63·6–69·7)	68·5 (64·7–72·1)	62·5 (60·3–64·9)	64·2 (61·7–66·6)	66·2 (63·3–69·1)
	Cambodia	72·2 (69·4–74·8)	74·2 (71·6–77·0)	77·3 (74·1–80·4)	67·0 (64·1–69·9)	69·1 (66·3–71·8)	72·5 (69·4–75·5)	62·5 (59·0–65·8)	64·2 (60·7–67·4)	66·2 (62·4–70·4)	59·6 (56·5–62·6)	61·3 (57·9–64·4)	63·7 (60·0–67·5)
	Indonesia	73·5 (71·2–75·5)	75·6 (73·1–77·8)	78·7 (76·0–81·7)	69·4 (67·0–71·7)	71·8 (69·4–74·1)	75·5 (72·6–78·5)	64·0 (60·7–67·0)	65·7 (62·3–68·6)	67·7 (63·7–71·6)	62·2 (59·4–65·0)	64·2 (61·2–67·0)	66·9 (63·5–70·0)
	Laos	70·5 (67·8–73·2)	74·1 (71·0–76·6)	78·1 (74·4–81·1)	65·9 (62·9–68·8)	69·8 (66·7–72·8)	74·0 (70·5–77·1)	61·5 (58·6–64·6)	64·4 (61·2–67·6)	67·0 (63·0–70·8)	59·1 (56·2–62·0)	62·4 (59·5–65·4)	65·4 (62·1–68·7)
	Malaysia	77·0 (76·8–77·2)	78·4 (77·8–78·9)	80·3 (78·7–81·5)	72·6 (72·3–72·9)	74·6 (73·8–75·3)	77·7 (75·7–79·3)	66·3 (63·1–69·0)	67·2 (64·0–69·9)	68·1 (64·4–71·3)	64·4 (62·0–66·4)	65·9 (63·5–68·0)	68·0 (64·9–70·7)
	Maldives	82·3 (80·8–83·7)	84·3 (82·6–85·9)	86·7 (83·8–89·1)	79·8 (78·1–81·6)	81·8 (79·8–83·6)	84·2 (81·4–86·6)	70·2 (66·9–73·4)	71·8 (68·2–75·0)	72·7 (68·5–76·7)	70·4 (67·8–73·2)	71·9 (69·1–74·7)	73·0 (69·4–76·4)
	Mauritius	78·1 (77·9–78·9)	79·5 (78·8–80·4)	82·1 (80·1–83·7)	71·8 (71·6–72·7)	73·5 (72·8–74·4)	76·7 (74·8–78·3)	66·6 (63·2–69·5)	67·5 (64·0–70·5)	68·9 (64·9–72·5)	63·2 (60·7–65·5)	64·4 (61·8–66·7)	66·5 (63·4–69·5)
	Myanmar	72·7 (69·7–74·9)	74·8 (71·4–77·0)	78·8 (74·6–81·8)	66·2 (62·6–68·8)	68·1 (63·9–70·5)	71·4 (67·0–74·6)	63·0 (59·8–66·0)	64·7 (61·2–67·8)	67·4 (63·1–71·2)	59·2 (55·9–61·8)	60·7 (57·2–63·6)	63·1 (59·1–66·5)
	Philippines	75·2 (73·4–77·0)	76·8 (74·6–78·5)	79·3 (76·9–81·6)	69·0 (66·5–71·5)	71·0 (68·5–73·7)	74·6 (71·7–77·6)	64·8 (61·6–67·9)	66·0 (62·8–69·1)	67·6 (64·0–71·0)	61·4 (58·6–64·4)	63·1 (60·1–66·2)	65·6 (62·2–69·3)
	Seychelles	77·2 (76·2–78·1)	78·6 (77·5–79·7)	80·0 (78·1–81·7)	71·4 (70·3–72·5)	73·8 (72·6–74·9)	75·7 (73·8–77·3)	66·1 (62·4–69·0)	67·4 (64·0–70·4)	67·5 (63·6–70·9)	63·2 (60·7–65·5)	65·1 (62·5–67·6)	65·7 (62·6–68·6)
	Sri Lanka	80·9 (77·4–84·0)	81·9 (78·2–85·2)	84·3 (80·6–87·7)	74·9 (69·8–79·3)	76·2 (71·6–80·5)	78·7 (73·9–83·0)	68·8 (64·9–72·7)	69·7 (65·6–73·4)	70·9 (66·8–75·1)	65·5 (61·5–69·7)	66·6 (62·1–70·7)	68·1 (64·2–72·3)
	Thailand	81·2 (78·8–83·6)	83·0 (80·6–85·4)	84·7 (82·0–87·4)	73·7 (70·5–76·8)	76·0 (72·7–79·2)	78·6 (75·1–82·0)	69·7 (65·9–72·8)	71·0 (67·1–74·5)	71·5 (67·3–75·3)	65·0 (61·7–68·3)	66·8 (63·4–70·2)	68·4 (64·8–72·2)
	Timor-Leste	72·1 (69·7–74·3)	74·7 (71·9–76·9)	78·8 (74·8–82·0)	69·1 (66·7–71·5)	71·2 (68·4–73·8)	74·9 (70·8–78·1)	62·6 (59·6–65·5)	64·7 (61·4–67·8)	67·5 (63·4–71·4)	60·7 (57·9–63·6)	62·6 (59·7–65·5)	65·2 (61·5–68·7)
	Viet Nam	78·8 (77·1–80·7)	80·2 (78·3–81·9)	81·9 (79·3–84·1)	70·4 (68·7–72·3)	71·4 (69·6–73·4)	72·1 (69·3–74·5)	68·6 (65·7–71·4)	69·6 (66·5–72·6)	70·6 (66·9–74·1)	63·4 (61·2–65·6)	64·1 (61·9–66·6)	64·5 (61·7–67·3)
**Sub-Saharan Africa**		**66·6 (64·9–68·0)**	**70·4 (68·3–72·3)**	**75·5 (71·6–78·2)**	**62·2 (60·3–64·0)**	**66·3 (64·1–68·2)**	**71·6 (67·7–74·2)**	**56·7 (53·7–59·5)**	**60·0 (56·6–63·3)**	**63·8 (59·8–68·0)**	**55·0 (52·4–57·2)**	**58·6 (55·9–61·2)**	**62·6 (58·8–66·1)**
Central sub- Saharan Africa		65·9 (63·4–68·2)	69·5 (65·8–72·4)	74·5 (69·6–77·8)	61·3 (58·5–63·8)	65·1 (61·1–67·9)	70·0 (64·8–73·4)	56·0 (52·9–59·1)	59·2 (55·1–62·5)	62·8 (58·1–66·9)	53·9 (51·1–56·8)	57·1 (53·7–60·2)	60·8 (56·6–64·5)
	Angola	66·5 (63·3–69·7)	69·7 (65·5–73·0)	75·3 (70·7–78·8)	62·2 (59·1–65·3)	65·5 (61·9–68·8)	70·6 (66·3–74·0)	56·6 (53·5–60·1)	59·4 (55·4–62·8)	63·2 (58·7–67·1)	54·7 (51·7–57·8)	57·5 (54·1–60·7)	61·0 (56·9–64·7)
	Central African Republic	55·9 (48·6–60·5)	61·9 (56·0–66·5)	68·3 (61·2–74·0)	49·2 (42·5–53·6)	55·0 (49·8–59·3)	61·1 (54·1–66·6)	47·7 (41·9–51·9)	52·7 (47·2–57·1)	57·5 (50·4–63·3)	43·5 (37·6–47·6)	48·4 (43·8–52·6)	53·3 (47·3–58·4)
	Congo (Brazzaville)	64·9 (61·5–67·8)	67·9 (64·0–71·1)	72·1 (67·9–75·8)	63·6 (60·1–66·3)	66·4 (62·6–69·4)	69·9 (65·3–73·5)	55·3 (51·7–58·6)	57·9 (54·0–61·8)	60·8 (56·2–65·3)	56·2 (52·8–59·1)	58·6 (54·8–61·6)	61·1 (56·5–64·9)
	DR Congo	66·5 (63·9–69·1)	70·1 (64·9–73·4)	74·6 (69·1–78·4)	61·8 (58·7–64·7)	65·5 (60·9–68·7)	70·2 (64·7–74·0)	56·4 (53·2–59·8)	59·6 (54·7–63·3)	63·1 (58·1–67·5)	54·3 (51·3–57·4)	57·4 (53·3–60·8)	61·1 (56·2–65·0)
	Equatorial Guinea	66·3 (61·0–70·9)	69·8 (64·4–74·6)	74·9 (69·1–79·6)	62·8 (58·2–66·6)	65·8 (61·0–69·8)	69·9 (64·7–74·2)	56·1 (51·5–60·6)	58·9 (53·4–63·8)	62·5 (56·9–67·7)	55·3 (51·1–59·1)	57·7 (52·9–61·7)	60·6 (55·1–64·9)
	Gabon	69·6 (65·9–72·8)	72·3 (68·3–75·9)	75·9 (72·1–79·7)	63·8 (60·1–67·0)	66·3 (62·7–69·6)	69·7 (65·8–73·0)	58·9 (54·9–62·7)	61·1 (56·7–65·2)	63·6 (59·1–67·9)	56·3 (52·7–59·6)	58·3 (54·7–61·8)	60·5 (56·6–64·4)
Eastern sub- Saharan Africa		67·3 (65·6–68·8)	71·5 (69·5–73·2)	76·5 (73·0–78·7)	62·8 (60·9–64·3)	67·2 (65·1–68·9)	72·4 (69·0–74·7)	57·6 (54·6–60·4)	61·2 (57·9–64·4)	65·0 (60·9–68·8)	55·6 (53·1–58·0)	59·4 (56·8–61·8)	63·4 (59·7–66·5)
	Burundi	64·6 (61·1–67·2)	70·3 (66·5–73·5)	75·6 (71·2–79·0)	59·6 (54·5–62·8)	66·2 (62·3–69·4)	71·2 (66·9–74·7)	56·0 (52·9–59·0)	60·7 (56·8–63·9)	64·8 (60·3–68·8)	52·9 (48·7–56·0)	58·4 (54·6–61·8)	62·5 (58·4–66·2)
	Comoros	69·7 (67·1–72·0)	72·0 (69·3–74·5)	75·8 (71·9–78·8)	67·6 (64·8–70·0)	69·9 (66·9–72·4)	73·5 (69·5–76·5)	59·9 (56·9–62·9)	62·0 (58·7–65·2)	64·6 (60·5–68·4)	59·9 (57·2–62·7)	61·9 (59·0–64·8)	64·3 (60·5–67·8)
	Djibouti	69·8 (65·4–73·1)	72·8 (68·8–76·3)	77·1 (72·7–80·6)	66·1 (62·0–69·5)	68·9 (65·0–72·2)	72·5 (68·0–76·0)	60·2 (56·1–64·0)	62·8 (58·7–66·7)	65·9 (61·3–70·4)	59·0 (55·3–62·5)	61·2 (57·4–64·9)	63·9 (59·6–68·1)
	Eritrea	66·0 (62·1–69·4)	69·5 (66·1–72·9)	74·3 (70·2–77·9)	60·4 (56·0–64·0)	64·1 (60·2–67·4)	69·1 (64·4–72·7)	57·0 (53·2–60·6)	60·0 (56·2–63·8)	63·7 (59·3–68·0)	53·3 (49·4–56·7)	56·3 (52·3–60·0)	60·1 (55·7–64·1)
	Ethiopia	70·4 (68·4–72·4)	74·0 (71·8–76·2)	78·6 (75·3–81·2)	66·6 (64·4–68·7)	70·6 (68·1–72·8)	75·5 (72·0–78·2)	60·2 (56·6–63·6)	63·4 (59·8–67·1)	66·7 (62·3–70·8)	58·6 (56·1–61·6)	61·9 (58·8–65·0)	65·3 (61·4–68·9)
	Kenya	70·6 (68·5–72·5)	73·9 (71·5–76·0)	77·6 (74·8–80·1)	65·4 (63·5–67·5)	69·1 (67·1–71·3)	73·4 (70·4–76·4)	60·4 (57·2–63·4)	63·3 (59·8–66·5)	66·0 (62·3–69·9)	58·1 (55·8–60·6)	61·3 (58·6–64·0)	64·7 (61·3–67·7)
	Madagascar	63·7 (61·0–66·7)	68·9 (66·0–71·7)	73·7 (69·7–77·2)	60·7 (57·7–64·0)	67·0 (63·6–70·2)	71·9 (67·9–75·3)	55·1 (52·0–58·2)	59·5 (56·0–62·9)	63·2 (59·0–67·3)	54·4 (51·4–57·5)	59·6 (56·3–62·9)	63·5 (59·6–67·3)
	Malawi	64·9 (61·8–67·7)	70·2 (66·5–73·8)	76·0 (71·7–80·1)	59·2 (56·6–61·7)	64·3 (61·1–67·1)	69·4 (65·2–72·8)	55·7 (52·5–59·1)	60·4 (56·7–64·0)	65·2 (60·8–69·8)	53·1 (50·7–55·7)	57·6 (54·6–60·5)	61·9 (58·2–65·3)
	Mozambique	63·0 (60·4–66·1)	69·3 (65·6–73·1)	76·0 (71·6–79·9)	56·9 (54·4–59·3)	62·4 (58·8–65·2)	68·7 (64·5–72·0)	52·5 (49·1–55·9)	58·2 (54·3–61·9)	63·3 (58·6–67·4)	50·3 (47·7–53·0)	55·2 (51·6–58·1)	60·3 (56·6–63·8)
	Rwanda	70·2 (67·4–72·8)	73·4 (70·4–76·2)	78·1 (74·5–80·8)	66·0 (63·4–68·8)	69·3 (66·4–72·2)	74·1 (70·6–77·0)	60·1 (57·3–63·4)	63·0 (59·8–66·3)	66·5 (62·7–70·1)	58·4 (55·6–61·4)	61·2 (58·2–64·3)	65·0 (61·6–68·4)
	Somalia	60·2 (56·0–63·8)	63·1 (58·8–67·1)	69·0 (60·7–74·5)	54·9 (50·4–59·0)	57·7 (52·7–62·1)	63·1 (55·5–68·5)	52·0 (48·0–55·6)	54·6 (50·2–59·1)	59·2 (52·1–65·0)	49·0 (44·9–52·8)	51·5 (46·7–55·6)	55·9 (49·4–61·1)
	South Sudan	60·2 (55·2–64·3)	64·5 (58·0–69·7)	71·7 (63·6–77·6)	55·2 (49·9–59·9)	59·7 (53·5–65·0)	66·5 (58·9–72·1)	51·0 (46·6–55·5)	54·8 (47·7–60·1)	60·7 (53·3–66·5)	48·5 (43·6–52·6)	52·4 (46·4–57·0)	58·0 (51·3–63·6)
	Tanzania	69·1 (66·7–71·2)	72·2 (69·5–74·5)	76·6 (73·3–79·2)	66·0 (63·4–68·3)	69·3 (66·5–71·6)	74·5 (71·1–77·0)	59·1 (55·9–62·1)	61·7 (58·1–64·9)	64·9 (61·0–68·5)	58·6 (55·7–61·3)	61·4 (58·5–64·1)	65·4 (61·7–68·5)
	Uganda	68·7 (65·3–71·0)	73·2 (70·1–75·7)	78·5 (74·8–81·0)	62·2 (59·0–64·5)	67·1 (63·7–69·5)	72·8 (69·4–75·1)	58·7 (55·4–61·9)	62·7 (58·9–66·0)	66·8 (62·7–70·3)	55·0 (52·0–57·7)	59·2 (56·0–62·1)	63·8 (60·6–66·9)
	Zambia	65·3 (61·4–69·0)	69·8 (66·0–73·6)	74·7 (70·6–78·8)	60·8 (56·8–64·0)	65·2 (61·5–68·6)	69·5 (65·7–73·2)	55·7 (51·8–59·4)	59·7 (55·4–63·6)	63·6 (59·2–68·1)	53·8 (49·9–57·1)	57·7 (54·0–61·0)	61·2 (57·4–64·9)
Southern sub-Saharan Africa		66·1 (64·9–67·3)	70·3 (69·0–71·6)	74·9 (72·8–76·7)	59·9 (58·5–61·2)	64·3 (62·7–65·6)	68·8 (66·4–70·8)	55·6 (52·6–58·4)	59·1 (55·9–62·2)	62·7 (58·9–66·2)	52·6 (50·4–54·7)	56·3 (53·8–58·8)	60·1 (57·1–63·0)
	Botswana	66·1 (63·3–68·7)	72·4 (70·0–74·7)	78·1 (75·1–80·5)	61·1 (58·5–63·3)	67·6 (65·0–69·8)	72·6 (69·7–75·1)	56·1 (52·8–59·5)	61·4 (57·9–64·8)	65·9 (61·8–69·6)	53·8 (51·2–56·4)	59·2 (56·2–62·0)	63·2 (59·8–66·5)
	Eswatini	59·8 (55·3–63·5)	66·5 (61·9–70·9)	72·9 (68·0–77·8)	53·6 (49·5–56·7)	59·3 (55·4–62·8)	64·3 (59·9–68·1)	51·1 (46·9–54·9)	56·7 (52·4–61·0)	61·8 (57·1–66·5)	47·6 (43·9–50·7)	52·5 (48·8–55·9)	56·7 (52·6–60·5)
	Lesotho	56·4 (53·5–59·2)	62·1 (59·0–65·3)	69·8 (65·6–73·5)	49·7 (47·3–52·1)	54·5 (51·9–57·1)	60·6 (56·6–63·7)	48·0 (44·8–51·1)	53·1 (50·0–56·4)	59·7 (55·8–63·6)	43·9 (41·5–46·4)	48·3 (45·7–51·0)	53·9 (50·5–57·2)
	Namibia	67·3 (63·9–70·5)	72·5 (68·6–76·1)	77·3 (73·1–80·9)	60·3 (56·8–63·1)	65·0 (61·3–68·0)	69·3 (65·4–72·7)	57·9 (54·4–61·4)	62·2 (58·2–66·2)	66·0 (61·4–70·3)	53·6 (50·3–56·8)	57·6 (53·9–60·9)	61·0 (57·0–64·7)
	South Africa	67·8 (66·9–68·8)	71·4 (70·4–72·3)	75·6 (73·9–77·0)	61·2 (60·1–62·3)	65·2 (64·0–66·4)	69·5 (67·5–71·1)	56·6 (53·4–59·4)	59·6 (56·1–62·5)	62·7 (58·8–66·1)	53·5 (51·2–55·7)	56·8 (54·3–59·1)	60·3 (57·5–63·0)
	Zimbabwe	61·1 (58·1–63·9)	65·9 (62·5–69·3)	71·0 (66·9–74·4)	56·3 (52·9–59·2)	61·0 (57·5–64·3)	65·7 (61·5–69·2)	52·8 (49·7–56·0)	57·1 (53·4–60·5)	61·3 (57·3–65·3)	50·4 (47·3–53·4)	54·6 (51·3–57·7)	58·6 (54·5–62·2)
Western sub-Saharan Africa		66·5 (64·6–68·4)	69·9 (67·2–72·5)	75·3 (70·9–78·9)	63·1 (60·7–65·3)	66·7 (63·7–69·3)	72·0 (67·4–75·3)	56·6 (53·8–59·7)	59·6 (55·8–63·3)	63·5 (59·1–68·1)	55·9 (53·1–58·4)	59·0 (55·7–62·1)	63·0 (58·2–67·0)
	Benin	68·2 (65·9–70·5)	71·0 (64·8–74·2)	75·4 (63·0–80·0)	63·3 (60·4–65·8)	66·0 (60·0–69·5)	70·9 (58·1–75·2)	58·1 (54·7–61·1)	60·4 (54·8–64·1)	63·4 (52·9–68·7)	56·2 (53·4–59·1)	58·4 (53·0–62·1)	61·7 (51·0–66·3)
	Burkina Faso	65·0 (61·8–67·5)	68·3 (58·8–72·1)	73·8 (61·6–78·4)	60·3 (57·3–62·9)	63·9 (57·4–67·7)	69·2 (57·0–73·8)	55·9 (52·4–59·1)	58·8 (51·2–62·9)	63·0 (53·5–68·4)	53·9 (50·8–56·8)	57·0 (51·6–60·9)	61·0 (51·5–65·7)
	Cabo Verde	78·3 (76·3–80·2)	80·0 (78·0–82·2)	82·2 (80·0–84·5)	69·6 (67·4–71·8)	71·6 (69·4–74·0)	74·5 (72·1–77·1)	66·8 (63·2–70·3)	68·3 (64·3–71·8)	69·2 (65·1–72·9)	62·0 (59·2–64·8)	63·7 (60·8–66·4)	65·3 (62·1–68·3)
	Cameroon	67·0 (63·3–70·2)	70·4 (65·1–74·5)	74·9 (70·1–78·9)	63·0 (59·6–66·0)	66·4 (62·0–69·9)	70·4 (64·5–74·4)	57·3 (53·4–61·1)	60·2 (54·9–65·0)	63·5 (58·3–68·5)	56·0 (52·7–59·2)	58·9 (54·3–62·6)	61·8 (56·3–66·0)
	Chad	62·3 (58·2–65·9)	65·8 (61·4–69·5)	73·5 (68·5–77·8)	59·2 (54·7–63·3)	62·7 (58·0–67·0)	69·7 (64·7–74·3)	53·2 (49·4–56·9)	56·3 (52·0–60·2)	62·2 (57·3–67·2)	52·4 (48·4–56·1)	55·5 (51·0–59·6)	61·0 (56·2–65·8)
	Côte d'Ivoire	67·8 (64·2–70·3)	71·7 (65·6–75·1)	77·0 (67·6–81·1)	62·9 (59·7–65·7)	66·9 (61·7–70·4)	72·4 (66·4–76·4)	57·8 (54·1–61·3)	61·1 (55·6–65·1)	64·8 (57·6–69·9)	55·8 (52·5–58·7)	59·2 (54·3–63·1)	63·0 (57·1–67·5)
	The Gambia	67·9 (65·2–70·4)	71·2 (68·5–74·0)	76·2 (72·8–79·3)	63·7 (61·1–66·4)	66·8 (64·0–69·5)	72·0 (68·2–75·4)	57·6 (54·2–60·9)	60·1 (56·6–63·6)	63·4 (59·2–67·4)	56·9 (54·4–59·6)	59·5 (56·6–62·3)	63·2 (59·5–66·5)
	Ghana	69·3 (66·6–72·0)	71·9 (67·6–75·1)	75·4 (69·9–79·2)	64·3 (61·7–66·7)	66·7 (62·7–69·6)	69·8 (63·5–73·5)	59·5 (56·5–62·9)	61·7 (57·3–65·4)	64·0 (58·0–68·4)	57·4 (54·9–60·0)	59·5 (55·8–62·6)	61·8 (56·4–65·7)
	Guinea	64·2 (60·2–67·6)	67·6 (60·3–71·9)	73·5 (65·0–78·2)	61·2 (57·4–65·1)	65·3 (58·8–69·7)	70·9 (61·5–75·7)	55·0 (50·9–58·7)	58·0 (51·3–62·5)	62·2 (54·8–67·7)	54·5 (51·0–58·1)	58·0 (52·1–62·3)	62·3 (53·1–67·4)
	Guinea-Bissau	61·6 (45·0–65·5)	67·0 (64·1–69·6)	72·9 (68·9–76·0)	55·9 (40·4–60·0)	61·3 (57·9–64·0)	67·4 (63·4–70·6)	53·1 (39·9–57·1)	57·5 (54·1–60·7)	61·7 (57·5–65·6)	50·2 (37·1–54·1)	54·7 (51·5–57·4)	59·4 (55·4–62·7)
	Liberia	66·1 (61·7–69·5)	69·3 (63·5–73·4)	74·0 (67·0–78·8)	64·6 (59·9–68·4)	67·7 (61·6–71·9)	72·2 (65·3–77·2)	54·9 (50·5–59·0)	57·7 (52·1–62·5)	61·4 (55·2–66·9)	56·2 (52·1–60·1)	58·9 (53·3–63·2)	62·5 (55·5–67·5)
	Mali	64·1 (61·5–66·6)	67·8 (64·0–70·9)	73·8 (68·3–77·7)	62·1 (59·5–64·7)	66·3 (61·5–69·5)	72·5 (67·9–76·2)	54·6 (51·4–57·7)	57·8 (53·8–61·4)	61·9 (57·0–66·4)	54·8 (51·9–57·6)	58·4 (53·7–61·7)	63·1 (58·4–66·9)
	Mauritania	70·7 (66·7–73·3)	73·6 (70·5–76·4)	76·7 (73·1–79·9)	69·6 (64·0–72·6)	72·8 (69·3–75·8)	75·5 (71·5–79·2)	60·8 (56·7–64·3)	63·3 (59·5–66·9)	65·1 (60·7–69·6)	62·1 (57·8–65·3)	64·6 (60·9–67·9)	66·1 (61·9–70·1)
	Niger	65·1 (61·1–68·5)	67·7 (61·7–71·8)	71·6 (63·2–76·4)	62·9 (58·4–66·6)	65·7 (57·6–70·4)	70·1 (63·0–75·0)	55·8 (52·0–59·8)	58·2 (53·1–62·5)	61·2 (53·8–66·5)	56·1 (52·2–59·9)	58·6 (52·0–63·2)	62·0 (55·6–66·8)
	Nigeria	66·9 (64·1–69·5)	70·4 (64·5–73·9)	76·2 (69·9–80·3)	64·1 (61·2–66·8)	67·8 (62·7–71·6)	73·7 (66·6–77·9)	56·6 (53·2–60·2)	59·7 (54·5–64·1)	64·0 (58·4–68·9)	56·6 (53·4–59·5)	59·9 (55·3–63·7)	64·3 (58·0–68·8)
	São Tomé and Príncipe	73·7 (71·5–75·6)	75·3 (73·1–77·2)	77·7 (75·1–79·9)	70·7 (68·7–72·4)	72·4 (70·3–74·1)	75·0 (72·8–76·9)	63·3 (60·0–66·4)	64·6 (61·2–67·7)	66·0 (62·2–69·7)	63·1 (60·3–65·7)	64·6 (61·9–67·1)	66·4 (63·3–69·3)
	Senegal	70·8 (67·9–73·1)	73·8 (70·8–76·3)	78·6 (75·1–81·5)	67·5 (64·7–70·0)	70·7 (67·9–73·3)	75·3 (72·0–78·4)	60·3 (56·8–63·9)	62·9 (59·1–66·6)	66·2 (62·0–70·4)	59·9 (56·7–62·9)	62·5 (59·2–65·7)	65·7 (62·1–69·4)
	Sierra Leone	63·9 (60·3–67·1)	67·4 (60·5–71·8)	73·6 (63·2–78·2)	61·8 (57·3–65·5)	65·7 (60·1–69·9)	72·2 (65·6–77·2)	54·7 (51·4–58·2)	57·6 (52·4–62·0)	62·3 (54·0–67·1)	54·6 (50·9–58·3)	58·0 (52·9–62·0)	63·2 (57·0–67·7)
	Togo	67·9 (64·1–71·0)	71·2 (66·6–75·0)	75·6 (68·4–80·1)	62·6 (58·6–65·9)	66·1 (61·3–70·0)	70·7 (64·5–75·4)	57·9 (53·8–61·7)	60·8 (55·8–65·4)	63·8 (57·3–69·2)	55·9 (52·2–59·4)	58·9 (54·0–62·9)	62·4 (56·2–67·0)

Estimates are listed as means (years) with 95% uncertainty intervals in parentheses. Rows with bold text indicate global and super-region results from the GBD location hierarchy. HALE=healthy life expectancy.

At the super-regional level, we forecast a convergence of life expectancy in 2050, whereby countries with lower life expectancy in 2022 are forecasted to see larger gains in the coming decades and countries with relatively high life expectancy in 2022 are forecasted to see slower growth. The highest life expectancy in 2050 in the reference scenario will be in the high-income super-region (83·3 years [95% UI 82·9–83·6]); followed by southeast Asia, east Asia, and Oceania (79·8 years [77·8–81·6]); and Latin America and the Caribbean (79·6 years [77·1–81·5]; appendix 2 table S2). Although it will remain the highest, life expectancy in the high-income super-region is forecasted to have the smallest increase across super-regions (from 81·4 years [81·2–81·5] in 2022 or 81·5 years [81·5–81·6 ] in 2019), while in sub-Saharan Africa, life expectancy will increase from 64·4 years (62·6–66·0) in 2022 (63·9 years [62·1–65·4] in 2019) to 73·6 years (69·4–76·2) in 2050—the largest increase.

Global increases in life expectancy from 2022 to 2050 are forecasted to be greater among males than females; 71·1 years (95% UI 70·1–72·0) to 76·0 years (73·2–77·9) for males (increase of 4·9 years [2·4–6·6]), and 76·2 years (75·4–77·0) to 80·5 years (77·4–82·6) for females (increase of 4·2 years [1·4–6·2]; table 1, figure 1). Life expectancy will remain highest in the high-income super-region in 2050 among both males and females (81·3 years [81·0–81·6] in males and 85·3 years [84·8–85·7] in females), although increases will be minimal for females in this super-region (1·4-year [1·0–1·8] change from 2022 to 2050). For both males and females, the super-region with the lowest life expectancy in 2050 but also the largest gains over the forecasted period will be sub-Saharan Africa.

We likewise forecast continued increases in global HALE in the reference scenario, from 62·6 years (95% UI 60·1–64·9) for males in 2022 to 66·0 years (62·6–69·3) in 2050, and 64·7 years (61·4–67·7) to 67·5 years (63·3–71·6) for females over the same forecasted period (table 1, appendix 2 figure S2). Super-regionally, HALE is forecasted to be highest in the high-income super-region in 2050 (70·8 years [67·3–73·8]), followed by 69·3 years (66·0–72·4) in southeast Asia, east Asia, and Oceania and 68·1 years (64·6–71·4) in central Europe, eastern Europe, and central Asia. Country-specific life expectancy and HALE forecasts can be found in table 1 and appendix 2 (figure S3).

#### Decomposition of life expectancy changes by cause

**Figure 2:**
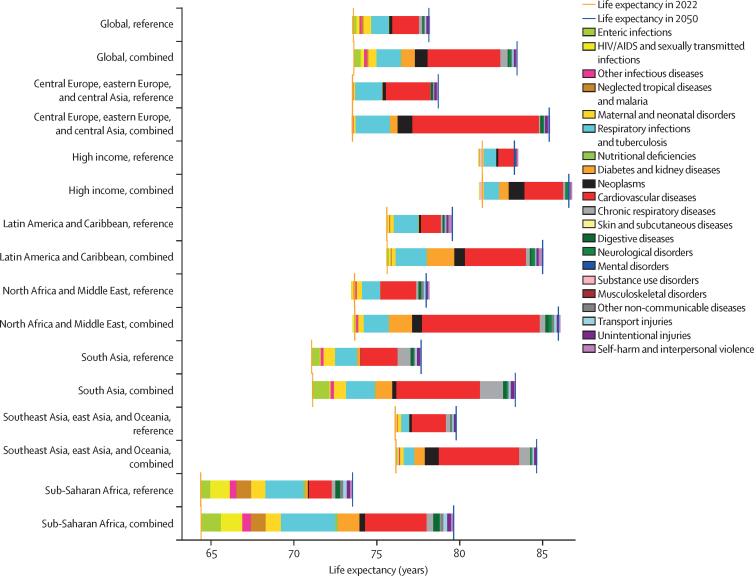
Decomposition of change in global and super-regional life expectancy, 2022–50, by Level 2 causes, for reference and combined scenarios Life expectancy at birth in 2022 is denoted by the orange vertical line, while life expectancy at birth in 2050 is denoted by the blue vertical line. The horizontal bars show the breakdown of causes of death trends by Level 2 GBD cause that have contributed to changes in life expectancy. Causes to the left of the 2022 life expectancy value indicate increases in mortality rates (thus declines in life expectancy), while causes to the right of the 2022 value represent reductions in mortality that have led to increases in life expectancy.

In the reference scenario, a decline in cardiovascular disease deaths was the leading Level 2 cause-specific contributor to increases in life expectancy between 2022 and 2050 globally (35·4% of total life expectancy gains) and in five of seven super-regions (figure 2). Declines in respiratory infections and tuberculosis deaths contributed the most to life expectancy gains in sub-Saharan Africa and Latin America and the Caribbean—largely due to COVID-19 declines—and the second-most in the other five super-regions and globally. At the global level, improvements in maternal and neonatal deaths were the third-leading contributor to life expectancy gains, followed, in descending order, by enteric infections, chronic respiratory diseases, neoplasms, and HIV/AIDS and sexually transmitted infections.

### Deaths and DALYs, reference scenario

#### Global trends

In the reference scenario, the numbers of global all-cause deaths and DALYs are forecasted to increase from 2022 to 2050, from 60·1 million (95% UI 57·0–63·2) deaths and 2·67 billion (2·39–2·97) DALYs in 2022 to 90·7 million (83·9–99·6) and 3·15 billion (2·72–3·60) in 2050 (table 2, appendix 2 table S4). At the same time, age-standardised rates are forecasted to decline, from 736·3 deaths (702·6–772·7) per 100 000 and 33 340·8 DALYs (30 241·6–36 798·4) per 100 000 in 2022 to 547·8 (467·9–670·7) and 26 421·3 (22 106·8–31 292·5) in 2050. All-age rates are forecasted to increase as the global population ages, from 756·3 deaths (723·0–791·7) and 33 548·3 DALYs (30 355·4–36 963·4) per 100 000 in 2022 to 967·2 (879·0–1087·6) and 33 576·8 (28 817·9–38 853·2) per 100 000 in 2050, respectively.

**Table 2:** Global cause-specific DALY counts and age-standardised DALY rates in 2022 and 2050 by scenario

		**All-age DALYs (in thousands)**						**Age-standardised DALY rate (per 100 000)**					
		Reference scenario, 2022	Reference scenario, 2050	Safer Environment scenario, 2050	Improved Behavioural and Metabolic Risks scenario, 2050	Improved Childhood Nutrition and Vaccination scenario, 2050	Combined scenario, 2050	Reference scenario, 2022	Reference scenario, 2050	Safer Environment scenario, 2050	Improved Behavioural and Metabolic Risks scenario, 2050	Improved Childhood Nutrition and Vaccination scenario, 2050	Combined scenario, 2050
**All causes**		**2 667 784·0 (2 391 576·5–2 966 825·2)**	**3 148 764·4 (2 720 385·3–3 600 864·1)**	**3 091 015·0 (2 669 273·6–3 531 277·2)**	**2 730 637·8 (2 352 253·0–3 149 258·5)**	**3 129 413·8 (2 700 814·7–3 575 590·5)**	**2 664 356·0 (2 291 924·2–3 069 069·1)**	**33 340·8 (30 241·6–36 798·4)**	**26 421·3 (22 106·8–31 292·5)**	**25 641·8 (21 447·8–30 261·0)**	**22 306·1 (18 623·7–26 272·7)**	**26 072·4 (21 790·1–30 953·0)**	**21 429·0 (17 912·3–25 250·8)**
**Communicable, maternal, neonatal, and nutritional diseases**		**707 296·6 (638 937·2–795 286·0)**	**448 240·9 (359 959·6–595 527·0)**	**396 758·2 (319 561·0–518 821·9)**	**467 091·4 (374 813·2–624 467·6)**	**427 696·0 (341 116·6–569 598·0)**	**398 112·7 (319 208·9–522 753·3)**	**9926·2 (8993·8–11 157·1)**	**5634·1 (4422·8–7525·2)**	**5056·2 (3977·0–6780·8)**	**5614·4 (4400·6–7507·4)**	**5281·6 (4138·8–7109·0)**	**4821·3 (3767·5–6596·2)**
	**HIV/AIDS and sexually transmitted infections**	**46 542·3 (41 042·8–54 213·5)**	**22 414·7 (17 060·3–36 408·6)**	**22 532·9 (17 143·4–36 539·3)**	**22 937·7 (17 440·9–37 224·7)**	**22 563·3 (17 128·9–36 553·5)**	**23 176·2 (17 599·5–37 526·8)**	**593·0 (517·0–711·4)**	**239·3 (178·0–392·1)**	**240·3 (178·7–393·4)**	**240·0 (178·5–393·1)**	**241·8 (179·5–395·4)**	**243·1 (180·4–397·2)**
	HIV/AIDS	38 841·8 (35 385·6–43 789·5)	18 416·2 (13 841·5–32 539·5)	18 480·5 (13 893·3–32 615·4)	18 895·6 (14 217·7–33 014·4)	18 421·6 (13 845·5–32 561·1)	18 961·6 (14 266·6–33 103·2)	474·7 (430·1–537·2)	179·8 (131·3–331·6)	180·0 (131·5–331·8)	180·5 (131·8–333·0)	179·8 (131·3–331·7)	180·7 (132·0–333·3)
	Sexually transmitted infections excluding HIV	7700·5 (3749·6–14 240·6)	3998·5 (2235·5–6650·0)	4052·4 (2259·8–6761·6)	4042·1 (2270·9–6688·9)	4141·6 (2297·2–6948·3)	4214·6 (2345·7–7054·2)	118·3 (56·5–225·4)	59·5 (30·3–105·2)	60·3 (30·7–107·0)	59·5 (30·3–105·2)	62·0 (31·4–110·5)	62·5 (31·6–111·5)
	**Respiratory infections and tuberculosis**	**227 541·4 (206 462·1–254 641·6)**	**119 966·9 (99 588·0–145 560·2)**	**104 121·6 (85 995·4–126 970·9)**	**128 739·7 (105 663·6–156 596·8)**	**107 597·4 (89 312·7–129 701·1)**	**102 503·9 (83 474·1–125 123·5)**	**2947·6 (2665·2–3300·7)**	**1127·4 (885·7–1428·5)**	**946·7 (747·5–1208·1)**	**1101·6 (861·5–1403·8)**	**926·9 (729·8–1184·2)**	**776·9 (604·9–1002·9)**
	Tuberculosis	41 652·9 (37 284·9–47 386·3)	24 826·6 (16 091·0–38 084·2)	24 510·5 (15 854·6–37 630·7)	26 688·5 (17 174·6–41 330·1)	25 042·5 (16 197·8–38 460·8)	26 441·9 (16 957·4–41 140·8)	508·8 (452·9–580·0)	216·8 (132·3–359·6)	214·2 (130·1–357·5)	219·1 (133·6–364·3)	220·5 (134·0–368·8)	219·0 (132·5–367·8)
	Lower respiratory infections	100 725·0 (88 301·5–113 278·5)	85 879·6 (73 497·0–100 079·0)	70 293·8 (60 844·6–81 503·9)	92 612·7 (78 736·0–108 412·4)	73 232·6 (63 588·3–83 671·3)	66 526·2 (56 760·1–76 247·3)	1434·0 (1259·3–1622·0)	797·0 (646·3–974·2)	618·2 (506·1–752·5)	768·9 (621·3–943·4)	592·0 (489·4–712·4)	443·0 (365·1–533·3)
	Upper respiratory infections	5675·4 (3164·3–8419·4)	5789·7 (3310·7–8503·7)	5825·2 (3322·4–8569·6)	5911·9 (3384·6–8683·1)	5840·5 (3322·8–8635·2)	5985·9 (3403·4–8869·9)	75·9 (41·8–113·7)	70·9 (39·0–104·2)	71·3 (39·0–105·1)	70·9 (39·0–104·3)	71·7 (39·1–106·3)	71·9 (39·2–106·9)
	Otitis media	2494·0 (1458·0–3998·5)	3446·5 (1955·9–5327·9)	3467·6 (1969·8–5351·7)	3501·7 (1987·3–5419·0)	3457·3 (1962·5–5342·0)	3524·9 (2000·4–5444·7)	32·5 (19·2–52·2)	42·4 (24·2–67·0)	42·7 (24·3–67·5)	42·4 (24·2–67·1)	42·5 (24·2–67·2)	42·7 (24·3–67·6)
	COVID-19	76 993·9 (65 624·6–98 919·5)	24·5 (0·0–127·6)	24·6 (0·0–127·8)	24·9 (0·0–129·3)	24·5 (0·0–127·7)	25·0 (0·1–129·6)	896·3 (761·4–1154·4)	0·3 (0·0–1·5)	0·3 (0·0–1·5)	0·3 (0·0–1·5)	0·3 (0·0–1·5)	0·3 (0·0–1·5)
	**Enteric infections**	**70 195·4 (56 860·6–84 333·1)**	**53 428·4 (34 536·1–81 329·3)**	**16 989·5 (10 619·5–25 702·0)**	**58 062·7 (37 228·4–89 074·8)**	**46 097·2 (29 753·1–69 193·2)**	**18 002·7 (11 181·1–27 459·7)**	**989·6 (805·0–1198·3)**	**594·9 (371·8–954·2)**	**201·5 (119·6–324·3)**	**597·5 (372·7–959·2)**	**461·8 (286·2–711·3)**	**196·5 (114·5–316·4)**
	Diarrhoeal diseases	57 378·8 (45 564·2–69 981·6)	45 335·0 (28 844·0–69 816·8)	8987·3 (5745·8–13 445·7)	49 931·4 (31 647·8–76 966·6)	37 684·5 (24 036·8–55 445·7)	9578·0 (5987·4–14 424·8)	805·9 (648·2–980·8)	469·4 (288·7–758·8)	77·7 (47·6–121·6)	471·9 (290·1–763·3)	330·9 (201·2–512·5)	66·1 (40·4–104·0)
	Typhoid and paratyphoid	7950·9 (4212·4–14 702·0)	3832·5 (1780·5–7449·2)	3778·5 (1757·8–7369·8)	3848·6 (1788·7–7475·5)	3919·2 (1817·8–7639·1)	3890·0 (1804·8–7603·3)	112·8 (60·0–204·8)	58·5 (27·1–111·8)	57·8 (26·5–110·8)	58·5 (27·1–111·9)	59·9 (27·5–114·7)	59·4 (27·0–114·0)
	Invasive non-typhoidal Salmonella	4756·1 (2675·3–7586·4)	4154·8 (1798·7–8262·4)	4117·1 (1786·4–8114·4)	4174·4 (1808·7–8301·5)	4386·8 (1897·2–8667·2)	4425·3 (1910·5–8723·8)	69·2 (38·1–109·9)	65·4 (27·9–128·9)	64·4 (27·5–127·5)	65·4 (27·9–128·9)	69·3 (29·5–137·1)	69·4 (29·7–137·7)
	Other intestinal infectious diseases	109·6 (77·9–151·8)	106·1 (72·2–155·8)	106·6 (72·7–155·3)	108·3 (74·0–158·5)	106·7 (72·6–156·7)	109·4 (74·5–159·2)	1·6 (1·1–2·3)	1·6 (1·1–2·4)	1·6 (1·1–2·4)	1·6 (1·1–2·4)	1·6 (1·1–2·4)	1·6 (1·1–2·4)
	**Neglected tropical diseases and malaria**	**67 422·9 (38 188·7–109 742·9)**	**49 334·8 (25 191·0–129 189·0)**	**51 877·8 (25 792·5–139 854·5)**	**50 264·1 (25 934·1–130 441·9)**	**53 437·0 (25 760·4–151 308·4)**	**55 714·2 (26 941·8–156 615·6)**	**955·4 (533·1–1554·0)**	**673·2 (304·1–1855·0)**	**712·6 (311·8–1999·6)**	**674·0 (304·7–1854·4)**	**744·6 (314·9–2268·6)**	**763·9 (319·2–2364·5)**
	Malaria	51 077·1 (22 642·1–93 943·1)	33 507·5 (9378·8–113 675·0)	35 854·5 (9762·9–123 395·4)	33 785·8 (9457·4–114 339·0)	37 388·6 (9820·3–135 650·5)	38 882·5 (10 269·8–140 255·6)	744·1 (324·7–1347·8)	497·0 (121·9–1694·4)	534·4 (127·6–1804·8)	497·4 (122·0–1693·3)	564·8 (129·5–2064·7)	582·6 (133·4–2147·8)
	Chagas disease	238·0 (203·5–278·9)	211·0 (176·4–249·8)	210·1 (175·6–248·8)	236·0 (195·4–284·6)	211·5 (176·9–250·3)	235·4 (194·9–283·8)	2·7 (2·3–3·2)	1·4 (1·2–1·7)	1·4 (1·1–1·7)	1·4 (1·2–1·7)	1·4 (1·2–1·7)	1·4 (1·2–1·7)
	Leishmaniasis	759·2 (462·3–1565·0)	1125·7 (694·4–1776·1)	1130·7 (697·2–1783·4)	1199·3 (742·2–1891·7)	1130·4 (696·5–1780·5)	1206·5 (746·2–1902·5)	9·9 (6·0–21·1)	11·8 (7·1–18·3)	11·8 (7·1–18·3)	11·9 (7·1–18·6)	11·8 (7·1–18·3)	12·0 (7·2–18·7)
	African trypanosomiasis	101·3 (25·0–391·4)	128·2 (45·1–354·4)	128·9 (45·3–356·3)	129·3 (45·5–357·9)	128·4 (45·1–354·7)	130·2 (45·7–359·8)	1·3 (0·3–5·0)	1·5 (0·5–4·1)	1·5 (0·5–4·1)	1·5 (0·5–4·1)	1·5 (0·5–4·1)	1·5 (0·5–4·1)
	Schistosomiasis	1735·2 (1048·4–2971·6)	1864·5 (951·9–3865·3)	1871·1 (955·7–3875·3)	1907·0 (966·4–3904·4)	1869·0 (953·9–3871·5)	1916·0 (971·7–3918·8)	21·6 (12·9–37·4)	20·6 (10·7–41·2)	20·7 (10·7–41·3)	20·7 (10·7–41·6)	20·7 (10·7–41·3)	20·7 (10·7–41·5)
	Cysticercosis	1246·7 (793·7–1835·8)	1798·3 (1192·3–2647·2)	1822·5 (1209·6–2680·0)	1981·9 (1310·9–2952·3)	1799·0 (1192·7–2648·0)	2009·0 (1330·6–2988·5)	14·6 (9·4–21·2)	13·3 (9·0–19·0)	13·3 (9·1–19·1)	13·3 (9·1–19·1)	13·3 (9·0–19·0)	13·3 (9·1–19·1)
	Cystic echinococcosis	105·9 (81·7–137·7)	119·4 (86·9–157·2)	120·1 (87·4–158·0)	124·6 (91·7–163·6)	120·0 (87·5–157·8)	125·7 (92·9–164·9)	1·3 (1·0–1·7)	1·2 (0·8–1·6)	1·2 (0·8–1·6)	1·2 (0·8–1·6)	1·2 (0·8–1·6)	1·2 (0·8–1·6)
	Lymphatic filariasis	1207·5 (685·2–2083·4)	238·9 (125·8–422·8)	240·1 (126·5–424·8)	245·5 (129·1–434·8)	239·1 (125·9–423·1)	246·7 (129·8–437·1)	15·0 (8·6–25·8)	2·5 (1·3–4·6)	2·5 (1·3–4·6)	2·6 (1·3–4·6)	2·5 (1·3–4·6)	2·6 (1·3–4·6)
	Onchocerciasis	1262·3 (708·3–1953·7)	1286·0 (725·0–1984·7)	1299·6 (733·4–2002·3)	1323·6 (750·5–2038·5)	1287·4 (725·6–1986·5)	1337·8 (759·7–2056·6)	15·6 (8·7–24·4)	13·2 (7·3–20·6)	13·3 (7·3–20·7)	13·3 (7·3–20·7)	13·2 (7·3–20·6)	13·4 (7·4–20·8)
	Trachoma	122·2 (78·8–173·6)	183·6 (119·9–264·9)	189·1 (123·7–271·7)	201·8 (132·6–287·9)	183·7 (119·9–265·0)	207·5 (136·9–295·5)	1·4 (0·9–2·0)	1·2 (0·8–1·8)	1·3 (0·8–1·8)	1·3 (0·8–1·8)	1·2 (0·8–1·8)	1·3 (0·8–1·8)
	Dengue	2098·1 (1088·7–3234·4)	2378·4 (1084·0–3871·4)	2382·3 (1086·3–3875·0)	2504·9 (1155·5–4051·4)	2377·3 (1084·5–3878·3)	2504·0 (1153·6–4041·0)	27·9 (14·7–42·7)	26·4 (11·4–43·8)	26·3 (11·4–43·7)	26·5 (11·5–43·9)	26·3 (11·4–43·6)	26·3 (11·4–43·5)
	Yellow fever	312·5 (112·0–703·2)	158·1 (62·1–441·8)	160·9 (62·9–458·9)	158·6 (62·3–443·5)	160·0 (62·7–453·4)	162·7 (63·4–467·8)	4·2 (1·5–9·4)	2·1 (0·8–6·5)	2·2 (0·8–6·7)	2·1 (0·8–6·5)	2·2 (0·8–6·7)	2·2 (0·8–6·9)
	Rabies	565·9 (313·3–837·0)	414·3 (144·1–800·6)	422·2 (145·7–813·4)	423·3 (148·5–813·7)	423·1 (146·2–816·5)	436·8 (151·2–843·0)	7·4 (4·1–10·9)	4·9 (1·6–9·9)	5·1 (1·6–10·1)	5·0 (1·6–9·9)	5·1 (1·6–10·2)	5·2 (1·7–10·5)
	Intestinal nematode infections	1350·8 (890·3–2014·7)	729·4 (423·5–1161·2)	736·3 (425·3–1204·5)	736·7 (428·2–1173·8)	737·4 (424·4–1209·8)	749·0 (430·7–1227·3)	18·4 (12·2–27·2)	10·1 (6·0–16·6)	10·2 (6·0–17·0)	10·2 (6·0–16·6)	10·3 (6·0–17·1)	10·3 (6·0–17·2)
	Food-borne trematodiases	986·3 (551·8–1627·2)	644·7 (377·2–1054·1)	646·8 (378·4–1057·5)	675·9 (395·8–1104·6)	644·8 (377·3–1054·3)	677·7 (396·8–1107·4)	11·5 (6·5–19·2)	5·2 (3·1–8·5)	5·2 (3·1–8·5)	5·2 (3·1–8·5)	5·2 (3·1–8·5)	5·2 (3·1–8·4)
	Leprosy	21·4 (13·8–31·8)	23·9 (14·5–36·1)	24·2 (14·6–36·5)	25·5 (15·4–38·2)	23·9 (14·5–36·1)	25·7 (15·6–38·6)	0·2 (0·2–0·4)	0·2 (0·1–0·3)	0·2 (0·1–0·3)	0·2 (0·1–0·3)	0·2 (0·1–0·3)	0·2 (0·1–0·3)
	Guinea worm disease	0·0 (0·0–0·0)	0·0 (0·0–0·0)	0·0 (0·0–0·0)	0·0 (0·0–0·0)	0·0 (0·0–0·0)	0·0 (0·0–0·0)	0·0 (0·0–0·0)	0·0 (0·0–0·0)	0·0 (0·0–0·0)	0·0 (0·0–0·0)	0·0 (0·0–0·0)	0·0 (0·0–0·0)
	Other neglected tropical diseases	4232·6 (3036·6–5541·9)	4522·9 (3215·2–6003·7)	4638·6 (3305·8–6163·9)	4604·3 (3278·6–6099·7)	4713·5 (3352·9–6262·1)	4861·0 (3472·5–6468·1)	58·3 (42·2–75·2)	60·4 (43·0–78·7)	62·1 (44·1–80·9)	60·4 (43·0–78·7)	63·7 (45·0–83·4)	64·6 (45·5–84·8)
	**Other infectious diseases**	**51 395·8 (38 796·1–68 098·5)**	**33 169·7 (23 013·0–47 905·8)**	**34 275·5 (23 715·3–50 139·3)**	**34 208·0 (23 640·0–49 327·4)**	**26 630·5 (18 191·6–38 573·5)**	**27 843·3 (18 954·3–40 441·2)**	**742·8 (551·3–988·2)**	**446·0 (298·6–662·3)**	**464·5 (309·0–695·7)**	**447·0 (299·2–664·0)**	**329·6 (219·8–488·0)**	**333·7 (221·9–495·7)**
	Meningitis	14 155·9 (11 341·7–18 167·3)	8432·2 (5680·4–12 785·1)	8680·3 (5775·7–13 222·3)	8663·3 (5867·9–13 104·2)	8200·4 (5398·4–12 563·5)	8515·4 (5570·9–13 053·2)	202·6 (160·7–264·4)	110·4 (69·4–174·0)	114·5 (71·3–182·4)	110·7 (69·6–174·3)	106·8 (66·4–172·4)	108·5 (67·0–176·1)
	Encephalitis	4956·7 (4144·2–5800·5)	5627·9 (3935·0–8077·8)	5684·5 (3976·9–8170·0)	5985·4 (4168·6–8605·9)	5770·3 (4036·2–8307·9)	6142·3 (4269·1–8911·1)	66·9 (55·6–78·1)	61·9 (41·6–91·0)	62·6 (41·9–92·4)	62·2 (41·8–91·6)	64·3 (42·8–94·8)	64·8 (42·9–96·0)
	Diphtheria	303·0 (196·1–426·2)	150·7 (86·8–245·5)	159·6 (91·3–264·9)	151·0 (87·0–245·8)	91·1 (61·0–129·7)	91·7 (61·5–130·6)	4·5 (2·9–6·3)	2·4 (1·4–3·9)	2·6 (1·5–4·2)	2·4 (1·4–3·9)	1·4 (0·9–2·0)	1·4 (0·9–2·0)
	Pertussis	11 970·3 (5808·4–20 775·8)	7615·7 (3598·5–13 716·7)	8205·3 (3880·6–14 773·7)	7620·4 (3600·9–13 725·3)	1687·4 (863·1–2878·3)	1726·3 (879·1–2939·3)	183·3 (89·0–318·0)	131·8 (61·0–239·6)	141·9 (66·1–259·1)	131·8 (61·0–239·7)	26·3 (13·5–46·0)	26·8 (13·8–46·9)
	Tetanus	1283·3 (595·5–2053·7)	388·6 (121·3–736·8)	387·2 (118·8–734·7)	396·9 (123·9–752·2)	283·9 (59·1–618·6)	286·1 (59·4–631·3)	18·6 (8·6–30·2)	5·1 (1·7–9·6)	5·1 (1·7–9·6)	5·2 (1·7–9·6)	3·2 (0·6–6·8)	3·1 (0·6–6·8)
	Measles	7845·2 (3060·2–16 965·7)	1063·6 (344·9–3551·2)	1147·4 (358·7–4103·0)	1064·3 (345·1–3552·9)	438·2 (245·3–774·3)	440·8 (244·9–788·6)	119·4 (46·7–257·3)	18·0 (5·7–60·4)	19·5 (5·9–68·9)	18·0 (5·7–60·4)	6·9 (4·0–12·1)	6·9 (4·0–12·3)
	Varicella and herpes zoster	877·1 (730·0–1033·0)	757·7 (428·2–1264·3)	773·7 (434·2–1298·8)	834·8 (470·1–1398·4)	784·7 (439·9–1323·8)	870·4 (485·6–1478·0)	12·1 (10·1–14·2)	8·3 (4·3–14·9)	8·5 (4·4–15·5)	8·3 (4·3–15·0)	8·8 (4·5–15·9)	8·9 (4·5–16·2)
	Acute hepatitis	4112·1 (3228·6–5358·7)	2443·0 (1682·3–3480·3)	2441·4 (1677·4–3480·4)	2522·5 (1727·8–3611·3)	2479·3 (1701·1–3548·0)	2547·3 (1738·4–3661·7)	54·8 (42·6–72·2)	27·8 (19·0–40·3)	27·9 (18·9–40·4)	28·0 (19·1–40·6)	28·4 (19·3–41·3)	28·5 (19·3–41·5)
	Other unspecified infectious diseases	5892·3 (4219·6–7981·5)	6690·4 (3979·2–10 469·3)	6796·2 (4019·1–10 629·9)	6969·4 (4136·8–10 989·8)	6895·2 (4066·9–10 774·5)	7222·9 (4237·5–11 342·7)	80·4 (57·1–110·1)	80·2 (45·5–128·8)	82·0 (46·2–132·1)	80·4 (45·6–129·3)	83·7 (46·7–135·9)	84·8 (47·2–138·2)
	**Maternal and neonatal disorders**	**195 460·3 (166 246·8–228 549·9)**	**122 550·9 (91 645·7–162 422·6)**	**119 206·2 (89 072·6–157 807·1)**	**122 792·4 (91 854·6–162 715·6)**	**124 634·4 (93 031·3–166 036·6)**	**121 176·3 (90 517·7–161 253·0)**	**3047·1 (2637·9–3505·5)**	**2025·1 (1605·2–2575·0)**	**1959·9 (1549·6–2493·0)**	**2025·2 (1605·2–2575·1)**	**2060·8 (1625·1–2636·9)**	**1989·8 (1566·8–2540·7)**
	Maternal disorders	11 212·2 (9584·0–13 246·0)	7025·6 (4145·3–11 462·1)	7006·4 (4141·4–11 406·2)	7028·8 (4145·7–11 471·8)	7051·5 (4162·0–11 508·8)	7030·2 (4155·4–11 451·9)	141·5 (120·9–166·8)	83·4 (49·6–135·0)	83·2 (49·5–135·0)	83·4 (49·6–135·0)	83·7 (49·8–135·4)	83·3 (49·6–135·3)
	Neonatal disorders	184 248·2 (156 806·0–216 056·7)	115 525·4 (87 192·4–150 867·0)	112 199·8 (84 638·7–146 433·2)	115 763·7 (87 404·1–151 155·0)	117 582·9 (88 588·1–154 533·6)	114 146·0 (86 018·1–149 794·2)	2905·7 (2513·7–3352·3)	1941·7 (1554·1–2441·9)	1876·8 (1500·1–2367·2)	1941·8 (1554·1–2442·1)	1977·1 (1571·4–2505·9)	1906·5 (1515·4–2421·0)
	**Nutritional deficiencies**	**48 738·6 (35 224·6–65 112·3)**	**47 375·5 (33 056·6–65 440·2)**	**47 754·7 (33 365·6–65 903·6)**	**50 086·7 (35 416·8–68 790·7)**	**46 736·2 (32 566·1–64 812·7)**	**49 696·3 (34 876·3–68 240·8)**	**650·7 (476·4–853·1)**	**528·2 (375·5–735·3)**	**530·6 (377·9–737·1)**	**529·1 (376·8–736·0)**	**516·0 (362·0–724·7)**	**517·3 (362·9–726·7)**
	Protein-energy malnutrition	11 558·2 (9704·0–13 756·4)	8177·1 (5558·9–12 274·8)	8307·4 (5606·0–12 610·5)	9405·6 (6340·0–14 369·4)	7490·1 (5222·5–10 739·1)	8718·5 (5961·6–12 908·4)	167·6 (140·5–198·5)	89·7 (57·2–143·7)	91·6 (58·0–148·2)	90·3 (57·6–144·8)	77·3 (51·7–116·0)	77·9 (51·8–117·5)
	Iodine deficiency	2256·1 (1175·8–3954·7)	2380·0 (1234·1–4215·6)	2388·8 (1239·0–4228·0)	2423·7 (1257·3–4285·3)	2381·3 (1234·8–4217·4)	2432·9 (1262·3–4297·8)	27·6 (14·4–48·4)	24·2 (12·7–42·4)	24·2 (12·7–42·4)	24·2 (12·7–42·4)	24·2 (12·7–42·4)	24·2 (12·7–42·4)
	Vitamin A deficiency	1083·2 (696·2–1547·2)	607·1 (392·8–899·6)	609·9 (394·5–904·1)	610·2 (394·6–902·7)	609·3 (394·2–903·2)	614·3 (397·2–909·6)	15·4 (9·9–21·9)	9·4 (6·0–13·4)	9·4 (6·0–13·4)	9·4 (6·0–13·4)	9·4 (6·0–13·4)	9·4 (6·0–13·5)
	Dietary iron deficiency	32 438·6 (21 496·5–46 880·2)	35 131·7 (23 207·7–50 957·6)	35 369·6 (23 354·2–51 328·6)	36 402·0 (24 024·1–52 749·4)	35 169·2 (23 227·7–51 019·5)	36 683·4 (24 190·8–53 199·6)	422·5 (281·3–607·6)	395·8 (262·6–560·6)	396·3 (263·0–561·4)	396·0 (262·8–560·8)	395·9 (262·7–560·8)	396·5 (263·2–561·5)
	Other nutritional deficiencies	1402·5 (1197·5–1670·9)	1079·6 (656·2–1676·5)	1079·0 (655·2–1680·3)	1245·3 (737·5–1949·2)	1086·4 (659·2–1689·5)	1247·2 (737·4–1956·7)	17·6 (15·2–20·9)	9·1 (5·4–14·8)	9·0 (5·3–14·7)	9·2 (5·4–15·1)	9·2 (5·4–15·0)	9·2 (5·4–15·2)
**Non-communicable diseases**		**1 713 594·1 (1 514 046·5–1 931 589·2)**	**2 442 221·9 (2 127 605·9–2 774 349·0)**	**2 435 156·2 (2 122 131·0–2 766 061·6)**	**1 988 801·4 (1 719 556·1–2 287 374·1)**	**2 443 219·5 (2 128 458·1–2 775 419·6)**	**1 990 529·7 (1 720 167·7–2 290 717·7)**	**20 350·5 (18 081·8–22 878·3)**	**18 354·3 (15 462·4–21 483·5)**	**18 161·3 (15 312·5–21 262·0)**	**14 253·9 (12 017·7–16 776·1)**	**18 356·7 (15 463·6–21 486·8)**	**14 178·0 (11 950·3–16 693·6)**
	**Neoplasms**	**255 459·1 (237 701·8–273 311·0)**	**388 098·6 (334 810·9–452 575·0)**	**389 095·1 (336 149·6–453 380·8)**	**340 635·1 (297 571·0–389 855·8)**	**388 148·4 (334 852·0–452 653·2)**	**341 220·1 (298 363·8–390 037·6)**	**2931·5 (2743·2–3119·4)**	**2680·2 (2401·0–3034·1)**	**2665·9 (2389·4–3017·4)**	**2169·8 (1964·4–2435·7)**	**2679·9 (2400·7–3033·9)**	**2159·2 (1955·5–2423·5)**
	Lip and oral cavity cancer	5968·3 (5365·1–6510·9)	10 336·8 (9200·3–11 417·6)	10 444·7 (9291·7–11 544·5)	9948·1 (8817·8–11 020·3)	10 337·9 (9201·4–11 418·7)	10 026·7 (8885·4–11 126·7)	67·7 (61·1–73·3)	74·6 (66·0–83·5)	74·9 (66·1–83·8)	66·8 (58·8–74·8)	74·6 (66·0–83·5)	66·9 (58·8–75·0)
	Nasopharynx cancer	2520·7 (2227·2–2826·1)	3719·5 (3053·8–4555·7)	3745·9 (3072·3–4590·2)	3538·5 (2856·1–4379·8)	3720·3 (3054·4–4556·6)	3557·0 (2867·5–4405·1)	28·8 (25·5–32·1)	29·2 (23·0–36·7)	29·2 (23·1–36·8)	26·5 (20·8–33·6)	29·2 (23·0–36·7)	26·5 (20·8–33·6)
	Other pharynx cancer	2894·1 (2627·1–3194·9)	5186·7 (4554·6–5961·7)	5231·8 (4591·0–6022·4)	4279·2 (3744·8–4898·5)	5187·2 (4555·1–5962·3)	4304·2 (3766·1–4926·7)	32·4 (29·6–35·4)	38·0 (33·5–43·1)	38·1 (33·6–43·2)	29·6 (25·9–33·6)	38·0 (33·5–43·1)	29·7 (26·0–33·6)
	Oesophageal cancer	13 156·7 (11 537·2–14 893·6)	20 064·8 (17 862·4–22 553·6)	20 281·5 (18 040·2–22 791·1)	17 387·2 (15 341·4–19 519·0)	20 061·6 (17 859·3–22 548·8)	17 515·8 (15 435·8–19 663·1)	147·6 (130·7–168·7)	132·0 (116·3–150·1)	132·3 (116·6–150·5)	103·1 (91·0–117·5)	132·0 (116·3–150·1)	103·1 (91·0–117·5)
	Stomach cancer	22 673·3 (20 057·2–25 765·2)	22 890·0 (20 118·0–25 659·1)	23 121·0 (20 309·8–25 904·7)	23 519·5 (20 414·5–26 310·2)	22 889·1 (20 118·2–25 658·9)	23 686·7 (20 540·7–26 474·1)	257·0 (227·5–290·5)	148·4 (130·1–165·5)	148·4 (130·1–165·5)	136·1 (119·6–151·7)	148·4 (130·1–165·5)	135·9 (119·4–151·5)
	Colon and rectum cancer	24 763·7 (22 662·4–26 510·9)	41 754·1 (29 927·6–55 439·1)	42 136·3 (30 225·9–55 920·6)	22 090·2 (16 070·5–29 183·3)	41 759·3 (29 929·6–55 448·1)	22 249·2 (16 195·8–29 382·4)	282·9 (260·1–303·0)	267·1 (202·6–345·6)	266·8 (202·2–345·4)	126·1 (94·5–164·6)	267·1 (202·6–345·5)	125·9 (94·3–164·4)
	Liver cancer	13 063·3 (11 697·8–14 751·3)	22 348·7 (18 124·3–28 392·8)	22 534·2 (18 265·5–28 657·4)	20 607·7 (16 526·8–26 460·5)	22 349·6 (18 125·4–28 393·5)	20 738·7 (16 622·4–26 651·2)	148·9 (135·1–168·9)	158·9 (125·3–210·3)	159·1 (125·5–210·5)	135·3 (106·7–179·1)	158·9 (125·3–210·2)	135·3 (106·7–179·1)
	Gallbladder and biliary tract cancer	3749·3 (3145·3–4255·9)	4529·9 (3472·0–5591·9)	4572·4 (3504·6–5642·4)	4409·2 (3415·0–5438·6)	4530·0 (3472·0–5592·5)	4440·2 (3443·4–5475·0)	42·7 (35·9–48·1)	28·5 (22·4–35·0)	28·5 (22·4–35·0)	24·7 (19·3–30·7)	28·5 (22·4–35·0)	24·6 (19·2–30·7)
	Pancreatic cancer	11 585·3 (10 621·8–12 559·4)	23 889·2 (17 685·0–31 413·2)	24 046·1 (17 811·3–31 609·2)	21 745·4 (16 289·7–28 448·5)	23 889·3 (17 684·8–31 413·3)	21 847·0 (16 368·9–28 571·4)	131·1 (120·7–142·2)	151·9 (118·1–196·0)	151·5 (117·7–195·7)	124·6 (95·8–161·4)	151·9 (118·1–196·0)	124·2 (95·5–161·0)
	Larynx cancer	3153·7 (2898·7–3420·4)	4056·2 (3634·9–4534·8)	4098·5 (3672·9–4584·1)	3057·8 (2769·3–3416·0)	4056·7 (3635·3–4535·4)	3081·6 (2790·6–3440·2)	35·3 (32·5–38·2)	27·725·2–30·7)	27·8 (25·2–30·8)	19·2 (17·2–21·3)	27·7 (25·1–30·7)	19·2 (17·2–21·4)
	Tracheal, bronchus, and lung cancer	46 421·4 (41 597·9–50 856·2)	64 261·0 (48 949·9–81 541·7)	62 523·4 (47 684·7–79 275·5)	45 442·7 (34 831·3–57 467·2)	64 257·4 (48 947·3–81 539·8)	44 059·2 (33 812·9–55 673·2)	522·0 (469·2–571·4)	402·2 (320·8–496·1)	387·4 (309·2–478·1)	255·6 (203·3–317·8)	402·1 (320·7–495·9)	245·6 (195·4–305·5)
	Malignant skin melanoma	1696·5 (1485·5–1855·3)	2524·7 (2126·0–2822·2)	2538·7 (2135·2–2839·5)	2826·0 (2380·4–3164·5)	2525·1 (2126·3–2822·6)	2839·5 (2389·4–3181·3)	19·6 (17·2–21·3)	17·9 (14·7–20·7)	17·9 (14·7–20·7)	18·2 (14·9–20·9)	17·9 (14·7–20·7)	18·1 (14·9–20·9)
	Non-melanoma skin cancer	1237·1 (1089·1–1374·2)	2644·7 (2117·0–3187·3)	2681·0 (2143·7–3228·6)	3313·9 (2644·0–3969·4)	2645·4 (2117·6–3188·3)	3352·2 (2678·0–4018·3)	14·4 (12·7–16·1)	15·7 (13·3–18·3)	15·6 (13·2–18·3)	16·3 (13·7–19·1)	15·7 (13·3–18·3)	16·2 (13·7–19·0)
	Soft tissue and other extraosseous sarcomas	1695·1 (1416·9–2116·9)	2576·8 (2117·1–3310·7)	2598·1 (2132·7–3340·1)	2797·9 (2292·2–3579·0)	2578·9 (2118·7–3314·2)	2819·1 (2308·0–3608·8)	20·5 (17·3–25·6)	21·9 (18·0–28·2)	22·0 (18·1–28·3)	22·2 (18·2–28·7)	22·0 (18·1–28·3)	22·3 (18·3–28·8)
	Malignant neoplasm of bone and articular cartilage	2575·1 (2097·4–2894·1)	4313·6 (2784·3–6138·2)	4345·3 (2805·6–6182·3)	4700·8 (3057·0–6662·8)	4316·0 (2785·9–6142·1)	4728·9 (3077·1–6702·0)	31·3 (25·9–35·3)	39·0 (25·5–56·2)	39·1 (25·6–56·4)	39·9 (26·1–57·6)	39·0 (25·5–56·2)	40·0 (26·2–57·7)
	Breast cancer	20 909·7 (19 403·4–22 462·0)	32 379·3 (24 527·0–40 894·0)	32 577·0 (24 693·7–41 136·1)	27 496·4 (21 025·6–34 699·2)	32 381·0 (24 528·0–40 896·4)	27 632·0 (21 152·8–34 859·4)	238·6 (223·2–254·2)	239·5 (186·9–300·6)	239·5 (186·8–300·7)	193·3 (149·9–243·2)	239·5 (186·9–300·6)	193·3 (149·8–243·2)
	Cervical cancer	9964·2 (9126·7–10 842·0)	12 420·7 (10 328·0–14 779·7)	12 503·9 (10 384·4–14 904·7)	12 553·5 (10 362·5–15 040·1)	12 421·7 (10 329·0–14 780·8)	12 619·6 (10 410·1–15 138·3)	114·0 (105·2–123·6)	99·4 (80·6–121·8)	99·6 (80·7–122·1)	96·0 (77·7–117·7)	99·4 (80·6–121·7)	96·2 (77·9–118·0)
	Uterine cancer	2600·5 (2321·9–2833·6)	3988·4 (3502·5–4490·4)	4009·5 (3516·4–4516·7)	2745·6 (2364·3–3145·6)	3988·5 (3502·5–4490·6)	2756·9 (2371·7–3159·6)	29·3 (26·5–32·1)	26·8 (23·1–30·9)	26·8 (23·1–30·8)	16·9 (14·6–19·6)	26·8 (23·1–30·9)	16·9 (14·5–19·5)
	Ovarian cancer	5279·4 (4744·8–5759·6)	9889·1 (7548·2–12 592·0)	9937·5 (7586·8–12 647·1)	9552·2 (7332·2–12 123·5)	9889·3 (7548·4–12 592·4)	9583·3 (7361·2–12 156·5)	59·9 (54·2–64·8)	72·4 (56·8–91·2)	72·3 (56·8–91·1)	65·8 (51·4–83·0)	72·4 (56·8–91·2)	65·8 (51·3–82·9)
	Prostate cancer	8282·3 (7242·0–8986·9)	18 364·3 (14 397·6–23 379·6)	18 670·4 (14 621·6–23 839·7)	22 039·2 (17 501·8–27 944·8)	18 371·5 (14 402·6–23 389·3)	22 362·9 (17 723·4–28 429·2)	96·0 (84·2–103·7)	103·2 (82·8–125·4)	103·4 (82·7–125·9)	105·3 (84·3–128·5)	103·2 (82·8–125·4)	105·5 (84·2–128·8)
	Testicular cancer	567·4 (530·8–606·0)	727·9 (556·5–924·1)	731·0 (559·2–927·9)	769·7 (590·7–973·5)	728·1 (556·7–924·4)	772·8 (593·4–977·5)	7·0 (6·5–7·4)	7·4 (5·7–9·4)	7·4 (5·7–9·4)	7·5 (5·8–9·5)	7·4 (5·7–9·4)	7·6 (5·8–9·5)
	Kidney cancer	4076·2 (3824·3–4320·6)	6846·3 (4991·7–9090·6)	6890·6 (5026·3–9144·4)	5583·6 (4106·3–7388·2)	6848·6 (4992·8–9093·9)	5613·7 (4128·5–7424·8)	47·3 (44·4–50·2)	47·7 (36·0–63·0)	47·7 (35·9–63·0)	35·9 (26·7–48·1)	47·7 (36·0–63·1)	35·8 (26·7–48·1)
	Bladder cancer	4445·3 (4101·6–4865·3)	7477·2 (6349·9–8865·9)	7571·0 (6429·6–8973·8)	6817·6 (5910·6–7978·1)	7478·8 (6350·9–8868·7)	6885·3 (5974·6–8063·1)	51·3 (47·2–55·9)	43·9 (39·3–50·2)	43·9 (39·3–50·2)	33·8 (30·4–38·5)	43·9 (39·3–50·2)	33·7 (30·2–38·4)
	Brain and CNS cancer	9043·1 (7854·5–10 429·6)	15 111·1 (13 073·6–17 390·8)	15 177·3 (13 126·7–17 470·8)	16 436·0 (14 171·6–18 869·2)	15 116·4 (13 077·4–17 396·1)	16 486·4 (14 208·4–18 930·5)	108·0 (93·3–123·5)	126·4 (107·0–144·4)	126·4 (106·9–144·3)	129·2 (109·1–147·8)	126·4 (107·0–144·4)	129·1 (109·0–147·7)
	Eye cancer	467·8 (345·4–572·1)	608·1 (446·0–744·5)	615·5 (451·9–754·4)	645·5 (475·4–788·3)	609·8 (447·0–746·1)	654·1 (481·7–799·1)	6·3 (4·5–7·8)	6·6 (4·7–8·2)	6·6 (4·7–8·3)	6·6 (4·7–8·2)	6·6 (4·7–8·2)	6·7 (4·7–8·3)
	Neuroblastoma and other peripheral nervous cell tumours	280·1 (224·0–338·0)	234·5 (147·3–343·1)	235·7 (148·0–344·9)	247·4 (157·9–358·0)	235·4 (147·7–344·5)	249·1 (158·9–360·6)	3·8 (3·1–4·7)	2·9 (1·8–4·3)	2·9 (1·8–4·4)	3·0 (1·8–4·4)	2·9 (1·8–4·4)	3·0 (1·8–4·4)
	Thyroid cancer	1268·8 (1140·3–1400·8)	2108·1 (1790·1–2441·0)	2126·2 (1806·0–2460·8)	2030·6 (1728·0–2340·5)	2108·4 (1790·3–2441·4)	2043·9 (1739·4–2354·2)	14·6 (13·2–16·1)	14·9 (12·9–17·0)	14·9 (13·0–17·0)	13·1 (11·4–15·0)	14·9 (12·9–17·0)	13·1 (11·4–15·0)
	Mesothelioma	698·8 (642·1–754·6)	1066·0 (920·9–1253·2)	1073·1 (927·2–1262·6)	1193·3 (1034·4–1395·8)	1066·1 (921·0–1253·4)	1199·4 (1039·9–1404·4)	8·0 (7·3–8·6)	7·1 (6·1–8·2)	7·0 (6·1–8·1)	7·2 (6·2–8·3)	7·1 (6·1–8·2)	7·2 (6·2–8·3)
	Hodgkin lymphoma	1201·1 (855·3–1600·5)	1289·7 (822·4–1713·1)	1297·6 (827·1–1724·7)	1355·2 (875·3–1798·2)	1290·9 (822·8–1715·5)	1363·5 (880·2–1812·2)	14·8 (10·5–19·5)	12·7 (7·6–17·5)	12·7 (7·7–17·5)	12·8 (7·7–17·6)	12·7 (7·6–17·5)	12·8 (7·8–17·7)
	Non-Hodgkin lymphoma	7859·6 (7267·6–8510·0)	12 019·8 (9531·7–14 915·4)	12 114·4 (9609·9–15 023·2)	12 571·2 (10 079·5–15 500·7)	12 024·9 (9535·2–14 922·3)	12 653·7 (10 152·7–15 595·0)	93·0 (85·8–101·2)	92·8 (75·5–114·3)	92·9 (75·6–114·4)	89·8 (73·0–110·7)	92·8 (75·5–114·3)	89·9 (73·0–110·8)
	Multiple myeloma	2640·3 (2316·0–2898·6)	4615·0 (3886·0–5350·1)	4647·1 (3914·4–5385·8)	4769·9 (4016·4–5511·7)	4615·3 (3886·2–5350·5)	4795·2 (4039·1–5543·6)	30·0 (26·3–33·1)	29·6 (25·2–34·1)	29·5 (25·1–34·0)	27·5 (23·3–32·0)	29·5 (25·2–34·1)	27·5 (23·3–32·0)
	Leukaemia	10 987·6 (9138·2–12 247·9)	13 163·3 (10 652·4–16 116·1)	13 240·7 (10 715·3–16 217·0)	12 111·8 (9849·7–14 756·1)	13 172·2 (10 657·5–16 127·9)	12 174·6 (9895·4–14 836·5)	135·5 (111·8–152·1)	114·6 (90·7–139·7)	114·5 (90·7–139·8)	101·1 (79·6–123·6)	114·6 (90·8–139·8)	101·1 (79·6–123·7)
	Other malignant neoplasms	6497·2 (5705·0–7185·8)	8598·6 (7506·3–9551·6)	8671·2 (7570·1–9632·8)	9588·6 (8319·8–10 663·3)	8603·0 (7510·0–9557·4)	9653·5 (8366·9–10 742·2)	77·4 (68·4–85·5)	66·6 (56·9–74·7)	66·7 (56·9–74·8)	68·2 (58·2–76·6)	66·7 (56·9–74·8)	68·3 (58·2–76·7)
	Other neoplasms	1236·2 (1062·9–1478·8)	2094·4 (1681·1–2650·7)	2106·2 (1690·7–2673·0)	2463·7 (1992·0–3125·1)	2094·7 (1681·3–2651·1)	2474·2 (2000·8–3138·0)	14·6 (12·6–17·5)	12·5 (10·4–15·7)	12·4 (10·3–15·6)	12·5 (10·2–15·9)	12·5 (10·4–15·7)	12·5 (10·2–15·8)
	**Cardiovascular diseases**	**424 524·6 (393 653·3–452 115·6)**	**508 147·5 (404 804·2–640 417·7)**	**498 375·0 (397 191·9–628 028·4)**	**234 866·0 (195 944·4–279 424·8)**	**508 319·2 (404 960·6–640 607·8)**	**234 314·3 (195 033·5–279 171·3)**	**4940·0 (4617·5–5232·7)**	**3215·2 (2412·3–4304·8)**	**3103·8 (2331·4–4149·2)**	**1246·1 (1019·8–1539·0)**	**3214·8 (2412·1–4304·4)**	**1226·2 (1005·2–1511·3)**
	Rheumatic heart disease	13 263·2 (11 413·3–15 853·5)	11 582·7 (8478·7–15 333·1)	11 734·9 (8567·5–15 549·9)	13 284·5 (9607·3–17 702·2)	11 588·2 (8482·5–15 339·6)	13 438·0 (9687·3–17 942·4)	158·4 (137·7–186·3)	97·6 (69·0–129·4)	97·9 (69·2–130·0)	101·3 (71·5–135·5)	97·6 (69·0–129·4)	101·5 (71·6–136·0)
	Ischaemic heart disease	185 630·7 (171 849·2–197 574·8)	186 515·6 (128 617·3–260 355·8)	181 054·8 (125 020·5–252 379·9)	33 581·8 (24 791·1–45 196·7)	186 569·9 (128 667·6–260 413·2)	33 033·3 (24 364·5–44 427·7)	2148·7 (2000·2–2278·3)	1175·7 (764·1–1789·5)	1122·6 (730·7–1708·0)	164·0 (115·9–232·3)	1175·5 (763·9–1789·3)	158·9 (112·6–224·3)
	Stroke	158 649·4 (145 916·5–170 944·9)	178 568·4 (149 132·3–211 490·0)	171 841·1 (143 626·0–203 651·1)	81 127·4 (66 657·9–96 661·8)	178 622·5 (149 179·1–211 549·1)	79 288·8 (65 064·1–94 723·3)	1836·3 (1700·2–1970·7)	1116·1 (889·4–1408·2)	1055·7 (841·8–1330·8)	417·3 (337·0–511·3)	1116·0 (889·3–1408·1)	401·7 (324·5–491·1)
	Hypertensive heart disease	25 952·9 (22 111·0–28 793·5)	60 527·5 (44 332·8–81 042·0)	61 655·7 (45 133·0–82 720·0)	33 848·7 (24 223·5–46 861·0)	60 548·9 (44 352·9–81 065·6)	34 494·3 (24 623·4–47 911·5)	303·2 (258·2–337·0)	367·8 (255·3–524·5)	369·3 (256·2–527·4)	159·4 (109·6–231·0)	367·7 (255·2–524·5)	159·8 (109·8–231·5)
	Non-rheumatic valvular heart disease	3297·4 (2937·3–3655·5)	6818·1 (5918·0–7681·1)	6894·5 (5977·6–7774·8)	8850·6 (7469·2–9913·7)	6820·0 (5919·4–7683·3)	8933·8 (7534·9–10 016·3)	40·3 (35·7–44·6)	37·8 (32·9–43·4)	37·5 (32·6–42·8)	38·5 (33·8–43·3)	37·8 (32·9–43·3)	38·2 (33·6–42·9)
	Cardiomyopathy and myocarditis	11 650·7 (10 638·2–12 583·0)	14 503·4 (12 866·1–16 141·2)	14 692·6 (13 016·4–16 349·0)	17 087·8 (15 263·5–18 826·5)	14 513·8 (12 874·9–16 154·0)	17 300·4 (15 428·9–19 078·1)	140·6 (128·5–151·6)	115·1 (102·5–126·7)	115·5 (102·8–127·3)	120·0 (107·2–131·9)	115·1 (102·6–126·7)	120·4 (107·5–132·4)
	Pulmonary arterial hypertension	651·4 (557·7–743·3)	1259·2 (844·7–1788·2)	1275·7 (855·0–1811·4)	1545·9 (1051·8–2203·6)	1260·3 (845·3–1790·1)	1562·9 (1063·8–2226·9)	8·3 (7·1–9·6)	9·9 (7·1–13·5)	9·9 (7·1–13·6)	10·5 (7·5–14·3)	9·9 (7·1–13·5)	10·5 (7·5–14·3)
	Atrial fibrillation and flutter	8549·2 (7039·2–10 311·5)	21 734·3 (17 682·0–26 481·6)	22 229·4 (18 096·1–27 062·5)	18 126·1 (14 600·6–21 769·9)	21 746·1 (17 691·8–26 496·2)	18 516·5 (14 946·5–22 243·7)	102·7 (84·9–122·3)	113·4 (93·4–134·6)	113·3 (93·3–134·5)	69·6 (57·7–82·8)	113·3 (93·4–134·6)	69·4 (57·5–82·5)
	Aortic aneurysm	3128·6 (2855·7–3372·4)	4516·4 (3655·1–5554·7)	4573·3 (3695·2–5623·9)	2949·3 (2392·4–3598·8)	4517·5 (3655·8–5556·4)	2984·5 (2425·7–3643·4)	36·3 (33·2–39·1)	28·4 (23·9–34·3)	28·4 (23·9–34·4)	14·9 (12·4–18·0)	28·4 (23·9–34·3)	14·9 (12·4–18·0)
	Lower extremity peripheral arterial disease	1586·3 (1260·9–2097·0)	3387·6 (2446·3–4532·5)	3440·3 (2480·6–4604·3)	2146·5 (1557·3–2878·4)	3388·6 (2446·8–4533·8)	2177·9 (1576·9–2914·3)	18·7 (15·0–24·7)	18·3 (14·2–24·0)	18·2 (14·1–23·9)	8·9 (6·8–11·8)	18·3 (14·2–24·0)	8·9 (6·8–11·8)
	Endocarditis	2120·4 (1835·1–2408·2)	4176·6 (3127·8–5327·1)	4222·4 (3155·6–5389·8)	4941·3 (3663·7–6326·0)	4178·6 (3129·1–5329·9)	4989·4 (3690·9–6400·6)	25·9 (22·3–29·3)	31·9 (22·3–44·3)	31·9 (22·3–44·4)	33·2 (23·4–45·9)	31·9 (22·3–44·3)	33·3 (23·4–46·0)
	Other cardiovascular and circulatory diseases	10 044·3 (8319·0–12 214·5)	14 557·6 (11 493·4–18 276·5)	14 760·4 (11 650·4–18 546·5)	17 376·1 (13 660·0–22 002·0)	14 564·8 (11 499·3–18 284·8)	17 594·5 (13 832·1–22 296·7)	120·6 (101·0–144·2)	103·3 (78·6–131·5)	103·6 (78·7–131·9)	108·5 (82·6–138·3)	103·3 (78·6–131·5)	108·7 (82·8–138·7)
	**Chronic respiratory diseases**	**107 582·5 (97 621·2–116 906·0)**	**163 649·9 (132 141·2–198 917·7)**	**156 454·8 (126 134·2–190 440·6)**	**157 255·4 (123 947·9–197 283·1)**	**163 730·1 (132 205·2–199 004·0)**	**149 934·5 (117 750·7–188 520·2)**	**1262·5 (1149·8–1368·3)**	**1004·1 (768·8–1308·5)**	**946·2 (725·0–1232·9)**	**826·2 (628·7–1083·5)**	**1004·1 (768·8–1308·5)**	**776·9 (592·2–1018·9)**
	Chronic obstructive pulmonary disease	79 656·0 (72 657·7–87 024·2)	136 833·4 (108 898·1–169 541·0)	129 322·5 (102 699·1–161 110·5)	130 977·8 (101 273·9–169 221·2)	136 894·6 (108 954·1–169 614·1)	123 358·0 (95 205·0–160 041·2)	924·4 (850·0–995·0)	788·6 (588·7–1049·1)	730·3 (545·0–974·1)	632·9 (465·9–853·4)	788·5 (588·7–1048·9)	583·2 (429·3–788·3)
	Pneumoconiosis	441·8 (387·5–508·4)	443·4 (376·7–526·0)	449·0 (381·5–534·1)	515·1 (435·7–609·6)	443·5 (376·8–526·1)	520·6 (439·9–617·3)	5·1 (4·5–5·9)	2·9 (2·4–3·4)	2·9 (2·4–3·4)	2·9 (2·4–3·5)	2·9 (2·4–3·4)	2·9 (2·4–3·5)
	Asthma	21 049·1 (16 560·3–26 262·1)	15 325·4 (11 308·9–19 975·3)	15 515·8 (11 436·0–20 214·6)	13 337·8 (9731·0–17 539·2)	15 340·7 (11 319·4–19 998·0)	13 512·4 (9851·4–17 828·1)	257·1 (202·2–320·8)	134·4 (94·3–184·7)	134·8 (94·6–185·2)	111·5 (77·1–154·6)	134·5 (94·4–184·8)	111·8 (77·3–155·1)
	Interstitial lung disease and pulmonary sarcoidosis	4137·5 (3457·6–4743·9)	7970·6 (6414·6–9430·9)	8069·8 (6493·2–9561·3)	9121·5 (7370·4–10 753·8)	7972·3 (6415·7–9432·7)	9218·2 (7441·0–10 879·3)	48·0 (40·7–54·8)	49·8 (39·8–58·7)	49·8 (39·7–58·8)	50·1 (39·9–59·4)	49·8 (39·8–58·7)	50·0 (39·8–59·4)
	Other chronic respiratory diseases	2298·1 (1918·0–2817·6)	3077·1 (2422·9–3943·3)	3097·6 (2436·3–3971·1)	3303·3 (2601·6–4206·6)	3079·1 (2423·9–3946·0)	3325·2 (2615·1–4241·8)	27·9 (23·4–33·6)	28·4 (22·7–35·5)	28·4 (22·7–35·6)	28·9 (23·1–36·0)	28·4 (22·7–35·5)	28·9 (23·1–36·0)
	**Digestive diseases**	**90 545·2 (82 312·0–99 541·2)**	**120 364·2 (101 896·8–140 782·1)**	**121 518·0 (102 760·0–142 229·8)**	**128 680·5 (108 070·8–151 493·3)**	**120 408·0 (101 937·2–140 841·5)**	**129 795·1 (108 803·3–152 888·2)**	**1064·4 (976·5–1165·3)**	**933·5 (772·1–1136·5)**	**935·3 (773·5–1139·0)**	**921·0 (761·5–1117·1)**	**933·6 (772·2–1136·8)**	**922·3 (762·3–1119·0)**
	Cirrhosis and other chronic liver diseases	46 683·2 (42 714·9–51 626·5)	59 227·9 (49 689·5–70 403·6)	59 722·0 (50 060·4–71 107·1)	65 124·9 (54 164·3–78 091·6)	59 243·2 (49 700·0–70 421·6)	65 579·2 (54 473·7–78 669·7)	540·5 (499·2–590·8)	472·2 (388·1–575·2)	473·6 (389·0–576·9)	488·6 (400·4–596·7)	472·3 (388·2–575·3)	489·4 (400·9–597·8)
	Upper digestive system diseases	15 231·2 (11 534·2–20 925·5)	18 047·1 (13 127·8–26 190·8)	18 219·8 (13 247·3–26 438·2)	19 242·5 (14 067·3–27 802·8)	18 052·9 (13 132·3–26 198·3)	19 412·3 (14 187·8–27 979·6)	178·8 (136·4–245·8)	145·6 (102·4–215·7)	145·8 (102·7–216·0)	143·9 (100·7–214·0)	145·6 (102·4–215·7)	144·1 (100·9–214·2)
	Appendicitis	1314·9 (1141·7–1530·4)	1016·8 (663·2–1490·9)	1026·7 (669·7–1509·4)	1106·0 (720·7–1635·6)	1017·4 (663·5–1492·2)	1116·5 (726·1–1654·8)	16·3 (14·2–18·9)	9·4 (5·8–14·4)	9·4 (5·8–14·5)	9·5 (5·9–14·7)	9·4 (5·8–14·4)	9·6 (5·9–14·7)
	Paralytic ileus and intestinal obstruction	6740·8 (5831·7–7487·5)	10 962·4 (8713·0–13 431·5)	11 122·1 (8819·2–13 630·6)	12 494·8 (9827·9–15 438·1)	10 971·8 (8720·6–13 442·6)	12 674·3 (9944·1–15 684·5)	84·5 (72·5–93·3)	86·5 (65·4–112·1)	86·9 (65·6–112·7)	87·7 (66·3–113·9)	86·6 (65·5–112·2)	88·1 (66·5–114·5)
	Inguinal, femoral, and abdominal hernia	2343·2 (1927·7–2855·1)	2748·9 (2148·5–3381·8)	2787·3 (2183·4–3431·0)	3135·7 (2460·7–3890·6)	2751·2 (2149·8–3385·0)	3178·3 (2491·8–3955·7)	29·5 (24·1–35·8)	22·9 (16·9–29·8)	22·9 (17·0–30·0)	23·4 (17·3–30·6)	22·9 (16·9–29·9)	23·5 (17·4–30·7)
	Inflammatory bowel disease	1522·5 (1305·3–1756·6)	2066·4 (1745·4–2399·0)	2083·2 (1758·6–2419·9)	2350·3 (1974·9–2741·7)	2067·0 (1746·0–2399·7)	2367·4 (1988·6–2763·2)	18·0 (15·5–20·7)	15·2 (12·5–18·2)	15·2 (12·5–18·1)	15·4 (12·6–18·3)	15·2 (12·5–18·2)	15·4 (12·6–18·3)
	Vascular intestinal disorders	1726·9 (1582·2–1868·1)	2849·6 (2434·0–3345·5)	2876·7 (2456·9–3384·9)	3425·5 (2937·2–4001·2)	2850·4 (2434·6–3346·3)	3453·1 (2959·4–4031·6)	20·4 (18·6–22·1)	17·3 (15·4–19·4)	17·2 (15·3–19·4)	17·5 (15·6–19·7)	17·3 (15·4–19·4)	17·4 (15·5–19·7)
	Gallbladder and biliary diseases	7814·4 (5817·5–10 511·5)	11 309·1 (8190·6–15 838·2)	11 411·8 (8263·1–15 986·8)	7680·9 (5494·9–10 840·0)	11 312·0 (8192·8–15 842·2)	7750·3 (5538·8–10 946·4)	91·5 (68·1–121·7)	77·2 (52·2–114·7)	77·1 (52·1–114·4)	46·1 (30·9–69·0)	77·2 (52·2–114·6)	46·0 (30·8–68·9)
	Pancreatitis	4115·1 (3613·1–4767·8)	5578·4 (4465·8–6883·8)	5619·5 (4494·6–6937·9)	6169·3 (4918·1–7651·2)	5579·6 (4466·7–6885·3)	6207·1 (4944·8–7705·2)	48·0 (42·4–55·5)	43·9 (34·2–55·7)	44·0 (34·3–55·8)	45·0 (35·1–57·2)	43·9 (34·2–55·7)	45·0 (35·1–57·3)
	Other digestive diseases	3052·9 (2711·3–3504·4)	6557·7 (5260·1–8290·0)	6648·9 (5322·6–8423·2)	7950·5 (6309·4–10 189·1)	6562·4 (5263·8–8297·6)	8056·5 (6381·3–10 354·4)	37·0 (32·9–42·4)	43·2 (32·5–58·6)	43·2 (32·4–58·5)	43·9 (33·1–59·3)	43·2 (32·5–58·6)	43·9 (33·1–59·3)
	**Neurological disorders**	**113 500·8 (69 182·8–178 907·6)**	**191 065·5 (124 466·0–306 168·9)**	**193 607·8 (126 243·6–310 716·3)**	**203 991·4 (134 735·2–319 728·8)**	**191 173·5 (124 544·9–306 305·4)**	**206 469·0 (136 465·3–323 427·1)**	**1392·5 (849·3–2187·2)**	**1380·1 (847·1–2166·6)**	**1378·4 (845·6–2165·1)**	**1296·2 (787·8–2063·6)**	**1380·0 (847·1–2166·6)**	**1294·5 (786·4–2060·7)**
	Alzheimer's disease and other dementias	37 068·3 (17 662·8–80 194·0)	90 156·7 (42 933·5–194 027·8)	92 129·3 (43 856·0–198 077·6)	95 674·3 (46 258·0–201 675·9)	90 209·6 (42 960·7–194 112·2)	97 559·6 (47 230·8–205 259·1)	458·4 (215·2–993·6)	445·3 (209·8–967·6)	443·4 (208·9–963·6)	354·1 (168·7–771·8)	445·1 (209·8–967·2)	352·2 (167·7–767·7)
	Parkinson's disease	7618·5 (6804·9–8379·5)	16 805·0 (14 916·7–19 032·9)	17 089·4 (15 157·9–19 369·3)	21 705·1 (19 159·4–24 451·2)	16 809·6 (14 920·0–19 038·4)	21 990·9 (19 412·4–24 749·9)	90·0 (80·9–98·5)	89·5 (79·6–99·7)	89·4 (79·6–99·6)	96·2 (85·7–106·9)	89·5 (79·6–99·6)	96·0 (85·5–106·6)
	Idiopathic epilepsy	13 951·3 (10 613·4–17 712·4)	18 604·7 (11 606·5–27 347·0)	18 749·0 (11 694·4–27 573·0)	19 683·0 (12 265·5–28 950·7)	18 627·4 (11 618·4–27 385·9)	19 844·5 (12 359·9–29 210·1)	177·4 (135·5–223·4)	189·2 (111·1–297·2)	189·7 (111·4–298·2)	190·5 (111·8–299·2)	189·4 (111·1–297·6)	191·1 (112·2–300·2)
	Multiple sclerosis	979·9 (832·8–1124·6)	1158·8 (957·6–1381·9)	1160·7 (959·1–1384·2)	1137·9 (936·9–1359·2)	1159·0 (957·8–1382·1)	1139·5 (938·3–1361·2)	11·3 (9·6–13·1)	9·6 (8·0–11·4)	9·6 (7·9–11·4)	9·0 (7·4–10·7)	9·6 (8·0–11·4)	9·0 (7·4–10·7)
	Motor neuron disease	1045·5 (953·8–1136·3)	1182·3 (1047·2–1344·3)	1183·9 (1048·5–1346·1)	1252·6 (1111·3–1422·8)	1182·4 (1047·2–1344·4)	1253·6 (1112·2–1424·0)	12·0 (11·0–13·1)	8·4 (7·5–9·4)	8·3 (7·5–9·4)	8·3 (7·4–9·3)	8·4 (7·5–9·4)	8·3 (7·4–9·3)
	Headache disorders	48 474·9 (10 002·5–101 907·9)	57 778·6 (12 868·7–119 696·5)	57 892·4 (12 897·7–119 906·9)	58 779·0 (13 165·0–121 695·0)	57 801·2 (12 871·7–119 740·1)	58 897·0 (13 196·0–121 913·7)	589·3 (117·4–1248·1)	587·2 (117·3–1245·9)	587·1 (117·3–1245·8)	586·9 (117·3–1245·4)	587·1 (117·3–1245·9)	586·8 (117·2–1245·2)
	Other neurological disorders	4362·2 (3584·4–5204·2)	5379·5 (3970·6–7140·4)	5403·1 (3989·1–7171·7)	5759·4 (4289·9–7595·7)	5384·3 (3974·3–7147·3)	5783·9 (4309·4–7628·9)	54·2 (44·7–64·5)	50·9 (36·4–67·8)	50·9 (36·3–67·8)	51·3 (36·6–68·3)	51·0 (36·4–67·8)	51·3 (36·6–68·3)
	**Mental disorders**	**143 542·8 (108 248·5–183 124·6)**	**181 202·8 (135 115·5–230 203·1)**	**181 771·8 (135 596·9–230 979·7)**	**186 227·4 (139 184·9–236 734·3)**	**181 277·1 (135 183·3–230 312·5)**	**186 812·8 (139 691·0–237 432·9)**	**1744·9 (1315·6–2220·6)**	**1786·1 (1338·9–2269·5)**	**1786·2 (1339·0–2269·7)**	**1785·8 (1338·6–2269·3)**	**1786·0 (1338·9–2269·4)**	**1785·9 (1338·6–2269·4)**
	Schizophrenia	14 983·5 (10 946·0–19 466·2)	18 704·9 (13 752·3–24 271·1)	18 737·4 (13 775·1–24 308·5)	19 013·1 (13 978·2–24 625·6)	18 707·9 (13 755·0–24 274·9)	19 041·9 (13 998·7–24 658·8)	178·1 (131·8–229·6)	179·0 (132·1–231·4)	179·0 (132·0–231·4)	178·9 (132·0–231·4)	179·0 (132·1–231·4)	178·9 (132·0–231·3)
	Depressive disorders	50 285·7 (35 108·5–68 483·9)	68 188·0 (47 508·6–91 416·8)	68 484·3 (47 732·4–91 812·7)	70 746·3 (49 369·5–94 920·0)	68 209·3 (47 523·8–91 447·6)	71 044·4 (49 590·7–95 325·8)	599·6 (415·8–816·3)	622·3 (431·0–847·8)	622·5 (431·1–848·1)	622·1 (430·8–847·6)	622·3 (431·0–847·8)	622·3 (430·9–847·9)
	Bipolar disorder	8102·3 (5206·9–11 637·8)	10 419·2 (6828·0–15 051·8)	10 441·6 (6845·4–15 080·7)	10 637·1 (6985·4–15 346·0)	10 422·3 (6830·1–15 056·2)	10 659·2 (7002·0–15 376·9)	97·6 (63·4–139·7)	102·2 (66·3–146·4)	102·1 (66·3–146·4)	102·1 (66·3–146·4)	102·1 (66·3–146·4)	102·1 (66·3–146·4)
	Anxiety disorders	36 235·2 (25 099·4–49 219·9)	44 752·0 (30 540·8–61 918·1)	44 865·7 (30 630·8–62 057·0)	45 843·7 (31 463·6–63 306·2)	44 771·3 (30 552·5–61 939·4)	45 962·5 (31 556·4–63 456·6)	442·0 (307·2–603·0)	448·8 (311·6–614·5)	448·7 (311·5–614·4)	448·3 (311·2–613·9)	448·8 (311·5–614·4)	448·2 (311·1–613·8)
	Eating disorders	3447·1 (2097·6–5362·6)	4779·8 (2808·5–7617·7)	4786·9 (2813·5–7629·5)	4804·6 (2826·2–7655·5)	4782·0 (2810·0–7621·4)	4812·5 (2831·6–7668·6)	43·8 (26·8–68·9)	57·1 (33·0–91·2)	57·2 (33·0–91·3)	57·4 (33·1–91·5)	57·1 (33·0–91·2)	57·4 (33·1–91·6)
	Autism spectrum disorders	11 625·5 (7979·7–16 246·2)	13 431·4 (9213·8–18 708·9)	13 460·1 (9232·5–18 752·3)	13 649·7 (9364·4–18 996·0)	13 441·7 (9220·1–18 724·0)	13 682·3 (9388·4–19 047·7)	147·6 (100·4–207·9)	152·6 (103·5–214·2)	152·6 (103·5–214·2)	152·6 (103·5–214·3)	152·6 (103·5–214·2)	152·6 (103·6–214·3)
	Attention-deficit hyperactivity disorder	1034·2 (565·2–1668·9)	929·1 (509·4–1553·1)	929·8 (509·8–1554·2)	931·0 (510·6–1555·4)	929·8 (509·8–1554·1)	932·2 (511·3–1557·1)	13·5 (7·3–21·7)	12·1 (6·6–19·4)	12·1 (6·5–19·4)	12·1 (6·6–19·4)	12·1 (6·5–19·4)	12·1 (6·5–19·4)
	Conduct disorder	4962·6 (2666·4–7798·7)	4668·3 (2550·7–7399·8)	4675·9 (2554·3–7412·0)	4669·5 (2551·3–7401·6)	4677·3 (2554·8–7414·0)	4683·5 (2557·9–7423·9)	66·7 (36·1–104·2)	68·6 (37·1–107·3)	68·6 (37·1–107·3)	68·6 (37·1–107·3)	68·6 (37·1–107·3)	68·6 (37·1–107·3)
	Idiopathic developmental intellectual disability	3788·0 (1783·1–6563·1)	3026·0 (1360·9–5304·7)	3032·5 (1364·1–5317·0)	3061·4 (1377·5–5367·8)	3028·6 (1362·1–5309·1)	3069·2 (1381·6–5382·7)	49·3 (23·3–83·6)	36·3 (16·4–62·2)	36·3 (16·4–62·2)	36·3 (16·4–62·2)	36·3 (16·4–62·2)	36·3 (16·4–62·2)
	Other mental disorders	9078·8 (5803·3–13 536·7)	12 304·2 (7948·6–18 350·1)	12 357·5 (7987·8–18 427·9)	12 871·1 (8341·7–19 116·3)	12 307·0 (7950·6–18 353·9)	12 925·0 (8382·2–19 195·4)	106·8 (68·4–160·9)	107·2 (68·6–161·0)	107·2 (68·7–161·0)	107·3 (68·7–161·1)	107·2 (68·6–161·0)	107·3 (68·7–161·1)
	**Substance use disorders**	**32 847·0 (26 942·4–39 248·1)**	**41 734·0 (34 493·1–49 791·0)**	**41 833·9 (34 567·9–49 917·8)**	**42 822·7 (35 331·7–51 082·6)**	**41 739·7 (34 497·4–49 798·6)**	**42 915·8 (35 400·5–51 203·1)**	**393·9 (324·5–469·6)**	**409·2 (337·8–485·6)**	**409·2 (337·9–485·6)**	**410·7 (339·4–487·5)**	**409·1 (337·8–485·5)**	**410·7 (339·4–487·5)**
	Alcohol use disorders	17 109·4 (13 520·6–21 686·8)	20 485·6 (15 681·1–26 112·9)	20 554·5 (15 728·4–26 211·0)	21 284·8 (16 249·3–27 086·9)	20 488·7 (15 683·0–26 116·7)	21 347·3 (16 292·3–27 165·5)	201·8 (160·4–253·7)	188·8 (144·6–240·9)	188·9 (144·6–241·1)	190·6 (145·9–243·2)	188·8 (144·6–240·9)	190·7 (145·9–243·3)
	Drug use disorders	15 737·6 (12 875·4–18 366·4)	21 248·4 (17 927·7–24 697·9)	21 279·4 (17 956·4–24 729·5)	21 537·8 (18 188·4–24 978·5)	21 251·0 (17 929·9–24 700·8)	21 568·5 (18 215·2–25 009·9)	192·1 (158·8–222·5)	220·3 (184·0–257·6)	220·3 (184·0–257·5)	220·1 (183·7–257·3)	220·3 (184·0–257·5)	220·0 (183·6–257·2)
	**Diabetes and kidney diseases**	**117 315·0 (103 732·2–134 310·1)**	**240 106·2 (192 546·5–287 801·5)**	**240 593·8 (192 722·4–288 574·3)**	**44 575·1 (34 559·2–55 151·3)**	**240 169·1 (192 594·4–287 876·5)**	**44 956·6 (34 823·1–55 655·7)**	**1352·0 (1202·0–1540·9)**	**1672·9 (1306·2–2086·5)**	**1661·0 (1296·1–2072·6)**	**319·4 (241·9–408·0)**	**1672·9 (1306·2–2086·6)**	**319·5 (241·9–408·4)**
	Diabetes mellitus	80 650·9 (67 415·6–96 905·8)	159 681·3 (125 414·6–202 412·9)	159 218·0 (124 814·7–202 310·1)	25 161·5 (17 911·3–34 935·6)	159 715·1 (125 441·5–202 453·3)	25 363·8 (18 060·7–35 233·3)	920·9 (776·1–1102·4)	1126·7 (871·5–1425·5)	1115·0 (861·9–1415·2)	180·0 (130·0–244·3)	1126·7 (871·5–1425·5)	180·2 (130·2–244·5)
	Chronic kidney disease	36 356·7 (33 379·6–38 945·8)	80 091·7 (61 552·2–102 856·2)	81 038·7 (62 184·8–104 161·0)	18 999·4 (13 758·4–25 600·6)	80 120·6 (61 574·8–102 893·8)	19 174·9 (13 866·1–25 859·3)	427·4 (394·7–456·5)	543·6 (392·6–750·1)	543·4 (392·3–750·1)	136·6 (93·2–201·4)	543·6 (392·6–750·2)	136·6 (93·2–201·5)
	Acute glomerulonephritis	307·4 (203·5–402·1)	333·2 (207·3–497·9)	337·0 (209·1–504·0)	414·2 (254·7–624·1)	333·4 (207·4–498·3)	417·9 (256·7–631·0)	3·7 (2·4–4·8)	2·6 (1·5–4·0)	2·6 (1·5–4·0)	2·7 (1·6–4·2)	2·6 (1·5–4·0)	2·7 (1·6–4·2)
	**Skin and subcutaneous diseases**	**42 167·9 (27 985·6–61 055·1)**	**50 818·7 (34 656·2–72 335·3)**	**51 076·5 (34 848·8–72 672·4)**	**53 537·0 (36 859·9–76 111·5)**	**50 860·7 (34 682·4–72 391·9)**	**53 832·2 (37 103·8–76 494·3)**	**533·8 (352·3–762·0)**	**529·9 (360·8–751·9)**	**529·9 (360·8–752·0)**	**530·9 (361·7–753·3)**	**530·0 (360·8–752·0)**	**530·9 (361·7–753·3)**
	Dermatitis	8204·1 (4902·2–12 986·6)	8864·7 (5428·8–13 697·6)	8889·9 (5444·2–13 738·2)	9106·0 (5583·4–14 082·5)	8871·2 (5432·2–13 708·0)	9134·5 (5600·3–14 127·6)	105·8 (62·3–169·0)	100·8 (59·6–160·5)	100·8 (59·6–160·4)	100·8 (59·5–160·4)	100·8 (59·6–160·5)	100·7 (59·5–160·3)
	Psoriasis	3715·7 (2711·0–4989·3)	4143·4 (3031·8–5541·9)	4153·8 (3040·3–5556·3)	4286·0 (3142·5–5721·5)	4144·6 (3032·8–5543·7)	4295·9 (3150·7–5734·5)	44·2 (32·0–59·0)	37·3 (27·0–49·9)	37·3 (27·0–49·8)	37·2 (26·9–49·7)	37·3 (27·0–49·9)	37·2 (26·9–49·7)
	Bacterial skin diseases	2258·5 (1909·8–2563·4)	5602·4 (4048·2–7573·0)	5674·6 (4092·9–7682·9)	6464·7 (4579·8–8936·0)	5608·3 (4052·5–7584·1)	6549·0 (4627·6–9085·5)	27·8 (23·4–31·6)	41·3 (28·0–58·1)	41·3 (28·0–58·2)	41·7 (28·4–58·9)	41·3 (28·0–58·1)	41·8 (28·4–59·1)
	Scabies	5330·9 (2920·1–8771·6)	5457·2 (2957·7–9078·4)	5476·2 (2967·7–9106·1)	5618·1 (3055·4–9319·2)	5461·1 (2959·6–9084·3)	5640·2 (3068·6–9351·8)	68·5 (37·8–113·5)	61·2 (33·9–101·0)	61·2 (33·9–101·0)	61·2 (33·9–101·0)	61·2 (33·9–101·0)	61·2 (33·9–101·0)
	Fungal skin diseases	3471·4 (1416·5–7191·5)	4901·2 (2003·3–10 040·1)	4933·7 (2018·1–10 105·6)	5215·6 (2152·2–10 652·2)	4906·1 (2005·2–10 050·8)	5254·0 (2169·9–10 729·6)	43·6 (18·0–89·4)	49·0 (20·1–100·6)	49·0 (20·1–100·7)	49·0 (20·1–100·6)	49·0 (20·1–100·6)	49·0 (20·1–100·7)
	Viral skin diseases	4214·0 (2683·3–6356·6)	4353·5 (2768·5–6497·4)	4362·7 (2774·3–6509·9)	4423·4 (2814·1–6593·2)	4357·7 (2771·0–6503·7)	4434·9 (2821·2–6609·5)	54·6 (34·8–82·0)	52·6 (33·6–78·7)	52·6 (33·6–78·7)	52·6 (33·5–78·6)	52·6 (33·6–78·7)	52·6 (33·5–78·6)
	Acne vulgaris	5195·9 (3257·7–8162·6)	5863·4 (3654·7–9391·1)	5872·1 (3659·2–9408·8)	5875·8 (3662·1–9412·5)	5871·8 (3659·5–9407·1)	5890·5 (3670·1–9441·4)	67·8 (42·7–106·6)	77·1 (48·4–121·7)	77·1 (48·4–121·7)	77·1 (48·4–121·6)	77·1 (48·4–121·7)	77·1 (48·4–121·7)
	Alopecia areata	576·6 (376·0–823·7)	673·5 (441·2–956·4)	674·8 (442·1–958·5)	685·1 (448·6–974·0)	673·8 (441·4–956·8)	686·4 (449·5–976·0)	7·0 (4·6–9·9)	6·8 (4·4–9·7)	6·8 (4·4–9·7)	6·8 (4·4–9·7)	6·8 (4·4–9·7)	6·8 (4·4–9·7)
	Pruritus	862·2 (419·1–1585·1)	1169·9 (563·4–2155·0)	1175·7 (566·3–2165·2)	1229·6 (595·5–2264·9)	1170·6 (563·7–2156·2)	1235·6 (598·7–2275·6)	10·5 (5·1–19·1)	10·7 (5·2–19·6)	10·7 (5·2–19·6)	10·7 (5·2–19·6)	10·7 (5·2–19·6)	10·7 (5·2–19·6)
	Urticaria	3998·5 (2622·8–5661·1)	4375·6 (2878·4–6243·0)	4386·2 (2885·6–6258·1)	4453·7 (2938·6–6356·9)	4379·6 (2880·8–6248·3)	4466·3 (2947·1–6374·8)	52·0 (34·2–74·5)	51·9 (34·1–74·1)	51·9 (34·1–74·1)	51·9 (34·1–74·1)	51·9 (34·1–74·1)	51·9 (34·1–74·1)
	Decubitus ulcer	813·5 (621·9–920·1)	1602·1 (1099·8–2129·7)	1635·1 (1123·6–2170·5)	1987·5 (1374·8–2606·9)	1603·0 (1100·4–2131·2)	2026·8 (1397·3–2651·6)	9·7 (7·5–11·0)	9·9 (7·0–12·5)	9·9 (7·0–12·5)	10·1 (7·2–12·8)	9·9 (7·0–12·5)	10·1 (7·2–12·8)
	Other skin and subcutaneous diseases	3526·7 (1790·1–6342·0)	3811·8 (1874·6–7048·9)	3841·8 (1892·8–7101·9)	4191·7 (2075·0–7717·0)	3813·1 (1875·3–7051·0)	4218·2 (2090·5–7761·7)	42·3 (21·6–75·5)	31·2 (15·4–56·5)	31·2 (15·4–56·5)	31·7 (15·6–57·3)	31·2 (15·4–56·5)	31·7 (15·6–57·3)
	**Sense organ diseases**	**78 774·4 (54 768·5–109 921·2)**	**132 542·8 (93 228·0–184 717·2)**	**134 035·3 (94 411·0–186 636·3)**	**147 607·2 (105 159·6–204 428·9)**	**132 597·3 (93 267·9–184 792·5)**	**149 210·9 (106 449·3–206 483·6)**	**916·4 (632·5–1275·1)**	**930·4 (644·5–1294·4)**	**931·3 (645·2–1295·5)**	**932·8 (646·7–1296·6)**	**930·5 (644·6–1294·4)**	**933·5 (647·2–1297·5)**
	Blindness and vision loss	29 688·4 (19 397·6–43 474·9)	50 978·7 (34 886·0–72 379·0)	51 625·5 (35 360·9–73 219·7)	56 960·9 (39 506·3–81 609·5)	50 998·2 (34 900·1–72 404·5)	57 666·4 (40 027·4–82 584·2)	343·8 (226·4–506·2)	356·0 (238·4–517·7)	356·7 (239·0–518·8)	357·9 (239·7–521·3)	356·0 (238·4–517·7)	358·5 (240·0–521·9)
	Age-related and other hearing loss	45 303·4 (31 657·1–63 442·2)	75 684·6 (53 374·8–104 941·7)	76 488·5 (53 926·3–106 040·3)	84 328·8 (59 660·3–116 475·7)	75 717·7 (53 397·2–104 980·6)	85 186·0 (60 241·3–117 653·0)	528·3 (366·8–735·9)	530·1 (369·3–736·5)	530·3 (369·5–736·7)	530·7 (369·9–737·3)	530·1 (369·4–736·5)	530·8 (370·0–737·4)
	Other sense organ diseases	3782·6 (2279·8–5731·3)	5879·4 (3552·2–8988·1)	5921·2 (3577·7–9046·5)	6317·6 (3810·4–9584·7)	5881·4 (3553·5–8990·8)	6358·5 (3833·4–9655·6)	44·3 (27·0–66·2)	44·3 (27·0–66·1)	44·3 (27·0–66·1)	44·2 (26·9–66·0)	44·3 (27·0–66·1)	44·2 (26·9–66·0)
	**Musculoskeletal disorders**	**165 090·1 (120 079·3–221 885·8)**	**266 231·0 (191 856·5–362 978·8)**	**267 517·8 (192 879·8–364 583·5)**	**279 907·3 (202 321·8–380 556·4)**	**266 284·6 (191 890·6–363 053·9)**	**281 152·9 (203 347·1–382 355·3)**	**1920·6 (1402·8–2560·8)**	**2176·3 (1598·7–2898·4)**	**2175·9 (1598·5–2898·0)**	**2172·6 (1596·1–2893·4)**	**2176·2 (1598·6–2898·3)**	**2172·2 (1595·9–2893·0)**
	Rheumatoid arthritis	3129·1 (2361·2–4049·7)	5219·7 (4008·8–6617·0)	5263·7 (4040·5–6663·6)	5557·0 (4294·6–6991·6)	5220·0 (4009·1–6617·3)	5592·5 (4317·4–7038·4)	36·0 (27·0–46·5)	36·3 (27·4–46·6)	36·3 (27·3–46·6)	35·0 (26·5–45·0)	36·3 (27·4–46·6)	35·0 (26·4–44·9)
	Osteoarthritis	21 772·4 (10 475·7–43 867·9)	36 585·2 (17 486·9–73 476·8)	36 875·2 (17 625·5–74 055·1)	39 959·7 (19 209·7–80 102·2)	36 594·4 (17 491·5–73 495·7)	40 254·6 (19 350·4–80 694·2)	245·5 (117·5–494·7)	243·2 (116·6–490·5)	243·0 (116·5–490·1)	242·1 (116·0–488·3)	243·2 (116·6–490·5)	242·0 (116·0–488·0)
	Low back pain	71 035·5 (50 395·4–95 305·2)	95 510·5 (67 892·9–129 066·1)	95 972·2 (68 218·3–129 696·9)	100 631·7 (71 568·7–135 783·0)	95 533·1 (67 908·5–129 096·9)	101 092·0 (71 931·8–136 366·4)	831·7 (598·0–1115·2)	788·2 (565·4–1058·0)	788·0 (565·2–1057·7)	786·9 (564·6–1056·2)	788·2 (565·4–1057·9)	786·7 (564·4–1056·0)
	Neck pain	20 704·0 (13 871·8–29 561·0)	28 136·7 (18 758·2–39 383·1)	28 237·8 (18 829·5–39 540·7)	29 263·5 (19 581·7–41 149·3)	28 143·6 (18 762·1–39 393·1)	29 362·2 (19 654·8–41 322·9)	242·8 (162·6–342·2)	244·3 (163·6–345·2)	244·2 (163·5–345·1)	244·1 (163·4–344·9)	244·3 (163·6–345·2)	244·1 (163·4–344·9)
	Gout	1784·6 (1210·5–2586·9)	2917·5 (1988·9–4231·2)	2939·4 (2005·9–4259·7)	3192·7 (2179·9–4592·2)	2918·4 (1989·5–4232·5)	3215·1 (2195·4–4619·8)	20·3 (13·8–29·0)	20·5 (13·9–29·1)	20·5 (13·9–29·1)	20·5 (14·0–29·2)	20·5 (13·9–29·1)	20·5 (14·0–29·2)
	Other musculoskeletal disorders	46 664·5 (32 524·3–64 105·1)	97 861·4 (66 752·1–136 313·8)	98 229·5 (67 015·9–136 780·8)	101 302·7 (69 333·4–140 621·2)	97 875·2 (66 762·5–136 333·3)	101 636·5 (69 578·0–141 038·8)	544·3 (382·4–739·5)	843·8 (582·7–1157·8)	844·0 (582·8–1157·9)	843·9 (583·0–1157·5)	843·8 (582·7–1157·7)	844·0 (583·1–1157·6)
	**Other non-communicable diseases**	**142 244·7 (115 702·7–174 894·8)**	**158 260·7 (125 457·6–202 567·1)**	**159 276·4 (126 295·5–203 894·5)**	**168 696·2 (133 699·3–216 095·7)**	**158 511·9 (125 696·8–202 871·7)**	**169 915·6 (134 739·0–217 576·4)**	**1898·0 (1582·8–2258·9)**	**1636·6 (1333·3–2052·5)**	**1638·2 (1334·6–2054·4)**	**1642·4 (1338·1–2059·9)**	**1639·5 (1336·0–2055·8)**	**1646·1 (1341·3–2064·3)**
	Congenital birth defects	51 631·5 (44 388·0–61 764·0)	34 867·8 (27 751·6–44 894·8)	34 995·5 (27 827·5–45 076·6)	35 023·1 (27 873·8–45 071·9)	35 044·5 (27 858·5–45 130·9)	35 282·5 (28 031·9–45 428·5)	789·8 (681·3–941·0)	566·1 (473·3–696·9)	567·3 (474·2–698·8)	566·4 (473·6–697·3)	568·6 (475·3–700·3)	569·4 (476·1–701·6)
	Urinary diseases and male infertility	11 567·2 (9972·1–13 257·0)	24 507·4 (21 091·8–28 297·5)	24 882·5 (21 405·6–28 754·4)	28 857·9 (24 540·7–33 508·1)	24 519·8 (21 101·0–28 310·8)	29 264·6 (24 893·9–34 000·5)	137·4 (119·1–156·6)	163·1 (134·8–194·0)	163·4 (135·0–194·4)	165·8 (136·9–197·4)	163·1 (134·8–194·0)	166·0 (137·1–197·8)
	Gynaecological diseases	27 775·9 (19 477·4–38 772·8)	28 977·0 (19 149·3–43 517·3)	29 036·2 (19 184·5–43 609·1)	29 483·8 (19 399·9–44 464·4)	28 983·0 (19 154·7–43 524·8)	29 543·1 (19 437·2–44 593·2)	335·7 (236·0–466·8)	291·7 (191·8–433·6)	291·7 (191·8–433·6)	291·8 (191·9–434·0)	291·7 (191·8–433·6)	291·9 (192·0–434·0)
	Haemoglobinopathies and haemolytic anaemias	11 967·3 (9309·9–15 327·0)	14 042·2 (10 630·9–18 265·7)	14 152·7 (10 716·4–18 401·6)	14 782·0 (11 152·7–19 064·4)	14 062·3 (10 645·3–18 295·8)	14 909·9 (11 249·9–19 215·8)	157·7 (123·2–198·8)	156·6 (120·4–201·3)	157·0 (120·6–201·8)	157·3 (120·9–202·1)	156·7 (120·5–201·5)	157·7 (121·2–202·6)
	Endocrine, metabolic, blood, and immune disorders	12 994·1 (9977·7–17 051·8)	18 616·6 (14 614·7–24 356·7)	18 727·2 (14 711·0–24 500·9)	20 486·6 (16 177·1–26 704·7)	18 625·7 (14 621·8–24 366·3)	20 598·9 (16 255·7–26 836·4)	157·5 (122·1–206·3)	155·8 (119·7–209·9)	155·8 (119·6–209·8)	157·8 (121·3–212·6)	155·9 (119·8–209·9)	157·8 (121·3–212·6)
	Oral disorders	23 631·2 (14 126·5–35 769·6)	36 058·3 (22 013·8–53 250·8)	36 293·0 (22 169·7–53 539·6)	38 870·9 (23 843·3–57 018·4)	36 070·6 (22 021·5–53 268·4)	39 115·5 (23 980·9–57 359·9)	276·9 (164·8–418·0)	281·6 (166·9–427·0)	281·4 (166·8–426·9)	281·6 (166·8–427·1)	281·6 (166·9–427·0)	281·4 (166·8–427·0)
	Sudden infant death syndrome	2677·5 (1572·7–3665·1)	1191·4 (677·3–1795·9)	1189·2 (675·0–1793·7)	1192·0 (677·7–1796·7)	1205·9 (686·2–1817·1)	1201·0 (681·9–1811·2)	43·0 (25·5–59·3)	21·7 (12·5–31·0)	21·6 (12·5–30·9)	21·7 (12·5–31·0)	22·0 (12·7–31·4)	21·8 (12·6–31·2)
**Injuries**		**246 893·3 (223 067·4–274 485·5)**	**258 301·6 (218 069·2–307 983·5)**	**259 100·7 (218 678·0–308 984·9)**	**274 745·0 (231 839·4–327 799·0)**	**258 498·3 (218 211·7–308 235·1)**	**275 713·6 (232 551·6–329 247·0)**	**3064·0 (2784·1–3364·8)**	**2432·8 (2023·3–3011·8)**	**2424·3 (2016·1–3001·0)**	**2437·7 (2026·7–3020·1)**	**2434·1 (2024·0–3013·9)**	**2429·7 (2019·5–3010·2)**
	**Transport injuries**	**69 623·7 (64 831·5–75 247·3)**	**68 342·6 (46 476·0–101 208·7)**	**68 122·1 (46 326·1–100 940·0)**	**70 577·3 (47 905·7–104 526·1)**	**68 395·7 (46 503·0–101 306·1)**	**70 363·7 (47 759·9–104 291·6)**	**857·9 (800·9–916·9)**	**690·9 (456·2–1082·2)**	**686·3 (453·2–1075·0)**	**692·9 (457·3–1085·8)**	**691·3 (456·4–1082·9)**	**688·5 (454·4–1079·0)**
	Road injuries	65 122·9 (60 600·9–70 289·7)	63 443·3 (41 742·7–96 520·0)	63 216·8 (41 586·2–96 186·5)	65 501·5 (43 051·3–99 797·5)	63 493·8 (41 764·9–96 626·2)	65 281·1 (42 884·0–99 492·7)	802·6 (749·3–858·0)	642·8 (408·4–1034·7)	638·3 (405·6–1027·6)	644·6 (409·5–1038·0)	643·2 (408·6–1035·5)	640·4 (406·7–1031·3)
	Other transport injuries	4500·8 (4114·6–4946·7)	4899·2 (4343·2–5591·7)	4905·3 (4351·6–5601·9)	5075·8 (4509·9–5797·8)	4902·0 (4345·9–5595·3)	5082·5 (4517·6–5806·6)	55·3 (50·9–60·2)	48·1 (42·9–54·6)	48·0 (42·8–54·5)	48·3 (43·0–54·7)	48·1 (42·9–54·6)	48·2 (42·9–54·6)
	**Unintentional injuries**	**107 749·3 (92 354·9–127 709·0)**	**119 386·0 (100 151·9–143 383·9)**	**120 566·9 (101 290·4–144 765·7)**	**131 711·6 (110 224·7–157 630·7)**	**119 489·7 (100 225·0–143 509·8)**	**133 046·4 (111 328·1–159 282·1)**	**1347·0 (1144·3–1594·9)**	**1011·6 (815·3–1261·2)**	**1011·3 (814·8–1261·5)**	**1012·4 (816·3–1262·8)**	**1012·4 (815·7–1262·8)**	**1012·3 (815·8–1263·8)**
	Falls	44 073·0 (36 207·2–53 430·8)	64 206·1 (51 831·2–77 966·1)	65 208·2 (52 747·3–79 093·0)	73 672·9 (59 865·2–89 284·5)	64 234·3 (51 851·4–78 001·5)	74 777·9 (61 067·1–90 529·4)	528·5 (438·3–632·2)	450·2 (373·6–541·7)	450·9 (374·4–542·7)	449·7 (372·8–540·8)	450·2 (373·6–541·8)	450·2 (373·4–541·5)
	Drowning	15 335·1 (13 682·0–17 154·1)	9023·5 (6629·0–12 494·4)	8993·7 (6608·6–12 460·1)	9314·7 (6859·7–12 910·8)	9043·8 (6638·3–12 536·1)	9294·7 (6842·5–12 891·3)	205·7 (184·3–229·9)	106·4 (75·1–154·7)	105·7 (74·5–153·6)	106·6 (75·2–154·9)	106·6 (75·2–155·1)	105·9 (74·7–154·1)
	Fire, heat, and hot substances	8396·7 (6448·7–10 026·0)	7190·6 (5567·3–8918·9)	7244·2 (5609·9–8984·8)	7543·5 (5848·7–9352·3)	7201·3 (5574·3–8933·7)	7605·9 (5897·6–9439·7)	106·8 (82·2–127·8)	72·1 (53·6–89·3)	72·3 (53·7–89·6)	72·3 (53·7–89·5)	72·2 (53·6–89·4)	72·5 (53·9–89·9)
	Poisonings	2804·5 (1996·9–3317·2)	1729·8 (1138·7–2163·7)	1740·0 (1145·2–2177·6)	1819·7 (1198·2–2273·5)	1732·1 (1139·8–2167·1)	1831·1 (1204·7–2292·6)	35·7 (25·4–42·7)	17·3 (11·1–22·7)	17·4 (11·1–22·8)	17·4 (11·2–22·8)	17·4 (11·1–22·8)	17·5 (11·2–22·9)
	Exposure to mechanical forces	10 319·7 (8148·6–12 978·3)	10 480·9 (7706·2–13 764·1)	10 468·9 (7695·6–13 753·7)	11 004·9 (8065·8–14 501·6)	10 486·9 (7709·9–13 775·5)	10 994·1 (8055·8–14 494·6)	125·4 (98·5–157·7)	97·7 (70·3–129·8)	97·2 (69·9–129·1)	98·0 (70·5–130·2)	97·7 (70·3–129·8)	97·5 (70·2–129·5)
	Adverse effects of medical treatment	4844·1 (4024·1–5534·2)	5075·9 (3353·4–7527·0)	5129·6 (3383·4–7617·9)	5466·5 (3607·6–8097·8)	5085·7 (3357·6–7548·9)	5529·2 (3643·7–8209·8)	63·6 (51·8–73·0)	49·5 (30·4–77·0)	49·6 (30·5–77·4)	49·7 (30·6–77·5)	49·6 (30·5–77·3)	49·9 (30·7–78·0)
	Animal contact	4902·5 (3923·7–6060·7)	6355·9 (3900·6–9947·2)	6390·3 (3918·6–10 010·8)	6640·0 (4072·3–10 415·0)	6366·2 (3906·1–9968·0)	6681·0 (4092·6–10 493·7)	62·6 (49·7–78·1)	63·8 (37·5–104·7)	63·8 (37·4–104·8)	64·1 (37·5–105·3)	63·9 (37·5–104·9)	64·2 (37·5–105·5)
	Foreign body	5591·6 (4309·9–6377·8)	5320·2 (3936·1–6923·8)	5357·3 (3959·2–6974·9)	5765·2 (4299·5–7450·2)	5327·4 (3941·3–6934·5)	5810·8 (4328·6–7515·7)	76·2 (57·8–87·3)	55·3 (38·2–74·6)	55·3 (38·2–74·6)	55·2 (38·1–74·4)	55·3 (38·2–74·7)	55·2 (38·1–74·5)
	Environmental heat and cold exposure	1675·7 (1196·7–2044·0)	1691·5 (1049·2–2461·4)	1705·7 (1054·8–2485·8)	1827·1 (1127·5–2674·6)	1692·5 (1049·5–2463·7)	1842·8 (1133·4–2700·7)	20·1 (14·3–24·5)	14·5 (8·6–22·1)	14·5 (8·6–22·1)	14·5 (8·7–22·1)	14·5 (8·6–22·1)	14·5 (8·7–22·2)
	Exposure to forces of nature	3510·2 (831·9–15 383·1)	3390·7 (888·1–13 534·4)	3403·9 (891·5–13 638·8)	3478·3 (912·1–13 859·4)	3394·7 (888·8–13 570·5)	3493·5 (916·0–13 966·3)	45·0 (10·5–202·2)	38·4 (9·5–163·7)	38·4 (9·5–164·5)	38·5 (9·5–164·8)	38·4 (9·5–164·0)	38·5 (9·5–165·6)
	Other unintentional injuries(internal)	6296·3 (4737·8–7972·0)	4920·9 (3605·1–6538·2)	4925·0 (3605·0–6549·2)	5178·6 (3784·2–6923·0)	4925·0 (3607·5–6543·2)	5185·3 (3785·5–6937·7)	77·4 (58·3–97·1)	46·4 (33·9–60·8)	46·2 (33·7–60·6)	46·5 (34·0–61·0)	46·5 (33·9–60·9)	46·4 (33·9–60·8)
	**Self-harm and interpersonal violence**	**69 520·2 (62 655·3–76 563·7)**	**70 573·0 (62 623·8–78 448·1)**	**70 411·7 (62 464·8–78 267·6)**	**72 456·1 (64 349·5–80 541·3)**	**70 612·8 (62 653·0–78 495·0)**	**72 303·5 (64 183·8–80 389·3)**	**859·0 (773·8–946·9)**	**730·3 (643·8–816·4)**	**726·6 (640·3–812·6)**	**732·4 (645·4–819·1)**	**730·4 (644·0–816·7)**	**728·8 (642·0–815·5)**
	Self-harm	33 520·0 (30 547·0–36 055·1)	33 181·3 (27 346·2–39 774·0)	33 124·7 (27 292·6–39 722·2)	34 304·4 (28 199·0–41 249·4)	33 190·2 (27 351·9–39 786·5)	34 240·6 (28 138·4–41 189·4)	407·0 (377·7–434·0)	324·2 (267·2–399·3)	322·6 (265·8–397·1)	325·1 (268·0–400·5)	324·2 (267·2–399·3)	323·4 (266·6–398·2)
	Interpersonal violence	26 839·1 (25 011·0–28 991·9)	24 501·3 (19 263·4–32 630·6)	24 364·2 (19 175·9–32 425·9)	24 904·2 (19 708·0–33 054·6)	24 521·7 (19 277·6–32 653·0)	24 778·7 (19 626·4–32 868·0)	334·7 (310·4–360·4)	263·8 (210·5–350·9)	261·8 (208·9–348·1)	264·2 (211·0–351·3)	264·0 (210·6–351·1)	262·2 (209·5–348·7)
	Conflict and terrorism	8183·8 (3257·2–13 572·5)	11 384·3 (5789·3–17 774·6)	11 414·4 (5809·5–17 820·1)	11 718·7 (5948·6–18 543·6)	11 393·6 (5794·7–17 789·9)	11 752·3 (5969·9–18 595·5)	105·1 (41·2–176·2)	125·4 (61·9–198·1)	125·5 (62·0–198·2)	126·2 (62·5–199·1)	125·5 (62·0–198·2)	126·3 (62·6–199·2)
	Police conflict and executions	977·4 (676·6–1600·0)	1506·1 (919·1–2590·5)	1508·5 (920·1–2595·5)	1528·8 (937·2–2623·0)	1507·3 (919·4–2593·3)	1531·9 (938·4–2629·6)	12·2 (8·3–20·0)	16·8 (9·9–28·9)	16·8 (9·9–29·0)	16·8 (9·9–29·0)	16·8 (9·9–28·9)	16·8 (9·9–29·0)

The five scenarios include the reference scenario, Safer Environment scenario, Improved Behavioural and Metabolic Risks scenario, Improved Childhood Nutrition and Vaccination scenario, and the combined scenario. Estimates are listed as means with 95% uncertainty intervals in parentheses. Rows with bold text indicate aggregated higher level causes from the GBD cause hierarchy. DALY=disability-adjusted life-year.

#### Leading causes of DALYs

**Figure 3:**
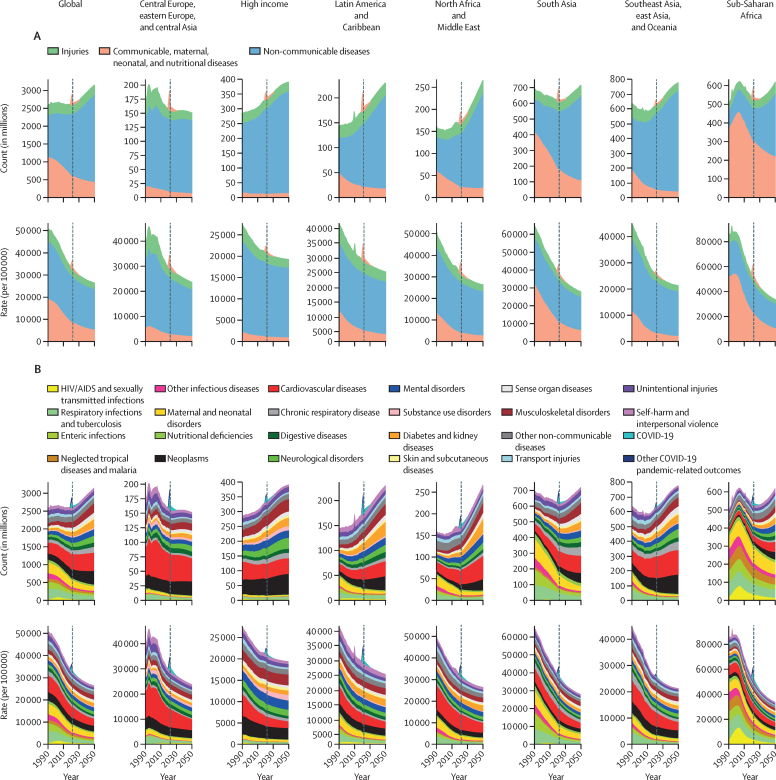
Global and super-regional DALY counts and age-standardised DALY rates by Level 1 (A) and Level 2 causes (B), 1990–2050 Forecasts are based on the reference scenario. The dashed vertical line indicates the year 2022 (the first forecast year). Note that scales are not anchored at zero and there are different scales across super-regions so that change over time is more visible.

**Figure 4:**
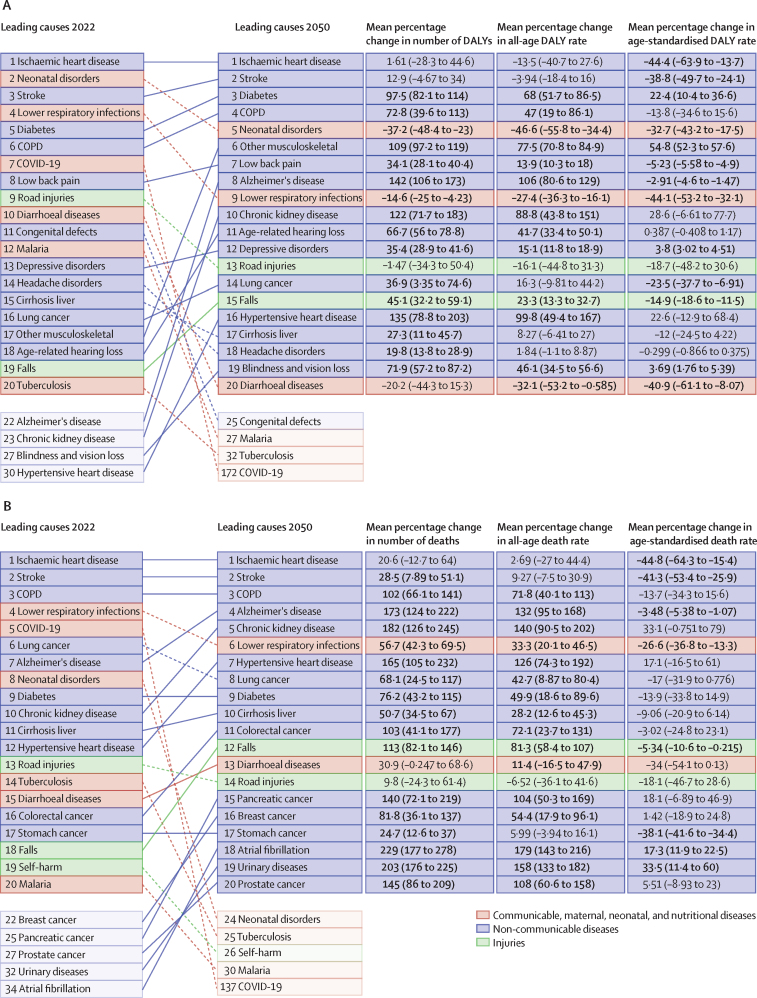
Leading 20 Level 3 causes of global DALYs (A) and deaths (B), and percentage change in number of DALYs (A) and deaths (B) and all-age and age-standardised DALY (A) and death (B) rates, 2022–50 The two main columns display the top 20 causes of DALYs and deaths in descending order for the specified year. Causes are coloured by Level 1 cause category and are connected by lines between time periods; solid lines represent an increase or lateral shift in ranking, dashed lines represent a decrease in rank. Bolded values indicate statistically significant changes from 2022 to 2050. Estimates are global and for the reference scenario. COPD=chronic obstructive pulmonary disease. DALY=disability-adjusted life-year.

**Figure 5:**
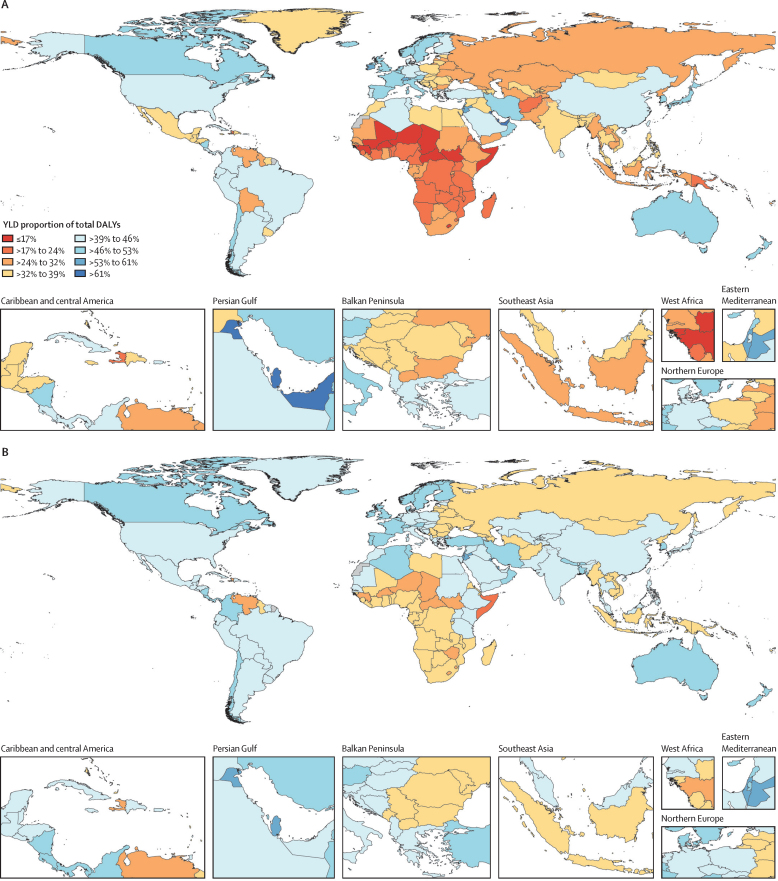
YLD proportion (%) of total DALYs by country and territory, 2022 (A) and 2050 (B) DALYs=disability-adjusted life-years. YLD=years lived with disability.

Our reference forecast predicts a continued shift in disease burden from CMNNs to NCDs, with 77·6% (95% UI 74·2–79·9) of global DALYs in 2050 coming from NCDs, an increase of 13·4% from 2022 levels (figure 3A). At the super-regional level, the largest shifts are forecasted to occur in sub-Saharan Africa (from 60·1% [56·8–63·1] of DALYs due to CMNNs in 2022 to just 35·8% [31·0–45·0] in 2050) and south Asia (from 31·7% [29·2–34·1] to 15·5% [13·7–17·5] over the forecasted period). The two largest Level 2 causes of the global increase in number of DALYs (both NCDs) were neoplasms (from 255·5 million [237·7–273·3] DALYs in 2022 to 388·1 million [334·8–452·6] in 2050) and diabetes and kidney disease (from 117·3 million [103·7–134·3] DALYs in 2022 to 240·1 million [192·6–287·8] DALYs in 2050; figure 3B, table 2). At the Level 3 cause-specific level, ischaemic heart disease is expected to remain the leading cause of total number of DALYs at the global level (185·6 million [171·9–197·6] DALYs in 2022 and 186·5 million [128·6–260·4] in 2050), while neonatal disorders will remain the leading cause of age-standardised DALY rates in 2050 (2905·7 age-standardised DALYs [2513·7–3352·3] per 100 000 in 2022 to 1941·7 [1554·1–2441·9] in 2050; figure 4A). Among the top ten causes of global DALYs in 2022, all three CMNN causes (neonatal disorders, lower respiratory infections, and diarrhoeal diseases; plus COVID-19, which the model dropped to zero DALYs by 2030) are expected to drop in ranking—from second, fourth, and tenth to the fifth, ninth, and 20th-leading causes—by 2050, while stroke (ranked third in 2022), diabetes (fifth), and chronic obstructive pulmonary disease (sixth) are forecasted to increase in ranking to second, third, and fourth, respectively. Another notable shift is from fatal to non-fatal burden, as measured by the proportion of DALYs due to YLDs (figure 5). The global proportion of DALYs due to YLDs is forecasted to increase from 33·8% (27·4–40·3) to 41·1% (33·9–48·1) from 2022 to 2050, a relative increase of 21·6% (11·4–30·4). The largest shift from fatal to non-fatal burden was forecasted for sub-Saharan Africa, from 20·1% (15·6–25·3) in 2022 to 35·6% (26·5–43·0) in 2050 (a relative increase in the proportion of DALYs due to YLDs of 77·3% [48·6–100·8]).

#### Leading causes of deaths

Reference scenario trends for the leading causes of death between 2022 and 2050 were similar to those of DALYs, with NCDs continuing to increase in prominence compared with CMNNs (figure 4B, appendix 2 table S4). Ischaemic heart disease is expected to remain the leading cause of death through to 2050 at the global level, followed in descending order by stroke and COPD. Among top-ten CMNN causes in 2022, lower respiratory infections and neonatal disorders are both expected to drop in ranking by 2050, from fourth and eighth to sixth and 24th, respectively. In contrast, Alzheimer's disease, chronic kidney disease, hypertensive heart disease, and colorectal cancer are all forecasted to rise in ranking over the forecasted period, from seventh, tenth, 12th, and 16th to fourth, fifth, seventh, and 11th, respectively. Country-specific leading causes of DALY and death results can be found in appendix 2 (figures S4–S8); country-specific leading causes of YLLs and YLDs are also shown in appendix 2 (figure S6).

#### Population and age structure, reference scenario

Our reference forecast shows a global increase in death and DALY counts (50·8% [95% UI 40·2–64·6] increase in number of deaths from 2022 to 2050 and 18·0% [9·4–28·8] increase in number of DALYs) as well as all-age rates (27·9% [17·2–41·8] increase in number of deaths per 100 000 from 2022 to 2050 and 0·1% [–8·4 to 10·9] increase in number of DALYs per 100 000), but a decline in age-standardised death and DALY rates (25·6% [10·6–35·3] decrease in the age-standardised death rate per 100 000 and 20·8% [8·3–28·9] decrease in age-standardised DALY rate per 100 000). This phenomenon is driven by shifts in the age structure of the global population between now and 2050, when we forecast 12·2% (11·3–13·0) of the global population will be older than 70 years, compared with 6·4% (6·3–6·5) in 2022 (appendix 2 figure S9). In absolute terms, this shift will be most pronounced in the southeast Asia, east Asia, and Oceania super-region, increasing from 7·4% (7·3–7·5) in 2022 to 18·4% (17·0–19·6) in 2050, while in relative terms, this shift will be most pronounced in north Africa and the Middle East, increasing from 3·3% (3·3–3·4) in 2022 to 9·2% (8·2–10·1) in 2050. Population growth will be most pronounced in the sub-Saharan Africa super-region (an 82·2% [71·4–94·0] increase from 2022 to 2050).

### Alternative scenarios

#### DALYs by scenario

**Figure 6:**
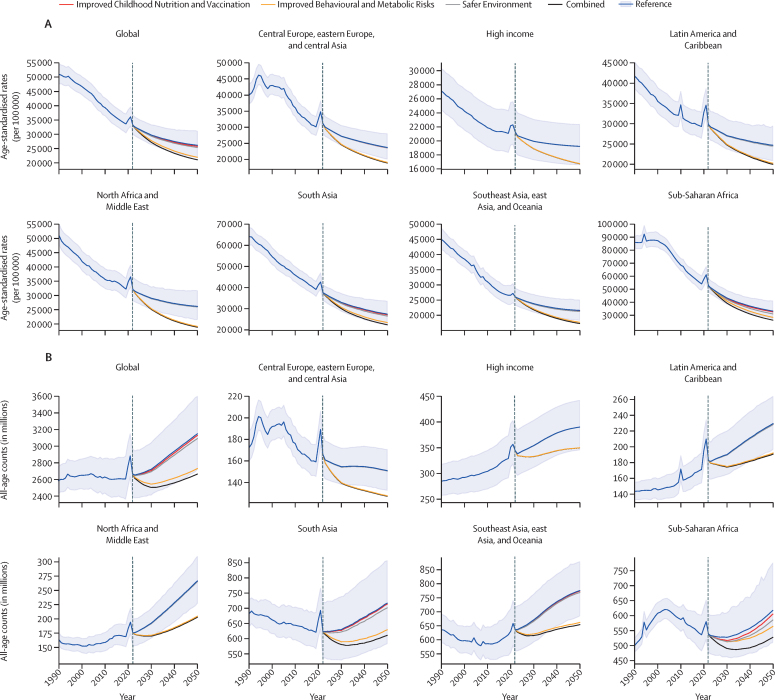
Global and super-regional all-cause age-standardised DALY rates (A) and all-age DALY counts (B), for the past and for five future scenarios, 1990–2050 The dashed vertical line indicates the year 2022 (the first forecast year). The blue shading indicates the 95% uncertainty interval for the reference scenario. The five scenarios include the reference scenario, Safer Environment scenario, Improved Behavioural and Metabolic Risks scenario, Improved Childhood Nutrition and Vaccination scenario, and the combined scenario. Note different scales were used across each super-region so that change over time is more visible. DALY=disability-adjusted life-year.

**Figure 7:**
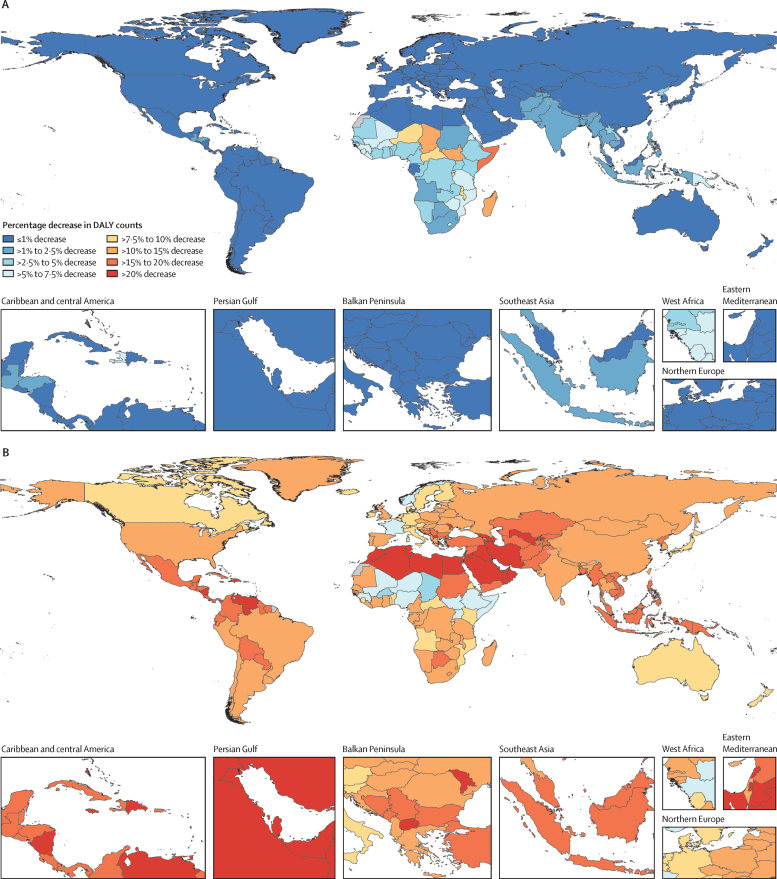

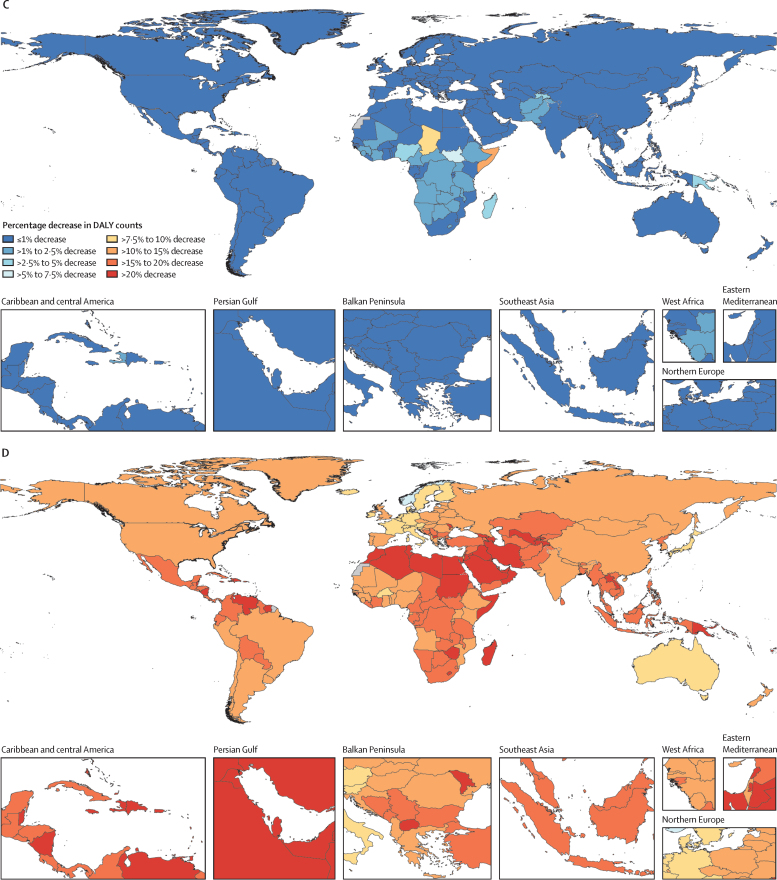
Percentage decrease in DALY counts in 2050 between the reference and four alternative scenarios by country and territory Alternative scenarios are as follows: Safer Environment scenario (A), Improved Behavioural and Metabolic Risks scenario (B), Improved Childhood Nutrition and Vaccination Scenario (C), and the combined scenario (D). DALY=disability-adjusted life-year.

We forecast large differences in future DALY burden between the alternative scenarios globally and across super-regions (table 2, figure 6A, B). Globally, the forecasted effects are strongest for the Improved Behavioural and Metabolic Risks scenario, with a 13·3% (95% UI 11·8–15·0) decrease in DALY counts in 2050 compared with the reference scenario. For the Safer Environment and Improved Childhood Nutrition and Vaccination scenarios, we forecasted decreases of 1·8% (1·4–2·4) and 0·6% (0·1–1·0), respectively, in 2050, compared with the reference scenario. The combined global impact of the three scenarios is forecasted to amount to 15·4% (13·5–17·5) fewer DALYs in 2050 than in the reference scenario. Across super-regions, the largest decrease in DALYs between the reference scenario and the Improved Behavioural and Metabolic Risks scenario was seen for north Africa and the Middle East (23·2% lower [20·2–26·5]) and the smallest for sub-Saharan Africa (8·7% lower [7·5–9·8]) and the high-income super-region (10·4% lower [9·6–11·2]). The Safer Environment scenario was forecasted to have the largest impact on DALYs in sub-Saharan Africa and south Asia (5·2% [3·5–6·8] and 2·1% [1·6–2·8] fewer DALYs compared with the reference scenario, respectively), while the smallest effects were seen in the high-income and central Europe, eastern Europe, and central Asia super-regions (0·1% [0·0–0·1] and 0·2% [0·2–0·3] lower, respectively). The Improved Childhood Nutrition and Vaccination scenario was forecasted to have by far its greatest impact in sub-Saharan Africa—a 2·0% (–0·6 to 3·6) decrease in DALYs—followed by south Asia, with a 0·5% (0·3–0·6) decrease. The impact of this scenario was smallest in the high-income super-region, with a decrease of 0·01% (0·01–0·02). The combined impact of the three scenarios ranged from a decrease of 10·4% (9·7–11·3) in the high-income super-region to a 23·9% (20·7–27·3) decrease in north Africa and the Middle East. Country-level results for the impact of the alternative scenarios on DALYs are shown on world maps in figure 7. Additional country-level scenario results for DALYs and deaths can be found in appendix 2 (figures S10–S13, tables S3–S4).

#### Life expectancy decomposition by scenario

Global life expectancy for all sexes combined was forecasted to increase by 4·6 years (95% UI 2·0–6·4) between 2022 and 2050 in the reference scenario and an additional 5·3 years (4·6–5·8) above the reference scenario under the combined alternative scenario (an increase of 127·9% [75·4–288·8] over the reference scenario). All seven super-regions were forecasted to experience substantial additional gains in life expectancy under the combined scenario, ranging from 3·3 additional years (3·1–3·4) compared with the reference scenario in the high-income super-region to 7·9 additional years (7·0–8·8) in north Africa and the Middle East. Globally and in all super-regions except sub-Saharan Africa and south Asia, the additional gain in the combined scenario more than doubled the gain in the reference scenario alone (ranging from 135·4% to 232·6%). In sub-Saharan Africa and south Asia, the additional gains were 6·1 years (5·3–6·7; a 67·9% increase from reference gain) and 5·6 years (4·8–6·2; a 91·9% increase), respectively. In the reference scenario, the largest Level 2 cause-specific gains in life expectancy were due to improvements in cardiovascular diseases in five of seven super-regions, with respiratory infections and tuberculosis (primarily COVID-19) declines causing the largest improvements in sub-Saharan Africa and Latin America and the Caribbean (figure 2). After cardiovascular disease gains (35·4% of the total global life expectancy gain), improvements in the GBD Level 2 causes that contributed most to global life expectancy gains in the reference scenario were, in descending order, respiratory infections and tuberculosis (primarily COVID-19), maternal and neonatal disorders, and enteric infections, contributing to 23·3%, 10·0%, and 4·2%, respectively, of the total gain. In the high-income super-region, the leading causes in the reference scenario after cardiovascular diseases were respiratory infections and tuberculosis (primarily COVID-19; 39·0% of total improvement in life expectancy), neoplasms (7·0%), and self-harm and interpersonal violence (4·7%), and in sub-Saharan Africa respiratory infections and tuberculosis gains were followed, in descending order, by cardiovascular diseases (14·9%), HIV/AIDS and other sexually transmitted infections (12·9%), and neglected tropical diseases and malaria (9·7%). The year 2022 was still marked by a considerable mortality burden from COVID-19, compared with the zero COVID-19 deaths we assume in 2050 (see Methods for details). In the combined scenario, improvements in cardiovascular diseases contributed to the largest gains in life expectancy across all seven super-regions, followed by respiratory infections and tuberculosis (primarily COVID-19) for three of seven super-regions. Additional country-level results for life expectancy decomposition for all scenarios can be found in appendix 2 (figure S14).

### Model performance

We used a skill metric to evaluate the performance of our forecasting model, for which a positive skill value indicates better performance than the baseline model (a simple model holding 2009 values constant in the future) while a negative skill value indicates worse performance than the baseline model. Skill values for all-cause mortality as well as the GBD Level 1 causes (NCDs, CMNNs, and injuries) were positive for out-of-sample predictions (appendix 1 table E). The skill value for all-cause mortality was 0·44 for males and 0·49 for females. Among Level 1 causes, the highest skill value was for CMNNs in females (0·75) and the lowest was for injuries in males (0·19). Among GBD Level 2 causes, neurological disorders in males had the highest skill value (0·74) and musculoskeletal disorders in females had the lowest skill value (–0·82). Five of 22 Level 2 causes had negative skill values, most notably mental disorders and musculoskeletal disorders, but the causes with negative skill values contributed just 11·81% of total deaths (appendix 1 table E).

Similarly, both male and female DALY out-of-sample model estimates had a positive skill value for all-cause DALYs and the GBD Level 1 causes (appendix 1 table F). All-cause DALY skill values were 0·58 for males and 0·66 for females. Among Level 1 causes, CMNNs in females had the highest skill value (0·78) while NCDs and injuries in males had equally low skill values (0·24). The highest skill value among Level 2 causes was for HIV/AIDS and other sexually transmitted infections in females (0·74), while the lowest skill value was for skin and subcutaneous diseases, also in females (–1·93).

## Discussion

### Main findings

This study presents comprehensive forecasts of future trends in disease burden in 204 countries and territories to the year 2050. Broadly, our reference scenario forecasts that age-standardised disease burden will continue to decline in the future, albeit at slower rates than during the three decades preceding the COVID-19 pandemic,^8^, ^16^ and with several notable cause-specific exceptions, including diabetes, hypertensive heart disease, and chronic kidney disease. While CMNN diseases will continue contributing to the overall disease burden across locations, the proportion of deaths and DALYs due to NCDs will increase substantially in most locations within the next generation. This shift reflects population ageing, improved prevention and treatment options for many CMNN diseases, and rising rates of obesity and metabolic disorders. That said, our reference scenario forecast of continued progress on CMNN disease burden is a reflection of historical trends in which CMNN prevention and treatment was invested in heavily. Thus, future progress will require that CMNNs remain a public health priority; if these diseases are neglected, the world will be unlikely to achieve the progress forecasted in our reference scenario.

Even with the large disruption in life expectancy caused by the COVID-19 pandemic, long-term trends in life expectancy are expected to continue improving in all regions in the coming decades, despite increases in exposure to high temperature and increases in BMI. Improvements in life expectancy in the coming years will be driven primarily by reductions in deaths from cardiovascular diseases and respiratory infections (partly due to short-term declines in COVID-19 because we use 2022 as the base year in the life expectancy decomposition analysis). Similar to trends in age-standardised disease burden, we forecast that improvements in life expectancy and HALE will be slower in the coming decades than from 1990 to 2019, and that differential progress will lead to narrower—but still persistent—life expectancy and HALE inequalities between locations in 2050 than we see in 2022. The slowdown of future growth in life expectancy that is most pronounced in the high-income super-region has already been observed and debated in the UK and the USA in the 5–10 years preceding the COVID-19 pandemic.^23^, ^24^ Increasing or stalled mortality trends have been observed both in midlife (deaths of despair due to suicide, drugs, and alcohol) and older age groups.^23^, ^24^, ^25^

Population growth will slow considerably by 2050 at the global level but will remain high in some of the poorest regions of the world. The highest rates of population growth in the coming decades will primarily be found in parts of sub-Saharan Africa; we forecast that population will increase by more than 80% in this super-region between 2022 and 2050. Pronounced increases in population growth in this super-region will pose considerable challenges to already strained health systems^26^, ^27^ and contribute to further food, water, and other resource scarcity in populations already particularly vulnerable to climate change.^21^, ^28^, ^29^, ^30^, ^31^ The interplay between population growth, limited health system infrastructure and capacity, and climate change is of great importance when considering how to address growing numbers of deaths and DALYs in parts of sub-Saharan Africa and in other vulnerable populations in the future. As a greater proportion of global deaths and DALYs occur in sub-Saharan Africa in the coming decades, due in large part to sustained population growth in this super-region,^2^ policy makers must consider how to sustain progress on declining age-standardised rates of disease burden in the face of greater strain on infrastructure, resources, health systems, and the environment.

### Impact of scenarios

In addition to our reference scenario, we also forecasted three risk group-specific and one combined alternative scenario that each illustrate the extreme ends of potential gains in human health that could be achieved if concerted efforts are made in the coming years to substantially reduce or eliminate exposure to different types of risk factors. While the scenarios reflect an unlikely magnitude of risk reduction—eg, it is unlikely that tobacco smoking will be eliminated completely by 2050—they nonetheless provide valuable insights to policy makers who want to understand the extent to which prioritising the prevention of exposure to a wide range of known and modifiable risks (the combined scenario) or to certain risks over others (by comparing each scenario) might impact health outcomes. We observed the largest scenario differences between the reference scenario and the Improved Behavioural and Metabolic Risks scenario both globally and across super-regions. The impacts of the two other scenarios compared with the reference scenario were much smaller. Our alternative scenarios thus suggest that globally and at the super-region level, policies that lead to substantial reductions in metabolic risk exposure and rates of tobacco smoking have the potential to reduce the global burden of disease to a greater extent than policies that lead to similarly substantial improvements in rates of childhood malnutrition and vaccination. Even in sub-Saharan Africa, where the impact of the Improved Childhood Nutrition and Vaccination scenario was largest among super-regions, the impacts of the Safer Environment and Improved Behavioural and Metabolic Risks scenarios were larger and largest, respectively. To fully understand the contrast between the reference and alternative scenarios, one must understand that the reference might be optimistic or pessimistic based on historical trends or investments in the relevant interventions (eg, considerable past progress on CMNN-related risks is reflected in continued progress in our forecasts). Thus, the seemingly moderate impact of this scenario relative to the gains already projected in the reference scenario reflects past successes in this area, and a failure to continue the interventions that led to past progress could lead to less future progress than we forecast.

That said, country-specific alternative scenarios demonstrate why targeted policy approaches that address the specific risk exposure and disease profile of a particular location are of great importance, as the effects of each scenario vary substantially by country and territory. Furthermore, risk exposure prevention is not equally feasible, simple, or cost-effective for all risk factors or in all settings. For example, while at the global level, policies that eliminate metabolic risk exposure might reduce disease burden more than policies that eliminate exposure to unsafe water, sanitation, and hygiene (WaSH), the scenarios do not consider the cost and feasibility of achieving equivalent risk reductions in one risk group or another (eg, the scenarios do not provide insights into how feasible bringing safe WaSH infrastructure to all households and communities worldwide would be compared with eliminating high systolic blood pressure worldwide). Finally, policy makers must consider the populations most impacted by policies that target certain risk factors over others, and the trade-offs of each. For example, risk factors like child growth failure primarily affect young, low-income populations, while risk factors like high LDL cholesterol primarily affect older, higher-income populations.

Our alternative scenarios demonstrate huge potential opportunities to alter the course of health in the 21st century through policy change. Much of the disease burden in the coming years will be determined by how much progress can be made towards reducing and ultimately eliminating exposure to well established risks and increasing access to well established health interventions (eg, statins for cholesterol-lowering and antihypertensive treatment). The risk factors and interventions in our alternative scenarios all have the capacity to be modified, meaning policy makers, governments, and populations around the world have a chance to drastically change the trajectory of human health by using the alternative scenarios in this study to help guide location-specific, population-specific policies and decisions that target the most impactful threats. Although providing population-specific prescriptions or evaluating economic feasibility is beyond the scope of this study, we have illustrated how our alternative scenarios can be used to inform and prioritise risk-specific policy change to maximise health gains in two examples below. Here, we use Chad and Germany as examples to demonstrate how the scenarios have different impacts depending on country-specific contexts, and how a policy maker might interpret different, country-specific alternative scenario forecasts and—in combination with other information such as current estimates of disease burden; feasibility, cost, and effectiveness assessments; and more—incorporate these forecasts into policy decision making.

First, in our Safer Environment and Improved Childhood Nutrition and Vaccination scenarios, we projected that Chad would experience approximately 13% and 9% fewer DALYs in 2050, respectively, if the scenario-specific risk factors were eliminated by that time compared with the reference forecast of disease burden (see appendix 2 table S3). In contrast, we can expect only 5% fewer DALYs than in the reference scenario if the included metabolic and behavioural risks were eliminated. Child growth failure was the leading contributor to risk-attributable DALYs in Chad in 2021—followed by low birthweight and short gestation, unsafe water, and particulate matter pollution—but all-age rates declined by more than 75% between 1990 and 2021,^9^ demonstrating that preventing and treating child growth failure in Chad remains critically important but also that the country has already seen considerable improvements towards this end. The findings from our alternative scenarios combined with estimates of disease burden from the recent past therefore suggest that continuing to prioritise and expand evidence-based and locally relevant interventions that improve childhood vaccination rates and further reduce rates of child growth failure, low birthweight and short gestation, unsafe WaSH, and particulate matter pollution could improve health outcomes in Chad considerably over the next several decades. Policies could include those that promote exclusive breastfeeding; support supplementation for infants whose nutrition from breastfeeding is insufficient; improve maternal nutrition; expand vitamin A deficiency detection and treatment interventions; improve infant and child access to WaSH infrastructure and primary health care—including health services equipped to detect early signs of child growth failure—and more.^32^, ^33^, ^34^ In contrast, failure to continue prioritising childhood malnutrition and vaccination in Chad in the coming decades could lead to reversals in the health progress that has already been made and is forecasted to continue.

Second, our Improved Behavioural and Metabolic Risks scenario suggests that Germany would experience approximately 9% fewer DALYs than expected in 2050 if high adult BMI, systolic blood pressure, LDL cholesterol, and fasting plasma glucose; tobacco smoking; and exposure to GBD dietary risk factors were eliminated by 2050. On the other hand, eliminating the risks in our Safer Environment and Improved Childhood Nutrition and Vaccination scenarios would have almost no effect on DALYs, with declines of just 0·05% and 0·01%, respectively, in 2050 compared with the reference scenario. Considering these projections and historical estimates that high systolic blood pressure, tobacco smoking, high BMI, and high fasting plasma glucose were the four leading contributors to risk-attributable DALYs in Germany in recent past years,^9^ there are considerable opportunities to improve health outcomes in Germany by placing greater emphasis on addressing the complex causes of metabolic risks, including individual behaviours, socioeconomic environments and other social determinants, and physical environments,^35^, ^36^, ^37^ as well as on tobacco smoking cessation and prevention programmes.^38^, ^39^, ^40^ Given Germany's commendable existing policies related to childhood vaccination,^41^ WaSH infrastructure, and air quality regulations,^42^ the greatest opportunities for further health improvements lie in targeted efforts that address specific risk factors contributing to metabolic risks. These policies should not, however, be de-emphasised, or continued progress could stall or even reverse.

An important strength of our alternative scenarios modelling framework is that it accounts for competing risks when contrasting the reference versus alternative scenarios.^43^ This is done by capturing different population dynamics between the scenarios. For example, in the Improved Behavioural and Metabolic Risks scenario, there are strong declines in exposure to behavioural (smoking and unhealthy diet) and metabolic risk factors, which leads to reductions in cause-specific mortality linked to those risks (such as lung cancer). Reductions in these mortality rates lead to forecasting a larger and older population in this alternative scenario compared with the reference scenario. This means the cause of death composition shifts to one of an older population. Accordingly, in the older ages we might see increases in causes unrelated to the scenario intervention, such as increases in rates of Alzheimer's deaths in older age groups alongside declines in cardiovascular disease deaths. These findings might seem paradoxical at first but are in fact a strength of the model in accounting for competing risks. Lastly, our scenarios estimate the avoidable burden that could be realised from elimination of key risk factors. However, these estimates do not account for the deaths avoided beyond the study period (2050) that are likely to be realised as population cohorts continue to age.

### Model performance

A positive skill metric indicates that a model being evaluated performs better than the baseline model, whereas a negative skill metric suggests the opposite. Both DALY and mortality model estimates had positive skill values for all-cause and for all three GBD Level 1 causes (appendix 1 tables E, F). There were several Level 2 causes that had negative skill values. For mortality, these included mental disorders and musculoskeletal disorders, which contributed just 0·23% of all deaths combined; for DALYs, these included skin and subcutaneous diseases and digestive diseases, which contributed 5·49% of all DALYs. The major limitation in skill evaluation is the short period (only 10 years) for validation due to the cause-specific data availability. To address negative skill values, we will revisit the corresponding models with negative skill and consider the addition of new covariates to improve model performance, as well as include data with longer range for the validation period when possible, which could result in higher skill values.

### Health threats not included in the framework

The world faces major potential threats to human health in the coming decades. These include climate change; antimicrobial resistance;^44^ new pandemics, either due to a novel virus or a more virulent SARS-CoV-2 variant; war and conflict; mass migration and internal displacement; food insecurity and famines, potentially at a scale some thought was history; and more. While this study incorporated more than 80 independent drivers known to influence population health, including all GBD 2019 fatal risk factors and select interventions, there are a number of important potential health threats that were not included in the current analysis.

First, while our framework does include models for natural disasters, war and conflict, and migration, these stochastic event models need improvement. Take, for example, natural disasters. Major natural disaster events do not regularly happen on an annual basis, and while we can infer from the past where they are most likely to occur in the future, we are unable to predict their exact timing and magnitude. Thus, predictions of stochastic events might be better captured by estimating the cumulative probability of the event using extreme value theory.^45^ Extreme value theory can likewise be useful for forecasting future pandemics. For example, extreme value theory has estimated that the annual probability of another pandemic of at least the magnitude of the COVID-19 pandemic is 2–3%.^46^ This equates to an 18–26% chance within a decade and 79–90% chance within the remainder of the 21st century. In future work, we will explore how we can utilise extreme value theory as a supplement to our current forecasting framework.

Second, while we include climate change in our framework through exposure to non-optimal temperatures and ambient particulate matter pollution (as modelled for CMIP6 climate scenarios^15^), there are additional pathways through which climate change is likely to impact population health in the coming decades. These pathways include impacts on migration, extreme weather events, vector-borne disease incidence and geography, sea level rise, drought, and crop failure. We have not yet incorporated these climate change-related events into our framework. As our reference forecast is a probabilistic estimate of the most likely future, we plan to use the probabilistic climate forecasts that were recently released by the Rhodium Group^47^ in the future. Their estimate of global temperature anomaly was 2·8°C (90% UI 2·0–4·0), very close to the Intergovernmental Panel on Climate Change estimate for their SSP2-4.5 scenario of 2·7°C,^21^ which we used in our reference scenario. Third, we did not include threats that are difficult to quantify due to lack of evidence on their potential impacts on health or the evolving magnitude of the risk. This includes malicious artificial intelligence, nuclear escalation, and bioterrorism. Similarly, we did not include health advancements that could potentially reduce disease burden but for which we also do not have sufficient evidence for their potential impact or scalability, such as mass use of GLP-1 agonists for the treatment of diabetes and obesity,^48^, ^49^ mass use of polypill formulations for the treatment of cardiovascular diseases,^50^, ^51^ or advancements in artificial intelligence. Fourth, we could not include new threats that the world has not yet observed and are so far unknown. For example, the world could not have predicted the health impacts of nuclear weapons before the weaponisation of nuclear energy.

### Limitations and future directions

As with any forecasting model, there are several uncertainties and limitations. Our framework depends on forecasting 81 drivers of health using past estimates from GBD and a collection of other health-related covariates estimated by IHME. As noted above, there are other drivers and potential threats to population health that we were not able to incorporate at this time. Our forecasts are also inherently limited by the data quality and limitations present in the GBD estimates themselves, which vary by cause, location, age, and sex.^7^, ^8^, ^9^, ^13^, ^52^ Estimates in data-sparse contexts will be reflected by wider uncertainty bounds in the forecast estimates, but these forecasts would also be improved if and as more data are made available to inform the historical estimates. In addition, while we use the GBD mediation matrix to account for risk factor mediation pathways—for example, how the effect of high BMI is mediated through systolic blood pressure and LDL cholesterol—the matrix is still incomplete in the sense that there are mediation pathways that we have not been able to include, often due to lack of solid data on the relationship between the distal risk factor and the mediator risk factor. We will work to expand and improve the mediation matrix in future studies. In addition, we have improved our methodology for estimating the impacts of smoking on cause-specific disease burden to capture the pack-year lagged effects that smoking has on mortality. We have not yet incorporated time lag effects for other risk factors where this might be appropriate, such as high BMI. As evidence becomes available to estimate the lagged effects of these changes at the population level, we will incorporate additional risk factors into this approach.

For the first time, we have provided estimates of YLDs and DALYs, computed from estimates of incidence and prevalence. These predictions are based primarily on modelling MIRs or MPRs using linear mixed-effects models with SDI as the main covariate. These models allow for the effects of all risk factors on cause-specific mortality to also influence non-fatal disease burden. For non-fatal diseases, we predict prevalence directly using similar SDI-based models. Future iterations of this work will refine these models and add additional cause-specific covariates and risk factors for non-fatal diseases, such as we have already done for forecasting diabetes and dementia prevalence.^53^, ^54^ In other cases, we noted implausible trend reversals in the future when prevalence or incidence modelling was based on the MPR or MIR models. Disconnecting these measures from the associated trends in mortality, and forecasting based only on trends in incidence or prevalence produced more plausible future trends. Our forecasts of YLDs were produced using average disability weights from GBD, thus assuming a static relationship between prevalence and disability over time. In cases where substantial improvements to treatment are made in the future, this relationship might not fully capture those improvements. We will also more closely explore the coherence of our estimates of cause-specific mortality, incidence, and prevalence rates as they run into the future.

Also for the first time, we have captured indirect impacts of the COVID-19 pandemic in our forecasting model, through disruptions in vaccine coverage, schooling (measured by educational attainment), and the economy (measured by income per capita). That said, data on COVID-19 cases and deaths in 2022–23 are still very limited due to reporting lags, and there remains great uncertainty around what the longer-term direct and indirect effects of the pandemic will be. In the absence of data that could indicate future trends in COVID-19 burden, we assumed that COVID-19 deaths and DALYs will decline linearly to zero between 2023 and 2030. Although speculative, there is basis for this assumption from the loss of virulence that has occurred to other coronaviruses now causing the common cold.^55^ As more data on the impacts of COVID-19 become available, we will revisit and revise our models accordingly.

The alternative scenarios included in this paper are intended to be illustrative of the potential health gains the world could make if substantial improvements are made to known risk factors, and the degree to which these benefits differentially play out by cause, age, sex, or geography. Computational constraints prevented us from producing alternative scenarios for each risk factor individually, but subsequent studies aim to understand the individual impacts of independent drivers on disease burden.

### Conclusion

Our reference forecasts of disease burden and life expectancy to 2050 indicate a continued, albeit slower, improvement in the health status of the world compared with the progress experienced from 1990 until the onset of the COVID-19 pandemic in 2020–21. If investment in CMNN disease prevention and treatment is maintained, the burden of disease will continue to decline for these causes and the proportion due to NCDs will continue to grow considerably. It is noteworthy that areas currently experiencing the highest disease burden are expected to see more pronounced health improvements. This will lead to a narrowing of health disparities across populations. Our alternative future scenarios demonstrate that major health improvements can be achieved by reducing exposure to established risk factors, offering an opportunity to alter the course of human health in the coming century through concerted risk factor prevention efforts. This is particularly true for countries currently facing high disease burden.

## Data sharing

To download GBD data used in these analyses, please visit the Global Health Data Exchange GBD 2021 website (https://ghdx.healthdata.org/gbd-2021/sources). To download forecasted estimates used in these analyses, please visit the GBD Foresight visualisation tool (https://vizhub.healthdata.org/gbd-foresight/).
