# Supplementary material for: Burden of disease scenarios for 204 countries and territories, 2022–2050: a forecasting analysis for the Global Burden of Disease Study 2021
Source: Lancet. 2024 May 18;403(10440):2204–56. doi: 10.1016/S0140-6736(24)00685-8 (PMC11121021; doi:10.1016/S0140-6736(24)00685-8)
Supplement: Supplementary appendix 1 [file mmc1.pdf]

THE LANCET

Supplementary appendix 1

This appendix formed part of the original submission and has been peer reviewed. We post it as supplied by the authors.

Supplement to: GBD 2021 Forecasting Collaborators. Burden of disease scenarios for 204 countries and territories, 2022–2050: a forecasting analysis for the Global Burden of Disease Study 2021. *Lancet* 2024; **403:** 2204–56.

Appendix 1: methods appendix to “Burden of disease scenarios for 204 countries and territories, 2022–2050: a forecasting analysis for the Global Burden of Disease Study 2021”

This appendix provides further methodological detail for “Burden of disease scenarios for 204 countries and territories, 2022–2050: a forecasting analysis for the Global Burden of Disease Study 2021”.

Preamble

This appendix provides further methodological detail for "Burden of disease scenarios for 204 countries and territories, 2022–2050: a forecasting analysis for the Global Burden of Disease Study 2021". This study complies with the Guidelines for Accurate and Transparent Reporting (GATHER) recommendations[1]. It includes detailed tables and information on data in an effort to maximise transparency in our estimation process and provide a comprehensive description of analytical steps. We intend this appendix to be a living document, to be updated with each iteration of the Global Burden of Disease Study.

Portions of this appendix have been reproduced or adapted from appendices for Foreman et al,.[2] Vollset et al,[3] GBD 2021 Fertility and Forecasting Collaborators,[4] Institute for Health Metrics and Evaluation (Financing Global Health 2021),[5] Murray et al.,[6] GBD 2021 Causes of Death Collaborators,[7] and GBD 2021 HIV Collaborators.[8] References are provided for reproduced or adapted sections.

Table of contents

Preamble 2
List of appendix tables 5
Section 1: GBD overview 6
Section 1.1: Study 2021 6
Section 1.2: Geographical locations of the analysis 6
Section 1.3: Time period of the analysis 6
Section 1.4: Statement of GATHER compliance 6
Section 1.5: List of abbreviations 6
Section 2: Overview of the Forecasting Framework 7
Section 2.1: Forecasting Independent Drivers 9
Section 2.1.1: Met need for contraception[4] 9
Section 2.1.2: Education 10
Section 2.1.3: Lag-distributed Income[5] 12
Section 2.1.4: Fertility[4] 12
Section 2.1.5: Vaccine Coverage[2] 13
Section 2.1.5.1 COVID-19 disruptions in vaccine coverage 14
Section 2.1.6: Computing SEVs, PAFs and scalars 14
Section 2.1.6.1: Risk Exposure (Summary Exposure Values) 16
Section 2.1.6.2: Risk Factors with Mediation 17
Section 2.1.6.3: Computing mediator SEVs 20
Section 2.1.6.4: Forecasting Direct Smoking Exposure 22
Section 2.1.6.5: PAF Mediation and Aggregation 23
Section 2.1.6.6: Directly Modelled PAFs 24
Section 2.1.6.7: Non-optimal temperature PAFs[6] 25
Section 2.1.6.8: Ambient particulate matter pollution PAFs 29
Section 2.1.6.9: Scalars 32
Section 2.2: Forecasting mortality[2,3] 32
Section 2.2.1: Cause-specific mortality models 32
Section 2.2.2: Aggregating to all-cause mortality 33
Section 2.2.2.1: Modelling latent trends in mortality 34
Section 2.2.3: Causes modelled outside of the 3-component framework 35
Section 2.2.3.1: HIV[3] 35
Forecasting ART Coverage 35

Forecasting the Transmission rate... 35
Section 2.2.3.2: Disasters, war and terrorism, legal interventions 37
Section 2.2.3.3: COVID-19 and other pandemic-related mortality (OPRM) estimation[7] 37
Section 2.2.4: YLLs 37
Section 2.3: Forecasting non-fatal disease burden 37
Section 2.3.1: MI, MP, PI ratios 37
Section 2.3.2: Prevalence-only models 37
Section 2.3.3: Producing forecasts of incidence and prevalence 38
Section 2.3.4: YLDs 38
Section 2.3.5: COVID-19 non-fatal estimation 38
Section 2.3.6: DALYs 38
Section 2.4: Forecasting population 39
Section 2.4.1: Forecasting migration[3] 39
Section 2.5: Forecasting Life expectancy and healthy life expectancy 39
Section 2.5.1: Life expectancy 39
Section 2.5.2: HALE 40
Section 3: Developing alternative scenarios 40
Section 4: Uncertainty interval estimation 41
Section 5: Model evaluation 42
Section 6: Post-processing of the results to align with GBD 2021 45
Section 7: References 46
Section 8: Tables 49

List of appendix tables

See section 8 for all appendix tables listed here.

Appendix Table S1: GBD location hierarchy used for forecasting with levels

Appendix Table S2: GATHER checklist

Appendix Table S3: Nonfatal modelling strategies for most detailed causes

Section 1: GBD overview

Section 1.1: Study 2021

The Global Burden of Diseases, Injuries, and Risk Factors Study (GBD) is a collaborative research effort aimed at estimating worldwide population, fertility, morbidity, and mortality. The GBD collaborator network draws on the expertise of over 10 000 contributors from around the world. For this paper, we estimated deaths, years of life lost, years lived with disability, disability adjusted life years, life expectancy and healthy life expectancy from 2022-2050 by cause, age, sex and location.

Section 1.2: Geographical locations of the analysis

We produced estimates for 204 countries and territories that were grouped into 21 regions and seven super-regions. A list of locations can be found in appendix 1 table S1 (section 8).

Section 1.3: Time period of the analysis

We estimated future disease burden from 2022 through 2050, using estimates from the Global Burden of Disease Study 2021.

Section 1.4: Statement of GATHER compliance

This study complies with the Guidelines for Accurate and Transparent Health Estimates Reporting (GATHER)[1] recommendations. We have documented the steps involved in our analytical procedures and detailed the data sources used. See appendix 1 table S2 (section 8) for the GATHER checklist. The GATHER recommendations can be found here: http://gather-statement.org/

Section 1.5: List of abbreviations

Abbreviation	Full phrase
**ARIMA**	Autoregressive Integrated Moving Average
**ARC**	Annualised rate of change
**ASFR**	Age-specific fertility rate
**CCF**	Completed cohort fertility
**CCMP**	Cohort-component method of projection
**CD4**	Clusters of differentiation 4
**CMIP6**	Coupled model intercomparison project phase 6
**DTP3**	diphtheria, tetanus toxoid, and pertussis (three doses)
**EPP-ASM**	Estimation projection package - age-sex model
**GBD**	Global Burden of Diseases, Injuries, and Risk Factors Study
**GDP**	Gross domestic product
**GenEM**	Generalized ensemble model
**Hib**	Haemophilus influenzae type b
**IHME**	Institute for Health Metrics and Evaluation
**IPCC**	Intergovernmental Panel on Climate Change

**MCV1**	Measles-containing-vaccine first-dose
**MCV1**	Measles-containing-vaccine second-dose
**MIR**	Mortality to incidence ratio
**MPR**	Mortality to prevalence ratio
**MR-BRT**	Meta-regression—Bayesian, regularised, trimmed
**LDI**	Lag distributed income
**PAF**	Population attributable fraction
**PCV3**	Pneumococcal conjugate vaccine (three doses)
**PIR**	Prevalence to incidence ratio
**RCP**	Representative Concentration Pathway
**RMSE**	Root mean-squared error
**ROC**	Rate of change
**SDGs**	Sustainable Development Goals
**SDI**	Socio-demographic Index
**SEV**	Summary exposure value
**SSP**	Shared socioeconomic pathway
**TFR**	Total fertility rate
**TMREL**	Theoretical minimum risk exposure value
**UI**	Uncertainty interval
**UNPD**	United Nations Population Division
**YLD**	Years lived with disability
**YLL**	Years of life lost

Section 2: Overview of the Forecasting Framework

The Institute for Health Metrics and Evaluation (IHME) forecasting framework uses estimates of disease burden, drivers of disease burden (such as risk exposure) and demographic indicators from the Global Burden of Diseases, Injuries, and Risk Factors Study (GBD). The modelling process is multi-staged. First, independent health drivers (section 2.1) are forecasted into the future. Second, these independent drivers, along with the Socioeconomic-demographic Index (SDI), a country-year specific composite index of fertility under 25 years, educational attainment, and GDP per capita, are utilized to forecast cause-specific mortality (section 2.2.1). All-cause mortality rate forecasts are obtained by aggregating cause-specific mortality, which is then utilized to generate life tables. Forecasts of cause-specific mortality are also used to generate forecasts of years of life lost (YLLs, section 2.2.4). Third, forecasts of incidence and prevalence (section 2.3) are obtained using a linear mixed effects regression model predicting mortality-to-incidence (MI) ratios or mortality-to-prevalence (MP) ratios, which are then applied to forecasts of cause specific mortality. For causes of disease burden which are non-fatal only (no deaths are observed), a

linear mixed effects model on prevalence is used to forecast prevalence directly. Forecasts of years lived with disability (YLDs, YLDs section) are then computed from average disability weights from GBD 2019 and forecasts of prevalence, and forecasts of healthy life expectancy are computed from forecasts of YLDs and reference life tables. Disability-adjusted life years (DALYs, section 2.3.6) are calculated as the sum of the YLLs and YLDs. Fourth, we compute population forecasts applying forecasts of mortality, fertility, migration, and sex ratio at birth to the GBD 2021 starting population (section 2.4). Finally, healthy life expectancy (HALE, section 2.5.2) is computed from forecasts of YLDs and life expectancy. Uncertainty in inputs and estimated model parameters are propagated by combining draw-level data from GBD 2021 with draws from the forecast-generating model incorporating, when feasible, parameter draws from estimated sampling or posterior distributions. In addition to a reference forecast, our framework allows us to produce alternative scenarios of disease burden, reflecting the potential impact of policies that modify drivers of health.

Many core methods used to forecast the independent drivers, risk factors, mortality, and demography indicators are described in detail in previous publications on forecasting by IHME's Future Health Scenarios Team and have been referenced as needed to provide additional detail.[2,3]

Appendix Figure A. Flowchart of the forecasting modelling framework.

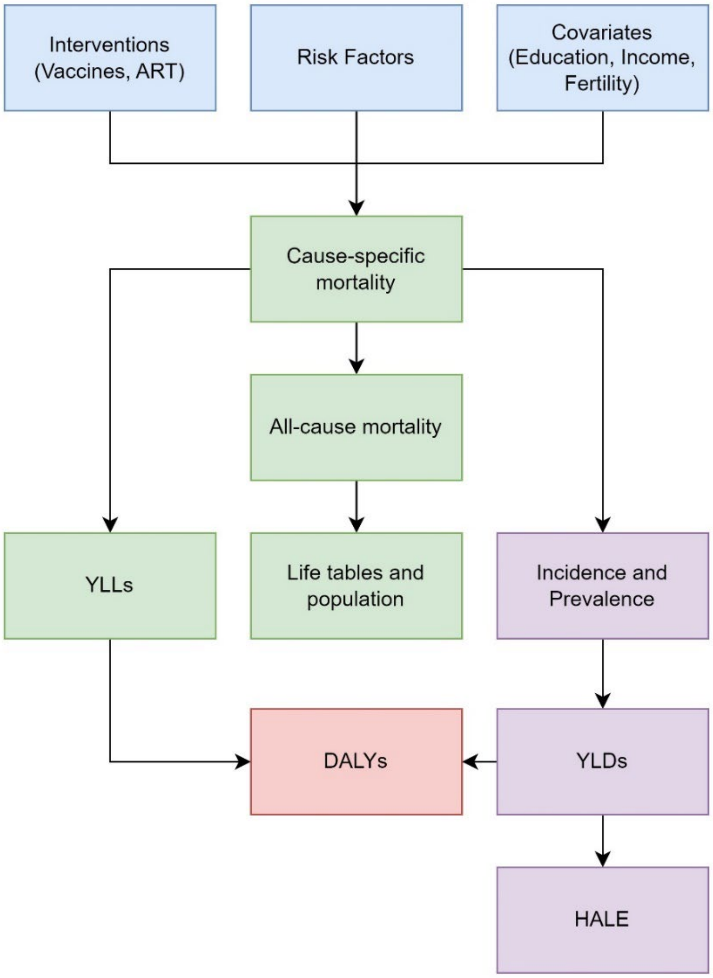

The following causes were modelled separately from the three component mortality model: HIV, COVID-19, forces of nature, conflict and terrorism, and executions and police conflict. These models are described in additional detail in the Causes modelled outside of the 3-component framework section.

Section 2.1: Forecasting Independent Drivers

Independent drivers of health include risk factors incorporated in the GBD 2019 comparative risk assessment in addition to sociodemographic determinants of health (met need for contraception, lag-distributed income, education, fertility) and additional interventions (such as vaccine coverage for select infectious diseases and anti-retroviral therapy for HIV).

Section 2.1.1: Met need for contraception[4]

We forecasted contraceptive met need by age and locations using an ensemble model. Met need was defined as the proportion of women—from among those aged 25–29 who are fertile and sexually active, who report not wanting children or more children or wanting to delay having a child—who are using or whose sexual partner is using a method of modern contraception. The ensemble model was comprised of

six annualised rate of change (ARC) sub-models with varying recency-weighting parameters (the higher the weight, the more weight given to recent years).

We calculated the age-standardised and location-specific annual change of the logit-transformed met need values. To account for the effect of noisy data, we replaced annual changes outside the 2.5th and 97.5th percentiles with those corresponding percentile-values. The weight of each sub-model was determined by running out-of-sample predictive validity, training each sub-model on data from 1990-2009 and validated based on 2010-2019 GBD estimates. We measured each child model's performance using root mean-squared error (RMSE) based on which we determined sampling weights of each child model. Then we produced the sub-model forecasts based on the 1990-2019 training dataset with 500 draws for each sub-model and sampled the draws according to the RMSE in the training dataset to obtain the final ensemble forecasts.

Section 2.1.2: Education

Educational attainment was forecasted using the methodology described in Foreman et al, 2018[2] with an added assumption that educational attainment (up to a maximum of 18 years of education) does not change after age 25 as in Vollset et al, 2020.[3] After age 25, we held forecasted education constant within each location- and sex-specific birth cohort (all individuals born in a certain year). This prevented implausible within-cohort changes in education during older age and was more congruent with our cohort-specific modelling approach for fertility forecasting (section 5), for which education was a key input.

Briefly, for age groups with a starting interval of 25 years or below, we computed age-, sex-, and location-specific annualised rates of change (AROCs) by a recency-weighted average of annual differences in logit space after scaling mean years of education (based on GBD 2019 estimates[9]) by 18 years. The recency-weighting parameters were chosen using cross-validation, where to reduce the potential for overfitting, we selected the parameter producing the smallest root-mean square error at least 5% greater than the minimum. These AROCs were applied to GBD 2019 draws to produce forecast draws, denoted $\widehat{EDU}_{lastd}$, of mean years of education for location l, age $a \leq 25$, sex s, future years $t = 2020, \ldots, 2100$, and draw d. For age groups with interval starts $a > 25$, the forecasted value was set to the previous value on the cohort trajectory, which is lagged in time by the age-group interval (5 years) due to the relationship

$$cohort\ birth = time - age. \tag{1}$$

Specifically, for age groups indexed by the interval start $a = 30, 35, \ldots, 95$ this is given by

$$\widehat{EDU}_{lastd} = \begin{cases} EDU_{l(a-5)s(t-5)d}, & t \leq 2019 + 5 \\ \widehat{EDU}_{l(a-5)s(t-5)d}, & t > 2019 + 5 \end{cases} \tag{2}$$

where $EDU_{l(a-5)s(t-5)d}$ and $\widehat{EDU}_{l(a-5)s(t-5)d}$ denote draws of past GBD and future forecasts, respectively.

In addition to modelling the impacts of the COVID-19 pandemic on GDP, IHME estimated the effects of disruptions in schooling on educational attainment. IHME collected daily school closure data from the Oxford COVID-19 Government Response Tracker, government mandate primary documents, and local and international news sources.[10] Closure data was split into primary and secondary education. The definition of a closure was "no in-person classroom activities for over 66% of students", though in most

cases a closure meant that all schools were closed. Daily closures were summed to annual counts and divided by 365 days to make proportions.

IHME extracted UNESCO data[11] to map primary and secondary closure data to specific ages in each country. GBD education data was split from five-year age groups to single-year age groups by linear interpolation prior to the application of the closure data.

Shocks were applied over the course of the cohort since school closures may affect a cohort over more than one year. School closure proportions were scaled to the amount of education students would have gained without shocks by multiplying by the one-year absolute change in education before the shocks were applied.

To account for online education, IHME extracted country-specific fixed broadband subscriptions per 100 people from the International Telecommunication Union (ITU),[12] and converted the statistic to per capita broadband subscriptions. IHME assumed that students can recover a maximum of half of their lost schooling through online education. Thus, the broadband data were scaled to be between zero and 0.5. The broadband data were then scaled again to the country-specific school closure proportions themselves. These values were added back to the closure proportions before they were applied to the education data as shocks.

To create long-term effects, a cumulative sum was applied to the scaled proportions over the full time series of the cohort. The cumulative values were then subtracted over the course of the whole cohort to generate education data with prolonged education disruptions. Data were subsequently converted back to period space, and the mean was taken over the single-year age groups to recreate the standard five-year age groups used for modelling.

Mean educational attainment over a cohort was defined as,

$$\bar{A} = \begin{pmatrix} a_1 \\ . \\ . \\ . \\ a_N \end{pmatrix} \tag{3}$$

Where α is the level of education attained by the cohort by a specific year.

Defining ρ_k as the fraction of education lost from group k, we define the cumulative shock value as,

$$c_i = \sum_{k=1}^{i} p_k \, , for\ i = 1, 2, \ldots, N \tag{4}$$

Thus,

$$\bar{C} = \begin{pmatrix} c_1 \\ . \\ . \\ . \\ c_N \end{pmatrix} \tag{5}$$

By subtraction, COVID-adjusted cohort educational attainment, $\bar{E}$, is:

$$\bar{E} = \bar{A} - \bar{C} \tag{6}$$

Section 2.1.3: Lag-distributed Income[5]

Lag distributed income (LDI) per capita, which is a moving average transformation of gross domestic product (GDP) per capita and is one of three components of SDI, was used in forecasting cause-specific mortality. LDI was computed by conducting a natural log transformation of the weighted average GDP with a 10-year lag.[13]

Data on retrospective GDP per capita was extracted from five leading sources of these estimates (World Bank, International Monetary Fund, United Nations, Penn World Tables, and Maddison) and used methods described by James and colleagues[14] to generate a single series of GDP per capita using Gaussian processes. The resulting series spans 204 countries from 1950 to 2023 and includes uncertainty bounds based on concordance or missingness of the input data.

GDP estimates in the short-term drew on predictions from several data sources which estimated the economic impacts of COVID-19 from 2021 to 2026, as well as the economic effects of the emerging conflict between Russia and Ukraine from 2022 to 2023.

GDP per working age adult was then calculated as follows:

$$GDP_{working\ population} = GDP_{total\ population} \times \frac{Working\ population\ (20\text{–}64\ years\ old)}{Total\ population} \tag{7}$$

Long-term GDP forecasts were created using GDP per worker, as compared to GDP per capita, because this improved out-of-sample predictive validity. Ensemble modelling techniques were implemented to generate 1000 projections from a broad set of models, and uncertainty was propagated across estimates. Models included indicators associated with ARIMA modelling, data recency weights, and a term for convergence to global growth rates. Model selection was determined by country-year-specific out-of-sample validation, as well as exclusion criteria such as statistical significance testing of estimated parameters, parameter estimates aligning with pre-determined causal priors, and observed growth rate from retrospective estimates. Projections from 2024 to 2100 were generated based on retrospective data through 2019.

Section 2.1.4: Fertility[4]

We produced forecasts of fertility using the updated modelling fertility framework[4] which differs from the published methods in Vollset et al, 2020.[3]

The crux of the model is forecasting a cumulative cohort fertility quantity, CCF50, out to the 2100 cohort, followed by unfolding it into age-specific fertility rates (ASFR). CCF50 is defined as the average number of children born to an individual female from an observed birth cohort (indexed by year of birth) if she lived to the end of her reproductive lifespan (from age 15 through 49).

The past CCF50 for birth cohorts from 1945 to 1972 were used to forecast CCF50 for birth cohorts from 1973 to 2100. In the updated methods, we utilized not only female education and proportion of met need for contraception estimates, but also under-5 mortality and urbanicity estimates as covariates in the CCF50 sub-models. We forecasted CCF50 using three sub-models (with 2, 3 and 4 covariates) to generate an ensemble model forecast where all three sub-models were equally weighted.

From forecasted CCF50, we then derived future age-specific fertility rates (ASFR) forecasts for years 2022 to 2100 using a combination of linear mixed effect model, spline interpolation, and $ARIMA_{(1,0,0)}$ on

residuals to estimate the age pattern of fertility for each cohort. Once the 15-49 ASFR values are obtained, we infer the 10-14 and 50-54 values based on their ratios to the rest of the age pattern during the last observed year (2021).

Forecasts for ASFR were used as a direct input to the populations forecasting model (section 2.4) and in forecasting future SDI, which is an input to cause-specific mortality (section 2.2) and migration models.

The detailed description of fertility forecasting methods can be found in GBD 2021 Fertility and Forecasting Collaborators.[4]

Section 2.1.5: Vaccine Coverage[2]

We produced vaccine coverage forecasts for 6 vaccines. These vaccines fell into two categories, those that had been introduced in all forecast locations by 2019 (diphtheria-tetanus-pertussis dose 3 [DTP3] and measles dose 1 [MCV1]), and those that had not been introduced in all forecast locations by 2019 (complete rotavirus vaccination [RotaC], pneumococcal conjugate vaccine dose 3 [PCV3], Haemophilus influenzae type B [Hib3], and measles dose 2 [MCV2]).

For DTP3 and MCV1, coverage for the reference scenario was assumed to follow a simple linear model in logit space using SDI as the covariate, represented by

$$logit(coverage) = \alpha + \beta * SDI \tag{8}$$

For vaccines that have more recently been introduced into global immunization programs – PCV3, RotaC, MCV2, and Hib3 – we modelled the relationship between coverage of these vaccines and either DTP3 or MCV1, in order to better capture expected relationships over time as these newer vaccines are introduced and scaled up. PCV3, RotaC, and Hib3 are generally given on similar schedules to DTP3; for these vaccines, we therefore modelled the ratio of their coverage to that of DTP3. We assumed that coverage of each of these vaccines would not exceed DTP3 coverage, given the comparatively broad and longstanding availability of DTP vaccines within global immunization programs and historical observations of these coverage relationships. For MCV2, we modelled coverage as a ratio to that of MCV1, given that receipt of at least one dose is a prerequisite for receipt of two or more doses.

For these newer vaccines, reference forecasts and scenarios were produced differently by location depending on whether a country had introduced the vaccine by the beginning of the forecasting period. For countries that had introduced a vaccine, scale-up curves of coverage were modelled as a function of healthcare access and quality using Spatio-Temporal Gaussian Process Regression (ST-GPR). For locations that had not yet introduced a vaccine, a Weibull regression analysis was performed using SDI, 2023 GAVI eligibility status (binary), and the corresponding vaccine coverage for DTP3 (or MCV1 for MCV2 modelling) as covariates to simulate introduction dates. The Weibull distribution assumes that the initial vaccine introduction into global immunization programs will be followed by a spike of introductions in many countries, then by a longer tail of introductions in remaining countries. Under this distribution, the likelihood of a vaccine being introduced in a country is assumed to increase over time, but to never reach precisely 100%. Country-specific introduction dates were then simulated using the parameterized Weibull distribution, where the reference scenario introduction date was assumed to be the median of the 1000 simulated introduction dates. Curves scaling up the coverage relative to the corresponding simple vaccine were produced for each simulated introduction year with a linear regression that uses income, education, and the corresponding simple vaccine coverage itself as covariates. The result of this process is a forecast both of introduction and subsequent scale-up, expressed as a ratio

relative to either DTP3 or MCV1. Last, the scale-up ratios produced in the preceding steps were then multiplied by DTP3 coverage (in the case of RotaC, PCV3, and Hib3) or by MCV1 coverage (in the case of MCV2) to produce final coverage estimates for these newer vaccines.

Section 2.1.5.1 COVID-19 disruptions in vaccine coverage

IHME estimated the short-term (2020–2021) effects in vaccination coverage due to the COVID-19 pandemic via administrative data on vaccine doses. To estimate disruptions in vaccine coverage, IHME used administrative vaccine coverage data collected through the 2022 Joint Reporting Form.[15] First, we assembled a "shock-free" time series of administrative vaccine coverage data, omitting country-year-vaccine data points for which countries reported stockouts or for which other known service delivery disruptions made sudden decreases in vaccine coverage plausible. In this step, we omitted all data points from 2020 and 2021 for all countries due to the COVID pandemic. Second, we then fit spatiotemporal Gaussian process regression (ST-GPR) models to this "shock-free" administrative time series, producing estimates of expected administrative coverage in the absence of disruptions. Third, we compared the reported administrative coverage to these expectations to estimate the magnitude of disruption implied by the administrative data for each country, vaccine, and year. Last, we used these estimated disruptions in administrative coverage to generate as covariates in our final ST-GPR coverage models, which were fit to survey data and bias-adjusted administrative data. If administrative data were missing in 2020 or 2021, we imputed disruptions using vaccine- and year-specific distributions of observed disruptions in countries with available administrative data, propagating uncertainty throughout this imputation process. This approach allowed us to leverage the magnitude of coverage disruptions implied by administrative data, while still adjusting for bias in this data.

Before using vaccine coverage as an input to the risk factor scalar computation (Section 2.1.6.9) for estimating cause-specific mortality, we used a rolling mean function to smooth both past and future values to mimic the lagged impacts of reduced vaccination coverage on subsequent changes to mortality rates. The value of year X was replaced by the mean of years [X-4, X]. For example, the smoothed 2020 values would be the mean of the values for years 2016 – 2020. In cases where the vaccine had not yet been introduced for all 5 years, the mean would only use post-introduction years. Continuing the example above, if the vaccine had only been introduced to a location in 2018, the smoothed value would be the mean of 2018–2020. These smoothed values were used only as inputs to the PAFs and scalars pipeline, such that distinct disruptions to vaccination coverage such as those observed during the COVID-19 pandemic were incorporated into the cause-specific mortality forecasts with lagged effects from reduced vaccination.

Section 2.1.6: Computing SEVs, PAFs and scalars

We modelled the future trajectories of risk factors via their summary exposure values (SEVs), which in turn generated population attributable fractions (PAFs) that were used to obtain cause-specific scalars for our mortality forecasts. As this is the primary conduit through which our scenarios are produced, we added several improvements over the risk factors pipeline of Vollset et al.[3] Firstly, we used the ensemble modelling approach instead of the weighted ARC approach for forecasting SEVs (section 2.1.6.1). Additionally, we accounted for mediation in the computation of mediator SEVs. This allowed us to model the direct or indirect contribution of a risk to mortality without incurring over-counting, such that changes in distal risk factors (leaf nodes) such as diets and smoking can be correctly reflected in mediators such as systolic blood pressure, LDL cholesterol, plasma glucose levels, etc., to ultimately lead to accurate estimation of PAFs (section 2.1.6.2). Appendix figure B provides the overview of our pipeline computing SEVs, PAFs and scalars.

Appendix Figure B. Overview of computing scalars.

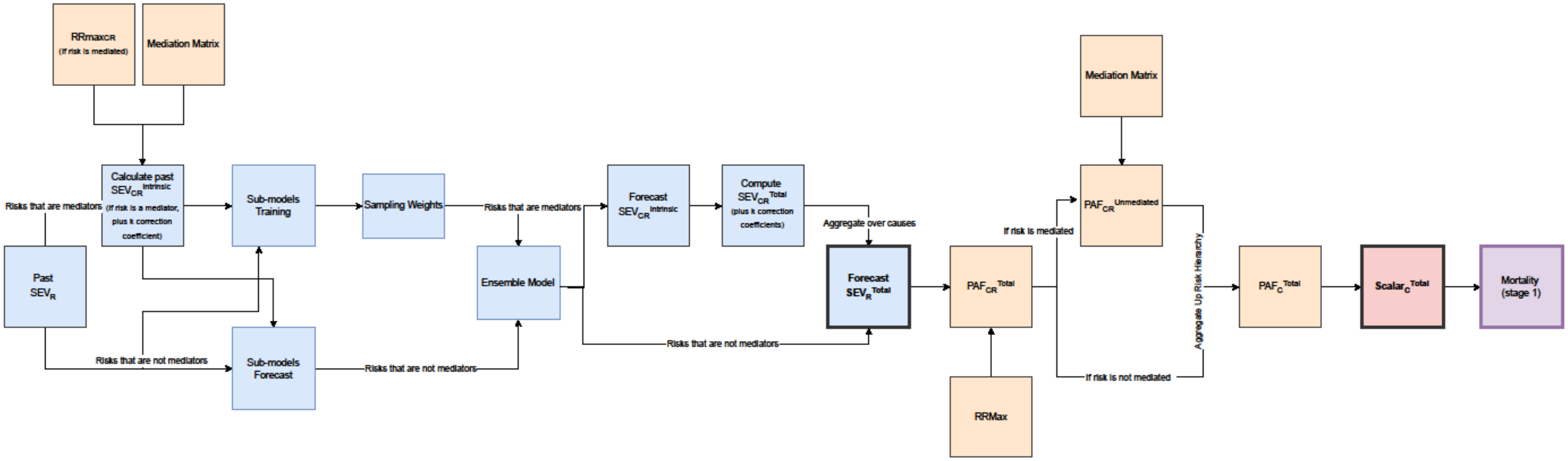

Section 2.1.6.1: Risk Exposure (Summary Exposure Values)

To forecast SEVs, we used a generalized ensemble modelling approach (GenEM) which utilized 12 different sub-models (or child models). For the child models, we employ two main modelling approaches: ARCs and a two-stage spline model based on Meta-Regression Bayesian Regularized Trimmed Tool (MR-BRT).[16] Each of these models had 6 different recency-weighting parameters[2] ranging from 0 to 2.5 (the higher the weight, the more weight is given to recent years[2]).

For the ARC child models, we calculated the age-standardized, sex-specific, and location-specific annual change of the logit-transformed SEV values. To account for the effect of noisy data, we replaced annual changes outside the 2.5th and 97.5th percentiles with those corresponding percentile-values. The two-stage MR-BRT child models used the first stage to fit age-standardized, sex-specific logit of the SEV on SDI:

$$logit(SEV_{c,s,t}) = \beta_0 + \beta_1 spline(SDI_{c,t}) + \varepsilon_{c,s,t} \,, \tag{9}$$

where $logit(SEV_{c,s,t})$ is the logit of the age-standardized SEV in country c, sex s and year t, β_0 is an intercept, β_1 is a coefficient matrix, $spline$ is the spline with five knots placed evenly across the distribution of SDI data and it assumes both right and left linear tails, and $\varepsilon_{c,s,t}$ is the residual. This is then followed by the second stage, where the logit of the residuals from the first stage was linearly modelled on time (year):

$$logit(\varepsilon_{c,s,t}) = year_t + \lambda + \Psi_{c,s,t} \,, \tag{10}$$

where λ is a fixed intercept value, and $\Psi_{c,s,t}$ is an error term.

The weight of each sub-model was defined by running out-of-sample predictive validity experiments. We trained each sub-model based on data from 1990-2009 and validated each sub-model based on 2010-2019 data. We measured each child model's performance using RMSE based on which we determined sampling weights of each child model.

We then produced the sub-model forecasts based on 1990-2019 training dataset (500 draws in each sub-model). For each ARC child models, we used the calculated annual change with corresponding recency-weighting parameter to produce 2020-2050 SEV forecasts. For the MR-BRT child models, we used forecast SDI values (500 draws) in addition to the recency weights to obtain forecasting values of SEVs based on the model fit.

We then obtained the final ensemble forecasts (500 draws) based on draws from the child models using the sampling weights from the out-of-sample experiments (appendix figure C).

Appendix Figure C. Ensemble modelling framework.

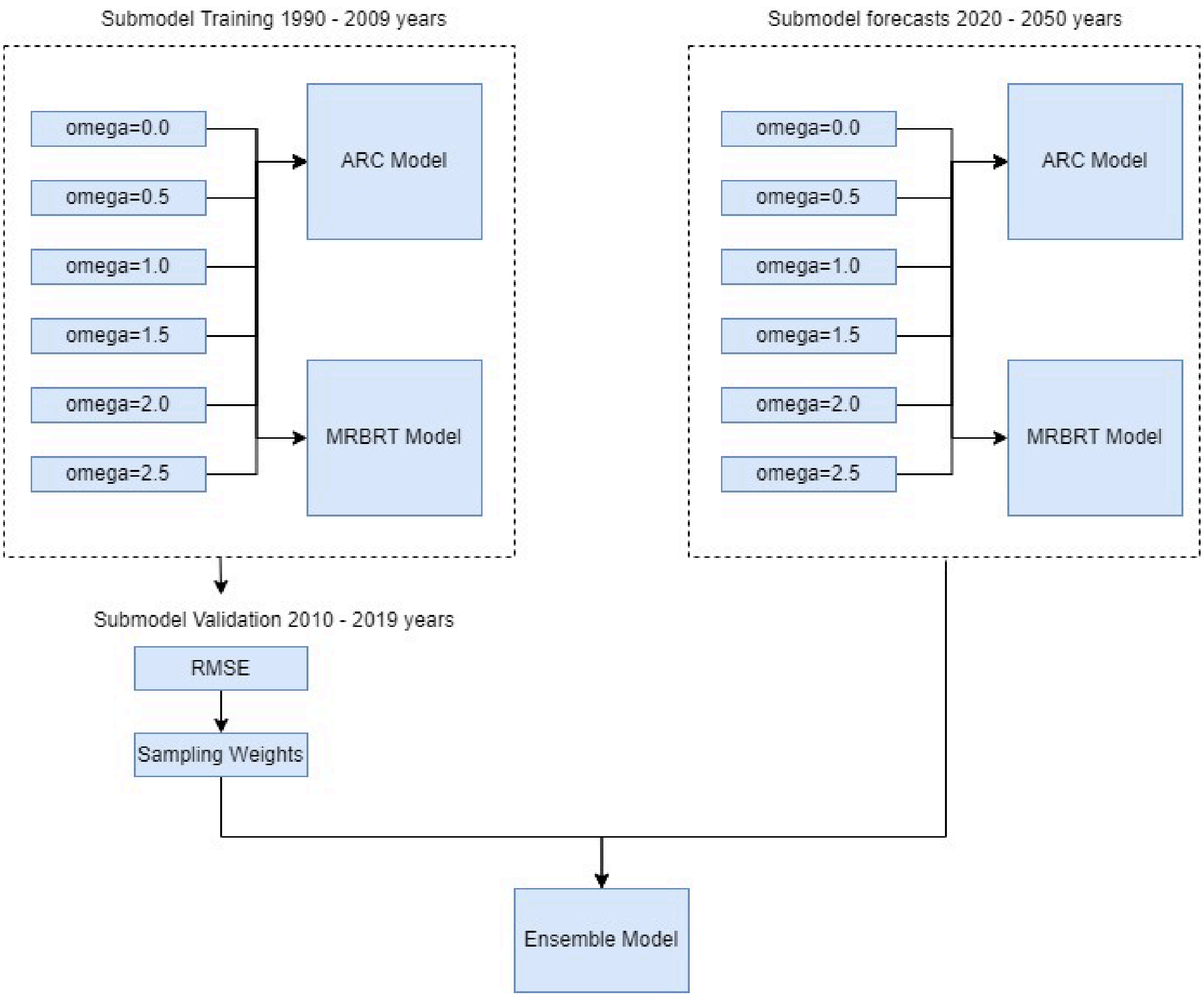

Section 2.1.6.2: Risk Factors with Mediation

A risk factor's summary exposure value (SEV) is a relative-risk weighted prevalence of risk exposure.[6] This risk contribution to mortality can be either direct (without mediation) or indirect (through a mediator risk).

We investigated mediation amongst risk factors for an outcome c by starting with the definition of PAF for a single risk r as

$$PAF_{cr} = \frac{P[c] - P[c \mid \bar{r}]}{P[c]}, \tag{11}$$

where $P[c]$ is the probability of outcome c in the population and $\bar{r}$ denotes the risk at the theoretical minimal risk exposure level (TMREL). Dropping the c subscript and implicitly indexing on r, one can extend the definition to a group of n risks

$$PAF_{1-n} = \frac{P[c] - P[c \mid \overline{r_{1-n}}]}{P[c]}, \tag{12}$$

where $\overline{r_{1-n}}$ indicates TMREL for all n risks. If the risks are independent[12], this can be decomposed into

$$PAF_{1-n} = 1 - \prod_{i=1}^{n}(1 - PAF_i), \tag{13}$$

or

$$1 - PAF_{1-n} = \prod_{i=1}^{n}(1 - PAF_i). \tag{14}$$

This formula embodies a directed acyclic graph (DAG) shown in appendix figure D, where the convergence of the distal risks onto the outcome is modelled by a product of their individual $(1 - PAF)$s.

Appendix Figure D. Causal DAG where each risk factor contributes directly to the outcome, for $n = 3$.

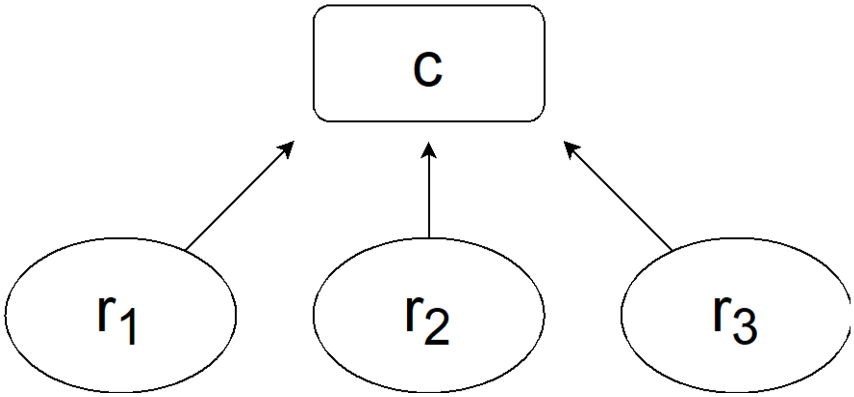

Oftentimes the distal risks not only provide direct contributions to the outcome, but also contribute indirectly via mediators. A schematic of such flow with one mediator is illustrated in appendix figure E, where the bold solid arrow from mediator risk m to the outcome c contains three components: the *intrinsic* contribution from m and the two indirect contributions from the distal risks r_i. We model such flow (illustrated by the solid arrows in appendix figure E) by rearranging the previous formula into

$$1 - PAF_m = (1 - PAF_m^I)\prod_{i=1}^{n}(1 - PAF_i^M), \tag{15}$$

where PAF_m is the total PAF from mediator m to the outcome, PAF_m^I captures the intrinsic contribution from m to c, and each PAF_i^M represents the effect from risk i that is mediated by m.

Appendix Figure E. Causal DAG where distal risks contribute both directly (broken lines) and indirectly (thin solid lines) via a mediator. The mediator's contribution summarizes three components: its intrinsic contribution, plus the two indirect distal contributions.

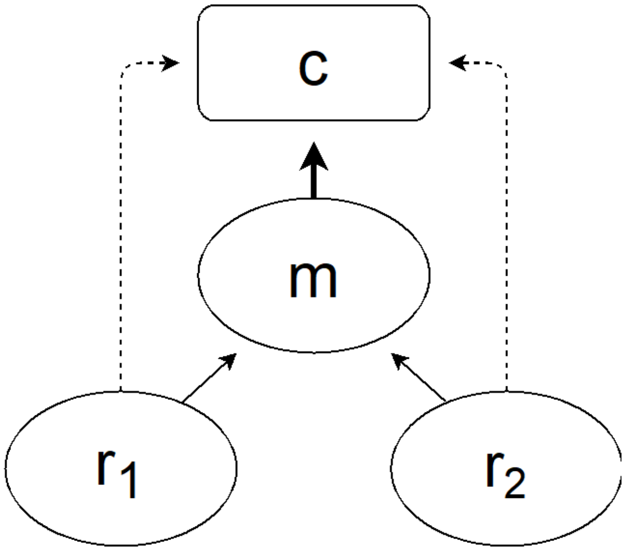

For computational convenience, we substitute an alternative[3] definition of PAF_i via SEV_i and maximum relative risk RR_i^{max}

$$PAF_i = 1 - \frac{1}{SEV_i\,(RR_i^{max} - 1) + 1} \tag{16}$$

into Eq. 15 to arrive at

$$(RR_m^{max} - 1)\,SEV_m + 1 = [(RR_m^{max} - 1)\,SEV_m^I + 1] \prod_{i=1}^{n} [\left(RR_i^{M,max} - 1\right) SEV_i + 1], \tag{17}$$

where

$$RR_i^{M,max} = (RR_i^{max} - 1)\,M_{im} + 1, \tag{18}$$

with M_{im} being the mediation factor of risk i through mediator m on the outcome. Please refer to Foreman *et al.*[2] for details on how Eq. 16 is executed in practice.

We interpret SEV_m^I as the *intrinsic* contribution of mediator m the outcome, without any contribution from the distal risks. Past values of SEV_m^I are computed via Eq. 17, then forecasted along with the distal risks, and then re-packaged together via Eq. 17 to arrive at the future total SEV_m of the mediators.

Due to the uncertainty in upstream quantities (M_{im}, $RR_{i,max}$, and SEV_i), one would occasionally observe $SEV_m^I < 0$ or $SEV_m > 1$ draw values from Eq. 17. They invariably occur due to too much contribution from the distal risks. Our approach to dealing with these wild draws starts with the definition of a "pinch factor"[2] k via a modified version of Eq. 17:

$$(RR_m^{max} - 1)\,SEV_m + 1 = [(RR_m^{max} - 1)\,SEV_m^I + 1] \prod_{i=1}^{n} [\,k\left(RR_i^{M,max} - 1\right) SEV_i + 1] \tag{19}$$

where k limits the distal flows to yield either $SEV_m^I = 0$ or $SEV_m = 1$. The value of k can be computed via standard Newton's method, further discussed in the following section. Note that $k = 1$ where the original SEV_m^I is within [0, 1].

Section 2.1.6.3: Computing mediator SEVs

The SEV pipeline begins with computing past SEV_m^I using Eq. 19 for every cause-mediator pair. There are a couple of computational constraints here. First, because the GBD does not store cause-risk specific past SEVs, the risk-only past SEV is used in Eq. 19 to compute the cause-risk intrinsic SEV. Second, due to the possibility of out-of-bound draws, a pinch factor k is inserted to ensure that all past $SEV_m^I < 0$ values are set to 0, as previously mentioned. The computation of k begins by defining $SEV_m^{I\,\prime}$ as the SEV_m^I where all negative values are set to 0. Then from Eq. 19 one may define

$$\frac{(RR_m^{max} - 1)\, SEV_m + 1}{(RR_m^{max} - 1)\, SEV_m^{I\,\prime} + 1} = \prod_{i=1}^{n} \left[k \left(RR_i^{M,max} - 1 \right) SEV_i + 1 \right] \tag{20}$$

where everything is known except k. Eq. 21 is of the form

$$\mathrm{b} = \prod_{i=1}^{n} (\mathrm{a_i}\, k + 1) \tag{21}$$

and can be solved by taking the natural logarithm of both sides and define the monotonic function

$$f(k) = ln(b) + \sum_{i=1}^{n} ln(a_i\, k + 1) \tag{22}$$

where k can then be solved using Newton's Method.

Equations 20-22 are employed again when one computes the future total SEV of a cause-mediator pair. This is because the aggregation on the right-hand side of Eq. 17 could lead to a final value of SEV_m that is greater than 1. Hence a new set of k values are computed for all future years. For self-consistency reasons, we require that last past year to set the upper bound of what future k values can be. In other words, for every trajectory (draw), we identify future k values that exceed their counterpart in the last past year and reset them to the last past year's k. Eqs. 20-22 ensure that all of our mediator SEV values lie within the [0, 1] bounds.

Once the future cause-mediator SEVs are computed, we calculate the global DALY-weighted average over the causes to obtain the risk-only SEV.

Appendix Figure F. A table representation of the relationships between distals, mediators, and causes, where each table is separated by mediator. Causes are shown across the x-axis and distals are shown across the y-axis. Gray squares represent a distal-mediator-cause combination with a mediation factor of 1 and green squares represent any mediation factor not equal to 1.

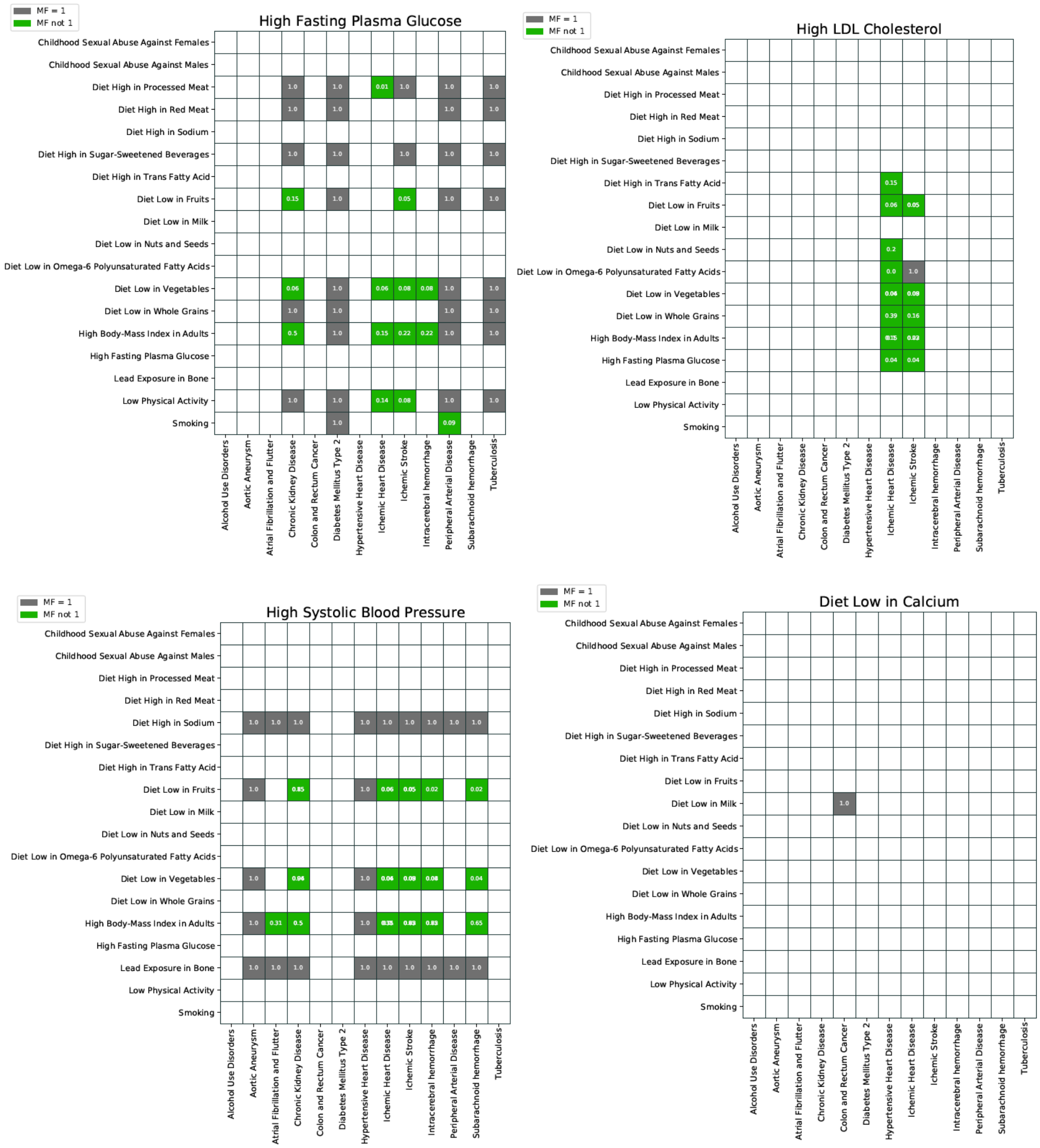

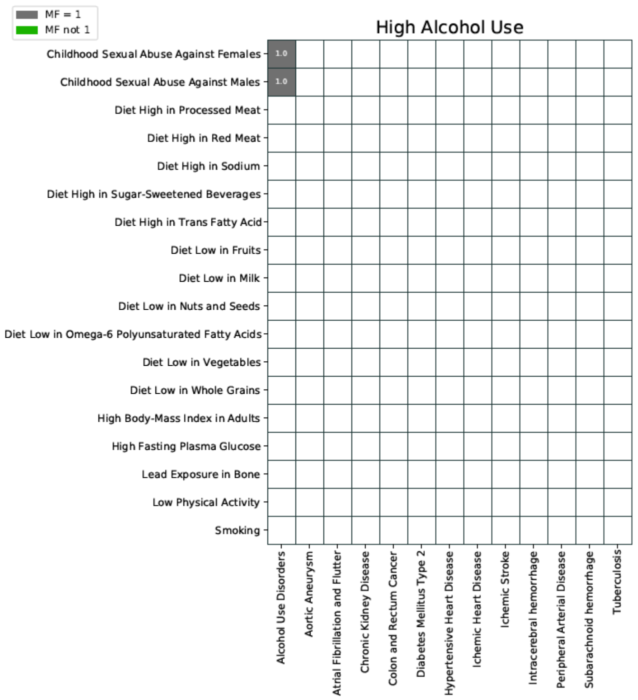

Section 2.1.6.4: Forecasting Direct Smoking Exposure

While most risk factor SEVs are forecast as described in the above sections, the complexity inherent in smoking exposure meant that forecasting smoking SEVs required a modified approach. Smoking SEVs computed within the GBD are composite measures of current, former, and never smoking prevalence, distributions of cigarettes smoked per day and pack-years smoked among current smokers, distributions of years since smoking cessation among former smokers, as well as the relative risks of smoking and risk reduction as a function of years since cessation for each of 32 fatal causes. To allow us to frame our reference and custom policy scenarios in policy-relevant units, we chose to forecast smoking prevalence rather than the smoking SEV. We then calculated future smoking cause-specific population attributable fractions (PAFs), from which we computed future SEVs.

To forecast smoking prevalence in the reference scenario, we first obtained current smoking and former smoking prevalence estimates for every location, sex, 5-year age group, and year from 1990 to 2019 from GBD 2021. We used the ensemble modelling framework described in section 2.1.6.1 to forecast each of these measures from 2020 to 2050. Importantly, the ensemble models we used consisted only of the six ARC child models. In the Improved Behavioural and Metabolic Risks scenario, we set smoking prevalence to zero for birth cohorts ages 0 to 19 in 2023. For older cohorts, we linearly reduced current smoking prevalence starting in 2023 to 0% by 2050.

Similarly, we obtained GBD 2021 estimates of the distributions of cigarette-equivalents smoked per day and pack-years among current smokers, and distributions of years since quitting among former smokers from 2019 to 2022. We assumed that the age-specific distributions of cigarette-equivalents smoked per day and pack-years among current smokers remained constant between 2022 and 2050. In the reference scenario, we assumed the 2022 age-specific distribution of years since quitting among former smokers

remained constant in the future. In the Improved Behavioural and Metabolic Risks scenario, we shifted the 2022 distribution of years since quitting for former smokers who quit in 2022 or earlier forward with every future year. We also created uniform distributions to model the years since quitting for former smokers who quit after 2022.

We also obtained cause-specific relative risk estimates for both current and former smokers from GBD 2021. To account for the effects of different mortality rates between current, former, and never smokers, we estimated the all-cause relative risks of mortality by smoking status. We computed exposure-weighted relative risks by location, age, sex, and cause in 2022 and aggregated these cause-specific relative risks across all causes to generate an all-cause relative risk of mortality. Finally, we computed the mortality rate among never smokers and used each of the mortality rates to adjust our prevalence estimates in every future year.

Using these inputs, we computed cause specific PAFs for 2019 through 2021 and at five-year intervals between 2022 and 2052, using the formula below, adapted from the GBD study (Eq. 23).

$$PAF = \frac{p(n) + p(f) \int \exp(x) * rr(x) + p(f') \int \exp(z) * rr(x) + p(c) \int \exp(y) * rr(y) - 1}{p(n) + p(f) \int \exp(x) * rr(x) + p(f') \int \exp(z) * rr(x) + p(c) \int \exp(y) * rr(y)} \quad (23)$$

Where $p(n)$ is the prevalence of never smokers, $p(f)$ is the prevalence of former smokers who quit in 2022 or earlier, $p(f')$ is the prevalence of former smokers who quit in 2023 or later, $exp(x)$ is a distribution of years since quitting among former smokers who quit in 2022 or earlier, $exp(z)$ is a uniform distribution of years since quitting among former smokers who quit in 2023 or later, $rr(x)$ is the relative risk for years since quitting, $p(c)$ is the prevalence of current smokers, $exp(y)$ is a distribution of cigarettes per smoker per day or pack-years, and $rr(y)$ is the relative risk for cigarettes per smoker per day or pack-years. These PAFs were then interpolated to obtain a full time series from 2019 to 2050 and used to compute all-cause SEVs using Eq. 24 below.

$$SEV = \frac{\frac{PAF_c}{1-PAF_c}}{RR_{max}-1} \quad (24)$$

where c is a cause. The causes for which we forecast smoking-attributable burden include all those estimated for the GBD 2019 study.[6]

Section 2.1.6.5: PAF Mediation and Aggregation

For cause of death c, Eq. 15 captures all contributions through the mediator m to its all-risk PAF, summarizing both the intrinsic effects of m as well as the mediated footprints of the distal risks. The dashed lines in Figure E, therefore, entail the *unmediated* influences of the distal risks to c. To model this indirect contribution, we start with the definition,

$$RR_j^{U,max} = 1 + \left(RR_j^{max} - 1\right) \prod_{m=1}^{M} \left(1 - MF_{jm}\right) \quad (25)$$

as the unmediated RR^{max} between distal risk j and cause c. The product term loops over all M mediators this distal risk contributes to.

We may then replace RR^{max} in Eq. 16 with Eq. 24 and define the unmediated PAF between distal risk j and cause c as

$$PAF_j^U = 1 - \frac{1}{SEV_j\left(RR_j^{U,max} - 1\right) + 1} \tag{26}$$

The all-risk PAF in Figure E is then modelled as $1 - (1 - PAF_m)\left(1 - PAF_{r_1}^U\right)\left(1 - PAF_{r_2}^U\right)$, with PAF_m being the only total PAF since only risk m does not have any indirect path to the outcome. As such, PAF_m accounts for the thick solid line in Figure E, which captures both the intrinsic contribution of m and the indirect effects of the two distal risks, while the broken lines denote the direct effects from the distal risks to the outcome and are signified by the unmediated PAFs.

When there are multiple risks along a given path, only the terminal mediator, which is the one closest to the outcome, contributes totally. All the other risks, including the in-between mediators, have their impacts mediated via Eqs. 24 and 25. One may henceforth generalize our model for the all-risk PAF as

$$1 - \mathrm{PAF}_{1-n} = \prod_{m=1}^{p}(1 - PAF_m)\prod_{\mathrm{i}=1}^{\mathrm{n-p}}\left(1 - \mathrm{PAF}_i^{\mathrm{U}}\right). \tag{27}$$

where m loops over the top-most (terminal) mediators and i loops over the distal risks and the lower-level mediators.

Section 2.1.6.6: Directly Modelled PAFs

Some of the PAF terms on the right-hand side of Eq. 26 are exceptions to our canonical pipeline due to custom GBD modelling for either the SEV or RR_{max}. We directly modelled these detailed cause-risk pairs via our ensemble model to complete the set of PAFs needed to perform the aggregation specified in Eq. 26. A list of directly modelled PAFs is given in appendix table A.

Appendix Table A. Directly modelled PAFs

Cause	Risk(s)
**Non-rotaviral enteritis**	Ambient particular matter pollution
**Rotaviral enteritis**	Ambient particular matter pollution
**Encephalitis**	Ambient particular matter pollution
**H influenzae type B meningitis**	Ambient particular matter pollution
**Non-pneumococcal non-H influenzae type B meningitis**	Ambient particular matter pollution
**Pneumococcal meningitis**	Ambient particular matter pollution
**Neonatal encephalopathy due to birth asphyxia and trauma**	Ambient particular matter pollution
**Haemolytic disease and other neonatal jaundice**	Ambient particular matter pollution
**Other neonatal disorders**<br>**Neonatal preterm birth**	Ambient particular matter pollution
**Neonatal sepsis and other neonatal infections**	Ambient particular matter pollution
**Otitis media**	Ambient particular matter pollution
**Sudden infant death syndrome**	Ambient particular matter pollution
**Upper respiratory infections**	Ambient particular matter pollution
**Non-venomous animal contact**	Occupational injuries, Low bone mineral Density, Smoking
**Venomous animal contact**	Occupational injuries

**Drowning**	Occupational injuries
**Falls**	Occupational injuries, Low bone mineral Density, Smoking
**Fire, heat, and hot substances**	Occupational injuries
**Unintentional firearm injuries**	Occupational injuries
**Other exposure to mechanical forces**	Occupational injuries, , Low bone mineral Density, Smoking
**Other unintentional injuries**	Occupational injuries
**Poisoning by carbon monoxide**	Occupational injuries
**Poisoning by other means**	Occupational injuries
**Other transport injuries**	Occupational injuries, Low bone mineral Density, Smoking
**Motorcyclist road injuries**	Occupational injuries, Low bone mineral Density, Smoking
**Motor vehicle road injuries**	Occupational injuries, Low bone mineral Density, Smoking
**Other road injuries**	Occupational injuries, Low bone mineral Density, Smoking
**Cyclist road injuries**	Occupational injuries, Low bone mineral Density, Smoking
**Pedestrian road injuries**	Occupational injuries, Low bone mineral Density, Smoking
**Pulmonary aspiration and foreign body in airway**	Occupational injuries
**Foreign body in other body part**	Occupational injuries
**Physical violence by firearm**	Intimate partner violence
**Physical violence by sharp object**	Intimate partner violence
**Physical violence by other means**	Intimate partner violence, Low bone mineral Density, Smoking
**Mesothelioma**	Occupational exposure to asbestos
**Liver cancer due to hepatitis B**	Injected drug use
**Liver cancer due to hepatitis C**	Injected drug use
**Cirrhosis and other chronic liver diseases due to hepatitis B**	Injected drug use
**Cirrhosis and other chronic liver diseases due to hepatitis C**	Injected drug use
**Acute hepatitis B**	Injected drug use
**Acute hepatitis C**	Injected drug use

Section 2.1.6.7: Non-optimal temperature PAFs[6]

Incorporation of the direct effects of non-optimal ambient temperature (heat and cold) are now incorporated as risk factors for forecasts of cause-specific mortality. Estimation of future population attributable fractions (PAFs) for non-optimal temperature leverage GBD 2021 estimation methods and forecasts of temperature from the CMIP-6 Shared Socioeconomic Pathways. These PAFs are then incorporated into the calculation of the all-risk PAF and scalar, described in section 2.1.6.5. Details for the methodology are described below.

Input data and modelling strategy for temperature

Case definition

The exposure of non-optimal temperature is defined as the same day exposure to ambient temperature that is either warmer or colder than the temperature associated with the minimum mortality risk. Specifically, we define the theoretical minimum risk exposure level (TMREL) for temperature as the temperature that is associated with the lowest overall mortality attributable to the risk, in a given location and temperature zone. Given varying exposure-response curves for different mean annual temperature zones, as well as spatially and temporally varying cause compositions, we estimate TMRELs by temperature zone and location and are not using a globally uniform TMREL. High temperature (heat) exposure is defined as exposure to temperatures warmer than this TMREL and low temperature (cold) is defined as temperatures colder than this TMREL.

Exposure

CMIP6

We estimated future temperature using Coupled Model Intercomparison Project Phase 6 (CMIP6) climate projections downloaded from the Copernicus Climate Change Service (C3S) Climate Data Store (CDS). CMIP6 data include a variety of experiments which each correspond to a combination of a Representative Concentration Pathway (RCP) and shared socio-economic pathways (SSP). We used temperature forecasts from three experiments: SSP1-RCP1.9, SSP2-RCP4.5, and SSP5-RCP 8.5, corresponding to optimistic, likely, and pessimistic trajectories. For each experiment we sampled from available models to incorporate between-model heterogeneity into our uncertainty. To ensure consistency between all CMIP6 models, and between GBD and FHS, we resampled CMIP 6 temperature rasters to match the resolution of the ERA5 temperature rasters used in the GBD Study, and implemented a baseline shift by day-of-year and pixel to ensure temporal continuity between the historical estimates from ERA5 and the forecasted estimates from CMIP6.

Population data

Population data for calculating population-weighted location means were derived from WorldPop, which is an open source project initiated in 2013.[17] Multi-temporal, globally consistent, high-resolution human population data at 1 km x 1 km resolution can be downloaded from http://www.worldpop.org.uk/ for 2000, 2005, 2010, 2015, and 2020. For the purpose of our work, we interpolated in-between the 5-year estimation bins to obtain annual data. Further, we extrapolated until 1990 by using the 2000-2005 growth rate for back-casting.

Exposure-response modelling for temperature

ERA5 data

We estimated exposure response curves by modelling the association between ambient temperature and cause-specific mortality. While we used forecasted temperature estimates to forecast future temperature-attributable burden, risk curve modelling requires temperature data that correspond to the dates and locations of deaths in our mortality datasets. We, therefore used historical exposure estimates from these ERA5 reanalysis dataset from the European Centre for Medium-Range Weather Forecasts (ECMWF) for risk curve modelling. ECMWF produced ERA5 estimates using their Integrated Forecast System (IFS). Hourly values of surface temperature are available for a spatial resolution of 0.25°x0.25°. Uncertainty estimates for these temperature values, i.e., the ensemble spread (standard deviation) is available for every 3 hours (00:00, 03:00, 06:00, 09:00, 12:00, 15:00, 18:00, 21:00) for a spatial resolution of 0.5°x0.5°. At the time of analysis, data were available from 1979 to June 2019.[18,19] We calculated daily averages of temperature and spread for each pixel and then assigned an uncertainty value to each daily temperature value. Based on the spread we derived 1,000 draws of each daily temperature pixel.

Mortality data

Deaths at the individual-level that included information regarding the cause (ie, ICD code), date, and the location at the second administrative level (admin2) or finer were collected from the GBD cause-of-death (CoD) database for vital registration data sources. We adapted the GBD standard procedure for garbage code redistribution to redistribute daily mortality data rather than annual data and mapped ICD causes to GBD causes for level 3. In total, we analysed 58.9 million deaths from eight different countries and 15,197 administrative units (table B). For Brazil, the data covers a period from 1999 to 2016 for 5,570 municipalities and 19.9 million deaths. For Chile, the data covers the period from 1990 to 1996 and 2009 to 2011 for 15 regions and 2.46 million deaths. For Colombia, the data covers a period from 2001 to 2005 for 1,125 municipalities and 0.95 million deaths. For Guatemala, the data covers a period from 2009 to 2016 for 333 municipalities and 0.49 million deaths. For Mexico, the data covers a period from 1996 to 2015 for 2,438 municipalities and 9.88 million deaths. For New Zealand, the data covers a period from 1988 to 2014 for 20 district health boards and 0.76 million deaths. For the United States, the data covers a period from 1980 to 1988 for 3,124 municipalities and 18.3 million deaths. For China, the data covers the year 2016 for 2,556 counties and 6.1 million deaths.

To estimate cause-specific mortality, based on average daily temperature and temperature zone (defined by mean annual temperature), we used a robust meta-regression framework MR-BRT (Bayesian, regularised, trimmed) tool.[20] We specified the functional dependence of outcome vs. average daily temperature and temperature zone as a 2-dimensional surface through a spline interface.

The use of trimming in a vast array of inference and machine learning problems is standard.[21–23] The use of high-dimensional splines has been proposed before,[24] but the methods used for estimation go beyond prior work, and we explain them below.

The functional relationship between any outcome y and input variables (t1, t2) models y as a linear combination of 2d spline basis elements. Each spline basis element is a product of individual basis elements for 1D splines for t1 and t2. Therefore, the inference problem looks for a combination of simple curvilinear 2D elements that fit the data while preserving smoothness across element boundaries. The MR-BRT tool also allows prior information to influence the shape of the spline, particularly in areas with sparse data.

For the purpose of modelling the relationship between mortality and mean annual and daily temperature we imposed monotonicity in the direction of daily temperature. For all J-shaped curves that depicted an increase in mortality above and below a threshold, we forced the curve to monotonically decrease at the lower end of the temperature distribution and to monotonically increase at the upper end. For all external causes that displayed a monotonic increase over the entire temperature range, we imposed monotonicity only in the direction of warmer temperatures. We placed 2 knots of degree 3 in the direction of mean annual temperature when fitting the surface. In the direction of daily mean temperature, we placed 3 knots of degree 3 for J-shaped causes and 2 knots of degree 1 for external causes that monotonically increase over temperature range. Figure G shows an example of a relative risk (RR) surface along daily and annual mean temperature for lower respiratory infection.

We estimated uncertainty using a two-step approach. First, we derived the uncertainty of the mean surface from the measurement error using the fit-retrofit error. Second, we added uncertainty from the random effects by sampling it separately from the cold and warm side.

Figure G: Log relative risk of death from lower respiratory infection along mean annual temperature (mean temp cat) and daily mean temperature (daily temp cat). The red dotted line depicts minimum mortality temperature along mean annual temperature zones. Green and blue lines depict isopleths, ie, lines of equal log RR of mortality.

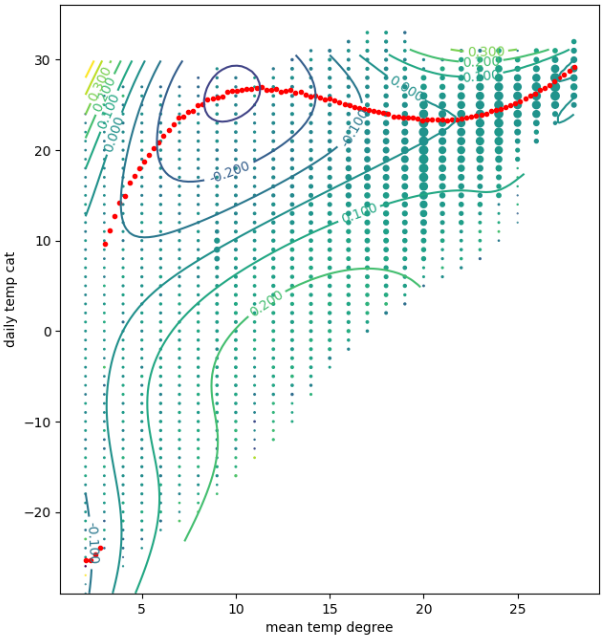

Table B: Data inputs for relative risks for non-optimal temperature

Input data	Relative risk
Source count (total)	112
Number of countries with data	8

Cause selection for temperature–outcome pairs

We excluded all causes with fewer than 100,000 deaths as well as causes of death that did not represent a particular entity but rather a summary category (eg, other cardiovascular diseases). Further, dementia and protein energy malnutrition were not considered in this analysis due to inconsistencies in data classification. The remaining causes were selected based on significance. For this, for each cause and each mean temperature zone we determined the widest range of consecutive daily temperatures with statistically significant relative risks, expressed as a percentage of the full range of daily temperatures in that mean temperature zone. Figure H gives an example of the temperature-mortality relationship for three selected slices (mean annual temperature of 6 °C, 17 °C and 21 °C). Significant areas along the exposure-response curves are marked in grey. We included all causes where at least 30% of zones had a consecutive significance range that spanned at least 5% of the full range of daily temperatures. Twelve causes met these criteria and were included as outcomes associated with non-optimal temperature: ischaemic heart disease, stroke, hypertensive heart disease, diabetes, chronic kidney disease, lower respiratory infection, chronic obstructive pulmonary disease, homicide, suicide, mechanical injuries, transport-related injuries, and drowning. See Burkhart et al.[25] for more methodological details.

Figure H: Selected exposure-response curves for the relationship between daily mean temperature and log RR of lower respiratory infection mortality for mean annual temperature categories of 6 °C, 17 °C and 21 °C. Temperatures where associations are significant are displayed in grey.

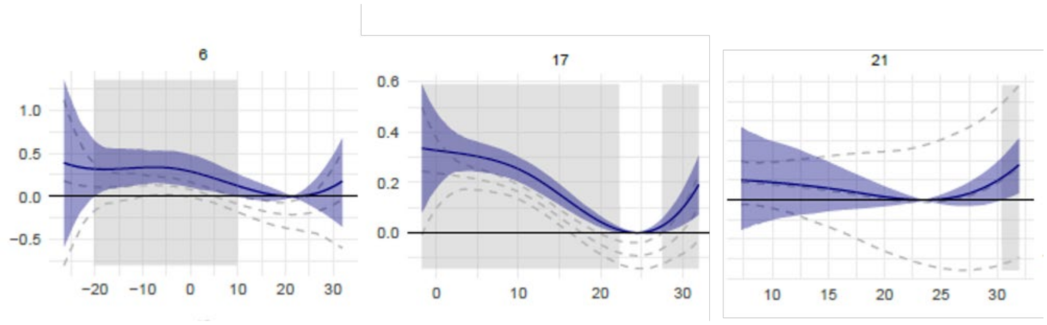

Theoretical minimum risk exposure level (TMREL) of temperature

For the purpose of this analysis, the TMREL was defined as the temperature associated with the lowest mortality for all included causes. We calculated a death-weighted average of the cause-specific exposure-response curves with the minimum of this average curve being the TMREL. This was done for each temperature zone and each of the 990 GBD most detailed locations using cause of death estimates produced for the GBD 2019 study. As climate zones or mean annual temperature can vary within a location, we calculated the TMREL for every mean annual temperature, assuming a consistent cause composition within a location.

Population attributable fractions for temperature

The population attributable fraction (PAF) was calculated for each temperature pixel and each day of the year (i.e., pixel-day). Subsequently, we population-weighted each pixel using the fraction of the population living in a given pixel relative to the GBD location. Depending on whether the daily mean temperature was below or above the TMREL, the effect was assigned to either low or high temperature. Daily population-weighted high and low temperature PAFs were then aggregated for the location and the year. Temperature effects can be either harmful or protective depending on whether the RR is above or below 1. For harmful temperature effects, ie, effects with a RR above 1, we used the following equation to derive PAFs: PAF=(RR-1)/RR; For temperature effects exhibiting a protective effect the equation was adapted by implementing the reverse RR: PAF=-((1/RR)-1)/(1/RR). The PAF associated with non-optimal temperature exposure is an aggregate of heat and cold effects in each location and year. We estimated the temperature attributable burden as the product of the total burden for that cause and the corresponding PAF for each GBD location, year, age group, and sex.

Section 2.1.6.8: Ambient particulate matter pollution PAFs

Incorporation of the effects of ambient particulate matter pollution utilize improved methods to that leverage the GBD 2021 estimation methods for air pollution attributable burden, alongside carbon emissions forecasts based on the CMIP-6 Shared Socioeconomic Pathway projections. These PAFs are then incorporated into the calculation of the all-risk PAF and scalar, described in section 2.1.6.5. Details for the methodology are described below.

Exposure to ambient particulate matter pollution

Definition

Exposure to ambient particulate matter pollution is defined as the population-weighted annual average mass concentration of particles with an aerodynamic diameter less than 2.5 micrometers ($PM_{2.5}$) in a cubic meter of air. This measurement is reported in $\mu g/m^3$.

Baseline Input data

Ambient air pollution exposure estimates use input data from multiple sources (Table C). These include satellite observations of aerosols in the atmosphere, ground monitor measurements, chemical transport model simulations, population estimates, and land-use data.

Table C: Data inputs for exposure for ambient particulate matter pollution

Input data	Exposure
Site-years (total)	5442
Number of countries with data	204
Number of GBD regions with data (out of 21 regions)	21
Number of GBD super-regions with data (out of 7 super-regions)	7

Theoretical minimum-risk exposure level for ambient particulate matter pollution

The theoretical minimum-risk exposure level (TMREL) was assigned a uniform distribution with lower/upper bounds given by the average of the minimum and 5th percentiles of outdoor air pollution cohort studies exposure distributions conducted in North America, with the assumption that current evidence was insufficient to precisely characterise the shape of the concentration-response function below the 5th percentile of the exposure distributions. The TMREL was defined as a uniform distribution rather than a fixed value in order to represent the uncertainty regarding the level at which the scientific evidence was consistent with adverse effects of exposure. The specific outdoor air pollution cohort studies selected for this averaging were based on the criteria that their 5th percentiles were less than that of the American Cancer Society Cancer Prevention II (CPSII) cohort's 5th percentile of 8.2 based on Turner et al. (2016).[26] This criterion was selected because GBD 2010 used the minimum, 5.8, and 5th percentile solely from the CPS II cohort. The resulting lower/upper bounds of the distribution for GBD 2020 were 2.4 and 5.9.

MR-BRT risk splines for ambient particulate matter pollution

To estimate relative risk curves for each of the $PM_{2.5}$ outcomes, we used the MR-BRT meta-regression tool to fit splines on the input datasets of OAP and HAP studies. We used the following functional form, where X and X_{CF} represent the range of exposure characterised by the effect size:

$$log\left(\frac{MRBRT(X)}{MRBRT(X_{CF})}\right) \sim \log\,(Published\ Effect\ Size) \qquad (28)$$

Several key updates were made to the model fitting methods. For each risk–outcome pair, model settings and priors were tested when fitting the MR-BRT splines. The final models used third-order splines with three interior knots and a constraint on the right-most segment forcing the fit to be linear rather than cubic. Splines were also constrained to be concave and monotonically increasing, the most biologically plausible shape for the $PM_{2.5}$ risk curve. We used an ensemble approach to generate final spline predictions, in which 50 different models were run with randomly placed knots, then weighted and combined based on a measure of fit that penalises excessive changes in the maximum derivative of the curve. Knots were free to be placed across the entire domain of the input exposure data. To prevent over-

fitting, on the non-linear segments, we implemented a Gaussian prior on the third derivative of mean 0 and variance 1e-4. On the linear segment, a stronger prior of mean 0 and variance 1e-6 was used to ensure that the risk curves do not continue to increase beyond the range of the data. 10% of all observations were trimmed during model fitting, in accordance with GBD protocol across risk factor teams.

Forecasted particulate matter concentrations

Global gridded air particulate matter forecasts were provided by Turnock et. al, 2020 for SSP1 1.9 and SSP4 4.5 with means and standard deviations across three different CMIP6 models (GFDL-ESM4, MRI-ESM2-0, and UKESM1-0-LL). For each scenario, draws were generated using a gamma probability distribution function, $p(x)$, in which with given standard deviation as σ, mean as u, and the Gamma function as $\Gamma(x)$ with x as the 1000 samples generated:

$$o = \frac{\sigma}{3} \tag{29}$$

$$v = o^2 \tag{30}$$

$$k = \frac{u^2}{2} \tag{31}$$

$$b = \frac{u}{v} \tag{32}$$

$$\theta = 1/b \tag{33}$$

$$p(x) = x^{k-1} * \frac{e^{-x/\theta}}{\theta^{k} * \Gamma(k)} \tag{34}$$

With generated samples, the forecasts are bilinearly re-gridded from 1 by 1 degree resolution to line up with GBD data on a 0.1 by 0.1-degree global grid. Past data for GBD air pollution differ from the Turnock et. al. data. For a smooth transition from GBD historical to forecasted PM, the difference for each cell between 2021 and each future year was calculated and applied to GBD 2021 estimates.

Population data

We obtained global population estimates from WorldPop[17] at 0.04167° by 0.04167° resolution for the year 2023. Aggregation to each 0.1° × 0.1° grid cell was accomplished by rescaling the raster grid to 0.1° resolution. These data were used in the PAF approach below.

Proportional PAF approach for ambient particulate matter pollution

Let Exp_{OAP} be the ambient $PM_{2.5}$ exposure level and Exp_{HAP} be the excess exposure for those who use solid fuel for cooking. Let P_{HAP} be the proportion of the population using solid fuel for cooking. We calculated PAFs at each 0.1° × 0.1° grid cell. We assumed that the distribution of those using solid fuel for cooking (HAP) was equivalent across all grid cells of the GBD location.

For the proportion of the population not exposed to HAP the relative risk was:

$$RR_{OAP} = MRBRT(z = Exp_{OAP})/MRBRT(z = TMREL), \tag{35}$$

And for those exposed to HAP, the relative risk was

$$RR_{HAP} = MRBRT(z = Exp_{OAP} + Exp_{HAP})/MRBRT(z = TMREL). \tag{36}$$

We then calculate a population-level RR and PAF for all particulate matter exposure:

$$RR_{PM} = RR_{OAP}(1 - P_{HAP}) + RR_{HAP}P_{HAP} \quad (37)$$

$$PAF_{PM} = \frac{RR_{PM}-1}{RR_{PM}} \quad (38)$$

We population weight the grid-cell level particulate matter PAFs to get a country-level PAF, and finally, we split this PAF based on the average exposure to each OAP and HAP:

$$PAF_{OAP} = \frac{Exp_{OAP}}{Exp_{OAP}+P_{HAP}*Exp_{HAP}} PAF_{PM}, \text{ and } PAF_{HAP} = \frac{P_{HAP}*Exp_{HAP}}{Exp_{OAP}+P_{HAP}*Exp_{HAP}} PAF_{PM}. \quad (40)$$

With this strategy, $PAF_{PM} = PAF_{HAP} + PAF_{OAP}$, and no burden is counted twice.

Section 2.1.6.9: Scalars

Once all risk PAFs are computed via Eq. 40, the outcome-specific scalars are,

$$S_c = \frac{1}{1 - PAF_c} \quad (41)$$

where $PAF_c = PAF_{1-n}$. This is identical to the formulation in Foreman et al[3].

Section 2.2: Forecasting mortality[2,3]

The study employed a comprehensive approach to model cause-specific mortality, encompassing 280 out of 285 causes and cause groups. This approach builds upon the framework established by Foreman et al, 2018[2] with certain adaptations. We utilized mixed-effect regression, incorporating the Socio Demographic Index (SDI), temporal factors, cause-specific covariates, and a risk factor scalar in separate models for males and females. For the remaining four causes (HIV, COVID-19, executions, and police conflict, as well as exposure to forces of nature, conflict, and terrorism), alternative methodologies were employed (see section 2.2.3). At a higher-level of cause hierarchy, forecasts were derived by aggregating from the most-detailed cause-specific mortality up to all-cause mortality. To capture latent trends not accounted for by the base model, a random walk with drift attenuation model was applied to all-cause mortality, while a random walk without drift model was used for causes in the lower cause hierarchy. Subsequently, forecasted mortality were converted into incidence and prevalence estimates for each of the 284 causes.

Section 2.2.1: Cause-specific mortality models

Our cause-specific model, applied to 280 of 285 causes and cause groups, is defined as

$$m^T = m^U \times S \quad (42)$$

Which we estimate in log as

$$ln(m^T) = ln(m^U) + \ln(S) + \epsilon \quad (43)$$

Where m^T is the total cause-specific mortality. The three components on the right-hand side are:

- The underlying (or risk deleted) mortality[27], m^U, which is formulated as a function of the Social Development Index (SDI), temporal factors, and other cause-specific covariates as deemed necessary.

- A risk factor scalar S that synthesises the collective effects of cause-specific risk factors, leveraging insights from the Global Burden of Disease (GBD) comparative risk assessment. This scalar corresponds to the ratio of total cause-specific mortality to underlying (risk-deleted) cause-specific mortality and facilitates the quantification of risk–outcome associations while accounting for the mediation of risk factors.[27] Additional details for the calculation of this risk factor scalar from population attributable fractions, please see Foreman et al, 2018 (appendix 1section 5).[2]
- Unexplained residual mortality, ϵ, accounting for any mortality not captured by the aforementioned components.

Specifically, the total mortality rate m_{ilast}^{T} for cause i, location l, age group a, and sex s at time t was decomposed in log space into an underlying mortality rate m_{ilast}^{U}, a risk factor scalar $\mathbb{S}_{ilast}$, and residual ϵ_{ilast} as

$$\ln\left(m_{ilast}^{T}\right) = \underbrace{\alpha_{ilas} + \beta_{is}\mathrm{SDI}_{lt} + \theta_{ias}\mathrm{t}}_{\ln\left(m_{ilast}^{U}\right)} + \ln(\mathbb{S}_{ilast}) + \epsilon_{ilast} \tag{44}$$

where for the ith cause and sth sex , $\alpha_{ilas} \sim \mathrm{N}\left(\beta_{\alpha,is}, \tau_{\alpha,is}^{2}\right)$ is a location-age-specific random intercept, β_{is} is a global fixed slope on SDI, and $\theta_{ias} \sim \mathrm{N}\left(\beta_{\theta,is}, \tau_{\theta,is}^{2}\right)$ is an age-specific random slope on the secular time trend. This model incorporates the option to apply Girosi-King type priors[2,28] on the log total cause-specific mortality rate. These priors serve the purpose of smoothing forecasts across age, time, and location by penalizing significant deviations in adjacent age groups, time intervals, and geographic areas. The residuals represent latent trends in total cause-specific mortality not captured that remain unexplained by risk factors, SDI, and global secular trends. Notably, our model diverges from the approach employed in Foreman et al, 2018[2] by omitting the use of a spline on SDI, a modification aimed at enhancing the long-term stability of forecasts. Further elucidation on the forecasting methodology—for the independent drivers used to compute SDI and the risk factor scalar is described in section 2.1.

The process of generating draws of non-latent cause-specific total mortality forecasts at time t proceeds through several stages. Initially, independent drivers (detailed in section 4) utilized for computing the forecasted risk factor scalar $\widehat{\mathbb{S}}_{ilastd}$ and covariate $\widehat{\mathrm{SDI}}_{ltd}$are incorporated, with d indexing the draw. To address the inherent uncertainty in model estimation, draws of the fixed and random effects were generated from a multivariate normal distribution with mean and variance matrix set to the estimates and their estimated joint covariance matrix, respectively, computed using the TMB package for R using the GBD estimates of past mortality and assuming $\epsilon_{ilast} \sim N\left(0, \sigma_{is}^{2}\right)$. Forecast draws of the underlying mortality, $\ln(\widehat{m_{ilastd}^{U}})$, are obtained by evaluating the model at these parameters and SDI forecast draws. The most-detailed cause-specific non-latent mortality forecast draws are obtained by combining draw-specific forecasts of the underlying mortality rate and risk factor scalar. These draws are used to generating mortality forecasts for higher-level causes and all-cause mortality forecasts, as elucidated in the following sections.

Section 2.2.2: Aggregating to all-cause mortality

Total mortality forecasts for upper-level causes and all-cause total mortality were derived by aggregating cause-specific non-latent mortality forecasts, which incorporate risk factors, SDI, and global secular trends. Additionally, forecasted latent trends, as described previously,[2] were incorporated into the forecasting process for each cause-level in the GBD hierarchy. At future times t, the age-, sex-, and location-specific all-cause non-latent mortality, denoted by m_{lastd}^{NL}, was forecasted (estimated) by aggregation of cause-specific estimates and forecasts (after exponentiation out of log space):

$$\widehat{m_{lastd}^{NL}} = \sum_{i=1}^{284} \exp\left[\ln(\widehat{m_{ilastd}^{U}}) + \ln(\mathbb{S}_{ilastd})\right] \quad (45)$$

Section 2.2.2.1: Modelling latent trends in mortality

The latent trends in all-cause mortality, referred to as the unexplained residual mortality at the all-cause level, were modelled using a random walk with attenuated drift. We employed the random walk with drift (RWD) methodology to forecast residuals, computed utilising equation 46. The historical drift was estimated through a linear regression model (equation 47), augmented with a slope attenuation function (equation 48). This refined approach aims to enhance the accuracy of residual forecasts and reduce the influence of latent trends over future time periods by adjusting drift estimation based on evolving slope patterns within the data. Similarly, latent trends for lower-level causes in the GBD cause hierarchy were modelled using a random walk without drift. This strategy aimed to minimize excessive variation for causes at the detailed cause-specific level.

Notably, past-time all-cause residuals were computed in log space (utilizing the mean of the reference scenario draws) as

$$\hat{\epsilon}_{last} = \frac{1}{D}\sum_d\left[\ln(m_{lastd}^{T}) - \ln(\widehat{m_{lastd}^{NL}})\right] \quad (46)$$

for $t = 1990, \dots, 2019$ where m_{lastd}^{T} is the GBD draw of all-cause total mortality for location l, age a, sex s, time t, and draw d. We estimated past time drift using the linear regression model with mean

$$\mathrm{E}(\hat{\epsilon}_{last}) = \gamma_{0,las} + \gamma_{1,las}t, \quad (47)$$

where $\gamma_{0,las}$ is an intercept and $\gamma_{1,las}$ is a slope on time for past times $t = 1990, \dots, 2019$. Beginning at the first forecasted year $t_0 = 2020$, future latent trend forecasts were generated with slope attenuation according to

$$\hat{\epsilon}_{las(t+1)} = \hat{\epsilon}_{last} + \gamma_{1,las}\exp[-0.1(t - t_0)] \quad (48)$$

This approach yielded more credible long-term forecasts compared to the ARIMA blend used by Foreman and colleagues, 2018.[2]

The ultimate forecasts for all-cause log total mortality and total log mortality for lower-level causes in the GBD hierarchy were formulated by incorporating latent trends drawn from the fitted random walk model, denoted by $\hat{\epsilon}_{lastd}$. These trends were integrated into the non-latent forecasts derived from our base model, with an adjustment made to accommodate the uncertainty associated with the input GBD estimates. Notably, estimation of both the non-latent and latent trends made use of past-time GBD estimates, which are means across the corresponding GBD draw distributions. To incorporate uncertainty in the supplied GBD estimates and ensure continuity between past and forecast means, we intercept-shifted the non-latent all-cause mortality forecast draws (in log space) by the draw-level residual in 2019 non-latent all-cause mortality. Specifically, the shifts were defined by

$$\hat{\zeta}_{lasd} = \ln(m_{las(2017)d}^{T}) - \ln(\widehat{m_{las(2017)d}^{T}}), \quad (49)$$

where $m_{las(2017)d}^{T}$ is the dth GBD draw of location-, age-, and sex-specific all-cause total mortality in 2019. In log space, the all-cause total mortality forecasts were computed as

$$\ln(\widehat{m_{lastd}^{T}}) = \ln(\widehat{m_{lastd}^{NL}}) + \hat{\epsilon}_{lastd} + \hat{\zeta}_{lasd}. \quad (50)$$

Since the log of the mean of a log normally distributed random variable is biased away from the mean ofthe corresponding normal distribution by an additive factor of the half the variance of the normal distribution, we applied the following bias correction in exponentiation to obtain final all-cause total mortality forecasts in the original mortality rate space:

$$\widehat{m^T_{lastd}} = \exp\left[\ln(\widehat{m^T_{lastd}}) + \frac{\hat{s}^2_{last}}{2}\right]. \quad (51)$$

where $\hat{s}^2_{last}$is the sample variance (across the draws) of $\ln(\widehat{m^T_{lastd}})$.

Section 2.2.3: Causes modelled outside of the 3-component framework

Five causes of death were modelled outside of the three-component model, due to their stochastic historical trends and in order to capture unique transmission dynamics of the HIV epidemic and the known impacts of HIV specific interventions. This follows the GBD cause of death estimation process to model these causes independently.[7]

Section 2.2.3.1: HIV[3]

Forecasting ART Coverage

In the past, ART coverage is projected using the spending on HIV care and treatment and ART price[29]. However, we found that some locations maintained flat coverage at lower levels into the future. We interrogated our ART forecasting methods to determine the reason. We started by assessing the capping system and the strength of the relationship between coverage and GDP/ART dose equivalents. ART dose was calculated using Spend/ART price, and we discovered that the Spending in some locations is zero.

To avoid using spending and to address the problem that ART projections maintain flat in the future, we projected the ART coverage using historical rate of change (ROC). The cap is 0.95 for all locations, CD4 counts, ages, and both sexes. We calculated ROCs using ART coverage from past 5 years by age, sex, and CD4 count. After projection, we found out that in the same age group and sex combination, the future trend of ART coverage by CD4 count differs. We wanted to force the future trend of ART coverage to be the same across CD4 count groups. So, we selected the largest ROC across all CD4 groups in each age-sex combination and apply this ROC to all CD4 groups. By doing so, the projected ART coverage has the same future trend in each age-sex combination.

Forecasting the Transmission rate

We explored various approaches to calculating and forecasting using the transmission rate. A few challenges came up related to differences when incidence is calculated within EPP-ASM compared to Spectrum. For this purpose, transmission rate = incidence hazard / prevalence.

In EPP-ASM, incidence is calculated at the beginning of each time loop, while in Spectrum it is at the end. This affected both the back-calculation of the "true" transmission rate and the forward calculation of incidence with the forecasted transmission rate. In the end, we realized that we were not interested in back calculating the true transmission rate, but the transmission rate that would reproduce new infections in Spectrum, the simulation we are currently using for forecasting. We then switched the input incidence for the transmission rate calculation from Spectrum/EPPASM output incidence to final GBD incidence.

Using the transmission rate in Spectrum required us to augment the code to calculate new infections using the transmission rate, prevalence, susceptible population, and ART coverage. The first year of the transmission rate-based calculation requires an epidemic seed, given that there are zero PLHIV to infect others. The parameter name used in the code is *iota*, and it operates just like incidence hazard (multiplied by the susceptible population). We included back-calculation of *iota* in the transmission rate calculation and set up Spectrum to pull the iota value from that file and use it to kick off the epidemic.

After back calculating the transmission rates and plotting them by region, we observed that the transmission rate generally exhibits a high starting value followed by a logistic decline, with random variation over the past ~15 years. This follows the r-hybrid functional form used to parameterize the time-trend for the transmission rate in EPP-ASM. R-hybrid combines a logistic function with a linear piecewise spline with a random-walk penalty. In general, high-income locations exhibited higher transmission rates (>0.3), and lower-income locations lower transmission rates (<0.3). We chose to use a simple global approach to forecasting after trying various super region-specific approaches and encountering issues of "runaway" epidemics (more to say about this below). We took the global median across all location-year values of the transmission rate in the last ten years (choosing this for its relative stability in contrast to the high variation in earlier periods) to get a global equilibrium transmission rate value to use as a target to converge towards over time.

The choice of a global equilibrium value followed experimentation where we used super-region equilibrium values. In higher income locations, we saw many forecasts that resulted in what we are calling "runaway" epidemics, where the effective reproductive rate is above one even in the presence of very high ART coverage and prevalence grows to greater than ten percent. This seems to partially be a result of the fact that the *entire* population is considered as potentially susceptible in these locations, even though the epidemics are dominated by higher-risk groups like injections drug users, sex workers, and men who have sex with men. Taking into consideration the true size of the susceptible population would be one approach to constraining new infections in the future, but this would represent a substantial change in methodology and would require some difficult calculations of the proportion of the population within in each high-risk group. This approach of estimating the size of high-risk groups and modelling them separately is taken in a few concentrated epidemics in Spectrum, but we are not clear on the quality of the estimation of the population sizes, nor the data available to support estimation. Instead, we can control the transmission rate and ART coverage. Therefore, we took a conservative approach and forecasted global convergence towards a lower global equilibrium and sought to increase ART coverage into the future.

To forecast location-specific trends in the transmission rate, we projected the location-specific trend over the last five years and took a shifting weighted average between that projection and the global equilibrium. We used a logistic weighting scheme that smoothly moved from fully weighting the location-specific projection to fully weighting the global equilibrium over a twenty-year window.

We averaged the location-specific projections with the global equilibrium value only in locations with increasing trend and where the projected transmission rate is larger than the global equilibrium value. If the location-specific projections decrease, even if the location-specific projections are higher than global equilibrium, it won't cause "runaway". And if the increasing location-specific projections are lower than global equilibrium in the twenty-year averaging window, there is no need to drag the projections up to global equilibrium. We cap the projection using the value 1.25 times transmission rate at extension year: 2021.

Projections of HIV incidence, prevalence, and mortality

In order to produce age- and sex-specific estimates of HIV incidence, prevalence, and mortality, we input projected transmission rates along with ART, PMTCT, and Cotrimoxazole coverage, as well as a number of other predicted demographic inputs, into the Spectrum model. Spectrum is a cohort component model originally developed by UNAIDs that we have modified to incorporate CD4-specific probability of treatment in addition to a number of other methods developments made for GBD.[30] Spectrum ages a population over time using demographic parameters while applying HIV incidence, disease progression, treatment coverage, and mortality. Our final results are age-, sex-, location-specific Spectrum outputs through 2100.

Section 2.2.3.2: Disasters, war and terrorism, legal interventions

Cause-specific mortality due to exposure to disasters, conflict and terrorism, as well as executions and police conflict were forecasted based on the methods described in Foreman et al, 2018.[2]

Section 2.2.3.3: COVID-19 and other pandemic-related mortality (OPRM) estimation[7]

To estimate excess mortality, we first developed a database of all-cause mortality by week and month after accounting for reporting lags, anomalies such as heat waves, and under-registration of death. Next, we developed an ensemble model to predict expected deaths in the absence of the COVID-19 pandemic for the years 2020 and 2021. In location and time combinations with data used for these models, excess mortality was estimated as observed mortality minus expected mortality. To estimate excess mortality for location-years without data, we developed a statistical model to directly predict the excess mortality due to COVID-19, using covariates that pertained to both the COVID-19 pandemic and background population-health-related metrics at the population level before SARS-CoV-2 emerged. Uncertainty was propagated through each step of this estimation procedure. Full details of the estimation of excess mortality, COVID-19 deaths, and OPRM are provided in appendix 1 of GBD 2021 Causes of Death Collaborators.[7] For the years 2023 through 2030, we assumed linear reductions in deaths from COVID-19.

Section 2.2.4: YLLs

YLLs were computed by multiplying the number of deaths by the reference life expectancy at the age of death. Forecasts for YLLs were computed from forecasted age-sex-location-specific mortality rates and the reference life table[31].

Section 2.3: Forecasting non-fatal disease burden

Non-fatal disease burden was forecasted via individually modelling 290 causes independently according to the properties of each cause. Briefly, mixed-effect models were used to model prevalence and incidence, either by modelling prevalence directly or by modelling the mortality-incidence (MI) ratio and mortality-prevalence (MP) ratio and using forecasted mortality to convert to incidence and prevalence. Causes modelled via the mortality-incidence ratio are converted to prevalence via a modelled prevalence-incidence (PI) ratio. Once forecasts of prevalence for all causes were obtained, they were converted to YLDs via average disability weights calculated as YLD divided by prevalence in 2019. These were then added to YLLs to obtain DALYs.

Section 2.3.1: MI, MP, PI ratios

Ratios of MI, MP, PI, and YLDs to YLLs are all modelled via:

$$\log\left(R_{a,s,l,y}\right) = \beta_0 + \beta_1 SDI_{l,y} + \pi_{0:a,s,l} + \pi_{1:a,s,l} SDI_{l,y} + \varepsilon_{a,s,l,y} \quad (52)$$

where $R_{a,s,l,y}$ is the age-sex-location-year specific ratio for a given cause, with the covariate $SDI_{l,y}$ being the location-year specific SDI. $\pi_{0:a,s,l}$ is the age-sex-location specific random intercept, $\pi_{1:a,s,l}$ is the age-sex-location specific slope on SDI, and $\varepsilon_{a,s,l,y}$is the residual term. A shift in log space is applied to each age-sex-location combination after prediction to align the value of the modelled value in the last year of GBD estimates with the GBD estimate for that year.

Section 2.3.2: Prevalence-only models

For causes where prevalence was directly modelled due to lack of mortality, the model was:

$$\text{logit}\left(P_{a,s,l,y}\right) = \beta_0 + \beta_1 SDI_{l,y} + \pi_{0:a,s,l} + \varepsilon_{a,s,l,y} \quad (53)$$

where $P_{a,s,l,y}$ is the age-sex-location-year specific prevalence of a given cause, with location-year specific SDI as the covariate for the fixed slope and $\pi_{0:a,s,l}$ as the age-sex-location specific random intercept.

Section 2.3.3: Producing forecasts of incidence and prevalence

After forecasts of the ratios were produced, the MI and MP ratios were converted to incidence and prevalence, respectively, via division of mortality forecasts by the corresponding ratio. These estimates were then converted into the "secondary" metric using the PI ratio. For instance, incidence forecasts multiplied by the PI ratio yields estimates of the prevalence for a given cause.

Section 2.3.4: YLDs

Once all the prevalence estimates were combined from the separate modelling paths (causes modelled primarily via prevalence directly, MP ratio, or MI ratio converted to prevalence via PI ratio), YLDs were produced by a simple multiplication of an average disability weight. The average disability weight was calculated as the division of GBD YLD estimates in 2019 by the prevalence in 2019 for a given cause. This assumed that disability weights for any given cause will remain constant from 2019 into the future, which will likely not be entirely accurate, but it is difficult to predict exactly how disability weights will change with evolving technologies and other societal shifts. A portion of YLDs were estimated using the modelled YLD-YLL ratio, which uses the same model as the rest of the ratios.

Section 2.3.5: COVID-19 non-fatal estimation

COVID-19 non-fatal estimation is split into two large components: estimating the acute sequela of COVID-19, and estimating the post-acute sequela among survivors of COVID-19.[32] First, estimates of daily infections, hospital admissions, ICU admissions, and deaths due to severe acute respiratory syndrome coronavirus 2 (SARS-CoV-2) infection were taken from the COVID-19 model of the Institute for Health Metrics and Evaluation (IHME). Infections were multiplied by the pooled estimate of the proportion of infections without symptoms, and deaths were subtracted from the estimate of symptomatic cases and to get estimates by age, sex, and country of symptomatic survivors of COVID-19 infection. Then, infections were followed through the disease course to obtain surviving cases of mild/moderate non-hospitalised, severe hospitalized, and critical ICU cases of COVID-19 at risk for post-acute symptoms.

Second, post-acute sequela were estimated. The proportions of symptomatic survivors with one or more of three symptom clusters of long COVID (with fatigue, cognitive problems, and shortness of breath as the key symptoms) were extracted from international cohort studies and two US medical record databases. Data from four cohort studies with individual case records available that did not report on excess risk of long COVID symptom clusters in comparison to controls or self-reported health status prior to COVID-19 were adjusted by the ratio of excess to total symptoms from six studies that reported both. Then, the proportions with long COVID symptom clusters by follow-up time since the end of the acute infection were estimated using a Bayesian meta-regression tool, separately for hospitalized and non-hospitalized cases. Subsequently, estimates from studies providing distributions of symptom cluster overlap and severity gradients of cognitive and respiratory problems were pooled. Finally, the global estimates of symptomatic COVID-19 survivors were multiplied by the proportions experiencing one or more of the symptom clusters at three months post infection.

Section 2.3.6: DALYs

DALYs were obtained for a given cause as the sum of YLDs and YLLs. Causes that only have YLLs or YLDs will have DALYs that are equal to the given measure.

Section 2.4: Forecasting population

Our methods for forecasting population, as well as the pipelines generating its requisite upstream inputs (life expectancy, ASFR, and sex ratio at birth), except migration, were directly inherited from Vollset *et al*.[3]

Section 2.4.1: Forecasting migration[3]

Forecasts of migration were adapted from those published in Vollset et al, 2020.[3] Revisions to the modelling approach are described below.

Net migration rates for each country or territory were forecasted as a function of natural population increase (NPI) and mortality resulting from natural disasters, wars, execution and police conflict, and terrorism (eg, events that can be considered cultural and/or economic shocks), utilizing past migration rates from UNPD[33,34] and GBD estimates of mortality.[9] Migration rates were converted to counts and age-sex split using Eurostat migrant population data and balanced across locations to achieve zero-net migration globally.

For country or territory l at year t, we fit the following multiple linear regression model for the migrate rate using least squares estimation with past times $t = 1990, 1991, \ldots, 2020, 2021$:

$$MIGR_l t = \beta_0 + \beta_N NPI_l t + \beta_s SHOCK_l t + \gamma_l + \varepsilon_l t, \tag{54}$$

where $MIGR_{lt}$is the UNPD migration rate estimate (annual net number of migrants per 1000 people); $NPI_{lt}$is the UNPD natural population increase estimate (difference between crude birth and death rates per 1000 people); $SHOCK_{lt}$ is the GBD mortality rate estimate aggregated over all ages and both sexes and summed across the causes natural disasters, war, terrorism, and legal interventions; γ_l is a location-specific random intercept; and ε_{lt} is a residual accounting for variation in location- and time-specific migration unexplained by the model.

We utilised a random walk with attenuated drift model to forecast the residuals. For each location, past-time residuals were computed as $\hat{\varepsilon}_{lt} = MIGR_{lt} - \widehat{MIGR}_{lt}$ and were used to fit the random walk model using the same approach described in section 2.2. Future forecasts of the migration rate, denoted $\widehat{MIGR}_{ltd}$, were generated by evaluating the estimated regression model at the UNPD forecasted estimates for NPI and our mean forecasts of SDI (section 4) and cause-specific mortality for natural disasters, war, terrorism and legal interventions (section 6.1.3) then adding this value to forecast draws of the residuals, $\hat{\varepsilon}_{ltd}$. Finally, to prevent implausibly extreme long-range trends in migration, migration rate forecast draws $\widehat{MIGR}_{ltd}$ were capped between -10 and 10, which approximates the 5th and 95th percentiles of past migration rates. For 21 countries where migration rates were implausibly low, we attenuated migration trends from 2021 to 0 net migration by 2050.

Migration rates were converted to counts by age and sex, and global net migration was balanced to 0 using methods described in Vollset et al.[3]

Section 2.5: Forecasting Life expectancy and healthy life expectancy

Section 2.5.1: Life expectancy

Life tables and life expectancy were calculated as in Vollset et al (2020)[35] with 23 age groups (early neonatal, late neonatal, post neonatal, 1-4 years, 5-9, … to 95 years and older). Older ages were handled as described in supplementary appendix (section 3.2) in Wang et al (2016).

Section 2.5.2: HALE

Healthy Life Expectancy (HALE) was calculated using the Sullivan method.[36] This entails first calculating adjusted person years lived in a given age interval (nLx_{adj}) using the formula:

$$nLx_{adj} = nLx * 100{,}000 * (1 - YLD_{rate}) \quad (55)$$

where YLD_{rate} is the rate of years lived with disability across all causes. Adjusted person-years lived in all this interval and all subsequent age intervals (Tx) is computed as

$$\sum_{a=x}^{max-age} adjusted\_nLa \quad (56)$$

Finally,

$$HALE = \frac{Tx_{adj}}{Ix} \quad (57)$$

where Ix is the number living at the beginning of the age interval of 100,000 born alive.

Section 3: Developing alternative scenarios

We produced DALY forecasts for the reference scenario and four alternative scenarios. The alternative scenarios were based on the full elimination by 2050 for the following risk factors: unsafe water, sanitation, handwashing (WaSH), temperature, and air pollution; behavioural and metabolic non-communicable disease (NCD) risk factors; nutrition risk factors and vaccination coverage (appendix table D). Additionally, we included a scenario that combined scenarios across all the above risk factors. The alternative scenario of full elimination by 2050 was defined as SEV for the corresponding risk factor to be reaching a value of zero by 2050 starting in 2022 with an exception for the smoking component.

To create the alternative smoking scenario, we linearly eliminated current smoking prevalence for each country between 2022 and 2050 for each birth cohort. We also did not allow new smoking initiation for any birth cohort, which resulted in no current or former smokers in birth cohorts that were younger than 10 years of age in 2022. As in the reference scenario, we assumed that the age-specific distributions of cigarettes per day among smokers and pack-years among smokers were constant between 2022 and 2050. Similarly, we maintained the existing distributions of years since quitting for former smokers as of 2022 or earlier, shifting them forward with each future year (i.e. individuals who had quit smoking 10 years prior in 2020 would have quit 20 years prior in 2030). As we decreased the proportion of current smokers in the population, the proportion of former smokers in the population increased. Since the proportion of the population that quit in every year after 2022 was held constant in the scenario, we assumed a uniform distribution of years since quitting for those individuals.

To account for the differences in risk of mortality among current, former, and never smokers, we used the cigarettes per day and pack-years distributions and the dose and cause-specific GBD relative risk curves to compute the cause specific exposure distribution weighted relative risk of mortality for each of the three groups. We then computed the mortality-rate weighted average of these cause-specific relative risks to obtain the all-cause relative risk of mortality for current smokers, former smokers, and never smokers, respectively. In each future year, we used these relative risk estimates to update the prevalence of current, former, and never smokers in the population. We then used these inputs to compute cause-specific population attributable fractions at five-year intervals between 2022 and 2052. We then interpolated to obtain a full time series of population attributable fractions between 2022 and 2050, from which we computed all-cause smoking SEVs.

Appendix Table D. Alternative scenarios description

Scenarios	Components	Targets
Reference	All drivers at reference	NA
	Ambient particulate matter pollution ($PM_{2.5}$)	SSP2-4.5
	Temperature	
Improved Environment	Household air pollution	Full elimination by 2050, linear decrease to 0 starting in 2023
	Drinking water	
	Sanitation	
	Handwashing	
	Ambient particulate matter pollution ($PM_{2.5}$)	SSP1-1.9
	Temperature	
Improved Behavioural and Metabolic Risks	Smoking	Full elimination of smoking prevalence by 2050, linear decrease to 0 starting in 2023; no new smoker initiation after 2022
	Diet	Full elimination by 2050, linear decrease to 0 starting in 2023
	BMI in adults	Full elimination by 2050, linear decrease to 0 starting in 2023
Improved Childhood Nutrition and Vaccination	Child wasting	Full elimination by 2050, linear decrease to 0 starting in 2023
	Child stunting	
	Child underweight	
	Iron deficiency	
	Vitamin A deficiency	
	Sub-optimal breast feeding	
	Vaccine coverage	Linear increase vaccine coverage to 100% in all locations for all 6 modelled vaccines by 2050 starting in 2023. For instances where the reference scenario is more optimistic than this alternative scenario, set the alternative scenario to reference.
Combined	Includes all components of above scenarios	Includes all targets of above scenarios.

Section 4: Uncertainty interval estimation

Uncertainty in data inputs, estimated model parameters, and bias-correction procedures were captured by generating 500 draws at the age-sex-location-year level for each of the measures carried through the many GBD multi-step estimation processes (population, mortality, migration, fertility,[3,37] risk factors,[38] causes of death,[31] and non-fatal estimation.[31] This approach captures uncertainty in each modelling stage and propagates it through the entire estimation process. Point estimates were computed as the mean of 500

draws from the corresponding final (posterior) draw distribution and 95% uncertainty limits (UIs) were computed from the 2.5 and 97.5 percentiles.

Section 5: Model evaluation

To evaluate the predictive validity of our results, we performed a 10-year holdout forecast through all components of the forecasting machinery. We then compared these 10-year forecasts to the actual values for those years in GBD 2019 results to evaluate out-of-sample predictions for mortality and DALY estimates. Specifically, we used the following skill metric[39] for validation period 2010–2019:

$$skill = 1 - \frac{RMSE(Model)}{RMSE(Baseline\ Model)} \quad (58)$$

where Model is the IHME forecasting model and Baseline Model is a simplistic model where value of 2009 year was held constant over 2010–2019. For each model, we calculated squared errors between observed and predicted mean values for each cause/sex/location/year and winsorized the squared errors at 95% level to remove outliers. To calculate RMSE values, we took a square root of an average of the winsorized squared errors across location/year. This skill metric was reported for both DALY and mortality estimates for males and females of all age group (age group id = 22) for level 0, 1, and 2 causes (appendix table E and F). A positive skill metric indicates that a model being evaluated performs better than the baseline model whereas a negative skill suggests the opposite.

Both level 0 and level 1 causes skill values were positive for mortality out-of-sample predictions (appendix table E). Skill value for mortality level 0 cause was 0.44 and 0.49 for male and female, respectively. The highest skill among level 1 causes was for communicable, maternal, neonatal, and nutritional diseases for female (0.75) and the lowest was for injuries among male (0.19). Among level 2 causes, neurological disorders cause had the highest skill value of 0.74 for male and musculoskeletal disorders cause had the lowest skill value of -0.82 for female. There were five Level 2 causes that had negative skills values (for mortality, these included mental disorders and musculoskeletal disorders), these five causes contributed just 11.81% of all deaths combined.

Similarly, both male and female DALY out-of-sample model estimates had a positive skill value for all level 0 and level 1 causes. DALY level 0 cause had skill values of 0.58 and 0.66 for male and female, respectively. Among level 1 causes, communicable, maternal, neonatal, and nutritional diseases had the highest skill value of 0.78 for female and non-communicable diseases and injuries had the lowest skill values of 0.24, respectively, both for male. The highest skill value among level 2 causes was HIV/AIDS and sexually transmitted infections for 0.74 for female and the lowest skill value was -1.93 for skin and subcutaneous diseases for female. Five of 22 Level 2 causes had negative skill values (these included skin and subcutaneous diseases and digestive diseases), but the causes with negative skill values contributed 20.73% of all DALYs.

The major limitation in skill evaluation is the short period (only 10 years) for validation due to the data availability. To address negative skill values, we will revisit the corresponding models with a negative skill and include data with longer range for validation period which will lead potentially to higher skill values.

Appendix Table E. Skill values for all age mortality estimates

Level	Cause name	Male	Female	Percent of total mortality (%)
0	All causes	0.44	0.49	100

Level	Cause name	Male	Female	Percent of total mortality (%)
1	Communicable, maternal, neonatal, and nutritional disorders	0.72	0.75	17.2
	Non-communicable diseases	0.36	0.36	74.24
	Injuries	0.19	0.34	8.56
2	Neglected tropical diseases	0.15	0.20	0.73
	Malaria	0.12	0.20	0.57
	Nutritional deficiencies	0.67	0.61	0.42
	Neoplasms	0.29	-0.05	19.23
	Cardiovascular diseases	0.1	0.08	30.20
	Chronic respiratory diseases	-0.12	-0.18	4.74
	Digestive diseases	-0.15	-0.03	4.86
	Neurological disorders	0.74	0.68	4.06
	Substance use disorders	0.14	0.20	0.56
	Diabetes and kidney diseases	0.44	0.37	5.27
	Other non-communicable diseases	-0.01	-0.21	1.98
	Skin and subcutaneous diseases	0.14	0.19	0.20
	Transport injuries	0.01	0.28	2.60
	Unintentional injuries	0.11	0.30	3.47
	Self-harm and interpersonal violence	0.26	0.37	2.49
	HIV/AIDS and sexually transmitted infections	0.60	0.70	1.57
	Respiratory infections and tuberculosis	0.51	0.41	6.25
	Enteric infections	0.54	0.56	3.07
	Other infectious diseases	0.64	0.63	1.37
	Maternal and neonatal disorders	0.55	0.62	3.80
	Mental disorders	-0.69	-0.47	0
	Musculoskeletal disorders	-0.04	-0.82	0.23

Appendix Table F. Skill values for all age DALY estimates

Level	Cause	Male	Female	Percent of total DALYs (%)
0	All causes	0.58	0.66	100
1	Communicable, maternal, neonatal, and nutritional disorders	0.76	0.78	25.37
	Non-communicable diseases	0.24	0.25	63.96
	Injuries	0.24	0.41	10.67
2	Neglected tropical diseases	0.26	0.32	1.76
	Malaria	0.18	0.25	1.07
	Nutritional deficiencies	0.72	0.57	1.81
	Neoplasms	0.10	-0.19	10.45
	Cardiovascular diseases	0	-0.08	14.2
	Chronic respiratory diseases	-0.26	-0.08	4.26
	Digestive diseases	-0.35	-0.28	3.71
	Neurological disorders	0.31	0.32	4.02
	Substance use disorders	0.01	0.08	1.44
	Diabetes and kidney diseases	0.28	0.11	4.15
	Other non-communicable diseases	-0.04	-0.31	5.86
	skin and subcutaneous diseases	-1.85	-1.93	1.78
	Transport injuries	0.06	0.33	3.46
	Unintentional injuries	0.20	0.25	4.30
	Self-harm and interpersonal violence	0.30	0.44	2.92
	HIV/AIDS and sexually transmitted infections	0.61	0.74	2.03
	Respiratory infections and tuberculosis	0.65	0.62	5.85
	Enteric infections	0.59	0.65	3.92
	Other infectious diseases	0.63	0.62	2.19
	Maternal and neonatal disorders	0.54	0.60	7.82

Level	Cause	Male	Female	Percent of total DALYs (%)
	Mental disorders	-0.20	0.05	5.12
	Musculoskeletal disorders	0.60	0.53	6.27

Section 6: Post-processing of the results to align with GBD 2021

In order to align forecasts with the most recently generated GBD 2021 results, we performed a shift on all burden measures (deaths, incidence, prevalence, YLLs, YLDs, DALYs) to align with GBD 2021 in the year 2021 ($t_0 = 2021$). We shifted prevalence in logit space to cap values between zero and one, while we shifted all other measures in log space. The shift had two main parts: a "reference" shift to align values in 2021 and a "scenario" shift to make the scenarios start diverging from reference in 2021.

For the "reference" shift D_{ref}, we calculated the difference using a blended approach of two methods by calculating both "relative" and "absolute" differences, $D_{relative}$ and $D_{absolute}$, respectively, for each location/sex/age at the draw level:

$$D_{ref} = \alpha * D_{relative} + (1 - \alpha) * D_{absolute}, \tag{59}$$

Where:

$$\alpha = \frac{1}{(1+e^{-k*(A_{t_0}+13.81)})} \tag{60}$$

α is a coefficient determining how much weight should go to each method, $D_{relative} = B_{t_{0,\ldots,}t_n} * \frac{C_{t_0}}{A_{t_0}}$ is a "relative" difference and $D_{absolute} = B_{t_{0,\ldots,}t_n} - (A_{t_0} - C_{t_0})$ is an "absolute" difference. Here, A_{t_0} is the reference scenario forecast in t_0 , $B_{t_{0,\ldots,}t_n}$ is the reference scenario forecasted values between t_0 and t_n, and C_{t_0} is GBD 2021 data in t_0.

We calculated the "relative" difference at the draw level by taking the ratio between the GBD 2021 data in 2021 (C_{t_0}) and the reference scenario forecast in 2021 (A_{t_0}) and then multiplying this ratio by the future forecasted values between t_0 and t_n years ($B_{t_{0,\ldots,}t_n}$). Additionally, we obtained the "absolute" difference at the draw level by first calculating the difference between the reference scenario forecasts in 2021 (A_{t_0}) and the GBD 2021 data in 2021 (C_{t_0}) , and then subtracting this difference from the future draw-level forecasted values between t_0 and t_n years ($B_{t_{0,\ldots,}t_n}$).We calculated α which is a weight given to the "relative" difference, and $(1 - \alpha)$ is a weight given to "absolute" difference. The weight α is given in a form of a logistic function with a value of 0.5 at 13.81 namely a rate of 1 per million, and k value being equal to 0.7. To account for special cases when we have extremely small values for A_{t_0}, we set α value to 0 at draw level for location/age/sex when A_{t_0} for a draw is less than 10^{-6}. Additionally, for 10 causes we also set α value to 0 if $B_{t_{0,\ldots,}t_n}$ at the mean level is higher than the maximum value of mean values of the past GBD 2021 data. The 10 causes include measles, malaria, African trypanosomiasis, gonococcal infection, cystic echinococcosis, other neglected tropical diseases, encephalitis, acute hepatitis B, exposure to forces of nature, and conflict and terrorism. Moreover, we set α value to 1 at draw level for location/age/sex when $log_{10}(\frac{A_{t_0}}{C_{t_0}}) \geq 1$, i.e. we use only the "relative" difference to avoid negative draw values. To get rid of negative draws being introduced when using an absolute shift, we set a

minimum value of 0 after all shifting is completed. These processes result in forecasts in which all scenarios align with GBD 2021 in the year 2021, along with alternative scenarios that diverge from the reference scenario in 2021.

Section 7: References

1 Stevens GA, Alkema L, Black RE, *et al.* Guidelines for accurate and transparent health estimates reporting: the GATHER statement. *PLoS medicine* 2016; **13**: e1002056.

2 Foreman KJ, Marquez N, Dolgert A, *et al.* Forecasting life expectancy, years of life lost, and all-cause and cause-specific mortality for 250 causes of death: reference and alternative scenarios for 2016–40 for 195 countries and territories. *The Lancet* 2018; **392**: 2052–90.

3 Vollset SE, Goren E, Yuan C-W, *et al.* Fertility, mortality, migration, and population scenarios for 195 countries and territories from 2017 to 2100: a forecasting analysis for the Global Burden of Disease Study. *The Lancet* 2020; **396**: 1285–306.

4 GBD 2021 Fertility and Forecasting Collaborators. Global fertility in 204 countries and territories, 1950–2021 with forecasts to 2100: a comprehensive demographic analysis for the Global Burden of Disease Study 2021. *The Lancet* 2024; published online March 20.

5 Institute for Health Metrics and Evaluation (IHME). Financing global health 2021: global health priorities in a time of change. Seattle, WA: IHME, 2023 https://www.healthdata.org/sites/default/files/files/policy_report/FGH/2023/FGH_2021.pdf.

6 Murray CJL, Aravkin AY, Zheng P, *et al.* Global burden of 87 risk factors in 204 countries and territories, 1990–2019: a systematic analysis for the Global Burden of Disease Study 2019. *The Lancet* 2020; **396**: 1223–49.

7 GBD 2021 Causes of Death Collaborators. Global burden of 288 causes of death and life expectancy decomposition in 204 countries and territories and 811 subnational locations, 1990–2021: a systematic analysis for the Global Burden of Disease Study 2021. *The Lancet* 2024; published online April 3.

8 GBD 2021 HIV Collaborators. Global, regional, and national burden of HIV/AIDS, 1990-2021, and forecasts to 2050, for 204 countries and territories: the Global Burden of Diseases Study 2021. *The Lancet* (drafting).

9 Wang H, Abbas KM, Abbasifard M, *et al.* Global age-sex-specific fertility, mortality, healthy life expectancy (HALE), and population estimates in 204 countries and territories, 1950–2019: a comprehensive demographic analysis for the Global Burden of Disease Study 2019. *The Lancet* 2020; **396**: 1160–203.

10 Hale T, Angrist N, Goldszmidt R, *et al.* A global panel database of pandemic policies (Oxford COVID-19 Government Response Tracker). *Nat Hum Behav* 2021; **5**: 529–38.

11 ISCED Mappings. 2016; published online Nov 22. https://uis.unesco.org/en/isced-mappings (accessed Dec 29, 2023).

12 International Telecommunication Union. Measuring digital development - Facts and figures 2021. Geneva, Switzerland: ITU, 2021 https://www.itu.int/itu-d/reports/statistics/facts-figures-2021/.

13 Micah AE, Dieleman JL, Kahn Case M, *et al.* Financing global health 2018: countries and programs in transition. Institute for Health Metrics and Evaluation, 2019.

14 James SL, Gubbins P, Murray CJ, Gakidou E. Developing a comprehensive time series of GDP per capita for 210 countries from 1950 to 2015. *Popul Health Metr* 2012; **10**: 12.

15 World Health Organization. Third round of the global pulse survey on continuity of essential health services during the COVID-19 pandemic: November–December 2021. Geneva, Switzerland: World Health Organization, 2022 https://www.who.int/publications-detail-redirect/WHO-2019-nCoV-EHS_continuity-survey-2022.1 (accessed July 27, 2022).

16 Zheng P, Barber R, Sorensen RJD, Murray CJL, Aravkin AY. Trimmed Constrained Mixed Effects Models: Formulations and Algorithms. *Journal of Computational and Graphical Statistics* 2021; **30**: 544–56.

17 Geography and Environmental Science, University of Southampton, WorldPop. Age and Sex Structures, Global Per Country 2000-2020. Southampton, United Kingdom: WorldPop, 2018.

18 Hersbach H, Bell B, Berrisford P, *et al.* Global reanalysis : goodbye Global reanalysis : goodbye ERA-Interim , hello. 2019. DOI:10.21957/vf291hehd7.

19 Copernicus Climate Change Service (C3S) (2017): ERA5: Fifth generation of ECMWF atmospheric reanalyses of the global climate. Copernicus Climate Change Service Climate Data Store (CDS), September 2019. .

20 Zheng P, Barber R, Sorensen RJD, Murray CJL, Aravkin AY. Trimmed Constrained Mixed Effects Models: Formulations and Algorithms. *Journal of Computational and Graphical Statistics* 2021; **30**: 544–56.

21 Rousseeuw PJ. Least Median of Squares Regression. *Journal of the American Statistical Association* 1984; **79**: 871–80.

22 Aravkin A, Davis D. Trimmed Statistical Estimation via Variance Reduction. *Mathematics of Operations Research* 2019; : moor.2019.0992.

23 Yang E, Lozano AC, Aravkin A. A general family of trimmed estimators for robust high-dimensional data analysis. *Electronic Journal of Statistics* 2018; **12**: 3519–53.

24 Pya N, Wood SN. Shape constrained additive models. *Statistics and Computing* 2015; **25**: 543–59.

25 Burkart KG, Brauer M, Aravkin AY, *et al.* Estimating the cause-specific relative risks of non-optimal temperature on daily mortality: a two-part modelling approach applied to the Global Burden of Disease Study. *Lancet* 2021; **398**: 685–97.

26 Turner MC, Jerrett M, Pope CA, *et al.* Long-term ozone exposure and mortality in a large prospective study. *Am J Respir Crit Care Med* 2016; **193**: 1134–42.

27 Stanaway JD, Afshin A, Gakidou E, *et al.* Global, regional, and national comparative risk assessment of 84 behavioural, environmental and occupational, and metabolic risks or clusters of risks for 195 countries and territories, 1990–2017: a systematic analysis for the Global Burden of Disease Study 2017. *The Lancet* 2018; **392**: 1923–94.

28 Girosi F, King G. Demographic forecasting. New Jersey: Princeton University Press, 2008.

29 Frank TD, Carter A, Jahagirdar D, *et al.* Global, regional, and national incidence, prevalence, and mortality of HIV, 1980–2017, and forecasts to 2030, for 195 countries and territories: a systematic analysis for the Global Burden of Diseases, Injuries, and Risk Factors Study 2017. *The Lancet HIV* 2019; **6**: e831–59.

30 Jahagirdar D, Walters MK, Novotney A, *et al.* Global, regional, and national sex-specific burden and control of the HIV epidemic, 1990–2019, for 204 countries and territories: the Global Burden of Diseases Study 2019. *The Lancet HIV* 2021; **8**: e633–51.

31 Vos T, Lim SS, Abbafati C, *et al.* Global burden of 369 diseases and injuries in 204 countries and territories, 1990–2019: a systematic analysis for the Global Burden of Disease Study 2019. *The Lancet* 2020; **396**: 1204–22.

32 Global Burden of Disease Long COVID Collaborators. Estimated Global Proportions of Individuals With Persistent Fatigue, Cognitive, and Respiratory Symptom Clusters Following Symptomatic COVID-19 in 2020 and 2021. *JAMA* 2022; **328**: 1604–15.

33 United Nations Department of Economic and Social Affairs, Population Division. World Population Prospects 2022: methodology of the United Nations population estimates and projections. New York: United Nations, 2022.

34 United Nations Department of Economic and Social Affairs, Population Division. World Population Prospects 2022: summary of results. 2022.

35 Vollset SE, Goren E, Yuan C-W, *et al.* Fertility, mortality, migration, and population scenarios for 195 countries and territories from 2017 to 2100: a forecasting analysis for the Global Burden of Disease Study. *The Lancet* 2020; **396**: 1285–306.

36 Sullivan DF. A single index of mortality and morbidity. *HSMHA Health Rep* 1971; **86**: 347–54.

37 Wang H, Naghavi M, Allen C, *et al.* Global, regional, and national life expectancy, all-cause mortality, and cause-specific mortality for 249 causes of death, 1980–2015: a systematic analysis for the Global Burden of Disease Study 2015. *The Lancet* 2016; **388**: 1459–544.

38 Murray CJL, Aravkin AY, Zheng P, *et al.* Global burden of 87 risk factors in 204 countries and territories, 1990–2019: a systematic analysis for the Global Burden of Disease Study 2019. *The Lancet* 2020; **396**: 1223–49.

39 Murphy AH. Skill scores based on the mean square error and their relationships to the correlation coefficient. *Monthly Weather Review* 1988; **116**: 2417–24.

40 Ezzati M, Hoorn SV, Rodgers A, *et al.* Estimates of global and regional potential health gains from reducing multiple major risk factors. *Lancet* 2003; **362**: 271–80.

Section 8: Tables

Appendix Table S1: GBD location hierarchy used for forecasting with levels	
**Location**	**Level**
Global	0
Central Europe, eastern Europe, and central Asia	1
Central Asia	2
Armenia	3
Azerbaijan	3
Georgia	3
Kazakhstan	3
Kyrgyzstan	3
Mongolia	3
Tajikistan	3
Turkmenistan	3
Uzbekistan	3
Central Europe	2
Albania	3
Bosnia and Herzegovina	3
Bulgaria	3
Croatia	3
Czechia	3
Hungary	3
Montenegro	3
North Macedonia	3
Poland	3
Romania	3
Serbia	3
Slovakia	3
Slovenia	3
Eastern Europe	2
Belarus	3
Estonia	3
Latvia	3
Lithuania	3
Moldova	3
Russia	3
Ukraine	3
High income	1
Australasia	2
Australia	3
New Zealand	3
High-income Asia Pacific	2
Brunei	3
Japan	3
South Korea	3
Singapore	3
High-income North America	2
Canada	3

Greenland	3
USA	3
Southern Latin America	2
Argentina	3
Chile	3
Uruguay	3
Western Europe	2
Andorra	3
Austria	3
Belgium	3
Cyprus	3
Denmark	3
Finland	3
France	3
Germany	3
Greece	3
Iceland	3
Ireland	3
Israel	3
Italy	3
Luxembourg	3
Malta	3
Monaco	3
Netherlands	3
Norway	3
Portugal	3
San Marino	3
Spain	3
Sweden	3
Switzerland	3
UK	3
Latin America and Caribbean	1
Andean Latin America	2
Bolivia	3
Ecuador	3
Peru	3
Caribbean	2
Antigua and Barbuda	3
The Bahamas	3
Barbados	3
Belize	3
Bermuda	3
Cuba	3
Dominica	3
Dominican Republic	3
Grenada	3
Guyana	3

Haiti	3
Jamaica	3
Puerto Rico	3
Saint Kitts and Nevis	3
Saint Lucia	3
Saint Vincent and the Grenadines	3
Suriname	3
Trinidad and Tobago	3
Virgin Islands	3
Central Latin America	2
Colombia	3
Costa Rica	3
El Salvador	3
Guatemala	3
Honduras	3
Mexico	3
Nicaragua	3
Panama	3
Venezuela	3
Tropical Latin America	2
Brazil	3
Paraguay	3
North Africa and Middle East	1
North Africa and Middle East	2
Afghanistan	3
Algeria	3
Bahrain	3
Egypt	3
Iran	3
Iraq	3
Jordan	3
Kuwait	3
Lebanon	3
Libya	3
Morocco	3
Oman	3
Palestine	3
Qatar	3
Saudi Arabia	3
Sudan	3
Syria	3
Tunisia	3
Türkiye	3
United Arab Emirates	3
Yemen	3
South Asia	1
South Asia	2

Bangladesh	3
Bhutan	3
India	3
Nepal	3
Pakistan	3
Southeast Asia, east Asia, and Oceania	1
East Asia	2
China	3
North Korea	3
Taiwan (province of China)	3
Oceania	2
American Samoa	3
Cook Islands	3
Fiji	3
Guam	3
Kiribati	3
Marshall Islands	3
Federated States of Micronesia	3
Nauru	3
Niue	3
Northern Mariana Islands	3
Palau	3
Papua New Guinea	3
Samoa	3
Solomon Islands	3
Tokelau	3
Tonga	3
Tuvalu	3
Vanuatu	3
Southeast Asia	2
Cambodia	3
Indonesia	3
Laos	3
Malaysia	3
Maldives	3
Mauritius	3
Myanmar	3
Philippines	3
Seychelles	3
Sri Lanka	3
Thailand	3
Timor-Leste	3
Viet Nam	3
Sub-Saharan Africa	1
Central sub-Saharan Africa	2
Angola	3
Central African Republic	3

Congo (Brazzaville)	3
DR Congo	3
Equatorial Guinea	3
Gabon	3
Eastern sub-Saharan Africa	2
Burundi	3
Comoros	3
Djibouti	3
Eritrea	3
Ethiopia	3
Kenya	3
Madagascar	3
Malawi	3
Mozambique	3
Rwanda	3
Somalia	3
South Sudan	3
Uganda	3
Tanzania	3
Zambia	3
Southern sub-Saharan Africa	2
Botswana	3
Eswatini	3
Lesotho	3
Namibia	3
South Africa	3
Zimbabwe	3
Western sub-Saharan Africa	2
Benin	3
Burkina Faso	3
Cabo Verde	3
Cameroon	3
Chad	3
Côte d'Ivoire	3
The Gambia	3
Ghana	3
Guinea	3
Guinea-Bissau	3
Liberia	3
Mali	3
Mauritania	3
Niger	3
Nigeria	3
São Tomé and Príncipe	3
Senegal	3
Sierra Leone	3
Togo	3

Gather Compliance

This study complies with the Guidelines for Accurate and Transparent Health Estimates Reporting (GATHER) recommendations. We have documented the steps involved in our analytical procedures and detailed the data sources in the GATHER checklist below. The GATHER recommendations can be found here: http://gather-statement.org/

Appendix Table S2. GATHER compliance

#	GATHER checklist item	Description of compliance	Reference
	Objectives and funding		
**1**	Define the indicator(s), populations (including age, sex, and geographic entities), and time period(s) for which estimates were made.	Narrative provided in the paper and appendix describing indicators, definitions, and populations	Main text (Methods - Overview) and appendix
**2**	List the funding sources for the work.	Funding sources listed in paper	Main text (Summary – Funding)
	Data Inputs		
	For all data inputs from multiple sources that are synthesized as part of the study:		
**3**	Describe how the data were identified and how the data were accessed.	Narrative provided in paper and appendix describing data seeking methods	Main text (Methods) and appendix
**4**	Specify the inclusion and exclusion criteria. Identify all ad-hoc exclusions.	Narrative provided in paper and appendix describing inclusion and exclusion criteria by data type	Main text (Methods) and appendix
**5**	Provide information on all included data sources and their main characteristics. For each data source used, report reference information or contact name/institution, population represented, data collection method, year(s) of data collection, sex and age range, diagnostic criteria or measurement method, and sample size, as relevant.	Narrative for data sources is provided in paper and appendix. Metadata for sources by geography are available through an online data source tool; information on metadata for UNPD data available in the appendix	Main text (Methods), appendix, and through the online sources tool, which includes all GBD data inputs used to produce the estimates used in the models for our forecasts: https://ghdx.healthdata.org/gbd-2021/sources
**6**	Identify and describe any categories of input data that have potentially important biases (e.g., based on characteristics listed in item 5).	Limitations of and biases in data included in paper	Main text (Discussion – Limitations)
	For data inputs that contribute to the analysis but were not synthesized as part of the study:		
**7**	Describe and give sources for any other data inputs.	Included in online data source tools	Online sources tool: https://ghdx.healthdata.org/gbd-2021/sources
	For all data inputs:		
**8**	Provide all data inputs in a file format from which data can be efficiently extracted (e.g., a spreadsheet rather than a PDF), including all relevant meta-data listed in item 5. For any data inputs that cannot be shared because of ethical or legal reasons, such as third-party ownership, provide a contact name or the name of the institution that retains the right to the data.	Downloads of input data are available through online data query tools	Online sources tool: https://ghdx.healthdata.org/gbd-2021/sources

	Data analysis		
**9**	Provide a conceptual overview of the data analysis method. A diagram may be helpful.	A brief overview of the overall methodological processes have been provided	Main text (Methods) and appendix
**10**	Provide a detailed description of all steps of the analysis, including mathematical formulae. This description should cover, as relevant, data cleaning, data pre-processing, data adjustments and weighting of data sources, and mathematical or statistical model(s).	Detailed descriptions of all steps of the analysis, as well as relevant mathematical formulae, have been provided	Main text (Methods) and appendix
**11**	Describe how candidate models were evaluated and how the final model(s) were selected.	Details on model evaluation and finalisation have been provided	Main text (Methods – Model performance) and appendix
**12**	Provide the results of an evaluation of model performance, if done, as well as the results of any relevant sensitivity analysis.	Details on evaluation of model performance have been provided	Main text (Results – Model performance) and appendix
**13**	Describe methods for calculating uncertainty of the estimates. State which sources of uncertainty were, and were not, accounted for in the uncertainty analysis.	Details on uncertainty calculations have been provided	Main text (Methods) and appendix
**14**	State how analytic or statistical source code used to generate estimates can be accessed.	Access statement and link provided	Code is provided in an online repository: https://ghdx.healthdata.org/gbd-2021/code
	Results and Discussion		
**15**	Provide published estimates in a file format from which data can be efficiently extracted.	Results are available to view and download through an online visualisation tool	Online tools: https://vizhub.healthdata.org/gbd-foresight/
**16**	Report a quantitative measure of the uncertainty of the estimates (e.g. uncertainty intervals).	Uncertainty intervals are provided with results	Main text (Results and Discussion) and online tools: https://vizhub.healthdata.org/gbd-foresight/
**17**	Interpret results in light of existing evidence. If updating a previous set of estimates, describe the reasons for changes in estimates.	Discussion of methodological differences between this study and existing evidence	Main text (Research in Context, Introduction, Methods, Discussion) and appendix
**18**	Discuss limitations of the estimates. Include a discussion of any modelling assumptions or data limitations that affect interpretation of the estimates.	Discussion of limitations was provided	Main text (Discussion – Limitations)

Appendix Table S3. Primary modelling strategies for incidence, prevalence and YLDs used for each cause. YLD, years lived with disability; MIR, mortality-incidence ratio, PIR, prevalence-incidence ratio; YLL, years of life lost; ARC, annualised rate of change; MPR, mortality-prevalence ratio.

Cause	Incidence	Prevalence	YLD
HIV/AIDS	Spectrum	Spectrum	YLD from Prevalence
Syphilis	MIR	PIR	YLD from Prevalence
Chlamydial infection	MIR	Direct Prevalence	YLD from Prevalence
Gonococcal infection	MIR	PIR	YLD from Prevalence
Trichomoniasis	PIR	Direct Prevalence	YLD from Prevalence
Genital herpes	PIR	Direct Prevalence	YLD from Prevalence
Other sexually transmitted infections	Not Modelled	MPR	YLD:YLL
Latent tuberculosis infection	Not Modelled	Direct Prevalence	Not Modelled
Tuberculosis Fatal	MIR	PIR	YLD from Prevalence
Pneumococcal pneumonia	MIR	PIR	YLD from Prevalence
H influenzae type B pneumonia	MIR	PIR	YLD from Prevalence
Non-pneumococcal non-H influenzae type B pneumonia	MIR	PIR	YLD from Prevalence
Upper respiratory infections	Direct Incidence	PIR	YLD from Prevalence
Otitis media	MIR	PIR	YLD from Prevalence
COVID-19	COVID	COVID	COVID

Rotaviral enteritis	MIR	PIR	YLD from Prevalence
Non-rotaviral enteritis	MIR	PIR	YLD from Prevalence
Typhoid fever	MIR	PIR	YLD from Prevalence
Paratyphoid fever	MIR	PIR	YLD from Prevalence
Invasive Non-typhoidal Salmonella (iNTS)	MIR	PIR	YLD from Prevalence
Other intestinal infectious diseases	Not Modelled	Not Modelled	YLD:YLL
Malaria	ARC	PIR	YLD from Prevalence
Chagas disease	PIR	ARC	YLD from Prevalence
Visceral leishmaniasis	PIR	ARC	YLD from Prevalence
Cutaneous and mucocutaneous leishmaniasis	PIR	ARC	YLD from Prevalence
African trypanosomiasis	PIR	ARC	YLD from Prevalence
Schistosomiasis	Not Modelled	ARC	YLD from Prevalence
Cysticercosis	Not Modelled	ARC	YLD from Prevalence
Cystic echinococcosis	PIR	ARC	YLD from Prevalence
Lymphatic filariasis	Not Modelled	ARC	YLD from Prevalence
Onchocerciasis	Not Modelled	ARC	YLD from Prevalence
Trachoma	Not Modelled	ARC	YLD from Prevalence

Dengue	MIR	ARC	YLD from Prevalence
Yellow fever	MIR	ARC	YLD from Prevalence
Rabies	MIR	ARC	YLD from Prevalence
Ascariasis	Not Modelled	ARC	YLD from Prevalence
Trichuriasis	Not Modelled	ARC	YLD from Prevalence
Hookworm disease	Not Modelled	ARC	YLD from Prevalence
Food-borne trematodiases	Not Modelled	ARC	YLD from Prevalence
Leprosy	PIR	ARC	YLD from Prevalence
Guinea worm disease	PIR	ARC	YLD from Prevalence
Other neglected tropical diseases	Not Modelled	ARC	YLD from Prevalence
Pneumococcal meningitis	MIR	PIR	YLD from Prevalence
H influenzae type B meningitis	MIR	PIR	YLD from Prevalence
Non-pneumococcal non-H influenzae type B meningitis	MIR	PIR	YLD from Prevalence
Encephalitis	MIR	PIR	YLD from Prevalence
Diphtheria	MIR	PIR	YLD from Prevalence
Whooping cough	MIR	PIR	YLD from Prevalence
Tetanus	MIR	PIR	YLD from Prevalence

Measles	PIR	Direct Prevalence	YLD from Prevalence
Varicella and herpes zoster	MIR	PIR	YLD from Prevalence
Acute hepatitis A	PIR	Direct Prevalence	YLD from Prevalence
Acute hepatitis B	PIR	MPR	YLD from Prevalence
Acute hepatitis C	PIR	MPR	YLD from Prevalence
Acute hepatitis E	Direct Incidence	PIR Congenital	YLD from Prevalence
Other unspecified infectious diseases	Not Modelled	MPR	YLD from Prevalence
Maternal hemorrhage	MIR	PIR	YLD from Prevalence
Maternal sepsis and other maternal infections	MIR	PIR	YLD from Prevalence
Maternal hypertensive disorders	MIR	PIR	YLD from Prevalence
Maternal obstructed labor and uterine rupture	MIR	PIR	YLD from Prevalence
Maternal abortion and miscarriage	MIR	PIR	YLD from Prevalence
Ectopic pregnancy	MIR	PIR	YLD from Prevalence
Other maternal disorders	Not Modelled	Not Modelled	YLD:YLL
Neonatal preterm birth	Direct Incidence	Direct Prevalence	YLD from Prevalence
Neonatal encephalopathy due to birth asphyxia and trauma	Direct Incidence	Direct Prevalence	YLD from Prevalence
Neonatal sepsis and other neonatal infections	Direct Incidence	PIR Congenital	YLD from Prevalence

Hemolytic disease and other neonatal jaundice	Direct Incidence	Direct Prevalence	YLD from Prevalence
Other neonatal disorders	Not Modelled	Not Modelled	YLD:YLL
Protein-energy malnutrition	Direct Incidence	PIR Congenital	YLD from Prevalence
Iodine deficiency	PIR	Direct Prevalence	YLD from Prevalence
Vitamin A deficiency	PIR	Direct Prevalence	YLD from Prevalence
Dietary iron deficiency	Not Modelled	Direct Prevalence	YLD from Prevalence
Other nutritional deficiencies	Not Modelled	Not Modelled	YLD:YLL
Lip and oral cavity cancer	MIR	PIR	YLD from Prevalence
Nasopharynx cancer	MIR	PIR	YLD from Prevalence
Other pharynx cancer	MIR	PIR	YLD from Prevalence
Esophageal cancer	MIR	PIR	YLD from Prevalence
Stomach cancer	MIR	PIR	YLD from Prevalence
Colon and rectum cancer	MIR	PIR	YLD from Prevalence
Liver cancer due to hepatitis B	MIR	PIR	YLD from Prevalence
Liver cancer due to hepatitis C	MIR	PIR	YLD from Prevalence
Liver cancer due to alcohol use	MIR	PIR	YLD from Prevalence
Liver cancer due to NASH	MIR	PIR	YLD from Prevalence

Hepatoblastoma	MIR	PIR	YLD from Prevalence
Liver cancer due to other causes (internal)	MIR	PIR	YLD from Prevalence
Gallbladder and biliary tract cancer	MIR	PIR	YLD from Prevalence
Pancreatic cancer	MIR	PIR	YLD from Prevalence
Larynx cancer	MIR	PIR	YLD from Prevalence
Tracheal, bronchus, and lung cancer	MIR	PIR	YLD from Prevalence
Malignant skin melanoma	MIR	PIR	YLD from Prevalence
Non-melanoma skin cancer (squamous-cell carcinoma)	MIR	PIR	YLD from Prevalence
Non-melanoma skin cancer (basal-cell carcinoma)	PIR	Direct Prevalence	YLD from Prevalence
Soft tissue and other extraosseous sarcomas	MIR	PIR	YLD from Prevalence
Malignant neoplasm of bone and articular cartilage	MIR	PIR	YLD from Prevalence
Breast cancer	MIR	PIR	YLD from Prevalence
Cervical cancer	MIR	PIR	YLD from Prevalence
Uterine cancer	MIR	PIR	YLD from Prevalence
Ovarian cancer	MIR	PIR	YLD from Prevalence
Prostate cancer	MIR	PIR	YLD from Prevalence
Testicular cancer	MIR	PIR	YLD from Prevalence

Kidney cancer	MIR	PIR	YLD from Prevalence
Bladder cancer	MIR	PIR	YLD from Prevalence
Brain and central nervous system cancer	MIR	PIR	YLD from Prevalence
Retinoblastoma	MIR	PIR	YLD from Prevalence
Other eye cancers	MIR	PIR	YLD from Prevalence
Neuroblastoma and other peripheral nervous cell tumors	MIR	PIR	YLD from Prevalence
Thyroid cancer	MIR	PIR	YLD from Prevalence
Mesothelioma	MIR	PIR	YLD from Prevalence
Hodgkin lymphoma	MIR	PIR	YLD from Prevalence
Burkitt lymphoma	MIR	PIR	YLD from Prevalence
Other non-Hodgkin lymphoma	MIR	PIR	YLD from Prevalence
Multiple myeloma	MIR	PIR	YLD from Prevalence
Acute lymphoid leukemia	MIR	PIR	YLD from Prevalence
Chronic lymphoid leukemia	MIR	PIR	YLD from Prevalence
Acute myeloid leukemia	MIR	PIR	YLD from Prevalence
Chronic myeloid leukemia	MIR	PIR	YLD from Prevalence
Other leukemia	MIR	PIR	YLD from Prevalence

Other malignant neoplasms (internal)	MIR	PIR	YLD from Prevalence
Myelodysplastic, myeloproliferative, and other hematopoietic neoplasms	MIR	PIR	YLD from Prevalence
Benign and in situ intestinal neoplasms	PIR	Direct Prevalence	Not Modelled
Benign and in situ cervical and uterine neoplasms	PIR	Direct Prevalence	Not Modelled
Other benign and in situ neoplasms	PIR	Direct Prevalence	Not Modelled
Rheumatic heart disease	PIR	Direct Prevalence	YLD from Prevalence
Ischemic heart disease	MIR	Direct Prevalence	YLD from Prevalence
Ischemic stroke	MIR	Direct Prevalence	YLD from Prevalence
Intracerebral hemorrhage	MIR	PIR	YLD from Prevalence
Subarachnoid hemorrhage	MIR	PIR	YLD from Prevalence
Hypertensive heart disease	Not Modelled	MPR	YLD from Prevalence
Non-rheumatic calcific aortic valve disease	PIR	Direct Prevalence	YLD from Prevalence
Non-rheumatic degenerative mitral valve disease	PIR	MPR	YLD from Prevalence
Other non-rheumatic valve diseases	Not Modelled	MPR	YLD from Prevalence
Myocarditis	MIR	PIR	YLD from Prevalence
Alcoholic cardiomyopathy	Not Modelled	MPR	YLD from Prevalence
Other cardiomyopathy	Not Modelled	MPR	YLD from Prevalence

Pulmonary Arterial Hypertension	Not Modelled	MPR	YLD from Prevalence
Atrial fibrillation and flutter	PIR	MPR	YLD from Prevalence
Peripheral artery disease	PIR	MPR	YLD from Prevalence
Endocarditis	MIR	PIR	YLD from Prevalence
Other cardiovascular and circulatory diseases (internal)	Not Modelled	MPR	YLD from Prevalence
Chronic obstructive pulmonary disease	PIR	MPR	YLD from Prevalence
Silicosis	PIR	MPR	YLD from Prevalence
Asbestosis	PIR	MPR	YLD from Prevalence
Coal workers pneumoconiosis	PIR	MPR	YLD from Prevalence
Other pneumoconiosis	PIR	MPR	YLD from Prevalence
Asthma	PIR	MPR	YLD from Prevalence
Interstitial lung disease and pulmonary sarcoidosis	PIR	MPR	YLD from Prevalence
Other chronic respiratory diseases	Not Modelled	Not Modelled	YLD:YLL
Cirrhosis and other chronic liver diseases due to hepatitis B	PIR	MPR	YLD from Prevalence
Cirrhosis and other chronic liver diseases due to hepatitis C	PIR	MPR	YLD from Prevalence
Cirrhosis and other chronic liver diseases due to alcohol use	PIR	MPR	YLD from Prevalence
Cirrhosis and other chronic liver diseases due to NAFLD	PIR	MPR	YLD from Prevalence

Cirrhosis and other chronic liver diseases due to other causes	PIR	MPR	YLD from Prevalence
Peptic ulcer disease	PIR	MPR	YLD from Prevalence
Gastritis and duodenitis	PIR	MPR	YLD from Prevalence
Gastroesophageal reflux disease	PIR	Direct Prevalence	YLD from Prevalence
Appendicitis	MIR	PIR	YLD from Prevalence
Paralytic ileus and intestinal obstruction	MIR	PIR	YLD from Prevalence
Inguinal, femoral, and abdominal hernia	PIR	MPR	YLD from Prevalence
Inflammatory bowel disease	PIR	MPR	YLD from Prevalence
Vascular intestinal disorders	MIR	PIR	YLD from Prevalence
Gallbladder and biliary diseases	PIR	MPR	YLD from Prevalence
Pancreatitis	PIR	MPR	YLD from Prevalence
Other digestive diseases	Not Modelled	Not Modelled	YLD:YLL
Alzheimer's disease and other dementias	PIR	MPR	YLD from Prevalence
Parkinson's disease	PIR	MPR	YLD from Prevalence
Idiopathic epilepsy	PIR	MPR	YLD from Prevalence
Multiple sclerosis	PIR	MPR	YLD from Prevalence
Motor neuron disease	PIR	MPR	YLD from Prevalence

Migraine	PIR	Direct Prevalence	YLD from Prevalence
Tension-type headache	PIR	Direct Prevalence	YLD from Prevalence
Other neurological disorders	Not Modelled	MPR	YLD from Prevalence
Schizophrenia	PIR	Direct Prevalence	YLD from Prevalence
Major depressive disorder	PIR	Direct Prevalence	YLD from Prevalence
Dysthymia	PIR	Direct Prevalence	YLD from Prevalence
Bipolar disorder	PIR	Direct Prevalence	YLD from Prevalence
Anxiety disorders	PIR	Direct Prevalence	YLD from Prevalence
Eating disorders	PIR	MPR	YLD from Prevalence
Autism spectrum disorders	Direct Incidence	Direct Prevalence	YLD from Prevalence
Attention-deficit/hyperactivity disorder	PIR	Direct Prevalence	YLD from Prevalence
Conduct disorder	PIR	Direct Prevalence	YLD from Prevalence
Idiopathic developmental intellectual disability	Not Modelled	Direct Prevalence	YLD from Prevalence
Other mental disorders	Not Modelled	Direct Prevalence	YLD from Prevalence
Alcohol use disorders	Direct Incidence	PIR Congenital	YLD from Prevalence
Opioid use disorders	PIR	Direct Prevalence	YLD from Prevalence
Cocaine use disorders	PIR	MPR	YLD from Prevalence

Amphetamine use disorders	PIR	MPR	YLD from Prevalence
Cannabis use disorders	PIR	Direct Prevalence	YLD from Prevalence
Other drug use disorders	PIR	MPR	YLD from Prevalence
Diabetes mellitus type 1	PIR	Direct Prevalence	YLD from Prevalence
Diabetes mellitus type 2	PIR	BMI covariate	YLD from Prevalence
Chronic kidney disease due to diabetes mellitus type 1	PIR	MPR	YLD from Prevalence
Chronic kidney disease due to diabetes mellitus type 2	PIR	MPR	YLD from Prevalence
Chronic kidney disease due to hypertension	PIR	MPR	YLD from Prevalence
Chronic kidney disease due to glomerulonephritis	PIR	MPR	YLD from Prevalence
Chronic kidney disease due to other and unspecified causes	PIR	MPR	YLD from Prevalence
Acute glomerulonephritis	MIR	PIR	YLD from Prevalence
Atopic dermatitis	PIR	Direct Prevalence	YLD from Prevalence
Contact dermatitis	PIR	Direct Prevalence	YLD from Prevalence
Seborrhoeic dermatitis	PIR	Direct Prevalence	YLD from Prevalence
Psoriasis	PIR	Direct Prevalence	YLD from Prevalence
Cellulitis	MIR	PIR	YLD from Prevalence
Pyoderma	MIR	PIR	YLD from Prevalence

Scabies	PIR	Direct Prevalence	YLD from Prevalence
Fungal skin diseases	PIR	Direct Prevalence	YLD from Prevalence
Viral skin diseases	PIR	Direct Prevalence	YLD from Prevalence
Acne vulgaris	PIR	Direct Prevalence	YLD from Prevalence
Alopecia areata	PIR	Direct Prevalence	YLD from Prevalence
Pruritus	PIR	Direct Prevalence	YLD from Prevalence
Urticaria	PIR	Direct Prevalence	YLD from Prevalence
Decubitus ulcer	MIR	PIR	YLD from Prevalence
Other skin and subcutaneous diseases	PIR	MPR	YLD:YLL
Glaucoma	Not Modelled	Direct Prevalence	YLD from Prevalence
Cataract	Not Modelled	Direct Prevalence	YLD from Prevalence
Age-related macular degeneration	Not Modelled	Direct Prevalence	YLD from Prevalence
Refraction disorders	Not Modelled	Direct Prevalence	YLD from Prevalence
Near vision loss	Not Modelled	Direct Prevalence	YLD from Prevalence
Other vision loss	Not Modelled	Direct Prevalence	YLD from Prevalence
Age-related and other hearing loss	Not Modelled	Direct Prevalence	YLD from Prevalence
Other sense organ diseases	Not Modelled	Direct Prevalence	YLD from Prevalence

Rheumatoid arthritis	PIR	MPR	YLD from Prevalence
Osteoarthritis hip	PIR	Direct Prevalence	YLD from Prevalence
Osteoarthritis knee	PIR	Direct Prevalence	YLD from Prevalence
Osteoarthritis hand	PIR	Direct Prevalence	YLD from Prevalence
Osteoarthritis other	PIR	Direct Prevalence	YLD from Prevalence
Low back pain	PIR	Direct Prevalence	YLD from Prevalence
Neck pain	PIR	Direct Prevalence	YLD from Prevalence
Gout	PIR	Direct Prevalence	YLD from Prevalence
Other musculoskeletal disorders	Not Modelled	Direct Prevalence	YLD from Prevalence
Neural tube defects	Direct Incidence	Direct Prevalence	YLD from Prevalence
Congenital heart anomalies	Direct Incidence	Direct Prevalence	YLD from Prevalence
Orofacial clefts	Direct Incidence	Direct Prevalence	YLD from Prevalence
Down syndrome	Direct Incidence	Direct Prevalence	YLD from Prevalence
Turner syndrome	Direct Incidence	Direct Prevalence	YLD from Prevalence
Klinefelter syndrome	Direct Incidence	Direct Prevalence	YLD from Prevalence
Other chromosomal abnormalities	Direct Incidence	Direct Prevalence	YLD from Prevalence
Congenital musculoskeletal and limb anomalies	Direct Incidence	Direct Prevalence	YLD from Prevalence

Urogenital congenital anomalies	Direct Incidence	Direct Prevalence	YLD from Prevalence
Digestive congenital anomalies	Direct Incidence	Direct Prevalence	YLD from Prevalence
Other congenital birth defects	Not Modelled	Direct Prevalence	YLD from Prevalence
Urinary tract infections and interstitial nephritis	MIR	PIR	YLD from Prevalence
Urolithiasis	MIR	PIR	YLD from Prevalence
Benign prostatic hyperplasia	PIR	Direct Prevalence	YLD from Prevalence
Male infertility	Not Modelled	Direct Prevalence	YLD from Prevalence
Other urinary diseases	Not Modelled	Not Modelled	YLD:YLL
Uterine fibroids	PIR	MPR	YLD from Prevalence
Polycystic ovarian syndrome	PIR	Direct Prevalence	YLD from Prevalence
Female infertility	Not Modelled	Direct Prevalence	YLD from Prevalence
Endometriosis	PIR	MPR	YLD from Prevalence
Genital prolapse	PIR	MPR	YLD from Prevalence
Premenstrual syndrome	PIR	Direct Prevalence	YLD from Prevalence
Other gynecological diseases	PIR	MPR	YLD from Prevalence
Thalassemias	Direct Incidence	Direct Prevalence	YLD from Prevalence
Thalassemias trait	Direct Incidence	Direct Prevalence	YLD from Prevalence

Sickle cell disorders	Direct Incidence	Direct Prevalence	YLD from Prevalence
Sickle cell trait	Direct Incidence	Direct Prevalence	YLD from Prevalence
G6PD deficiency	Direct Incidence	Direct Prevalence	YLD from Prevalence
G6PD trait	Direct Incidence	Direct Prevalence	YLD from Prevalence
Other hemoglobinopathies and hemolytic anemias	Not Modelled	MPR	YLD from Prevalence
Endocrine, metabolic, blood, and immune disorders	Not Modelled	MPR	YLD from Prevalence
Caries of deciduous teeth	PIR	Direct Prevalence	YLD from Prevalence
Caries of permanent teeth	PIR	Direct Prevalence	YLD from Prevalence
Periodontal diseases	PIR	Direct Prevalence	YLD from Prevalence
Edentulism	PIR	Direct Prevalence	YLD from Prevalence
Other oral disorders	Not Modelled	Direct Prevalence	YLD from Prevalence
Pedestrian road injuries	MIR	PIR	YLD from Prevalence
Cyclist road injuries	MIR	PIR	YLD from Prevalence
Motorcyclist road injuries	MIR	PIR	YLD from Prevalence
Motor vehicle road injuries	MIR	PIR	YLD from Prevalence
Other road injuries	MIR	PIR	YLD from Prevalence
Other transport injuries	MIR	PIR	YLD from Prevalence

Falls	MIR	PIR	YLD from Prevalence
Drowning	MIR	PIR	YLD from Prevalence
Fire, heat, and hot substances	MIR	PIR	YLD from Prevalence
Poisoning by carbon monoxide	MIR	PIR	YLD from Prevalence
Poisoning by other means	MIR	PIR	YLD from Prevalence
Unintentional firearm injuries	MIR	PIR	YLD from Prevalence
Other exposure to mechanical forces	MIR	PIR	YLD from Prevalence
Adverse effects of medical treatment	MIR	PIR	YLD from Prevalence
Venomous animal contact	MIR	PIR	YLD from Prevalence
Non-venomous animal contact	MIR	PIR	YLD from Prevalence
Pulmonary aspiration and foreign body in airway	MIR	PIR	YLD from Prevalence
Foreign body in eyes	PIR	Direct Prevalence	YLD from Prevalence
Foreign body in other body part	MIR	PIR	YLD from Prevalence
Environmental heat and cold exposure	MIR	PIR	YLD from Prevalence
Exposure to forces of nature	MIR	PIR	YLD from Prevalence
Other unintentional injuries	MIR	PIR	YLD from Prevalence
Self-harm by firearm	MIR	PIR	YLD from Prevalence

Self-harm by other specified means	MIR	PIR	YLD from Prevalence
Physical violence by firearm	MIR	PIR	YLD from Prevalence
Physical violence by sharp object	MIR	PIR	YLD from Prevalence
Sexual violence	Not Modelled	Direct Prevalence	YLD from Prevalence
Physical violence by other means	MIR	PIR	YLD from Prevalence
Conflict and terrorism	PIR	MPR	YLD from Prevalence
Executions and police conflict	MIR	PIR	YLD from Prevalence
