# Supplementary material for: Burden of disease scenarios for 204 countries and territories, 2022–2050: a forecasting analysis for the Global Burden of Disease Study 2021
Source: Lancet. 2024 May 18;403(10440):2204–56. doi: 10.1016/S0140-6736(24)00685-8 (PMC11121021; doi:10.1016/S0140-6736(24)00685-8)
Supplement: Supplementary appendix 3 [file mmc3.pdf]

THE LANCET

Supplementary appendix 3

This appendix formed part of the original submission and has been peer reviewed. We post it as supplied by the authors.

Supplement to: GBD 2021 Forecasting Collaborators. Burden of disease scenarios for 204 countries and territories, 2022–2050: a forecasting analysis for the Global Burden of Disease Study 2021. *Lancet* 2024; **403:** 2204–56.

Appendix 3: Authorship appendix to "Burden of disease scenarios for 204 countries and territories, 2022–2050: a forecasting analysis for the Global Burden of Disease Study 2021"

This appendix provides further authorship detail for "Burden of disease scenarios for 204 countries and territories, 2022–2050: a forecasting analysis for the Global Burden of Disease Study 2021."

Table of contents

GBD 2021 Forecasting Collaborators **2**

Affiliations **7**

Authors' Contributions **32**

Managing the overall research enterprise 32

Writing the first draft of the manuscript 32

Primary responsibility for applying analytical methods to produce estimates 32

Primary responsibility for seeking, cataloguing, extracting, or cleaning data; designing or coding figures and tables 32

Providing data or critical feedback on data sources 32

Developing methods or computational machinery 34

Providing critical feedback on methods or results 34

Drafting the work or revising it critically for important intellectual content 38

Managing the estimation or publications process 41

GBD 2021 Forecasting Collaborators

Stein Emil Vollset*, Hazim S Ababneh, Yohannes Habtegiorgis Abate, Cristiana Abbafati, Rouzbeh Abbasgholizadeh, Mohammadreza Abbasian, Hedayat Abbastabar, Abdallah H A Abd Al Magied, Samar Abd ElHafeez, Atef Abdelkader, Michael Abdelmasseh, Sherief Abd-Elsalam, Parsa Abdi, Mohammad Abdollahi, Meriem Abdoun, Auwal Abdullahi, Mesfin Abebe, Olumide Abiodun, Richard Gyan Aboagye, Hassan Abolhassani, Mohamed Abouzid, Girma Beressa Aboye, Lucas Guimarães Abreu, Abdorrahim Absalan, Hasan Abualruz, Bilyaminu Abubakar, Hana Jihad Jihad Abukhadijah, Giovanni Addolorato, Victor Adekanmbi, Charles Oluwaseun Adetunji, Juliana Bunmi Adetunji, Temitayo Esther Adeyeoluwa, Rishan Adha, Ripon Kumar Adhikary, Qorinah Estiningtyas Sakilah Adnani, Leticia Akua Adzigbli, Fatemeh Afrashteh, Muhammad Sohail Afzal, Saira Afzal, Faith Agbozo, Antonella Agodi, Anurag Agrawal, Williams Agyemang-Duah, Bright Opoku Ahinkorah, Austin J Ahlstrom, Aqeel Ahmad, Firdos Ahmad, Muayyad M Ahmad, Sajjad Ahmad, Shahzaib Ahmad, Anisuddin Ahmed, Ayman Ahmed, Haroon Ahmed, Safoora Ahmed, Syed Anees Ahmed, Karolina Akinosoglou, Mohammed Ahmed Akkaif, Ashley E Akrami, Ema Akter, Salah Al Awaidy, Syed Mahfuz Al Hasan, Amjad S Al Mosa, Omar Al Ta'ani, Omar Ali Mohammed Al Zaabi, Fares Alahdab, Muaaz M Alajlani, Yazan Al-Ajlouni, Samer O Alalalmeh, Ziyad Al-Aly, Khurshid Alam, Noore Alam, Tahiya Alam, Zufishan Alam, Rasmieh Mustafa Al-amer, Fahad Mashhour Alanezi, Turki M Alanzi, Almaza Albakri, Wafa A Aldhaleei, Robert W Aldridge, Seyedeh Yasaman Alemohammad, Yihun Mulugeta Alemu, Adel Ali Saeed Al-Gheethi, Mohammed Khaled Al-Hanawi, Abid Ali, Amjad Ali, Iman Ali, Mohammed Usman Ali, Rafat Ali, Syed Shujait Shujait Ali, Victor Ekoche Ali, Waad Ali, Akram Al-Ibraheem, Gianfranco Alicandro, Sheikh Mohammad Alif, Syed Mohamed Aljunid, François Alla, Joseph Uy Almazan, Hesham M Al-Mekhlafi, Ahmed Yaseen Alqutaibi, Ahmad Alrawashdeh, Sahel Majed Alrousan, Salman Khalifah Al-Sabah, Mohammed A Alsabri, Zaid Altaany, Ala'a B Al-Tammemi, Jaffar A Al-Tawfiq, Khalid A Altirkawi, Deborah Oyine Aluh, Nelson Alvis-Guzman, Mohammad Sami Al-Wardat, Yaser Mohammed Al-Worafi, Hany Aly, Mohammad Sharif Alyahya, Karem H Alzoubi, Walid Al-Zyoud, Reza Amani, Edward Kwabena Ameyaw, Tarek Tawfik Amin, Alireza Amindarolzarbi, Sohrab Amiri, Mohammad Hosein Amirzade-Iranaq, Hubert Amu, Dickson A Amugsi, Robert Ancuceanu, Deanna Anderlini, David B Anderson, Pedro Prata Andrade, Catalina Liliana Andrei, Tudorel Andrei, Erick Adrian Andrews, Abhishek Anil, Sneha Anil, Amir Anoushiravani, Catherine M Antony, Ernoiz Antriyandarti, Boluwatife Stephen Anuoluwa, Saeid Anvari, Anayochukwu Edward Anyasodor, Francis Appiah, Michele Aquilano, Juan Pablo Arab, Jalal Arabloo, Elshaimaa A Arafa, Mosab Arafat, Aleksandr Y Aravkin, Ali Ardekani, Demelash Areda, Brhane Berhe Aregawi, Abdulfatai Aremu, Hany Ariffin, Mesay Arkew, Keivan Armani, Anton A Artamonov, Ashokan Arumugam, Mohammad Asghari-Jafarabadi, Charlie Ashbaugh, Thomas Astell-Burt, Seyyed Shamsadin Athari, Prince Atorkey, Maha Moh'd Wahbi Atout, Avinash Aujayeb, Marcel Ausloos, Hamzeh Awad, Adedapo Wasiu Awotidebe, Haleh Ayatollahi, Jose L Ayuso-Mateos, Sina Azadnajafabad, Fahad Khan Azeez, Rui M S Azevedo, Muhammad Badar, Soroush Baghdadi, Mahboube Bagheri, Nasser Bagheri, Ruhai Bai, Jennifer L Baker, Abdulaziz T Bako, Senthilkumar Balakrishnan, Wondu Feyisa Balcha, Ovidiu Constantin Baltatu, Martina Barchitta, Erfan Bardideh, Suzanne Lyn Barker-Collo, Till Winfried Bärnighausen, Hiba Jawdat Barqawi, Sandra Barteit, Afisu Basiru, João Diogo Basso, Mohammad-Mahdi Bastan, Sanjay Basu, Matteo Bauckneht, Bernhard T Baune, Mohsen Bayati, Nebiyou Simegnew Bayileyegn, Amir Hossein Behnoush, Payam Behzadi, Maryam Beiranvand, Olorunjuwon Omolaja Bello, Luis Belo, Apostolos Beloukas, Maryam Bemanalizadeh, Isabela M Bensenor, Habib Benzian, Azizullah Beran, Zombor Berezvai, Robert S Bernstein, Paulo J G Bettencourt, Kebede A Beyene, Melak Gedamu Beyene, Devidas S Bhagat, Akshaya Srikanth Bhagavathula, Neeraj Bhala, Dinesh Bhandari, Ravi Bharadwaj, Nikha Bhardwaj, Pankaj Bhardwaj, Ashish Bhargava, Sonu Bhaskar, Vivek Bhat, Natalia V Bhattacharjee, Gurjit Kaur Bhatti, Jasvinder Singh Bhatti, Manpreet S Bhatti, Mohiuddin Ahmed Bhuiyan, Catherine Bisignano, Bijit Biswas, Tone Bjørge, Virginia Bodolica, Aadam Olalekan Bodunrin, Milad Bonakdar Hashemi, Berrak Bora Basara, Hamed Borhany, Samuel Adolf Bosoka, Alejandro Botero Carvajal, Souad Bouaoud, Soufiane Boufous, Christopher Boxe, Edward J Boyko, Oliver J Brady, Dejana Braithwaite, Michael Brauer, Javier Brazo-Sayavera, Hermann Brenner, Colin Stewart Brown, Annie J Browne, Traolach Brugha, Dana Bryazka, Norma B Bulamu, Danilo Buonsenso, Katrin Burkart, Richard A Burns, Reinhard Busse, Yasser Bustanji, Zahid A Butt, Florentino Luciano Caetano dos Santos, Mehtap Çakmak Barsbay, Daniela Calina, Luciana Aparecida Campos, Shujin Cao, Angelo Capodici, Rosario Cárdenas, Giulia Carreras, Andrea Carugno, Márcia Carvalho, Joao Mauricio Castaldelli-Maia, Giulio Castelpietra, Maria Sofia Cattaruzza, Arthur Caye, Luca Cegolon, Francieli Cembranel, Edina Cenko, Ester Cerin, Steven J Chadban, Joshua Chadwick, Chiranjib Chakraborty, Sandip Chakraborty, Julian Chalek, Jeffrey Shi Kai Chan, Rama Mohan Chandika, Sara Chandy, Jaykaran Charan, Anis Ahmad Chaudhary, Akhilanand Chaurasia, An-Tian Chen, Haowei Chen, Meng Xuan Chen, Simiao Chen, Nicolas Cherbuin, Gerald Chi, Fatemeh Chichagi, Odgerel Chimed-Ochir, Ritesh Chimoriya, Patrick R Ching, Jesus Lorenzo Chirinos-Caceres, Abdulaal

Chitheer, Daniel Youngwhan Cho, William C S Cho, Dong-Woo Choi, Bryan Chong, Chean Lin Chong, Hitesh Chopra, Dinh-Toi Chu, Eric Chung, Muhammad Chutiyami, Justin T Clayton, Rebecca M Cogen, Aaron J Cohen, Alyssa Columbus, Haley Comfort, Joao Conde, Jon T Connolly, Ezra E K Cooper, Samuele Cortese, Natália Cruz-Martins, Alanna Gomes da Silva, Omid Dadras, Xiaochen Dai, Zhaoli Dai, Bronte E Dalton, Giovanni Damiani, Lalit Dandona, Rakhi Dandona, Jai K Das, Saswati Das, Subasish Das, Nihar Ranjan Dash, Kairat Davletov, Fernando Pio De la Hoz, Diego De Leo, Shayom Debopadhaya, Ivan Delgado-Enciso, Edgar Denova-Gutiérrez, Nikolaos Dervenis, Hardik Dineshbhai Desai, Vinoth Gnana Chellaiyan Devanbu, Syed Masudur Rahman Dewan, Kuldeep Dhama, Amol S Dhane, Sameer Dhingra, Diana Dias da Silva, Daniel Diaz, Luis Antonio Diaz, Michael J Diaz, Adriana Dima, Delaney D Ding, Thao Huynh Phuong Do, Camila Bruneli do Prado, Masoud Dodangeh, Milad Dodangeh, Phidelia Theresa Doegah, Sushil Dohare, Wanyue Dong, Mario D'Oria, Rajkumar Doshi, Robert Kokou Dowou, Haneil Larson Dsouza, Viola Dsouza, John Dube, Samuel C Dumith, Bruce B Duncan, Andre Rodrigues Duraes, Senbagam Duraisamy, Oyewole Christopher Durojaiye, Anar Dushpanova, Sulagna Dutta, Paulina Agnieszka Dzianach, Arkadiusz Marian Dziedzic, Ejemai Eboreime, Alireza Ebrahimi, Mohammad Ebrahimi Kalan, Hisham Atan Edinur, Ferry Efendi, Terje Andreas Eikemo, Ebrahim Eini, Temitope Cyrus Ekundayo, Rabie Adel El Arab, Iman El Sayed, Osman Elamin, Noha Mousaad Elemam, Ghada Metwally Tawfik ElGohary, Muhammed Elhadi, Omar Abdelsadek Abdou Elmeligy, Adel B Elmoselhi, Mohammed Elshaer, Ibrahim Elsohaby, Mohd. Elmagzoub Eltahir, Theophilus I Emeto, Babak Eshrati, Majid Eslami, Zahra Esmaeili, Natalia Fabin, Adeniyi Francis Fagbamigbe, Omotayo Francis Fagbule, Luca Falzone, Mohammad Fareed, Carla Sofia e Sá Farinha, MoezAlIslam Ezzat Mahmoud Faris, Andre Faro, Kiana Fasihi, Ali Fatehizadeh, Nelsensius Klau Fauk, Timur Fazylov, Valery L Feigin, Ginenus Fekadu, Xiaoqi Feng, Seyed-Mohammad Fereshtehnejad, Pietro Ferrara, Nuno Ferreira, Belete Sewasew Firew, Florian Fischer, Ida Fitriana, Joanne Flavel, Luisa S Flor, Morenike Oluwatoyin Folayan, Kristen Marie Foley, Marco Fonzo, Lisa M Force, Matteo Foschi, Alberto Freitas, Ni Kadek Yuni Fridayani, Kai Glenn Fukutaki, João M Furtado, Blima Fux, Peter Andras Gaal, Muktar A Gadanya, Silvano Gallus, Balasankar Ganesan, Mohammad Arfat Ganiyani, Rupesh K Gautam, Tilaye Gebru Gebi, Miglas W Gebregergis, Mesfin Gebrehiwot, Lemma Getacher, Genanew K A Getahun, Peter W Gething, Delaram J Ghadimi, Fataneh Ghadirian, Sadegh Ghafarian, Khalid Yaser Ghailan, MohammadReza Ghasemi, Ghazal Ghasempour Dabaghi, Ramy Mohamed Ghazy, Sama Ghoba, Ehsan Gholami, Ali Gholamrezanezhad, Nasim Gholizadeh, Mahsa Ghorbani, Pooyan Ghorbani Vajargah, Elena Ghotbi, Artyom Urievich Gil, Tiffany K Gill, Alem Girmay, James C Glasbey, Ekaterina Vladimirovna Glushkova, Elena V Gnedovskaya, Laszlo Göbölös, Mohamad Goldust, Pouya Goleij, Davide Golinelli, Sameer Vali Gopalani, Alessandra C Goulart, Mahdi Gouravani, Anmol Goyal, Michal Grivna, Giuseppe Grosso, Giovanni Guarducci, Mohammed Ibrahim Mohialdeen Gubari, Stefano Guicciardi, Rafael Alves Guimarães, Snigdha Gulati, David Gulisashvili, Damitha Asanga Gunawardane, Cui Guo, Anish Kumar Gupta, Rahul Gupta, Rajeev Gupta, Renu Gupta, Sapna Gupta, Vijai Kumar Gupta, Annie Haakenstad, Najah R Hadi, Nils Haep, Abdul Hafiz, Dariush Haghmorad, Demewoz Haile, Adel Hajj Ali, Ali Hajj Ali, Arvin Haj-Mirzaian, Esam S Halboub, Sebastian Haller, Rabih Halwani, Kanaan Hamagharib Abdullah, Nadia M Hamdy, Rifat Hamoudi, Nasrin Hanifi, Graeme J Hankey, Zaim Anan Haq, Md Rabiul Haque, Harapan Harapan, Arief Hargono, Josep Maria Haro, Ahmed I Hasaballah, S. M. Mahmudul Hasan, Mohammad Hasanian, Md Saquib Hasnain, Amr Hassan, Johannes Haubold, Simon I Hay, Jeffrey J Hebert, Omar E Hegazi, Mohammad Heidari, Mehdi Hemmati, Claire A Henson, Brenda Yuliana Herrera-Serna, Claudiu Herteliu, Majid Heydari, Kamal Hezam, Irma Hidayana, Yuta Hiraike, Nguyen Quoc Hoan, Ramesh Holla, Praveen Hoogar, Nobuyuki Horita, Md Mahbub Hossain, Hassan Hosseinzadeh, Mehdi Hosseinzadeh, Mihaela Hostiuc, Sorin Hostiuc, Chengxi Hu, Junjie Huang, Michael Hultström, Tsegaye Gebreyes Hundie, Aliza J Hunt, Kiavash Hushmandi, Javid Hussain, M. Azhar Hussain, Nawfal R Hussein, Hong-Han Huynh, Bing-Fang Hwang, Segun Emmanuel Ibitoye, Pulwasha Maria Iftikhar, Adalia I Ikiroma, Paul Chukwudi Ikwegbue, Irena M Ilic, Milena D Ilic, Mustapha Immurana, Mustafa Alhaji Isa, Md. Rabiul Islam, Sheikh Mohammed Shariful Islam, Faisal Ismail, Nahlah Elkudssiah Ismail, Gaetano Isola, Masao Iwagami, Ihoghosa Osamuyi Iyamu, Louis Jacob, Kathryn H Jacobsen, Morteza Jafarinia, Kasra Jahankhani, Nader Jahanmehr, Nityanand Jain, Ammar Abdulrahman Jairoun, Dr Ruchi Jakhmola Mani, Safayet Jamil, Roland Dominic G Jamora, Abubakar Ibrahim Jatau, Sabzali Javadov, Tahereh Javaheri, Shubha Jayaram, Sun Ha Jee, Jayakumar Jeganathan, Heng Jiang, Mohammad Jokar, Jost B Jonas, Nitin Joseph, Charity Ehimwenma Joshua, Mikk Jürisson, Vaishali K, Ali Kabir, Zubair Kabir, Vidya Kadashetti, Laleh R Kalankesh, Sanjay Kalra, Ashwin Kamath, Rajesh Kamath, Arun Kamireddy, Mona Kanaan, Tanuj Kanchan, Edmund Wedam Kanmiki, Kehinde Kazeem Kanmodi, Sushil Kumar Kansal, Asima Karim, Samad Karkhah, Faizan Zaffar Kashoo, Hengameh Kasraei, Molly B Kassel, Srinivasa Vittal Katikireddi, Joonas H Kauppila, Harkiran Kaur, Gbenga A Kayode, Foad Kazemi, Sina Kazemian, Fassikaw Kebede, Evie Shoshannah Kendal, Emmanuelle Kesse-Guyot, Shahram Khademvatan, Himanshu Khajuria, Amirmohammad Khalaji, Asaad Khalid, Nauman Khalid, Alireza Khalilian, Faham Khamesipour, Fayaz Khan, Mohammad Jobair Khan, Moien AB Khan, Shaghayegh

Khanmohammadi, Khaled Khatab, Haitham Khatatbeh, Moawiah Mohammad Khatatbeh, Mahalaqua Nazli Khatib, Hamid Reza Khayat Kashani, Khalid A Kheirallah, Manoj Khokhar, Moein Khormali, Zahra Khorrami, Atulya Aman Khosla, Majid Khosravi, Mahmood Khosrowjerdi, Jagdish Khubchandani, Zemene Demelash Kifle, Grace Kim, Julie Sojin Kim, Min Seo Kim, Yun Jin Kim, Ruth W Kimokoti, Adnan Kisa, Sezer Kisa, Luke D Knibbs, Ann Kristin Skrindo Knudsen, Sonali Kochhar, Ali-Asghar Kolahi, Farzad Kompani, Gerbrand Koren, Oleksii Korzh, Kewal Krishan, Varun Krishna, Vijay Krishnamoorthy, Burcu Kucuk Bicer, Md Abdul Kuddus, Mohammed Kuddus, Ilari Kuitunen, Omar Kujan, Mukhtar Kulimbet, Vishnutheertha Kulkarni, G Anil Kumar, Harish Kumar, Nithin Kumar, Rakesh Kumar, Vijay Kumar, Amartya Kundu, Dian Kusuma, Frank Kyei-Arthur, Ville Kytö, Hmwe Hmwe Kyu, Carlo La Vecchia, Ben Lacey, Muhammad Awwal Ladan, Lucie Laflamme, Chandrakant Lahariya, Daphne Teck Ching Lai, Ratilal Lalloo, Tea Lallukka, Judit Lám, Qing Lan, Tuo Lan, Iván Landires, Francesco Lanfranchi, Berthold Langguth, Van Charles Lansingh, Ariane Laplante-Lévesque, Bagher Larijani, Anders O Larsson, Savita Lasrado, Paolo Lauriola, Hilary R Lawlor, Huu-Hoai Le, Long Khanh Dao Le, Nhi Huu Hanh Le, Thao Thi Thu Le, Trang Diep Thanh Le, Janet L Leasher, Doo Woong Lee, Munjae Lee, Paul H Lee, Sang-woong Lee, Seung Won Lee, Shaun Wen Huey Lee, Yo Han Lee, James Leigh, Elvynna Leong, Ming-Chieh Li, Massimo Libra, Virendra S Ligade, Lee-Ling Lim, Stephen S Lim, Liknaw Workie Limenh, Daniel Lindholm, Paulina A Lindstedt, Stefan Listl, Gang Liu, Shiwei Liu, Shuke Liu, Xiaofeng Liu, Xuefeng Liu, Erand Llanaj, Rubén López-Bueno, José Francisco López-Gil, Arianna Maever Loreche, Paulo A Lotufo, Rafael Lozano, Jailos Lubinda, Giancarlo Lucchetti, Lisha Luo, Jay B Lusk, Lei Lv, Hawraz Ibrahim M Amin, Zheng Feei Ma, Kelsey Lynn Maass, Nikolaos Machairas, Monika Machoy, Áurea M Madureira-Carvalho, Hassan Magdy Abd El Razek, Azzam A Maghazachi, D. R. Mahadeshwara Prasad, Mehrdad Mahalleh, Phetole Walter Mahasha, Mansour Adam Mahmoud, Elham Mahmoudi, Golnaz Mahmoudvand, Maureen Makama, Elaheh Malakan Rad, Kashish Malhotra, Ahmad Azam Malik, Deborah Carvalho Malta, Yosef Manla, Ali Mansour, Mohammad Hadi Mansouri, Pejman Mansouri, Vahid Mansouri, Marjan Mansourian, Mohammad Ali Mansournia, Bishnu P Marasini, Hamid Reza Marateb, Joemer C Maravilla, Parham Mardi, Abdoljalal Marjani, Hamed Markazi Moghadam, Carlos Alberto Marrugo Arnedo, Gabriel Martinez, Ramon Martinez-Piedra, Francisco Rogerlândio Martins-Melo, Miquel Martorell, Wolfgang Marx, Roy Rillera Marzo, Sahar Masoudi, Yasith Mathangasinghe, Alexander G Mathioudakis, Medha Mathur, Navgeet Mathur, Neeta Mathur, Fernanda Penido Matozinhos, Jishanth Mattumpuram, Richard James Maude, Andrea Maugeri, Mahsa Mayeli, Mohsen Mazidi, Antonio Mazzotti, John J McGrath, Martin McKee, Anna Laura W McKowen, Michael A McPhail, Steven M McPhail, Asim Mehmood, Kamran Mehrabani-Zeinabad, Sepideh Mehravar, Tesfahun Mekene Meto, Endalkachew Belayneh Melese, Max Alberto Mendez Mendez-Lopez, Walter Mendoza, Ritesh G Menezes, George A Mensah, Laverne G Mensah, Alexios-Fotios A Mentis, Sultan Ayoub Meo, Atte Meretoja, Tuomo J Meretoja, Abera M Mersha, Tomislav Mestrovic, Kukulege Chamila Dinushi Mettananda, Sachith Mettananda, Adquate Mhlanga, Laurette Mhlanga, Tomasz Miazgowski, Irmina Maria Michalek, Ana Carolina Micheletti Gomide Nogueira de Sá, Ted R Miller, Le Huu Nhat Minh, Alireza Mirahmadi, Antonio Mirijello, Erkin M Mirrakhimov, Roya Mirzaei, Philip B Mitchell, Chaitanya Mittal, Madeline E Moberg, Atousa Moghadam Fard, Seyedehfatemeh Mohajelin, Ashraf Mohamadkhani, Ahmed Ismail Mohamed, Jama Mohamed, Mouhand F H Mohamed, Nouh Saad Mohamed, Ameen Mosa Mohammad, Soheil Mohammadi, Hussen Mohammed, Mustapha Mohammed, Shafiu Mohammed, Ali H Mokdad, Mariam Molokhia, Shaher Mohammad Momani, Sara Momtazmanesh, Lorenzo Monasta, Stefania Mondello, Mohammad Ali Moni, Fateme Montazeri, AmirAli Moodi Ghalibaf, Maryam Moradi, Yousef Moradi, Paula Moraga, Lidia Morawska, Rafael Silveira Moreira, Negar Morovatdar, Shane Douglas Morrison, Abbas Mosapour, Jonathan F Mosser, Elias Mossialos, Rohith Motappa, Vincent Mougin, Parsa Mousavi, Matías Mrejen, Sumaira Mubarik, Ulrich Otto Mueller, Francesk Mulita, Kavita Munjal, Efrén Murillo-Zamora, Khaled M Musallam, Ana-Maria Musina, Ghulam Mustafa, Woojae Myung, Ayoub Nafei, Ahamarshan Jayaraman Nagarajan, Pirouz Naghavi, Ganesh R Naik, Gurudatta Naik, Firzan Nainu, Soroush Najdaghi, Noureddin Nakhostin Ansari, Vinay Nangia, Sreenivas Narasimha Swamy, Shumaila Nargus, Delaram Narimani Davani, Bruno Ramos Nascimento, Gustavo G Nascimento, Abdallah Y Naser, Abdulqadir J Nashwan, Zuhair S Natto, Javaid Nauman, Samidi N K Navaratna, Muhammad Naveed, Nawsherwan , Biswa Prakash Nayak, Vinod C Nayak, Hadush Negash, Ionut Negoi, Ruxandra Irina Negoi, Seyed Aria Nejadghaderi, Chakib Nejjari, Soroush Nematollahi, Henok Biresaw Netsere, Marie Ng, Georges Nguefack-Tsague, Josephine W Ngunjiri, Anh Hoang Nguyen, Dang H Nguyen, Duc Hoang Nguyen, Hau Thi Hien Nguyen, Nhan Nguyen, Nhien Ngoc Y Nguyen, Phat Tuan Nguyen, QuynhAnh P Nguyen, Van Thanh Nguyen, Duc Nguyen Tran Minh, Robina Khan Niazi, Yeshambel T Nigatu, Mahdieh Niknam, Ali Nikoobar, Amin Reza Nikpoor, Nasrin Nikravangolsefid, Efaq Ali Noman, Shuhei Nomura, Syed Toukir Ahmed Noor, Nafise Noroozi, Mehran Nouri, Majid Nozari, Chisom Adaobi Nri-Ezedi, George Ntaios, Mengistu H Nunemo, Dieta Nurrika, Jerry John Nutor, Chimezie Igwegbe Nzoputam, Ogochukwu Janet Nzoputam, Bogdan Oancea, Kehinde O Obamiro, Ismail A Odetokun, Michael Safo Oduro, Oluwaseun Adeolu Ogundijo, Adesola Adenike Ogunfowokan, Abiola Ogunkoya,

Ayodipupo Sikiru Oguntade, In-Hwan Oh, Tolulope R Ojo-Akosile, Hassan Okati-Aliabad, Akinkunmi Paul Okekunle, Osaretin Christabel Okonji, Andrew T Olagunju, Matthew Idowu Olatubi, Gláucia Maria Moraes Oliveira, Bolajoko Olubukunola Olusanya, Jacob Olusegun Olusanya, Yinka Doris Oluwafemi, Hany A Omar, Goran Latif Omer, Sokking Ong, Sandersan Onie, Obinna E Onwujekwe, Abdulahi Opejin Opejin, Michal Ordak, Verner N Orish, Alberto Ortiz, Esteban Ortiz-Prado, Wael M S Osman, Sergej M Ostojic, Samuel M Ostroff, Uchechukwu Levi Osuagwu, Adrian Otoiu, Stanislav S Otstavnov, Amel Ouyahia, Mayowa O Owolabi, Oyetunde T Oyeyemi, Ahmad Ozair, Mahesh Padukudru P A, Alicia Padron-Monedero, Jagadish Rao Padubidri, Pramod Kumar Pal, Tamás Palicz, Feng Pan, Hai-Feng Pan, Songhomitra Panda-Jonas, Anamika Pandey, Victoria Pando-Robles, Helena Ullyartha Pangaribuan, Georgios D Panos, Leonidas D Panos, Ioannis Pantazopoulos, Anca Mihaela Pantea Stoian, Romil R Parikh, Eun-Kee Park, Seoyeon Park, Sungchul Park, Nicholas Parsons, Ashwaghosha Parthasarathi, Maja Pasovic, Roberto Passera, Jay Patel, Aslam Ramjan Pathan, Shankargouda Patil, Dimitrios Patoulias, Shrikant Pawar, Hamidreza Pazoki Toroudi, Spencer A Pease, Amy E Peden, Paolo Pedersini, Umberto Pensato, Veincent Christian Filipino Pepito, Prince Peprah, Marcos Pereira, Maria Odete Pereira, Arokiasamy Perianayagam, Norberto Perico, Simone Perna, Konrad Pesudovs, Fanny Emily Petermann-Rocha, Hoang Tran Pham, Anil K Philip, Michael R Phillips, Manon Pigeolet, Michael A Piradov, Enrico Pisoni, Evgenii Plotnikov, Dimitri Poddighe, Roman V Polibin, Ramesh Poluru, Ville T Ponkilainen, Djordje S Popovic, Maarten J Postma, Ahmad Pour-Rashidi, Disha Prabhu, Sergio I Prada, Jalandhar Pradhan, Pranil Man Singh Pradhan, Akila Prashant, Elton Junio Sady Prates, Tina Priscilla, Hery Purnobasuki, Bharathi M Purohit, Jagadeesh Puvvula, Nameer Hashim Qasim, Ibrahim Qattea, Asma Saleem Qazi, Gangzhen Qian, Mehrdad Rabiee Rad, Venkatraman Radhakrishnan, Hadi Raeisi Shahraki, Quinn Rafferty, Alberto Raggi, Cat Raggi, Nasiru Raheem, Fakher Rahim, Md Jillur Rahim, Sarvenaz Rahimibarghani, Md Mijanur Mijanur Rahman Rahman, Mosiur Rahman, Muhammad Aziz Rahman, Tafhimur Rahman, Amir Masoud Rahmani, Mohammad Rahmanian, Nazanin Rahmanian, Rahem Rahmati, Setyaningrum Rahmawaty, Diego Raimondo, Adarsh Raja, Prashant Rajput, Majed Ramadan, Shakthi Kumaran Ramasamy, Sheena Ramazanu, Pramod W Ramteke, Kritika Rana, Rishabh Kumar Rana, Chhabi Lal Ranabhat, Amey Rane, Chythra R Rao, Mithun Rao, Davide Rasella, Vahid Rashedi, Ahmed Mustafa Rashid, Ashkan Rasouli-Saravani, Prateek Rastogi, Azad Rasul, Devarajan Rathish, Giridhara Rathnaiah Babu, Santosh Kumar Rauniyar, Ramin Ravangard, David Laith Rawaf, Salman Rawaf, Rabail Zehra Raza, Elrashdy Moustafa Mohamed Redwan, Lennart Reifels, Marissa B Reitsma, Giuseppe Remuzzi, Kannan RR Rengasamy, Bhageerathy Reshmi, Serge Resnikoff, Stefano Restaino, Luis Felipe Reyes, Nazila Rezaei, Negar Rezaei, Zahra Sadat Rezaei, Mohsen Rezaeian, Taeho Gregory Rhee, Jennifer Rickard, Toshana Robalik, Hannah Elizabeth Robinson-Oden, Hermano Alexandre Lima Rocha, Mónica Rodrigues, Jefferson Antonio Buendia Rodriguez, Leonardo Roever, Debby Syahru Romadlon, Luca Ronfani, Moustaq karim khan Rony, Gholamreza Roshandel, Kunle Rotimi, Himanshu Sekhar Rout, Bedanta Roy, Enrico Rubagotti, Guilherme de Andrade Ruela, Susan Fred Rumisha, Tilleye Runghien, Michele Russo, Aly M A Saad, Korosh Saber, Maha Mohamed Saber-Ayad, Cameron John Sabet, Siamak Sabour, Perminder S Sachdev, Adam Saddler, Bashdar Abuzed Sadee, Masoumeh Sadeghi, Mohammad Reza Saeb, Umar Saeed, Sher Zaman Safi, Rajesh Sagar, Alireza Saghafi, Dominic Sagoe, Amirhossein Sahebkar, Pragyan Monalisa Sahoo, Mirza Rizwan Sajid, Nasir Salam, Payman Salamati, Afeez Abolarinwa Salami, Mohamed A Saleh, Leili Salehi, Marwa Rashad Salem, Aanuoluwa James Salemcity, Sohrab Salimi, Hossein Samadi Kafil, Saad Samargandy, Yoseph Leonardo Samodra, Abdallah M Samy, Juan Sanabria, Francesca Sanna, Milena M Santric-Milicevic, Bruno Piassi Sao Jose, Sivan Yegnanarayana Iyer Saraswathy, Aswini Saravanan, Rodrigo Sarmiento-Suárez, Gargi Sachin Sarode, Sachin C Sarode, Benn Sartorius, Maheswar Satpathy, Abu Sayeed, Nikolaos Scarmeas, Benedikt Michael Schaarschmidt, Christophe Schinckus, Art Schuermans, Austin E Schumacher, Aletta Elisabeth Schutte, David C Schwebel, Falk Schwendicke, Siddharthan Selvaraj, Mohammad H Semreen, Sabyasachi Senapati, Pallav Sengupta, Subramanian Senthilkumaran, Dragos Serban, Yashendra Sethi, Allen Seylani, Mahan Shafie, Pritik A Shah, Ataollah Shahbandi, Samiah Shahid, Wajeehah Shahid, Hamid R Shahsavari, Moyad Jamal Shahwan, Masood Ali Shaikh, Ali S Shalash, Ali Shamekh, Muhammad Aaqib Shamim, Mohd Shanawaz, Abhishek Shankar, Mohammed Shannawaz, Medha Sharath, Sadaf Sharfaei, Amin Sharifan, Javad Sharifi-Rad, Anupam Sharma, Manoj Sharma, Saurab Sharma, Vishal Sharma, Rajesh P Shastry, Maryam Shayan, Shashank Shekhar, Rekha R Shenoy, Mahabalesh Shetty, Pavanchand H Shetty, Premalatha K Shetty, Peilin Shi, Amir Shiani, Mika Shigematsu, Tariku Shimels, Rahman Shiri, Aminu Shittu, Ivy Shiue, K M Shivakumar, Sina Shool, Seyed Afshin Shorofi, Sunil Shrestha, Kerem Shuval, Yafei Si, Emmanuel Edwar Siddig, Jaspreet Kaur Sidhu, João Pedro Silva, Luís Manuel Lopes Rodrigues Silva, Soraia Silva, Thales Philipe R Silva, Colin R Simpson, Kyle E Simpson, Abhinav Singh, Balbir Bagicha Singh, Baljinder Singh, Harmanjit Singh, Jasbir Singh, Paramdeep Singh, Puneetpal Singh, Søren T Skou, Georgia Smith, Farrukh Sobia, Bogdan Socea, Shipra Solanki, Hamidreza Soleimani, Sameh S M Soliman, Yi Song, Ireneous N Soyiri, Michael Spartalis, Sandra Spearman, Chandrashekhar T Sreeramareddy, Jeffrey D Stanaway, Muhammad Haroon Stanikzai, Antonina V Starodubova, Dan J Stein, Caitlyn Steiner, Paschalis

Steiropoulos, Leo Stockfelt, Mark A Stokes, Kurt Straif, Narayan Subedi, Rizwan Suliankatchi Abdulkader, Abida Sultana, Jing Sun, Johan Sundström, Chandan Kumar Swain, Lukasz Szarpak, Mindy D Szeto, Payam Tabaee Damavandi, Rafael Tabarés-Seisdedos, Ozra Tabatabaei Malazy, Seyed-Amir Tabatabaeizadeh, Shima Tabatabai, Karen M Tabb, Celine Tabche, Mohammad Tabish, Yasaman Taheri Abkenar, Moslem Taheri Soodejani, Jabeen Taiba, Iman M Talaat, Jacques Lukenze Tamuzi, Ker-Kan Tan, Haosu Tang, Nathan Y Tat, Razieh Tavakoli Oliaee, Seyed Mohammad Tavangar, Nuno Taveira, Abdelghani Tbakhi, Hadi Tehrani, Mohamad-Hani Temsah, Masayuki Teramoto, Behailu Terefe Tesfaye, Enoch Teye-Kwadjo, Pugazhenthan Thangaraju, Kavumpurathu Raman Thankappan, Rekha Thapar, Rasiah Thayakaran, Sathish Thirunavukkarasu, Nihal Thomas, Lau Caspar Thygesen, Jansje Henny Vera Ticoalu, Dinesh Timalsena, Tenaw Yimer Tiruye, Krishna Tiwari, Sojit Tomo, Marcello Tonelli, Roman Topor-Madry, Mathilde Touvier, Marcos Roberto Tovani-Palone, An Thien Tran, Jasmine T Tran, Nghia Minh Tran, Thang Huu Tran, Domenico Trico, Samuel Joseph Tromans, Thien Tan Tri Tai Truyen, Aristidis Tsatsakis, Evangelia Eirini Tsermpini, Munkhtuya Tumurkhuu, Steven T Turnock, Arit Udoh, Atta Ullah, Saeed Ullah, Sana Ullah, Srikanth Umakanthan, Muhammad Umar, Shehu Salihu Umar, Brigid Unim, Bhaskaran Unnikrishnan, Era Upadhyay, Jibrin Sammani Usman, Sanaz Vahdati, Asokan Govindaraj Vaithinathan, Omid Vakili, Rohollah Valizadeh, Jef Van den Eynde, Priya Vart, Shoban Babu Varthya, Tommi Juhani Vasankari, Milena Vasic, Narayanaswamy Venketasubramanian, Massimiliano Veroux, Georgios-Ioannis Verras, Dominique Vervoort, Mathavaswami Vijayageetha, Jorge Hugo Villafañe, Manish Vinayak, Francesco S Violante, Sergey Konstantinovitch Vladimirov, Vasily Vlassov, Bay Vo, Karn Vohra, Theo Vos, Abdul Wadood Wadood, Yasir Waheed, Fang Wang, Shaopan Wang, Shu Wang, Yanqing Wang, Yanzhong Wang, Yuan-Pang Wang, Mary Njeri Wanjau, Muhammad Waqas, Paul Ward, Abdul Waris, Emebet Gashaw Wassie, Stefanie Watson, Marcia R Weaver, Kosala Gayan Weerakoon, Robert G Weintraub, Haftom Legese Legese Weldetinsaa, Katherine M Wells, Yi Feng Wen, Ronny Westerman, Taweewat Wiangkham, Dakshitha Praneeth Wickramasinghe, Evi Widowati, Marcin W Wojewodzic, Dawit Habte Woldeyes, Axel Walter Wolf, Charles D A Wolfe, Chenkai Wu, Dongze Wu, Felicia Wu, Jiayuan Wu, Zenghong Wu, Sarah Wulf Hanson, Hong Xiao, Suowen Xu, Rakesh Yadav, Kazumasa Yamagishi, Danting Yang, Yuichiro Yano, Amir Yarahmadi, Iman Yazdani Nia, Pengpeng Ye, Renjulal Yesodharan, Subah Abderehim Yesuf, Saber Yezli, Arzu Yiğit, Vahit Yiğit, Zeamanuel Anteneh Yigzaw, Dehui Yin, Paul Yip, Naohiro Yonemoto, Yuyi You, Mustafa Z Younis, Chuanhua Yu, Elaine A Yu, Yong Yu, Chun-Wei Yuan, Hadiza Yusuf, Uzma Zafar, Nima Zafari, Mondal Hasan Zahid, Fathiah Zakham, Nazar Zaki, Taddese Alemu Zerfu, Haijun Zhang, Jingya Zhang, Liqun Zhang, Yunquan Zhang, Zhiqiang Zhang, Xiu-Ju George Zhao, Yang Zhao, Zhongyi Zhao, Chenwen Zhong, Bolun Zhou, Juexiao Zhou, Shangcheng Zhou, Bin Zhu, Abzal Zhumagaliuly, Magdalena Zielińska, Ghazal Zoghi, Alimuddin Zumla, Sa'ed H Zyoud, Samer H Zyoud, Amanda E Smith**, and Christopher J L Murray**.

*First author
**Joint senior authors

Affiliations

Institute for Health Metrics and Evaluation (Prof S E Vollset DrPH, A J Ahlstrom MSc, T Alam MPH, E A Andrews MSc, C M Antony MA, A Y Aravkin PhD, C Ashbaugh MA, N V Bhattacharjee PhD, C Bisignano MPH, Prof M Brauer DSc, D Bryazka BA, K Burkart PhD, S Cao MS, J Chalek BS, J T Clayton BS, R M Cogen BA, A J Cohen DSc, H Comfort MPH, J T Connolly BS, E E K Cooper PhD, X Dai PhD, B E Dalton BA, Prof L Dandona MD, Prof R Dandona PhD, Prof V L Feigin PhD, L S Flor MPH, L M Force MD, S Ghoba MS, A Haakenstad ScD, D Haile PhD, Prof S I Hay FMedSci, C A Henson MPH, M B Kassel BA, J S Kim MS, H H Kyu PhD, H R Lawlor BA, Prof S S Lim PhD, P A Lindstedt MPH, Prof R Lozano MD, K L Maass PhD, A W McKowen MA, T Mestrovic PhD, M E Moberg MS, A H Mokdad PhD, J F Mosser MD, V Mougin BA, Q P Nguyen BS, S M Ostroff PhD, M Pasovic MEd, S A Pease BS, Q Rafferty BA, C Raggi MS, M B Reitsma BS, T Robalik BSc, H E Robinson-Oden MLIS, T Runghien MSc, A E Schumacher PhD, K E Simpson BS, G Smith MS, S Spearman MS, J D Stanaway PhD, C Steiner MPH, Prof T Vos PhD, S Watson MS, Prof M R Weaver PhD, K M Wells BA, S Wulf Hanson PhD, C Yuan PhD, A E Smith MPA, Prof C J L Murray DPhil), Department of Health Metrics Sciences, School of Medicine (Prof S E Vollset DrPH, A Y Aravkin PhD, K Burkart PhD, X Dai PhD, Prof R Dandona PhD, L S Flor MPH, L M Force MD, Prof S I Hay FMedSci, J S Kim MS, H H Kyu PhD, Prof S S Lim PhD, Prof R Lozano MD, A H Mokdad PhD, B Sartorius PhD, J D Stanaway PhD, Prof T Vos PhD, Prof M R Weaver PhD, Prof C J L Murray DPhil), Department of Applied Mathematics (A J Ahlstrom MSc, A Y Aravkin PhD), School of Medicine (Prof E J Boyko MD), Division of Pediatric Hematology-Oncology (L M Force MD), Department of Global Health (S Kochhar MD), Department of Anesthesiology & Pain Medicine (V Krishnamoorthy MD), Division of Plastic and Reconstructive Surgery (S D Morrison MD), Henry M Jackson School of International Studies (S M Ostroff PhD), University of Washington, Seattle, WA, USA; GBD Collaborating Unit (Prof S E Vollset DrPH), Department of Disease Burden (A S Knudsen PhD), Norwegian Institute of Public Health, Bergen, Norway; Department of Radiation Oncology (H S Ababneh MD), Department of Orthopaedic Surgery (A Ebrahimi MD), Department of Radiology (A Haj-Mirzaian MD, X Liu PhD), Division of Cardiology (D H Nguyen BS), Cardiovascular Research Center (A Schuermans BSc), Massachusetts General Hospital, Boston, MA, USA; Department of Clinical Governance and Quality Improvement (Y H Abate MSc), Aleta Wondo Hospital, Aleta Wondo, Ethiopia; Department of Juridical and Economic Studies (C Abbafati PhD), Department of Public Health and Infectious Diseases (M S Cattaruzza PhD), La Sapienza University, Rome, Italy; Doheny Eye Institute (R Abbasgholizadeh MD), Radiology Department (N Nguyen BSc), University of California Los Angeles, Los Angeles, CA, USA; Department of Orthopedic Surgery (M Abbasian MD), T.H. Chan School of Public Health (Prof T W Bärnighausen MD, P M S Pradhan MD), Center for Primary Care (S Basu PhD), Harvard Business School (F Caetano dos Santos PhD), Division of Cardiovascular Medicine (G Chi MD), Department of Health Policy and Oral Epidemiology (Z S Natto DrPH), Department of Global Health and Social Medicine (M Pigeolet MD), Beth Israel Deaconess Medical Center (S Sharfaei MD), Harvard University, Boston, MA, USA; Department of Orthopaedic Surgery (M Abbasian MD), Urology Department (M Bonakdar Hashemi MD), Internal Medicine Department of SBMU (H Borhany MD), School of Medicine (D J Ghadimi MD, F Montazeri MD, S Nejadghaderi MD), Psychiatric Nursing and Management Department (F Ghadirian PhD), Department of Medical Genetics (M Ghasemi PhD), Center for Comprehensive Genetic Services (M Ghasemi PhD), Obstetrics and Gynecology Department (E Ghotbi MD), Obesity Research Center (A Haj-Mirzaian MD), Department of Immunology (K Jahankhani MSc, A Rasouli-Saravani PhD), Department of Health Policy and Management (N Jahanmehr PhD), Safety Promotion and Injury Prevention Research Center (N Jahanmehr PhD), Department of Neurosurgery (H Khayat Kashani MD), Ophthalmic Epidemiology Research Center (Z Khorrami PhD), Social Determinants of Health Research Center (A Kolahi MD, A Nikoobar DipSc), Department of Orthopedics (A Mirahmadi MD), Research Center for Social Determinants of Health (M Niknam PhD), Student Research Committee (M Rahmanian MD), Department of Epidemiology (S Sabour PhD), Department of Anesthesiology (S Salimi MD), Ophthalmic Research Center (ORC) (M Shayan MD), Emergency Department (S Shool MD), Department of Medical Education (S Tabatabai PhD), Shahid Beheshti University of Medical Sciences, Tehran, Iran; Advanced Diagnostic and Interventional Radiology Research Center (H Abbastabar PhD), The Institute of Pharmaceutical Sciences (TIPS) (Prof M Abdollahi PhD), School of Pharmacy (Prof M Abdollahi PhD), Research Center for Immunodeficiencies (H Abolhassani PhD), Universal Scientific Education and Research Network (USERN) (M Amirzade-Iranaq DDS), Digestive Diseases Research Institute (A Anoushiravani MD, V Mansouri MD, S Masoudi MSc, A Mohamadkhani PhD), Non-communicable Diseases Research Center (S Azadnajafabad MD, M Bastan MD, S Momtazmanesh MD, F Montazeri MD, P Mousavi MD, N Rezaei MD, N Rezaei PhD), School of Medicine (A Behnoush BS, M Gouravani MD, A Khalaji BS, S Khanmohammadi MD, M Mayeli MD, S Mohammadi MD, S Momtazmanesh MD), Department of Pediatric Neurology (M Bemanalizadeh MD), Department of Scientific Research (F Chichagi MD),

Iranian Research Center for HIV/AIDS (O Dadras DrPH), Department of Cardiology (Z Esmaeili MD, M Mahalleh MD, E Mahmoudi MD, P Mansouri MD), Department of Ophthalmology (S Ghafarian MD), Cardiac Primary Prevention Research Center (S Kazemian MD), Department of Cardiac Electrophysiology (S Kazemian MD), Center for Research and Training in Skin Diseases and Leprosy (F Khamesipour PhD), Sina Trauma and Surgery Research Center (M Khormali MD, Prof P Salamati MD, S Shool MD), Children's Medical Center (F Kompani MD), Endocrinology and Metabolism Research Institute (Prof B Larijani FACE, N Rezaei PhD, O Tabatabaei Malazy PhD), Department of Pediatric Cardiology (Prof E Malakan Rad MD), Department of Epidemiology and Biostatistics (M Mansournia PhD), Water Quality Research Center (R Mirzaei PhD), NeuroTRACT Association (A Moghadam Fard MD), Department of Physiotherapy (Prof N Nakhostin Ansari PhD), Research Center for War-affected People (Prof N Nakhostin Ansari PhD), Department of Cardiovascular Diseases (S Nematollahi MD), Department of Pharmacology (N Noroozi DVM), Department of Neurosurgery (A Pour-Rashidi MD), Department of Physical Medicine and Rehabilitation (S Rahimibarghani MD), Department of Neurology (M Shafie MD), Department of Medicine (A Shahbandi MD), Department of Pharmaceutical Care (A Sharifan PharmD), Research Center for Rational Use of Drugs (A Sharifan PharmD), Department of Pathology (Prof S Tavangar MD), Tehran University of Medical Sciences, Tehran, Iran; College of Pharmacy (A H A Abd Al Magied BPharm), Department of Mathematics and Sciences (A Abdelkader PhD), Department of Clinical Sciences (S O Alalalmeh BPharm, Prof E A Arafa PhD, O E Hegazi BPharm), College of Medicine (S Dutta PhD), Department of Education (M Eltahir PhD), Nonlinear Dynamics Research Center (NDRC) (Prof S M Momani PhD), Center for Medical and Bio-Allied Health Sciences Research (Prof M J Shahwan PhD, S H Zyoud PhD), Ajman University, Ajman, United Arab Emirates; Department of Epidemiology (S Abd ElHafeez DrPH), Biomedical Informatics and Medical Statistics Department (I El Sayed PhD), Pediatric Dentistry and Dental Public Health Department (Prof O A A Elmeligy PhD), Department of Tropical Health and Parasitology (R M Ghazy PhD), Department of Pathology (Prof I M Talaat PhD), Alexandria University, Alexandria, Egypt; Department of Surgery (M Abdelmasseh MD, Prof J Sanabria MD), Marshall University, Huntington, WV, USA; Department of Tropical Medicine and Infectious Diseases (S Abd-Elsalam PhD), Tanta University, Tanta, Egypt; Department of Medicine (P Abdi BEng), Memorial University, St. John's, NL, Canada; Department of Medicine (Prof M Abdoun BMedSc), University of Setif Algeria, Setif, Algeria; Department of Physiotherapy (A Abdullahi PhD, A W Awotidebe PhD, J S Usman PhD), Department of Community Medicine (Prof M A Gadanya FMCPH), Department of Nursing Science (M Ladan PhD), Bayero University Kano, Kano, Nigeria; Department of Rehabilitation Sciences (A Abdullahi PhD, M U Ali MSc, M Khan MPH, J S Usman PhD), Hong Kong Polytechnic University, Hong Kong, China; Department of Midwifery (M Abebe MSc), Dilla University, Dilla, Ethiopia; Department of Community Medicine (O Abiodun MPH), Babcock University, Ilishan-Remo, Nigeria; Department of Family and Community Health (R G Aboagye MPH), Department of Epidemiology and Biostatistics (L A Adzigbli BSc, S A Bosoka Mphil, R K Dowou MPhil), Department of Population and Behavioural Sciences (H Amu PhD), Institute of Health Research (P T Doegah PhD, M Immurana PhD), Department of Microbiology and Immunology (V N Orish PhD), University of Health and Allied Sciences, Ho, Ghana; Department of Medical Biochemistry and Biophysics (H Abolhassani PhD), Department of Neurobiology, Care Sciences, and Society (S Fereshtehnejad PhD), Department of Molecular Medicine and Surgery (Prof J H Kauppila MD), Department of Global Public Health (Prof L Laflamme PhD), Karolinska Institute, Stockholm, Sweden; Department of Physical Pharmacy and Pharmacokinetics (M Abouzid PharmD), Poznan University of Medical Sciences, Poznan, Poland; Department of Public Health (G B Aboye MSc), Madda Walabu University, Addis Ababa, Ethiopia; Department of Nutrition and Dietetics (G B Aboye MSc), Jimma University, Addis Ababa, Ethiopia; Department of Pediatric Dentistry (Prof L G Abreu PhD), School of Nursing (A G da Silva PhD), Department of Maternal and Child Nursing and Public Health (Prof D C Malta PhD, Prof A C Micheletti Gomide Nogueira de Sá MSc, E J S Prates BS), Department of Maternal and Public Health (Prof F P Matozinhos PhD), Department of Clinical Medicine (Prof B R Nascimento PhD), Clinical Hospital (Prof B R Nascimento PhD), Department of Applied Nursing (Prof M O Pereira PhD), Department of Infectious Diseases and Tropical Medicine (B P Sao Jose PhD), Vaccination Research Observatory (T R Silva PhD), Federal University of Minas Gerais, Belo Horizonte, Brazil; Medical Laboratory Sciences (A Absalan PhD), Khomein University of Medical Sciences, Khomein, Iran; Department of Research and Development (A Absalan PhD), Satras Biotechnology Company, Tehran, Iran; Department of Nursing (H Abualruz PhD), Al Zaytoonah University of Jordan, Amman, Jordan; Department of Pharmacology and Toxicology (B Abubakar PhD), Department of Veterinary Public Health and Preventive Medicine (A Shittu MSc), Usmanu Danfodiyo University, Sokoto, Sokoto, Nigeria; Nigerian Institute of Medical Research (B Abubakar PhD), Nigerian Institute of Medical Research, Lagos, Nigeria; Department of Research (H J J Abukhadijah MPH), Department of Nursing Education and Research (A J Nashwan MSc), Hamad Medical Corporation, Doha, Qatar; Internal Medicine and Alcohol Related Disease Unit (Prof G Addolorato MD), Department of Woman and Child Health and Public Health (D Buonsenso MD), Fondazione Policlinico

Universitario A. Gemelli IRCCS (Agostino Gemelli University Polyclinic IRCCS), Rome, Italy; Department of Medical and Surgical Sciences (Prof G Addolorato MD), Università Cattolica di Roma (Catholic University of Rome), Rome, Italy; Department of Obstetrics and Gynecology (V Adekanmbi PhD), University of Texas Medical Branch, Galveston, TX, USA; Department of Microbiology (Prof C O Adetunji PhD), Edo State University Uzairue, Iyamho, Nigeria; Department of Biochemistry (J B Adetunji PhD), Osun State University, Osogbo, Nigeria; Department of Biosciences and Biotechnology (T E Adeyeoluwa PhD, O T Oyeyemi PhD), Department of Environmental and Occupational Health (B S Anuoluwa MPH), Department of Microbiology (O O Bello PhD, Y D Oluwafemi PhD), Department of Biological Sciences (T C Ekundayo PhD), Department of Biochemistry (A J Salemcity PhD), University of Medical Sciences, Ondo, Ondo, Nigeria; Department of Veterinary Medicine (T E Adeyeoluwa PhD), Department of Epidemiology and Medical Statistics (A F Fagbamigbe PhD), Department of Periodontology and Community Dentistry (O F Fagbule FWACS), Department of Health Promotion and Education (S E Ibitoye MPH, A Ogunkoya MPH), Department of Veterinary Public Health and Preventive Medicine (O A Ogundijo MSc), College of Medicine (A P Okekunle PhD), Department of Medicine (Prof M O Owolabi DrM), University of Ibadan, Ibadan, Nigeria; Department of Business Administration (R Adha PhD), Muhammadiyah University of Mataram, Mataram, Indonesia; Department of Fisheries and Marine Bioscience (R K Adhikary MS), Jashore University of Science and Technology, Jashore, Bangladesh; Research School of Population Health (R K Adhikary MS, N Bagheri PhD, R A Burns PhD, Prof N Cherbuin PhD), National Centre for Epidemiology and Population Health (Y Alemu MPH), Australian National University, Canberra, ACT, Australia; Faculty of Medicine (Q E S Adnani PhD), Center of Excellence in Higher Education for Pharmaceutical Care Innovation (Prof M J Postma PhD), Universitas Padjadjaran, Bandung, Indonesia; Department of Medicine (F Afrashteh MD), Health Management and Economics Research Center (J Arabloo PhD, H Ayatollahi PhD), Department of Health Information Management (H Ayatollahi PhD), School of Medicine (M Bastan MD, M Dodangeh MD), Preventive Medicine and Public Health Research Center (B Eshrati PhD), Minimally Invasive Surgery Research Center (A Kabir MD), Eye Research Center (H Kasraei MD), Department of Health Economics (M Khosravi PhD), Comprehensive Research Laboratory (R Mirzaei PhD), Department of Physiology (H Pazoki Toroudi PhD), Physiology Research Center (H Pazoki Toroudi PhD), Iran University of Medical Sciences, Tehran, Iran (M Moradi MD); Department of Life Sciences (M S Afzal PhD), University of Management and Technology, Lahore, Pakistan; Department of Community Medicine (Prof S Afzal PhD), King Edward Memorial Hospital, Lahore, Pakistan; Department of Public Health (Prof S Afzal PhD), Public Health Institute, Lahore, Pakistan; Family and Community Health Department (F Agbozo PhD), University of Health and Allied Sciences, Hohoe, Ghana; Department of Global and Public Health (F Agbozo PhD), McGill University, Montreal, Quebec, Canada; Department of Medical and Surgical Sciences and Advanced Technologies "GF Ingrassia" (Prof A Agodi PhD, M Barchitta PhD, A Maugeri PhD, Prof M Veroux PhD), Department of Biomedical and Biotechnological Sciences (L Falzone PhD, G Grosso PhD, Prof M Libra PhD), Department of General Surgery and Medical-Surgical Specialties (Prof G Isola PhD), University of Catania, Catania, Italy; Trivedi School of Biosciences (Prof A Agrawal PhD), Ashoka University, Sonipat, India; Section of General Internal Medicine (Prof A Agrawal PhD), Baylor College of Medicine, Houston, TX, USA; Department of Geography and Planning (W Agyemang-Duah MSc), Department of Biomedical and Molecular Sciences (A Nikpoor PhD), Queen's University, Kingston, ON, Canada; School of Public Health (B O Ahinkorah MPhil), School of Nursing and Midwifery (M Chutiyami PhD), University of Technology Sydney, Sydney, NSW, Australia; Department of Medical Biochemistry (A Ahmad PhD), Department of Pediatrics (Prof G Mustafa MD), Department of Pharmacology (A R Pathan PhD, M Tabish MPharm), Shaqra University, Shaqra, Saudi Arabia; College of Medicine (F Ahmad PhD, Prof R Halwani PhD, Prof R Hamoudi PhD, M A Saleh PhD), Department of Pharmacy Practice and Pharmacotherapeutics (Prof K H Alzoubi PhD, Prof H A Omar PhD), Department of Physiotherapy (A Arumugam PhD), Clinical Sciences Department (H J Barqawi MPhil, N R Dash MD, Prof R Halwani PhD, Prof A A Maghazachi PhD, M M Saber-Ayad MD, Prof I M Talaat PhD), Department of Basic Biomedical Sciences (Y Bustanji PhD), Sharjah Institute for Medical Research (N M Elemam PhD), Basic Medical Sciences Department (A B Elmoselhi PhD), Research Institute of Medical & Health Sciences (A B Elmoselhi PhD, Prof M H Semreen PhD), Department of Clinical Nutrition and Dietetics (M E M Faris PhD), Department of Finance and Economics (Prof M Hussain PhD), Department of Basic Medical Sciences (A Karim PhD), College of Pharmacy (Prof M H Semreen PhD), Department of Medicinal Chemistry (S S M Soliman PhD), University of Sharjah, Sharjah, United Arab Emirates (K A Altirkawi MD); Department of Clinical Nursing (Prof M M Ahmad PhD), University of Jordan, Amman, Jordan; Department of Health and Biological Sciences (S Ahmad PhD), Abasyn University, Peshawar, Pakistan; Department of Natural Sciences (S Ahmad PhD), Gilbert and Rose-Marie Chagoury School of Medicine (L Roever PhD), Lebanese American University, Beirut, Lebanon; Department of Medical Oncology (S Ahmad MD), Department of Medicine (M Ganiyani MD), Miami Cancer Institute, Miami, FL, USA; Department of Community Medicine and Preventive Health (S Ahmad MD), King Edward Medical

University Lahore, Lahore, Pakistan; Maternal and Child Health Division (A Ahmed MS), International Centre for Diarrhoeal Disease Research, Bangladesh (icddr,b), Dhaka, Bangladesh; Department of Women's and Children's Health (A Ahmed MS), Department of Surgical Sciences (M Hultström PhD), Department of Medical Cell Biology (M Hultström PhD), Department of Medical Sciences (Prof A O Larsson PhD, D Lindholm MD, Prof J Sundström PhD), Uppsala University, Uppsala, Sweden; Institute of Endemic Diseases (A Ahmed MSc), Unit of Basic Medical Sciences (E E Siddig MD), University of Khartoum, Khartoum, Sudan; Swiss Tropical and Public Health Institute (A Ahmed MSc), University of Basel, Basel, Switzerland; Department of Biosciences (H Ahmed PhD), COMSATS Institute of Information Technology, Islamabad, Pakistan; Department of Biochemistry (S Ahmed BSc), Jamia Hamdard, Delhi, India; Brody School of Medicine (S Ahmed PhD), Department of Computer Science (A O Bodunrin MSc), Department of Geography (A Opejin MS), Department of Physiology (M Tumurkhuu PhD), East Carolina University, Greenville, NC, USA; Department of Internal Medicine (K Akinosoglou PhD), University of Patras, Patras, Greece; Department of Internal Medicine and Infectious Diseases (K Akinosoglou PhD), University General Hospital of Patras, Patras, Greece; Department of Cardiology (M A Akkaif PhD), Fudan University, Shanghai, China; Chicago College of Osteopathic Medicine (A E Akrami BS), Midwestern University, Downers Grove, IL, USA; Feinberg School of Medicine (A E Akrami BS), Department of Preventive Medicine (L Mhlanga PhD), Northwestern University, Chicago, IL, USA; Department of Maternal and Child Health (E Akter MSc), Health System and Population Studies Division (S Jamil BPharm), Maternal and Child Health Division (S Noor MS, A Sayeed MSc), International Centre for Diarrhoeal Disease Research, Bangladesh, Dhaka, Bangladesh; Communicable Diseases Adviser to Health Affairs (S Al Awaidy MSc), HQ, Ministry of Health, Muscat, Oman; ME'NA-ISN Chairperson, Muscat, Oman (S Al Awaidy MSc); Division of Public Health Sciences (S Al Hasan PhD), John T. Milliken Department of Internal Medicine (Z Al-Aly MD), Washington University School of Medicine, St Louis, MO, USA; Department of Medicine (A S Al Mosa MD), Department of Health Information Management and Technology (T M Alanzi PhD), Division of Forensic Medicine (Prof R G Menezes MD), Imam Abdulrahman Bin Faisal University, Dammam, Saudi Arabia (F M Alanezi PhD); Department of Internal Medicine (O Al Ta'ani MD), Allegheny Health Network, Pittsburgh, PA, USA; Department of Adult Health and Critical Care (O A Al Zaabi PhD), Department of Geography (W Ali PhD), Sultan Qaboos University, Muscat, Oman; McWilliams School of Biomedical Informatics (F Alahdab MD), UTHealth, Houston, TX, USA; Department of Biomedical Informatics, Biostatistics, and Epidemiology (F Alahdab MD), University of Missouri, Columbia, MO, USA; Faculty of Pharmacy (Prof M M Alajlani PhD), Al-Sham Private University, Damascus, Syria; School of Medicine (Y Al-Ajlouni MD), New York Medical College, Valhalla, NY, USA; Department of Epidemiology (Y Al-Ajlouni MD), Columbia University, New York, USA; Clinical Epidemiology Center (Z Al-Aly MD), US Department of Veterans Affairs (VA), St Louis, MO, USA; Murdoch Business School (K Alam PhD), Murdoch University, Perth, WA, Australia; Prevention Division (N Alam MPH), Department of Medicine (V Kulkarni MS), Digital Health and Informatics Directorate (Prof S M McPhail PhD), Queensland Health, Brisbane, QLD, Australia; Centre for Environment and Population Health (N Alam MPH), Griffith University, Nathan, QLD, Australia; Institute of Public Health (Z Alam PhD), College of Medicine and Health Sciences (Prof M Grivna PhD, J Nauman PhD), Family Medicine Department (M A Khan MSc), Department of Computer Science and Software Engineering (Prof N Zaki PhD), United Arab Emirates University, Al Ain, United Arab Emirates; School of Nursing (R M Al-amer PhD), Department of Basic Medical Sciences (M M Khatatbeh PhD), Yarmouk University, Irbid, Jordan; School of Nursing and Midwifery (R M Al-amer PhD), Translational Health Research Institute (R Chimoriya PhD), Department of Engineering (G R Naik PhD), Western Sydney University, Sydney, NSW, Australia; Department of Medicine (A Albakri MD), Royal Jordanian Medical Services, Amman, Jordan; Division of Gastroenterology and Hepatology (W A Aldhaleei MD), Mayo Clinic, Jacksonville, FL, USA; Institute of Health Informatics (R W Aldridge PhD), Surgical Biotechnology, Royal Free Hospital, UCL (Prof R Hamoudi PhD), Institute of Cardiovascular Science (A S Oguntade MSc), Department of Geography (K Vohra PhD), Department of Infection (Prof A Zumla PhD), University College London, London, UK; Robert Stempel College of Public Health and Social Work (S Alemohammad MD), Department of Epidemiology (M Ebrahimi Kalan MSc), Florida International University, Miami, FL, USA; Department of Epidemiology and Biostatistics (Y Alemu MPH), Department of Midwifery (W F Balcha MSc), Department of Pediatrics and Child Health (B S Firew MD), Department of Anatomy, Histology, and Embryology (D H Woldeyes MSc), Department of Health Promotion and Behavioral Science (Z A Yigzaw MPH), Bahir Dar University, Bahir Dar, Ethiopia; Global Centre for Environmental Remediation (A A S Al-Gheethi PhD), School of Medicine and Public Health (P Atorkey MPhil), University of Newcastle, Newcastle, NSW, Australia; Cooperative Research Centre for Contamination Assessment and Remediation of the Environment, Newcastle, NSW, Australia (A A S Al-Gheethi PhD); Department of Health Services and Hospital Administration (M K Al-Hanawi PhD), Health Economics Research Group (M K Al-Hanawi PhD), Department of Pediatric Dentistry (Prof O A A Elmeligy PhD), Department of Physical Therapy (F Khan

PhD), Rabigh Faculty of Medicine (A A Malik PhD), Department of Dental Public Health (Z S Natto DrPH), Department of Community Medicine (S Samargandy PhD), King Abdulaziz University, Jeddah, Saudi Arabia; Department of Zoology (A Ali PhD), Department of Biochemistry (Prof A W Wadood PhD), Abdul Wali Khan University Mardan, Mardan, Pakistan; Department of Biotechnology and Genetic Engineering (A Ali PhD, M Waqas PhD), Hazara University Mansehra, Mansehra, Pakistan; School of Medicine (I Ali BS), Department of Radiology and Radiological Science (A Amindarolzarbi MD), Department of Biostatistics (A Columbus MS), Department of Epidemiology (T G Hundie MD), Russell H. Morgan Department of Radiology and Radiological Science (A Kamireddy MD), Department of Neurosurgery (F Kazemi MD), Bloomberg School of Public Health (A Ozair MD), Department of Health Policy and Management (D Vervoort MD), The Russell H. Morgan Department of Radiology and Radiological Science (I Yazdani Nia MD), Department of International Health (H Zhang MS), Johns Hopkins University, Baltimore, MD, USA; Department of Medical Rehabilitation (Physiotherapy) (M U Ali MSc), Department of Microbiology (M A Isa PhD), Department of Clinical Pharmacy and Pharmacy Administration (H Yusuf PhD), University of Maiduguri, Maiduguri, Nigeria; Department of Biosciences (R Ali MPhil, N Salam PhD), Jamia Millia Islamia, New Delhi, India; Center for Biotechnology and Microbiology (S S Ali PhD), University of Swat, Swat, Pakistan; Department of Medical Laboratory Sciences (V E Ali BSc), Clinical Pharmacy and Pharmacy Management (D O Aluh MSc), University of Nigeria Nsukka, Nsukka, Nigeria; Department of Nuclear Medicine (Prof A Al-Ibraheem MD), Department of Cell Therapy and Applied Genomics (A Tbakhi MD), King Hussein Cancer Center, Amman, Jordan; Department of Diagnostic Radiology and Nuclear Medicine (Prof A Al-Ibraheem MD), Department of Biopharmaceutics and Clinical Pharmacy (Y Bustanji PhD), Department of Mathematics (Prof S M Momani PhD), The University of Jordan, Amman, Jordan; Department of Pathophysiology and Transplantation (G Alicandro PhD), Università degli Studi di Milano, Milan, Italy; Cystic Fibrosis Center (G Alicandro PhD), Fondazione IRCCS Ospedale Maggiore Policlinico, Milan, Italy; School of Public Health and Preventive Medicine (S M Alif PhD), School of Public Health and Preventative Medicine (Prof M Asghari-Jafarabadi PhD), Monash University, Melbourne, VIC, Australia; Department of Health Policy and Management (Prof S M Aljunid PhD), Department of Surgery (S K Al-Sabah MD), Kuwait University, Kuwait, Kuwait; International Centre for Casemix and Clinical Coding (Prof S M Aljunid PhD), National University of Malaysia, Bandar Tun Razak, Malaysia; Bordeaux School of Public Health (Prof F Alla PhD), University of Bordeaux, Bordeaux, France; Department of Medicine (J U Almazan PhD, Prof D Poddighe PhD), Nazarbayev University, Astana, Kazakhstan; Department of Parasitology (Prof H M Al-Mekhlafi PhD), Department of Paediatrics (Prof H Ariffin MD), University of Malaya Medical Centre (Prof H Ariffin MD), Department of Medicine (L Lim MRCP), University of Malaya, Kuala Lumpur, Malaysia; Department of Parasitology (Prof H M Al-Mekhlafi PhD), Sana'a University, Sana'a, Yemen; Department of Prosthodontics and Implant Dentistry (A Alqutaibi PhD), Taibah University, Medinah, Saudi Arabia; Department of Prosthodontics (A Alqutaibi PhD), Ibb University, Ibb, Yemen; Department of Allied Medical Sciences (A Alrawashdeh PhD), Department of Rehabilitation Sciences (M S Al-Wardat PhD), Department of Clinical Pharmacy (Prof K H Alzoubi PhD), Department of Public Health (Prof K A Kheirallah PhD), Jordan University of Science and Technology, Irbid, Jordan; Macro-Fiscal Policy Department (S M Alrousan PhD), Ministry of Finance, Dubai, United Arab Emirates; Jaber Al Ahmad Al Sabah Hospital (S K Al-Sabah MD), Ministry of Health, Kuwait, Kuwait; Department of Emergency Medicine (M A Alsabri MD), Sana'a University, Sanaa, Yemen; Department of Basic Medical Sciences (Z Altaany PhD), Yarmouk Univeristy, Irbid, Jordan; Research, Policy and Training Directorate (A Al-Tammemi MPH), Jordan Center for Disease Control, Amman, Jordan; Applied Science Research Center (A Al-Tammemi MPH), Applied Science Private University, Amman, Jordan; Department of Specialty Internal Medicine (Prof J A Al-Tawfiq MD), Johns Hopkins Aramco Healthcare, Dhahran, Saudi Arabia; Department of Medicine (Prof J A Al-Tawfiq MD), Indiana University School of Medicine, Indianapolis, IN, USA; Lisbon Institute of Global Mental Health (D O Aluh MSc), Nova Medical School (J Conde PhD), Nova University of Lisbon, Lisbon, Portugal; Research Group in Hospital Management and Health Policies (Prof N Alvis-Guzman PhD), Universidad de la Costa, Barranquilla, Colombia; Research Group in Health Economics (Prof N Alvis-Guzman PhD, Prof C A Marrugo Arnedo MSc), University of Cartagena, Cartagena, Colombia; Department of Medical Sciences (Prof Y M Al-Worafi PhD), Azal University for Human Development, Sana'a, Yemen; Department of Clinical Sciences (Prof Y M Al-Worafi PhD), University of Science and Technology of Fujairah, Fujairah, United Arab Emirates; Department of Pediatrics (Prof H Aly MD), Heart, Vascular, and Thoracic Institute (A Hajj Ali MD), Lerner Research Institute (X Liu PhD), Department of Cardiovascular Medicine (S Shekhar MD), Cleveland Clinic, Cleveland, OH, USA; Faculty of Medicine (Prof M S Alyahya PhD), Jordan University of Science and Technology, Alramtha, Jordan; Department of Biomedical Engineering (W Al-Zyoud PhD), German Jordanian University, Amman, Jordan; Interdisciplinary Graduate Program in Human Toxicology (R Amani DVM), University of Iowa, Iowa City, IA, USA; Health Policy Research Center (R Amani DVM, A Ardekani MD, H Kasraei MD, M Nouri PhD), Health Human Resources Research Center

(M Bayati PhD), Shiraz Neuroscience Research Center (M Jafarinia PhD, R Tavakoli Oliaee PhD), Department of Community Nutrition (M Nouri PhD), Department of Epidemiology and Biostatistics (H Raeisi Shahraki PhD), Department of Health Services Management (R Ravangard PhD), Department of Clinical Biochemistry (O Vakili PhD), Department of Medicine (A Yarahmadi PhD), Shiraz University of Medical Sciences, Shiraz, Iran; School of Graduate Studies (E K Ameyaw MPhil), Lingnan University, Hong Kong, China; Public Health and Community Medicine Department (Prof T T Amin MD), Department of Neurology (A Hassan MD), Cairo University, Cairo, Egypt; Medicine, Quran and Hadith Research Center (S Amiri PhD), Nephrology and Urology Research Center (K Hushmandi PhD), Baqiyatallah University of Medical Sciences, Tehran, Iran; Department of Maternal and Child Wellbeing (D A Amugsi PhD), African Population and Health Research Center, Nairobi, Kenya; Faculty of Pharmacy (Prof R Ancuceanu PhD), Department of Cardiology (C Andrei PhD), Department of Internal Medicine (M Hostiuc PhD), Department of Legal Medicine and Bioethics (S Hostiuc PhD), Department of General Surgery (I Negoi PhD, D Serban PhD, B Socea PhD), Department of Anatomy and Embryology (R I Negoi PhD), Department of Diabetes, Nutrition and Metabolic Diseases (A Pantea Stoian PhD), Carol Davila University of Medicine and Pharmacy, Bucharest, Romania; Centre for Sensorimotor Performance (D Anderlini MD), Department of Urology (Prof E Chung MD), Institute for Social Science Research (E Kanmiki MPH, J C Maravilla PhD), School of Dentistry (R Lalloo PhD), Queensland Brain Institute (Prof J J McGrath MD), School of Health and Rehabilitation Sciences (M Moni PhD), Faculty of Medicine (B Sartorius PhD), The University of Queensland, Brisbane, QLD, Australia; Neurology Department (D Anderlini MD), Royal Brisbane and Women's Hospital, Brisbane, QLD, Australia; Faculty of Medicine (D B Anderson PhD), School of Architecture, Design, and Planning (Prof T Astell-Burt PhD), Charles Perkins Centre (Prof S J Chadban PhD), Westmead Clinical School (R Chimoriya PhD), School of Pharmacy and Charles Perkins Centre (Z Dai PhD), Sydney Medical School (S Islam PhD), School of Public Health (L D Knibbs PhD), Asbestos Diseases Research Institute (J Leigh MD), School of Chemical & Biomolecular Engineering (E A Noman PhD), The Daffodil Centre (M M R Rahman PhD), School of Veterinary Science (B B Singh PhD), Save Sight Institute (Y You PhD), University of Sydney, Sydney, NSW, Australia; Department of Health Care Management (P P Andrade MD, Prof R Busse PhD, S Mohammed PhD), Technical University of Berlin, Berlin, Germany; European University, Lisbon, Portugal (P P Andrade MD); Department of Statistics and Econometrics (Prof T Andrei PhD, Prof M Ausloos PhD, Prof C Herteliu PhD, A Otoiu PhD), Faculty of Management (A Dima PhD), Bucharest University of Economic Studies, Bucharest, Romania; Department of Pharmacology (A Anil MD, J Charan MD, M Shamim MBBS, K Tiwari MBBS, S B Varthya MD), Department of Anatomy (Prof N Bhardwaj MD, R Gupta MD), Department of Community Medicine and Family Medicine (P Bhardwaj MD), School of Public Health (P Bhardwaj MD), Department of Forensic Medicine and Toxicology (T Kanchan MD), Department of Biochemistry (M Khokhar PhD, S Tomo MD), Department of Pharmacology and Research (A Saravanan MD), All India Institute of Medical Sciences, Jodhpur, India; All India Institute of Medical Sciences, Bhubaneswar, India (A Anil MD); Department of Obstetrics and Gynecology (S Anil MBBS), Ernakulam Medical Centre, Palarivattom, Kochi, India; Agribusiness Study Program (E Antriyandarti DrAgrSc), Sebelas Maret University, Surakarta, Indonesia; Regenerative Medicine, Organ Procurement and Transplantation Multi-diciplinary Center (S Anvari MD), Department of Medical-Surgical Nursing (P Ghorbani Vajargah MSc, S Karkhah MSc), Inflammatory Lung Diseases Research Center (N Rahmanian PhD), Guilan University of Medical Sciences, Rasht, Iran; School of Dentistry and Medical Sciences (A E Anyasodor PhD), Charles Sturt University, Orange, NSW, Australia; Department of Social Sciences (F Appiah MPhil), Berekum College of Education, Berekum, Ghana; School of Public Health (F Appiah MPhil), Kwame Nkrumah University of Science and Technology, Kumasi, Ghana; Radiation Oncology Mater Olbia Hospital (M Aquilano MD), Independent Consultant, Olbia, Italy; Department of Medicine (J Arab MD), Western University, London, ON, Canada; Gastroenterology Department (J Arab MD), Department of Gastroenterology (L A Diaz MD), Pontifical Catholic University of Chile, Santiago, Chile; Faculty of Pharmacy (Prof E A Arafa PhD), Department of Pharmacology and Toxicology (Prof H A Omar PhD), Beni-Suef University, Beni-Suef, Egypt; College of Pharmacy (M Arafat PhD), Al Ain University, Abu Dhabi, United Arab Emirates; College of Art and Science (D Areda PhD), Ottawa University, Surprise, AZ, USA; School of Life Sciences (D Areda PhD), Arizona State University, Tempe, AZ, USA; College of Medicine and Health Sciences (B B Aregawi PhD), Department of Midwifery (M W Gebregergis MSc), Department of Medical Laboratory Sciences (H Negash MSc), Medical Laboratory (H L L Weldetinsaa MSc), Adigrat University, Adigrat, Ethiopia; Department of Veterinary Pharmacology and Toxicology (A Aremu PhD), Department of Veterinary Physiology and Biochemistry (A Basiru PhD), Department of Veterinary Public Health and Preventive Medicine (I A Odetokun PhD), University of Ilorin, Ilorin, Nigeria; Department of Medical Laboratory Sciences (M Arkew MSc), Health Sciences Department of Oncology Nursing (T G Gebi MSc), Haramaya University, Harar, Ethiopia; School of Public Health (K Armani PhD, S Basu PhD), Department of Infection (C S Brown MD), Department of Surgery and Cancer (Prof E Mossialos PhD), WHO Collaborating Centre for Public Health Education and Training

(D L Rawaf MRCS), Department of Primary Care and Public Health (Prof S Rawaf MLIS, C Tabche MSc), Imperial College London, London, UK; Faculty of Pharmaceutical Sciences (K Armani PhD), UCSI University, Kuala Lumpur, Malaysia; Department of Biophysics (A A Artamonov PhD), Russian Academy of Sciences, Moscow, Russia; Department of Community Medicine and Rehabilitation (A Arumugam PhD), Umeå University, Umea, Sweden; Cabrini Research (Prof M Asghari-Jafarabadi PhD), Cabrini Health, Malvern, VIC, Australia; Department of Immunology (S Athari PhD), Department of Critical Care and Emergency Nursing (N Hanifi PhD), Zanjan University of Medical Sciences, Zanjan, Iran; Hunter New England Population Health, Wallsend, NSW, Australia (P Atorkey MPhil); Faculty of Nursing (M M W Atout PhD), Philadelphia University, Amman, Jordan; Northumbria HealthCare NHS Foundation Trust, Newcastle upon Tyne, UK (A Aujayeb MBBS); School of Business (Prof M Ausloos PhD), Department of Health Sciences (Prof T Brugha MD, P H Lee PhD, S J Tromans PhD), University of Leicester, Leicester, UK; Department of Health Sciences (H Awad PhD), Higher College of Technology, Abu Dhabi, United Arab Emirates; School of Nursing and Public Health (A W Awotidebe PhD), University of KwaZulu-Natal, Durban, South Africa; Department of Psychiatry (Prof J L Ayuso-Mateos PhD), Department of Medicine (Prof A Ortiz MD), Autonomous University of Madrid, Madrid, Spain; Biomedical Research Networking Center for Mental Health Network (CIBERSAM) (Prof J L Ayuso-Mateos PhD), National School of Public Health (A Padron-Monedero PhD), Institute of Health Carlos III, Madrid, Spain; Department of Health Informatics (F K Azeez MSc), Jazan University, Jazan Saudi Arabia, Saudi Arabia; Department of Community Health (F K Azeez MSc), National University of Malaysia, Kuala Lumpur, Malaysia; Department of Sciences (Prof R M S Azevedo PhD), Therapeutic and Diagnostic Technologies Department (Prof N Cruz-Martins PhD), Toxicology Research Unit (TOXRUN) (Prof D Dias da Silva PhD, Á M Madureira-Carvalho PhD), Cooperativa de Ensino Superior Politécnico e Universitário (Polytechnic and University Higher Education Cooperative), Gandra, Portugal; Gomal Center of Biochemistry and Biotechnology (M Badar PhD), Gomal University, Dera Ismail Khan, Pakistan; Division of Orthopaedics (S Baghdadi MD), Children's Hospital of Philadelphia, Philadelphia, PA, USA; Department of food science and technology (M Bagheri PhD), Shahid Bahonar university of Kerman, Kerman, Iran; Health Research Institute (N Bagheri PhD), University of Canberra, Canberra, ACT, Australia; School of Public Affairs (R Bai MD), Nanjing University of Science and Technology, Nanjing, China; Center for Clinical Research and Prevention (J L Baker PhD), Bispebjerg University Hospital, Frederiksberg, Denmark; Department of Neurosurgery (A T Bako PhD), Houston Methodist Hospital, Houston, TX, USA; Division of Biological Sciences (S Balakrishnan PhD), Tamil Nadu State Council for Science and Technology, Chennai, India; Center of Innovation, Technology and Education (CITE) (Prof O C Baltatu PhD), Institute of Biomedical Engineering (Prof L A Campos PhD), Anhembi Morumbi University, Sao Jose dos Campos, Brazil; Dental Research Center (E Bardideh DDS), Orthodontics Department (M Ghorbani DDS), Clinical Research Development Unit (N Morovatdar MD), Applied Biomedical Research Center (A Sahebkar PhD), Biotechnology Research Center (A Sahebkar PhD), Department of Health Education and Health Promotion (H Tehrani PhD), Department of Medicine (A Yarahmadi PhD), Department of Medical Genetics (N Zafari MD), Mashhad University of Medical Sciences, Mashhad, Iran; School of Psychology (Prof S L Barker-Collo PhD), School of Pharmacy (K A Beyene PhD), University of Auckland, Auckland, New Zealand; Heidelberg Institute of Global Health (HIGH) (Prof T W Bärnighausen MD, S Barteit PhD, S Chen DSc), Heidelberg University, Heidelberg, Germany; Faculty of Pharmacy (J D Basso PharmD, S Silva MSc), Coimbra Chemistry Centre (J D Basso PharmD), Department of Geography and Demography (M Rodrigues PhD), Coimbra Institute for Biomedical Imaging and Translational Research (S Silva MSc), University of Coimbra, Coimbra, Portugal; Department of Health Sciences (DISSAL) (M Bauckneht PhD), University of Genoa, Genova, Italy; Department of Nuclear Medicine (M Bauckneht PhD), IRCCS Ospedale Policlinico San Martino, Genova, Italy; Department of Psychiatry (Prof B T Baune PhD), University of Münster, Münster, Germany; Department of Psychiatry (Prof B T Baune PhD), Melbourne Medical School, Melbourne, VIC, Australia; Department of Surgery (N S Bayileyegn MD), Institute of Health Science (A I Mohamed MSc), Department of Clinical Pharmacy (B Tesfaye MSc), Jimma University, Jimma, Ethiopia; Endocrinology and Metabolism Research Institute (A Khalaji BS), Department of Epidemiology (S Khanmohammadi MD, S Nejadghaderi MD, H Soleimani MD), Non-Communicable Diseases Research Center (NCDRC), Tehran, Iran (A Behnoush BS); Department of Microbiology (P Behzadi PhD), Islamic Azad University, Tehran, Iran; Division of Pulmonary, Critical Care, and Sleep (M Beiranvand PhD), University of Florida, Jacksonville, FL, USA; Department of Biological Sciences (L Belo PhD), Research Unit on Applied Molecular Biosciences (UCIBIO) (L Belo PhD, Prof D Dias da Silva PhD, J P Silva PhD), Associated Laboratory for Green Chemistry (LAQV) (M Carvalho PhD), Institute for Research and Innovation in Health (Prof N Cruz-Martins PhD), Department of Community Medicine, Information and Health Decision Sciences (A Freitas PhD), Laboratório de Farmacognosia (LAQV) (Associated Laboratory for Green Chemistry (Á M Madureira-Carvalho PhD), University of Porto, Porto, Portugal; Department of Biomedical Sciences (Prof A Beloukas PhD), University of West Attica, Athens, Greece; Institute of Infection and Global Health (Prof A Beloukas PhD), University of

Liverpool, Liverpool, UK; Department of Pediatrics (M Bemanalizadeh MD), Department of Environmental Health Engineering (A Fatehizadeh PhD), School of Medicine (G Ghasempour Dabaghi MD, M Rabiee Rad MD), Cardiology Department (M Mansouri MD), Department of Epidemiology and Biostatistics (Prof M Mansourian PhD), Cardiac Rehabilitation Research Center (K Mehrabani-Zeinabad PhD, Prof M Sadeghi MD), Heart Failure Research Center (S Najdaghi MD, D Narimani Davani MD), Neuroscience Research Center (S Najdaghi MD), Department of Medical Physics (K Saber PhD), Department of Clinical Biochemistry (O Vakili PhD), Isfahan University of Medical Sciences, Isfahan, Iran; Department of Internal Medicine (I M Bensenor PhD), Department of Psychiatry (Prof J Castaldelli-Maia PhD, Y Wang PhD), Department of Epidemiology (Prof A C Goulart PhD), University of São Paulo, São Paulo, Brazil; Department of Epidemiology and Health Promotion (Prof H Benzian PhD), Department of Child and Adolescent Psychiatry (Prof S Cortese PhD), New York University, New York, NY, USA; School of Medicine (A Beran MD, J T Tran BS), Indiana University, Indianapolis, IN, USA; Institute of Marketing (Z Berezvai PhD), Corvinus University of Budapest, Budapest, Hungary; Competition Economics and Market Research Section (Z Berezvai PhD), Hungarian Competition Authority, Budapest, Hungary; Hubert Department of Global Health (R S Bernstein MD), Department of Gynecology and Obstetrics (T R Ojo-Akosile MPH), Department of Family and Preventive Medicine (S Thirunavukkarasu PhD), Emory University, Atlanta, GA, USA; Butte County Department of Public Health, Chico, CA, USA (R S Bernstein MD); Faculty of Medicine (P J G Bettencourt PhD), Catholic University of Portugal, Rio de Mouro, Portugal; Department of Pharmaceutical and Administrative Sciences (K A Beyene PhD), University of Health Sciences and Pharmacy in St. Louis, St Louis, MO, USA; School of Pharmacy (M G Beyene MSc), Addis Ababa University, Addis Ababa, Ethiopia; Department of Forensic Chemistry (D S Bhagat PhD), Government Institute of Forensic Science, Aurangabad, Aurangabad, India; Department of Public Health (A S Bhagavathula PhD), North Dakota State University, Fargo, ND, USA; Institutes of Applied Health Research and Translational Medicine (N Bhala PhD), Queen Elizabeth Hospital Birmingham, Birmingham, UK; Institute of Applied Health Research (N Bhala PhD, K Malhotra MBBS, R Thayakaran PhD), NIHR Global Health Research Unit on Global Surgery (J C Glasbey MSc), University of Birmingham, Birmingham, UK; School of Nursing and Midwifery (D Bhandari PhD), Australian Regenerative Medicine Institute (Y Mathangasinghe MD), Monash University, Clayton, VIC, Australia; Public Health Research Laboratory (D Bhandari PhD), Department of Biotechnology (B P Marasini PhD), Department of Community Medicine (P M S Pradhan MD), Central Department of Public Health (N Subedi MPH), Tribhuvan University, Kathmandu, Nepal; Department of Medicine (R Bharadwaj PhD), University of Massachusetts Medical School, Worcester, MA, USA; Department of Internal Medicine (A Bhargava MD), Wayne State University, Detroit, MI, USA; Global Health Neurology Lab (S Bhaskar PhD), NSW Brain Clot Bank, Sydney, NSW, Australia; Department of Neurology and Neurophysiology (S Bhaskar PhD), South West Sydney Local Heath District and Liverpool Hospital, Sydney, NSW, Australia; Department of Internal Medicine (V Bhat MBBS), St. John's National Academy of Health Sciences, Bangalore, India; Medical Lab Technology (G K Bhatti PhD), University Centre for Research and Development (S Kalra DM), Chandigarh University, Mohali, India; Department of Human Genetics and Molecular Medicine (Prof J S Bhatti PhD, S Senapati PhD), Central University of Punjab, Bathinda, India; Department of Botanical and Environmental Sciences (Prof M S Bhatti PhD), Guru Nanak Dev University, Amritsar, India; Department of Pharmacy (Prof M A Bhuiyan PhD, S Dewan PhD), University of Asia Pacific, Dhaka, Bangladesh; Department of Community and Family Medicine (B Biswas MD), All India Institute of Medical Sciences, Deoghar, India; Department of Global Public Health and Primary Care (Prof T Bjørge PhD), Department of Psychosocial Science (D Sagoe PhD), University of Bergen, Bergen, Norway; Department of Research (M W Wojewodzic PhD), Cancer Registry of Norway, Oslo, Norway (Prof T Bjørge PhD); School of Business Administration (Prof V Bodolica PhD), American University of Sharjah, Sharjah, United Arab Emirates; General Directorate of Health Information Systems (B Bora Basara PhD), Ministry of Health, Ankara, Turkiye; Disease Surveillance Department (S A Bosoka Mphil), Ghana Health Service, Ho, Ghana; Facultad de Salud (Faculty of Health) (Prof A Botero Carvajal MSc), Universidad Santiago de Cali (Santiago de Cali University), Cali, Colombia; Department of Medicine (Prof S Bouaoud MD), Faculty of Medicine (Prof A Ouyahia PhD), University Ferhat Abbas of Setif, Setif, Algeria; Department of Epidemiology and Preventive Medicine (Prof S Bouaoud MD), University Hospital Saadna Abdenour, Setif, Algeria; Transport and Road Safety (TARS) Research Centre (S Boufous PhD), School of Population Health (X Feng PhD), School of Psychiatry (Prof P B Mitchell MD), Centre for Research Excellence in Suicide Prevention (S Onie PhD), School of Public Health and Community Medicine (A E Peden PhD, Prof A E Schutte PhD), Centre for Primary Health Care and Equity (CPHCE) (P Peprah MSc), School of Optometry and Vision Science (Prof K Pesudovs PhD, Prof S Resnikoff MD), Faculty of Medicine and Health (S Sharma PhD), School of Risk and Actuarial Studies (Y Si PhD), The George Institute for Global Health (P Ye MPH), University of New South Wales, Sydney, NSW, Australia; Department of Earth, Environment, and Equity (C Boxe PhD), Howard University, Washington, DC, USA; General Medicine Service (Prof E J Boyko MD),

Department of Veterans Affairs, Seattle, WA, USA; Department of Infectious Disease Epidemiology (O J Brady PhD), MSc Epidemiology Programme (A Hafiz PhD), Department of Non-Communicable Disease Epidemiology (M Iwagami PhD), Department of Health Services Research and Policy (Prof M McKee DSc), London School of Hygiene & Tropical Medicine, London, UK; Department of Epidemiology (D Braithwaite PhD, D D Ding BS, D Yang MPH), College of Medicine (M J Diaz BS), Department of Computer and Information Science and Engineering (P Naghavi MSc), Biology and Emerging Pathogens Institute (M H Zahid PhD), University of Florida, Gainesville, FL, USA; Cancer Population Sciences Program (D Braithwaite PhD), University of Florida Health Cancer Center, Gainesville, FL, USA; School of Population and Public Health (Prof M Brauer DSc, I O Iyamu MD), University of British Columbia, Vancouver, BC, Canada; Department of Sports and Computer Science (J Brazo-Sayavera PhD), Universidad Pablo de Olavid (Pablo de Olavide University), Seville, Spain; Division of Clinical Epidemiology and Aging Research (Prof H Brenner MD), German Cancer Research Center, Heidelberg, Germany; HCAI, Fungal, AMR, AMU, & Sepsis Division (C S Brown MD), United Kingdom Health Security Agency, London, UK; Big Data Institute (A J Browne MPH), Nuffield Department of Population Health (B Lacey PhD), Nuffield Department of Medicine (Prof R J Maude PhD, B Sartorius PhD), Health Economics Research Centre (Prof J A B Rodriguez PhD), University of Oxford, Oxford, UK; Flinders Health and Medical Research Institute (N B Bulamu PhD), College of Nursing and Health Sciences (K M Foley MPH), College of Medicine and Public Health (G R Naik PhD), Department of Nursing and Health Sciences (S Shorofi PhD), Flinders University, Adelaide, SA, Australia; Global Health Research Institute (D Buonsenso MD), Università Cattolica del Sacro Cuore (Catholic University of Sacred Heart), Rome, Italy; School of Public Health Sciences (Z A Butt PhD), University of Waterloo, Waterloo, ON, Canada; Al Shifa School of Public Health (Z A Butt PhD), Al Shifa Trust Eye Hospital, Rawalpindi, Pakistan; Faculty of Health Sciences (M Çakmak Barsbay PhD), Ankara University, Ankara, Turkiye; Department of Clinical Pharmacy (Prof D Calina PhD), University of Medicine and Pharmacy of Craiova, Craiova, Romania; Department of Biomedical and Neuromotor Sciences (A Capodici MD, S Guicciardi MD, A Mazzotti PhD), Department of Medical and Surgical Sciences (E Cenko MD, Prof F S Violante MD), University of Bologna, Bologna, Italy; Management and Healthcare (EMbeDS) (A Capodici MD), Sant'Anna School of Advanced Studies, Pisa, Italy; Department of Health Care (Prof R Cárdenas DSc), Metropolitan Autonomous University, Mexico City, Mexico; Institute for Cancer Research, Prevention and Clinical Network, Florence, Italy (G Carreras PhD); Dermatology Unit (A Carugno MD), Azienda Socio Sanitaria Territoriale Papa Giovanni XXIII, Bergamo, Italy; Instituto de Investigação, Inovação e Desenvolvimento (Institute of Research Innovation and Development) (M Carvalho PhD), University Fernando Pessoa, Porto, Portugal; Department of Medicine (G Castelpietra PhD), University of Udine, Udine, Italy; Department of Mental Health (G Castelpietra PhD), Healthcare Agency "Friuli Occidentale", Pordenone, Italy; Department of Psychiatry (A Caye PhD), Postgraduate Program in Epidemiology (Prof B B Duncan MD), Federal University of Rio Grande do Sul, Porto Alegre, Brazil; Department of Medical, Surgical, and Health Sciences (L Cegolon PhD, Prof M D'Oria MD), University of Trieste, Trieste, Italy; Public Health Unit (L Cegolon PhD), University Health Agency Giuliano-Isontina (ASUGI), Trieste, Italy; Department of Nutrition (Prof F Cembranel DSc), Federal University of Santa Catarina, Florianópolis, Brazil; Mary MacKillop Institute for Health Research (Prof E Cerin PhD), Australian Catholic University, Melbourne, VIC, Australia; School of Public Health (Prof E Cerin PhD), Department of Urban Planning and Design (C Guo PhD), Centre for Suicide Research and Prevention (Prof P Yip PhD), Department of Social Work and Social Administration (Prof P Yip PhD), University of Hong Kong, Hong Kong, China; Department of Renal Medicine (Prof S J Chadban PhD), Royal Prince Alfred Hospital, Sydney, NSW, Australia; ICMR School of Public Health (J Chadwick MD), National Institute of Epidemiology, Chennai, India; Department of Biotechnology (Prof C Chakraborty PhD), Adamas University, Kolkata, India; Department of Skeletal Aging and Orthopedic Surgery (Prof C Chakraborty PhD), Hallym University, Chuncheon, South Korea; State Disease Investigation Laboratory (S Chakraborty MVSc), Animal Resources Development Department, Agartala, India; Heart Failure and Structural Heart Disease Unit (J Chan MBChB), Cardiovascular Analytics Group, Hong Kong, China; Department of Clinical Nutrition (R M Chandika PhD), Department of Epidemiology (S Dohare MD, K Y Ghailan PhD), Department of Maxillofacial Surgery and Diagnostic Sciences (E S Halboub PhD), Health Research Center (Prof A Khalid PhD), Department of Health Informatics (A Mehmood PhD), Department of Health Education and Promotion (M Shanawaz MD), Health Education and Promotion (F Sobia PhD), Jazan University, Jazan, Saudi Arabia; Department of Pediatrics (S Chandy PhD), The Childs Trust Medical Research Foundation (CTMRF), Chennai, India; Department of Biology (A A Chaudhary PhD), Al-Imam Mohammad Ibn Saud Islamic University, Riyadh, Saudi Arabia; Department of Oral Medicine and Radiology (A Chaurasia MD), King George's Medical University, Lucknow, India; Fuwai Hospital (A Chen PhD), Chinese Academy of Medical Sciences & Peking Union Medical College, Beijing, China; Department of Computer Science (A Chen PhD), University of Texas Austin, Austin, TX, USA; Clinical Research Center (H Chen MB), Southern Medical University, Guangzhou, China; University of Michigan, Ann Arbor, MI, USA (M

Chen BDS); Department of Public Health and Health Policy (O Chimed-Ochir PhD), Hiroshima University, Hiroshima, Japan; Division of Infectious Diseases (P R Ching MD), Virginia Commonwealth University, Richmond, VA, USA; Department of Public Health, Administration, and Social Sciences (J L Chirinos-Caceres DrPH), Cayetano Heredia University, Lima, Peru; Iraq Field Epidemiology Training Program (I-FETP) (A Chitheer MD), Ministry of Health, Baghdad, Iraq; Division of Plastic Surgery (D Y Cho MD), Children's Hospital of Philadelphia, Philadelphia, PA, USA; Department of Clinical Oncology (W C S Cho PhD), Queen Elizabeth Hospital, Hong Kong, China; Cancer Big Data Center (D Choi PhD), National Cancer Center, Goyang, South Korea; Department of Medicine (B Chong MBBS), School of Medicine (M Ng PhD), Leadership Institute for Global Health Transformation (LIGHT) (S Ramazanu PhD), Department of Surgery (K Tan PhD), Yong Loo Lin School of Medicine (Prof N Venketasubramanian MBBS), National University of Singapore, Singapore; RIPAS Hospital (C Chong MD), School of Digital Science (D T C Lai PhD), Institute of Applied Data Analytics (D T C Lai PhD), Faculty of Science (E Leong PhD), Universiti Brunei Darussalam, Bandar Seri Begawan, Brunei; Department of Biosciences (H Chopra PhD), Center for Global Health Research (M Fareed PhD), Saveetha Dental College and Hospitals (M R Tovani-Palone PhD), Saveetha Institute of Medical and Technical Sciences (SIMATS), Chennai, India; Center for Biomedicine and Community Health (D Chu PhD), Viet Nam National University-International School, Hanoi, Viet Nam; Department of AndroUrology (Prof E Chung MD), AndroUrology Centre, Brisbane, QLD, Australia; Health Effects Institute, Boston, MA, USA (A J Cohen DSc); School of Psychology (Prof S Cortese PhD), Faculty of Medicine (R Thayakaran PhD), University of Southampton, Southampton, UK; School of Population Health (Z Dai PhD), School of Psychiatry (Prof P S Sachdev MD), University of New South Wales, Kensington, NSW, Australia; IRCCS Istituto Ortopedico Galeazzi (G Damiani MD), Department of Clinical Sciences and Community Health (Prof C La Vecchia MD), University of Milan, Milan, Italy; Department of Dermatology (G Damiani MD), Lerner College of Medicine (L Göbölös PhD), Department of Quantitative Health Science (X Liu PhD), Department of Neonatology (I Qattea MD), Department of Nutrition and Preventive Medicine (Prof J Sanabria MD), Case Western Reserve University, Cleveland, OH, USA; Public Health Foundation of India, Gurugram, India (Prof L Dandona MD, Prof R Dandona PhD, G Kumar PhD, A Pandey PhD); Indian Council of Medical Research, New Delhi, India (Prof L Dandona MD); Division of Women and Child Health (J K Das MD), Aga Khan University, Karachi, Pakistan; Department of Biochemistry (S Das MD), Ministry of Health and Welfare, New Delhi, India; Ingram School of Engineering (S Das PhD), Texas State University, San Marcos, TX, USA; Health Research Institute (K Davletov PhD, M Kulimbet MSc), Laboratory of Experimental Medicine (T Fazylov MD), Atchabarov Scientific Research Institute of Fundamental and Applied Medicine (M Kulimbet MSc), Department of Public Health (A Zhumagaliuly MD), Kazakh National Medical University, Almaty, Kazakhstan; Department of Public Health (Prof F P De la Hoz PhD), National University of Colombia, Bogota, Colombia; Australian Institute for Suicide Research and Prevention (Prof D De Leo DSc), Griffith University, Mount Gravatt, QLD, Australia; Medical College (S Debopadhaya BS), Albany Medical College, Albany, NY, USA; School of Medicine (I Delgado-Enciso DSc), University of Colima, Colima, Mexico; Department of Research (I Delgado-Enciso DSc), Colima State Health Services, Colima, Mexico; Center for Nutrition and Health Research (E Denova-Gutiérrez DSc), Infectious Disease Research Center (Prof V Pando-Robles PhD), National Institute of Public Health, Cuernavaca, Mexico; St Paul's Eye Unit (N Dervenis MD), Royal Liverpool University Hospital, Liverpool, UK; Department of Ophthalmology (N Dervenis MD), Second Department of Cardiology (D Patoulias PhD), Aristotle University of Thessaloniki, Thessaloniki, Greece; Graduate Medical Education Department (H D Desai MD), Gujarat Adani Institute of Medical Sciences, Bhuj, India; Department of Community Medicine (V G C Devanbu MD), Chettinad Academy of Research and Education, Chennai, India; Pharmacology Department (S Dewan PhD), Center for Life Sciences Research Bangladesh, Dhaka, Bangladesh; Division of Pathology (K Dhama PhD), ICAR-Indian Veterinary Research Institute, Bareilly, India; Research and Development Cell (A S Dhane MBA), Dr. D. Y. Patil Vidyapeeth, Pune (Deemed to be University), Maharashtra, India, Pune, India; Department of Pharmacy Practice (S Dhingra PhD), National Institute of Pharmaceutical Education and Research, Hajipur, India; Faculty of Science (Prof D Diaz PhD), School of Medicine (Prof R Lozano MD), National Autonomous University of Mexico, Mexico City, Mexico; Department of Medicine (T H Do MD), Can Tho University of Medicine and Pharmacy, Can Tho, Viet Nam; Center for Health Sciences (C B do Prado MSc), Federal University of Espírito Santo, Vitória, Brazil; Department of Biostatistics (M Dodangeh Mcom), Independent Consultant, Tehran, Iran; School of Elderly Care Services and Management (W Dong MD), Nanjing University of Chinese Medicine, Nanjing, China; Cardio-Thoraco-Vascular Department (Prof M D'Oria MD), Azienda Sanitaria Universitaria Giuliano Isontina, Trieste, Italy; Department of Cardiology (R Doshi MD), St. Joseph's University Medical Center, Paterson, NJ, USA; Department of Forensic Medicine and Toxicology (H L Dsouza MD, V Krishna MD, Prof P Rastogi MD, P H Shetty MD), Department of General Medicine (J Jeganathan MD), Department of Community Medicine (N Joseph MD, N Kumar MD, R Motappa MD, R Thapar MD), Department of Oral and Maxillofacial Surgery (Prof P K

Shetty MDS), Kasturba Medical College, Mangalore (Prof B Unnikrishnan MD), Manipal Academy of Higher Education, Mangalore, India; Department of Forensic Medicine and Toxicology (H L Dsouza MD), Kasturba Medical College Mangalore, Mangalore, India; Prasanna School of Public Heath (PSPH) (V Dsouza MSc), Kasturba Medical College, Mangalore (R Holla MD, A Kamath MD, M Rao MD), Department of Physiotherapy (Prof V K PhD), Prasanna School of Public health (R Kamath MHA), Department of Pharmacy Management (V S Ligade PhD), Department of Forensic Medicine and Toxicology (Prof V C Nayak MD), Department of Community Medicine (C R Rao MD), Department of Health Information Management (B Reshmi PhD), Department of Pharmacology (R R Shenoy PhD), Manipal Academy of Higher Education, Manipal, India; Office of Institutional Analysis (J Dube MA), University of Windsor, Windsor, ON, Canada; Post-graduate Program in Health Sciences (S C Dumith PhD), Federal University of Rio Grande, Rio Grande, Brazil; School of Medicine (Prof A R Duraes PhD), Institute of Collective Health (Prof M Pereira PhD, Prof D Rasella PhD), Federal University of Bahia, Salvador, Brazil; Department of Internal Medicine (Prof A R Duraes PhD), Escola Bahiana de Medicina e Saúde Pública, Salvador, Brazil; Department of Biotechnology (S Duraisamy PhD), SRM Institute of Science and Technology, Kattankulathur, India; Department of Infection and Tropical Medicine (O C Durojaiye MPH), University of Sheffield, Sheffield, UK; Health Research Institute (A Dushpanova PhD), Al Farabi Kazakh National University, Almaty, Kazakhstan; Child Health Analytics Research Program (P A Dzianach PhD, Prof P W Gething PhD, F Sanna PhD), Geospatial Health and Development Team (J Lubinda PhD, A Saddler PhD), The Malaria Atlas Project (M A McPhail PhD, S F Rumisha PhD), Telethon Kids Institute, Perth, WA, Australia; Department of Conservative Dentistry with Endodontics (A M Dziedzic DSc), Medical University of Silesia, Katowice, Poland; Department of Psychiatry (E Eboreime PhD, E Tsermpini PhD), Dalhousie University, Halifax, NS, Canada; Department of Psychiatry (E Eboreime PhD), University of Alberta, Edmonton, AB, Canada; School of Health Sciences (H A Edinur PhD), University of Science Malaysia, Kubang Kerian, Malaysia; Department of Community Health Nursing (F Efendi PhD), Department of Epidemiology (A Hargono DMD), Department of Biology (Prof H Purnobasuki PhD), Universitas Airlangga (Airlangga University), Surabaya, Indonesia; School of Nursing and Midwifery (F Efendi PhD, M Rahman PhD), Department of Public Health (H Jiang PhD), La Trobe University, Melbourne, VIC, Australia; Centre for Global Health Inequalities Research (CHAIN) (Prof T Eikemo PhD), Department of Circulation and Medical Imaging (J Nauman PhD), Norwegian University of Science and Technology, Trondheim, Norway; Department of Orthodontics (E Eini DDS), Ahvaz Jundishapur University of Medical Sciences, Ahvaz, Iran; AlMoosa College of Health Science, Al Ahsa, Saudi Arabia (R A El Arab MSc); Neurosurgery Department (O Elamin MD), Jordan Hospital and Medical Centre, Amman, Jordan; Department of Internal Medicine and Hematology Unit (Prof G M T ElGohary MD), Biochemistry Department (Prof N M Hamdy PhD), Department of Entomology (A M Samy PhD), Medical Ain Shams Research Institute (MASRI) (A M Samy PhD), Neurology Department (Prof A S Shalash PhD), Ain Shams University, Cairo, Egypt; Section of Adult Hematology (Prof G M T ElGohary MD), Department of Physiology (Prof S A Meo PhD), Pediatric Intensive Care Unit (M Temsah MD), King Saud University, Riyadh, Saudi Arabia; Faculty of Medicine (M Elhadi MD), University of Tripoli, Tripoli, Libya; Department of Clinical Pathology (M Elshaer MD), Mansoura Faculty of Medicine (H Magdy Abd El Razek MD), Faculty of Pharmacy (M A Saleh PhD), Mansoura University, Mansoura, Egypt; Department of Infectious Diseases and Public Health (I Elsohaby PhD), Department of Biomedical Sciences (A Waris MS), City University of Hong Kong, Hong Kong, China; Department of Animal Medicine (I Elsohaby PhD), Cardiovascular Department (Prof A M A Saad MD), Zagazig University, Zagazig, Egypt; Department of Public Health and Tropical Medicine (T I Emeto PhD), College of Public Health, Medical, and Veterinary Sciences (A E Peden PhD), James Cook University, Townsville, QLD, Australia (K O Obamiro PhD); Department of Bacteriology and Virology (M Eslami PhD), Cancer Research Center (M Eslami PhD, D Haghmorad PhD), Semnan University of Medical Sciences, Semnan, Iran; Independent Consultant, Bologna, Italy (N Fabin MD); Research Centre for Healthcare and Community (A F Fagbamigbe PhD), Coventry University, Coventry, UK; Department of Periodontology and Community Dentistry (O F Fagbule FWACS), Department of Medicine (A S Oguntade MSc, Prof M O Owolabi DrM), Department of Oral and Maxillofacial Surgery (A A Salami BDS), University College Hospital, Ibadan, Ibadan, Nigeria; Epidemiology and Biostatistics Unit (L Falzone PhD), IRCCS Pascale, Naples, Italy; Dissemination Division (C S e Farinha MSc), National Institute of Statistics, Lisbon, Portugal; Activity Planning and Control Unit (C S e Farinha MSc), Directorate-General of Health (DGS), Lisbon, Portugal; Department of Psychology (Prof A Faro PhD), Federal University of Sergipe, São Cristóvão, Brazil; Department of Medicinal Chemistry (K Fasihi PharmD), Hamadan University of Medical Sciences, Hamadan, Iran; Department of Public Health, Equity, and Human Flourishing (K M Foley MPH), Centre for Health Policy Research (Prof P Ward PhD), Torrens University Australia, Adelaide, SA, Australia (N K Fauk MSc); Institute of Resource Governance and Social Change, Kupang, Indonesia (N K Fauk MSc); National Institute for Stroke and Applied Neurosciences (Prof V L Feigin PhD), Auckland University of Technology, Auckland, New Zealand; Third Department of Neurology (E V Gnedovskaya

PhD), Research Center of Neurology, Moscow, Russia (Prof V L Feigin PhD, Prof M A Piradov DSc); School of Pharmacy (G Fekadu MSc), Jockey Club School of Public Health and Primary Care (J Huang MD, C Zhong MD), Department of Medicine and Therapeutics (L Lim MRCP), The Chinese University of Hong Kong, Hong Kong, China; Department of Pharmacy (G Fekadu MSc), Wollega University, Nekemte, Ethiopia; National Institute of Environmental Health (X Feng PhD), National Center for Chronic and Noncommunicable Disease Control and Prevention (P Ye MPH), Chinese Center for Disease Control and Prevention, Beijing, China (Prof S Liu PhD); Division of Neurology (S Fereshtehnejad PhD), University of Toronto, Toronto, ON, Canada; Research Center on Public Health (P Ferrara MD), University of Milan Bicocca, Monza, Italy; Department of Social Sciences (Prof N Ferreira PhD), University of Nicosia, Nicosia, Cyprus; Institute of Public Health (F Fischer PhD), Charité Medical University Berlin, Berlin, Germany; Department of Pharmacology, Faculty of Veterinary Medicine (I Fitriana PhD), Gadjah Mada University, Yogyakarta, Indonesia; School of Social Sciences (J Flavel PhD), Stretton Health Equity, Adelaide, SA, Australia; Department of Child Dental Health (Prof M O Folayan FWACS), Department of Nursing Science (Prof A A Ogunfowokan PhD), Obafemi Awolowo University, Ile Ife, Nigeria; Clinical Science Department (Prof M O Folayan FWACS), Nigerian Institute of Medical Research, Yaba, Nigeria; Department of Cardiac, Thoracic, Vascular Sciences and Public Health (M Fonzo MD), University of Padova, Padova, Italy; Department of Biotechnological and Applied Clinical Sciences (DISCAB) (M Foschi MD), University of L'Aquila, L'Aquila, Italy; Department of Neuroscience (M Foschi MD), Hospital Santa Maria delle Croci, Ravenna, Italy; Center for Health Technology and Services Research (CINTESIS), Porto, Portugal (A Freitas PhD); Graduate Institute of Injury Prevention and Control (N Y Fridayani MSc), International Master Program for Translational Science (H Huynh BS), International Ph.D. Program in Medicine (L Minh MD), Research Center for Artificial Intelligence in Medicine (L Minh MD), School of Public Health (Y L Samodra MPH, Y L Samodra MPH), Taipei Medical University, Taipei, Taiwan; Department of Public Health (K G Fukutaki MPH), Independent Consultant, Seattle, WA, USA; Division of Ophthalmology (J M Furtado MD), University of São Paulo, Ribeirão Preto, Brazil; Department of Pathology (Prof B Fux PhD), Federal University of Espirito Santo, Vitória, Brazil; Health Services Management Training Centre (P A Gaal PhD, J Lám PhD, T Palicz MD), Semmelweis University, Budapest, Hungary; Department of Applied Social Sciences (P A Gaal PhD), Sapientia Hungarian University of Transylvania, Târgu-Mureş, Romania; Department of Community Medicine (Prof M A Gadanya FMCPH), Aminu Kano Teaching Hospital, Kano, Nigeria; Department of Environmental Health Sciences (S Gallus DSc), Mario Negri Institute for Pharmacological Research, Milan, Italy; Institute of Health and Wellbeing (B Ganesan PhD), Federation University, Churchill, VIC, Australia; Department of General Medicine (M Ganiyani MD), Grant Medical College & Sir J.J. Group of Hospitals, Mumbai, India; Department of Pharmacology (Prof R K Gautam PhD), Indore Institute of Pharmacy, Indore, India; Department of Environmental Health (M Gebrehiwot DSc), Wollo University, Dessie, Ethiopia; Department of Public Health (L Getacher MPH), Debre Berhan University, Debre Berhan, Ethiopia; Department of Public Health (G K A Getahun MPH), Menelik II Medical and Health Science College, Addis Ababa, Ethiopia; School of Population Health (Prof P W Gething PhD), School of Public Health (T R Miller PhD), Curtin University, Perth, WA, Australia; Center of Health Management (K Y Ghailan PhD), Aden University, Aden, Yemen; Department of Electrical and Computer Engineering (E Gholami PhD), University of California Davis, Davis, CA, USA; Department of Radiology (A Gholamrezanezhad MD), University of Southern California, Los Angeles, CA, USA; Department of Dermatology (N Gholizadeh MD), Department of Medical-Surgical Nursing (S Shorofi PhD), Mazandaran University of Medical Sciences, Sari, Iran; NCD Surveillance Unit (A U Gil PhD), World Health Organization (WHO), Moscow, Russia; Institute for Leadership and Health Management (A U Gil PhD), Moscow Medical Academy, Moscow, Russia; Adelaide Medical School (T K Gill PhD), School of Public Health (K Malhotra MBBS), University of Adelaide, Adelaide, SA, Australia; Department of Nursing (A Girmay MSc), Aksum University, Aksum, Ethiopia; Department of Epidemiology and Evidence-Based Medicine (E V Glushkova PhD, R V Polibin PhD), I.M. Sechenov First Moscow State Medical University, Moscow, Russia; Department of Cardiac Surgery (L Göbölös PhD), Cleveland Clinic Abu Dhabi, Abu Dhabi, United Arab Emirates; Department of Dermatology (M Goldust MD), Department of Radiology and Biomedical Imaging (X Liu PhD), Department of Genetics (S Pawar PhD), Department of Psychiatry (T G Rhee PhD), Yale University, New Haven, CT, USA; Department of Genetics (P Goleij MSc), Sana Institute of Higher Education, Sari, Iran; Universal Scientific Education and Research Network (USERN) (P Goleij MSc), Department of Speech Therapy (A Shiani PhD), Kermanshah University of Medical Sciences, Kermanshah, Iran; Department of Life Sciences (D Golinelli MD), Link Campus University, Rome, Italy; Hudson College of Public Health (S V Gopalani MPH), University of Oklahoma Health Sciences Center, Oklahoma City, OK, USA; Department of Health and Social Affairs (S V Gopalani MPH), Government of the Federated States of Micronesia, Palikir, Federated States of Micronesia; Blood and Marrow Transplantation and Cellular Therapy Program (A Goyal MD), Stanford University, Palo Alto, CA, USA; Department of Public Health and Preventive Medicine (Prof M Grivna PhD), Charles University, Prague,

Czech Republic; Post Graduate School of Public Health (G Guarducci MD), University of Siena, Siena, Italy; Department of Family and Community Medicine (M I M Gubari PhD), University Of Sulaimani, Sulaimani, Iraq; Health Directorate (S Guicciardi MD), Local Health Authority of Bologna, Bologna, Italy; Faculty of Nursing (Prof R A Guimarães PhD), Federal University of Goiás, Goiânia, Brazil; Department of General Surgery (S Gulati MD), Dignity Health, Phoenix, AZ, USA; Diagnostic Radiology and Nuclear Medicine (D Gulisashvili MD), University of Maryland, Baltimore, MD, USA; Department of Community Medicine (D A Gunawardane MD), University of Peradeniya, Kandy, Sri Lanka; Department of Internal Medicine (A K Gupta PharmD), Shree Guru Gobind Singh Tricentenary University, Gurugram, India; Non-communicable Division (NCD) (A K Gupta PharmD), Indian Council of Medical Research, Delhi, India; Department of Cardiology (R Gupta MD), Lehigh Valley Health Network, Allentown, PA, USA; Department of Preventive Cardiology (Prof R Gupta MD), Eternal Heart Care Centre & Research Institute, Jaipur, India; Department of Medicine (Prof R Gupta MD), Mahatma Gandhi University Medical Sciences, Jaipur, India; Department of Toxicology (S Gupta MSc), Shriram Institute for Industrial Research, Delhi, India; School of Biotechnology (V Gupta PhD), Dublin City University, Glasnevin, Ireland; Department of Global Health and Population (A Haakenstad ScD), T.H. Chan School of Public Health, Boston, MA, USA; Department of Clinical Pharmacology and Medicine (Prof N R Hadi PhD), University of Kufa, Najaf, Iraq; Department of Surgery (N Haep MD), Charité University Medical Center Berlin, Berlin, Germany; Clinician Scientist Program (N Haep MD), Berlin Institute of Health, Berlin, Germany; College of Medicine (A Hafiz PhD), Umm AL Qura University, Makkah, Saudi Arabia; Department of Immunology (D Haghmorad PhD), Semnan University of Medical Sciences, Semnan, Iran, Semnan, Iran; Faculty of Medicine (A Hajj Ali BS), American University of Beirut, Beirut, Lebanon; Department of Infectious Disease Epidemiology (S Haller MD), Robert Koch Institute, Berlin, Germany; Department of Public Health (S Haller MD), Charité Institute of Public Health, Berlin, Germany; College of Law and Political Science (K Hamagharib Abdullah PhD), University of Human Development, Sulaimaniya, Iraq; Centre for Neuromuscular and Neurological Disorders (Prof G J Hankey MD), Dental School (O Kujan PhD), The University of Western Australia, Perth, WA, Australia; Perron Institute for Neurological and Translational Science, Perth, WA, Australia (Prof G J Hankey MD); The Warren Alpert Medical School (Z A Haq BA), Department of Internal Medicine (M F H Mohamed MSc), Brown University, Providence, RI, USA; Department of Population Sciences (Prof M Haque PhD), University of Dhaka, Dhaka, Bangladesh; Medical Research Unit (H Harapan PhD), Universitas Syiah Kuala, Banda Aceh, Indonesia; Research Unit (J M Haro MD), University of Barcelona, Barcelona, Spain; Biomedical Research Networking Center for Mental Health Network (CiberSAM), Barcelona, Spain (J M Haro MD); Department of Zoology and Entomology (A I Hasaballah PhD), Al-Azhar University, Cairo, Egypt; Department of Biomedical Engineering and Public Health (S Hasan PhD), World University of Bangladesh, Dhaka, Bangladesh; Department of Radiology (M Hasanian MD), Arak University of Medical Sciences, Arak, Iran; Department of Pharmacy (Prof M S Hasnain PhD), Palamau Institute of Pharmacy, Daltonganj, India; Department of Diagnostic and Interventional Radiology and Neuroradiology (J Haubold MD, Prof B M Schaarschmidt MD), Institute of Artificial Intelligence in Medicine (J Haubold MD), University Hospital Essen, Essen, Germany; Faculty of Kinesiology (Prof J J Hebert PhD), University of New Brunswick, Fredericton, NB, Canada; School of Allied Health (Prof J J Hebert PhD), Murdoch University, Murdoch, WA, Australia; Community-Oriented Nursing Midwifery Research Center (M Heidari PhD), Students Research Committee (R Rahmati MD), Shahrekord University of Medical Sciences, Shahrekord, Iran; Department of Medicine (M Hemmati MD), MedStar Health, Columbia, MD, USA; Department of Medicine (M Hemmati MD), Georgetown University, Washington DC, DC, USA; Departamento de Salud Oral (Department of Oral Health) (B Y Herrera-Serna PhD), Universidad Autónoma de Manizales (Autonomous University of Manizales), Manizales, Colombia; School of Business (Prof C Herteliu PhD), London South Bank University, London, UK; National Agency for Strategic Research in medical education (M Heydari PhD), Ministry of Health and Medical Education, Tehran, Iran; Department of Microbiology (K Hezam PhD), Taiz University, Taiz, Yemen; School of Medicine (K Hezam PhD), Nankai University, Tianjin, China; Department of of Population Health (I Hidayana PhD), Hofstra University, Hempstead, NY, USA; Division for Health Service Promotion (Y Hiraike PhD), Department of Global Health Policy (S Nomura PhD, S K Rauniyar PhD), University of Tokyo, Tokyo, Japan; School of Dentistry (N Q Hoan DDS), Department of Allergy, Immunology and Dermatology (D H Nguyen MD), Hanoi Medical University, Hanoi, Viet Nam; School of Social Sciences (P Hoogar PhD), The Apollo University, Chittoor, India; Department of Pulmonology (N Horita PhD), Yokohama City University, Yokohama, Japan; National Human Genome Research Institute (NHGRI) (N Horita PhD), Center for Translation Research and Implementation Science (G A Mensah MD), National Institutes of Health, Bethesda, MD, USA; Social and Environmental Health Research (M Hossain MPH), Nature Study Society of Bangladesh, Khulna, Bangladesh; Department of Health Promotion and Community Health Sciences (M Hossain MPH), Texas A&M University, College Station, TX, USA; School of Health and Society (H Hosseinzadeh PhD), University of Wollongong, Wollongong, NSW, Australia; Institute of Research and

Development (Prof M Hosseinzadeh PhD), Faculty of Medicine (H T H Nguyen MD), Institue for Research and Training in Medicine, Biology and Pharmacy (H T H Nguyen MD), Duy Tan University, Da Nang, Viet Nam; Department of Computer Science (Prof M Hosseinzadeh PhD), University of Human Development, Sulaymaniyah, Iraq; Department of Clinical Legal Medicine (S Hostiuc PhD), National Institute of Legal Medicine Mina Minovici, Bucharest, Romania; Department of Psychology (C Hu PhD), Tsinghua University, Beijing, China; Centre for Mental Health Research (A J Hunt PhD), Australian National University, Canberra, NSW, Australia; History and Philosophy of Science (A J Hunt PhD), University of Sydney, Sydney, New South Wales, Australia; Department of Biological Sciences and Chemistry (Prof J Hussain PhD), School of Pharmacy (A K Philip PhD), Natural and Medical Sciences Research Center (A Ullah MS, S Ullah MSc, M Waqas PhD), University of Nizwa, Nizwa, Oman; Department of Social Sciences and Business (Prof M Hussain PhD), Roskilde University, Roskilde, Denmark; Department of Biomolecular Sciences (N R Hussein PhD), University of Zakho, Zakho, Iraq; Department of Occupational Safety and Health (Prof B Hwang PhD), China Medical University, Taichung, Taiwan; Department of Occupational Therapy (Prof B Hwang PhD), Asia University, Taiwan, Taichung, Taiwan; Health Policy and Management Department (P M Iftikhar MD), City University of New York, New York, NY, USA; Department of Clinical Effectiveness (A I Ikiroma PhD), NHS National Services Scotland, Edinburgh, Scotland; Department of Pathology (P C Ikwegbue MSc), Department of Medicine (G A Mensah MD), University of Cape Town, Cape Town, South Africa; Faculty of Medicine (I M Ilic PhD, Prof M M Santric-Milicevic PhD), School of Public Health and Health Management (Prof M M Santric-Milicevic PhD), University of Belgrade, Belgrade, Serbia; Department of Epidemiology (Prof M D Ilic PhD), University of Kragujevac, Kragujevac, Serbia; Department of Biotechnology (M A Isa PhD), Sharda University, Greater Noida, India; School of Pharmacy (M R Islam PhD), BRAC University, Dhaka, Bangladesh; Institute for Physical Activity and Nutrition (S Islam PhD), Department of Psychology (M A Stokes PhD), Deakin University, Burwood, VIC, Australia; Clinical Laboratory (F Ismail PhD), Tobruk University, Tobruk, Libya; Department of Blood Transmitted Diseases (F Ismail PhD), National Center for Disease Control, Tobruk, Libya; Department of Clinical Pharmacy & Pharmacy Practice (Prof N Ismail PhD), Asian Institute of Medicine, Science and Technology, Kedah, Malaysia; Malaysian Academy of Pharmacy, Puchong, Malaysia (Prof N Ismail PhD); Department of Health Services Research (M Iwagami PhD), Research and Development Center for Health Services (Prof K Yamagishi MD), University of Tsukuba, Tsukuba, Japan; Knowledge Translation Program (I O Iyamu MD), Centre for Health Evaluation and Outcome Sciences, Vancouver, BC, Canada; Research and Development Unit (L Jacob MD), Biomedical Research Networking Center for Mental Health Network (CiberSAM), Sant Boi de Llobregat, Spain; Faculty of Medicine (L Jacob MD), University of Versailles Saint-Quentin-en-Yvelines, Montigny-le-Bretonneux, France; Department of Health Studies (K H Jacobsen PhD), University of Richmond, Richmond, VA, USA; Statistics Unit (N Jain MD), Riga Stradins University, Riga, Latvia; Department of Health and Safety (A A Jairoun PhD), Dubai Municipality, Dubai, United Arab Emirates; Amity Institute of Biotechnology (D Jakhmola Mani PhD), Amity Institute of Forensic Sciences (H Khajuria PhD, B P Nayak PhD), Amity Institute of Pharmacy (K Munjal PhD), Amity Institute of Public Health (M Shannawaz PhD), Amity University, Noida, India; Department of Public Health (S Jamil BPharm), Daffodil International University, Dhaka, Bangladesh; Department of Neurosciences (Prof R G Jamora PhD), National Institutes of Health (A Loreche BS), University of the Philippines Manila, Manila, Philippines; Institute for Neurosciences (Prof R G Jamora PhD), St. Luke's Medical Center, Bonifacio Global City, Philippines; School of Pharmacy and Pharmacology (A Jatau PhD), Menzies Institute for Medical Research (F Pan PhD), University of Tasmania, Hobart, TAS, Australia; Department of Physiology (Prof S Javadov PhD), University of Puerto Rico Medical Sciences Campus, San Juan, Puerto Rico; Health Informatic Lab (T Javaheri PhD), Boston University, Boston, MA, USA; Department of Biochemistry (Prof S Jayaram MD), Government Medical College, Mysuru, India; Department of Epidemiology and Health Promotion (Prof S Jee PhD), Institute of Health Services Research (D Lee BS), Department of Public Health (D Lee BS), Yonsei University, Seoul, South Korea; Melbourne School of Population and Global Health (H Jiang PhD, L Reifels PhD), School of Health Sciences (A Meretoja MD), University of Melbourne, Melbourne, VIC, Australia; Zoonoses Research Center (M Jokar DVM), Islamic Azad University, Karaj, Iran; Department of Clinical Sciences (M Jokar DVM), Jahrom University of Medical Sciences, Jahrom, Iran; Institute of Molecular and Clinical Ophthalmology Basel, Basel, Switzerland (Prof J B Jonas MD); Department of Ophthalmology (Prof J B Jonas MD), Heidelberg University, Mannheim, Germany; Department of Economics (C E Joshua BSc), National Open University, Benin City, Nigeria; Institute of Family Medicine and Public Health (M Jürisson PhD), University of Tartu, Tartu, Estonia; School of Public Health (Z Kabir PhD), University College Cork, Cork, Ireland; Department of Oral and Maxillofacial Pathology (V Kadashetti MDS), Department of Public Health Dentistry (Prof K M Shivakumar PhD), Krishna Vishwa Vidyapeeth (Deemed to be University), Karad, India; Social Determinants of Health Research Center (L R Kalankesh PhD), Gonabad University of Medical Sciences, Gonabad, Iran; Department of Endocrinology (S Kalra DM), Bharti Hospital Karnal, Karnal, India; Care and Public Health

Research Institute (CAPHRI) (R Kamath MHA), Maastricht University, Maastricht, Netherlands; Department of Health Sciences (Prof M Kanaan PhD), University of York, York, UK; Regional Institute for Population Studies (E Kanmiki MPH), University of Ghana, Accra, Ghana; Faculty of Dentistry (K K Kanmodi MPH), University of Puthisastra, Phnom Penh, Cambodia; Office of the Executive Director (K K Kanmodi MPH), Campaign for Health and Neck Cancer Education (CHANCE) Programme (A A Salami BDS), Cephas Health Research Initiative Inc, Ibadan, Nigeria; Dr. S S Bhatnagar University Institute of Chemical Engg. & Technology (Prof S K Kansal PhD), Department of Anthropology (Prof K Krishan PhD), Institute of Forensic Science and Criminology (V Sharma PhD), Panjab University, Chandigarh, India; Department of Physical Therapy and Health Rehabilitation (F Z Kashoo MSc), Majmaah University, Majmaah, Saudi Arabia; MRC/CSO Social and Public Health Sciences Unit (S V Katikireddi PhD), School of Cardiovascular and Metabolic Health (F E Petermann-Rocha PhD), University of Glasgow, Glasgow, UK; Surgery Research Unit (Prof J H Kauppila MD), Center for Environmental and Respiratory Health Research (I Shiue PhD), Martti Ahtisaari Institute (I Shiue PhD), University of Oulu, Oulu, Finland; Public Health Foundation of India, New Delhi, India (H Kaur MPH); International Research Center of Excellence (G A Kayode PhD), Institute of Human Virology Nigeria, Abuja, Nigeria; Julius Centre for Health Sciences and Primary Care (G A Kayode PhD), Copernicus Institute of Sustainable Development (G Koren PhD), Utrecht University, Utrecht, Netherlands; Department of Public Health (F Kebede MPH), Woldia University, Woldia, Ethiopia; Department of Health Sciences and Biostatistics (E S Kendal PhD), Swinburne University of Technology, Hawthorn, VIC, Australia; Department of Human Nutrition (E Kesse-Guyot PhD), National Research Institute for Agriculture, Food and Environment, Jouy-en-Josas, France; University Sorbonne Paris Nord (E Kesse-Guyot PhD), Department of Health, Medicine and Human Biology (M Touvier PhD), Sorbonne Paris Nord University, Bobigny, France; Department of Parasitology and Mycology (Prof S Khademvatan PhD), Urmia University of Medical Sciences, Urmia, Iran (R Valizadeh PhD); College of Health Sciences (N Khalid PhD), Abu Dhabi University, Adu Dhabi, United Arab Emirates; Department of Biostatistics (Prof A Khalilian PhD), Mazandaran University of Medical Sciences, Mazandaran, Iran; Research Center for Hydatid Disease in Iran (F Khamesipour PhD), Kerman University of Medical Sciences, Kerman, Iran; Primary Care Department (M A Khan MSc), NHS North West London, London, UK; College of Health, Wellbeing and Life Sciences (Prof K Khatab PhD), Sheffield Hallam University, Sheffield, UK; College of Arts and Sciences (Prof K Khatab PhD), Ohio University, Zanesville, OH, USA; Faculty of Nursing (H Khatatbeh PhD), Jerash University, Jerash, Jordan; Global Consortium for Public Health Research (Prof M Khatib PhD), Datta Meghe Institute of Higher Education and Research, Wardha, India; Department of Intenal Medicine (A A Khosla MD), Corewell Health East William Beaumont University Hospital, Royal Oak, MI, USA; Department of Medical Oncology (A A Khosla MD), Miami Cancer Institute, Miami, Florida, USA; Department of Health Management and Economics (M Khosravi PhD), Clinical Research Development Center (CRDC) (A Saghafi MD), Qom University of Medical Sciences, Qom, Iran; Research Department (M Khosrowjerdi PhD), Inland Norway University of Applied Sciences, Elverum, Norway; Department of Public Health (Prof J Khubchandani PhD), New Mexico State University, Las Cruces, NM, USA; Department of Pharmacology (Z D Kifle MSc), Department of Pharmaceutics (L W Limenh MSc), Department of Internal Medicine (E B Melese MD), School of Nursing (H B Netsere MS), University of Gondar, Gondar, Ethiopia; Department of Pediatrics (G Kim MD), Case Western Reserve University School of Medicine, Cleveland, OH, USA; Division of Pediatric Hospital Medicine (G Kim MD), University Hospitals Rainbow Babies and Children's Hospital, Cleveland, OH, USA; Cardiovascular Disease Initiative (M Kim MD), Broad Institute of MIT and Harvard, Cambridge, MA, USA; School of Traditional Chinese Medicine (Y Kim PhD), Xiamen University Malaysia, Sepang, Malaysia; Millennium Prevention, Westwood, MA, USA (R W Kimokoti MD); School of Health Sciences (Prof A Kisa PhD), Kristiania University College, Oslo, Norway; Department of International Health and Sustainable Development (Prof A Kisa PhD), Tulane University, New Orleans, LA, USA; Department of Nursing and Health Promotion (S Kisa PhD), Faculty of Health Sciences (Prof A W Wolf PhD), Oslo Metropolitan University, Oslo, Norway; Global Healthcare Consulting, New Delhi, India (S Kochhar MD); Department of General Practice (Prof O Korzh DSc), Kharkiv National Medical University, Kharkiv, Ukraine; Department of Anesthesiology (V Krishnamoorthy MD), Department of Population Health Sciences (J B Lusk MD), Duke Global Health Institute (C Wu PhD), Duke University, Durham, NC, USA; Faculty of Medicine (B Kucuk Bicer PhD), Gazi University, Ankara, Turkiye; Department of Mathematics (M Kuddus PhD), Department of Population Science and Human Resource Development (M Rahman DrPH), University of Rajshahi, Rajshahi, Bangladesh; Department of Biochemistry (Prof M Kuddus PhD), College of Public Health & Health Informatics (R Kumar PhD), University of Hail, Hail, Saudi Arabia; Department of Pediatrics (I Kuitunen PhD), Kuopio University Hospital, Kuopio, Finland; Institute of Clinical Medicine (I Kuitunen PhD), University of Eastern Finland, Kuopio, Finland; Department of Food Technology (Prof H Kumar PhD), Shri Vishwakarma Skill University, Palwal, India; Department of Biotechnology (Prof H Kumar PhD), Amity Institute of Biotechnology (E Upadhyay PhD), Amity University

Rajasthan, Jaipur, India; Department of Economics (V Kumar PhD), Parul University, Gujarat, Vadodara, India; Centre for Studies in Economics and Planning (V Kumar PhD), Central University of Gujarat, Gandhinagar, India; Division of Cardiovascular Medicine (A Kundu MD), University of Kentucky, Lexington, KY, USA; Department of Health Services Research and Management (D Kusuma DSc), City University of London, London, UK; Faculty of Public Health (D Kusuma DSc), University of Indonesia, Depok, Indonesia; Department of Environment and Public Health (F Kyei-Arthur PhD), University of Environment and Sustainable Development, Somanya, Ghana; Clinical Research Center (V Kytö MD), Turku University Hospital, Turku, Finland; Heart Center (V Kytö MD), University of Turku, Turku, Finland; National Institute for Health Research (NIHR) Oxford Biomedical Research Centre, Oxford, UK (B Lacey PhD); Institute for Social and Health Sciences (Prof L Laflamme PhD), University of South Africa, Pretoria, South Africa; Department of Health Policy and Strategy (Prof C Lahariya MD), Foundation for People-centric Health Systems, New Delhi, India; SD Gupta School of Public Health (Prof C Lahariya MD), Indian Institute of Health Management Research University, Jaipur, India; Department of Public Health (Prof T Lallukka PhD), Department of Virology (F Zakham PhD), University of Helsinki, Helsinki, Finland (T J Meretoja MD); NEVES Society for Patient Safety, Budapest, Hungary (J Lám PhD); Division of Cancer Epidemiology and Genetics (Q Lan PhD), National Cancer Institute, Rockville, MD, USA; Department of Surgery (T Lan PhD), Washington University in St. Louis, St Louis, MO, USA; Unit of Genetics and Public Health (Prof I Landires MD), Institute of Medical Sciences, Las Tablas, Panama; Ministry of Health, Herrera, Panama (Prof I Landires MD); Department of Health Sciences (DISSAL) (F Lanfranchi MD), University of Genoa, Genoa, Italy; Department of Psychiatry and Psychotherapy (B Langguth PhD), University of Regensburg, Regensburg, Germany; Chief Medical Office (Prof V C Lansingh PhD), HelpMeSee, New York, NY, USA; Mexican Institute of Ophthalmology, Queretaro, Mexico (Prof V C Lansingh PhD); Department of Behavioural Sciences and Learning (A Laplante-Lévesque PhD), Linköping University, Linköping, Sweden; Department of Clinical Chemistry and Pharmacology (Prof A O Larsson PhD), Uppsala University Hospital, Uppsala, Sweden; Department of Otorhinolaryngology (S Lasrado MS), Father Muller Medical College, Mangalore, India; International Society Doctors for the Environment, Arezzo, Italy (P Lauriola MD); Faculty of Medicine (H Le MD, N Le MD), Department of General Medicine (V T Nguyen MD), Department of Internal Medicine (T H Tran MD), University of Medicine and Pharmacy at Ho Chi Minh City, Ho Chi Minh City, Viet Nam (T T Le MD, T D T Le MD); Cardiovascular Research Department (H Le MD), Department of Cardiovascular Research (N Le MD), Methodist Hospital, Merrillville, IN, USA; Health Economics Division (L K D Le PhD), Monash University, Burwood, VIC, Australia; Independent Consultant, Ho Chi Minh City, Viet Nam (T D T Le MD); College of Optometry (J L Leasher OD), Nova Southeastern University, Fort Lauderdale, FL, USA; Department of Medical Science (M Lee PhD), Ajou University School of Medicine, Suwon, South Korea; Pattern Recognition and Machine Learning Lab (Prof S Lee PhD), Gachon University, Seongnam, South Korea; Department of Precision Medicine (Prof S W Lee MD), Sungkyunkwan University, Suwon-si, South Korea; School of Pharmacy (S W H Lee PhD), Monash University, Bandar Sunway, Malaysia; School of Pharmacy (S W H Lee PhD), Taylor's University Lakeside Campus, Subang Jaya, Malaysia; Department of Preventive Medicine (Prof Y Lee PhD), Department of Health Policy and Management (S Park PhD), Korea University, Seoul, South Korea; Department of Health Promotion and Health Education (M Li PhD), National Taiwan Normal University, Taipei, Taiwan; Department of Medicine (D Lindholm MD), Norrtälje hospital (Tiohundra), Norrtälje, Sweden; Department of Dentistry (Prof S Listl PhD), Radboud University, Nijmegen, Netherlands; Department of Translational Health Economics (Prof S Listl PhD), Heidelberg University Hospital, Heidelberg, Germany; School of Life Sciences (G Liu PhD), University of Technology Sydney, Ultimo, NSW, Australia; Department of Cardiology (S Liu MSc), Guiqian International General Hospital, Guiyang, China; Department of Molecular Epidemiology (E Llanaj PhD), German Institute of Human Nutrition Potsdam-Rehbrücke, Potsdam, Germany; German Center for Diabetes Research (DZD), München-Neuherberg, Germany (E Llanaj PhD); Department of Physical Medicine and Nursing (R López-Bueno PhD), University of Zaragoza, Zaragoza, Spain; Department of Musculoskeletal disorders (R López-Bueno PhD), National Research Centre for the Working Environment, Copenhagen, Denmark; One Health Research Group (J López-Gil PhD), Universidad de Las Américas, Quito, Ecuador; School of Medicine and Public Health (A Loreche BS), Center for Research and Innovation (V F Pepito MSc), Ateneo De Manila University, Pasig City, Philippines; Department of Medicine (Prof P A Lotufo DrPH), University of Sao Paulo, São Paulo, Brazil; School of Medicine (Prof G Lucchetti PhD), Federal University of Juiz de Fora, Juiz de Fora, Brazil; Center for Evidence-Based and Translational Medicine (L Luo MPH), Department of Epidemiology and Biostatistics (Prof C Yu PhD), School of Health Sciences (X G Zhao PhD), Wuhan University, Wuhan, China; Clinical Data Science and Evidence (L Lv PhD), Novo Nordisk, Plainsboro, NJ, USA; Department of Chemistry (H I M Amin PhD), Department of Food Technology (B A Sadee PhD), Salahaddin University-Erbil, Erbil, Iraq; Department of Medical Biochemical Analysis (H I M Amin PhD), Department of Nutrition and Dietetics (B A Sadee PhD), Cihan University-Erbil, Erbil, Iraq; Centre for Public

Health and Wellbeing (Z Ma PhD), University of the West of England, Bristol, UK; 2nd Department of Propaedeutic Surgery (N Machairas PhD), 3rd Department of Cardiology (M Spartalis PhD), University of Athens, Athens, Greece; Periodontal Department (Prof M Machoy PhD), Department of Propedeutics of Internal Diseases & Arterial Hypertension (Prof T Miazgowski MD), Pomeranian Medical University, Szczecin, Poland; Department of Forensic Medicine and Toxicology (D Mahadeshwara Prasad MD), Mysore Medical College & Research Institute, Mysooru, India; Department of Health & Family Welfare (D Mahadeshwara Prasad MD), Government of Karnataka, Bangalore, India; Grants, Innovation and Product Development Unit (P W Mahasha PhD), Risk and Resilience in Mental Disorders Unit (Prof D J Stein MD), South African Medical Research Council, Cape Town, South Africa; Department of Nuclear Medicine (P W Mahasha PhD), University of Pretoria, Pretoria, South Africa; Department of Clinical and Hospital Pharmacy (M A Mahmoud PhD), Taibah University, Al-Madinah Al-Munawarrah, Saudi Arabia; USERN Office (G Mahmoudvand MD), Lorestan University of Medical Sciences, Khorramabad, Iran; Maternal, Child and Adolescent Health Program (M Makama PhD), Burnet Institute, Melbourne, VIC, Australia; School of Public Health and Preventive Medicine (M Makama PhD), Monash University, Melbourne, Victoria, Australia; Rama Medical College Hospital and Research Centre, Uttar Pradesh, India (K Malhotra MBBS); University Institute of Public Health (A A Malik PhD, S Nargus PhD), Institute of Molecular Biology and Biotechnology (S Shahid PhD), Research Centre for Health Sciences (RCHS) (S Shahid PhD), Department of Physics (W Shahid PhD), Lahore Business School (M Umar MBA), The University of Lahore, Lahore, Pakistan; Smidt Heart Institute (Y Manla MD), Cedars-Sinai Medical Center, Los Angeles, CA, USA; Information and Communication Technology Research Pole (Lab-STICC) (Prof A Mansour PhD), ENSTA Bretagne, Brest, France; Research Department (B P Marasini PhD), Nepal Health Research Council, Kathmandu, Nepal; Biomedical Engineering Research Center (CREB) (H Marateb PhD), Universitat Politècnica de Catalunya (Barcelona Tech - UPC), Barcelona, Spain; Department of Artificial Intelligence (H Marateb PhD), Smart University of Medical Sciences, Tehran, Iran; Non-communicable Diseases Research Center (P Mardi MD), Research Center for Health, Safety and Environment (Prof L Salehi PhD), Alborz University of Medical Sciences, Karaj, Iran; Department of Biochemistry (A Marjani PhD), Golestan Research Center of Gastroenterology and Hepatology (G Roshandel PhD), Golestan University of Medical Sciences, Gorgan, Iran; Institute of Labour Economics (H Markazi Moghadam PhD), Leibniz University Hannover, Hannover, Germany; RGS Econ (H Markazi Moghadam PhD), Ruhr Graduate School in Economics, Essen, Germany; Department of Health Economics (Prof C A Marrugo Arnedo MSc), Mayor University, Cartagena, Colombia; Department of Economics (Prof G Martinez PhD), Autonomous Technology Institute of Mexico, Mexico City, Mexico; Noncommunicable Diseases and Mental Health Department (R Martinez-Piedra BSc), Pan American Health Organization, Washington, DC, USA; Campus Fortaleza (F R Martins-Melo PhD), Federal Institute of Education, Science and Technology of Ceará, Fortaleza, Brazil; Department of Nutrition and Dietetics (M Martorell PhD), University of Concepcion, Concepción, Chile; Centre for Healthy Living (M Martorell PhD), University of Concepción, Concepción, Chile; Institute for Mental and Physical Health and Clinical Translation (IMPACT) (W Marx PhD), Deakin University, Geelong, VIC, Australia; Faculty of Humanities and Health Sciences (Prof R R Marzo MD), Curtin University, Malaysia, Sarawak, Malaysia; Jeffrey Cheah School of Medicine and Health Sciences (Prof R R Marzo MD), Monash University, Subang Jaya, Malaysia; Department of Anatomy, Genetics and Biomedical Informatics (Y Mathangasinghe MD), Postgraduate Institute of Medicine (S N K Navaratna MD), Department of Surgery (D P Wickramasinghe MD), University of Colombo, Colombo, Sri Lanka; Division of Immunology, Immunity to Infection and Respiratory Medicine (A G Mathioudakis PhD), University of Manchester, Manchester, UK; North West Lung Centre (A G Mathioudakis PhD), Manchester University NHS Foundation Trust, Manchester, UK; Department of Community Medicine (M Mathur MD), Department of General Medicine (N Mathur MD), Geetanjali Medical College and Hospital, Udaipur, India; Community Medicine Department (N Mathur MD), Apollo Hospital, Hyderabad, India; Department of Medicine (J Mattumpuram MD), University of Louisville, Louisville, KY, USA; Department of Epidemiology (Prof R J Maude PhD), Mahidol Oxford Tropical Medicine Research Unit, Bangkok, Thailand; Department of Twin Research and Genetic Epidemiology (M Mazidi PhD), Faculty of Life Sciences and Medicine (M Molokhia PhD), School of Population Health and Environmental Sciences (Y Wang PhD, Prof C D A Wolfe MD), King's College London, London, UK; Orthopedic Trauma Pathology Department (A Mazzotti PhD), IRCCS, Bologna, Italy; National Centre for Register-based Research (Prof J J McGrath MD), Aarhus University, Aarhus, Denmark; Australian Centre for Health Services Innovation (Prof S M McPhail PhD), Queensland University of Technology, Kelvin Grove, QLD, Australia; Department of Medicine (S Mehravar MD), Cedar Associates, Los Angeles, CA, USA; Department of Public Health (T Mekene Meto MPH), Arba Minch University, Arbaminch, Ethiopia; Department of Medical Oncology and Hematology (M A M Mendez-Lopez PhD), Kantonsspital St. Gallen, St. Gallen, Switzerland; Peru Country Office (W Mendoza MD), United Nations Population Fund (UNFPA), Lima, Peru; Eunice Kennedy Shriver National Institute of Child Health and Human Development (L G Mensah

MD), National Institute of Health, Bethesda, MD, USA; International Dx Department (A A Mentis MD), BGI Genomics, Copenhagen, Denmark; Neurology Unit (A Meretoja MD), Breast Surgery Unit (T J Meretoja MD), Helsinki University Hospital, Helsinki, Finland; Department of Nursing (A M Mersha MSc), Arba Minch University, Arba Minch, Ethiopia; University Centre Varazdin (T Mestrovic PhD), University North, Varazdin, Croatia; Department of Pharmacology (Prof K D Mettananda PhD), Department of Paediatrics (Prof S Mettananda DPhil), University of Kelaniya, Ragama, Sri Lanka; Clinical Medicine Department (Prof K D Mettananda PhD), North Colombo Teadhing Hospital, Ragama, Sri Lanka; University Paediatrics Unit (Prof S Mettananda DPhil), Colombo North Teaching Hospital, Ragama, Sri Lanka; Stritch School of Medicine (A Mhlanga PhD), Loyola University Chicago, Chicago, IL, USA; South African Centre for Epidemiological Modelling and Analysis (SACEMA) (L Mhlanga PhD), Department of Epidemiology (J L Tamuzi MSc), Department of Industrial Psychology (E Teye-Kwadjo PhD), Stellenbosch University, Cape Town, South Africa; Department of Epidemiology (I Michalek PhD), National Cancer Registry (I Michalek PhD), Maria Sklodowska-Curie National Research Institute of Oncology, Warsaw, Poland; Pacific Institute for Research & Evaluation, Calverton, MD, USA (T R Miller PhD); Department of Medical Sciences (A Mirijello MD), IRCCS Casa Sollievo della Sofferenza General Hospital, San Giovanni Rotondo, Italy; Internal Medicine Programme (Prof E M Mirrakhimov PhD), Kyrgyz State Medical Academy, Bishkek, Kyrgyzstan; Department of Atherosclerosis and Coronary Heart Disease (Prof E M Mirrakhimov PhD), National Center of Cardiology and Internal Disease, Bishkek, Kyrgyzstan; Department of Forensic Medicine and Toxicology (C Mittal MD), Dr. B. C. Roy Multi-Specialty Medical Research Centre, Kharagpur, India; Universal Scientific Education and Research Network (USERN) (A Moghadam Fard MD), Universal Scientific Education and Research Network (USERN), Tehran, Iran; Department of Psychology (S Mohajelin MA), Aston University, Birmingham, UK; College of Health Science (A I Mohamed MSc), College of Applied and Natural Science (J Mohamed MSc), University of Hargeisa, Hargeisa, Somalia; Molecular Biology Unit (N S Mohamed MSc), Bio-Statistical and Molecular Biology Department (N S Mohamed MSc), Sirius Training and Research Centre, Khartoum, Sudan; College of Medicine (Prof A M Mohammad MD), University of Duhok, Duhok, Iraq; Department of Public Health (H Mohammed MPH), Dire Dawa University, Dire Dawa, Ethiopia; QU Health (M Mohammed PhD), Social and Economic Survey Research Institute (Prof A Perianayagam PhD), Department of Population Medicine (Prof G Rathnaiah Babu PhD), Qatar University, Doha, Qatar; Health Systems and Policy Research Unit (S Mohammed PhD), Department of Community Medicine (S S Umar FWACS), Ahmadu Bello University, Zaria, Nigeria; Clinical Epidemiology and Public Health Research Unit (L Monasta DSc, L Ronfani PhD), Burlo Garofolo Institute for Maternal and Child Health, Trieste, Italy; Department of Biomedical and Dental Sciences and Morphofunctional Imaging (Prof S Mondello MD), Messina University, Messina, Italy; Faculty of Medicine (A Moodi Ghalibaf MD), Birjand University of Medical Sciences, Birjand, Iran; Department of Epidemiology and Biostatistics (Y Moradi PhD), Kurdistan University of Medical Sciences, Sanandaj, Iran; Computer, Electrical, and Mathematical Sciences and Engineering Division (P Moraga PhD), King Abdullah University of Science and Technology, Thuwal, Saudi Arabia; International Laboratory for Air Quality and Health (Prof L Morawska PhD), Queensland University of Technology, Brisbane, QLD, Australia; Department of Public Health (Prof R S Moreira PhD), Oswaldo Cruz Foundation, Recife, Brazil; Department of Public Health (Prof R S Moreira PhD), Federal University of Pernambuco, Recife, Brazil; Department of Clinical Biochemistry (A Mosapour PhD), Babol University of Medical Sciences, Babol, Iran; Department of Clinical Biochemistry (A Mosapour PhD), Tarbiat Modares University, Tehran, Iran; Department of Health Policy (Prof E Mossialos PhD), London School of Economics and Political Science, London, UK; Department of Economics (M Mrejen PhD), Fluminense Federal University, Rio de Janeiro, Brazil; Unit of Pharmacotherapy, Epidemiology and Economy (S Mubarik PhD), University Medical Center Groningen (Prof M J Postma PhD), Department of Internal Medicine (P Vart PhD), University of Groningen, Groningen, Netherlands; Competence Center of Mortality-Follow-Up of the German National Cohort (R Westerman DSc), Federal Institute for Population Research, Wiesbaden, Germany (Prof U O Mueller MD); Center for Population and Health, Wiesbaden, Germany (Prof U O Mueller MD); Department of Surgery (F Mulita PhD, G Verras MD), General University Hospital of Patras, Patras, Greece; Faculty of Medicine (F Mulita PhD), Department of Internal Medicine (G Ntaios PhD), Department of Emergency Medicine (I Pantazopoulos PhD), University of Thessaly, Larissa, Greece; Clinical Epidemiology Research Unit (E Murillo-Zamora PhD), Mexican Institute of Social Security, Villa de Alvarez, Mexico; Postgraduate in Medical Sciences (E Murillo-Zamora PhD), Universidad de Colima, Colima, Mexico; Research and Innovation Department (Prof K M Musallam MD), Burjeel Medical City, Abu Dhabi, United Arab Emirates; Surgery Department (A Musina MD), University of Medicine and Pharmacy Grigore T. Popa, Iasi, Romania; Second Surgical Unit (A Musina MD), Regional Institute of Oncology Iasi, Iasi, Romania; Department of Pediatrics & Pediatric Pulmonology (Prof G Mustafa MD), Institute of Mother & Child Care, Multan, Pakistan; Department of Neuropsychiatry (W Myung PhD), Department of Food and Nutrition (A P Okekunle PhD), Seoul National University, Seoul, South Korea;

Department of Neuropsychiatry (W Myung PhD), Seoul National University Bundang Hospital, Seongnam, South Korea; Elderly Health Research Center (A Nafei PhD), Research and Academic Institution, Tehran, Iran; Research and Analytics Department (A J Nagarajan MTech), Initiative for Financing Health and Human Development, Chennai, India; Department of Research and Analytics (A J Nagarajan MTech), Bioinsilico Technologies, Chennai, India; Comprehensive Cancer Center (G Naik MPH), Department of Health Policy and Organization (M Rahim MA), Department of Health Services Administration (M Rahim MA), Department of Psychology (D C Schwebel PhD), University of Alabama at Birmingham, Birmingham, AL, USA; Faculty of Pharmacy (F Nainu PhD), Hasanuddin University, Makassar, Indonesia; Suraj Eye Institute, Nagpur, India (V Nangia MD); Mysore Medical College and Research Institute (Prof S Narasimha Swamy MD), Government Medical College, Mysore, India; National Dental Research Institute Singapore (G G Nascimento PhD), Duke-NUS Medical School, Singapore; Department of Applied Pharmaceutical Sciences and Clinical Pharmacy (A Y Naser PhD), Isra University, Amman, Jordan; Department of Community Medicine (S N K Navaratna MD), University of Peradeniya, Peradeniya, Sri Lanka; Department of Biotechnology (M Naveed PhD), University of Central Punjab, Lahore, Pakistan; School of Medicine (N PhD), Xiamen University, Xiamen, China; Department of General Surgery (I Negoi PhD), Fourth Department of General Surgery (D Serban PhD), Emergency University Hospital Bucharest, Bucharest, Romania; Department of Cardiology (R I Negoi PhD), Cardio-Aid, Bucharest, Romania; Faculty of Medicine (Prof C Nejjari PhD), Euromed University of Fes, Fez, Morocco; Faculty of Medicine (Prof C Nejjari PhD), University Sidi Mohammed Ben Abdellah, Fez, Morocco; College of Medicine and Health Sciences (H B Netsere MS), Bahir Dar University, Gondar, Ethiopia; Department of Public Health (G Nguefack-Tsague PhD), University of Yaoundé I, Yaoundé, Cameroon; Department of Biological Sciences (J W Ngunjiri DrPH), University of Embu, Embu, Kenya; Department of General Medicine (A H Nguyen MD), Thai Binh University of Medicine and Pharmacy, Thai Binh City, Viet Nam; Department of Medical Engineering (D H Nguyen BS), University of South Florida, Tampa, FL, USA; Cardiovascular Laboratory (D H Nguyen MD), Methodist Hospital, Merrillville, Merrillville, IN, USA; Department of Pediatrics (N N Nguyen MD), Children 2 Hospital, Ho Chi Minh City, Viet Nam; Faculty of Medicine (N N Nguyen MD), School of Medicine (H Pham MD), Pham Ngoc Thach University of Medicine, Ho Chi Minh City, Viet Nam; Department of Surgery (P T Nguyen MD), Danang Family Hospital, Danang, Viet Nam; Molecular Neuroscience Research Center (D Nguyen Tran Minh MD), Shiga University of Medical Science, Shiga, Japan; International Islamic University Islamabad, Islamabad, Pakistan (R K Niazi PhD); Institute for Mental Health and Policy (Y T Nigatu PhD), Centre for Addiction and Mental Health, Toronto, ON, Canada; Department of Nephrology and Hypertension (N Nikravangolsefid MD), Department of Informatics and Radiology (S Vahdati MD), Mayo Clinic, Rochester, MN, USA; Faculty of Applied Sciences and Technology (E A Noman PhD), Universiti Tun Hussein Onn Malaysia, Johor, Malaysia; Department of Health Policy and Management (S Nomura PhD), Keio University, Tokyo, Japan; Department of Statistics (S Noor MS), Shahjalal University of Science and Technology, Sylhet, Bangladesh; School of Health (M Nozari PhD), Bam University of Medical Sciences, Bam, Iran; Department of Paediatrics (C A Nri-Ezedi MD), Nnamdi Azikiwe University, Awka, Nigeria; Public Health Department (M H Nunemo MPH), Wachemo University, Addis Ababa, Ethiopia; Department of Public Health (D Nurrika PhD), Banten School of Health Science, South Tangerang, Indonesia; Ministry of Research, Technology and Higher Education (D Nurrika PhD), Higher Education Service Institutions (LL-DIKTI) Region IV, Bandung, Indonesia; School of Nursing (J Nutor PhD), Department of Epidemiology and Biostatistics (M Teramoto MD), University of California San Francisco, San Francisco, CA, USA; Center of Excellence in Reproductive Health Innovation (CERHI) (C I Nzoputam MPH), University of Benin, Benin City, Nigeria; Department of Physiology (O J Nzoputam PhD), University of Benin, Edo, Nigeria; Department of Physiology (O J Nzoputam PhD), Benson Idahosa University, Benin City, Nigeria; Department of Applied Economics and Quantitative Analysis (Prof B Oancea PhD), University of Bucharest, Bucharest, Romania; PSSM Data Sciences (M Oduro PhD), Pfizer Inc., Groton, CT, USA; Department of Preventive Medicine (I Oh PhD), Kyung Hee University, Seoul, South Korea; Health Promotion Research Center (H Okati-Aliabad PhD), Zahedan University of Medical Sciences, Zahedan, Iran; School of Pharmacy (O C Okonji MSc), University of the Western Cape, Cape Town, South Africa; Department of Psychiatry and Behavioural Neurosciences (A T Olagunju MD), McMaster University, Hamilton, ON, Canada; Department of Psychiatry (A T Olagunju MD), University of Lagos, Lagos, Nigeria; Department of Nursing Science (M I Olatubi PhD), Bowen University, Iwo, Nigeria; Cardiology Department (G M M Oliveira PhD), Federal University of Rio de Janeiro, Rio de Janeiro, Brazil; Centre for Healthy Start Initiative, Lagos, Nigeria (B O Olusanya PhD, J O Olusanya MBA); Surgery Department (G L Omer MD), Sulaimani University, Sulaimani, Iraq; ENT Department (G L Omer MD), Tor Vergata University of Rome, Rome, Italy; Non-communicable Disease Prevention Unit (S Ong FAMS), Ministry of Health, Bandar Seri Begawan, Brunei; Early Detection & Cancer Prevention Services (S Ong FAMS), Pantai Jerudong Specialist Centre, Bandar Seri Begawan, Brunei; Faculty of Psychology (S Onie PhD), Universitas Airlangga, Surabaya, Indonesia; Department of Pharmacology and

Therapeutics (Prof O E Onwujekwe PhD), University of Nigeria Nsukka, Enugu, Nigeria; Department of Pharmacotherapy and Pharmaceutical Care (M Ordak PhD), Department of Biochemistry and Pharmacogenomics (M Zielińska MPharm), Medical University of Warsaw, Warsaw, Poland; Sickle Cell Unit (V N Orish PhD), Ho Teaching Hospital, Ho Municipality, Ghana; Department of Nephrology and Hypertension (Prof A Ortiz MD), The Institute for Health Research Foundation Jiménez Díaz University Hospital, Madrid, Spain; One Health Global Research Group (Prof E Ortiz-Prado PhD), Universidad Diego Portales, Quito, Ecuador; Department of Biology (W M S Osman PhD), Khalifa University, Abu Dhabi, United Arab Emirates; Department of Biomedical Sciences (Prof S M Ostojic PhD), Department of Internal Medicine (D S Popovic PhD), University of Novi Sad, Novi Sad, Serbia; School of Medicine (U L Osuagwu PhD), Translational Health Research Institute (K Rana PhD), Western Sydney University, Campbelltown, NSW, Australia; Department of Optometry and Vision Science (U L Osuagwu PhD), University of KwaZulu-Natal, KwaZulu-Natal, South Africa; Laboratory of Public Health Indicators Analysis and Health Digitalization (S S Otstavnov PhD), Department of Information Technologies and Management (S K Vladimirov PhD), Moscow Institute of Physics and Technology, Dolgoprudny, Russia; Department of Project Management (S S Otstavnov PhD), Department of Health Care Administration and Economics (Prof V Vlassov MD), National Research University Higher School of Economics, Moscow, Russia; Division of Infectious Diseases (Prof A Ouyahia PhD), University Hospital of Setif, Setif, Algeria; Miami Cancer Institute (A Ozair MD), Baptist Health South Florida, Miami, Florida, USA; Department of Respiratory Medicine (Prof M P P A DNB), Jagadguru Sri Shivarathreeswara University, Mysore, India; Department of Forensic Medicine and Toxicology (J Padubidri MD), Kasturba Medical College, Mangalore, Mangalore, India; Department of Neurology (Prof P K Pal DM), National Institute of Mental Health and Neurosciences, Bangalore, India; Hungarian Health Management Association (T Palicz MD), Hungarian Health Management Association, Budapest, Hungary; Department of Epidemiology and Biostatistics (Prof H Pan PhD), Anhui Medical University, Hefei, China; Privatpraxis, Heidelberg, Germany (S Panda-Jonas MD); National Research and Innovation Agency Republic of Indonesia (BRIN), Jakarta, Indonesia (H U Pangaribuan MSc); Department of Ophthalmology (G D Panos PhD), Nottingham University Hospitals Queen's Medical Centre Campus, Nottingham, UK; Division of Ophthalmology & Visual Sciences (G D Panos PhD), University of Nottingham, Nottingham, UK; Department of Neurology (L D Panos MD), Department of Emergency Medicine (I Pantazopoulos PhD), University of Bern, Bern, Switzerland; Department of Epidemiology and Community Health (R R Parikh MD), Department of Surgery (J Rickard MD), University of Minnesota, Minneapolis, MN, USA; Department of Medical Humanities and Social Medicine (Prof E Park PhD), Kosin University, Busan, South Korea; Department of Biomedical Data Science (S Park MD), Department of Radiology (S Ramasamy MD), Stanford University, Stanford, CA, USA; School of Psychological Sciences (N Parsons PhD), Monash University, Melbourne, Australia; Center for Pharmacoepidemiology and Treatment Science (A Parthasarathi MD), Rutgers University, New Brunswick, NJ, USA; Research Center (A Parthasarathi MD), Allergy Asthma and Chest Center, Mysore, India; Department of Medical Sciences (R Passera PhD), University of Torino, Torino, Italy; Department of Imaging (R Passera PhD), AOU Città della Salute e della Scienza di Torino, Torino, Italy; Global Health Governance Programme (J Patel BSc), Usher Institute (Prof C R Simpson PhD), College of Medicine and Veterinary Medicine (G Verras MD), Public Health Department (T A Zerfu PhD), University of Edinburgh, Edinburgh, UK; School of Dentistry (J Patel BSc), University of Leeds, Leeds, UK; Research Consultancy (A R Pathan PhD), Author Gate Publications, Malegaon, India; College of Dental Medicine (Prof S Patil PhD), Roseman University of Health Sciences, South Jordan, UT, USA; Centre of Molecular Medicine and Diagnostics (COMManD) (Prof S Patil PhD), Saveetha Dental College and Hospitals (K Rengasamy PhD), Saveetha University, Chennai, India; Second Department of Internal Medicine (D Patoulias PhD), European Interbalkan Medical Center, Thessaloniki, Greece; Clinical Research Department (P Pedersini MSc), IRCCS Fondazione Don Carlo Gnocchi, Milan, Italy; Department of Neurology (U Pensato MD), IRCCS Humanitas Research Hospital, Milan, Italy; Mario Negri Institute for Pharmacological Research, Bergamo, Italy (N Perico MD, Prof G Remuzzi MD); Department of Biology (Prof S Perna PhD), University of Bahrain, Sakir, Bahrain; Facultad de Medicina (F E Petermann-Rocha PhD), Diego Portales University, Santiago, Chile; Shanghai Mental Health Center (Prof M R Phillips MD), Shanghai Jiao Tong University, Shanghai, China; Department of Psychiatry (Prof M R Phillips MD), Department of Neurology (Prof N Scarmeas PhD), Columbia University, New York, NY, USA; Department of Pediatric Orthopedic Surgery (M Pigeolet MD), Hôpital Necker - Enfants Malades, Paris, France; Air and Climate Unit (E Pisoni PhD), European Commission, Ispra, Italy; Research School of Chemistry and Applied Biomedical Sciences (E Plotnikov PhD), Tomsk Polytechnic University, Tomsk, Russia; Mental Health Research Institute (E Plotnikov PhD), Tomsk National Research Medical Center of the Russian Academy of Sciences, Tomsk, Russia; Clinical Academic Department of Pediatrics (Prof D Poddighe PhD), University Medical Center (UMC), Astana, Kazakhstan; Department of Data Management and Analysis (R Poluru PhD), The INCLEN Trust International, New Delhi, India; Department of Ortopedics and Traumatology (V T Ponkilainen PhD), University of

Tampere, Tampere, Finland; Clinic for Endocrinology, Diabetes and Metabolic Disorders (D S Popovic PhD), Clinical Center of Vojvodina, Novi Sad, Serbia; Independent Research Scholar, San Diego, CA, USA (D Prabhu PhD); Centro de Investigaciones Clinicas (Clinical Research Center) (S I Prada PhD), Fundación Valle del Lili, Cali, Colombia; Humanities and Social Sciences (Prof J Pradhan PhD), National Institute of Technology Rourkela, Rourkela, India; Department of Biochemistry (Prof A Prashant PhD), Jagadguru Sri Shivarathreeswara University, Mysuru, India; Department of Dermatology, Venereology and Leprosy-DVL (Prof T Priscilla MD), Apollo Institute of Medical Sciences and Research, Hyderabad, India; Centre for Dental Education and Research (B M Purohit MDS), Department of Psychiatry (Prof R Sagar MD), Department of Radiation Oncology (A Shankar MD), All India Institute of Medical Sciences, New Delhi, India; Department of Biostatistics Epidemiology and Informatics (J Puvvula PhD), University of Pennsylvania, Philadelphia, PA, USA; Cihan University Sulaimaniya Research Center (N H Qasim PhD), Cihan University Sulaimaniya, Sulaimaniya, Iraq; Department of Biological Sciences (A S Qazi PhD, R Z Raza PhD), National University of Medical Sciences (NUMS), Rawalpindi, Pakistan; Department of Cardiology (G Qian MS), Third Military Medical University, Chongqing, China; Cardiovascular Research Center (M Rabiee Rad MD), Isfahan Cariovascular Research Institute, Isfahan, Iran; Department of Medical Oncology (Prof V Radhakrishnan MD), Cancer Institute (W.I.A), Chennai, India; UO Neurologia, Salute Pubblica e Disabilità (A Raggi PhD), Fondazione IRCCS Istituto Neurologico Carlo Besta, Milan, Italy; Pathology Department (N Raheem FMCPath), Mobiddo Adama University Teaching Hospital -Yola, Yola, Nigeria; Department of Health Sciences (Prof F Rahim PhD), Cihan University-Sulaymaniyah, Sulaimaniya, Iraq; Cihan University Sulaimaniya Research Center (CUSRC), Sulaymaniyah, Iraq (Prof F Rahim PhD); School of Medicine and Public Health (M M R Rahman PhD), The University of Newcastle, Wollongong, NSW, Australia; Institute of Health and Wellbeing (M Rahman PhD), Federation University Australia, Berwick, VIC, Australia; College of Communication and Information Sciences (T Rahman MCom), The University of Alabama, Tuscaloosa, AL, USA; Future Technology Research Center (A Rahmani PhD), National Yunlin University of Science and Technology, Yunlin, Taiwan; Student Research Committee (N Rahmanian PhD), Student Research Committee, Rasht, Iran; Department of Nutrition Science (S Rahmawaty PhD), Muhammadiyah University of Surakarta, Surakarta, Indonesia; Department "Hospital of women and children", IRCCS Azienda Ospedaliero-Universitaria di Bologna (D Raimondo PhD), Occupational Health Unit (Prof F S Violante MD), Sant'Orsola Malpighi Hospital, Bologna, Italy; Department of Cardiology (A Raja MD), Dow University of Health Sciences, Karachi, Pakistan; Centre for Chronic Disease Control, New Delhi, India (P Rajput PhD); Population Health (M Ramadan DrPH), King Abdullah International Medical Research Center, Jeddah, Saudi Arabia; Laboratory (Prof P W Ramteke PhD), Mure Memorial Hospital, Nagpur, India; Department of Molecular Biology & Genetic Engineering (Prof P W Ramteke PhD), RTM Nagpur University, Nagpur, India; Department of Community Medicine (R K Rana MD), Shaheed Nirmal Mahto Medical College and Hospital, Dhanbad, India; Research Department (C L Ranabhat PhD), Policy Research Institute, Kathmandu, Nepal; Health and Public Policy Department (C L Ranabhat PhD), Global Center for Research and Development, Kathmandu, Nepal; Health Economics and Outcomes Research Department (A Rane MS), Agios Pharmaceuticals, Cambridge, MA, USA; Department of Pharmaceutical Economics and Policy (A Rane MS), Massachusetts College of Pharmacy and Health Sciences, Boston, MA, USA; University of Social Welfare and Rehabilitation Sciences, Tehran, Iran (V Rashedi PhD); Department of Medicine (A M Rashid MD), Jinnah Sindh Medical University, Karachi, Pakistan; Department of Geography (A Rasul PhD), Soran University, Soran, Iraq; Department of Family Medicine (Prof D Rathish MPH), Department of Parasitology (Prof K G Weerakoon PhD), Rajarata University of Sri Lanka, Anuradhapura, Sri Lanka; Inovus Medical, St Helens, UK (D L Rawaf MRCS); Academic Public Health England (Prof S Rawaf MLIS), Public Health England, London, UK; Department of Biological Sciences (Prof E M M Redwan PhD), King Abdulaziz University, Jeddah, Egypt; Department of Protein Research (Prof E M M Redwan PhD), Research and Academic Institution, Alexandria, Egypt; Centre for Excellence in Pharmaceutical Sciences (K Rengasamy PhD), North-West University, Potchefstroom, South Africa; Brien Holden Vision Institute, Sydney, NSW, Australia (Prof S Resnikoff MD); Department of Obstetrics and Gynecology (S Restaino MD), Azienda Sanitaria Universitaria Friuli Centrale - Udine -, Udine, Italy; Unisabana Center for Translational Science (L F Reyes PhD), Universidad de La Sabana (Savannah University), Chia, Colombia; Critical Care Department (L F Reyes PhD), Clinica Universidad De La Sabana (Savannah University Clinic), Chia, Colombia; Clinical Pathology and Cancer Diagnosis (Z Rezaei MD), Karolinska University Hospital, Stockholm, Sweden; Department of Epidemiology and Biostatistics (Prof M Rezaeian PhD), Rafsanjan University of Medical Sciences, Rafsanjan, Iran; Department of Public Health Sciences (T G Rhee PhD), University of Connecticut, Farmington, CT, USA; Department of Surgery (J Rickard MD), University Teaching Hospital of Kigali, Kigali, Rwanda; Community Health Department (Prof H A L Rocha PhD), Federal University of Ceará, Fortaleza, Brazil; Department of Pharmacology and Toxicology (Prof J A B Rodriguez PhD), Universidad de Antioquia, Medellin, Colombia; Department of Clinical Research (L Roever PhD), Federal University of Uberlândia, Uberlândia, Brazil;

Faculty of Nursing (D S Romadlon PhD), Chulalongkorn University, Bangkok, Thailand; Department of Nursing (M Rony MPH), Directorate General of Health Services, Gazipur, Bangladesh; Technical Department (K Rotimi MSc), Malaria Consortium, Abuja, Nigeria; Public Health Pharmacy (K Rotimi MSc), West African Postgraduate College of Pharmacists, Lagos, Nigeria; Department of Analytical and Applied Economics (Prof H S Rout PhD, P M Sahoo MA, C K Swain MPhil), UGC Centre of Advanced Study in Psychology (M Satpathy PhD), Utkal University, Bhubaneswar, India; Faculty of Medicine (B Roy PhD), Quest International University Perak, Ipoh, Malaysia; Centro de Investigación Palmira (Palmira Research Center) (E Rubagotti PhD), Corporación Colombiana de Investigación Agropecuaria AGROSAVIA (Colombian Agricultural Research Corporation), Bogota, Colombia; Advanced Campus Governador Valadares (Prof G d Ruela MSc), Juiz de For a Federal University, Governador Valadares, Brazil; Nursing Department (Prof G d Ruela MSc), Universidade Presidente Antônio Carlos (President Antônio Carlos University), Governador Valadares, Brazil; Department of Health Statistics (S F Rumisha PhD), National Institute for Medical Research, Dar es Salaam, Tanzania; Nuffield Department of Medicine (T Runghien MSc), Oxford University, Oxford, UK; Department of Cardiology (M Russo PhD), S. Maria dei Battuti Hospital, AULSS 2 Veneto, Conegliano, Italy; Department of Medical Pharmacology (M M Saber-Ayad MD), Public Health and Community Medicine Department (M R Salem MD), Cairo University, Giza, Egypt; Department of Medicine (C J Sabet MA), Georgetown University, Washington, DC, USA; Neuropsychiatric Institute (Prof P S Sachdev MD), Prince of Wales Hospital, Randwick, NSW, Australia; Department of Pharmaceutical Chemistry (Prof M R Saeb PhD), Medical University of Gdańsk, Gdańsk, Poland; Multidisciplinary Laboratory (Prof U Saeed PhD), Foundation University, Islamabad, Pakistan; International Center of Medical Sciences Research (ICMSR), Islamabad, Pakistan (Prof U Saeed PhD); Faculty of Medicine, Bioscience and Nursing (S Z Safi PhD), MAHSA University, Selangor, Malaysia; Interdisciplinary Research Centre in Biomedical Materials (IRCBM) (S Z Safi PhD), COMSATS Institute of Information Technology, Lahore, Pakistan; Department of Statistics (M R Sajid PhD), University of Gujrat, Gujrat, Pakistan; Department of Health Education and Promotion (Prof L Salehi PhD), A.C.S. Medical College and Hospital, Karaj, Iran; Drug Applied Research Center (H Samadi Kafil PhD), Faculty of Medicine (A Shamekh MD), Aging Research Institute (A Shamekh MD), Tabriz University of Medical Sciences, Tabriz, Iran; Independent Consultant, Thiruvananthapuram, India (S Y Saraswathy PhD); Indira Gandhi Medical College and Research Institute, Puducherry, India (A Saravanan MD); Department of Health and Society (Prof R Sarmiento-Suárez MPH), University of Applied and Environmental Sciences, Bogota, Colombia; National School of Public Health (Prof R Sarmiento-Suárez MPH), Carlos III Health Institute, Madrid, Spain; Department of Oral Pathology and Microbiology (Prof G S Sarode PhD, Prof S C Sarode PhD), Dr. D. Y. Patil University, Pune, India; Udyam-Global Association for Sustainable Development, Bhubaneswar, India (M Satpathy PhD); Department of Post-Harvest Technology and Marketing (A Sayeed MSc), Patuakhali Science and Technology University, Patuakhali, Bangladesh; Department of Neurology (Prof N Scarmeas PhD), National and Kapodistrian University of Athens, Athens, Greece; Faculty of Business and Computing (Prof C Schinckus PhD), University of the Fraser Valley, Abbotsford, BC, Canada; Department of Finance (Prof C Schinckus PhD), International School of Management, Paris, France; Department of Cardiovascular Sciences (A Schuermans BSc, J Van den Eynde BSc), Katholieke Universiteit Leuven, Leuven, Belgium; The George Institute for Global Health, Sydney, NSW, Australia (Prof A E Schutte PhD, Prof J Sundström PhD); Clinic for Conservative Dentistry and Periodontology (Prof F Schwendicke PhD), University Hospital of the Ludwig-Maximilians-University Munich, Munich, Germany; Faculty of Dentistry (S Selvaraj PhD), AIMST University, Bedong, Malaysia; Department of Biomedical Sciences (P Sengupta PhD), Gulf Medical University, Ajman, United Arab Emirates; Emergency Department (S Senthilkumaran MD), Manian Medical Centre, Erode, India; Department of Medicine and Surgery (Y Sethi MBBS), Government Doon Medical College, Dehradun, India; National Heart, Lung, and Blood Institute (A Seylani BS), National Institute of Health, Rockville, MD, USA; Department of Infectious Diseases and Microbiology (P A Shah MBBS), Rajiv Gandhi University of Health Sciences, Bangalore, India; HepatoPancreatoBiliary Surgery and Liver Transplant Department (P A Shah MBBS), Healthcare Global Limited Cancer Care Hospital, Bangalore, India; Department of Chemistry (H Shahsavari PhD), Institute for Advanced Studies in Basic Sciences (IASBS), Zanjan, Iran; Independent Consultant, Karachi, Pakistan (M A Shaikh MD); Department of Medicine (M Sharath MBBS), Bangalore Medical College and Research Institute, Bangalore, India; Department of Clinical Review and Safety (S Sharfaei MD), Baim Institute for Clinical Research, Boston, MA, USA; Faculty of Medicine (J Sharifi-Rad PhD), Facultad de Medicina, Universidad del Azuay, Cuenca, Ecuador; Department of Hemato-oncology (A Sharma MD), Fortis Hospital, Noida, India; Department of Social and Behavioral Health (Prof M Sharma PhD), University of Nevada Las Vegas, Las Vegas, NV, USA; Department of Physiotherapy (S Sharma PhD), Kathmandu University, Dhulikhel, Nepal; Division of Microbiology and Biotechnology (R P Shastry PhD), Yenepoya Research Center, Mangalore, India; Department of Ophthalmology (M Shayan MD), Harvard Medical School, Boston, MA, USA; Forensic Medicine& Toxicology ,Forensic odontology (Prof M Shetty MD), Nitte University, Mangalore, India;

Friedman School of Nutrition Science and Policy (P Shi PhD), Tufts University, Boston, MA, USA; National Institute of Infectious Diseases, Tokyo, Japan (M Shigematsu PhD); Department of Pharmacology (T Shimels MSc), Saint Paul's Hospital Millennium Medical College, Addis Ababa, Ethiopia; Finnish Institute of Occupational Health, Helsinki, Finland (R Shiri PhD); School of Pharmacy (S Shrestha PharmD), Monash University, Selangor, Malaysia; The Cooper Institute, Dallas, TX, USA (K Shuval PhD); Department of Medical Microbiology and Infectious Diseases (E E Siddig MD), Erasmus University, Rotterdam, Netherlands; Department of Pharmacology (J K Sidhu MD), Amrita School of Medicine, Faridabad, India; Center of Potential and Innovation of Natural Resources (Prof L M R Silva PhD), Polytechnic Institute of Guarda, Guarda, Portugal; Health Sciences Research Centre (Prof L M R Silva PhD), University of Beira Interior, Covilhã, Portugal; Department of Nursing in Women's Health (T R Silva PhD), Federal University of São Paulo, São Paulo, Brazil; School of Health (Prof C R Simpson PhD), Victoria University of Wellington, Wellington, New Zealand; Department of Dentistry (A Singh MD), All India Institute of Medical Sciences, Bhopal, India; School of Public Health and Zoonoses (B B Singh PhD), Guru Angad Dev Veterinary & Animal Sciences University, Ludhiana, India; Department of Biochemistry (B Singh PhD), Central University of Punbjab, Bathinda, India; Department of Pharmacology (H Singh DM), Government Medical College and Hospital, Chandigarh, India; Department of Paediatrics (J Singh MD), All India Institute of Medical Sciences, Bilaspur, India; Department of Radiodiagnosis (P Singh MD), All India Institute of Medical Sciences, Bathinda, India; Department of Human Genetics (P Singh PhD), Punjabi University, Patiala, Patiala, India; Department of Sports Science and Clinical Biomechanics (Prof S T Skou PhD), University of Southern Denmark, Odense, Denmark; Department of Physiotherapy and Occupational Therapy (Prof S T Skou PhD), Næstved-Slagelse-Ringsted Hospitals, Slagelse, Denmark; Department of Surgery (B Socea PhD), "Sf. Pantelimon" Emergency Clinical Hospital Bucharest, Bucharest, Romania; Department of Biochemistry (S Solanki MD), American University of Integrative Sciences, Barbados; Institute of Child and Adolescent Health (Y Song PhD), School of Public Health (H Zhang MS), Peking University, Beijing, China; Hull York Medical School (I N Soyiri PhD), University of Hull, Hull City, UK; Division of Community Medicine (C T Sreeramareddy MD), International Medical University, Kuala Lumpur, Malaysia; Public Health Department (M Stanikzai MPH), Kandahar University, Kandahar, Afghanistan; Nutrition and Dietetics Department (A V Starodubova DSc), Federal Research Institute of Nutrition, Biotechnology and Food Safety, Moscow, Russia; Department of Internal Disease (A V Starodubova DSc), Pirogov Russian National Research Medical University, Moscow, Russia; Department of Medicine (P Steiropoulos MD), Democritus University of Thrace, Alexandroupolis, Greece; Occupational and Environmental Medicine Department (L Stockfelt PhD), Institute of Health and Care Sciences (Prof A W Wolf PhD), University of Gothenburg, Gothenburg, Sweden; Schiller Institute (Prof K Straif PhD), Boston College, Boston, MA, USA; Barcelona Institute for Global Health, Barcelona, Spain (Prof K Straif PhD); School of Exercise and Nutrition Sciences (N Subedi MPH), Deakin University, Melbourne, VIC, Australia; National Institute of Epidemiology (R Suliankatchi Abdulkader MD), Indian Council of Medical Research, Chennai, India; Mental Health Research (A Sultana MD), Independent Consultant, Khulna, Bangladesh; Division of Global Mental Health (A Sultana MD), EviSyn Health, Khulna, Bangladesh; Rural Health Research Institute (Prof J Sun PhD), Charles Sturt University, Bathurst, NSW, Australia; Institute of Integrated Intelligence and Systems (Prof J Sun PhD), Griffith University, QLD, Australia; Department of Clinical Outcomes (Prof L Szarpak PhD), Maria Sklodowska-Curie Medical Academy, Warsaw, Poland; Department of Clinical Research and Development (Prof L Szarpak PhD), LUXMED Group, Warsaw, Poland; Department of Dermatology (M D Szeto BS), University of Colorado, Aurora, CO, USA; Department of Neurology (P Tabaee Damavandi MD), Neurocenter of Southern Switzerland (NSI), Lugano, Switzerland; Department of Medicine (Prof R Tabarés-Seisdedos PhD), University of Valencia, Valencia, Spain; Carlos III Health Institute (Prof R Tabarés-Seisdedos PhD), Biomedical Research Networking Center for Mental Health Network (CiberSAM), Madrid, Spain; Department of Basic Medical Sciences (S Tabatabaeizadeh PhD), Department of Internal Medicine (S Tabatabaeizadeh PhD), Islamic Azad University, Mashhad, Iran; School of Social Work (Prof K M Tabb PhD), University of Illinois, Urbana, IL, USA; Living Systems Institute (Y Taheri Abkenar PharmD), Department of Health and Community Sciences (A Udoh PhD), University of Exeter, Exeter, UK; Department of Biostatistics and Epidemiology (M Taheri Soodejani PhD), Shahid Sadoughi University of Medical Sciences, Yazd, Iran; Department of Environmental, Agricultural and Occupational Health (J Taiba MPH), University of Nebraska Medical Center, Omaha, NE, USA; Department of Medicine (J L Tamuzi MSc), Northlands Medical Group, Omuthiya, Namibia; State Key Laboratory of Numerical Modeling for Atmospheric Sciences and Geophysical Fluid Dynamics (LASG) (H Tang PhD), Chinese Academy of Sciences, Beijing, China; Department of Economics (N Y Tat MS), Rice University, Houston, TX, USA; Department of Research and Innovation (N Y Tat MS), Enventure Medical Innovation, Houston, TX, USA; University Institute "Egas Moniz", Monte da Caparica, Portugal (Prof N Taveira PhD); Research Institute for Medicines (Prof N Taveira PhD), Universidade de Lisboa, Lisbon, Portugal; Department of Psychology (E Teye-Kwadjo PhD), University of Ghana, Legon, Ghana;

Department of Pharmacology (P Thangaraju MD), All India Institute of Medical Sciences, Raipur, India; Public Health Department (Prof K R Thankappan MD), Amrita Institute of Medical Sciences, Kochi, India; Department of Endocrinology, Diabetes and Metabolism (Prof N Thomas PhD), Christian Medical College and Hospital (CMC), Vellore, India; National Institute of Public Health (Prof L C Thygesen PhD), University of Southern Denmark, Copenhagen, Denmark; Faculty of Public Health (J H V Ticoalu MPH), Universitas Sam Ratulangi, Manado, Indonesia; Department of Research and Development (D Timalsena MSc), Dhulikhel Hospital Kathmandu University Hospital, Dhulikhel, Nepal; Department of Community Health (D Timalsena MSc), Pratyanashil Community Development Society (PRAYAS-Nepal), Dhading Besi, Nepal; Department of Allied Health and Human Performance (T Y Tiruye PhD), University of South Australia, Adelaide, SA, Australia; Public Health Department (T Y Tiruye PhD), Department of Human Nutrition and Food Sciences (E G Wassie MSc), Debre Markos University, Debre Markos, Ethiopia; Department of Medicine (Prof M Tonelli MD), University of Calgary, Calgary, AB, Canada; Institute of Public Health (R Topor-Madry PhD), Jagiellonian University Medical College, Kraków, Poland; Agency for Health Technology Assessment and Tariff System, Warsaw, Poland (R Topor-Madry PhD); Nutritional Epidemiology Research Team (EREN) (M Touvier PhD), National Institute for Health and Medical Research (INSERM), Paris, France; SRM College of Pharmacy (M R Tovani-Palone PhD), SRM Institute of Science and Technology (SRMIST), Chennai, India; Department of Medicine (A T Tran MD), Pham Ngoc Thach University of Medicine, Ho Chi Minh city, Viet Nam; Department of Health (N M Tran MD), Children's Hospital 1, Ho Chi Minh City, Viet Nam; Department of Business Analytics (T H Tran MD), University of Massachusetts Dartmouth, Dartmouth, Massachusetts, USA; Department of Surgical, Medical, Molecular Pathology and Critical Care Medicine (D Trico MD), University of Pisa, Pisa, Italy; Adult Learning Disability Service (S J Tromans PhD), Leicestershire Partnership National Health Service Trust, Leicester, UK; School of Medicine (T T Truyen MD), Nam Can Tho University, Can Tho, Viet Nam; Department of Medicine (Prof A Tsatsakis DSc), University of Crete, Heraklion, Greece; Met Office Hadley Centre, Exeter, UK (S T Turnock PhD); International Center for Chemical and Biological Sciences (S Ullah MSc), University of Karachi, Karachi, Pakistan; Department of Biochemistry and Molecular Biology (S Ullah PhD), Department of Pathology (S Ullah PhD), The University of Texas Medical Branch at Galveston, Galveston, TX, USA; Department of Paraclinical Sciences (S Umakanthan MD), The University of the West Indies, St. Augustine, Trinidad and Tobago; Department of Oncology (S S Umar FWACS), Federal Medical Centre, Gusau, Nigeria; Department of Cardiovascular, Endocrine-metabolic Diseases and Aging (B Unim PhD), National Institute of Health, Rome, Italy; College of Health and Sport Sciences (A G Vaithinathan MSc), University of Bahrain, Salmanya, Bahrain; UKK Institute, Tampere, Finland (Prof T J Vasankari MD); Faculty of Medicine and Health Technology (Prof T J Vasankari MD), Tampere University, Tampere, Finland; Institute of Public Health of Serbia, Belgrade, Serbia (M Vasic PhD); Raffles Neuroscience Centre (Prof N Venketasubramanian MBBS), Raffles Hospital, Singapore; Department of Community Medicine (M Vijayageetha MD), All India Institute of Medical Sciences, Nagpur, India; Department of Physiotherapy (J H Villafañe PhD), Universidad Europea de Madrid, Villaviciosa de Odón, Spain; Department of Cardiology (M Vinayak MD), Icahn School of Medicine at Mount Sinai, New York, NY, USA; Department of Molecular Epidemiology (S K Vladimirov PhD), Research Institute for Systems Biology and Medicine, Moscow, Russia; Faculty of Information Technology (B Vo PhD), HUTECH University, Ho Chi Minh City, Viet Nam; Office of Research, Innovation, and Commercialization (ORIC) (Prof Y Waheed PhD), Shaheed Zulfiqar Ali Bhutto Medical University (SZABMU), Islamabad, Pakistan; Gilbert and Rose-Marie Chagoury School of Medicine (Prof Y Waheed PhD), Lebanese American University, Byblos, Lebanon; School of Public Health (F Wang PhD, D Yin DrPH), Xuzhou Medical University, Xuzhou, China; Department of Artificial Intelligence (S Wang PhD), Xiamen University Malaysia, Xiamen, China; Department of Neurosurgery (S Wang MD), Capital Medical University, Beijing, China; Department of Neurosurgery (S Wang MD), Beijing Tiantan Hospital, Beijing, China; Department of Basic Biomedical Sciences (Y Wang MD), Shandong University, Jinan, China; Department of Neuroscience (Y Wang MD), Mount Sinai Health System, New York, USA; School of Medicine and Dentistry (Public Health) (M N Wanjau MA), Griffith University, Gold Coast, QLD, Australia; School of Nursing Sciences (M N Wanjau MA), University of Nairobi, Nairobi, Kenya; Cardiology Department (Prof R G Weintraub MB), Royal Children's Hospital, Melbourne, VIC, Australia; Department of Critical Care and Neurosciences (Prof R G Weintraub MB), Murdoch Childrens Research Institute, Parkville, VIC, Australia; Key Laboratory of Shaanxi Province for Craniofacial Precision Medicine Research (Y Wen PhD), School of Public Policy and Administration (J Zhang BA), Xi'an Jiaotong University, Xi'an, China; Department of Physical Therapy (T Wiangkham PhD), Naresuan University, Phitsanulok, Thailand; Department of Public Health (E Widowati PhD), Semarang State University, Semarang, Indonesia; Department of Chemical Toxicology (M W Wojewodzic PhD), Norwegian Institute of Public Health, Oslo, Norway; NIHR-Biomedical Research Centre (Prof C D A Wolfe MD), Guy's and St. Thomas' Hospital and Kings College London, London, UK; Global Health Research Center (C Wu PhD), Duke Kunshan University,

Kunshan, China; Department of Rheumatology and Immunology (D Wu PhD), Sichuan Provincial People's Hospital, Chengdu, China; Department of Food Science and Human Nutrition (Prof F Wu PhD), Michigan State University, East Lansing, MI, USA; Affiliated Hospital of Guangdong Medical University (J Wu MD), Guangdong Medical University, Zhanjiang, China; Division of Gastroenterology (Prof Z Wu PhD), Huazhong University of Science and Technology, Wuhan, China; School of Public Health (H Xiao PhD), Zhejiang University, Zhejiang, China; Department of Public Health Science (H Xiao PhD), Fred Hutchinson Cancer Research Center, Seattle, WA, USA; Department of Endocrinology (Prof S Xu PhD), University of Science and Technology of China, Hefei, China; School of Medicine (Prof S Xu PhD), University of Rochester, Rochester, NY, USA; Department of Cardiology (Prof R Yadav MD), All India Institute of Medical Sciences, Delhi, India; Graduate School of Medicine (Prof K Yamagishi MD), Osaka University, Suita, Japan; Faculty of Medicine (Y Yano MD), Department of Public Health (Prof N Yonemoto PhD), Juntendo University, Tokyo, Japan; Manipal College of Nursing (R Yesodharan MSc), Manipal Academy of Higher Education, Udupi, India; Department of Family Medicine (S A Yesuf MSc), St. Peter's Specialized Hospital, Addis Ababa, Ethiopia; Independent Consultant, Addis Ababa, Ethiopia (S A Yesuf MSc); Biostatics, Epidemiology, and Science Computing Department (S Yezli PhD), King Faisal Specialist Hospital & Research Center, Riyadh, Saudi Arabia; Department of Health Management (A Yiğit PhD, V Yiğit PhD), Süleyman Demirel Üniversitesi, Isparta, Turkiye; Department of Biostatistics (Prof N Yonemoto PhD), University of Toyama School of Medicine, Toyama, Japan; Macquarie Medical School (Y You PhD), Macquarie University, Sydney, NSW, Australia; Department of Health Policy and Management (Prof M Z Younis PhD), Jackson State University, Jackson, MS, USA; School of Business & Economics (Prof M Z Younis PhD), Universiti Putra Malaysia (University of Putra Malaysia), Kuala Lumpur, Malaysia; Department of Epidemiology (E A Yu PhD), Vitalant Research Institute, San Francisco, CA, USA; Laboratory Medicine (E A Yu PhD), University of California San Francisco, San Francisco, California, USA; School of Public Health and Management (Y Yu MS), Hubei University of Medicine, Shiyan, China; Department of Pathology (U Zafar MD), Rutgers University, Livingston, NJ, USA; Faculty of Medicine and Health Sciences (F Zakham PhD), Hodeidah University, Hodeidah, Yemen; International Food Policy Research Institute (IFPRI), Addis Ababa, Ethiopia (T A Zerfu PhD); Medical Oncology Department of Gastrointestinal Cancer (L Zhang MS), Cancer Hospital of Dalian University of Technology, Shenyang, China; School of Biomedical Engineering, Faculty of Medicine (L Zhang MS), Dalian University of Technology, Dalian, China; School of Public Health (Y Zhang PhD), Hubei Province Key Laboratory of Occupational Hazard Identification and Control (Y Zhang PhD), Wuhan University of Science and Technology, Wuhan, China; Tianjin Medical University General Hospital (Z Zhang MD), Tianjin Centers for Disease Control and Prevention, Tianjin, China; School of Biology and Pharmaceutical Engineering (X G Zhao PhD), Wuhan Polytechnic University, Wuhan, China; Department of Basic Medicine (Y Zhao BS), Army Medical University, Chongqing, China; Department of Health Management (Z Zhao PhD), Shengjing Hospital of China Medical University, Shenyang, China; Department of Thoracic Surgery (B Zhou BMedSc), Peking Union Medical College, Beijing, China; Computational Bioscience Research Center (J Zhou PhD), King Abdullah University of Science and Technology, Jeddah, Saudi Arabia; School of Humanities and Management (Prof S Zhou PhD), Zhejiang Chinese Medical University, Hangzhou, China; School of Public Health and Emergency Management (B Zhu PhD), Southern University of Science and Technology, Shenzhen, China; Endocrinology and Metabolism Research Center (G Zoghi MD), Hormozgan University of Medical Sciences, Bandar Abbas, Iran; NIHR-Biomedical Research Centre (Prof A Zumla PhD), University College London Hospitals, London, UK; Department of Clinical and Community Pharmacy (Prof S H Zyoud PhD), Clinical Research Centre (Prof S H Zyoud PhD), An-Najah National University, Nablus, Palestine; School of Physics (S H Zyoud PhD), Universiti Sains Malaysia (University of Science Malaysia), Penang, Malaysia.

Authors' Contributions

Managing the overall research enterprise

Simon I Hay, Paulina A Lindstedt, Christopher J L Murray, Amanda E Smith, Caitlyn Steiner, and Stein Emil Vollset

Writing the first draft of the manuscript

Natalia Bhattacharjee, Catherine Bisignano, Paulina A Lindstedt, Christopher J L Murray, Amanda E Smith, Stein Emil Vollset, and Chun Wei Yuan

Primary responsibility for applying analytical methods to produce estimates

Dana Bryazka, Shujin Cao, Julian Chalek, Bronte Dalton, Cat Raggi, Amanda E Smith, Stein Emil Vollset, and Chun Wei Yuan

Primary responsibility for seeking, cataloguing, extracting, or cleaning data; designing or coding figures and tables

Austin Ahlstrom, Julie Kim, QuynhAnh Nguyen, Georgia Smith, and Stein Emil Vollset

Providing data or critical feedback on data sources

Yohannes Habtegiorgis Abate, Cristiana Abbafati, Mohammadreza Abbasian, Hedayat Abbastabar, Samar Abd ElHafeez, Parsa Abdi, Auwal Abdullahi, Mesfin Abebe, Richard Gyan Aboagye, Hassan Abolhassani, Girma Beressa Aboye, Lucas Guimarães Abreu, Giovanni Addolorato, Victor Adekanmbi, Rishan Adha, Qorinah Estiningtyas Sakilah Adnani, Leticia Akua Adzigbli, Muhammad Sohail Afzal, Saira Afzal, Antonella Agodi, Bright Opoku Ahinkorah, Muayyad M Ahmad, Sajjad Ahmad, Ayman Ahmed, Haroon Ahmed, Safoora Ahmed, Salah Al Awaidy, Fares Alahdab, Muaaz M Alajlani, Noore Alam, Fahad Mashhour Alanezi, Turki M Alanzi, Almaza Albakri, Robert W Aldridge, Abid Ali, Iman Ali, Syed Shujait Shujait Ali, Sheikh Mohammad Alif, Syed Mohamed Aljunid, François Alla, Ala'a B. Al-Tammemi, Nelson Alvis-Guzman, Mohammad Sami Al-Wardat, Hany Aly, Edward Kwabena Ameyaw, Alireza Amindarolzarbi, Hubert Amu, Deanna Anderlini, Pedro Prata Andrade, Tudorel Andrei, Amir Anoushiravani, Saeid Anvari, Michele Aquilano, Jalal Arabloo, Elshaimaa A Arafa, Demelash Areda, Hany Ariffin, Anton A Artamonov, Seyyed Shamsadin Athari, Prince Atorkey, Marcel Ausloos, Jose L. Ayuso-Mateos, Abdulaziz T Bako, Senthilkumar Balakrishnan, Ovidiu Constantin Baltatu, Martina Barchitta, Till Winfried Bärnighausen, Hiba Jawdat Barqawi, Mohammad-Mahdi Bastan, Sanjay Basu, Nebiyou Simegnew Bayileyegn, Olorunjuwon Omolaja Bello, Apostolos Beloukas, Akshaya Srikanth Bhagavathula, Dinesh Bhandari, Ravi Bharadwaj, Sonu Bhaskar, Vivek Bhat, Gurjit Kaur Bhatti, Jasvinder Singh Bhatti, Bijit Biswas, Virginia Bodolica, Aadam Olalekan Bodunrin, Hamed Borhany, Souad Bouaoud, Soufiane Boufous, Christopher Boxe, Dejana Braithwaite, Traolach Brugha, Dana Bryazka, Danilo Buonsenso, Katrin Burkart, Florentino Luciano Caetano dos Santos, Joao Mauricio Castaldelli-Maia, Luca Cegolon, Francieli Cembranel, Joshua Chadwick, Chiranjib Chakraborty, Rama Mohan Chandika, Sara Chandy, Akhilanand Chaurasia, Abdulaal Chitheer, William C S Cho, Dong-Woo Choi, Bryan Chong, Chean Lin Chong, Hitesh Chopra, Dinh-Toi Chu, Eric Chung, Alyssa Columbus, Haley Comfort, Joao Conde, Samuele Cortese, Natália Cruz-Martins, Xiaochen Dai, Giovanni Damiani, Lalit Dandona, Rakhi Dandona, Saswati Das, Fernando Pio De la Hoz, Nikolaos Dervenis, Hardik Dineshbhai Desai, Vinoth Gnana Chellaiyan Devanbu, Sameer Dhingra, Diana Dias da Silva, Michael J Diaz, Thao Huynh Phuong Do, Masoud Dodangeh, Milad Dodangeh, Rajkumar Doshi, Robert Kokou Dowou, Haneil Larson Dsouza, Bruce B Duncan, Andre Rodrigues Duraes, Senbagam Duraisamy, Anar Dushpanova, Hisham Atan Edinur, Ebrahim Eini, Temitope Cyrus Ekundayo, Rabie Adel El Arab, Osman Elamin, Mohammed Elshaer, Adeniyi Francis Fagbamigbe, Luca Falzone, Andre Faro, Kiana Fasihi, Ali Fatehizadeh, Timur Fazylov, Seyed-Mohammad Fereshtehnejad, Pietro Ferrara, Belete Sewasew Firew, Luisa S Flor, Morenike Oluwatoyin Folayan, João M Furtado, Peter Andras Gaal, Muktar A Gadanya, Silvano Gallus, Balasankar Ganesan, Tilaye Gebru Gebi, Lemma Getacher, Peter W Gething, Sama Ghoba, Ehsan Gholami, Pooyan Ghorbani Vajargah, Alem Girmay, Ekaterina Vladimirovna Glushkova, Laszlo Göbölös, Pouya Goleij, Sameer Vali Gopalani, Giuseppe Grosso, Rafael Alves Guimarães, Anish Kumar Gupta, Rajeev Gupta, Sapna Gupta, Vijai Kumar Gupta, Najah R Hadi, Nils Haep, Abdul Hafiz, Demewoz Haile, Adel Hajj Ali, Arvin Haj-Mirzaian, Rabih Halwani, Kanaan Hamagharib Abdullah, Nadia M Hamdy, Rifat Hamoudi, Zaim Anan Haq, Harapan Harapan, Arief Hargono, Josep Maria Haro, Ahmed I. Hasaballah, Mohammad Hasanian, Johannes Haubold, Simon I Hay, Jeffrey J. Hebert, Mehdi Hemmati, Claire A Henson, Claudiu Herteliu, Irma Hidayana, Nguyen Quoc Hoan, Praveen Hoogar, Nobuyuki Horita, Md Mahbub Hossain, Mehdi

Hosseinzadeh, Chengxi Hu, Michael Hultström, Aliza J Hunt, M. Azhar Hussain, Nawfal R Hussein, Hong-Han Huynh, Segun Emmanuel Ibitoye, Pulwasha Maria Iftikhar, Sheikh Mohammed Shariful Islam, Nahlah Elkudssiah Ismail, Gaetano Isola, Kathryn H Jacobsen, Morteza Jafarinia, Nader Jahanmehr, Nityanand Jain, Tahereh Javaheri, Shubha Jayaram, Heng Jiang, Jost B. Jonas, Charity Ehimwenma Joshua, Mikk Jürisson, Vaishali K, Zubair Kabir, Vidya Kadashetti, Laleh R Kalankesh, Samad Karkhah, Faizan Zaffar Kashoo, Srinivasa Vittal Katikireddi, Harkiran Kaur, Gbenga A Kayode, Fassikaw Kebede, Himanshu Khajuria, Nauman Khalid, Faham Khamesipour, Moien AB Khan, Khaled Khatab, Mahalaqua Nazli Khatib, Manoj Khokhar, Atulya Aman Khosla, Majid Khosravi, Jagdish Khubchandani, Zemene Demelash Kifle, Grace Kim, Min Seo Kim, Yun Jin Kim, Adnan Kisa, Sezer Kisa, Luke D Knibbs, Ann Kristin Skrindo Knudsen, Sonali Kochhar, Gerbrand Koren, Oleksii Korzh, Kewal Krishan, Varun Krishna, Vijay Krishnamoorthy, Mohammed Kuddus, Omar Kujan, Mukhtar Kulimbet, G Anil Kumar, Dian Kusuma, Ville Kytö, Hmwe Hmwe Kyu, Muhammad Awwal Ladan, Chandrakant Lahariya, Iván Landires, Savita Lasrado, Hilary R Lawlor, Huu-Hoai Le, Nhi Huu Hanh Le, Thao Thi Thu Le, Trang Diep Thanh Le, Janet L Leasher, Munjae Lee, Sang-woong Lee, Seung Won Lee, Shaun Wen Huey Lee, James Leigh, Massimo Libra, Virendra S Ligade, Lee-Ling Lim, Stephen S Lim, Gang Liu, Shiwei Liu, Xuefeng Liu, Erand Llanaj, Rubén López-Bueno, José Francisco López-Gil, Paulo A Lotufo, Rafael Lozano, Jailos Lubinda, Zheng Feei Ma, Kelsey Lynn Maass, Monika Machoy, Hassan Magdy Abd El Razek, Azzam A Maghazachi, Mehrdad Mahalleh, Elaheh Malakan Rad, Kashish Malhotra, Deborah Carvalho Malta, Mohammad Ali Mansournia, Joemer C Maravilla, Parham Mardi, Abdoljalal Marjani, Francisco Rogerlândio Martins-Melo, Roy Rillera Marzo, Alexander G Mathioudakis, Medha Mathur, Navgeet Mathur, Neeta Mathur, Jishanth Mattumpuram, Anna Laura W McKowen, Michael A McPhail, Asim Mehmood, Tesfahun Mekene Meto, Walter Mendoza, Ritesh G Menezes, Atte Meretoja, Sachith Mettananda, Tomasz Miazgowski, Irmina Maria Michalek, Le Huu Nhat Minh, Antonio Mirijello, Erkin M Mirrakhimov, Chaitanya Mittal, Madeline E Moberg, Soheil Mohammadi, Mustapha Mohammed, Shafiu Mohammed, Ali H Mokdad, Mariam Molokhia, Sara Momtazmanesh, Lorenzo Monasta, Mohammad Ali Moni, Maryam Moradi, Yousef Moradi, Jonathan F Mosser, Rohith Motappa, Vincent Mougin, Sumaira Mubarik, Ulrich Otto Mueller, Francesk Mulita, Efrén Murillo-Zamora, Christopher J L Murray, Ahamarshan Jayaraman Nagarajan, Ganesh R Naik, Sreenivas Narasimha Swamy, Shumaila Nargus, Bruno Ramos Nascimento, Zuhair S Natto, Biswa Prakash Nayak, Ionut Negoi, Ruxandra Irina Negoi, Henok Biresaw Netsere, Georges Nguefack-Tsague, Josephine W Ngunjiri, Anh Hoang Nguyen, Dang H Nguyen, Duc Hoang Nguyen, Hau Thi Hien Nguyen, Nhan Nguyen, Nhien Ngoc Y Nguyen, Phat Tuan Nguyen, QuynhAnh P Nguyen, Van Thanh Nguyen, Robina Khan Niazi, Shuhei Nomura, Mehran Nouri, Jerry John Nutor, Chimezie Igwegbe Nzoputam, Ogochukwu Janet Nzoputam, Bogdan Oancea, Kehinde O Obamiro, Ayodipupo Sikiru Oguntade, Akinkunmi Paul Okekunle, Osaretin Christabel Okonji, Andrew T Olagunju, Gláucia Maria Moraes Oliveira, Bolajoko Olubukunola Olusanya, Jacob Olusegun Olusanya, Yinka Doris Oluwafemi, Hany A Omar, Sokking Ong, Sandersan Onie, Obinna E Onwujekwe, Abdulahi Opejin Opejin, Alberto Ortiz, Sergej M Ostojic, Uchechukwu Levi Osuagwu, Adrian Otoiu, Amel Ouyahia, Mahesh Padukudru P A, Songhomitra Panda-Jonas, Anamika Pandey, Helena Ullyartha Pangaribuan, Leonidas D Panos, Romil R Parikh, Sungchul Park, Ashwaghosha Parthasarathi, Maja Pasovic, Jay Patel, Aslam Ramjan Pathan, Shankargouda Patil, Shrikant Pawar, Spencer A Pease, Paolo Pedersini, Prince Peprah, Maria Odete Pereira, Arokiasamy Perianayagam, Simone Perna, Konrad Pesudovs, Hoang Tran Pham, Anil K Philip, Ramesh Poluru, Maarten J Postma, Sergio I Prada, Jalandhar Pradhan, Elton Junio Sady Prates, Tina Priscilla, Jagadeesh Puvvula, Asma Saleem Qazi, Alberto Raggi, Nasiru Raheem, Fakher Rahim, Md Jillur Rahim, Sarvenaz Rahimibarghani, Tafhimur Rahman, Amir Masoud Rahmani, Mohammad Rahmanian, Adarsh Raja, Shakthi Kumaran Ramasamy, Sheena Ramazanu, Chhabi Lal Ranabhat, Chythra R Rao, Mithun Rao, Vahid Rashedi, Ahmed Mustafa Rashid, Prateek Rastogi, Santosh Kumar Rauniyar, Ramin Ravangard, Salman Rawaf, Lennart Reifels, Kannan RR Rengasamy, Bhageerathy Reshmi, Luis Felipe Reyes, Negar Rezaei, Taeho Gregory Rhee, Hannah Elizabeth Robinson-Oden, Mónica Rodrigues, Jefferson Antonio Buendia Rodriguez, Leonardo Roever, Luca Ronfani, Moustaq karim khan Rony, Gholamreza Roshandel, Kunle Rotimi, Himanshu Sekhar Rout, Enrico Rubagotti, Susan Fred Rumisha, Tilleye Runghien, Aly M A Saad, Korosh Saber, Cameron John Sabet, Siamak Sabour, Perminder S Sachdev, Adam Saddler, Bashdar Abuzed Sadee, Umar Saeed, Rajesh Sagar, Mirza Rizwan Sajid, Afeez Abolarinwa Salami, Marwa Rashad Salem, Hossein Samadi Kafil, Abdallah M Samy, Juan Sanabria, Francesca Sanna, Milena M Santric-Milicevic, Maheswar Satpathy, David C Schwebel, Subramanian Senthilkumaran, Dragos Serban, Yashendra Sethi, Pritik A Shah, Masood Ali Shaikh, Ali Shamekh, Muhammad Aaqib Shamim, Abhishek Shankar, Mohammed Shannawaz, Medha Sharath, Amin Sharifan, Javad Sharifi-Rad, Vishal Sharma, Rajesh P Shastry, Peilin Shi, Aminu Shittu, K M Shivakumar, Sina Shool, Sunil Shrestha, Luís Manuel Lopes Rodrigues Silva, Abhinav Singh, Baljinder Singh, Harmanjit Singh, Paramdeep Singh, Sameh S M Soliman, Ireneous N Soyiri, Michael Spartalis, Chandrashekhar T Sreeramareddy, Muhammad Haroon Stanikzai, Mark A Stokes, Rizwan Suliankatchi Abdulkader, Abida Sultana, Johan Sundström, Chandan Kumar Swain, Lukasz Szarpak, Mindy D

Szeto, Rafael Tabarés-Seisdedos, Shima Tabatabai, Karen M Tabb, Mohammad Tabish, Yasaman Taheri Abkenar, Moslem Taheri Soodejani, Ker-Kan Tan, Nuno Taveira, Pugazhenthan Thangaraju, Nihal Thomas, Lau Caspar Thygesen, Marcos Roberto Tovani-Palone, Jasmine T Tran, Domenico Trico, Steven T Turnock, Sana Ullah, Srikanth Umakanthan, Bhaskaran Unnikrishnan, Era Upadhyay, Jibrin Sammani Usman, Jef Van den Eynde, Shoban Babu Varthya, Tommi Juhani Vasankari, Narayanaswamy Venketasubramanian, Georgios-Ioannis Verras, Sergey Konstantinovitch Vladimirov, Vasily Vlassov, Bay Vo, Stein Emil Vollset, Theo Vos, Abdul Wadood Wadood, Yasir Waheed, Shu Wang, Abdul Waris, Stefanie Watson, Ronny Westerman, Taweewat Wiangkham, Dakshitha Praneeth Wickramasinghe, Charles D A Wolfe, Felicia Wu, Sarah Wulf Hanson, Suowen Xu, Kazumasa Yamagishi, Iman Yazdani Nia, Pengpeng Ye, Zeamanuel Anteneh Yigzaw, Naohiro Yonemoto, Mustafa Z Younis, Chuanhua Yu, Yong Yu, Taddese Alemu Zerfu, Yang Zhao, Juexiao Zhou, Shangcheng Zhou, Abzal Zhumagaliuly, Magdalena Zielińska, Alimuddin Zumla.

Developing methods or computational machinery

Cristiana Abbafati, Qorinah Estiningtyas Sakilah Adnani, Saira Afzal, Bright Opoku Ahinkorah, Austin J Ahlstrom, Safoora Ahmed, Mohammad Sami Al-Wardat, Walid Al-Zyoud, Hubert Amu, Erick Adrian Andrews, Jalal Arabloo, Aleksandr Y Aravkin, Charlie Ashbaugh, Mohammad-Mahdi Bastan, Akshaya Srikanth Bhagavathula, Natalia V Bhattacharjee, Aadam Olalekan Bodunrin, Hamed Borhany, Michael Brauer, Dana Bryazka, Shujin Cao, Julian Chalek, William C S Cho, Justin T Clayton, Rebecca M Cogen, Ezra E K Cooper, Xiaochen Dai, Bronte E Dalton, Giovanni Damiani, Hardik Dineshbhai Desai, Rabie Adel El Arab, Iman El Sayed, Adeniyi Francis Fagbamigbe, Carla Sofia e Sá Farinha, Ali Fatehizadeh, Luisa S Flor, Kai Glenn Fukutaki, Peter W Gething, Ehsan Gholami, Pooyan Ghorbani Vajargah, Alem Girmay, Demewoz Haile, Adel Hajj Ali, Arvin Haj-Mirzaian, Kanaan Hamagharib Abdullah, Simon I Hay, Claire A Henson, Mehdi Hosseinzadeh, Hong-Han Huynh, Gaetano Isola, Morteza Jafarinia, Safayet Jamil, Tahereh Javaheri, Charity Ehimwenma Joshua, Laleh R Kalankesh, Samad Karkhah, Faizan Zaffar Kashoo, Fassikaw Kebede, Mahalaqua Nazli Khatib, Atulya Aman Khosla, Majid Khosravi, Adnan Kisa, Vijay Kumar, Chandrakant Lahariya, Huu-Hoai Le, Nhi Huu Hanh Le, Thao Thi Thu Le, Munjae Lee, Sang-woong Lee, James Leigh, Paulina A Lindstedt, Erand Llanaj, Kelsey Lynn Maass, Hassan Magdy Abd El Razek, D R Mahadeshwara Prasad, Abdoljalal Marjani, Navgeet Mathur, Andrea Maugeri, Michael A McPhail, Le Huu Nhat Minh, Madeline E Moberg, Shafiu Mohammed, Ali H Mokdad, Mohammad Ali Moni, Yousef Moradi, Jonathan F Mosser, Vincent Mougin, Francesk Mulita, Christopher J L Murray, Bruno Ramos Nascimento, Josephine W Ngunjiri, Anh Hoang Nguyen, Nhan Nguyen, Phat Tuan Nguyen, Van Thanh Nguyen, Robina Khan Niazi, Mehran Nouri, Matthew Idowu Olatubi, Abdulahi Opejin Opejin, Michal Ordak, Amel Ouyahia, Hoang Tran Pham, Hadi Raeisi Shahraki, Cat Raggi, Amir Masoud Rahmani, Chhabi Lal Ranabhat, Giridhara Rathnaiah Babu, Marissa B Reitsma, Toshana Robalik, Mónica Rodrigues, Himanshu Sekhar Rout, Enrico Rubagotti, Susan Fred Rumisha, Tilleye Runghien, Korosh Saber, Adam Saddler, Umar Saeed, Abdallah M Samy, Francesca Sanna, Maheswar Satpathy, Austin E Schumacher, Mohammad H Semreen, Yashendra Sethi, Mohammed Shannawaz, Javad Sharifi-Rad, Vishal Sharma, Kyle E Simpson, Amanda E Smith, Georgia Smith, Yi Song, Michael Spartalis, Sandra Spearman, Muhammad Haroon Stanikzai, Chandan Kumar Swain, Yasaman Taheri Abkenar, Razieh Tavakoli Oliaee, Roman Topor-Madry, Bay Vo, Stein Emil Vollset, Theo Vos, Abdul Wadood Wadood, Ronny Westerman, Zenghong Wu, Sarah Wulf Hanson, Iman Yazdani Nia, Chun-Wei Yuan, Yang Zhao.

Providing critical feedback on methods or results

Hazim S Ababneh, Yohannes Habtegiorgis Abate, Cristiana Abbafati, Rouzbeh Abbasgholizadeh, Mohammadreza Abbasian, Hedayat Abbastabar, Abdallah H A Abd Al Magied, Samar Abd ElHafeez, Atef Abdelkader, Michael Abdelmasseh, Sherief Abd-Elsalam, Parsa Abdi, Mohammad Abdollahi, Meriem Abdoun, Auwal Abdullahi, Mesfin Abebe, Richard Gyan Aboagye, Hassan Abolhassani, Mohamed Abouzid, Girma Beressa Aboye, Lucas Guimarães Abreu, Abdorrahim Absalan, Hasan Abualruz, Bilyaminu Abubakar, Hana Jihad Jihad Abukhadijah, Victor Adekanmbi, Charles Oluwaseun Adetunji, Juliana Bunmi Adetunji, Temitayo Esther Adeyeoluwa, Rishan Adha, Ripon Kumar Adhikary, Qorinah Estiningtyas Sakilah Adnani, Leticia Akua Adzigbli, Fatemeh Afrashteh, Muhammad Sohail Afzal, Saira Afzal, Faith Agbozo, Antonella Agodi, Anurag Agrawal, Williams Agyemang-Duah, Bright Opoku Ahinkorah, Aqeel Ahmad, Firdos Ahmad, Muayyad M Ahmad, Sajjad Ahmad, Shahzaib Ahmad, Anisuddin Ahmed, Ayman Ahmed, Haroon Ahmed, Syed Anees Ahmed, Karolina Akinosoglou, Mohammed Ahmed Akkaif, Ashley E Akrami, Ema Akter, Salah Al Awaidy, Syed Mahfuz Al Hasan, Amjad S Al Mosa, Omar Al Ta'ani, Omar Ali Mohammed Al Zaabi, Fares Alahdab, Muaaz M Alajlani, Yazan Al-Ajlouni, Samer O. Alalalmeh, Ziyad Al-Aly, Khurshid Alam, Noore Alam, Zufishan Alam, Rasmieh Mustafa Al-amer, Fahad Mashhour Alanezi, Turki M Alanzi, Almaza Albakri, Seyedeh Yasaman Alemohammad, Yihun Mulugeta

Alemu, Adel Ali Saeed Al-Gheethi, Mohammed Khaled Al-Hanawi, Abid Ali, Iman Ali, Mohammed Usman Ali, Rafat Ali, Syed Shujait Shujait Ali, Victor Ekoche Ali, Akram Al-Ibraheem, Gianfranco Alicandro, Sheikh Mohammad Alif, Syed Mohamed Aljunid, Joseph Uy Almazan, Hesham M Al-Mekhlafi, Ahmed Yaseen Alqutaibi, Sahel Majed Alrousan, Salman Khalifah Al-Sabah, Mohammed A Alsabri, Zaid Altaany, Ala'a B Al-Tammemi, Jaffar A Al-Tawfiq, Khalid A Altirkawi, Nelson Alvis-Guzman, Mohammad Sami Al-Wardat, Yaser Mohammed Al-Worafi, Hany Aly, Mohammad Sharif Alyahya, Karem H Alzoubi, Walid Al-Zyoud, Reza Amani, Edward Kwabena Ameyaw, Tarek Tawfik Amin, Alireza Amindarolzarbi, Sohrab Amiri, Hubert Amu, Dickson A Amugsi, Robert Ancuceanu, Deanna Anderlini, David B Anderson, Pedro Prata Andrade, Catalina Liliana Andrei, Tudorel Andrei, Amir Anoushiravani, Ernoiz Antriyandarti, Boluwatife Stephen Anuoluwa, Saeid Anvari, Anayochukwu Edward Anyasodor, Francis Appiah, Michele Aquilano, Juan Pablo Arab, Jalal Arabloo, Elshaimaa A Arafa, Mosab Arafat, Brhane Berhe Aregawi, Hany Ariffin, Mesay Arkew, Keivan Armani, Anton A Artamonov, Ashokan Arumugam, Mohammad Asghari-Jafarabadi, Charlie Ashbaugh, Thomas Astell-Burt, Seyyed Shamsadin Athari, Prince Atorkey, Maha Moh'd Wahbi Atout, Avinash Aujayeb, Marcel Ausloos, Hamzeh Awad, Sina Azadnajafabad, Fahad Khan Azeez, Rui M S Azevedo, Muhammad Badar, Soroush Baghdadi, Mahboube Bagheri, Nasser Bagheri, Ruhai Bai, Jennifer L Baker, Abdulaziz T Bako, Senthilkumar Balakrishnan, Wondu Feyisa Balcha, Ovidiu Constantin Baltatu, Martina Barchitta, Suzanne Lyn Barker-Collo, Till Winfried Bärnighausen, Hiba Jawdat Barqawi, Sandra Barteit, João Diogo Basso, Mohammad-Mahdi Bastan, Sanjay Basu, Matteo Bauckneht, Bernhard T Baune, Mohsen Bayati, Nebiyou Simegnew Bayileyegn, Amir Hossein Behnoush, Payam Behzadi, Olorunjuwon Omolaja Bello, Luis Belo, Apostolos Beloukas, Maryam Bemanalizadeh, Isabela M Bensenor, Habib Benzian, Azizullah Beran, Zombor Berezvai, Robert S Bernstein, Devidas S Bhagat, Akshaya Srikanth Bhagavathula, Neeraj Bhala, Dinesh Bhandari, Ravi Bharadwaj, Nikha Bhardwaj, Pankaj Bhardwaj, Ashish Bhargava, Sonu Bhaskar, Vivek Bhat, Natalia V Bhattacharjee, Gurjit Kaur Bhatti, Jasvinder Singh Bhatti, Manpreet S Bhatti, Mohiuddin Ahmed Bhuiyan, Bijit Biswas, Virginia Bodolica, Aadam Olalekan Bodunrin, Milad Bonakdar Hashemi, Hamed Borhany, Samuel Adolf Bosoka, Alejandro Botero Carvajal, Souad Bouaoud, Christopher Boxe, Oliver J Brady, Dejana Braithwaite, Michael Brauer, Javier Brazo-Sayavera, Hermann Brenner, Colin Stewart Brown, Annie J Browne, Traolach Brugha, Dana Bryazka, Norma B Bulamu, Danilo Buonsenso, Katrin Burkart, Richard A Burns, Reinhard Busse, Yasser Bustanji, Zahid A Butt, Florentino Luciano Caetano dos Santos, Mehtap Çakmak Barsbay, Luciana Aparecida Campos, Shujin Cao, Angelo Capodici, Rosario Cárdenas, Márcia Carvalho, Joao Mauricio Castaldelli-Maia, Luca Cegolon, Francieli Cembranel, Edina Cenko, Ester Cerin, Steven J Chadban, Joshua Chadwick, Chiranjib Chakraborty, Rama Mohan Chandika, Sara Chandy, Jaykaran Charan, Akhilanand Chaurasia, An-Tian Chen, Haowei Chen, Meng Xuan Chen, Nicolas Cherbuin, Fatemeh Chichagi, Odgerel Chimed-Ochir, Ritesh Chimoriya, Jesus Lorenzo Chirinos-Caceres, Daniel Youngwhan Cho, William C S Cho, Dong-Woo Choi, Bryan Chong, Chean Lin Chong, Hitesh Chopra, Dinh-Toi Chu, Eric Chung, Muhammad Chutiyami, Aaron J Cohen, Alyssa Columbus, Joao Conde, Samuele Cortese, Natália Cruz-Martins, Alanna Gomes da Silva, Omid Dadras, Xiaochen Dai, Zhaoli Dai, Giovanni Damiani, Lalit Dandona, Rakhi Dandona, Jai K Das, Saswati Das, Subasish Das, Nihar Ranjan Dash, Kairat Davletov, Fernando Pio De la Hoz, Diego De Leo, Shayom Debopadhaya, Ivan Delgado-Enciso, Nikolaos Dervenis, Hardik Dineshbhai Desai, Vinoth Gnana Chellaiyan Devanbu, Kuldeep Dhama, Amol S Dhane, Sameer Dhingra, Diana Dias da Silva, Daniel Diaz, Michael J Diaz, Adriana Dima, Delaney D Ding, Thao Huynh Phuong Do, Camila Bruneli do Prado, Masoud Dodangeh, Milad Dodangeh, Mario D'Oria, Rajkumar Doshi, Robert Kokou Dowou, Haneil Larson Dsouza, John Dube, Samuel C Dumith, Senbagam Duraisamy, Oyewole Christopher Durojaiye, Anar Dushpanova, Sulagna Dutta, Arkadiusz Marian Dziedzic, Ejemai Eboreime, Alireza Ebrahimi, Mohammad Ebrahimi Kalan, Hisham Atan Edinur, Ferry Efendi, Terje Andreas Eikemo, Ebrahim Eini, Temitope Cyrus Ekundayo, Rabie Adel El Arab, Iman El Sayed, Osman Elamin, Noha Mousaad Elemam, Ghada Metwally Tawfik ElGohary, Muhammed Elhadi, Omar Abdelsadek Abdou Elmeligy, Adel B Elmoselhi, Mohammed Elshaer, Ibrahim Elsohaby, Mohd. Elmagzoub Eltahir, Theophilus I Emeto, Babak Eshrati, Majid Eslami, Zahra Esmaeili, Natalia Fabin, Adeniyi Francis Fagbamigbe, Omotayo Francis Fagbule, Luca Falzone, Mohammad Fareed, Carla Sofia e Sá Farinha, Andre Faro, Kiana Fasihi, Ali Fatehizadeh, Valery L Feigin, Ginenus Fekadu, Xiaoqi Feng, Seyed-Mohammad Fereshtehnejad, Pietro Ferrara, Belete Sewasew Firew, Florian Fischer, Ida Fitriana, Joanne Flavel, Luisa S Flor, Morenike Oluwatoyin Folayan, Kristen Marie Foley, Lisa M Force, Matteo Foschi, Alberto Freitas, Ni Kadek Yuni Fridayani, Blima Fux, Peter Andras Gaal, Muktar A. Gadanya, Balasankar Ganesan, Mohammad Arfat Ganiyani, Rupesh K Gautam, Tilaye Gebru Gebi, Miglas W Gebregergis, Mesfin Gebrehiwot, Lemma Getacher, Genanew K A Getahun, Delaram J Ghadimi, Khalid Yaser Ghailan, Ghazal Ghasempour Dabaghi, Ramy Mohamed Ghazy, Ehsan Gholami, Ali Gholamrezanezhad, Pooyan Ghorbani Vajargah, Elena Ghotbi, Artyom Urievich Gil, Tiffany K Gill, Alem Girmay, James C Glasbey, Ekaterina Vladimirovna Glushkova, Elena V Gnedovskaya, Laszlo Göbölös, Mohamad Goldust, Davide Golinelli, Alessandra C Goulart, Anmol Goyal, Michal Grivna, Giuseppe Grosso, Mohammed Ibrahim Mohialdeen Gubari, Stefano

Guicciardi, Rafael Alves Guimarães, Snigdha Gulati, David Gulisashvili, Damitha Asanga Gunawardane, Cui Guo, Anish Kumar Gupta, Rahul Gupta, Rajeev Gupta, Sapna Gupta, Vijai Kumar Gupta, Annie Haakenstad, Najah R Hadi, Nils Haep, Abdul Hafiz, Dariush Haghmorad, Demewoz Haile, Adel Hajj Ali, Ali Hajj Ali, Arvin Haj-Mirzaian, Sebastian Haller, Rabih Halwani, Kanaan Hamagharib Abdullah, Nadia M Hamdy, Rifat Hamoudi, Nasrin Hanifi, Zaim Anan Haq, Md Rabiul Haque, Harapan Harapan, Ahmed I Hasaballah, S M Mahmudul Hasan, Md Saquib Hasnain, Johannes Haubold, Simon I Hay, Omar E Hegazi, Mohammad Heidari, Mehdi Hemmati, Claire A Henson, Brenda Yuliana Herrera-Serna, Claudiu Herteliu, Majid Heydari, Kamal Hezam, Irma Hidayana, Yuta Hiraike, Nguyen Quoc Hoan, Ramesh Holla, Praveen Hoogar, Nobuyuki Horita, Md Mahbub Hossain, Hassan Hosseinzadeh, Mehdi Hosseinzadeh, Mihaela Hostiuc, Sorin Hostiuc, Chengxi Hu, Michael Hultström, Tsegaye Gebreyes Hundie, Aliza J Hunt, Kiavash Hushmandi, Javid Hussain, M Azhar Hussain, Nawfal R Hussein, Hong-Han Huynh, Bing-Fang Hwang, Segun Emmanuel Ibitoye, Irena M Ilic, Milena D Ilic, Mustapha Immurana, Mustafa Alhaji Isa, Md Rabiul Islam, Sheikh Mohammed Shariful Islam, Faisal Ismail, Nahlah Elkudssiah Ismail, Gaetano Isola, Masao Iwagami, Ihoghosa Osamuyi Iyamu, Louis Jacob, Kathryn H Jacobsen, Morteza Jafarinia, Nader Jahanmehr, Nityanand Jain, Ammar Abdulrahman Jairoun, Safayet Jamil, Roland Dominic G Jamora, Abubakar Ibrahim Jatau, Sabzali Javadov, Tahereh Javaheri, Shubha Jayaram, Sun Ha Jee, Jayakumar Jeganathan, Heng Jiang, Mohammad Jokar, Jost B Jonas, Nitin Joseph, Charity Ehimwenma Joshua, Mikk Jürisson, Vaishali K, Ali Kabir, Zubair Kabir, Vidya Kadashetti, Laleh R Kalankesh, Ashwin Kamath, Rajesh Kamath, Mona Kanaan, Tanuj Kanchan, Edmund Wedam Kanmiki, Kehinde Kazeem Kanmodi, Sushil Kumar Kansal, Samad Karkhah, Faizan Zaffar Kashoo, Srinivasa Vittal Katikireddi, Joonas H Kauppila, Harkiran Kaur, Gbenga A Kayode, Foad Kazemi, Sina Kazemian, Fassikaw Kebede, Evie Shoshannah Kendal, Emmanuelle Kesse-Guyot, Himanshu Khajuria, Amirmohammad Khalaji, Asaad Khalid, Nauman Khalid, Alireza Khalilian, Faham Khamesipour, Fayaz Khan, Mohammad Jobair Khan, Moien AB Khan, Shaghayegh Khanmohammadi, Khaled Khatab, Haitham Khatatbeh, Moawiah Mohammad Khatatbeh, Mahalaqua Nazli Khatib, Manoj Khokhar, Moein Khormali, Zahra Khorrami, Atulya Aman Khosla, Majid Khosravi, Jagdish Khubchandani, Zemene Demelash Kifle, Grace Kim, Min Seo Kim, Yun Jin Kim, Ruth W Kimokoti, Adnan Kisa, Sezer Kisa, Luke D Knibbs, Ann Kristin Skrindo Knudsen, Sonali Kochhar, Ali-Asghar Kolahi, Farzad Kompani, Gerbrand Koren, Oleksii Korzh, Kewal Krishan, Varun Krishna, Vijay Krishnamoorthy, Md Abdul Kuddus, Mohammed Kuddus, Ilari Kuitunen, Omar Kujan, Mukhtar Kulimbet, Vishnutheertha Kulkarni, G Anil Kumar, Harish Kumar, Nithin Kumar, Vijay Kumar, Amartya Kundu, Dian Kusuma, Frank Kyei-Arthur, Ville Kytö, Hmwe Hmwe Kyu, Carlo La Vecchia, Muhammad Awwal Ladan, Chandrakant Lahariya, Daphne Teck Ching Lai, Tea Lallukka, Judit Lám, Qing Lan, Tuo Lan, Iván Landires, Francesco Lanfranchi, Van Charles Lansingh, Bagher Larijani, Savita Lasrado, Paolo Lauriola, Huu-Hoai Le, Long Khanh Dao Le, Nhi Huu Hanh Le, Thao Thi Thu Le, Trang Diep Thanh Le, Janet L Leasher, Doo Woong Lee, Munjae Lee, Sang-woong Lee, Seung Won Lee, Shaun Wen Huey Lee, Yo Han Lee, James Leigh, Elvynna Leong, Ming-Chieh Li, Massimo Libra, Virendra S Ligade, Lee-Ling Lim, Stephen S Lim, Liknaw Workie Limenh, Daniel Lindholm, Paulina A Lindstedt, Stefan Listl, Gang Liu, Shuke Liu, Xiaofeng Liu, Xuefeng Liu, Erand Llanaj, Rubén López-Bueno, José Francisco López-Gil, Arianna Maever Loreche, Paulo A Lotufo, Jailos Lubinda, Giancarlo Lucchetti, Lisha Luo, Jay B Lusk, Lei Lv, Zheng Feei Ma, Nikolaos Machairas, Monika Machoy, Áurea M Madureira-Carvalho, Hassan Magdy Abd El Razek, Azzam A Maghazachi, D R Mahadeshwara Prasad, Mehrdad Mahalleh, Phetole Walter Mahasha, Mansour Adam Mahmoud, Elham Mahmoudi, Golnaz Mahmoudvand, Maureen Makama, Elaheh Malakan Rad, Kashish Malhotra, Ahmad Azam Malik, Deborah Carvalho Malta, Yosef Manla, Ali Mansour, Mohammad Hadi Mansouri, Pejman Mansouri, Vahid Mansouri, Marjan Mansourian, Mohammad Ali Mansournia, Bishnu P Marasini, Hamid Reza Marateb, Joemer C Maravilla, Parham Mardi, Abdoljalal Marjani, Hamed Markazi Moghadam, Carlos Alberto Marrugo Arnedo, Ramon Martinez-Piedra, Francisco Rogerlândio Martins-Melo, Miquel Martorell, Roy Rillera Marzo, Sahar Masoudi, Yasith Mathangasinghe, Alexander G Mathioudakis, Medha Mathur, Navgeet Mathur, Neeta Mathur, Fernanda Penido Matozinhos, Jishanth Mattumpuram, Richard James Maude, Andrea Maugeri, Mahsa Mayeli, Mohsen Mazidi, Martin McKee, Anna Laura W McKowen, Michael A McPhail, Steven M McPhail, Asim Mehmood, Kamran Mehrabani-Zeinabad, Sepideh Mehravar, Tesfahun Mekene Meto, Endalkachew Belayneh Melese, Max Alberto Mendez Mendez-Lopez, Walter Mendoza, Ritesh G Menezes, George A Mensah, Alexios-Fotios A Mentis, Sultan Ayoub Meo, Atte Meretoja, Tuomo J Meretoja, Abera M Mersha, Tomislav Mestrovic, Kukulege Chamila Dinushi Mettananda, Sachith Mettananda, Adquate Mhlanga, Laurette Mhlanga, Tomasz Miazgowski, Irmina Maria Michalek, Ana Carolina Micheletti Gomide Nogueira de Sá, Ted R Miller, Le Huu Nhat Minh, Alireza Mirahmadi, Erkin M Mirrakhimov, Roya Mirzaei, Philip B Mitchell, Chaitanya Mittal, Madeline E Moberg, Atousa Moghadam Fard, Seyedehfatemeh Mohajelin, Ashraf Mohamadkhani, Ahmed Ismail Mohamed, Jama Mohamed, Mouhand F H Mohamed, Nouh Saad Mohamed, Ameen Mosa Mohammad, Soheil Mohammadi, Mustapha Mohammed, Shafiu Mohammed, Ali H Mokdad, Mariam Molokhia, Shaher Mohammad Momani, Sara Momtazmanesh, Stefania

Mondello, Mohammad Ali Moni, Fateme Montazeri, AmirAli Moodi Ghalibaf, Maryam Moradi, Yousef Moradi, Paula Moraga, Lidia Morawska, Rafael Silveira Moreira, Negar Morovatdar, Jonathan F Mosser, Elias Mossialos, Rohith Motappa, Sumaira Mubarik, Ulrich Otto Mueller, Francesk Mulita, Kavita Munjal, Efrén Murillo-Zamora, Christopher J L Murray, Khaled M Musallam, Ana-Maria Musina, Ghulam Mustafa, Woojae Myung, Ayoub Nafei, Ahamarshan Jayaraman Nagarajan, Pirouz Naghavi, Ganesh R Naik, Gurudatta Naik, Firzan Nainu, Vinay Nangia, Sreenivas Narasimha Swamy, Shumaila Nargus, Bruno Ramos Nascimento, Abdallah Y Naser, Abdulqadir J Nashwan, Zuhair S Natto, Javaid Nauman, Samidi N K Navaratna, Muhammad Naveed, Biswa Prakash Nayak, Vinod C Nayak, Hadush Negash, Ionut Negoi, Ruxandra Irina Negoi, Seyed Aria Nejadghaderi, Chakib Nejjari, Soroush Nematollahi, Henok Biresaw Netsere, Marie Ng, Georges Nguefack-Tsague, Josephine W Ngunjiri, Anh Hoang Nguyen, Dang H Nguyen, Duc Hoang Nguyen, Nhan Nguyen, Nhien Ngoc Y Nguyen, Phat Tuan Nguyen, Van Thanh Nguyen, Duc Nguyen Tran Minh, Robina Khan Niazi, Yeshambel T Nigatu, Ali Nikoobar, Amin Reza Nikpoor, Nasrin Nikravangolsefid, Efaq Ali Noman, Shuhei Nomura, Syed Toukir Ahmed Noor, Nafise Noroozi, Mehran Nouri, Majid Nozari, Chisom Adaobi Nri-Ezedi, George Ntaios, Mengistu H Nunemo, Dieta Nurrika, Jerry John Nutor, Chimezie Igwegbe Nzoputam, Ogochukwu Janet Nzoputam, Bogdan Oancea, Kehinde O Obamiro, Ismail A. Odetokun, Michael Safo Oduro, Oluwaseun Adeolu Ogundijo, Ayodipupo Sikiru Oguntade, In-Hwan Oh, Tolulope R Ojo-Akosile, Hassan Okati-Aliabad, Akinkunmi Paul Okekunle, Osaretin Christabel Okonji, Andrew T Olagunju, Matthew Idowu Olatubi, Gláucia Maria Moraes Oliveira, Bolajoko Olubukunola Olusanya, Jacob Olusegun Olusanya, Yinka Doris Oluwafemi, Hany A Omar, Goran Latif Omer, Sokking Ong, Obinna E Onwujekwe, Abdulahi Opejin Opejin, Michal Ordak, Alberto Ortiz, Esteban Ortiz-Prado, Wael M S Osman, Sergej M Ostojic, Samuel M Ostroff, Uchechukwu Levi Osuagwu, Adrian Otoiu, Stanislav S Otstavnov, Amel Ouyahia, Mayowa O Owolabi, Oyetunde T Oyeyemi, Ahmad Ozair, Mahesh Padukudru P A, Pramod Kumar Pal, Feng Pan, Hai-Feng Pan, Songhomitra Panda-Jonas, Anamika Pandey, Helena Ullyartha Pangaribuan, Georgios D Panos, Leonidas D Panos, Ioannis Pantazopoulos, Anca Mihaela Pantea Stoian, Romil R Parikh, Eun-Kee Park, Seoyeon Park, Sungchul Park, Nicholas Parsons, Ashwaghosha Parthasarathi, Maja Pasovic, Roberto Passera, Jay Patel, Aslam Ramjan Pathan, Shankargouda Patil, Dimitrios Patoulias, Shrikant Pawar, Amy E Peden, Paolo Pedersini, Veincent Christian Filipino Pepito, Prince Peprah, Marcos Pereira, Maria Odete Pereira, Arokiasamy Perianayagam, Konrad Pesudovs, Fanny Emily Petermann-Rocha, Hoang Tran Pham, Anil K Philip, Michael R Phillips, Manon Pigeolet, Michael A Piradov, Evgenii Plotnikov, Roman V Polibin, Ramesh Poluru, Ville T Ponkilainen, Maarten J Postma, Ahmad Pour-Rashidi, Disha Prabhu, Sergio I Prada, Jalandhar Pradhan, Pranil Man Singh Pradhan, Akila Prashant, Elton Junio Sady Prates, Tina Priscilla, Hery Purnobasuki, Bharathi M Purohit, Jagadeesh Puvvula, Nameer Hashim Qasim, Ibrahim Qattea, Asma Saleem Qazi, Gangzhen Qian, Mehrdad Rabiee Rad, Venkatraman Radhakrishnan, Hadi Raeisi Shahraki, Quinn Rafferty, Cat Raggi, Fakher Rahim, Md Jillur Rahim, Sarvenaz Rahimibarghani, Md Mijanur Mijanur Rahman Rahman, Mosiur Rahman, Muhammad Aziz Rahman, Tafhimur Rahman, Amir Masoud Rahmani, Mohammad Rahmanian, Nazanin Rahmanian, Rahem Rahmati, Setyaningrum Rahmawaty, Diego Raimondo, Adarsh Raja, Prashant Rajput, Shakthi Kumaran Ramasamy, Pramod W Ramteke, Kritika Rana, Rishabh Kumar Rana, Chhabi Lal Ranabhat, Amey Rane, Chythra R Rao, Mithun Rao, Vahid Rashedi, Ahmed Mustafa Rashid, Prateek Rastogi, Azad Rasul, Devarajan Rathish, Giridhara Rathnaiah Babu, Santosh Kumar Rauniyar, Ramin Ravangard, David Laith Rawaf, Salman Rawaf, Rabail Zehra Raza, Elrashdy Moustafa Mohamed Redwan, Lennart Reifels, Marissa B Reitsma, Kannan RR Rengasamy, Bhageerathy Reshmi, Serge Resnikoff, Stefano Restaino, Luis Felipe Reyes, Nazila Rezaei, Mohsen Rezaeian, Taeho Gregory Rhee, Jennifer Rickard, Hermano Alexandre Lima Rocha, Mónica Rodrigues, Jefferson Antonio Buendia Rodriguez, Leonardo Roever, Debby Syahru Romadlon, Moustaq karim khan Rony, Kunle Rotimi, Himanshu Sekhar Rout, Enrico Rubagotti, Tilleye Runghien, Michele Russo, Aly M A Saad, Korosh Saber, Maha Mohamed Saber-Ayad, Cameron John Sabet, Siamak Sabour, Bashdar Abuzed Sadee, Mohammad Reza Saeb, Umar Saeed, Sher Zaman Safi, Rajesh Sagar, Pragyan Monalisa Sahoo, Mirza Rizwan Sajid, Payman Salamati, Afeez Abolarinwa Salami, Mohamed A Saleh, Marwa Rashad Salem, Hossein Samadi Kafil, Saad Samargandy, Yoseph Leonardo Samodra, Abdallah M Samy, Juan Sanabria, Milena M Santric-Milicevic, Sivan Yegnanarayana Iyer Saraswathy, Rodrigo Sarmiento-Suárez, Gargi Sachin Sarode, Sachin C Sarode, Benn Sartorius, Maheswar Satpathy, Abu Sayeed, Nikolaos Scarmeas, Benedikt Michael Schaarschmidt, Christophe Schinckus, Art Schuermans, Aletta Elisabeth Schutte, David C Schwebel, Falk Schwendicke, Sabyasachi Senapati, Pallav Sengupta, Subramanian Senthilkumaran, Dragos Serban, Yashendra Sethi, Mahan Shafie, Pritik A Shah, Ataollah Shahbandi, Samiah Shahid, Wajeehah Shahid, Hamid R Shahsavari, Moyad Jamal Shahwan, Masood Ali Shaikh, Ali S Shalash, Ali Shamekh, Mohd Shanawaz, Abhishek Shankar, Mohammed Shannawaz, Medha Sharath, Sadaf Sharfaei, Amin Sharifan, Javad Sharifi-Rad, Anupam Sharma, Saurab Sharma, Vishal Sharma, Rajesh P Shastry, Rekha R Shenoy, Amir Shiani, Mika Shigematsu, Tariku Shimels, Rahman Shiri, Aminu Shittu, Ivy Shiue, K M Shivakumar, Sina Shool, Seyed Afshin Shorofi, Sunil Shrestha, Kerem Shuval, Emmanuel Edwar Siddig, Jaspreet Kaur Sidhu, Luís

Manuel Lopes Rodrigues Silva, Soraia Silva, Thales Philipe R Silva, Abhinav Singh, Balbir Bagicha Singh, Baljinder Singh, Harmanjit Singh, Jasbir Singh, Paramdeep Singh, Puneetpal Singh, Søren T Skou, Amanda E Smith, Farrukh Sobia, Hamidreza Soleimani, Sameh S M Soliman, Yi Song, Ireneous N Soyiri, Michael Spartalis, Chandrashekhar T Sreeramareddy, Jeffrey D Stanaway, Muhammad Haroon Stanikzai, Antonina V Starodubova, Leo Stockfelt, Mark A Stokes, Kurt Straif, Rizwan Suliankatchi Abdulkader, Abida Sultana, Jing Sun, Chandan Kumar Swain, Lukasz Szarpak, Mindy D Szeto, Payam Tabaee Damavandi, Rafael Tabarés-Seisdedos, Ozra Tabatabaei Malazy, Seyed-Amir Tabatabaeizadeh, Shima Tabatabai, Celine Tabche, Mohammad Tabish, Yasaman Taheri Abkenar, Jabeen Taiba, Iman M Talaat, Jacques Lukenze Tamuzi, Ker-Kan Tan, Haosu Tang, Razieh Tavakoli Oliaee, Seyed Mohammad Tavangar, Nuno Taveira, Abdelghani Tbakhi, Mohamad-Hani Temsah, Masayuki Teramoto, Behailu Terefe Tesfaye, Enoch Teye-Kwadjo, Pugazhenthan Thangaraju, Kavumpurathu Raman Thankappan, Rekha Thapar, Rasiah Thayakaran, Sathish Thirunavukkarasu, Nihal Thomas, Lau Caspar Thygesen, Jansje Henny Vera Ticoalu, Dinesh Timalsena, Tenaw Yimer Tiruye, Sojit Tomo, Marcello Tonelli, Roman Topor-Madry, Mathilde Touvier, Marcos Roberto Tovani-Palone, An Thien Tran, Jasmine T Tran, Nghia Minh Tran, Thang Huu Tran, Domenico Trico, Samuel Joseph Tromans, Evangelia Eirini Tsermpini, Munkhtuya Tumurkhuu, Arit Udoh, Atta Ullah, Saeed Ullah, Sana Ullah, Srikanth Umakanthan, Muhammad Umar, Shehu Salihu Umar, Bhaskaran Unnikrishnan, Era Upadhyay, Jibrin Sammani Usman, Sanaz Vahdati, Omid Vakili, Rohollah Valizadeh, Jef Van den Eynde, Priya Vart, Shoban Babu Varthya, Milena Vasic, Narayanaswamy Venketasubramanian, Massimiliano Veroux, Georgios-Ioannis Verras, Dominique Vervoort, Mathavaswami Vijayageetha, Jorge Hugo Villafañe, Manish Vinayak, Francesco S Violante, Bay Vo, Karn Vohra, Stein Emil Vollset, Theo Vos, Abdul Wadood Wadood, Yasir Waheed, Fang Wang, Shaopan Wang, Shu Wang, Yanqing Wang, Yanzhong Wang, Yuan-Pang Wang, Mary Njeri Wanjau, Muhammad Waqas, Paul Ward, Abdul Waris, Emebet Gashaw Wassie, Kosala Gayan Weerakoon, Haftom Legese Legese Weldetinsaa, Yi Feng Wen, Ronny Westerman, Taweewat Wiangkham, Dakshitha Praneeth Wickramasinghe, Evi Widowati, Marcin W Wojewodzic, Axel Walter Wolf, Charles D A Wolfe, Chenkai Wu, Dongze Wu, Felicia Wu, Jiayuan Wu, Sarah Wulf Hanson, Hong Xiao, Danting Yang, Yuichiro Yano, Amir Yarahmadi, Iman Yazdani Nia, Pengpeng Ye, Renjulal Yesodharan, Subah Abderehim Yesuf, Saber Yezli, Arzu Yiğit, Vahit Yiğit, Zeamanuel Anteneh Yigzaw, Dehui Yin, Paul Yip, Naohiro Yonemoto, Yuyi You, Mustafa Z Younis, Chuanhua Yu, Elaine A Yu, Yong Yu, Chun-Wei Yuan, Hadiza Yusuf, Uzma Zafar, Nima Zafari, Mondal Hasan Zahid, Fathiah Zakham, Nazar Zaki, Taddese Alemu Zerfu, Haijun Zhang, Jingya Zhang, Liqun Zhang, Yunquan Zhang, Zhiqiang Zhang, Yang Zhao, Zhongyi Zhao, Chenwen Zhong, Bolun Zhou, Juexiao Zhou, Bin Zhu, Abzal Zhumagaliuly, Magdalena Zielińska, Ghazal Zoghi, Alimuddin Zumla, Sa'ed H Zyoud, Samer H Zyoud.

Drafting the work or revising it critically for important intellectual content

Hazim S Ababneh, Yohannes Habtegiorgis Abate, Cristiana Abbafati, Hedayat Abbastabar, Abdallah H A Abd Al Magied, Samar Abd ElHafeez, Atef Abdelkader, Michael Abdelmasseh, Sherief Abd-Elsalam, Parsa Abdi, Mohammad Abdollahi, Auwal Abdullahi, Olumide Abiodun, Hassan Abolhassani, Mohamed Abouzid, Girma Beressa Aboye, Bilyaminu Abubakar, Hana Jihad Jihad Abukhadijah, Giovanni Addolorato, Victor Adekanmbi, Charles Oluwaseun Adetunji, Juliana Bunmi Adetunji, Ripon Kumar Adhikary, Qorinah Estiningtyas Sakilah Adnani, Fatemeh Afrashteh, Muhammad Sohail Afzal, Saira Afzal, Faith Agbozo, Antonella Agodi, Bright Opoku Ahinkorah, Firdos Ahmad, Muayyad M Ahmad, Anisuddin Ahmed, Ayman Ahmed, Haroon Ahmed, Safoora Ahmed, Syed Anees Ahmed, Mohammed Ahmed Akkaif, Ashley E Akrami, Ema Akter, Amjad S Al Mosa, Omar Al Ta'ani, Omar Ali Mohammed Al Zaabi, Fares Alahdab, Muaaz M Alajlani, Yazan Al-Ajlouni, Samer O Alalalmeh, Khurshid Alam, Zufishan Alam, Rasmieh Mustafa Al-amer, Almaza Albakri, Wafa A Aldhaleei, Seyedeh Yasaman Alemohammad, Mohammed Khaled Al-Hanawi, Abid Ali, Amjad Ali, Mohammed Usman Ali, Rafat Ali, Syed Shujait Shujait Ali, Victor Ekoche Ali, Waad Ali, Akram Al-Ibraheem, Gianfranco Alicandro, Joseph Uy Almazan, Ahmad Alrawashdeh, Sahel Majed Alrousan, Salman Khalifah Al-Sabah, Mohammed A Alsabri, Zaid Altaany, Ala'a B Al-Tammemi, Jaffar A Al-Tawfiq, Deborah Oyine Aluh, Nelson Alvis-Guzman, Mohammad Sami Al-Wardat, Yaser Mohammed Al-Worafi, Hany Aly, Mohammad Sharif Alyahya, Karem H Alzoubi, Walid Al-Zyoud, Reza Amani, Tarek Tawfik Amin, Alireza Amindarolzarbi, Sohrab Amiri, Mohammad Hosein Amirzade-Iranaq, Hubert Amu, Dickson A Amugsi, Robert Ancuceanu, Deanna Anderlini, David B Anderson, Pedro Prata Andrade, Catalina Liliana Andrei, Abhishek Anil, Sneha Anil, Amir Anoushiravani, Catherine M Antony, Ernoiz Antriyandarti, Boluwatife Stephen Anuoluwa, Saeid Anvari, Anayochukwu Edward Anyasodor, Francis Appiah, Michele Aquilano, Juan Pablo Arab, Jalal Arabloo, Elshaimaa A Arafa, Mosab Arafat, Ali Ardekani, Abdulfatai Aremu, Hany Ariffin, Mesay Arkew, Ashokan Arumugam, Seyyed Shamsadin Athari, Prince Atorkey, Maha Moh'd Wahbi Atout, Avinash Aujayeb, Marcel Ausloos, Adedapo Wasiu Awotidebe, Haleh Ayatollahi, Sina Azadnajafabad, Rui M S Azevedo, Muhammad Badar, Soroush Baghdadi, Ruhai Bai, Jennifer L

Baker, Abdulaziz T Bako, Senthilkumar Balakrishnan, Wondu Feyisa Balcha, Ovidiu Constantin Baltatu, Martina Barchitta, Erfan Bardideh, Suzanne Lyn Barker-Collo, Till Winfried Bärnighausen, Hiba Jawdat Barqawi, Sandra Barteit, Afisu Basiru, João Diogo Basso, Mohammad-Mahdi Bastan, Sanjay Basu, Matteo Bauckneht, Bernhard T Baune, Amir Hossein Behnoush, Maryam Beiranvand, Olorunjuwon Omolaja Bello, Luis Belo, Apostolos Beloukas, Maryam Bemanalizadeh, Isabela M Bensenor, Habib Benzian, Azizullah Beran, Paulo J G Bettencourt, Kebede A Beyene, Melak Gedamu Beyene, Devidas S Bhagat, Akshaya Srikanth Bhagavathula, Dinesh Bhandari, Ravi Bharadwaj, Ashish Bhargava, Sonu Bhaskar, Vivek Bhat, Natalia V Bhattacharjee, Gurjit Kaur Bhatti, Jasvinder Singh Bhatti, Manpreet S Bhatti, Mohiuddin Ahmed Bhuiyan, Catherine Bisignano, Bijit Biswas, Tone Bjørge, Virginia Bodolica, Aadam Olalekan Bodunrin, Milad Bonakdar Hashemi, Berrak Bora Basara, Hamed Borhany, Alejandro Botero Carvajal, Souad Bouaoud, Soufiane Boufous, Christopher Boxe, Edward J Boyko, Oliver J Brady, Dejana Braithwaite, Hermann Brenner, Colin Stewart Brown, Traolach Brugha, Dana Bryazka, Norma B Bulamu, Danilo Buonsenso, Richard A Burns, Yasser Bustanji, Florentino Luciano Caetano dos Santos, Mehtap Çakmak Barsbay, Daniela Calina, Luciana Aparecida Campos, Shujin Cao, Angelo Capodici, Giulia Carreras, Andrea Carugno, Márcia Carvalho, Joao Mauricio Castaldelli-Maia, Giulio Castelpietra, Maria Sofia Cattaruzza, Arthur Caye, Luca Cegolon, Francieli Cembranel, Edina Cenko, Ester Cerin, Steven J Chadban, Joshua Chadwick, Chiranjib Chakraborty, Sandip Chakraborty, Jeffrey Shi Kai Chan, Rama Mohan Chandika, Sara Chandy, Anis Ahmad Chaudhary, Akhilanand Chaurasia, An-Tian Chen, Haowei Chen, Meng Xuan Chen, Simiao Chen, Nicolas Cherbuin, Gerald Chi, Fatemeh Chichagi, Ritesh Chimoriya, Patrick R Ching, Jesus Lorenzo Chirinos-Caceres, Daniel Youngwhan Cho, William C S Cho, Bryan Chong, Hitesh Chopra, Dinh-Toi Chu, Eric Chung, Muhammad Chutiyami, Alyssa Columbus, Joao Conde, Samuele Cortese, Natália Cruz-Martins, Alanna Gomes da Silva, Xiaochen Dai, Zhaoli Dai, Nihar Ranjan Dash, Ivan Delgado-Enciso, Edgar Denova-Gutiérrez, Nikolaos Dervenis, Hardik Dineshbhai Desai, Syed Masudur Rahman Dewan, Amol S Dhane, Sameer Dhingra, Diana Dias da Silva, Daniel Diaz, Luis Antonio Diaz, Michael J Diaz, Adriana Dima, Delaney D Ding, Camila Bruneli do Prado, Masoud Dodangeh, Milad Dodangeh, Phidelia Theresa Doegah, Sushil Dohare, Wanyue Dong, Mario D'Oria, Rajkumar Doshi, Robert Kokou Dowou, Haneil Larson Dsouza, Viola Dsouza, John Dube, Samuel C. Dumith, Bruce B Duncan, Senbagam Duraisamy, Oyewole Christopher Durojaiye, Sulagna Dutta, Paulina Agnieszka Dzianach, Arkadiusz Marian Dziedzic, Ejemai Eboreime, Ebrahim Eini, Rabie Adel El Arab, Iman El Sayed, Noha Mousaad Elemam, Ghada Metwally Tawfik ElGohary, Muhammed Elhadi, Omar Abdelsadek Abdou Elmeligy, Adel B Elmoselhi, Mohammed Elshaer, Ibrahim Elsohaby, Mohd. Elmagzoub Eltahir, Theophilus I Emeto, Majid Eslami, Zahra Esmaeili, Natalia Fabin, Adeniyi Francis Fagbamigbe, Omotayo Francis Fagbule, Luca Falzone, Mohammad Fareed, MoezAlIslam Ezzat Mahmoud Faris, Andre Faro, Kiana Fasihi, Ali Fatehizadeh, Nelsensius Klau Fauk, Valery L Feigin, Seyed-Mohammad Fereshtehnejad, Nuno Ferreira, Belete Sewasew Firew, Florian Fischer, Ida Fitriana, Joanne Flavel, Morenike Oluwatoyin Folayan, Kristen Marie Foley, Marco Fonzo, Matteo Foschi, Alberto Freitas, Ni Kadek Yuni Fridayani, Blima Fux, Peter Andras Gaal, Muktar A Gadanya, Silvano Gallus, Balasankar Ganesan, Mohammad Arfat Ganiyani, Rupesh K. Gautam, Tilaye Gebru Gebi, Miglas W Gebregergis, Lemma Getacher, Delaram J Ghadimi, Fataneh Ghadirian, Sadegh Ghafarian, Khalid Yaser Ghailan, MohammadReza Ghasemi, Ghazal Ghasempour Dabaghi, Ramy Mohamed Ghazy, Ehsan Gholami, Nasim Gholizadeh, Mahsa Ghorbani, Pooyan Ghorbani Vajargah, Elena Ghotbi, Tiffany K Gill, Alem Girmay, James C Glasbey, Elena V Gnedovskaya, Laszlo Göbölös, Mohamad Goldust, Davide Golinelli, Sameer Vali Gopalani, Alessandra C Goulart, Mahdi Gouravani, Anmol Goyal, Michal Grivna, Giovanni Guarducci, Stefano Guicciardi, Rafael Alves Guimarães, Snigdha Gulati, David Gulisashvili, Damitha Asanga Gunawardane, Cui Guo, Rahul Gupta, Rajeev Gupta, Renu Gupta, Sapna Gupta, Najah R Hadi, Nils Haep, Abdul Hafiz, Dariush Haghmorad, Adel Hajj Ali, Ali Hajj Ali, Arvin Haj-Mirzaian, Esam S Halboub, Rabih Halwani, Nadia M Hamdy, Rifat Hamoudi, Nasrin Hanifi, Graeme J Hankey, Zaim Anan Haq, Harapan Harapan, Josep Maria Haro, Ahmed I Hasaballah, S M Mahmudul Hasan, Md Saquib Hasnain, Amr Hassan, Johannes Haubold, Simon I Hay, Jeffrey J Hebert, Omar E Hegazi, Mohammad Heidari, Mehdi Hemmati, Claudiu Herteliu, Kamal Hezam, Yuta Hiraike, Nguyen Quoc Hoan, Ramesh Holla, Md Mahbub Hossain, Sorin Hostiuc, Junjie Huang, Michael Hultström, Kiavash Hushmandi, Javid Hussain, M Azhar Hussain, Hong-Han Huynh, Segun Emmanuel Ibitoye, Pulwasha Maria Iftikhar, Adalia I Ikiroma, Paul Chukwudi Ikwegbue, Irena M Ilic, Milena D Ilic, Mustapha Immurana, Mustafa Alhaji Isa, Md Rabiul Islam, Sheikh Mohammed Shariful Islam, Faisal Ismail, Nahlah Elkudssiah Ismail, Gaetano Isola, Ihoghosa Osamuyi Iyamu, Louis Jacob, Kathryn H Jacobsen, Morteza Jafarinia, Kasra Jahankhani, Nader Jahanmehr, Nityanand Jain, Dr Ruchi Jakhmola Mani, Safayet Jamil, Abubakar Ibrahim Jatau, Shubha Jayaram, Jost B Jonas, Nitin Joseph, Charity Ehimwenma Joshua, Mikk Jürisson, Ali Kabir, Vidya Kadashetti, Laleh R Kalankesh, Sanjay Kalra, Rajesh Kamath, Arun Kamireddy, Mona Kanaan, Edmund Wedam Kanmiki, Kehinde Kazeem Kanmodi, Sushil Kumar Kansal, Asima Karim, Samad Karkhah, Faizan Zaffar Kashoo, Hengameh Kasraei, Srinivasa Vittal Katikireddi, Joonas H Kauppila, Gbenga A Kayode, Foad Kazemi, Sina Kazemian, Fassikaw Kebede, Emmanuelle Kesse-Guyot, Shahram Khademvatan,

Himanshu Khajuria, Amirmohammad Khalaji, Asaad Khalid, Nauman Khalid, Fayaz Khan, Mohammad Jobair Khan, Moien AB Khan, Shaghayegh Khanmohammadi, Khaled Khatab, Haitham Khatatbeh, Moawiah Mohammad Khatatbeh, Mahalaqua Nazli Khatib, Hamid Reza Khayat Kashani, Khalid A Kheirallah, Atulya Aman Khosla, Majid Khosravi, Mahmood Khosrowjerdi, Grace Kim, Julie Sojin Kim, Min Seo Kim, Yun Jin Kim, Adnan Kisa, Sezer Kisa, Ann Kristin Skrindo Knudsen, Sonali Kochhar, Farzad Kompani, Oleksii Korzh, Kewal Krishan, Varun Krishna, Burcu Kucuk Bicer, Md Abdul Kuddus, Ilari Kuitunen, Mukhtar Kulimbet, Vishnutheertha Kulkarni, Rakesh Kumar, Vijay Kumar, Amartya Kundu, Dian Kusuma, Ville Kytö, Carlo La Vecchia, Ben Lacey, Muhammad Awwal Ladan, Lucie Laflamme, Chandrakant Lahariya, Ratilal Lalloo, Tea Lallukka, Judit Lám, Iván Landires, Berthold Langguth, Ariane Laplante-Lévesque, Bagher Larijani, Anders O Larsson, Savita Lasrado, Huu-Hoai Le, Long Khanh Dao Le, Nhi Huu Hanh Le, Thao Thi Thu Le, Doo Woong Lee, Munjae Lee, Paul H Lee, Elvynna Leong, Massimo Libra, Lee-Ling Lim, Daniel Lindholm, Paulina A Lindstedt, Stefan Listl, Xuefeng Liu, Erand Llanaj, Rubén López-Bueno, José Francisco López-Gil, Paulo A Lotufo, Giancarlo Lucchetti, Jay B Lusk, Hawraz Ibrahim M Amin, Zheng Feei Ma, Nikolaos Machairas, Monika Machoy, Áurea M Madureira-Carvalho, Hassan Magdy Abd El Razek, Elham Mahmoudi, Golnaz Mahmoudvand, Elaheh Malakan Rad, Kashish Malhotra, Ahmad Azam Malik, Deborah Carvalho Malta, Vahid Mansouri, Marjan Mansourian, Hamid Reza Marateb, Parham Mardi, Abdoljalal Marjani, Gabriel Martinez, Ramon Martinez-Piedra, Francisco Rogerlândio Martins-Melo, Miquel Martorell, Wolfgang Marx, Roy Rillera Marzo, Yasith Mathangasinghe, Alexander G Mathioudakis, Medha Mathur, Navgeet Mathur, Neeta Mathur, Fernanda Penido Matozinhos, Jishanth Mattumpuram, Andrea Maugeri, Mahsa Mayeli, Antonio Mazzotti, John J McGrath, Anna Laura W McKowen, Steven M McPhail, Asim Mehmood, Kamran Mehrabani-Zeinabad, Tesfahun Mekene Meto, Endalkachew Belayneh Melese, Max Alberto Mendez Mendez-Lopez, Walter Mendoza, Ritesh G Menezes, George A Mensah, Laverne G Mensah, Alexios-Fotios A Mentis, Sultan Ayoub Meo, Atte Meretoja, Tuomo J Meretoja, Tomislav Mestrovic, Kukulege Chamila Dinushi Mettananda, Sachith Mettananda, Tomasz Miazgowski, Irmina Maria Michalek, Ana Carolina Micheletti Gomide Nogueira de Sá, Ted R Miller, Le Huu Nhat Minh, Alireza Mirahmadi, Antonio Mirijello, Philip B Mitchell, Chaitanya Mittal, Atousa Moghadam Fard, Seyedehfatemeh Mohajelin, Mouhand F H Mohamed, Nouh Saad Mohamed, Ameen Mosa Mohammad, Soheil Mohammadi, Hussen Mohammed, Mustapha Mohammed, Shafiu Mohammed, Ali H Mokdad, Mariam Molokhia, Shaher Mohammad Momani, Sara Momtazmanesh, Lorenzo Monasta, Stefania Mondello, Mohammad Ali Moni, Fateme Montazeri, AmirAli Moodi Ghalibaf, Maryam Moradi, Yousef Moradi, Paula Moraga, Rafael Silveira Moreira, Shane Douglas Morrison, Abbas Mosapour, Parsa Mousavi, Matías Mrejen, Ulrich Otto Mueller, Francesk Mulita, Christopher J L Murray, Khaled M Musallam, Ana-Maria Musina, Ghulam Mustafa, Ayoub Nafei, Ahamarshan Jayaraman Nagarajan, Soroush Najdaghi, Noureddin Nakhostin Ansari, Sreenivas Narasimha Swamy, Shumaila Nargus, Delaram Narimani Davani, Bruno Ramos Nascimento, Gustavo G Nascimento, Abdallah Y Naser, Abdulqadir J Nashwan, Zuhair S Natto, Javaid Nauman, Samidi N K Navaratna, Muhammad Naveed, Biswa Prakash Nayak, Vinod C Nayak, Hadush Negash, Ionut Negoi, Ruxandra Irina Negoi, Seyed Aria Nejadghaderi, Soroush Nematollahi, Henok Biresaw Netsere, Georges Nguefack-Tsague, Josephine W. Ngunjiri, Anh Hoang Nguyen, Dang H Nguyen, Duc Hoang Nguyen, Hau Thi Hien Nguyen, Nhan Nguyen, Nhien Ngoc Y Nguyen, Phat Tuan Nguyen, QuynhAnh P Nguyen, Van Thanh Nguyen, Duc Nguyen Tran Minh, Robina Khan Niazi, Yeshambel T Nigatu, Mahdieh Niknam, Amin Reza Nikpoor, Nasrin Nikravangolsefid, Nawsherwan Not applicable, Mehran Nouri, Chisom Adaobi Nri-Ezedi, George Ntaios, Jerry John Nutor, Chimezie Igwegbe Nzoputam, Ogochukwu Janet Nzoputam, Bogdan Oancea, Kehinde O Obamiro, Ismail A. Odetokun, Oluwaseun Adeolu Ogundijo, Adesola Adenike Ogunfowokan, Abiola Ogunkoya, Ayodipupo Sikiru Oguntade, Akinkunmi Paul Okekunle, Osaretin Christabel Okonji, Andrew T Olagunju, Matthew Idowu Olatubi, Bolajoko Olubukunola Olusanya, Jacob Olusegun Olusanya, Hany A Omar, Sandersan Onie, Obinna E Onwujekwe, Abdulahi Opejin Opejin, Michal Ordak, Verner N. Orish, Alberto Ortiz, Esteban Ortiz-Prado, Wael M. S. Osman, Sergej M Ostojic, Samuel M Ostroff, Uchechukwu Levi Osuagwu, Adrian Otoiu, Amel Ouyahia, Mayowa O Owolabi, Oyetunde T Oyeyemi, Mahesh Padukudru P A, Alicia Padron-Monedero, Jagadish Rao Padubidri, Tamás Palicz, Feng Pan, Songhomitra Panda-Jonas, Victoria Pando-Robles, Leonidas D Panos, Ioannis Pantazopoulos, Anca Mihaela Pantea Stoian, Romil R Parikh, Nicholas Parsons, Ashwaghosha Parthasarathi, Maja Pasovic, Roberto Passera, Jay Patel, Shankargouda Patil, Dimitrios Patoulias, Shrikant Pawar, Hamidreza Pazoki Toroudi, Amy E Peden, Paolo Pedersini, Umberto Pensato, Veincent Christian Filipino Pepito, Marcos Pereira, Maria Odete Pereira, Arokiasamy Perianayagam, Norberto Perico, Konrad Pesudovs, Fanny Emily Petermann-Rocha, Hoang Tran Pham, Anil K. Philip, Michael R Phillips, Michael A Piradov, Enrico Pisoni, Dimitri Poddighe, Ramesh Poluru, Ville T Ponkilainen, Djordje S Popovic, Maarten J Postma, Sergio I Prada, Jalandhar Pradhan, Pranil Man Singh Pradhan, Akila Prashant, Elton Junio Sady Prates, Tina Priscilla, Nameer Hashim Qasim, Ibrahim Qattea, Asma Saleem Qazi, Mehrdad Rabiee Rad, Venkatraman Radhakrishnan, Hadi Raeisi Shahraki, Alberto Raggi, Cat Raggi, Fakher Rahim, Md Mijanur Mijanur Rahman Rahman, Mohammad Rahmanian, Rahem Rahmati,

Diego Raimondo, Adarsh Raja, Prashant Rajput, Majed Ramadan, Shakthi Kumaran Ramasamy, Kritika Rana, Rishabh Kumar Rana, Chhabi Lal Ranabhat, Chythra R Rao, Mithun Rao, Davide Rasella, Vahid Rashedi, Ahmed Mustafa Rashid, Ashkan Rasouli-Saravani, Devarajan Rathish, Giridhara Rathnaiah Babu, Santosh Kumar Rauniyar, Ramin Ravangard, David Laith Rawaf, Salman Rawaf, Rabail Zehra Raza, Elrashdy Moustafa Mohamed Redwan, Giuseppe Remuzzi, Bhageerathy Reshmi, Serge Resnikoff, Luis Felipe Reyes, Nazila Rezaei, Zahra Sadat Rezaei, Taeho Gregory Rhee, Jennifer Rickard, Hermano Alexandre Lima Rocha, Mónica Rodrigues, Jefferson Antonio Buendia Rodriguez, Leonardo Roever, Debby Syahru Romadlon, Luca Ronfani, Moustaq karim khan Rony, Gholamreza Roshandel, Himanshu Sekhar Rout, Bedanta Roy, Enrico Rubagotti, Guilherme de Andrade Ruela, Susan Fred Rumisha, Michele Russo, Aly M A Saad, Korosh Saber, Maha Mohamed Saber-Ayad, Cameron John Sabet, Siamak Sabour, Perminder S Sachdev, Bashdar Abuzed Sadee, Masoumeh Sadeghi, Umar Saeed, Rajesh Sagar, Alireza Saghafi, Dominic Sagoe, Amirhossein Sahebkar, Pragyan Monalisa Sahoo, Mirza Rizwan Sajid, Nasir Salam, Afeez Abolarinwa Salami, Leili Salehi, Marwa Rashad Salem, Aanuoluwa James Salemcity, Sohrab Salimi, Saad Samargandy, Abdallah M Samy, Juan Sanabria, Milena M. Santric-Milicevic, Bruno Piassi Sao Jose, Aswini Saravanan, Rodrigo Sarmiento-Suárez, Gargi Sachin Sarode, Sachin C Sarode, Maheswar Satpathy, Abu Sayeed, Nikolaos Scarmeas, Benedikt Michael Schaarschmidt, Christophe Schinckus, Art Schuermans, Aletta Elisabeth Schutte, David C Schwebel, Falk Schwendicke, Siddharthan Selvaraj, Mohammad H. Semreen, Sabyasachi Senapati, Pallav Sengupta, Dragos Serban, Yashendra Sethi, Allen Seylani, Mahan Shafie, Pritik A Shah, Samiah Shahid, Moyad Jamal Shahwan, Ali S Shalash, Muhammad Aaqib Shamim, Mohd Shanawaz, Abhishek Shankar, Mohammed Shannawaz, Medha Sharath, Sadaf Sharfaei, Amin Sharifan, Javad Sharifi-Rad, Anupam Sharma, Manoj Sharma, Saurab Sharma, Vishal Sharma, Rajesh P. Shastry, Maryam Shayan, Shashank Shekhar, Rekha R Shenoy, Mahabalesh Shetty, Pavanchand H Shetty, Premalatha K Shetty, Mika Shigematsu, Aminu Shittu, K M Shivakumar, Sina Shool, Seyed Afshin Shorofi, Yafei Si, Emmanuel Edwar Siddig, Jaspreet Kaur Sidhu, João Pedro Silva, Soraia Silva, Thales Philipe R Silva, Colin R Simpson, Abhinav Singh, Balbir Bagicha Singh, Jasbir Singh, Paramdeep Singh, Puneetpal Singh, Søren T. Skou, Amanda E Smith, Bogdan Socea, Shipra Solanki, Hamidreza Soleimani, Sameh S. M. Soliman, Yi Song, Ireneous N Soyiri, Michael Spartalis, Chandrashekhar T Sreeramareddy, Muhammad Haroon Stanikzai, Dan J Stein, Paschalis Steiropoulos, Leo Stockfelt, Mark A Stokes, Narayan Subedi, Abida Sultana, Johan Sundström, Chandan Kumar Swain, Lukasz Szarpak, Mindy D Szeto, Payam Tabaee Damavandi, Ozra Tabatabaei Malazy, Seyed-Amir Tabatabaeizadeh, Shima Tabatabai, Karen M Tabb, Celine Tabche, Mohammad Tabish, Yasaman Taheri Abkenar, Iman M. Talaat, Jacques Lukenze Tamuzi, Ker-Kan Tan, Nathan Y. Tat, Razieh Tavakoli Oliaee, Seyed Mohammad Tavangar, Nuno Taveira, Hadi Tehrani, Mohamad-Hani Temsah, Masayuki Teramoto, Pugazhenthan Thangaraju, Sathish Thirunavukkarasu, Nihal Thomas, Lau Caspar Thygesen, Dinesh Timalsena, Tenaw Yimer Tiruye, Krishna Tiwari, Marcello Tonelli, Roman Topor-Madry, Mathilde Touvier, Marcos Roberto Tovani-Palone, An Thien Tran, Jasmine T Tran, Nghia Minh Tran, Thang Huu Tran, Domenico Trico, Samuel Joseph Tromans, Thien Tan Tri Tai Truyen, Aristidis Tsatsakis, Evangelia Eirini Tsermpini, Arit Udoh, Srikanth Umakanthan, Shehu Salihu Umar, Brigid Unim, Bhaskaran Unnikrishnan, Era Upadhyay, Jibrin Sammani Usman, Sanaz Vahdati, Asokan Govindaraj Vaithinathan, Omid Vakili, Jef Van den Eynde, Priya Vart, Shoban Babu Varthya, Tommi Juhani Vasankari, Narayanaswamy Venketasubramanian, Massimiliano Veroux, Georgios-Ioannis Verras, Dominique Vervoort, Jorge Hugo Villafañe, Manish Vinayak, Francesco S Violante, Vasily Vlassov, Stein Emil Vollset, Theo Vos, Abdul Wadood Wadood, Fang Wang, Shaopan Wang, Shu Wang, Yanzhong Wang, Yuan-Pang Wang, Paul Ward, Emebet Gashaw Wassie, Marcia R Weaver, Kosala Gayan Weerakoon, Robert G Weintraub, Ronny Westerman, Taweewat Wiangkham, Dakshitha Praneeth Wickramasinghe, Marcin W Wojewodzic, Dawit Habte Woldeyes, Axel Walter Wolf, Charles D A Wolfe, Dongze Wu, Hong Xiao, Rakesh Yadav, Kazumasa Yamagishi, Danting Yang, Yuichiro Yano, Amir Yarahmadi, Iman Yazdani Nia, Saber Yezli, Arzu Yiğit, Vahit Yiğit, Naohiro Yonemoto, Elaine A Yu, Chun-Wei Yuan, Hadiza Yusuf, Uzma Zafar, Nima Zafari, Mondal Hasan Zahid, Haijun Zhang, Jingya Zhang, Zhiqiang Zhang, Xiu-Ju George Zhao, Yang Zhao, Zhongyi Zhao, Chenwen Zhong, Abzal Zhumagaliuly, Magdalena Zielińska, Ghazal Zoghi, Alimuddin Zumla, Sa'ed H Zyoud, and Samer H Zyoud

Managing the estimation or publications process

Saira Afzal, Muaaz M Alajlani, Tahiya Alam, Catherine M Antony, Sonu Bhaskar, Catherine Bisignano, Milad Bonakdar Hashemi, Francieli Cembranel, Jon T Connolly, Xiaochen Dai, Robert Kokou Dowou, Omotayo Francis Fagbule, Ali Fatehizadeh, Pooyan Ghorbani Vajargah, Alem Girmay, Abdul Hafiz, Simon I Hay, Hong-Han Huynh, Morteza Jafarinia, Samad Karkhah, Molly B Kassel, Fassikaw Kebede, Mahalaqua Nazli Khatib, Majid Khosravi, Chandrakant Lahariya, Huu-Hoai Le, Nhi Huu Hanh Le, Thao Thi Thu Le, Paulina A Lindstedt, Erand Llanaj, Hassan Magdy Abd El Razek, Abdoljalal Marjani, Navgeet Mathur, Anna Laura W McKowen, Le Huu Nhat Minh, Ali H Mokdad, Christopher J L Murray, Nhan Nguyen, Phat Tuan Nguyen, Van Thanh Nguyen, Bolajoko

Olubukunola Olusanya, Jacob Olusegun Olusanya, Mahesh Padukudru P A, Maja Pasovic, Hoang Tran Pham, Jalandhar Pradhan, Hadi Raeisi Shahraki, Giridhara Rathnaiah Babu, Mónica Rodrigues, Aly M A Saad, Korosh Saber, Cameron John Sabet, Bashdar Abuzed Sadee, Rajesh Sagar, Abdallah M Samy, Maheswar Satpathy, Amanda E Smith, Michael Spartalis, Caitlyn Steiner, Yasaman Taheri Abkenar, Razieh Tavakoli Oliaee, Shoban Babu Varthya, Stein Emil Vollset, Katherine M Wells, Iman Yazdani Nia, and Yang Zhao
