# Supplementary material for: Burden of disease scenarios for 204 countries and territories, 2022–2050: a forecasting analysis for the Global Burden of Disease Study 2021
Source: Lancet. 2024 May 18;403(10440):2204–56. doi: 10.1016/S0140-6736(24)00685-8 (PMC11121021; doi:10.1016/S0140-6736(24)00685-8)
Supplement: Supplementary appendix 2 [file mmc2.pdf]

THE LANCET

Supplementary appendix 2

This appendix formed part of the original submission and has been peer reviewed. We post it as supplied by the authors.

Supplement to: GBD 2021 Forecasting Collaborators. Burden of disease scenarios for 204 countries and territories, 2022–2050: a forecasting analysis for the Global Burden of Disease Study 2021. *Lancet* 2024; **403:** 2204–56.

Appendix 2: supplementary results appendix to "Burden of disease scenarios for 204 countries and territories, 2022–2050: a forecasting analysis for the Global Burden of Disease Study 2021"

This supplement provides additional figures and tables containing more detailed results for **"**Burden of disease scenarios for 204 countries and territories, 2022–2050: a forecasting analysis for the Global Burden of Disease Study 2021**".**

These and other results are available to view and download in GBD Foresight, an interactive forecasting visualisation tool.

Figures

Supplemental Results Figure S1. Select risk factor Summary Exposure Values (SEVs) by global and GBD super region and scenario, 1990-2050……………………………………………………………………………..3

Supplemental Results Figure S2. Global and super-regional healthy life expectancy, 1990–2050 for females (A) and males (B)…………………………………………………………………………………………………...11

Supplemental Results Figure S3. Life expectancy and HALE by sex and location, 1990-2050……………………..12

Supplemental Results Figure S4. Age-standardised DALY rates and all age DALY counts and by Level 1 and Level 2 causes, 1990–2050…………………………………………………………...…………………………....245

Supplemental Results Figure S5. Age-standardised death rates and all age death counts and by Level 1 and Level 2 causes, 1990–2050…………………………………………..……………………………………………478

Supplemental Results Figure S6. Cause level 2 area plots for 4 burden measures for reference scenario by location, 1990-2050…..…………………………………………………………………………………..………..…..711

Supplemental Results Figure S7. Leading 20 level 3 causes of DALYs, and percentage change in number of DALYs and all-age and age-standardised DALY rates by location, 2022 and 2050………………….……………….……..944

Supplemental Results Figure S8. Leading 20 level 3 causes of deaths, and percentage change in number of deaths and all-age and age-standardised death rates by location, 2022 and 2050……………………………….………...1177

Supplemental Results Figure S9. Population aging by global and GBD super region, 1990-2050…………….….1410

Supplemental Results Figure S10. All-cause age standardised DALY rates (A) and all age DALY counts (B), for the past and for five future scenarios, 1990–2050……..…………………………………………………….….…..1411

Supplemental Results Figure S11. All-cause age standardised death rates (A) and all age death counts (B), for the past and for five future scenarios, 1990–2050………………………………………………..……….…...1644

Supplemental Results Figure S12. All cause and top NCD causes age standardized DALY rates and all age counts by scenario and location, 1990-2050……………………………………………………………………………….1877

Supplemental Results Figure S13. All cause and top NCD causes age standardized death rates and all age counts by scenario and location, 1990-2050……………………………………………………………………….….…2110

Supplemental Results Figure S14. Decomposition of change in life expectancy by location and level 2 causes plus COVID-19 for reference and alternative scenarios, 2022–2050…………………………………………………...2343

Tables

Supplemental Results Table S1. Annual change in life expectancy in pre-COVID pandemic years (1990-2019) and post-COVID pandemic years (2022-2050) by global and super region for females, males and both sexes………2547

Supplemental Results Table S2. Life expectancy and healthy life expectancy (HALE) in 2022 and 2050 (reference scenario) by location for both sexes……………………………………………………………………….…….2548

Supplemental Results Table S3. Percent decrease in DALY counts by scenario compared to reference in 2050…2560

Supplemental Results Table S4. Cause-specific death counts and age-standardised death rates in 2022 and 2050 by scenario……………………………………………………………………………………………………….2570

Supplemental Results Figure S1. Select risk factor Summary Exposure Values (SEVs) by global and GBD super region and scenario, 1990-2050

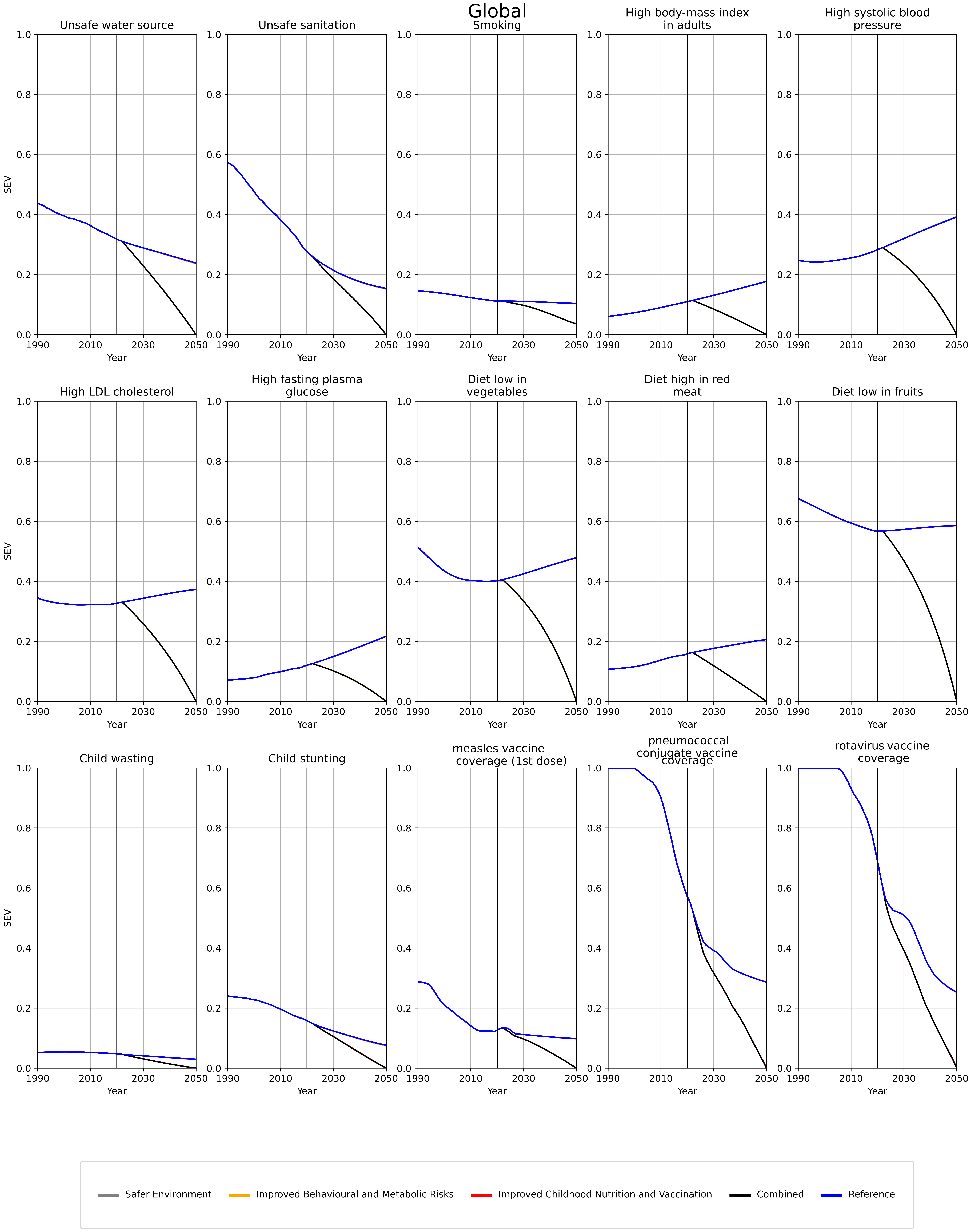

Supplemental Results Figure S1. Select risk factor Summary Exposure Values (SEVs) by global and GBD super region and scenario, 1990-2050

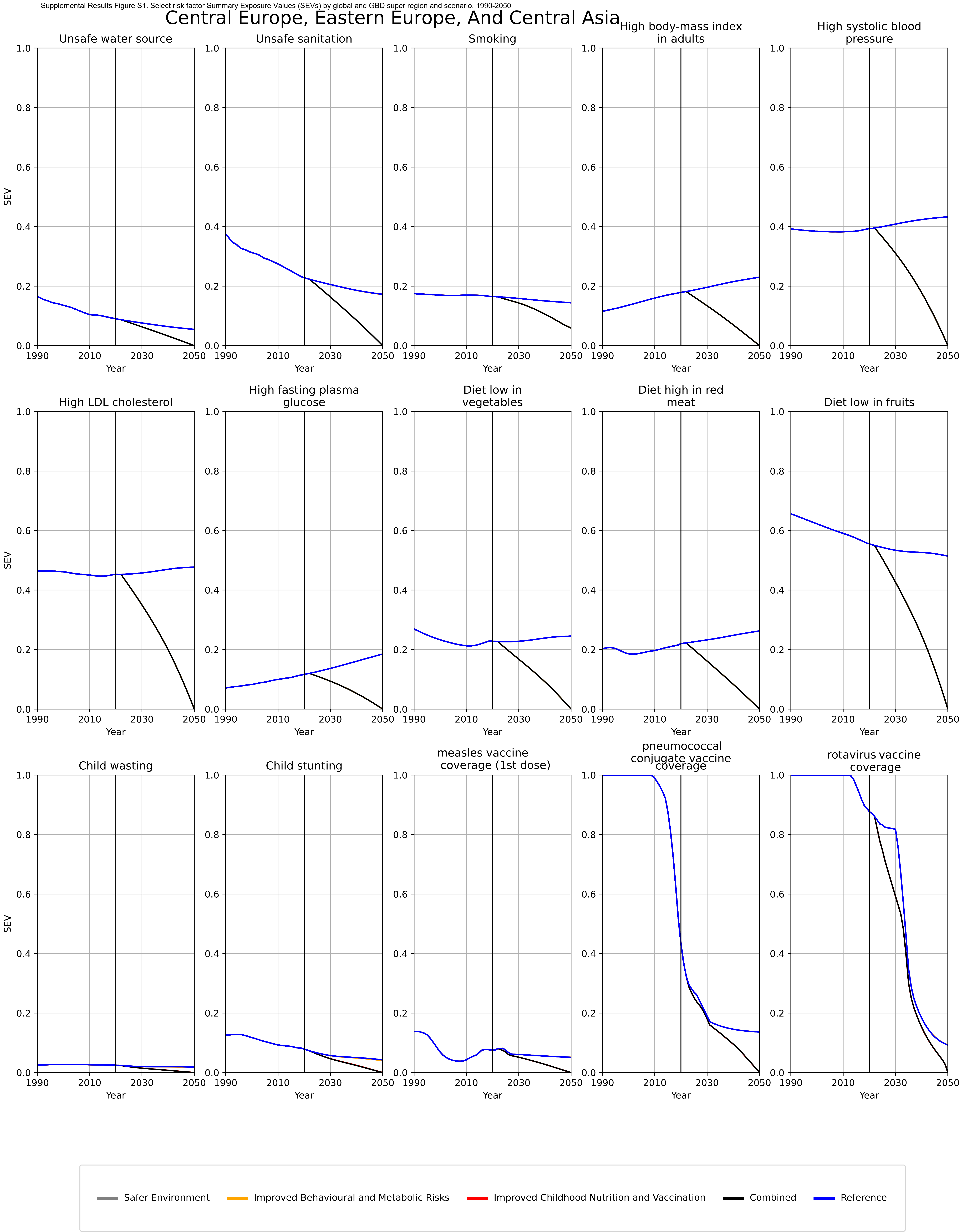

Supplemental Results Figure S1. Select risk factor Summary Exposure Values (SEVs) by global and GBD super region and scenario, 1990-2050

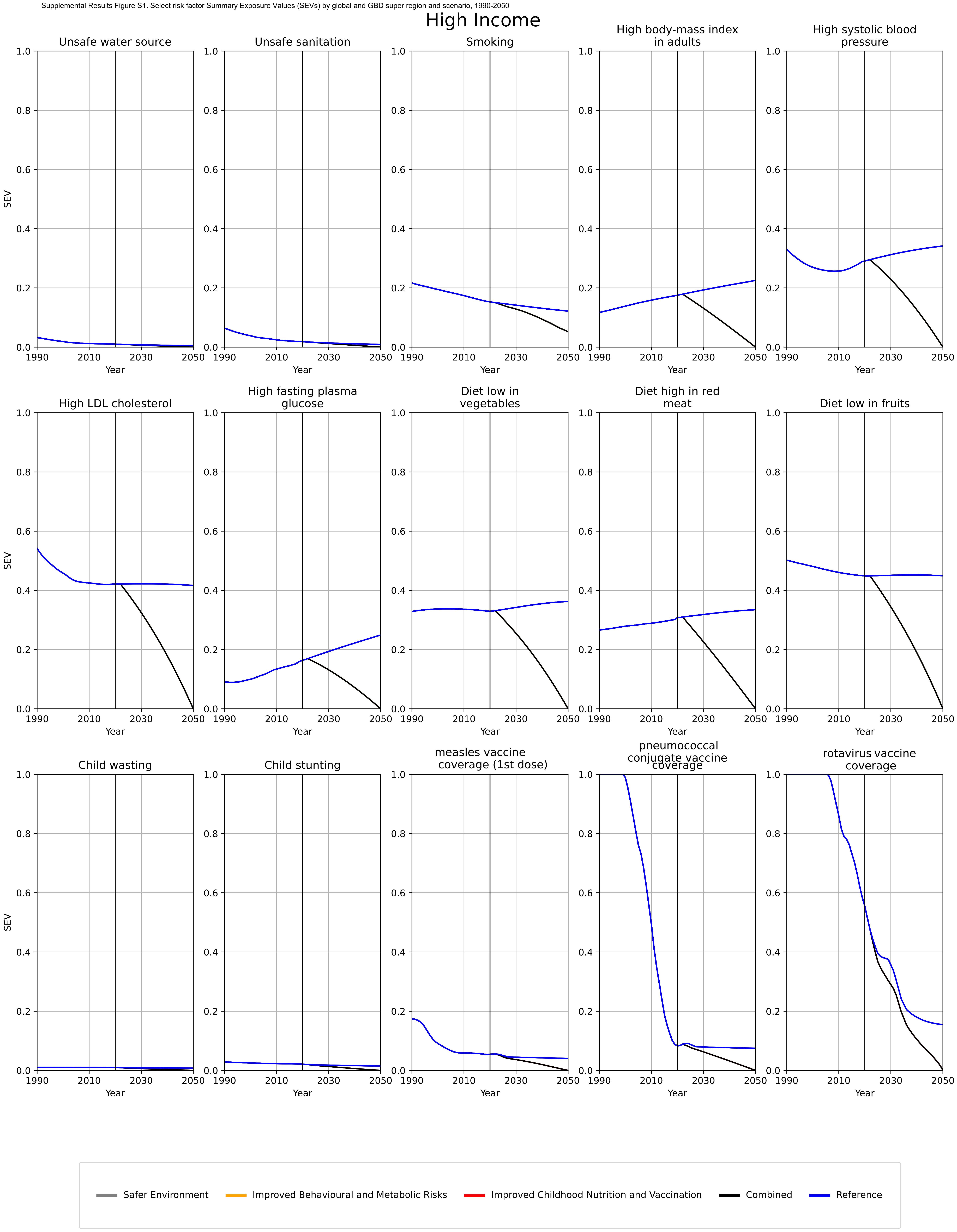

Supplemental Results Figure S1. Select risk factor Summary Exposure Values (SEVs) by global and GBD super region and scenario, 1990-2050

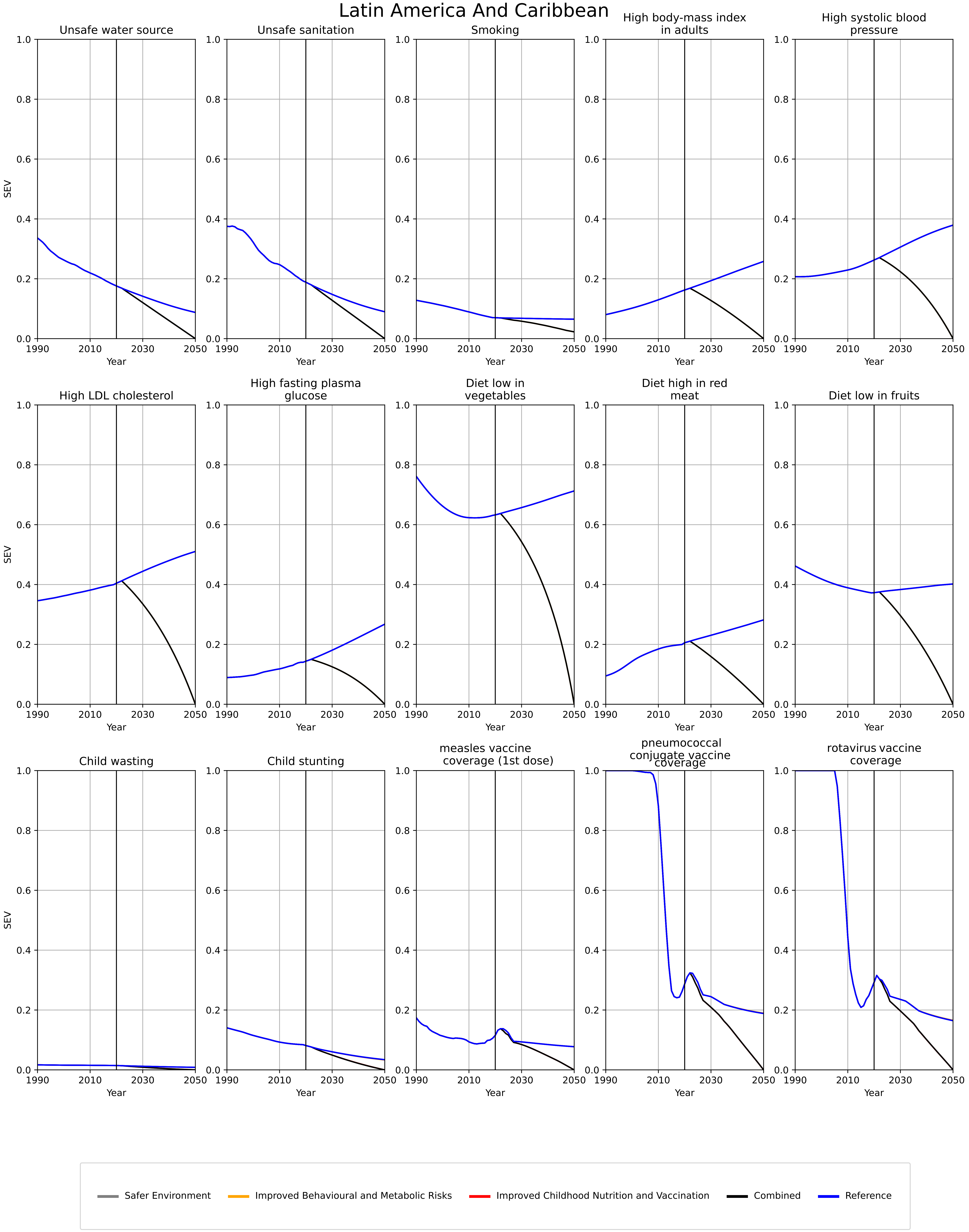

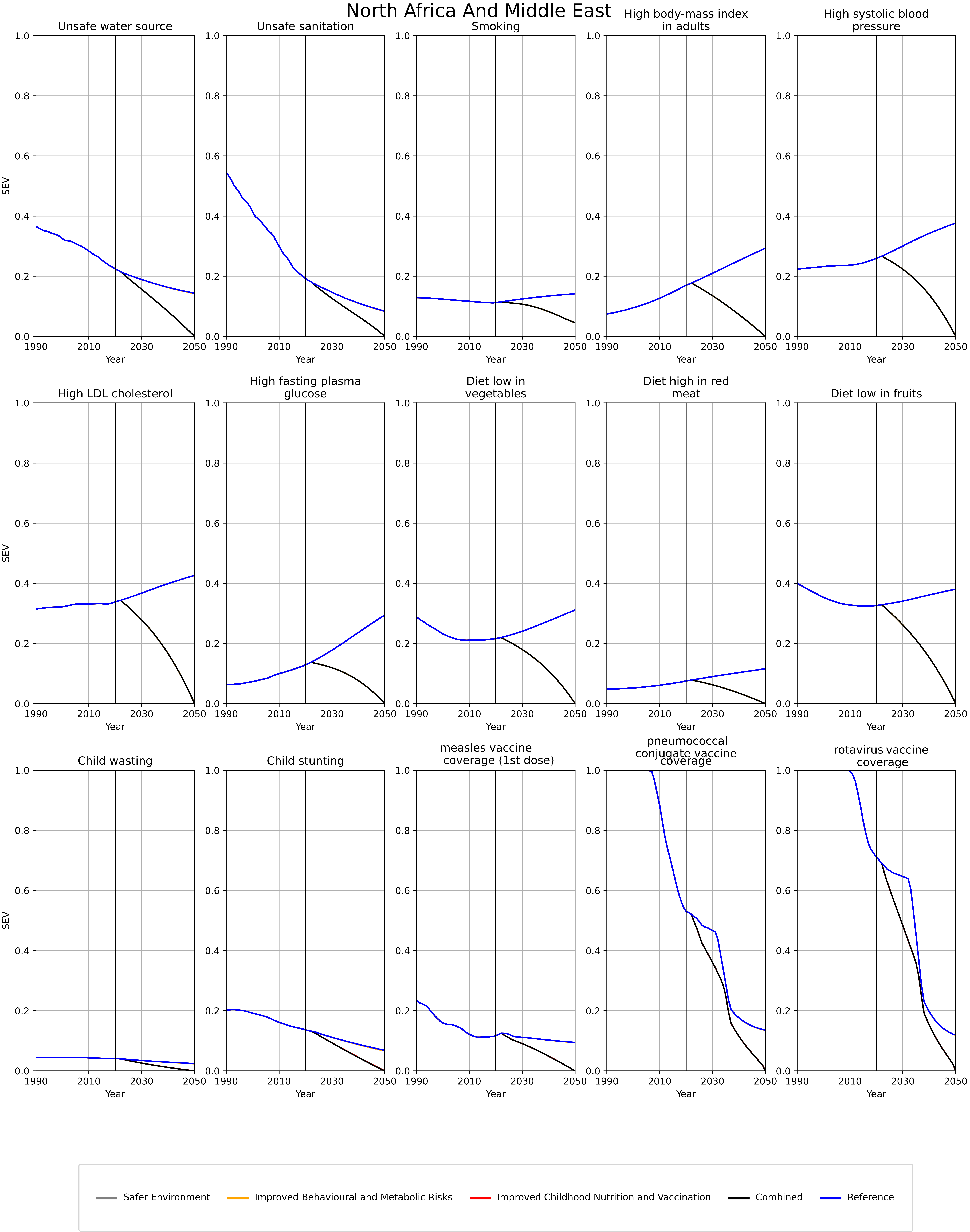
North Africa And Middle East
Unsafe water source
Unsafe sanitation
Smoking
High body-mass index in adults
High systolic blood pressure
High LDL cholesterol
High fasting plasma glucose
Diet low in vegetables
Diet high in red meat
Diet low in fruits
Child wasting
Child stunting
measles vaccine coverage (1st dose)
pneumococcal conjugate vaccine coverage
rotavirus vaccine coverage
SEV
Year
0.0
0.2
0.4
0.6
0.8
1.0
1990
2010
2030
2050
Safer Environment
Improved Behavioural and Metabolic Risks
Improved Childhood Nutrition and Vaccination
Combined
Reference

Supplemental Results Figure S1. Select risk factor Summary Exposure Values (SEVs) by global and GBD super region and scenario, 1990-2050

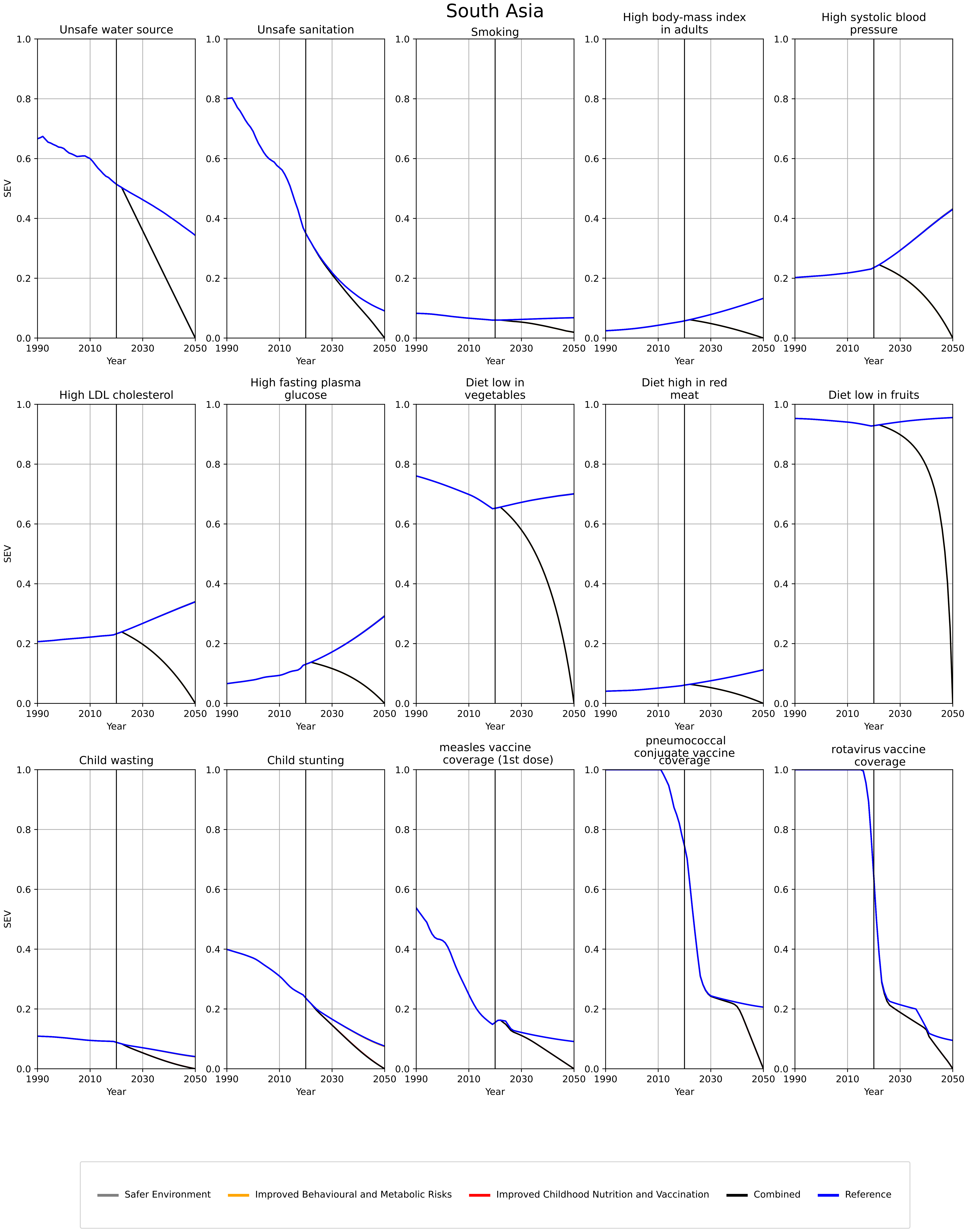

Supplemental Results Figure S1. Select risk factor Summary Exposure Values (SEVs) by global and GBD super region and scenario, 1990-2050

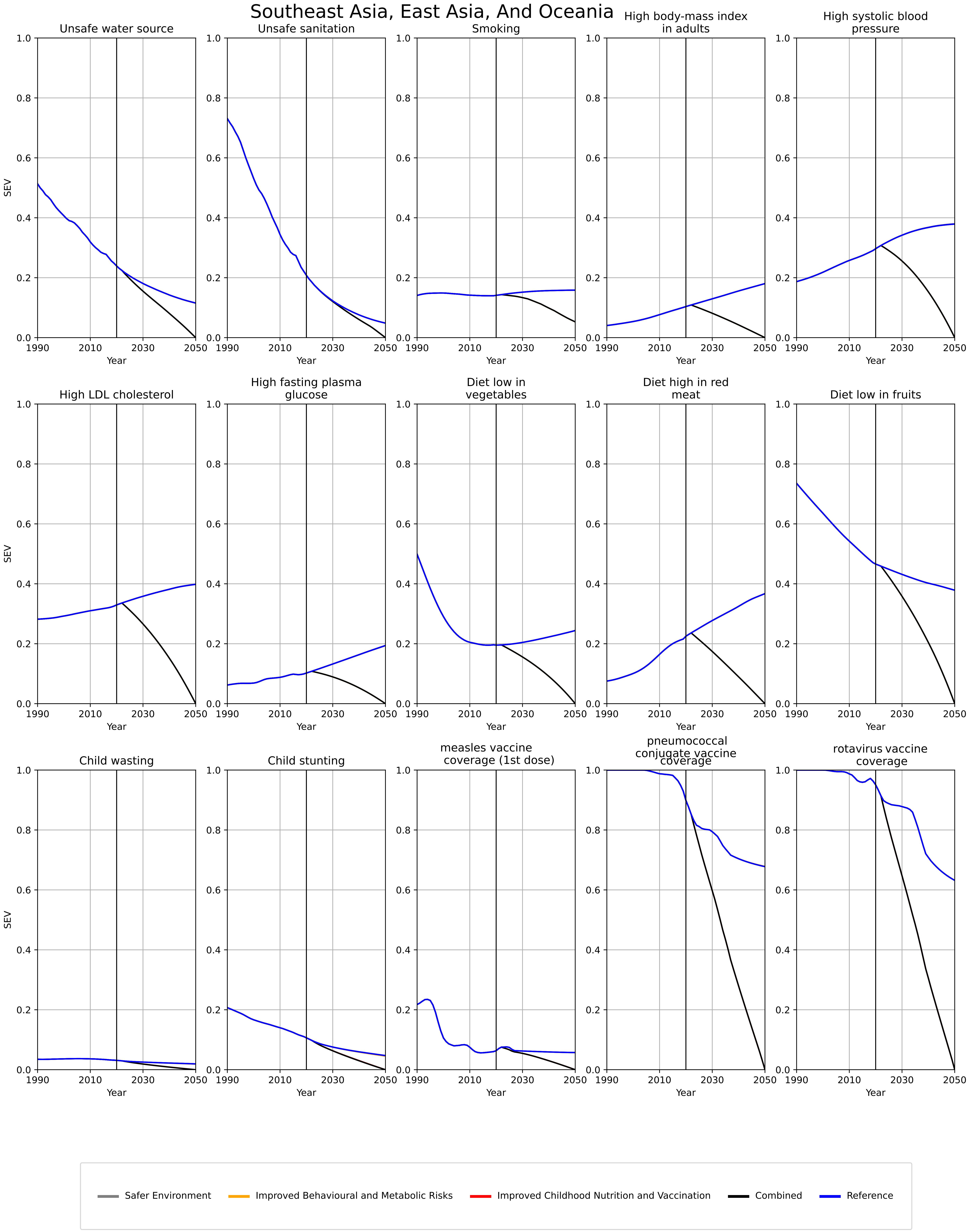

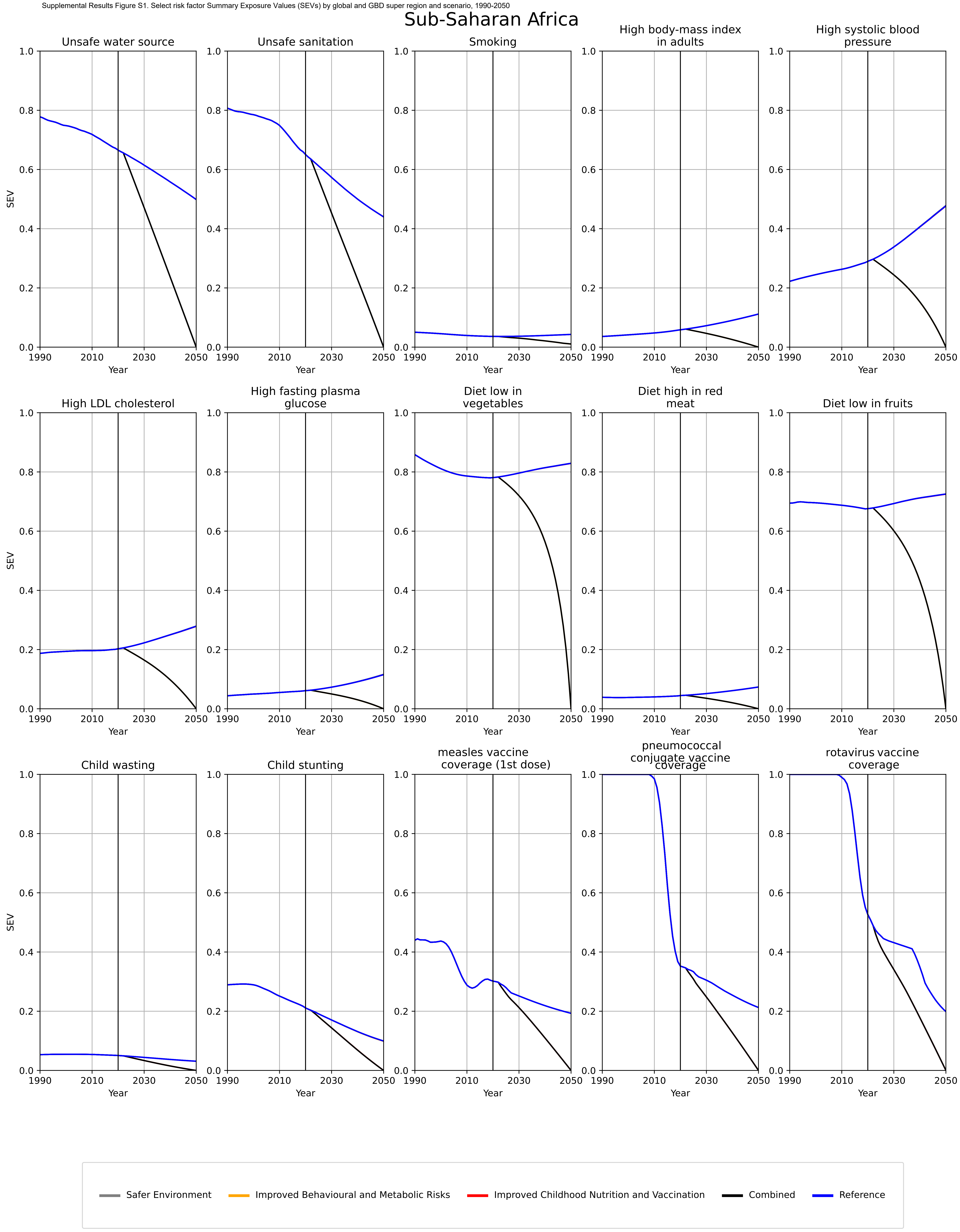
Sub-Saharan Africa
Unsafe water source
Unsafe sanitation
Smoking
High body-mass index in adults
High systolic blood pressure
High LDL cholesterol
High fasting plasma glucose
Diet low in vegetables
Diet high in red meat
Diet low in fruits
Child wasting
Child stunting
measles vaccine coverage (1st dose)
pneumococcal conjugate vaccine coverage
rotavirus vaccine coverage
SEV
Year
1.0
0.8
0.6
0.4
0.2
0.0
1990
2010
2030
2050
Safer Environment
Improved Behavioural and Metabolic Risks
Improved Childhood Nutrition and Vaccination
Combined
Reference

Supplemental Results Figure S2. Global and super-regional healthy life expectancy, 1990–2050 for females (A) and males (B)

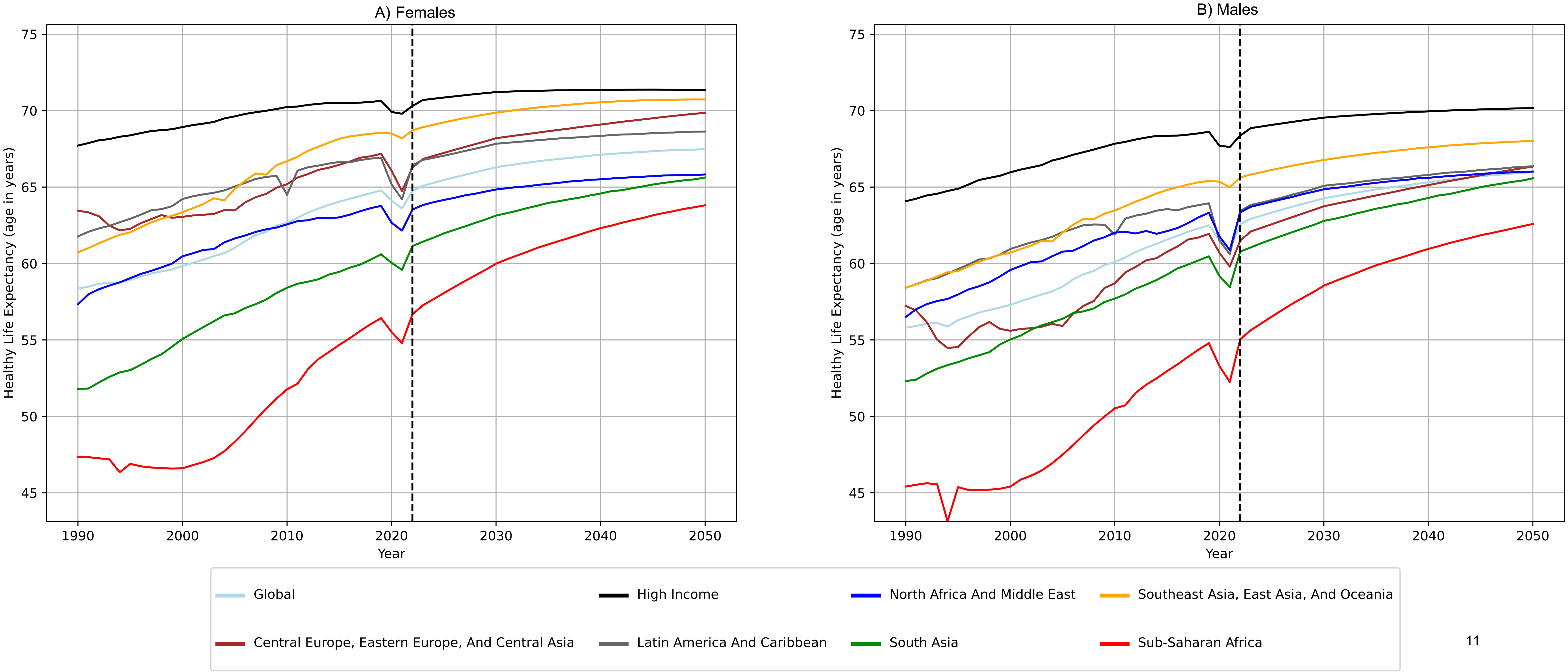

Armenia

Supplemental Results Figure S3. Life expectancy and HALE by sex and location, 1990-2050

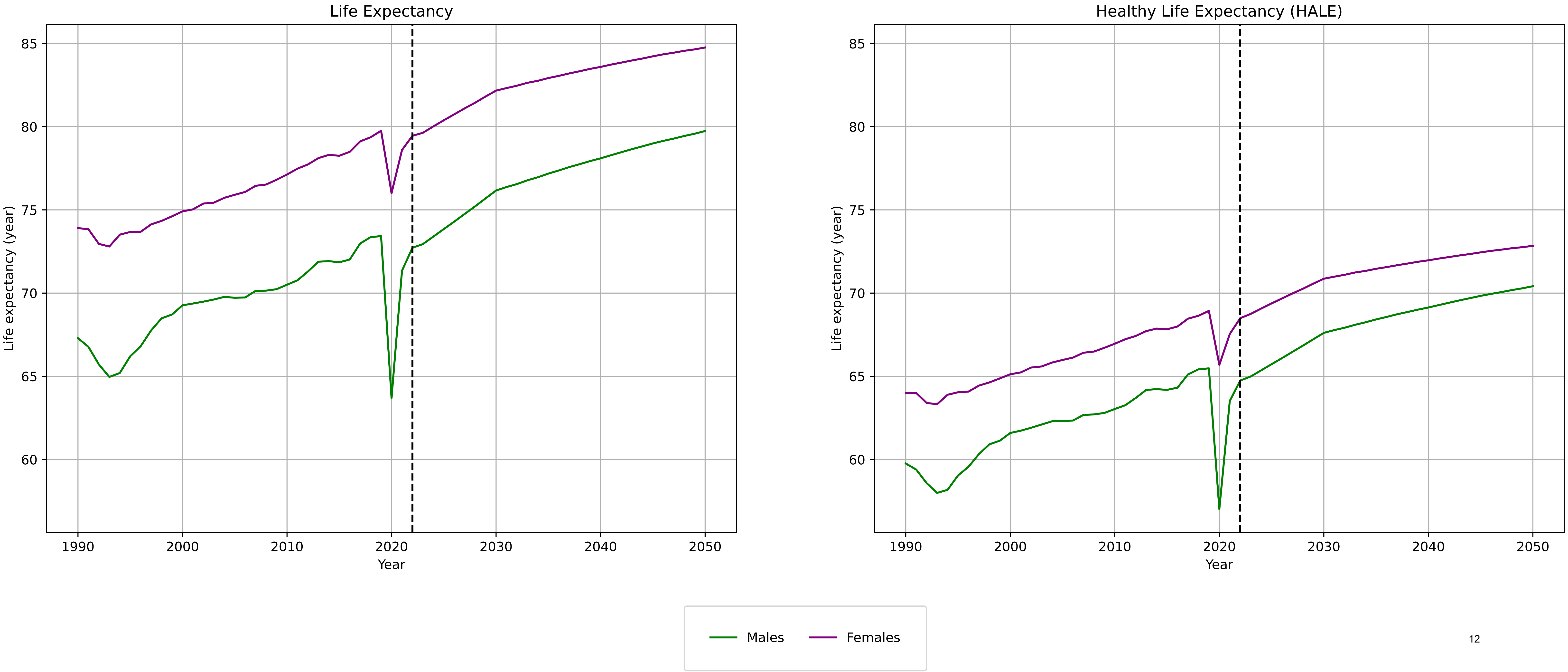

Azerbaijan

Supplemental Results Figure S3. Life expectancy and HALE by sex and location, 1990-2050

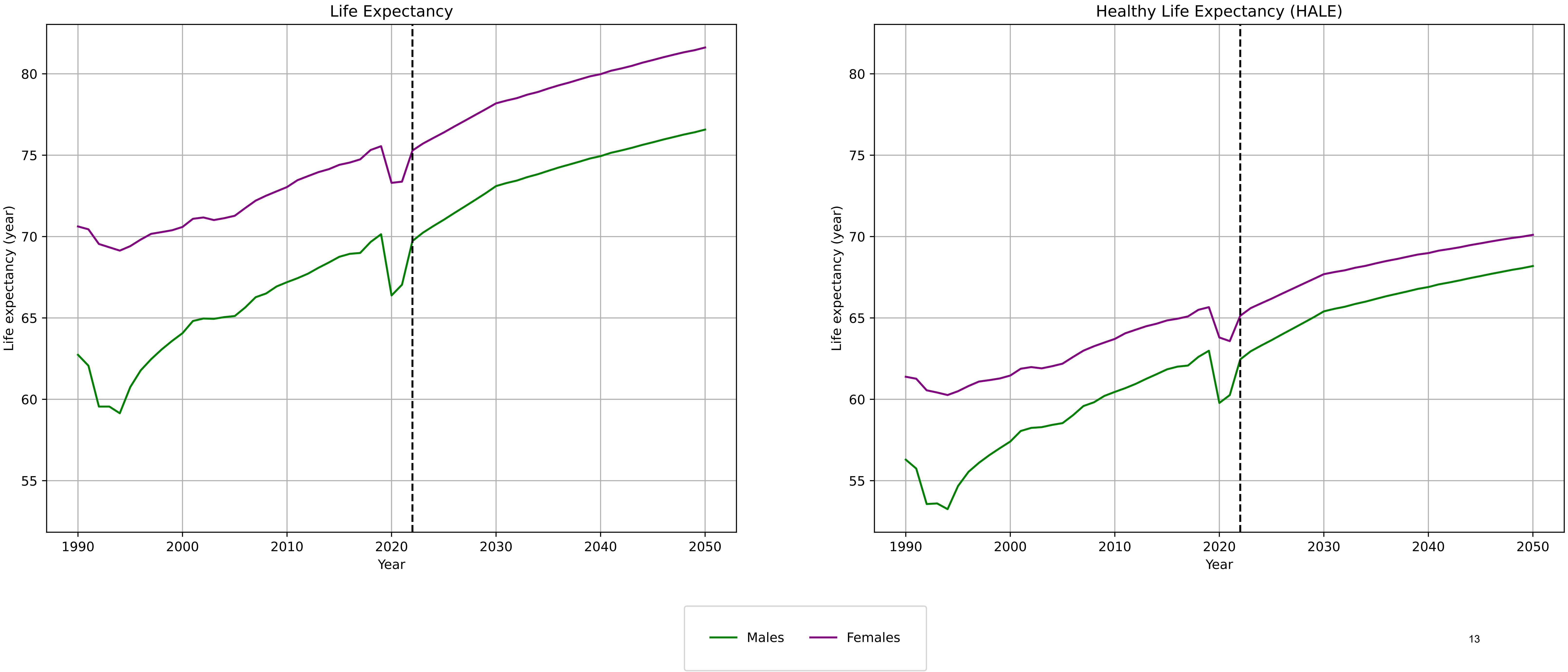

Georgia

Supplemental Results Figure S3. Life expectancy and HALE by sex and location, 1990-2050

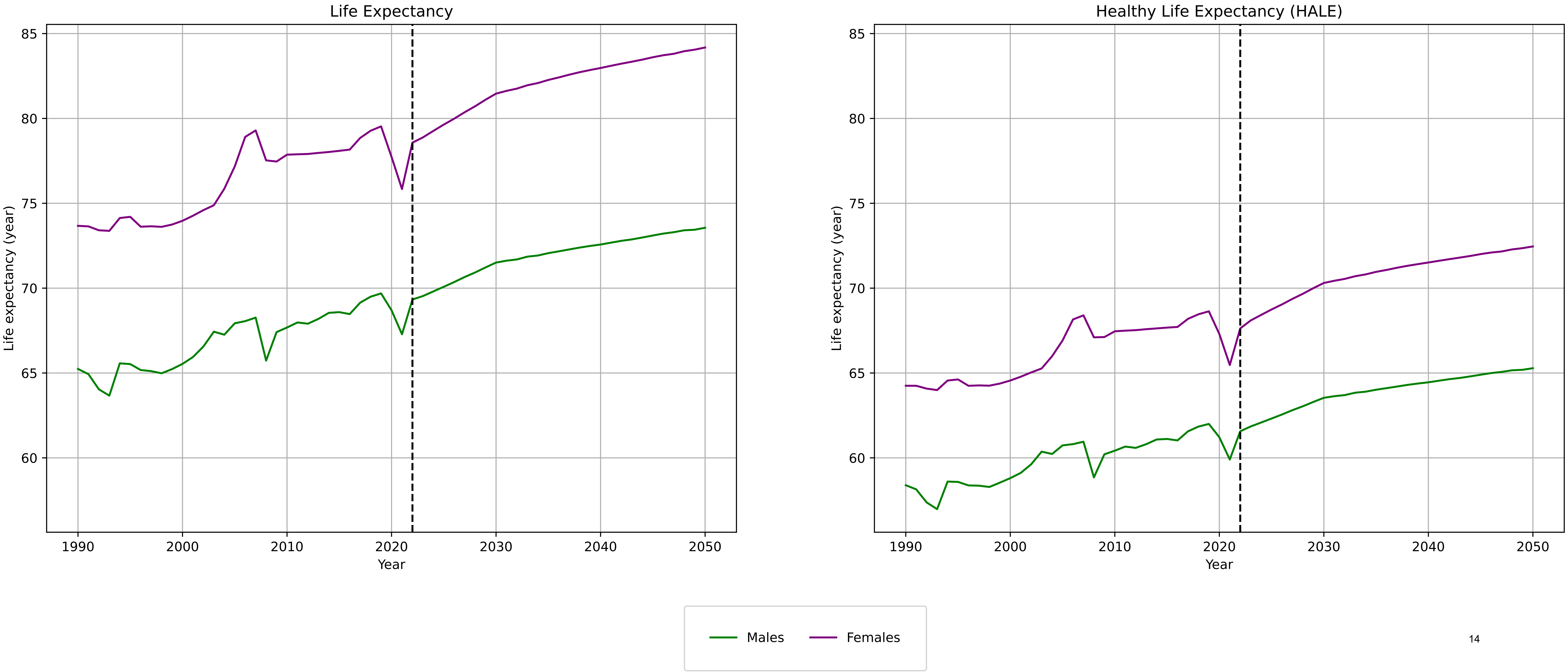

Supplemental Results Figure S3. Life expectancy and HALE by sex and location, 1990-2050

Kazakhstan

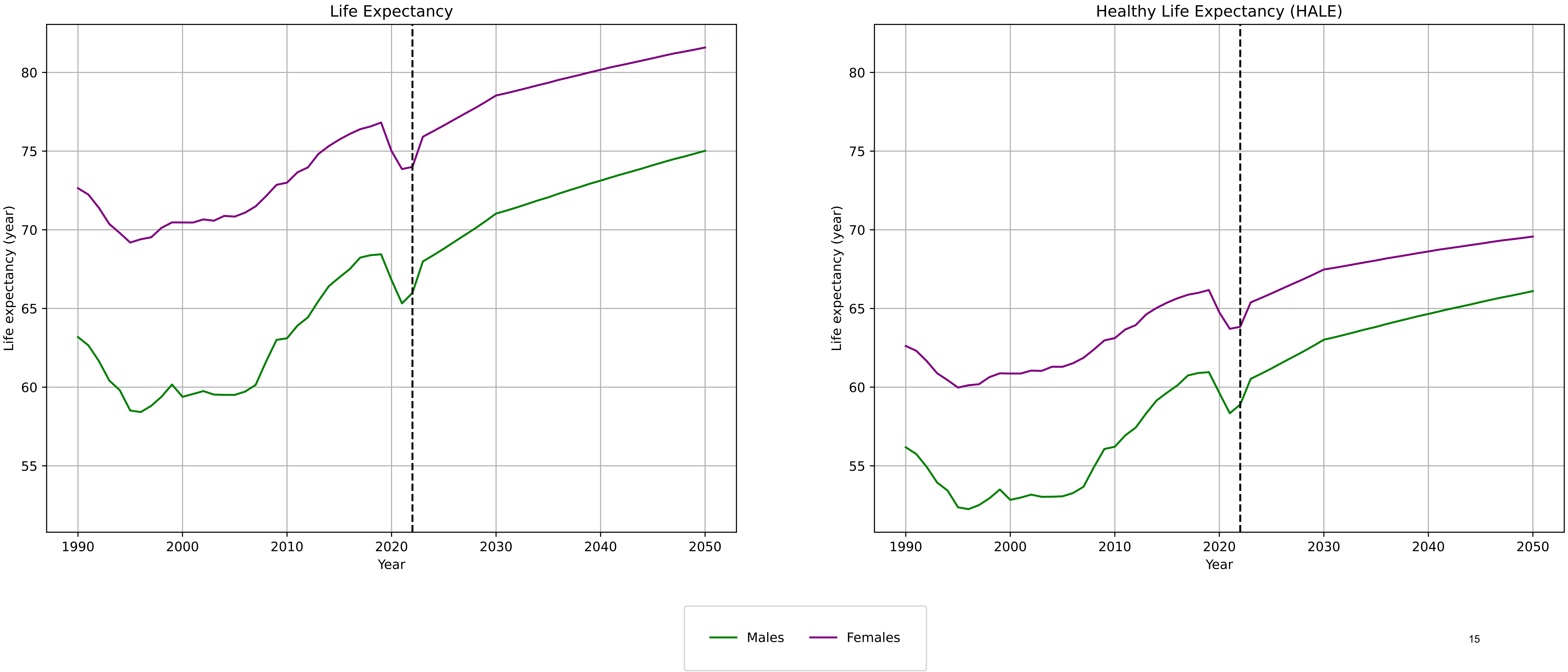

Supplemental Results Figure S3. Life expectancy and HALE by sex and location, 1990-2050

Kyrgyzstan

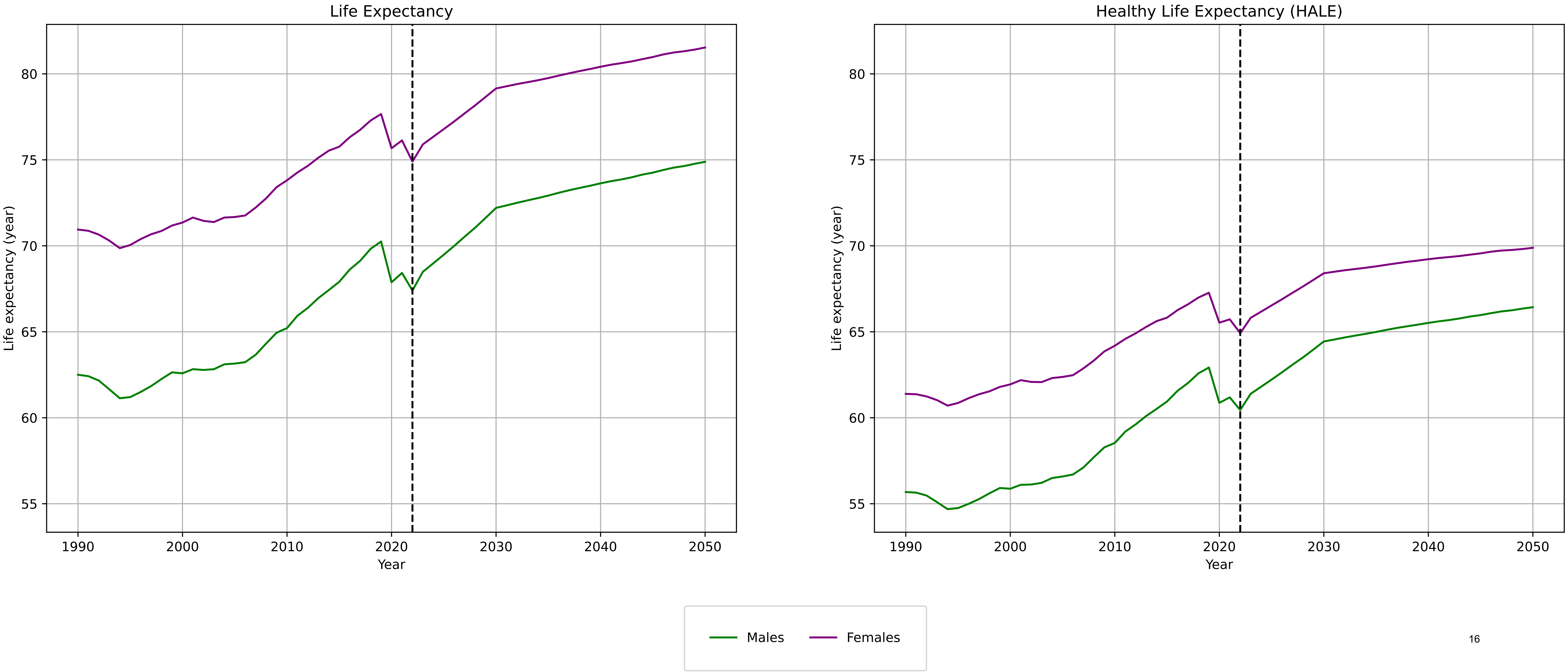

Supplemental Results Figure S3. Life expectancy and HALE by sex and location, 1990-2050

Mongolia

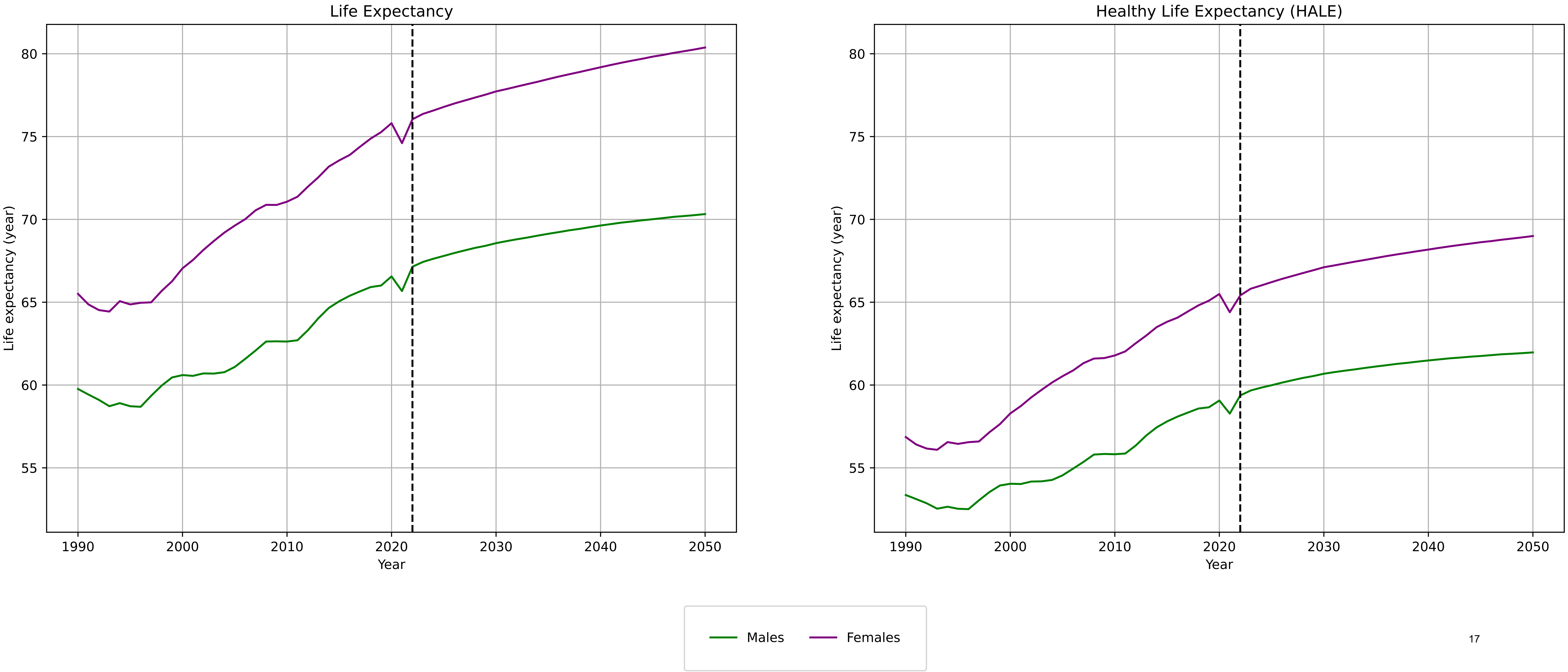

Tajikistan

Supplemental Results Figure S3. Life expectancy and HALE by sex and location, 1990-2050

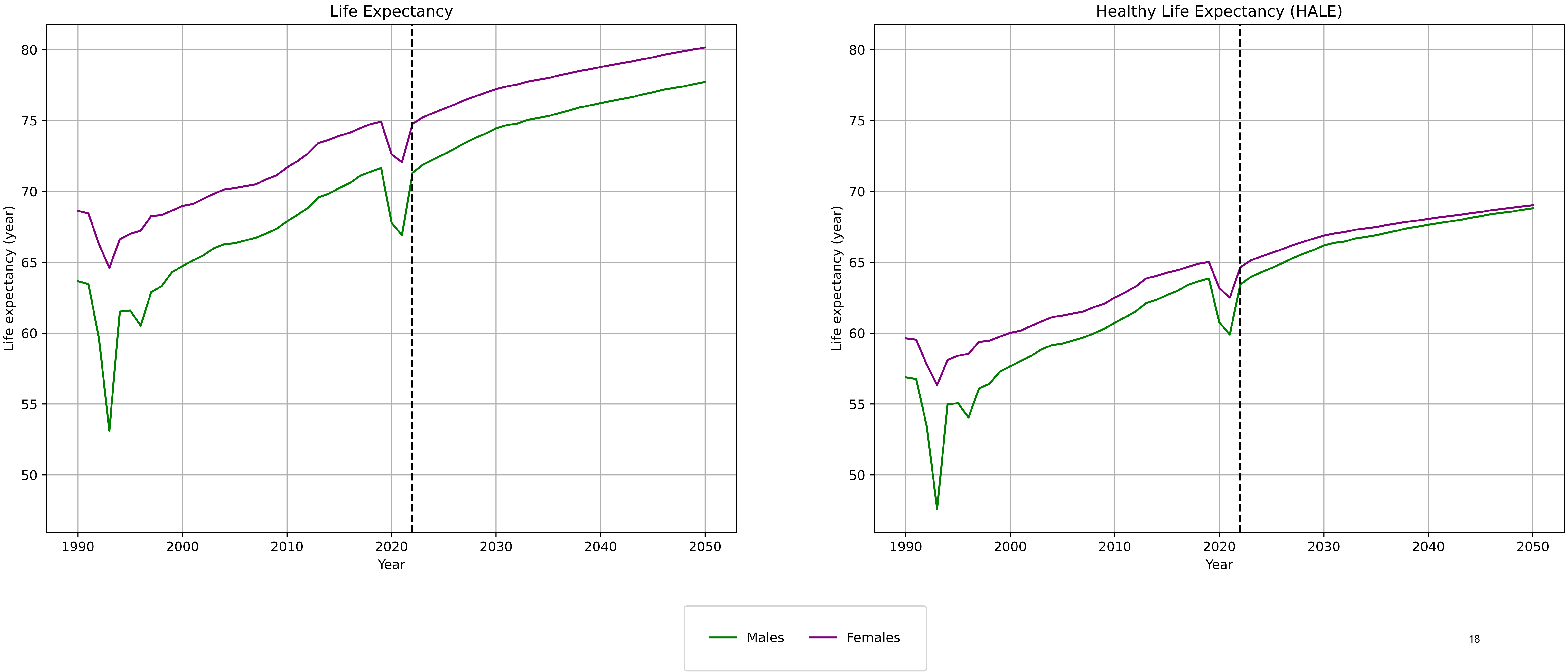

Turkmenistan

Supplemental Results Figure S3. Life expectancy and HALE by sex and location, 1990-2050

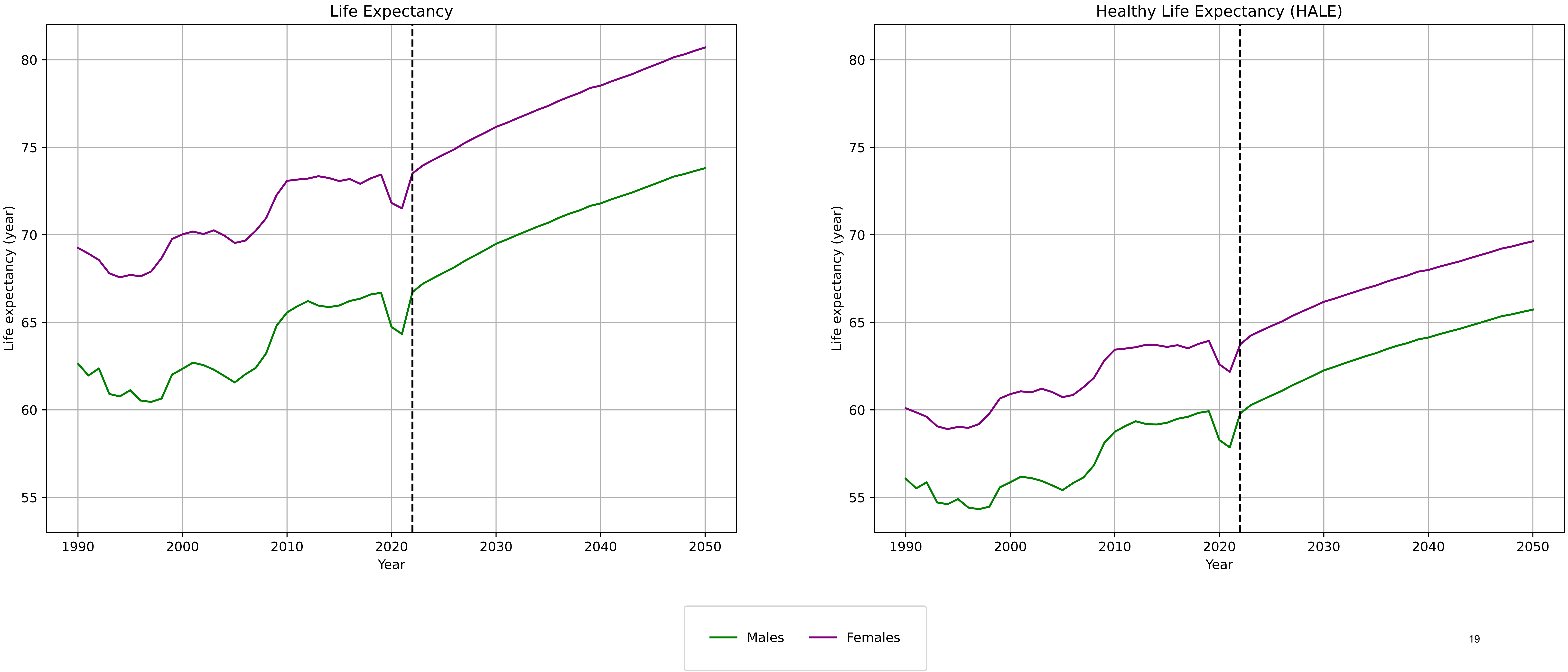

Uzbekistan

Supplemental Results Figure S3. Life expectancy and HALE by sex and location, 1990-2050

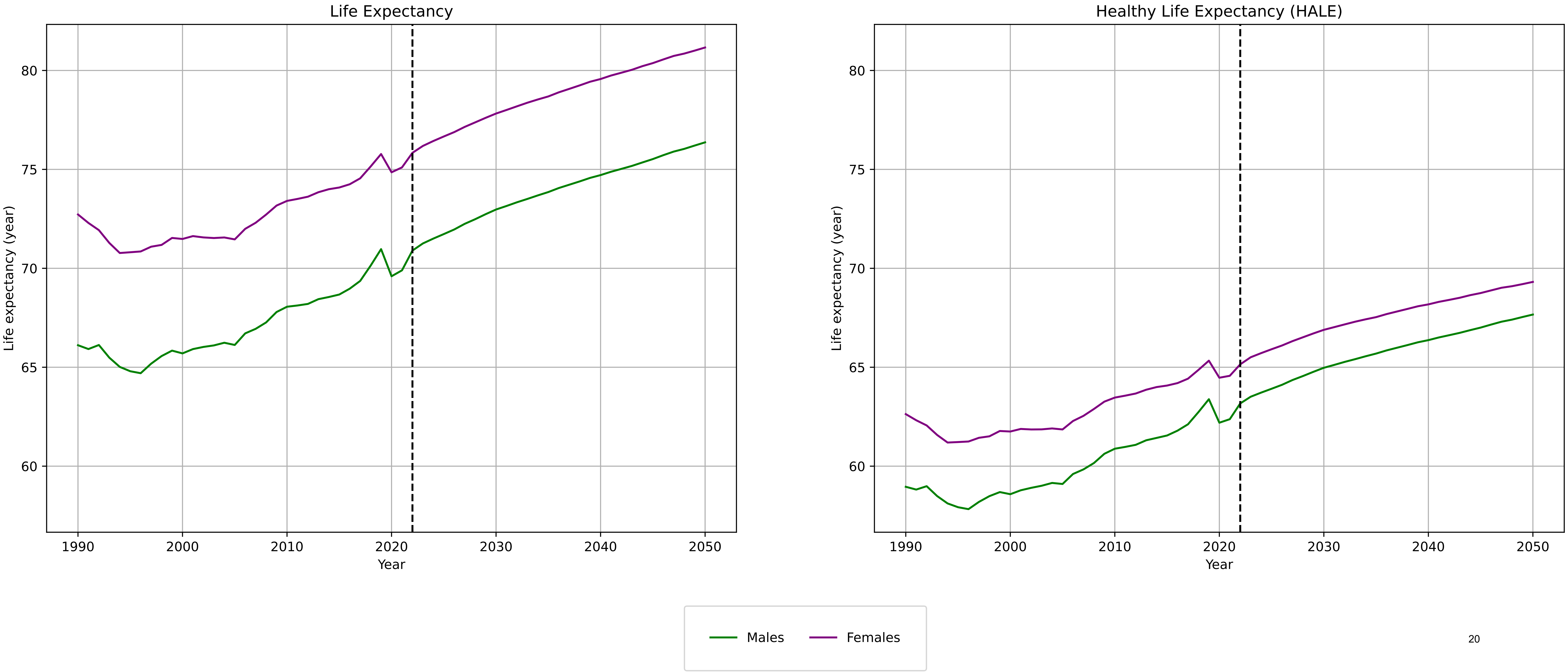

Albania

Supplemental Results Figure S3. Life expectancy and HALE by sex and location, 1990-2050

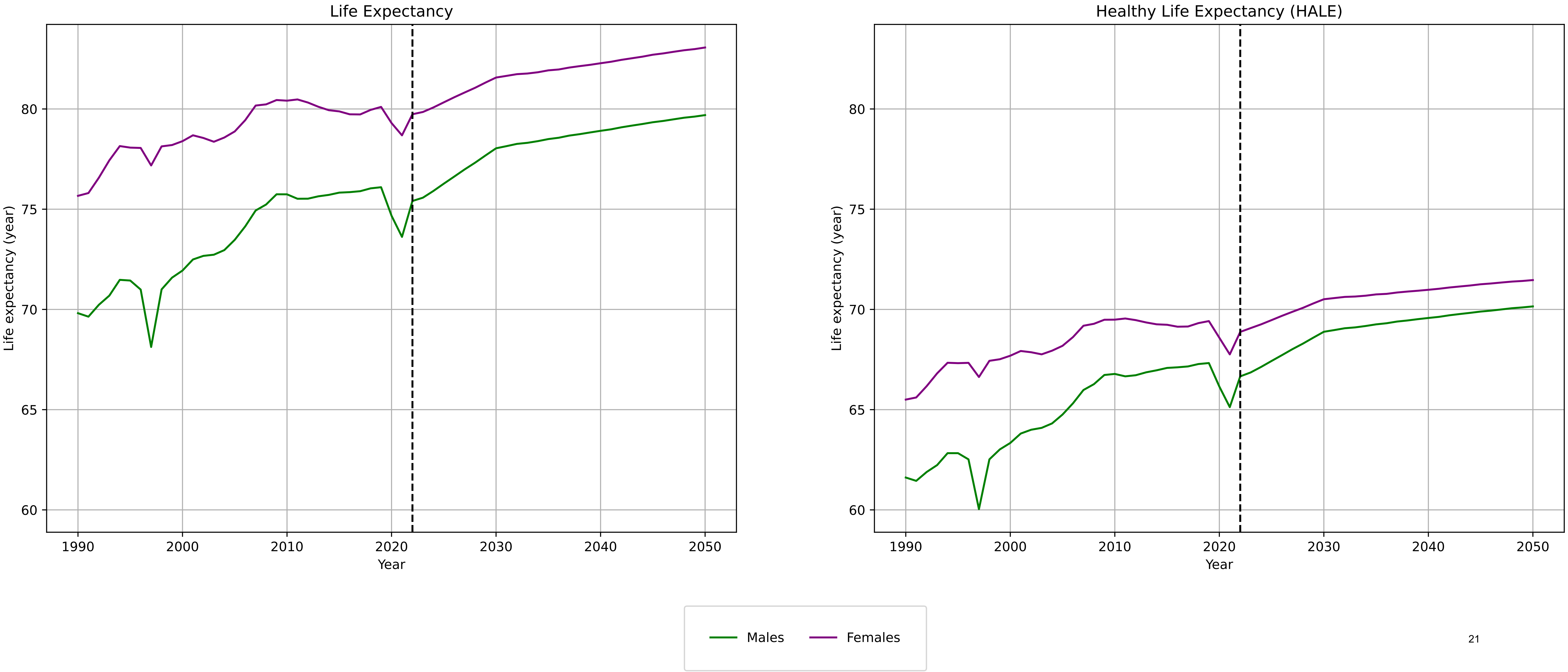

Bosnia And Herzegovina

Supplemental Results Figure S3. Life expectancy and HALE by sex and location, 1990-2050

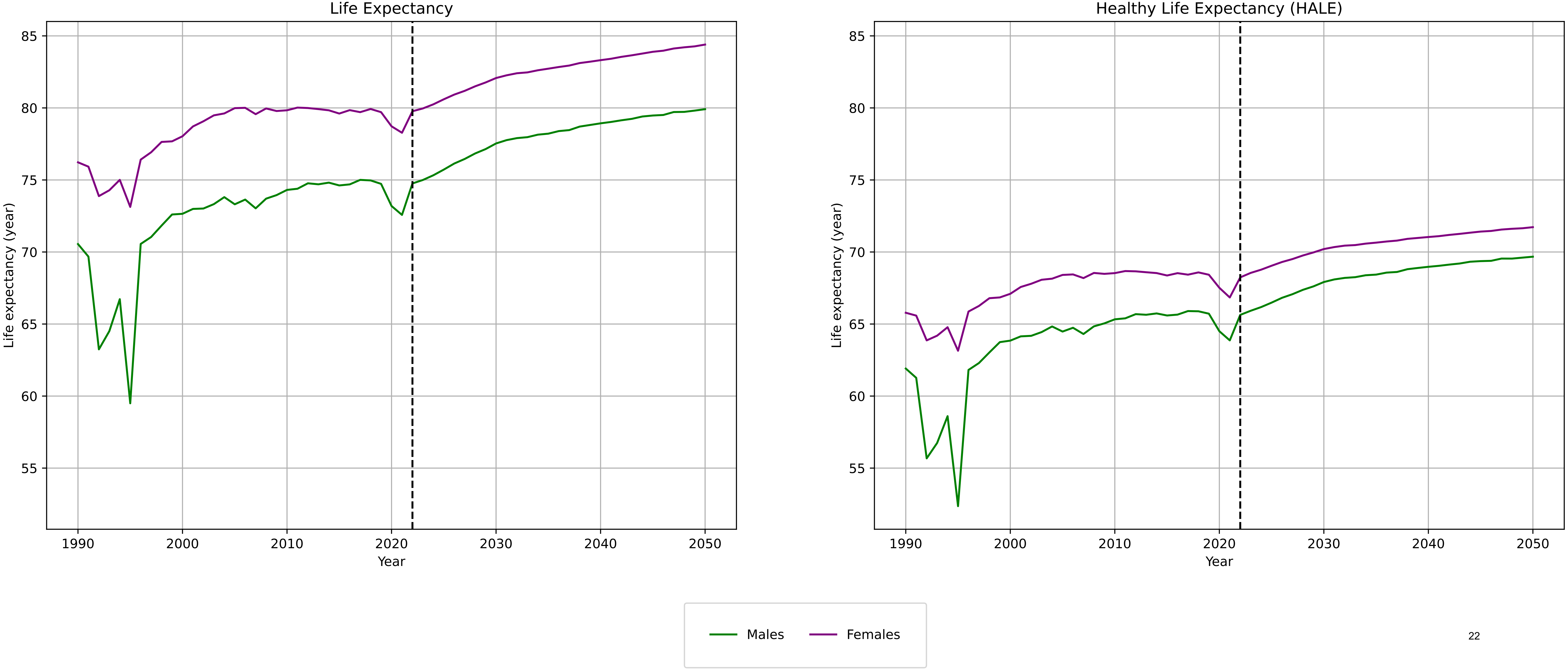

Bulgaria

Supplemental Results Figure S3. Life expectancy and HALE by sex and location, 1990-2050

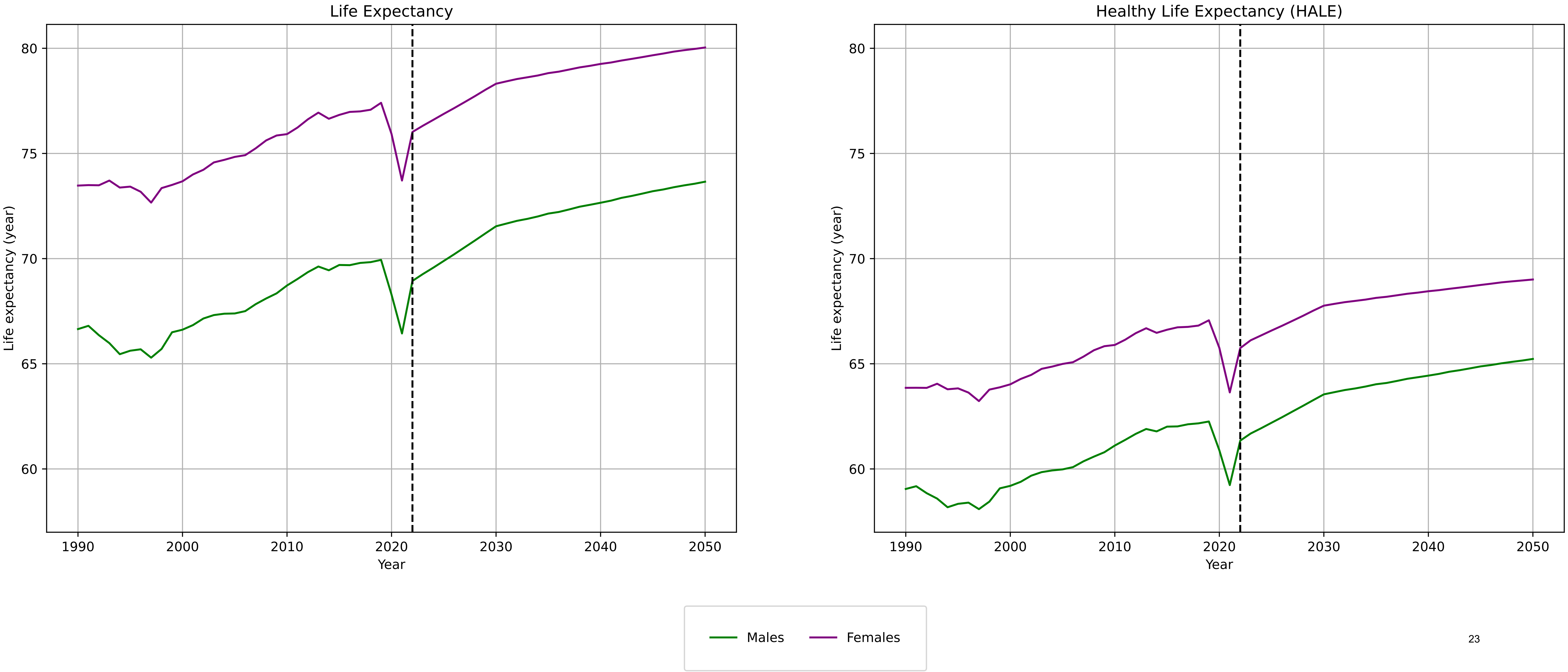

Croatia

Supplemental Results Figure S3. Life expectancy and HALE by sex and location, 1990-2050

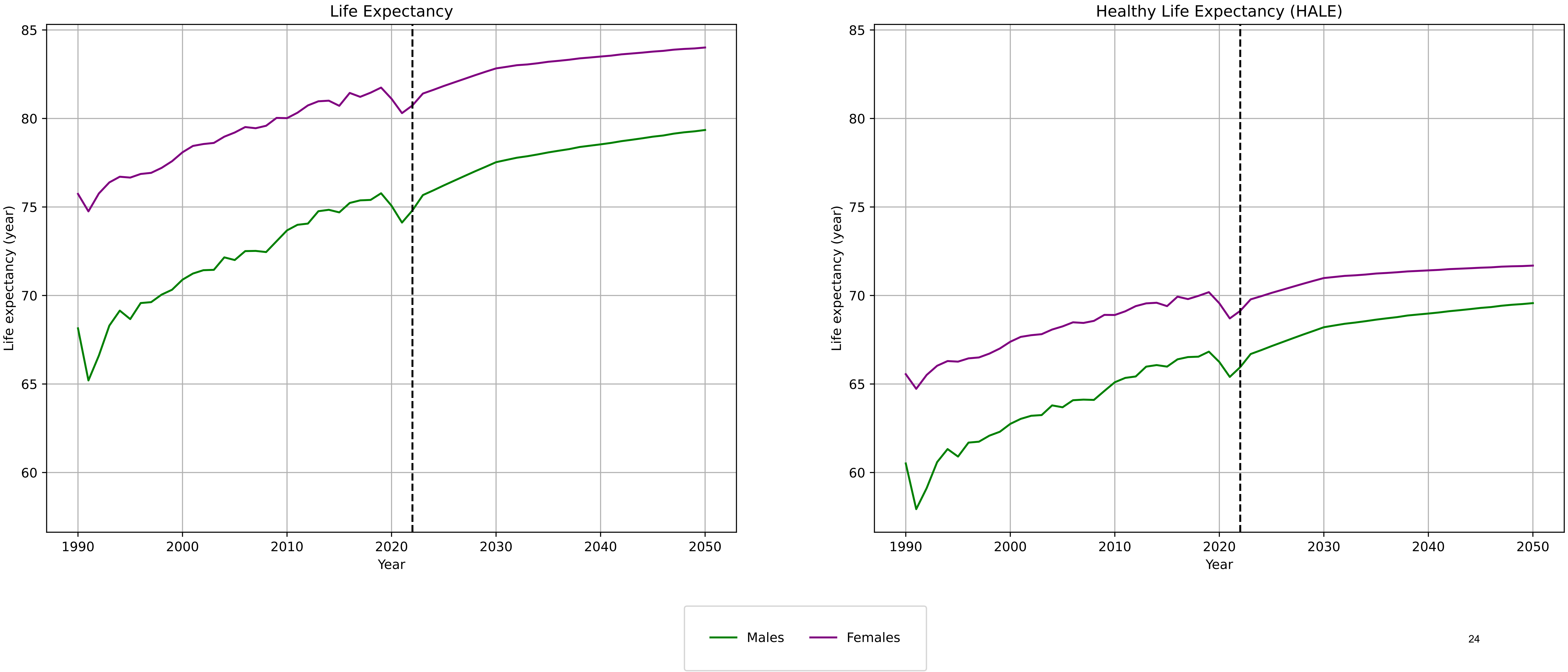

Czechia

Supplemental Results Figure S3. Life expectancy and HALE by sex and location, 1990-2050

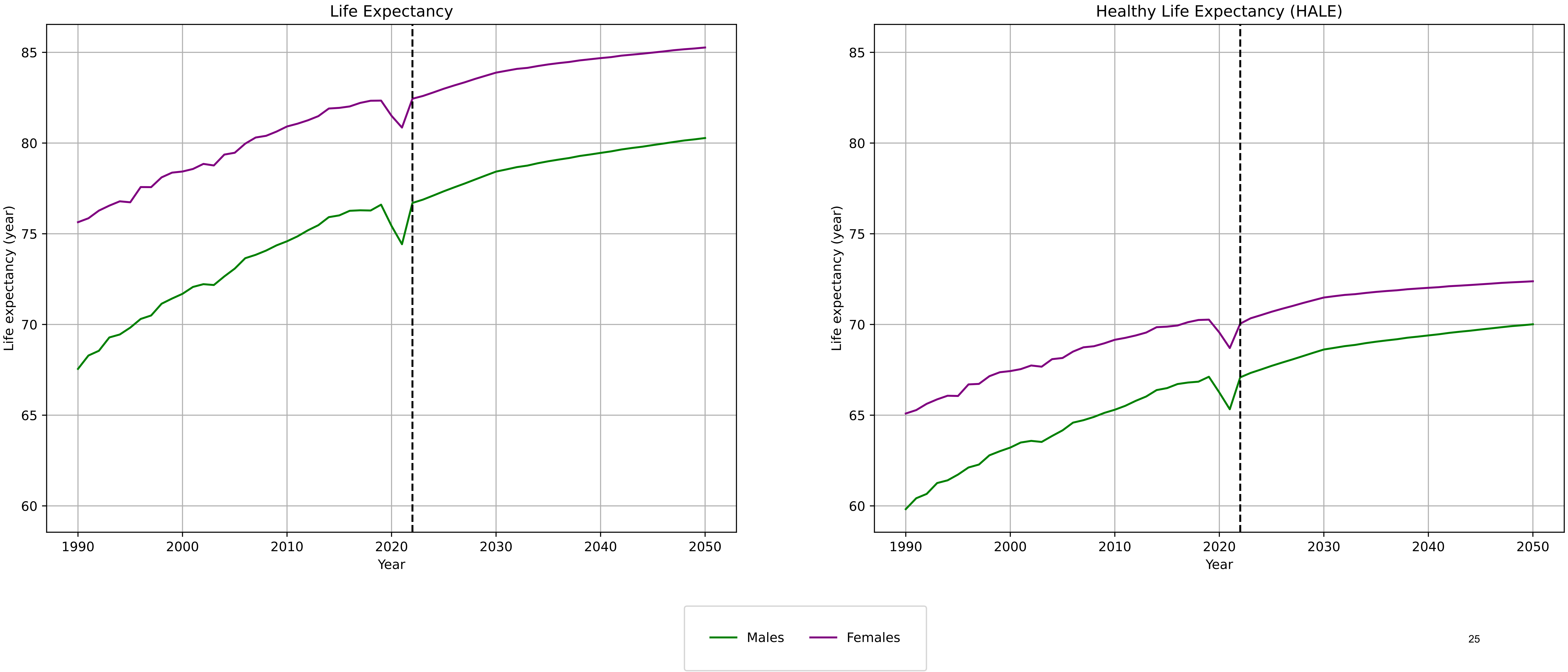

Hungary

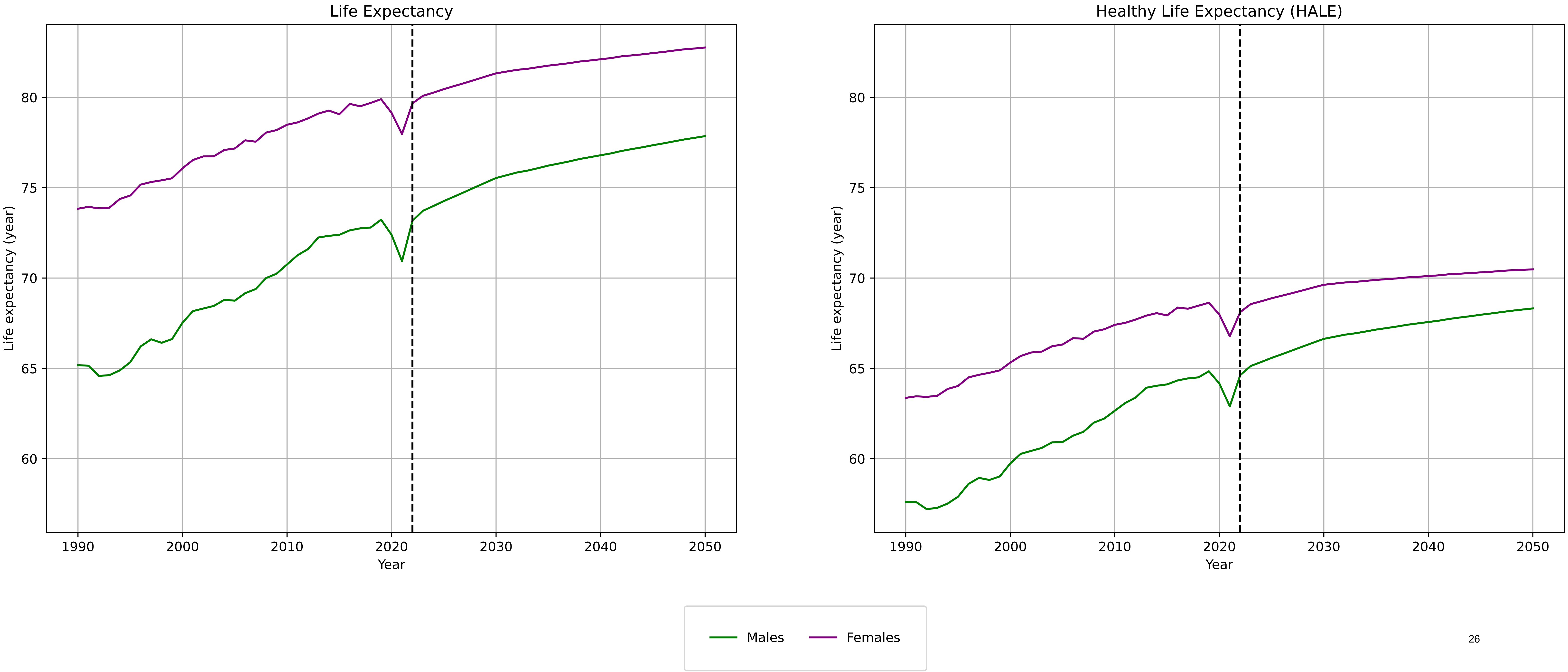
Life Expectancy
Healthy Life Expectancy (HALE)
Life expectancy (year)
Year
60
65
70
75
80
1990
2000
2010
2020
2030
2040
2050
Males
Females

Montenegro

Supplemental Results Figure S3. Life expectancy and HALE by sex and location, 1990-2050

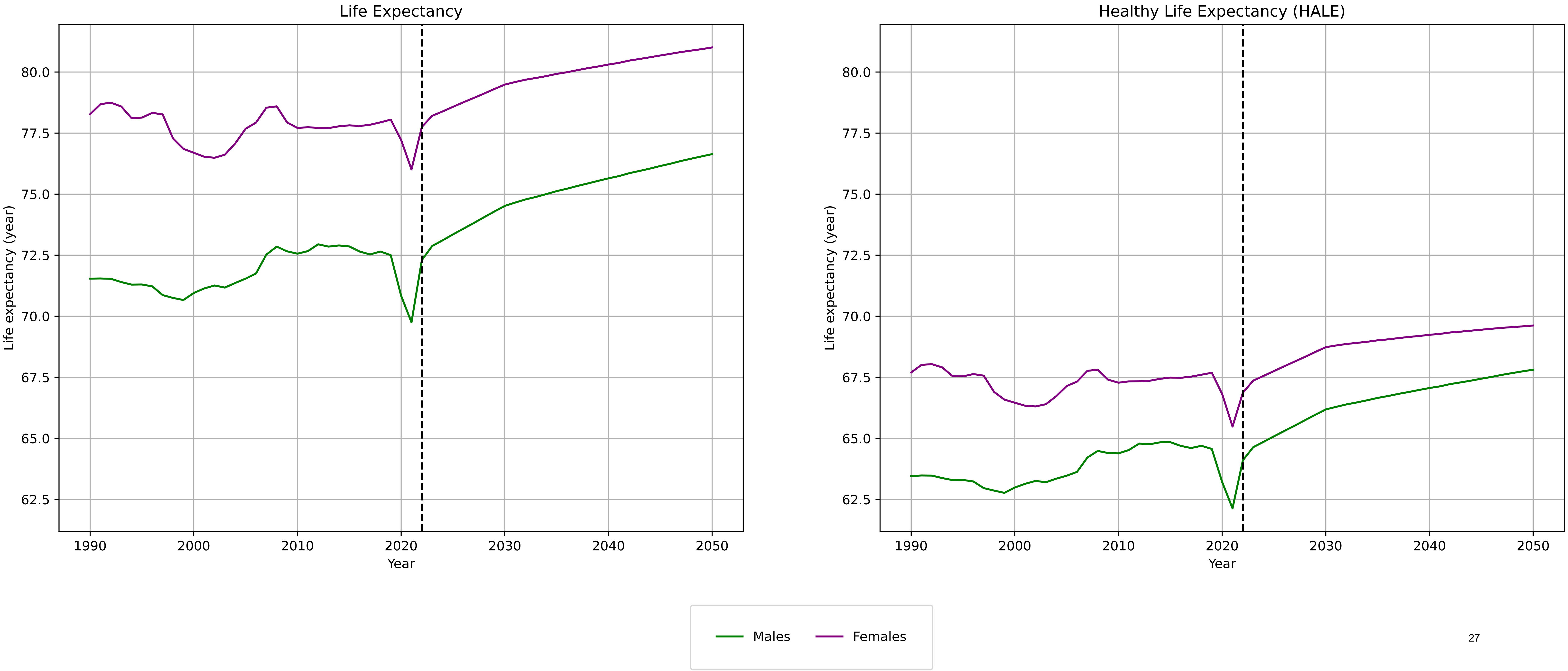

North Macedonia

Supplemental Results Figure S3. Life expectancy and HALE by sex and location, 1990-2050

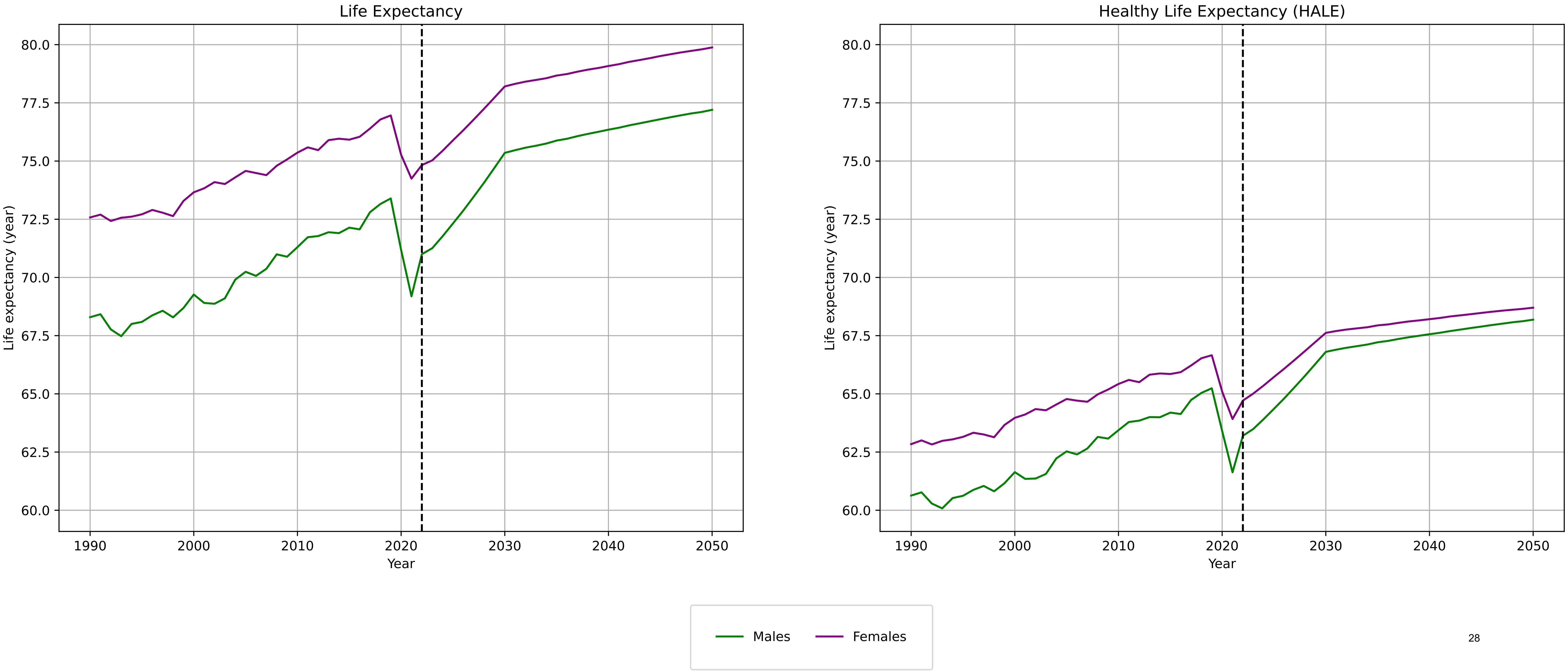

Poland

Supplemental Results Figure S3. Life expectancy and HALE by sex and location, 1990-2050

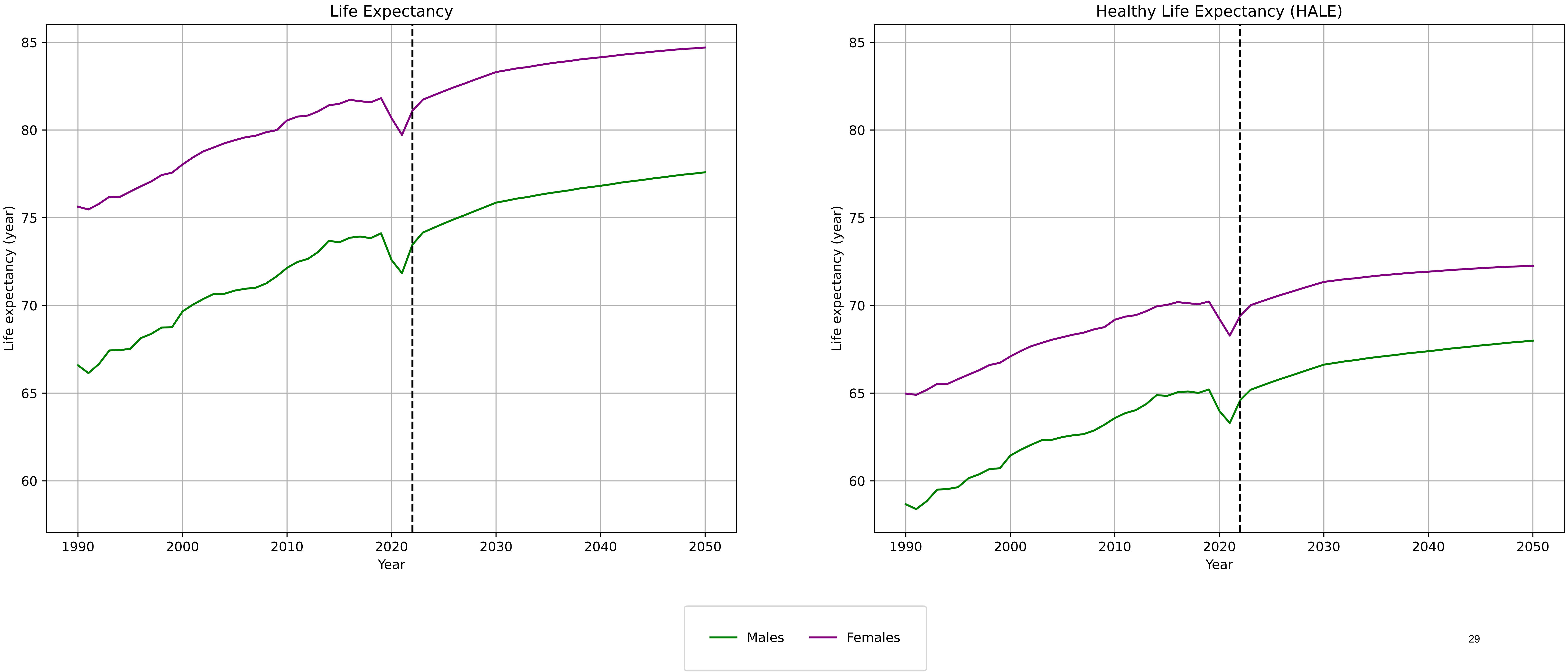

Supplemental Results Figure S3. Life expectancy and HALE by sex and location, 1990-2050

Romania

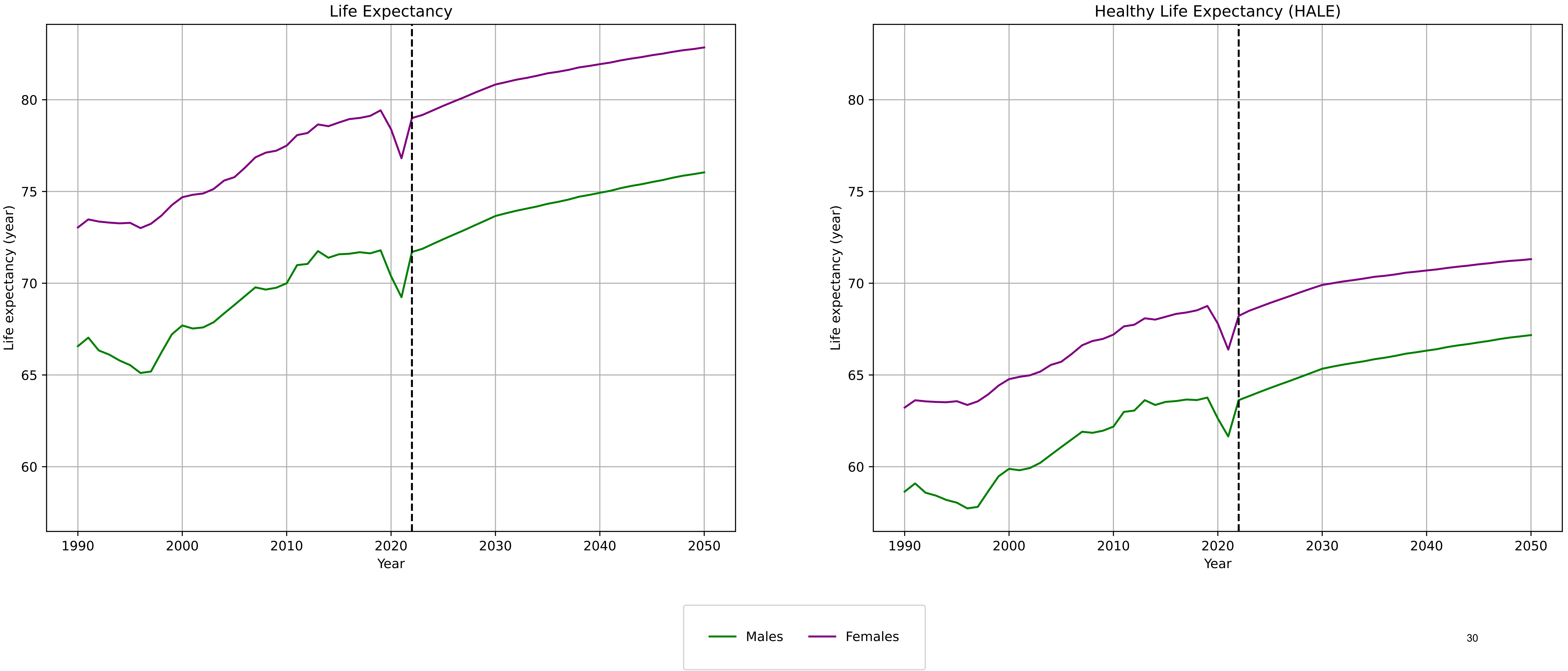

Serbia

Supplemental Results Figure S3. Life expectancy and HALE by sex and location, 1990-2050

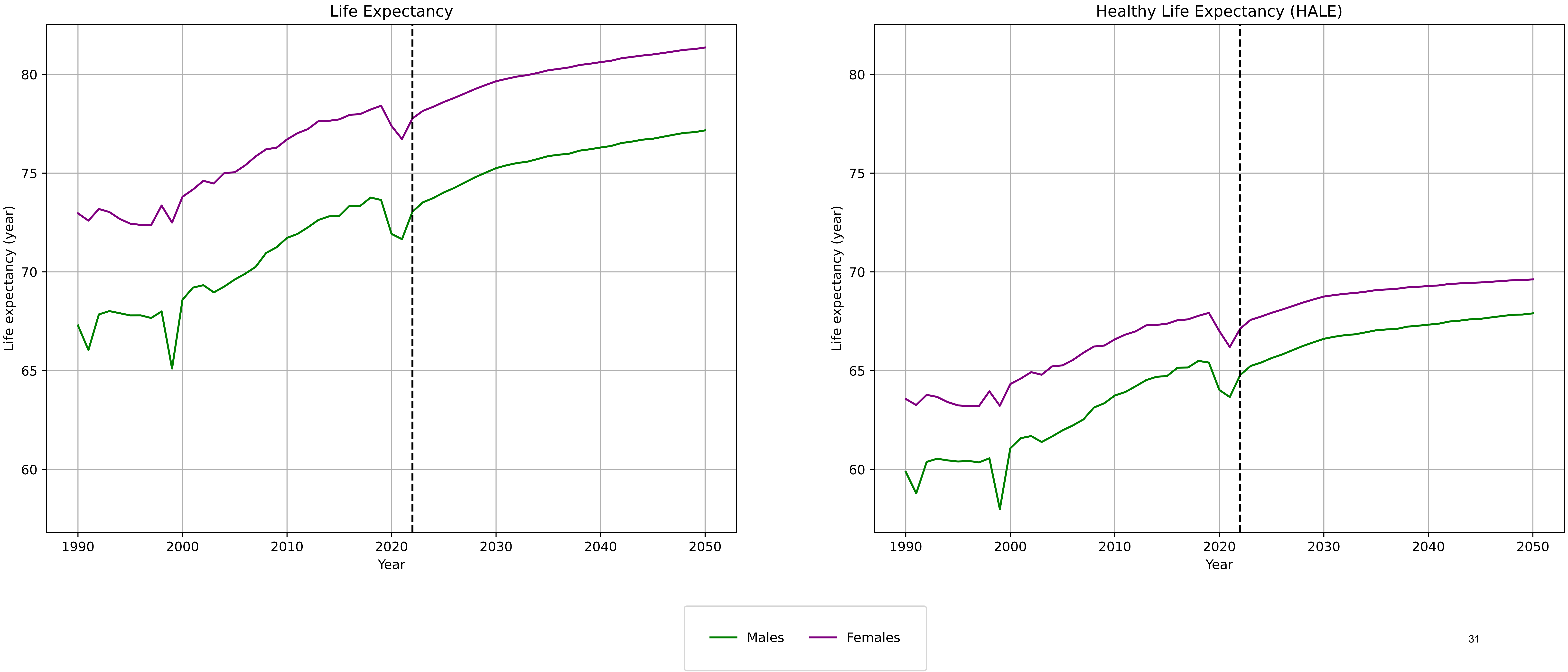

Slovakia

Supplemental Results Figure S3. Life expectancy and HALE by sex and location, 1990-2050

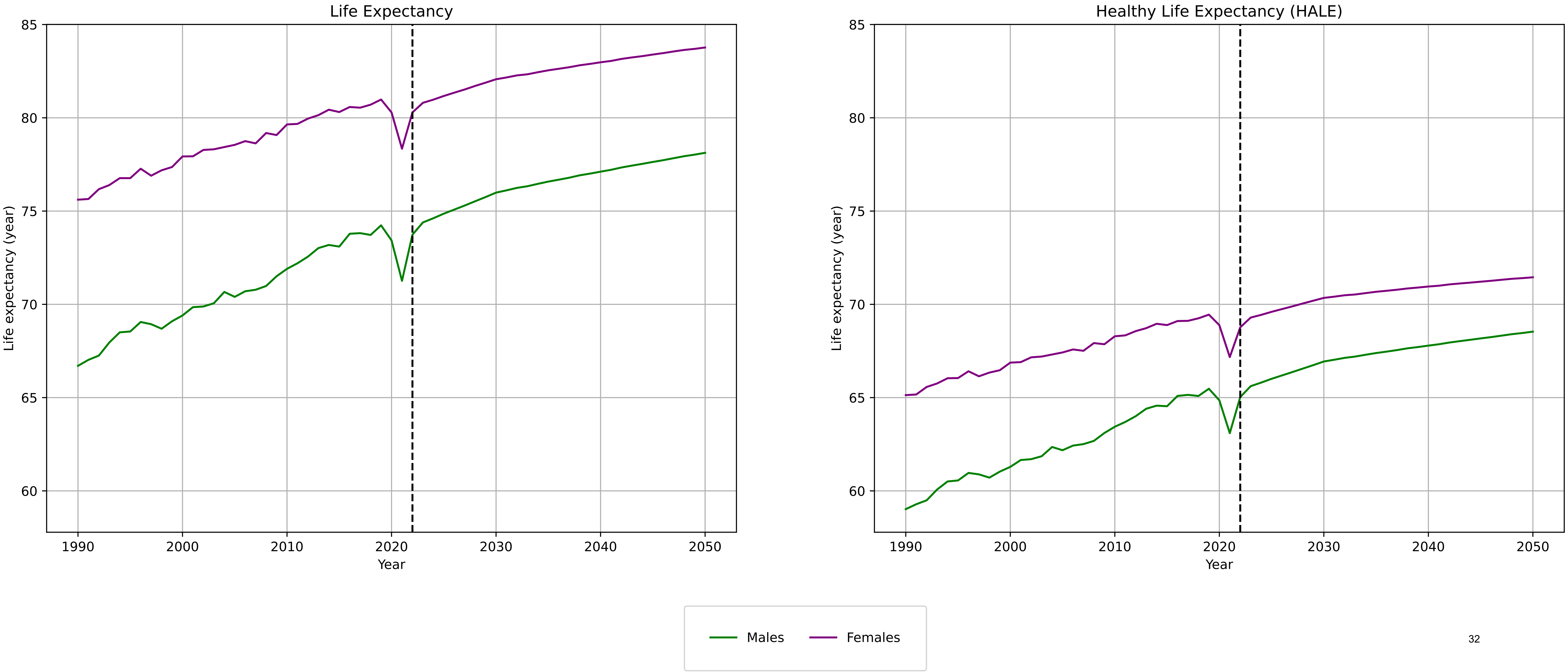

Slovenia

Supplemental Results Figure S3. Life expectancy and HALE by sex and location, 1990-2050

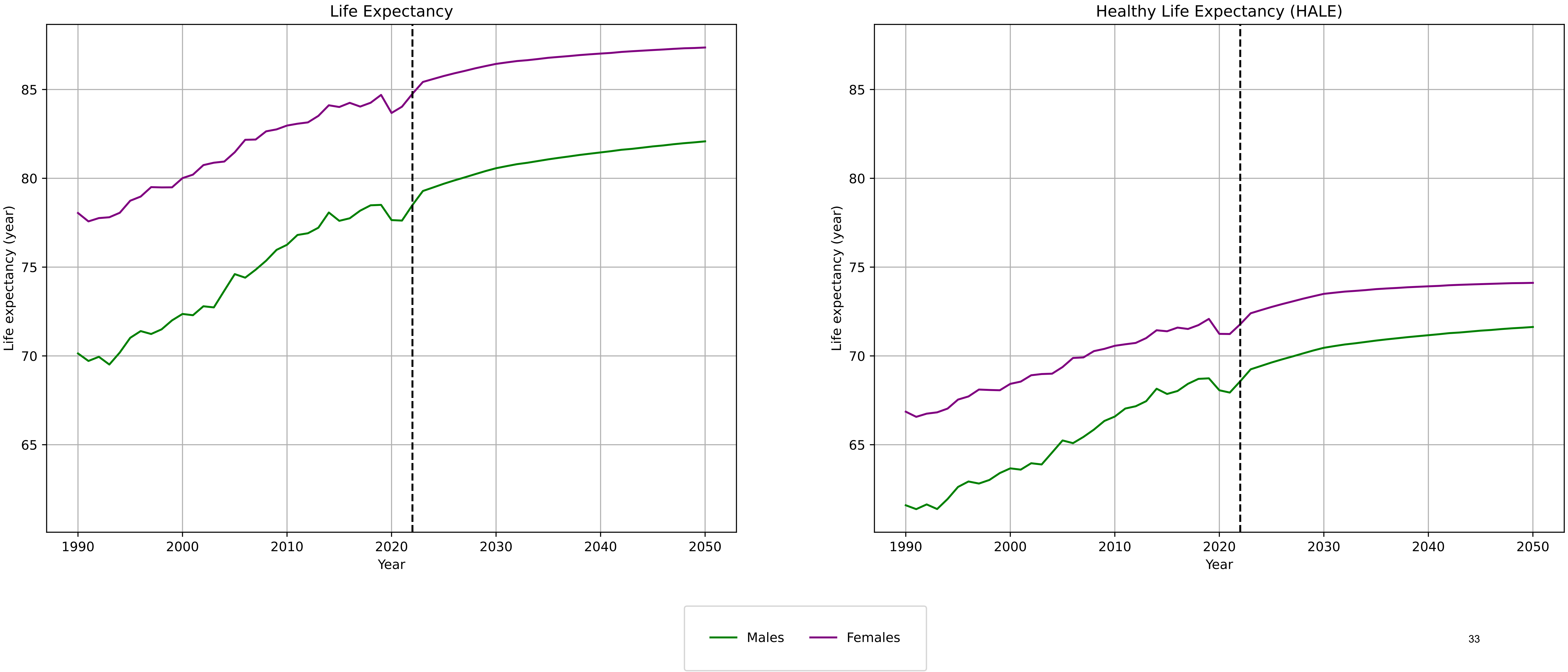

Belarus

Supplemental Results Figure S3. Life expectancy and HALE by sex and location, 1990-2050

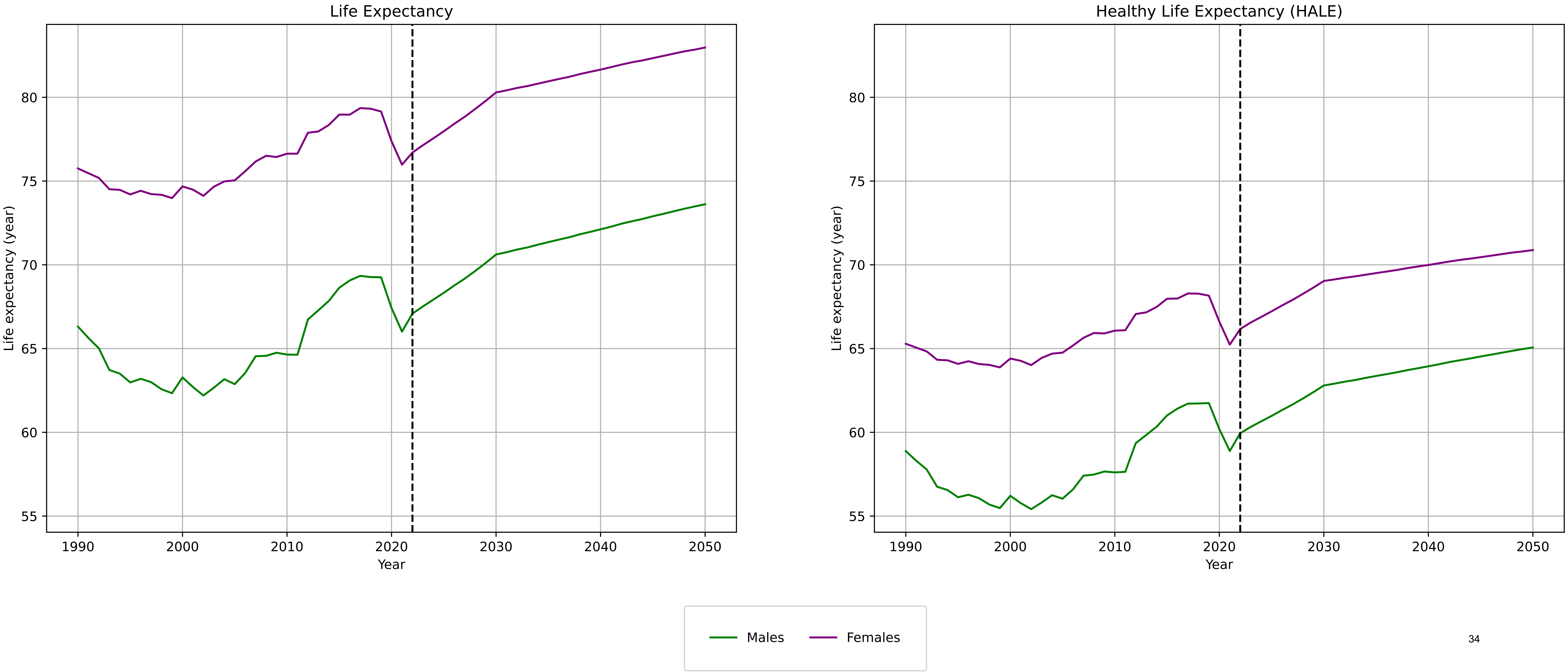

Supplemental Results Figure S3. Life expectancy and HALE by sex and location, 1990-2050

Estonia

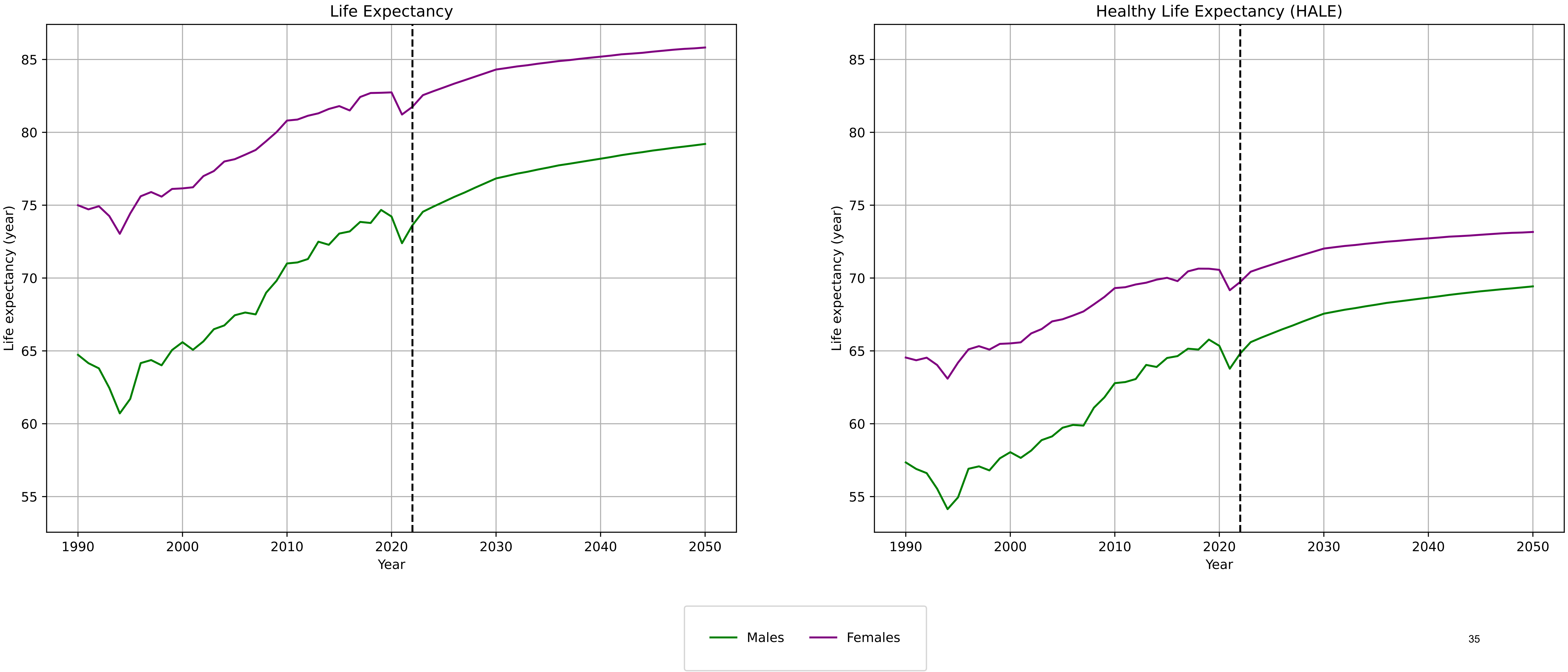

Latvia

Supplemental Results Figure S3. Life expectancy and HALE by sex and location, 1990-2050

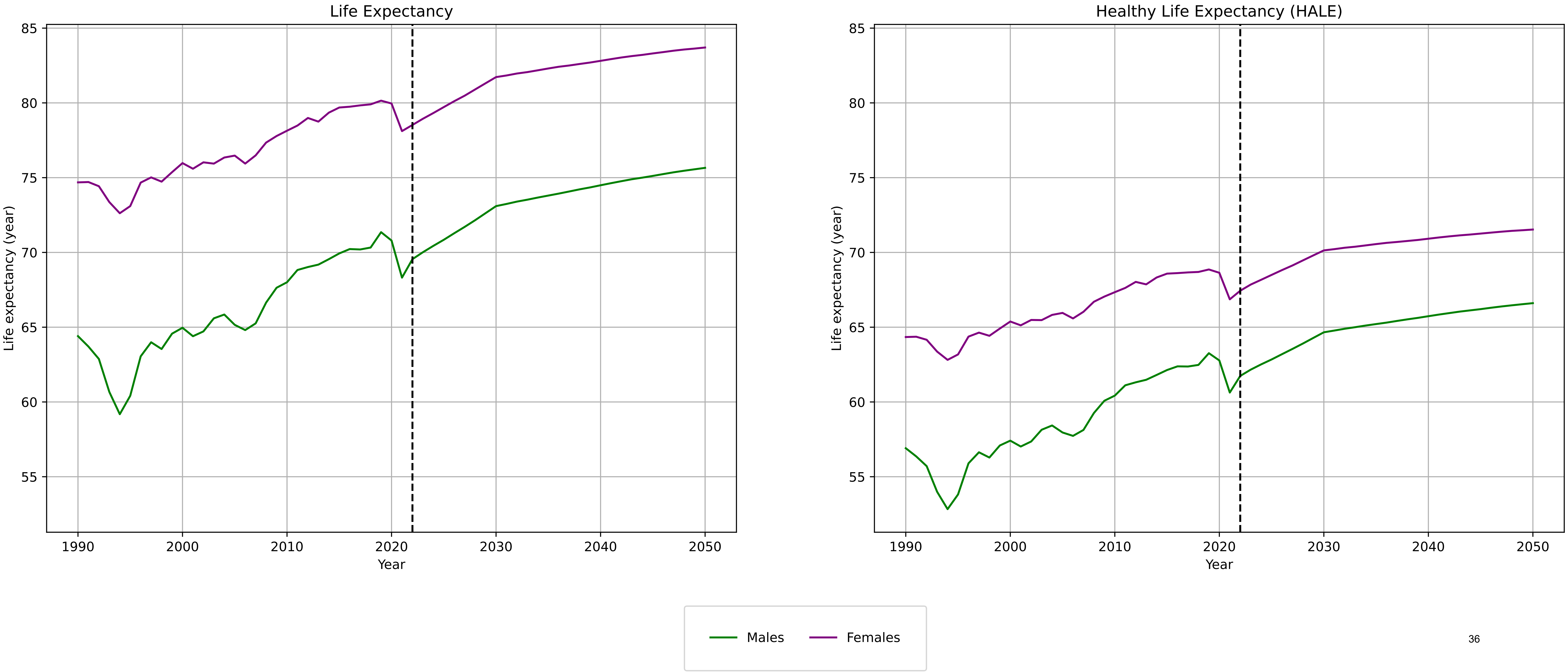

Lithuania

Supplemental Results Figure S3. Life expectancy and HALE by sex and location, 1990-2050

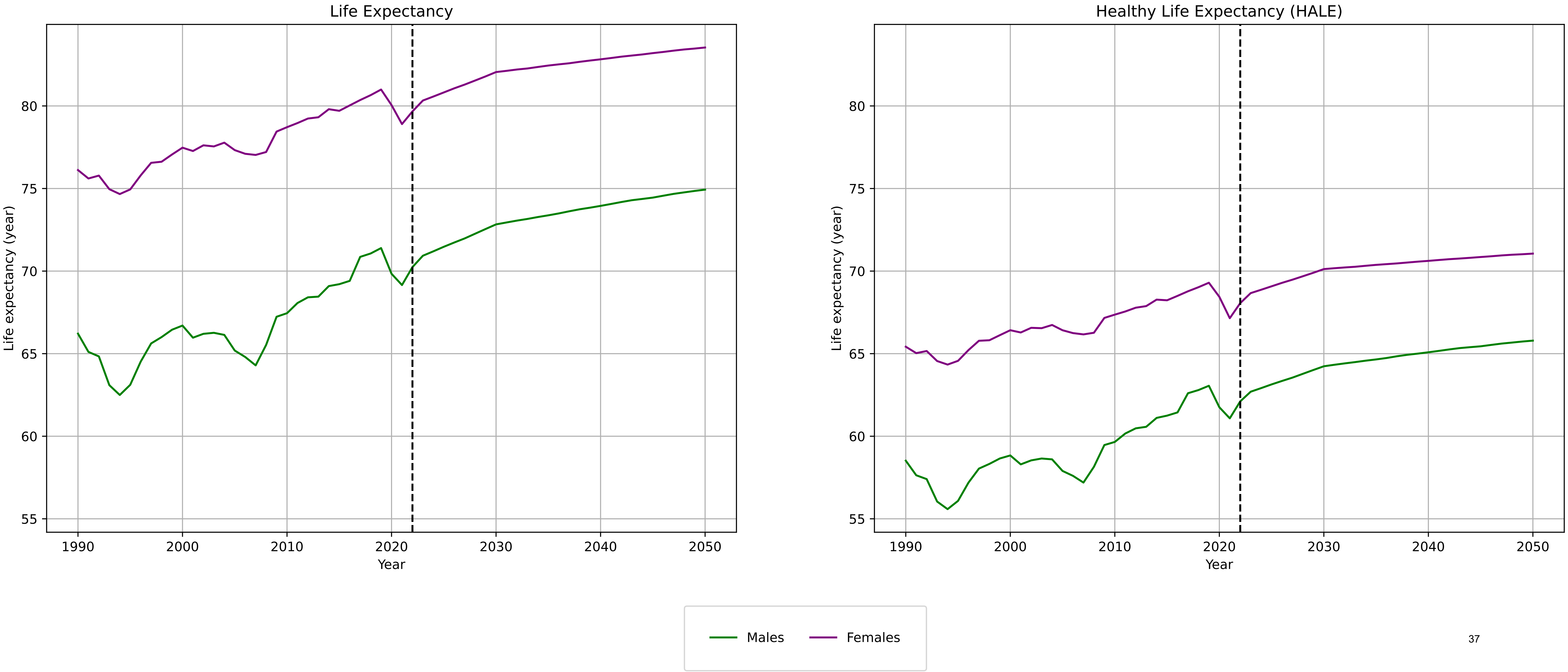

Moldova

Supplemental Results Figure S3. Life expectancy and HALE by sex and location, 1990-2050

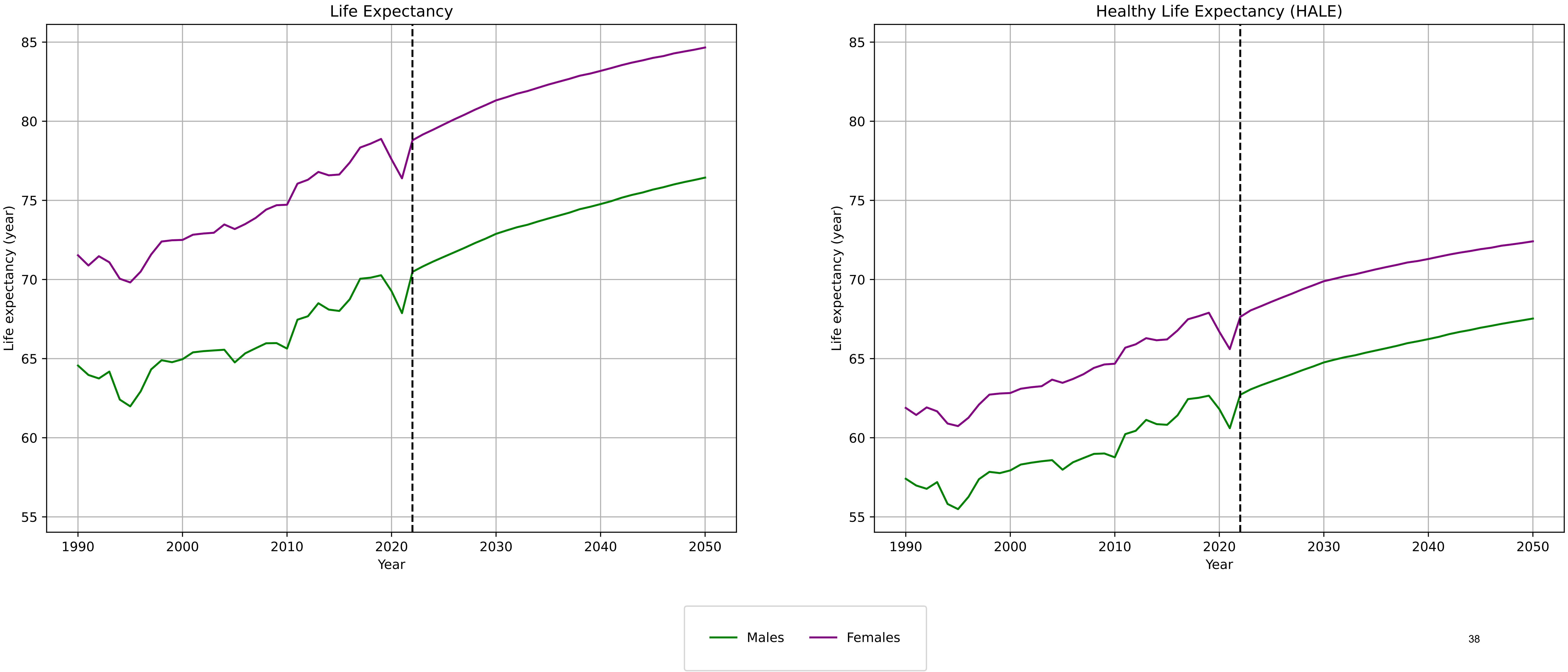

Russia

Supplemental Results Figure S3. Life expectancy and HALE by sex and location, 1990-2050

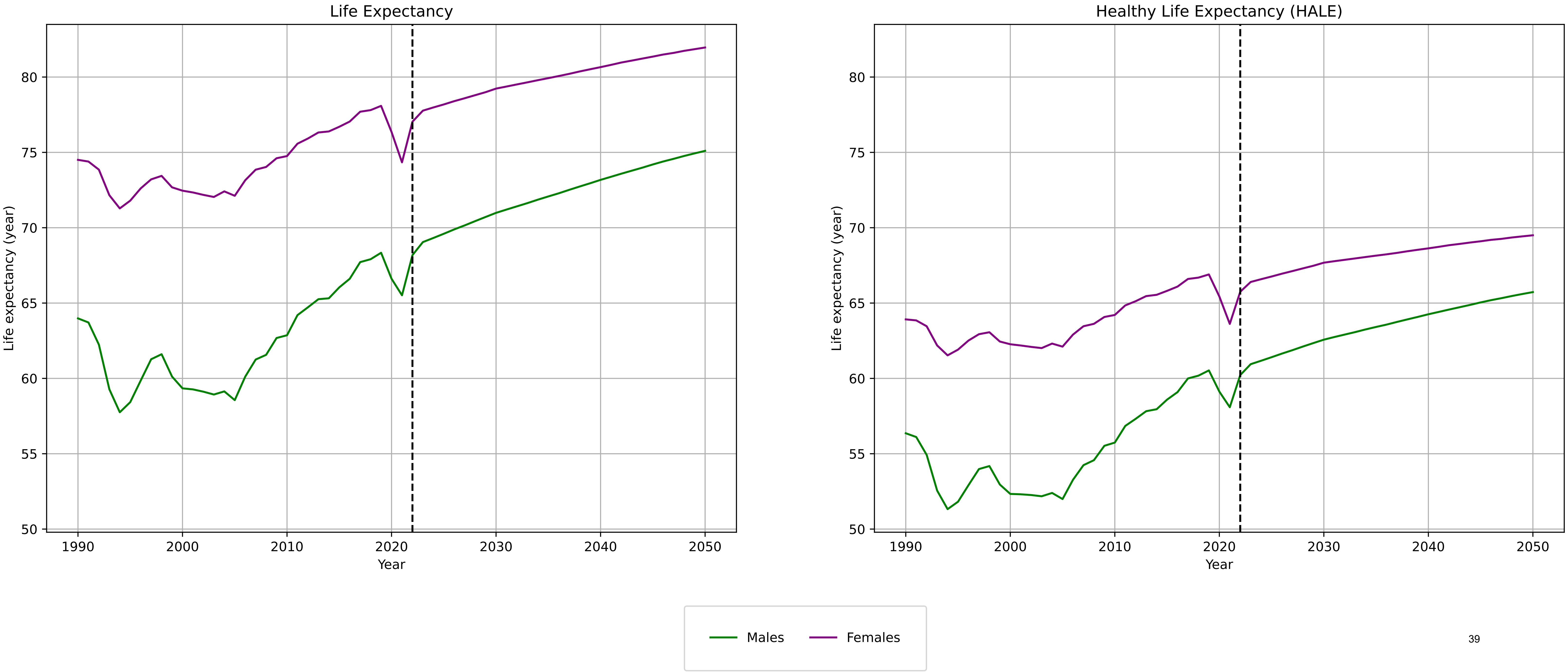

Supplemental Results Figure S3. Life expectancy and HALE by sex and location, 1990-2050

Ukraine

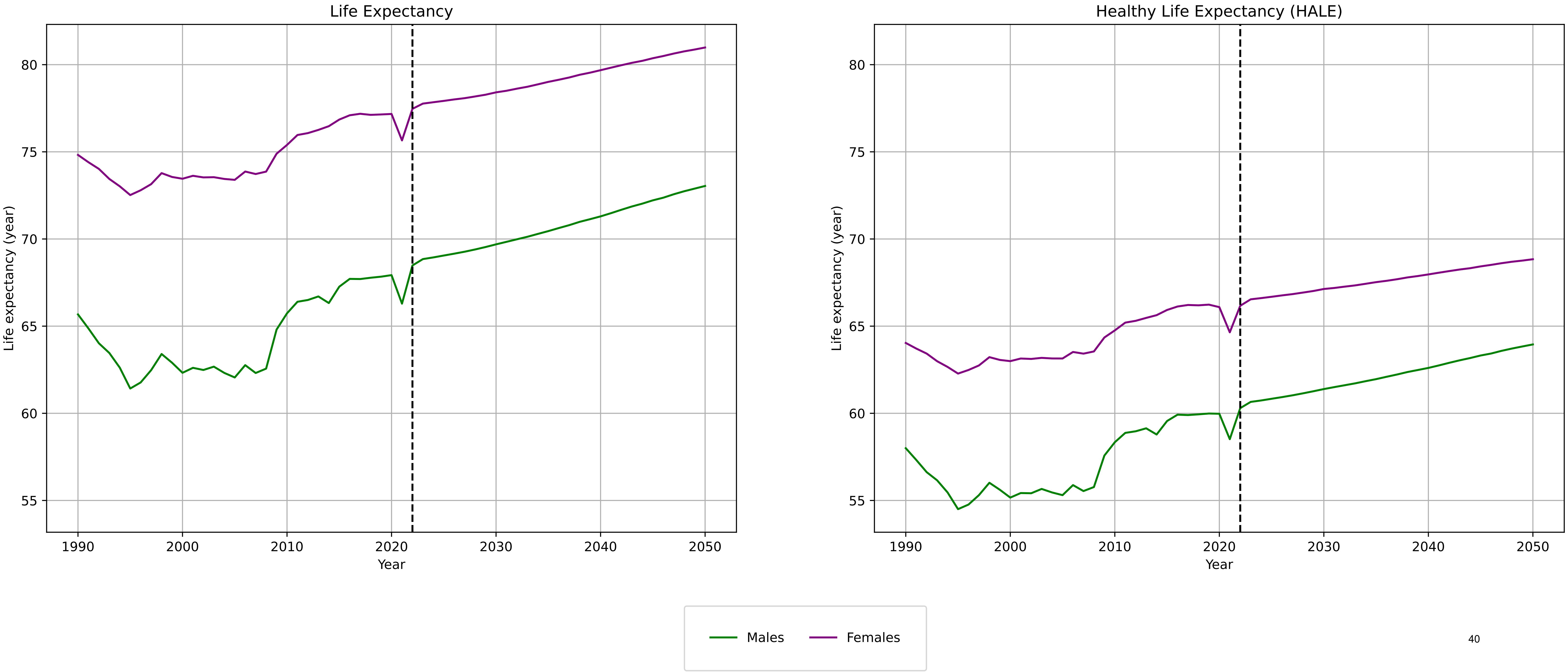

Australia

Supplemental Results Figure S3. Life expectancy and HALE by sex and location, 1990-2050

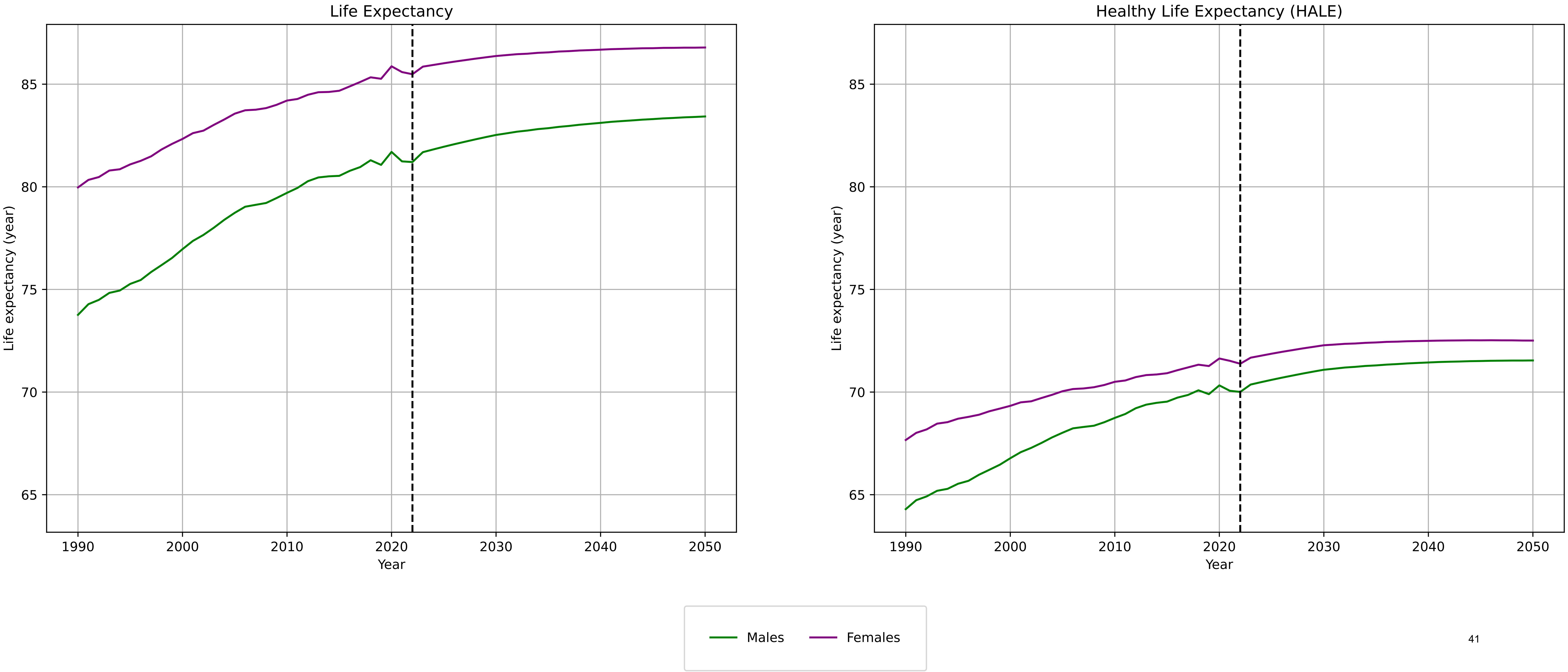

New Zealand

Supplemental Results Figure S3. Life expectancy and HALE by sex and location, 1990-2050

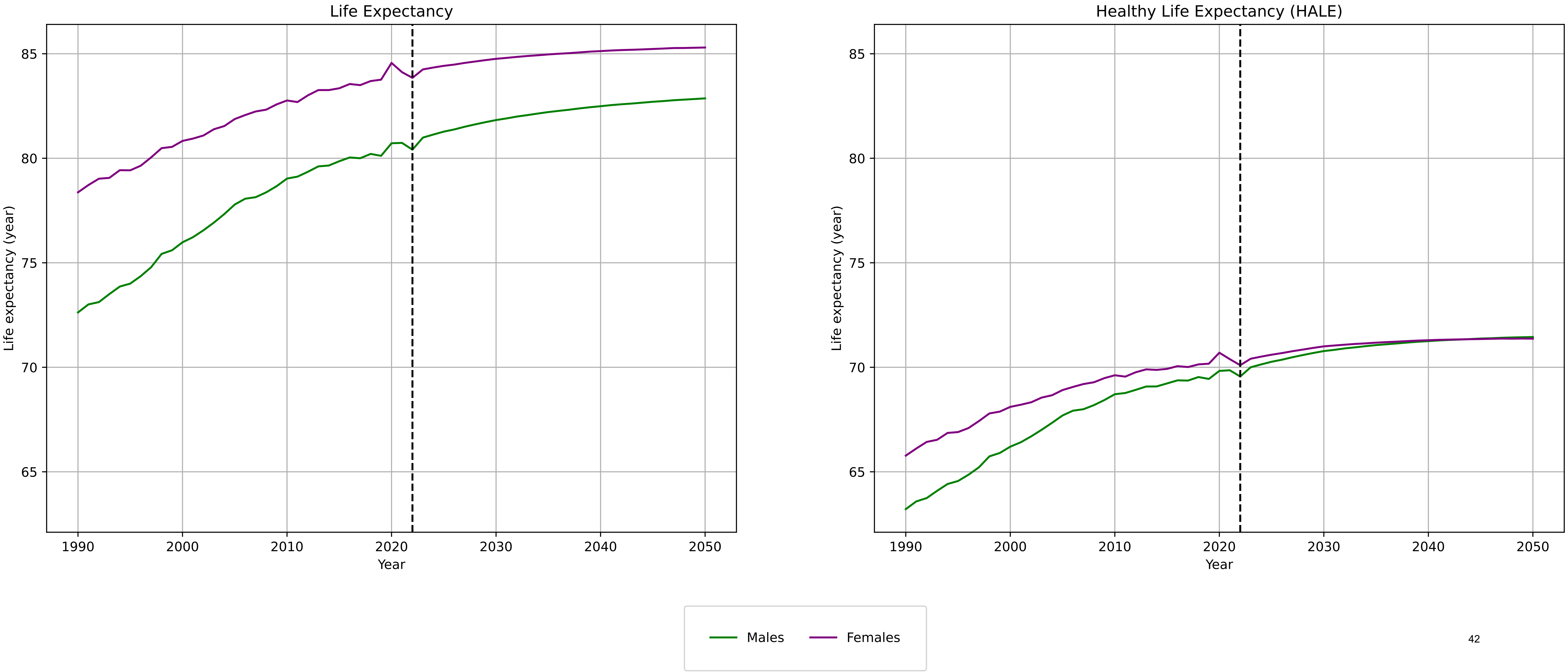

Brunei

Supplemental Results Figure S3. Life expectancy and HALE by sex and location, 1990-2050

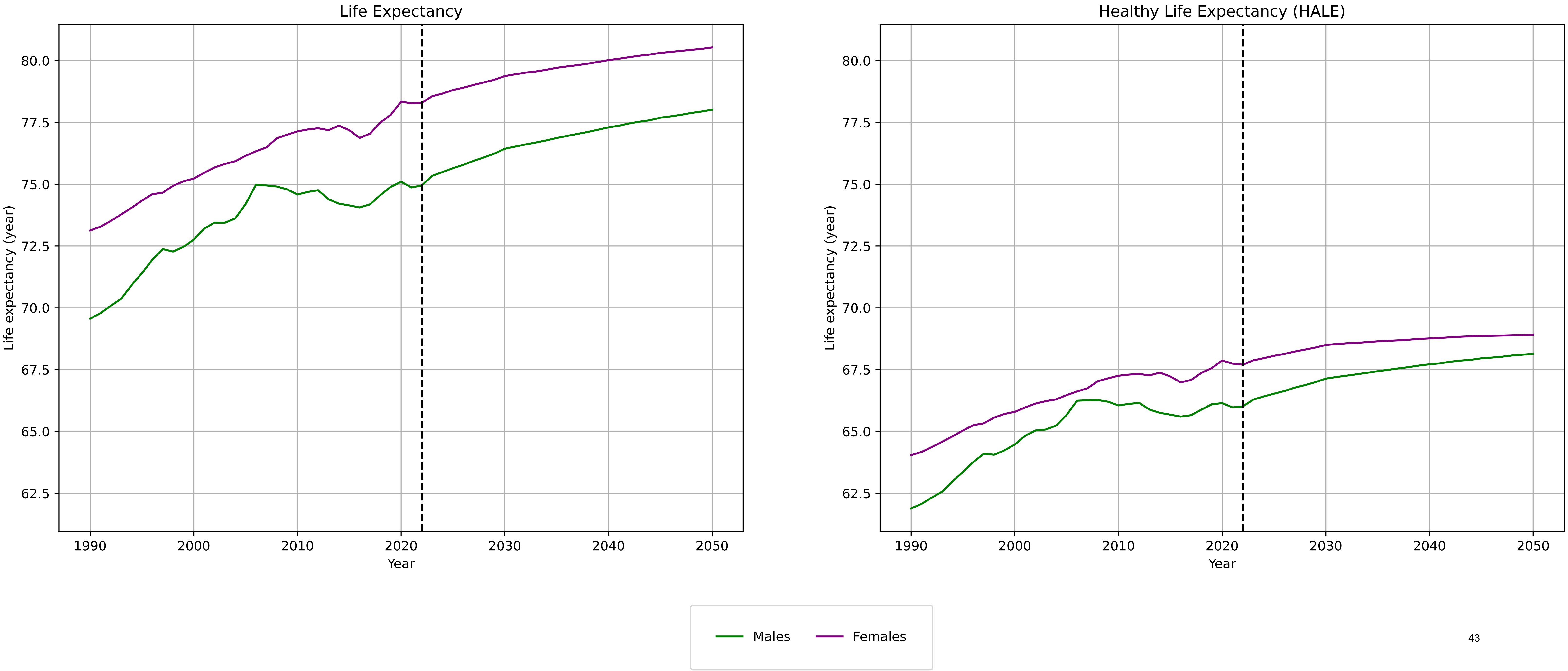

Supplemental Results Figure S3. Life expectancy and HALE by sex and location, 1990-2050

Japan

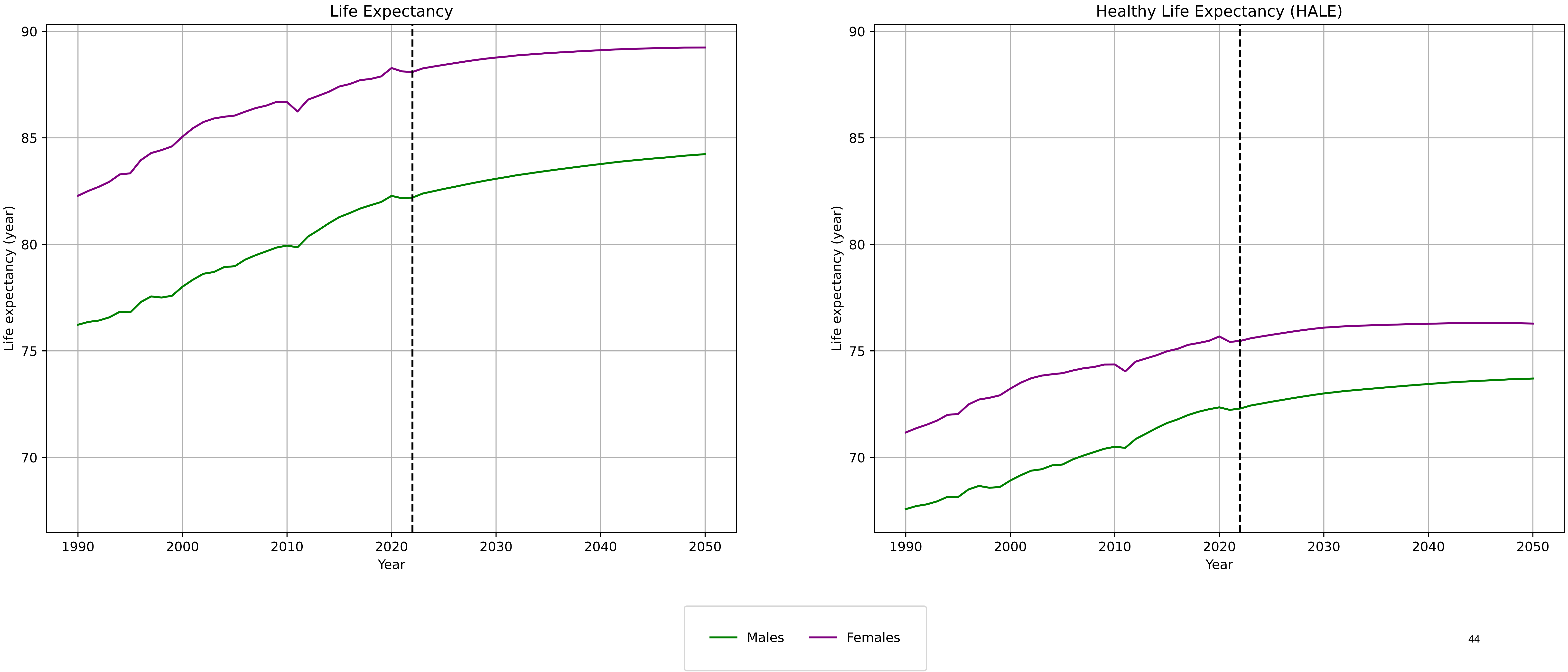

Supplemental Results Figure S3. Life expectancy and HALE by sex and location, 1990-2050

South Korea

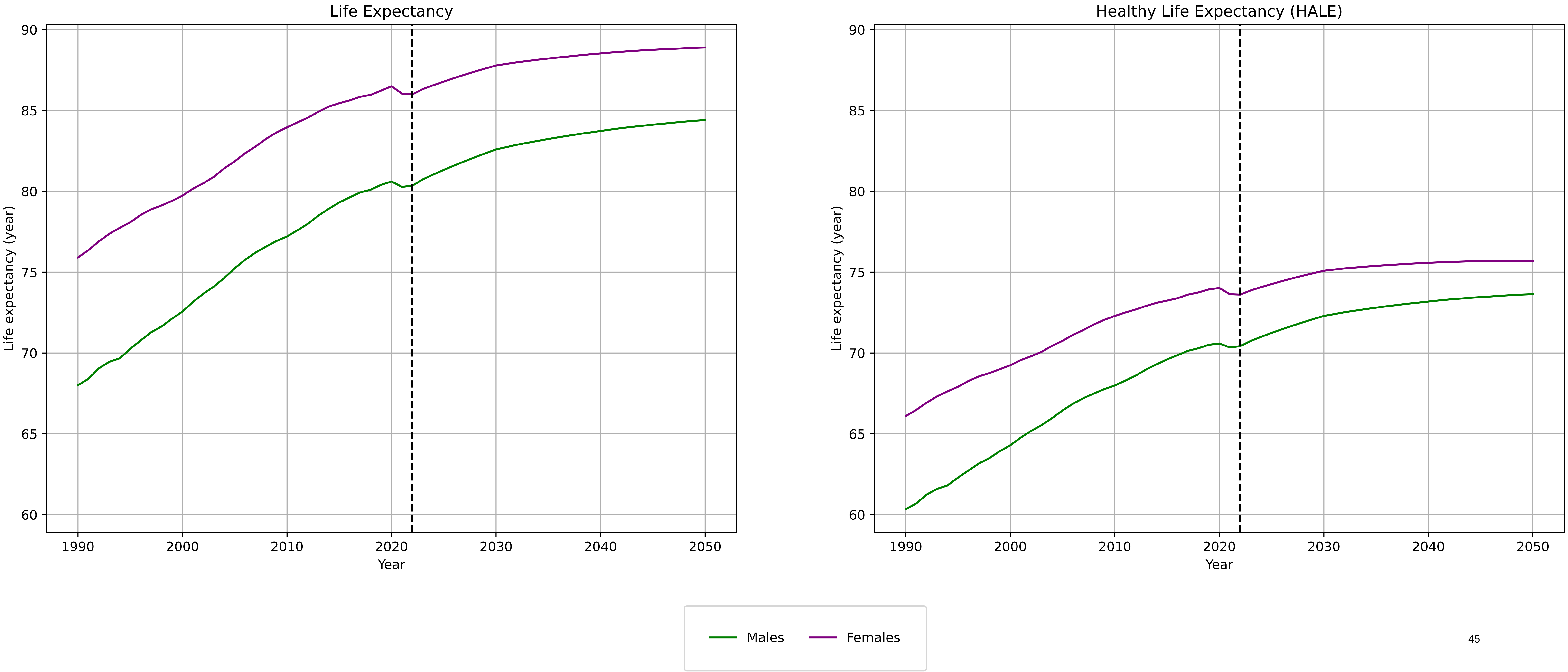

Supplemental Results Figure S3. Life expectancy and HALE by sex and location, 1990-2050

Singapore

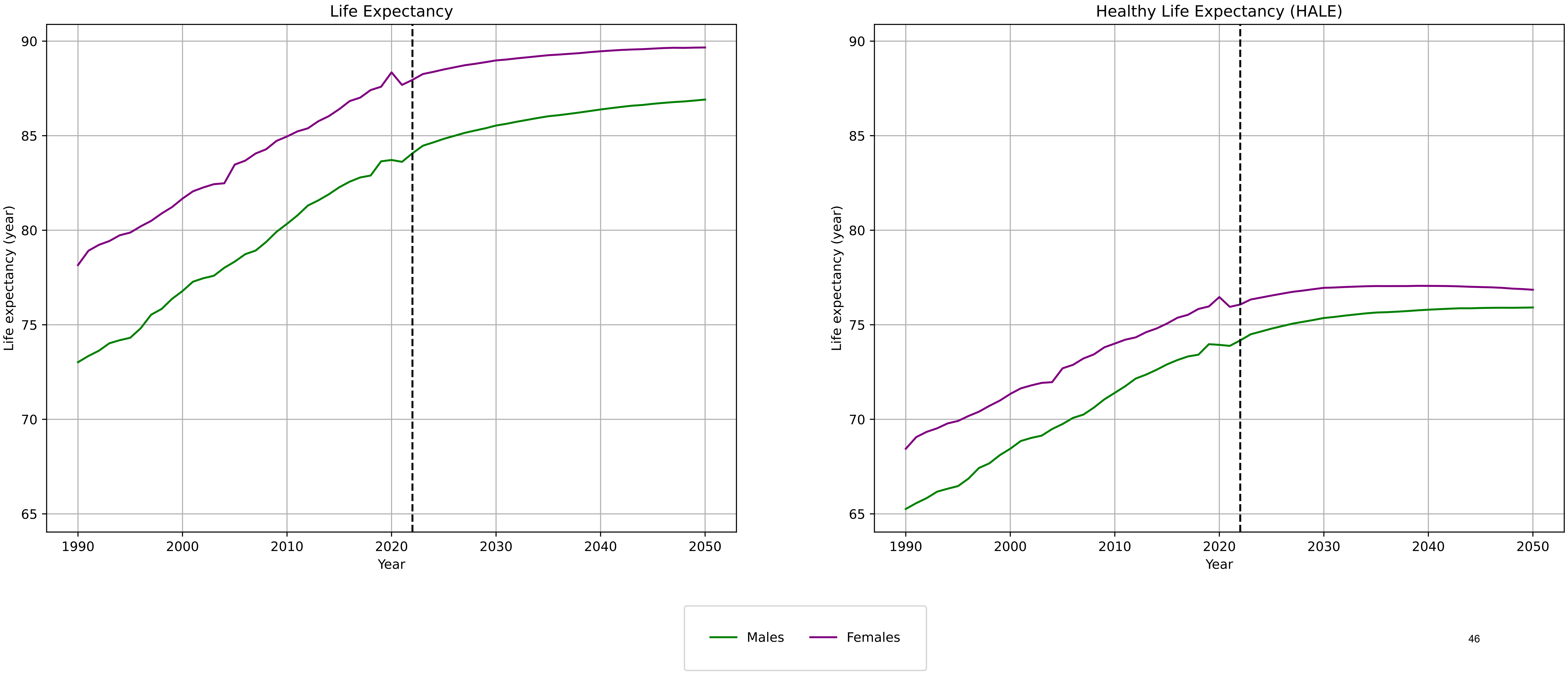

Canada

Supplemental Results Figure S3. Life expectancy and HALE by sex and location, 1990-2050

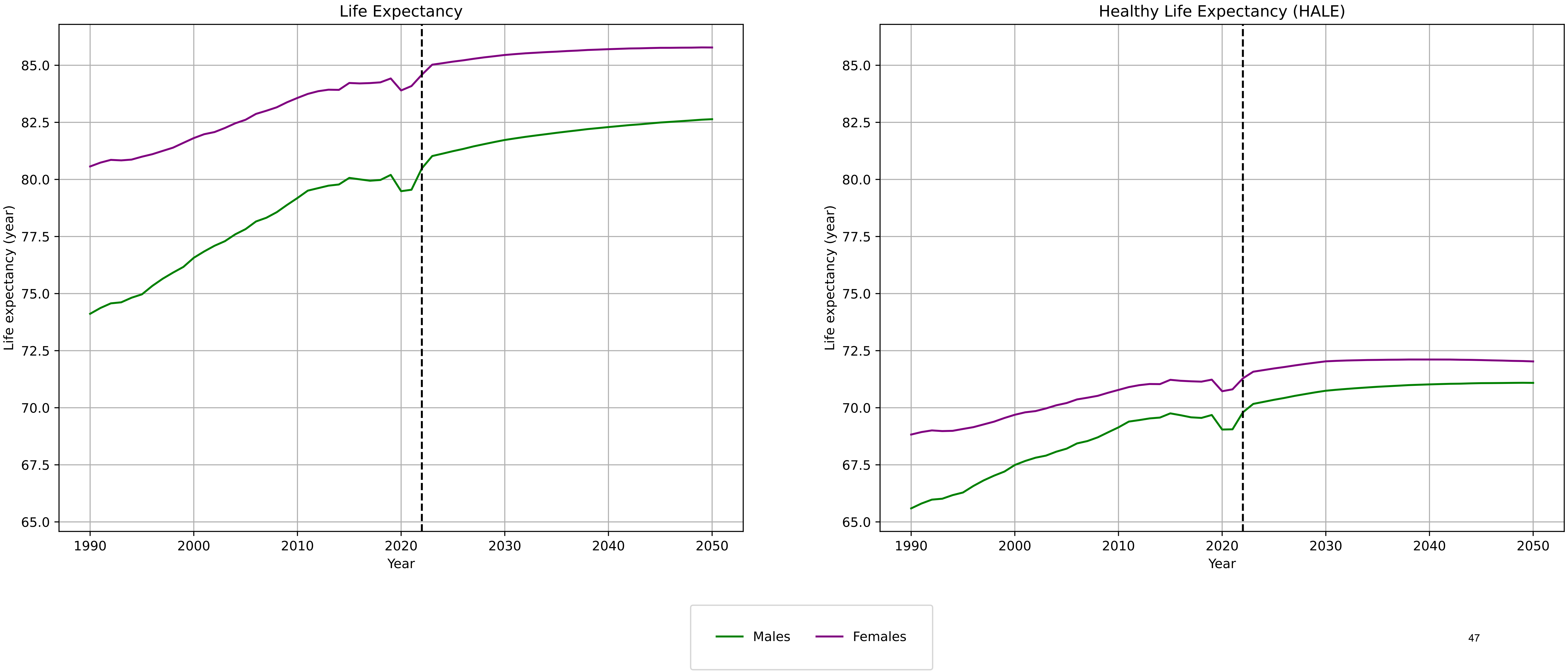

Supplemental Results Figure S3. Life expectancy and HALE by sex and location, 1990-2050

Greenland

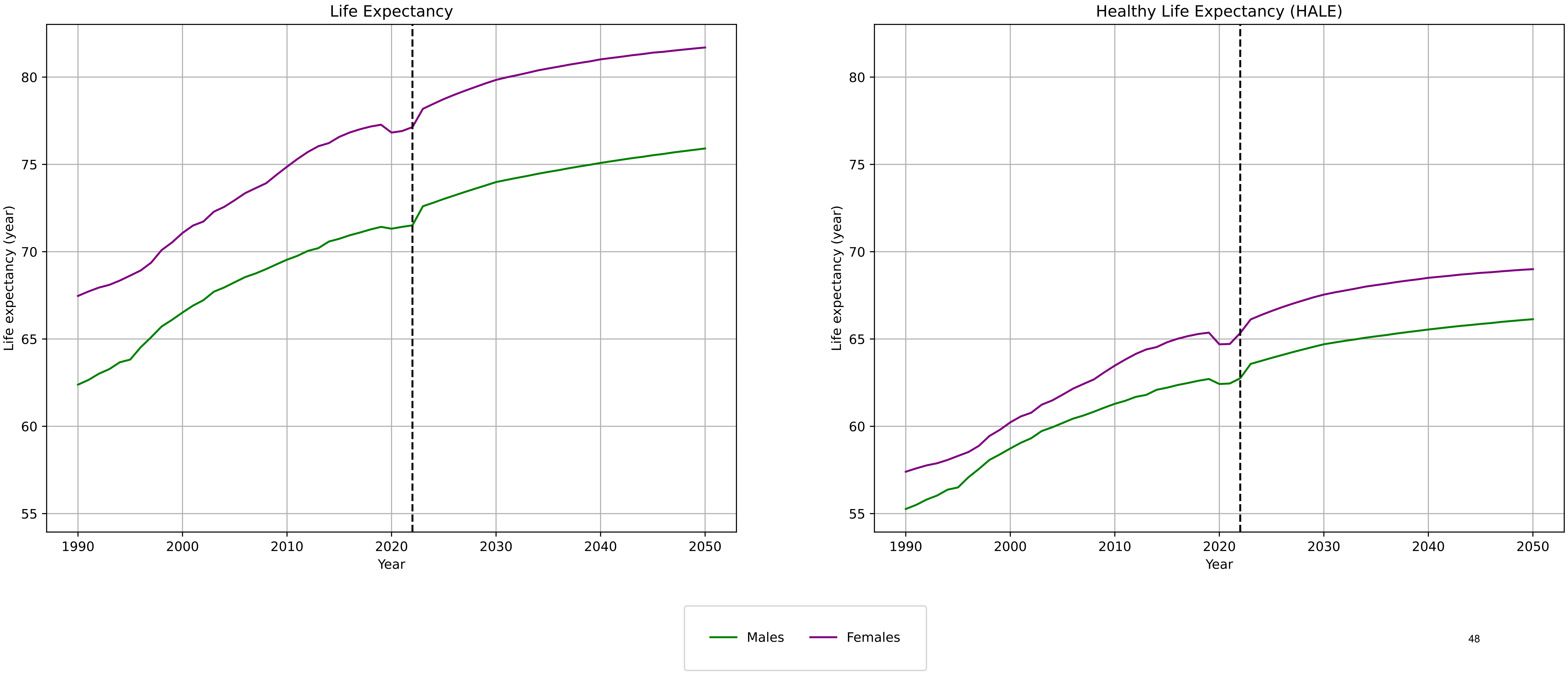

Supplemental Results Figure S3. Life expectancy and HALE by sex and location, 1990-2050

USA

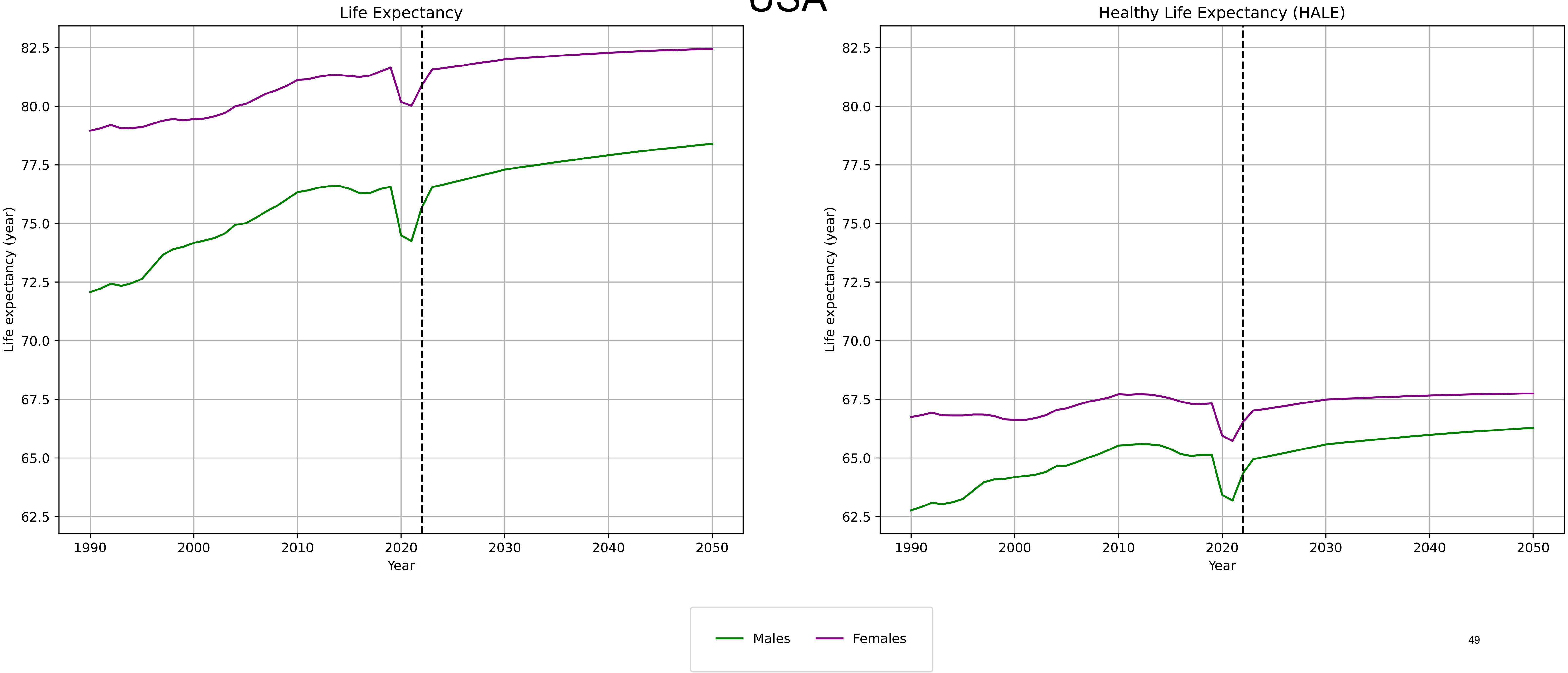

Argentina

Supplemental Results Figure S3. Life expectancy and HALE by sex and location, 1990-2050

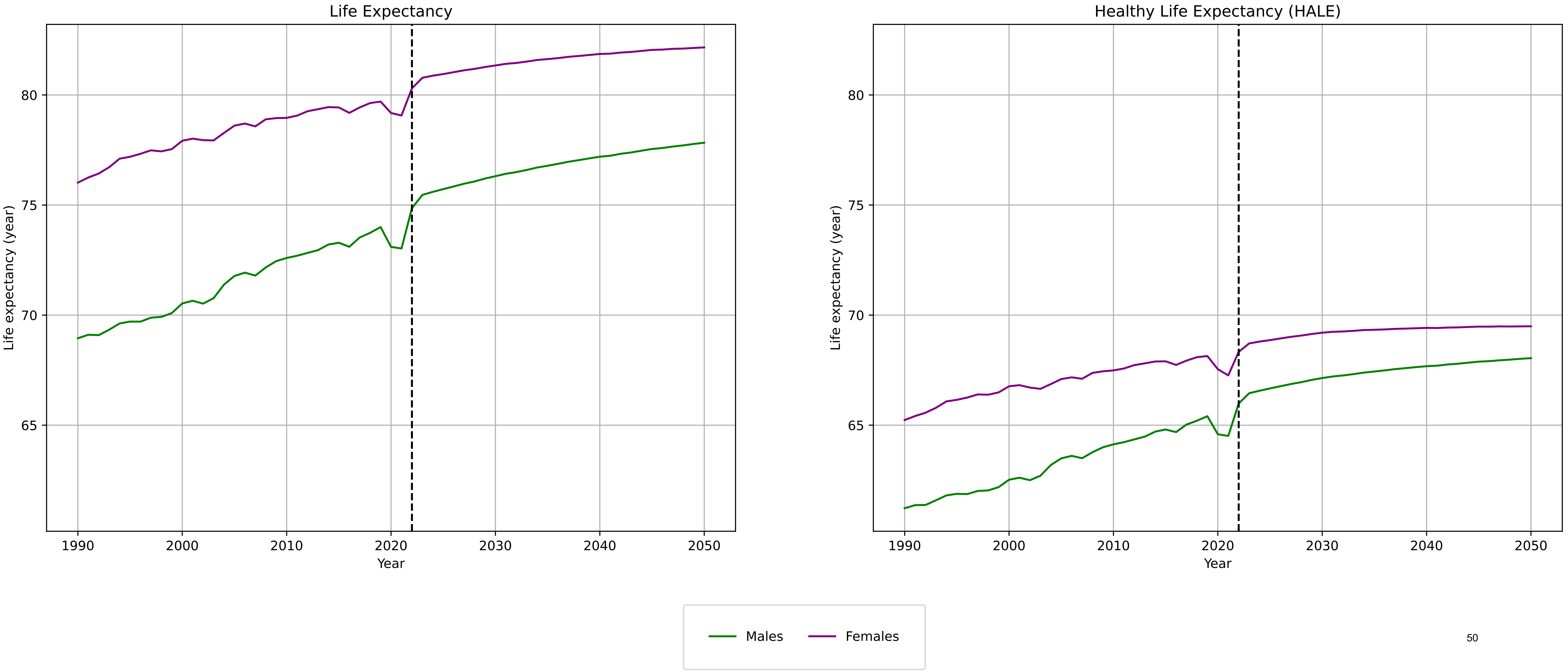

Supplemental Results Figure S3. Life expectancy and HALE by sex and location, 1990-2050

Chile

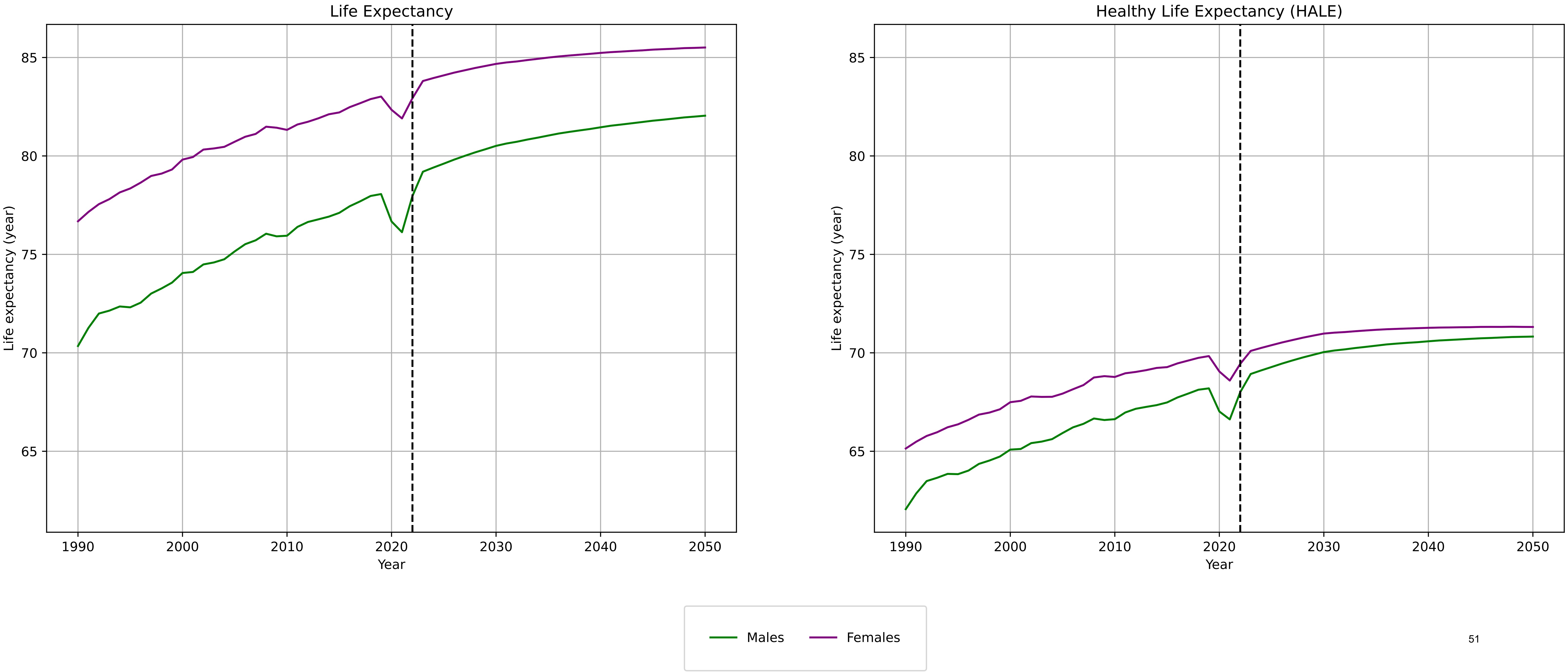

Supplemental Results Figure S3. Life expectancy and HALE by sex and location, 1990-2050

Uruguay

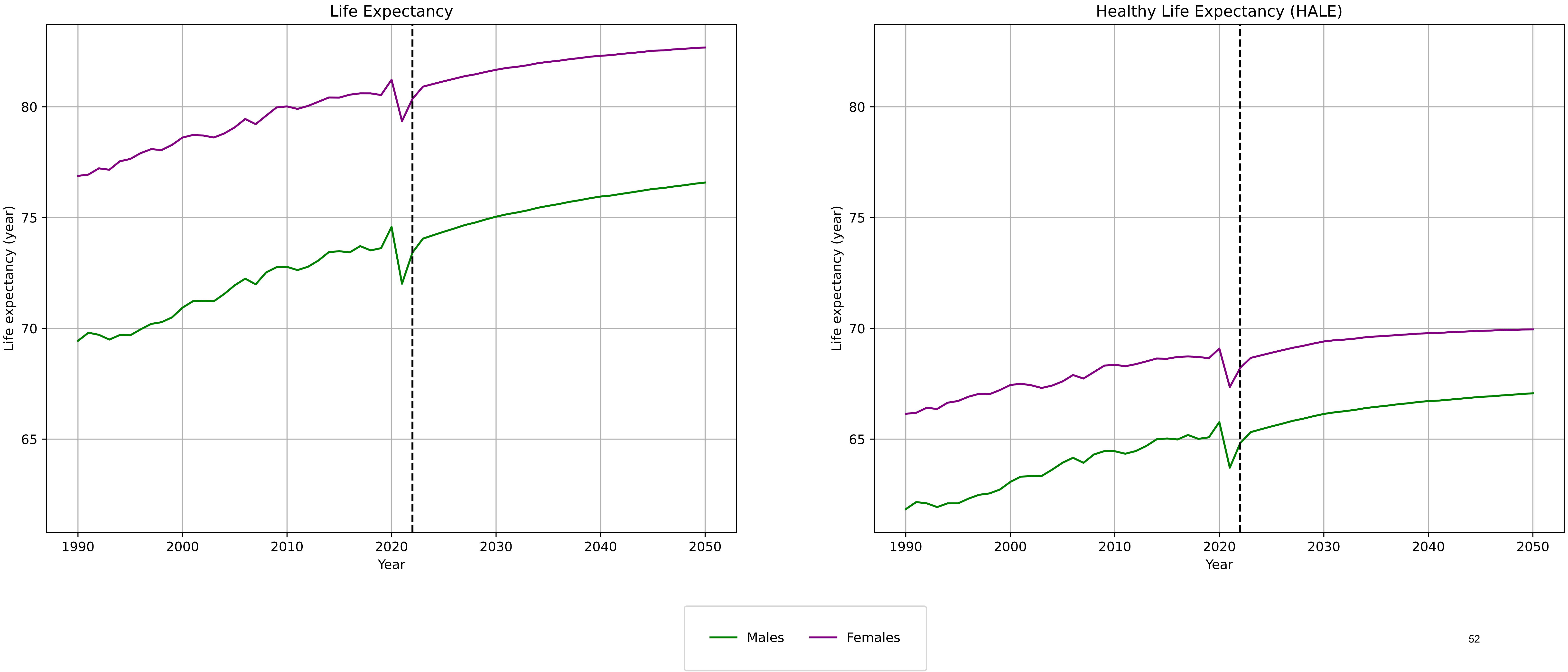

Andorra

Supplemental Results Figure S3. Life expectancy and HALE by sex and location, 1990-2050

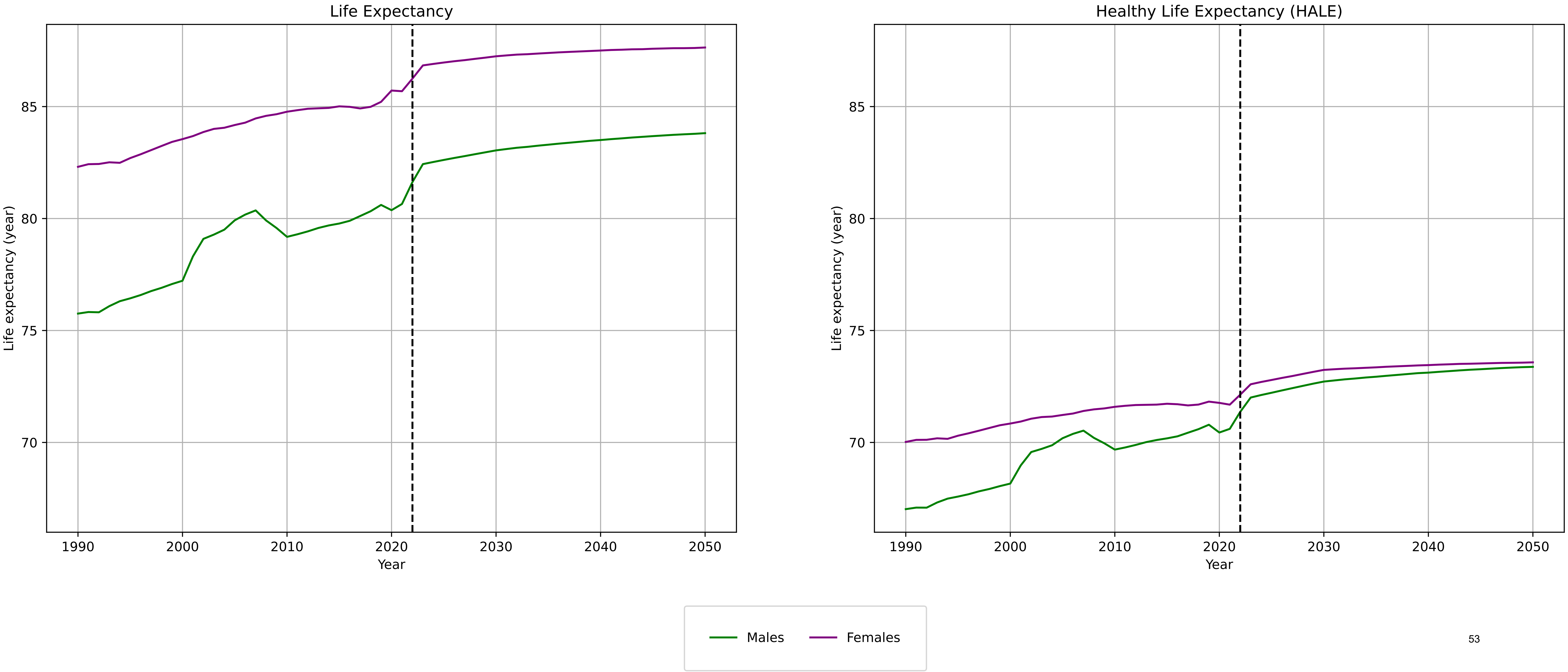

Supplemental Results Figure S3. Life expectancy and HALE by sex and location, 1990-2050

Austria

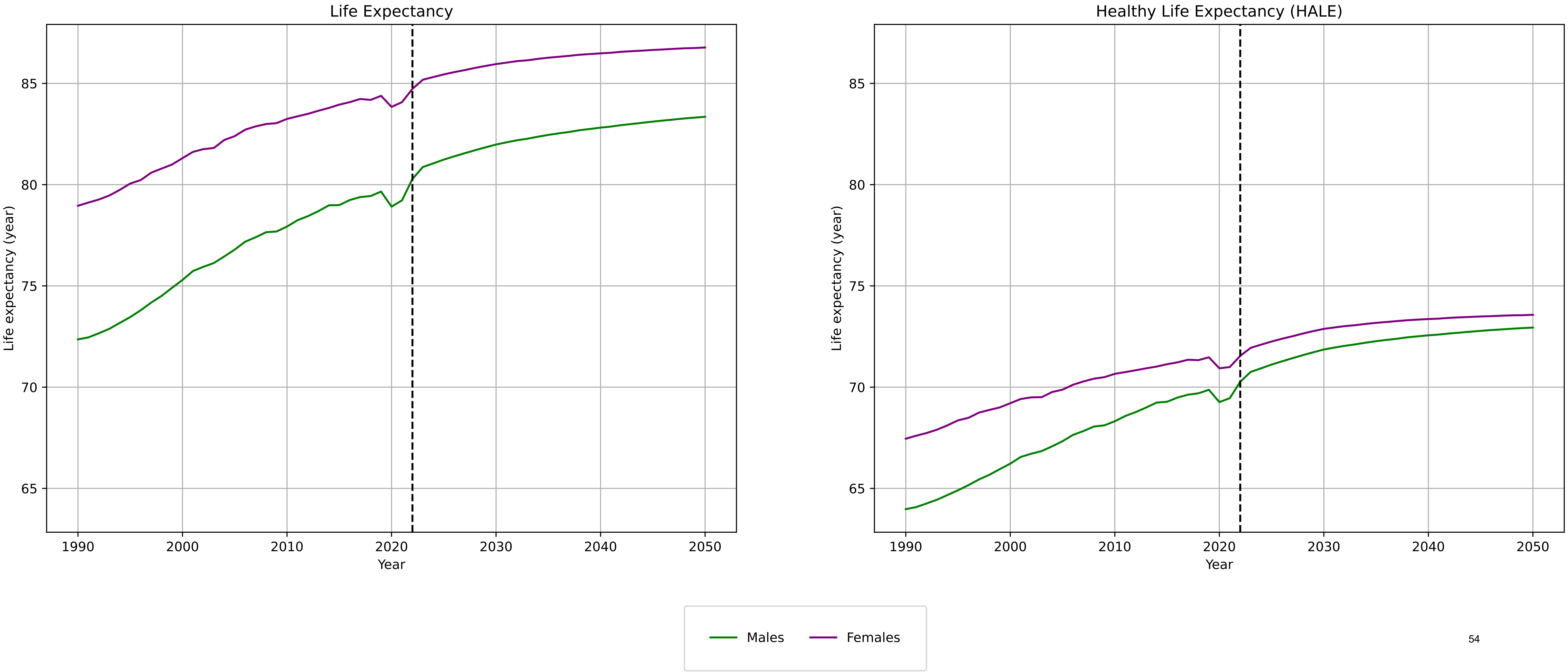

Belgium

Supplemental Results Figure S3. Life expectancy and HALE by sex and location, 1990-2050

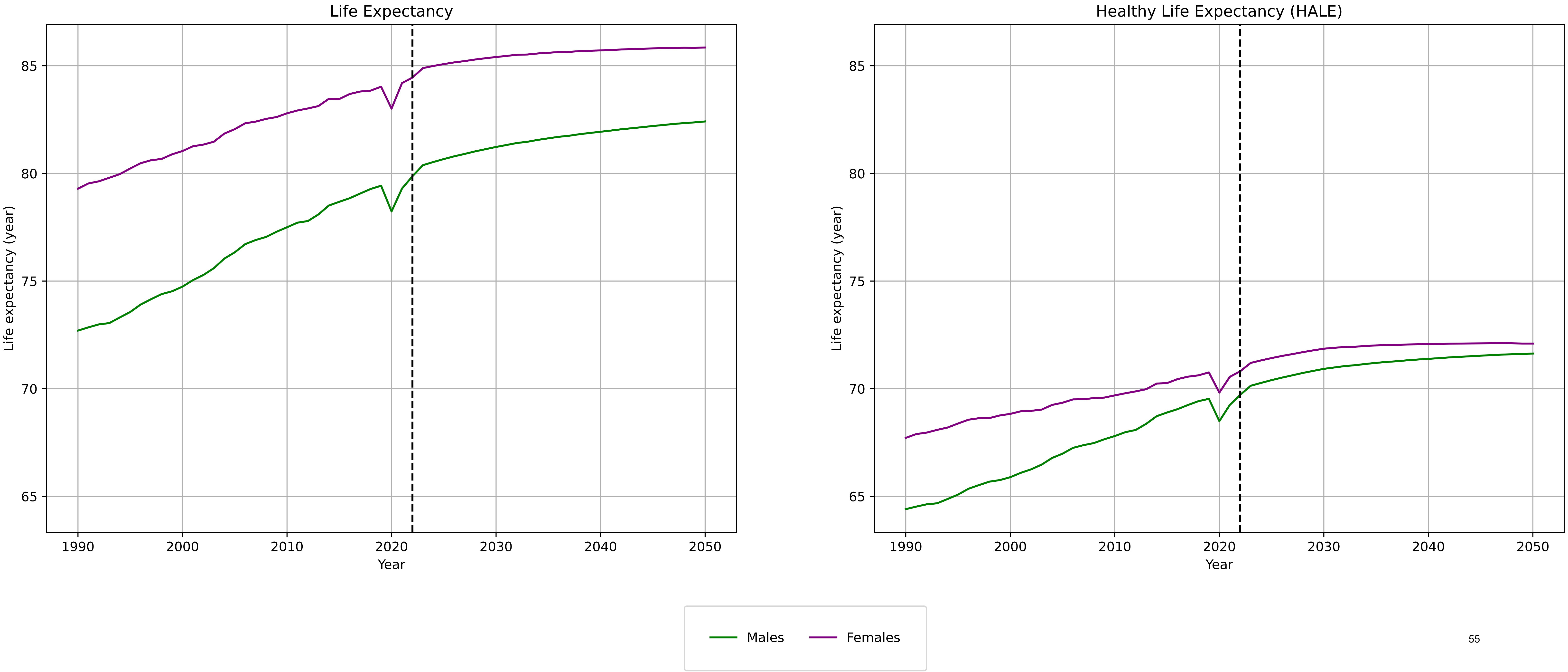

Cyprus

Supplemental Results Figure S3. Life expectancy and HALE by sex and location, 1990-2050

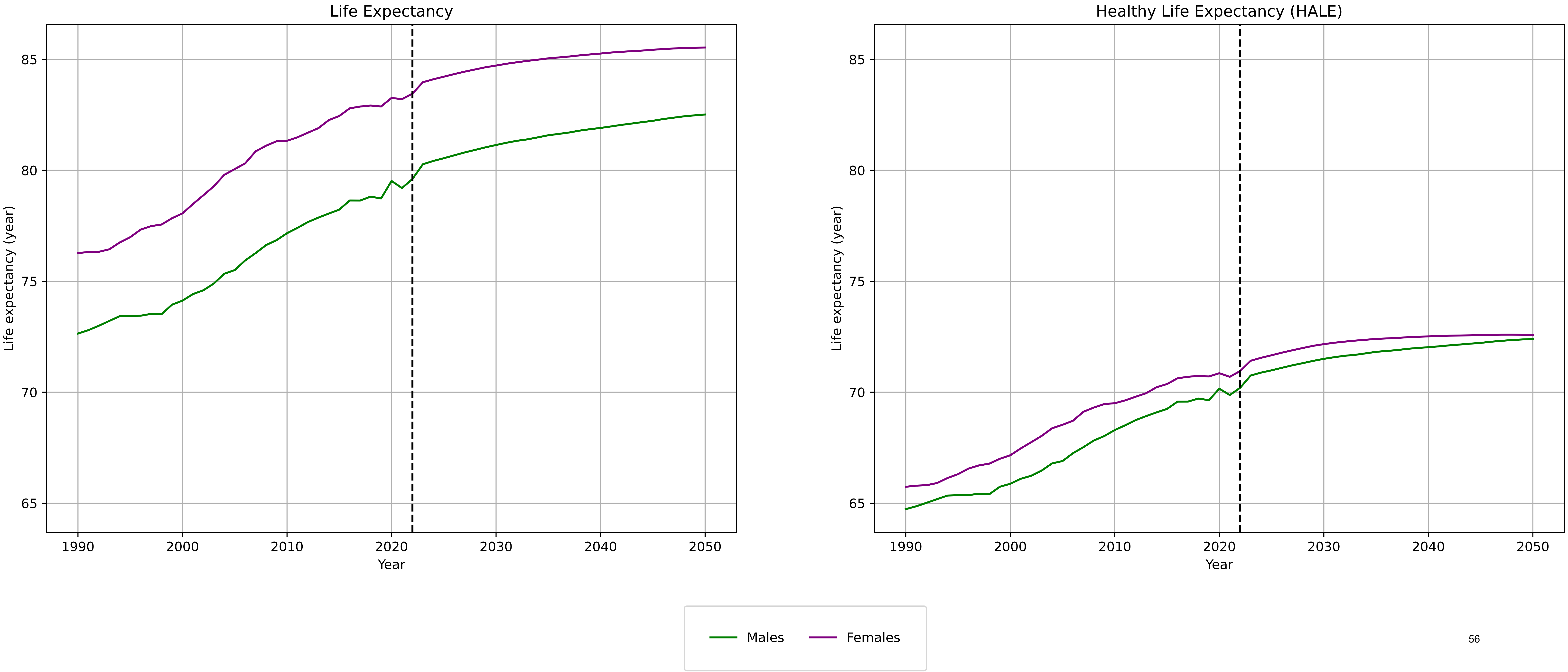

Denmark

Supplemental Results Figure S3. Life expectancy and HALE by sex and location, 1990-2050

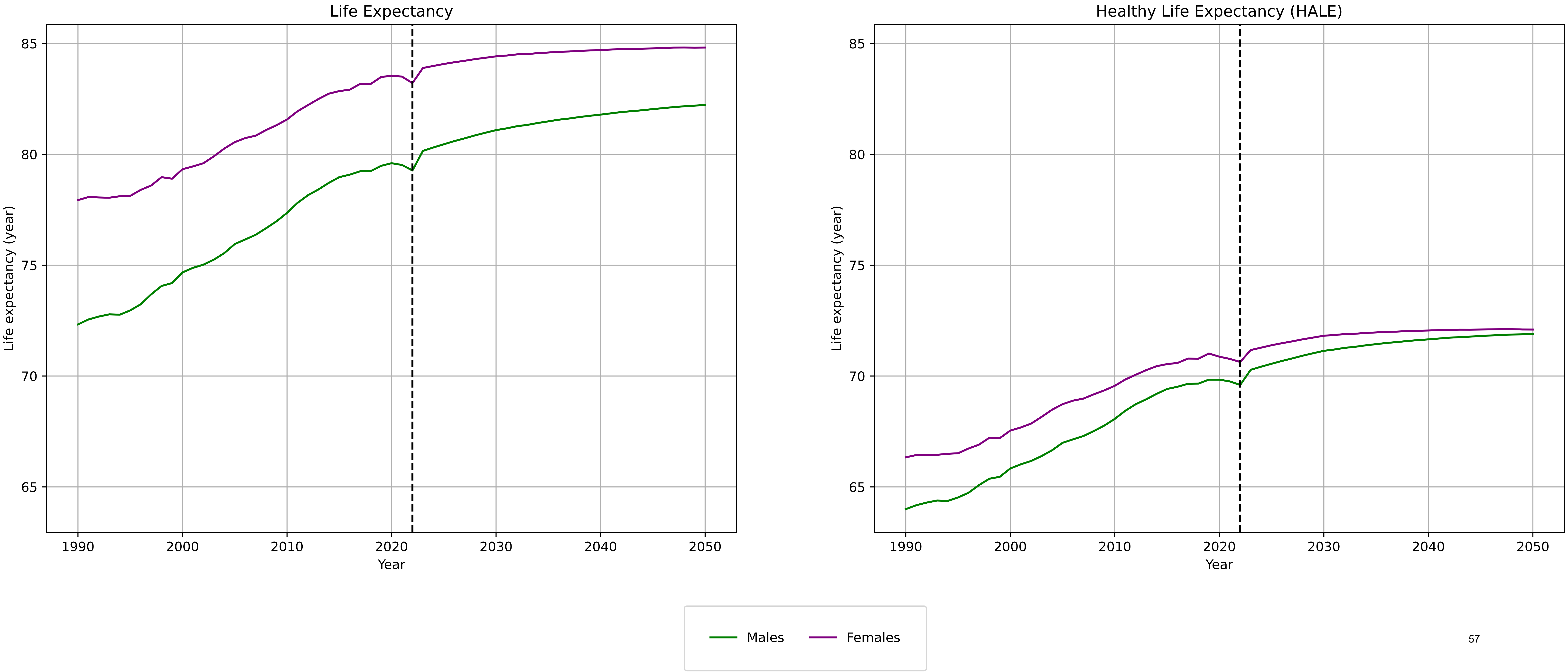

Finland

Supplemental Results Figure S3. Life expectancy and HALE by sex and location, 1990-2050

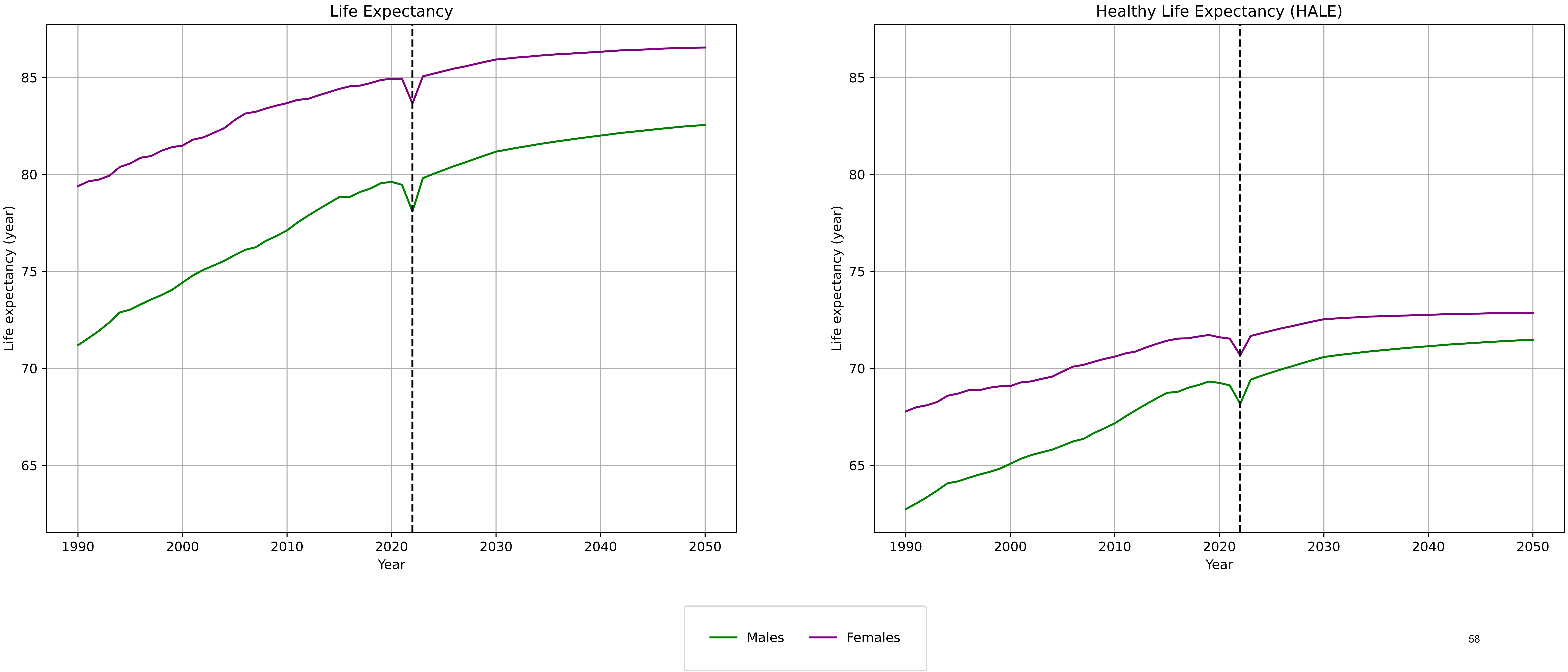

Supplemental Results Figure S3. Life expectancy and HALE by sex and location, 1990-2050

France

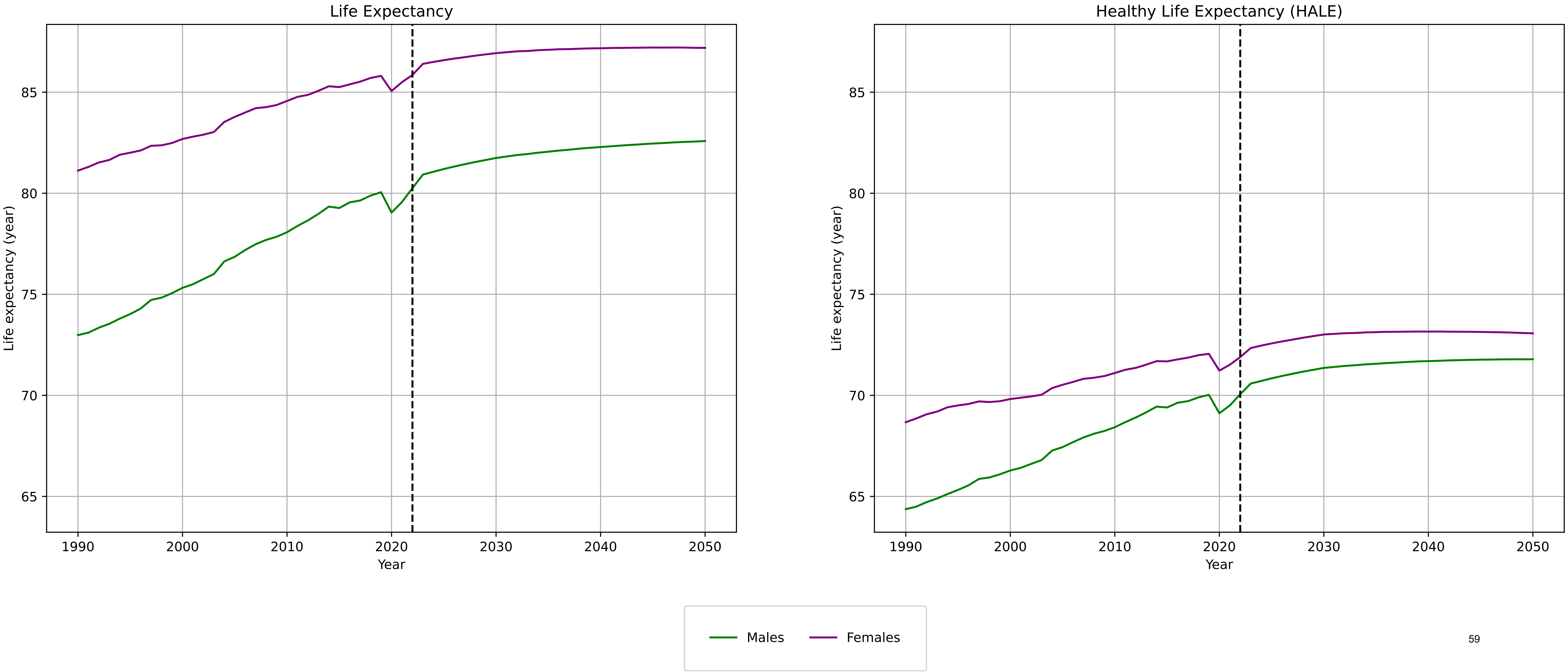

Supplemental Results Figure S3. Life expectancy and HALE by sex and location, 1990-2050

Germany

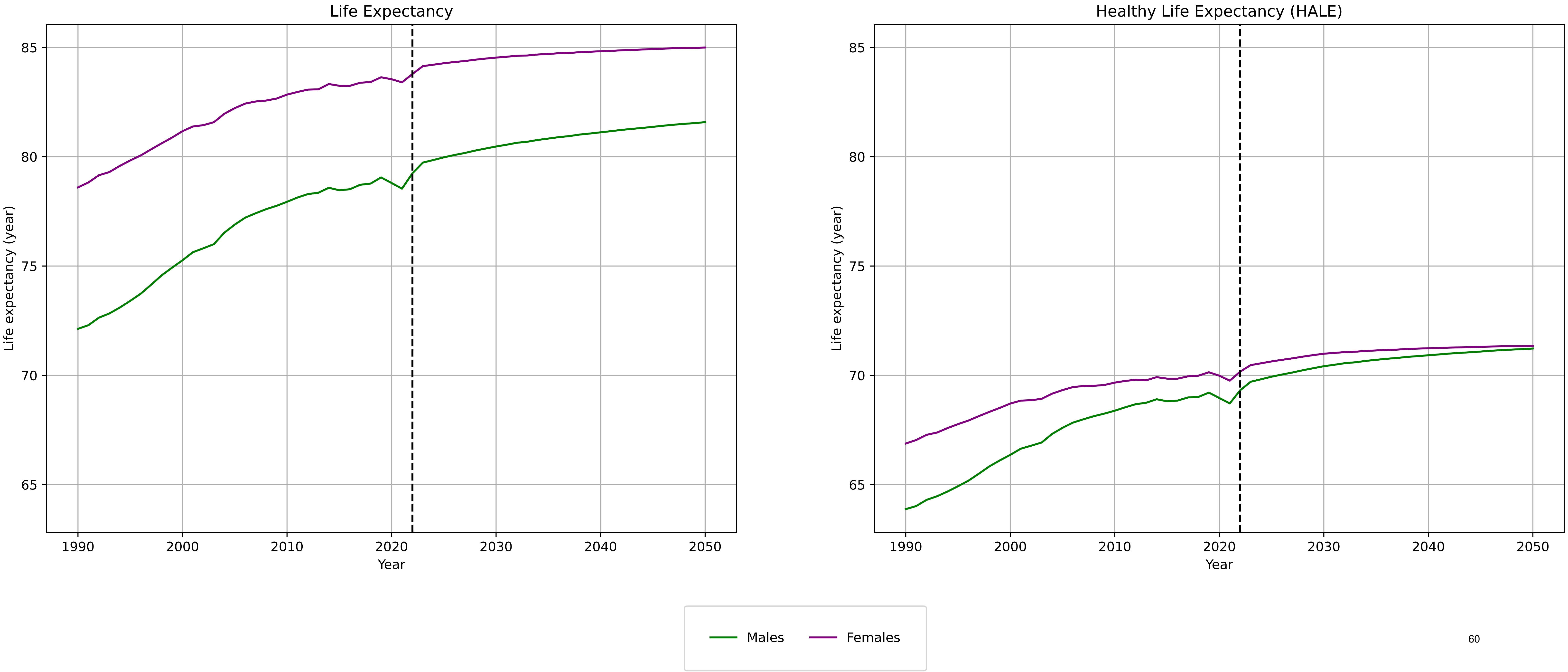

Supplemental Results Figure S3. Life expectancy and HALE by sex and location, 1990-2050

Greece

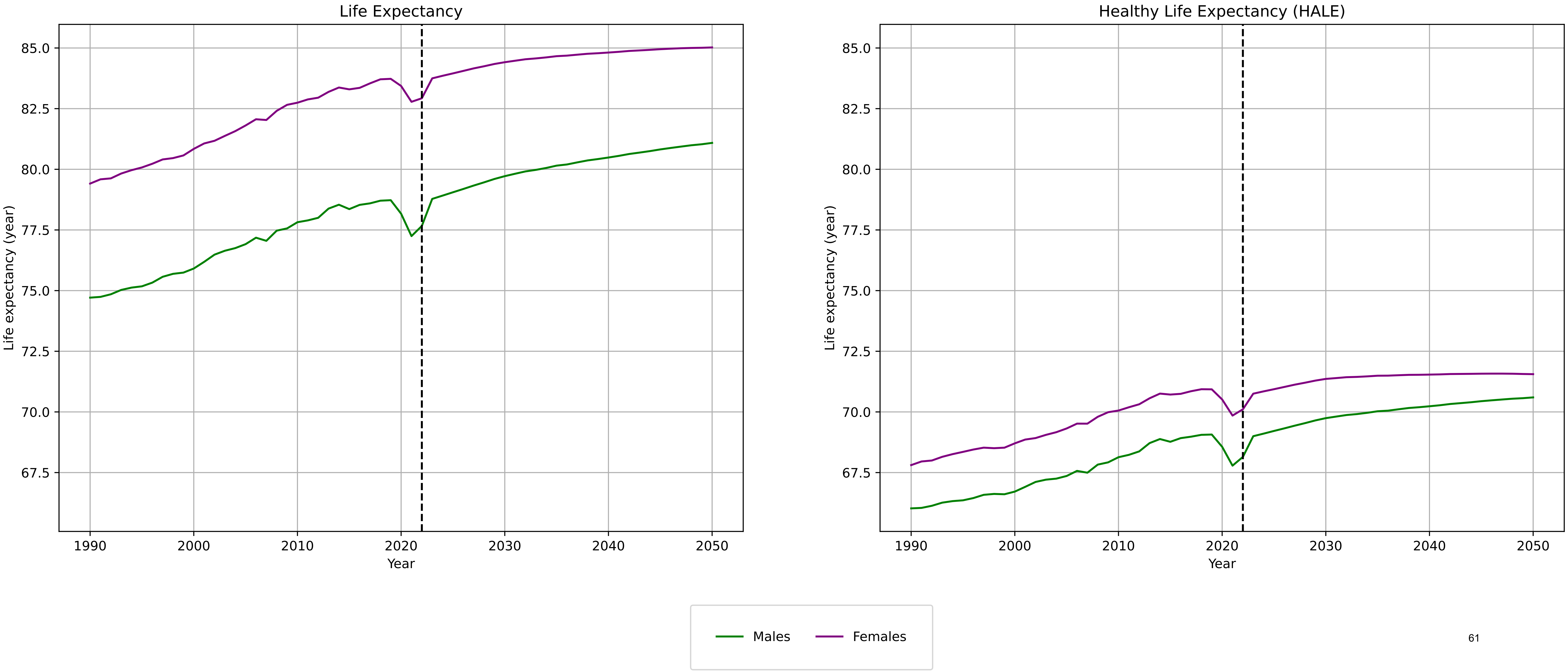

Iceland

Supplemental Results Figure S3. Life expectancy and HALE by sex and location, 1990-2050

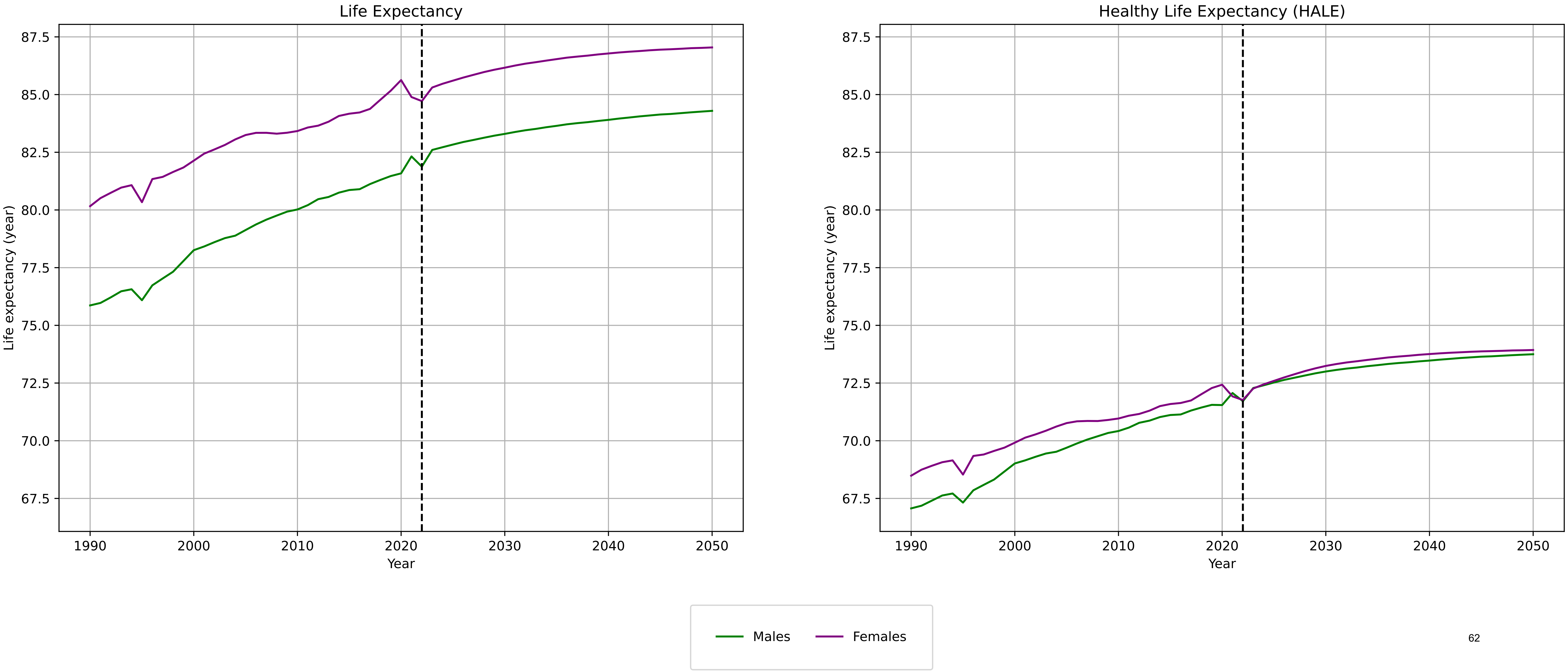

Ireland

Supplemental Results Figure S3. Life expectancy and HALE by sex and location, 1990-2050

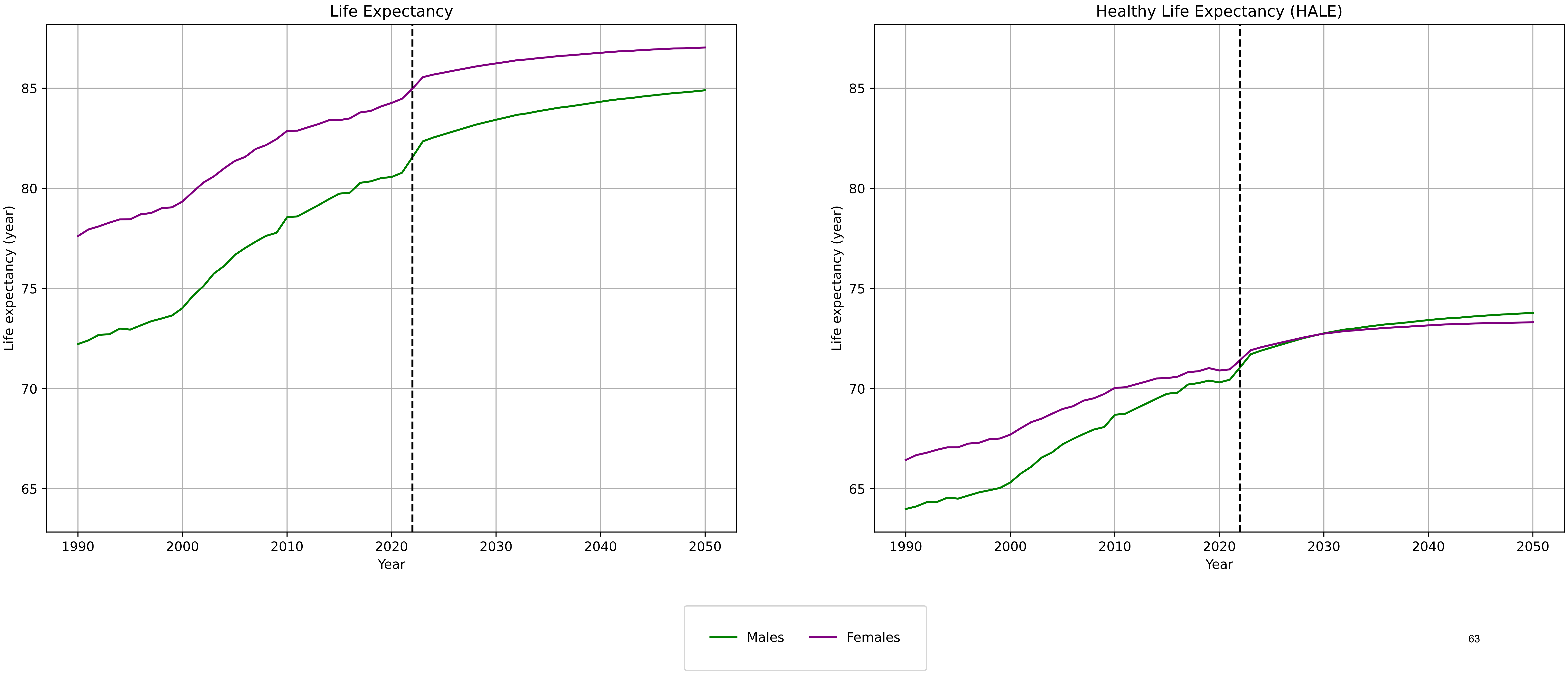

Israel

Supplemental Results Figure S3. Life expectancy and HALE by sex and location, 1990-2050

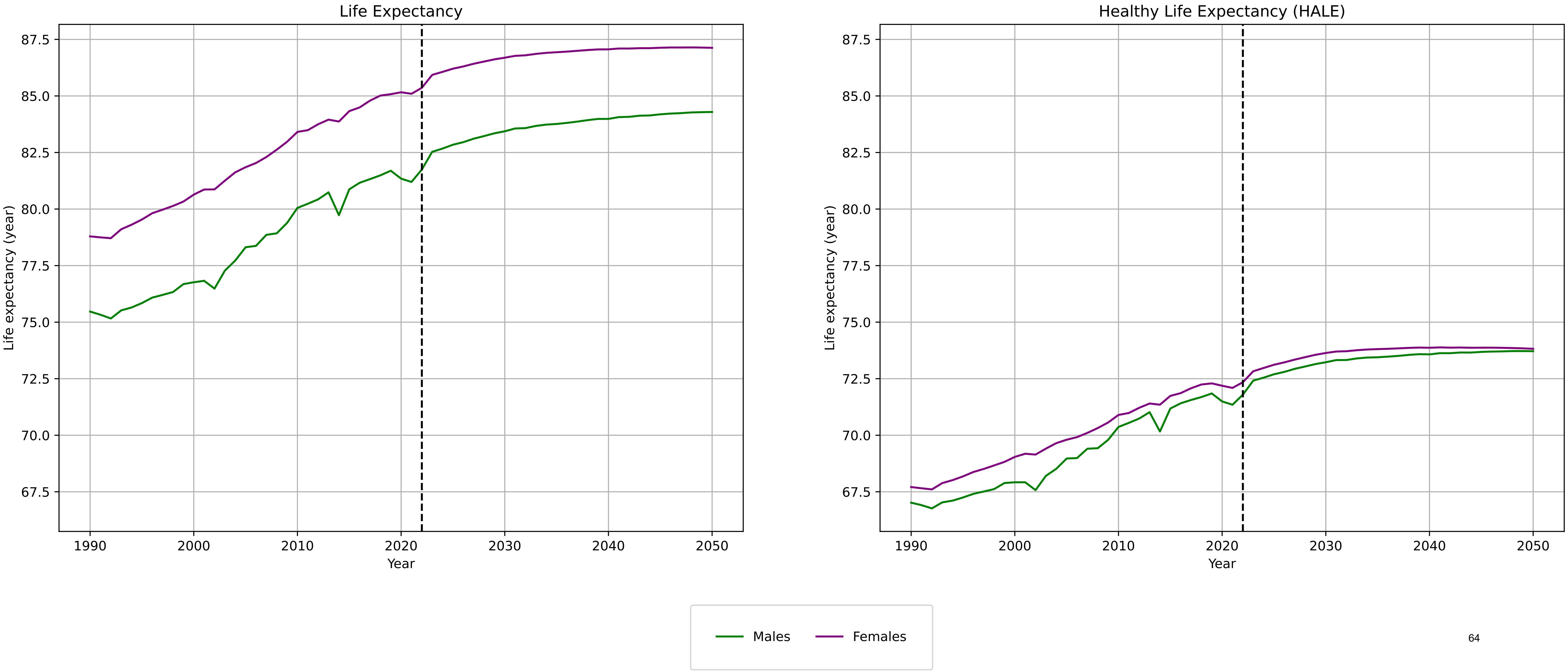

Italy

Supplemental Results Figure S3. Life expectancy and HALE by sex and location, 1990-2050

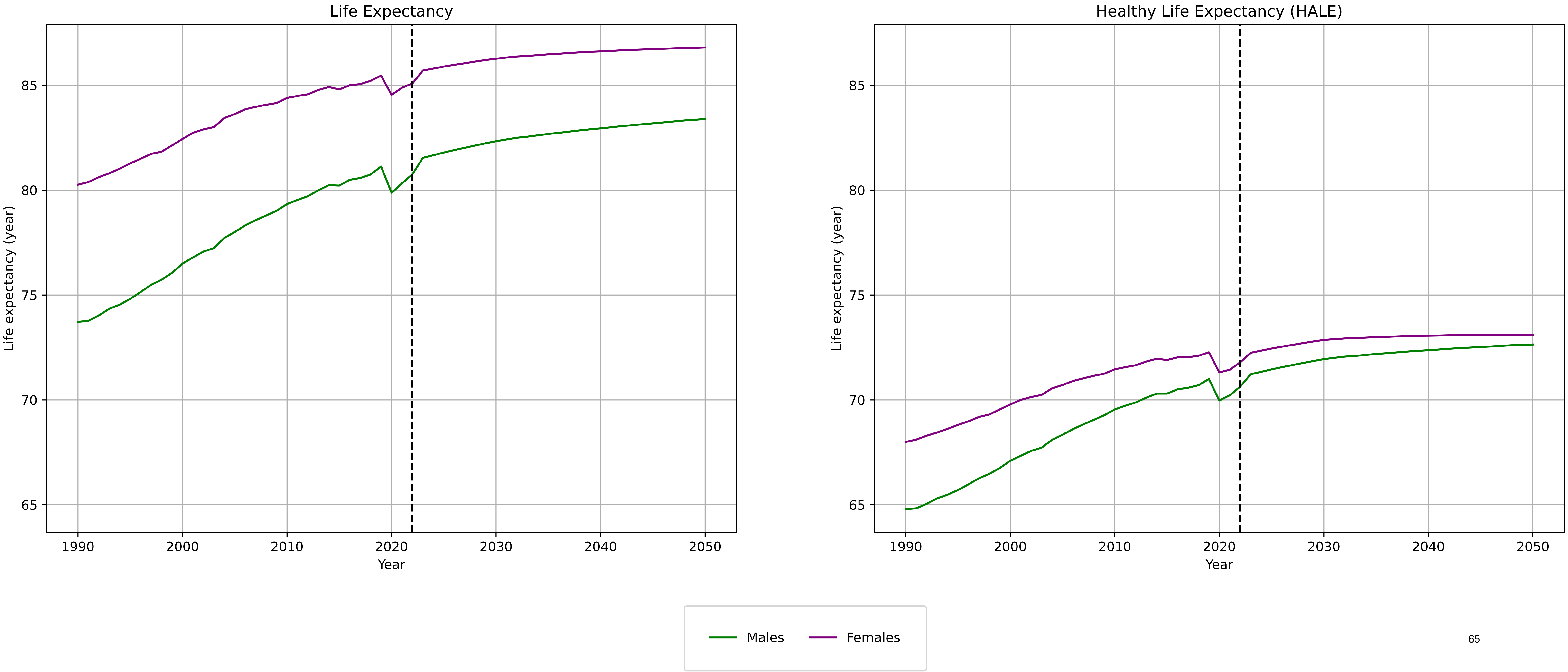

Luxembourg

Supplemental Results Figure S3. Life expectancy and HALE by sex and location, 1990-2050

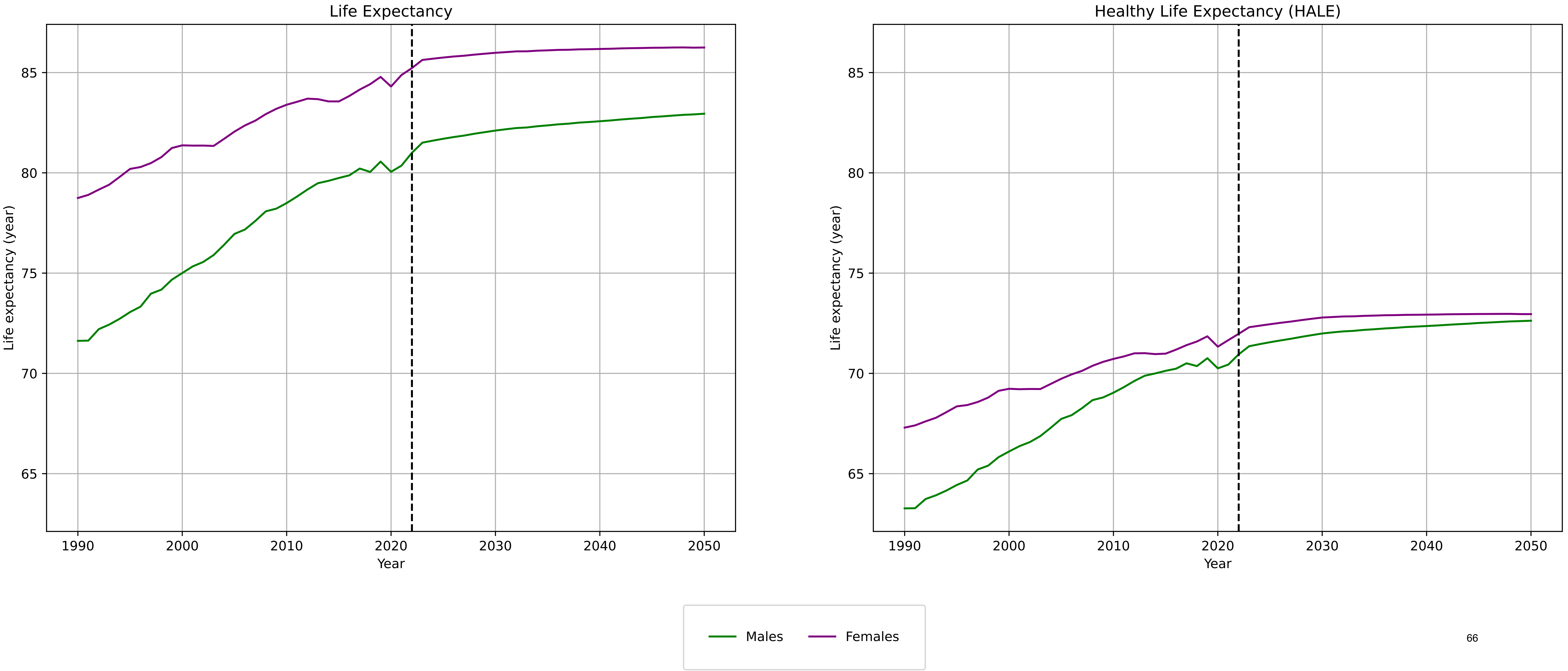

Supplemental Results Figure S3. Life expectancy and HALE by sex and location, 1990-2050

Malta

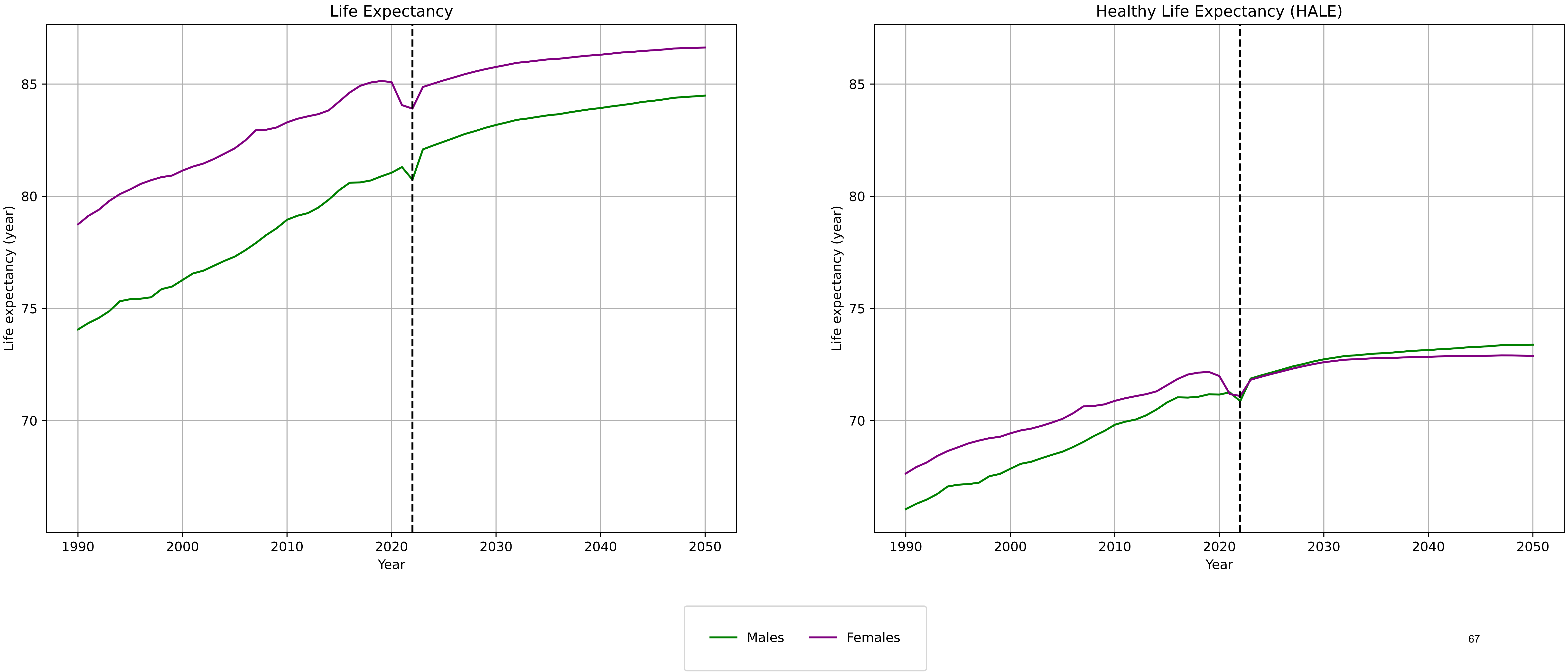

Monaco

Supplemental Results Figure S3. Life expectancy and HALE by sex and location, 1990-2050

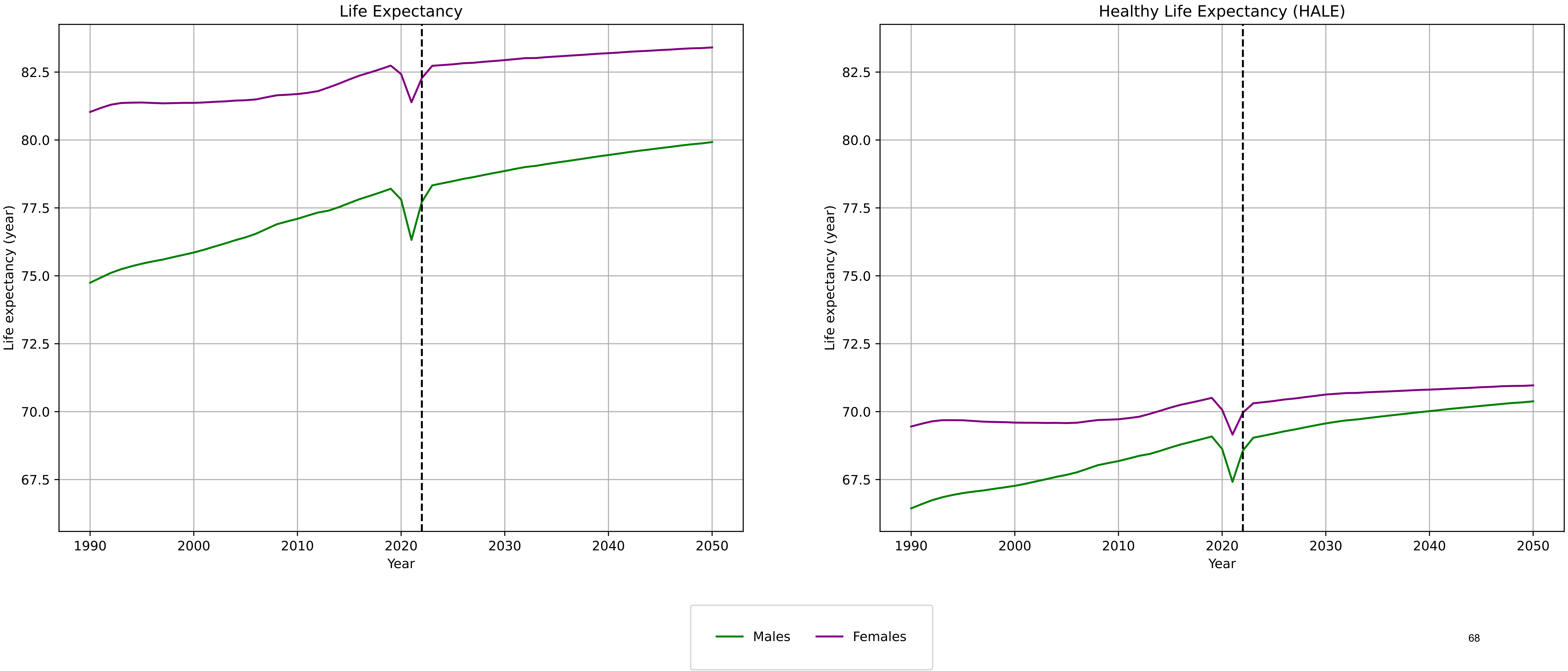

Netherlands

Supplemental Results Figure S3. Life expectancy and HALE by sex and location, 1990-2050

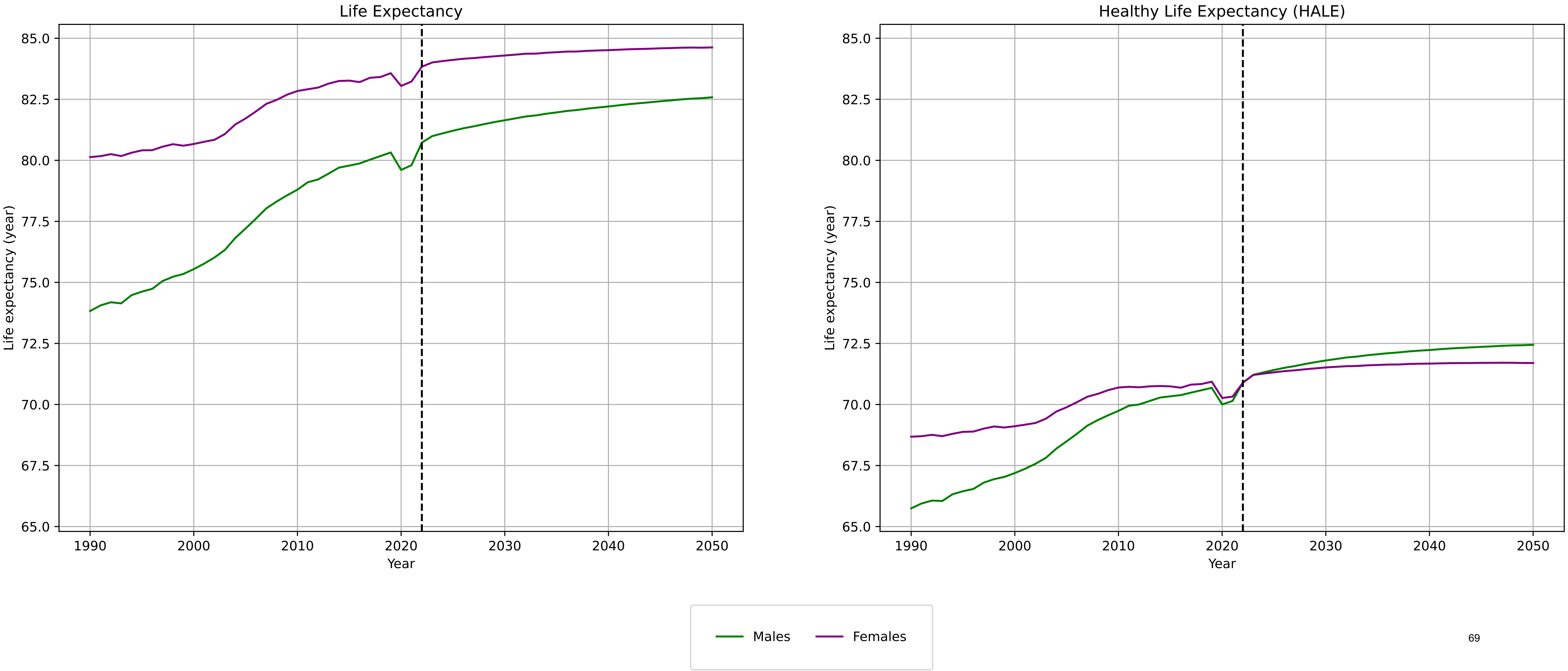

Supplemental Results Figure S3. Life expectancy and HALE by sex and location, 1990-2050

Norway

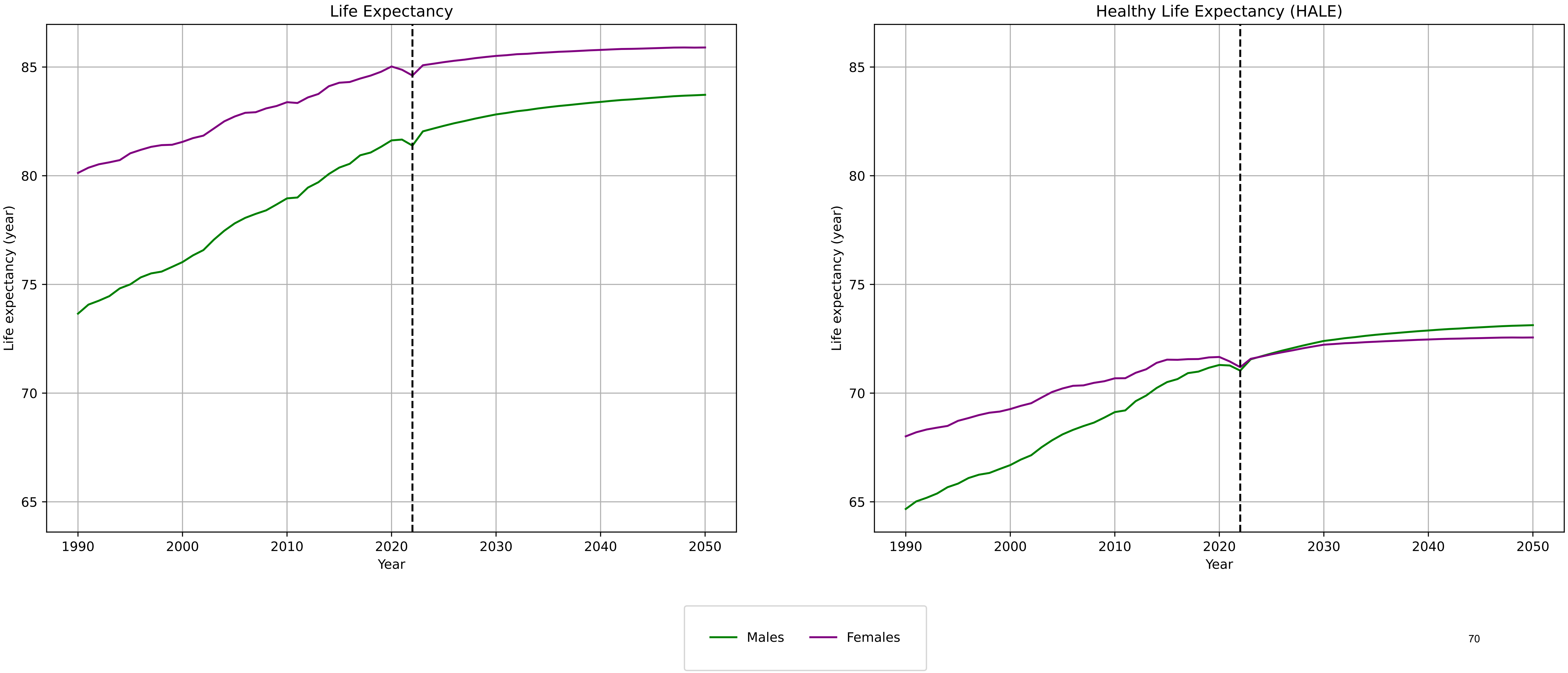

Supplemental Results Figure S3. Life expectancy and HALE by sex and location, 1990-2050

Portugal

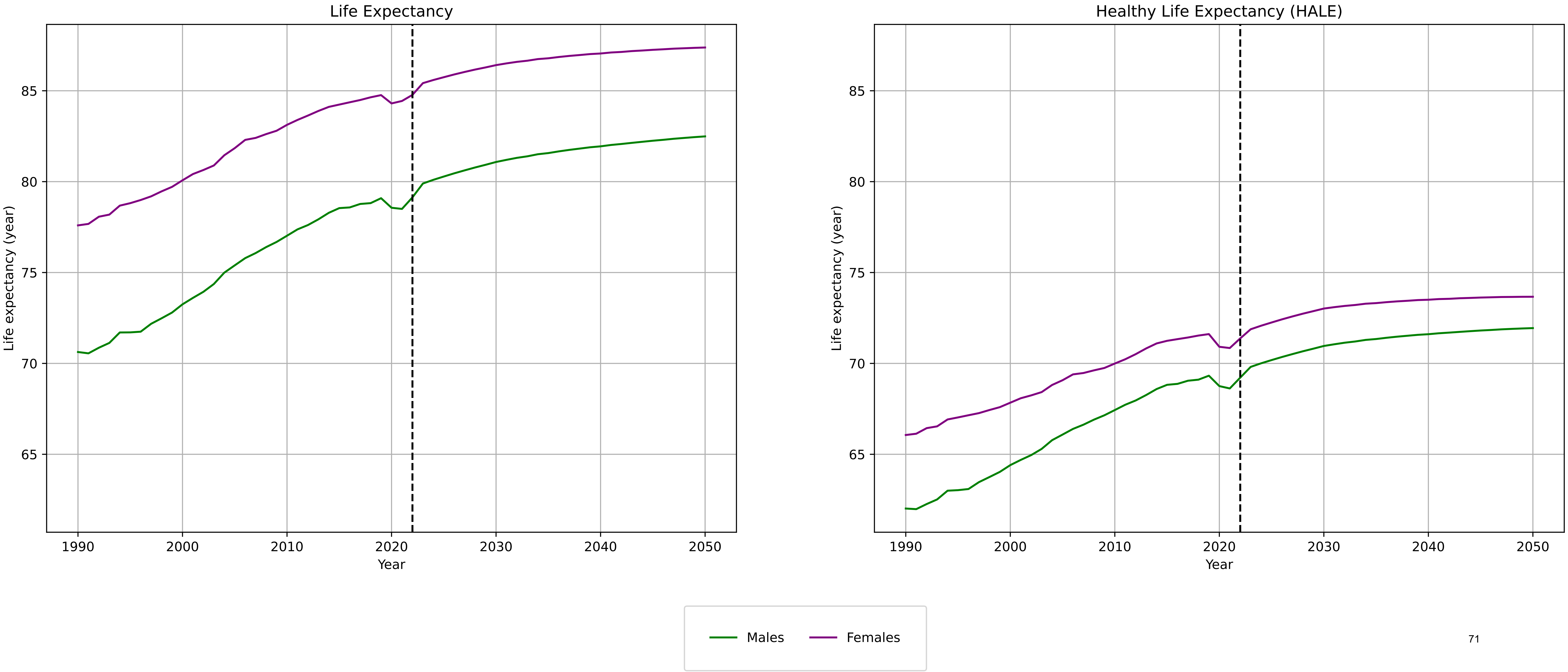

Supplemental Results Figure S3. Life expectancy and HALE by sex and location, 1990-2050

San Marino

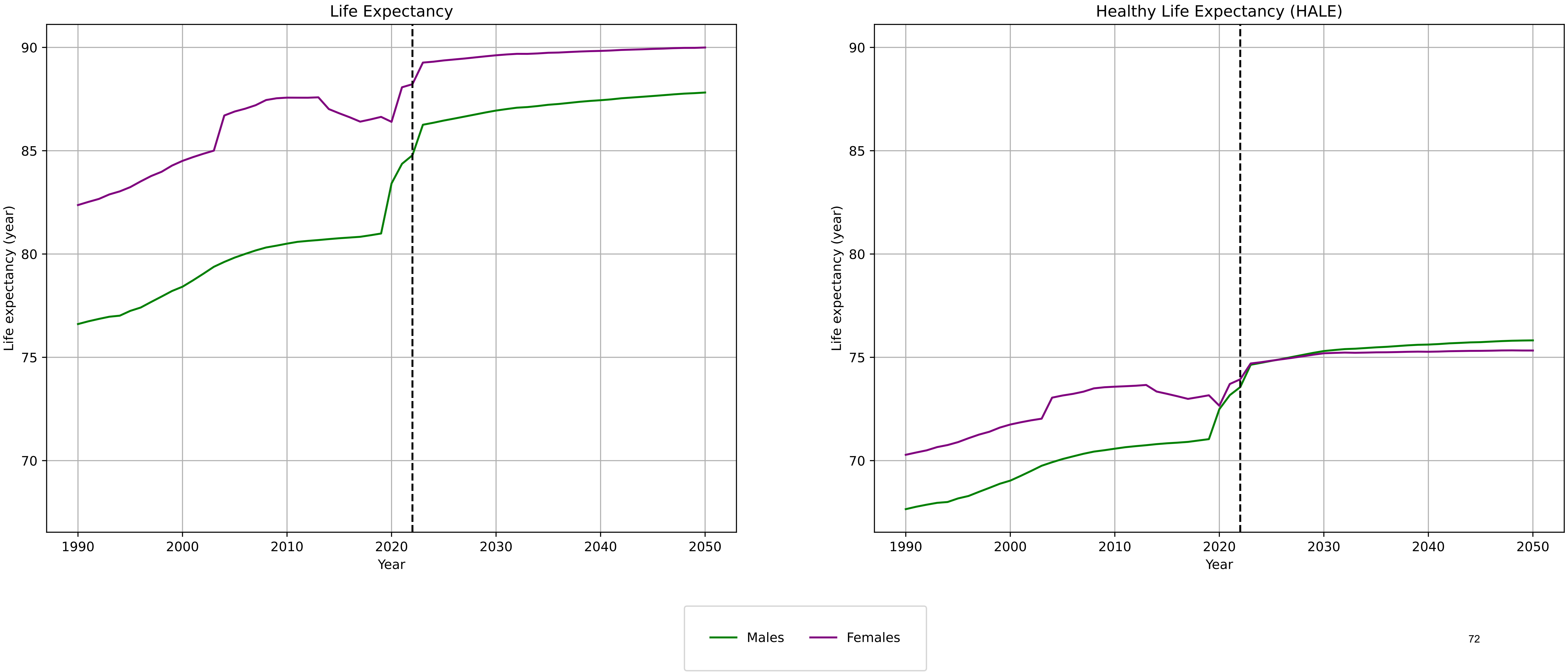

Spain

Supplemental Results Figure S3. Life expectancy and HALE by sex and location, 1990-2050

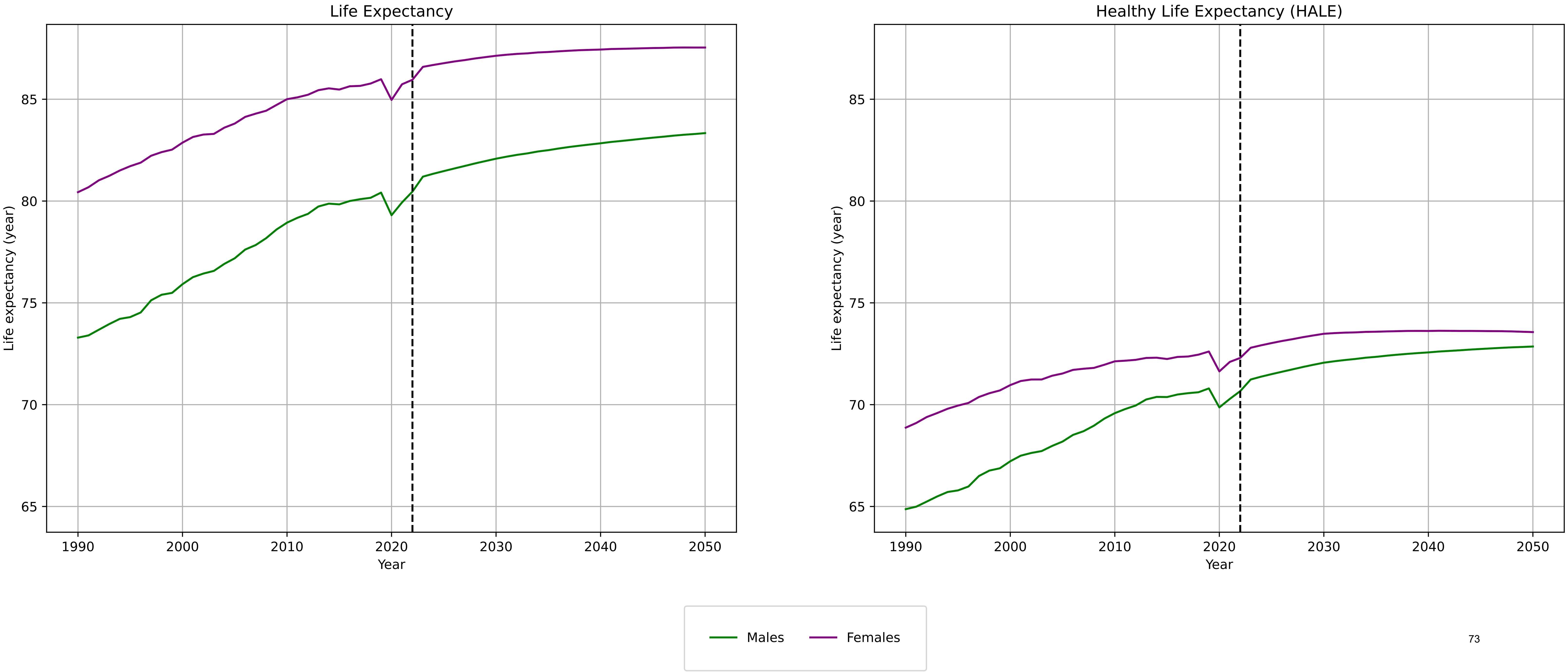

Sweden

Supplemental Results Figure S3. Life expectancy and HALE by sex and location, 1990-2050

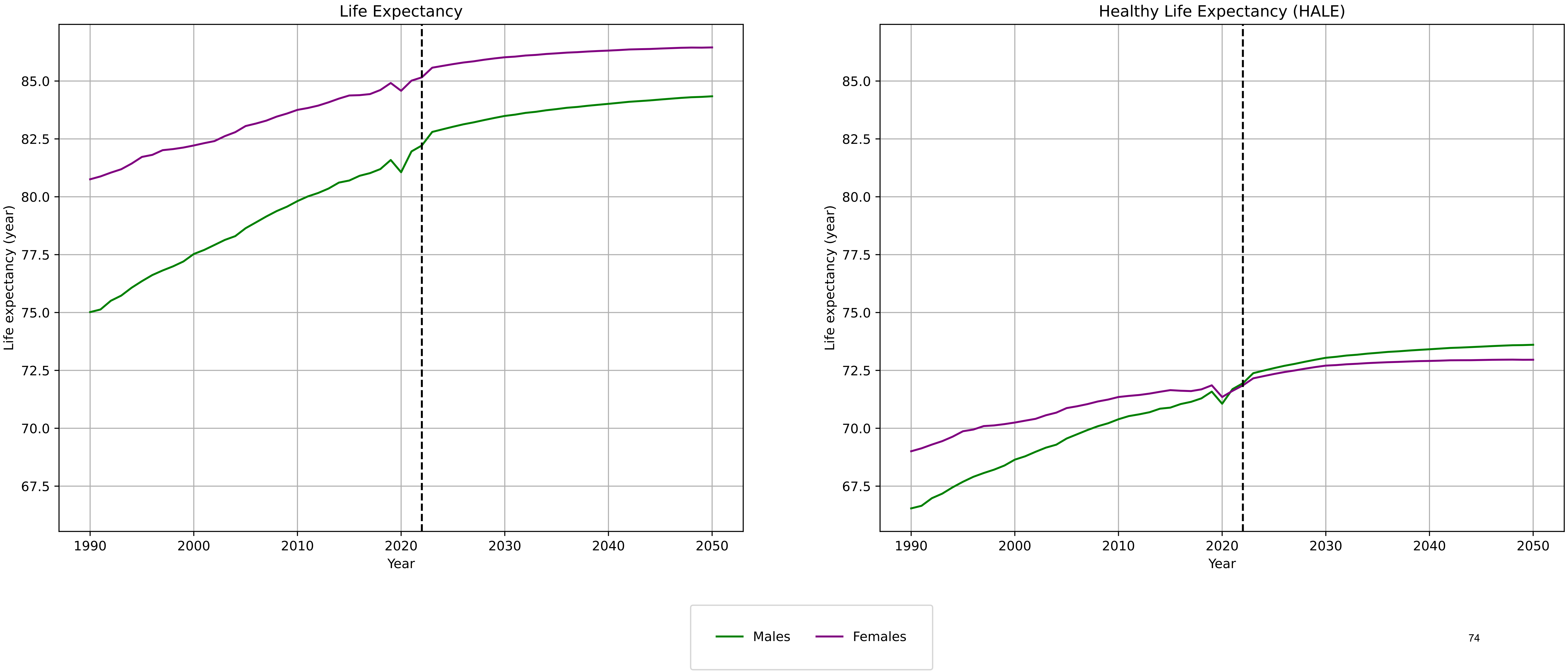

Switzerland

Supplemental Results Figure S3. Life expectancy and HALE by sex and location, 1990-2050

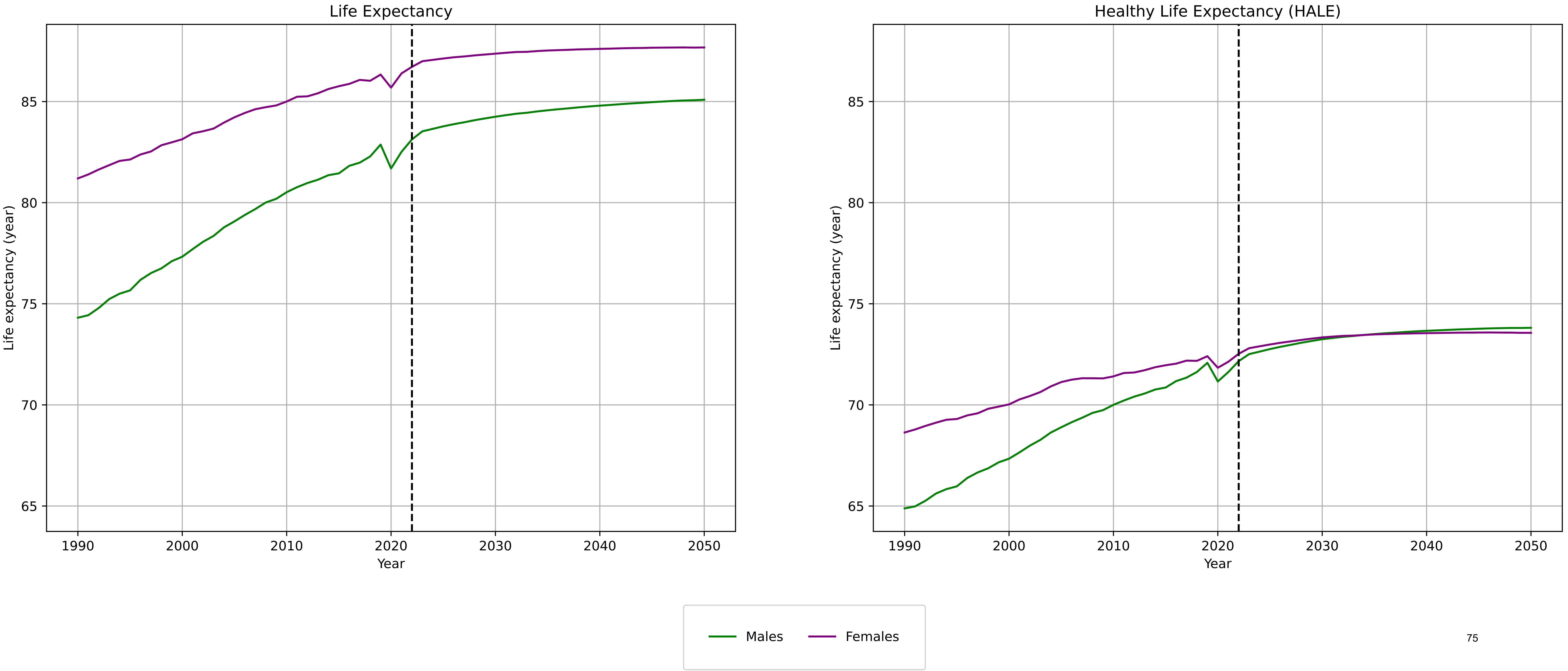

Supplemental Results Figure S3. Life expectancy and HALE by sex and location, 1990-2050

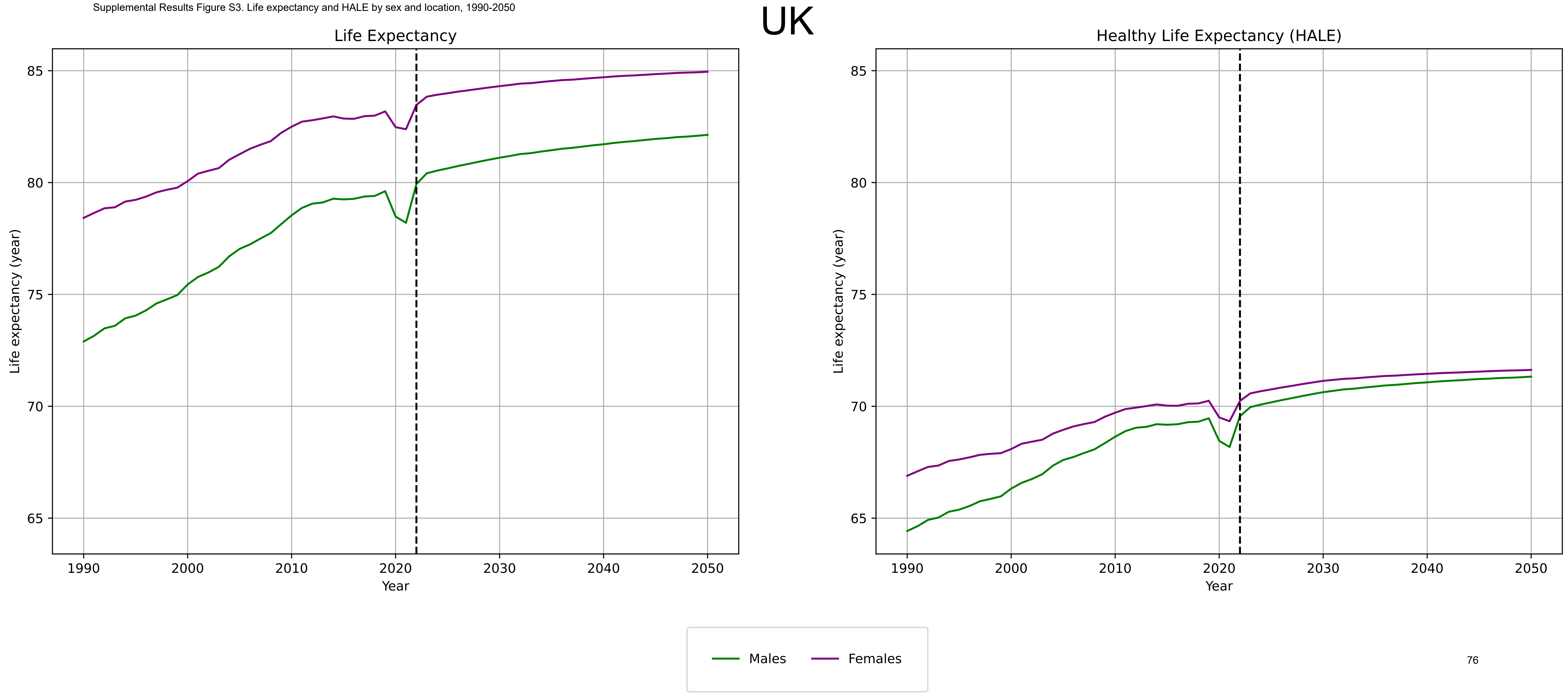

Bolivia

Supplemental Results Figure S3. Life expectancy and HALE by sex and location, 1990-2050

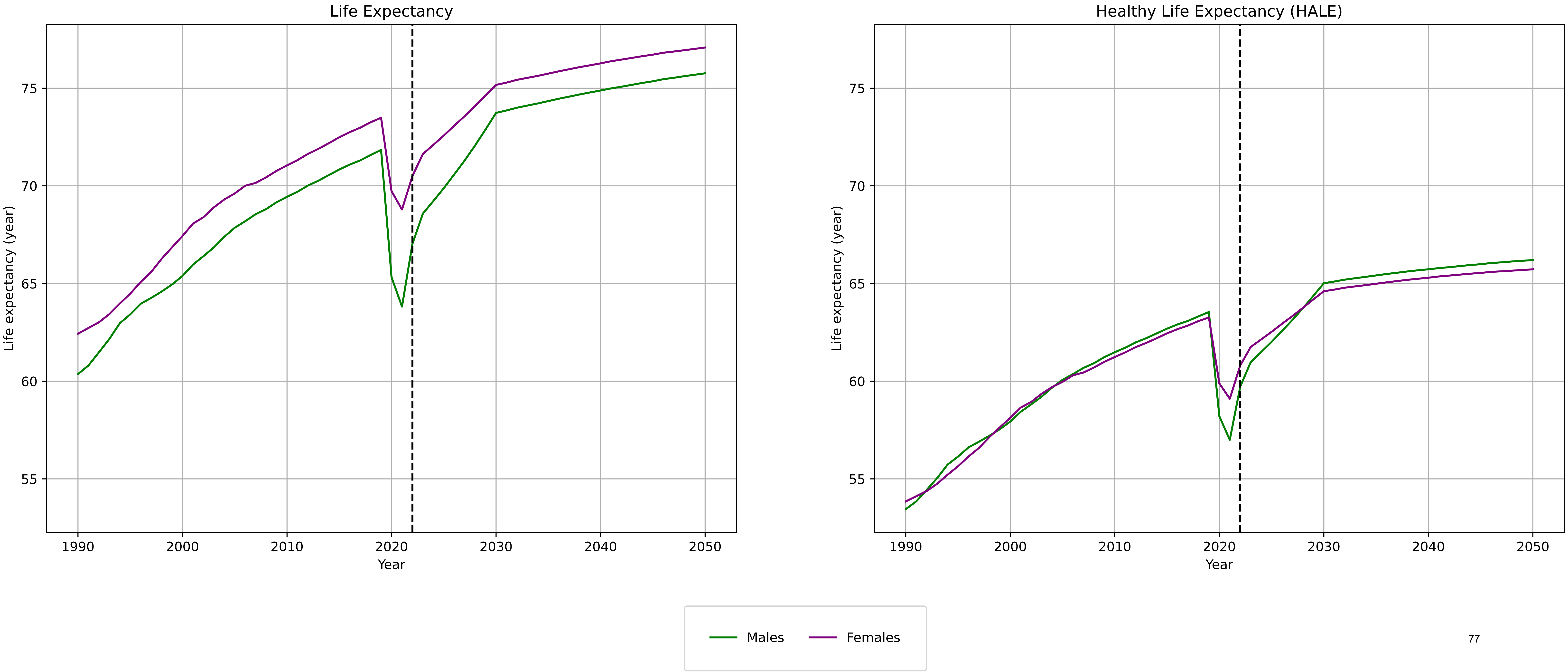

Ecuador

Supplemental Results Figure S3. Life expectancy and HALE by sex and location, 1990-2050

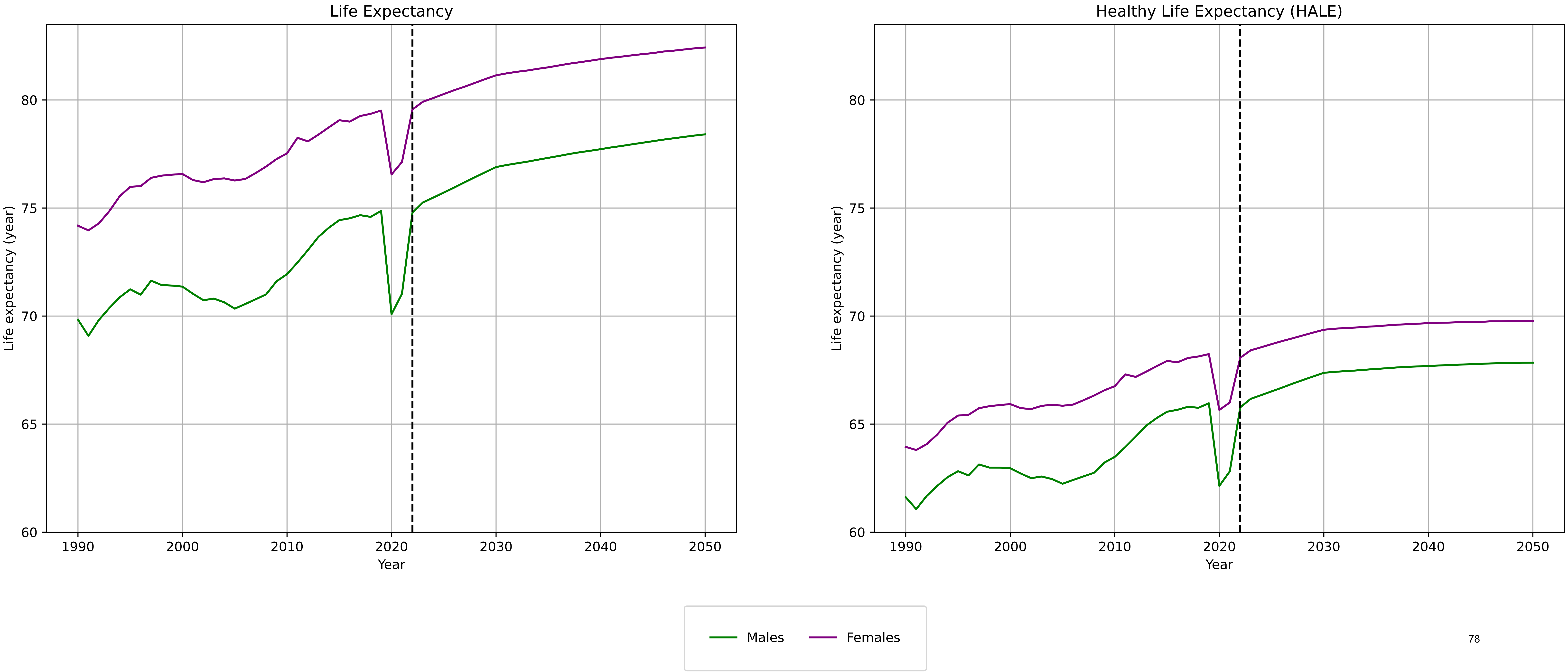

Peru

Supplemental Results Figure S3. Life expectancy and HALE by sex and location, 1990-2050

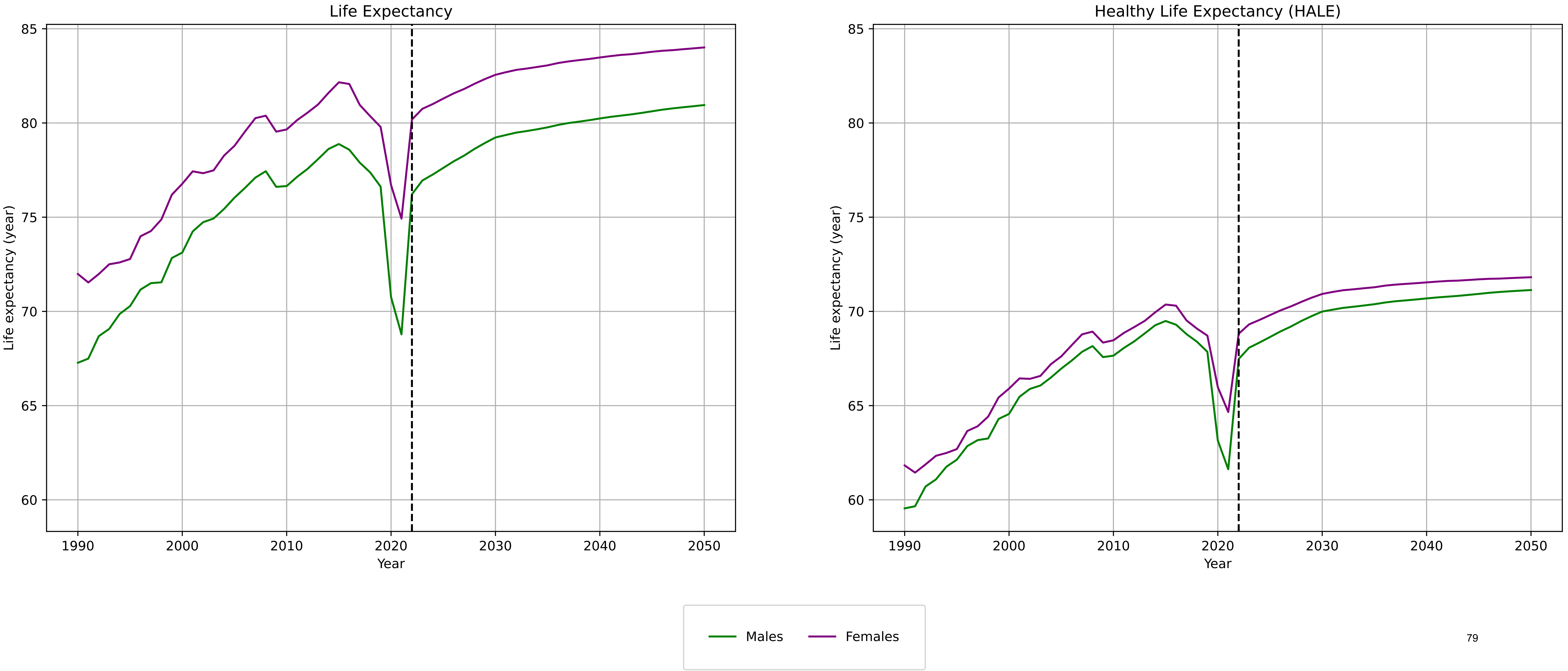

Supplemental Results Figure S3. Life expectancy and HALE by sex and location, 1990-2050

Antigua And Barbuda

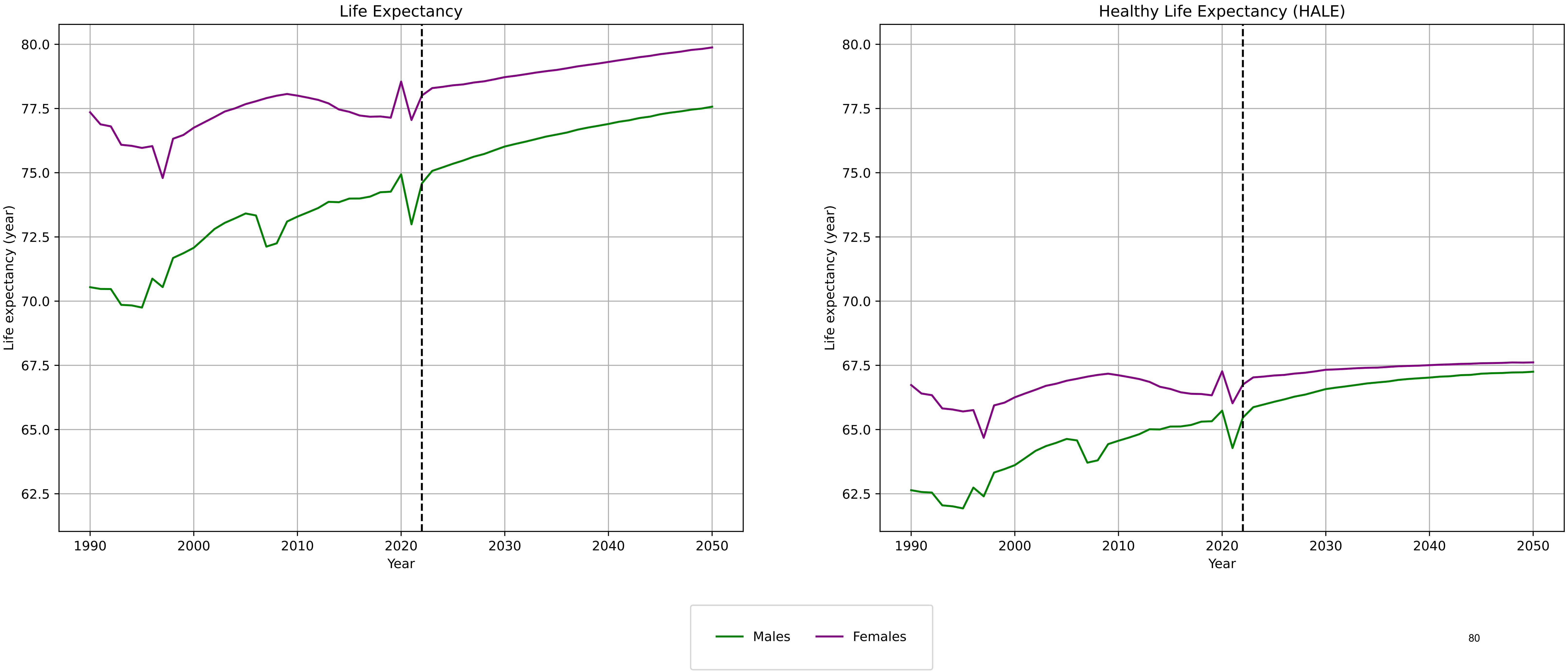

The Bahamas

Supplemental Results Figure S3. Life expectancy and HALE by sex and location, 1990-2050

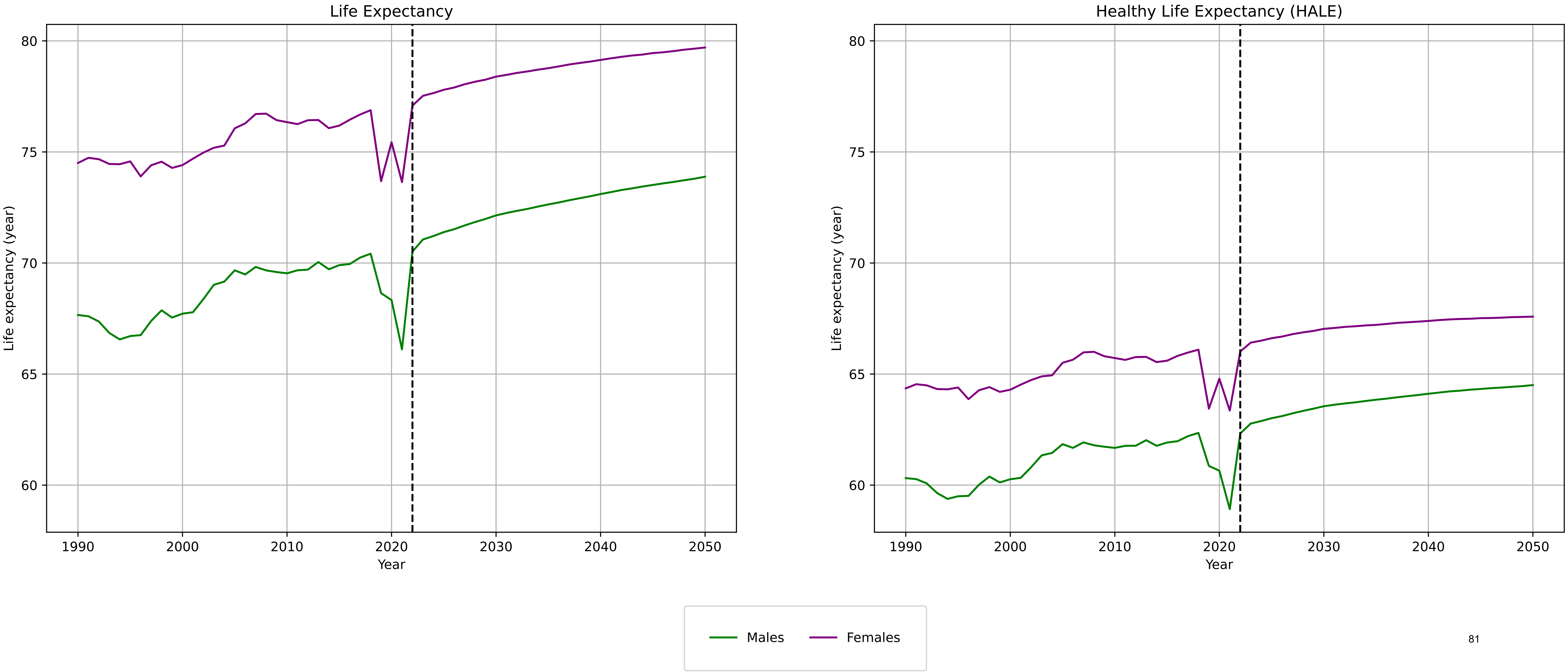

Barbados

Supplemental Results Figure S3. Life expectancy and HALE by sex and location, 1990-2050

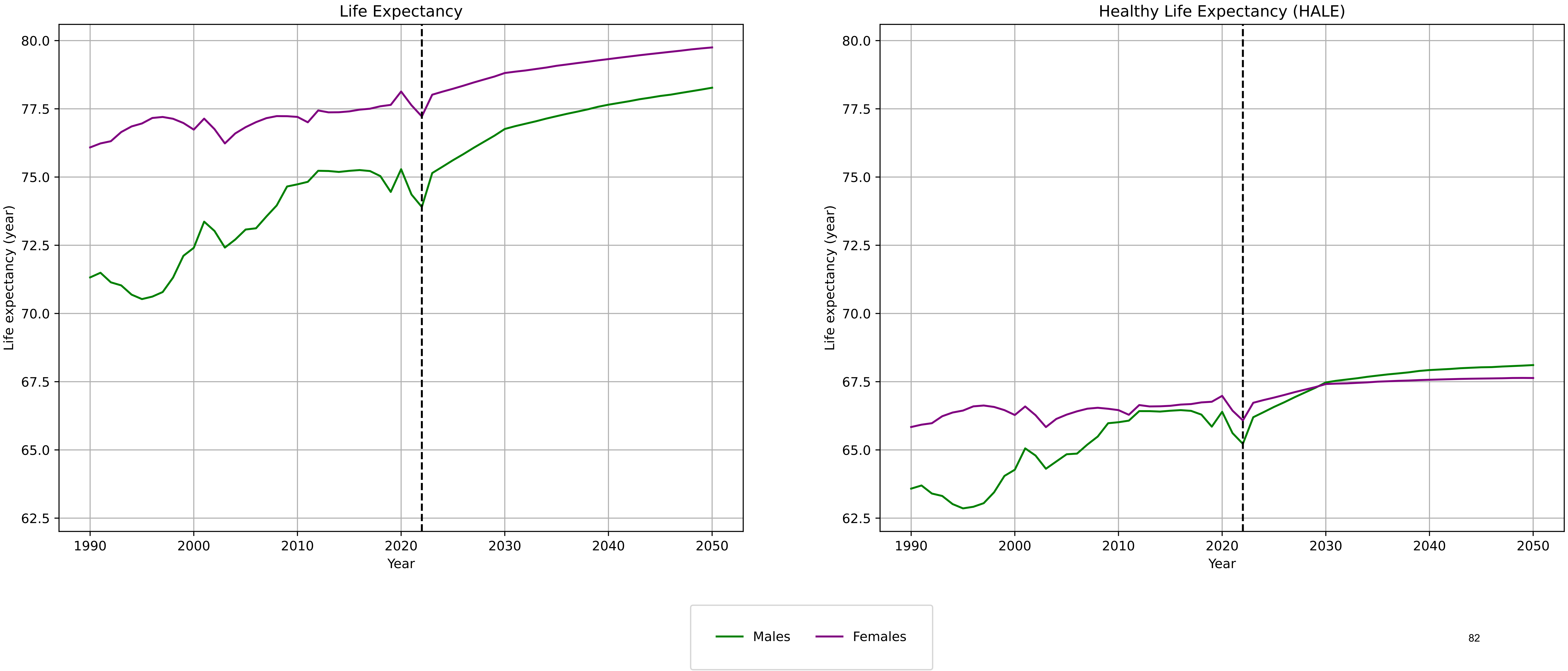

Belize

Supplemental Results Figure S3. Life expectancy and HALE by sex and location, 1990-2050

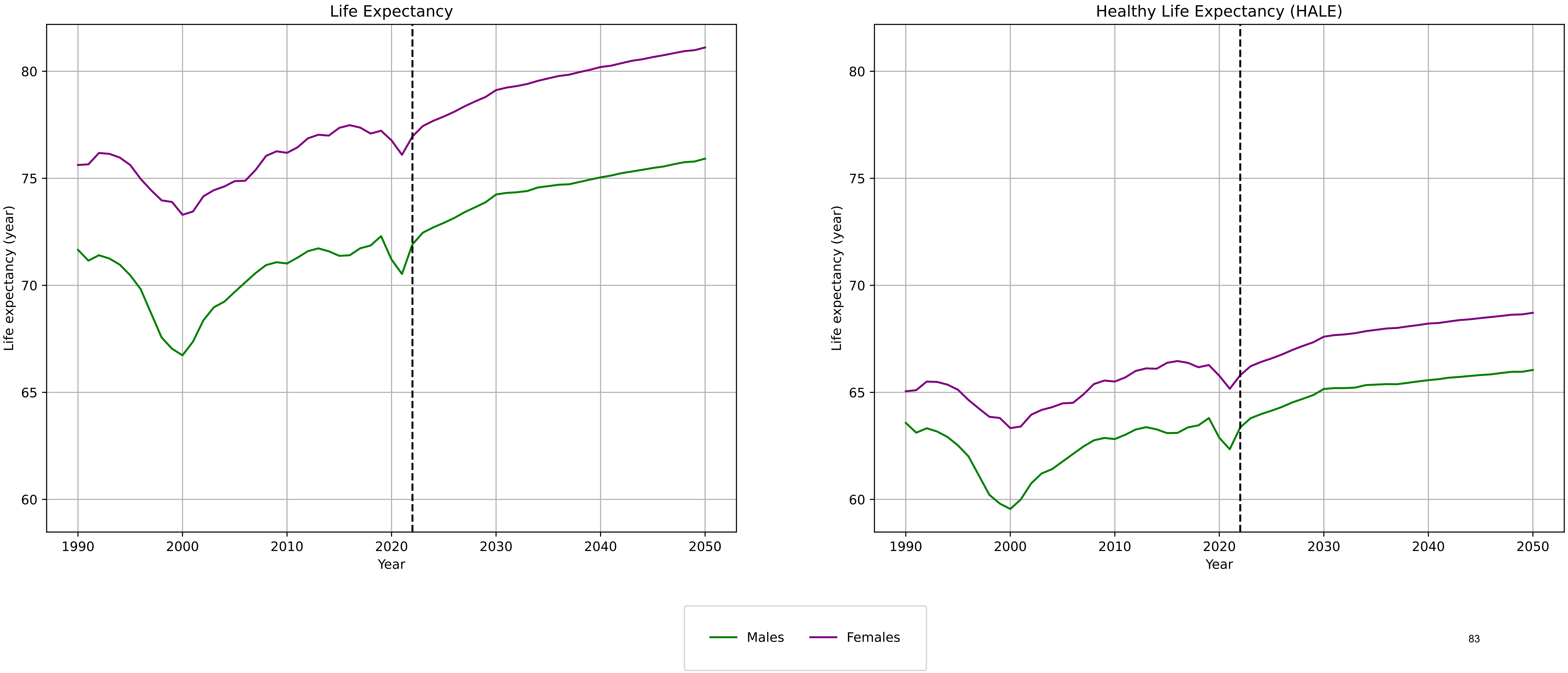

Bermuda

Supplemental Results Figure S3. Life expectancy and HALE by sex and location, 1990-2050

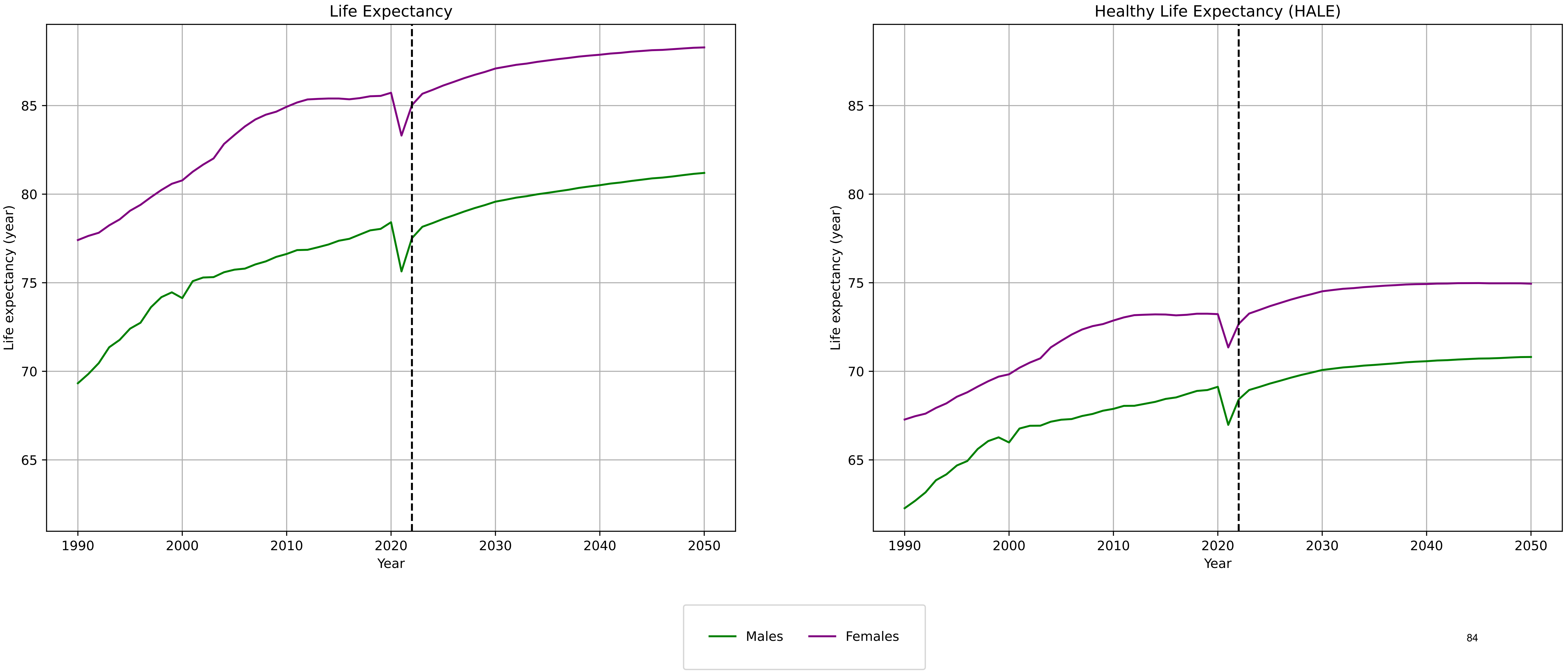

Cuba

Supplemental Results Figure S3. Life expectancy and HALE by sex and location, 1990-2050

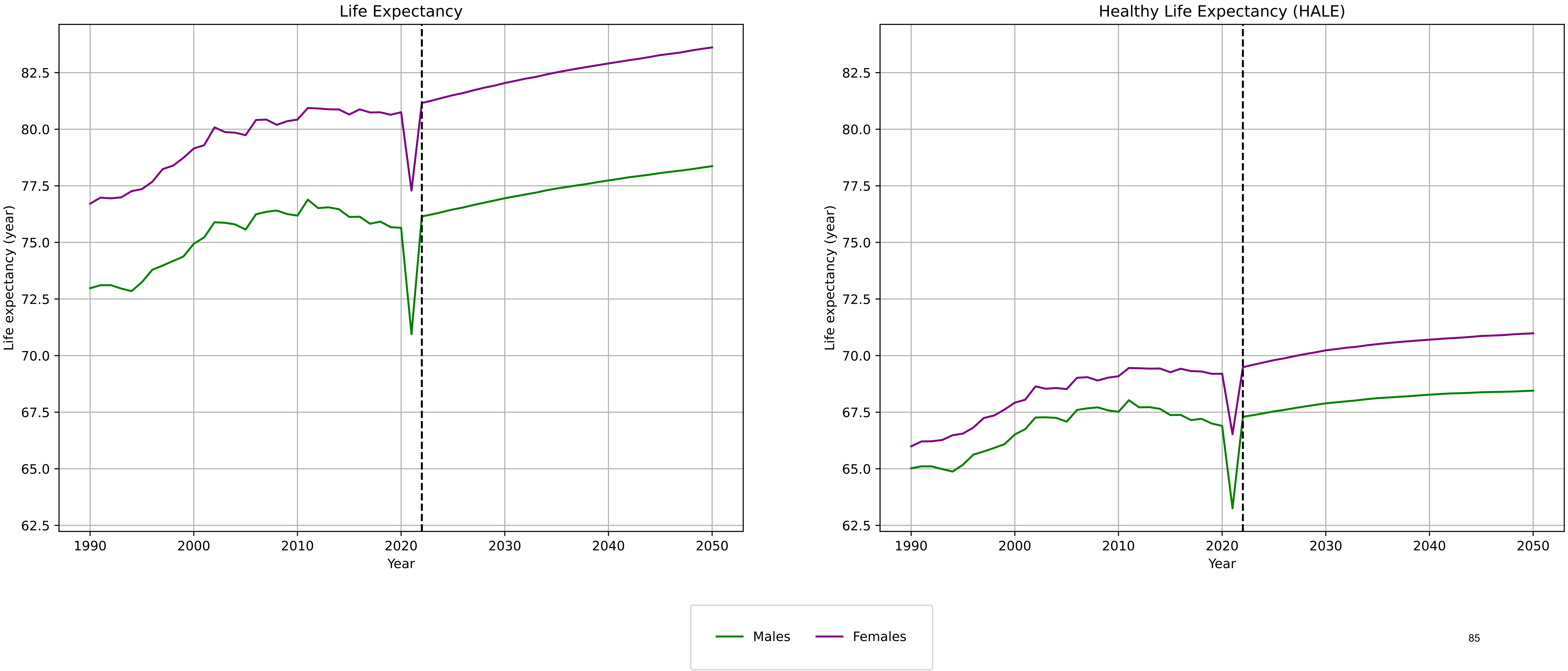

Dominica

Supplemental Results Figure S3. Life expectancy and HALE by sex and location, 1990-2050

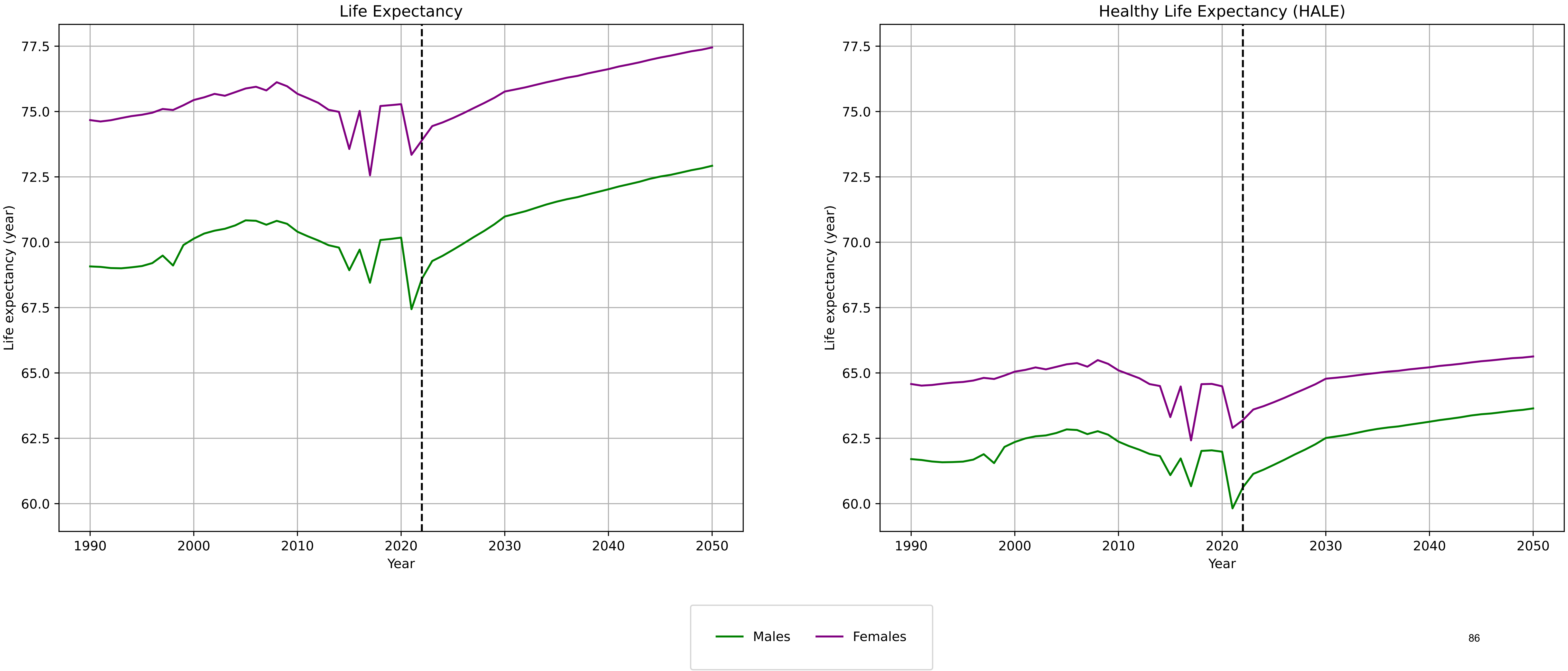

Dominican Republic

Supplemental Results Figure S3. Life expectancy and HALE by sex and location, 1990-2050

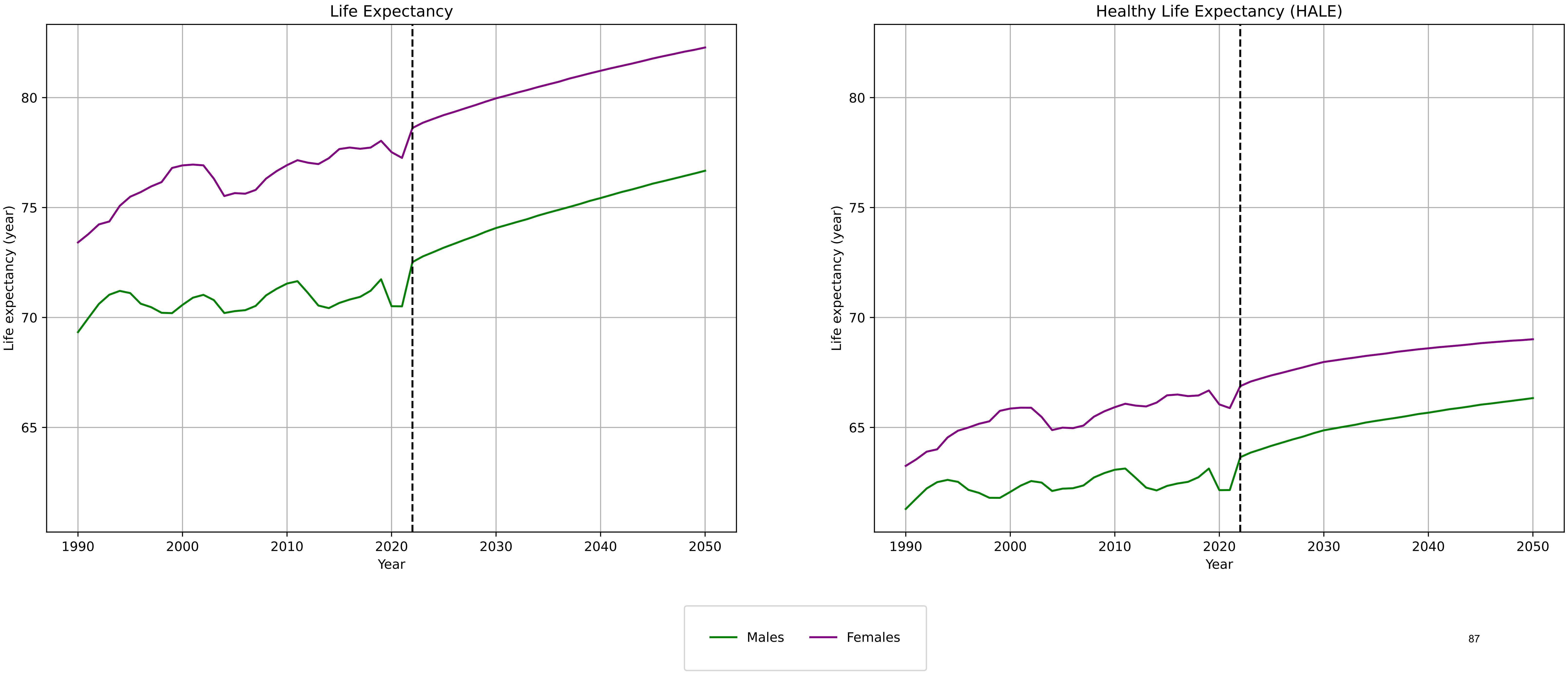

Grenada

Supplemental Results Figure S3. Life expectancy and HALE by sex and location, 1990-2050

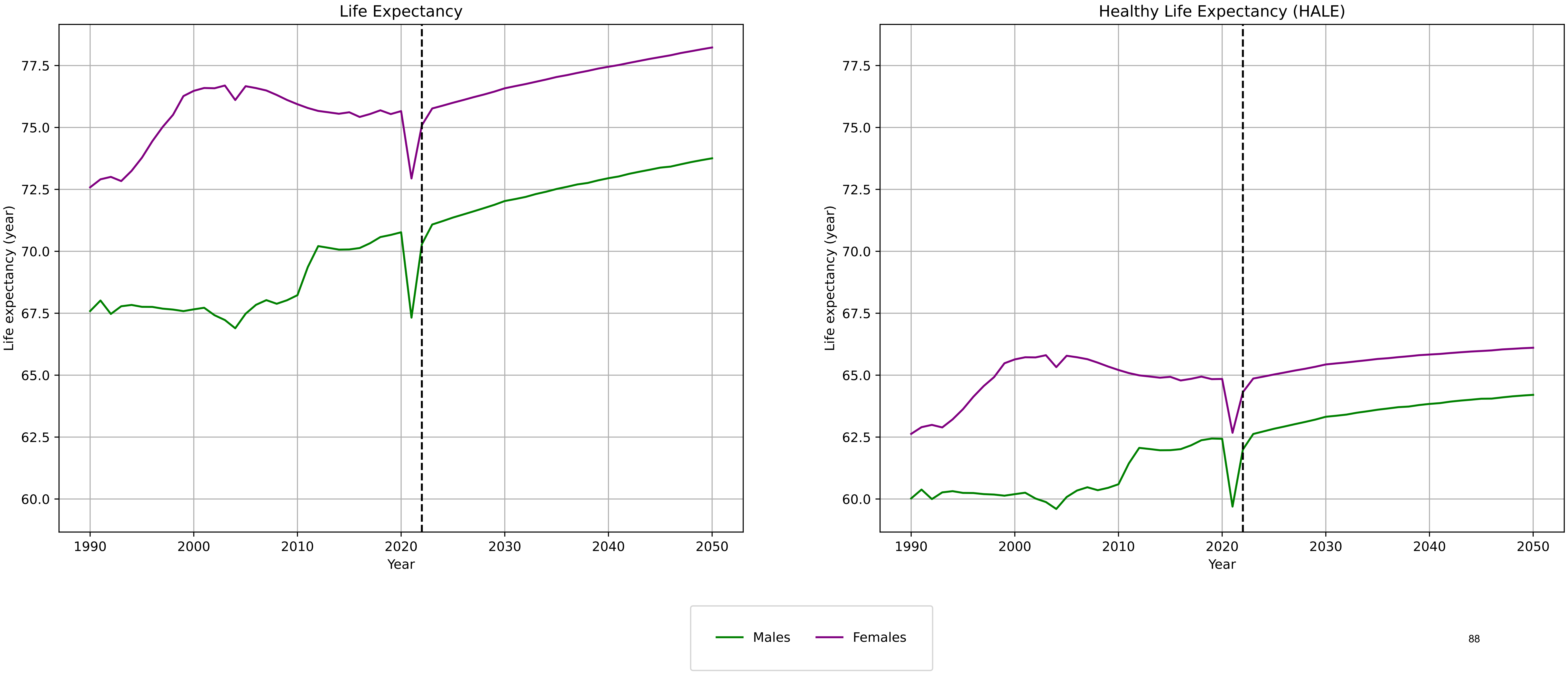

Supplemental Results Figure S3. Life expectancy and HALE by sex and location, 1990-2050

Guyana

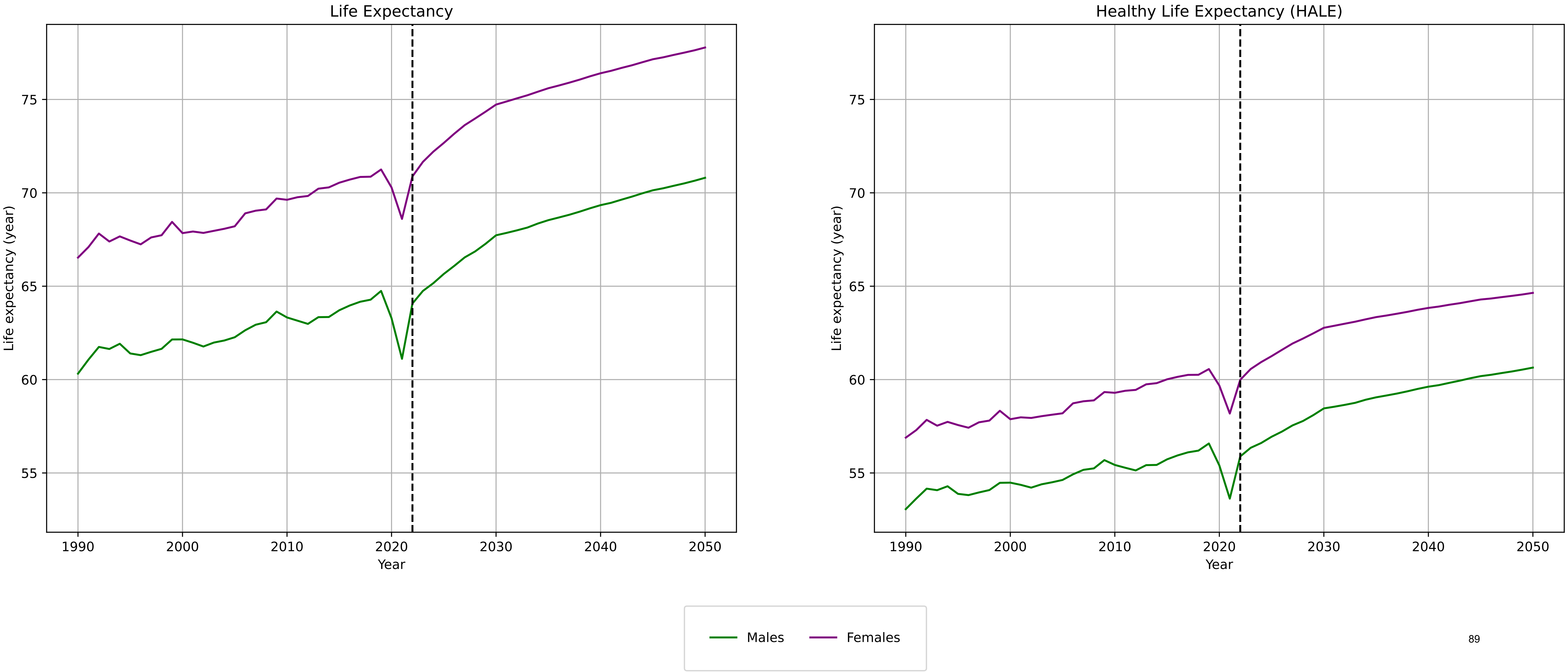

Haiti

Supplemental Results Figure S3. Life expectancy and HALE by sex and location, 1990-2050

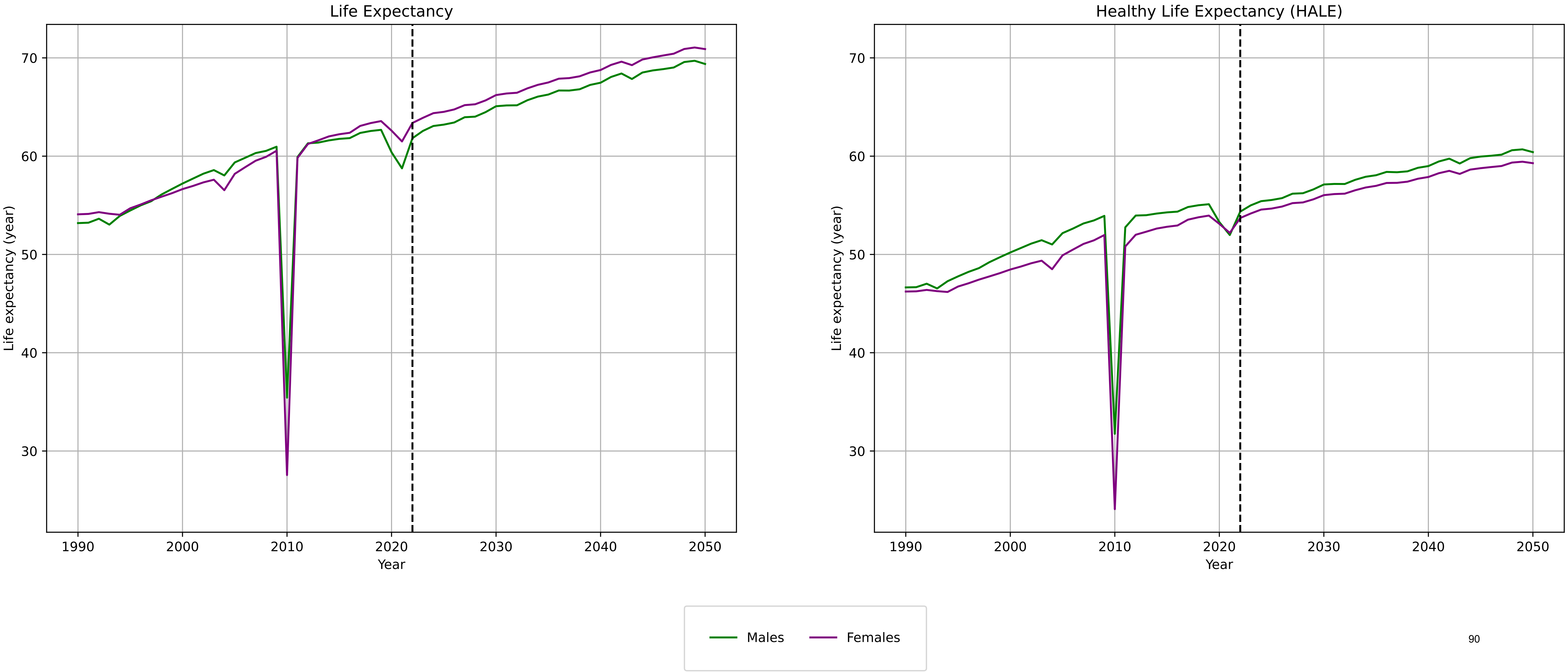

Jamaica

Supplemental Results Figure S3. Life expectancy and HALE by sex and location, 1990-2050

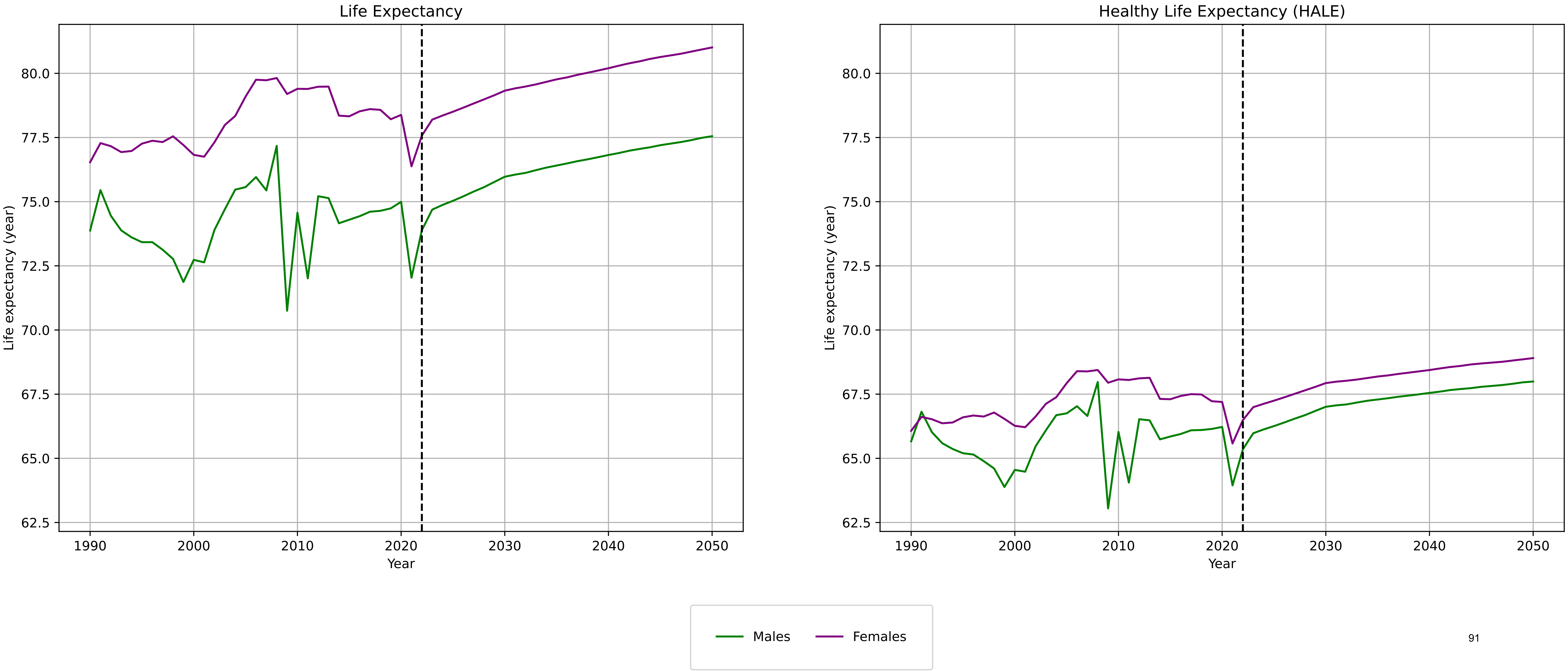

Puerto Rico

Supplemental Results Figure S3. Life expectancy and HALE by sex and location, 1990-2050

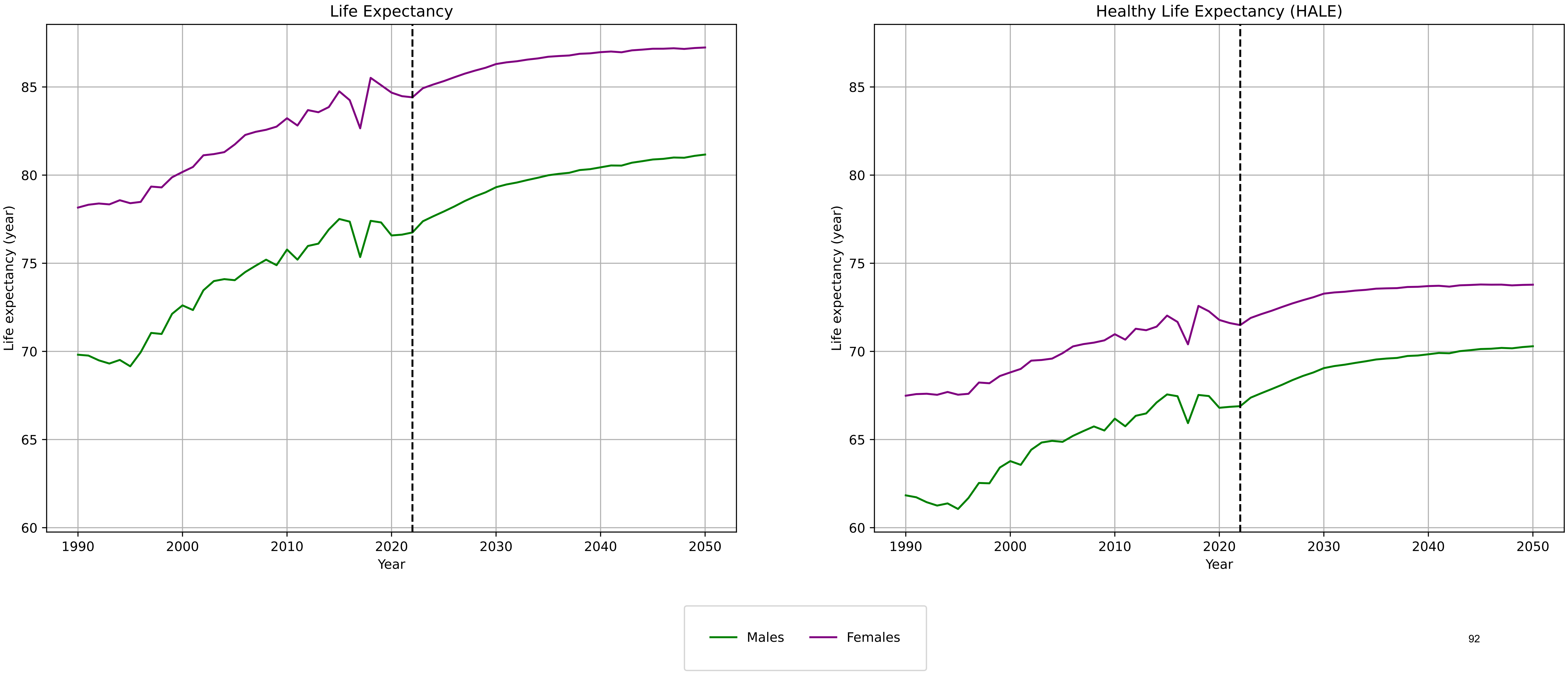

Saint Kitts And Nevis

Supplemental Results Figure S3. Life expectancy and HALE by sex and location, 1990-2050

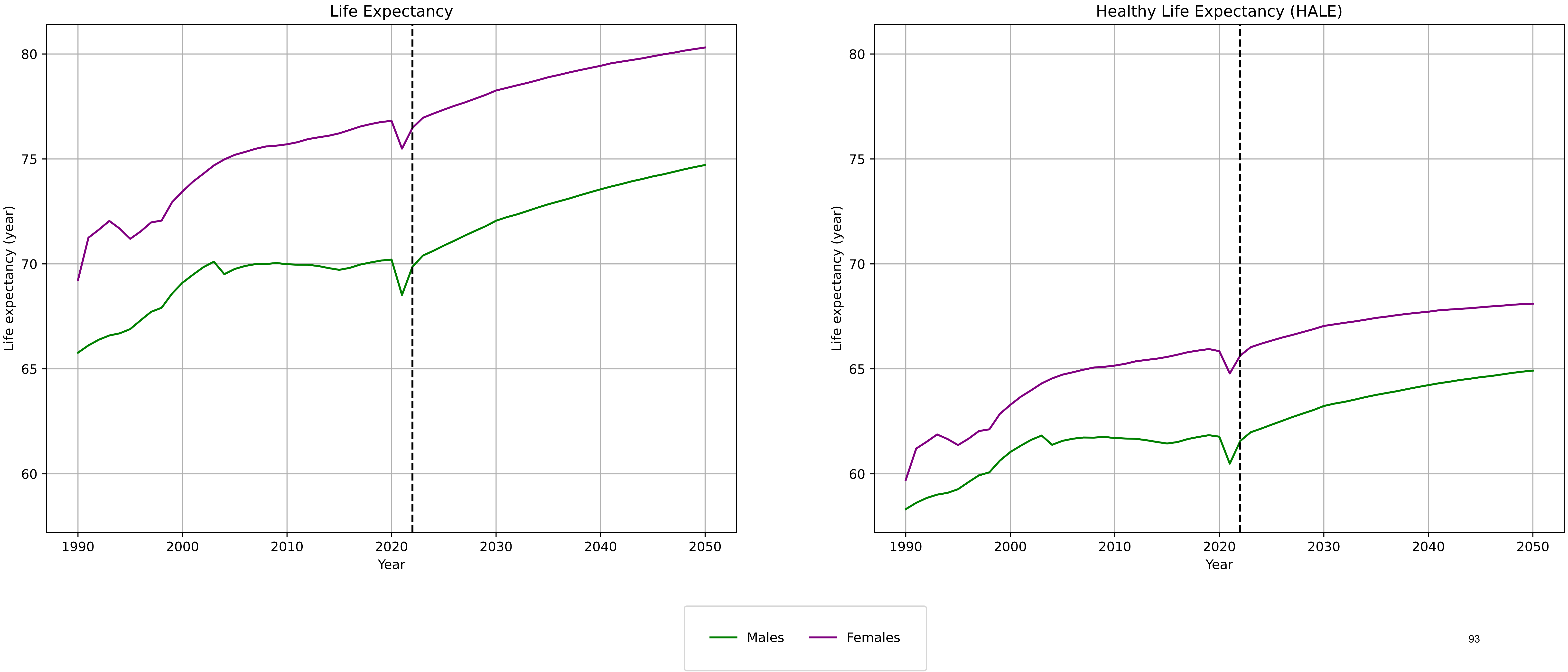

Saint Lucia

Supplemental Results Figure S3. Life expectancy and HALE by sex and location, 1990-2050

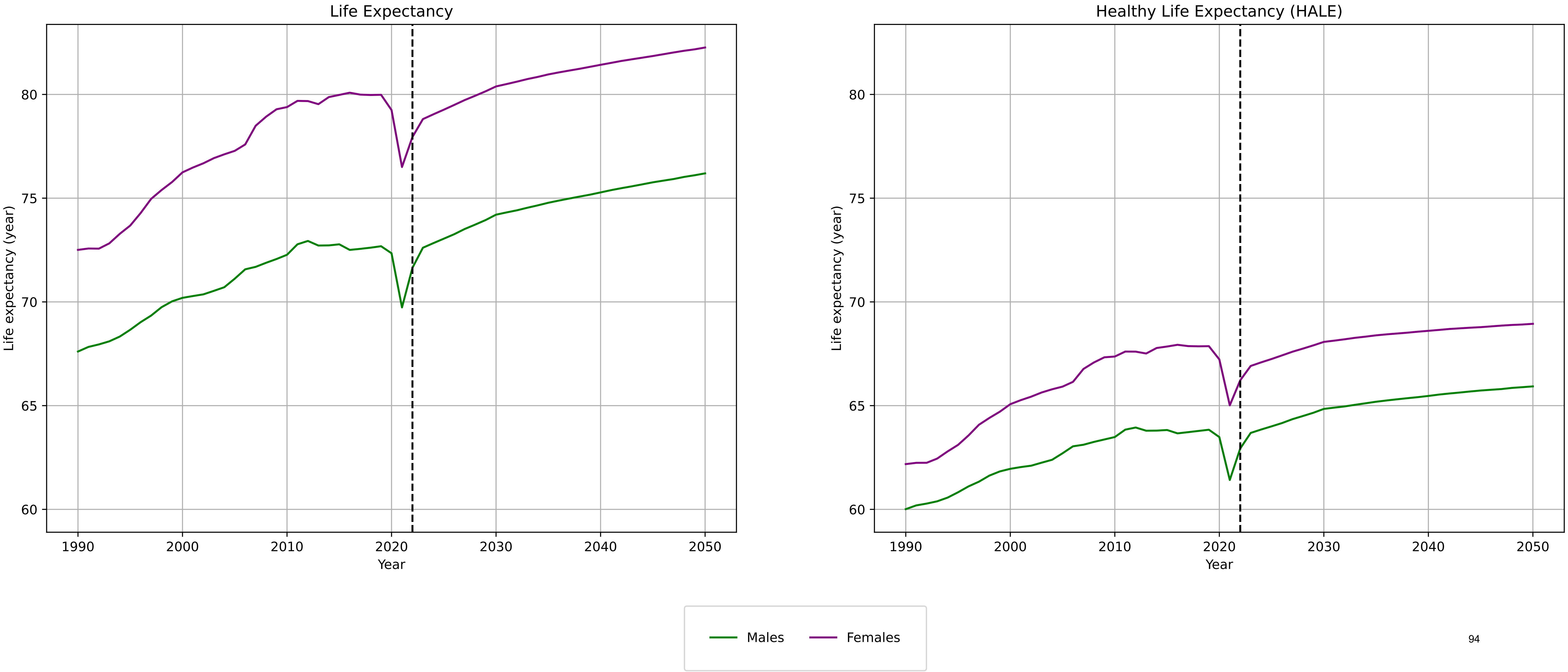

Supplemental Results Figure S3. Life expectancy and HALE by sex and location, 1990-2050

Saint Vincent And The Grenadines

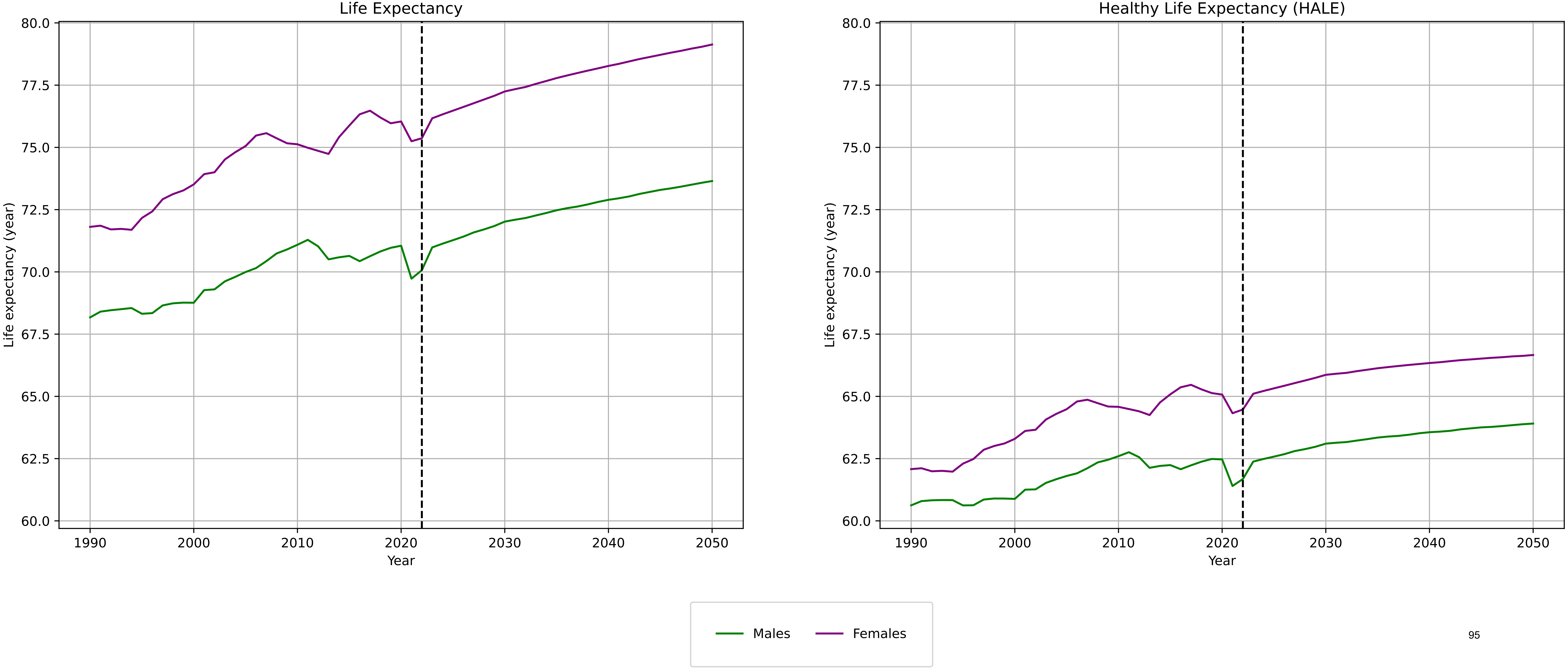

Suriname

Supplemental Results Figure S3. Life expectancy and HALE by sex and location, 1990-2050

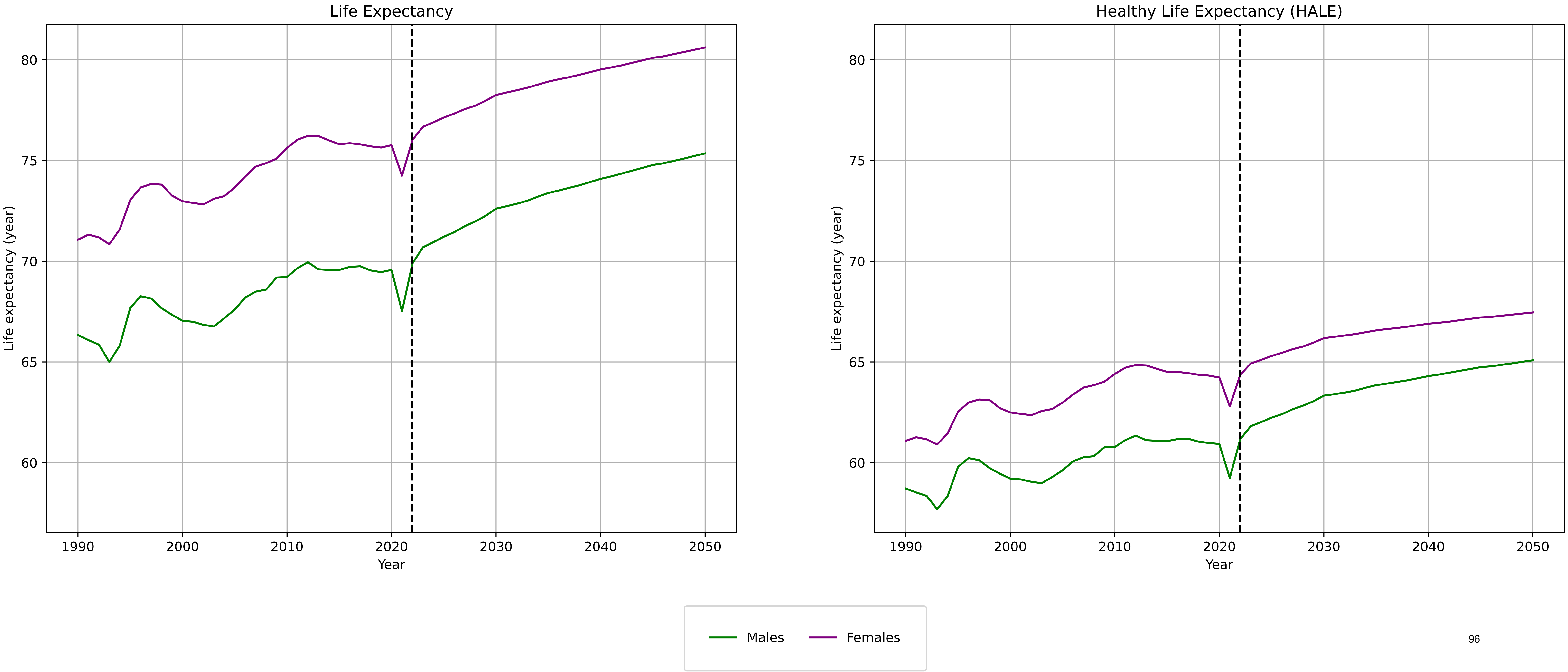

Trinidad And Tobago

Supplemental Results Figure S3. Life expectancy and HALE by sex and location, 1990-2050

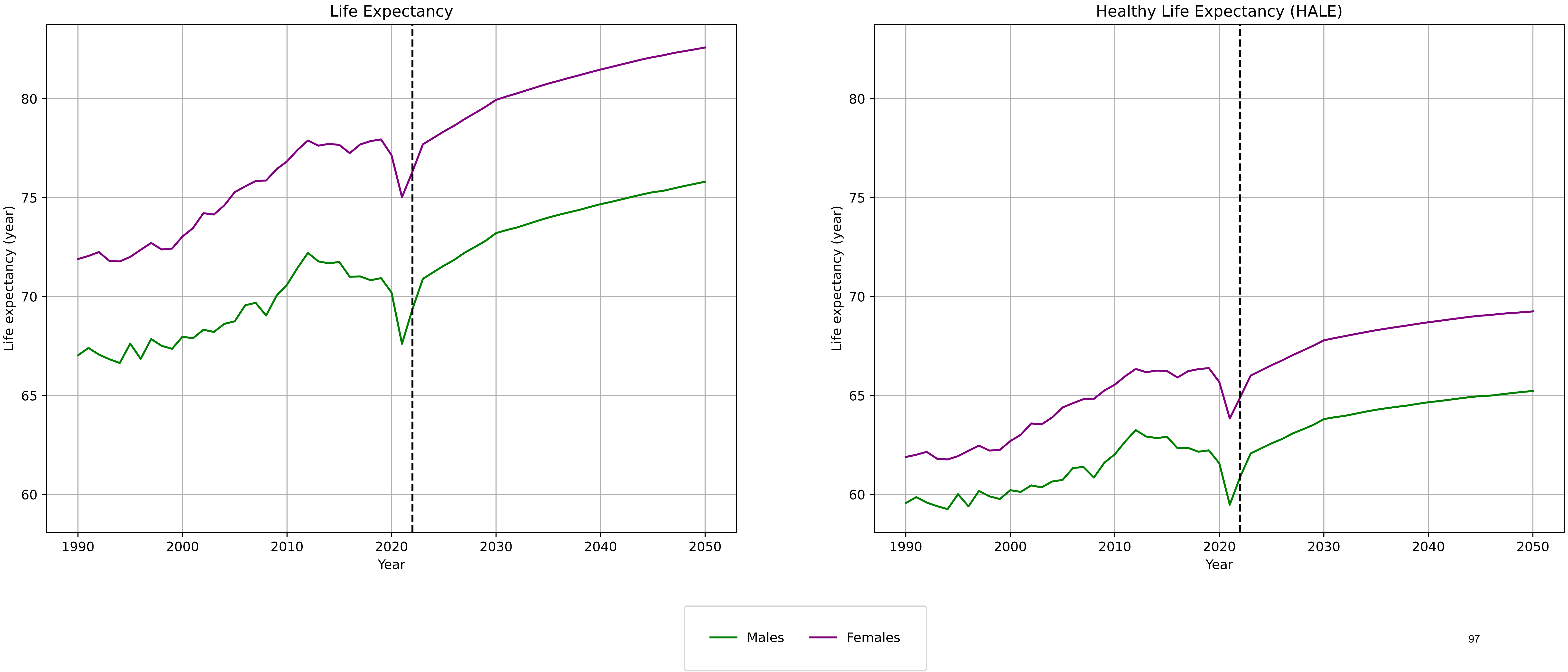

Virgin Islands

Supplemental Results Figure S3. Life expectancy and HALE by sex and location, 1990-2050

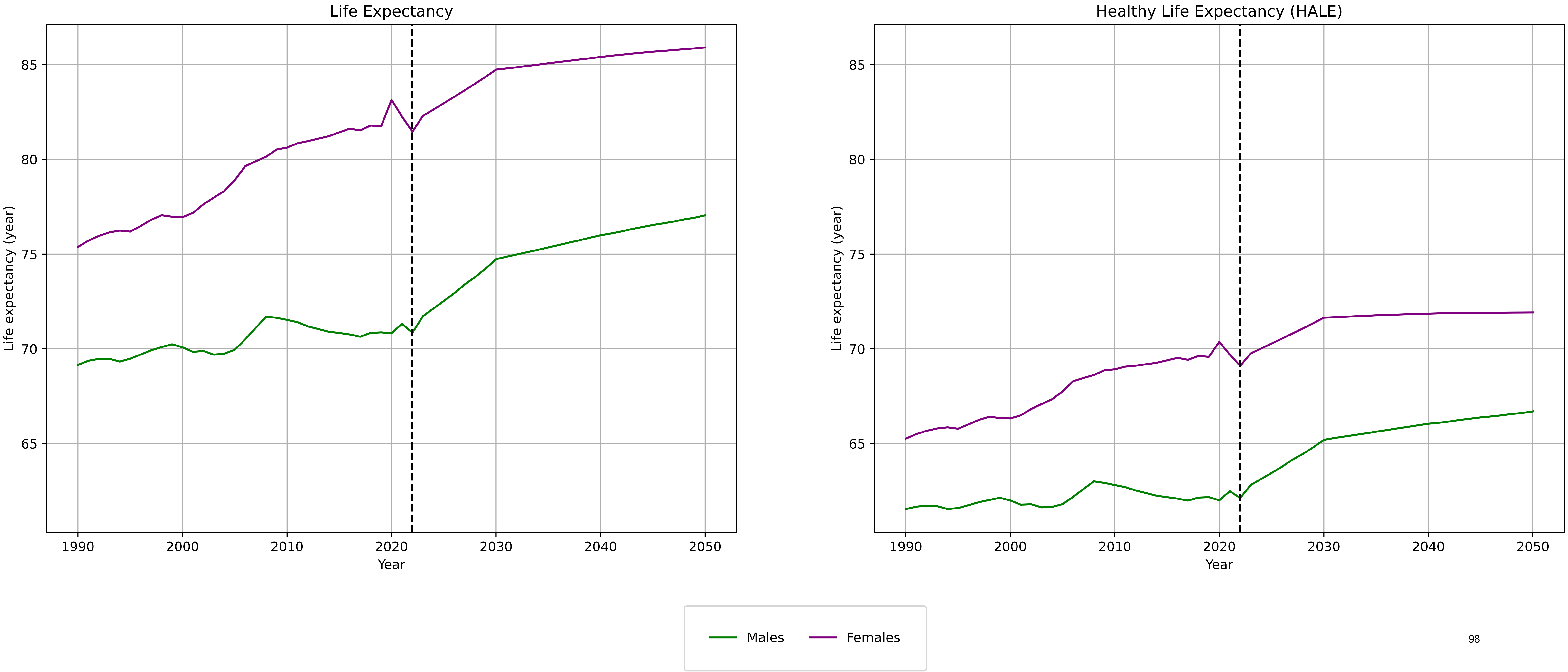

Colombia

Supplemental Results Figure S3. Life expectancy and HALE by sex and location, 1990-2050

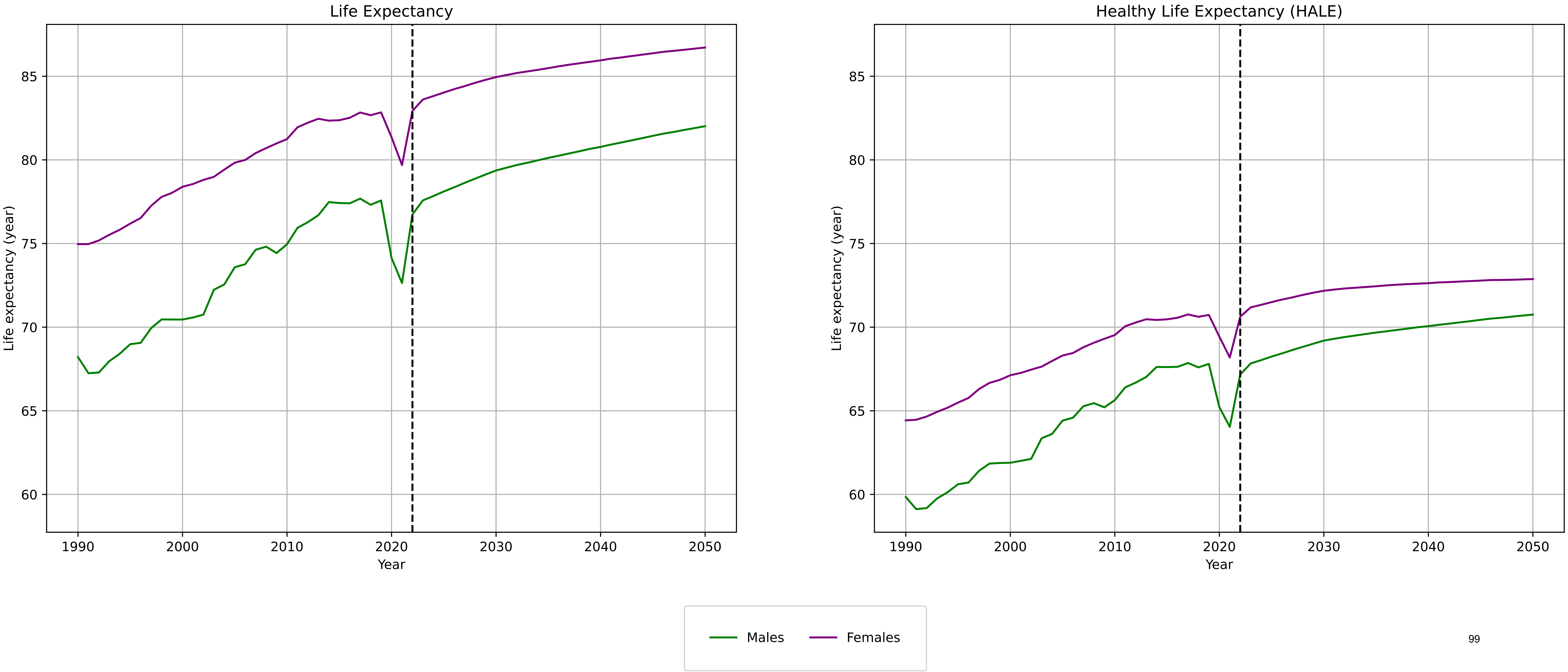

Supplemental Results Figure S3. Life expectancy and HALE by sex and location, 1990-2050

Costa Rica

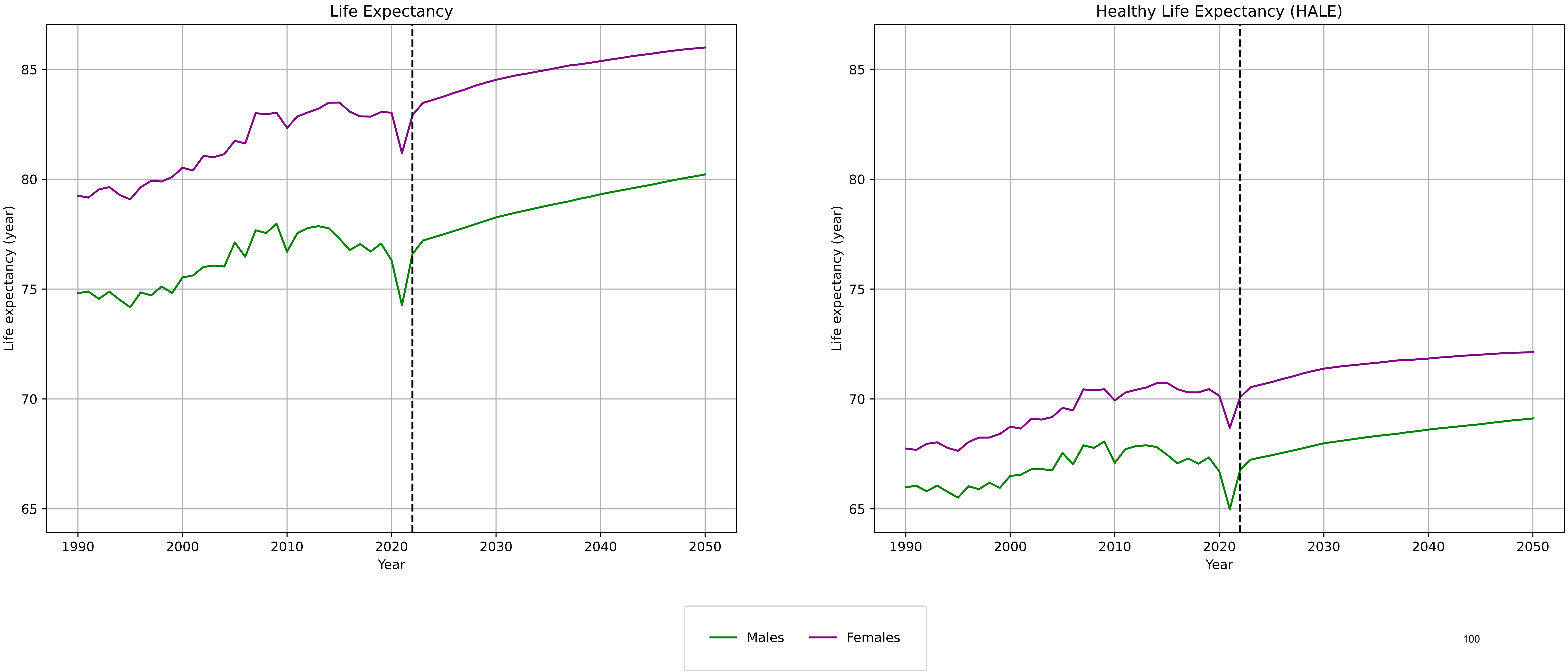

El Salvador

Supplemental Results Figure S3. Life expectancy and HALE by sex and location, 1990-2050

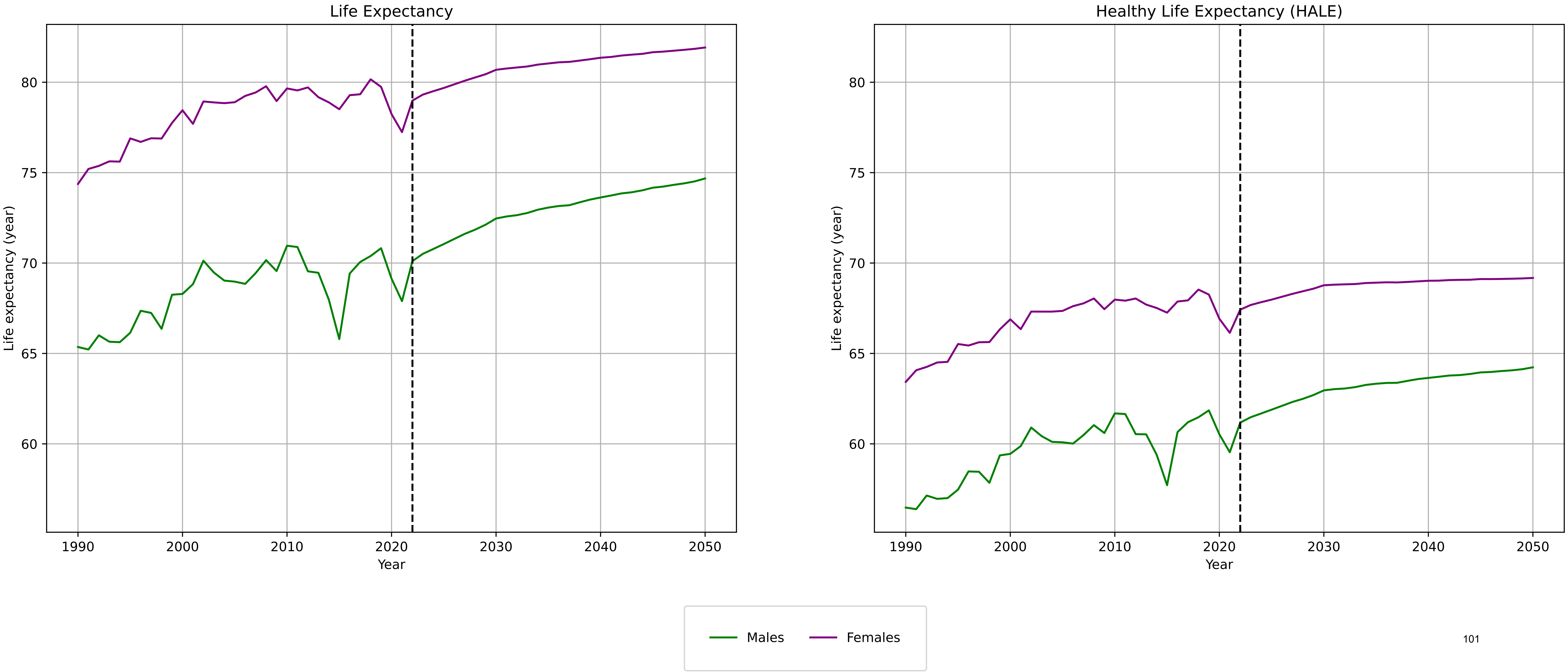

Guatemala

Supplemental Results Figure S3. Life expectancy and HALE by sex and location, 1990-2050

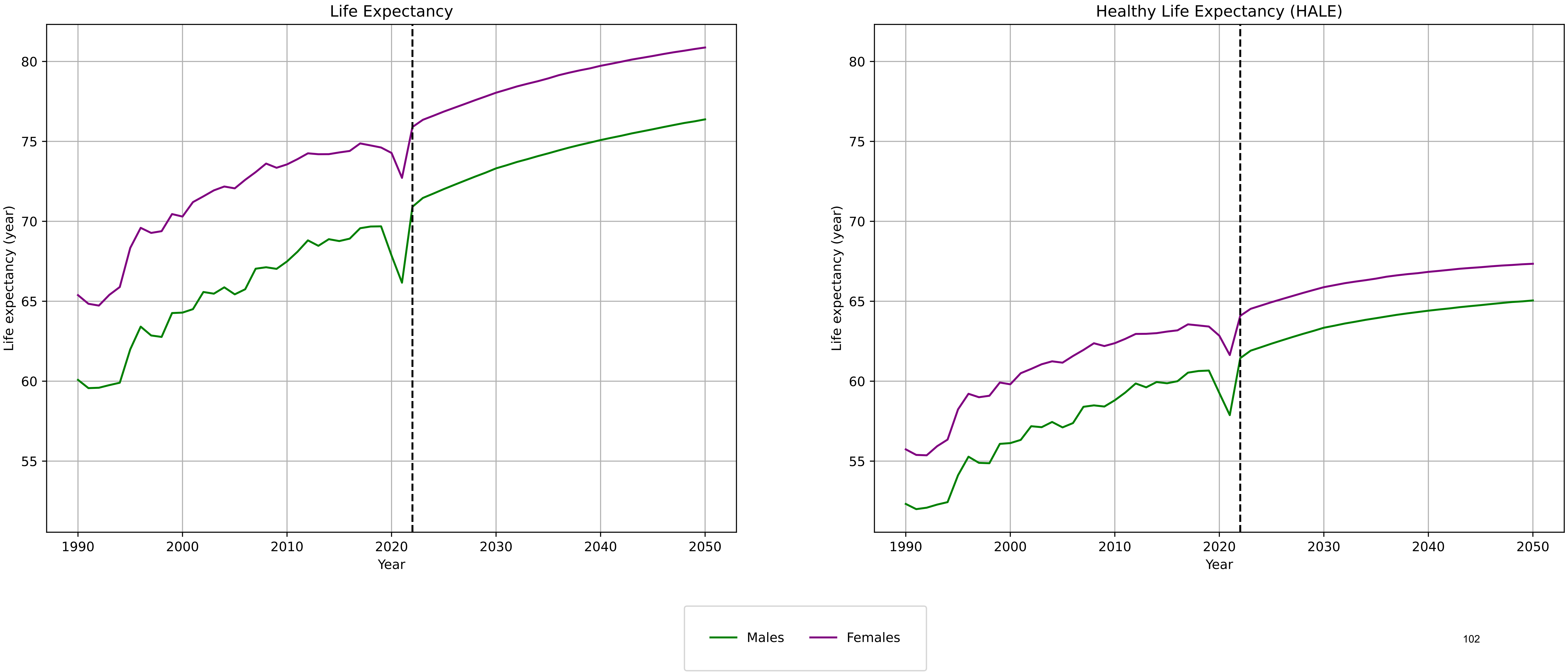

Honduras

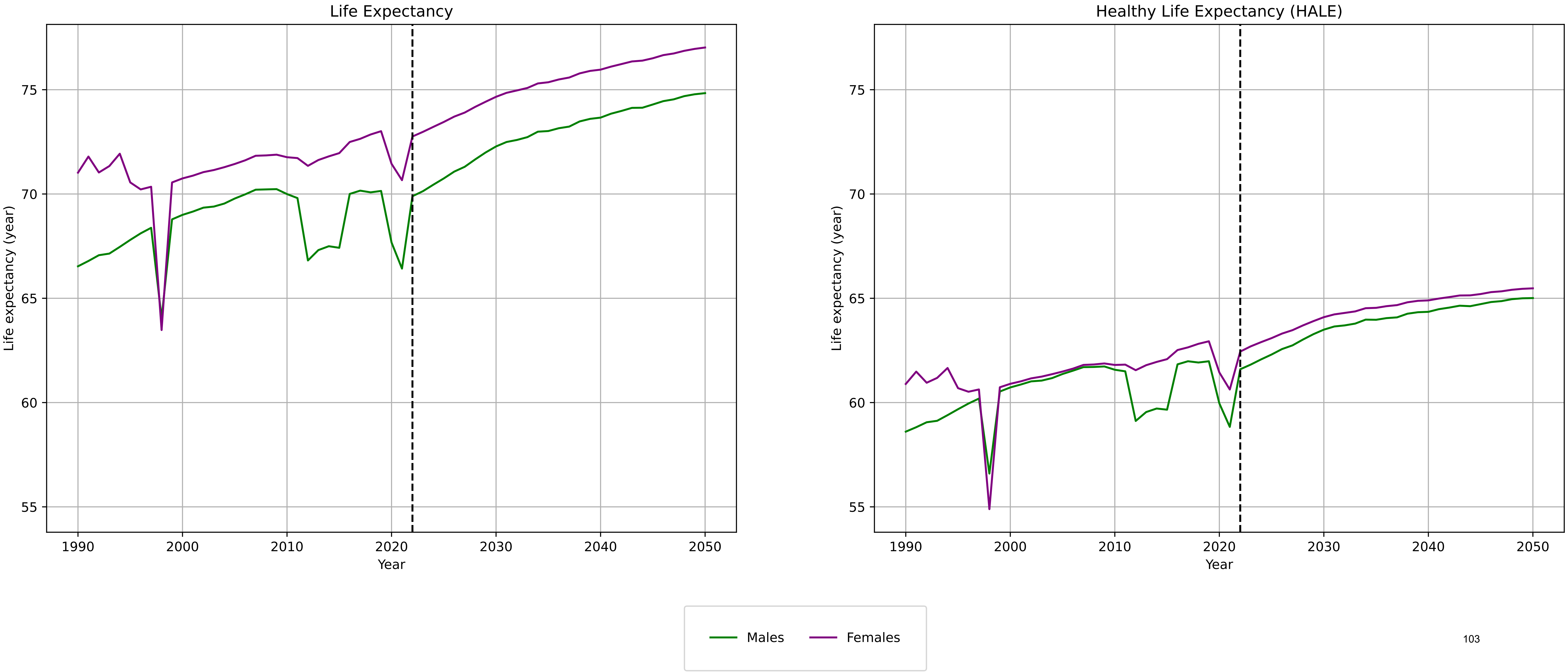
Life Expectancy
Healthy Life Expectancy (HALE)
Life expectancy (year)
Year
55
60
65
70
75
1990
2000
2010
2020
2030
2040
2050
Males
Females

Mexico

Supplemental Results Figure S3. Life expectancy and HALE by sex and location, 1990-2050

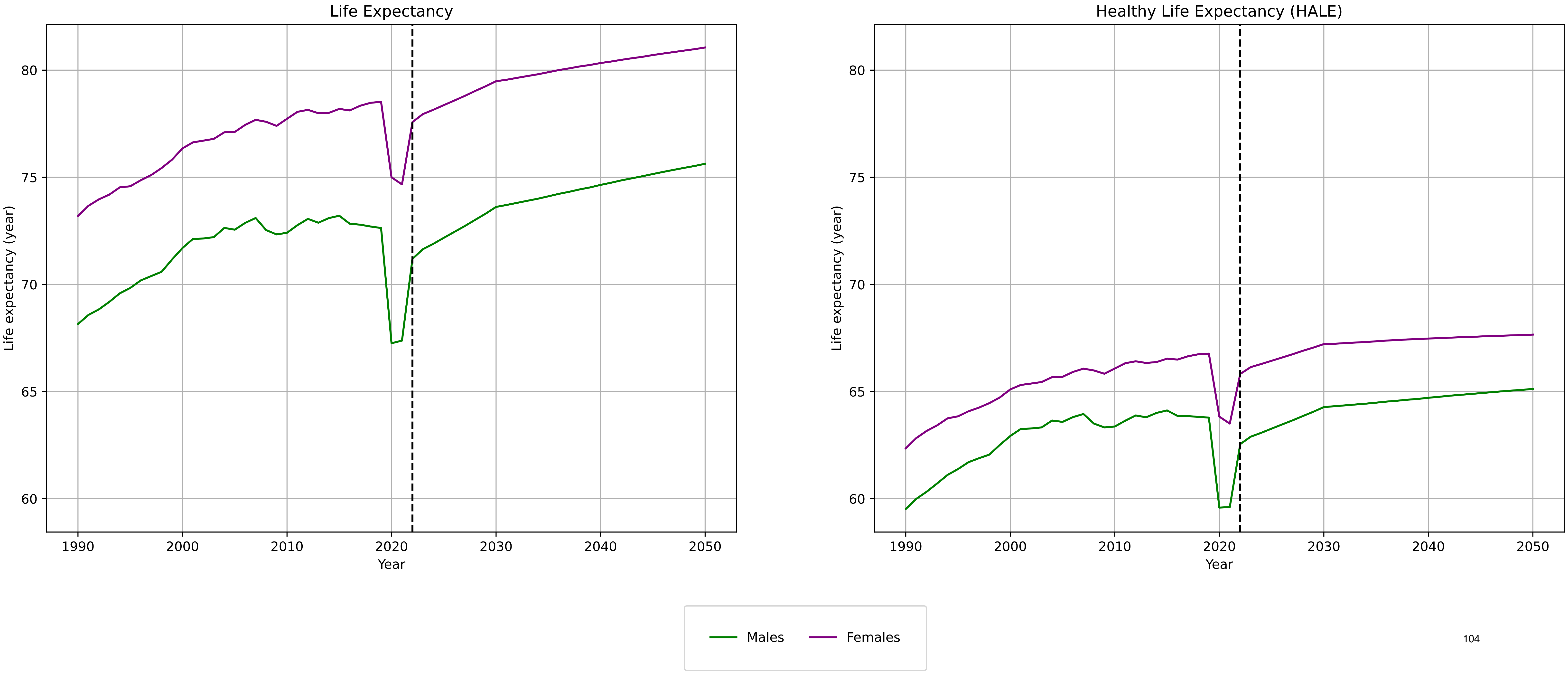

Supplemental Results Figure S3. Life expectancy and HALE by sex and location, 1990-2050

Nicaragua

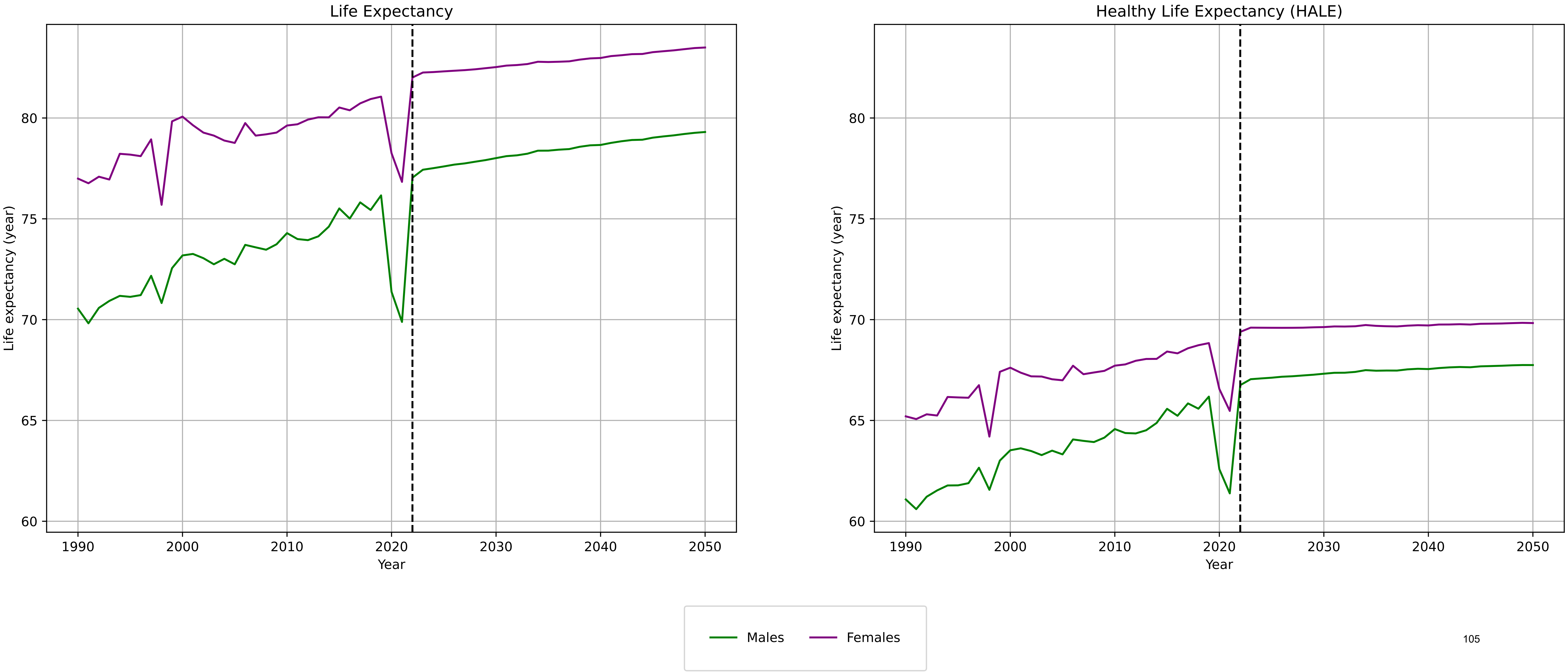

Panama

Supplemental Results Figure S3. Life expectancy and HALE by sex and location, 1990-2050

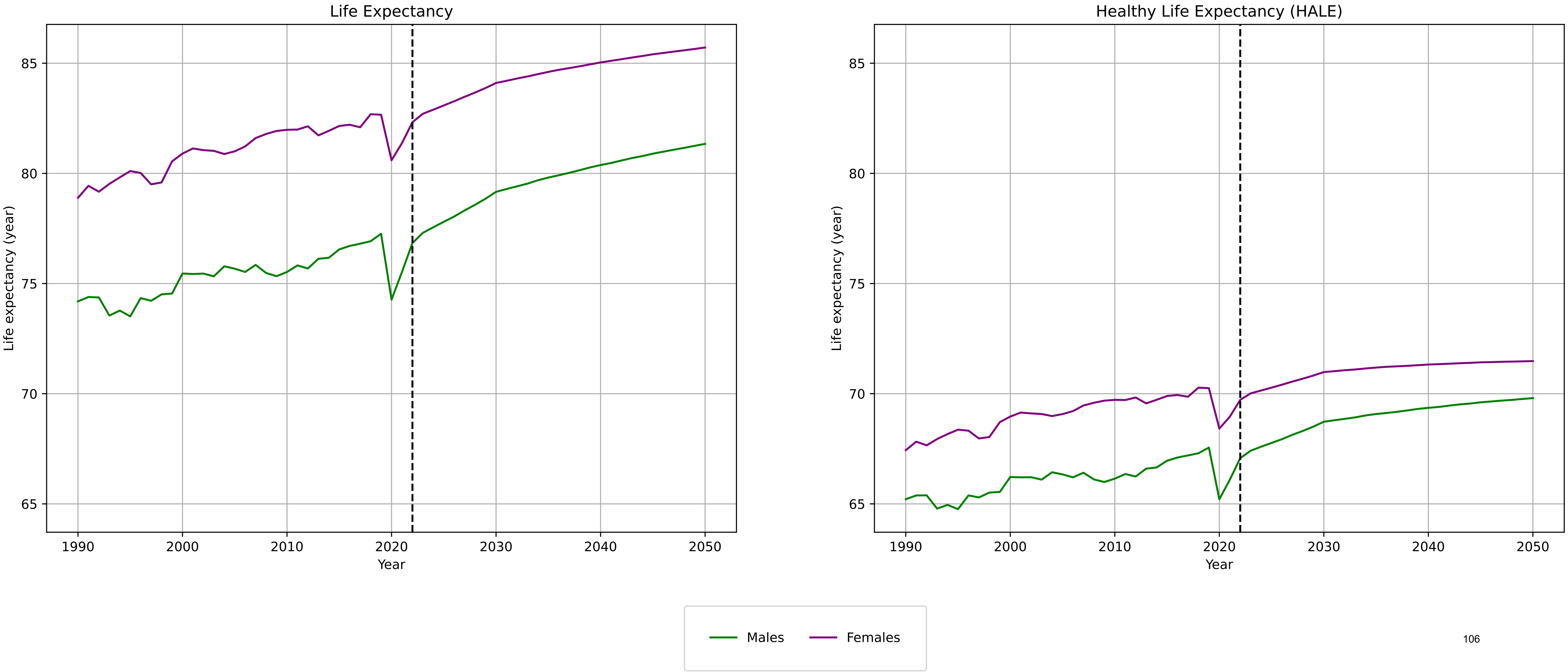

Supplemental Results Figure S3. Life expectancy and HALE by sex and location, 1990-2050

Venezuela

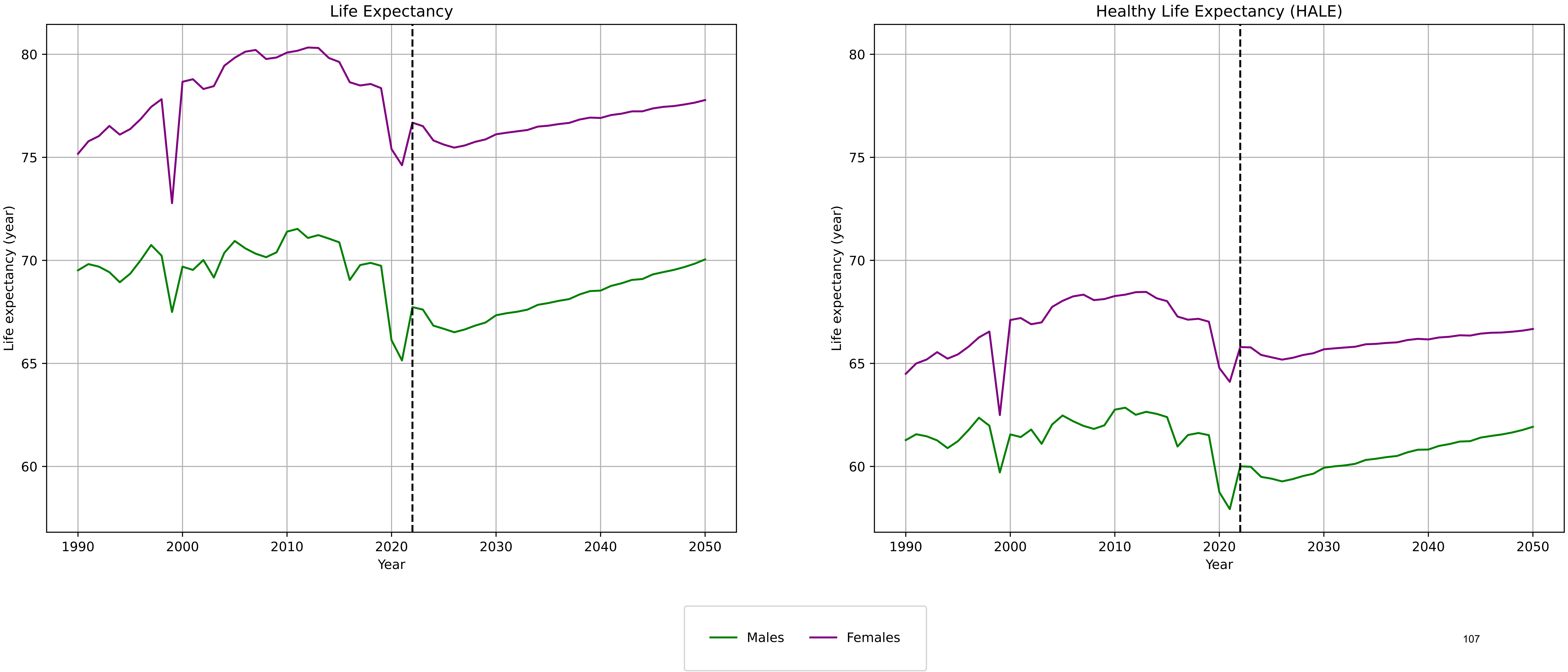

Brazil

Supplemental Results Figure S3. Life expectancy and HALE by sex and location, 1990-2050

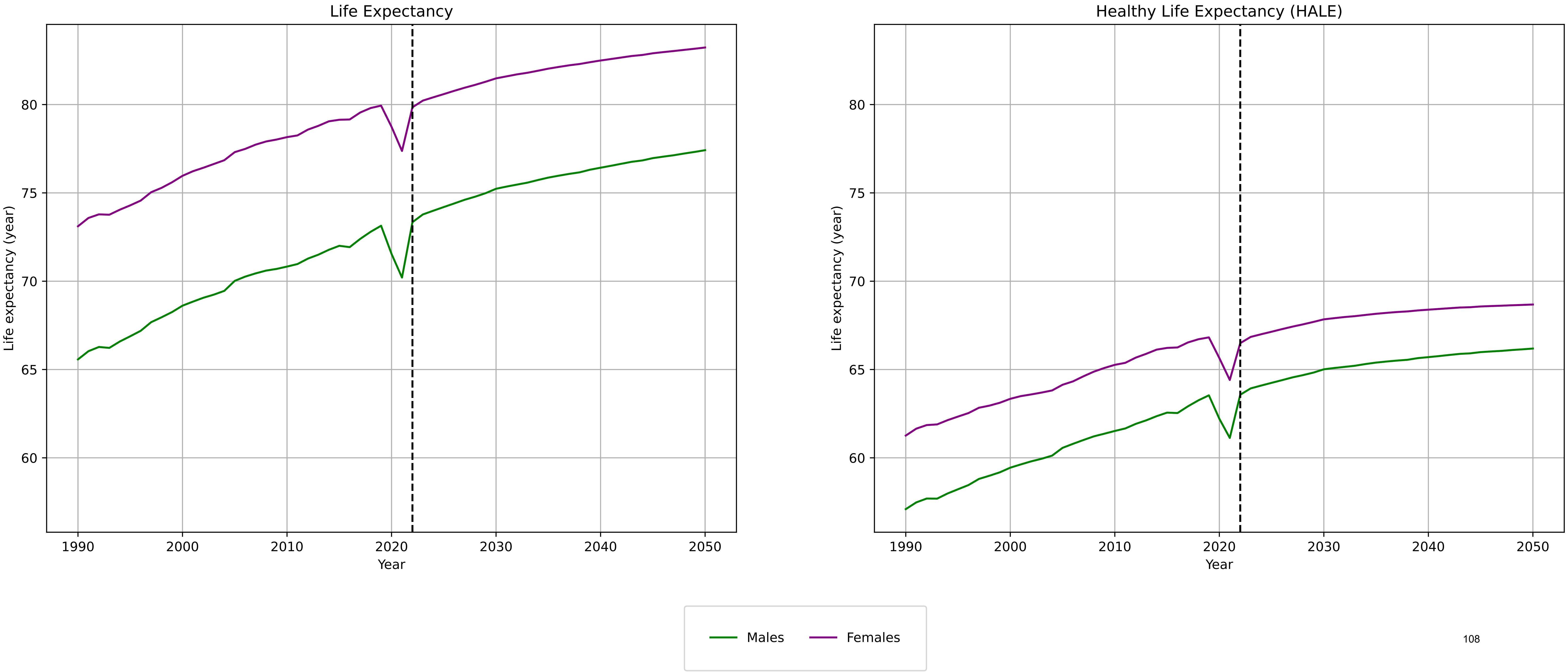

Paraguay

Supplemental Results Figure S3. Life expectancy and HALE by sex and location, 1990-2050

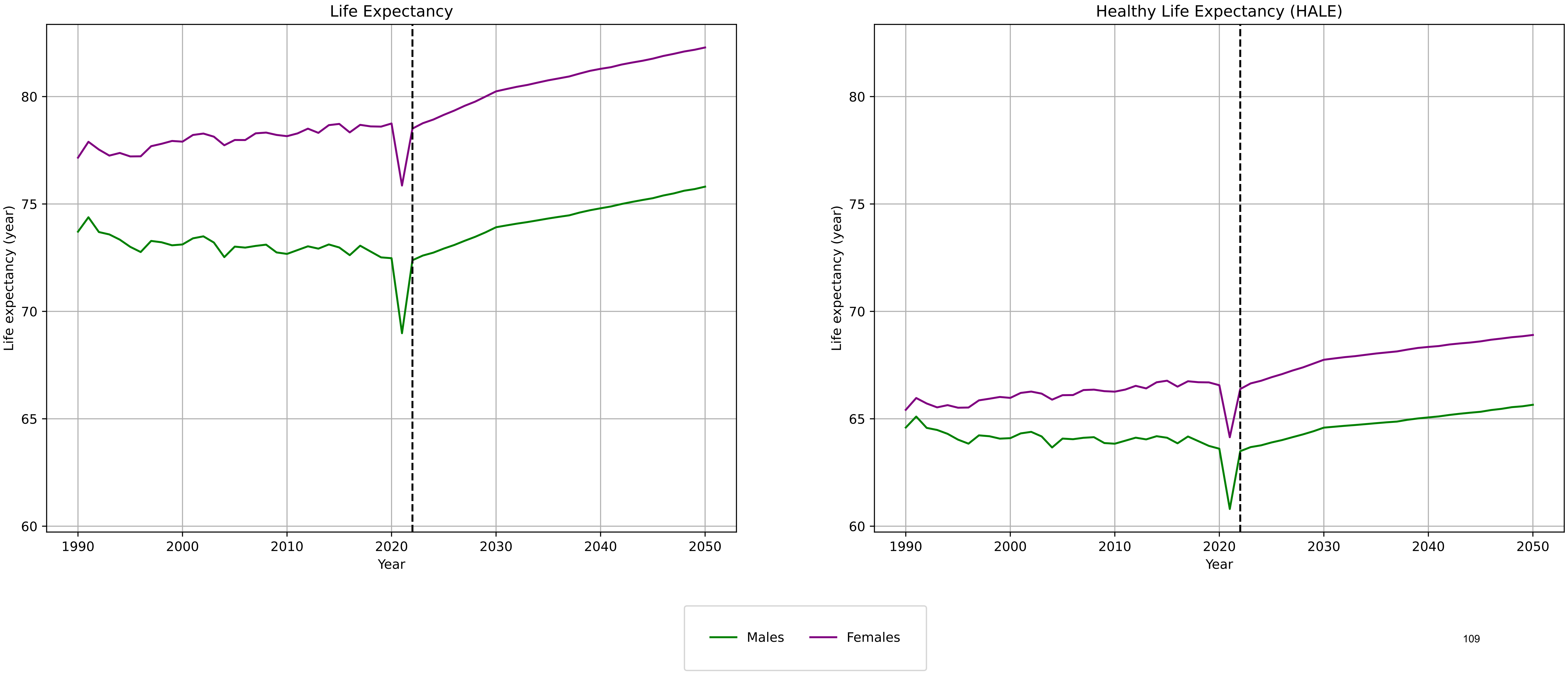

Supplemental Results Figure S3. Life expectancy and HALE by sex and location, 1990-2050

Afghanistan

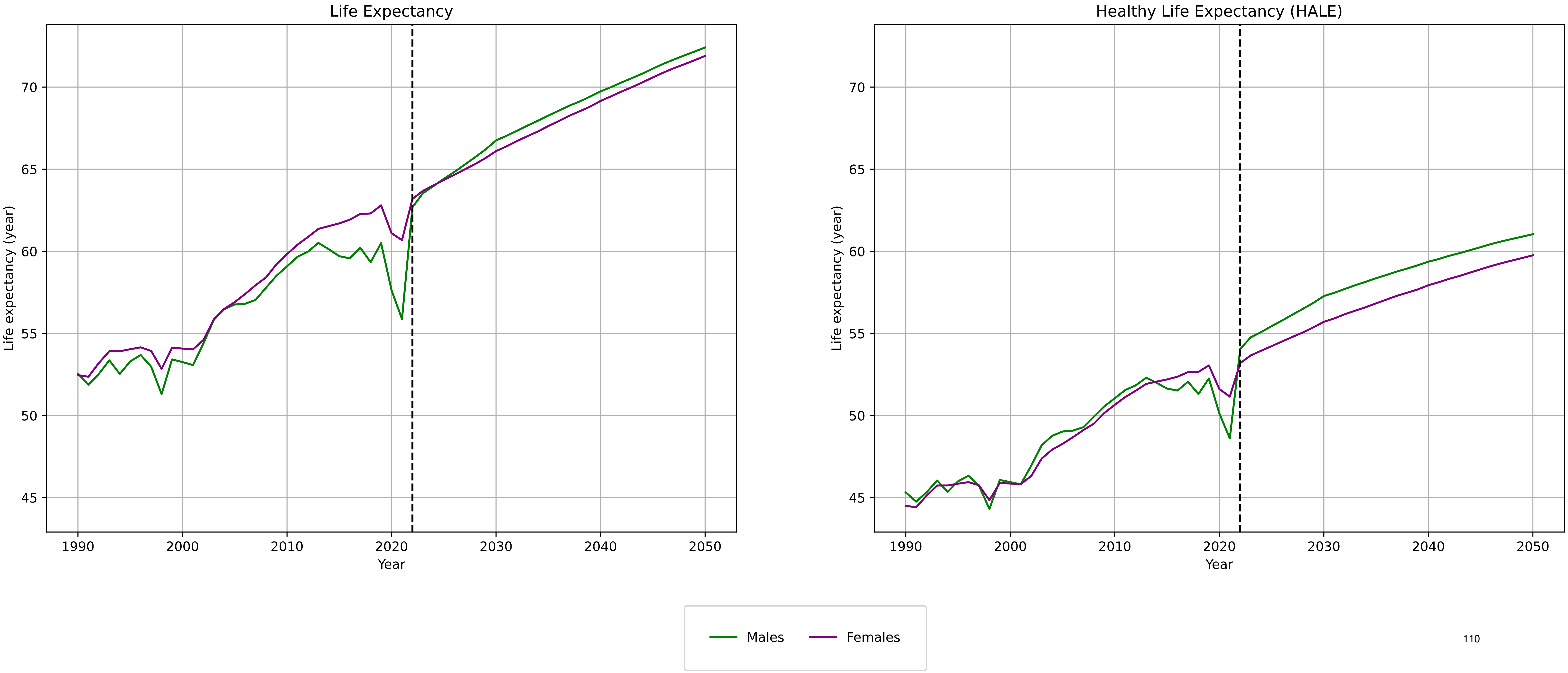

Algeria

Supplemental Results Figure S3. Life expectancy and HALE by sex and location, 1990-2050

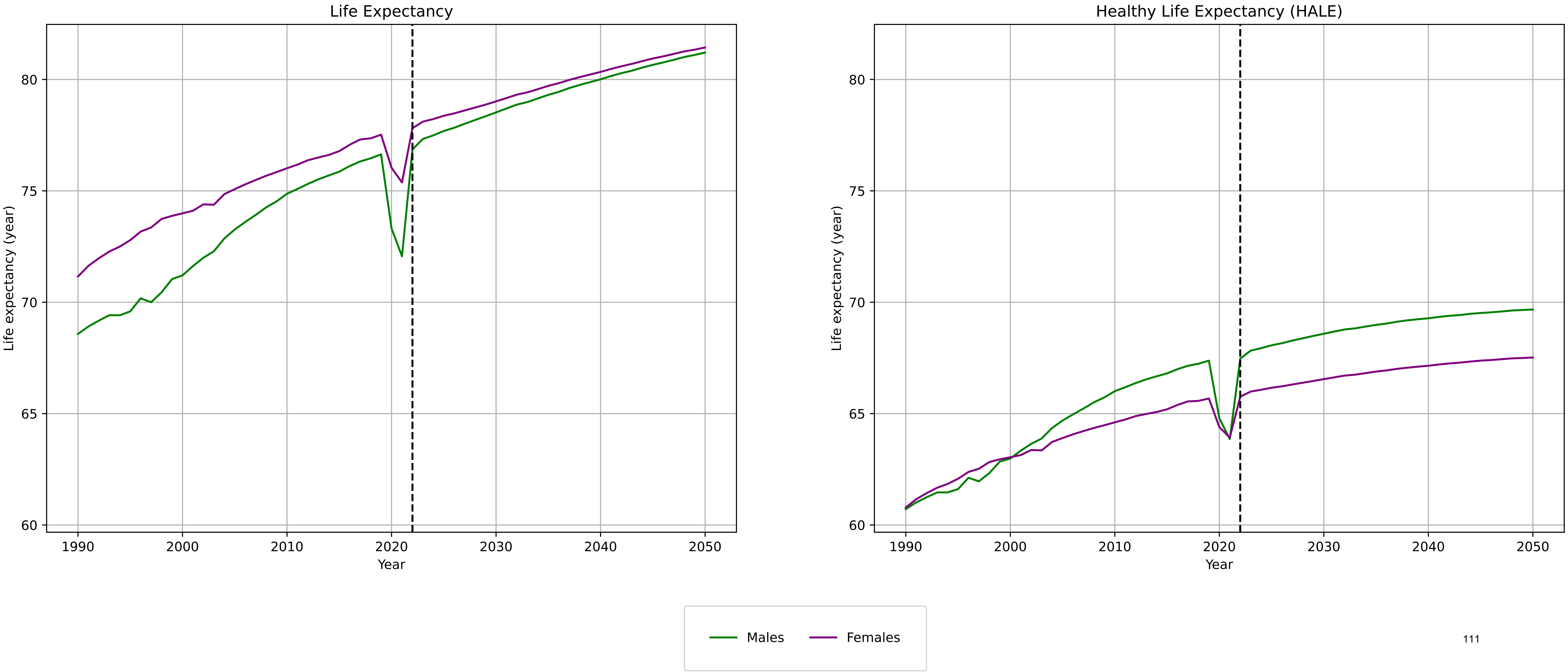

Bahrain

Supplemental Results Figure S3. Life expectancy and HALE by sex and location, 1990-2050

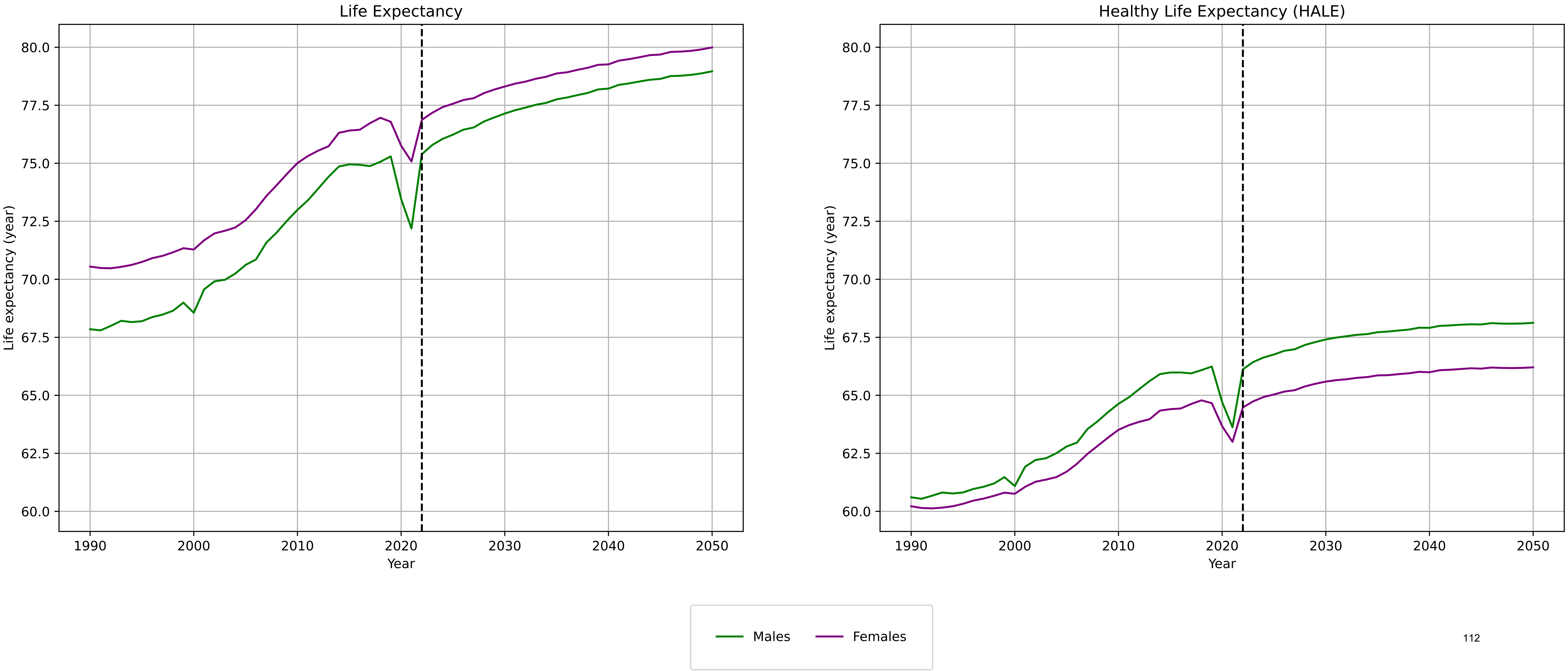

Supplemental Results Figure S3. Life expectancy and HALE by sex and location, 1990-2050

Egypt

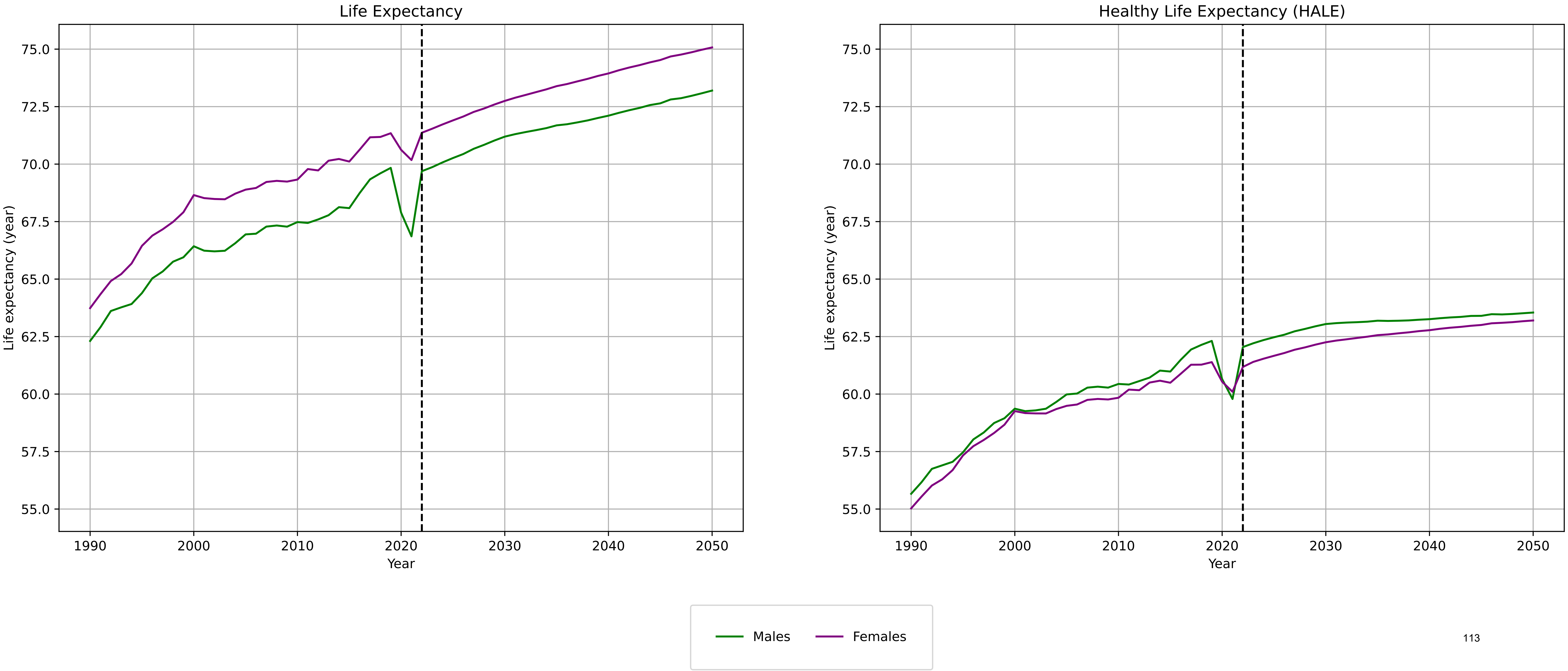

Supplemental Results Figure S3. Life expectancy and HALE by sex and location, 1990-2050

Iran

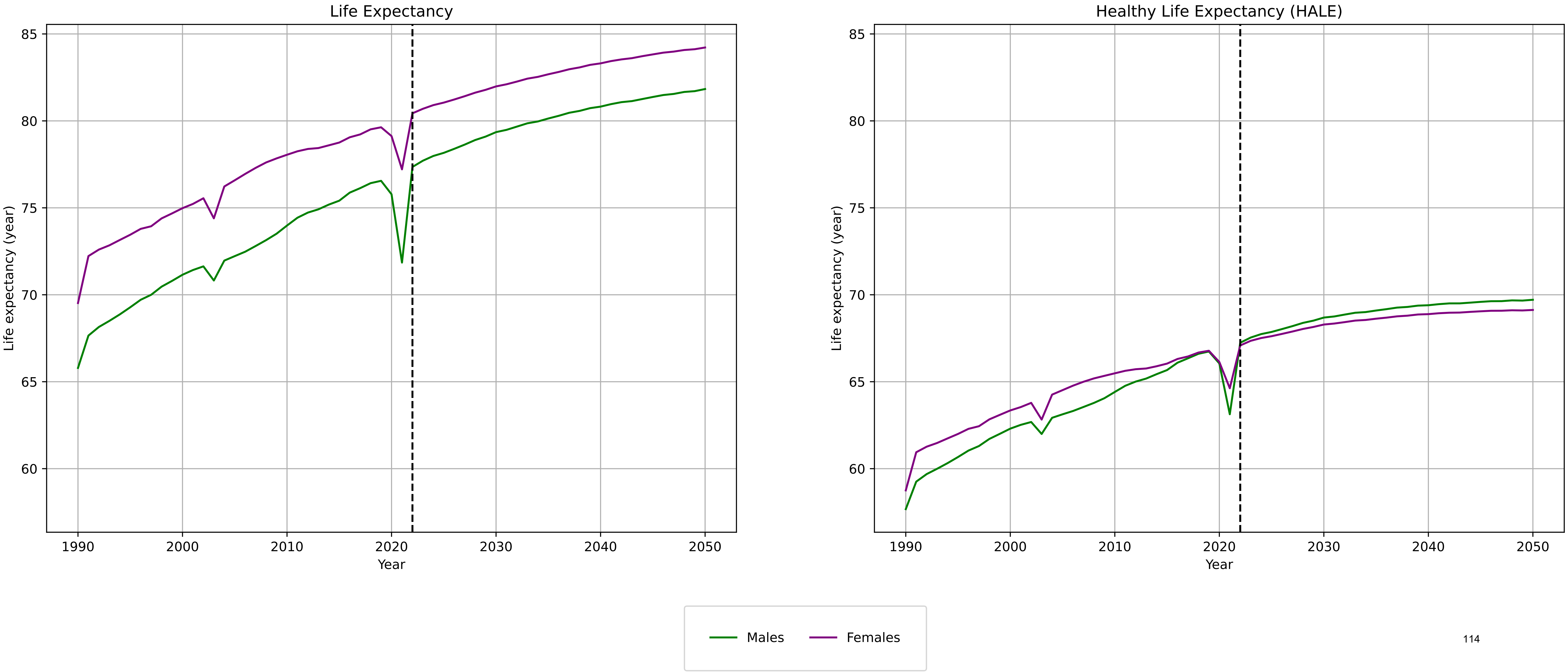

Iraq

Supplemental Results Figure S3. Life expectancy and HALE by sex and location, 1990-2050

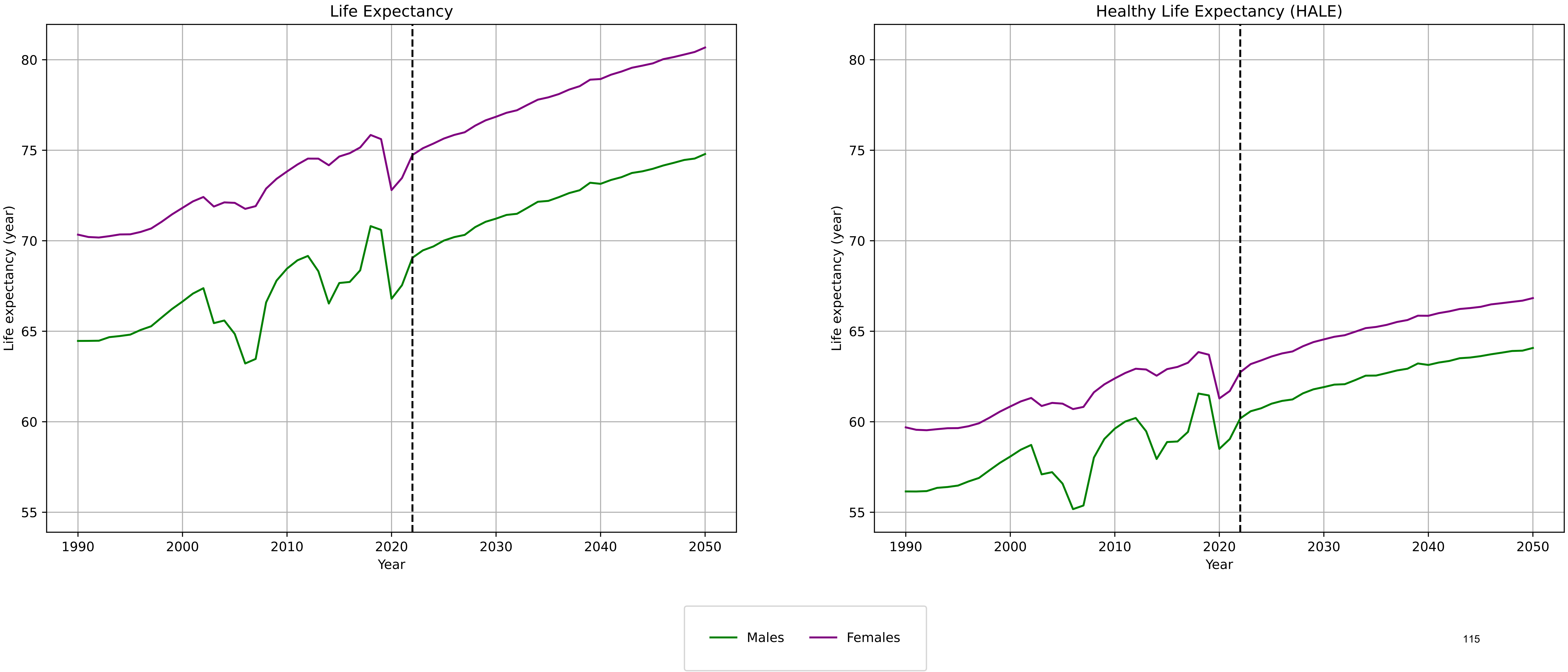

Supplemental Results Figure S3. Life expectancy and HALE by sex and location, 1990-2050

Jordan

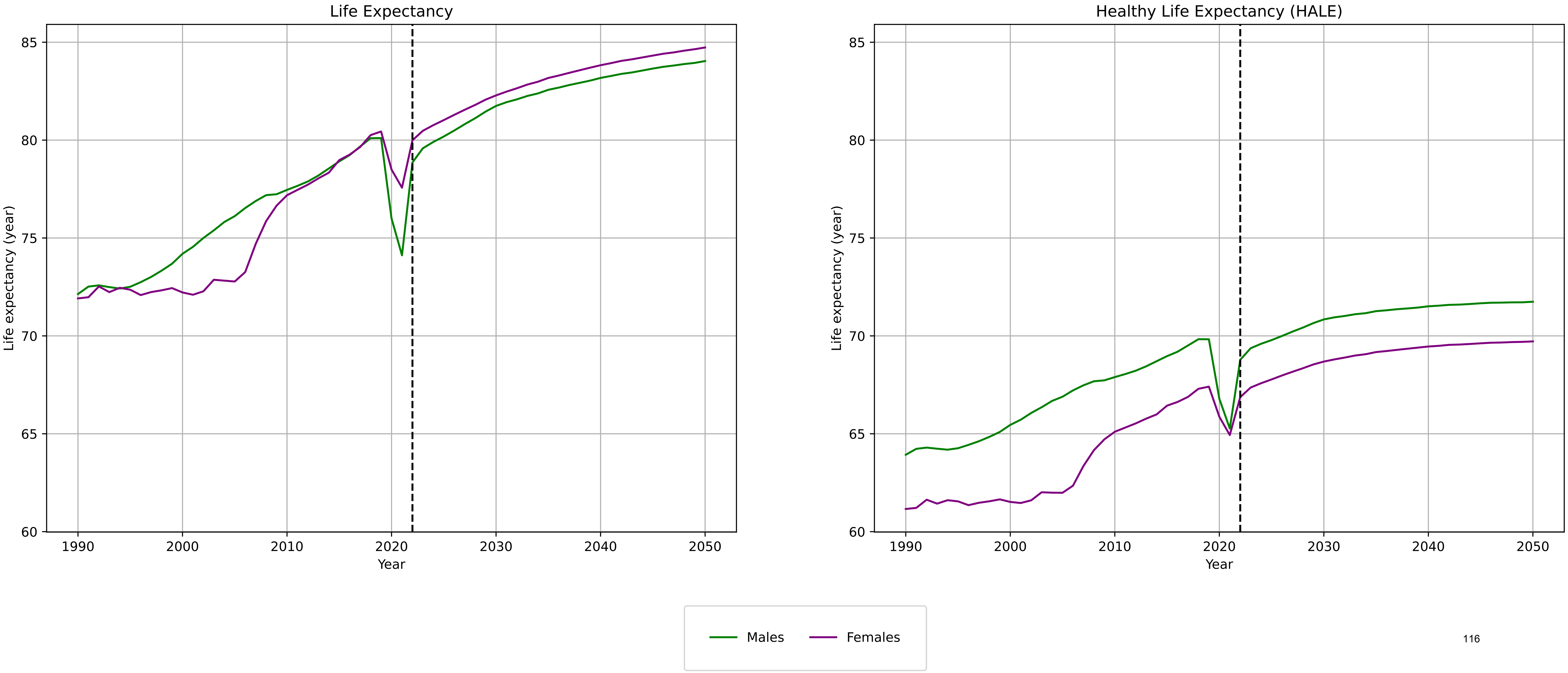

Supplemental Results Figure S3. Life expectancy and HALE by sex and location, 1990-2050

Kuwait

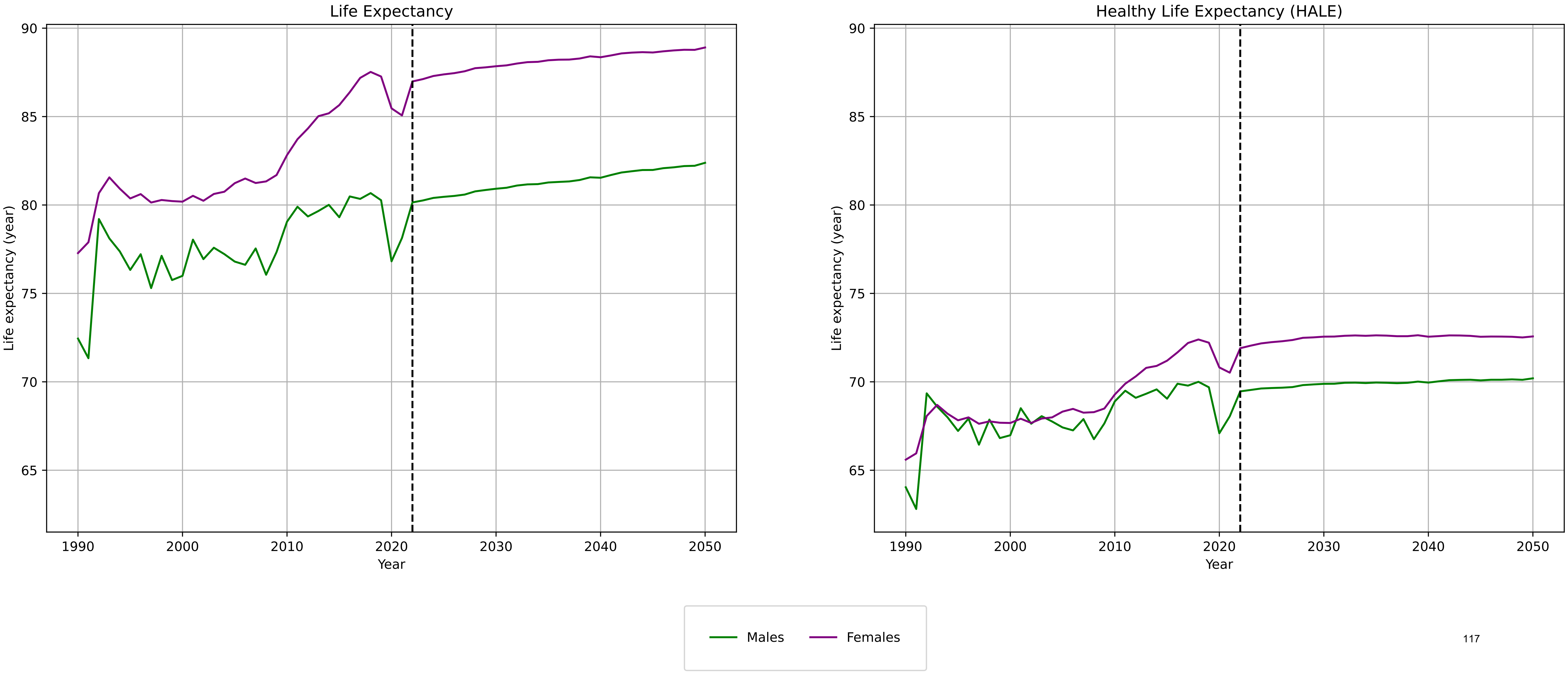

Supplemental Results Figure S3. Life expectancy and HALE by sex and location, 1990-2050

Lebanon

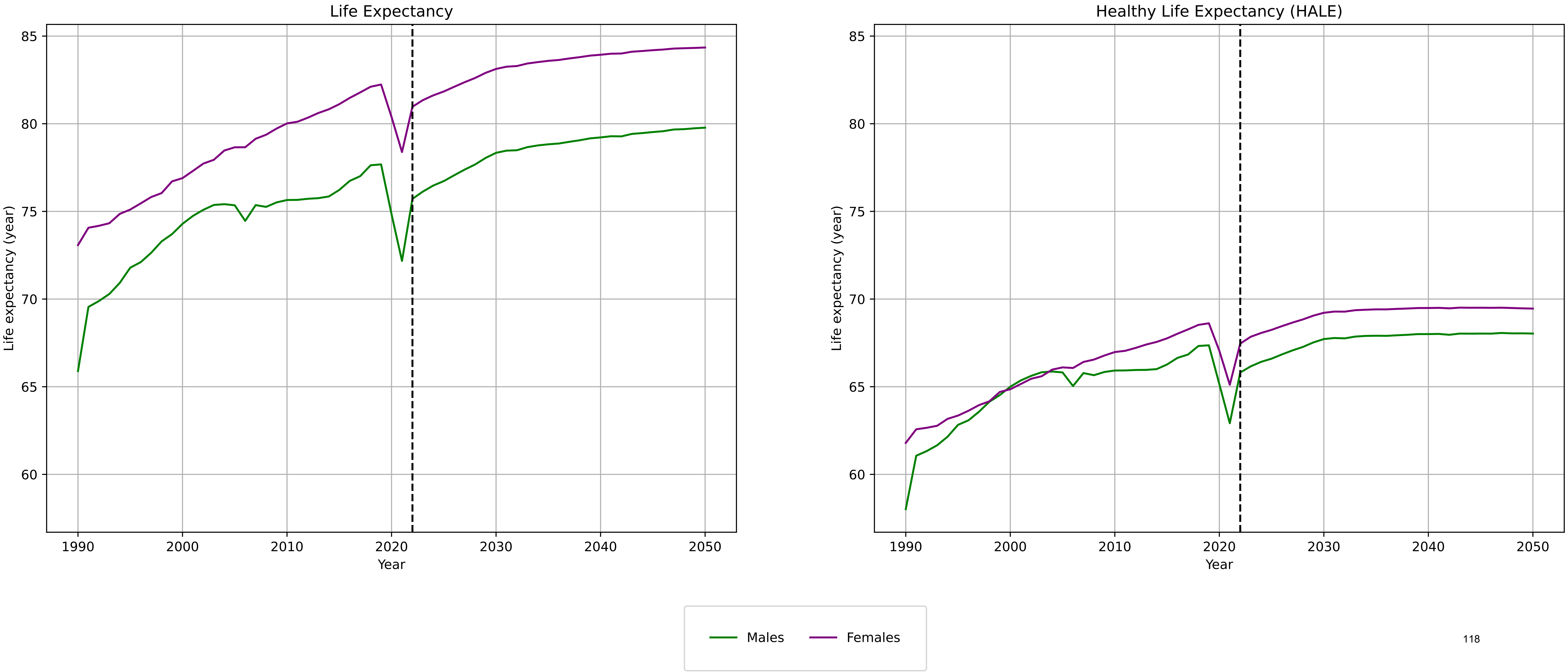

Libya

Supplemental Results Figure S3. Life expectancy and HALE by sex and location, 1990-2050

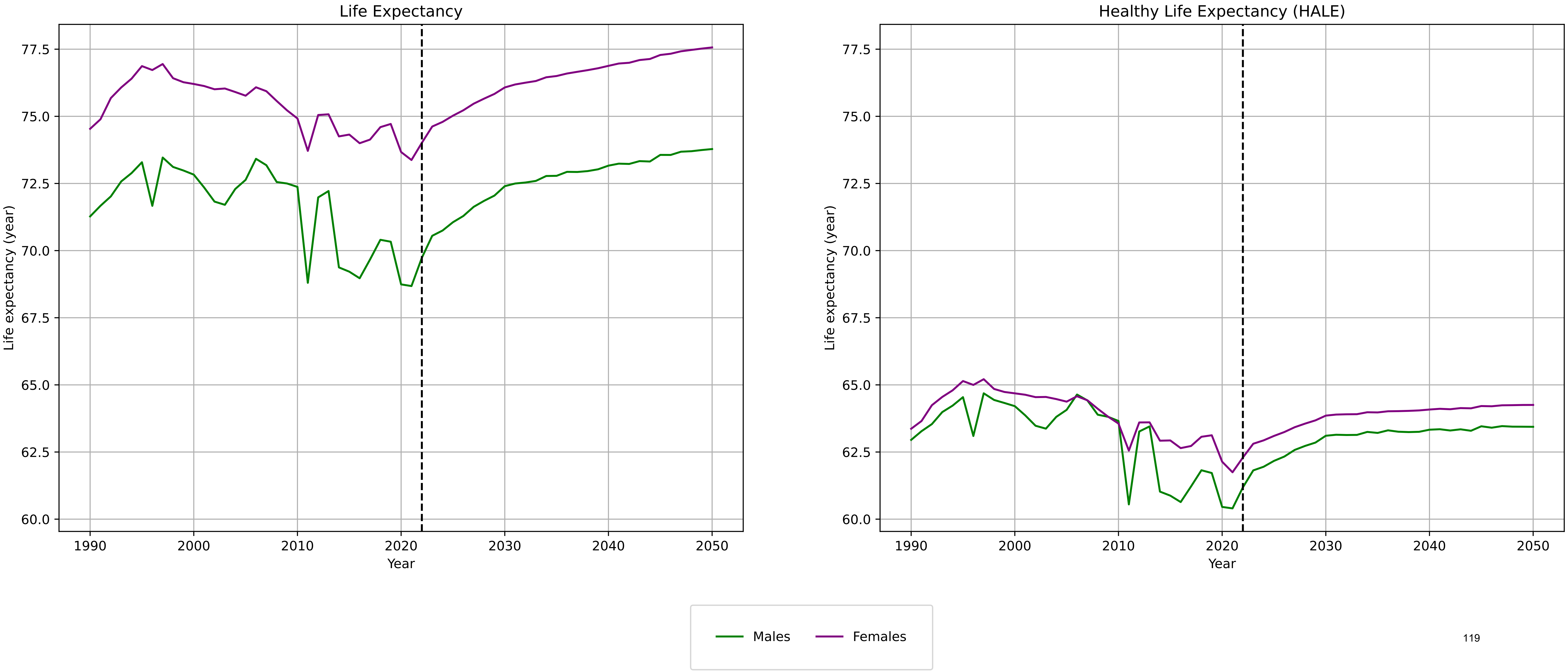

Supplemental Results Figure S3. Life expectancy and HALE by sex and location, 1990-2050

Morocco

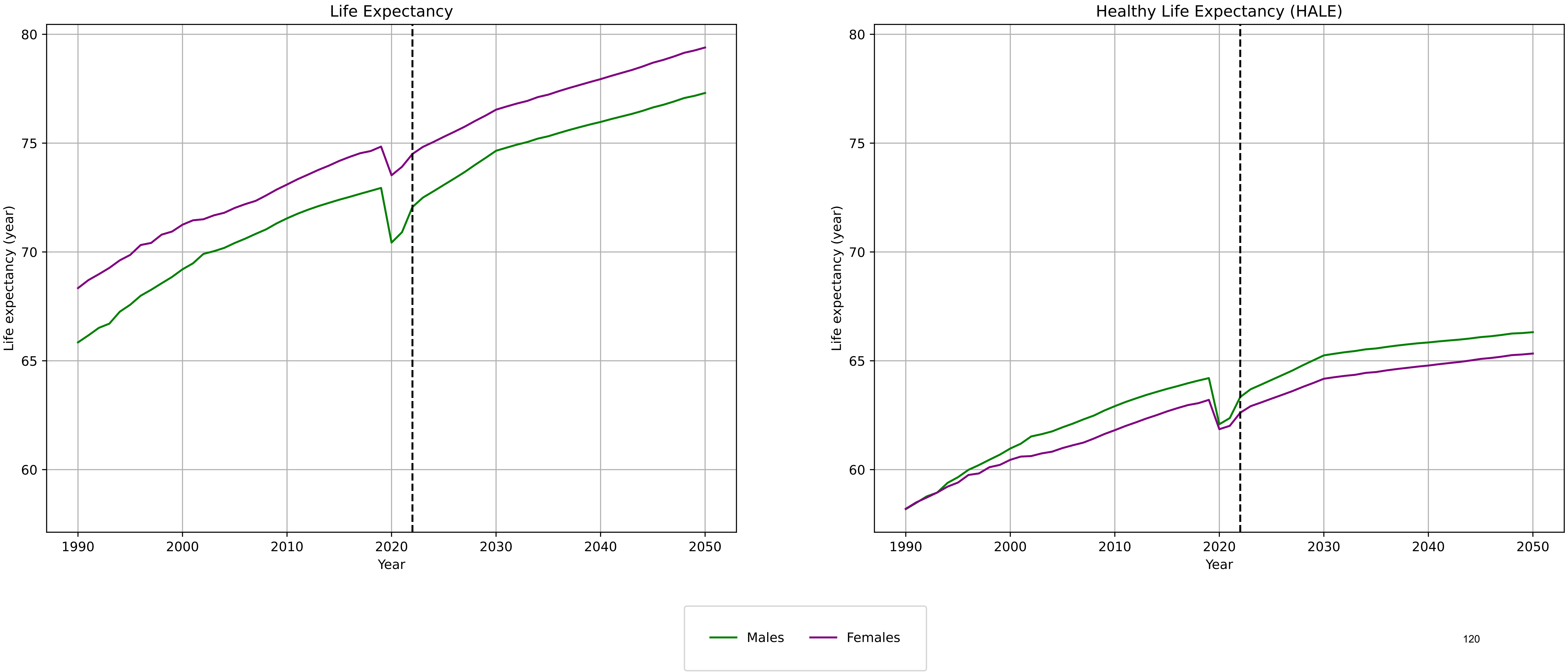

Oman

Supplemental Results Figure S3. Life expectancy and HALE by sex and location, 1990-2050

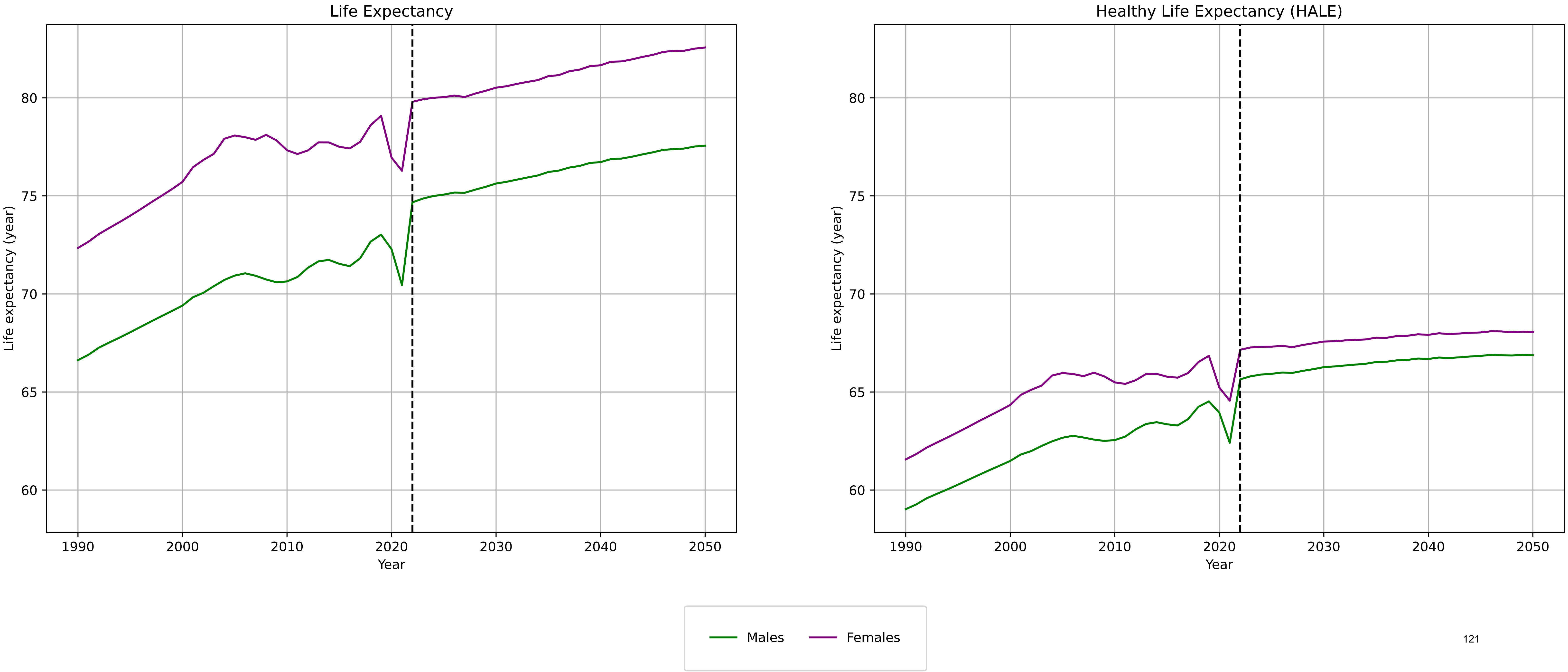

Palestine

Supplemental Results Figure S3. Life expectancy and HALE by sex and location, 1990-2050

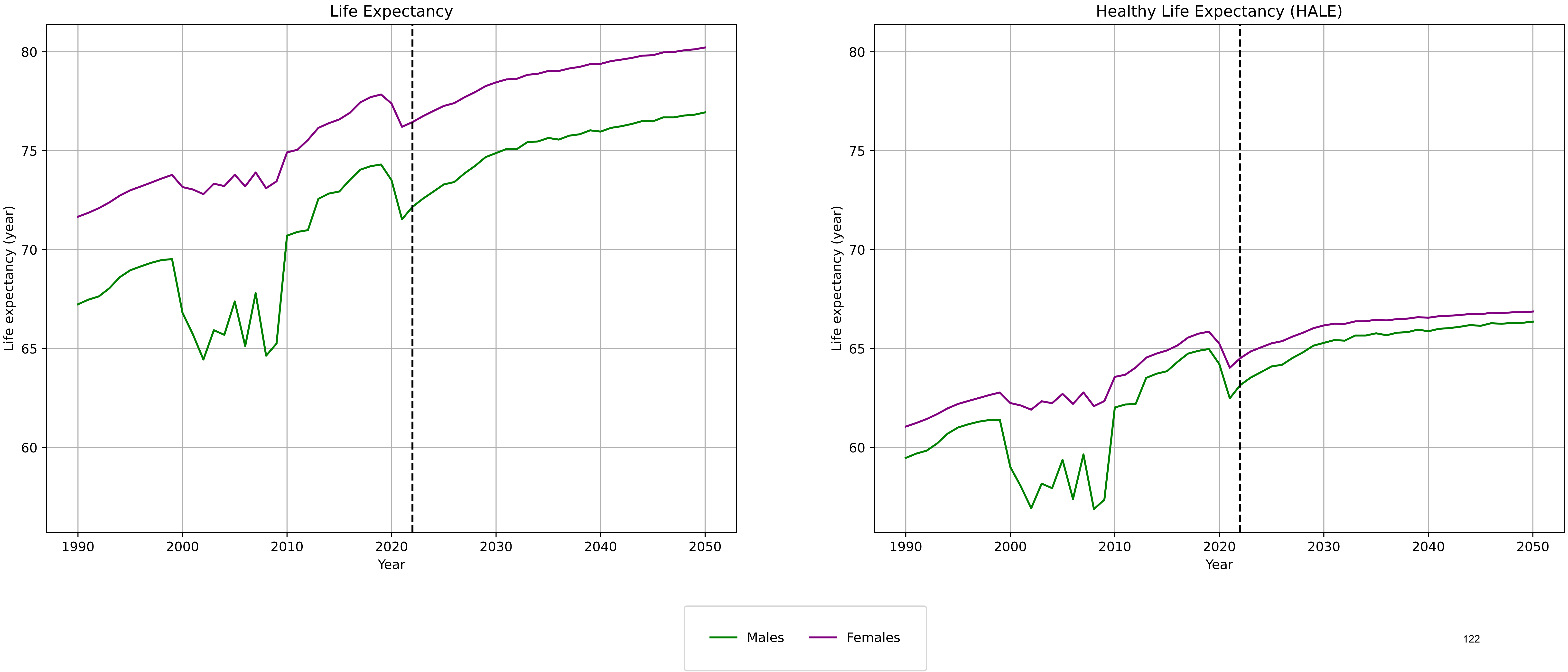

Qatar

Supplemental Results Figure S3. Life expectancy and HALE by sex and location, 1990-2050

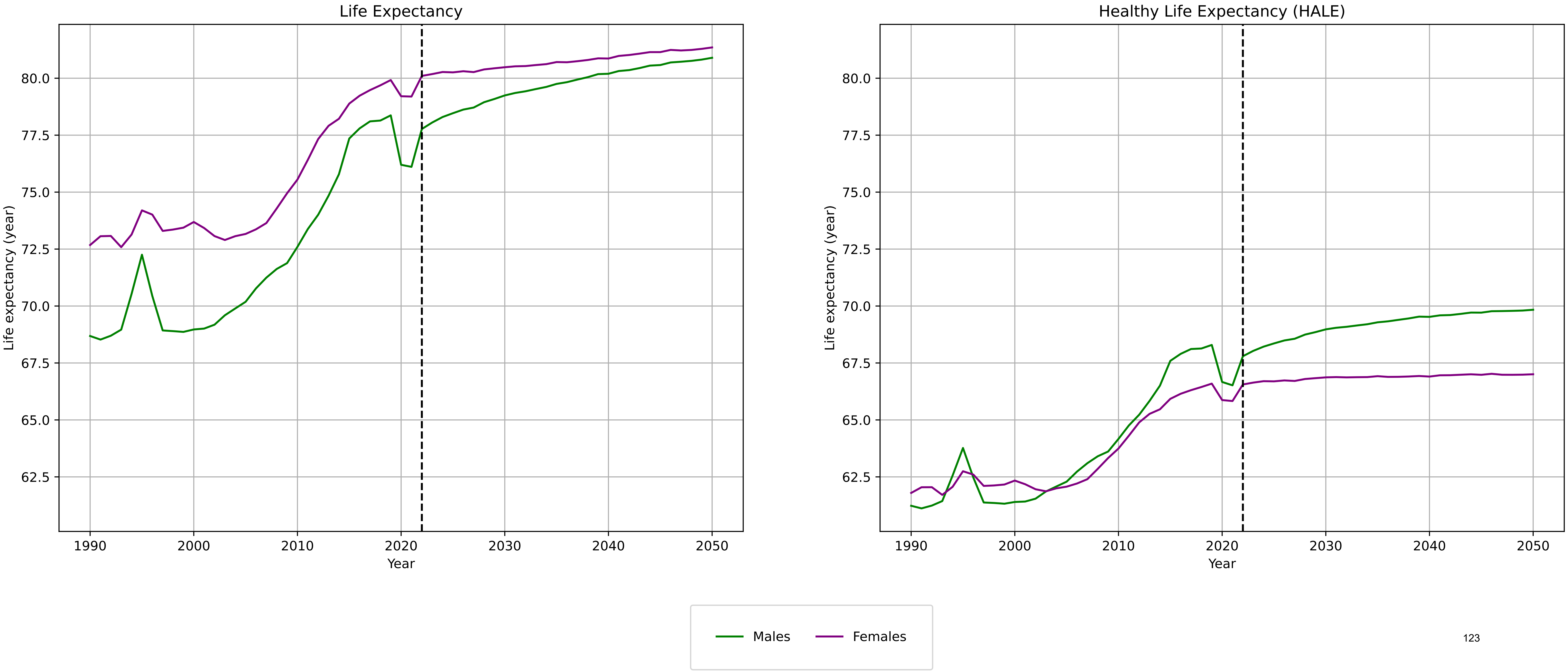

Saudi Arabia

Supplemental Results Figure S3. Life expectancy and HALE by sex and location, 1990-2050

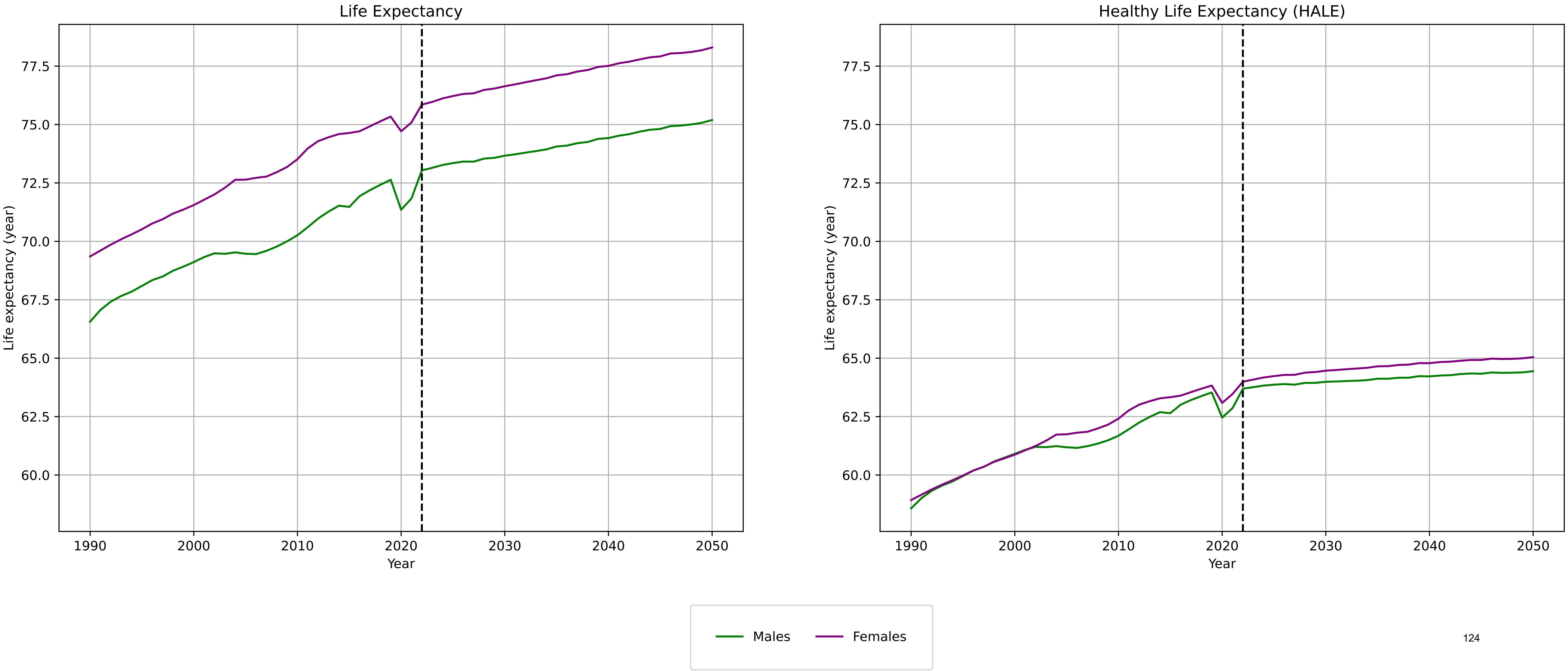

Sudan

Supplemental Results Figure S3. Life expectancy and HALE by sex and location, 1990-2050

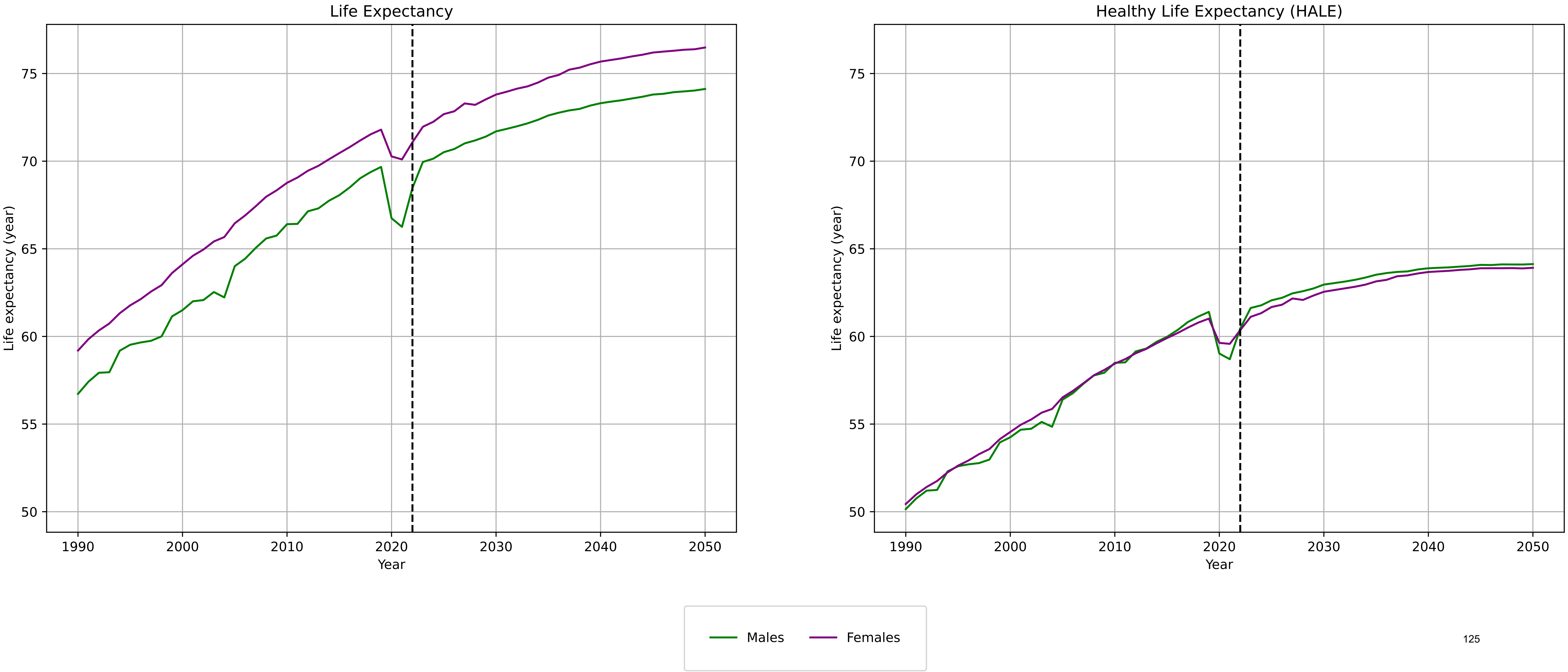

Supplemental Results Figure S3. Life expectancy and HALE by sex and location, 1990-2050

Syria

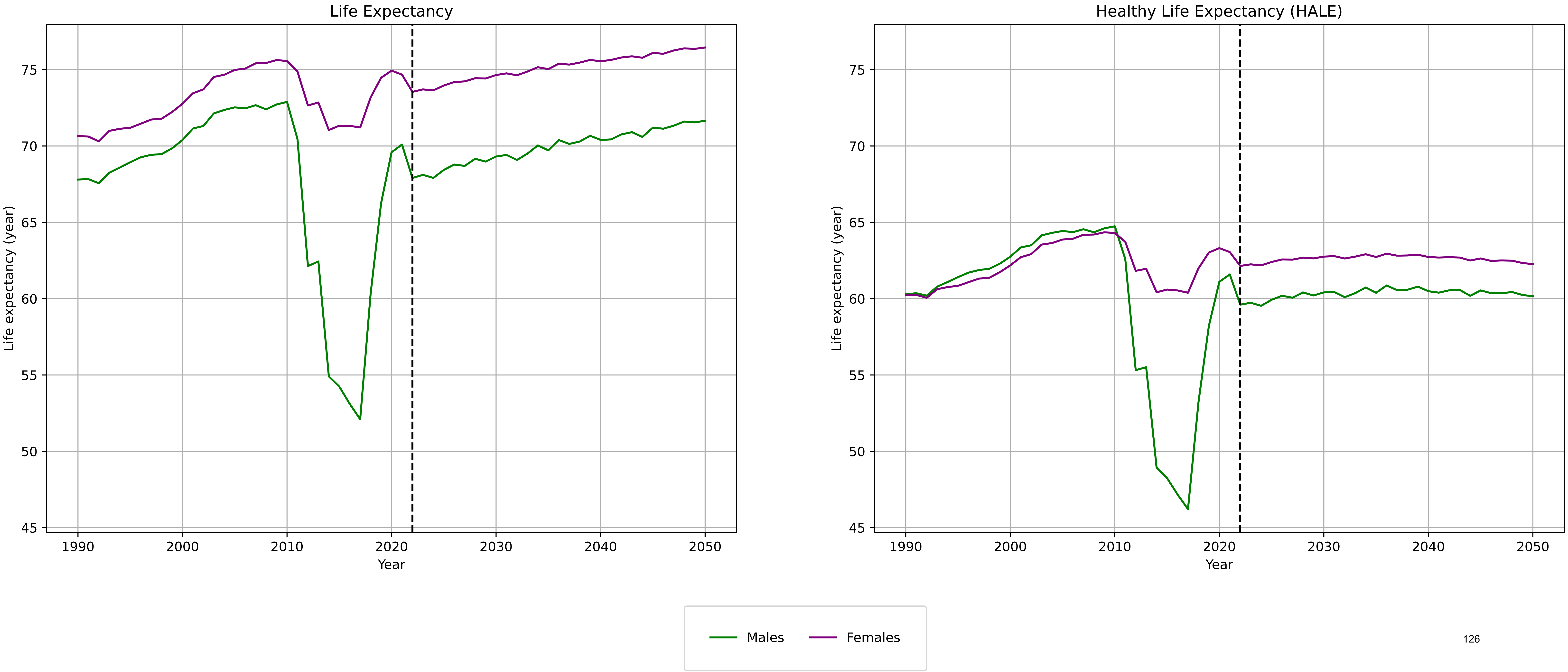

Tunisia

Supplemental Results Figure S3. Life expectancy and HALE by sex and location, 1990-2050

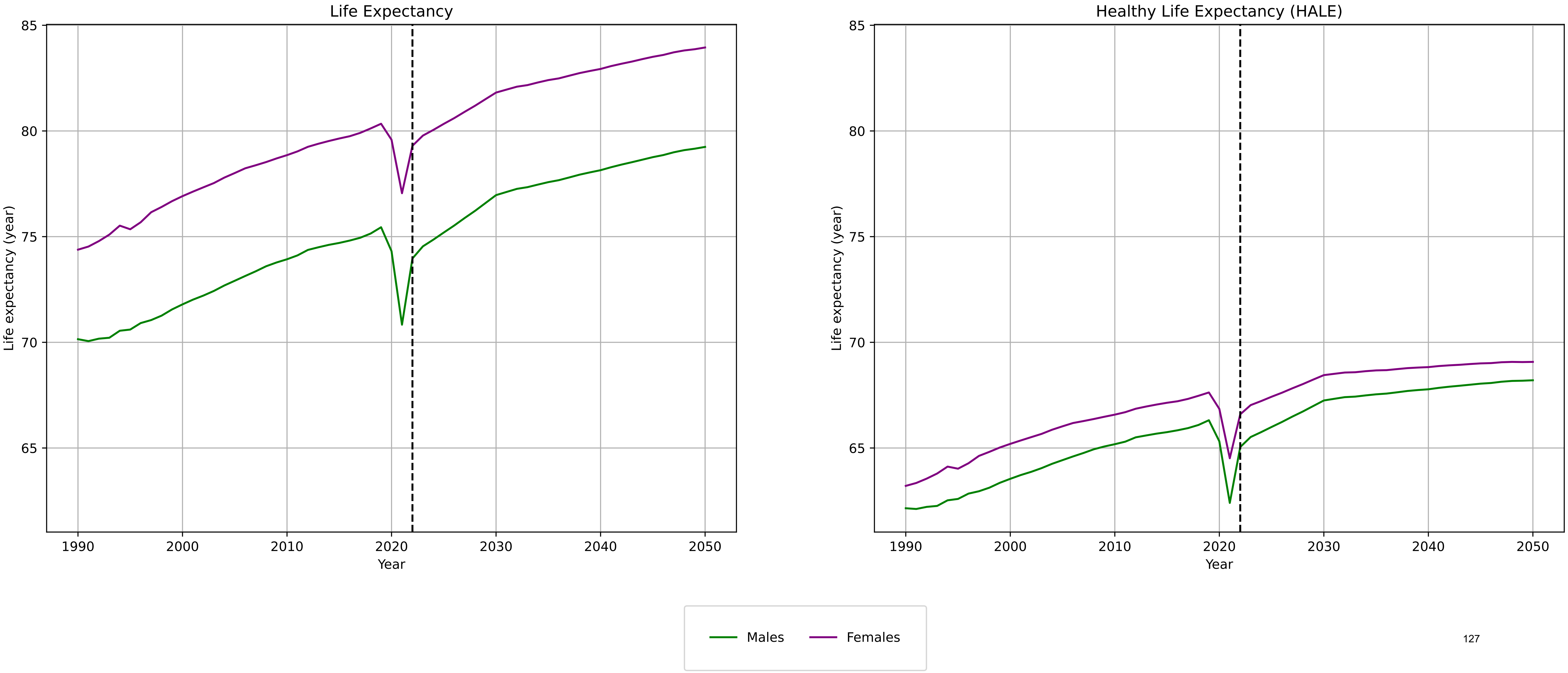

Türkiye

Supplemental Results Figure S3. Life expectancy and HALE by sex and location, 1990-2050

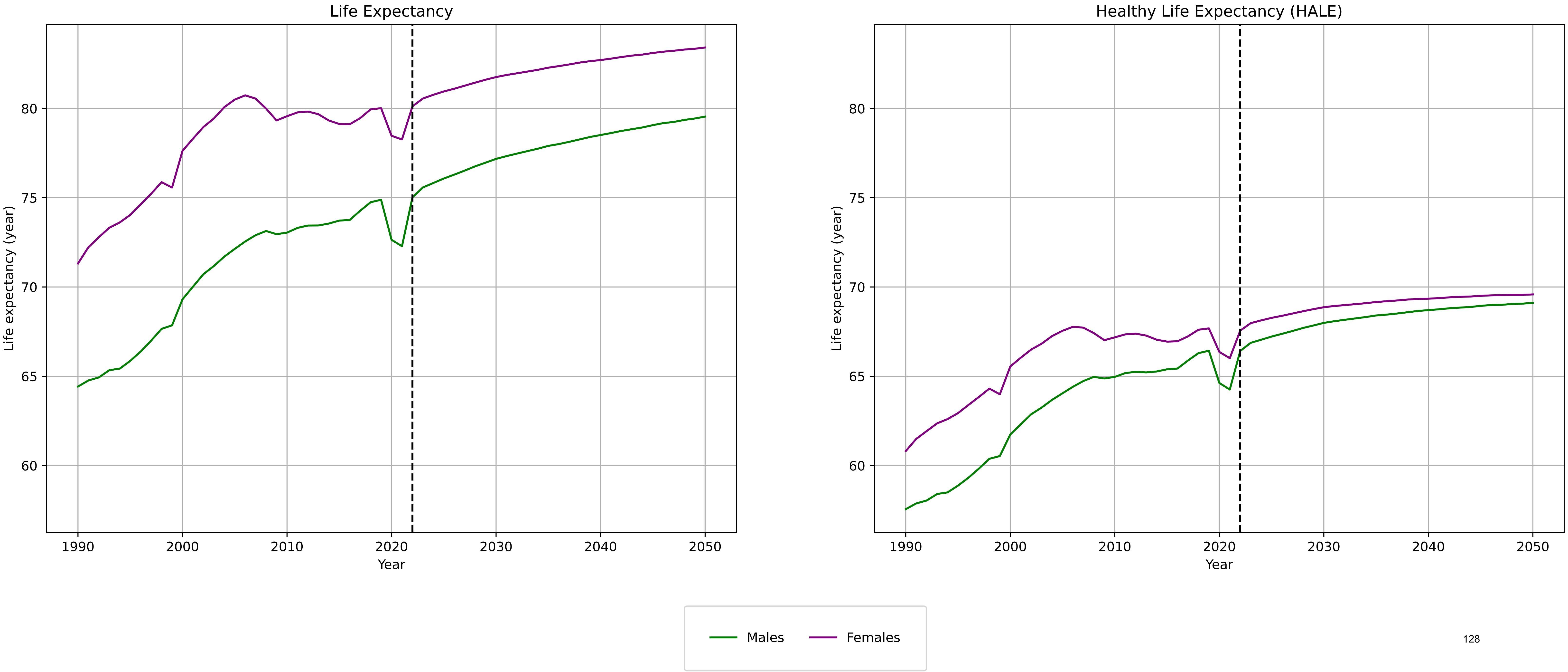

United Arab Emirates

Supplemental Results Figure S3. Life expectancy and HALE by sex and location, 1990-2050

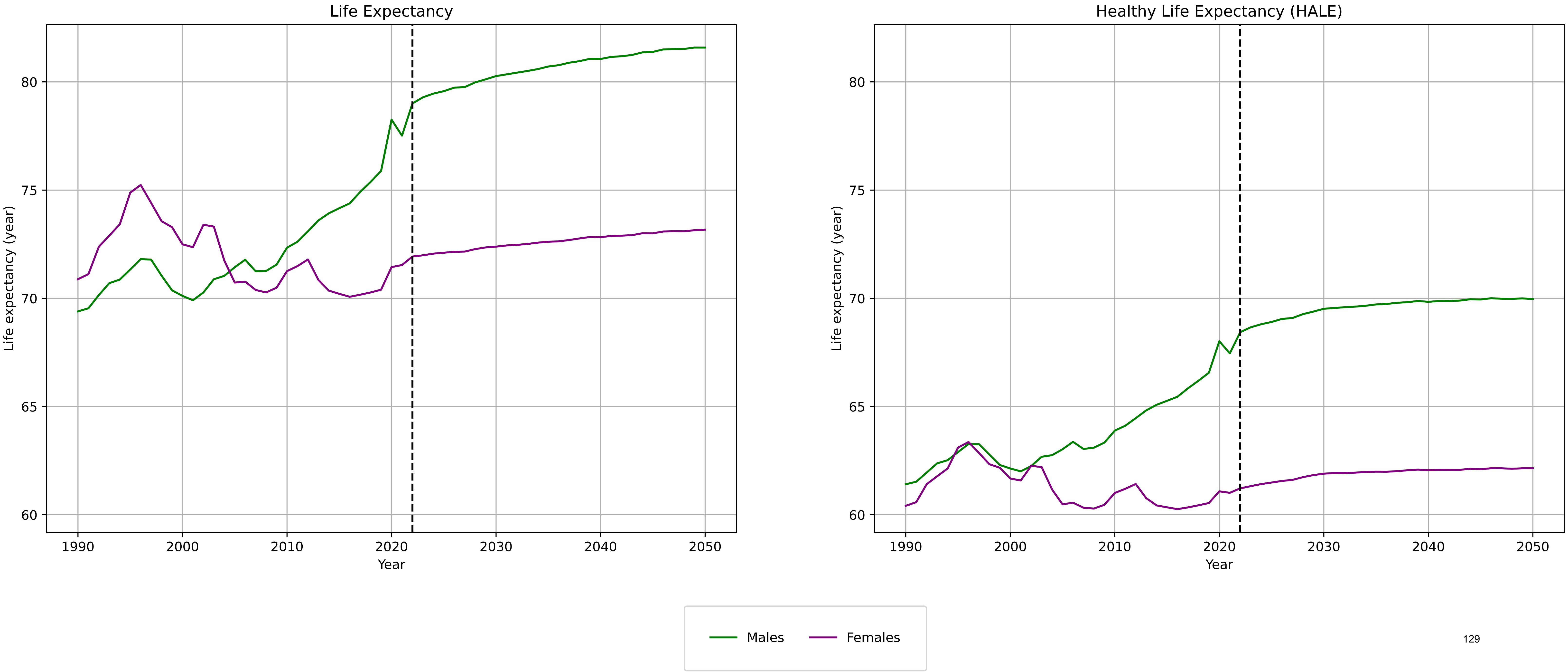

Yemen

Supplemental Results Figure S3. Life expectancy and HALE by sex and location, 1990-2050

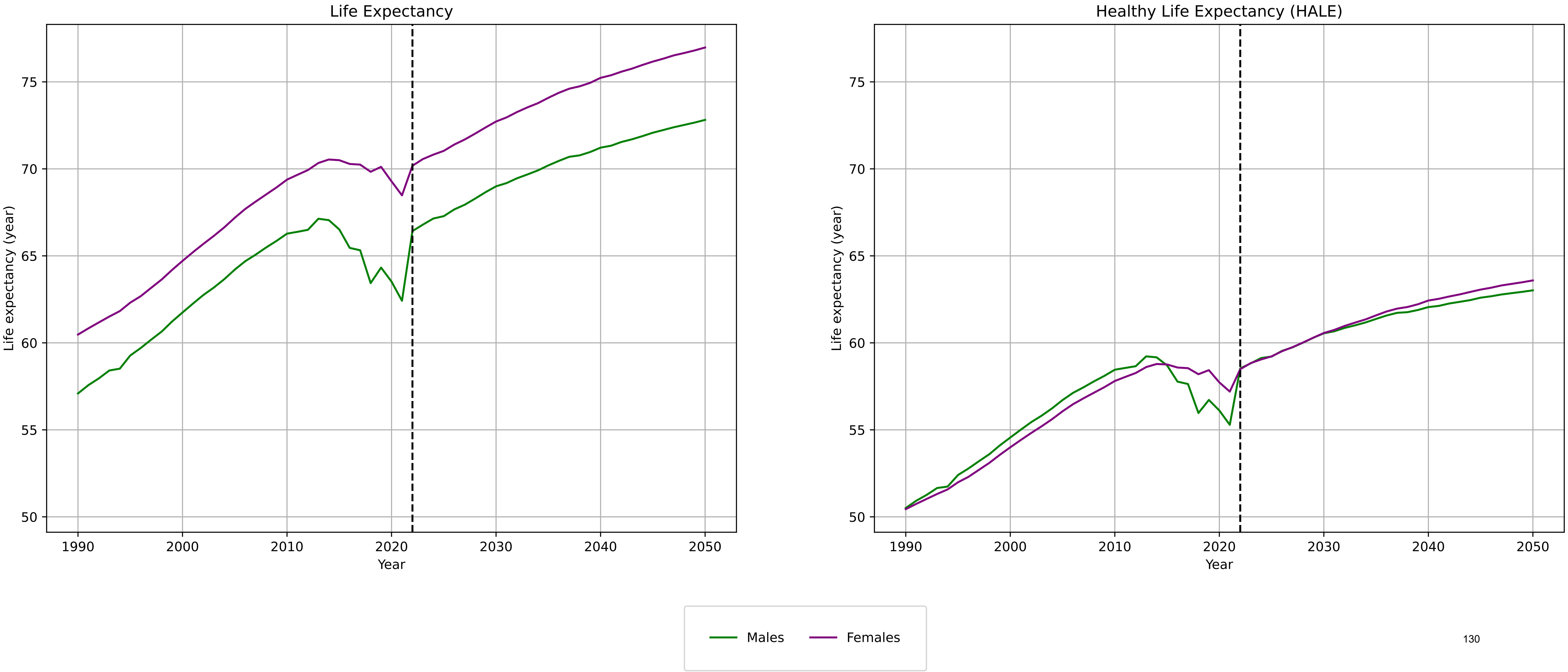

Bangladesh

Supplemental Results Figure S3. Life expectancy and HALE by sex and location, 1990-2050

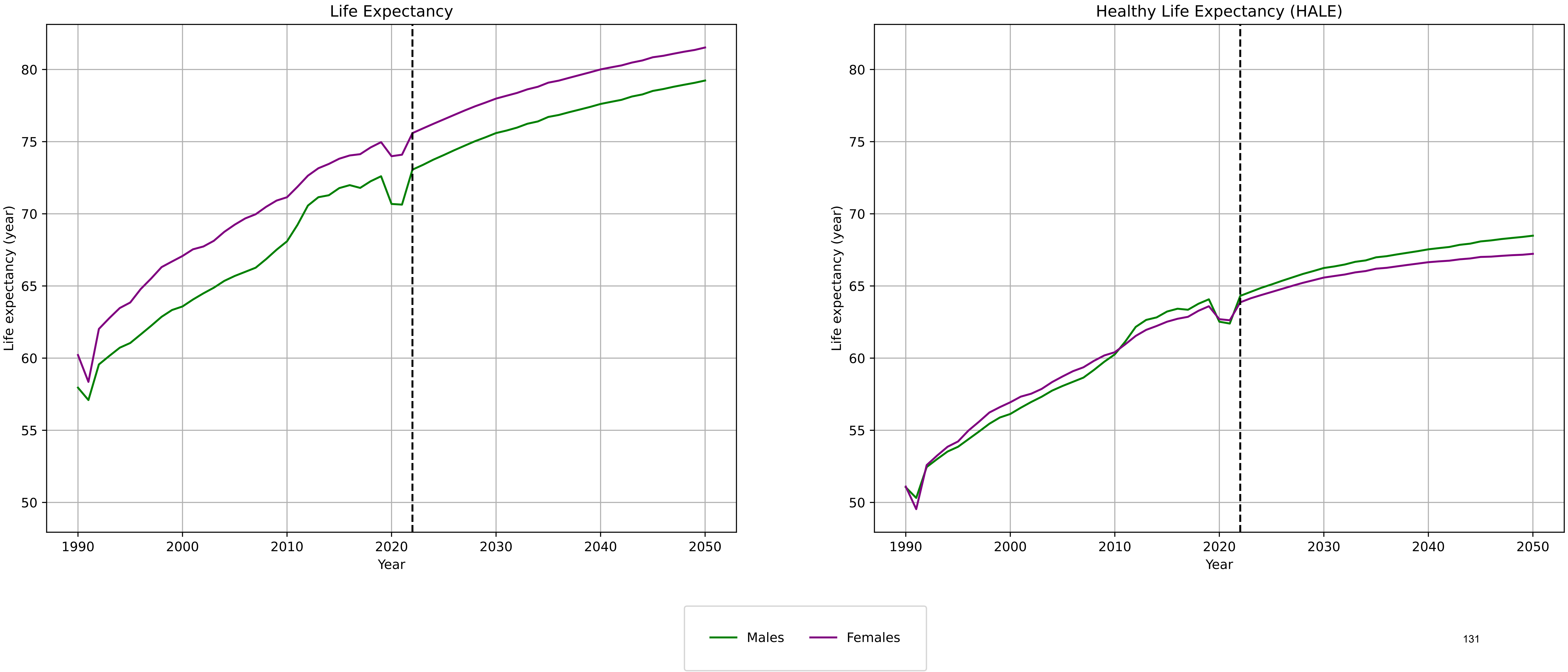

Bhutan

Supplemental Results Figure S3. Life expectancy and HALE by sex and location, 1990-2050

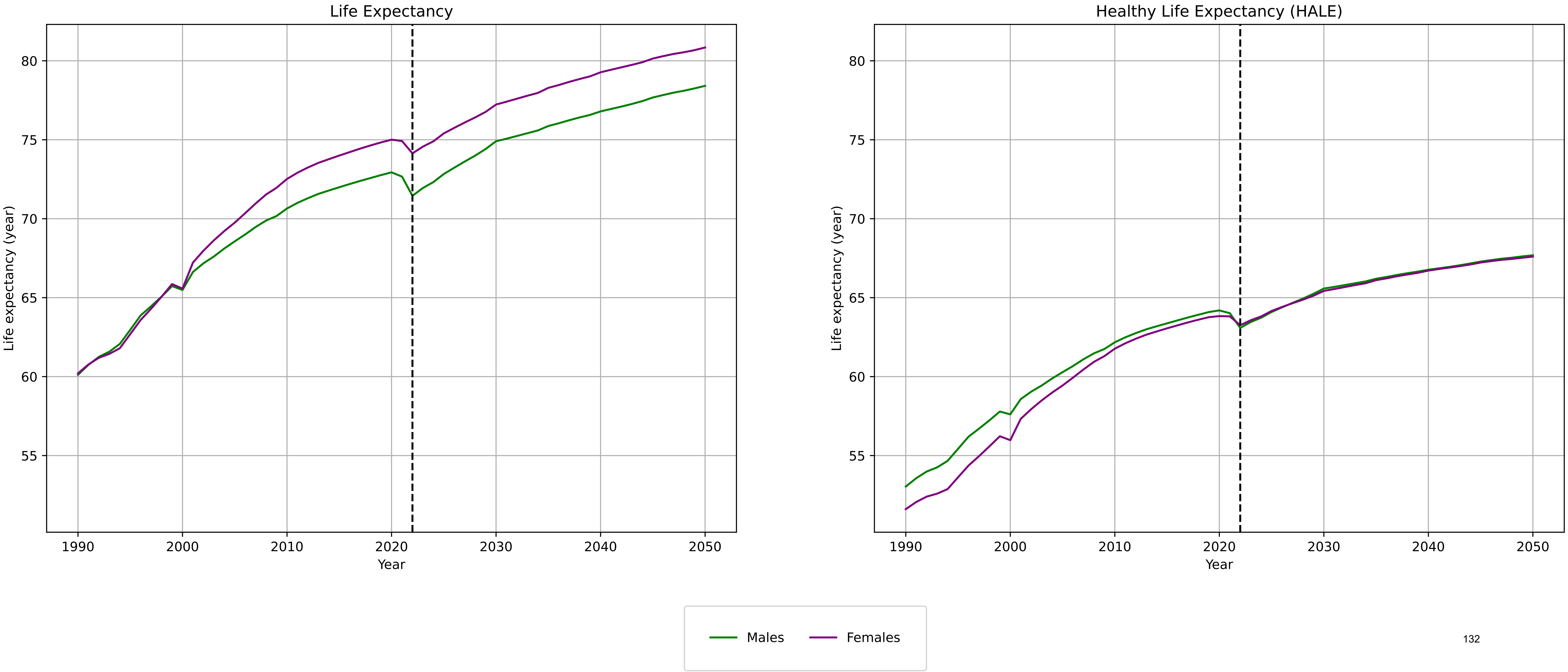

India

Supplemental Results Figure S3. Life expectancy and HALE by sex and location, 1990-2050

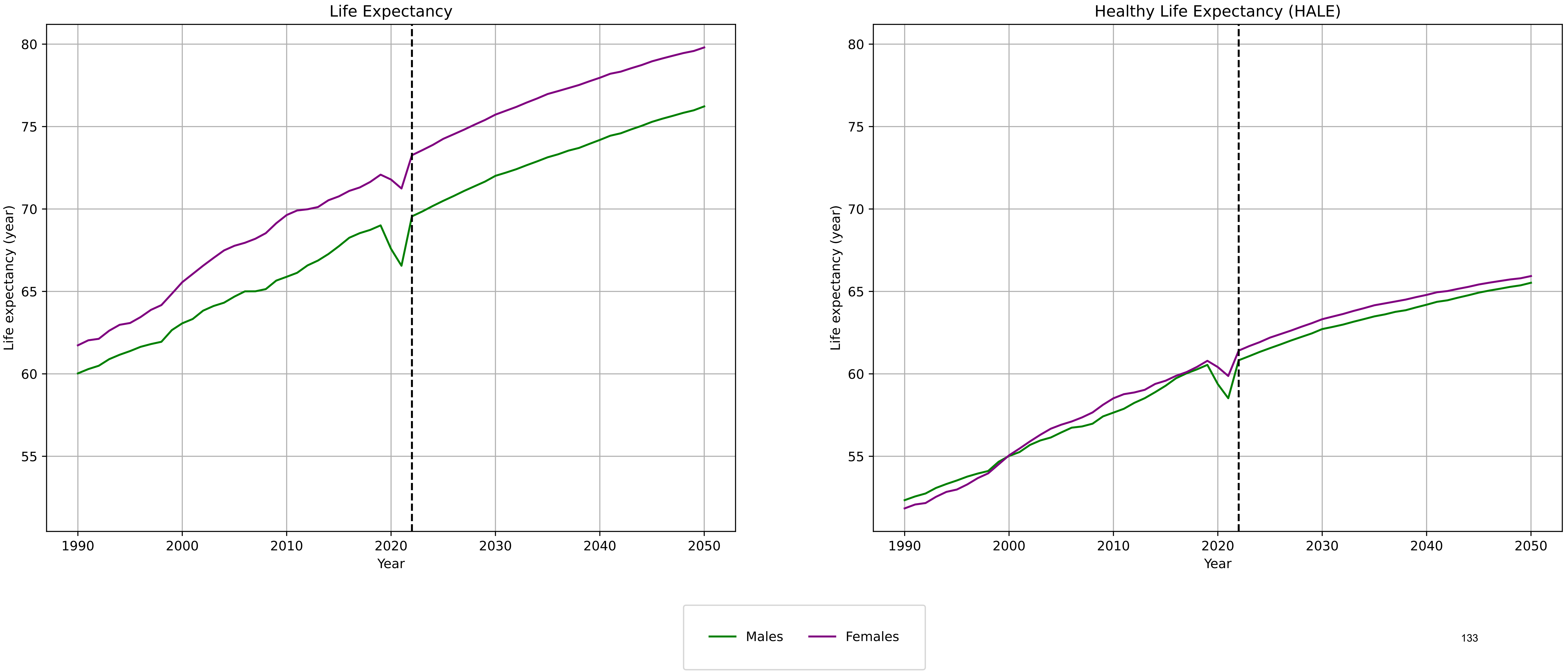

Nepal

Supplemental Results Figure S3. Life expectancy and HALE by sex and location, 1990-2050

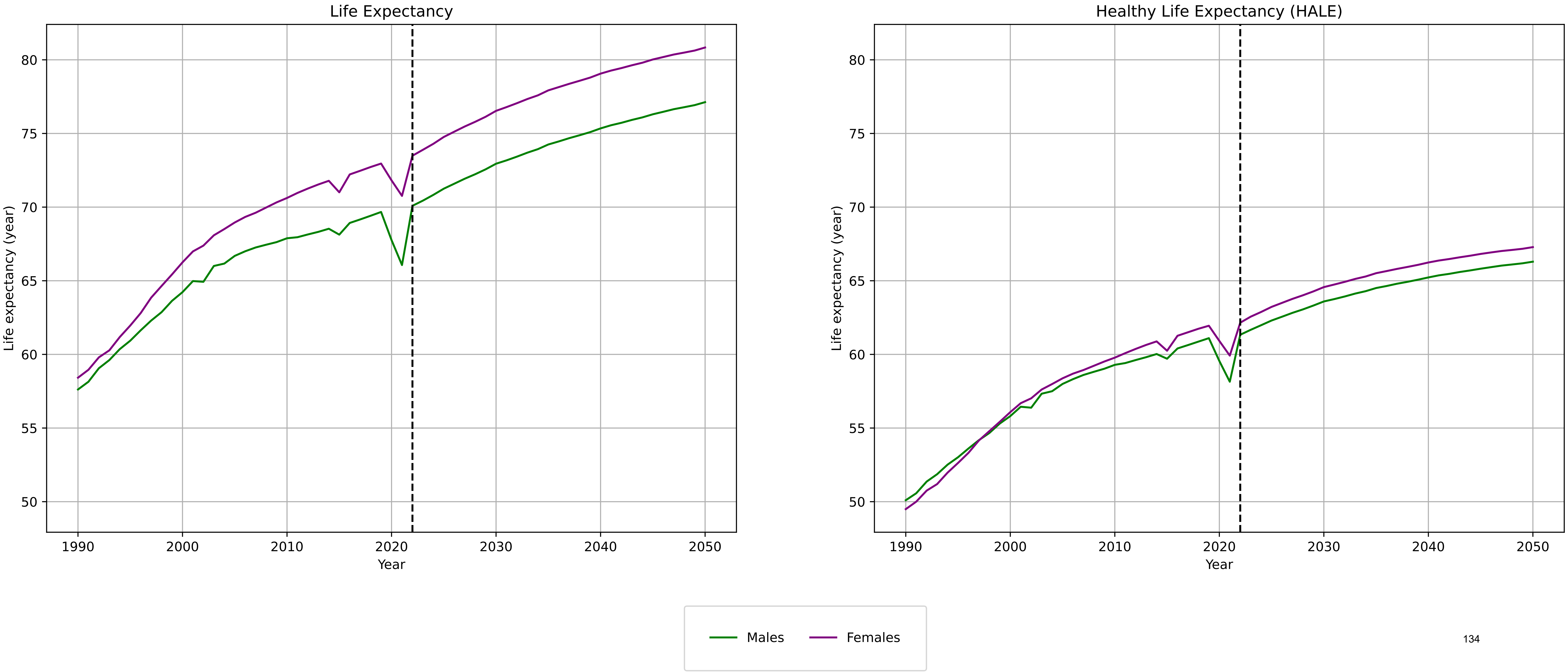

Supplemental Results Figure S3. Life expectancy and HALE by sex and location, 1990-2050

Pakistan

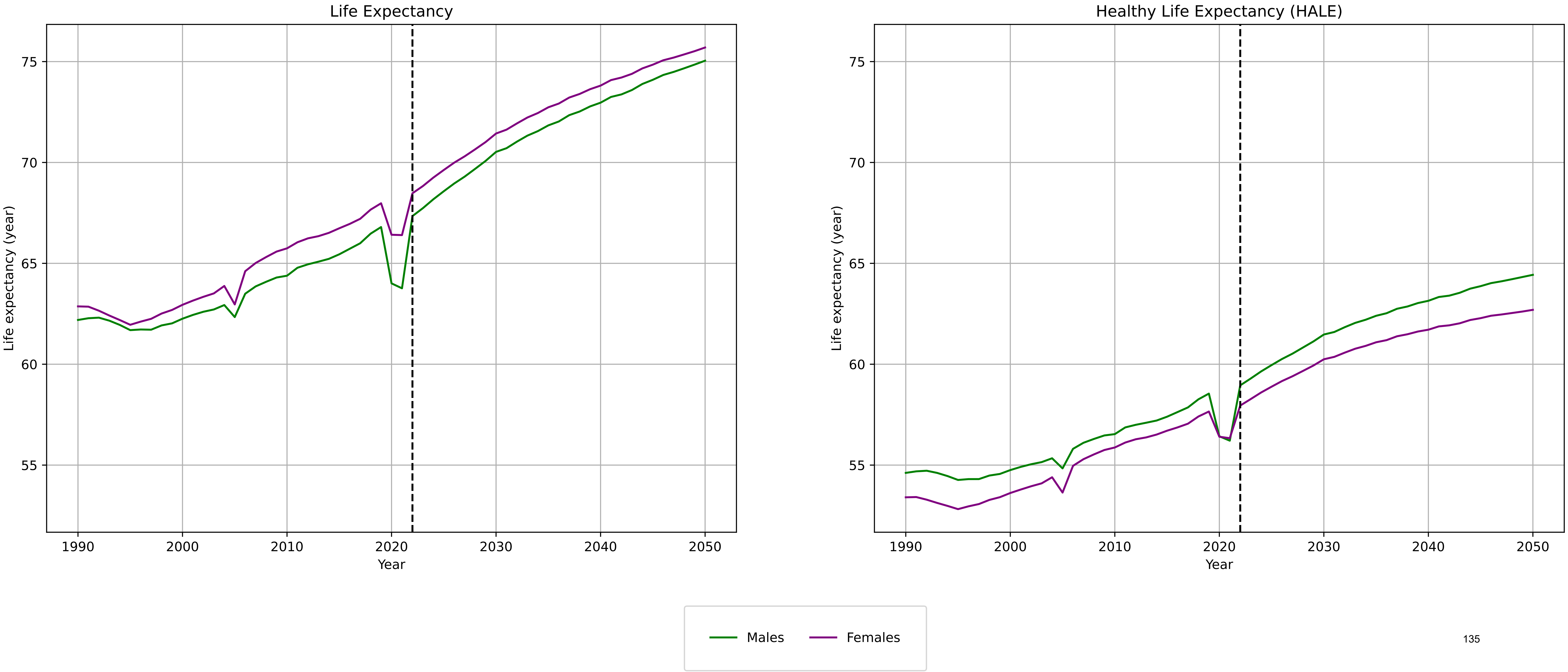

China

Supplemental Results Figure S3. Life expectancy and HALE by sex and location, 1990-2050

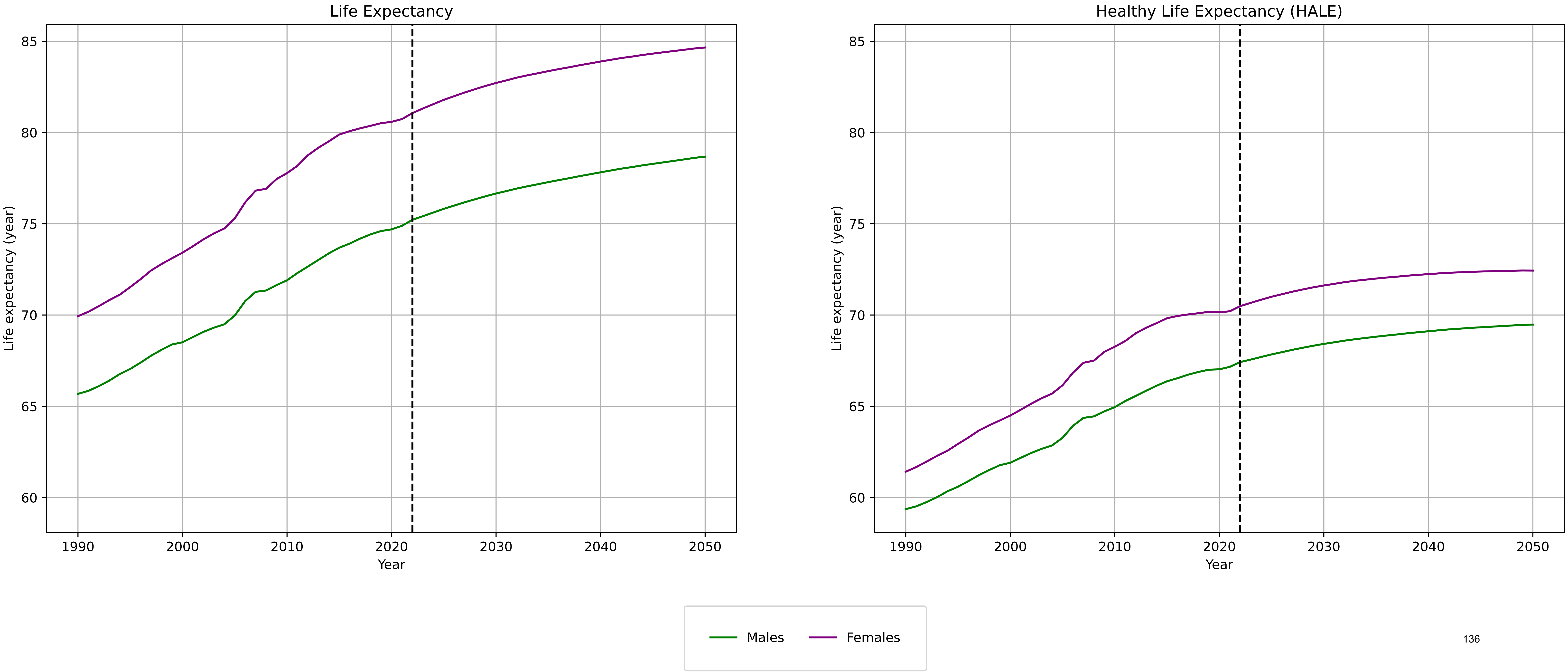

Supplemental Results Figure S3. Life expectancy and HALE by sex and location, 1990-2050

North Korea

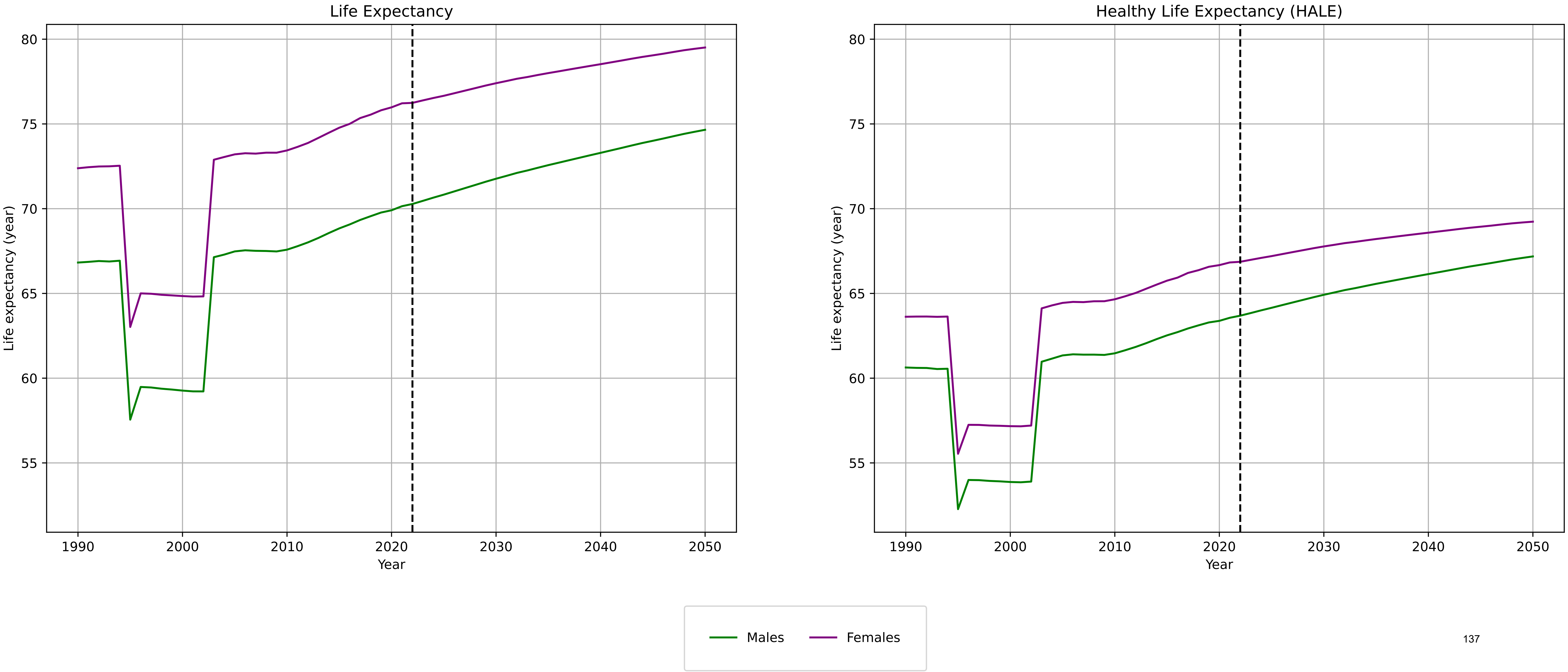

Supplemental Results Figure S3. Life expectancy and HALE by sex and location, 1990-2050

Taiwan (Province Of China)

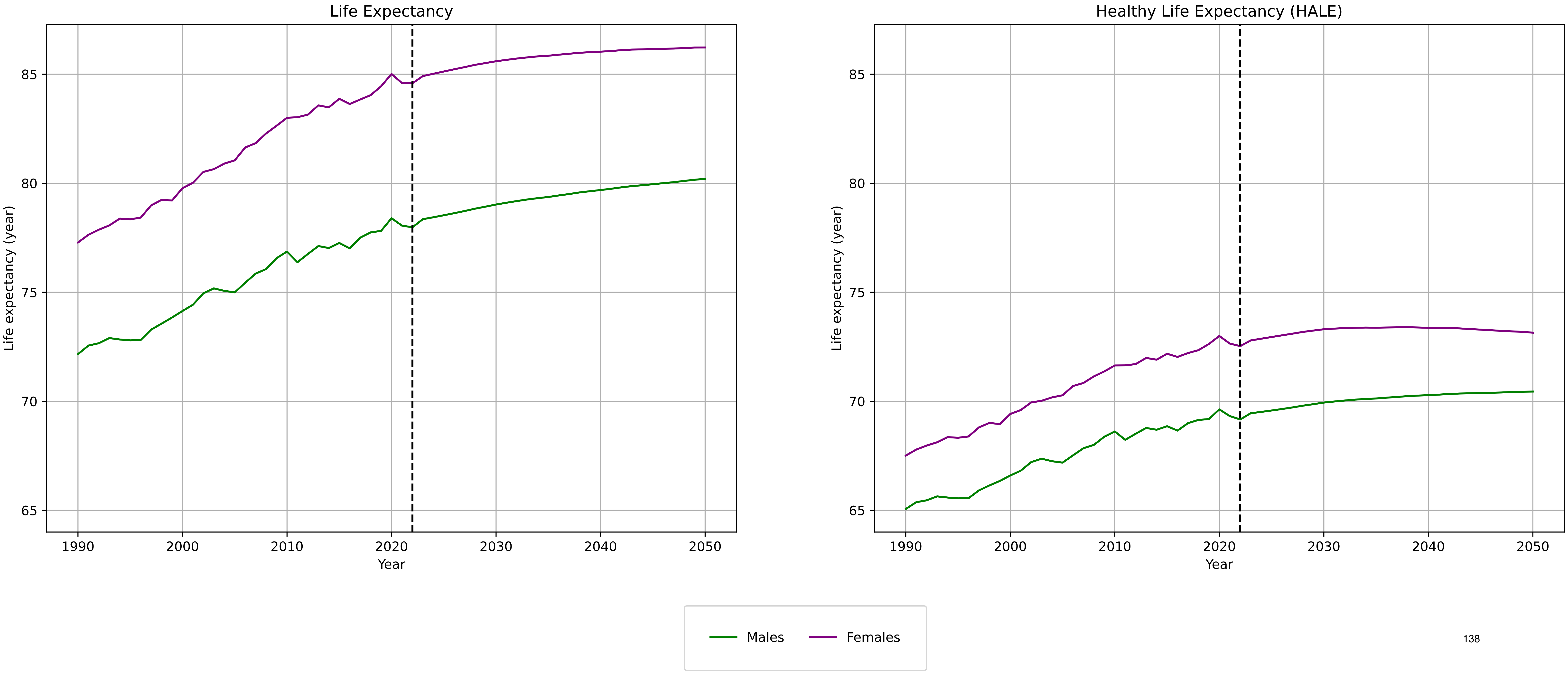

American Samoa

Supplemental Results Figure S3. Life expectancy and HALE by sex and location, 1990-2050

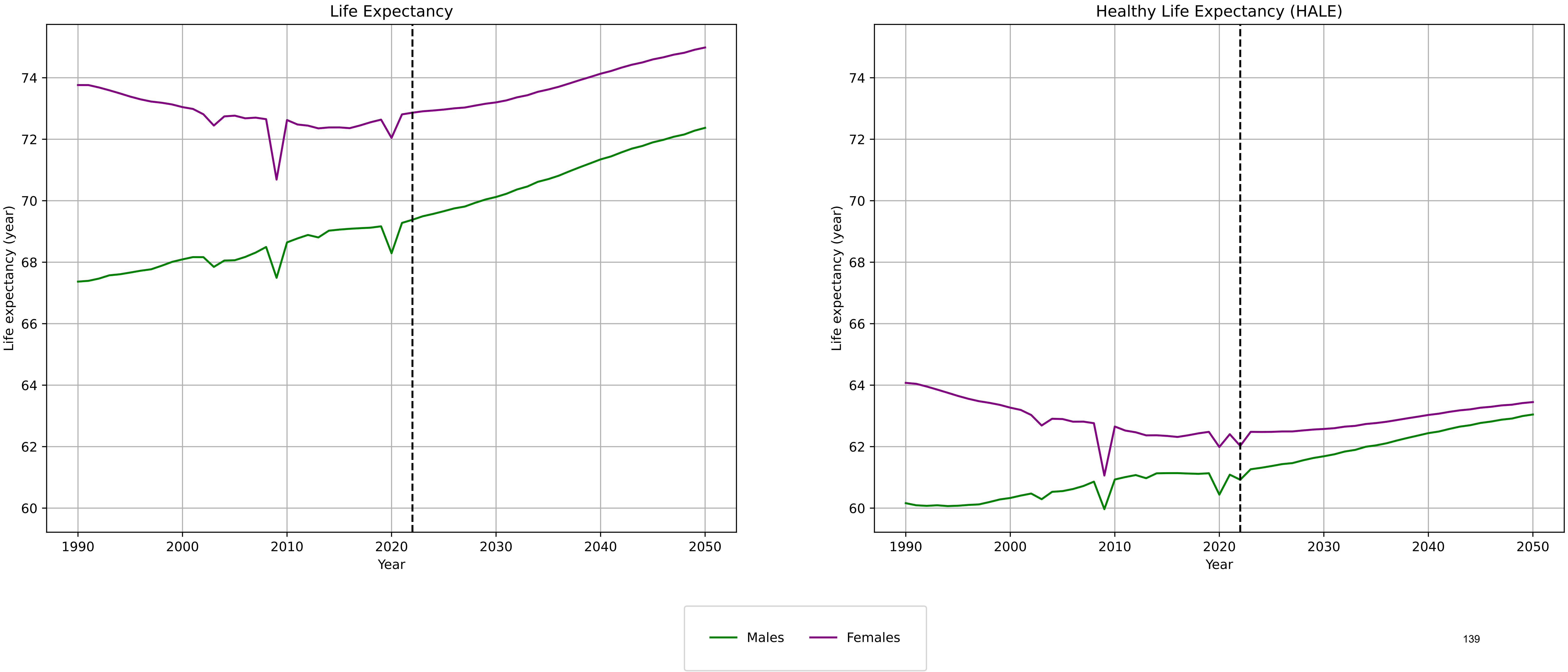

Cook Islands

Supplemental Results Figure S3. Life expectancy and HALE by sex and location, 1990-2050

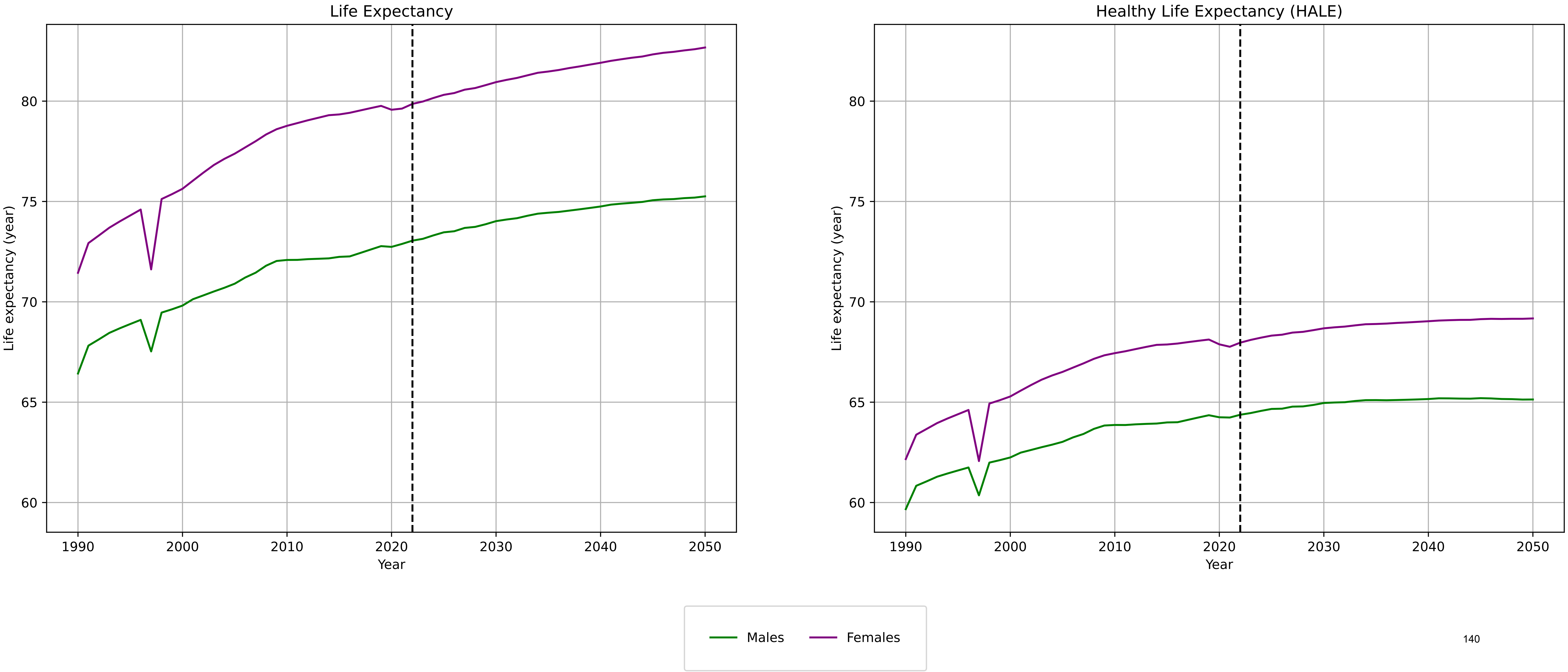

Fiji

Supplemental Results Figure S3. Life expectancy and HALE by sex and location, 1990-2050

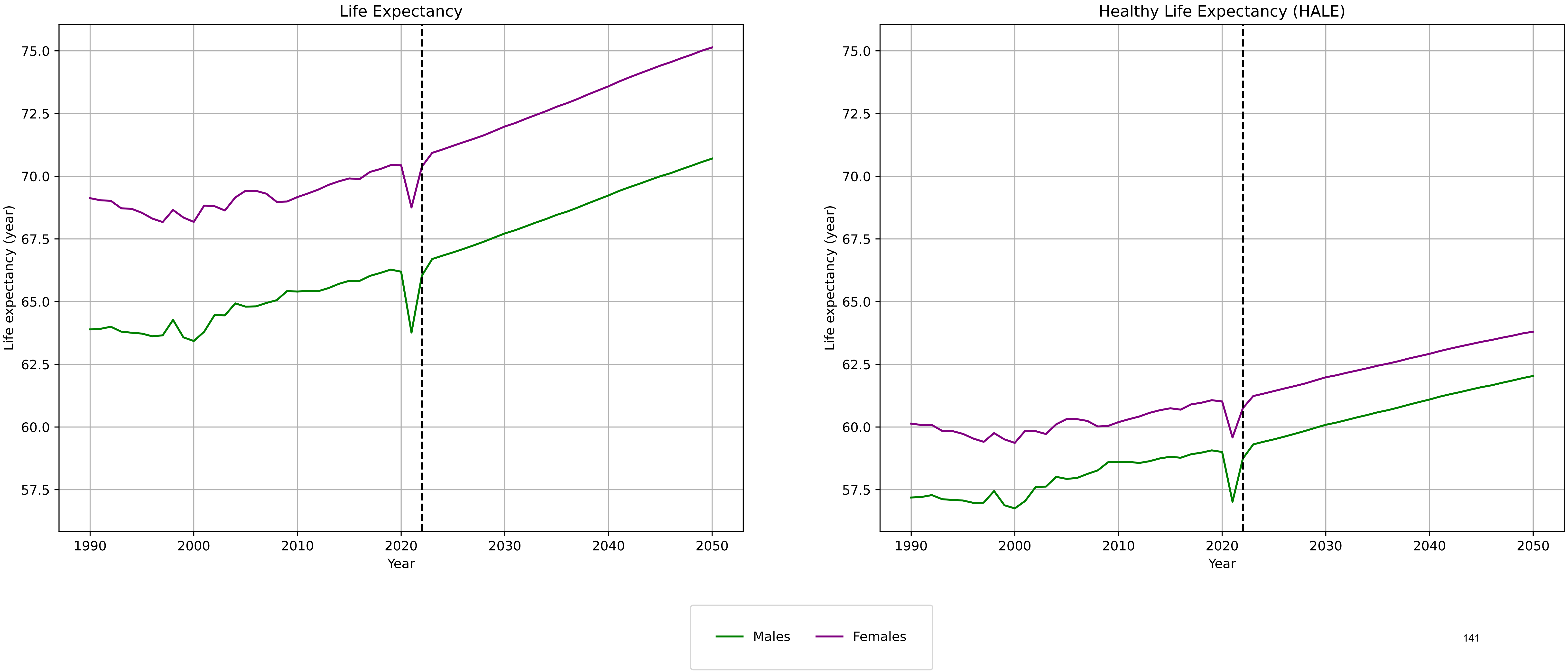

Supplemental Results Figure S3. Life expectancy and HALE by sex and location, 1990-2050

Guam

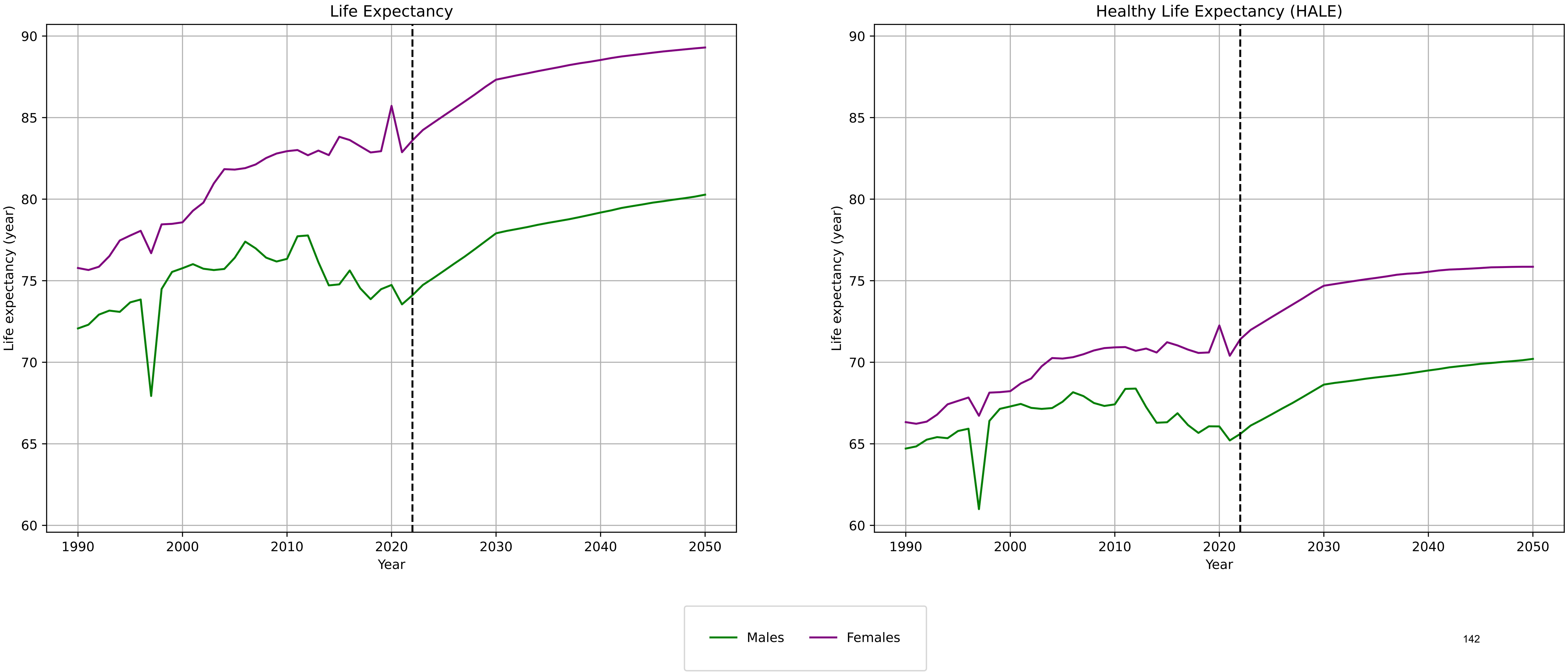

Supplemental Results Figure S3. Life expectancy and HALE by sex and location, 1990-2050

Kiribati

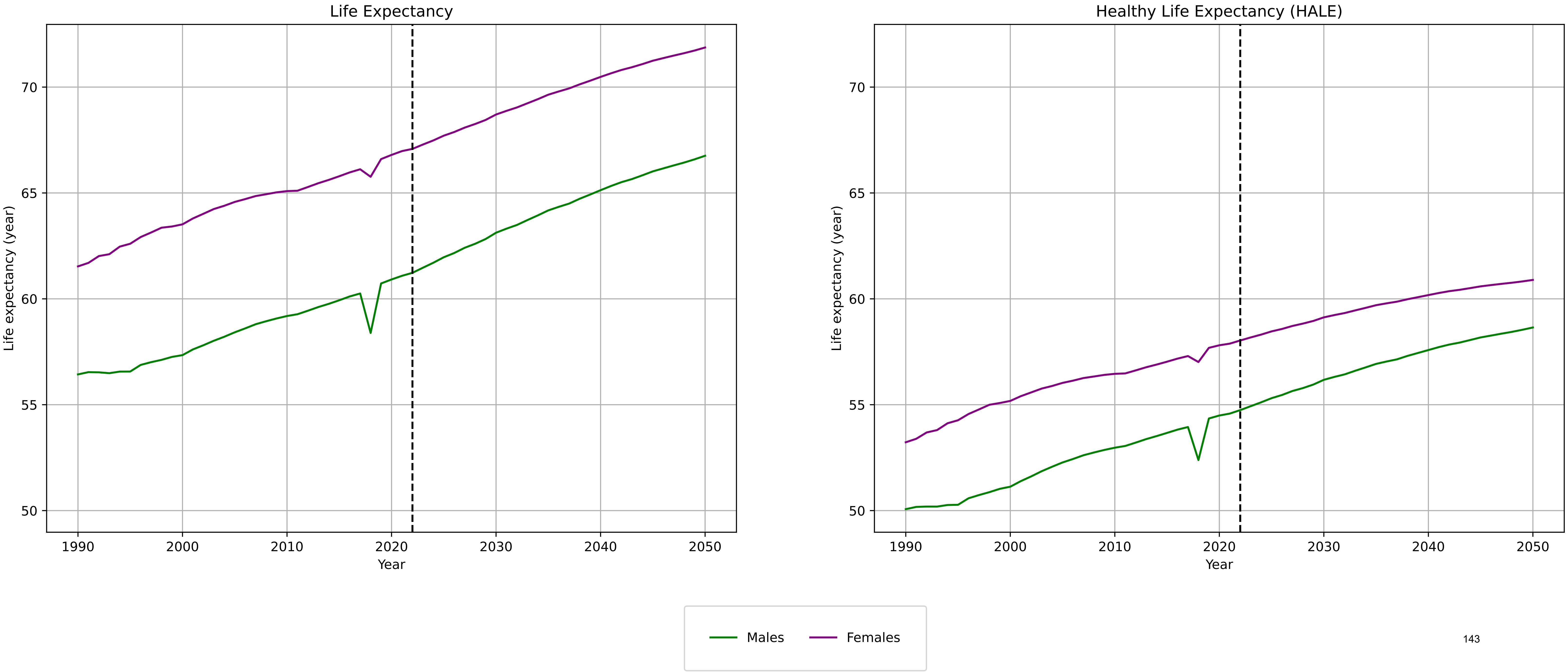

Marshall Islands

Supplemental Results Figure S3. Life expectancy and HALE by sex and location, 1990-2050

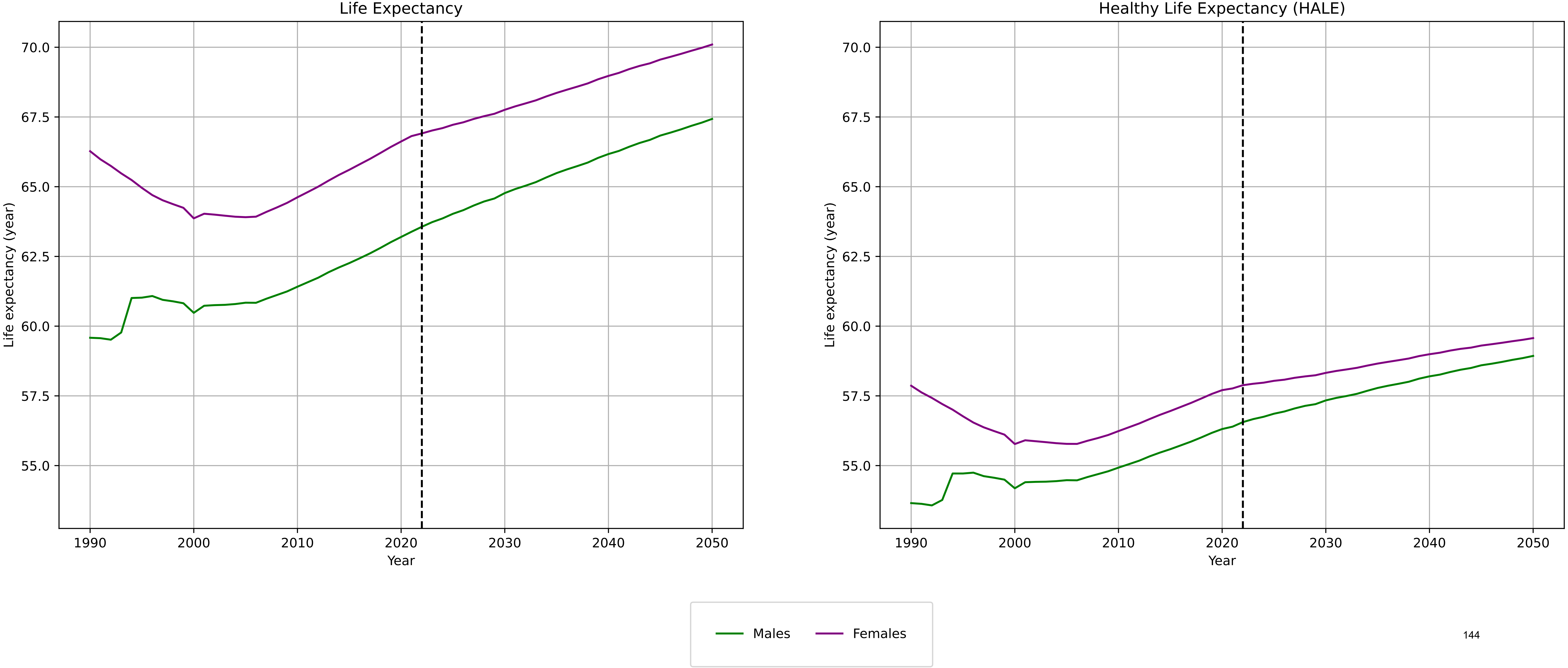

Supplemental Results Figure S3. Life expectancy and HALE by sex and location, 1990-2050

Federated States Of Micronesia

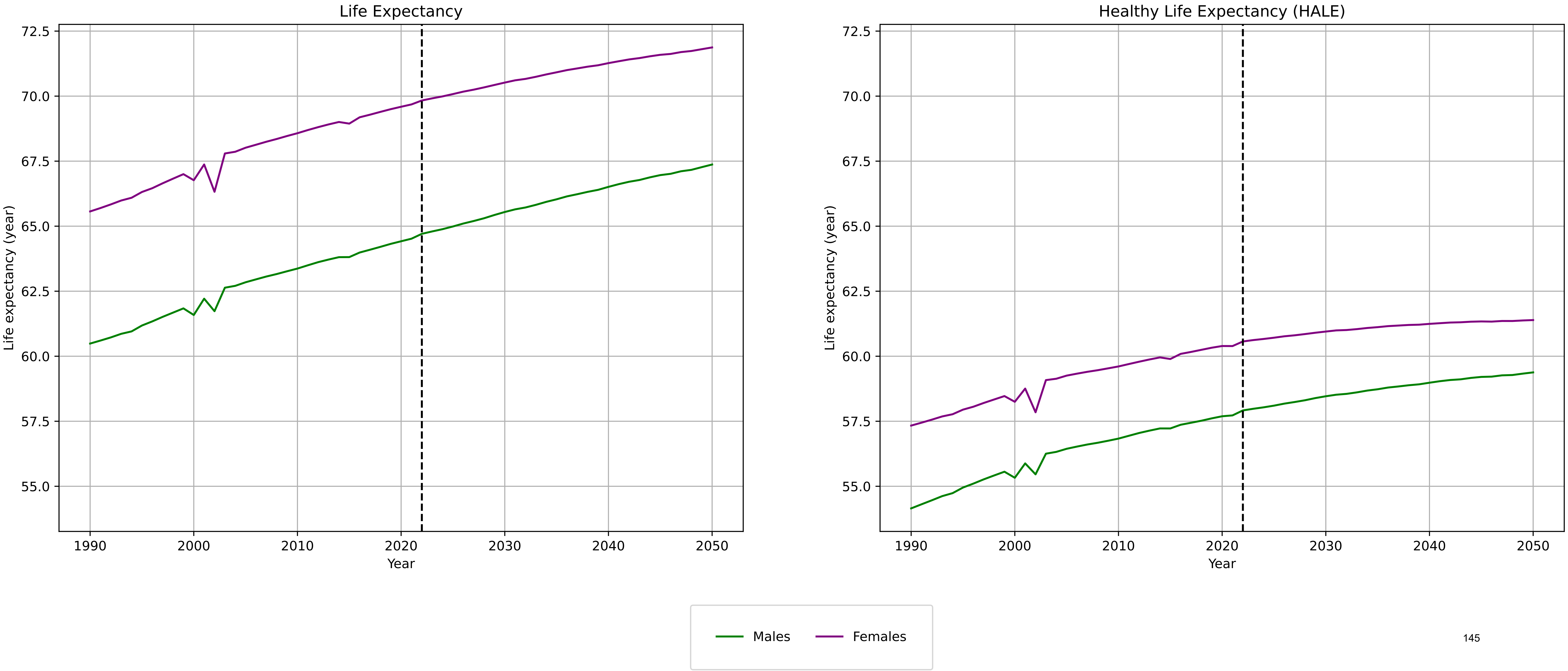

Supplemental Results Figure S3. Life expectancy and HALE by sex and location, 1990-2050

Nauru

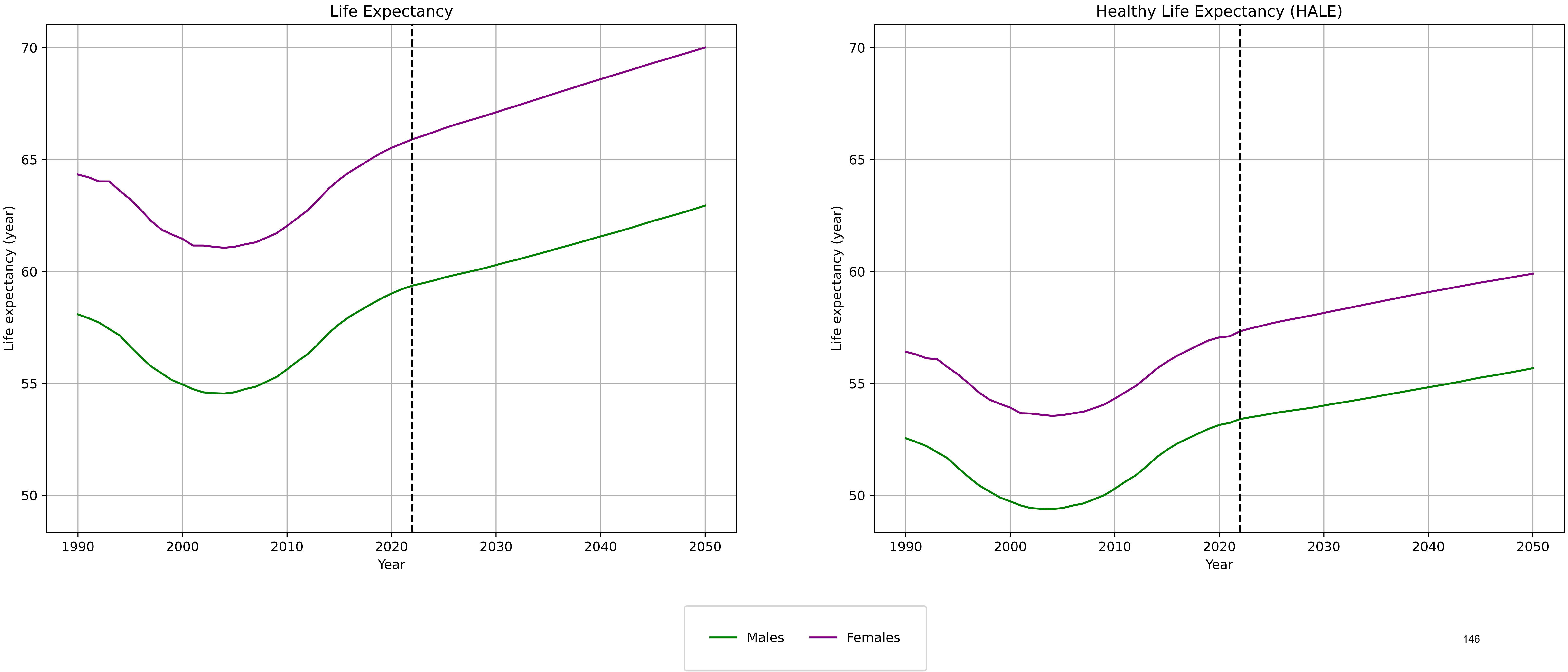

Niue

Supplemental Results Figure S3. Life expectancy and HALE by sex and location, 1990-2050

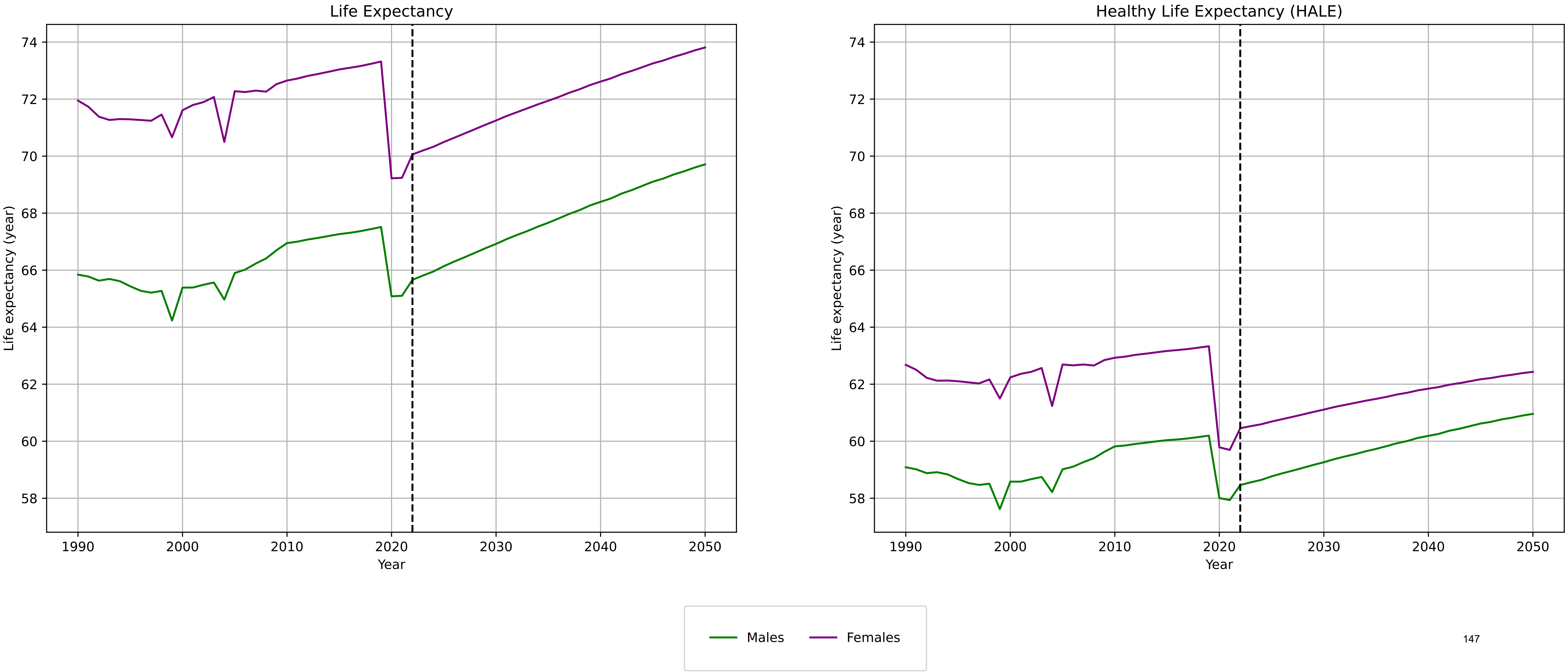

Northern Mariana Islands

Supplemental Results Figure S3. Life expectancy and HALE by sex and location, 1990-2050

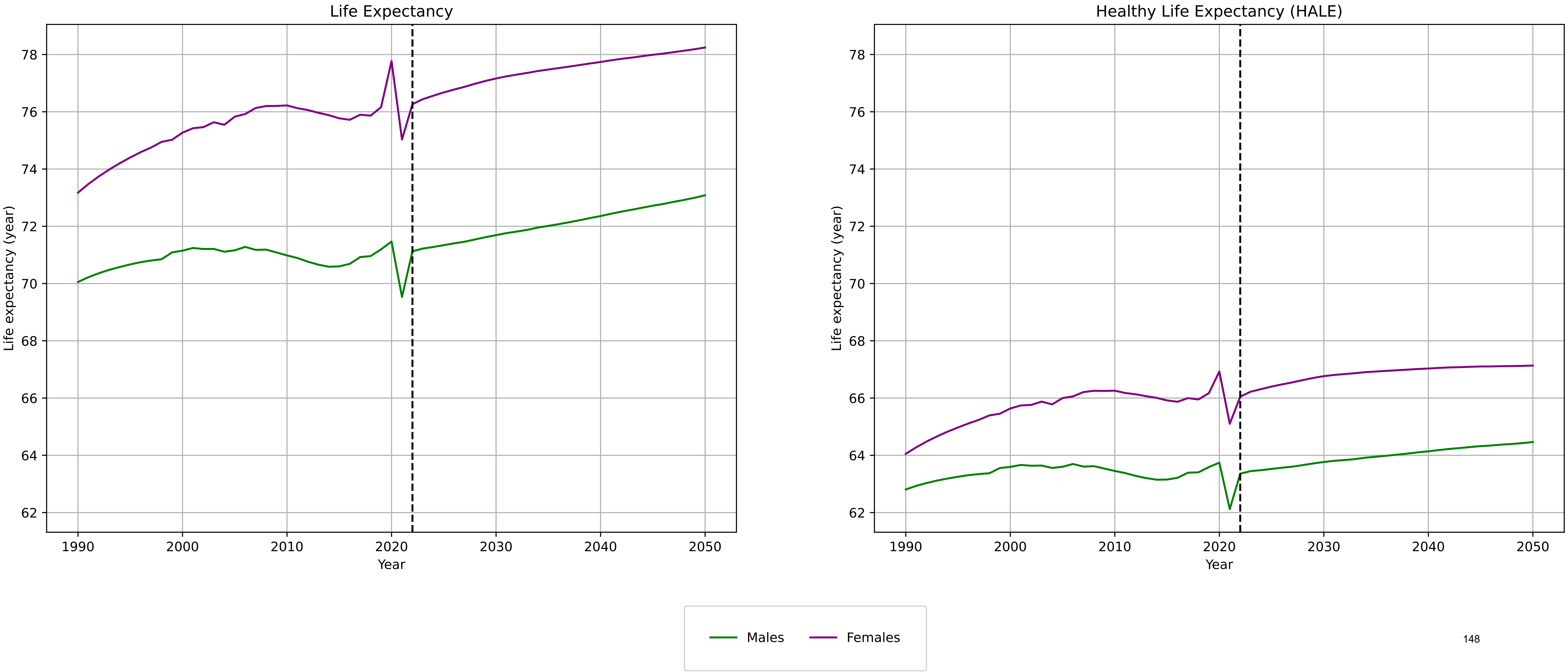

Palau

Supplemental Results Figure S3. Life expectancy and HALE by sex and location, 1990-2050

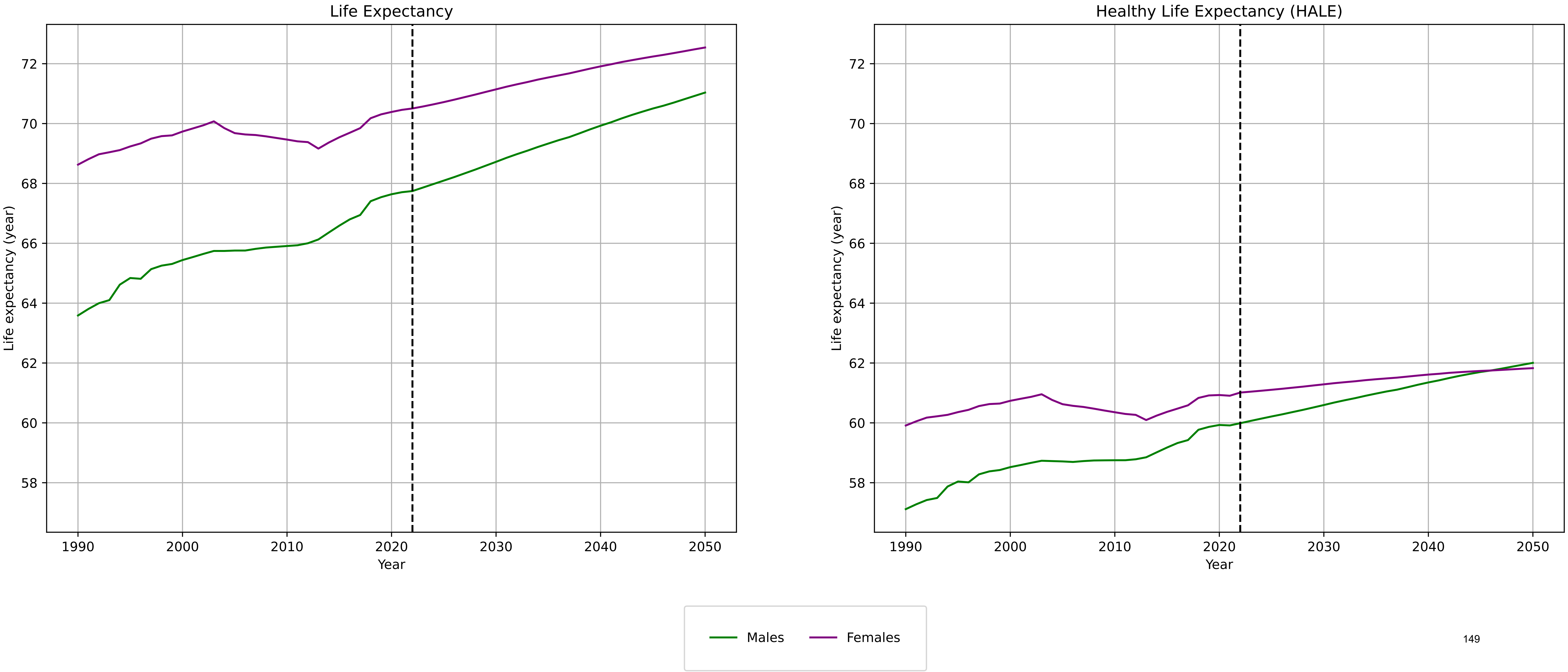

Papua New Guinea

Supplemental Results Figure S3. Life expectancy and HALE by sex and location, 1990-2050

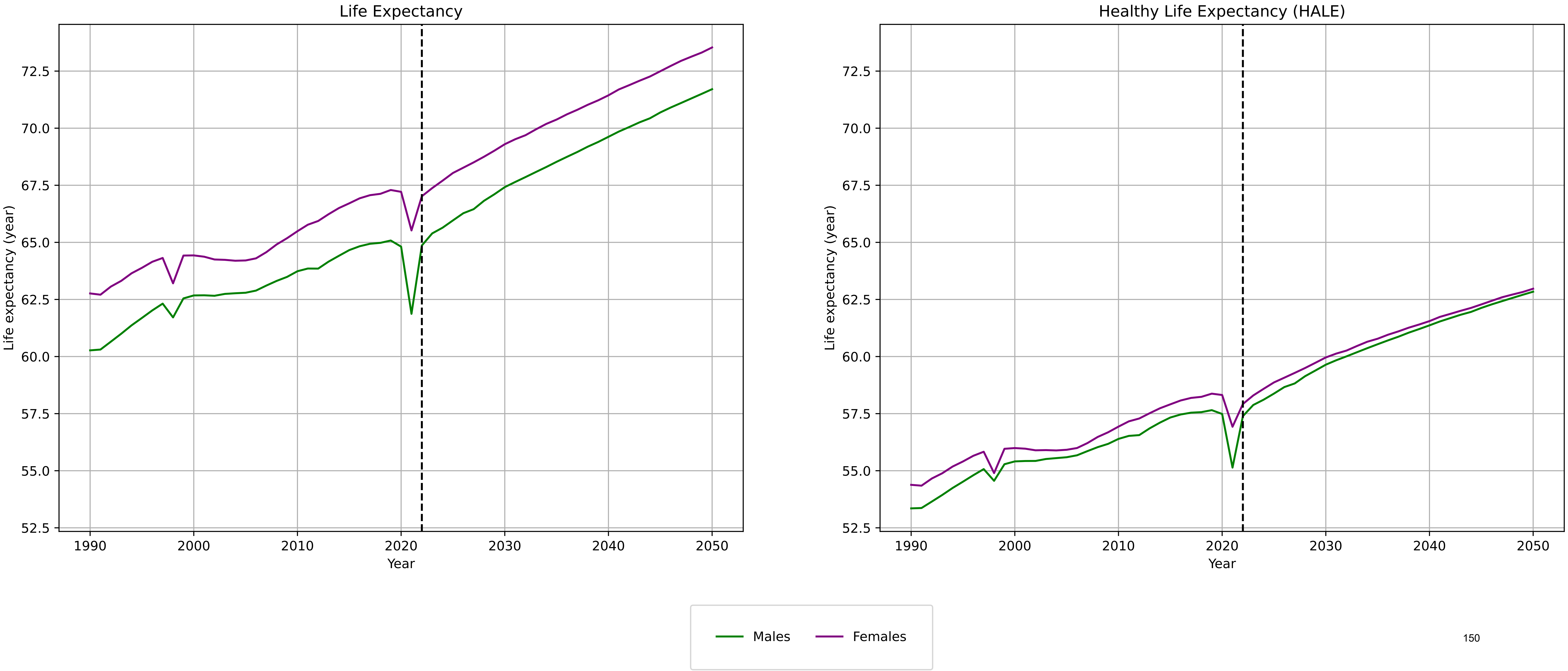

Samoa

Supplemental Results Figure S3. Life expectancy and HALE by sex and location, 1990-2050

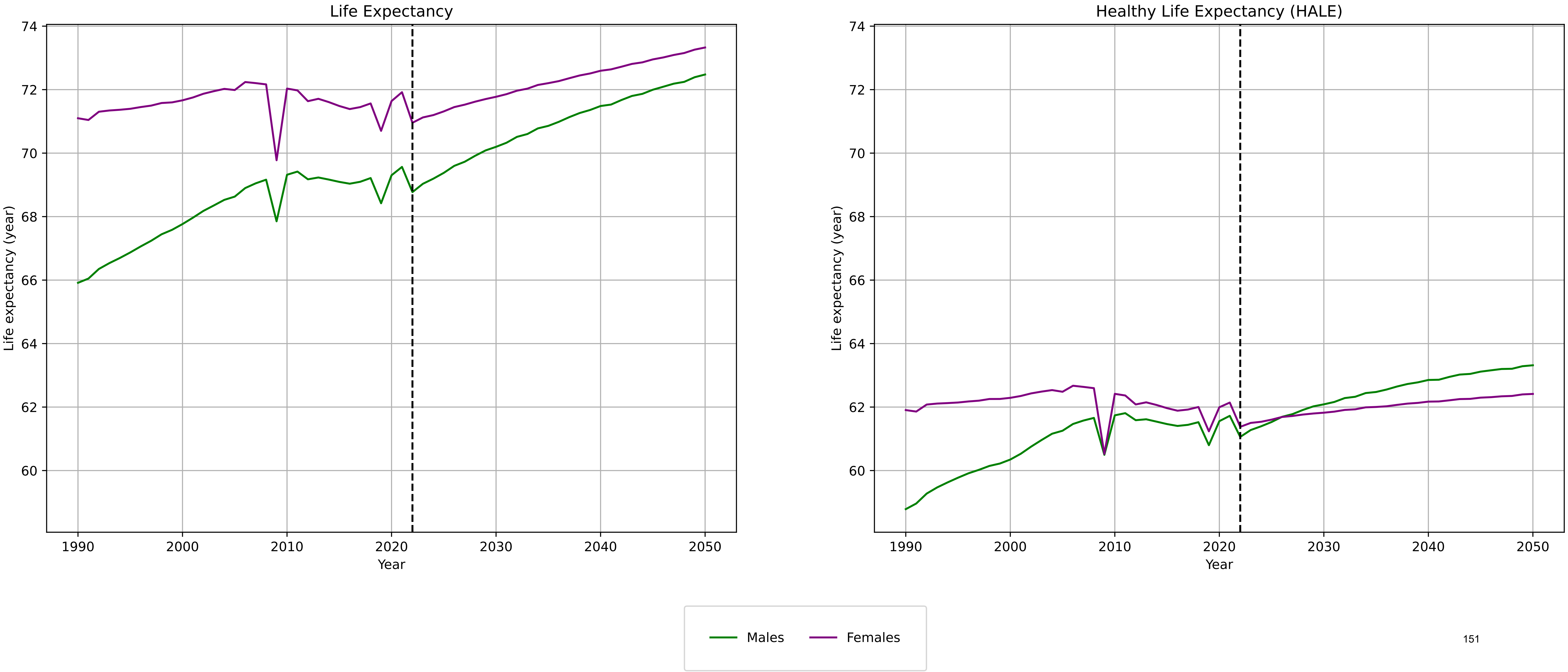

Solomon Islands

Supplemental Results Figure S3. Life expectancy and HALE by sex and location, 1990-2050

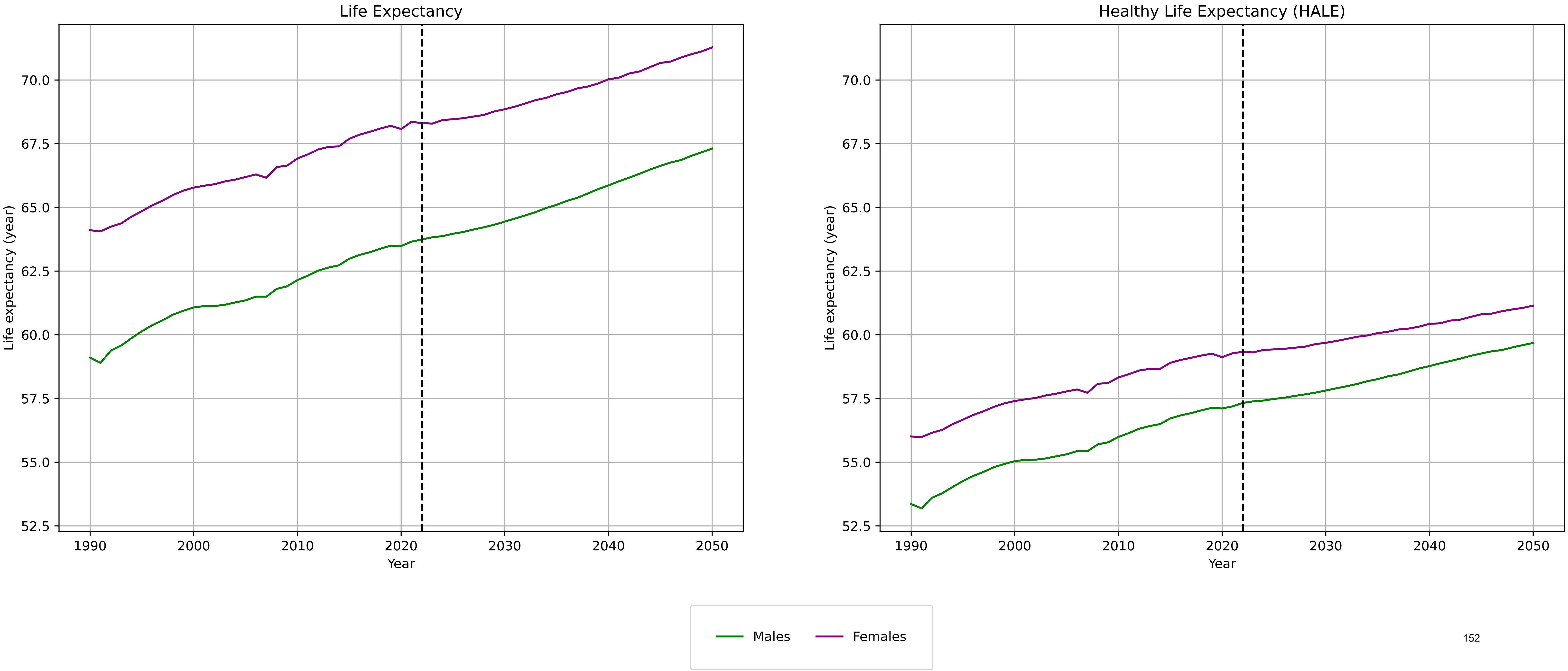

Tokelau

Supplemental Results Figure S3. Life expectancy and HALE by sex and location, 1990-2050

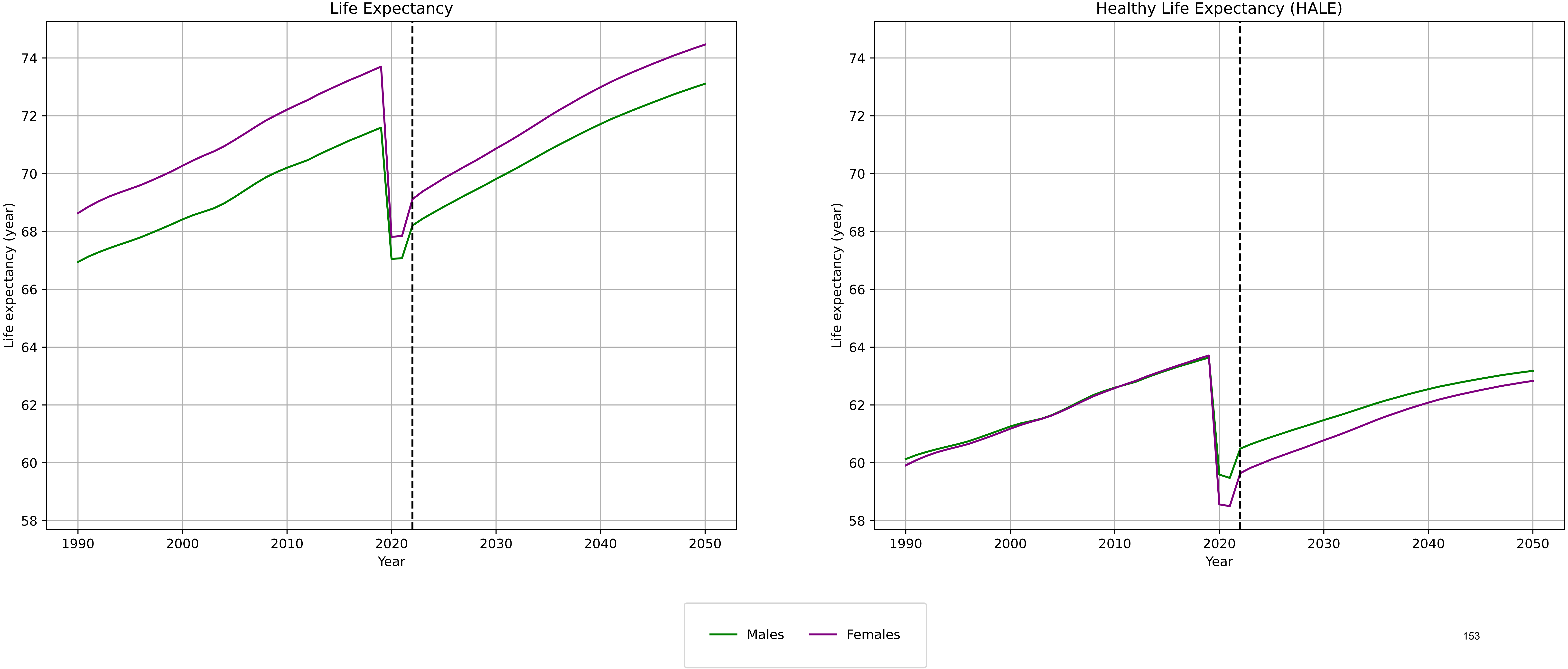

Tonga

Supplemental Results Figure S3. Life expectancy and HALE by sex and location, 1990-2050

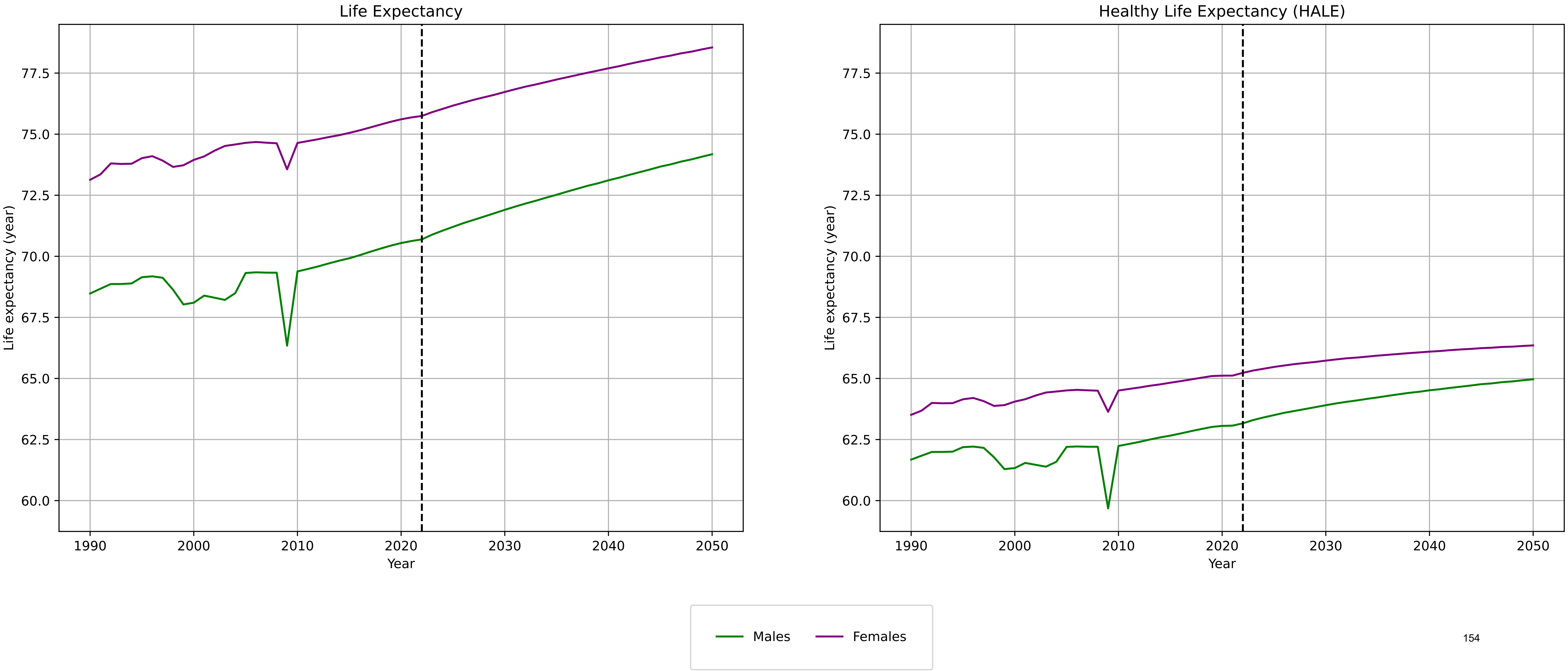

Tuvalu

Supplemental Results Figure S3. Life expectancy and HALE by sex and location, 1990-2050

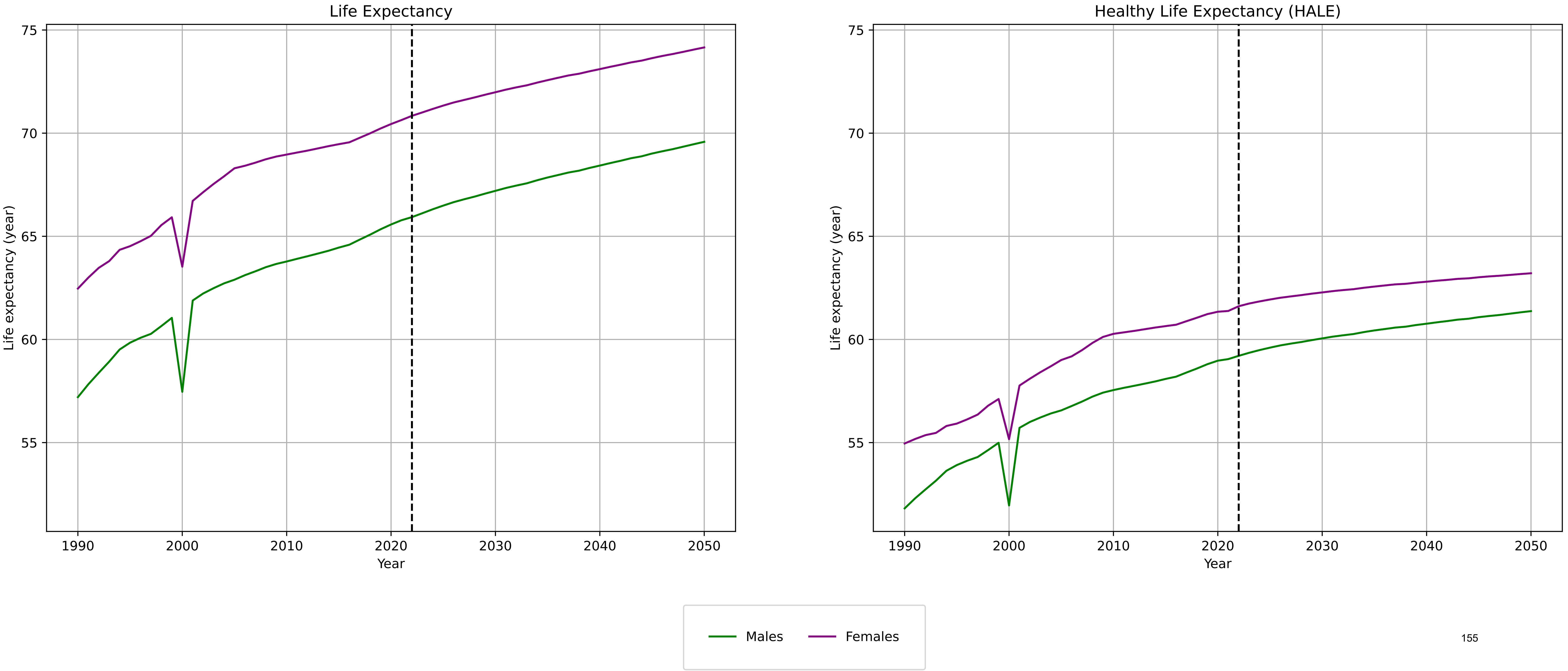

Vanuatu

Supplemental Results Figure S3. Life expectancy and HALE by sex and location, 1990-2050

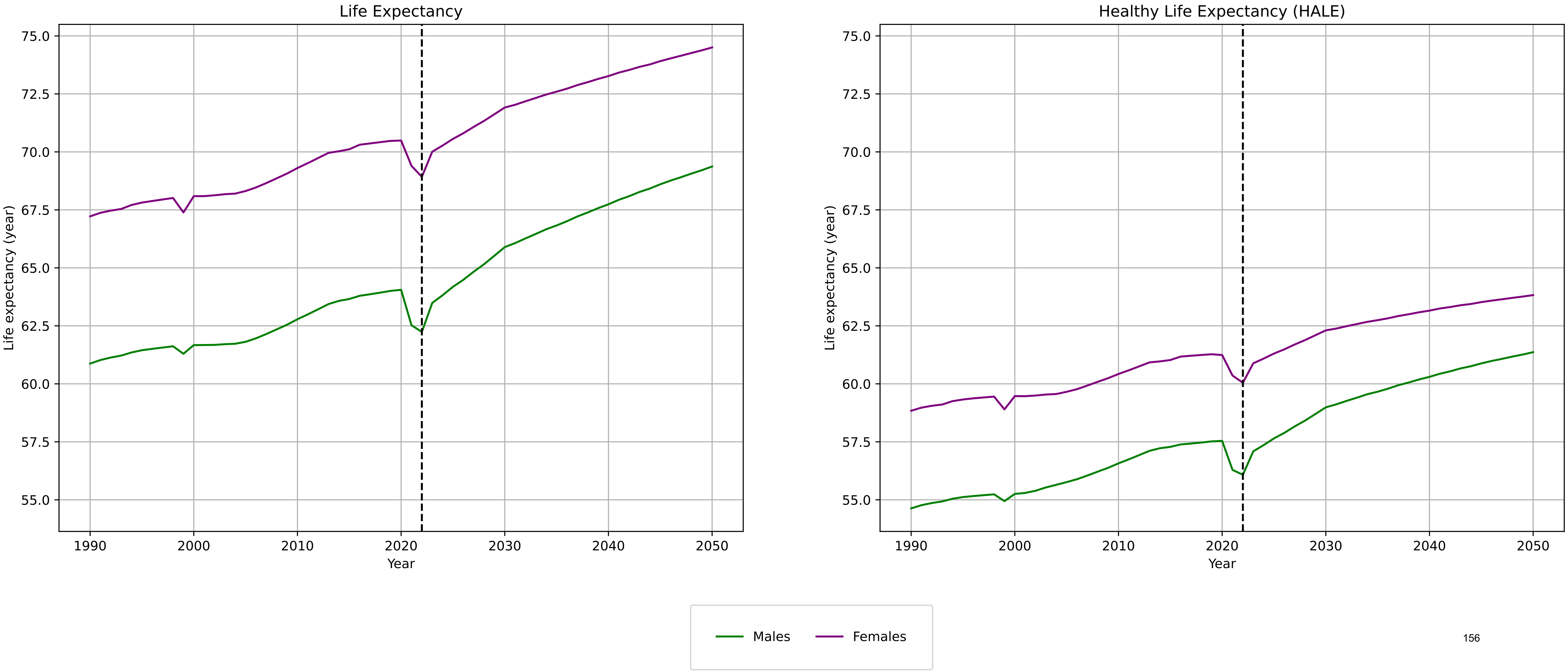

Cambodia

Supplemental Results Figure S3. Life expectancy and HALE by sex and location, 1990-2050

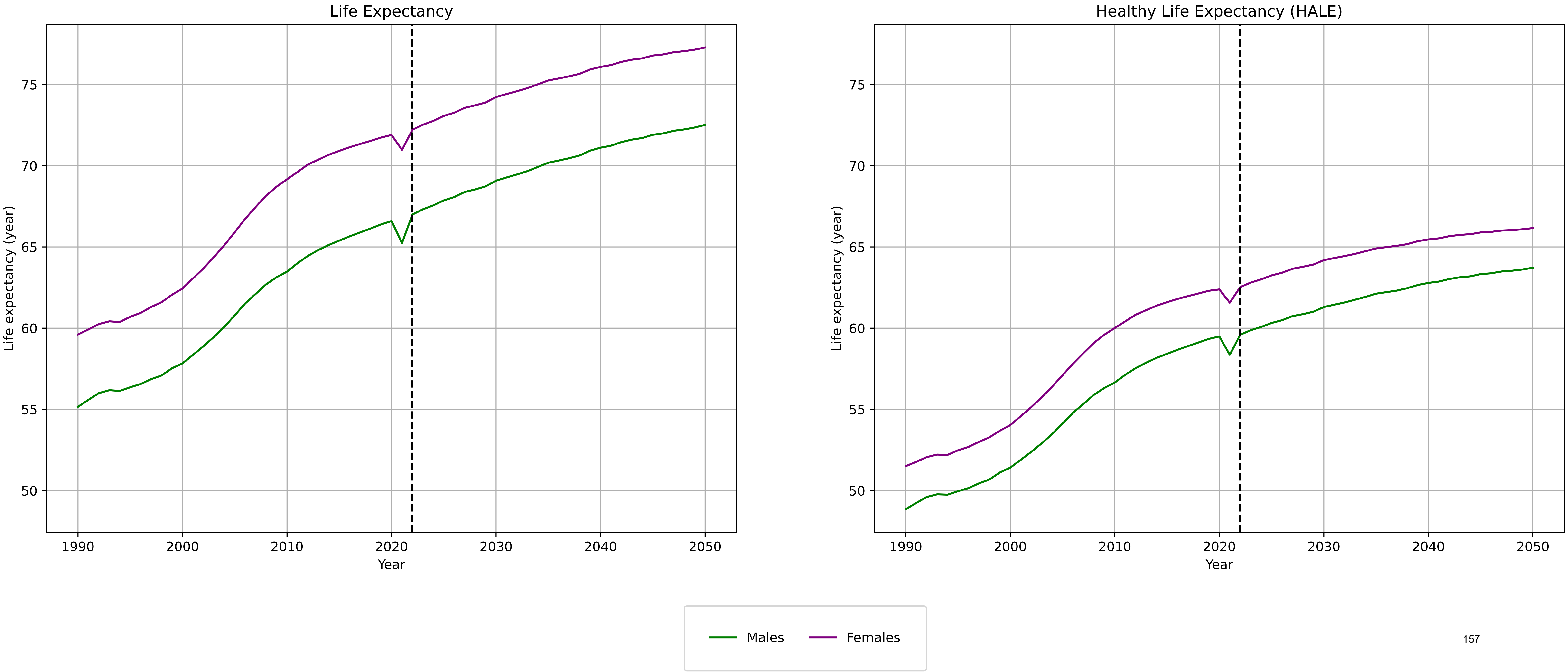

Indonesia

Supplemental Results Figure S3. Life expectancy and HALE by sex and location, 1990-2050

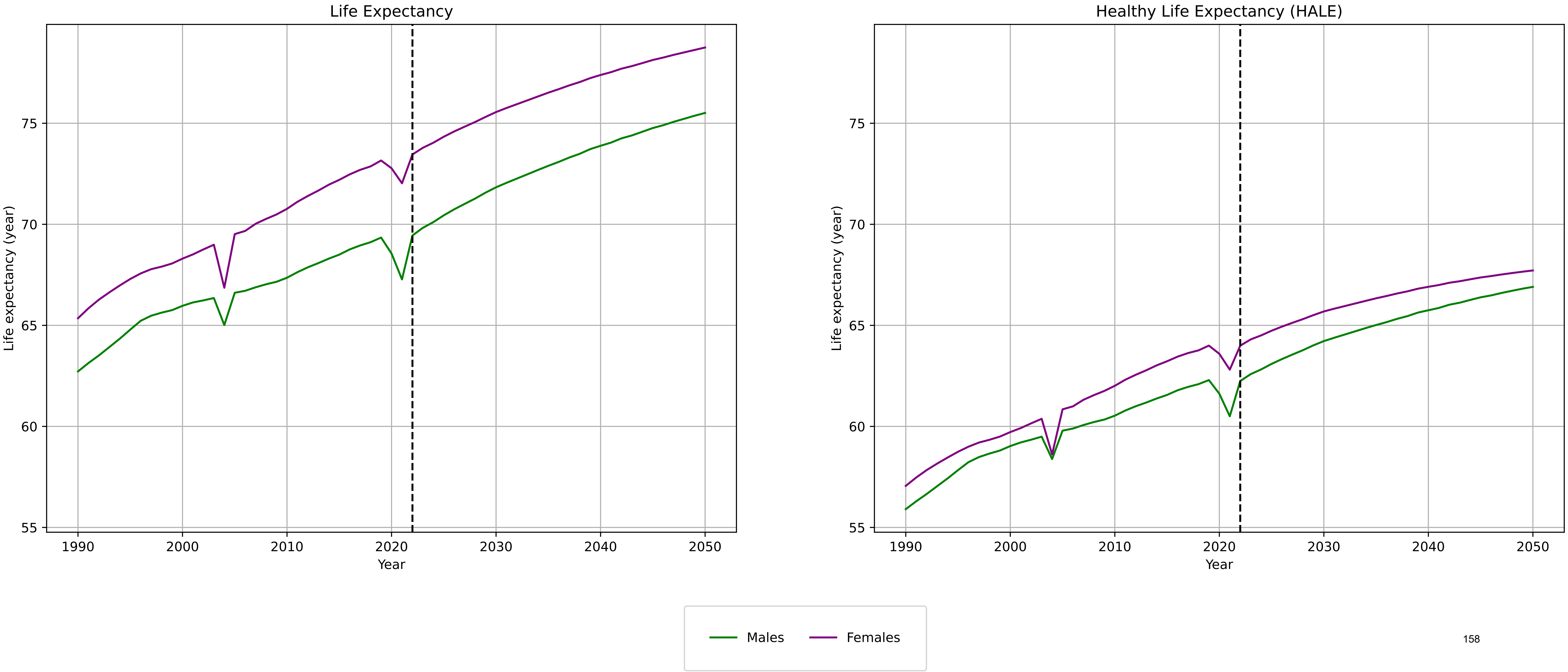

Laos

Supplemental Results Figure S3. Life expectancy and HALE by sex and location, 1990-2050

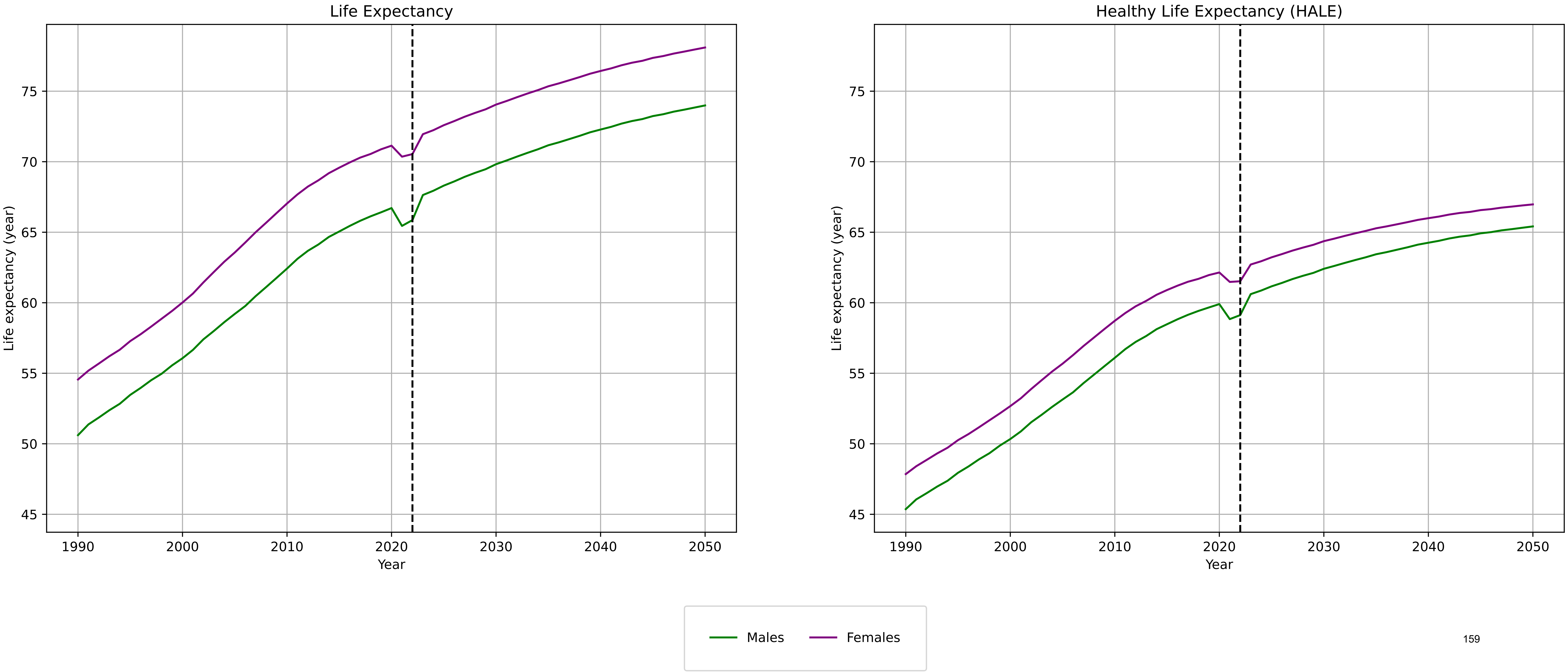

Supplemental Results Figure S3. Life expectancy and HALE by sex and location, 1990-2050

Malaysia

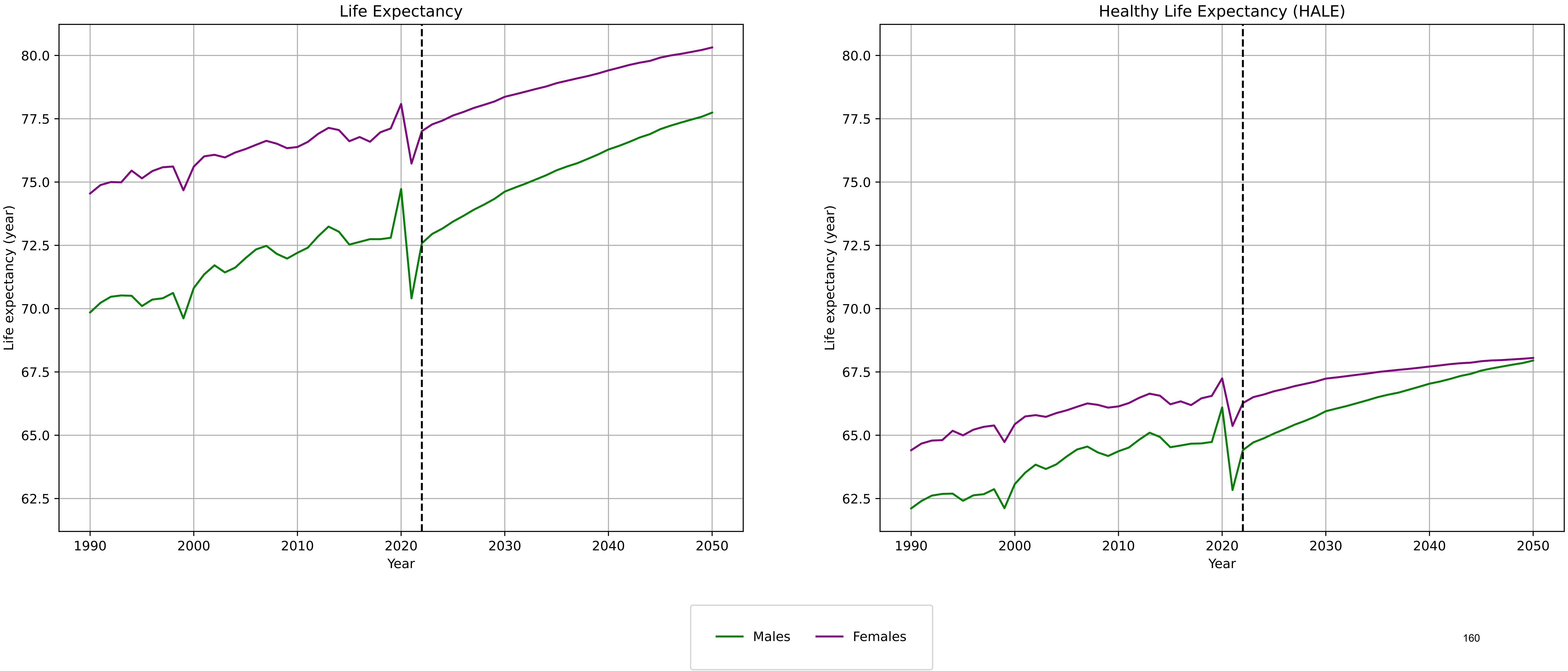

Maldives

Supplemental Results Figure S3. Life expectancy and HALE by sex and location, 1990-2050

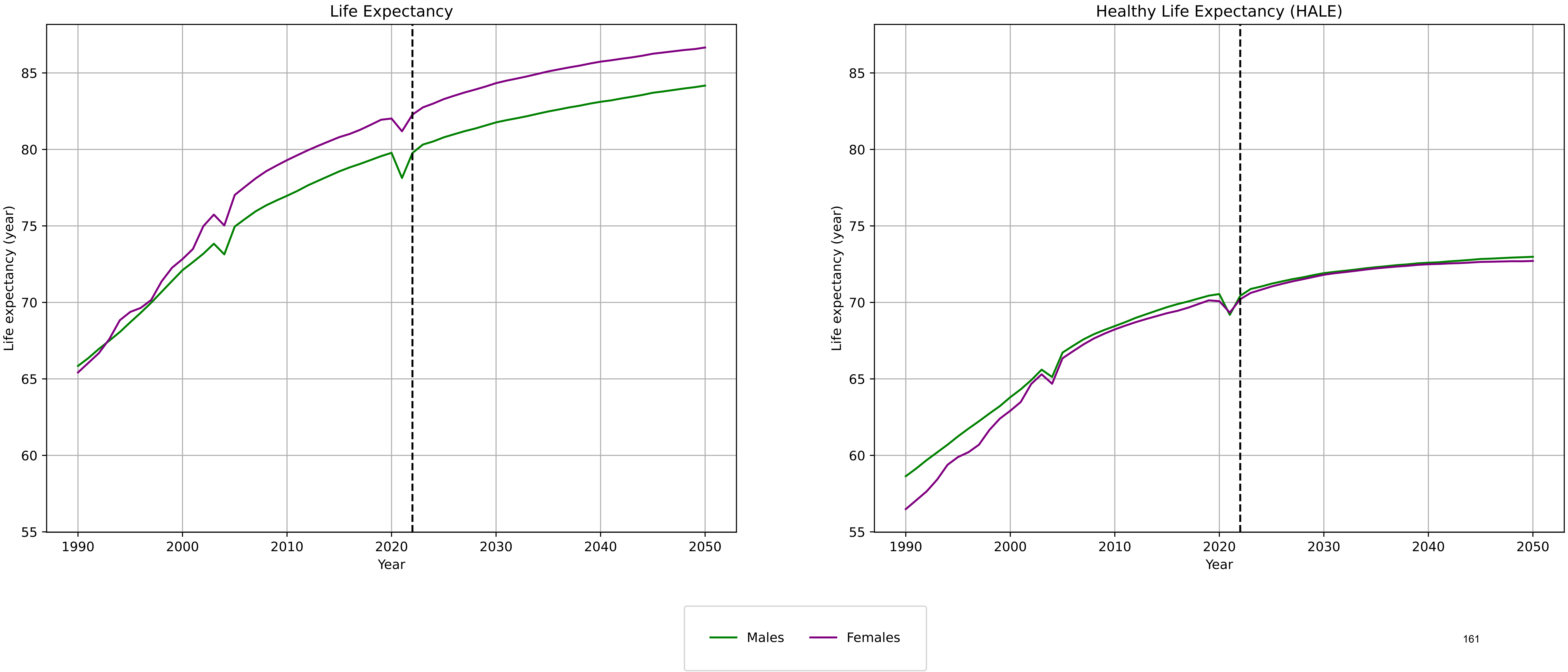

Supplemental Results Figure S3. Life expectancy and HALE by sex and location, 1990-2050

Mauritius

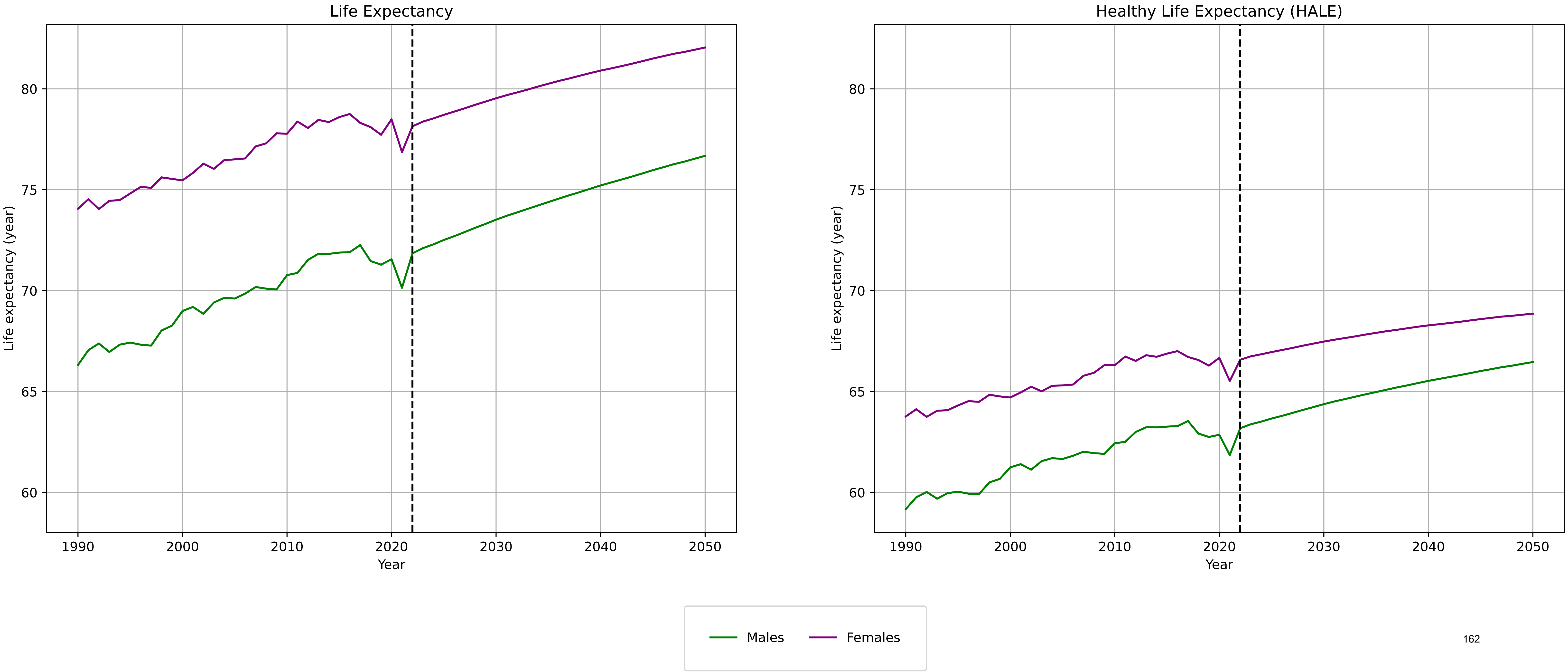

Myanmar

Supplemental Results Figure S3. Life expectancy and HALE by sex and location, 1990-2050

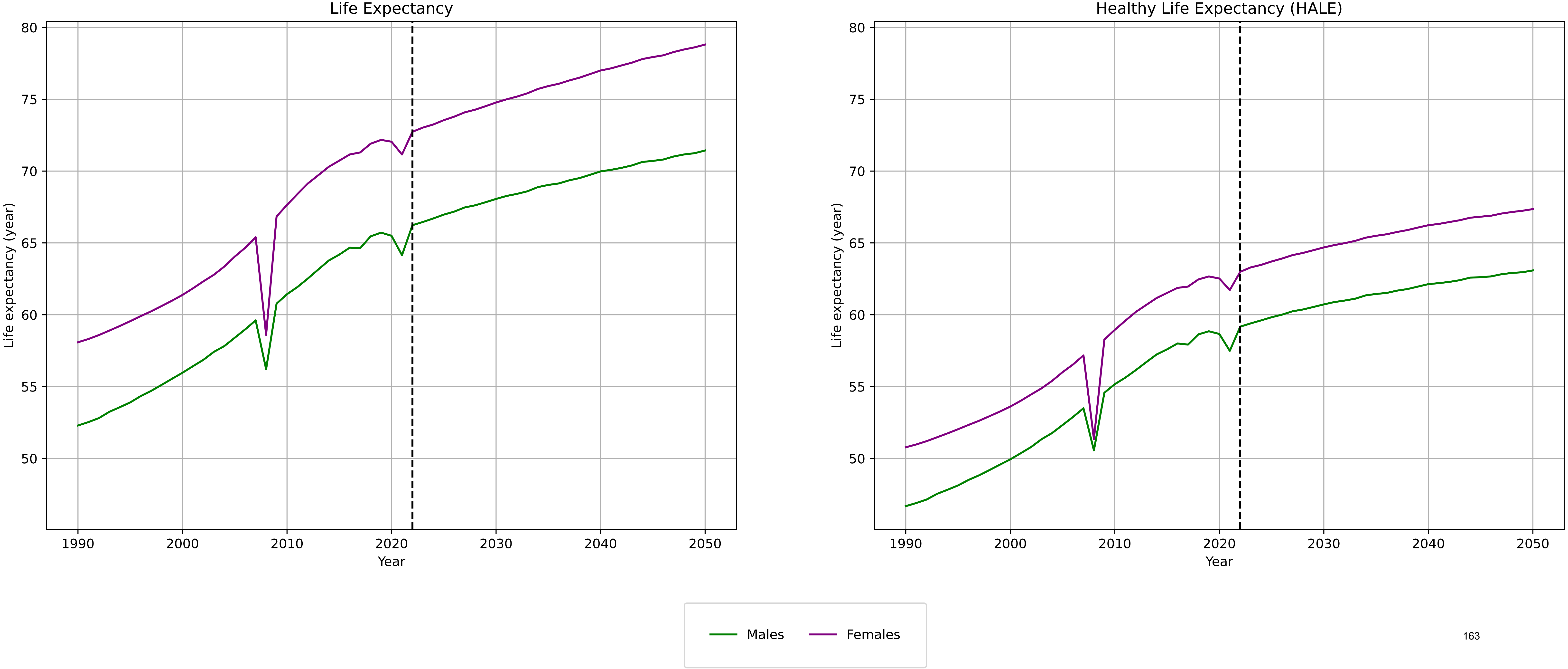

Supplemental Results Figure S3. Life expectancy and HALE by sex and location, 1990-2050

Philippines

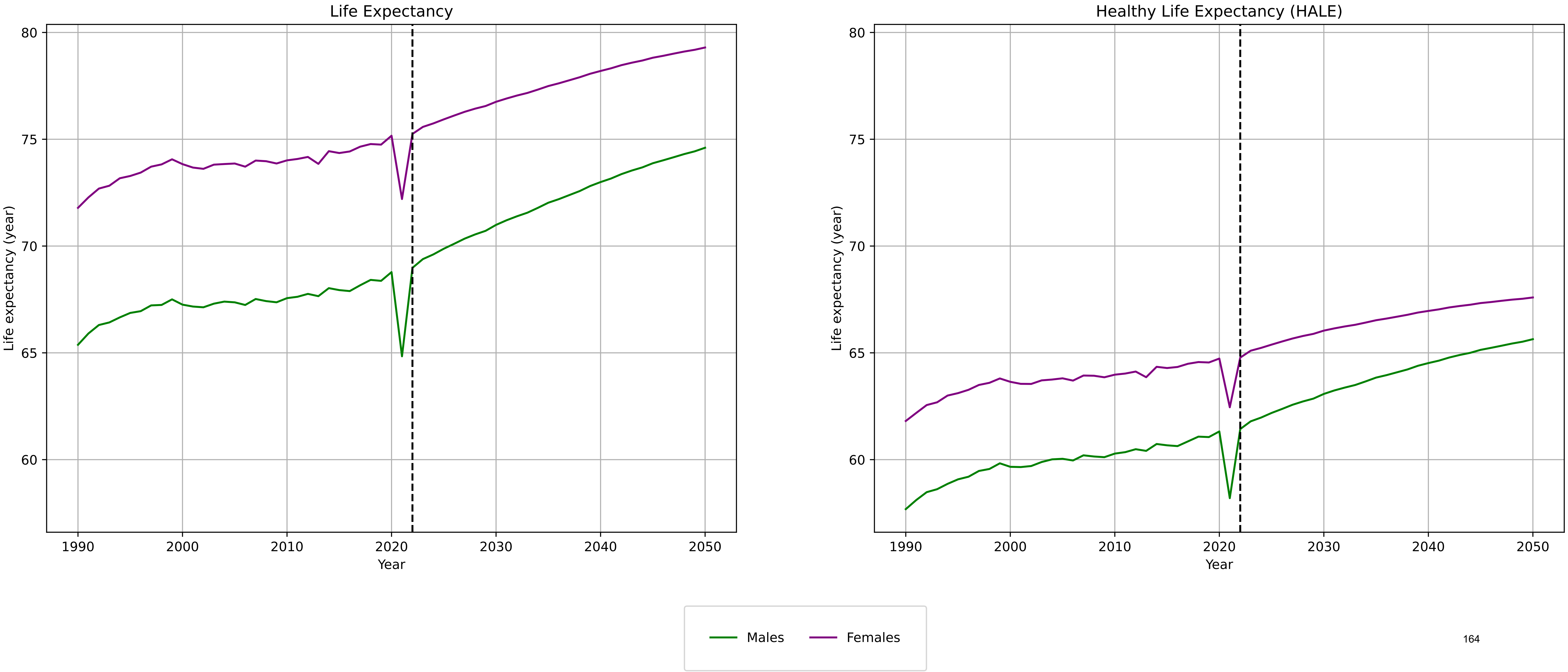

Seychelles

Supplemental Results Figure S3. Life expectancy and HALE by sex and location, 1990-2050

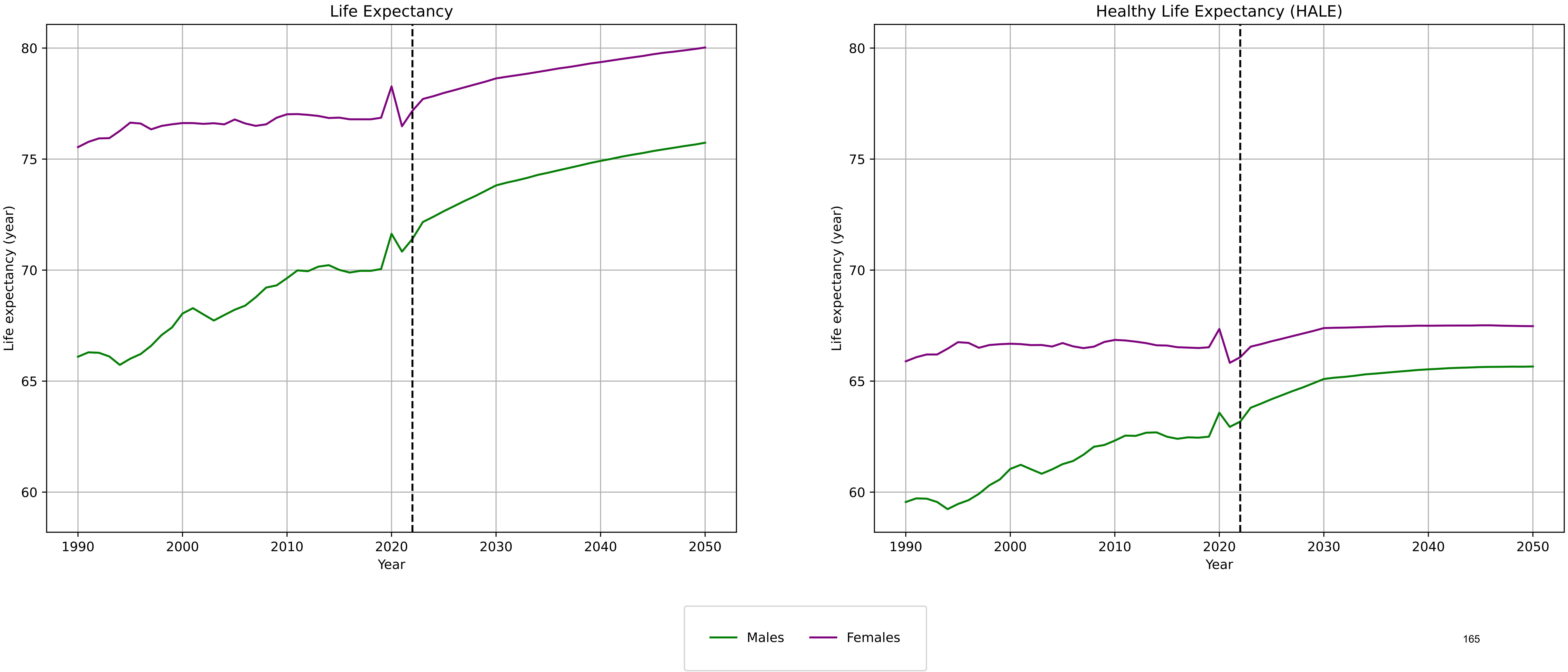

Sri Lanka

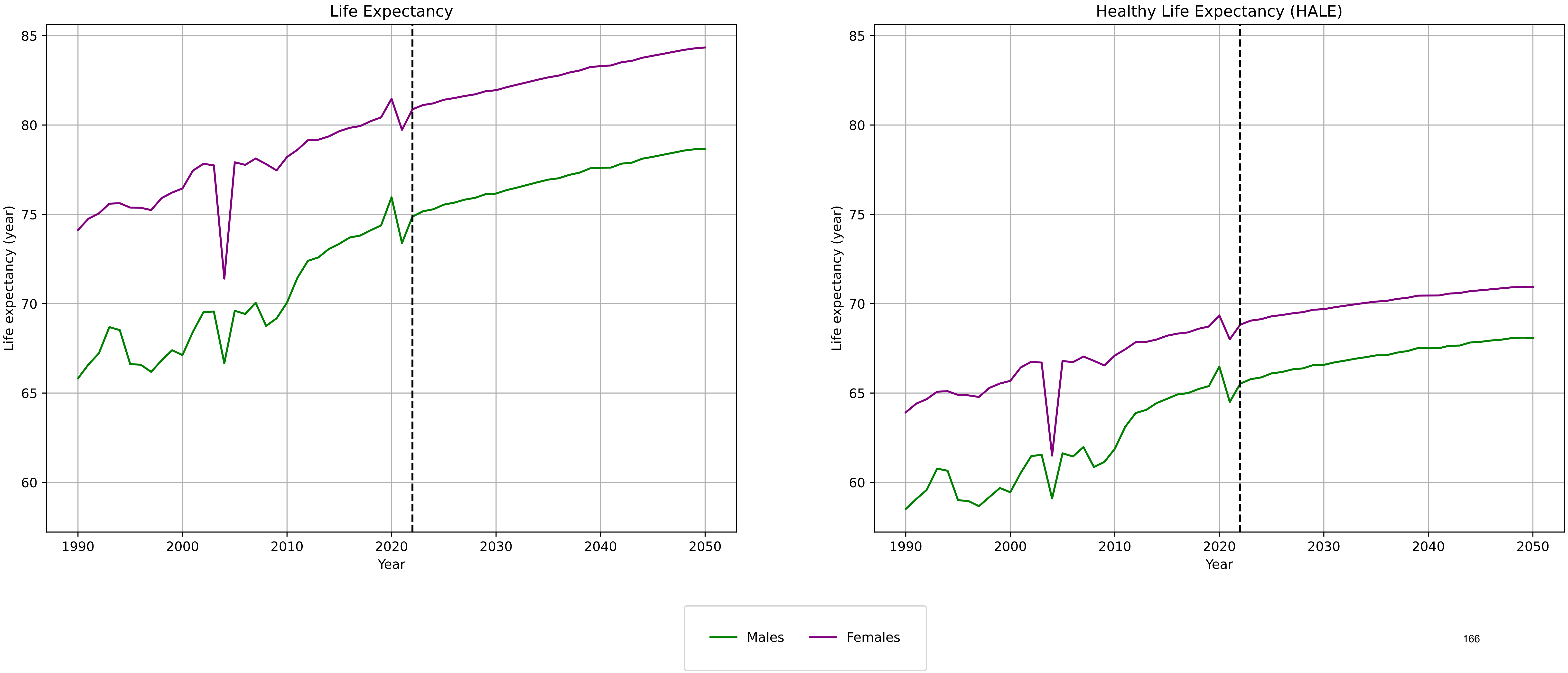
Life Expectancy
Healthy Life Expectancy (HALE)
Life expectancy (year)
Year
85
80
75
70
65
60
1990
2000
2010
2020
2030
2040
2050
Males
Females

Thailand

Supplemental Results Figure S3. Life expectancy and HALE by sex and location, 1990-2050

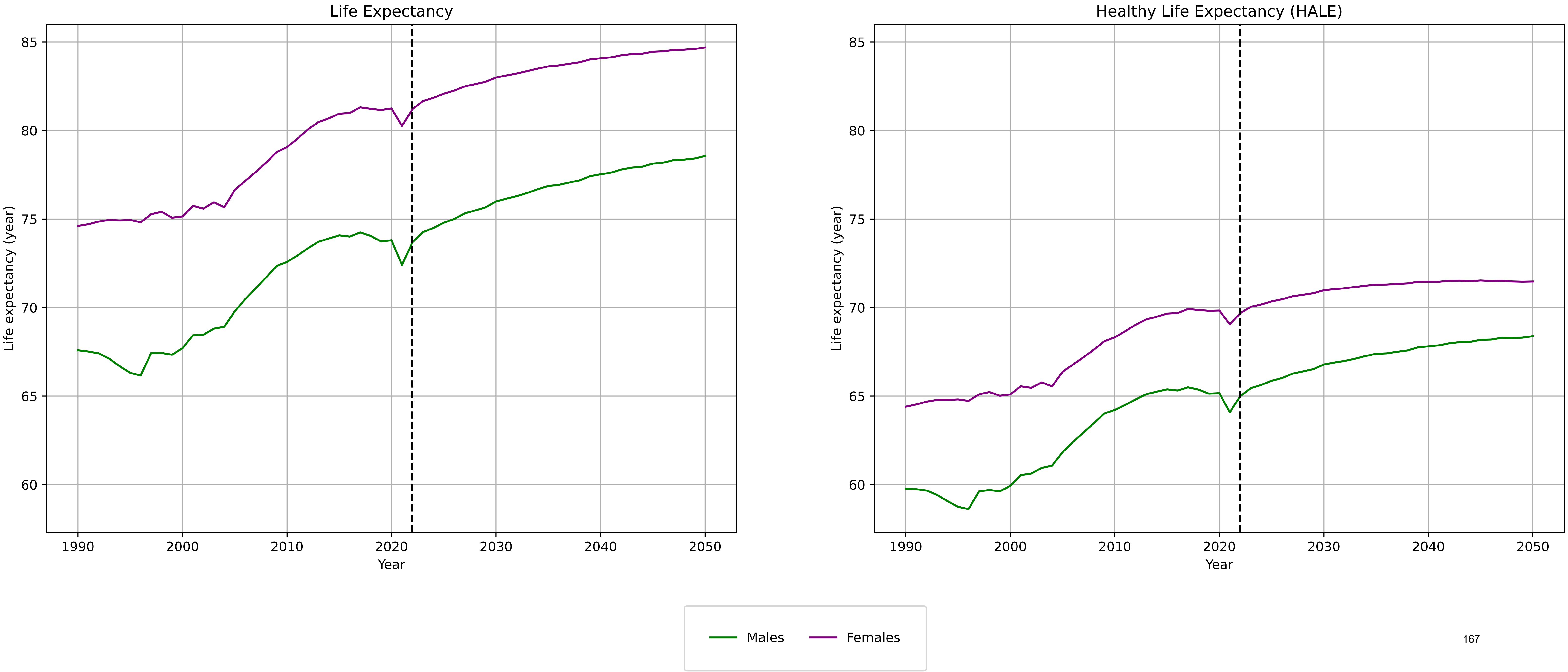

Timor-Leste

Supplemental Results Figure S3. Life expectancy and HALE by sex and location, 1990-2050

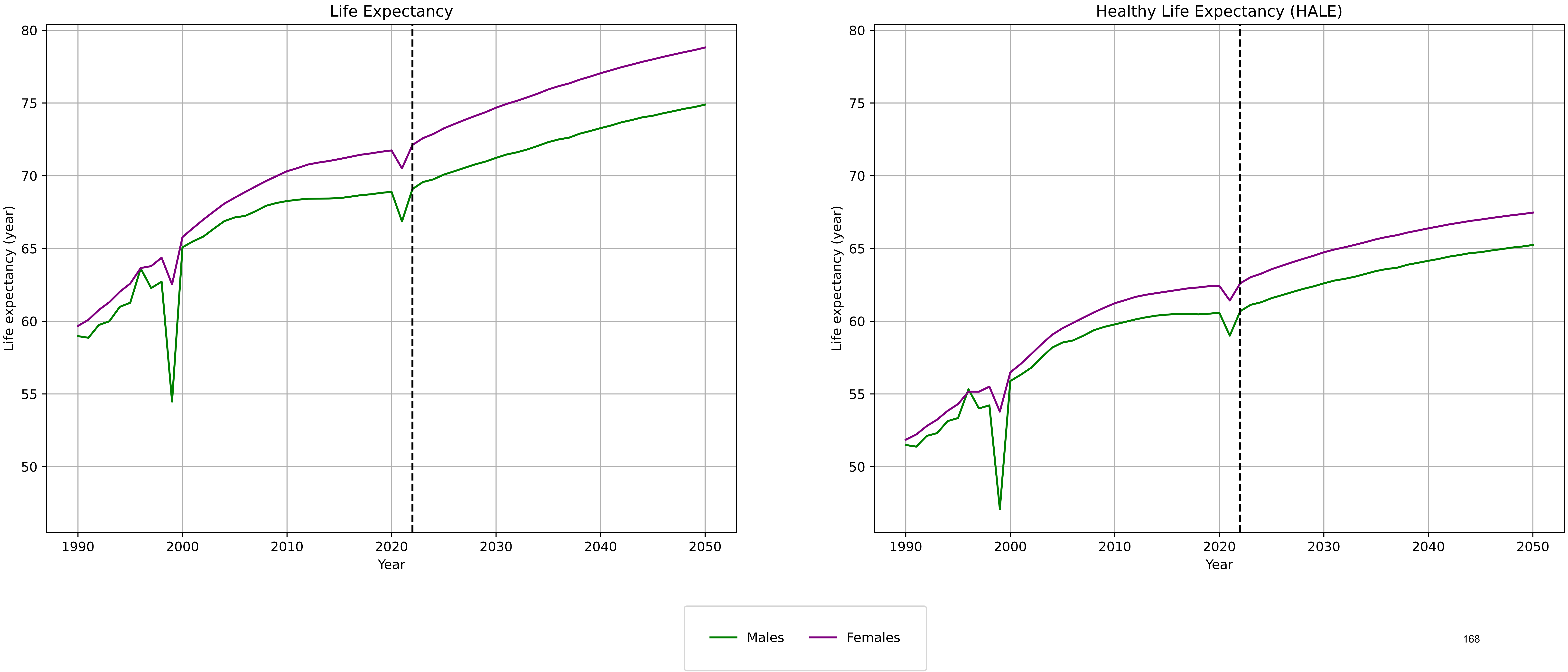

Viet Nam

Supplemental Results Figure S3. Life expectancy and HALE by sex and location, 1990-2050

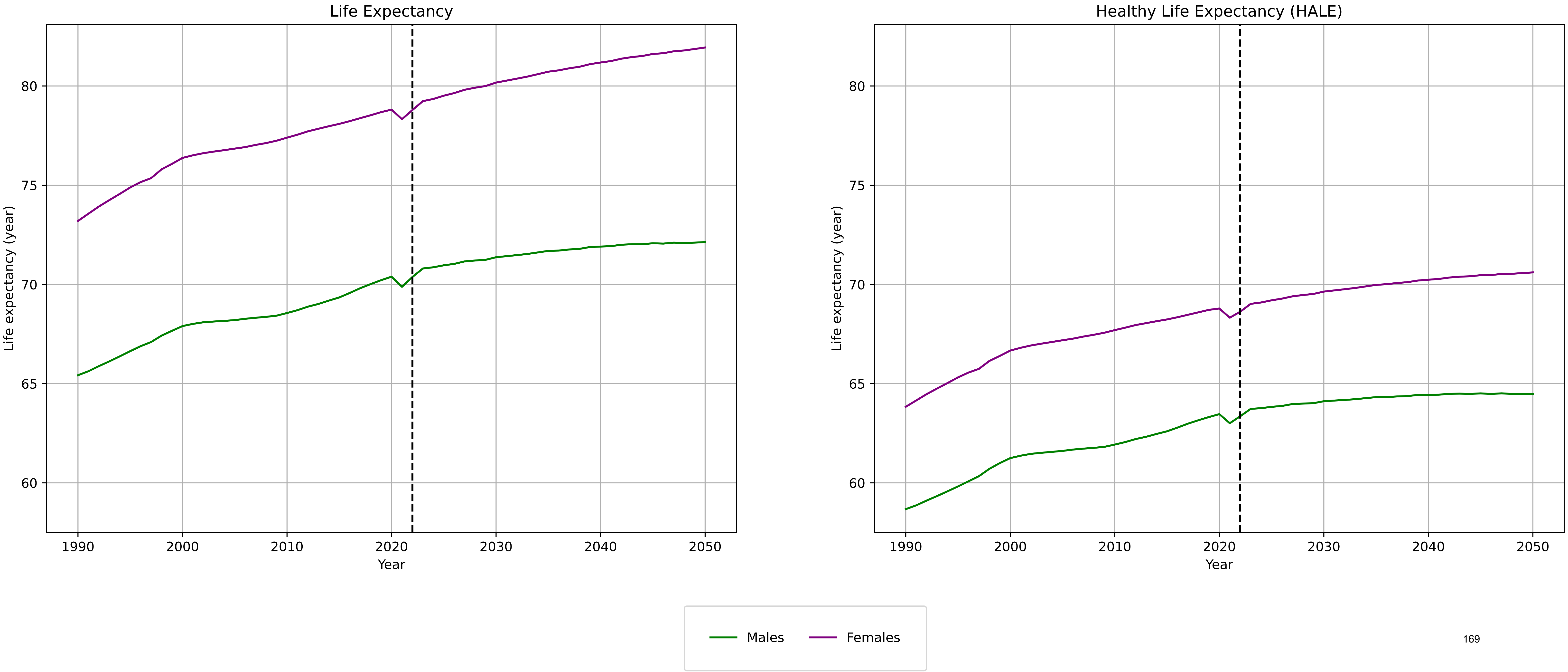

Angola

Supplemental Results Figure S3. Life expectancy and HALE by sex and location, 1990-2050

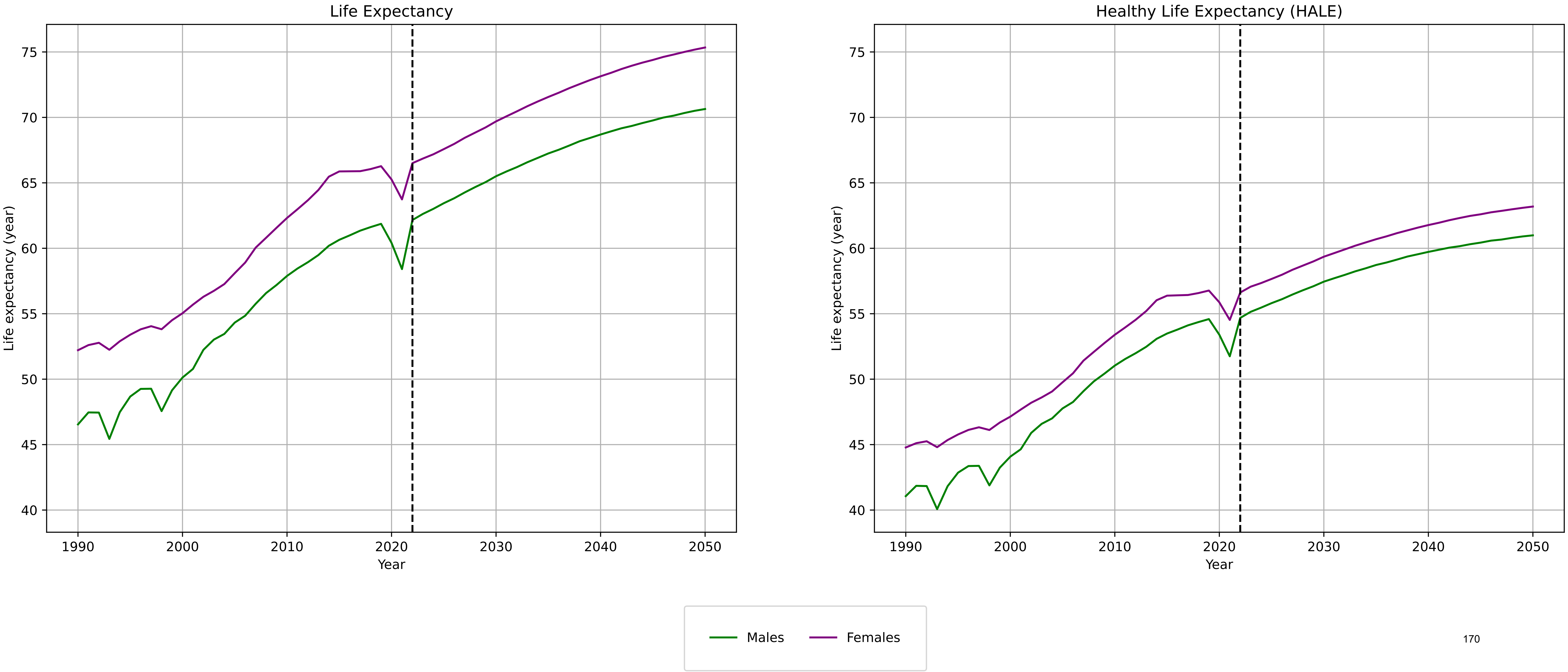

Central African Republic

Supplemental Results Figure S3. Life expectancy and HALE by sex and location, 1990-2050

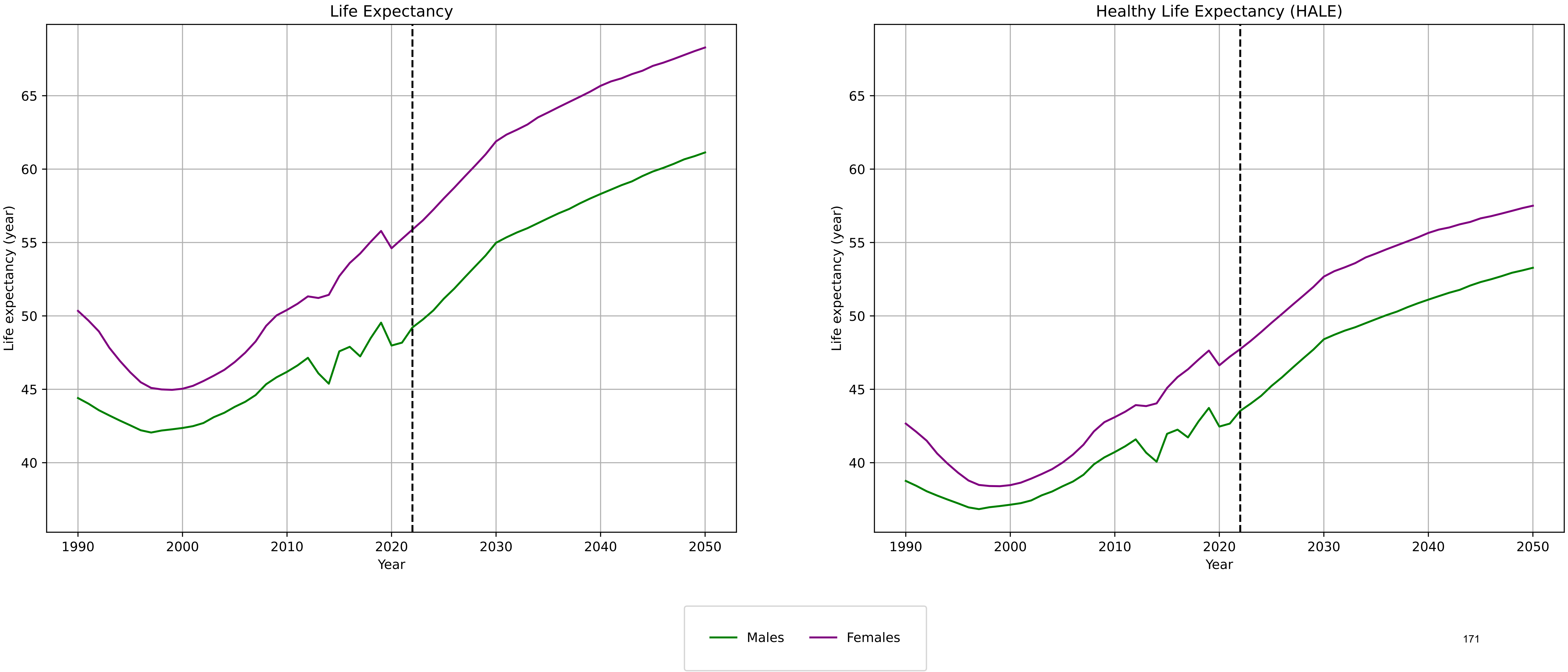

Congo (Brazzaville)

Supplemental Results Figure S3. Life expectancy and HALE by sex and location, 1990-2050

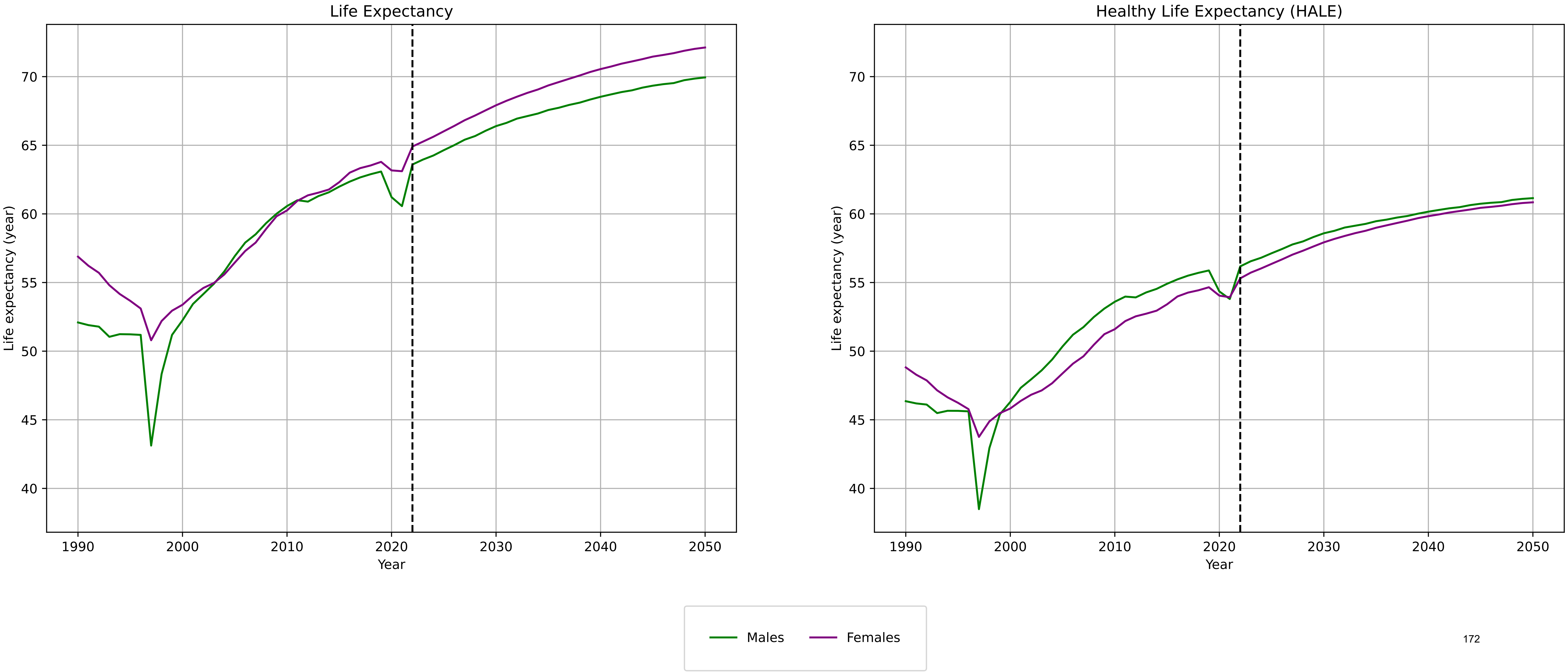

Supplemental Results Figure S3. Life expectancy and HALE by sex and location, 1990-2050

DR Congo

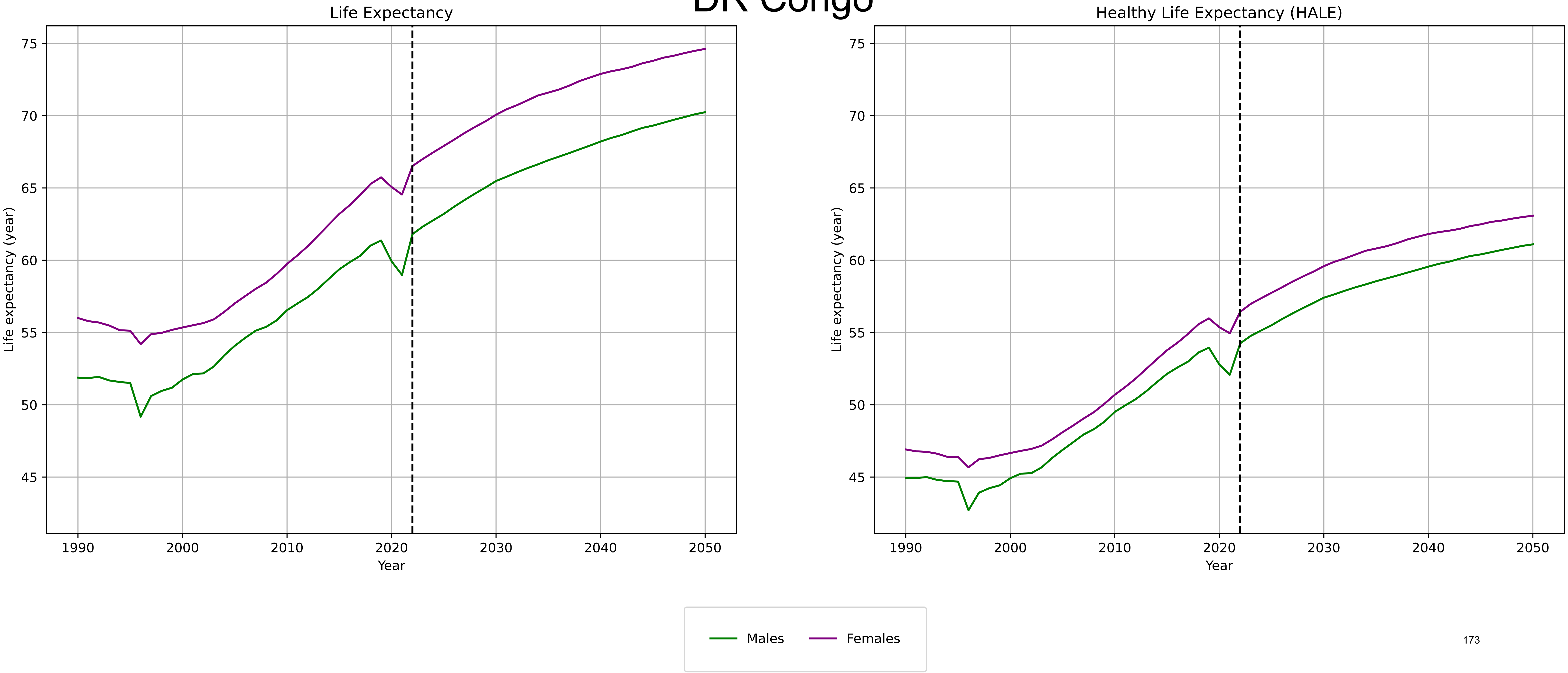

Equatorial Guinea

Supplemental Results Figure S3. Life expectancy and HALE by sex and location, 1990-2050

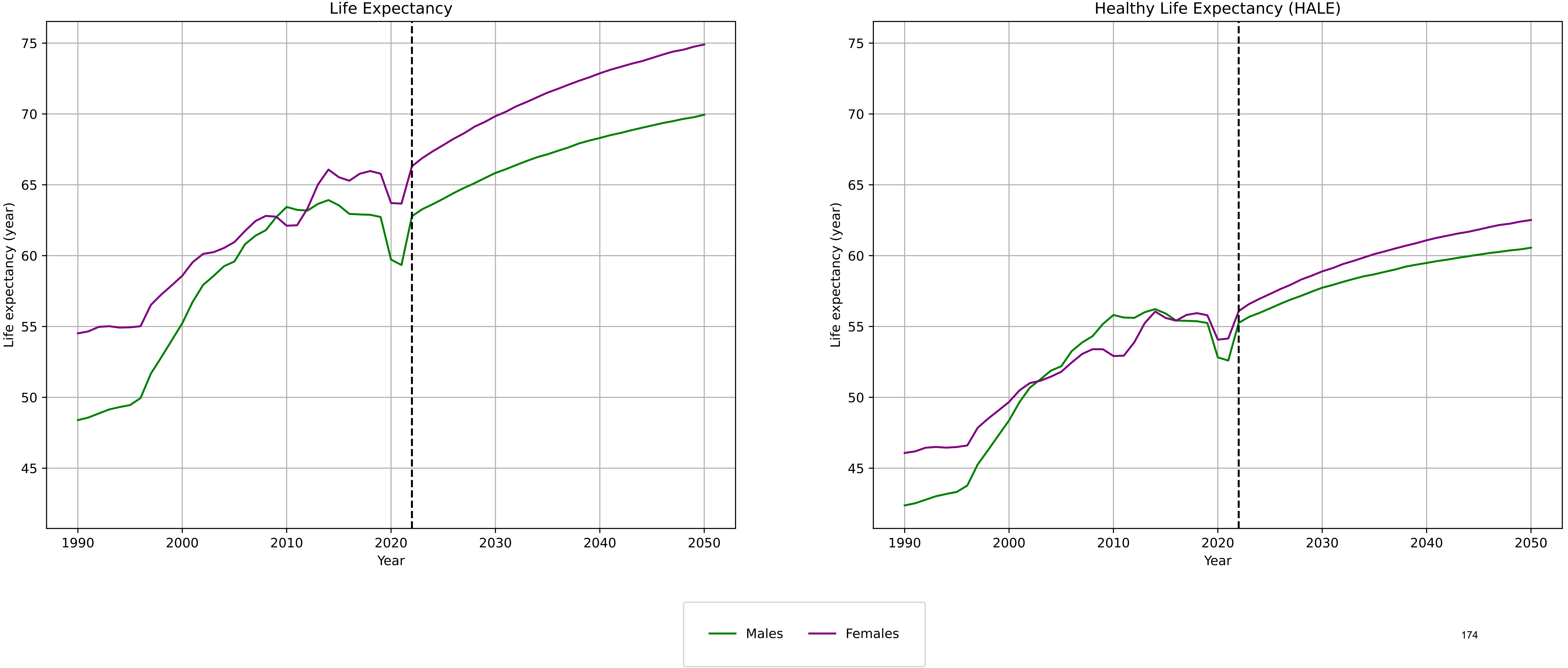

Gabon

Supplemental Results Figure S3. Life expectancy and HALE by sex and location, 1990-2050

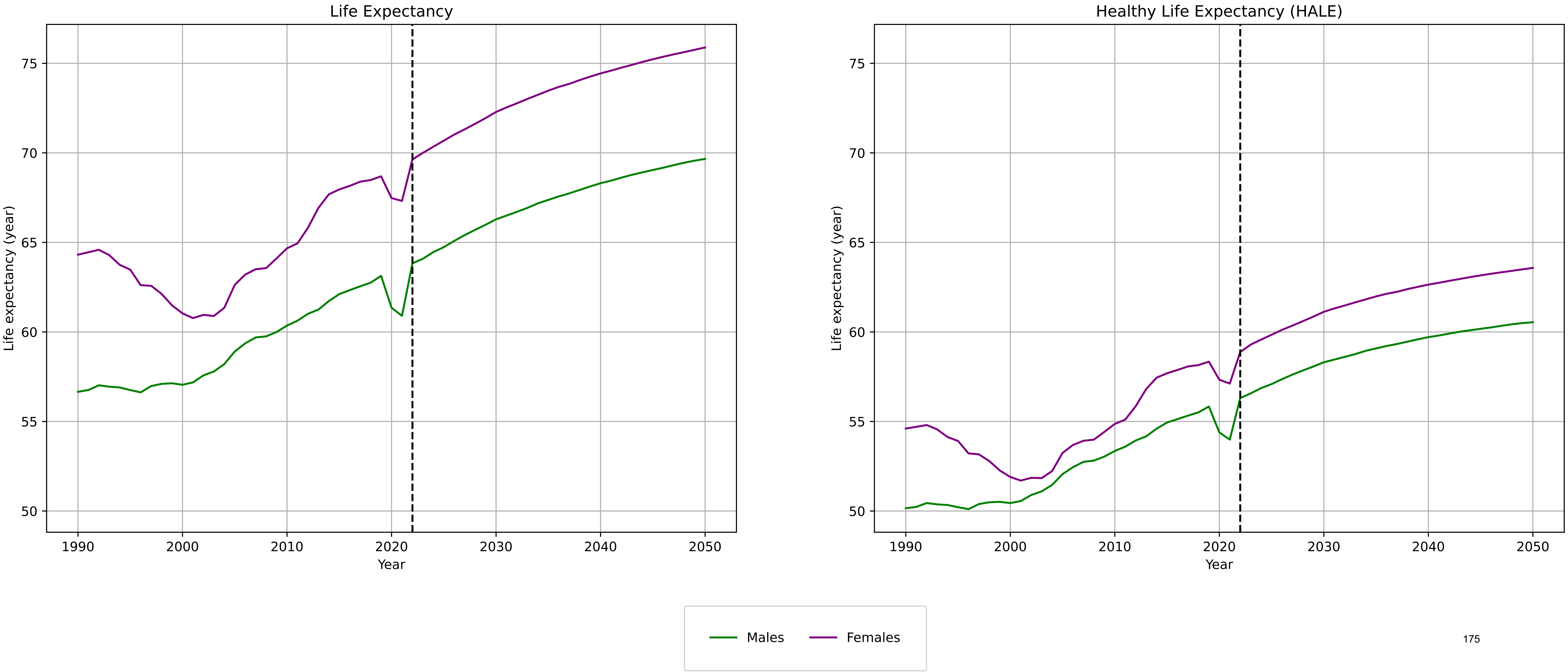

Burundi

Supplemental Results Figure S3. Life expectancy and HALE by sex and location, 1990-2050

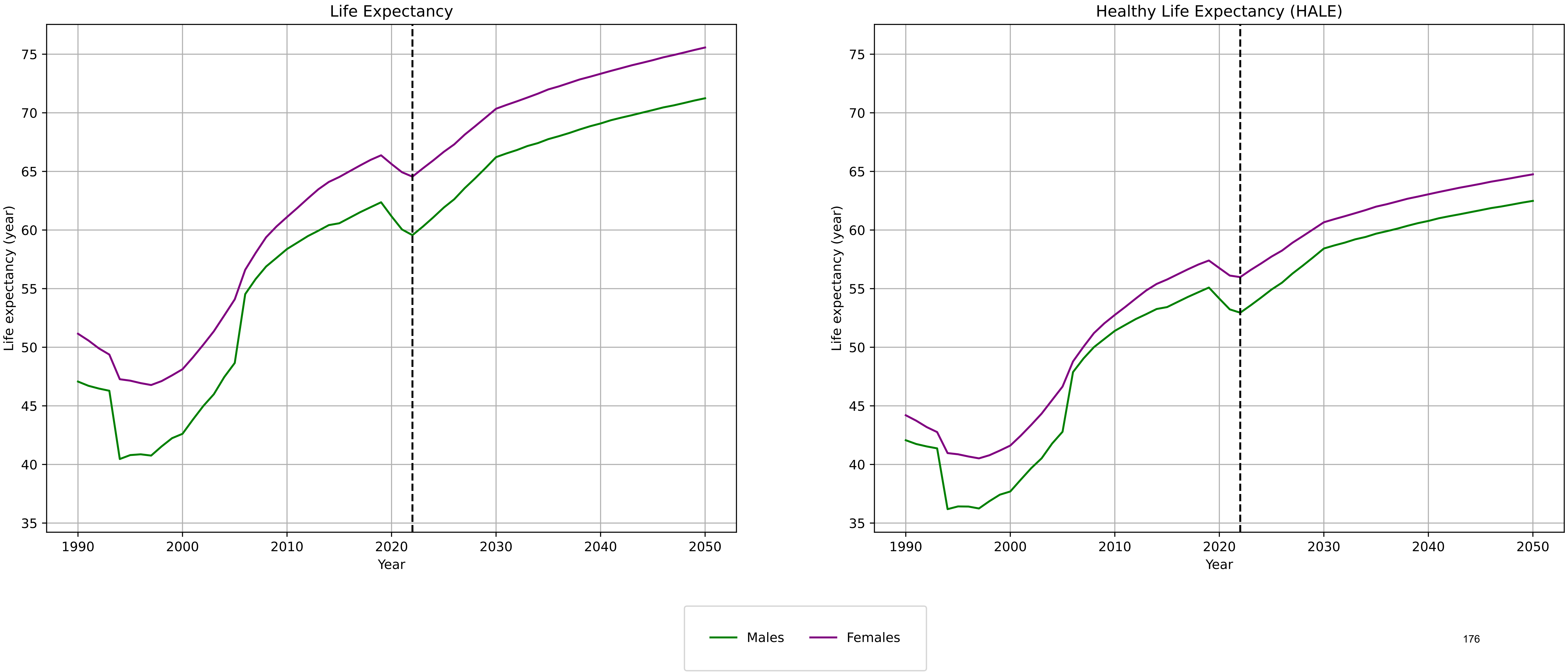

Comoros

Supplemental Results Figure S3. Life expectancy and HALE by sex and location, 1990-2050

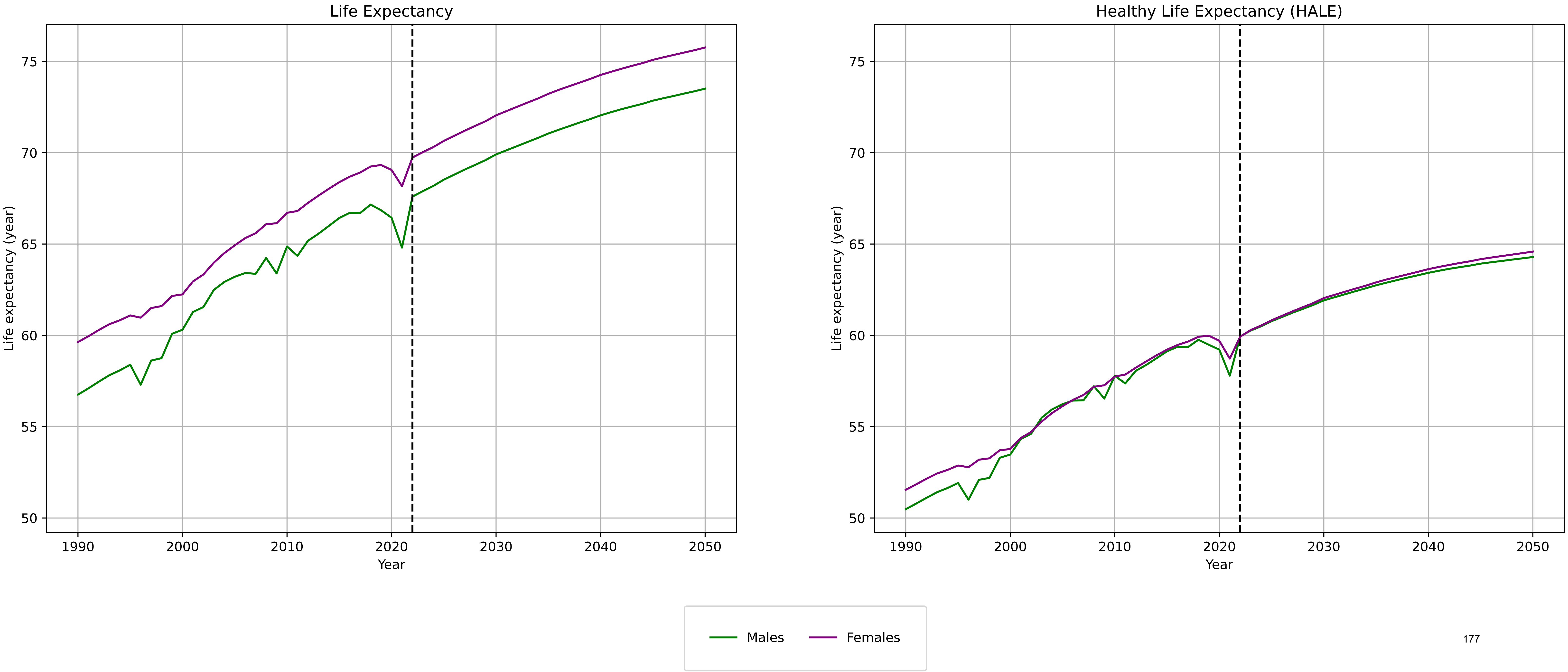

Supplemental Results Figure S3. Life expectancy and HALE by sex and location, 1990-2050

Djibouti

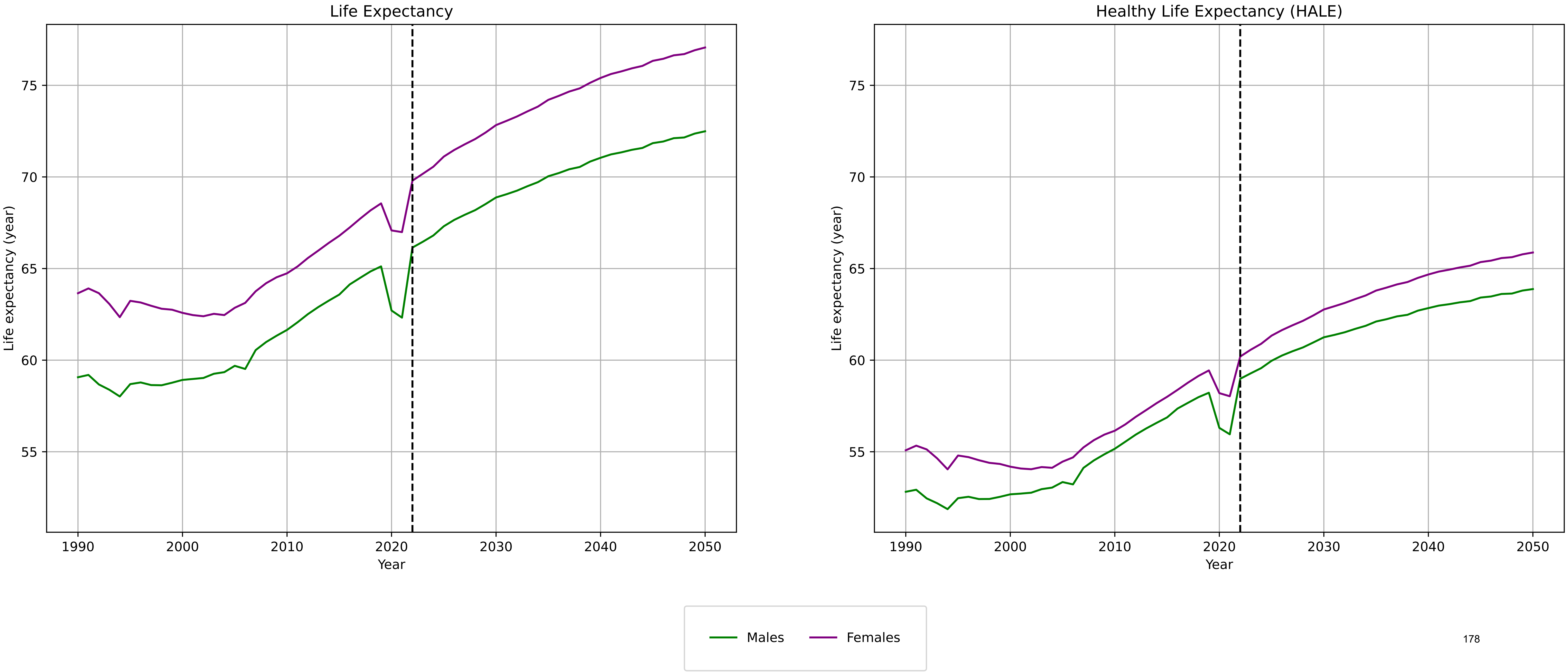

Eritrea

Supplemental Results Figure S3. Life expectancy and HALE by sex and location, 1990-2050

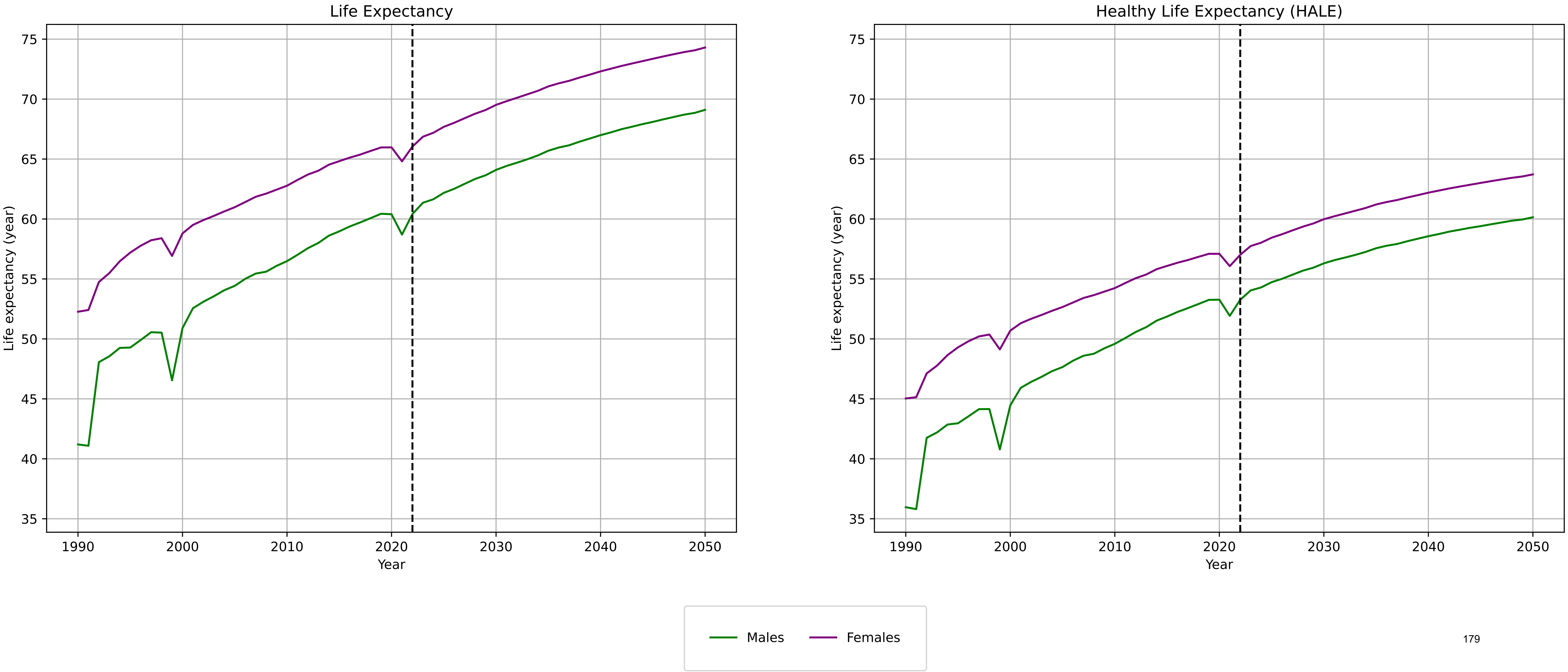

Supplemental Results Figure S3. Life expectancy and HALE by sex and location, 1990-2050

Ethiopia

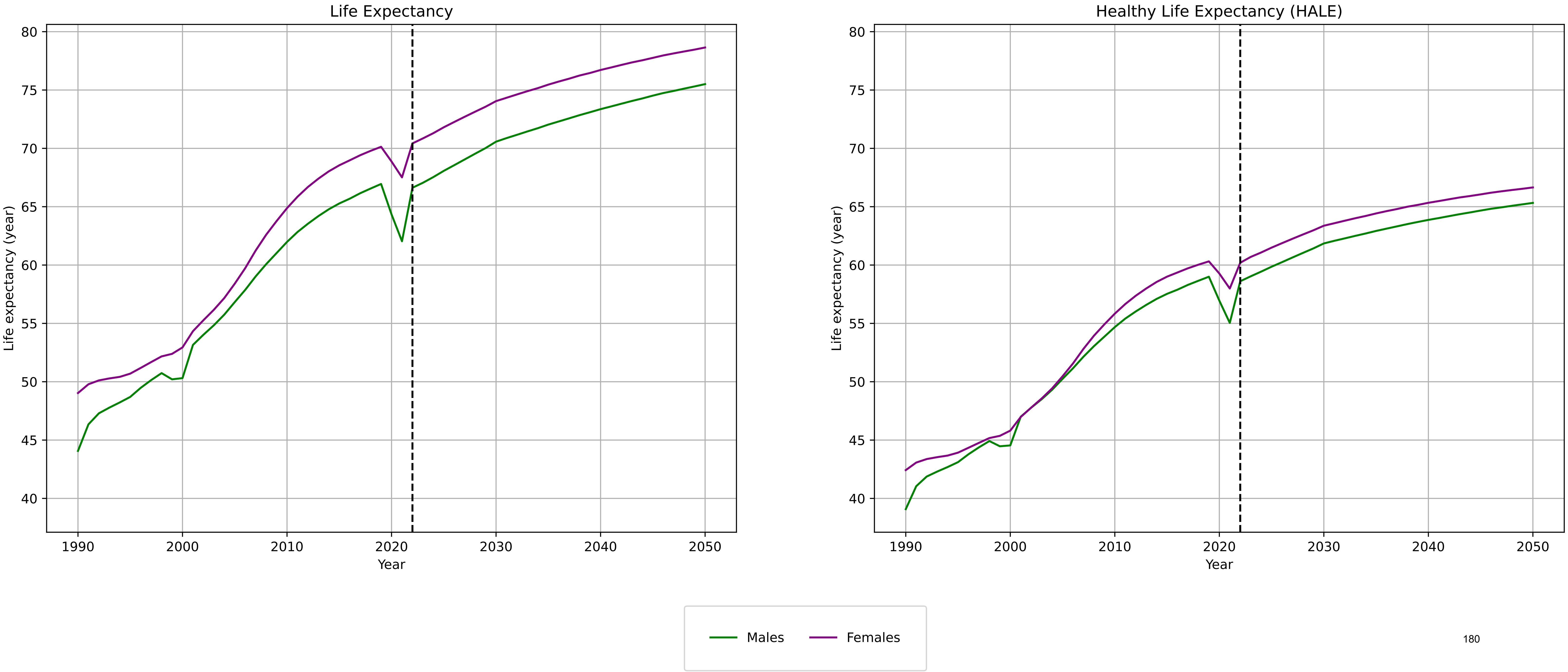

Kenya

Supplemental Results Figure S3. Life expectancy and HALE by sex and location, 1990-2050

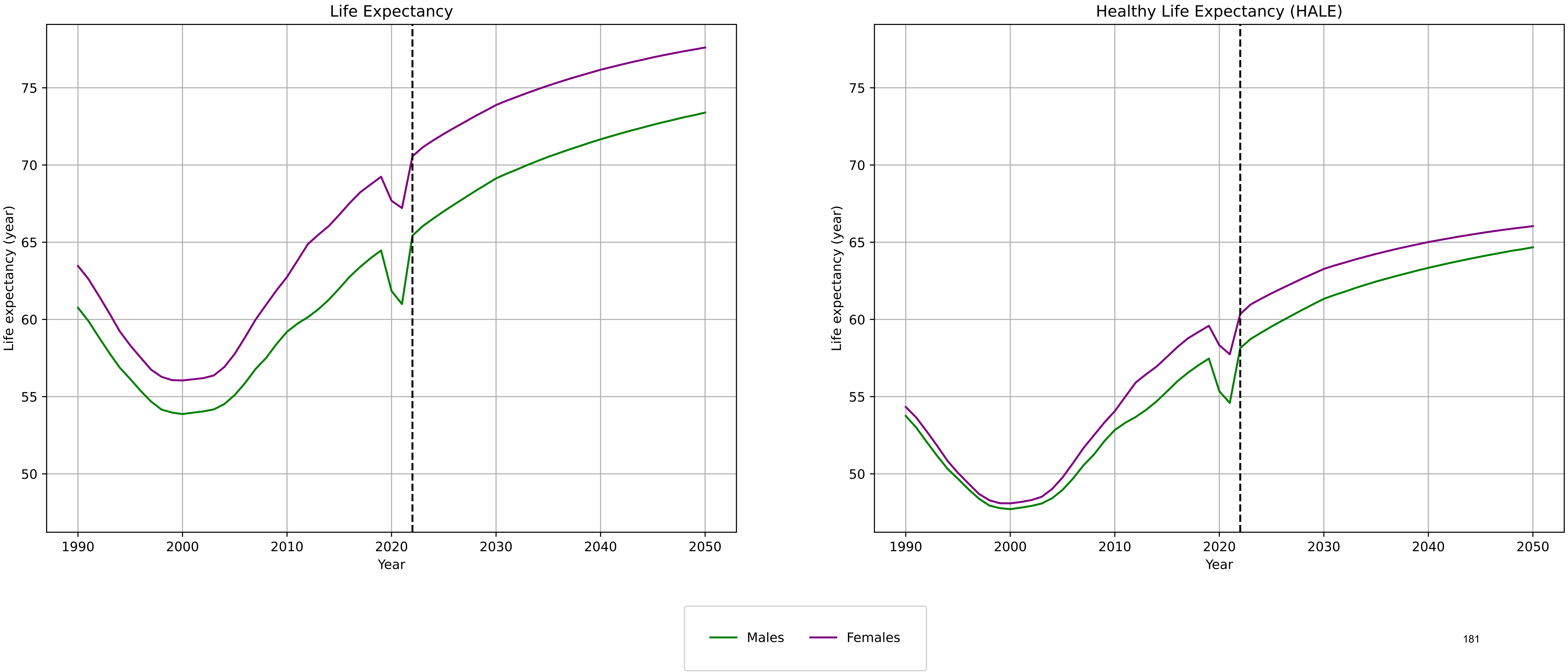

Madagascar

Supplemental Results Figure S3. Life expectancy and HALE by sex and location, 1990-2050

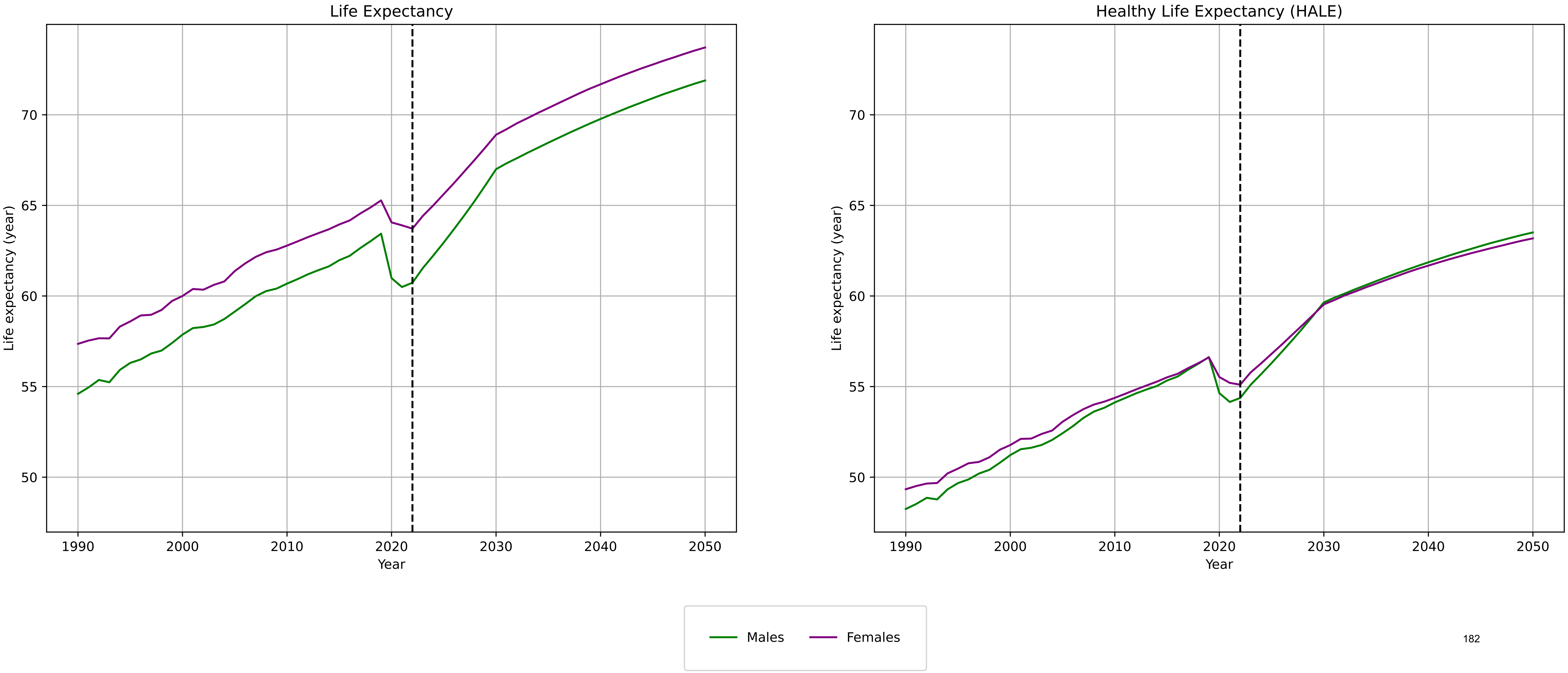

Malawi

Supplemental Results Figure S3. Life expectancy and HALE by sex and location, 1990-2050

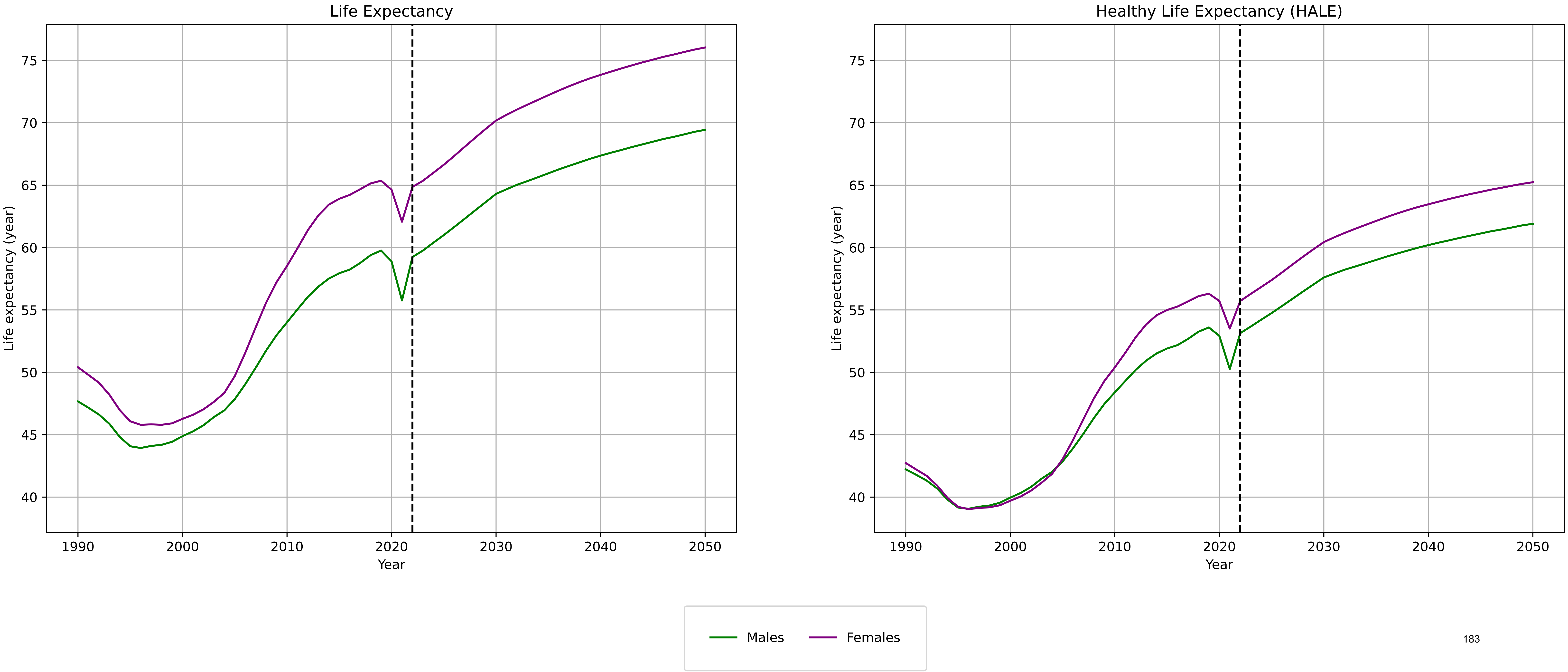

Supplemental Results Figure S3. Life expectancy and HALE by sex and location, 1990-2050

Mozambique

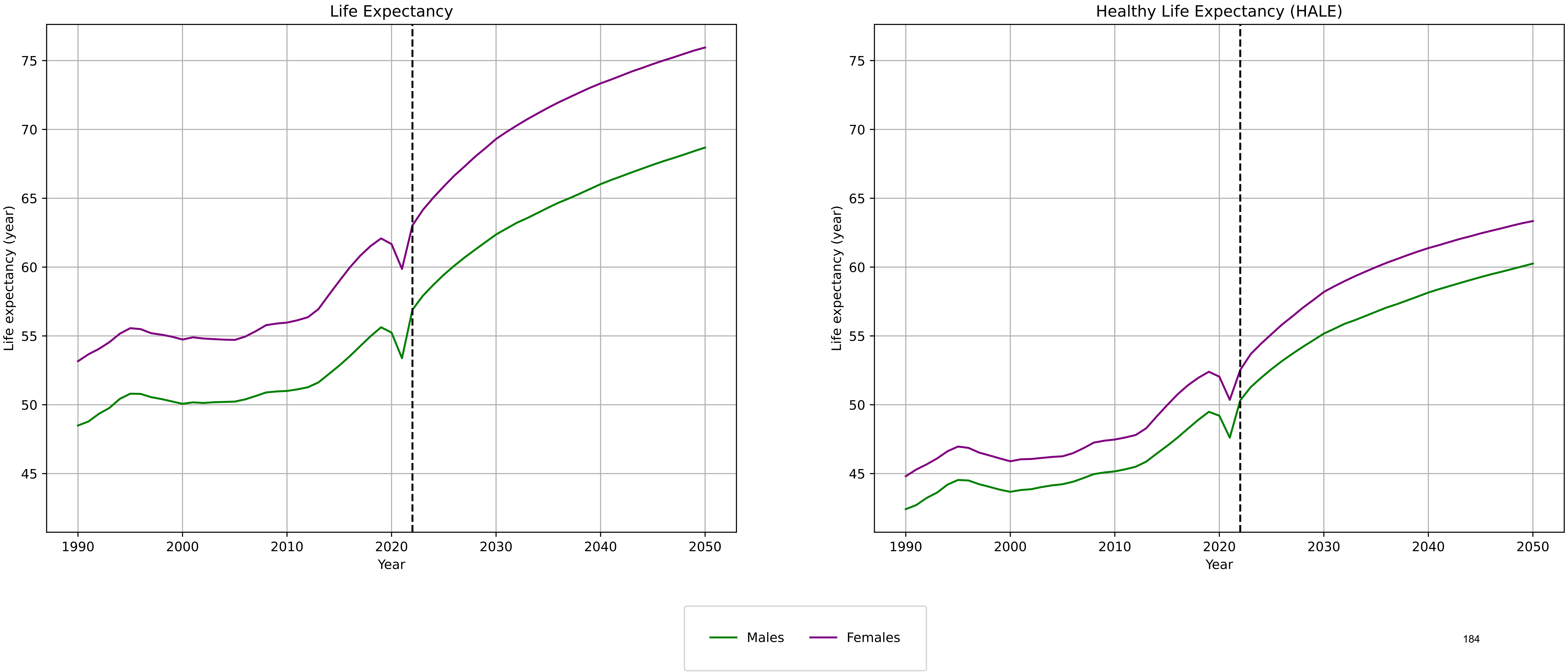

Rwanda

Supplemental Results Figure S3. Life expectancy and HALE by sex and location, 1990-2050

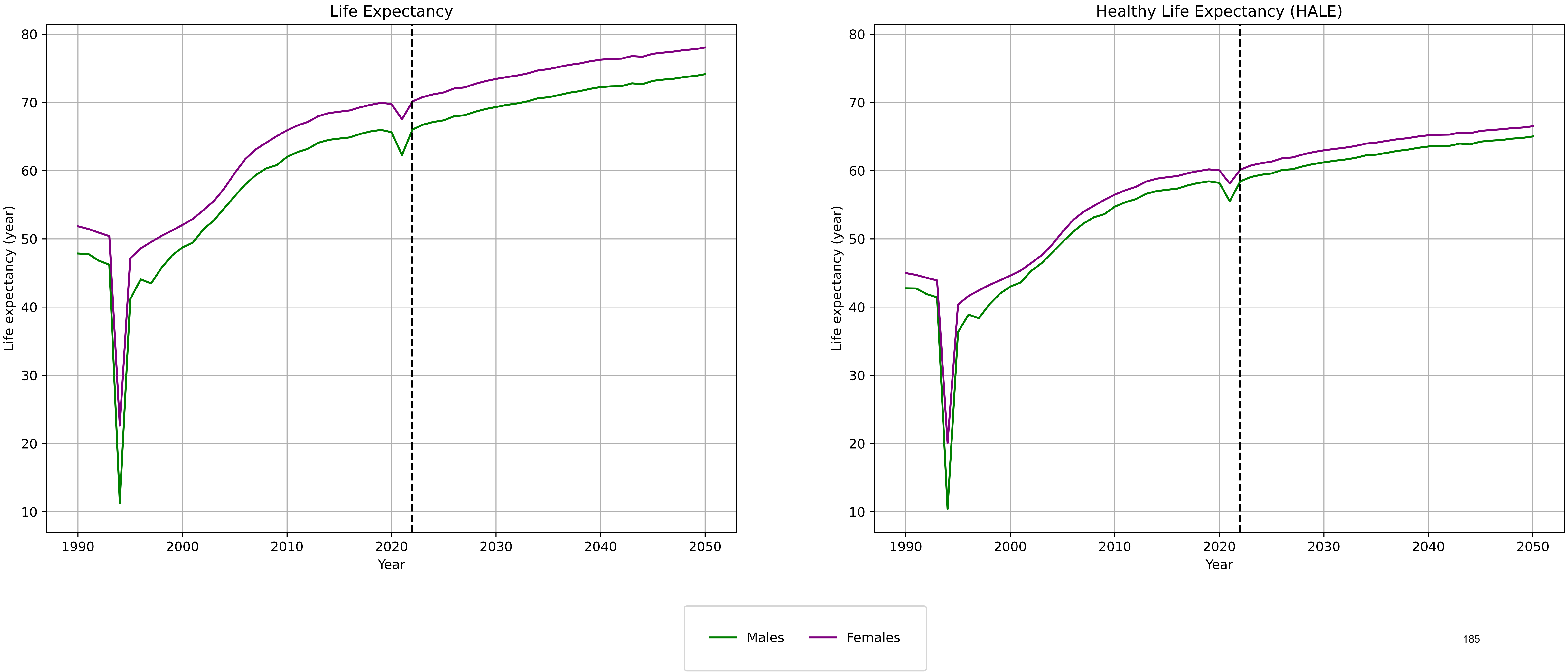

Supplemental Results Figure S3. Life expectancy and HALE by sex and location, 1990-2050

Somalia

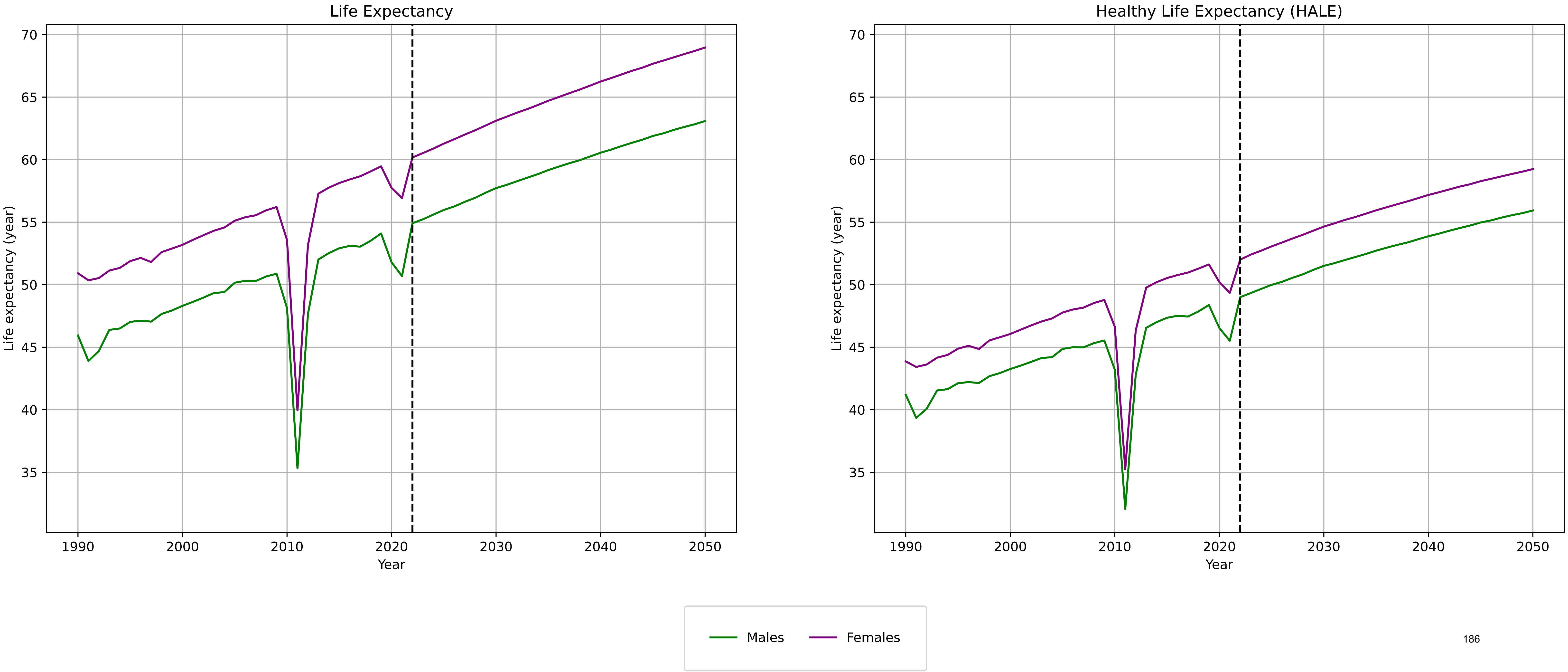

Supplemental Results Figure S3. Life expectancy and HALE by sex and location, 1990-2050

South Sudan

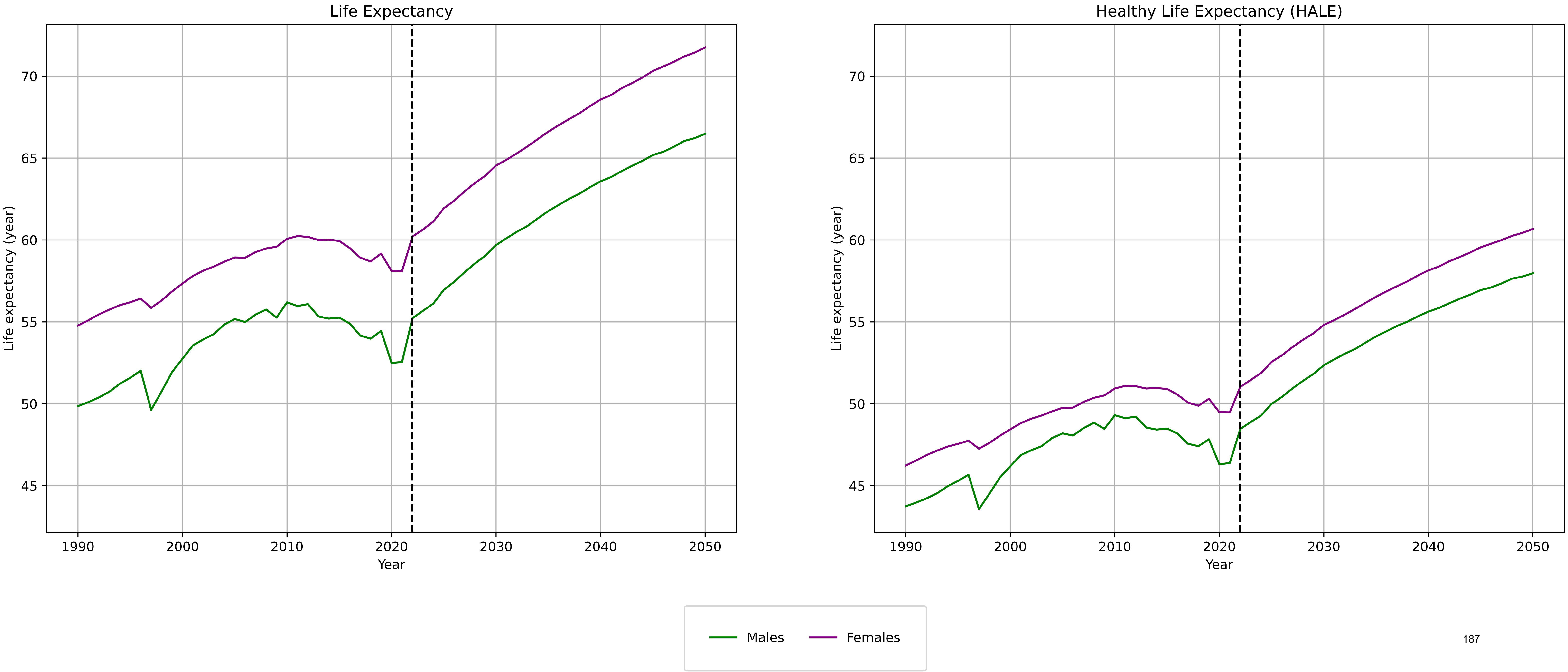

Uganda

Supplemental Results Figure S3. Life expectancy and HALE by sex and location, 1990-2050

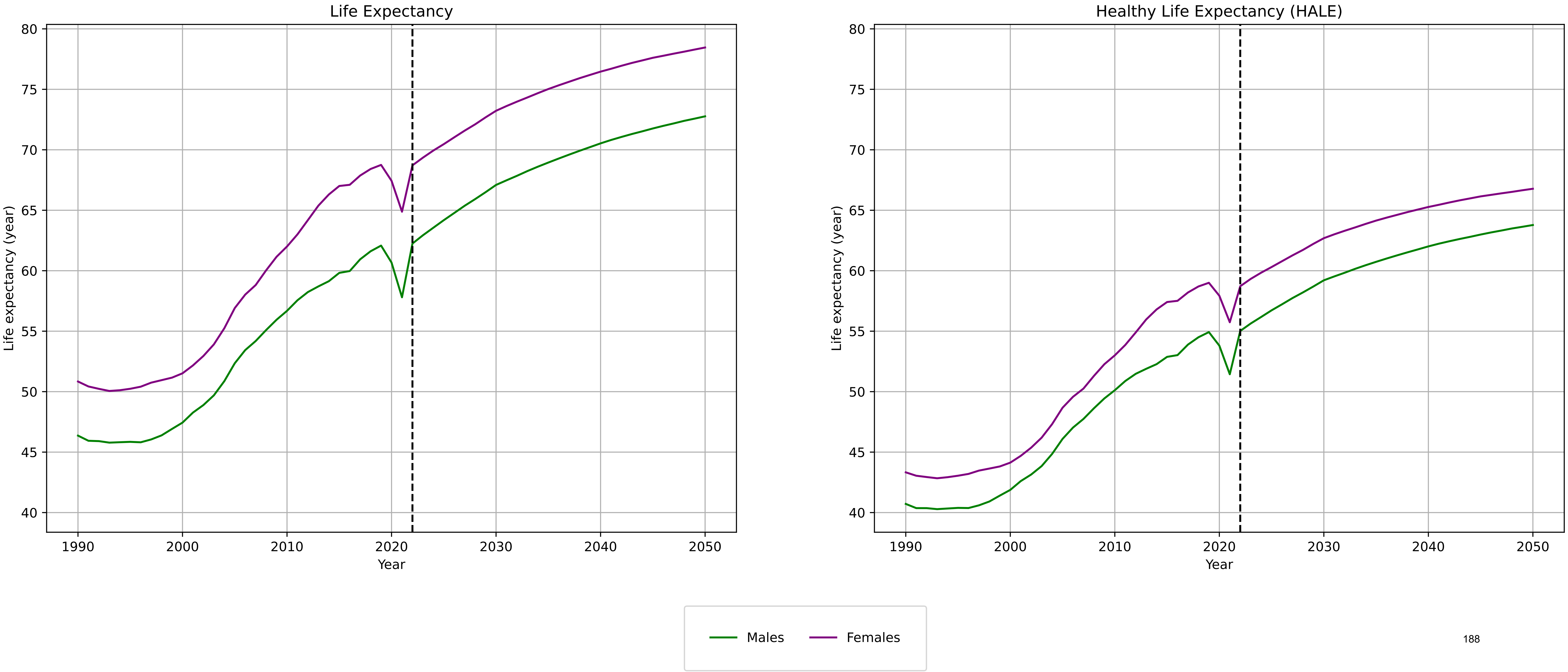

Tanzania

Supplemental Results Figure S3. Life expectancy and HALE by sex and location, 1990-2050

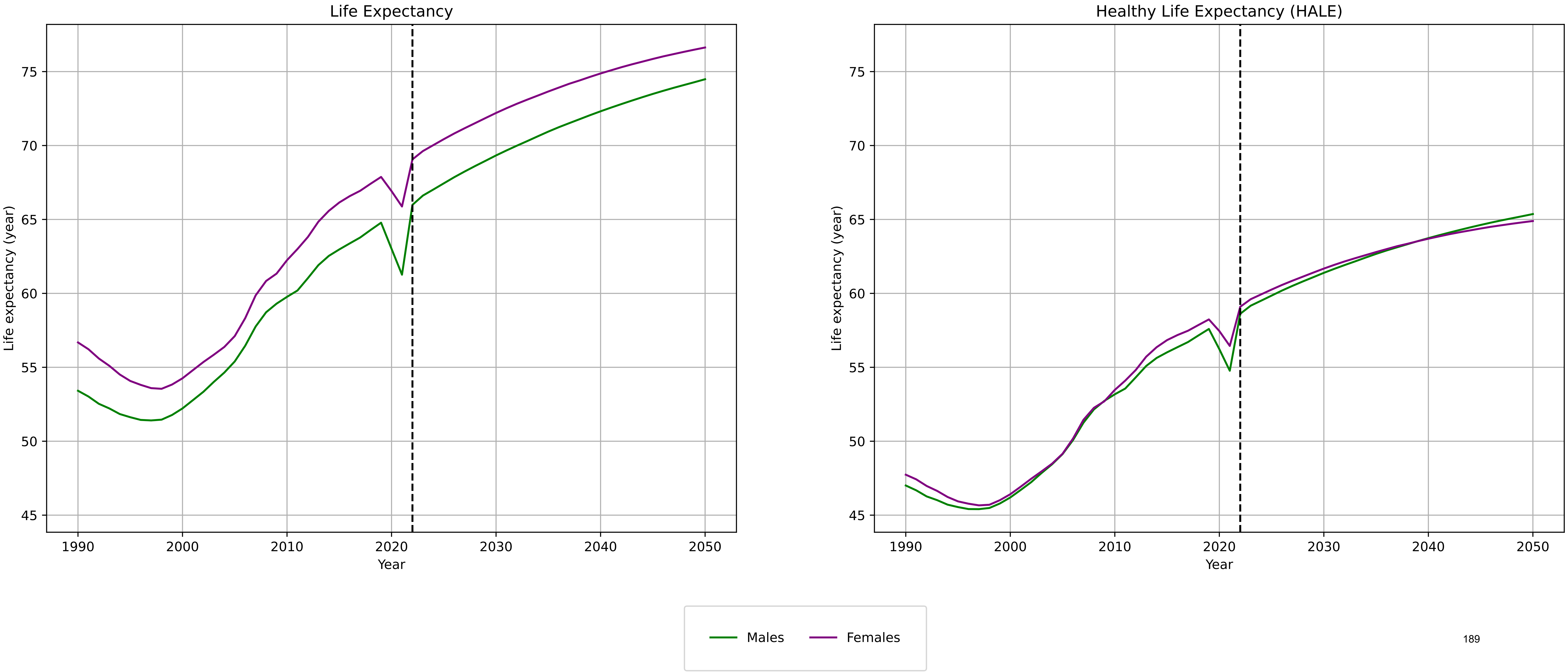

Supplemental Results Figure S3. Life expectancy and HALE by sex and location, 1990-2050

Zambia

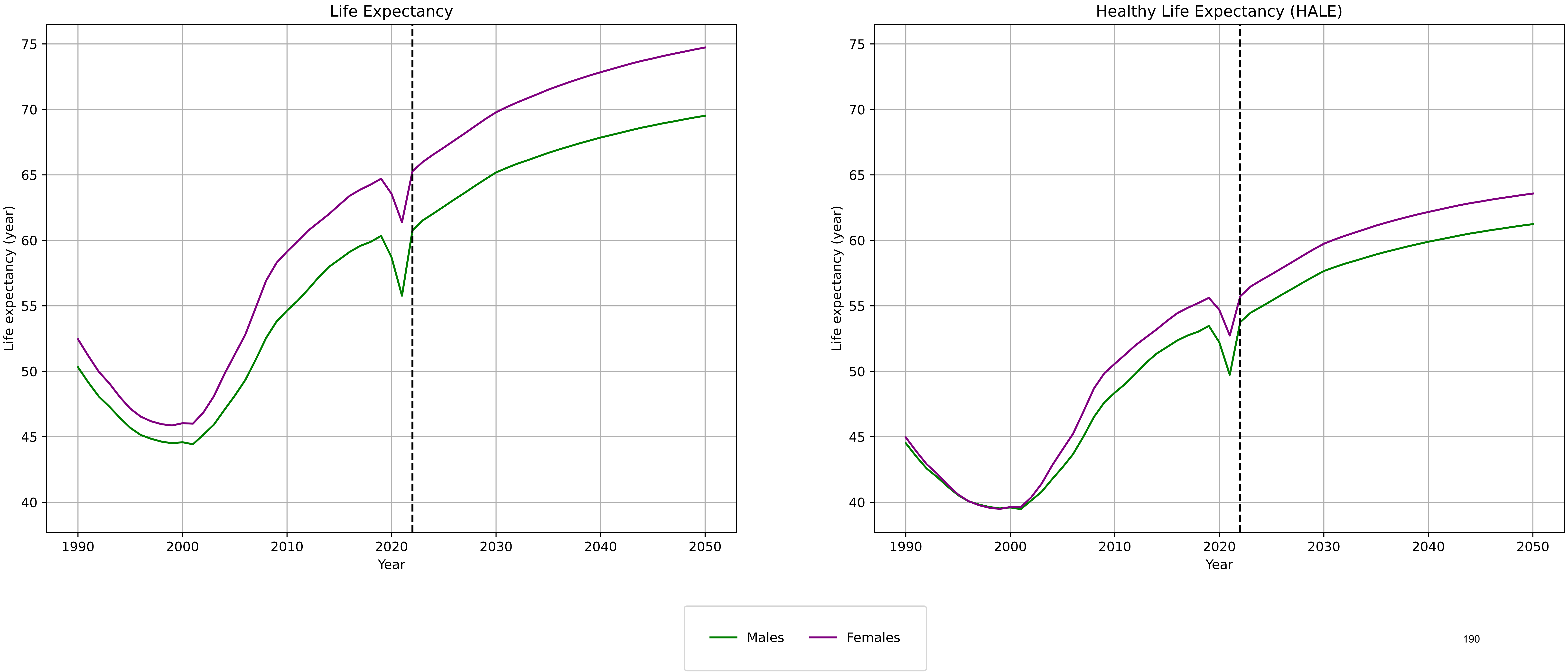

Botswana

Supplemental Results Figure S3. Life expectancy and HALE by sex and location, 1990-2050

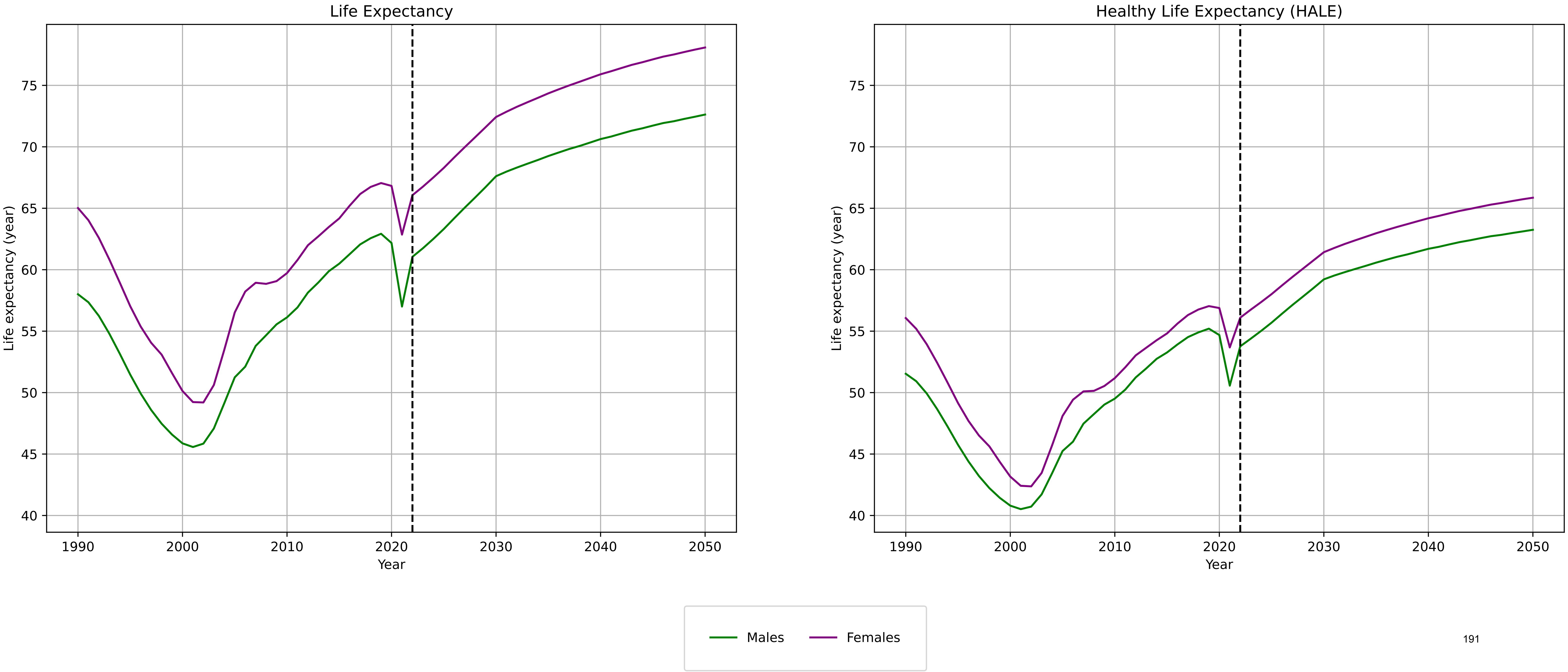

Eswatini

Supplemental Results Figure S3. Life expectancy and HALE by sex and location, 1990-2050

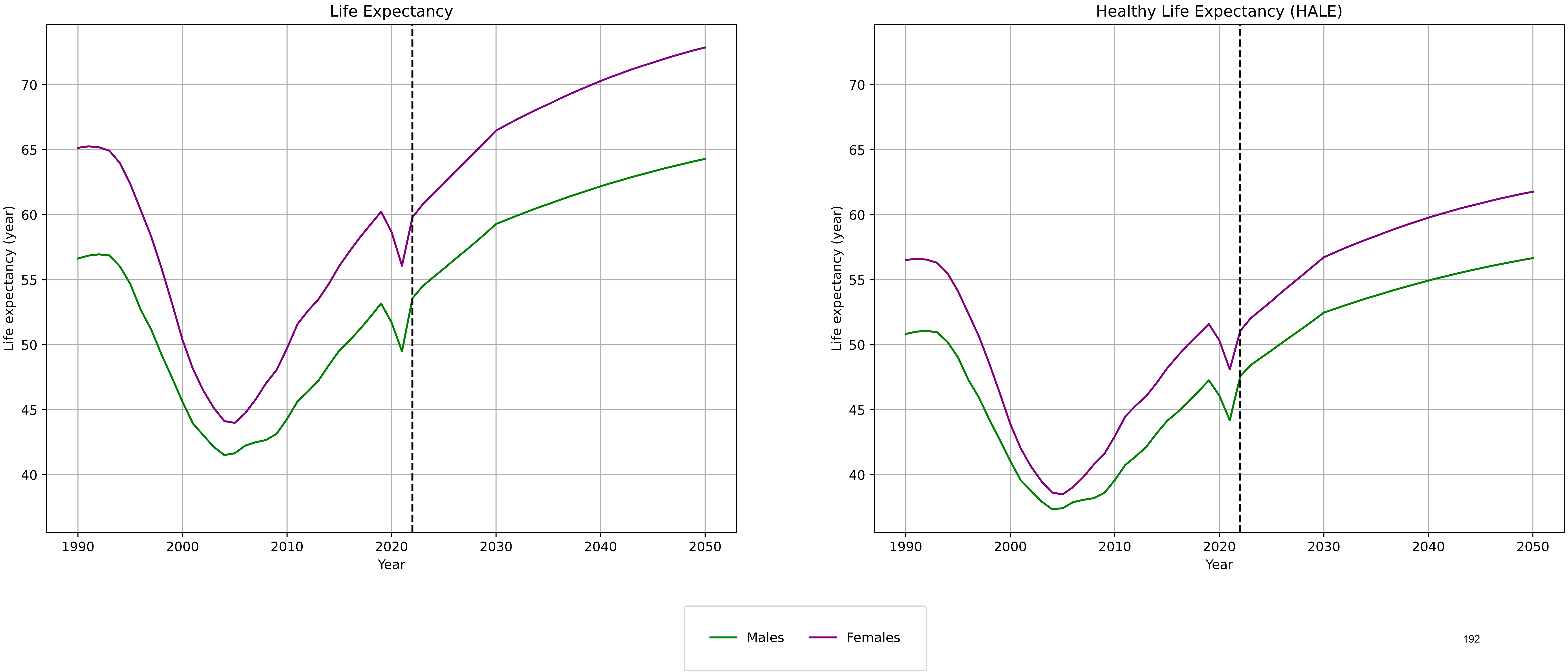

Supplemental Results Figure S3. Life expectancy and HALE by sex and location, 1990-2050

Lesotho

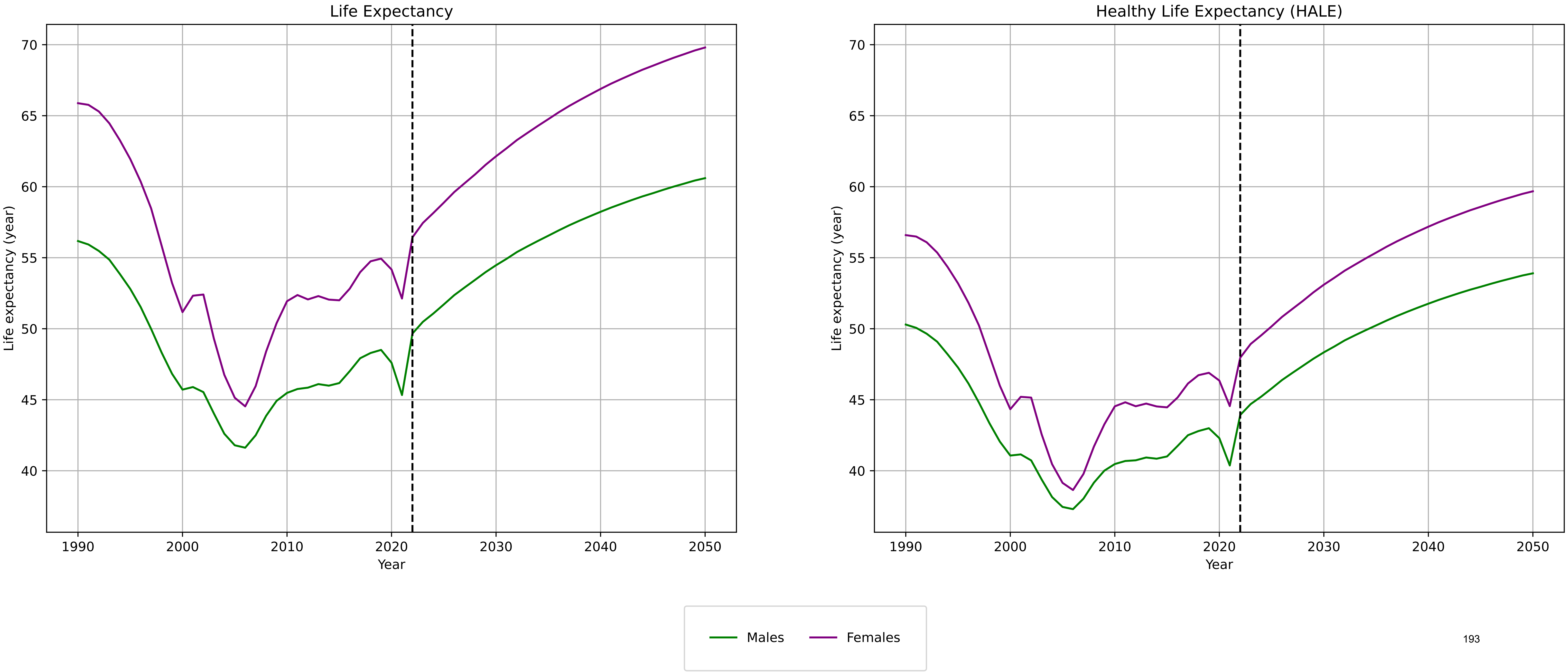

Namibia

Supplemental Results Figure S3. Life expectancy and HALE by sex and location, 1990-2050

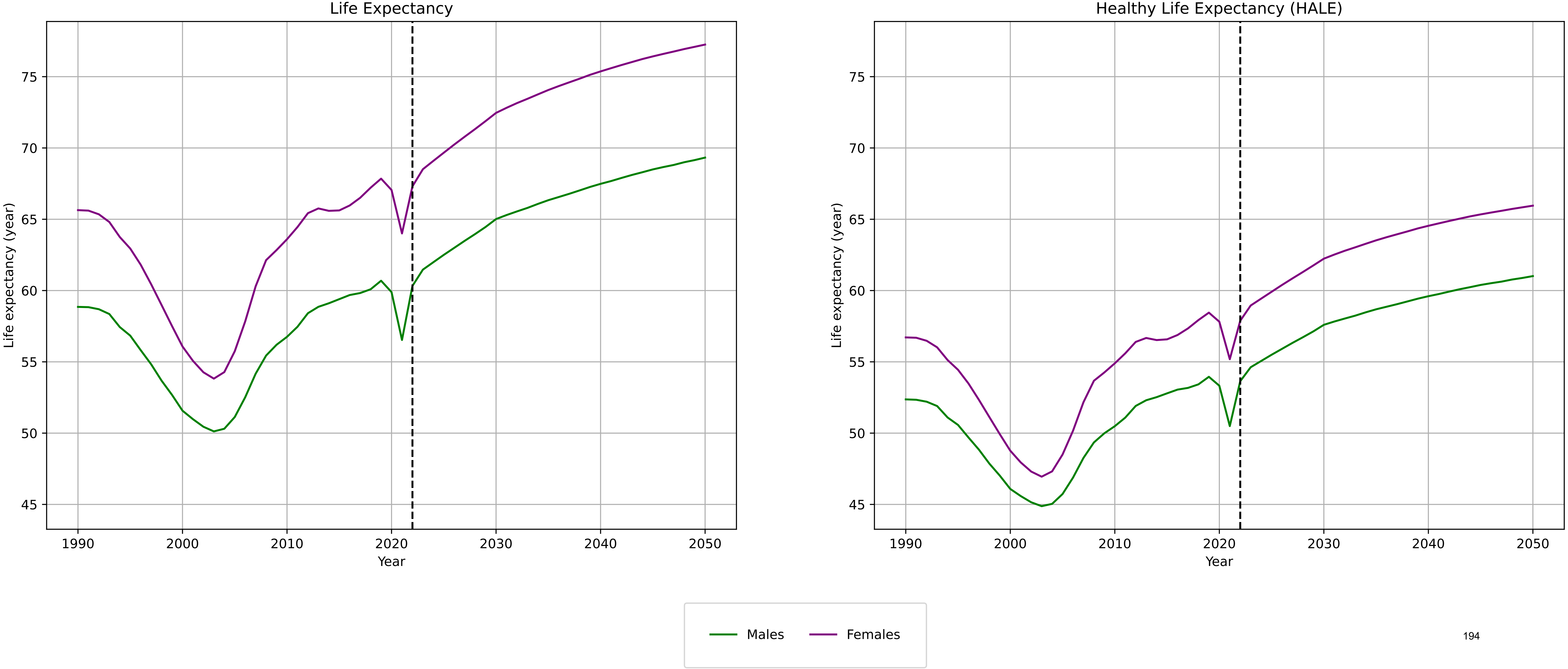

South Africa

Supplemental Results Figure S3. Life expectancy and HALE by sex and location, 1990-2050

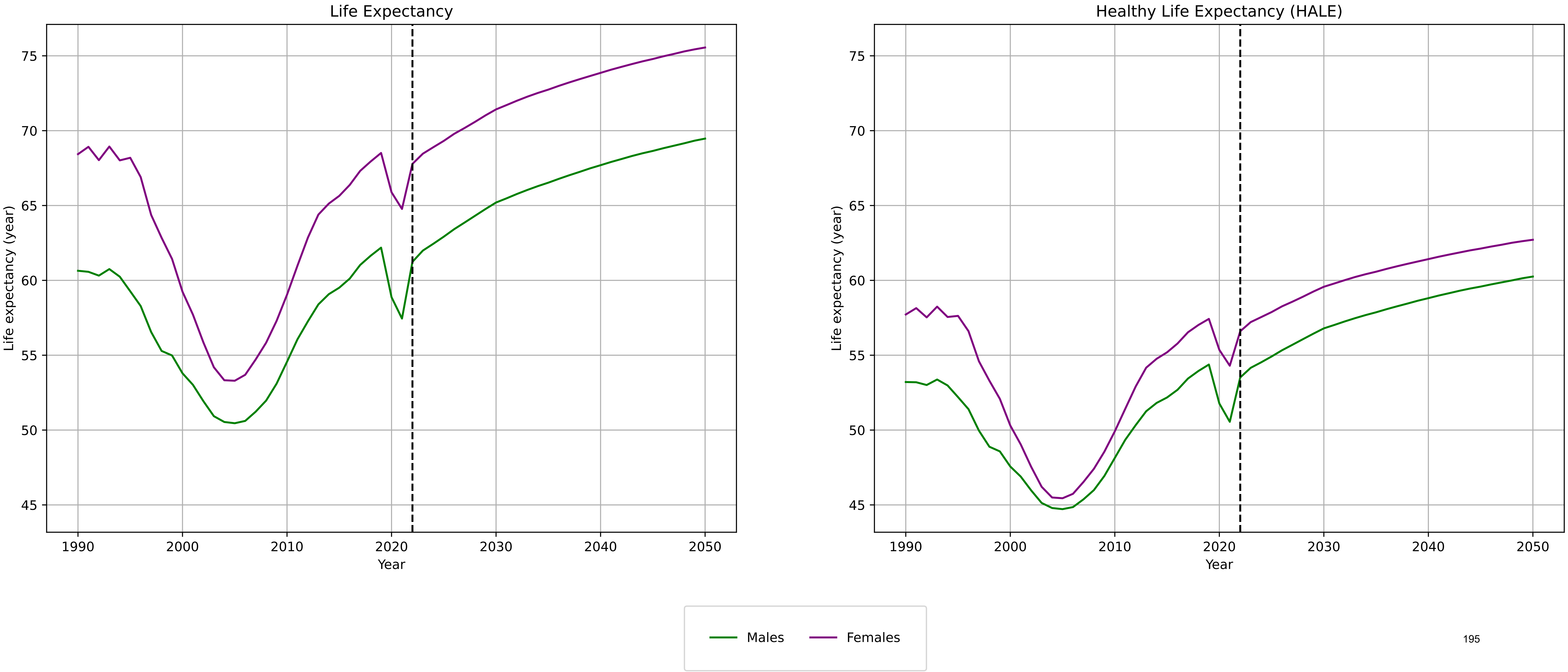

Supplemental Results Figure S3. Life expectancy and HALE by sex and location, 1990-2050

Zimbabwe

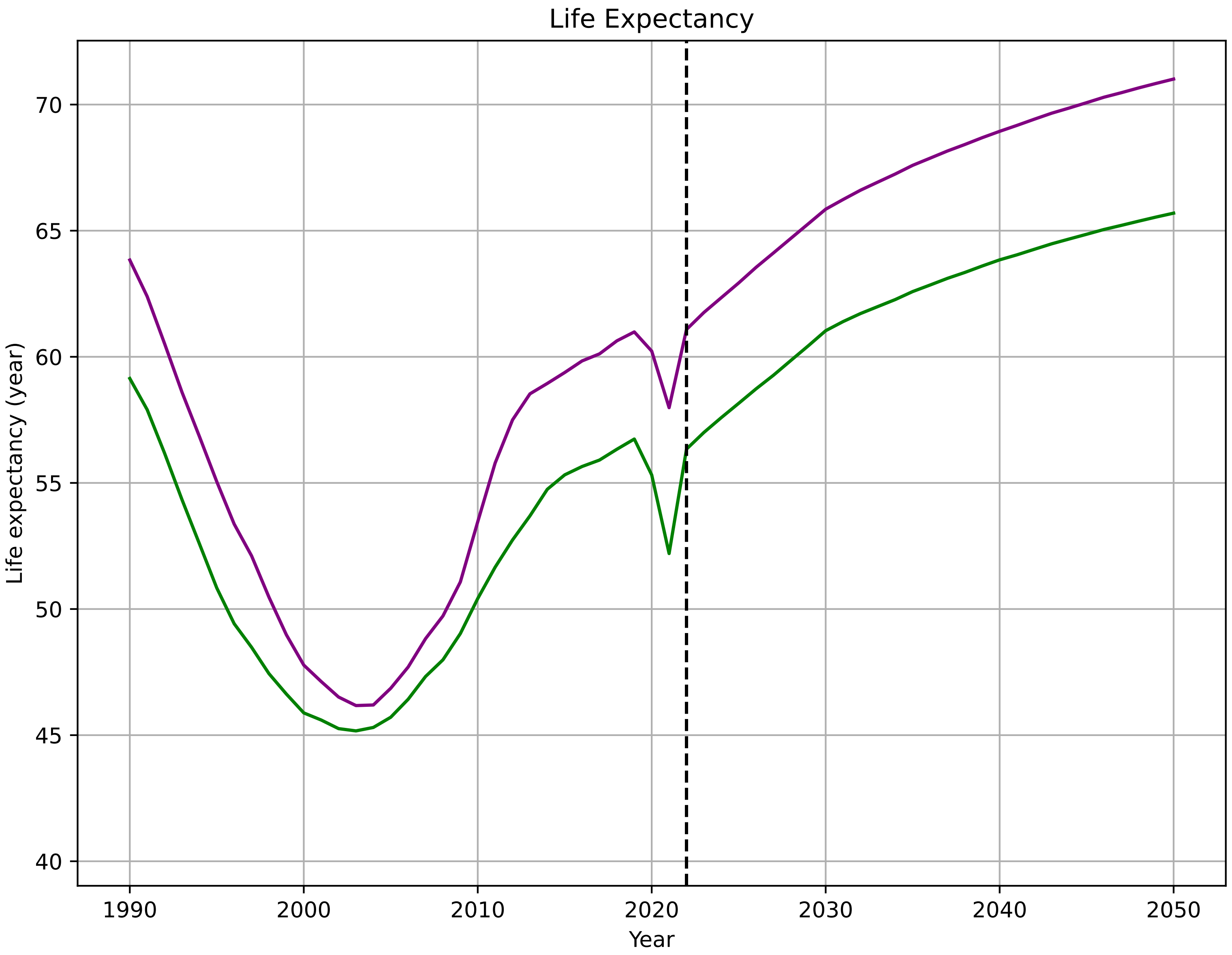

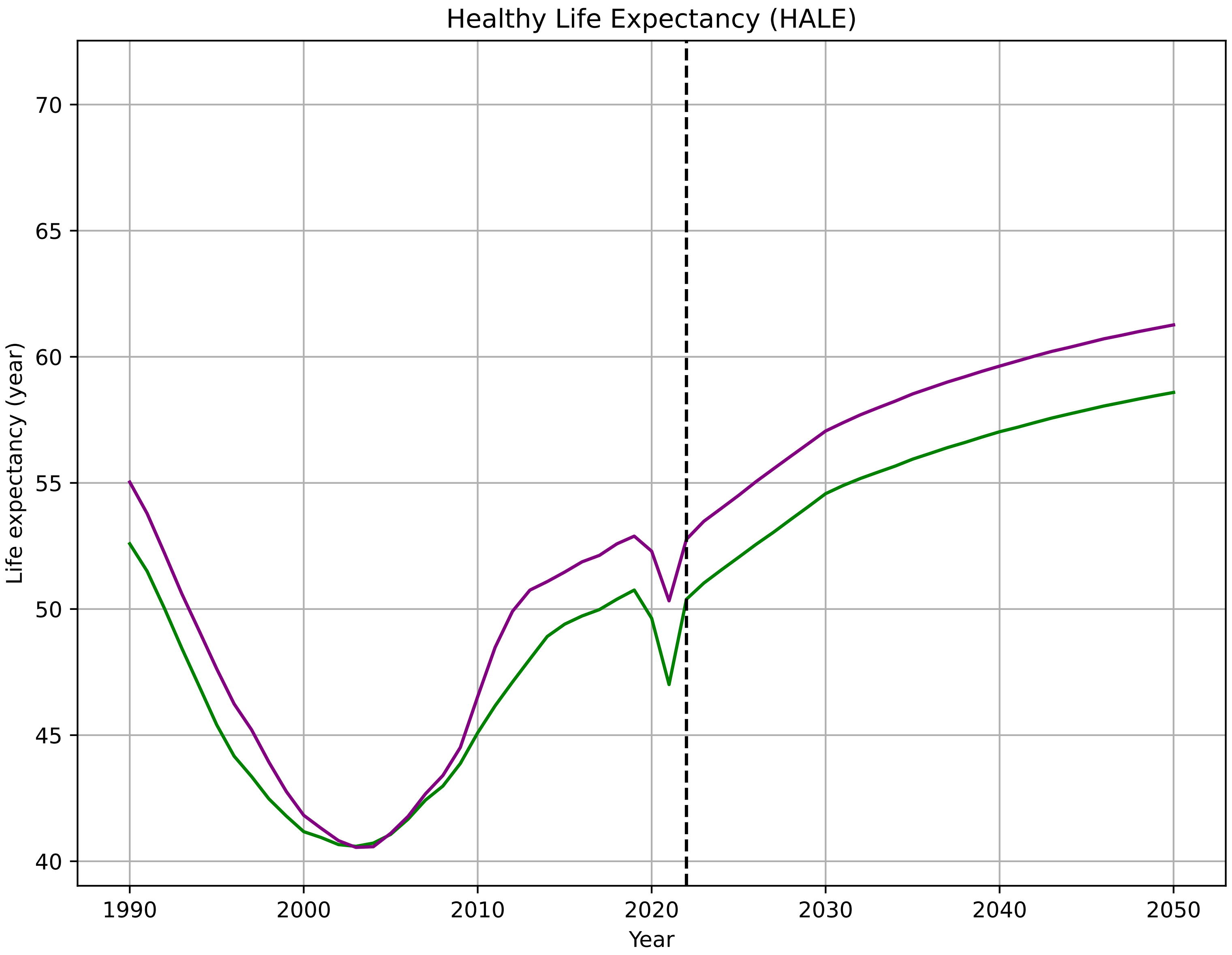

Males Females

Supplemental Results Figure S3. Life expectancy and HALE by sex and location, 1990-2050

Benin

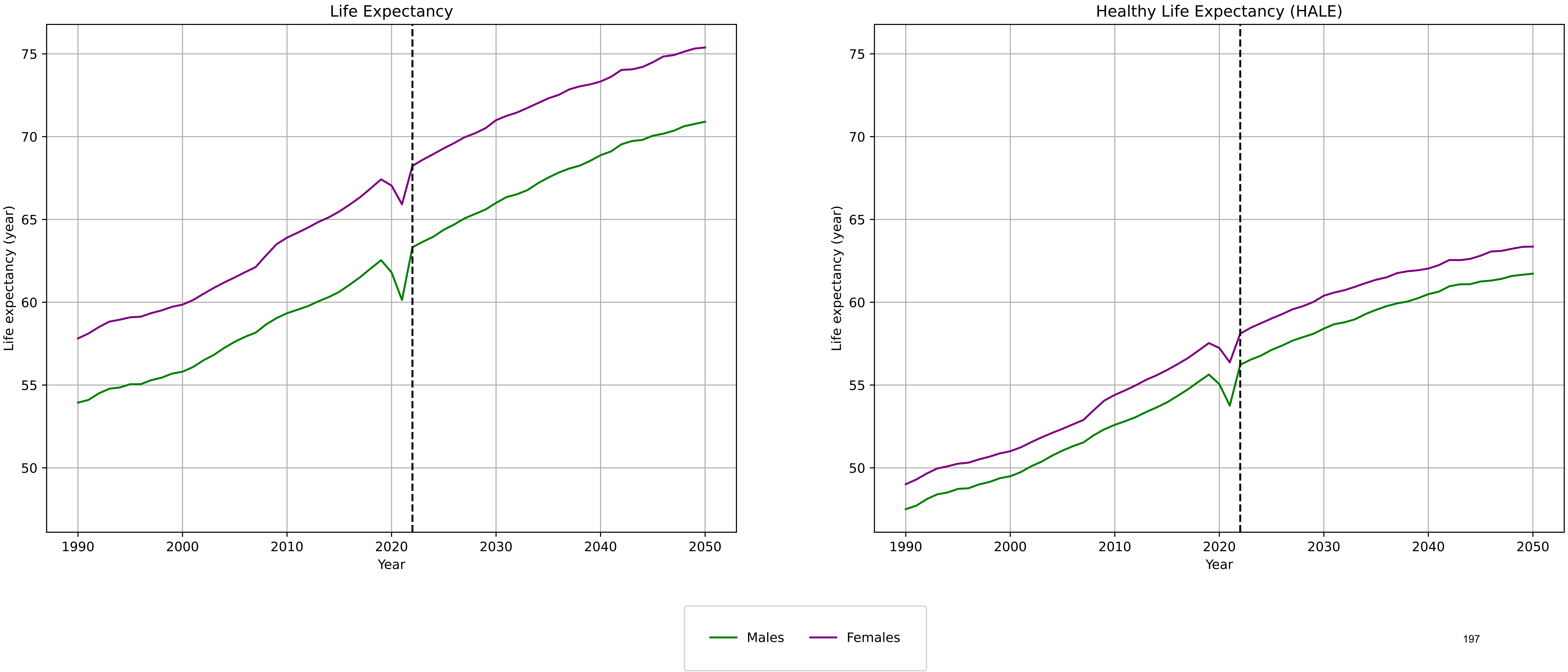

Burkina Faso

Supplemental Results Figure S3. Life expectancy and HALE by sex and location, 1990-2050

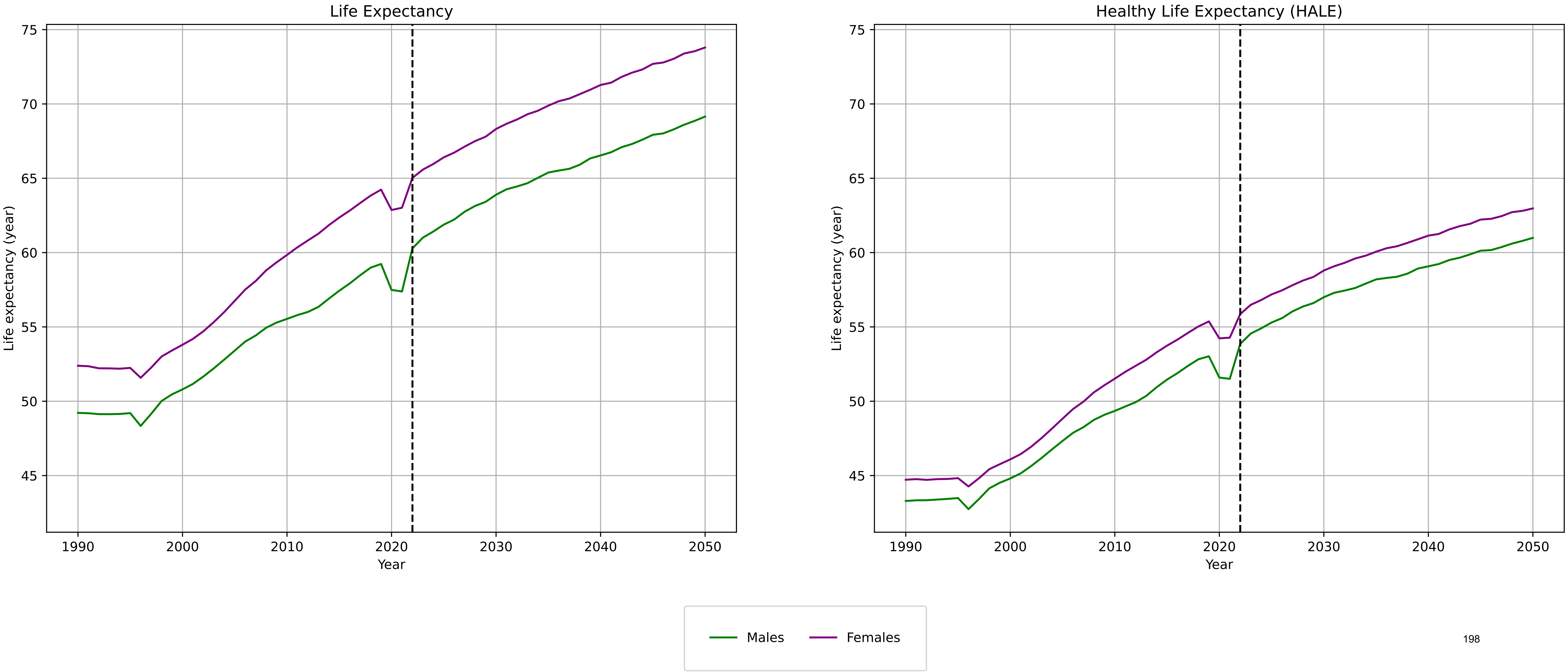

Cabo Verde

Supplemental Results Figure S3. Life expectancy and HALE by sex and location, 1990-2050

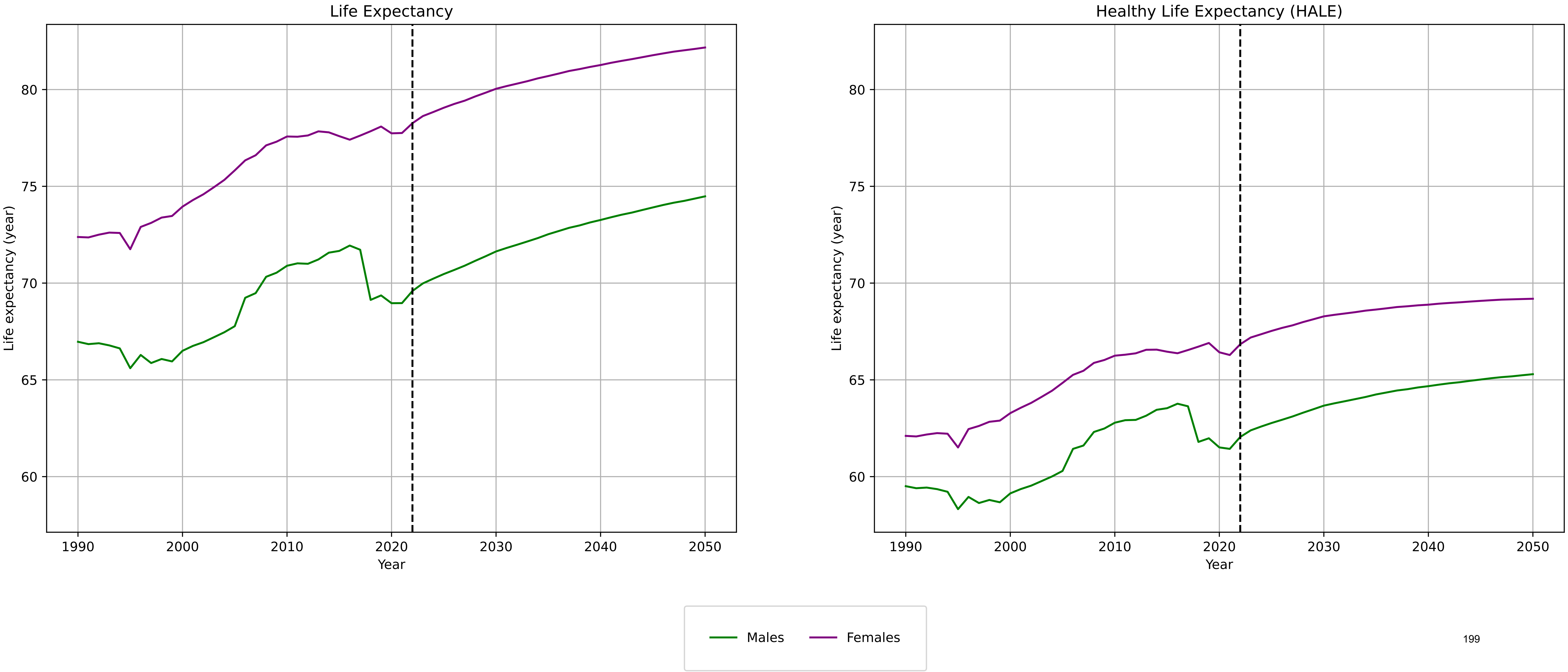

Supplemental Results Figure S3. Life expectancy and HALE by sex and location, 1990-2050

Cameroon

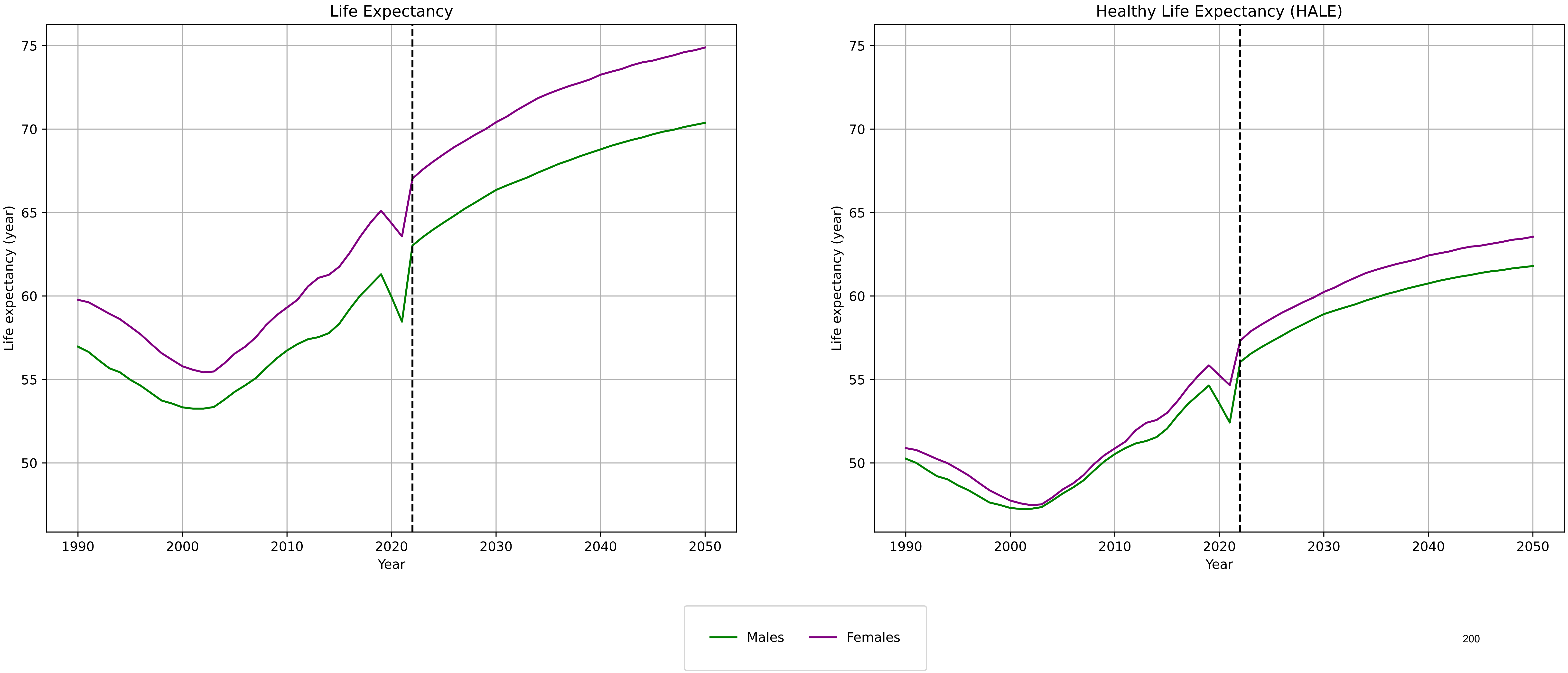

Supplemental Results Figure S3. Life expectancy and HALE by sex and location, 1990-2050

Chad

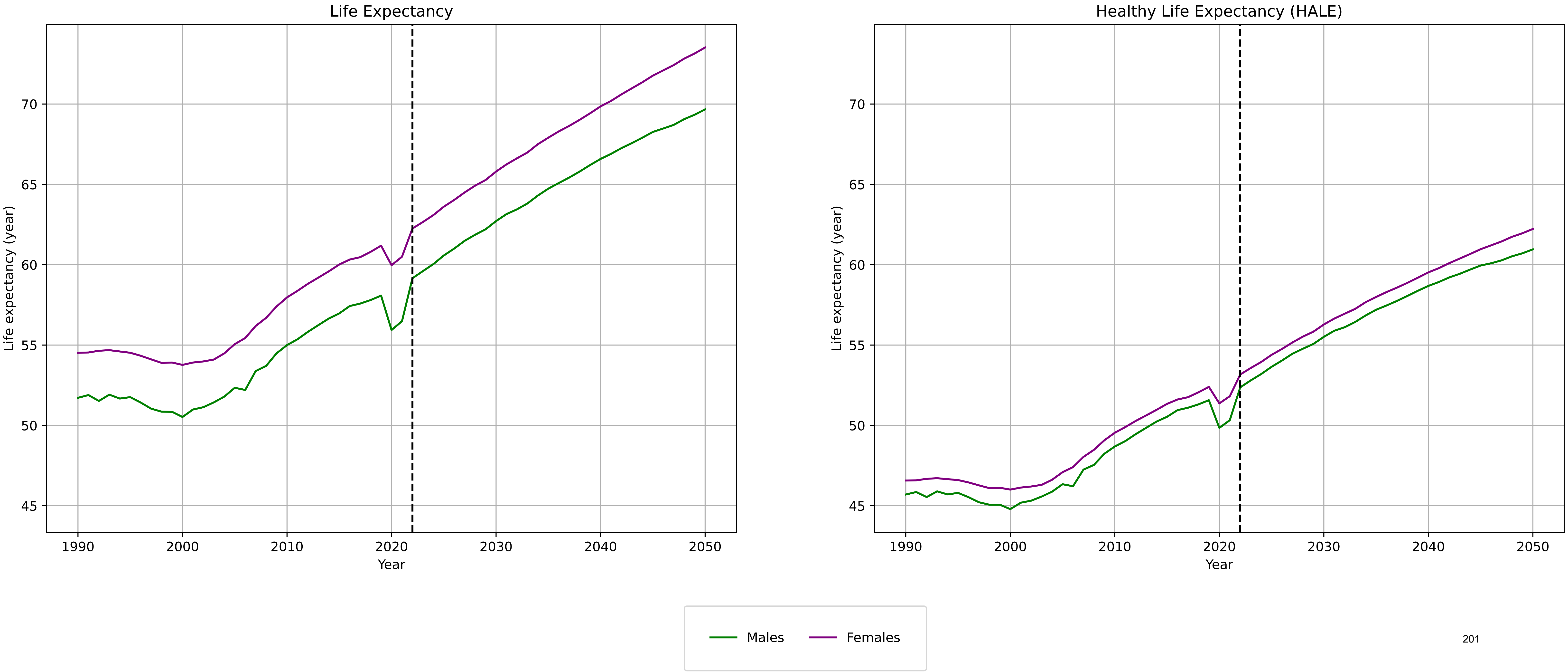

Côte D'Ivoire

Supplemental Results Figure S3. Life expectancy and HALE by sex and location, 1990-2050

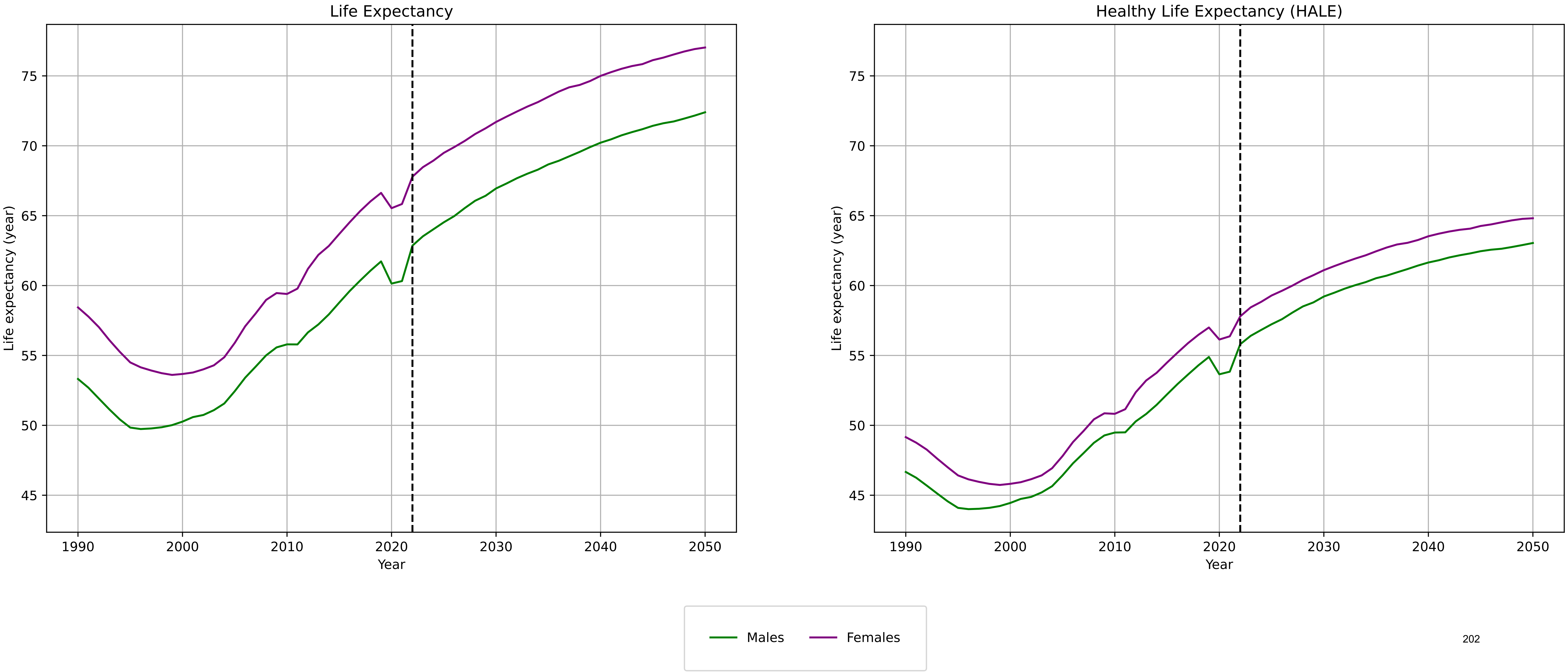

Supplemental Results Figure S3. Life expectancy and HALE by sex and location, 1990-2050

The Gambia

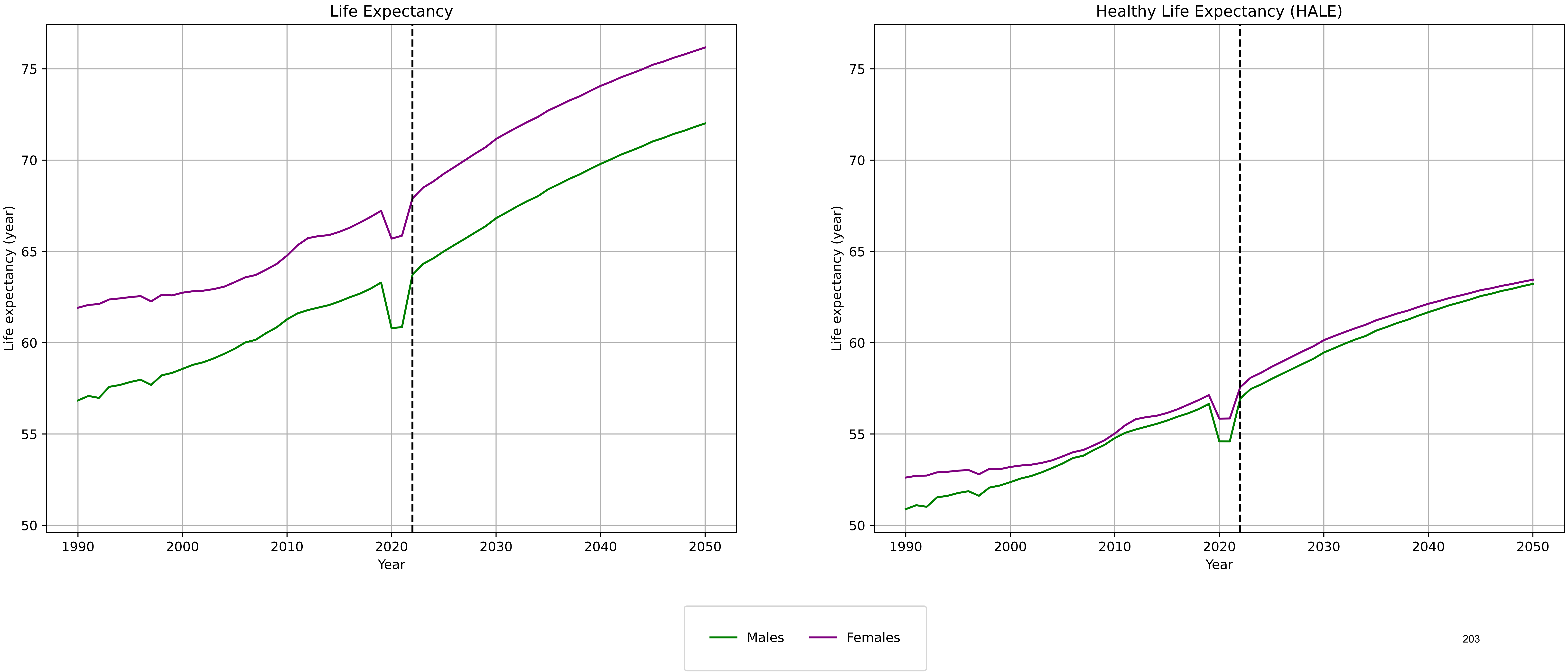

Supplemental Results Figure S3. Life expectancy and HALE by sex and location, 1990-2050

Ghana

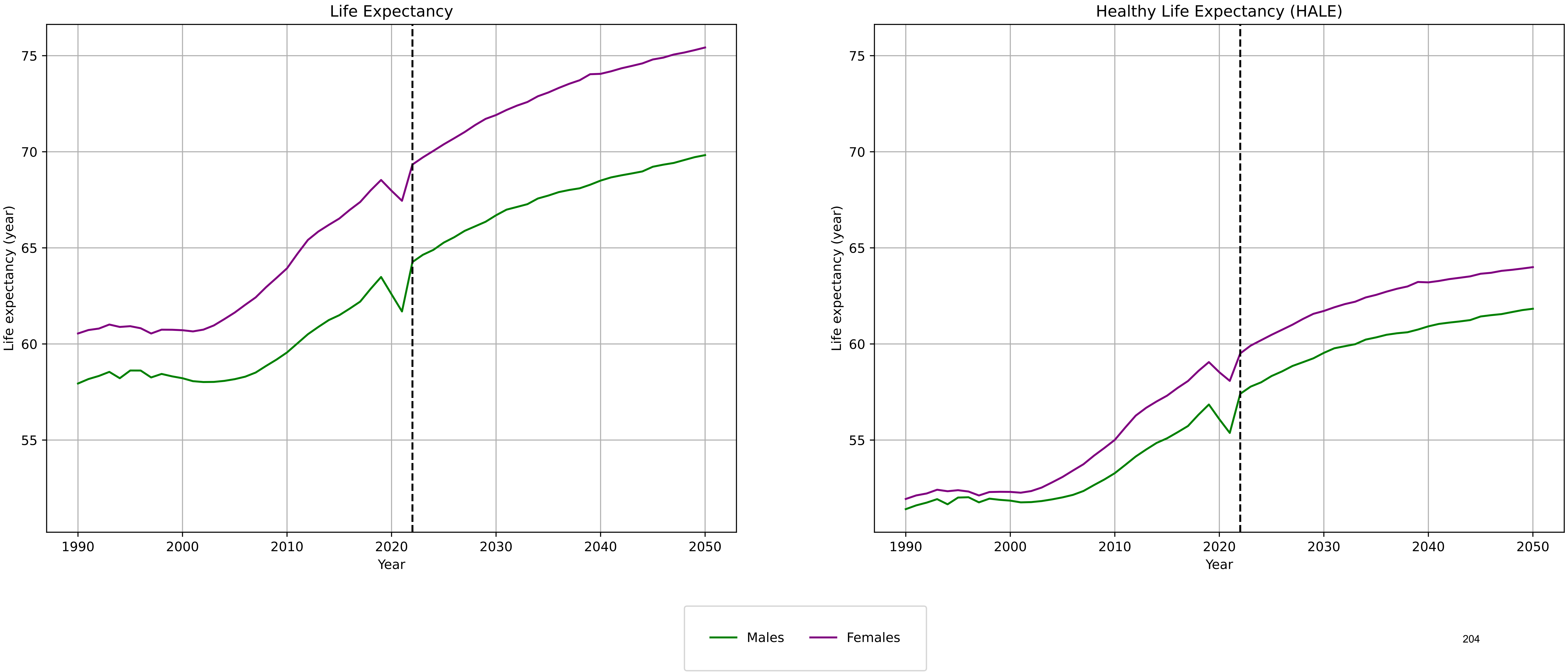

Guinea

Supplemental Results Figure S3. Life expectancy and HALE by sex and location, 1990-2050

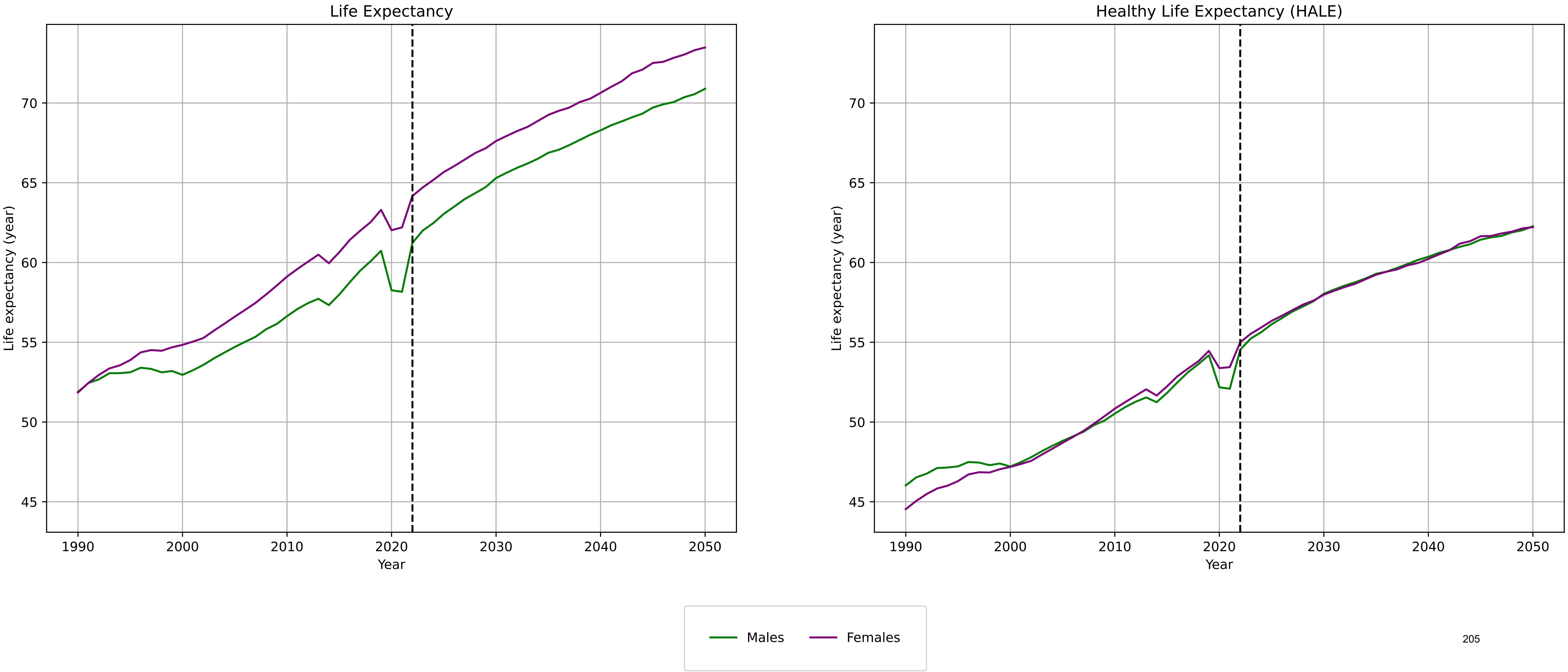

Guinea-Bissau

Supplemental Results Figure S3. Life expectancy and HALE by sex and location, 1990-2050

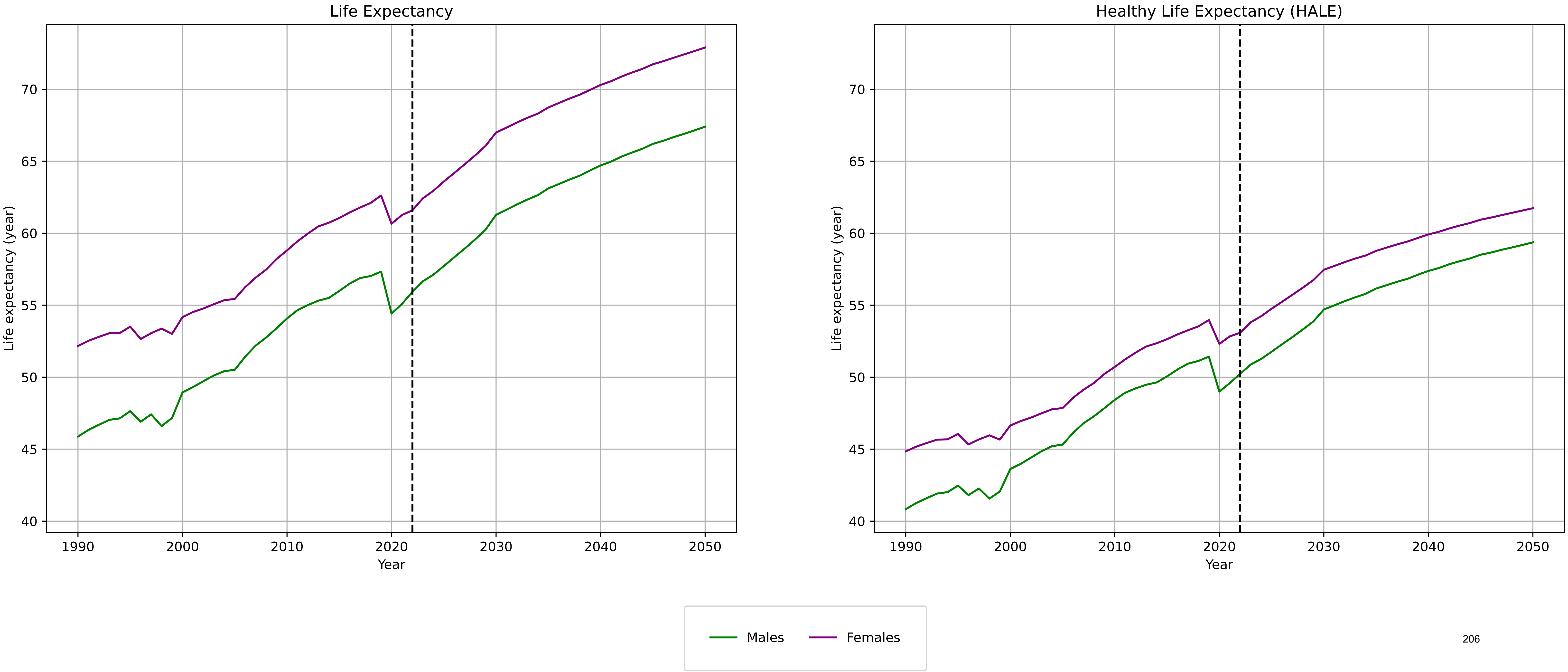

Liberia

Supplemental Results Figure S3. Life expectancy and HALE by sex and location, 1990-2050

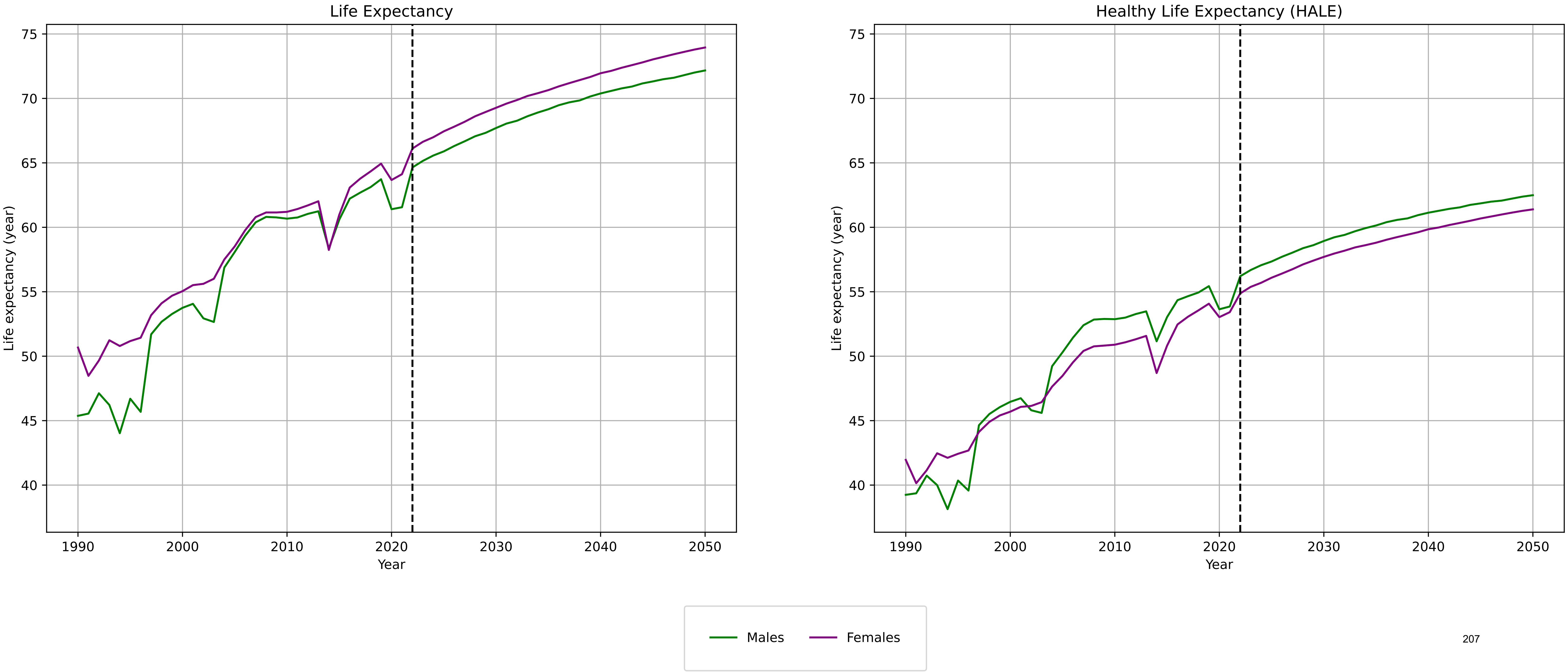

Mali

Supplemental Results Figure S3. Life expectancy and HALE by sex and location, 1990-2050

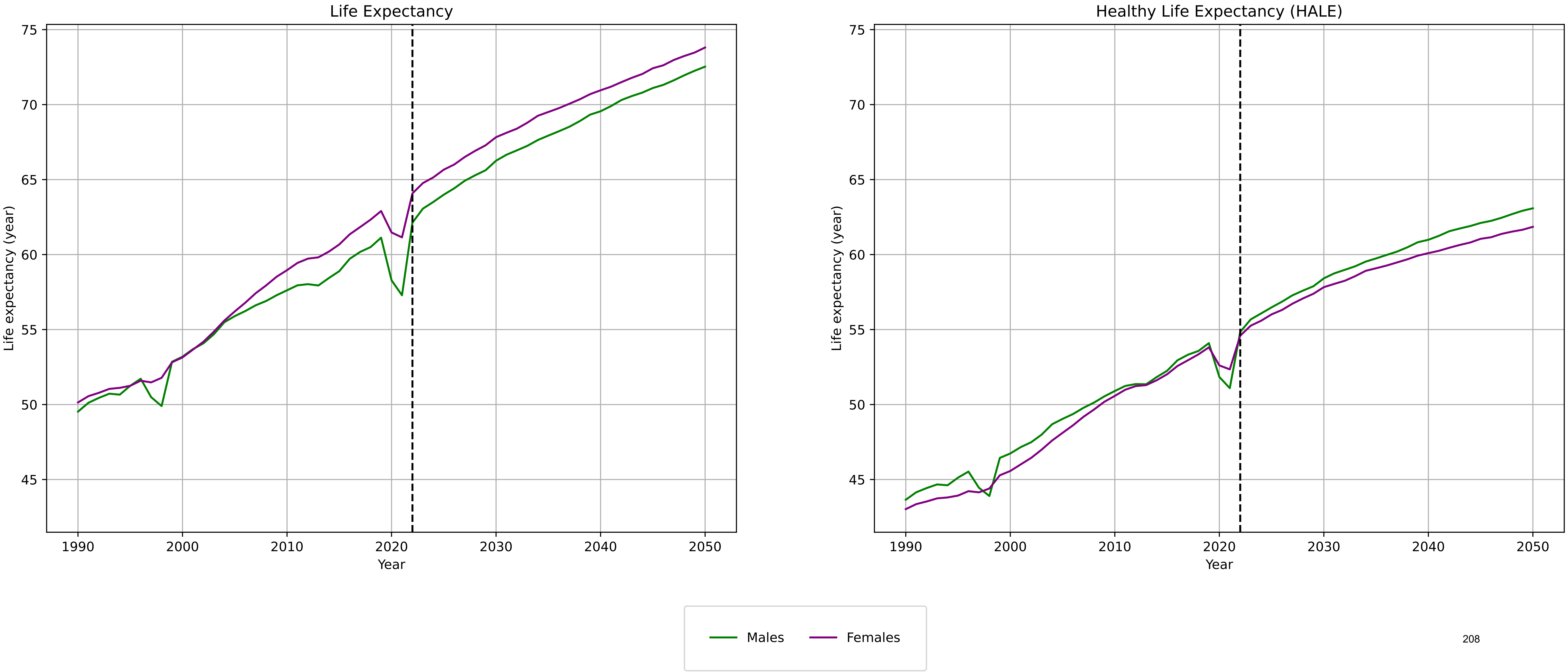

Supplemental Results Figure S3. Life expectancy and HALE by sex and location, 1990-2050

Mauritania

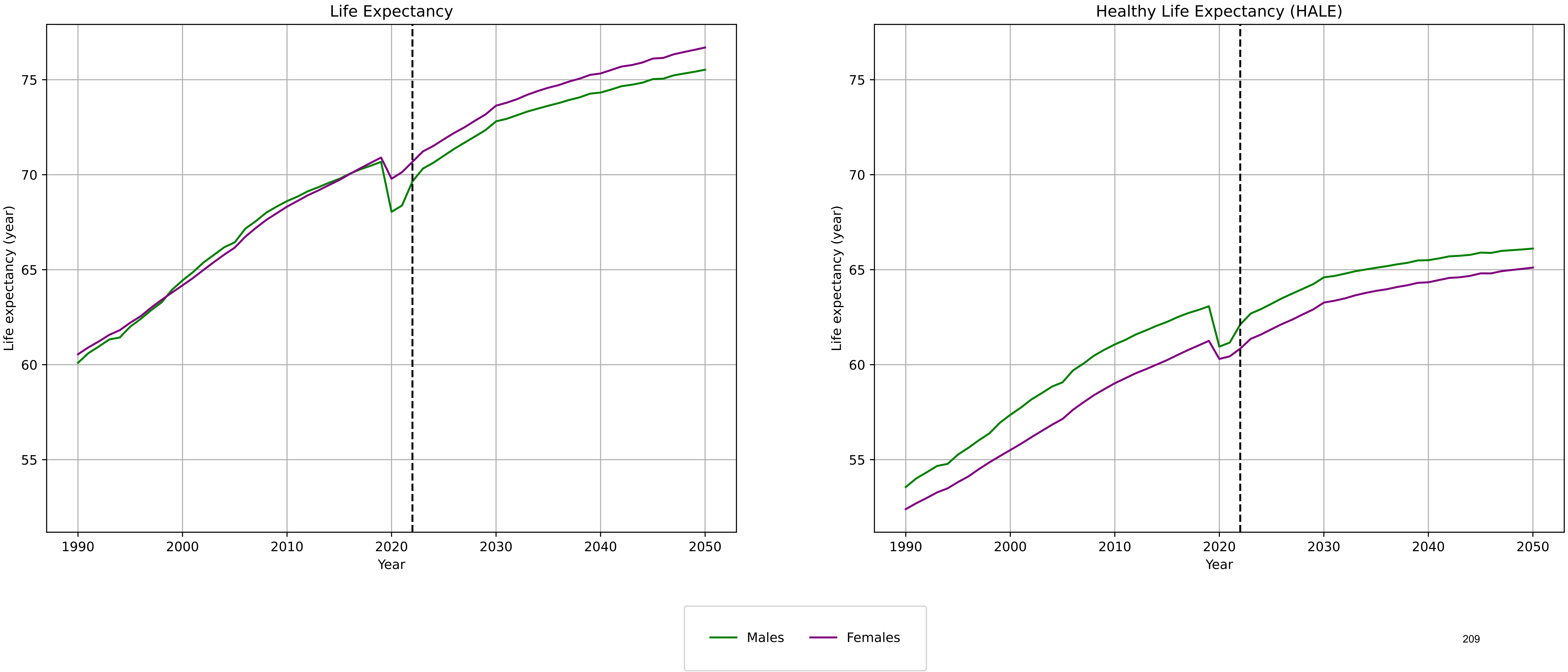

Supplemental Results Figure S3. Life expectancy and HALE by sex and location, 1990-2050

Niger

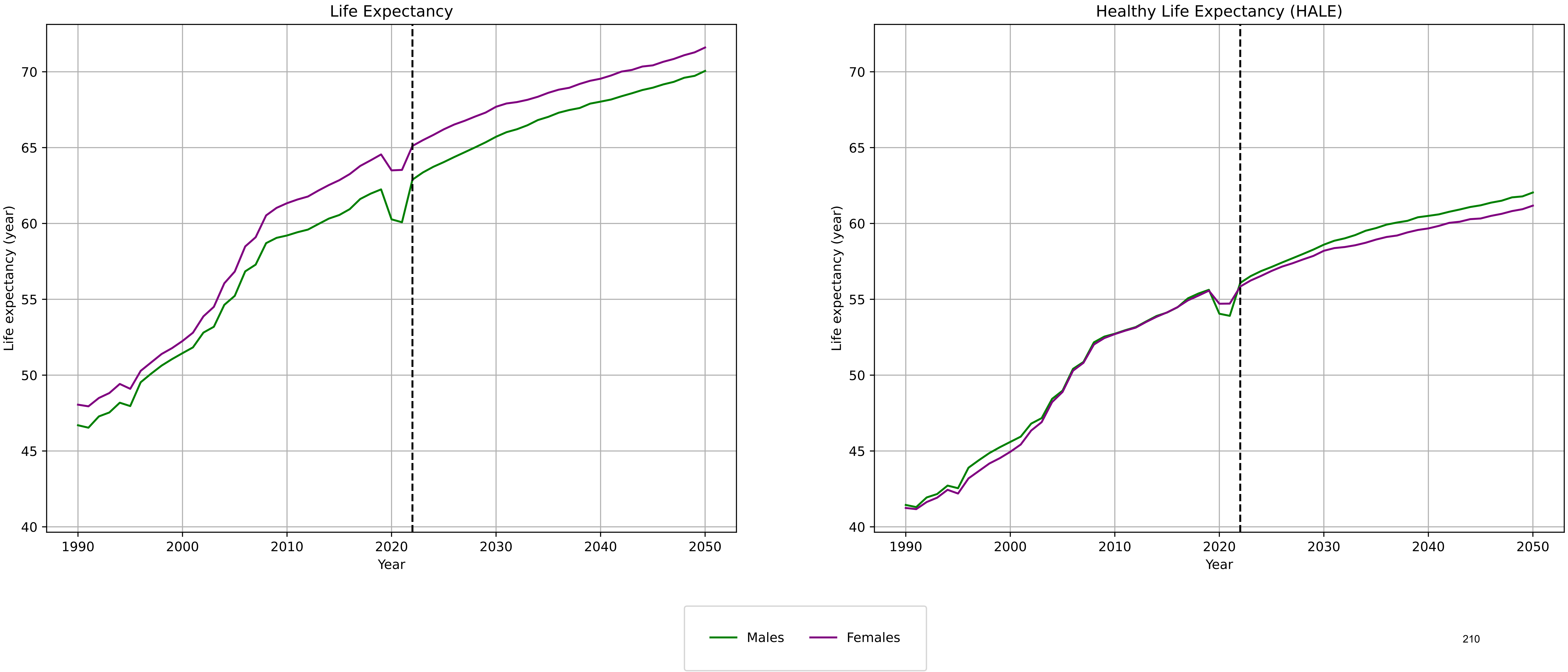

Nigeria

Supplemental Results Figure S3. Life expectancy and HALE by sex and location, 1990-2050

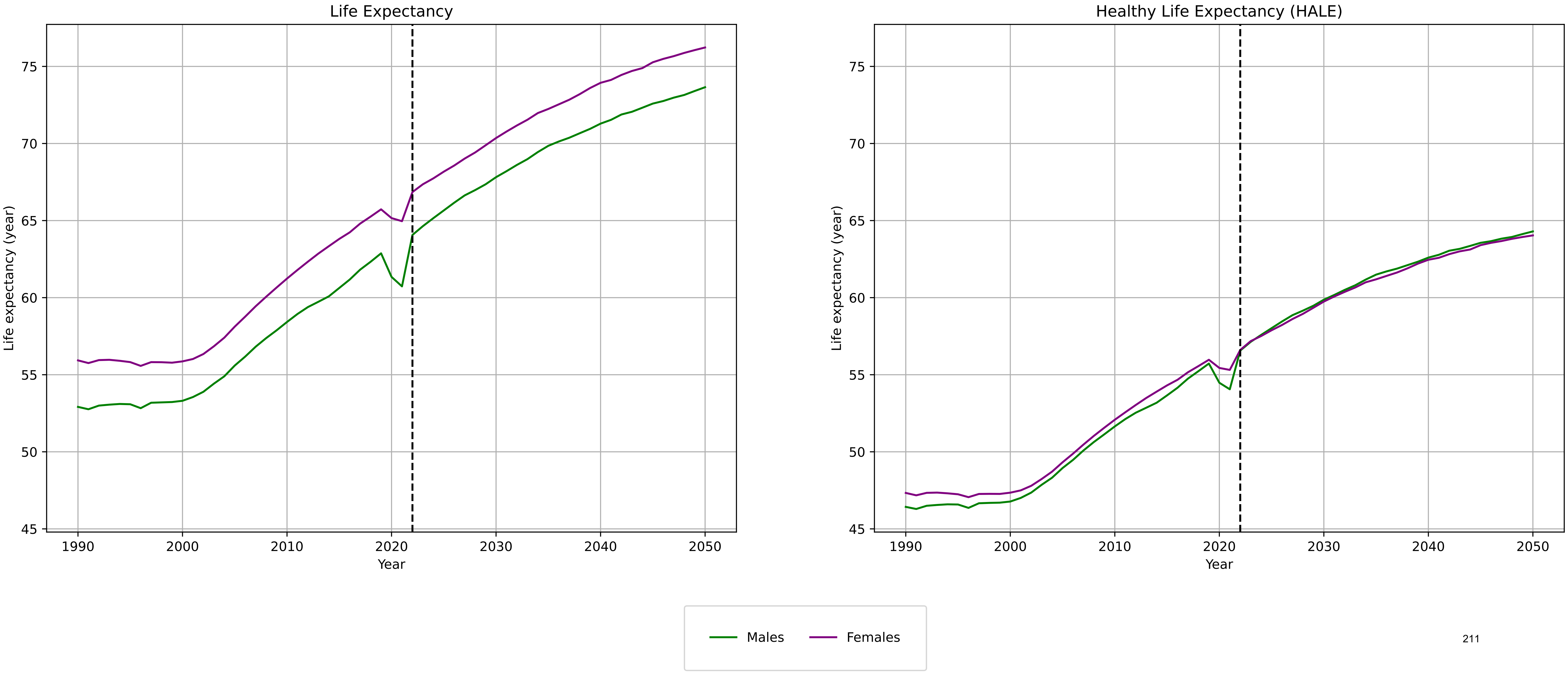

São Tomé And Príncipe

Supplemental Results Figure S3. Life expectancy and HALE by sex and location, 1990-2050

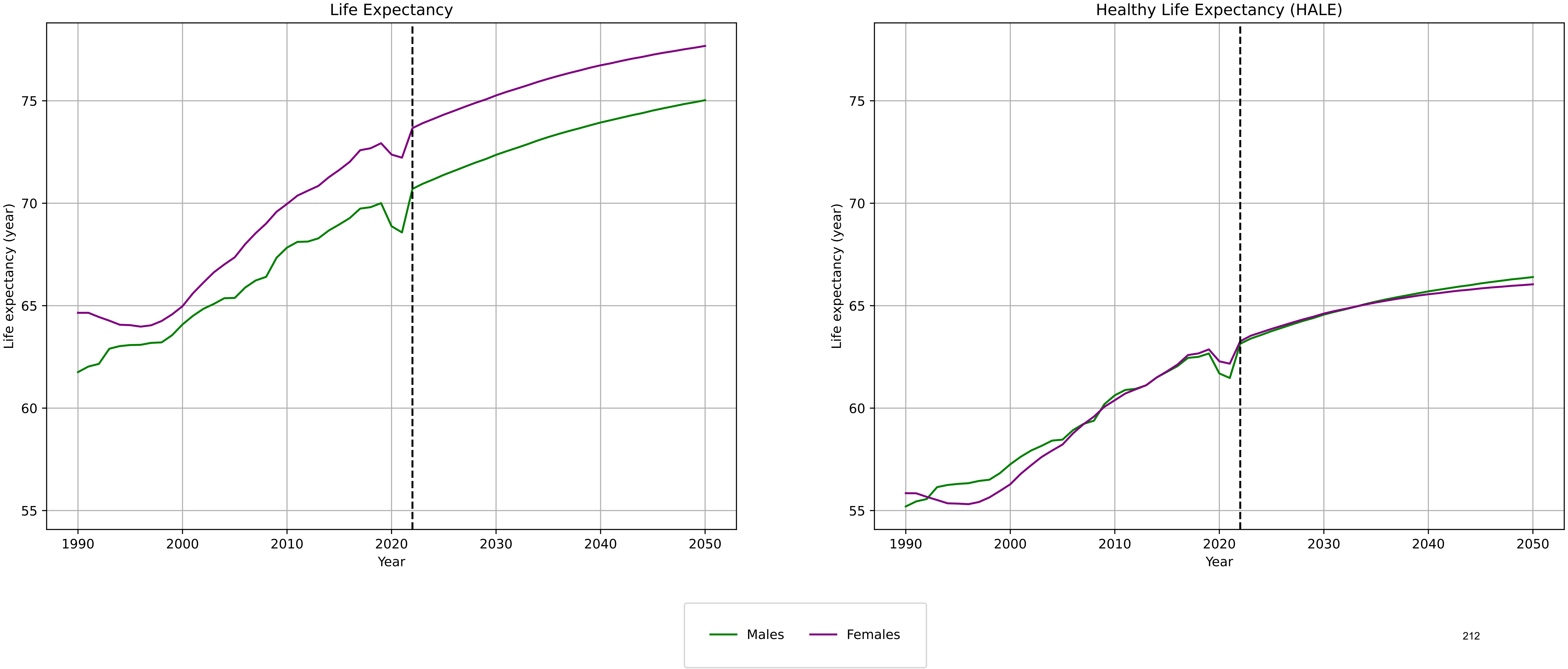

Supplemental Results Figure S3. Life expectancy and HALE by sex and location, 1990-2050

Senegal

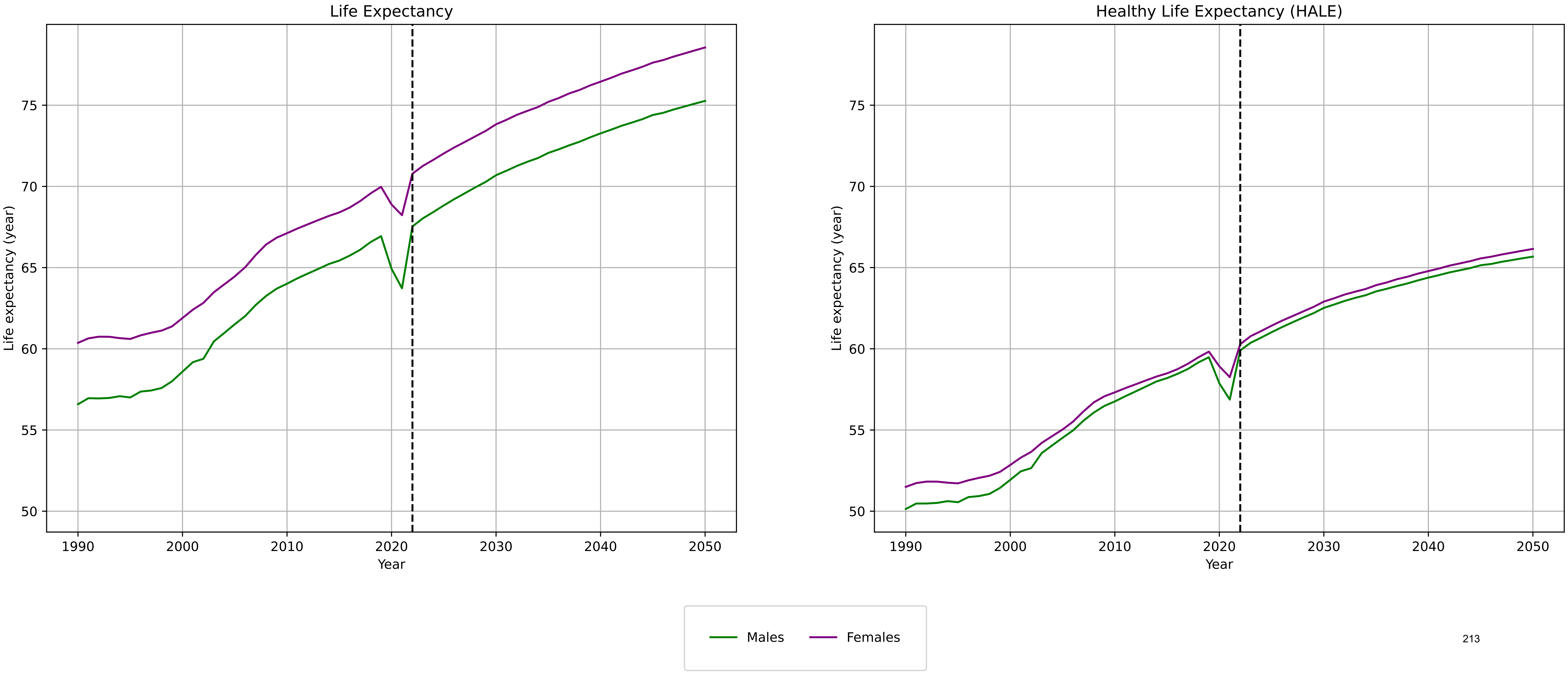

Sierra Leone

Supplemental Results Figure S3. Life expectancy and HALE by sex and location, 1990-2050

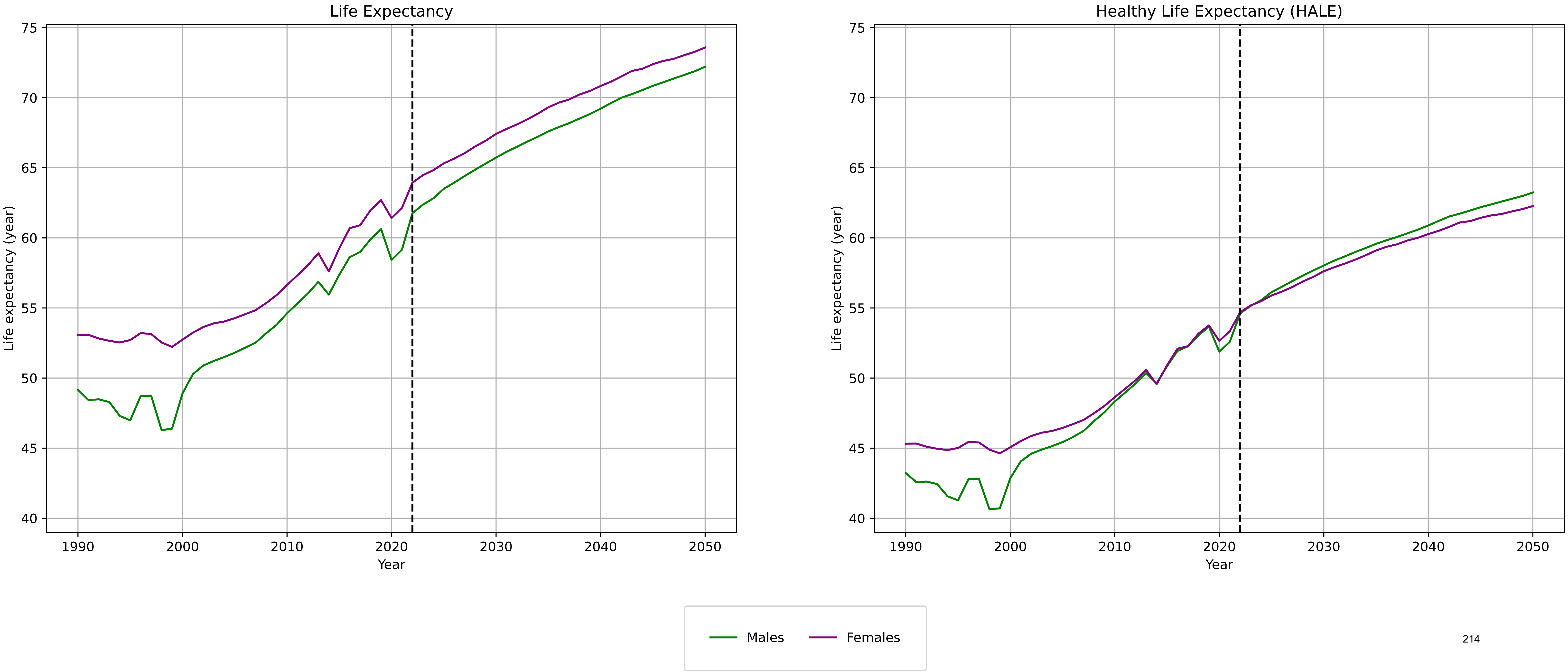

Togo

Supplemental Results Figure S3. Life expectancy and HALE by sex and location, 1990-2050

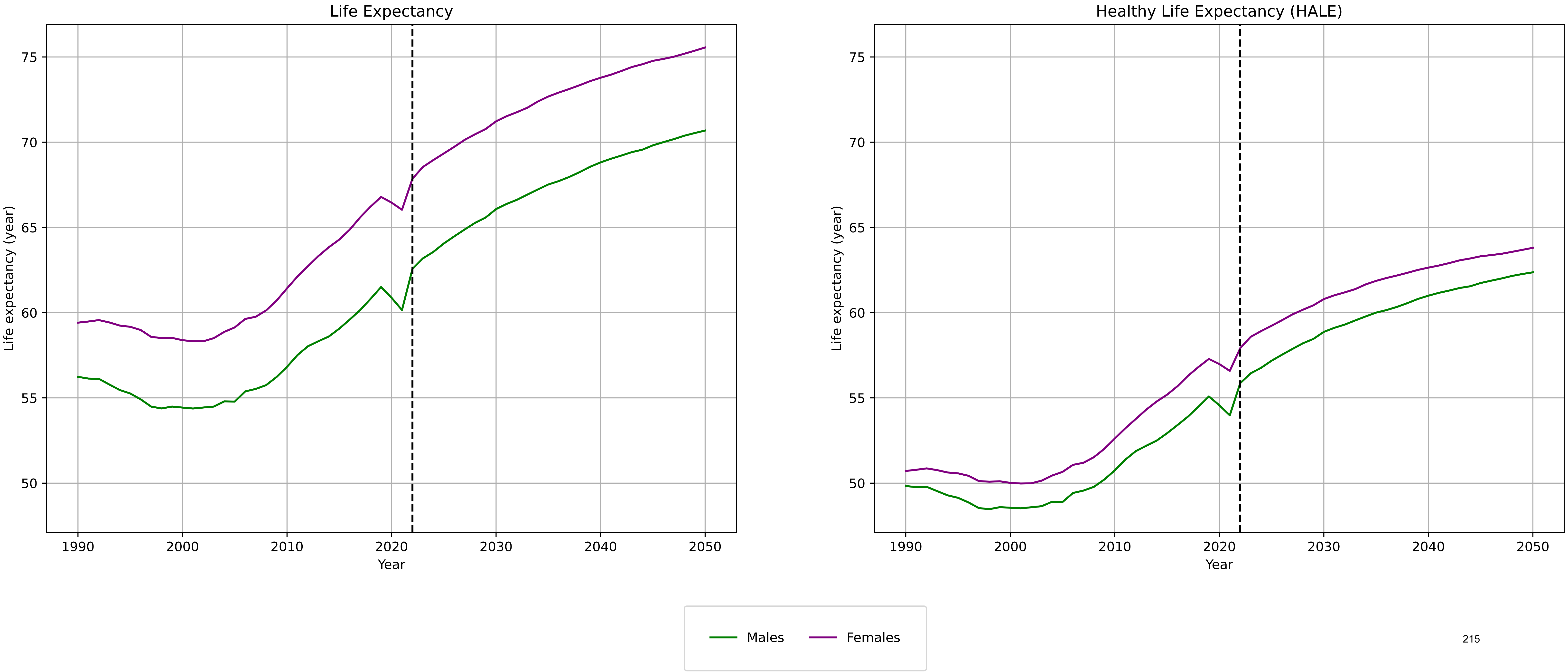

Global

Supplemental Results Figure S3. Life expectancy and HALE by sex and location, 1990-2050

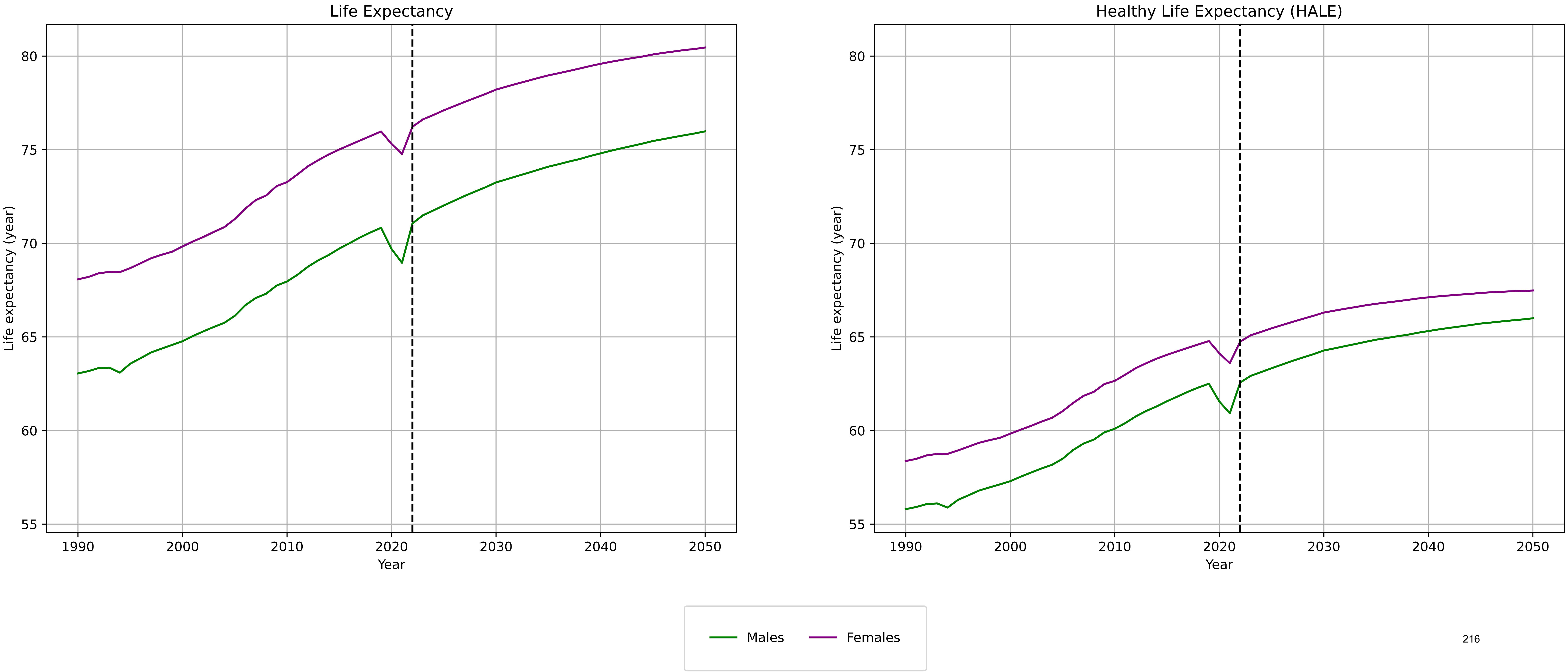

Supplemental Results Figure S3. Life expectancy and HALE by sex and location, 1990-2050

Central Europe, Eastern Europe, And Central Asia

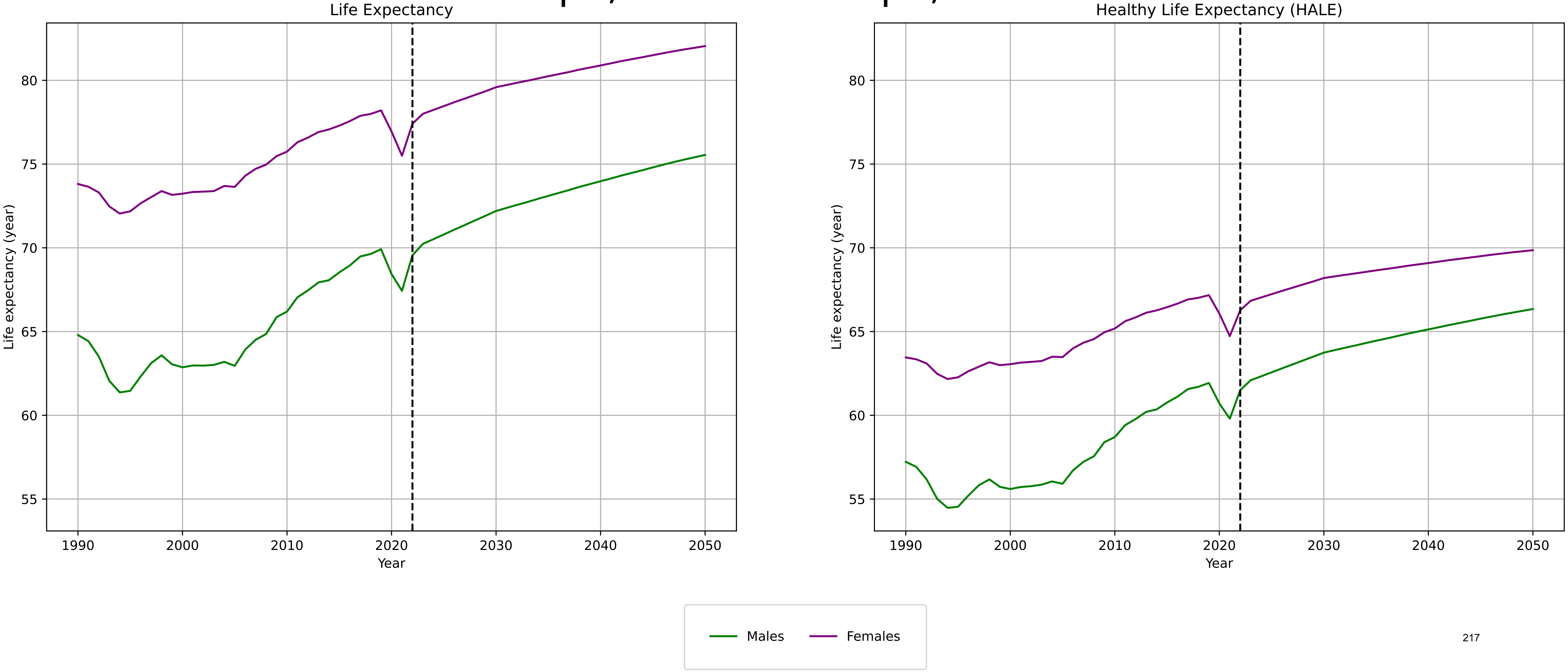

Supplemental Results Figure S3. Life expectancy and HALE by sex and location, 1990-2050

High Income

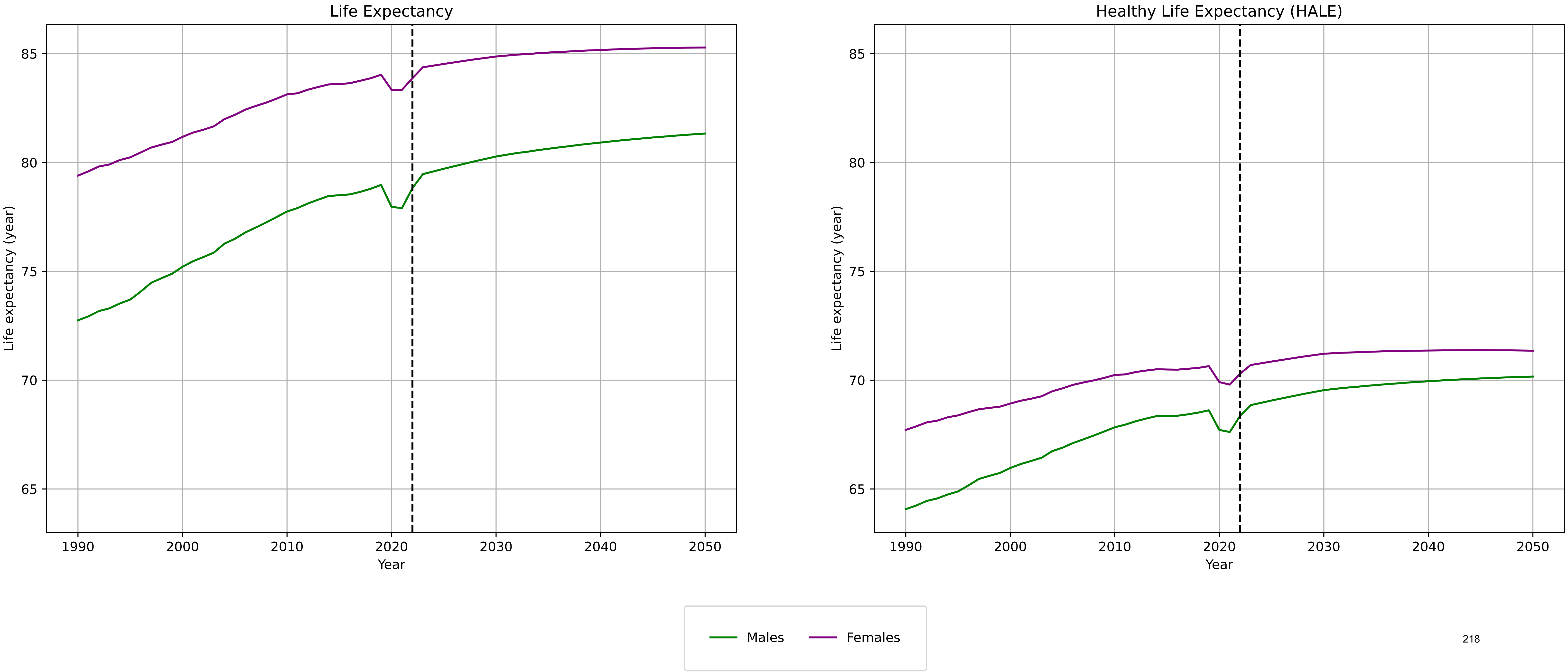

Supplemental Results Figure S3. Life expectancy and HALE by sex and location, 1990-2050

Latin America And Caribbean

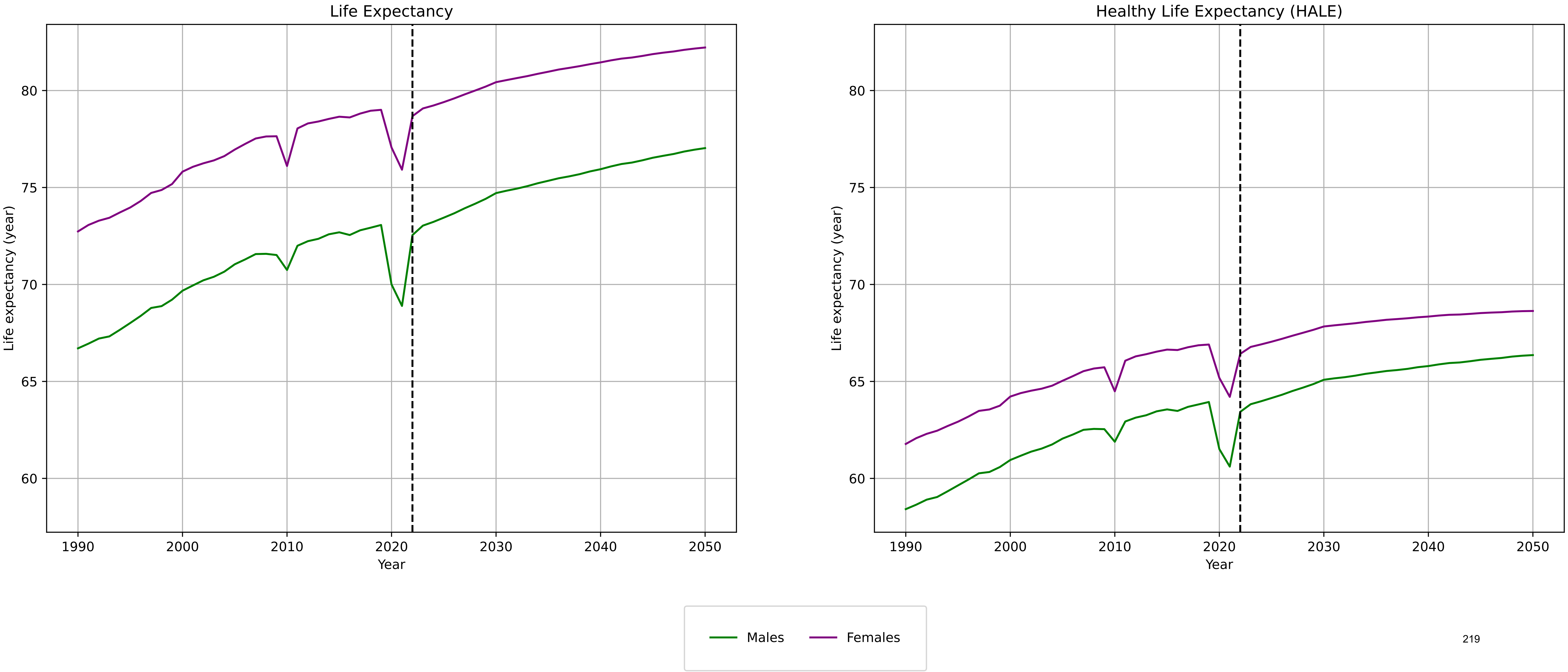

Supplemental Results Figure S3. Life expectancy and HALE by sex and location, 1990-2050

North Africa And Middle East

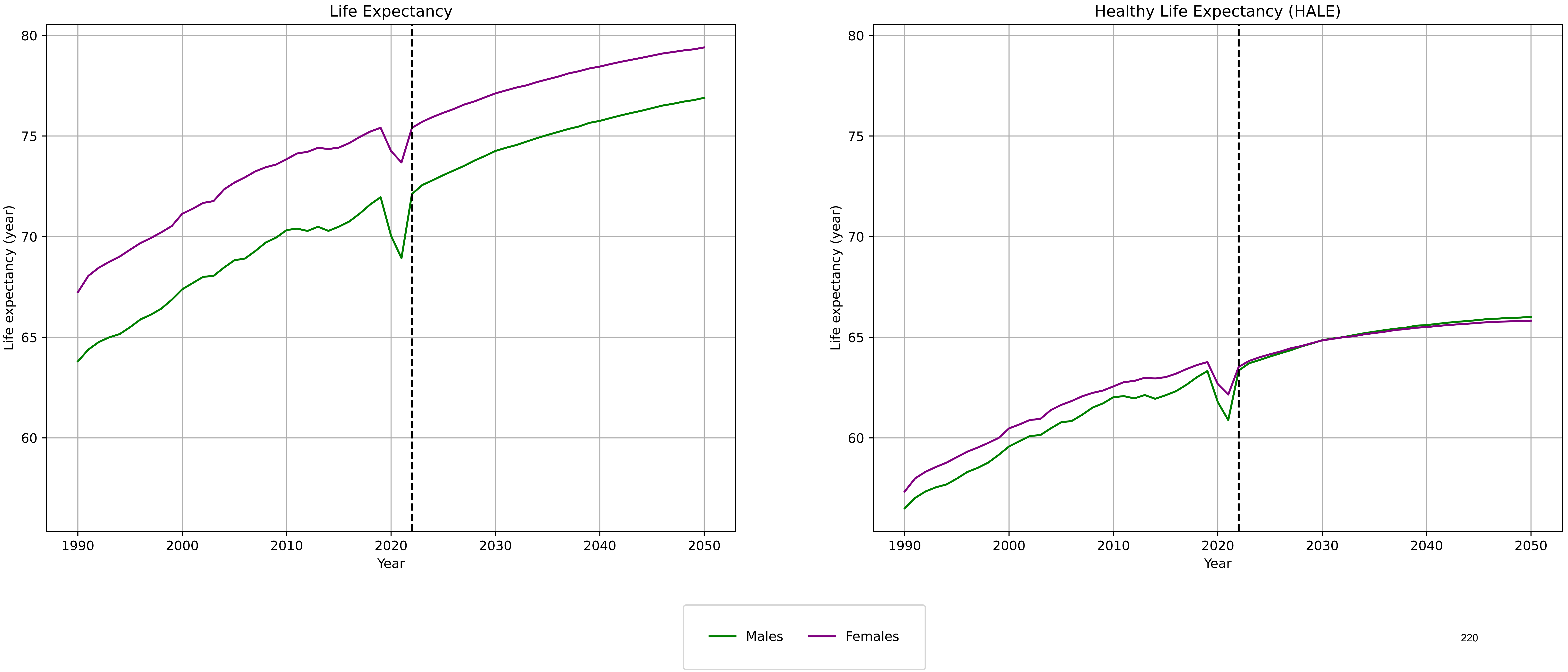

Supplemental Results Figure S3. Life expectancy and HALE by sex and location, 1990-2050

South Asia

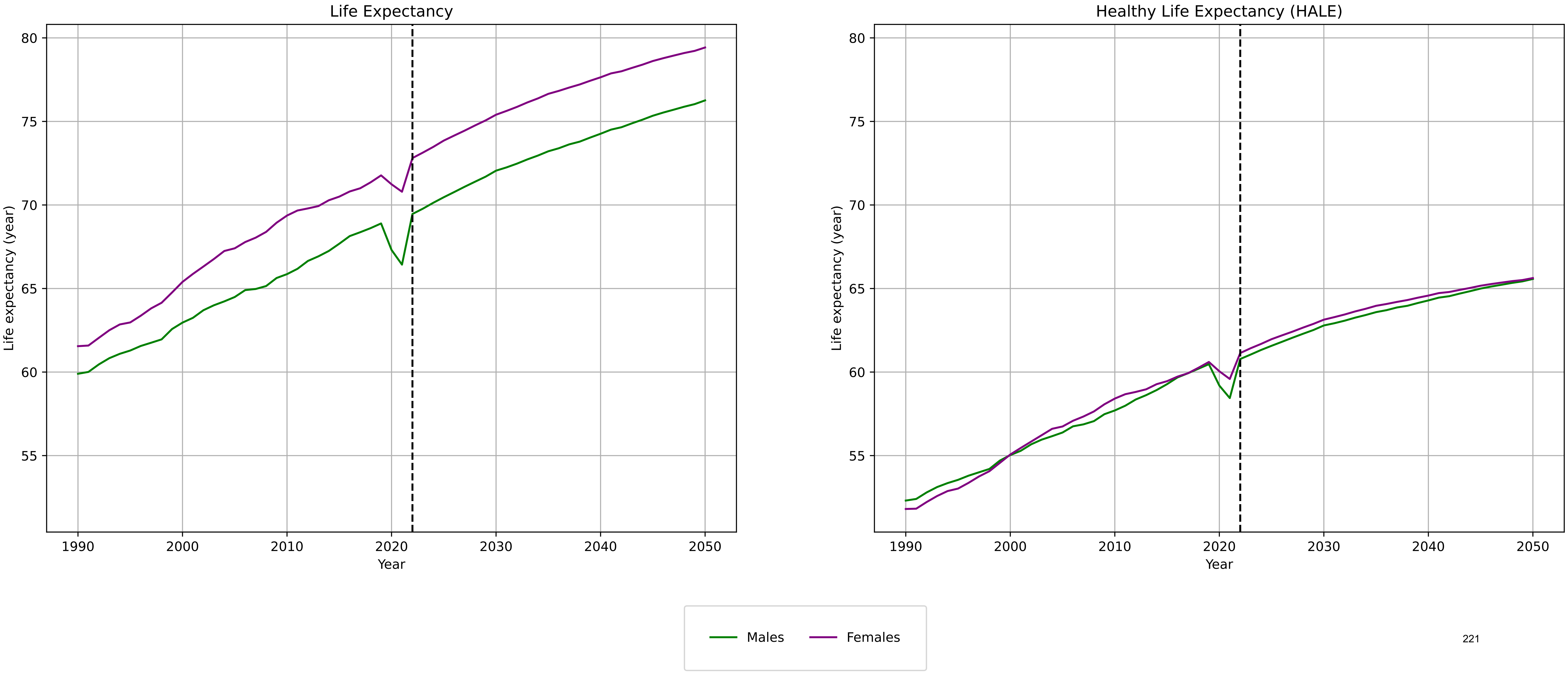

Supplemental Results Figure S3. Life expectancy and HALE by sex and location, 1990-2050

Southeast Asia, East Asia, And Oceania

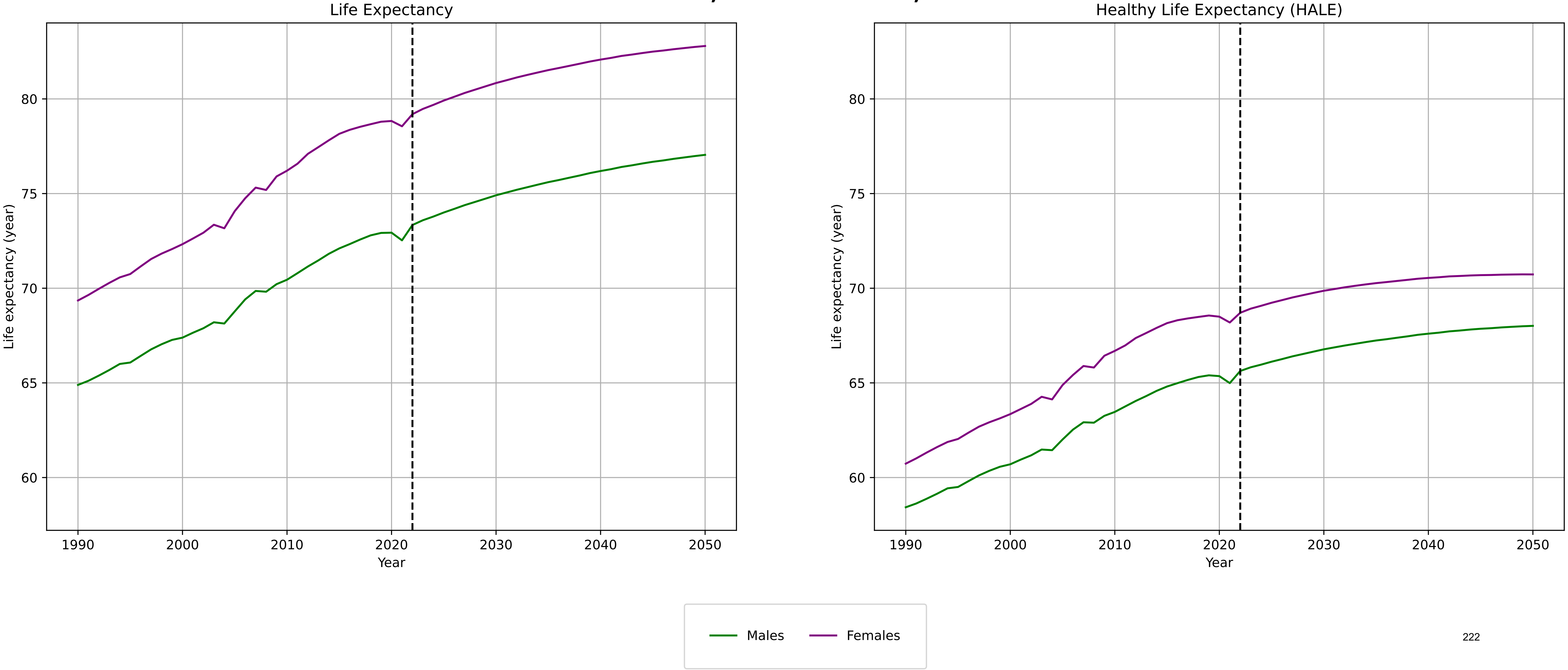

Sub-Saharan Africa

Supplemental Results Figure S3. Life expectancy and HALE by sex and location, 1990-2050

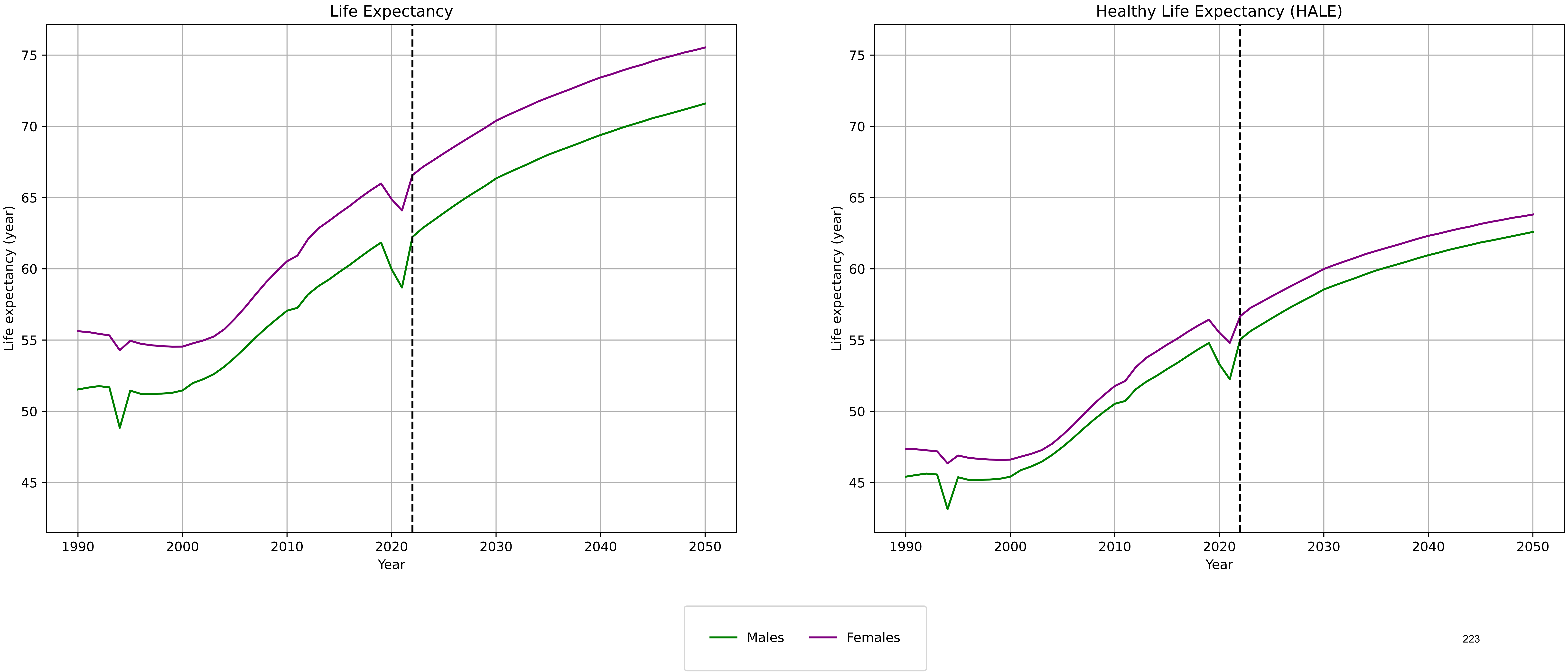

Central Asia

Supplemental Results Figure S3. Life expectancy and HALE by sex and location, 1990-2050

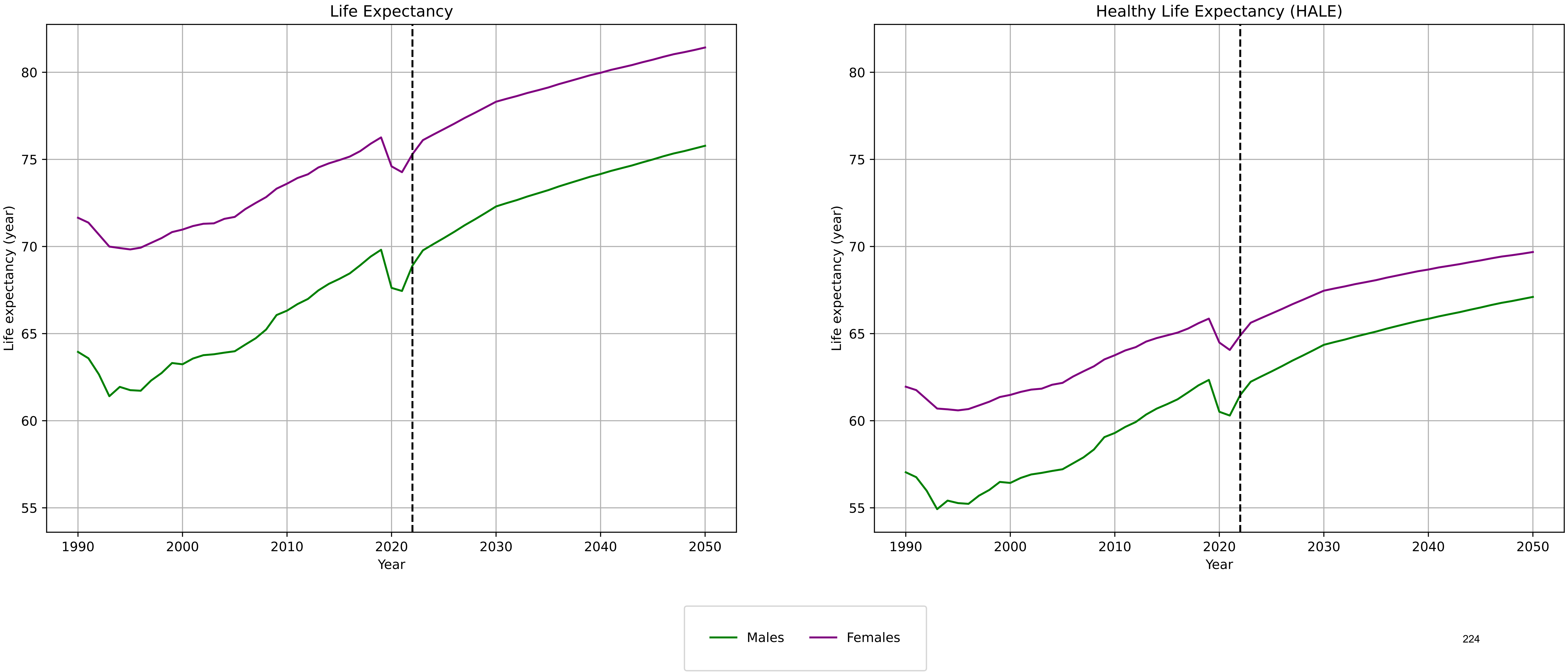

Central Europe

Supplemental Results Figure S3. Life expectancy and HALE by sex and location, 1990-2050

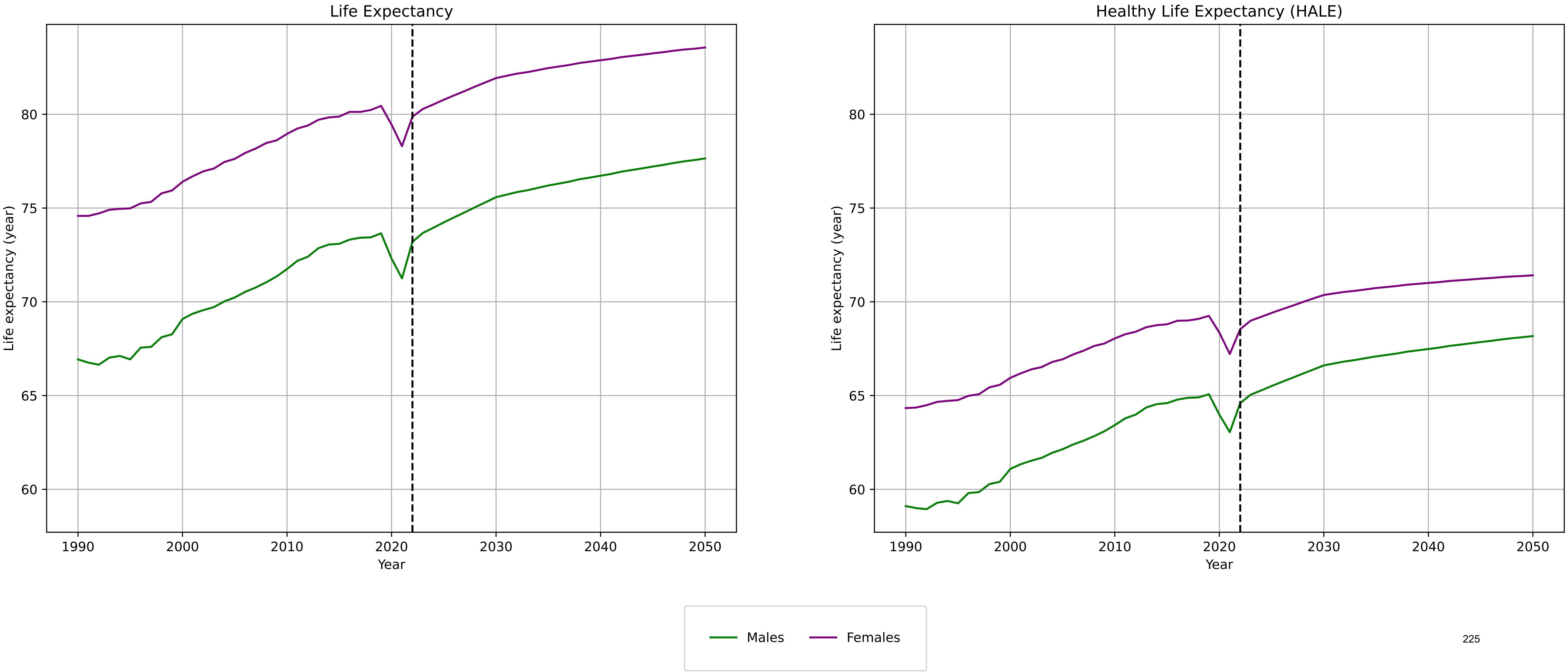

Supplemental Results Figure S3. Life expectancy and HALE by sex and location, 1990-2050

Eastern Europe

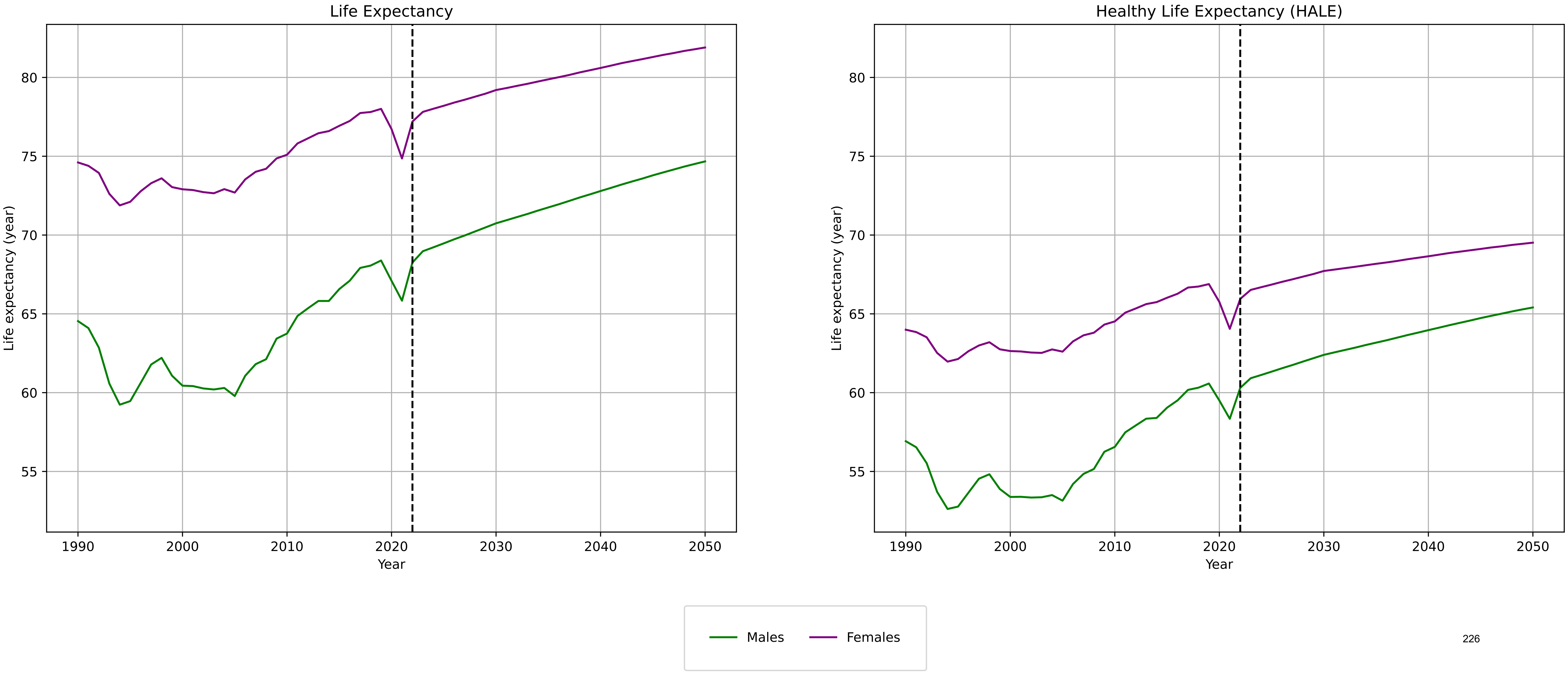

Australasia

Supplemental Results Figure S3. Life expectancy and HALE by sex and location, 1990-2050

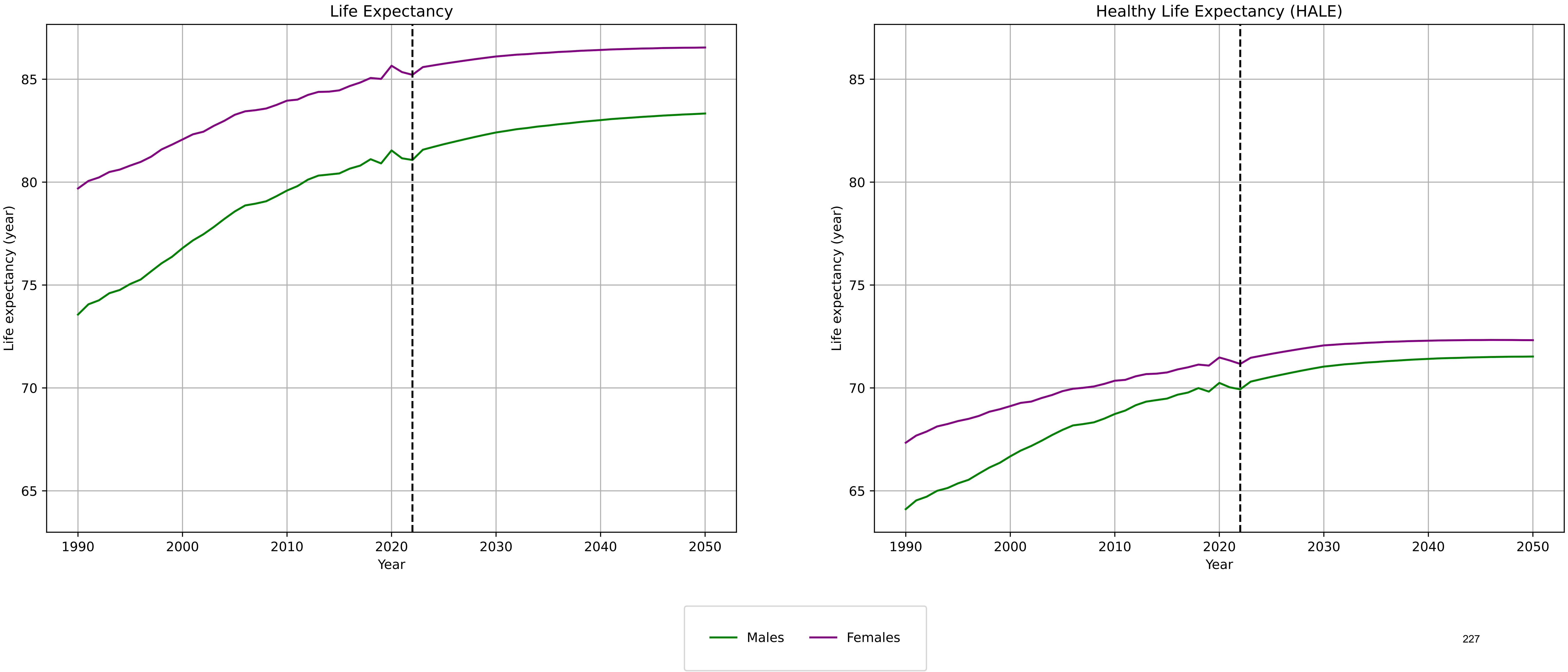

High-Income Asia Pacific

Supplemental Results Figure S3. Life expectancy and HALE by sex and location, 1990-2050

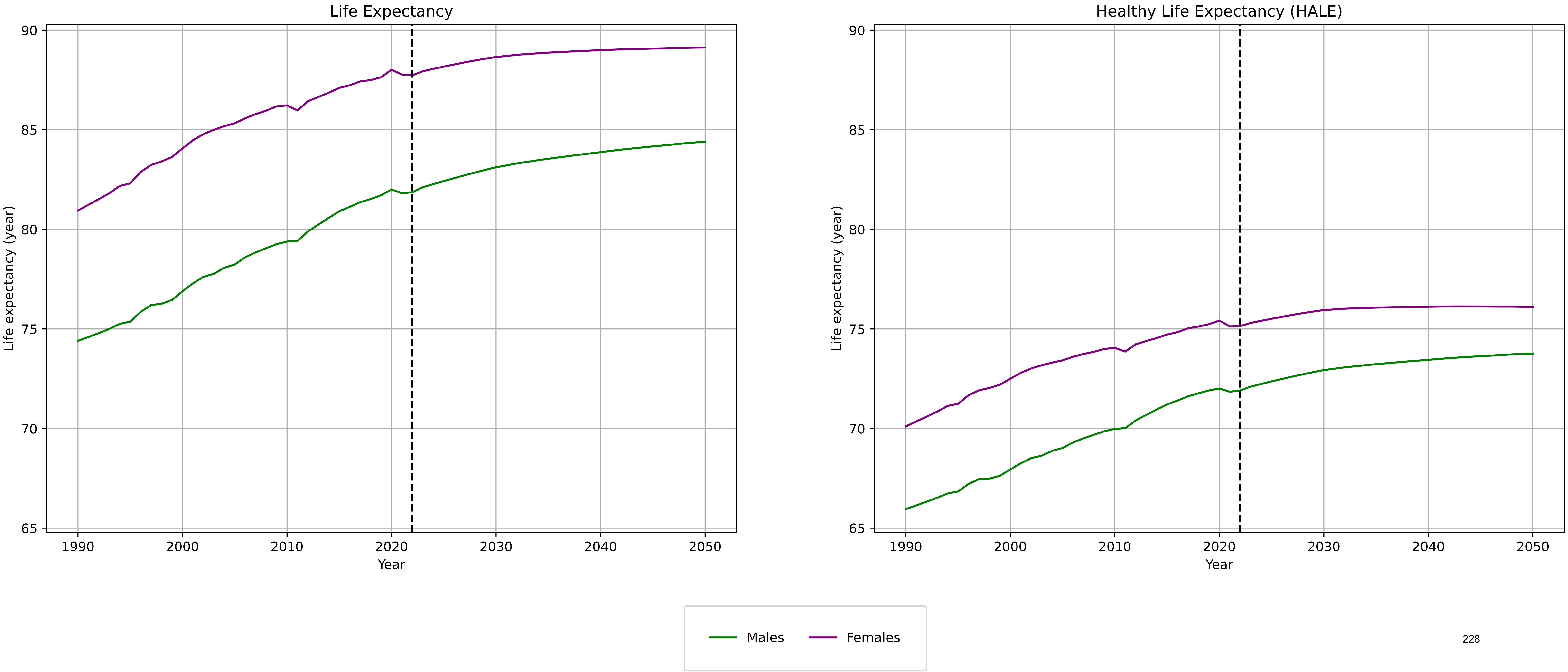

Supplemental Results Figure S3. Life expectancy and HALE by sex and location, 1990-2050

High-Income North America

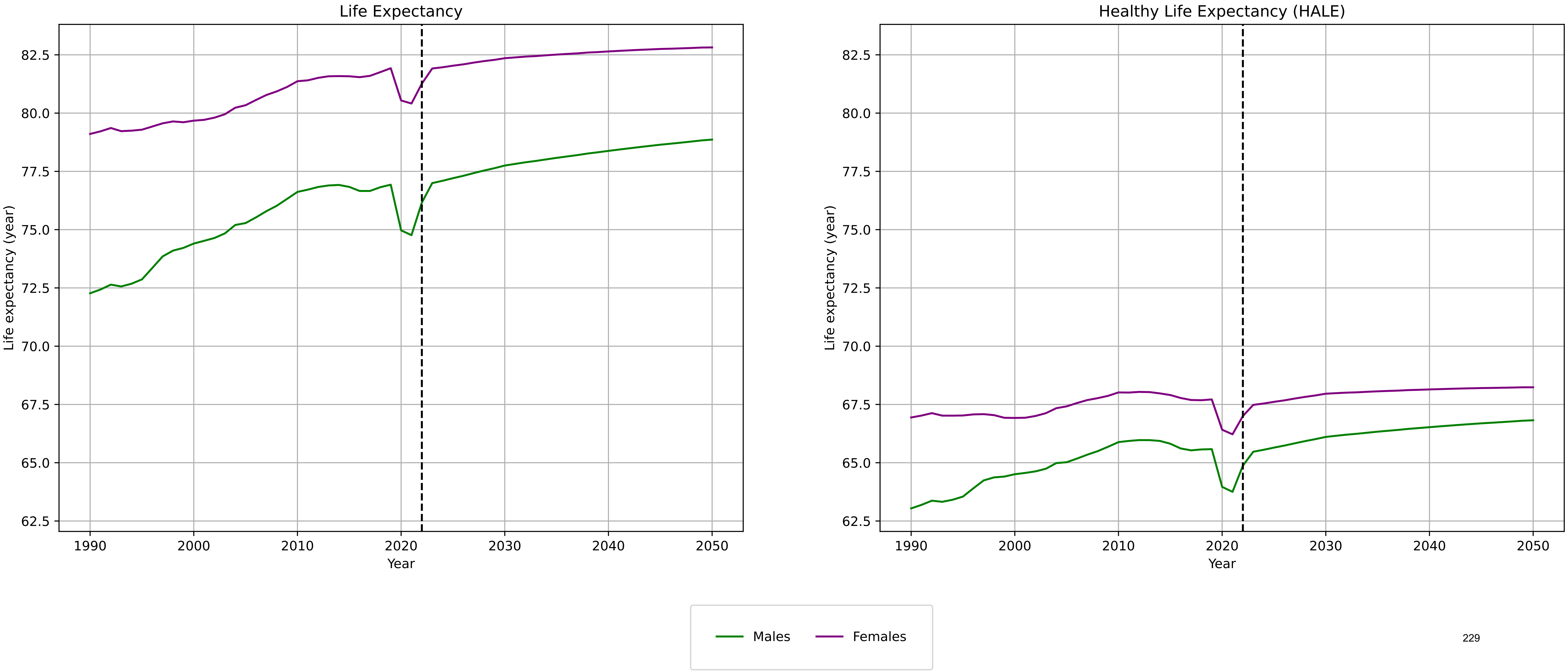

Supplemental Results Figure S3. Life expectancy and HALE by sex and location, 1990-2050

Southern Latin America

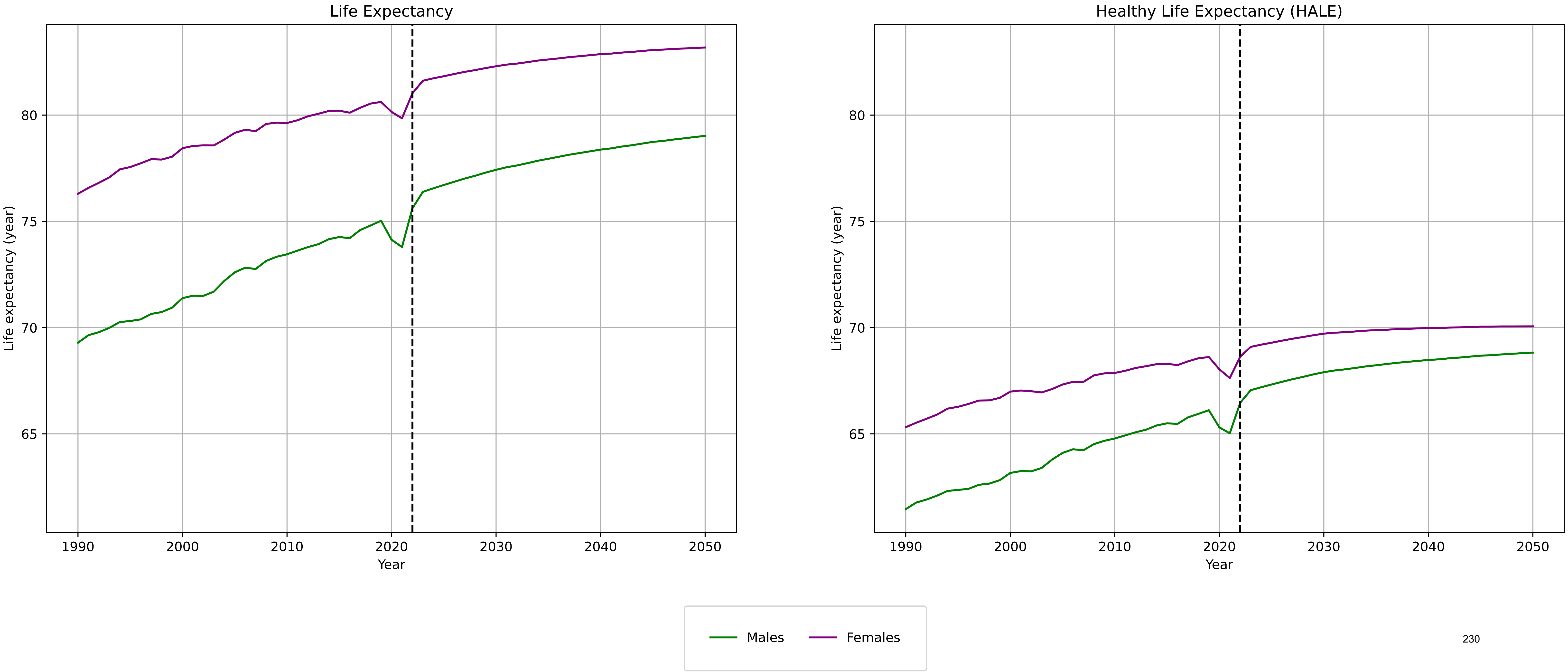

Supplemental Results Figure S3. Life expectancy and HALE by sex and location, 1990-2050

Western Europe

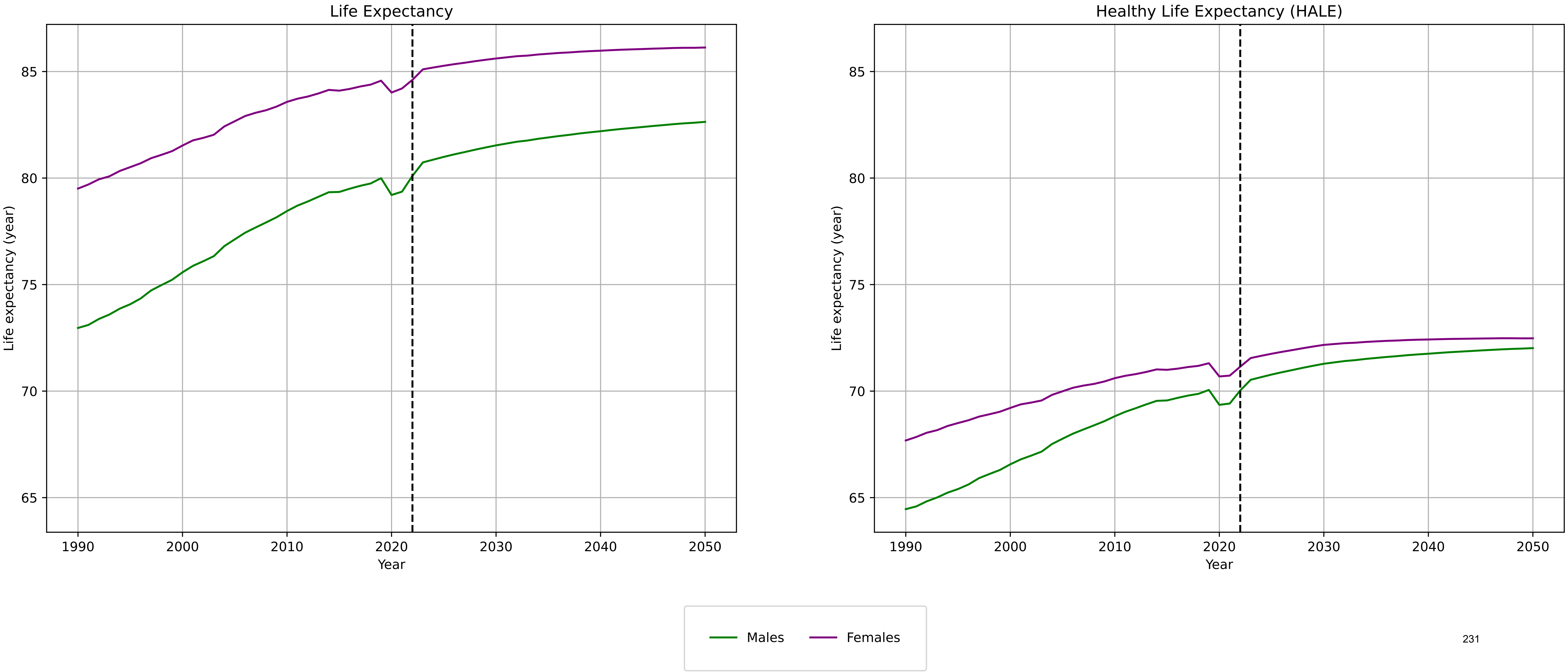

Andean Latin America

Supplemental Results Figure S3. Life expectancy and HALE by sex and location, 1990-2050

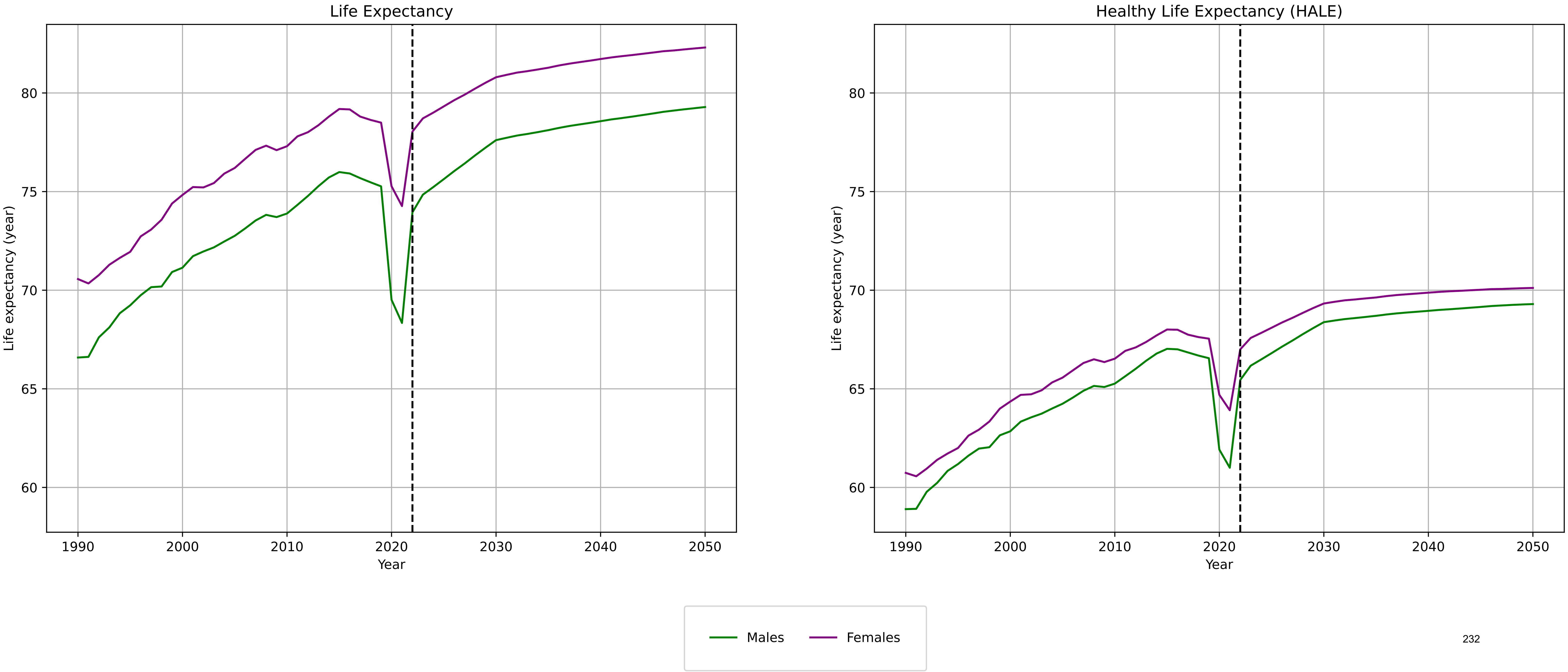

Supplemental Results Figure S3. Life expectancy and HALE by sex and location, 1990-2050

Caribbean

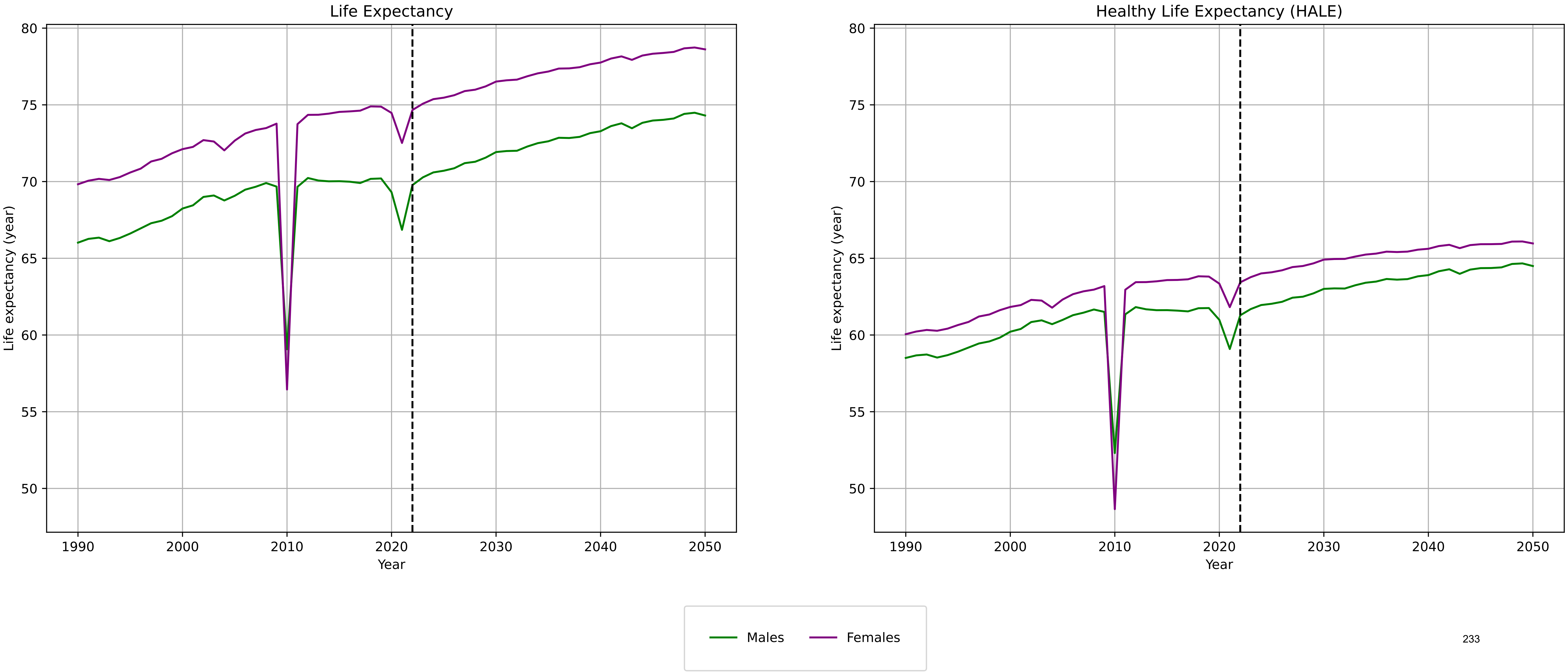

Central Latin America

Supplemental Results Figure S3. Life expectancy and HALE by sex and location, 1990-2050

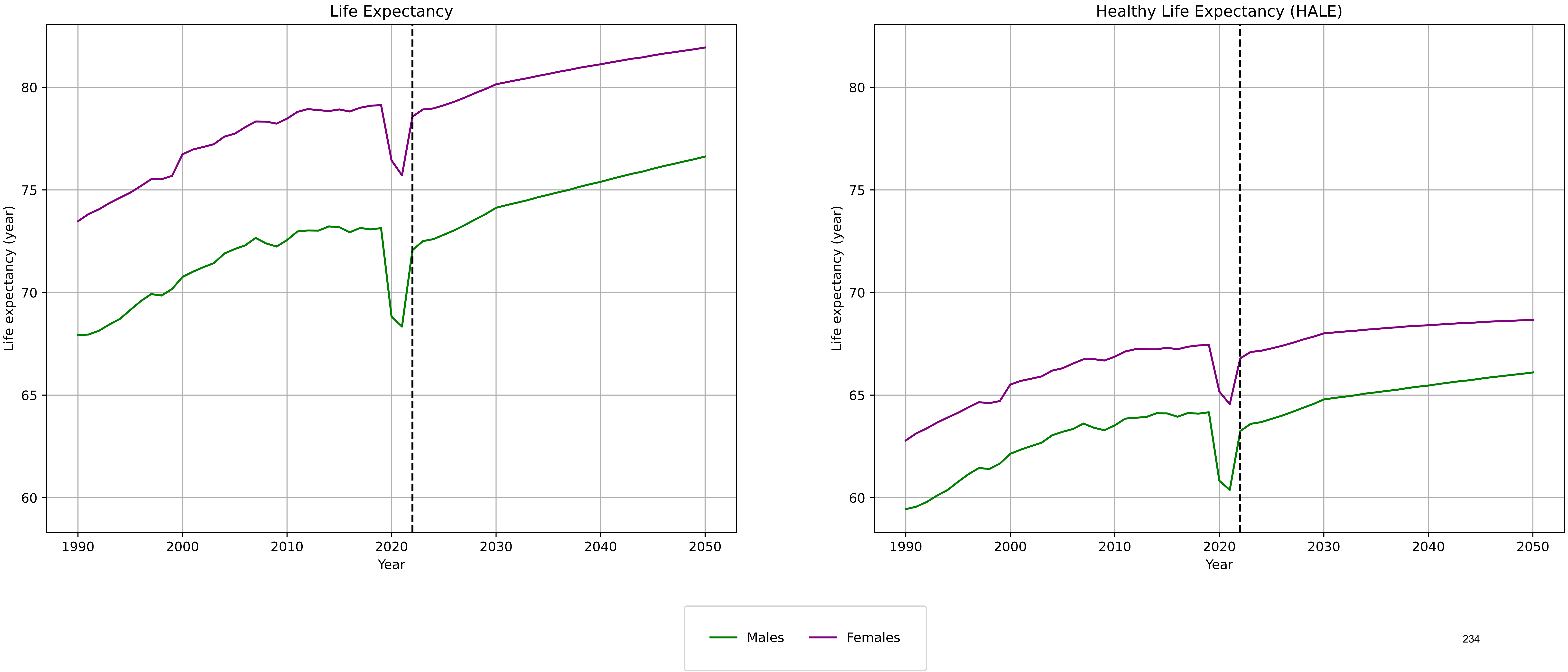

Tropical Latin America

Supplemental Results Figure S3. Life expectancy and HALE by sex and location, 1990-2050

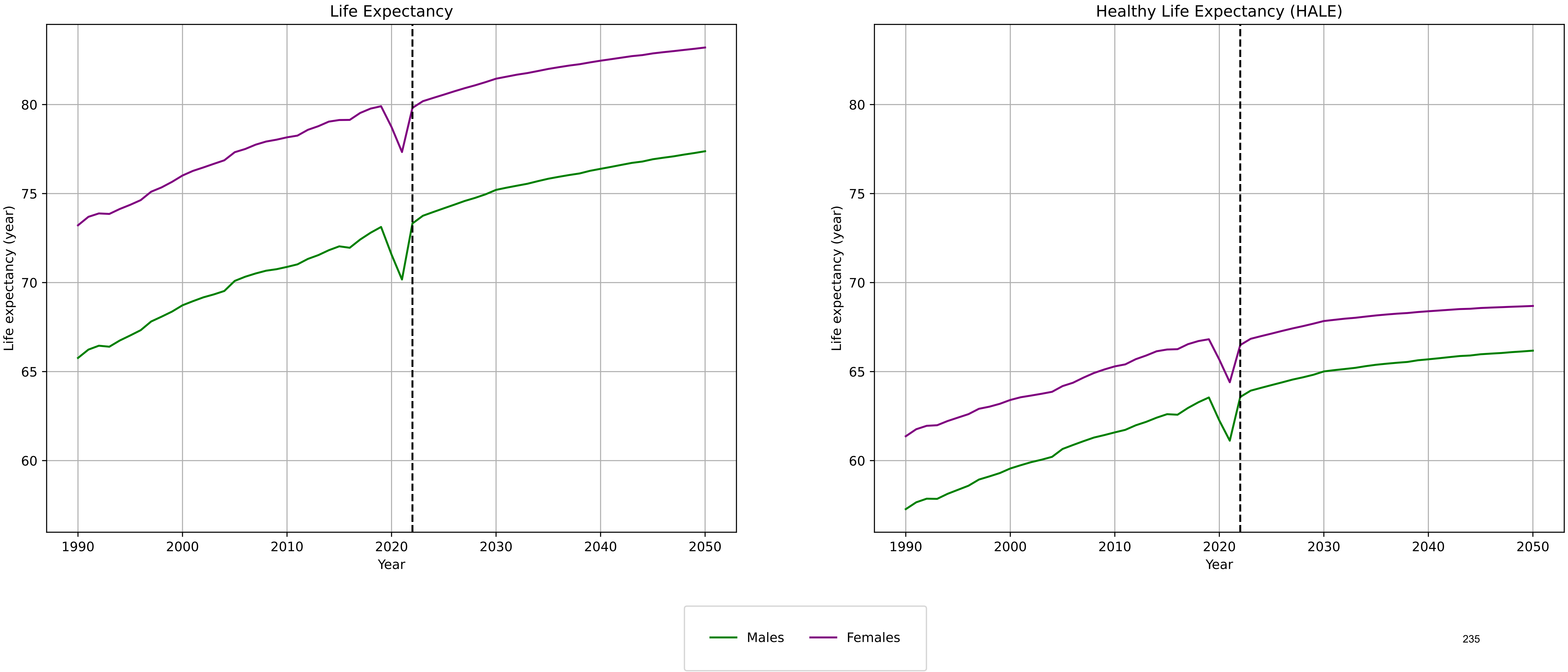

North Africa And Middle East

Supplemental Results Figure S3. Life expectancy and HALE by sex and location, 1990-2050

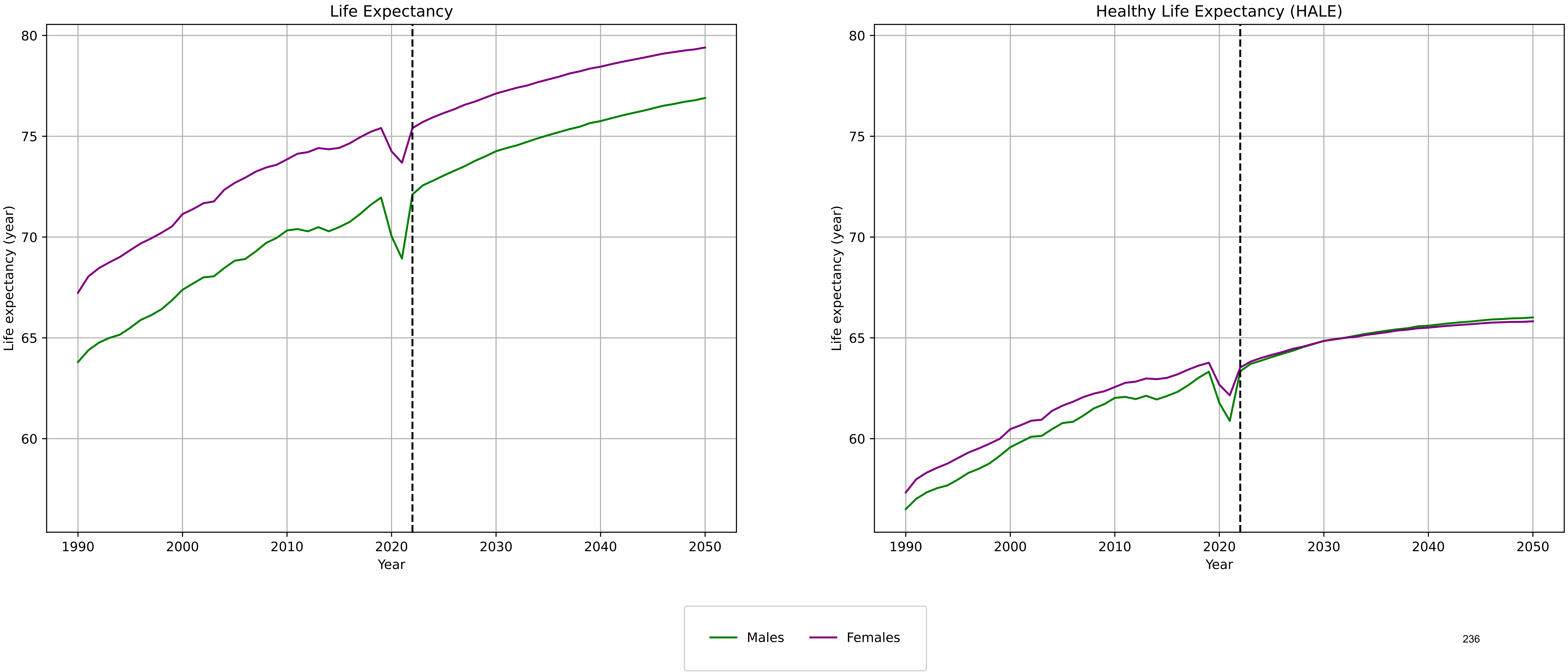

Supplemental Results Figure S3. Life expectancy and HALE by sex and location, 1990-2050

South Asia

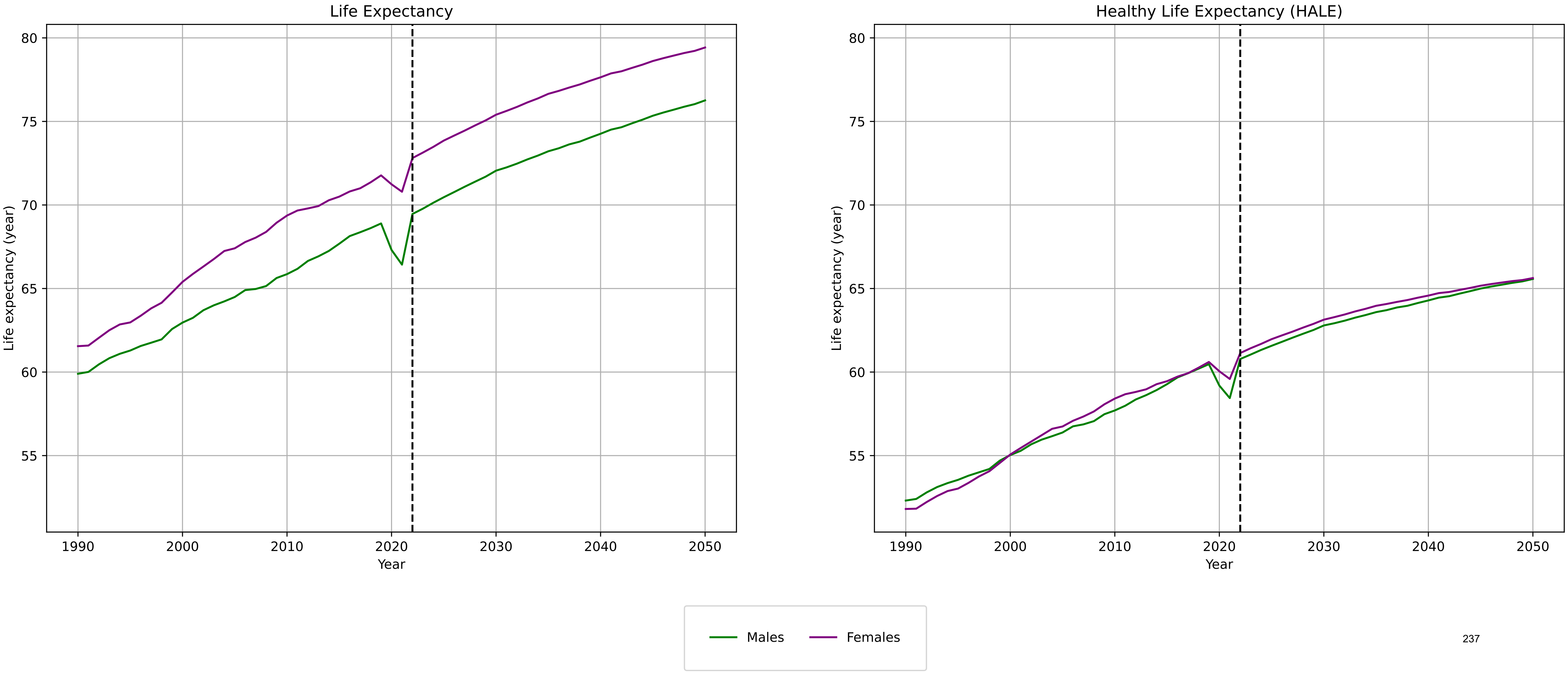

Supplemental Results Figure S3. Life expectancy and HALE by sex and location, 1990-2050

East Asia

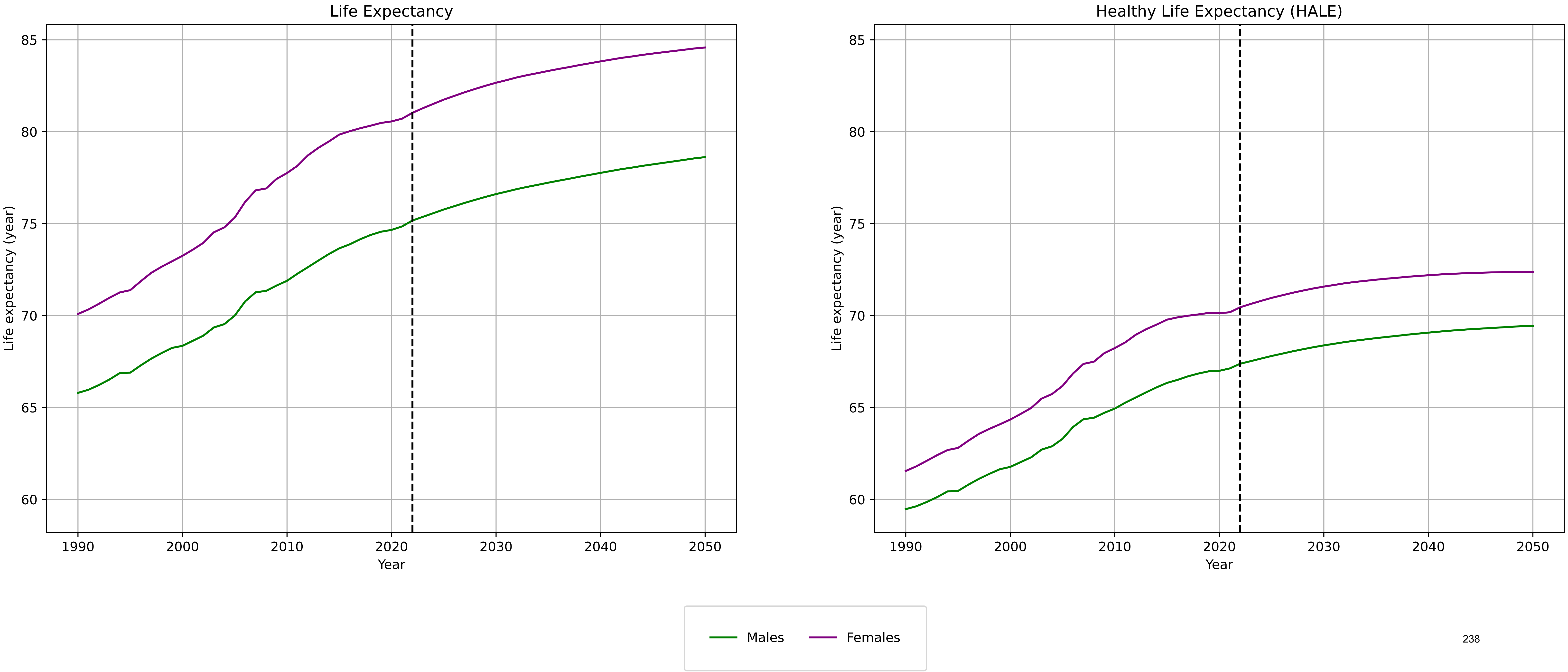

Oceania

Supplemental Results Figure S3. Life expectancy and HALE by sex and location, 1990-2050

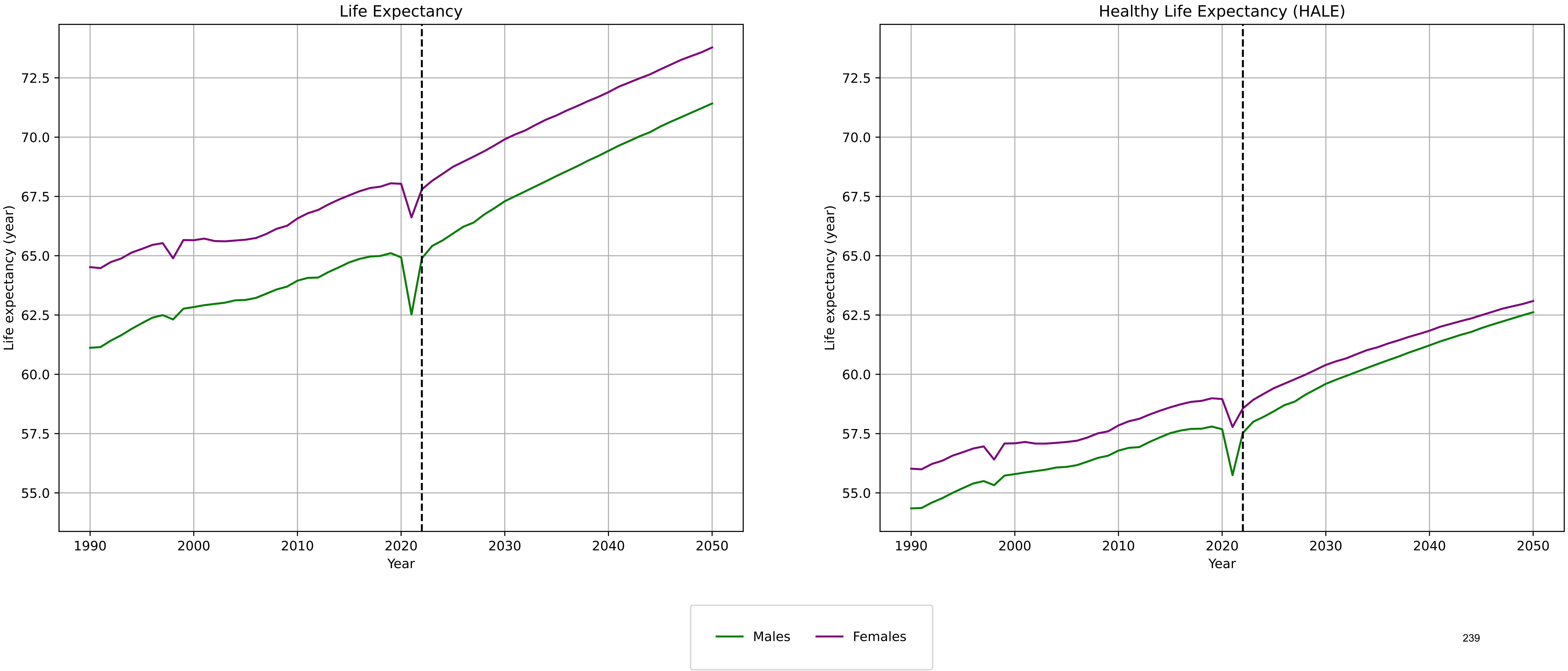

Southeast Asia

Supplemental Results Figure S3. Life expectancy and HALE by sex and location, 1990-2050

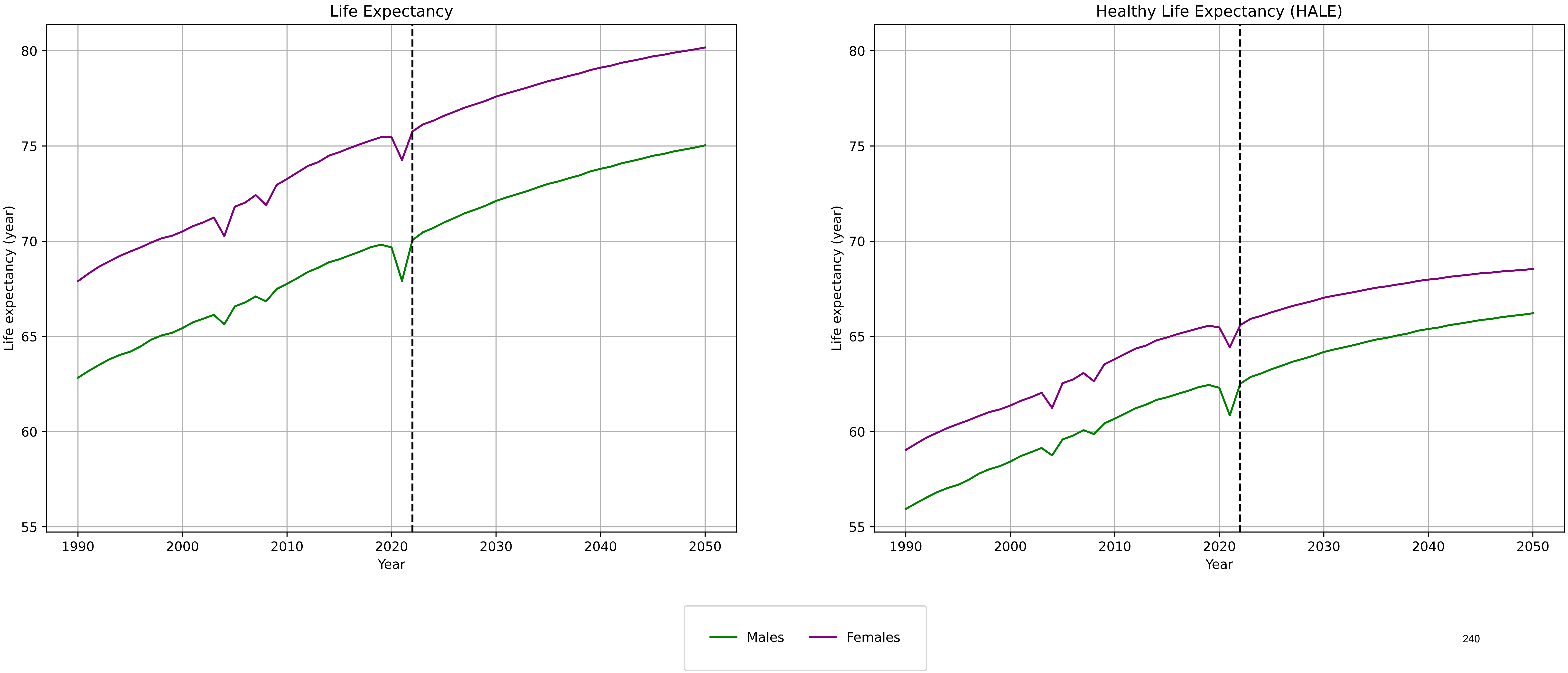

Supplemental Results Figure S3. Life expectancy and HALE by sex and location, 1990-2050

Central Sub-Saharan Africa

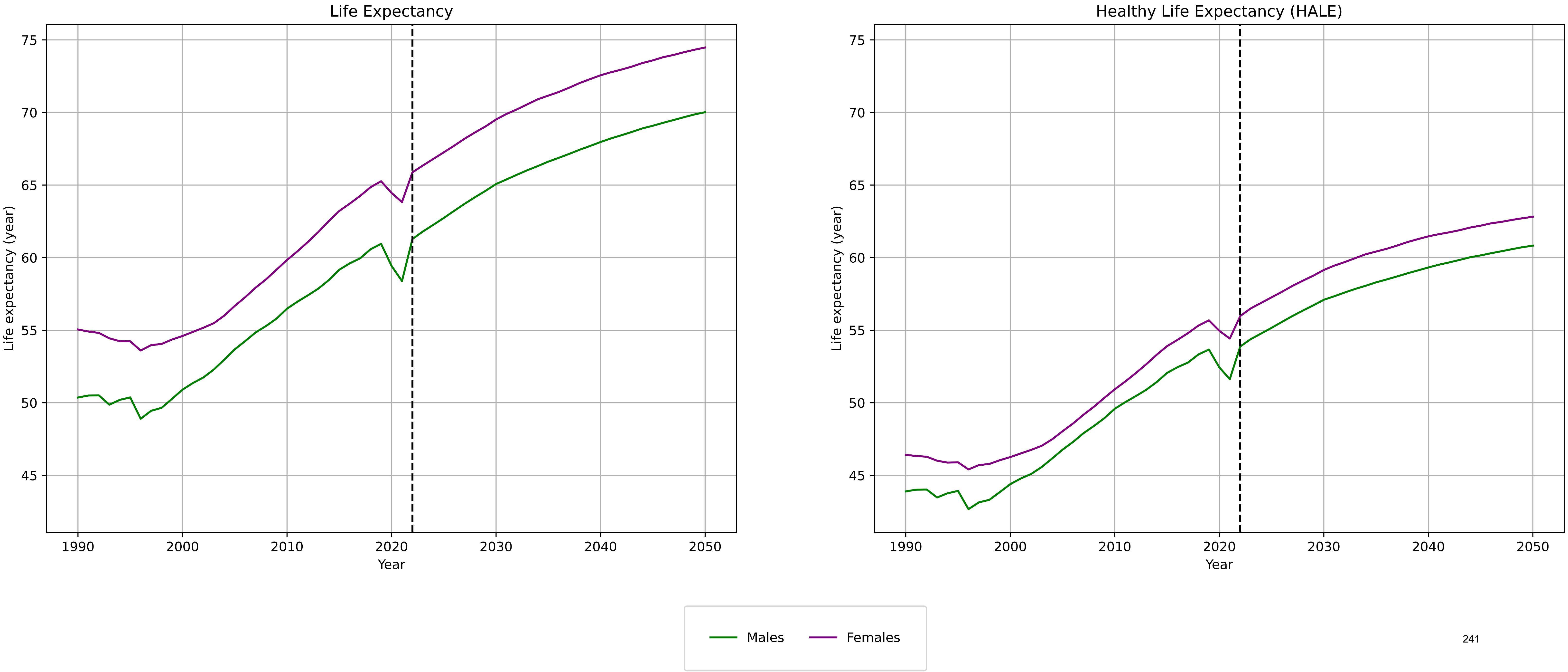

Supplemental Results Figure S3. Life expectancy and HALE by sex and location, 1990-2050

Eastern Sub-Saharan Africa

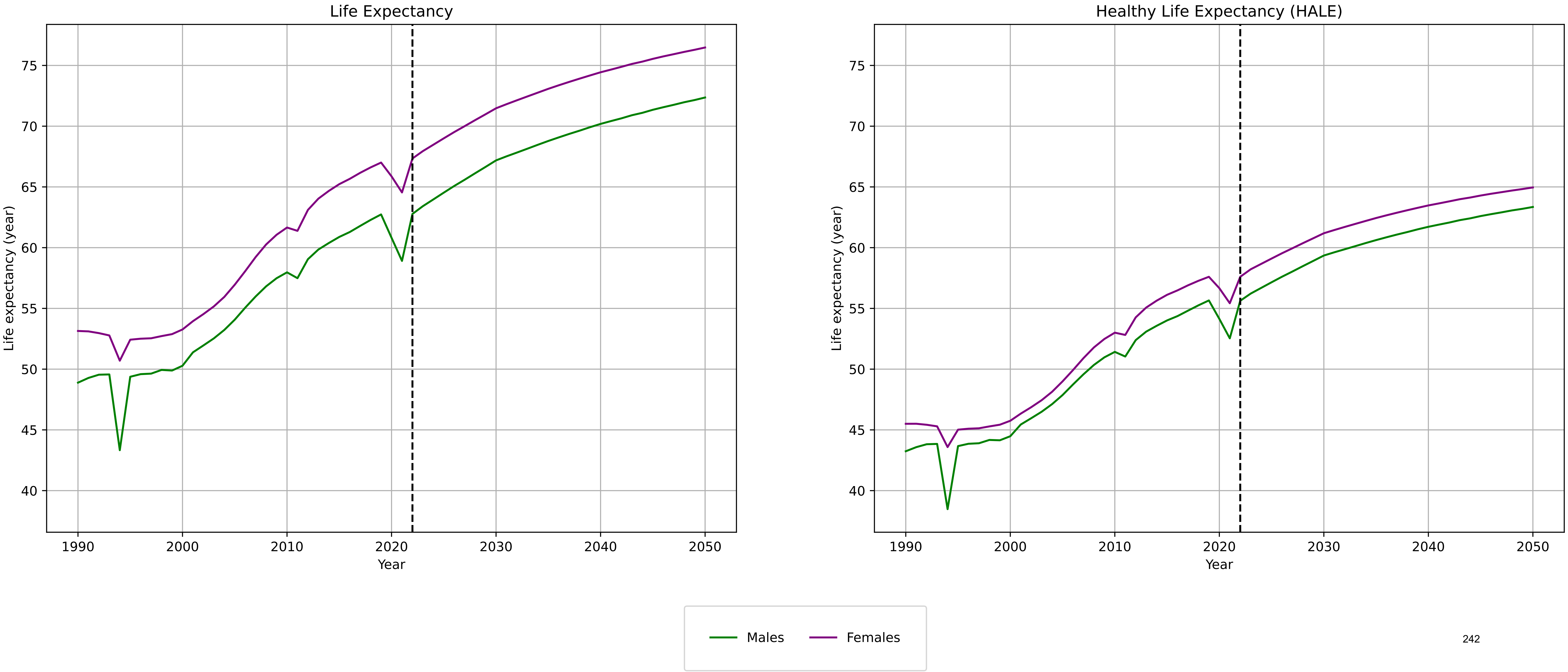

Supplemental Results Figure S3. Life expectancy and HALE by sex and location, 1990-2050

Southern Sub-Saharan Africa

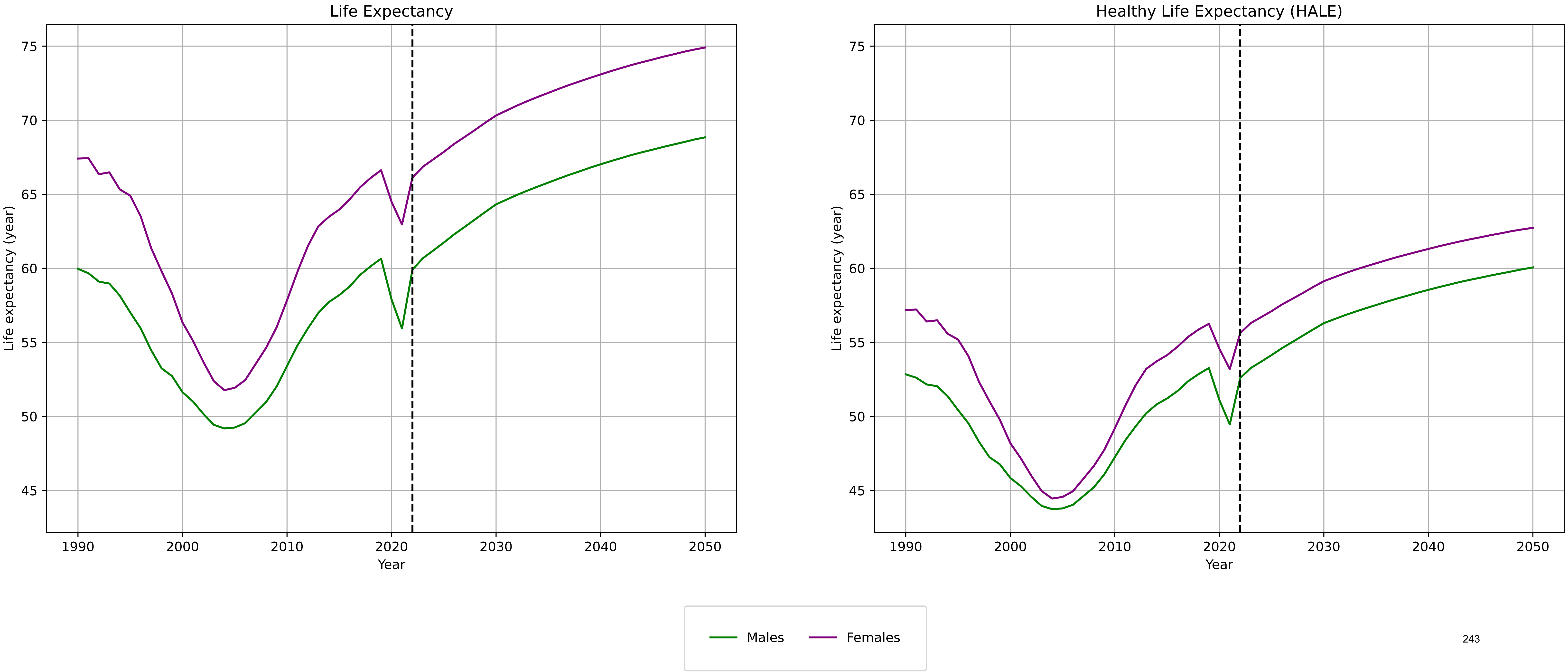

Supplemental Results Figure S3. Life expectancy and HALE by sex and location, 1990-2050

Western Sub-Saharan Africa

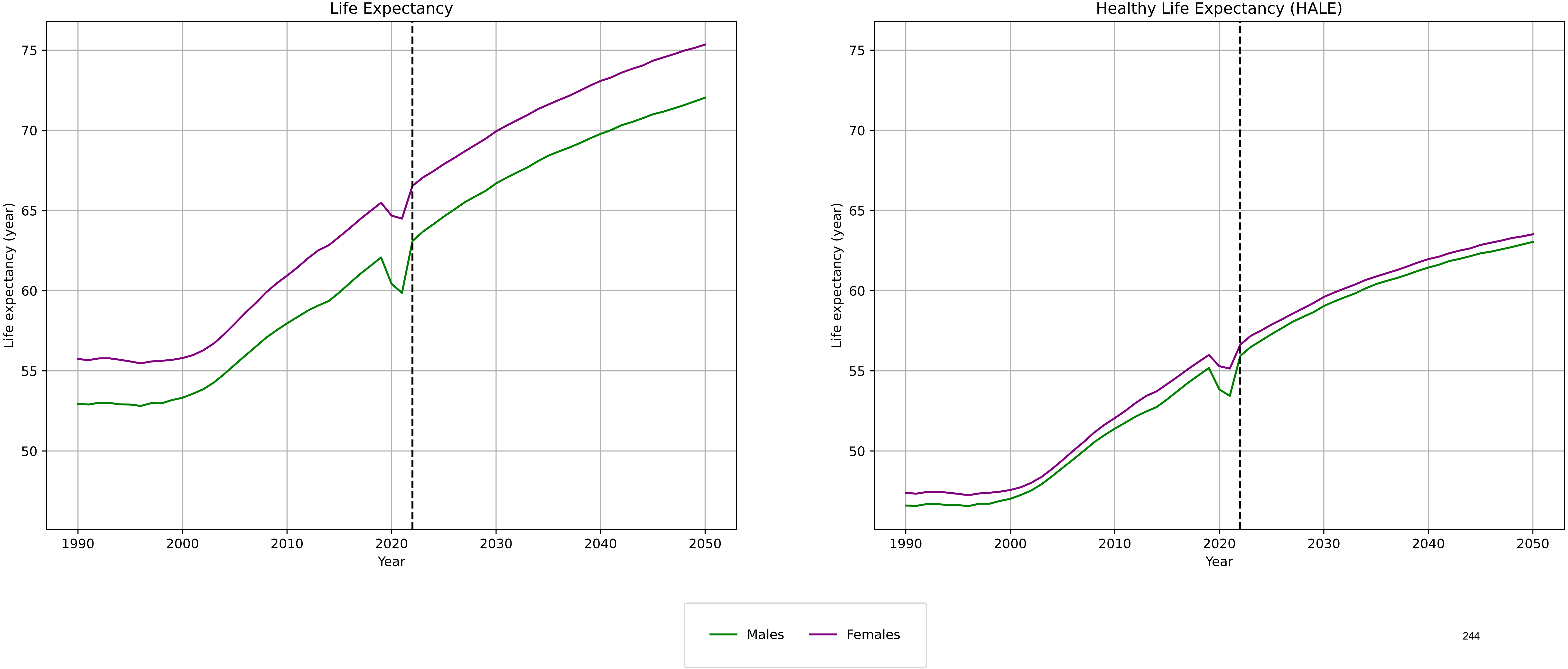

Armenia

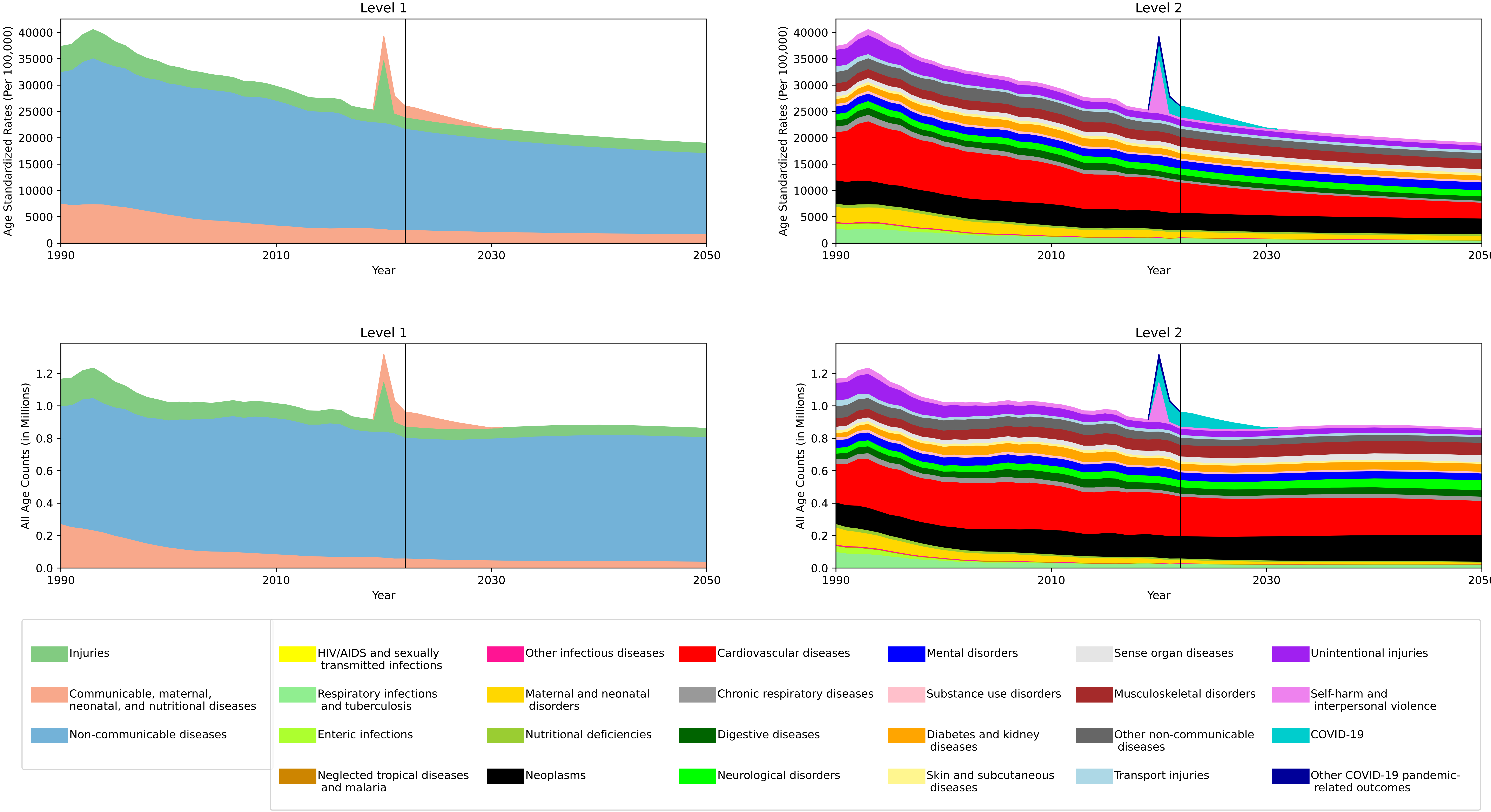
Level 1
Level 2
Age Standardized Rates (Per 100,000)
All Age Counts (in Millions)
Year
1990
2010
2030
2050
Injuries
Communicable, maternal, neonatal, and nutritional diseases
Non-communicable diseases
HIV/AIDS and sexually transmitted infections
Respiratory infections and tuberculosis
Enteric infections
Neglected tropical diseases and malaria
Other infectious diseases
Maternal and neonatal disorders
Nutritional deficiencies
Neoplasms
Cardiovascular diseases
Chronic respiratory diseases
Digestive diseases
Neurological disorders
Mental disorders
Substance use disorders
Diabetes and kidney diseases
Skin and subcutaneous diseases
Sense organ diseases
Musculoskeletal disorders
Other non-communicable diseases
Transport injuries
Unintentional injuries
Self-harm and interpersonal violence
COVID-19
Other COVID-19 pandemic-related outcomes

Azerbaijan

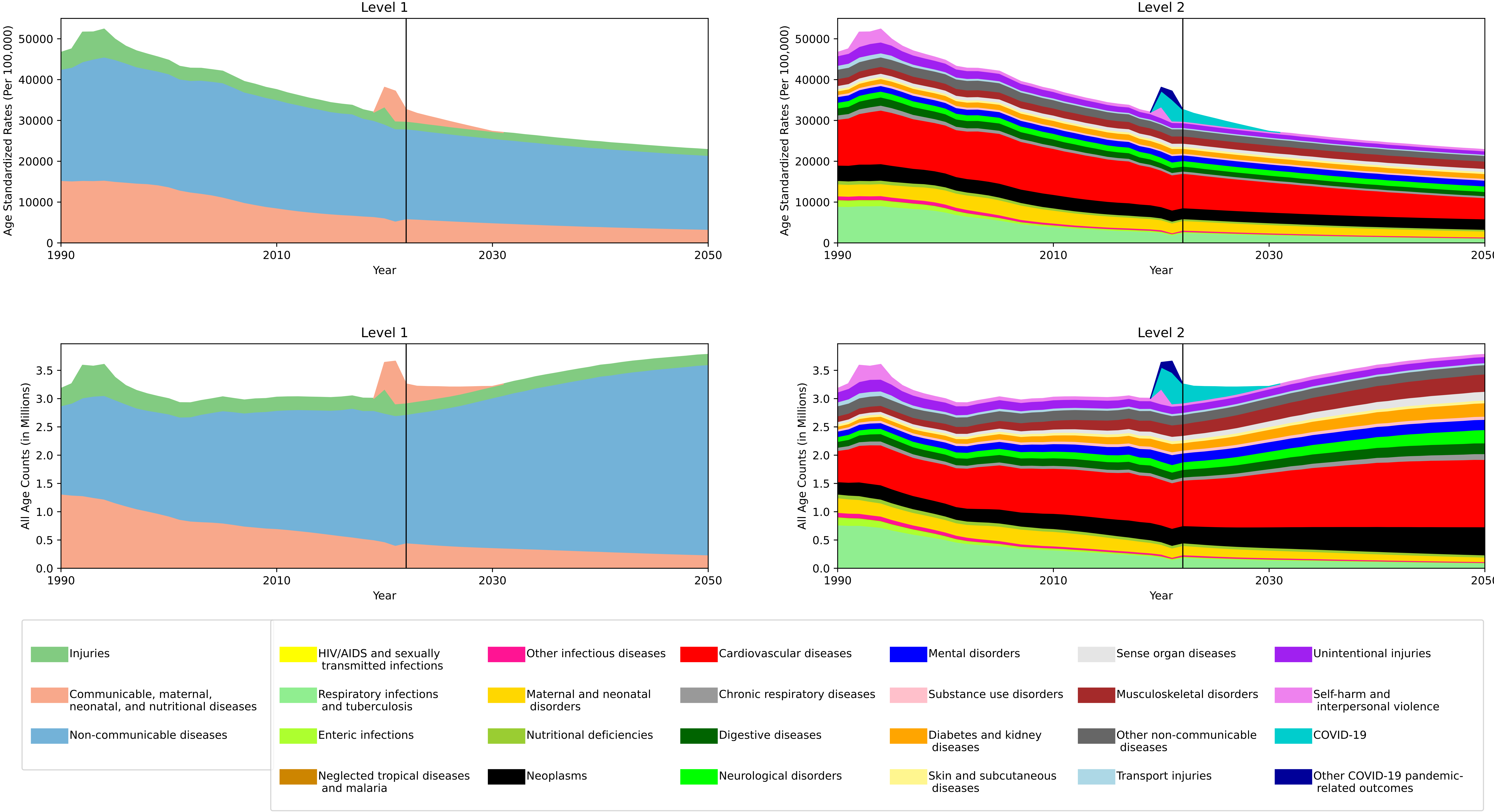
Level 1
Level 2
Age Standardized Rates (Per 100,000)
All Age Counts (in Millions)
Year
Injuries
Communicable, maternal, neonatal, and nutritional diseases
Non-communicable diseases
HIV/AIDS and sexually transmitted infections
Respiratory infections and tuberculosis
Enteric infections
Neglected tropical diseases and malaria
Other infectious diseases
Maternal and neonatal disorders
Nutritional deficiencies
Neoplasms
Cardiovascular diseases
Chronic respiratory diseases
Digestive diseases
Neurological disorders
Mental disorders
Substance use disorders
Diabetes and kidney diseases
Skin and subcutaneous diseases
Sense organ diseases
Musculoskeletal disorders
Other non-communicable diseases
Transport injuries
Unintentional injuries
Self-harm and interpersonal violence
COVID-19
Other COVID-19 pandemic-related outcomes

Georgia

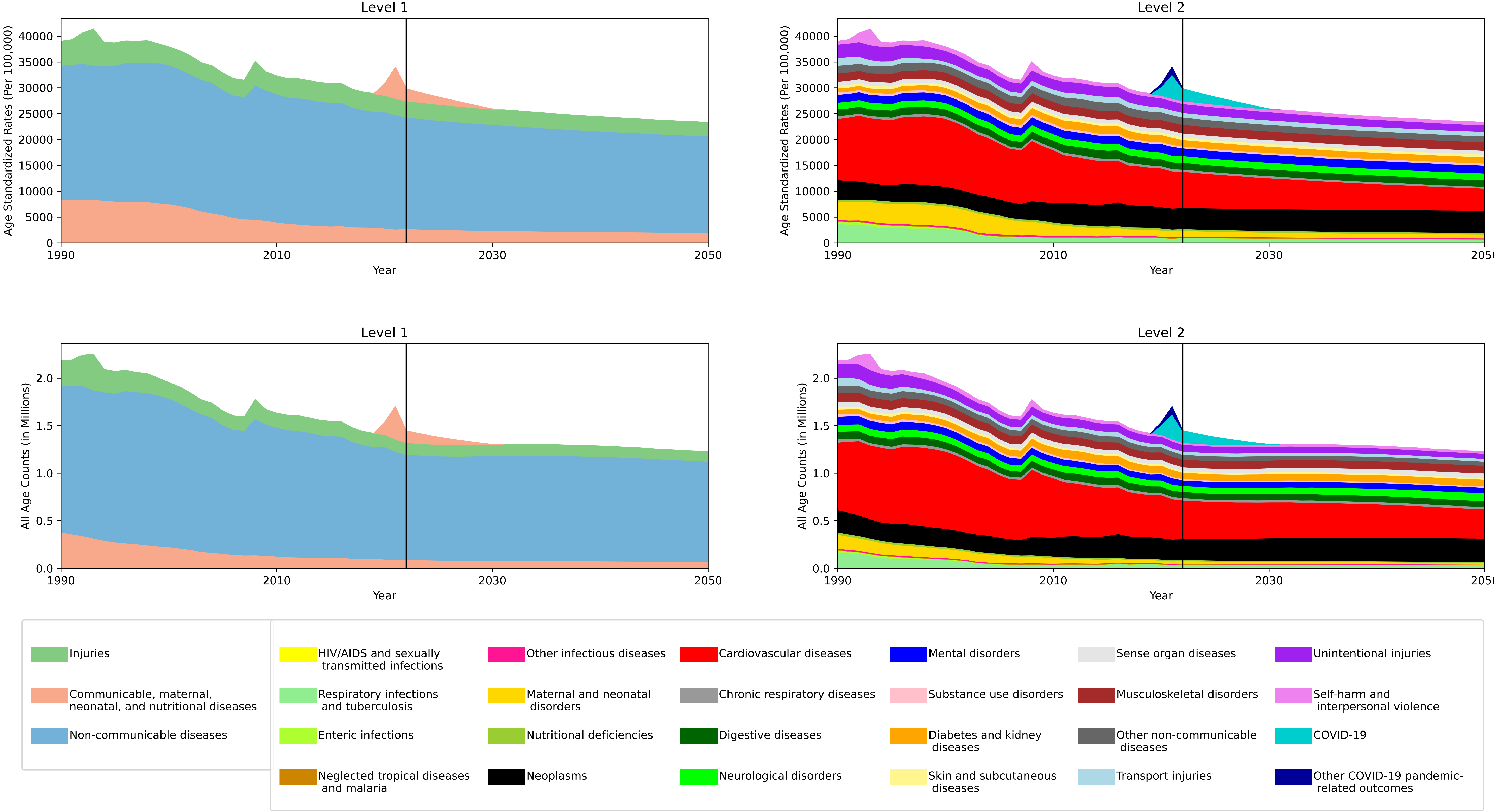
Level 1
Level 2
Age Standardized Rates (Per 100,000)
All Age Counts (in Millions)
Year
1990
2010
2030
2050
Injuries
Communicable, maternal, neonatal, and nutritional diseases
Non-communicable diseases
HIV/AIDS and sexually transmitted infections
Respiratory infections and tuberculosis
Enteric infections
Neglected tropical diseases and malaria
Other infectious diseases
Maternal and neonatal disorders
Nutritional deficiencies
Neoplasms
Cardiovascular diseases
Chronic respiratory diseases
Digestive diseases
Neurological disorders
Mental disorders
Substance use disorders
Diabetes and kidney diseases
Skin and subcutaneous diseases
Sense organ diseases
Musculoskeletal disorders
Other non-communicable diseases
Transport injuries
Unintentional injuries
Self-harm and interpersonal violence
COVID-19
Other COVID-19 pandemic-related outcomes

Kazakhstan

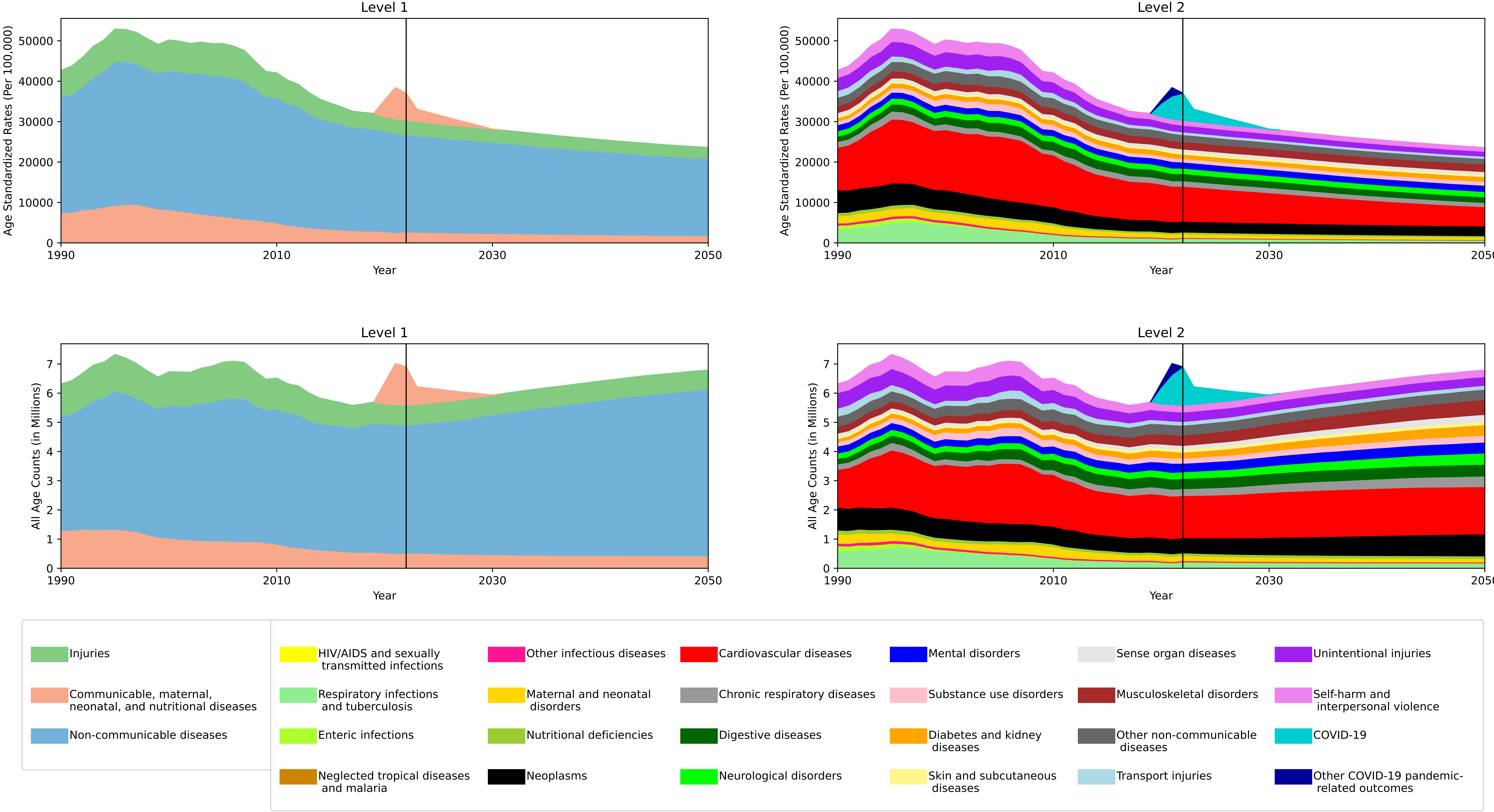
Level 1
Level 2
Age Standardized Rates (Per 100,000)
All Age Counts (in Millions)
Year
0
10000
20000
30000
40000
50000
1
2
3
4
5
6
7
1990
2010
2030
2050
Injuries
Communicable, maternal, neonatal, and nutritional diseases
Non-communicable diseases
HIV/AIDS and sexually transmitted infections
Respiratory infections and tuberculosis
Enteric infections
Neglected tropical diseases and malaria
Other infectious diseases
Maternal and neonatal disorders
Nutritional deficiencies
Neoplasms
Cardiovascular diseases
Chronic respiratory diseases
Digestive diseases
Neurological disorders
Mental disorders
Substance use disorders
Diabetes and kidney diseases
Skin and subcutaneous diseases
Sense organ diseases
Musculoskeletal disorders
Other non-communicable diseases
Transport injuries
Unintentional injuries
Self-harm and interpersonal violence
COVID-19
Other COVID-19 pandemic-related outcomes

Kyrgyzstan

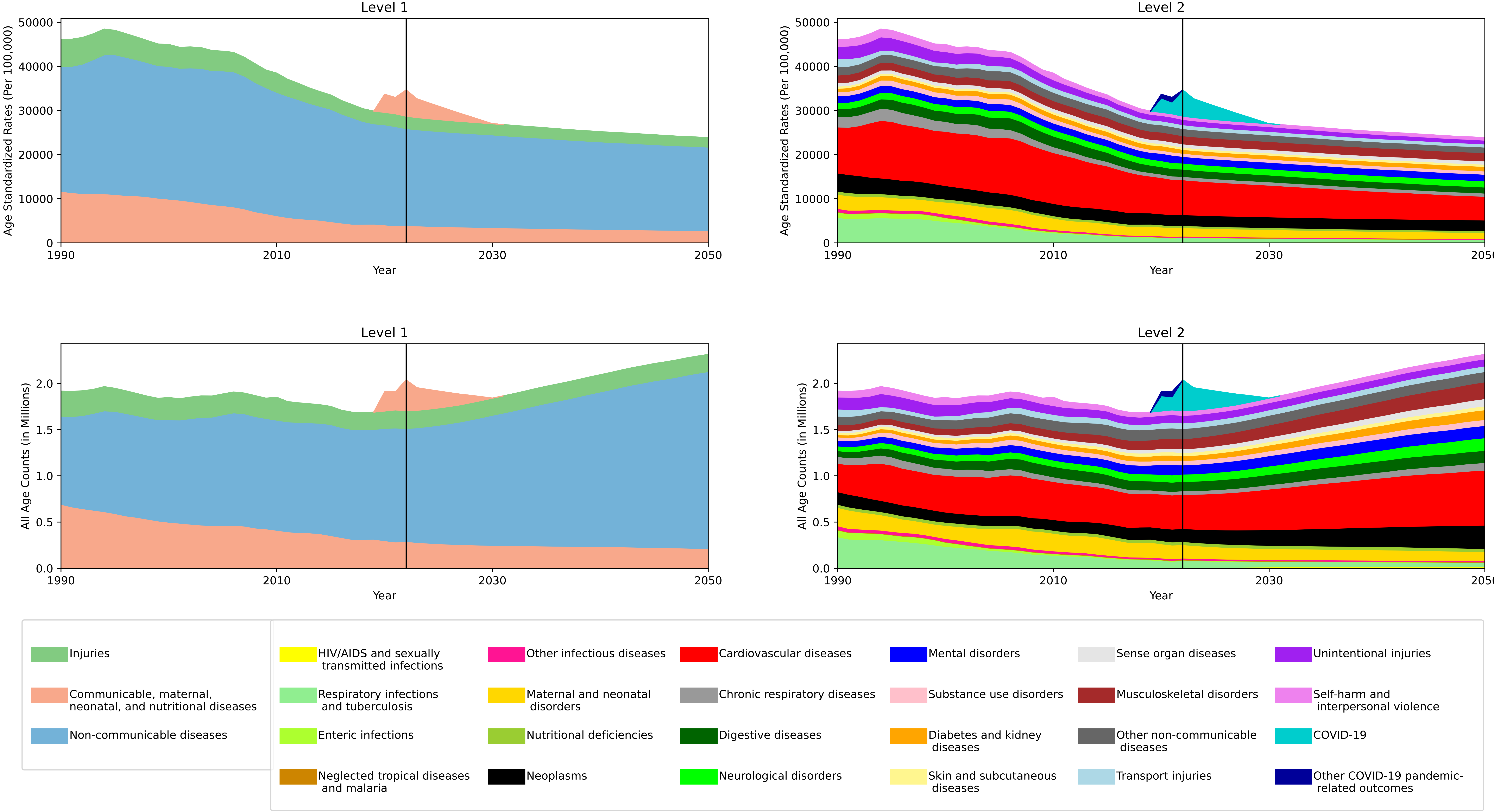
Level 1
Level 2
Age Standardized Rates (Per 100,000)
All Age Counts (in Millions)
Year
0
10000
20000
30000
40000
50000
0.0
0.5
1.0
1.5
2.0
1990
2010
2030
2050
Injuries
Communicable, maternal, neonatal, and nutritional diseases
Non-communicable diseases
HIV/AIDS and sexually transmitted infections
Respiratory infections and tuberculosis
Enteric infections
Neglected tropical diseases and malaria
Other infectious diseases
Maternal and neonatal disorders
Nutritional deficiencies
Neoplasms
Cardiovascular diseases
Chronic respiratory diseases
Digestive diseases
Neurological disorders
Mental disorders
Substance use disorders
Diabetes and kidney diseases
Skin and subcutaneous diseases
Sense organ diseases
Musculoskeletal disorders
Other non-communicable diseases
Transport injuries
Unintentional injuries
Self-harm and interpersonal violence
COVID-19
Other COVID-19 pandemic-related outcomes

Mongolia

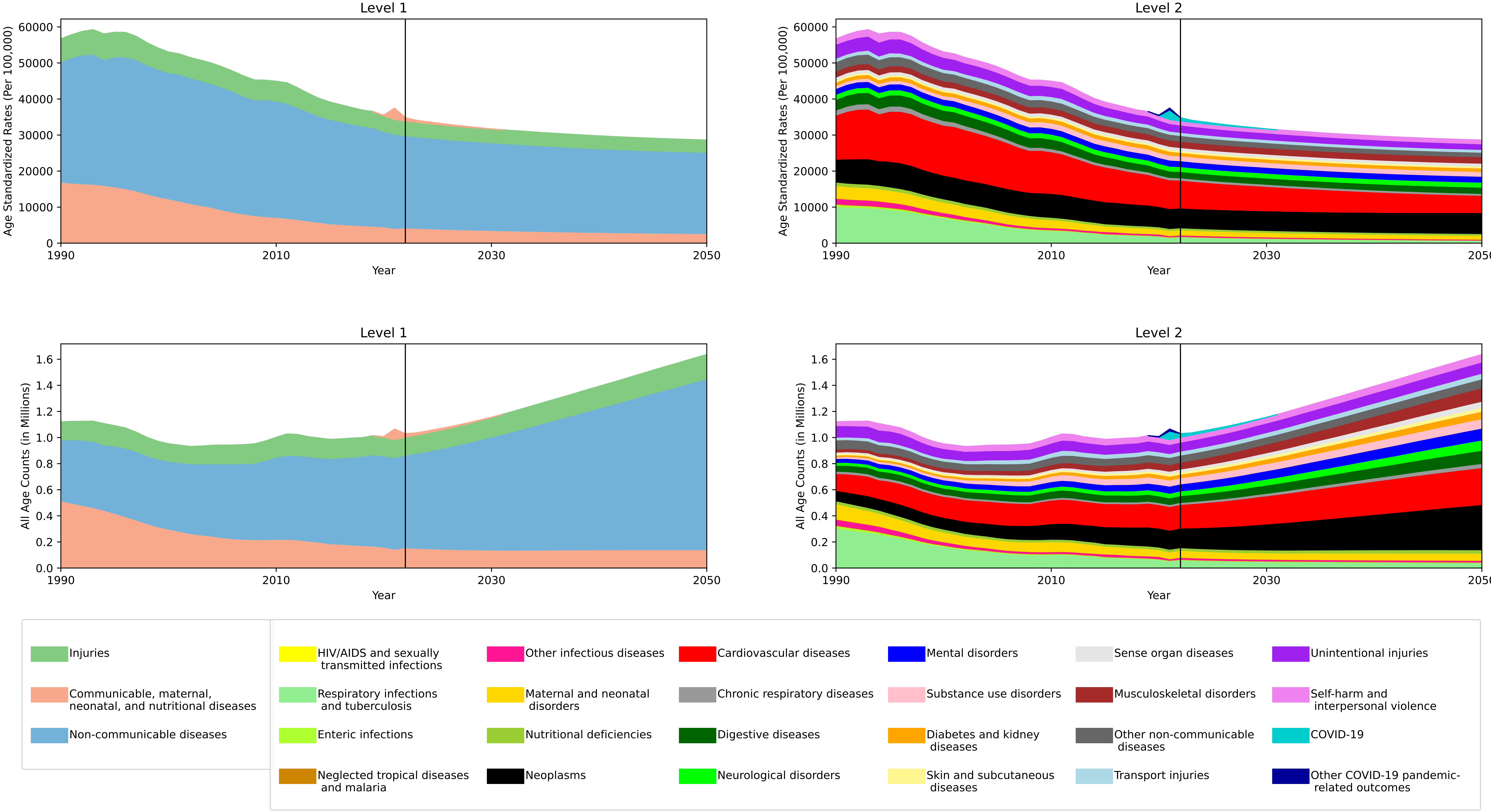
Level 1
Level 2
Age Standardized Rates (Per 100,000)
All Age Counts (in Millions)
Year
1990
2010
2030
2050
Injuries
Communicable, maternal, neonatal, and nutritional diseases
Non-communicable diseases
HIV/AIDS and sexually transmitted infections
Respiratory infections and tuberculosis
Enteric infections
Neglected tropical diseases and malaria
Other infectious diseases
Maternal and neonatal disorders
Nutritional deficiencies
Neoplasms
Cardiovascular diseases
Chronic respiratory diseases
Digestive diseases
Neurological disorders
Mental disorders
Substance use disorders
Diabetes and kidney diseases
Skin and subcutaneous diseases
Sense organ diseases
Musculoskeletal disorders
Other non-communicable diseases
Transport injuries
Unintentional injuries
Self-harm and interpersonal violence
COVID-19
Other COVID-19 pandemic-related outcomes

Tajikistan

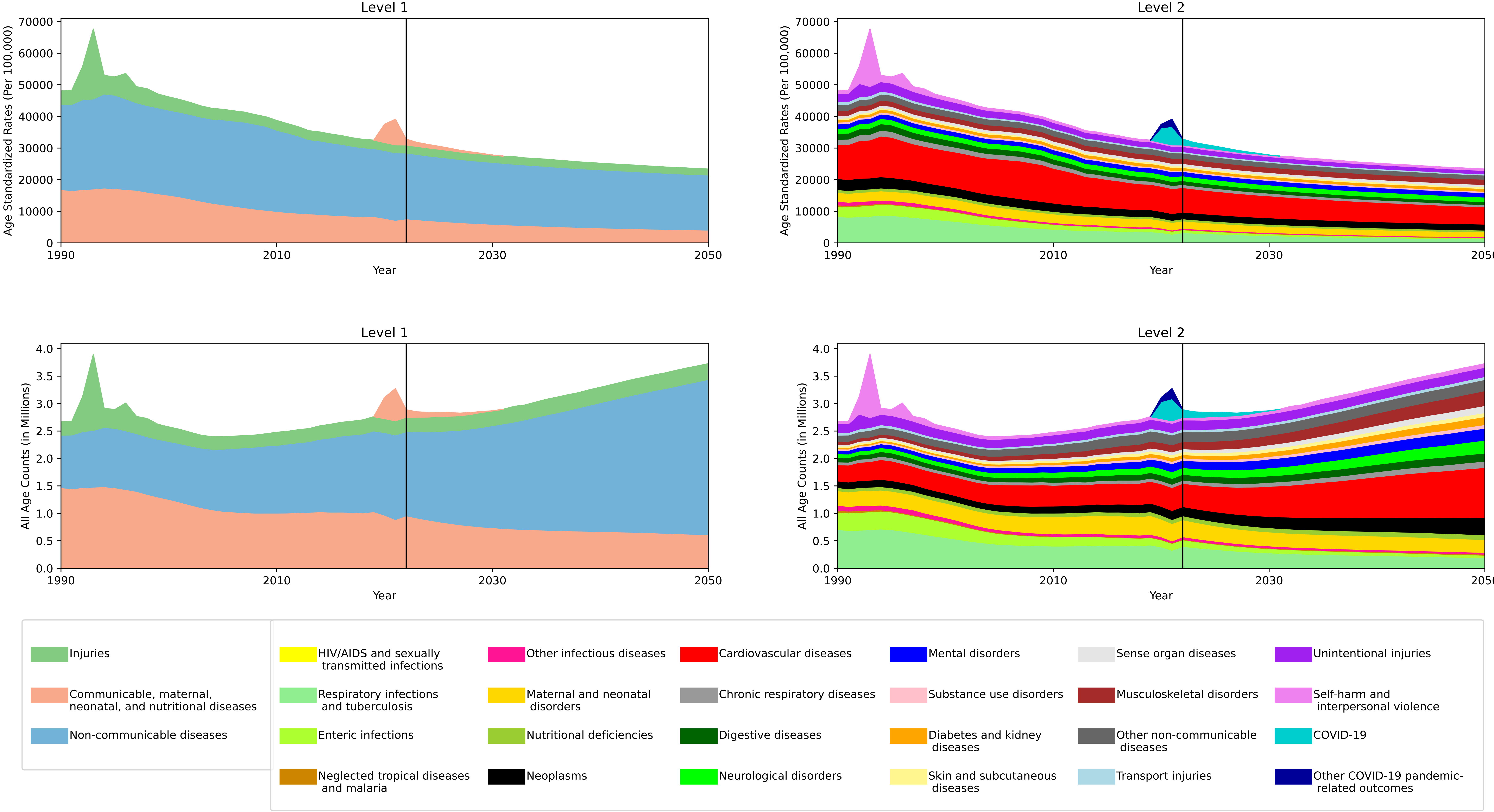
Level 1
Level 2
Age Standardized Rates (Per 100,000)
All Age Counts (in Millions)
Year
1990
2010
2030
2050
Injuries
Communicable, maternal, neonatal, and nutritional diseases
Non-communicable diseases
HIV/AIDS and sexually transmitted infections
Respiratory infections and tuberculosis
Enteric infections
Neglected tropical diseases and malaria
Other infectious diseases
Maternal and neonatal disorders
Nutritional deficiencies
Neoplasms
Cardiovascular diseases
Chronic respiratory diseases
Digestive diseases
Neurological disorders
Mental disorders
Substance use disorders
Diabetes and kidney diseases
Skin and subcutaneous diseases
Sense organ diseases
Musculoskeletal disorders
Other non-communicable diseases
Transport injuries
Unintentional injuries
Self-harm and interpersonal violence
COVID-19
Other COVID-19 pandemic-related outcomes

Turkmenistan

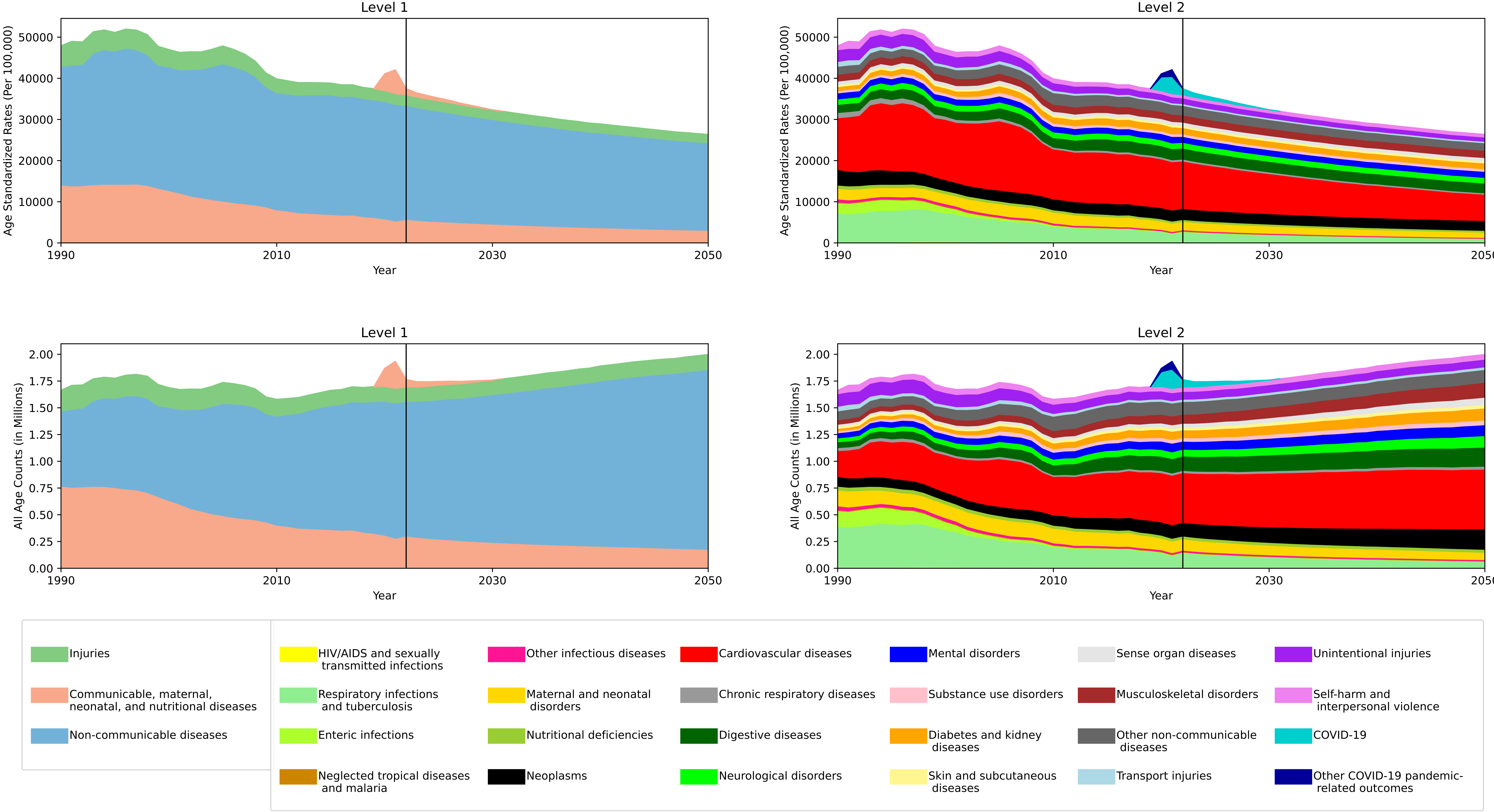

Uzbekistan

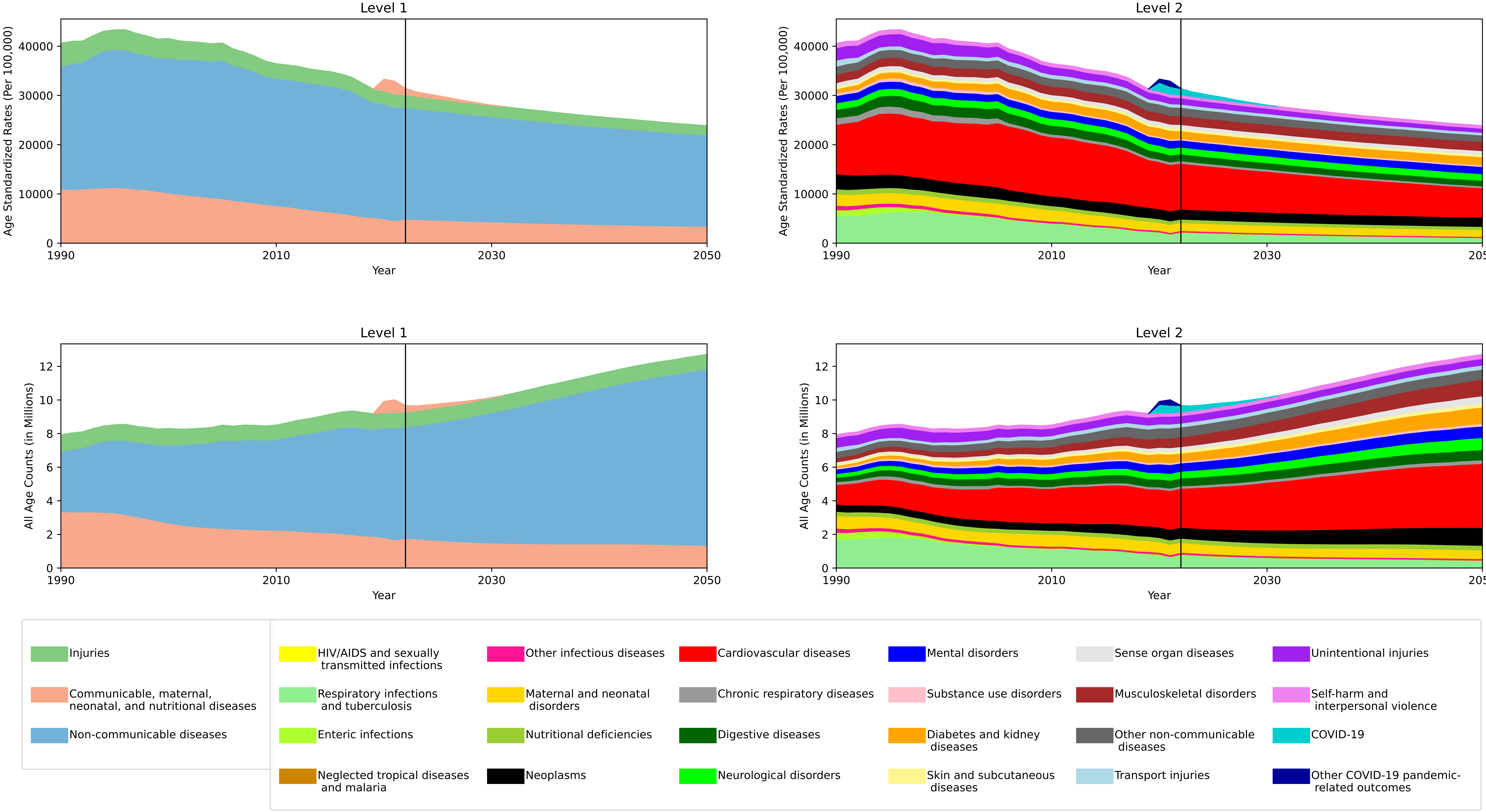
Level 1
Level 2
Age Standardized Rates (Per 100,000)
All Age Counts (in Millions)
Year
1990
2010
2030
2050
Injuries
Communicable, maternal, neonatal, and nutritional diseases
Non-communicable diseases
HIV/AIDS and sexually transmitted infections
Respiratory infections and tuberculosis
Enteric infections
Neglected tropical diseases and malaria
Other infectious diseases
Maternal and neonatal disorders
Nutritional deficiencies
Neoplasms
Cardiovascular diseases
Chronic respiratory diseases
Digestive diseases
Neurological disorders
Mental disorders
Substance use disorders
Diabetes and kidney diseases
Skin and subcutaneous diseases
Sense organ diseases
Musculoskeletal disorders
Other non-communicable diseases
Transport injuries
Unintentional injuries
Self-harm and interpersonal violence
COVID-19
Other COVID-19 pandemic-related outcomes

Albania

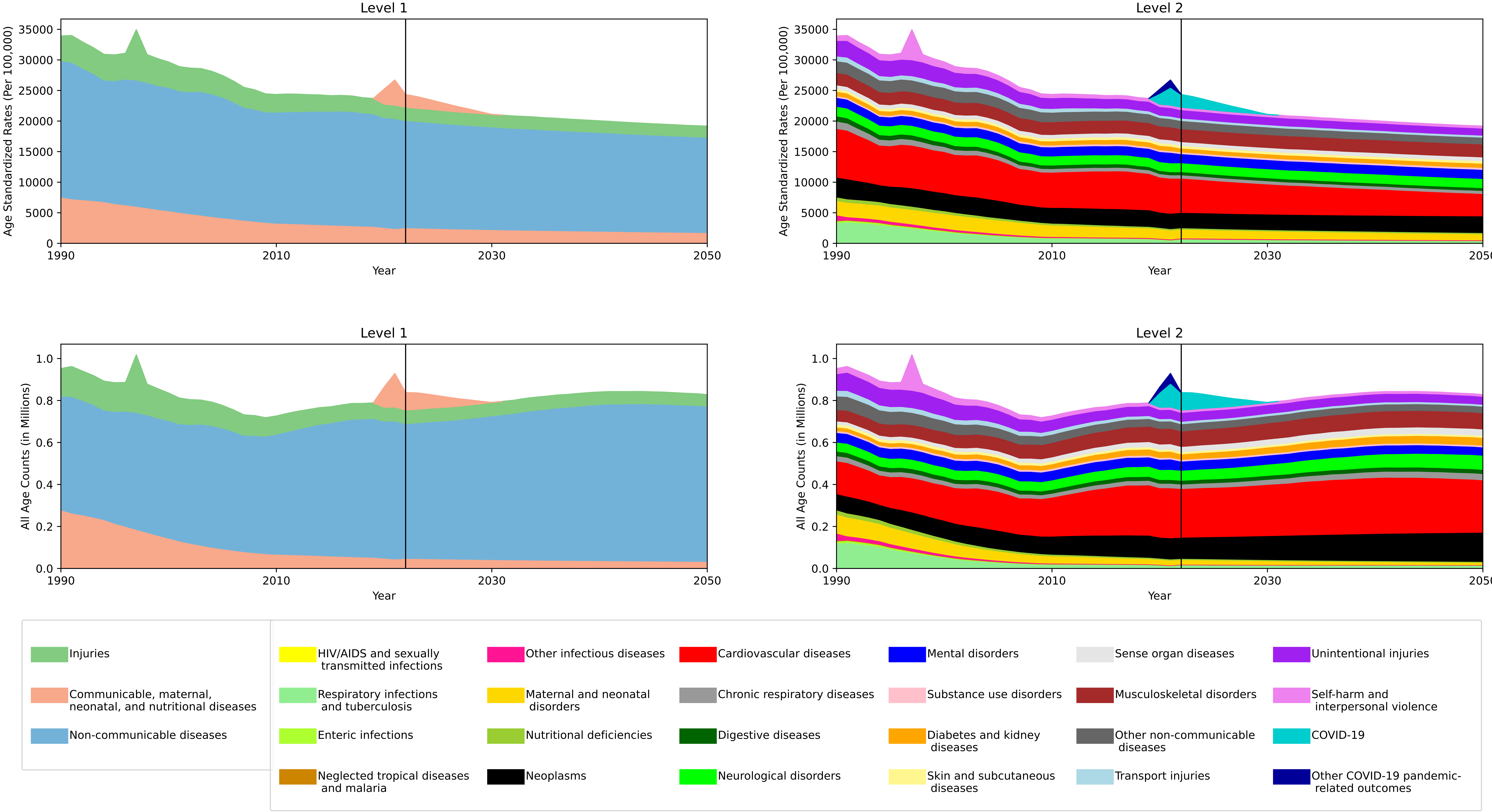
Level 1
Level 2
Age Standardized Rates (Per 100,000)
All Age Counts (in Millions)
Year
1990
2010
2030
2050
0
5000
10000
15000
20000
25000
30000
35000
0.0
0.2
0.4
0.6
0.8
1.0
Injuries
Communicable, maternal, neonatal, and nutritional diseases
Non-communicable diseases
HIV/AIDS and sexually transmitted infections
Respiratory infections and tuberculosis
Enteric infections
Neglected tropical diseases and malaria
Other infectious diseases
Maternal and neonatal disorders
Nutritional deficiencies
Neoplasms
Cardiovascular diseases
Chronic respiratory diseases
Digestive diseases
Neurological disorders
Mental disorders
Substance use disorders
Diabetes and kidney diseases
Skin and subcutaneous diseases
Sense organ diseases
Musculoskeletal disorders
Other non-communicable diseases
Transport injuries
Unintentional injuries
Self-harm and interpersonal violence
COVID-19
Other COVID-19 pandemic-related outcomes

Bosnia And Herzegovina

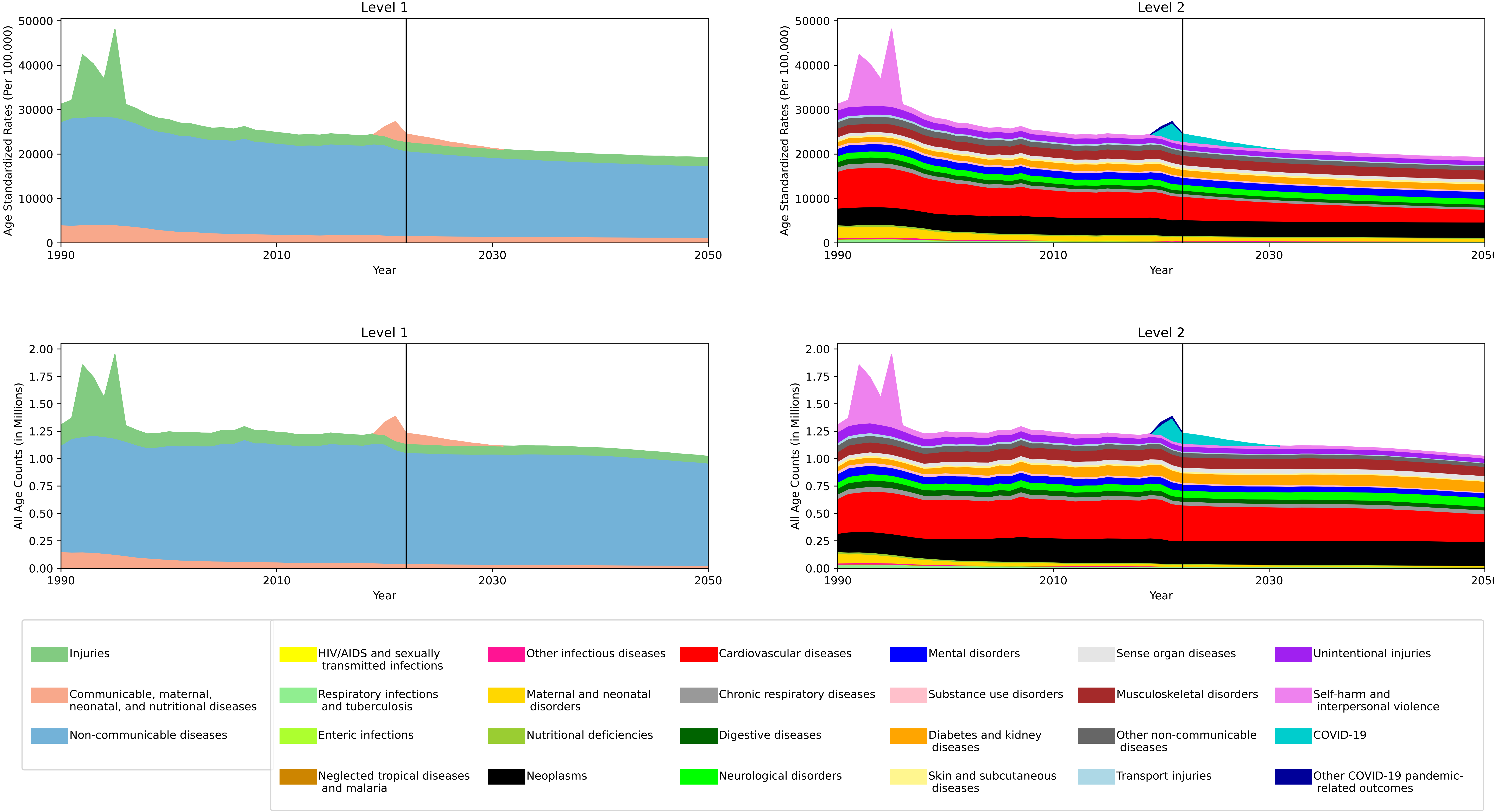

Bulgaria

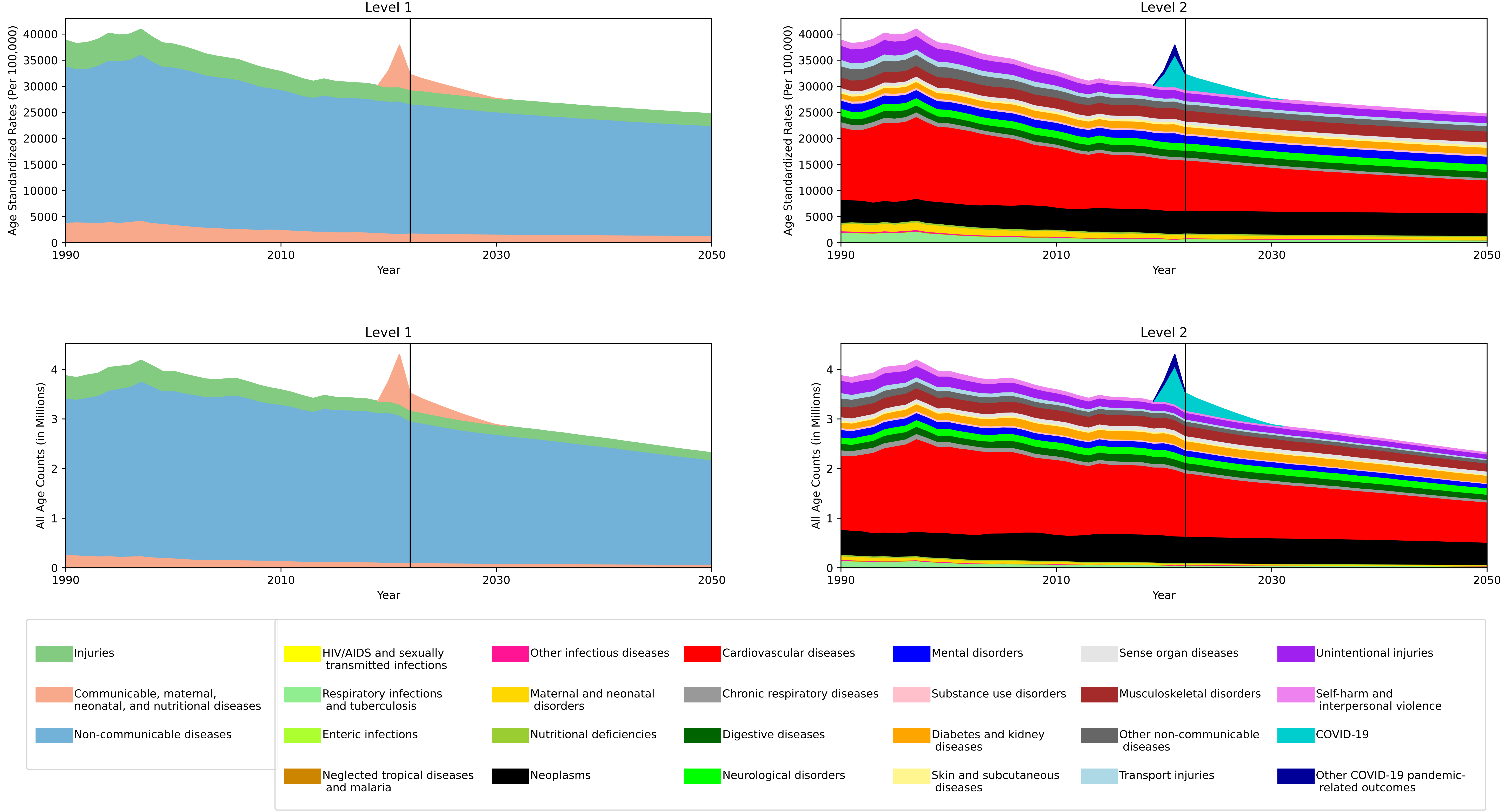
Level 1
Level 2
Age Standardized Rates (Per 100,000)
All Age Counts (in Millions)
Year
1990
2010
2030
2050
Injuries
Communicable, maternal, neonatal, and nutritional diseases
Non-communicable diseases
HIV/AIDS and sexually transmitted infections
Respiratory infections and tuberculosis
Enteric infections
Neglected tropical diseases and malaria
Other infectious diseases
Maternal and neonatal disorders
Nutritional deficiencies
Neoplasms
Cardiovascular diseases
Chronic respiratory diseases
Digestive diseases
Neurological disorders
Mental disorders
Substance use disorders
Diabetes and kidney diseases
Skin and subcutaneous diseases
Sense organ diseases
Musculoskeletal disorders
Other non-communicable diseases
Transport injuries
Unintentional injuries
Self-harm and interpersonal violence
COVID-19
Other COVID-19 pandemic-related outcomes

Croatia

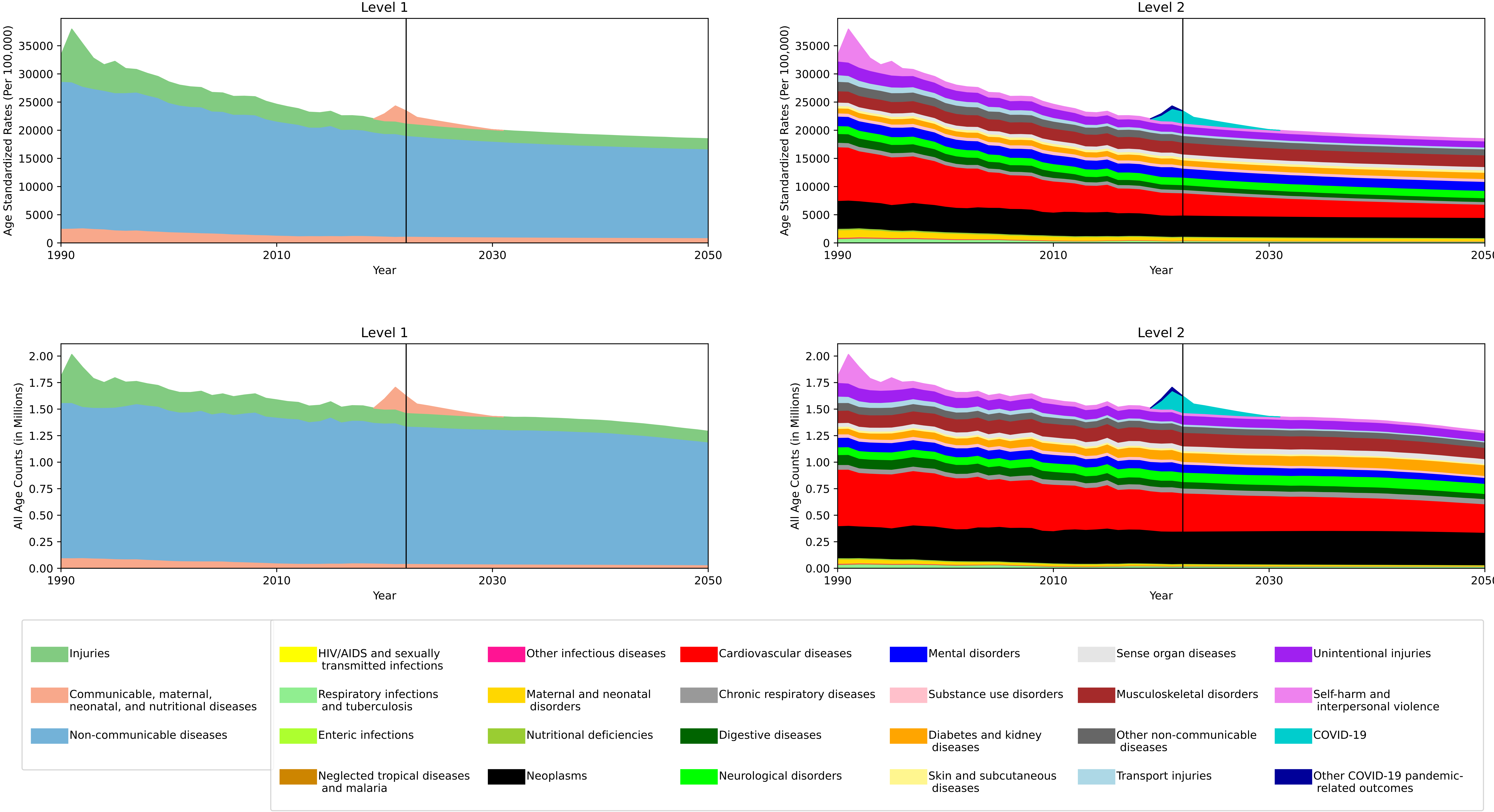
Level 1
Level 2
Age Standardized Rates (Per 100,000)
All Age Counts (in Millions)
Year
1990
2010
2030
2050
Injuries
Communicable, maternal, neonatal, and nutritional diseases
Non-communicable diseases
HIV/AIDS and sexually transmitted infections
Respiratory infections and tuberculosis
Enteric infections
Neglected tropical diseases and malaria
Other infectious diseases
Maternal and neonatal disorders
Nutritional deficiencies
Neoplasms
Cardiovascular diseases
Chronic respiratory diseases
Digestive diseases
Neurological disorders
Mental disorders
Substance use disorders
Diabetes and kidney diseases
Skin and subcutaneous diseases
Sense organ diseases
Musculoskeletal disorders
Other non-communicable diseases
Transport injuries
Unintentional injuries
Self-harm and interpersonal violence
COVID-19
Other COVID-19 pandemic-related outcomes

Czechia

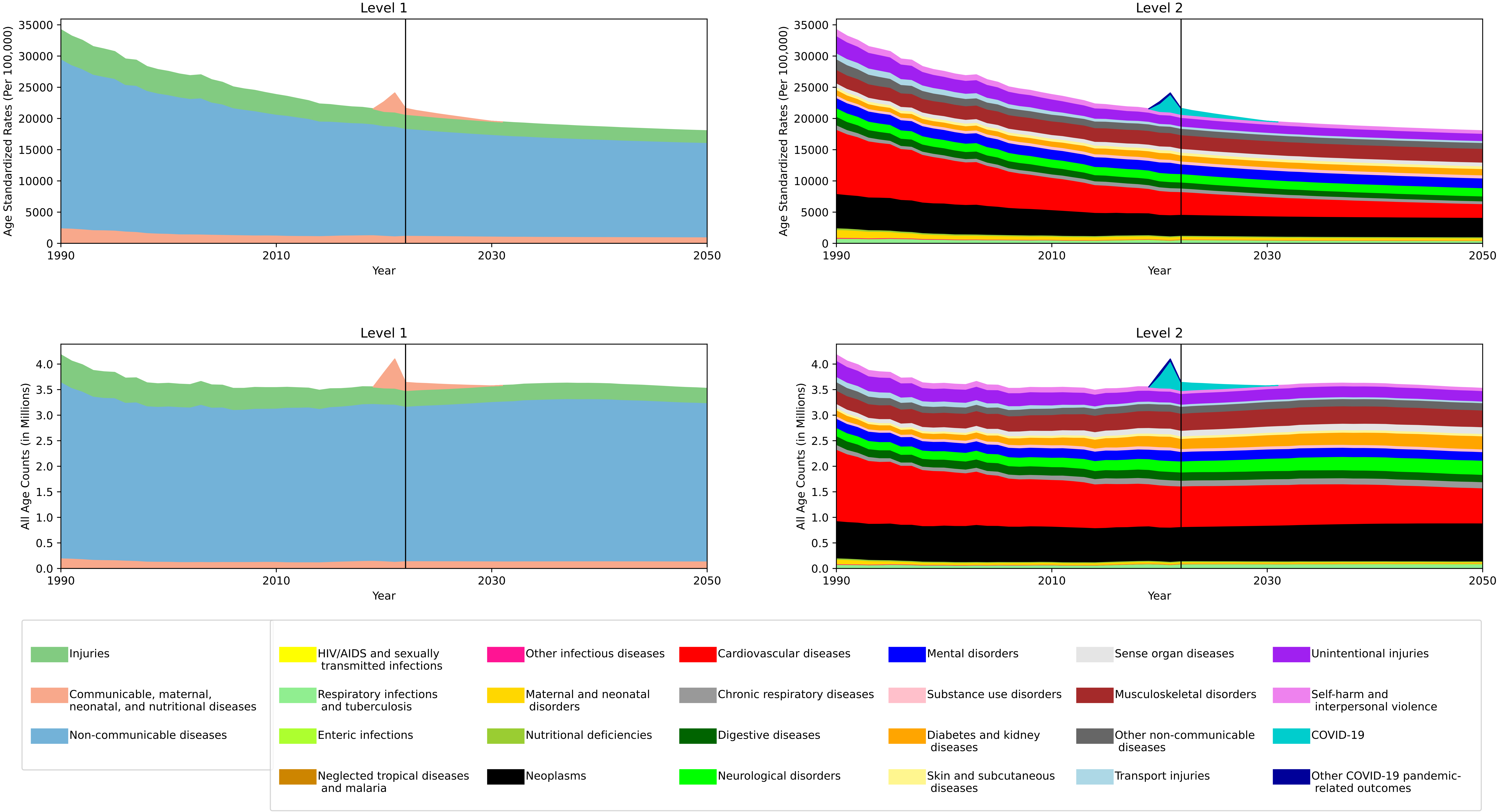
Level 1
Level 2
Age Standardized Rates (Per 100,000)
All Age Counts (in Millions)
Year
1990
2010
2030
2050
Injuries
Communicable, maternal, neonatal, and nutritional diseases
Non-communicable diseases
HIV/AIDS and sexually transmitted infections
Respiratory infections and tuberculosis
Enteric infections
Neglected tropical diseases and malaria
Other infectious diseases
Maternal and neonatal disorders
Nutritional deficiencies
Neoplasms
Cardiovascular diseases
Chronic respiratory diseases
Digestive diseases
Neurological disorders
Mental disorders
Substance use disorders
Diabetes and kidney diseases
Skin and subcutaneous diseases
Sense organ diseases
Musculoskeletal disorders
Other non-communicable diseases
Transport injuries
Unintentional injuries
Self-harm and interpersonal violence
COVID-19
Other COVID-19 pandemic-related outcomes

Hungary

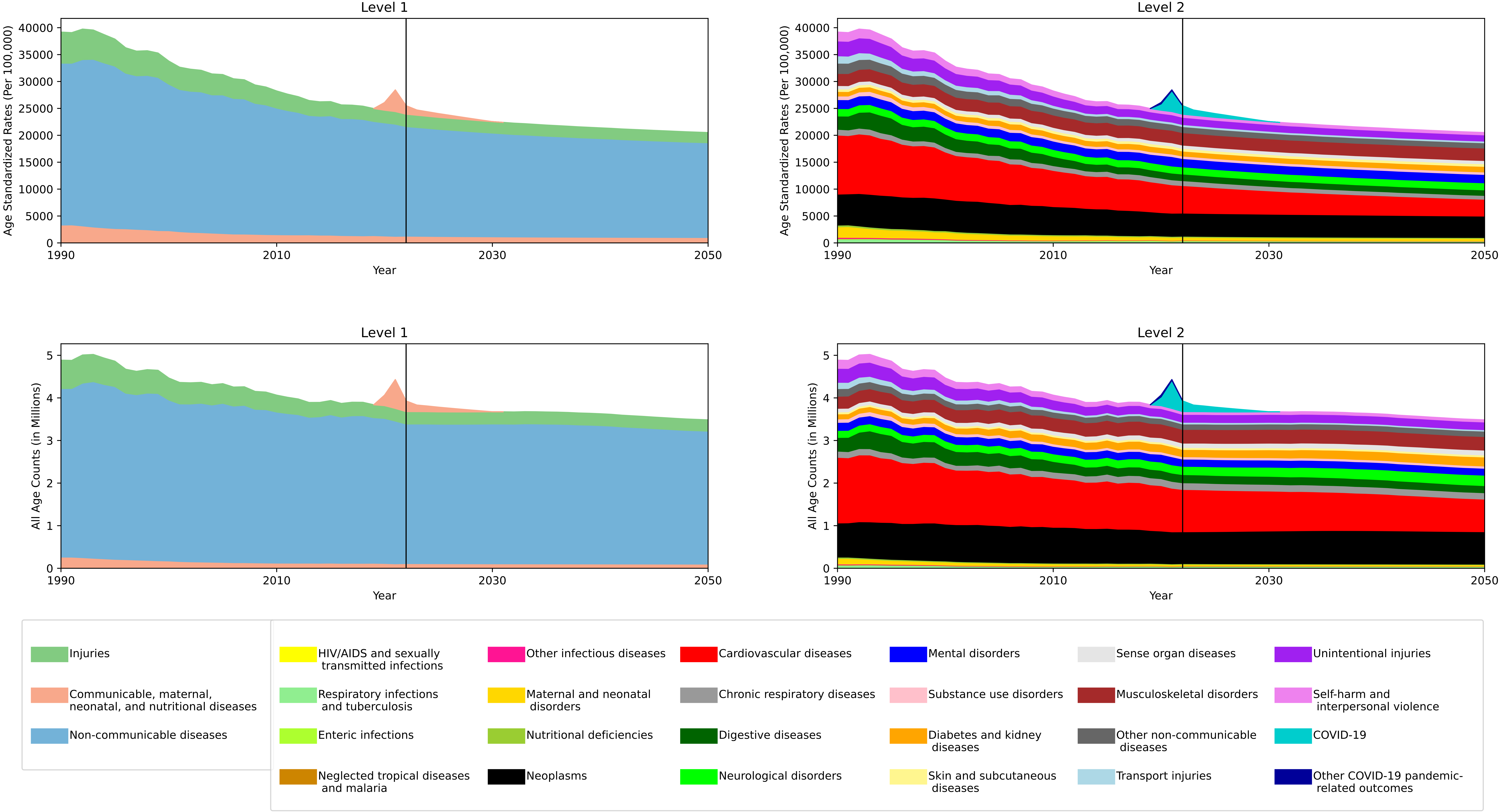
Level 1
Level 2
Age Standardized Rates (Per 100,000)
All Age Counts (in Millions)
Year
1990
2010
2030
2050
Injuries
Communicable, maternal, neonatal, and nutritional diseases
Non-communicable diseases
HIV/AIDS and sexually transmitted infections
Respiratory infections and tuberculosis
Enteric infections
Neglected tropical diseases and malaria
Other infectious diseases
Maternal and neonatal disorders
Nutritional deficiencies
Neoplasms
Cardiovascular diseases
Chronic respiratory diseases
Digestive diseases
Neurological disorders
Mental disorders
Substance use disorders
Diabetes and kidney diseases
Skin and subcutaneous diseases
Sense organ diseases
Musculoskeletal disorders
Other non-communicable diseases
Transport injuries
Unintentional injuries
Self-harm and interpersonal violence
COVID-19
Other COVID-19 pandemic-related outcomes

Montenegro

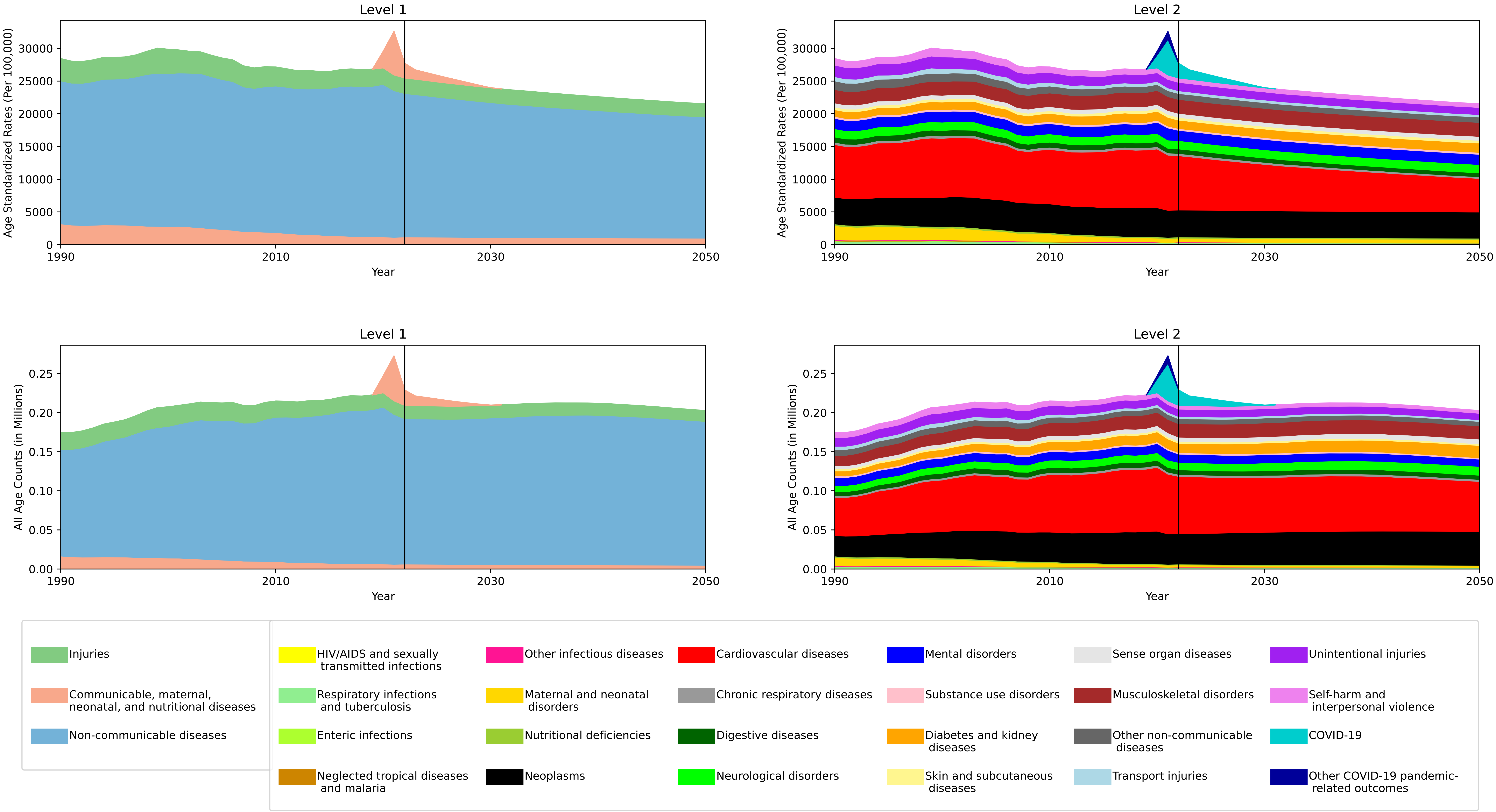
Level 1
Level 2
Age Standardized Rates (Per 100,000)
All Age Counts (in Millions)
Year
1990
2010
2030
2050
30000
25000
20000
15000
10000
5000
0
0.25
0.20
0.15
0.10
0.05
0.00
Injuries
Communicable, maternal, neonatal, and nutritional diseases
Non-communicable diseases
HIV/AIDS and sexually transmitted infections
Respiratory infections and tuberculosis
Enteric infections
Neglected tropical diseases and malaria
Other infectious diseases
Maternal and neonatal disorders
Nutritional deficiencies
Neoplasms
Cardiovascular diseases
Chronic respiratory diseases
Digestive diseases
Neurological disorders
Mental disorders
Substance use disorders
Diabetes and kidney diseases
Skin and subcutaneous diseases
Sense organ diseases
Musculoskeletal disorders
Other non-communicable diseases
Transport injuries
Unintentional injuries
Self-harm and interpersonal violence
COVID-19
Other COVID-19 pandemic-related outcomes

North Macedonia

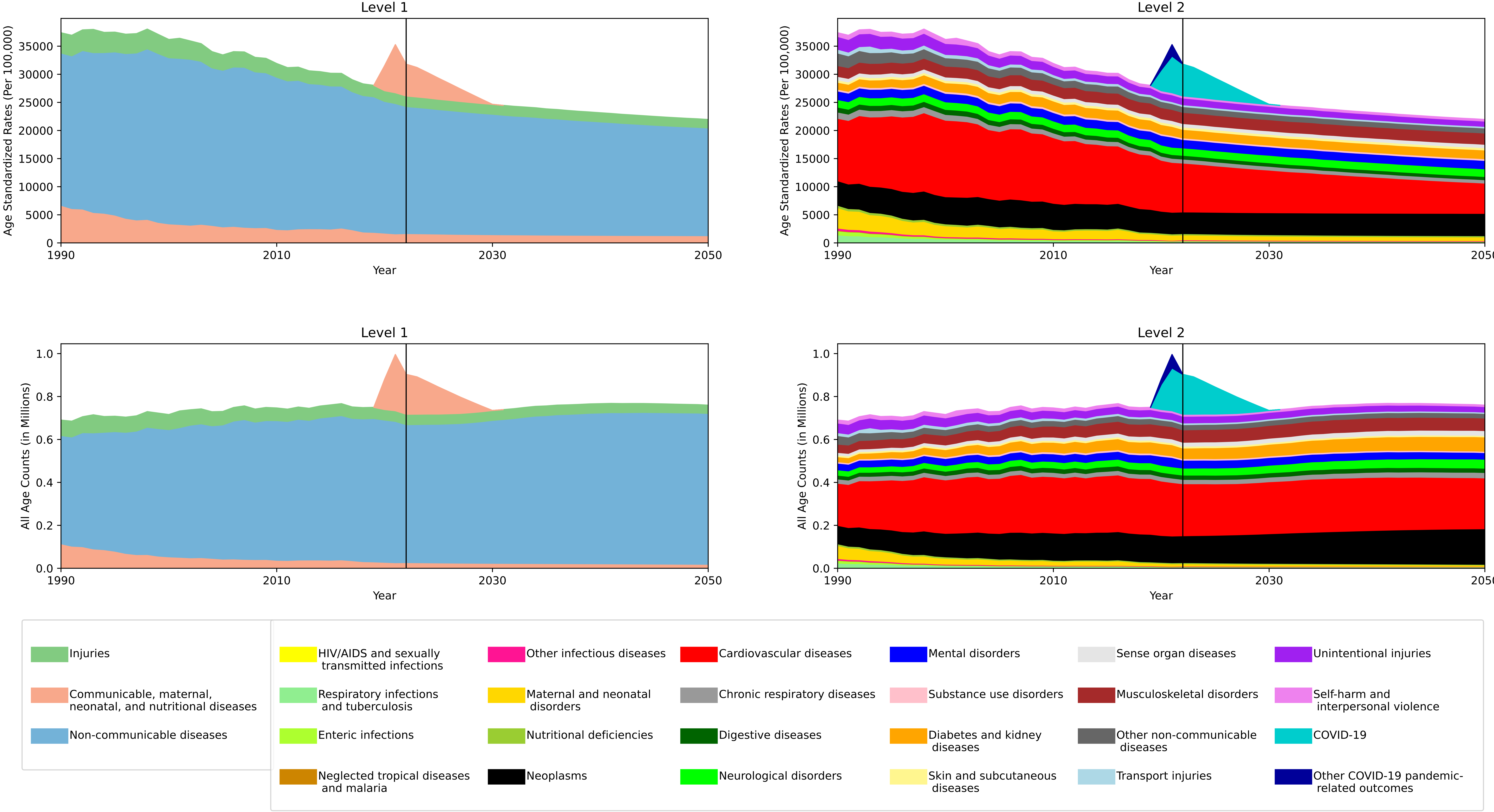

Poland

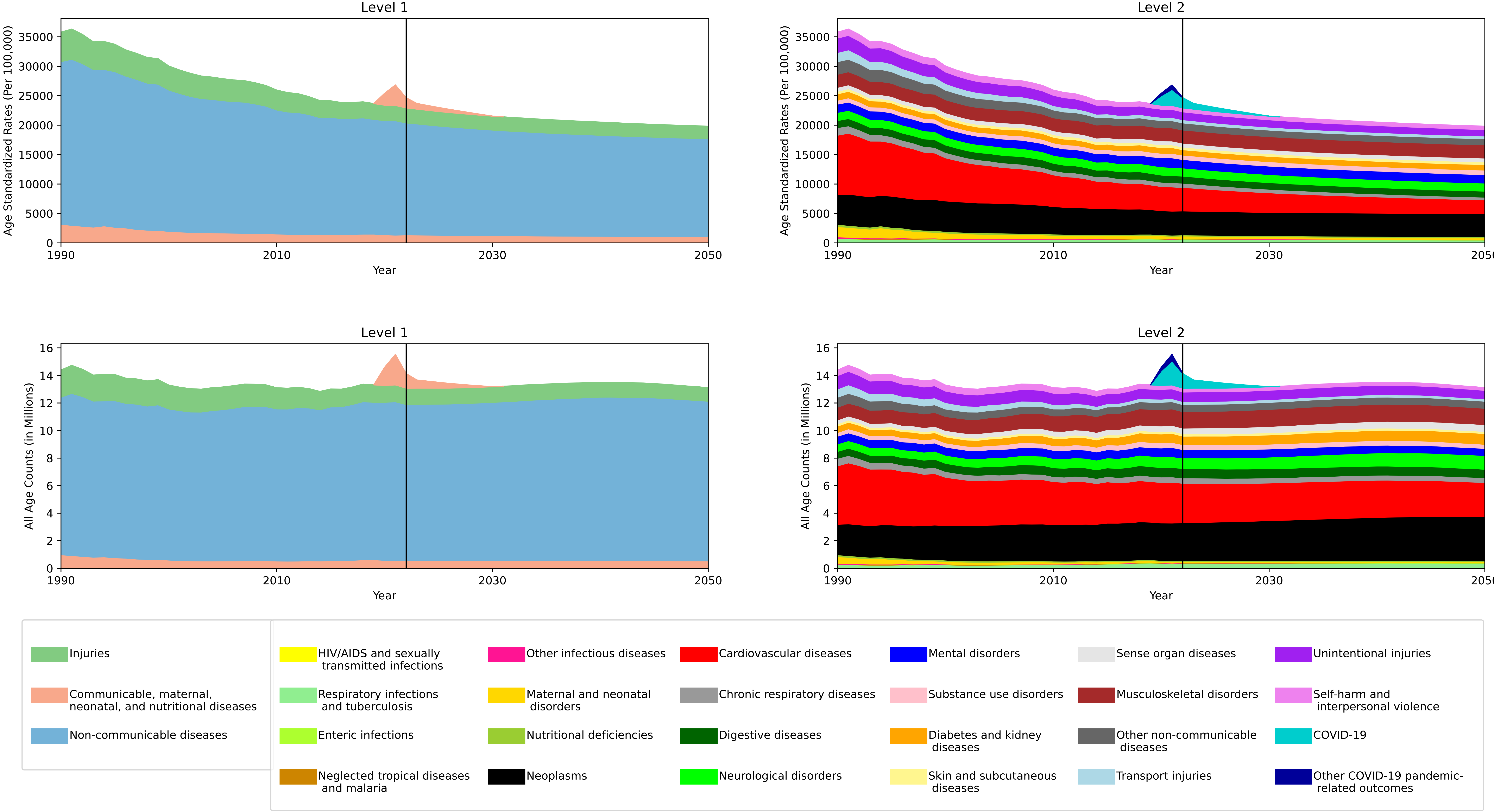
Level 1
Level 2
Age Standardized Rates (Per 100,000)
All Age Counts (in Millions)
Year
1990
2010
2030
2050
Injuries
Communicable, maternal, neonatal, and nutritional diseases
Non-communicable diseases
HIV/AIDS and sexually transmitted infections
Respiratory infections and tuberculosis
Enteric infections
Neglected tropical diseases and malaria
Other infectious diseases
Maternal and neonatal disorders
Nutritional deficiencies
Neoplasms
Cardiovascular diseases
Chronic respiratory diseases
Digestive diseases
Neurological disorders
Mental disorders
Substance use disorders
Diabetes and kidney diseases
Skin and subcutaneous diseases
Sense organ diseases
Musculoskeletal disorders
Other non-communicable diseases
Transport injuries
Unintentional injuries
Self-harm and interpersonal violence
COVID-19
Other COVID-19 pandemic-related outcomes

Romania

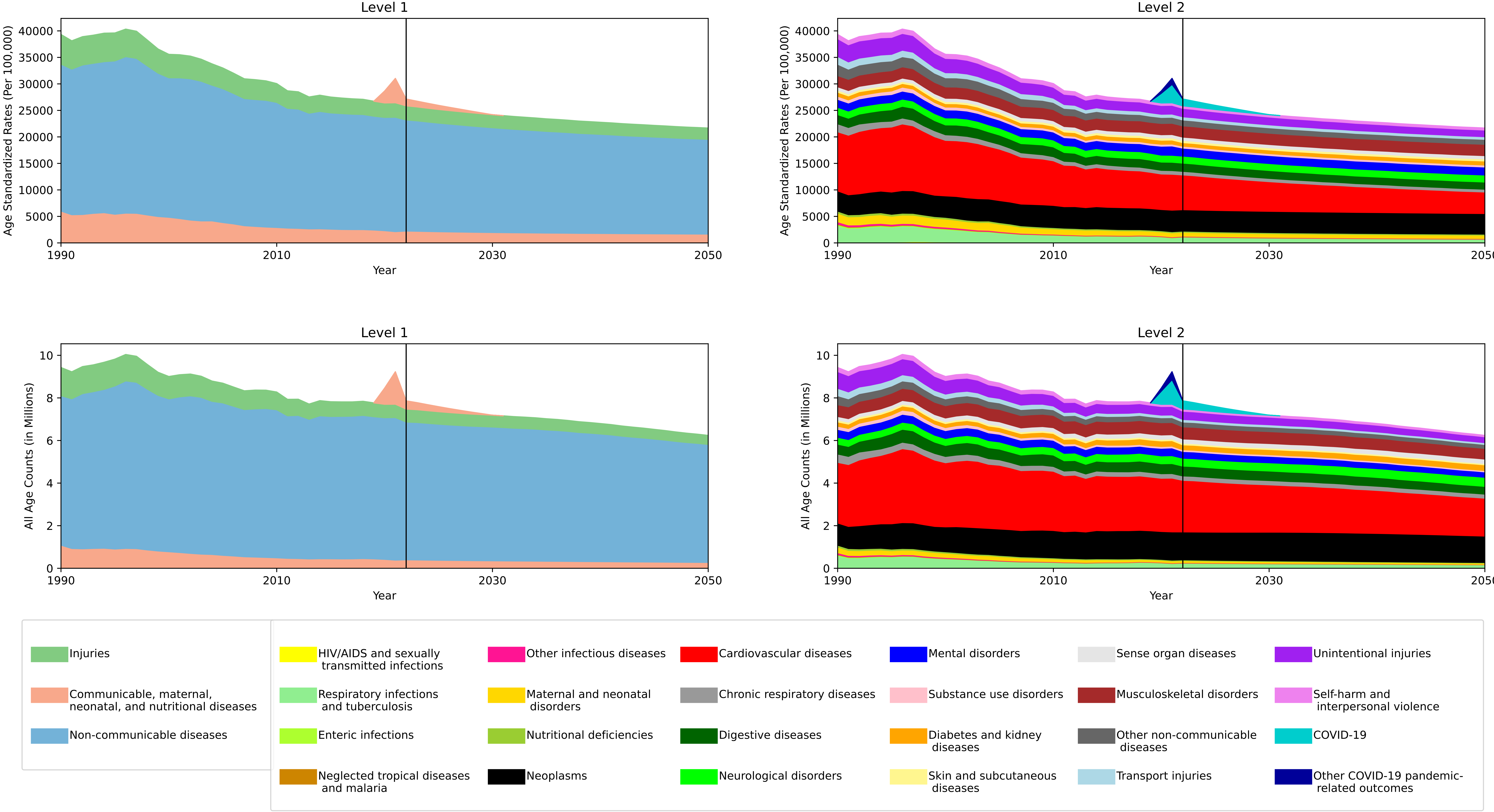
Level 1
Level 2
Age Standardized Rates (Per 100,000)
All Age Counts (in Millions)
Year
1990
2010
2030
2050
40000
35000
30000
25000
20000
15000
10000
5000
0
10
8
6
4
2
Injuries
Communicable, maternal, neonatal, and nutritional diseases
Non-communicable diseases
HIV/AIDS and sexually transmitted infections
Respiratory infections and tuberculosis
Enteric infections
Neglected tropical diseases and malaria
Other infectious diseases
Maternal and neonatal disorders
Nutritional deficiencies
Neoplasms
Cardiovascular diseases
Chronic respiratory diseases
Digestive diseases
Neurological disorders
Mental disorders
Substance use disorders
Diabetes and kidney diseases
Skin and subcutaneous diseases
Sense organ diseases
Musculoskeletal disorders
Other non-communicable diseases
Transport injuries
Unintentional injuries
Self-harm and interpersonal violence
COVID-19
Other COVID-19 pandemic-related outcomes

Serbia

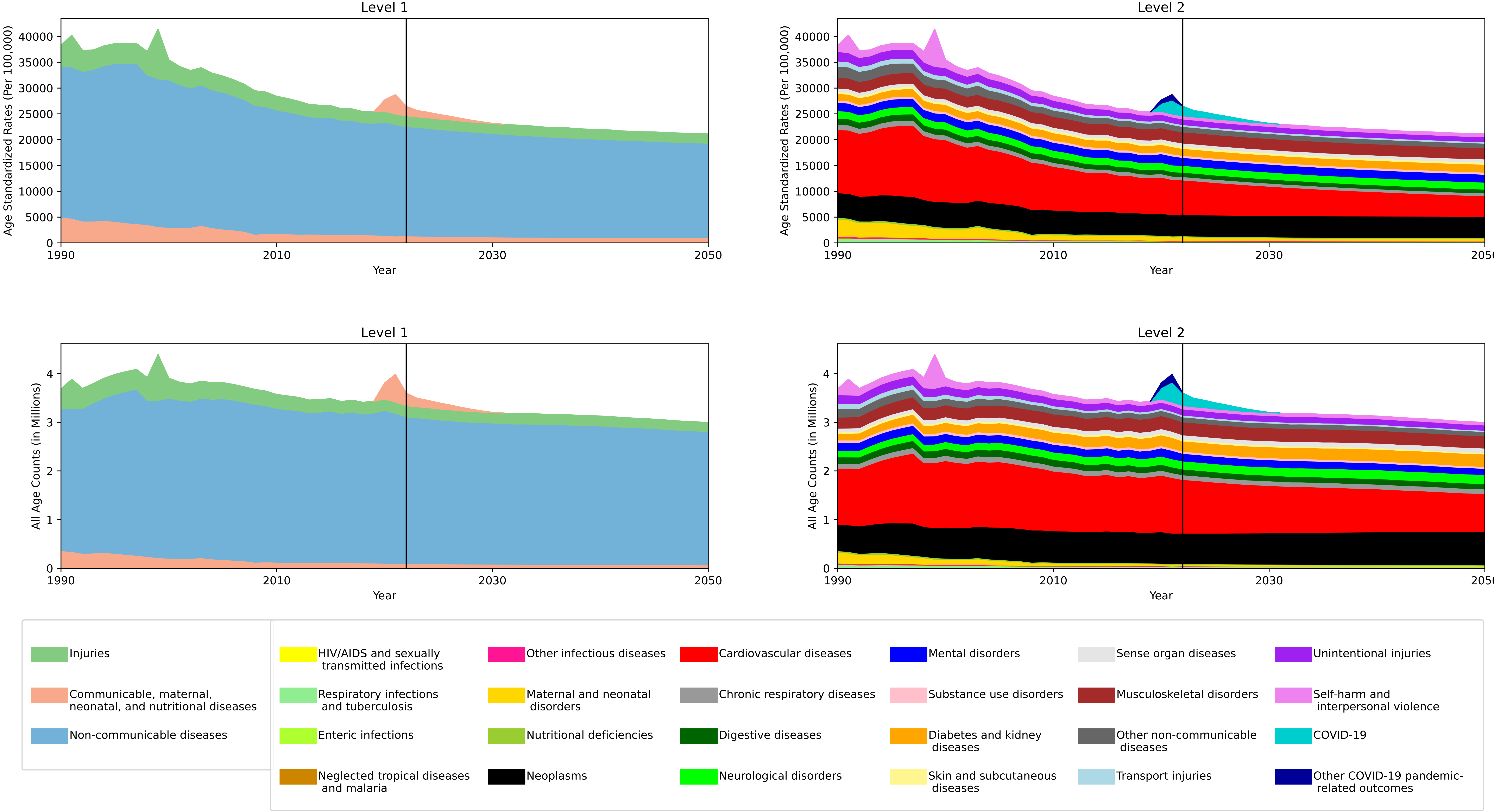
Level 1
Level 2
Age Standardized Rates (Per 100,000)
All Age Counts (in Millions)
Year
Injuries
Communicable, maternal, neonatal, and nutritional diseases
Non-communicable diseases
HIV/AIDS and sexually transmitted infections
Respiratory infections and tuberculosis
Enteric infections
Neglected tropical diseases and malaria
Other infectious diseases
Maternal and neonatal disorders
Nutritional deficiencies
Neoplasms
Cardiovascular diseases
Chronic respiratory diseases
Digestive diseases
Neurological disorders
Mental disorders
Substance use disorders
Diabetes and kidney diseases
Skin and subcutaneous diseases
Sense organ diseases
Musculoskeletal disorders
Other non-communicable diseases
Transport injuries
Unintentional injuries
Self-harm and interpersonal violence
COVID-19
Other COVID-19 pandemic-related outcomes

Slovakia

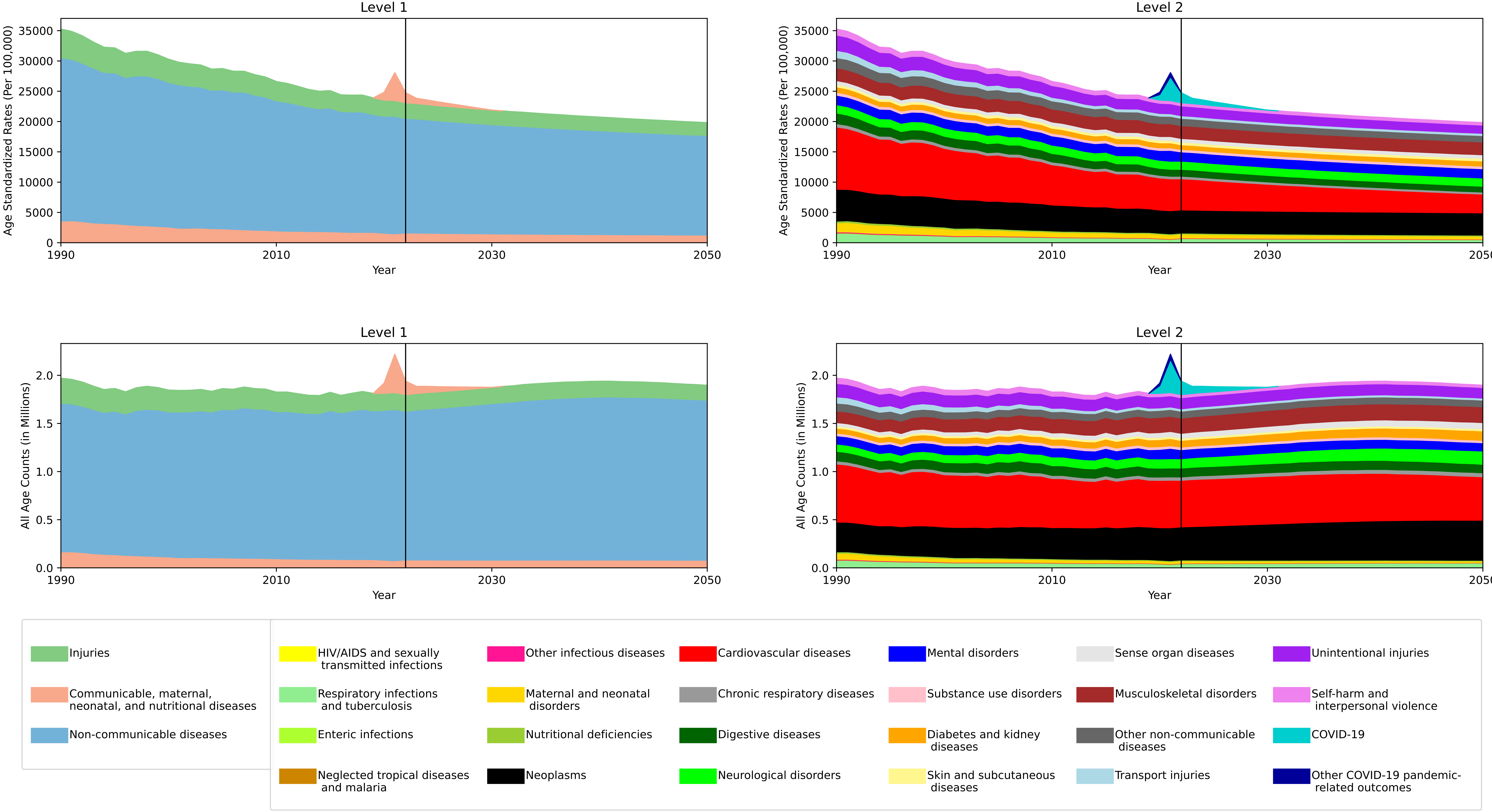
Level 1
Level 2
Age Standardized Rates (Per 100,000)
All Age Counts (in Millions)
Year
0
5000
10000
15000
20000
25000
30000
35000
0.0
0.5
1.0
1.5
2.0
1990
2010
2030
2050
Injuries
Communicable, maternal, neonatal, and nutritional diseases
Non-communicable diseases
HIV/AIDS and sexually transmitted infections
Respiratory infections and tuberculosis
Enteric infections
Neglected tropical diseases and malaria
Other infectious diseases
Maternal and neonatal disorders
Nutritional deficiencies
Neoplasms
Cardiovascular diseases
Chronic respiratory diseases
Digestive diseases
Neurological disorders
Mental disorders
Substance use disorders
Diabetes and kidney diseases
Skin and subcutaneous diseases
Sense organ diseases
Musculoskeletal disorders
Other non-communicable diseases
Transport injuries
Unintentional injuries
Self-harm and interpersonal violence
COVID-19
Other COVID-19 pandemic-related outcomes

Slovenia

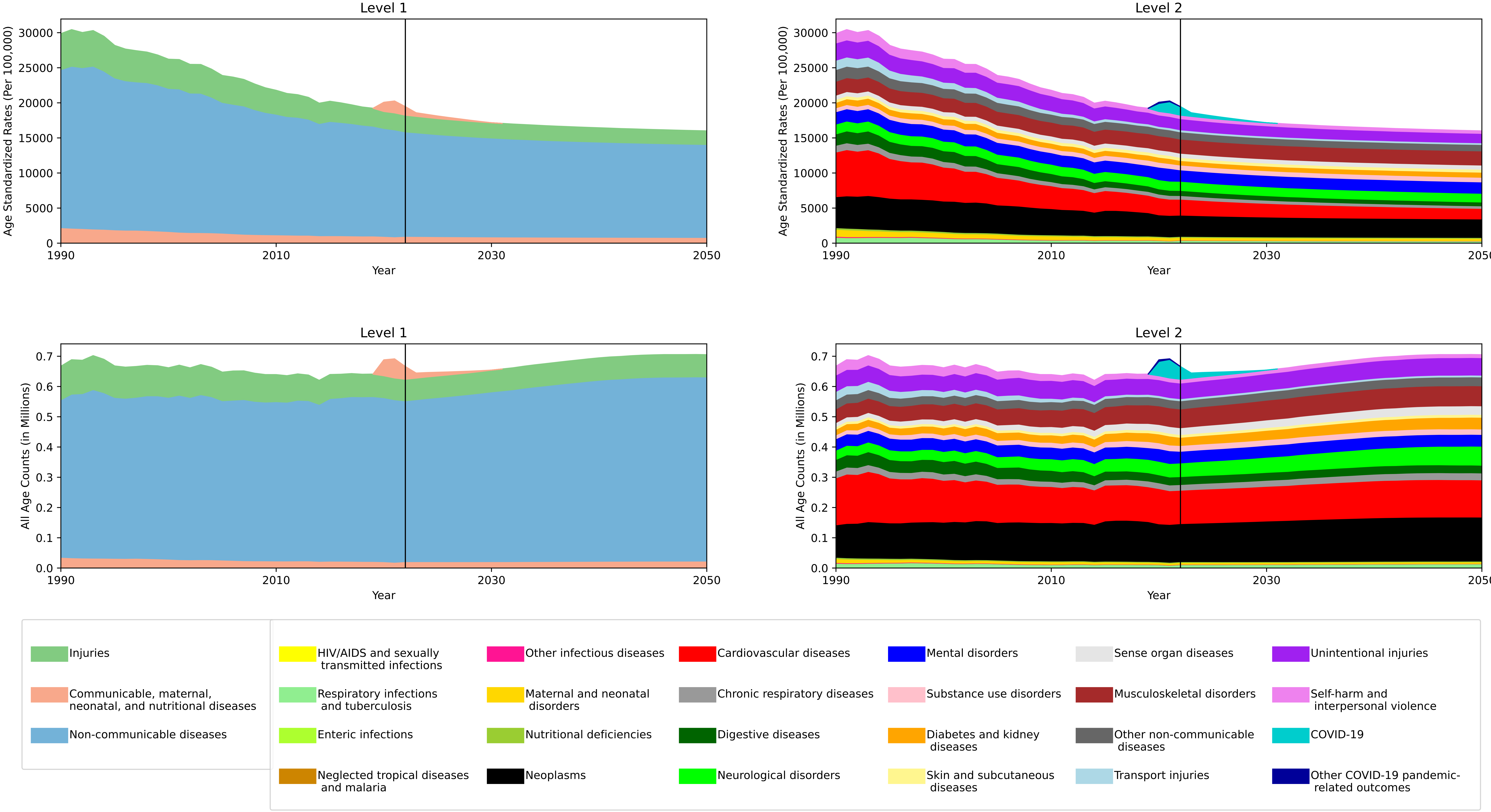
Level 1
Level 2
Age Standardized Rates (Per 100,000)
All Age Counts (in Millions)
Year
0
5000
10000
15000
20000
25000
30000
0.0
0.1
0.2
0.3
0.4
0.5
0.6
0.7
1990
2010
2030
2050
Injuries
Communicable, maternal, neonatal, and nutritional diseases
Non-communicable diseases
HIV/AIDS and sexually transmitted infections
Respiratory infections and tuberculosis
Enteric infections
Neglected tropical diseases and malaria
Other infectious diseases
Maternal and neonatal disorders
Nutritional deficiencies
Neoplasms
Cardiovascular diseases
Chronic respiratory diseases
Digestive diseases
Neurological disorders
Mental disorders
Substance use disorders
Diabetes and kidney diseases
Skin and subcutaneous diseases
Sense organ diseases
Musculoskeletal disorders
Other non-communicable diseases
Transport injuries
Unintentional injuries
Self-harm and interpersonal violence
COVID-19
Other COVID-19 pandemic-related outcomes

Belarus

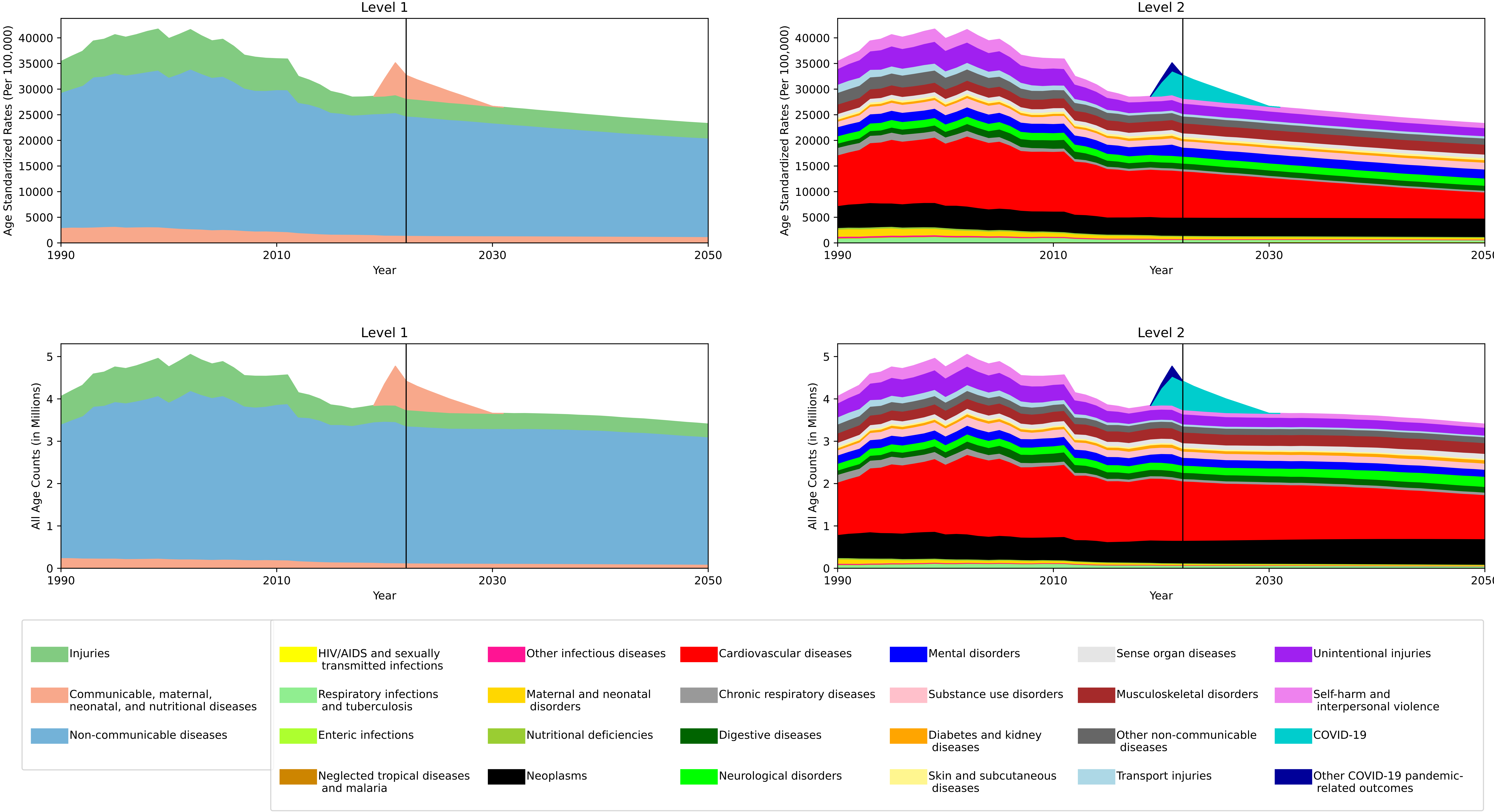
Level 1
Level 2
Age Standardized Rates (Per 100,000)
All Age Counts (in Millions)
Year
1990
2010
2030
2050
Injuries
Communicable, maternal, neonatal, and nutritional diseases
Non-communicable diseases
HIV/AIDS and sexually transmitted infections
Respiratory infections and tuberculosis
Enteric infections
Neglected tropical diseases and malaria
Other infectious diseases
Maternal and neonatal disorders
Nutritional deficiencies
Neoplasms
Cardiovascular diseases
Chronic respiratory diseases
Digestive diseases
Neurological disorders
Mental disorders
Substance use disorders
Diabetes and kidney diseases
Skin and subcutaneous diseases
Sense organ diseases
Musculoskeletal disorders
Other non-communicable diseases
Transport injuries
Unintentional injuries
Self-harm and interpersonal violence
COVID-19
Other COVID-19 pandemic-related outcomes

Estonia

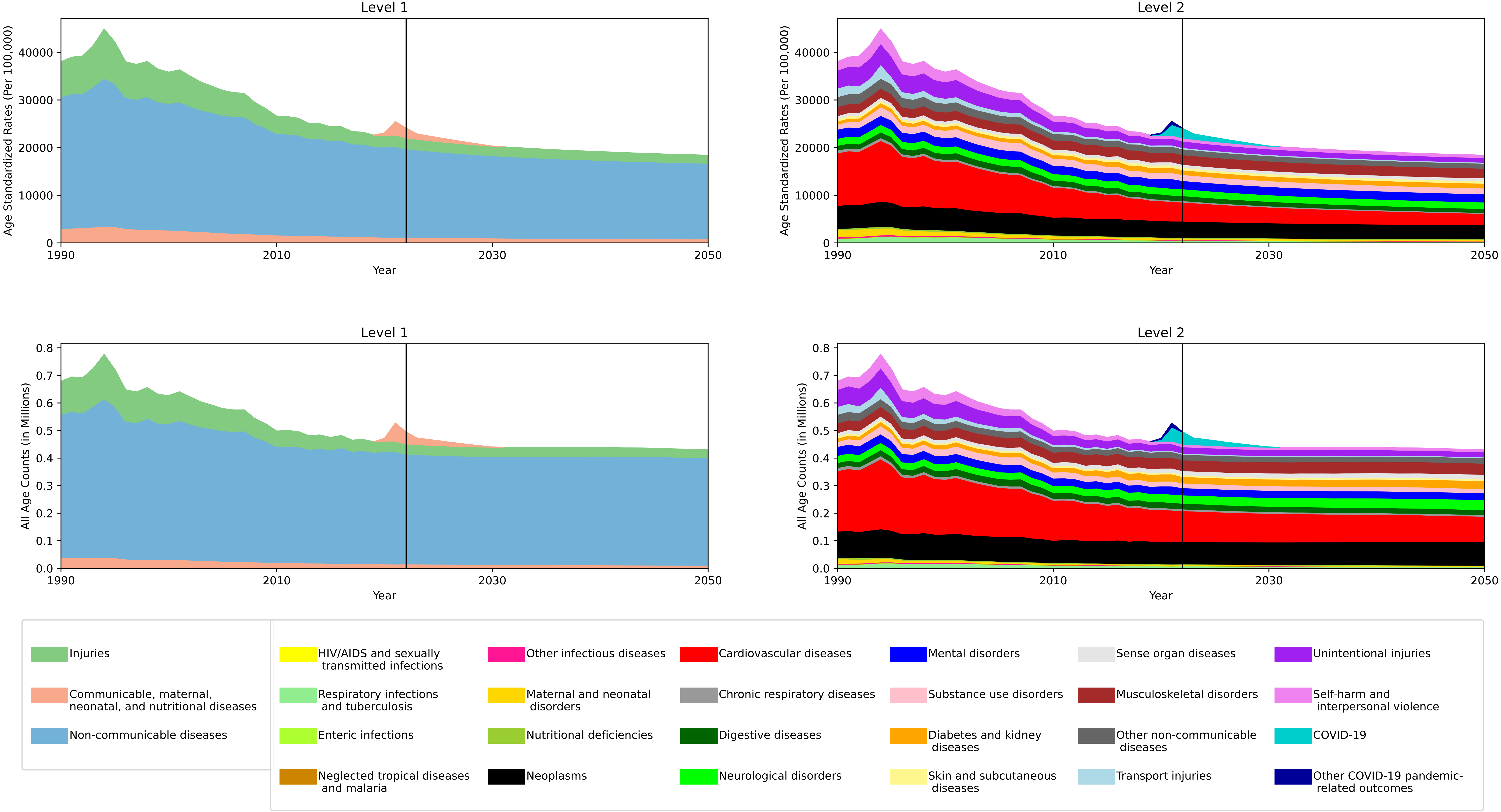
Level 1
Level 2
Age Standardized Rates (Per 100,000)
All Age Counts (in Millions)
Year
1990
2010
2030
2050
Injuries
Communicable, maternal, neonatal, and nutritional diseases
Non-communicable diseases
HIV/AIDS and sexually transmitted infections
Respiratory infections and tuberculosis
Enteric infections
Neglected tropical diseases and malaria
Other infectious diseases
Maternal and neonatal disorders
Nutritional deficiencies
Neoplasms
Cardiovascular diseases
Chronic respiratory diseases
Digestive diseases
Neurological disorders
Mental disorders
Substance use disorders
Diabetes and kidney diseases
Skin and subcutaneous diseases
Sense organ diseases
Musculoskeletal disorders
Other non-communicable diseases
Transport injuries
Unintentional injuries
Self-harm and interpersonal violence
COVID-19
Other COVID-19 pandemic-related outcomes

Latvia

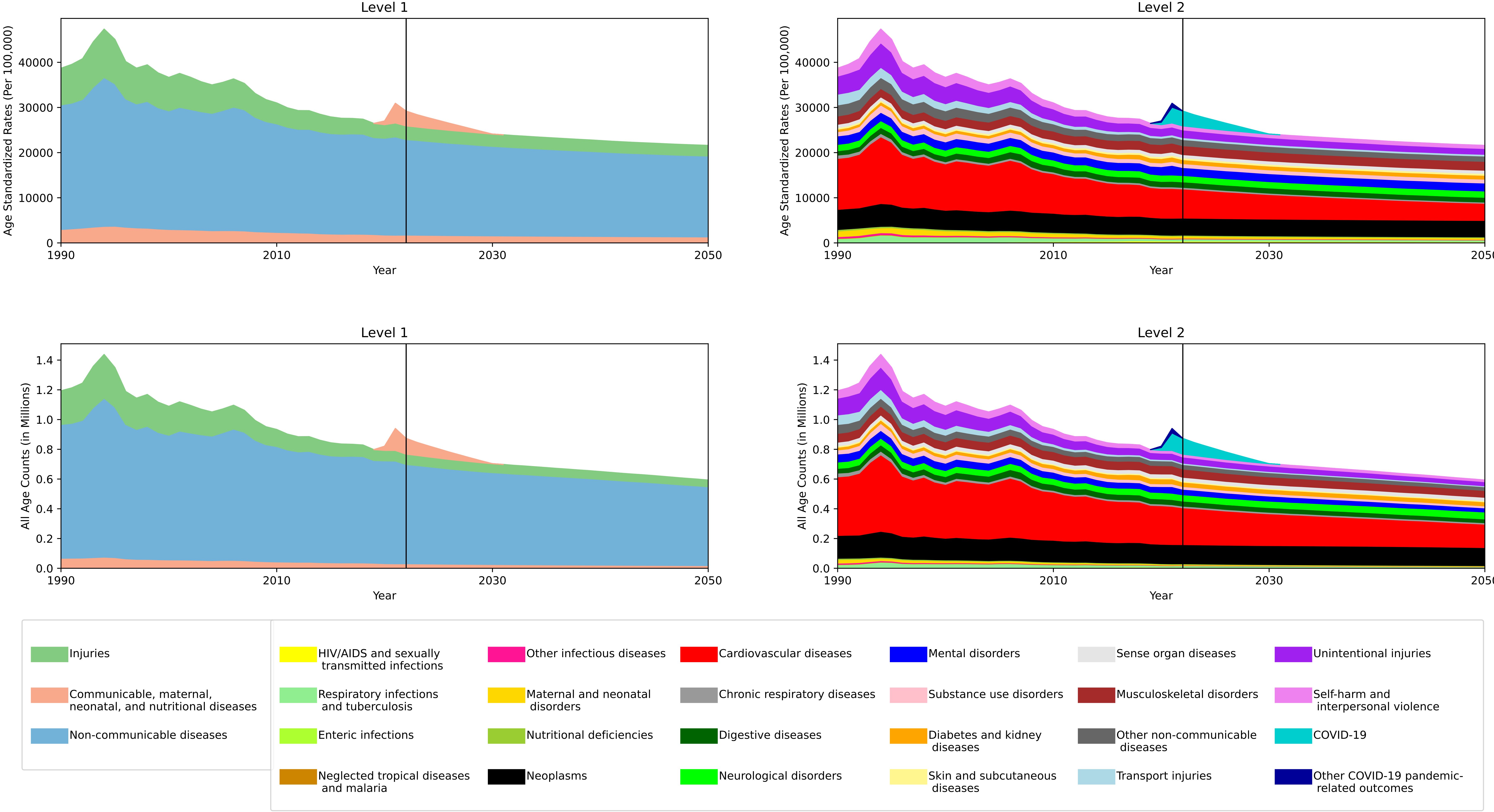
Level 1
Level 2
Age Standardized Rates (Per 100,000)
All Age Counts (in Millions)
Year
1990
2010
2030
2050
40000
30000
20000
10000
0
1.4
1.2
1.0
0.8
0.6
0.4
0.2
0.0
Injuries
Communicable, maternal, neonatal, and nutritional diseases
Non-communicable diseases
HIV/AIDS and sexually transmitted infections
Respiratory infections and tuberculosis
Enteric infections
Neglected tropical diseases and malaria
Other infectious diseases
Maternal and neonatal disorders
Nutritional deficiencies
Neoplasms
Cardiovascular diseases
Chronic respiratory diseases
Digestive diseases
Neurological disorders
Mental disorders
Substance use disorders
Diabetes and kidney diseases
Skin and subcutaneous diseases
Sense organ diseases
Musculoskeletal disorders
Other non-communicable diseases
Transport injuries
Unintentional injuries
Self-harm and interpersonal violence
COVID-19
Other COVID-19 pandemic-related outcomes

Lithuania

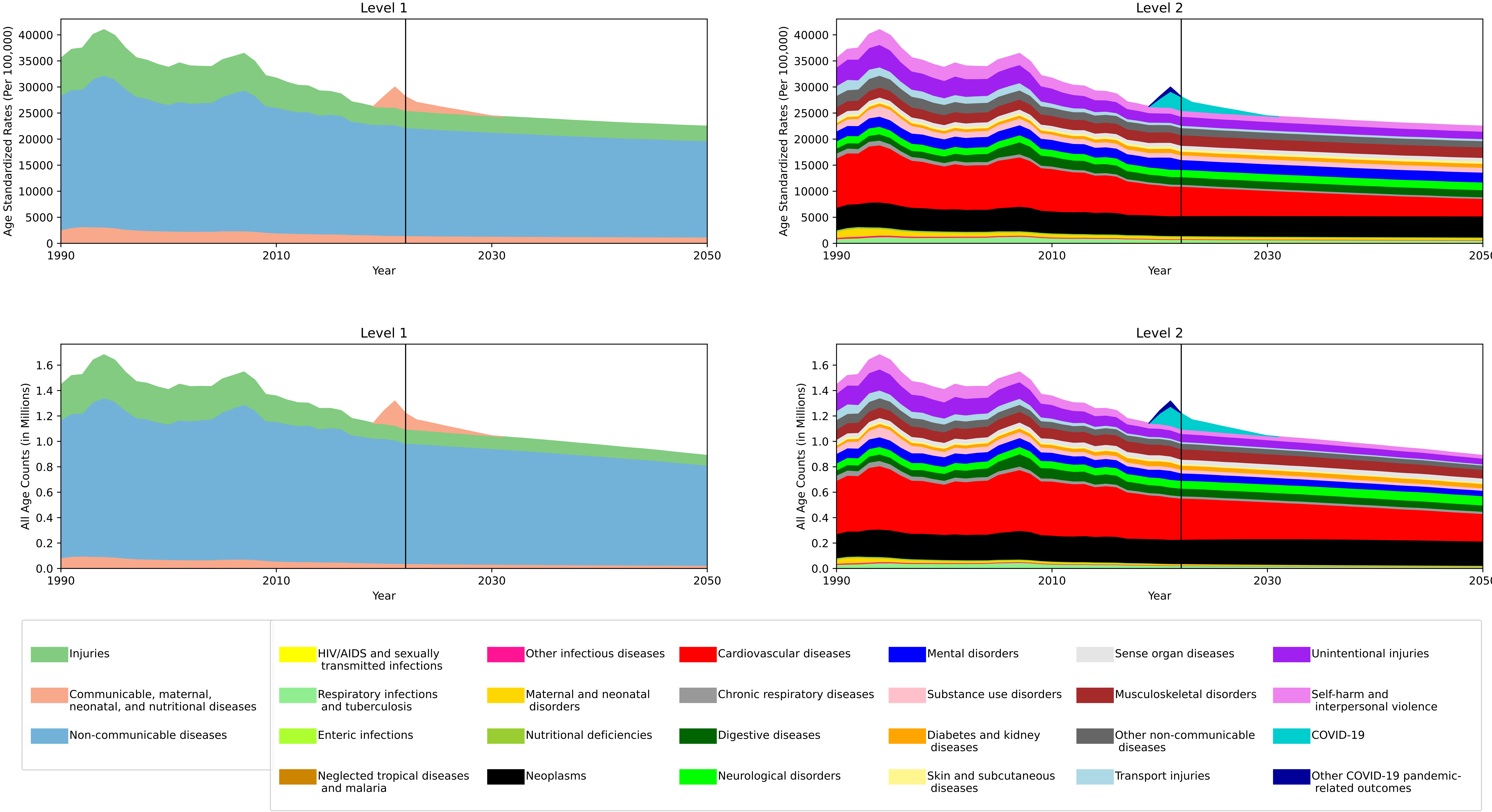
Level 1
Level 2
Age Standardized Rates (Per 100,000)
All Age Counts (in Millions)
Year
0
5000
10000
15000
20000
25000
30000
35000
40000
0.0
0.2
0.4
0.6
0.8
1.0
1.2
1.4
1.6
1990
2010
2030
2050
Injuries
Communicable, maternal, neonatal, and nutritional diseases
Non-communicable diseases
HIV/AIDS and sexually transmitted infections
Respiratory infections and tuberculosis
Enteric infections
Neglected tropical diseases and malaria
Other infectious diseases
Maternal and neonatal disorders
Nutritional deficiencies
Neoplasms
Cardiovascular diseases
Chronic respiratory diseases
Digestive diseases
Neurological disorders
Mental disorders
Substance use disorders
Diabetes and kidney diseases
Skin and subcutaneous diseases
Sense organ diseases
Musculoskeletal disorders
Other non-communicable diseases
Transport injuries
Unintentional injuries
Self-harm and interpersonal violence
COVID-19
Other COVID-19 pandemic-related outcomes

Moldova

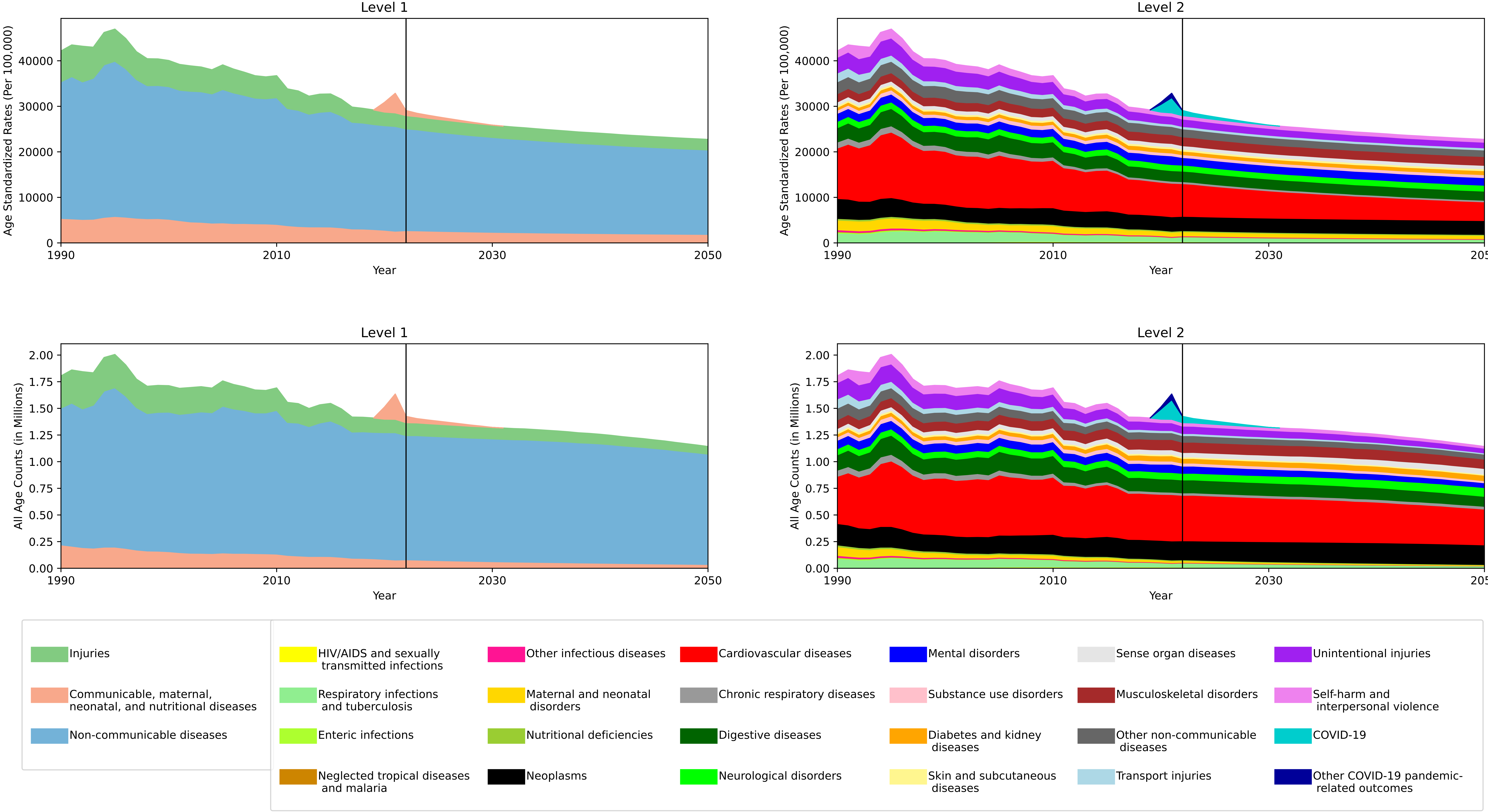
Level 1
Level 2
Age Standardized Rates (Per 100,000)
All Age Counts (in Millions)
Year
1990
2010
2030
2050
0
10000
20000
30000
40000
0.00
0.25
0.50
0.75
1.00
1.25
1.50
1.75
2.00
Injuries
Communicable, maternal, neonatal, and nutritional diseases
Non-communicable diseases
HIV/AIDS and sexually transmitted infections
Respiratory infections and tuberculosis
Enteric infections
Neglected tropical diseases and malaria
Other infectious diseases
Maternal and neonatal disorders
Nutritional deficiencies
Neoplasms
Cardiovascular diseases
Chronic respiratory diseases
Digestive diseases
Neurological disorders
Mental disorders
Substance use disorders
Diabetes and kidney diseases
Skin and subcutaneous diseases
Sense organ diseases
Musculoskeletal disorders
Other non-communicable diseases
Transport injuries
Unintentional injuries
Self-harm and interpersonal violence
COVID-19
Other COVID-19 pandemic-related outcomes

Russia

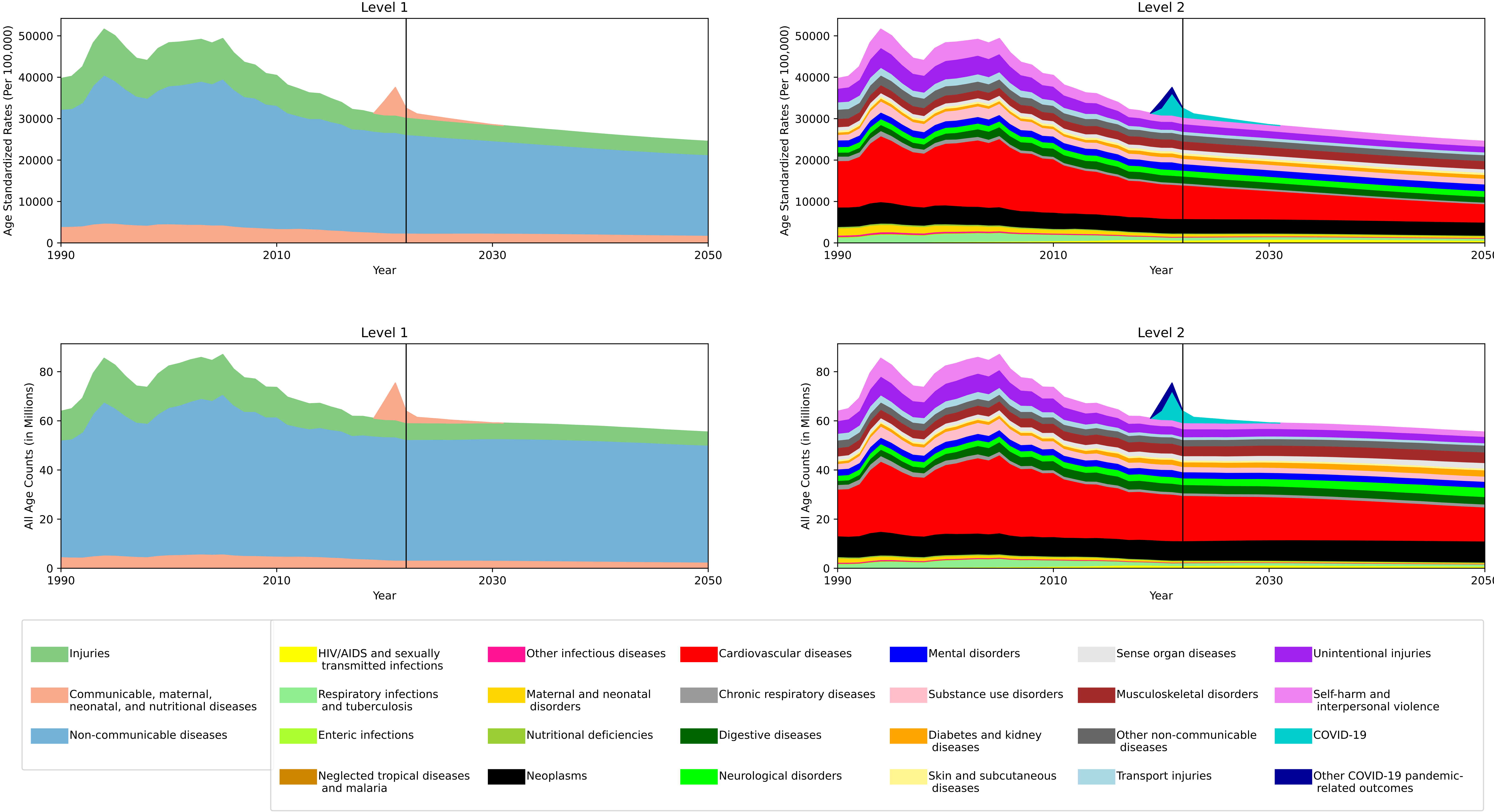
Level 1
Level 2
Age Standardized Rates (Per 100,000)
All Age Counts (in Millions)
Year
1990
2010
2030
2050
Injuries
Communicable, maternal, neonatal, and nutritional diseases
Non-communicable diseases
HIV/AIDS and sexually transmitted infections
Respiratory infections and tuberculosis
Enteric infections
Neglected tropical diseases and malaria
Other infectious diseases
Maternal and neonatal disorders
Nutritional deficiencies
Neoplasms
Cardiovascular diseases
Chronic respiratory diseases
Digestive diseases
Neurological disorders
Mental disorders
Substance use disorders
Diabetes and kidney diseases
Skin and subcutaneous diseases
Sense organ diseases
Musculoskeletal disorders
Other non-communicable diseases
Transport injuries
Unintentional injuries
Self-harm and interpersonal violence
COVID-19
Other COVID-19 pandemic-related outcomes

Ukraine

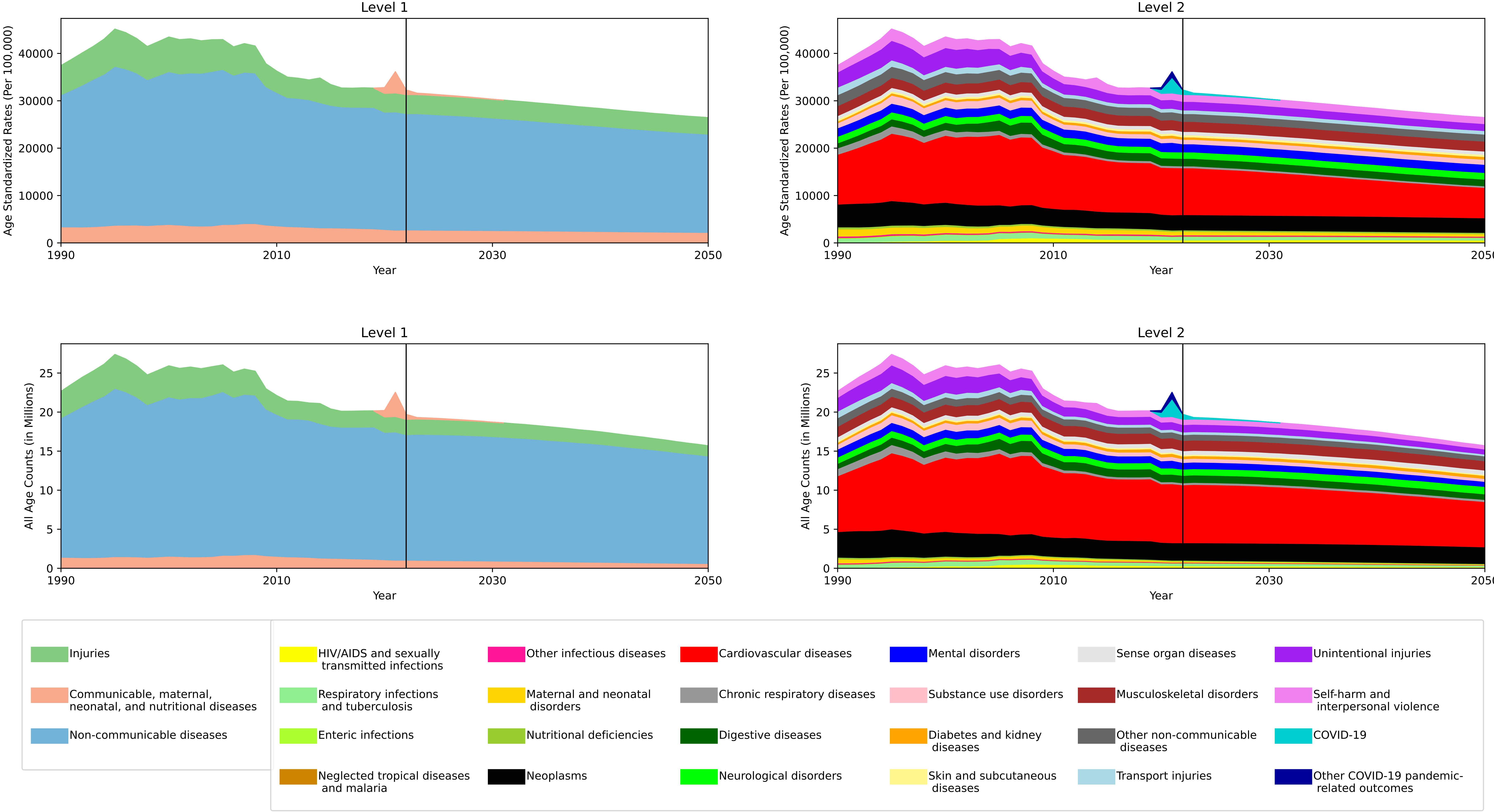

Level 1
Level 2
Age Standardized Rates (Per 100,000)
All Age Counts (in Millions)
Year
0
10000
20000
30000
40000
5
10
15
20
25
1990
2010
2030
2050
Injuries
Communicable, maternal, neonatal, and nutritional diseases
Non-communicable diseases
HIV/AIDS and sexually transmitted infections
Respiratory infections and tuberculosis
Enteric infections
Neglected tropical diseases and malaria
Other infectious diseases
Maternal and neonatal disorders
Nutritional deficiencies
Neoplasms
Cardiovascular diseases
Chronic respiratory diseases
Digestive diseases
Neurological disorders
Mental disorders
Substance use disorders
Diabetes and kidney diseases
Skin and subcutaneous diseases
Sense organ diseases
Musculoskeletal disorders
Other non-communicable diseases
Transport injuries
Unintentional injuries
Self-harm and interpersonal violence
COVID-19
Other COVID-19 pandemic-related outcomes

Australia

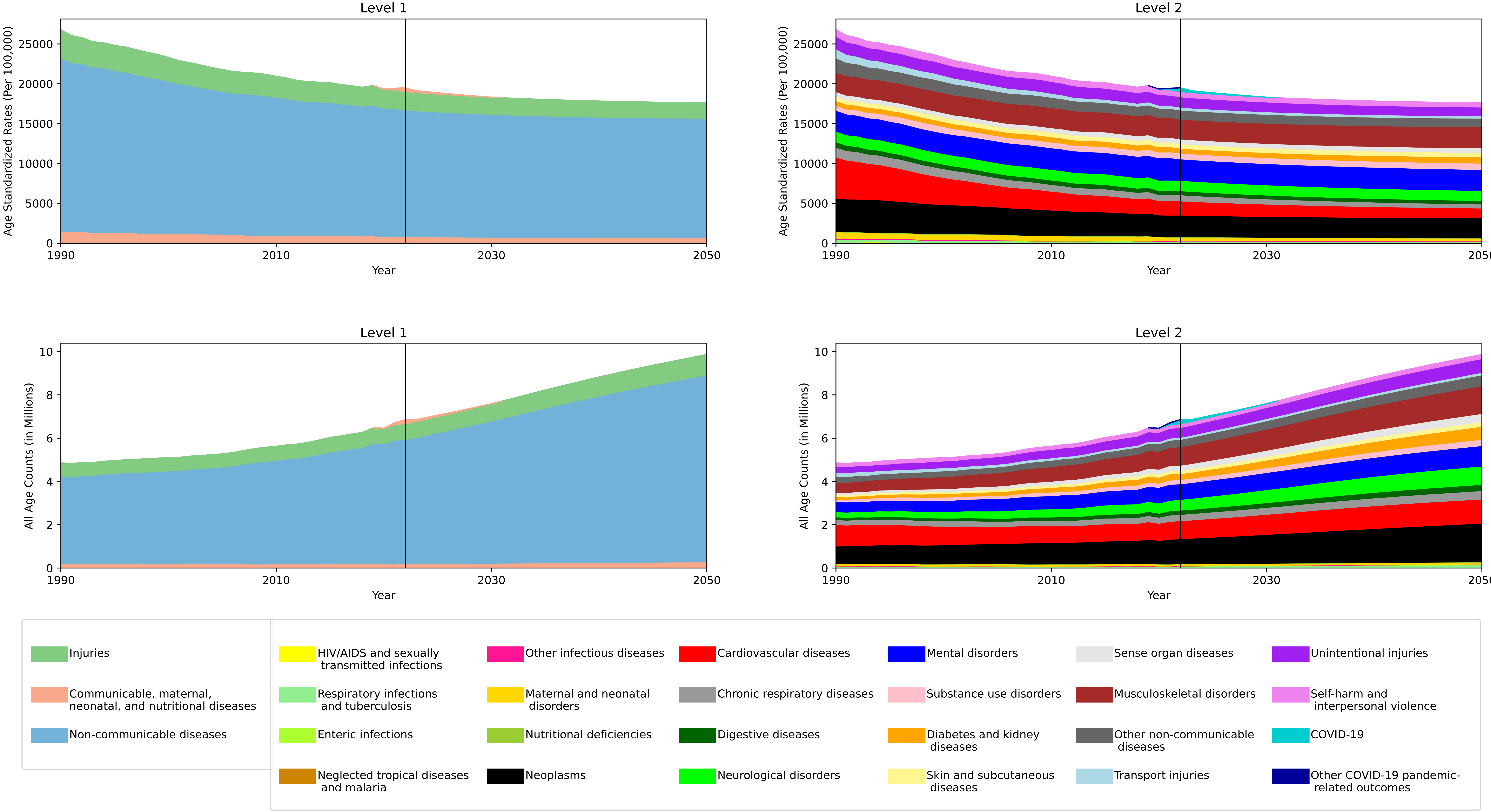

Level 1
Level 2
Age Standardized Rates (Per 100,000)
All Age Counts (in Millions)
Year
1990
2010
2030
2050
Injuries
Communicable, maternal, neonatal, and nutritional diseases
Non-communicable diseases
HIV/AIDS and sexually transmitted infections
Respiratory infections and tuberculosis
Enteric infections
Neglected tropical diseases and malaria
Other infectious diseases
Maternal and neonatal disorders
Nutritional deficiencies
Neoplasms
Cardiovascular diseases
Chronic respiratory diseases
Digestive diseases
Neurological disorders
Mental disorders
Substance use disorders
Diabetes and kidney diseases
Skin and subcutaneous diseases
Sense organ diseases
Musculoskeletal disorders
Other non-communicable diseases
Transport injuries
Unintentional injuries
Self-harm and interpersonal violence
COVID-19
Other COVID-19 pandemic-related outcomes

New Zealand

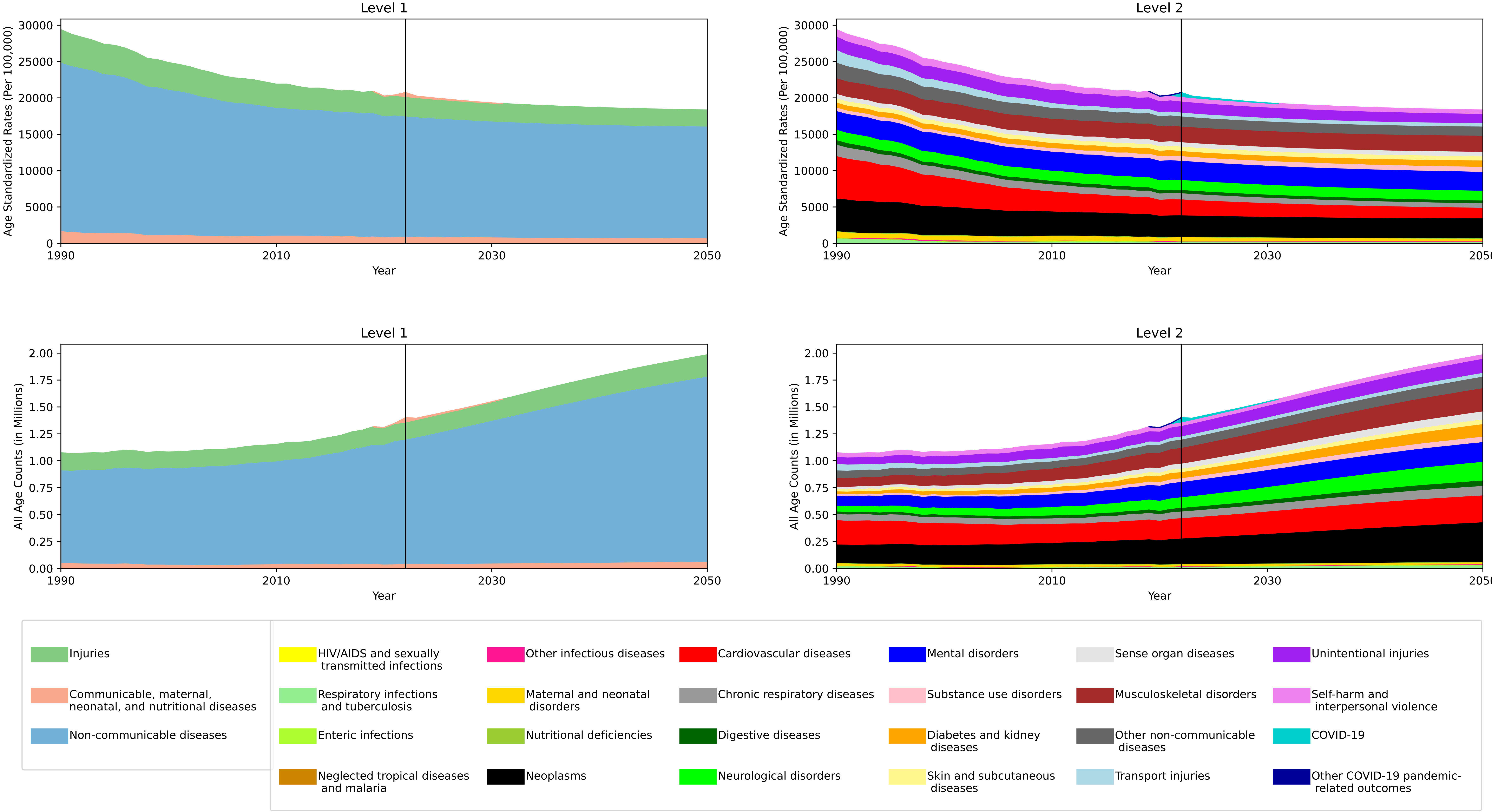
Level 1
Level 2
Age Standardized Rates (Per 100,000)
30000
25000
20000
15000
10000
5000
0
All Age Counts (in Millions)
2.00
1.75
1.50
1.25
1.00
0.75
0.50
0.25
0.00
1990
2010
2030
2050
Year
Injuries
Communicable, maternal, neonatal, and nutritional diseases
Non-communicable diseases
HIV/AIDS and sexually transmitted infections
Respiratory infections and tuberculosis
Enteric infections
Neglected tropical diseases and malaria
Other infectious diseases
Maternal and neonatal disorders
Nutritional deficiencies
Neoplasms
Cardiovascular diseases
Chronic respiratory diseases
Digestive diseases
Neurological disorders
Mental disorders
Substance use disorders
Diabetes and kidney diseases
Skin and subcutaneous diseases
Sense organ diseases
Musculoskeletal disorders
Other non-communicable diseases
Transport injuries
Unintentional injuries
Self-harm and interpersonal violence
COVID-19
Other COVID-19 pandemic-related outcomes

Brunei

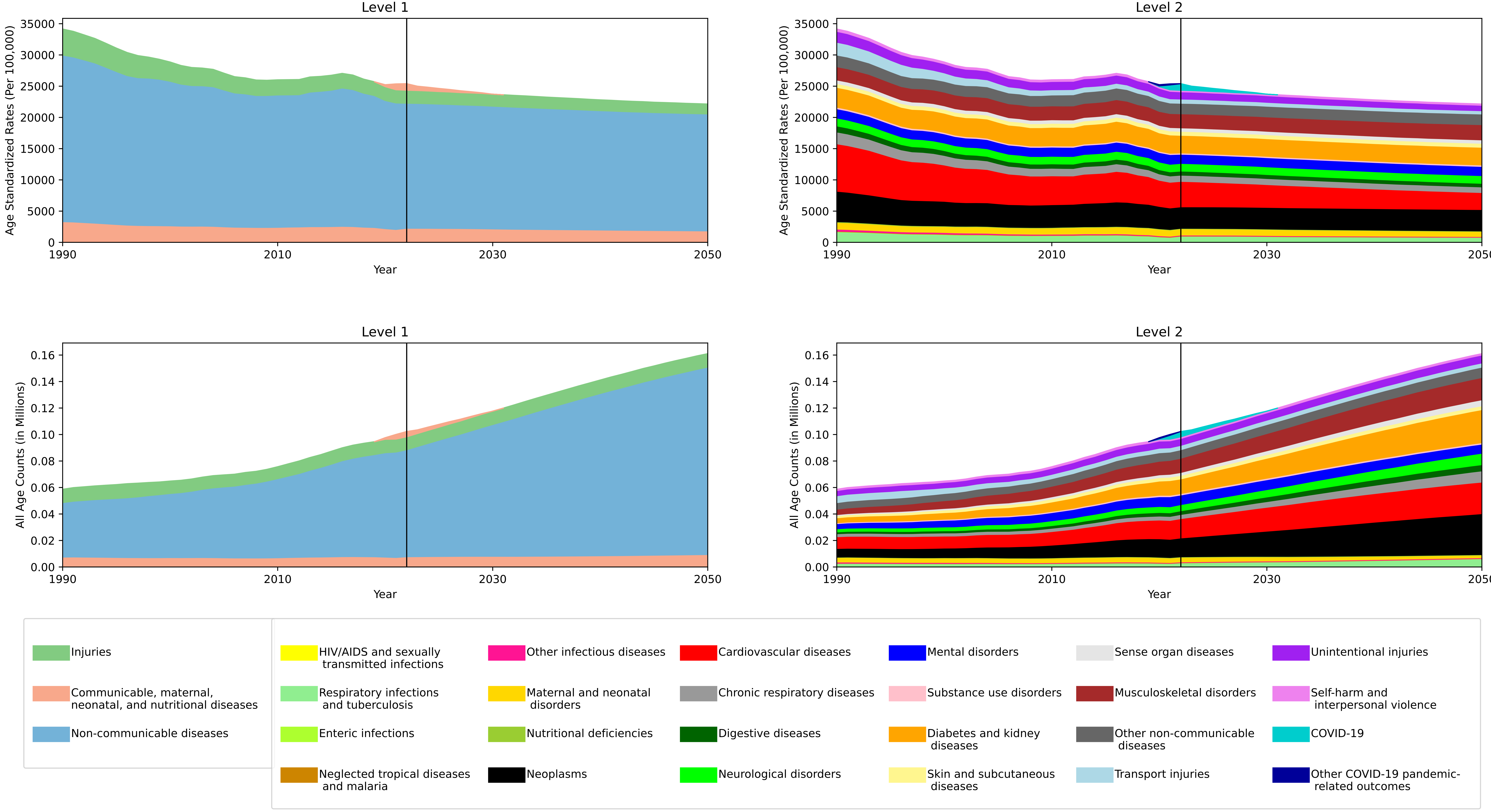
Level 1
Level 2
Age Standardized Rates (Per 100,000)
All Age Counts (in Millions)
Year
1990
2010
2030
2050
Injuries
Communicable, maternal, neonatal, and nutritional diseases
Non-communicable diseases
HIV/AIDS and sexually transmitted infections
Respiratory infections and tuberculosis
Enteric infections
Neglected tropical diseases and malaria
Other infectious diseases
Maternal and neonatal disorders
Nutritional deficiencies
Neoplasms
Cardiovascular diseases
Chronic respiratory diseases
Digestive diseases
Neurological disorders
Mental disorders
Substance use disorders
Diabetes and kidney diseases
Skin and subcutaneous diseases
Sense organ diseases
Musculoskeletal disorders
Other non-communicable diseases
Transport injuries
Unintentional injuries
Self-harm and interpersonal violence
COVID-19
Other COVID-19 pandemic-related outcomes

Japan

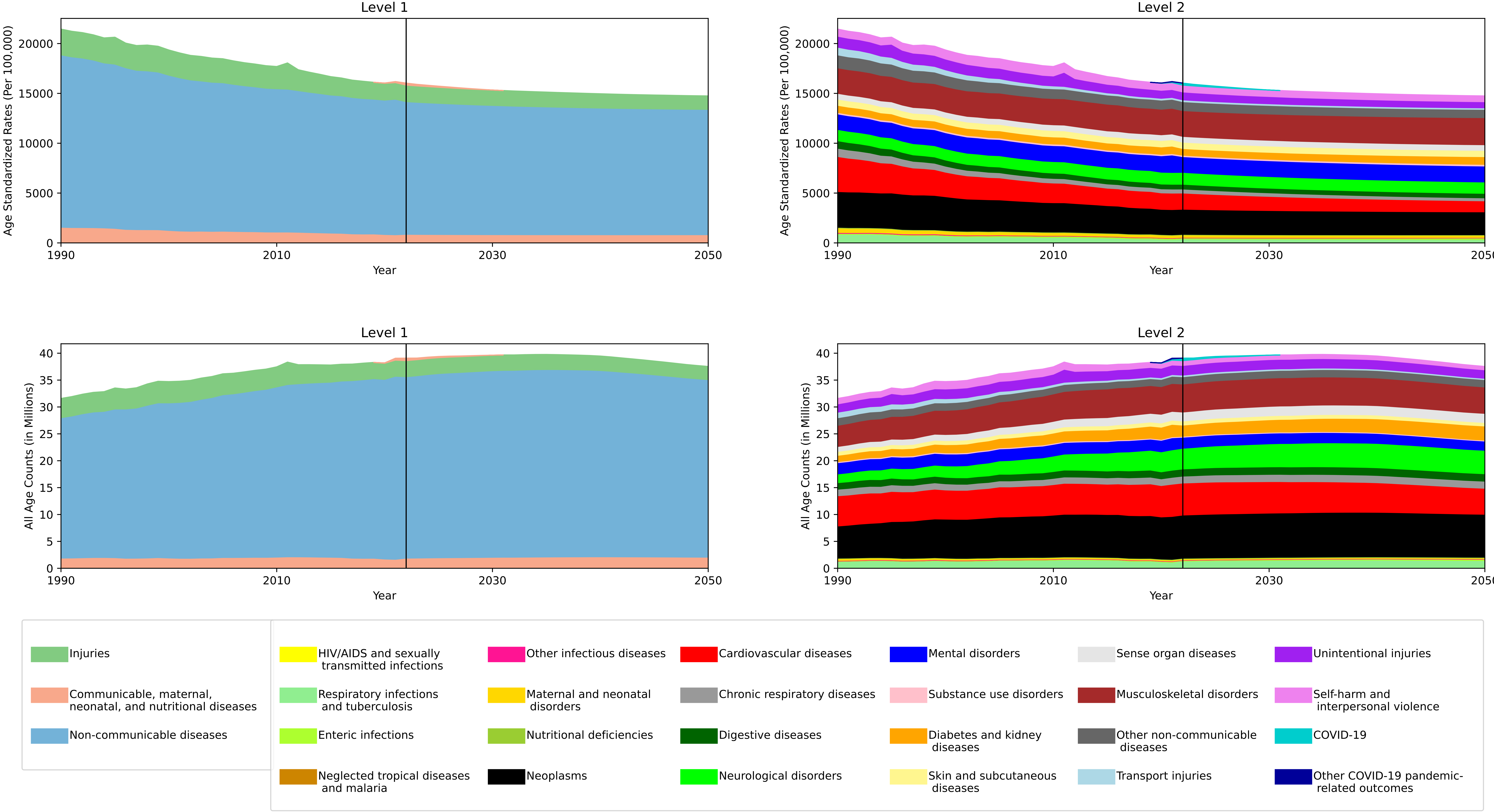
Level 1
Level 2
Age Standardized Rates (Per 100,000)
All Age Counts (in Millions)
Year
Injuries
Communicable, maternal, neonatal, and nutritional diseases
Non-communicable diseases
HIV/AIDS and sexually transmitted infections
Respiratory infections and tuberculosis
Enteric infections
Neglected tropical diseases and malaria
Other infectious diseases
Maternal and neonatal disorders
Nutritional deficiencies
Neoplasms
Cardiovascular diseases
Chronic respiratory diseases
Digestive diseases
Neurological disorders
Mental disorders
Substance use disorders
Diabetes and kidney diseases
Skin and subcutaneous diseases
Sense organ diseases
Musculoskeletal disorders
Other non-communicable diseases
Transport injuries
Unintentional injuries
Self-harm and interpersonal violence
COVID-19
Other COVID-19 pandemic-related outcomes

South Korea

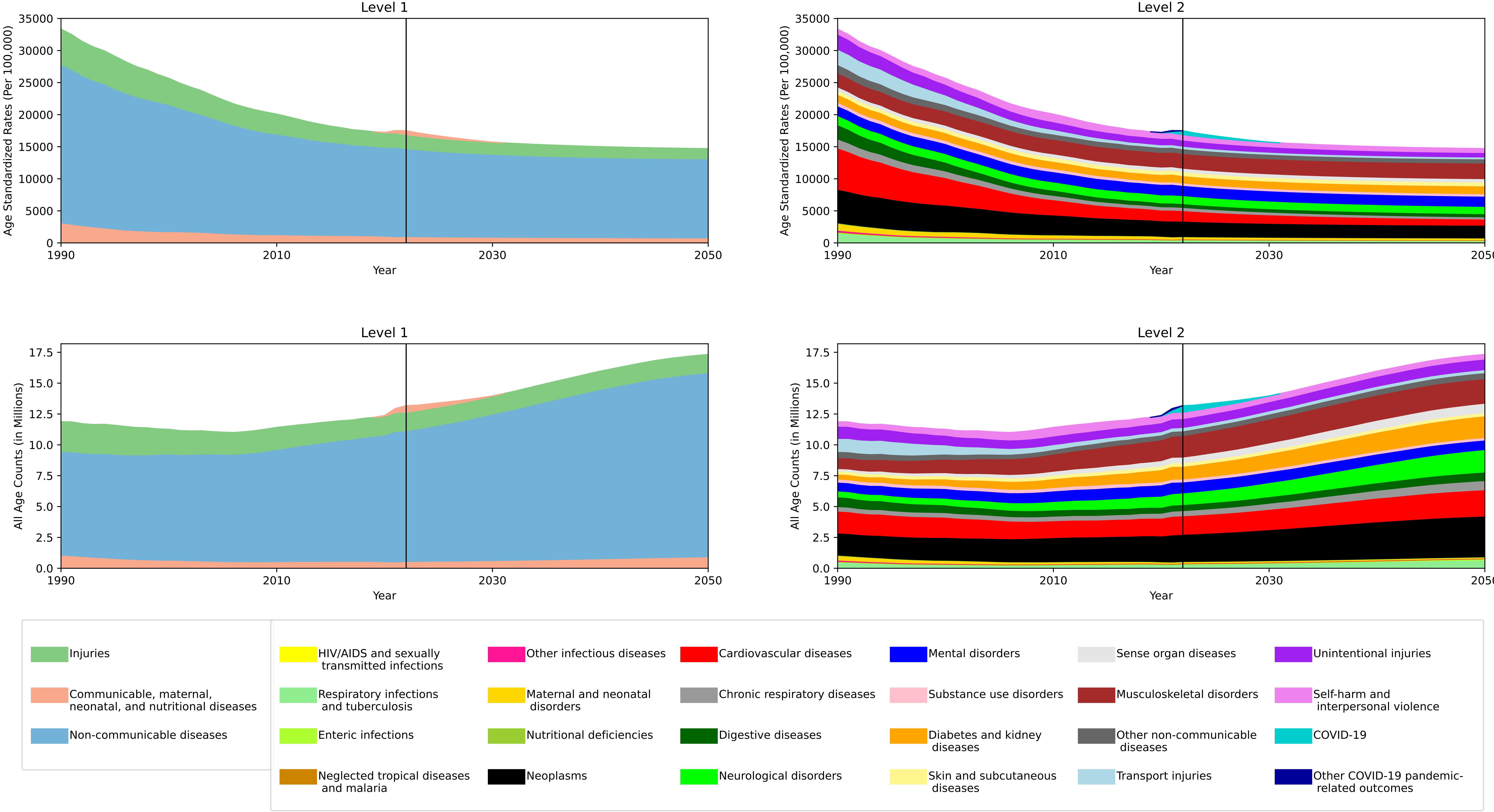
Level 1
Level 2
Age Standardized Rates (Per 100,000)
All Age Counts (in Millions)
Year
1990
2010
2030
2050
35000
30000
25000
20000
15000
10000
5000
0
17.5
15.0
12.5
10.0
7.5
5.0
2.5
0.0
Injuries
Communicable, maternal, neonatal, and nutritional diseases
Non-communicable diseases
HIV/AIDS and sexually transmitted infections
Respiratory infections and tuberculosis
Enteric infections
Neglected tropical diseases and malaria
Other infectious diseases
Maternal and neonatal disorders
Nutritional deficiencies
Neoplasms
Cardiovascular diseases
Chronic respiratory diseases
Digestive diseases
Neurological disorders
Mental disorders
Substance use disorders
Diabetes and kidney diseases
Skin and subcutaneous diseases
Sense organ diseases
Musculoskeletal disorders
Other non-communicable diseases
Transport injuries
Unintentional injuries
Self-harm and interpersonal violence
COVID-19
Other COVID-19 pandemic-related outcomes

Supplemental Results Figure S4. Age-standardised DALY rates and all age DALY counts and by Level 1 and Level 2 causes, 1990–2050

Singapore

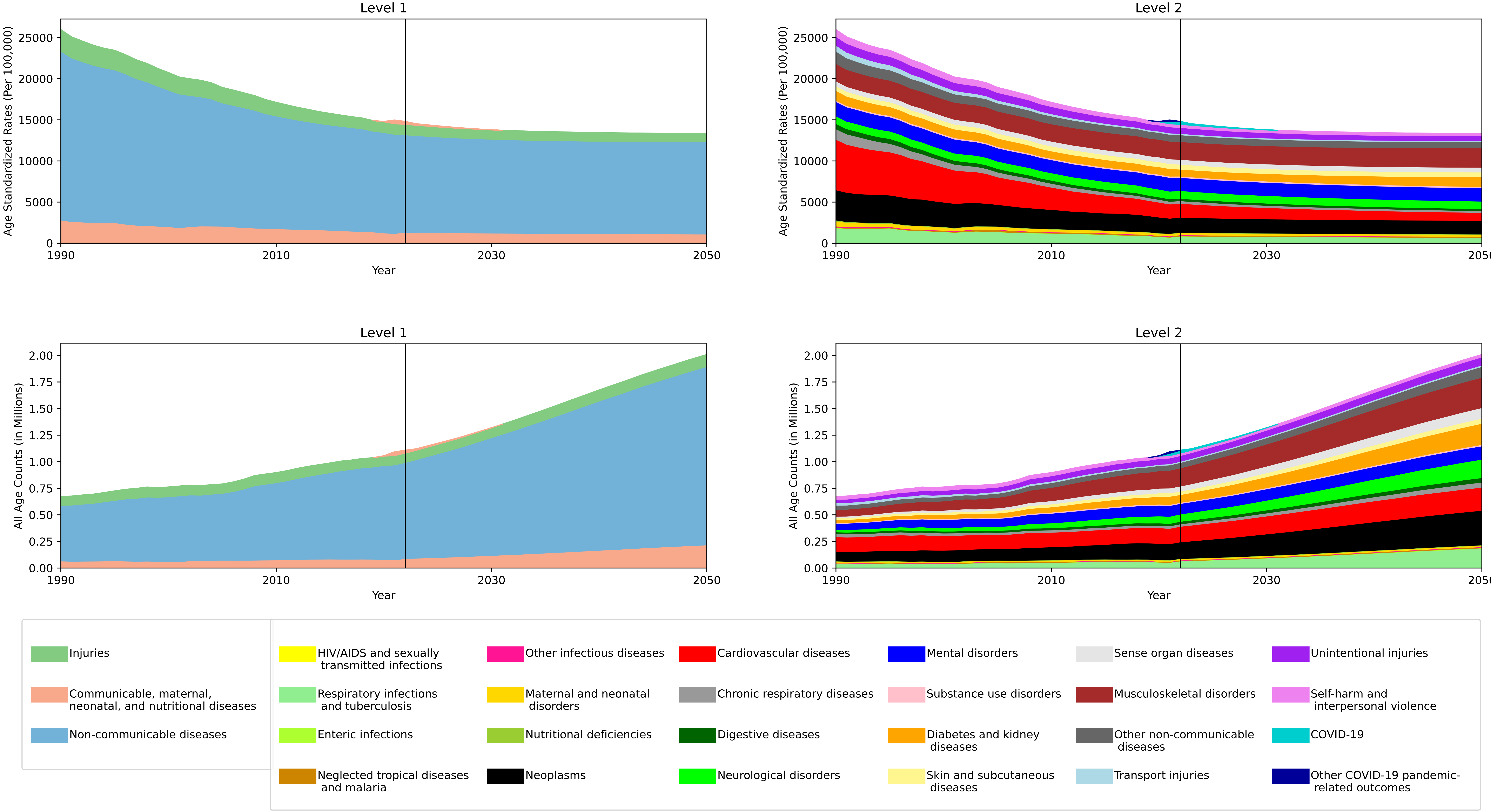

Canada

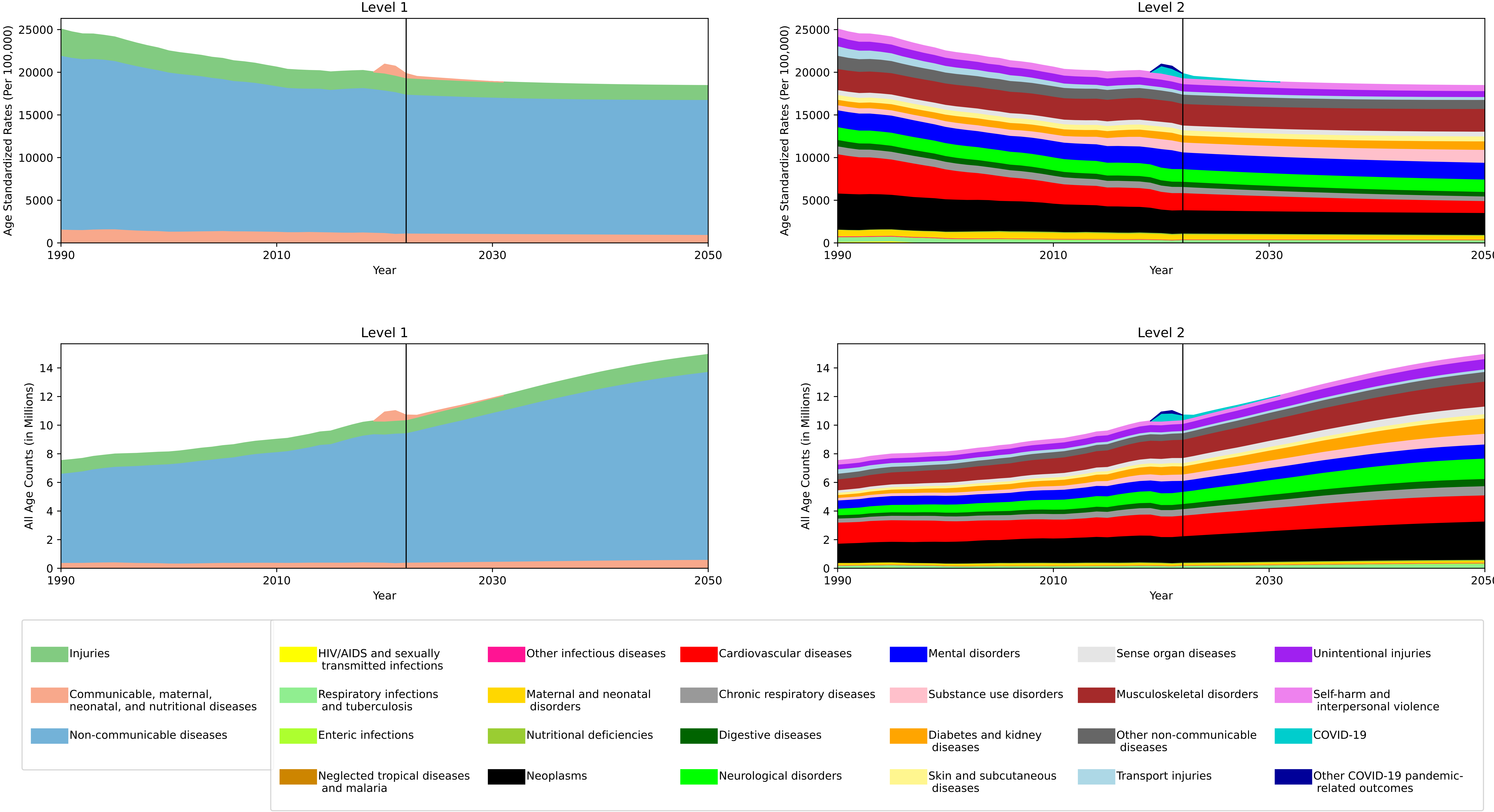
Level 1
Level 2
Age Standardized Rates (Per 100,000)
All Age Counts (in Millions)
Year
1990
2010
2030
2050
Injuries
Communicable, maternal, neonatal, and nutritional diseases
Non-communicable diseases
HIV/AIDS and sexually transmitted infections
Respiratory infections and tuberculosis
Enteric infections
Neglected tropical diseases and malaria
Other infectious diseases
Maternal and neonatal disorders
Nutritional deficiencies
Neoplasms
Cardiovascular diseases
Chronic respiratory diseases
Digestive diseases
Neurological disorders
Mental disorders
Substance use disorders
Diabetes and kidney diseases
Skin and subcutaneous diseases
Sense organ diseases
Musculoskeletal disorders
Other non-communicable diseases
Transport injuries
Unintentional injuries
Self-harm and interpersonal violence
COVID-19
Other COVID-19 pandemic-related outcomes

Greenland

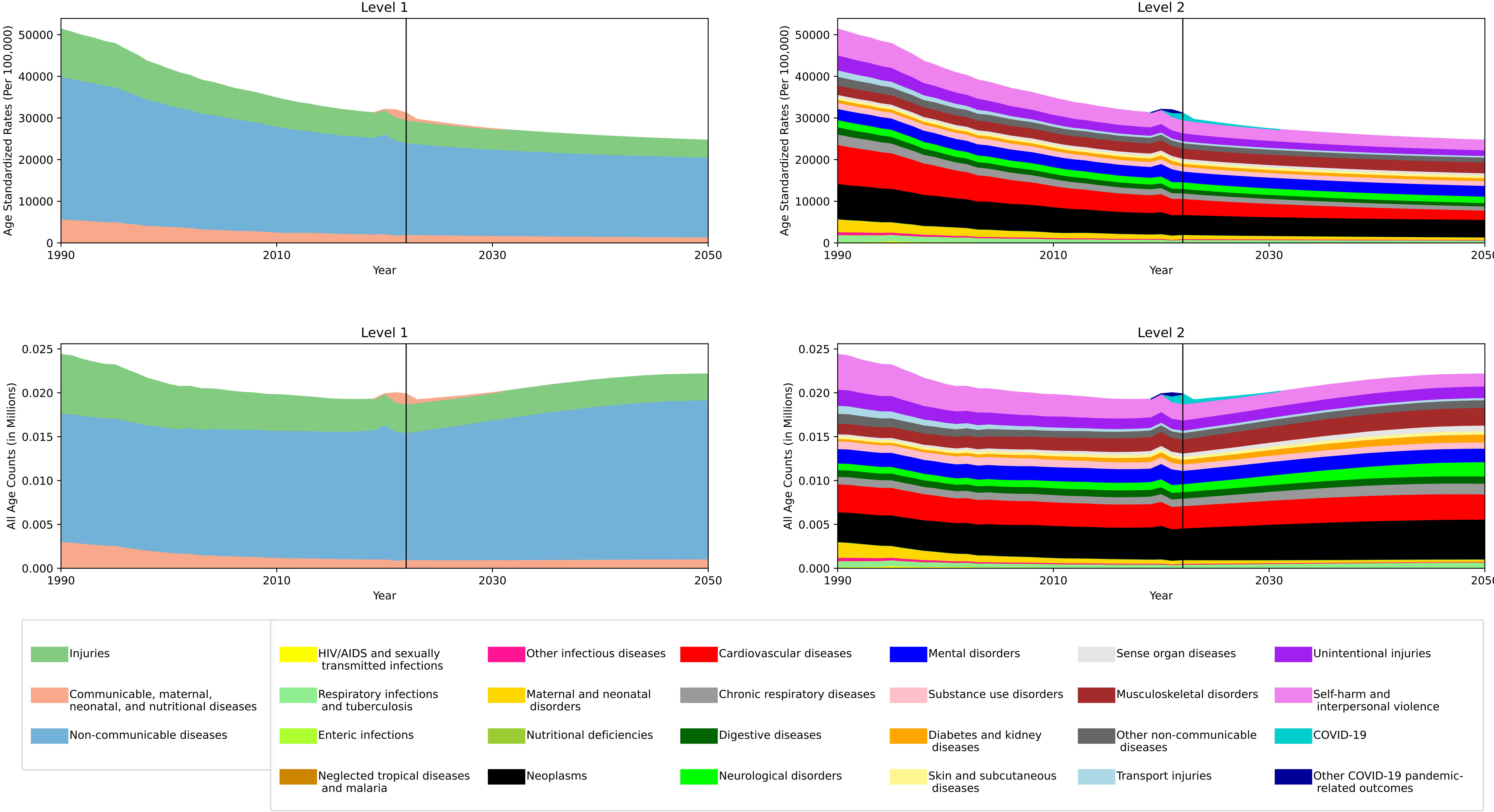
Level 1
Level 2
Age Standardized Rates (Per 100,000)
All Age Counts (in Millions)
Year
Injuries
Communicable, maternal, neonatal, and nutritional diseases
Non-communicable diseases
HIV/AIDS and sexually transmitted infections
Respiratory infections and tuberculosis
Enteric infections
Neglected tropical diseases and malaria
Other infectious diseases
Maternal and neonatal disorders
Nutritional deficiencies
Neoplasms
Cardiovascular diseases
Chronic respiratory diseases
Digestive diseases
Neurological disorders
Mental disorders
Substance use disorders
Diabetes and kidney diseases
Skin and subcutaneous diseases
Sense organ diseases
Musculoskeletal disorders
Other non-communicable diseases
Transport injuries
Unintentional injuries
Self-harm and interpersonal violence
COVID-19
Other COVID-19 pandemic-related outcomes

USA

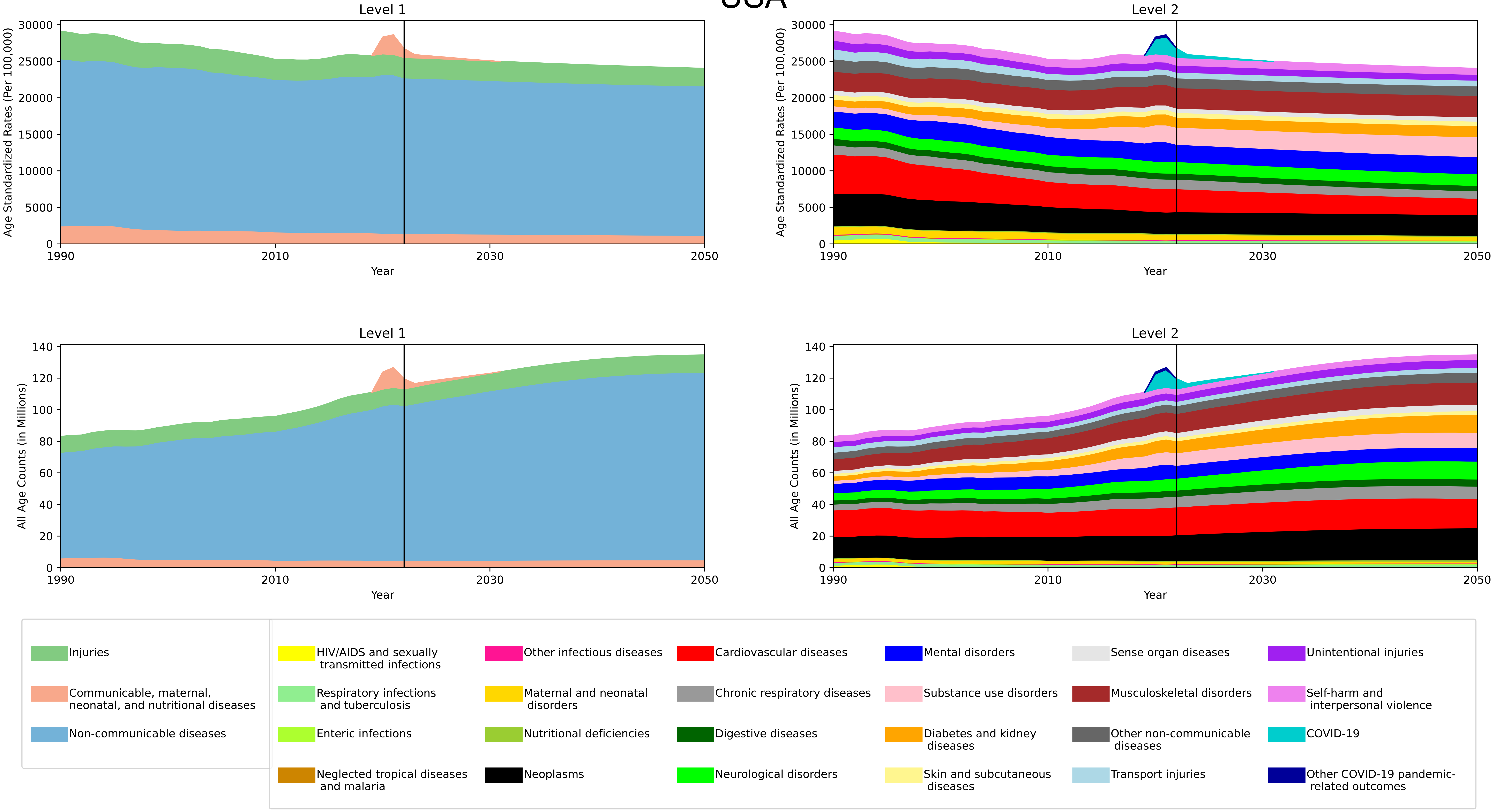
Level 1
Level 2
Age Standardized Rates (Per 100,000)
All Age Counts (in Millions)
Year
Injuries
Communicable, maternal, neonatal, and nutritional diseases
Non-communicable diseases
HIV/AIDS and sexually transmitted infections
Respiratory infections and tuberculosis
Enteric infections
Neglected tropical diseases and malaria
Other infectious diseases
Maternal and neonatal disorders
Nutritional deficiencies
Neoplasms
Cardiovascular diseases
Chronic respiratory diseases
Digestive diseases
Neurological disorders
Mental disorders
Substance use disorders
Diabetes and kidney diseases
Skin and subcutaneous diseases
Sense organ diseases
Musculoskeletal disorders
Other non-communicable diseases
Transport injuries
Unintentional injuries
Self-harm and interpersonal violence
COVID-19
Other COVID-19 pandemic-related outcomes

Argentina

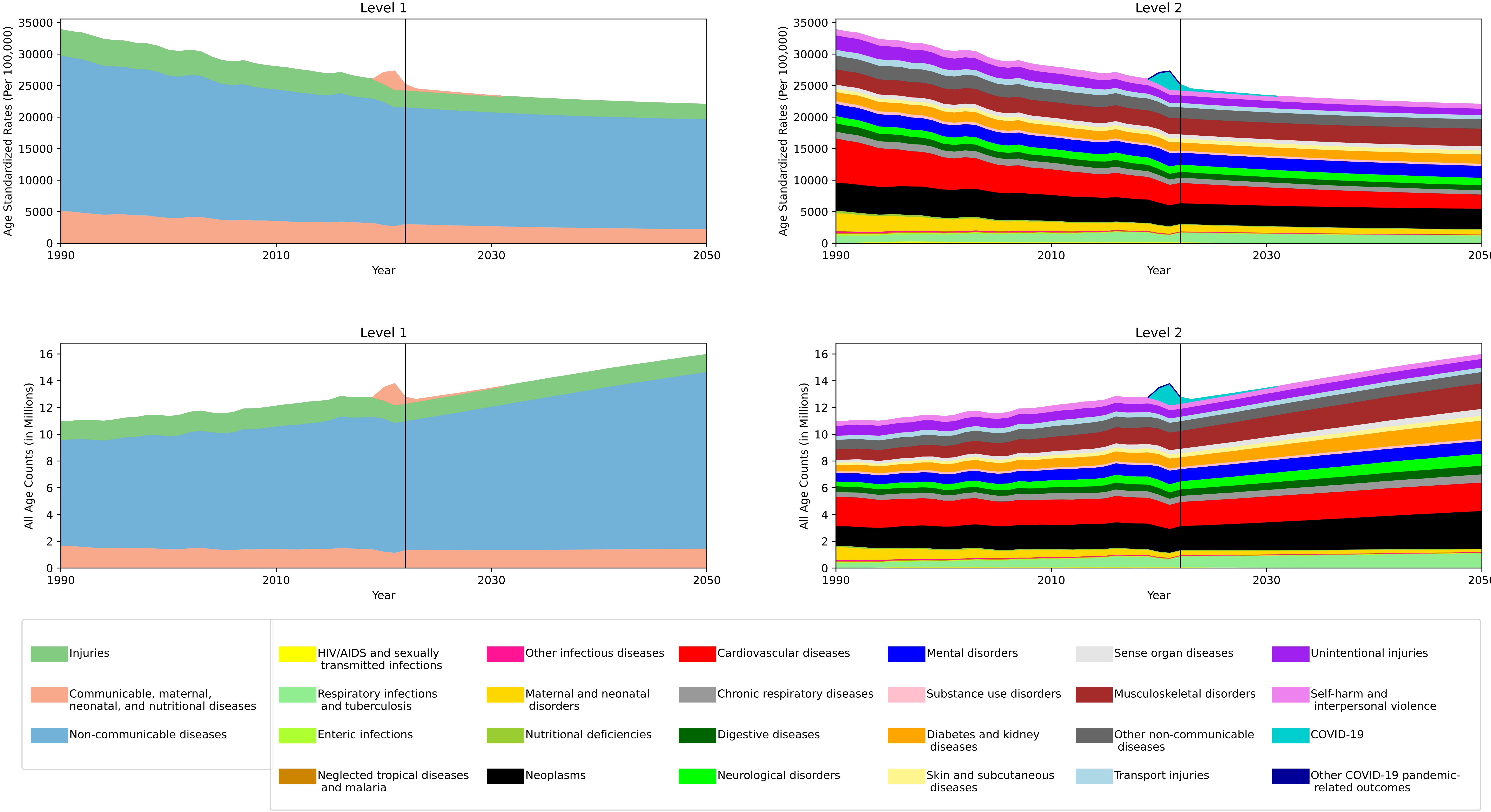
Level 1
Level 2
Age Standardized Rates (Per 100,000)
All Age Counts (in Millions)
Year
1990
2010
2030
2050
0
5000
10000
15000
20000
25000
30000
35000
2
4
6
8
10
12
14
16
Injuries
Communicable, maternal, neonatal, and nutritional diseases
Non-communicable diseases
HIV/AIDS and sexually transmitted infections
Respiratory infections and tuberculosis
Enteric infections
Neglected tropical diseases and malaria
Other infectious diseases
Maternal and neonatal disorders
Nutritional deficiencies
Neoplasms
Cardiovascular diseases
Chronic respiratory diseases
Digestive diseases
Neurological disorders
Mental disorders
Substance use disorders
Diabetes and kidney diseases
Skin and subcutaneous diseases
Sense organ diseases
Musculoskeletal disorders
Other non-communicable diseases
Transport injuries
Unintentional injuries
Self-harm and interpersonal violence
COVID-19
Other COVID-19 pandemic-related outcomes

Chile

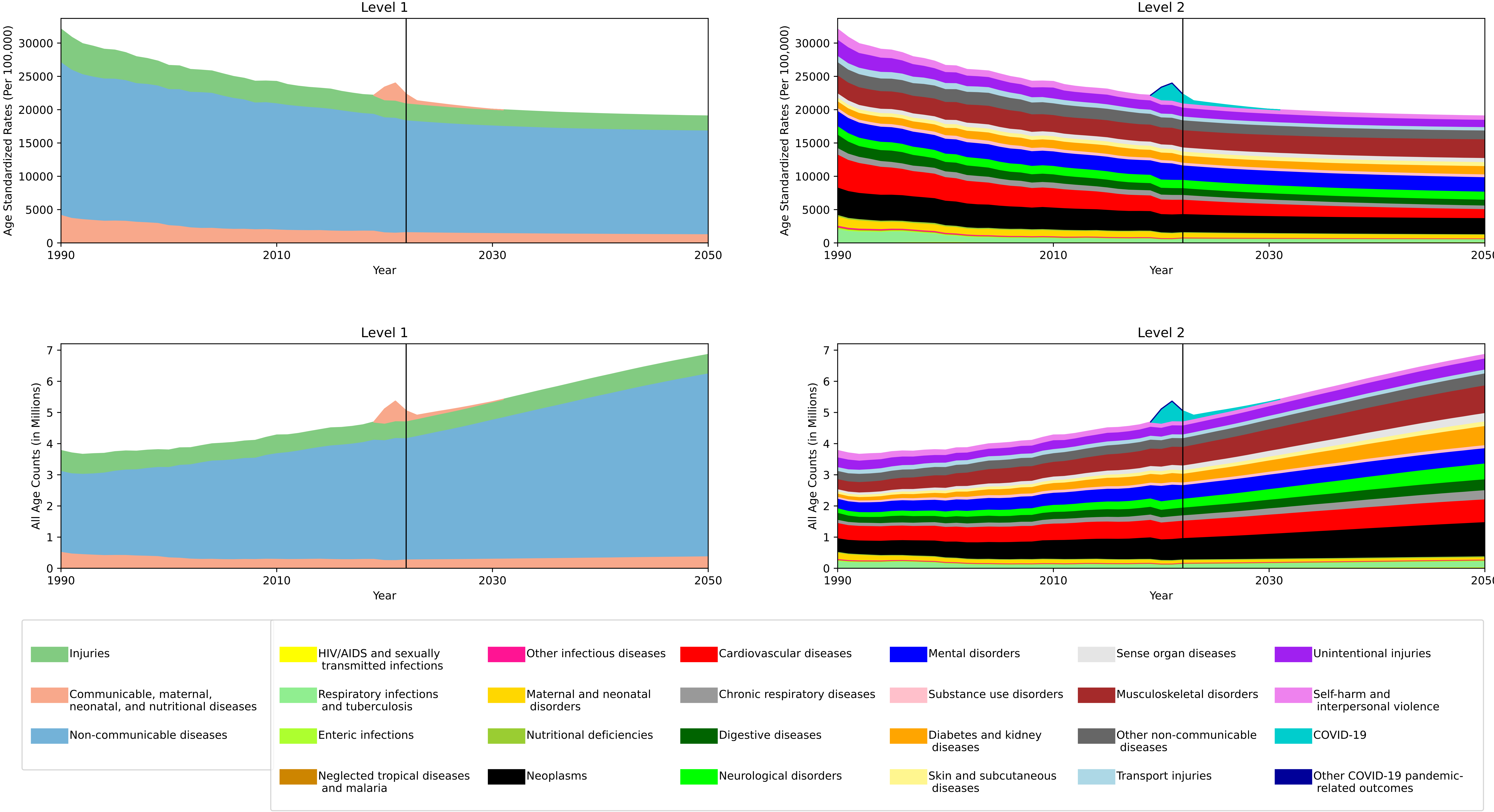
Level 1
Level 2
Age Standardized Rates (Per 100,000)
All Age Counts (in Millions)
Year
1990
2010
2030
2050
Injuries
Communicable, maternal, neonatal, and nutritional diseases
Non-communicable diseases
HIV/AIDS and sexually transmitted infections
Respiratory infections and tuberculosis
Enteric infections
Neglected tropical diseases and malaria
Other infectious diseases
Maternal and neonatal disorders
Nutritional deficiencies
Neoplasms
Cardiovascular diseases
Chronic respiratory diseases
Digestive diseases
Neurological disorders
Mental disorders
Substance use disorders
Diabetes and kidney diseases
Skin and subcutaneous diseases
Sense organ diseases
Musculoskeletal disorders
Other non-communicable diseases
Transport injuries
Unintentional injuries
Self-harm and interpersonal violence
COVID-19
Other COVID-19 pandemic-related outcomes

Uruguay

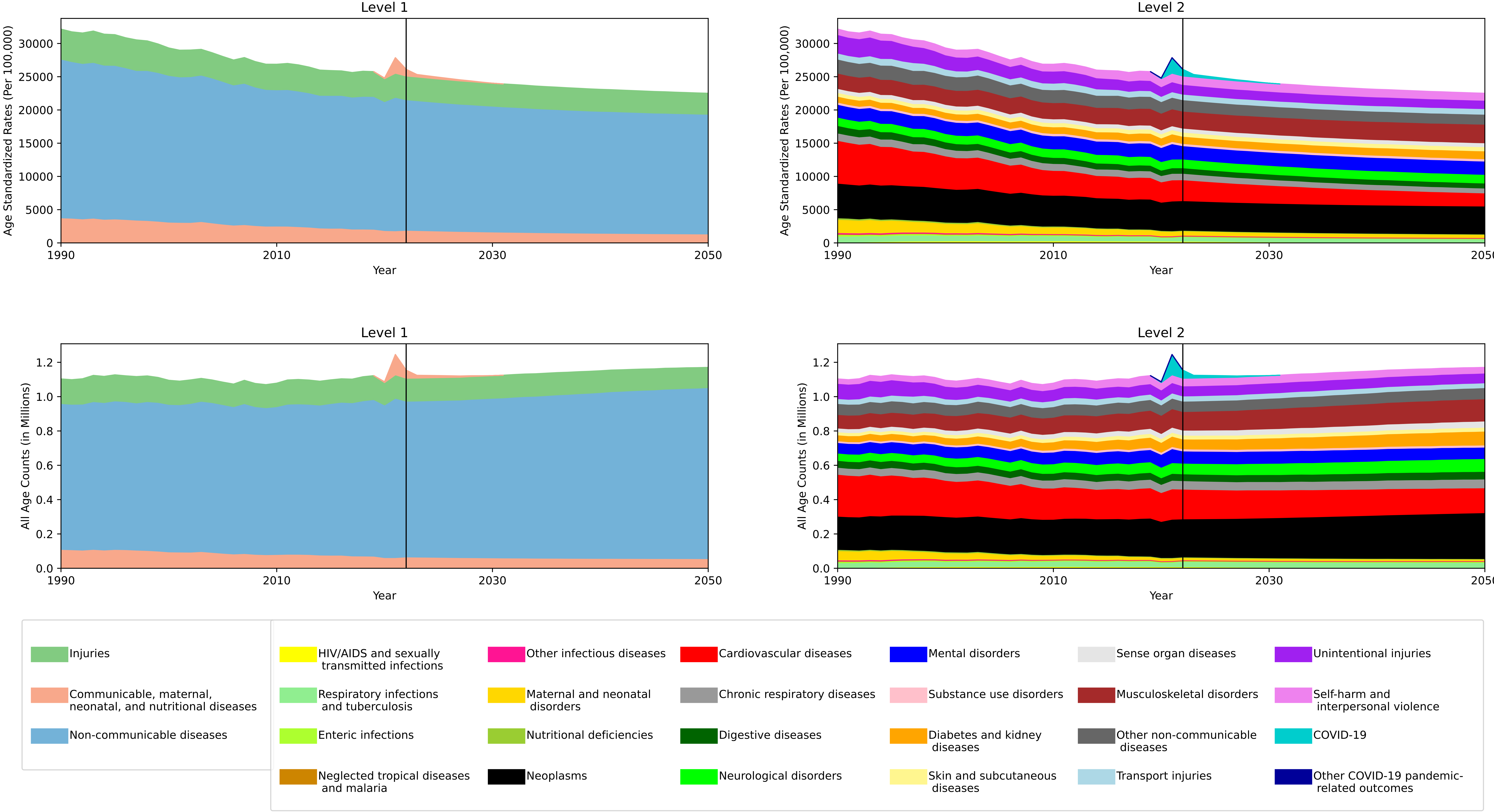
Level 1
Level 2
Age Standardized Rates (Per 100,000)
All Age Counts (in Millions)
Year
1990
2010
2030
2050
Injuries
Communicable, maternal, neonatal, and nutritional diseases
Non-communicable diseases
HIV/AIDS and sexually transmitted infections
Respiratory infections and tuberculosis
Enteric infections
Neglected tropical diseases and malaria
Other infectious diseases
Maternal and neonatal disorders
Nutritional deficiencies
Neoplasms
Cardiovascular diseases
Chronic respiratory diseases
Digestive diseases
Neurological disorders
Mental disorders
Substance use disorders
Diabetes and kidney diseases
Skin and subcutaneous diseases
Sense organ diseases
Musculoskeletal disorders
Other non-communicable diseases
Transport injuries
Unintentional injuries
Self-harm and interpersonal violence
COVID-19
Other COVID-19 pandemic-related outcomes

Andorra

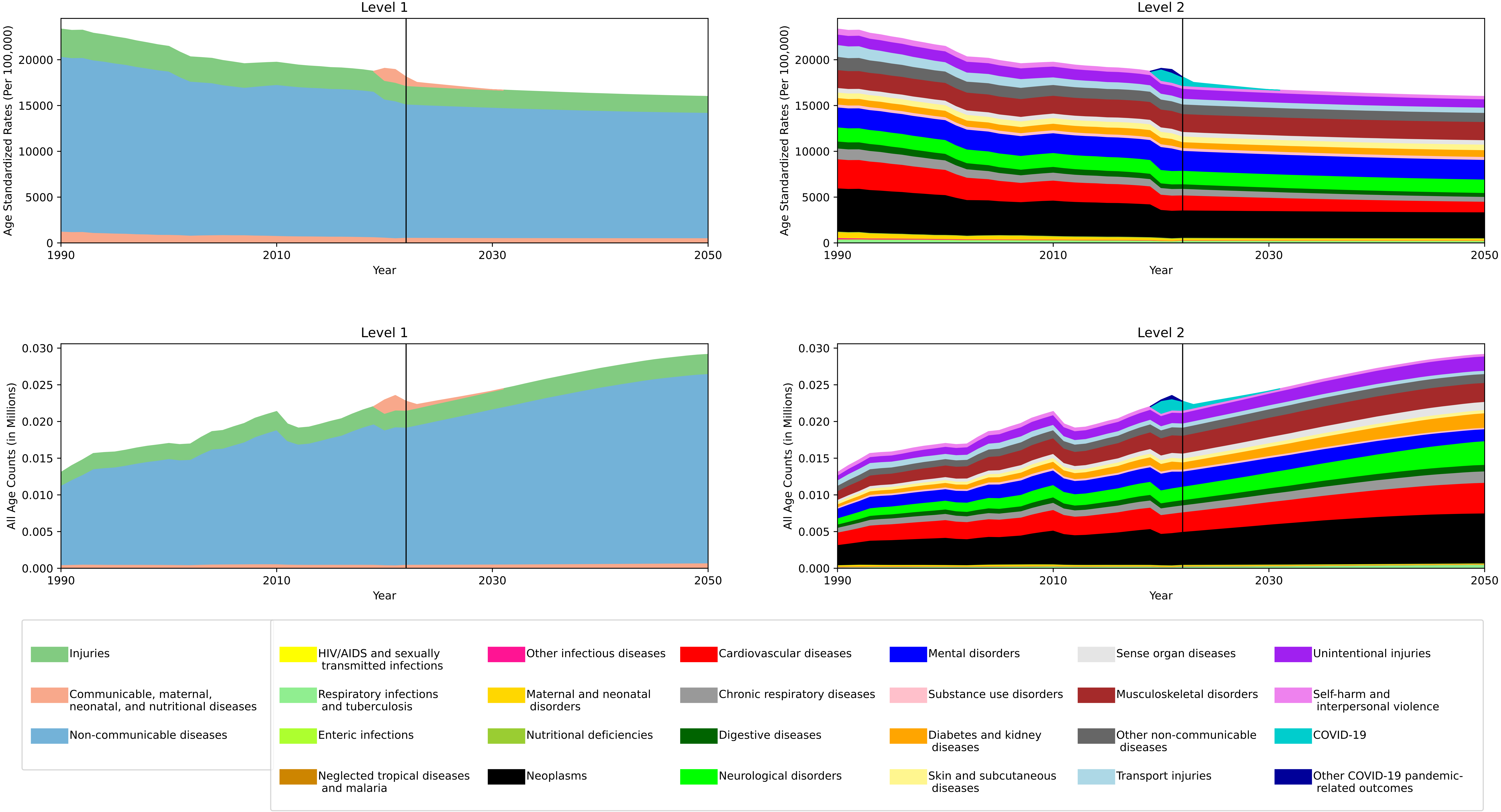
Level 1
Level 2
Age Standardized Rates (Per 100,000)
All Age Counts (in Millions)
Year
1990
2010
2030
2050
0
5000
10000
15000
20000
0.000
0.005
0.010
0.015
0.020
0.025
0.030
Injuries
Communicable, maternal, neonatal, and nutritional diseases
Non-communicable diseases
HIV/AIDS and sexually transmitted infections
Respiratory infections and tuberculosis
Enteric infections
Neglected tropical diseases and malaria
Other infectious diseases
Maternal and neonatal disorders
Nutritional deficiencies
Neoplasms
Cardiovascular diseases
Chronic respiratory diseases
Digestive diseases
Neurological disorders
Mental disorders
Substance use disorders
Diabetes and kidney diseases
Skin and subcutaneous diseases
Sense organ diseases
Musculoskeletal disorders
Other non-communicable diseases
Transport injuries
Unintentional injuries
Self-harm and interpersonal violence
COVID-19
Other COVID-19 pandemic-related outcomes

Austria

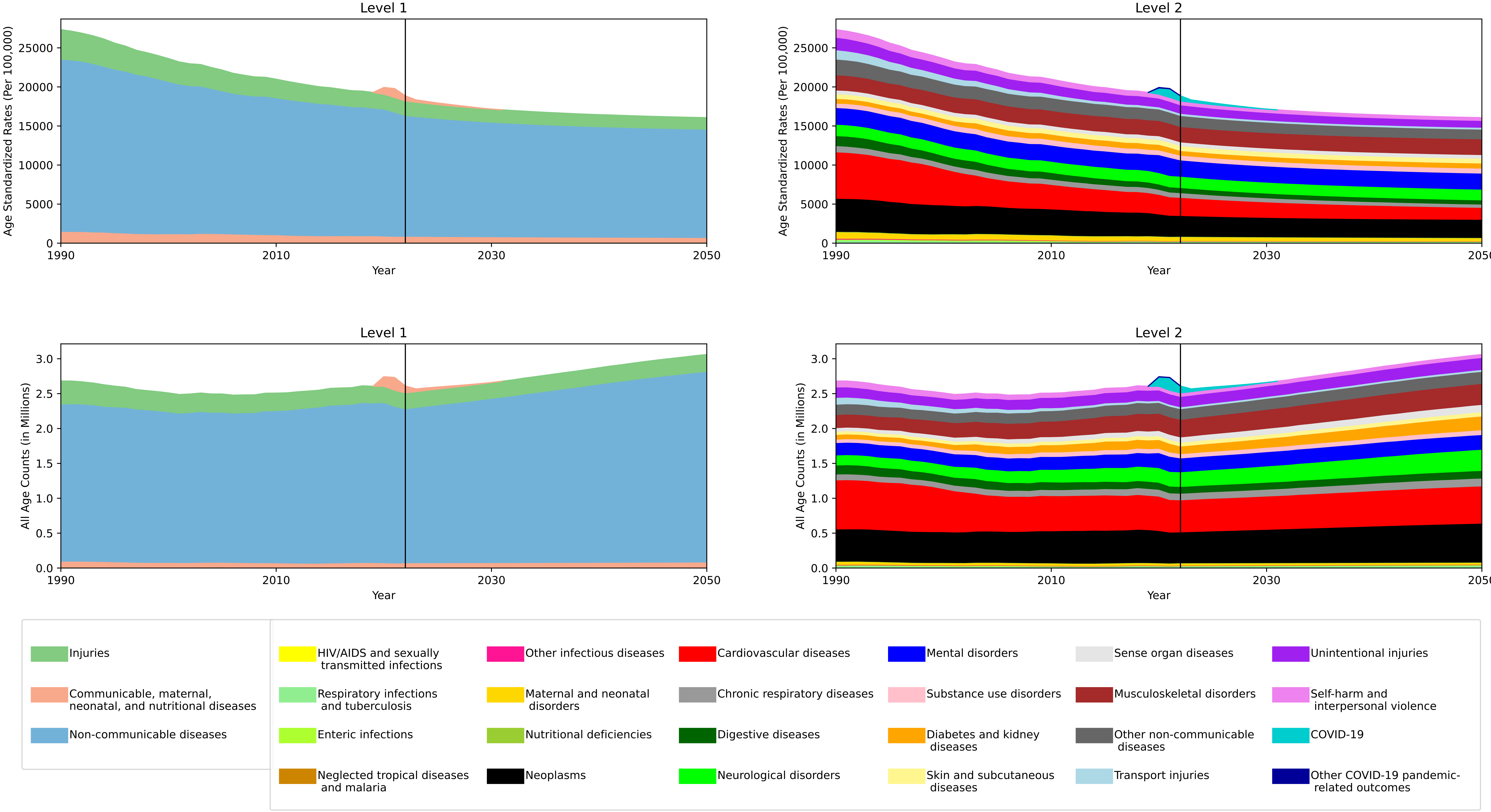
Level 1
Level 2
Age Standardized Rates (Per 100,000)
All Age Counts (in Millions)
Year
Injuries
Communicable, maternal, neonatal, and nutritional diseases
Non-communicable diseases
HIV/AIDS and sexually transmitted infections
Respiratory infections and tuberculosis
Enteric infections
Neglected tropical diseases and malaria
Other infectious diseases
Maternal and neonatal disorders
Nutritional deficiencies
Neoplasms
Cardiovascular diseases
Chronic respiratory diseases
Digestive diseases
Neurological disorders
Mental disorders
Substance use disorders
Diabetes and kidney diseases
Skin and subcutaneous diseases
Sense organ diseases
Musculoskeletal disorders
Other non-communicable diseases
Transport injuries
Unintentional injuries
Self-harm and interpersonal violence
COVID-19
Other COVID-19 pandemic-related outcomes

Belgium

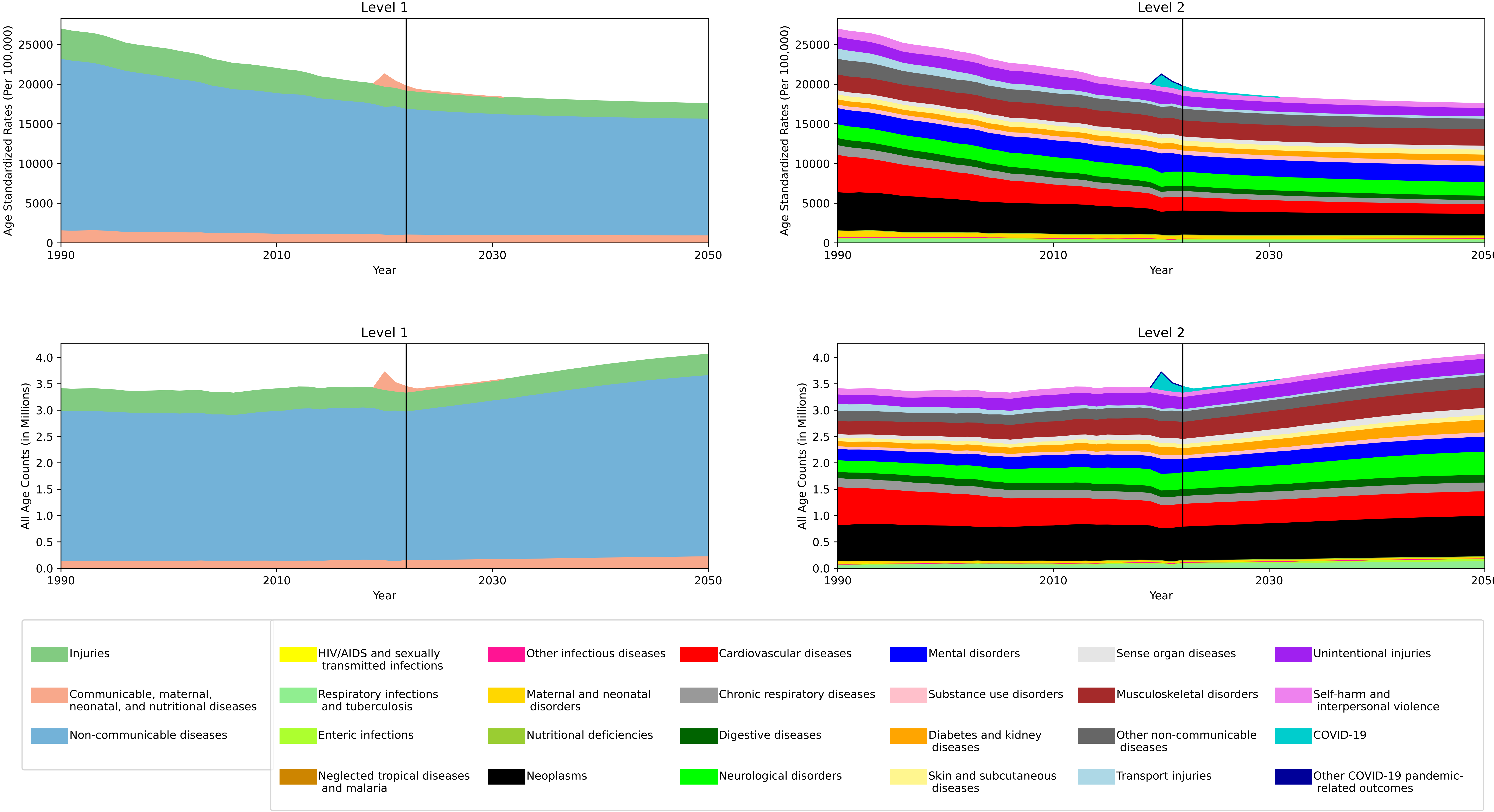
Level 1
Level 2
Age Standardized Rates (Per 100,000)
All Age Counts (in Millions)
Year
1990
2010
2030
2050
0
5000
10000
15000
20000
25000
0.0
0.5
1.0
1.5
2.0
2.5
3.0
3.5
4.0
Injuries
Communicable, maternal, neonatal, and nutritional diseases
Non-communicable diseases
HIV/AIDS and sexually transmitted infections
Respiratory infections and tuberculosis
Enteric infections
Neglected tropical diseases and malaria
Other infectious diseases
Maternal and neonatal disorders
Nutritional deficiencies
Neoplasms
Cardiovascular diseases
Chronic respiratory diseases
Digestive diseases
Neurological disorders
Mental disorders
Substance use disorders
Diabetes and kidney diseases
Skin and subcutaneous diseases
Sense organ diseases
Musculoskeletal disorders
Other non-communicable diseases
Transport injuries
Unintentional injuries
Self-harm and interpersonal violence
COVID-19
Other COVID-19 pandemic-related outcomes

Cyprus

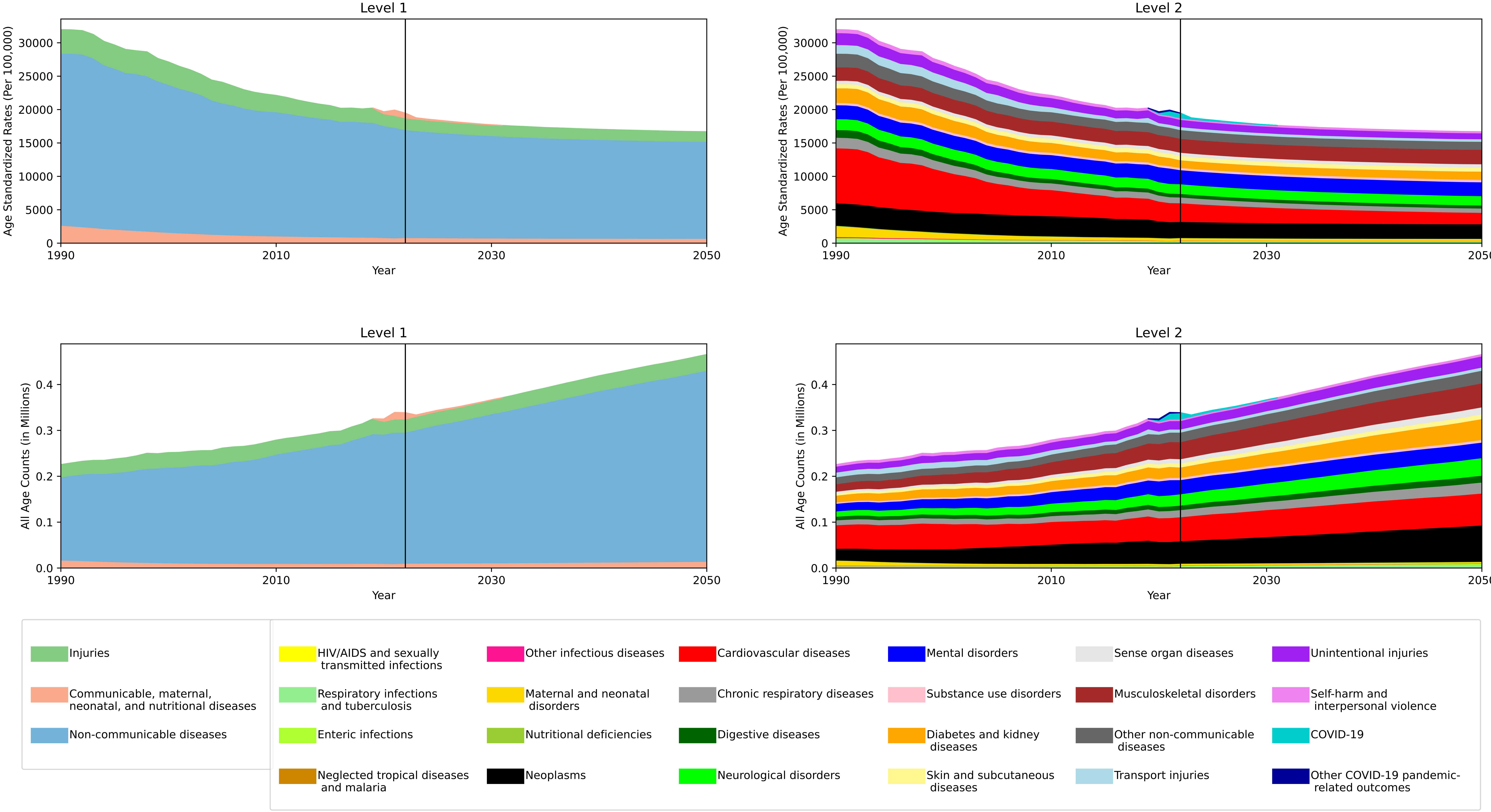
Level 1
Level 2
Age Standardized Rates (Per 100,000)
All Age Counts (in Millions)
Year
1990
2010
2030
2050
Injuries
Communicable, maternal, neonatal, and nutritional diseases
Non-communicable diseases
HIV/AIDS and sexually transmitted infections
Respiratory infections and tuberculosis
Enteric infections
Neglected tropical diseases and malaria
Other infectious diseases
Maternal and neonatal disorders
Nutritional deficiencies
Neoplasms
Cardiovascular diseases
Chronic respiratory diseases
Digestive diseases
Neurological disorders
Mental disorders
Substance use disorders
Diabetes and kidney diseases
Skin and subcutaneous diseases
Sense organ diseases
Musculoskeletal disorders
Other non-communicable diseases
Transport injuries
Unintentional injuries
Self-harm and interpersonal violence
COVID-19
Other COVID-19 pandemic-related outcomes

Denmark

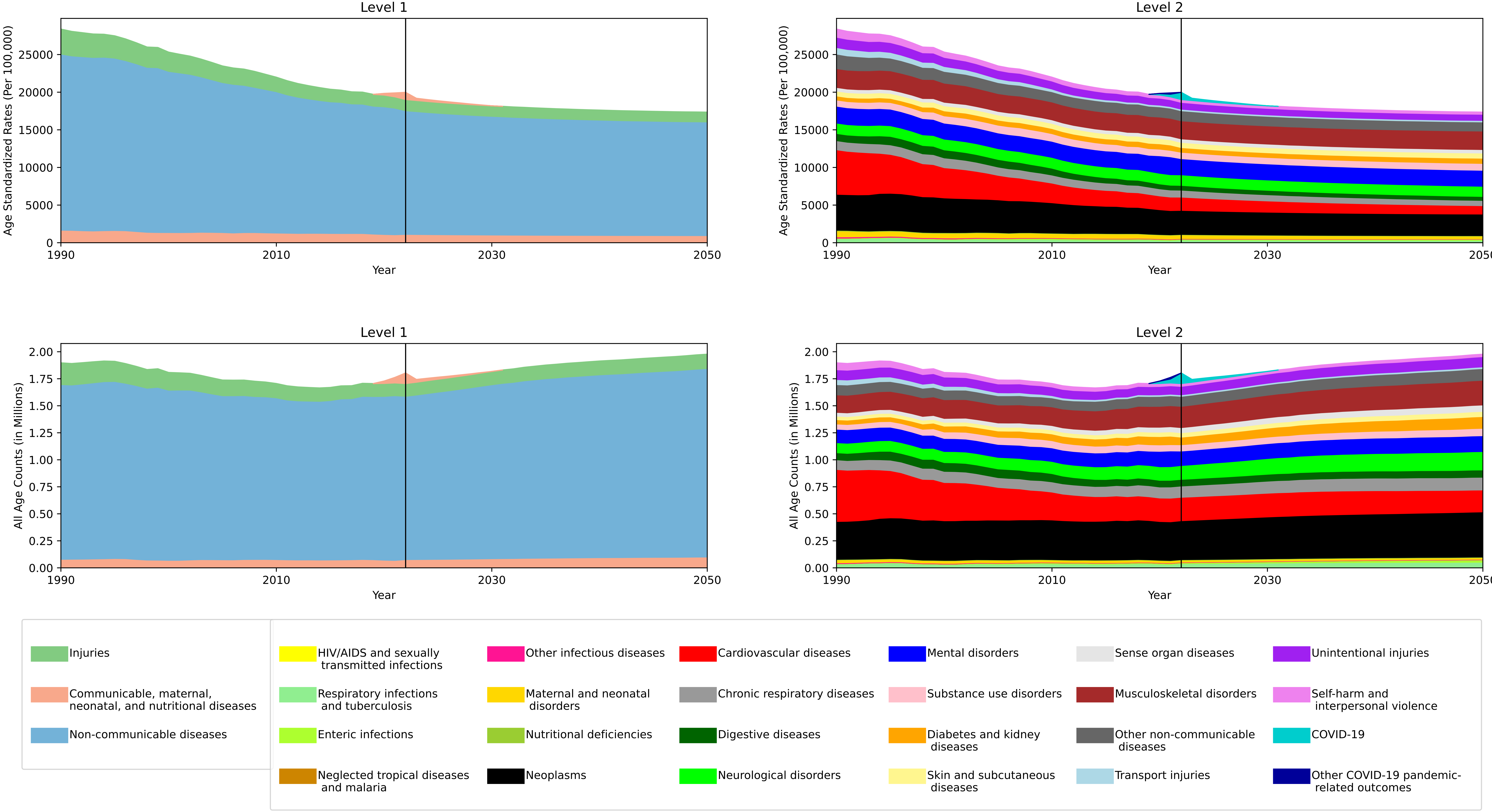
Level 1
Level 2
Age Standardized Rates (Per 100,000)
All Age Counts (in Millions)
Year
1990
2010
2030
2050
Injuries
Communicable, maternal, neonatal, and nutritional diseases
Non-communicable diseases
HIV/AIDS and sexually transmitted infections
Respiratory infections and tuberculosis
Enteric infections
Neglected tropical diseases and malaria
Other infectious diseases
Maternal and neonatal disorders
Nutritional deficiencies
Neoplasms
Cardiovascular diseases
Chronic respiratory diseases
Digestive diseases
Neurological disorders
Mental disorders
Substance use disorders
Diabetes and kidney diseases
Skin and subcutaneous diseases
Sense organ diseases
Musculoskeletal disorders
Other non-communicable diseases
Transport injuries
Unintentional injuries
Self-harm and interpersonal violence
COVID-19
Other COVID-19 pandemic-related outcomes

Finland

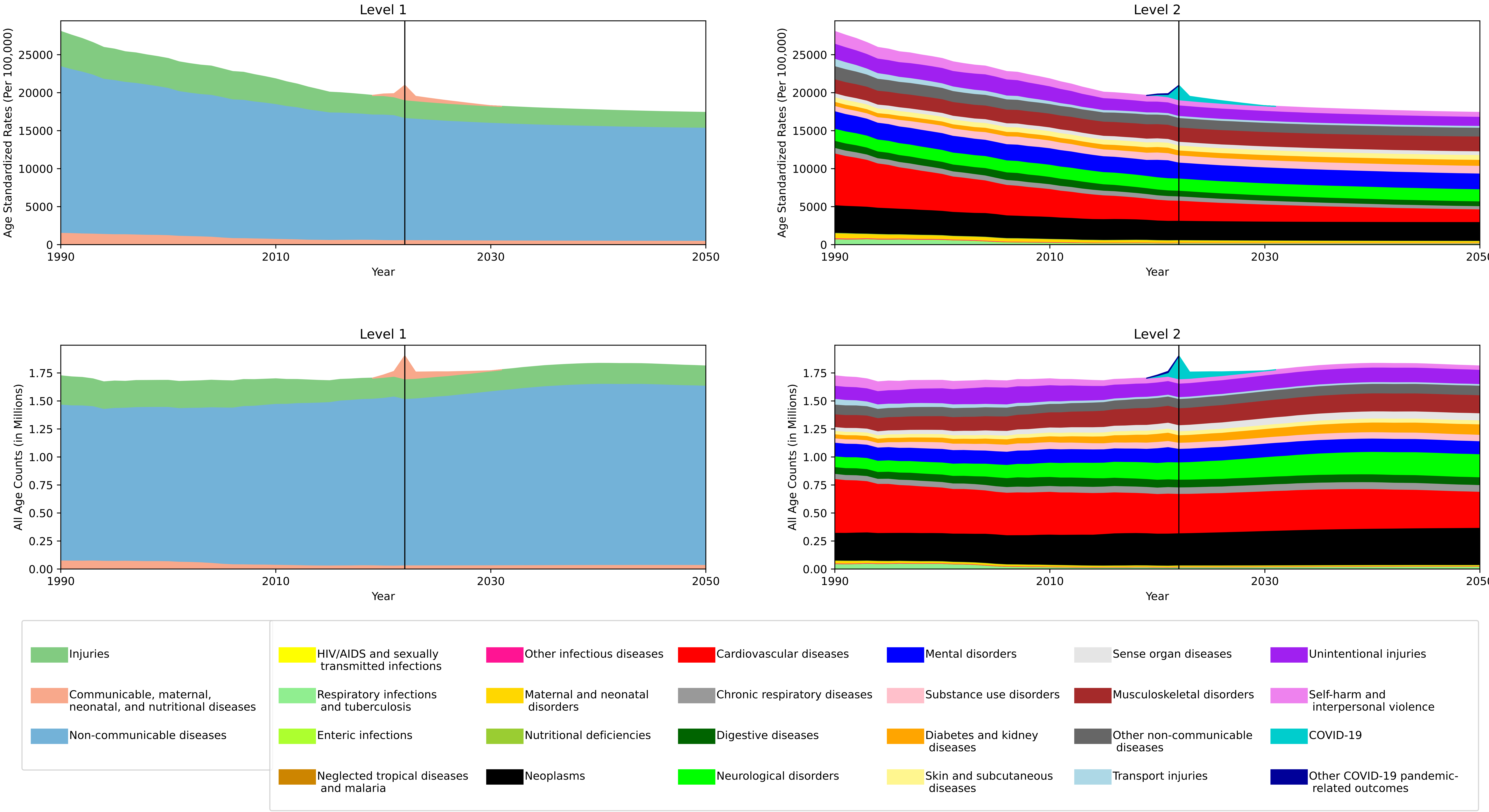
Level 1
Level 2
Age Standardized Rates (Per 100,000)
All Age Counts (in Millions)
Year
1990
2010
2030
2050
Injuries
Communicable, maternal, neonatal, and nutritional diseases
Non-communicable diseases
HIV/AIDS and sexually transmitted infections
Respiratory infections and tuberculosis
Enteric infections
Neglected tropical diseases and malaria
Other infectious diseases
Maternal and neonatal disorders
Nutritional deficiencies
Neoplasms
Cardiovascular diseases
Chronic respiratory diseases
Digestive diseases
Neurological disorders
Mental disorders
Substance use disorders
Diabetes and kidney diseases
Skin and subcutaneous diseases
Sense organ diseases
Musculoskeletal disorders
Other non-communicable diseases
Transport injuries
Unintentional injuries
Self-harm and interpersonal violence
COVID-19
Other COVID-19 pandemic-related outcomes

France

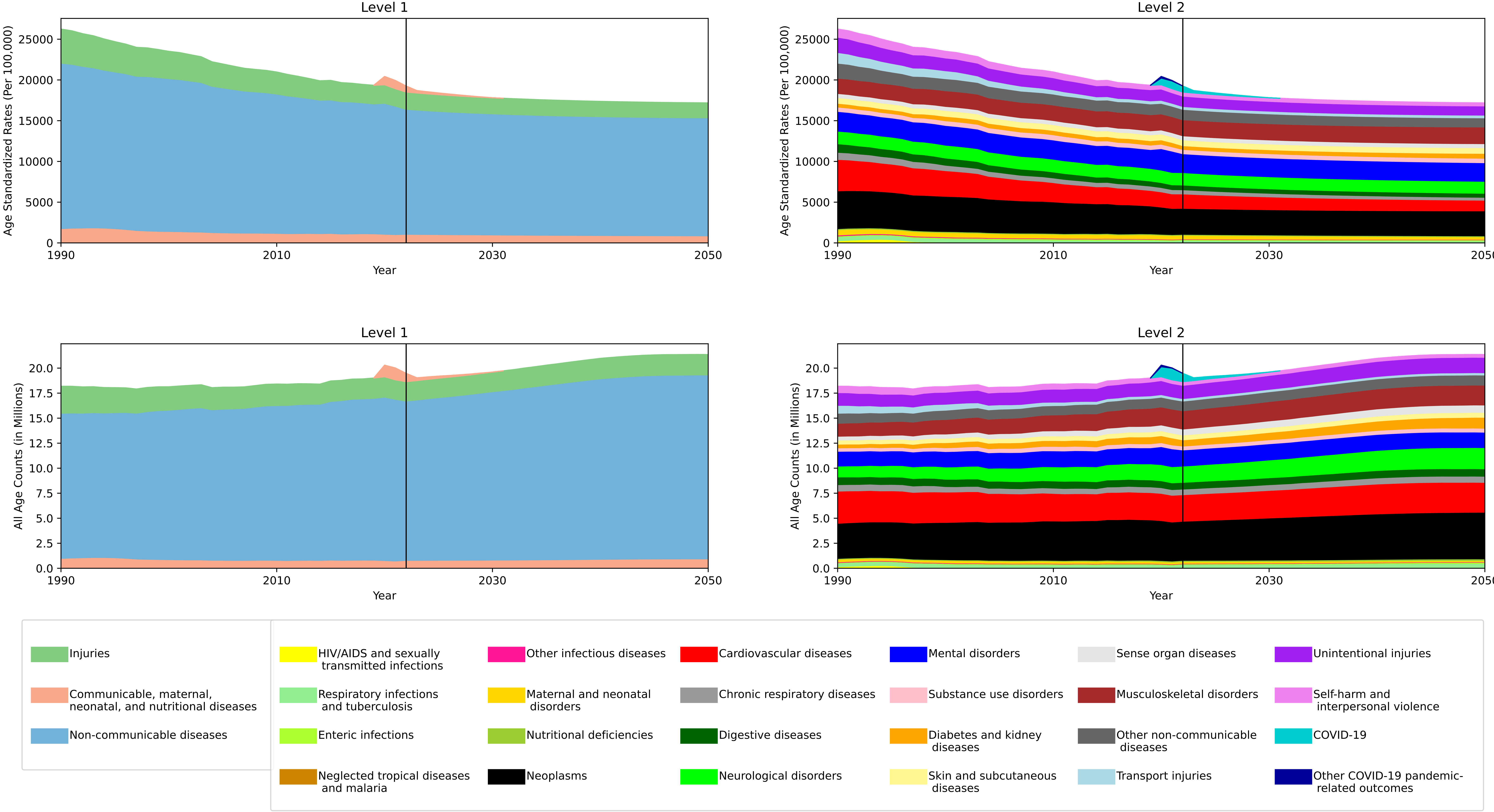
Level 1
Level 2
Age Standardized Rates (Per 100,000)
All Age Counts (in Millions)
Year
1990
2010
2030
2050
Injuries
Communicable, maternal, neonatal, and nutritional diseases
Non-communicable diseases
HIV/AIDS and sexually transmitted infections
Respiratory infections and tuberculosis
Enteric infections
Neglected tropical diseases and malaria
Other infectious diseases
Maternal and neonatal disorders
Nutritional deficiencies
Neoplasms
Cardiovascular diseases
Chronic respiratory diseases
Digestive diseases
Neurological disorders
Mental disorders
Substance use disorders
Diabetes and kidney diseases
Skin and subcutaneous diseases
Sense organ diseases
Musculoskeletal disorders
Other non-communicable diseases
Transport injuries
Unintentional injuries
Self-harm and interpersonal violence
COVID-19
Other COVID-19 pandemic-related outcomes

Supplemental Results Figure S4. Age-standardised DALY rates and all age DALY counts and by Level 1 and Level 2 causes, 1990–2050

Germany

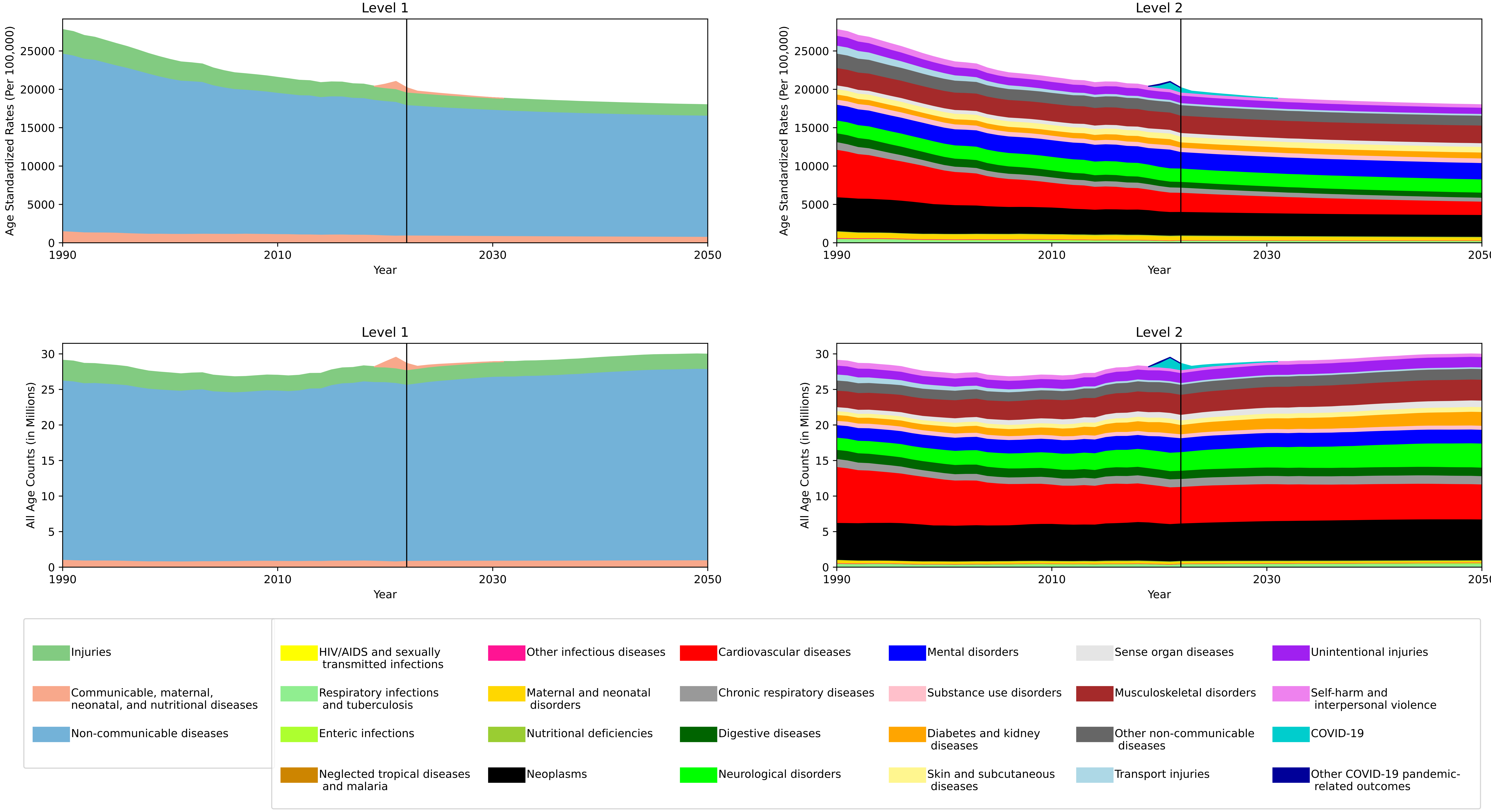

Greece

Supplemental Results Figure S4. Age-standardised DALY rates and all age DALY counts and by Level 1 and Level 2 causes, 1990–2050

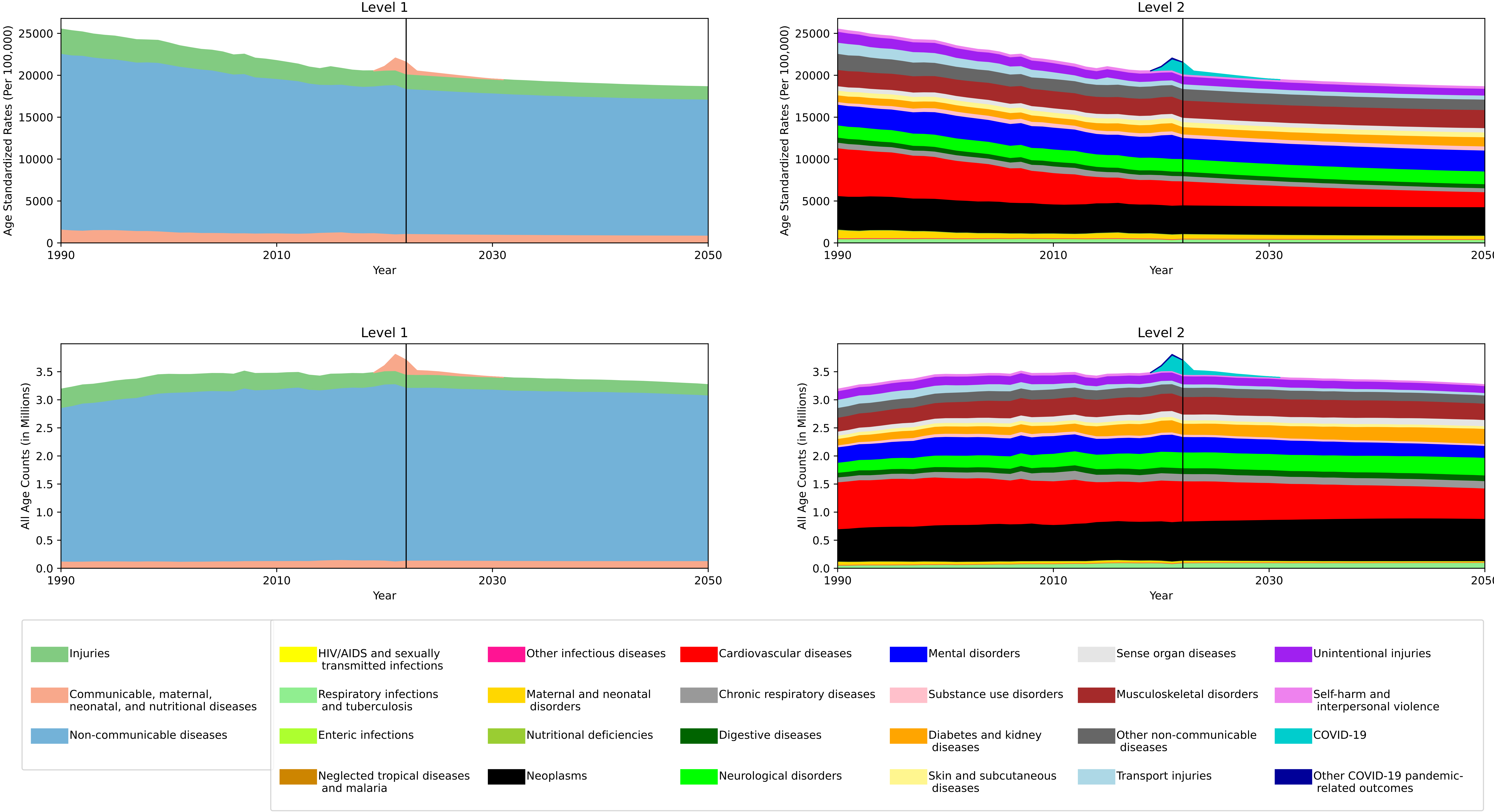

Iceland

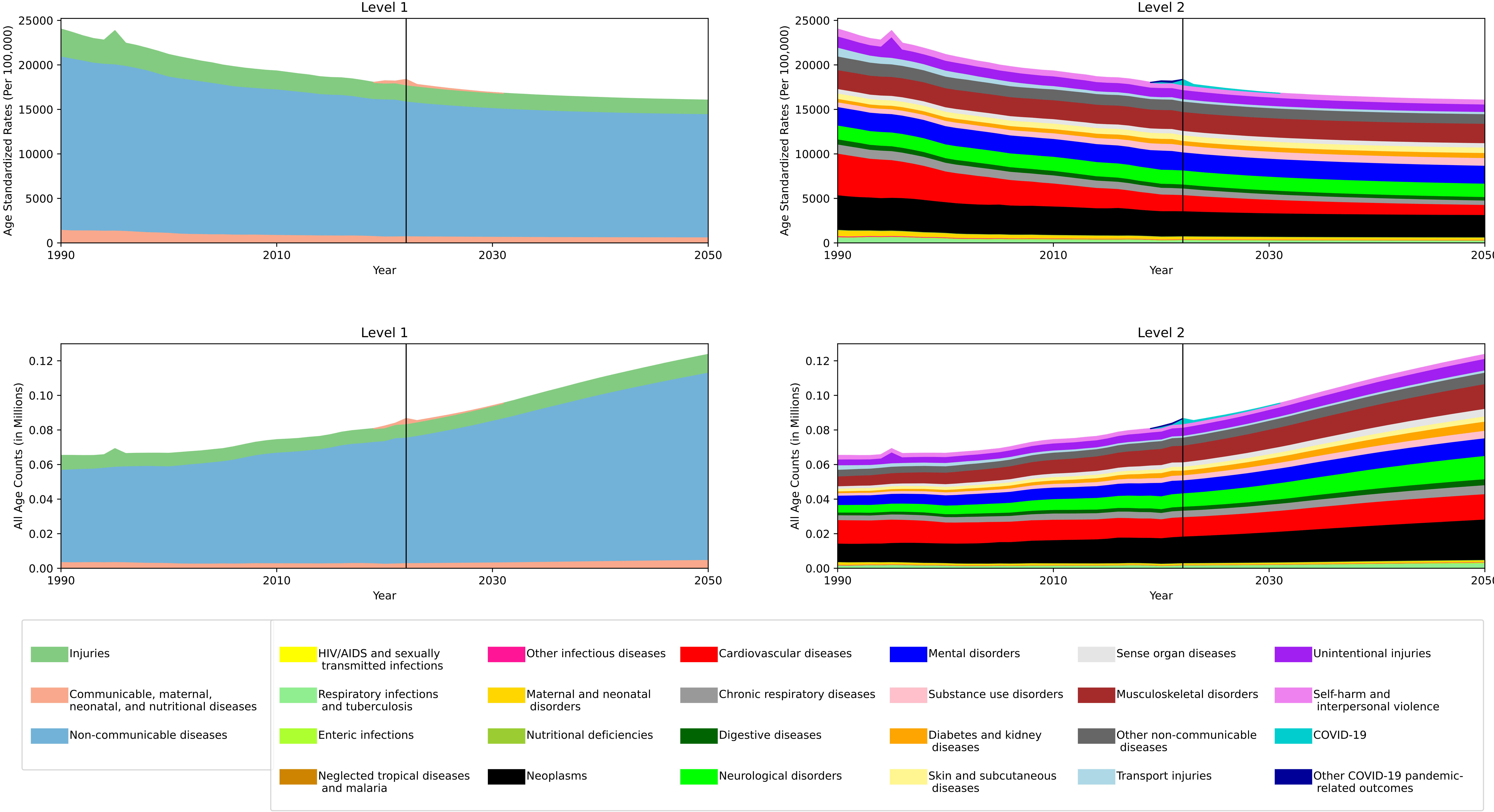
Level 1
Level 2
Age Standardized Rates (Per 100,000)
All Age Counts (in Millions)
Year
Injuries
Communicable, maternal, neonatal, and nutritional diseases
Non-communicable diseases
HIV/AIDS and sexually transmitted infections
Respiratory infections and tuberculosis
Enteric infections
Neglected tropical diseases and malaria
Other infectious diseases
Maternal and neonatal disorders
Nutritional deficiencies
Neoplasms
Cardiovascular diseases
Chronic respiratory diseases
Digestive diseases
Neurological disorders
Mental disorders
Substance use disorders
Diabetes and kidney diseases
Skin and subcutaneous diseases
Sense organ diseases
Musculoskeletal disorders
Other non-communicable diseases
Transport injuries
Unintentional injuries
Self-harm and interpersonal violence
COVID-19
Other COVID-19 pandemic-related outcomes

Ireland

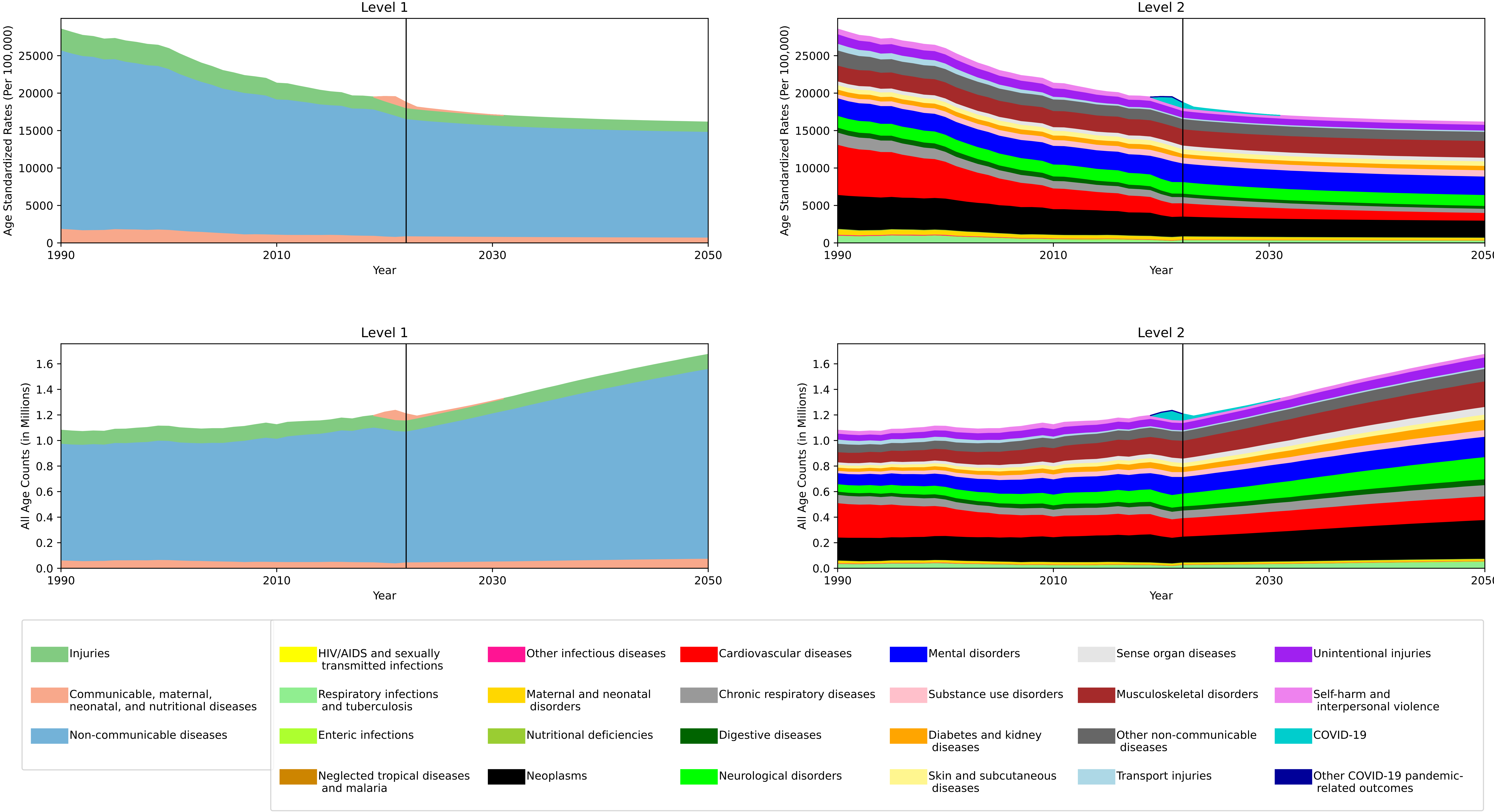
Level 1
Level 2
Age Standardized Rates (Per 100,000)
All Age Counts (in Millions)
Year
1990
2010
2030
2050
Injuries
Communicable, maternal, neonatal, and nutritional diseases
Non-communicable diseases
HIV/AIDS and sexually transmitted infections
Respiratory infections and tuberculosis
Enteric infections
Neglected tropical diseases and malaria
Other infectious diseases
Maternal and neonatal disorders
Nutritional deficiencies
Neoplasms
Cardiovascular diseases
Chronic respiratory diseases
Digestive diseases
Neurological disorders
Mental disorders
Substance use disorders
Diabetes and kidney diseases
Skin and subcutaneous diseases
Sense organ diseases
Musculoskeletal disorders
Other non-communicable diseases
Transport injuries
Unintentional injuries
Self-harm and interpersonal violence
COVID-19
Other COVID-19 pandemic-related outcomes

Israel

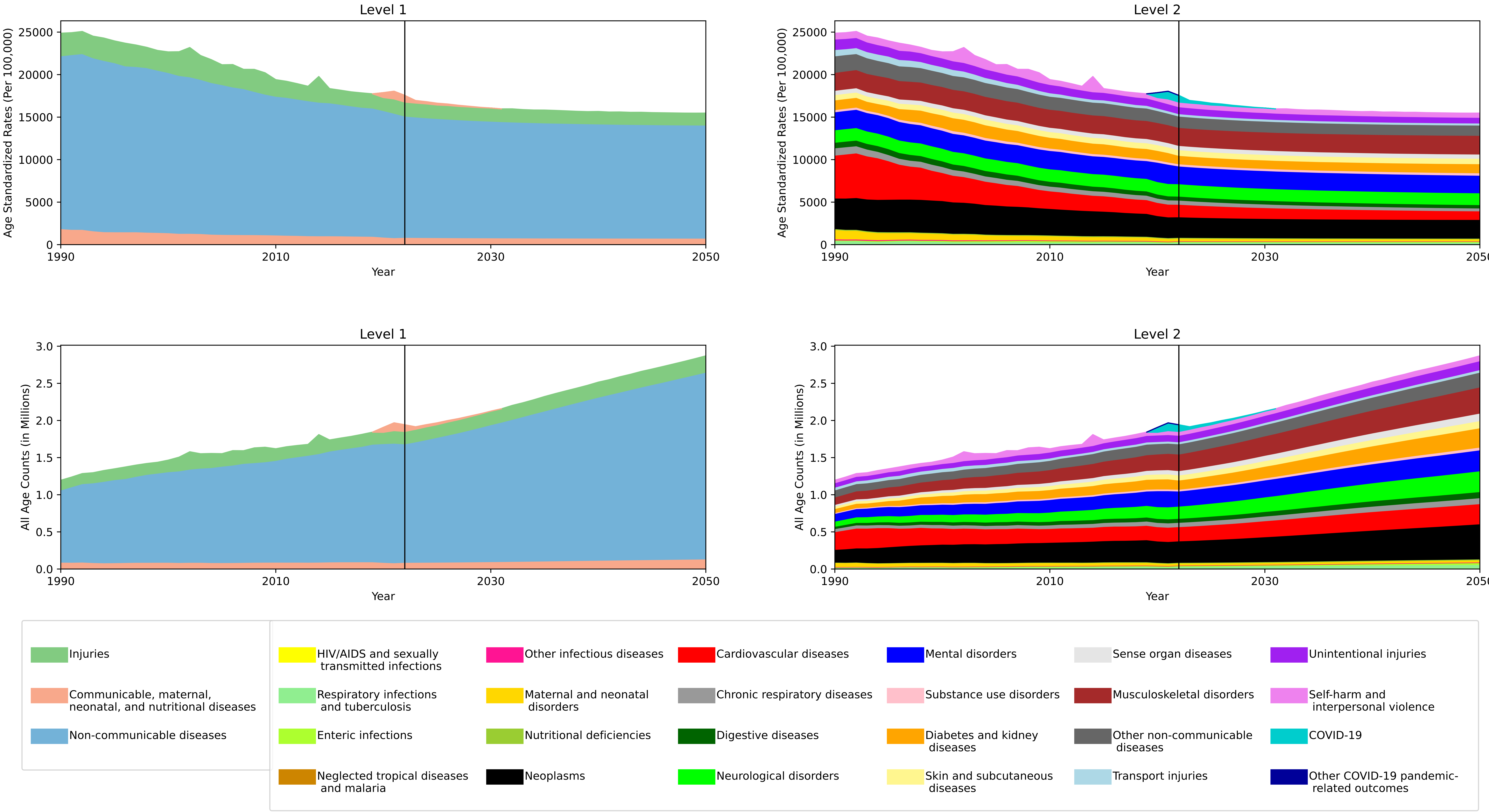
Level 1
Level 2
Age Standardized Rates (Per 100,000)
All Age Counts (in Millions)
Year
1990
2010
2030
2050
Injuries
Communicable, maternal, neonatal, and nutritional diseases
Non-communicable diseases
HIV/AIDS and sexually transmitted infections
Respiratory infections and tuberculosis
Enteric infections
Neglected tropical diseases and malaria
Other infectious diseases
Maternal and neonatal disorders
Nutritional deficiencies
Neoplasms
Cardiovascular diseases
Chronic respiratory diseases
Digestive diseases
Neurological disorders
Mental disorders
Substance use disorders
Diabetes and kidney diseases
Skin and subcutaneous diseases
Sense organ diseases
Musculoskeletal disorders
Other non-communicable diseases
Transport injuries
Unintentional injuries
Self-harm and interpersonal violence
COVID-19
Other COVID-19 pandemic-related outcomes

Italy

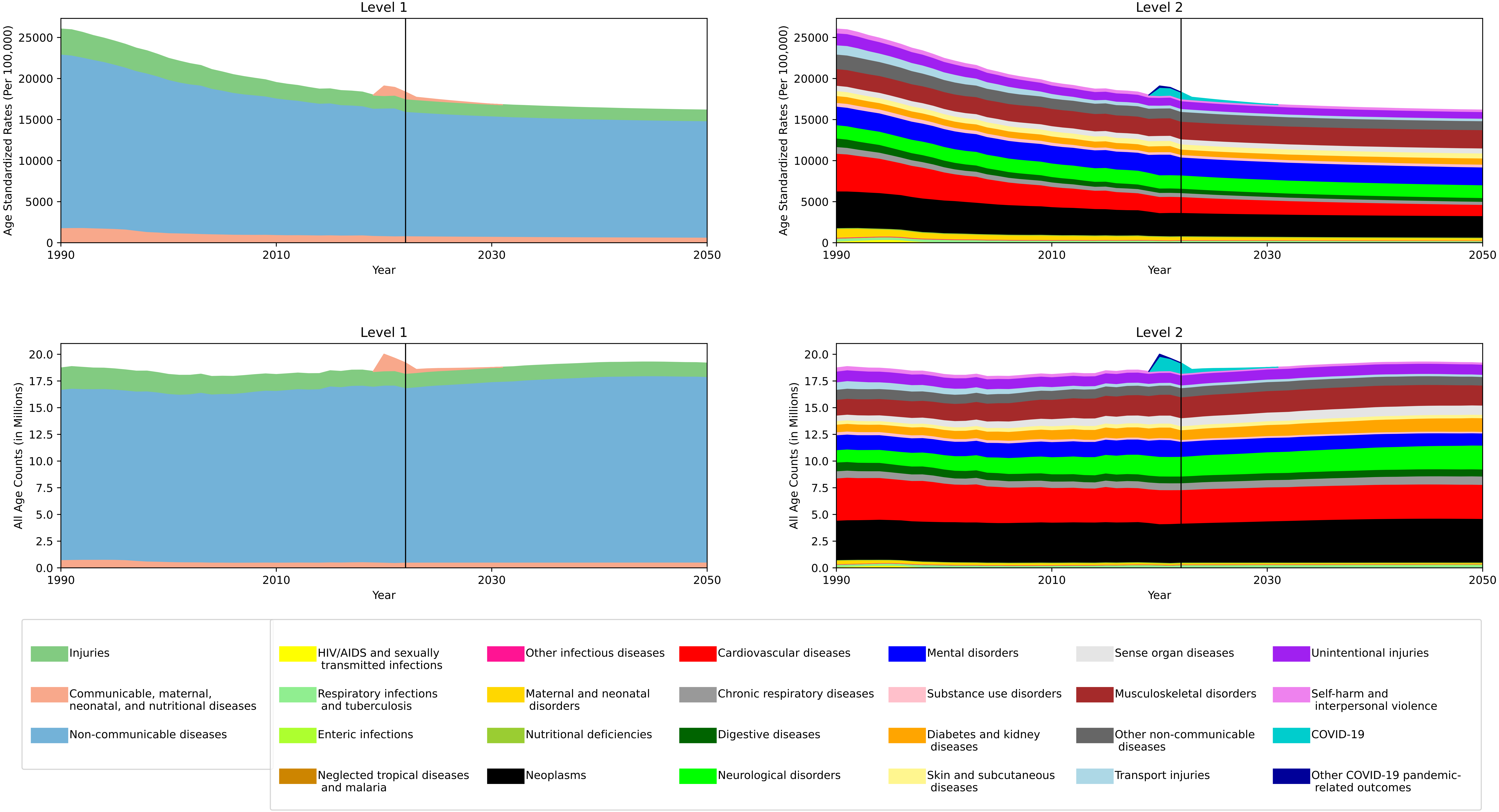

Level 1
Level 2
Age Standardized Rates (Per 100,000)
All Age Counts (in Millions)
Year
1990
2010
2030
2050
0
5000
10000
15000
20000
25000
0.0
2.5
5.0
7.5
10.0
12.5
15.0
17.5
20.0
Injuries
Communicable, maternal, neonatal, and nutritional diseases
Non-communicable diseases
HIV/AIDS and sexually transmitted infections
Respiratory infections and tuberculosis
Enteric infections
Neglected tropical diseases and malaria
Other infectious diseases
Maternal and neonatal disorders
Nutritional deficiencies
Neoplasms
Cardiovascular diseases
Chronic respiratory diseases
Digestive diseases
Neurological disorders
Mental disorders
Substance use disorders
Diabetes and kidney diseases
Skin and subcutaneous diseases
Sense organ diseases
Musculoskeletal disorders
Other non-communicable diseases
Transport injuries
Unintentional injuries
Self-harm and interpersonal violence
COVID-19
Other COVID-19 pandemic-related outcomes

Luxembourg

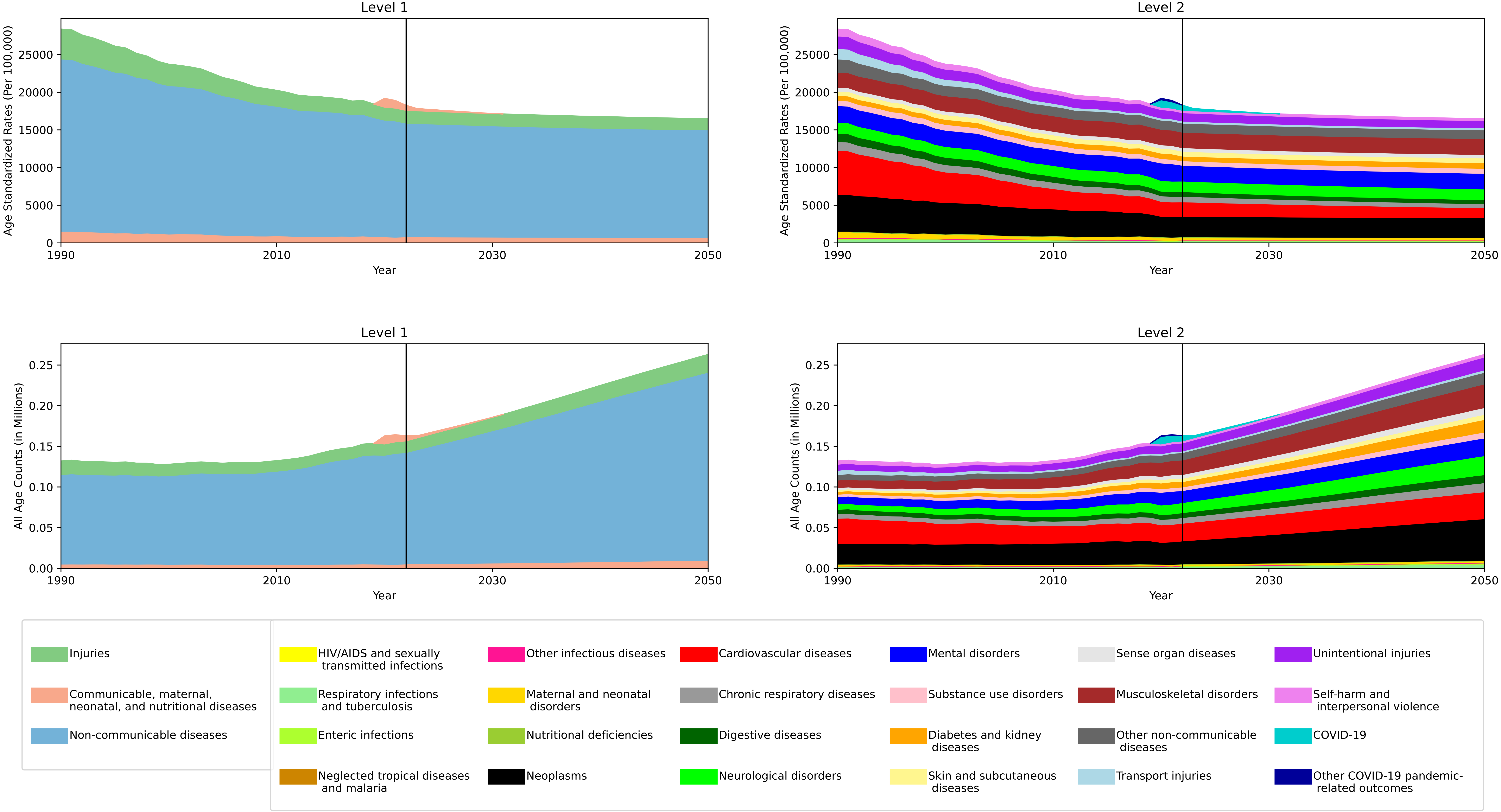
Level 1
Level 2
Age Standardized Rates (Per 100,000)
All Age Counts (in Millions)
Year
0
5000
10000
15000
20000
25000
0.00
0.05
0.10
0.15
0.20
0.25
1990
2010
2030
2050
Injuries
Communicable, maternal, neonatal, and nutritional diseases
Non-communicable diseases
HIV/AIDS and sexually transmitted infections
Respiratory infections and tuberculosis
Enteric infections
Neglected tropical diseases and malaria
Other infectious diseases
Maternal and neonatal disorders
Nutritional deficiencies
Neoplasms
Cardiovascular diseases
Chronic respiratory diseases
Digestive diseases
Neurological disorders
Mental disorders
Substance use disorders
Diabetes and kidney diseases
Skin and subcutaneous diseases
Sense organ diseases
Musculoskeletal disorders
Other non-communicable diseases
Transport injuries
Unintentional injuries
Self-harm and interpersonal violence
COVID-19
Other COVID-19 pandemic-related outcomes

Malta

Supplemental Results Figure S4. Age-standardised DALY rates and all age DALY counts and by Level 1 and Level 2 causes, 1990–2050

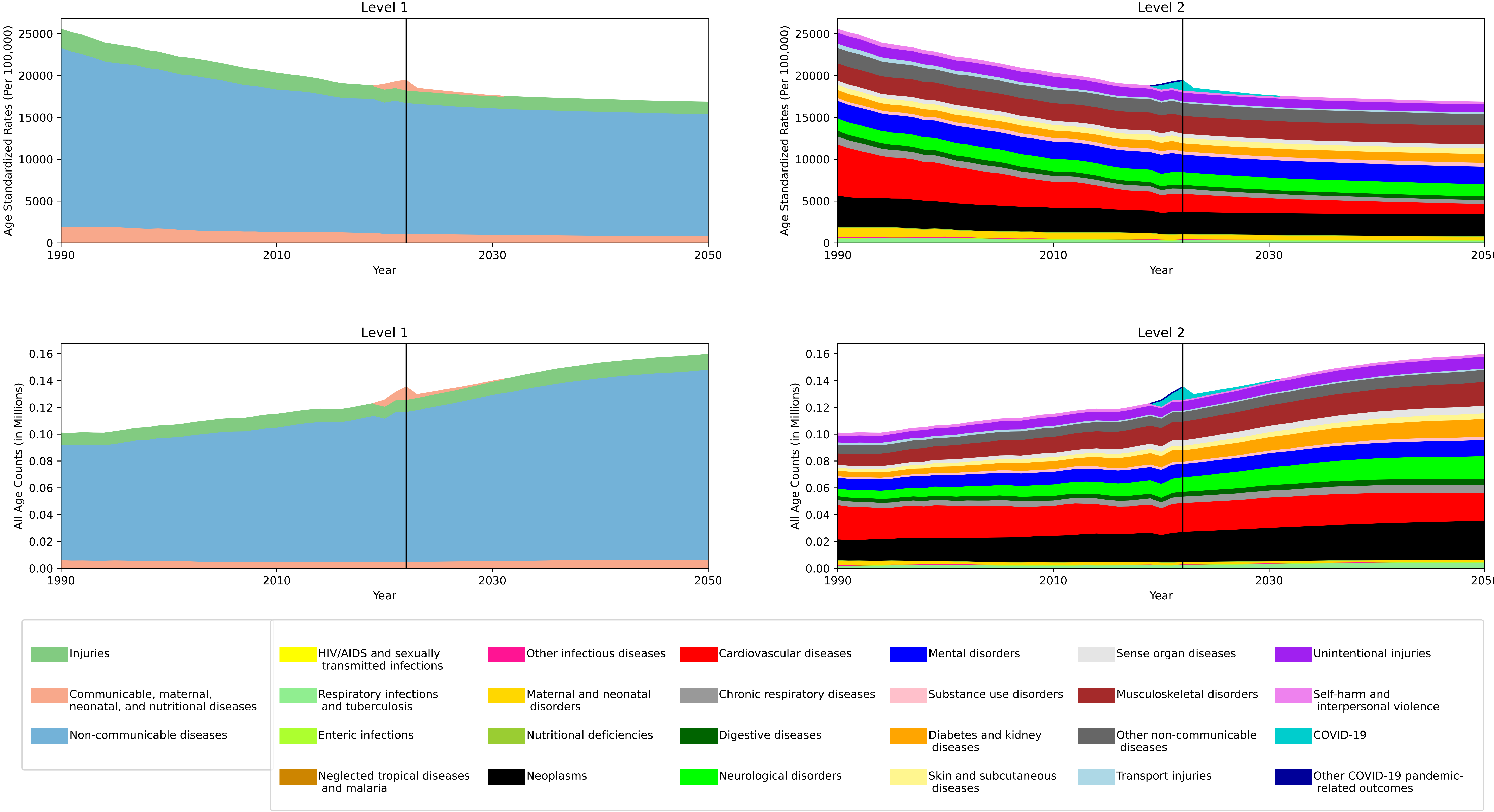

Monaco

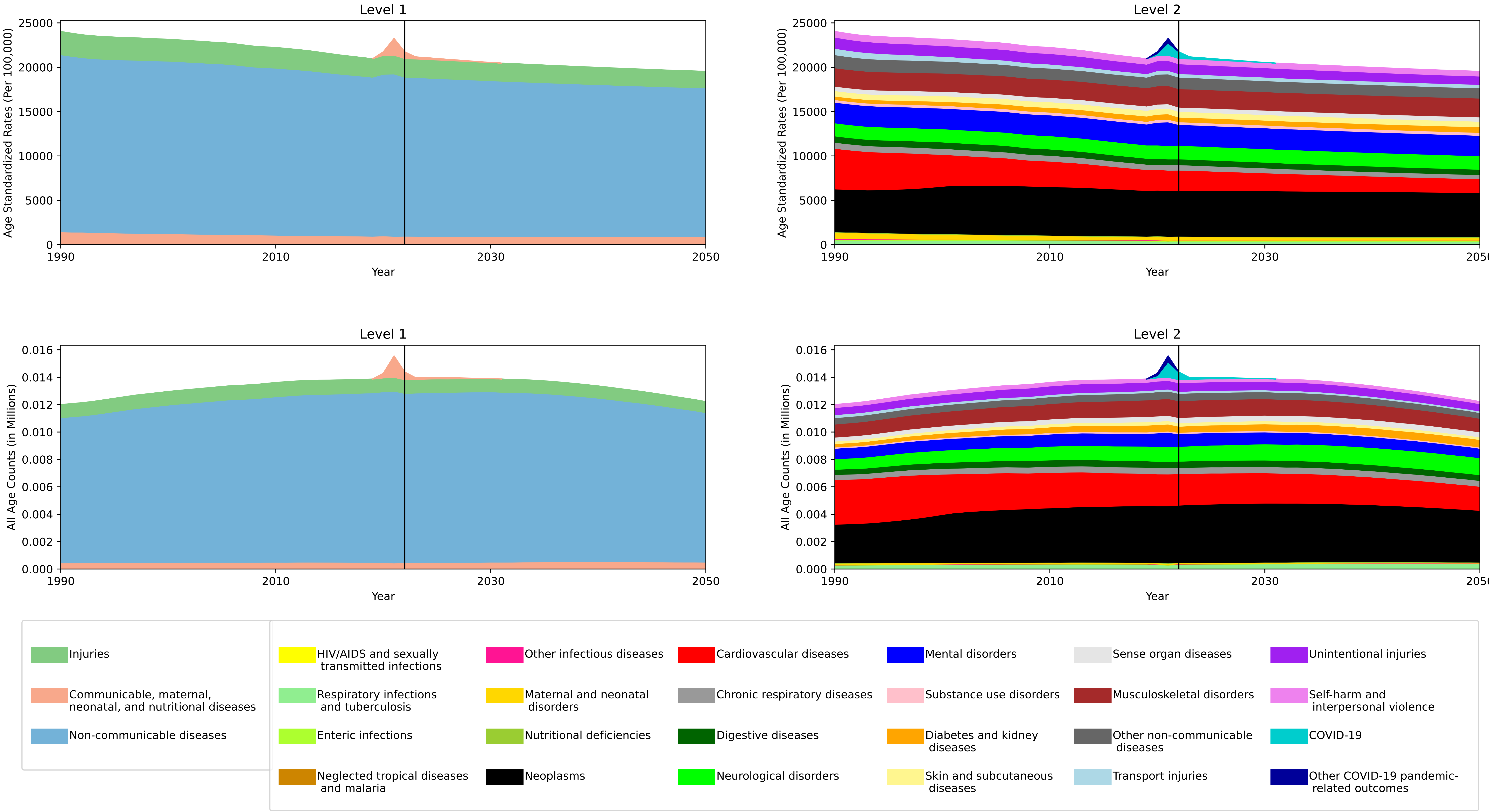
Level 1
Age Standardized Rates (Per 100,000)
Year
Level 2
Age Standardized Rates (Per 100,000)
Year
Level 1
All Age Counts (in Millions)
Year
Level 2
All Age Counts (in Millions)
Year
Injuries
Communicable, maternal, neonatal, and nutritional diseases
Non-communicable diseases
HIV/AIDS and sexually transmitted infections
Respiratory infections and tuberculosis
Enteric infections
Neglected tropical diseases and malaria
Other infectious diseases
Maternal and neonatal disorders
Nutritional deficiencies
Neoplasms
Cardiovascular diseases
Chronic respiratory diseases
Digestive diseases
Neurological disorders
Mental disorders
Substance use disorders
Diabetes and kidney diseases
Skin and subcutaneous diseases
Sense organ diseases
Musculoskeletal disorders
Other non-communicable diseases
Transport injuries
Unintentional injuries
Self-harm and interpersonal violence
COVID-19
Other COVID-19 pandemic-related outcomes

Netherlands

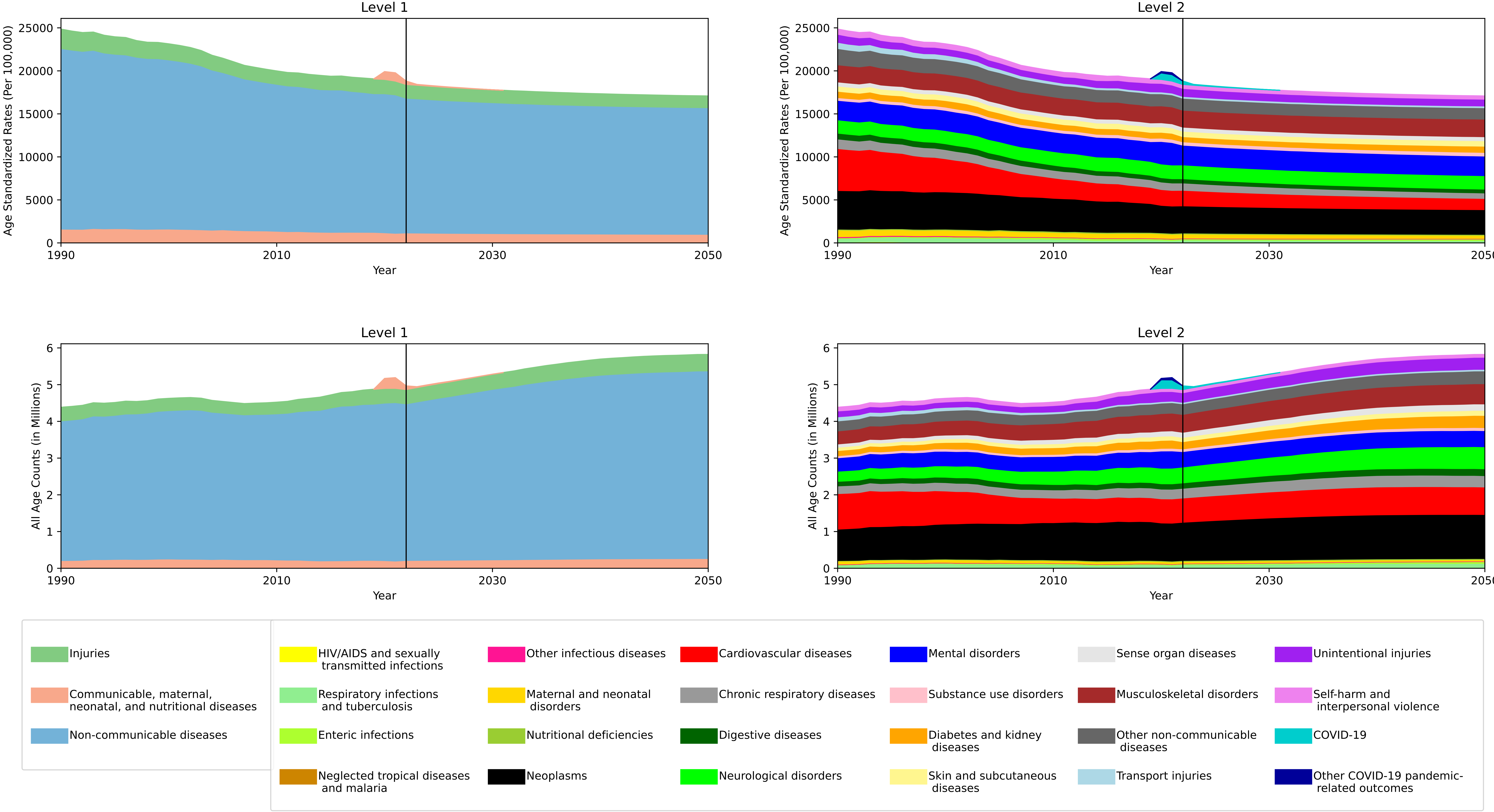

Norway

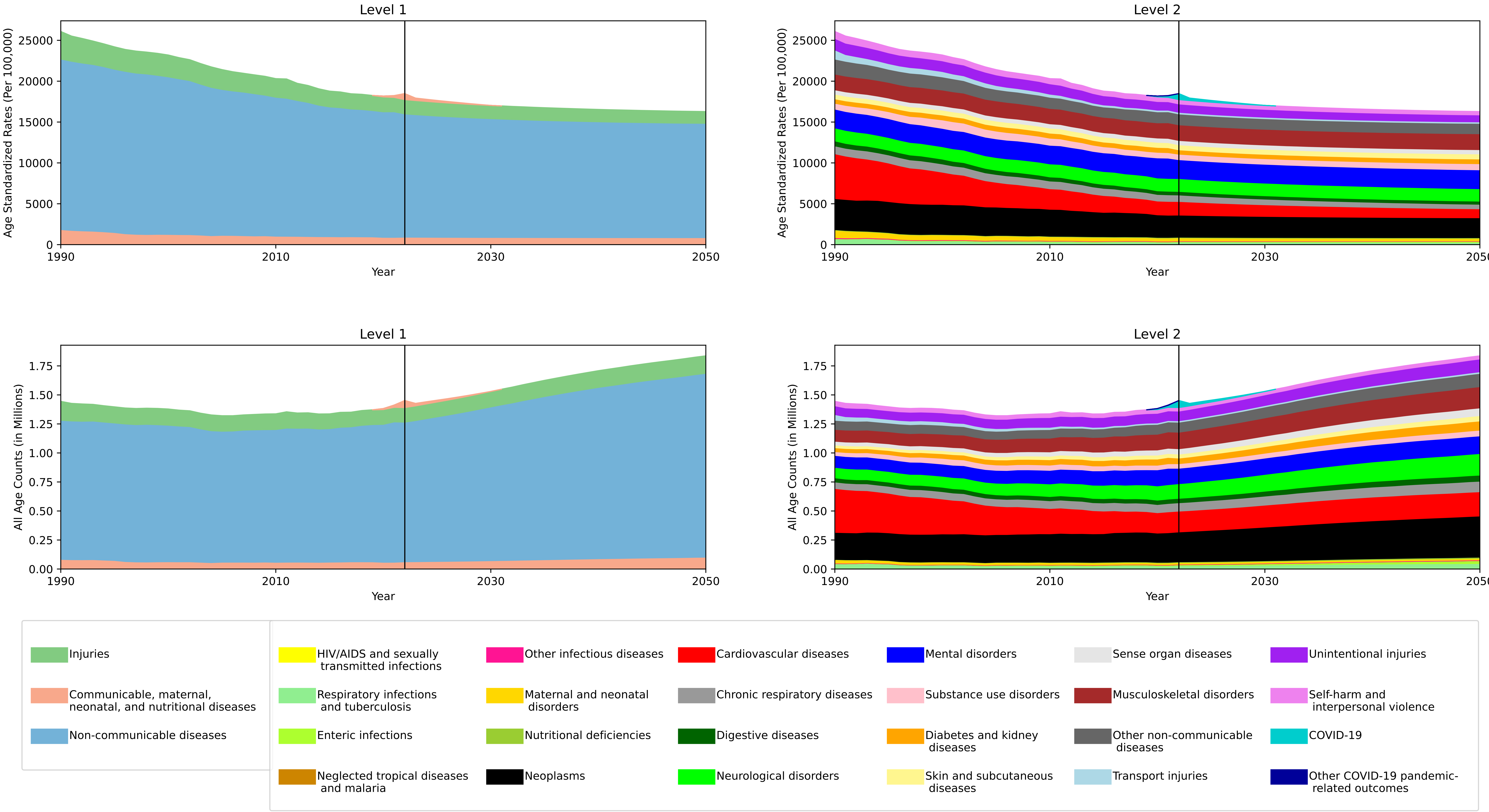
Level 1
Level 2
Age Standardized Rates (Per 100,000)
All Age Counts (in Millions)
Year
1990
2010
2030
2050
Injuries
Communicable, maternal, neonatal, and nutritional diseases
Non-communicable diseases
HIV/AIDS and sexually transmitted infections
Respiratory infections and tuberculosis
Enteric infections
Neglected tropical diseases and malaria
Other infectious diseases
Maternal and neonatal disorders
Nutritional deficiencies
Neoplasms
Cardiovascular diseases
Chronic respiratory diseases
Digestive diseases
Neurological disorders
Mental disorders
Substance use disorders
Diabetes and kidney diseases
Skin and subcutaneous diseases
Sense organ diseases
Musculoskeletal disorders
Other non-communicable diseases
Transport injuries
Unintentional injuries
Self-harm and interpersonal violence
COVID-19
Other COVID-19 pandemic-related outcomes

Portugal

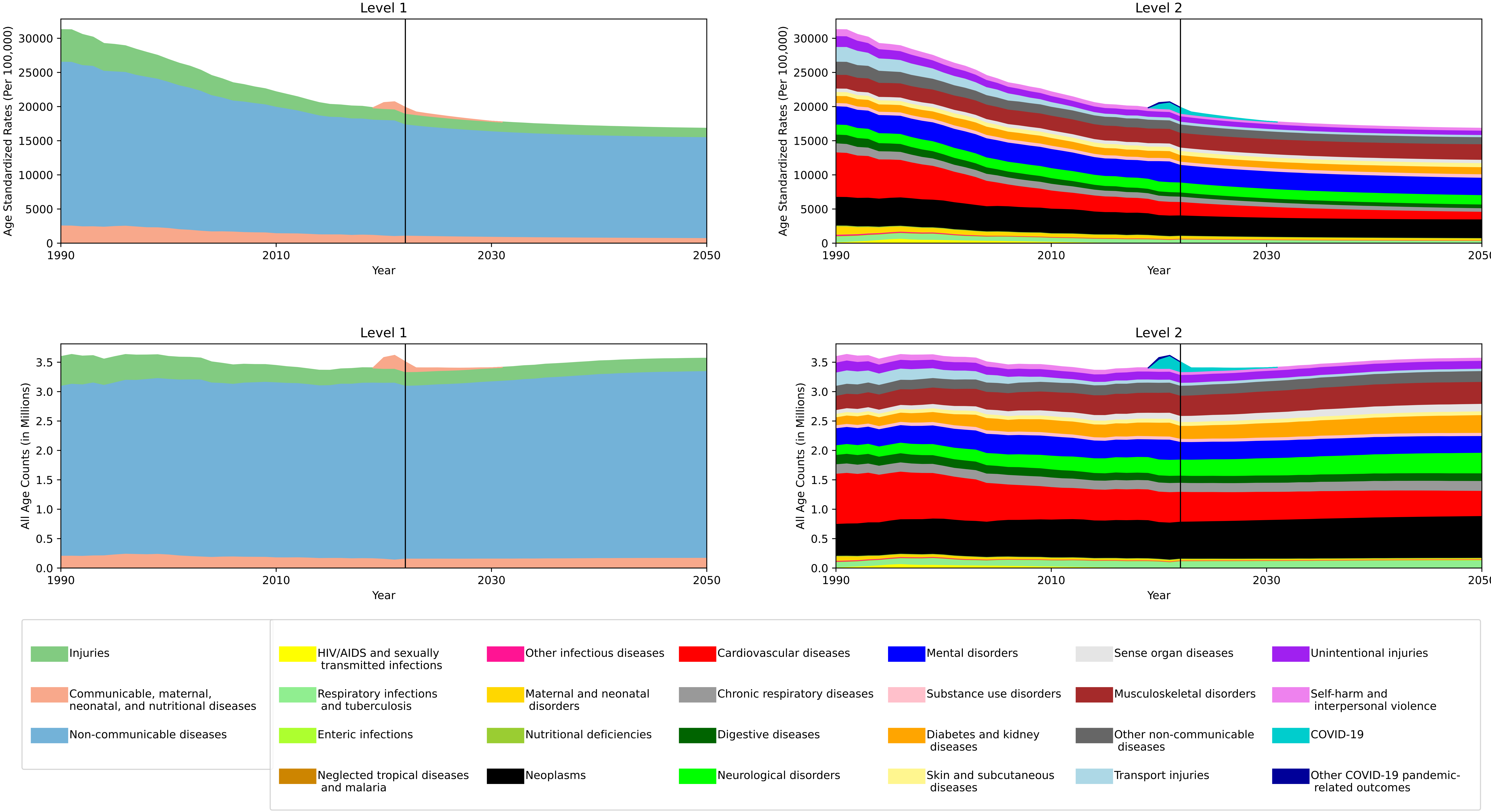
Level 1
Level 2
Age Standardized Rates (Per 100,000)
All Age Counts (in Millions)
Year
1990
2010
2030
2050
Injuries
Communicable, maternal, neonatal, and nutritional diseases
Non-communicable diseases
HIV/AIDS and sexually transmitted infections
Respiratory infections and tuberculosis
Enteric infections
Neglected tropical diseases and malaria
Other infectious diseases
Maternal and neonatal disorders
Nutritional deficiencies
Neoplasms
Cardiovascular diseases
Chronic respiratory diseases
Digestive diseases
Neurological disorders
Mental disorders
Substance use disorders
Diabetes and kidney diseases
Skin and subcutaneous diseases
Sense organ diseases
Musculoskeletal disorders
Other non-communicable diseases
Transport injuries
Unintentional injuries
Self-harm and interpersonal violence
COVID-19
Other COVID-19 pandemic-related outcomes

San Marino

Supplemental Results Figure S4. Age-standardised DALY rates and all age DALY counts and by Level 1 and Level 2 causes, 1990–2050

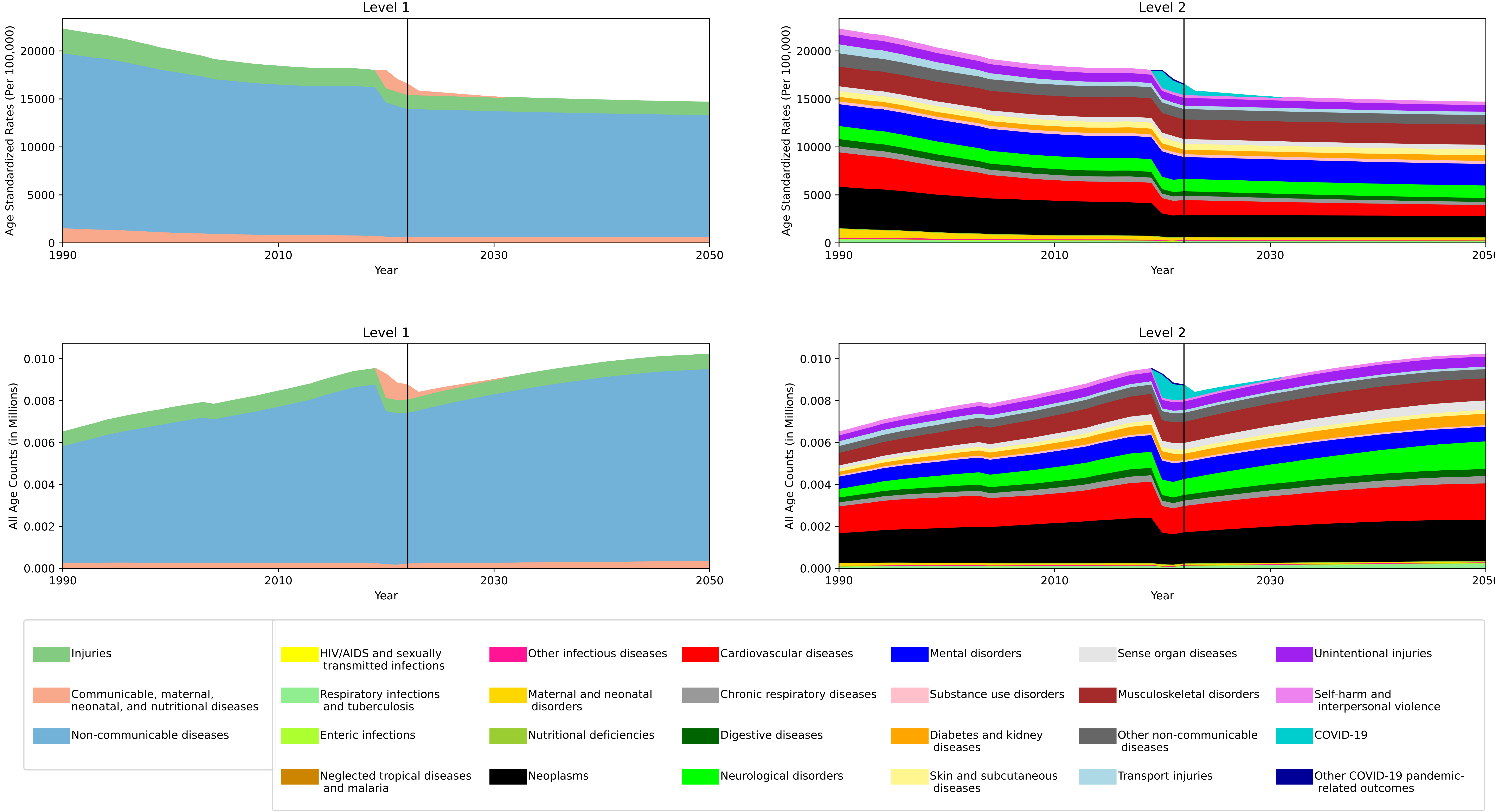

Spain

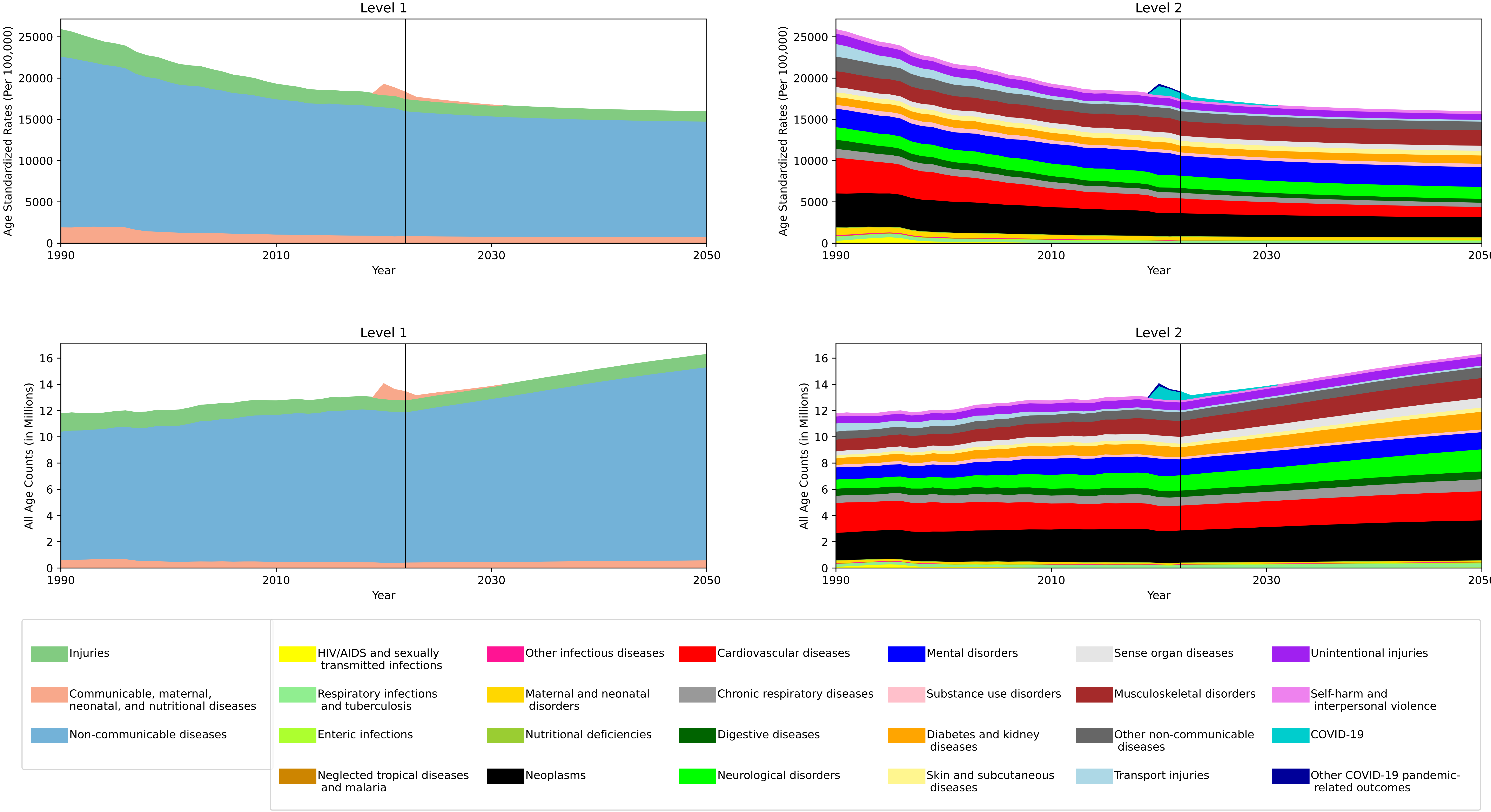
Level 1
Level 2
Age Standardized Rates (Per 100,000)
All Age Counts (in Millions)
Year
1990
2010
2030
2050
0
5000
10000
15000
20000
25000
2
4
6
8
10
12
14
16
Injuries
Communicable, maternal, neonatal, and nutritional diseases
Non-communicable diseases
HIV/AIDS and sexually transmitted infections
Respiratory infections and tuberculosis
Enteric infections
Neglected tropical diseases and malaria
Other infectious diseases
Maternal and neonatal disorders
Nutritional deficiencies
Neoplasms
Cardiovascular diseases
Chronic respiratory diseases
Digestive diseases
Neurological disorders
Mental disorders
Substance use disorders
Diabetes and kidney diseases
Skin and subcutaneous diseases
Sense organ diseases
Musculoskeletal disorders
Other non-communicable diseases
Transport injuries
Unintentional injuries
Self-harm and interpersonal violence
COVID-19
Other COVID-19 pandemic-related outcomes

Sweden

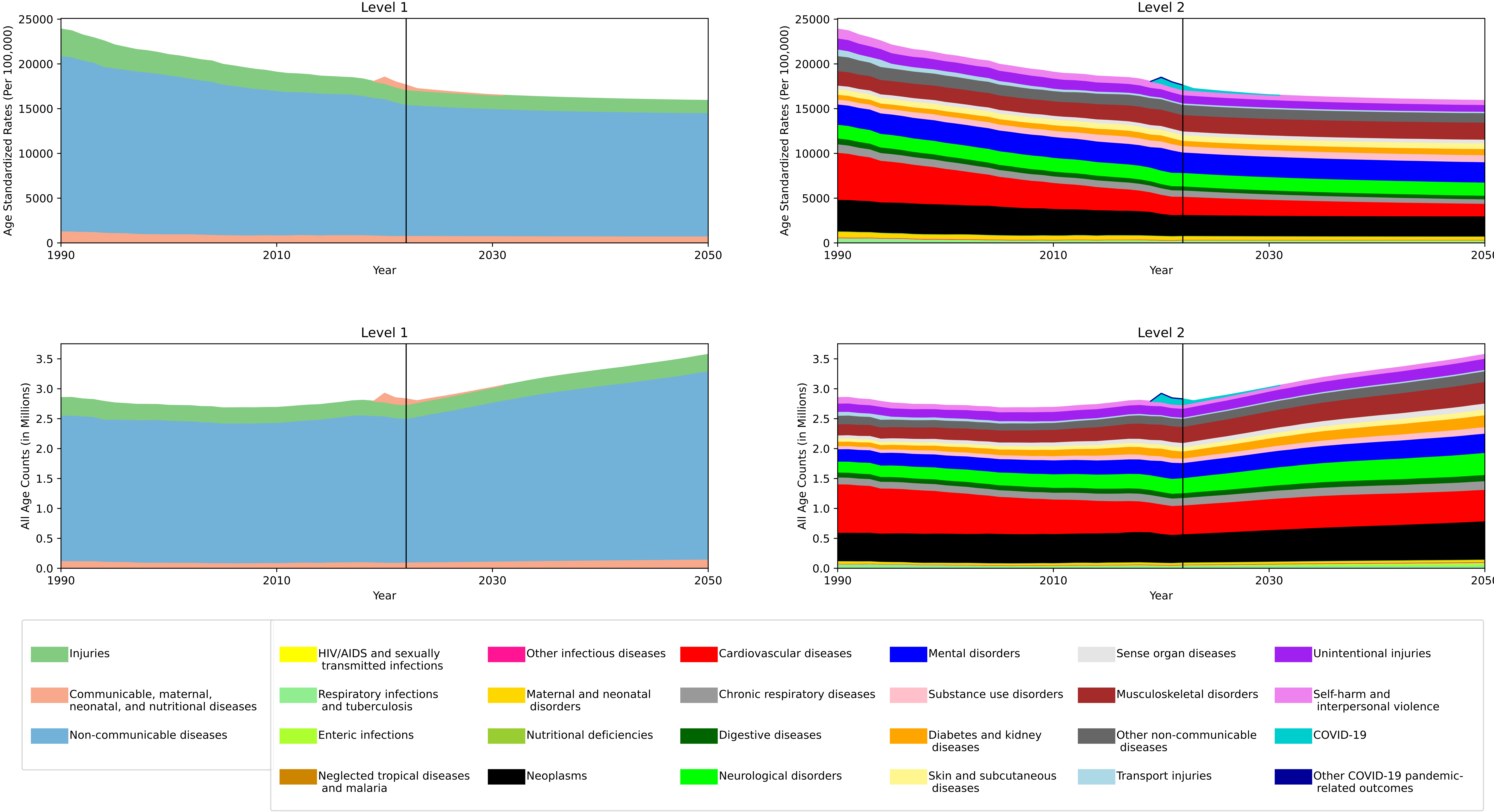
Level 1
Level 2
Age Standardized Rates (Per 100,000)
All Age Counts (in Millions)
Year
Injuries
Communicable, maternal, neonatal, and nutritional diseases
Non-communicable diseases
HIV/AIDS and sexually transmitted infections
Respiratory infections and tuberculosis
Enteric infections
Neglected tropical diseases and malaria
Other infectious diseases
Maternal and neonatal disorders
Nutritional deficiencies
Neoplasms
Cardiovascular diseases
Chronic respiratory diseases
Digestive diseases
Neurological disorders
Mental disorders
Substance use disorders
Diabetes and kidney diseases
Skin and subcutaneous diseases
Sense organ diseases
Musculoskeletal disorders
Other non-communicable diseases
Transport injuries
Unintentional injuries
Self-harm and interpersonal violence
COVID-19
Other COVID-19 pandemic-related outcomes

Switzerland

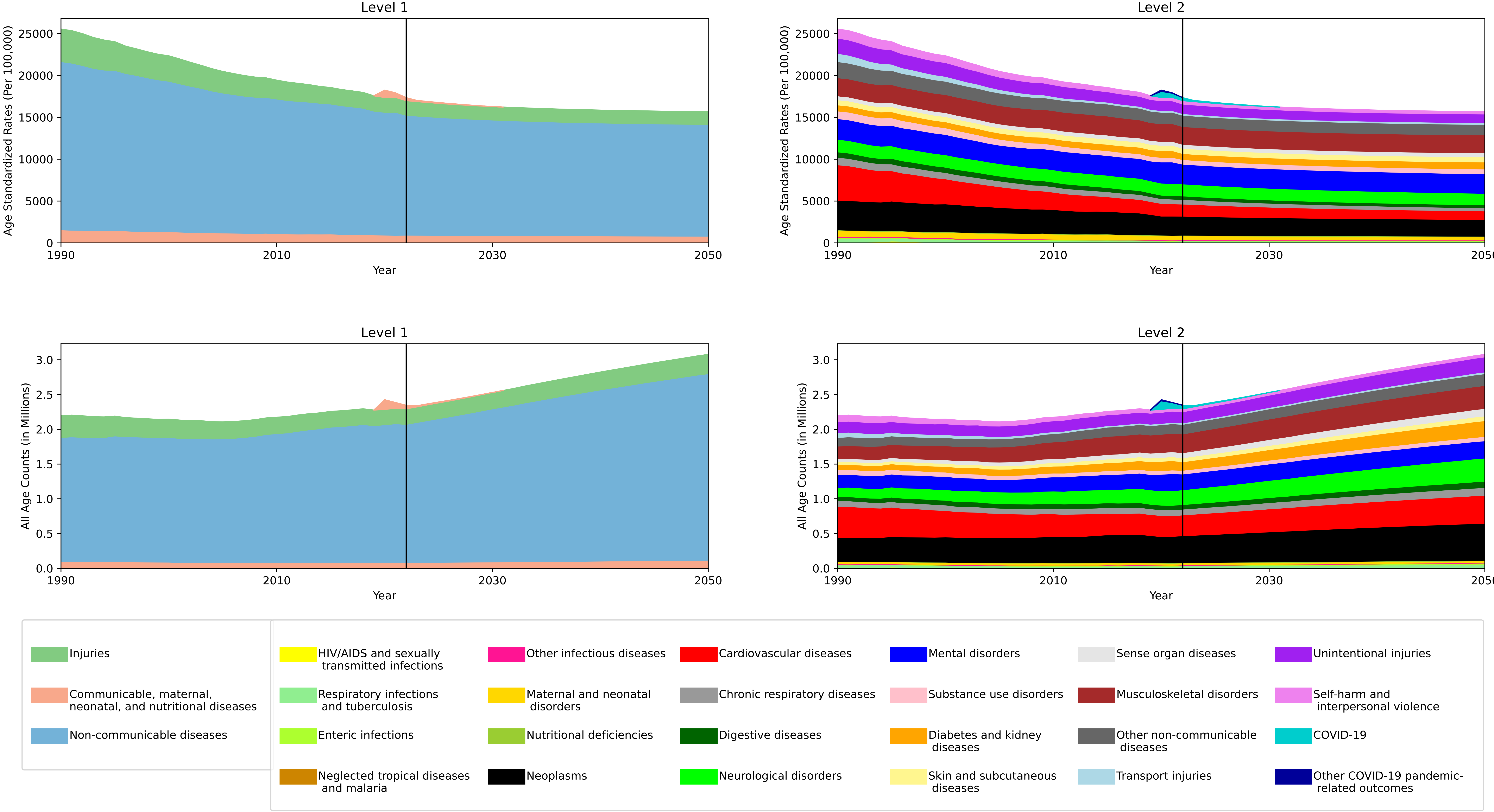

UK

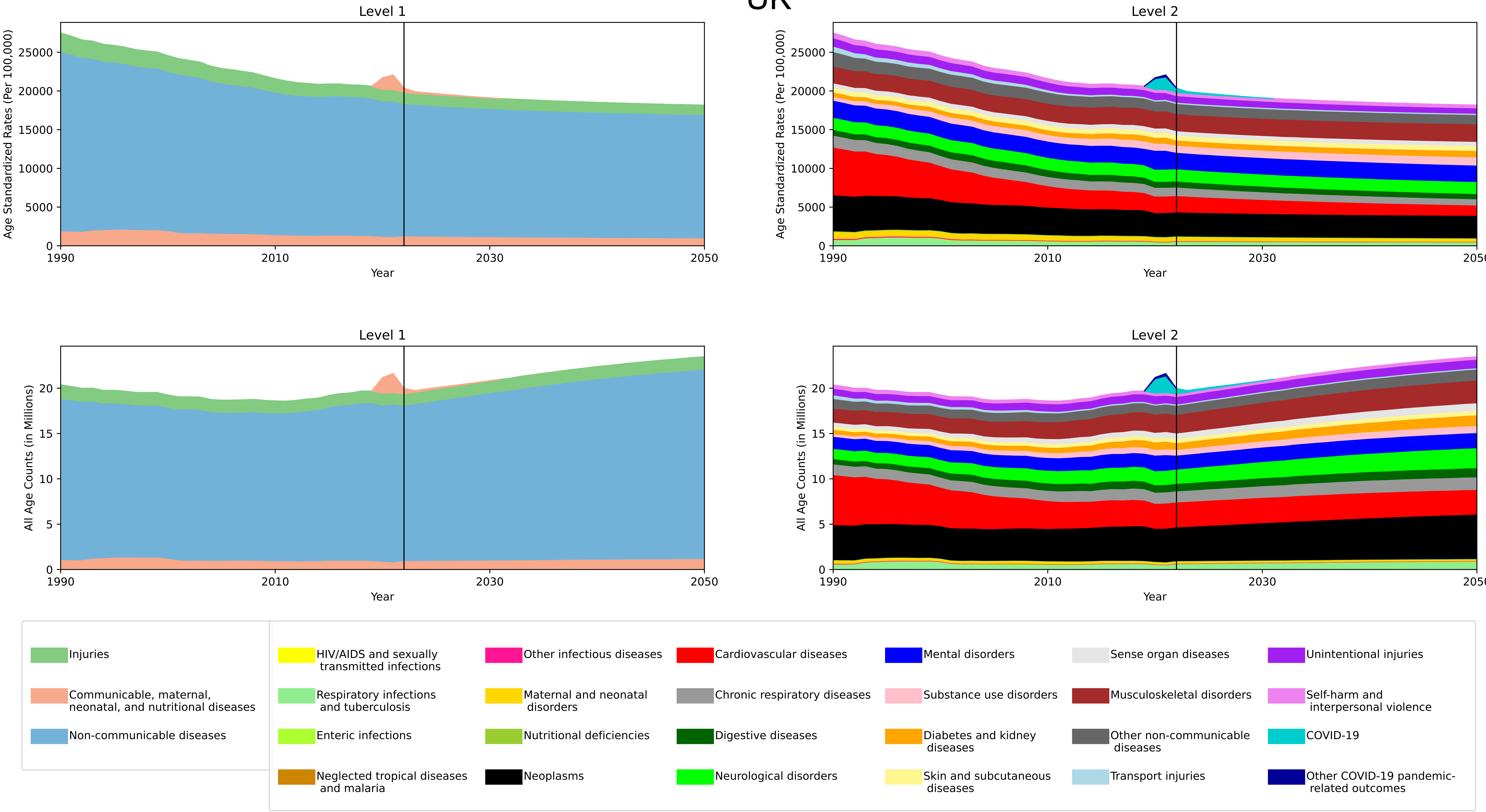
Level 1
Age Standardized Rates (Per 100,000)
25000
20000
15000
10000
5000
0
1990
2010
2030
2050
Year
Level 2
Age Standardized Rates (Per 100,000)
Level 1
All Age Counts (in Millions)
20
15
10
5
0
Level 2
Injuries
Communicable, maternal, neonatal, and nutritional diseases
Non-communicable diseases
HIV/AIDS and sexually transmitted infections
Respiratory infections and tuberculosis
Enteric infections
Neglected tropical diseases and malaria
Other infectious diseases
Maternal and neonatal disorders
Nutritional deficiencies
Neoplasms
Cardiovascular diseases
Chronic respiratory diseases
Digestive diseases
Neurological disorders
Mental disorders
Substance use disorders
Diabetes and kidney diseases
Skin and subcutaneous diseases
Sense organ diseases
Musculoskeletal disorders
Other non-communicable diseases
Transport injuries
Unintentional injuries
Self-harm and interpersonal violence
COVID-19
Other COVID-19 pandemic-related outcomes

Bolivia

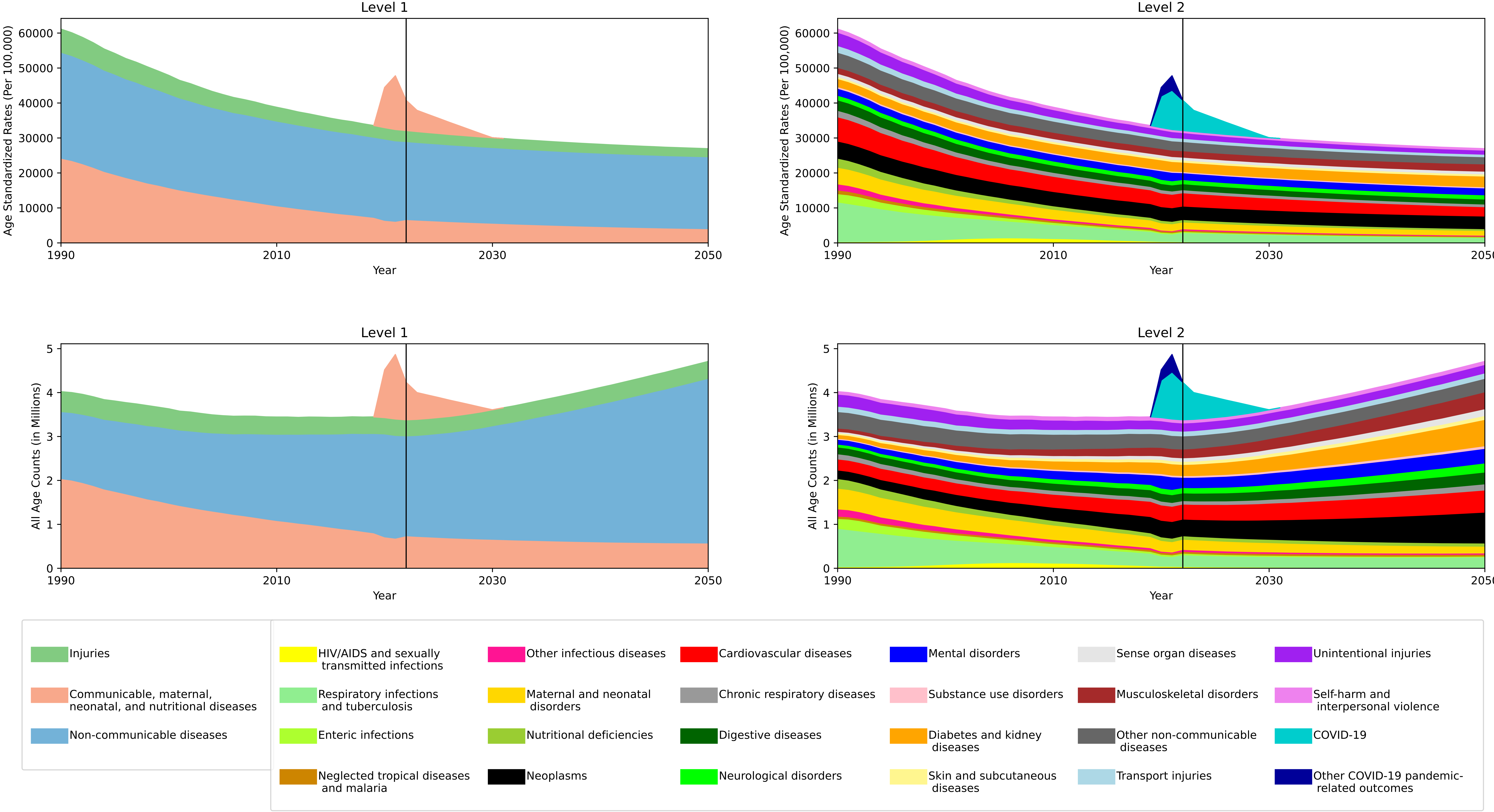
Level 1
Level 2
Age Standardized Rates (Per 100,000)
All Age Counts (in Millions)
Year
1990
2010
2030
2050
Injuries
Communicable, maternal, neonatal, and nutritional diseases
Non-communicable diseases
HIV/AIDS and sexually transmitted infections
Respiratory infections and tuberculosis
Enteric infections
Neglected tropical diseases and malaria
Other infectious diseases
Maternal and neonatal disorders
Nutritional deficiencies
Neoplasms
Cardiovascular diseases
Chronic respiratory diseases
Digestive diseases
Neurological disorders
Mental disorders
Substance use disorders
Diabetes and kidney diseases
Skin and subcutaneous diseases
Sense organ diseases
Musculoskeletal disorders
Other non-communicable diseases
Transport injuries
Unintentional injuries
Self-harm and interpersonal violence
COVID-19
Other COVID-19 pandemic-related outcomes

Ecuador

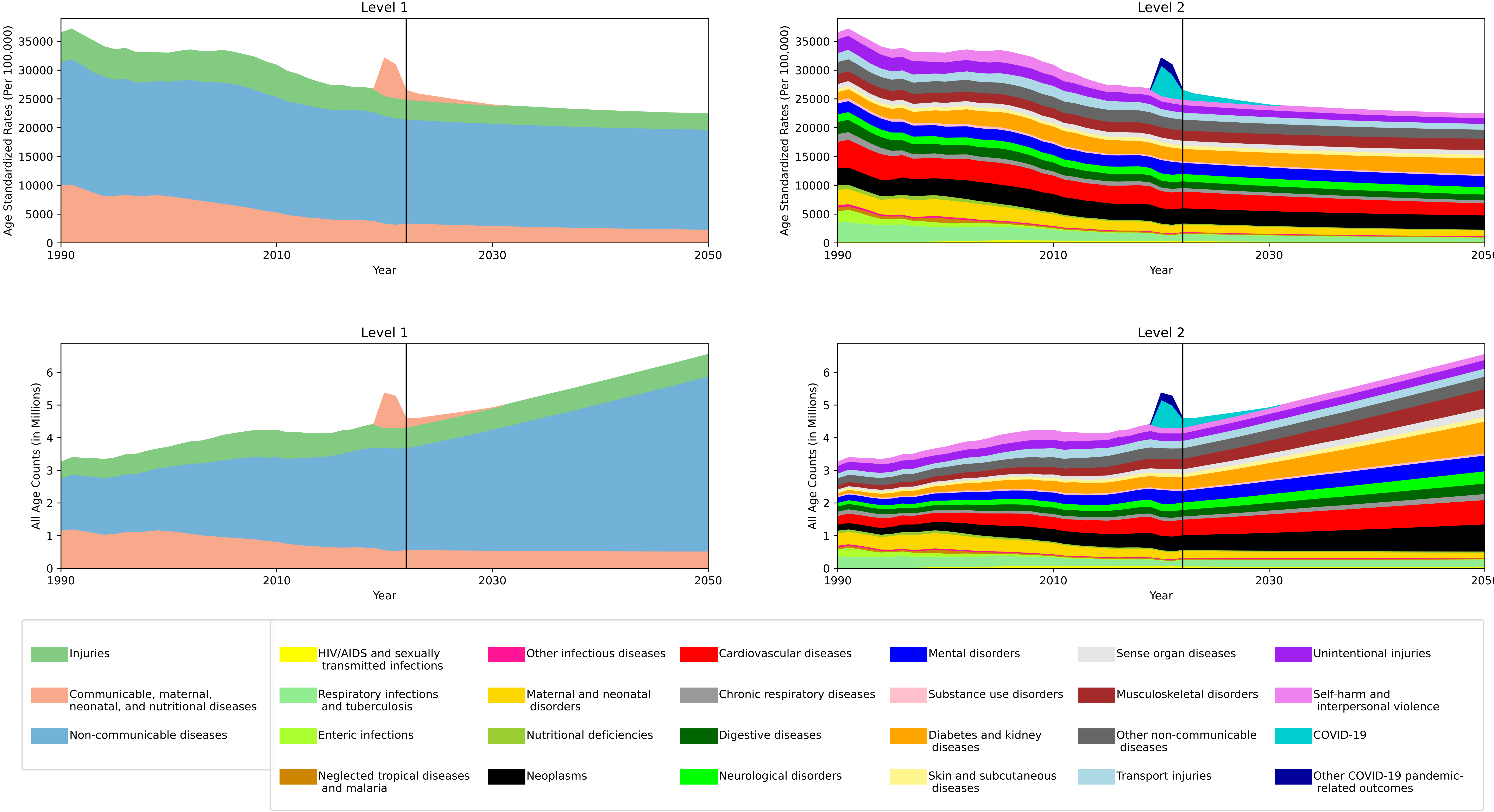
Level 1
Level 2
Age Standardized Rates (Per 100,000)
All Age Counts (in Millions)
Year
Injuries
Communicable, maternal, neonatal, and nutritional diseases
Non-communicable diseases
HIV/AIDS and sexually transmitted infections
Respiratory infections and tuberculosis
Enteric infections
Neglected tropical diseases and malaria
Other infectious diseases
Maternal and neonatal disorders
Nutritional deficiencies
Neoplasms
Cardiovascular diseases
Chronic respiratory diseases
Digestive diseases
Neurological disorders
Mental disorders
Substance use disorders
Diabetes and kidney diseases
Skin and subcutaneous diseases
Sense organ diseases
Musculoskeletal disorders
Other non-communicable diseases
Transport injuries
Unintentional injuries
Self-harm and interpersonal violence
COVID-19
Other COVID-19 pandemic-related outcomes

Peru

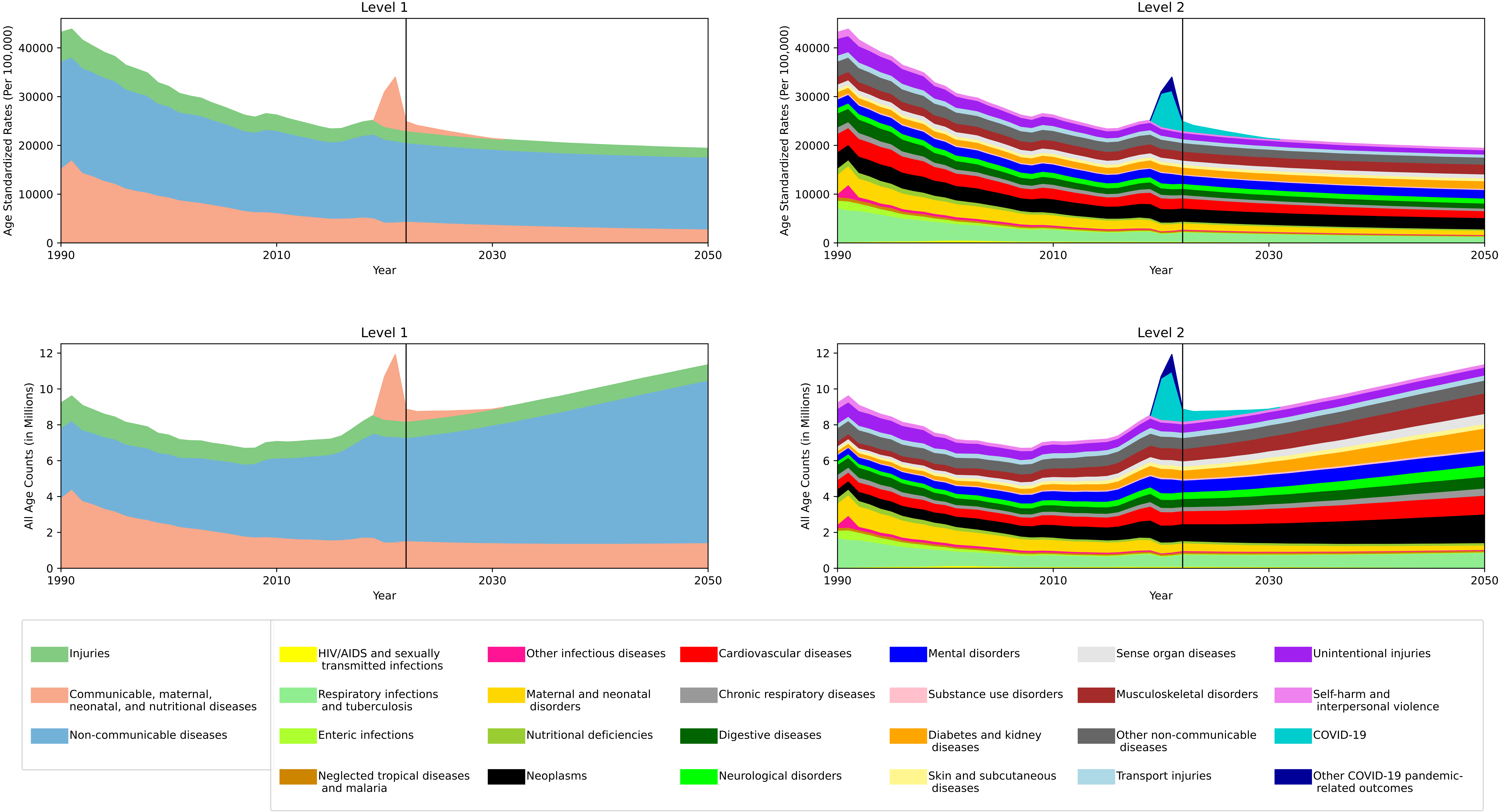

Level 1
Level 2
Age Standardized Rates (Per 100,000)
All Age Counts (in Millions)
Year
1990
2010
2030
2050
0
10000
20000
30000
40000
2
4
6
8
10
12
Injuries
Communicable, maternal, neonatal, and nutritional diseases
Non-communicable diseases
HIV/AIDS and sexually transmitted infections
Respiratory infections and tuberculosis
Enteric infections
Neglected tropical diseases and malaria
Other infectious diseases
Maternal and neonatal disorders
Nutritional deficiencies
Neoplasms
Cardiovascular diseases
Chronic respiratory diseases
Digestive diseases
Neurological disorders
Mental disorders
Substance use disorders
Diabetes and kidney diseases
Skin and subcutaneous diseases
Sense organ diseases
Musculoskeletal disorders
Other non-communicable diseases
Transport injuries
Unintentional injuries
Self-harm and interpersonal violence
COVID-19
Other COVID-19 pandemic-related outcomes

Antigua And Barbuda

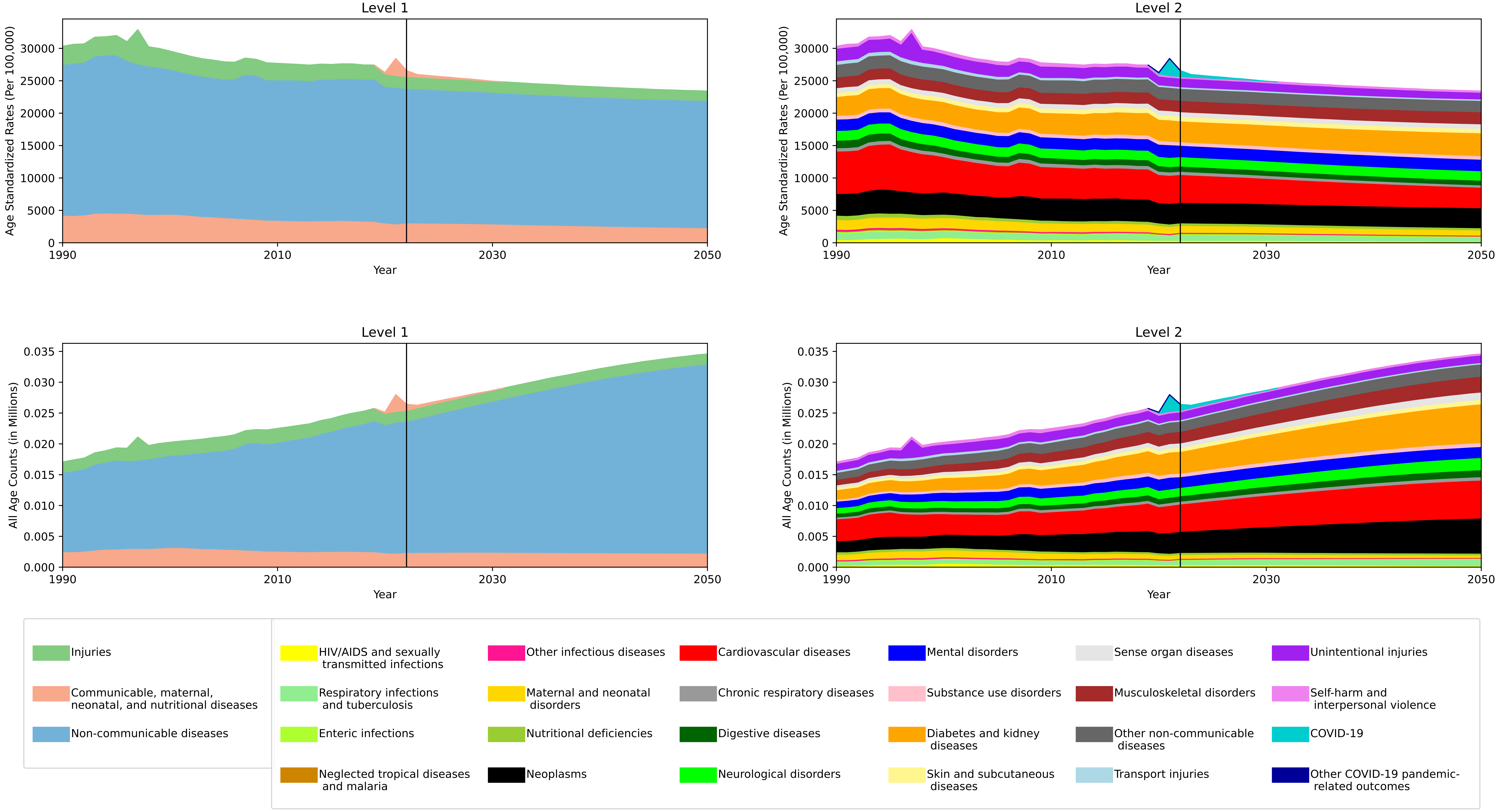

The Bahamas

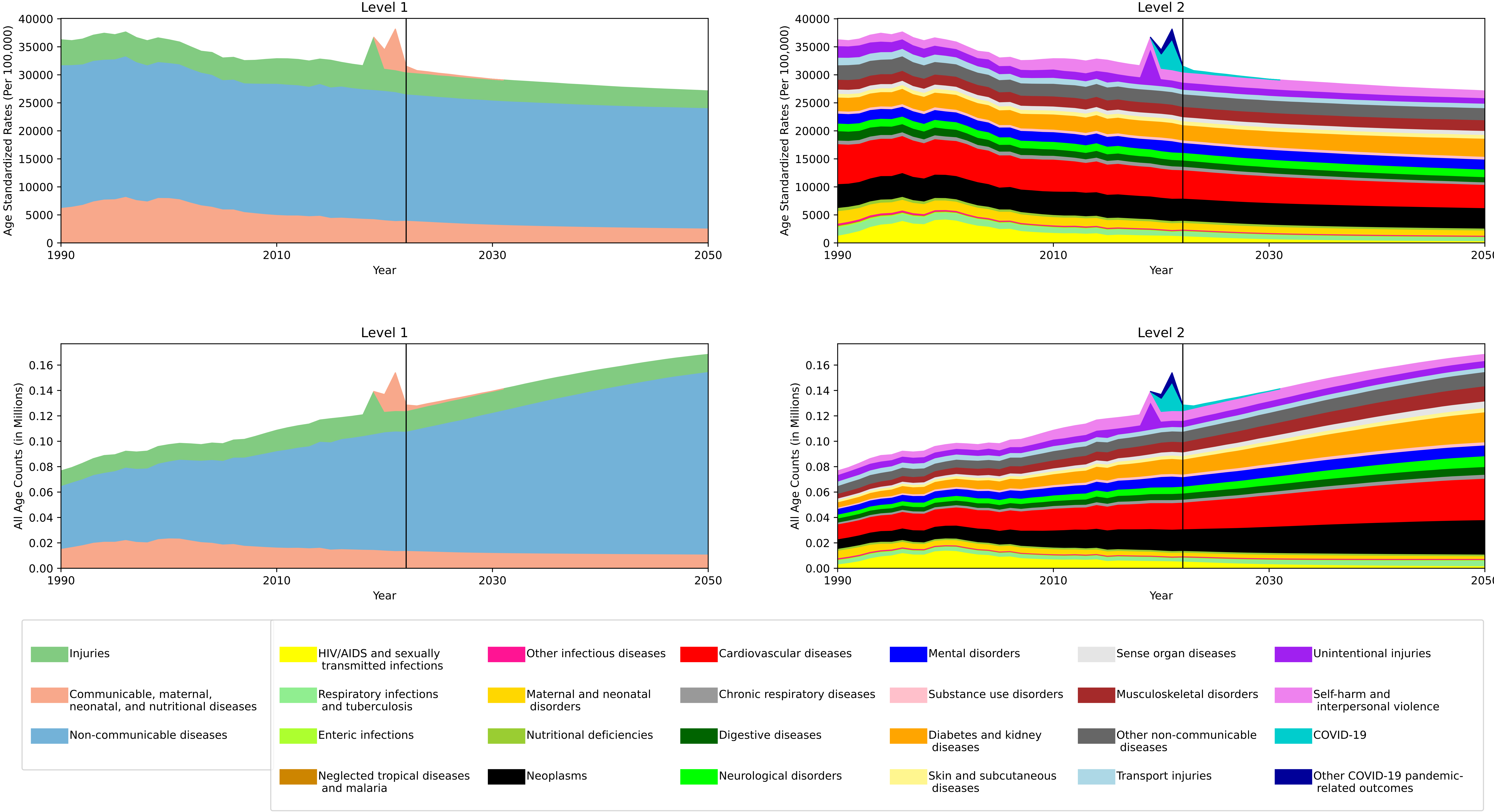
Level 1
Level 2
Age Standardized Rates (Per 100,000)
All Age Counts (in Millions)
Year
1990
2010
2030
2050
0
5000
10000
15000
20000
25000
30000
35000
40000
0.00
0.02
0.04
0.06
0.08
0.10
0.12
0.14
0.16
Injuries
Communicable, maternal, neonatal, and nutritional diseases
Non-communicable diseases
HIV/AIDS and sexually transmitted infections
Respiratory infections and tuberculosis
Enteric infections
Neglected tropical diseases and malaria
Other infectious diseases
Maternal and neonatal disorders
Nutritional deficiencies
Neoplasms
Cardiovascular diseases
Chronic respiratory diseases
Digestive diseases
Neurological disorders
Mental disorders
Substance use disorders
Diabetes and kidney diseases
Skin and subcutaneous diseases
Sense organ diseases
Musculoskeletal disorders
Other non-communicable diseases
Transport injuries
Unintentional injuries
Self-harm and interpersonal violence
COVID-19
Other COVID-19 pandemic-related outcomes

Barbados

Supplemental Results Figure S4. Age-standardised DALY rates and all age DALY counts and by Level 1 and Level 2 causes, 1990–2050

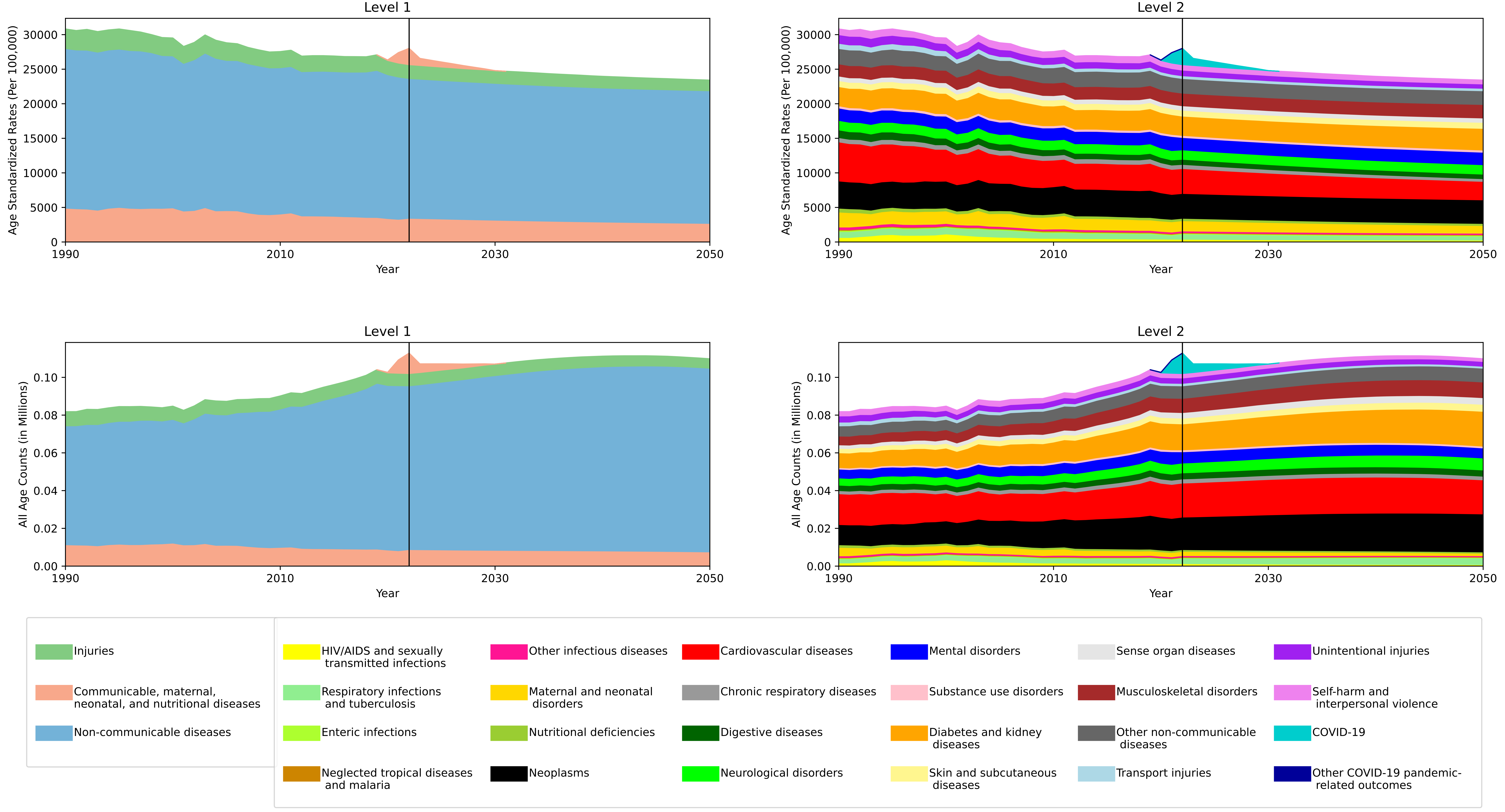

Belize

Supplemental Results Figure S4. Age-standardised DALY rates and all age DALY counts and by Level 1 and Level 2 causes, 1990–2050

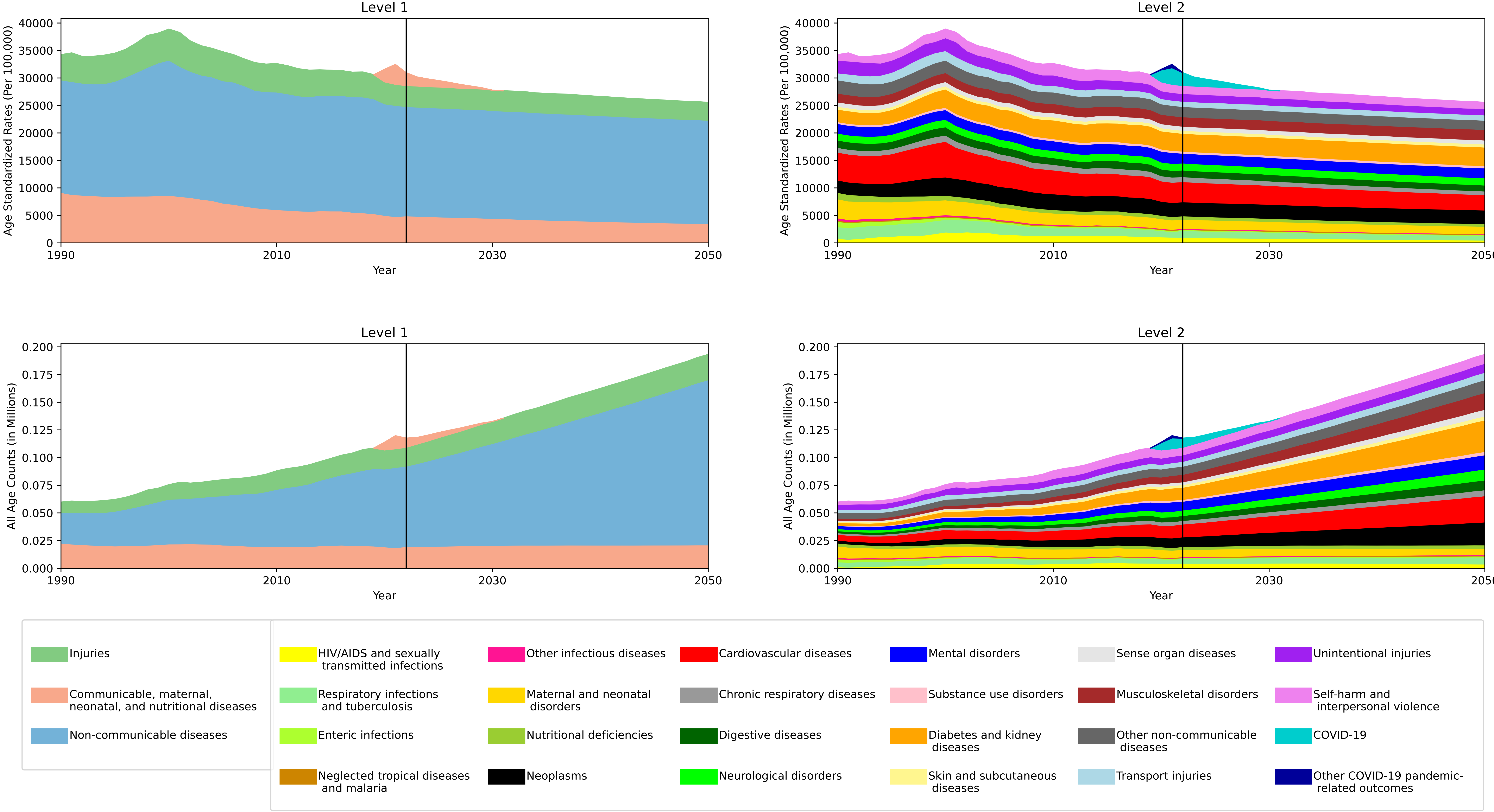

Bermuda

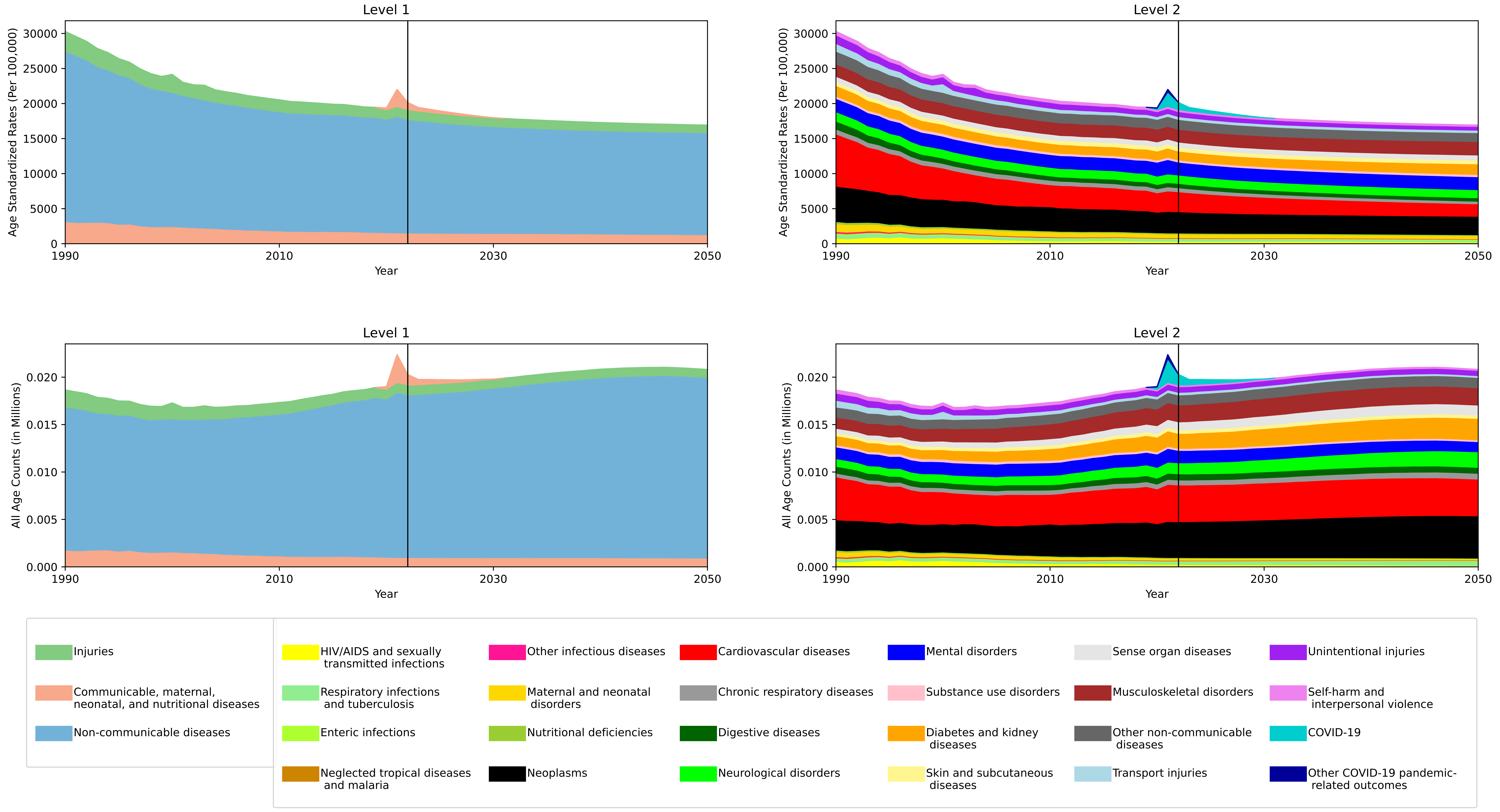

Level 1
Level 2
Age Standardized Rates (Per 100,000)
All Age Counts (in Millions)
Year
1990
2010
2030
2050
Injuries
Communicable, maternal, neonatal, and nutritional diseases
Non-communicable diseases
HIV/AIDS and sexually transmitted infections
Respiratory infections and tuberculosis
Enteric infections
Neglected tropical diseases and malaria
Other infectious diseases
Maternal and neonatal disorders
Nutritional deficiencies
Neoplasms
Cardiovascular diseases
Chronic respiratory diseases
Digestive diseases
Neurological disorders
Mental disorders
Substance use disorders
Diabetes and kidney diseases
Skin and subcutaneous diseases
Sense organ diseases
Musculoskeletal disorders
Other non-communicable diseases
Transport injuries
Unintentional injuries
Self-harm and interpersonal violence
COVID-19
Other COVID-19 pandemic-related outcomes

Cuba

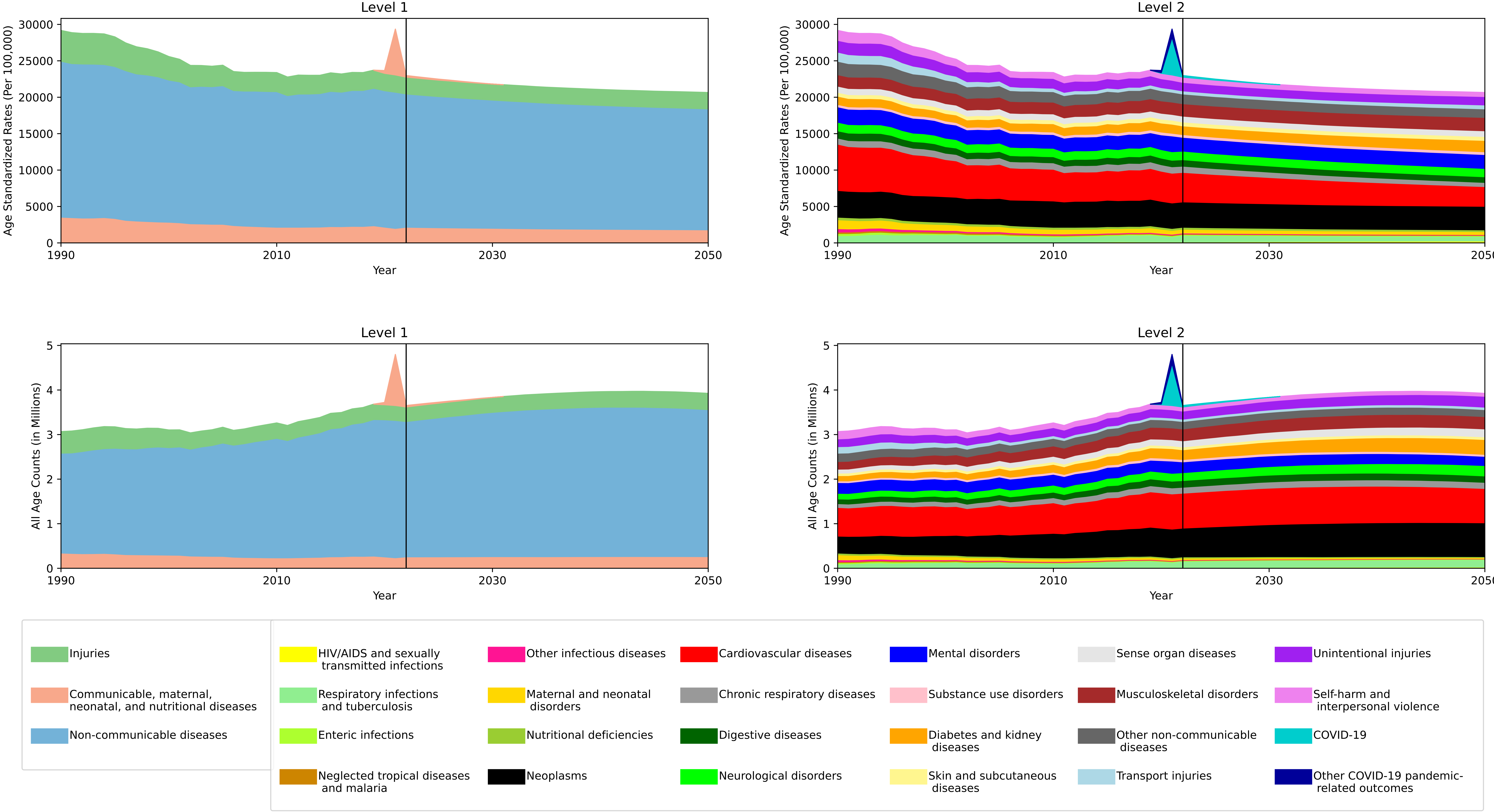
Level 1
Level 2
Age Standardized Rates (Per 100,000)
All Age Counts (in Millions)
Year
1990
2010
2030
2050
30000
25000
20000
15000
10000
5000
0
5
4
3
2
1
Injuries
Communicable, maternal, neonatal, and nutritional diseases
Non-communicable diseases
HIV/AIDS and sexually transmitted infections
Respiratory infections and tuberculosis
Enteric infections
Neglected tropical diseases and malaria
Other infectious diseases
Maternal and neonatal disorders
Nutritional deficiencies
Neoplasms
Cardiovascular diseases
Chronic respiratory diseases
Digestive diseases
Neurological disorders
Mental disorders
Substance use disorders
Diabetes and kidney diseases
Skin and subcutaneous diseases
Sense organ diseases
Musculoskeletal disorders
Other non-communicable diseases
Transport injuries
Unintentional injuries
Self-harm and interpersonal violence
COVID-19
Other COVID-19 pandemic-related outcomes

Dominica

Supplemental Results Figure S4. Age-standardised DALY rates and all age DALY counts and by Level 1 and Level 2 causes, 1990–2050

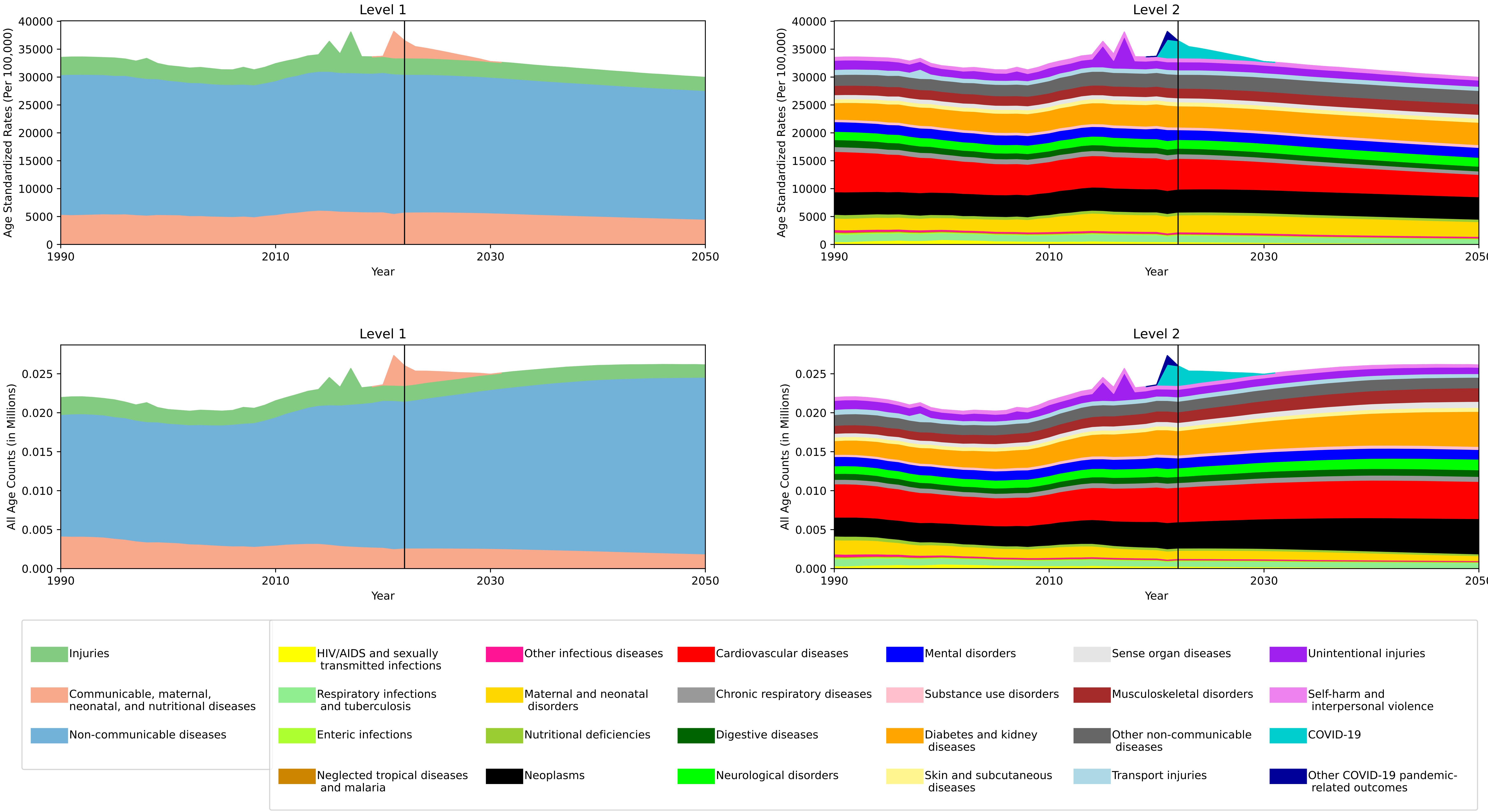

Dominican Republic

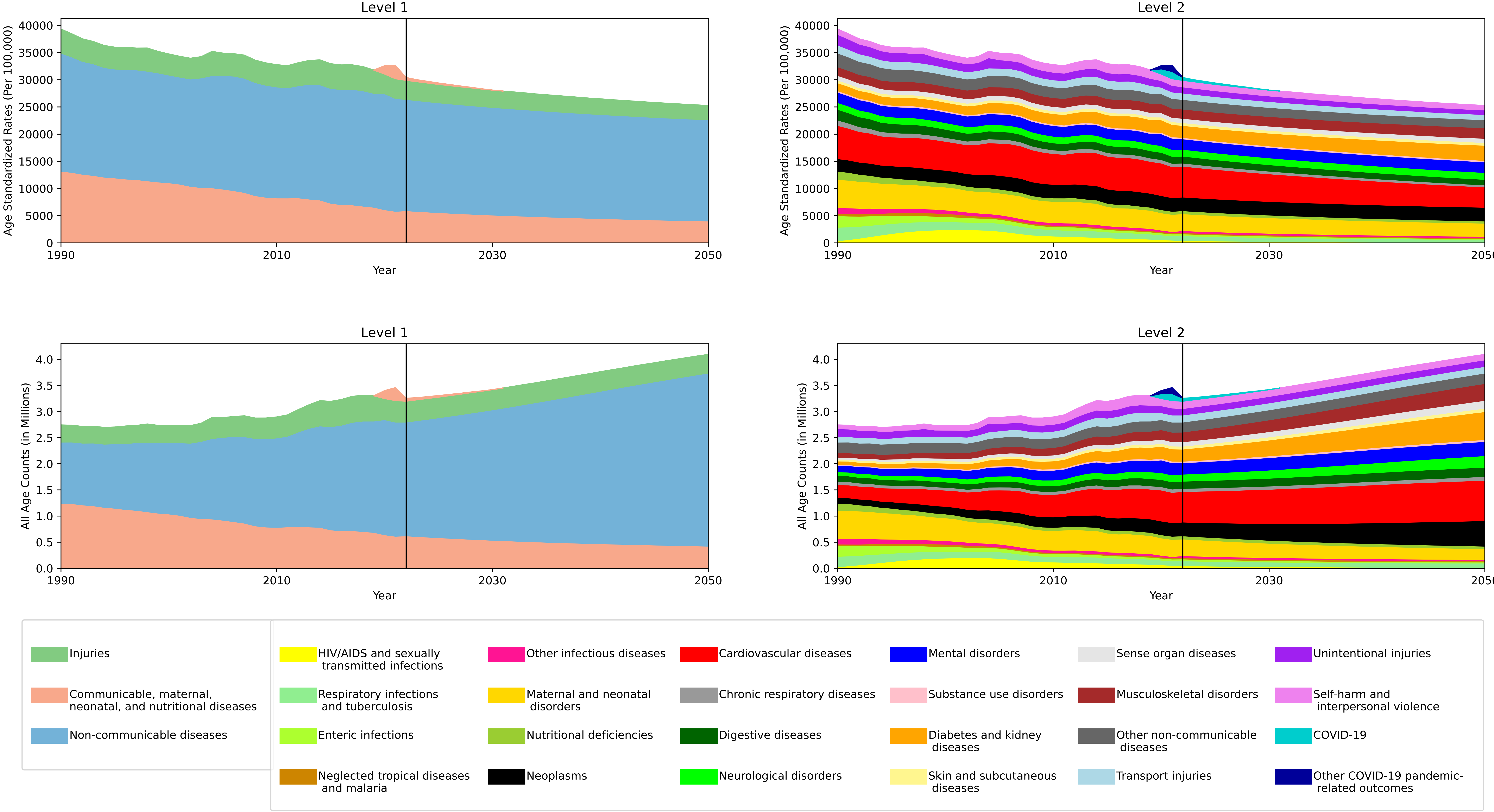

Grenada

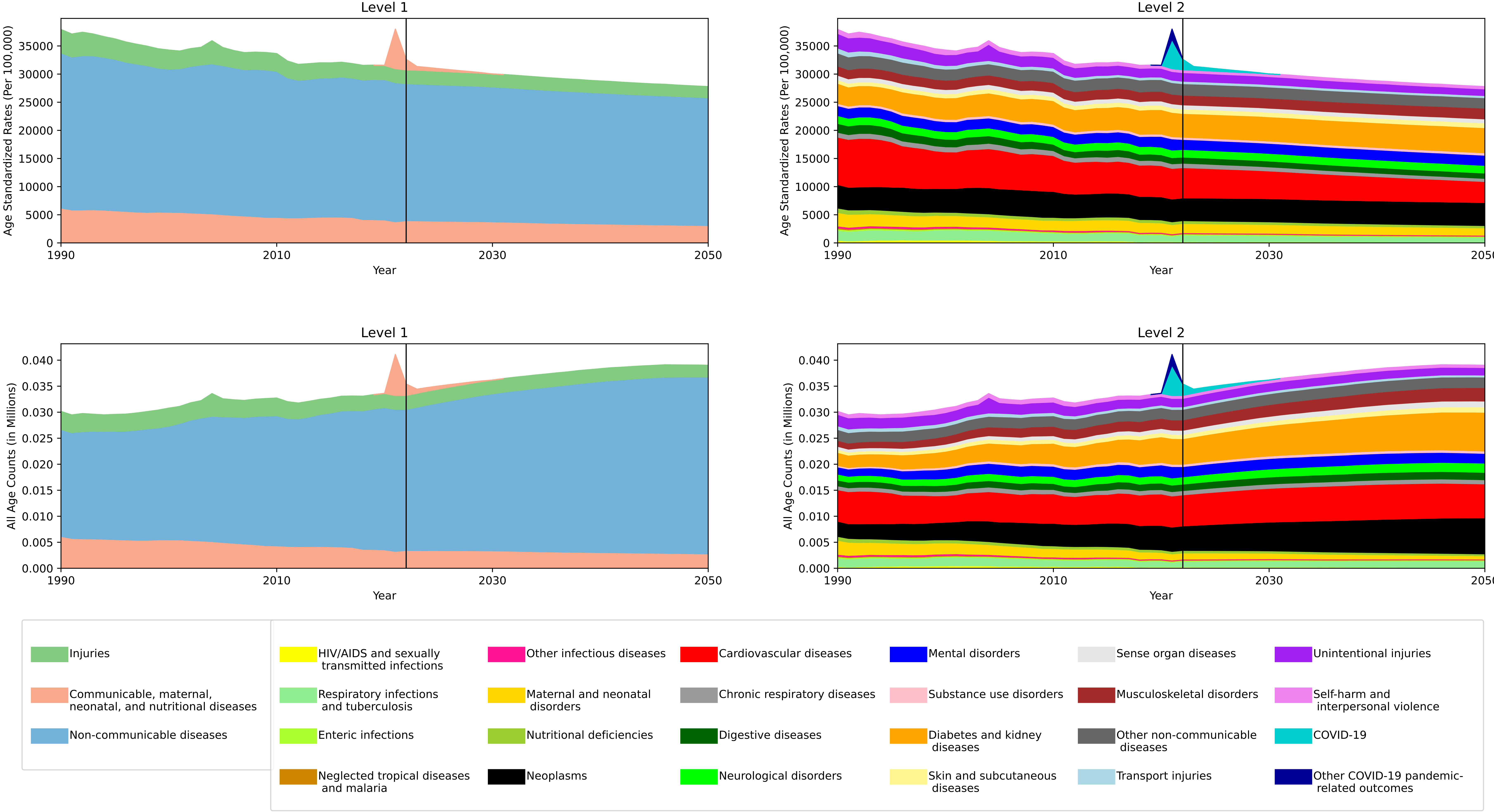

Level 1
Level 2
Age Standardized Rates (Per 100,000)
All Age Counts (in Millions)
Year
1990
2010
2030
2050
Injuries
Communicable, maternal, neonatal, and nutritional diseases
Non-communicable diseases
HIV/AIDS and sexually transmitted infections
Respiratory infections and tuberculosis
Enteric infections
Neglected tropical diseases and malaria
Other infectious diseases
Maternal and neonatal disorders
Nutritional deficiencies
Neoplasms
Cardiovascular diseases
Chronic respiratory diseases
Digestive diseases
Neurological disorders
Mental disorders
Substance use disorders
Diabetes and kidney diseases
Skin and subcutaneous diseases
Sense organ diseases
Musculoskeletal disorders
Other non-communicable diseases
Transport injuries
Unintentional injuries
Self-harm and interpersonal violence
COVID-19
Other COVID-19 pandemic-related outcomes

Guyana

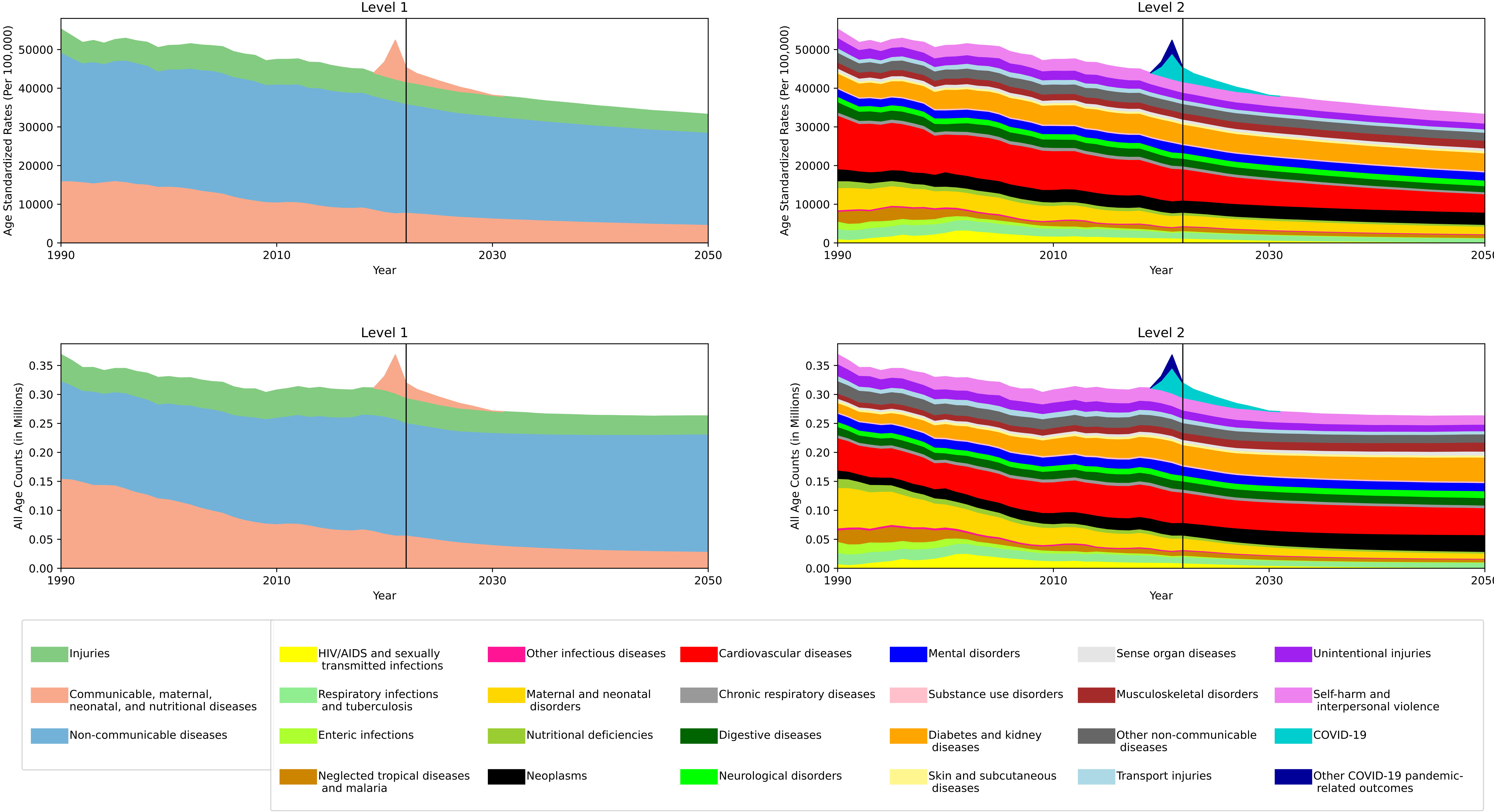
Level 1
Level 2
Age Standardized Rates (Per 100,000)
All Age Counts (in Millions)
Year
1990
2010
2030
2050
Injuries
Communicable, maternal, neonatal, and nutritional diseases
Non-communicable diseases
HIV/AIDS and sexually transmitted infections
Respiratory infections and tuberculosis
Enteric infections
Neglected tropical diseases and malaria
Other infectious diseases
Maternal and neonatal disorders
Nutritional deficiencies
Neoplasms
Cardiovascular diseases
Chronic respiratory diseases
Digestive diseases
Neurological disorders
Mental disorders
Substance use disorders
Diabetes and kidney diseases
Skin and subcutaneous diseases
Sense organ diseases
Musculoskeletal disorders
Other non-communicable diseases
Transport injuries
Unintentional injuries
Self-harm and interpersonal violence
COVID-19
Other COVID-19 pandemic-related outcomes

Haiti

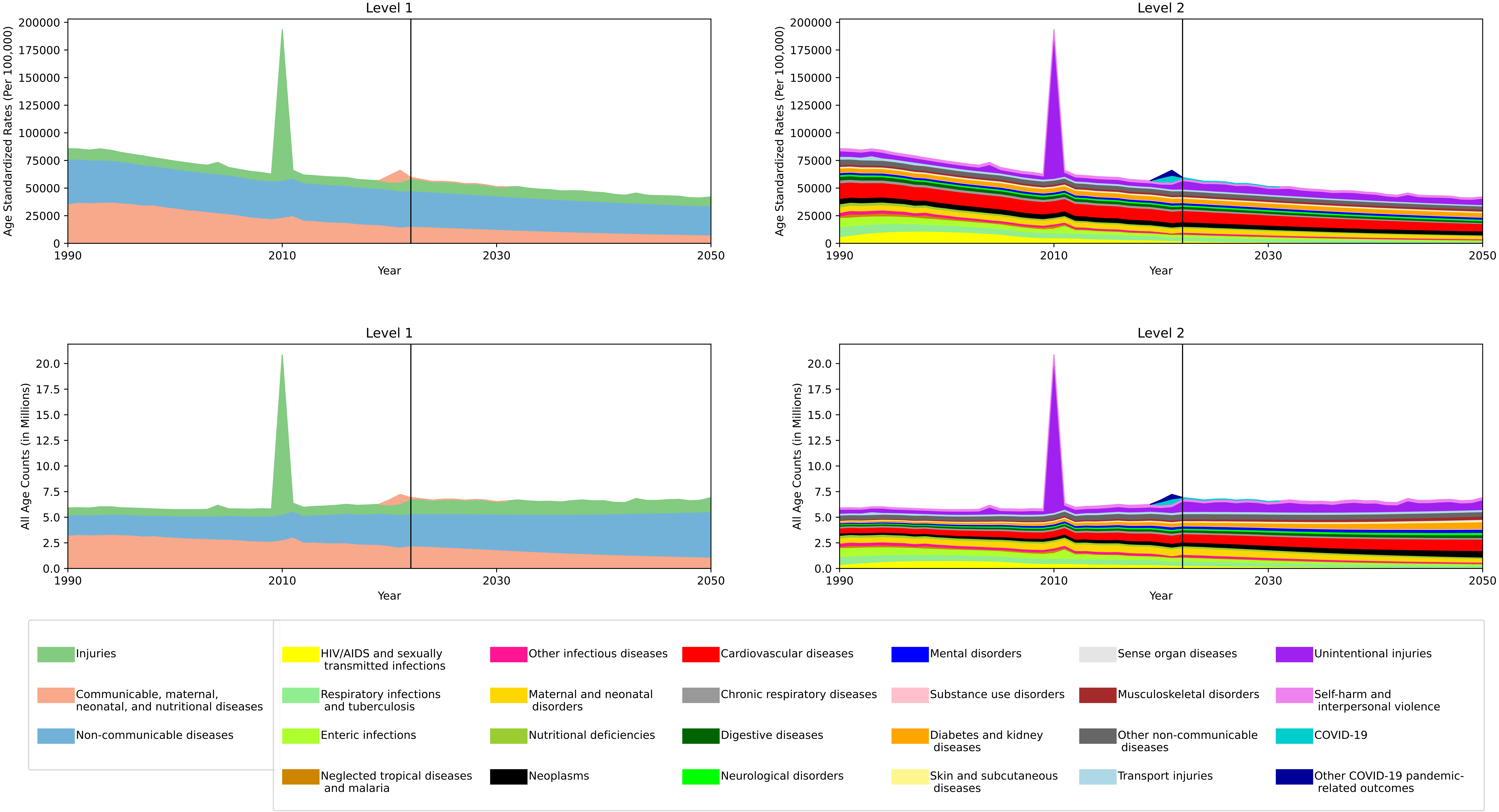
Level 1
Level 2
Age Standardized Rates (Per 100,000)
All Age Counts (in Millions)
Year
0
25000
50000
75000
100000
125000
150000
175000
200000
0.0
2.5
5.0
7.5
10.0
12.5
15.0
17.5
20.0
1990
2010
2030
2050
Injuries
Communicable, maternal, neonatal, and nutritional diseases
Non-communicable diseases
HIV/AIDS and sexually transmitted infections
Respiratory infections and tuberculosis
Enteric infections
Neglected tropical diseases and malaria
Other infectious diseases
Maternal and neonatal disorders
Nutritional deficiencies
Neoplasms
Cardiovascular diseases
Chronic respiratory diseases
Digestive diseases
Neurological disorders
Mental disorders
Substance use disorders
Diabetes and kidney diseases
Skin and subcutaneous diseases
Sense organ diseases
Musculoskeletal disorders
Other non-communicable diseases
Transport injuries
Unintentional injuries
Self-harm and interpersonal violence
COVID-19
Other COVID-19 pandemic-related outcomes

Jamaica

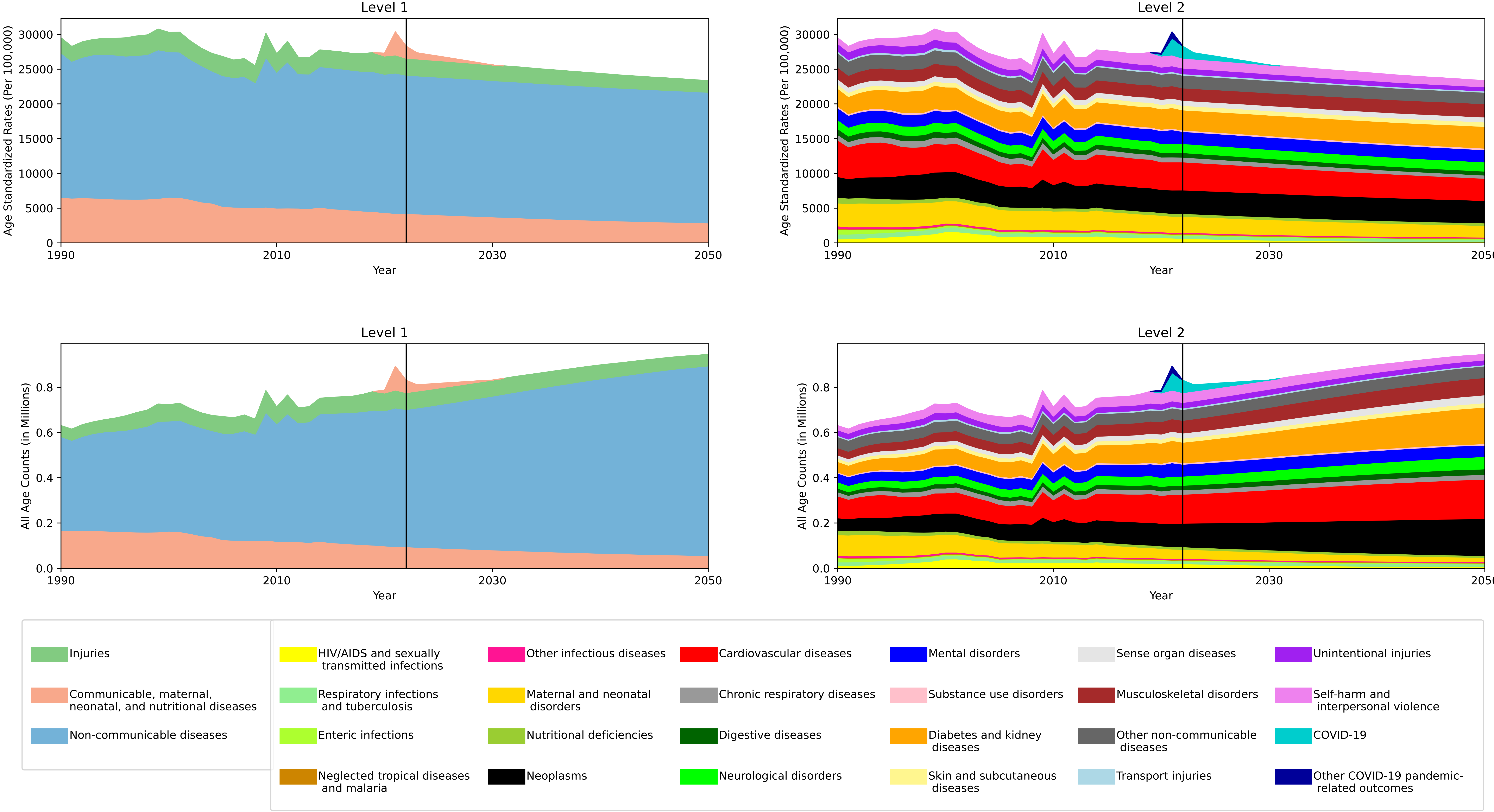
Level 1
Level 2
Age Standardized Rates (Per 100,000)
All Age Counts (in Millions)
Year
1990
2010
2030
2050
0
5000
10000
15000
20000
25000
30000
0.0
0.2
0.4
0.6
0.8
Injuries
Communicable, maternal, neonatal, and nutritional diseases
Non-communicable diseases
HIV/AIDS and sexually transmitted infections
Respiratory infections and tuberculosis
Enteric infections
Neglected tropical diseases and malaria
Other infectious diseases
Maternal and neonatal disorders
Nutritional deficiencies
Neoplasms
Cardiovascular diseases
Chronic respiratory diseases
Digestive diseases
Neurological disorders
Mental disorders
Substance use disorders
Diabetes and kidney diseases
Skin and subcutaneous diseases
Sense organ diseases
Musculoskeletal disorders
Other non-communicable diseases
Transport injuries
Unintentional injuries
Self-harm and interpersonal violence
COVID-19
Other COVID-19 pandemic-related outcomes

Supplemental Results Figure S4. Age-standardised DALY rates and all age DALY counts and by Level 1 and Level 2 causes, 1990–2050

Puerto Rico

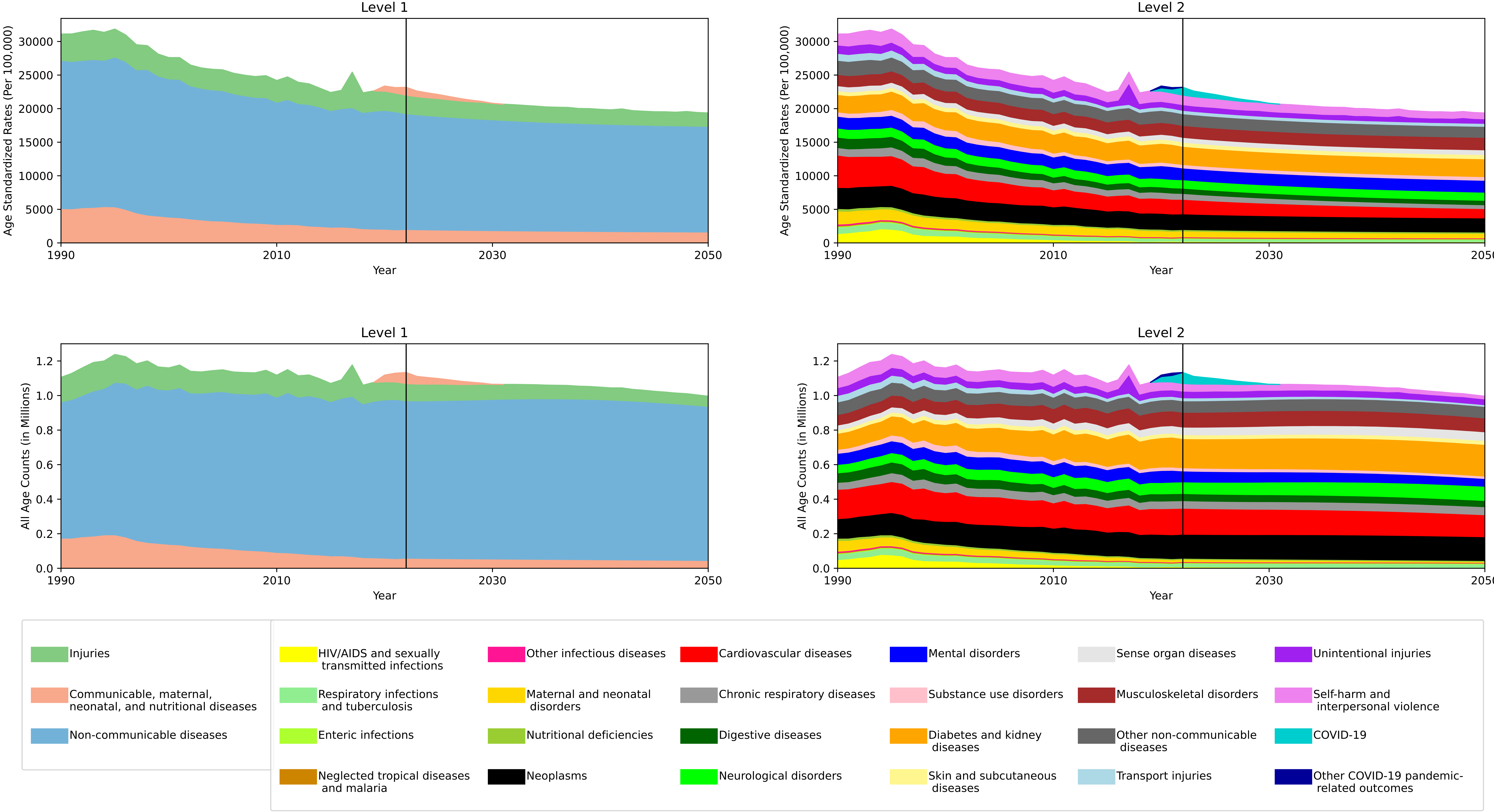

Saint Kitts And Nevis

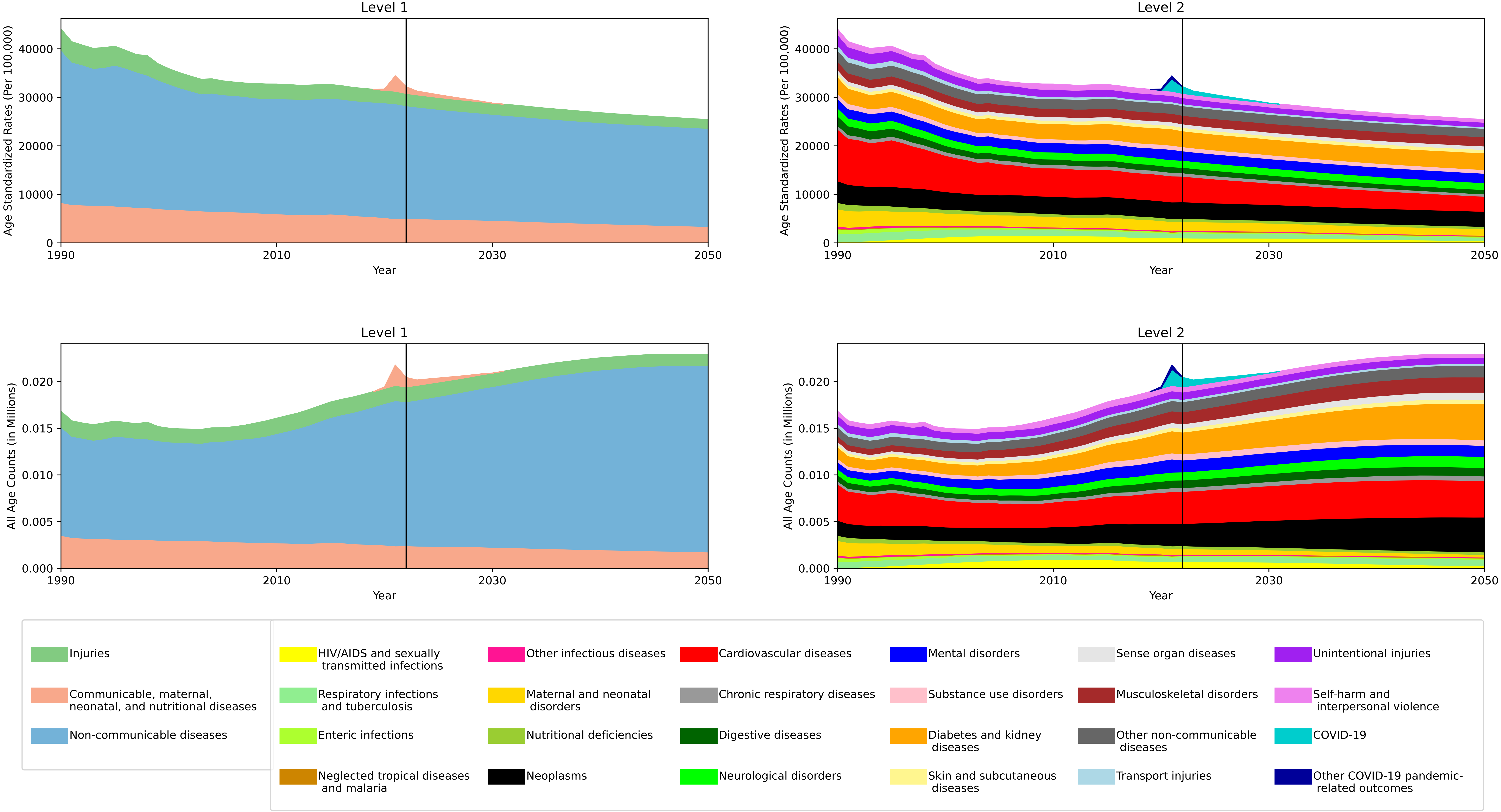

Saint Lucia

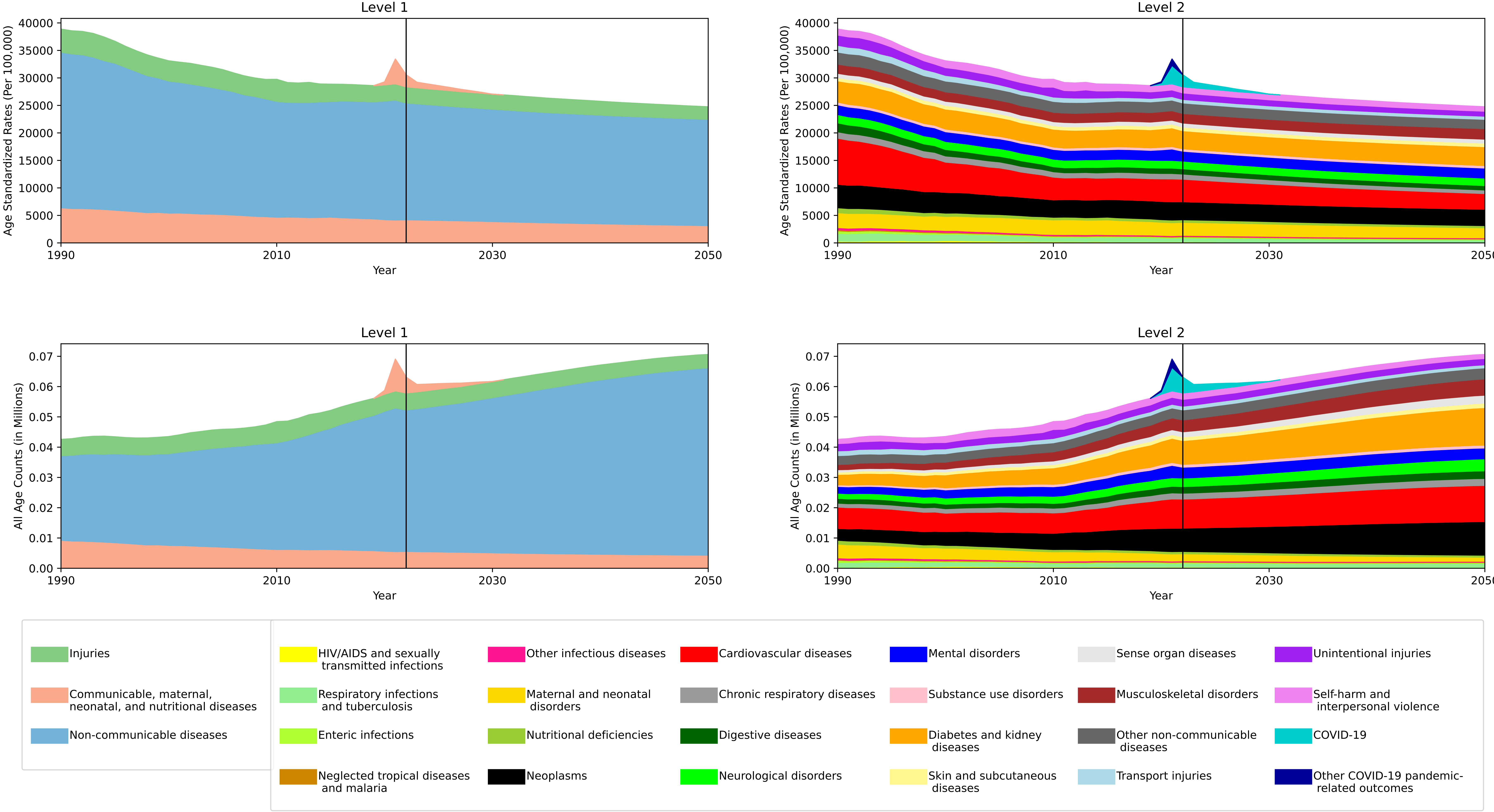
Level 1
Level 2
Age Standardized Rates (Per 100,000)
All Age Counts (in Millions)
Year
1990
2010
2030
2050
Injuries
Communicable, maternal, neonatal, and nutritional diseases
Non-communicable diseases
HIV/AIDS and sexually transmitted infections
Respiratory infections and tuberculosis
Enteric infections
Neglected tropical diseases and malaria
Other infectious diseases
Maternal and neonatal disorders
Nutritional deficiencies
Neoplasms
Cardiovascular diseases
Chronic respiratory diseases
Digestive diseases
Neurological disorders
Mental disorders
Substance use disorders
Diabetes and kidney diseases
Skin and subcutaneous diseases
Sense organ diseases
Musculoskeletal disorders
Other non-communicable diseases
Transport injuries
Unintentional injuries
Self-harm and interpersonal violence
COVID-19
Other COVID-19 pandemic-related outcomes

Saint Vincent And The Grenadines

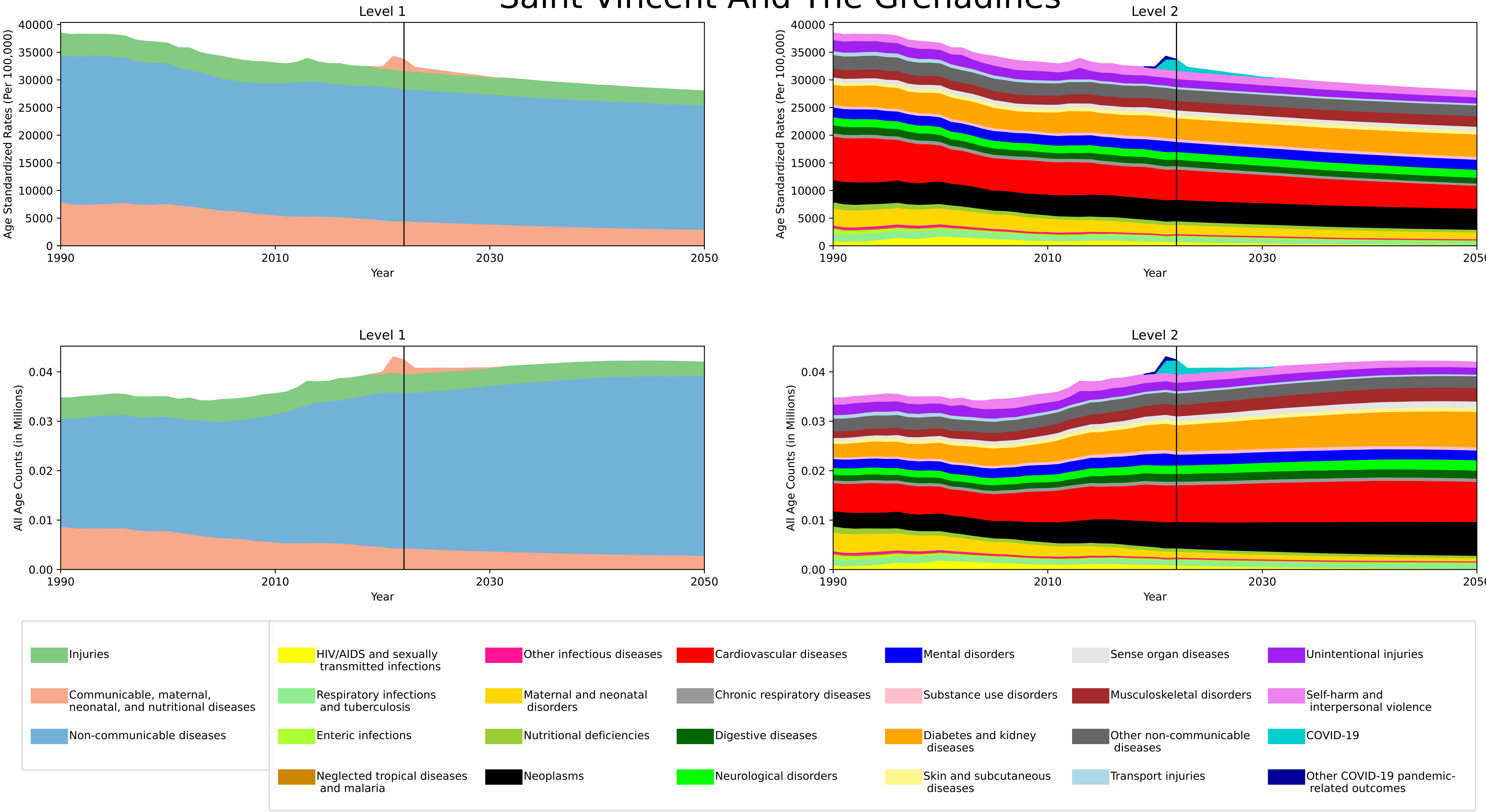

Suriname

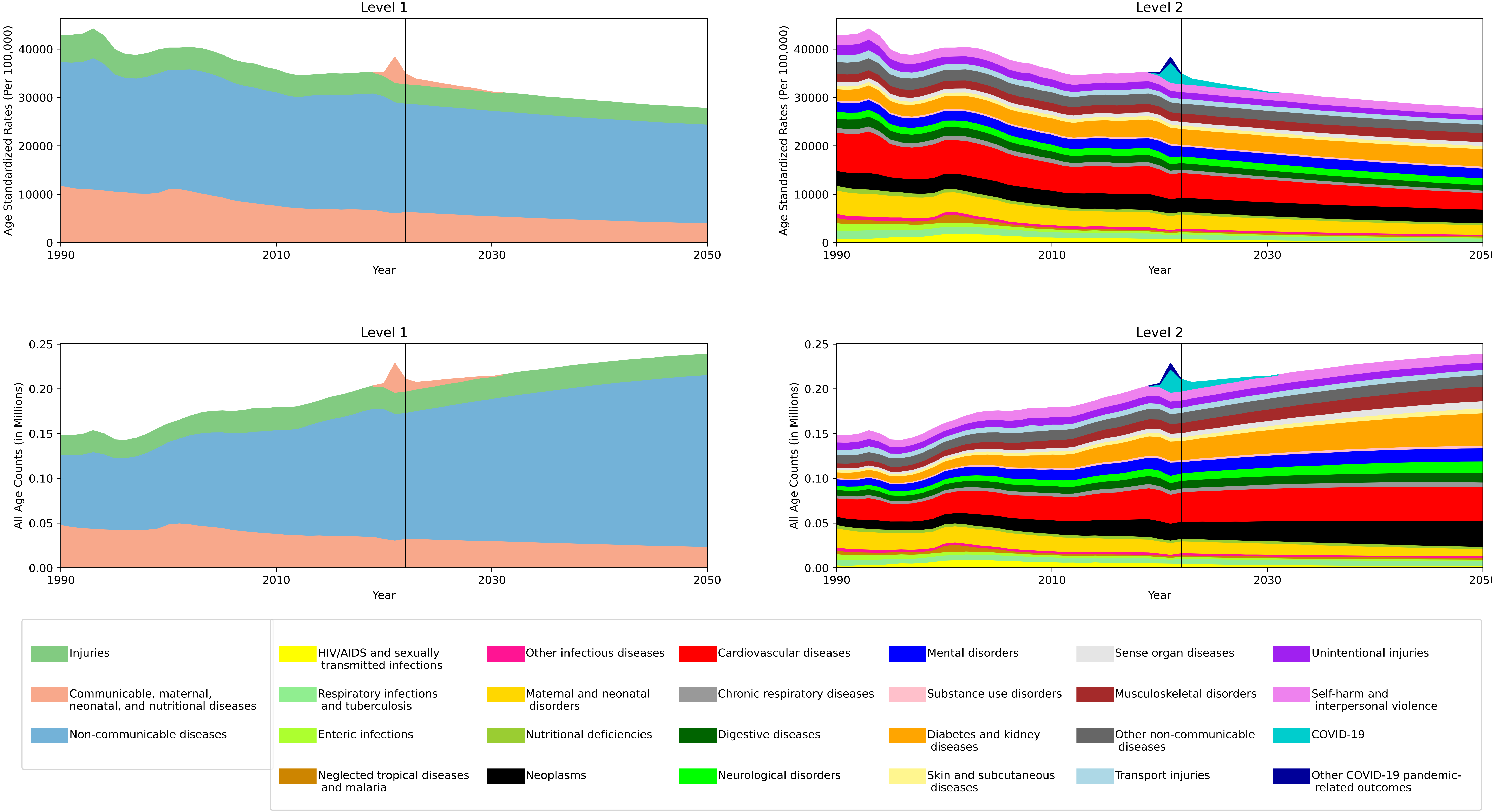
Level 1
Level 2
Age Standardized Rates (Per 100,000)
All Age Counts (in Millions)
Year
1990
2010
2030
2050
0
10000
20000
30000
40000
0.00
0.05
0.10
0.15
0.20
0.25
Injuries
Communicable, maternal, neonatal, and nutritional diseases
Non-communicable diseases
HIV/AIDS and sexually transmitted infections
Respiratory infections and tuberculosis
Enteric infections
Neglected tropical diseases and malaria
Other infectious diseases
Maternal and neonatal disorders
Nutritional deficiencies
Neoplasms
Cardiovascular diseases
Chronic respiratory diseases
Digestive diseases
Neurological disorders
Mental disorders
Substance use disorders
Diabetes and kidney diseases
Skin and subcutaneous diseases
Sense organ diseases
Musculoskeletal disorders
Other non-communicable diseases
Transport injuries
Unintentional injuries
Self-harm and interpersonal violence
COVID-19
Other COVID-19 pandemic-related outcomes

Trinidad And Tobago

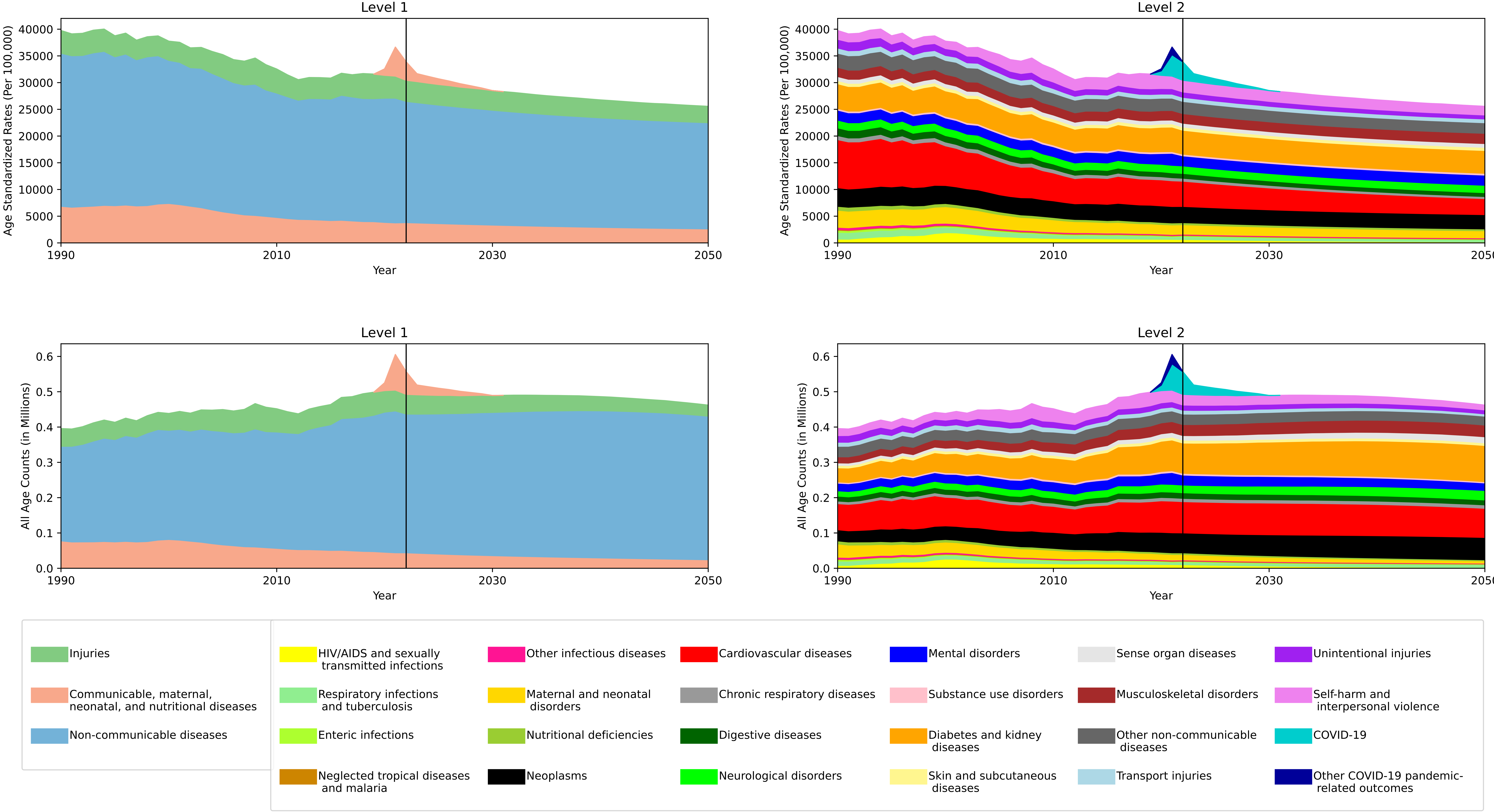

Virgin Islands

Supplemental Results Figure S4. Age-standardised DALY rates and all age DALY counts and by Level 1 and Level 2 causes, 1990–2050

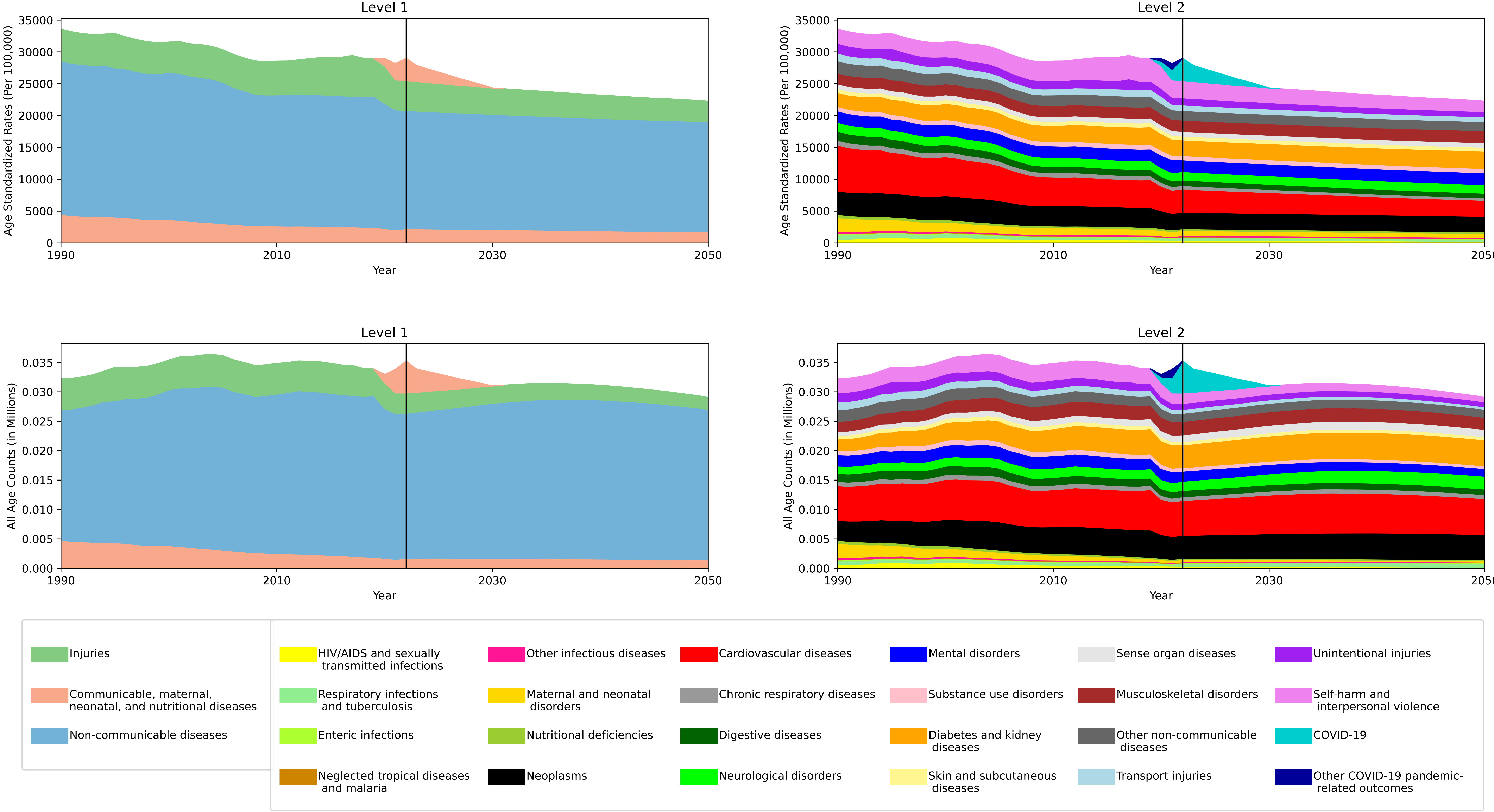

Colombia

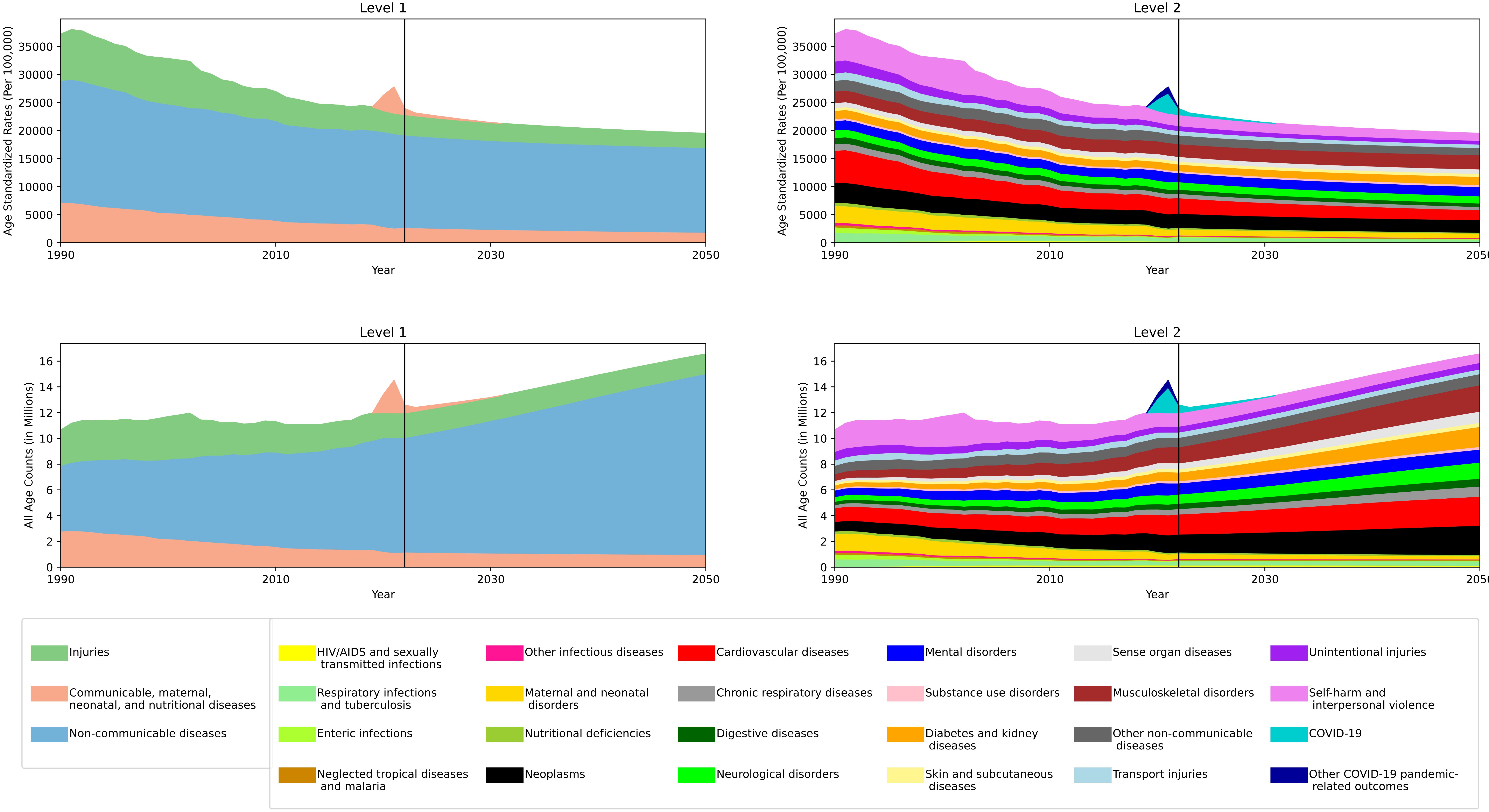
Level 1
Level 2
Age Standardized Rates (Per 100,000)
All Age Counts (in Millions)
Year
1990
2010
2030
2050
35000
30000
25000
20000
15000
10000
5000
0
16
14
12
10
8
6
4
2
Injuries
Communicable, maternal, neonatal, and nutritional diseases
Non-communicable diseases
HIV/AIDS and sexually transmitted infections
Respiratory infections and tuberculosis
Enteric infections
Neglected tropical diseases and malaria
Other infectious diseases
Maternal and neonatal disorders
Nutritional deficiencies
Neoplasms
Cardiovascular diseases
Chronic respiratory diseases
Digestive diseases
Neurological disorders
Mental disorders
Substance use disorders
Diabetes and kidney diseases
Skin and subcutaneous diseases
Sense organ diseases
Musculoskeletal disorders
Other non-communicable diseases
Transport injuries
Unintentional injuries
Self-harm and interpersonal violence
COVID-19
Other COVID-19 pandemic-related outcomes

Costa Rica

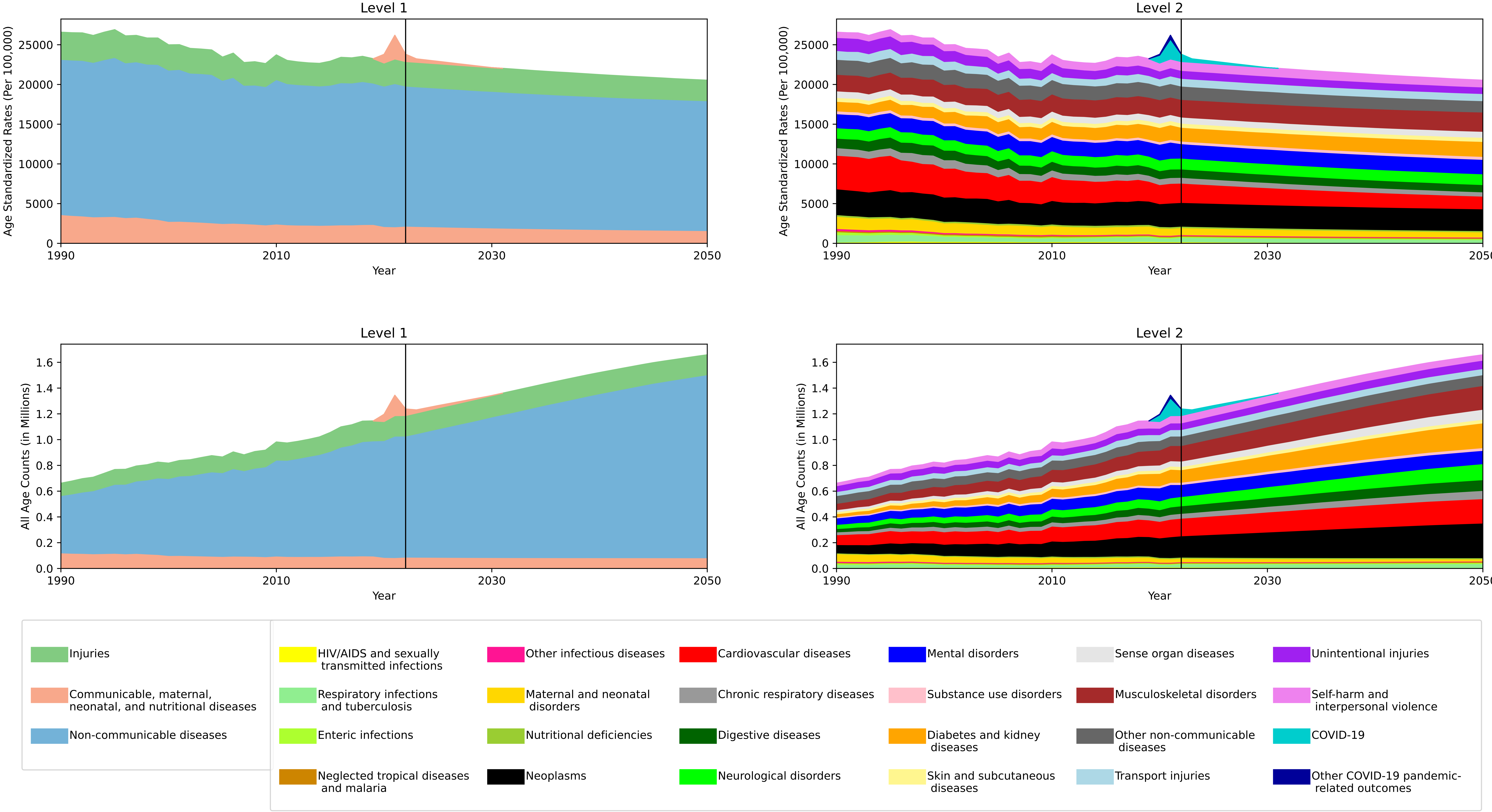
Level 1
Level 2
Age Standardized Rates (Per 100,000)
All Age Counts (in Millions)
Year
0
5000
10000
15000
20000
25000
0.0
0.2
0.4
0.6
0.8
1.0
1.2
1.4
1.6
1990
2010
2030
2050
Injuries
Communicable, maternal, neonatal, and nutritional diseases
Non-communicable diseases
HIV/AIDS and sexually transmitted infections
Respiratory infections and tuberculosis
Enteric infections
Neglected tropical diseases and malaria
Other infectious diseases
Maternal and neonatal disorders
Nutritional deficiencies
Neoplasms
Cardiovascular diseases
Chronic respiratory diseases
Digestive diseases
Neurological disorders
Mental disorders
Substance use disorders
Diabetes and kidney diseases
Skin and subcutaneous diseases
Sense organ diseases
Musculoskeletal disorders
Other non-communicable diseases
Transport injuries
Unintentional injuries
Self-harm and interpersonal violence
COVID-19
Other COVID-19 pandemic-related outcomes

El Salvador

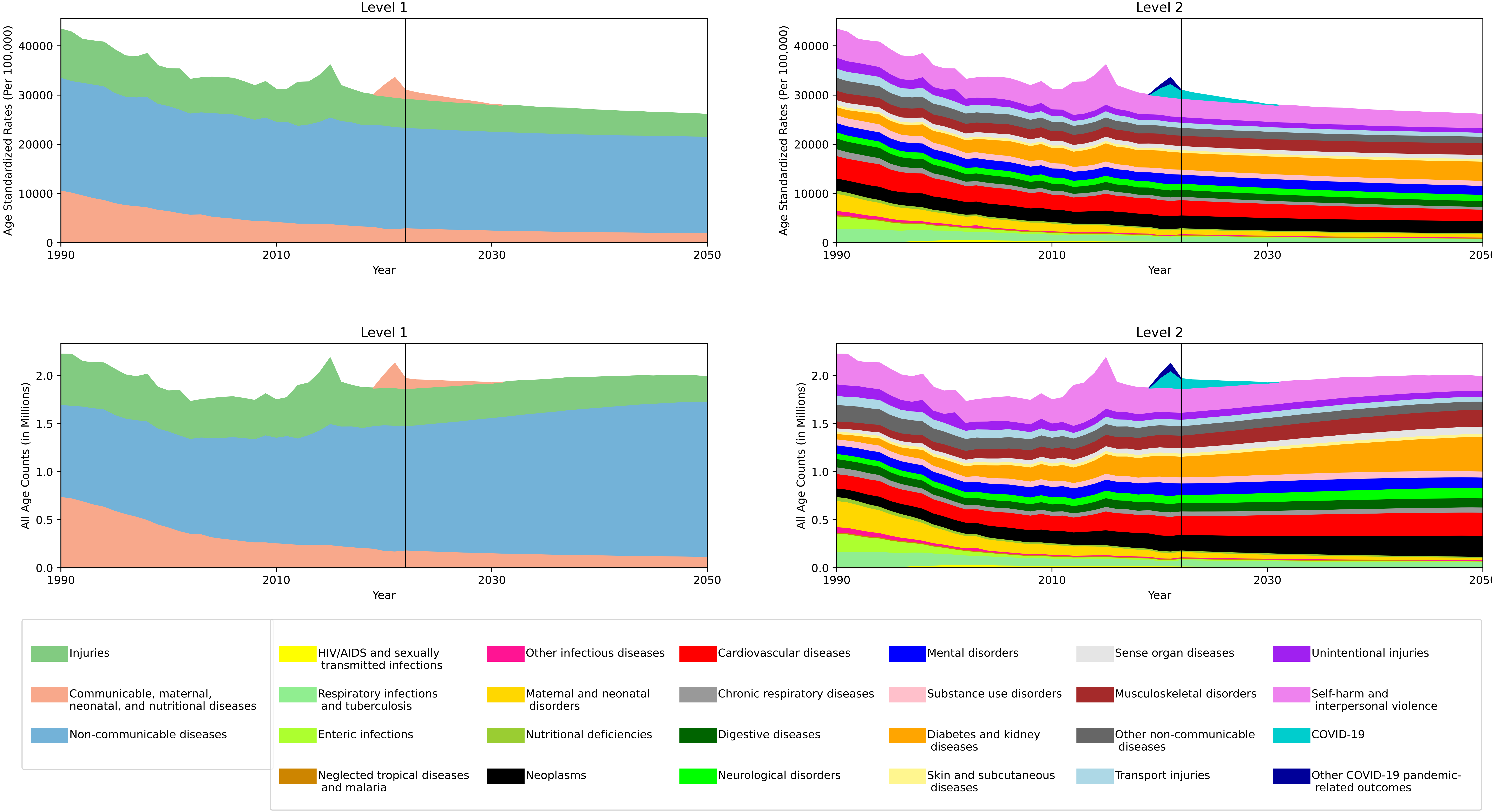
Level 1
Level 2
Age Standardized Rates (Per 100,000)
All Age Counts (in Millions)
Year
1990
2010
2030
2050
Injuries
Communicable, maternal, neonatal, and nutritional diseases
Non-communicable diseases
HIV/AIDS and sexually transmitted infections
Respiratory infections and tuberculosis
Enteric infections
Neglected tropical diseases and malaria
Other infectious diseases
Maternal and neonatal disorders
Nutritional deficiencies
Neoplasms
Cardiovascular diseases
Chronic respiratory diseases
Digestive diseases
Neurological disorders
Mental disorders
Substance use disorders
Diabetes and kidney diseases
Skin and subcutaneous diseases
Sense organ diseases
Musculoskeletal disorders
Other non-communicable diseases
Transport injuries
Unintentional injuries
Self-harm and interpersonal violence
COVID-19
Other COVID-19 pandemic-related outcomes

Guatemala

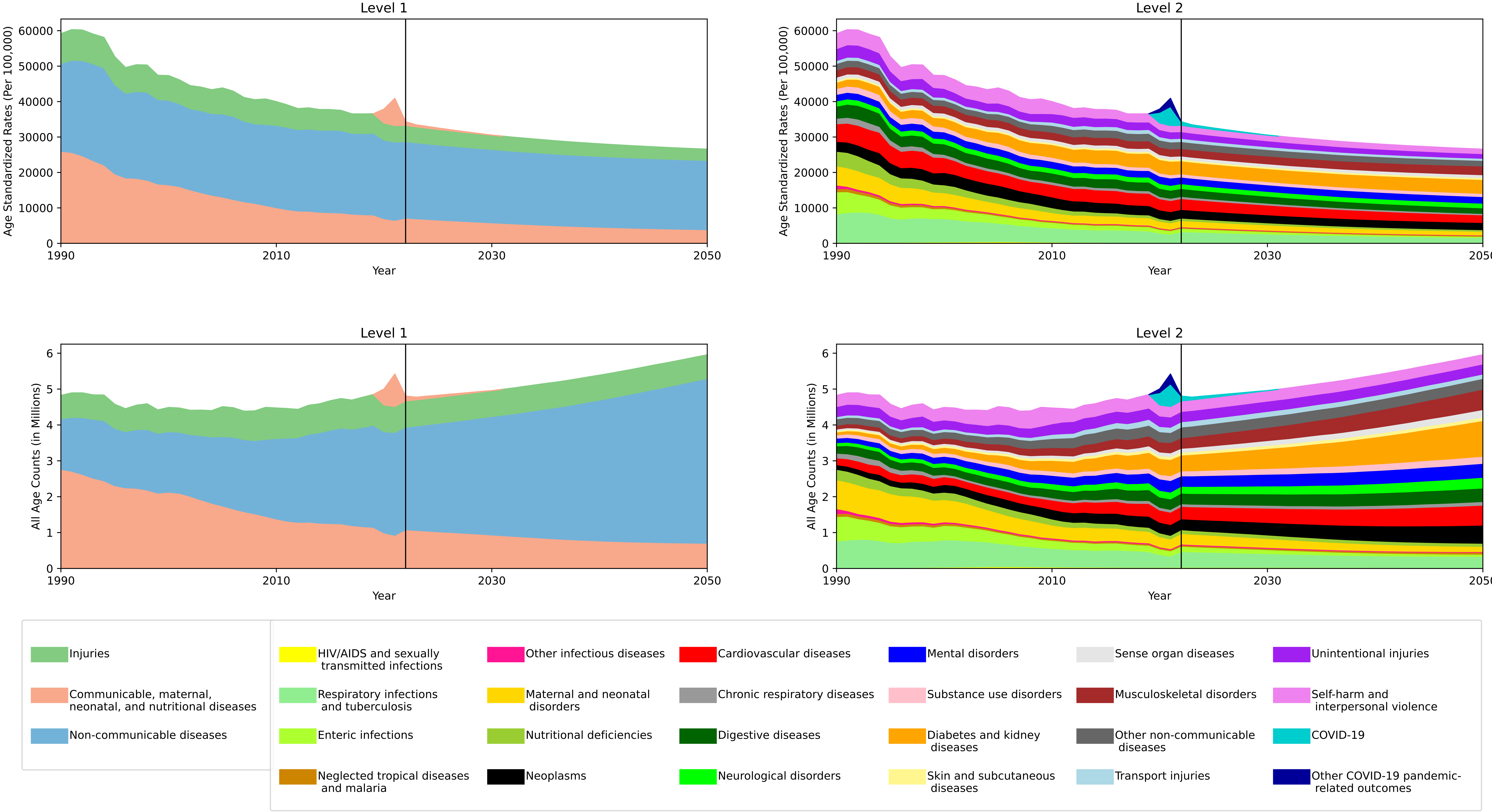
Level 1
Level 2
Age Standardized Rates (Per 100,000)
All Age Counts (in Millions)
Year
1990
2010
2030
2050
Injuries
Communicable, maternal, neonatal, and nutritional diseases
Non-communicable diseases
HIV/AIDS and sexually transmitted infections
Respiratory infections and tuberculosis
Enteric infections
Neglected tropical diseases and malaria
Other infectious diseases
Maternal and neonatal disorders
Nutritional deficiencies
Neoplasms
Cardiovascular diseases
Chronic respiratory diseases
Digestive diseases
Neurological disorders
Mental disorders
Substance use disorders
Diabetes and kidney diseases
Skin and subcutaneous diseases
Sense organ diseases
Musculoskeletal disorders
Other non-communicable diseases
Transport injuries
Unintentional injuries
Self-harm and interpersonal violence
COVID-19
Other COVID-19 pandemic-related outcomes

Honduras

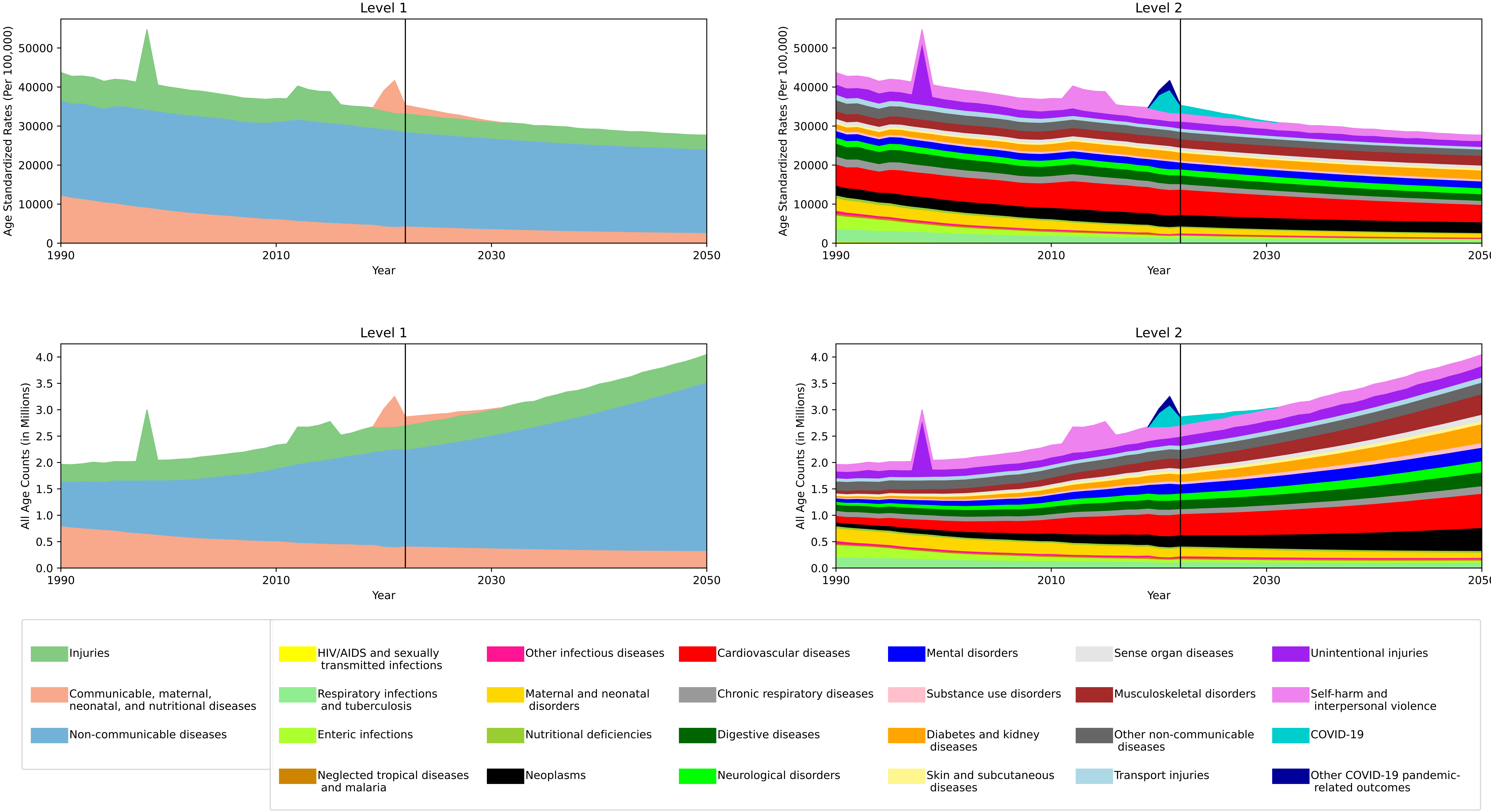
Level 1
Level 2
Age Standardized Rates (Per 100,000)
All Age Counts (in Millions)
Year
1990
2010
2030
2050
0
10000
20000
30000
40000
50000
0.0
0.5
1.0
1.5
2.0
2.5
3.0
3.5
4.0
Injuries
Communicable, maternal, neonatal, and nutritional diseases
Non-communicable diseases
HIV/AIDS and sexually transmitted infections
Respiratory infections and tuberculosis
Enteric infections
Neglected tropical diseases and malaria
Other infectious diseases
Maternal and neonatal disorders
Nutritional deficiencies
Neoplasms
Cardiovascular diseases
Chronic respiratory diseases
Digestive diseases
Neurological disorders
Mental disorders
Substance use disorders
Diabetes and kidney diseases
Skin and subcutaneous diseases
Sense organ diseases
Musculoskeletal disorders
Other non-communicable diseases
Transport injuries
Unintentional injuries
Self-harm and interpersonal violence
COVID-19
Other COVID-19 pandemic-related outcomes

Mexico

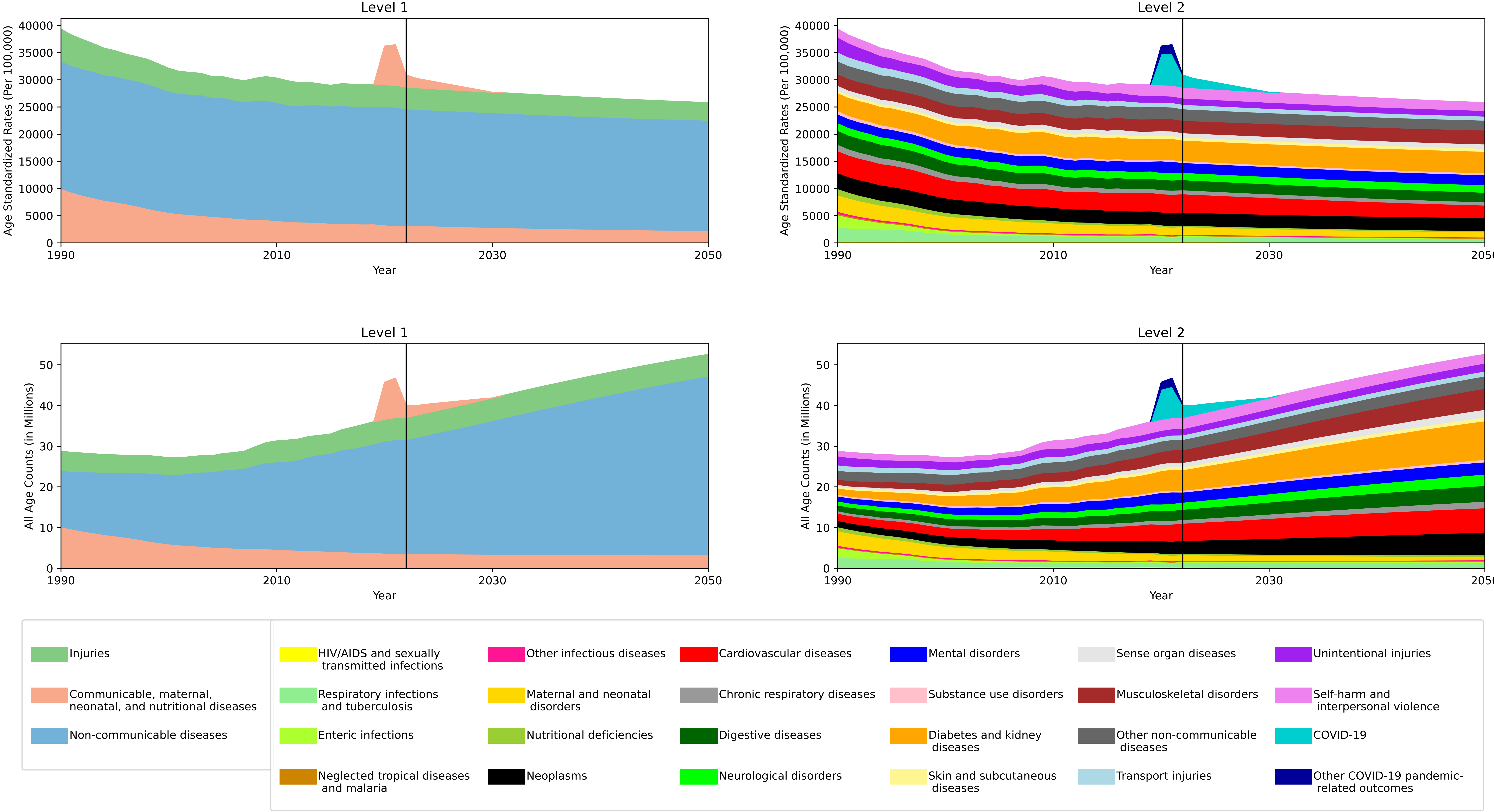
Level 1
Level 2
Age Standardized Rates (Per 100,000)
All Age Counts (in Millions)
Year
1990
2010
2030
2050
Injuries
Communicable, maternal, neonatal, and nutritional diseases
Non-communicable diseases
HIV/AIDS and sexually transmitted infections
Respiratory infections and tuberculosis
Enteric infections
Neglected tropical diseases and malaria
Other infectious diseases
Maternal and neonatal disorders
Nutritional deficiencies
Neoplasms
Cardiovascular diseases
Chronic respiratory diseases
Digestive diseases
Neurological disorders
Mental disorders
Substance use disorders
Diabetes and kidney diseases
Skin and subcutaneous diseases
Sense organ diseases
Musculoskeletal disorders
Other non-communicable diseases
Transport injuries
Unintentional injuries
Self-harm and interpersonal violence
COVID-19
Other COVID-19 pandemic-related outcomes

Nicaragua

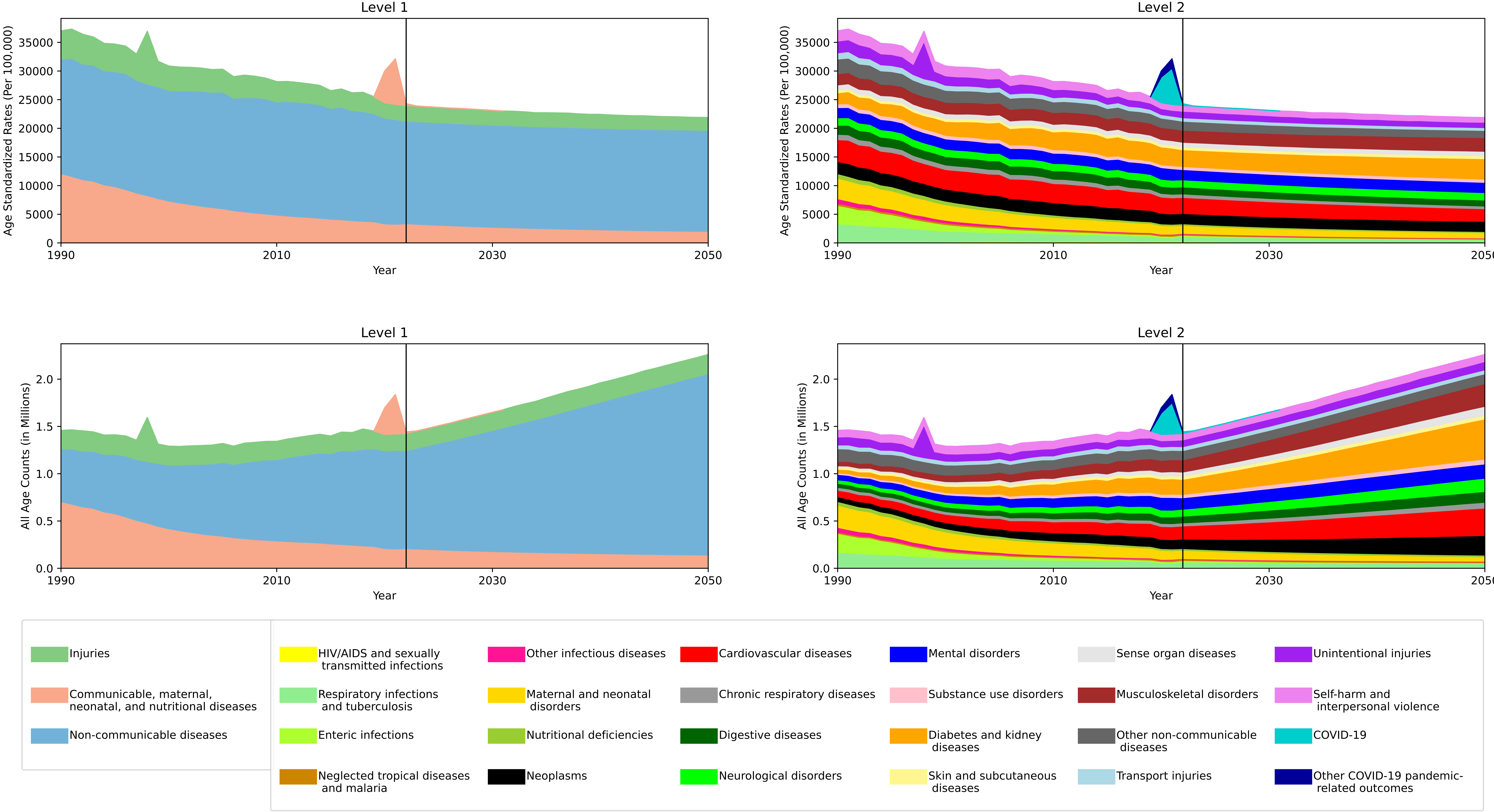
Level 1
Level 2
Age Standardized Rates (Per 100,000)
All Age Counts (in Millions)
Year
1990
2010
2030
2050
Injuries
Communicable, maternal, neonatal, and nutritional diseases
Non-communicable diseases
HIV/AIDS and sexually transmitted infections
Respiratory infections and tuberculosis
Enteric infections
Neglected tropical diseases and malaria
Other infectious diseases
Maternal and neonatal disorders
Nutritional deficiencies
Neoplasms
Cardiovascular diseases
Chronic respiratory diseases
Digestive diseases
Neurological disorders
Mental disorders
Substance use disorders
Diabetes and kidney diseases
Skin and subcutaneous diseases
Sense organ diseases
Musculoskeletal disorders
Other non-communicable diseases
Transport injuries
Unintentional injuries
Self-harm and interpersonal violence
COVID-19
Other COVID-19 pandemic-related outcomes

Panama

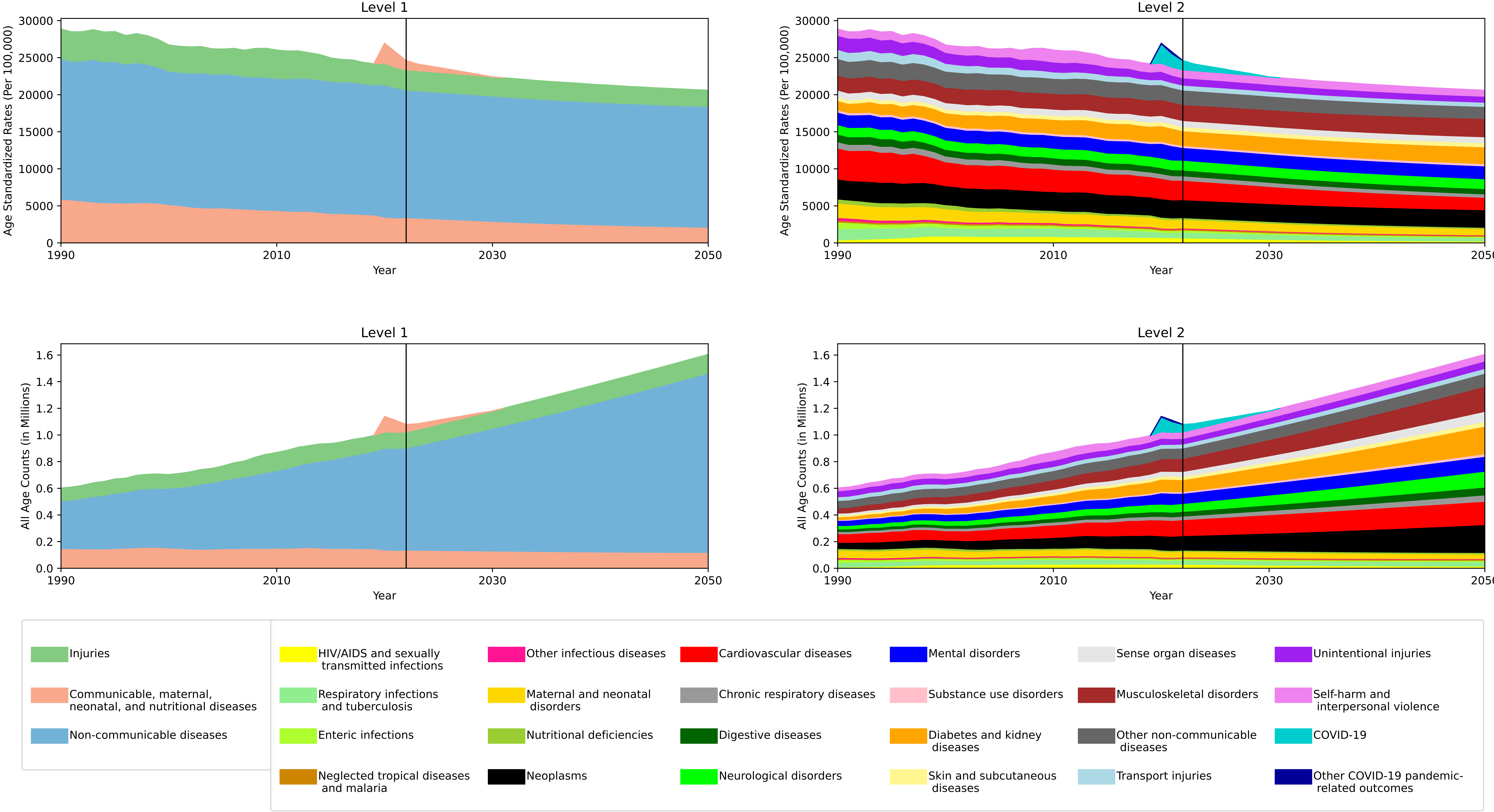
Level 1
Level 2
Age Standardized Rates (Per 100,000)
All Age Counts (in Millions)
Year
1990
2010
2030
2050
Injuries
Communicable, maternal, neonatal, and nutritional diseases
Non-communicable diseases
HIV/AIDS and sexually transmitted infections
Respiratory infections and tuberculosis
Enteric infections
Neglected tropical diseases and malaria
Other infectious diseases
Maternal and neonatal disorders
Nutritional deficiencies
Neoplasms
Cardiovascular diseases
Chronic respiratory diseases
Digestive diseases
Neurological disorders
Mental disorders
Substance use disorders
Diabetes and kidney diseases
Skin and subcutaneous diseases
Sense organ diseases
Musculoskeletal disorders
Other non-communicable diseases
Transport injuries
Unintentional injuries
Self-harm and interpersonal violence
COVID-19
Other COVID-19 pandemic-related outcomes

Venezuela

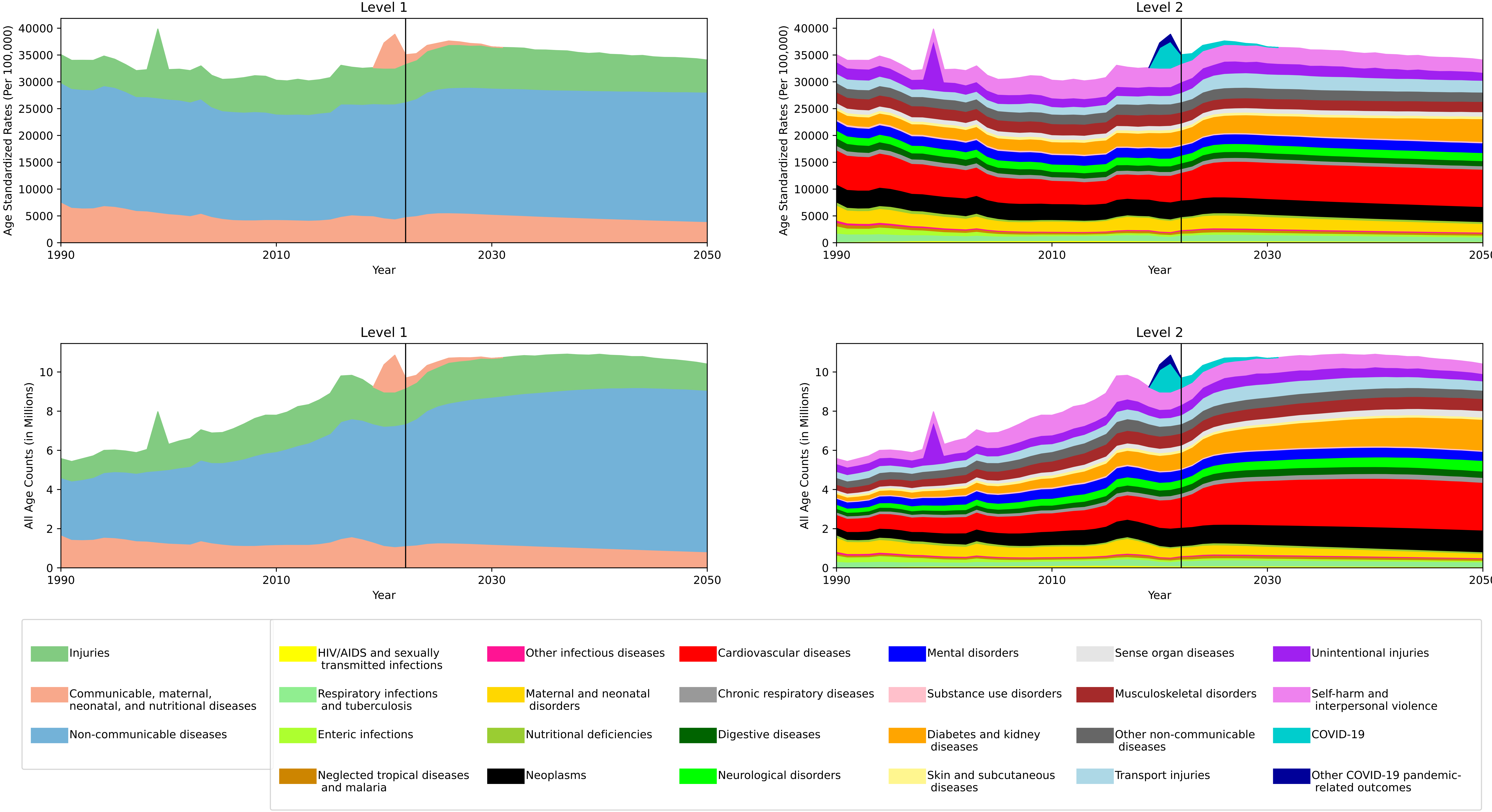
Level 1
Level 2
Age Standardized Rates (Per 100,000)
All Age Counts (in Millions)
Year
1990
2010
2030
2050
Injuries
Communicable, maternal, neonatal, and nutritional diseases
Non-communicable diseases
HIV/AIDS and sexually transmitted infections
Respiratory infections and tuberculosis
Enteric infections
Neglected tropical diseases and malaria
Other infectious diseases
Maternal and neonatal disorders
Nutritional deficiencies
Neoplasms
Cardiovascular diseases
Chronic respiratory diseases
Digestive diseases
Neurological disorders
Mental disorders
Substance use disorders
Diabetes and kidney diseases
Skin and subcutaneous diseases
Sense organ diseases
Musculoskeletal disorders
Other non-communicable diseases
Transport injuries
Unintentional injuries
Self-harm and interpersonal violence
COVID-19
Other COVID-19 pandemic-related outcomes

Brazil

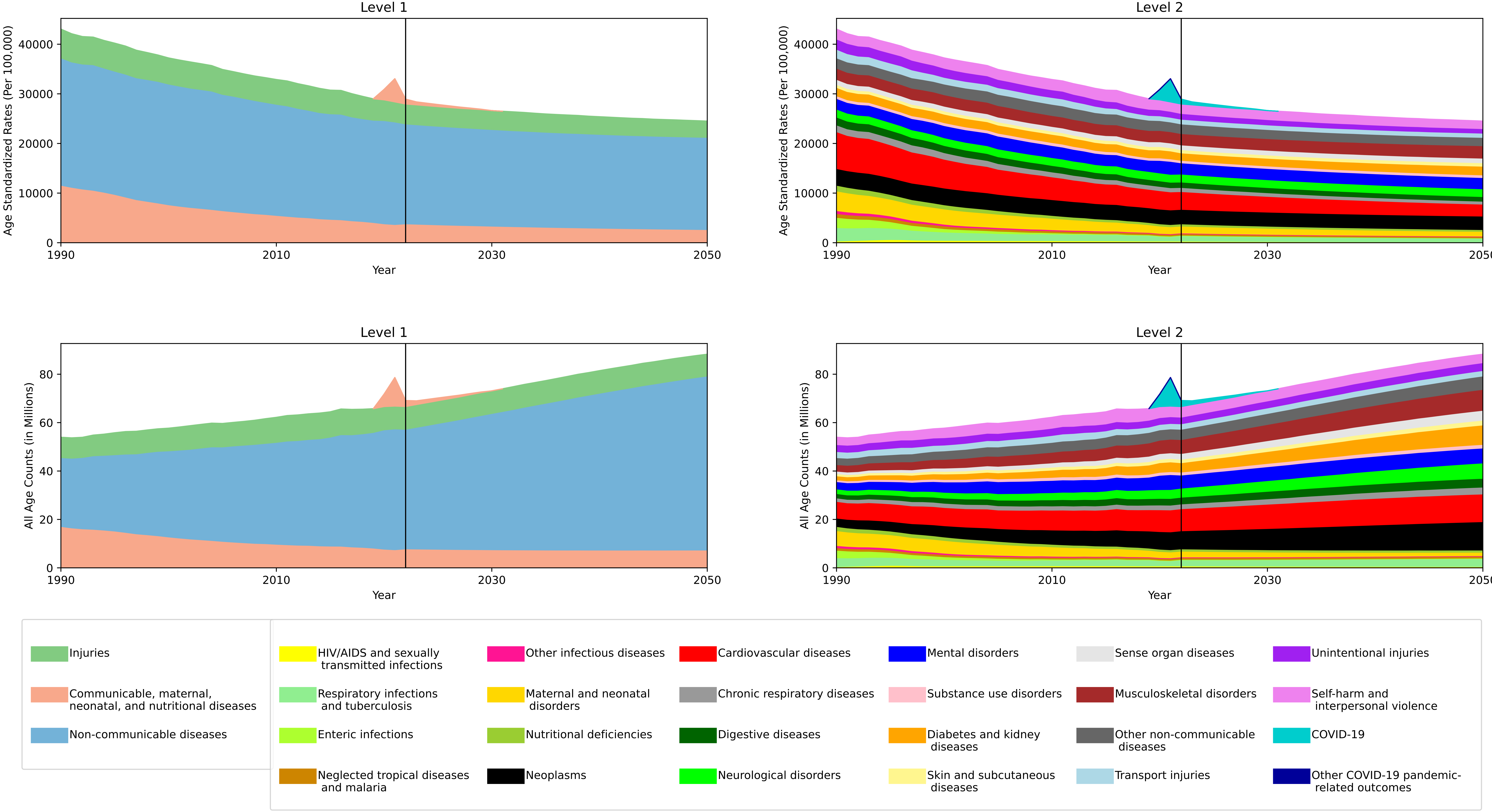
Level 1
Level 2
Age Standardized Rates (Per 100,000)
All Age Counts (in Millions)
Year
1990
2010
2030
2050
40000
30000
20000
10000
0
80
60
40
20
Injuries
Communicable, maternal, neonatal, and nutritional diseases
Non-communicable diseases
HIV/AIDS and sexually transmitted infections
Respiratory infections and tuberculosis
Enteric infections
Neglected tropical diseases and malaria
Other infectious diseases
Maternal and neonatal disorders
Nutritional deficiencies
Neoplasms
Cardiovascular diseases
Chronic respiratory diseases
Digestive diseases
Neurological disorders
Mental disorders
Substance use disorders
Diabetes and kidney diseases
Skin and subcutaneous diseases
Sense organ diseases
Musculoskeletal disorders
Other non-communicable diseases
Transport injuries
Unintentional injuries
Self-harm and interpersonal violence
COVID-19
Other COVID-19 pandemic-related outcomes

Paraguay

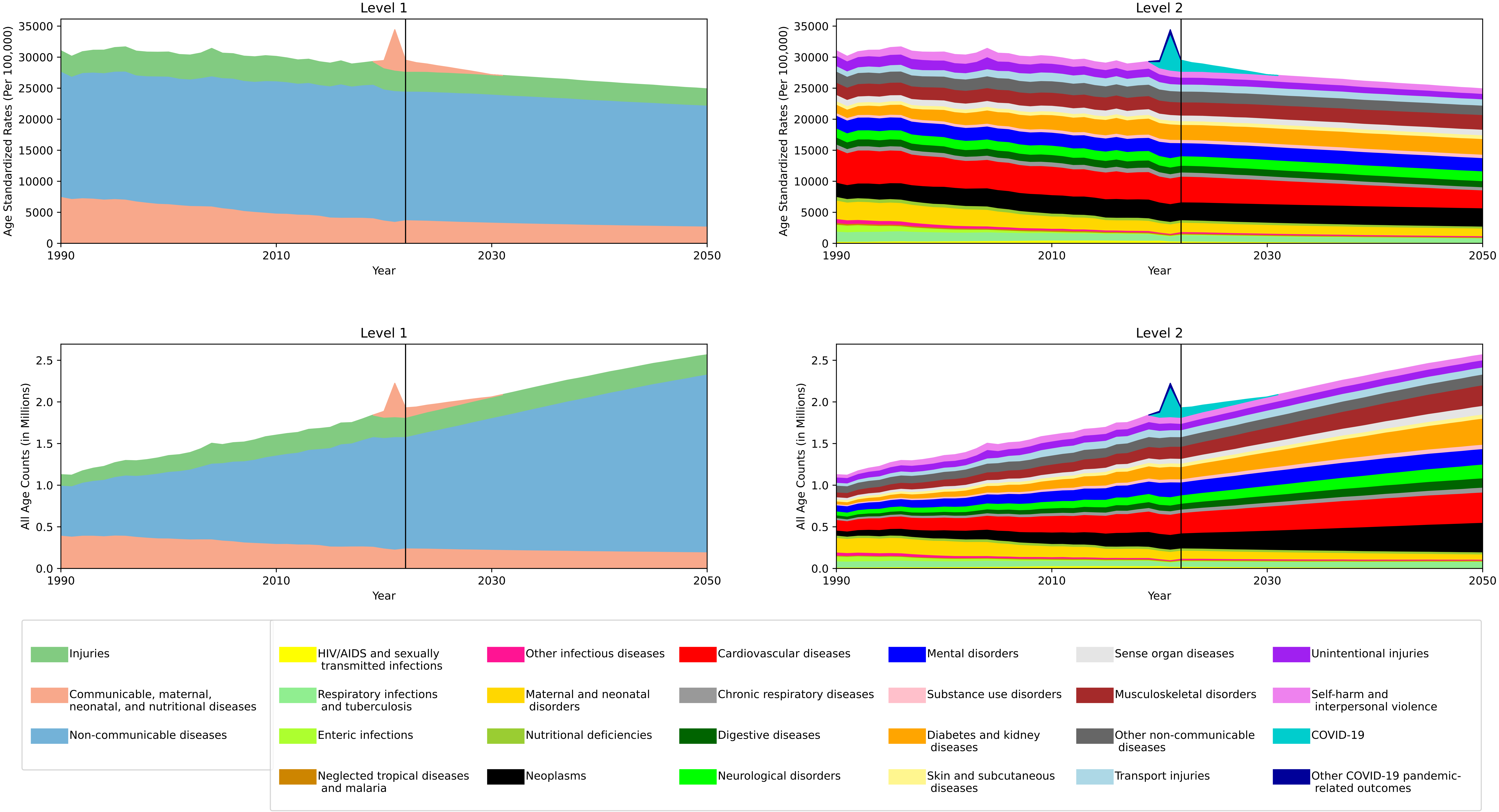
Level 1
Level 2
Age Standardized Rates (Per 100,000)
All Age Counts (in Millions)
Year
1990
2010
2030
2050
35000
30000
25000
20000
15000
10000
5000
0
2.5
2.0
1.5
1.0
0.5
0.0
Injuries
Communicable, maternal, neonatal, and nutritional diseases
Non-communicable diseases
HIV/AIDS and sexually transmitted infections
Respiratory infections and tuberculosis
Enteric infections
Neglected tropical diseases and malaria
Other infectious diseases
Maternal and neonatal disorders
Nutritional deficiencies
Neoplasms
Cardiovascular diseases
Chronic respiratory diseases
Digestive diseases
Neurological disorders
Mental disorders
Substance use disorders
Diabetes and kidney diseases
Skin and subcutaneous diseases
Sense organ diseases
Musculoskeletal disorders
Other non-communicable diseases
Transport injuries
Unintentional injuries
Self-harm and interpersonal violence
COVID-19
Other COVID-19 pandemic-related outcomes

Afghanistan

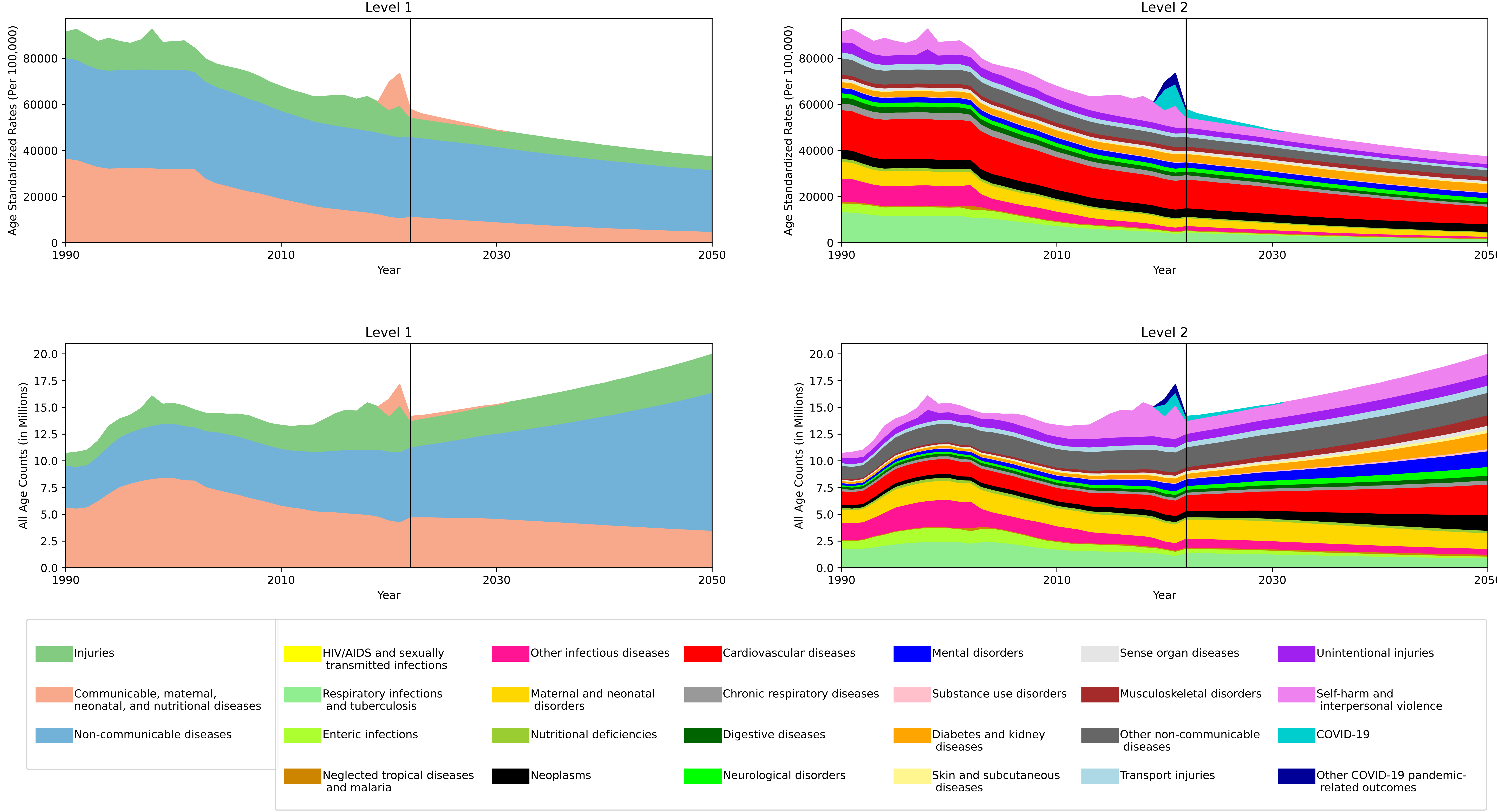

Algeria

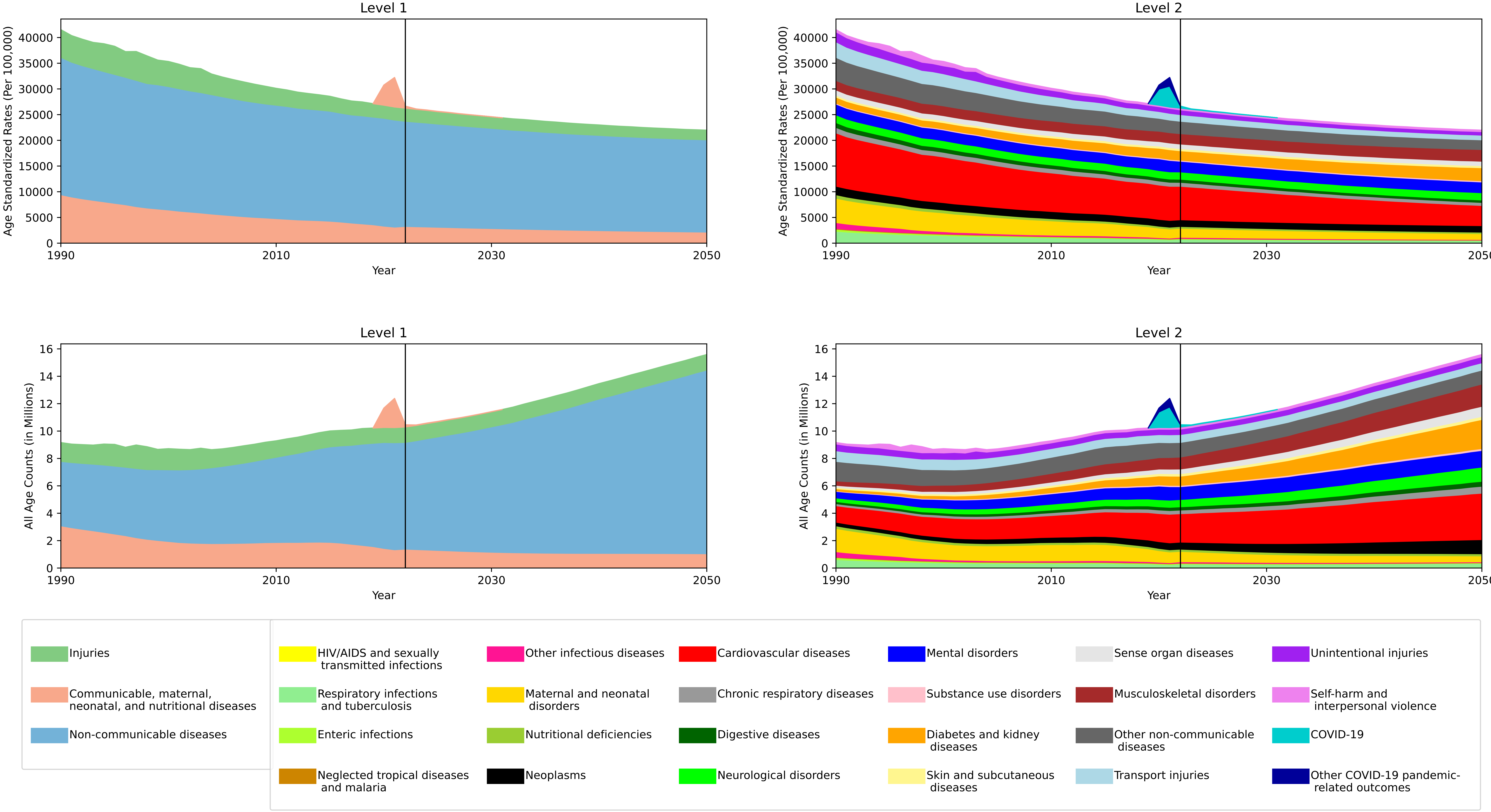
Level 1
Level 2
Age Standardized Rates (Per 100,000)
All Age Counts (in Millions)
Year
Injuries
Communicable, maternal, neonatal, and nutritional diseases
Non-communicable diseases
HIV/AIDS and sexually transmitted infections
Respiratory infections and tuberculosis
Enteric infections
Neglected tropical diseases and malaria
Other infectious diseases
Maternal and neonatal disorders
Nutritional deficiencies
Neoplasms
Cardiovascular diseases
Chronic respiratory diseases
Digestive diseases
Neurological disorders
Mental disorders
Substance use disorders
Diabetes and kidney diseases
Skin and subcutaneous diseases
Sense organ diseases
Musculoskeletal disorders
Other non-communicable diseases
Transport injuries
Unintentional injuries
Self-harm and interpersonal violence
COVID-19
Other COVID-19 pandemic-related outcomes

Bahrain

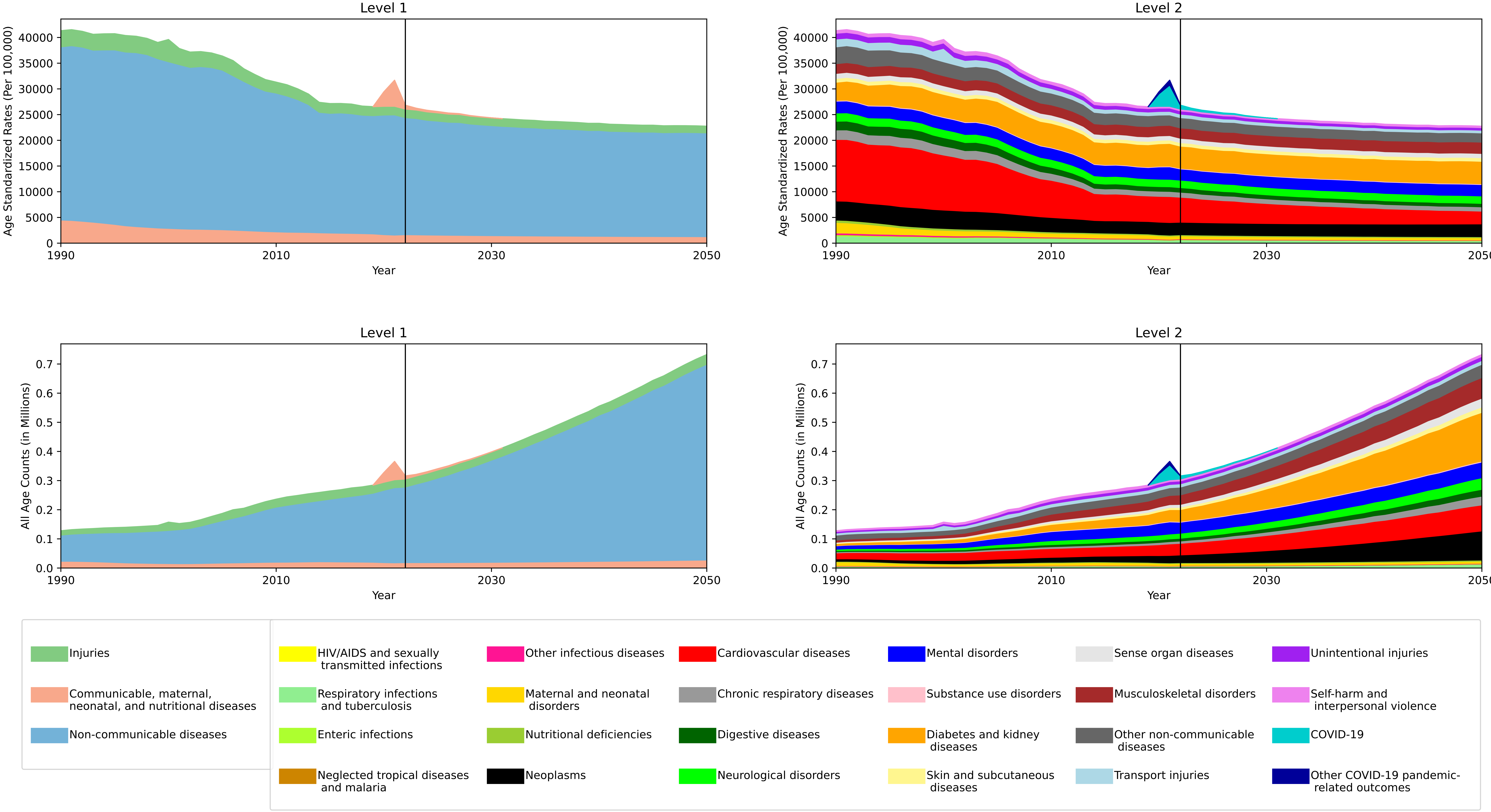
Level 1
Level 2
Age Standardized Rates (Per 100,000)
All Age Counts (in Millions)
Year
1990
2010
2030
2050
40000
35000
30000
25000
20000
15000
10000
5000
0
0.7
0.6
0.5
0.4
0.3
0.2
0.1
0.0
Injuries
Communicable, maternal, neonatal, and nutritional diseases
Non-communicable diseases
HIV/AIDS and sexually transmitted infections
Respiratory infections and tuberculosis
Enteric infections
Neglected tropical diseases and malaria
Other infectious diseases
Maternal and neonatal disorders
Nutritional deficiencies
Neoplasms
Cardiovascular diseases
Chronic respiratory diseases
Digestive diseases
Neurological disorders
Mental disorders
Substance use disorders
Diabetes and kidney diseases
Skin and subcutaneous diseases
Sense organ diseases
Musculoskeletal disorders
Other non-communicable diseases
Transport injuries
Unintentional injuries
Self-harm and interpersonal violence
COVID-19
Other COVID-19 pandemic-related outcomes

Supplemental Results Figure S4. Age-standardised DALY rates and all age DALY counts and by Level 1 and Level 2 causes, 1990–2050

Egypt

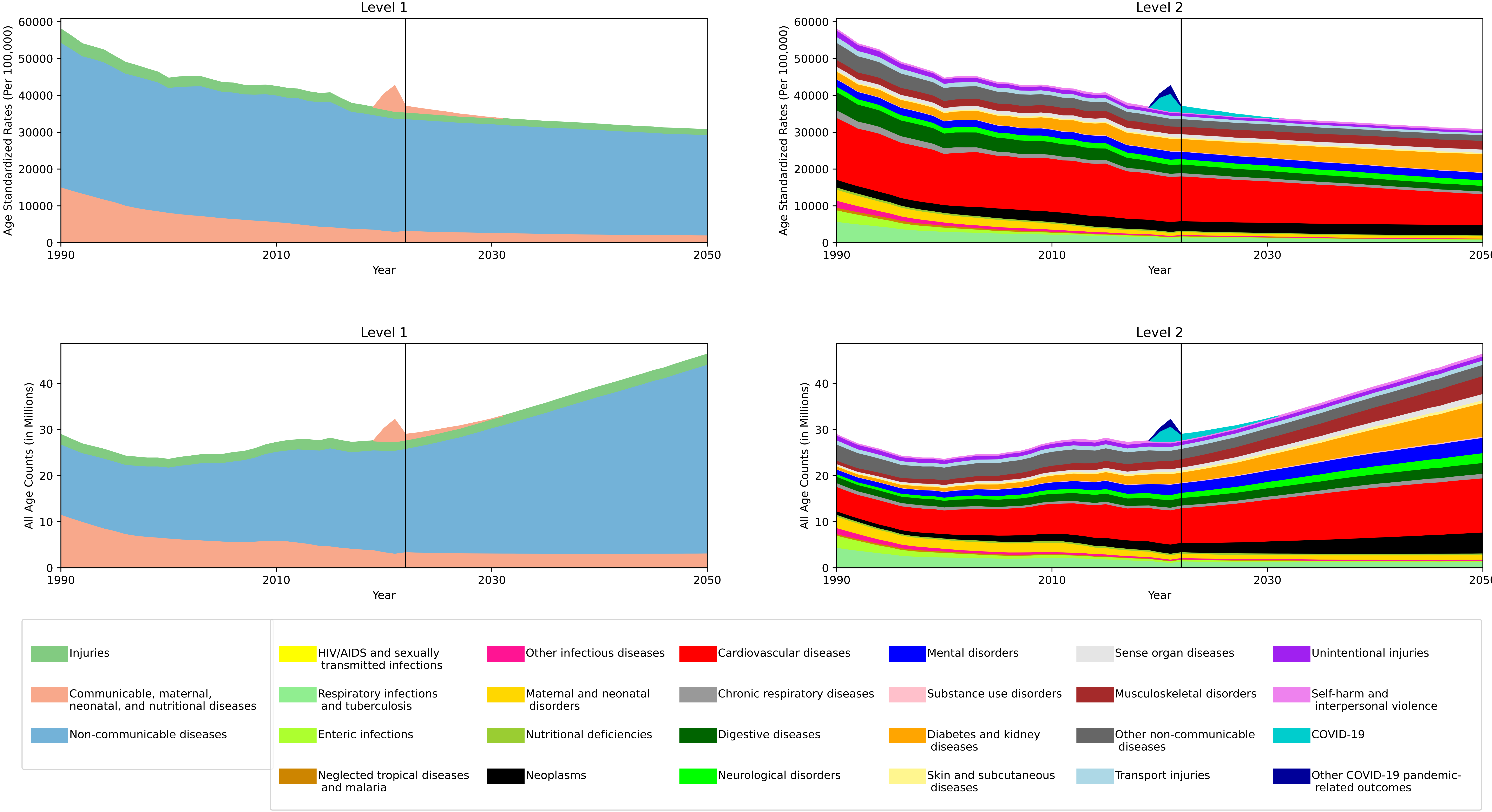

Iran

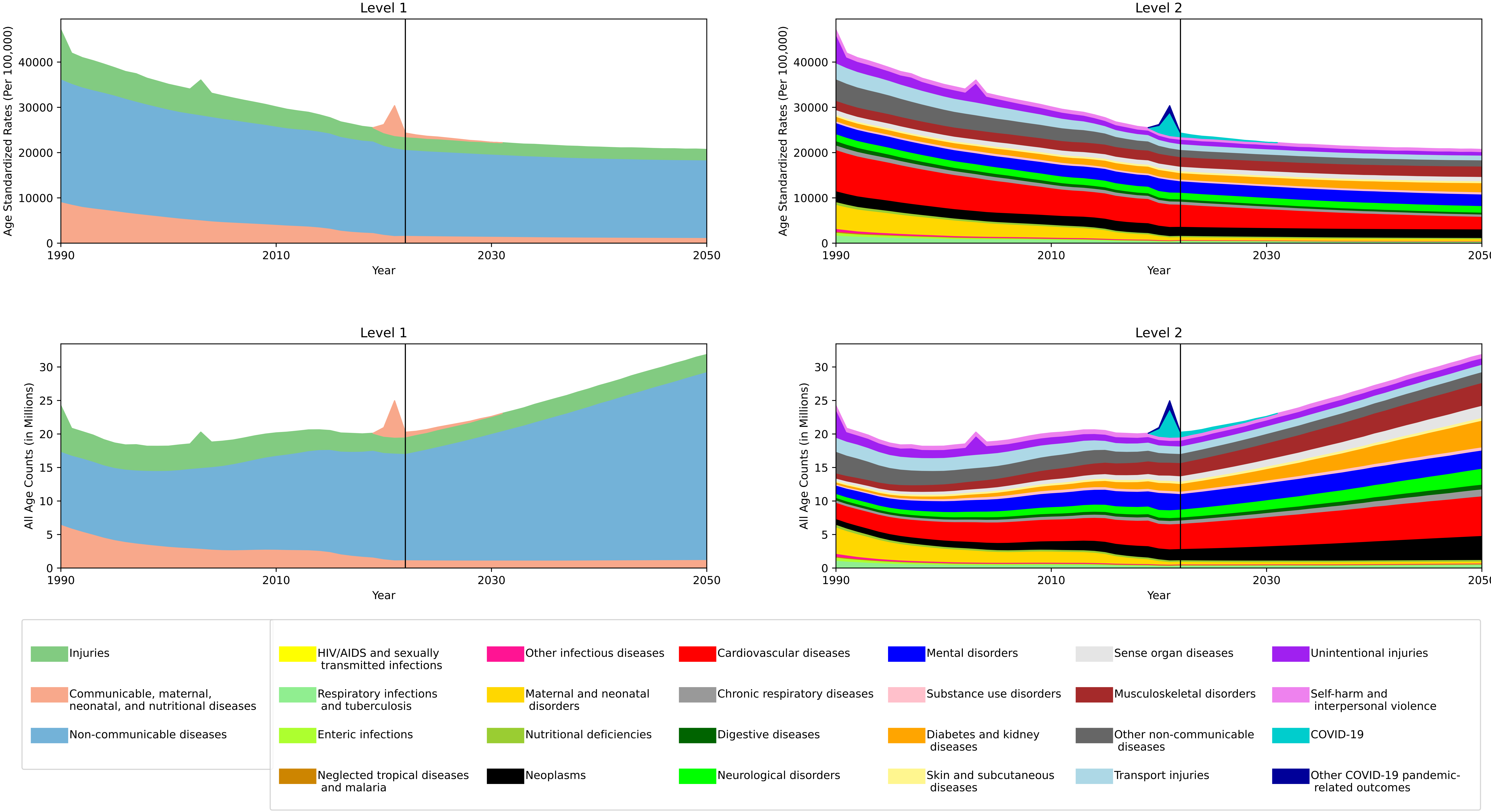
Level 1
Level 2
Age Standardized Rates (Per 100,000)
All Age Counts (in Millions)
Year
1990
2010
2030
2050
Injuries
Communicable, maternal, neonatal, and nutritional diseases
Non-communicable diseases
HIV/AIDS and sexually transmitted infections
Respiratory infections and tuberculosis
Enteric infections
Neglected tropical diseases and malaria
Other infectious diseases
Maternal and neonatal disorders
Nutritional deficiencies
Neoplasms
Cardiovascular diseases
Chronic respiratory diseases
Digestive diseases
Neurological disorders
Mental disorders
Substance use disorders
Diabetes and kidney diseases
Skin and subcutaneous diseases
Sense organ diseases
Musculoskeletal disorders
Other non-communicable diseases
Transport injuries
Unintentional injuries
Self-harm and interpersonal violence
COVID-19
Other COVID-19 pandemic-related outcomes

Iraq

Supplemental Results Figure S4. Age-standardised DALY rates and all age DALY counts and by Level 1 and Level 2 causes, 1990–2050

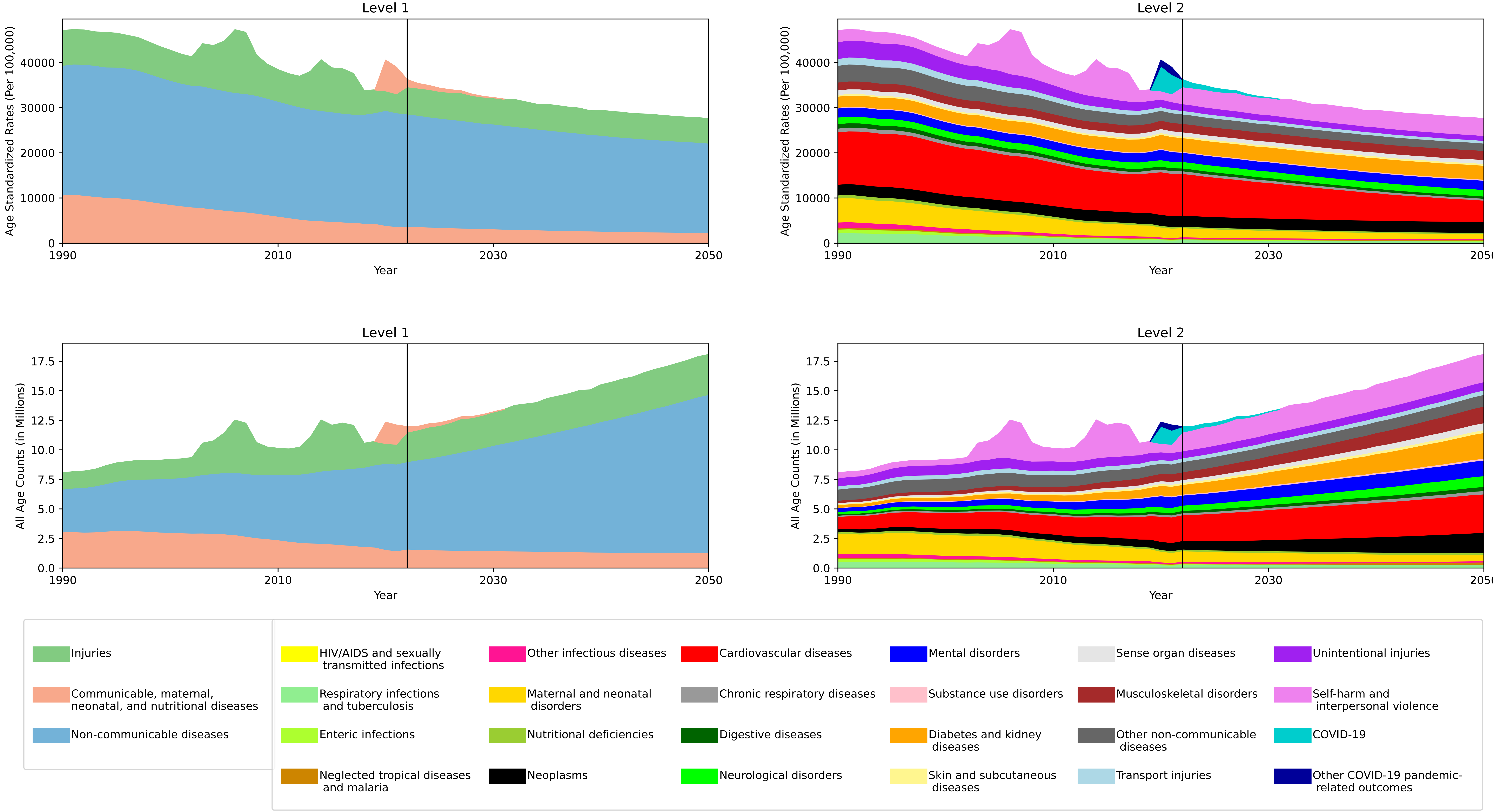

Jordan

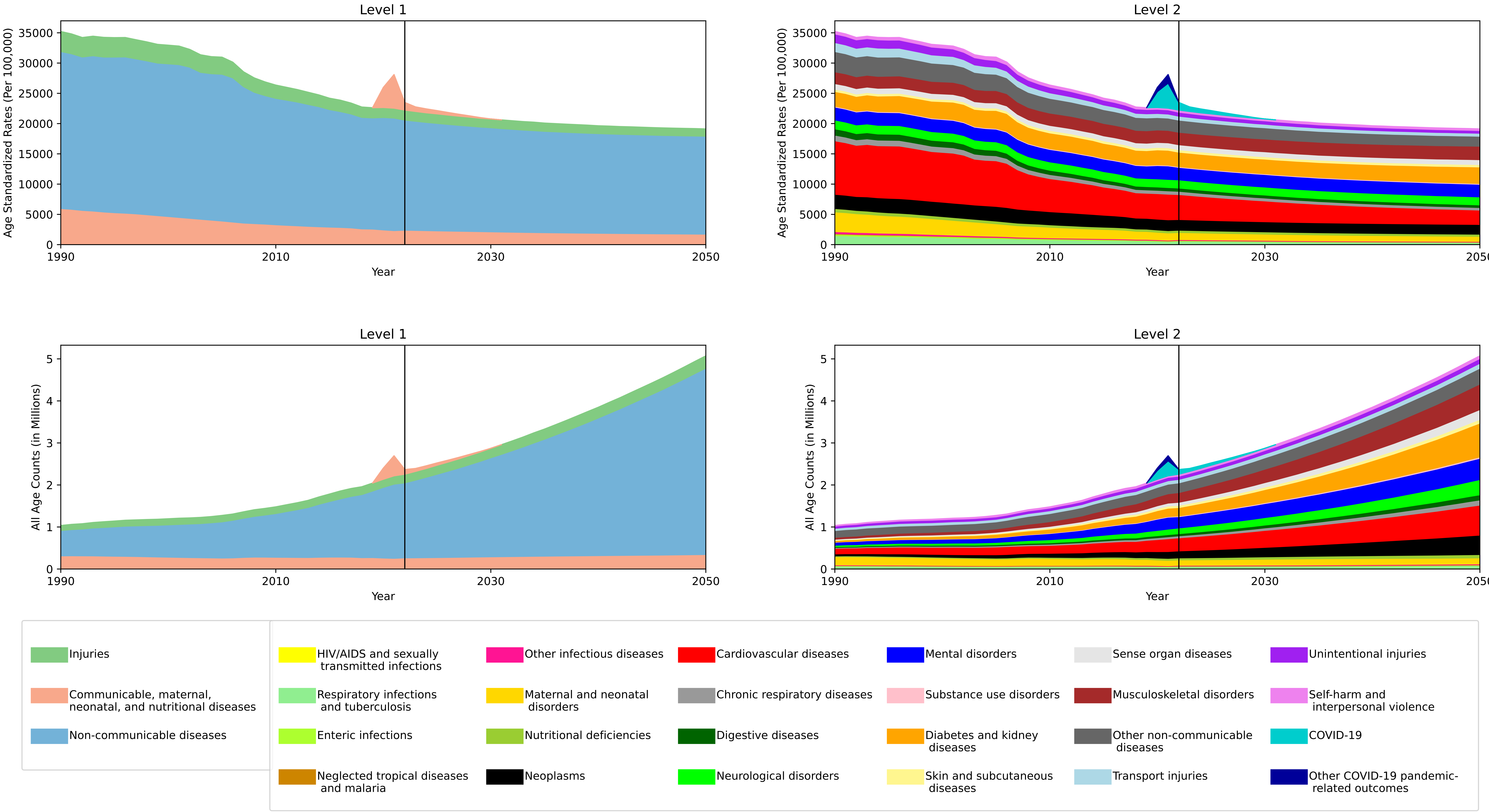
Level 1
Level 2
Age Standardized Rates (Per 100,000)
All Age Counts (in Millions)
Year
1990
2010
2030
2050
35000
30000
25000
20000
15000
10000
5000
0
5
4
3
2
1
Injuries
Communicable, maternal, neonatal, and nutritional diseases
Non-communicable diseases
HIV/AIDS and sexually transmitted infections
Respiratory infections and tuberculosis
Enteric infections
Neglected tropical diseases and malaria
Other infectious diseases
Maternal and neonatal disorders
Nutritional deficiencies
Neoplasms
Cardiovascular diseases
Chronic respiratory diseases
Digestive diseases
Neurological disorders
Mental disorders
Substance use disorders
Diabetes and kidney diseases
Skin and subcutaneous diseases
Sense organ diseases
Musculoskeletal disorders
Other non-communicable diseases
Transport injuries
Unintentional injuries
Self-harm and interpersonal violence
COVID-19
Other COVID-19 pandemic-related outcomes

Kuwait

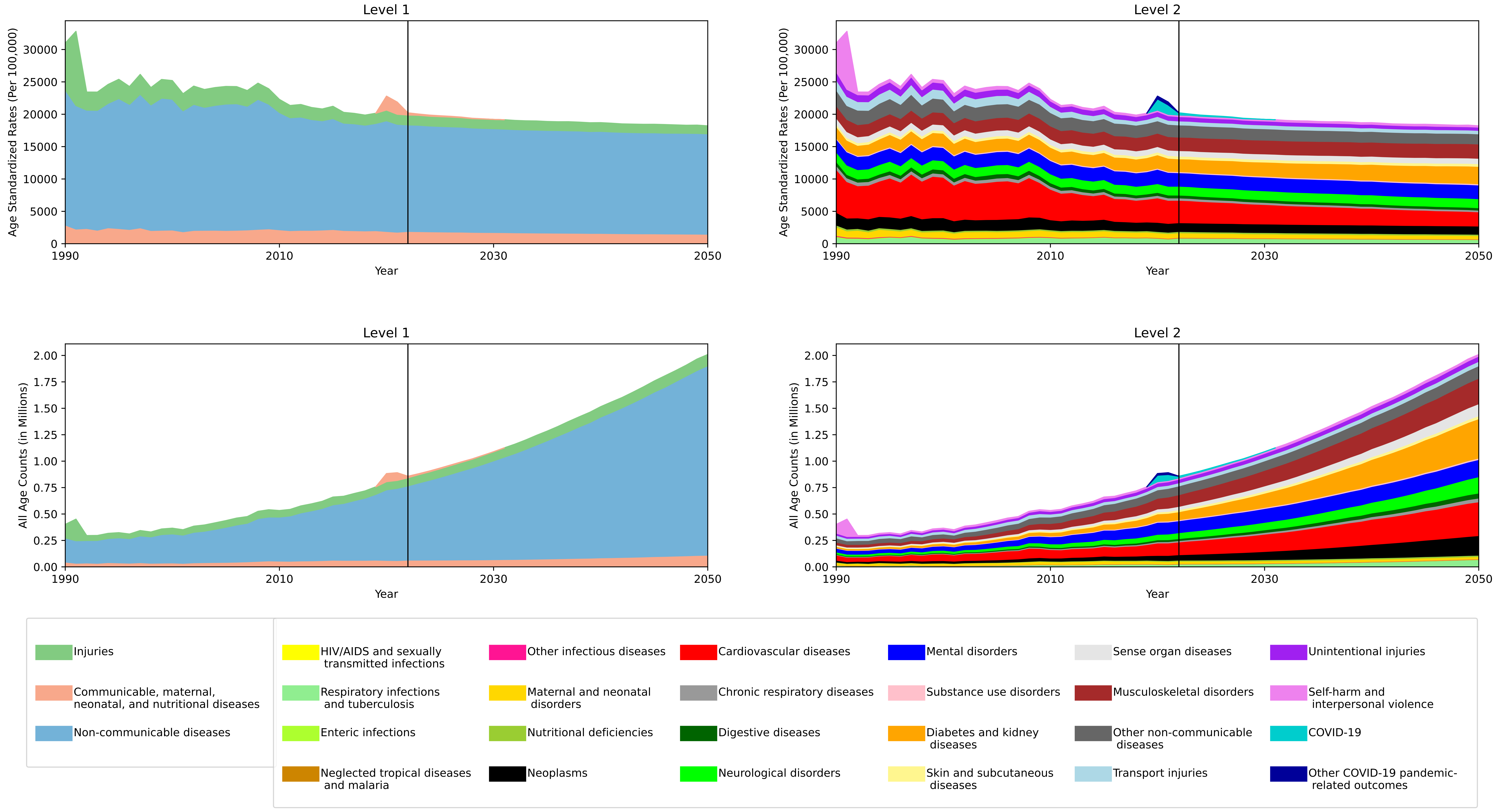
Level 1
Level 2
Age Standardized Rates (Per 100,000)
All Age Counts (in Millions)
Year
1990
2010
2030
2050
Injuries
Communicable, maternal, neonatal, and nutritional diseases
Non-communicable diseases
HIV/AIDS and sexually transmitted infections
Respiratory infections and tuberculosis
Enteric infections
Neglected tropical diseases and malaria
Other infectious diseases
Maternal and neonatal disorders
Nutritional deficiencies
Neoplasms
Cardiovascular diseases
Chronic respiratory diseases
Digestive diseases
Neurological disorders
Mental disorders
Substance use disorders
Diabetes and kidney diseases
Skin and subcutaneous diseases
Sense organ diseases
Musculoskeletal disorders
Other non-communicable diseases
Transport injuries
Unintentional injuries
Self-harm and interpersonal violence
COVID-19
Other COVID-19 pandemic-related outcomes

Lebanon

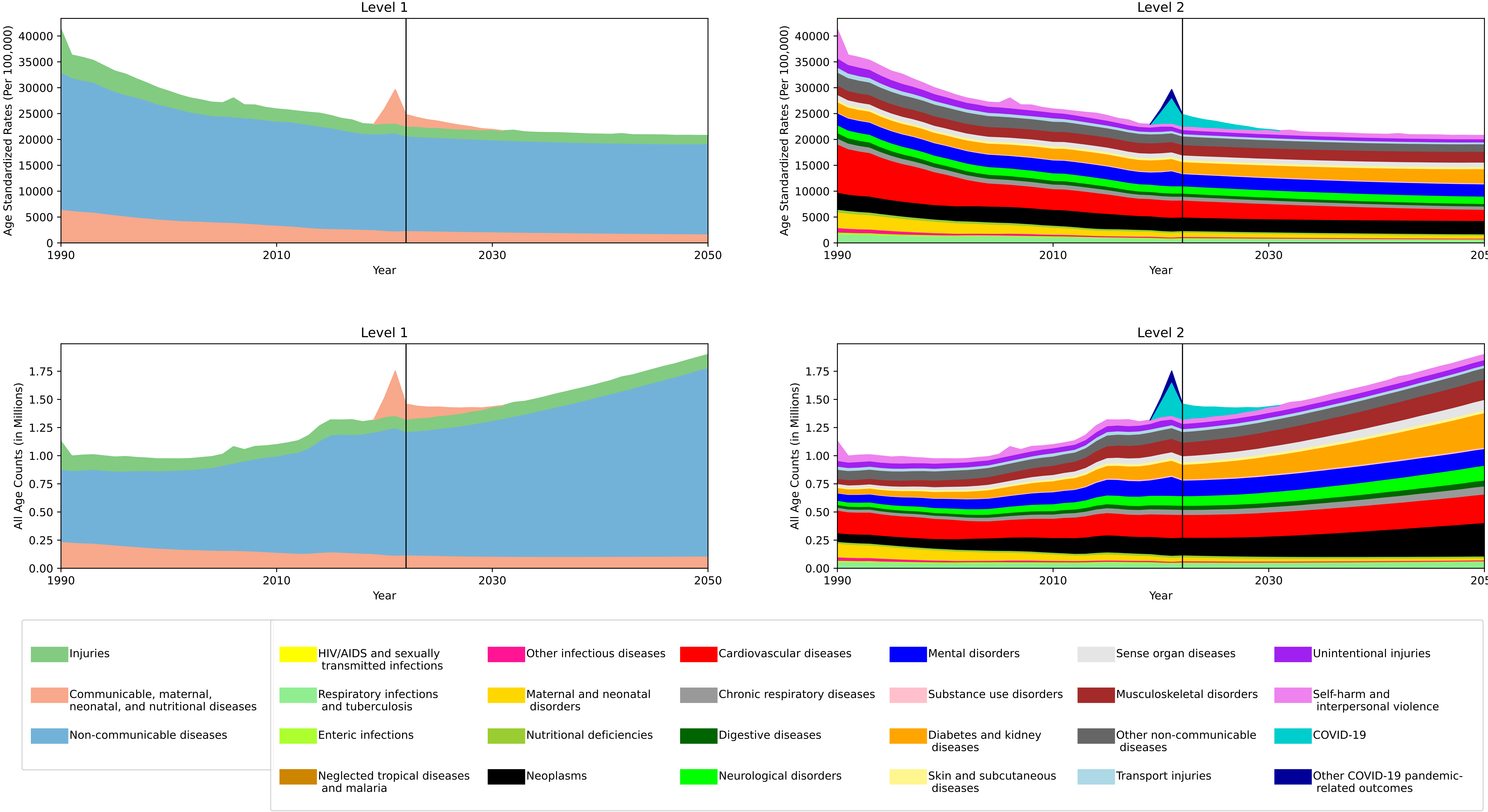
Level 1
Level 2
Age Standardized Rates (Per 100,000)
All Age Counts (in Millions)
Year
1990
2010
2030
2050
Injuries
Communicable, maternal, neonatal, and nutritional diseases
Non-communicable diseases
HIV/AIDS and sexually transmitted infections
Respiratory infections and tuberculosis
Enteric infections
Neglected tropical diseases and malaria
Other infectious diseases
Maternal and neonatal disorders
Nutritional deficiencies
Neoplasms
Cardiovascular diseases
Chronic respiratory diseases
Digestive diseases
Neurological disorders
Mental disorders
Substance use disorders
Diabetes and kidney diseases
Skin and subcutaneous diseases
Sense organ diseases
Musculoskeletal disorders
Other non-communicable diseases
Transport injuries
Unintentional injuries
Self-harm and interpersonal violence
COVID-19
Other COVID-19 pandemic-related outcomes

Libya

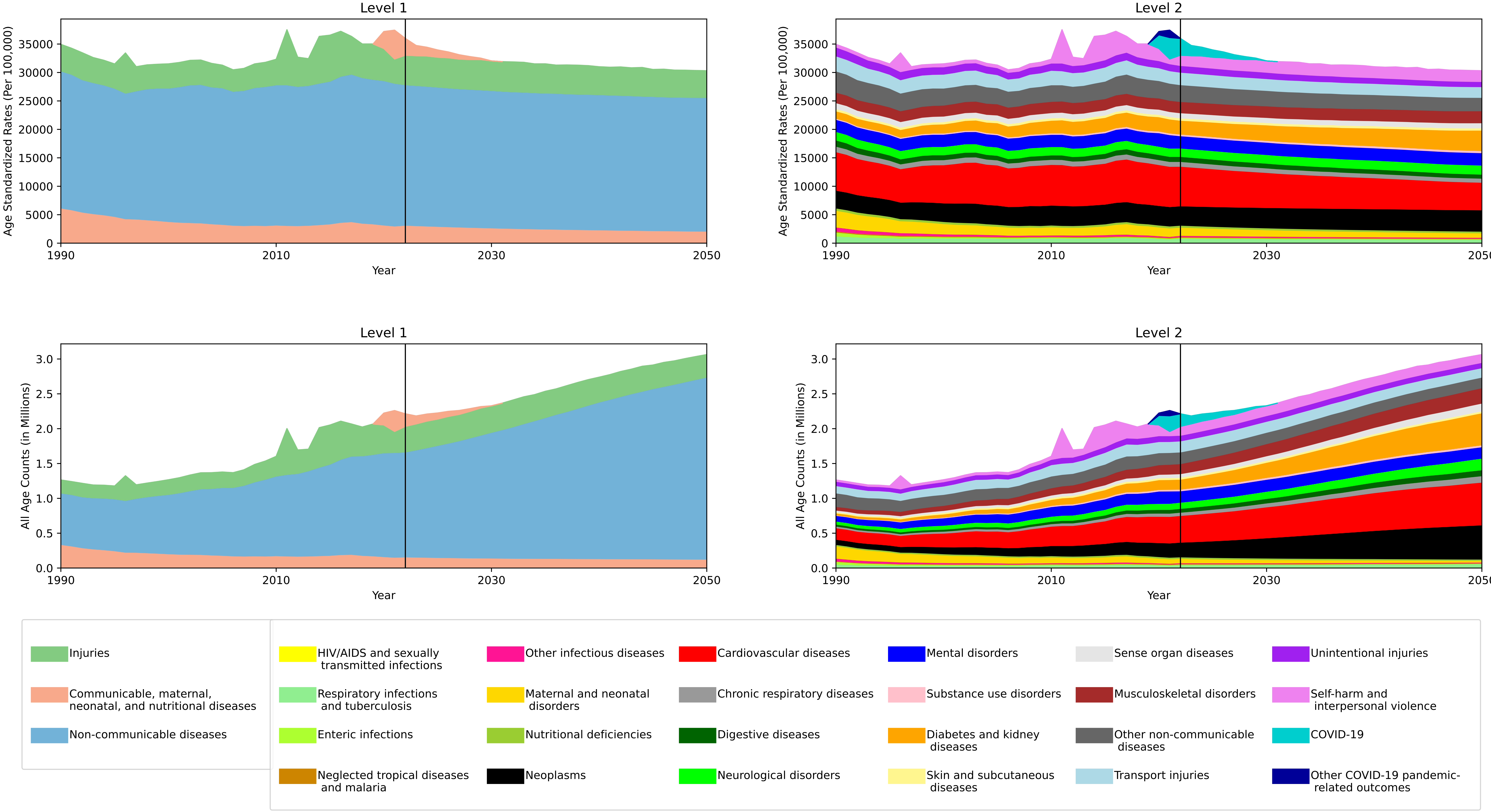
Level 1
Level 2
Age Standardized Rates (Per 100,000)
All Age Counts (in Millions)
Year
1990
2010
2030
2050
Injuries
Communicable, maternal, neonatal, and nutritional diseases
Non-communicable diseases
HIV/AIDS and sexually transmitted infections
Respiratory infections and tuberculosis
Enteric infections
Neglected tropical diseases and malaria
Other infectious diseases
Maternal and neonatal disorders
Nutritional deficiencies
Neoplasms
Cardiovascular diseases
Chronic respiratory diseases
Digestive diseases
Neurological disorders
Mental disorders
Substance use disorders
Diabetes and kidney diseases
Skin and subcutaneous diseases
Sense organ diseases
Musculoskeletal disorders
Other non-communicable diseases
Transport injuries
Unintentional injuries
Self-harm and interpersonal violence
COVID-19
Other COVID-19 pandemic-related outcomes

Morocco

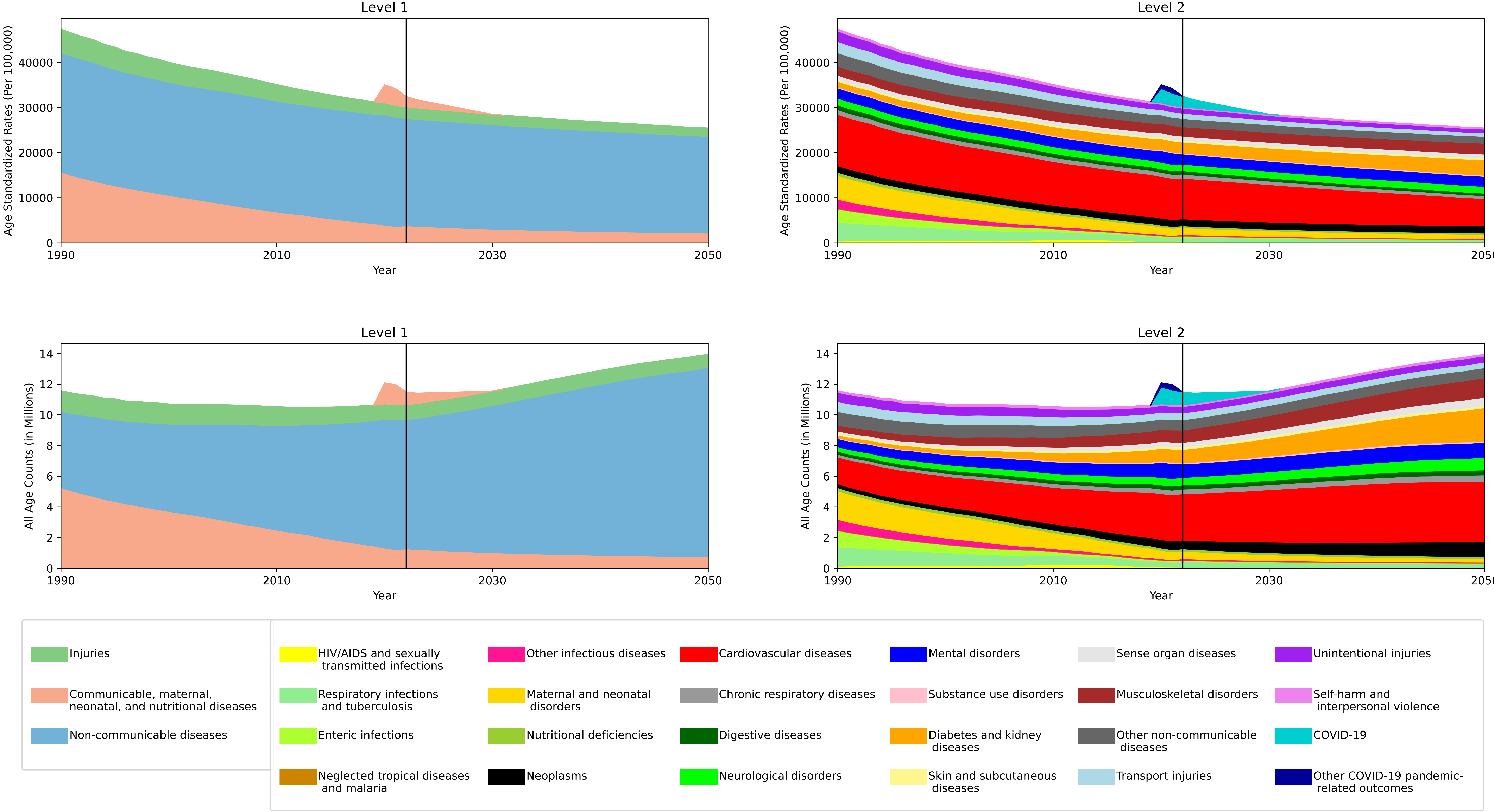
Level 1
Level 2
Age Standardized Rates (Per 100,000)
All Age Counts (in Millions)
Year
1990
2010
2030
2050
0
10000
20000
30000
40000
2
4
6
8
10
12
14
Injuries
Communicable, maternal, neonatal, and nutritional diseases
Non-communicable diseases
HIV/AIDS and sexually transmitted infections
Respiratory infections and tuberculosis
Enteric infections
Neglected tropical diseases and malaria
Other infectious diseases
Maternal and neonatal disorders
Nutritional deficiencies
Neoplasms
Cardiovascular diseases
Chronic respiratory diseases
Digestive diseases
Neurological disorders
Mental disorders
Substance use disorders
Diabetes and kidney diseases
Skin and subcutaneous diseases
Sense organ diseases
Musculoskeletal disorders
Other non-communicable diseases
Transport injuries
Unintentional injuries
Self-harm and interpersonal violence
COVID-19
Other COVID-19 pandemic-related outcomes

Oman

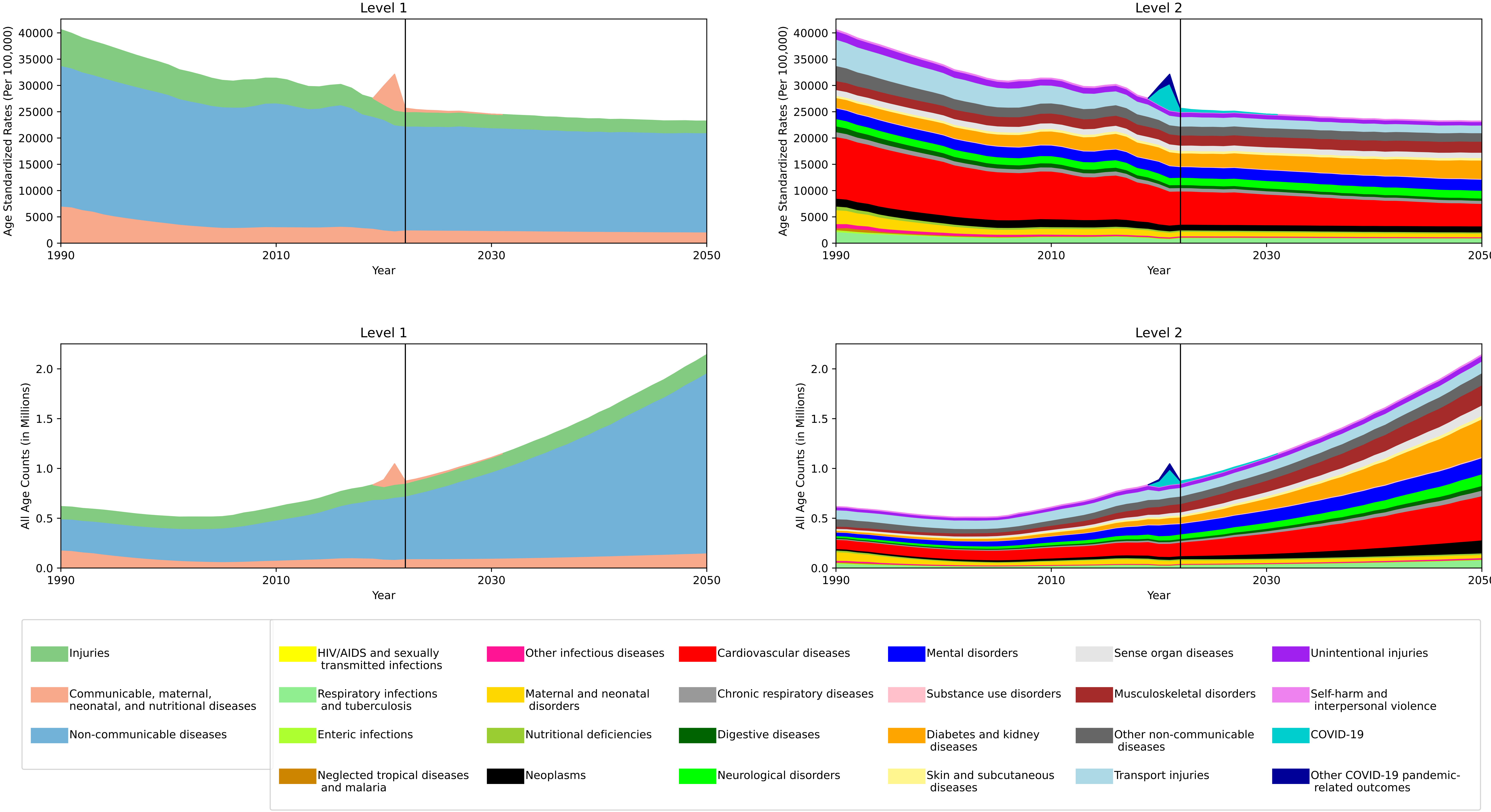
Level 1
Level 2
Age Standardized Rates (Per 100,000)
All Age Counts (in Millions)
Year
1990
2010
2030
2050
40000
35000
30000
25000
20000
15000
10000
5000
0
2.0
1.5
1.0
0.5
0.0
Injuries
Communicable, maternal, neonatal, and nutritional diseases
Non-communicable diseases
HIV/AIDS and sexually transmitted infections
Respiratory infections and tuberculosis
Enteric infections
Neglected tropical diseases and malaria
Other infectious diseases
Maternal and neonatal disorders
Nutritional deficiencies
Neoplasms
Cardiovascular diseases
Chronic respiratory diseases
Digestive diseases
Neurological disorders
Mental disorders
Substance use disorders
Diabetes and kidney diseases
Skin and subcutaneous diseases
Sense organ diseases
Musculoskeletal disorders
Other non-communicable diseases
Transport injuries
Unintentional injuries
Self-harm and interpersonal violence
COVID-19
Other COVID-19 pandemic-related outcomes

Palestine

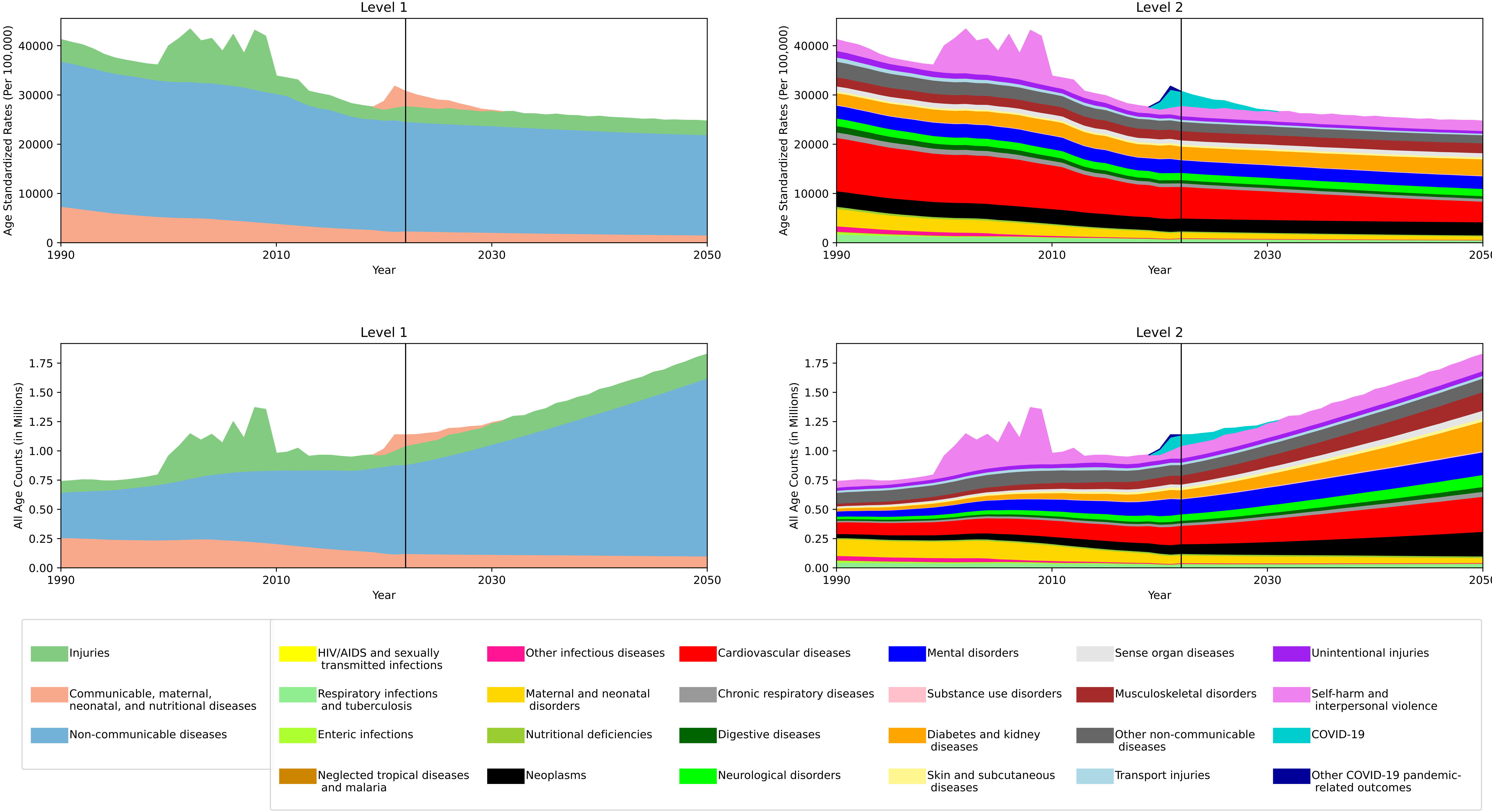
Level 1
Level 2
Age Standardized Rates (Per 100,000)
All Age Counts (in Millions)
Year
0
10000
20000
30000
40000
0.00
0.25
0.50
0.75
1.00
1.25
1.50
1.75
1990
2010
2030
2050
Injuries
Communicable, maternal, neonatal, and nutritional diseases
Non-communicable diseases
HIV/AIDS and sexually transmitted infections
Respiratory infections and tuberculosis
Enteric infections
Neglected tropical diseases and malaria
Other infectious diseases
Maternal and neonatal disorders
Nutritional deficiencies
Neoplasms
Cardiovascular diseases
Chronic respiratory diseases
Digestive diseases
Neurological disorders
Mental disorders
Substance use disorders
Diabetes and kidney diseases
Skin and subcutaneous diseases
Sense organ diseases
Musculoskeletal disorders
Other non-communicable diseases
Transport injuries
Unintentional injuries
Self-harm and interpersonal violence
COVID-19
Other COVID-19 pandemic-related outcomes

Qatar

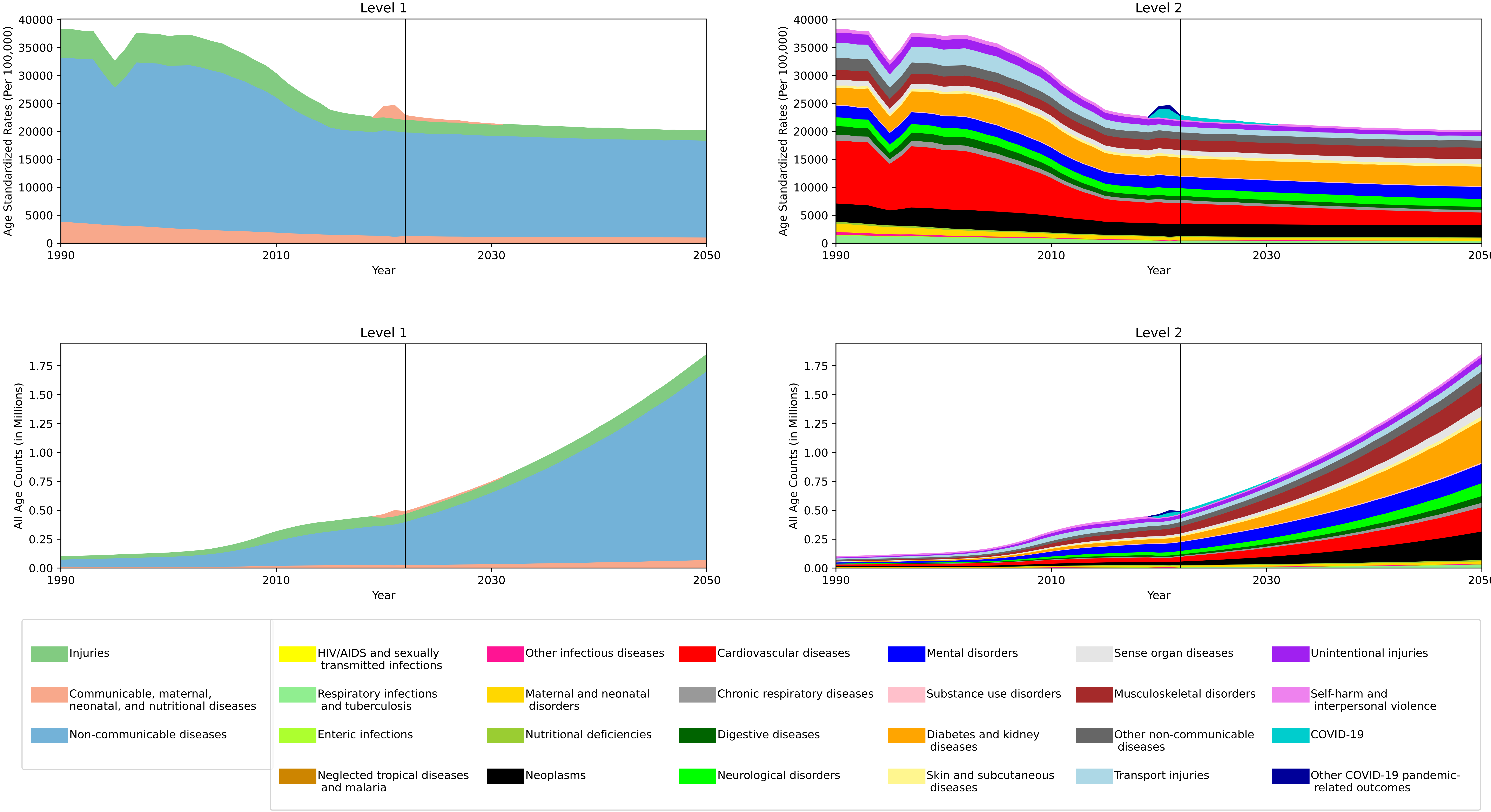
Level 1
Level 2
Age Standardized Rates (Per 100,000)
All Age Counts (in Millions)
Year
1990
2010
2030
2050
0
5000
10000
15000
20000
25000
30000
35000
40000
0.00
0.25
0.50
0.75
1.00
1.25
1.50
1.75
Injuries
Communicable, maternal, neonatal, and nutritional diseases
Non-communicable diseases
HIV/AIDS and sexually transmitted infections
Respiratory infections and tuberculosis
Enteric infections
Neglected tropical diseases and malaria
Other infectious diseases
Maternal and neonatal disorders
Nutritional deficiencies
Neoplasms
Cardiovascular diseases
Chronic respiratory diseases
Digestive diseases
Neurological disorders
Mental disorders
Substance use disorders
Diabetes and kidney diseases
Skin and subcutaneous diseases
Sense organ diseases
Musculoskeletal disorders
Other non-communicable diseases
Transport injuries
Unintentional injuries
Self-harm and interpersonal violence
COVID-19
Other COVID-19 pandemic-related outcomes

Saudi Arabia

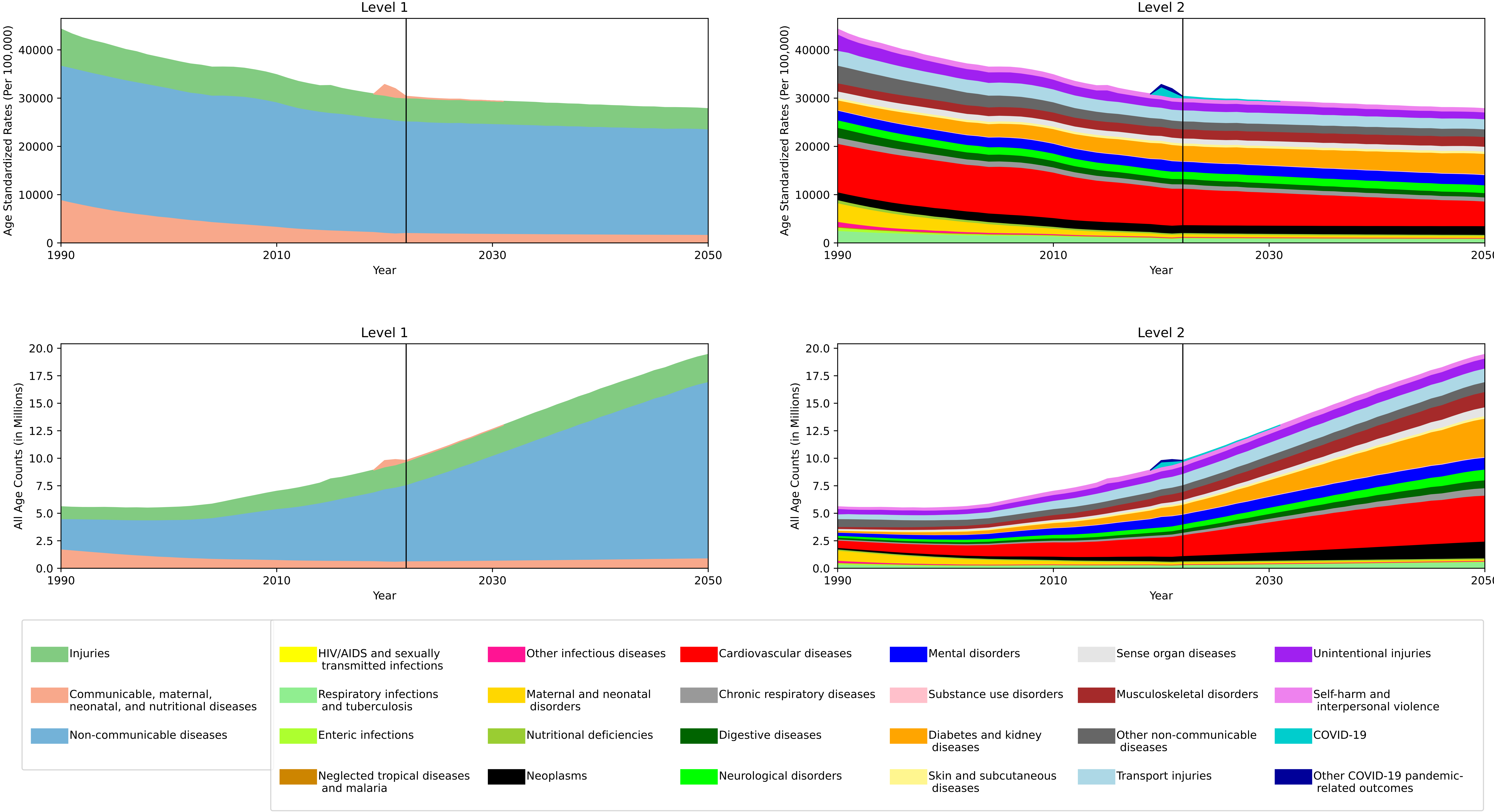

Sudan

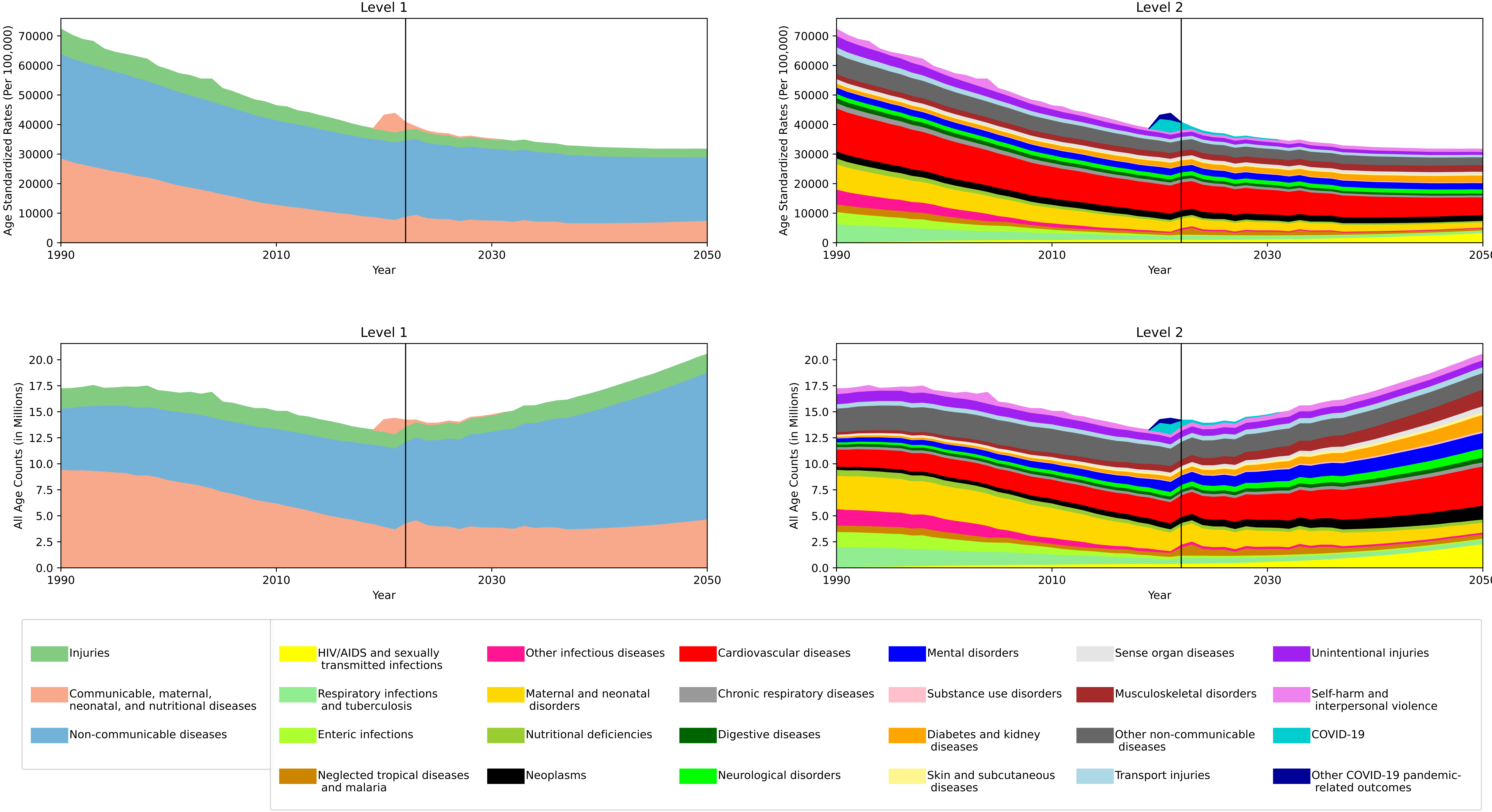

Level 1
Level 2
Age Standardized Rates (Per 100,000)
All Age Counts (in Millions)
Year
1990
2010
2030
2050
Injuries
Communicable, maternal, neonatal, and nutritional diseases
Non-communicable diseases
HIV/AIDS and sexually transmitted infections
Respiratory infections and tuberculosis
Enteric infections
Neglected tropical diseases and malaria
Other infectious diseases
Maternal and neonatal disorders
Nutritional deficiencies
Neoplasms
Cardiovascular diseases
Chronic respiratory diseases
Digestive diseases
Neurological disorders
Mental disorders
Substance use disorders
Diabetes and kidney diseases
Skin and subcutaneous diseases
Sense organ diseases
Musculoskeletal disorders
Other non-communicable diseases
Transport injuries
Unintentional injuries
Self-harm and interpersonal violence
COVID-19
Other COVID-19 pandemic-related outcomes

Syria

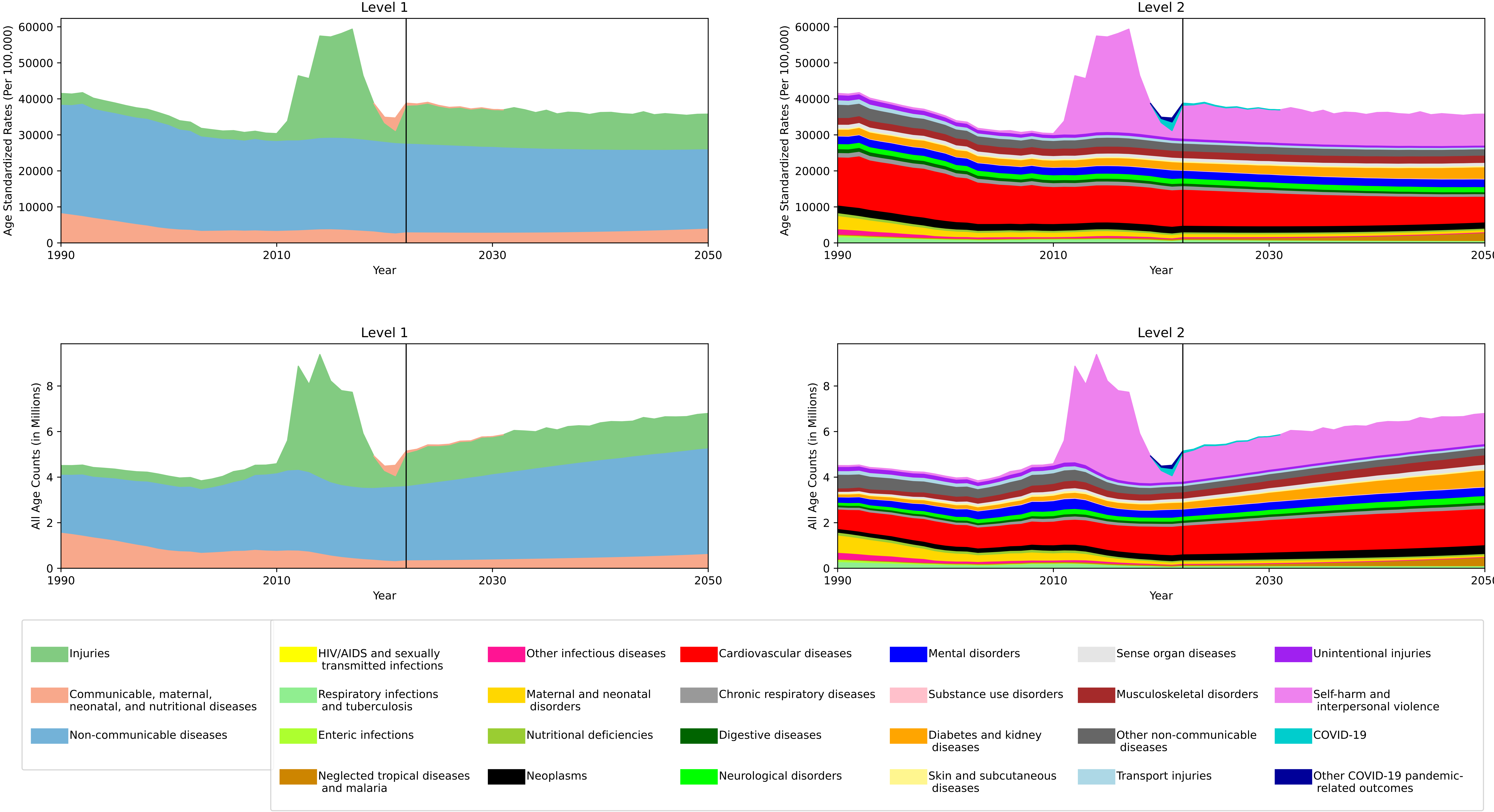

Level 1
Level 2
Age Standardized Rates (Per 100,000)
All Age Counts (in Millions)
Year
1990
2010
2030
2050
Injuries
Communicable, maternal, neonatal, and nutritional diseases
Non-communicable diseases
HIV/AIDS and sexually transmitted infections
Respiratory infections and tuberculosis
Enteric infections
Neglected tropical diseases and malaria
Other infectious diseases
Maternal and neonatal disorders
Nutritional deficiencies
Neoplasms
Cardiovascular diseases
Chronic respiratory diseases
Digestive diseases
Neurological disorders
Mental disorders
Substance use disorders
Diabetes and kidney diseases
Skin and subcutaneous diseases
Sense organ diseases
Musculoskeletal disorders
Other non-communicable diseases
Transport injuries
Unintentional injuries
Self-harm and interpersonal violence
COVID-19
Other COVID-19 pandemic-related outcomes

Tunisia

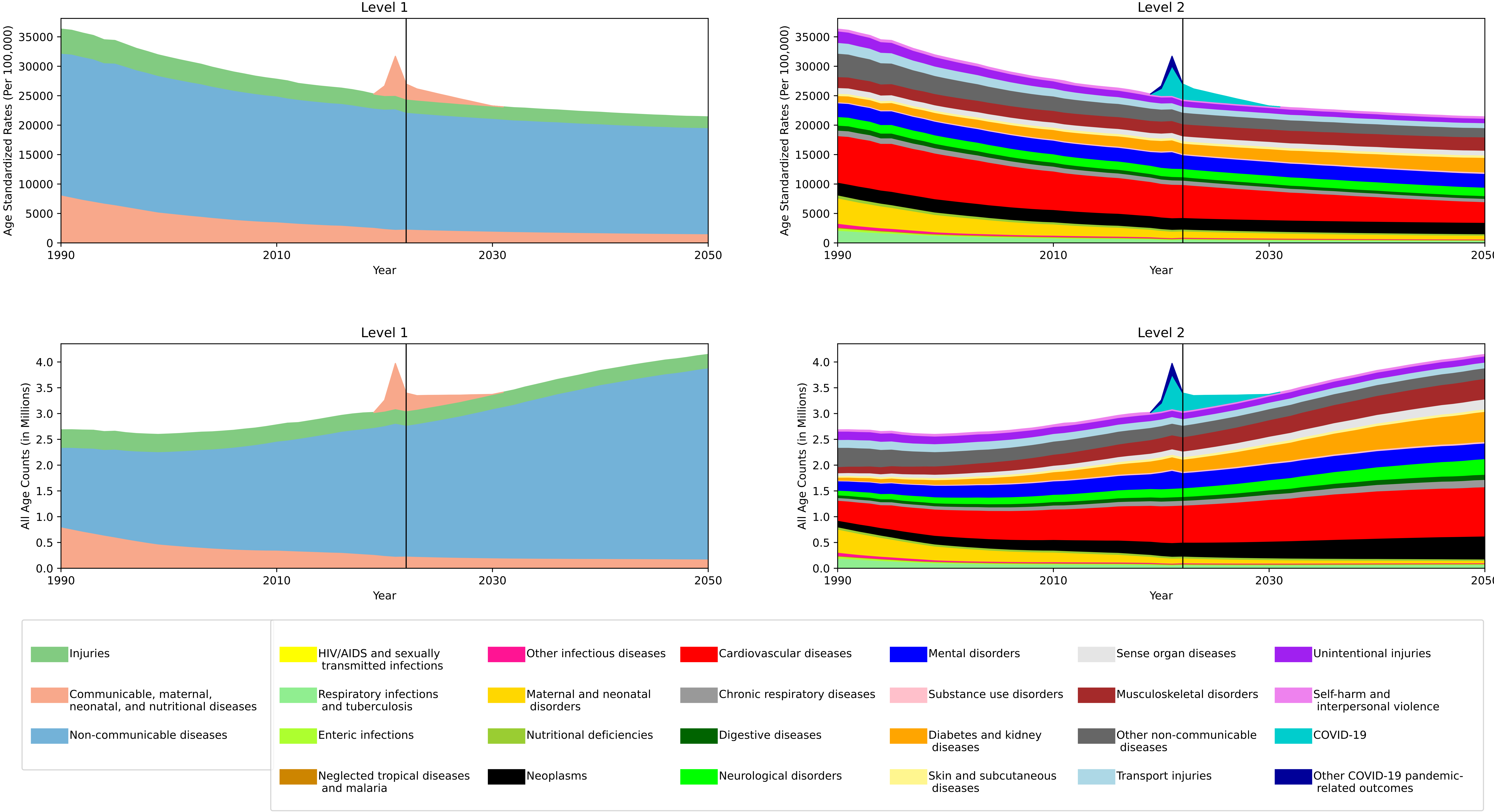
Level 1
Level 2
Age Standardized Rates (Per 100,000)
All Age Counts (in Millions)
Year
1990
2010
2030
2050
Injuries
Communicable, maternal, neonatal, and nutritional diseases
Non-communicable diseases
HIV/AIDS and sexually transmitted infections
Respiratory infections and tuberculosis
Enteric infections
Neglected tropical diseases and malaria
Other infectious diseases
Maternal and neonatal disorders
Nutritional deficiencies
Neoplasms
Cardiovascular diseases
Chronic respiratory diseases
Digestive diseases
Neurological disorders
Mental disorders
Substance use disorders
Diabetes and kidney diseases
Skin and subcutaneous diseases
Sense organ diseases
Musculoskeletal disorders
Other non-communicable diseases
Transport injuries
Unintentional injuries
Self-harm and interpersonal violence
COVID-19
Other COVID-19 pandemic-related outcomes

Türkiye

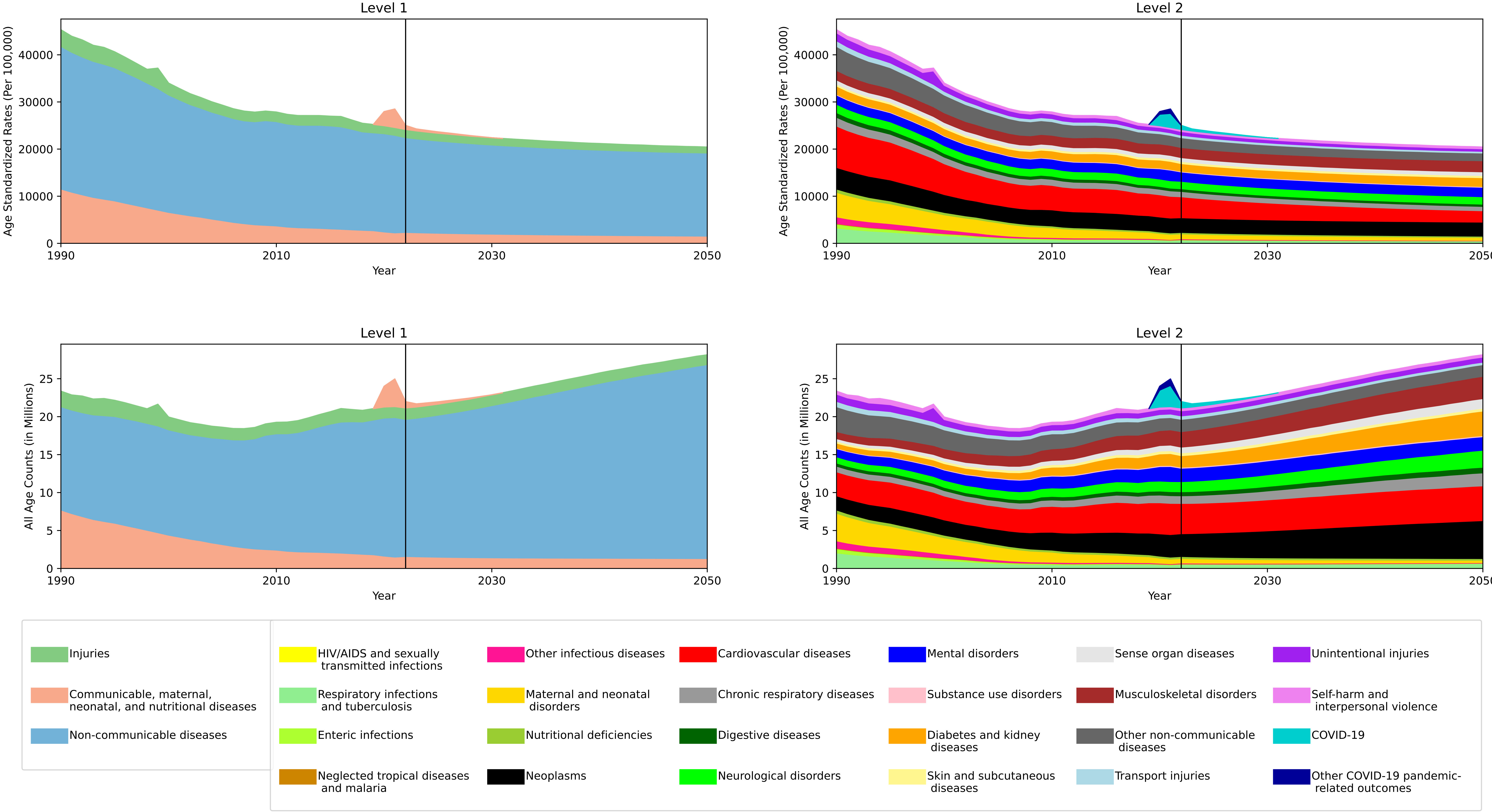
Level 1
Level 2
Age Standardized Rates (Per 100,000)
All Age Counts (in Millions)
Year
1990
2010
2030
2050
Injuries
Communicable, maternal, neonatal, and nutritional diseases
Non-communicable diseases
HIV/AIDS and sexually transmitted infections
Respiratory infections and tuberculosis
Enteric infections
Neglected tropical diseases and malaria
Other infectious diseases
Maternal and neonatal disorders
Nutritional deficiencies
Neoplasms
Cardiovascular diseases
Chronic respiratory diseases
Digestive diseases
Neurological disorders
Mental disorders
Substance use disorders
Diabetes and kidney diseases
Skin and subcutaneous diseases
Sense organ diseases
Musculoskeletal disorders
Other non-communicable diseases
Transport injuries
Unintentional injuries
Self-harm and interpersonal violence
COVID-19
Other COVID-19 pandemic-related outcomes

United Arab Emirates

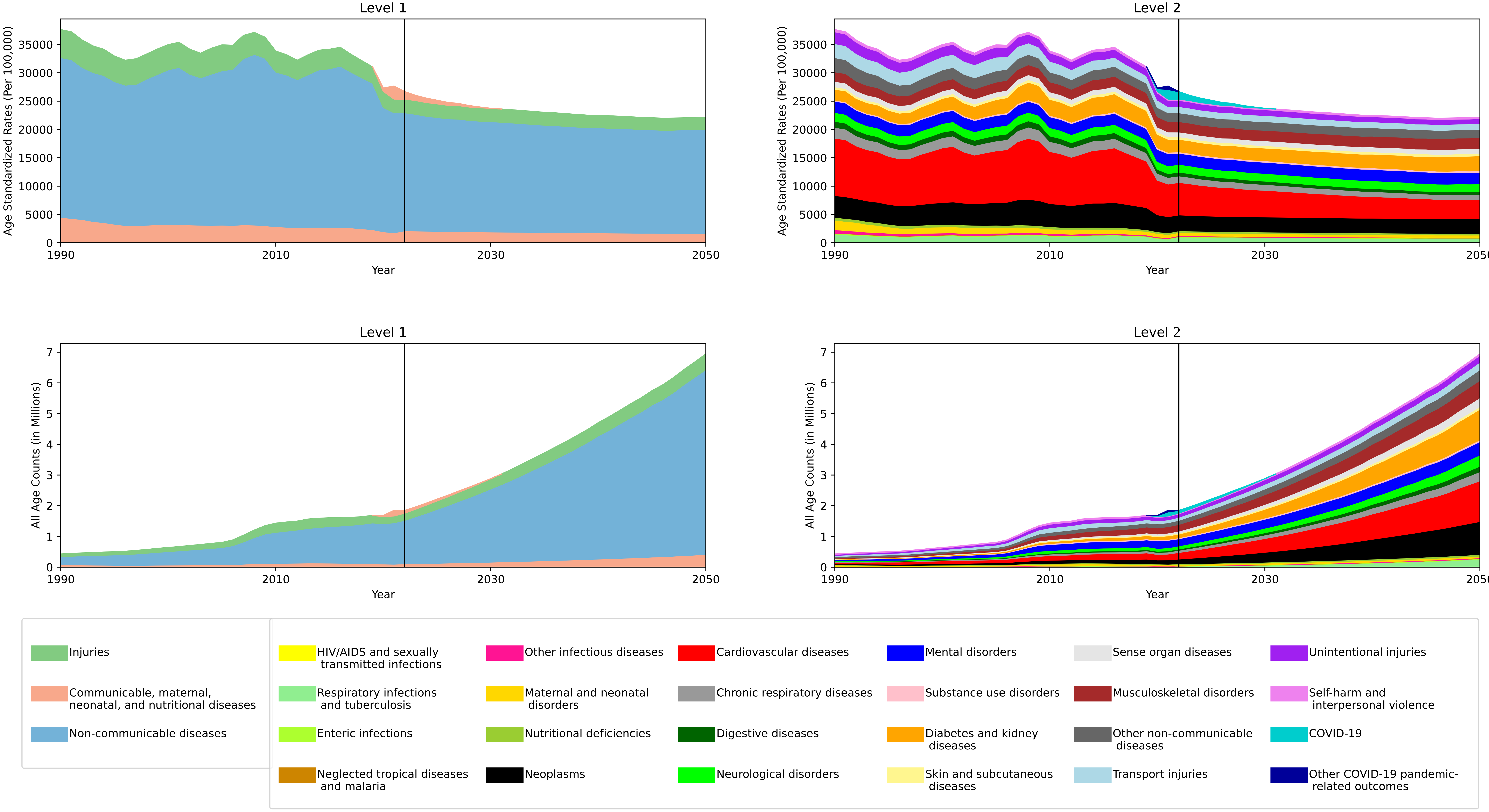

Yemen

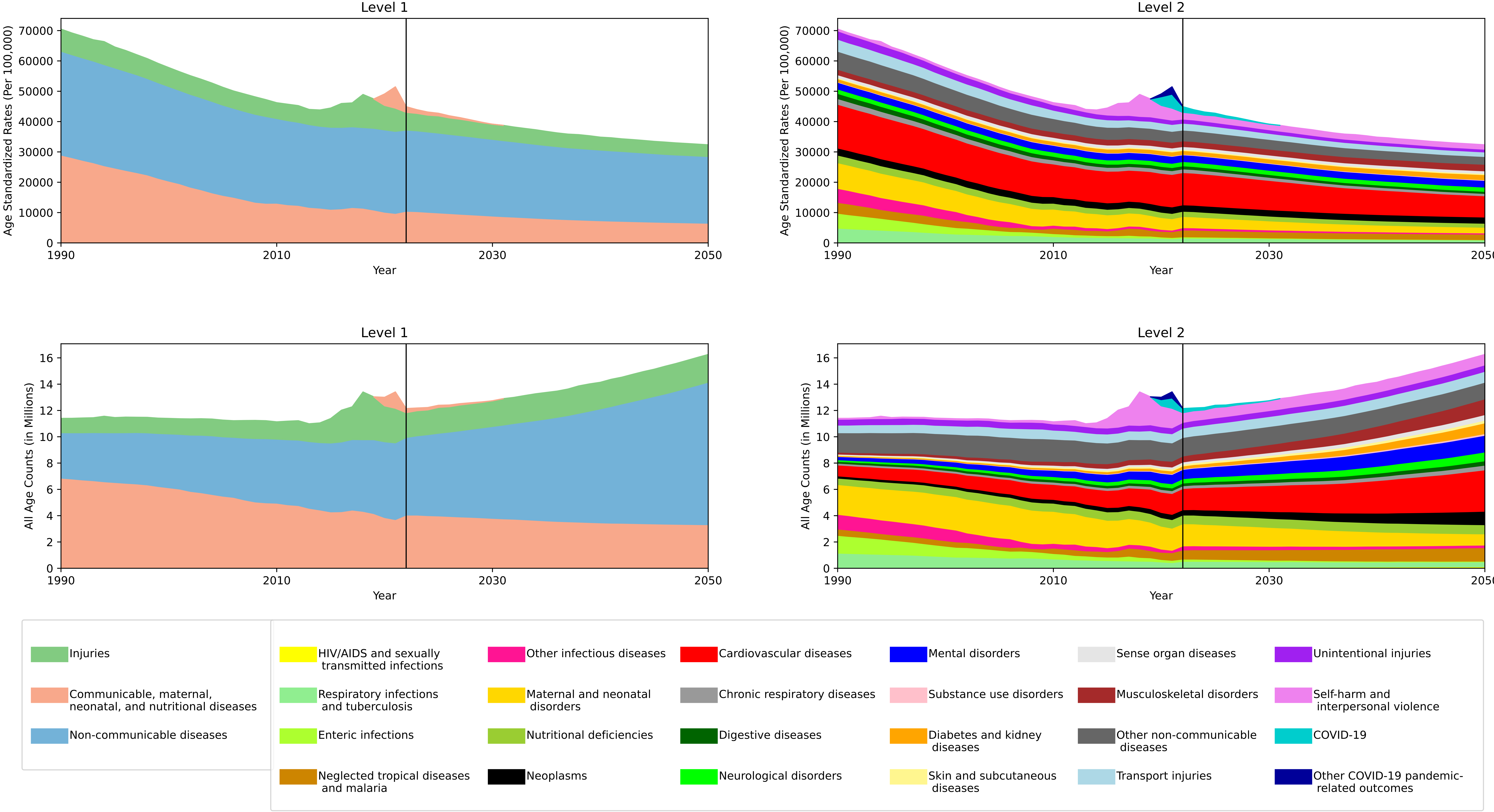
Level 1
Level 2
Age Standardized Rates (Per 100,000)
All Age Counts (in Millions)
Year
1990
2010
2030
2050
Injuries
Communicable, maternal, neonatal, and nutritional diseases
Non-communicable diseases
HIV/AIDS and sexually transmitted infections
Respiratory infections and tuberculosis
Enteric infections
Neglected tropical diseases and malaria
Other infectious diseases
Maternal and neonatal disorders
Nutritional deficiencies
Neoplasms
Cardiovascular diseases
Chronic respiratory diseases
Digestive diseases
Neurological disorders
Mental disorders
Substance use disorders
Diabetes and kidney diseases
Skin and subcutaneous diseases
Sense organ diseases
Musculoskeletal disorders
Other non-communicable diseases
Transport injuries
Unintentional injuries
Self-harm and interpersonal violence
COVID-19
Other COVID-19 pandemic-related outcomes

Bangladesh

Supplemental Results Figure S4. Age-standardised DALY rates and all age DALY counts and by Level 1 and Level 2 causes, 1990–2050

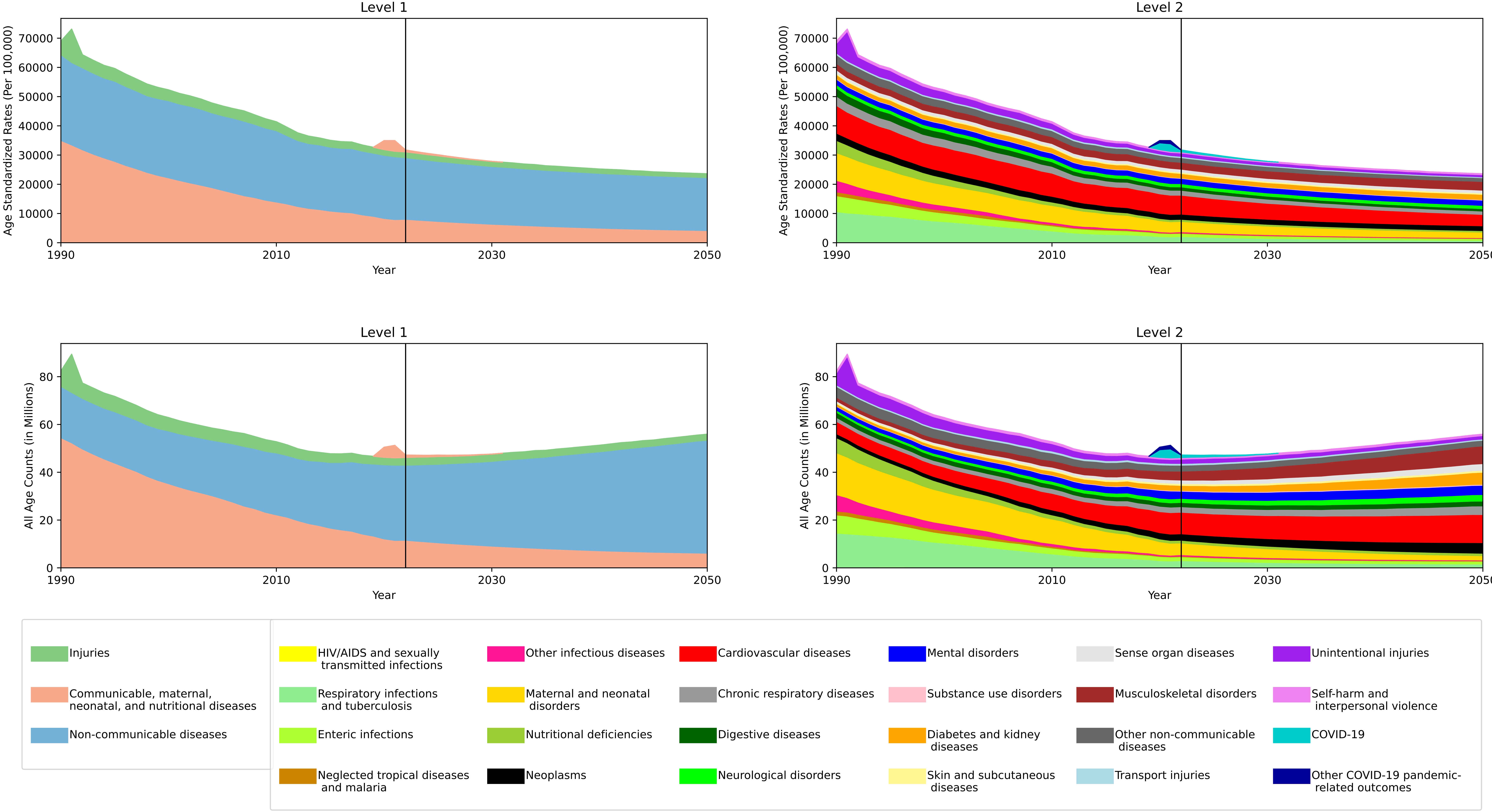

Bhutan

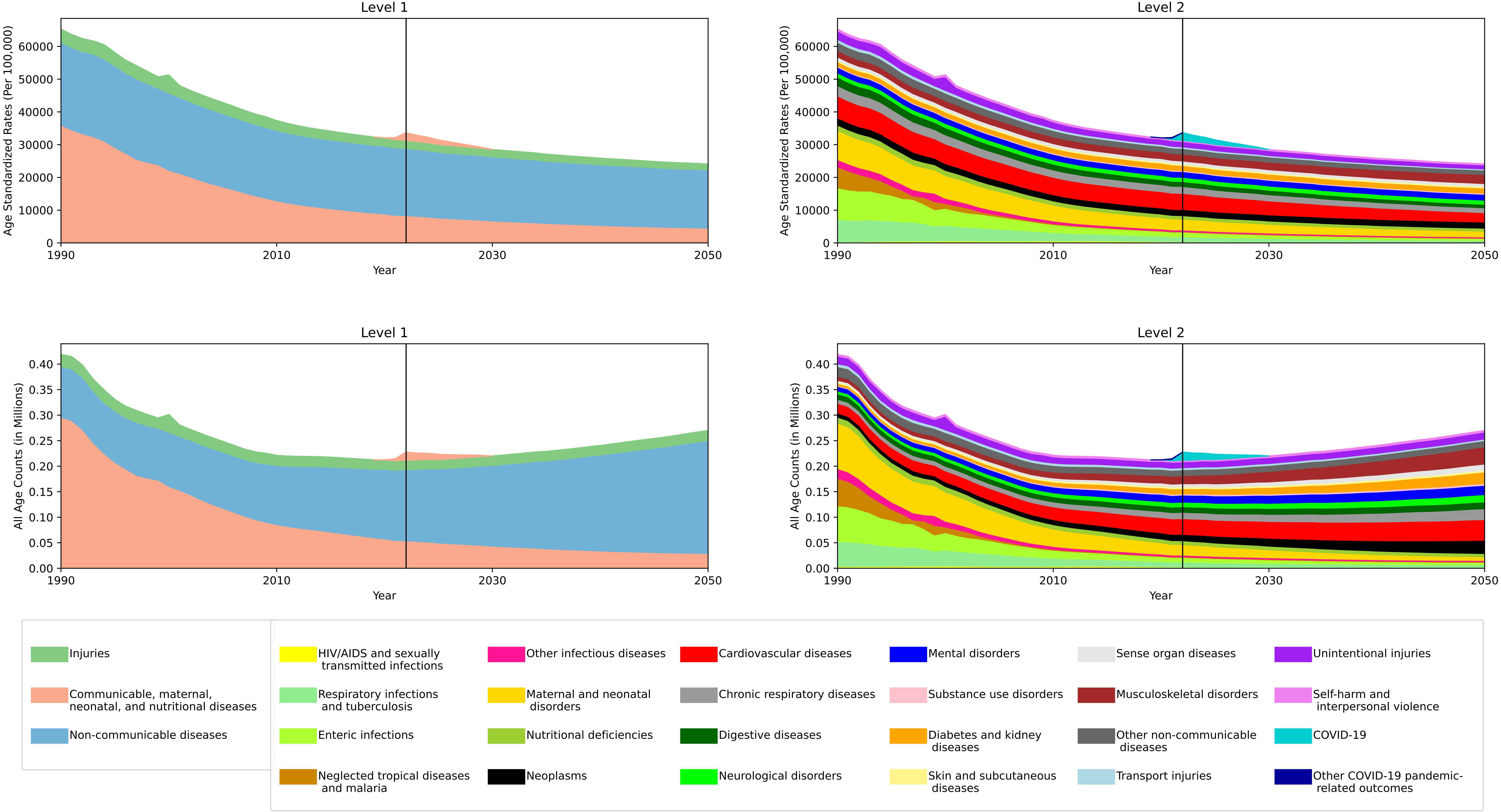
Level 1
Level 2
Age Standardized Rates (Per 100,000)
All Age Counts (in Millions)
Year
1990
2010
2030
2050
Injuries
Communicable, maternal, neonatal, and nutritional diseases
Non-communicable diseases
HIV/AIDS and sexually transmitted infections
Respiratory infections and tuberculosis
Enteric infections
Neglected tropical diseases and malaria
Other infectious diseases
Maternal and neonatal disorders
Nutritional deficiencies
Neoplasms
Cardiovascular diseases
Chronic respiratory diseases
Digestive diseases
Neurological disorders
Mental disorders
Substance use disorders
Diabetes and kidney diseases
Skin and subcutaneous diseases
Sense organ diseases
Musculoskeletal disorders
Other non-communicable diseases
Transport injuries
Unintentional injuries
Self-harm and interpersonal violence
COVID-19
Other COVID-19 pandemic-related outcomes

India

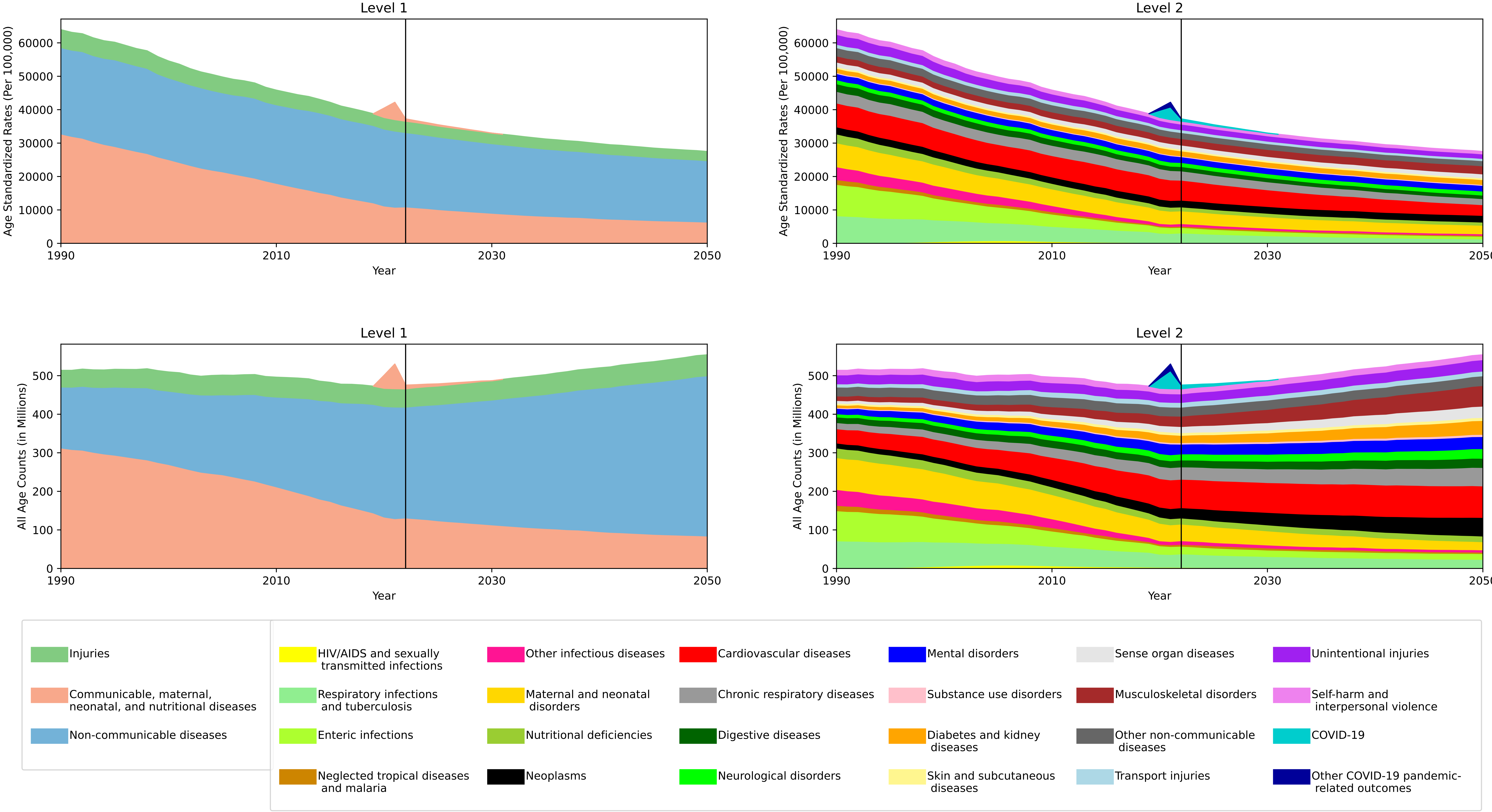
Level 1
Level 2
Age Standardized Rates (Per 100,000)
All Age Counts (in Millions)
Year
1990
2010
2030
2050
0
10000
20000
30000
40000
50000
60000
100
200
300
400
500
Injuries
Communicable, maternal, neonatal, and nutritional diseases
Non-communicable diseases
HIV/AIDS and sexually transmitted infections
Respiratory infections and tuberculosis
Enteric infections
Neglected tropical diseases and malaria
Other infectious diseases
Maternal and neonatal disorders
Nutritional deficiencies
Neoplasms
Cardiovascular diseases
Chronic respiratory diseases
Digestive diseases
Neurological disorders
Mental disorders
Substance use disorders
Diabetes and kidney diseases
Skin and subcutaneous diseases
Sense organ diseases
Musculoskeletal disorders
Other non-communicable diseases
Transport injuries
Unintentional injuries
Self-harm and interpersonal violence
COVID-19
Other COVID-19 pandemic-related outcomes

Nepal

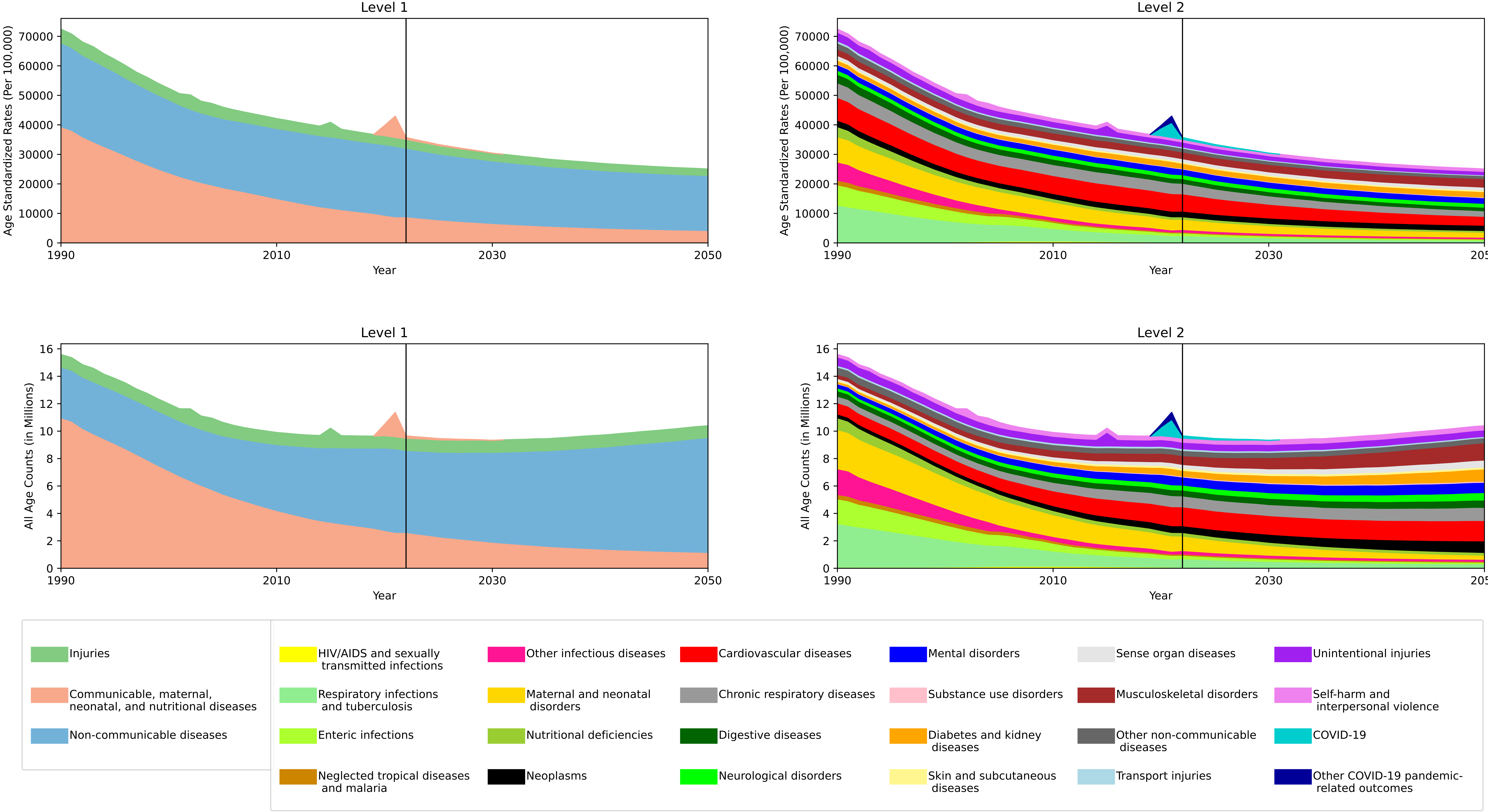
Level 1
Level 2
Age Standardized Rates (Per 100,000)
All Age Counts (in Millions)
Year
1990
2010
2030
2050
Injuries
Communicable, maternal, neonatal, and nutritional diseases
Non-communicable diseases
HIV/AIDS and sexually transmitted infections
Respiratory infections and tuberculosis
Enteric infections
Neglected tropical diseases and malaria
Other infectious diseases
Maternal and neonatal disorders
Nutritional deficiencies
Neoplasms
Cardiovascular diseases
Chronic respiratory diseases
Digestive diseases
Neurological disorders
Mental disorders
Substance use disorders
Diabetes and kidney diseases
Skin and subcutaneous diseases
Sense organ diseases
Musculoskeletal disorders
Other non-communicable diseases
Transport injuries
Unintentional injuries
Self-harm and interpersonal violence
COVID-19
Other COVID-19 pandemic-related outcomes

Pakistan

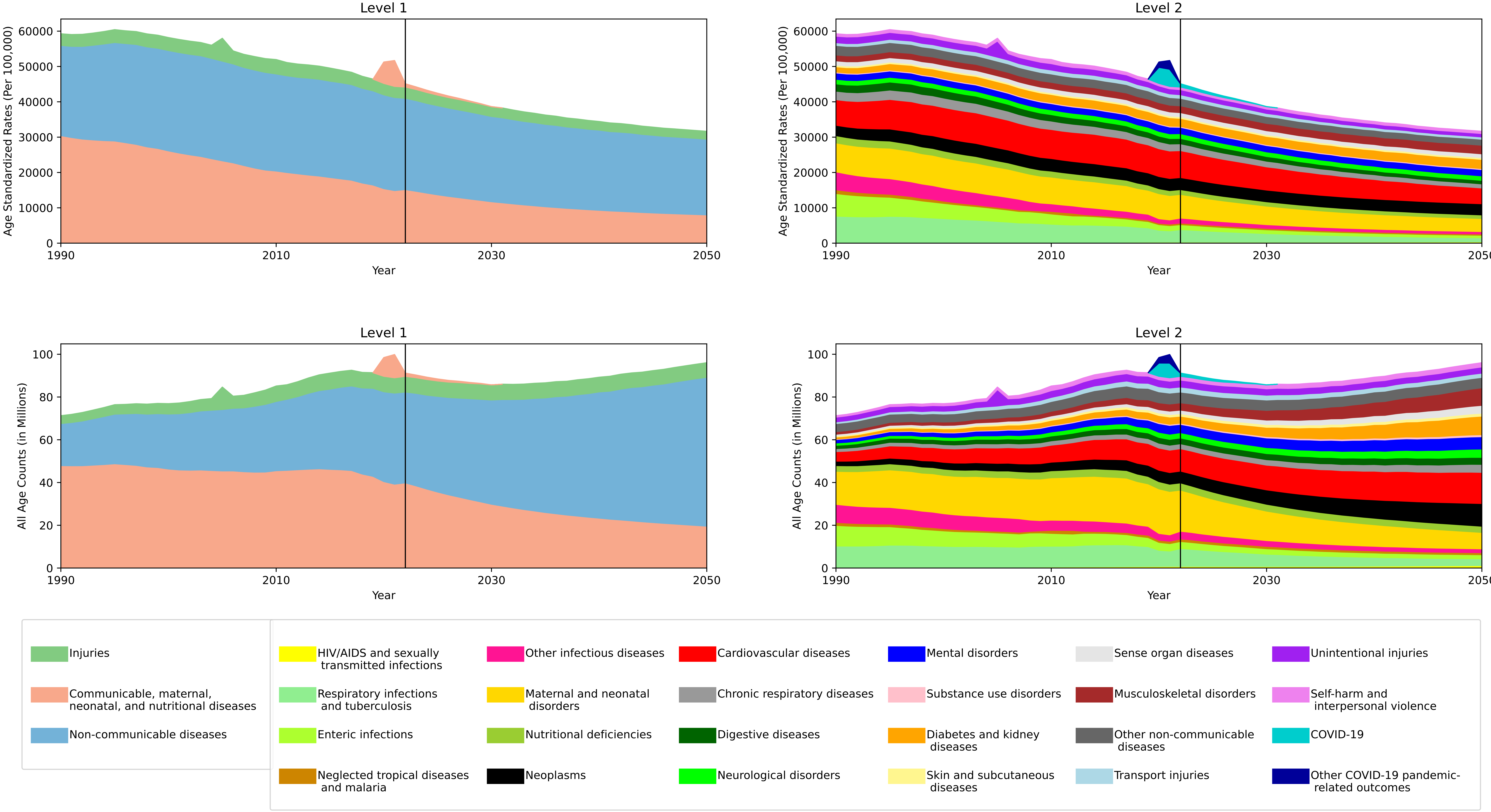
Level 1
Level 2
Age Standardized Rates (Per 100,000)
All Age Counts (in Millions)
Year
0
10000
20000
30000
40000
50000
60000
20
40
60
80
100
1990
2010
2030
2050
Injuries
Communicable, maternal, neonatal, and nutritional diseases
Non-communicable diseases
HIV/AIDS and sexually transmitted infections
Respiratory infections and tuberculosis
Enteric infections
Neglected tropical diseases and malaria
Other infectious diseases
Maternal and neonatal disorders
Nutritional deficiencies
Neoplasms
Cardiovascular diseases
Chronic respiratory diseases
Digestive diseases
Neurological disorders
Mental disorders
Substance use disorders
Diabetes and kidney diseases
Skin and subcutaneous diseases
Sense organ diseases
Musculoskeletal disorders
Other non-communicable diseases
Transport injuries
Unintentional injuries
Self-harm and interpersonal violence
COVID-19
Other COVID-19 pandemic-related outcomes

China

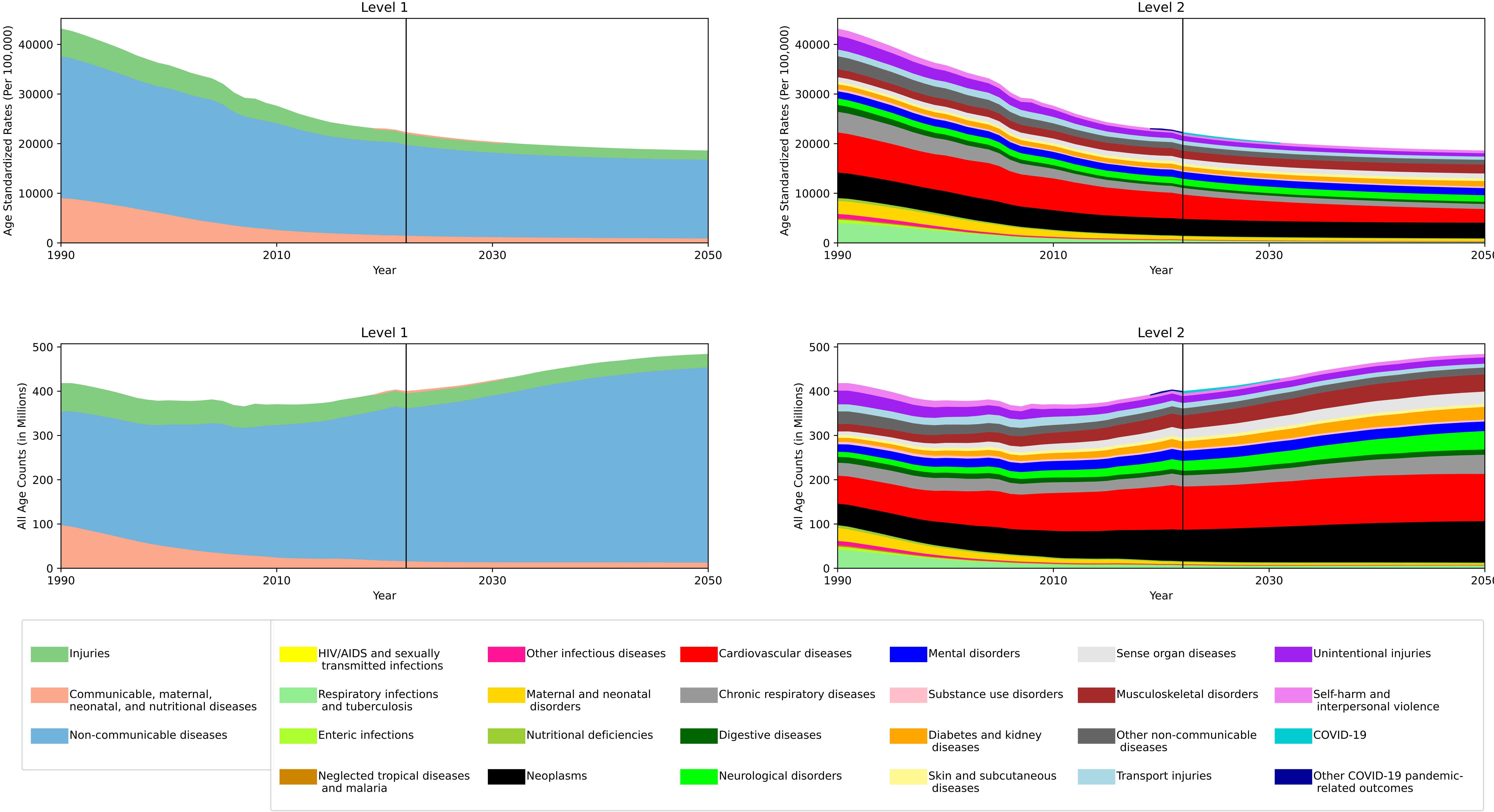
Level 1
Level 2
Age Standardized Rates (Per 100,000)
All Age Counts (in Millions)
Year
1990
2010
2030
2050
Injuries
Communicable, maternal, neonatal, and nutritional diseases
Non-communicable diseases
HIV/AIDS and sexually transmitted infections
Respiratory infections and tuberculosis
Enteric infections
Neglected tropical diseases and malaria
Other infectious diseases
Maternal and neonatal disorders
Nutritional deficiencies
Neoplasms
Cardiovascular diseases
Chronic respiratory diseases
Digestive diseases
Neurological disorders
Mental disorders
Substance use disorders
Diabetes and kidney diseases
Skin and subcutaneous diseases
Sense organ diseases
Musculoskeletal disorders
Other non-communicable diseases
Transport injuries
Unintentional injuries
Self-harm and interpersonal violence
COVID-19
Other COVID-19 pandemic-related outcomes

North Korea

Supplemental Results Figure S4. Age-standardised DALY rates and all age DALY counts and by Level 1 and Level 2 causes, 1990–2050

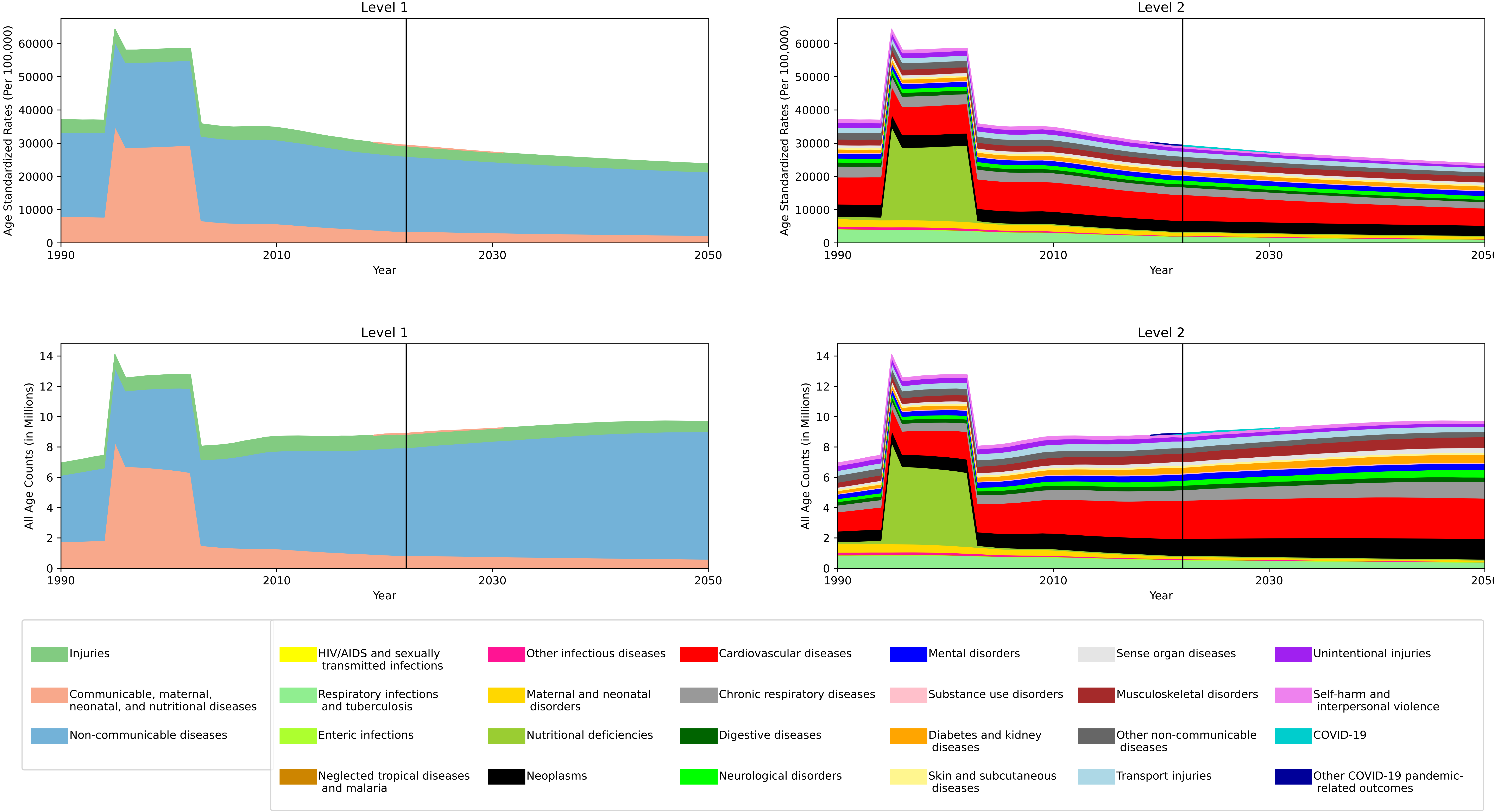

Supplemental Results Figure S4. Age-standardised DALY rates and all age DALY counts and by Level 1 and Level 2 causes, 1990–2050

Taiwan (Province Of China)

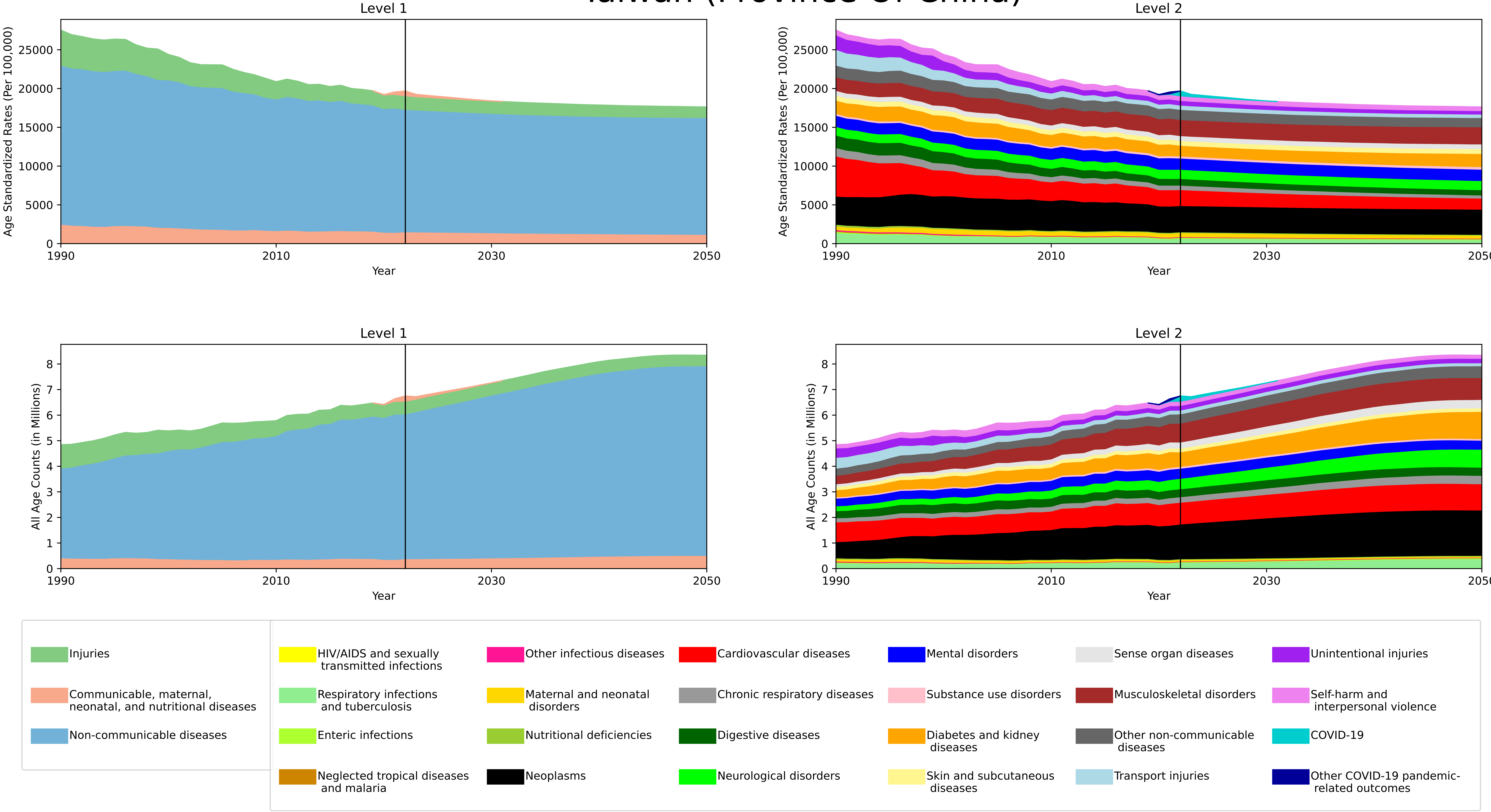

American Samoa

Supplemental Results Figure S4. Age-standardised DALY rates and all age DALY counts and by Level 1 and Level 2 causes, 1990–2050

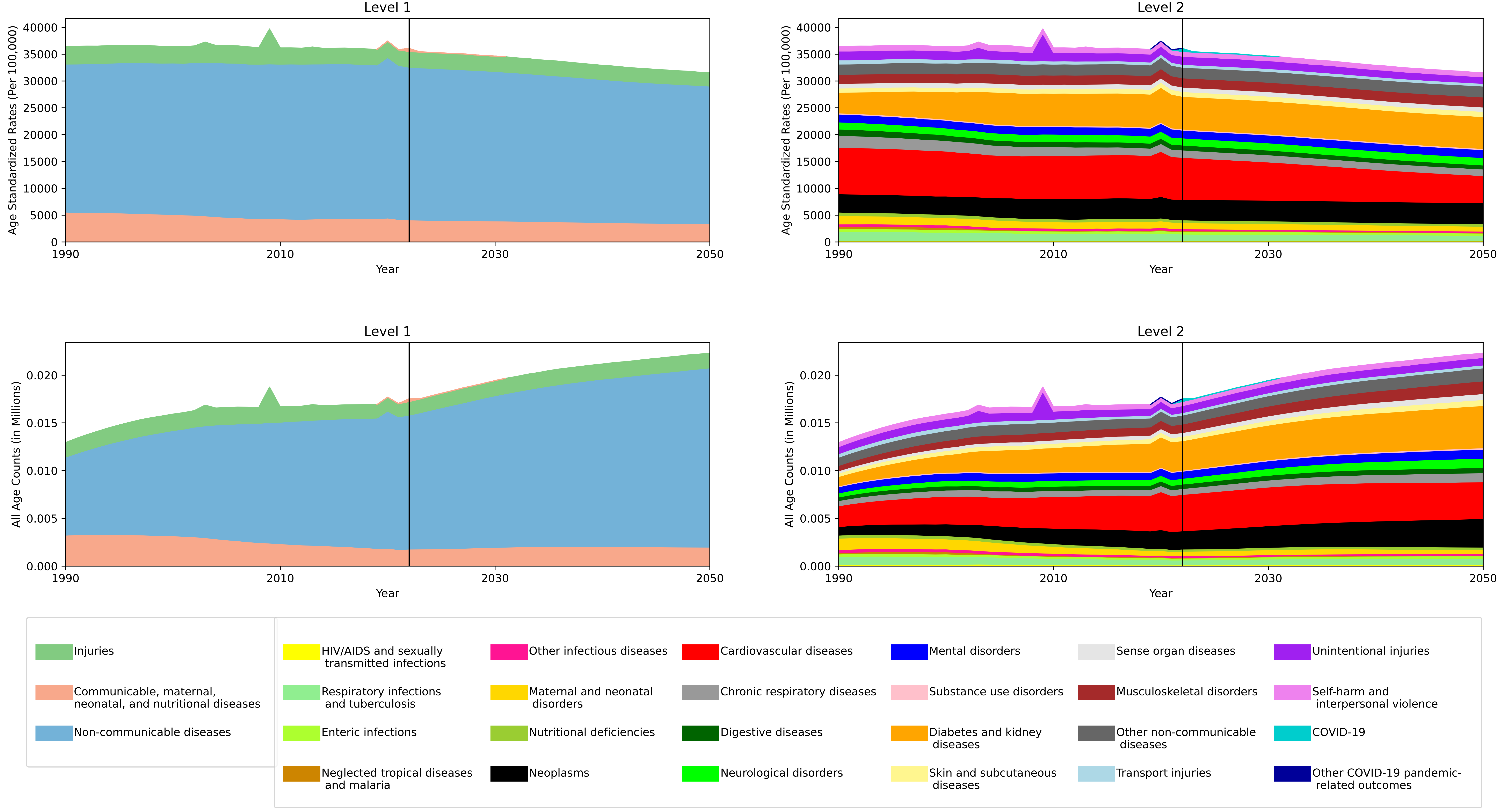

Cook Islands

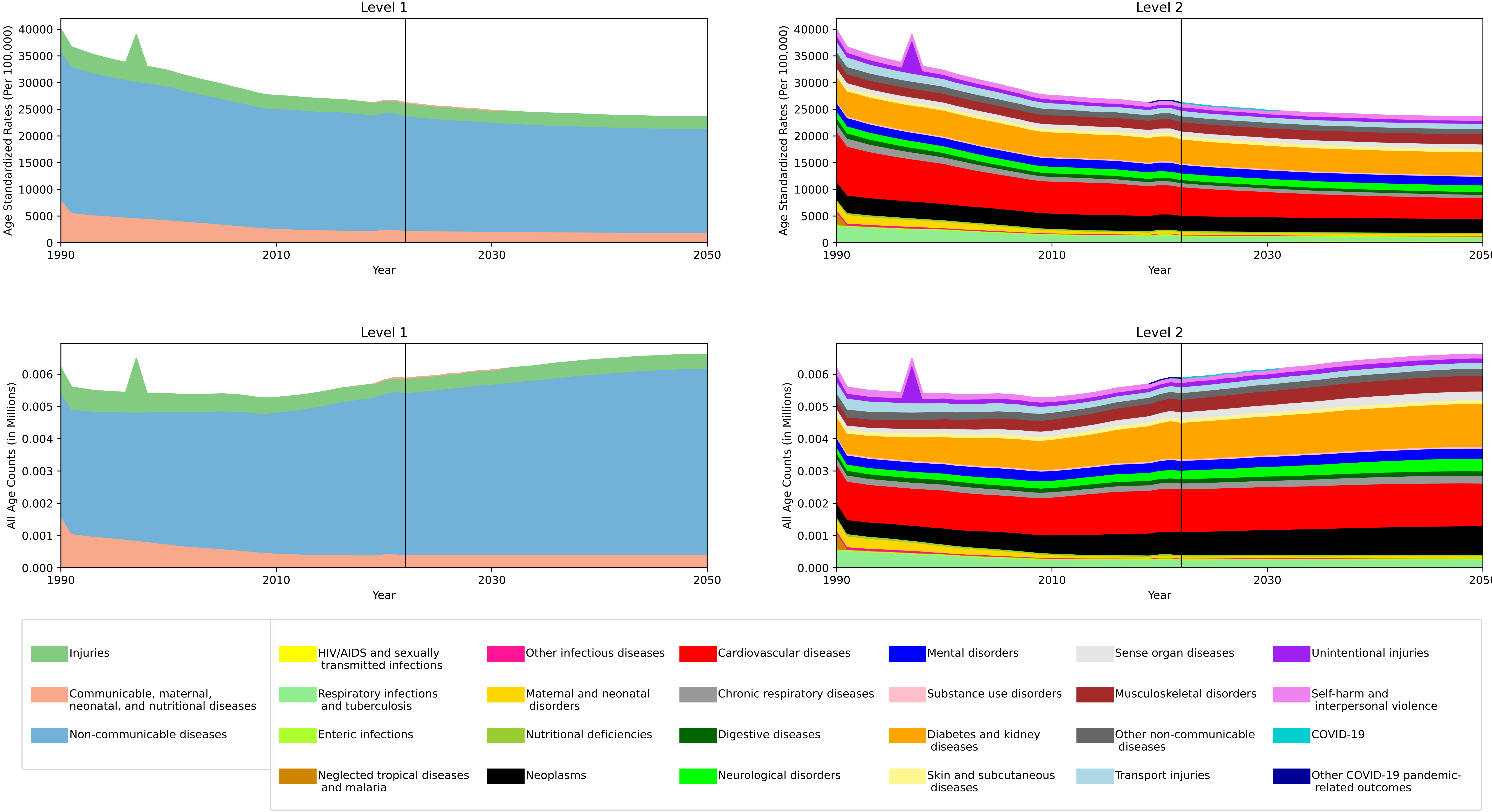

Fiji

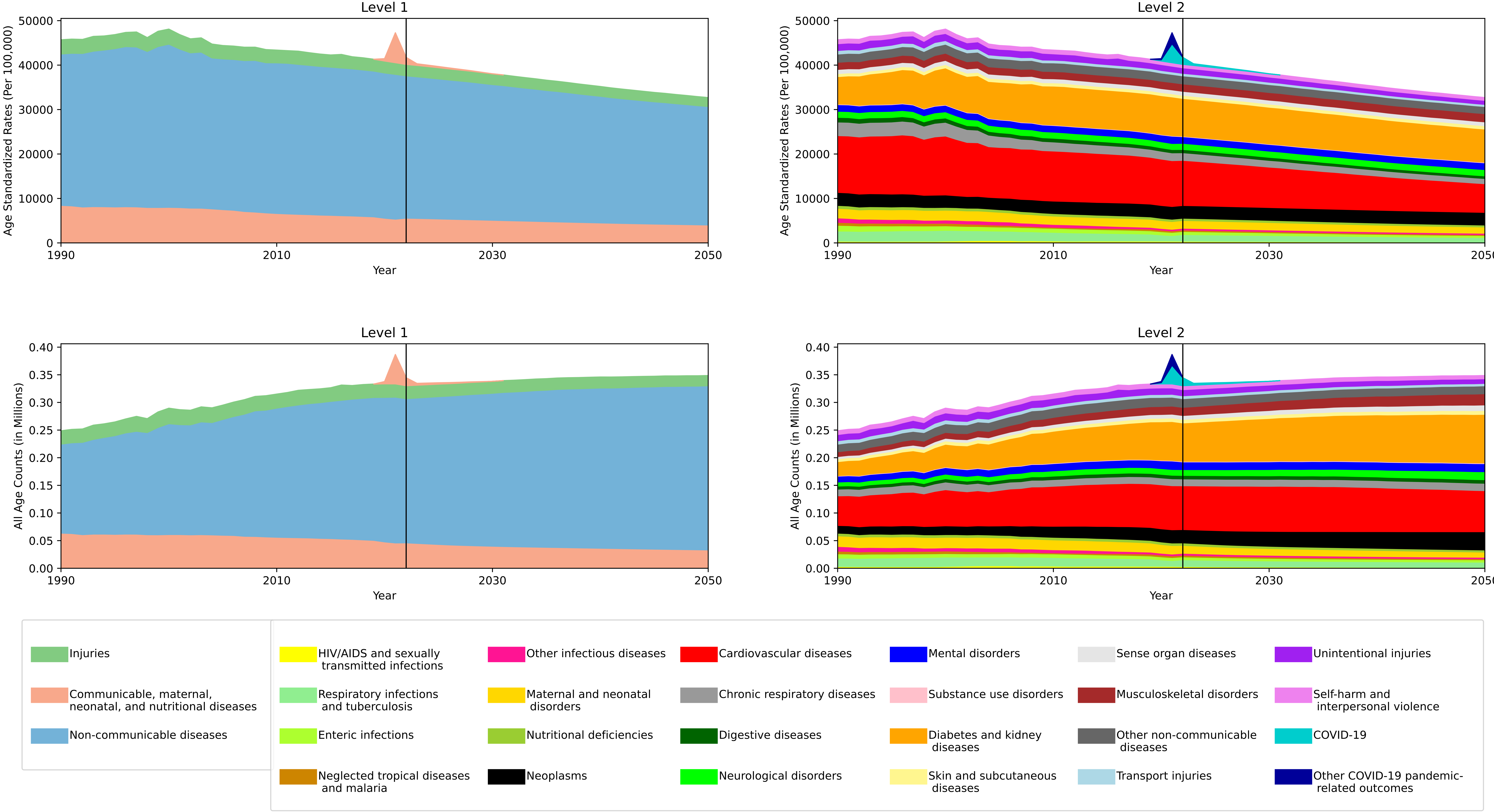

Level 1
Level 2
Age Standardized Rates (Per 100,000)
All Age Counts (in Millions)
Year
1990
2010
2030
2050
Injuries
Communicable, maternal, neonatal, and nutritional diseases
Non-communicable diseases
HIV/AIDS and sexually transmitted infections
Respiratory infections and tuberculosis
Enteric infections
Neglected tropical diseases and malaria
Other infectious diseases
Maternal and neonatal disorders
Nutritional deficiencies
Neoplasms
Cardiovascular diseases
Chronic respiratory diseases
Digestive diseases
Neurological disorders
Mental disorders
Substance use disorders
Diabetes and kidney diseases
Skin and subcutaneous diseases
Sense organ diseases
Musculoskeletal disorders
Other non-communicable diseases
Transport injuries
Unintentional injuries
Self-harm and interpersonal violence
COVID-19
Other COVID-19 pandemic-related outcomes

Guam

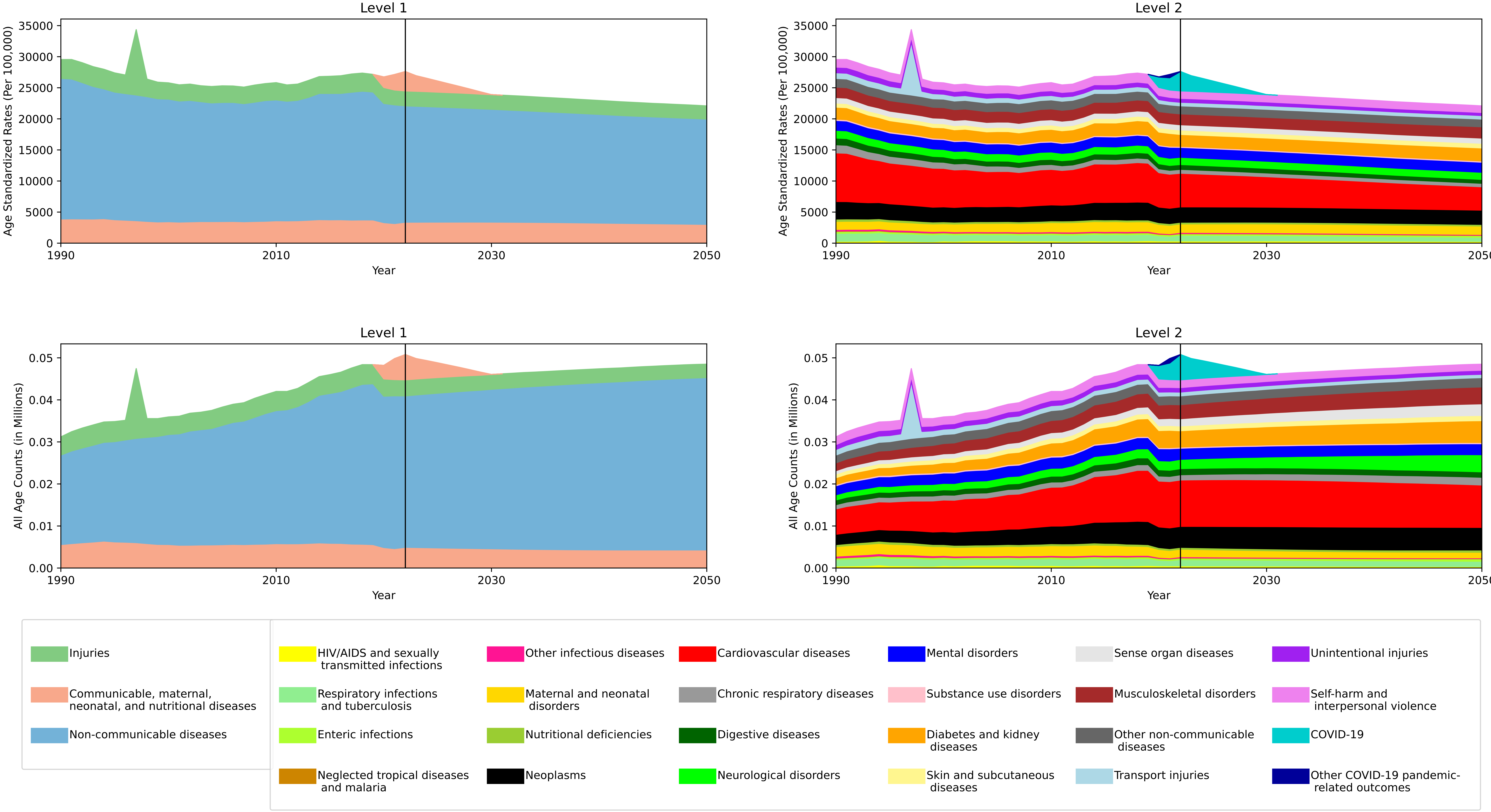
Level 1
Level 2
Age Standardized Rates (Per 100,000)
All Age Counts (in Millions)
Year
1990
2010
2030
2050
35000
30000
25000
20000
15000
10000
5000
0
0.05
0.04
0.03
0.02
0.01
0.00
Injuries
Communicable, maternal, neonatal, and nutritional diseases
Non-communicable diseases
HIV/AIDS and sexually transmitted infections
Respiratory infections and tuberculosis
Enteric infections
Neglected tropical diseases and malaria
Other infectious diseases
Maternal and neonatal disorders
Nutritional deficiencies
Neoplasms
Cardiovascular diseases
Chronic respiratory diseases
Digestive diseases
Neurological disorders
Mental disorders
Substance use disorders
Diabetes and kidney diseases
Skin and subcutaneous diseases
Sense organ diseases
Musculoskeletal disorders
Other non-communicable diseases
Transport injuries
Unintentional injuries
Self-harm and interpersonal violence
COVID-19
Other COVID-19 pandemic-related outcomes

Kiribati

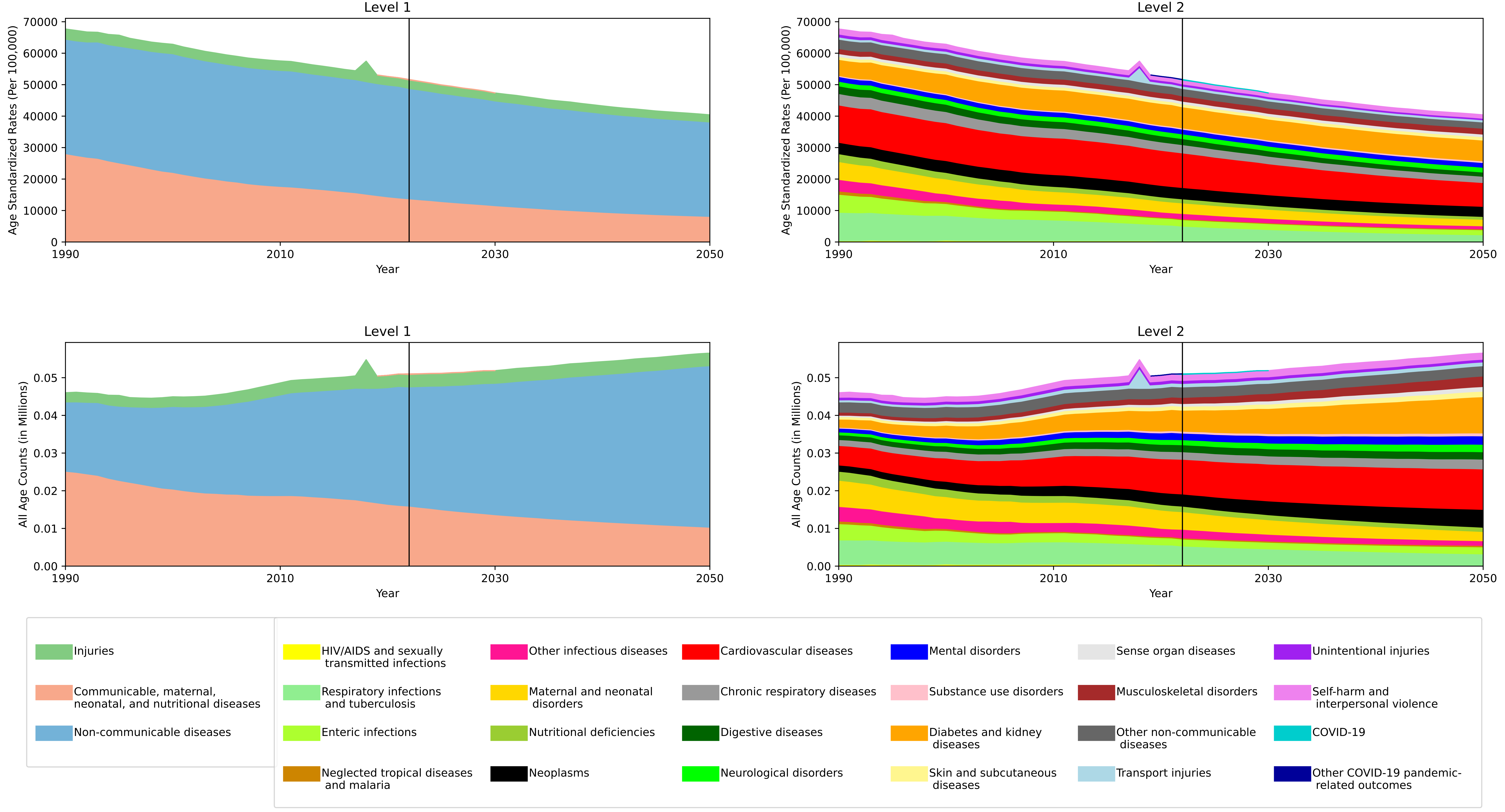
Level 1
Level 2
Age Standardized Rates (Per 100,000)
All Age Counts (in Millions)
Year
0
10000
20000
30000
40000
50000
60000
70000
0.00
0.01
0.02
0.03
0.04
0.05
1990
2010
2030
2050
Injuries
Communicable, maternal, neonatal, and nutritional diseases
Non-communicable diseases
HIV/AIDS and sexually transmitted infections
Respiratory infections and tuberculosis
Enteric infections
Neglected tropical diseases and malaria
Other infectious diseases
Maternal and neonatal disorders
Nutritional deficiencies
Neoplasms
Cardiovascular diseases
Chronic respiratory diseases
Digestive diseases
Neurological disorders
Mental disorders
Substance use disorders
Diabetes and kidney diseases
Skin and subcutaneous diseases
Sense organ diseases
Musculoskeletal disorders
Other non-communicable diseases
Transport injuries
Unintentional injuries
Self-harm and interpersonal violence
COVID-19
Other COVID-19 pandemic-related outcomes

Marshall Islands

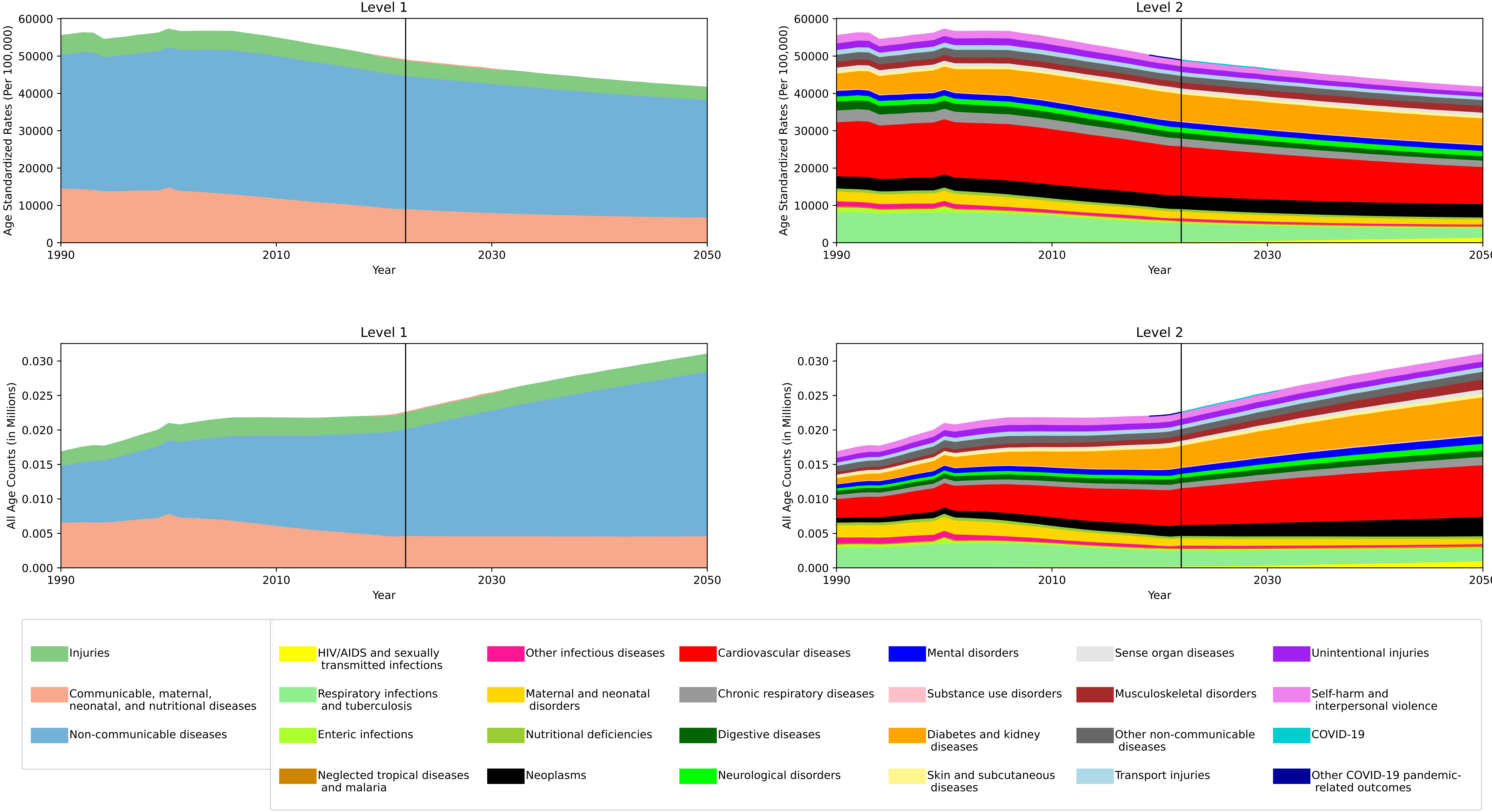

Federated States Of Micronesia

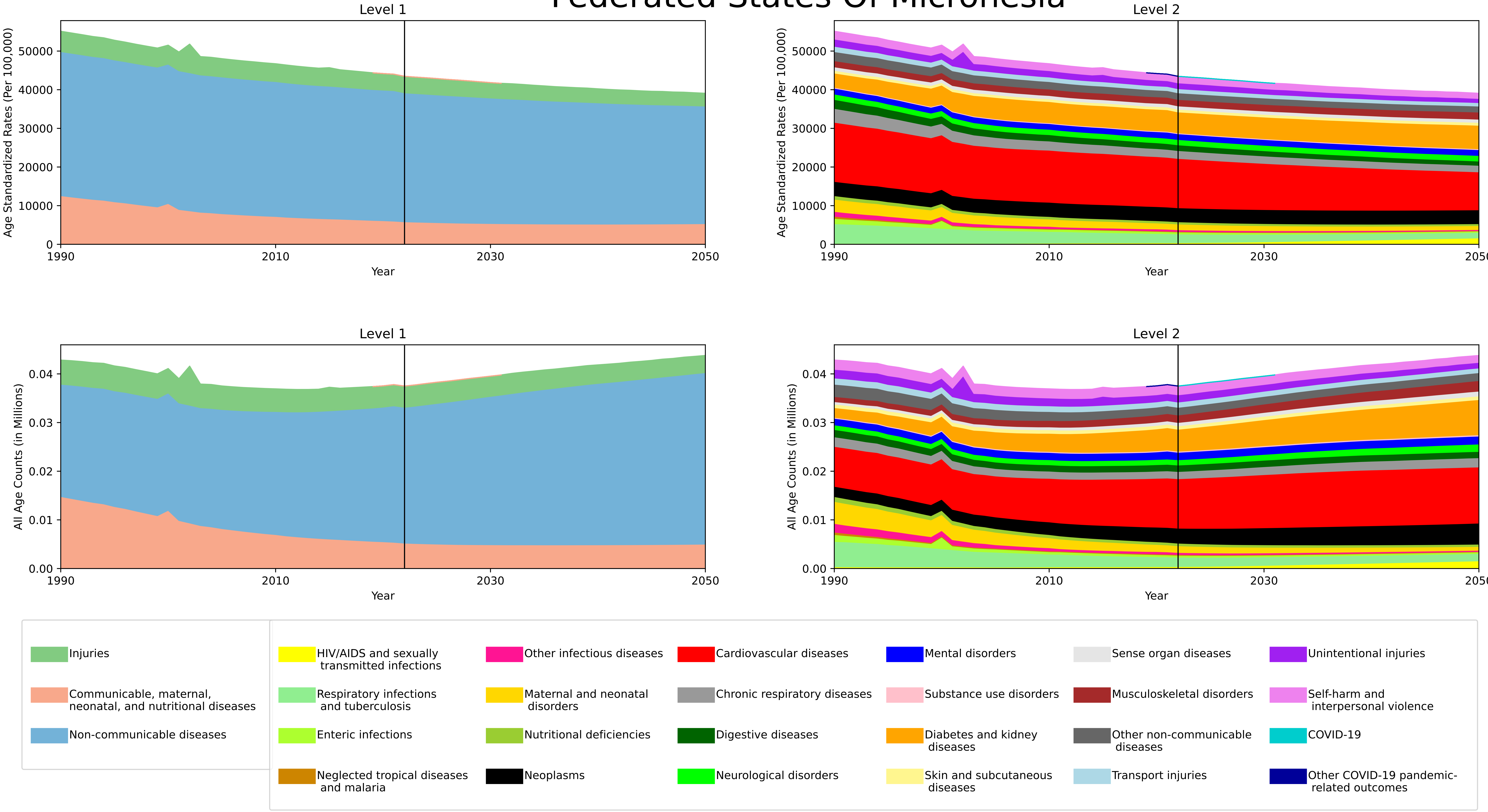

Nauru

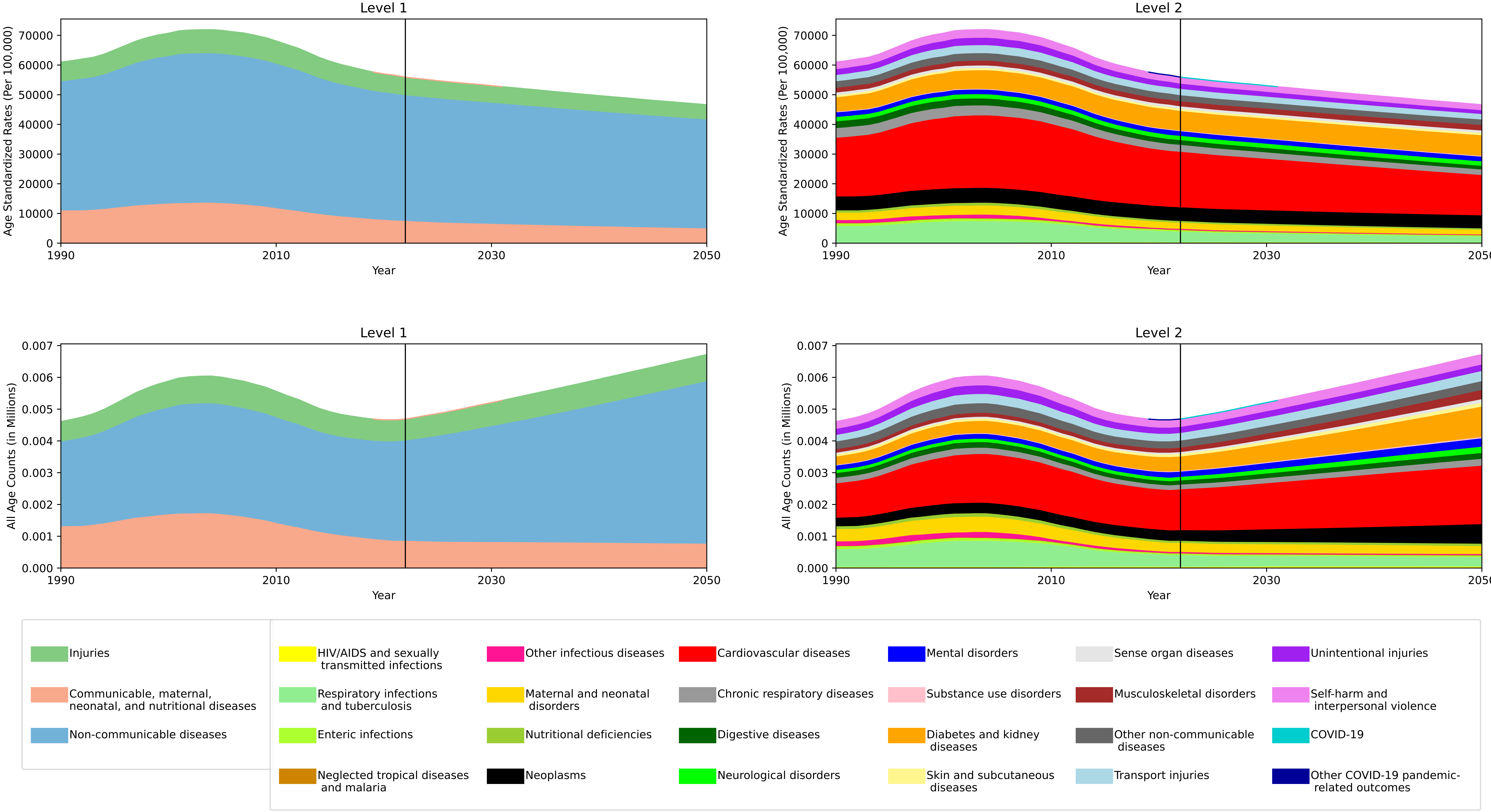
Level 1
Level 2
Age Standardized Rates (Per 100,000)
All Age Counts (in Millions)
Year
1990
2010
2030
2050
70000
60000
50000
40000
30000
20000
10000
0
0.007
0.006
0.005
0.004
0.003
0.002
0.001
0.000
Injuries
Communicable, maternal, neonatal, and nutritional diseases
Non-communicable diseases
HIV/AIDS and sexually transmitted infections
Respiratory infections and tuberculosis
Enteric infections
Neglected tropical diseases and malaria
Other infectious diseases
Maternal and neonatal disorders
Nutritional deficiencies
Neoplasms
Cardiovascular diseases
Chronic respiratory diseases
Digestive diseases
Neurological disorders
Mental disorders
Substance use disorders
Diabetes and kidney diseases
Skin and subcutaneous diseases
Sense organ diseases
Musculoskeletal disorders
Other non-communicable diseases
Transport injuries
Unintentional injuries
Self-harm and interpersonal violence
COVID-19
Other COVID-19 pandemic-related outcomes

Niue

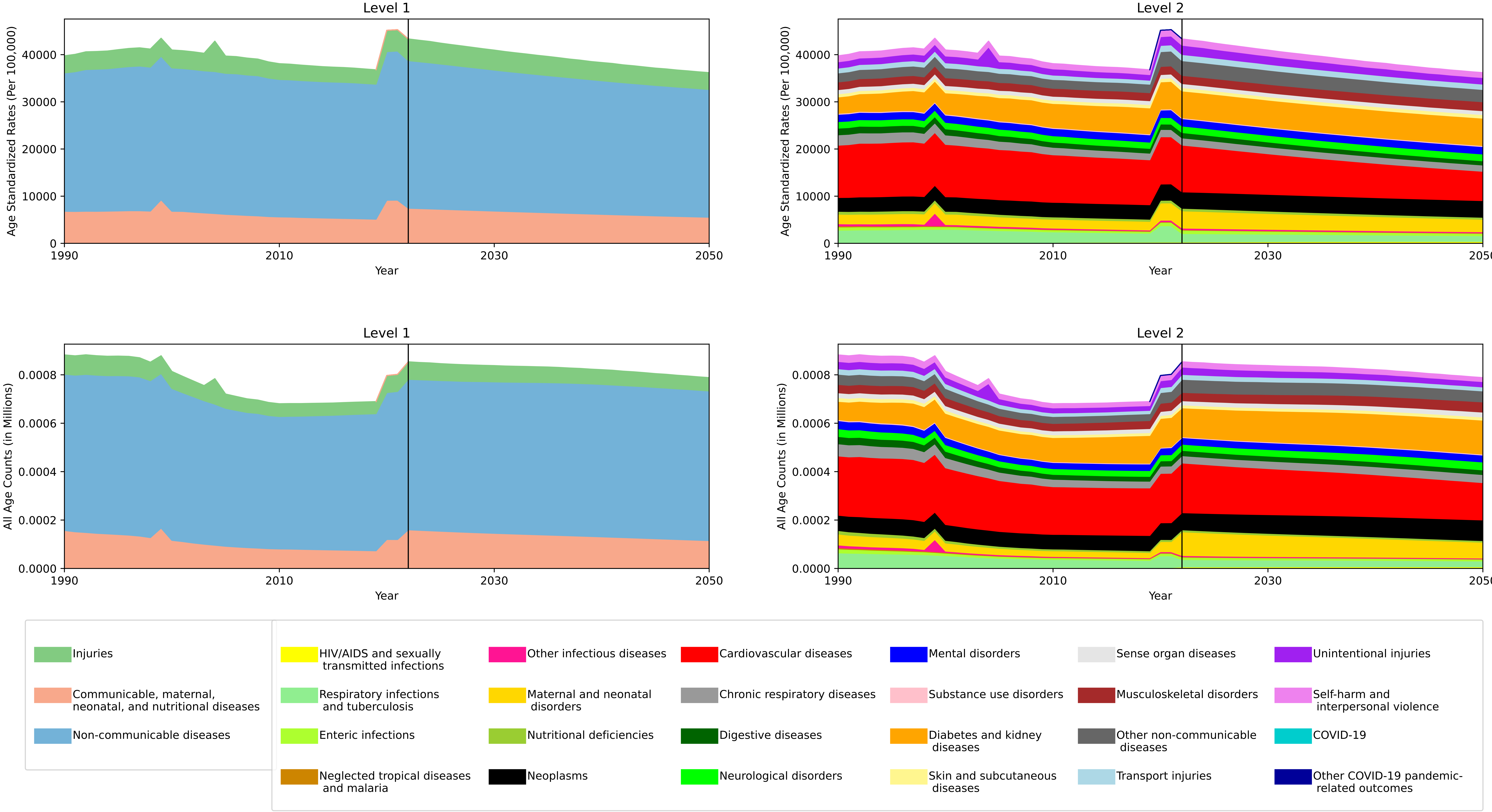
Level 1
Level 2
Age Standardized Rates (Per 100,000)
All Age Counts (in Millions)
Year
1990
2010
2030
2050
0
10000
20000
30000
40000
0.0000
0.0002
0.0004
0.0006
0.0008
Injuries
Communicable, maternal, neonatal, and nutritional diseases
Non-communicable diseases
HIV/AIDS and sexually transmitted infections
Respiratory infections and tuberculosis
Enteric infections
Neglected tropical diseases and malaria
Other infectious diseases
Maternal and neonatal disorders
Nutritional deficiencies
Neoplasms
Cardiovascular diseases
Chronic respiratory diseases
Digestive diseases
Neurological disorders
Mental disorders
Substance use disorders
Diabetes and kidney diseases
Skin and subcutaneous diseases
Sense organ diseases
Musculoskeletal disorders
Other non-communicable diseases
Transport injuries
Unintentional injuries
Self-harm and interpersonal violence
COVID-19
Other COVID-19 pandemic-related outcomes

Supplemental Results Figure S4. Age-standardised DALY rates and all age DALY counts and by Level 1 and Level 2 causes, 1990–2050

Northern Mariana Islands

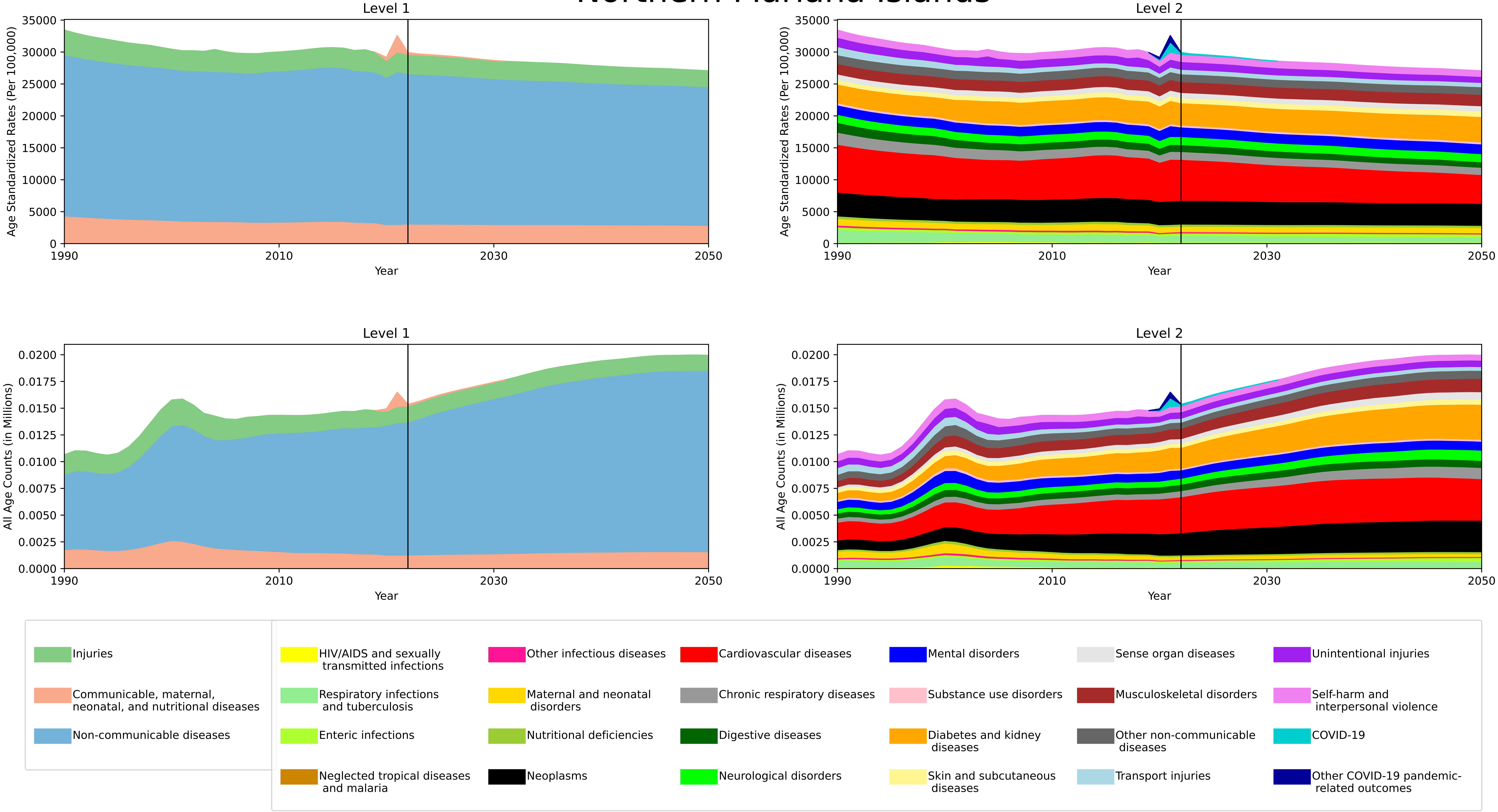

Palau

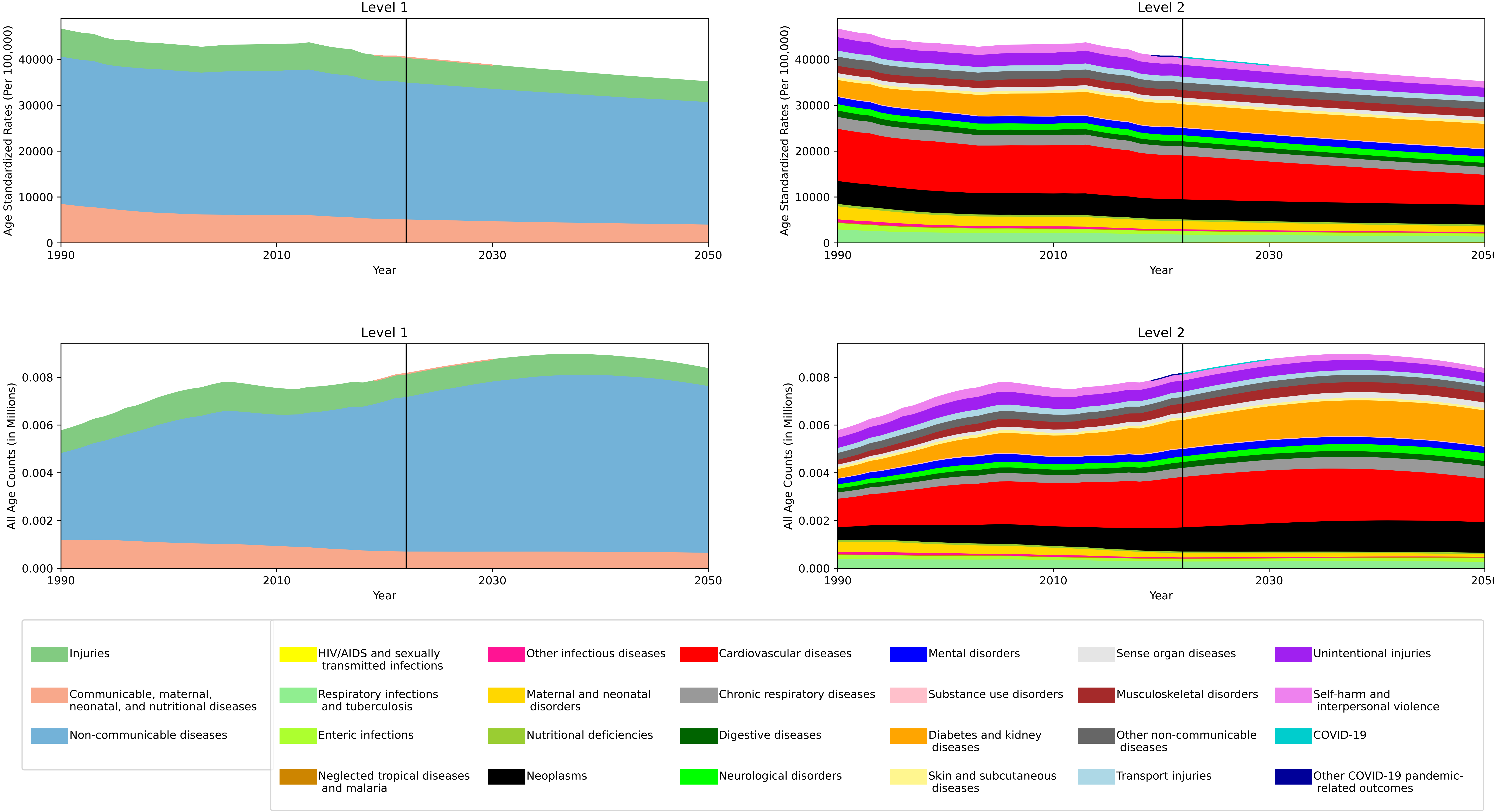
Level 1
Level 2
Age Standardized Rates (Per 100,000)
All Age Counts (in Millions)
Year
Injuries
Communicable, maternal, neonatal, and nutritional diseases
Non-communicable diseases
HIV/AIDS and sexually transmitted infections
Respiratory infections and tuberculosis
Enteric infections
Neglected tropical diseases and malaria
Other infectious diseases
Maternal and neonatal disorders
Nutritional deficiencies
Neoplasms
Cardiovascular diseases
Chronic respiratory diseases
Digestive diseases
Neurological disorders
Mental disorders
Substance use disorders
Diabetes and kidney diseases
Skin and subcutaneous diseases
Sense organ diseases
Musculoskeletal disorders
Other non-communicable diseases
Transport injuries
Unintentional injuries
Self-harm and interpersonal violence
COVID-19
Other COVID-19 pandemic-related outcomes

Papua New Guinea

Supplemental Results Figure S4. Age-standardised DALY rates and all age DALY counts and by Level 1 and Level 2 causes, 1990–2050

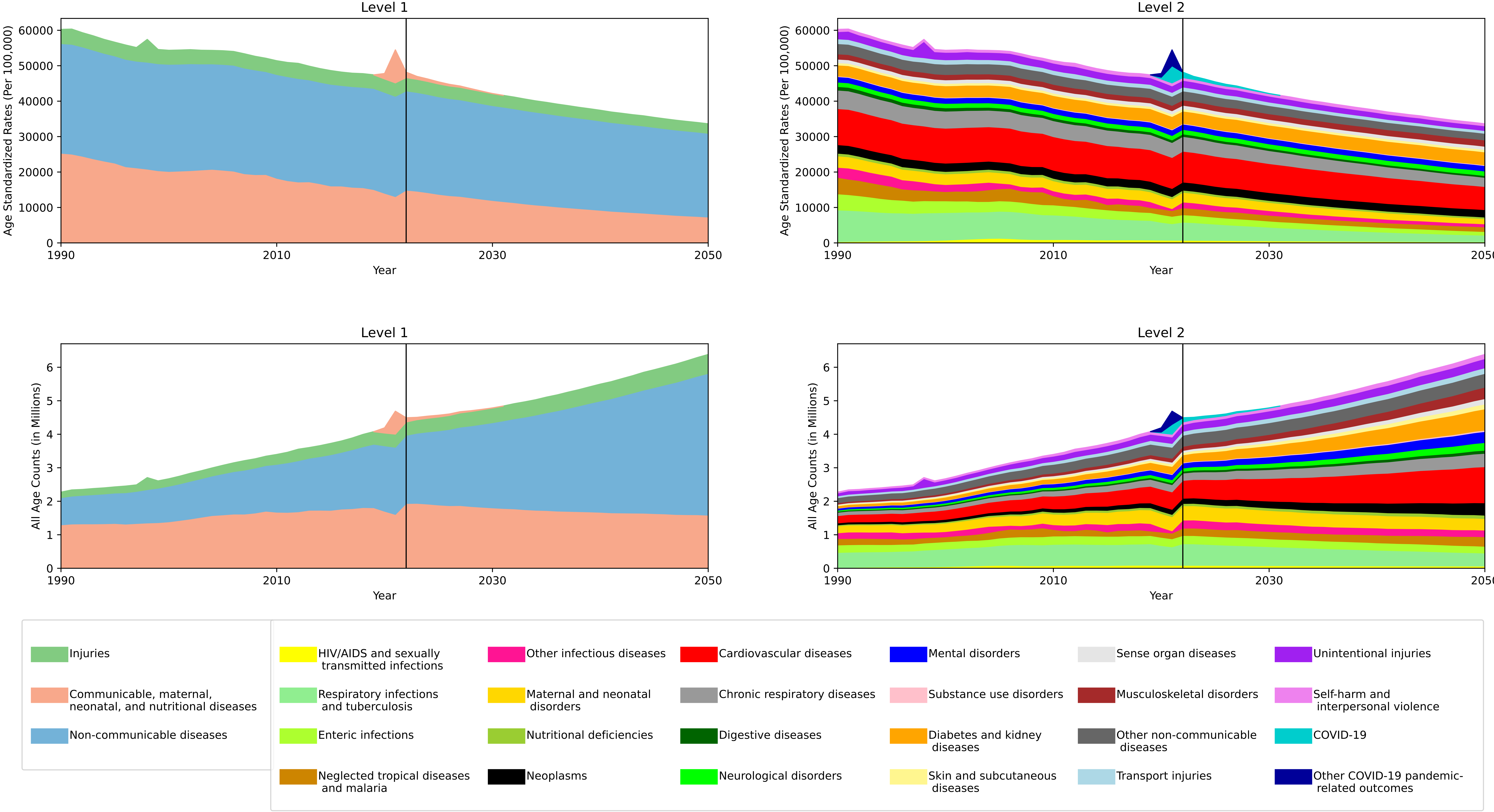

Samoa

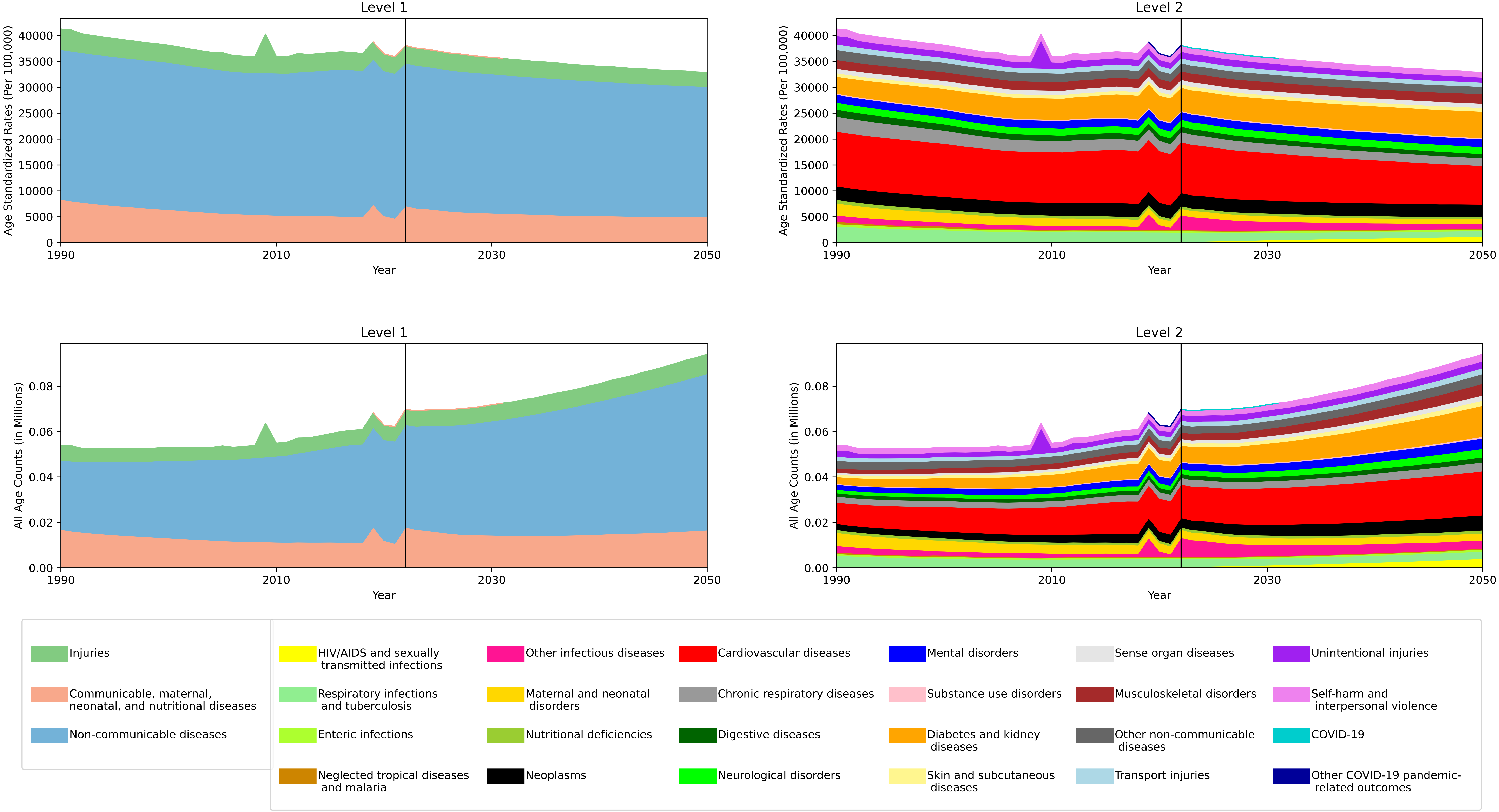
Level 1
Level 2
Age Standardized Rates (Per 100,000)
All Age Counts (in Millions)
Year
1990
2010
2030
2050
Injuries
Communicable, maternal, neonatal, and nutritional diseases
Non-communicable diseases
HIV/AIDS and sexually transmitted infections
Respiratory infections and tuberculosis
Enteric infections
Neglected tropical diseases and malaria
Other infectious diseases
Maternal and neonatal disorders
Nutritional deficiencies
Neoplasms
Cardiovascular diseases
Chronic respiratory diseases
Digestive diseases
Neurological disorders
Mental disorders
Substance use disorders
Diabetes and kidney diseases
Skin and subcutaneous diseases
Sense organ diseases
Musculoskeletal disorders
Other non-communicable diseases
Transport injuries
Unintentional injuries
Self-harm and interpersonal violence
COVID-19
Other COVID-19 pandemic-related outcomes

Solomon Islands

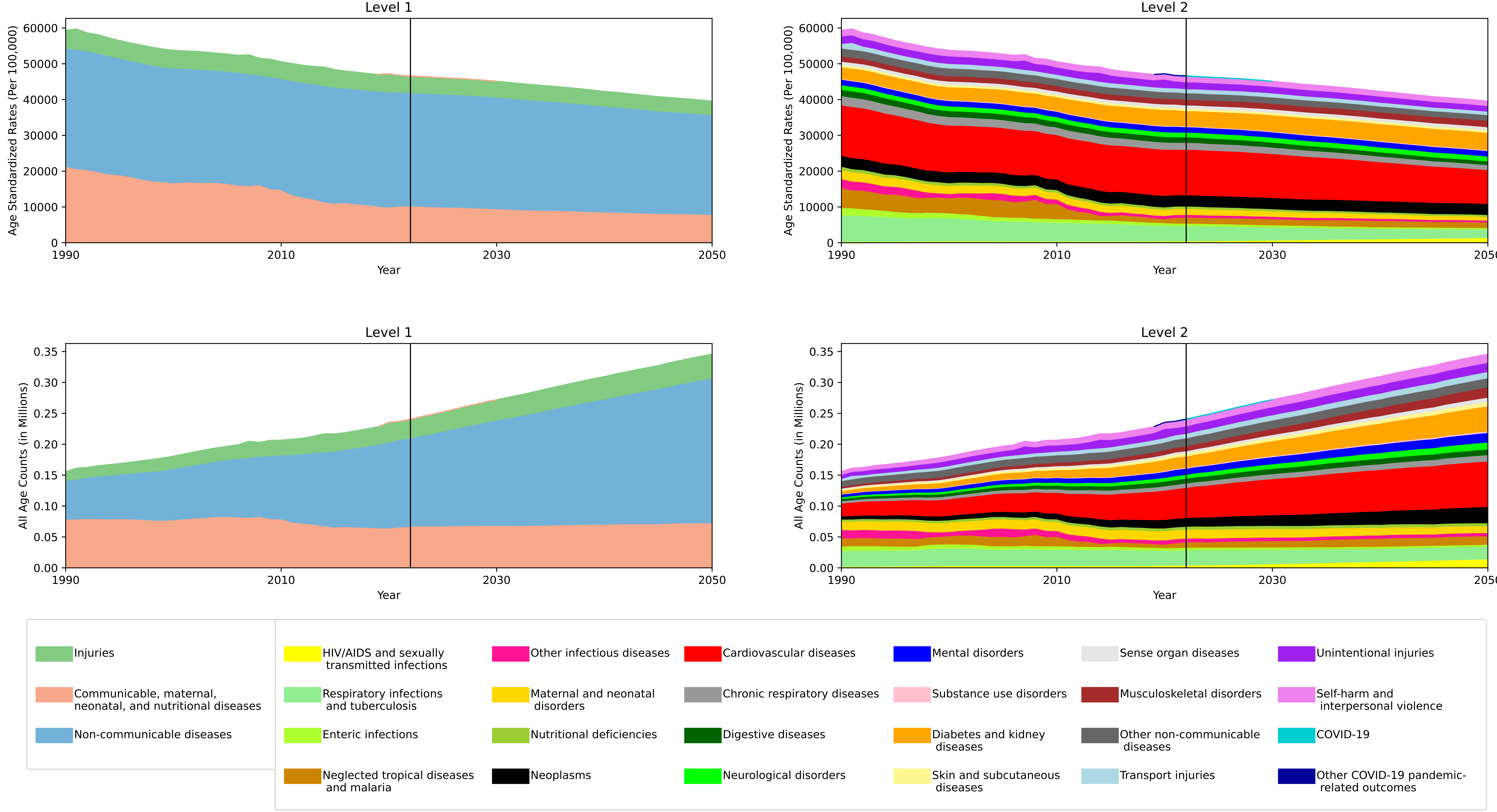

Tokelau

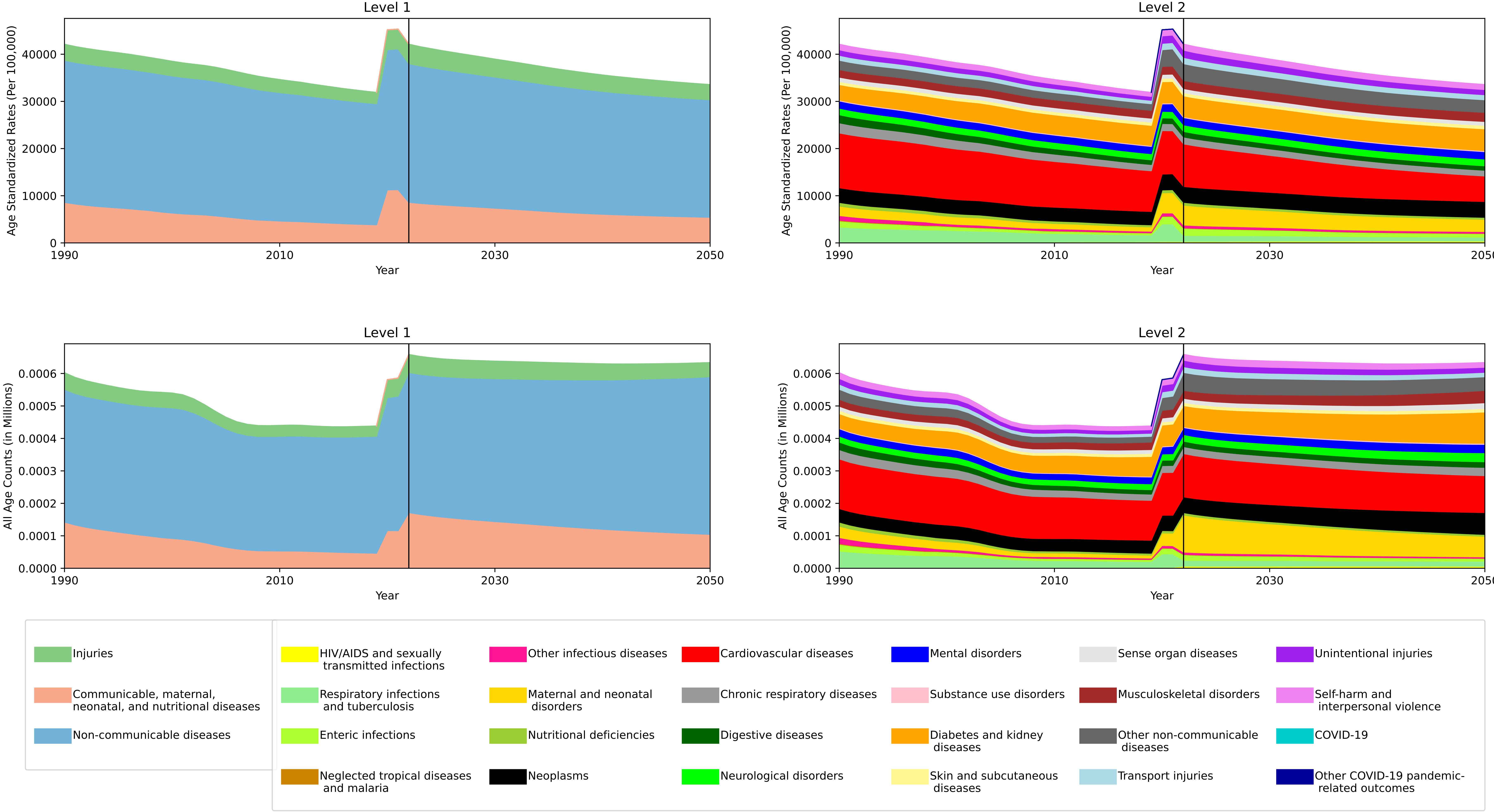

Level 1
Level 2
Age Standardized Rates (Per 100,000)
All Age Counts (in Millions)
Year
1990
2010
2030
2050
Injuries
Communicable, maternal, neonatal, and nutritional diseases
Non-communicable diseases
HIV/AIDS and sexually transmitted infections
Respiratory infections and tuberculosis
Enteric infections
Neglected tropical diseases and malaria
Other infectious diseases
Maternal and neonatal disorders
Nutritional deficiencies
Neoplasms
Cardiovascular diseases
Chronic respiratory diseases
Digestive diseases
Neurological disorders
Mental disorders
Substance use disorders
Diabetes and kidney diseases
Skin and subcutaneous diseases
Sense organ diseases
Musculoskeletal disorders
Other non-communicable diseases
Transport injuries
Unintentional injuries
Self-harm and interpersonal violence
COVID-19
Other COVID-19 pandemic-related outcomes

Tonga

Supplemental Results Figure S4. Age-standardised DALY rates and all age DALY counts and by Level 1 and Level 2 causes, 1990–2050

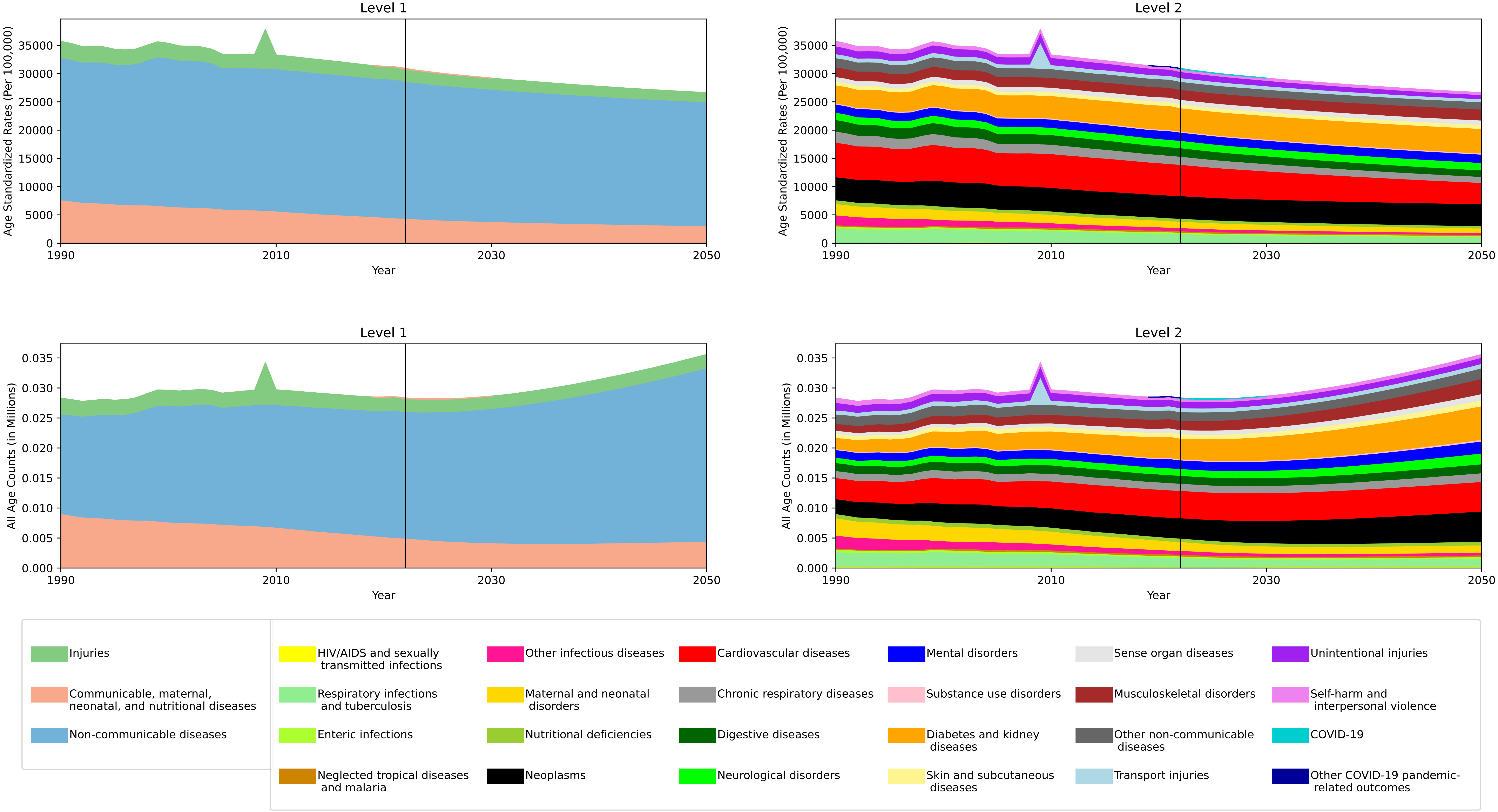

Tuvalu

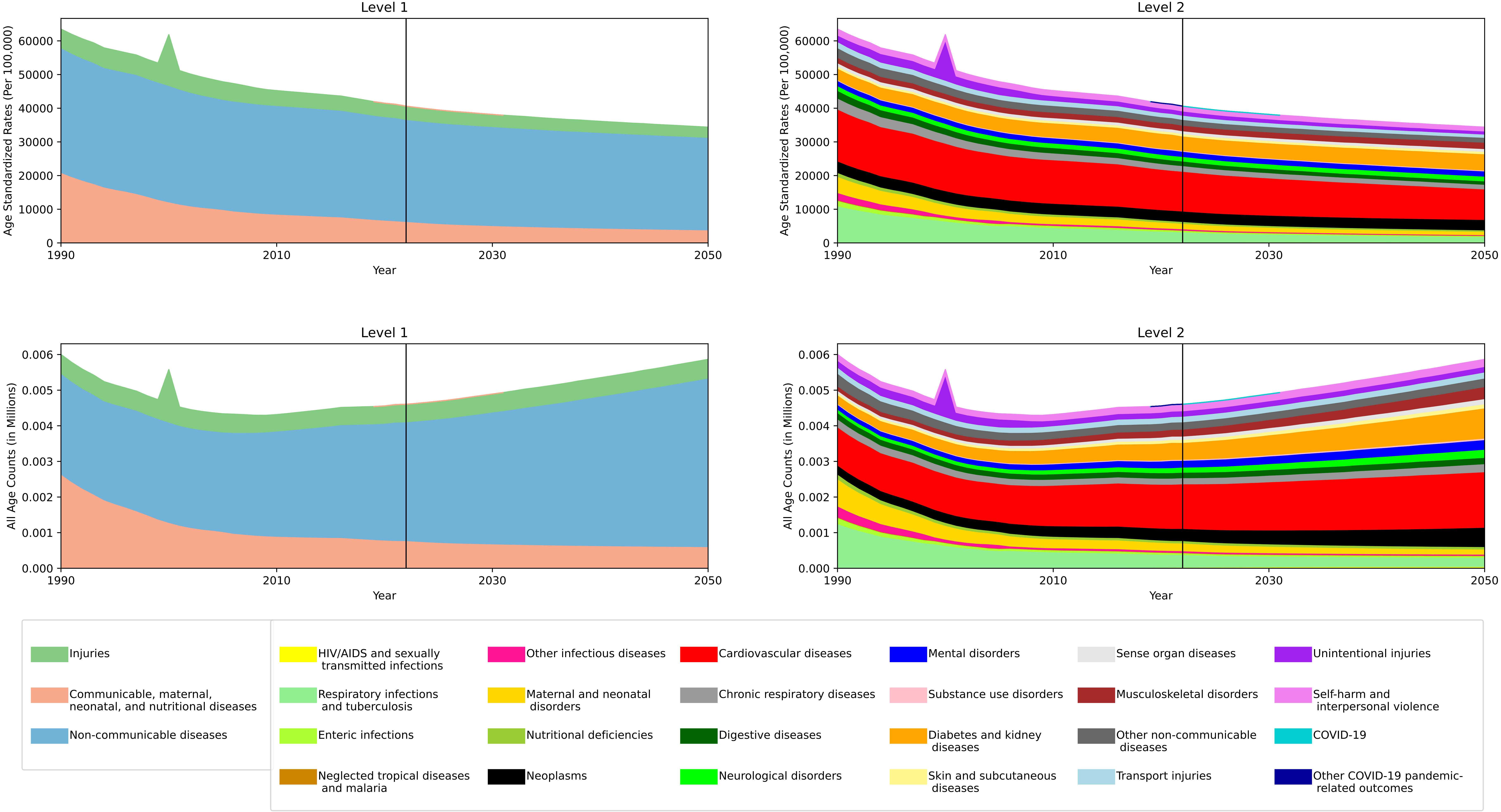
Level 1
Level 2
Age Standardized Rates (Per 100,000)
All Age Counts (in Millions)
Year
1990
2010
2030
2050
0
10000
20000
30000
40000
50000
60000
0.000
0.001
0.002
0.003
0.004
0.005
0.006
Injuries
Communicable, maternal, neonatal, and nutritional diseases
Non-communicable diseases
HIV/AIDS and sexually transmitted infections
Respiratory infections and tuberculosis
Enteric infections
Neglected tropical diseases and malaria
Other infectious diseases
Maternal and neonatal disorders
Nutritional deficiencies
Neoplasms
Cardiovascular diseases
Chronic respiratory diseases
Digestive diseases
Neurological disorders
Mental disorders
Substance use disorders
Diabetes and kidney diseases
Skin and subcutaneous diseases
Sense organ diseases
Musculoskeletal disorders
Other non-communicable diseases
Transport injuries
Unintentional injuries
Self-harm and interpersonal violence
COVID-19
Other COVID-19 pandemic-related outcomes

Vanuatu

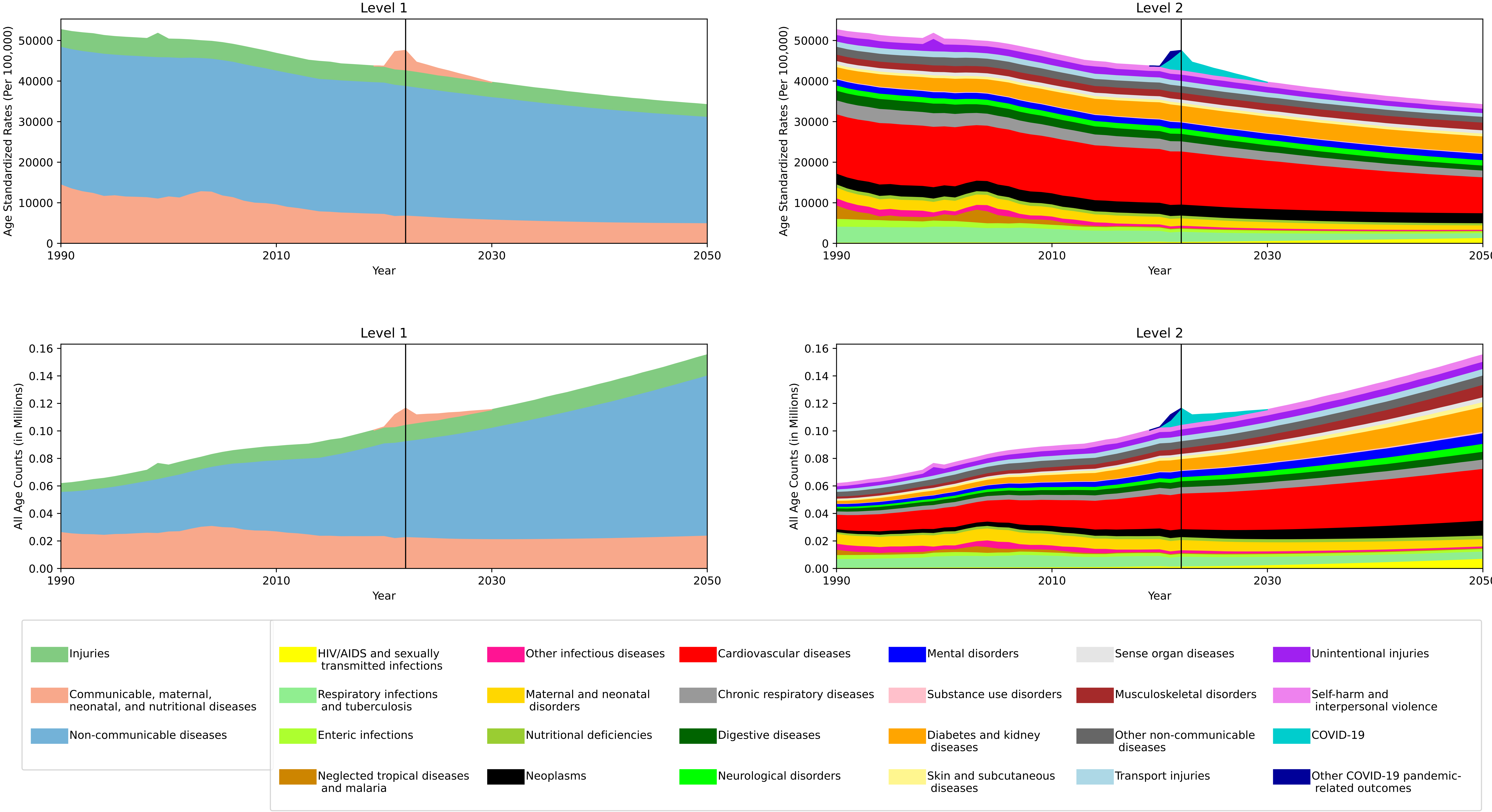
Level 1
Level 2
Age Standardized Rates (Per 100,000)
All Age Counts (in Millions)
Year
1990
2010
2030
2050
Injuries
Communicable, maternal, neonatal, and nutritional diseases
Non-communicable diseases
HIV/AIDS and sexually transmitted infections
Respiratory infections and tuberculosis
Enteric infections
Neglected tropical diseases and malaria
Other infectious diseases
Maternal and neonatal disorders
Nutritional deficiencies
Neoplasms
Cardiovascular diseases
Chronic respiratory diseases
Digestive diseases
Neurological disorders
Mental disorders
Substance use disorders
Diabetes and kidney diseases
Skin and subcutaneous diseases
Sense organ diseases
Musculoskeletal disorders
Other non-communicable diseases
Transport injuries
Unintentional injuries
Self-harm and interpersonal violence
COVID-19
Other COVID-19 pandemic-related outcomes

Cambodia

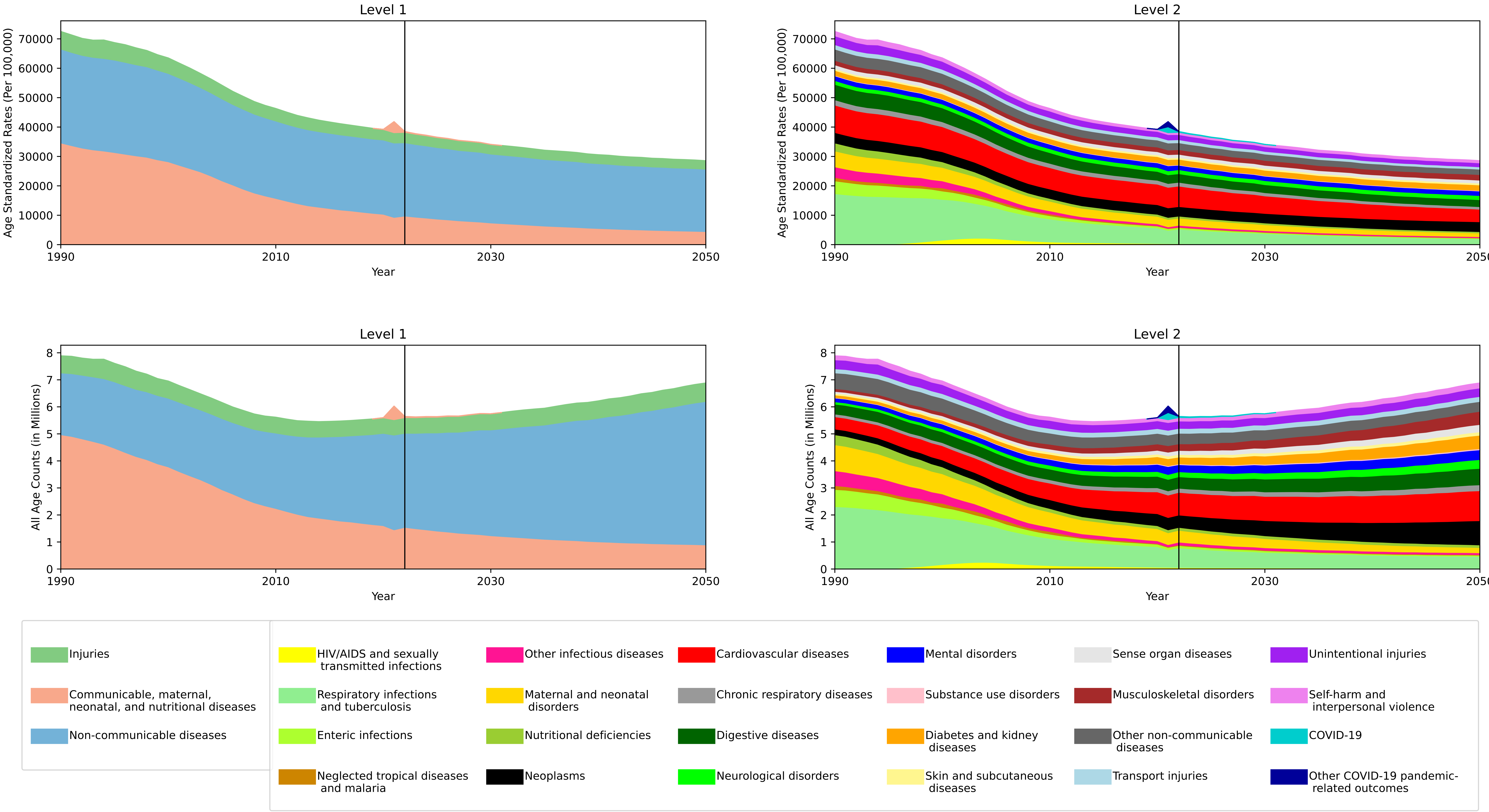
Level 1
Level 2
Age Standardized Rates (Per 100,000)
All Age Counts (in Millions)
Year
1990
2010
2030
2050
Injuries
Communicable, maternal, neonatal, and nutritional diseases
Non-communicable diseases
HIV/AIDS and sexually transmitted infections
Respiratory infections and tuberculosis
Enteric infections
Neglected tropical diseases and malaria
Other infectious diseases
Maternal and neonatal disorders
Nutritional deficiencies
Neoplasms
Cardiovascular diseases
Chronic respiratory diseases
Digestive diseases
Neurological disorders
Mental disorders
Substance use disorders
Diabetes and kidney diseases
Skin and subcutaneous diseases
Sense organ diseases
Musculoskeletal disorders
Other non-communicable diseases
Transport injuries
Unintentional injuries
Self-harm and interpersonal violence
COVID-19
Other COVID-19 pandemic-related outcomes

Indonesia

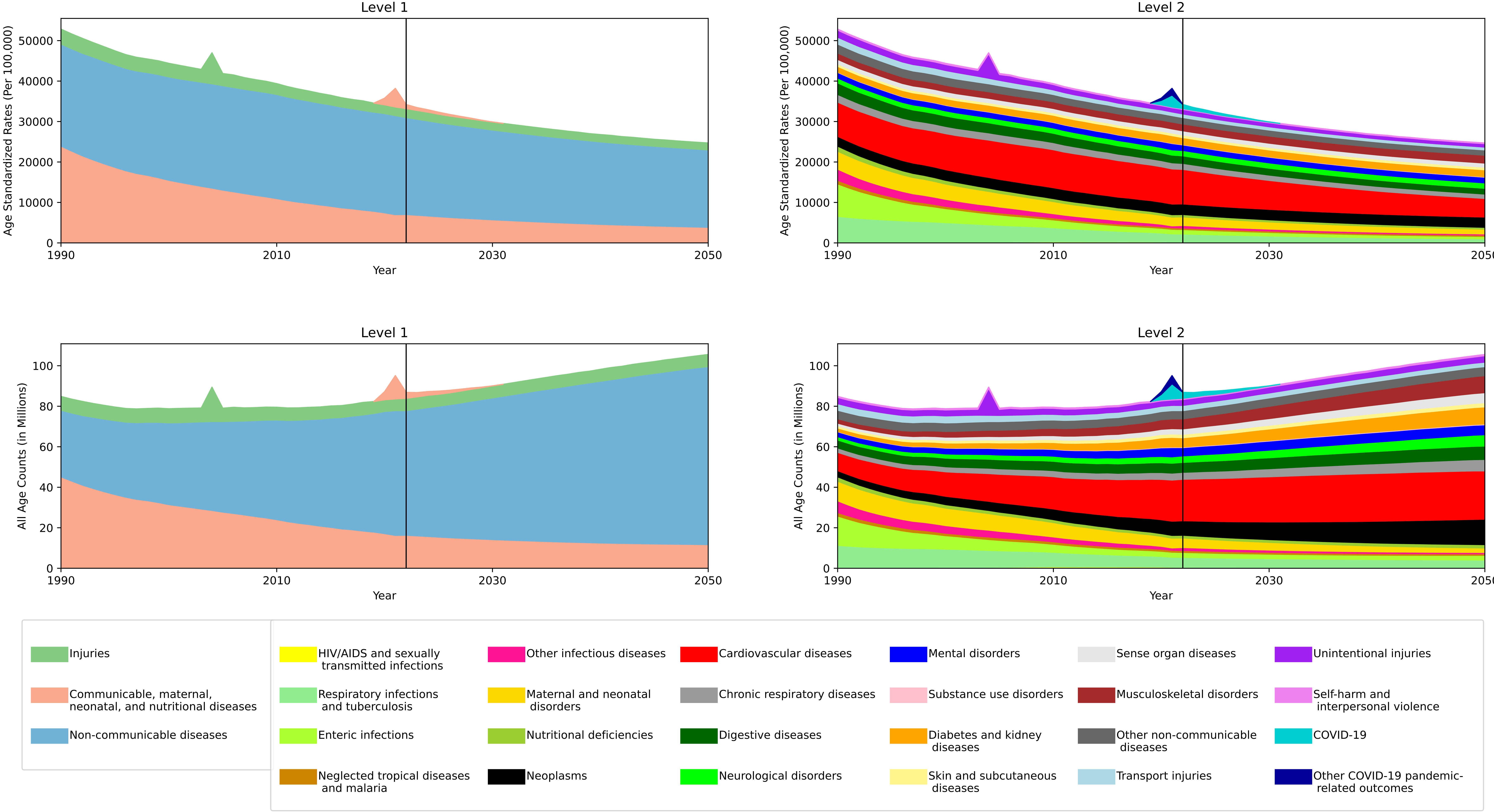
Level 1
Level 2
Age Standardized Rates (Per 100,000)
All Age Counts (in Millions)
Year
1990
2010
2030
2050
Injuries
Communicable, maternal, neonatal, and nutritional diseases
Non-communicable diseases
HIV/AIDS and sexually transmitted infections
Respiratory infections and tuberculosis
Enteric infections
Neglected tropical diseases and malaria
Other infectious diseases
Maternal and neonatal disorders
Nutritional deficiencies
Neoplasms
Cardiovascular diseases
Chronic respiratory diseases
Digestive diseases
Neurological disorders
Mental disorders
Substance use disorders
Diabetes and kidney diseases
Skin and subcutaneous diseases
Sense organ diseases
Musculoskeletal disorders
Other non-communicable diseases
Transport injuries
Unintentional injuries
Self-harm and interpersonal violence
COVID-19
Other COVID-19 pandemic-related outcomes

Laos

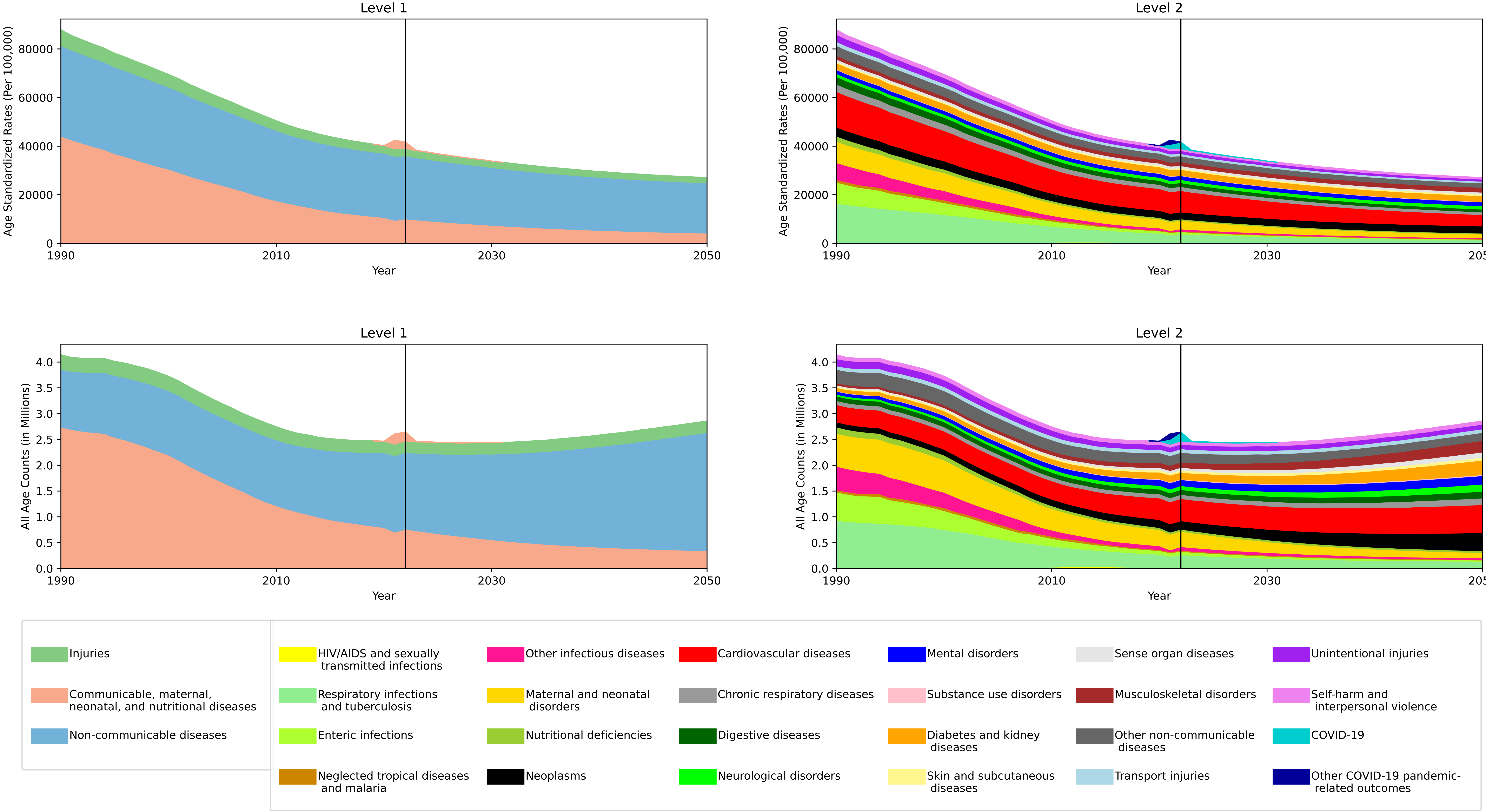
Level 1
Level 2
Age Standardized Rates (Per 100,000)
All Age Counts (in Millions)
Year
Injuries
Communicable, maternal, neonatal, and nutritional diseases
Non-communicable diseases
HIV/AIDS and sexually transmitted infections
Respiratory infections and tuberculosis
Enteric infections
Neglected tropical diseases and malaria
Other infectious diseases
Maternal and neonatal disorders
Nutritional deficiencies
Neoplasms
Cardiovascular diseases
Chronic respiratory diseases
Digestive diseases
Neurological disorders
Mental disorders
Substance use disorders
Diabetes and kidney diseases
Skin and subcutaneous diseases
Sense organ diseases
Musculoskeletal disorders
Other non-communicable diseases
Transport injuries
Unintentional injuries
Self-harm and interpersonal violence
COVID-19
Other COVID-19 pandemic-related outcomes

Malaysia

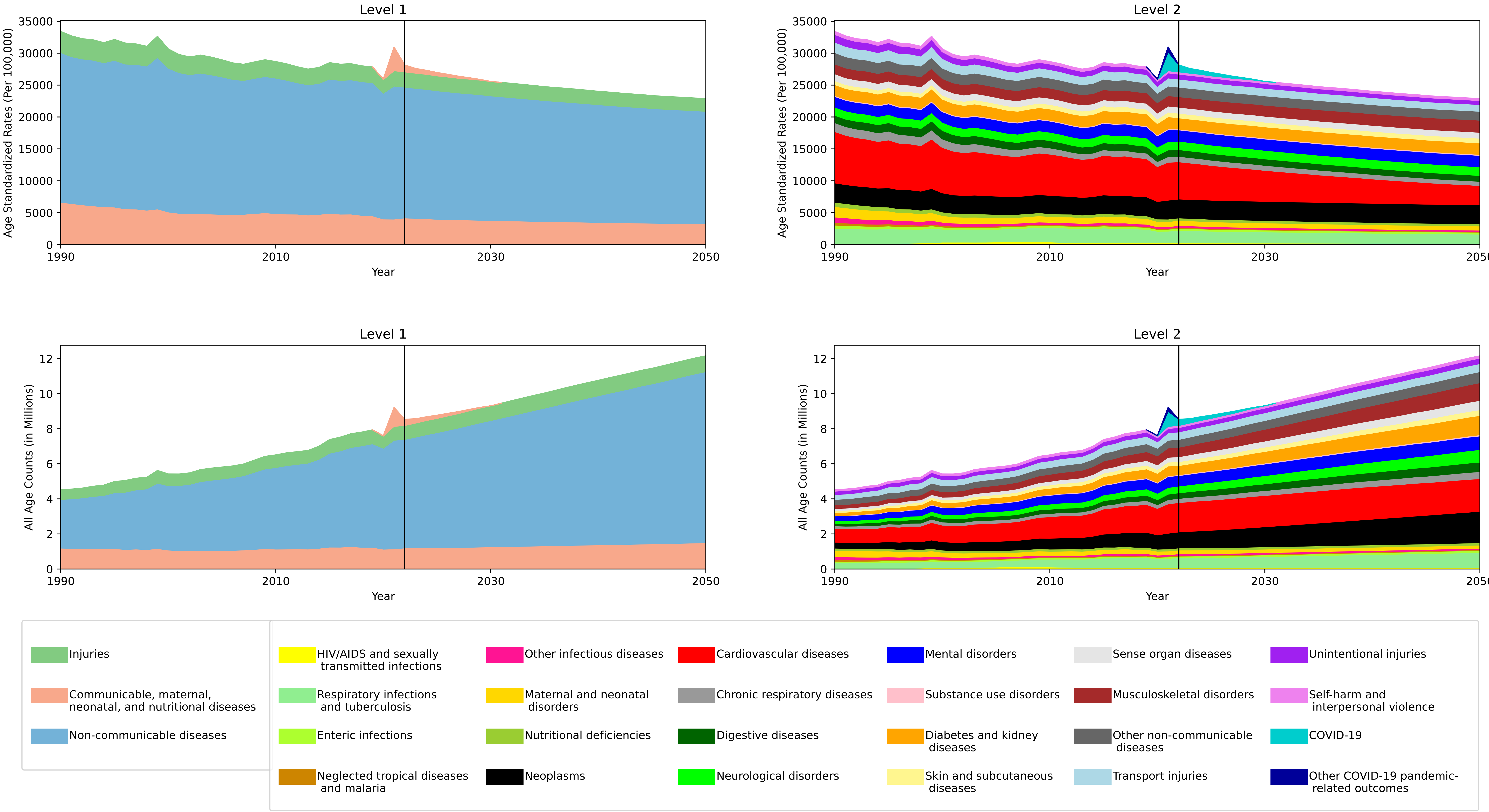
Level 1
Level 2
Age Standardized Rates (Per 100,000)
All Age Counts (in Millions)
Year
Injuries
Communicable, maternal, neonatal, and nutritional diseases
Non-communicable diseases
HIV/AIDS and sexually transmitted infections
Respiratory infections and tuberculosis
Enteric infections
Neglected tropical diseases and malaria
Other infectious diseases
Maternal and neonatal disorders
Nutritional deficiencies
Neoplasms
Cardiovascular diseases
Chronic respiratory diseases
Digestive diseases
Neurological disorders
Mental disorders
Substance use disorders
Diabetes and kidney diseases
Skin and subcutaneous diseases
Sense organ diseases
Musculoskeletal disorders
Other non-communicable diseases
Transport injuries
Unintentional injuries
Self-harm and interpersonal violence
COVID-19
Other COVID-19 pandemic-related outcomes

Maldives

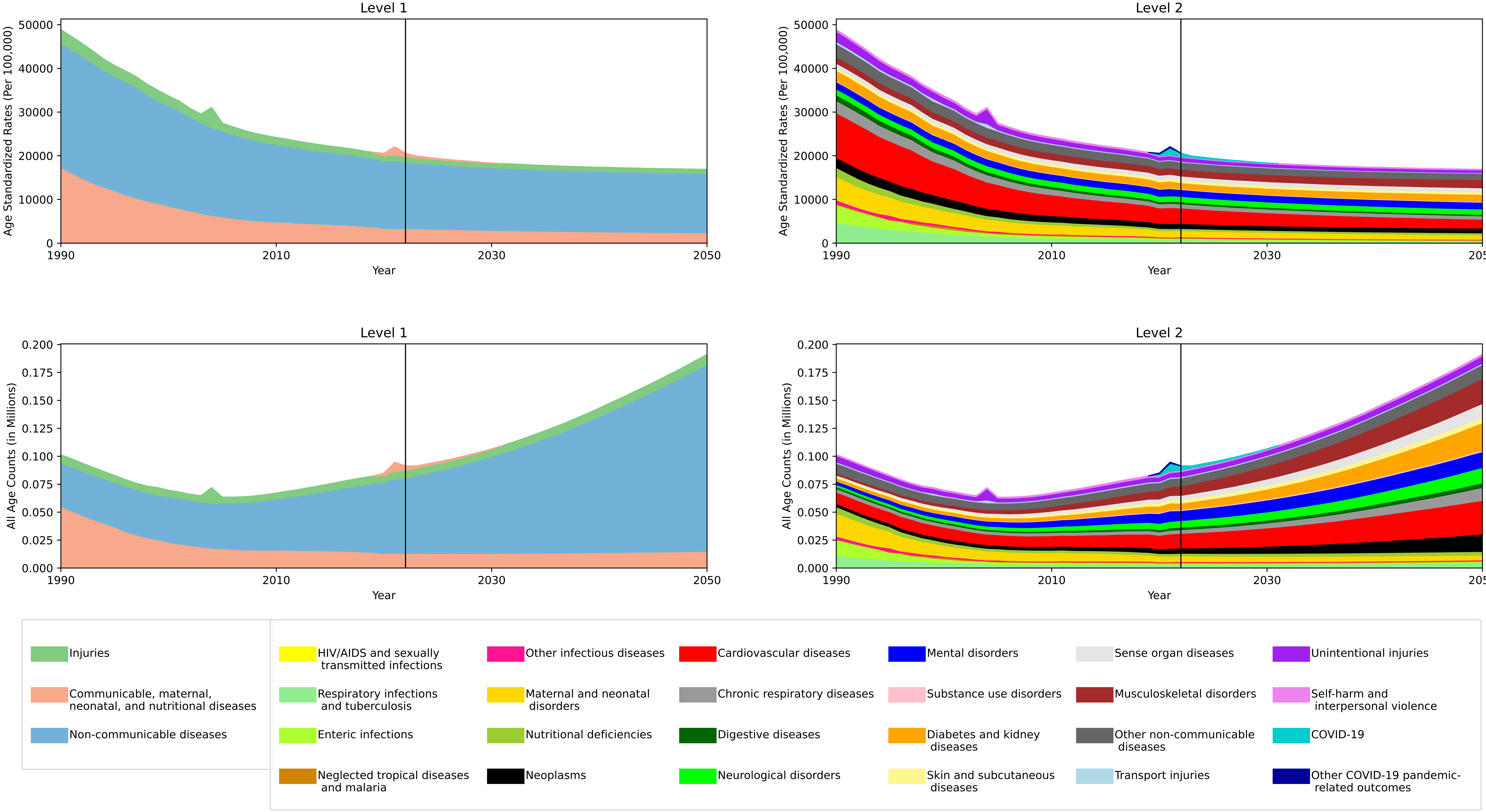
Level 1
Level 2
Age Standardized Rates (Per 100,000)
All Age Counts (in Millions)
Year
Injuries
Communicable, maternal, neonatal, and nutritional diseases
Non-communicable diseases
HIV/AIDS and sexually transmitted infections
Respiratory infections and tuberculosis
Enteric infections
Neglected tropical diseases and malaria
Other infectious diseases
Maternal and neonatal disorders
Nutritional deficiencies
Neoplasms
Cardiovascular diseases
Chronic respiratory diseases
Digestive diseases
Neurological disorders
Mental disorders
Substance use disorders
Diabetes and kidney diseases
Skin and subcutaneous diseases
Sense organ diseases
Musculoskeletal disorders
Other non-communicable diseases
Transport injuries
Unintentional injuries
Self-harm and interpersonal violence
COVID-19
Other COVID-19 pandemic-related outcomes

Mauritius

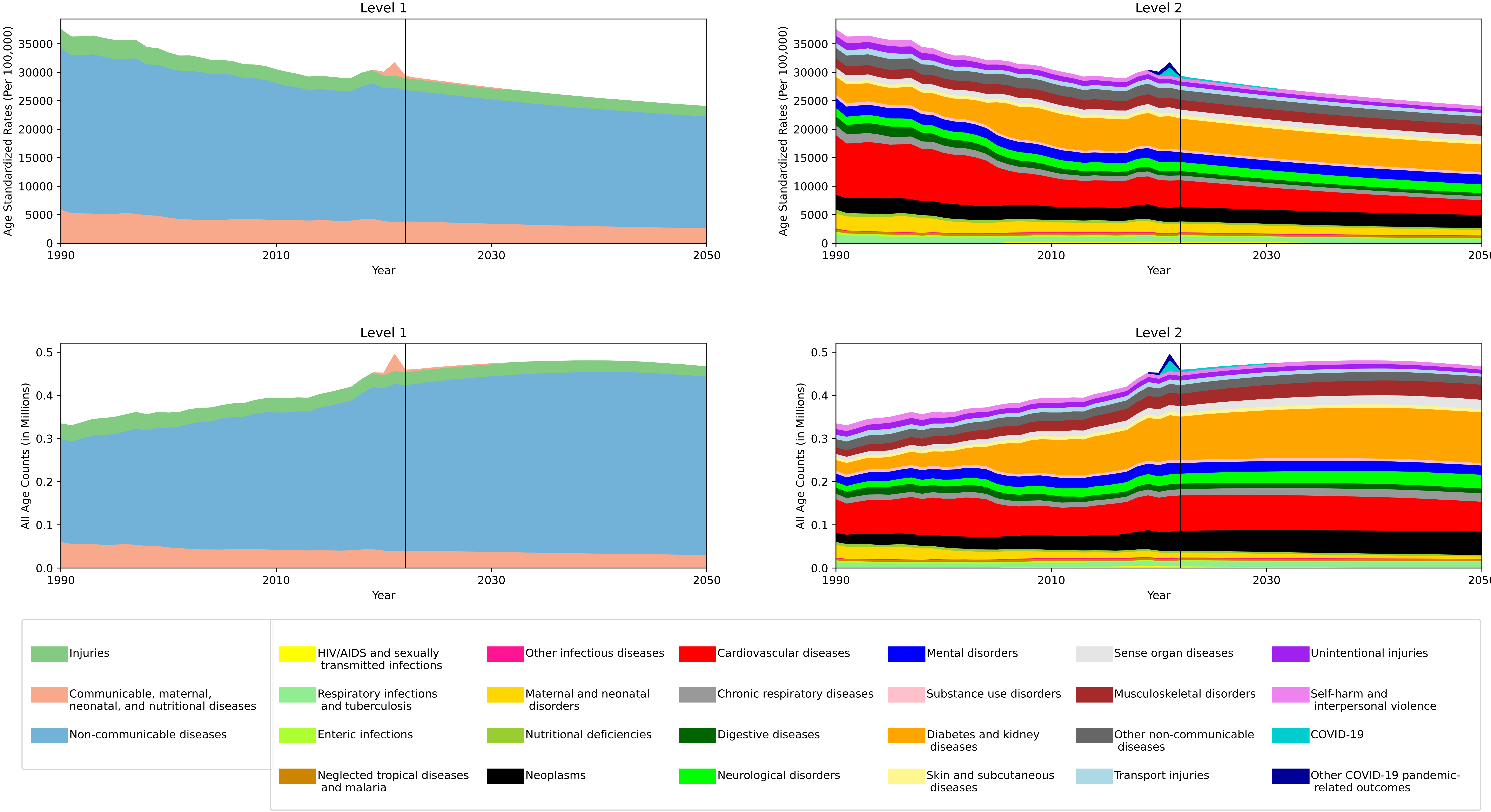
Level 1
Level 2
Age Standardized Rates (Per 100,000)
All Age Counts (in Millions)
Year
1990
2010
2030
2050
Injuries
Communicable, maternal, neonatal, and nutritional diseases
Non-communicable diseases
HIV/AIDS and sexually transmitted infections
Respiratory infections and tuberculosis
Enteric infections
Neglected tropical diseases and malaria
Other infectious diseases
Maternal and neonatal disorders
Nutritional deficiencies
Neoplasms
Cardiovascular diseases
Chronic respiratory diseases
Digestive diseases
Neurological disorders
Mental disorders
Substance use disorders
Diabetes and kidney diseases
Skin and subcutaneous diseases
Sense organ diseases
Musculoskeletal disorders
Other non-communicable diseases
Transport injuries
Unintentional injuries
Self-harm and interpersonal violence
COVID-19
Other COVID-19 pandemic-related outcomes

Myanmar

Supplemental Results Figure S4. Age-standardised DALY rates and all age DALY counts and by Level 1 and Level 2 causes, 1990–2050

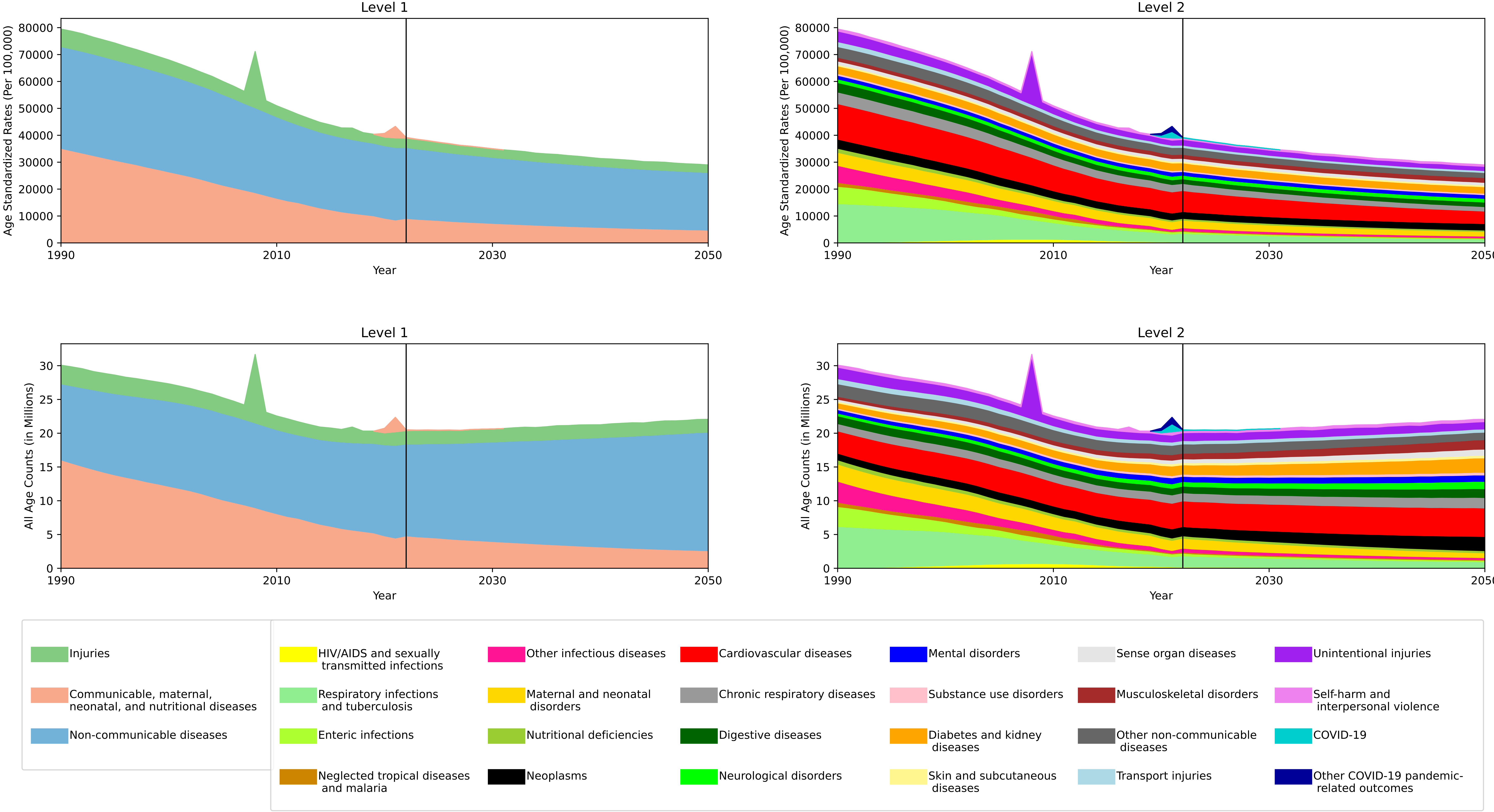

Philippines

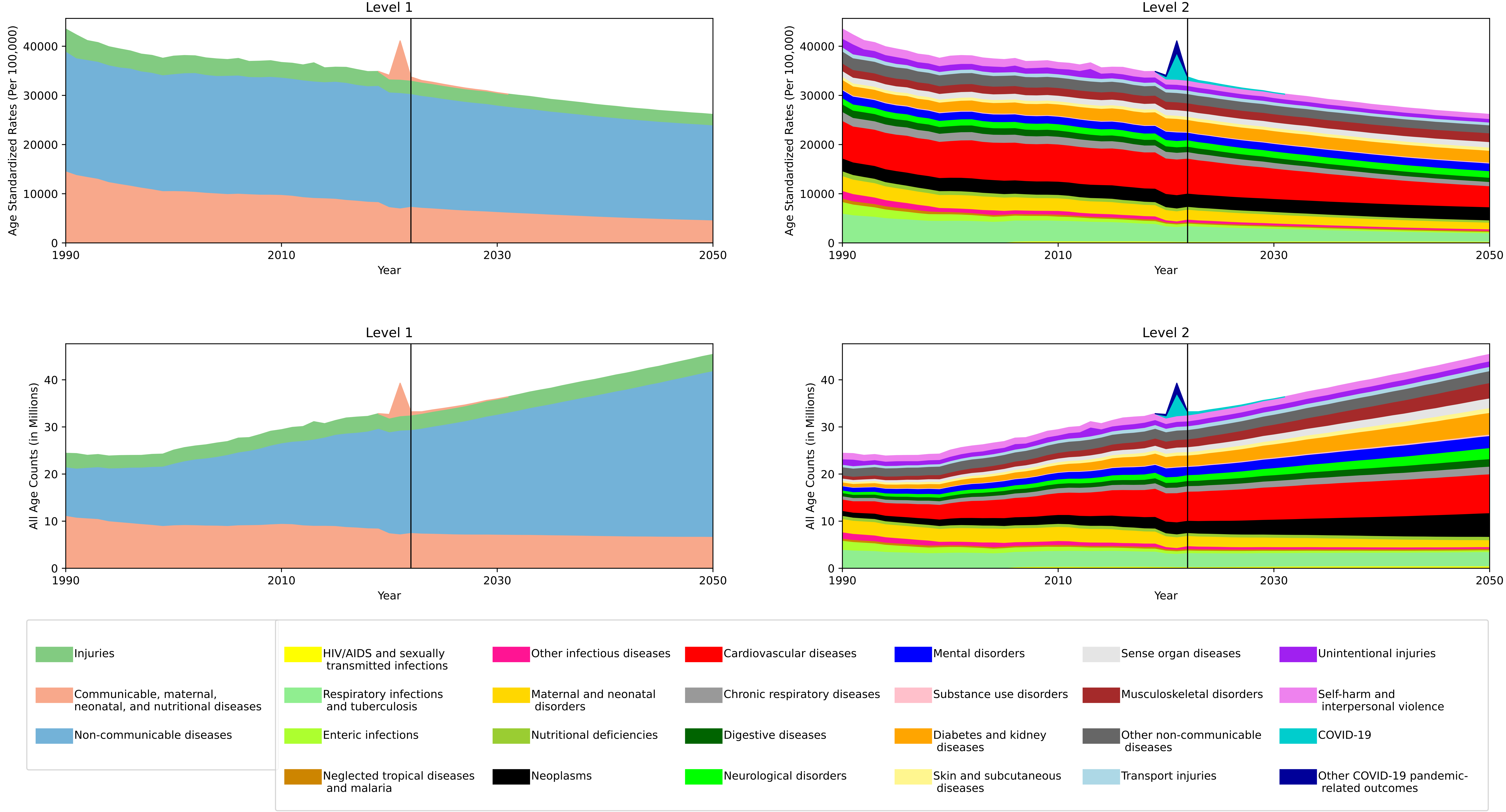

Seychelles

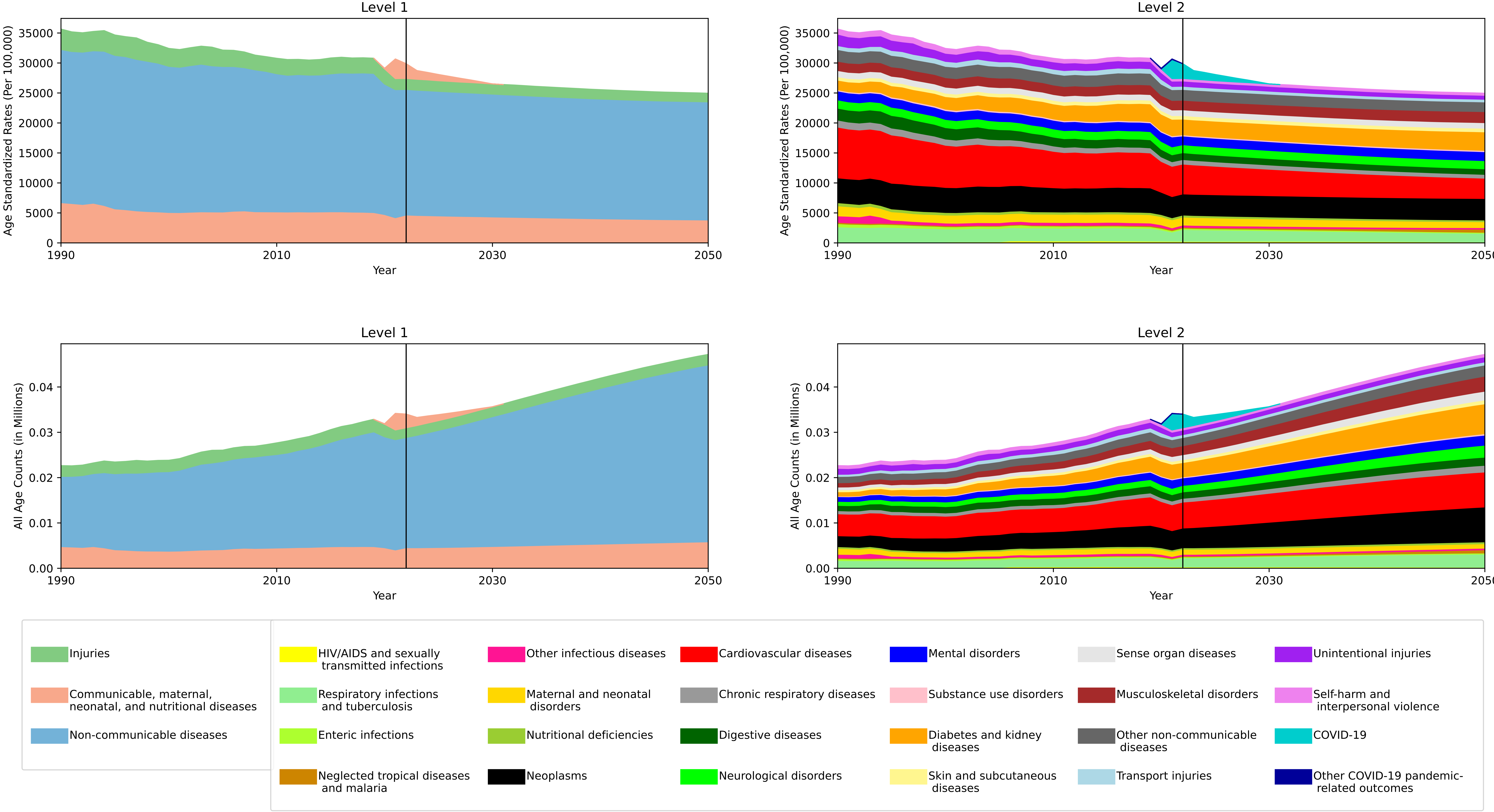
Level 1
Level 2
Age Standardized Rates (Per 100,000)
All Age Counts (in Millions)
Year
1990
2010
2030
2050
35000
30000
25000
20000
15000
10000
5000
0
0.04
0.03
0.02
0.01
0.00
Injuries
Communicable, maternal, neonatal, and nutritional diseases
Non-communicable diseases
HIV/AIDS and sexually transmitted infections
Respiratory infections and tuberculosis
Enteric infections
Neglected tropical diseases and malaria
Other infectious diseases
Maternal and neonatal disorders
Nutritional deficiencies
Neoplasms
Cardiovascular diseases
Chronic respiratory diseases
Digestive diseases
Neurological disorders
Mental disorders
Substance use disorders
Diabetes and kidney diseases
Skin and subcutaneous diseases
Sense organ diseases
Musculoskeletal disorders
Other non-communicable diseases
Transport injuries
Unintentional injuries
Self-harm and interpersonal violence
COVID-19
Other COVID-19 pandemic-related outcomes

Sri Lanka

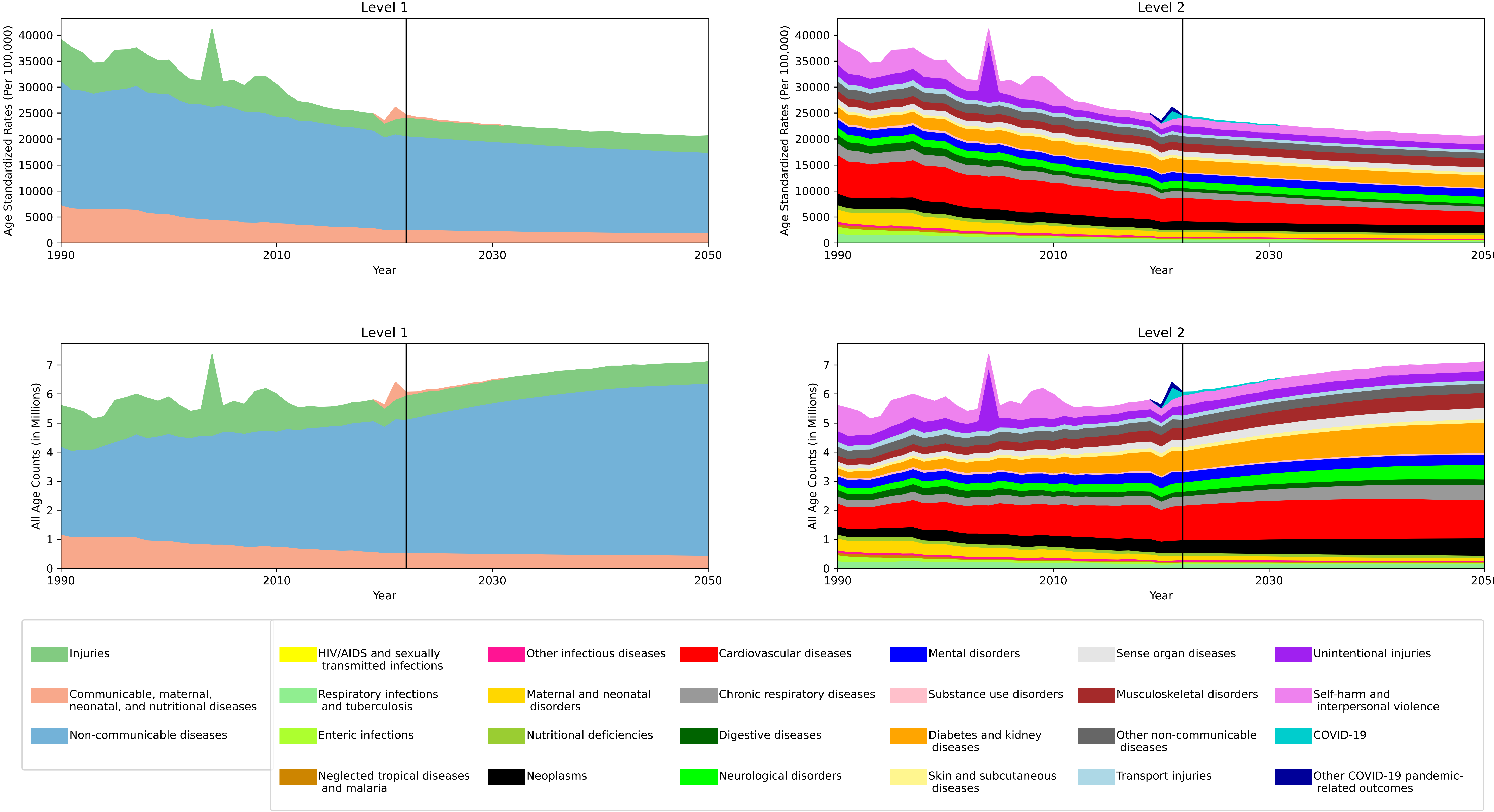
Level 1
Level 2
Age Standardized Rates (Per 100,000)
All Age Counts (in Millions)
Year
1990
2010
2030
2050
Injuries
Communicable, maternal, neonatal, and nutritional diseases
Non-communicable diseases
HIV/AIDS and sexually transmitted infections
Respiratory infections and tuberculosis
Enteric infections
Neglected tropical diseases and malaria
Other infectious diseases
Maternal and neonatal disorders
Nutritional deficiencies
Neoplasms
Cardiovascular diseases
Chronic respiratory diseases
Digestive diseases
Neurological disorders
Mental disorders
Substance use disorders
Diabetes and kidney diseases
Skin and subcutaneous diseases
Sense organ diseases
Musculoskeletal disorders
Other non-communicable diseases
Transport injuries
Unintentional injuries
Self-harm and interpersonal violence
COVID-19
Other COVID-19 pandemic-related outcomes

Supplemental Results Figure S4. Age-standardised DALY rates and all age DALY counts and by Level 1 and Level 2 causes, 1990–2050

Thailand

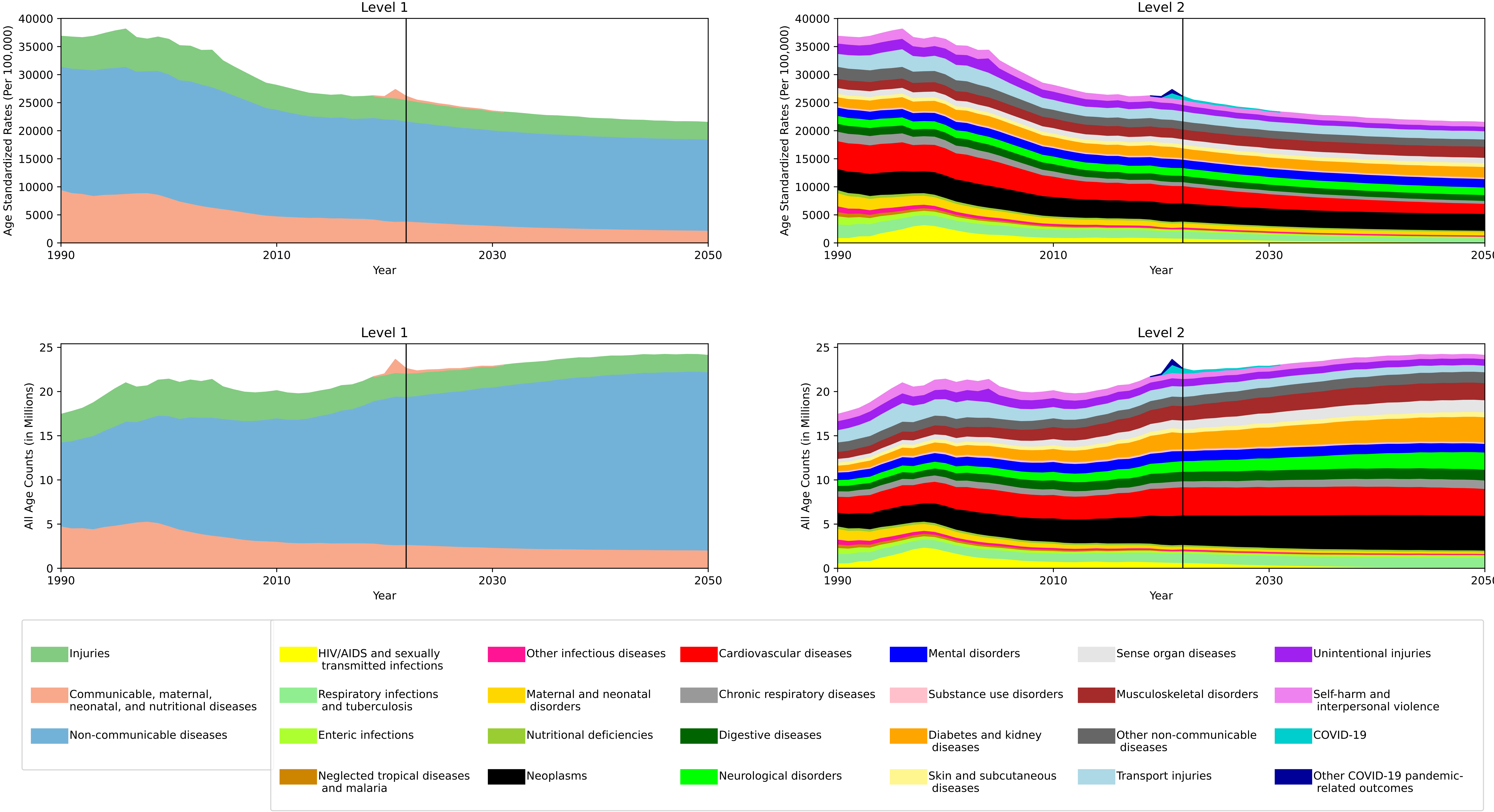

Timor-Leste

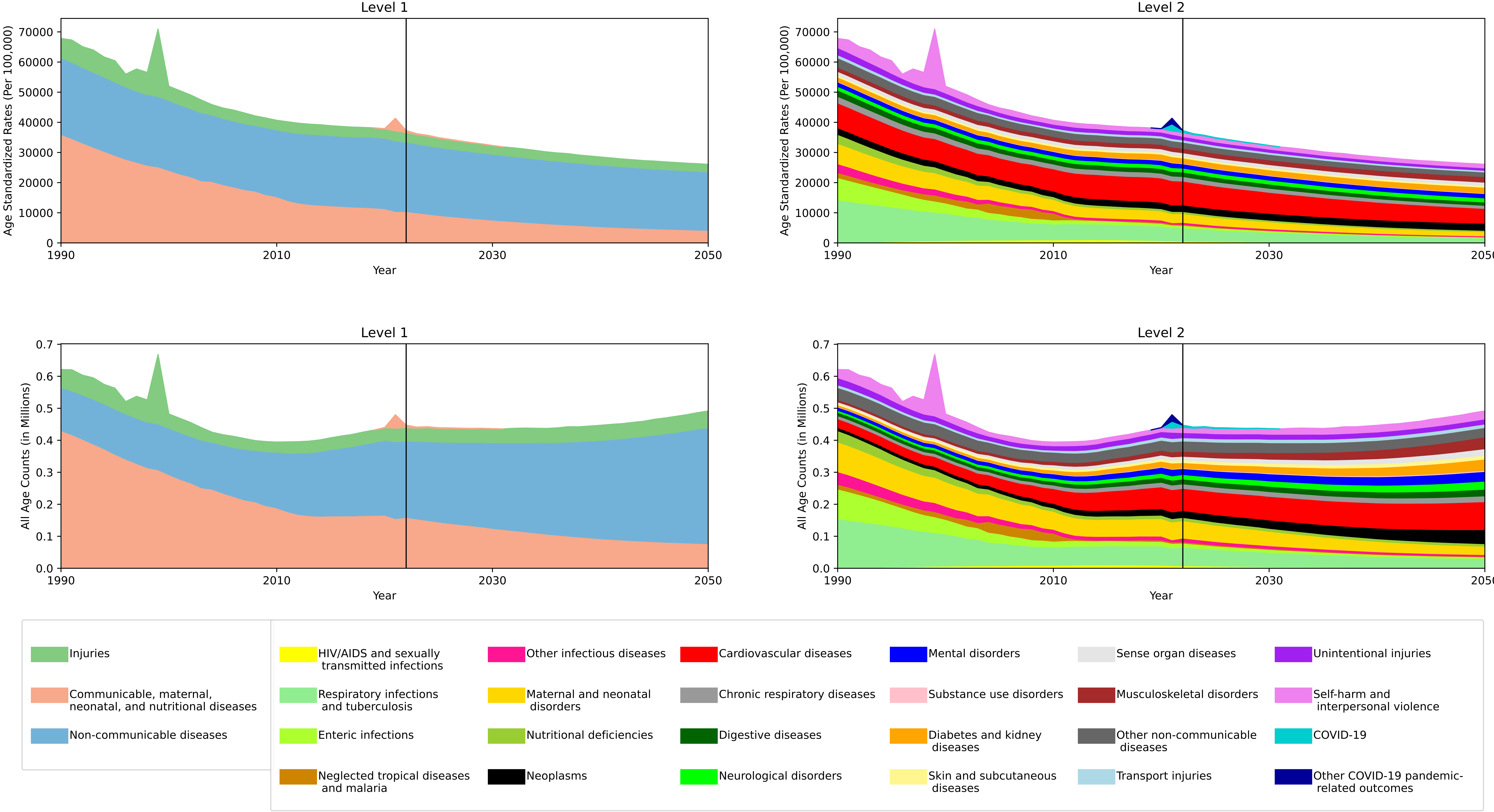
Level 1
Age Standardized Rates (Per 100,000)
Year
Level 2
Age Standardized Rates (Per 100,000)
Year
Level 1
All Age Counts (in Millions)
Year
Level 2
All Age Counts (in Millions)
Year
Injuries
Communicable, maternal, neonatal, and nutritional diseases
Non-communicable diseases
HIV/AIDS and sexually transmitted infections
Respiratory infections and tuberculosis
Enteric infections
Neglected tropical diseases and malaria
Other infectious diseases
Maternal and neonatal disorders
Nutritional deficiencies
Neoplasms
Cardiovascular diseases
Chronic respiratory diseases
Digestive diseases
Neurological disorders
Mental disorders
Substance use disorders
Diabetes and kidney diseases
Skin and subcutaneous diseases
Sense organ diseases
Musculoskeletal disorders
Other non-communicable diseases
Transport injuries
Unintentional injuries
Self-harm and interpersonal violence
COVID-19
Other COVID-19 pandemic-related outcomes

Viet Nam

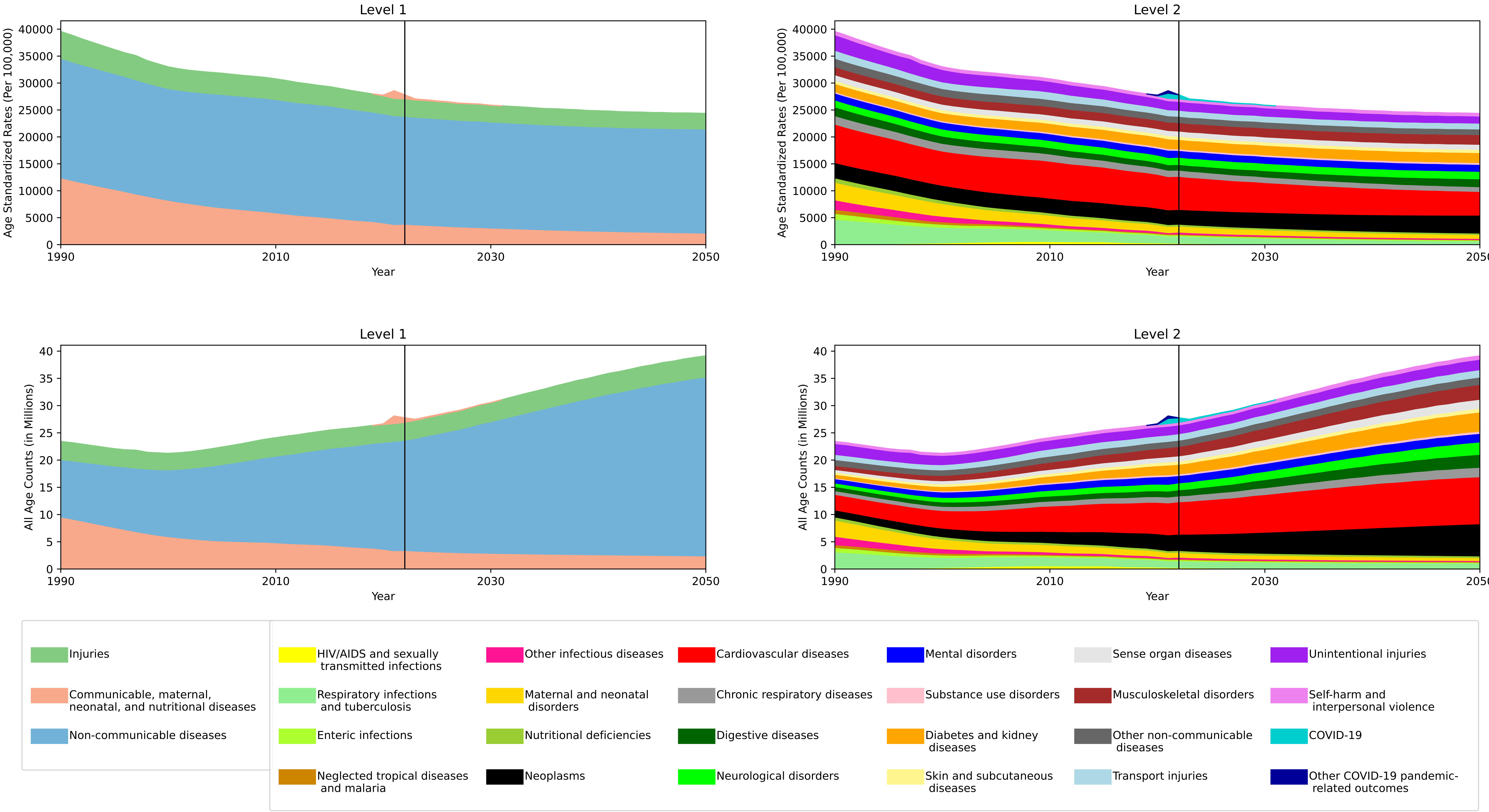

Level 1
Level 2
Age Standardized Rates (Per 100,000)
All Age Counts (in Millions)
Year
1990
2010
2030
2050
Injuries
Communicable, maternal, neonatal, and nutritional diseases
Non-communicable diseases
HIV/AIDS and sexually transmitted infections
Respiratory infections and tuberculosis
Enteric infections
Neglected tropical diseases and malaria
Other infectious diseases
Maternal and neonatal disorders
Nutritional deficiencies
Neoplasms
Cardiovascular diseases
Chronic respiratory diseases
Digestive diseases
Neurological disorders
Mental disorders
Substance use disorders
Diabetes and kidney diseases
Skin and subcutaneous diseases
Sense organ diseases
Musculoskeletal disorders
Other non-communicable diseases
Transport injuries
Unintentional injuries
Self-harm and interpersonal violence
COVID-19
Other COVID-19 pandemic-related outcomes

Angola

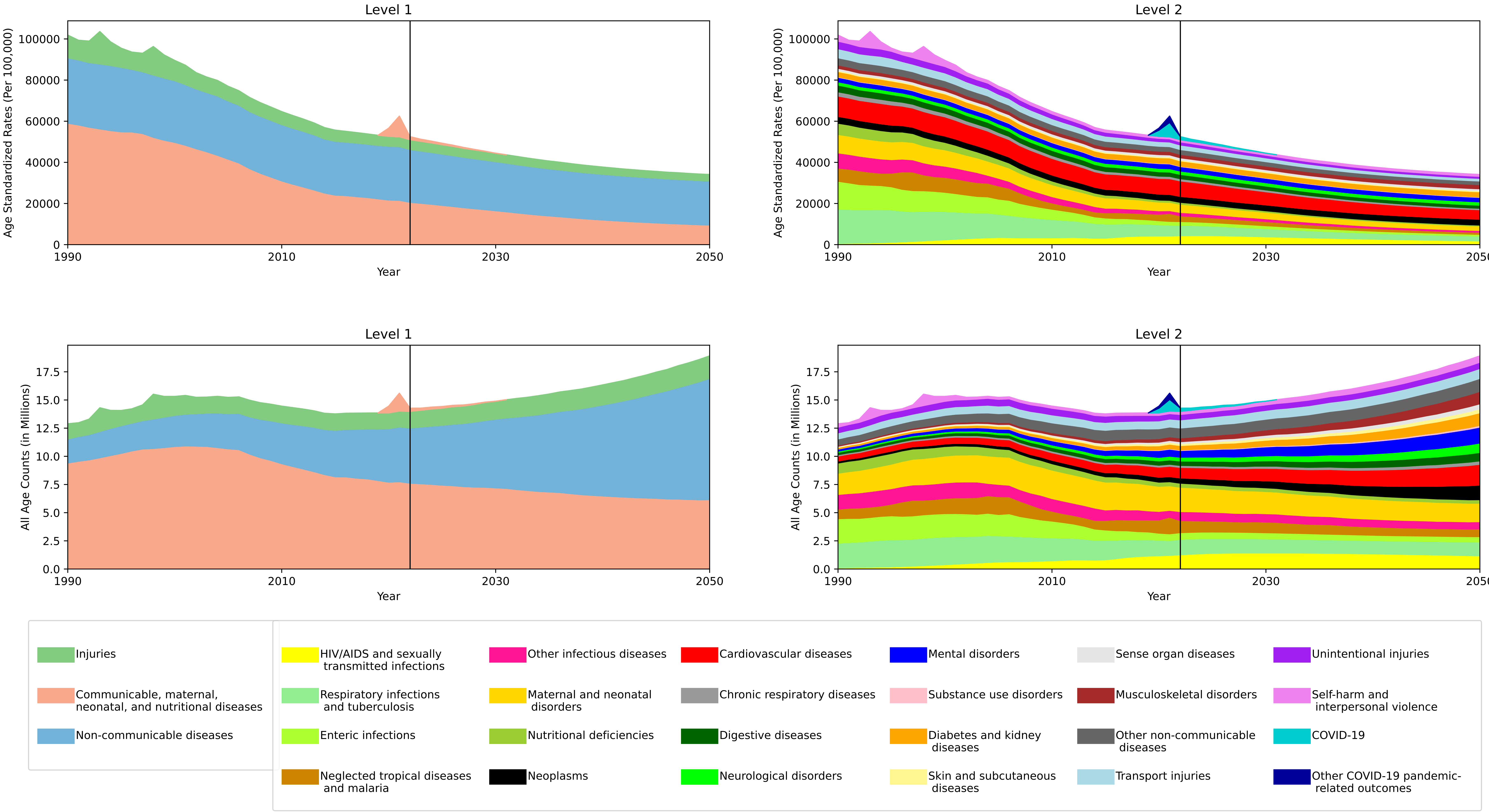
Level 1
Level 2
Age Standardized Rates (Per 100,000)
All Age Counts (in Millions)
Year
1990
2010
2030
2050
Injuries
Communicable, maternal, neonatal, and nutritional diseases
Non-communicable diseases
HIV/AIDS and sexually transmitted infections
Respiratory infections and tuberculosis
Enteric infections
Neglected tropical diseases and malaria
Other infectious diseases
Maternal and neonatal disorders
Nutritional deficiencies
Neoplasms
Cardiovascular diseases
Chronic respiratory diseases
Digestive diseases
Neurological disorders
Mental disorders
Substance use disorders
Diabetes and kidney diseases
Skin and subcutaneous diseases
Sense organ diseases
Musculoskeletal disorders
Other non-communicable diseases
Transport injuries
Unintentional injuries
Self-harm and interpersonal violence
COVID-19
Other COVID-19 pandemic-related outcomes

Supplemental Results Figure S4. Age-standardised DALY rates and all age DALY counts and by Level 1 and Level 2 causes, 1990–2050

Central African Republic

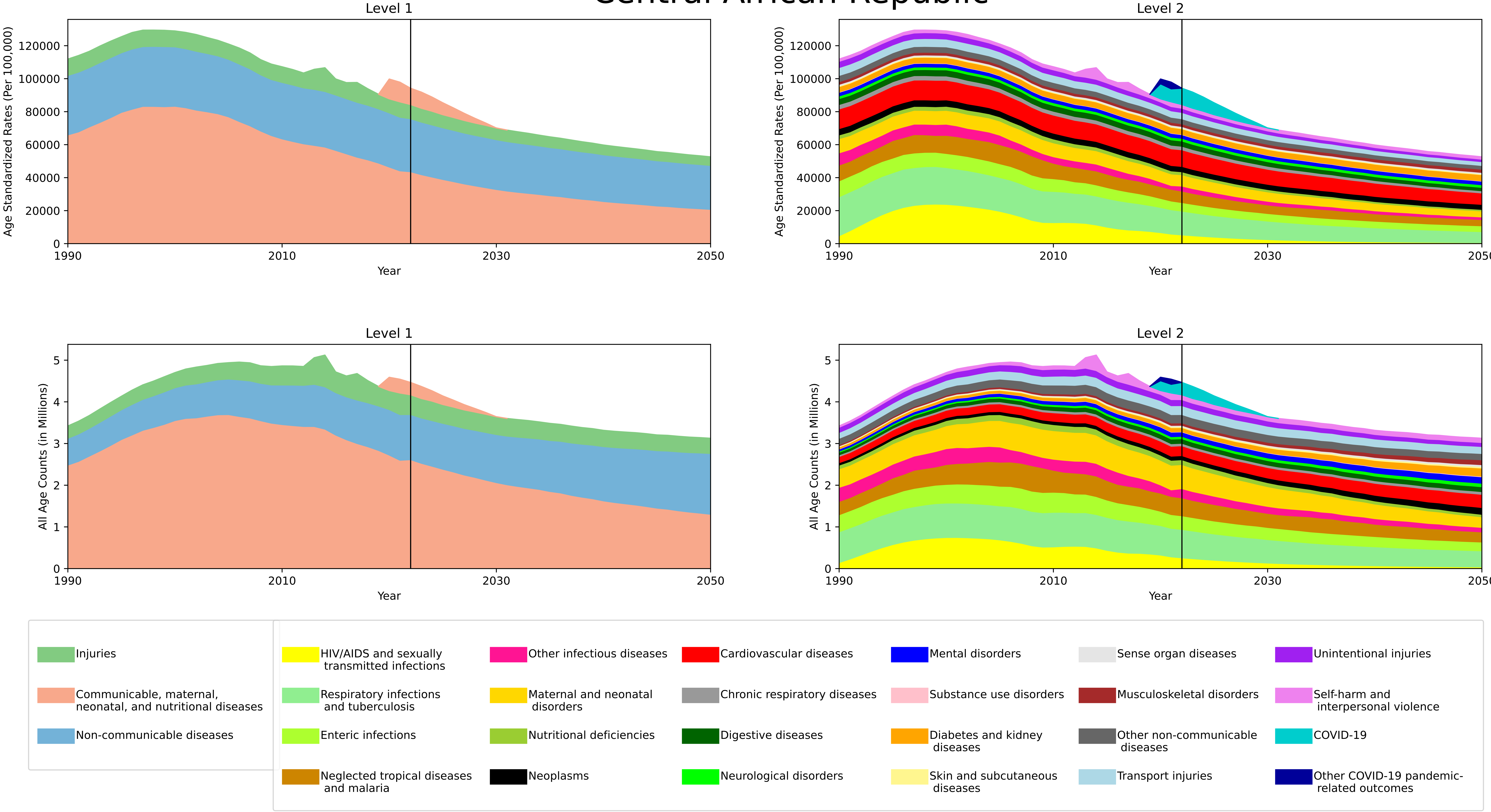

Congo (Brazzaville)

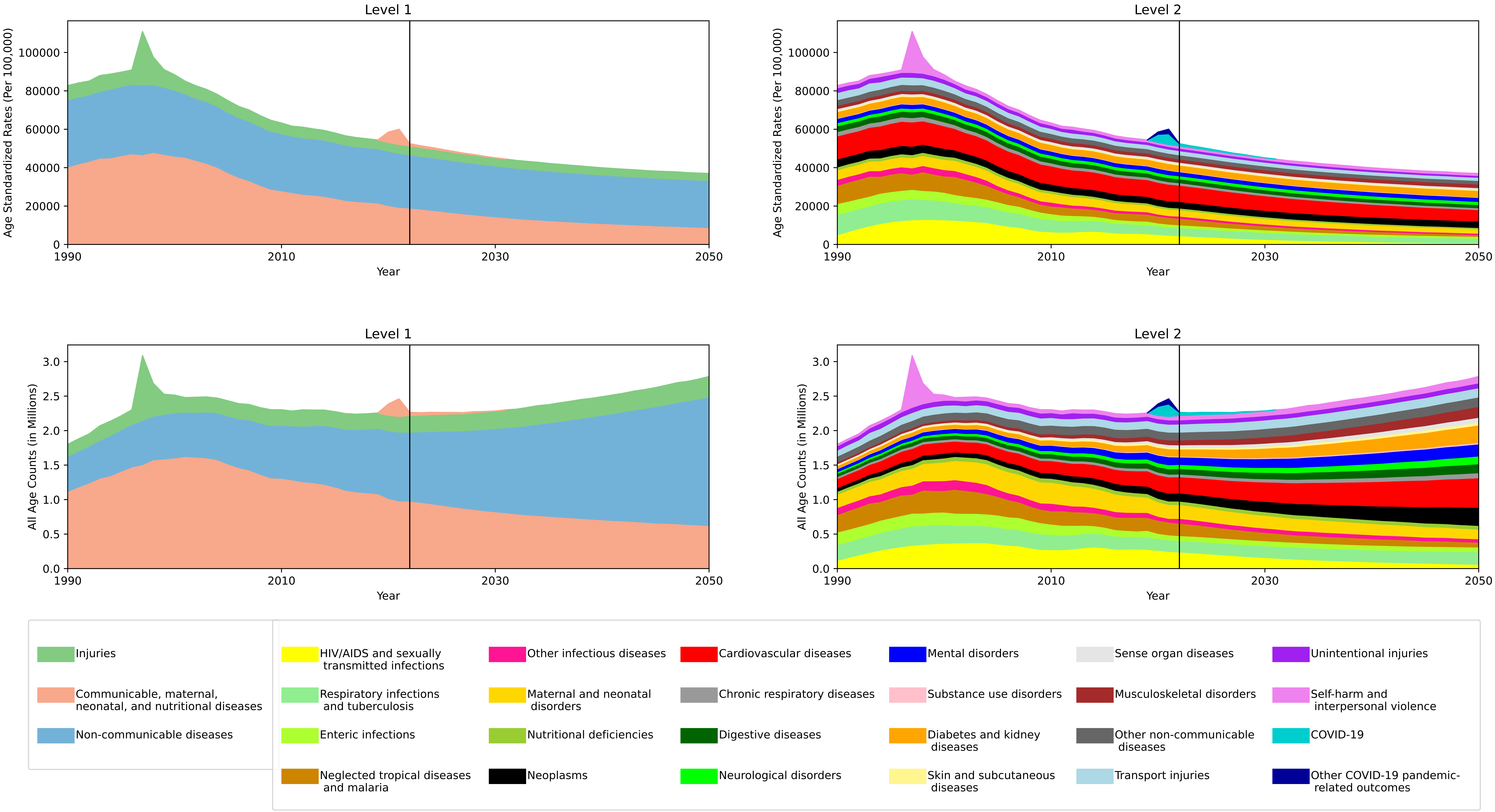

DR Congo

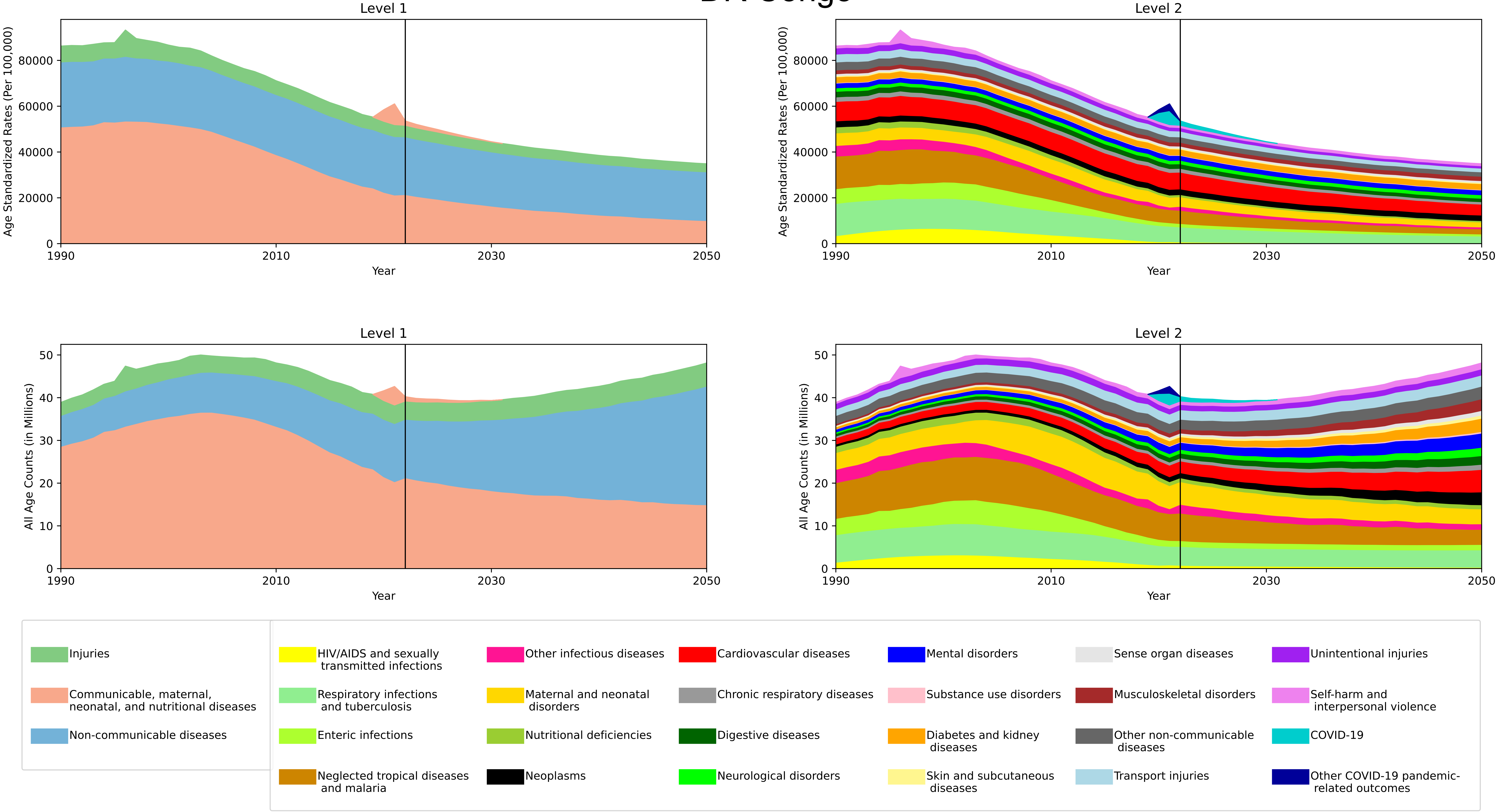
Level 1
Level 2
Age Standardized Rates (Per 100,000)
All Age Counts (in Millions)
Year
1990
2010
2030
2050
Injuries
Communicable, maternal, neonatal, and nutritional diseases
Non-communicable diseases
HIV/AIDS and sexually transmitted infections
Respiratory infections and tuberculosis
Enteric infections
Neglected tropical diseases and malaria
Other infectious diseases
Maternal and neonatal disorders
Nutritional deficiencies
Neoplasms
Cardiovascular diseases
Chronic respiratory diseases
Digestive diseases
Neurological disorders
Mental disorders
Substance use disorders
Diabetes and kidney diseases
Skin and subcutaneous diseases
Sense organ diseases
Musculoskeletal disorders
Other non-communicable diseases
Transport injuries
Unintentional injuries
Self-harm and interpersonal violence
COVID-19
Other COVID-19 pandemic-related outcomes

Equatorial Guinea

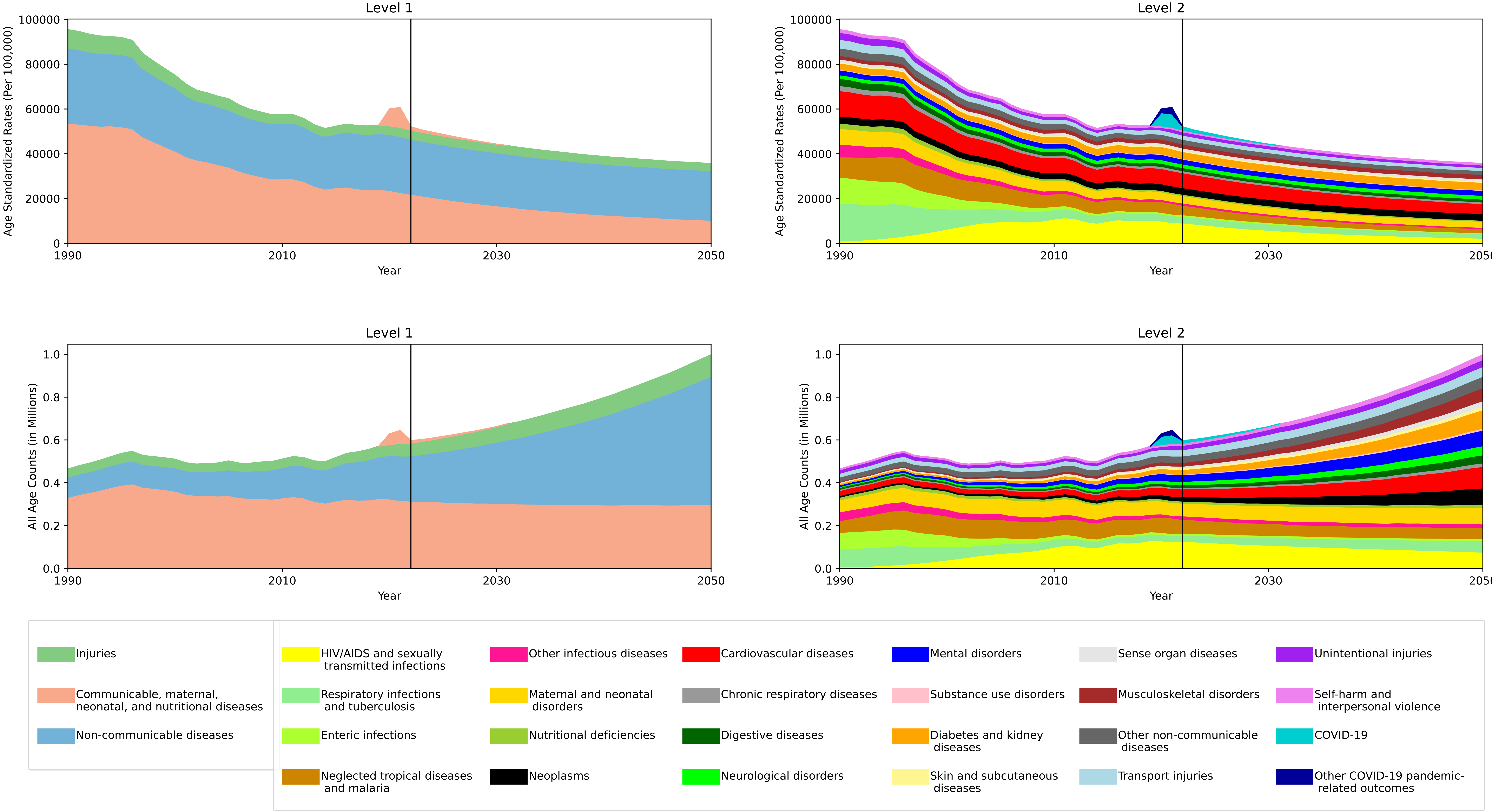

Gabon

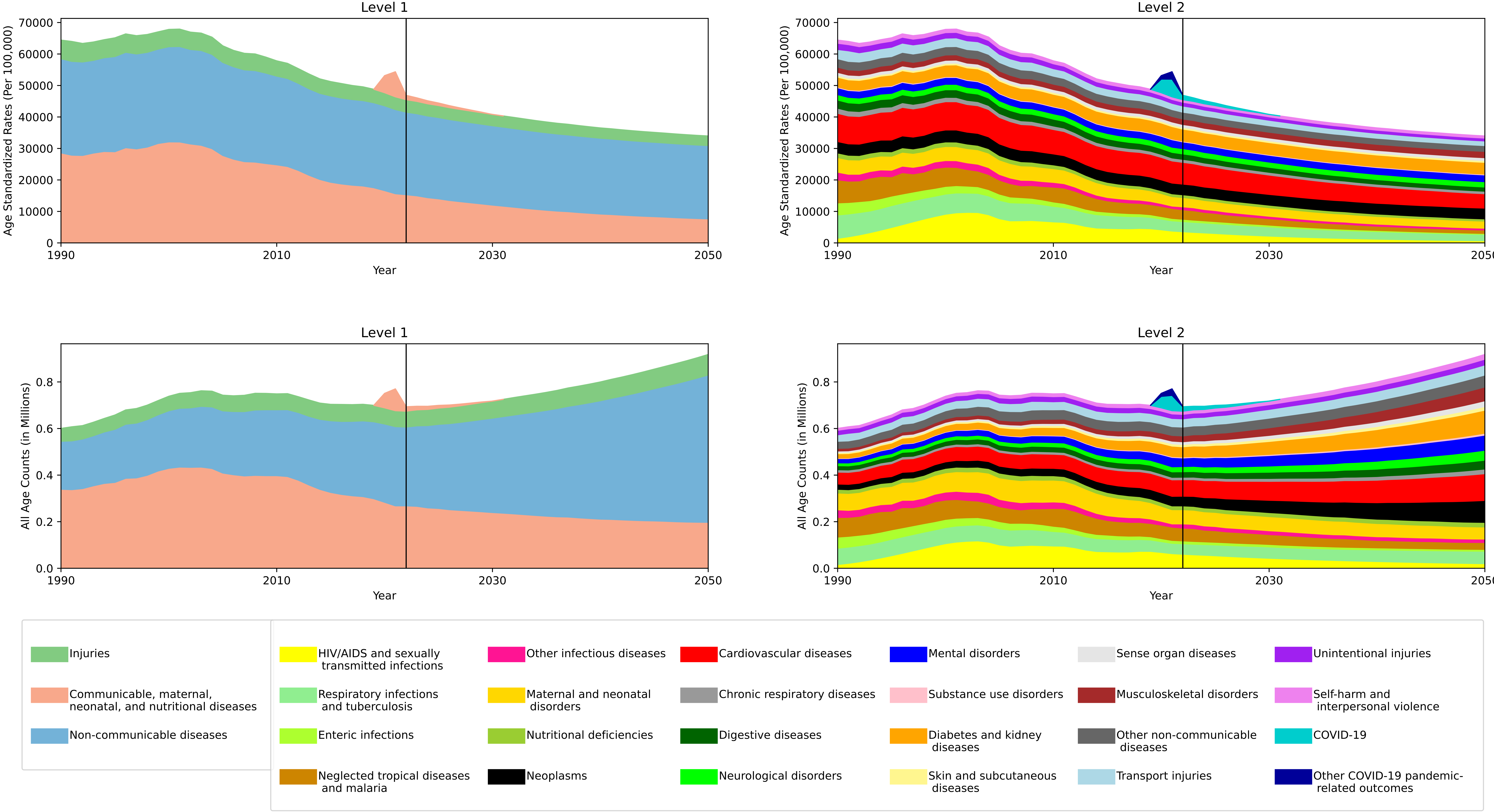
Level 1
Level 2
Age Standardized Rates (Per 100,000)
All Age Counts (in Millions)
Year
70000
60000
50000
40000
30000
20000
10000
0
0.8
0.6
0.4
0.2
0.0
1990
2010
2030
2050
Injuries
Communicable, maternal, neonatal, and nutritional diseases
Non-communicable diseases
HIV/AIDS and sexually transmitted infections
Respiratory infections and tuberculosis
Enteric infections
Neglected tropical diseases and malaria
Other infectious diseases
Maternal and neonatal disorders
Nutritional deficiencies
Neoplasms
Cardiovascular diseases
Chronic respiratory diseases
Digestive diseases
Neurological disorders
Mental disorders
Substance use disorders
Diabetes and kidney diseases
Skin and subcutaneous diseases
Sense organ diseases
Musculoskeletal disorders
Other non-communicable diseases
Transport injuries
Unintentional injuries
Self-harm and interpersonal violence
COVID-19
Other COVID-19 pandemic-related outcomes

Burundi

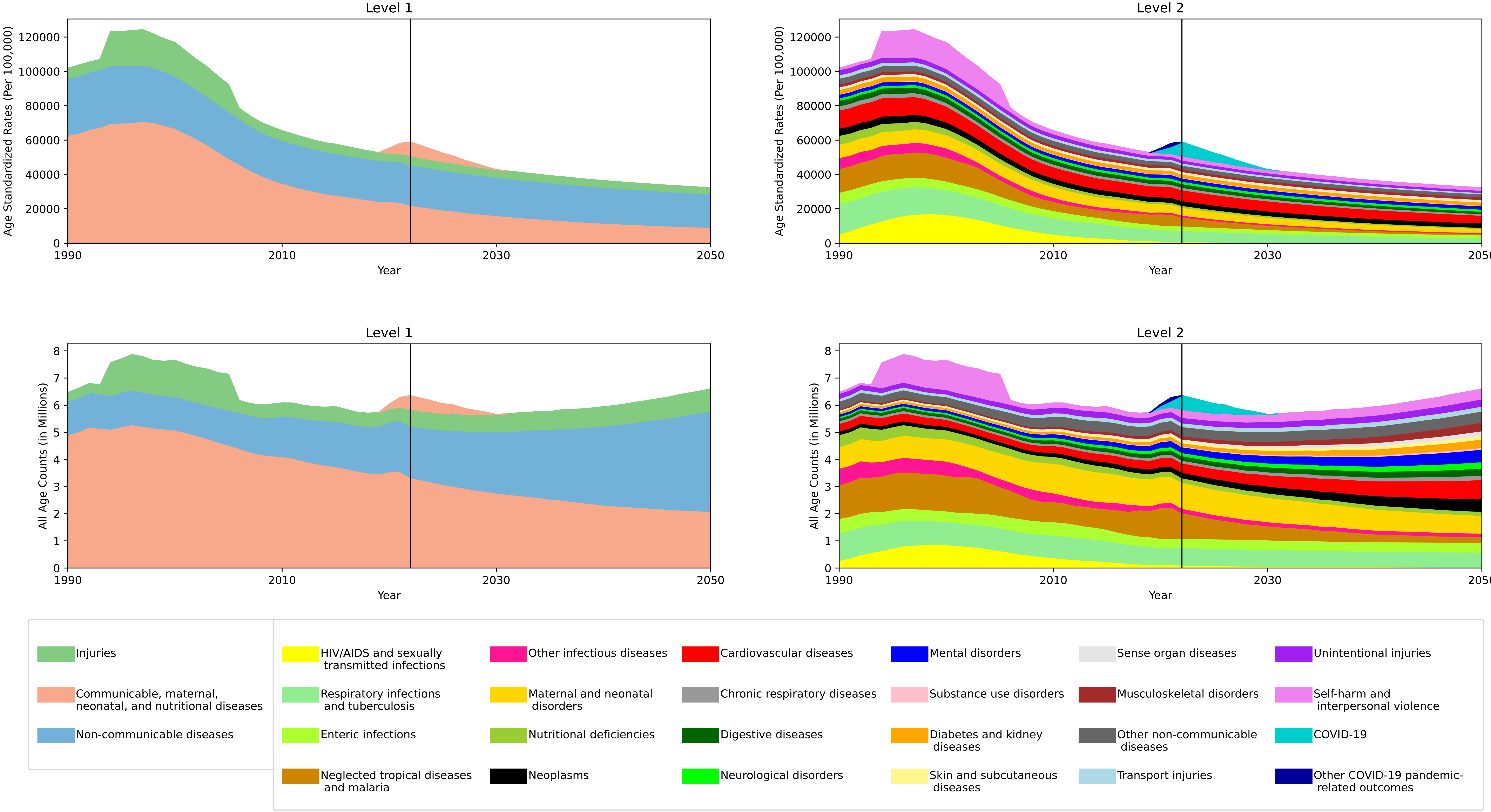
Level 1
Level 2
Age Standardized Rates (Per 100,000)
0
20000
40000
60000
80000
100000
120000
1990
2010
2030
2050
Year
Level 1
Level 2
All Age Counts (in Millions)
0
1
2
3
4
5
6
7
8
Injuries
Communicable, maternal, neonatal, and nutritional diseases
Non-communicable diseases
HIV/AIDS and sexually transmitted infections
Respiratory infections and tuberculosis
Enteric infections
Neglected tropical diseases and malaria
Other infectious diseases
Maternal and neonatal disorders
Nutritional deficiencies
Neoplasms
Cardiovascular diseases
Chronic respiratory diseases
Digestive diseases
Neurological disorders
Mental disorders
Substance use disorders
Diabetes and kidney diseases
Skin and subcutaneous diseases
Sense organ diseases
Musculoskeletal disorders
Other non-communicable diseases
Transport injuries
Unintentional injuries
Self-harm and interpersonal violence
COVID-19
Other COVID-19 pandemic-related outcomes

Comoros

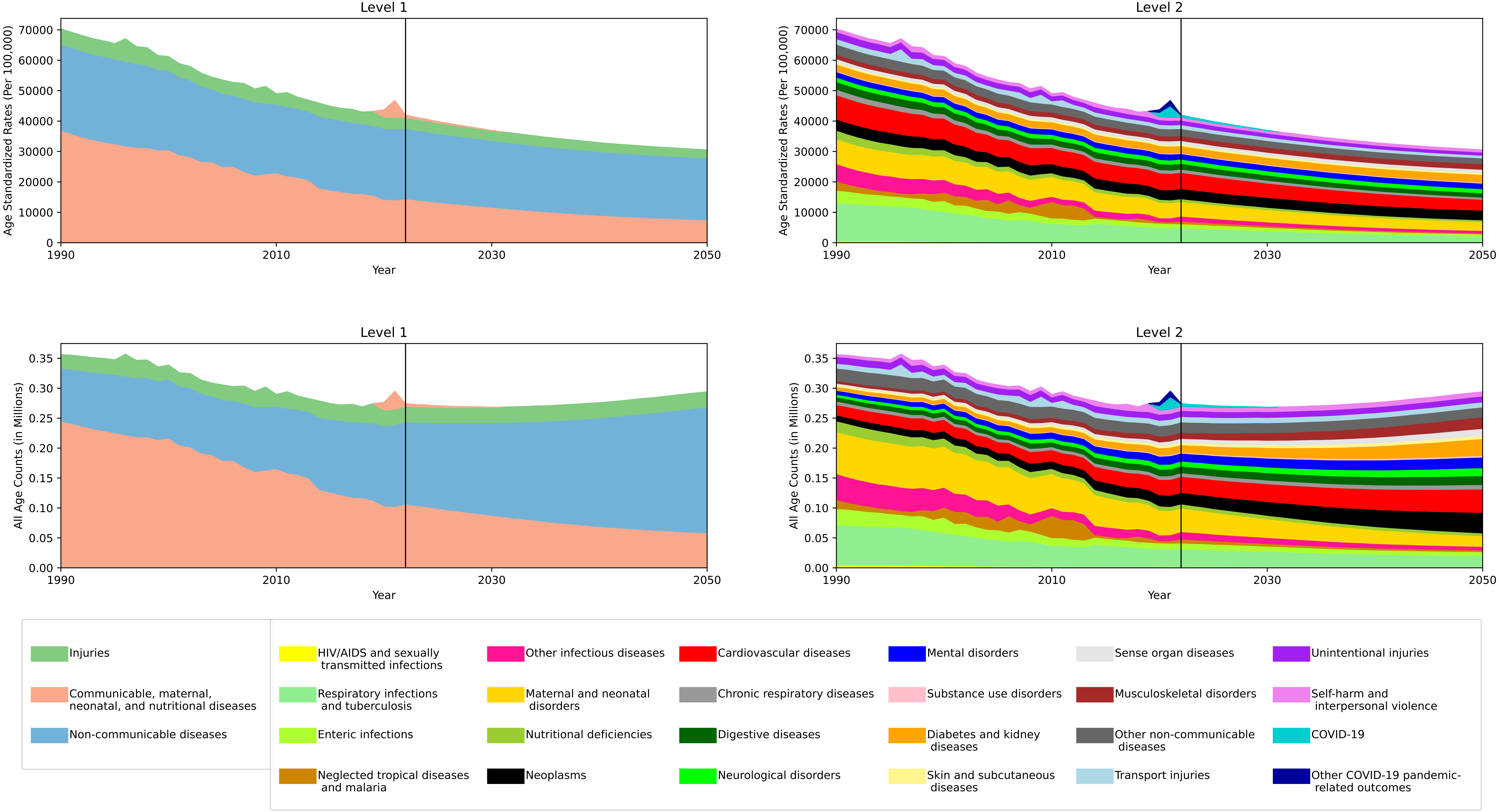
Level 1
Level 2
Age Standardized Rates (Per 100,000)
All Age Counts (in Millions)
Year
1990
2010
2030
2050
Injuries
Communicable, maternal, neonatal, and nutritional diseases
Non-communicable diseases
HIV/AIDS and sexually transmitted infections
Respiratory infections and tuberculosis
Enteric infections
Neglected tropical diseases and malaria
Other infectious diseases
Maternal and neonatal disorders
Nutritional deficiencies
Neoplasms
Cardiovascular diseases
Chronic respiratory diseases
Digestive diseases
Neurological disorders
Mental disorders
Substance use disorders
Diabetes and kidney diseases
Skin and subcutaneous diseases
Sense organ diseases
Musculoskeletal disorders
Other non-communicable diseases
Transport injuries
Unintentional injuries
Self-harm and interpersonal violence
COVID-19
Other COVID-19 pandemic-related outcomes

Djibouti

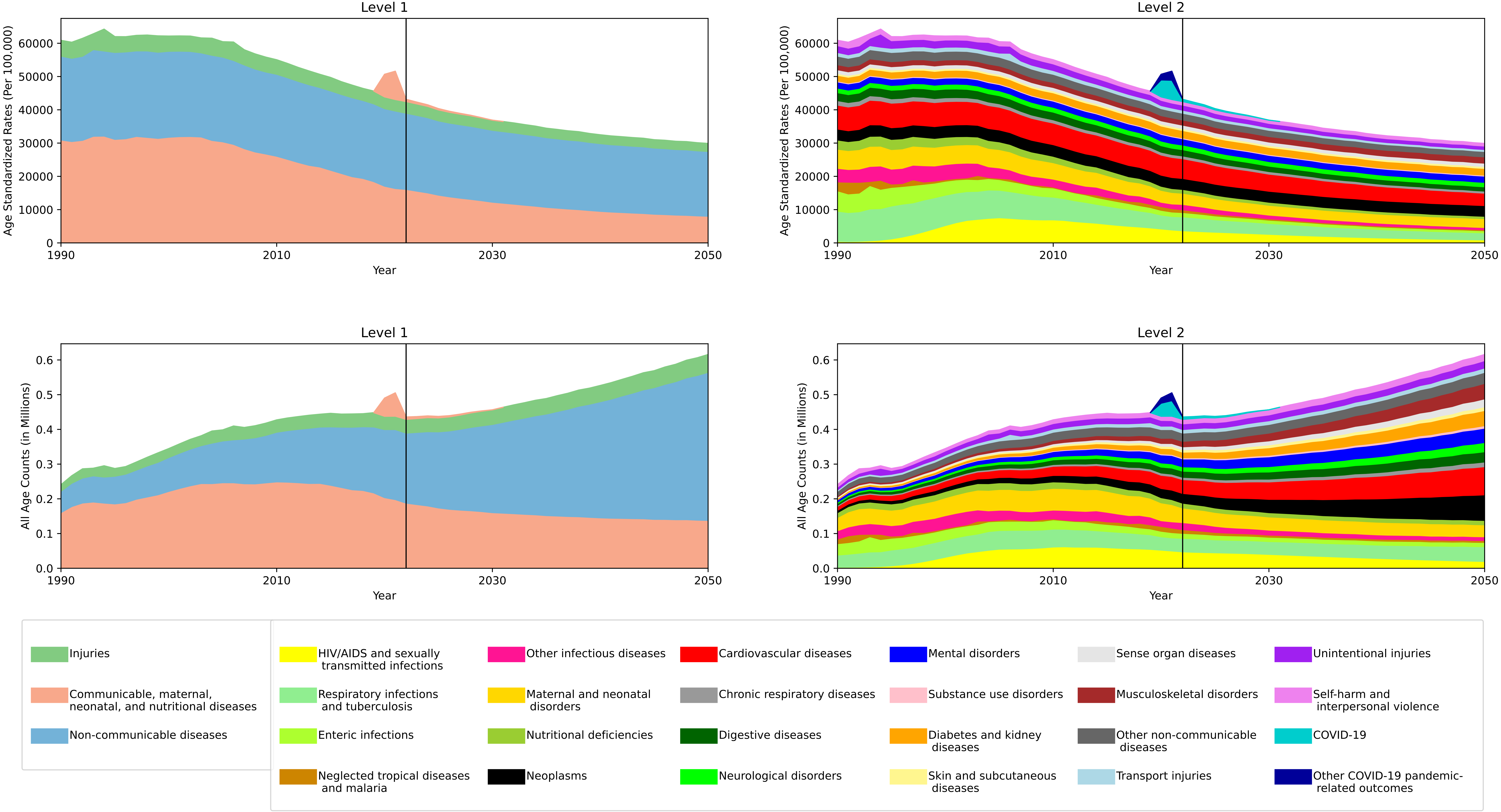
Level 1
Level 2
Age Standardized Rates (Per 100,000)
All Age Counts (in Millions)
Year
1990
2010
2030
2050
0
10000
20000
30000
40000
50000
60000
0.0
0.1
0.2
0.3
0.4
0.5
0.6
Injuries
Communicable, maternal, neonatal, and nutritional diseases
Non-communicable diseases
HIV/AIDS and sexually transmitted infections
Respiratory infections and tuberculosis
Enteric infections
Neglected tropical diseases and malaria
Other infectious diseases
Maternal and neonatal disorders
Nutritional deficiencies
Neoplasms
Cardiovascular diseases
Chronic respiratory diseases
Digestive diseases
Neurological disorders
Mental disorders
Substance use disorders
Diabetes and kidney diseases
Skin and subcutaneous diseases
Sense organ diseases
Musculoskeletal disorders
Other non-communicable diseases
Transport injuries
Unintentional injuries
Self-harm and interpersonal violence
COVID-19
Other COVID-19 pandemic-related outcomes

Eritrea

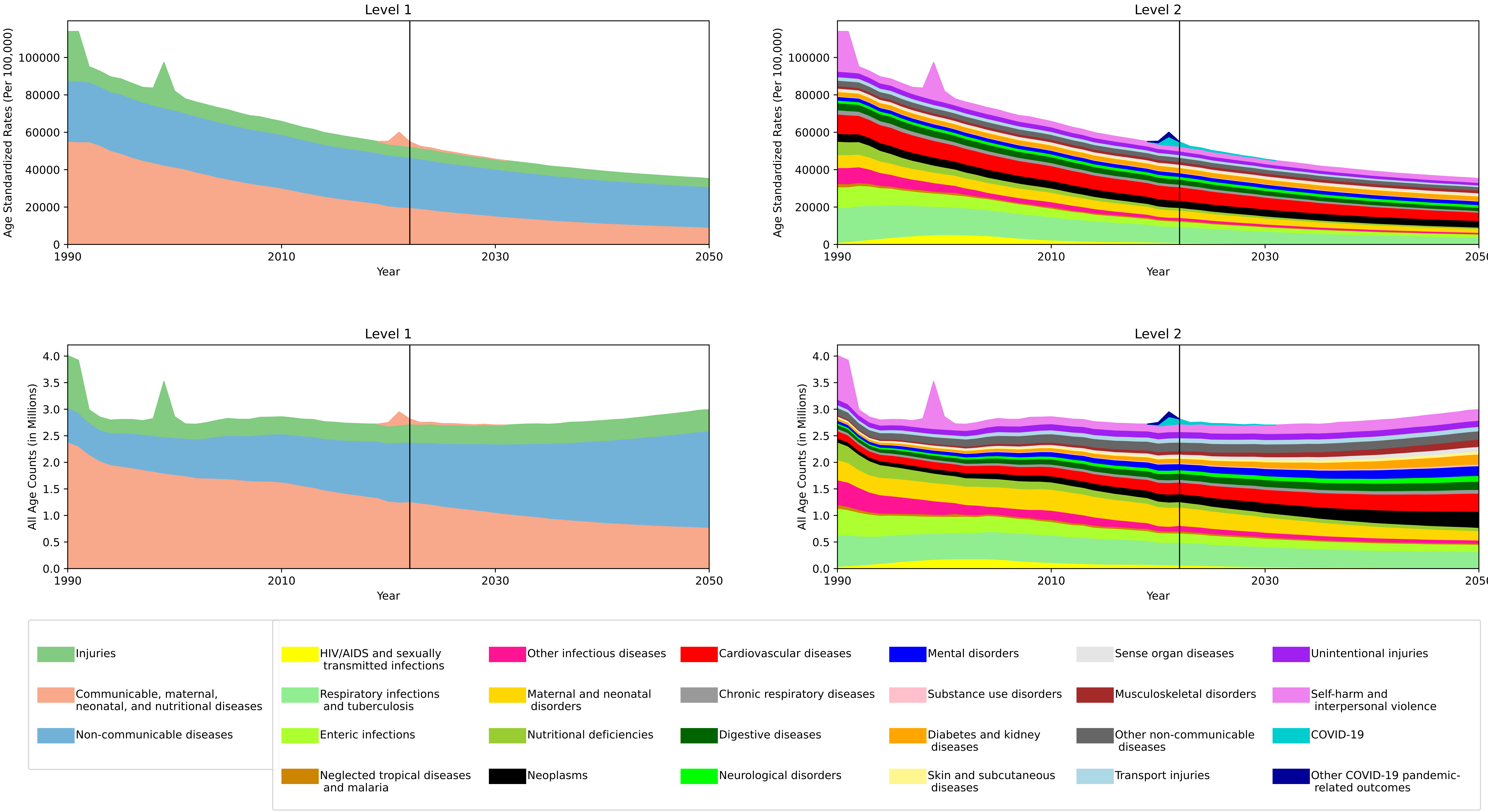
Level 1
Level 2
Age Standardized Rates (Per 100,000)
All Age Counts (in Millions)
Year
1990
2010
2030
2050
Injuries
Communicable, maternal, neonatal, and nutritional diseases
Non-communicable diseases
HIV/AIDS and sexually transmitted infections
Respiratory infections and tuberculosis
Enteric infections
Neglected tropical diseases and malaria
Other infectious diseases
Maternal and neonatal disorders
Nutritional deficiencies
Neoplasms
Cardiovascular diseases
Chronic respiratory diseases
Digestive diseases
Neurological disorders
Mental disorders
Substance use disorders
Diabetes and kidney diseases
Skin and subcutaneous diseases
Sense organ diseases
Musculoskeletal disorders
Other non-communicable diseases
Transport injuries
Unintentional injuries
Self-harm and interpersonal violence
COVID-19
Other COVID-19 pandemic-related outcomes

Ethiopia

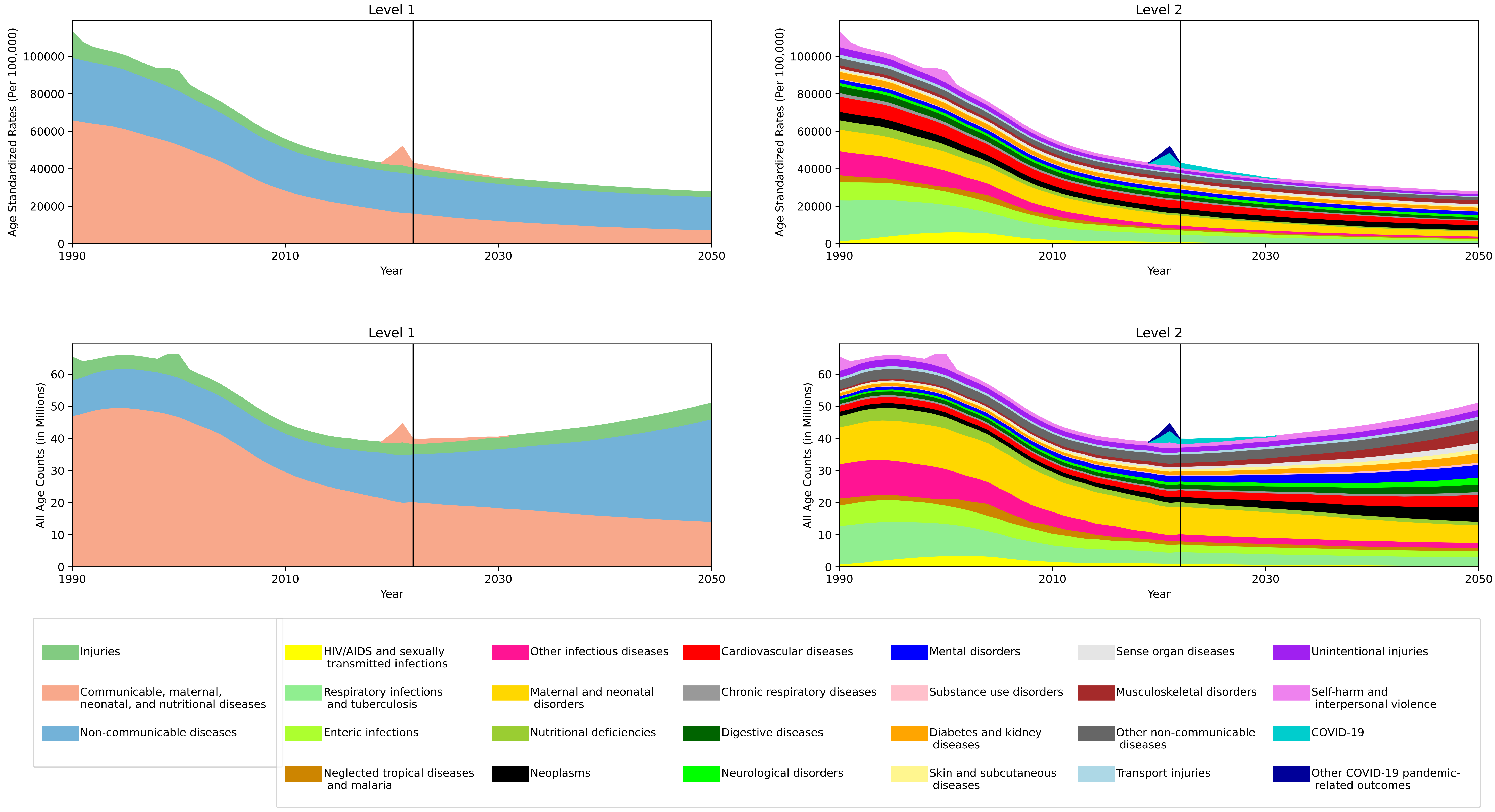
Level 1
Level 2
Age Standardized Rates (Per 100,000)
All Age Counts (in Millions)
Year
1990
2010
2030
2050
Injuries
Communicable, maternal, neonatal, and nutritional diseases
Non-communicable diseases
HIV/AIDS and sexually transmitted infections
Respiratory infections and tuberculosis
Enteric infections
Neglected tropical diseases and malaria
Other infectious diseases
Maternal and neonatal disorders
Nutritional deficiencies
Neoplasms
Cardiovascular diseases
Chronic respiratory diseases
Digestive diseases
Neurological disorders
Mental disorders
Substance use disorders
Diabetes and kidney diseases
Skin and subcutaneous diseases
Sense organ diseases
Musculoskeletal disorders
Other non-communicable diseases
Transport injuries
Unintentional injuries
Self-harm and interpersonal violence
COVID-19
Other COVID-19 pandemic-related outcomes

Kenya

Supplemental Results Figure S4. Age-standardised DALY rates and all age DALY counts and by Level 1 and Level 2 causes, 1990–2050

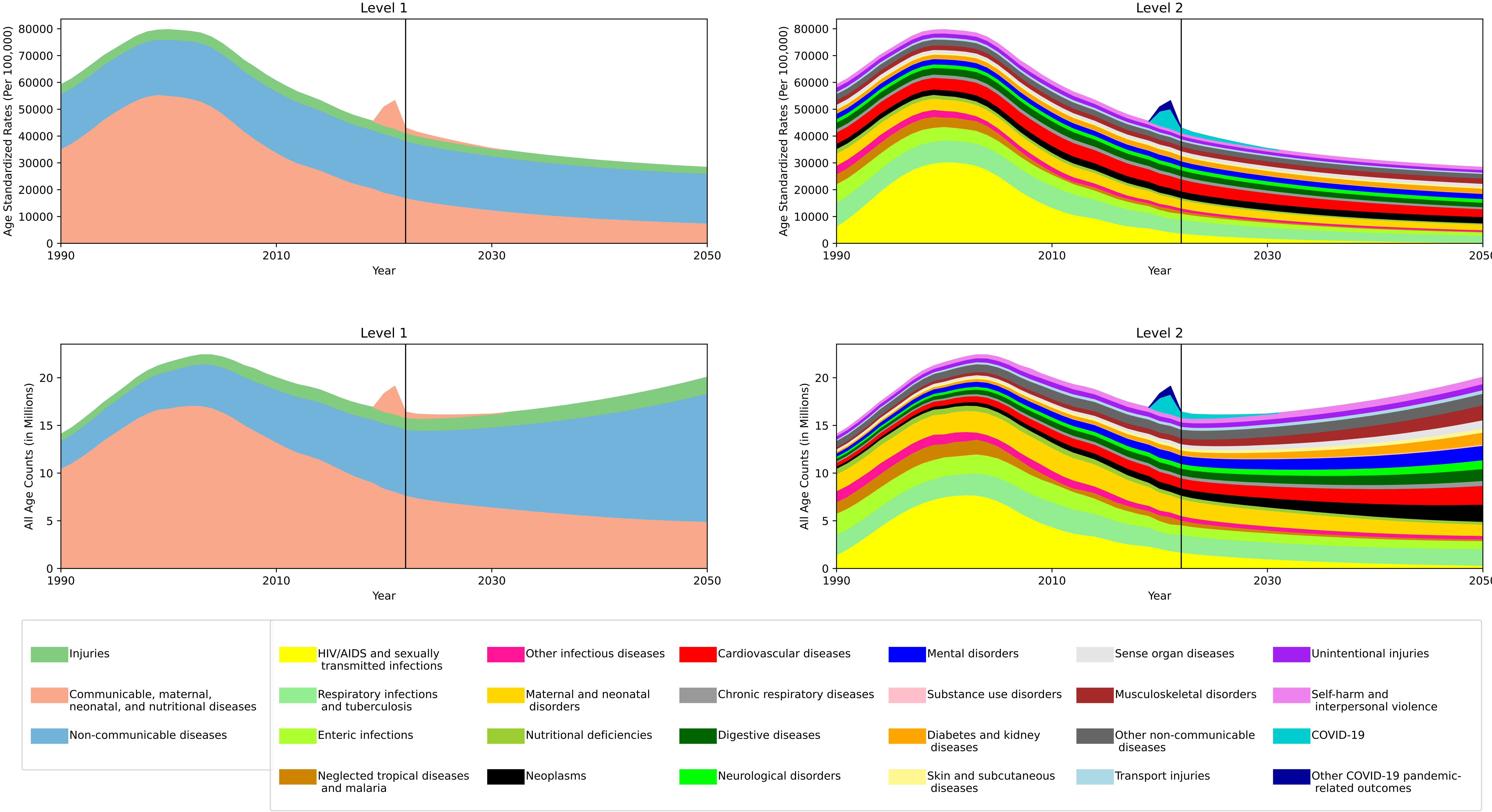

Madagascar

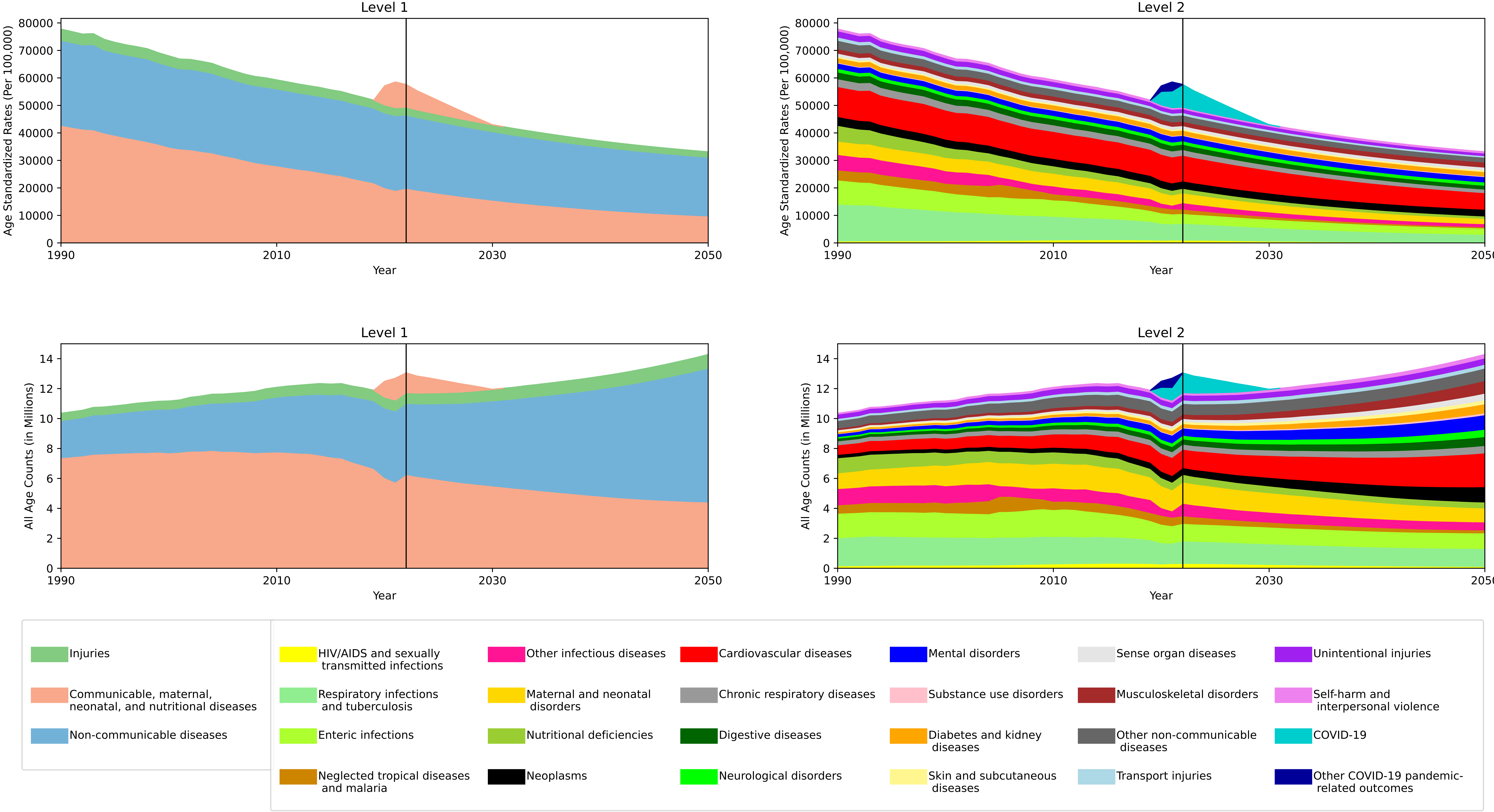
Level 1
Level 2
Age Standardized Rates (Per 100,000)
All Age Counts (in Millions)
Year
1990
2010
2030
2050
Injuries
Communicable, maternal, neonatal, and nutritional diseases
Non-communicable diseases
HIV/AIDS and sexually transmitted infections
Respiratory infections and tuberculosis
Enteric infections
Neglected tropical diseases and malaria
Other infectious diseases
Maternal and neonatal disorders
Nutritional deficiencies
Neoplasms
Cardiovascular diseases
Chronic respiratory diseases
Digestive diseases
Neurological disorders
Mental disorders
Substance use disorders
Diabetes and kidney diseases
Skin and subcutaneous diseases
Sense organ diseases
Musculoskeletal disorders
Other non-communicable diseases
Transport injuries
Unintentional injuries
Self-harm and interpersonal violence
COVID-19
Other COVID-19 pandemic-related outcomes

Malawi

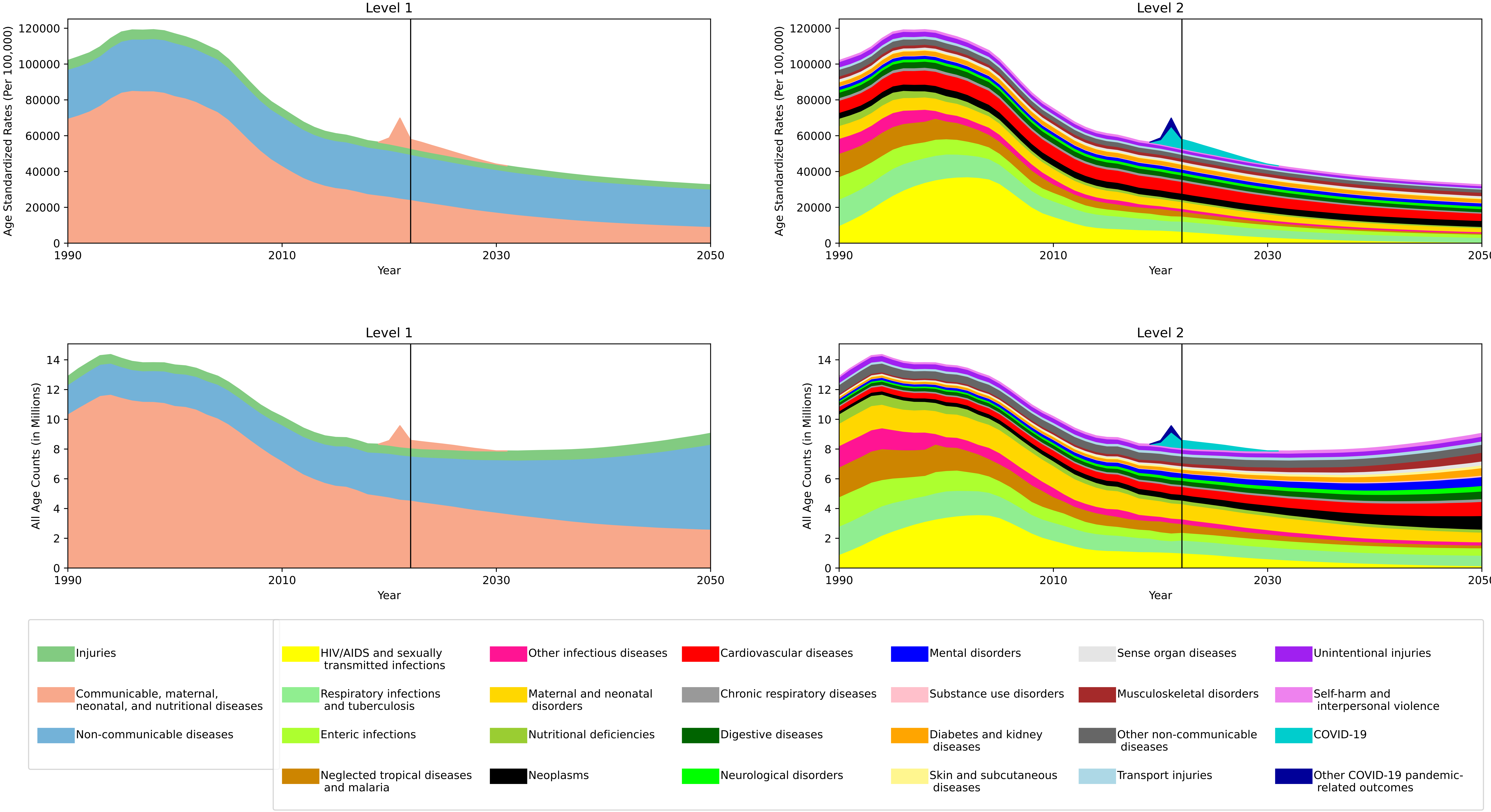
Level 1
Age Standardized Rates (Per 100,000)
Year
Level 2
Age Standardized Rates (Per 100,000)
Year
Level 1
All Age Counts (in Millions)
Year
Level 2
All Age Counts (in Millions)
Year
Injuries
Communicable, maternal, neonatal, and nutritional diseases
Non-communicable diseases
HIV/AIDS and sexually transmitted infections
Respiratory infections and tuberculosis
Enteric infections
Neglected tropical diseases and malaria
Other infectious diseases
Maternal and neonatal disorders
Nutritional deficiencies
Neoplasms
Cardiovascular diseases
Chronic respiratory diseases
Digestive diseases
Neurological disorders
Mental disorders
Substance use disorders
Diabetes and kidney diseases
Skin and subcutaneous diseases
Sense organ diseases
Musculoskeletal disorders
Other non-communicable diseases
Transport injuries
Unintentional injuries
Self-harm and interpersonal violence
COVID-19
Other COVID-19 pandemic-related outcomes

Mozambique

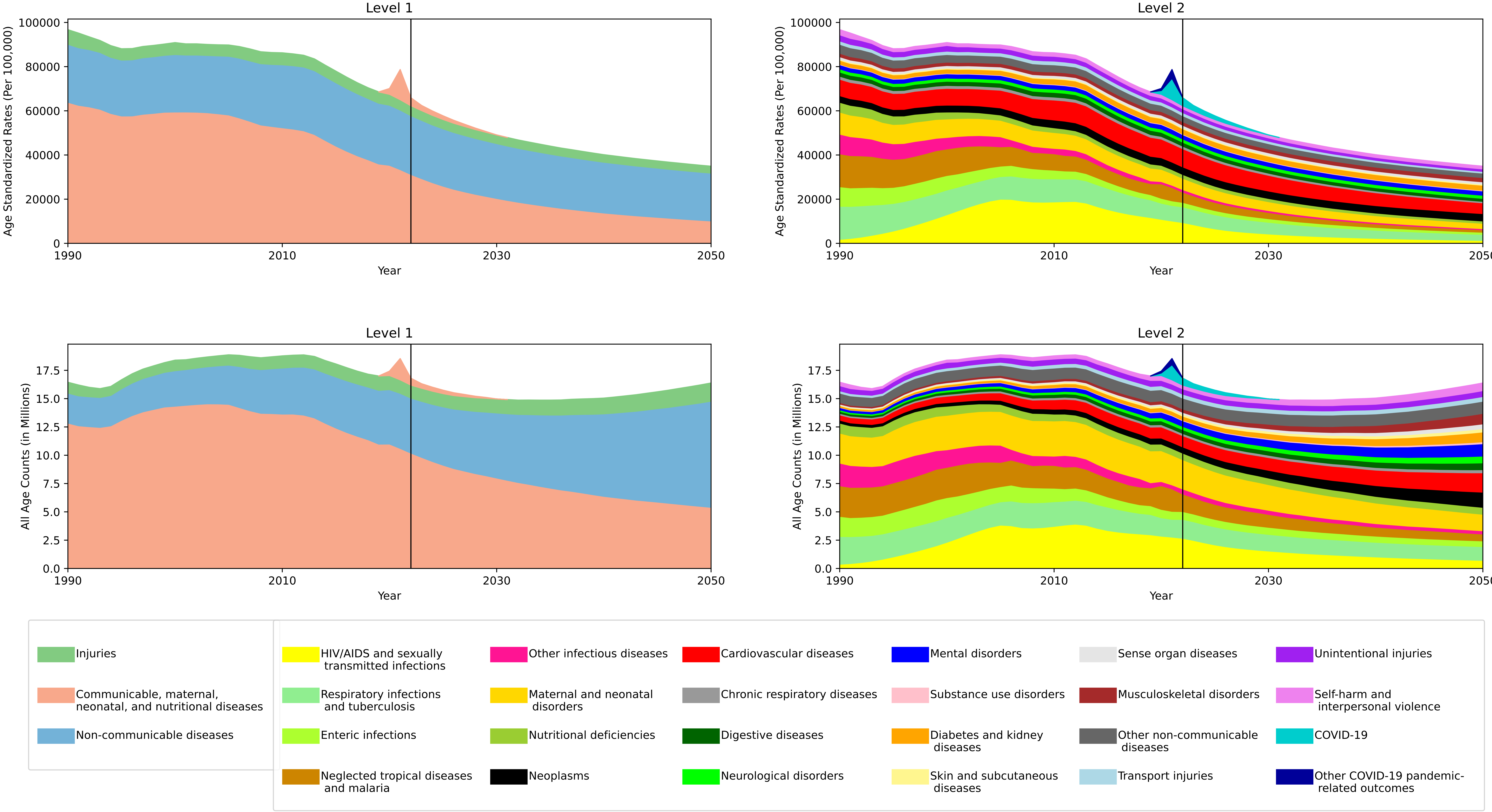
Level 1
Level 2
Age Standardized Rates (Per 100,000)
All Age Counts (in Millions)
Year
Injuries
Communicable, maternal, neonatal, and nutritional diseases
Non-communicable diseases
HIV/AIDS and sexually transmitted infections
Respiratory infections and tuberculosis
Enteric infections
Neglected tropical diseases and malaria
Other infectious diseases
Maternal and neonatal disorders
Nutritional deficiencies
Neoplasms
Cardiovascular diseases
Chronic respiratory diseases
Digestive diseases
Neurological disorders
Mental disorders
Substance use disorders
Diabetes and kidney diseases
Skin and subcutaneous diseases
Sense organ diseases
Musculoskeletal disorders
Other non-communicable diseases
Transport injuries
Unintentional injuries
Self-harm and interpersonal violence
COVID-19
Other COVID-19 pandemic-related outcomes

Rwanda

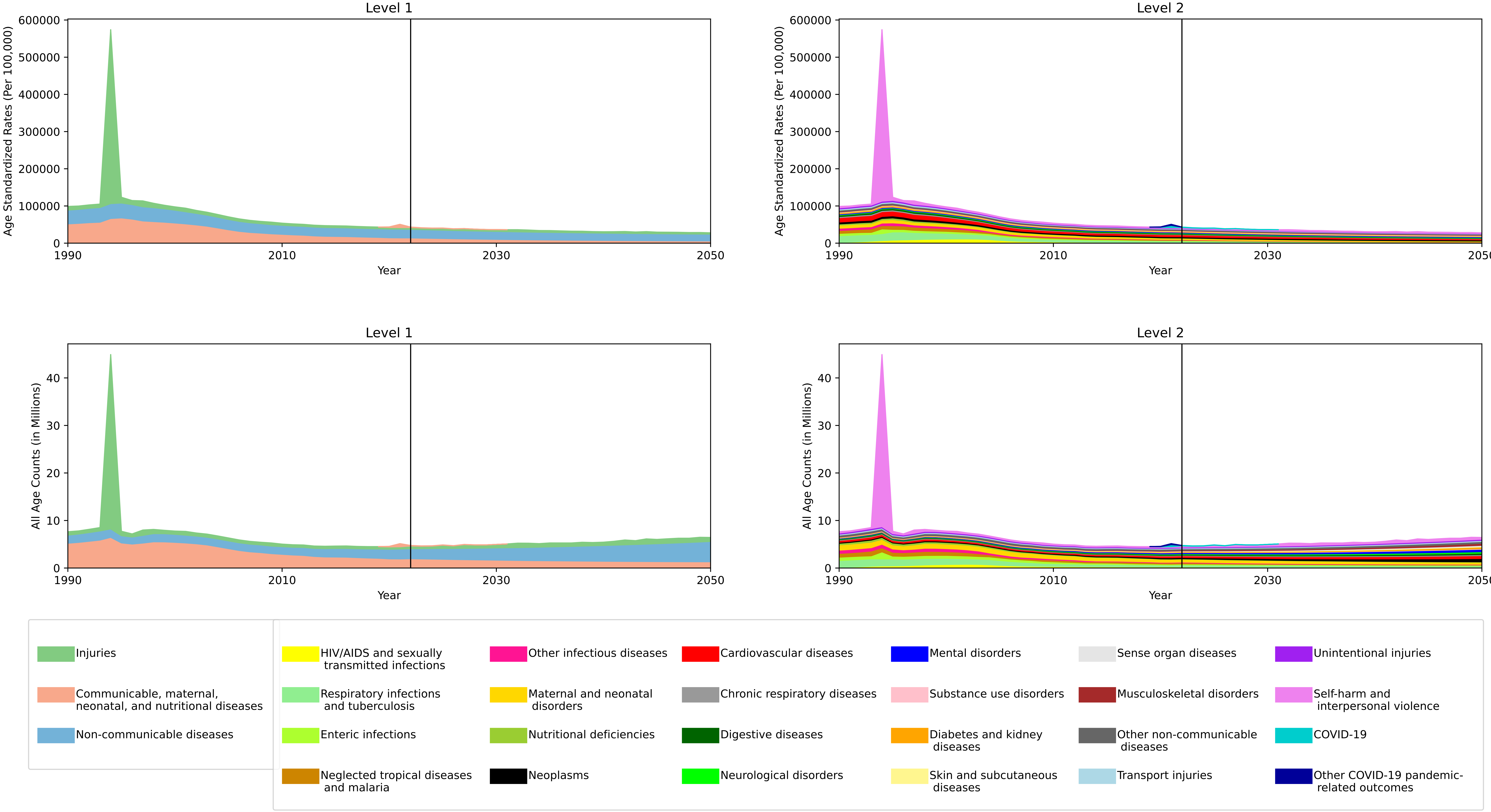
Level 1
Level 2
Age Standardized Rates (Per 100,000)
All Age Counts (in Millions)
Year
600000
500000
400000
300000
200000
100000
0
40
30
20
10
1990
2010
2030
2050
Injuries
Communicable, maternal, neonatal, and nutritional diseases
Non-communicable diseases
HIV/AIDS and sexually transmitted infections
Respiratory infections and tuberculosis
Enteric infections
Neglected tropical diseases and malaria
Other infectious diseases
Maternal and neonatal disorders
Nutritional deficiencies
Neoplasms
Cardiovascular diseases
Chronic respiratory diseases
Digestive diseases
Neurological disorders
Mental disorders
Substance use disorders
Diabetes and kidney diseases
Skin and subcutaneous diseases
Sense organ diseases
Musculoskeletal disorders
Other non-communicable diseases
Transport injuries
Unintentional injuries
Self-harm and interpersonal violence
COVID-19
Other COVID-19 pandemic-related outcomes

Somalia

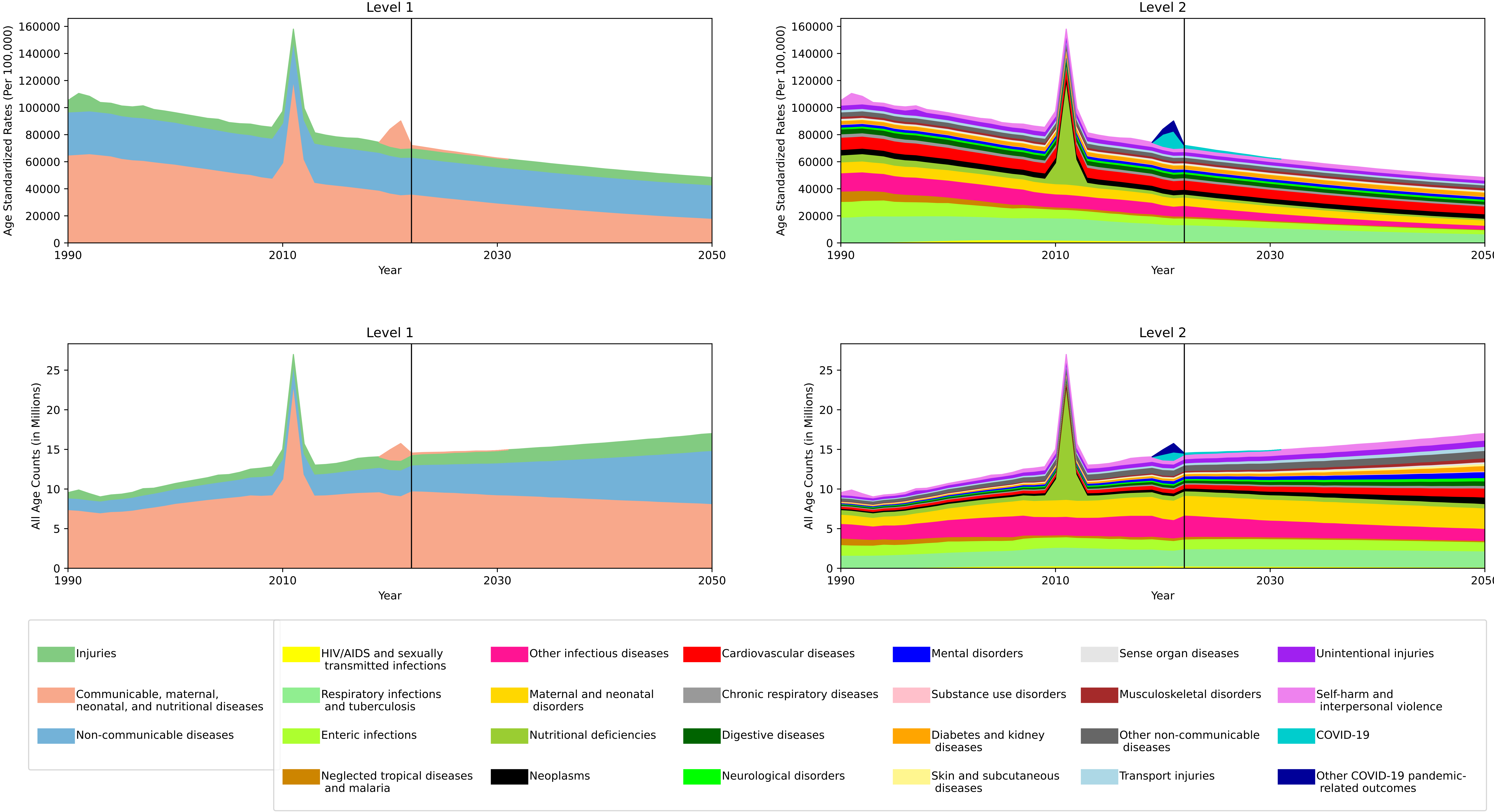
Level 1
Level 2
Age Standardized Rates (Per 100,000)
All Age Counts (in Millions)
Year
Injuries
Communicable, maternal, neonatal, and nutritional diseases
Non-communicable diseases
HIV/AIDS and sexually transmitted infections
Respiratory infections and tuberculosis
Enteric infections
Neglected tropical diseases and malaria
Other infectious diseases
Maternal and neonatal disorders
Nutritional deficiencies
Neoplasms
Cardiovascular diseases
Chronic respiratory diseases
Digestive diseases
Neurological disorders
Mental disorders
Substance use disorders
Diabetes and kidney diseases
Skin and subcutaneous diseases
Sense organ diseases
Musculoskeletal disorders
Other non-communicable diseases
Transport injuries
Unintentional injuries
Self-harm and interpersonal violence
COVID-19
Other COVID-19 pandemic-related outcomes

South Sudan

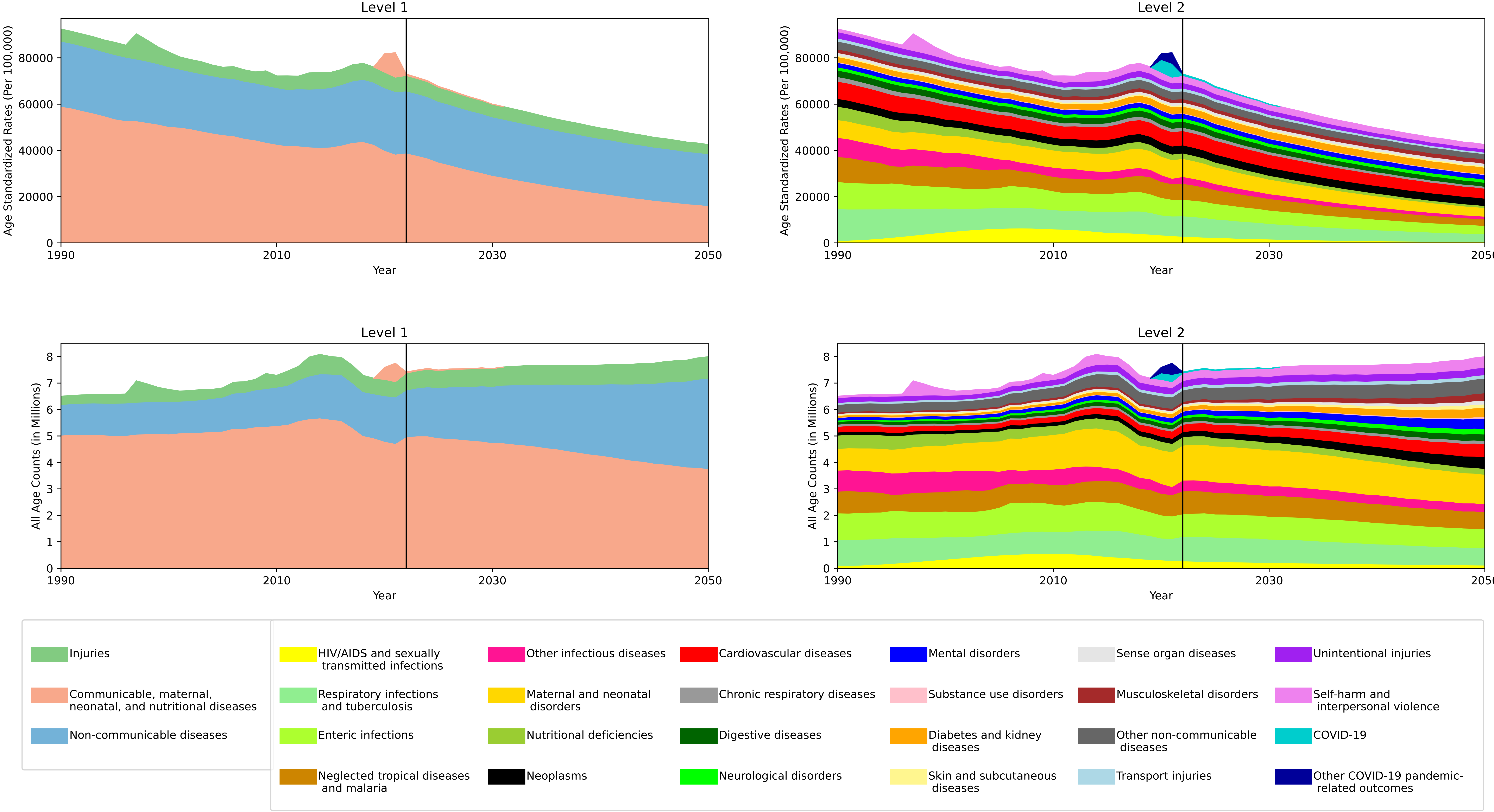
Level 1
Level 2
Age Standardized Rates (Per 100,000)
All Age Counts (in Millions)
Year
0
20000
40000
60000
80000
1
2
3
4
5
6
7
8
1990
2010
2030
2050
Injuries
Communicable, maternal, neonatal, and nutritional diseases
Non-communicable diseases
HIV/AIDS and sexually transmitted infections
Respiratory infections and tuberculosis
Enteric infections
Neglected tropical diseases and malaria
Other infectious diseases
Maternal and neonatal disorders
Nutritional deficiencies
Neoplasms
Cardiovascular diseases
Chronic respiratory diseases
Digestive diseases
Neurological disorders
Mental disorders
Substance use disorders
Diabetes and kidney diseases
Skin and subcutaneous diseases
Sense organ diseases
Musculoskeletal disorders
Other non-communicable diseases
Transport injuries
Unintentional injuries
Self-harm and interpersonal violence
COVID-19
Other COVID-19 pandemic-related outcomes

Uganda

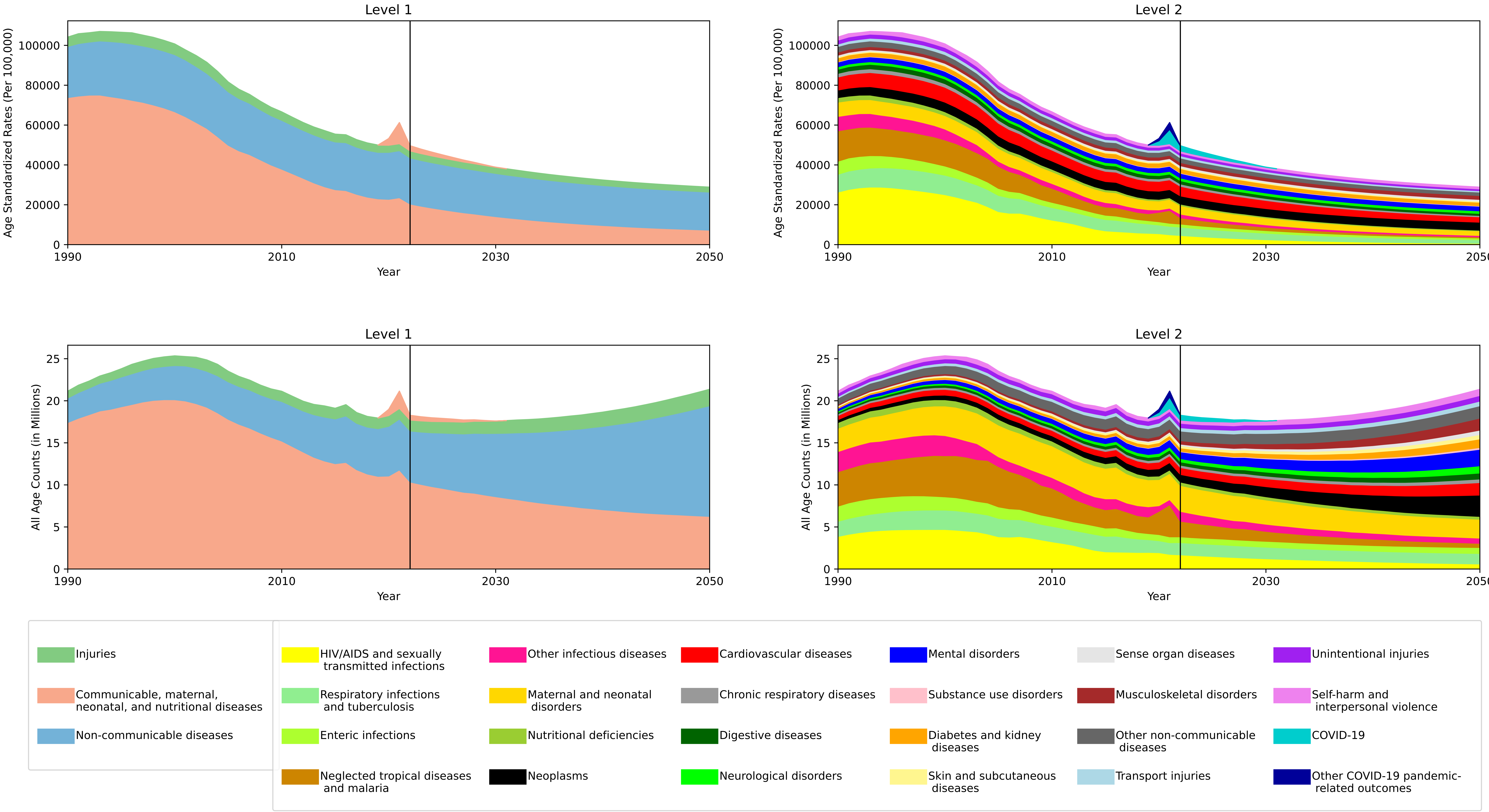
Level 1
Level 2
Age Standardized Rates (Per 100,000)
All Age Counts (in Millions)
Year
1990
2010
2030
2050
0
20000
40000
60000
80000
100000
5
10
15
20
25
Injuries
Communicable, maternal, neonatal, and nutritional diseases
Non-communicable diseases
HIV/AIDS and sexually transmitted infections
Respiratory infections and tuberculosis
Enteric infections
Neglected tropical diseases and malaria
Other infectious diseases
Maternal and neonatal disorders
Nutritional deficiencies
Neoplasms
Cardiovascular diseases
Chronic respiratory diseases
Digestive diseases
Neurological disorders
Mental disorders
Substance use disorders
Diabetes and kidney diseases
Skin and subcutaneous diseases
Sense organ diseases
Musculoskeletal disorders
Other non-communicable diseases
Transport injuries
Unintentional injuries
Self-harm and interpersonal violence
COVID-19
Other COVID-19 pandemic-related outcomes

Tanzania

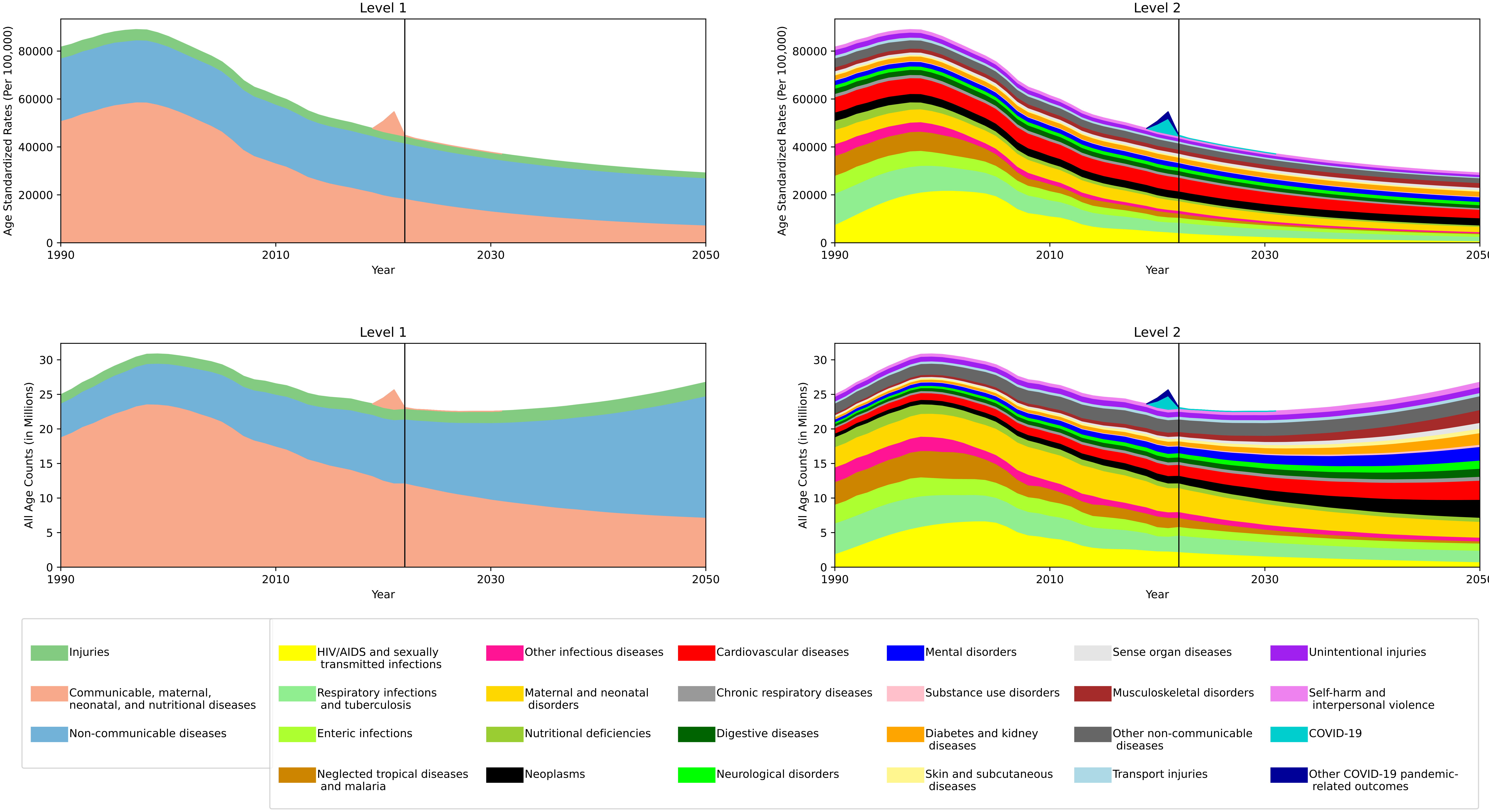
Level 1
Level 2
Age Standardized Rates (Per 100,000)
All Age Counts (in Millions)
Year
1990
2010
2030
2050
0
20000
40000
60000
80000
0
5
10
15
20
25
30
Injuries
Communicable, maternal, neonatal, and nutritional diseases
Non-communicable diseases
HIV/AIDS and sexually transmitted infections
Respiratory infections and tuberculosis
Enteric infections
Neglected tropical diseases and malaria
Other infectious diseases
Maternal and neonatal disorders
Nutritional deficiencies
Neoplasms
Cardiovascular diseases
Chronic respiratory diseases
Digestive diseases
Neurological disorders
Mental disorders
Substance use disorders
Diabetes and kidney diseases
Skin and subcutaneous diseases
Sense organ diseases
Musculoskeletal disorders
Other non-communicable diseases
Transport injuries
Unintentional injuries
Self-harm and interpersonal violence
COVID-19
Other COVID-19 pandemic-related outcomes

Zambia

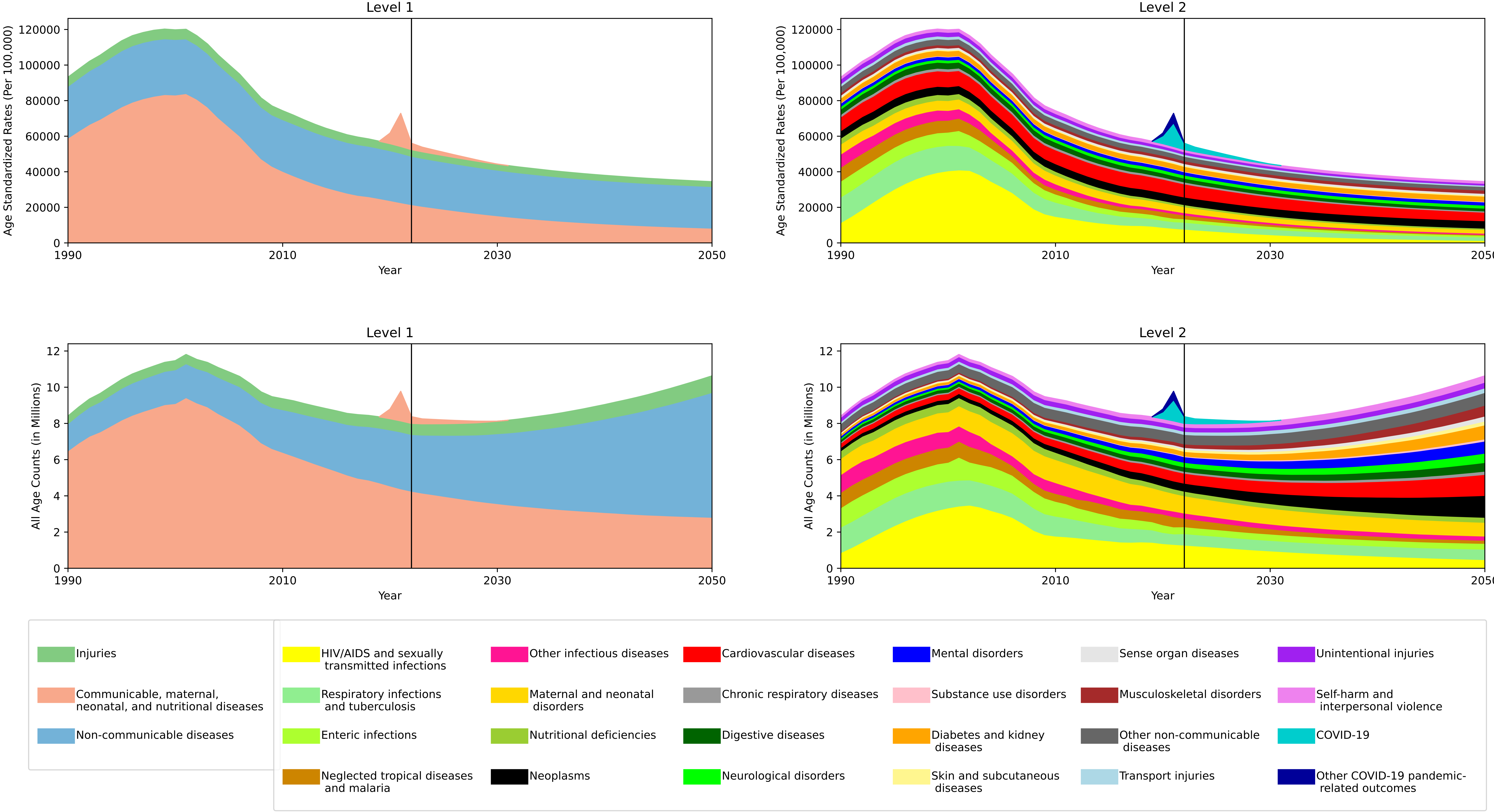
Level 1
Level 2
Age Standardized Rates (Per 100,000)
All Age Counts (in Millions)
Year
1990
2010
2030
2050
Injuries
Communicable, maternal, neonatal, and nutritional diseases
Non-communicable diseases
HIV/AIDS and sexually transmitted infections
Respiratory infections and tuberculosis
Enteric infections
Neglected tropical diseases and malaria
Other infectious diseases
Maternal and neonatal disorders
Nutritional deficiencies
Neoplasms
Cardiovascular diseases
Chronic respiratory diseases
Digestive diseases
Neurological disorders
Mental disorders
Substance use disorders
Diabetes and kidney diseases
Skin and subcutaneous diseases
Sense organ diseases
Musculoskeletal disorders
Other non-communicable diseases
Transport injuries
Unintentional injuries
Self-harm and interpersonal violence
COVID-19
Other COVID-19 pandemic-related outcomes

Botswana

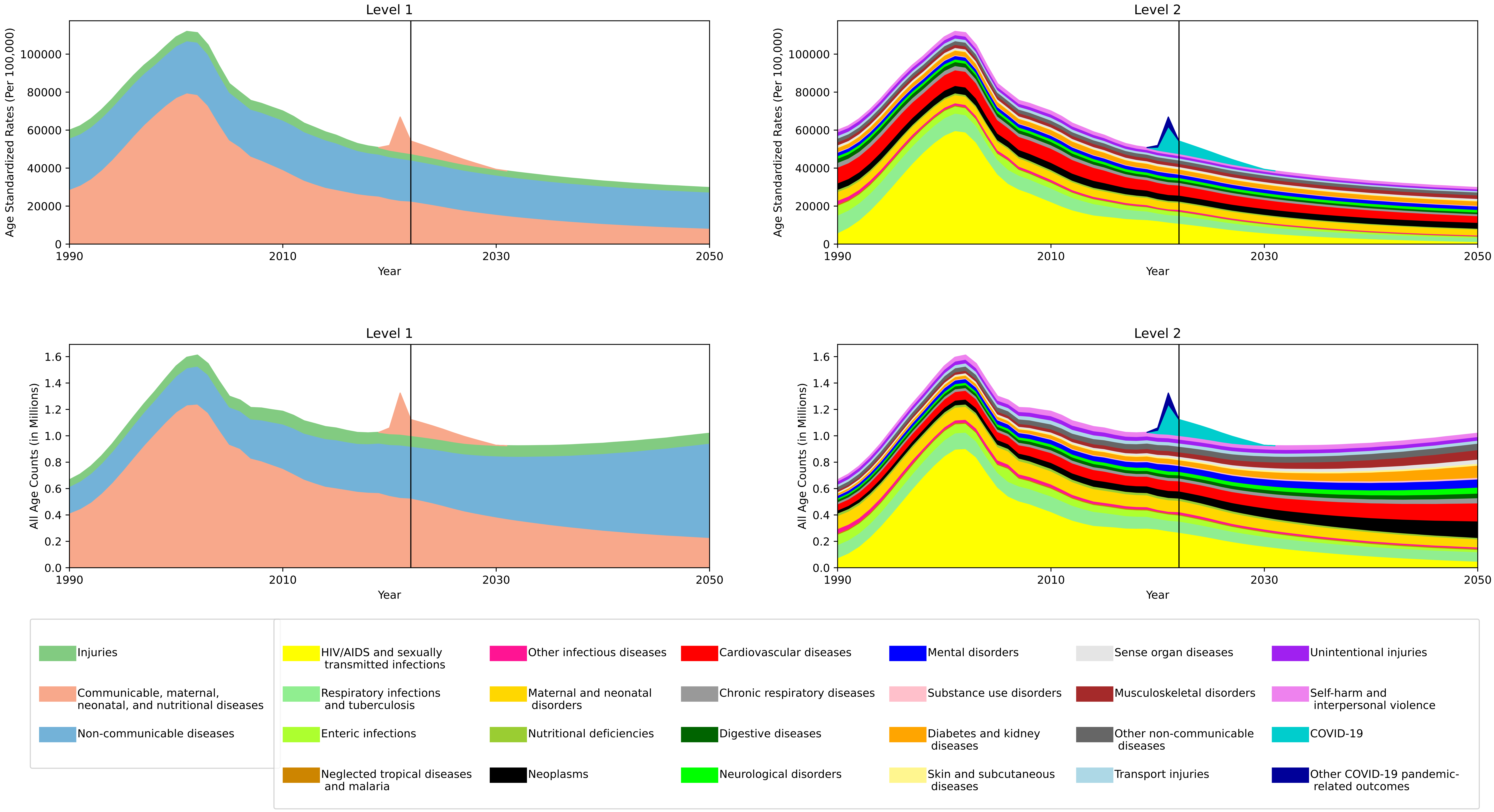
Level 1
Level 2
Age Standardized Rates (Per 100,000)
All Age Counts (in Millions)
Year
1990
2010
2030
2050
0
20000
40000
60000
80000
100000
0.0
0.2
0.4
0.6
0.8
1.0
1.2
1.4
1.6
Injuries
Communicable, maternal, neonatal, and nutritional diseases
Non-communicable diseases
HIV/AIDS and sexually transmitted infections
Respiratory infections and tuberculosis
Enteric infections
Neglected tropical diseases and malaria
Other infectious diseases
Maternal and neonatal disorders
Nutritional deficiencies
Neoplasms
Cardiovascular diseases
Chronic respiratory diseases
Digestive diseases
Neurological disorders
Mental disorders
Substance use disorders
Diabetes and kidney diseases
Skin and subcutaneous diseases
Sense organ diseases
Musculoskeletal disorders
Other non-communicable diseases
Transport injuries
Unintentional injuries
Self-harm and interpersonal violence
COVID-19
Other COVID-19 pandemic-related outcomes

Eswatini

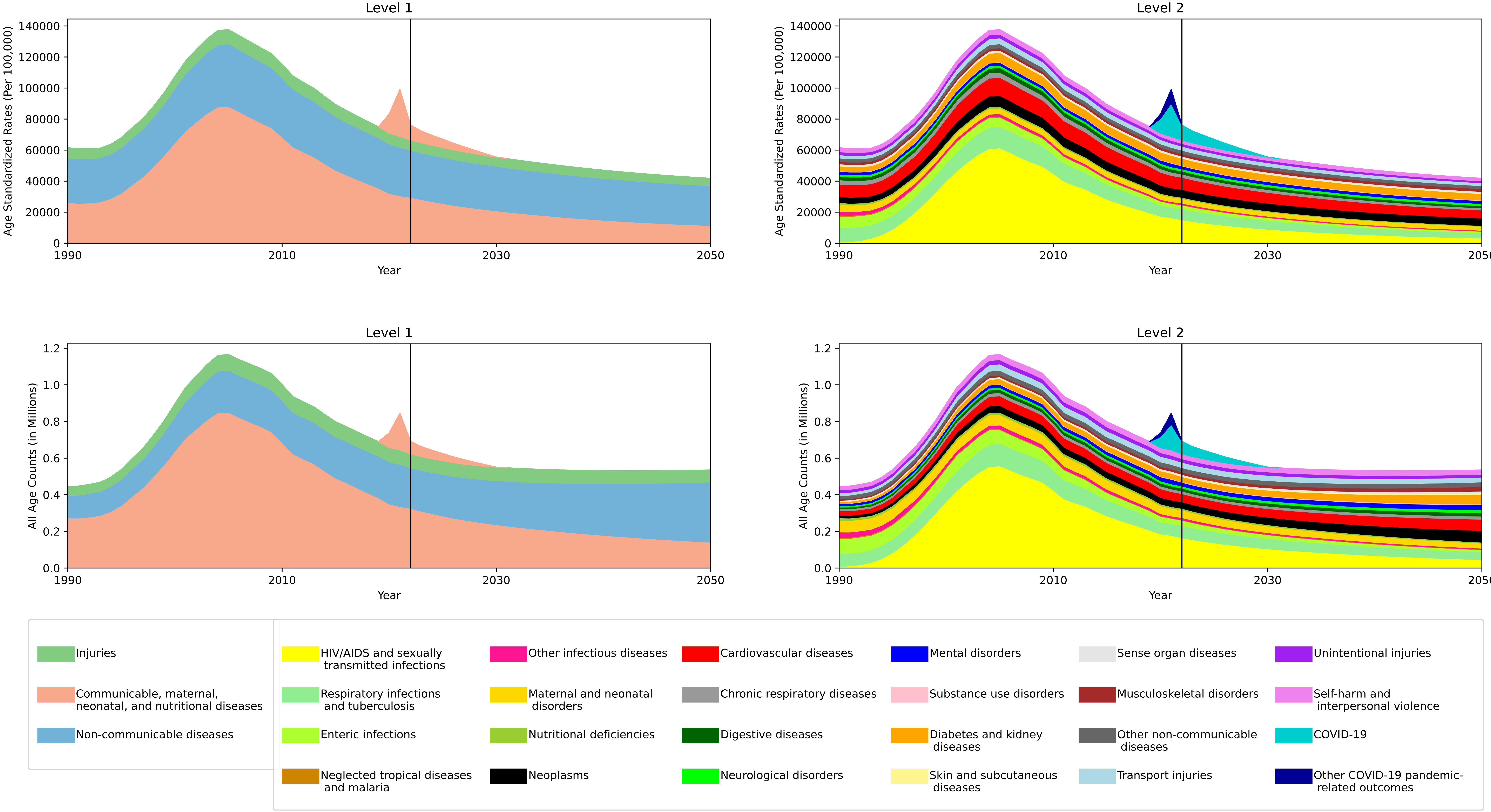
Level 1
Level 2
Age Standardized Rates (Per 100,000)
All Age Counts (in Millions)
Year
1990
2010
2030
2050
Injuries
Communicable, maternal, neonatal, and nutritional diseases
Non-communicable diseases
HIV/AIDS and sexually transmitted infections
Respiratory infections and tuberculosis
Enteric infections
Neglected tropical diseases and malaria
Other infectious diseases
Maternal and neonatal disorders
Nutritional deficiencies
Neoplasms
Cardiovascular diseases
Chronic respiratory diseases
Digestive diseases
Neurological disorders
Mental disorders
Substance use disorders
Diabetes and kidney diseases
Skin and subcutaneous diseases
Sense organ diseases
Musculoskeletal disorders
Other non-communicable diseases
Transport injuries
Unintentional injuries
Self-harm and interpersonal violence
COVID-19
Other COVID-19 pandemic-related outcomes

Lesotho

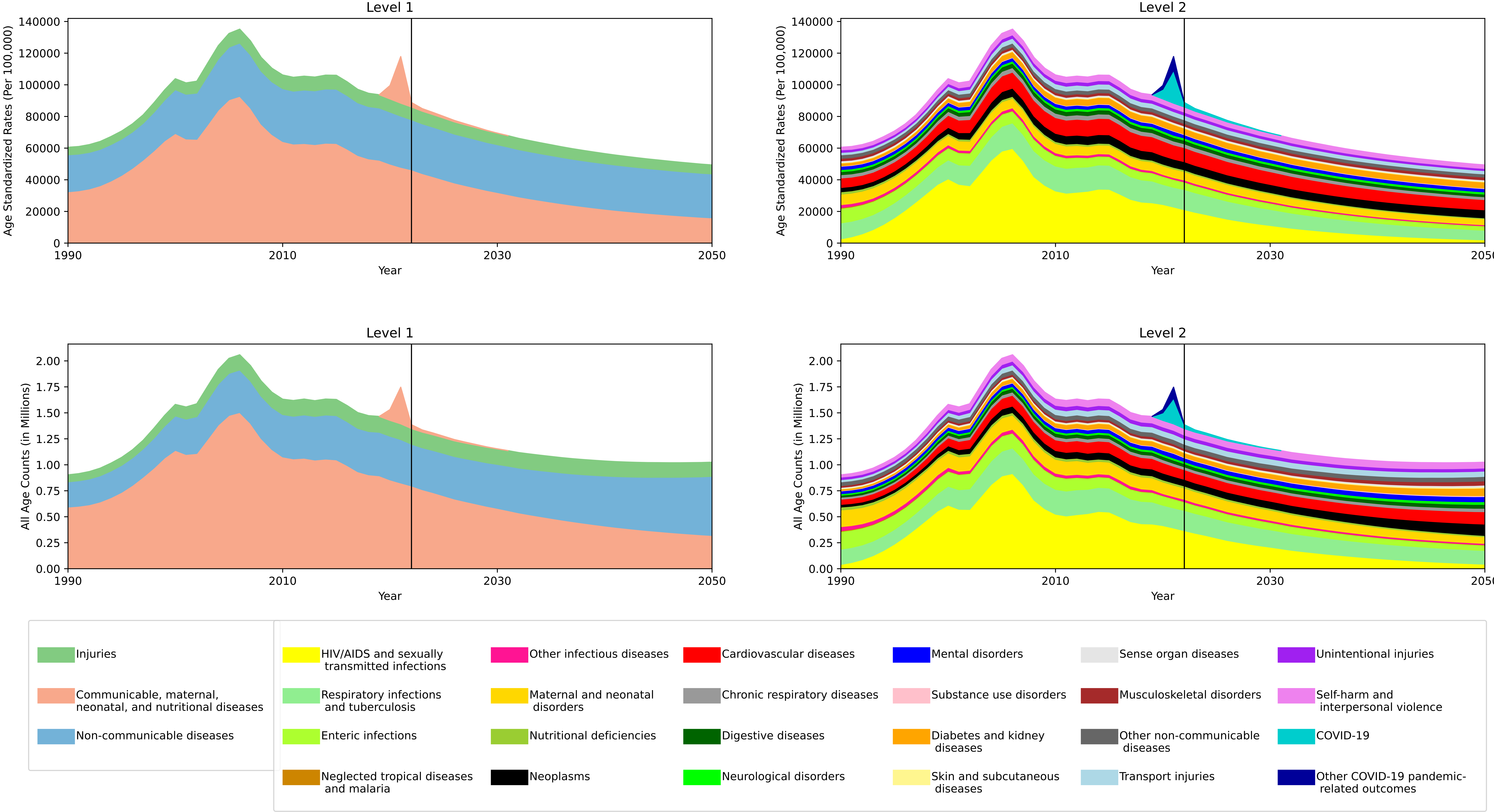

Level 1
Level 2
Age Standardized Rates (Per 100,000)
All Age Counts (in Millions)
Year
1990
2010
2030
2050
0
20000
40000
60000
80000
100000
120000
140000
0.00
0.25
0.50
0.75
1.00
1.25
1.50
1.75
2.00
Injuries
Communicable, maternal, neonatal, and nutritional diseases
Non-communicable diseases
HIV/AIDS and sexually transmitted infections
Respiratory infections and tuberculosis
Enteric infections
Neglected tropical diseases and malaria
Other infectious diseases
Maternal and neonatal disorders
Nutritional deficiencies
Neoplasms
Cardiovascular diseases
Chronic respiratory diseases
Digestive diseases
Neurological disorders
Mental disorders
Substance use disorders
Diabetes and kidney diseases
Skin and subcutaneous diseases
Sense organ diseases
Musculoskeletal disorders
Other non-communicable diseases
Transport injuries
Unintentional injuries
Self-harm and interpersonal violence
COVID-19
Other COVID-19 pandemic-related outcomes

Namibia

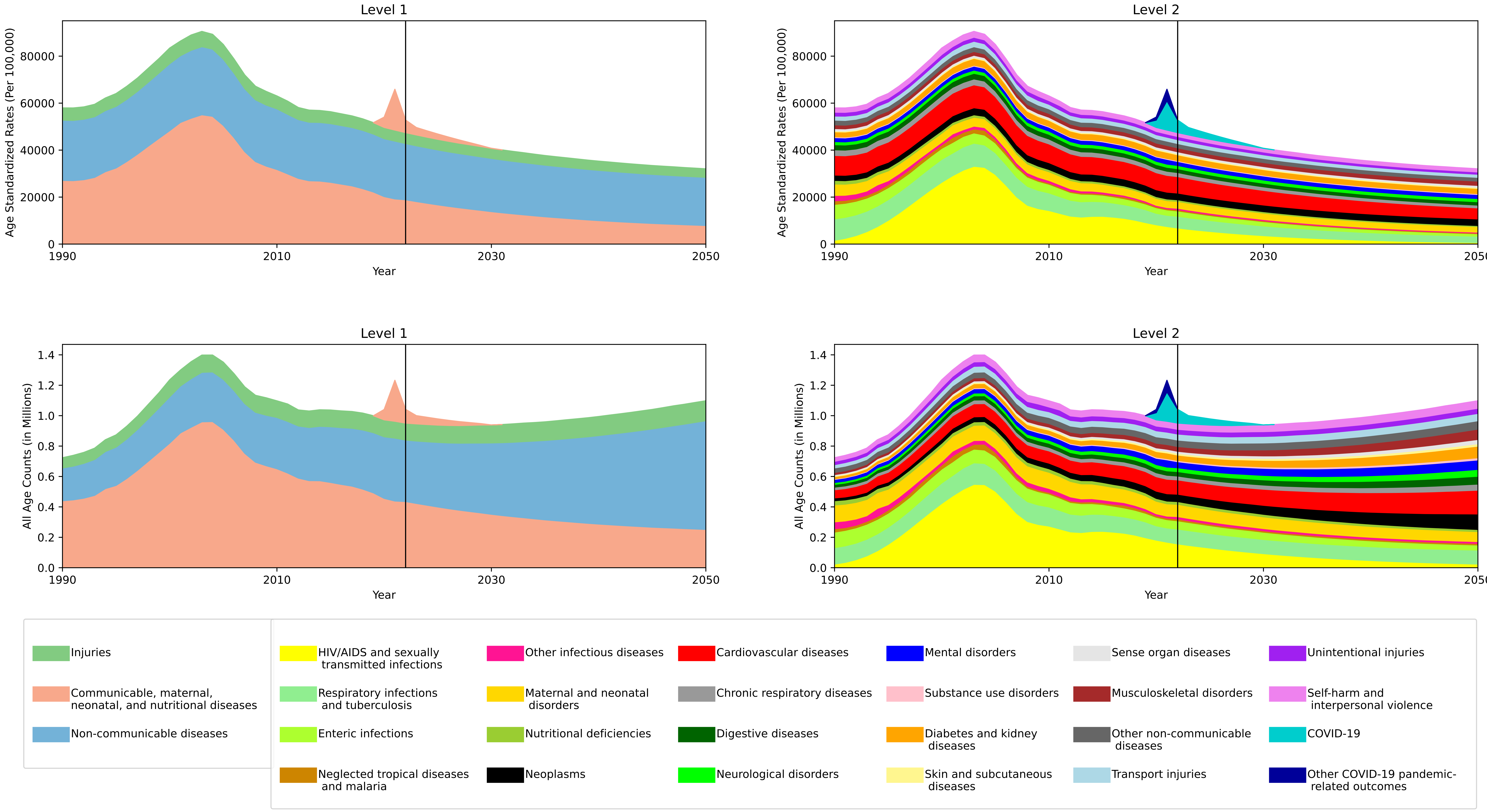
Level 1
Level 2
Age Standardized Rates (Per 100,000)
All Age Counts (in Millions)
Year
0
20000
40000
60000
80000
0.0
0.2
0.4
0.6
0.8
1.0
1.2
1.4
1990
2010
2030
2050
Injuries
Communicable, maternal, neonatal, and nutritional diseases
Non-communicable diseases
HIV/AIDS and sexually transmitted infections
Respiratory infections and tuberculosis
Enteric infections
Neglected tropical diseases and malaria
Other infectious diseases
Maternal and neonatal disorders
Nutritional deficiencies
Neoplasms
Cardiovascular diseases
Chronic respiratory diseases
Digestive diseases
Neurological disorders
Mental disorders
Substance use disorders
Diabetes and kidney diseases
Skin and subcutaneous diseases
Sense organ diseases
Musculoskeletal disorders
Other non-communicable diseases
Transport injuries
Unintentional injuries
Self-harm and interpersonal violence
COVID-19
Other COVID-19 pandemic-related outcomes

South Africa

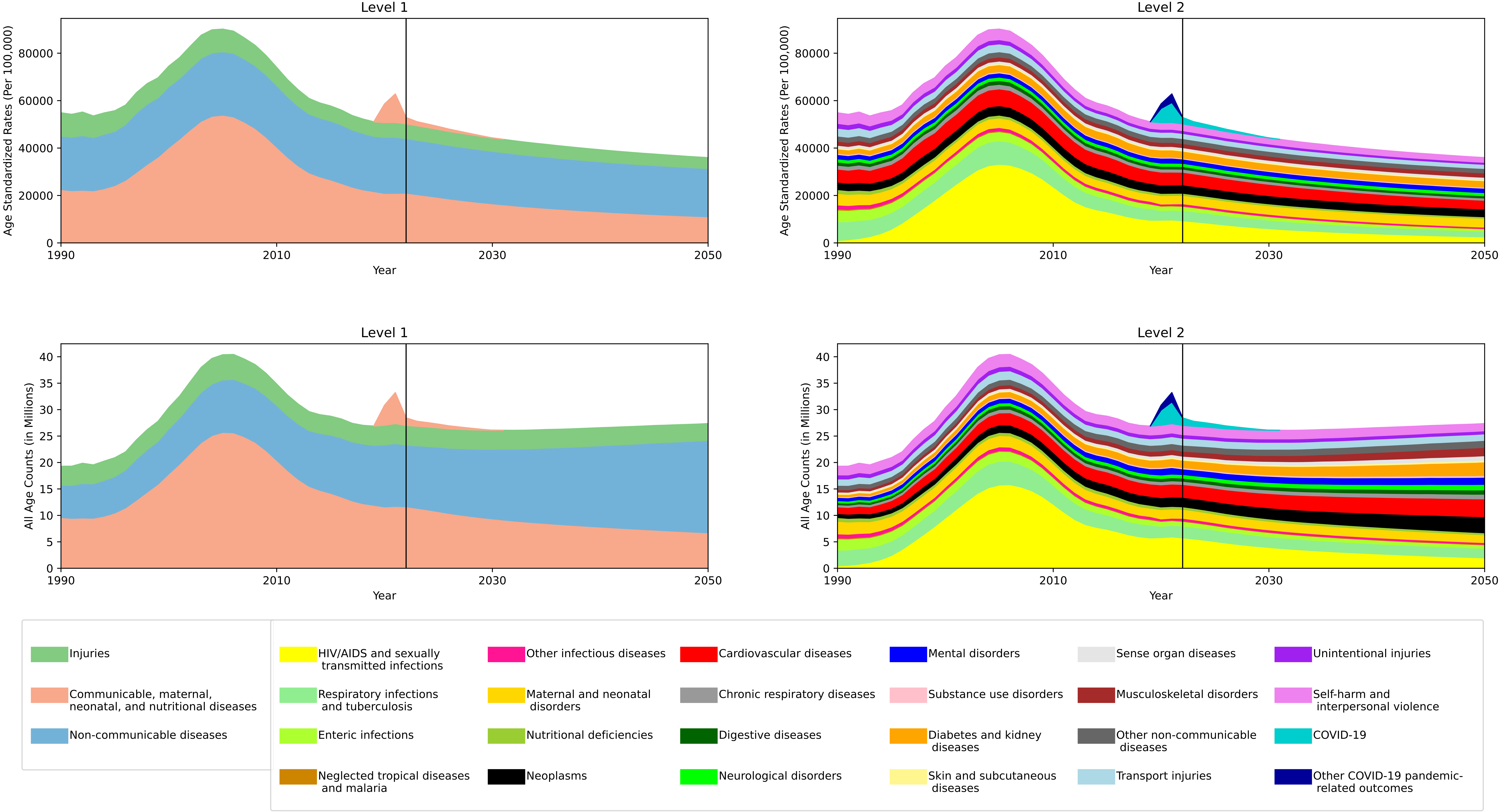

Zimbabwe

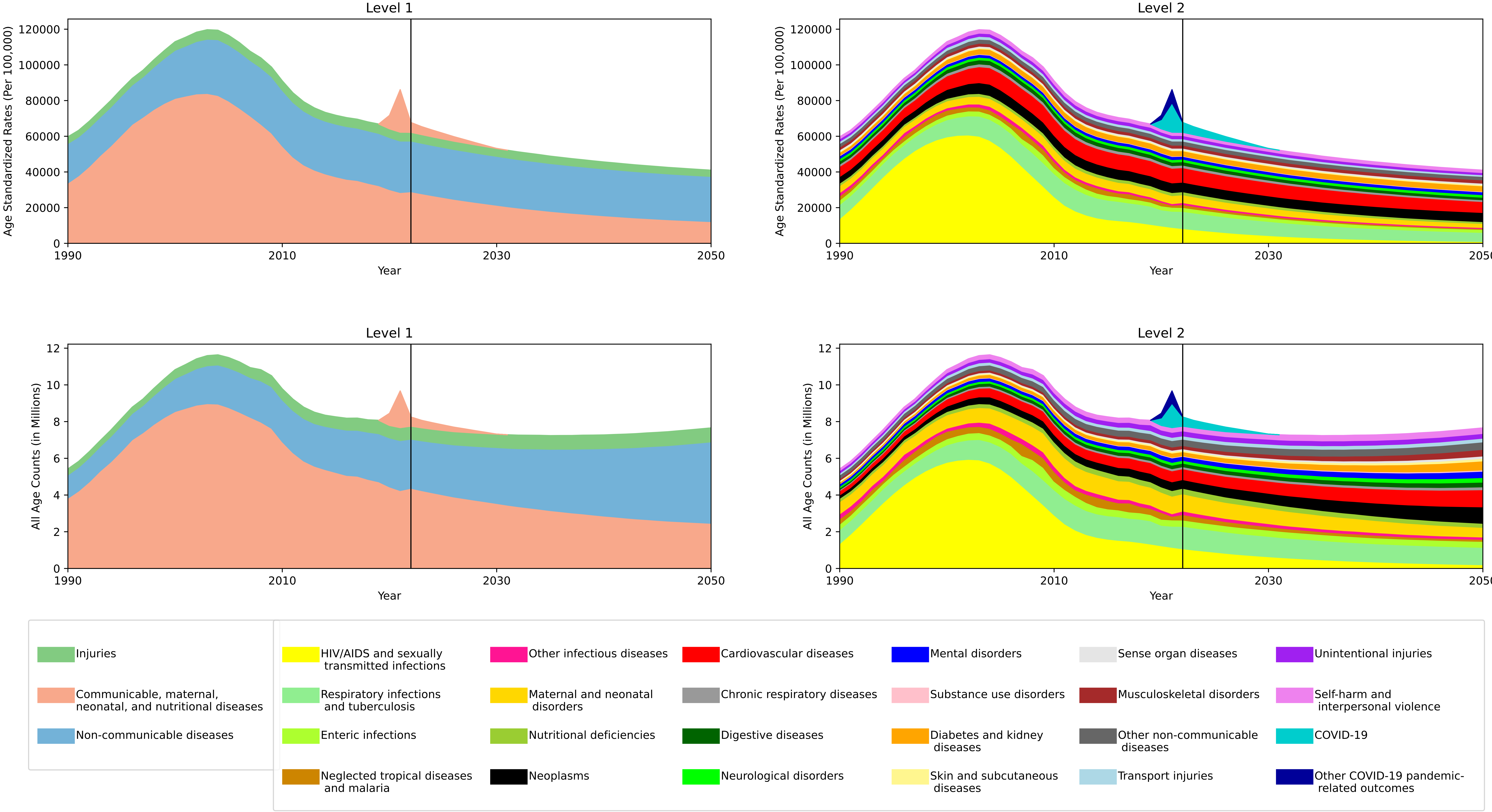
Level 1
Age Standardized Rates (Per 100,000)
Year
Level 2
Age Standardized Rates (Per 100,000)
Year
Level 1
All Age Counts (in Millions)
Year
Level 2
All Age Counts (in Millions)
Year
Injuries
Communicable, maternal, neonatal, and nutritional diseases
Non-communicable diseases
HIV/AIDS and sexually transmitted infections
Respiratory infections and tuberculosis
Enteric infections
Neglected tropical diseases and malaria
Other infectious diseases
Maternal and neonatal disorders
Nutritional deficiencies
Neoplasms
Cardiovascular diseases
Chronic respiratory diseases
Digestive diseases
Neurological disorders
Mental disorders
Substance use disorders
Diabetes and kidney diseases
Skin and subcutaneous diseases
Sense organ diseases
Musculoskeletal disorders
Other non-communicable diseases
Transport injuries
Unintentional injuries
Self-harm and interpersonal violence
COVID-19
Other COVID-19 pandemic-related outcomes

Benin

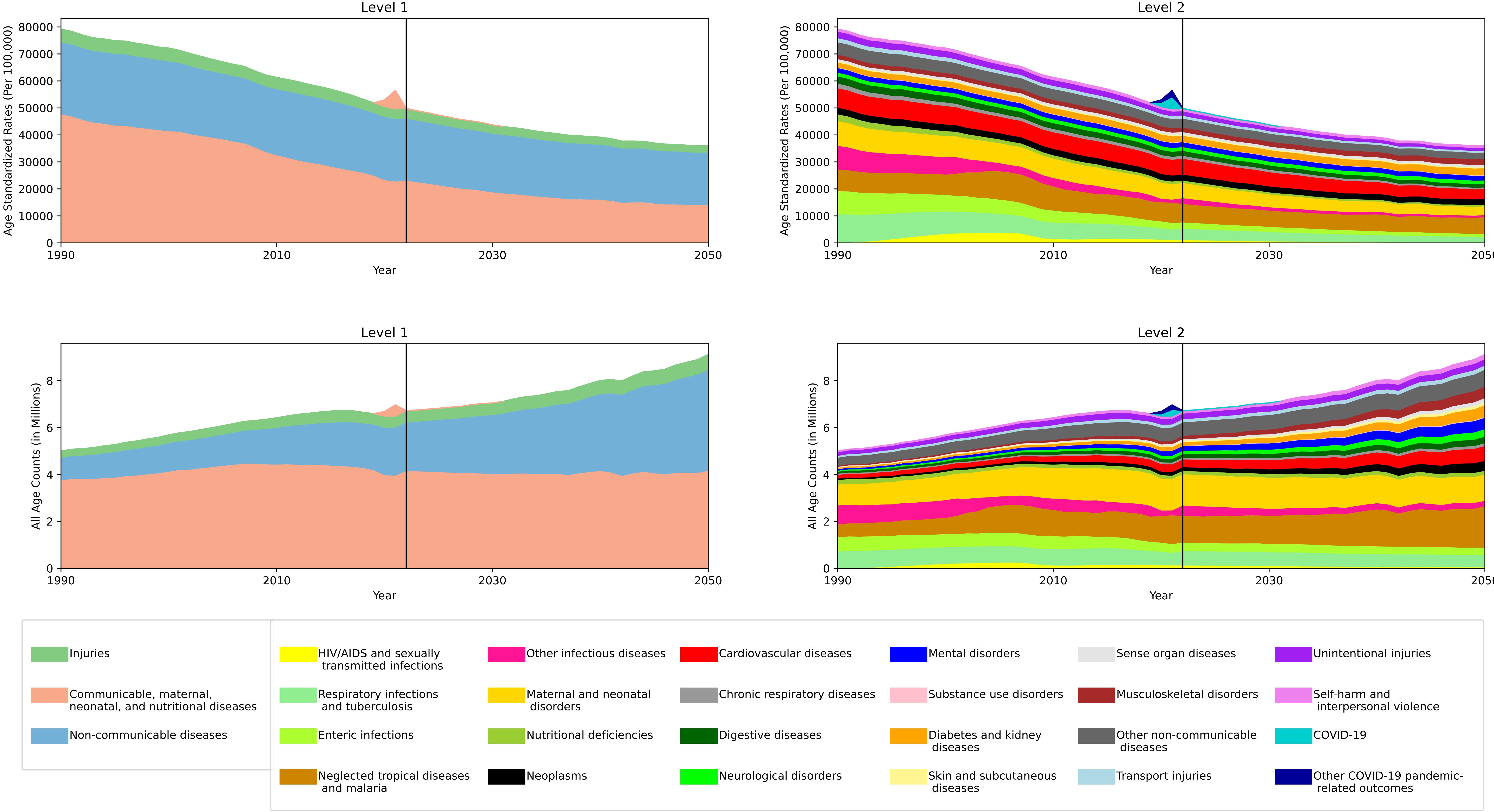
Level 1
Age Standardized Rates (Per 100,000)
Year
Level 2
Age Standardized Rates (Per 100,000)
Year
Level 1
All Age Counts (in Millions)
Year
Level 2
All Age Counts (in Millions)
Year
Injuries
Communicable, maternal, neonatal, and nutritional diseases
Non-communicable diseases
HIV/AIDS and sexually transmitted infections
Respiratory infections and tuberculosis
Enteric infections
Neglected tropical diseases and malaria
Other infectious diseases
Maternal and neonatal disorders
Nutritional deficiencies
Neoplasms
Cardiovascular diseases
Chronic respiratory diseases
Digestive diseases
Neurological disorders
Mental disorders
Substance use disorders
Diabetes and kidney diseases
Skin and subcutaneous diseases
Sense organ diseases
Musculoskeletal disorders
Other non-communicable diseases
Transport injuries
Unintentional injuries
Self-harm and interpersonal violence
COVID-19
Other COVID-19 pandemic-related outcomes

Burkina Faso

Supplemental Results Figure S4. Age-standardised DALY rates and all age DALY counts and by Level 1 and Level 2 causes, 1990–2050

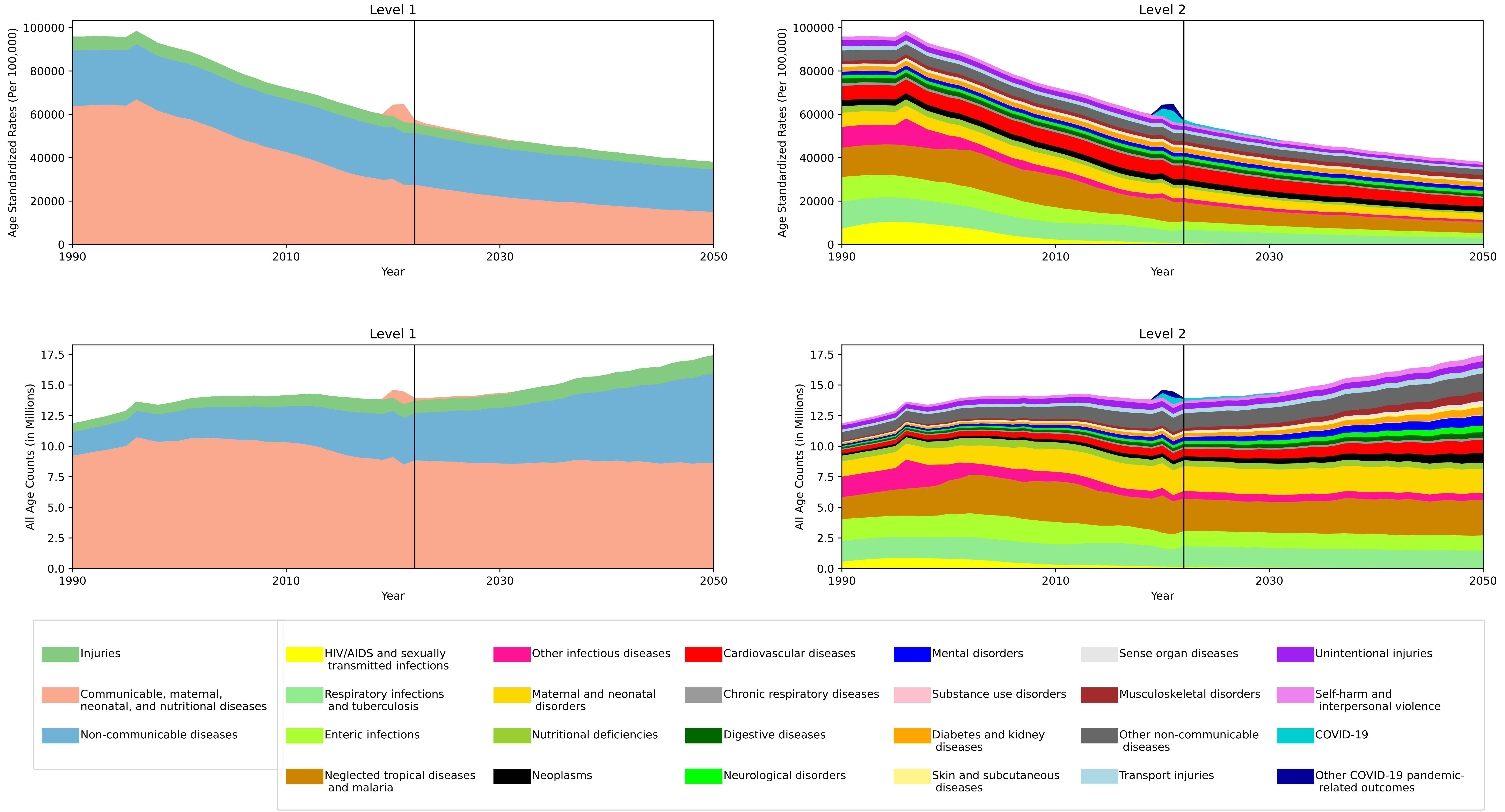

Cabo Verde

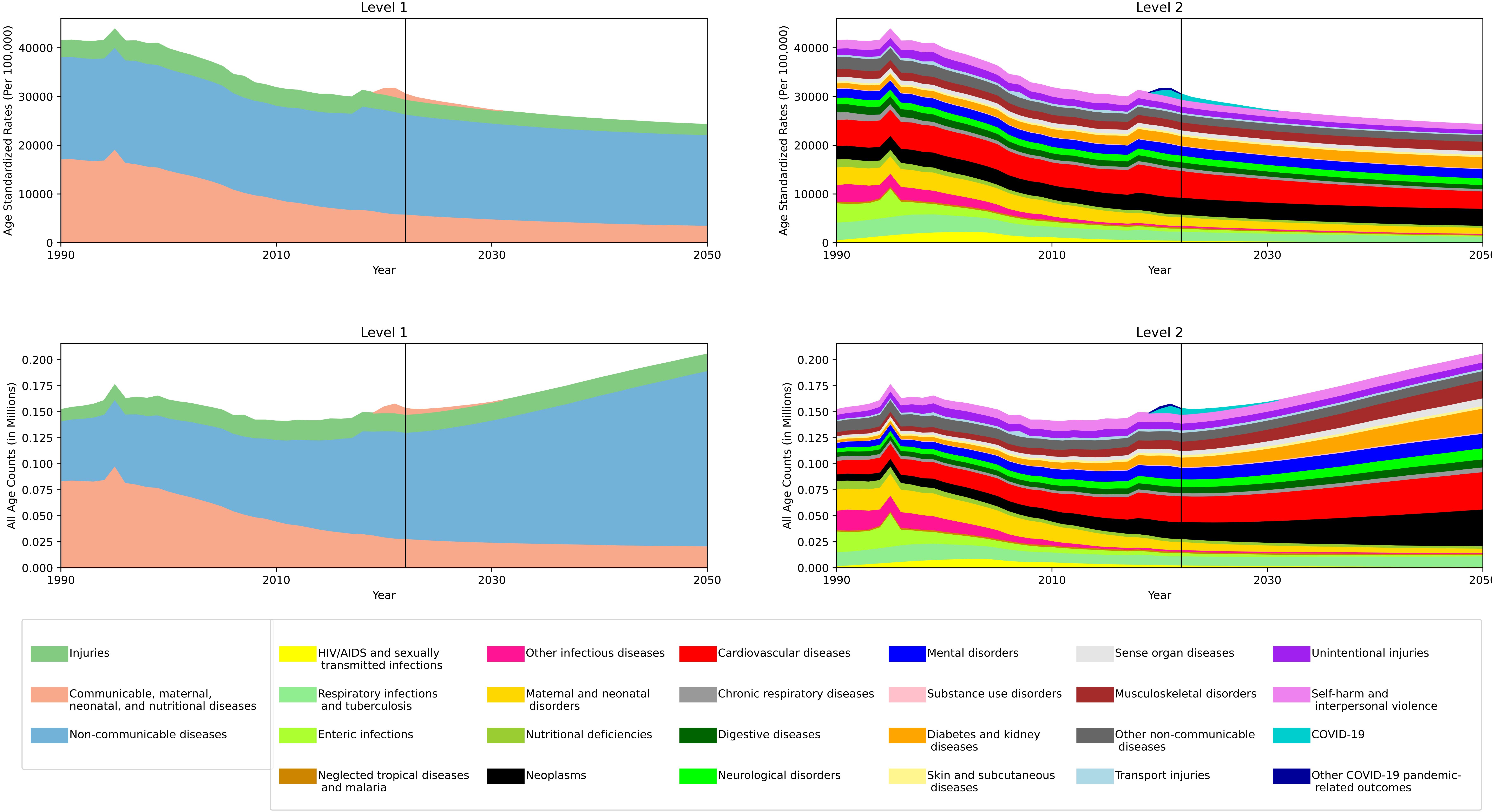
Level 1
Level 2
Age Standardized Rates (Per 100,000)
All Age Counts (in Millions)
Year
Injuries
Communicable, maternal, neonatal, and nutritional diseases
Non-communicable diseases
HIV/AIDS and sexually transmitted infections
Respiratory infections and tuberculosis
Enteric infections
Neglected tropical diseases and malaria
Other infectious diseases
Maternal and neonatal disorders
Nutritional deficiencies
Neoplasms
Cardiovascular diseases
Chronic respiratory diseases
Digestive diseases
Neurological disorders
Mental disorders
Substance use disorders
Diabetes and kidney diseases
Skin and subcutaneous diseases
Sense organ diseases
Musculoskeletal disorders
Other non-communicable diseases
Transport injuries
Unintentional injuries
Self-harm and interpersonal violence
COVID-19
Other COVID-19 pandemic-related outcomes

Cameroon

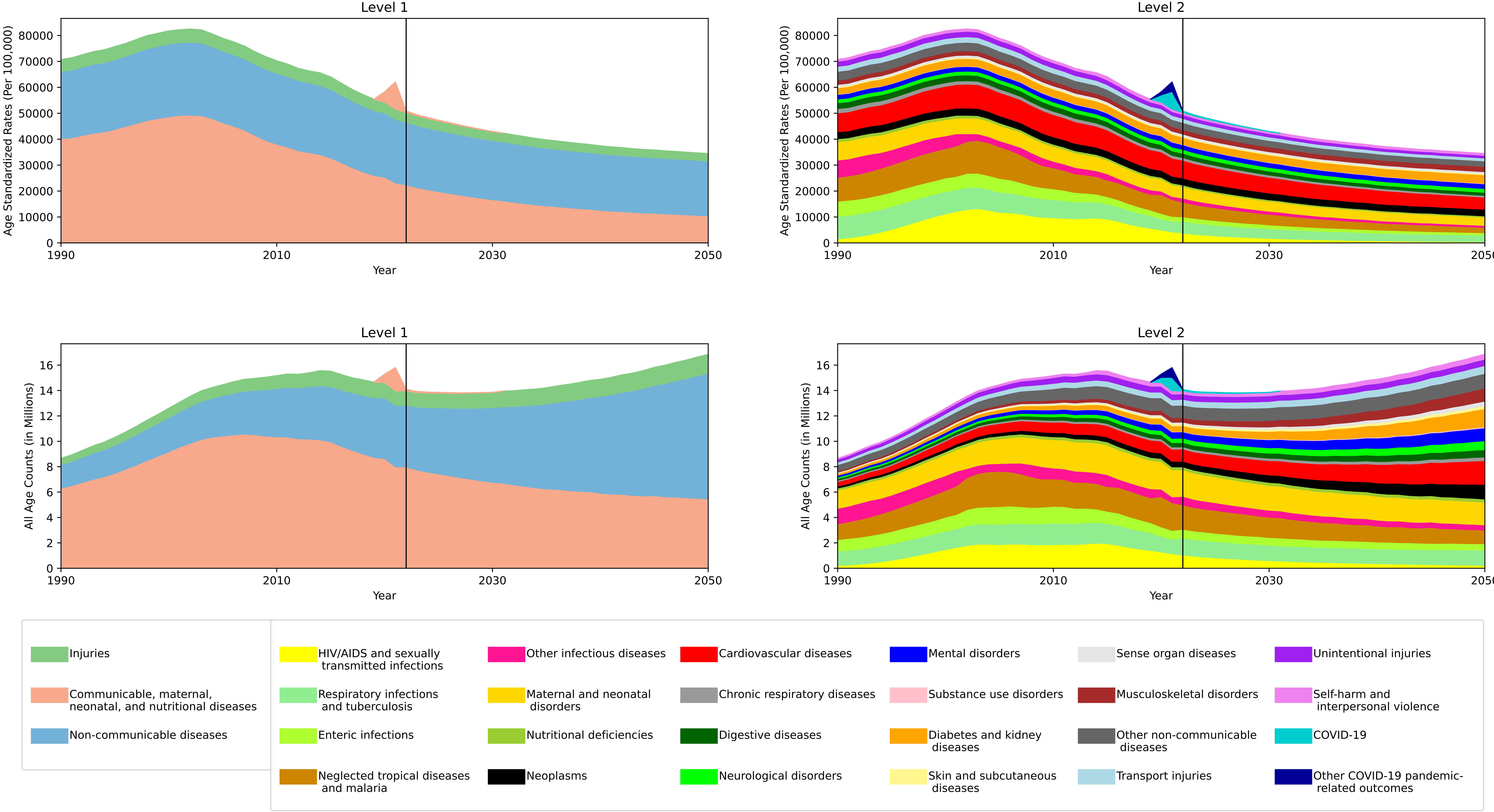
Level 1
Level 2
Age Standardized Rates (Per 100,000)
All Age Counts (in Millions)
Year
1990
2010
2030
2050
Injuries
Communicable, maternal, neonatal, and nutritional diseases
Non-communicable diseases
HIV/AIDS and sexually transmitted infections
Respiratory infections and tuberculosis
Enteric infections
Neglected tropical diseases and malaria
Other infectious diseases
Maternal and neonatal disorders
Nutritional deficiencies
Neoplasms
Cardiovascular diseases
Chronic respiratory diseases
Digestive diseases
Neurological disorders
Mental disorders
Substance use disorders
Diabetes and kidney diseases
Skin and subcutaneous diseases
Sense organ diseases
Musculoskeletal disorders
Other non-communicable diseases
Transport injuries
Unintentional injuries
Self-harm and interpersonal violence
COVID-19
Other COVID-19 pandemic-related outcomes

Chad

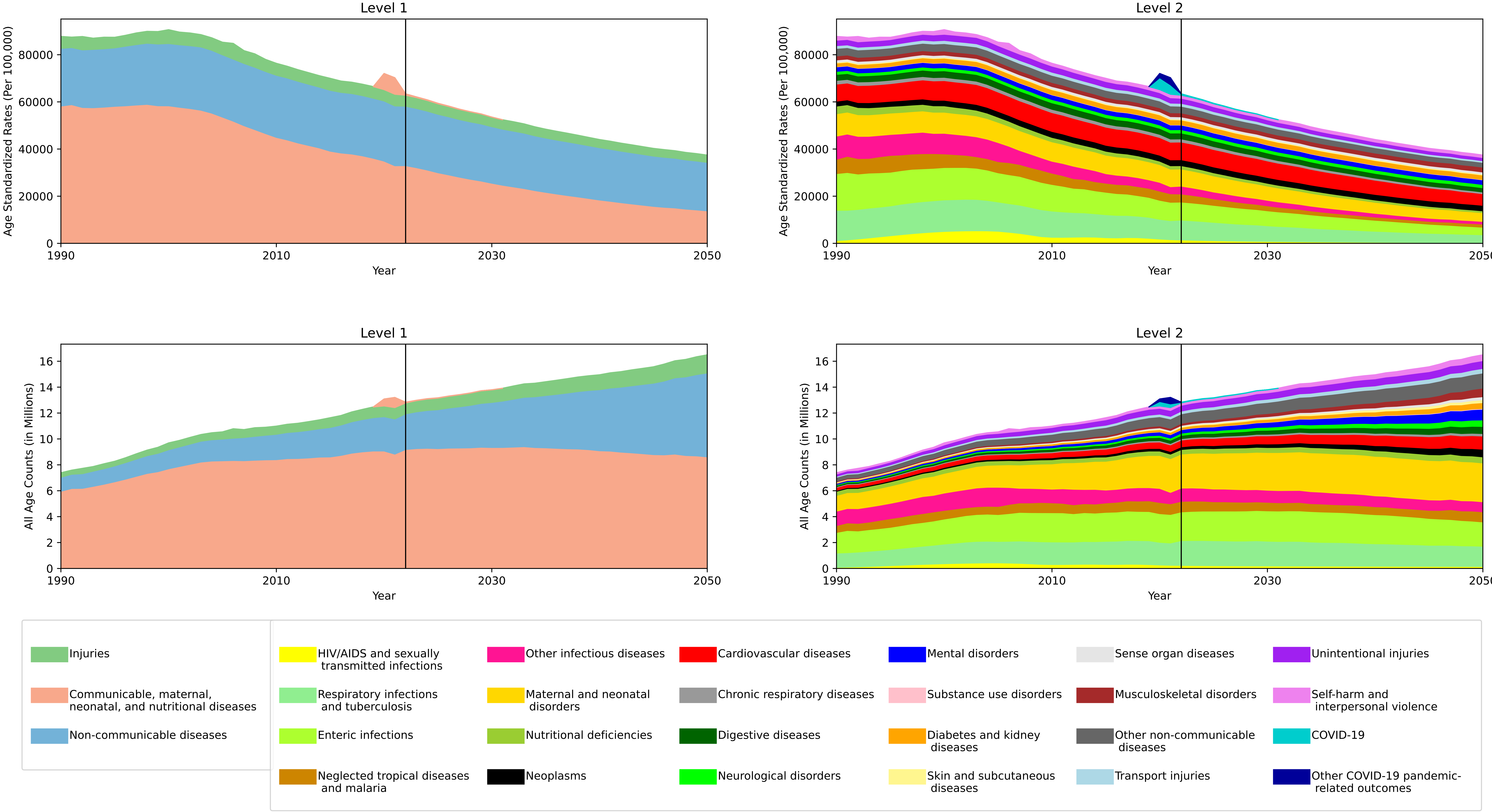
Level 1
Level 2
Age Standardized Rates (Per 100,000)
All Age Counts (in Millions)
Year
1990
2010
2030
2050
0
20000
40000
60000
80000
2
4
6
8
10
12
14
16
Injuries
Communicable, maternal, neonatal, and nutritional diseases
Non-communicable diseases
HIV/AIDS and sexually transmitted infections
Respiratory infections and tuberculosis
Enteric infections
Neglected tropical diseases and malaria
Other infectious diseases
Maternal and neonatal disorders
Nutritional deficiencies
Neoplasms
Cardiovascular diseases
Chronic respiratory diseases
Digestive diseases
Neurological disorders
Mental disorders
Substance use disorders
Diabetes and kidney diseases
Skin and subcutaneous diseases
Sense organ diseases
Musculoskeletal disorders
Other non-communicable diseases
Transport injuries
Unintentional injuries
Self-harm and interpersonal violence
COVID-19
Other COVID-19 pandemic-related outcomes

Côte D'Ivoire

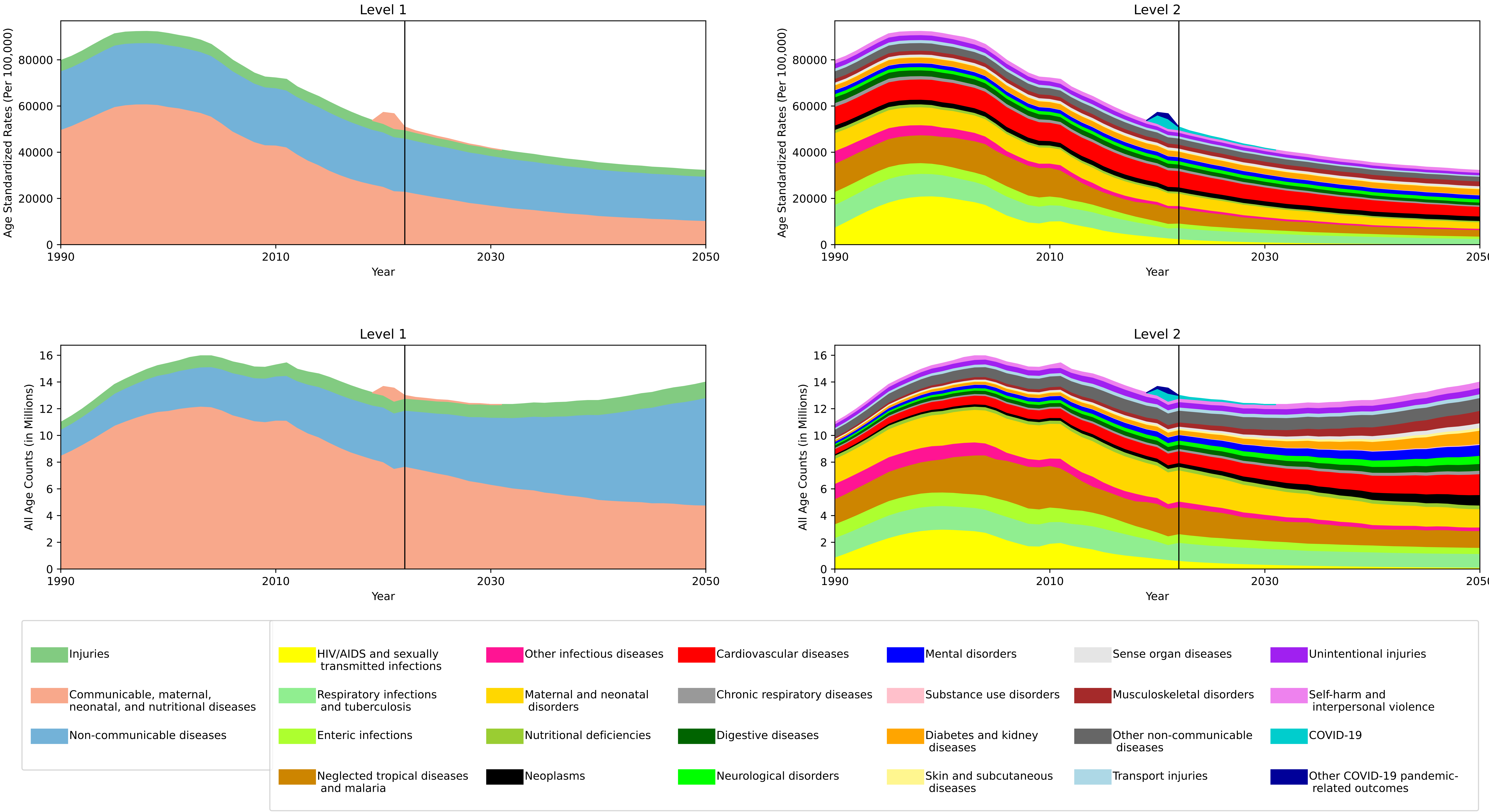

The Gambia

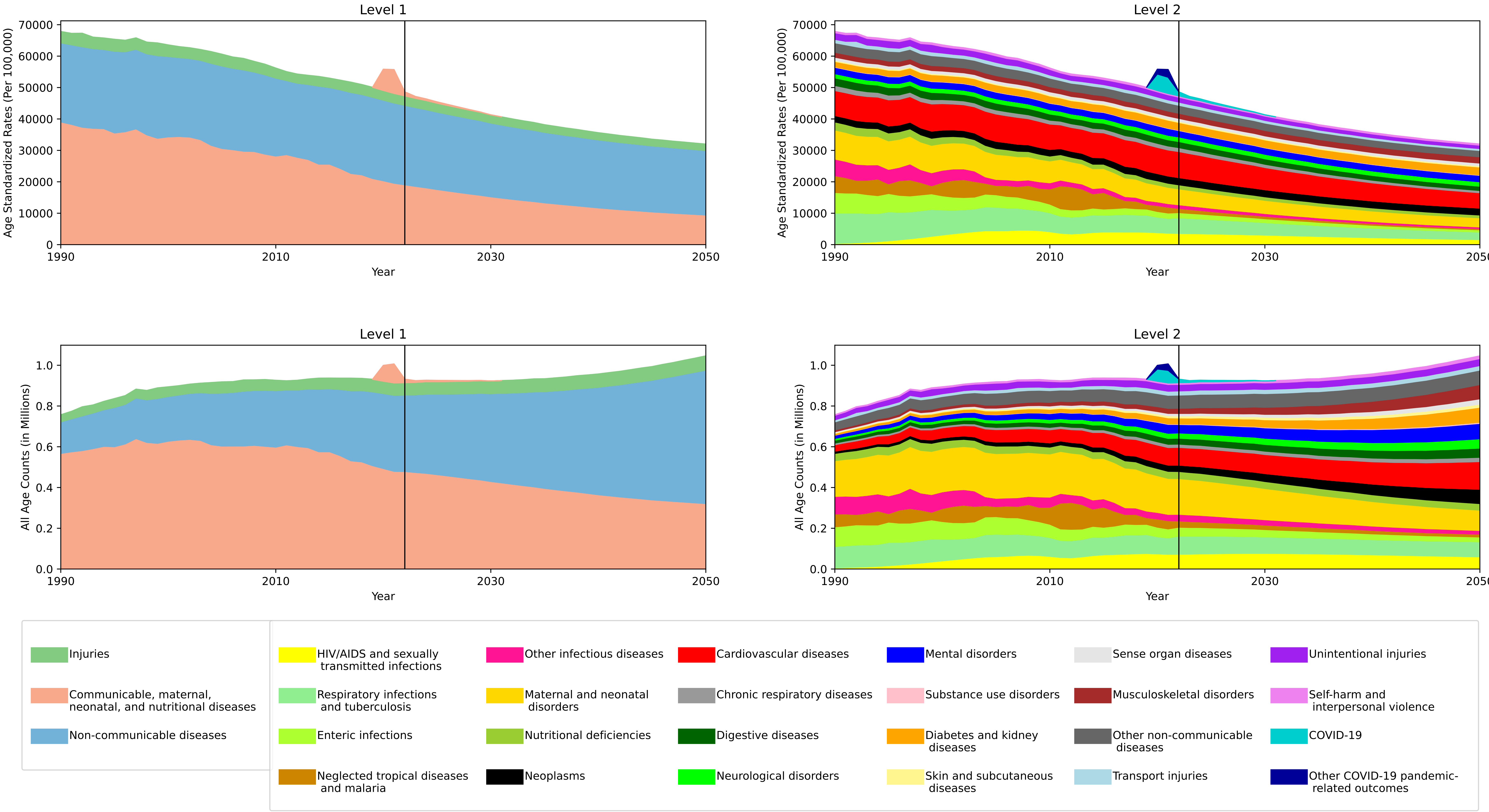
Level 1
Level 2
Age Standardized Rates (Per 100,000)
All Age Counts (in Millions)
Year
1990
2010
2030
2050
Injuries
Communicable, maternal, neonatal, and nutritional diseases
Non-communicable diseases
HIV/AIDS and sexually transmitted infections
Respiratory infections and tuberculosis
Enteric infections
Neglected tropical diseases and malaria
Other infectious diseases
Maternal and neonatal disorders
Nutritional deficiencies
Neoplasms
Cardiovascular diseases
Chronic respiratory diseases
Digestive diseases
Neurological disorders
Mental disorders
Substance use disorders
Diabetes and kidney diseases
Skin and subcutaneous diseases
Sense organ diseases
Musculoskeletal disorders
Other non-communicable diseases
Transport injuries
Unintentional injuries
Self-harm and interpersonal violence
COVID-19
Other COVID-19 pandemic-related outcomes

Ghana

Supplemental Results Figure S4. Age-standardised DALY rates and all age DALY counts and by Level 1 and Level 2 causes, 1990–2050

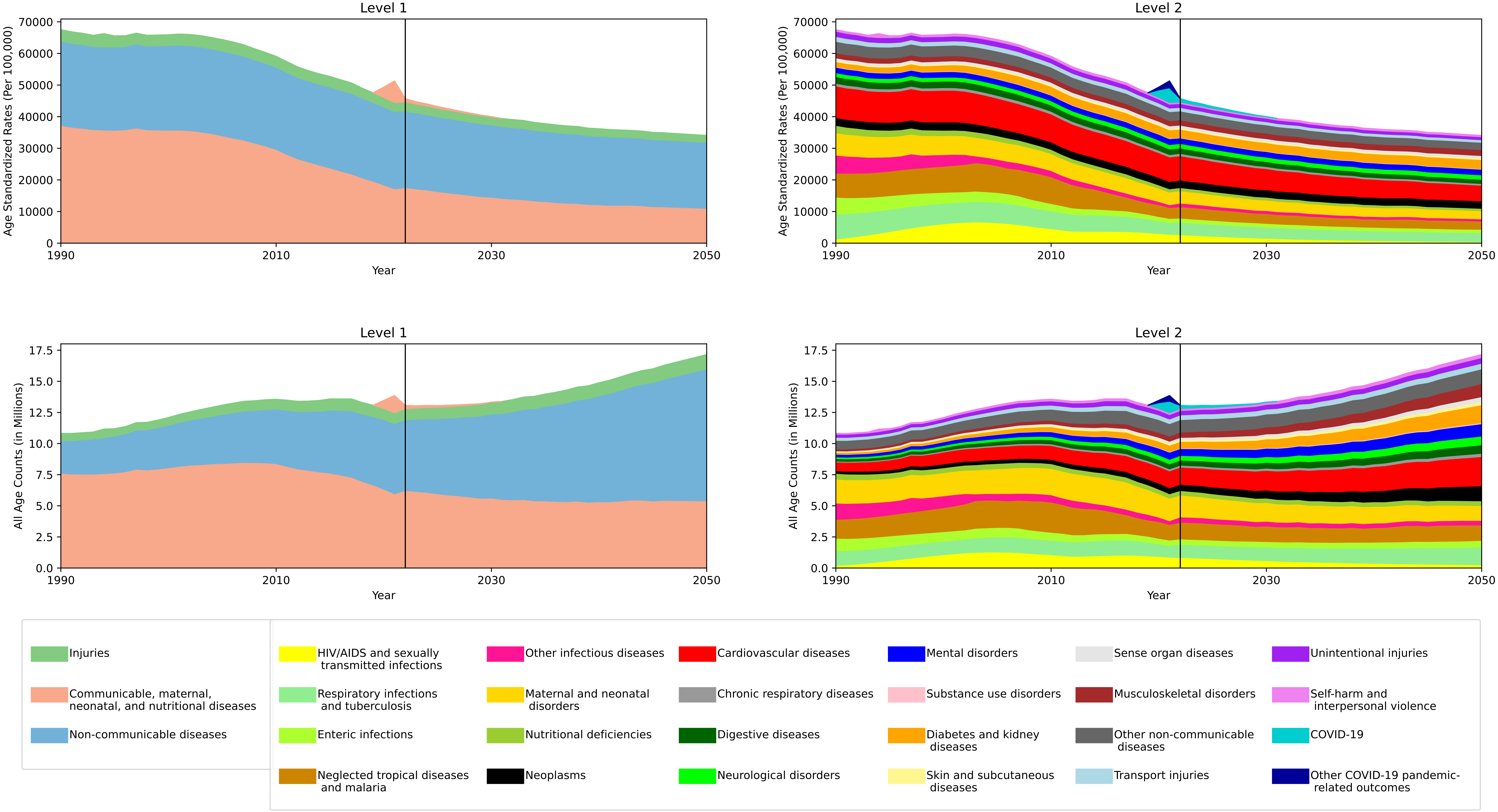

Guinea

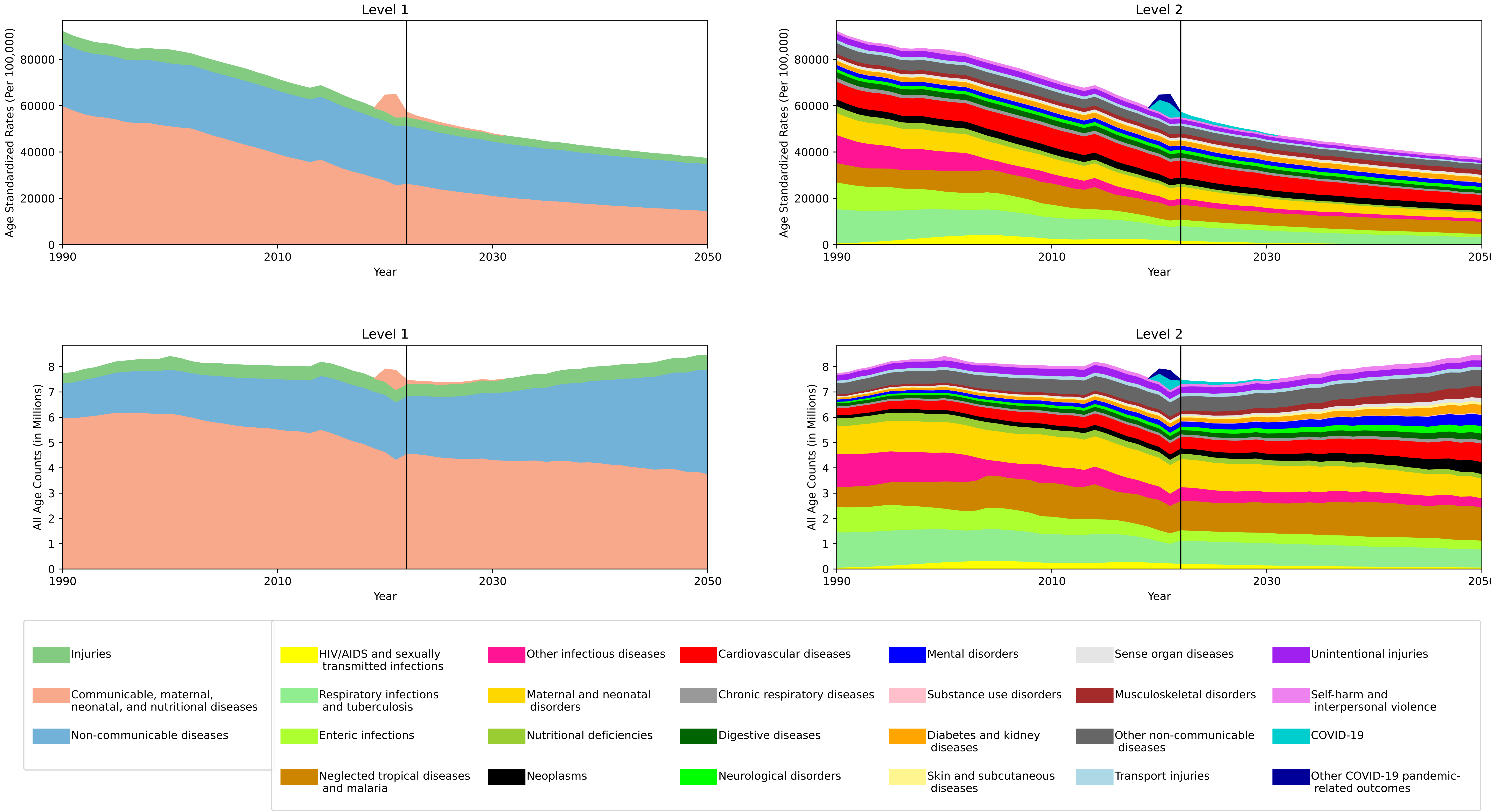

Level 1
Level 2
Age Standardized Rates (Per 100,000)
All Age Counts (in Millions)
Year
0
20000
40000
60000
80000
0
1
2
3
4
5
6
7
8
1990
2010
2030
2050
Injuries
Communicable, maternal, neonatal, and nutritional diseases
Non-communicable diseases
HIV/AIDS and sexually transmitted infections
Respiratory infections and tuberculosis
Enteric infections
Neglected tropical diseases and malaria
Other infectious diseases
Maternal and neonatal disorders
Nutritional deficiencies
Neoplasms
Cardiovascular diseases
Chronic respiratory diseases
Digestive diseases
Neurological disorders
Mental disorders
Substance use disorders
Diabetes and kidney diseases
Skin and subcutaneous diseases
Sense organ diseases
Musculoskeletal disorders
Other non-communicable diseases
Transport injuries
Unintentional injuries
Self-harm and interpersonal violence
COVID-19
Other COVID-19 pandemic-related outcomes

Guinea-Bissau

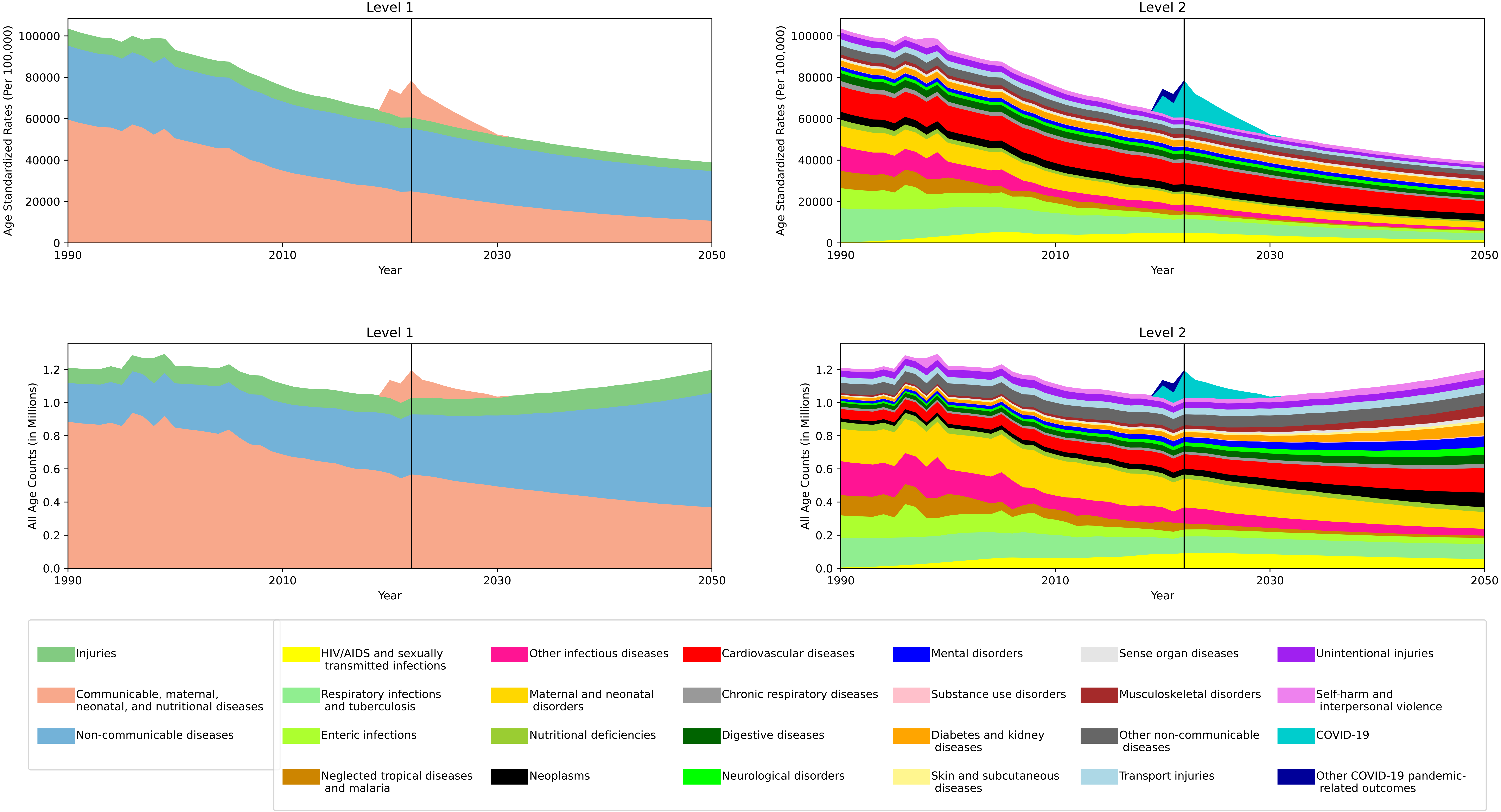

Liberia

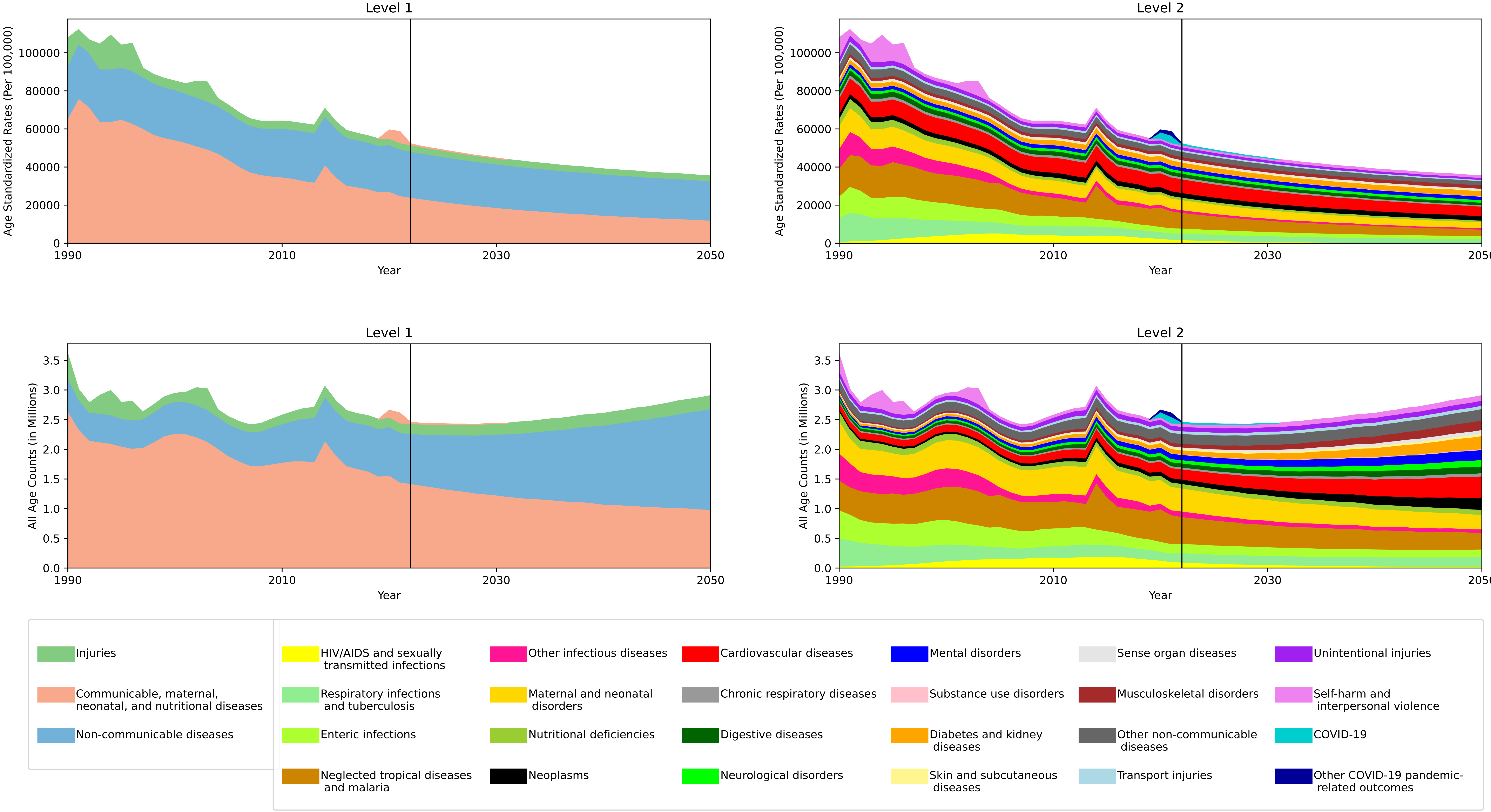
Level 1
Level 2
Age Standardized Rates (Per 100,000)
All Age Counts (in Millions)
Year
Injuries
Communicable, maternal, neonatal, and nutritional diseases
Non-communicable diseases
HIV/AIDS and sexually transmitted infections
Respiratory infections and tuberculosis
Enteric infections
Neglected tropical diseases and malaria
Other infectious diseases
Maternal and neonatal disorders
Nutritional deficiencies
Neoplasms
Cardiovascular diseases
Chronic respiratory diseases
Digestive diseases
Neurological disorders
Mental disorders
Substance use disorders
Diabetes and kidney diseases
Skin and subcutaneous diseases
Sense organ diseases
Musculoskeletal disorders
Other non-communicable diseases
Transport injuries
Unintentional injuries
Self-harm and interpersonal violence
COVID-19
Other COVID-19 pandemic-related outcomes

Mali

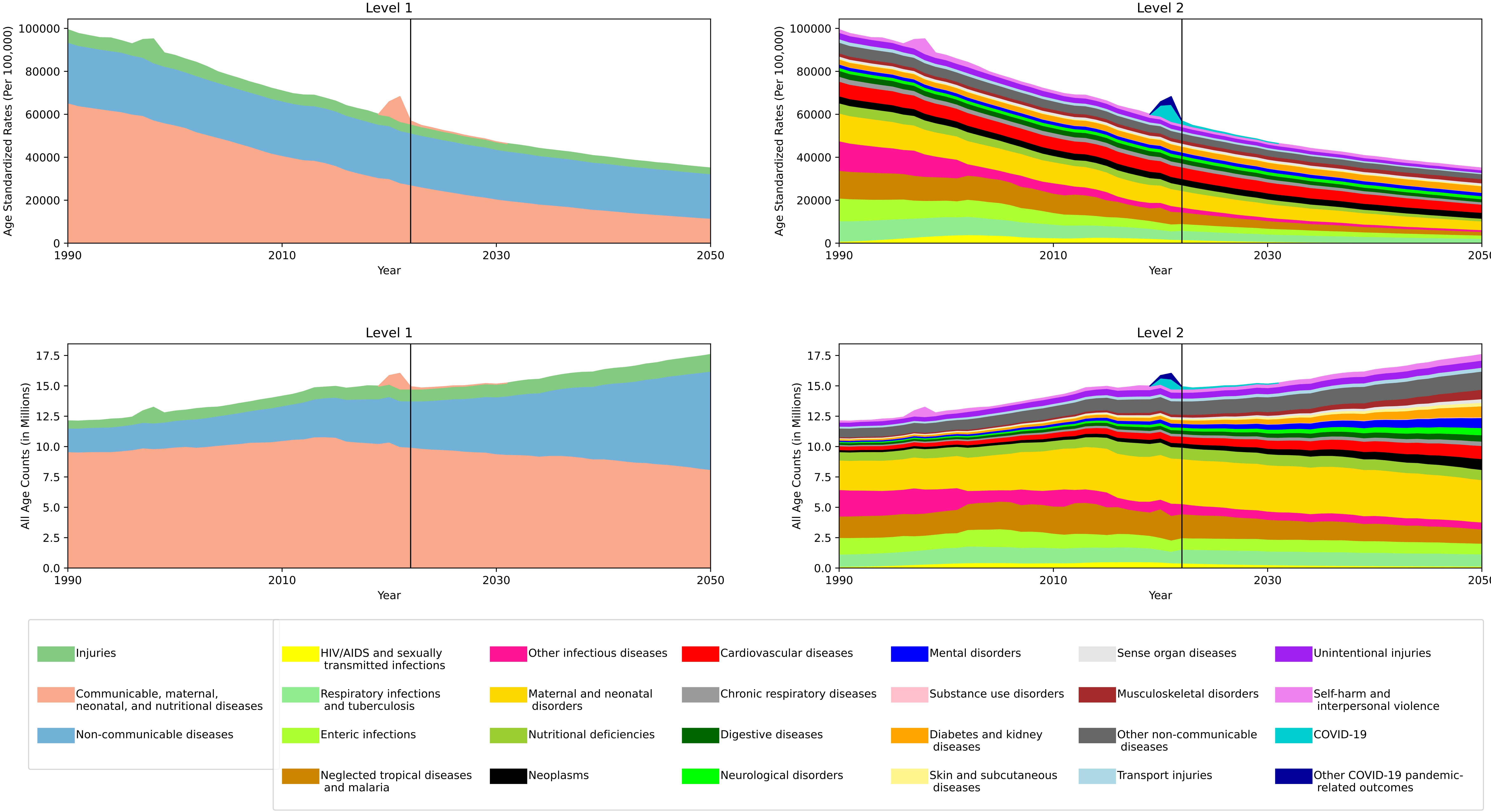
Level 1
Level 2
Age Standardized Rates (Per 100,000)
All Age Counts (in Millions)
Year
1990
2010
2030
2050
0
20000
40000
60000
80000
100000
0.0
2.5
5.0
7.5
10.0
12.5
15.0
17.5
Injuries
Communicable, maternal, neonatal, and nutritional diseases
Non-communicable diseases
HIV/AIDS and sexually transmitted infections
Respiratory infections and tuberculosis
Enteric infections
Neglected tropical diseases and malaria
Other infectious diseases
Maternal and neonatal disorders
Nutritional deficiencies
Neoplasms
Cardiovascular diseases
Chronic respiratory diseases
Digestive diseases
Neurological disorders
Mental disorders
Substance use disorders
Diabetes and kidney diseases
Skin and subcutaneous diseases
Sense organ diseases
Musculoskeletal disorders
Other non-communicable diseases
Transport injuries
Unintentional injuries
Self-harm and interpersonal violence
COVID-19
Other COVID-19 pandemic-related outcomes

Mauritania

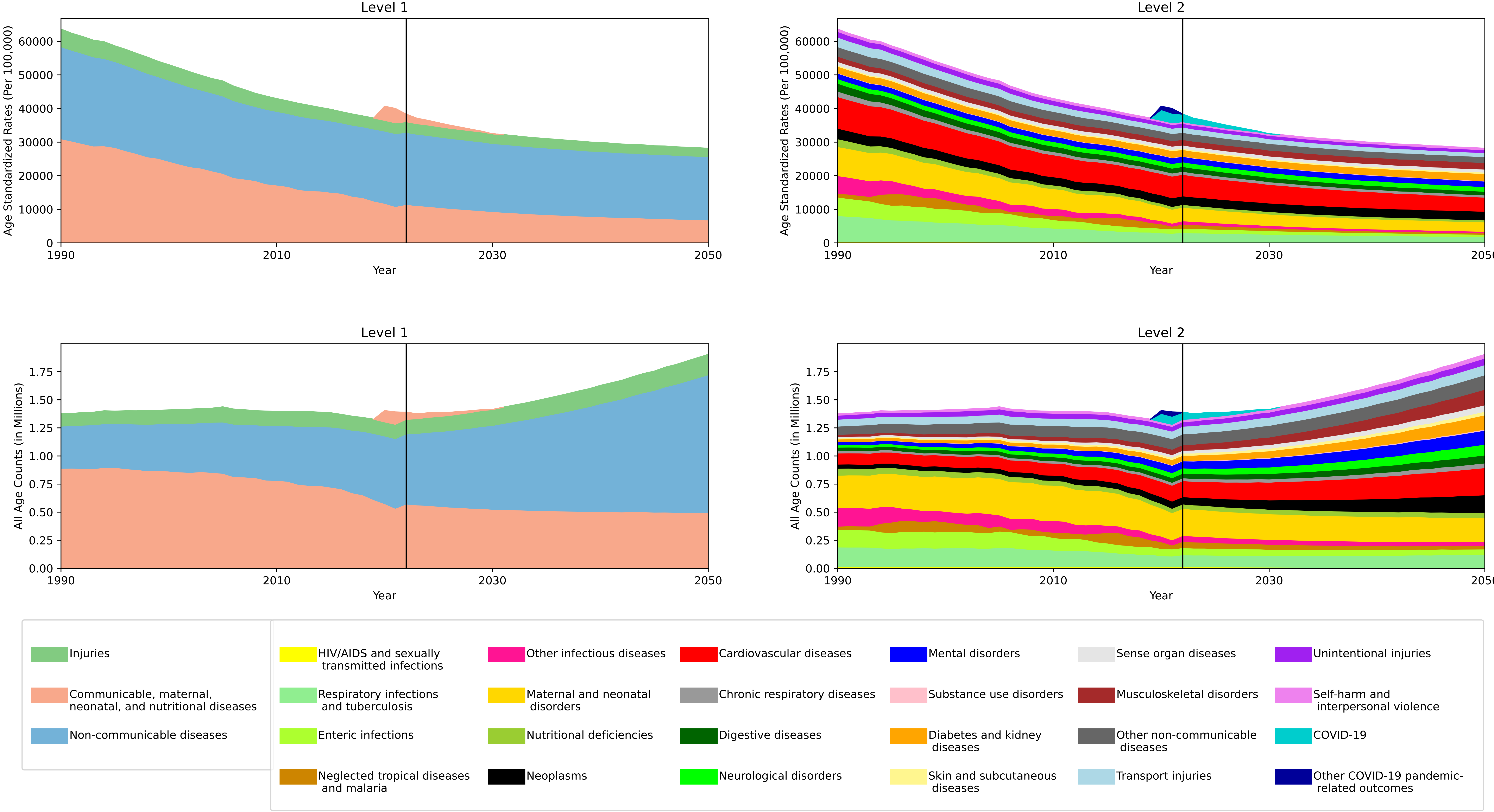

Level 1
Level 2
Age Standardized Rates (Per 100,000)
All Age Counts (in Millions)
Year
1990
2010
2030
2050
Injuries
Communicable, maternal, neonatal, and nutritional diseases
Non-communicable diseases
HIV/AIDS and sexually transmitted infections
Respiratory infections and tuberculosis
Enteric infections
Neglected tropical diseases and malaria
Other infectious diseases
Maternal and neonatal disorders
Nutritional deficiencies
Neoplasms
Cardiovascular diseases
Chronic respiratory diseases
Digestive diseases
Neurological disorders
Mental disorders
Substance use disorders
Diabetes and kidney diseases
Skin and subcutaneous diseases
Sense organ diseases
Musculoskeletal disorders
Other non-communicable diseases
Transport injuries
Unintentional injuries
Self-harm and interpersonal violence
COVID-19
Other COVID-19 pandemic-related outcomes

Niger

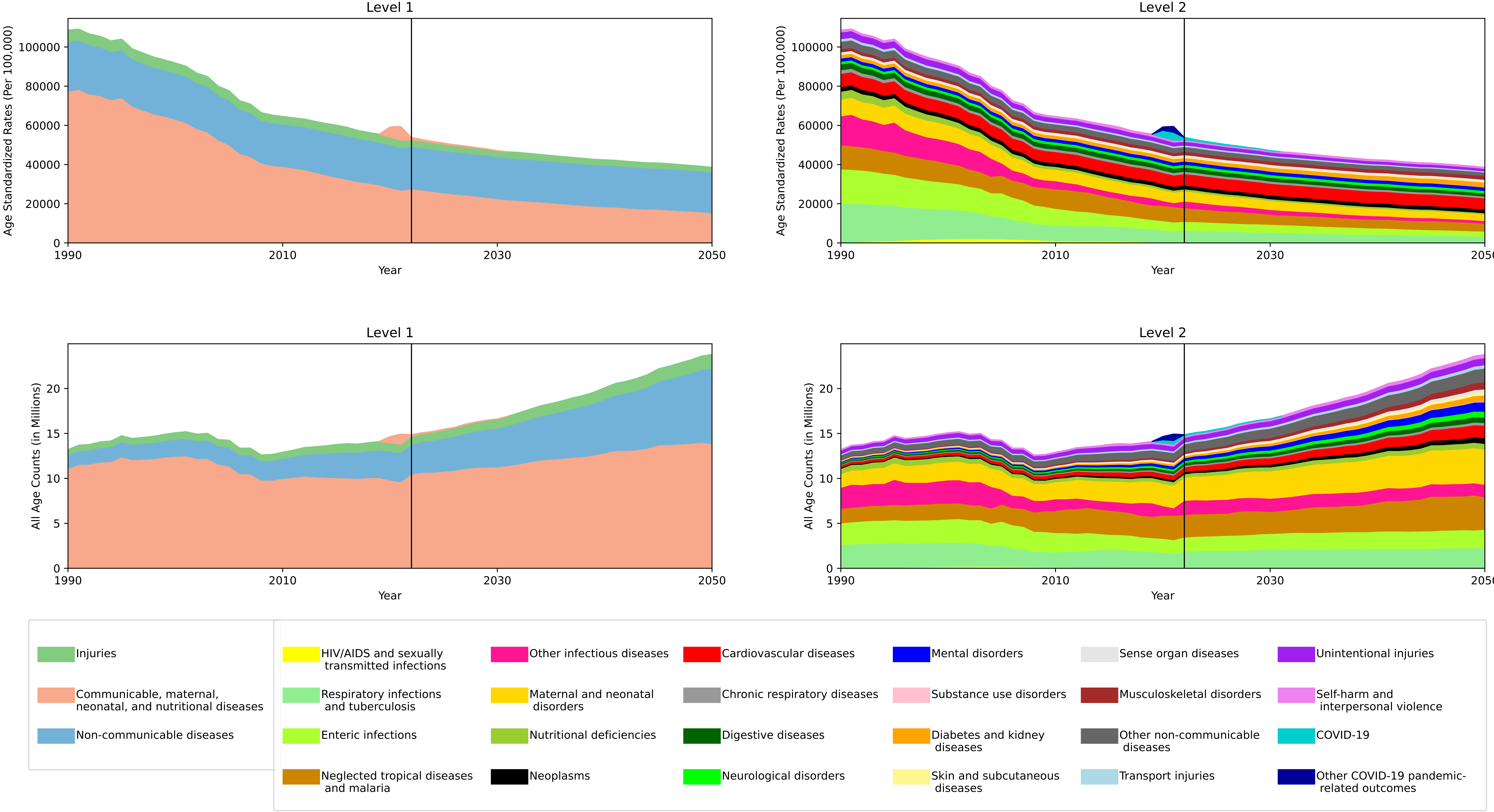
Level 1
Level 2
Age Standardized Rates (Per 100,000)
All Age Counts (in Millions)
Year
1990
2010
2030
2050
Injuries
Communicable, maternal, neonatal, and nutritional diseases
Non-communicable diseases
HIV/AIDS and sexually transmitted infections
Respiratory infections and tuberculosis
Enteric infections
Neglected tropical diseases and malaria
Other infectious diseases
Maternal and neonatal disorders
Nutritional deficiencies
Neoplasms
Cardiovascular diseases
Chronic respiratory diseases
Digestive diseases
Neurological disorders
Mental disorders
Substance use disorders
Diabetes and kidney diseases
Skin and subcutaneous diseases
Sense organ diseases
Musculoskeletal disorders
Other non-communicable diseases
Transport injuries
Unintentional injuries
Self-harm and interpersonal violence
COVID-19
Other COVID-19 pandemic-related outcomes

Nigeria

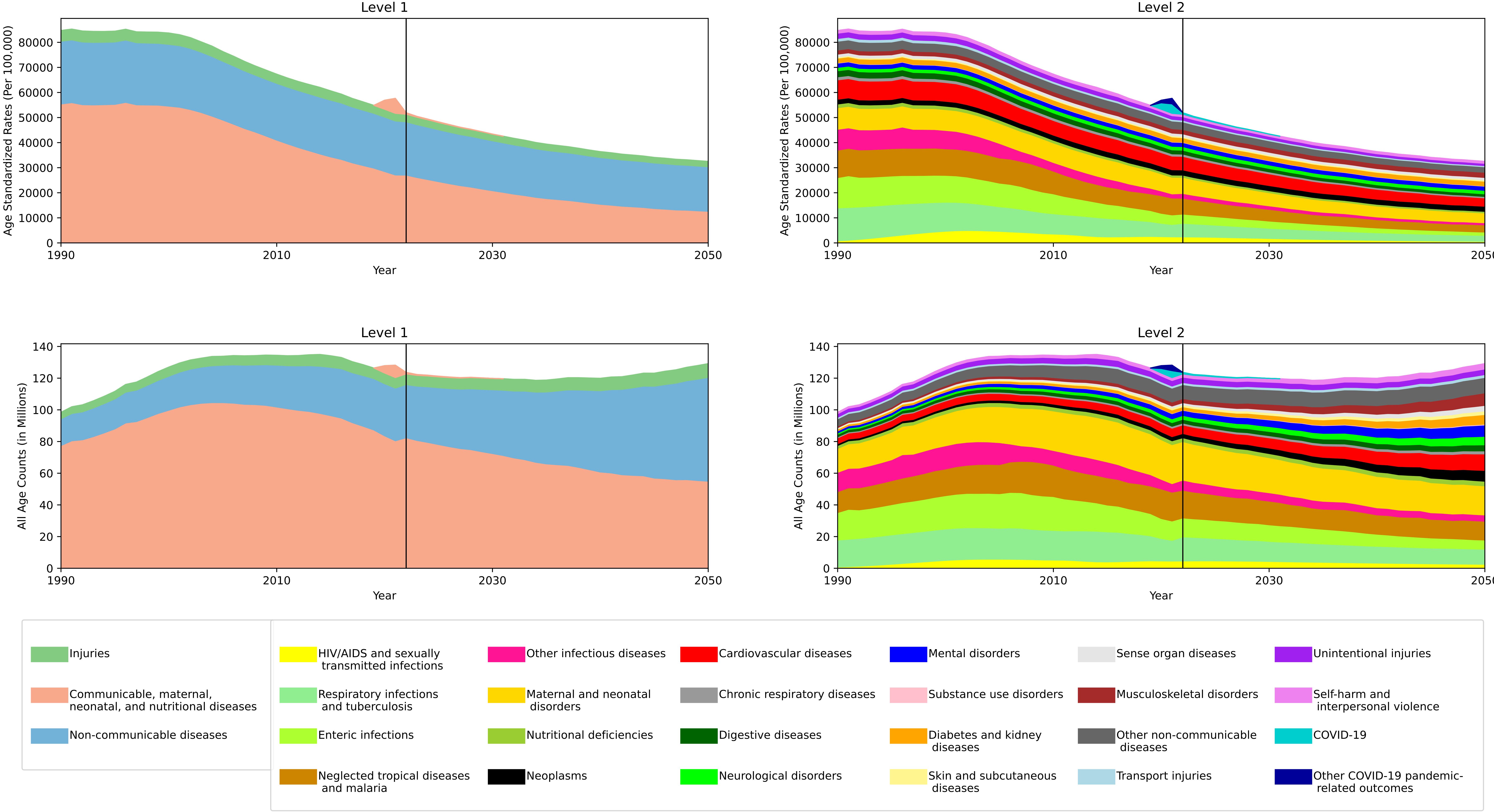
Level 1
Level 2
Age Standardized Rates (Per 100,000)
All Age Counts (in Millions)
Year
Injuries
Communicable, maternal, neonatal, and nutritional diseases
Non-communicable diseases
HIV/AIDS and sexually transmitted infections
Respiratory infections and tuberculosis
Enteric infections
Neglected tropical diseases and malaria
Other infectious diseases
Maternal and neonatal disorders
Nutritional deficiencies
Neoplasms
Cardiovascular diseases
Chronic respiratory diseases
Digestive diseases
Neurological disorders
Mental disorders
Substance use disorders
Diabetes and kidney diseases
Skin and subcutaneous diseases
Sense organ diseases
Musculoskeletal disorders
Other non-communicable diseases
Transport injuries
Unintentional injuries
Self-harm and interpersonal violence
COVID-19
Other COVID-19 pandemic-related outcomes

Supplemental Results Figure S4. Age-standardised DALY rates and all age DALY counts and by Level 1 and Level 2 causes, 1990–2050

São Tomé And Príncipe

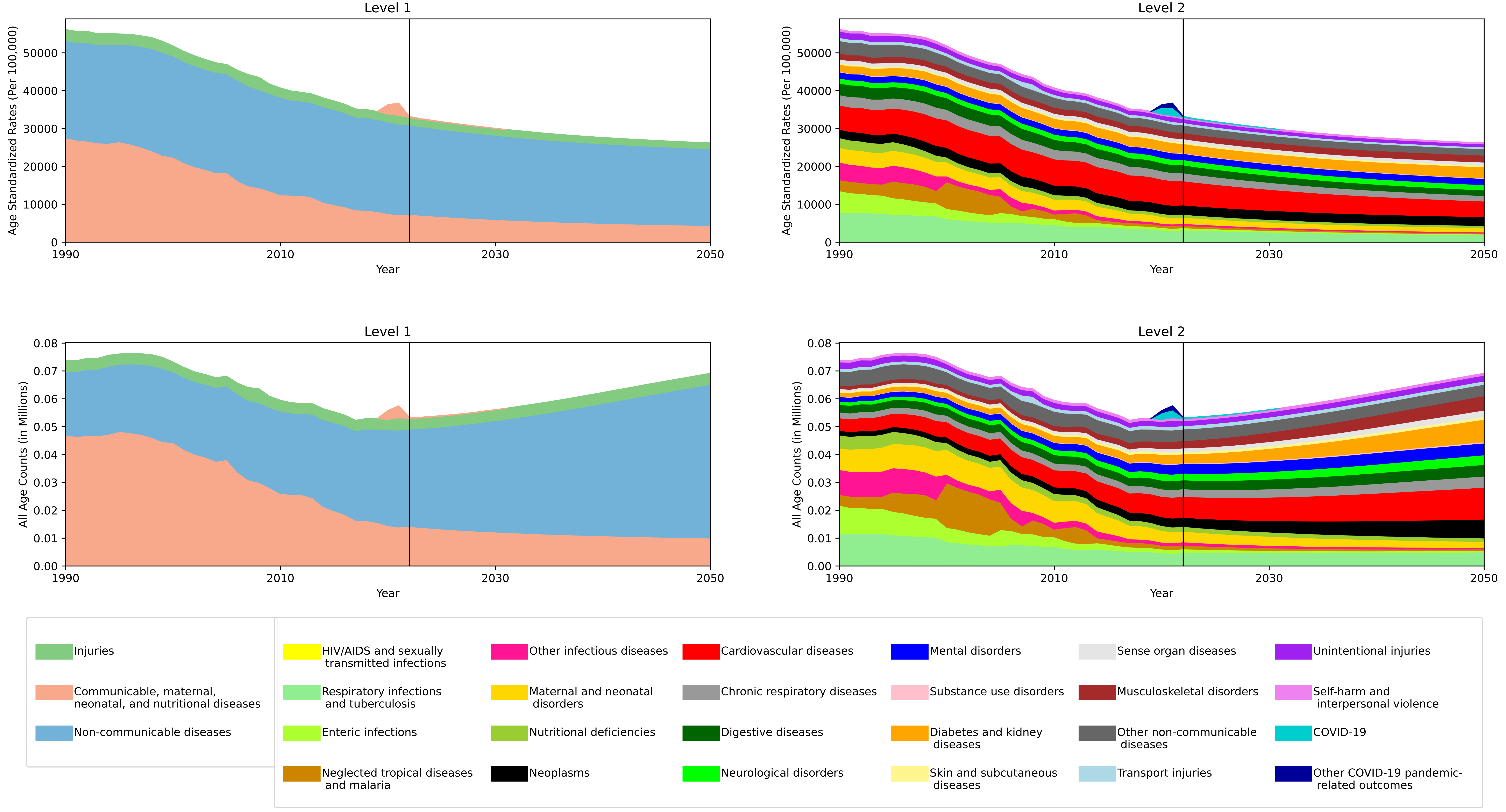

Senegal

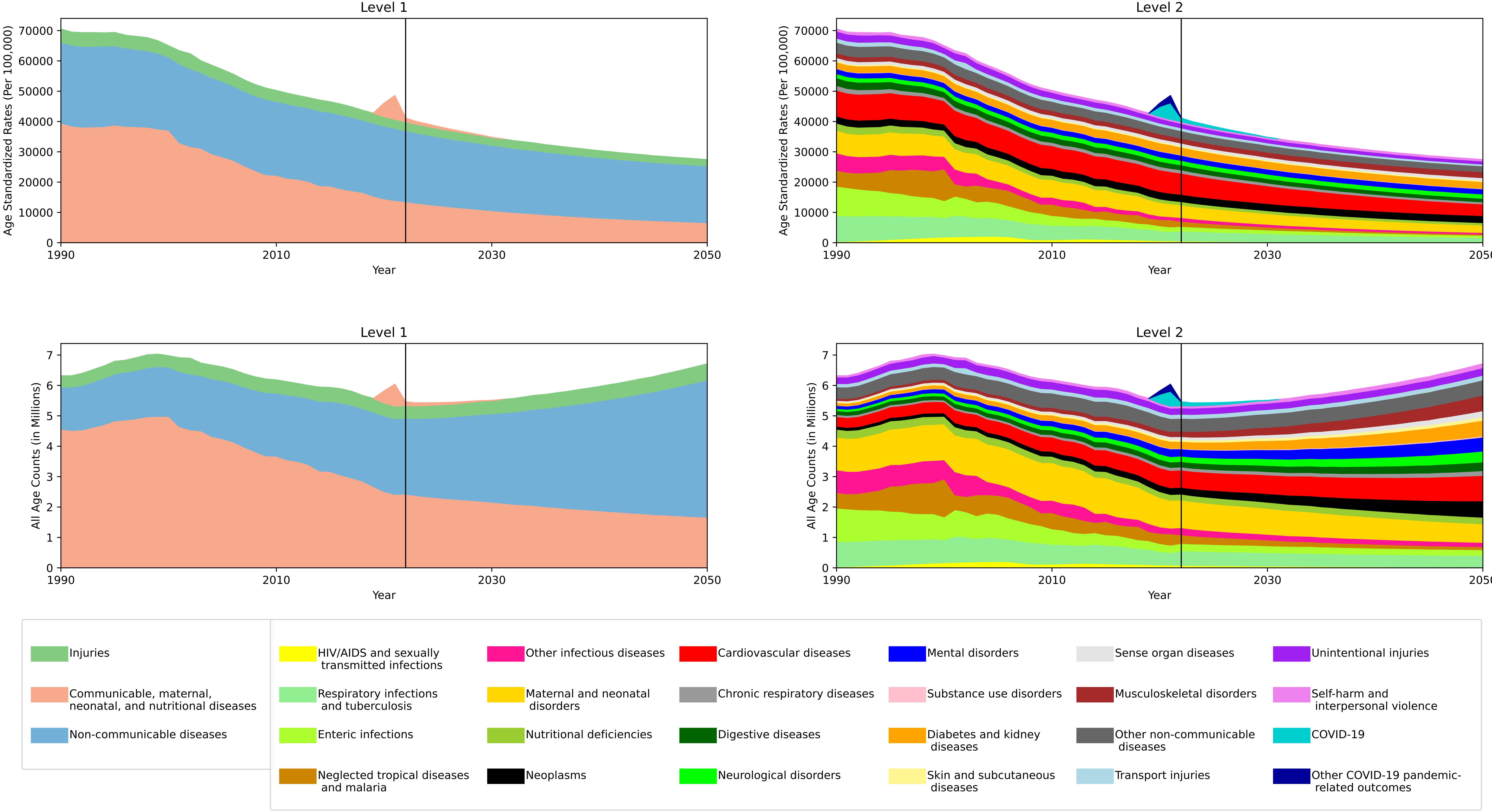
Level 1
Level 2
Age Standardized Rates (Per 100,000)
All Age Counts (in Millions)
Year
1990
2010
2030
2050
Injuries
Communicable, maternal, neonatal, and nutritional diseases
Non-communicable diseases
HIV/AIDS and sexually transmitted infections
Respiratory infections and tuberculosis
Enteric infections
Neglected tropical diseases and malaria
Other infectious diseases
Maternal and neonatal disorders
Nutritional deficiencies
Neoplasms
Cardiovascular diseases
Chronic respiratory diseases
Digestive diseases
Neurological disorders
Mental disorders
Substance use disorders
Diabetes and kidney diseases
Skin and subcutaneous diseases
Sense organ diseases
Musculoskeletal disorders
Other non-communicable diseases
Transport injuries
Unintentional injuries
Self-harm and interpersonal violence
COVID-19
Other COVID-19 pandemic-related outcomes

Sierra Leone

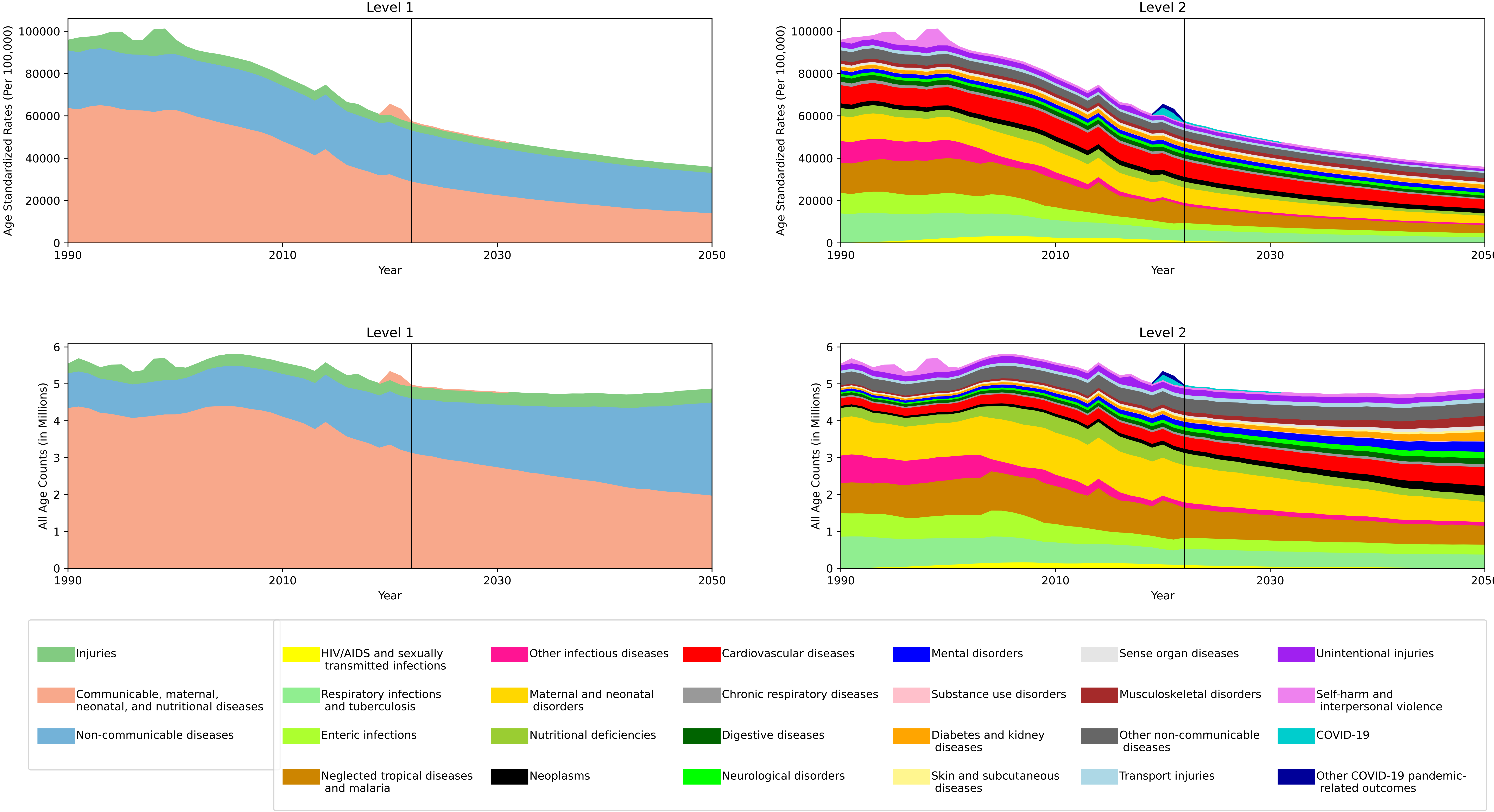

Supplemental Results Figure S4. Age-standardised DALY rates and all age DALY counts and by Level 1 and Level 2 causes, 1990–2050

Togo

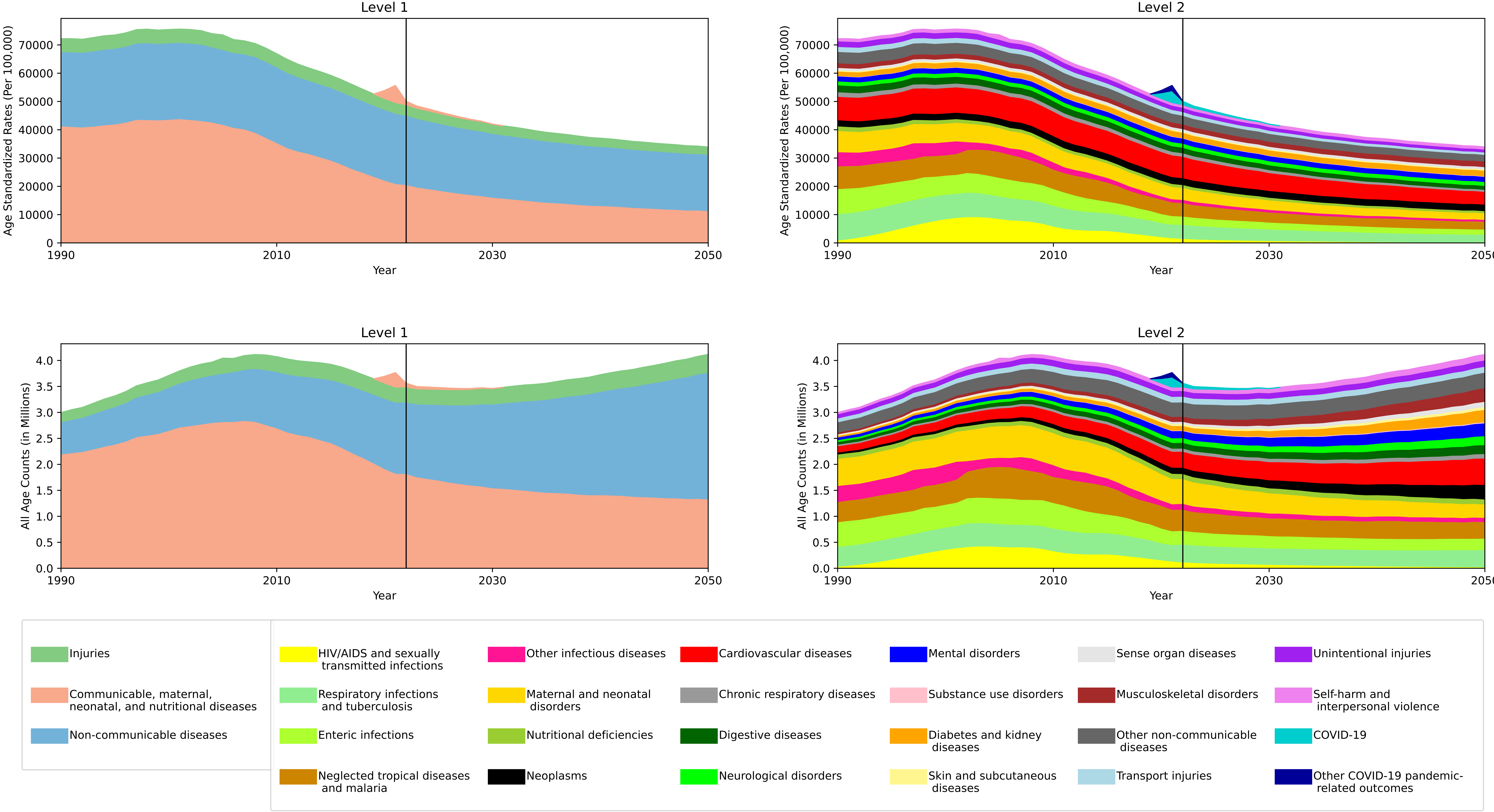

Global

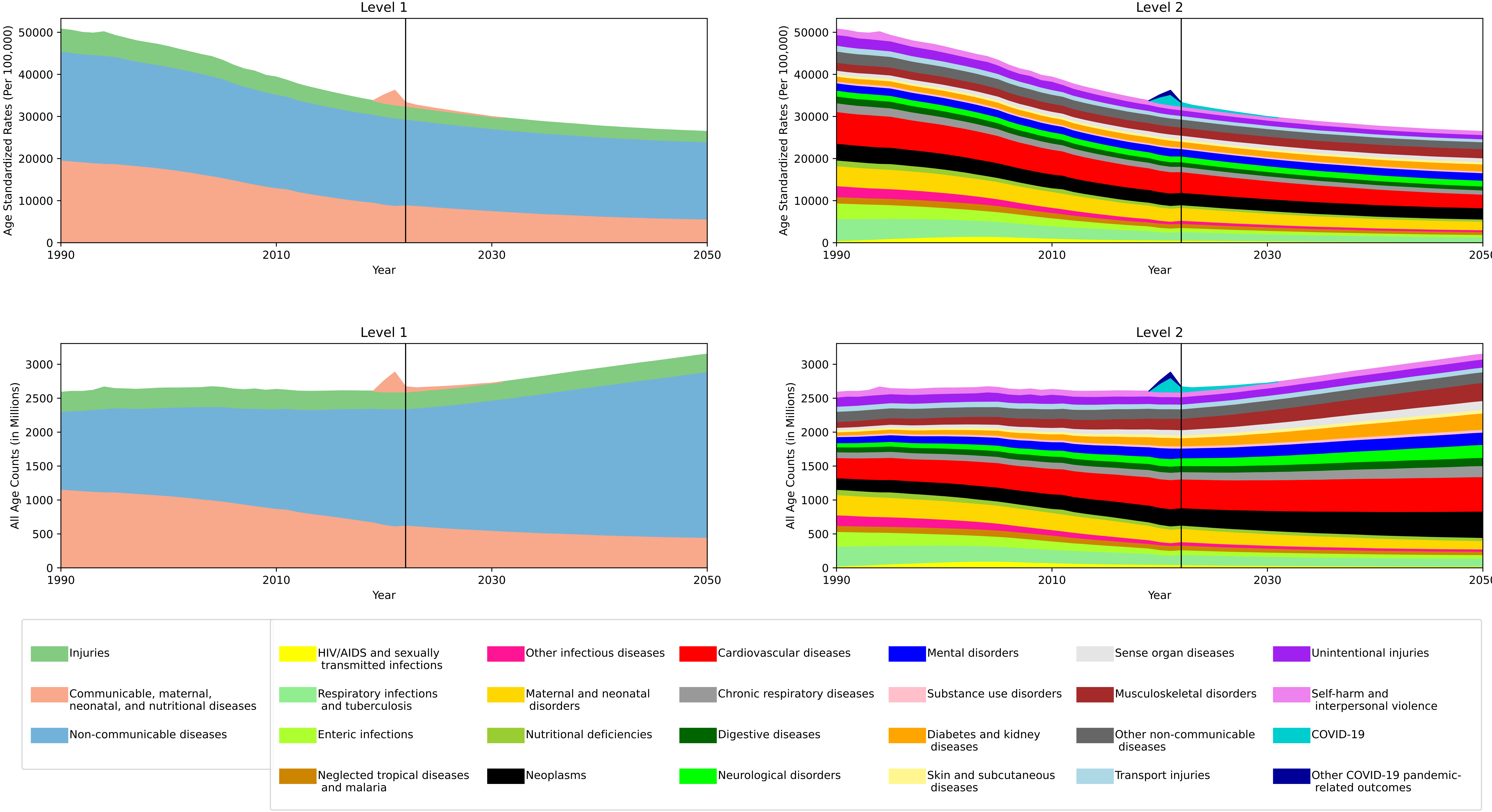
Level 1
Age Standardized Rates (Per 100,000)
Year
Level 2
Age Standardized Rates (Per 100,000)
Year
Level 1
All Age Counts (in Millions)
Year
Level 2
All Age Counts (in Millions)
Year
Injuries
Communicable, maternal, neonatal, and nutritional diseases
Non-communicable diseases
HIV/AIDS and sexually transmitted infections
Respiratory infections and tuberculosis
Enteric infections
Neglected tropical diseases and malaria
Other infectious diseases
Maternal and neonatal disorders
Nutritional deficiencies
Neoplasms
Cardiovascular diseases
Chronic respiratory diseases
Digestive diseases
Neurological disorders
Mental disorders
Substance use disorders
Diabetes and kidney diseases
Skin and subcutaneous diseases
Sense organ diseases
Musculoskeletal disorders
Other non-communicable diseases
Transport injuries
Unintentional injuries
Self-harm and interpersonal violence
COVID-19
Other COVID-19 pandemic-related outcomes

Supplemental Results Figure S4. Age-standardised DALY rates and all age DALY counts and by Level 1 and Level 2 causes, 1990–2050

Central Europe, Eastern Europe, And Central Asia

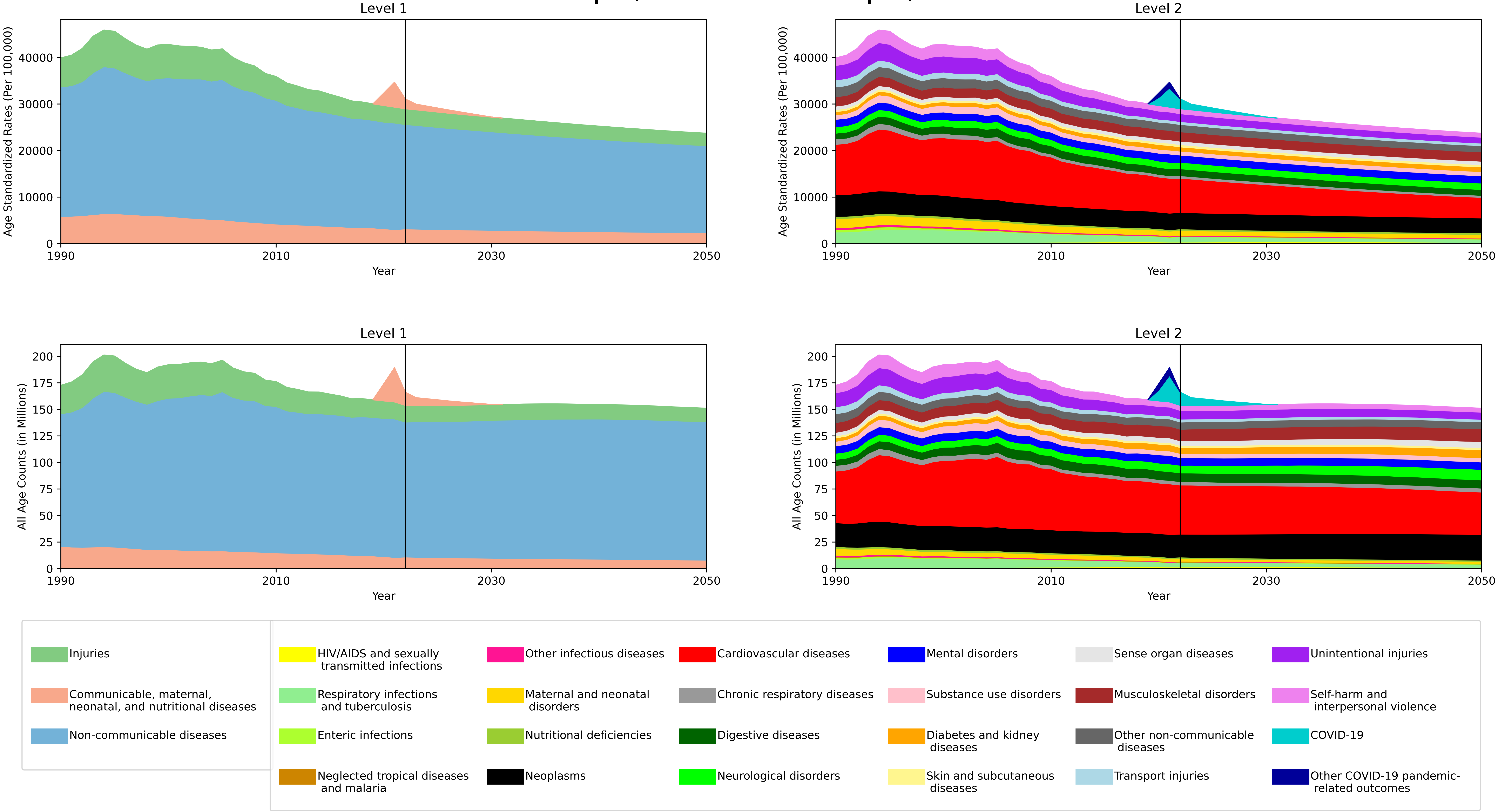

High Income

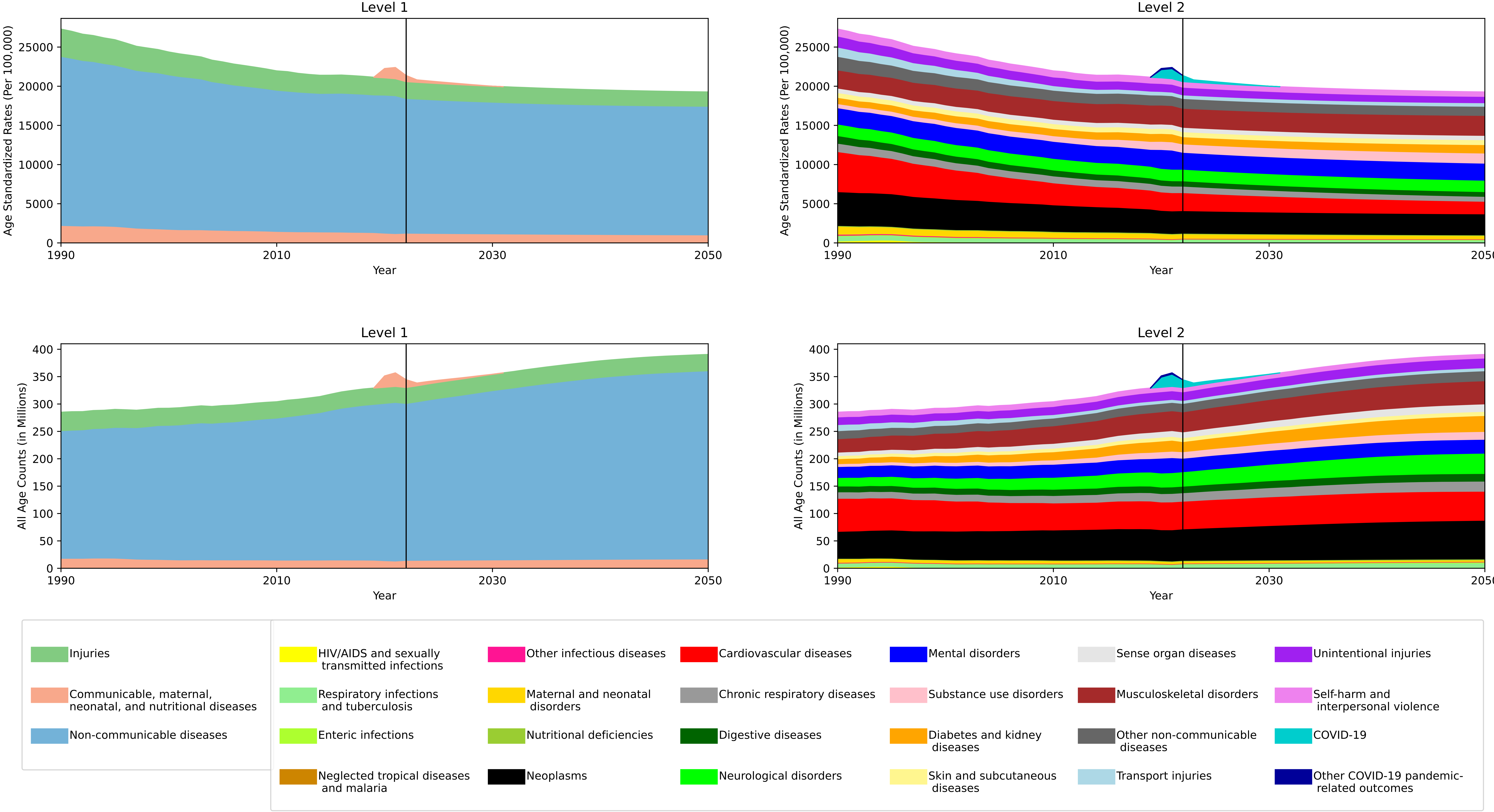
Level 1
Age Standardized Rates (Per 100,000)
Year
Level 2
Age Standardized Rates (Per 100,000)
Year
Level 1
All Age Counts (in Millions)
Year
Level 2
All Age Counts (in Millions)
Year
Injuries
Communicable, maternal, neonatal, and nutritional diseases
Non-communicable diseases
HIV/AIDS and sexually transmitted infections
Respiratory infections and tuberculosis
Enteric infections
Neglected tropical diseases and malaria
Other infectious diseases
Maternal and neonatal disorders
Nutritional deficiencies
Neoplasms
Cardiovascular diseases
Chronic respiratory diseases
Digestive diseases
Neurological disorders
Mental disorders
Substance use disorders
Diabetes and kidney diseases
Skin and subcutaneous diseases
Sense organ diseases
Musculoskeletal disorders
Other non-communicable diseases
Transport injuries
Unintentional injuries
Self-harm and interpersonal violence
COVID-19
Other COVID-19 pandemic-related outcomes

Supplemental Results Figure S4. Age-standardised DALY rates and all age DALY counts and by Level 1 and Level 2 causes, 1990–2050

Latin America And Caribbean

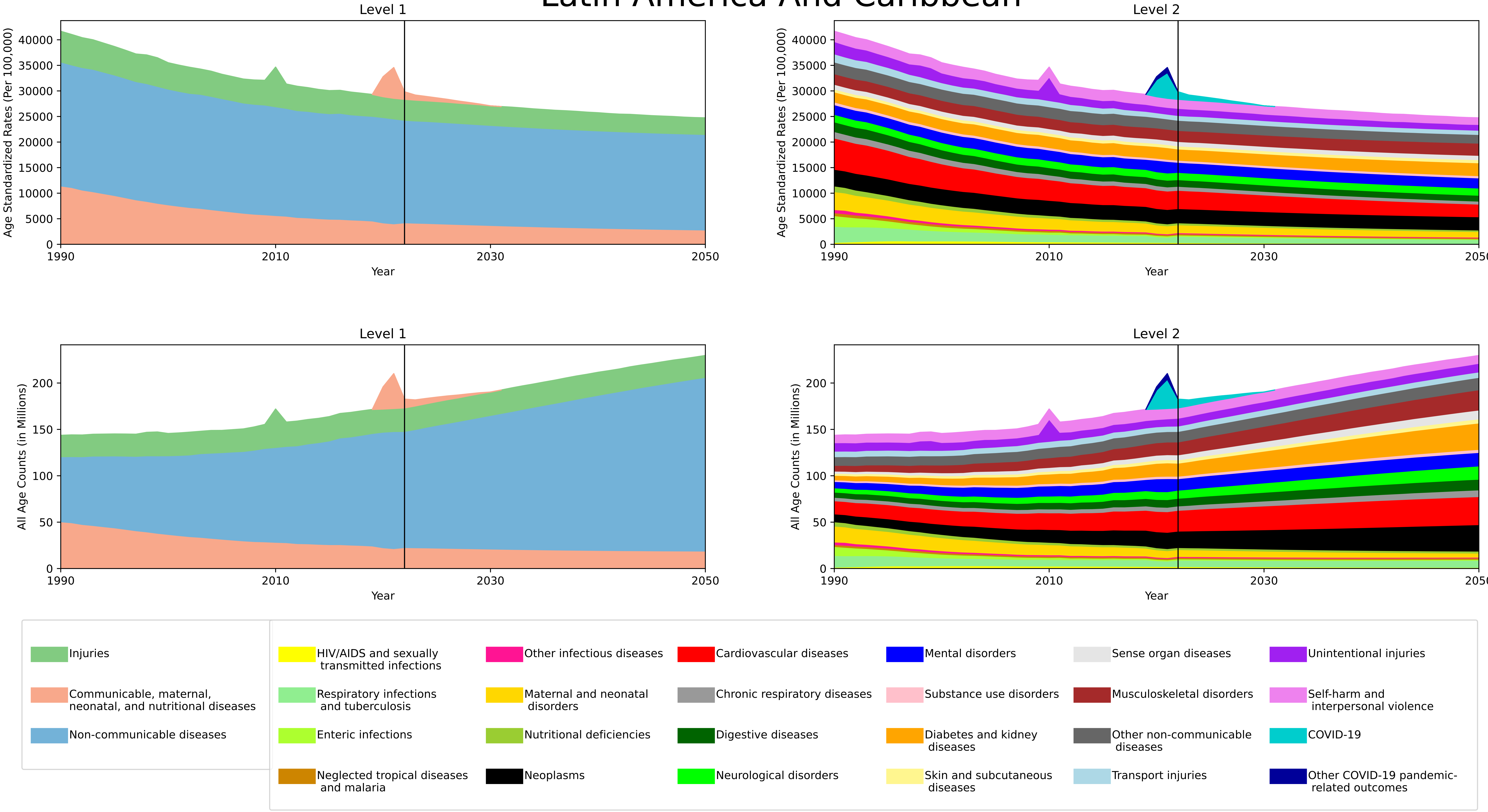

Supplemental Results Figure S4. Age-standardised DALY rates and all age DALY counts and by Level 1 and Level 2 causes, 1990–2050

North Africa And Middle East

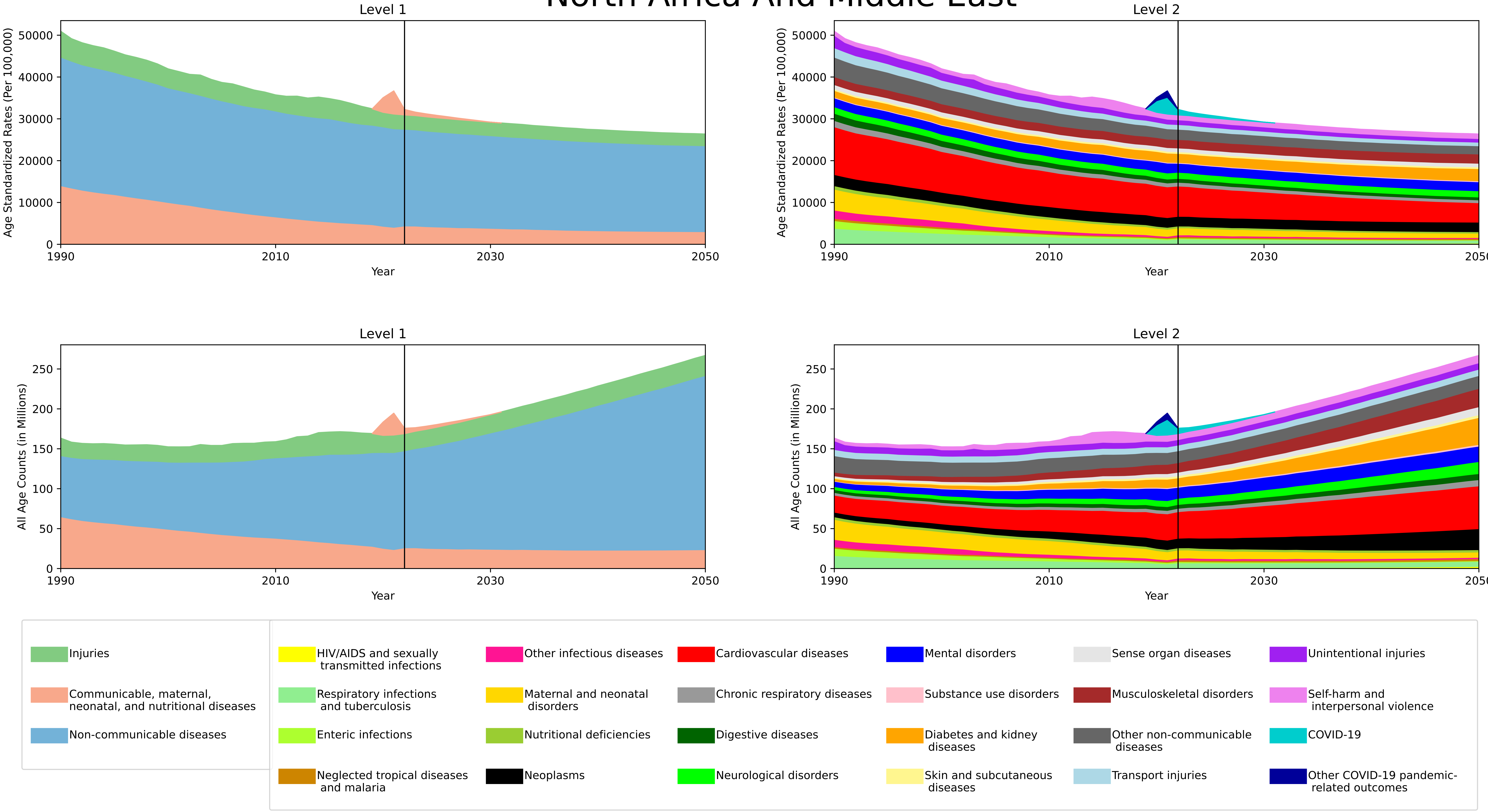

South Asia

Supplemental Results Figure S4. Age-standardised DALY rates and all age DALY counts and by Level 1 and Level 2 causes, 1990–2050

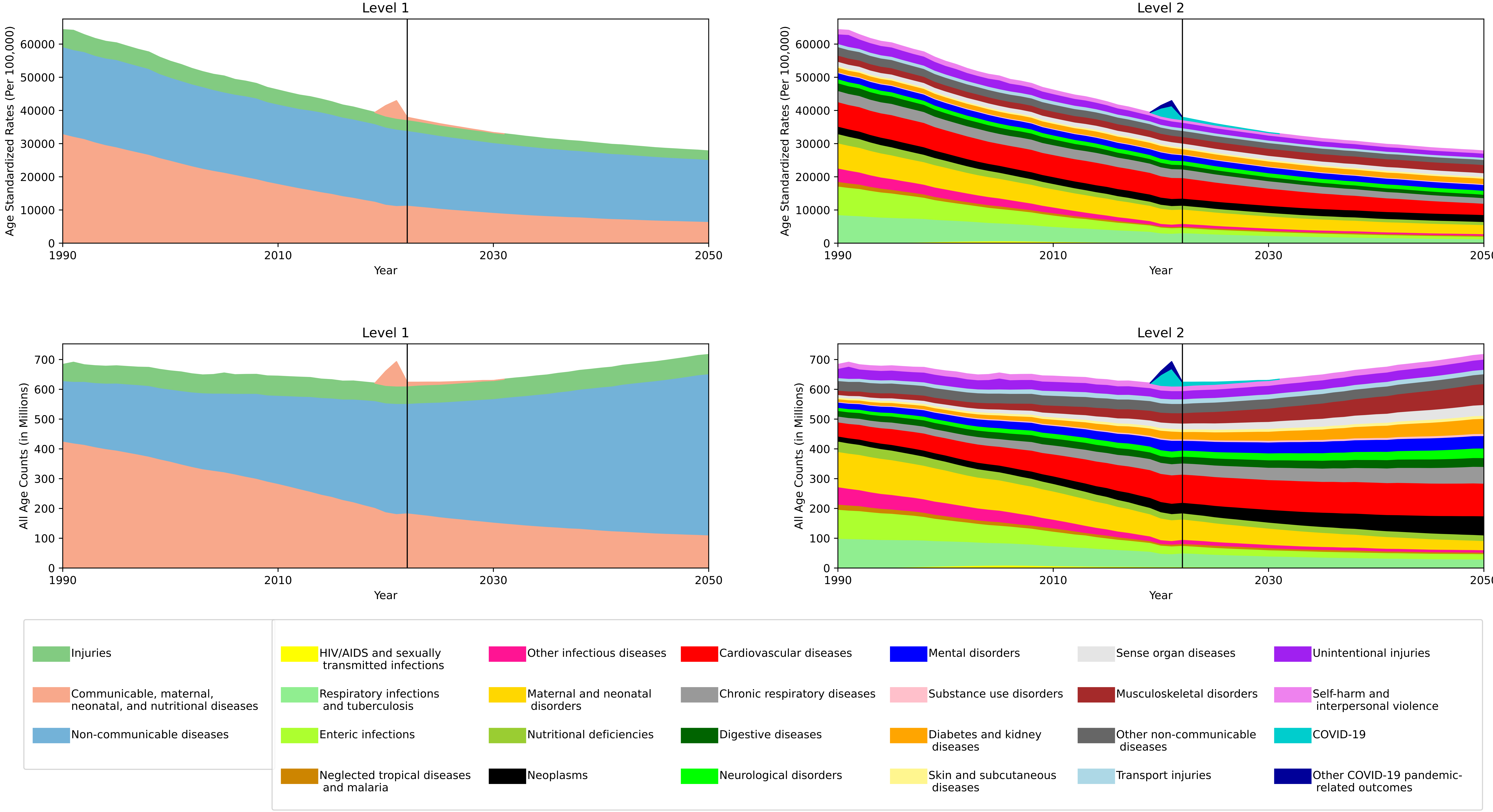

Supplemental Results Figure S4. Age-standardised DALY rates and all age DALY counts and by Level 1 and Level 2 causes, 1990–2050

Southeast Asia, East Asia, And Oceania

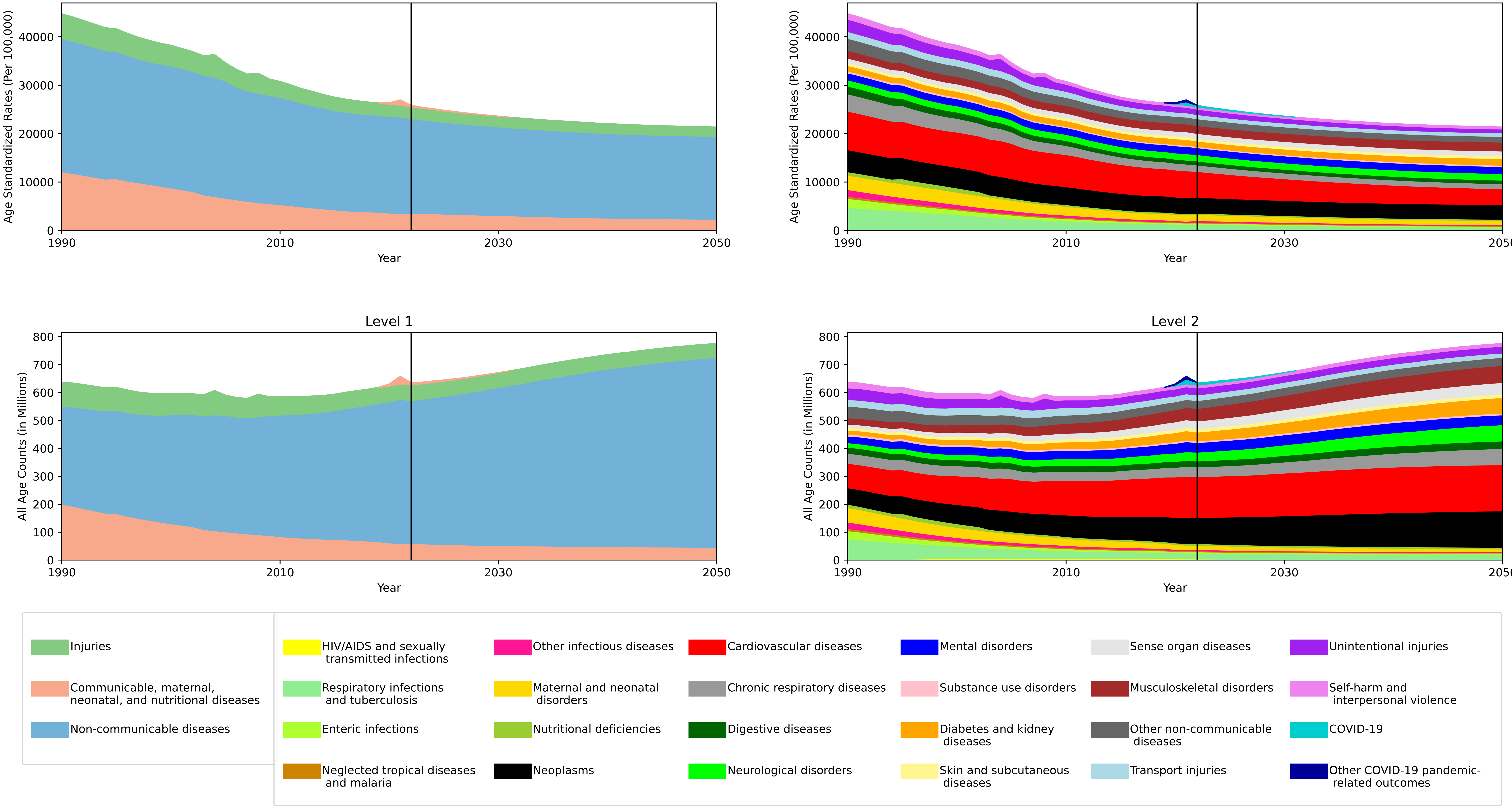

Supplemental Results Figure S4. Age-standardised DALY rates and all age DALY counts and by Level 1 and Level 2 causes, 1990–2050

Sub-Saharan Africa

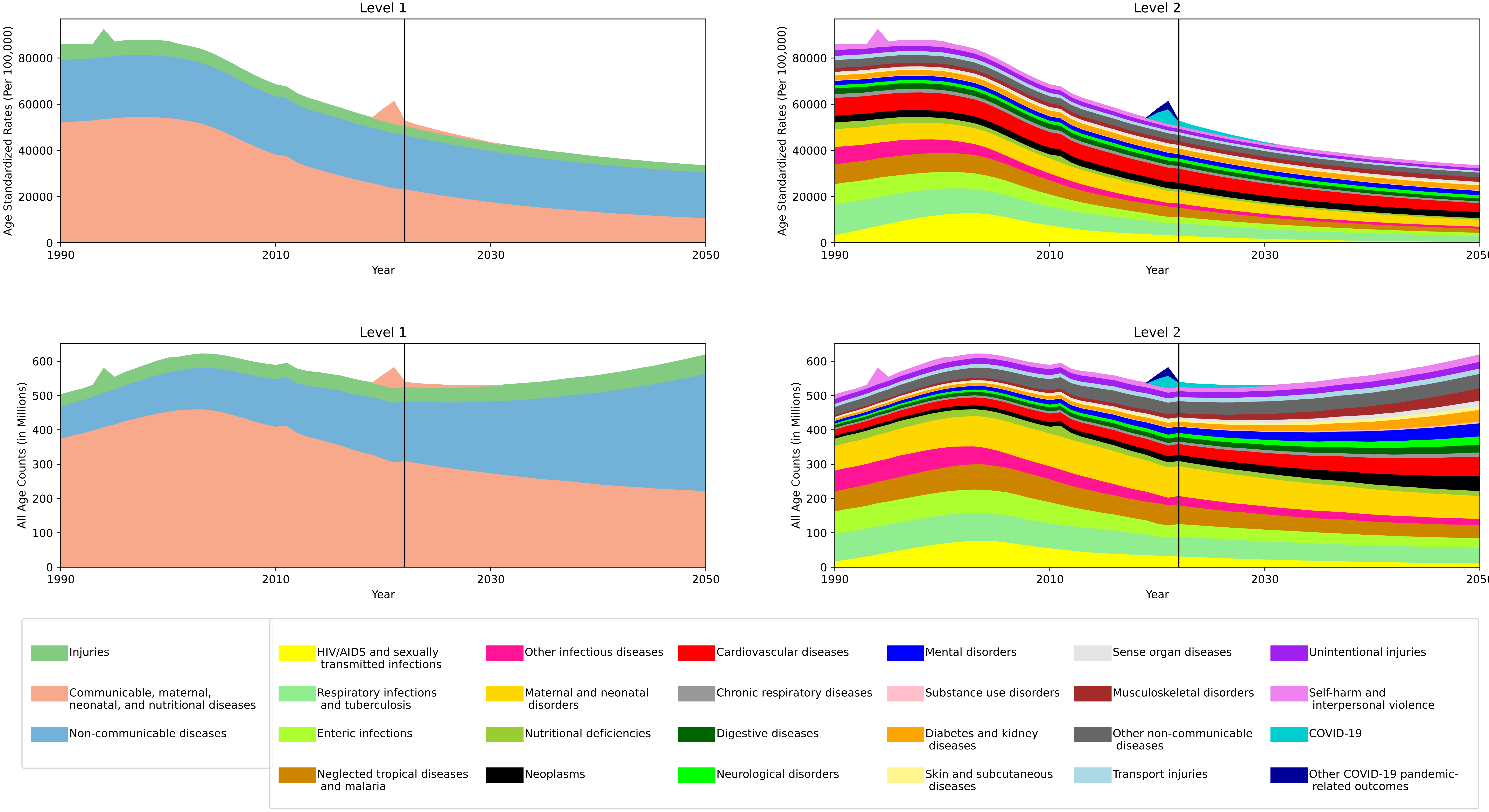

Central Asia

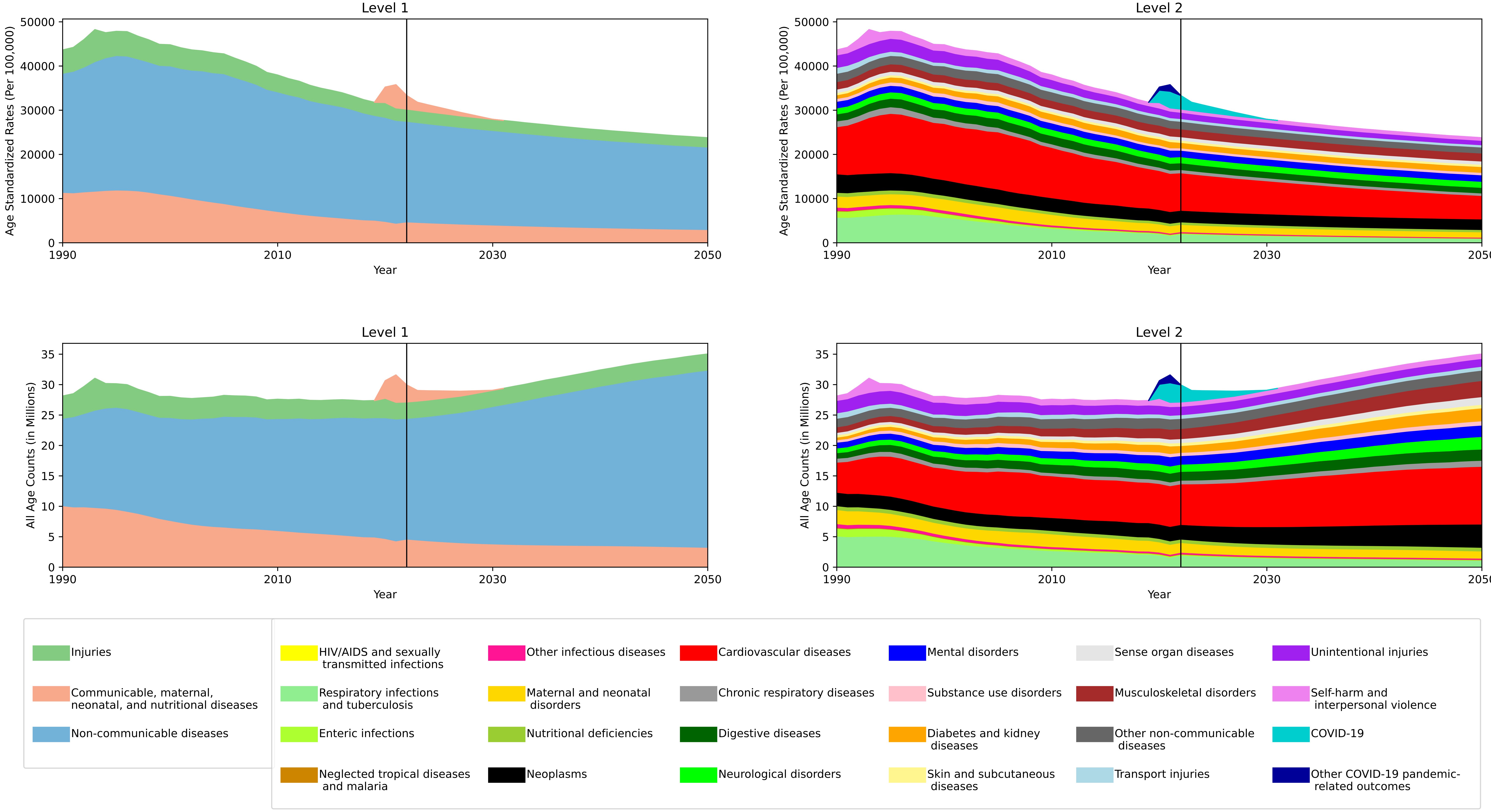

Central Europe

Supplemental Results Figure S4. Age-standardised DALY rates and all age DALY counts and by Level 1 and Level 2 causes, 1990–2050

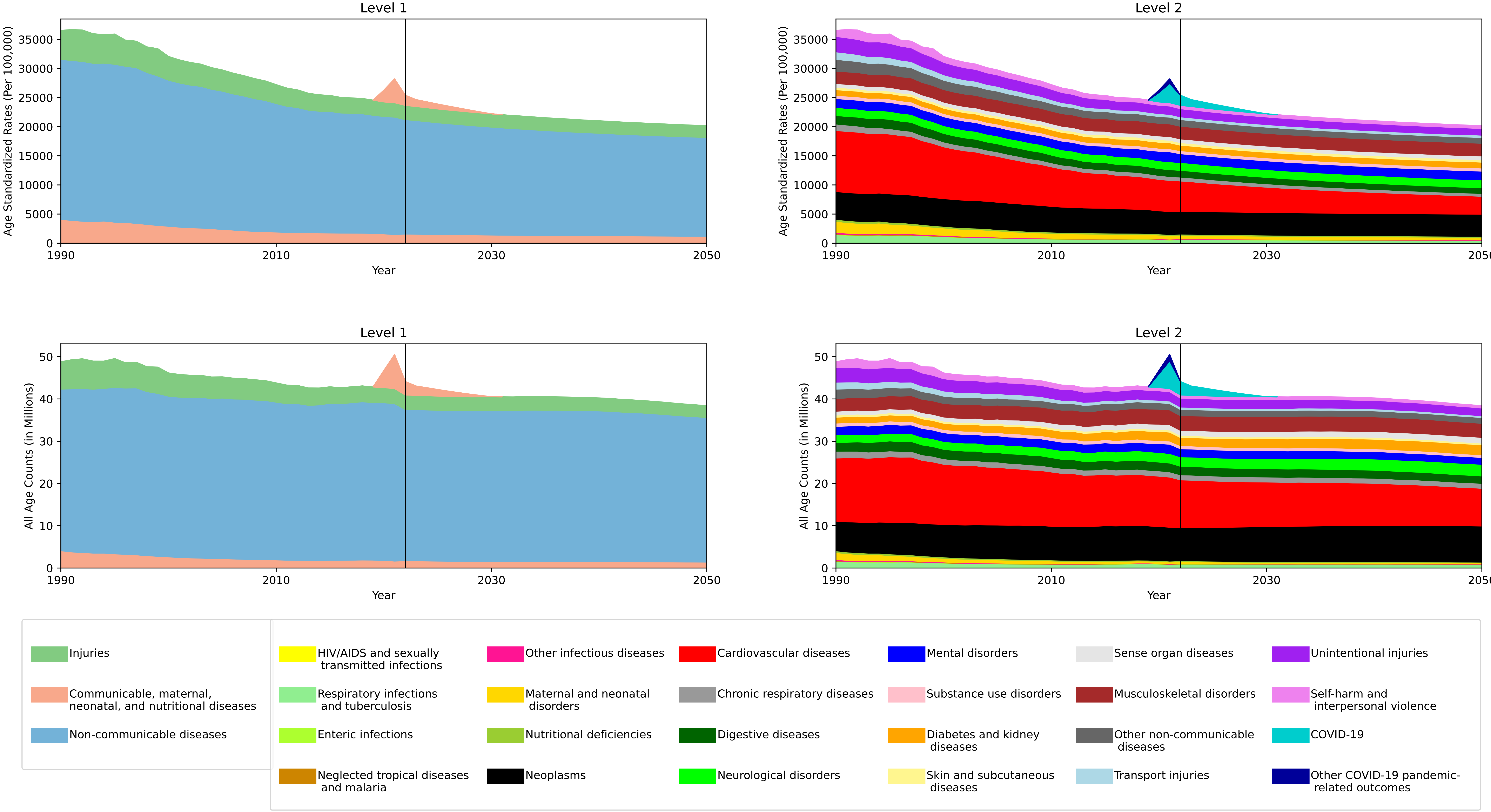

Eastern Europe

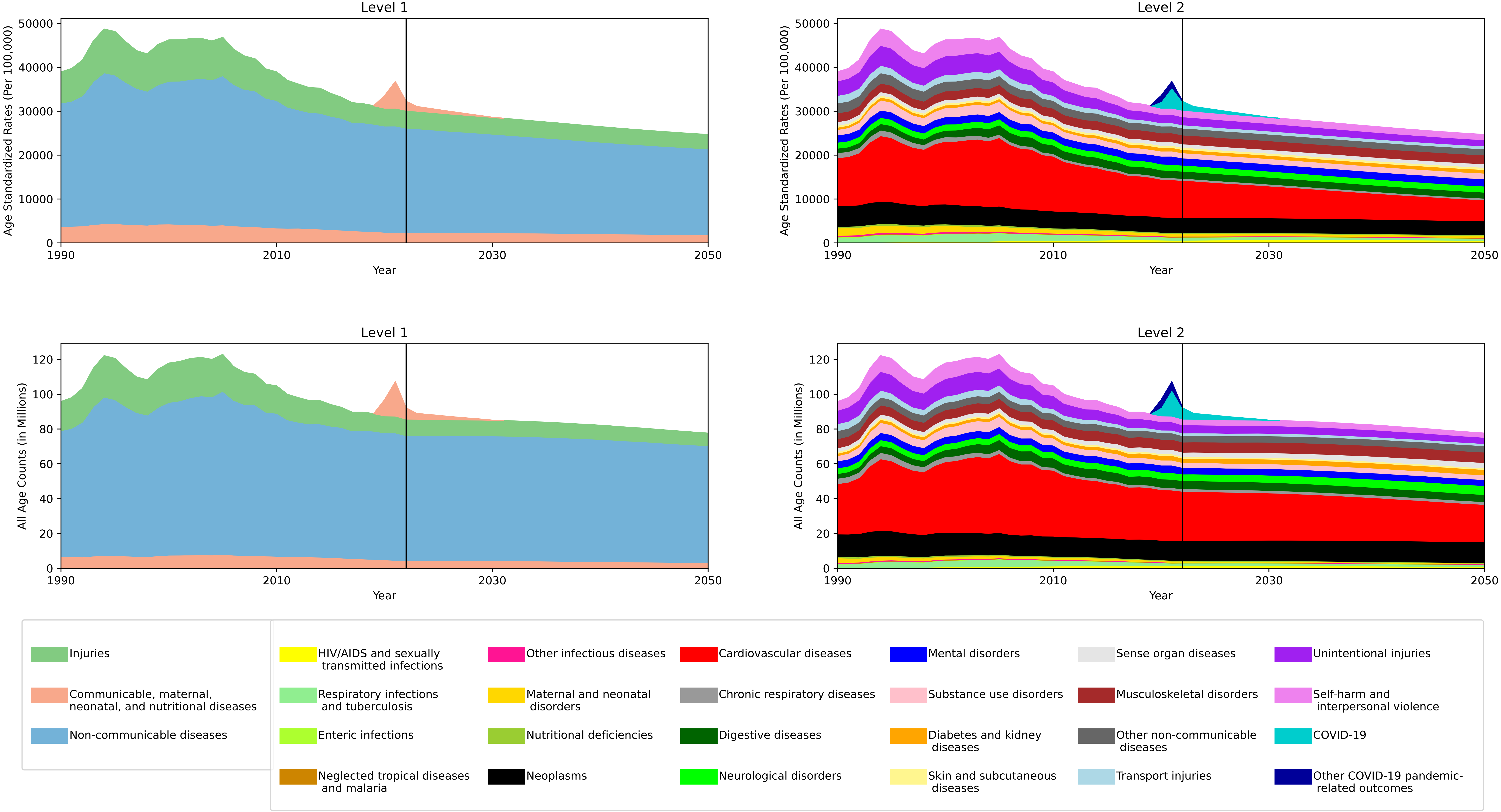

Australasia

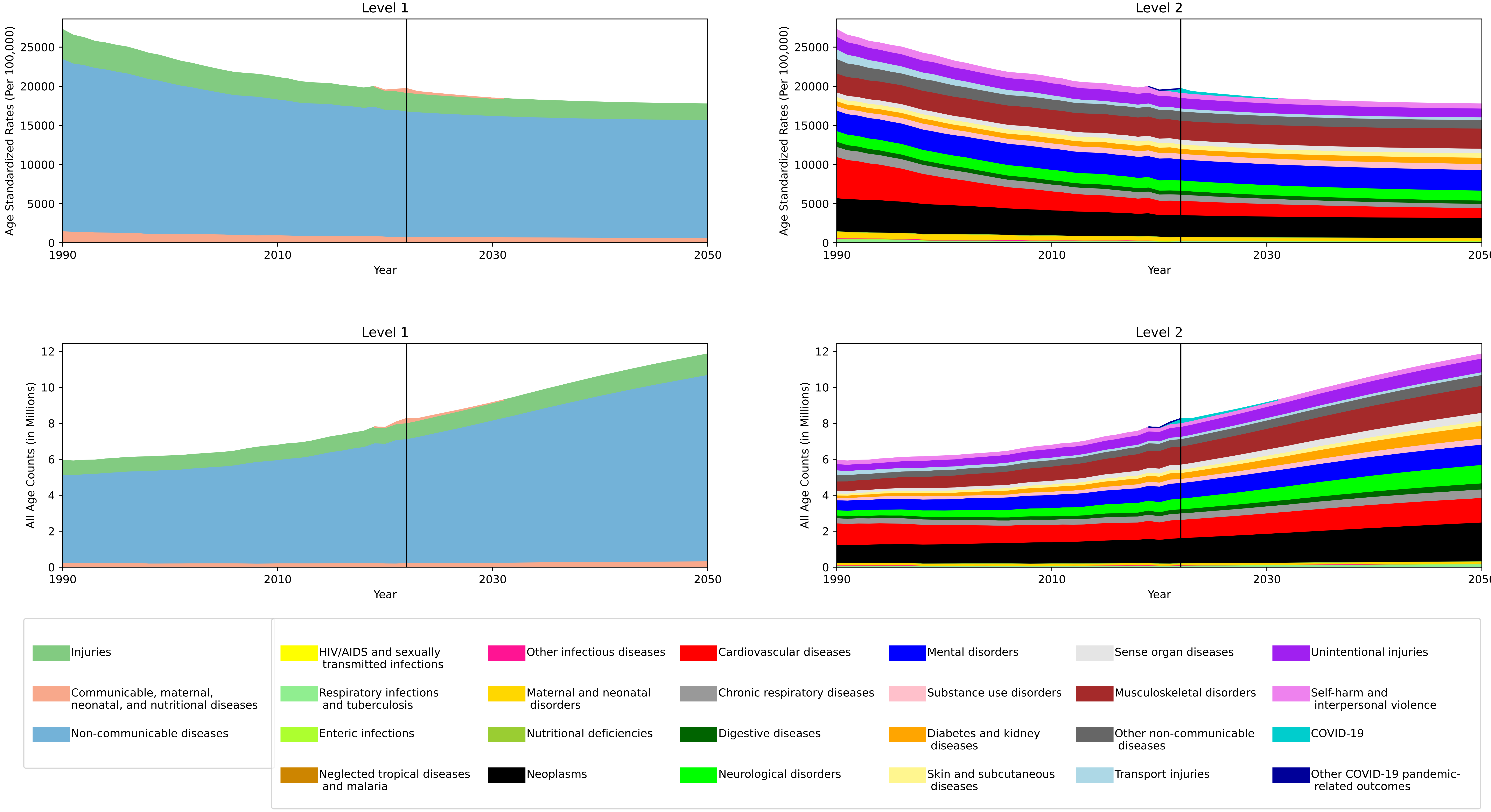

Supplemental Results Figure S4. Age-standardised DALY rates and all age DALY counts and by Level 1 and Level 2 causes, 1990–2050

High-Income Asia Pacific

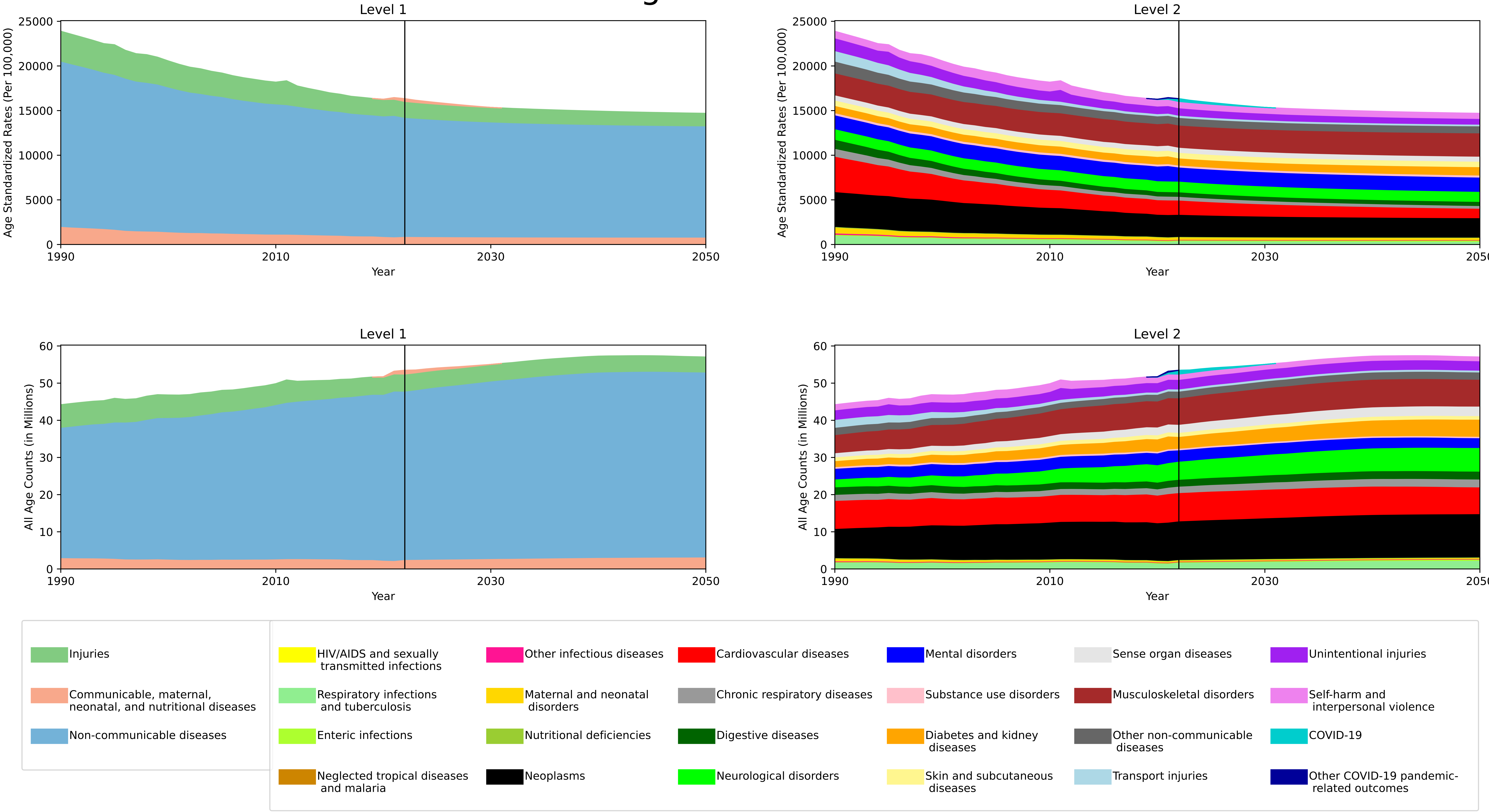

High-Income North America

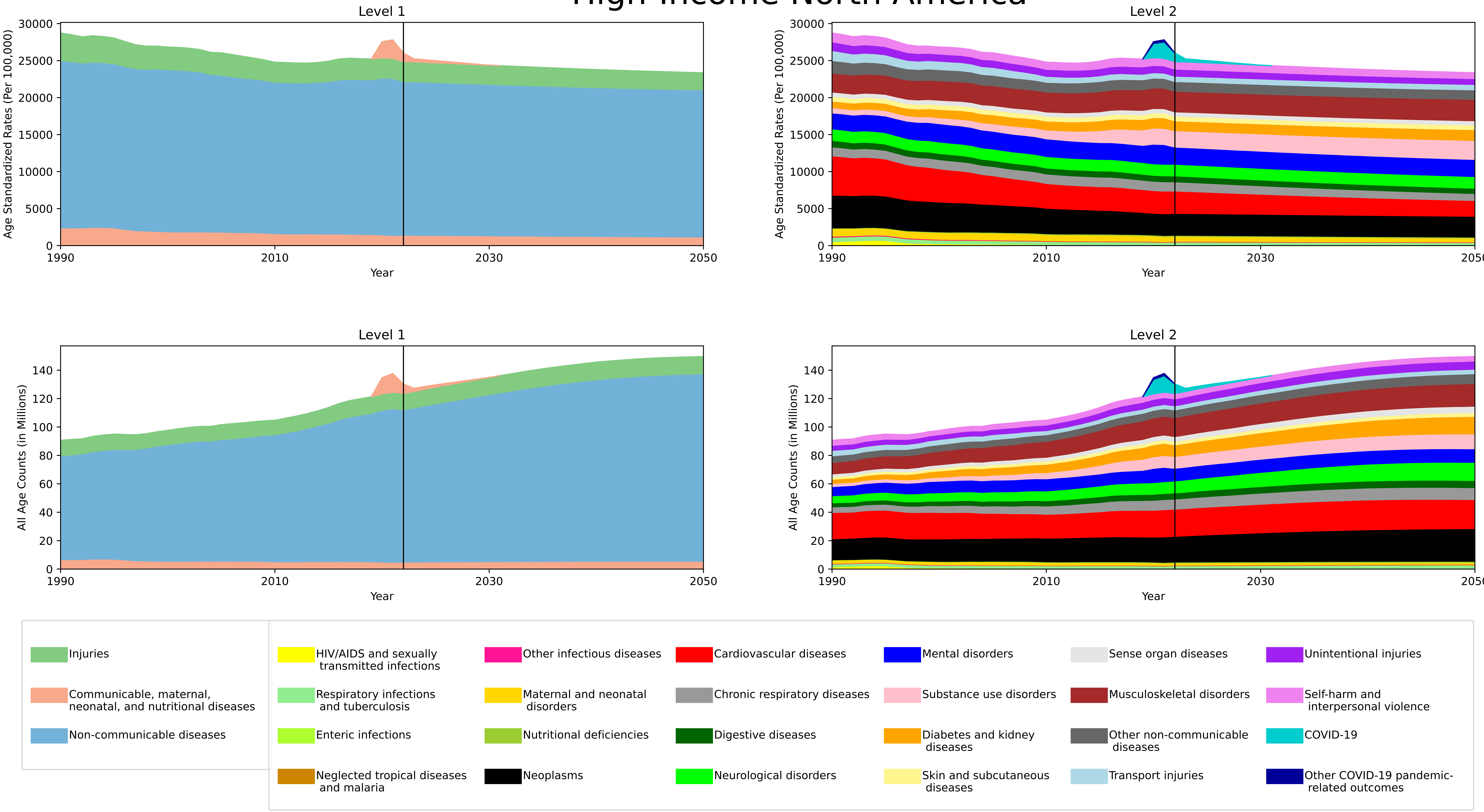

Southern Latin America

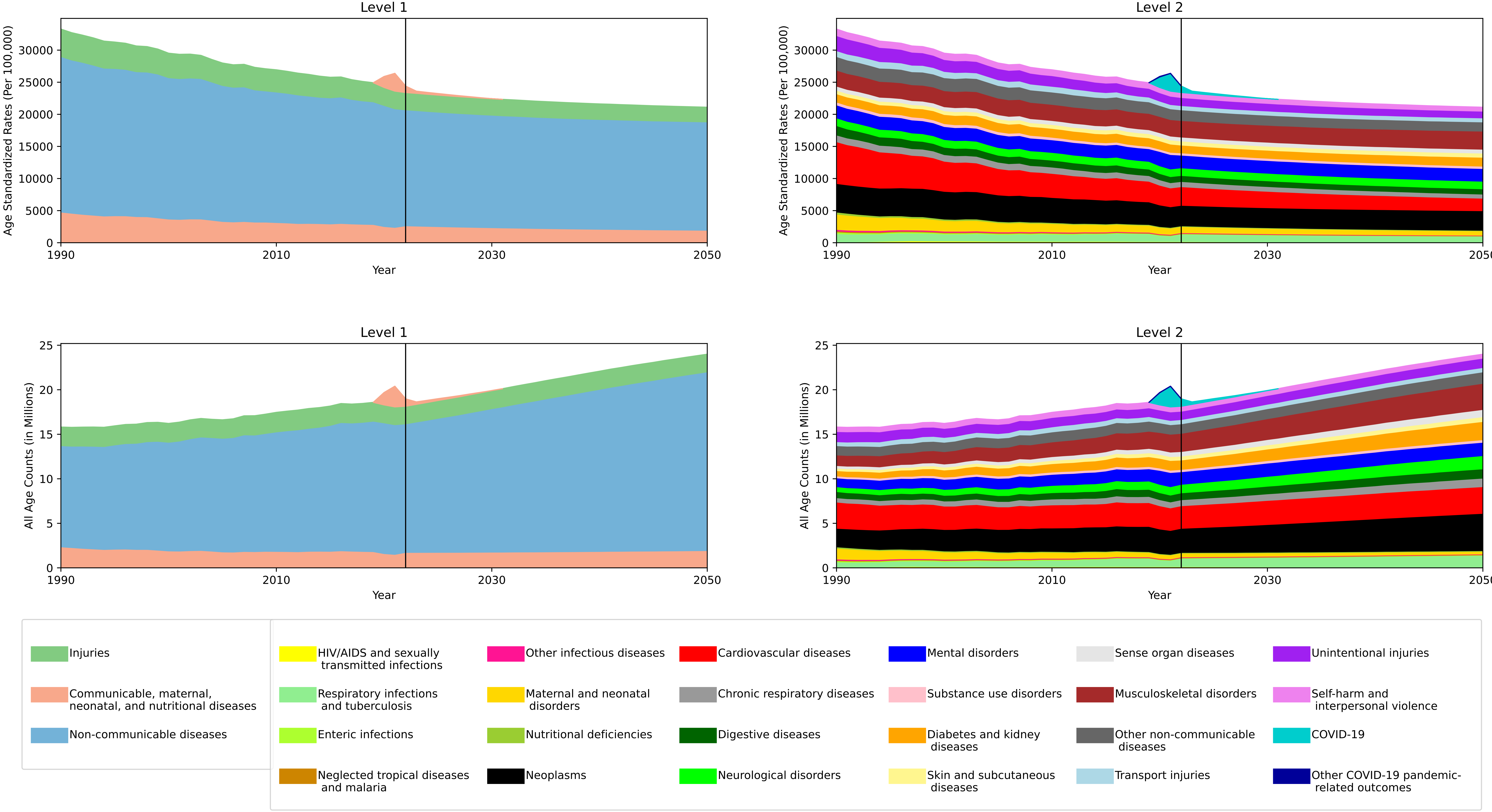

Western Europe

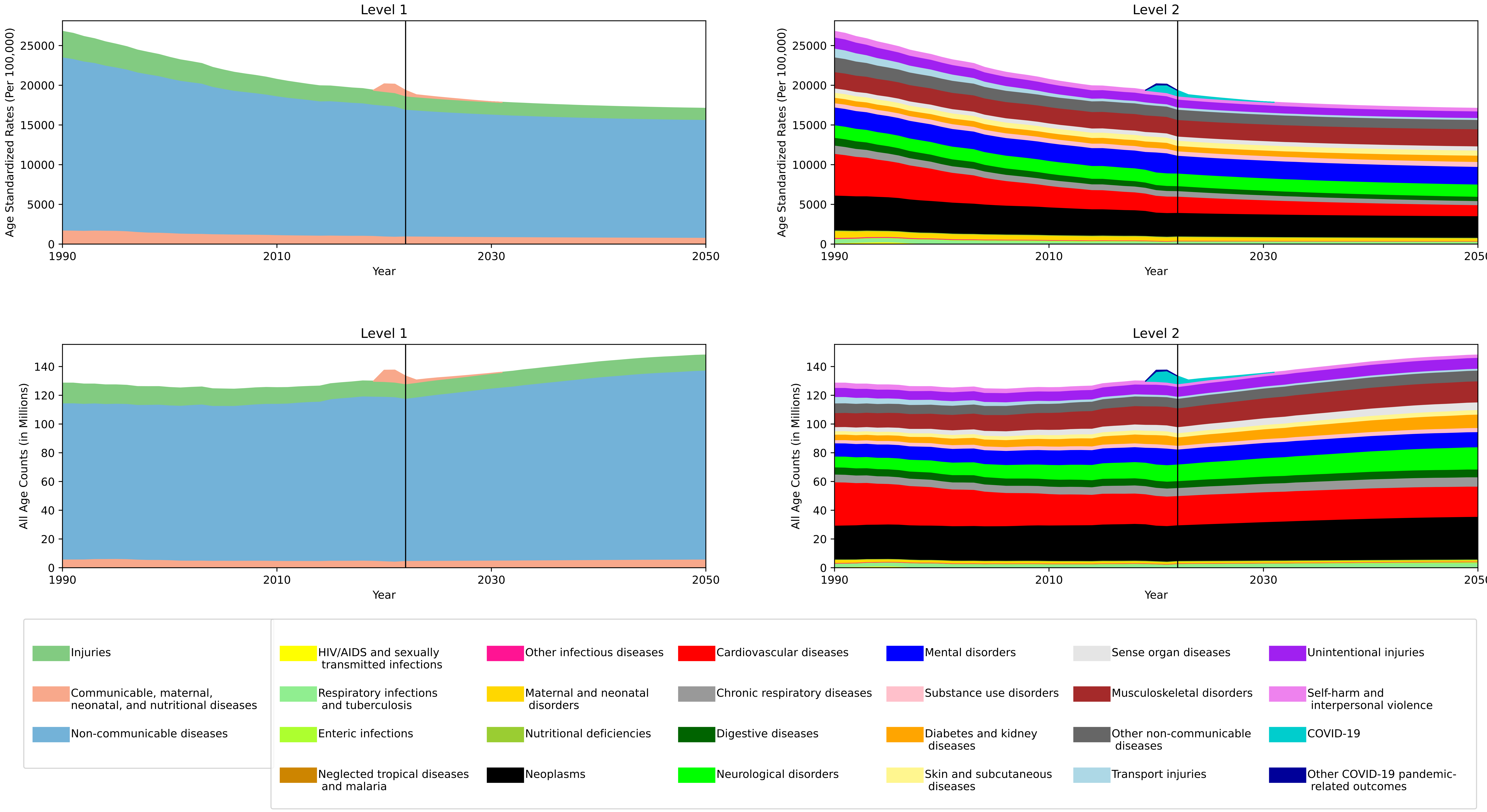

Supplemental Results Figure S4. Age-standardised DALY rates and all age DALY counts and by Level 1 and Level 2 causes, 1990–2050

Andean Latin America

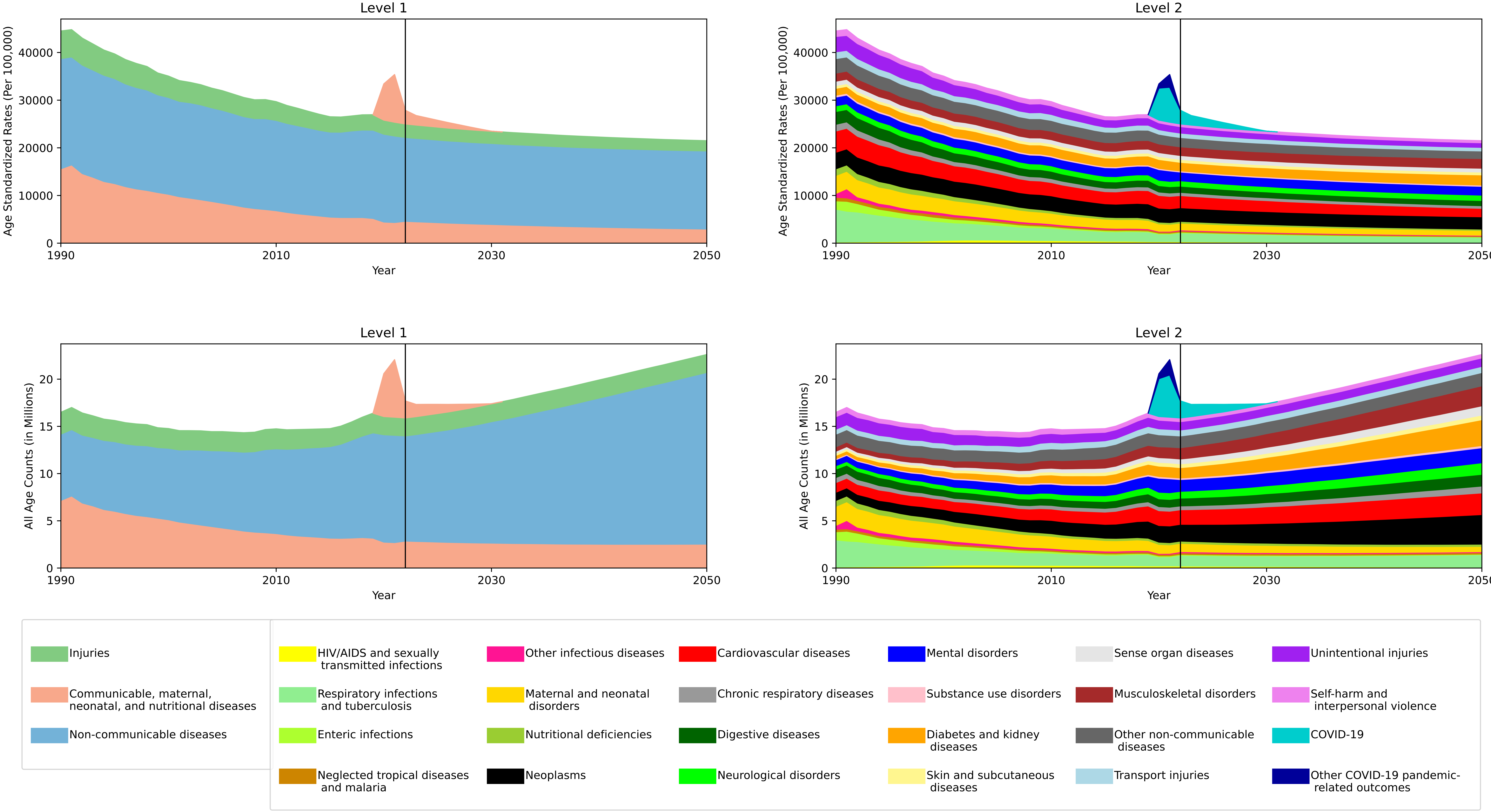

Caribbean

Supplemental Results Figure S4. Age-standardised DALY rates and all age DALY counts and by Level 1 and Level 2 causes, 1990–2050

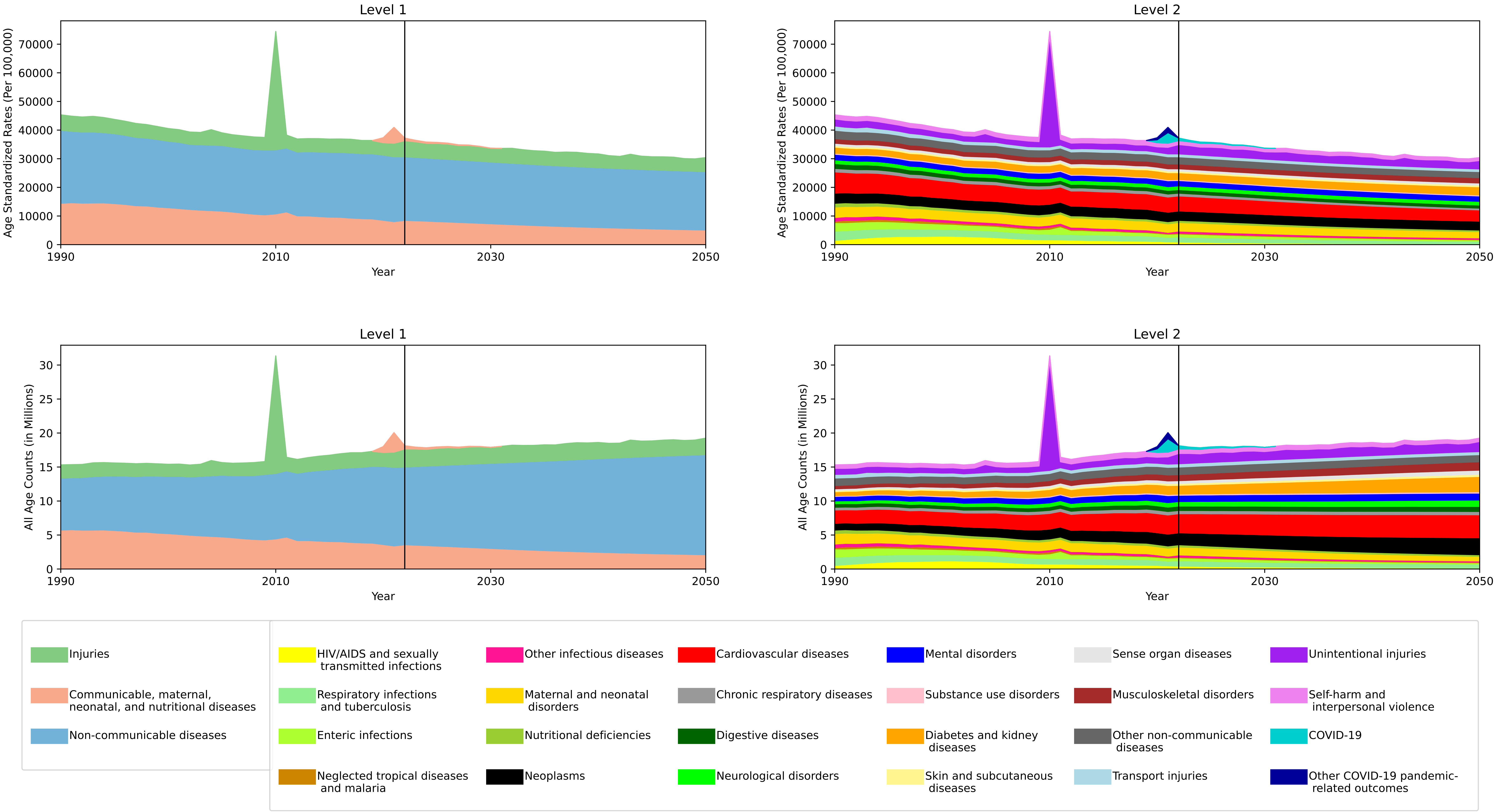

Supplemental Results Figure S4. Age-standardised DALY rates and all age DALY counts and by Level 1 and Level 2 causes, 1990–2050

Central Latin America

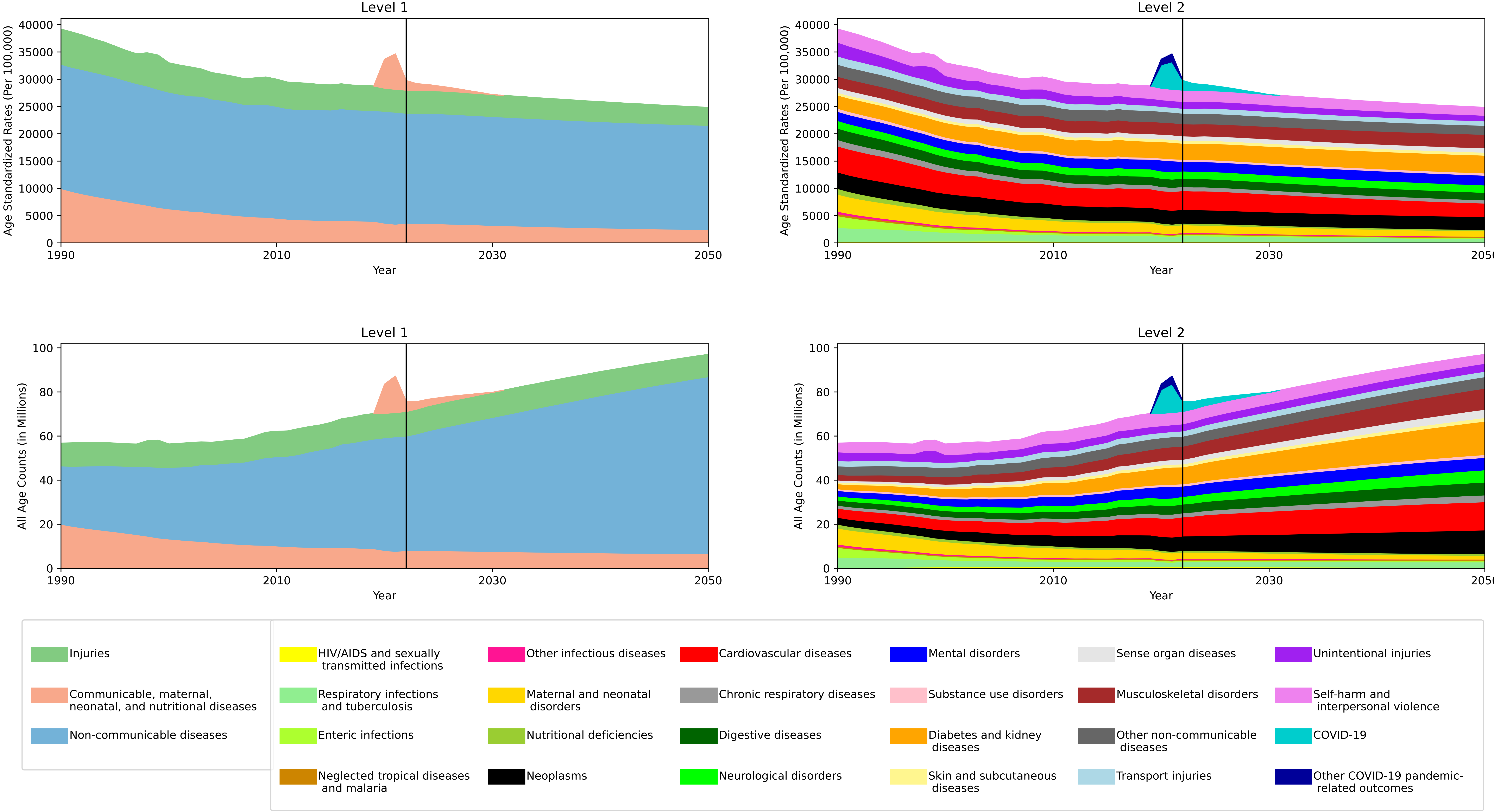

Supplemental Results Figure S4. Age-standardised DALY rates and all age DALY counts and by Level 1 and Level 2 causes, 1990–2050

Tropical Latin America

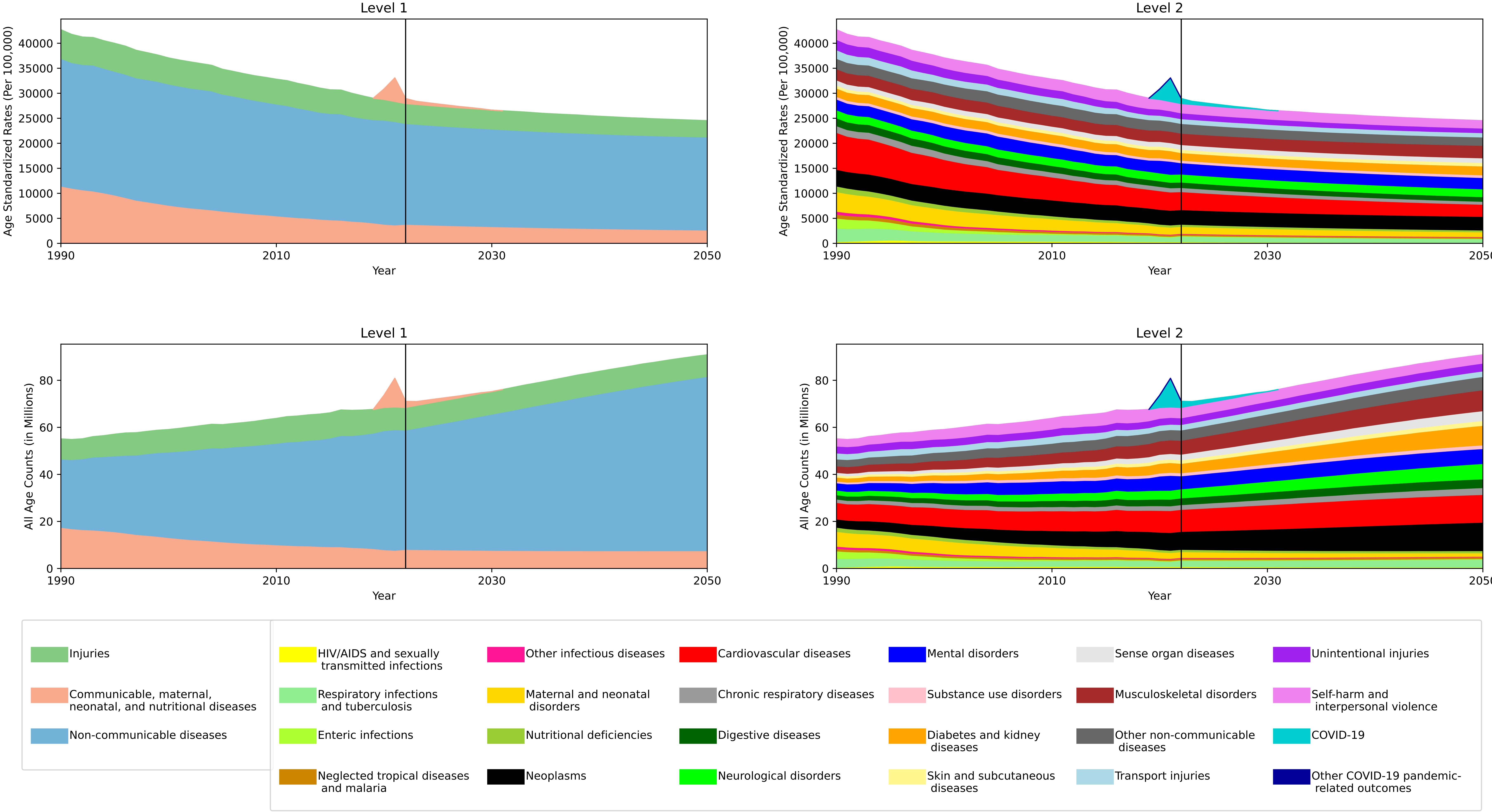

Supplemental Results Figure S4. Age-standardised DALY rates and all age DALY counts and by Level 1 and Level 2 causes, 1990–2050

North Africa And Middle East

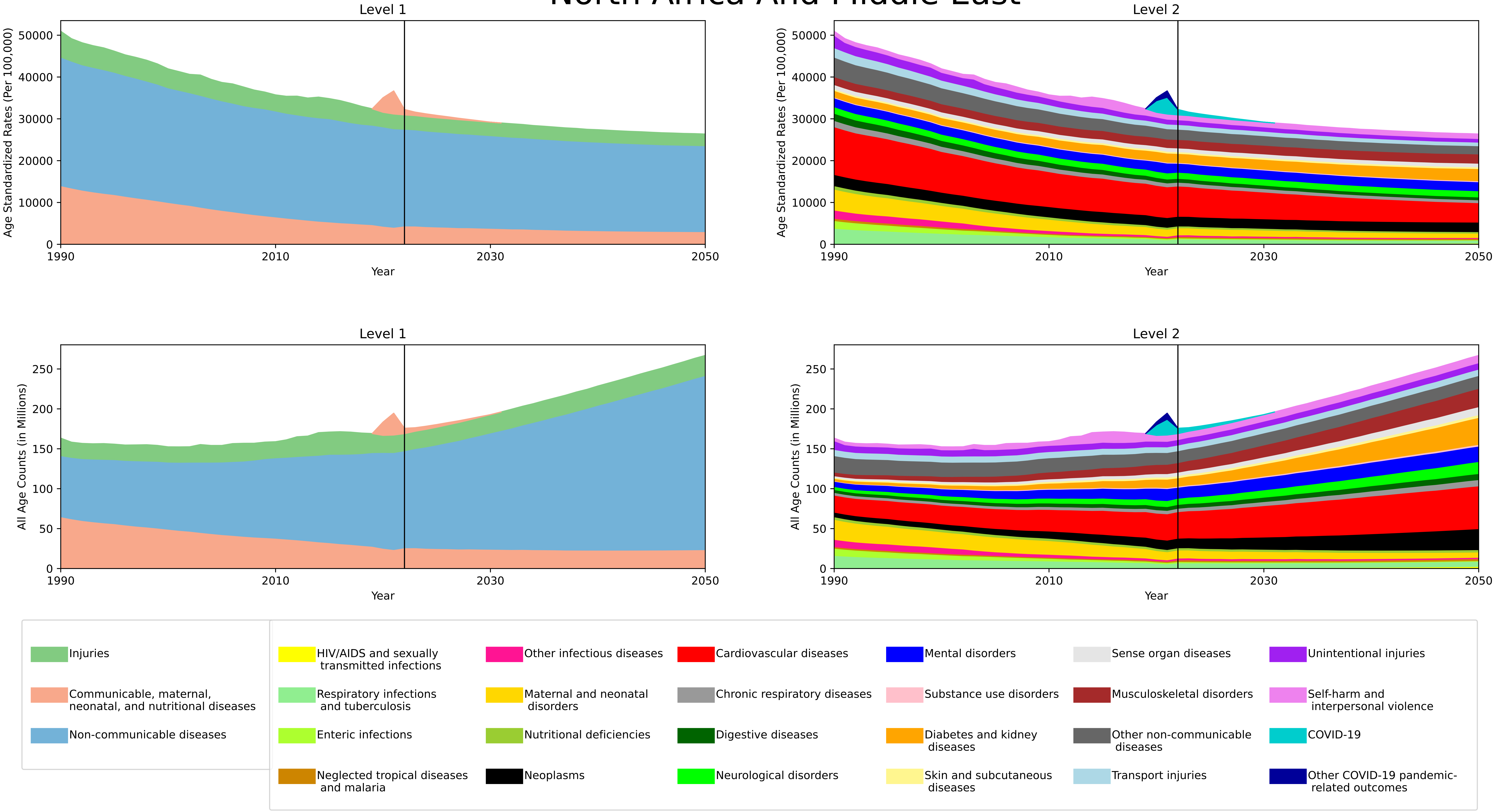

South Asia

Supplemental Results Figure S4. Age-standardised DALY rates and all age DALY counts and by Level 1 and Level 2 causes, 1990–2050

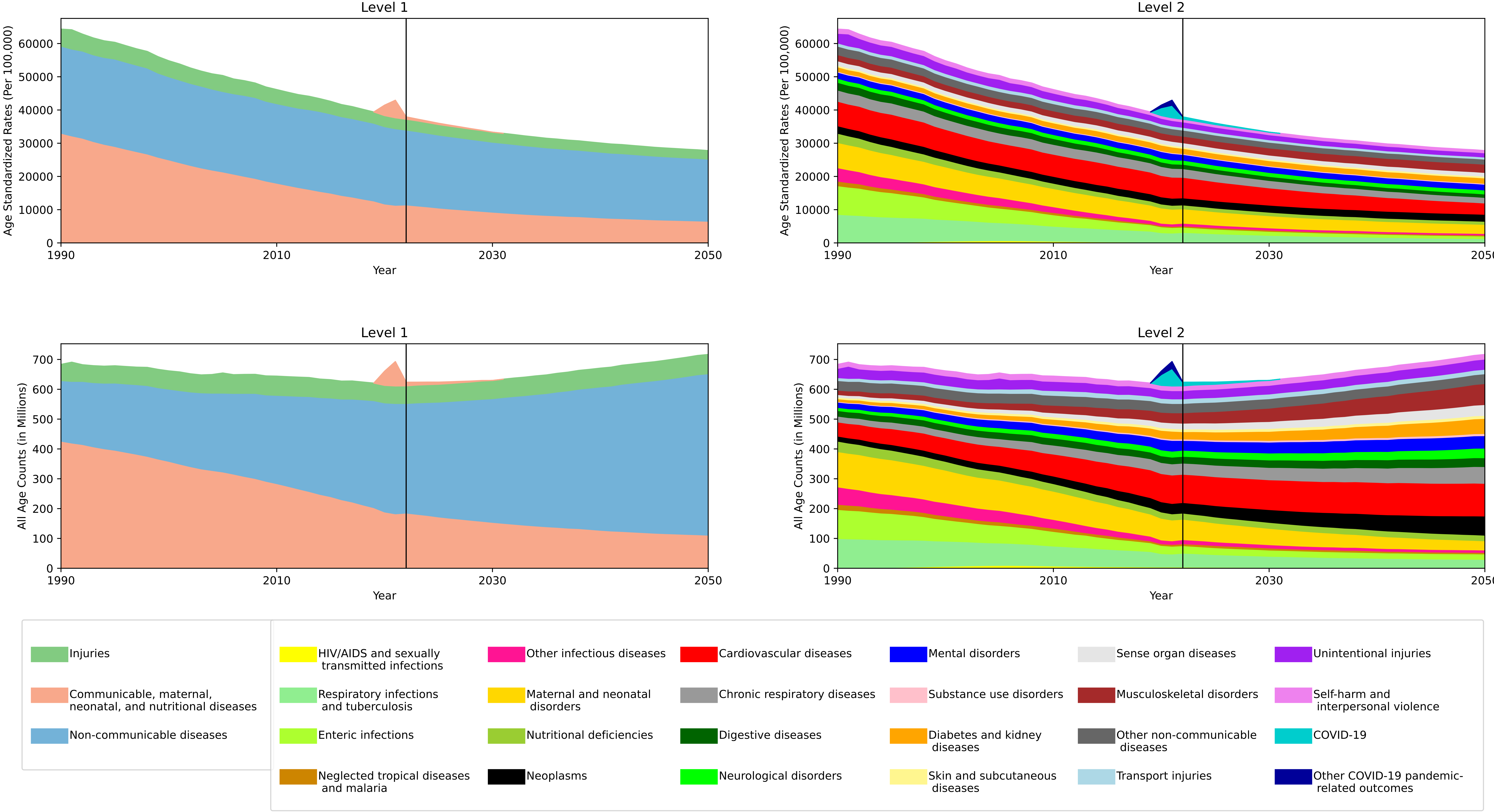

East Asia

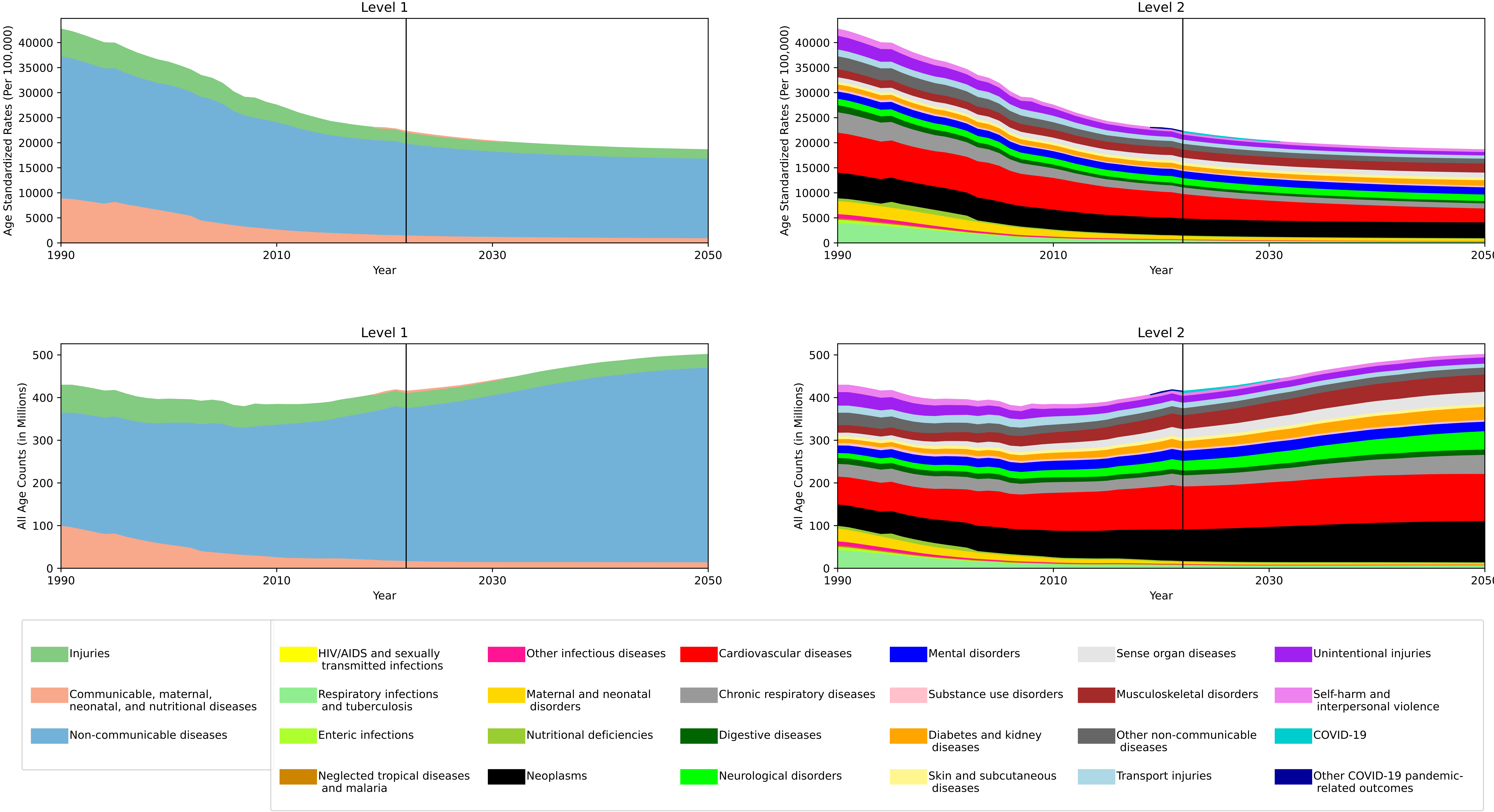
Level 1
Level 2
Age Standardized Rates (Per 100,000)
All Age Counts (in Millions)
Year
1990
2010
2030
2050
Injuries
Communicable, maternal, neonatal, and nutritional diseases
Non-communicable diseases
HIV/AIDS and sexually transmitted infections
Respiratory infections and tuberculosis
Enteric infections
Neglected tropical diseases and malaria
Other infectious diseases
Maternal and neonatal disorders
Nutritional deficiencies
Neoplasms
Cardiovascular diseases
Chronic respiratory diseases
Digestive diseases
Neurological disorders
Mental disorders
Substance use disorders
Diabetes and kidney diseases
Skin and subcutaneous diseases
Sense organ diseases
Musculoskeletal disorders
Other non-communicable diseases
Transport injuries
Unintentional injuries
Self-harm and interpersonal violence
COVID-19
Other COVID-19 pandemic-related outcomes

Oceania

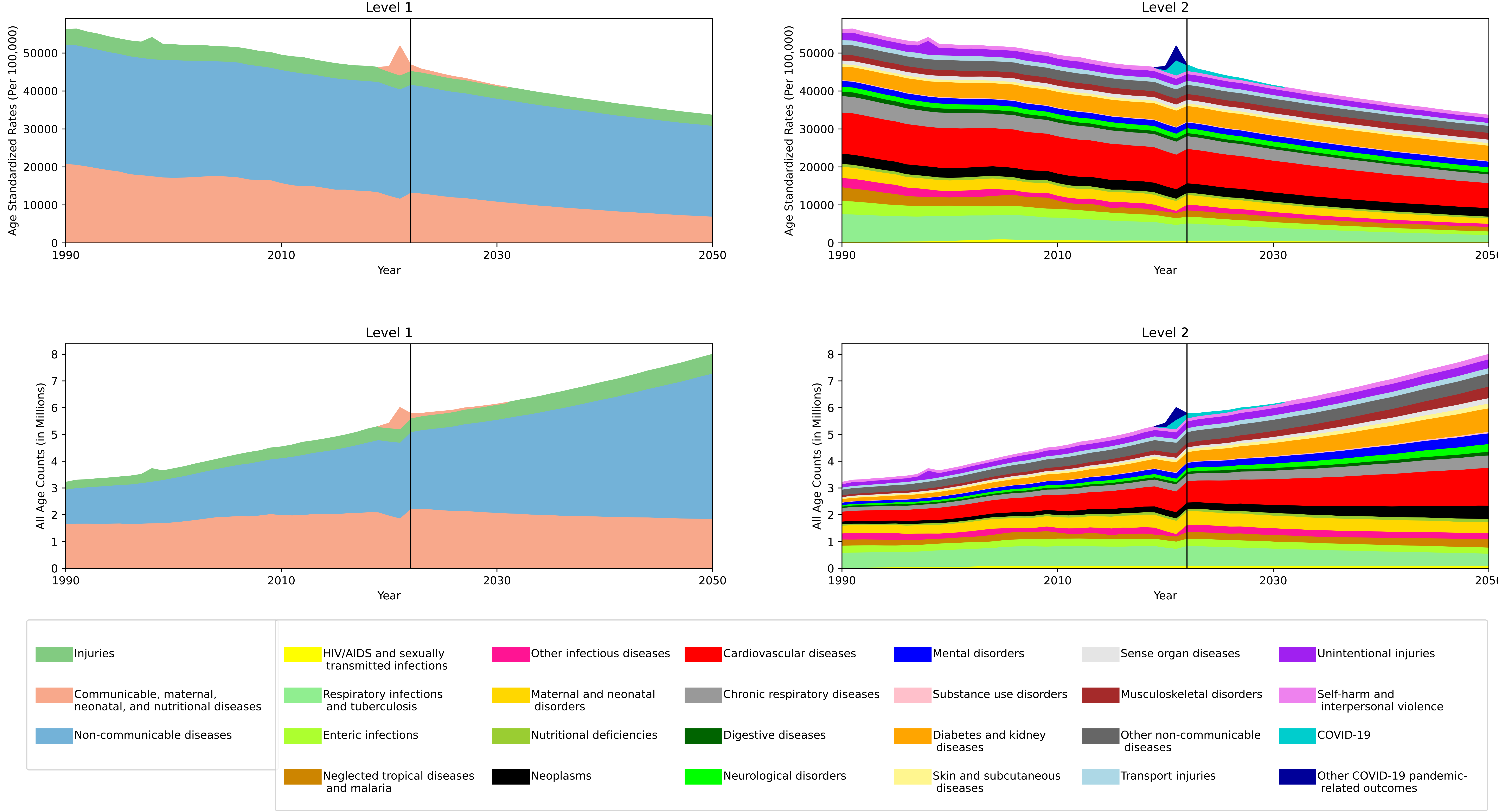
Level 1
Level 2
Age Standardized Rates (Per 100,000)
All Age Counts (in Millions)
Year
0
10000
20000
30000
40000
50000
1
2
3
4
5
6
7
8
1990
2010
2030
2050
Injuries
Communicable, maternal, neonatal, and nutritional diseases
Non-communicable diseases
HIV/AIDS and sexually transmitted infections
Respiratory infections and tuberculosis
Enteric infections
Neglected tropical diseases and malaria
Other infectious diseases
Maternal and neonatal disorders
Nutritional deficiencies
Neoplasms
Cardiovascular diseases
Chronic respiratory diseases
Digestive diseases
Neurological disorders
Mental disorders
Substance use disorders
Diabetes and kidney diseases
Skin and subcutaneous diseases
Sense organ diseases
Musculoskeletal disorders
Other non-communicable diseases
Transport injuries
Unintentional injuries
Self-harm and interpersonal violence
COVID-19
Other COVID-19 pandemic-related outcomes

Southeast Asia

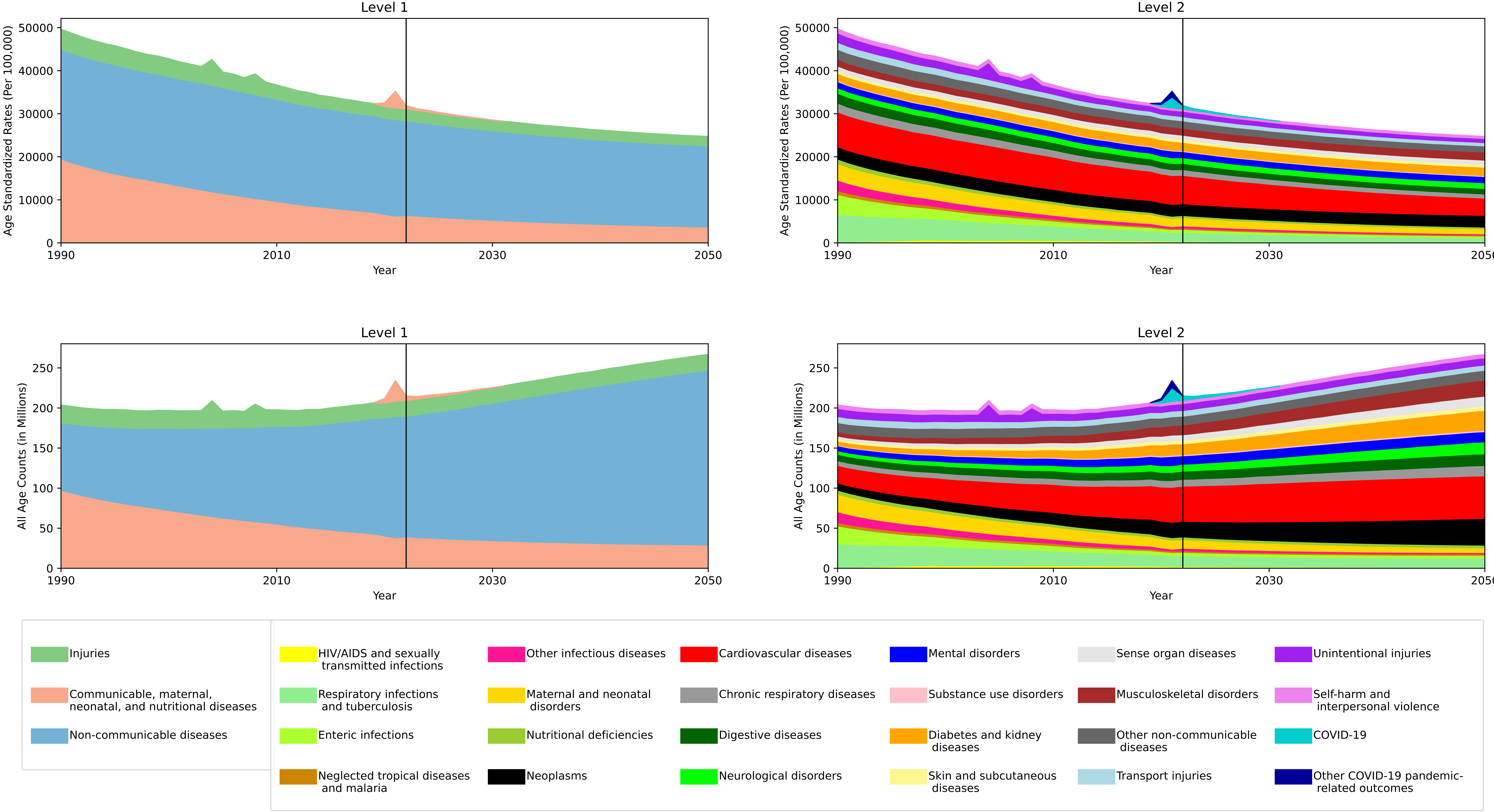

Supplemental Results Figure S4. Age-standardised DALY rates and all age DALY counts and by Level 1 and Level 2 causes, 1990–2050

Central Sub-Saharan Africa

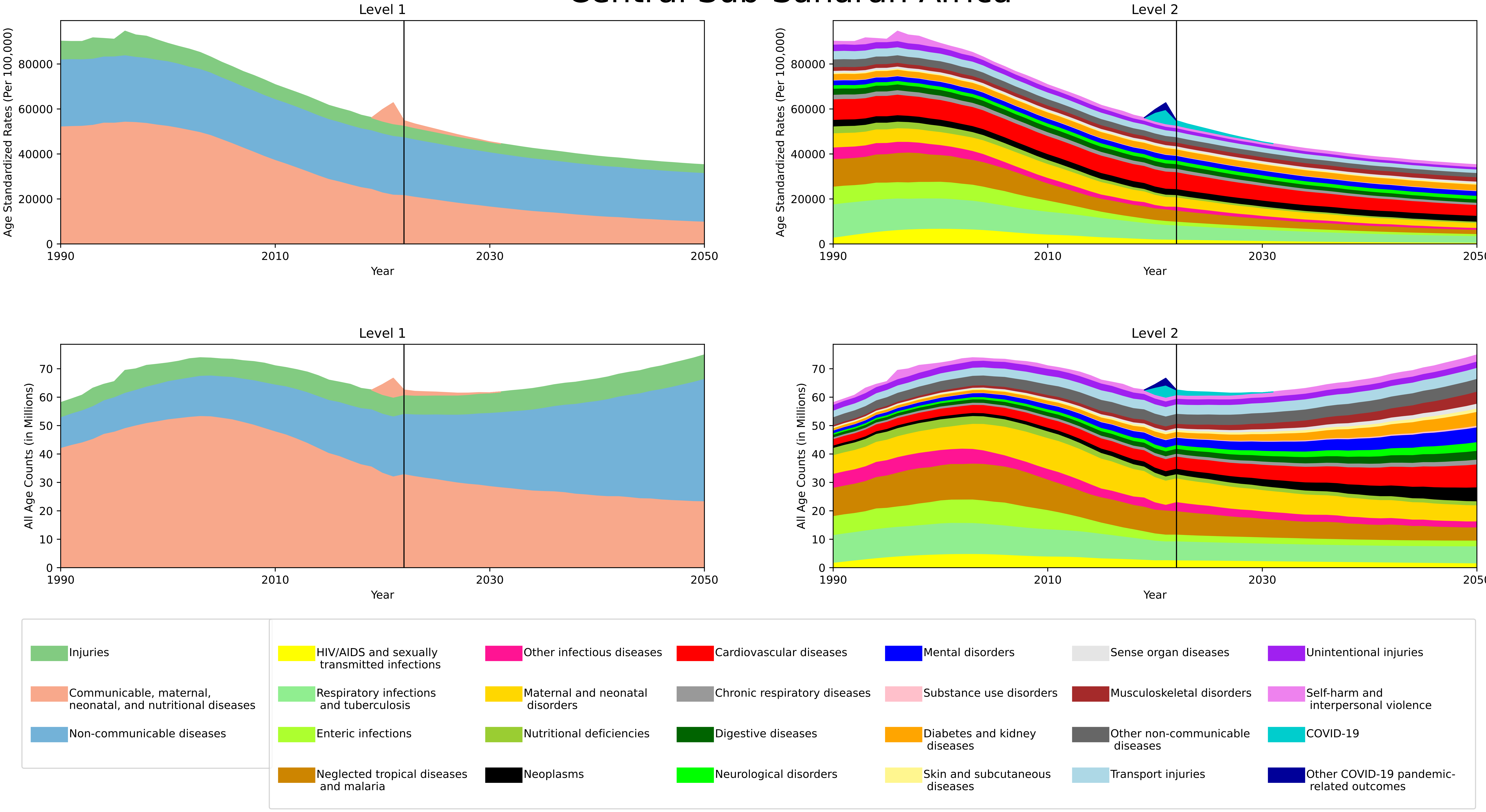

Supplemental Results Figure S4. Age-standardised DALY rates and all age DALY counts and by Level 1 and Level 2 causes, 1990–2050

Eastern Sub-Saharan Africa

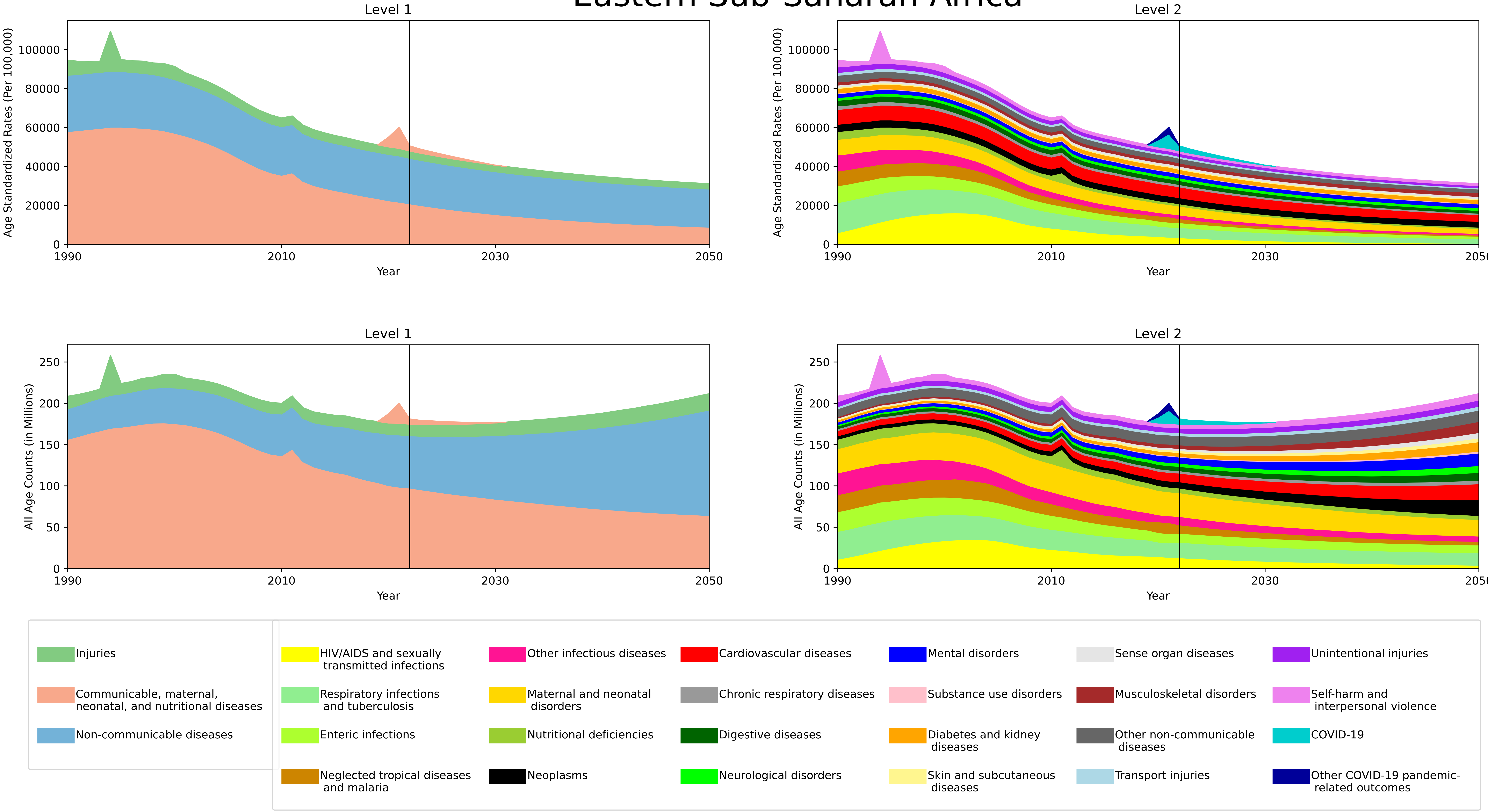

Supplemental Results Figure S4. Age-standardised DALY rates and all age DALY counts and by Level 1 and Level 2 causes, 1990–2050

Southern Sub-Saharan Africa

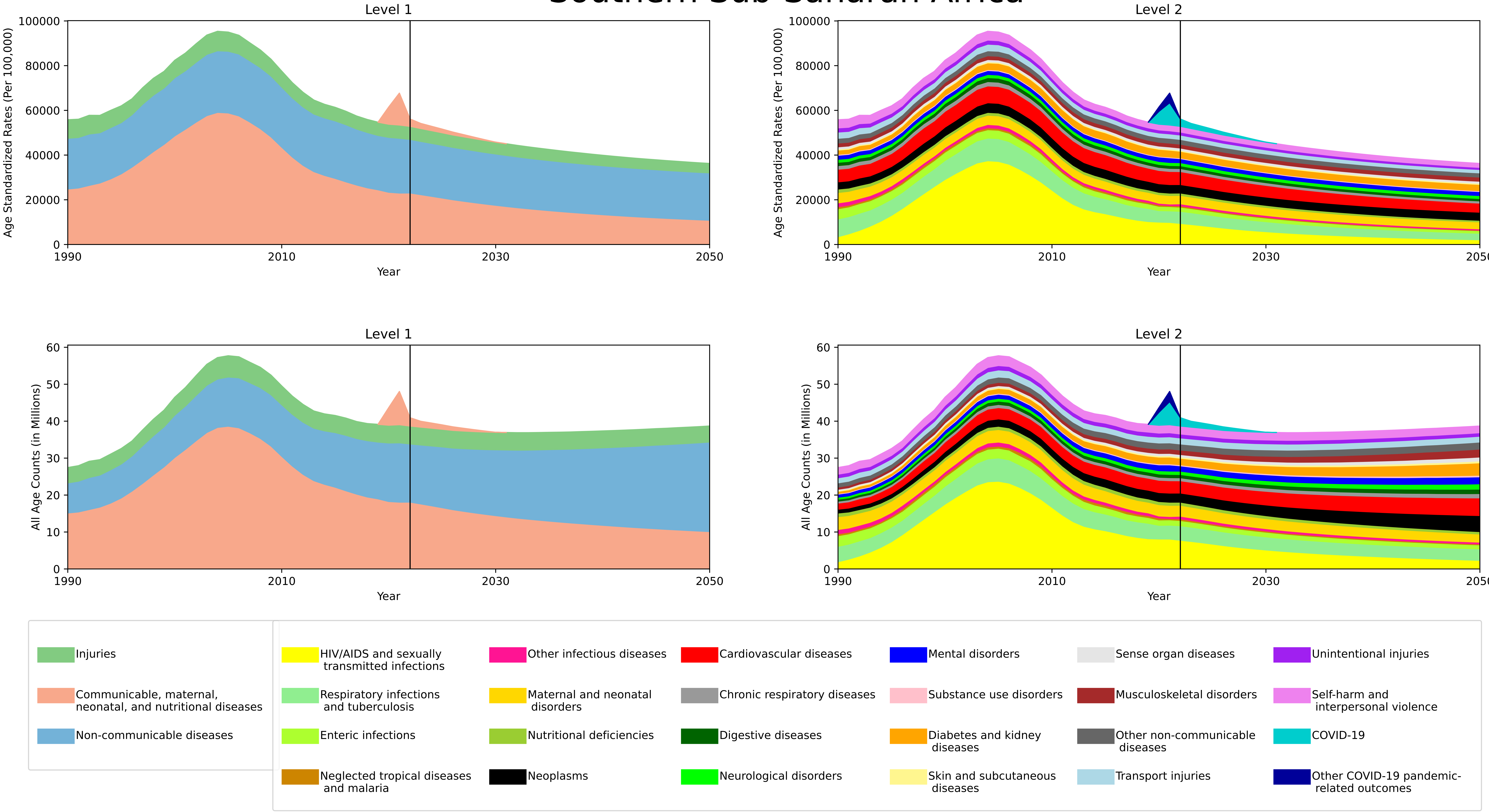

Supplemental Results Figure S4. Age-standardised DALY rates and all age DALY counts and by Level 1 and Level 2 causes, 1990–2050

Western Sub-Saharan Africa

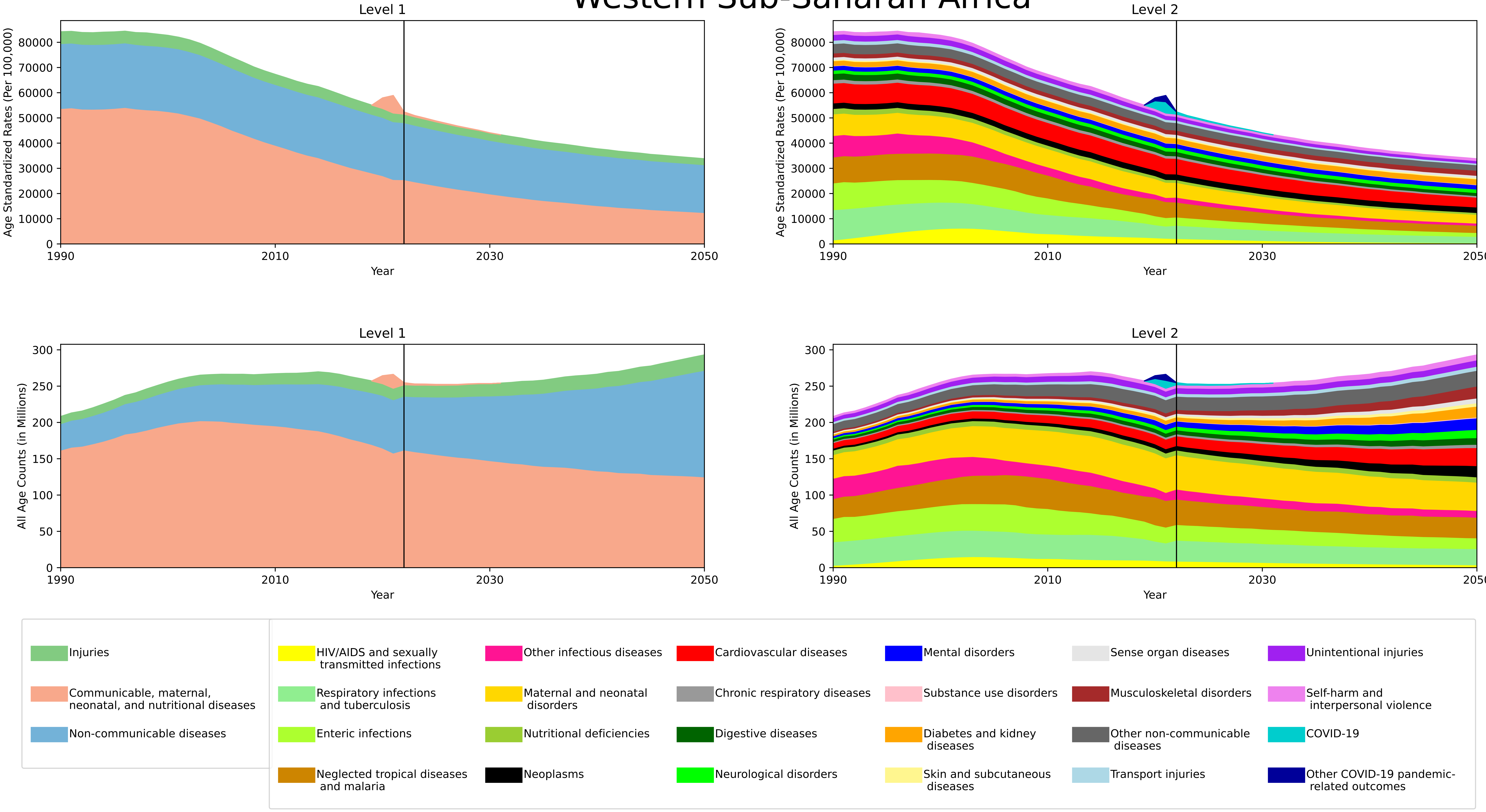

Armenia

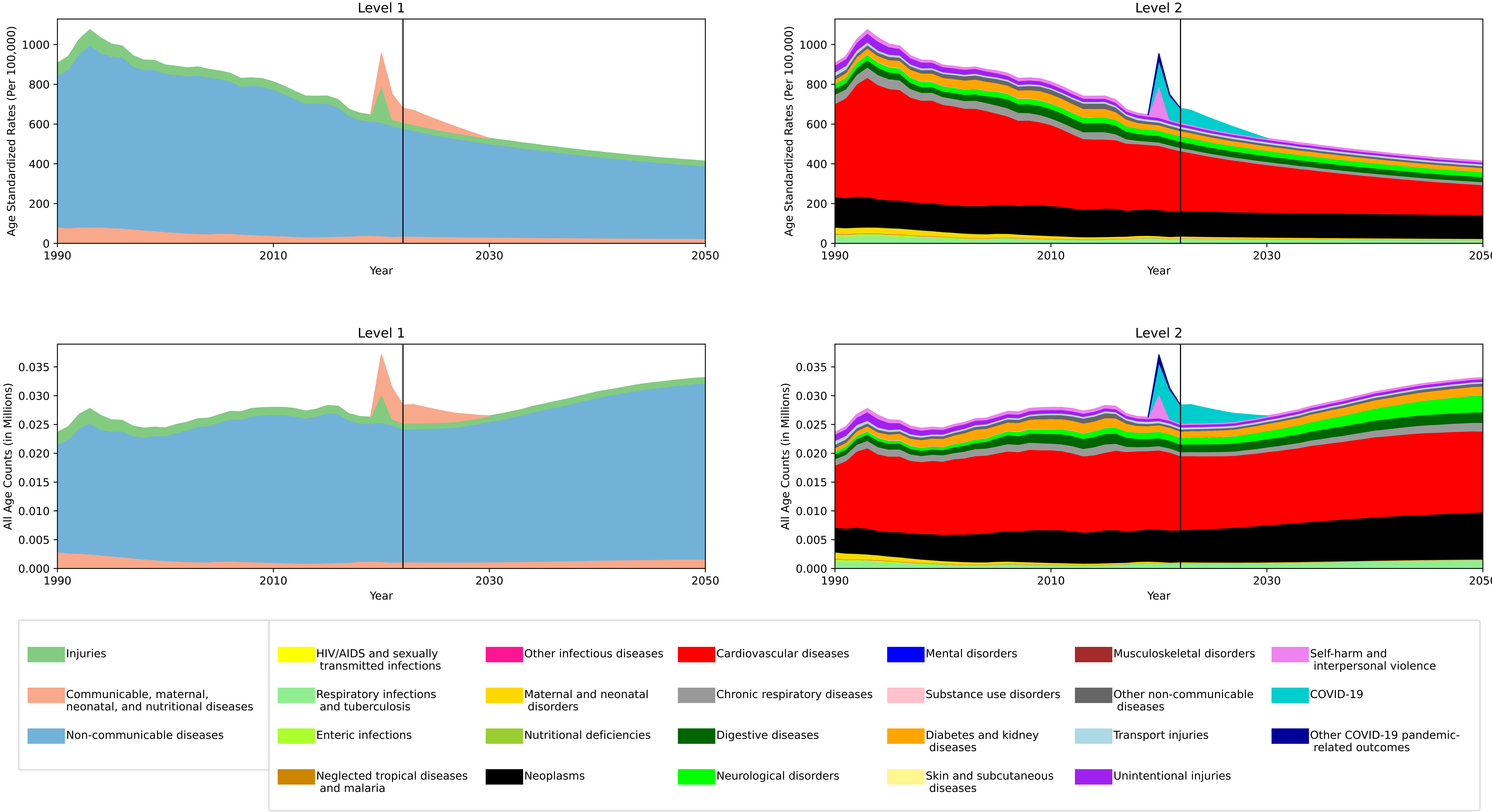
Level 1
Level 2
Age Standardized Rates (Per 100,000)
All Age Counts (in Millions)
Year
1990
2010
2030
2050
0
200
400
600
800
1000
0.000
0.005
0.010
0.015
0.020
0.025
0.030
0.035
Injuries
Communicable, maternal, neonatal, and nutritional diseases
Non-communicable diseases
HIV/AIDS and sexually transmitted infections
Respiratory infections and tuberculosis
Enteric infections
Neglected tropical diseases and malaria
Other infectious diseases
Maternal and neonatal disorders
Nutritional deficiencies
Neoplasms
Cardiovascular diseases
Chronic respiratory diseases
Digestive diseases
Neurological disorders
Mental disorders
Substance use disorders
Diabetes and kidney diseases
Skin and subcutaneous diseases
Musculoskeletal disorders
Other non-communicable diseases
Transport injuries
Unintentional injuries
Self-harm and interpersonal violence
COVID-19
Other COVID-19 pandemic-related outcomes

Azerbaijan

Supplemental Results Figure S5. Age-standardised death rates and all age death counts and by Level 1 and Level 2 causes, 1990–2050

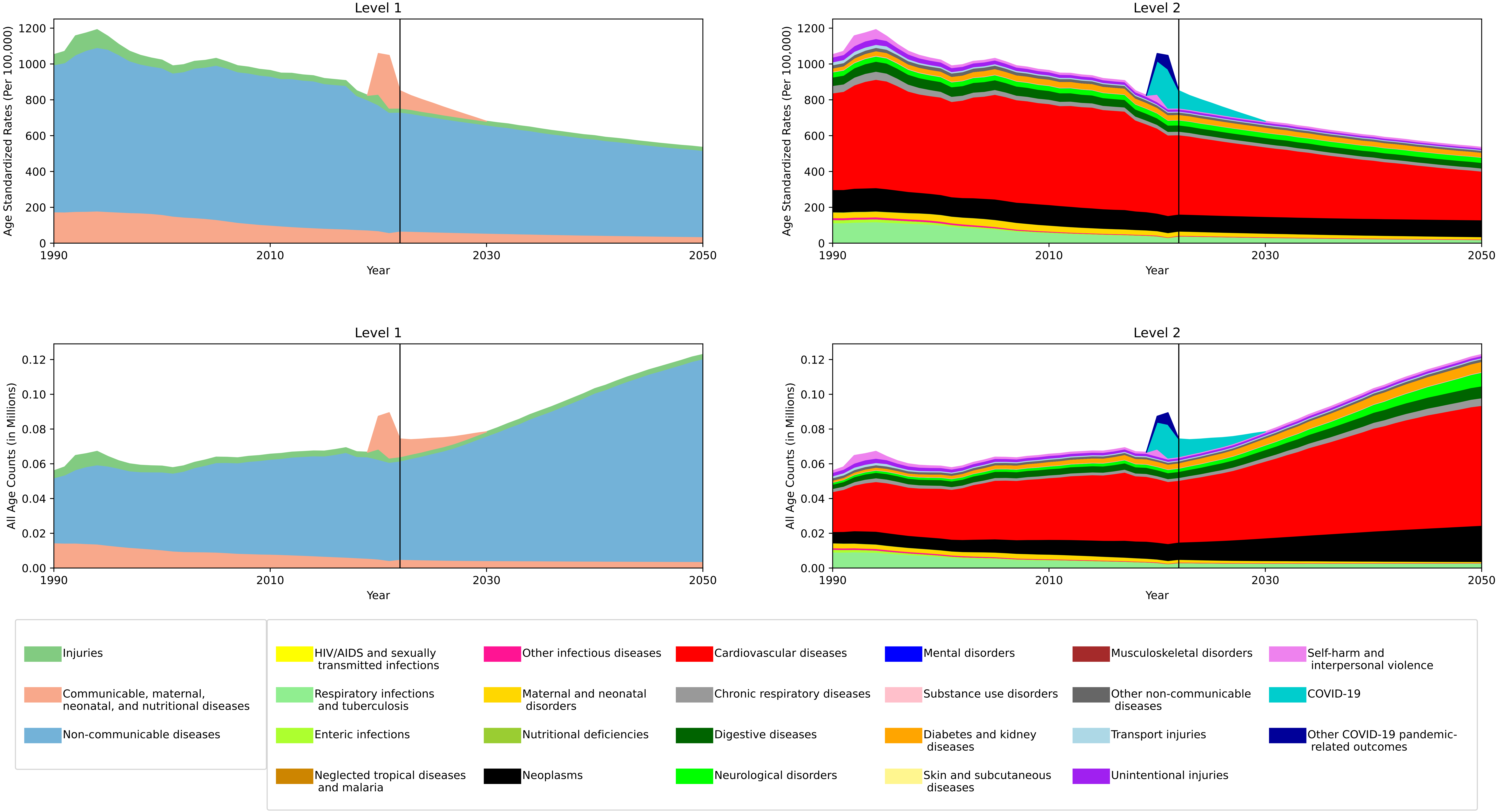

Georgia

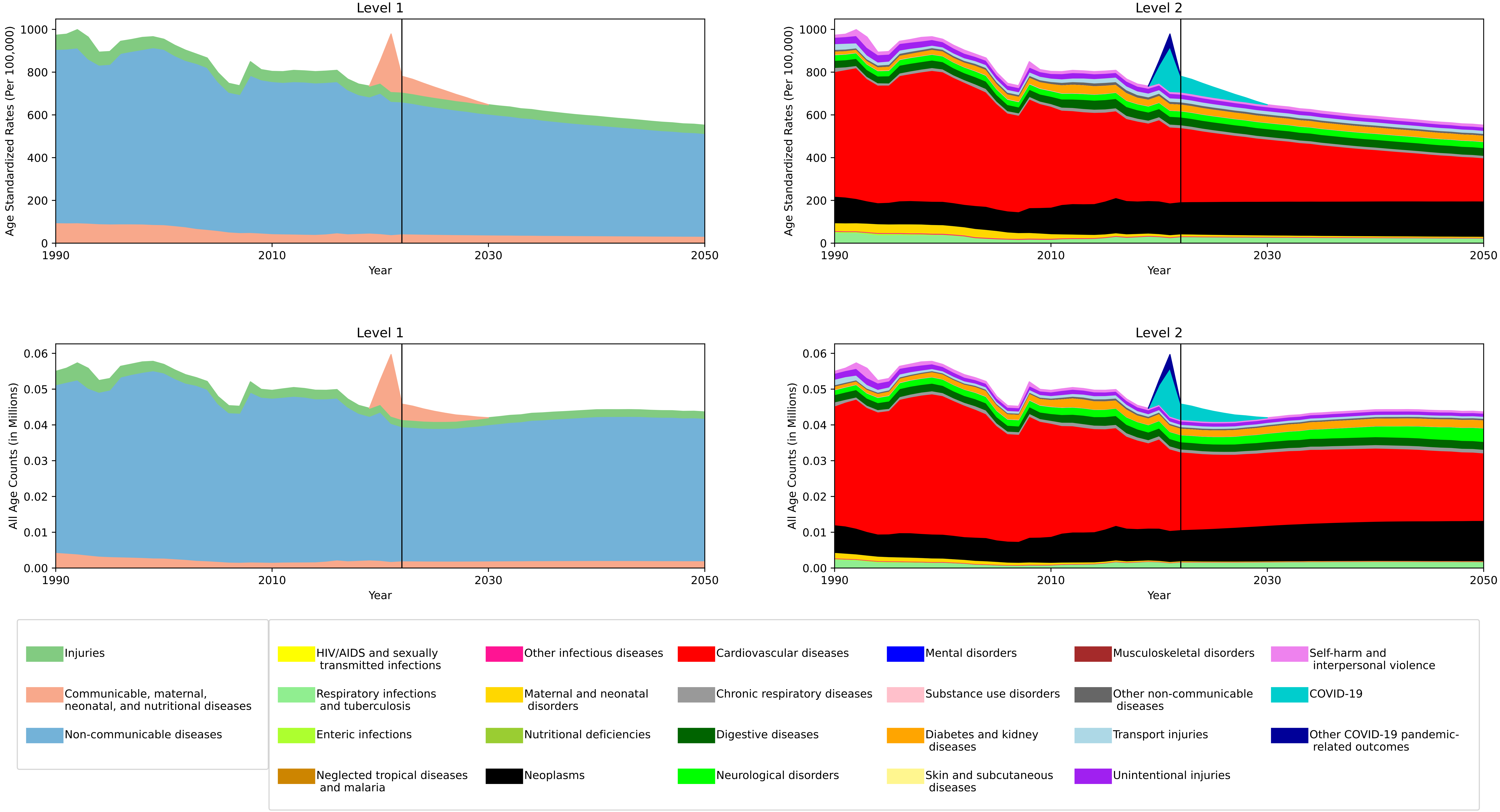
Level 1
Level 2
Age Standardized Rates (Per 100,000)
All Age Counts (in Millions)
Year
1990
2010
2030
2050
0
200
400
600
800
1000
0.00
0.01
0.02
0.03
0.04
0.05
0.06
Injuries
Communicable, maternal, neonatal, and nutritional diseases
Non-communicable diseases
HIV/AIDS and sexually transmitted infections
Respiratory infections and tuberculosis
Enteric infections
Neglected tropical diseases and malaria
Other infectious diseases
Maternal and neonatal disorders
Nutritional deficiencies
Neoplasms
Cardiovascular diseases
Chronic respiratory diseases
Digestive diseases
Neurological disorders
Mental disorders
Substance use disorders
Diabetes and kidney diseases
Skin and subcutaneous diseases
Musculoskeletal disorders
Other non-communicable diseases
Transport injuries
Unintentional injuries
Self-harm and interpersonal violence
COVID-19
Other COVID-19 pandemic-related outcomes

Kazakhstan

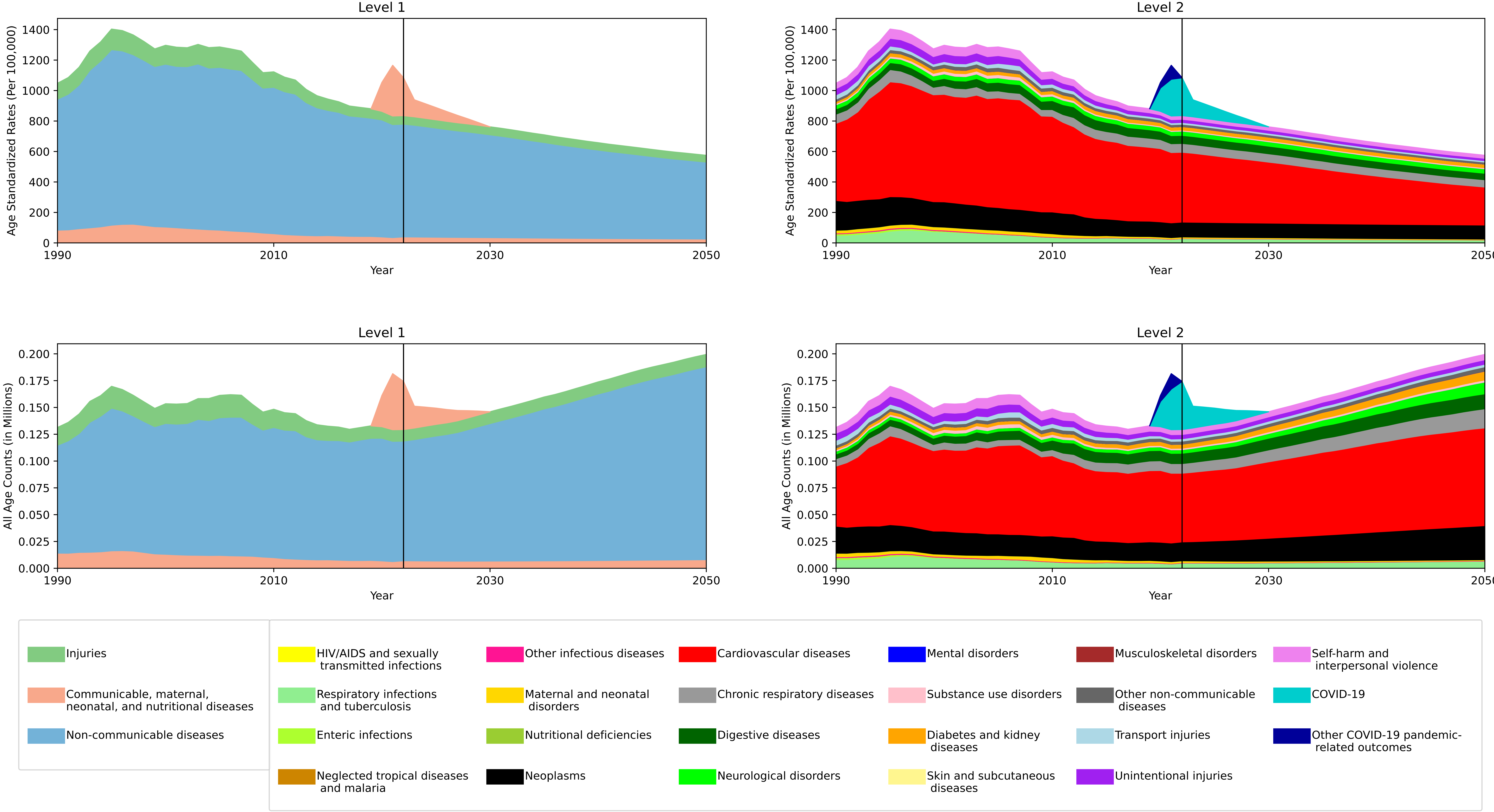
Level 1
Level 2
Age Standardized Rates (Per 100,000)
All Age Counts (in Millions)
Year
1990
2010
2030
2050
Injuries
Communicable, maternal, neonatal, and nutritional diseases
Non-communicable diseases
HIV/AIDS and sexually transmitted infections
Respiratory infections and tuberculosis
Enteric infections
Neglected tropical diseases and malaria
Other infectious diseases
Maternal and neonatal disorders
Nutritional deficiencies
Neoplasms
Cardiovascular diseases
Chronic respiratory diseases
Digestive diseases
Neurological disorders
Mental disorders
Substance use disorders
Diabetes and kidney diseases
Skin and subcutaneous diseases
Musculoskeletal disorders
Other non-communicable diseases
Transport injuries
Unintentional injuries
Self-harm and interpersonal violence
COVID-19
Other COVID-19 pandemic-related outcomes

Kyrgyzstan

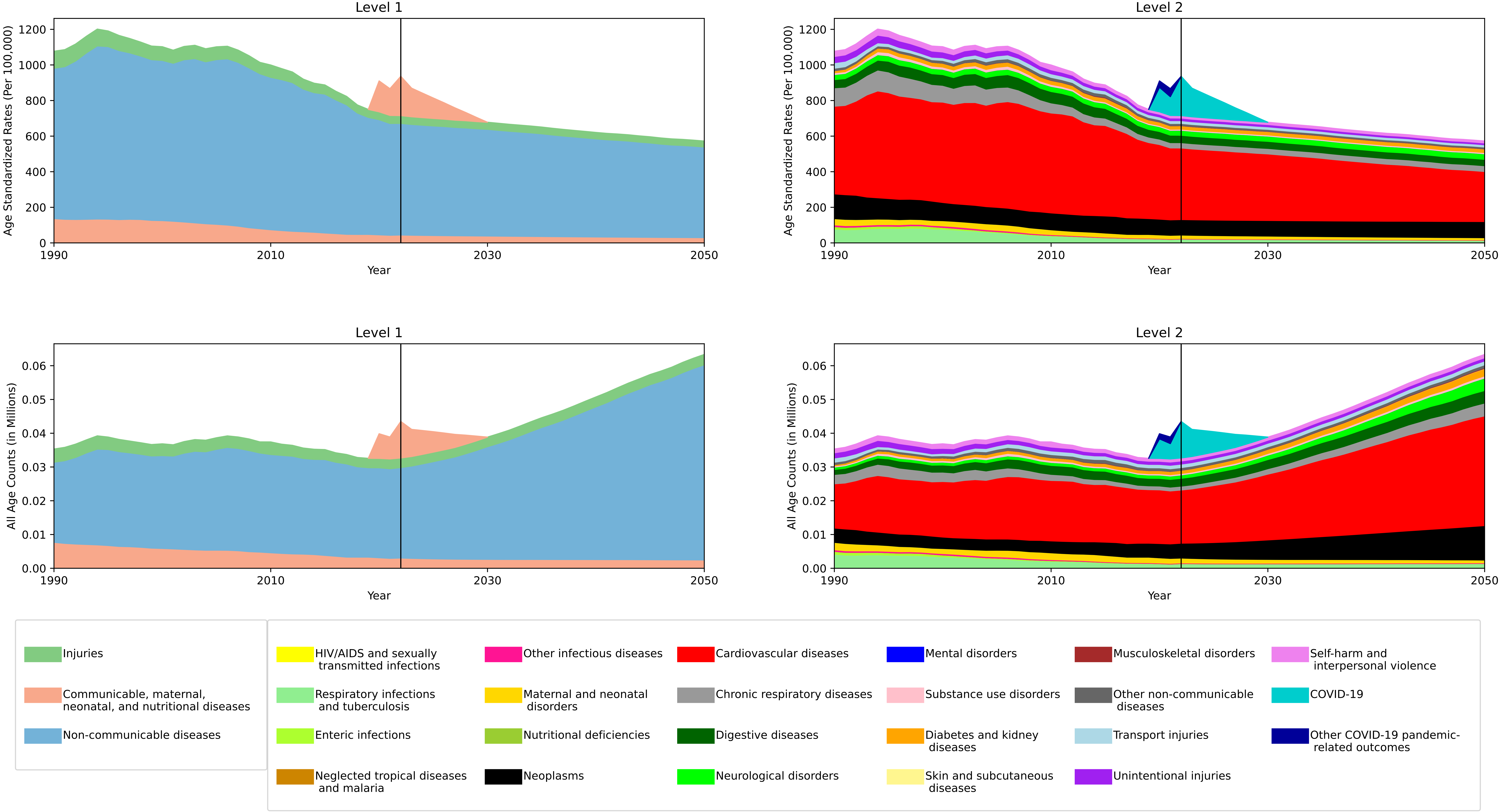
Level 1
Level 2
Age Standardized Rates (Per 100,000)
All Age Counts (in Millions)
Year
0
200
400
600
800
1000
1200
0.00
0.01
0.02
0.03
0.04
0.05
0.06
1990
2010
2030
2050
Injuries
Communicable, maternal, neonatal, and nutritional diseases
Non-communicable diseases
HIV/AIDS and sexually transmitted infections
Respiratory infections and tuberculosis
Enteric infections
Neglected tropical diseases and malaria
Other infectious diseases
Maternal and neonatal disorders
Nutritional deficiencies
Neoplasms
Cardiovascular diseases
Chronic respiratory diseases
Digestive diseases
Neurological disorders
Mental disorders
Substance use disorders
Diabetes and kidney diseases
Skin and subcutaneous diseases
Musculoskeletal disorders
Other non-communicable diseases
Transport injuries
Unintentional injuries
Self-harm and interpersonal violence
COVID-19
Other COVID-19 pandemic-related outcomes

Mongolia

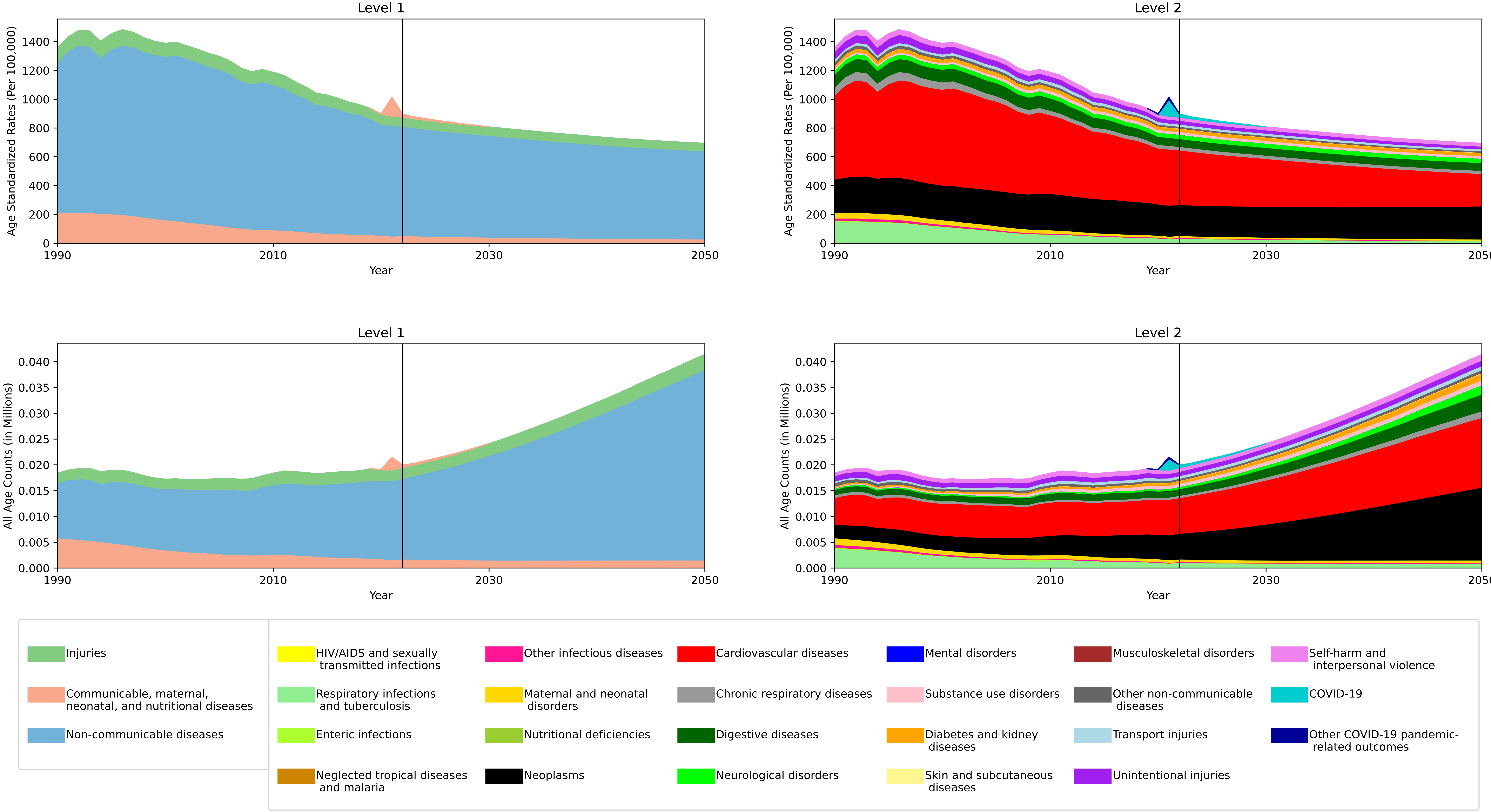
Level 1
Level 2
Age Standardized Rates (Per 100,000)
All Age Counts (in Millions)
Year
Injuries
Communicable, maternal, neonatal, and nutritional diseases
Non-communicable diseases
HIV/AIDS and sexually transmitted infections
Respiratory infections and tuberculosis
Enteric infections
Neglected tropical diseases and malaria
Other infectious diseases
Maternal and neonatal disorders
Nutritional deficiencies
Neoplasms
Cardiovascular diseases
Chronic respiratory diseases
Digestive diseases
Neurological disorders
Mental disorders
Substance use disorders
Diabetes and kidney diseases
Skin and subcutaneous diseases
Musculoskeletal disorders
Other non-communicable diseases
Transport injuries
Unintentional injuries
Self-harm and interpersonal violence
COVID-19
Other COVID-19 pandemic-related outcomes

Tajikistan

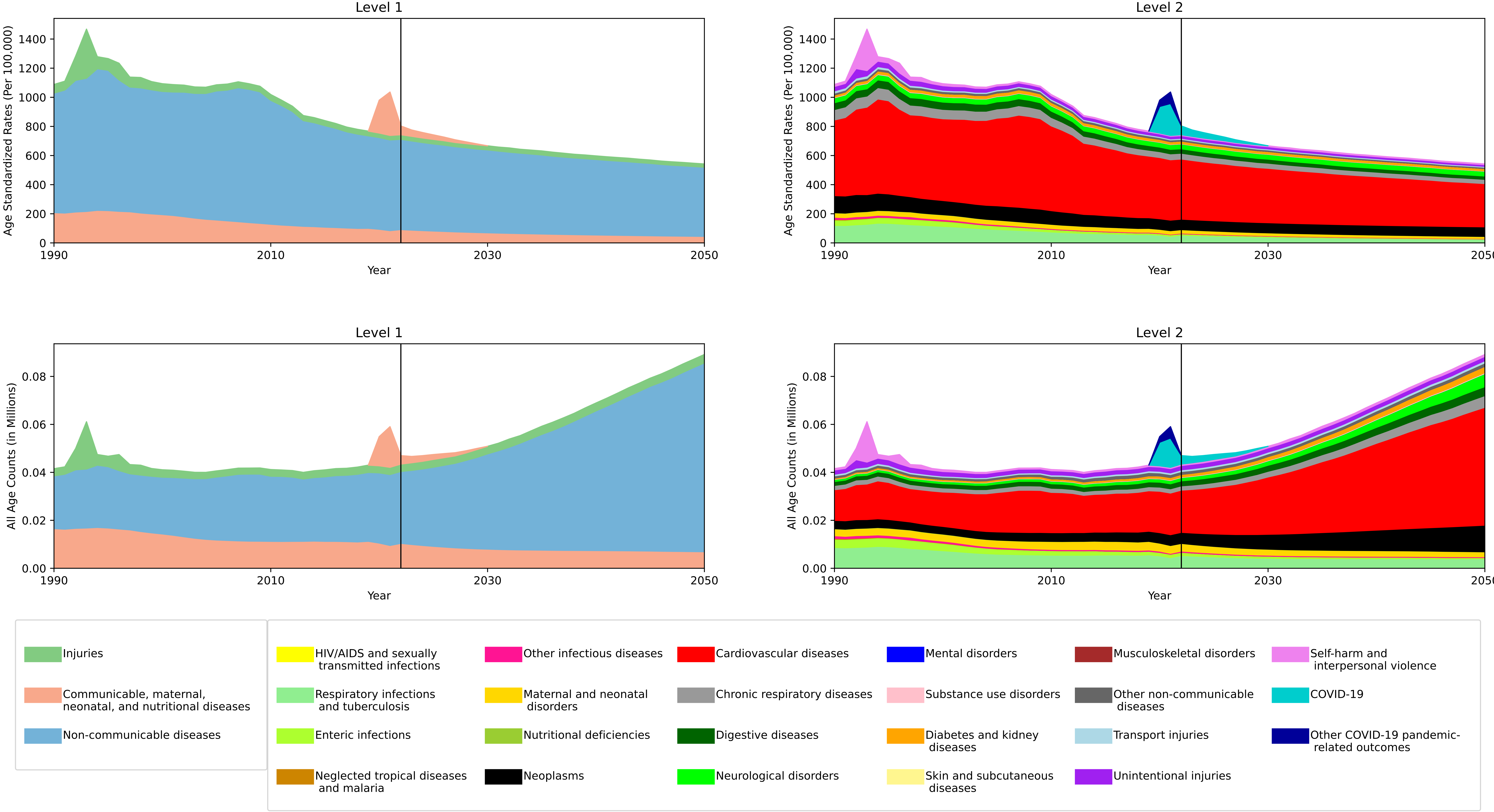
Level 1
Level 2
Age Standardized Rates (Per 100,000)
All Age Counts (in Millions)
Year
1990
2010
2030
2050
Injuries
Communicable, maternal, neonatal, and nutritional diseases
Non-communicable diseases
HIV/AIDS and sexually transmitted infections
Respiratory infections and tuberculosis
Enteric infections
Neglected tropical diseases and malaria
Other infectious diseases
Maternal and neonatal disorders
Nutritional deficiencies
Neoplasms
Cardiovascular diseases
Chronic respiratory diseases
Digestive diseases
Neurological disorders
Mental disorders
Substance use disorders
Diabetes and kidney diseases
Skin and subcutaneous diseases
Musculoskeletal disorders
Other non-communicable diseases
Transport injuries
Unintentional injuries
Self-harm and interpersonal violence
COVID-19
Other COVID-19 pandemic-related outcomes

Turkmenistan

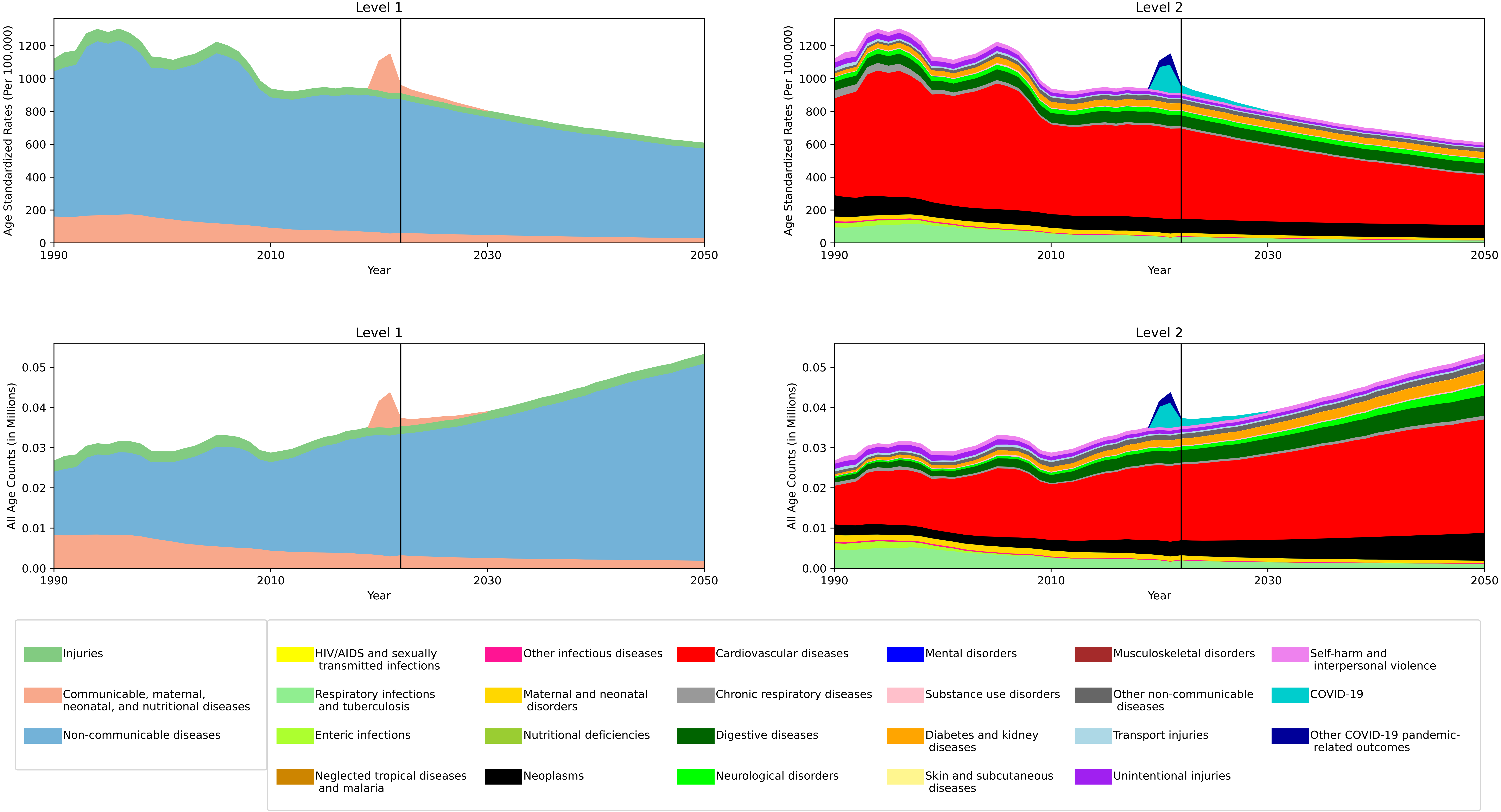

Uzbekistan

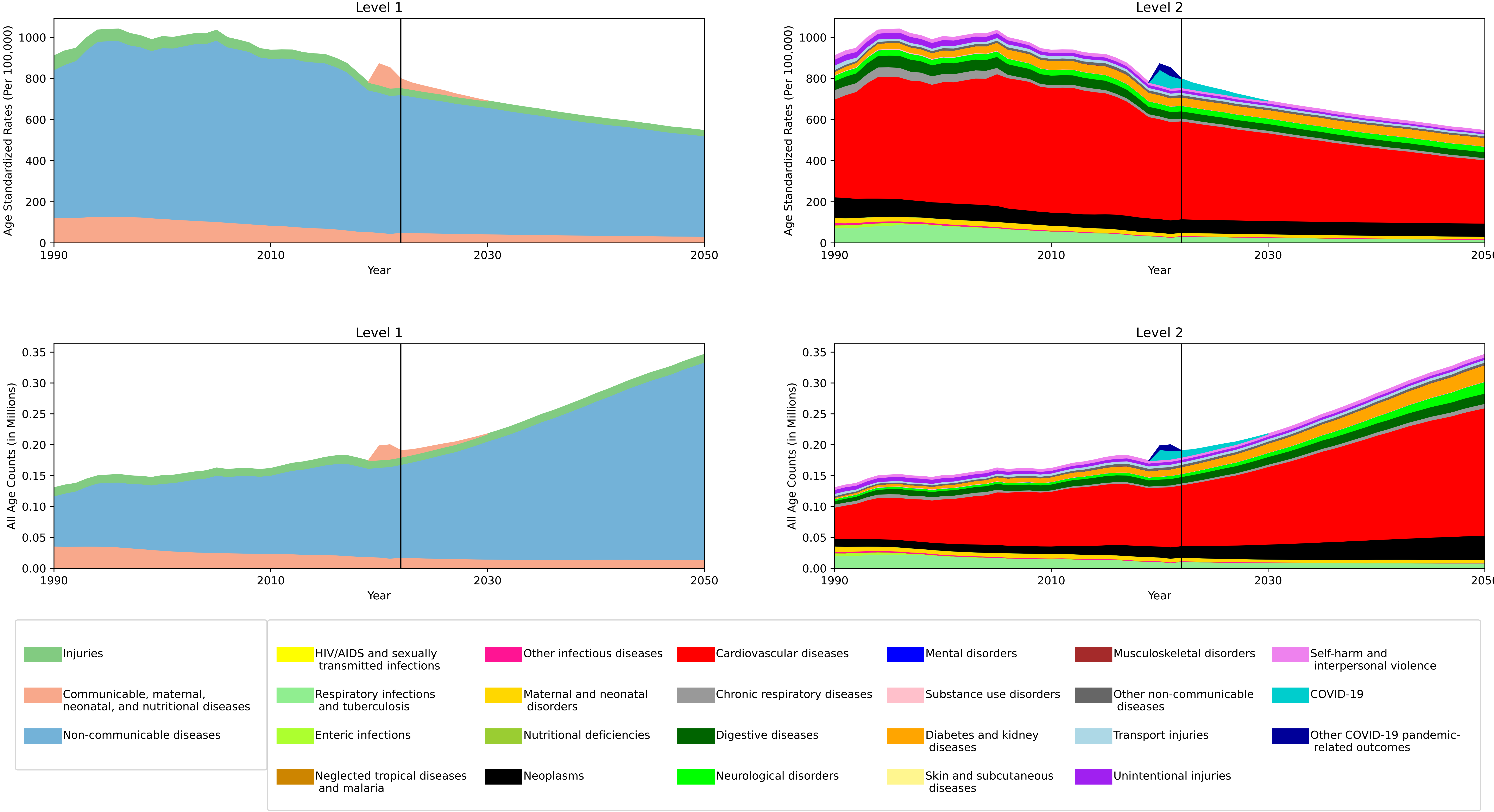

Level 1
Level 2
Age Standardized Rates (Per 100,000)
All Age Counts (in Millions)
Year
1990
2010
2030
2050
Injuries
Communicable, maternal, neonatal, and nutritional diseases
Non-communicable diseases
HIV/AIDS and sexually transmitted infections
Respiratory infections and tuberculosis
Enteric infections
Neglected tropical diseases and malaria
Other infectious diseases
Maternal and neonatal disorders
Nutritional deficiencies
Neoplasms
Cardiovascular diseases
Chronic respiratory diseases
Digestive diseases
Neurological disorders
Mental disorders
Substance use disorders
Diabetes and kidney diseases
Skin and subcutaneous diseases
Musculoskeletal disorders
Other non-communicable diseases
Transport injuries
Unintentional injuries
Self-harm and interpersonal violence
COVID-19
Other COVID-19 pandemic-related outcomes

Albania

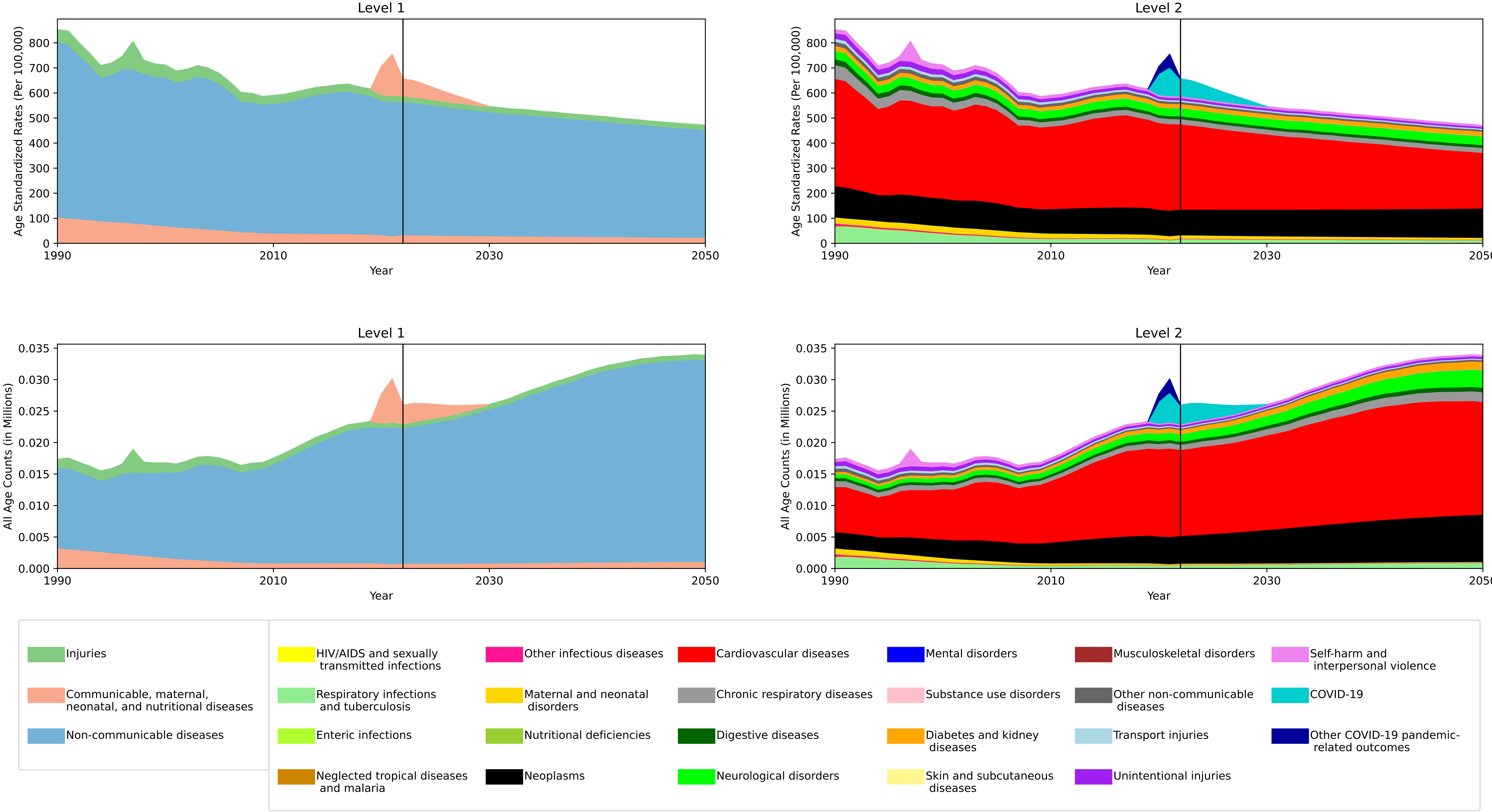
Level 1
Level 2
Age Standardized Rates (Per 100,000)
All Age Counts (in Millions)
Year
1990
2010
2030
2050
0
100
200
300
400
500
600
700
800
0.000
0.005
0.010
0.015
0.020
0.025
0.030
0.035
Injuries
Communicable, maternal, neonatal, and nutritional diseases
Non-communicable diseases
HIV/AIDS and sexually transmitted infections
Respiratory infections and tuberculosis
Enteric infections
Neglected tropical diseases and malaria
Other infectious diseases
Maternal and neonatal disorders
Nutritional deficiencies
Neoplasms
Cardiovascular diseases
Chronic respiratory diseases
Digestive diseases
Neurological disorders
Mental disorders
Substance use disorders
Diabetes and kidney diseases
Skin and subcutaneous diseases
Musculoskeletal disorders
Other non-communicable diseases
Transport injuries
Unintentional injuries
Self-harm and interpersonal violence
COVID-19
Other COVID-19 pandemic-related outcomes

Bosnia And Herzegovina

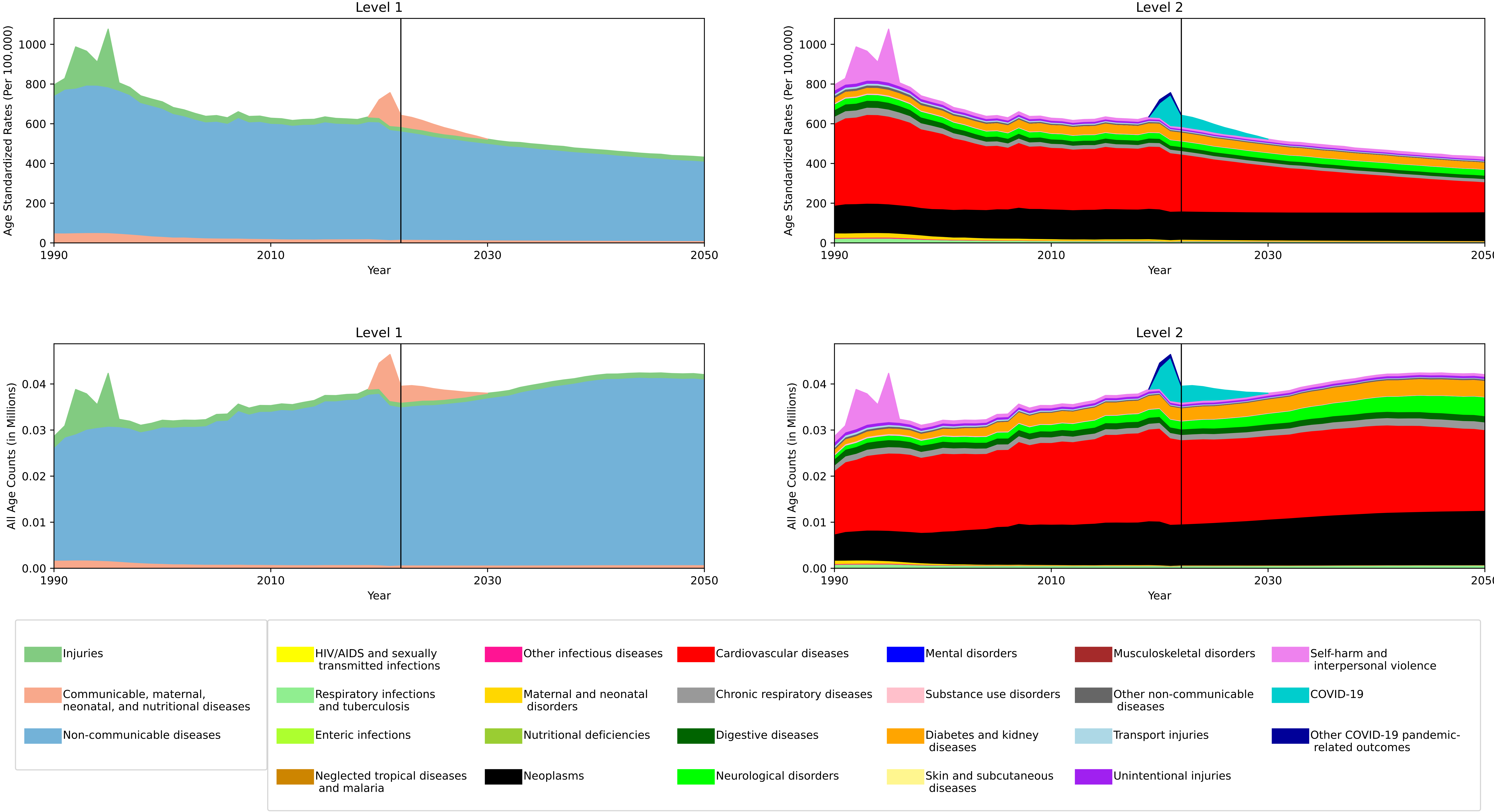

Bulgaria

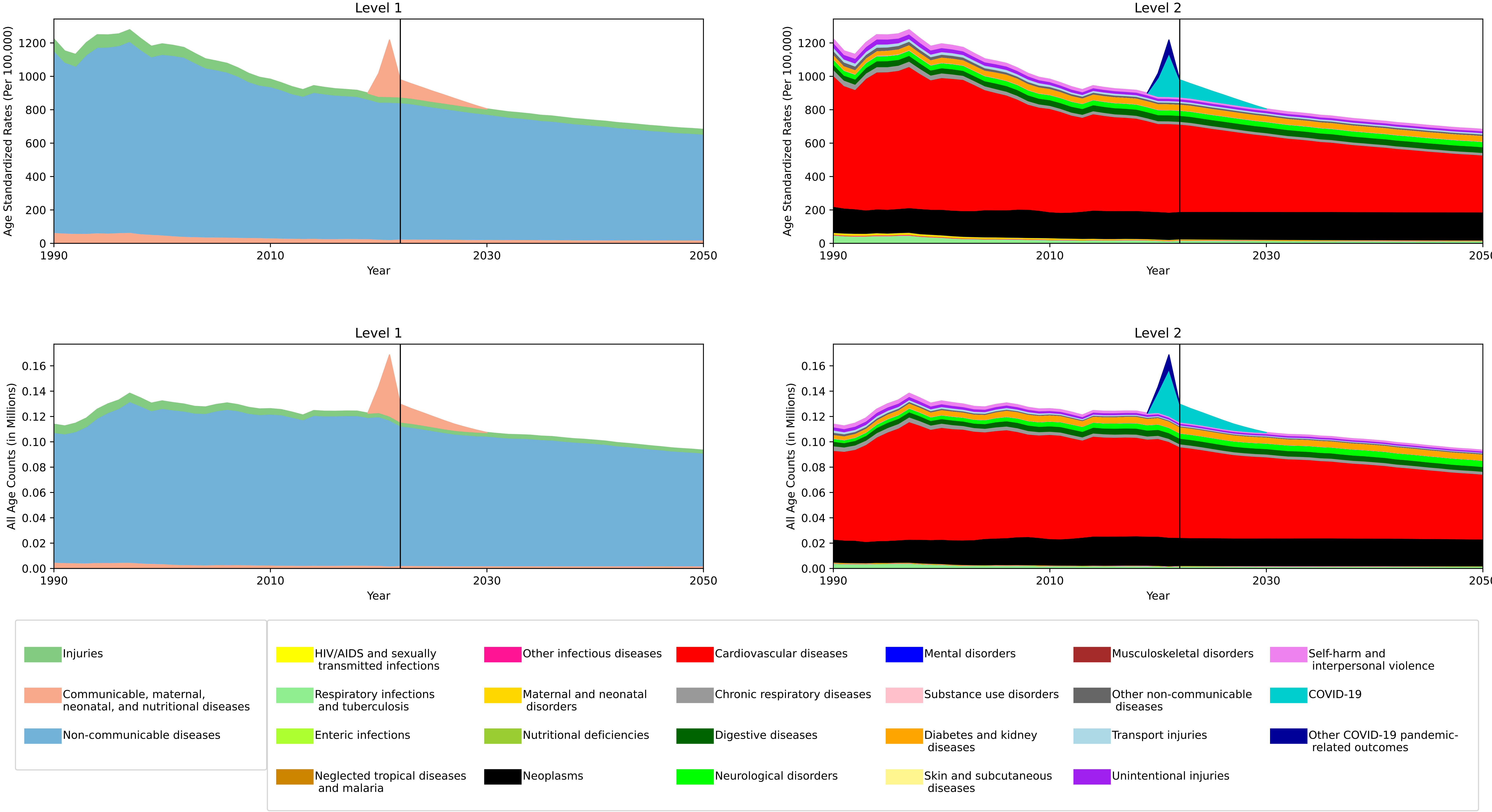
Level 1
Level 2
Age Standardized Rates (Per 100,000)
All Age Counts (in Millions)
Year
1990
2010
2030
2050
Injuries
Communicable, maternal, neonatal, and nutritional diseases
Non-communicable diseases
HIV/AIDS and sexually transmitted infections
Respiratory infections and tuberculosis
Enteric infections
Neglected tropical diseases and malaria
Other infectious diseases
Maternal and neonatal disorders
Nutritional deficiencies
Neoplasms
Cardiovascular diseases
Chronic respiratory diseases
Digestive diseases
Neurological disorders
Mental disorders
Substance use disorders
Diabetes and kidney diseases
Skin and subcutaneous diseases
Musculoskeletal disorders
Other non-communicable diseases
Transport injuries
Unintentional injuries
Self-harm and interpersonal violence
COVID-19
Other COVID-19 pandemic-related outcomes

Croatia

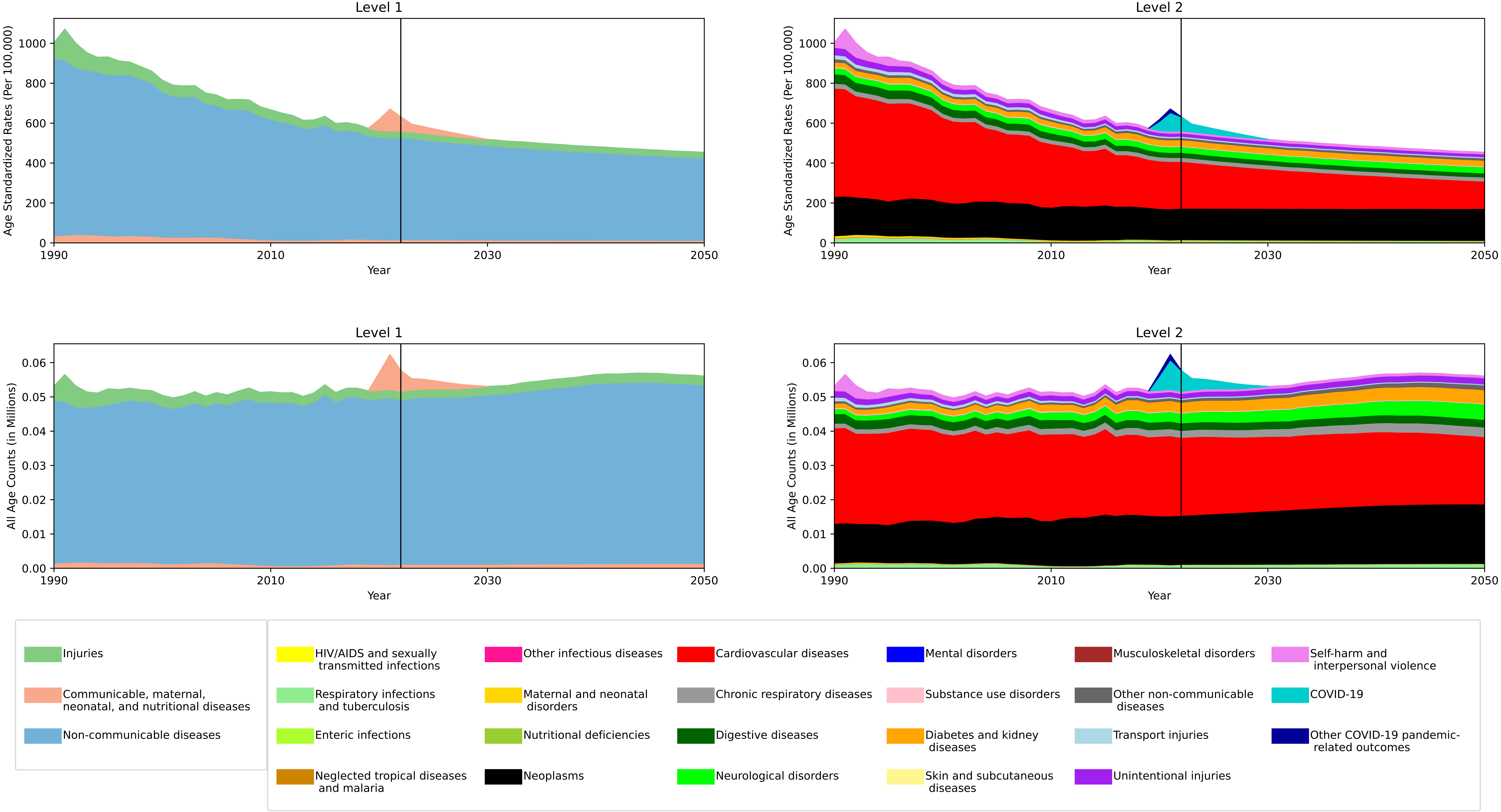

Level 1
Level 2
Age Standardized Rates (Per 100,000)
All Age Counts (in Millions)
Year
Injuries
Communicable, maternal, neonatal, and nutritional diseases
Non-communicable diseases
HIV/AIDS and sexually transmitted infections
Respiratory infections and tuberculosis
Enteric infections
Neglected tropical diseases and malaria
Other infectious diseases
Maternal and neonatal disorders
Nutritional deficiencies
Neoplasms
Cardiovascular diseases
Chronic respiratory diseases
Digestive diseases
Neurological disorders
Mental disorders
Substance use disorders
Diabetes and kidney diseases
Skin and subcutaneous diseases
Musculoskeletal disorders
Other non-communicable diseases
Transport injuries
Unintentional injuries
Self-harm and interpersonal violence
COVID-19
Other COVID-19 pandemic-related outcomes

Czechia

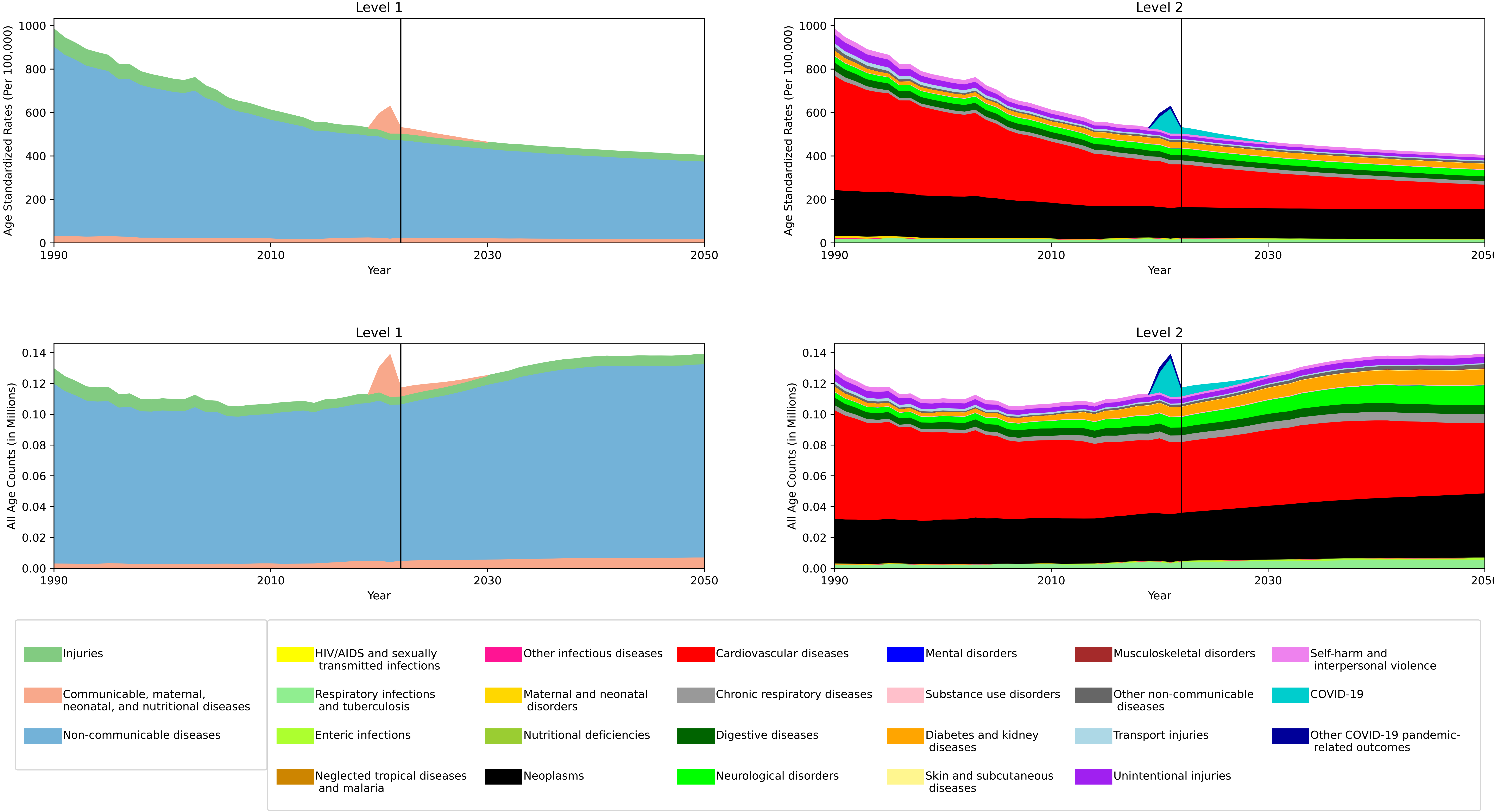
Level 1
Age Standardized Rates (Per 100,000)
Year
Level 2
Age Standardized Rates (Per 100,000)
Year
Level 1
All Age Counts (in Millions)
Year
Level 2
All Age Counts (in Millions)
Year
Injuries
Communicable, maternal, neonatal, and nutritional diseases
Non-communicable diseases
HIV/AIDS and sexually transmitted infections
Respiratory infections and tuberculosis
Enteric infections
Neglected tropical diseases and malaria
Other infectious diseases
Maternal and neonatal disorders
Nutritional deficiencies
Neoplasms
Cardiovascular diseases
Chronic respiratory diseases
Digestive diseases
Neurological disorders
Mental disorders
Substance use disorders
Diabetes and kidney diseases
Skin and subcutaneous diseases
Musculoskeletal disorders
Other non-communicable diseases
Transport injuries
Unintentional injuries
Self-harm and interpersonal violence
COVID-19
Other COVID-19 pandemic-related outcomes

Hungary

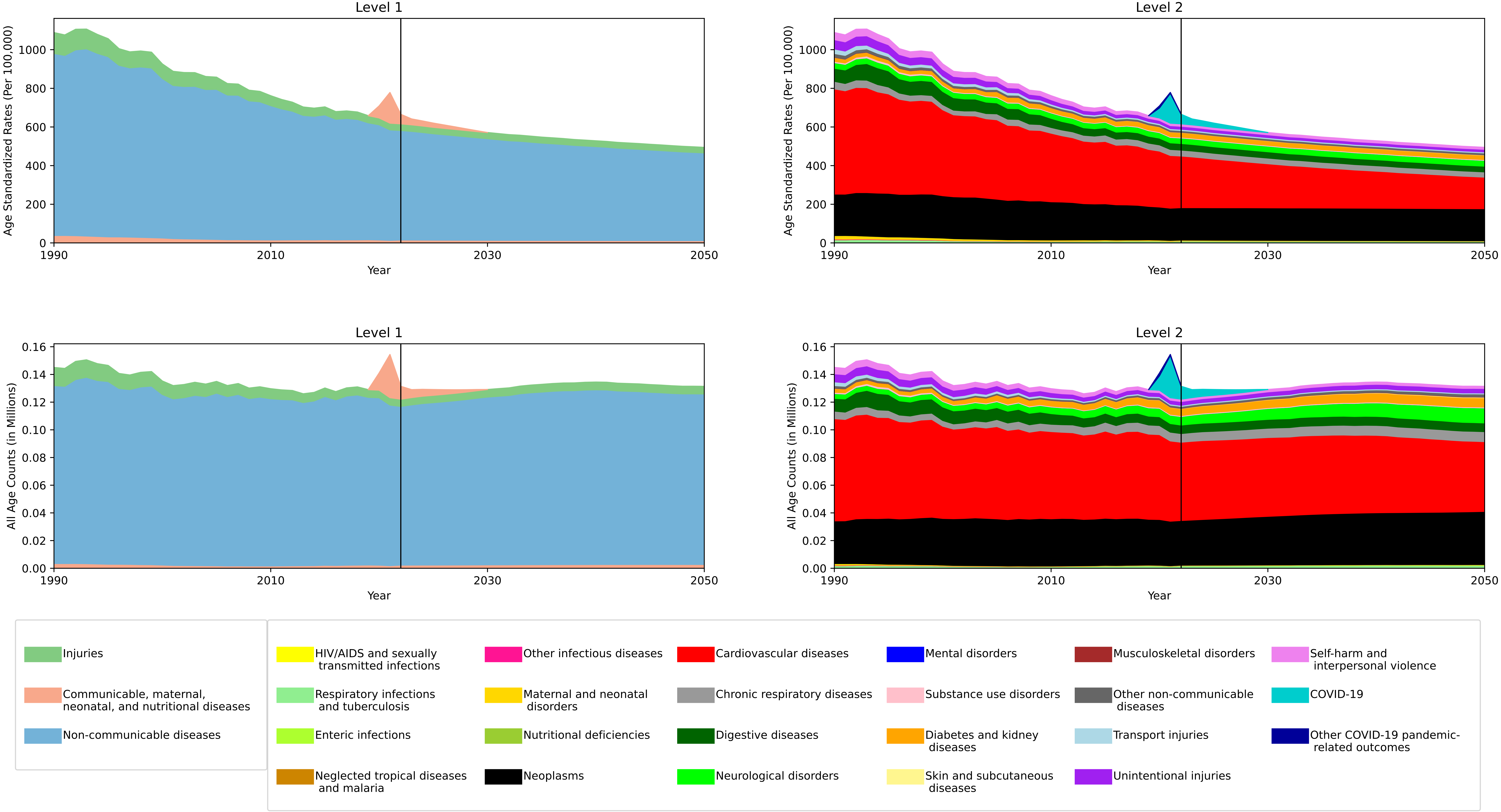
Level 1
Level 2
Age Standardized Rates (Per 100,000)
All Age Counts (in Millions)
Year
1990
2010
2030
2050
0
200
400
600
800
1000
0.00
0.02
0.04
0.06
0.08
0.10
0.12
0.14
0.16
Injuries
Communicable, maternal, neonatal, and nutritional diseases
Non-communicable diseases
HIV/AIDS and sexually transmitted infections
Respiratory infections and tuberculosis
Enteric infections
Neglected tropical diseases and malaria
Other infectious diseases
Maternal and neonatal disorders
Nutritional deficiencies
Neoplasms
Cardiovascular diseases
Chronic respiratory diseases
Digestive diseases
Neurological disorders
Mental disorders
Substance use disorders
Diabetes and kidney diseases
Skin and subcutaneous diseases
Musculoskeletal disorders
Other non-communicable diseases
Transport injuries
Unintentional injuries
Self-harm and interpersonal violence
COVID-19
Other COVID-19 pandemic-related outcomes

Montenegro

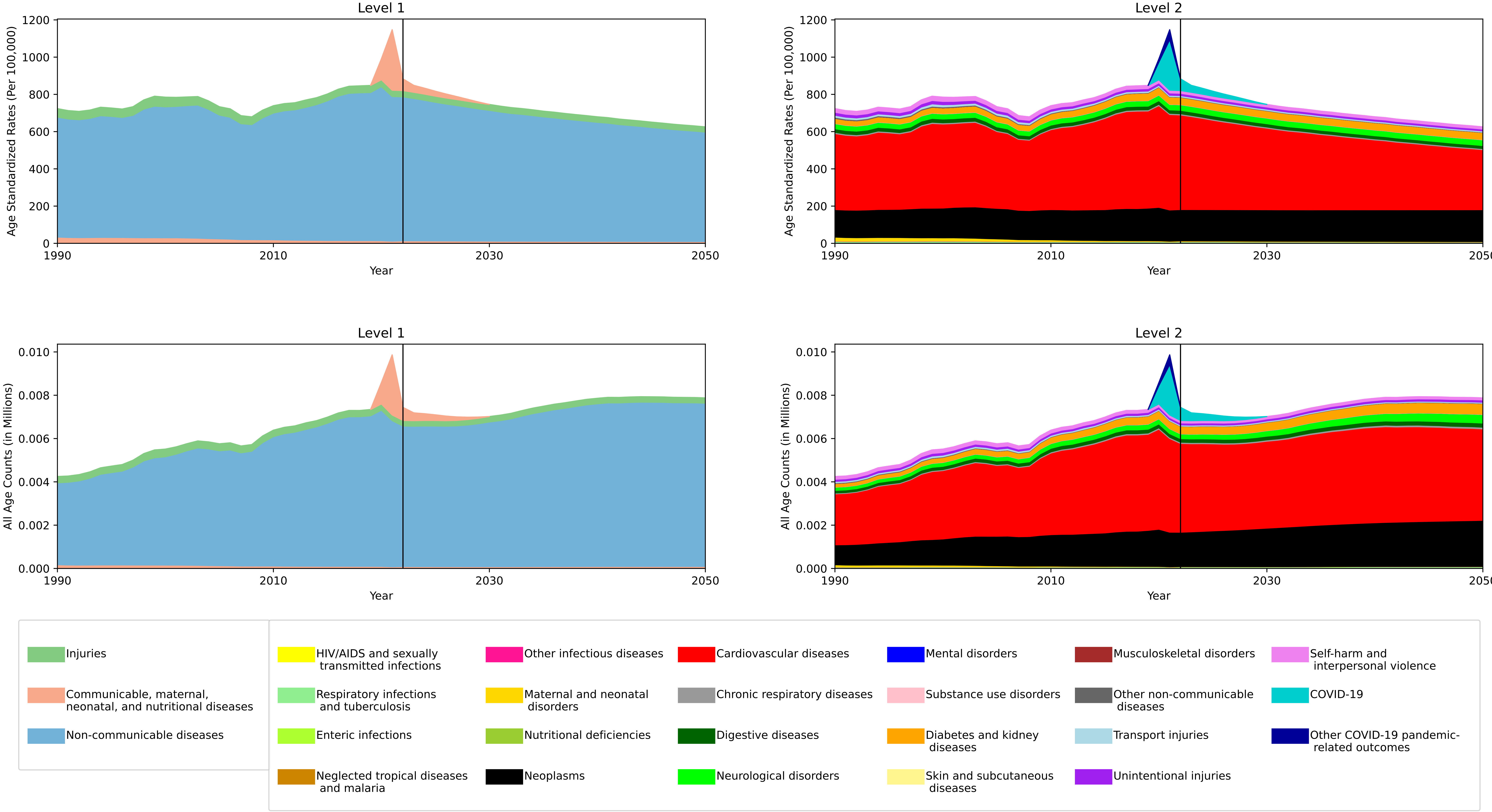

Level 1
Level 2
Age Standardized Rates (Per 100,000)
All Age Counts (in Millions)
Year
1990
2010
2030
2050
0
200
400
600
800
1000
1200
0.000
0.002
0.004
0.006
0.008
0.010
Injuries
Communicable, maternal, neonatal, and nutritional diseases
Non-communicable diseases
HIV/AIDS and sexually transmitted infections
Respiratory infections and tuberculosis
Enteric infections
Neglected tropical diseases and malaria
Other infectious diseases
Maternal and neonatal disorders
Nutritional deficiencies
Neoplasms
Cardiovascular diseases
Chronic respiratory diseases
Digestive diseases
Neurological disorders
Mental disorders
Substance use disorders
Diabetes and kidney diseases
Skin and subcutaneous diseases
Musculoskeletal disorders
Other non-communicable diseases
Transport injuries
Unintentional injuries
Self-harm and interpersonal violence
COVID-19
Other COVID-19 pandemic-related outcomes

North Macedonia

Supplemental Results Figure S5. Age-standardised death rates and all age death counts and by Level 1 and Level 2 causes, 1990–2050

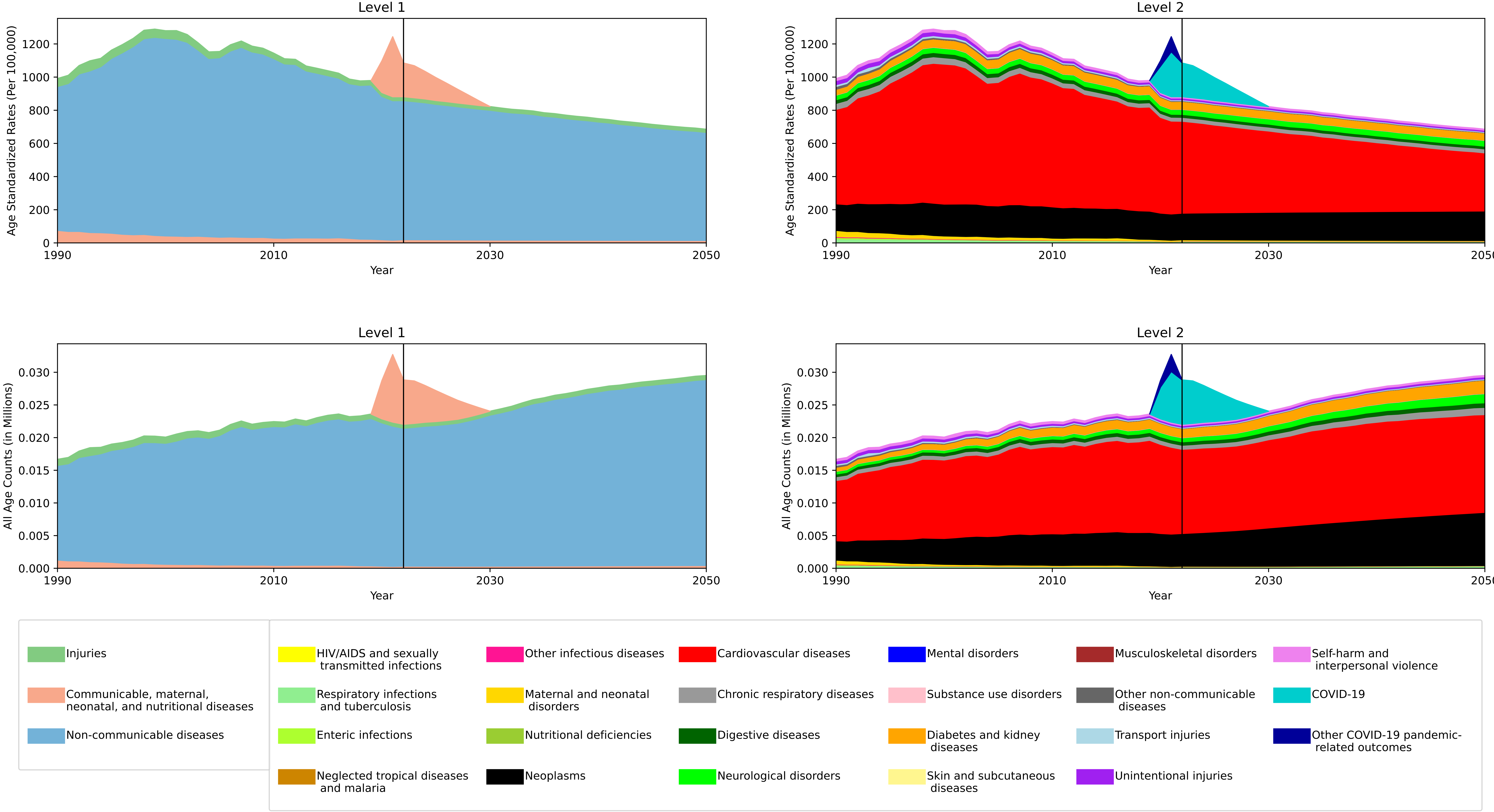

Poland

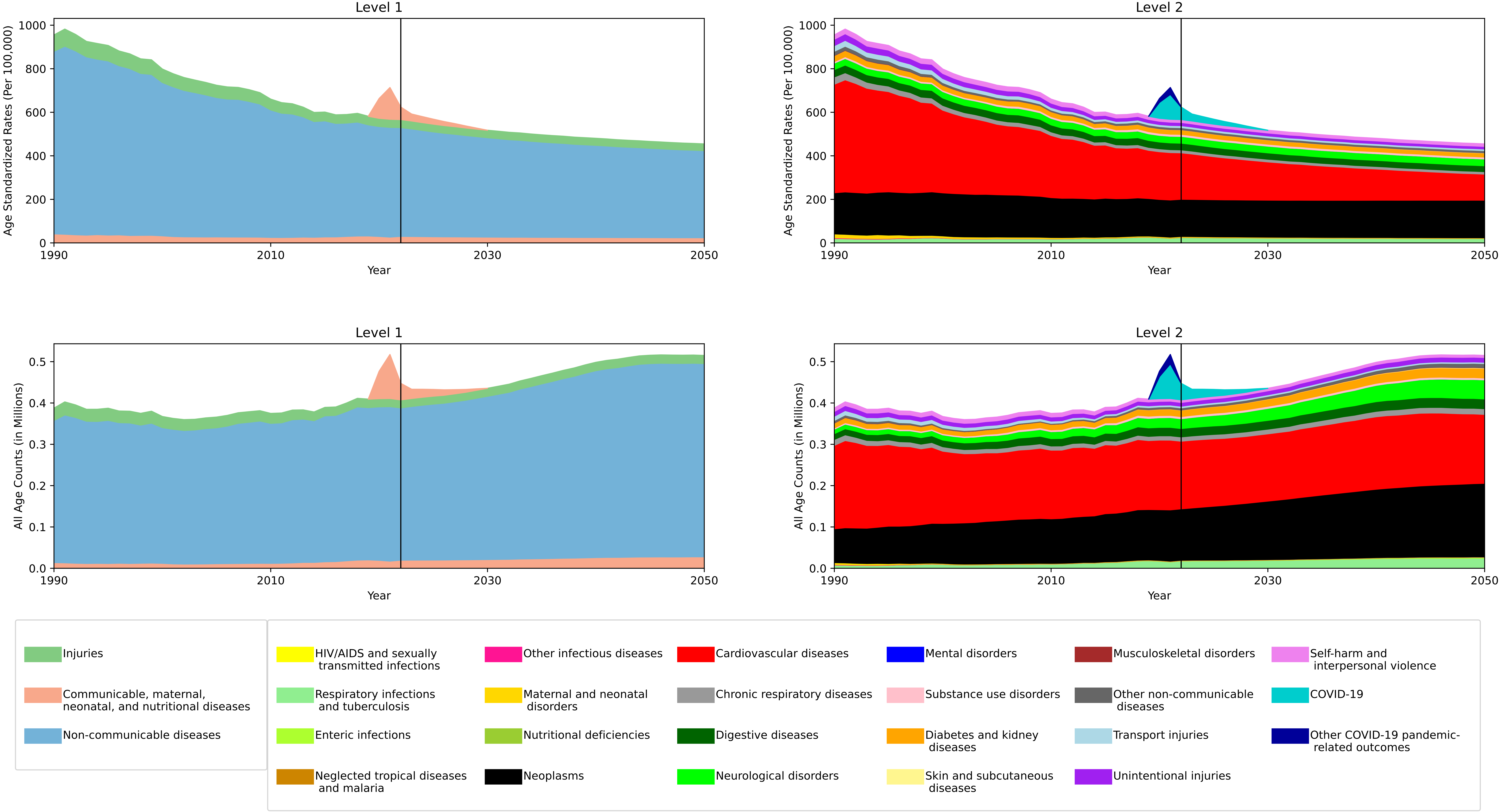
Level 1
Level 2
Age Standardized Rates (Per 100,000)
All Age Counts (in Millions)
Year
0
200
400
600
800
1000
0.0
0.1
0.2
0.3
0.4
0.5
1990
2010
2030
2050
Injuries
Communicable, maternal, neonatal, and nutritional diseases
Non-communicable diseases
HIV/AIDS and sexually transmitted infections
Respiratory infections and tuberculosis
Enteric infections
Neglected tropical diseases and malaria
Other infectious diseases
Maternal and neonatal disorders
Nutritional deficiencies
Neoplasms
Cardiovascular diseases
Chronic respiratory diseases
Digestive diseases
Neurological disorders
Mental disorders
Substance use disorders
Diabetes and kidney diseases
Skin and subcutaneous diseases
Musculoskeletal disorders
Other non-communicable diseases
Transport injuries
Unintentional injuries
Self-harm and interpersonal violence
COVID-19
Other COVID-19 pandemic-related outcomes

Romania

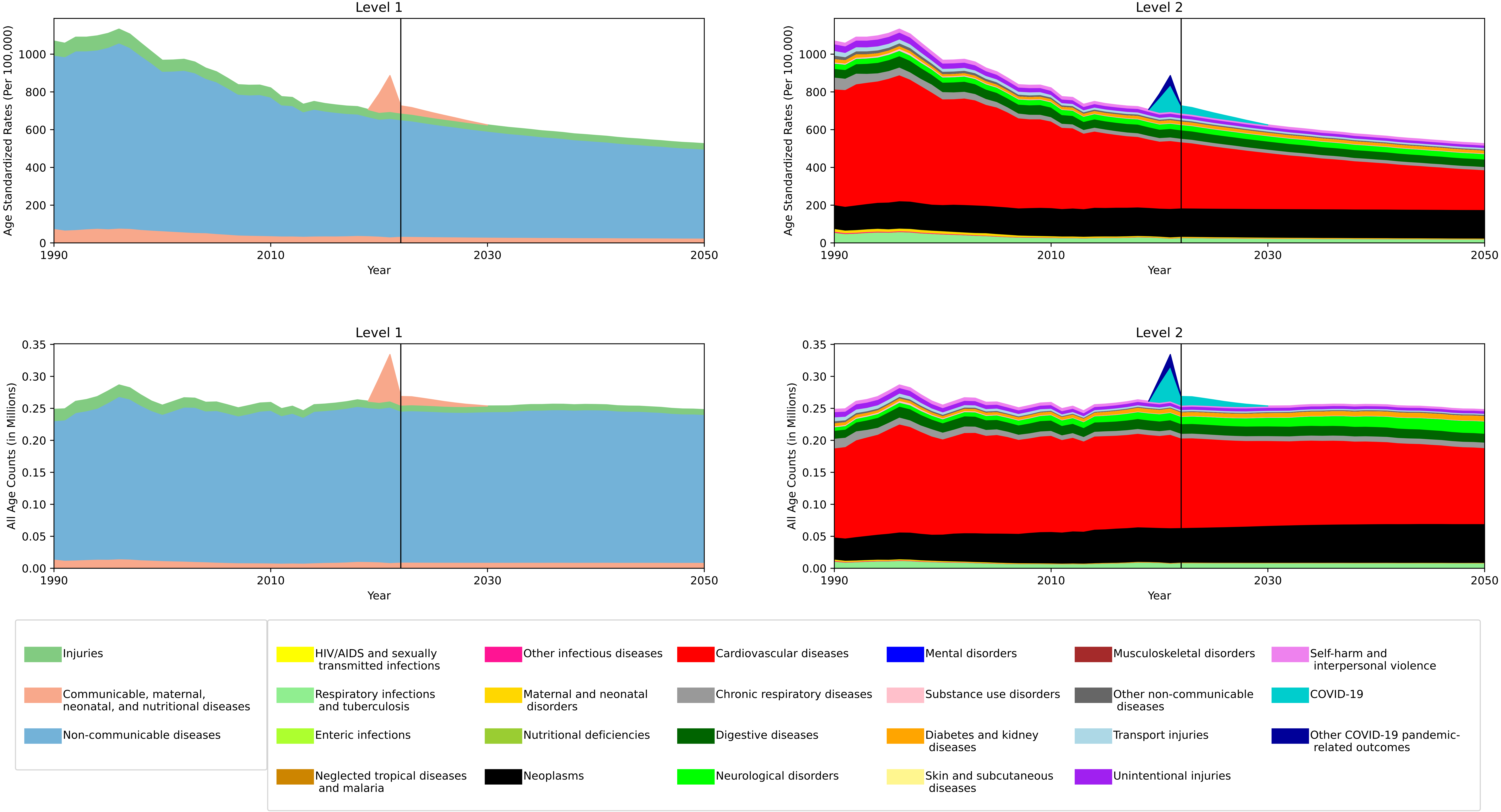
Level 1
Level 2
Age Standardized Rates (Per 100,000)
All Age Counts (in Millions)
Year
1990
2010
2030
2050
0
200
400
600
800
1000
0.00
0.05
0.10
0.15
0.20
0.25
0.30
0.35
Injuries
Communicable, maternal, neonatal, and nutritional diseases
Non-communicable diseases
HIV/AIDS and sexually transmitted infections
Respiratory infections and tuberculosis
Enteric infections
Neglected tropical diseases and malaria
Other infectious diseases
Maternal and neonatal disorders
Nutritional deficiencies
Neoplasms
Cardiovascular diseases
Chronic respiratory diseases
Digestive diseases
Neurological disorders
Mental disorders
Substance use disorders
Diabetes and kidney diseases
Skin and subcutaneous diseases
Musculoskeletal disorders
Other non-communicable diseases
Transport injuries
Unintentional injuries
Self-harm and interpersonal violence
COVID-19
Other COVID-19 pandemic-related outcomes

Serbia

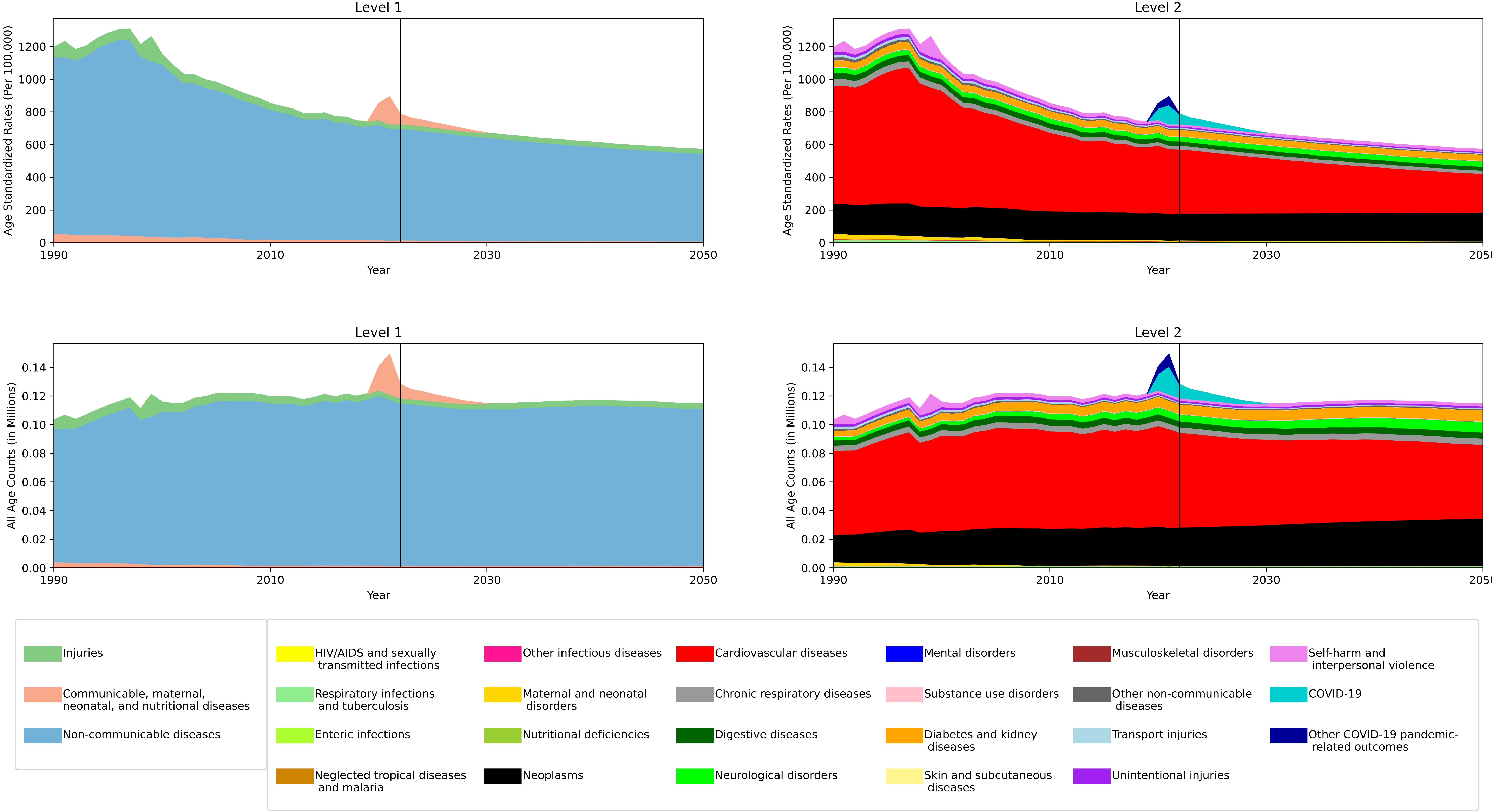

Level 1
Level 2
Age Standardized Rates (Per 100,000)
All Age Counts (in Millions)
Year
1990
2010
2030
2050
0
200
400
600
800
1000
1200
0.00
0.02
0.04
0.06
0.08
0.10
0.12
0.14
Injuries
Communicable, maternal, neonatal, and nutritional diseases
Non-communicable diseases
HIV/AIDS and sexually transmitted infections
Respiratory infections and tuberculosis
Enteric infections
Neglected tropical diseases and malaria
Other infectious diseases
Maternal and neonatal disorders
Nutritional deficiencies
Neoplasms
Cardiovascular diseases
Chronic respiratory diseases
Digestive diseases
Neurological disorders
Mental disorders
Substance use disorders
Diabetes and kidney diseases
Skin and subcutaneous diseases
Musculoskeletal disorders
Other non-communicable diseases
Transport injuries
Unintentional injuries
Self-harm and interpersonal violence
COVID-19
Other COVID-19 pandemic-related outcomes

Slovakia

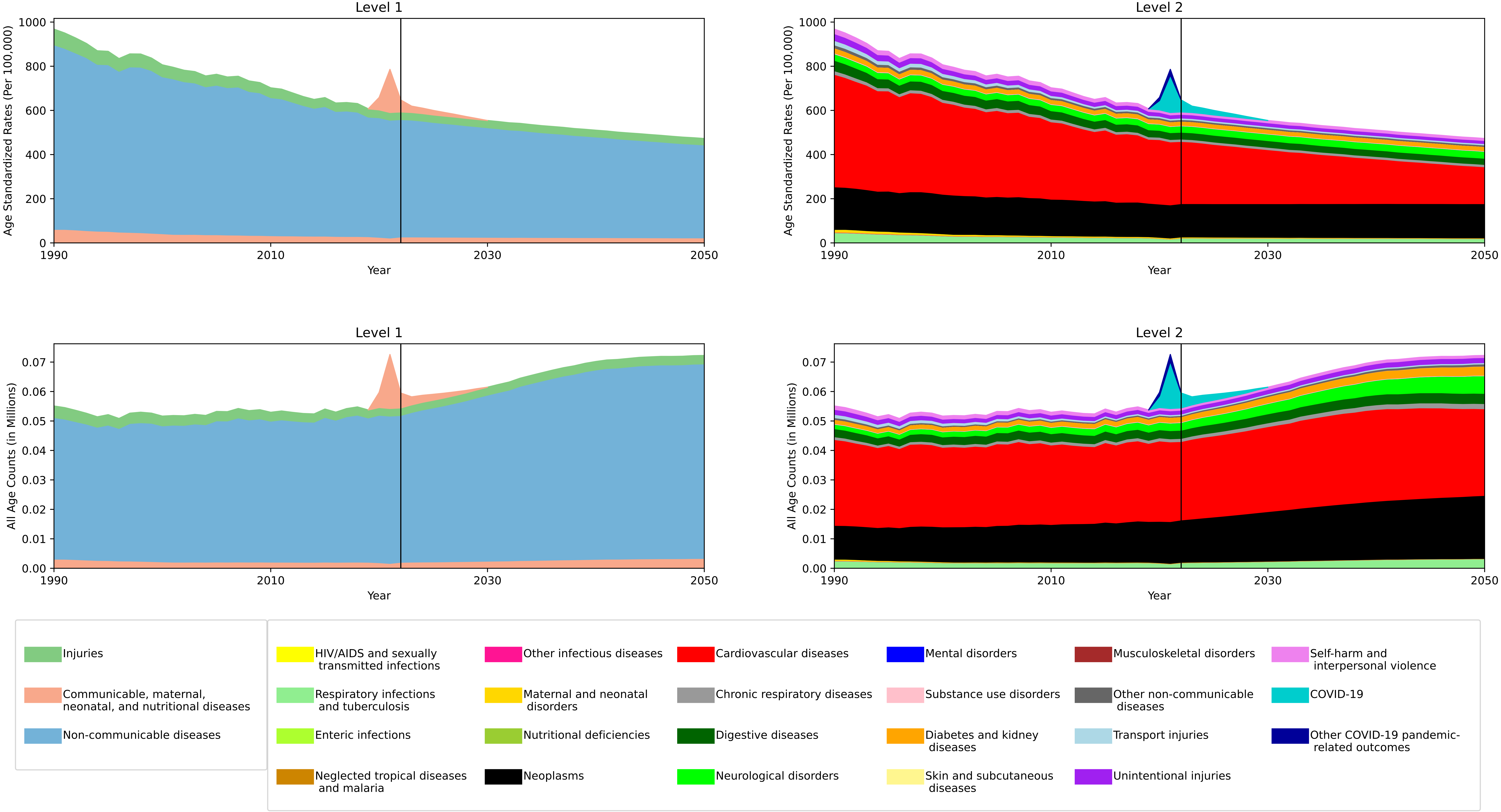
Level 1
Level 2
Age Standardized Rates (Per 100,000)
All Age Counts (in Millions)
Year
1990
2010
2030
2050
Injuries
Communicable, maternal, neonatal, and nutritional diseases
Non-communicable diseases
HIV/AIDS and sexually transmitted infections
Respiratory infections and tuberculosis
Enteric infections
Neglected tropical diseases and malaria
Other infectious diseases
Maternal and neonatal disorders
Nutritional deficiencies
Neoplasms
Cardiovascular diseases
Chronic respiratory diseases
Digestive diseases
Neurological disorders
Mental disorders
Substance use disorders
Diabetes and kidney diseases
Skin and subcutaneous diseases
Musculoskeletal disorders
Other non-communicable diseases
Transport injuries
Unintentional injuries
Self-harm and interpersonal violence
COVID-19
Other COVID-19 pandemic-related outcomes

Slovenia

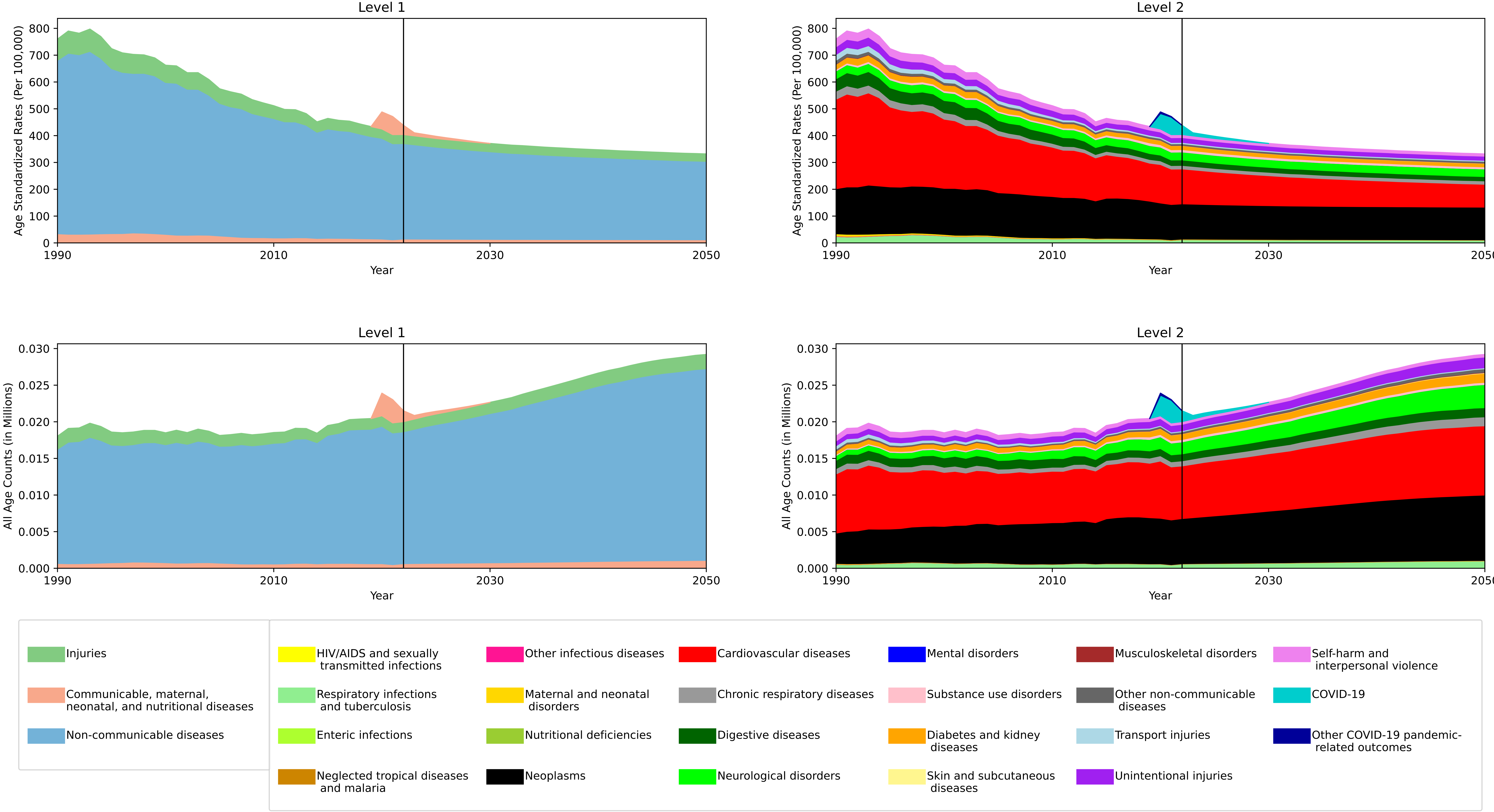
Level 1
Level 2
Age Standardized Rates (Per 100,000)
All Age Counts (in Millions)
Year
Injuries
Communicable, maternal, neonatal, and nutritional diseases
Non-communicable diseases
HIV/AIDS and sexually transmitted infections
Respiratory infections and tuberculosis
Enteric infections
Neglected tropical diseases and malaria
Other infectious diseases
Maternal and neonatal disorders
Nutritional deficiencies
Neoplasms
Cardiovascular diseases
Chronic respiratory diseases
Digestive diseases
Neurological disorders
Mental disorders
Substance use disorders
Diabetes and kidney diseases
Skin and subcutaneous diseases
Musculoskeletal disorders
Other non-communicable diseases
Transport injuries
Unintentional injuries
Self-harm and interpersonal violence
COVID-19
Other COVID-19 pandemic-related outcomes

Belarus

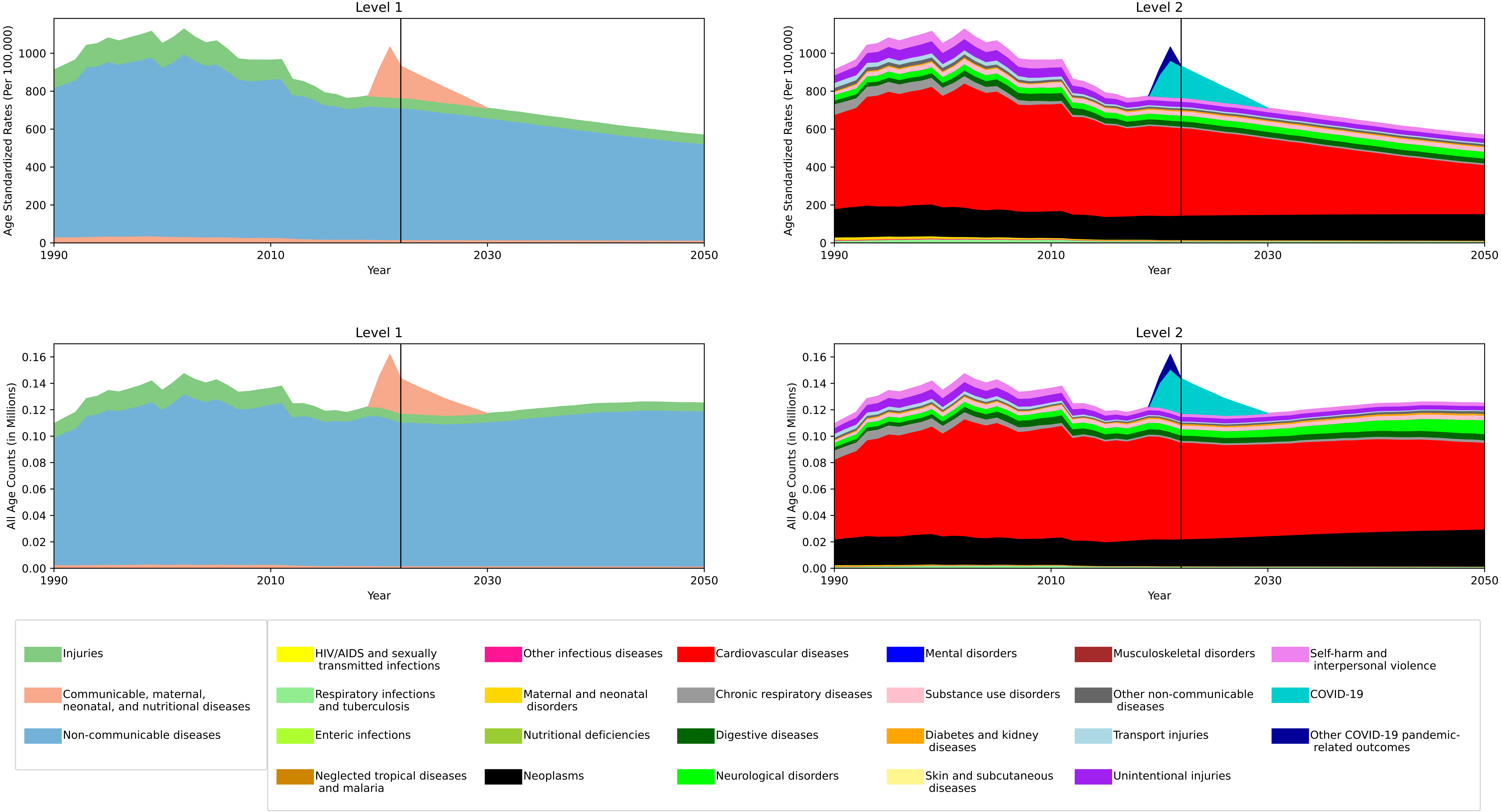
Level 1
Level 2
Age Standardized Rates (Per 100,000)
All Age Counts (in Millions)
Year
1990
2010
2030
2050
0
200
400
600
800
1000
0.00
0.02
0.04
0.06
0.08
0.10
0.12
0.14
0.16
Injuries
Communicable, maternal, neonatal, and nutritional diseases
Non-communicable diseases
HIV/AIDS and sexually transmitted infections
Respiratory infections and tuberculosis
Enteric infections
Neglected tropical diseases and malaria
Other infectious diseases
Maternal and neonatal disorders
Nutritional deficiencies
Neoplasms
Cardiovascular diseases
Chronic respiratory diseases
Digestive diseases
Neurological disorders
Mental disorders
Substance use disorders
Diabetes and kidney diseases
Skin and subcutaneous diseases
Musculoskeletal disorders
Other non-communicable diseases
Transport injuries
Unintentional injuries
Self-harm and interpersonal violence
COVID-19
Other COVID-19 pandemic-related outcomes

Estonia

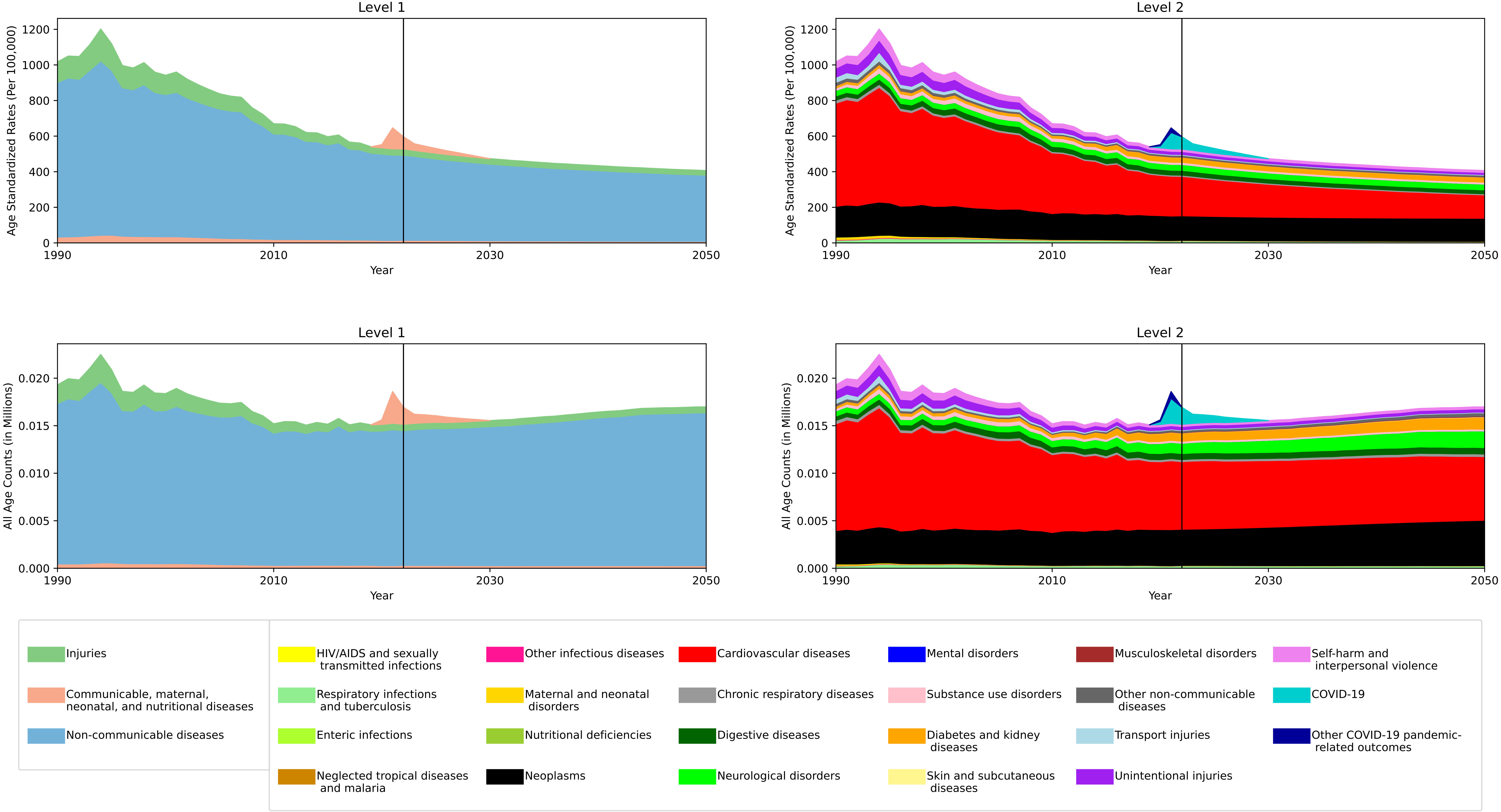
Level 1
Age Standardized Rates (Per 100,000)
Year
Level 2
Age Standardized Rates (Per 100,000)
Year
Level 1
All Age Counts (in Millions)
Year
Level 2
All Age Counts (in Millions)
Year
Injuries
Communicable, maternal, neonatal, and nutritional diseases
Non-communicable diseases
HIV/AIDS and sexually transmitted infections
Respiratory infections and tuberculosis
Enteric infections
Neglected tropical diseases and malaria
Other infectious diseases
Maternal and neonatal disorders
Nutritional deficiencies
Neoplasms
Cardiovascular diseases
Chronic respiratory diseases
Digestive diseases
Neurological disorders
Mental disorders
Substance use disorders
Diabetes and kidney diseases
Skin and subcutaneous diseases
Musculoskeletal disorders
Other non-communicable diseases
Transport injuries
Unintentional injuries
Self-harm and interpersonal violence
COVID-19
Other COVID-19 pandemic-related outcomes

Latvia

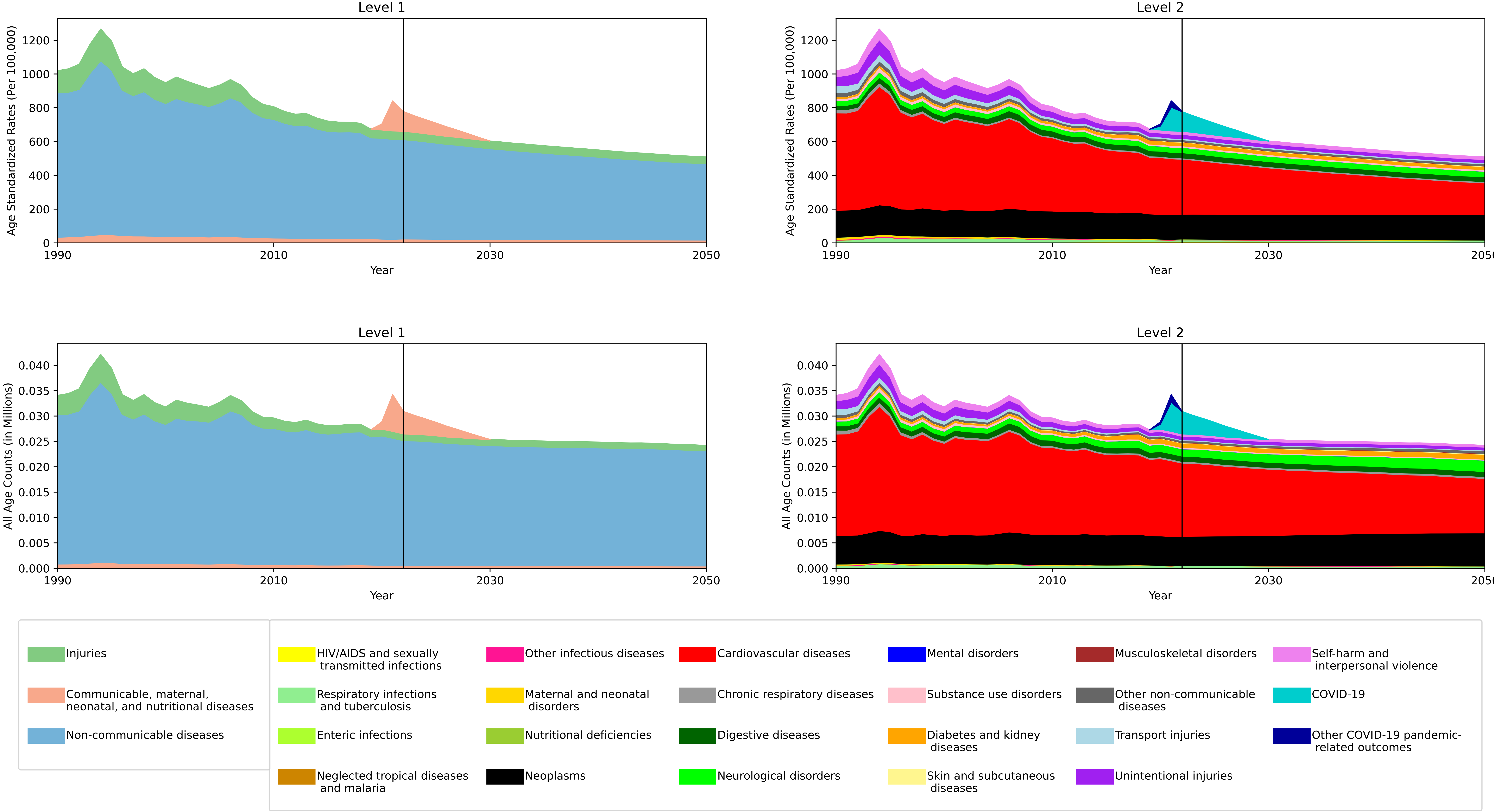
Level 1
Level 2
Level 1
Level 2
Age Standardized Rates (Per 100,000)
All Age Counts (in Millions)
Year
1990
2010
2030
2050
Injuries
Communicable, maternal, neonatal, and nutritional diseases
Non-communicable diseases
HIV/AIDS and sexually transmitted infections
Respiratory infections and tuberculosis
Enteric infections
Neglected tropical diseases and malaria
Other infectious diseases
Maternal and neonatal disorders
Nutritional deficiencies
Neoplasms
Cardiovascular diseases
Chronic respiratory diseases
Digestive diseases
Neurological disorders
Mental disorders
Substance use disorders
Diabetes and kidney diseases
Skin and subcutaneous diseases
Musculoskeletal disorders
Other non-communicable diseases
Transport injuries
Unintentional injuries
Self-harm and interpersonal violence
COVID-19
Other COVID-19 pandemic-related outcomes

Lithuania

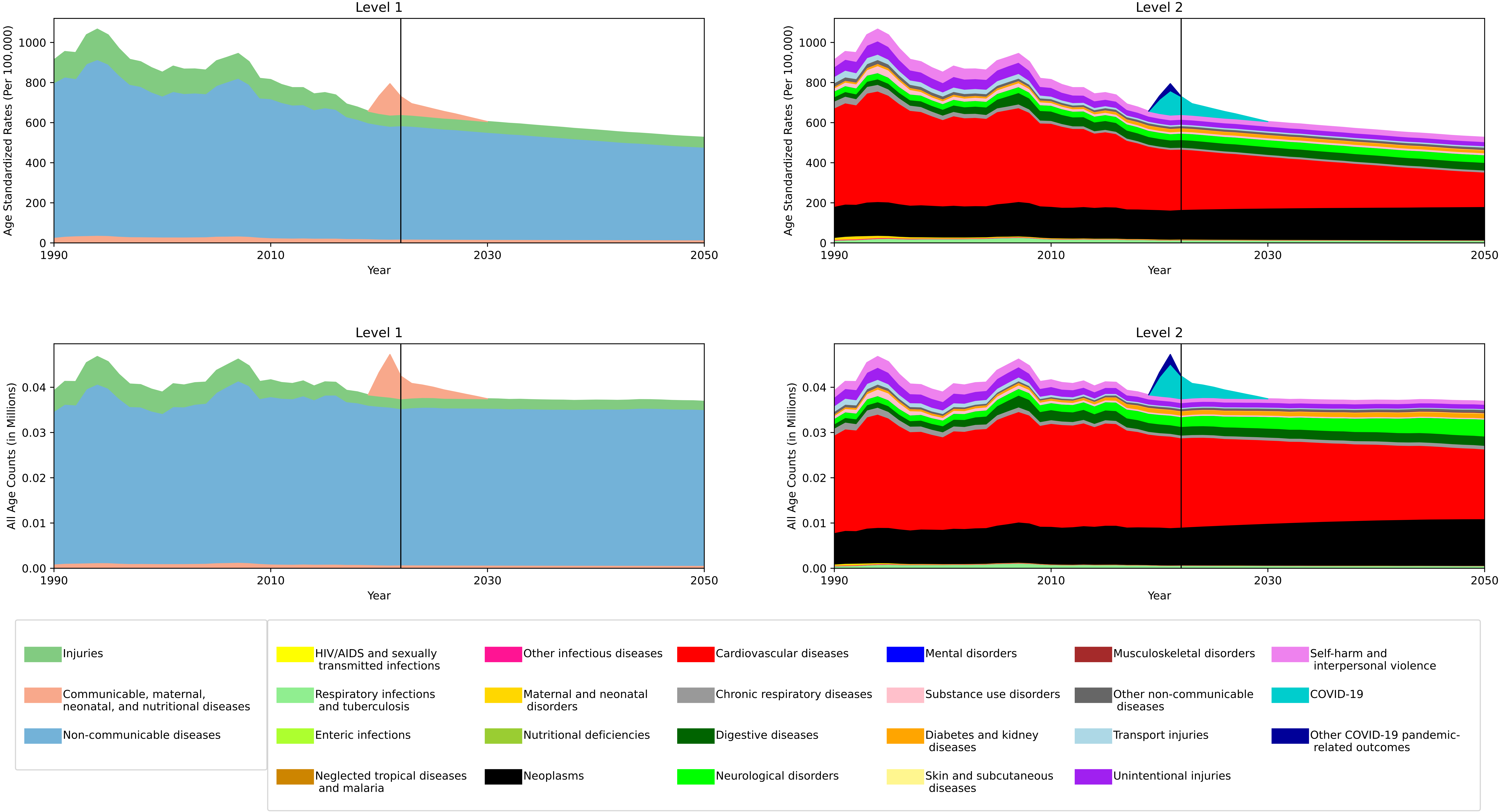
Level 1
Level 2
Age Standardized Rates (Per 100,000)
All Age Counts (in Millions)
Year
1990
2010
2030
2050
Injuries
Communicable, maternal, neonatal, and nutritional diseases
Non-communicable diseases
HIV/AIDS and sexually transmitted infections
Respiratory infections and tuberculosis
Enteric infections
Neglected tropical diseases and malaria
Other infectious diseases
Maternal and neonatal disorders
Nutritional deficiencies
Neoplasms
Cardiovascular diseases
Chronic respiratory diseases
Digestive diseases
Neurological disorders
Mental disorders
Substance use disorders
Diabetes and kidney diseases
Skin and subcutaneous diseases
Musculoskeletal disorders
Other non-communicable diseases
Transport injuries
Unintentional injuries
Self-harm and interpersonal violence
COVID-19
Other COVID-19 pandemic-related outcomes

Moldova

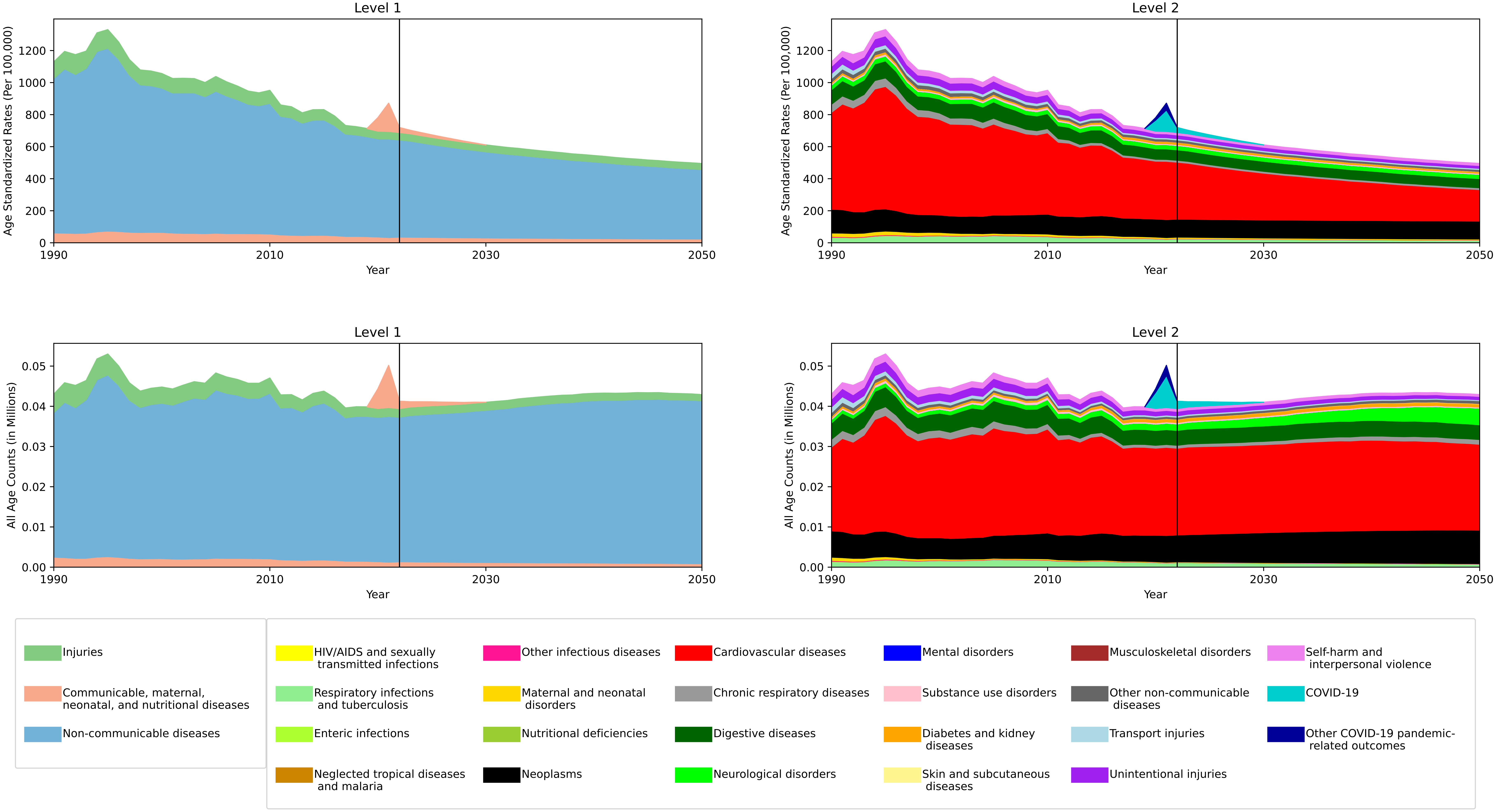
Level 1
Level 2
Age Standardized Rates (Per 100,000)
All Age Counts (in Millions)
Year
1990
2010
2030
2050
0
200
400
600
800
1000
1200
0.00
0.01
0.02
0.03
0.04
0.05
Injuries
Communicable, maternal, neonatal, and nutritional diseases
Non-communicable diseases
HIV/AIDS and sexually transmitted infections
Respiratory infections and tuberculosis
Enteric infections
Neglected tropical diseases and malaria
Other infectious diseases
Maternal and neonatal disorders
Nutritional deficiencies
Neoplasms
Cardiovascular diseases
Chronic respiratory diseases
Digestive diseases
Neurological disorders
Mental disorders
Substance use disorders
Diabetes and kidney diseases
Skin and subcutaneous diseases
Musculoskeletal disorders
Other non-communicable diseases
Transport injuries
Unintentional injuries
Self-harm and interpersonal violence
COVID-19
Other COVID-19 pandemic-related outcomes

Russia

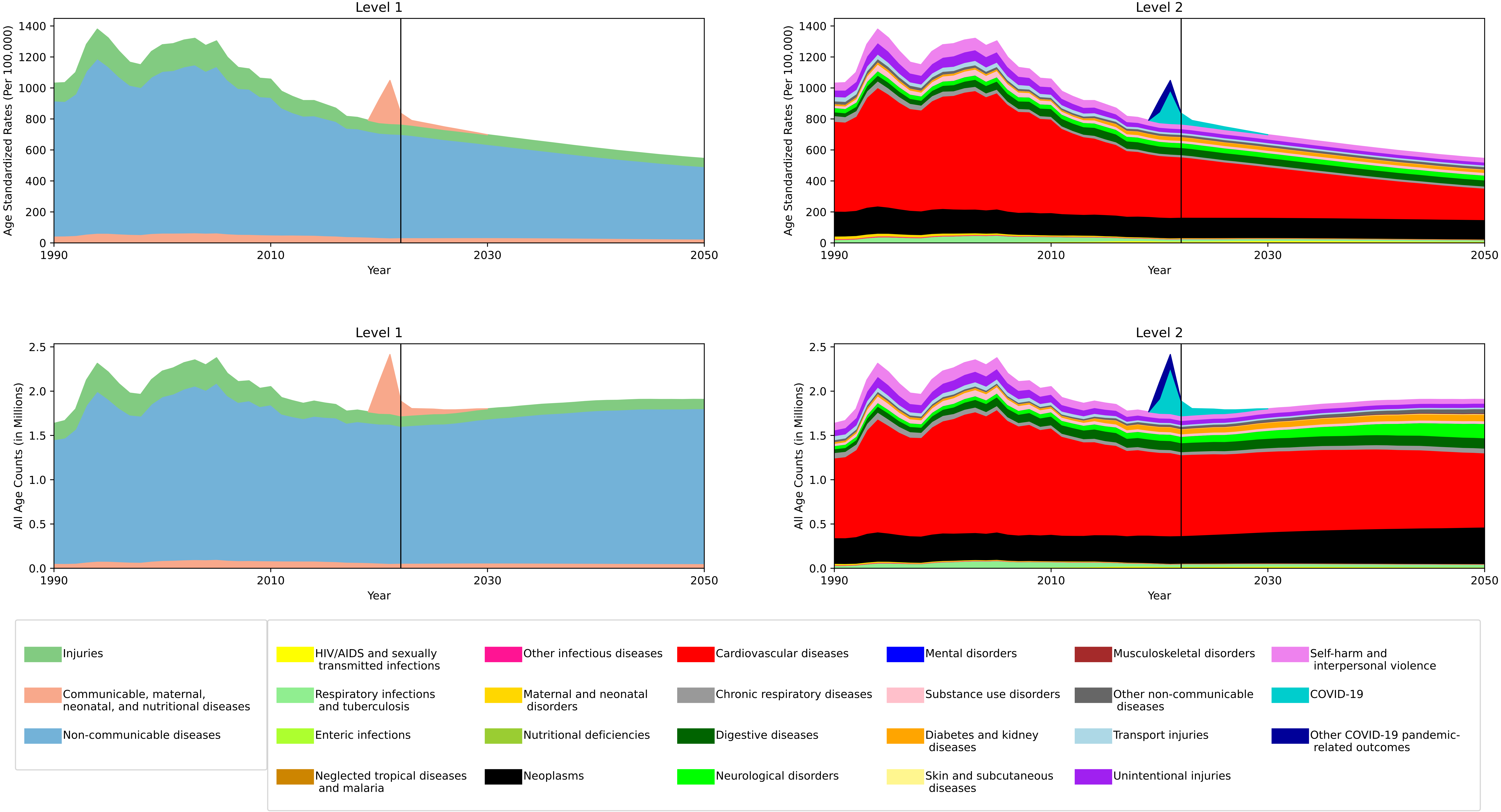
Level 1
Level 2
Age Standardized Rates (Per 100,000)
All Age Counts (in Millions)
Year
1990
2010
2030
2050
Injuries
Communicable, maternal, neonatal, and nutritional diseases
Non-communicable diseases
HIV/AIDS and sexually transmitted infections
Respiratory infections and tuberculosis
Enteric infections
Neglected tropical diseases and malaria
Other infectious diseases
Maternal and neonatal disorders
Nutritional deficiencies
Neoplasms
Cardiovascular diseases
Chronic respiratory diseases
Digestive diseases
Neurological disorders
Mental disorders
Substance use disorders
Diabetes and kidney diseases
Skin and subcutaneous diseases
Musculoskeletal disorders
Other non-communicable diseases
Transport injuries
Unintentional injuries
Self-harm and interpersonal violence
COVID-19
Other COVID-19 pandemic-related outcomes

Ukraine

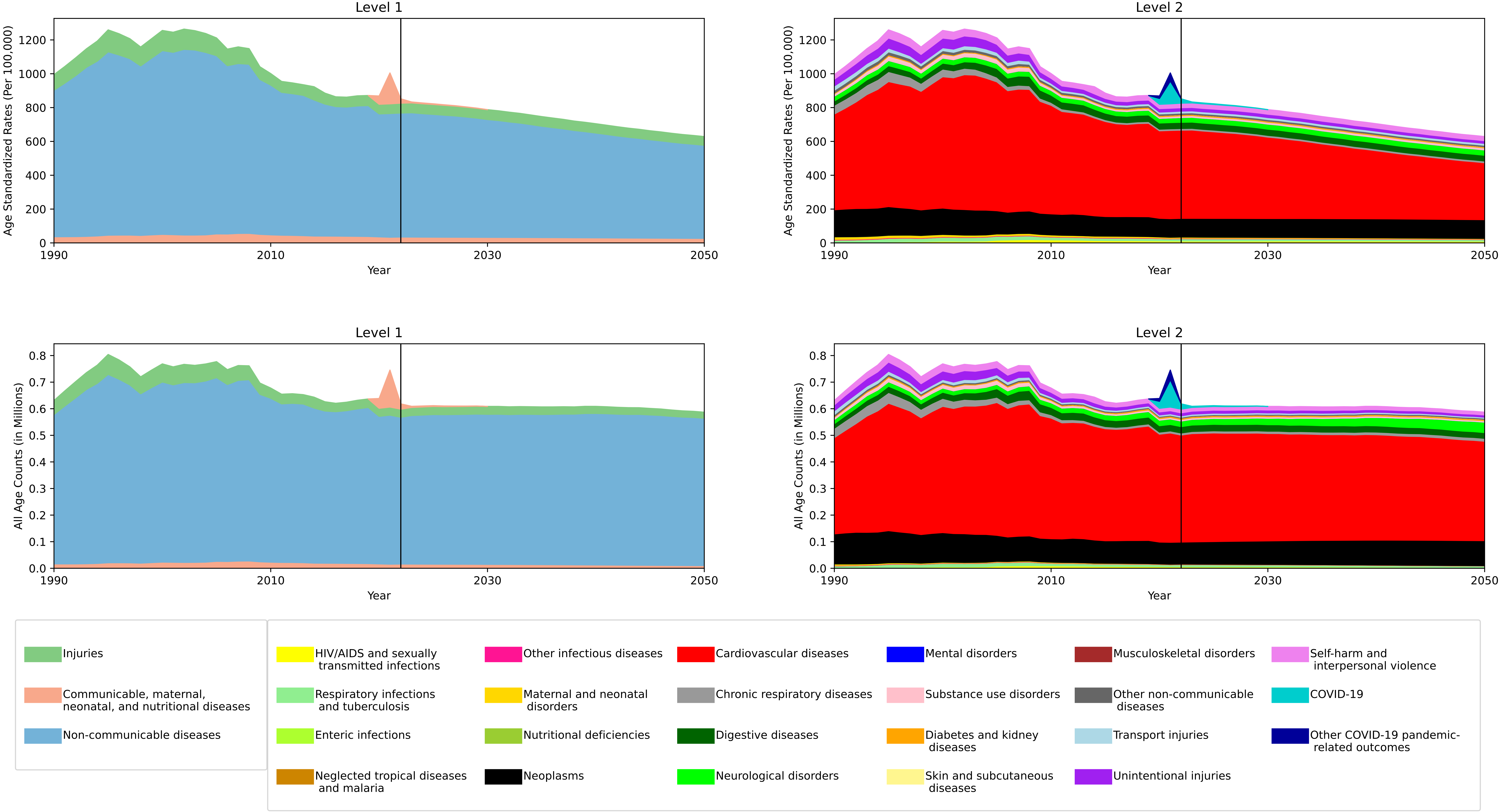
Level 1
Level 2
Age Standardized Rates (Per 100,000)
All Age Counts (in Millions)
Year
1990
2010
2030
2050
Injuries
Communicable, maternal, neonatal, and nutritional diseases
Non-communicable diseases
HIV/AIDS and sexually transmitted infections
Respiratory infections and tuberculosis
Enteric infections
Neglected tropical diseases and malaria
Other infectious diseases
Maternal and neonatal disorders
Nutritional deficiencies
Neoplasms
Cardiovascular diseases
Chronic respiratory diseases
Digestive diseases
Neurological disorders
Mental disorders
Substance use disorders
Diabetes and kidney diseases
Skin and subcutaneous diseases
Musculoskeletal disorders
Other non-communicable diseases
Transport injuries
Unintentional injuries
Self-harm and interpersonal violence
COVID-19
Other COVID-19 pandemic-related outcomes

Australia

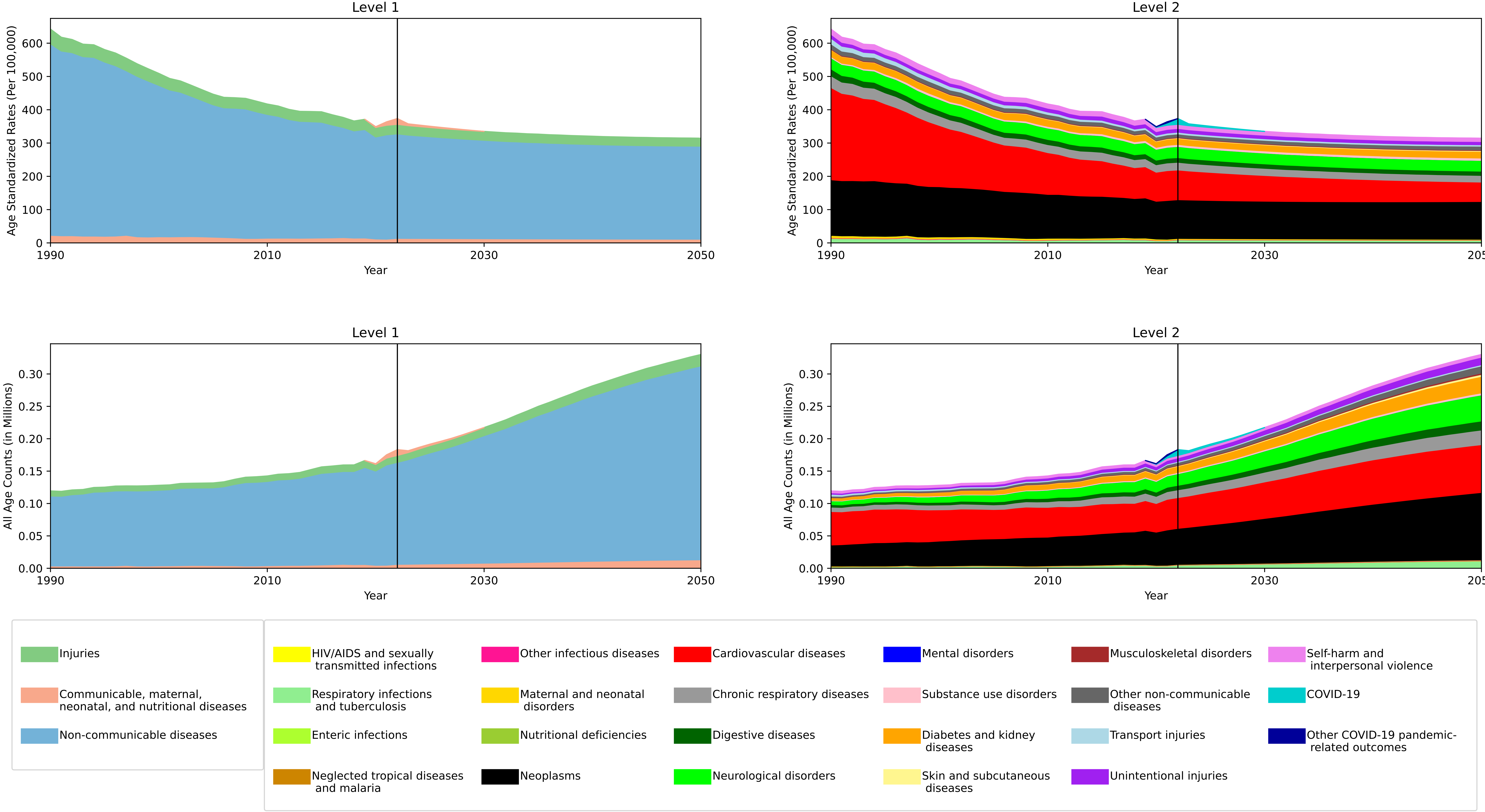
Level 1
Level 2
Age Standardized Rates (Per 100,000)
All Age Counts (in Millions)
Year
Injuries
Communicable, maternal, neonatal, and nutritional diseases
Non-communicable diseases
HIV/AIDS and sexually transmitted infections
Respiratory infections and tuberculosis
Enteric infections
Neglected tropical diseases and malaria
Other infectious diseases
Maternal and neonatal disorders
Nutritional deficiencies
Neoplasms
Cardiovascular diseases
Chronic respiratory diseases
Digestive diseases
Neurological disorders
Mental disorders
Substance use disorders
Diabetes and kidney diseases
Skin and subcutaneous diseases
Musculoskeletal disorders
Other non-communicable diseases
Transport injuries
Unintentional injuries
Self-harm and interpersonal violence
COVID-19
Other COVID-19 pandemic-related outcomes

New Zealand

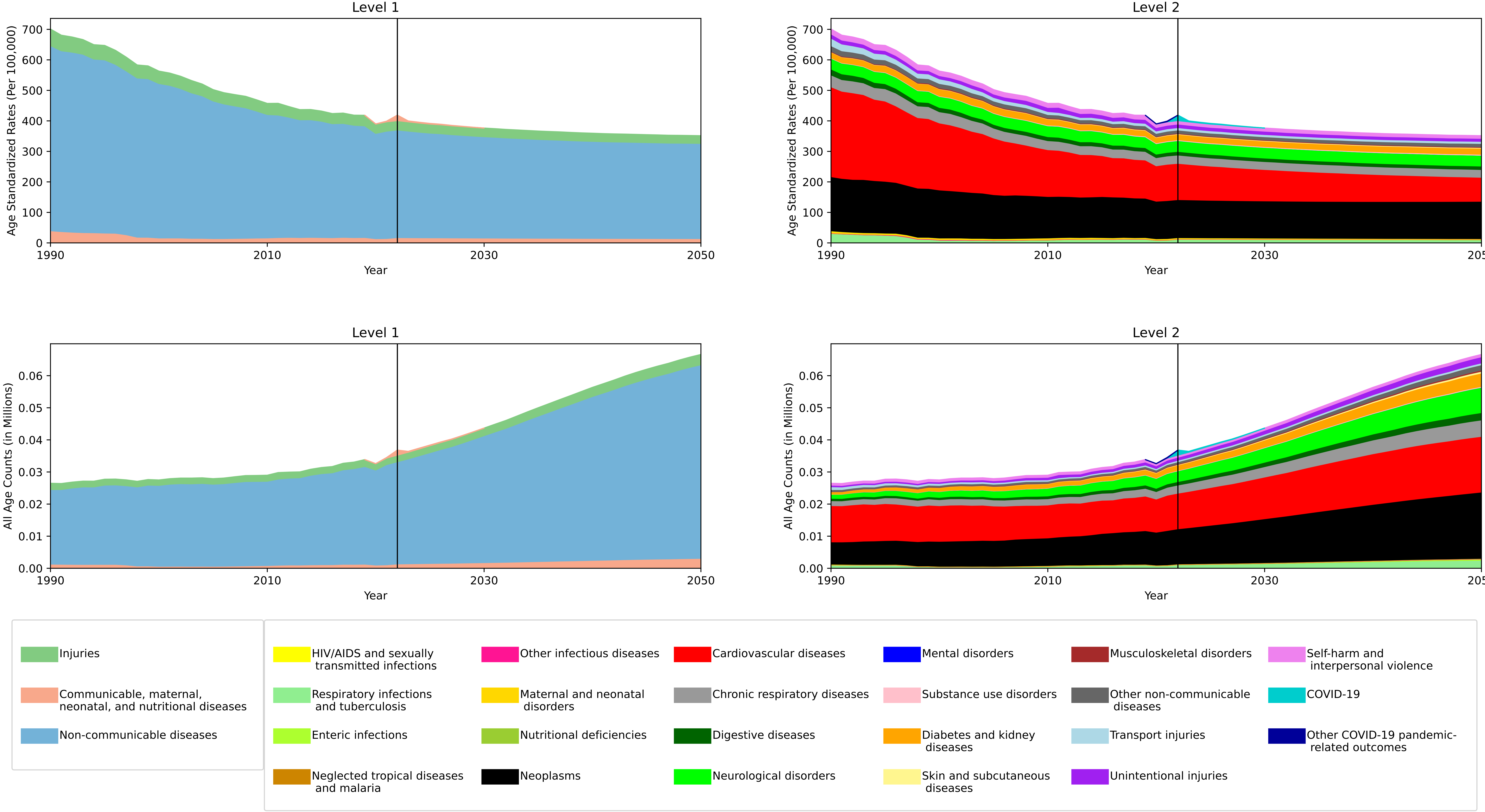
Level 1
Level 2
Age Standardized Rates (Per 100,000)
All Age Counts (in Millions)
Year
1990
2010
2030
2050
Injuries
Communicable, maternal, neonatal, and nutritional diseases
Non-communicable diseases
HIV/AIDS and sexually transmitted infections
Respiratory infections and tuberculosis
Enteric infections
Neglected tropical diseases and malaria
Other infectious diseases
Maternal and neonatal disorders
Nutritional deficiencies
Neoplasms
Cardiovascular diseases
Chronic respiratory diseases
Digestive diseases
Neurological disorders
Mental disorders
Substance use disorders
Diabetes and kidney diseases
Skin and subcutaneous diseases
Musculoskeletal disorders
Other non-communicable diseases
Transport injuries
Unintentional injuries
Self-harm and interpersonal violence
COVID-19
Other COVID-19 pandemic-related outcomes

Brunei

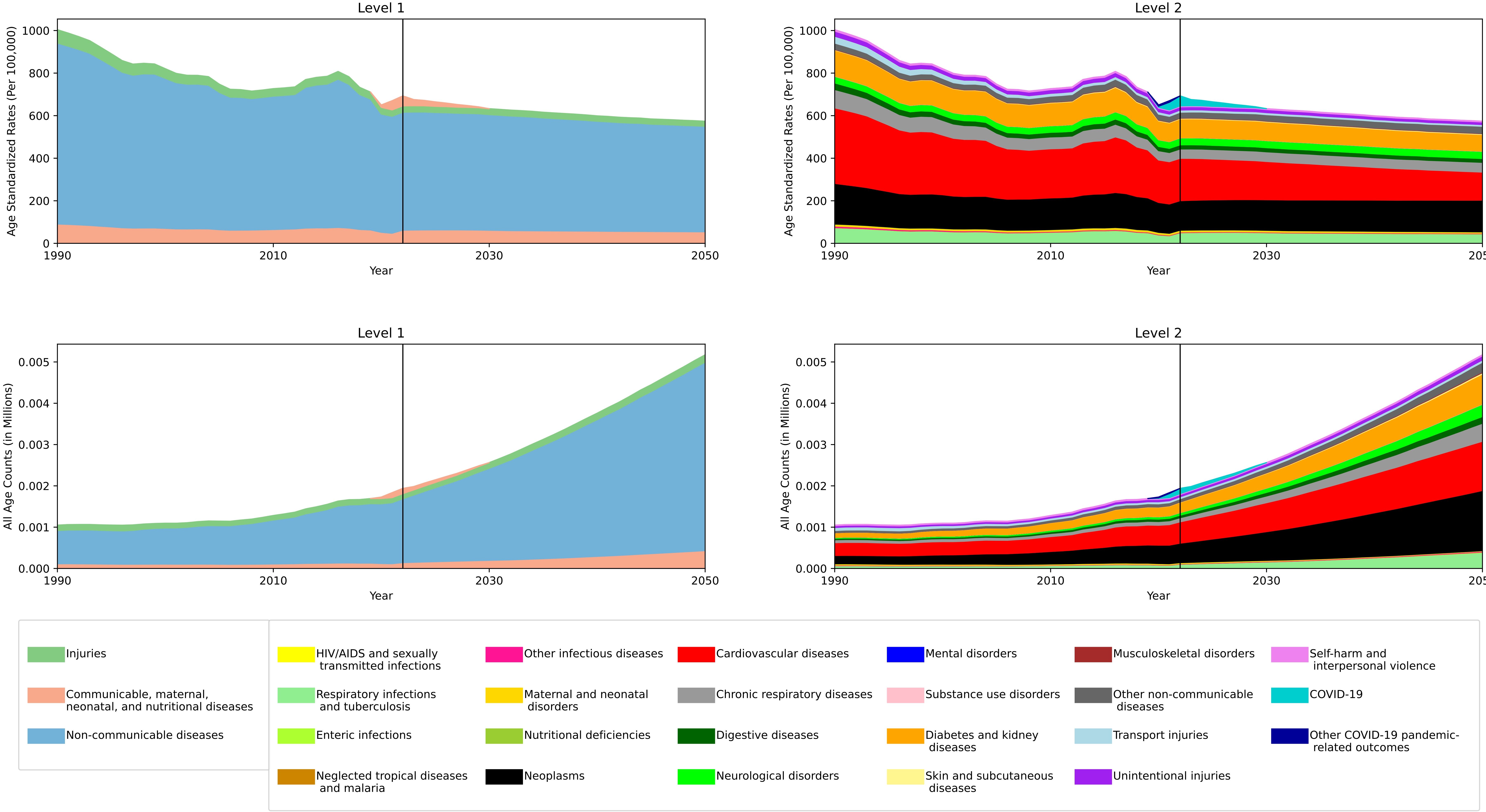
Level 1
Level 2
Age Standardized Rates (Per 100,000)
All Age Counts (in Millions)
Year
1990
2010
2030
2050
Injuries
Communicable, maternal, neonatal, and nutritional diseases
Non-communicable diseases
HIV/AIDS and sexually transmitted infections
Respiratory infections and tuberculosis
Enteric infections
Neglected tropical diseases and malaria
Other infectious diseases
Maternal and neonatal disorders
Nutritional deficiencies
Neoplasms
Cardiovascular diseases
Chronic respiratory diseases
Digestive diseases
Neurological disorders
Mental disorders
Substance use disorders
Diabetes and kidney diseases
Skin and subcutaneous diseases
Musculoskeletal disorders
Other non-communicable diseases
Transport injuries
Unintentional injuries
Self-harm and interpersonal violence
COVID-19
Other COVID-19 pandemic-related outcomes

Japan

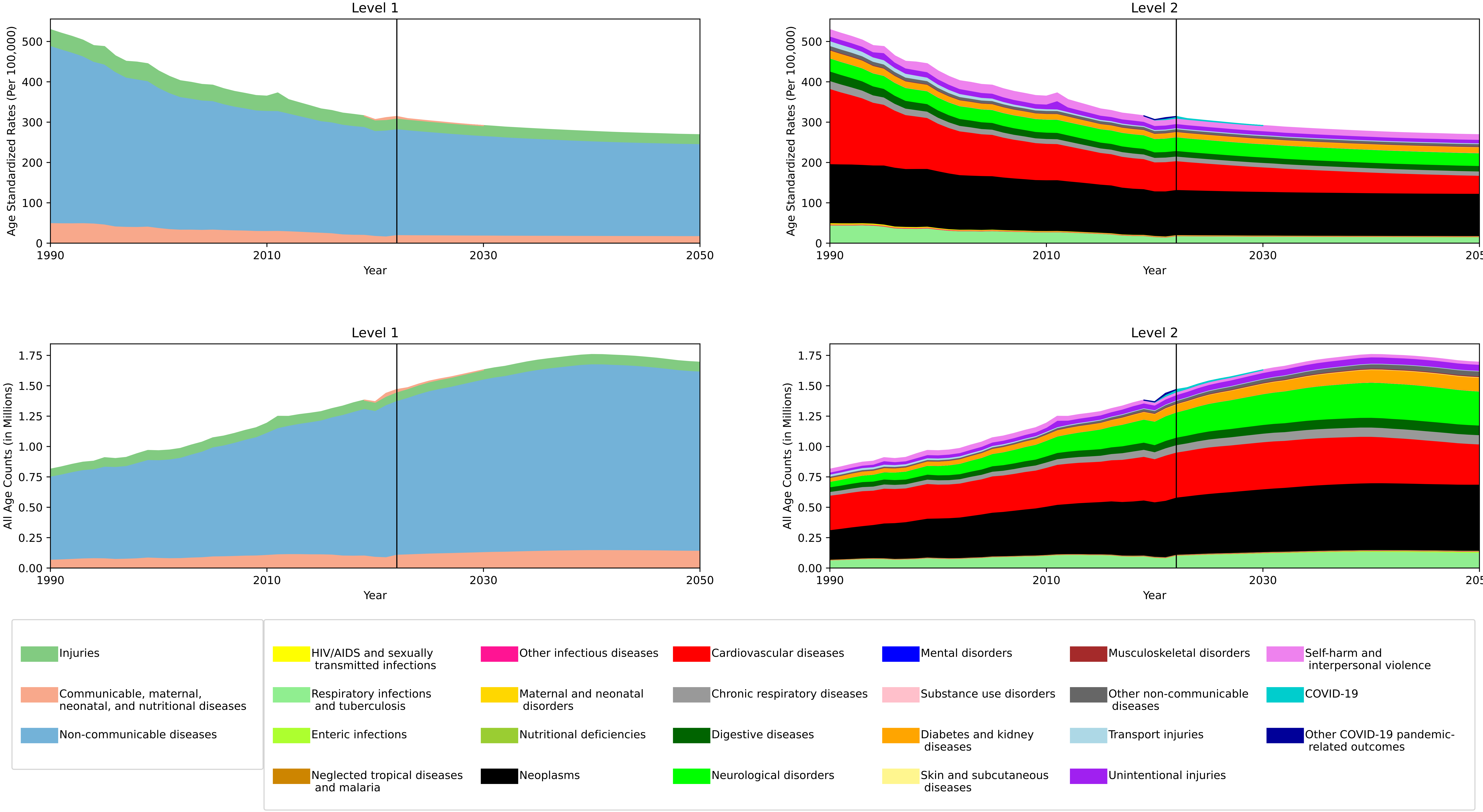
Level 1
Level 2
Age Standardized Rates (Per 100,000)
All Age Counts (in Millions)
Year
1990
2010
2030
2050
0
100
200
300
400
500
0.00
0.25
0.50
0.75
1.00
1.25
1.50
1.75
Injuries
Communicable, maternal, neonatal, and nutritional diseases
Non-communicable diseases
HIV/AIDS and sexually transmitted infections
Respiratory infections and tuberculosis
Enteric infections
Neglected tropical diseases and malaria
Other infectious diseases
Maternal and neonatal disorders
Nutritional deficiencies
Neoplasms
Cardiovascular diseases
Chronic respiratory diseases
Digestive diseases
Neurological disorders
Mental disorders
Substance use disorders
Diabetes and kidney diseases
Skin and subcutaneous diseases
Musculoskeletal disorders
Other non-communicable diseases
Transport injuries
Unintentional injuries
Self-harm and interpersonal violence
COVID-19
Other COVID-19 pandemic-related outcomes

South Korea

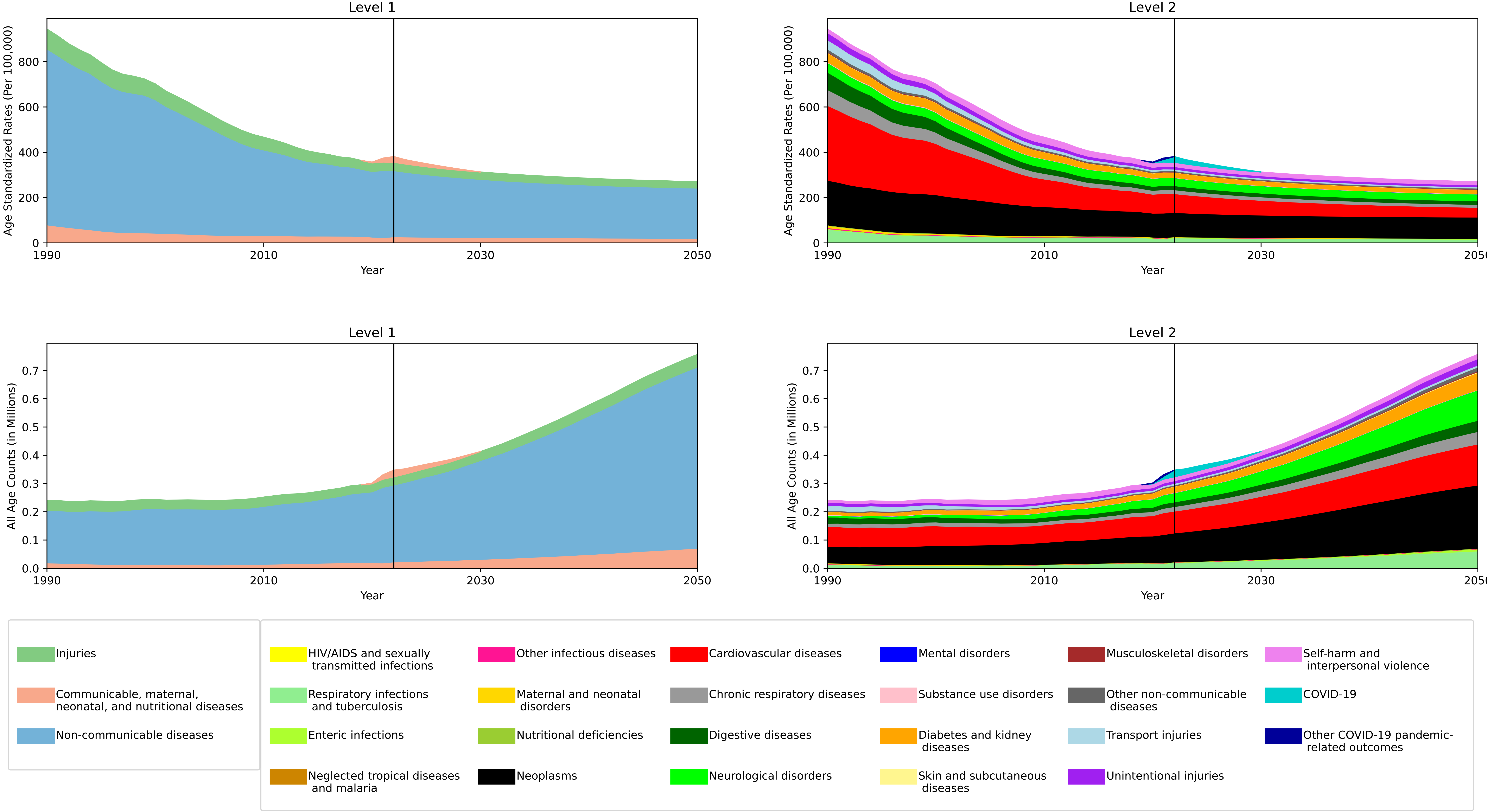
Level 1
Level 2
Age Standardized Rates (Per 100,000)
All Age Counts (in Millions)
Year
Injuries
Communicable, maternal, neonatal, and nutritional diseases
Non-communicable diseases
HIV/AIDS and sexually transmitted infections
Respiratory infections and tuberculosis
Enteric infections
Neglected tropical diseases and malaria
Other infectious diseases
Maternal and neonatal disorders
Nutritional deficiencies
Neoplasms
Cardiovascular diseases
Chronic respiratory diseases
Digestive diseases
Neurological disorders
Mental disorders
Substance use disorders
Diabetes and kidney diseases
Skin and subcutaneous diseases
Musculoskeletal disorders
Other non-communicable diseases
Transport injuries
Unintentional injuries
Self-harm and interpersonal violence
COVID-19
Other COVID-19 pandemic-related outcomes

Singapore

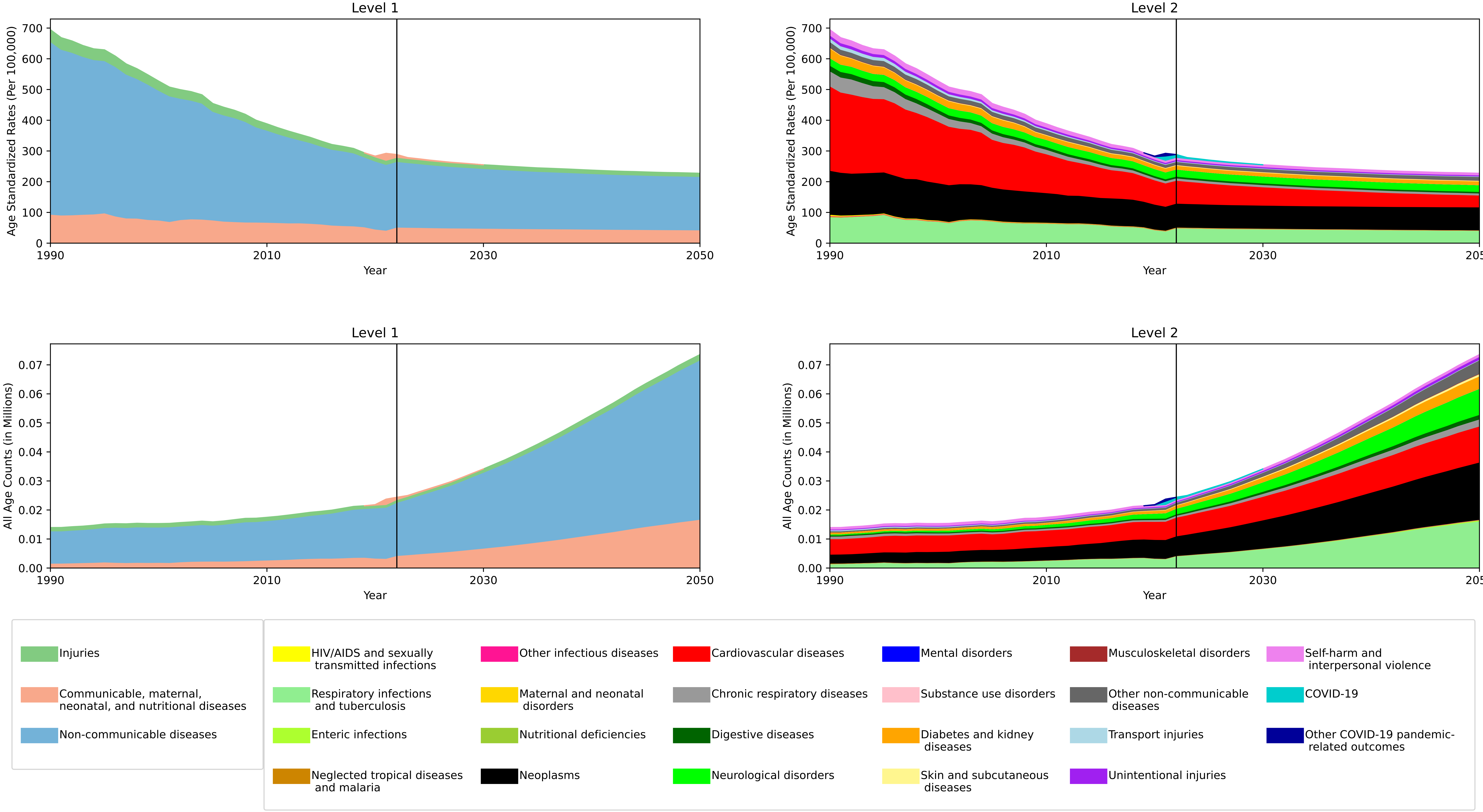
Level 1
Level 2
Age Standardized Rates (Per 100,000)
All Age Counts (in Millions)
Year
1990
2010
2030
2050
Injuries
Communicable, maternal, neonatal, and nutritional diseases
Non-communicable diseases
HIV/AIDS and sexually transmitted infections
Respiratory infections and tuberculosis
Enteric infections
Neglected tropical diseases and malaria
Other infectious diseases
Maternal and neonatal disorders
Nutritional deficiencies
Neoplasms
Cardiovascular diseases
Chronic respiratory diseases
Digestive diseases
Neurological disorders
Mental disorders
Substance use disorders
Diabetes and kidney diseases
Skin and subcutaneous diseases
Musculoskeletal disorders
Other non-communicable diseases
Transport injuries
Unintentional injuries
Self-harm and interpersonal violence
COVID-19
Other COVID-19 pandemic-related outcomes

Canada

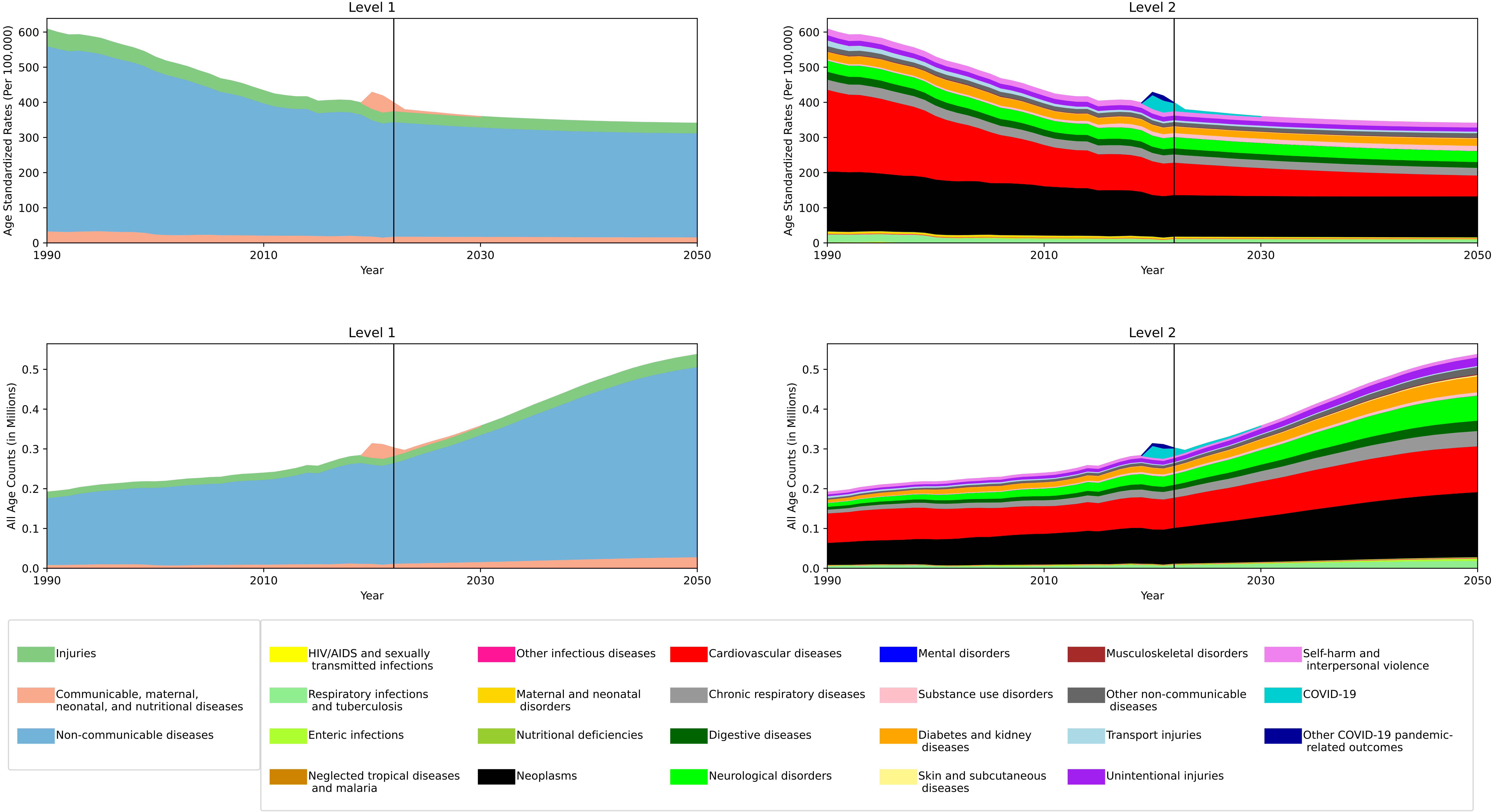
Level 1
Level 2
Age Standardized Rates (Per 100,000)
All Age Counts (in Millions)
Year
1990
2010
2030
2050
Injuries
Communicable, maternal, neonatal, and nutritional diseases
Non-communicable diseases
HIV/AIDS and sexually transmitted infections
Respiratory infections and tuberculosis
Enteric infections
Neglected tropical diseases and malaria
Other infectious diseases
Maternal and neonatal disorders
Nutritional deficiencies
Neoplasms
Cardiovascular diseases
Chronic respiratory diseases
Digestive diseases
Neurological disorders
Mental disorders
Substance use disorders
Diabetes and kidney diseases
Skin and subcutaneous diseases
Musculoskeletal disorders
Other non-communicable diseases
Transport injuries
Unintentional injuries
Self-harm and interpersonal violence
COVID-19
Other COVID-19 pandemic-related outcomes

Greenland

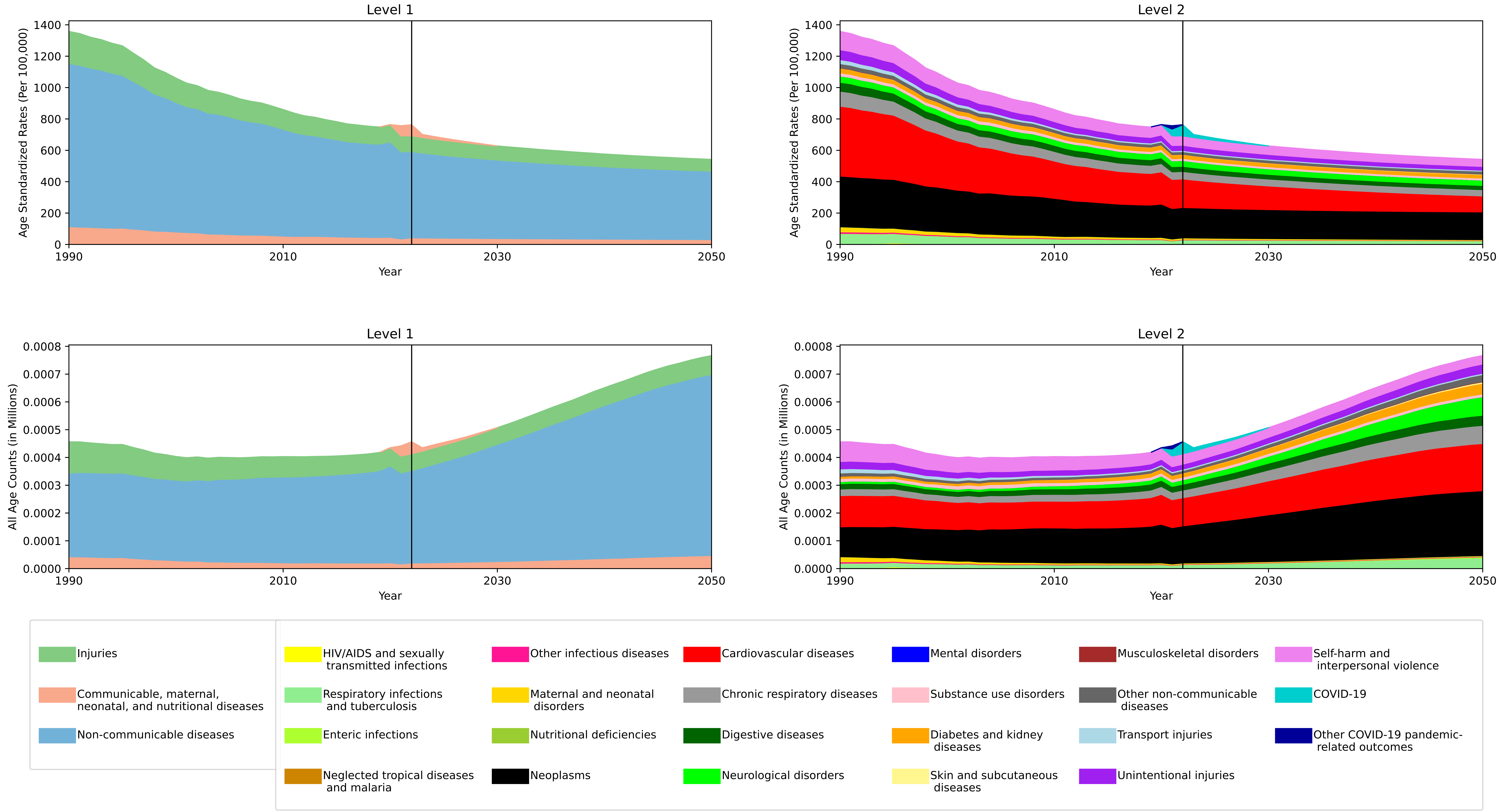
Level 1
Level 2
Age Standardized Rates (Per 100,000)
All Age Counts (in Millions)
Year
1990
2010
2030
2050
Injuries
Communicable, maternal, neonatal, and nutritional diseases
Non-communicable diseases
HIV/AIDS and sexually transmitted infections
Respiratory infections and tuberculosis
Enteric infections
Neglected tropical diseases and malaria
Other infectious diseases
Maternal and neonatal disorders
Nutritional deficiencies
Neoplasms
Cardiovascular diseases
Chronic respiratory diseases
Digestive diseases
Neurological disorders
Mental disorders
Substance use disorders
Diabetes and kidney diseases
Skin and subcutaneous diseases
Musculoskeletal disorders
Other non-communicable diseases
Transport injuries
Unintentional injuries
Self-harm and interpersonal violence
COVID-19
Other COVID-19 pandemic-related outcomes

USA

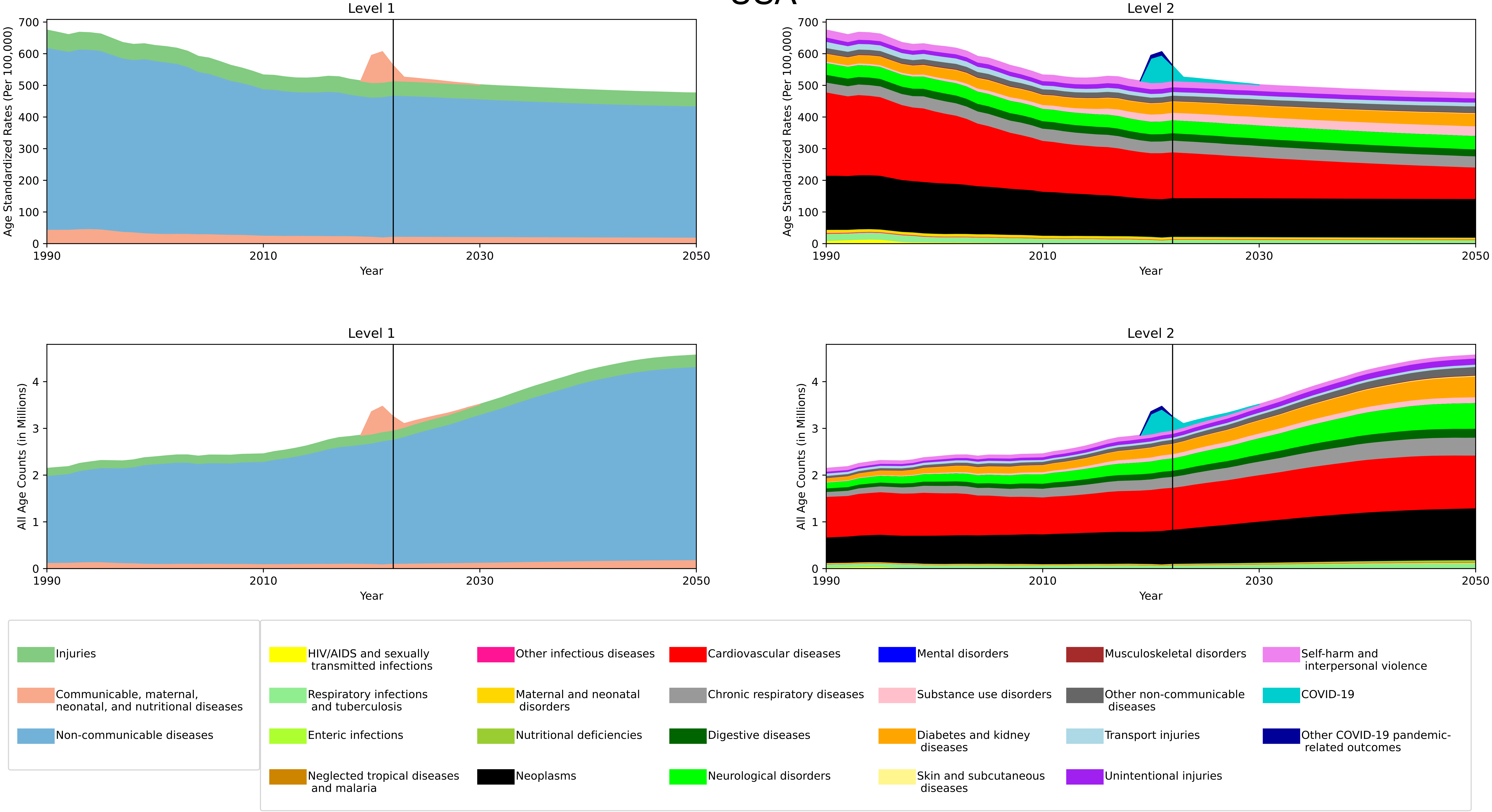
Level 1
Level 2
Age Standardized Rates (Per 100,000)
All Age Counts (in Millions)
Year
0
100
200
300
400
500
600
700
1
2
3
4
1990
2010
2030
2050
Injuries
Communicable, maternal, neonatal, and nutritional diseases
Non-communicable diseases
HIV/AIDS and sexually transmitted infections
Respiratory infections and tuberculosis
Enteric infections
Neglected tropical diseases and malaria
Other infectious diseases
Maternal and neonatal disorders
Nutritional deficiencies
Neoplasms
Cardiovascular diseases
Chronic respiratory diseases
Digestive diseases
Neurological disorders
Mental disorders
Substance use disorders
Diabetes and kidney diseases
Skin and subcutaneous diseases
Musculoskeletal disorders
Other non-communicable diseases
Transport injuries
Unintentional injuries
Self-harm and interpersonal violence
COVID-19
Other COVID-19 pandemic-related outcomes

Argentina

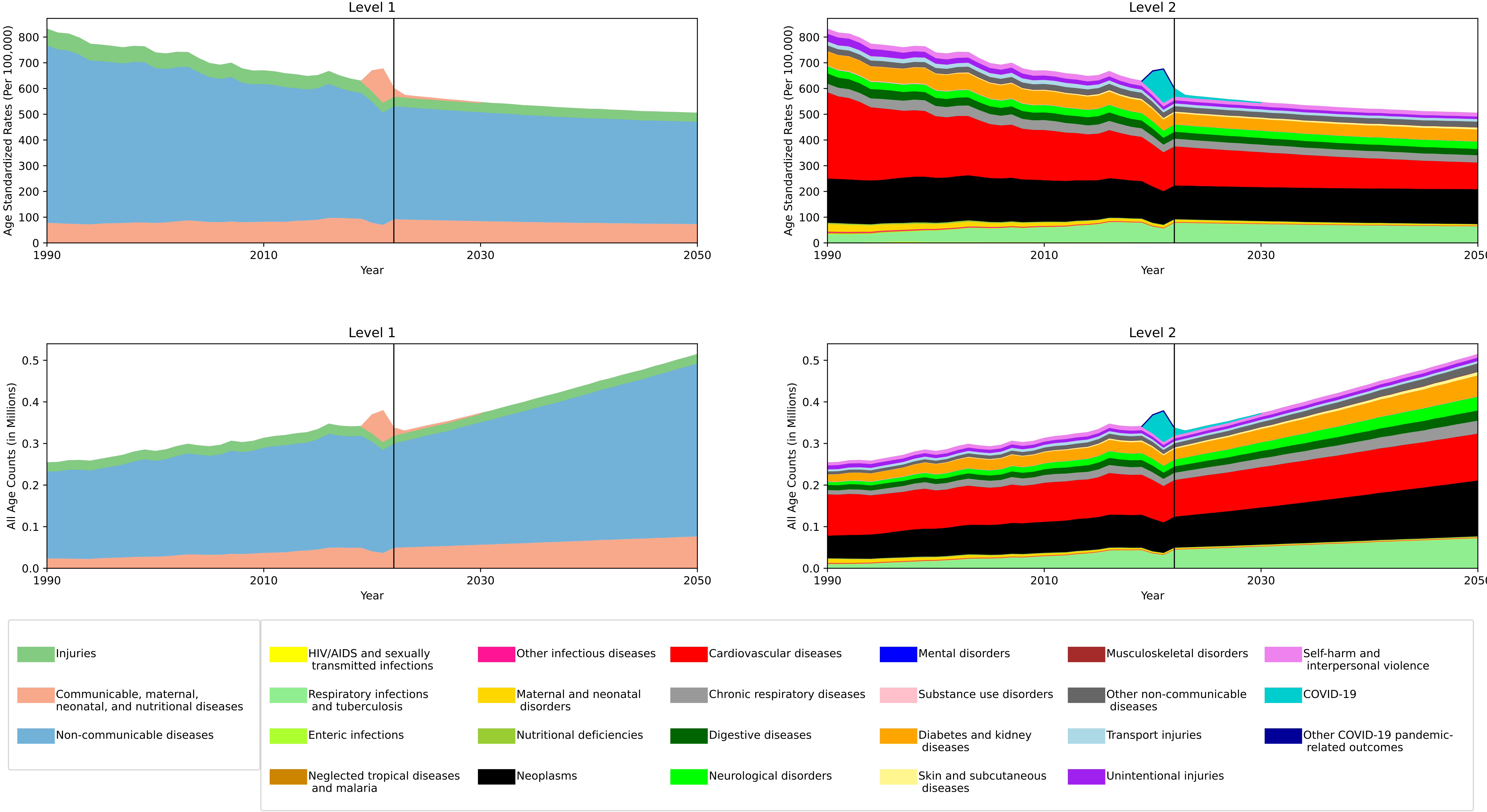
Level 1
Level 2
Age Standardized Rates (Per 100,000)
All Age Counts (in Millions)
Year
1990
2010
2030
2050
0
100
200
300
400
500
600
700
800
0.0
0.1
0.2
0.3
0.4
0.5
Injuries
Communicable, maternal, neonatal, and nutritional diseases
Non-communicable diseases
HIV/AIDS and sexually transmitted infections
Respiratory infections and tuberculosis
Enteric infections
Neglected tropical diseases and malaria
Other infectious diseases
Maternal and neonatal disorders
Nutritional deficiencies
Neoplasms
Cardiovascular diseases
Chronic respiratory diseases
Digestive diseases
Neurological disorders
Mental disorders
Substance use disorders
Diabetes and kidney diseases
Skin and subcutaneous diseases
Musculoskeletal disorders
Other non-communicable diseases
Transport injuries
Unintentional injuries
Self-harm and interpersonal violence
COVID-19
Other COVID-19 pandemic-related outcomes

Chile

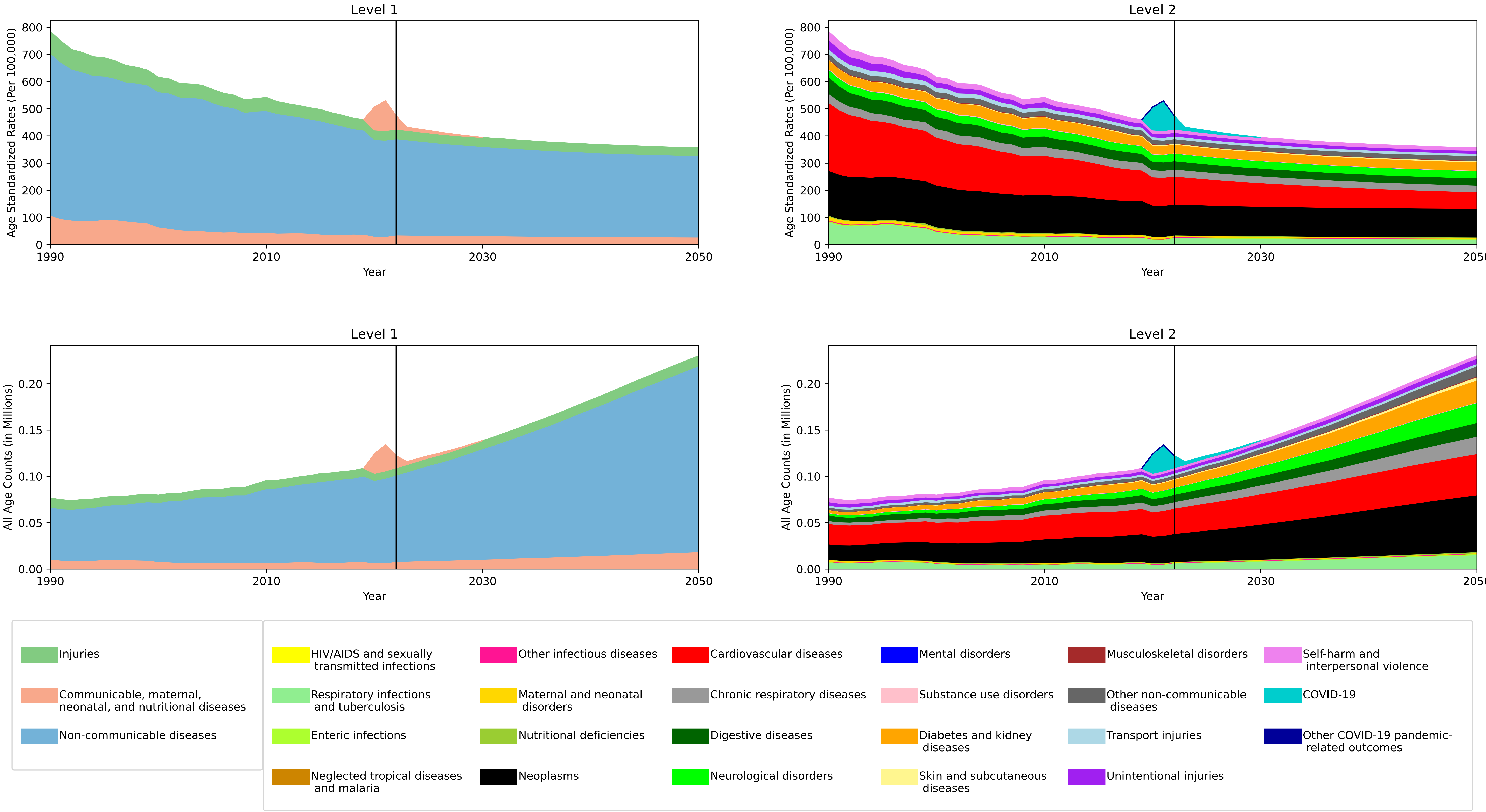
Level 1
Level 2
Age Standardized Rates (Per 100,000)
All Age Counts (in Millions)
Year
1990
2010
2030
2050
Injuries
Communicable, maternal, neonatal, and nutritional diseases
Non-communicable diseases
HIV/AIDS and sexually transmitted infections
Respiratory infections and tuberculosis
Enteric infections
Neglected tropical diseases and malaria
Other infectious diseases
Maternal and neonatal disorders
Nutritional deficiencies
Neoplasms
Cardiovascular diseases
Chronic respiratory diseases
Digestive diseases
Neurological disorders
Mental disorders
Substance use disorders
Diabetes and kidney diseases
Skin and subcutaneous diseases
Musculoskeletal disorders
Other non-communicable diseases
Transport injuries
Unintentional injuries
Self-harm and interpersonal violence
COVID-19
Other COVID-19 pandemic-related outcomes

Uruguay

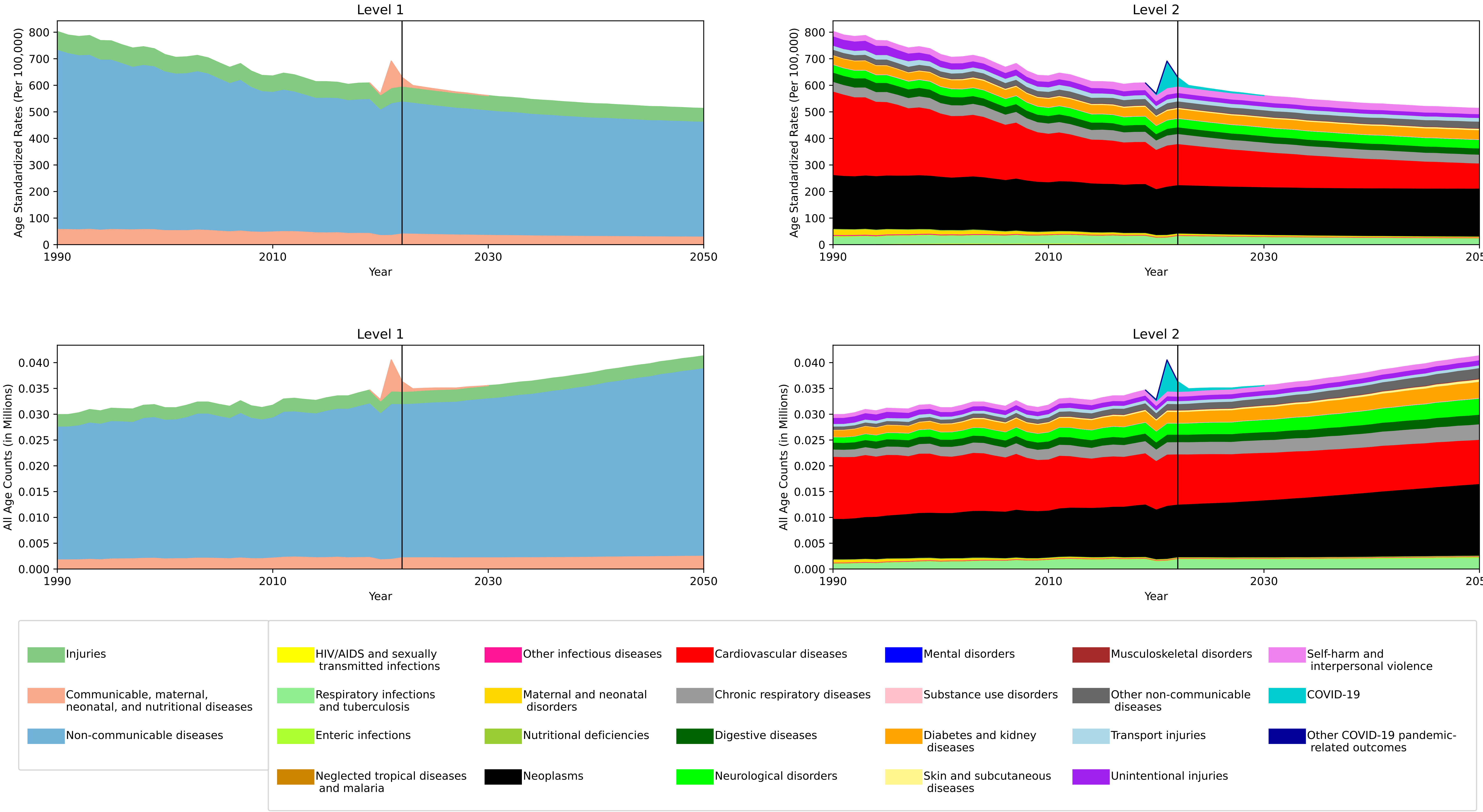
Level 1
Level 2
Age Standardized Rates (Per 100,000)
All Age Counts (in Millions)
Year
1990
2010
2030
2050
Injuries
Communicable, maternal, neonatal, and nutritional diseases
Non-communicable diseases
HIV/AIDS and sexually transmitted infections
Respiratory infections and tuberculosis
Enteric infections
Neglected tropical diseases and malaria
Other infectious diseases
Maternal and neonatal disorders
Nutritional deficiencies
Neoplasms
Cardiovascular diseases
Chronic respiratory diseases
Digestive diseases
Neurological disorders
Mental disorders
Substance use disorders
Diabetes and kidney diseases
Skin and subcutaneous diseases
Musculoskeletal disorders
Other non-communicable diseases
Transport injuries
Unintentional injuries
Self-harm and interpersonal violence
COVID-19
Other COVID-19 pandemic-related outcomes

Andorra

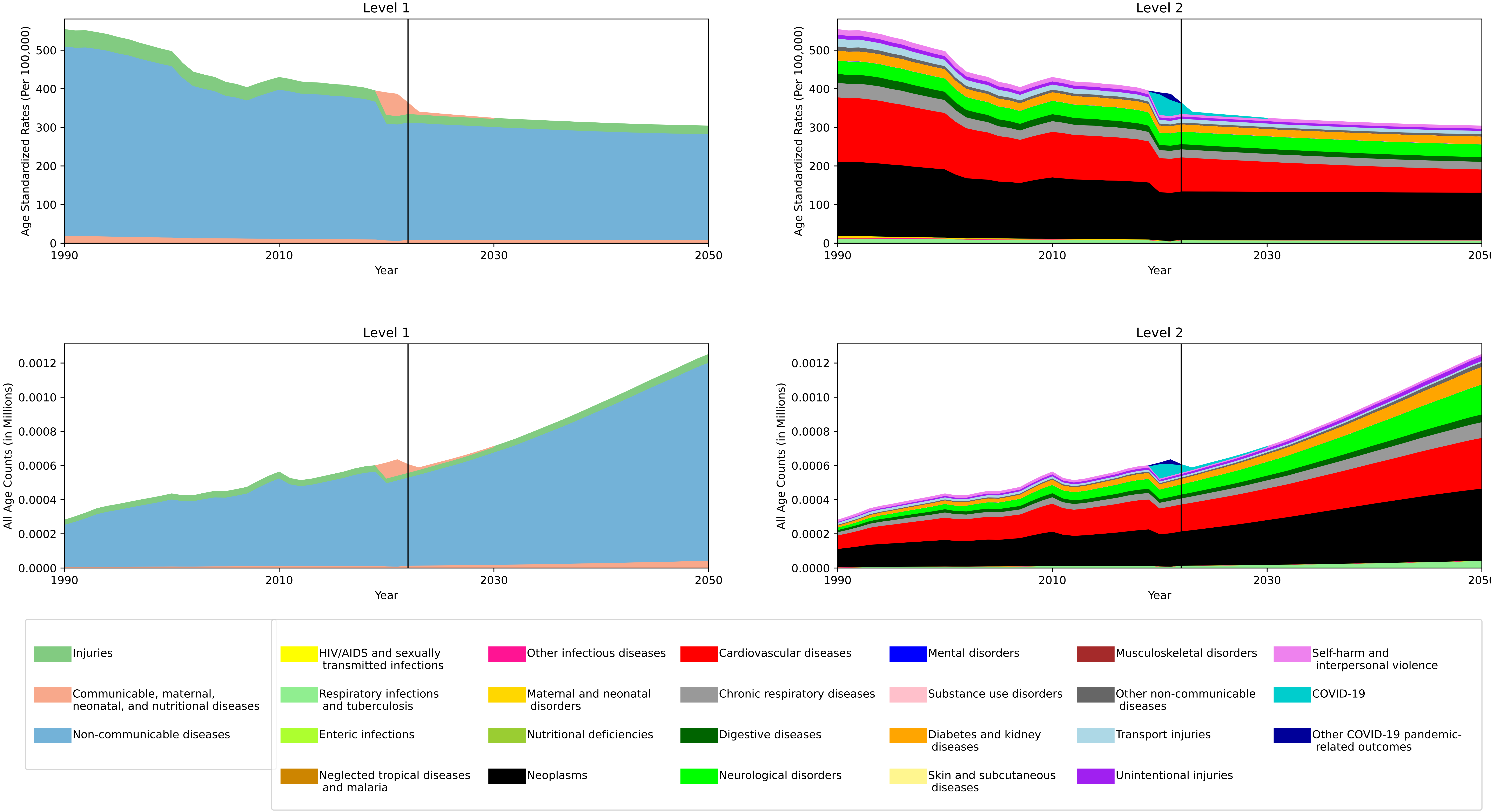
Level 1
Level 2
Age Standardized Rates (Per 100,000)
All Age Counts (in Millions)
Year
1990
2010
2030
2050
0
100
200
300
400
500
0.0000
0.0002
0.0004
0.0006
0.0008
0.0010
0.0012
Injuries
Communicable, maternal, neonatal, and nutritional diseases
Non-communicable diseases
HIV/AIDS and sexually transmitted infections
Respiratory infections and tuberculosis
Enteric infections
Neglected tropical diseases and malaria
Other infectious diseases
Maternal and neonatal disorders
Nutritional deficiencies
Neoplasms
Cardiovascular diseases
Chronic respiratory diseases
Digestive diseases
Neurological disorders
Mental disorders
Substance use disorders
Diabetes and kidney diseases
Skin and subcutaneous diseases
Musculoskeletal disorders
Other non-communicable diseases
Transport injuries
Unintentional injuries
Self-harm and interpersonal violence
COVID-19
Other COVID-19 pandemic-related outcomes

Austria

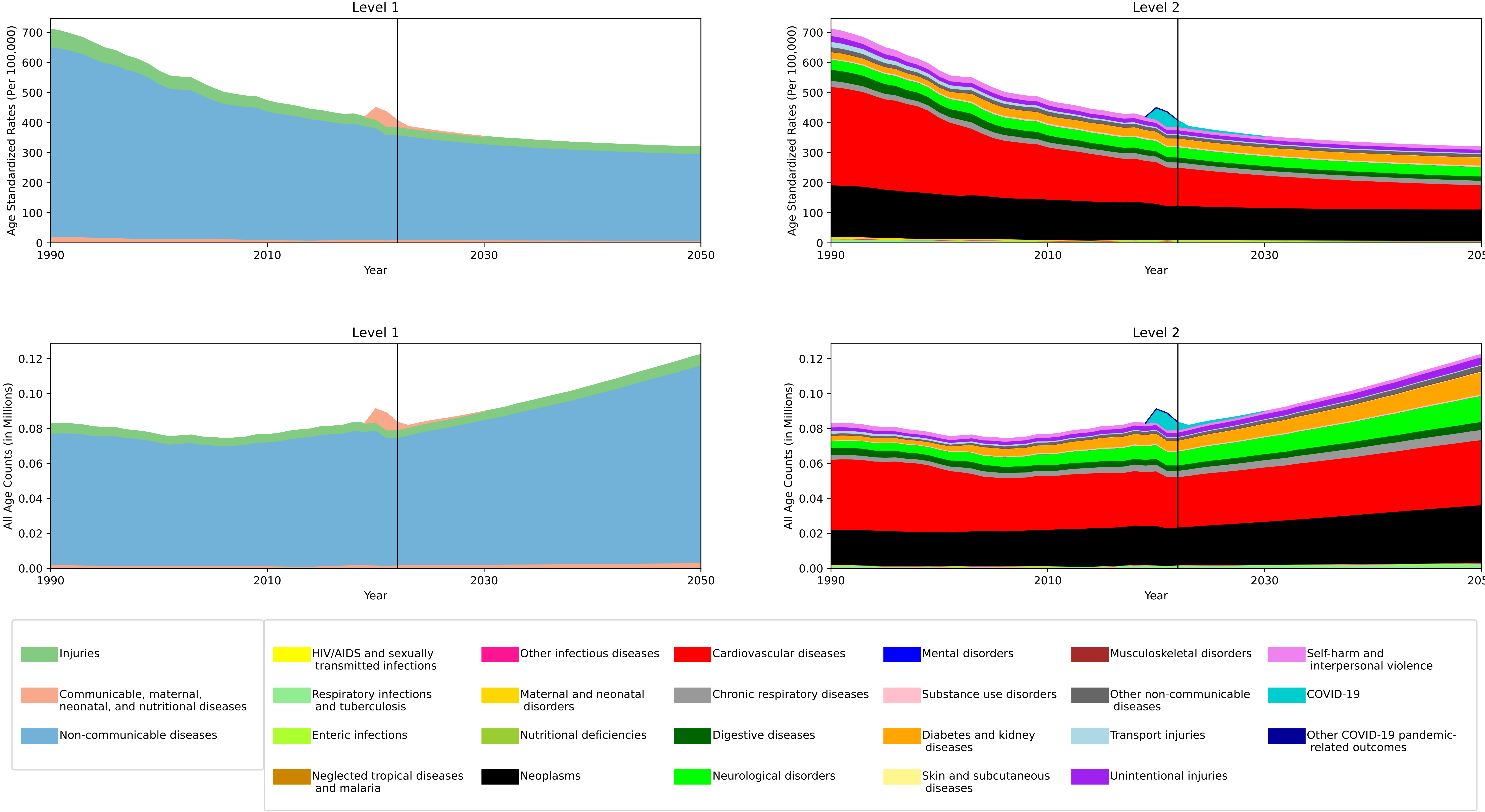
Level 1
Level 2
Age Standardized Rates (Per 100,000)
All Age Counts (in Millions)
Year
Injuries
Communicable, maternal, neonatal, and nutritional diseases
Non-communicable diseases
HIV/AIDS and sexually transmitted infections
Respiratory infections and tuberculosis
Enteric infections
Neglected tropical diseases and malaria
Other infectious diseases
Maternal and neonatal disorders
Nutritional deficiencies
Neoplasms
Cardiovascular diseases
Chronic respiratory diseases
Digestive diseases
Neurological disorders
Mental disorders
Substance use disorders
Diabetes and kidney diseases
Skin and subcutaneous diseases
Musculoskeletal disorders
Other non-communicable diseases
Transport injuries
Unintentional injuries
Self-harm and interpersonal violence
COVID-19
Other COVID-19 pandemic-related outcomes

Belgium

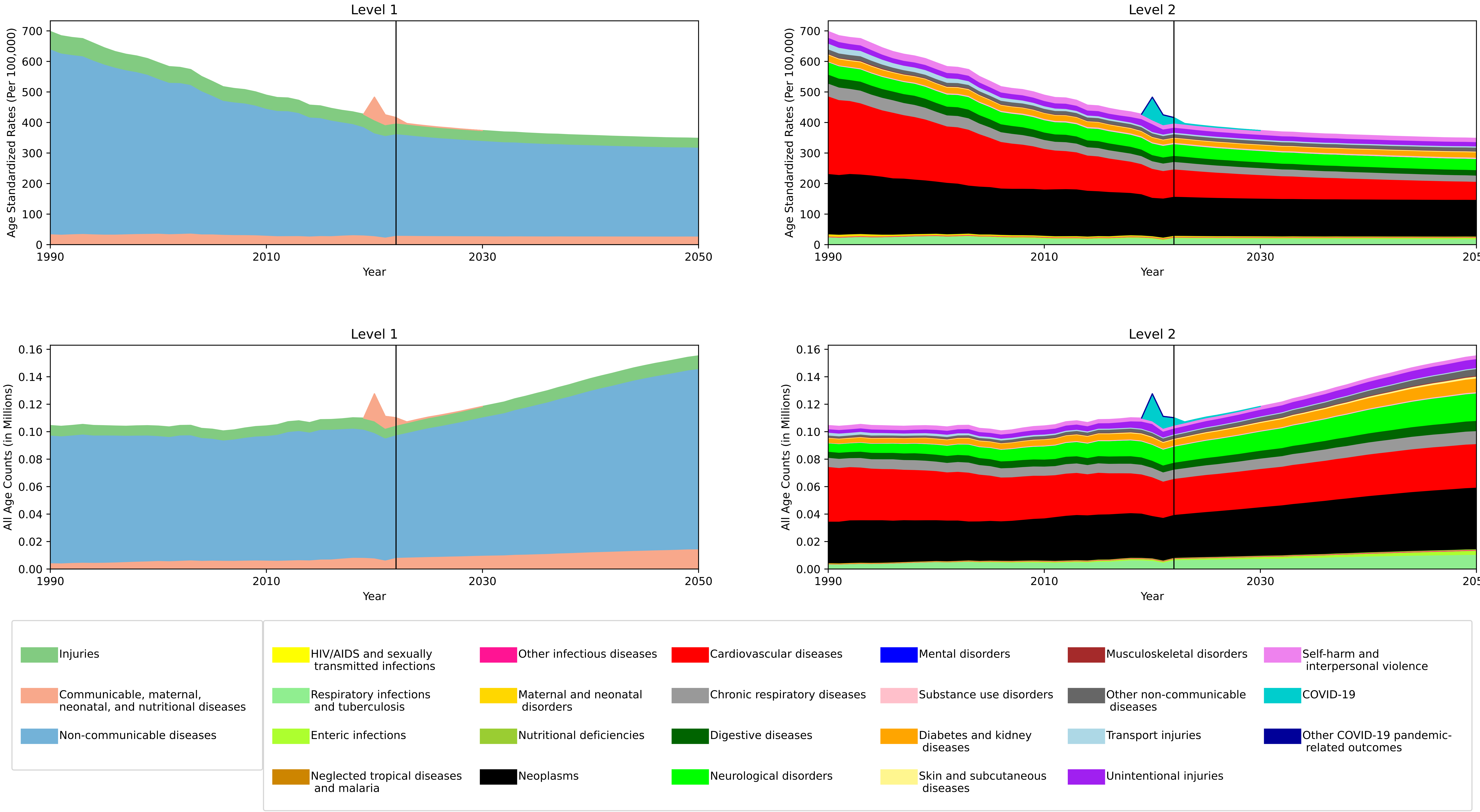
Level 1
Level 2
Age Standardized Rates (Per 100,000)
All Age Counts (in Millions)
Year
1990
2010
2030
2050
Injuries
Communicable, maternal, neonatal, and nutritional diseases
Non-communicable diseases
HIV/AIDS and sexually transmitted infections
Respiratory infections and tuberculosis
Enteric infections
Neglected tropical diseases and malaria
Other infectious diseases
Maternal and neonatal disorders
Nutritional deficiencies
Neoplasms
Cardiovascular diseases
Chronic respiratory diseases
Digestive diseases
Neurological disorders
Mental disorders
Substance use disorders
Diabetes and kidney diseases
Skin and subcutaneous diseases
Musculoskeletal disorders
Other non-communicable diseases
Transport injuries
Unintentional injuries
Self-harm and interpersonal violence
COVID-19
Other COVID-19 pandemic-related outcomes

Cyprus

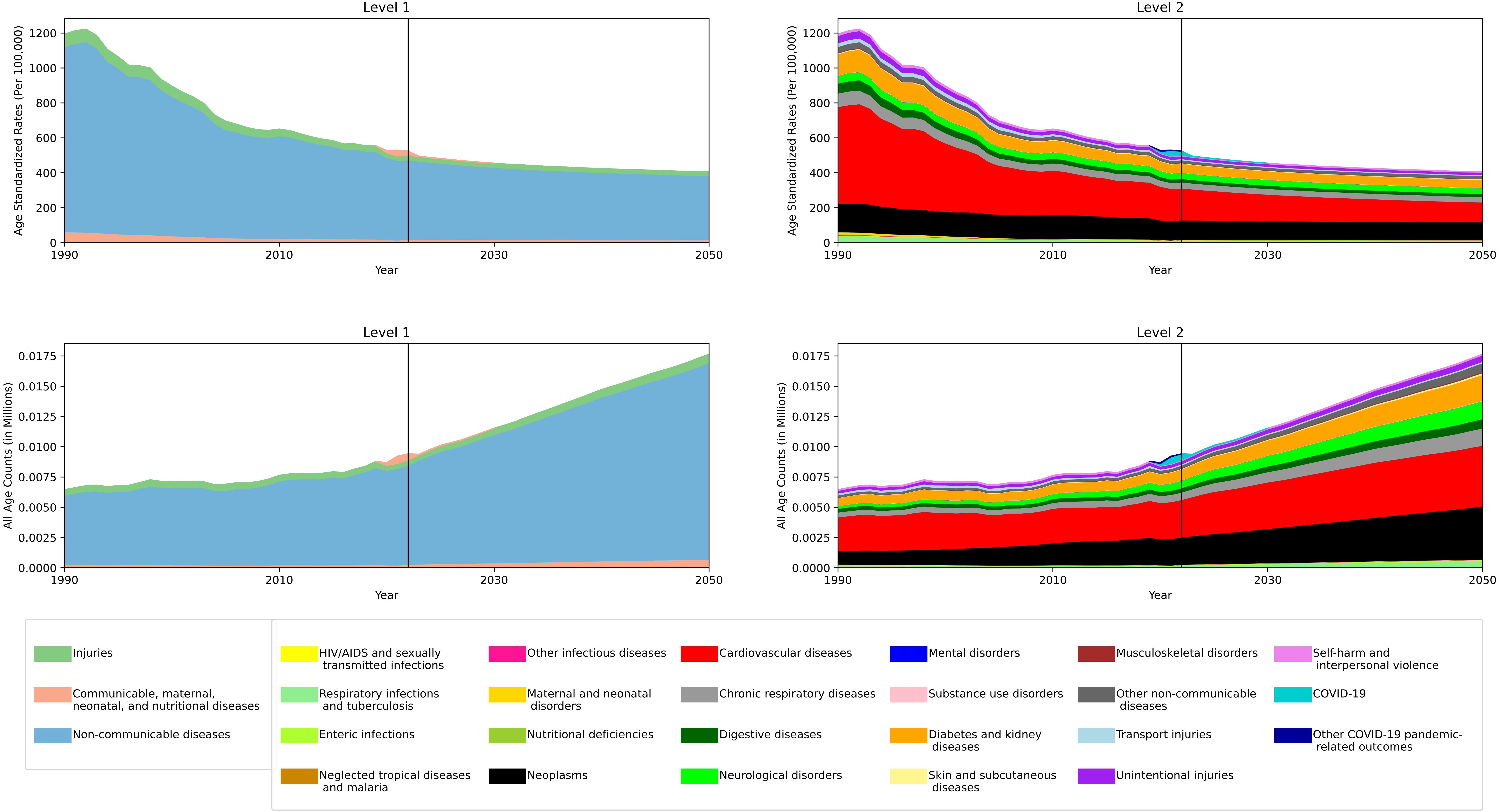
Level 1
Age Standardized Rates (Per 100,000)
1200
1000
800
600
400
200
0
1990
2010
2030
2050
Year
Level 2
Age Standardized Rates (Per 100,000)
Level 1
All Age Counts (in Millions)
0.0175
0.0150
0.0125
0.0100
0.0075
0.0050
0.0025
0.0000
Level 2
All Age Counts (in Millions)
Injuries
Communicable, maternal, neonatal, and nutritional diseases
Non-communicable diseases
HIV/AIDS and sexually transmitted infections
Respiratory infections and tuberculosis
Enteric infections
Neglected tropical diseases and malaria
Other infectious diseases
Maternal and neonatal disorders
Nutritional deficiencies
Neoplasms
Cardiovascular diseases
Chronic respiratory diseases
Digestive diseases
Neurological disorders
Mental disorders
Substance use disorders
Diabetes and kidney diseases
Skin and subcutaneous diseases
Musculoskeletal disorders
Other non-communicable diseases
Transport injuries
Unintentional injuries
Self-harm and interpersonal violence
COVID-19
Other COVID-19 pandemic-related outcomes

Denmark

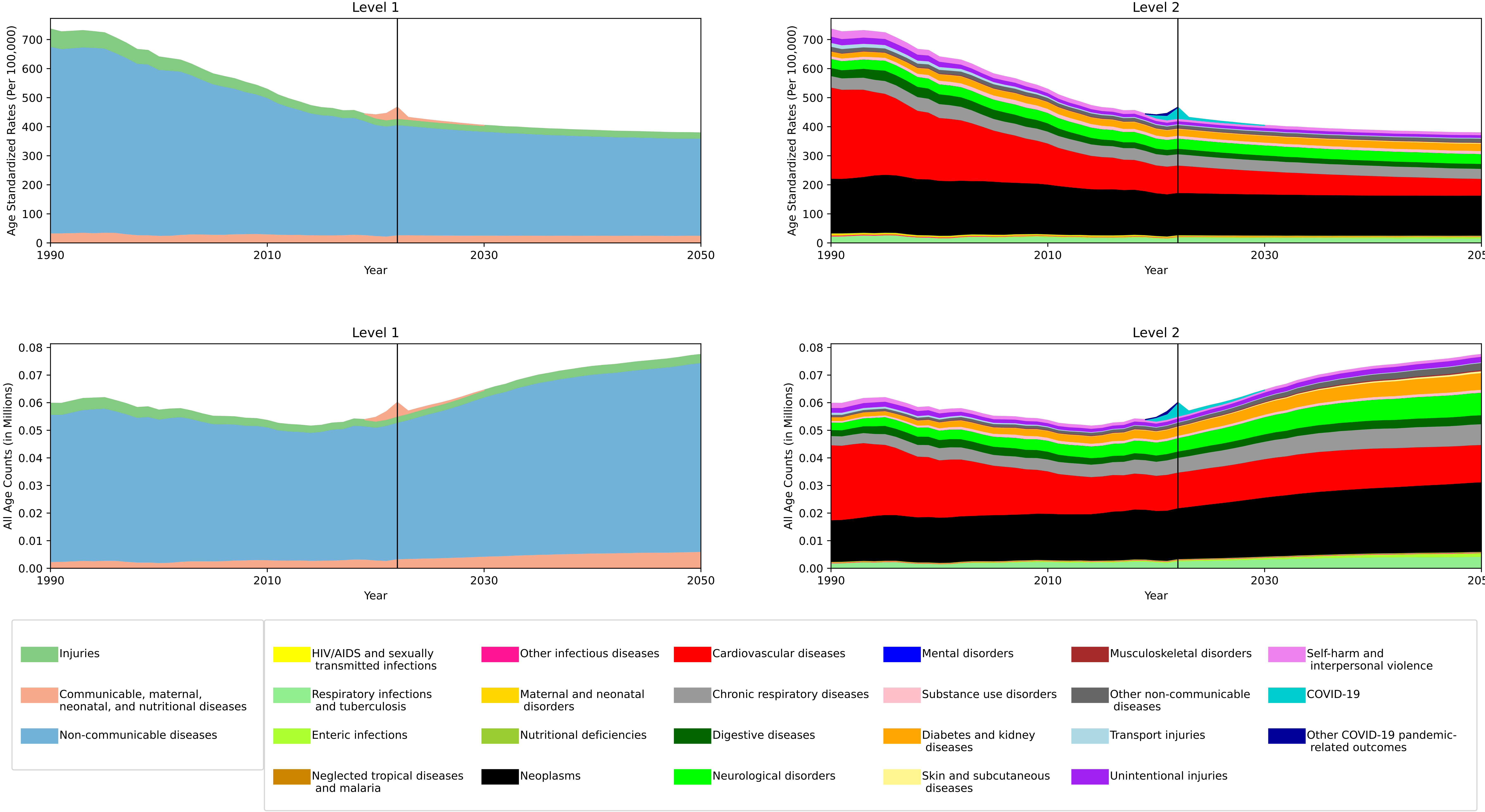
Level 1
Level 2
Age Standardized Rates (Per 100,000)
All Age Counts (in Millions)
Year
1990
2010
2030
2050
Injuries
Communicable, maternal, neonatal, and nutritional diseases
Non-communicable diseases
HIV/AIDS and sexually transmitted infections
Respiratory infections and tuberculosis
Enteric infections
Neglected tropical diseases and malaria
Other infectious diseases
Maternal and neonatal disorders
Nutritional deficiencies
Neoplasms
Cardiovascular diseases
Chronic respiratory diseases
Digestive diseases
Neurological disorders
Mental disorders
Substance use disorders
Diabetes and kidney diseases
Skin and subcutaneous diseases
Musculoskeletal disorders
Other non-communicable diseases
Transport injuries
Unintentional injuries
Self-harm and interpersonal violence
COVID-19
Other COVID-19 pandemic-related outcomes

Finland

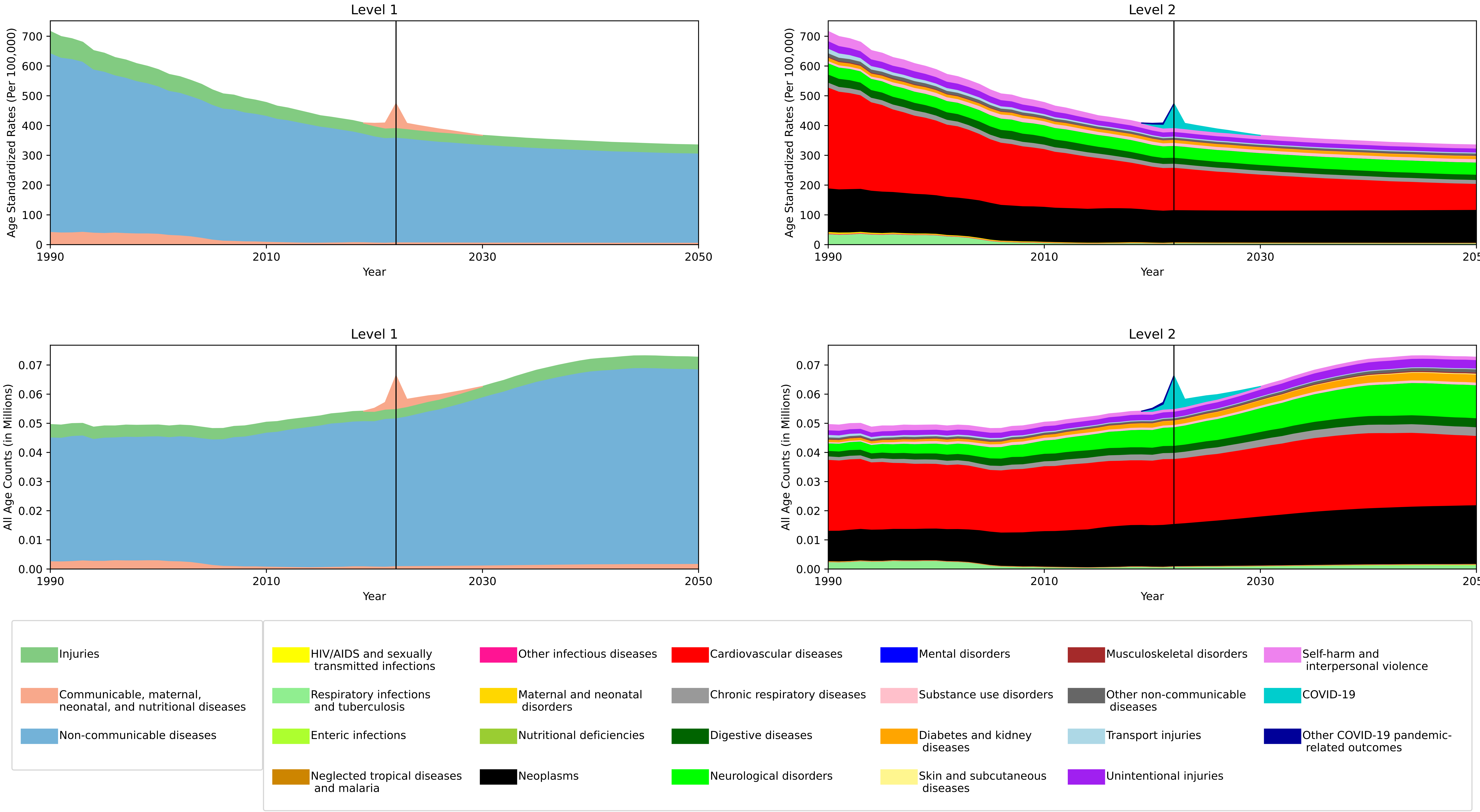
Level 1
Level 2
Age Standardized Rates (Per 100,000)
All Age Counts (in Millions)
Year
1990
2010
2030
2050
Injuries
Communicable, maternal, neonatal, and nutritional diseases
Non-communicable diseases
HIV/AIDS and sexually transmitted infections
Respiratory infections and tuberculosis
Enteric infections
Neglected tropical diseases and malaria
Other infectious diseases
Maternal and neonatal disorders
Nutritional deficiencies
Neoplasms
Cardiovascular diseases
Chronic respiratory diseases
Digestive diseases
Neurological disorders
Mental disorders
Substance use disorders
Diabetes and kidney diseases
Skin and subcutaneous diseases
Musculoskeletal disorders
Other non-communicable diseases
Transport injuries
Unintentional injuries
Self-harm and interpersonal violence
COVID-19
Other COVID-19 pandemic-related outcomes

France

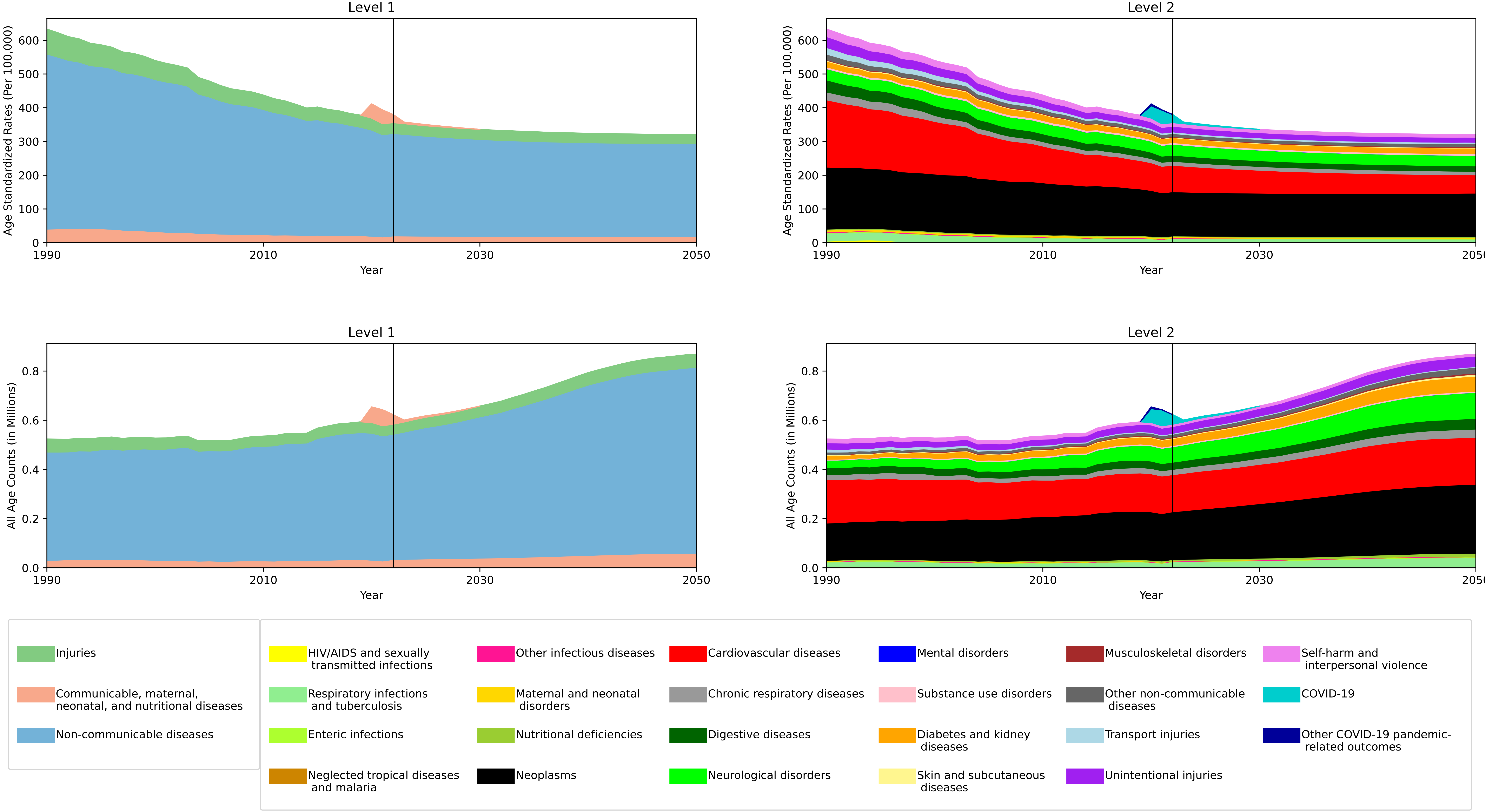
Level 1
Level 2
Age Standardized Rates (Per 100,000)
All Age Counts (in Millions)
Year
0
100
200
300
400
500
600
0.0
0.2
0.4
0.6
0.8
1990
2010
2030
2050
Injuries
Communicable, maternal, neonatal, and nutritional diseases
Non-communicable diseases
HIV/AIDS and sexually transmitted infections
Respiratory infections and tuberculosis
Enteric infections
Neglected tropical diseases and malaria
Other infectious diseases
Maternal and neonatal disorders
Nutritional deficiencies
Neoplasms
Cardiovascular diseases
Chronic respiratory diseases
Digestive diseases
Neurological disorders
Mental disorders
Substance use disorders
Diabetes and kidney diseases
Skin and subcutaneous diseases
Musculoskeletal disorders
Other non-communicable diseases
Transport injuries
Unintentional injuries
Self-harm and interpersonal violence
COVID-19
Other COVID-19 pandemic-related outcomes

Germany

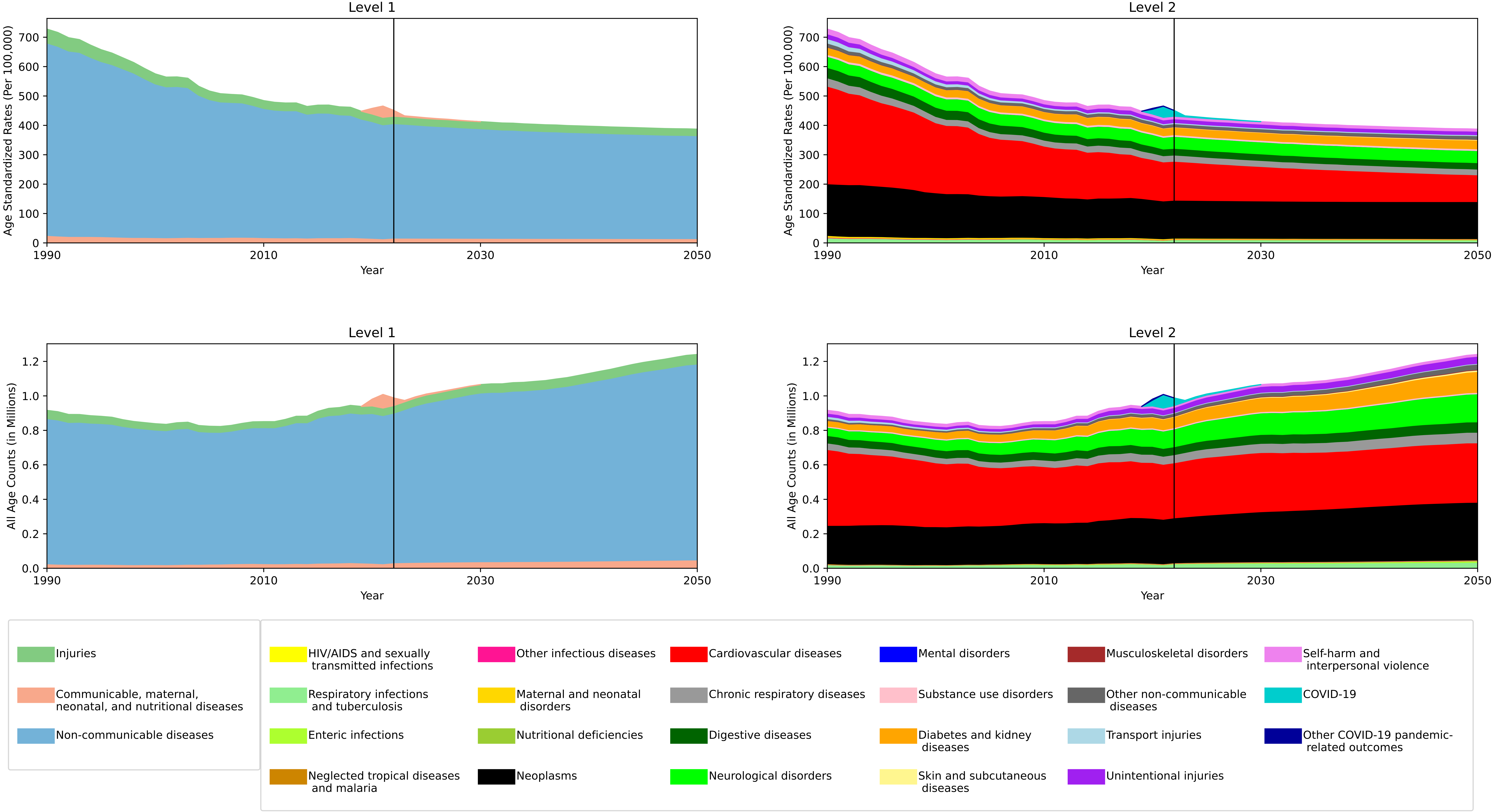
Level 1
Level 2
Age Standardized Rates (Per 100,000)
All Age Counts (in Millions)
Year
0
100
200
300
400
500
600
700
0.0
0.2
0.4
0.6
0.8
1.0
1.2
1990
2010
2030
2050
Injuries
Communicable, maternal, neonatal, and nutritional diseases
Non-communicable diseases
HIV/AIDS and sexually transmitted infections
Respiratory infections and tuberculosis
Enteric infections
Neglected tropical diseases and malaria
Other infectious diseases
Maternal and neonatal disorders
Nutritional deficiencies
Neoplasms
Cardiovascular diseases
Chronic respiratory diseases
Digestive diseases
Neurological disorders
Mental disorders
Substance use disorders
Diabetes and kidney diseases
Skin and subcutaneous diseases
Musculoskeletal disorders
Other non-communicable diseases
Transport injuries
Unintentional injuries
Self-harm and interpersonal violence
COVID-19
Other COVID-19 pandemic-related outcomes

Greece

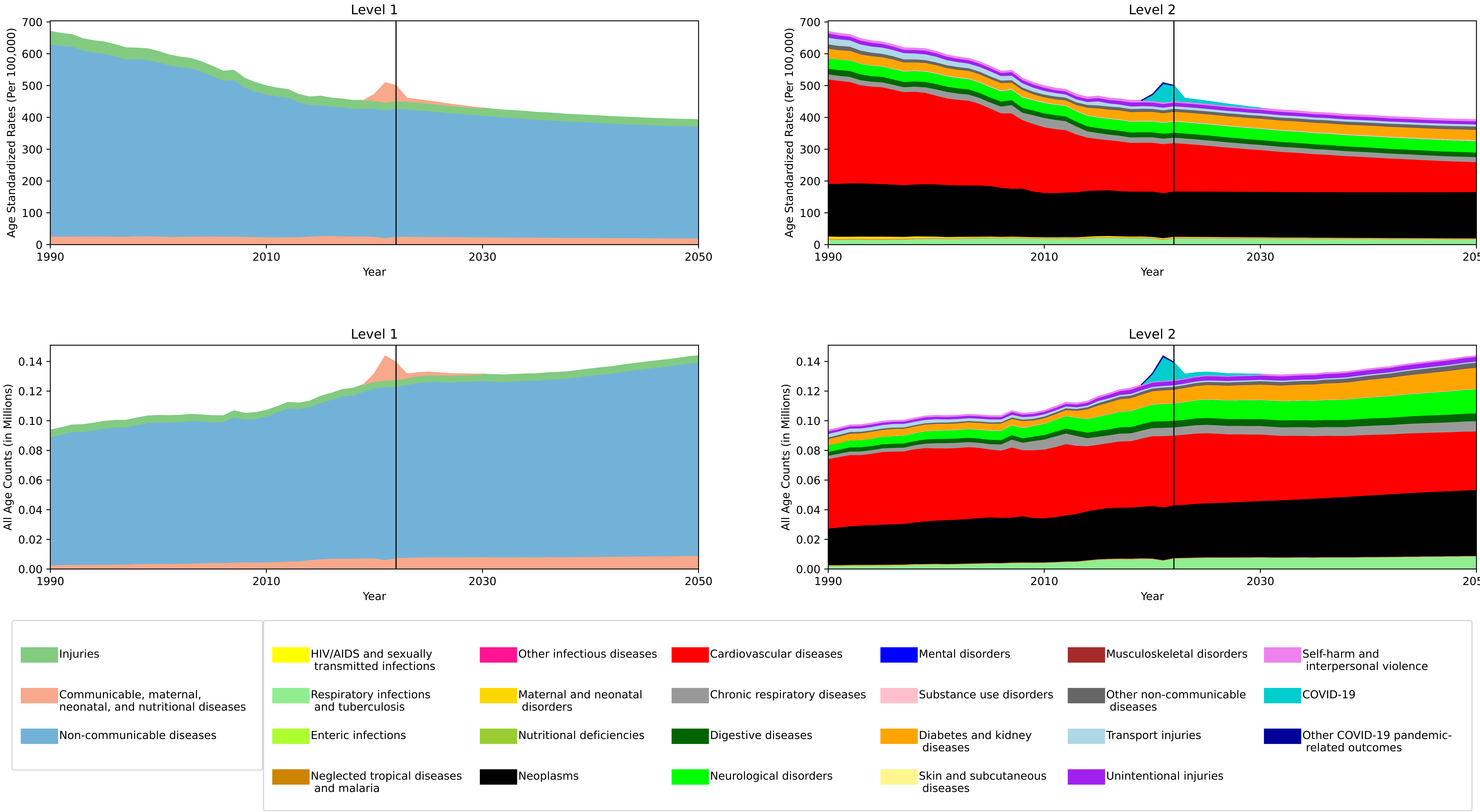
Level 1
Level 2
Age Standardized Rates (Per 100,000)
All Age Counts (in Millions)
Year
0
100
200
300
400
500
600
700
0.00
0.02
0.04
0.06
0.08
0.10
0.12
0.14
1990
2010
2030
2050
Injuries
Communicable, maternal, neonatal, and nutritional diseases
Non-communicable diseases
HIV/AIDS and sexually transmitted infections
Respiratory infections and tuberculosis
Enteric infections
Neglected tropical diseases and malaria
Other infectious diseases
Maternal and neonatal disorders
Nutritional deficiencies
Neoplasms
Cardiovascular diseases
Chronic respiratory diseases
Digestive diseases
Neurological disorders
Mental disorders
Substance use disorders
Diabetes and kidney diseases
Skin and subcutaneous diseases
Musculoskeletal disorders
Other non-communicable diseases
Transport injuries
Unintentional injuries
Self-harm and interpersonal violence
COVID-19
Other COVID-19 pandemic-related outcomes

Iceland

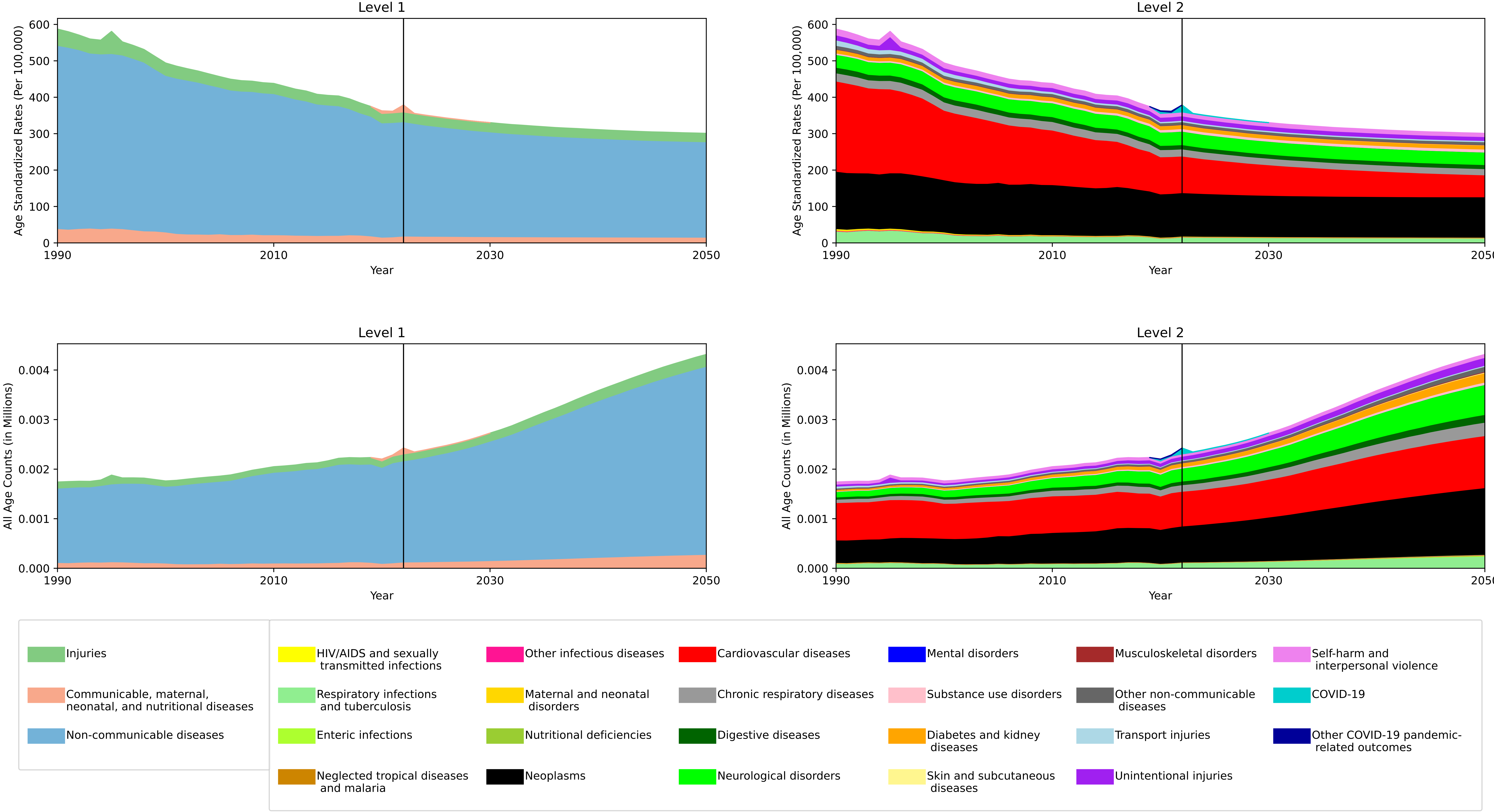
Level 1
Age Standardized Rates (Per 100,000)
Year
Level 2
All Age Counts (in Millions)
Injuries
Communicable, maternal, neonatal, and nutritional diseases
Non-communicable diseases
HIV/AIDS and sexually transmitted infections
Respiratory infections and tuberculosis
Enteric infections
Neglected tropical diseases and malaria
Other infectious diseases
Maternal and neonatal disorders
Nutritional deficiencies
Neoplasms
Cardiovascular diseases
Chronic respiratory diseases
Digestive diseases
Neurological disorders
Mental disorders
Substance use disorders
Diabetes and kidney diseases
Skin and subcutaneous diseases
Musculoskeletal disorders
Other non-communicable diseases
Transport injuries
Unintentional injuries
Self-harm and interpersonal violence
COVID-19
Other COVID-19 pandemic-related outcomes

Ireland

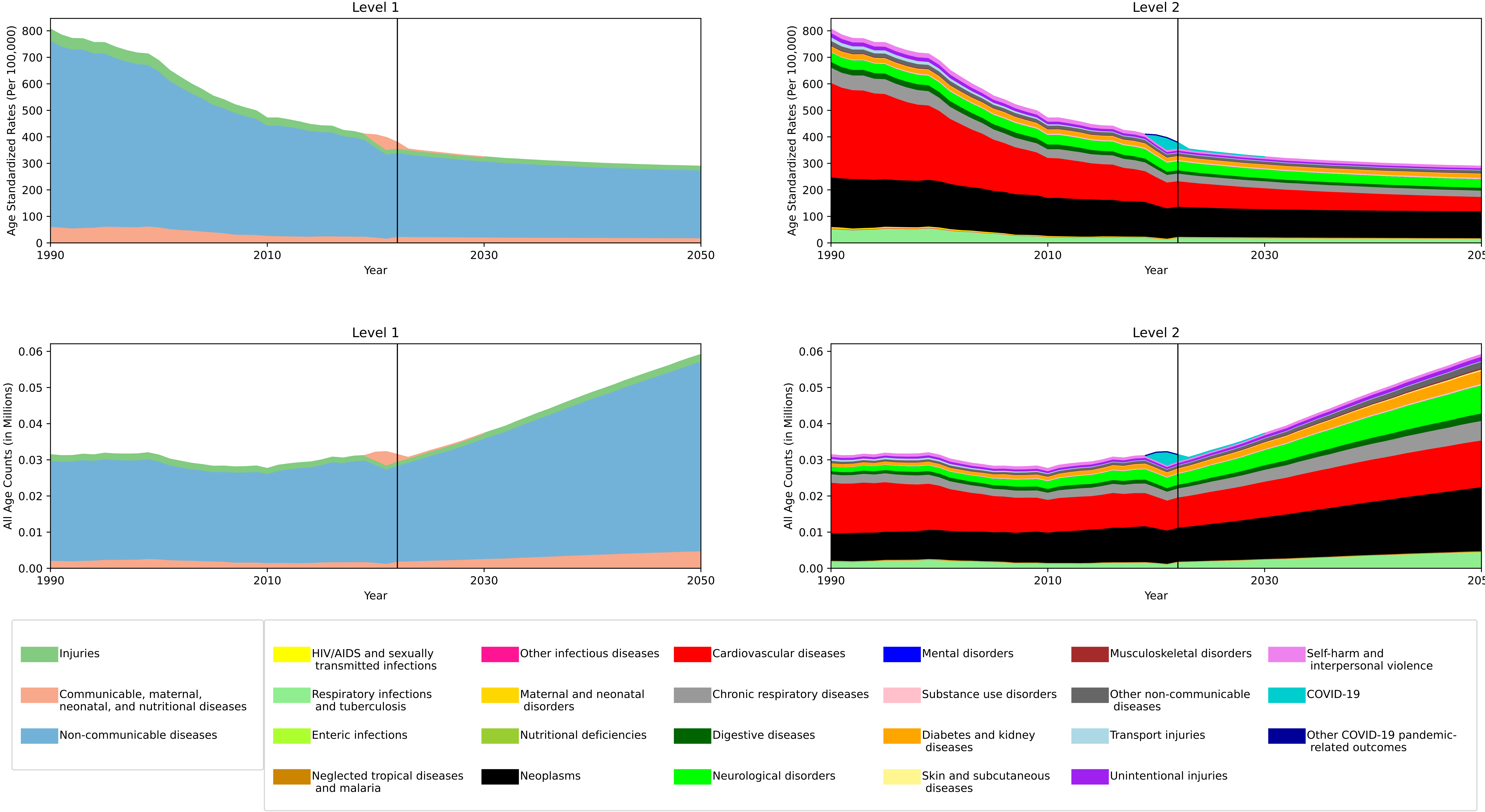
Level 1
Level 2
Age Standardized Rates (Per 100,000)
All Age Counts (in Millions)
Year
1990
2010
2030
2050
Injuries
Communicable, maternal, neonatal, and nutritional diseases
Non-communicable diseases
HIV/AIDS and sexually transmitted infections
Respiratory infections and tuberculosis
Enteric infections
Neglected tropical diseases and malaria
Other infectious diseases
Maternal and neonatal disorders
Nutritional deficiencies
Neoplasms
Cardiovascular diseases
Chronic respiratory diseases
Digestive diseases
Neurological disorders
Mental disorders
Substance use disorders
Diabetes and kidney diseases
Skin and subcutaneous diseases
Musculoskeletal disorders
Other non-communicable diseases
Transport injuries
Unintentional injuries
Self-harm and interpersonal violence
COVID-19
Other COVID-19 pandemic-related outcomes

Israel

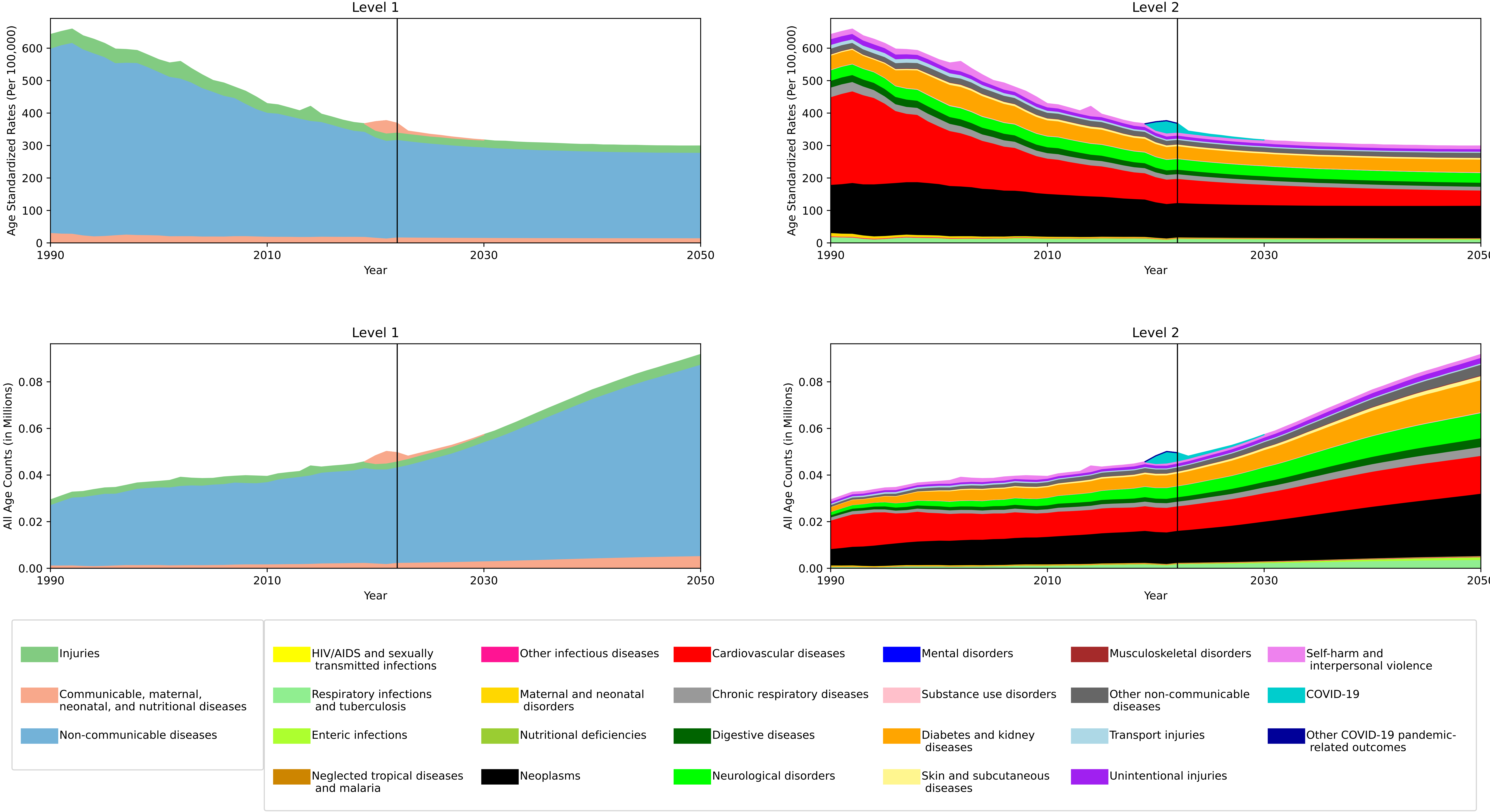
Level 1
Level 2
Age Standardized Rates (Per 100,000)
All Age Counts (in Millions)
Year
Injuries
Communicable, maternal, neonatal, and nutritional diseases
Non-communicable diseases
HIV/AIDS and sexually transmitted infections
Respiratory infections and tuberculosis
Enteric infections
Neglected tropical diseases and malaria
Other infectious diseases
Maternal and neonatal disorders
Nutritional deficiencies
Neoplasms
Cardiovascular diseases
Chronic respiratory diseases
Digestive diseases
Neurological disorders
Mental disorders
Substance use disorders
Diabetes and kidney diseases
Skin and subcutaneous diseases
Musculoskeletal disorders
Other non-communicable diseases
Transport injuries
Unintentional injuries
Self-harm and interpersonal violence
COVID-19
Other COVID-19 pandemic-related outcomes

Italy

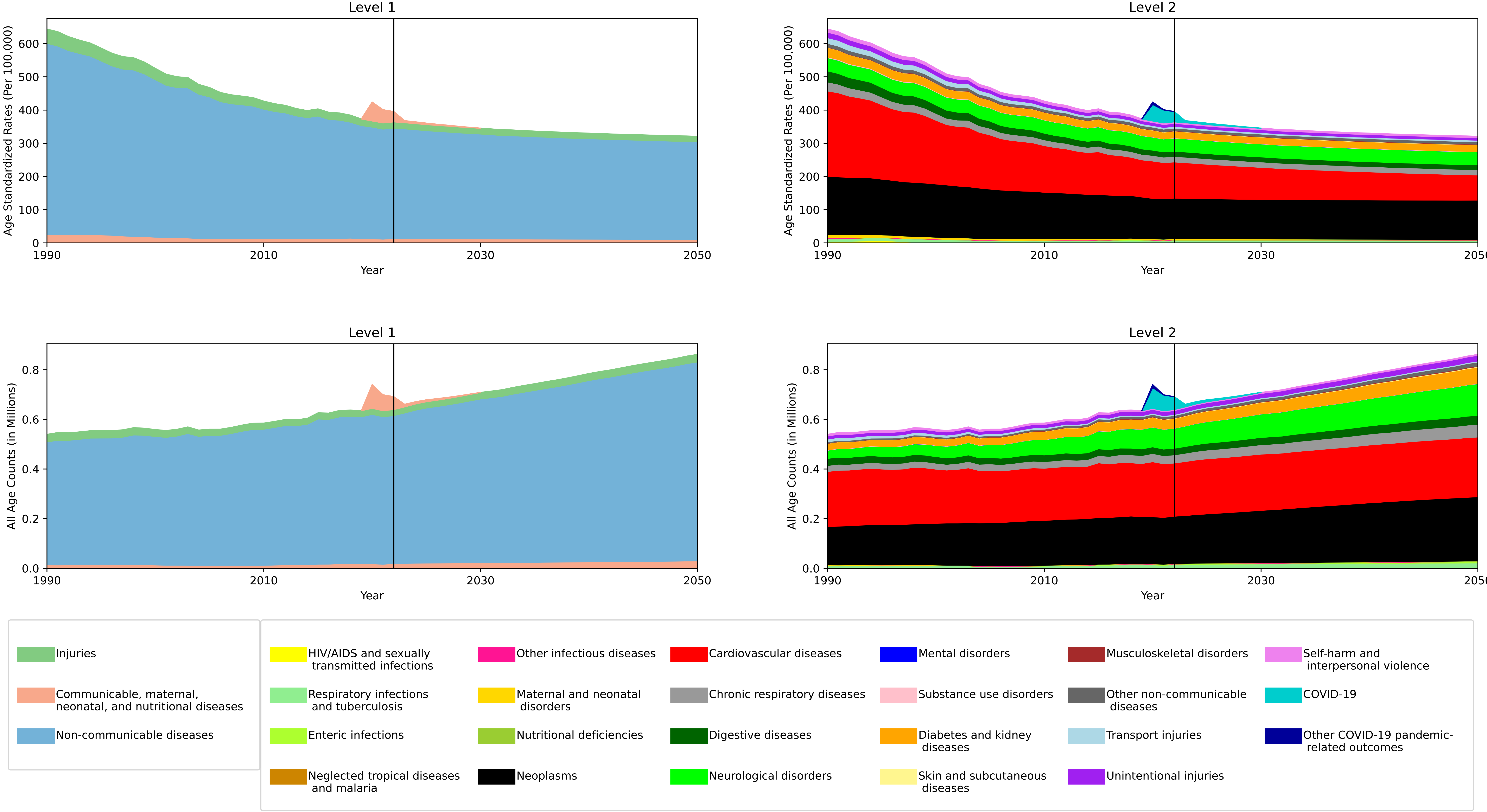
Level 1
Level 2
Age Standardized Rates (Per 100,000)
All Age Counts (in Millions)
Year
1990
2010
2030
2050
0
100
200
300
400
500
600
0.0
0.2
0.4
0.6
0.8
Injuries
Communicable, maternal, neonatal, and nutritional diseases
Non-communicable diseases
HIV/AIDS and sexually transmitted infections
Respiratory infections and tuberculosis
Enteric infections
Neglected tropical diseases and malaria
Other infectious diseases
Maternal and neonatal disorders
Nutritional deficiencies
Neoplasms
Cardiovascular diseases
Chronic respiratory diseases
Digestive diseases
Neurological disorders
Mental disorders
Substance use disorders
Diabetes and kidney diseases
Skin and subcutaneous diseases
Musculoskeletal disorders
Other non-communicable diseases
Transport injuries
Unintentional injuries
Self-harm and interpersonal violence
COVID-19
Other COVID-19 pandemic-related outcomes

Luxembourg

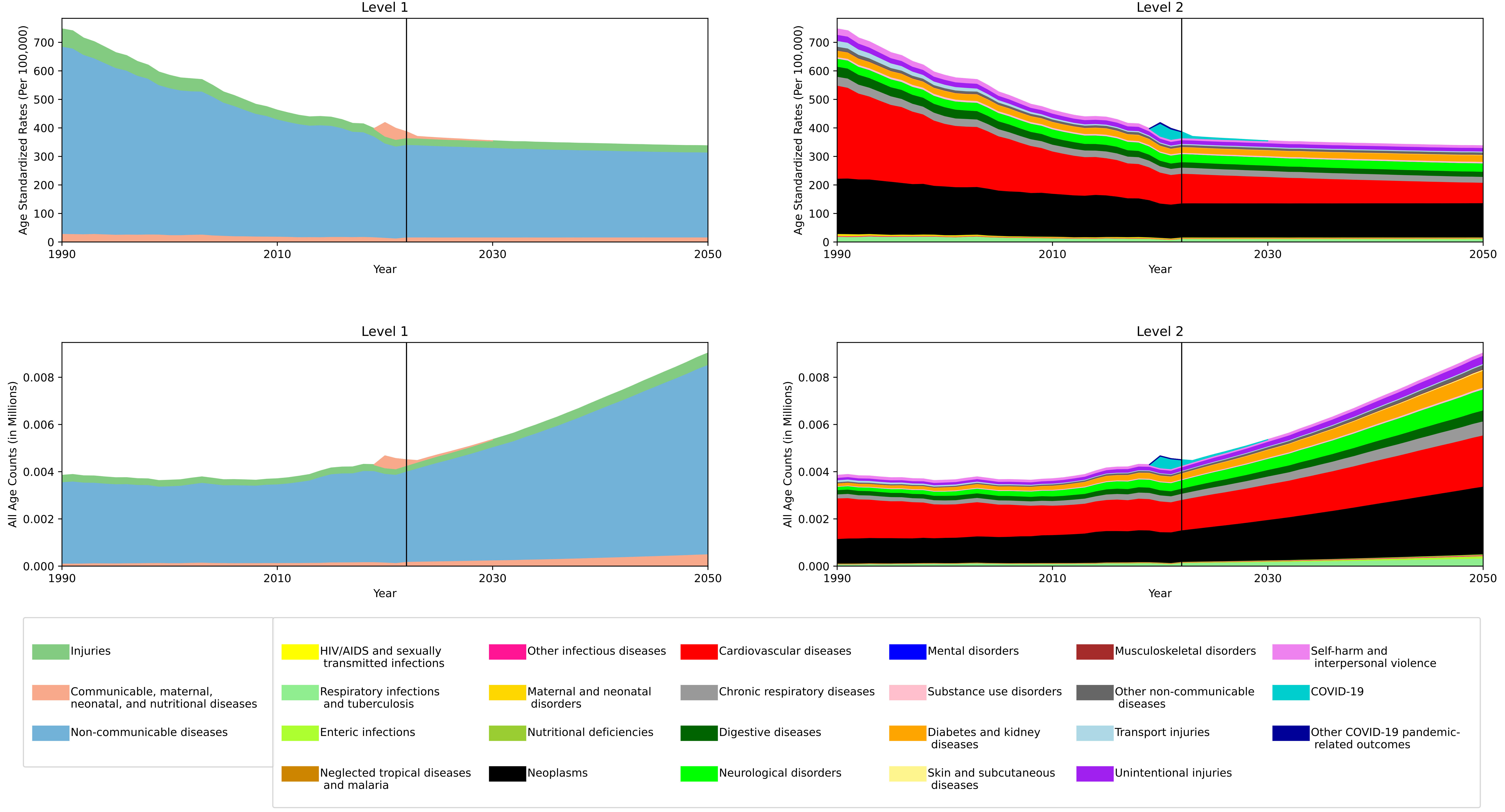
Level 1
Level 2
Age Standardized Rates (Per 100,000)
All Age Counts (in Millions)
Year
1990
2010
2030
2050
0
100
200
300
400
500
600
700
0.000
0.002
0.004
0.006
0.008
Injuries
Communicable, maternal, neonatal, and nutritional diseases
Non-communicable diseases
HIV/AIDS and sexually transmitted infections
Respiratory infections and tuberculosis
Enteric infections
Neglected tropical diseases and malaria
Other infectious diseases
Maternal and neonatal disorders
Nutritional deficiencies
Neoplasms
Cardiovascular diseases
Chronic respiratory diseases
Digestive diseases
Neurological disorders
Mental disorders
Substance use disorders
Diabetes and kidney diseases
Skin and subcutaneous diseases
Musculoskeletal disorders
Other non-communicable diseases
Transport injuries
Unintentional injuries
Self-harm and interpersonal violence
COVID-19
Other COVID-19 pandemic-related outcomes

Malta

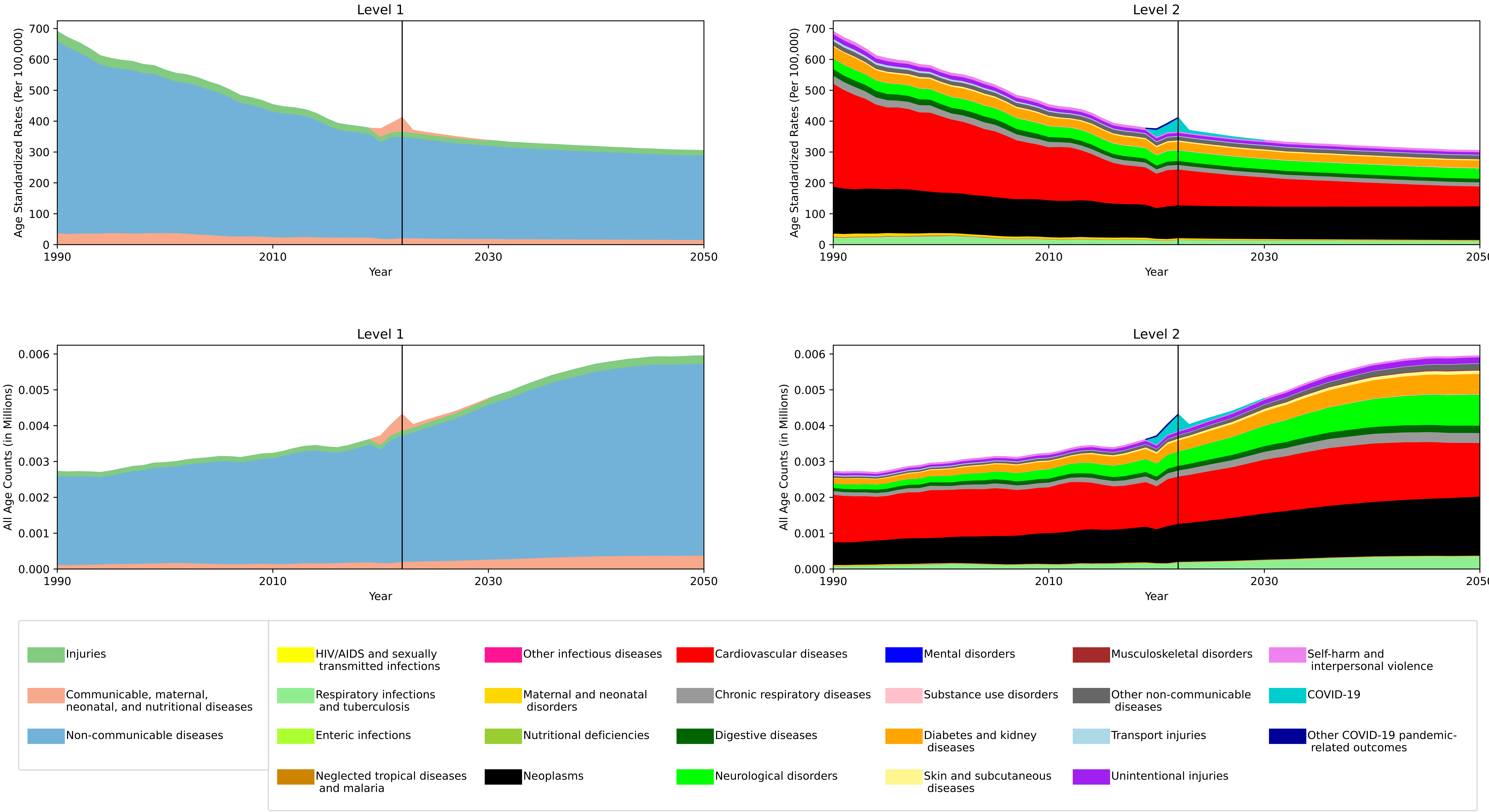
Level 1
Level 2
Age Standardized Rates (Per 100,000)
All Age Counts (in Millions)
Year
1990
2010
2030
2050
Injuries
Communicable, maternal, neonatal, and nutritional diseases
Non-communicable diseases
HIV/AIDS and sexually transmitted infections
Respiratory infections and tuberculosis
Enteric infections
Neglected tropical diseases and malaria
Other infectious diseases
Maternal and neonatal disorders
Nutritional deficiencies
Neoplasms
Cardiovascular diseases
Chronic respiratory diseases
Digestive diseases
Neurological disorders
Mental disorders
Substance use disorders
Diabetes and kidney diseases
Skin and subcutaneous diseases
Musculoskeletal disorders
Other non-communicable diseases
Transport injuries
Unintentional injuries
Self-harm and interpersonal violence
COVID-19
Other COVID-19 pandemic-related outcomes

Monaco

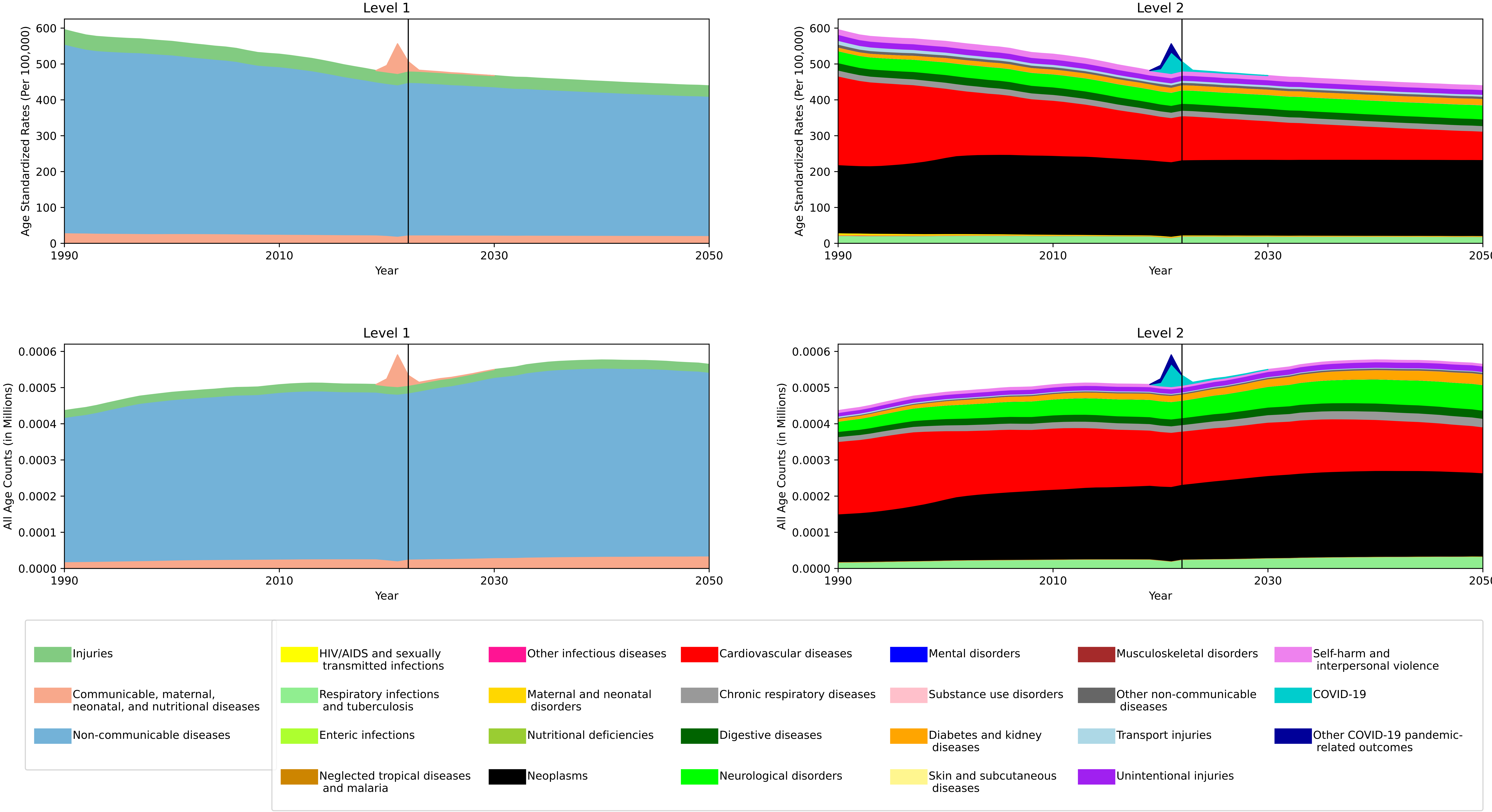
Level 1
Level 2
Age Standardized Rates (Per 100,000)
All Age Counts (in Millions)
Year
1990
2010
2030
2050
0
100
200
300
400
500
600
0.0000
0.0001
0.0002
0.0003
0.0004
0.0005
0.0006
Injuries
Communicable, maternal, neonatal, and nutritional diseases
Non-communicable diseases
HIV/AIDS and sexually transmitted infections
Respiratory infections and tuberculosis
Enteric infections
Neglected tropical diseases and malaria
Other infectious diseases
Maternal and neonatal disorders
Nutritional deficiencies
Neoplasms
Cardiovascular diseases
Chronic respiratory diseases
Digestive diseases
Neurological disorders
Mental disorders
Substance use disorders
Diabetes and kidney diseases
Skin and subcutaneous diseases
Musculoskeletal disorders
Other non-communicable diseases
Transport injuries
Unintentional injuries
Self-harm and interpersonal violence
COVID-19
Other COVID-19 pandemic-related outcomes

Netherlands

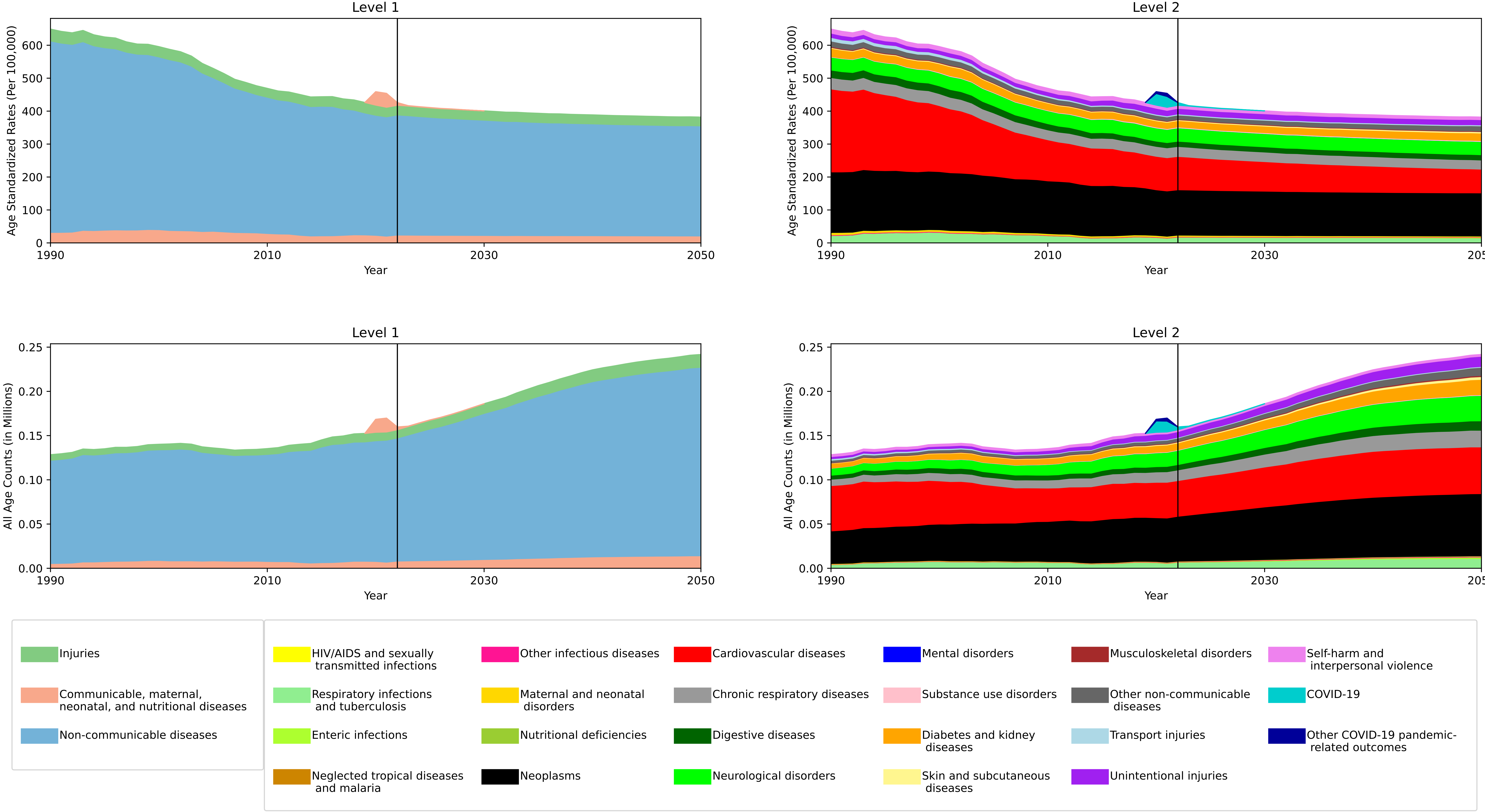

Norway

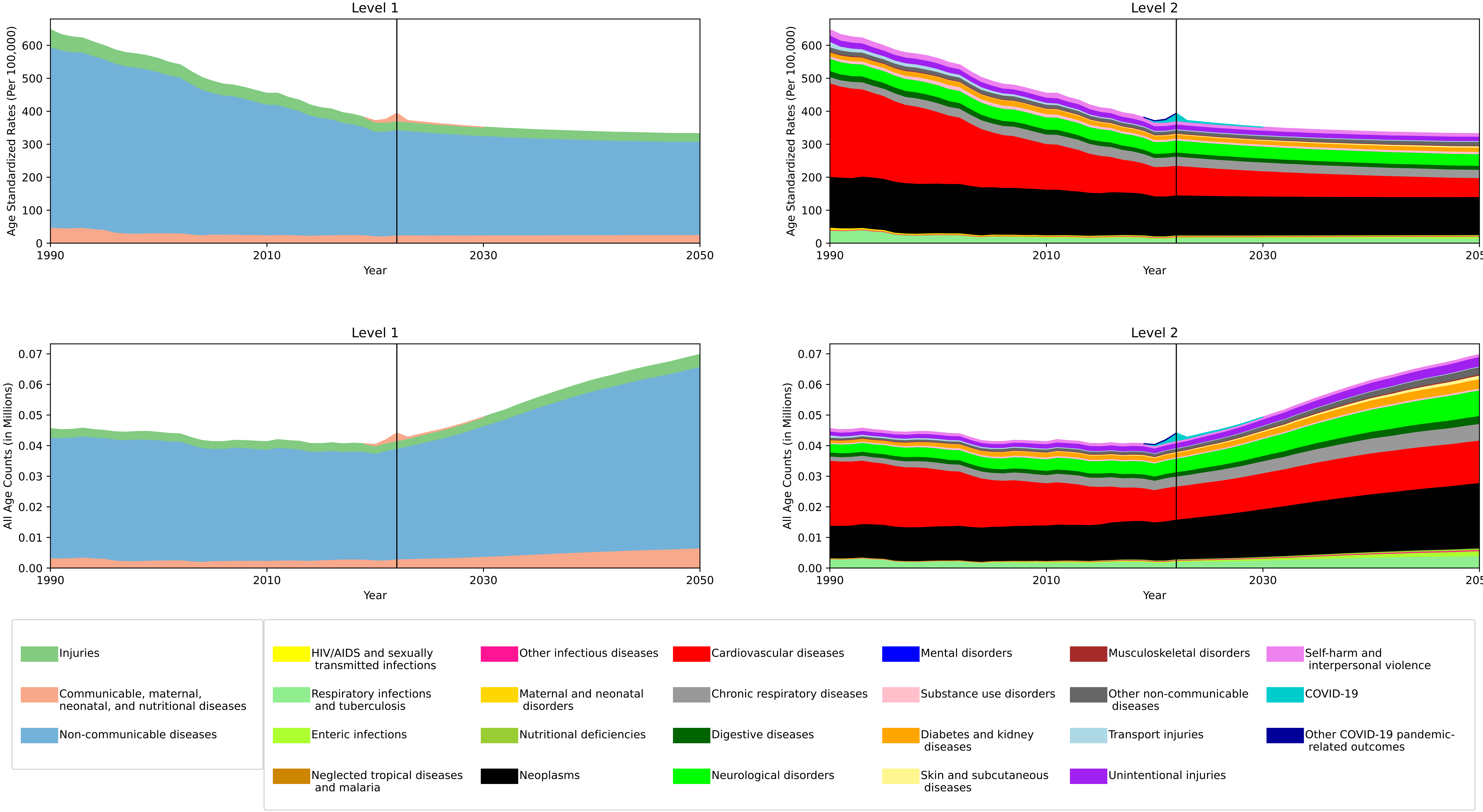
Level 1
Level 2
Age Standardized Rates (Per 100,000)
All Age Counts (in Millions)
Year
1990
2010
2030
2050
0
100
200
300
400
500
600
0.00
0.01
0.02
0.03
0.04
0.05
0.06
0.07
Injuries
Communicable, maternal, neonatal, and nutritional diseases
Non-communicable diseases
HIV/AIDS and sexually transmitted infections
Respiratory infections and tuberculosis
Enteric infections
Neglected tropical diseases and malaria
Other infectious diseases
Maternal and neonatal disorders
Nutritional deficiencies
Neoplasms
Cardiovascular diseases
Chronic respiratory diseases
Digestive diseases
Neurological disorders
Mental disorders
Substance use disorders
Diabetes and kidney diseases
Skin and subcutaneous diseases
Musculoskeletal disorders
Other non-communicable diseases
Transport injuries
Unintentional injuries
Self-harm and interpersonal violence
COVID-19
Other COVID-19 pandemic-related outcomes

Portugal

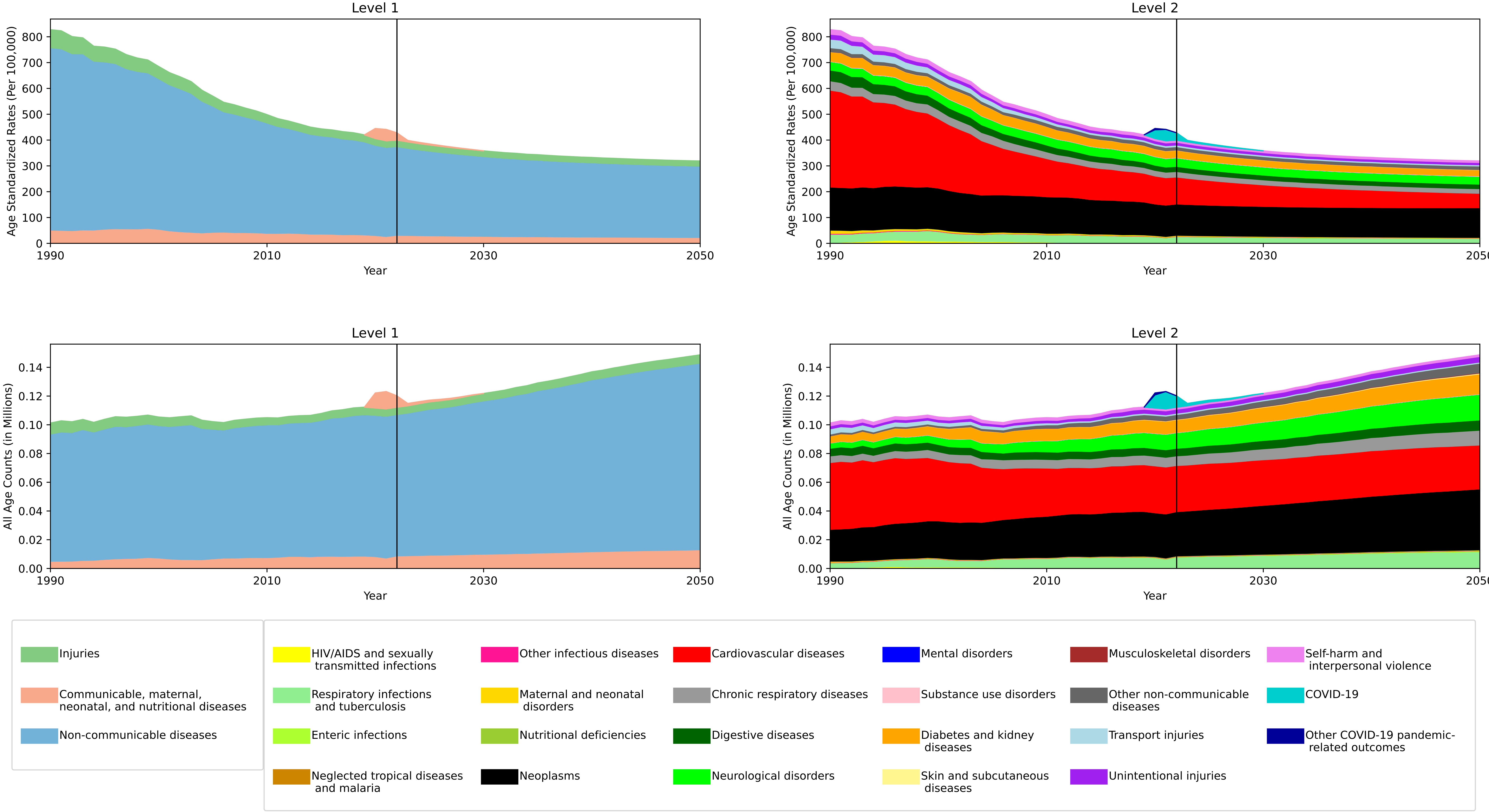
Level 1
Level 2
Age Standardized Rates (Per 100,000)
All Age Counts (in Millions)
Year
Injuries
Communicable, maternal, neonatal, and nutritional diseases
Non-communicable diseases
HIV/AIDS and sexually transmitted infections
Respiratory infections and tuberculosis
Enteric infections
Neglected tropical diseases and malaria
Other infectious diseases
Maternal and neonatal disorders
Nutritional deficiencies
Neoplasms
Cardiovascular diseases
Chronic respiratory diseases
Digestive diseases
Neurological disorders
Mental disorders
Substance use disorders
Diabetes and kidney diseases
Skin and subcutaneous diseases
Musculoskeletal disorders
Other non-communicable diseases
Transport injuries
Unintentional injuries
Self-harm and interpersonal violence
COVID-19
Other COVID-19 pandemic-related outcomes

San Marino

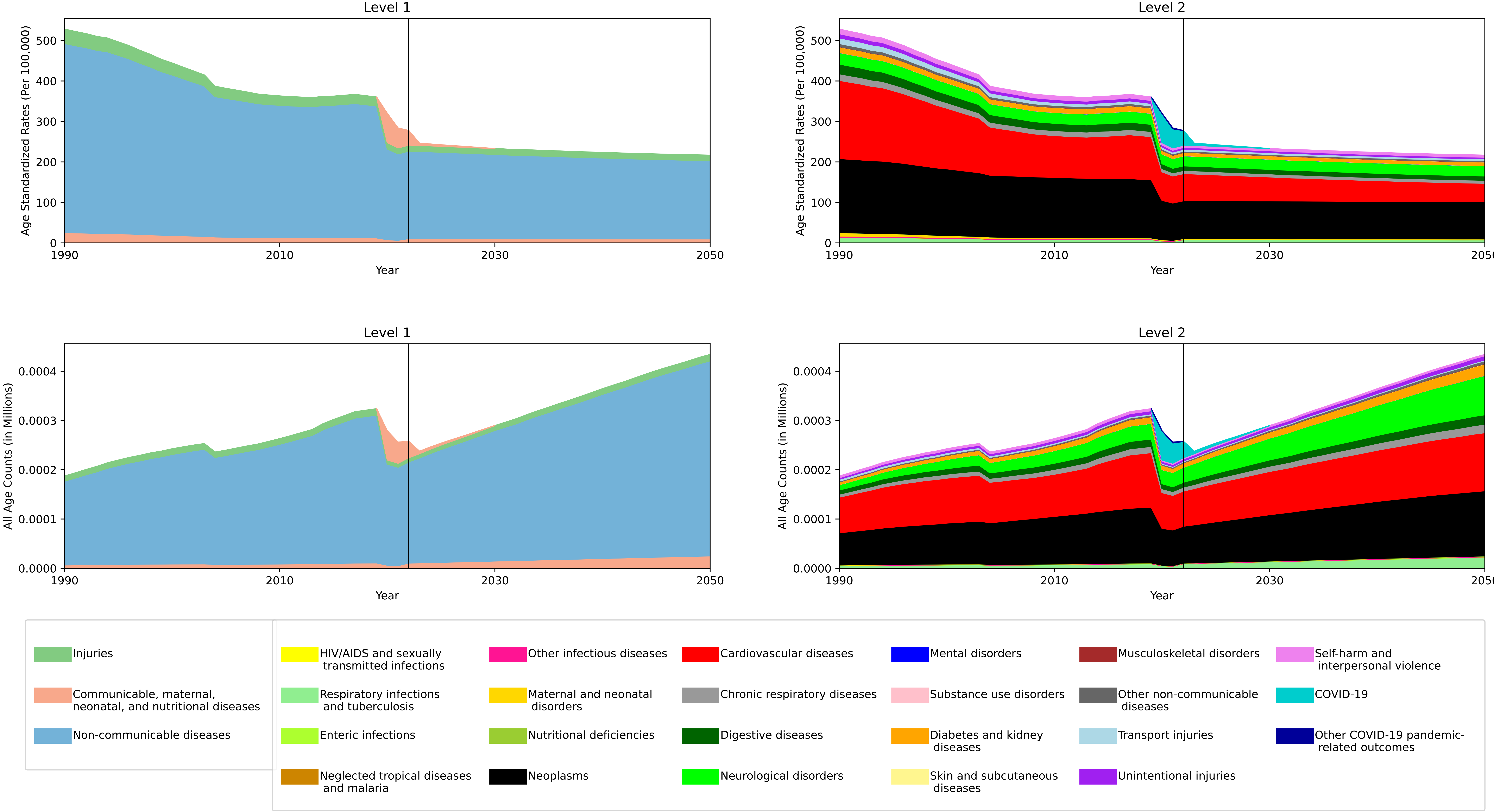
Level 1
Level 2
Age Standardized Rates (Per 100,000)
All Age Counts (in Millions)
Year
1990
2010
2030
2050
0
100
200
300
400
500
0.0000
0.0001
0.0002
0.0003
0.0004
Injuries
Communicable, maternal, neonatal, and nutritional diseases
Non-communicable diseases
HIV/AIDS and sexually transmitted infections
Respiratory infections and tuberculosis
Enteric infections
Neglected tropical diseases and malaria
Other infectious diseases
Maternal and neonatal disorders
Nutritional deficiencies
Neoplasms
Cardiovascular diseases
Chronic respiratory diseases
Digestive diseases
Neurological disorders
Mental disorders
Substance use disorders
Diabetes and kidney diseases
Skin and subcutaneous diseases
Musculoskeletal disorders
Other non-communicable diseases
Transport injuries
Unintentional injuries
Self-harm and interpersonal violence
COVID-19
Other COVID-19 pandemic-related outcomes

Spain

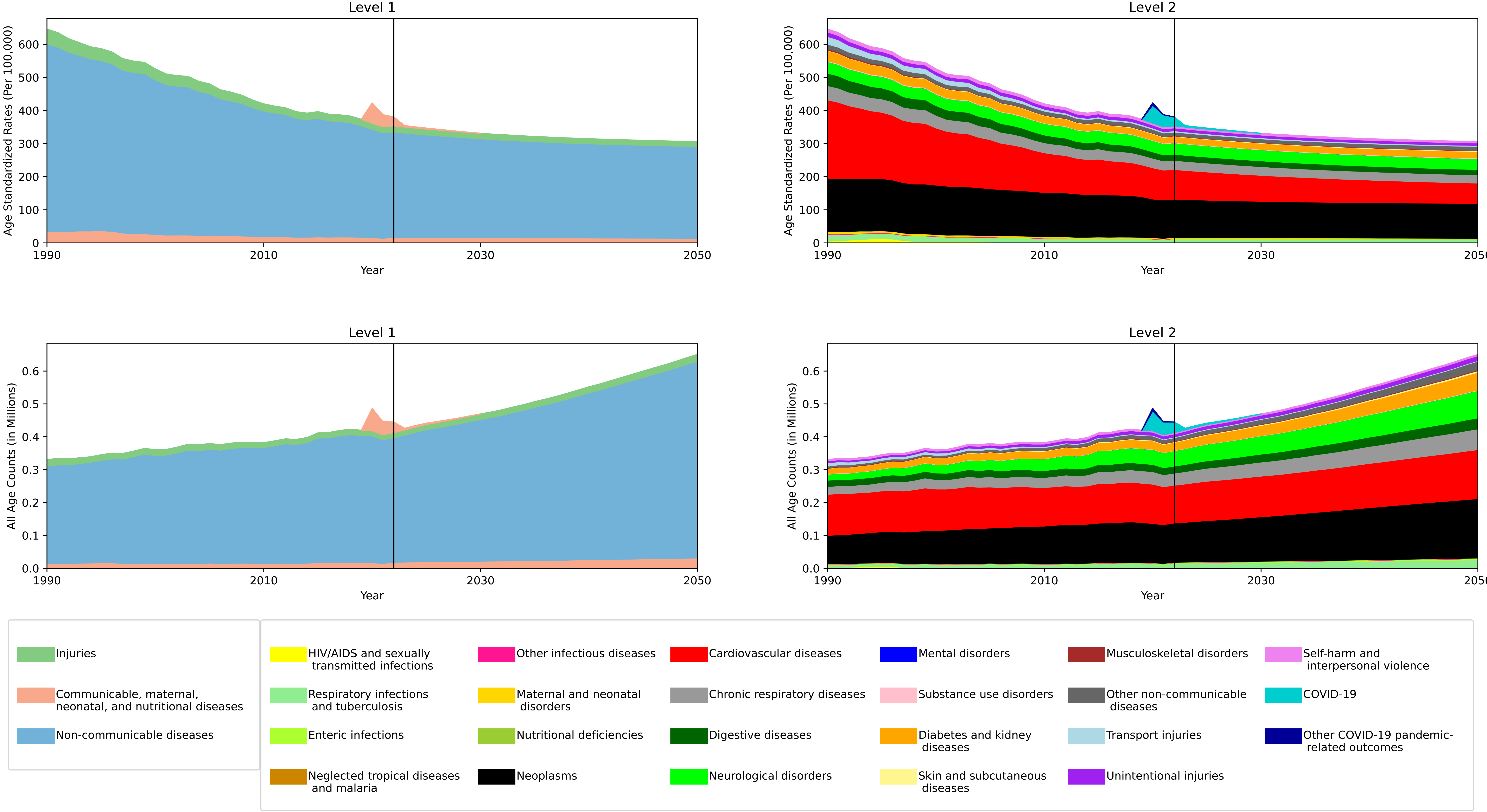

Level 1
Level 2
Age Standardized Rates (Per 100,000)
All Age Counts (in Millions)
Year
1990
2010
2030
2050
Injuries
Communicable, maternal, neonatal, and nutritional diseases
Non-communicable diseases
HIV/AIDS and sexually transmitted infections
Respiratory infections and tuberculosis
Enteric infections
Neglected tropical diseases and malaria
Other infectious diseases
Maternal and neonatal disorders
Nutritional deficiencies
Neoplasms
Cardiovascular diseases
Chronic respiratory diseases
Digestive diseases
Neurological disorders
Mental disorders
Substance use disorders
Diabetes and kidney diseases
Skin and subcutaneous diseases
Musculoskeletal disorders
Other non-communicable diseases
Transport injuries
Unintentional injuries
Self-harm and interpersonal violence
COVID-19
Other COVID-19 pandemic-related outcomes

Sweden

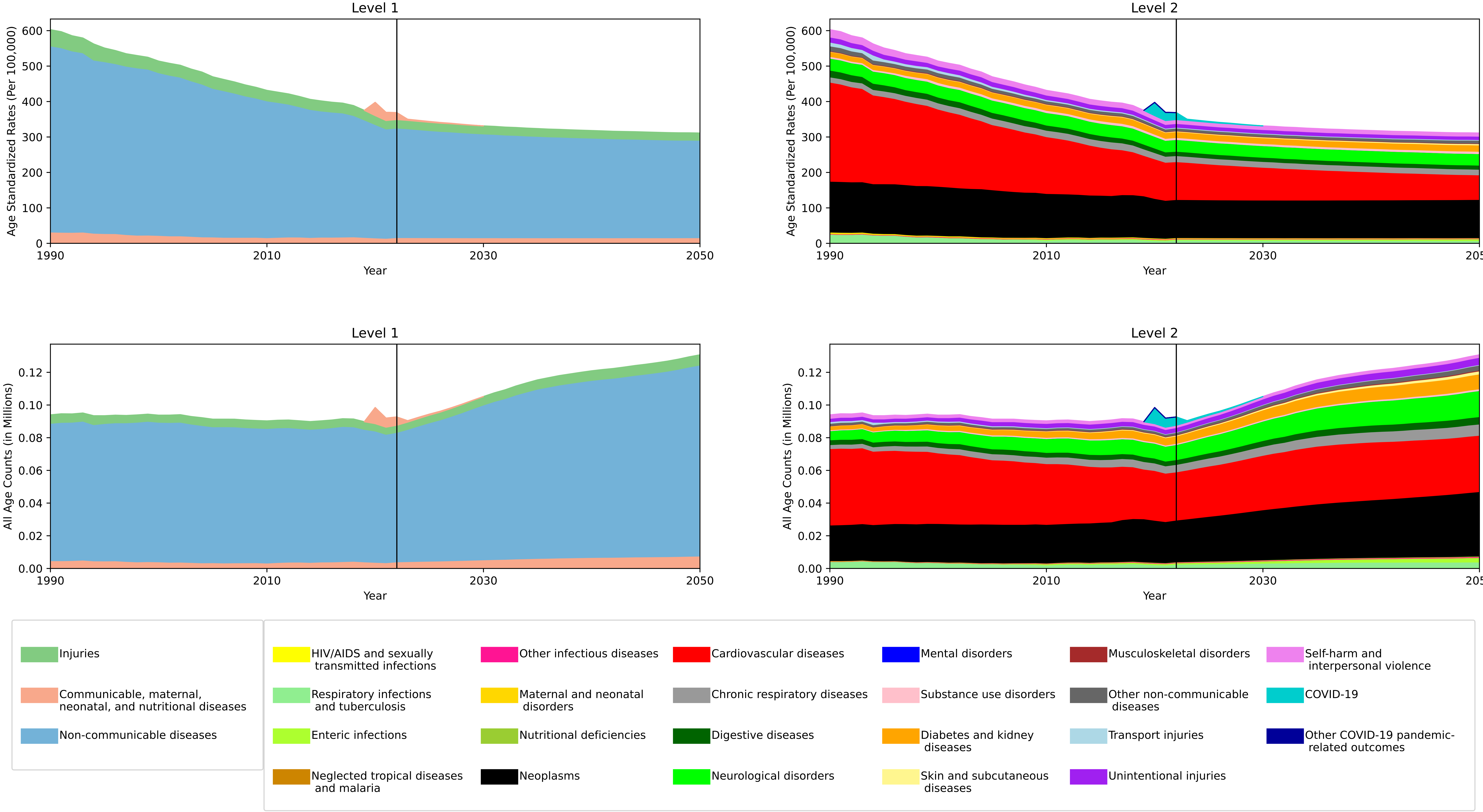
Level 1
Level 2
Age Standardized Rates (Per 100,000)
All Age Counts (in Millions)
Year
1990
2010
2030
2050
Injuries
Communicable, maternal, neonatal, and nutritional diseases
Non-communicable diseases
HIV/AIDS and sexually transmitted infections
Respiratory infections and tuberculosis
Enteric infections
Neglected tropical diseases and malaria
Other infectious diseases
Maternal and neonatal disorders
Nutritional deficiencies
Neoplasms
Cardiovascular diseases
Chronic respiratory diseases
Digestive diseases
Neurological disorders
Mental disorders
Substance use disorders
Diabetes and kidney diseases
Skin and subcutaneous diseases
Musculoskeletal disorders
Other non-communicable diseases
Transport injuries
Unintentional injuries
Self-harm and interpersonal violence
COVID-19
Other COVID-19 pandemic-related outcomes

Switzerland

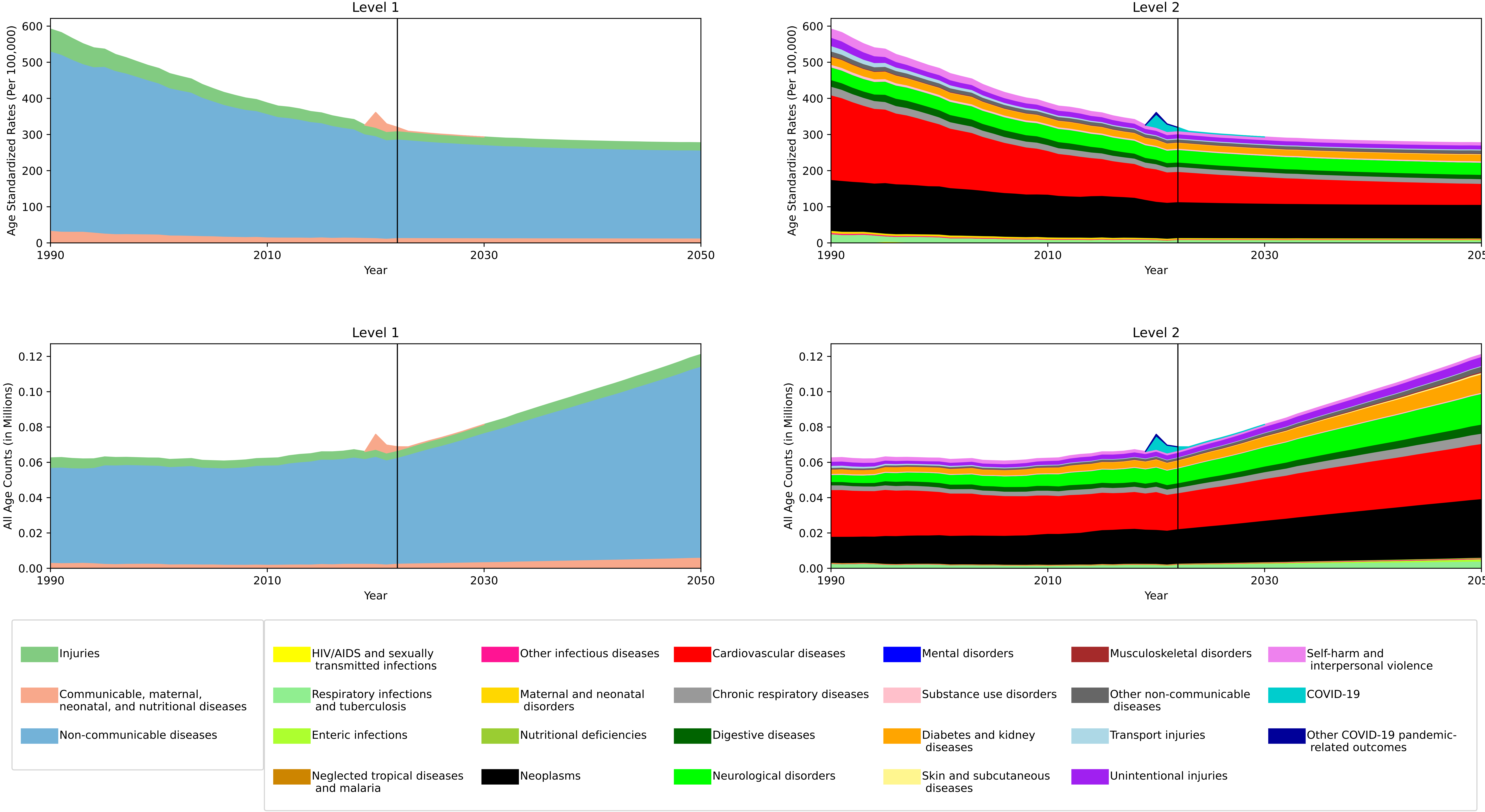

UK

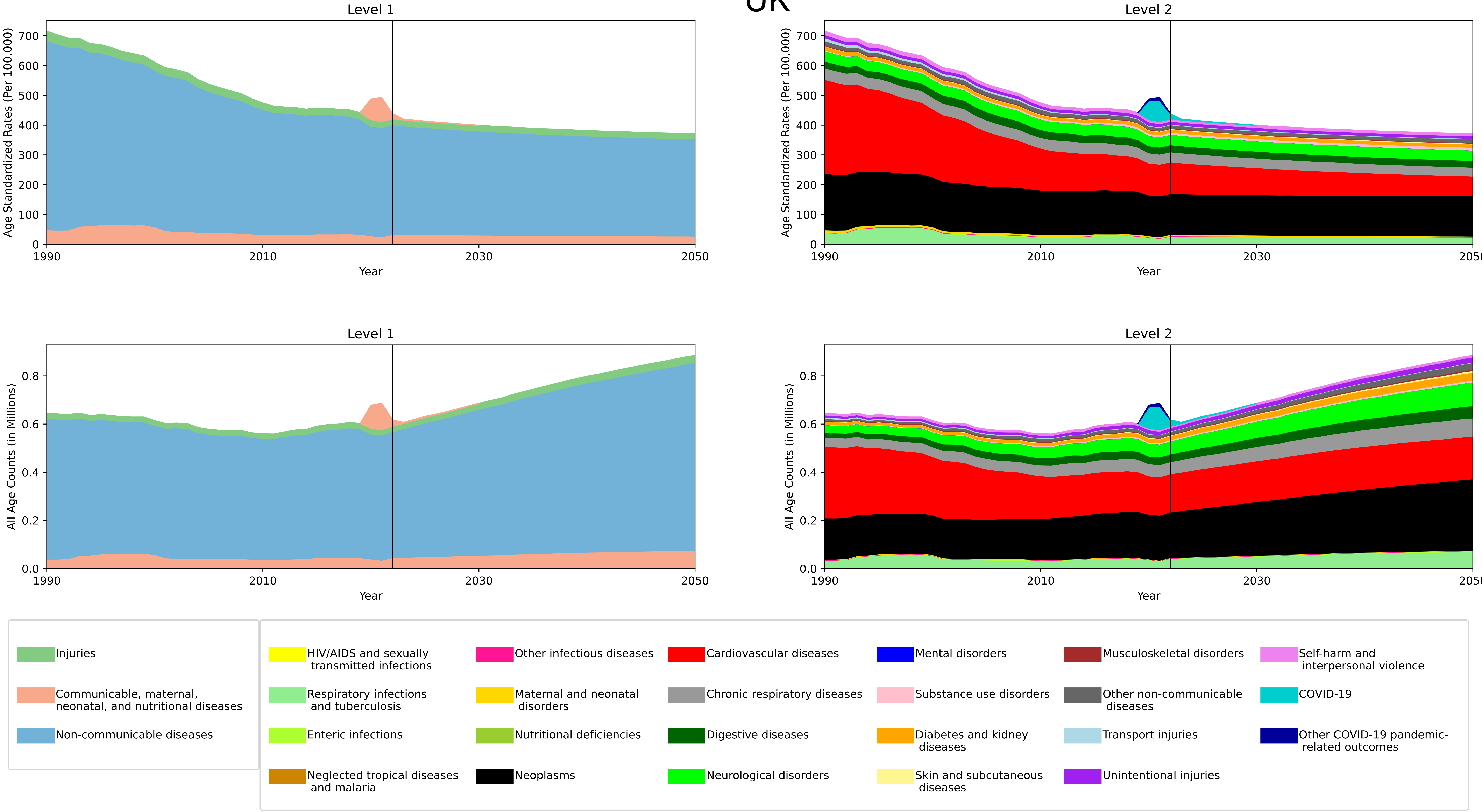
Level 1
Age Standardized Rates (Per 100,000)
Year
Level 2
Age Standardized Rates (Per 100,000)
Year
Level 1
All Age Counts (in Millions)
Year
Level 2
All Age Counts (in Millions)
Year
Injuries
Communicable, maternal, neonatal, and nutritional diseases
Non-communicable diseases
HIV/AIDS and sexually transmitted infections
Respiratory infections and tuberculosis
Enteric infections
Neglected tropical diseases and malaria
Other infectious diseases
Maternal and neonatal disorders
Nutritional deficiencies
Neoplasms
Cardiovascular diseases
Chronic respiratory diseases
Digestive diseases
Neurological disorders
Mental disorders
Substance use disorders
Diabetes and kidney diseases
Skin and subcutaneous diseases
Musculoskeletal disorders
Other non-communicable diseases
Transport injuries
Unintentional injuries
Self-harm and interpersonal violence
COVID-19
Other COVID-19 pandemic-related outcomes

Bolivia

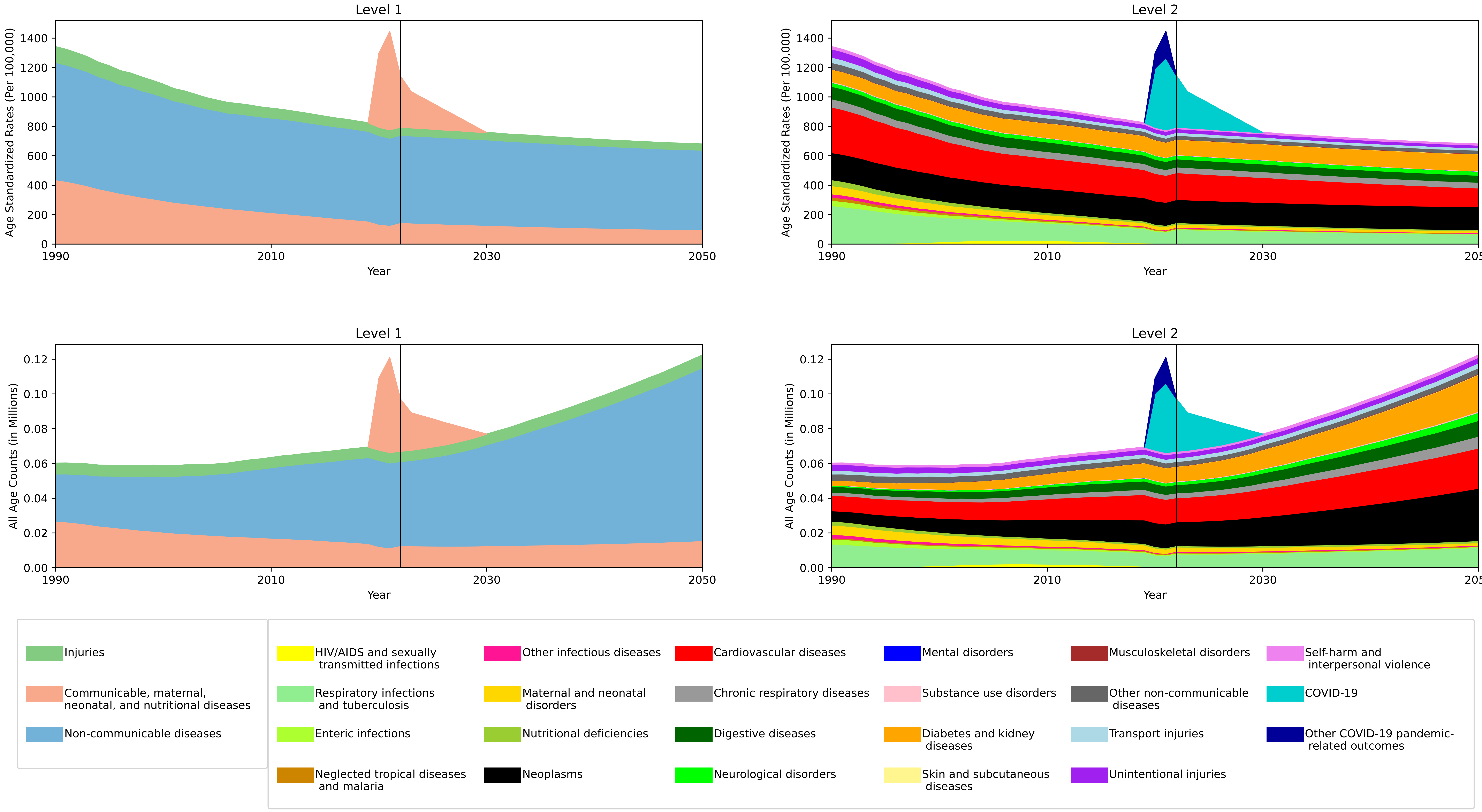
Level 1
Level 2
Age Standardized Rates (Per 100,000)
All Age Counts (in Millions)
Year
1990
2010
2030
2050
Injuries
Communicable, maternal, neonatal, and nutritional diseases
Non-communicable diseases
HIV/AIDS and sexually transmitted infections
Respiratory infections and tuberculosis
Enteric infections
Neglected tropical diseases and malaria
Other infectious diseases
Maternal and neonatal disorders
Nutritional deficiencies
Neoplasms
Cardiovascular diseases
Chronic respiratory diseases
Digestive diseases
Neurological disorders
Mental disorders
Substance use disorders
Diabetes and kidney diseases
Skin and subcutaneous diseases
Musculoskeletal disorders
Other non-communicable diseases
Transport injuries
Unintentional injuries
Self-harm and interpersonal violence
COVID-19
Other COVID-19 pandemic-related outcomes

Ecuador

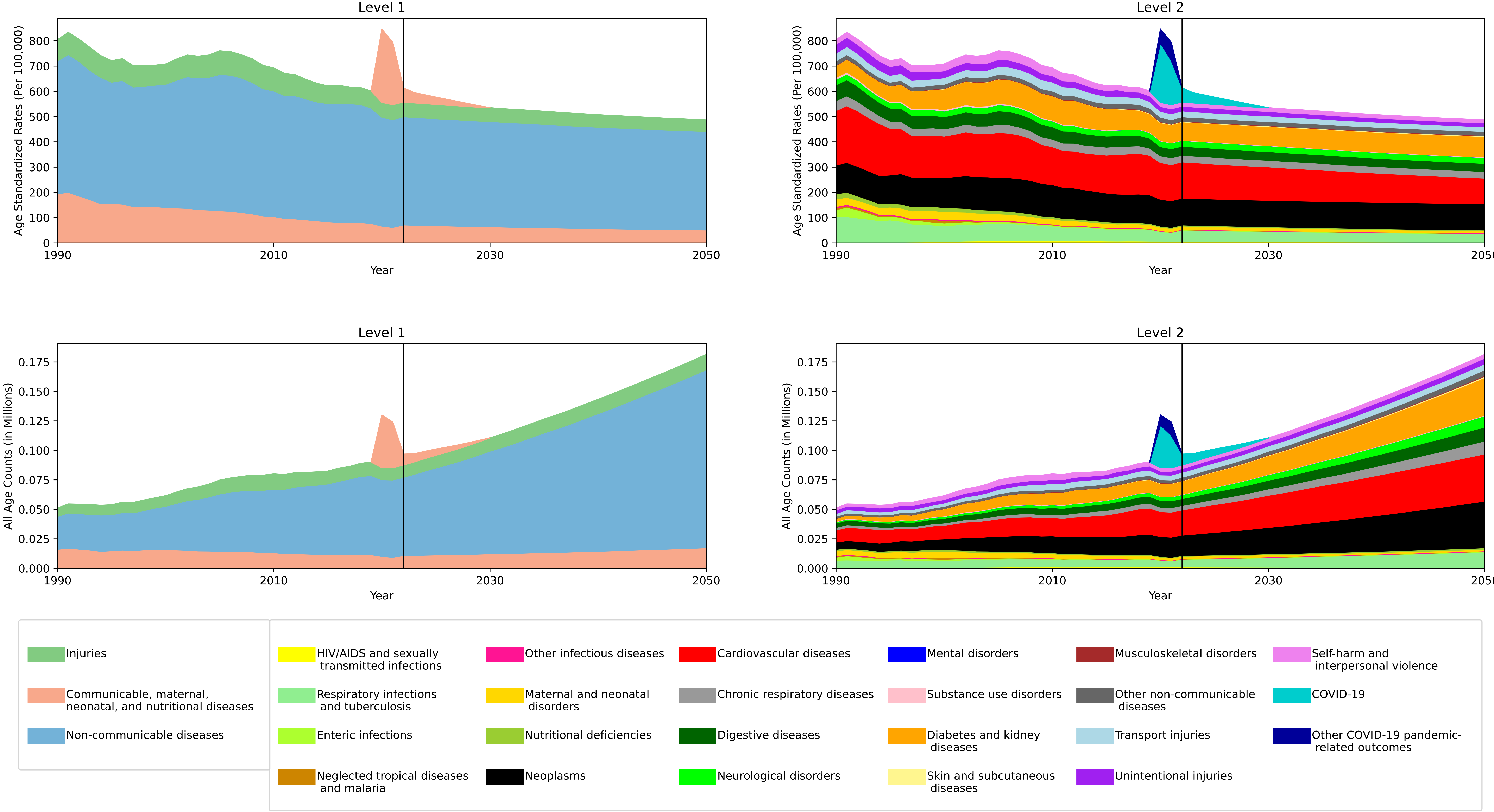
Level 1
Level 2
Age Standardized Rates (Per 100,000)
All Age Counts (in Millions)
Year
1990
2010
2030
2050
0
100
200
300
400
500
600
700
800
0.000
0.025
0.050
0.075
0.100
0.125
0.150
0.175
Injuries
Communicable, maternal, neonatal, and nutritional diseases
Non-communicable diseases
HIV/AIDS and sexually transmitted infections
Respiratory infections and tuberculosis
Enteric infections
Neglected tropical diseases and malaria
Other infectious diseases
Maternal and neonatal disorders
Nutritional deficiencies
Neoplasms
Cardiovascular diseases
Chronic respiratory diseases
Digestive diseases
Neurological disorders
Mental disorders
Substance use disorders
Diabetes and kidney diseases
Skin and subcutaneous diseases
Musculoskeletal disorders
Other non-communicable diseases
Transport injuries
Unintentional injuries
Self-harm and interpersonal violence
COVID-19
Other COVID-19 pandemic-related outcomes

Peru

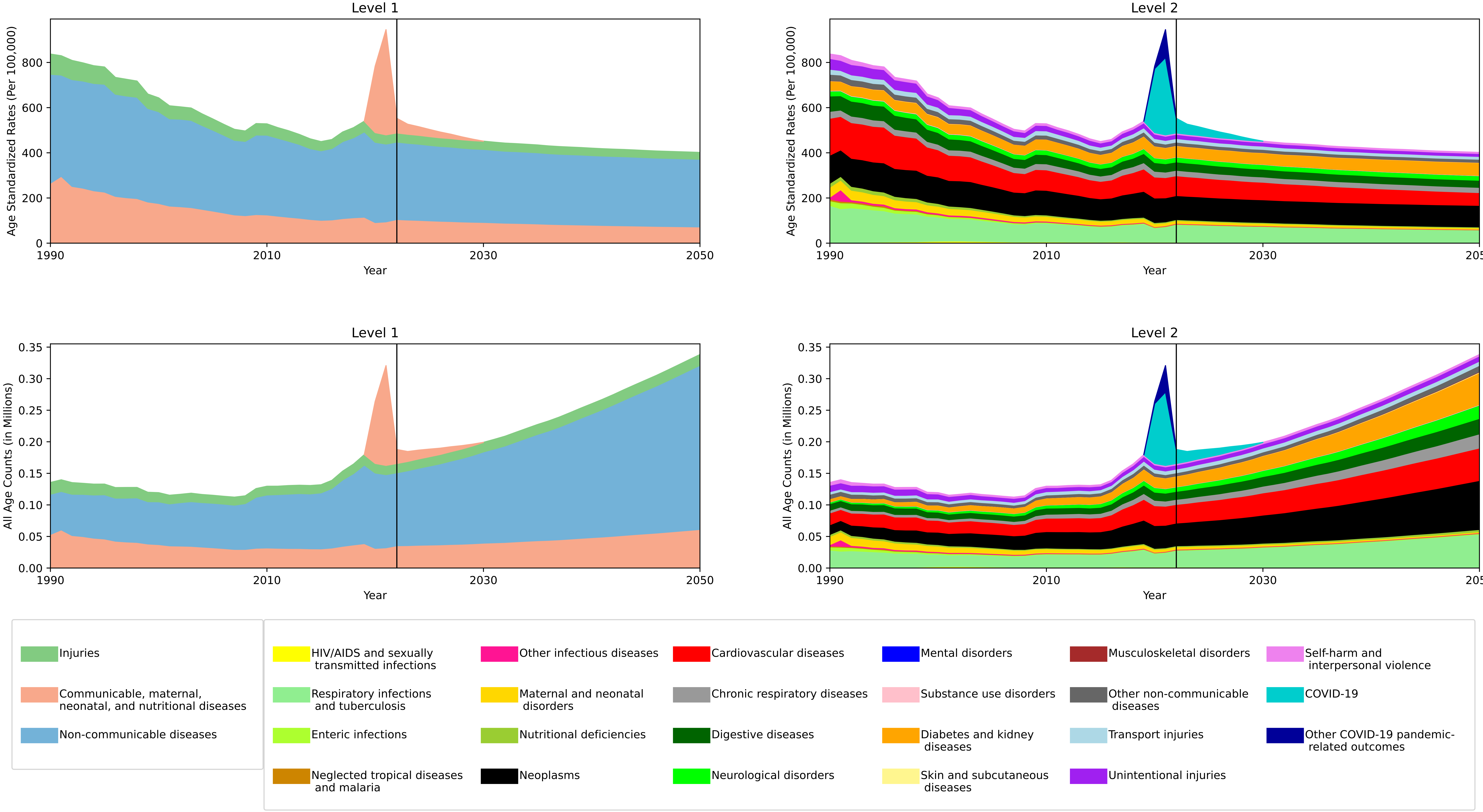
Level 1
Level 2
Age Standardized Rates (Per 100,000)
All Age Counts (in Millions)
Year
Injuries
Communicable, maternal, neonatal, and nutritional diseases
Non-communicable diseases
HIV/AIDS and sexually transmitted infections
Respiratory infections and tuberculosis
Enteric infections
Neglected tropical diseases and malaria
Other infectious diseases
Maternal and neonatal disorders
Nutritional deficiencies
Neoplasms
Cardiovascular diseases
Chronic respiratory diseases
Digestive diseases
Neurological disorders
Mental disorders
Substance use disorders
Diabetes and kidney diseases
Skin and subcutaneous diseases
Musculoskeletal disorders
Other non-communicable diseases
Transport injuries
Unintentional injuries
Self-harm and interpersonal violence
COVID-19
Other COVID-19 pandemic-related outcomes

Antigua And Barbuda

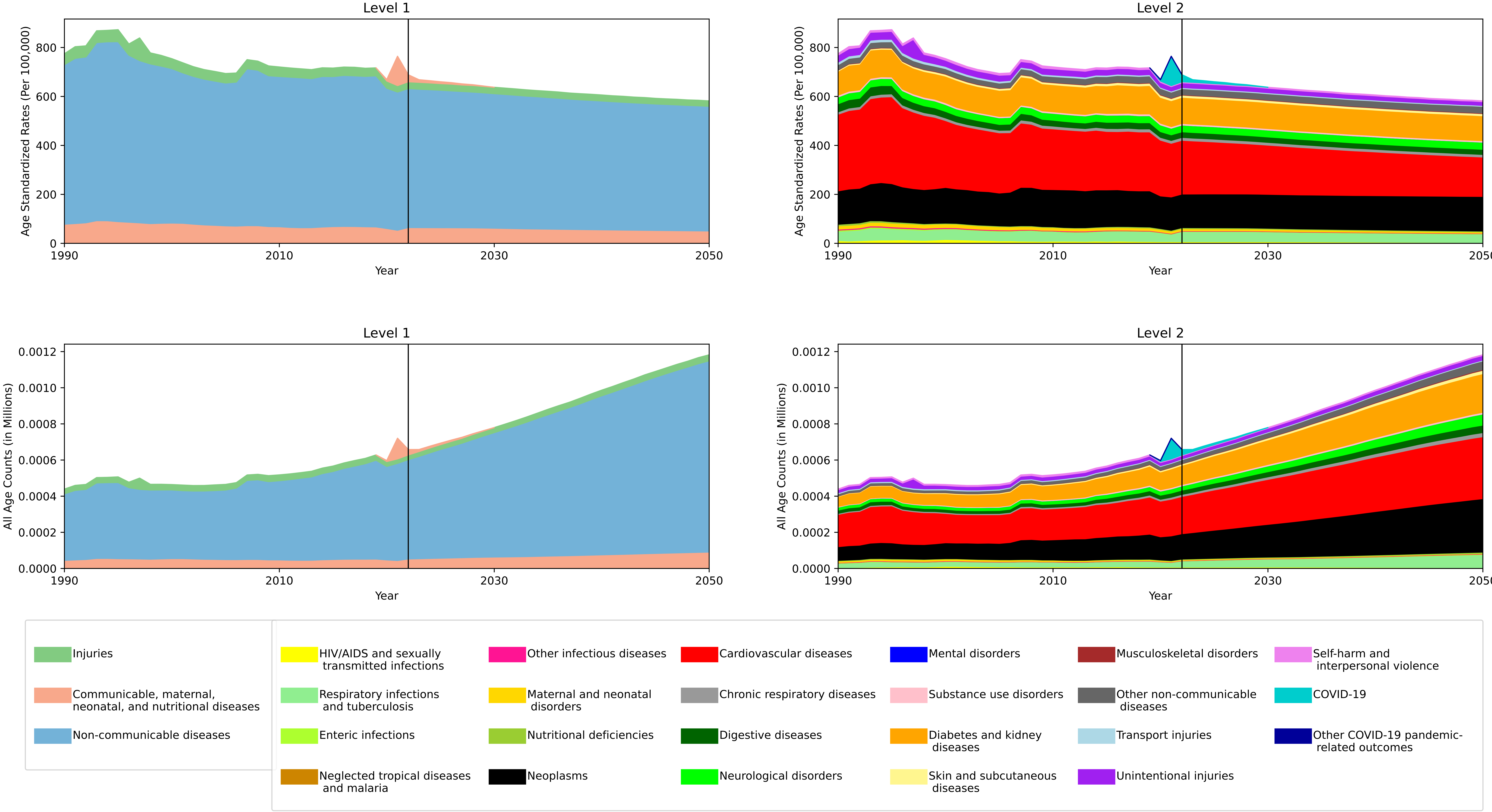

The Bahamas

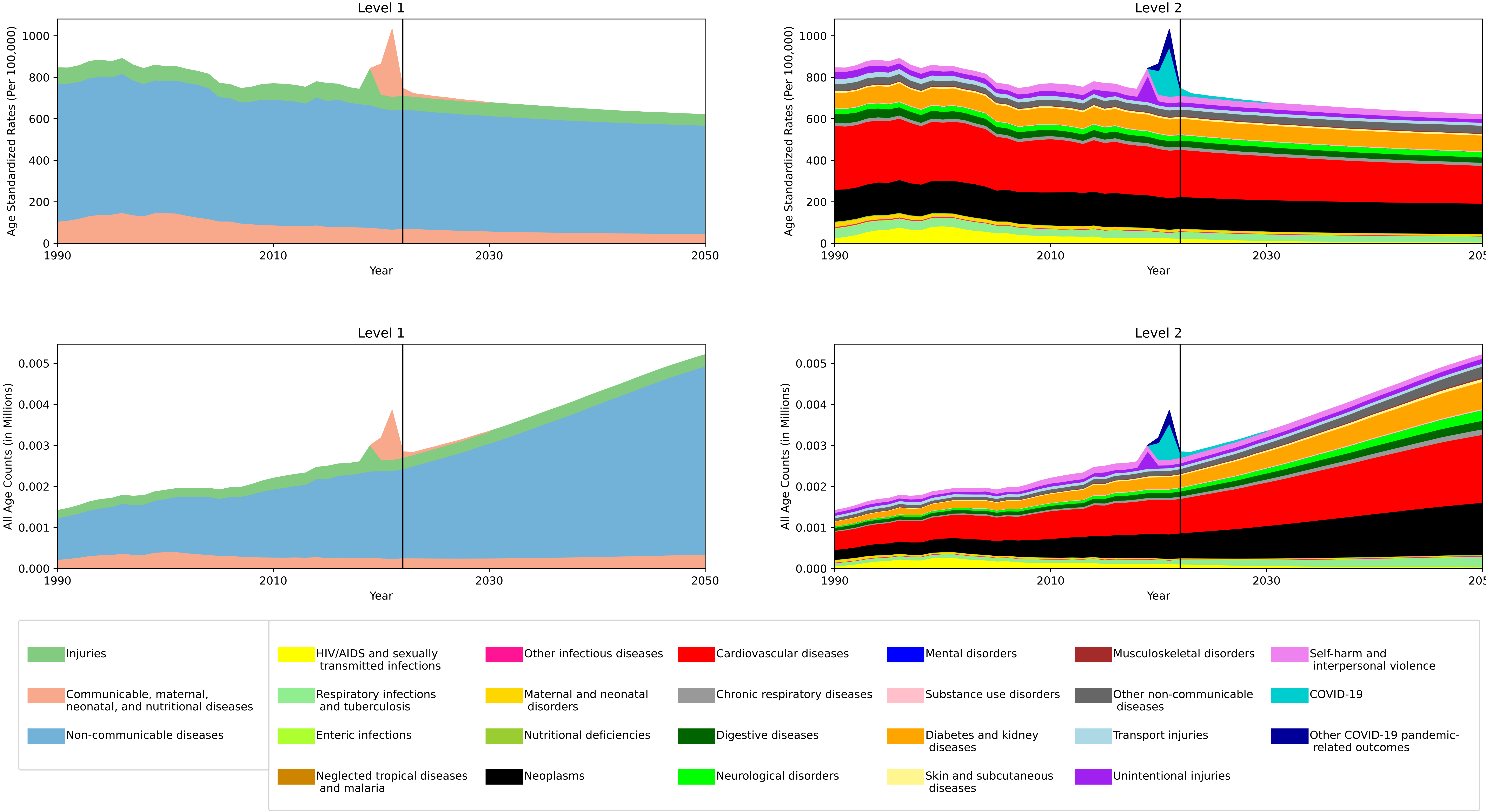
Level 1
Level 2
Age Standardized Rates (Per 100,000)
All Age Counts (in Millions)
Year
1990
2010
2030
2050
Injuries
Communicable, maternal, neonatal, and nutritional diseases
Non-communicable diseases
HIV/AIDS and sexually transmitted infections
Respiratory infections and tuberculosis
Enteric infections
Neglected tropical diseases and malaria
Other infectious diseases
Maternal and neonatal disorders
Nutritional deficiencies
Neoplasms
Cardiovascular diseases
Chronic respiratory diseases
Digestive diseases
Neurological disorders
Mental disorders
Substance use disorders
Diabetes and kidney diseases
Skin and subcutaneous diseases
Musculoskeletal disorders
Other non-communicable diseases
Transport injuries
Unintentional injuries
Self-harm and interpersonal violence
COVID-19
Other COVID-19 pandemic-related outcomes

Barbados

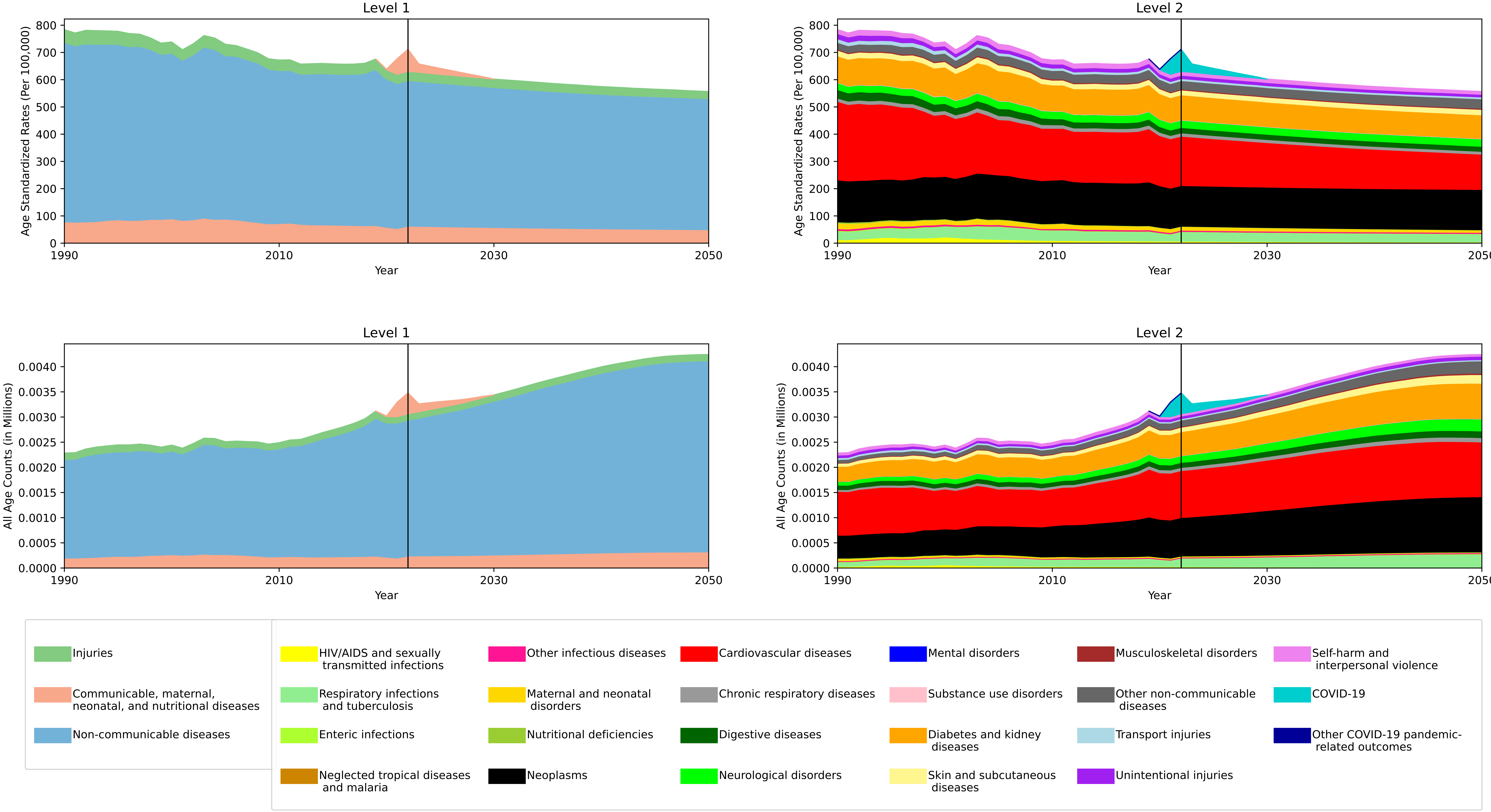
Level 1
Level 2
Age Standardized Rates (Per 100,000)
All Age Counts (in Millions)
Year
1990
2010
2030
2050
Injuries
Communicable, maternal, neonatal, and nutritional diseases
Non-communicable diseases
HIV/AIDS and sexually transmitted infections
Respiratory infections and tuberculosis
Enteric infections
Neglected tropical diseases and malaria
Other infectious diseases
Maternal and neonatal disorders
Nutritional deficiencies
Neoplasms
Cardiovascular diseases
Chronic respiratory diseases
Digestive diseases
Neurological disorders
Mental disorders
Substance use disorders
Diabetes and kidney diseases
Skin and subcutaneous diseases
Musculoskeletal disorders
Other non-communicable diseases
Transport injuries
Unintentional injuries
Self-harm and interpersonal violence
COVID-19
Other COVID-19 pandemic-related outcomes

Belize

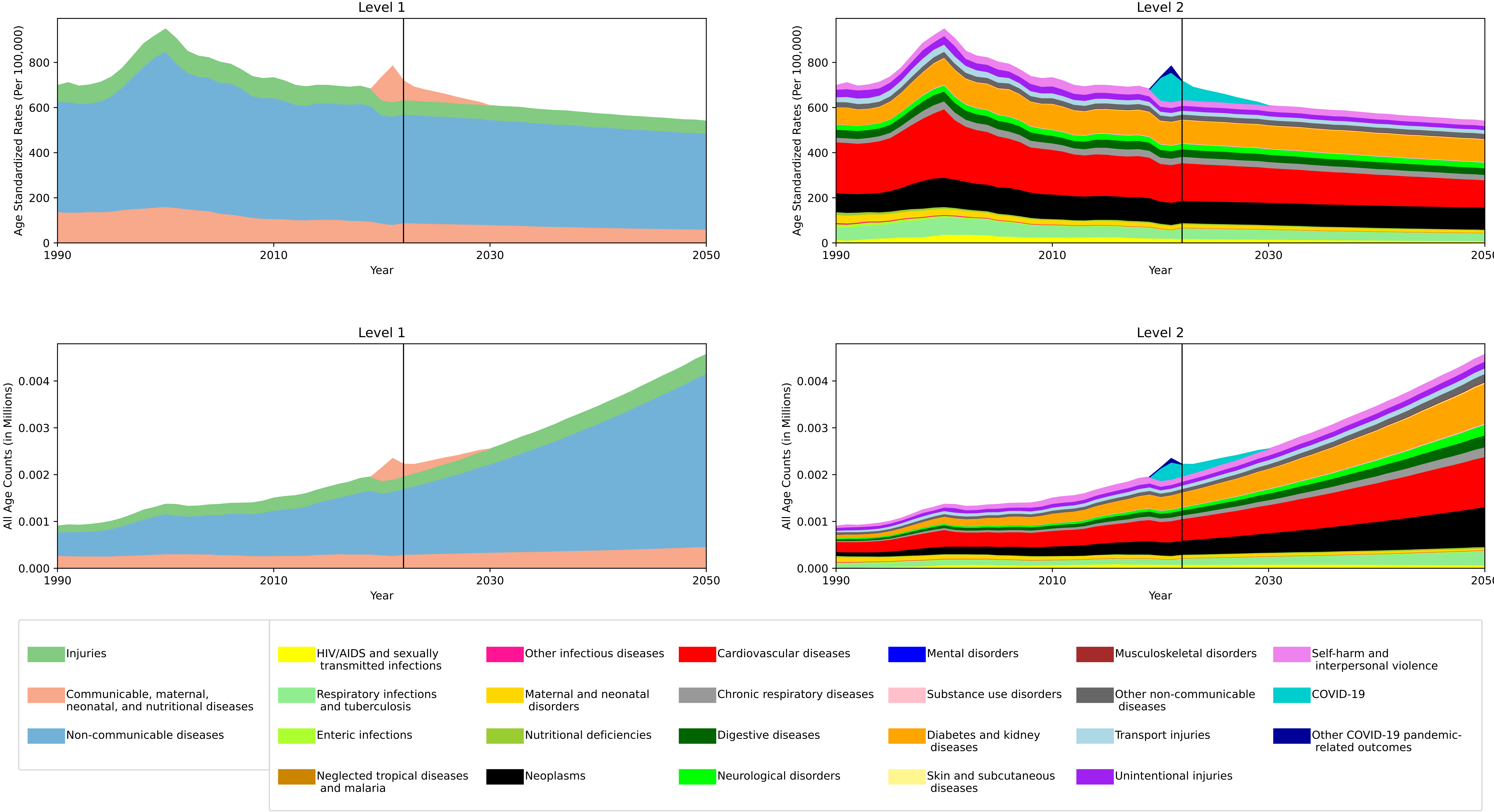

Level 1
Level 2
Age Standardized Rates (Per 100,000)
All Age Counts (in Millions)
Year
1990
2010
2030
2050
Injuries
Communicable, maternal, neonatal, and nutritional diseases
Non-communicable diseases
HIV/AIDS and sexually transmitted infections
Respiratory infections and tuberculosis
Enteric infections
Neglected tropical diseases and malaria
Other infectious diseases
Maternal and neonatal disorders
Nutritional deficiencies
Neoplasms
Cardiovascular diseases
Chronic respiratory diseases
Digestive diseases
Neurological disorders
Mental disorders
Substance use disorders
Diabetes and kidney diseases
Skin and subcutaneous diseases
Musculoskeletal disorders
Other non-communicable diseases
Transport injuries
Unintentional injuries
Self-harm and interpersonal violence
COVID-19
Other COVID-19 pandemic-related outcomes

Bermuda

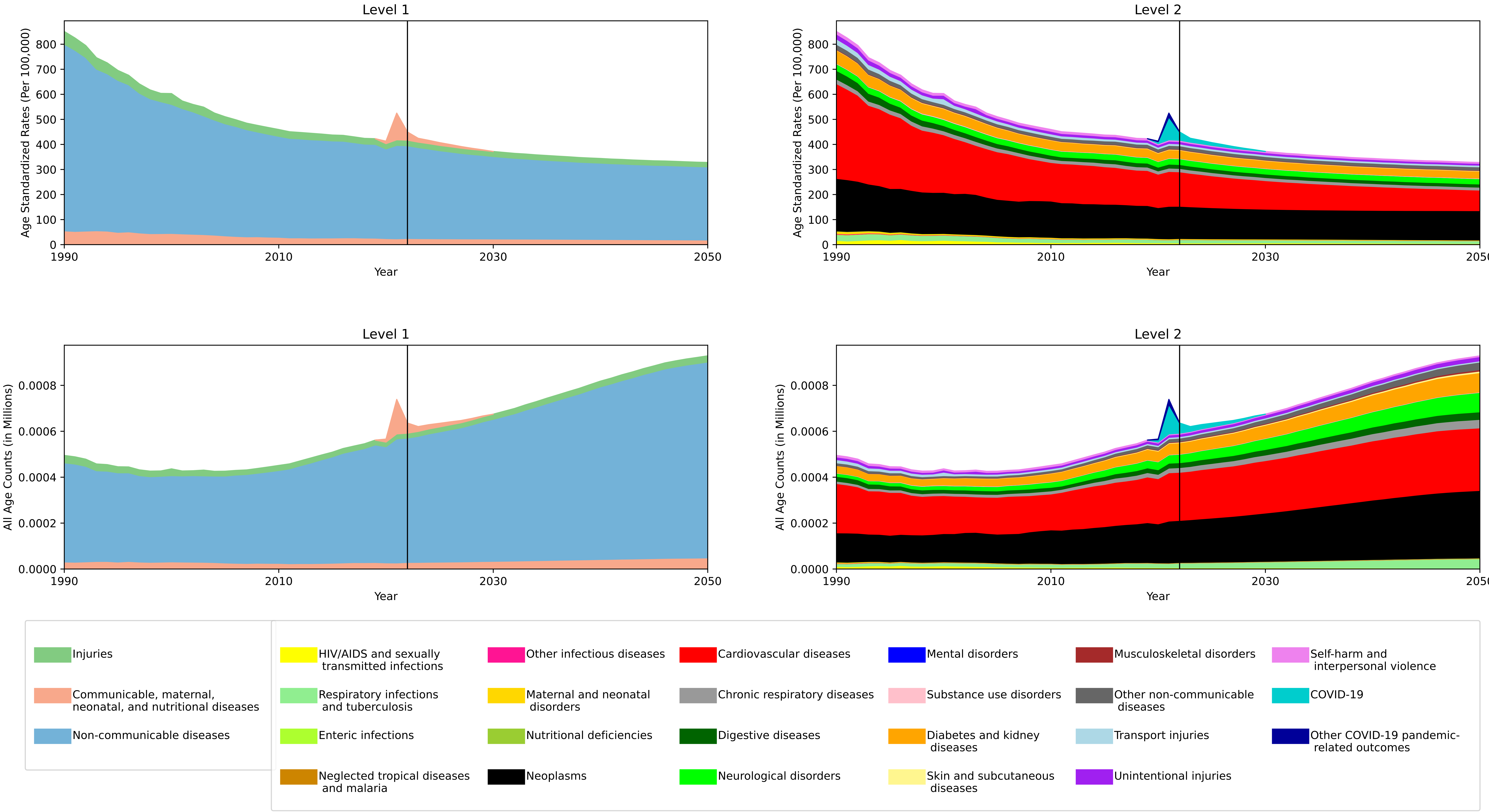
Level 1
Level 2
Age Standardized Rates (Per 100,000)
All Age Counts (in Millions)
Year
1990
2010
2030
2050
Injuries
Communicable, maternal, neonatal, and nutritional diseases
Non-communicable diseases
HIV/AIDS and sexually transmitted infections
Respiratory infections and tuberculosis
Enteric infections
Neglected tropical diseases and malaria
Other infectious diseases
Maternal and neonatal disorders
Nutritional deficiencies
Neoplasms
Cardiovascular diseases
Chronic respiratory diseases
Digestive diseases
Neurological disorders
Mental disorders
Substance use disorders
Diabetes and kidney diseases
Skin and subcutaneous diseases
Musculoskeletal disorders
Other non-communicable diseases
Transport injuries
Unintentional injuries
Self-harm and interpersonal violence
COVID-19
Other COVID-19 pandemic-related outcomes

Cuba

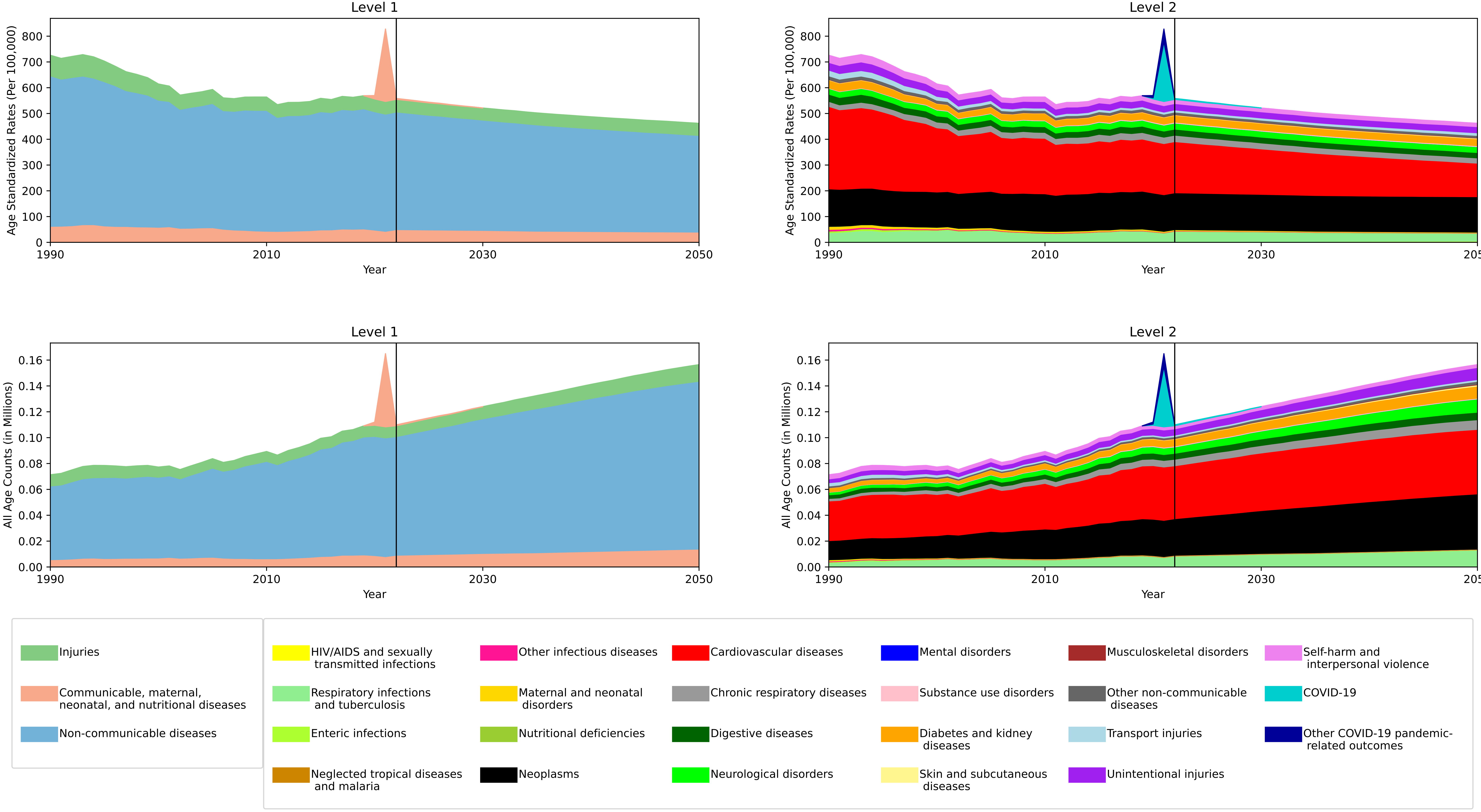
Level 1
Level 2
Age Standardized Rates (Per 100,000)
All Age Counts (in Millions)
Year
1990
2010
2030
2050
Injuries
Communicable, maternal, neonatal, and nutritional diseases
Non-communicable diseases
HIV/AIDS and sexually transmitted infections
Respiratory infections and tuberculosis
Enteric infections
Neglected tropical diseases and malaria
Other infectious diseases
Maternal and neonatal disorders
Nutritional deficiencies
Neoplasms
Cardiovascular diseases
Chronic respiratory diseases
Digestive diseases
Neurological disorders
Mental disorders
Substance use disorders
Diabetes and kidney diseases
Skin and subcutaneous diseases
Musculoskeletal disorders
Other non-communicable diseases
Transport injuries
Unintentional injuries
Self-harm and interpersonal violence
COVID-19
Other COVID-19 pandemic-related outcomes

Dominica

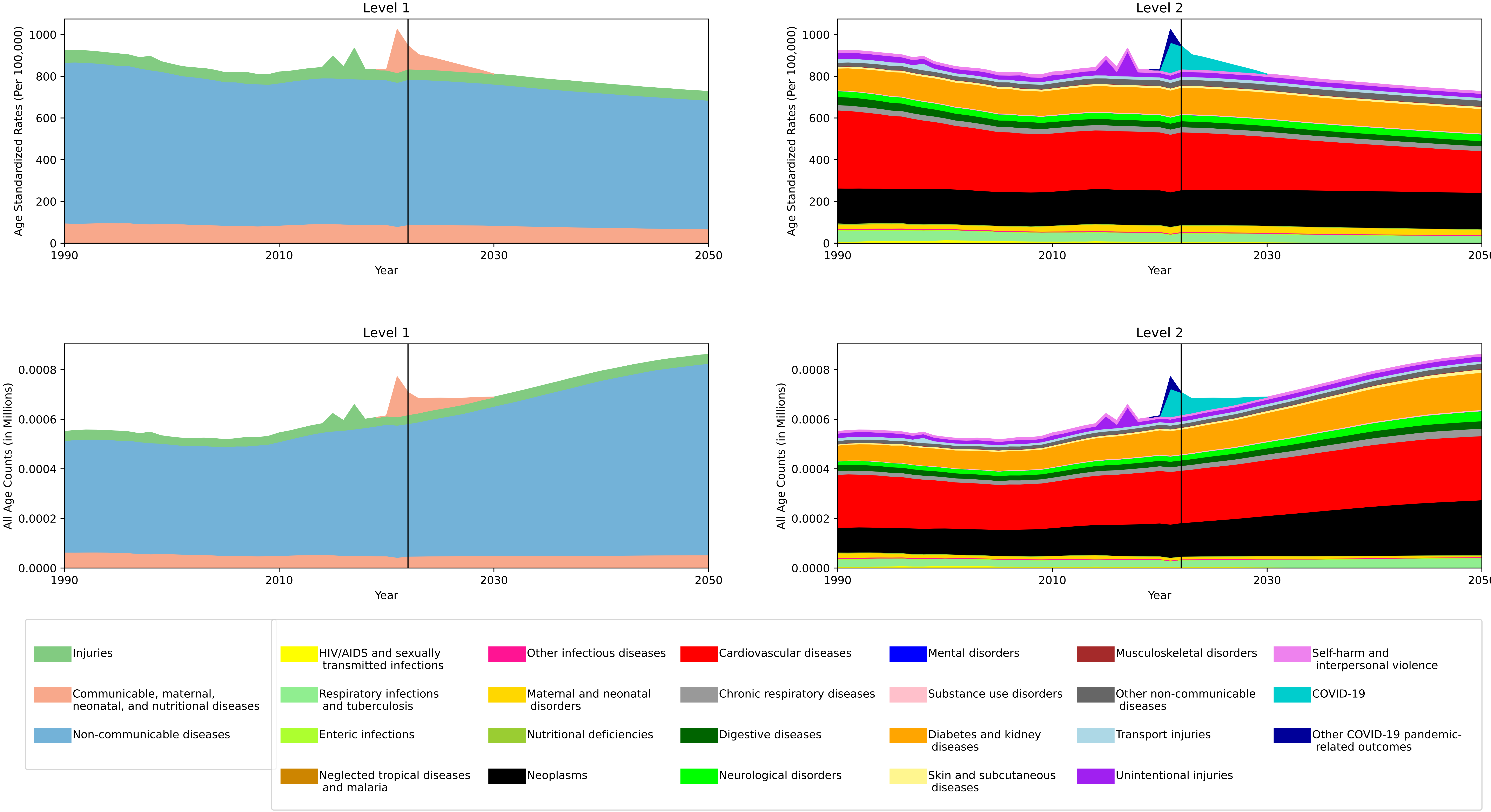
Level 1
Level 2
Age Standardized Rates (Per 100,000)
All Age Counts (in Millions)
Year
1990
2010
2030
2050
Injuries
Communicable, maternal, neonatal, and nutritional diseases
Non-communicable diseases
HIV/AIDS and sexually transmitted infections
Respiratory infections and tuberculosis
Enteric infections
Neglected tropical diseases and malaria
Other infectious diseases
Maternal and neonatal disorders
Nutritional deficiencies
Neoplasms
Cardiovascular diseases
Chronic respiratory diseases
Digestive diseases
Neurological disorders
Mental disorders
Substance use disorders
Diabetes and kidney diseases
Skin and subcutaneous diseases
Musculoskeletal disorders
Other non-communicable diseases
Transport injuries
Unintentional injuries
Self-harm and interpersonal violence
COVID-19
Other COVID-19 pandemic-related outcomes

Dominican Republic

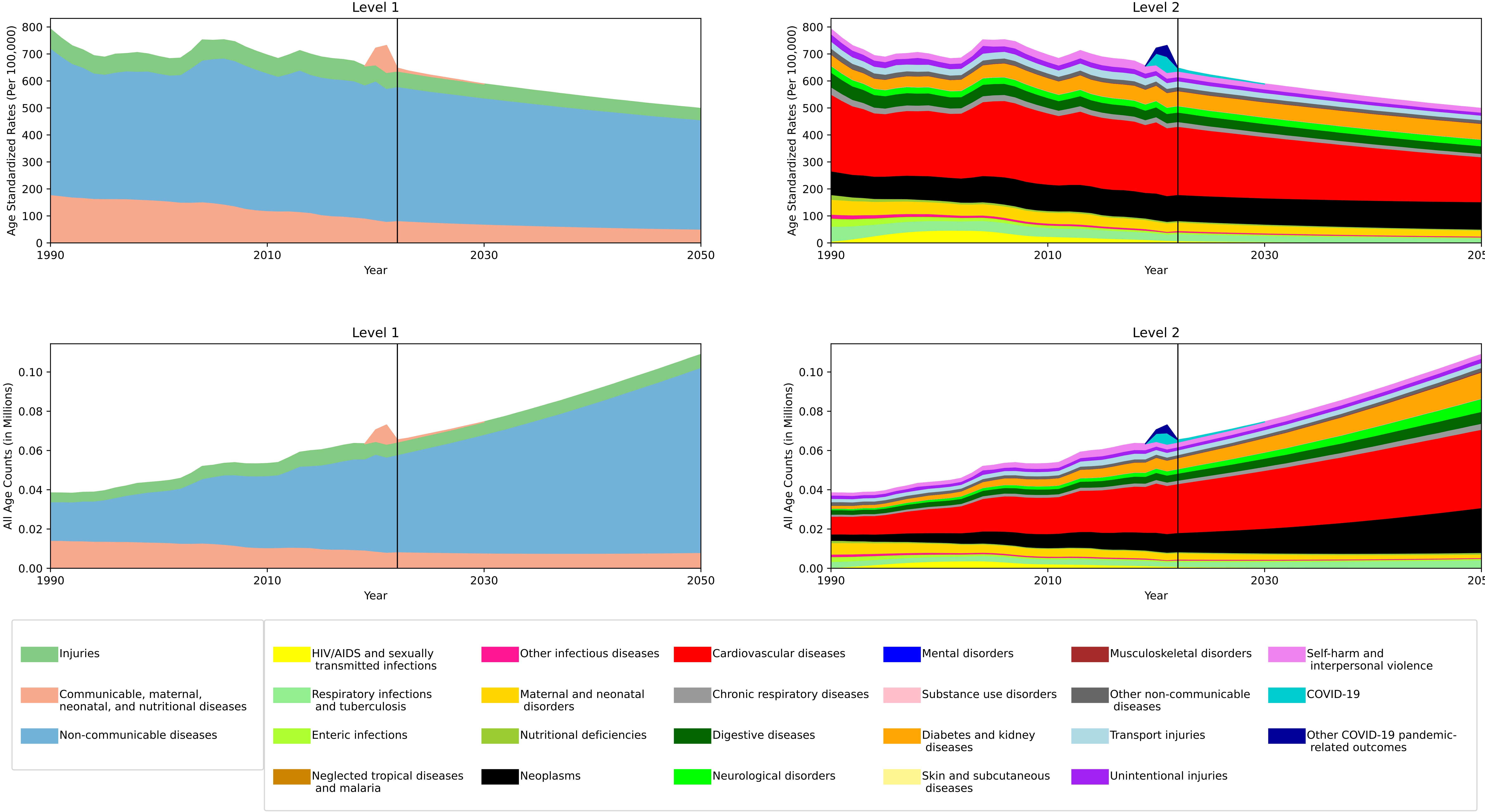

Grenada

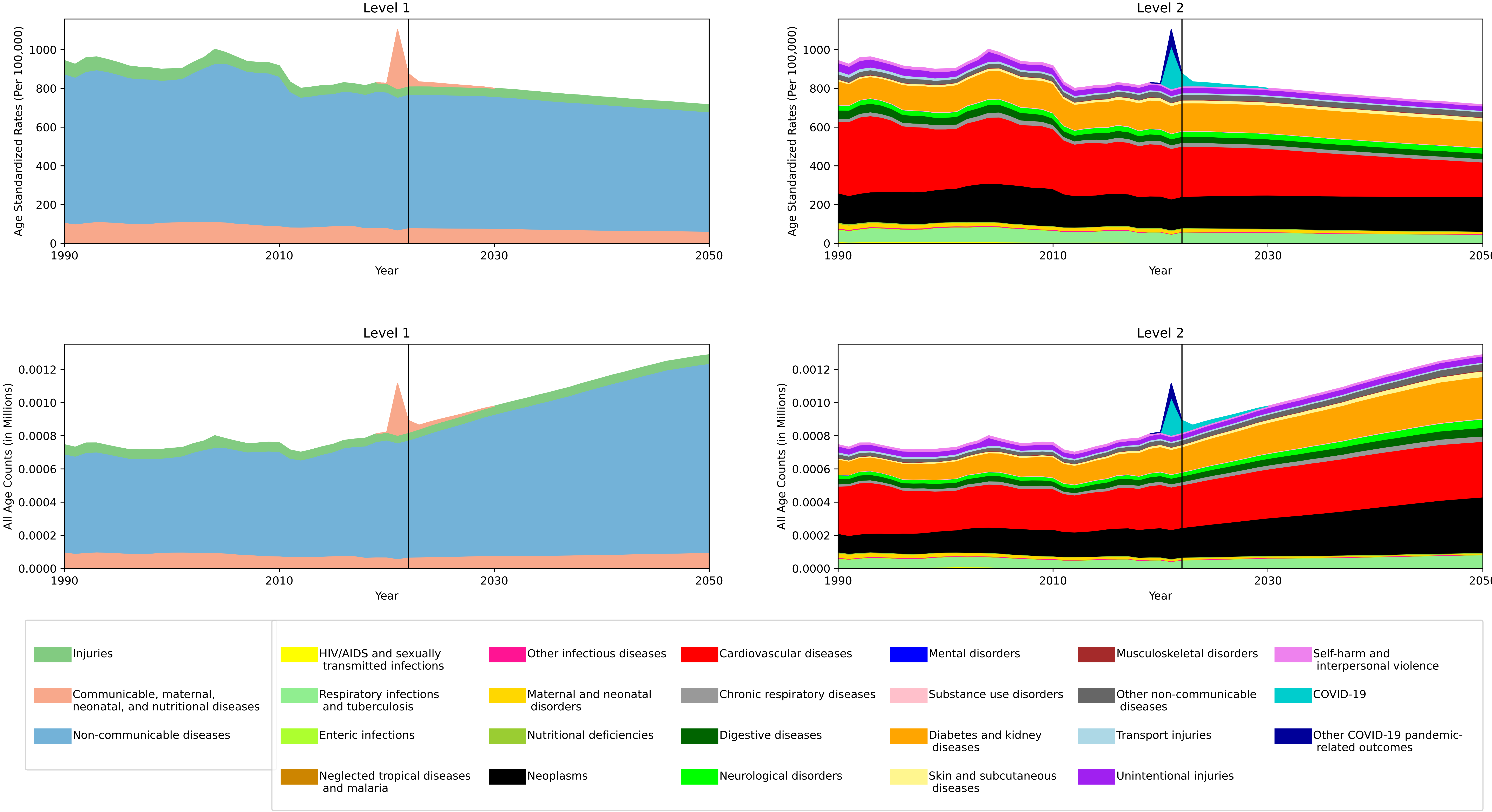
Level 1
Age Standardized Rates (Per 100,000)
Year
Level 2
Age Standardized Rates (Per 100,000)
Year
Level 1
All Age Counts (in Millions)
Year
Level 2
All Age Counts (in Millions)
Year
Injuries
Communicable, maternal, neonatal, and nutritional diseases
Non-communicable diseases
HIV/AIDS and sexually transmitted infections
Respiratory infections and tuberculosis
Enteric infections
Neglected tropical diseases and malaria
Other infectious diseases
Maternal and neonatal disorders
Nutritional deficiencies
Neoplasms
Cardiovascular diseases
Chronic respiratory diseases
Digestive diseases
Neurological disorders
Mental disorders
Substance use disorders
Diabetes and kidney diseases
Skin and subcutaneous diseases
Musculoskeletal disorders
Other non-communicable diseases
Transport injuries
Unintentional injuries
Self-harm and interpersonal violence
COVID-19
Other COVID-19 pandemic-related outcomes

Guyana

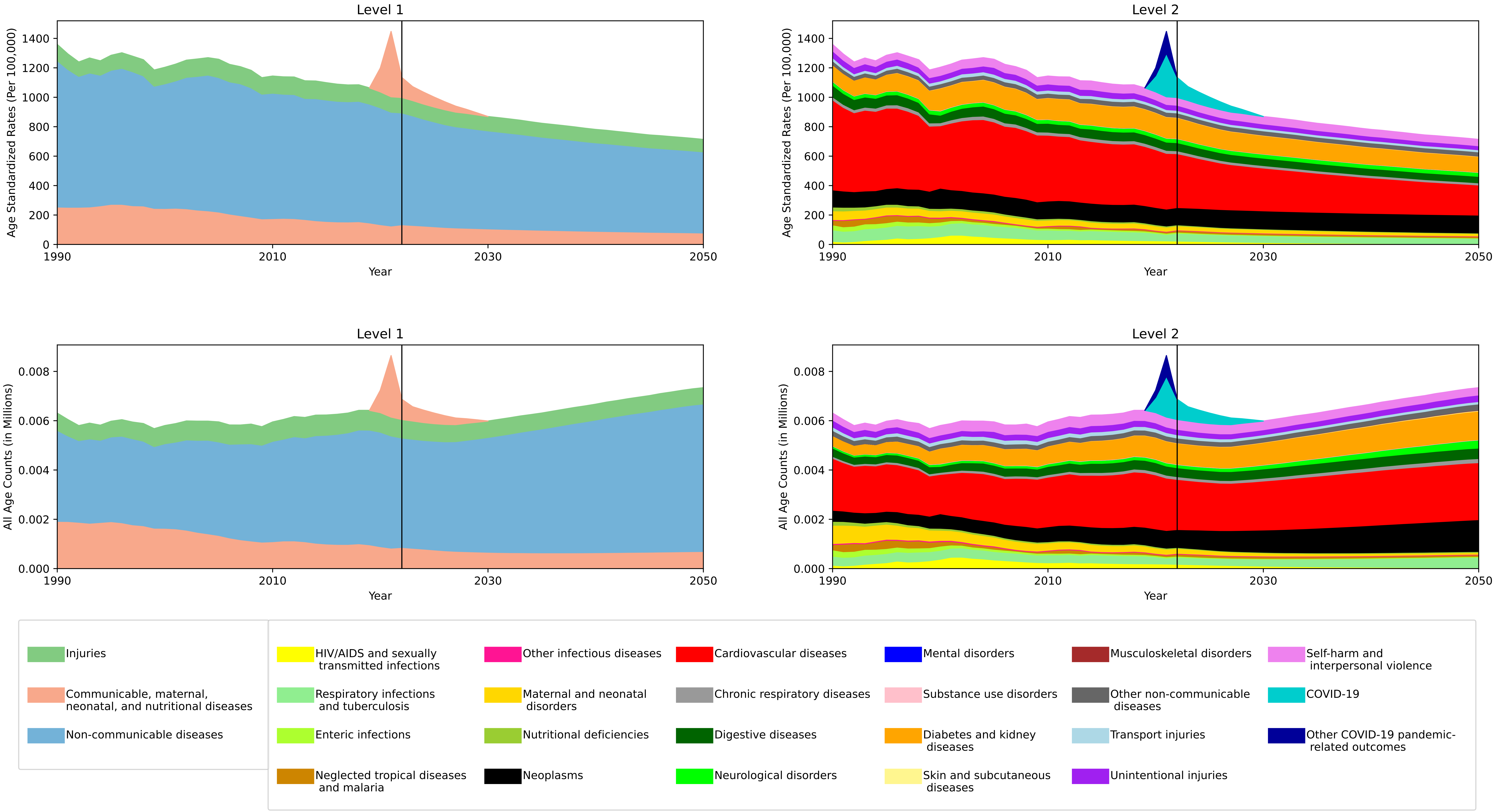
Level 1
Level 2
Age Standardized Rates (Per 100,000)
All Age Counts (in Millions)
Year
1990
2010
2030
2050
0
200
400
600
800
1000
1200
1400
0.000
0.002
0.004
0.006
0.008
Injuries
Communicable, maternal, neonatal, and nutritional diseases
Non-communicable diseases
HIV/AIDS and sexually transmitted infections
Respiratory infections and tuberculosis
Enteric infections
Neglected tropical diseases and malaria
Other infectious diseases
Maternal and neonatal disorders
Nutritional deficiencies
Neoplasms
Cardiovascular diseases
Chronic respiratory diseases
Digestive diseases
Neurological disorders
Mental disorders
Substance use disorders
Diabetes and kidney diseases
Skin and subcutaneous diseases
Musculoskeletal disorders
Other non-communicable diseases
Transport injuries
Unintentional injuries
Self-harm and interpersonal violence
COVID-19
Other COVID-19 pandemic-related outcomes

Haiti

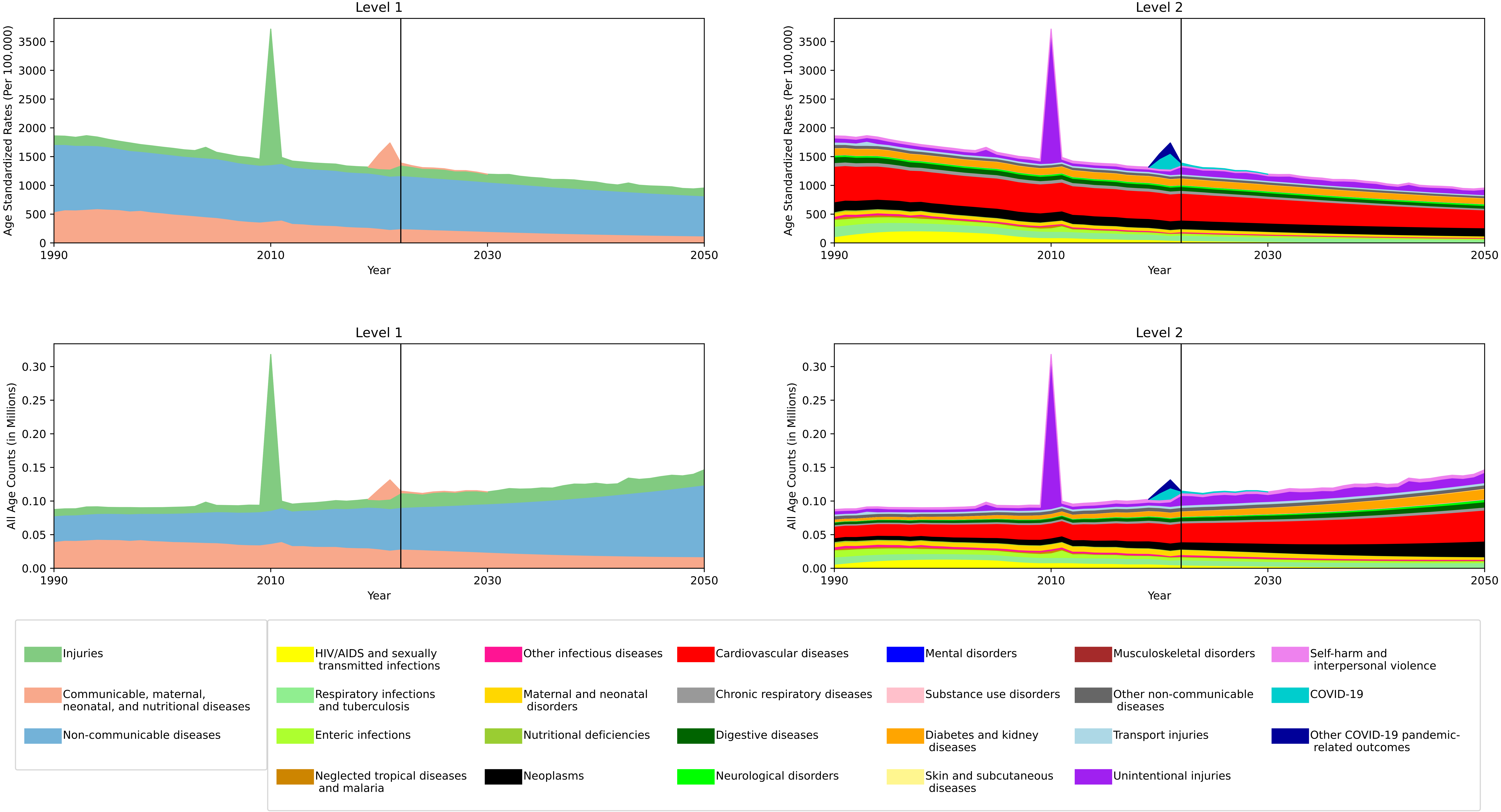
Level 1
Level 2
Age Standardized Rates (Per 100,000)
All Age Counts (in Millions)
Year
1990
2010
2030
2050
0
500
1000
1500
2000
2500
3000
3500
0.00
0.05
0.10
0.15
0.20
0.25
0.30
Injuries
Communicable, maternal, neonatal, and nutritional diseases
Non-communicable diseases
HIV/AIDS and sexually transmitted infections
Respiratory infections and tuberculosis
Enteric infections
Neglected tropical diseases and malaria
Other infectious diseases
Maternal and neonatal disorders
Nutritional deficiencies
Neoplasms
Cardiovascular diseases
Chronic respiratory diseases
Digestive diseases
Neurological disorders
Mental disorders
Substance use disorders
Diabetes and kidney diseases
Skin and subcutaneous diseases
Musculoskeletal disorders
Other non-communicable diseases
Transport injuries
Unintentional injuries
Self-harm and interpersonal violence
COVID-19
Other COVID-19 pandemic-related outcomes

Jamaica

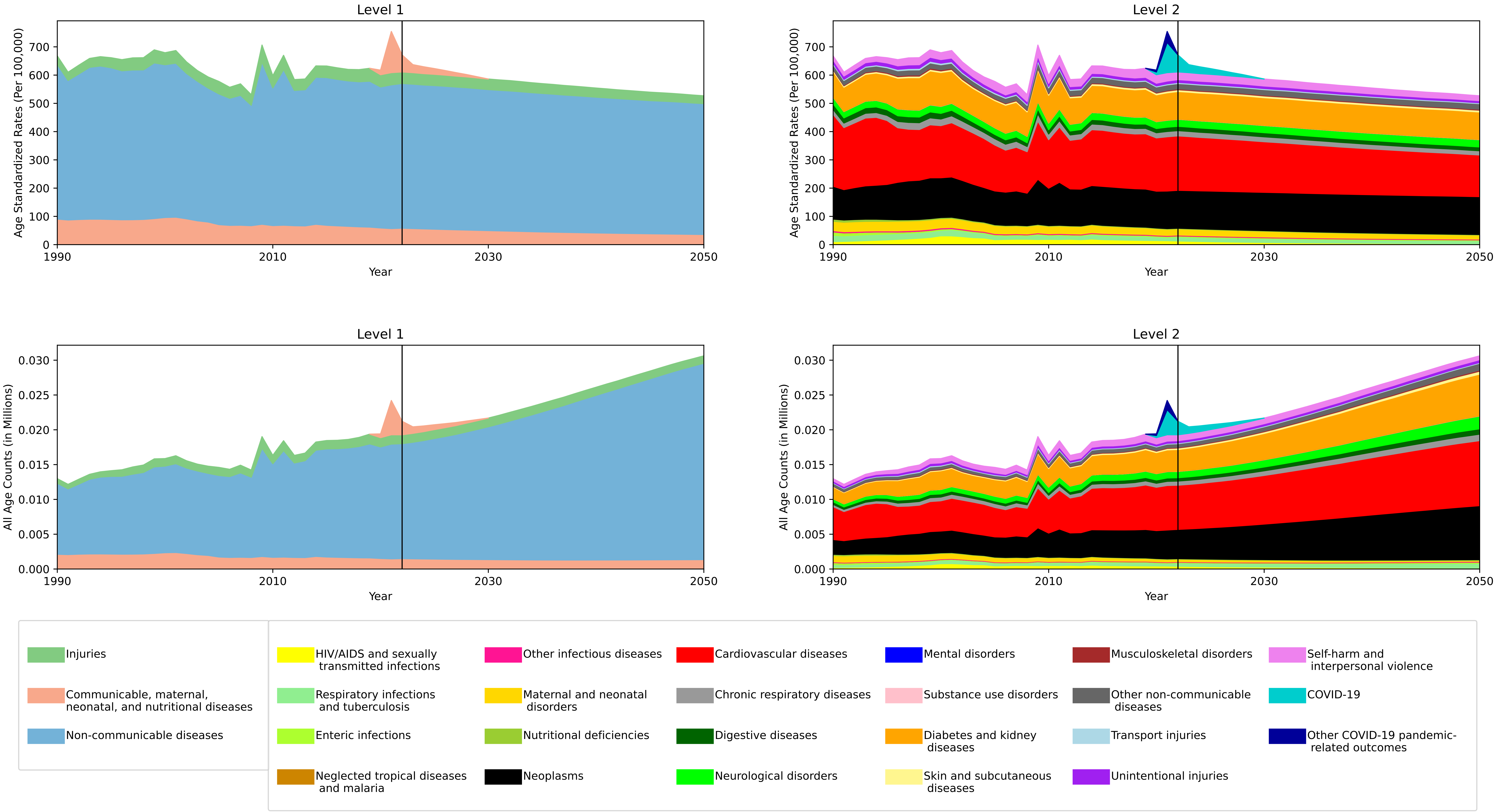
Level 1
Level 2
Age Standardized Rates (Per 100,000)
All Age Counts (in Millions)
Year
1990
2010
2030
2050
Injuries
Communicable, maternal, neonatal, and nutritional diseases
Non-communicable diseases
HIV/AIDS and sexually transmitted infections
Respiratory infections and tuberculosis
Enteric infections
Neglected tropical diseases and malaria
Other infectious diseases
Maternal and neonatal disorders
Nutritional deficiencies
Neoplasms
Cardiovascular diseases
Chronic respiratory diseases
Digestive diseases
Neurological disorders
Mental disorders
Substance use disorders
Diabetes and kidney diseases
Skin and subcutaneous diseases
Musculoskeletal disorders
Other non-communicable diseases
Transport injuries
Unintentional injuries
Self-harm and interpersonal violence
COVID-19
Other COVID-19 pandemic-related outcomes

Puerto Rico

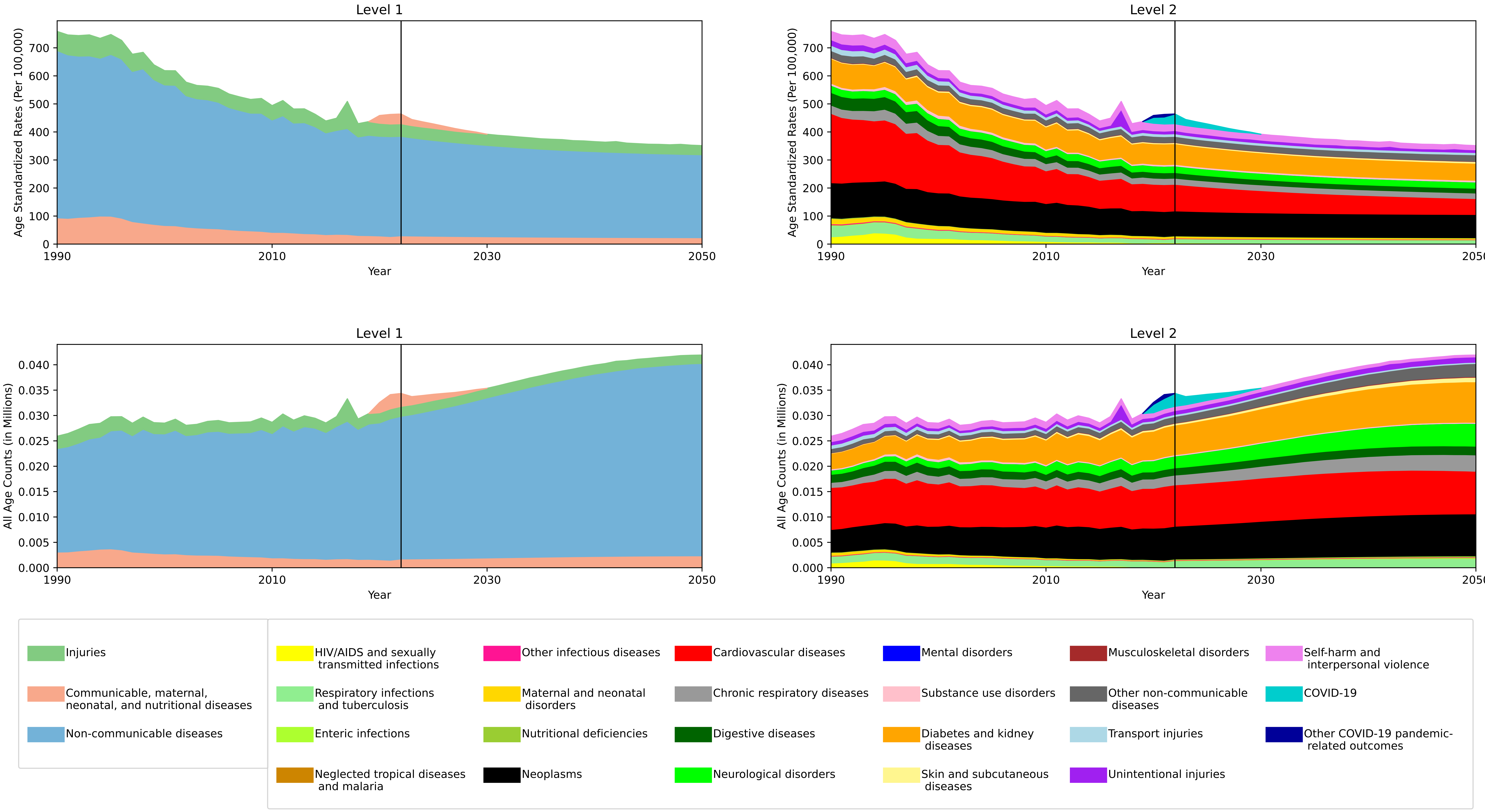

Level 1
Level 2
Age Standardized Rates (Per 100,000)
All Age Counts (in Millions)
Year
1990
2010
2030
2050
Injuries
Communicable, maternal, neonatal, and nutritional diseases
Non-communicable diseases
HIV/AIDS and sexually transmitted infections
Respiratory infections and tuberculosis
Enteric infections
Neglected tropical diseases and malaria
Other infectious diseases
Maternal and neonatal disorders
Nutritional deficiencies
Neoplasms
Cardiovascular diseases
Chronic respiratory diseases
Digestive diseases
Neurological disorders
Mental disorders
Substance use disorders
Diabetes and kidney diseases
Skin and subcutaneous diseases
Musculoskeletal disorders
Other non-communicable diseases
Transport injuries
Unintentional injuries
Self-harm and interpersonal violence
COVID-19
Other COVID-19 pandemic-related outcomes

Saint Kitts And Nevis

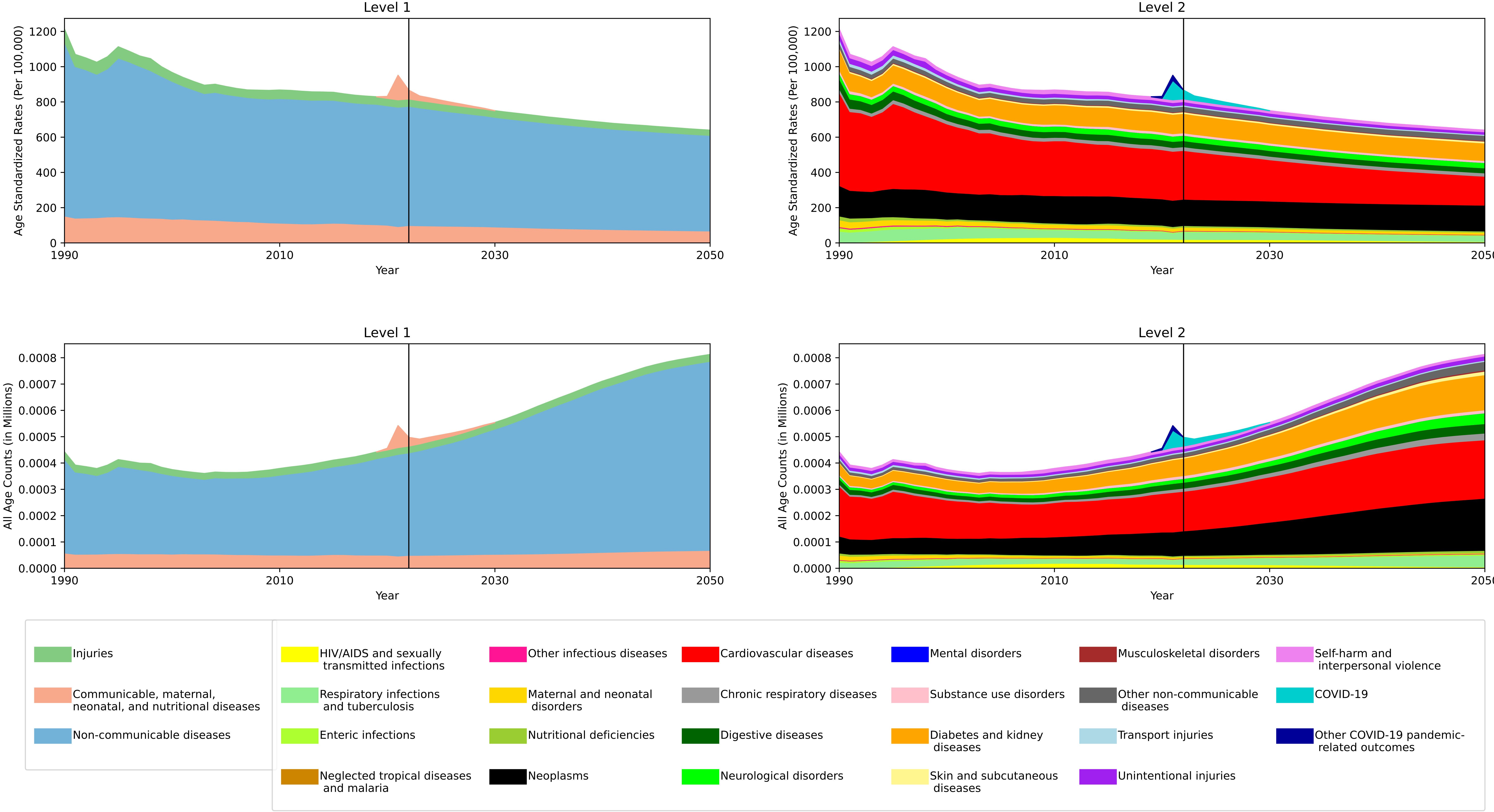

Saint Lucia

Supplemental Results Figure S5. Age-standardised death rates and all age death counts and by Level 1 and Level 2 causes, 1990–2050

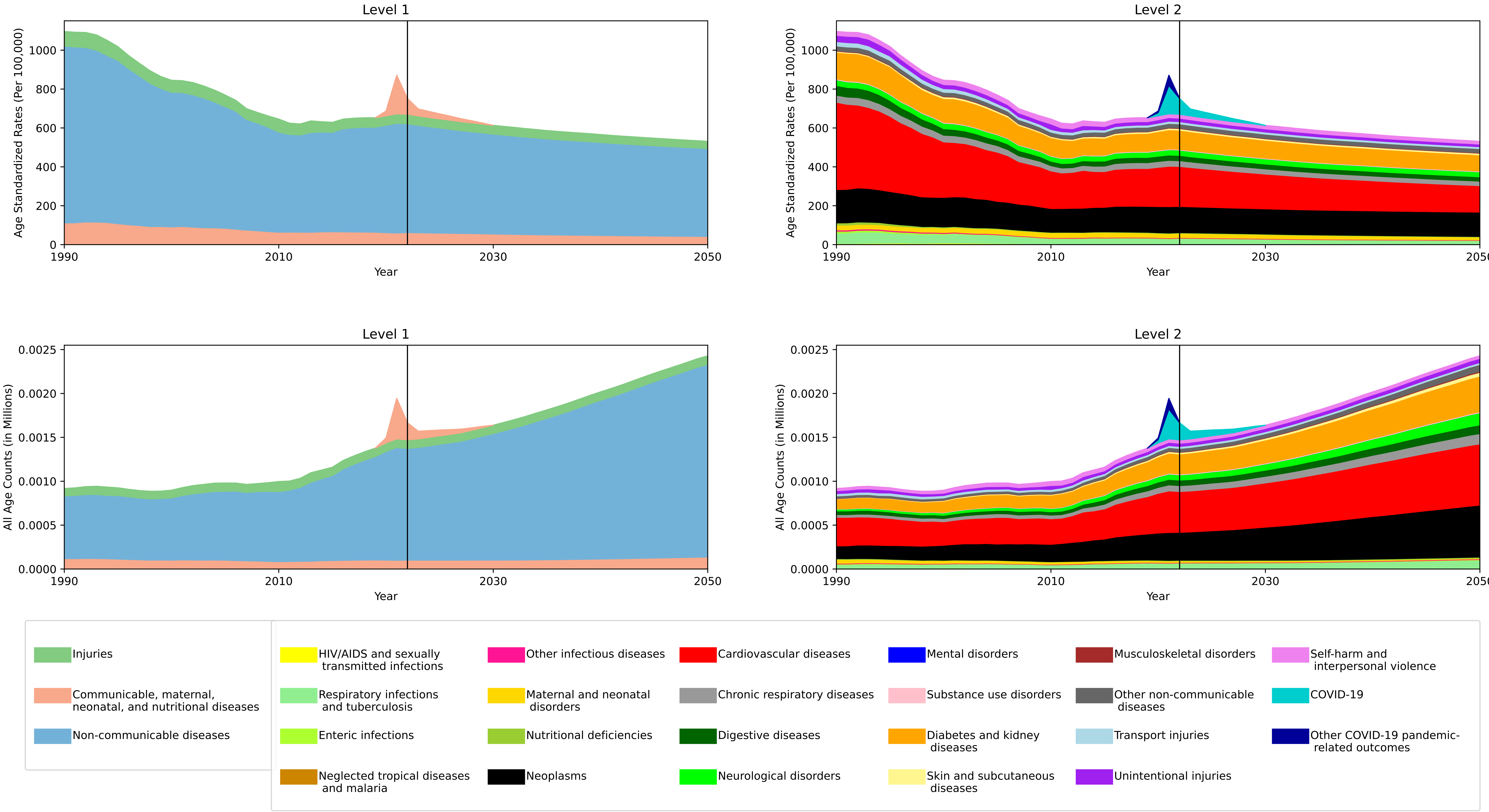

Saint Vincent And The Grenadines

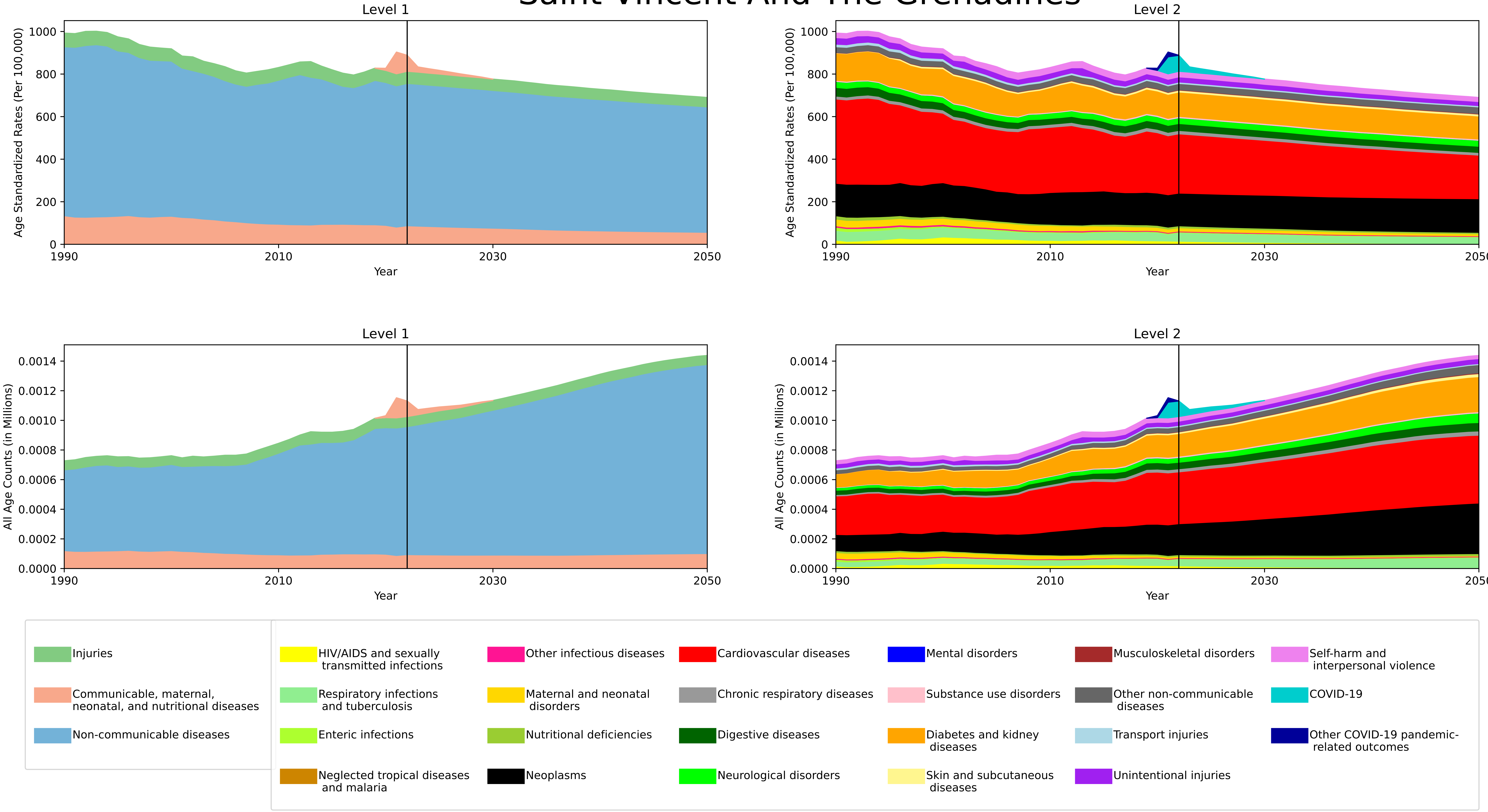

Suriname

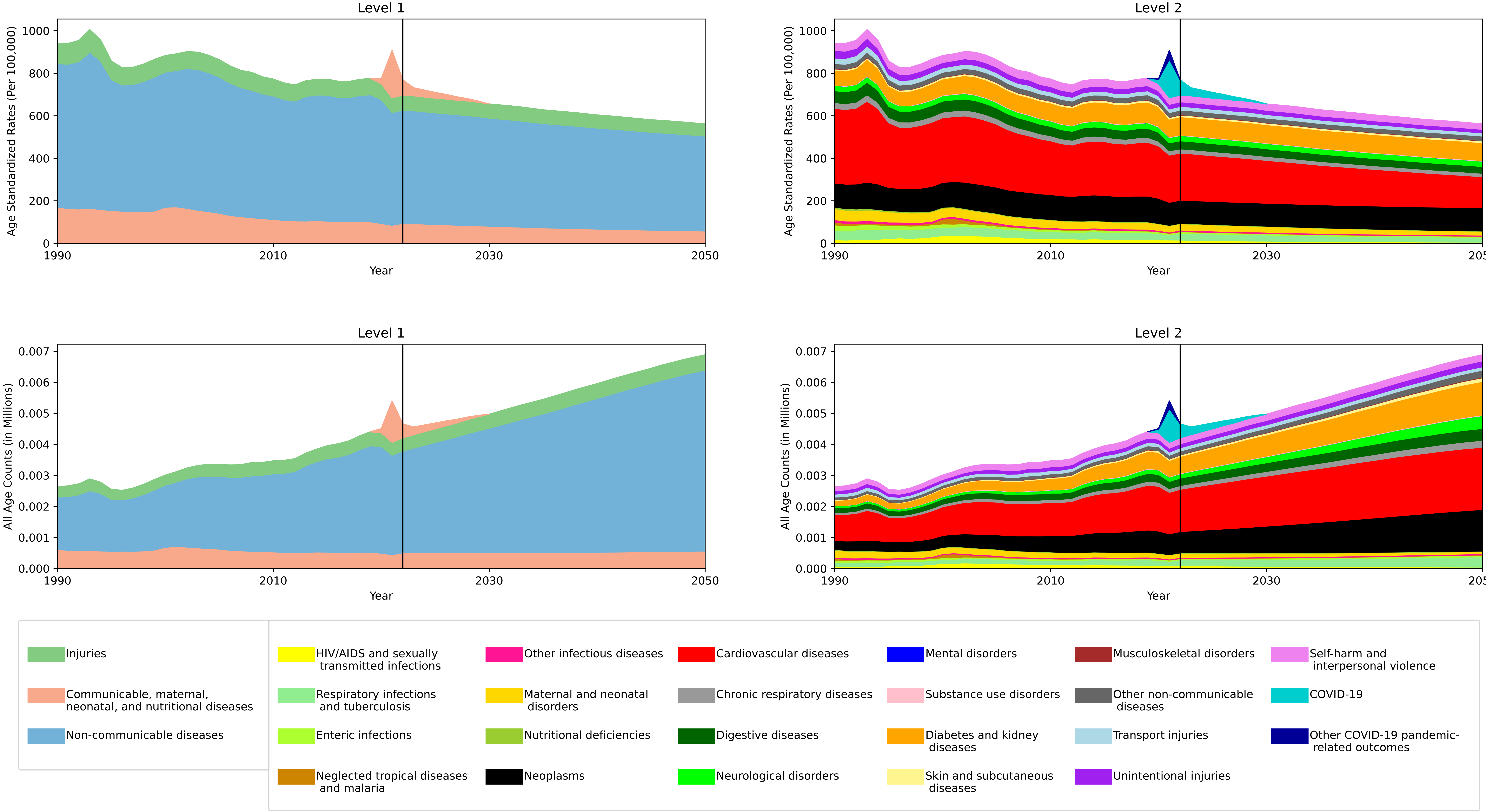
Level 1
Level 2
Age Standardized Rates (Per 100,000)
All Age Counts (in Millions)
Year
1990
2010
2030
2050
0
200
400
600
800
1000
0.000
0.001
0.002
0.003
0.004
0.005
0.006
0.007
Injuries
Communicable, maternal, neonatal, and nutritional diseases
Non-communicable diseases
HIV/AIDS and sexually transmitted infections
Respiratory infections and tuberculosis
Enteric infections
Neglected tropical diseases and malaria
Other infectious diseases
Maternal and neonatal disorders
Nutritional deficiencies
Neoplasms
Cardiovascular diseases
Chronic respiratory diseases
Digestive diseases
Neurological disorders
Mental disorders
Substance use disorders
Diabetes and kidney diseases
Skin and subcutaneous diseases
Musculoskeletal disorders
Other non-communicable diseases
Transport injuries
Unintentional injuries
Self-harm and interpersonal violence
COVID-19
Other COVID-19 pandemic-related outcomes

Trinidad And Tobago

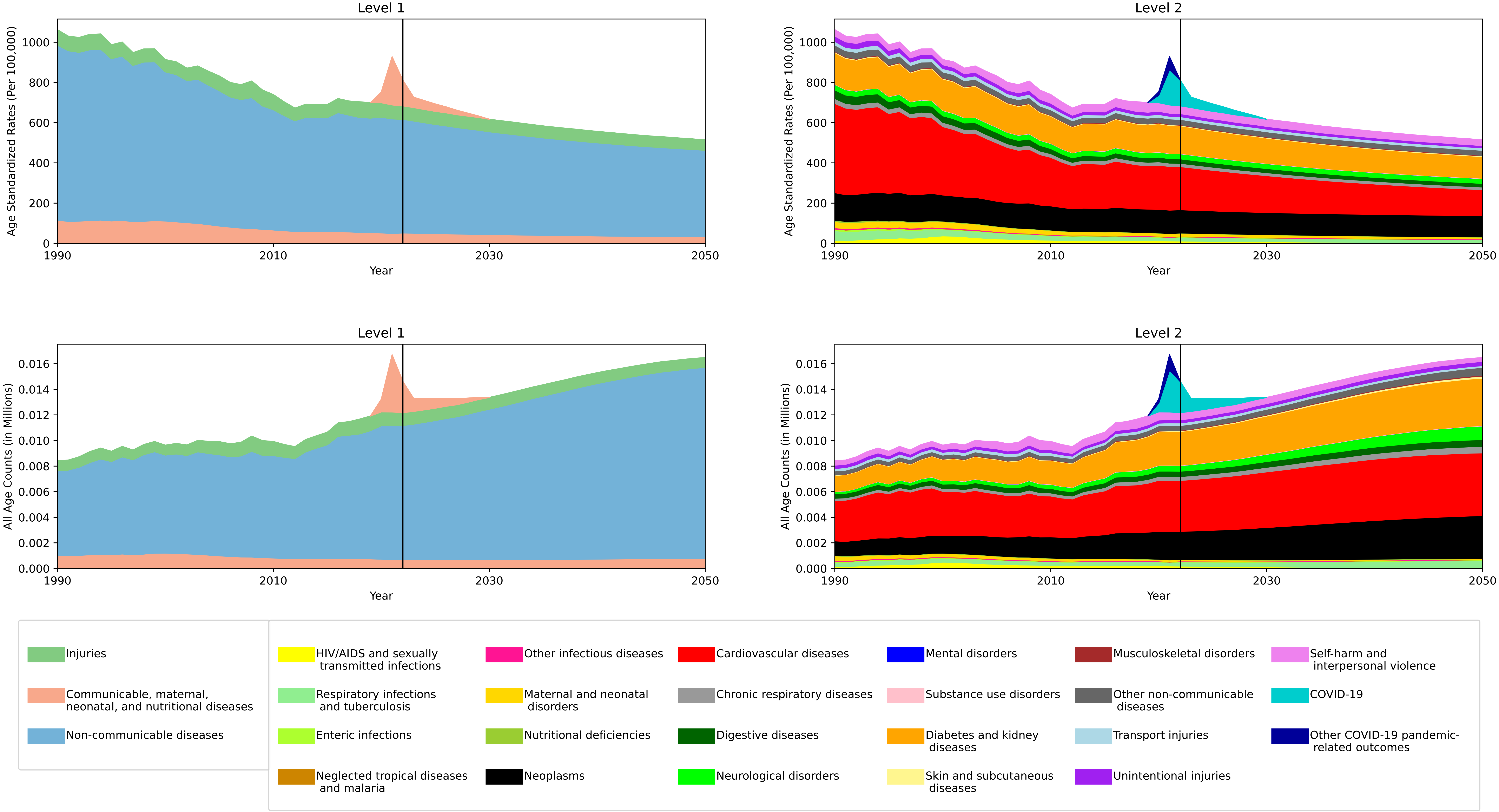

Virgin Islands

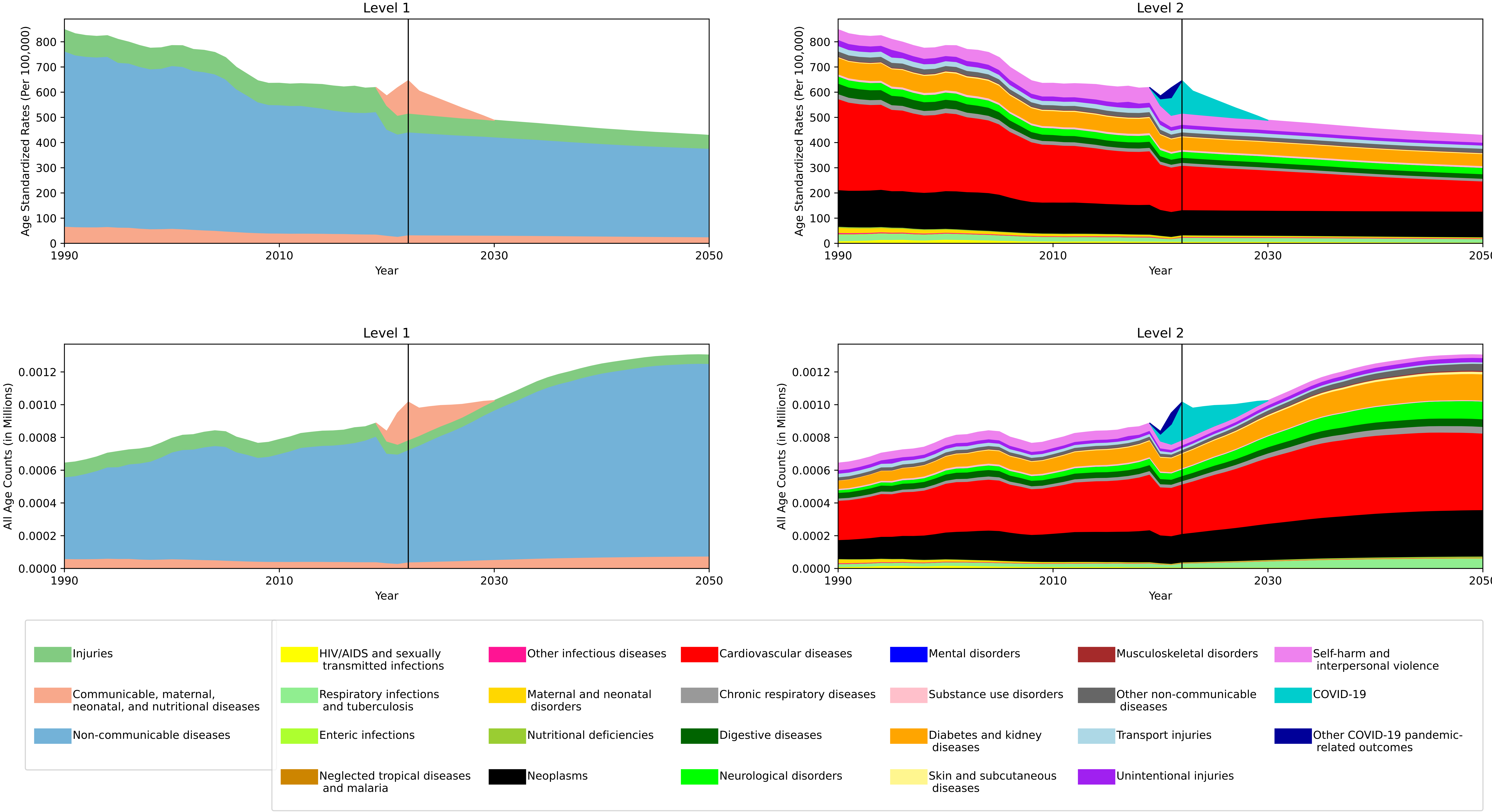

Colombia

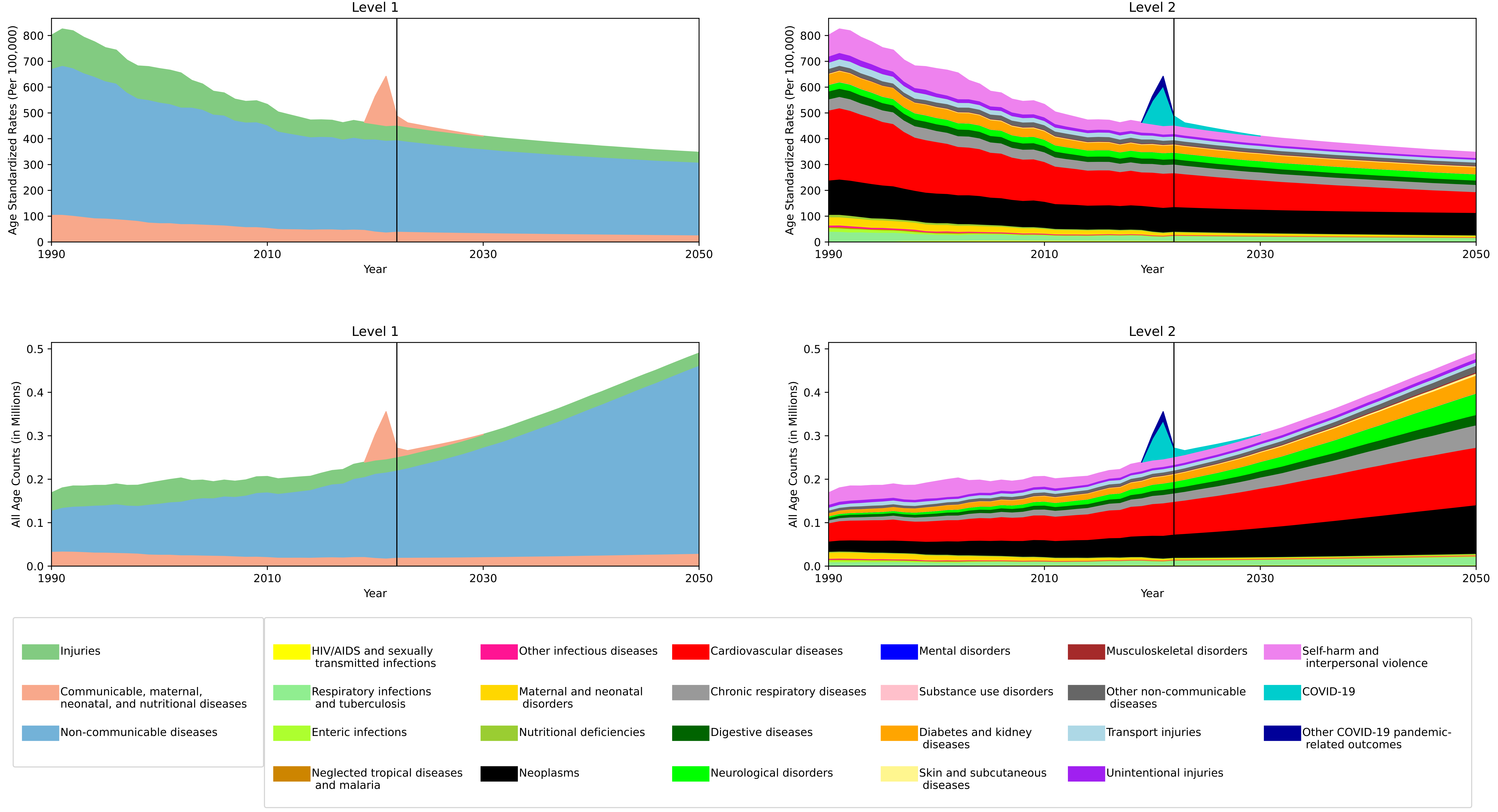
Level 1
Level 2
Age Standardized Rates (Per 100,000)
All Age Counts (in Millions)
Year
1990
2010
2030
2050
Injuries
Communicable, maternal, neonatal, and nutritional diseases
Non-communicable diseases
HIV/AIDS and sexually transmitted infections
Respiratory infections and tuberculosis
Enteric infections
Neglected tropical diseases and malaria
Other infectious diseases
Maternal and neonatal disorders
Nutritional deficiencies
Neoplasms
Cardiovascular diseases
Chronic respiratory diseases
Digestive diseases
Neurological disorders
Mental disorders
Substance use disorders
Diabetes and kidney diseases
Skin and subcutaneous diseases
Musculoskeletal disorders
Other non-communicable diseases
Transport injuries
Unintentional injuries
Self-harm and interpersonal violence
COVID-19
Other COVID-19 pandemic-related outcomes

Costa Rica

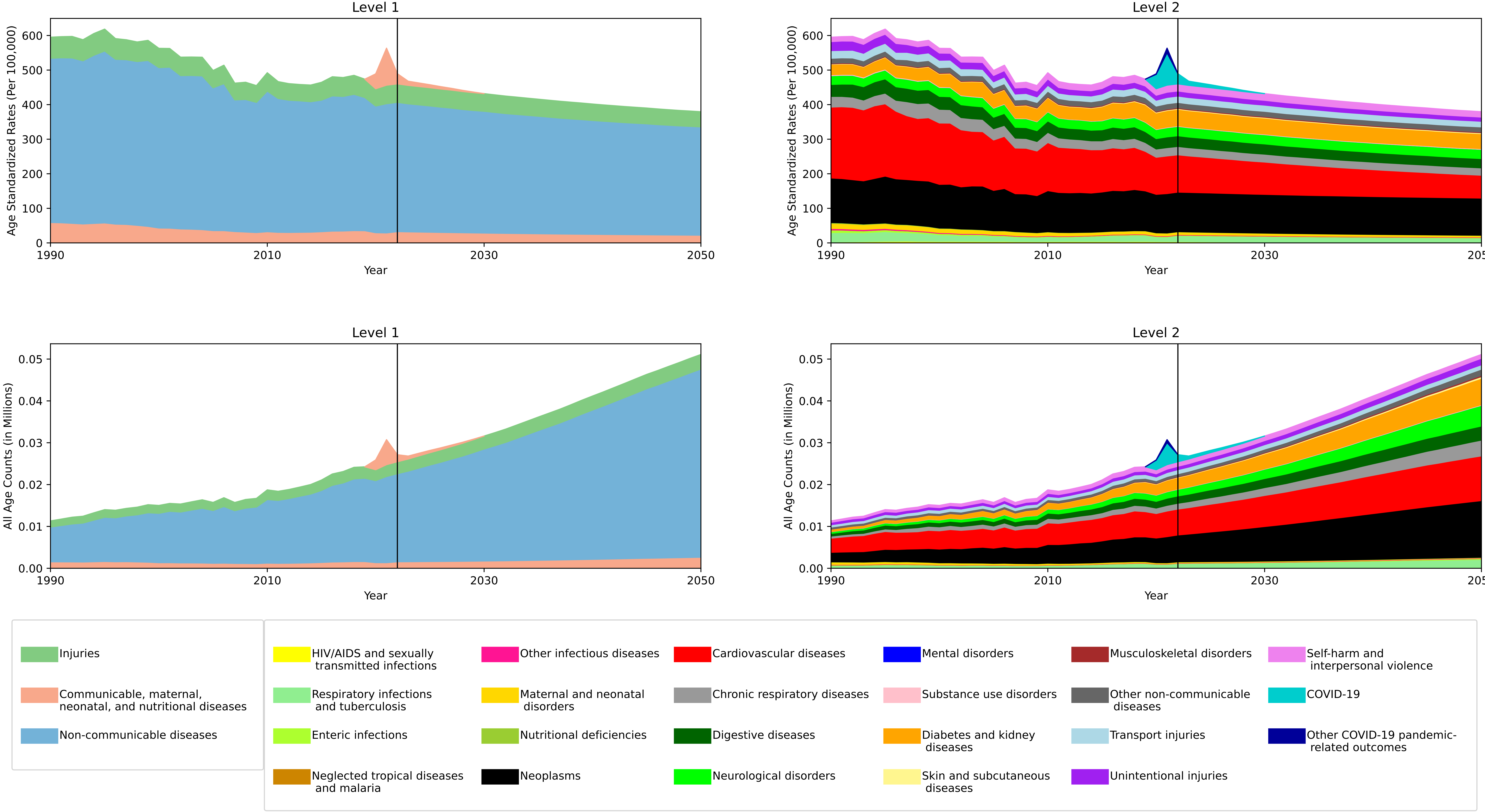
Level 1
Age Standardized Rates (Per 100,000)
0
100
200
300
400
500
600
1990
2010
2030
2050
Year
Level 2
Age Standardized Rates (Per 100,000)
Level 1
All Age Counts (in Millions)
0.00
0.01
0.02
0.03
0.04
0.05
Level 2
All Age Counts (in Millions)
Injuries
Communicable, maternal, neonatal, and nutritional diseases
Non-communicable diseases
HIV/AIDS and sexually transmitted infections
Respiratory infections and tuberculosis
Enteric infections
Neglected tropical diseases and malaria
Other infectious diseases
Maternal and neonatal disorders
Nutritional deficiencies
Neoplasms
Cardiovascular diseases
Chronic respiratory diseases
Digestive diseases
Neurological disorders
Mental disorders
Substance use disorders
Diabetes and kidney diseases
Skin and subcutaneous diseases
Musculoskeletal disorders
Other non-communicable diseases
Transport injuries
Unintentional injuries
Self-harm and interpersonal violence
COVID-19
Other COVID-19 pandemic-related outcomes

El Salvador

Supplemental Results Figure S5. Age-standardised death rates and all age death counts and by Level 1 and Level 2 causes, 1990–2050

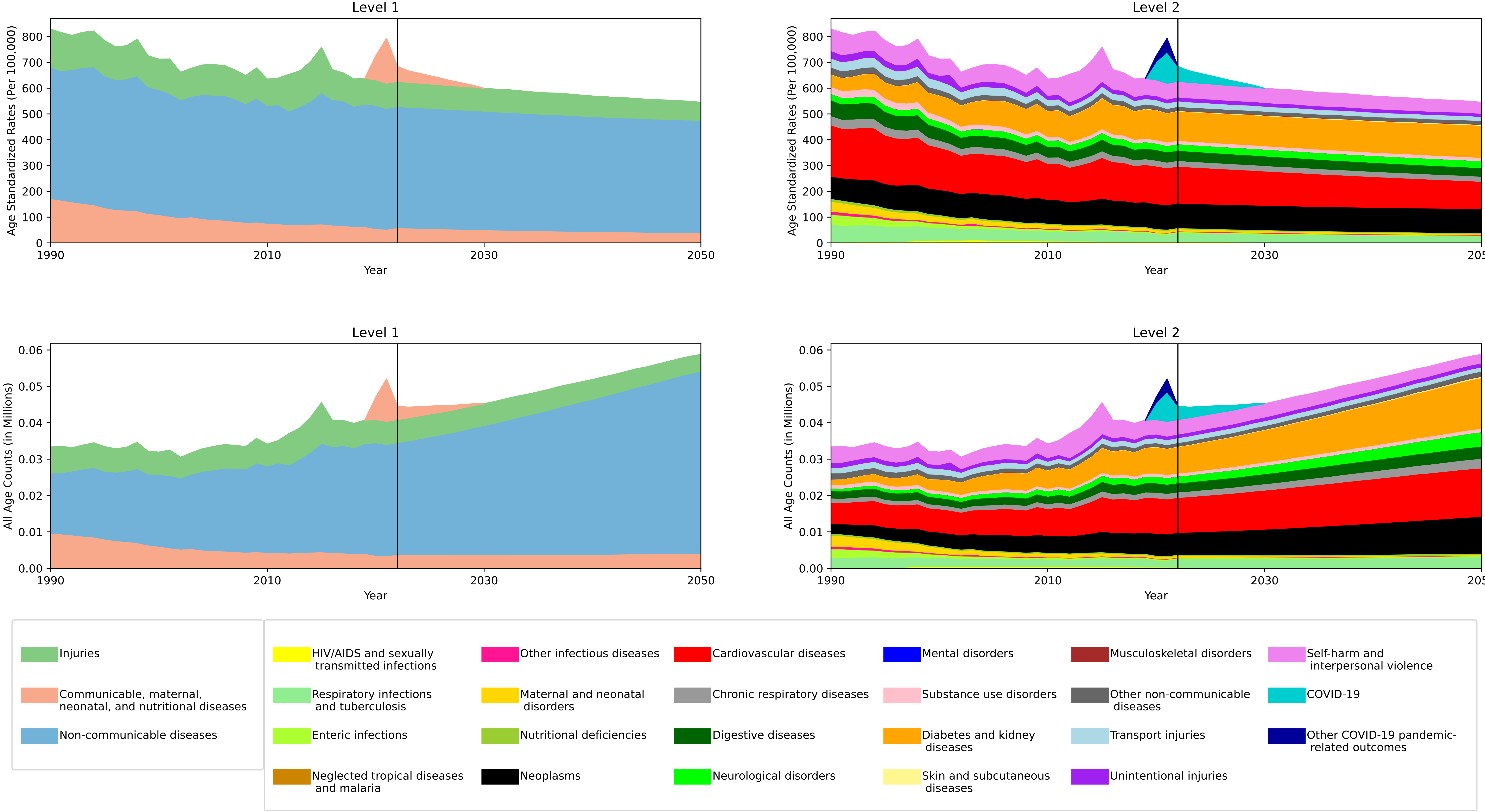

Guatemala

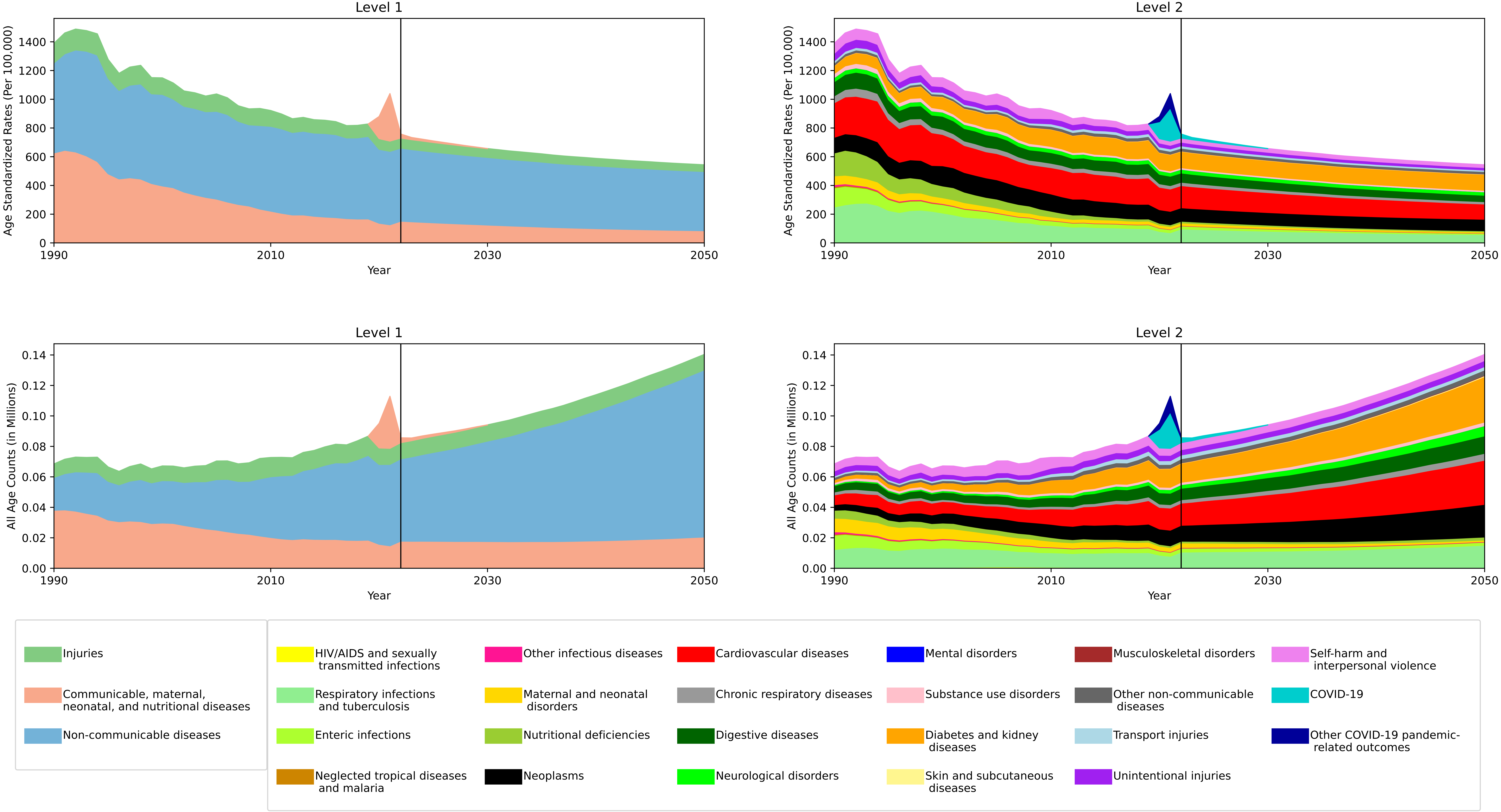
Level 1
Age Standardized Rates (Per 100,000)
Year
Level 2
Age Standardized Rates (Per 100,000)
Year
Level 1
All Age Counts (in Millions)
Year
Level 2
All Age Counts (in Millions)
Year
Injuries
Communicable, maternal, neonatal, and nutritional diseases
Non-communicable diseases
HIV/AIDS and sexually transmitted infections
Respiratory infections and tuberculosis
Enteric infections
Neglected tropical diseases and malaria
Other infectious diseases
Maternal and neonatal disorders
Nutritional deficiencies
Neoplasms
Cardiovascular diseases
Chronic respiratory diseases
Digestive diseases
Neurological disorders
Mental disorders
Substance use disorders
Diabetes and kidney diseases
Skin and subcutaneous diseases
Musculoskeletal disorders
Other non-communicable diseases
Transport injuries
Unintentional injuries
Self-harm and interpersonal violence
COVID-19
Other COVID-19 pandemic-related outcomes

Honduras

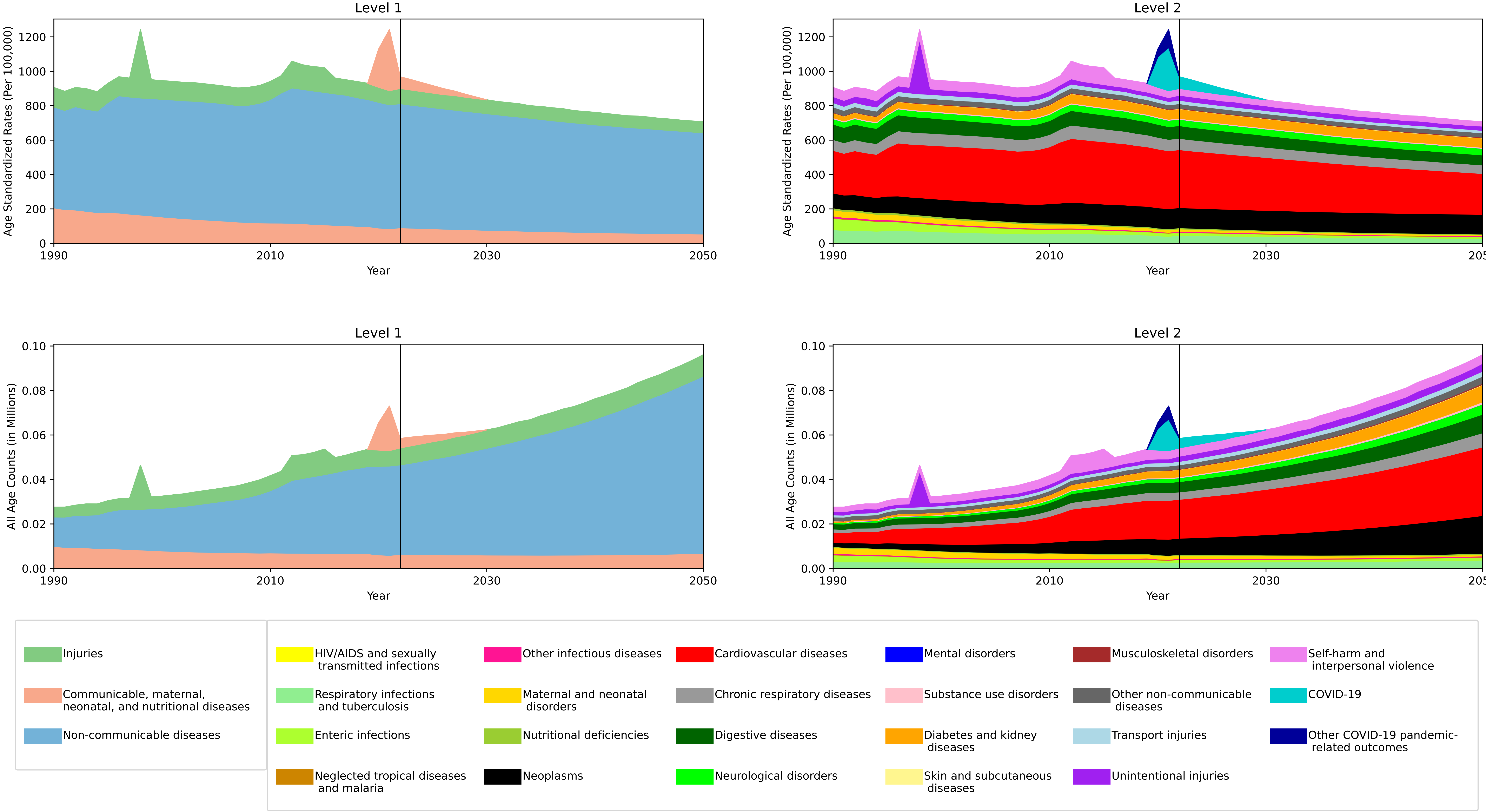
Level 1
Level 2
Age Standardized Rates (Per 100,000)
All Age Counts (in Millions)
Year
1990
2010
2030
2050
0
200
400
600
800
1000
1200
0.00
0.02
0.04
0.06
0.08
0.10
Injuries
Communicable, maternal, neonatal, and nutritional diseases
Non-communicable diseases
HIV/AIDS and sexually transmitted infections
Respiratory infections and tuberculosis
Enteric infections
Neglected tropical diseases and malaria
Other infectious diseases
Maternal and neonatal disorders
Nutritional deficiencies
Neoplasms
Cardiovascular diseases
Chronic respiratory diseases
Digestive diseases
Neurological disorders
Mental disorders
Substance use disorders
Diabetes and kidney diseases
Skin and subcutaneous diseases
Musculoskeletal disorders
Other non-communicable diseases
Transport injuries
Unintentional injuries
Self-harm and interpersonal violence
COVID-19
Other COVID-19 pandemic-related outcomes

Mexico

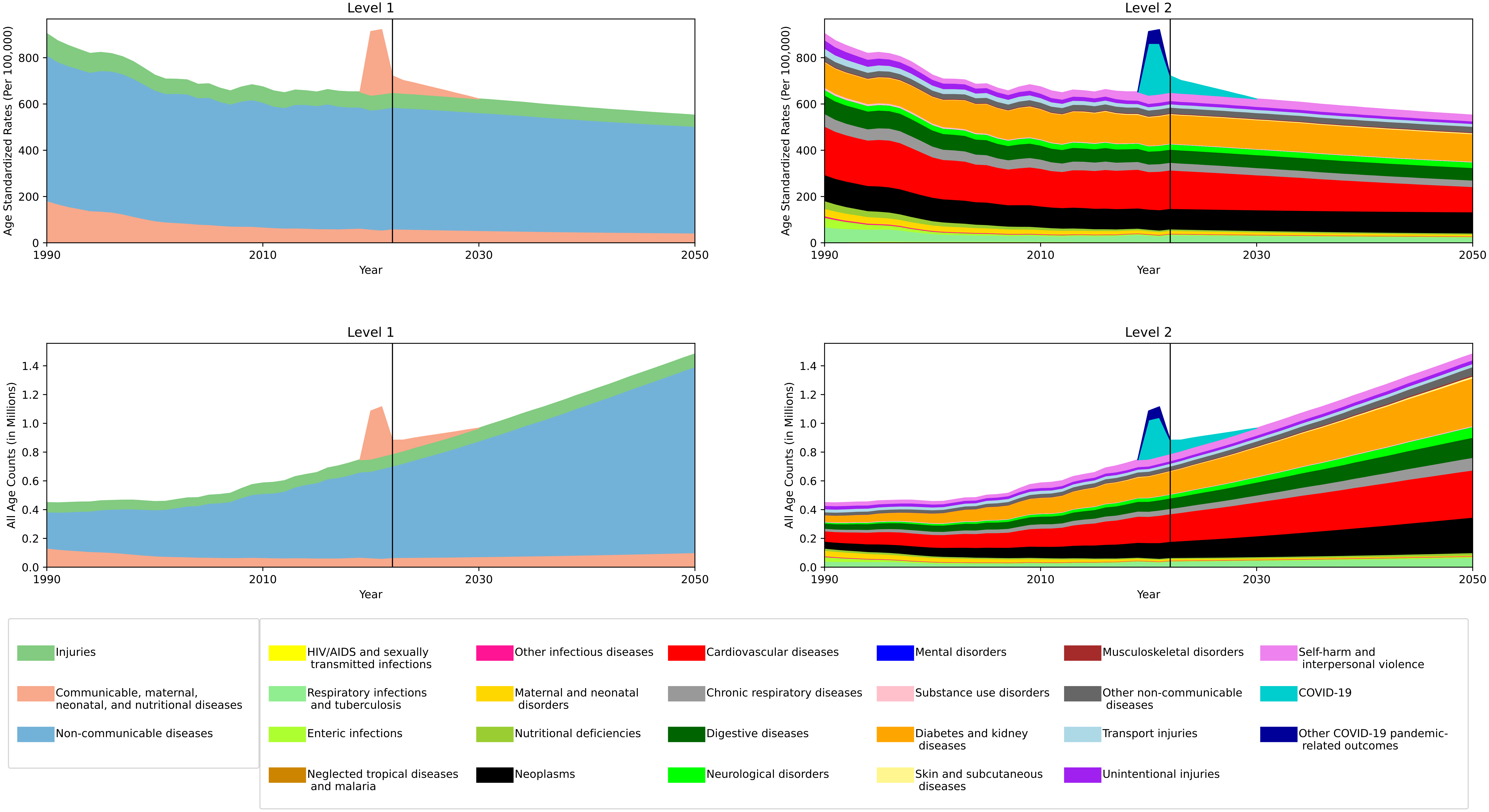
Level 1
Level 2
Age Standardized Rates (Per 100,000)
All Age Counts (in Millions)
Year
1990
2010
2030
2050
Injuries
Communicable, maternal, neonatal, and nutritional diseases
Non-communicable diseases
HIV/AIDS and sexually transmitted infections
Respiratory infections and tuberculosis
Enteric infections
Neglected tropical diseases and malaria
Other infectious diseases
Maternal and neonatal disorders
Nutritional deficiencies
Neoplasms
Cardiovascular diseases
Chronic respiratory diseases
Digestive diseases
Neurological disorders
Mental disorders
Substance use disorders
Diabetes and kidney diseases
Skin and subcutaneous diseases
Musculoskeletal disorders
Other non-communicable diseases
Transport injuries
Unintentional injuries
Self-harm and interpersonal violence
COVID-19
Other COVID-19 pandemic-related outcomes

Nicaragua

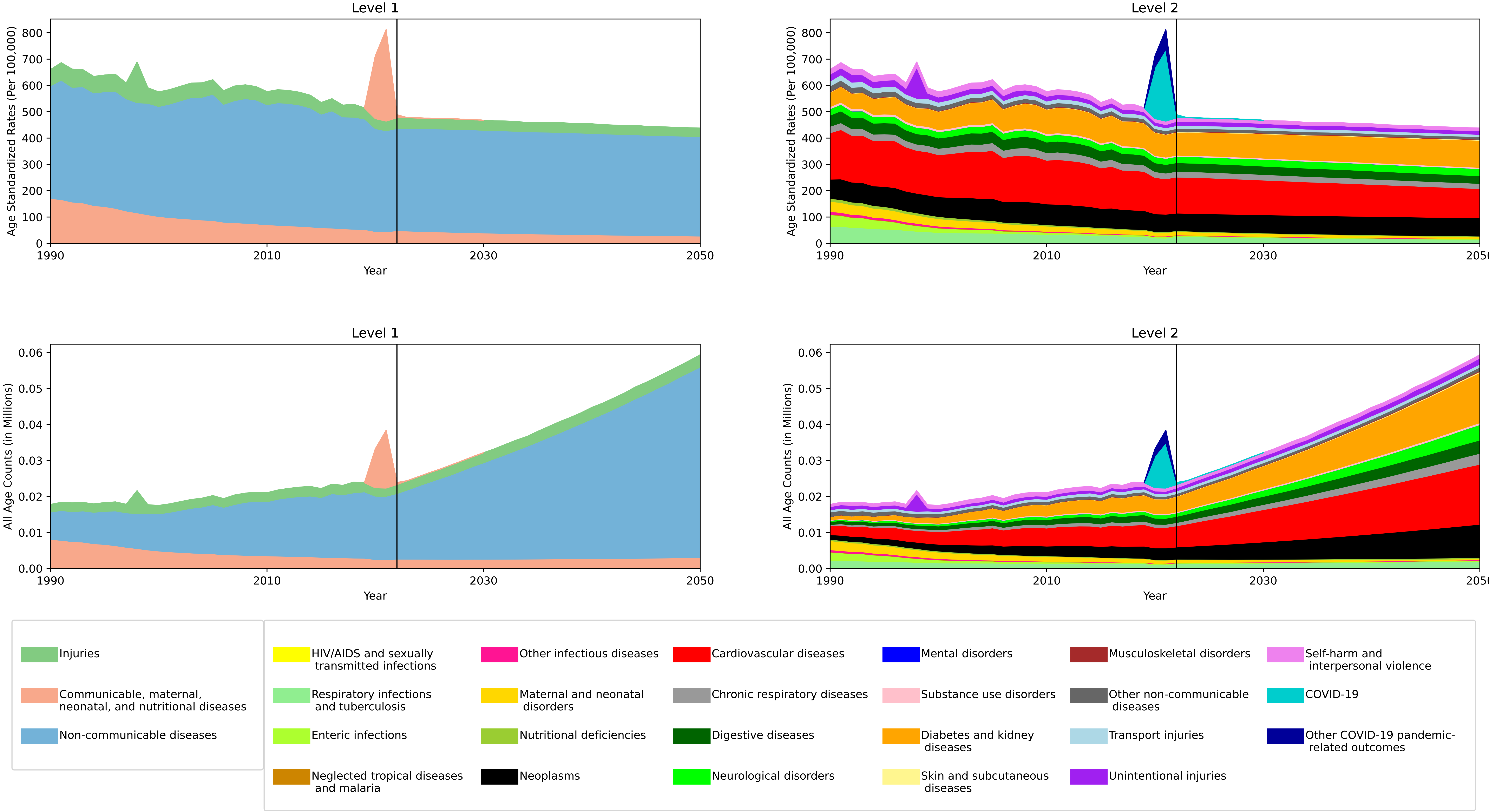
Level 1
Level 2
Age Standardized Rates (Per 100,000)
All Age Counts (in Millions)
Year
1990
2010
2030
2050
Injuries
Communicable, maternal, neonatal, and nutritional diseases
Non-communicable diseases
HIV/AIDS and sexually transmitted infections
Respiratory infections and tuberculosis
Enteric infections
Neglected tropical diseases and malaria
Other infectious diseases
Maternal and neonatal disorders
Nutritional deficiencies
Neoplasms
Cardiovascular diseases
Chronic respiratory diseases
Digestive diseases
Neurological disorders
Mental disorders
Substance use disorders
Diabetes and kidney diseases
Skin and subcutaneous diseases
Musculoskeletal disorders
Other non-communicable diseases
Transport injuries
Unintentional injuries
Self-harm and interpersonal violence
COVID-19
Other COVID-19 pandemic-related outcomes

Panama

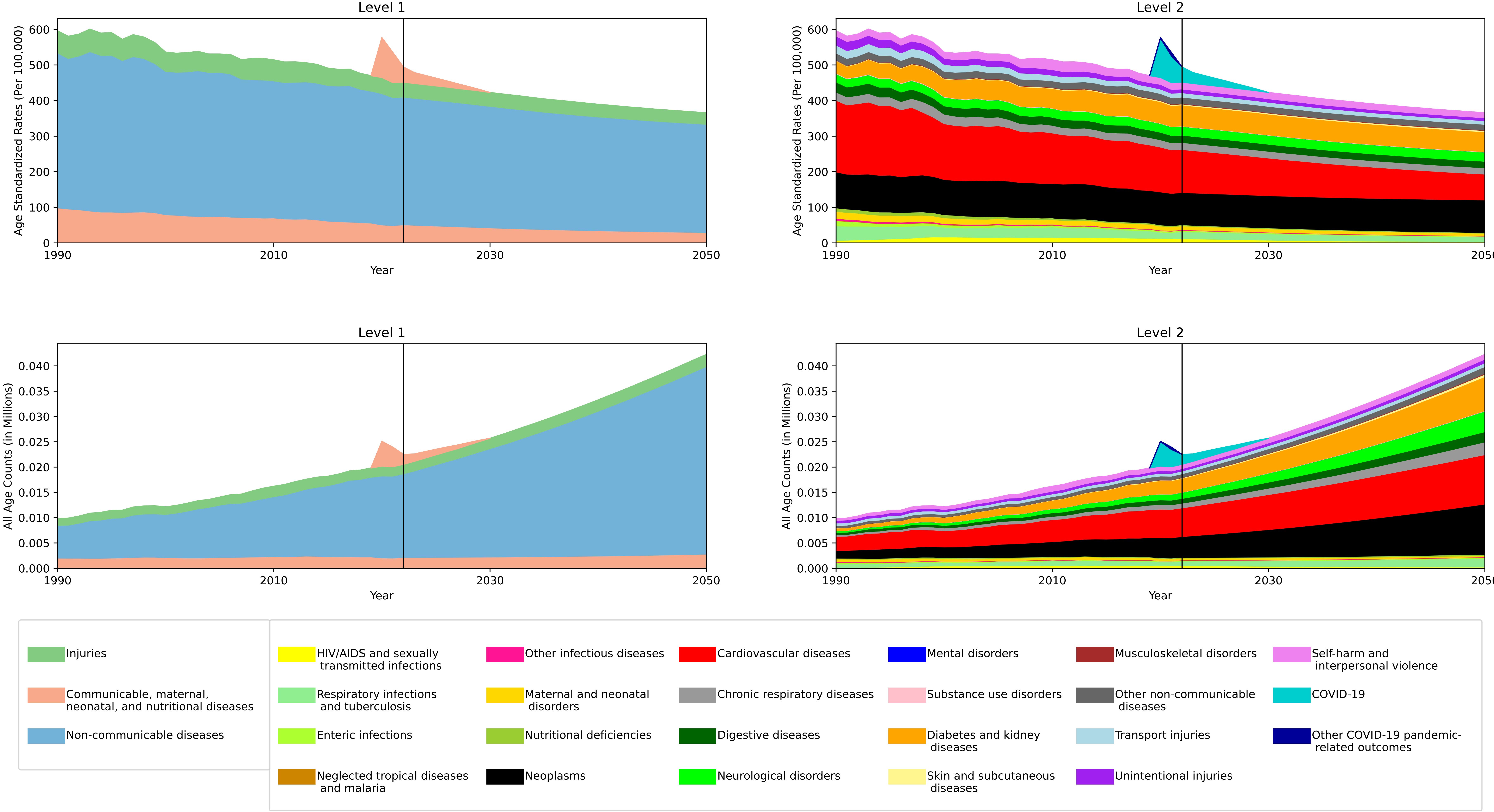
Level 1
Level 2
Age Standardized Rates (Per 100,000)
All Age Counts (in Millions)
Year
1990
2010
2030
2050
0
100
200
300
400
500
600
0.000
0.005
0.010
0.015
0.020
0.025
0.030
0.035
0.040
Injuries
Communicable, maternal, neonatal, and nutritional diseases
Non-communicable diseases
HIV/AIDS and sexually transmitted infections
Respiratory infections and tuberculosis
Enteric infections
Neglected tropical diseases and malaria
Other infectious diseases
Maternal and neonatal disorders
Nutritional deficiencies
Neoplasms
Cardiovascular diseases
Chronic respiratory diseases
Digestive diseases
Neurological disorders
Mental disorders
Substance use disorders
Diabetes and kidney diseases
Skin and subcutaneous diseases
Musculoskeletal disorders
Other non-communicable diseases
Transport injuries
Unintentional injuries
Self-harm and interpersonal violence
COVID-19
Other COVID-19 pandemic-related outcomes

Supplemental Results Figure S5. Age-standardised death rates and all age death counts and by Level 1 and Level 2 causes, 1990–2050

Venezuela

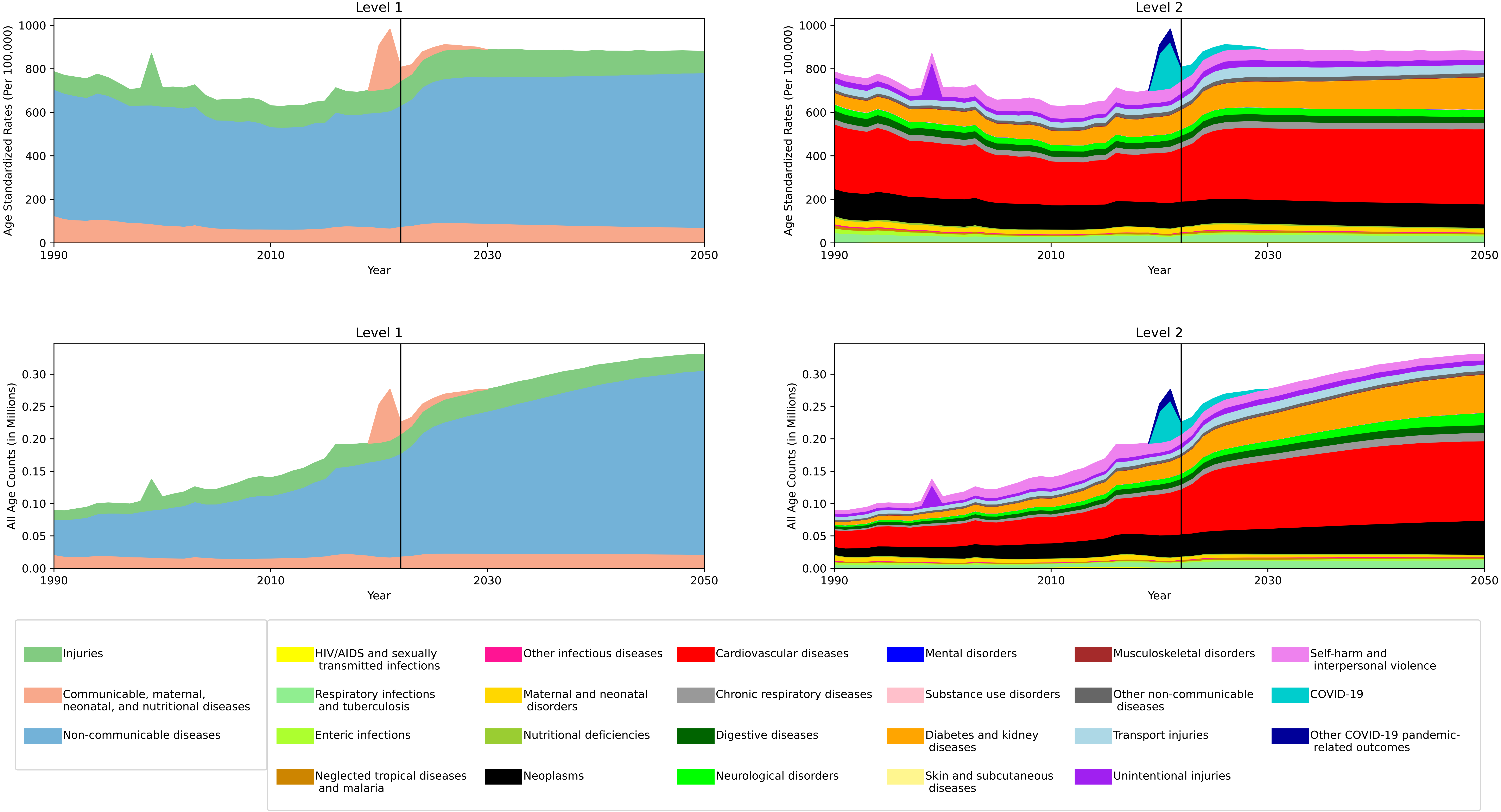

Brazil

Supplemental Results Figure S5. Age-standardised death rates and all age death counts and by Level 1 and Level 2 causes, 1990–2050

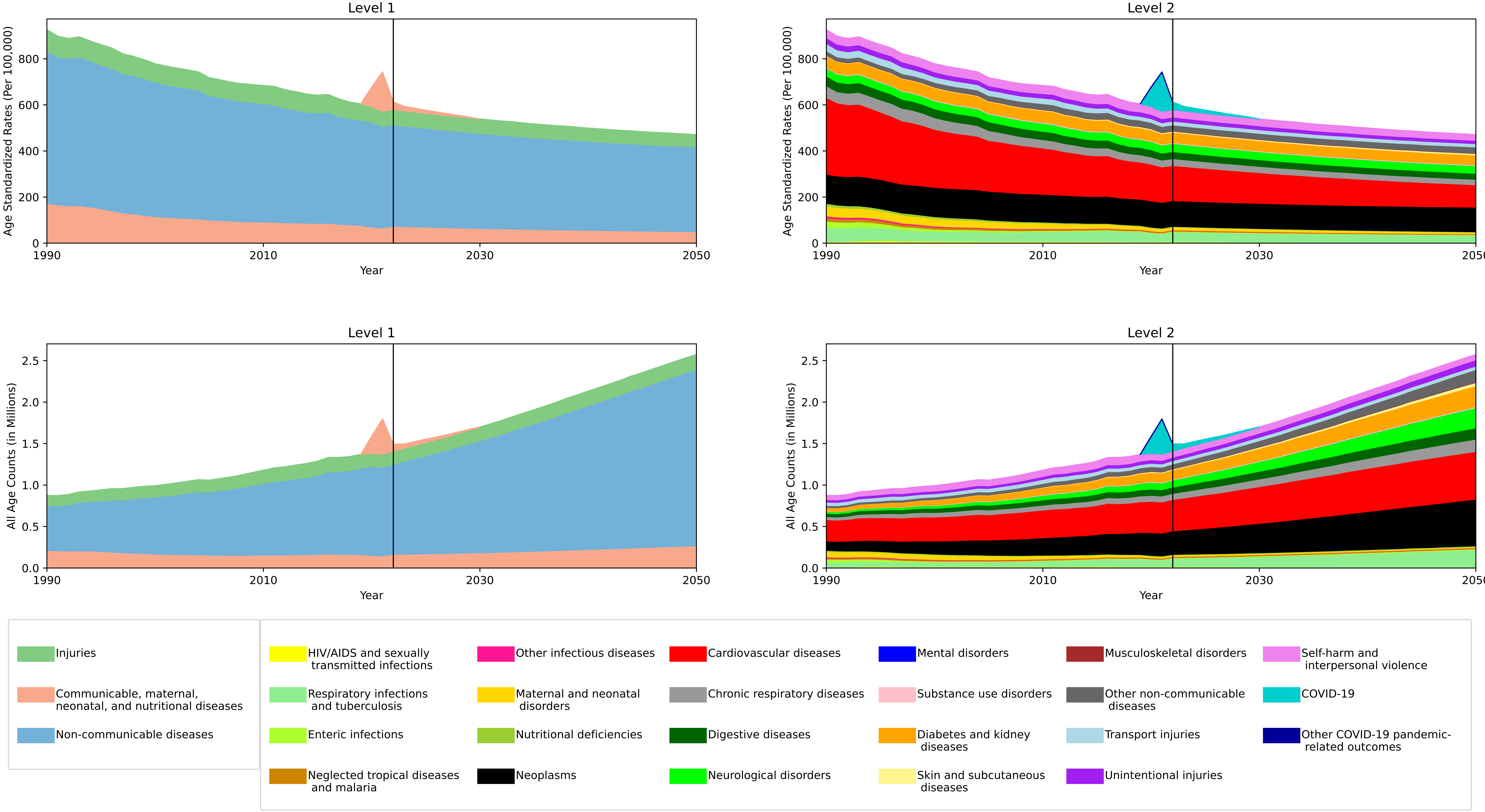

Paraguay

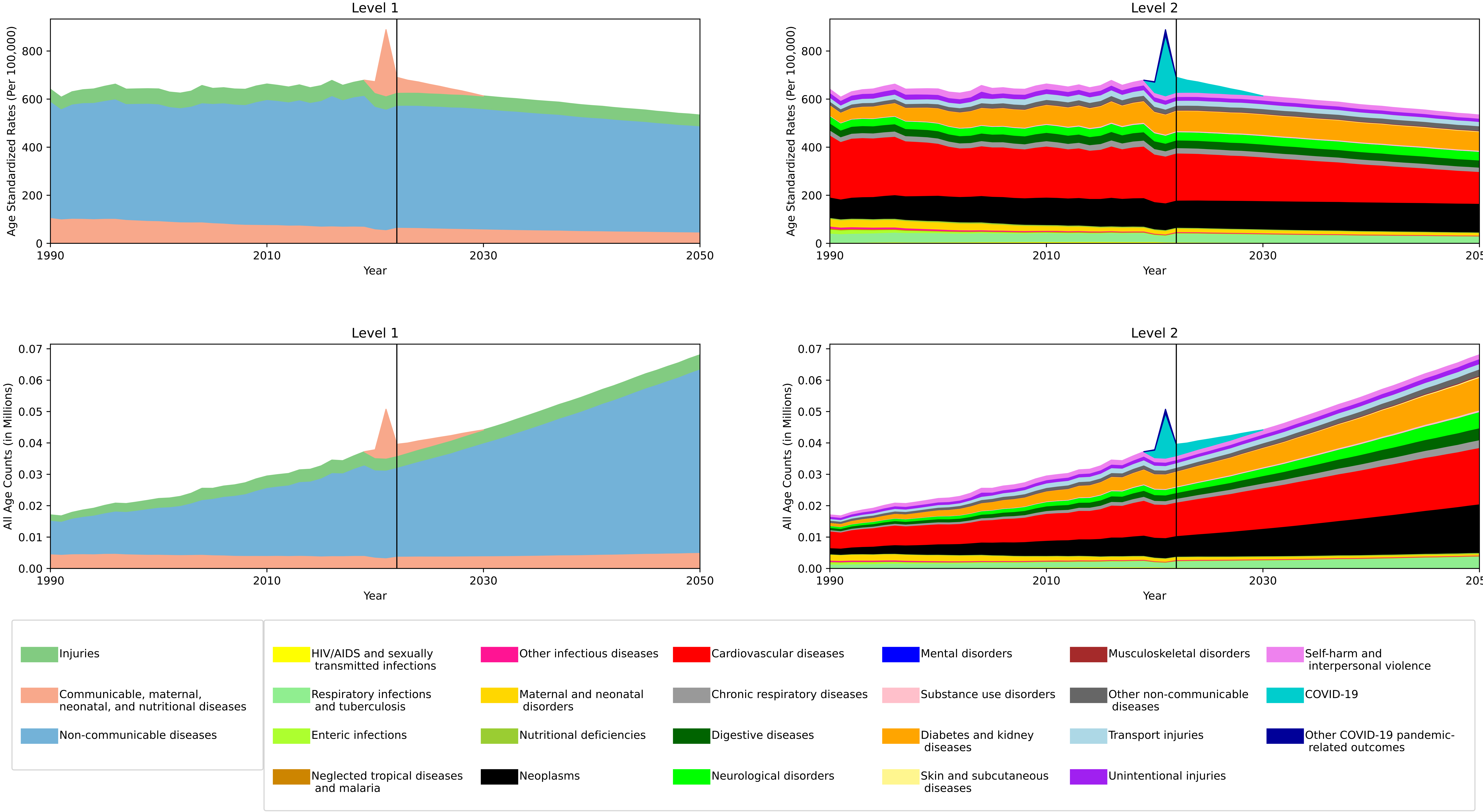
Level 1
Level 2
Age Standardized Rates (Per 100,000)
All Age Counts (in Millions)
Year
0
200
400
600
800
0.00
0.01
0.02
0.03
0.04
0.05
0.06
0.07
1990
2010
2030
2050
Injuries
Communicable, maternal, neonatal, and nutritional diseases
Non-communicable diseases
HIV/AIDS and sexually transmitted infections
Respiratory infections and tuberculosis
Enteric infections
Neglected tropical diseases and malaria
Other infectious diseases
Maternal and neonatal disorders
Nutritional deficiencies
Neoplasms
Cardiovascular diseases
Chronic respiratory diseases
Digestive diseases
Neurological disorders
Mental disorders
Substance use disorders
Diabetes and kidney diseases
Skin and subcutaneous diseases
Musculoskeletal disorders
Other non-communicable diseases
Transport injuries
Unintentional injuries
Self-harm and interpersonal violence
COVID-19
Other COVID-19 pandemic-related outcomes

Afghanistan

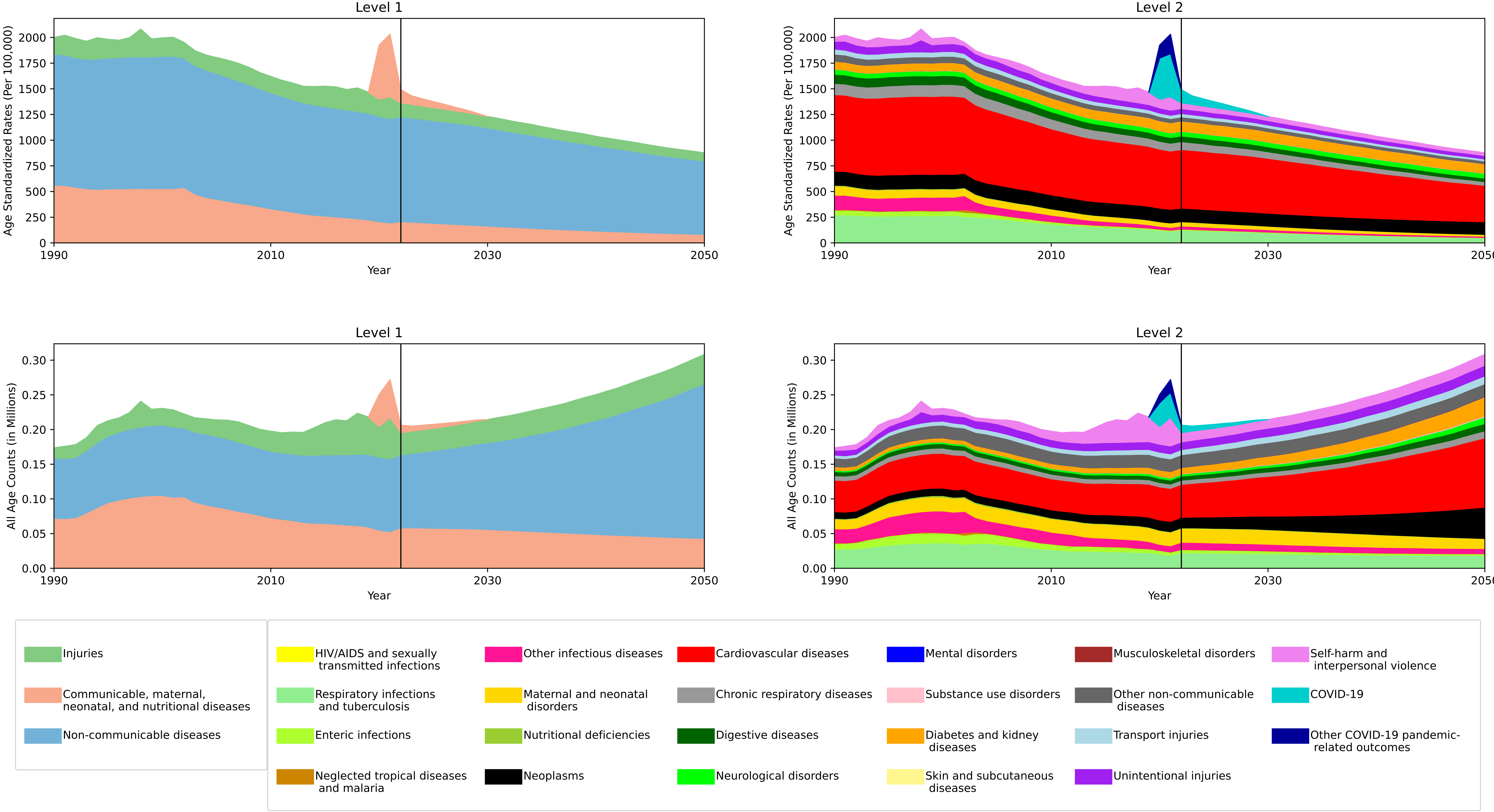

Algeria

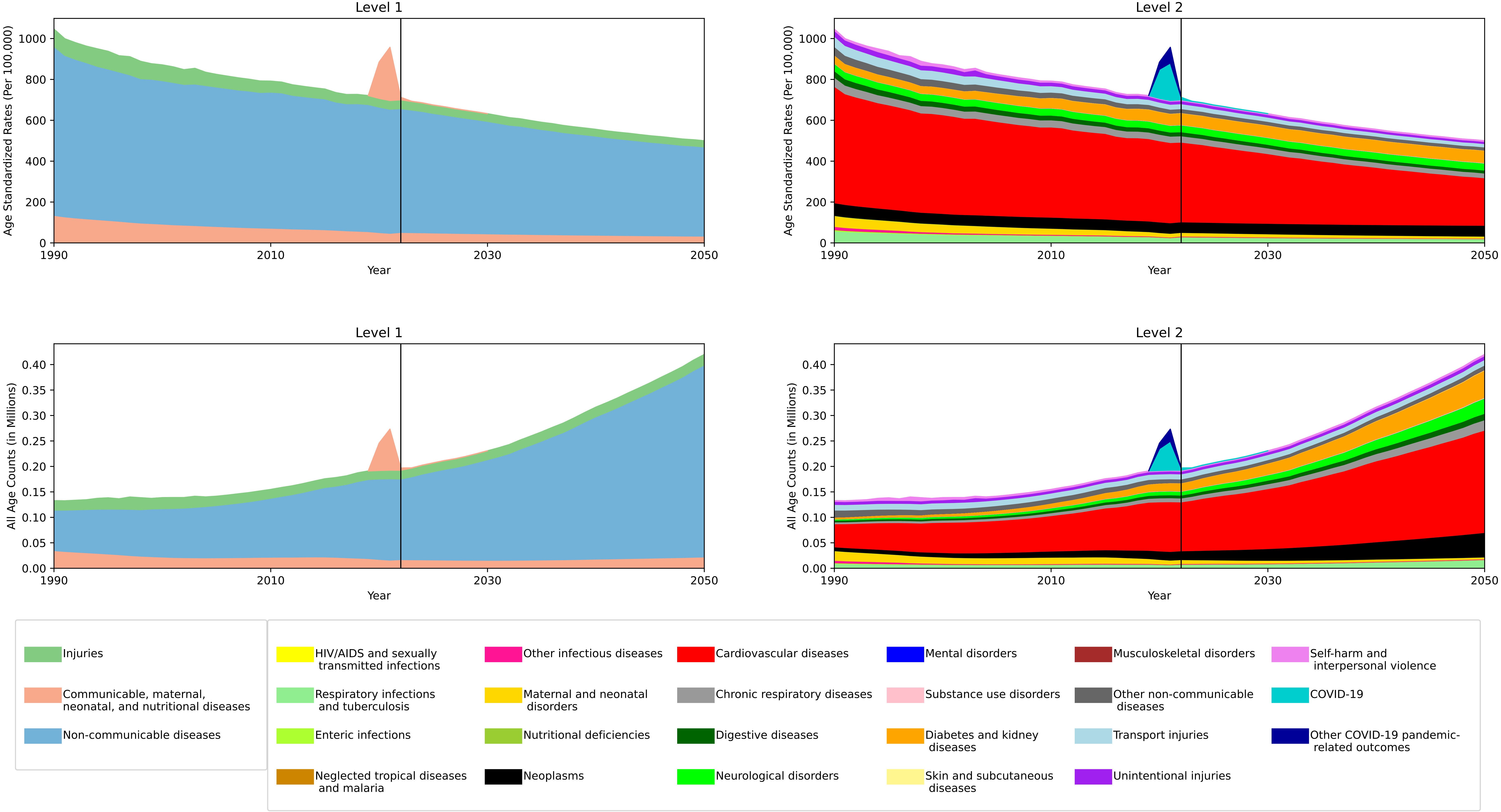
Level 1
Age Standardized Rates (Per 100,000)
Year
Level 2
Age Standardized Rates (Per 100,000)
Year
Level 1
All Age Counts (in Millions)
Year
Level 2
All Age Counts (in Millions)
Year
Injuries
Communicable, maternal, neonatal, and nutritional diseases
Non-communicable diseases
HIV/AIDS and sexually transmitted infections
Respiratory infections and tuberculosis
Enteric infections
Neglected tropical diseases and malaria
Other infectious diseases
Maternal and neonatal disorders
Nutritional deficiencies
Neoplasms
Cardiovascular diseases
Chronic respiratory diseases
Digestive diseases
Neurological disorders
Mental disorders
Substance use disorders
Diabetes and kidney diseases
Skin and subcutaneous diseases
Musculoskeletal disorders
Other non-communicable diseases
Transport injuries
Unintentional injuries
Self-harm and interpersonal violence
COVID-19
Other COVID-19 pandemic-related outcomes

Bahrain

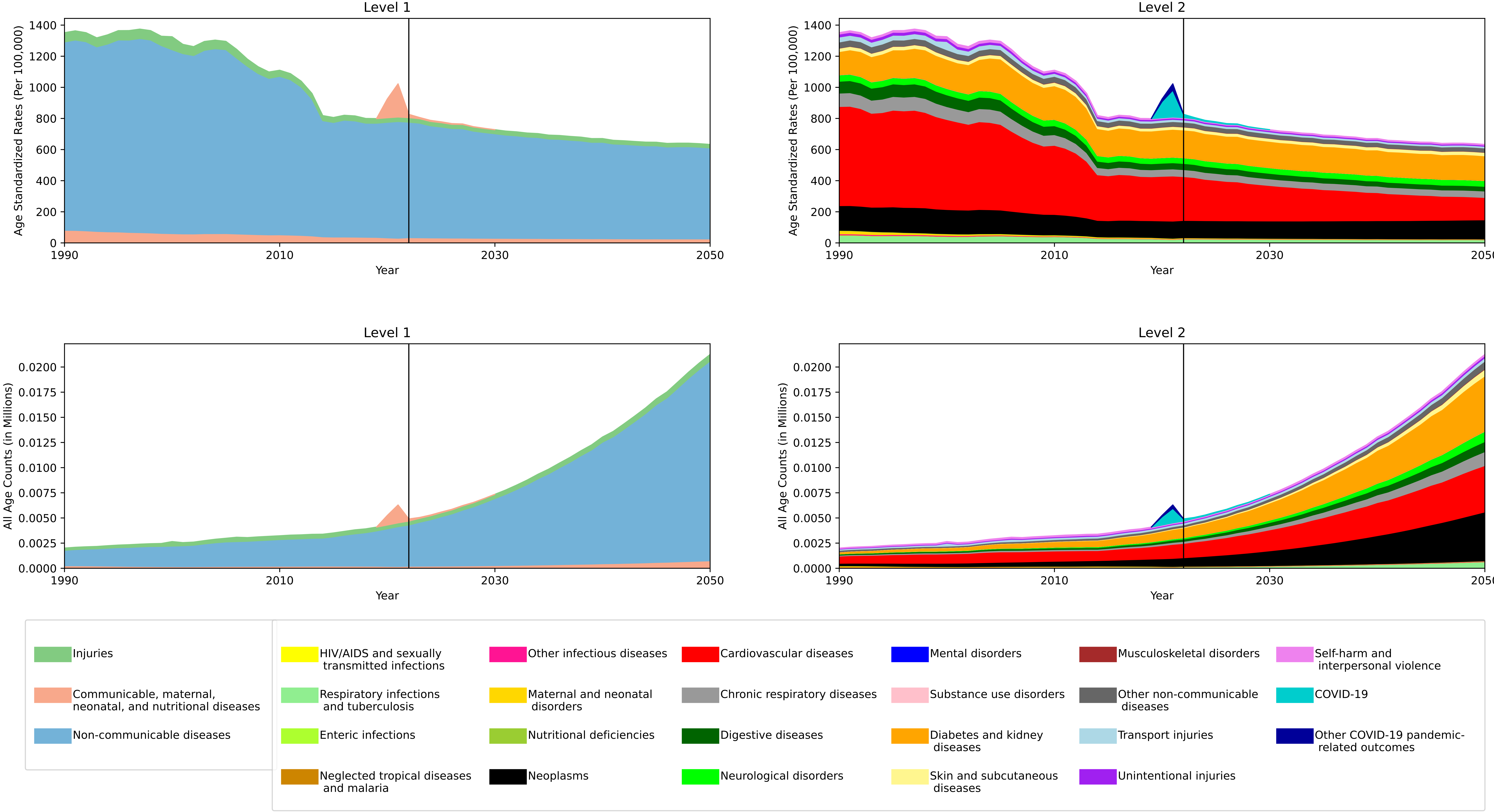
Level 1
Level 2
Age Standardized Rates (Per 100,000)
All Age Counts (in Millions)
Year
1990
2010
2030
2050
0
200
400
600
800
1000
1200
1400
0.0000
0.0025
0.0050
0.0075
0.0100
0.0125
0.0150
0.0175
0.0200
Injuries
Communicable, maternal, neonatal, and nutritional diseases
Non-communicable diseases
HIV/AIDS and sexually transmitted infections
Respiratory infections and tuberculosis
Enteric infections
Neglected tropical diseases and malaria
Other infectious diseases
Maternal and neonatal disorders
Nutritional deficiencies
Neoplasms
Cardiovascular diseases
Chronic respiratory diseases
Digestive diseases
Neurological disorders
Mental disorders
Substance use disorders
Diabetes and kidney diseases
Skin and subcutaneous diseases
Musculoskeletal disorders
Other non-communicable diseases
Transport injuries
Unintentional injuries
Self-harm and interpersonal violence
COVID-19
Other COVID-19 pandemic-related outcomes

Egypt

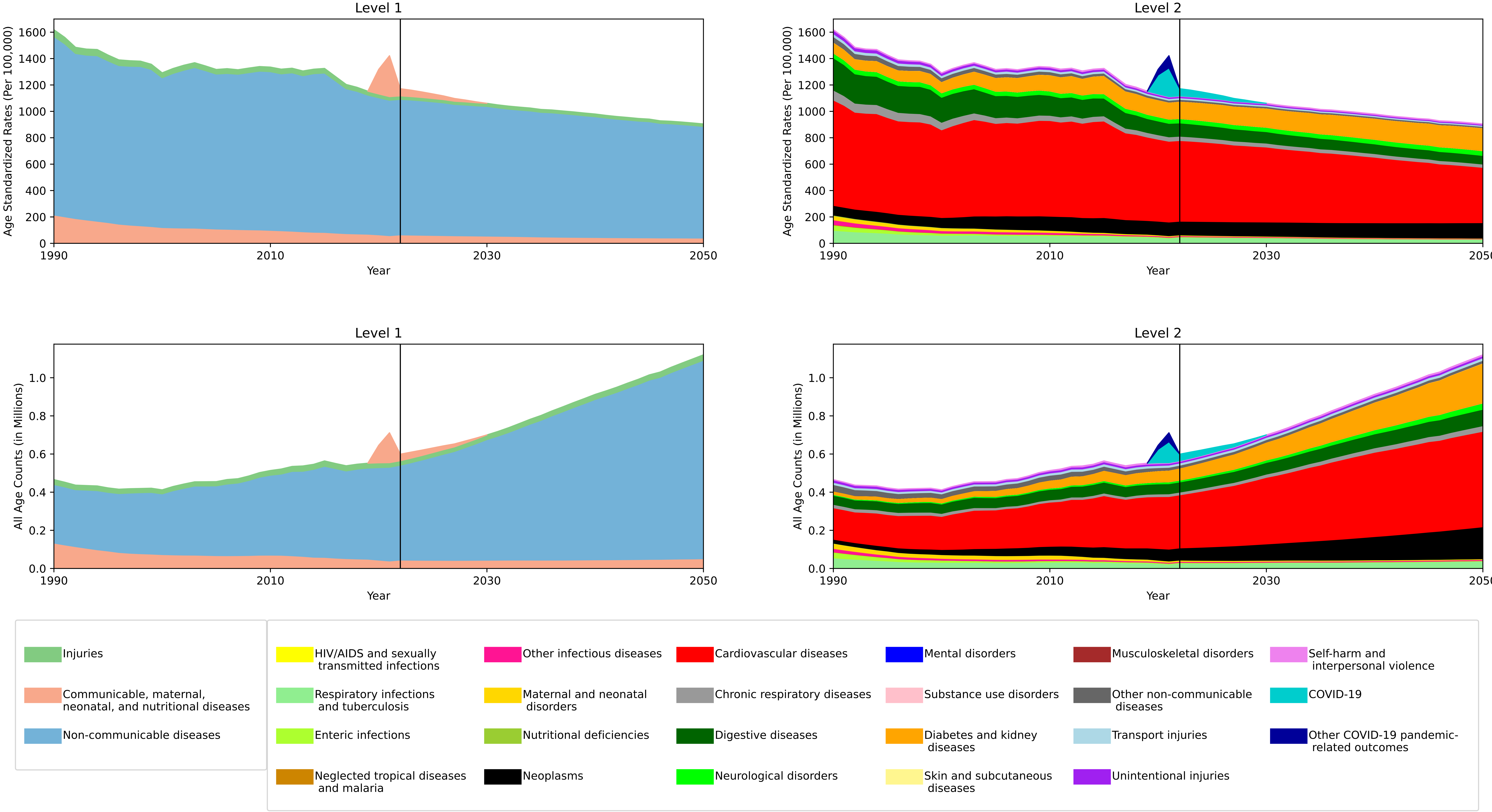
Level 1
Level 2
Age Standardized Rates (Per 100,000)
All Age Counts (in Millions)
Year
1990
2010
2030
2050
Injuries
Communicable, maternal, neonatal, and nutritional diseases
Non-communicable diseases
HIV/AIDS and sexually transmitted infections
Respiratory infections and tuberculosis
Enteric infections
Neglected tropical diseases and malaria
Other infectious diseases
Maternal and neonatal disorders
Nutritional deficiencies
Neoplasms
Cardiovascular diseases
Chronic respiratory diseases
Digestive diseases
Neurological disorders
Mental disorders
Substance use disorders
Diabetes and kidney diseases
Skin and subcutaneous diseases
Musculoskeletal disorders
Other non-communicable diseases
Transport injuries
Unintentional injuries
Self-harm and interpersonal violence
COVID-19
Other COVID-19 pandemic-related outcomes

Iran

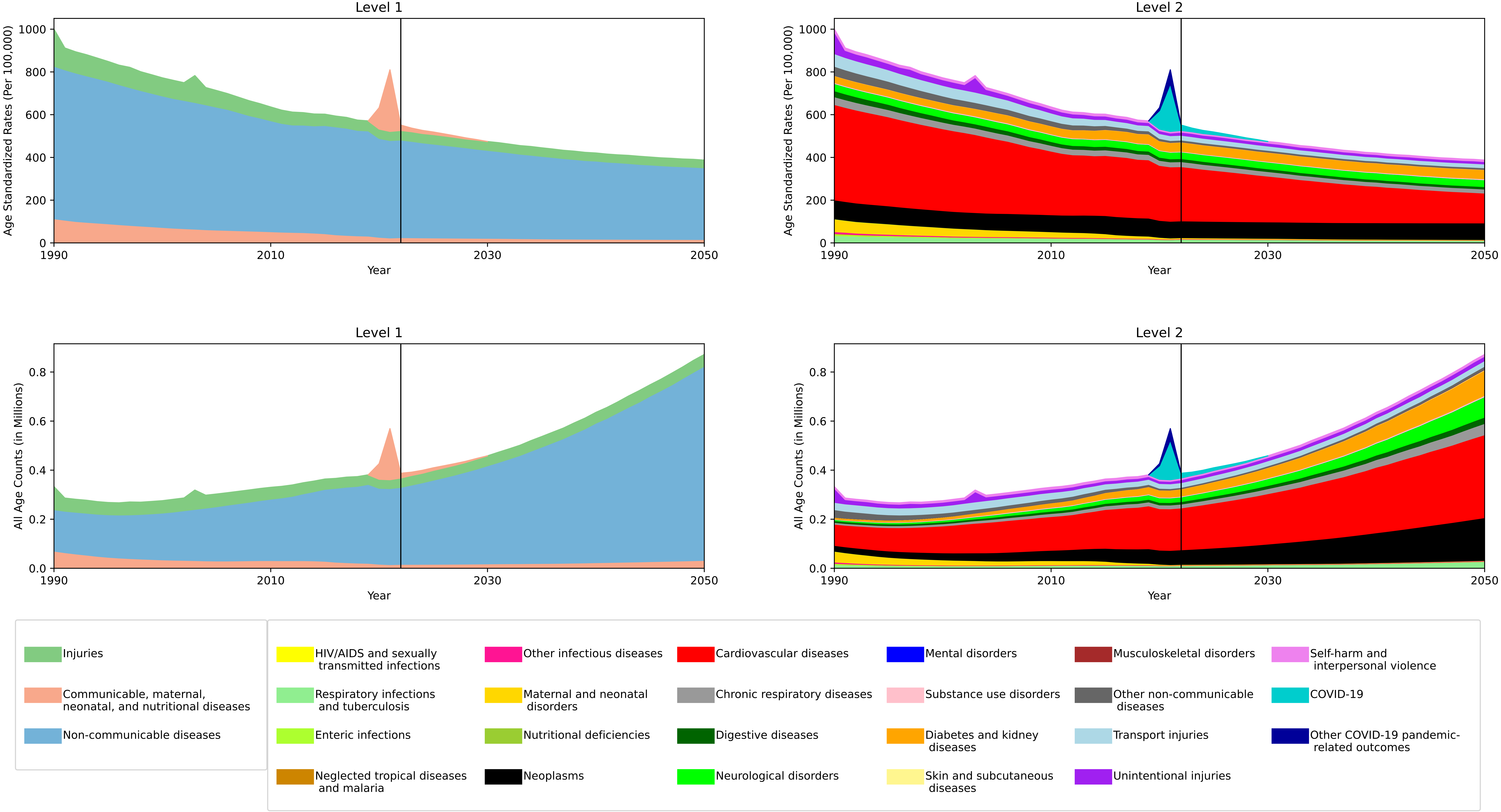
Level 1
Level 2
Age Standardized Rates (Per 100,000)
All Age Counts (in Millions)
Year
1990
2010
2030
2050
Injuries
Communicable, maternal, neonatal, and nutritional diseases
Non-communicable diseases
HIV/AIDS and sexually transmitted infections
Respiratory infections and tuberculosis
Enteric infections
Neglected tropical diseases and malaria
Other infectious diseases
Maternal and neonatal disorders
Nutritional deficiencies
Neoplasms
Cardiovascular diseases
Chronic respiratory diseases
Digestive diseases
Neurological disorders
Mental disorders
Substance use disorders
Diabetes and kidney diseases
Skin and subcutaneous diseases
Musculoskeletal disorders
Other non-communicable diseases
Transport injuries
Unintentional injuries
Self-harm and interpersonal violence
COVID-19
Other COVID-19 pandemic-related outcomes

Iraq

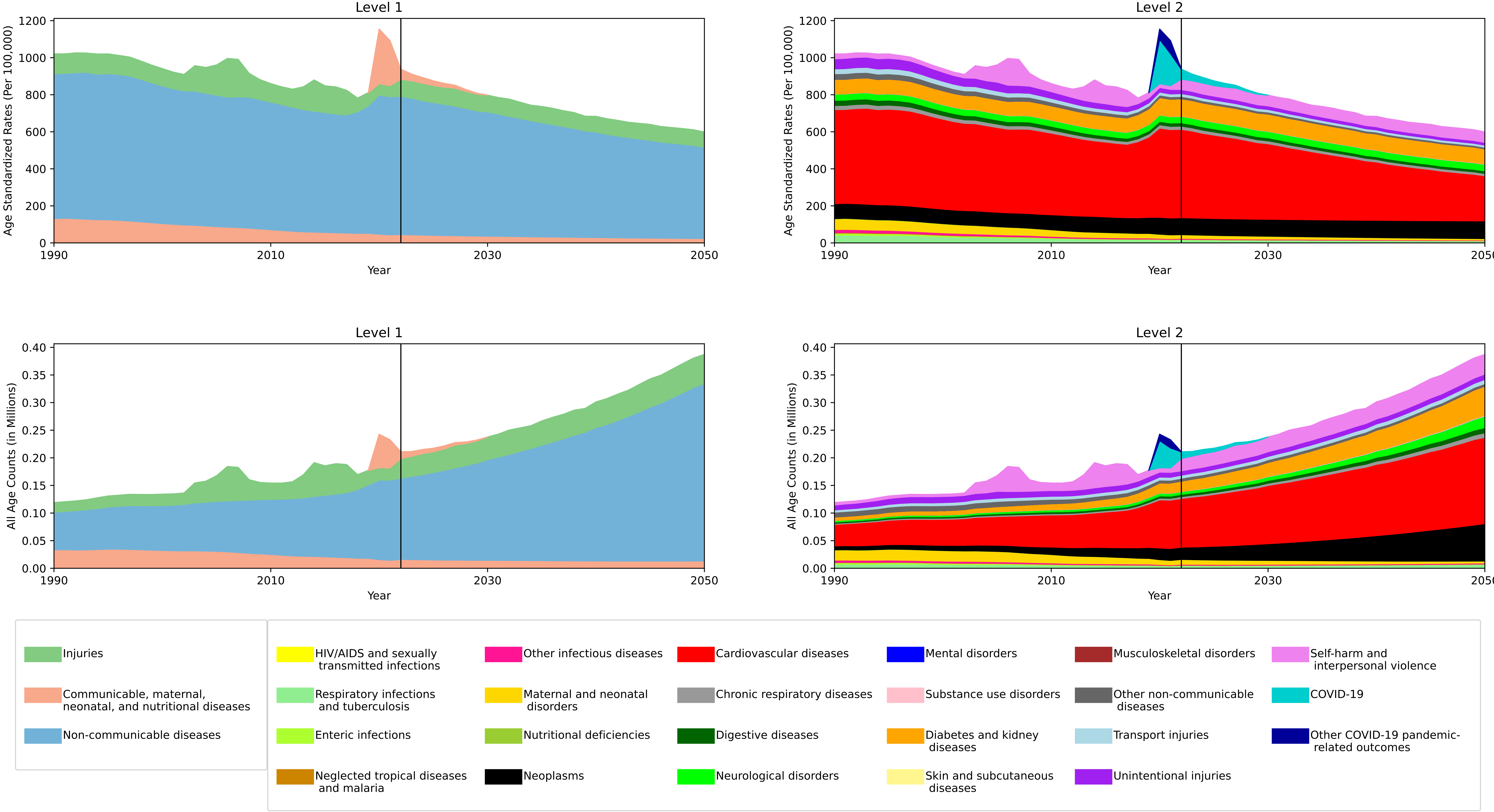
Level 1
Age Standardized Rates (Per 100,000)
Year
Level 2
Age Standardized Rates (Per 100,000)
Year
Level 1
All Age Counts (in Millions)
Year
Level 2
All Age Counts (in Millions)
Year
Injuries
Communicable, maternal, neonatal, and nutritional diseases
Non-communicable diseases
HIV/AIDS and sexually transmitted infections
Respiratory infections and tuberculosis
Enteric infections
Neglected tropical diseases and malaria
Other infectious diseases
Maternal and neonatal disorders
Nutritional deficiencies
Neoplasms
Cardiovascular diseases
Chronic respiratory diseases
Digestive diseases
Neurological disorders
Mental disorders
Substance use disorders
Diabetes and kidney diseases
Skin and subcutaneous diseases
Musculoskeletal disorders
Other non-communicable diseases
Transport injuries
Unintentional injuries
Self-harm and interpersonal violence
COVID-19
Other COVID-19 pandemic-related outcomes

Jordan

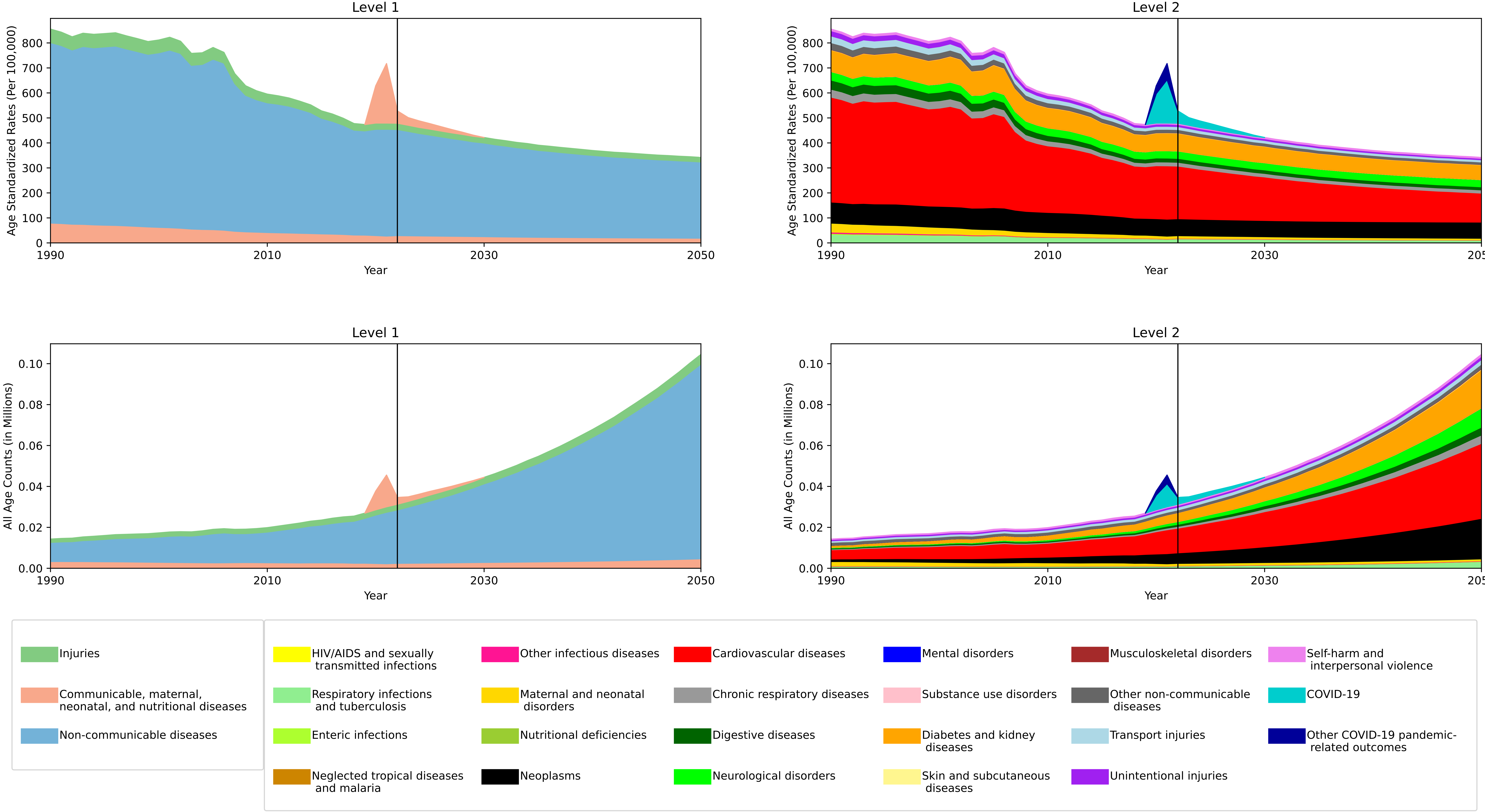
Level 1
Age Standardized Rates (Per 100,000)
Year
Level 2
Age Standardized Rates (Per 100,000)
Year
Level 1
All Age Counts (in Millions)
Year
Level 2
All Age Counts (in Millions)
Year
Injuries
Communicable, maternal, neonatal, and nutritional diseases
Non-communicable diseases
HIV/AIDS and sexually transmitted infections
Respiratory infections and tuberculosis
Enteric infections
Neglected tropical diseases and malaria
Other infectious diseases
Maternal and neonatal disorders
Nutritional deficiencies
Neoplasms
Cardiovascular diseases
Chronic respiratory diseases
Digestive diseases
Neurological disorders
Mental disorders
Substance use disorders
Diabetes and kidney diseases
Skin and subcutaneous diseases
Musculoskeletal disorders
Other non-communicable diseases
Transport injuries
Unintentional injuries
Self-harm and interpersonal violence
COVID-19
Other COVID-19 pandemic-related outcomes

Kuwait

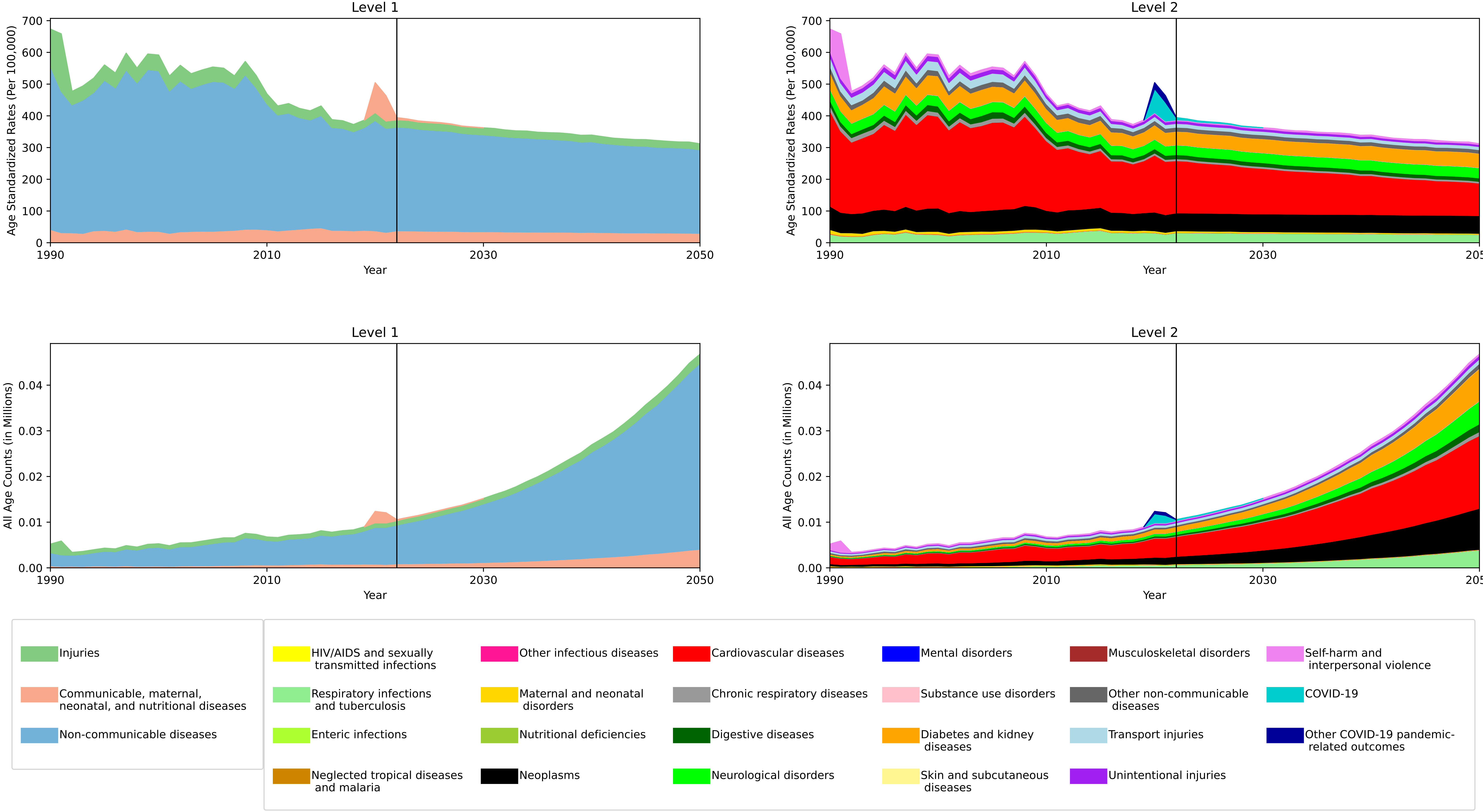
Level 1
Age Standardized Rates (Per 100,000)
Year
Level 2
Age Standardized Rates (Per 100,000)
Year
Level 1
All Age Counts (in Millions)
Year
Level 2
All Age Counts (in Millions)
Year
Injuries
Communicable, maternal, neonatal, and nutritional diseases
Non-communicable diseases
HIV/AIDS and sexually transmitted infections
Respiratory infections and tuberculosis
Enteric infections
Neglected tropical diseases and malaria
Other infectious diseases
Maternal and neonatal disorders
Nutritional deficiencies
Neoplasms
Cardiovascular diseases
Chronic respiratory diseases
Digestive diseases
Neurological disorders
Mental disorders
Substance use disorders
Diabetes and kidney diseases
Skin and subcutaneous diseases
Musculoskeletal disorders
Other non-communicable diseases
Transport injuries
Unintentional injuries
Self-harm and interpersonal violence
COVID-19
Other COVID-19 pandemic-related outcomes

Lebanon

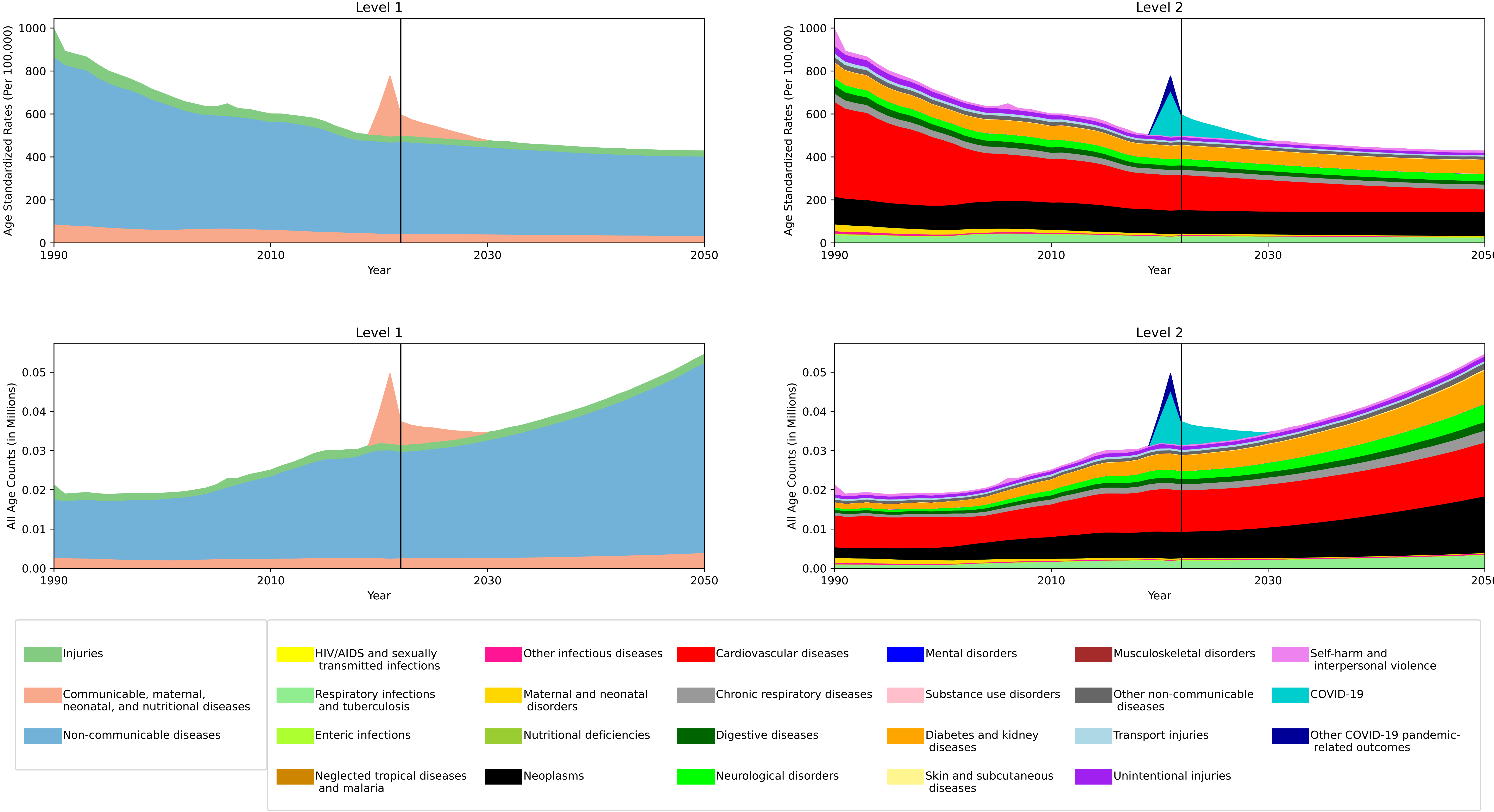
Level 1
Level 2
Age Standardized Rates (Per 100,000)
All Age Counts (in Millions)
Year
0
200
400
600
800
1000
0.00
0.01
0.02
0.03
0.04
0.05
1990
2010
2030
2050
Injuries
Communicable, maternal, neonatal, and nutritional diseases
Non-communicable diseases
HIV/AIDS and sexually transmitted infections
Respiratory infections and tuberculosis
Enteric infections
Neglected tropical diseases and malaria
Other infectious diseases
Maternal and neonatal disorders
Nutritional deficiencies
Neoplasms
Cardiovascular diseases
Chronic respiratory diseases
Digestive diseases
Neurological disorders
Mental disorders
Substance use disorders
Diabetes and kidney diseases
Skin and subcutaneous diseases
Musculoskeletal disorders
Other non-communicable diseases
Transport injuries
Unintentional injuries
Self-harm and interpersonal violence
COVID-19
Other COVID-19 pandemic-related outcomes

Libya

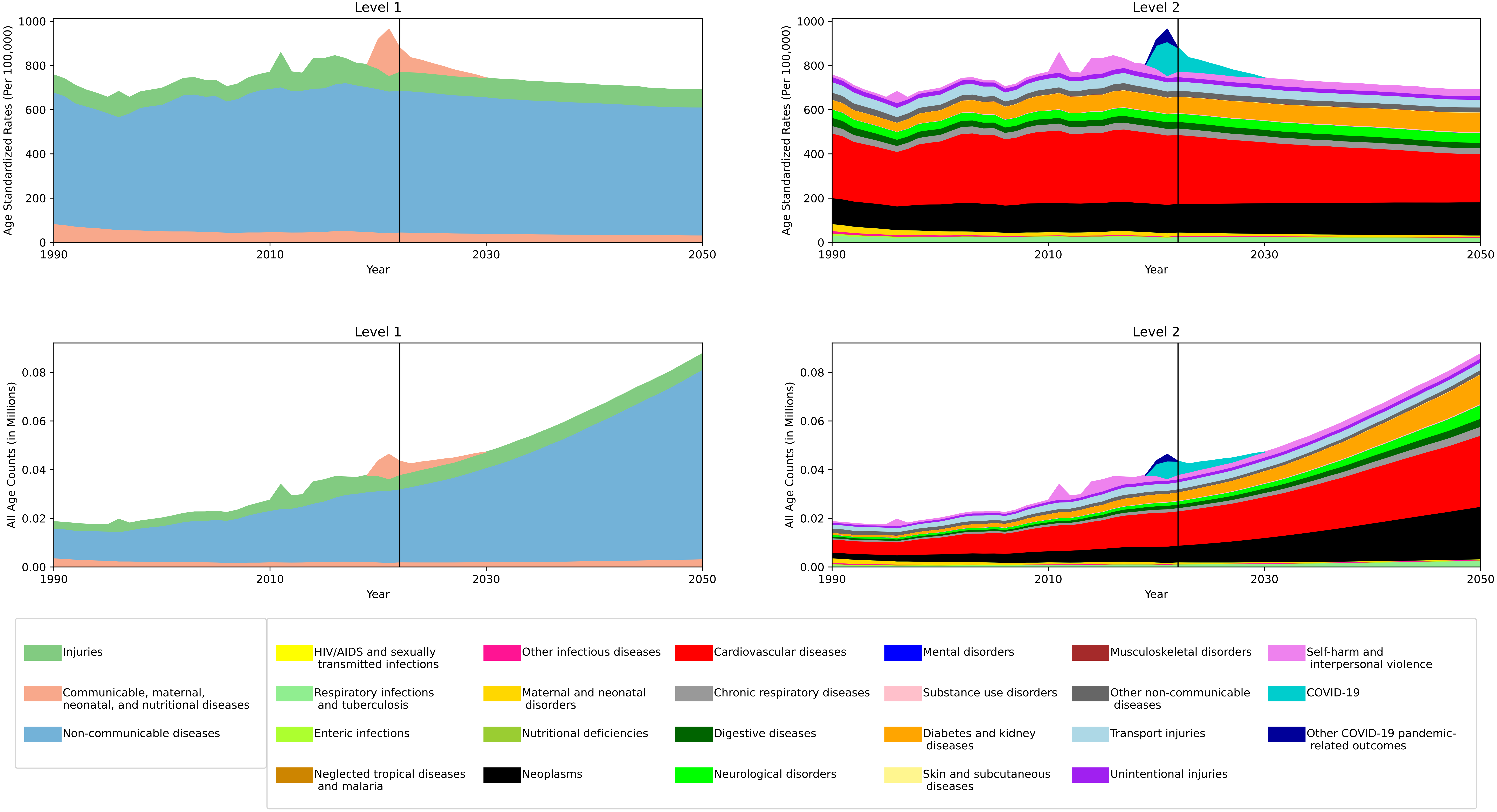
Level 1
Level 2
Age Standardized Rates (Per 100,000)
All Age Counts (in Millions)
Year
Injuries
Communicable, maternal, neonatal, and nutritional diseases
Non-communicable diseases
HIV/AIDS and sexually transmitted infections
Respiratory infections and tuberculosis
Enteric infections
Neglected tropical diseases and malaria
Other infectious diseases
Maternal and neonatal disorders
Nutritional deficiencies
Neoplasms
Cardiovascular diseases
Chronic respiratory diseases
Digestive diseases
Neurological disorders
Mental disorders
Substance use disorders
Diabetes and kidney diseases
Skin and subcutaneous diseases
Musculoskeletal disorders
Other non-communicable diseases
Transport injuries
Unintentional injuries
Self-harm and interpersonal violence
COVID-19
Other COVID-19 pandemic-related outcomes

Morocco

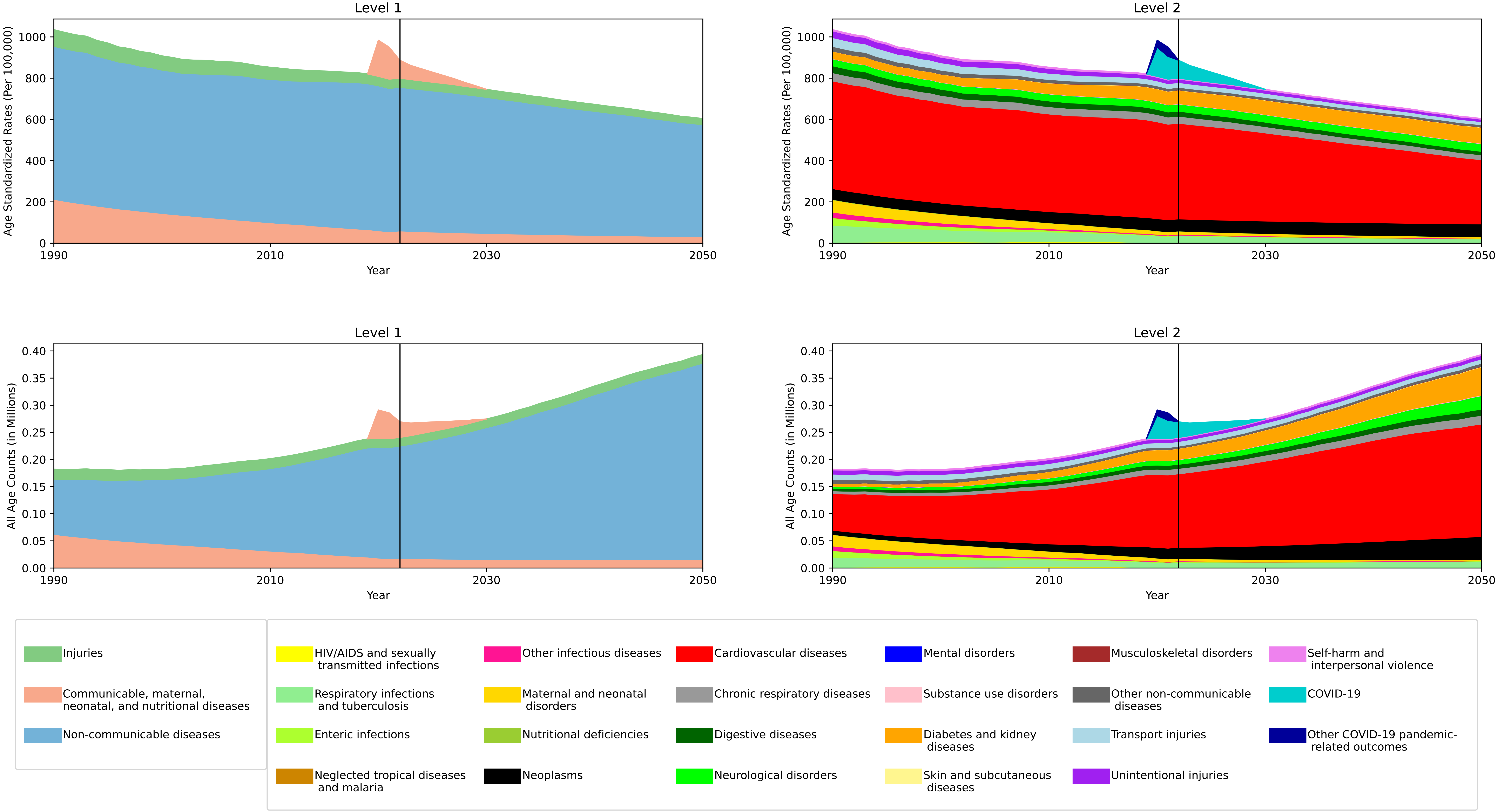
Level 1
Level 2
Age Standardized Rates (Per 100,000)
All Age Counts (in Millions)
Year
1990
2010
2030
2050
0
200
400
600
800
1000
0.00
0.05
0.10
0.15
0.20
0.25
0.30
0.35
0.40
Injuries
Communicable, maternal, neonatal, and nutritional diseases
Non-communicable diseases
HIV/AIDS and sexually transmitted infections
Respiratory infections and tuberculosis
Enteric infections
Neglected tropical diseases and malaria
Other infectious diseases
Maternal and neonatal disorders
Nutritional deficiencies
Neoplasms
Cardiovascular diseases
Chronic respiratory diseases
Digestive diseases
Neurological disorders
Mental disorders
Substance use disorders
Diabetes and kidney diseases
Skin and subcutaneous diseases
Musculoskeletal disorders
Other non-communicable diseases
Transport injuries
Unintentional injuries
Self-harm and interpersonal violence
COVID-19
Other COVID-19 pandemic-related outcomes

Oman

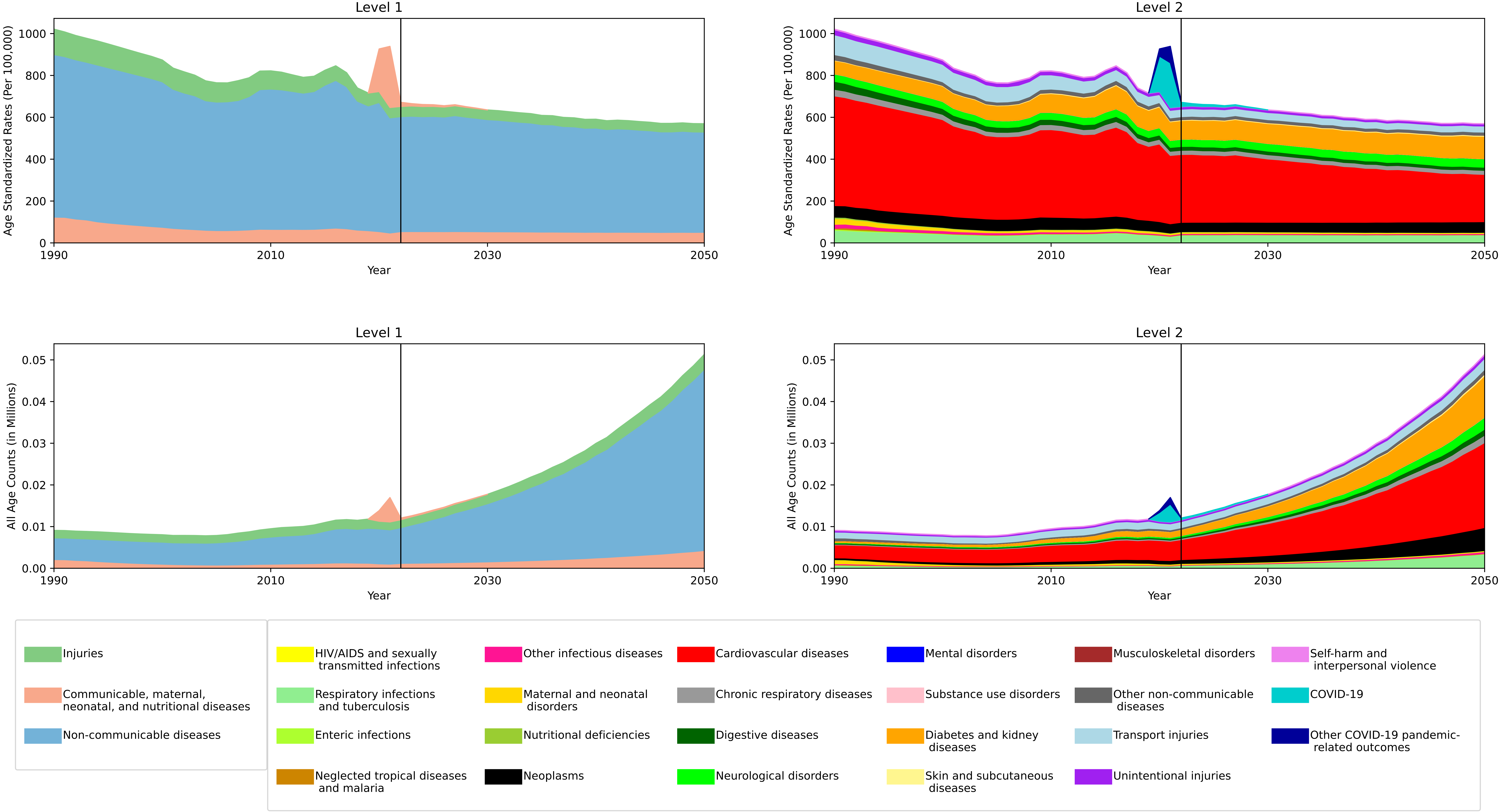
Level 1
Level 2
Age Standardized Rates (Per 100,000)
All Age Counts (in Millions)
Year
Injuries
Communicable, maternal, neonatal, and nutritional diseases
Non-communicable diseases
HIV/AIDS and sexually transmitted infections
Respiratory infections and tuberculosis
Enteric infections
Neglected tropical diseases and malaria
Other infectious diseases
Maternal and neonatal disorders
Nutritional deficiencies
Neoplasms
Cardiovascular diseases
Chronic respiratory diseases
Digestive diseases
Neurological disorders
Mental disorders
Substance use disorders
Diabetes and kidney diseases
Skin and subcutaneous diseases
Musculoskeletal disorders
Other non-communicable diseases
Transport injuries
Unintentional injuries
Self-harm and interpersonal violence
COVID-19
Other COVID-19 pandemic-related outcomes

Palestine

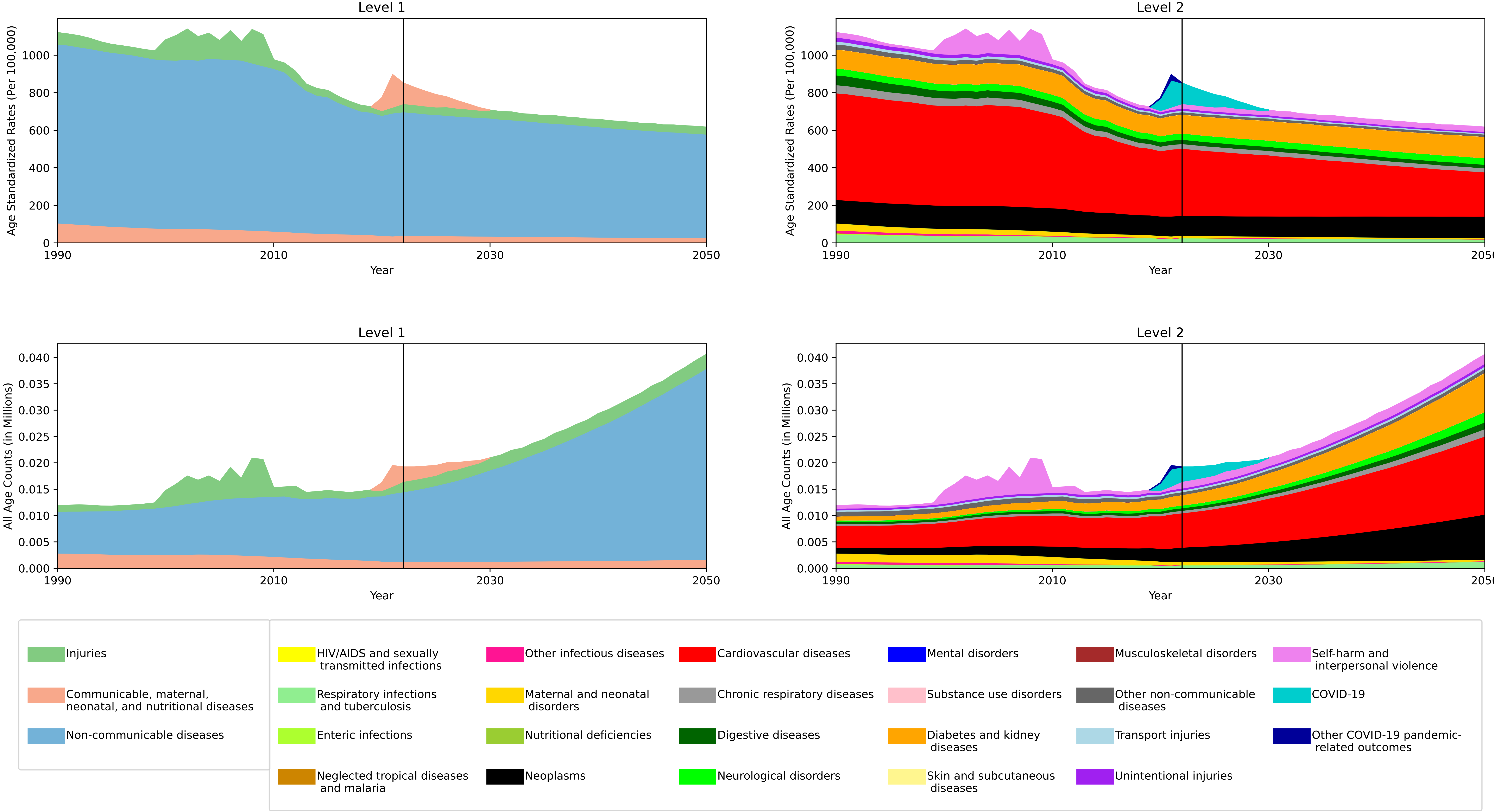
Level 1
Level 2
Age Standardized Rates (Per 100,000)
All Age Counts (in Millions)
Year
1990
2010
2030
2050
Injuries
Communicable, maternal, neonatal, and nutritional diseases
Non-communicable diseases
HIV/AIDS and sexually transmitted infections
Respiratory infections and tuberculosis
Enteric infections
Neglected tropical diseases and malaria
Other infectious diseases
Maternal and neonatal disorders
Nutritional deficiencies
Neoplasms
Cardiovascular diseases
Chronic respiratory diseases
Digestive diseases
Neurological disorders
Mental disorders
Substance use disorders
Diabetes and kidney diseases
Skin and subcutaneous diseases
Musculoskeletal disorders
Other non-communicable diseases
Transport injuries
Unintentional injuries
Self-harm and interpersonal violence
COVID-19
Other COVID-19 pandemic-related outcomes

Qatar

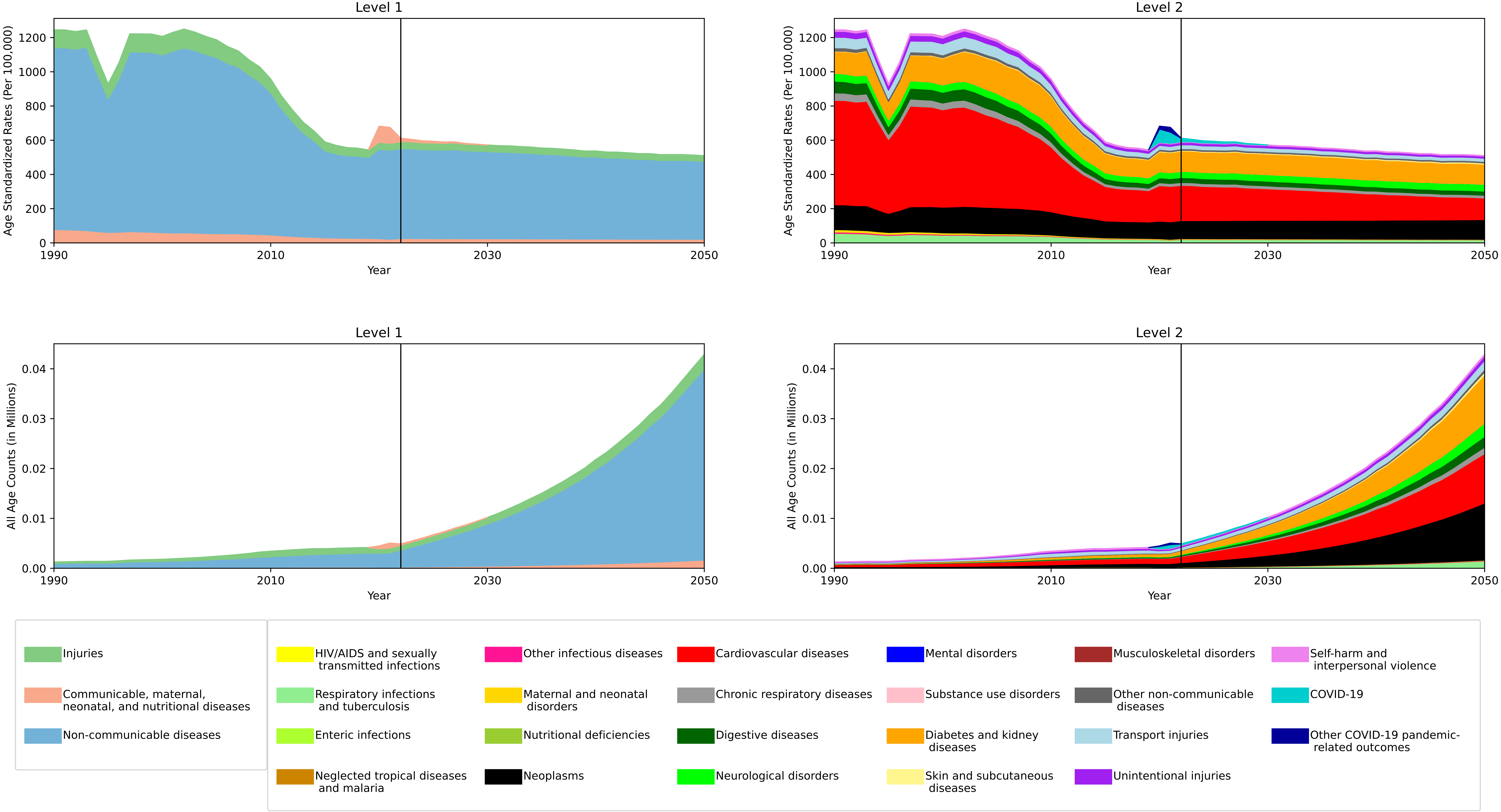
Level 1
Level 2
Age Standardized Rates (Per 100,000)
All Age Counts (in Millions)
Year
1990
2010
2030
2050
0
200
400
600
800
1000
1200
0.00
0.01
0.02
0.03
0.04
Injuries
Communicable, maternal, neonatal, and nutritional diseases
Non-communicable diseases
HIV/AIDS and sexually transmitted infections
Respiratory infections and tuberculosis
Enteric infections
Neglected tropical diseases and malaria
Other infectious diseases
Maternal and neonatal disorders
Nutritional deficiencies
Neoplasms
Cardiovascular diseases
Chronic respiratory diseases
Digestive diseases
Neurological disorders
Mental disorders
Substance use disorders
Diabetes and kidney diseases
Skin and subcutaneous diseases
Musculoskeletal disorders
Other non-communicable diseases
Transport injuries
Unintentional injuries
Self-harm and interpersonal violence
COVID-19
Other COVID-19 pandemic-related outcomes

Saudi Arabia

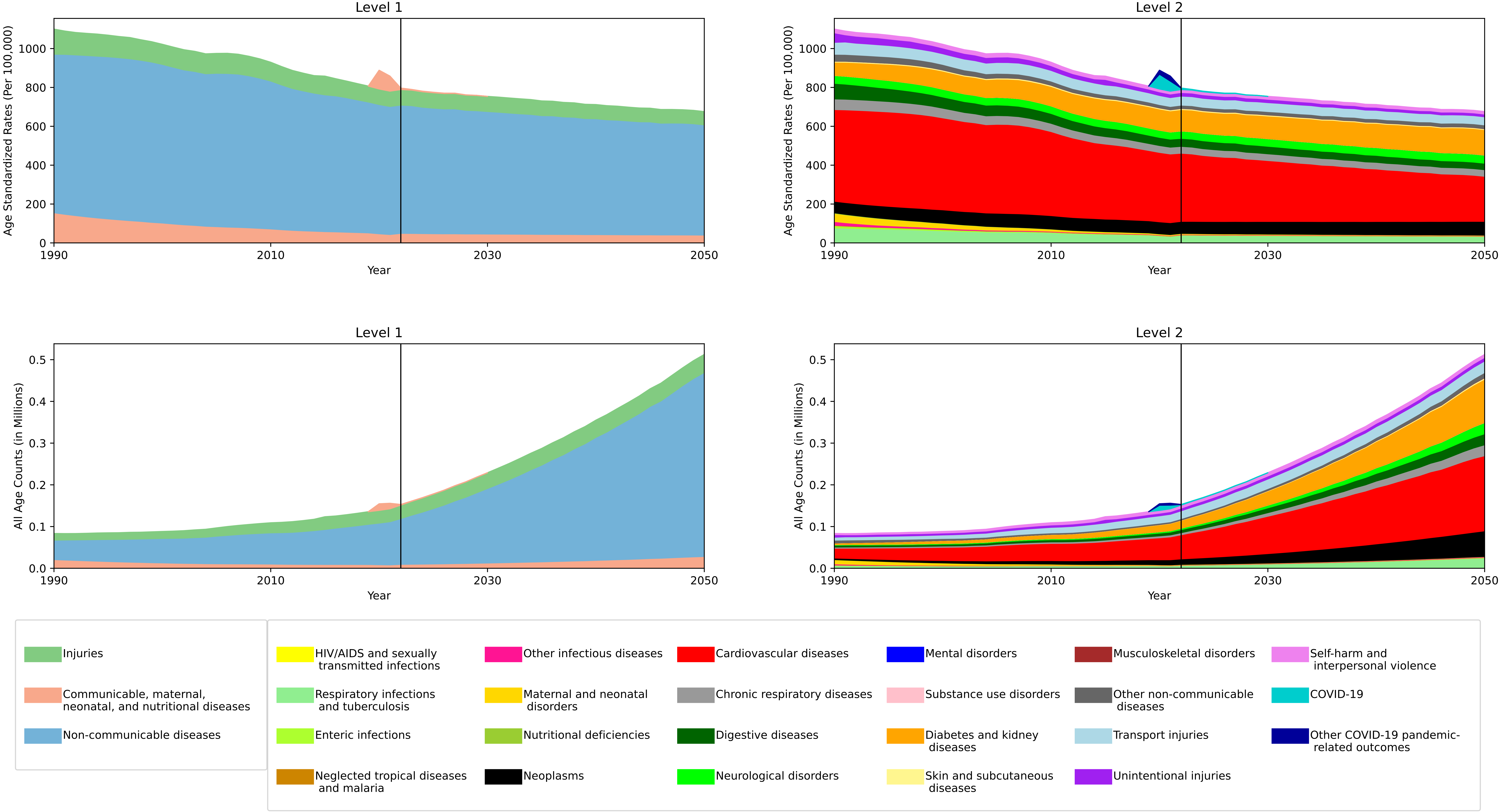

Sudan

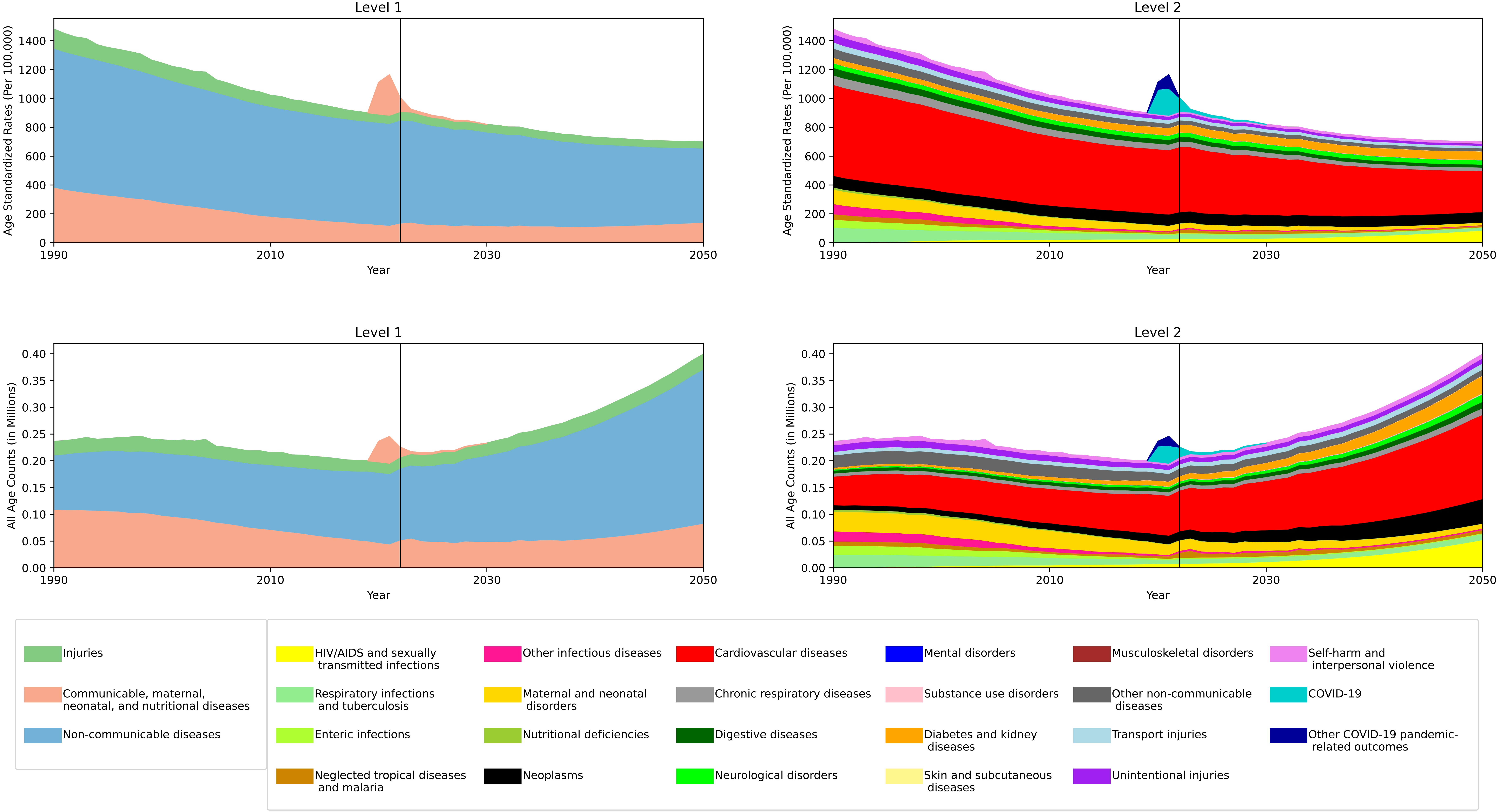
Level 1
Level 2
Age Standardized Rates (Per 100,000)
All Age Counts (in Millions)
Year
1990
2010
2030
2050
0
200
400
600
800
1000
1200
1400
0.00
0.05
0.10
0.15
0.20
0.25
0.30
0.35
0.40
Injuries
Communicable, maternal, neonatal, and nutritional diseases
Non-communicable diseases
HIV/AIDS and sexually transmitted infections
Respiratory infections and tuberculosis
Enteric infections
Neglected tropical diseases and malaria
Other infectious diseases
Maternal and neonatal disorders
Nutritional deficiencies
Neoplasms
Cardiovascular diseases
Chronic respiratory diseases
Digestive diseases
Neurological disorders
Mental disorders
Substance use disorders
Diabetes and kidney diseases
Skin and subcutaneous diseases
Musculoskeletal disorders
Other non-communicable diseases
Transport injuries
Unintentional injuries
Self-harm and interpersonal violence
COVID-19
Other COVID-19 pandemic-related outcomes

Syria

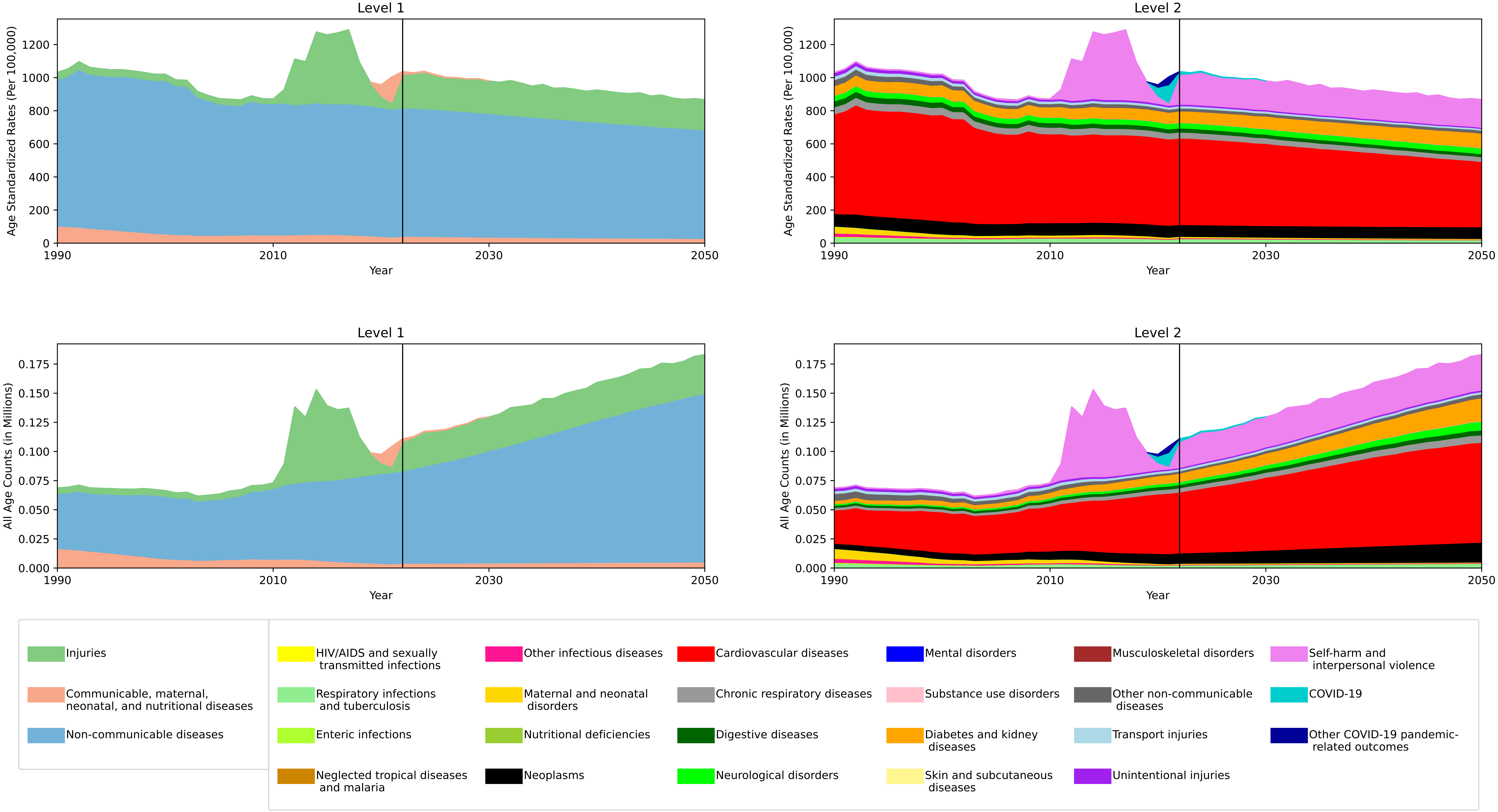
Level 1
Level 2
Age Standardized Rates (Per 100,000)
All Age Counts (in Millions)
Year
1990
2010
2030
2050
0
200
400
600
800
1000
1200
0.000
0.025
0.050
0.075
0.100
0.125
0.150
0.175
Injuries
Communicable, maternal, neonatal, and nutritional diseases
Non-communicable diseases
HIV/AIDS and sexually transmitted infections
Respiratory infections and tuberculosis
Enteric infections
Neglected tropical diseases and malaria
Other infectious diseases
Maternal and neonatal disorders
Nutritional deficiencies
Neoplasms
Cardiovascular diseases
Chronic respiratory diseases
Digestive diseases
Neurological disorders
Mental disorders
Substance use disorders
Diabetes and kidney diseases
Skin and subcutaneous diseases
Musculoskeletal disorders
Other non-communicable diseases
Transport injuries
Unintentional injuries
Self-harm and interpersonal violence
COVID-19
Other COVID-19 pandemic-related outcomes

Tunisia

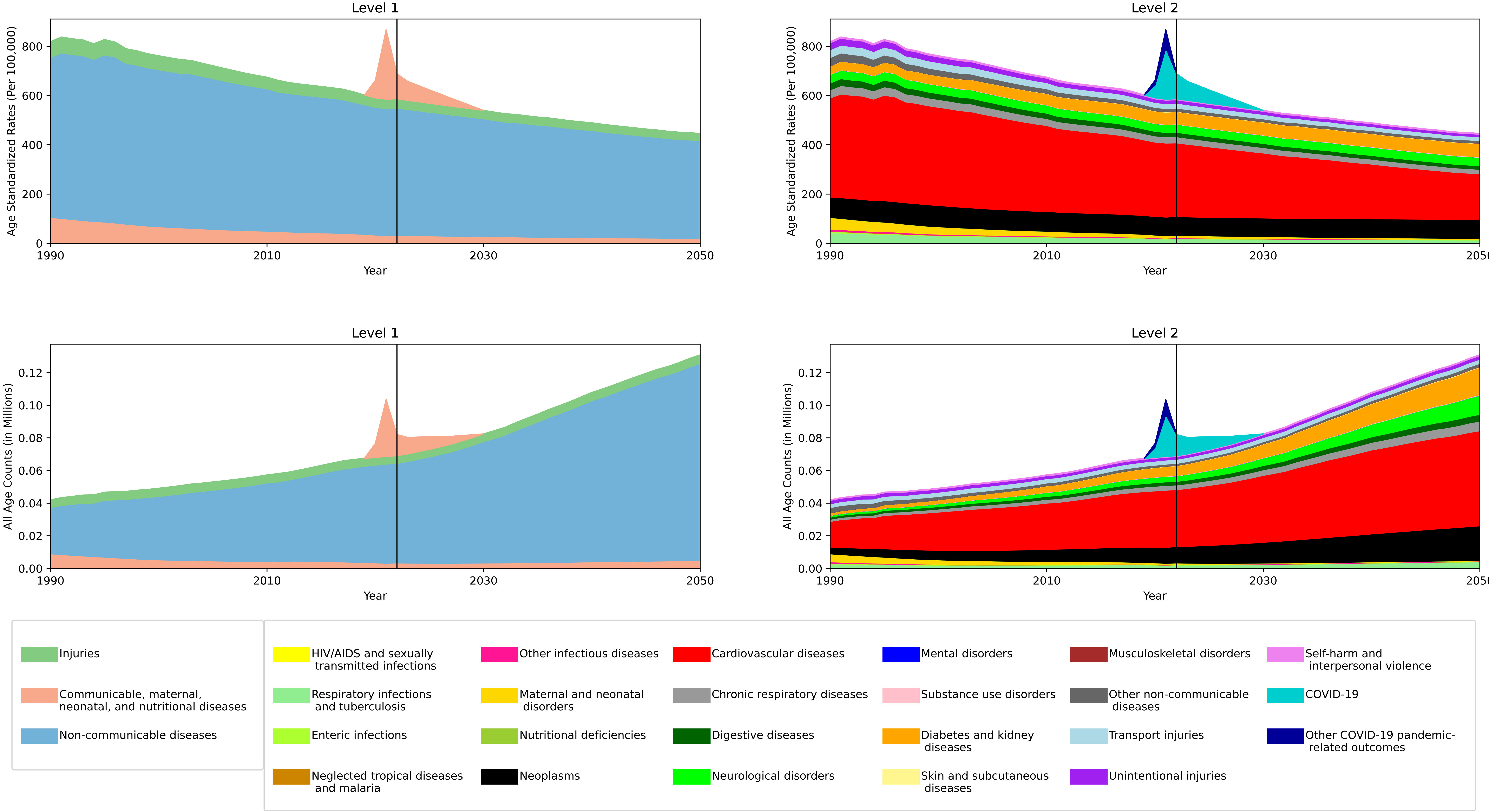
Level 1
Level 2
Age Standardized Rates (Per 100,000)
All Age Counts (in Millions)
Year
1990
2010
2030
2050
0
200
400
600
800
0.00
0.02
0.04
0.06
0.08
0.10
0.12
Injuries
Communicable, maternal, neonatal, and nutritional diseases
Non-communicable diseases
HIV/AIDS and sexually transmitted infections
Respiratory infections and tuberculosis
Enteric infections
Neglected tropical diseases and malaria
Other infectious diseases
Maternal and neonatal disorders
Nutritional deficiencies
Neoplasms
Cardiovascular diseases
Chronic respiratory diseases
Digestive diseases
Neurological disorders
Mental disorders
Substance use disorders
Diabetes and kidney diseases
Skin and subcutaneous diseases
Musculoskeletal disorders
Other non-communicable diseases
Transport injuries
Unintentional injuries
Self-harm and interpersonal violence
COVID-19
Other COVID-19 pandemic-related outcomes

Türkiye

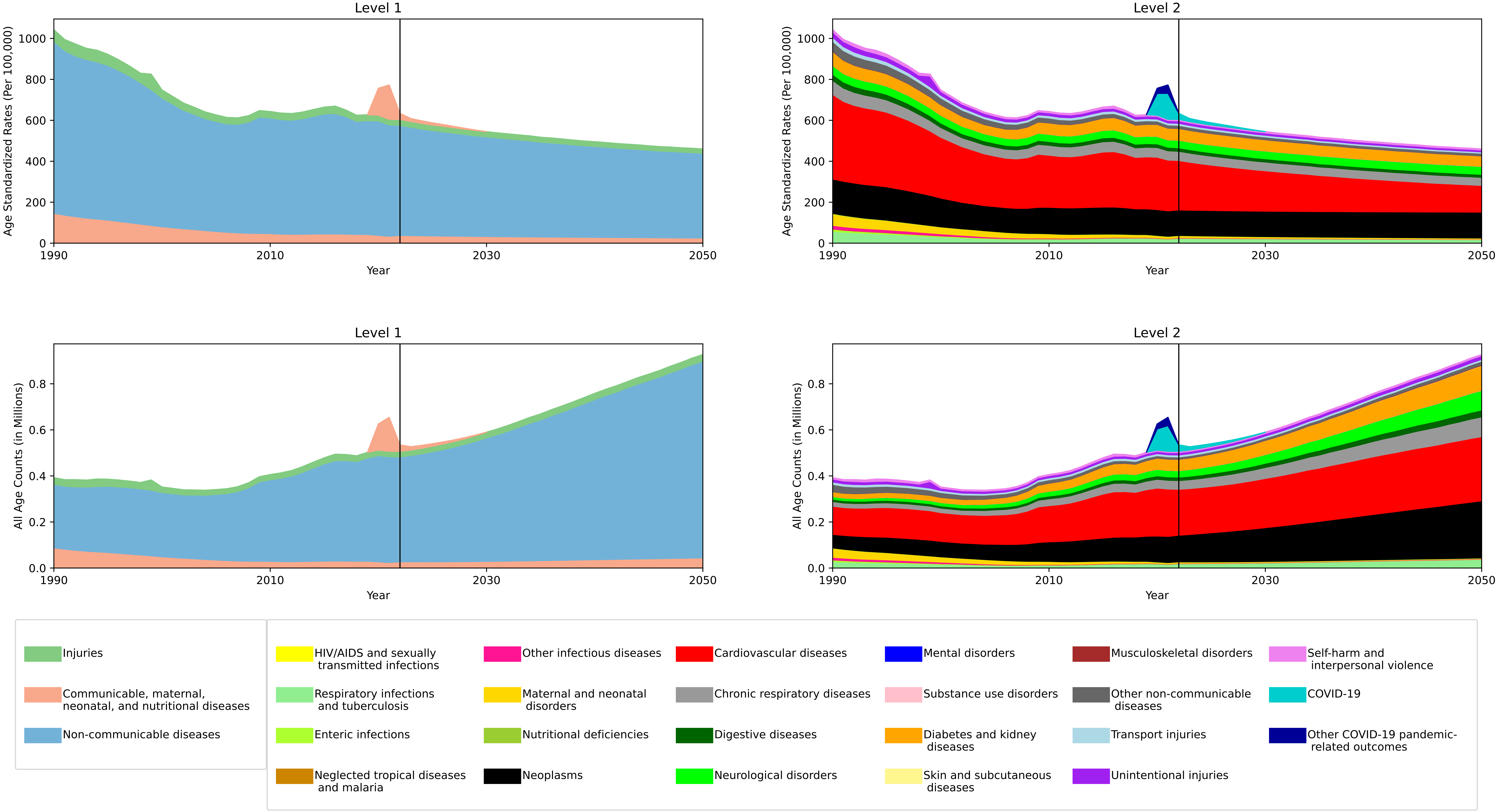
Level 1
Level 2
Age Standardized Rates (Per 100,000)
All Age Counts (in Millions)
Year
1990
2010
2030
2050
0
200
400
600
800
1000
0.0
0.2
0.4
0.6
0.8
Injuries
Communicable, maternal, neonatal, and nutritional diseases
Non-communicable diseases
HIV/AIDS and sexually transmitted infections
Respiratory infections and tuberculosis
Enteric infections
Neglected tropical diseases and malaria
Other infectious diseases
Maternal and neonatal disorders
Nutritional deficiencies
Neoplasms
Cardiovascular diseases
Chronic respiratory diseases
Digestive diseases
Neurological disorders
Mental disorders
Substance use disorders
Diabetes and kidney diseases
Skin and subcutaneous diseases
Musculoskeletal disorders
Other non-communicable diseases
Transport injuries
Unintentional injuries
Self-harm and interpersonal violence
COVID-19
Other COVID-19 pandemic-related outcomes

United Arab Emirates

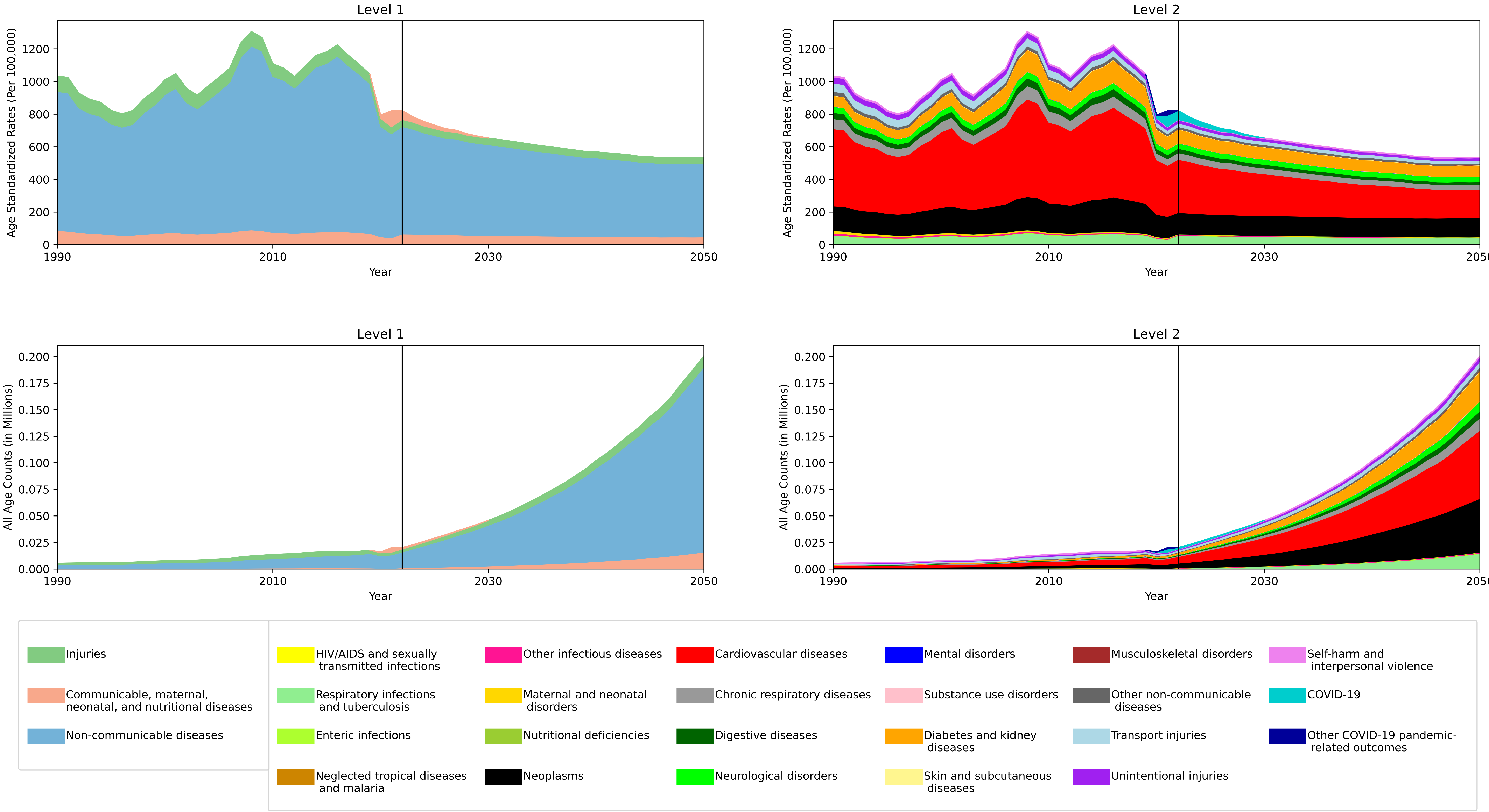

Yemen

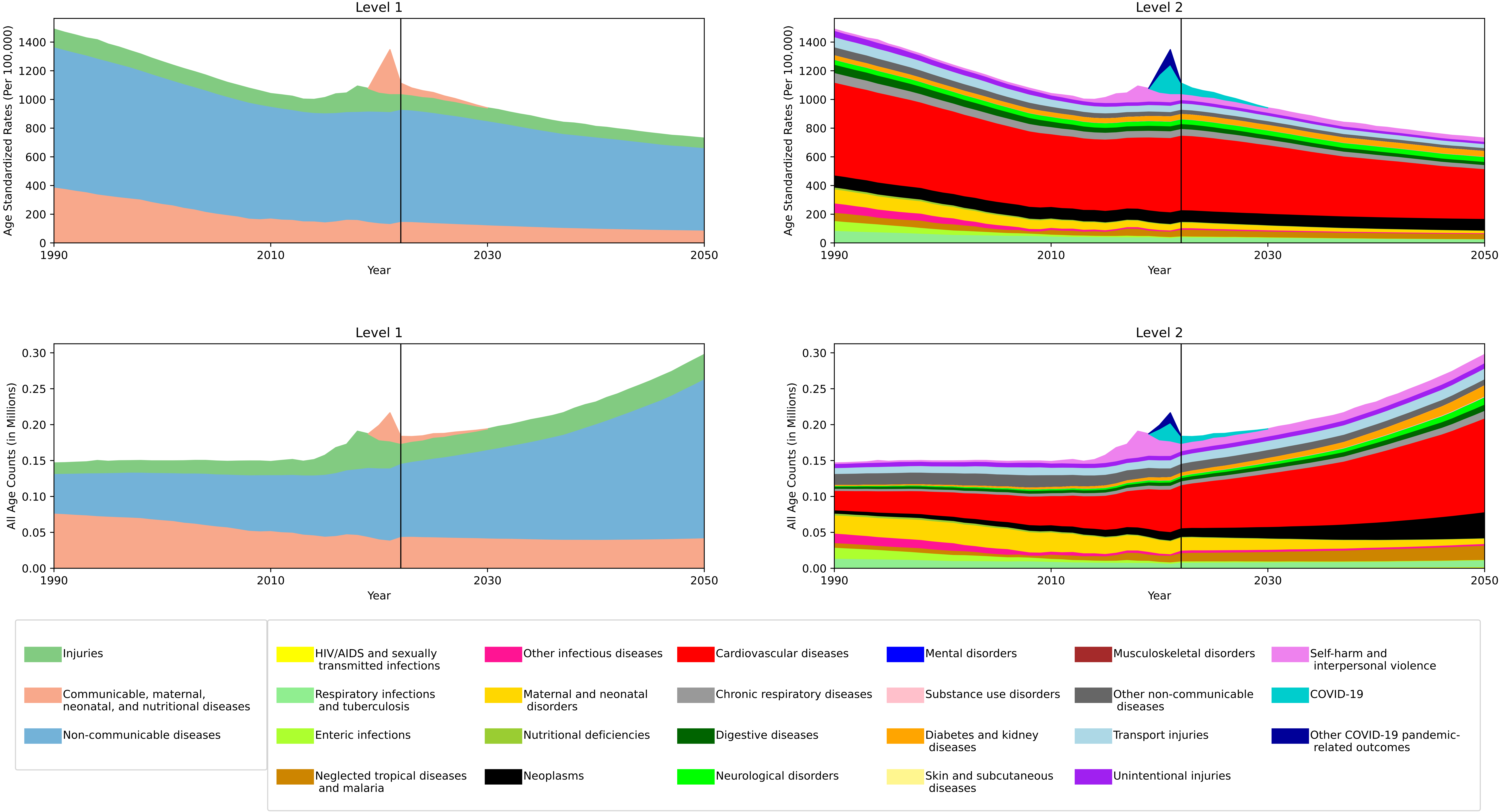
Level 1
Level 2
Age Standardized Rates (Per 100,000)
All Age Counts (in Millions)
Year
Injuries
Communicable, maternal, neonatal, and nutritional diseases
Non-communicable diseases
HIV/AIDS and sexually transmitted infections
Respiratory infections and tuberculosis
Enteric infections
Neglected tropical diseases and malaria
Other infectious diseases
Maternal and neonatal disorders
Nutritional deficiencies
Neoplasms
Cardiovascular diseases
Chronic respiratory diseases
Digestive diseases
Neurological disorders
Mental disorders
Substance use disorders
Diabetes and kidney diseases
Skin and subcutaneous diseases
Musculoskeletal disorders
Other non-communicable diseases
Transport injuries
Unintentional injuries
Self-harm and interpersonal violence
COVID-19
Other COVID-19 pandemic-related outcomes

Bangladesh

Supplemental Results Figure S5. Age-standardised death rates and all age death counts and by Level 1 and Level 2 causes, 1990–2050

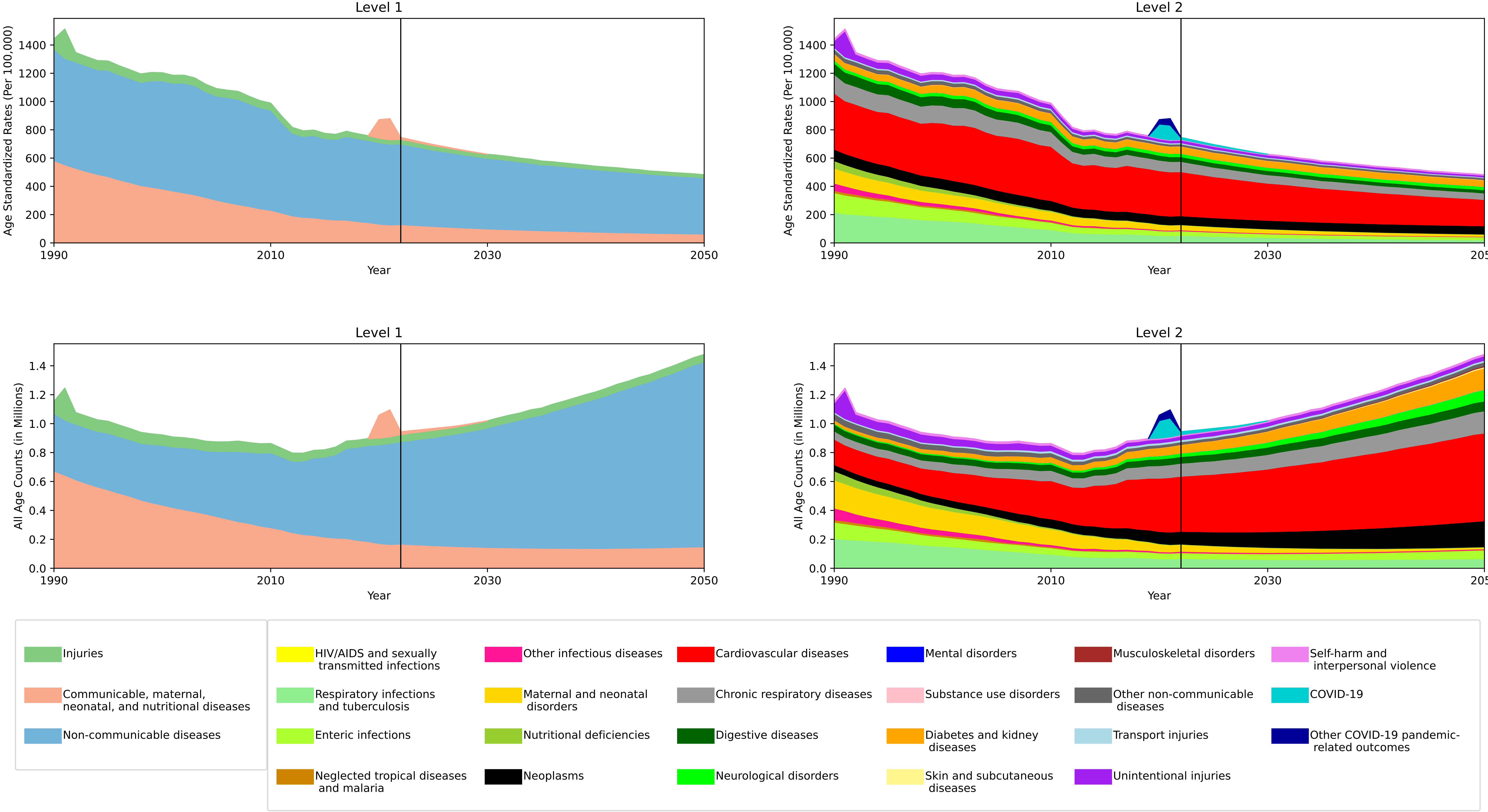

Bhutan

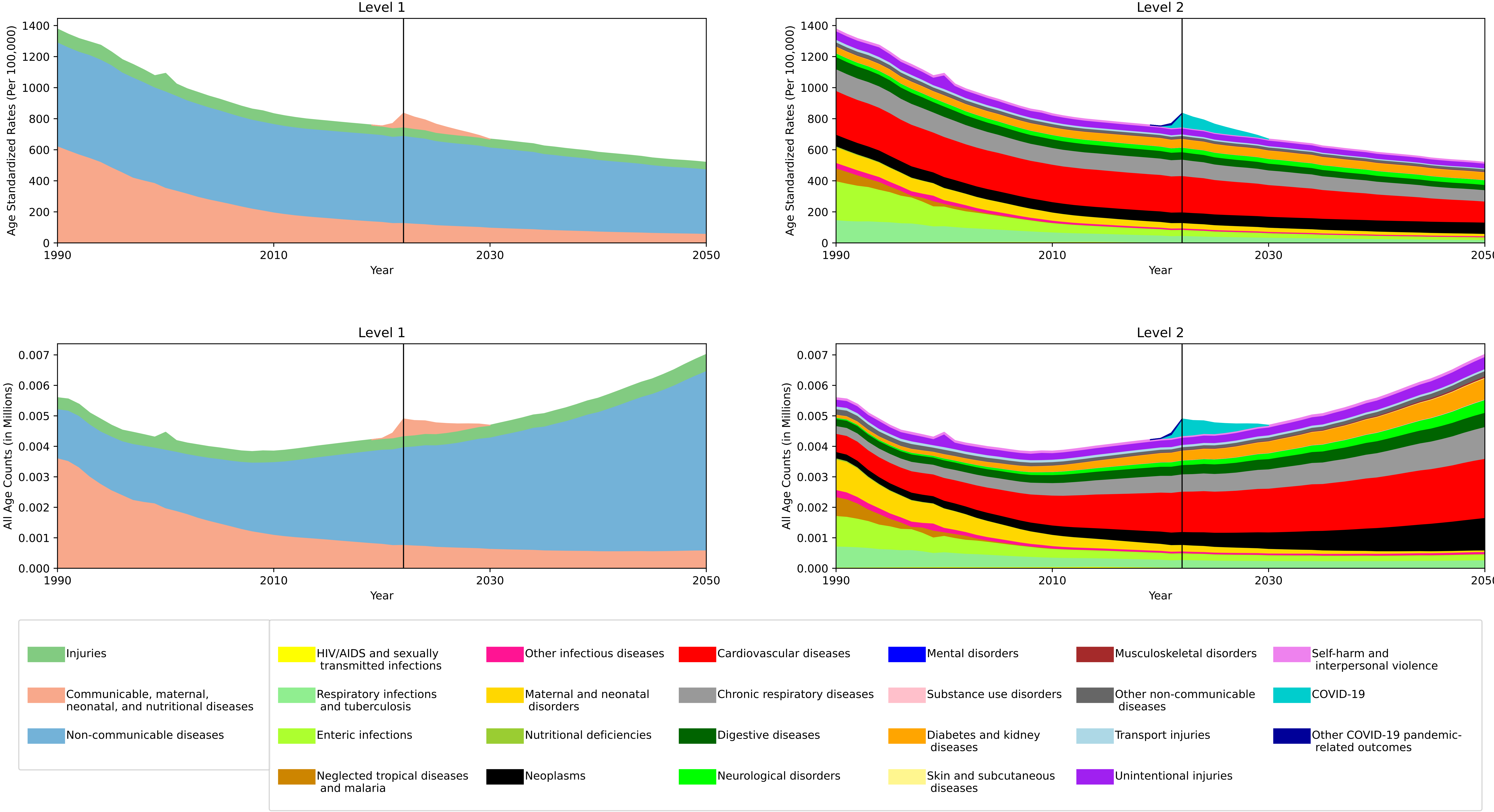
Level 1
Level 2
Age Standardized Rates (Per 100,000)
All Age Counts (in Millions)
Year
1990
2010
2030
2050
Injuries
Communicable, maternal, neonatal, and nutritional diseases
Non-communicable diseases
HIV/AIDS and sexually transmitted infections
Respiratory infections and tuberculosis
Enteric infections
Neglected tropical diseases and malaria
Other infectious diseases
Maternal and neonatal disorders
Nutritional deficiencies
Neoplasms
Cardiovascular diseases
Chronic respiratory diseases
Digestive diseases
Neurological disorders
Mental disorders
Substance use disorders
Diabetes and kidney diseases
Skin and subcutaneous diseases
Musculoskeletal disorders
Other non-communicable diseases
Transport injuries
Unintentional injuries
Self-harm and interpersonal violence
COVID-19
Other COVID-19 pandemic-related outcomes

India

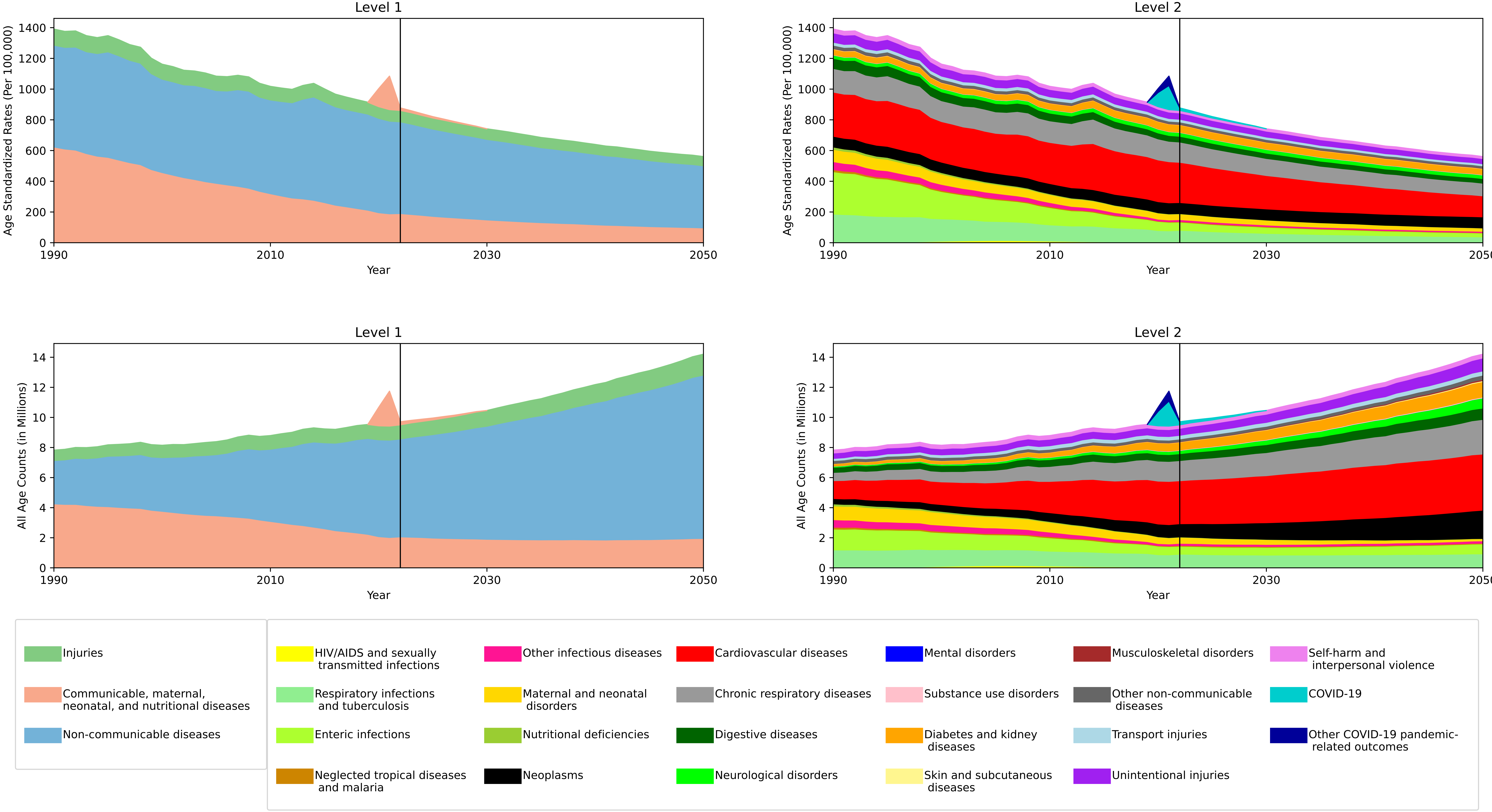
Level 1
Level 2
Age Standardized Rates (Per 100,000)
All Age Counts (in Millions)
Year
1990
2010
2030
2050
Injuries
Communicable, maternal, neonatal, and nutritional diseases
Non-communicable diseases
HIV/AIDS and sexually transmitted infections
Respiratory infections and tuberculosis
Enteric infections
Neglected tropical diseases and malaria
Other infectious diseases
Maternal and neonatal disorders
Nutritional deficiencies
Neoplasms
Cardiovascular diseases
Chronic respiratory diseases
Digestive diseases
Neurological disorders
Mental disorders
Substance use disorders
Diabetes and kidney diseases
Skin and subcutaneous diseases
Musculoskeletal disorders
Other non-communicable diseases
Transport injuries
Unintentional injuries
Self-harm and interpersonal violence
COVID-19
Other COVID-19 pandemic-related outcomes

Supplemental Results Figure S5. Age-standardised death rates and all age death counts and by Level 1 and Level 2 causes, 1990–2050

Nepal

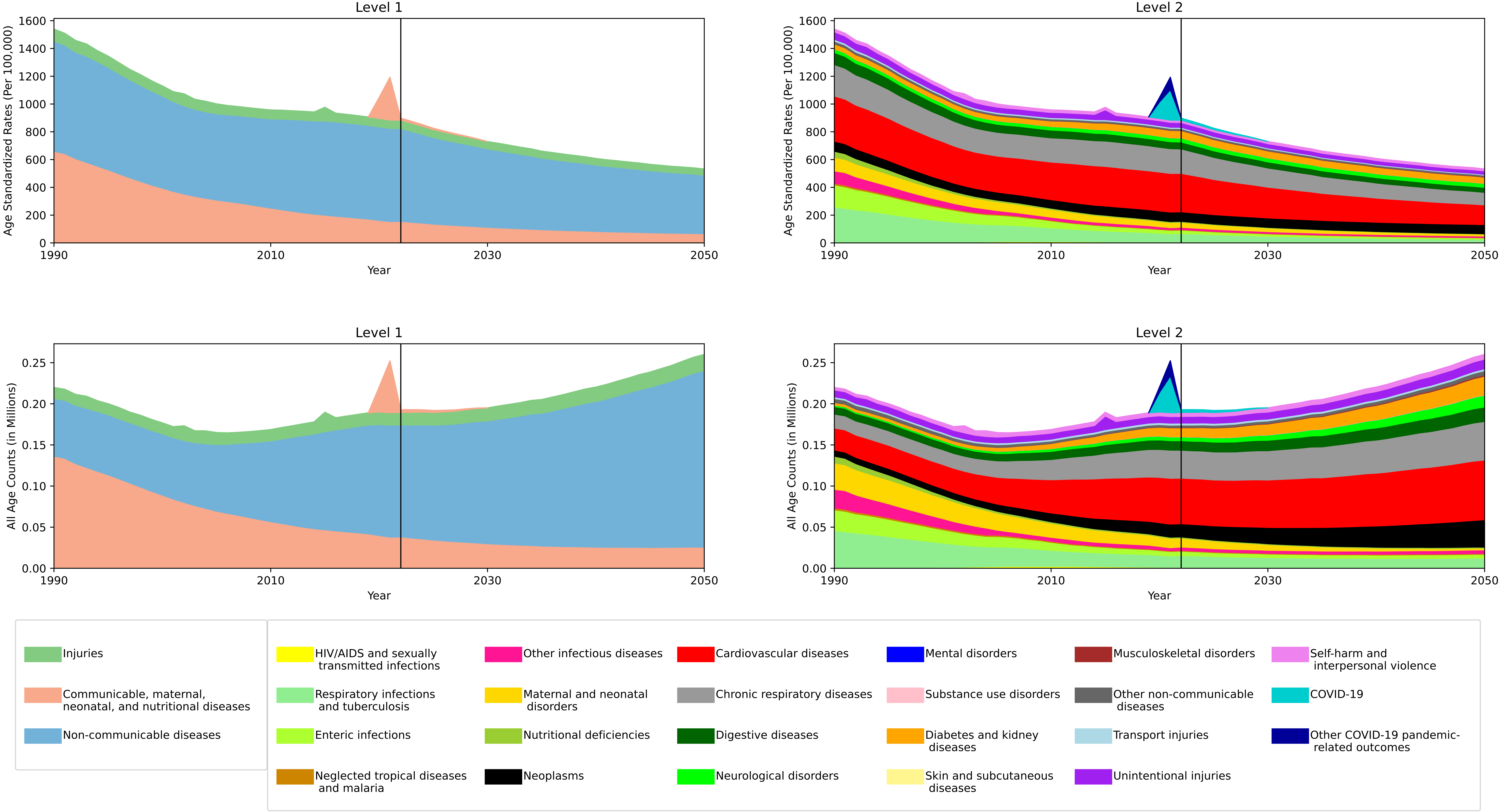

Pakistan

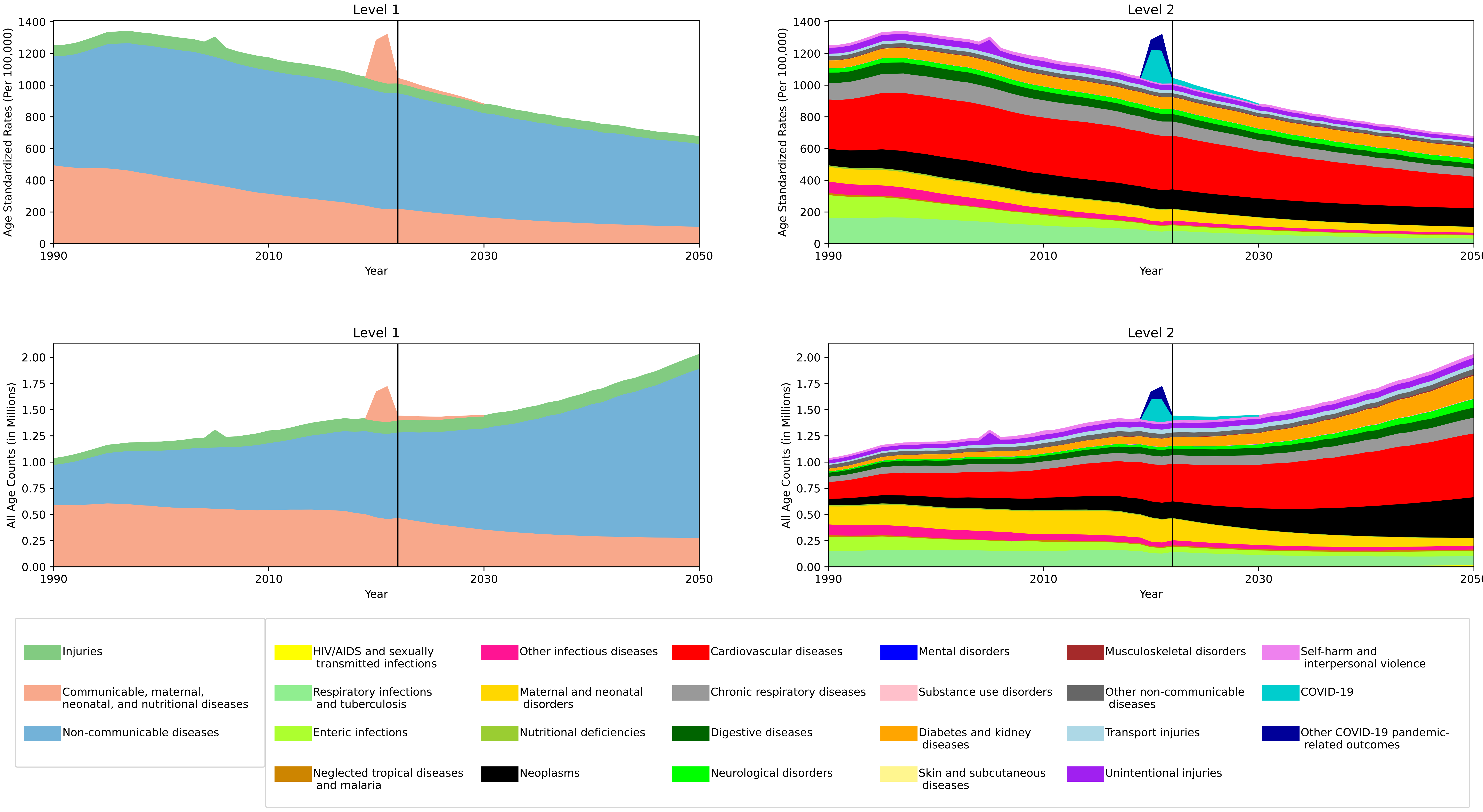
Level 1
Level 2
Age Standardized Rates (Per 100,000)
All Age Counts (in Millions)
Year
1990
2010
2030
2050
Injuries
Communicable, maternal, neonatal, and nutritional diseases
Non-communicable diseases
HIV/AIDS and sexually transmitted infections
Respiratory infections and tuberculosis
Enteric infections
Neglected tropical diseases and malaria
Other infectious diseases
Maternal and neonatal disorders
Nutritional deficiencies
Neoplasms
Cardiovascular diseases
Chronic respiratory diseases
Digestive diseases
Neurological disorders
Mental disorders
Substance use disorders
Diabetes and kidney diseases
Skin and subcutaneous diseases
Musculoskeletal disorders
Other non-communicable diseases
Transport injuries
Unintentional injuries
Self-harm and interpersonal violence
COVID-19
Other COVID-19 pandemic-related outcomes

China

Supplemental Results Figure S5. Age-standardised death rates and all age death counts and by Level 1 and Level 2 causes, 1990–2050

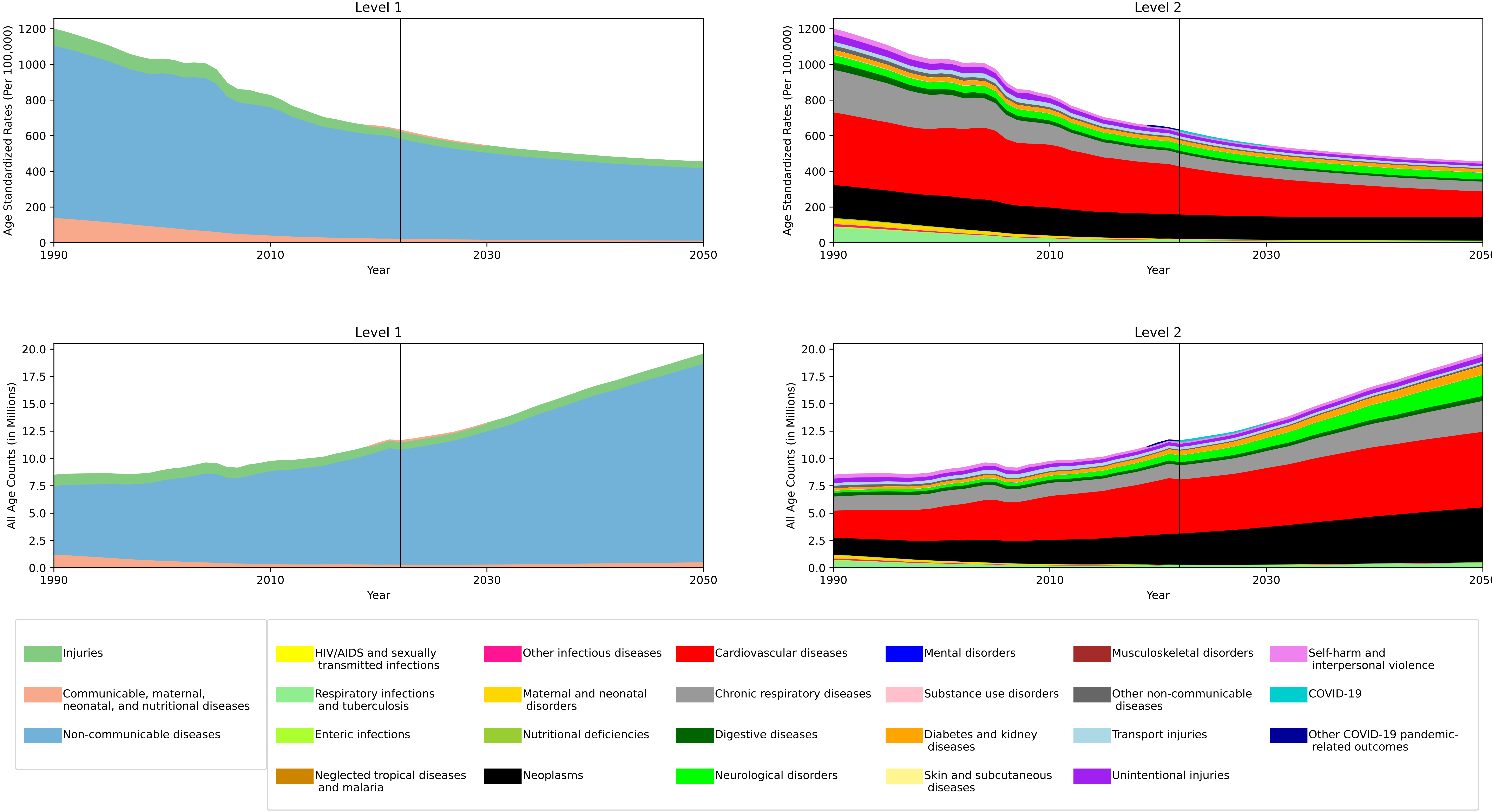

North Korea

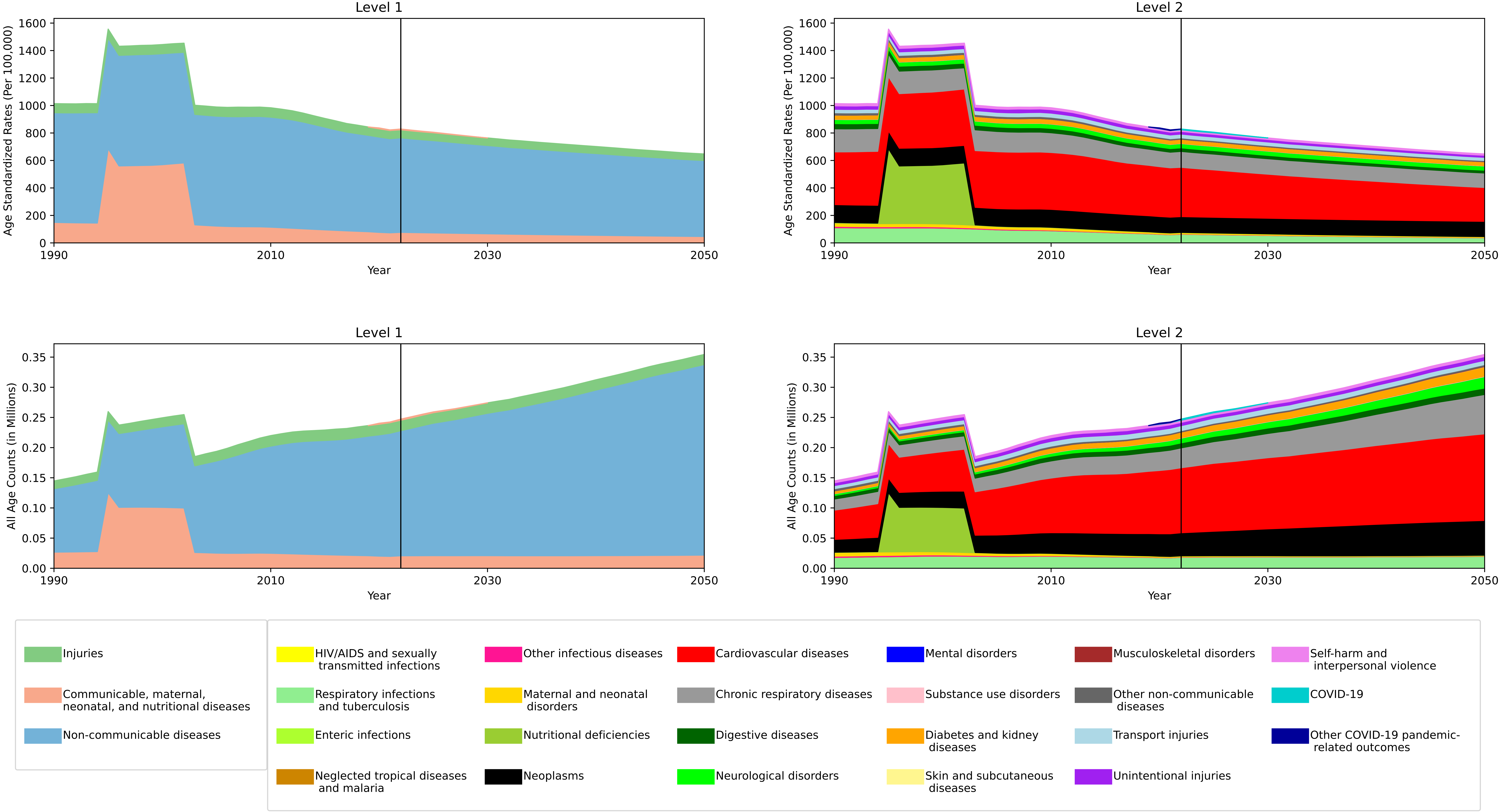
Level 1
Age Standardized Rates (Per 100,000)
Year
Level 2
Age Standardized Rates (Per 100,000)
Year
Level 1
All Age Counts (in Millions)
Year
Level 2
All Age Counts (in Millions)
Year
Injuries
Communicable, maternal, neonatal, and nutritional diseases
Non-communicable diseases
HIV/AIDS and sexually transmitted infections
Respiratory infections and tuberculosis
Enteric infections
Neglected tropical diseases and malaria
Other infectious diseases
Maternal and neonatal disorders
Nutritional deficiencies
Neoplasms
Cardiovascular diseases
Chronic respiratory diseases
Digestive diseases
Neurological disorders
Mental disorders
Substance use disorders
Diabetes and kidney diseases
Skin and subcutaneous diseases
Musculoskeletal disorders
Other non-communicable diseases
Transport injuries
Unintentional injuries
Self-harm and interpersonal violence
COVID-19
Other COVID-19 pandemic-related outcomes

Supplemental Results Figure S5. Age-standardised death rates and all age death counts and by Level 1 and Level 2 causes, 1990–2050

Taiwan (Province Of China)

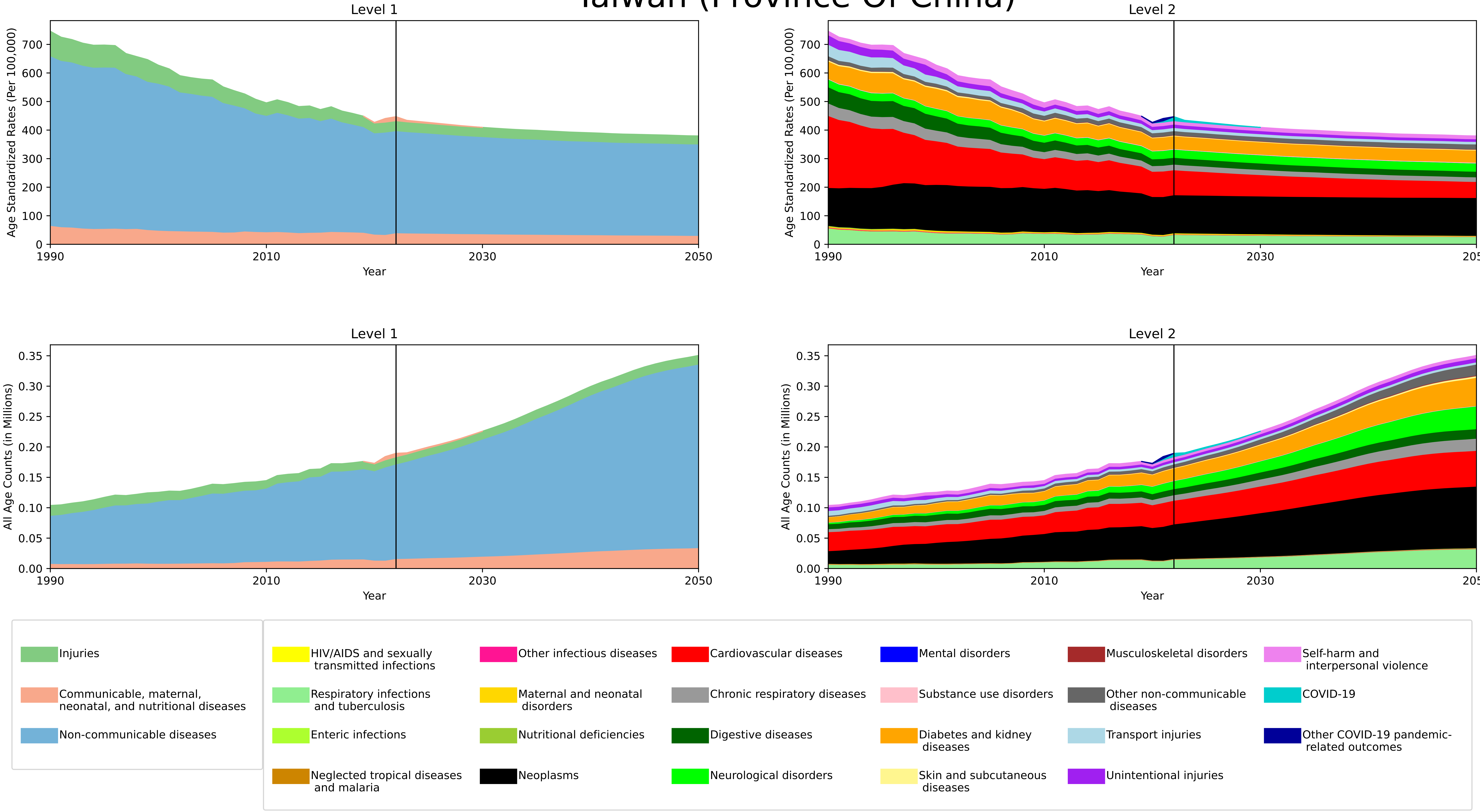

American Samoa

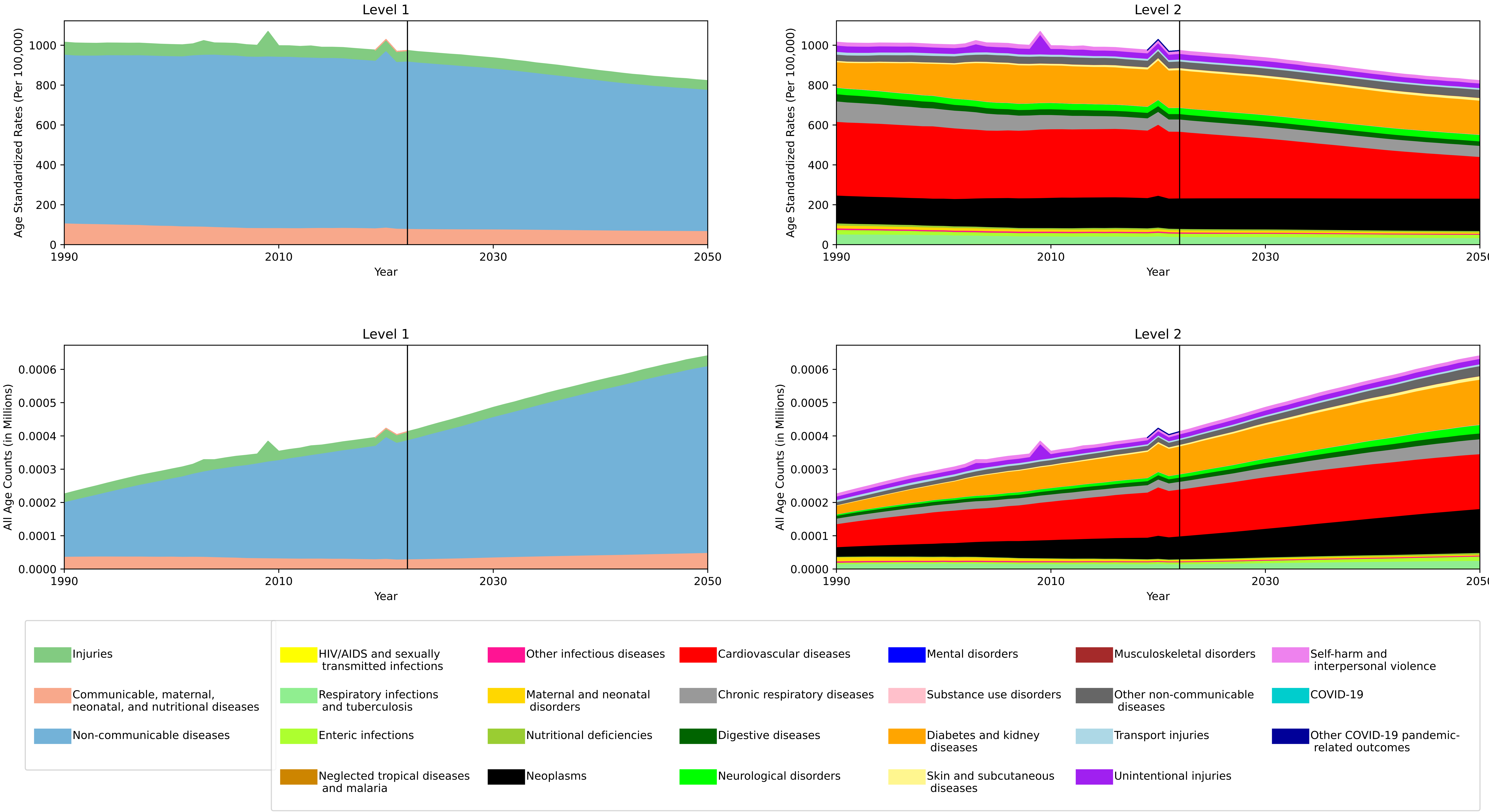

Cook Islands

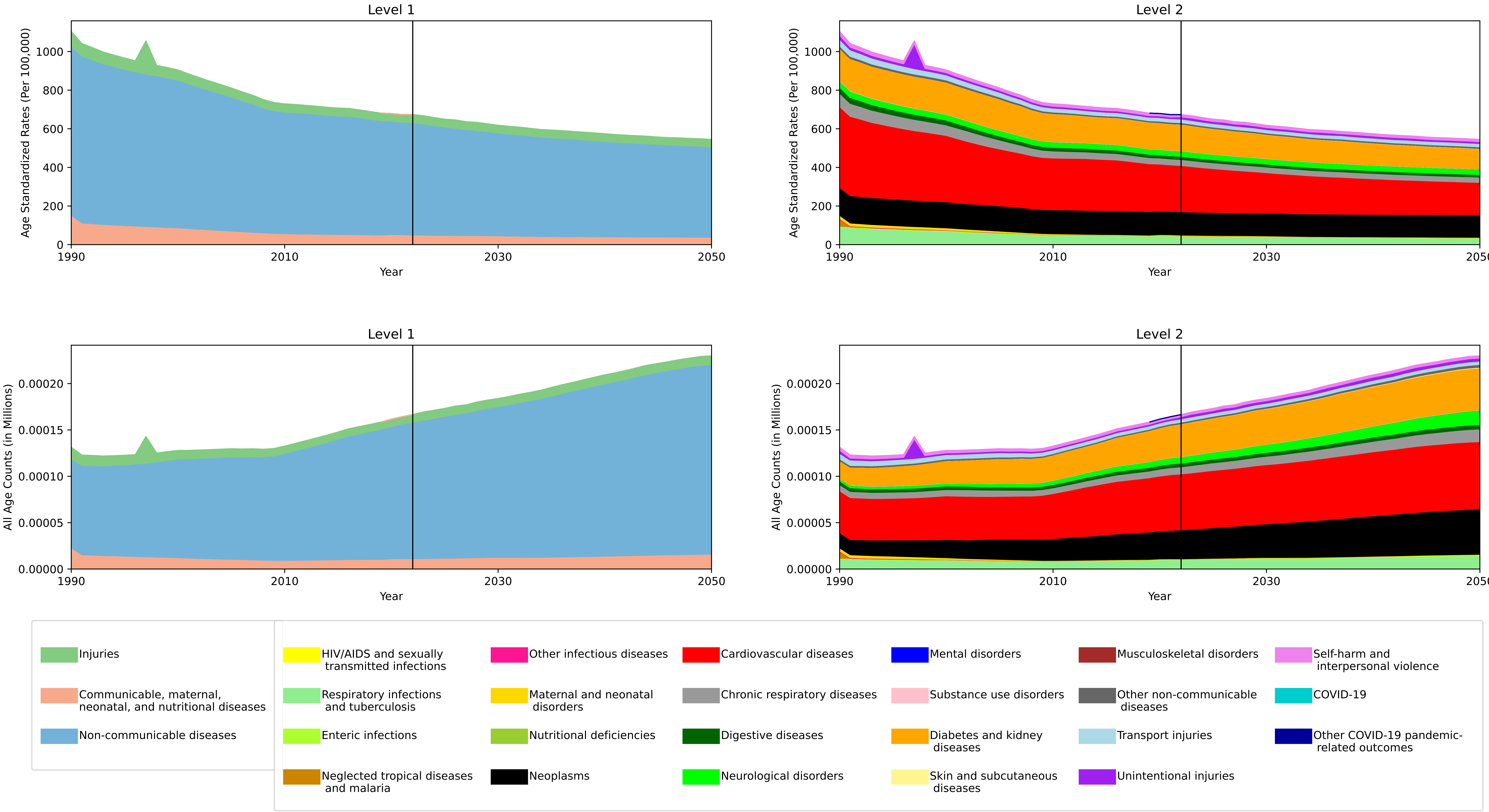

Fiji

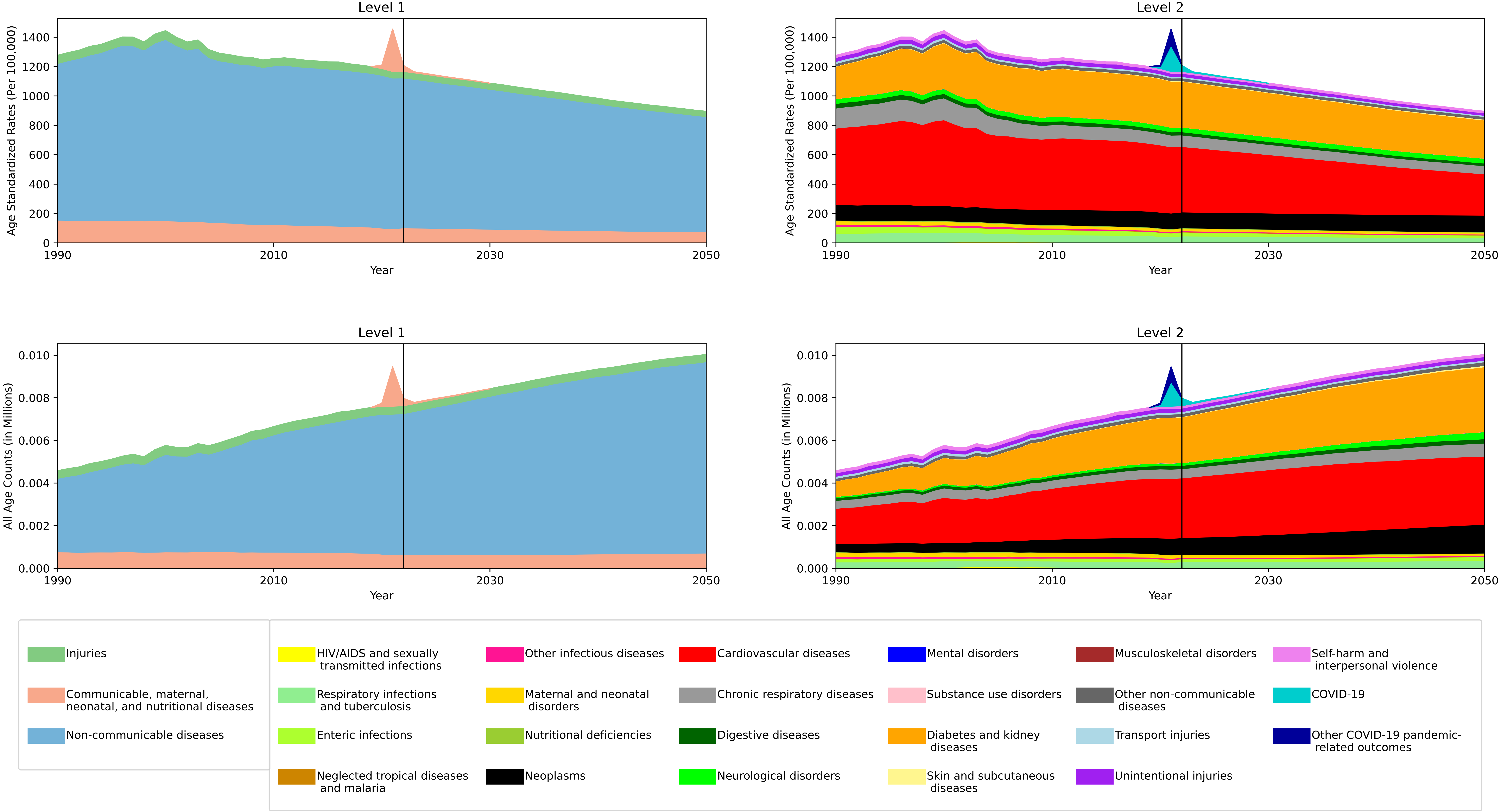
Level 1
Level 2
Age Standardized Rates (Per 100,000)
All Age Counts (in Millions)
Year
1990
2010
2030
2050
Injuries
Communicable, maternal, neonatal, and nutritional diseases
Non-communicable diseases
HIV/AIDS and sexually transmitted infections
Respiratory infections and tuberculosis
Enteric infections
Neglected tropical diseases and malaria
Other infectious diseases
Maternal and neonatal disorders
Nutritional deficiencies
Neoplasms
Cardiovascular diseases
Chronic respiratory diseases
Digestive diseases
Neurological disorders
Mental disorders
Substance use disorders
Diabetes and kidney diseases
Skin and subcutaneous diseases
Musculoskeletal disorders
Other non-communicable diseases
Transport injuries
Unintentional injuries
Self-harm and interpersonal violence
COVID-19
Other COVID-19 pandemic-related outcomes

Guam

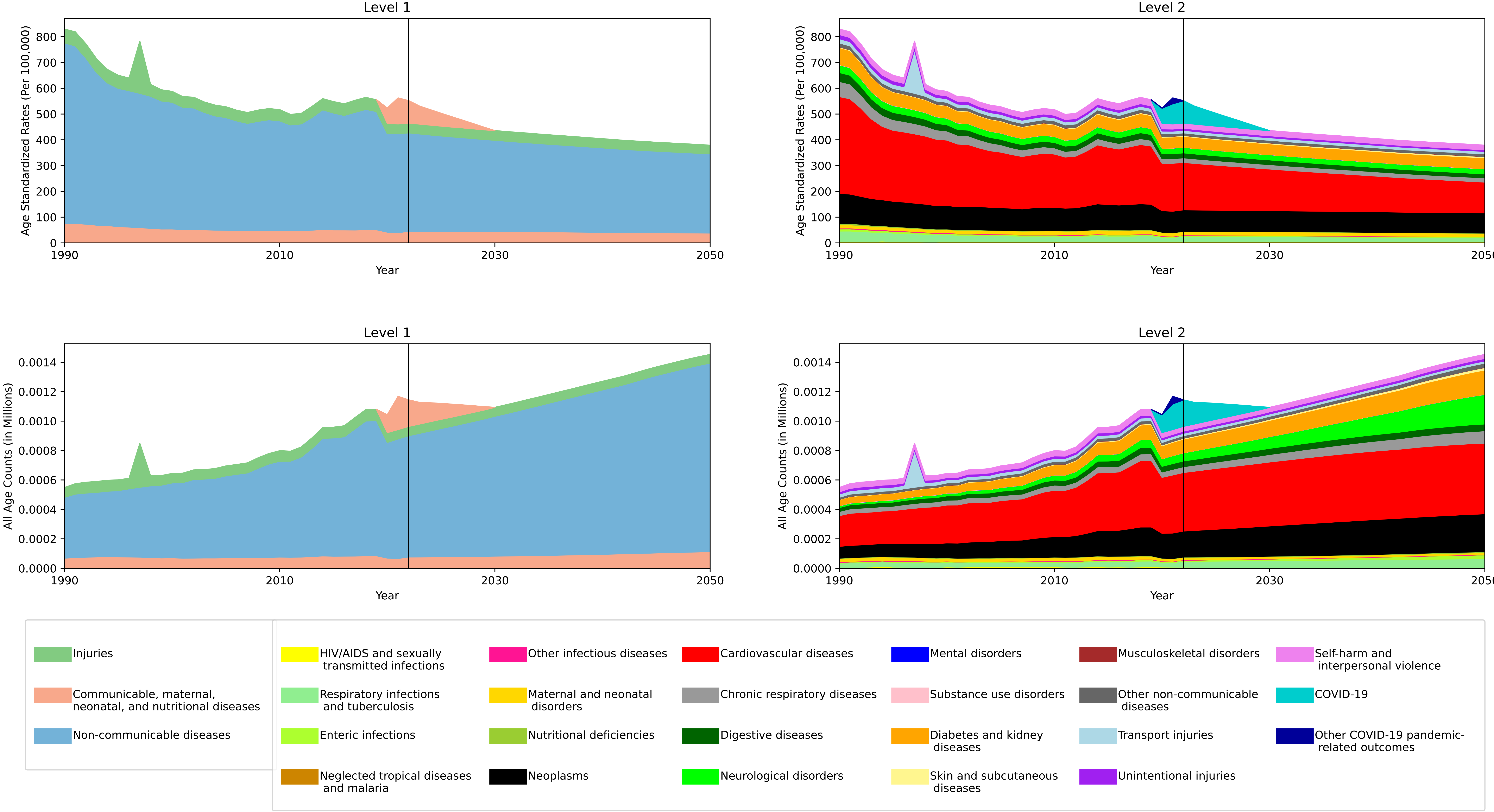
Level 1
Level 2
Age Standardized Rates (Per 100,000)
All Age Counts (in Millions)
Year
1990
2010
2030
2050
Injuries
Communicable, maternal, neonatal, and nutritional diseases
Non-communicable diseases
HIV/AIDS and sexually transmitted infections
Respiratory infections and tuberculosis
Enteric infections
Neglected tropical diseases and malaria
Other infectious diseases
Maternal and neonatal disorders
Nutritional deficiencies
Neoplasms
Cardiovascular diseases
Chronic respiratory diseases
Digestive diseases
Neurological disorders
Mental disorders
Substance use disorders
Diabetes and kidney diseases
Skin and subcutaneous diseases
Musculoskeletal disorders
Other non-communicable diseases
Transport injuries
Unintentional injuries
Self-harm and interpersonal violence
COVID-19
Other COVID-19 pandemic-related outcomes

Kiribati

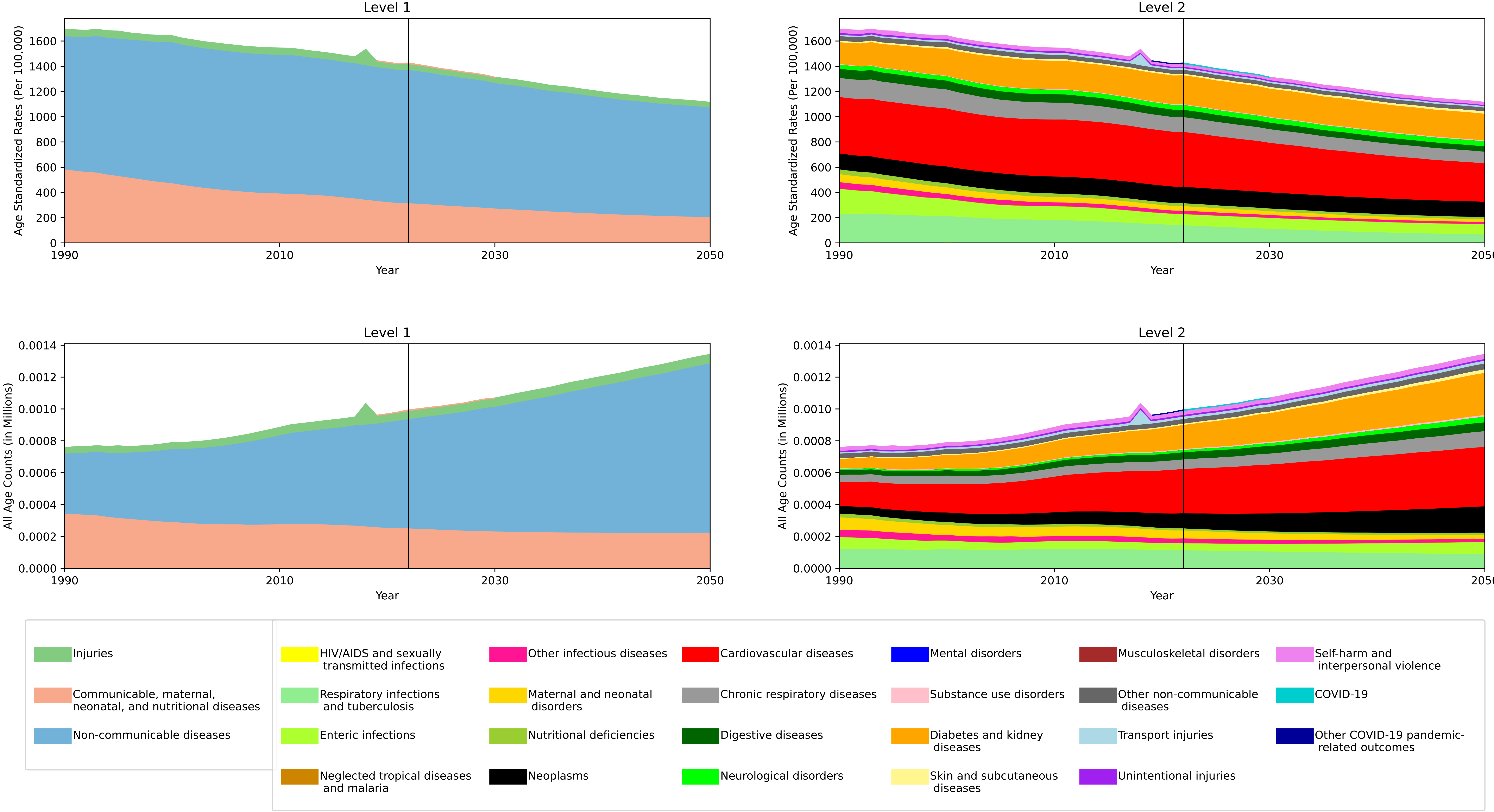
Level 1
Age Standardized Rates (Per 100,000)
Year
Level 2
Age Standardized Rates (Per 100,000)
Year
Level 1
All Age Counts (in Millions)
Year
Level 2
All Age Counts (in Millions)
Year
Injuries
Communicable, maternal, neonatal, and nutritional diseases
Non-communicable diseases
HIV/AIDS and sexually transmitted infections
Respiratory infections and tuberculosis
Enteric infections
Neglected tropical diseases and malaria
Other infectious diseases
Maternal and neonatal disorders
Nutritional deficiencies
Neoplasms
Cardiovascular diseases
Chronic respiratory diseases
Digestive diseases
Neurological disorders
Mental disorders
Substance use disorders
Diabetes and kidney diseases
Skin and subcutaneous diseases
Musculoskeletal disorders
Other non-communicable diseases
Transport injuries
Unintentional injuries
Self-harm and interpersonal violence
COVID-19
Other COVID-19 pandemic-related outcomes

Marshall Islands

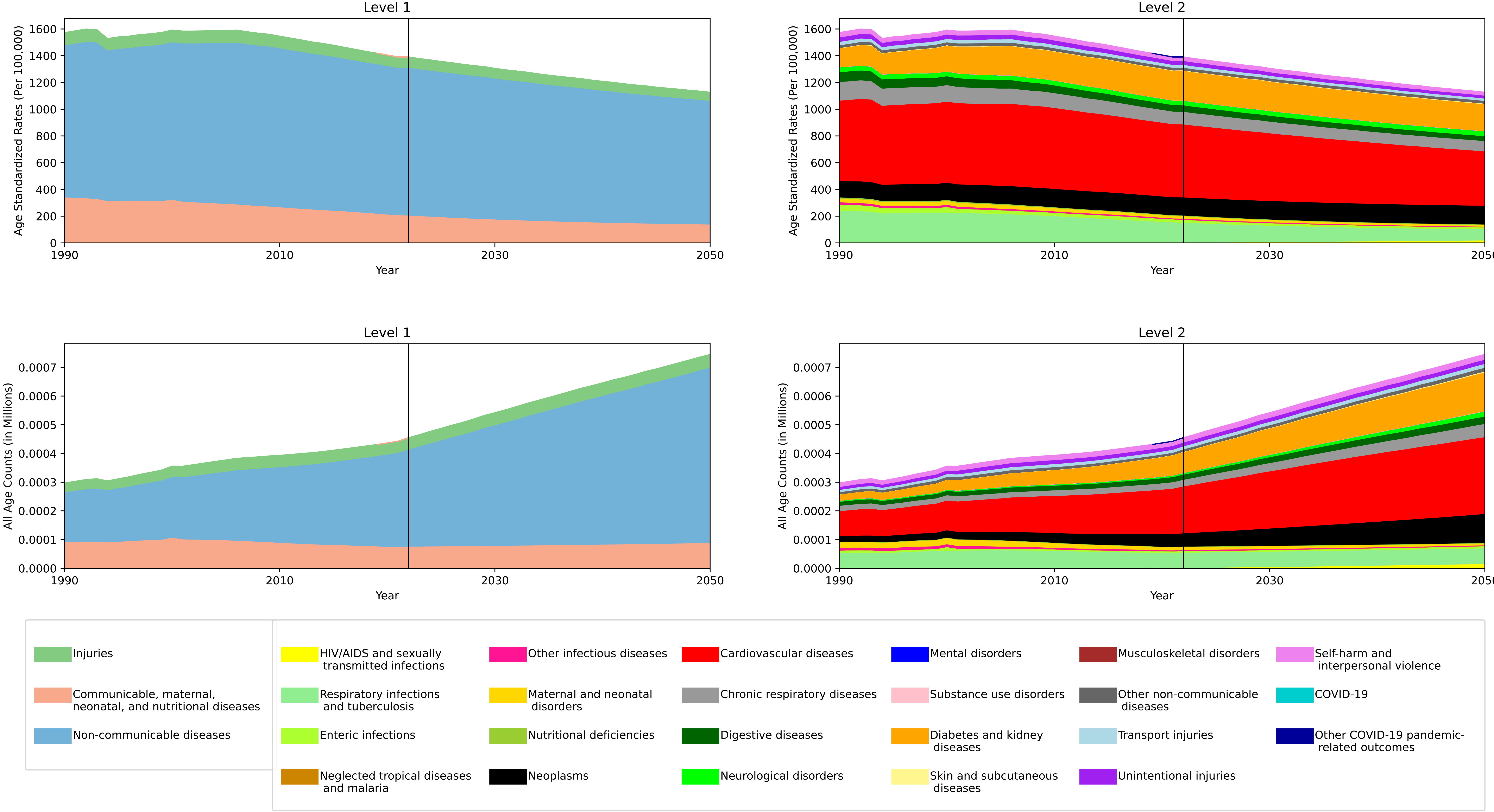

Supplemental Results Figure S5. Age-standardised death rates and all age death counts and by Level 1 and Level 2 causes, 1990–2050

Federated States Of Micronesia

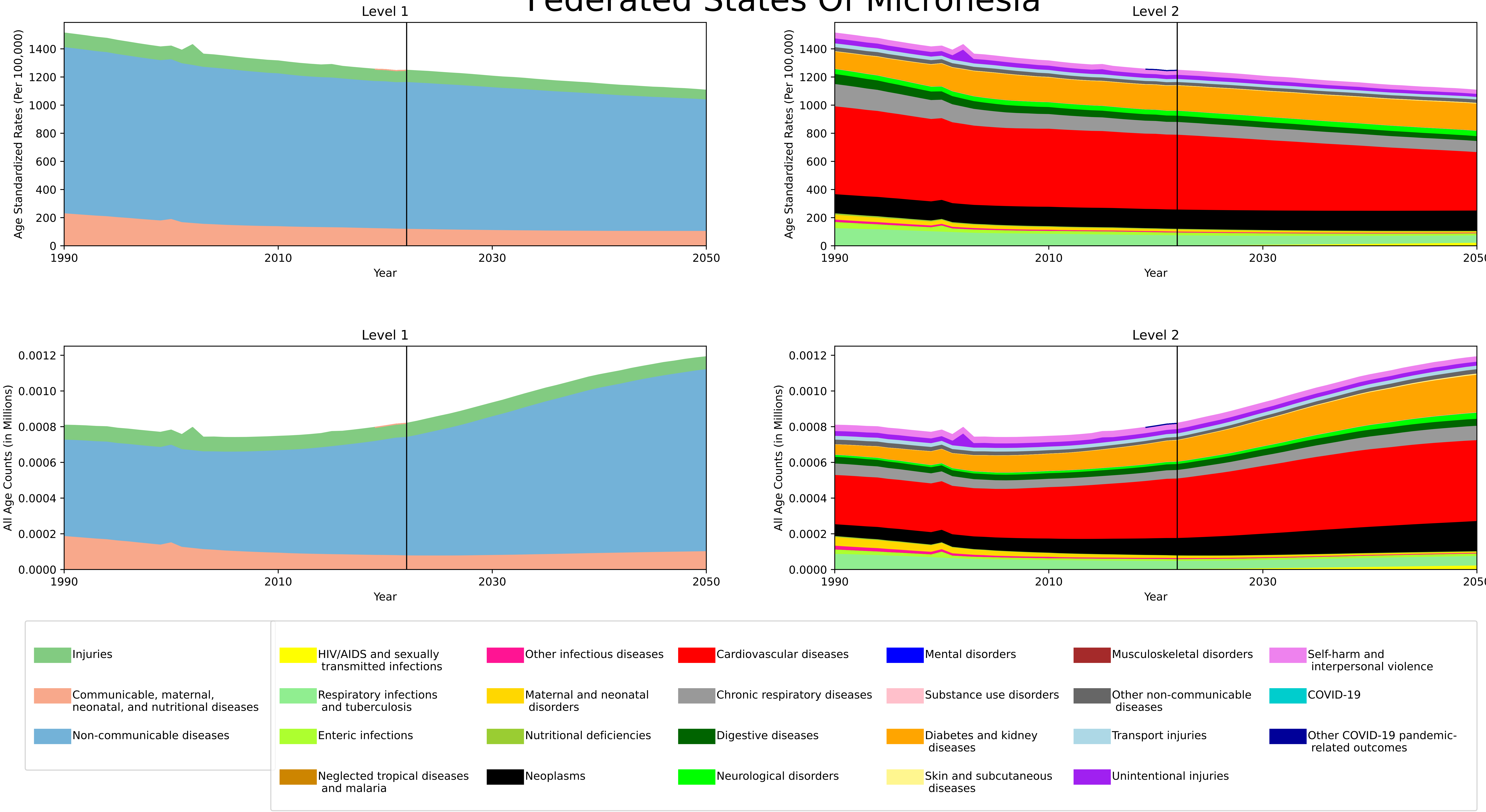

Nauru

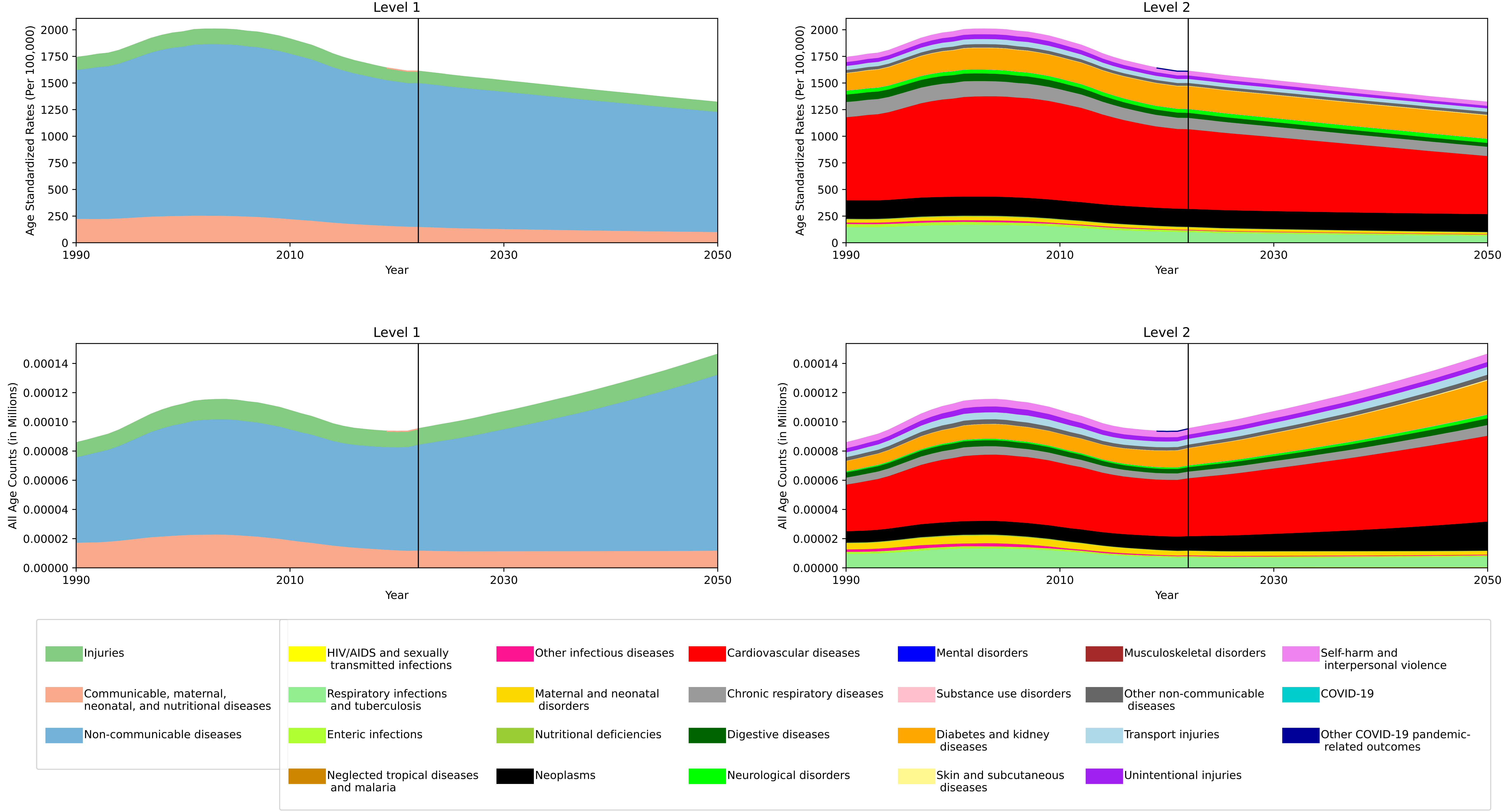
Level 1
Age Standardized Rates (Per 100,000)
Year
Level 2
Age Standardized Rates (Per 100,000)
Year
Level 1
All Age Counts (in Millions)
Year
Level 2
All Age Counts (in Millions)
Year
Injuries
Communicable, maternal, neonatal, and nutritional diseases
Non-communicable diseases
HIV/AIDS and sexually transmitted infections
Respiratory infections and tuberculosis
Enteric infections
Neglected tropical diseases and malaria
Other infectious diseases
Maternal and neonatal disorders
Nutritional deficiencies
Neoplasms
Cardiovascular diseases
Chronic respiratory diseases
Digestive diseases
Neurological disorders
Mental disorders
Substance use disorders
Diabetes and kidney diseases
Skin and subcutaneous diseases
Musculoskeletal disorders
Other non-communicable diseases
Transport injuries
Unintentional injuries
Self-harm and interpersonal violence
COVID-19
Other COVID-19 pandemic-related outcomes

Niue

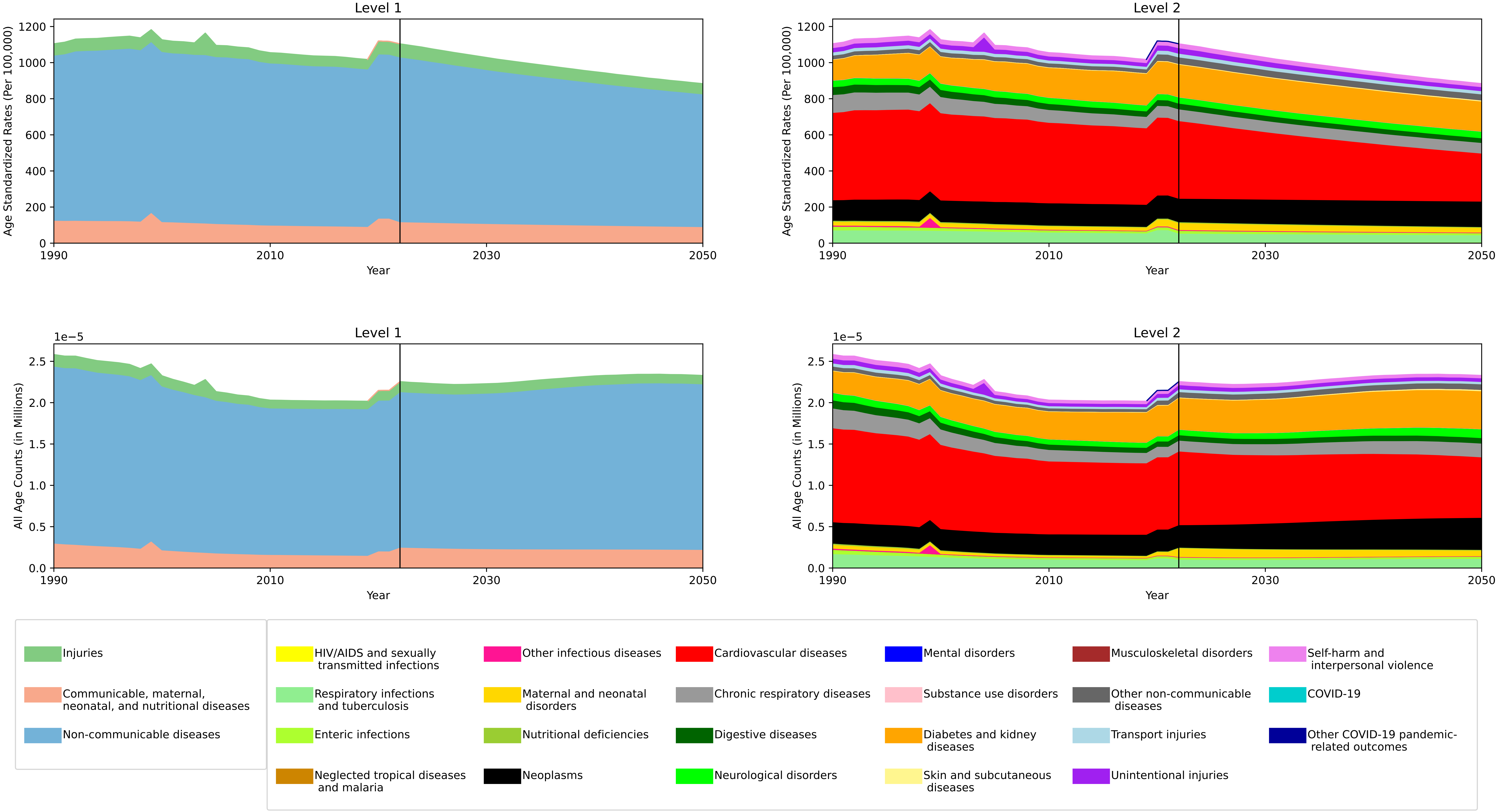
Level 1
Level 2
Age Standardized Rates (Per 100,000)
All Age Counts (in Millions)
1e−5
Year
1990
2010
2030
2050
Injuries
Communicable, maternal, neonatal, and nutritional diseases
Non-communicable diseases
HIV/AIDS and sexually transmitted infections
Respiratory infections and tuberculosis
Enteric infections
Neglected tropical diseases and malaria
Other infectious diseases
Maternal and neonatal disorders
Nutritional deficiencies
Neoplasms
Cardiovascular diseases
Chronic respiratory diseases
Digestive diseases
Neurological disorders
Mental disorders
Substance use disorders
Diabetes and kidney diseases
Skin and subcutaneous diseases
Musculoskeletal disorders
Other non-communicable diseases
Transport injuries
Unintentional injuries
Self-harm and interpersonal violence
COVID-19
Other COVID-19 pandemic-related outcomes

Northern Mariana Islands

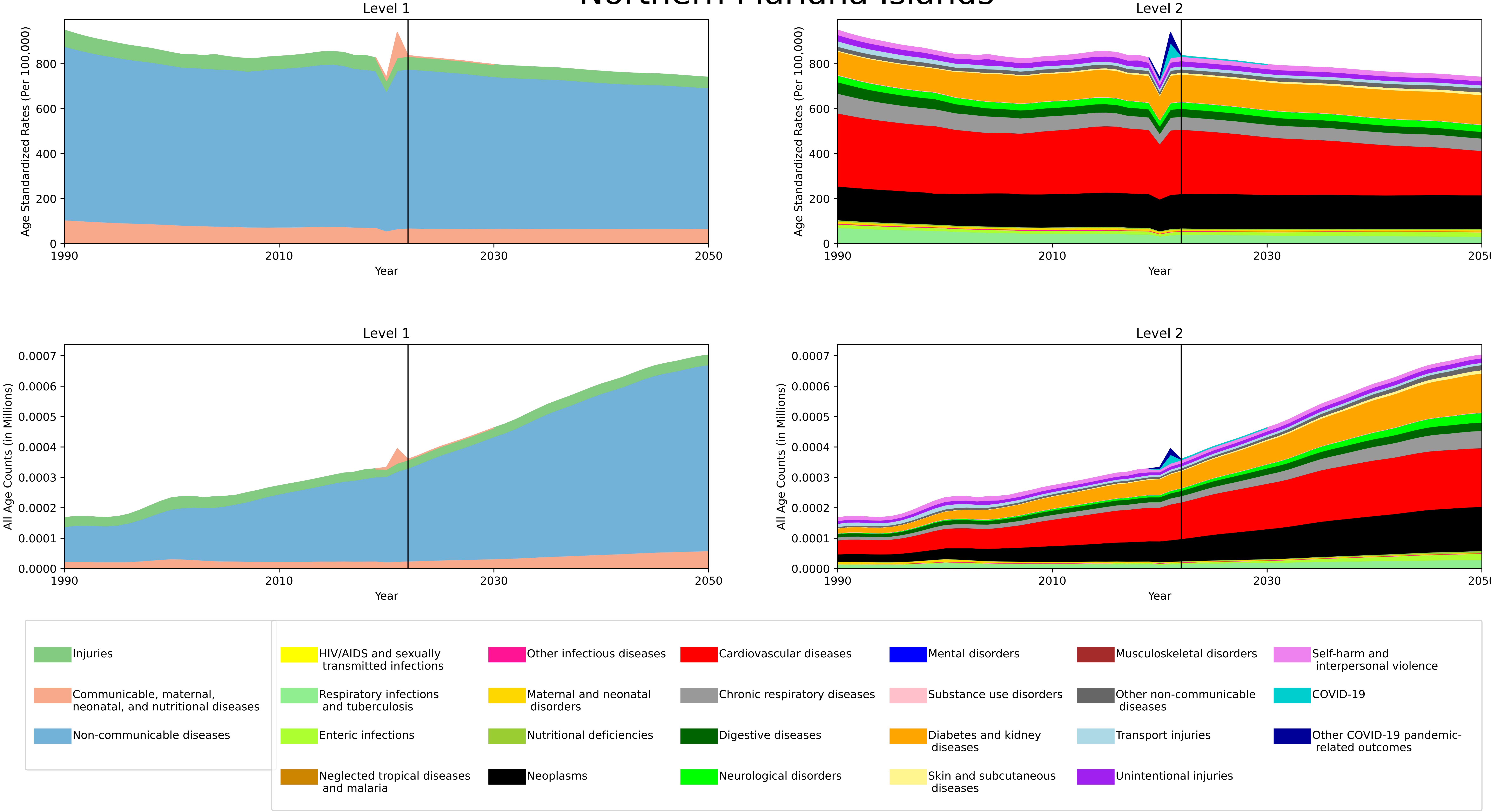

Palau

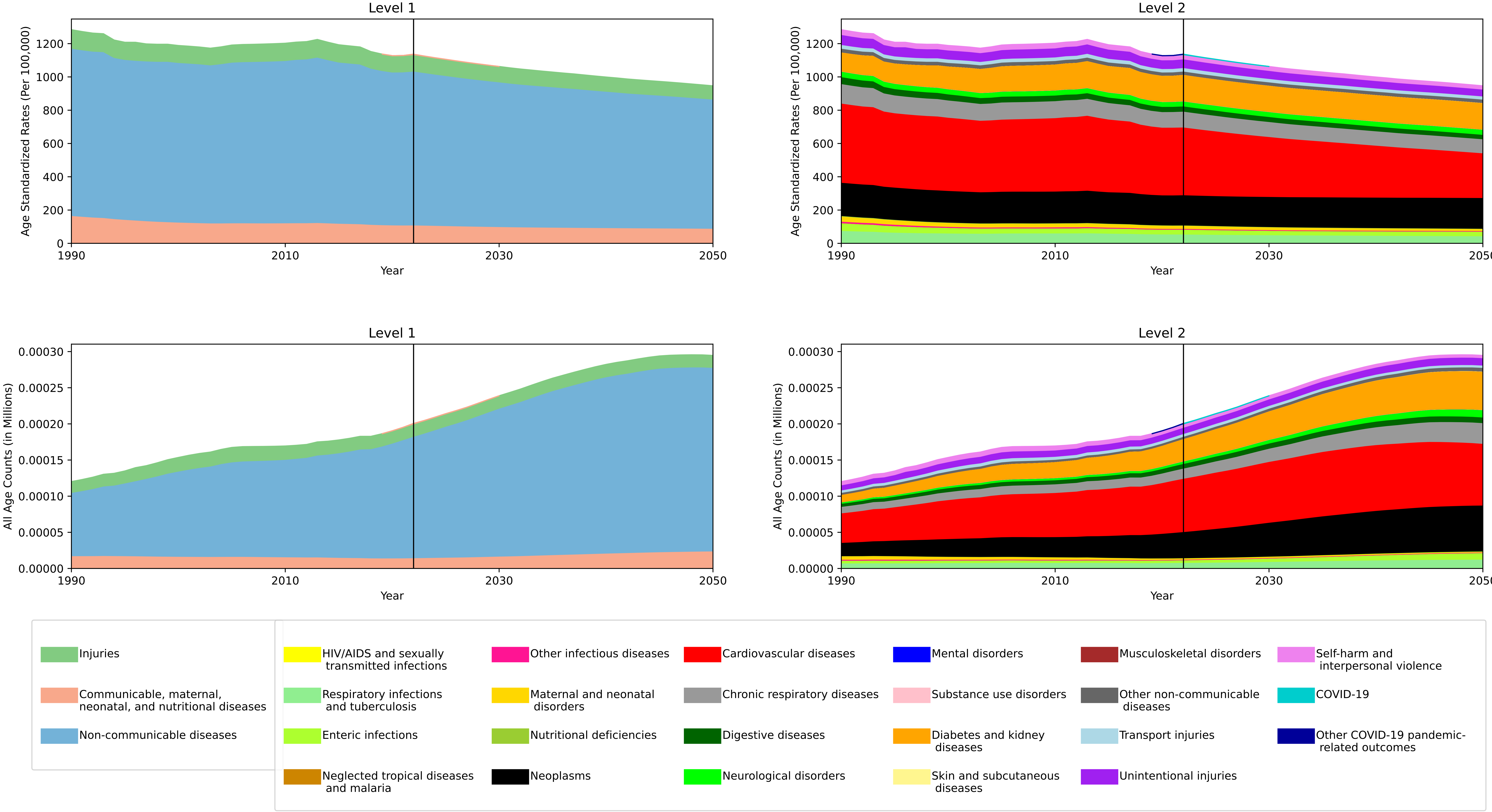
Level 1
Level 2
Age Standardized Rates (Per 100,000)
All Age Counts (in Millions)
Year
1990
2010
2030
2050
Injuries
Communicable, maternal, neonatal, and nutritional diseases
Non-communicable diseases
HIV/AIDS and sexually transmitted infections
Respiratory infections and tuberculosis
Enteric infections
Neglected tropical diseases and malaria
Other infectious diseases
Maternal and neonatal disorders
Nutritional deficiencies
Neoplasms
Cardiovascular diseases
Chronic respiratory diseases
Digestive diseases
Neurological disorders
Mental disorders
Substance use disorders
Diabetes and kidney diseases
Skin and subcutaneous diseases
Musculoskeletal disorders
Other non-communicable diseases
Transport injuries
Unintentional injuries
Self-harm and interpersonal violence
COVID-19
Other COVID-19 pandemic-related outcomes

Papua New Guinea

Supplemental Results Figure S5. Age-standardised death rates and all age death counts and by Level 1 and Level 2 causes, 1990–2050

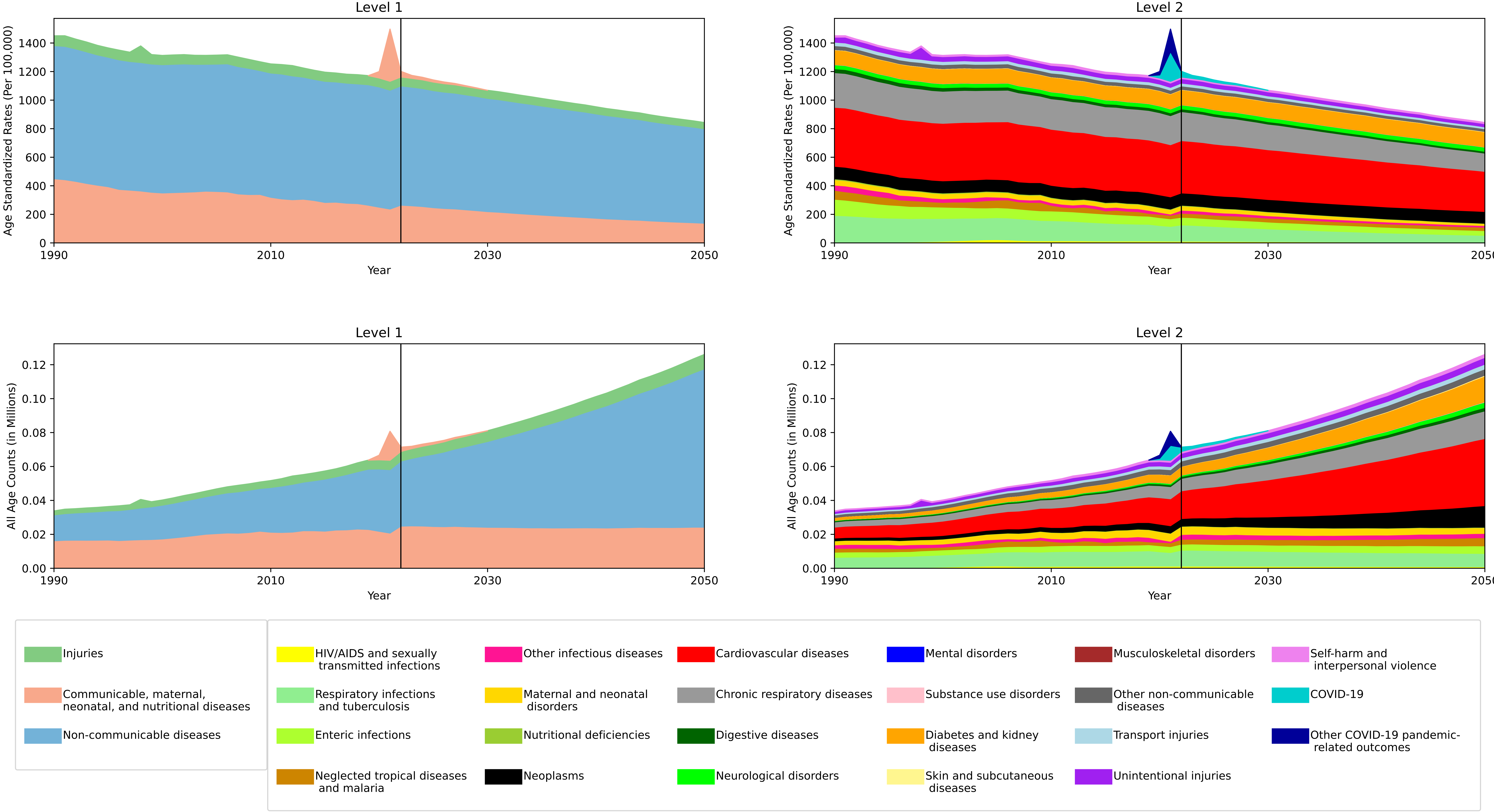

Samoa

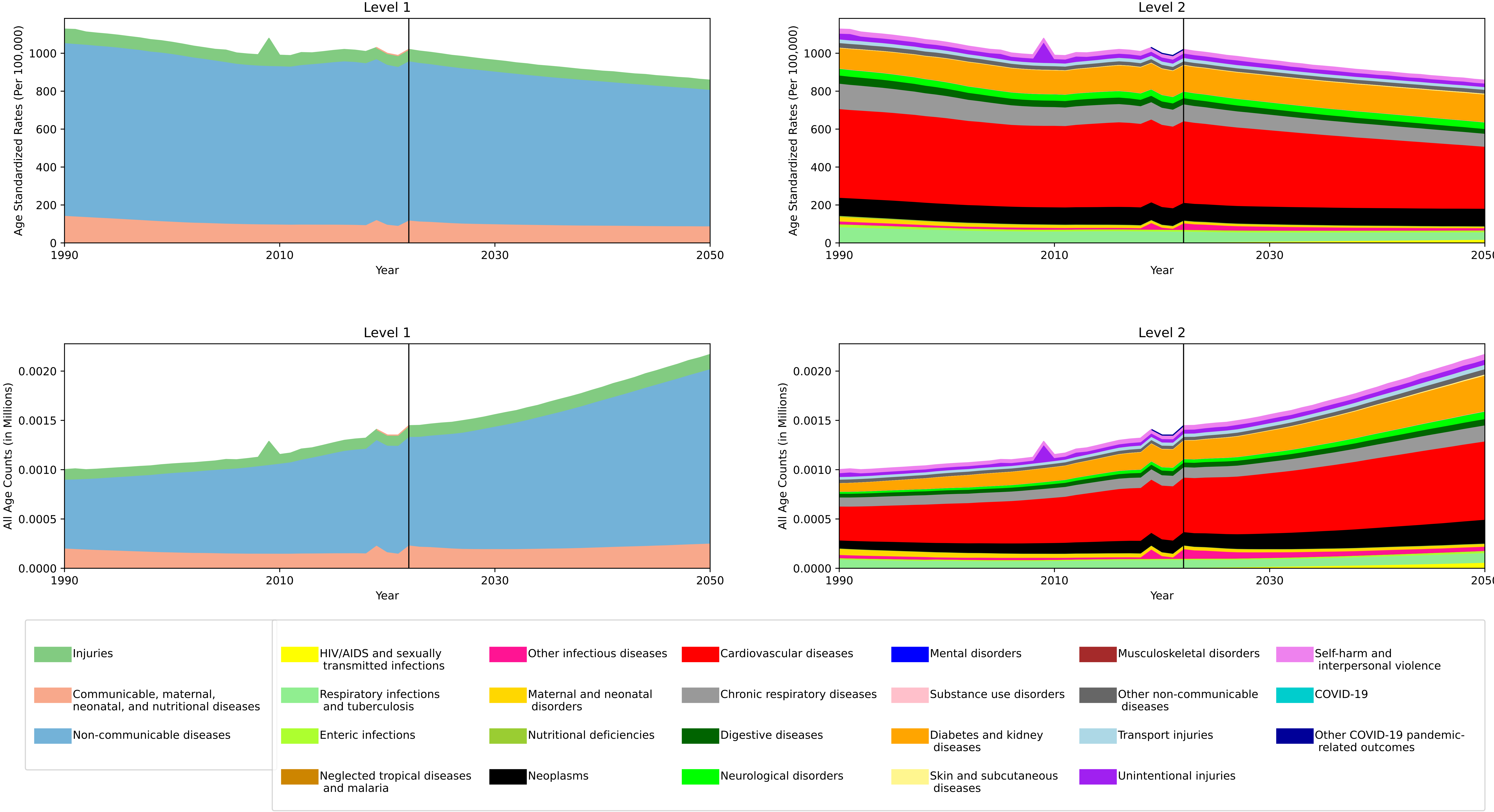
Level 1
Level 2
Age Standardized Rates (Per 100,000)
All Age Counts (in Millions)
Year
1990
2010
2030
2050
0
200
400
600
800
1000
0.0000
0.0005
0.0010
0.0015
0.0020
Injuries
Communicable, maternal, neonatal, and nutritional diseases
Non-communicable diseases
HIV/AIDS and sexually transmitted infections
Respiratory infections and tuberculosis
Enteric infections
Neglected tropical diseases and malaria
Other infectious diseases
Maternal and neonatal disorders
Nutritional deficiencies
Neoplasms
Cardiovascular diseases
Chronic respiratory diseases
Digestive diseases
Neurological disorders
Mental disorders
Substance use disorders
Diabetes and kidney diseases
Skin and subcutaneous diseases
Musculoskeletal disorders
Other non-communicable diseases
Transport injuries
Unintentional injuries
Self-harm and interpersonal violence
COVID-19
Other COVID-19 pandemic-related outcomes

Solomon Islands

Supplemental Results Figure S5. Age-standardised death rates and all age death counts and by Level 1 and Level 2 causes, 1990–2050

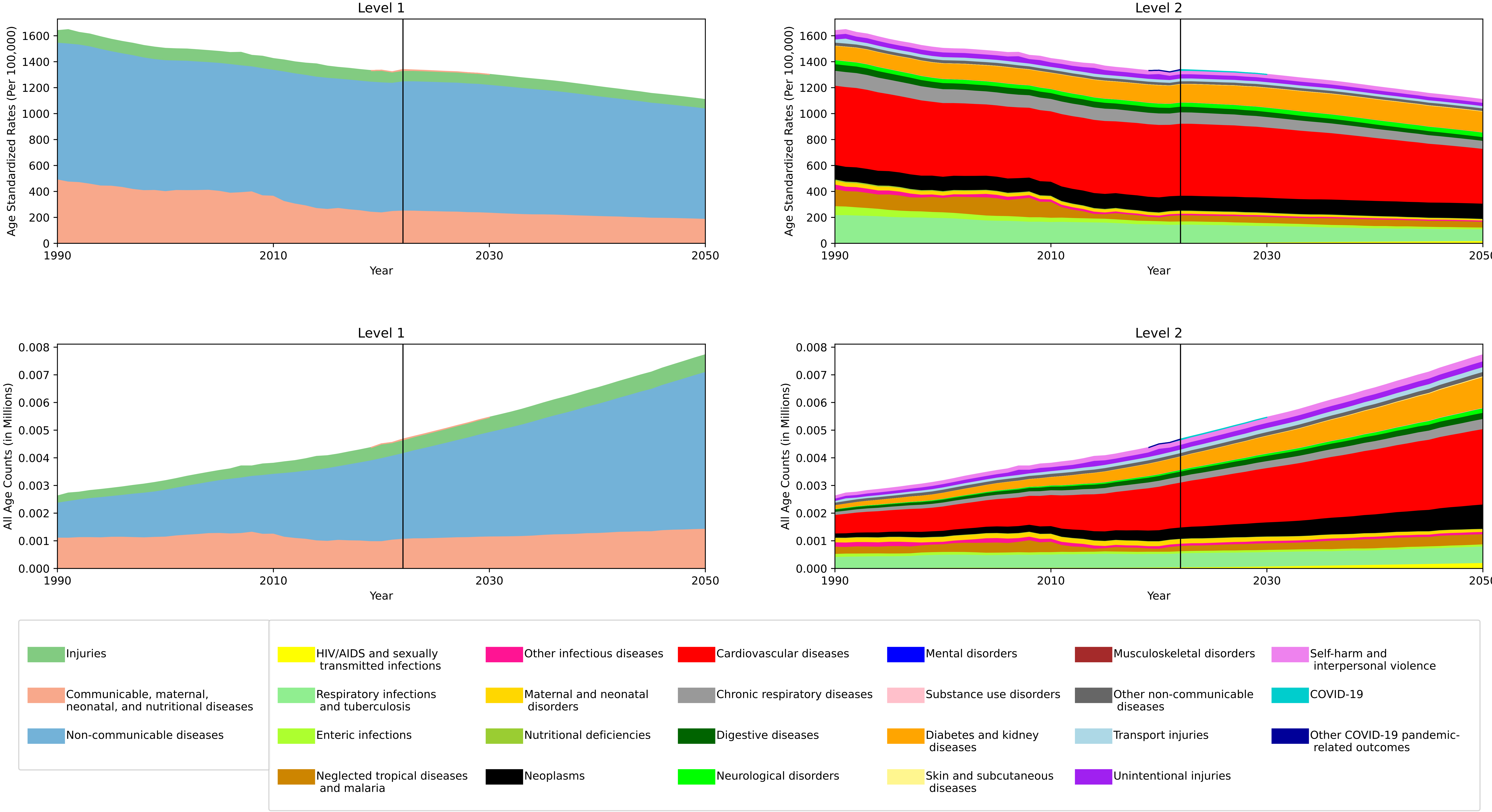

Tokelau

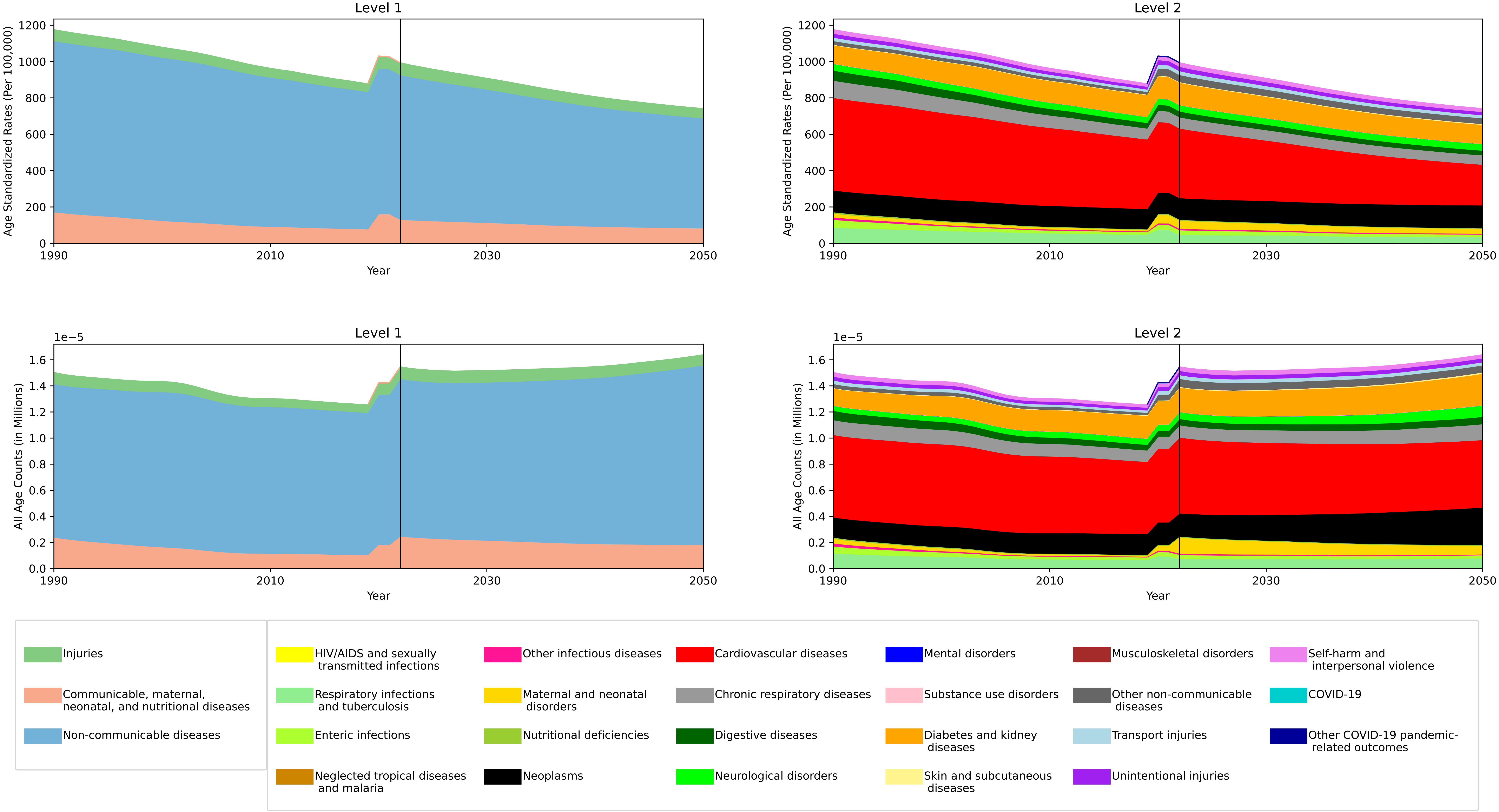
Level 1
Level 2
Age Standardized Rates (Per 100,000)
All Age Counts (in Millions)
1e−5
Year
1990
2010
2030
2050
Injuries
Communicable, maternal, neonatal, and nutritional diseases
Non-communicable diseases
HIV/AIDS and sexually transmitted infections
Respiratory infections and tuberculosis
Enteric infections
Neglected tropical diseases and malaria
Other infectious diseases
Maternal and neonatal disorders
Nutritional deficiencies
Neoplasms
Cardiovascular diseases
Chronic respiratory diseases
Digestive diseases
Neurological disorders
Mental disorders
Substance use disorders
Diabetes and kidney diseases
Skin and subcutaneous diseases
Musculoskeletal disorders
Other non-communicable diseases
Transport injuries
Unintentional injuries
Self-harm and interpersonal violence
COVID-19
Other COVID-19 pandemic-related outcomes

Tonga

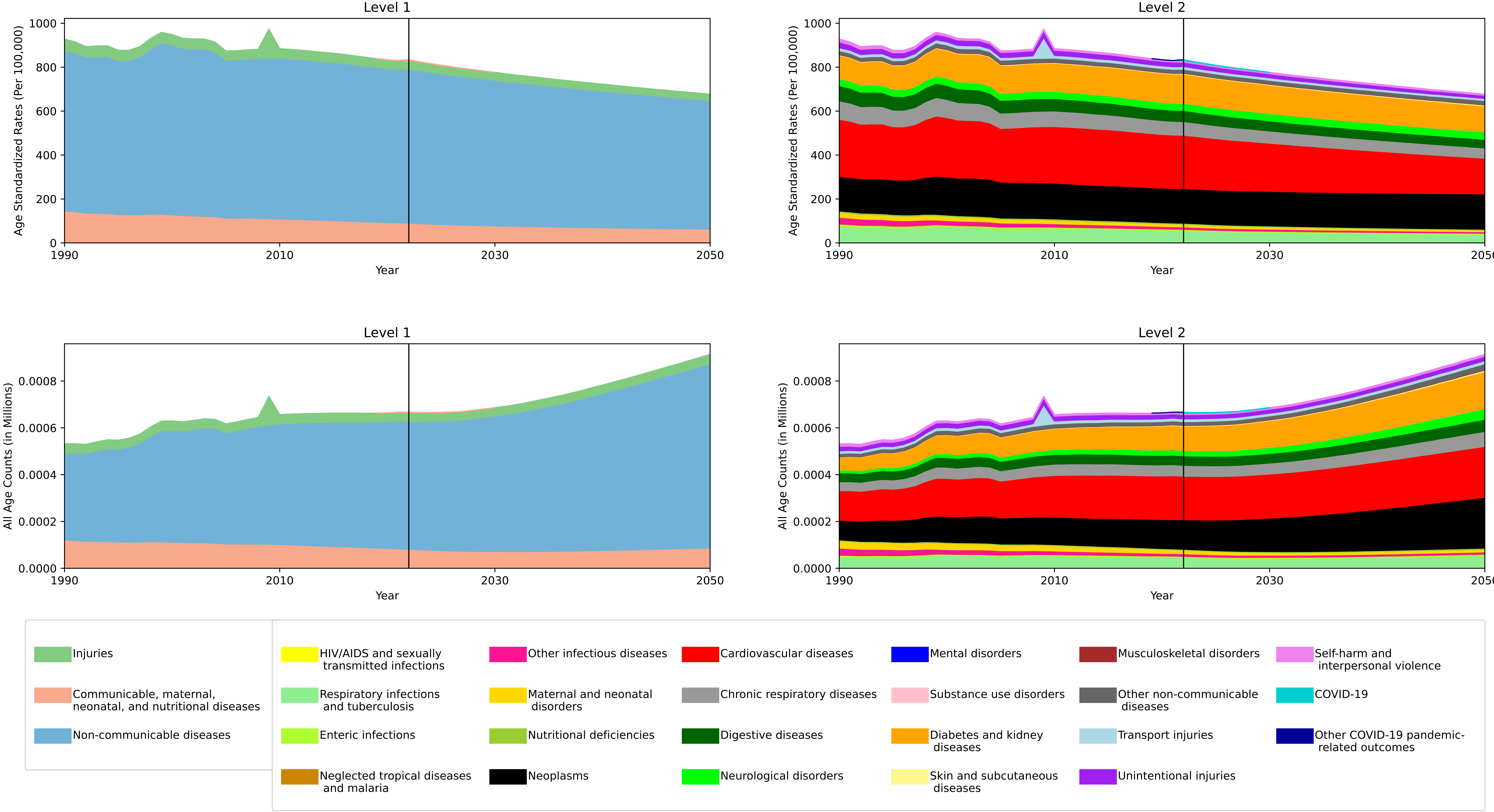
Level 1
Level 2
Age Standardized Rates (Per 100,000)
All Age Counts (in Millions)
Year
1990
2010
2030
2050
0
200
400
600
800
1000
0.0000
0.0002
0.0004
0.0006
0.0008
Injuries
Communicable, maternal, neonatal, and nutritional diseases
Non-communicable diseases
HIV/AIDS and sexually transmitted infections
Respiratory infections and tuberculosis
Enteric infections
Neglected tropical diseases and malaria
Other infectious diseases
Maternal and neonatal disorders
Nutritional deficiencies
Neoplasms
Cardiovascular diseases
Chronic respiratory diseases
Digestive diseases
Neurological disorders
Mental disorders
Substance use disorders
Diabetes and kidney diseases
Skin and subcutaneous diseases
Musculoskeletal disorders
Other non-communicable diseases
Transport injuries
Unintentional injuries
Self-harm and interpersonal violence
COVID-19
Other COVID-19 pandemic-related outcomes

Tuvalu

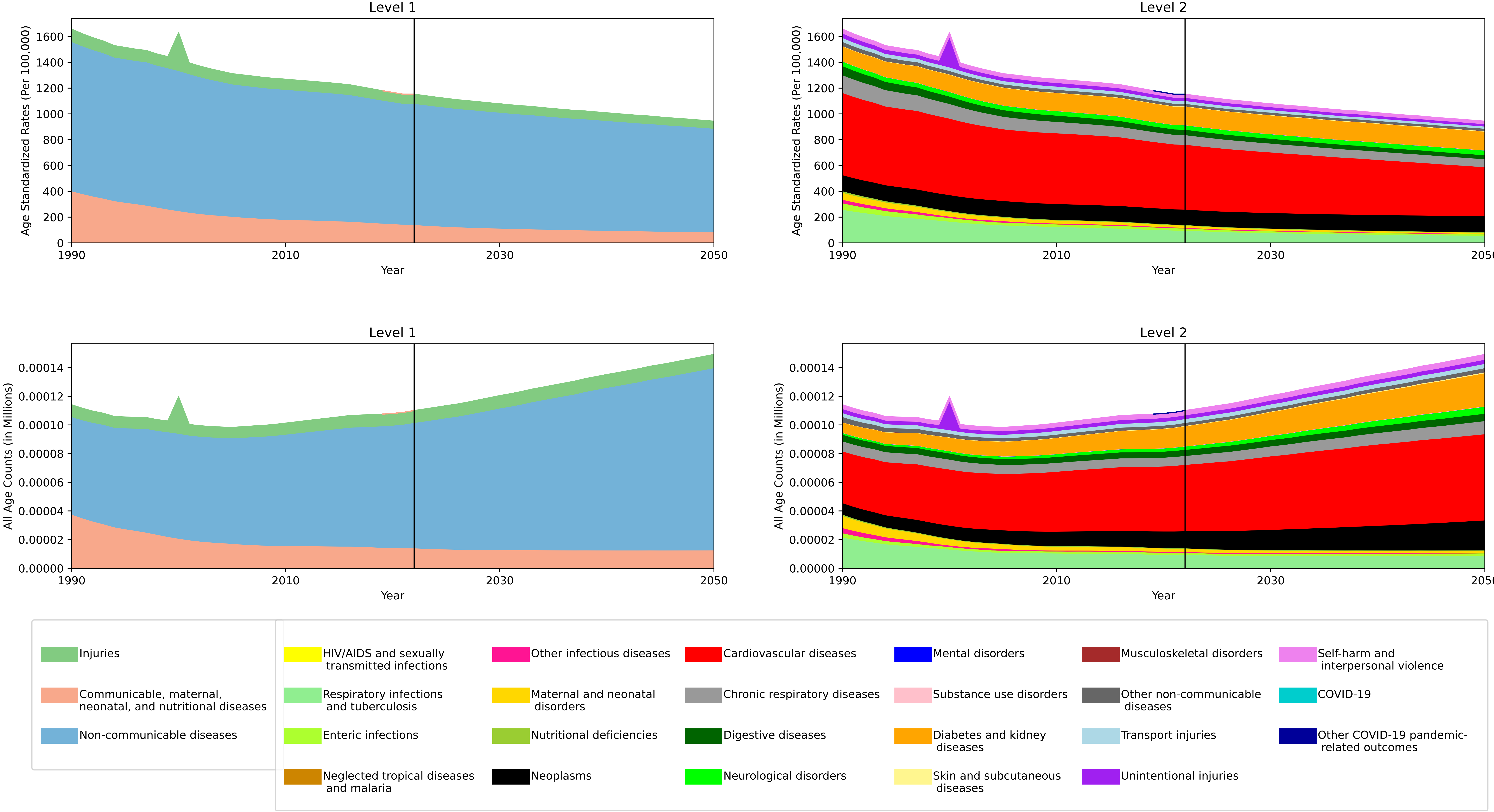
Level 1
Age Standardized Rates (Per 100,000)
0
200
400
600
800
1000
1200
1400
1600
1990
2010
2030
2050
Year
Level 2
Age Standardized Rates (Per 100,000)
Level 1
All Age Counts (in Millions)
0.00000
0.00002
0.00004
0.00006
0.00008
0.00010
0.00012
0.00014
Level 2
All Age Counts (in Millions)
Injuries
Communicable, maternal, neonatal, and nutritional diseases
Non-communicable diseases
HIV/AIDS and sexually transmitted infections
Respiratory infections and tuberculosis
Enteric infections
Neglected tropical diseases and malaria
Other infectious diseases
Maternal and neonatal disorders
Nutritional deficiencies
Neoplasms
Cardiovascular diseases
Chronic respiratory diseases
Digestive diseases
Neurological disorders
Mental disorders
Substance use disorders
Diabetes and kidney diseases
Skin and subcutaneous diseases
Musculoskeletal disorders
Other non-communicable diseases
Transport injuries
Unintentional injuries
Self-harm and interpersonal violence
COVID-19
Other COVID-19 pandemic-related outcomes

Vanuatu

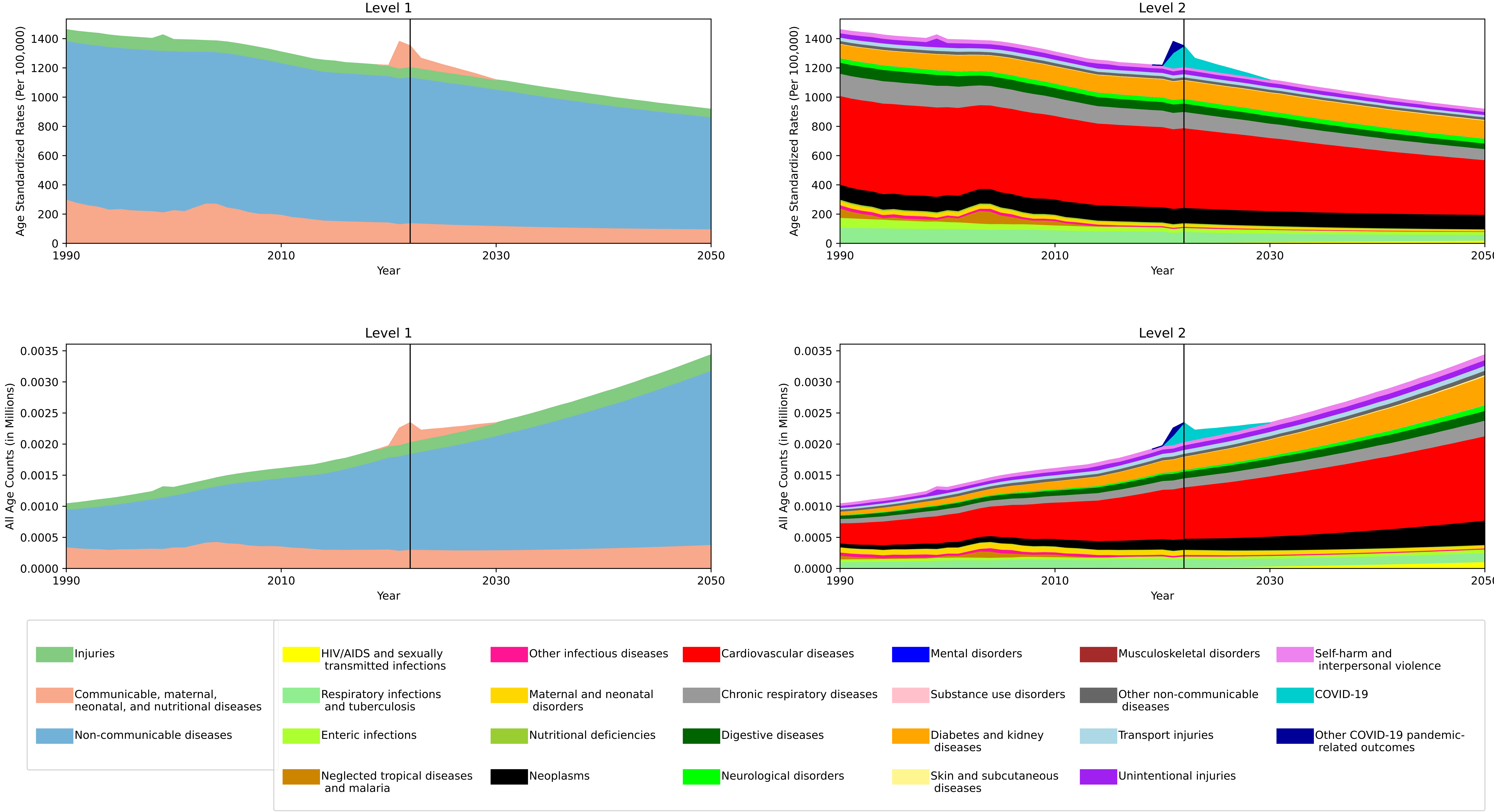
Level 1
Level 2
Age Standardized Rates (Per 100,000)
All Age Counts (in Millions)
Year
0
200
400
600
800
1000
1200
1400
0.0000
0.0005
0.0010
0.0015
0.0020
0.0025
0.0030
0.0035
1990
2010
2030
2050
Injuries
Communicable, maternal, neonatal, and nutritional diseases
Non-communicable diseases
HIV/AIDS and sexually transmitted infections
Respiratory infections and tuberculosis
Enteric infections
Neglected tropical diseases and malaria
Other infectious diseases
Maternal and neonatal disorders
Nutritional deficiencies
Neoplasms
Cardiovascular diseases
Chronic respiratory diseases
Digestive diseases
Neurological disorders
Mental disorders
Substance use disorders
Diabetes and kidney diseases
Skin and subcutaneous diseases
Musculoskeletal disorders
Other non-communicable diseases
Transport injuries
Unintentional injuries
Self-harm and interpersonal violence
COVID-19
Other COVID-19 pandemic-related outcomes

Supplemental Results Figure S5. Age-standardised death rates and all age death counts and by Level 1 and Level 2 causes, 1990–2050

Cambodia

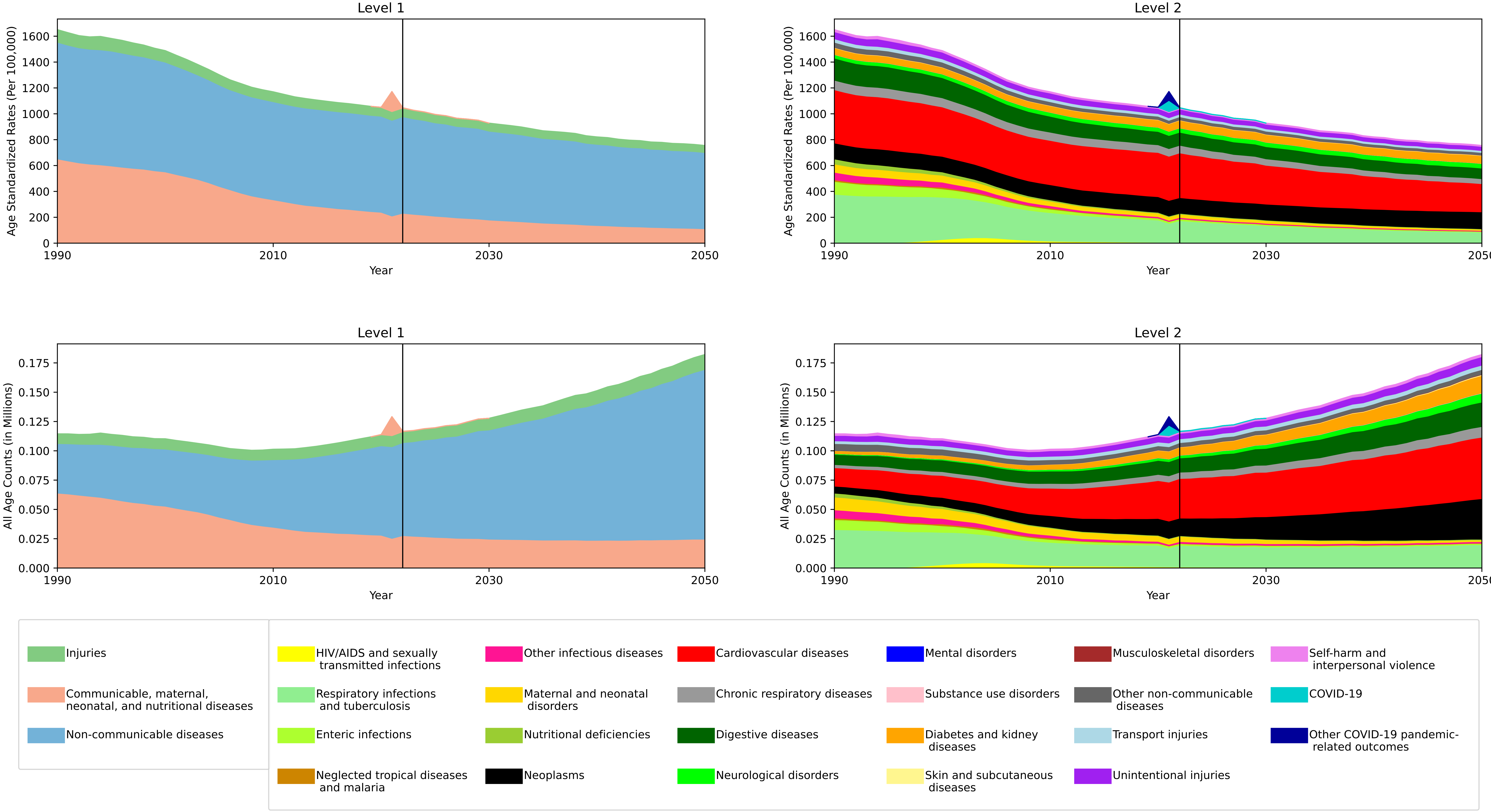

Indonesia

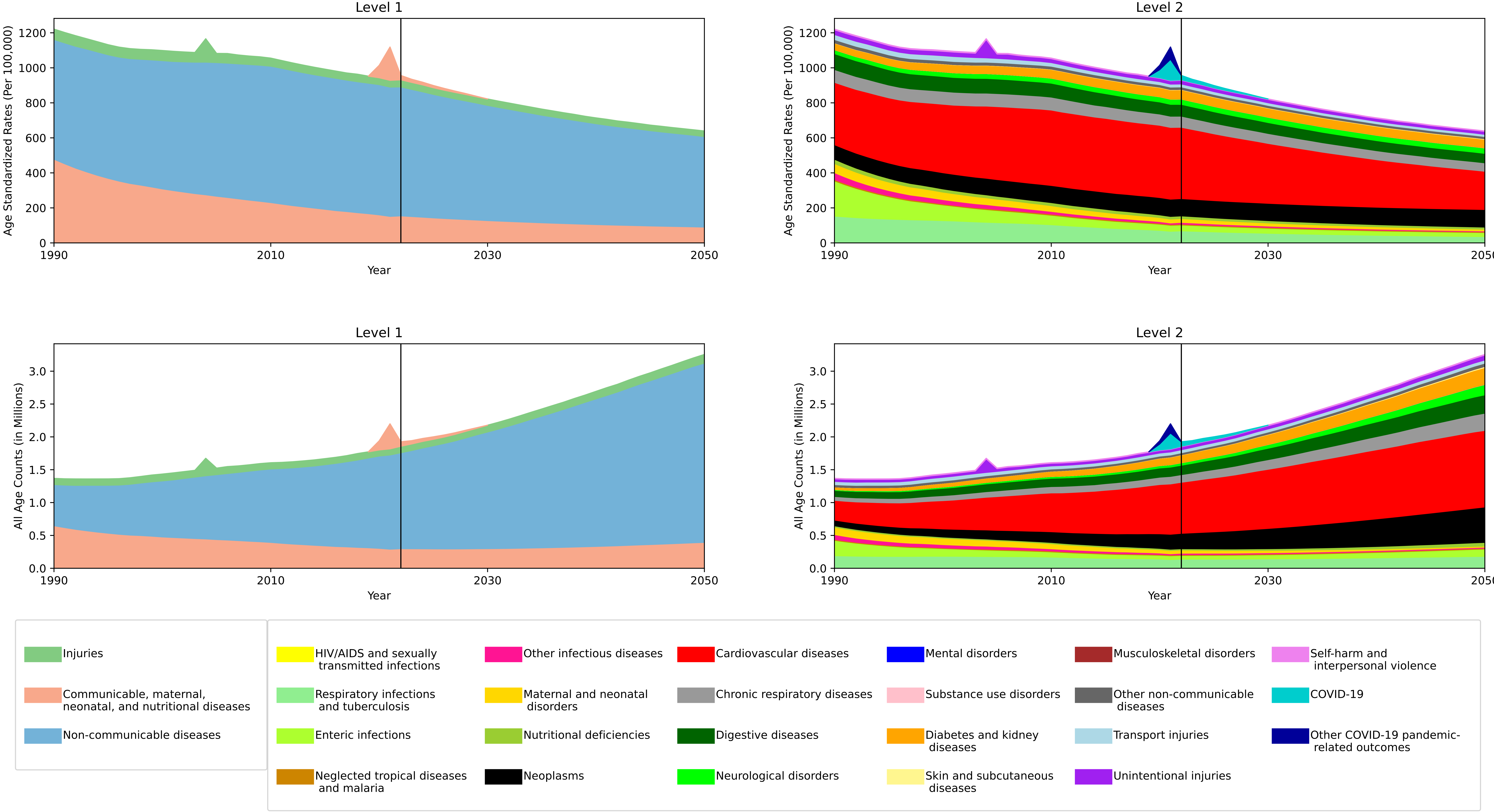
Level 1
Level 2
Age Standardized Rates (Per 100,000)
All Age Counts (in Millions)
Year
1990
2010
2030
2050
Injuries
Communicable, maternal, neonatal, and nutritional diseases
Non-communicable diseases
HIV/AIDS and sexually transmitted infections
Respiratory infections and tuberculosis
Enteric infections
Neglected tropical diseases and malaria
Other infectious diseases
Maternal and neonatal disorders
Nutritional deficiencies
Neoplasms
Cardiovascular diseases
Chronic respiratory diseases
Digestive diseases
Neurological disorders
Mental disorders
Substance use disorders
Diabetes and kidney diseases
Skin and subcutaneous diseases
Musculoskeletal disorders
Other non-communicable diseases
Transport injuries
Unintentional injuries
Self-harm and interpersonal violence
COVID-19
Other COVID-19 pandemic-related outcomes

Laos

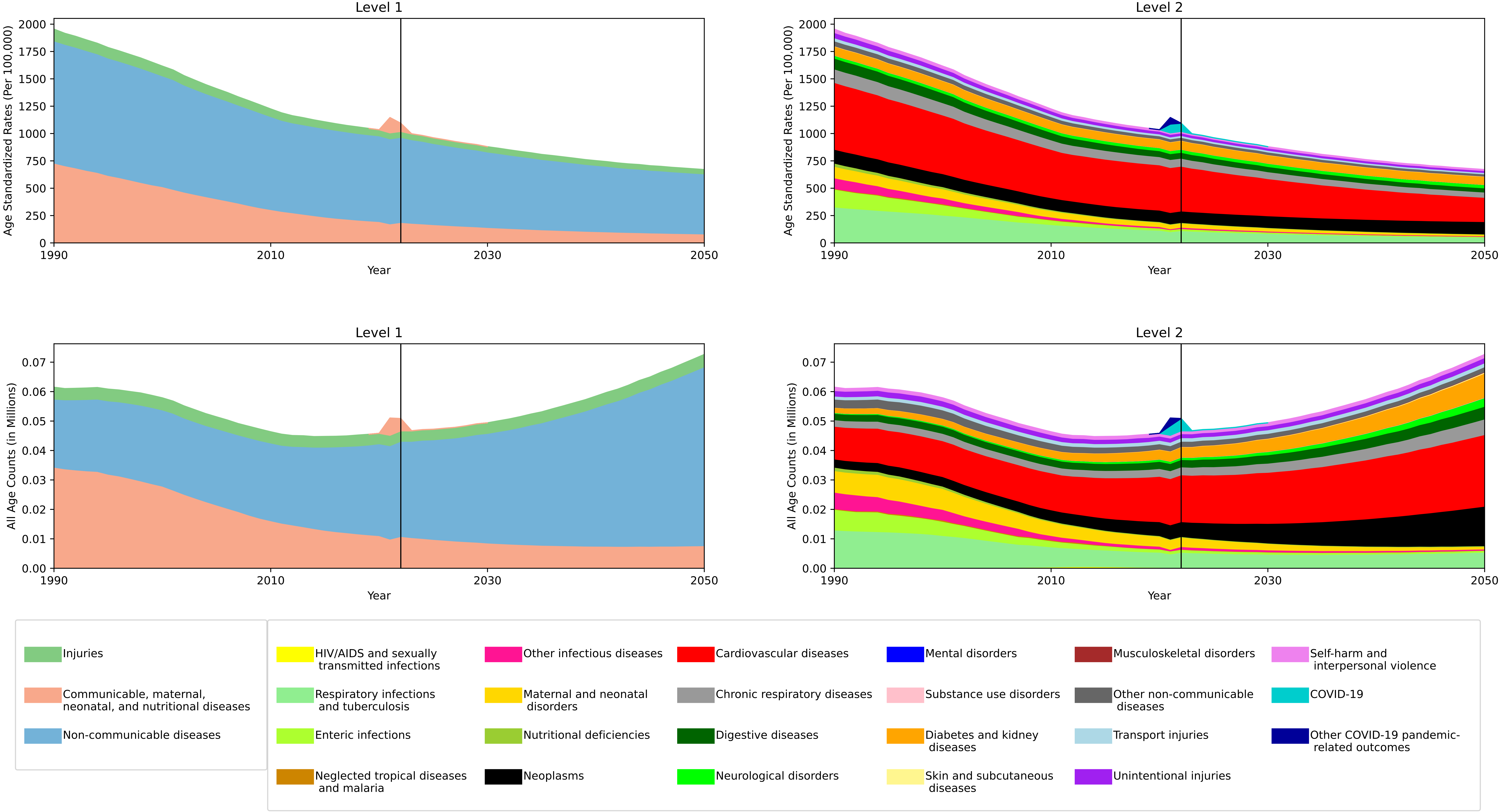
Level 1
Level 2
Age Standardized Rates (Per 100,000)
All Age Counts (in Millions)
Year
0
250
500
750
1000
1250
1500
1750
2000
0.00
0.01
0.02
0.03
0.04
0.05
0.06
0.07
1990
2010
2030
2050
Injuries
Communicable, maternal, neonatal, and nutritional diseases
Non-communicable diseases
HIV/AIDS and sexually transmitted infections
Respiratory infections and tuberculosis
Enteric infections
Neglected tropical diseases and malaria
Other infectious diseases
Maternal and neonatal disorders
Nutritional deficiencies
Neoplasms
Cardiovascular diseases
Chronic respiratory diseases
Digestive diseases
Neurological disorders
Mental disorders
Substance use disorders
Diabetes and kidney diseases
Skin and subcutaneous diseases
Musculoskeletal disorders
Other non-communicable diseases
Transport injuries
Unintentional injuries
Self-harm and interpersonal violence
COVID-19
Other COVID-19 pandemic-related outcomes

Malaysia

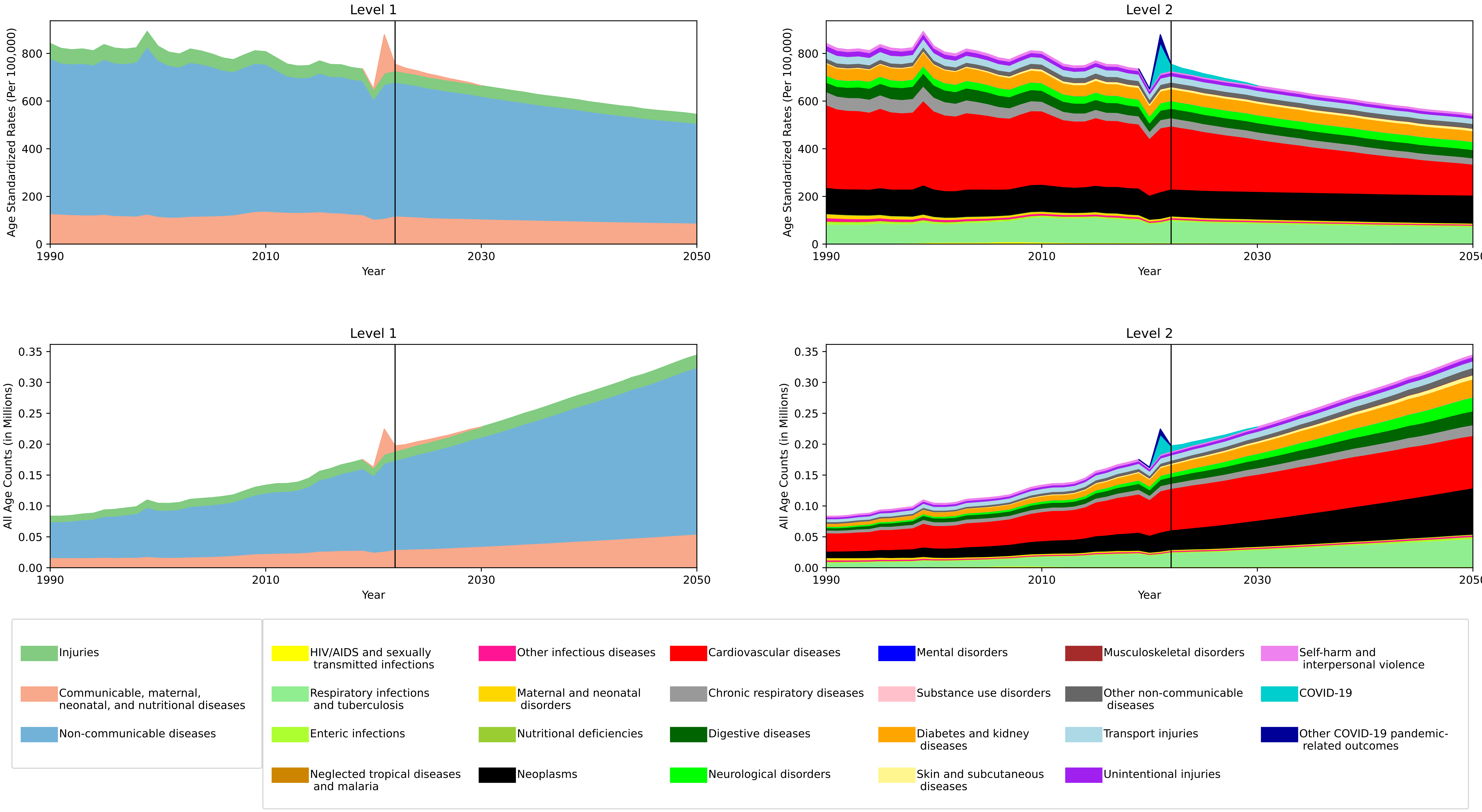
Level 1
Level 2
Age Standardized Rates (Per 100,000)
All Age Counts (in Millions)
Year
1990
2010
2030
2050
0
200
400
600
800
0.00
0.05
0.10
0.15
0.20
0.25
0.30
0.35
Injuries
Communicable, maternal, neonatal, and nutritional diseases
Non-communicable diseases
HIV/AIDS and sexually transmitted infections
Respiratory infections and tuberculosis
Enteric infections
Neglected tropical diseases and malaria
Other infectious diseases
Maternal and neonatal disorders
Nutritional deficiencies
Neoplasms
Cardiovascular diseases
Chronic respiratory diseases
Digestive diseases
Neurological disorders
Mental disorders
Substance use disorders
Diabetes and kidney diseases
Skin and subcutaneous diseases
Musculoskeletal disorders
Other non-communicable diseases
Transport injuries
Unintentional injuries
Self-harm and interpersonal violence
COVID-19
Other COVID-19 pandemic-related outcomes

Maldives

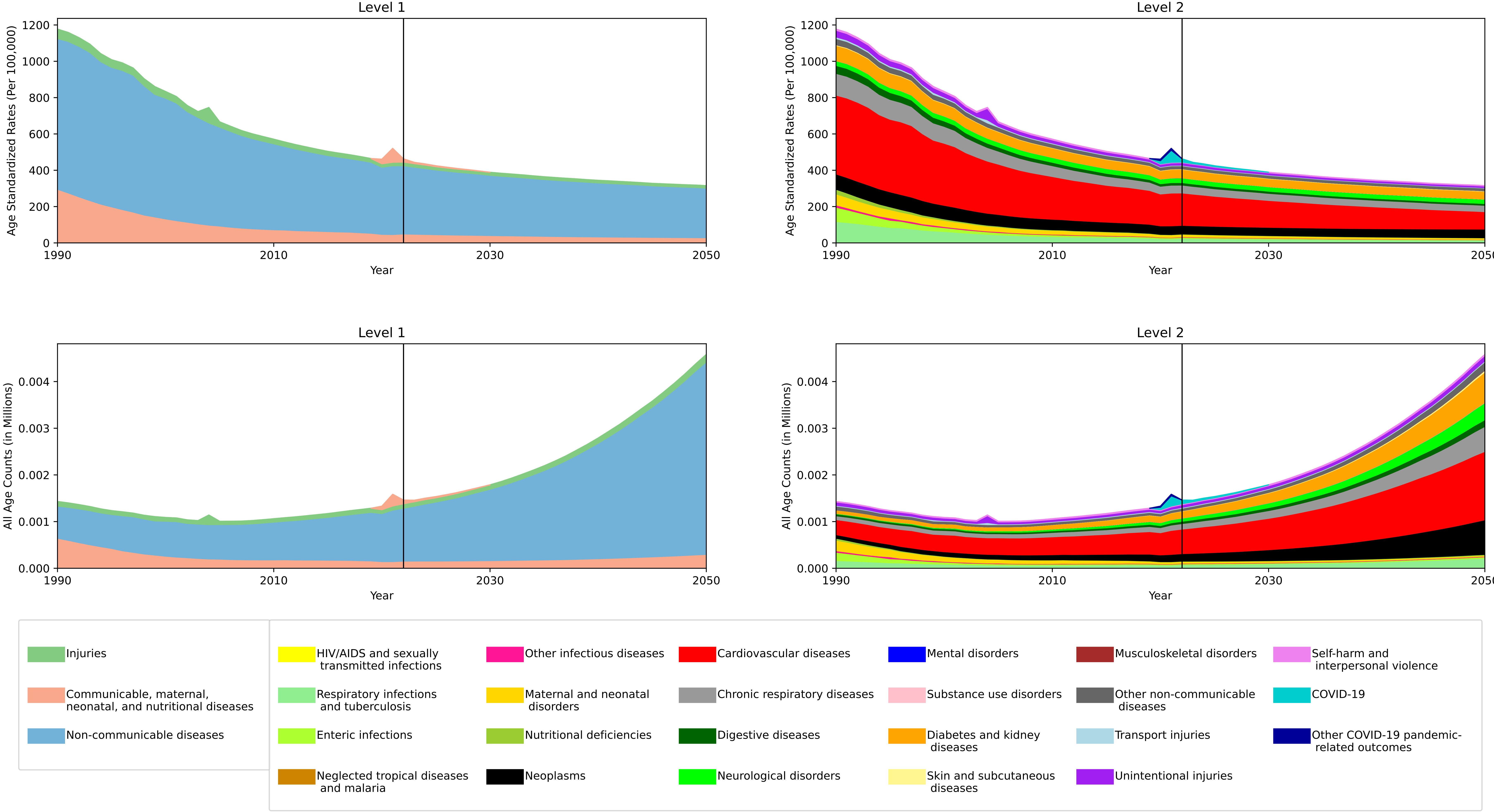
Level 1
Level 2
Age Standardized Rates (Per 100,000)
All Age Counts (in Millions)
Year
1990
2010
2030
2050
Injuries
Communicable, maternal, neonatal, and nutritional diseases
Non-communicable diseases
HIV/AIDS and sexually transmitted infections
Respiratory infections and tuberculosis
Enteric infections
Neglected tropical diseases and malaria
Other infectious diseases
Maternal and neonatal disorders
Nutritional deficiencies
Neoplasms
Cardiovascular diseases
Chronic respiratory diseases
Digestive diseases
Neurological disorders
Mental disorders
Substance use disorders
Diabetes and kidney diseases
Skin and subcutaneous diseases
Musculoskeletal disorders
Other non-communicable diseases
Transport injuries
Unintentional injuries
Self-harm and interpersonal violence
COVID-19
Other COVID-19 pandemic-related outcomes

Mauritius

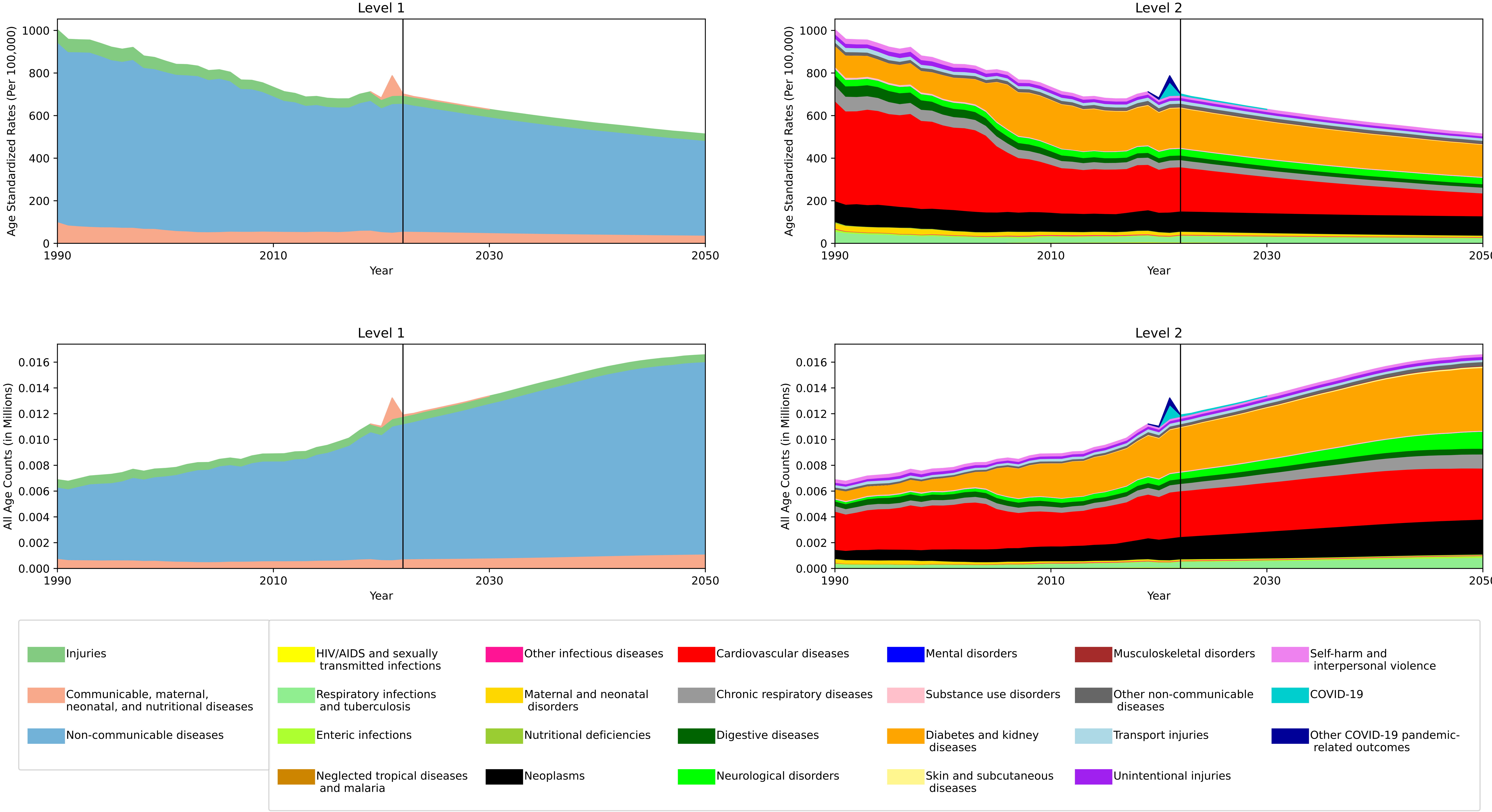
Level 1
Level 2
Age Standardized Rates (Per 100,000)
All Age Counts (in Millions)
Year
Injuries
Communicable, maternal, neonatal, and nutritional diseases
Non-communicable diseases
HIV/AIDS and sexually transmitted infections
Respiratory infections and tuberculosis
Enteric infections
Neglected tropical diseases and malaria
Other infectious diseases
Maternal and neonatal disorders
Nutritional deficiencies
Neoplasms
Cardiovascular diseases
Chronic respiratory diseases
Digestive diseases
Neurological disorders
Mental disorders
Substance use disorders
Diabetes and kidney diseases
Skin and subcutaneous diseases
Musculoskeletal disorders
Other non-communicable diseases
Transport injuries
Unintentional injuries
Self-harm and interpersonal violence
COVID-19
Other COVID-19 pandemic-related outcomes

Myanmar

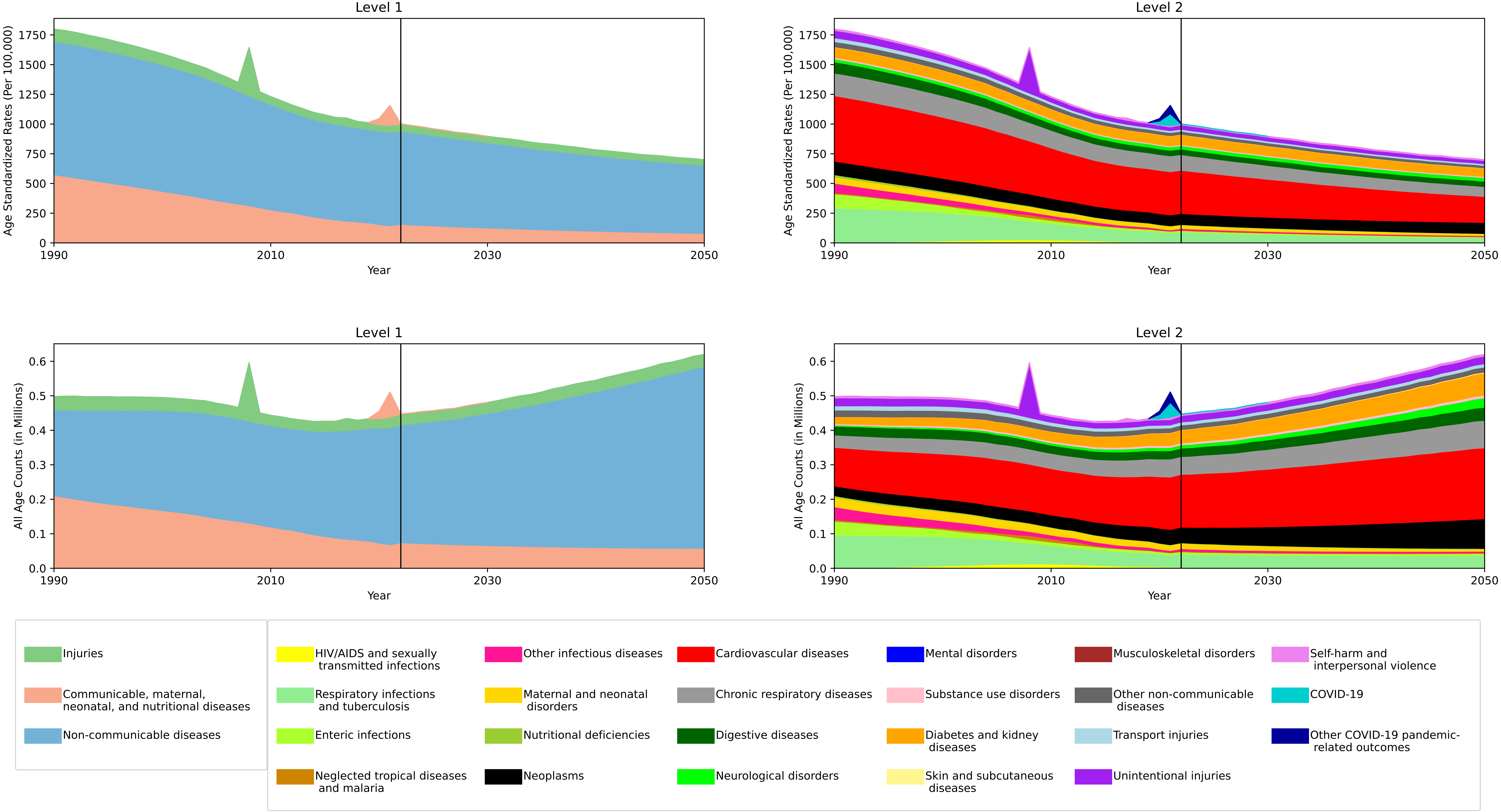
Level 1
Level 2
Age Standardized Rates (Per 100,000)
All Age Counts (in Millions)
Year
1990
2010
2030
2050
Injuries
Communicable, maternal, neonatal, and nutritional diseases
Non-communicable diseases
HIV/AIDS and sexually transmitted infections
Respiratory infections and tuberculosis
Enteric infections
Neglected tropical diseases and malaria
Other infectious diseases
Maternal and neonatal disorders
Nutritional deficiencies
Neoplasms
Cardiovascular diseases
Chronic respiratory diseases
Digestive diseases
Neurological disorders
Mental disorders
Substance use disorders
Diabetes and kidney diseases
Skin and subcutaneous diseases
Musculoskeletal disorders
Other non-communicable diseases
Transport injuries
Unintentional injuries
Self-harm and interpersonal violence
COVID-19
Other COVID-19 pandemic-related outcomes

Philippines

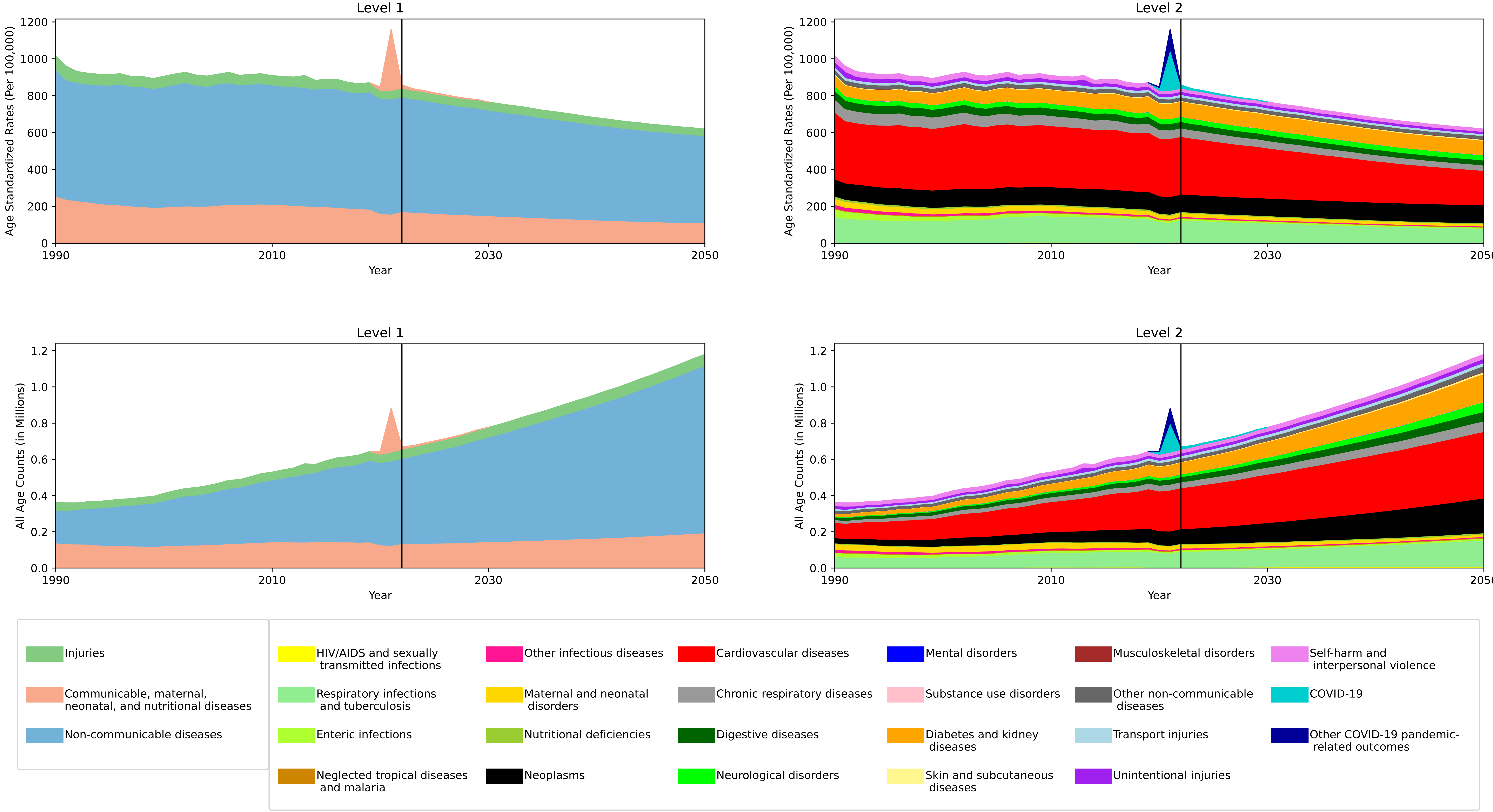

Seychelles

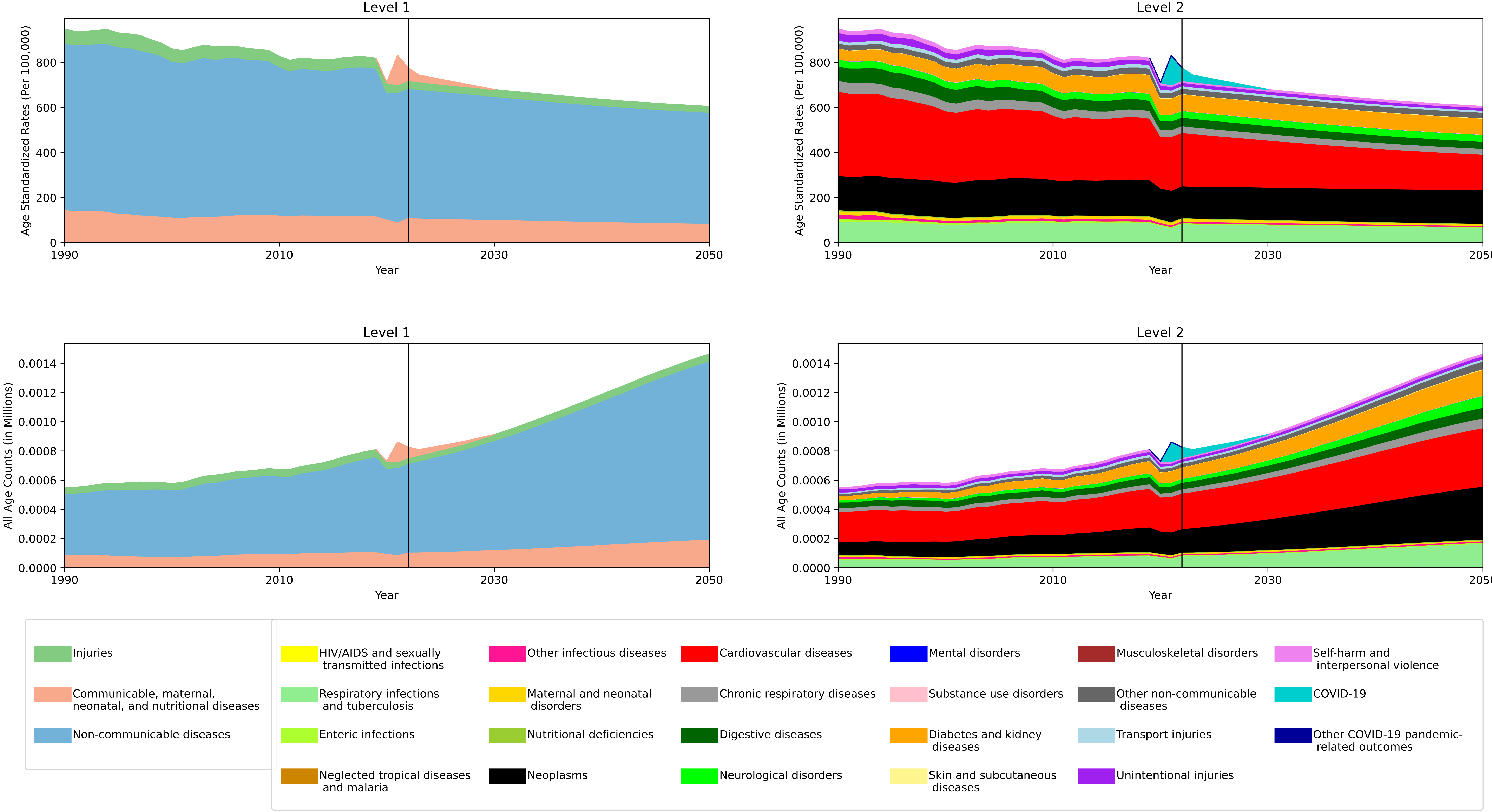
Level 1
Level 2
Age Standardized Rates (Per 100,000)
All Age Counts (in Millions)
Year
0
200
400
600
800
0.0000
0.0002
0.0004
0.0006
0.0008
0.0010
0.0012
0.0014
1990
2010
2030
2050
Injuries
Communicable, maternal, neonatal, and nutritional diseases
Non-communicable diseases
HIV/AIDS and sexually transmitted infections
Respiratory infections and tuberculosis
Enteric infections
Neglected tropical diseases and malaria
Other infectious diseases
Maternal and neonatal disorders
Nutritional deficiencies
Neoplasms
Cardiovascular diseases
Chronic respiratory diseases
Digestive diseases
Neurological disorders
Mental disorders
Substance use disorders
Diabetes and kidney diseases
Skin and subcutaneous diseases
Musculoskeletal disorders
Other non-communicable diseases
Transport injuries
Unintentional injuries
Self-harm and interpersonal violence
COVID-19
Other COVID-19 pandemic-related outcomes

Sri Lanka

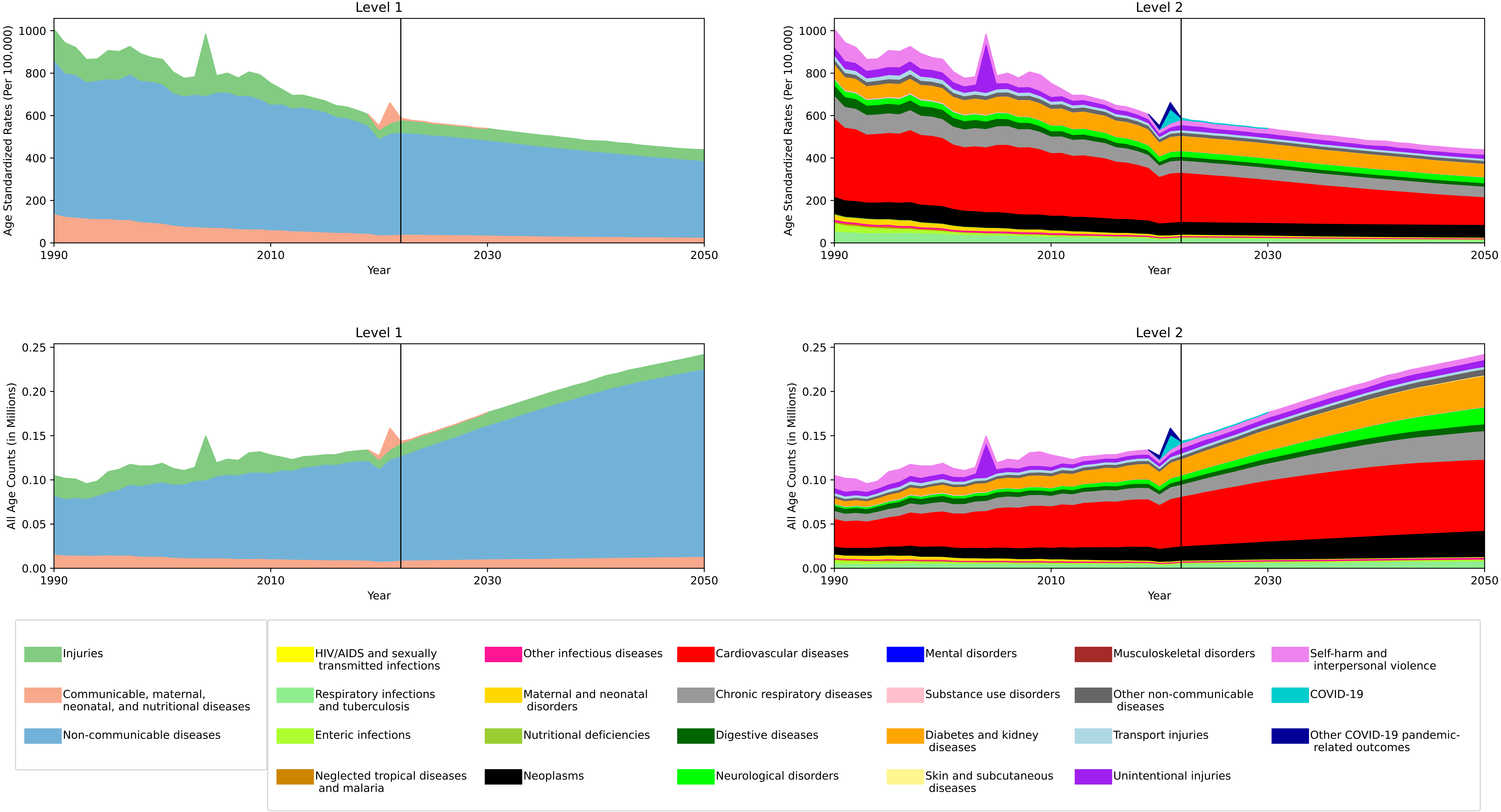
Level 1
Level 2
Age Standardized Rates (Per 100,000)
All Age Counts (in Millions)
Year
1990
2010
2030
2050
Injuries
Communicable, maternal, neonatal, and nutritional diseases
Non-communicable diseases
HIV/AIDS and sexually transmitted infections
Respiratory infections and tuberculosis
Enteric infections
Neglected tropical diseases and malaria
Other infectious diseases
Maternal and neonatal disorders
Nutritional deficiencies
Neoplasms
Cardiovascular diseases
Chronic respiratory diseases
Digestive diseases
Neurological disorders
Mental disorders
Substance use disorders
Diabetes and kidney diseases
Skin and subcutaneous diseases
Musculoskeletal disorders
Other non-communicable diseases
Transport injuries
Unintentional injuries
Self-harm and interpersonal violence
COVID-19
Other COVID-19 pandemic-related outcomes

Thailand

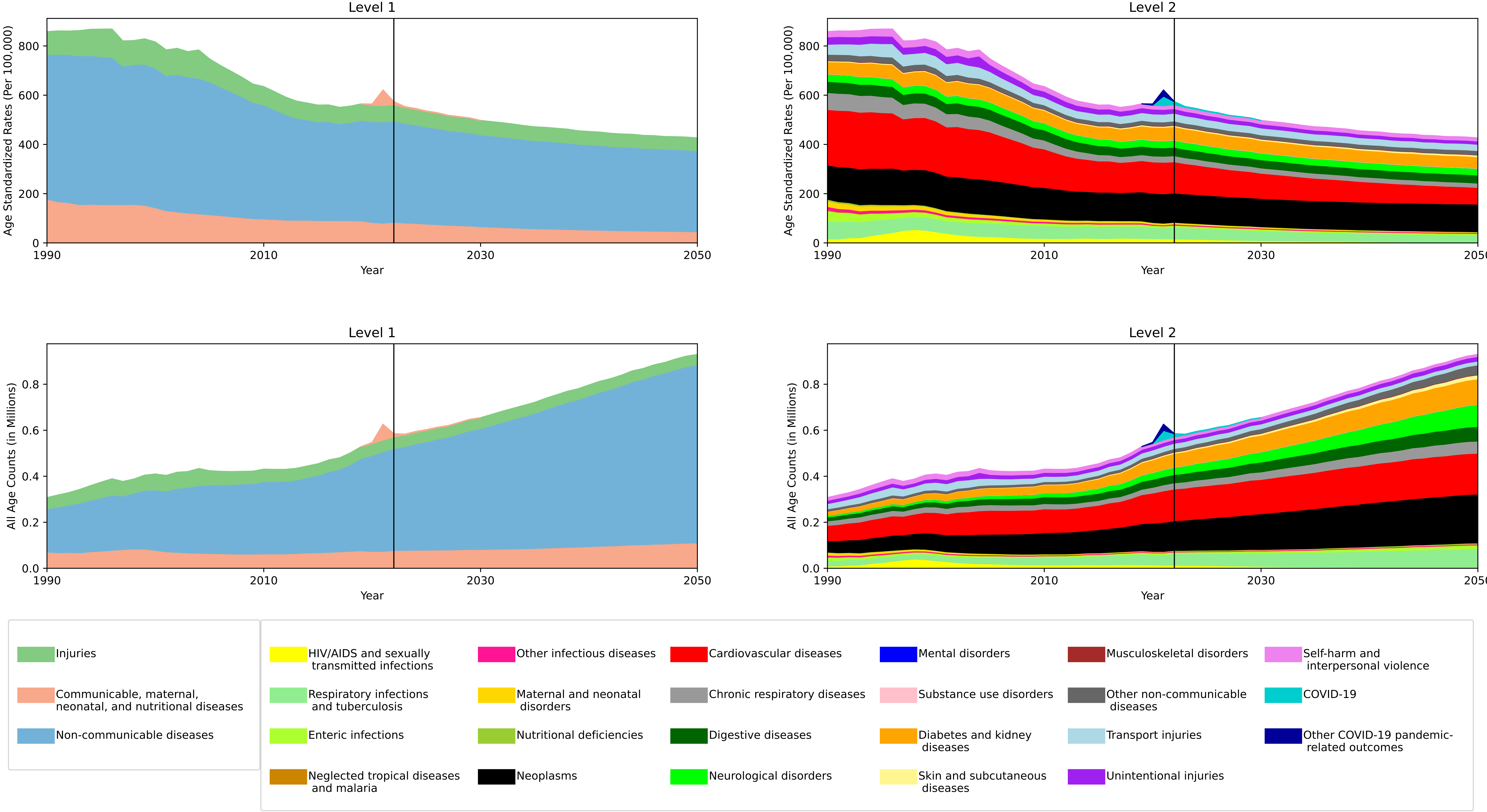
Level 1
Age Standardized Rates (Per 100,000)
Year
Level 2
Age Standardized Rates (Per 100,000)
Year
Level 1
All Age Counts (in Millions)
Year
Level 2
All Age Counts (in Millions)
Year
Injuries
Communicable, maternal, neonatal, and nutritional diseases
Non-communicable diseases
HIV/AIDS and sexually transmitted infections
Respiratory infections and tuberculosis
Enteric infections
Neglected tropical diseases and malaria
Other infectious diseases
Maternal and neonatal disorders
Nutritional deficiencies
Neoplasms
Cardiovascular diseases
Chronic respiratory diseases
Digestive diseases
Neurological disorders
Mental disorders
Substance use disorders
Diabetes and kidney diseases
Skin and subcutaneous diseases
Musculoskeletal disorders
Other non-communicable diseases
Transport injuries
Unintentional injuries
Self-harm and interpersonal violence
COVID-19
Other COVID-19 pandemic-related outcomes

Timor-Leste

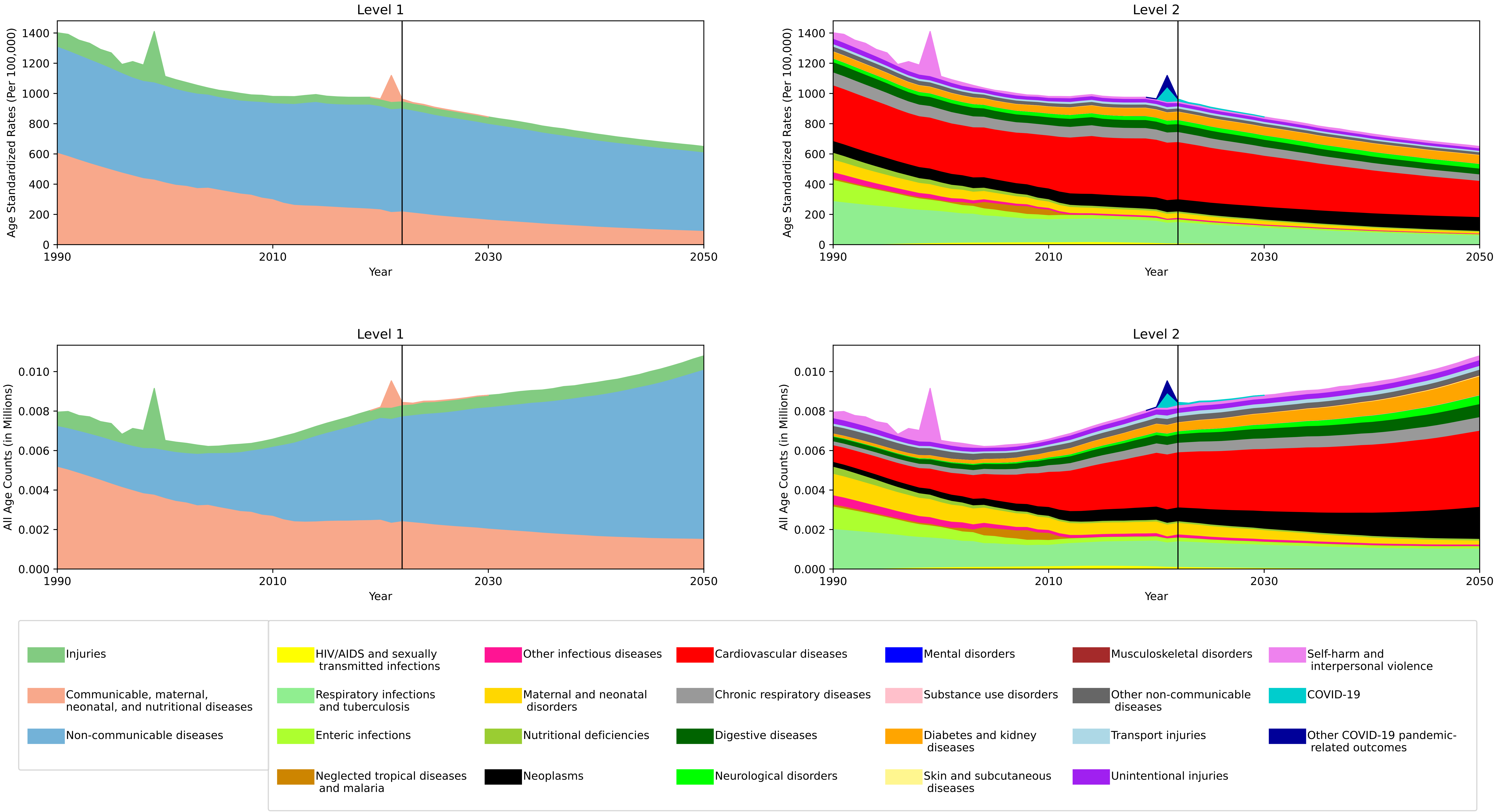
Level 1
Level 2
Age Standardized Rates (Per 100,000)
All Age Counts (in Millions)
Year
1990
2010
2030
2050
0
200
400
600
800
1000
1200
1400
0.000
0.002
0.004
0.006
0.008
0.010
Injuries
Communicable, maternal, neonatal, and nutritional diseases
Non-communicable diseases
HIV/AIDS and sexually transmitted infections
Respiratory infections and tuberculosis
Enteric infections
Neglected tropical diseases and malaria
Other infectious diseases
Maternal and neonatal disorders
Nutritional deficiencies
Neoplasms
Cardiovascular diseases
Chronic respiratory diseases
Digestive diseases
Neurological disorders
Mental disorders
Substance use disorders
Diabetes and kidney diseases
Skin and subcutaneous diseases
Musculoskeletal disorders
Other non-communicable diseases
Transport injuries
Unintentional injuries
Self-harm and interpersonal violence
COVID-19
Other COVID-19 pandemic-related outcomes

Viet Nam

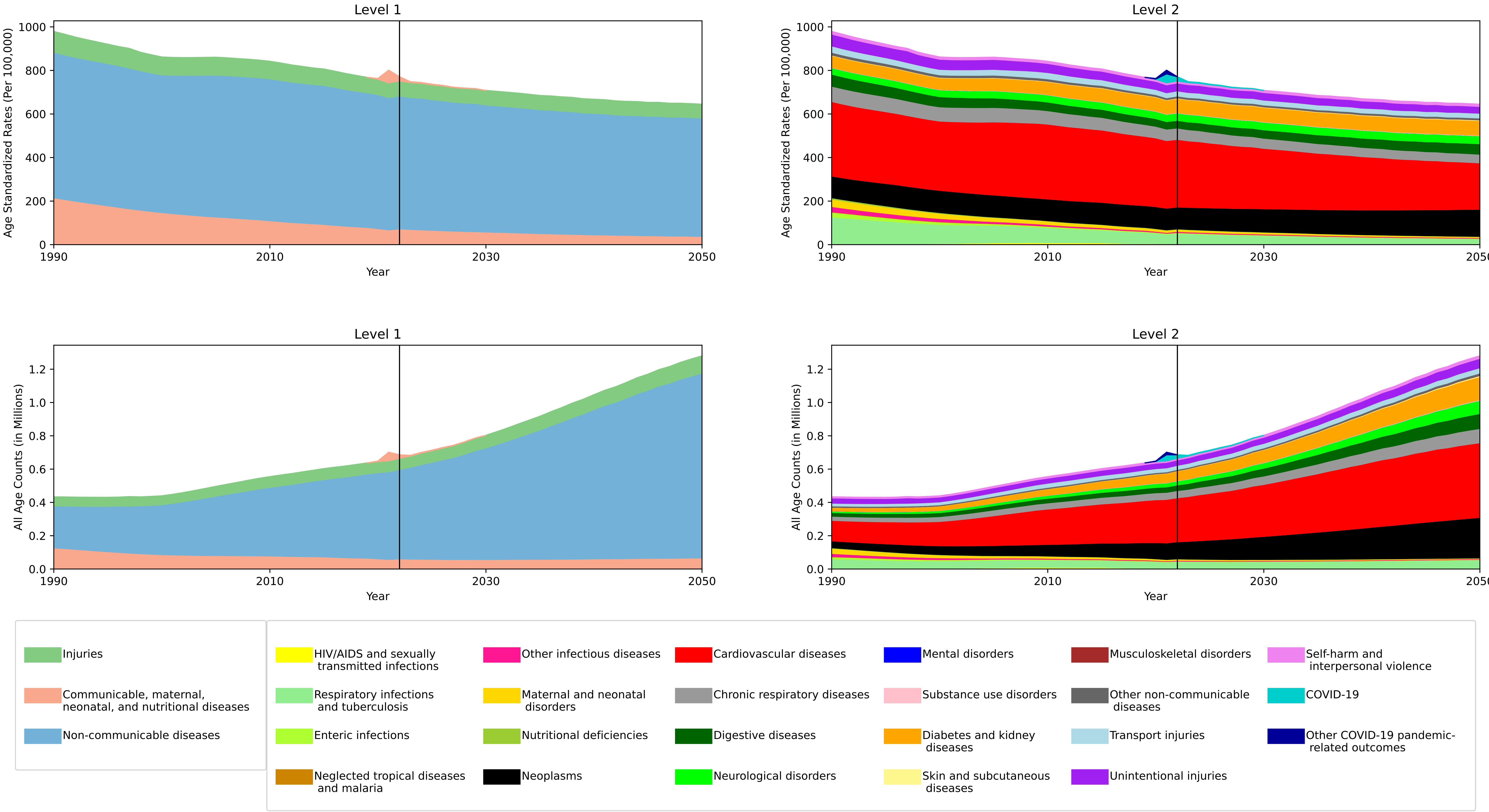
Level 1
Level 2
Age Standardized Rates (Per 100,000)
All Age Counts (in Millions)
Year
1990
2010
2030
2050
Injuries
Communicable, maternal, neonatal, and nutritional diseases
Non-communicable diseases
HIV/AIDS and sexually transmitted infections
Respiratory infections and tuberculosis
Enteric infections
Neglected tropical diseases and malaria
Other infectious diseases
Maternal and neonatal disorders
Nutritional deficiencies
Neoplasms
Cardiovascular diseases
Chronic respiratory diseases
Digestive diseases
Neurological disorders
Mental disorders
Substance use disorders
Diabetes and kidney diseases
Skin and subcutaneous diseases
Musculoskeletal disorders
Other non-communicable diseases
Transport injuries
Unintentional injuries
Self-harm and interpersonal violence
COVID-19
Other COVID-19 pandemic-related outcomes

Angola

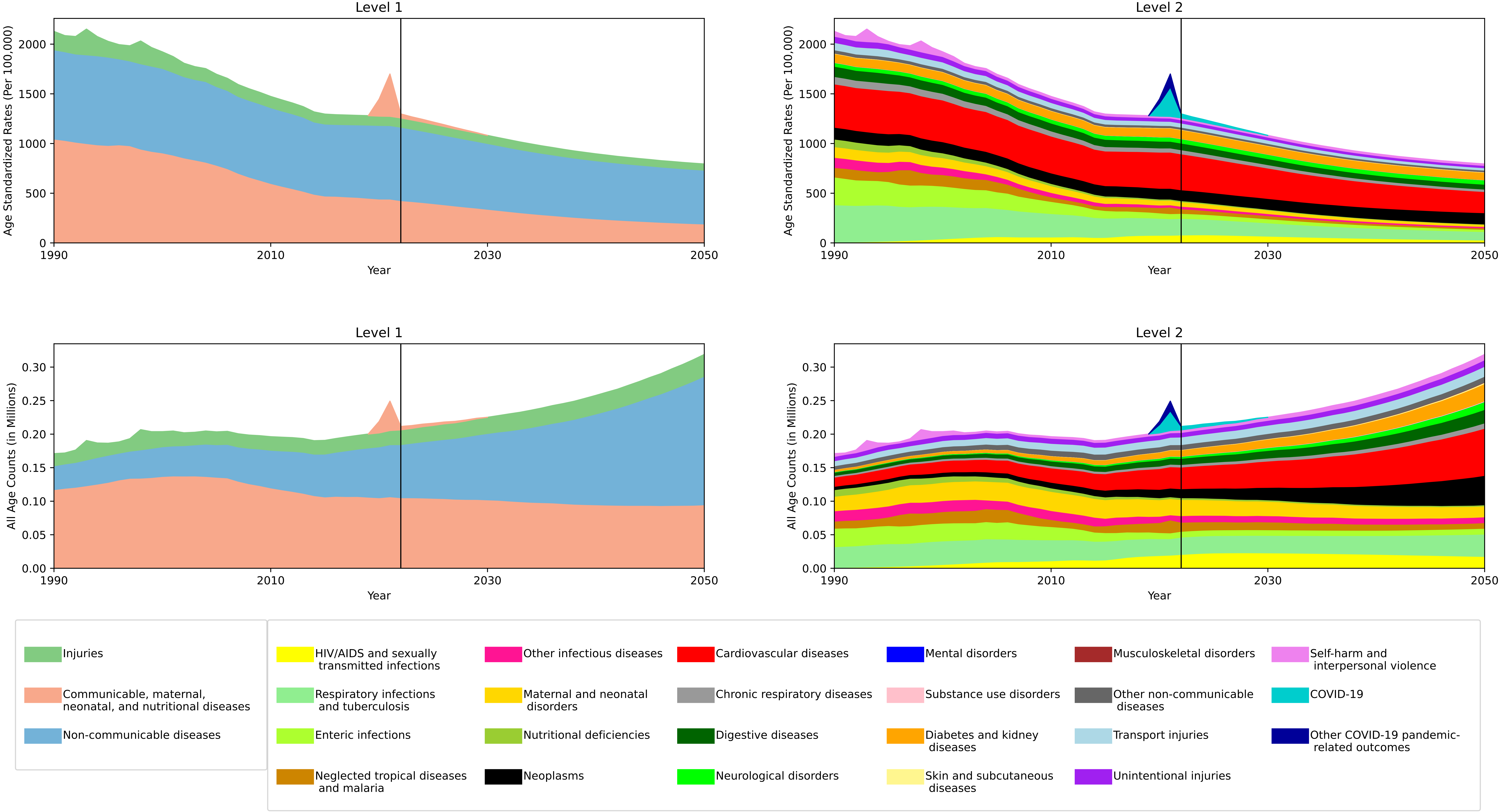
Level 1
Level 2
Age Standardized Rates (Per 100,000)
All Age Counts (in Millions)
Year
1990
2010
2030
2050
0
500
1000
1500
2000
0.00
0.05
0.10
0.15
0.20
0.25
0.30
Injuries
Communicable, maternal, neonatal, and nutritional diseases
Non-communicable diseases
HIV/AIDS and sexually transmitted infections
Respiratory infections and tuberculosis
Enteric infections
Neglected tropical diseases and malaria
Other infectious diseases
Maternal and neonatal disorders
Nutritional deficiencies
Neoplasms
Cardiovascular diseases
Chronic respiratory diseases
Digestive diseases
Neurological disorders
Mental disorders
Substance use disorders
Diabetes and kidney diseases
Skin and subcutaneous diseases
Musculoskeletal disorders
Other non-communicable diseases
Transport injuries
Unintentional injuries
Self-harm and interpersonal violence
COVID-19
Other COVID-19 pandemic-related outcomes

Central African Republic

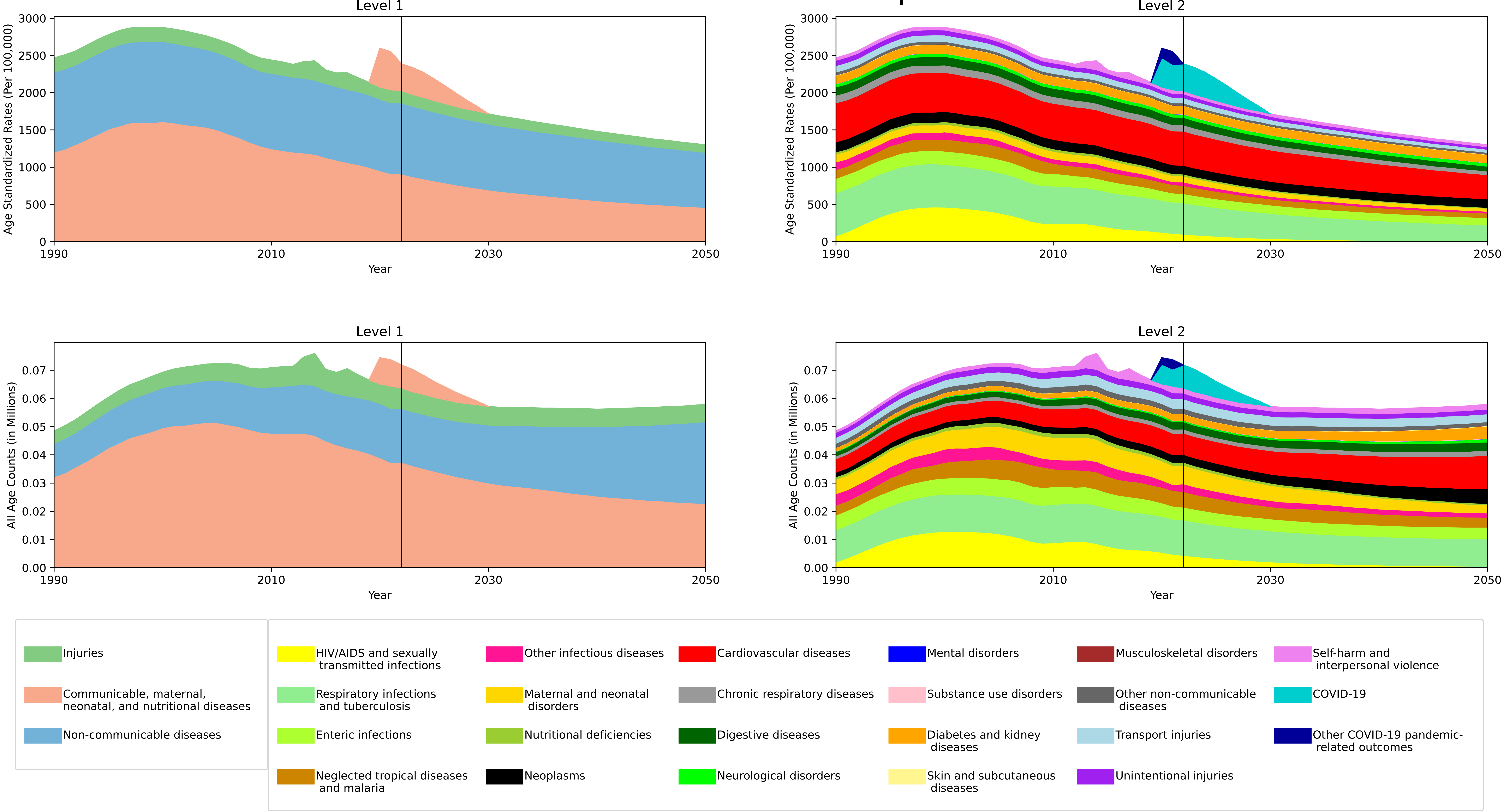

Congo (Brazzaville)

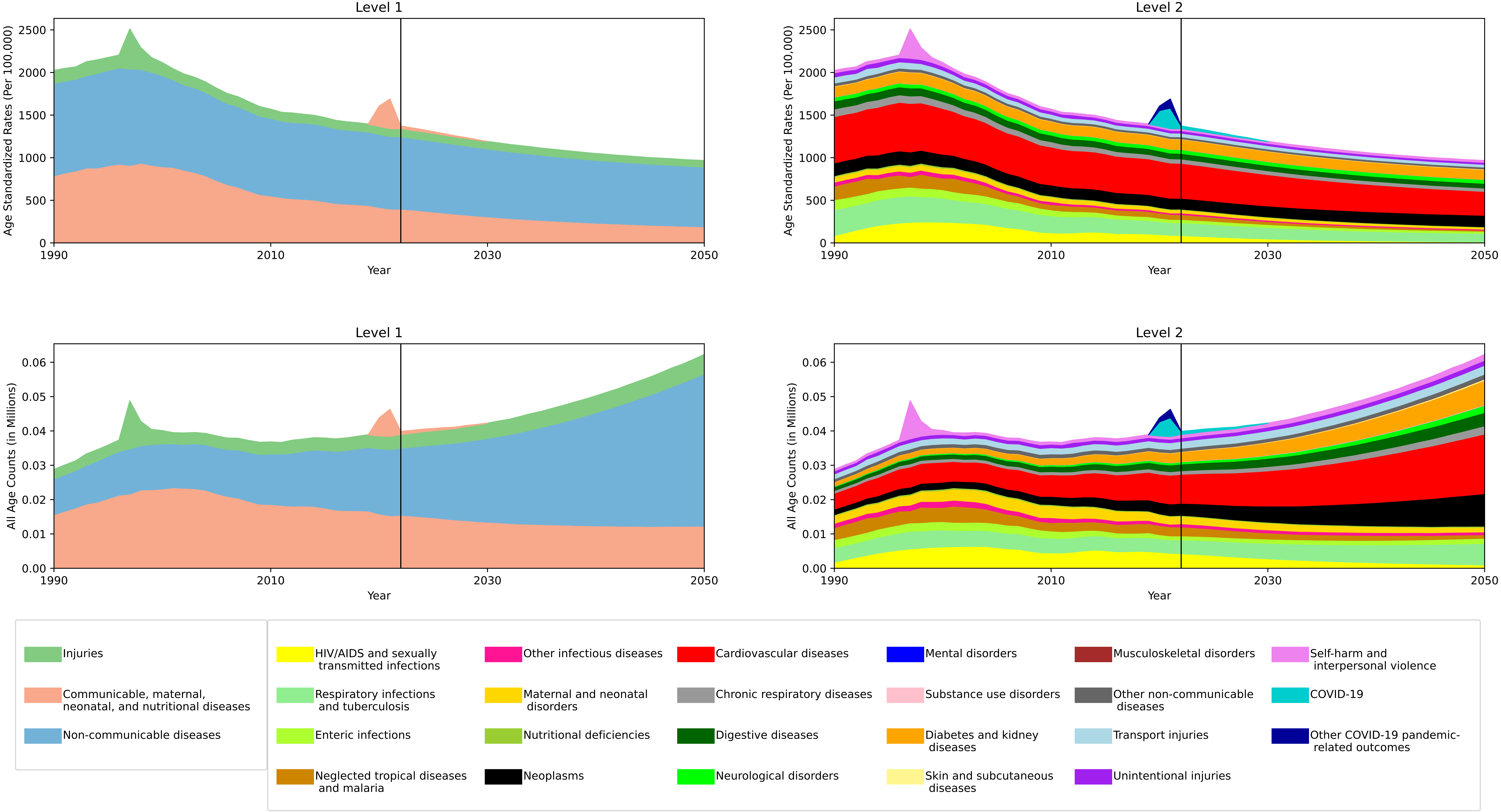

DR Congo

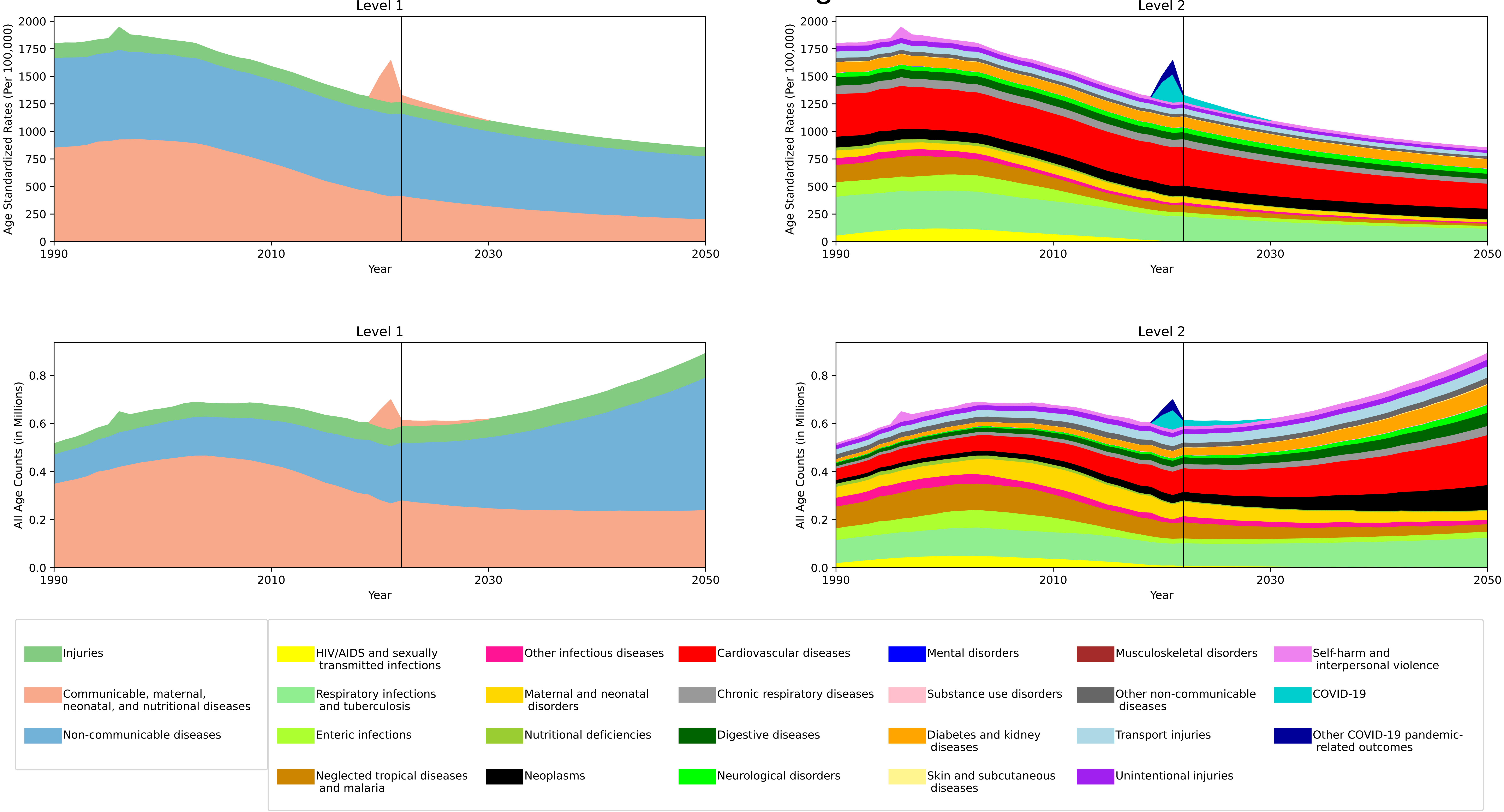
Level 1
Level 2
Age Standardized Rates (Per 100,000)
All Age Counts (in Millions)
Year
1990
2010
2030
2050
Injuries
Communicable, maternal, neonatal, and nutritional diseases
Non-communicable diseases
HIV/AIDS and sexually transmitted infections
Respiratory infections and tuberculosis
Enteric infections
Neglected tropical diseases and malaria
Other infectious diseases
Maternal and neonatal disorders
Nutritional deficiencies
Neoplasms
Cardiovascular diseases
Chronic respiratory diseases
Digestive diseases
Neurological disorders
Mental disorders
Substance use disorders
Diabetes and kidney diseases
Skin and subcutaneous diseases
Musculoskeletal disorders
Other non-communicable diseases
Transport injuries
Unintentional injuries
Self-harm and interpersonal violence
COVID-19
Other COVID-19 pandemic-related outcomes

Equatorial Guinea

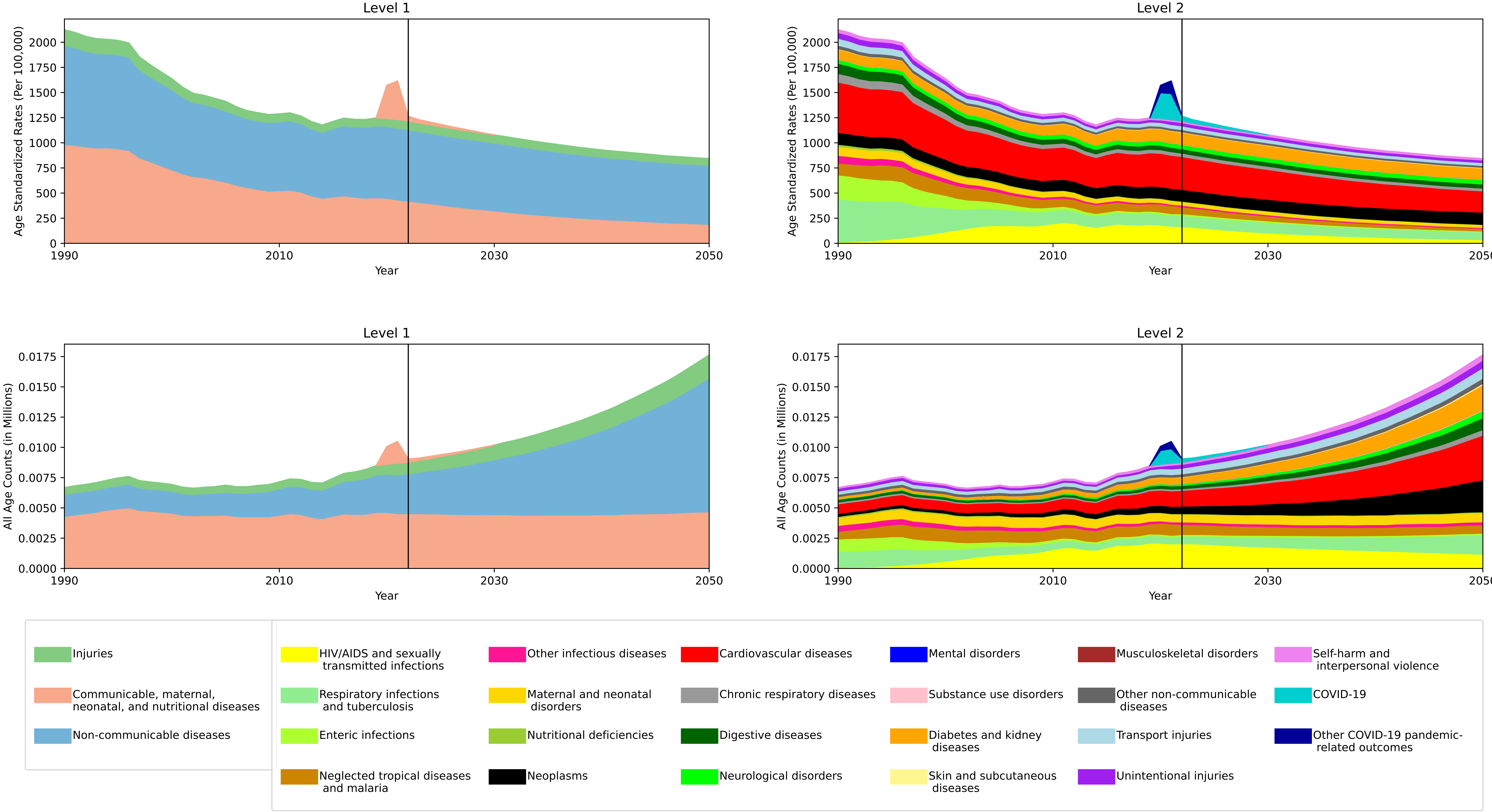

Gabon

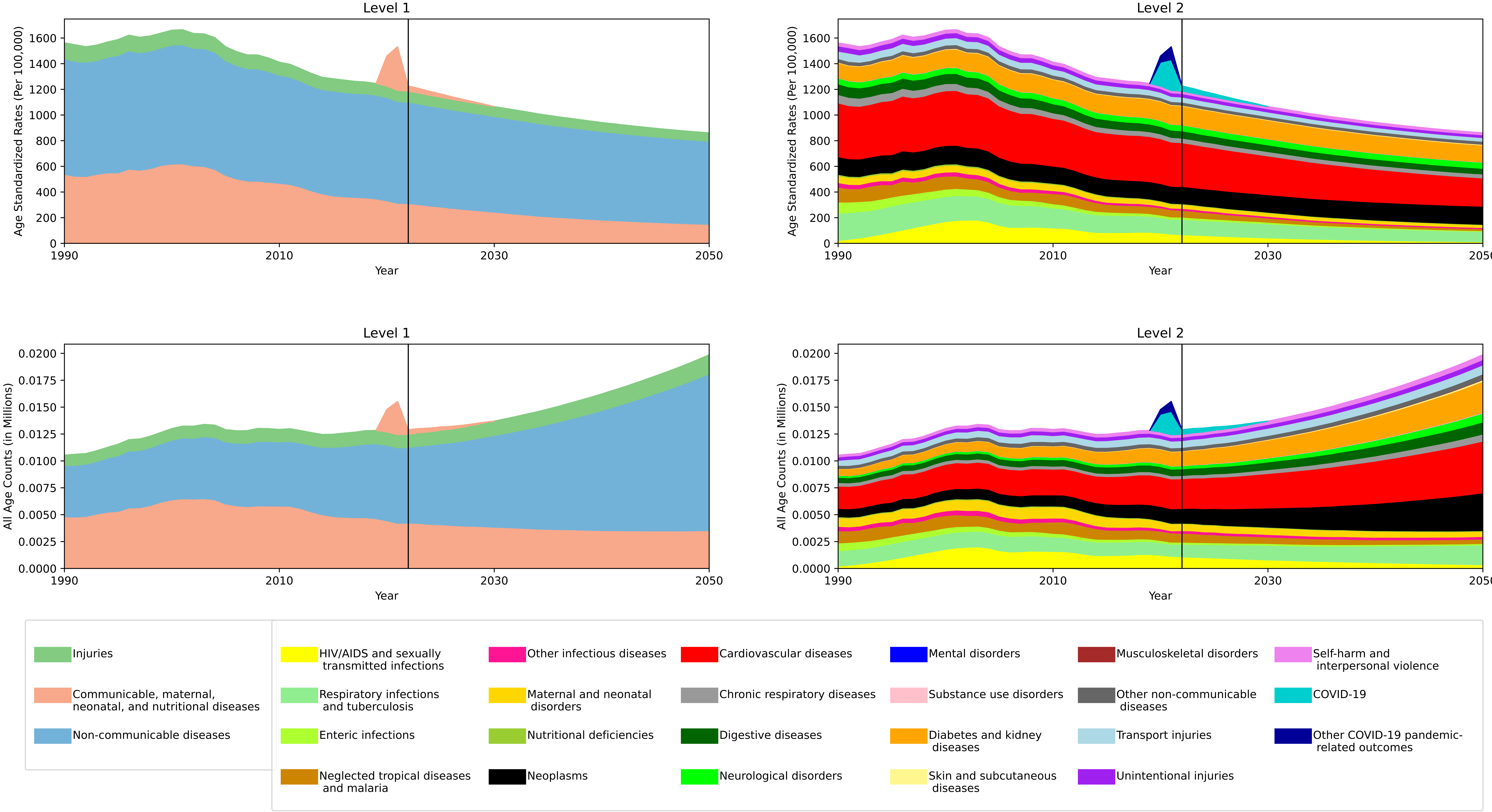
Level 1
Level 2
Age Standardized Rates (Per 100,000)
All Age Counts (in Millions)
Year
1990
2010
2030
2050
0
200
400
600
800
1000
1200
1400
1600
0.0000
0.0025
0.0050
0.0075
0.0100
0.0125
0.0150
0.0175
0.0200
Injuries
Communicable, maternal, neonatal, and nutritional diseases
Non-communicable diseases
HIV/AIDS and sexually transmitted infections
Respiratory infections and tuberculosis
Enteric infections
Neglected tropical diseases and malaria
Other infectious diseases
Maternal and neonatal disorders
Nutritional deficiencies
Neoplasms
Cardiovascular diseases
Chronic respiratory diseases
Digestive diseases
Neurological disorders
Mental disorders
Substance use disorders
Diabetes and kidney diseases
Skin and subcutaneous diseases
Musculoskeletal disorders
Other non-communicable diseases
Transport injuries
Unintentional injuries
Self-harm and interpersonal violence
COVID-19
Other COVID-19 pandemic-related outcomes

Burundi

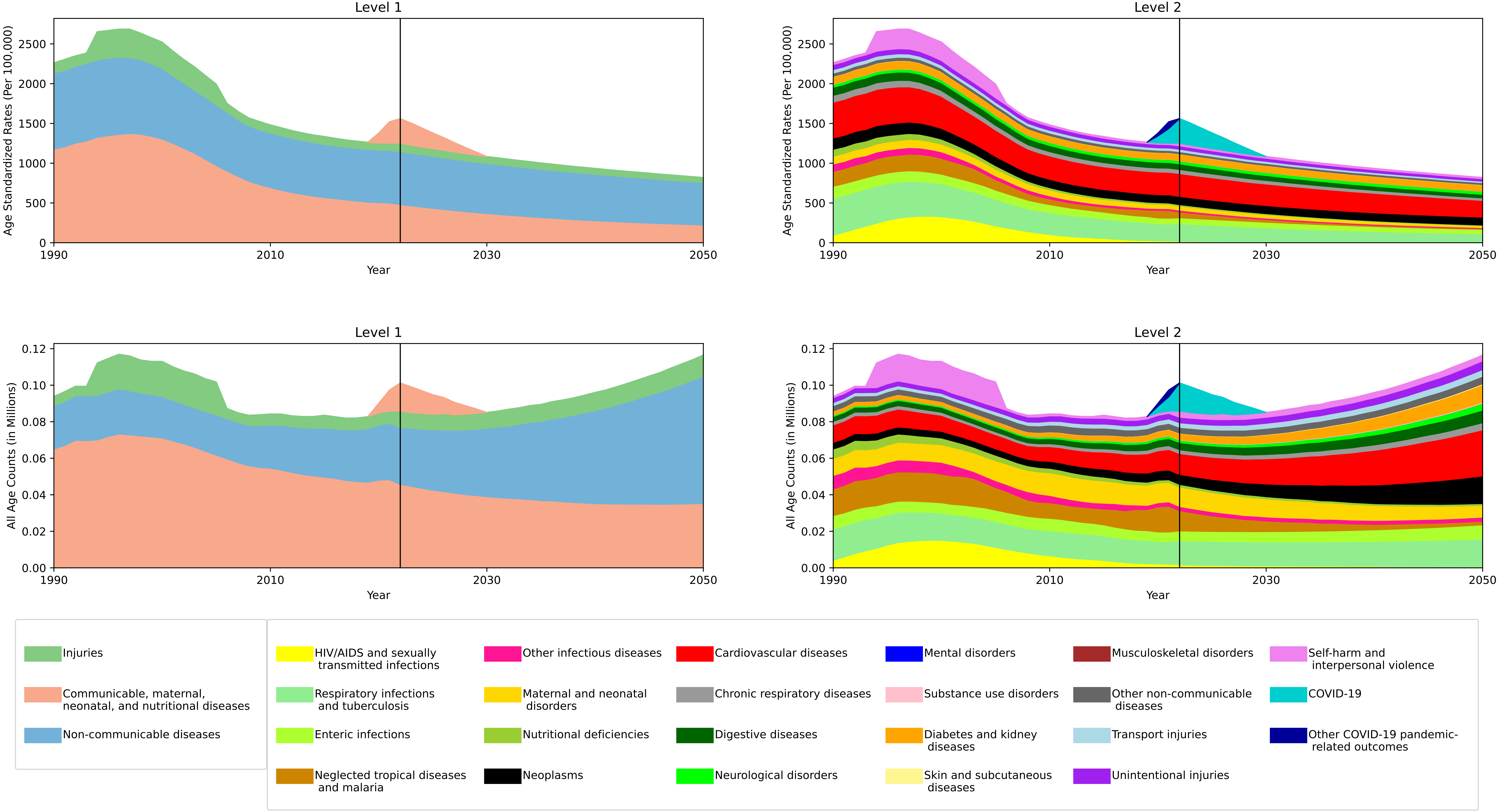
Level 1
Level 2
Age Standardized Rates (Per 100,000)
All Age Counts (in Millions)
Year
1990
2010
2030
2050
0
500
1000
1500
2000
2500
0.00
0.02
0.04
0.06
0.08
0.10
0.12
Injuries
Communicable, maternal, neonatal, and nutritional diseases
Non-communicable diseases
HIV/AIDS and sexually transmitted infections
Respiratory infections and tuberculosis
Enteric infections
Neglected tropical diseases and malaria
Other infectious diseases
Maternal and neonatal disorders
Nutritional deficiencies
Neoplasms
Cardiovascular diseases
Chronic respiratory diseases
Digestive diseases
Neurological disorders
Mental disorders
Substance use disorders
Diabetes and kidney diseases
Skin and subcutaneous diseases
Musculoskeletal disorders
Other non-communicable diseases
Transport injuries
Unintentional injuries
Self-harm and interpersonal violence
COVID-19
Other COVID-19 pandemic-related outcomes

Comoros

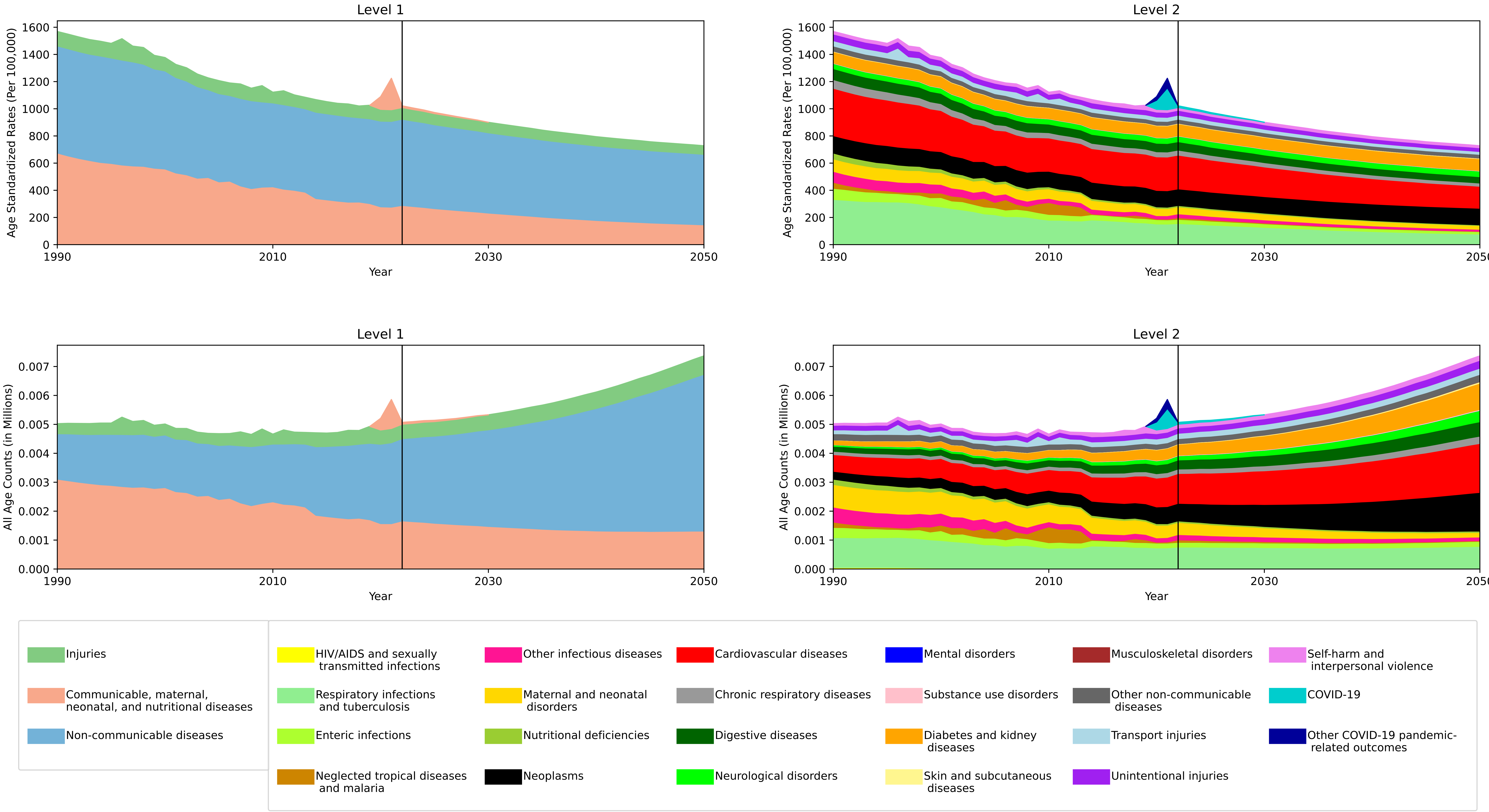

Level 1
Level 2
Age Standardized Rates (Per 100,000)
All Age Counts (in Millions)
Year
1990
2010
2030
2050
Injuries
Communicable, maternal, neonatal, and nutritional diseases
Non-communicable diseases
HIV/AIDS and sexually transmitted infections
Respiratory infections and tuberculosis
Enteric infections
Neglected tropical diseases and malaria
Other infectious diseases
Maternal and neonatal disorders
Nutritional deficiencies
Neoplasms
Cardiovascular diseases
Chronic respiratory diseases
Digestive diseases
Neurological disorders
Mental disorders
Substance use disorders
Diabetes and kidney diseases
Skin and subcutaneous diseases
Musculoskeletal disorders
Other non-communicable diseases
Transport injuries
Unintentional injuries
Self-harm and interpersonal violence
COVID-19
Other COVID-19 pandemic-related outcomes

Djibouti

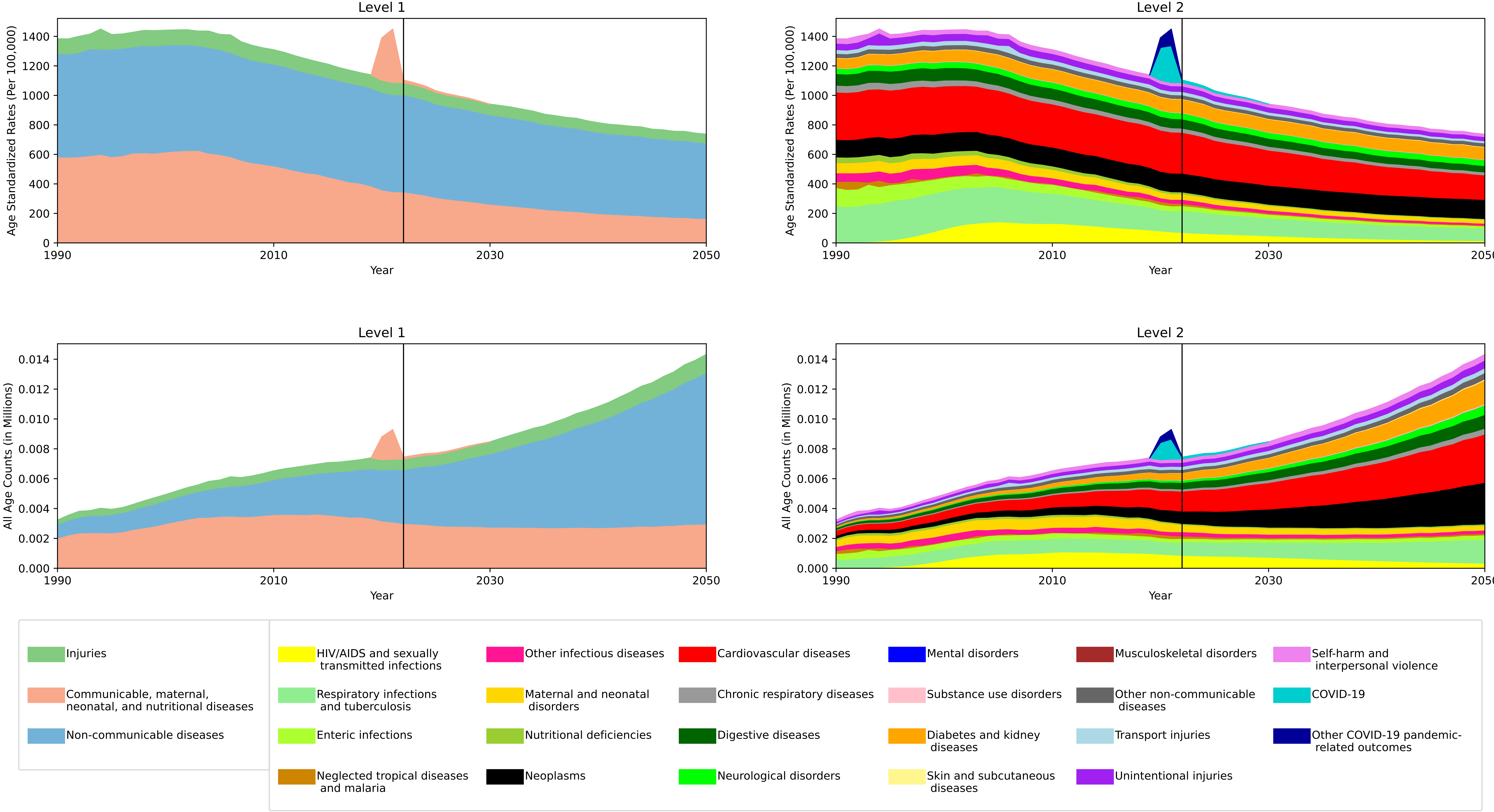
Level 1
Level 2
Level 1
Level 2
Age Standardized Rates (Per 100,000)
All Age Counts (in Millions)
Year
1990
2010
2030
2050
Injuries
Communicable, maternal, neonatal, and nutritional diseases
Non-communicable diseases
HIV/AIDS and sexually transmitted infections
Respiratory infections and tuberculosis
Enteric infections
Neglected tropical diseases and malaria
Other infectious diseases
Maternal and neonatal disorders
Nutritional deficiencies
Neoplasms
Cardiovascular diseases
Chronic respiratory diseases
Digestive diseases
Neurological disorders
Mental disorders
Substance use disorders
Diabetes and kidney diseases
Skin and subcutaneous diseases
Musculoskeletal disorders
Other non-communicable diseases
Transport injuries
Unintentional injuries
Self-harm and interpersonal violence
COVID-19
Other COVID-19 pandemic-related outcomes

Eritrea

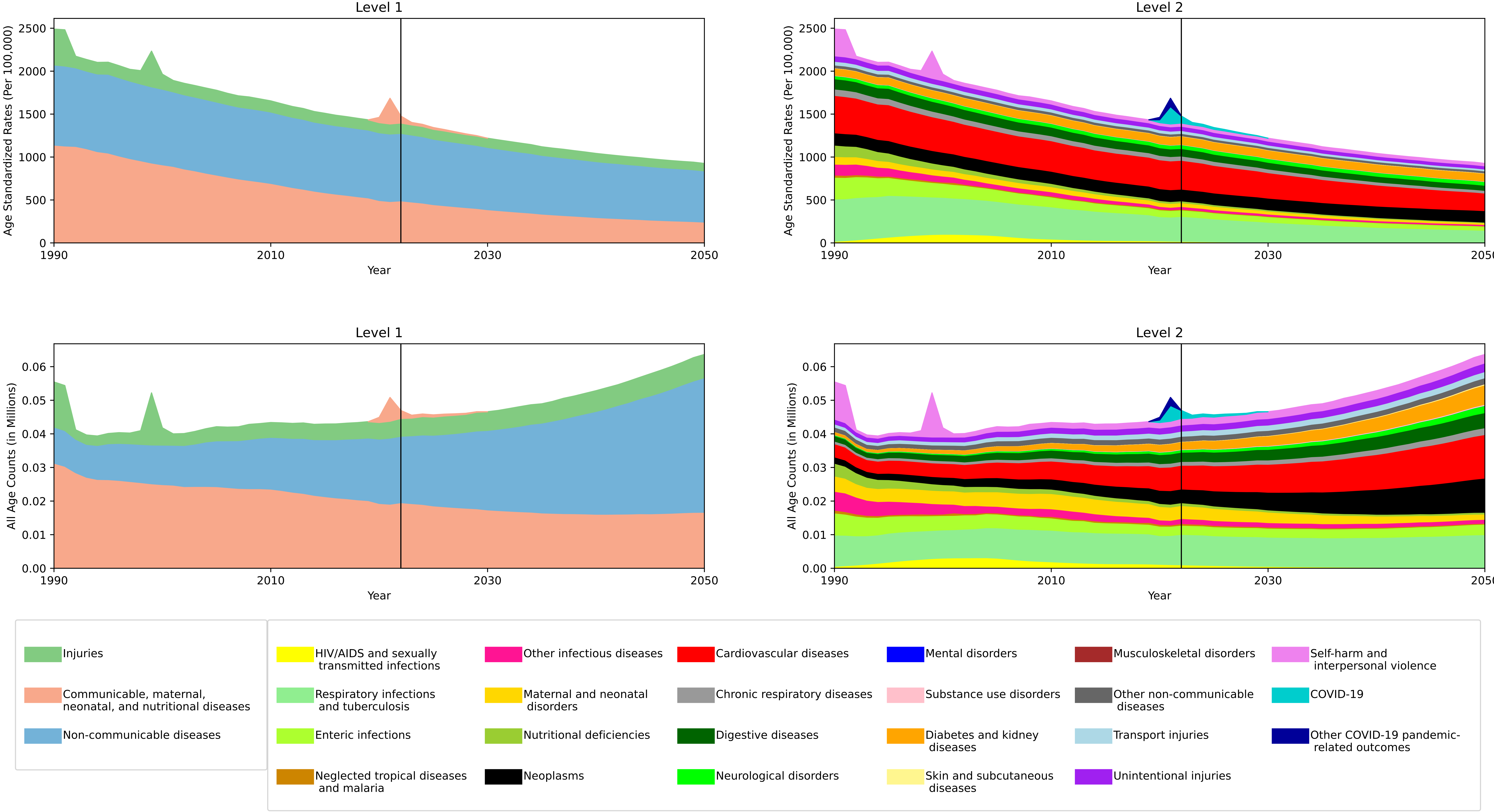
Level 1
Level 2
Age Standardized Rates (Per 100,000)
All Age Counts (in Millions)
Year
1990
2010
2030
2050
Injuries
Communicable, maternal, neonatal, and nutritional diseases
Non-communicable diseases
HIV/AIDS and sexually transmitted infections
Respiratory infections and tuberculosis
Enteric infections
Neglected tropical diseases and malaria
Other infectious diseases
Maternal and neonatal disorders
Nutritional deficiencies
Neoplasms
Cardiovascular diseases
Chronic respiratory diseases
Digestive diseases
Neurological disorders
Mental disorders
Substance use disorders
Diabetes and kidney diseases
Skin and subcutaneous diseases
Musculoskeletal disorders
Other non-communicable diseases
Transport injuries
Unintentional injuries
Self-harm and interpersonal violence
COVID-19
Other COVID-19 pandemic-related outcomes

Ethiopia

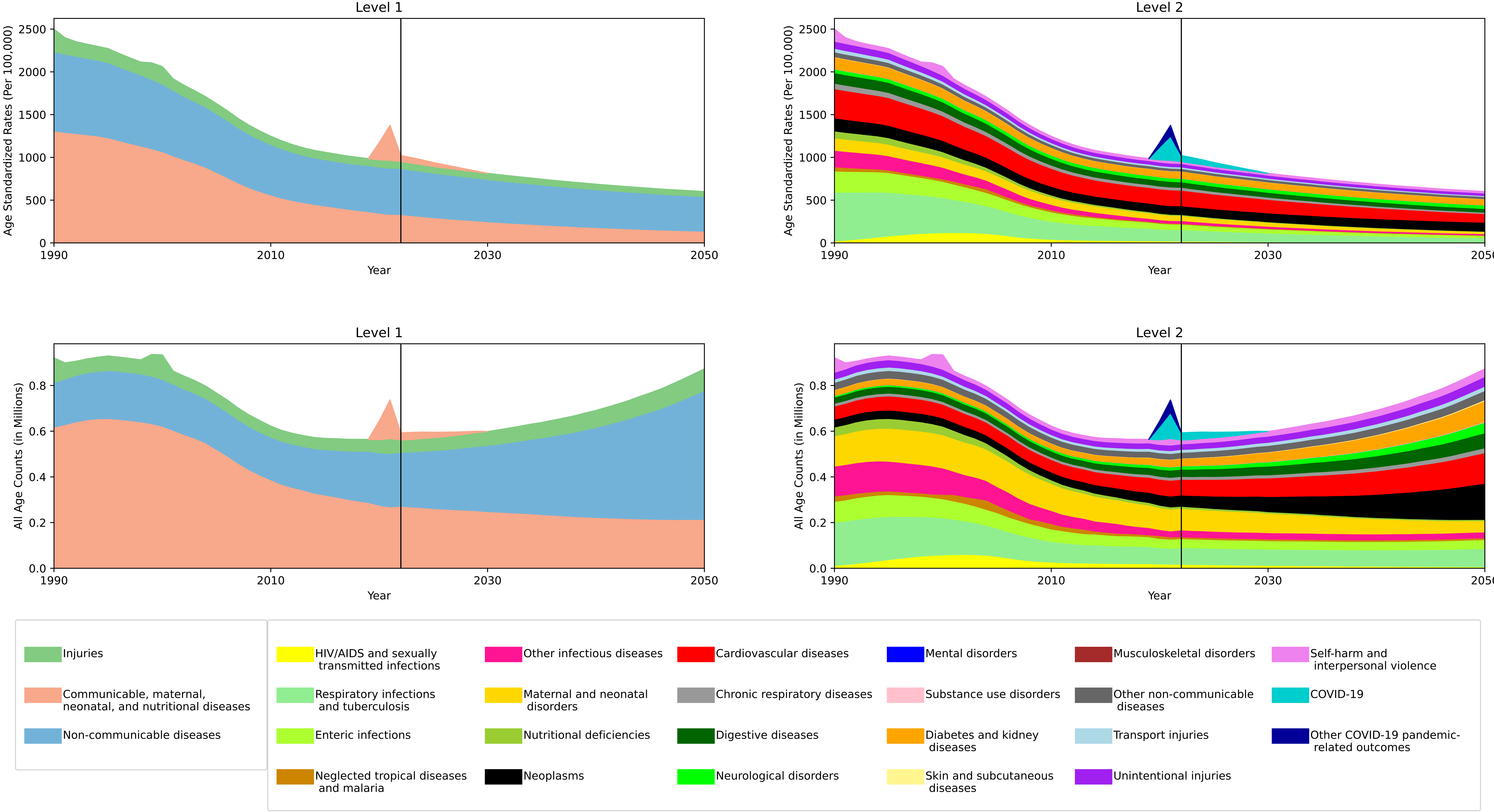
Level 1
Level 2
Age Standardized Rates (Per 100,000)
All Age Counts (in Millions)
Year
1990
2010
2030
2050
Injuries
Communicable, maternal, neonatal, and nutritional diseases
Non-communicable diseases
HIV/AIDS and sexually transmitted infections
Respiratory infections and tuberculosis
Enteric infections
Neglected tropical diseases and malaria
Other infectious diseases
Maternal and neonatal disorders
Nutritional deficiencies
Neoplasms
Cardiovascular diseases
Chronic respiratory diseases
Digestive diseases
Neurological disorders
Mental disorders
Substance use disorders
Diabetes and kidney diseases
Skin and subcutaneous diseases
Musculoskeletal disorders
Other non-communicable diseases
Transport injuries
Unintentional injuries
Self-harm and interpersonal violence
COVID-19
Other COVID-19 pandemic-related outcomes

Kenya

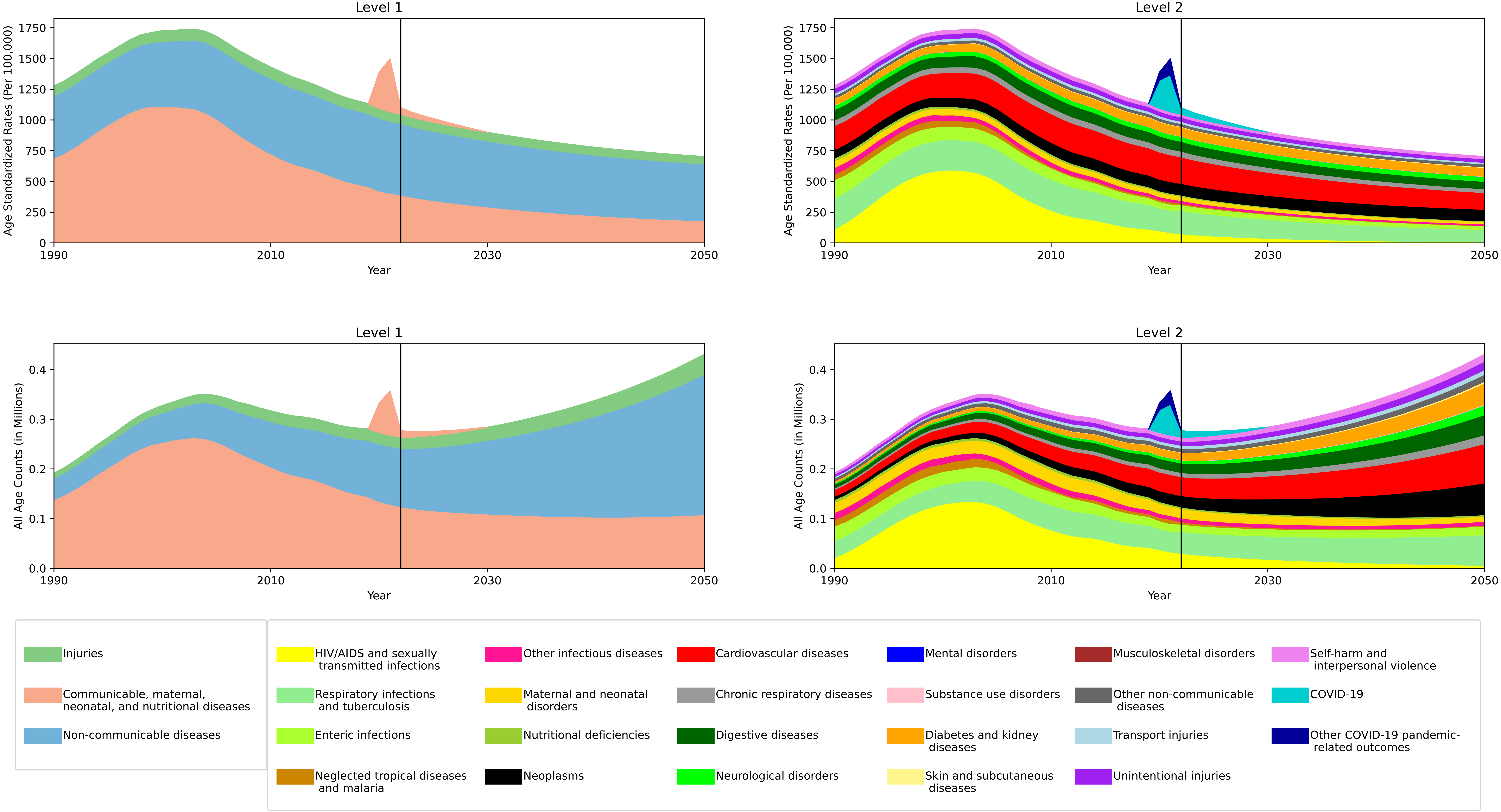
Level 1
Level 2
Age Standardized Rates (Per 100,000)
All Age Counts (in Millions)
Year
1990
2010
2030
2050
0
250
500
750
1000
1250
1500
1750
0.0
0.1
0.2
0.3
0.4
Injuries
Communicable, maternal, neonatal, and nutritional diseases
Non-communicable diseases
HIV/AIDS and sexually transmitted infections
Respiratory infections and tuberculosis
Enteric infections
Neglected tropical diseases and malaria
Other infectious diseases
Maternal and neonatal disorders
Nutritional deficiencies
Neoplasms
Cardiovascular diseases
Chronic respiratory diseases
Digestive diseases
Neurological disorders
Mental disorders
Substance use disorders
Diabetes and kidney diseases
Skin and subcutaneous diseases
Musculoskeletal disorders
Other non-communicable diseases
Transport injuries
Unintentional injuries
Self-harm and interpersonal violence
COVID-19
Other COVID-19 pandemic-related outcomes

Madagascar

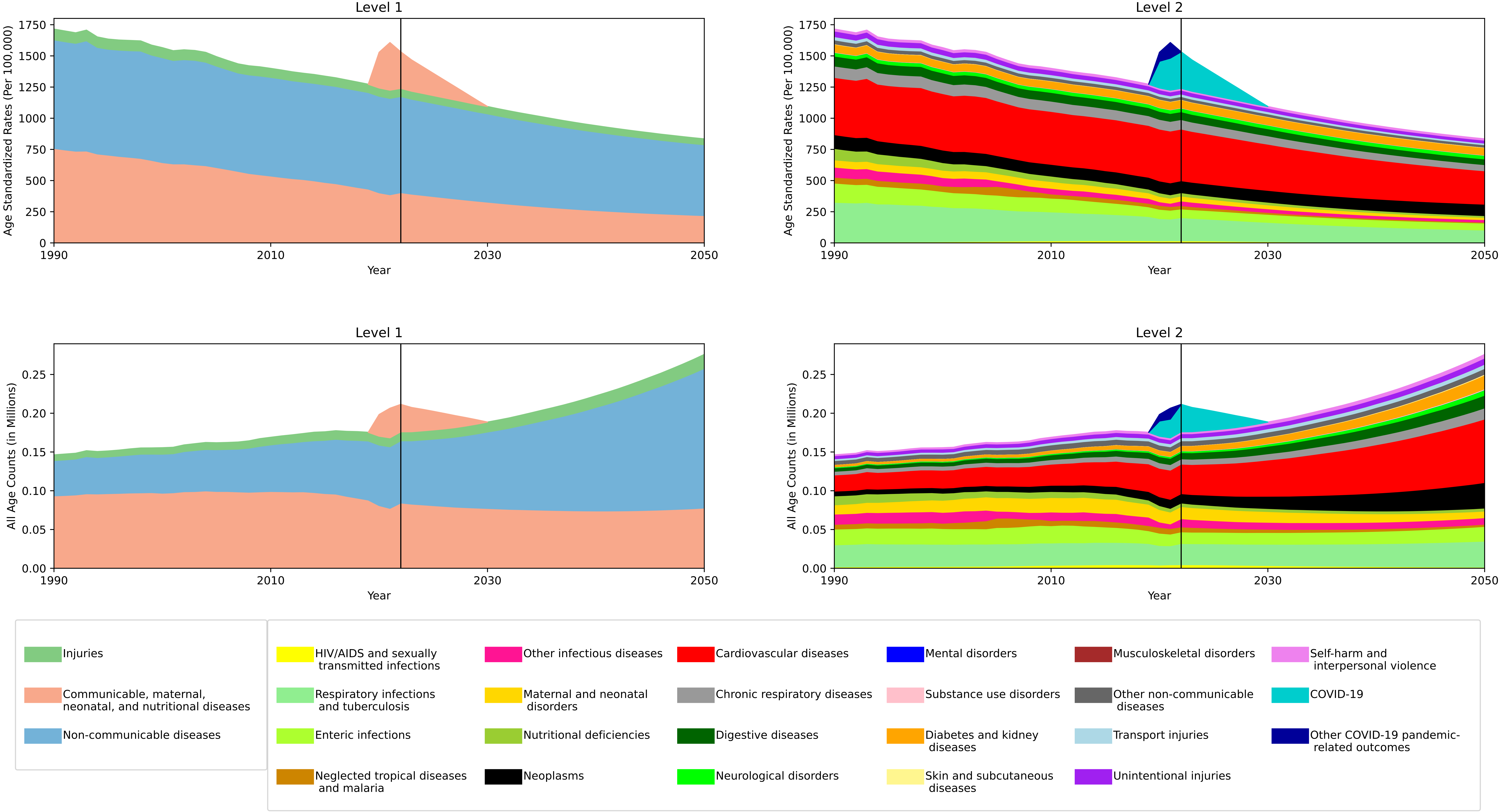
Level 1
Level 2
Age Standardized Rates (Per 100,000)
All Age Counts (in Millions)
Year
1750
1500
1250
1000
750
500
250
0
0.25
0.20
0.15
0.10
0.05
0.00
1990
2010
2030
2050
Injuries
Communicable, maternal, neonatal, and nutritional diseases
Non-communicable diseases
HIV/AIDS and sexually transmitted infections
Respiratory infections and tuberculosis
Enteric infections
Neglected tropical diseases and malaria
Other infectious diseases
Maternal and neonatal disorders
Nutritional deficiencies
Neoplasms
Cardiovascular diseases
Chronic respiratory diseases
Digestive diseases
Neurological disorders
Mental disorders
Substance use disorders
Diabetes and kidney diseases
Skin and subcutaneous diseases
Musculoskeletal disorders
Other non-communicable diseases
Transport injuries
Unintentional injuries
Self-harm and interpersonal violence
COVID-19
Other COVID-19 pandemic-related outcomes

Malawi

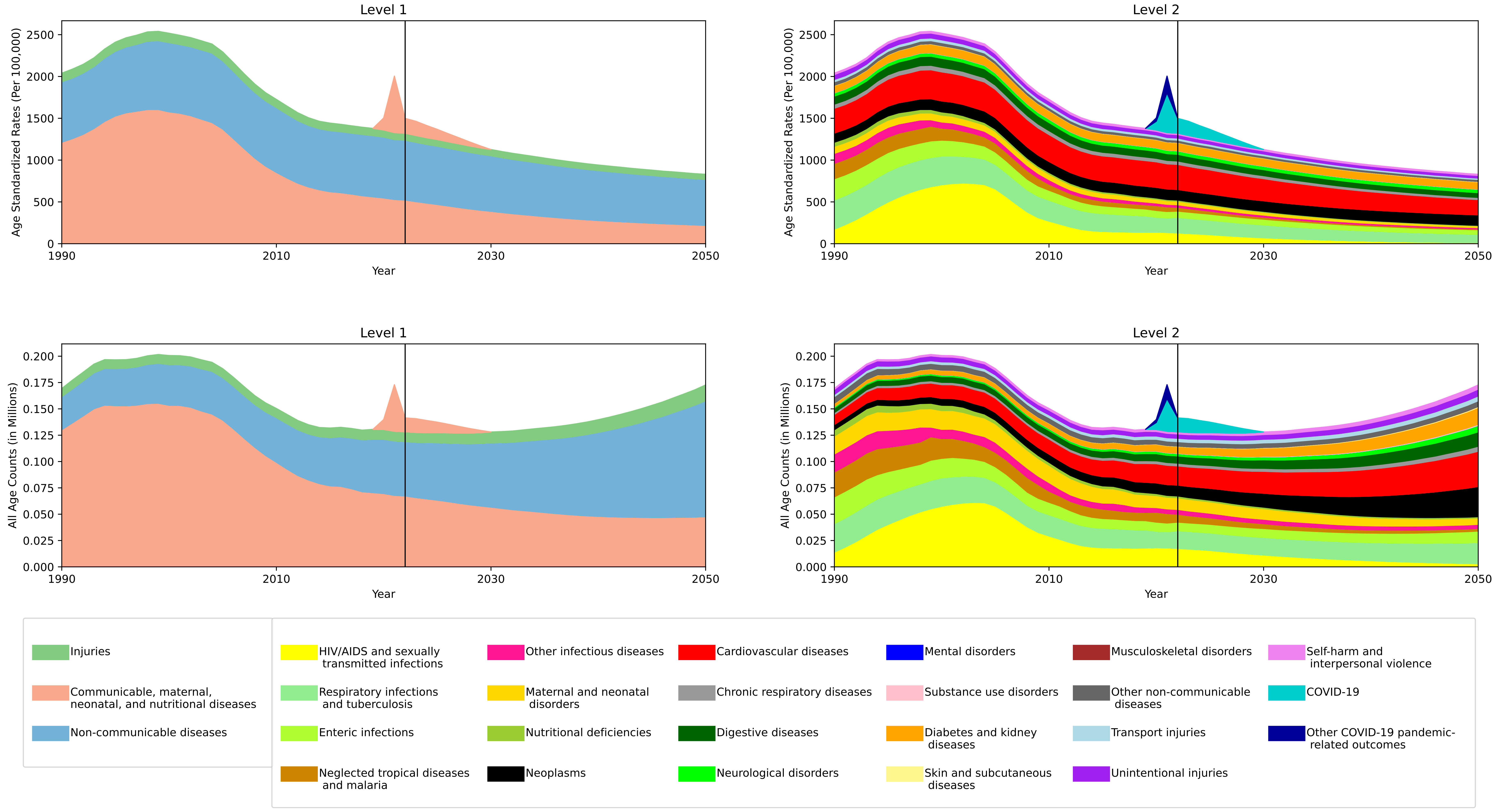
Level 1
Level 2
Age Standardized Rates (Per 100,000)
All Age Counts (in Millions)
Year
Injuries
Communicable, maternal, neonatal, and nutritional diseases
Non-communicable diseases
HIV/AIDS and sexually transmitted infections
Respiratory infections and tuberculosis
Enteric infections
Neglected tropical diseases and malaria
Other infectious diseases
Maternal and neonatal disorders
Nutritional deficiencies
Neoplasms
Cardiovascular diseases
Chronic respiratory diseases
Digestive diseases
Neurological disorders
Mental disorders
Substance use disorders
Diabetes and kidney diseases
Skin and subcutaneous diseases
Musculoskeletal disorders
Other non-communicable diseases
Transport injuries
Unintentional injuries
Self-harm and interpersonal violence
COVID-19
Other COVID-19 pandemic-related outcomes

Mozambique

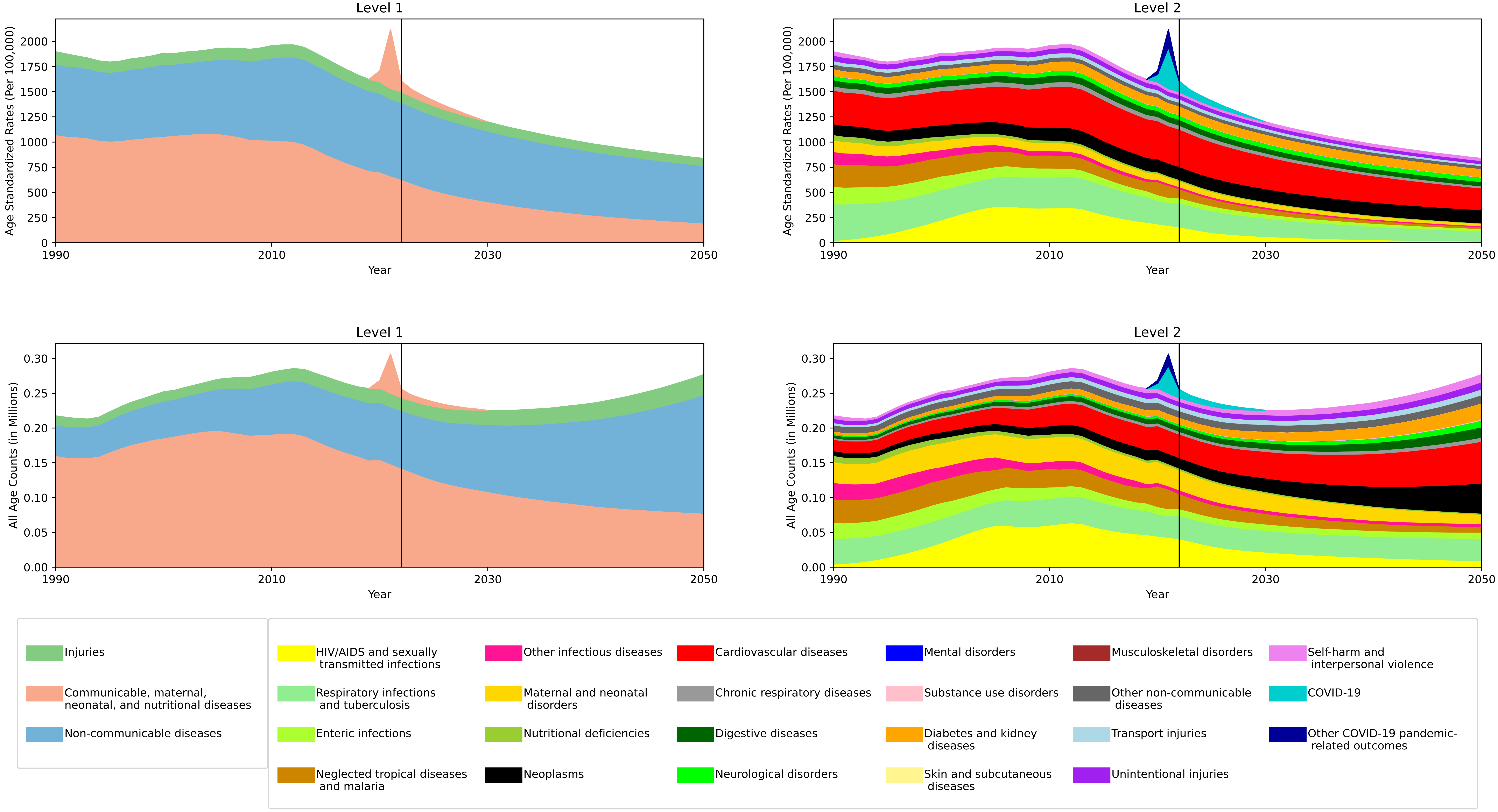
Level 1
Level 2
Age Standardized Rates (Per 100,000)
All Age Counts (in Millions)
Year
1990
2010
2030
2050
0
250
500
750
1000
1250
1500
1750
2000
0.00
0.05
0.10
0.15
0.20
0.25
0.30
Injuries
Communicable, maternal, neonatal, and nutritional diseases
Non-communicable diseases
HIV/AIDS and sexually transmitted infections
Respiratory infections and tuberculosis
Enteric infections
Neglected tropical diseases and malaria
Other infectious diseases
Maternal and neonatal disorders
Nutritional deficiencies
Neoplasms
Cardiovascular diseases
Chronic respiratory diseases
Digestive diseases
Neurological disorders
Mental disorders
Substance use disorders
Diabetes and kidney diseases
Skin and subcutaneous diseases
Musculoskeletal disorders
Other non-communicable diseases
Transport injuries
Unintentional injuries
Self-harm and interpersonal violence
COVID-19
Other COVID-19 pandemic-related outcomes

Rwanda

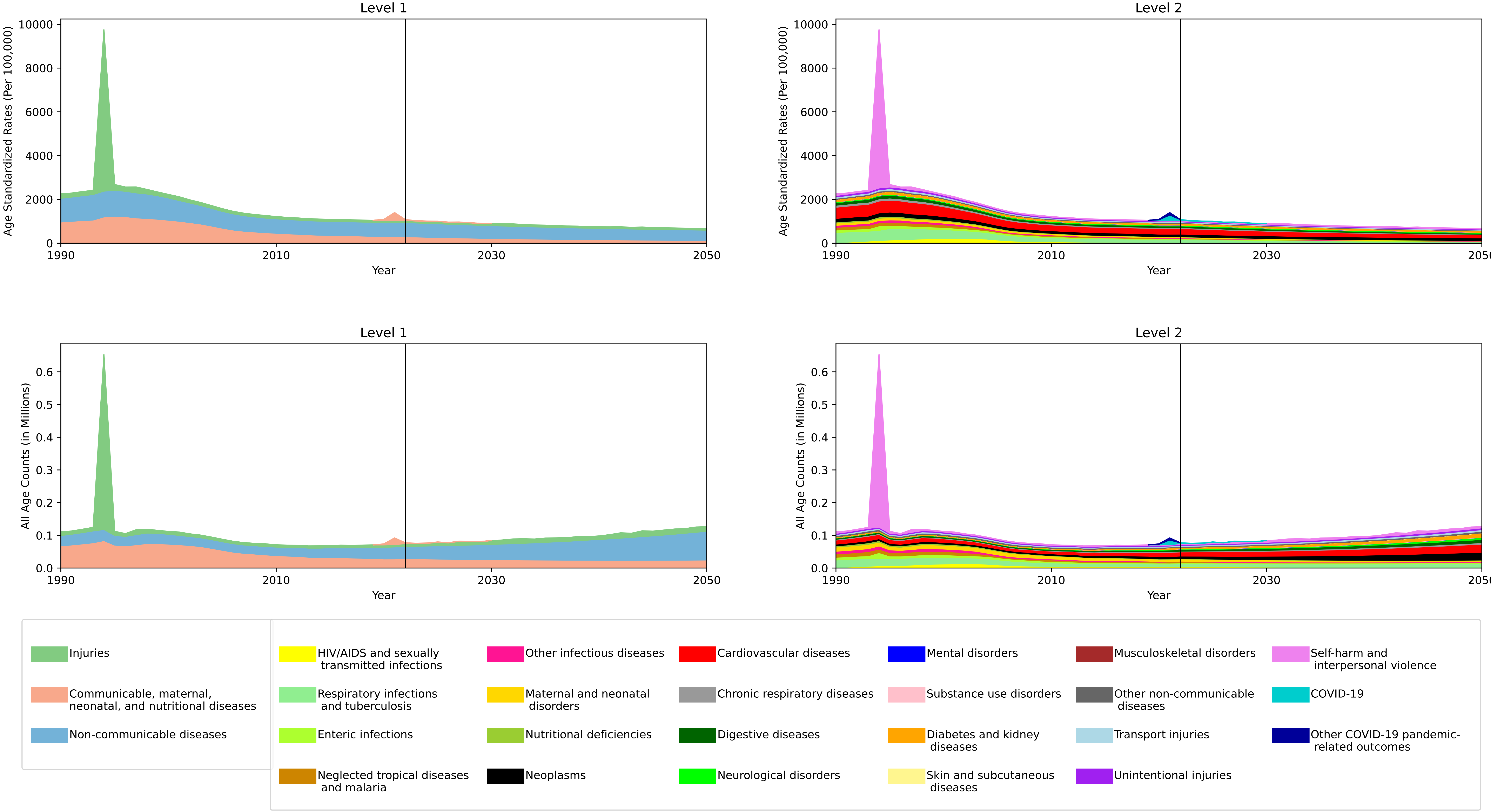
Level 1
Level 2
Age Standardized Rates (Per 100,000)
All Age Counts (in Millions)
Year
0
2000
4000
6000
8000
10000
0.0
0.1
0.2
0.3
0.4
0.5
0.6
1990
2010
2030
2050
Injuries
Communicable, maternal, neonatal, and nutritional diseases
Non-communicable diseases
HIV/AIDS and sexually transmitted infections
Respiratory infections and tuberculosis
Enteric infections
Neglected tropical diseases and malaria
Other infectious diseases
Maternal and neonatal disorders
Nutritional deficiencies
Neoplasms
Cardiovascular diseases
Chronic respiratory diseases
Digestive diseases
Neurological disorders
Mental disorders
Substance use disorders
Diabetes and kidney diseases
Skin and subcutaneous diseases
Musculoskeletal disorders
Other non-communicable diseases
Transport injuries
Unintentional injuries
Self-harm and interpersonal violence
COVID-19
Other COVID-19 pandemic-related outcomes

Somalia

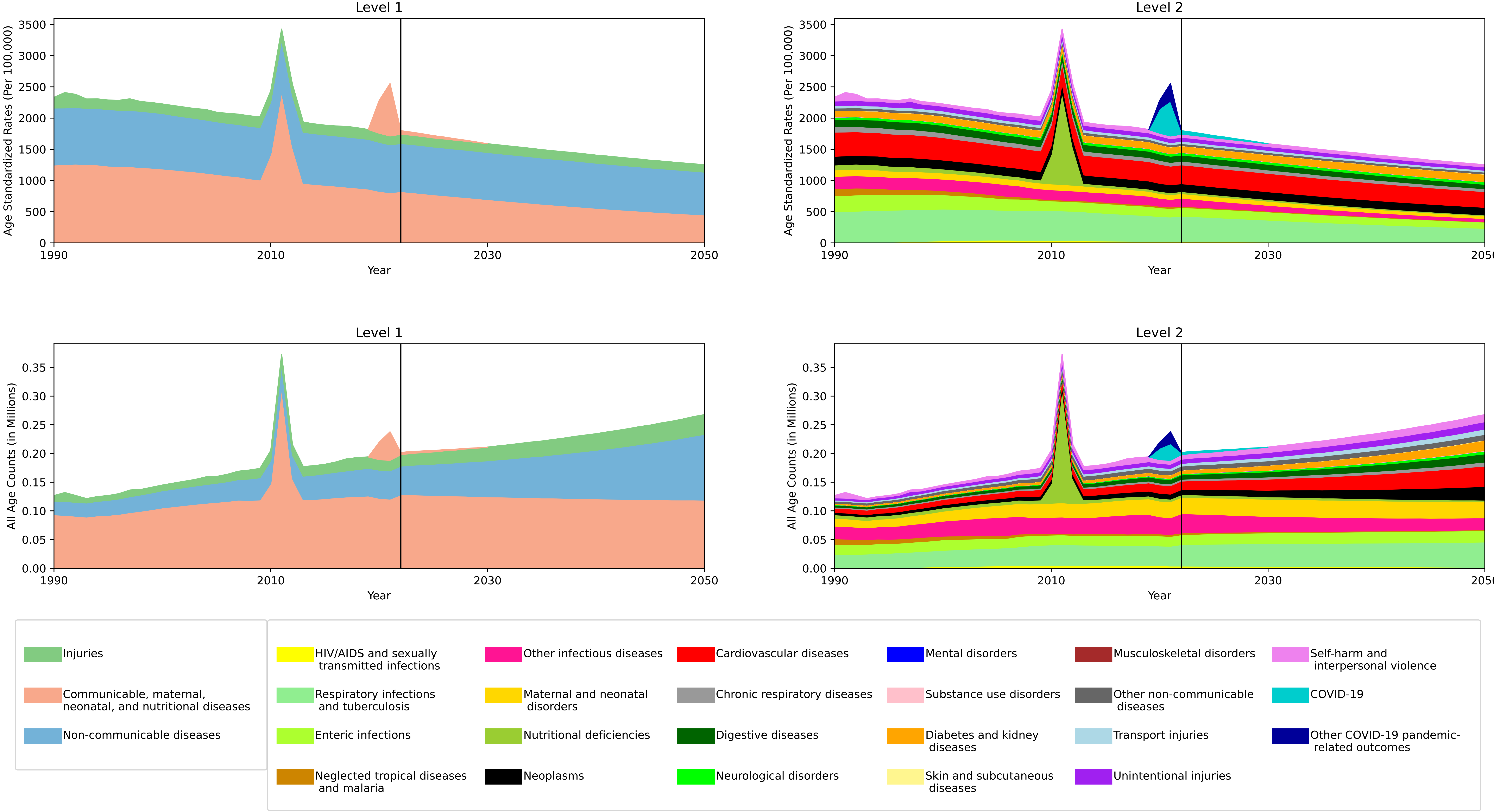
Level 1
Level 2
Age Standardized Rates (Per 100,000)
All Age Counts (in Millions)
Year
1990
2010
2030
2050
Injuries
Communicable, maternal, neonatal, and nutritional diseases
Non-communicable diseases
HIV/AIDS and sexually transmitted infections
Respiratory infections and tuberculosis
Enteric infections
Neglected tropical diseases and malaria
Other infectious diseases
Maternal and neonatal disorders
Nutritional deficiencies
Neoplasms
Cardiovascular diseases
Chronic respiratory diseases
Digestive diseases
Neurological disorders
Mental disorders
Substance use disorders
Diabetes and kidney diseases
Skin and subcutaneous diseases
Musculoskeletal disorders
Other non-communicable diseases
Transport injuries
Unintentional injuries
Self-harm and interpersonal violence
COVID-19
Other COVID-19 pandemic-related outcomes

South Sudan

Supplemental Results Figure S5. Age-standardised death rates and all age death counts and by Level 1 and Level 2 causes, 1990–2050

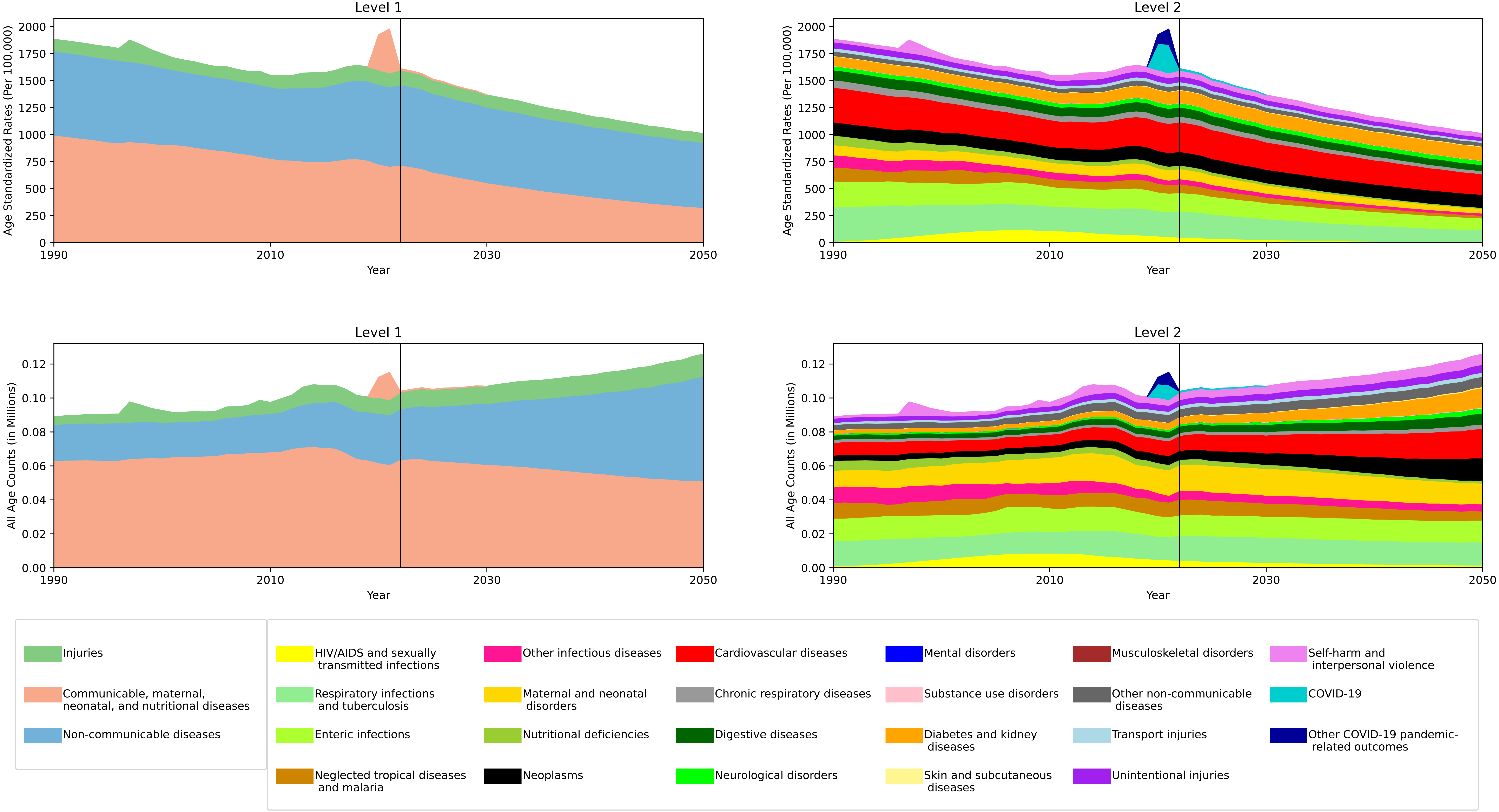

Uganda

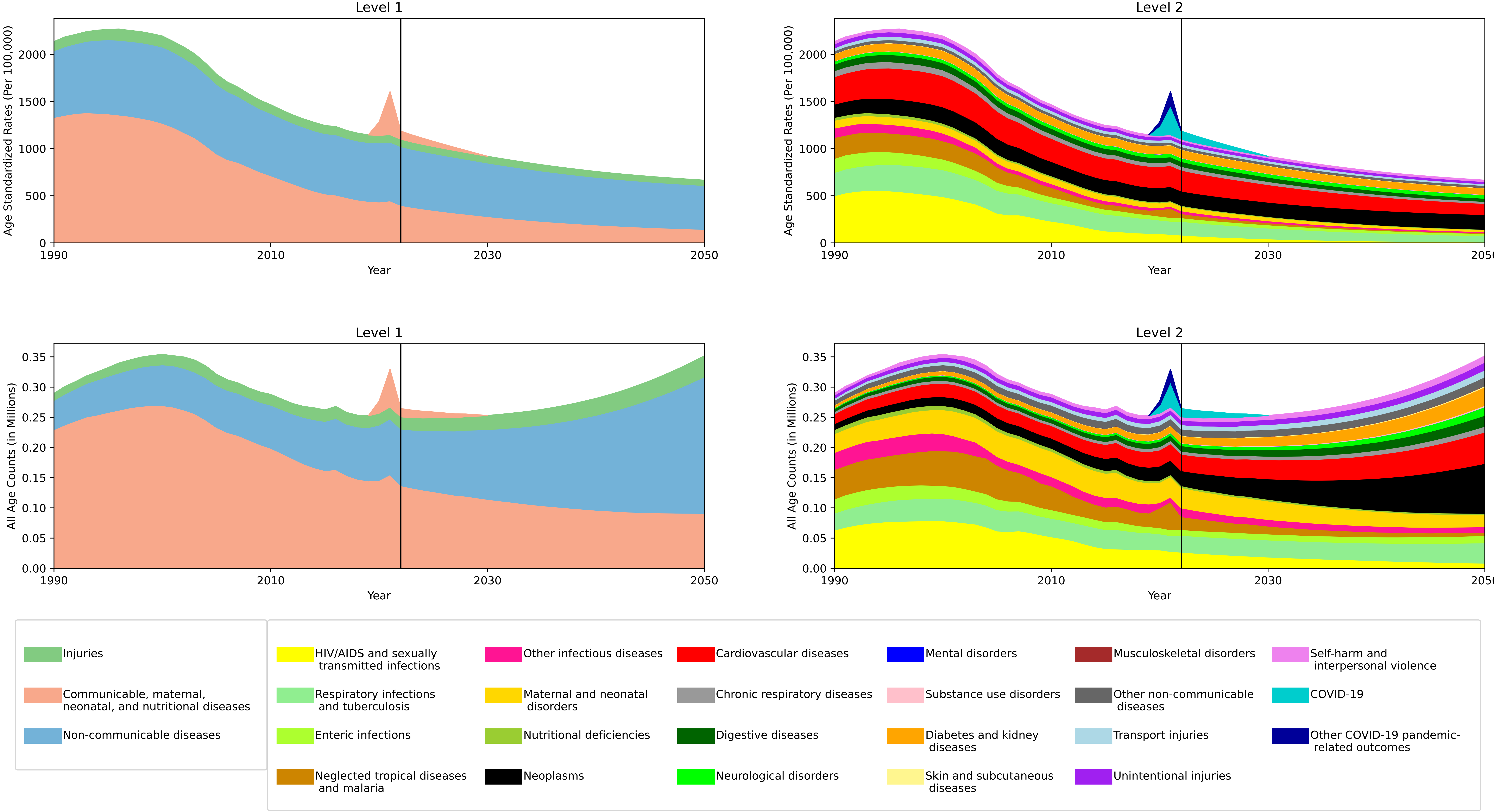
Level 1
Level 2
Age Standardized Rates (Per 100,000)
All Age Counts (in Millions)
Year
1990
2010
2030
2050
Injuries
Communicable, maternal, neonatal, and nutritional diseases
Non-communicable diseases
HIV/AIDS and sexually transmitted infections
Respiratory infections and tuberculosis
Enteric infections
Neglected tropical diseases and malaria
Other infectious diseases
Maternal and neonatal disorders
Nutritional deficiencies
Neoplasms
Cardiovascular diseases
Chronic respiratory diseases
Digestive diseases
Neurological disorders
Mental disorders
Substance use disorders
Diabetes and kidney diseases
Skin and subcutaneous diseases
Musculoskeletal disorders
Other non-communicable diseases
Transport injuries
Unintentional injuries
Self-harm and interpersonal violence
COVID-19
Other COVID-19 pandemic-related outcomes

Tanzania

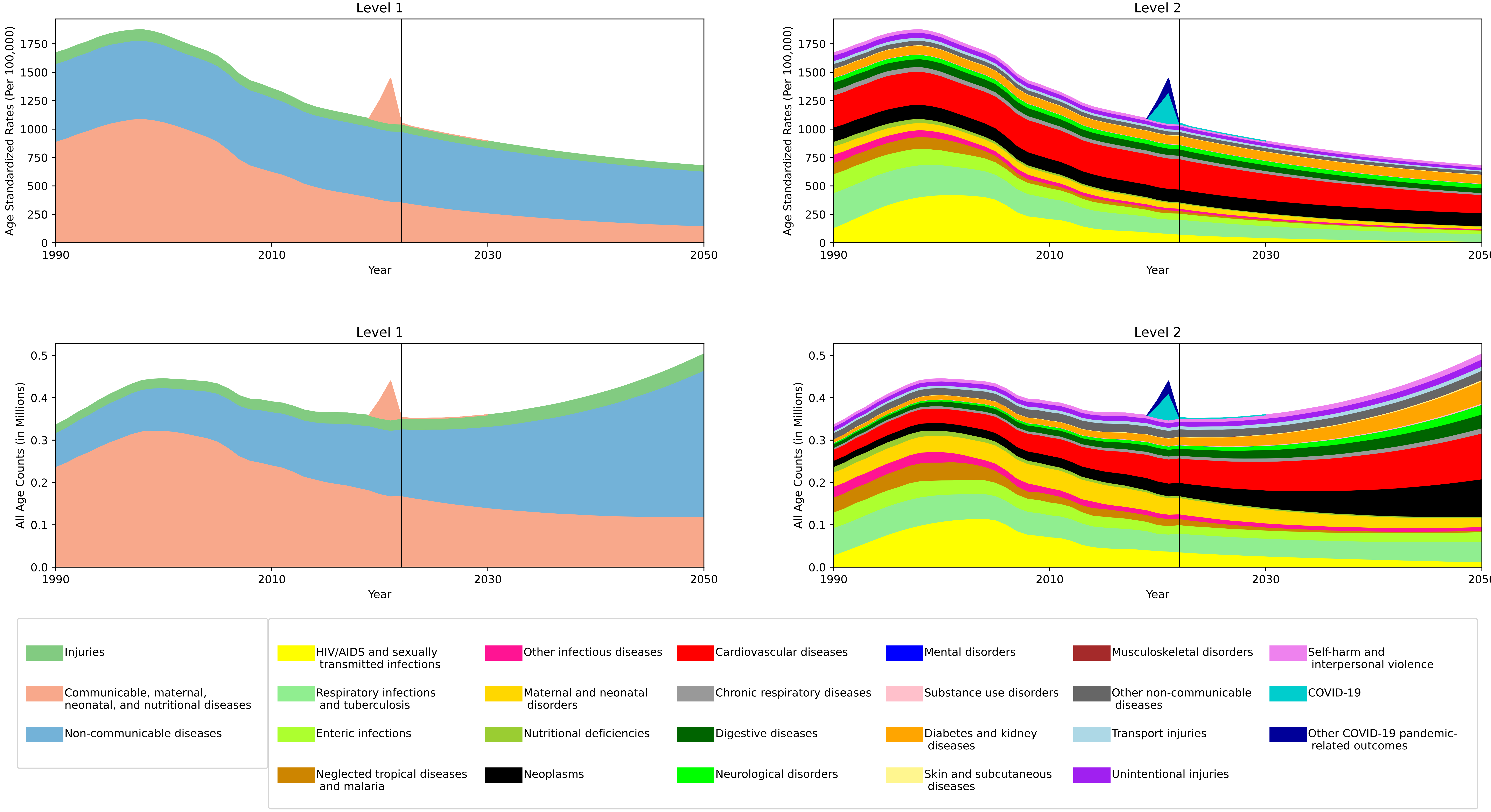
Level 1
Level 2
Age Standardized Rates (Per 100,000)
All Age Counts (in Millions)
Year
1990
2010
2030
2050
0
250
500
750
1000
1250
1500
1750
0.0
0.1
0.2
0.3
0.4
0.5
Injuries
Communicable, maternal, neonatal, and nutritional diseases
Non-communicable diseases
HIV/AIDS and sexually transmitted infections
Respiratory infections and tuberculosis
Enteric infections
Neglected tropical diseases and malaria
Other infectious diseases
Maternal and neonatal disorders
Nutritional deficiencies
Neoplasms
Cardiovascular diseases
Chronic respiratory diseases
Digestive diseases
Neurological disorders
Mental disorders
Substance use disorders
Diabetes and kidney diseases
Skin and subcutaneous diseases
Musculoskeletal disorders
Other non-communicable diseases
Transport injuries
Unintentional injuries
Self-harm and interpersonal violence
COVID-19
Other COVID-19 pandemic-related outcomes

Zambia

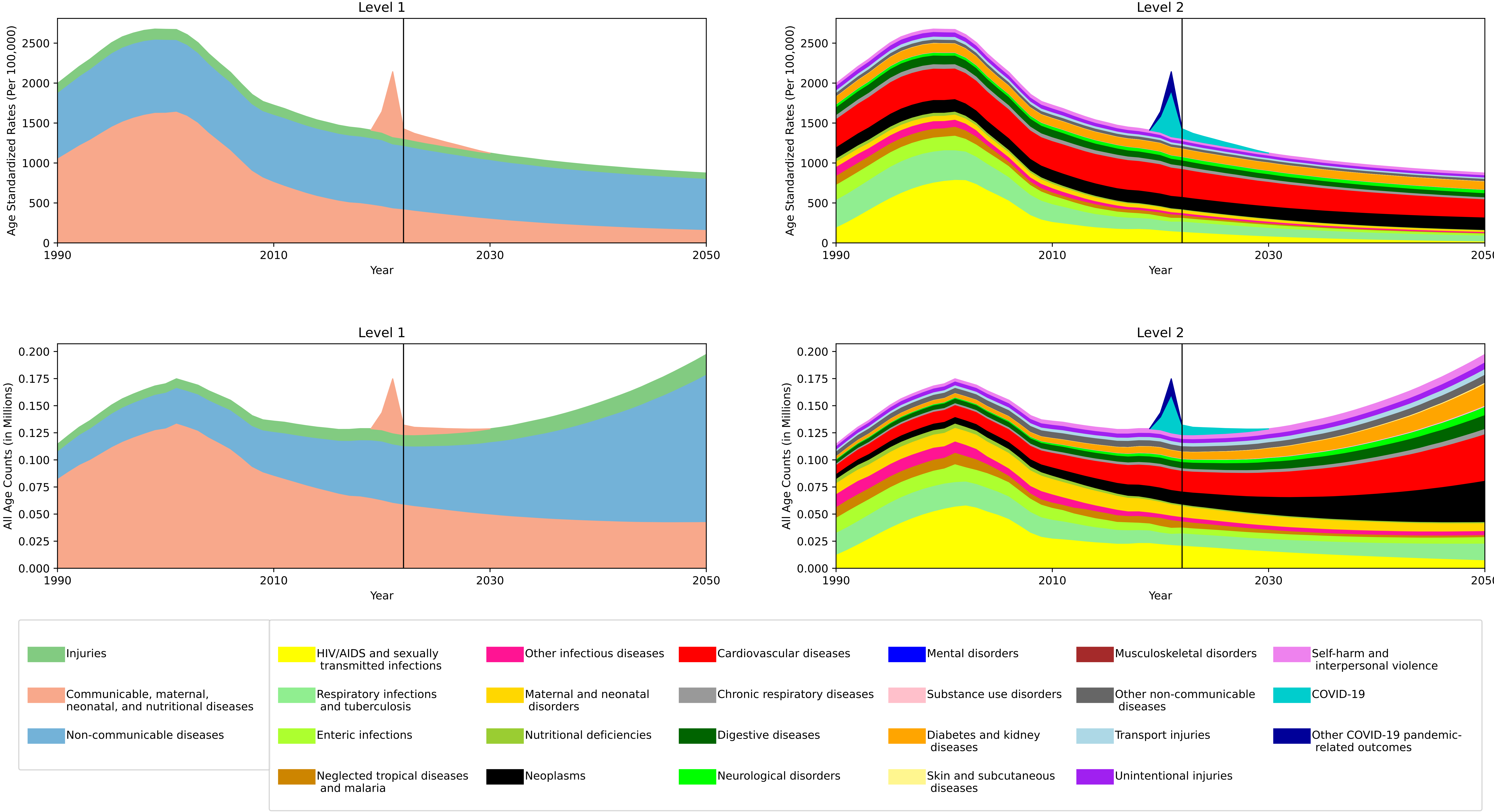
Level 1
Level 2
Age Standardized Rates (Per 100,000)
All Age Counts (in Millions)
Year
1990
2010
2030
2050
Injuries
Communicable, maternal, neonatal, and nutritional diseases
Non-communicable diseases
HIV/AIDS and sexually transmitted infections
Respiratory infections and tuberculosis
Enteric infections
Neglected tropical diseases and malaria
Other infectious diseases
Maternal and neonatal disorders
Nutritional deficiencies
Neoplasms
Cardiovascular diseases
Chronic respiratory diseases
Digestive diseases
Neurological disorders
Mental disorders
Substance use disorders
Diabetes and kidney diseases
Skin and subcutaneous diseases
Musculoskeletal disorders
Other non-communicable diseases
Transport injuries
Unintentional injuries
Self-harm and interpersonal violence
COVID-19
Other COVID-19 pandemic-related outcomes

Botswana

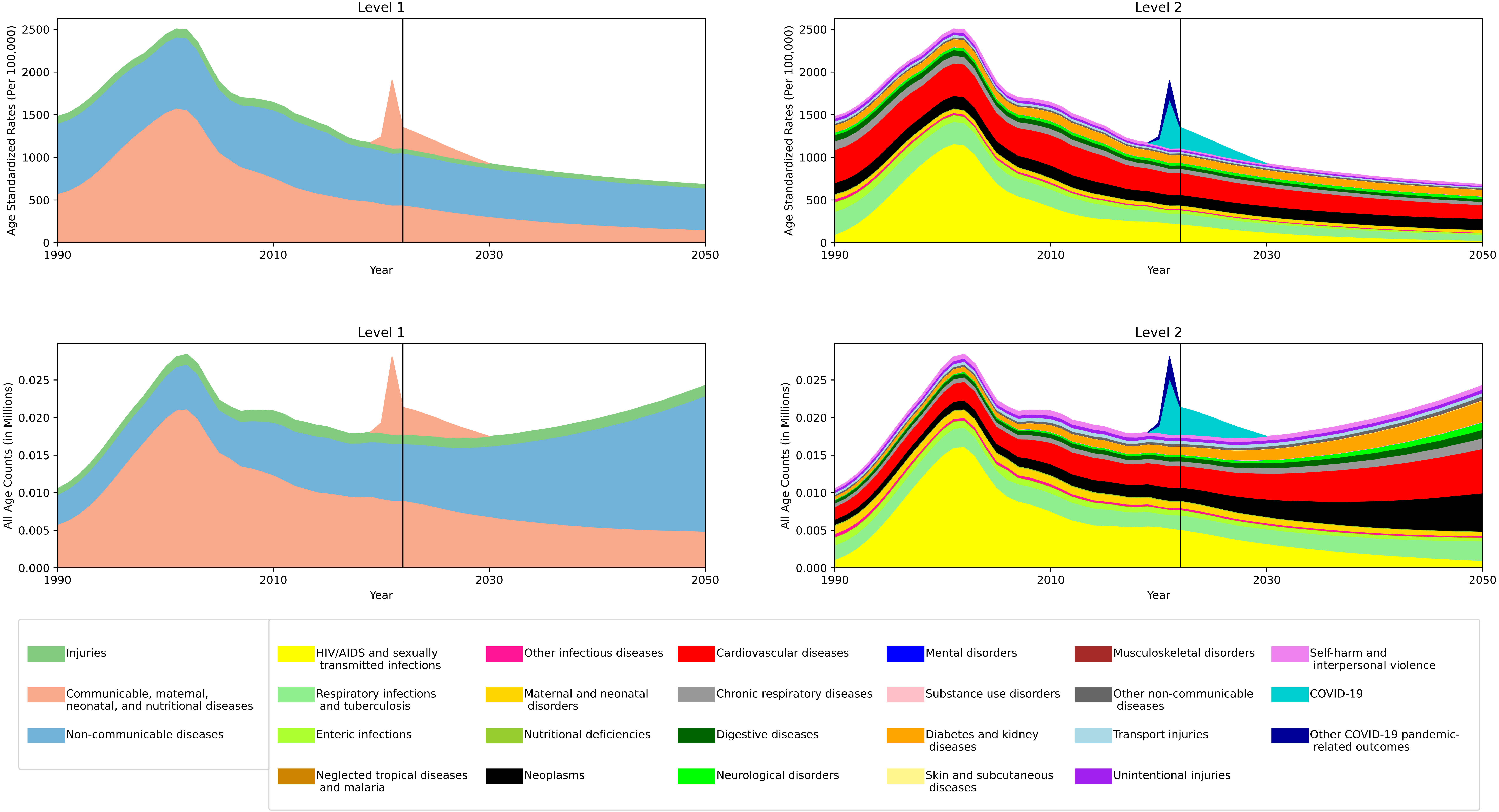
Level 1
Level 2
Age Standardized Rates (Per 100,000)
All Age Counts (in Millions)
Year
0
500
1000
1500
2000
2500
0.000
0.005
0.010
0.015
0.020
0.025
1990
2010
2030
2050
Injuries
Communicable, maternal, neonatal, and nutritional diseases
Non-communicable diseases
HIV/AIDS and sexually transmitted infections
Respiratory infections and tuberculosis
Enteric infections
Neglected tropical diseases and malaria
Other infectious diseases
Maternal and neonatal disorders
Nutritional deficiencies
Neoplasms
Cardiovascular diseases
Chronic respiratory diseases
Digestive diseases
Neurological disorders
Mental disorders
Substance use disorders
Diabetes and kidney diseases
Skin and subcutaneous diseases
Musculoskeletal disorders
Other non-communicable diseases
Transport injuries
Unintentional injuries
Self-harm and interpersonal violence
COVID-19
Other COVID-19 pandemic-related outcomes

Eswatini

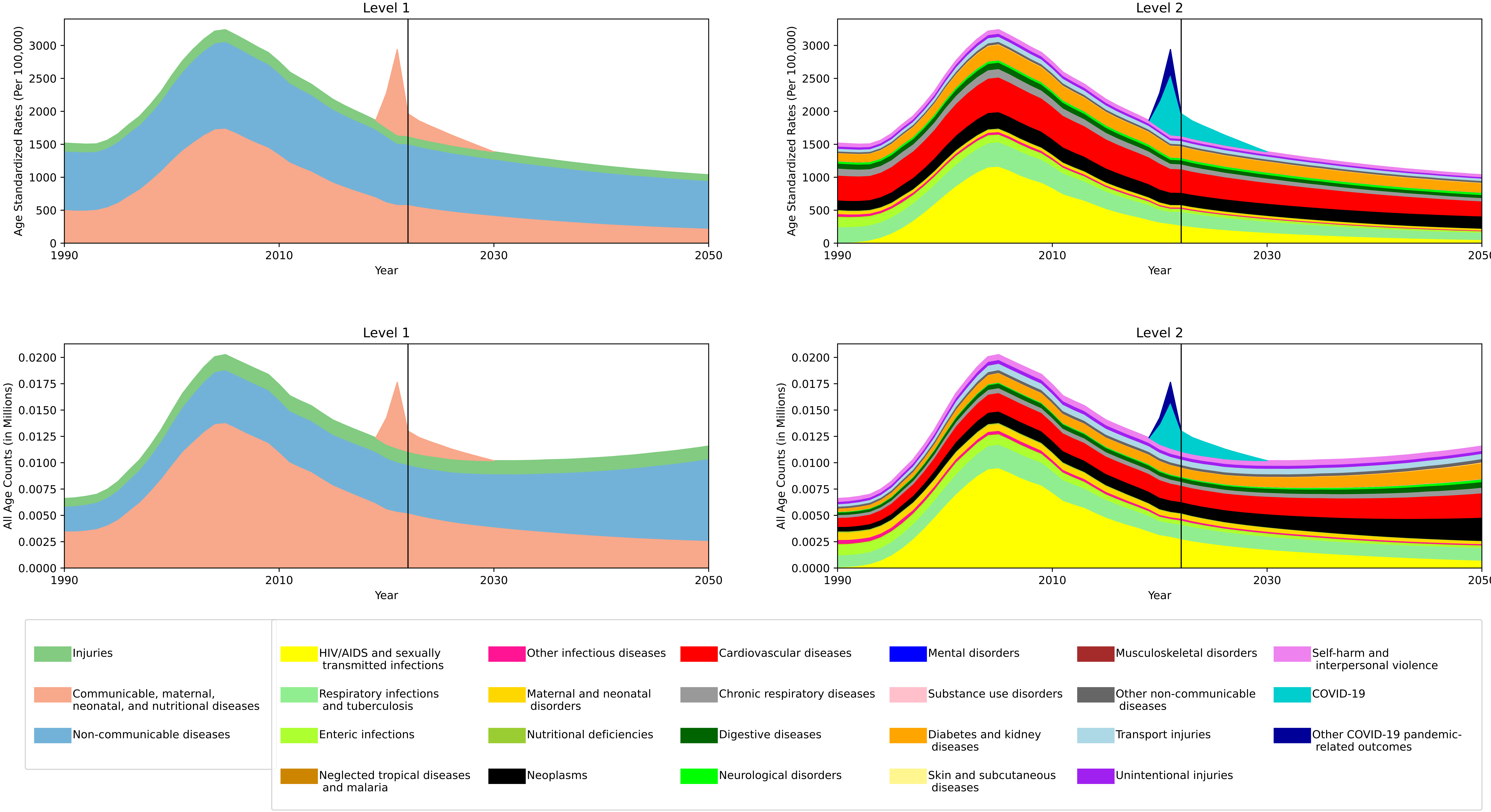
Level 1
Level 2
Age Standardized Rates (Per 100,000)
All Age Counts (in Millions)
Year
1990
2010
2030
2050
0
500
1000
1500
2000
2500
3000
0.0000
0.0025
0.0050
0.0075
0.0100
0.0125
0.0150
0.0175
0.0200
Injuries
Communicable, maternal, neonatal, and nutritional diseases
Non-communicable diseases
HIV/AIDS and sexually transmitted infections
Respiratory infections and tuberculosis
Enteric infections
Neglected tropical diseases and malaria
Other infectious diseases
Maternal and neonatal disorders
Nutritional deficiencies
Neoplasms
Cardiovascular diseases
Chronic respiratory diseases
Digestive diseases
Neurological disorders
Mental disorders
Substance use disorders
Diabetes and kidney diseases
Skin and subcutaneous diseases
Musculoskeletal disorders
Other non-communicable diseases
Transport injuries
Unintentional injuries
Self-harm and interpersonal violence
COVID-19
Other COVID-19 pandemic-related outcomes

Lesotho

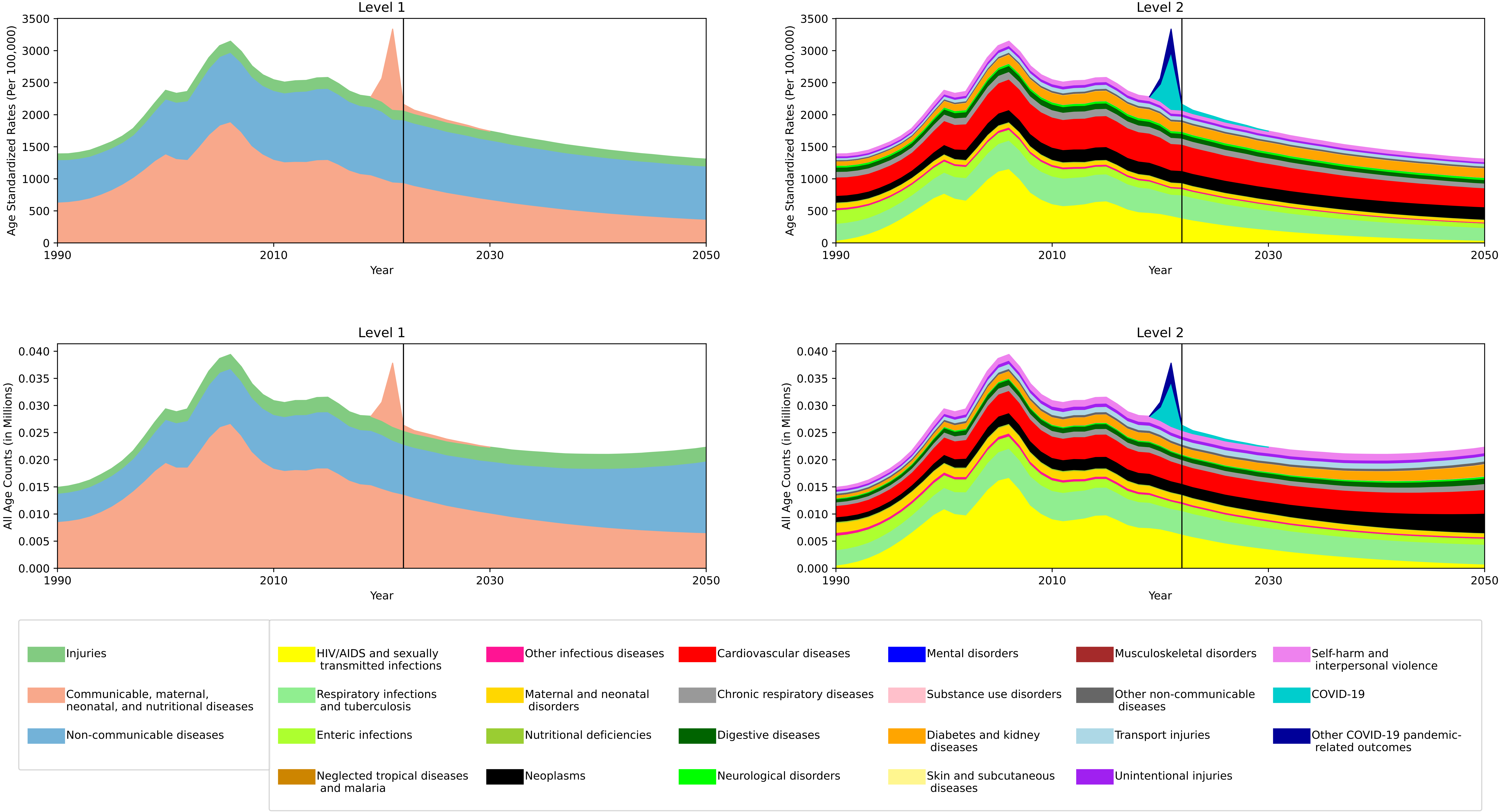
Level 1
Age Standardized Rates (Per 100,000)
Year
Level 2
Age Standardized Rates (Per 100,000)
Year
Level 1
All Age Counts (in Millions)
Year
Level 2
All Age Counts (in Millions)
Year
Injuries
Communicable, maternal, neonatal, and nutritional diseases
Non-communicable diseases
HIV/AIDS and sexually transmitted infections
Respiratory infections and tuberculosis
Enteric infections
Neglected tropical diseases and malaria
Other infectious diseases
Maternal and neonatal disorders
Nutritional deficiencies
Neoplasms
Cardiovascular diseases
Chronic respiratory diseases
Digestive diseases
Neurological disorders
Mental disorders
Substance use disorders
Diabetes and kidney diseases
Skin and subcutaneous diseases
Musculoskeletal disorders
Other non-communicable diseases
Transport injuries
Unintentional injuries
Self-harm and interpersonal violence
COVID-19
Other COVID-19 pandemic-related outcomes

Namibia

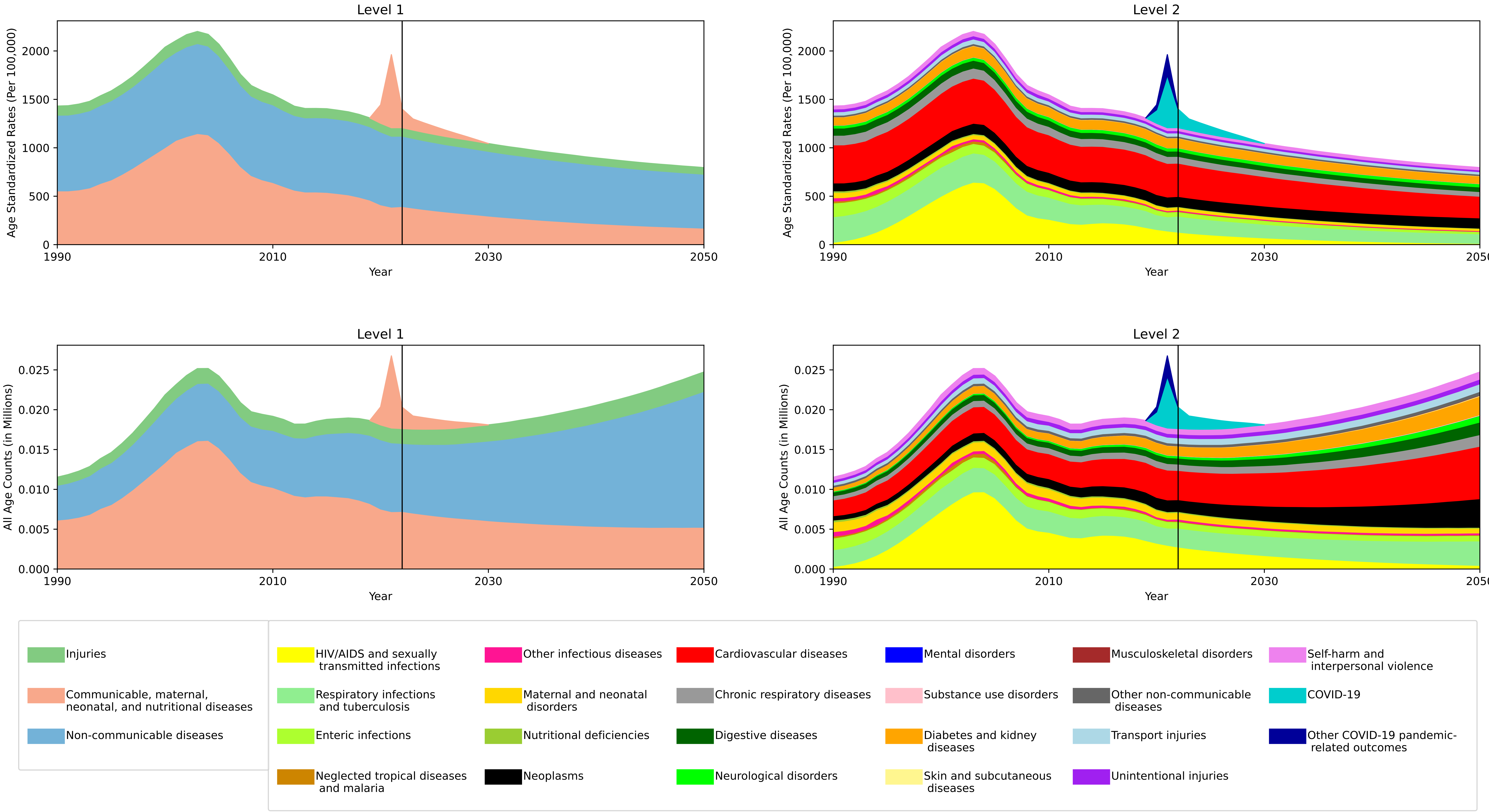
Level 1
Level 2
Age Standardized Rates (Per 100,000)
All Age Counts (in Millions)
Year
1990
2010
2030
2050
0
500
1000
1500
2000
0.000
0.005
0.010
0.015
0.020
0.025
Injuries
Communicable, maternal, neonatal, and nutritional diseases
Non-communicable diseases
HIV/AIDS and sexually transmitted infections
Respiratory infections and tuberculosis
Enteric infections
Neglected tropical diseases and malaria
Other infectious diseases
Maternal and neonatal disorders
Nutritional deficiencies
Neoplasms
Cardiovascular diseases
Chronic respiratory diseases
Digestive diseases
Neurological disorders
Mental disorders
Substance use disorders
Diabetes and kidney diseases
Skin and subcutaneous diseases
Musculoskeletal disorders
Other non-communicable diseases
Transport injuries
Unintentional injuries
Self-harm and interpersonal violence
COVID-19
Other COVID-19 pandemic-related outcomes

South Africa

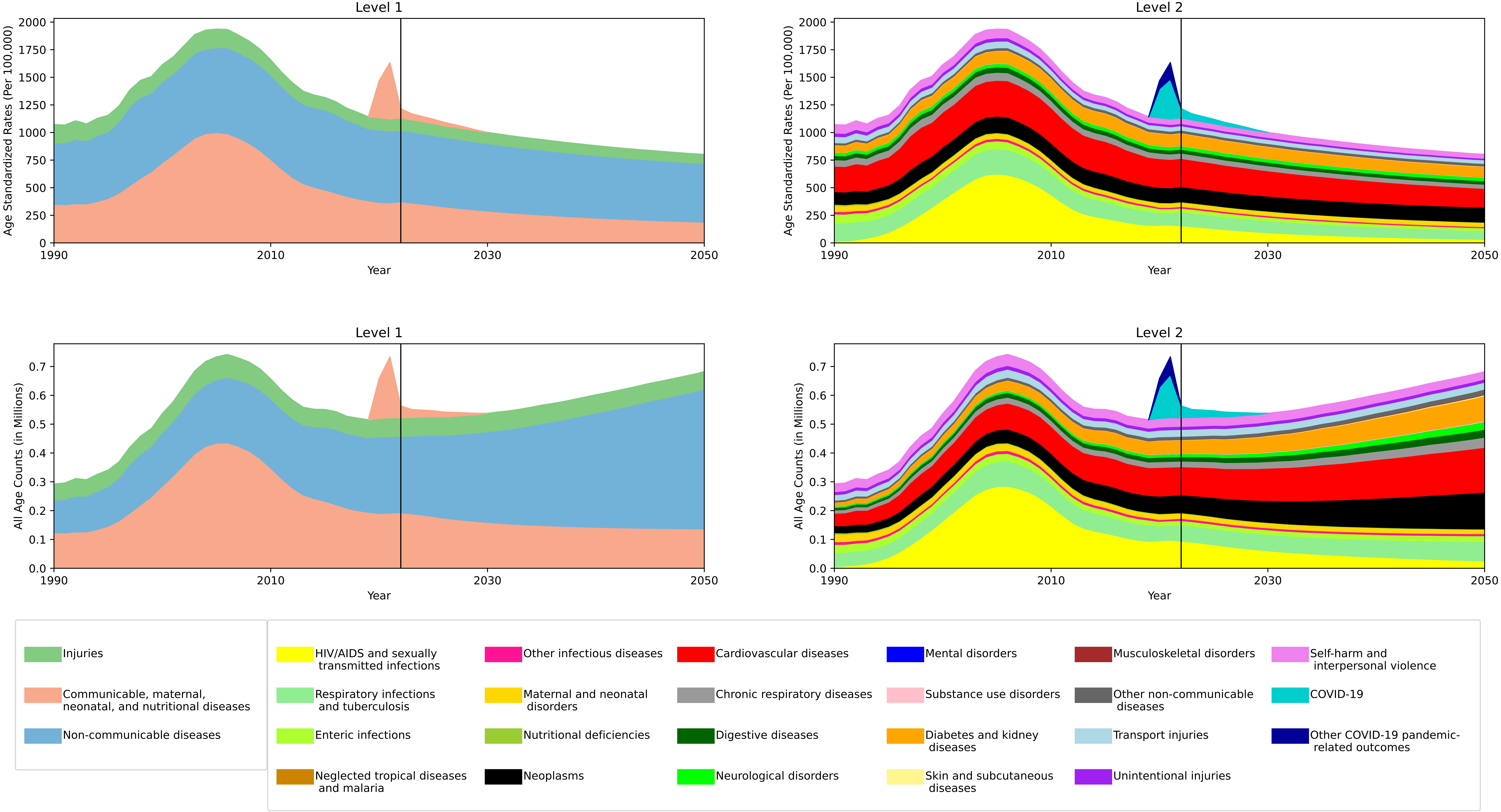

Zimbabwe

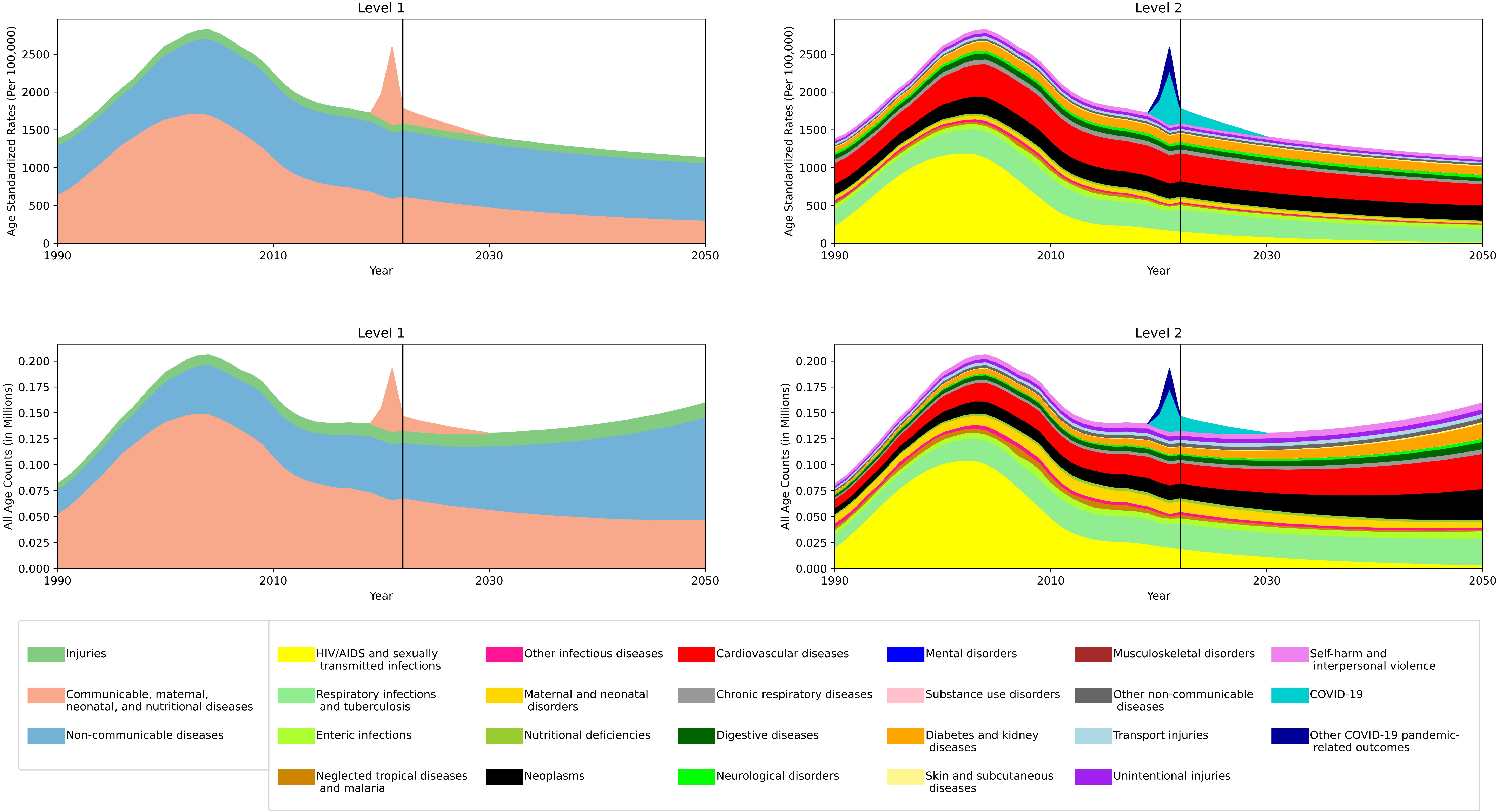
Level 1
Level 2
Age Standardized Rates (Per 100,000)
All Age Counts (in Millions)
Year
Injuries
Communicable, maternal, neonatal, and nutritional diseases
Non-communicable diseases
HIV/AIDS and sexually transmitted infections
Respiratory infections and tuberculosis
Enteric infections
Neglected tropical diseases and malaria
Other infectious diseases
Maternal and neonatal disorders
Nutritional deficiencies
Neoplasms
Cardiovascular diseases
Chronic respiratory diseases
Digestive diseases
Neurological disorders
Mental disorders
Substance use disorders
Diabetes and kidney diseases
Skin and subcutaneous diseases
Musculoskeletal disorders
Other non-communicable diseases
Transport injuries
Unintentional injuries
Self-harm and interpersonal violence
COVID-19
Other COVID-19 pandemic-related outcomes

Benin

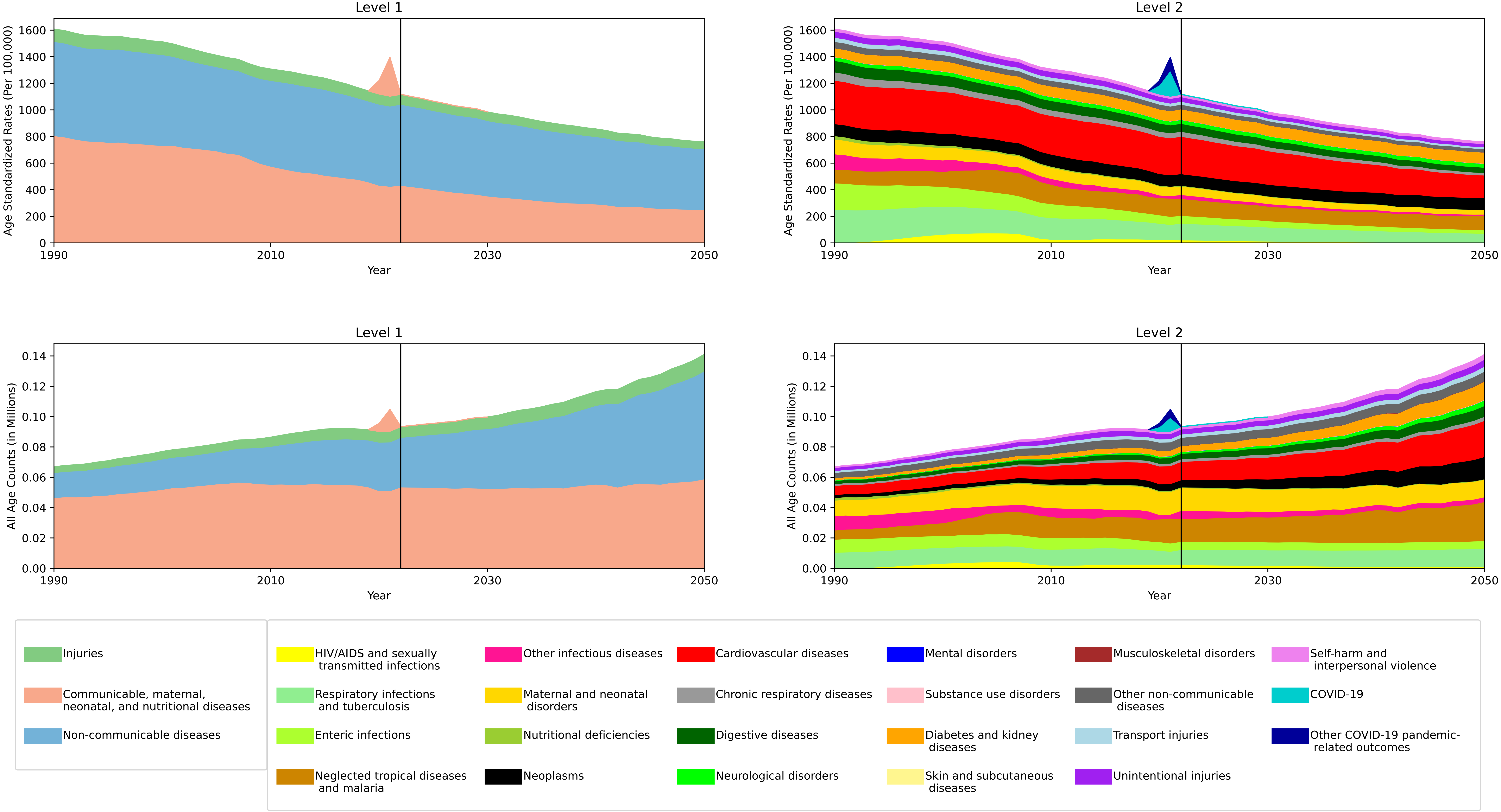
Level 1
Level 2
Age Standardized Rates (Per 100,000)
All Age Counts (in Millions)
Year
0
200
400
600
800
1000
1200
1400
1600
0.00
0.02
0.04
0.06
0.08
0.10
0.12
0.14
1990
2010
2030
2050
Injuries
Communicable, maternal, neonatal, and nutritional diseases
Non-communicable diseases
HIV/AIDS and sexually transmitted infections
Respiratory infections and tuberculosis
Enteric infections
Neglected tropical diseases and malaria
Other infectious diseases
Maternal and neonatal disorders
Nutritional deficiencies
Neoplasms
Cardiovascular diseases
Chronic respiratory diseases
Digestive diseases
Neurological disorders
Mental disorders
Substance use disorders
Diabetes and kidney diseases
Skin and subcutaneous diseases
Musculoskeletal disorders
Other non-communicable diseases
Transport injuries
Unintentional injuries
Self-harm and interpersonal violence
COVID-19
Other COVID-19 pandemic-related outcomes

Burkina Faso

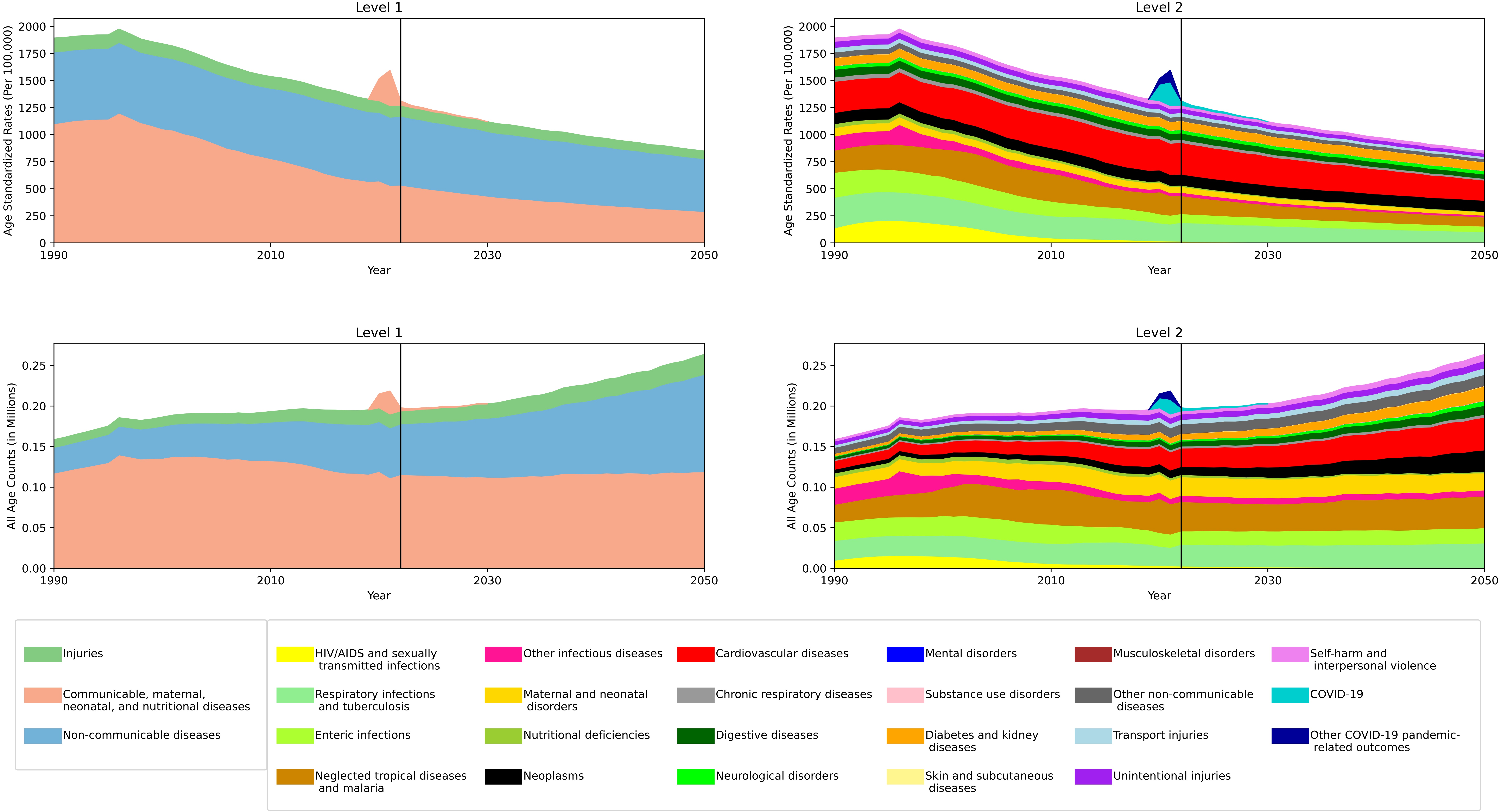

Cabo Verde

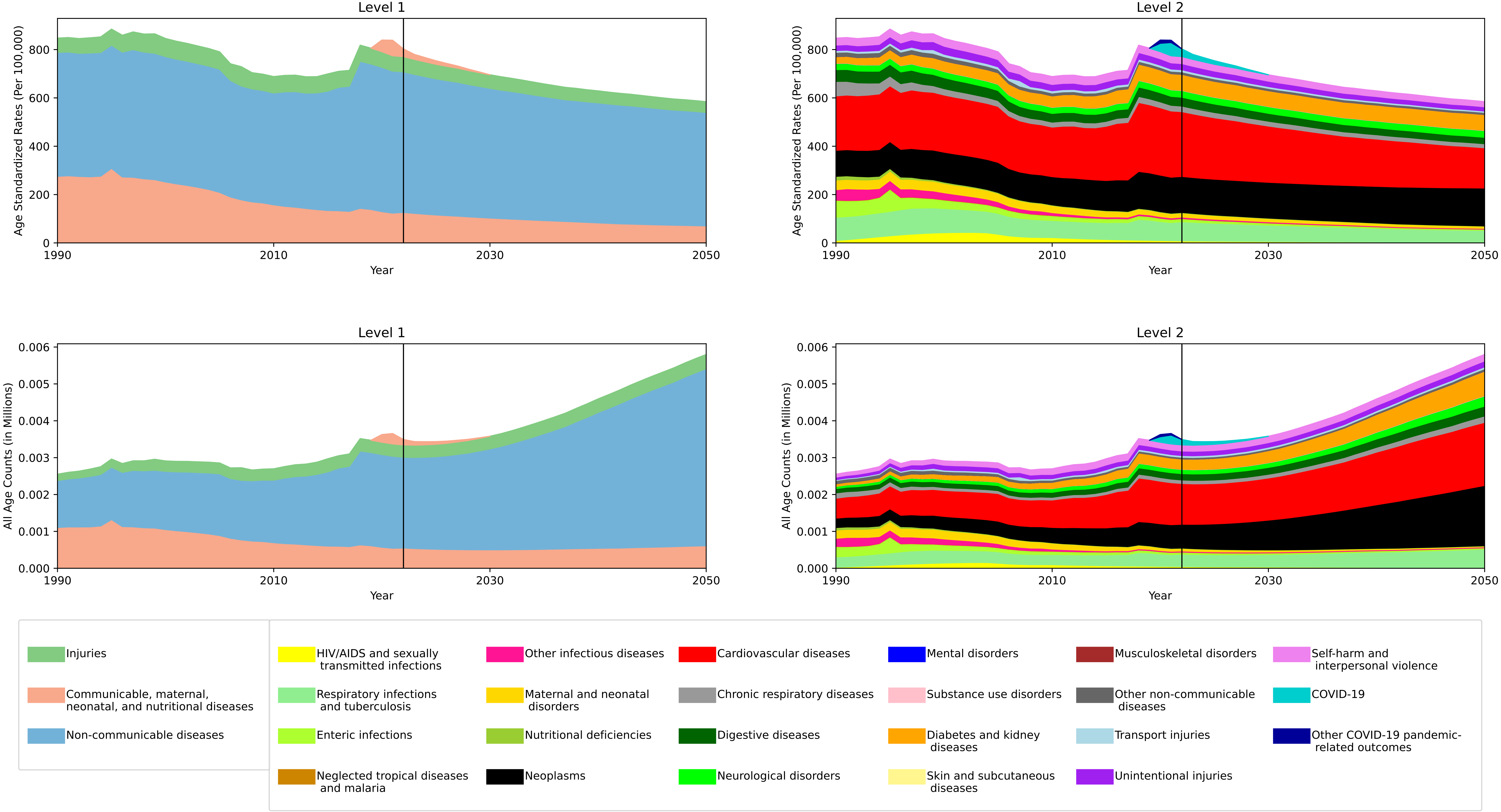
Level 1
Level 2
Age Standardized Rates (Per 100,000)
All Age Counts (in Millions)
Year
1990
2010
2030
2050
Injuries
Communicable, maternal, neonatal, and nutritional diseases
Non-communicable diseases
HIV/AIDS and sexually transmitted infections
Respiratory infections and tuberculosis
Enteric infections
Neglected tropical diseases and malaria
Other infectious diseases
Maternal and neonatal disorders
Nutritional deficiencies
Neoplasms
Cardiovascular diseases
Chronic respiratory diseases
Digestive diseases
Neurological disorders
Mental disorders
Substance use disorders
Diabetes and kidney diseases
Skin and subcutaneous diseases
Musculoskeletal disorders
Other non-communicable diseases
Transport injuries
Unintentional injuries
Self-harm and interpersonal violence
COVID-19
Other COVID-19 pandemic-related outcomes

Cameroon

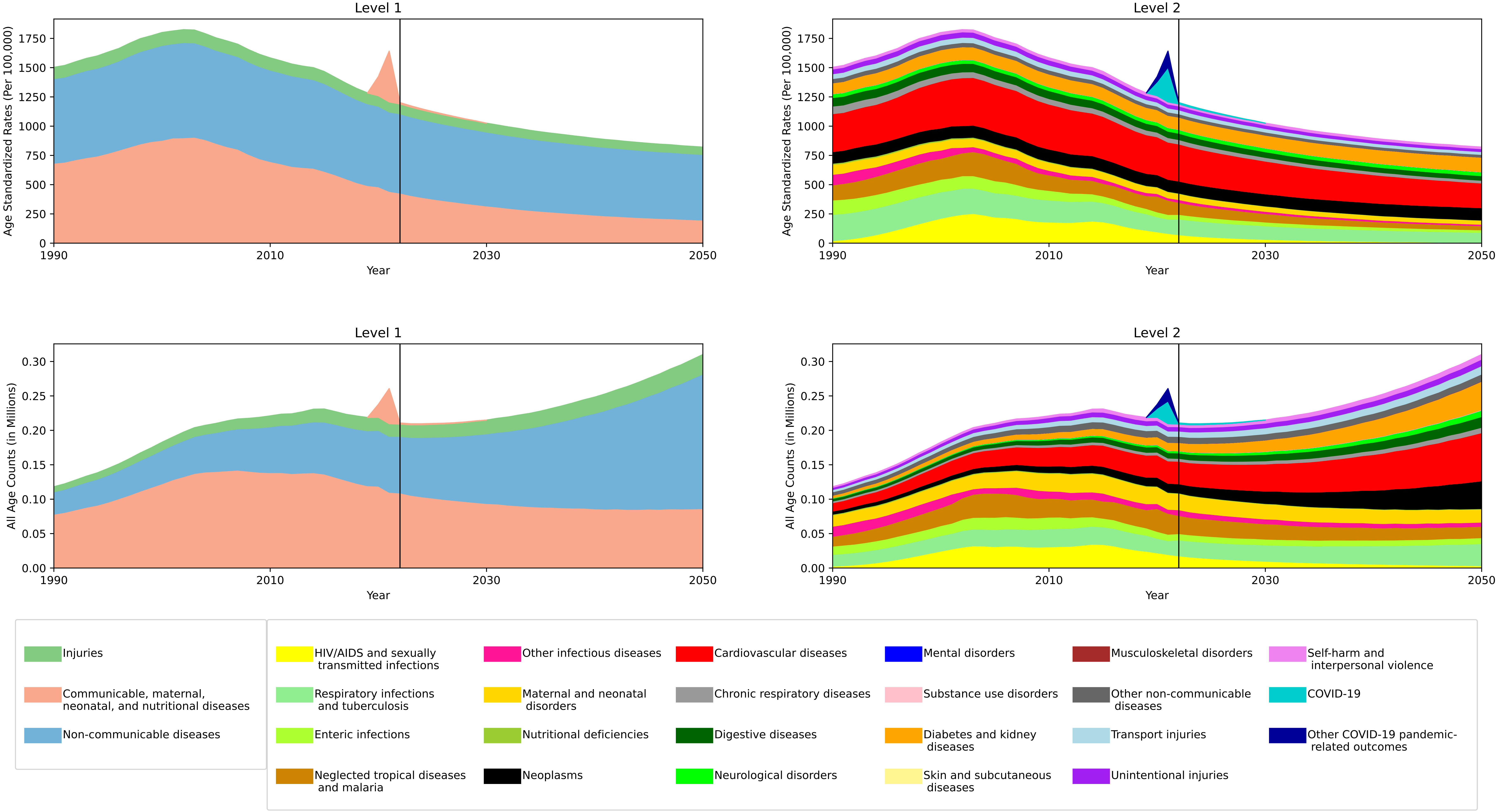
Level 1
Level 2
Age Standardized Rates (Per 100,000)
All Age Counts (in Millions)
Year
1990
2010
2030
2050
0
250
500
750
1000
1250
1500
1750
0.00
0.05
0.10
0.15
0.20
0.25
0.30
Injuries
Communicable, maternal, neonatal, and nutritional diseases
Non-communicable diseases
HIV/AIDS and sexually transmitted infections
Respiratory infections and tuberculosis
Enteric infections
Neglected tropical diseases and malaria
Other infectious diseases
Maternal and neonatal disorders
Nutritional deficiencies
Neoplasms
Cardiovascular diseases
Chronic respiratory diseases
Digestive diseases
Neurological disorders
Mental disorders
Substance use disorders
Diabetes and kidney diseases
Skin and subcutaneous diseases
Musculoskeletal disorders
Other non-communicable diseases
Transport injuries
Unintentional injuries
Self-harm and interpersonal violence
COVID-19
Other COVID-19 pandemic-related outcomes

Chad

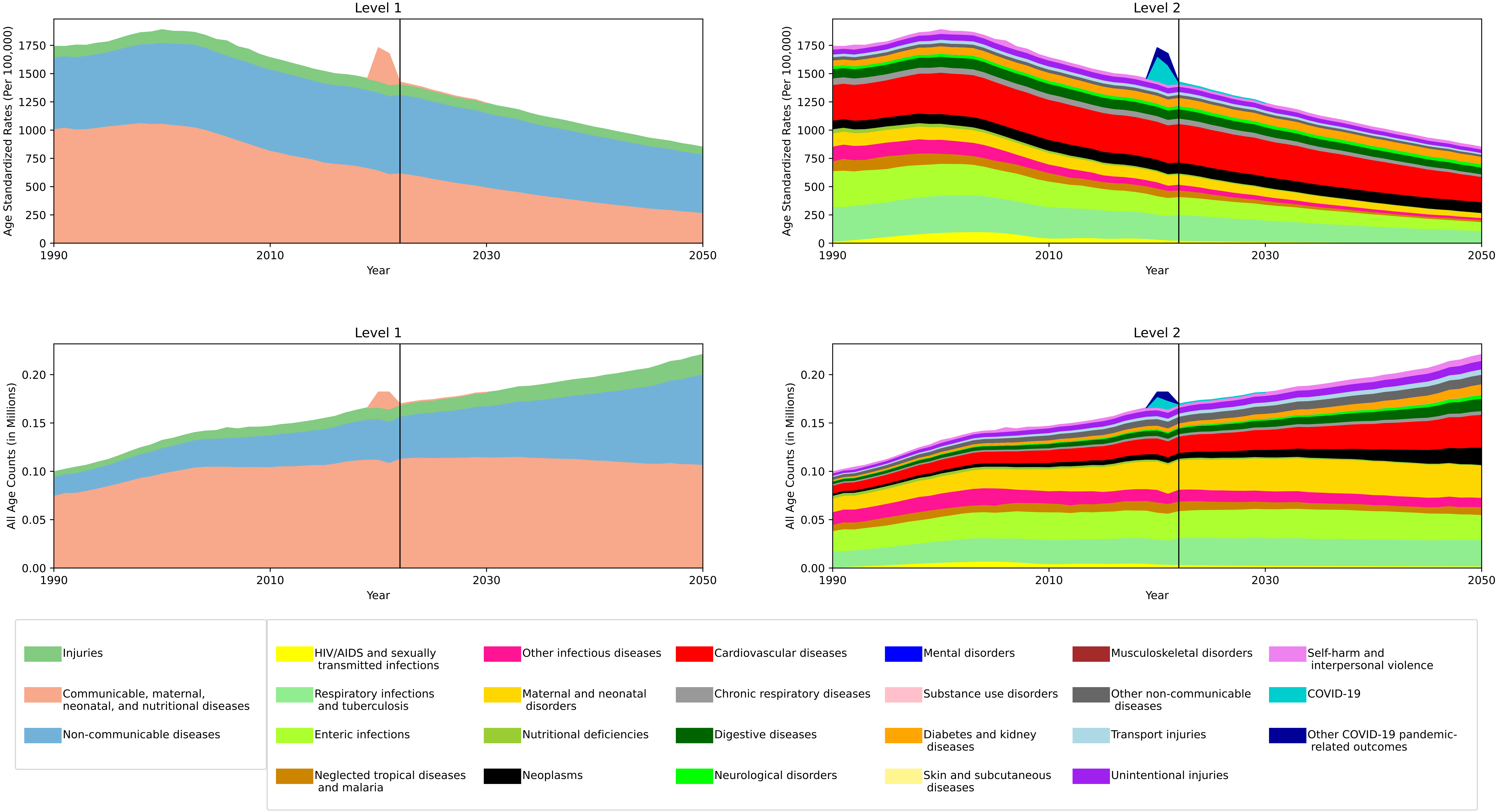
Level 1
Level 2
Age Standardized Rates (Per 100,000)
All Age Counts (in Millions)
Year
1990
2010
2030
2050
Injuries
Communicable, maternal, neonatal, and nutritional diseases
Non-communicable diseases
HIV/AIDS and sexually transmitted infections
Respiratory infections and tuberculosis
Enteric infections
Neglected tropical diseases and malaria
Other infectious diseases
Maternal and neonatal disorders
Nutritional deficiencies
Neoplasms
Cardiovascular diseases
Chronic respiratory diseases
Digestive diseases
Neurological disorders
Mental disorders
Substance use disorders
Diabetes and kidney diseases
Skin and subcutaneous diseases
Musculoskeletal disorders
Other non-communicable diseases
Transport injuries
Unintentional injuries
Self-harm and interpersonal violence
COVID-19
Other COVID-19 pandemic-related outcomes

Côte D'Ivoire

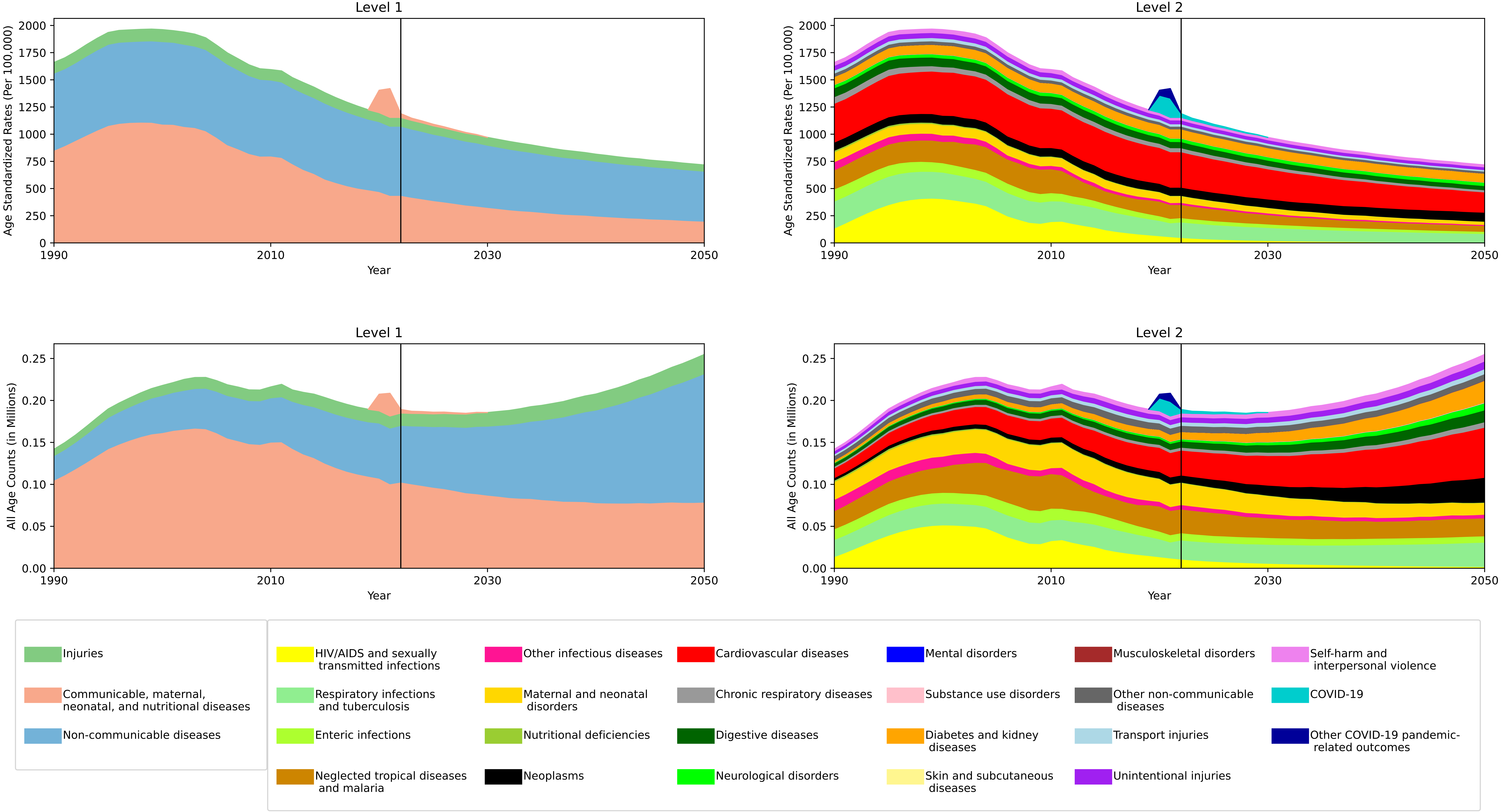

The Gambia

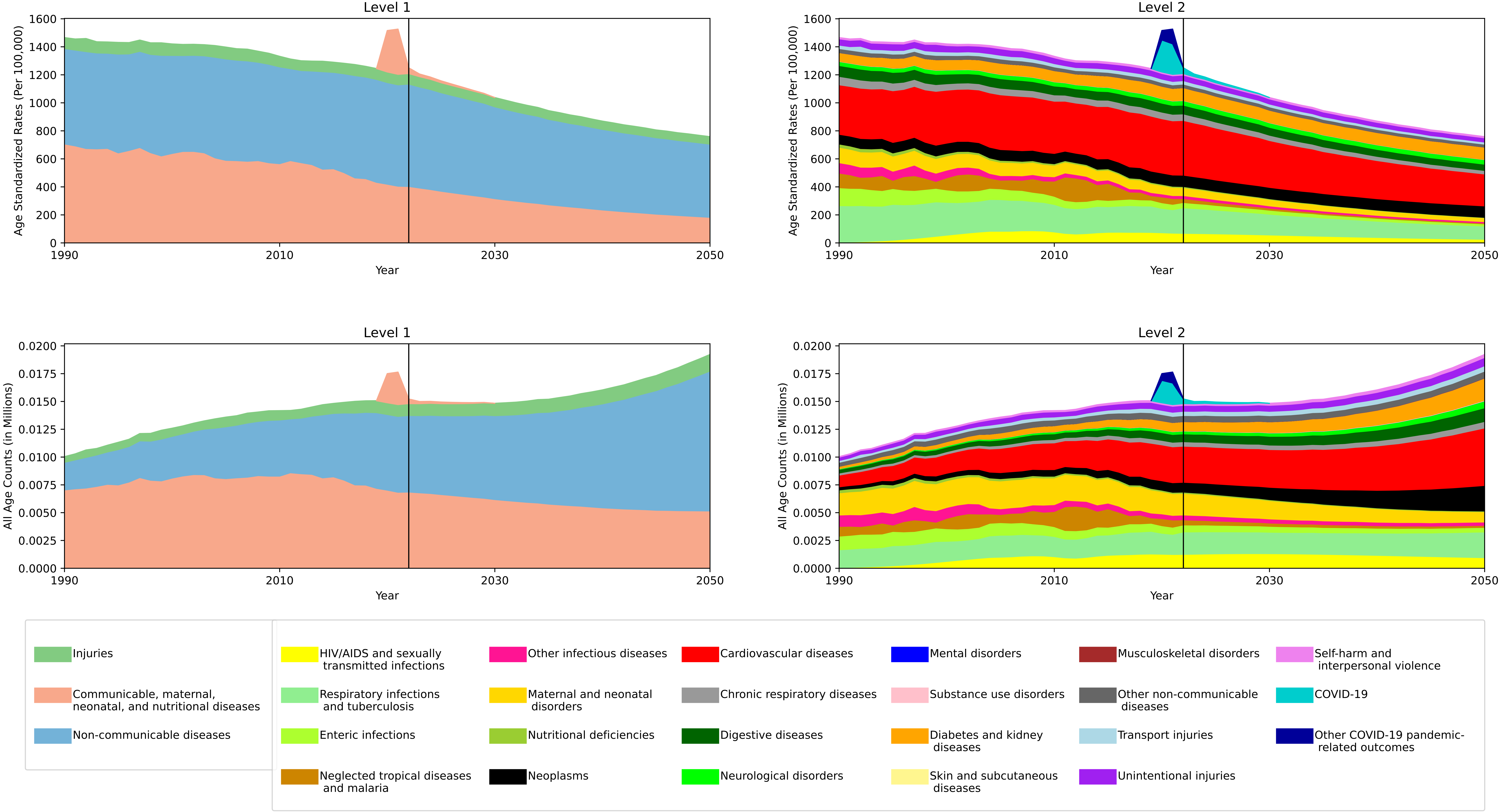

Level 1
Level 2
Age Standardized Rates (Per 100,000)
All Age Counts (in Millions)
Year
1990
2010
2030
2050
Injuries
Communicable, maternal, neonatal, and nutritional diseases
Non-communicable diseases
HIV/AIDS and sexually transmitted infections
Respiratory infections and tuberculosis
Enteric infections
Neglected tropical diseases and malaria
Other infectious diseases
Maternal and neonatal disorders
Nutritional deficiencies
Neoplasms
Cardiovascular diseases
Chronic respiratory diseases
Digestive diseases
Neurological disorders
Mental disorders
Substance use disorders
Diabetes and kidney diseases
Skin and subcutaneous diseases
Musculoskeletal disorders
Other non-communicable diseases
Transport injuries
Unintentional injuries
Self-harm and interpersonal violence
COVID-19
Other COVID-19 pandemic-related outcomes

Ghana

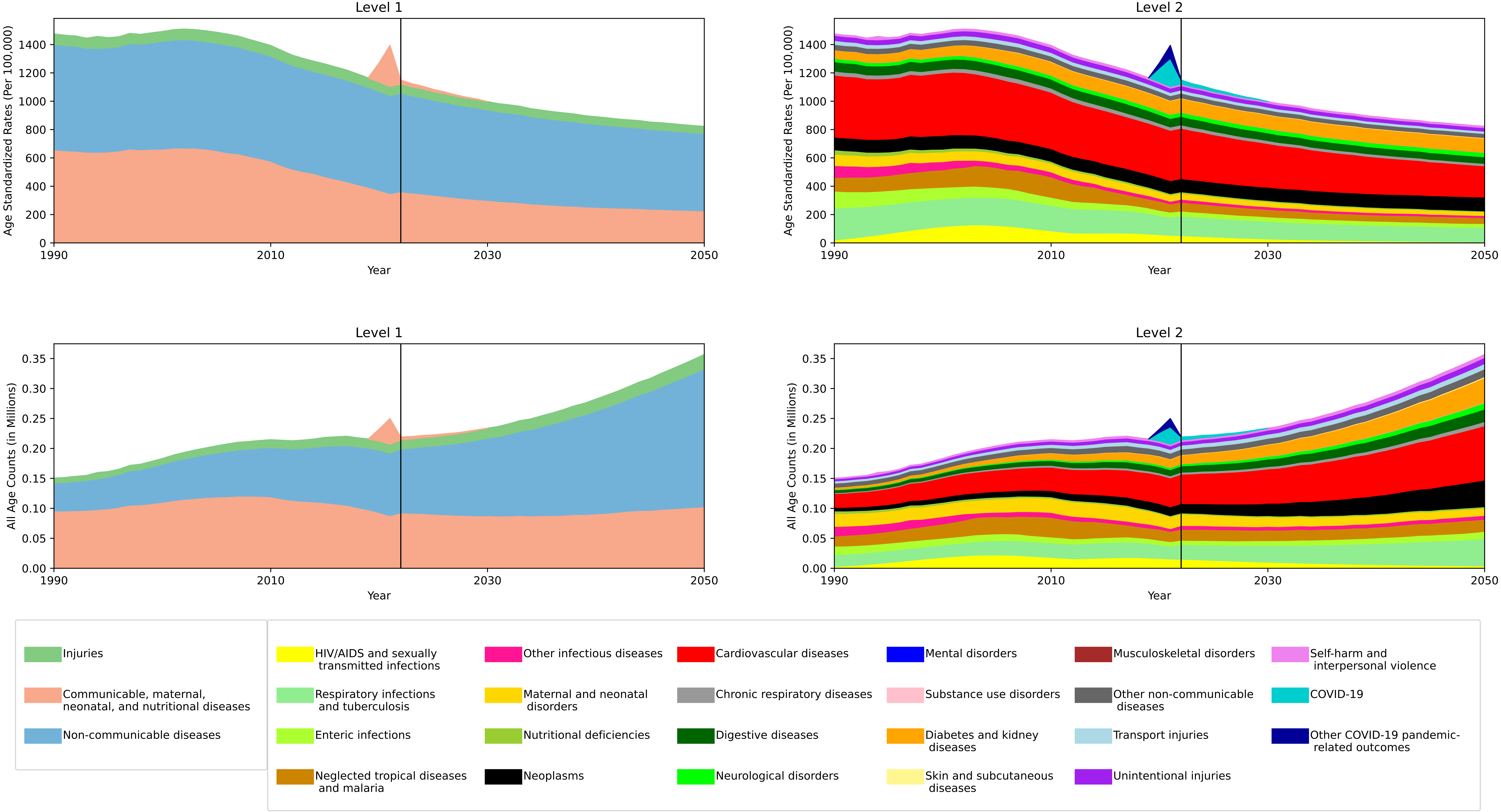
Level 1
Level 2
Age Standardized Rates (Per 100,000)
All Age Counts (in Millions)
Year
1990
2010
2030
2050
Injuries
Communicable, maternal, neonatal, and nutritional diseases
Non-communicable diseases
HIV/AIDS and sexually transmitted infections
Respiratory infections and tuberculosis
Enteric infections
Neglected tropical diseases and malaria
Other infectious diseases
Maternal and neonatal disorders
Nutritional deficiencies
Neoplasms
Cardiovascular diseases
Chronic respiratory diseases
Digestive diseases
Neurological disorders
Mental disorders
Substance use disorders
Diabetes and kidney diseases
Skin and subcutaneous diseases
Musculoskeletal disorders
Other non-communicable diseases
Transport injuries
Unintentional injuries
Self-harm and interpersonal violence
COVID-19
Other COVID-19 pandemic-related outcomes

Guinea

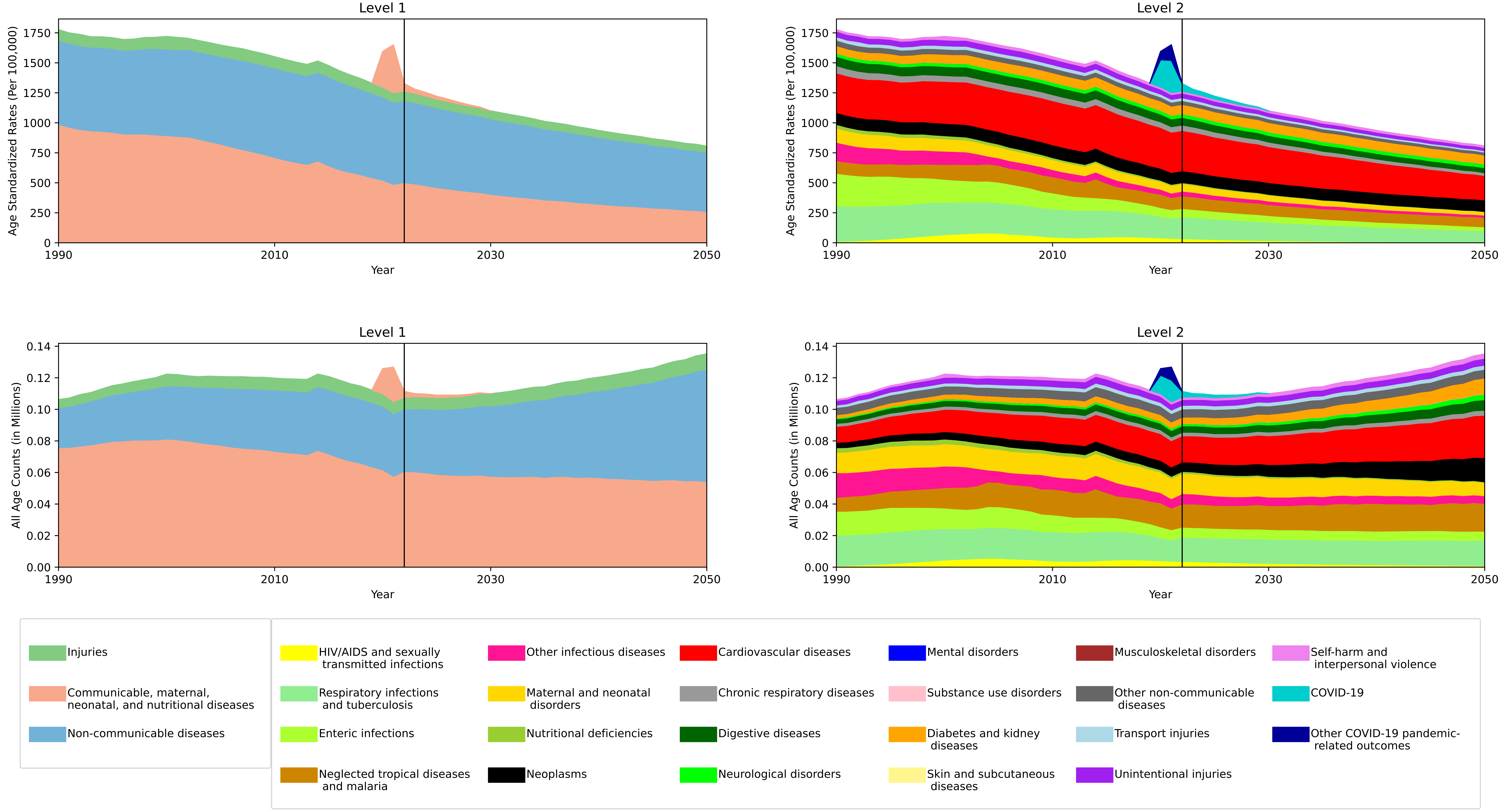
Level 1
Level 2
Level 1
Level 2
Age Standardized Rates (Per 100,000)
All Age Counts (in Millions)
Year
0
250
500
750
1000
1250
1500
1750
0.00
0.02
0.04
0.06
0.08
0.10
0.12
0.14
1990
2010
2030
2050
Injuries
Communicable, maternal, neonatal, and nutritional diseases
Non-communicable diseases
HIV/AIDS and sexually transmitted infections
Respiratory infections and tuberculosis
Enteric infections
Neglected tropical diseases and malaria
Other infectious diseases
Maternal and neonatal disorders
Nutritional deficiencies
Neoplasms
Cardiovascular diseases
Chronic respiratory diseases
Digestive diseases
Neurological disorders
Mental disorders
Substance use disorders
Diabetes and kidney diseases
Skin and subcutaneous diseases
Musculoskeletal disorders
Other non-communicable diseases
Transport injuries
Unintentional injuries
Self-harm and interpersonal violence
COVID-19
Other COVID-19 pandemic-related outcomes

Guinea-Bissau

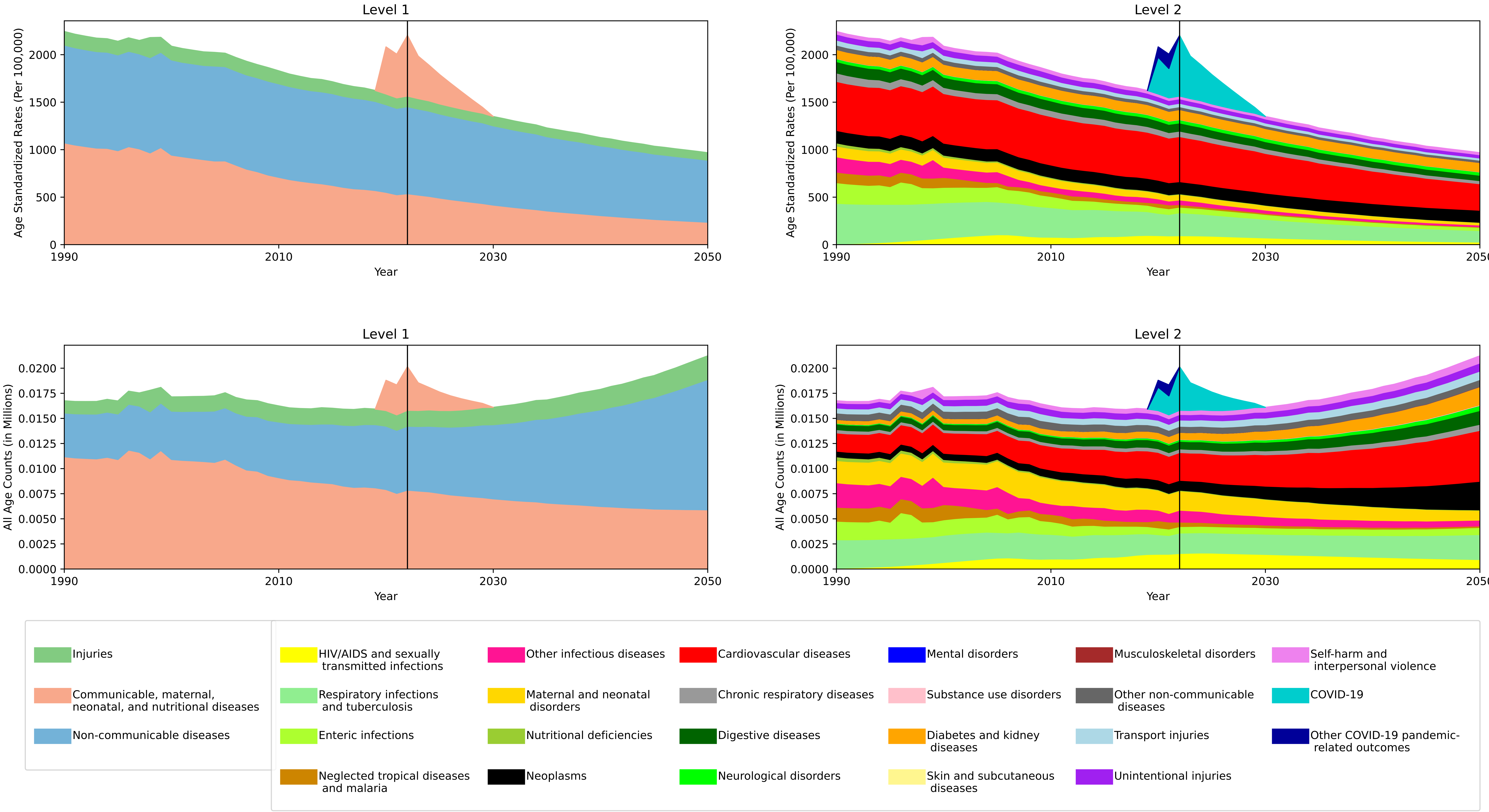

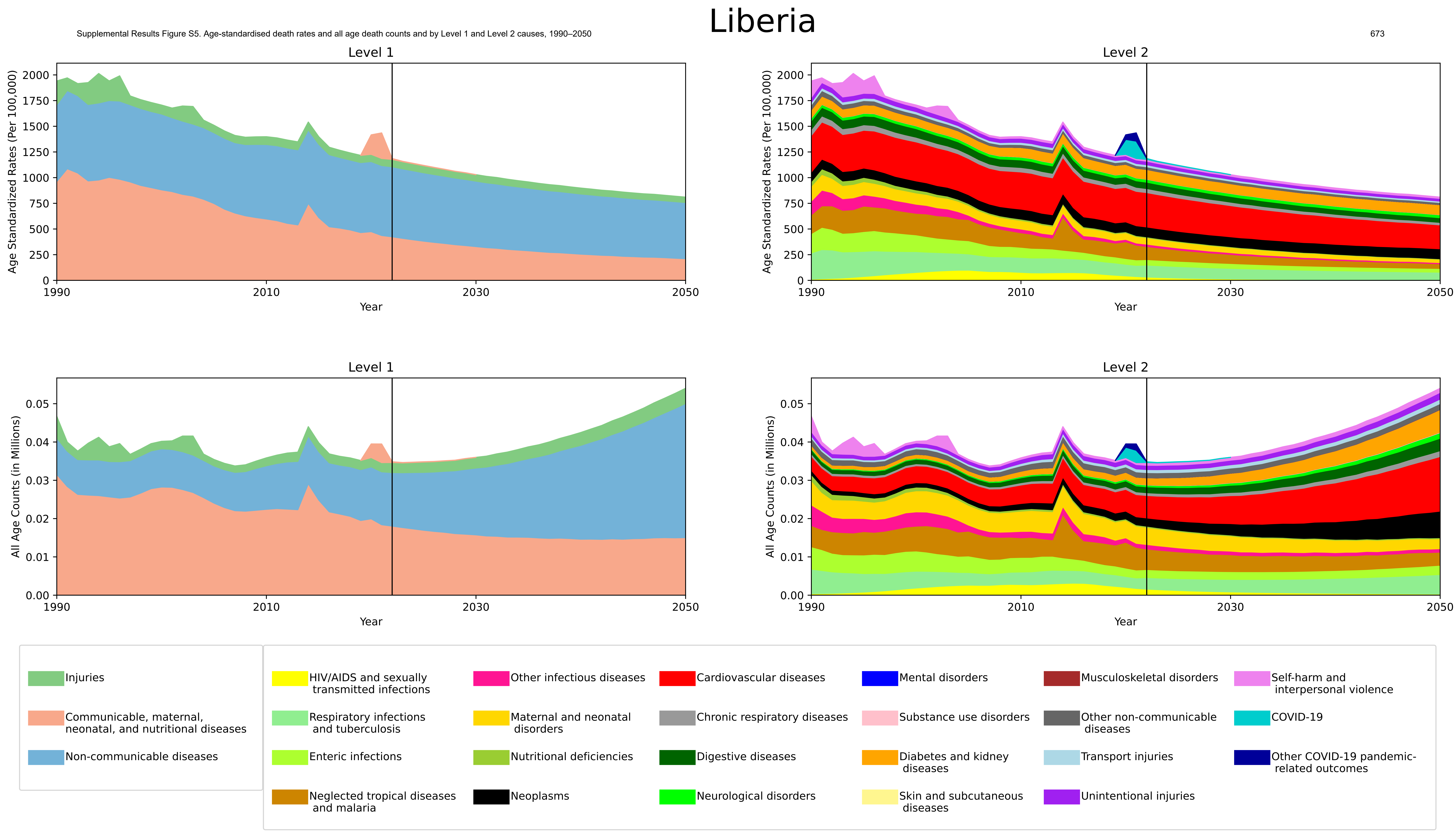
Liberia
Level 1
Level 2
Age Standardized Rates (Per 100,000)
All Age Counts (in Millions)
Year
1990
2010
2030
2050
Injuries
Communicable, maternal, neonatal, and nutritional diseases
Non-communicable diseases
HIV/AIDS and sexually transmitted infections
Respiratory infections and tuberculosis
Enteric infections
Neglected tropical diseases and malaria
Other infectious diseases
Maternal and neonatal disorders
Nutritional deficiencies
Neoplasms
Cardiovascular diseases
Chronic respiratory diseases
Digestive diseases
Neurological disorders
Mental disorders
Substance use disorders
Diabetes and kidney diseases
Skin and subcutaneous diseases
Musculoskeletal disorders
Other non-communicable diseases
Transport injuries
Unintentional injuries
Self-harm and interpersonal violence
COVID-19
Other COVID-19 pandemic-related outcomes

Mali

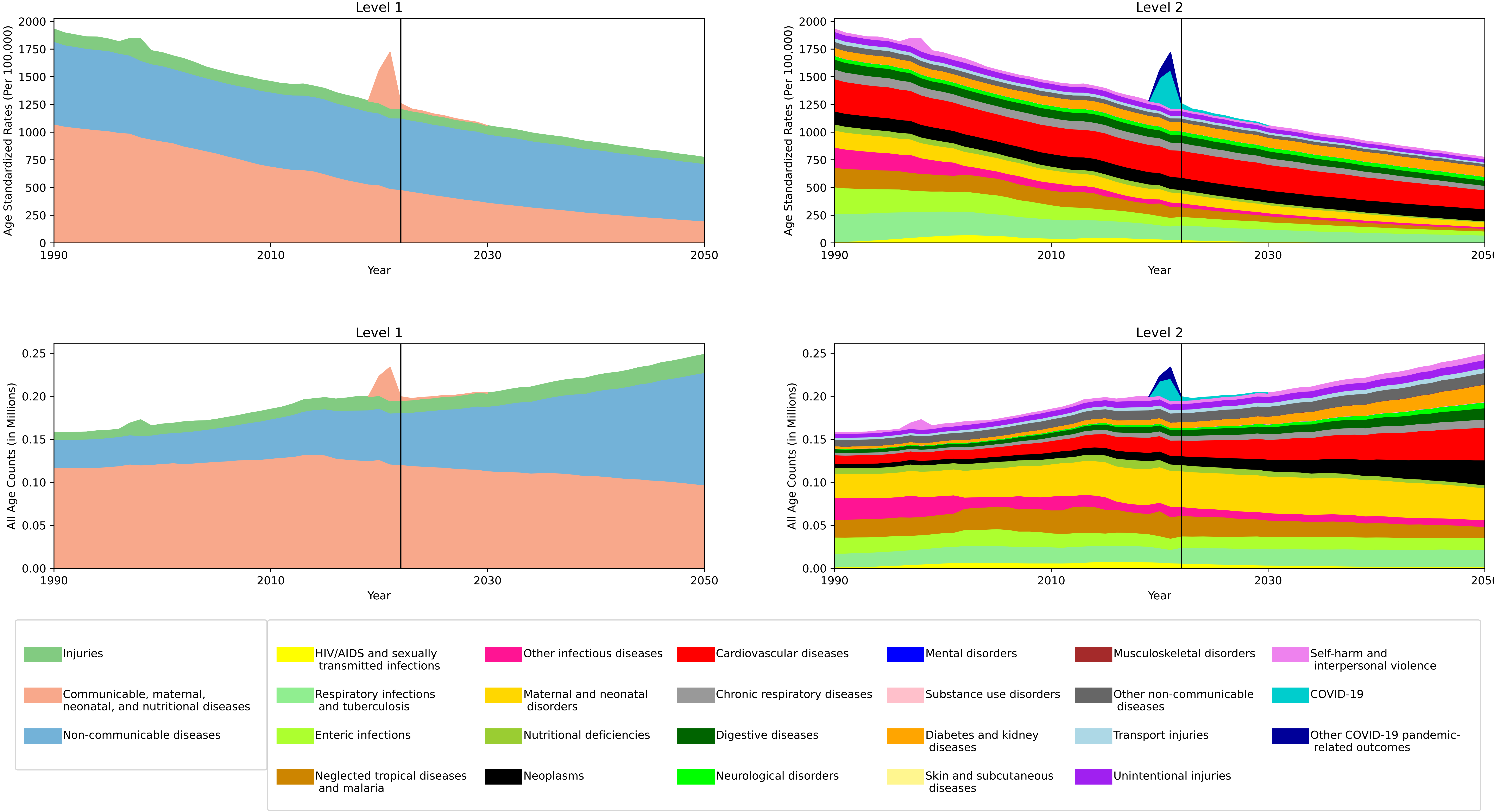
Level 1
Level 2
Age Standardized Rates (Per 100,000)
All Age Counts (in Millions)
Year
1990
2010
2030
2050
0
250
500
750
1000
1250
1500
1750
2000
0.00
0.05
0.10
0.15
0.20
0.25
Injuries
Communicable, maternal, neonatal, and nutritional diseases
Non-communicable diseases
HIV/AIDS and sexually transmitted infections
Respiratory infections and tuberculosis
Enteric infections
Neglected tropical diseases and malaria
Other infectious diseases
Maternal and neonatal disorders
Nutritional deficiencies
Neoplasms
Cardiovascular diseases
Chronic respiratory diseases
Digestive diseases
Neurological disorders
Mental disorders
Substance use disorders
Diabetes and kidney diseases
Skin and subcutaneous diseases
Musculoskeletal disorders
Other non-communicable diseases
Transport injuries
Unintentional injuries
Self-harm and interpersonal violence
COVID-19
Other COVID-19 pandemic-related outcomes

Mauritania

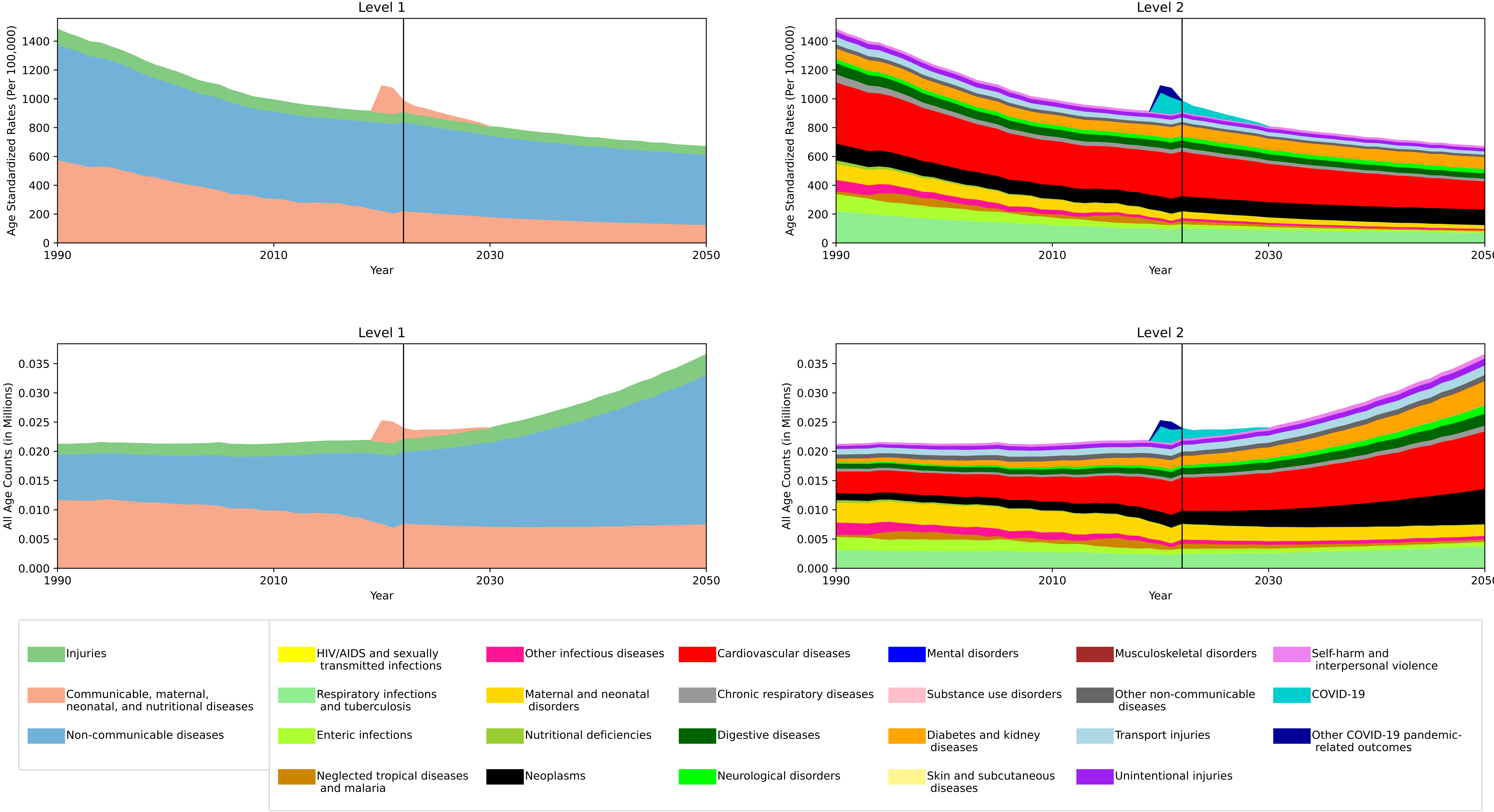
Level 1
Level 2
Age Standardized Rates (Per 100,000)
All Age Counts (in Millions)
Year
1990
2010
2030
2050
0
200
400
600
800
1000
1200
1400
0.000
0.005
0.010
0.015
0.020
0.025
0.030
0.035
Injuries
Communicable, maternal, neonatal, and nutritional diseases
Non-communicable diseases
HIV/AIDS and sexually transmitted infections
Respiratory infections and tuberculosis
Enteric infections
Neglected tropical diseases and malaria
Other infectious diseases
Maternal and neonatal disorders
Nutritional deficiencies
Neoplasms
Cardiovascular diseases
Chronic respiratory diseases
Digestive diseases
Neurological disorders
Mental disorders
Substance use disorders
Diabetes and kidney diseases
Skin and subcutaneous diseases
Musculoskeletal disorders
Other non-communicable diseases
Transport injuries
Unintentional injuries
Self-harm and interpersonal violence
COVID-19
Other COVID-19 pandemic-related outcomes

Niger

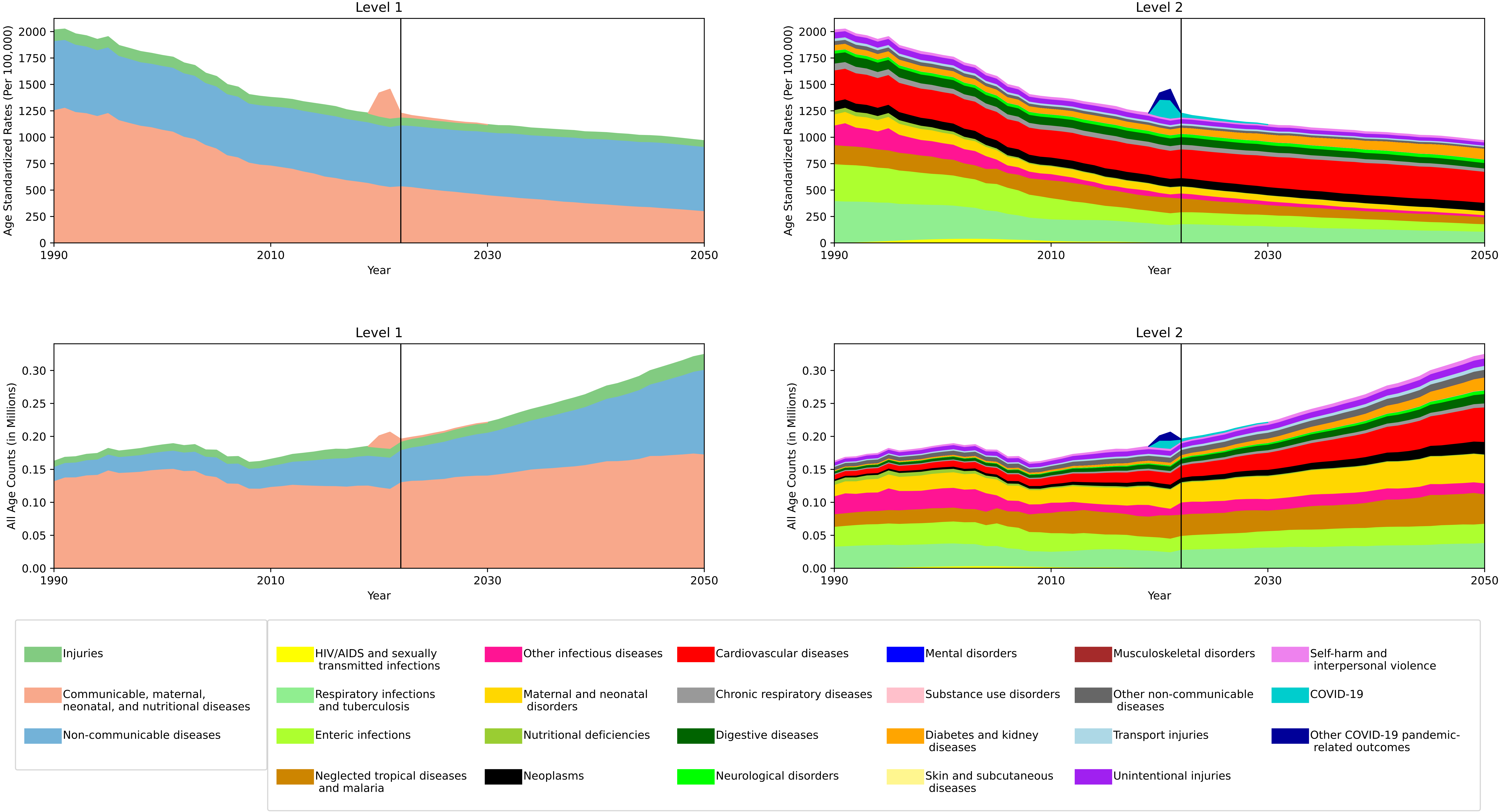
Level 1
Level 2
Age Standardized Rates (Per 100,000)
All Age Counts (in Millions)
Year
1990
2010
2030
2050
0
250
500
750
1000
1250
1500
1750
2000
0.00
0.05
0.10
0.15
0.20
0.25
0.30
Injuries
Communicable, maternal, neonatal, and nutritional diseases
Non-communicable diseases
HIV/AIDS and sexually transmitted infections
Respiratory infections and tuberculosis
Enteric infections
Neglected tropical diseases and malaria
Other infectious diseases
Maternal and neonatal disorders
Nutritional deficiencies
Neoplasms
Cardiovascular diseases
Chronic respiratory diseases
Digestive diseases
Neurological disorders
Mental disorders
Substance use disorders
Diabetes and kidney diseases
Skin and subcutaneous diseases
Musculoskeletal disorders
Other non-communicable diseases
Transport injuries
Unintentional injuries
Self-harm and interpersonal violence
COVID-19
Other COVID-19 pandemic-related outcomes

Nigeria

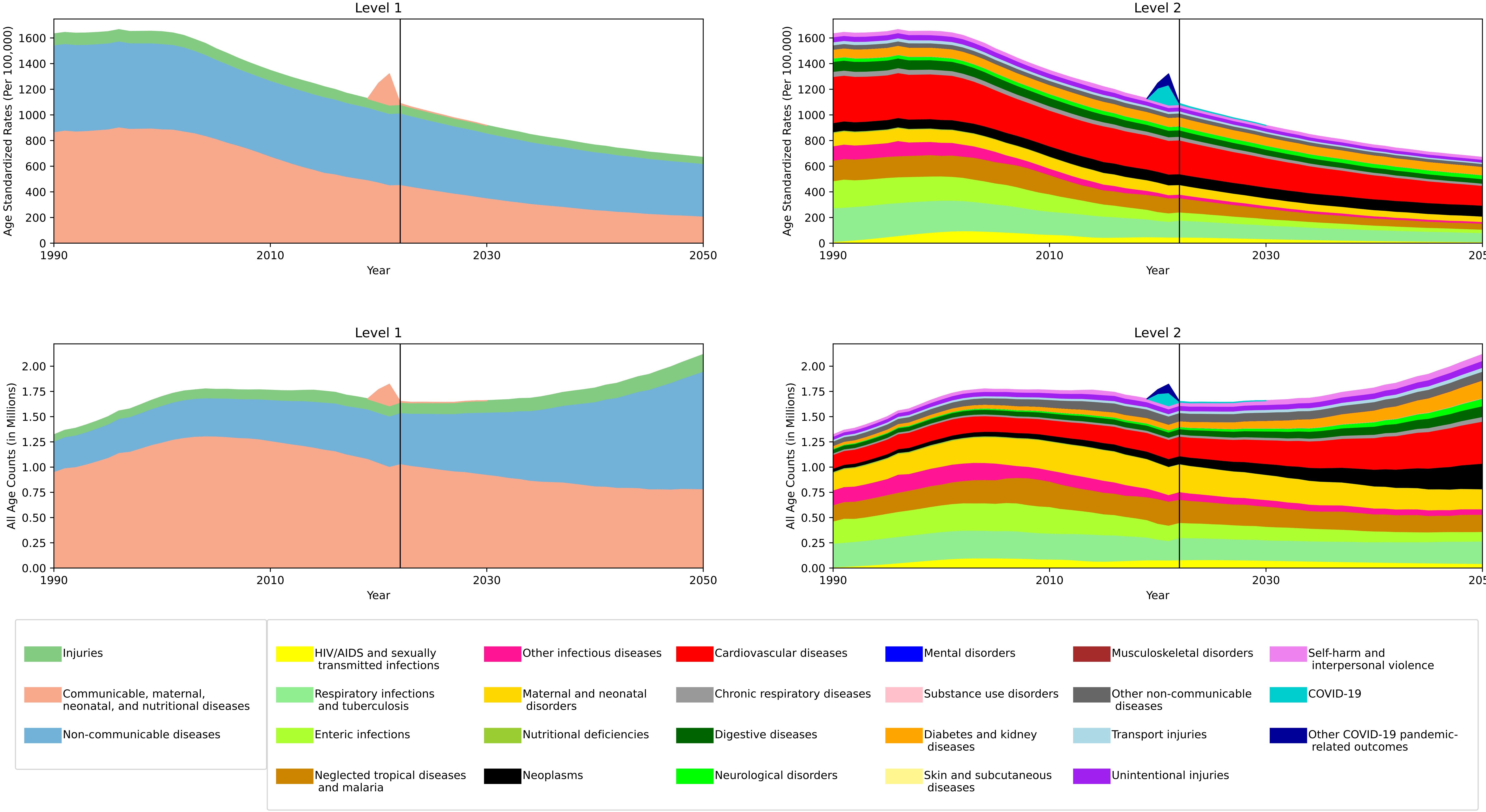
Level 1
Level 2
Age Standardized Rates (Per 100,000)
All Age Counts (in Millions)
Year
Injuries
Communicable, maternal, neonatal, and nutritional diseases
Non-communicable diseases
HIV/AIDS and sexually transmitted infections
Respiratory infections and tuberculosis
Enteric infections
Neglected tropical diseases and malaria
Other infectious diseases
Maternal and neonatal disorders
Nutritional deficiencies
Neoplasms
Cardiovascular diseases
Chronic respiratory diseases
Digestive diseases
Neurological disorders
Mental disorders
Substance use disorders
Diabetes and kidney diseases
Skin and subcutaneous diseases
Musculoskeletal disorders
Other non-communicable diseases
Transport injuries
Unintentional injuries
Self-harm and interpersonal violence
COVID-19
Other COVID-19 pandemic-related outcomes

São Tomé And Príncipe

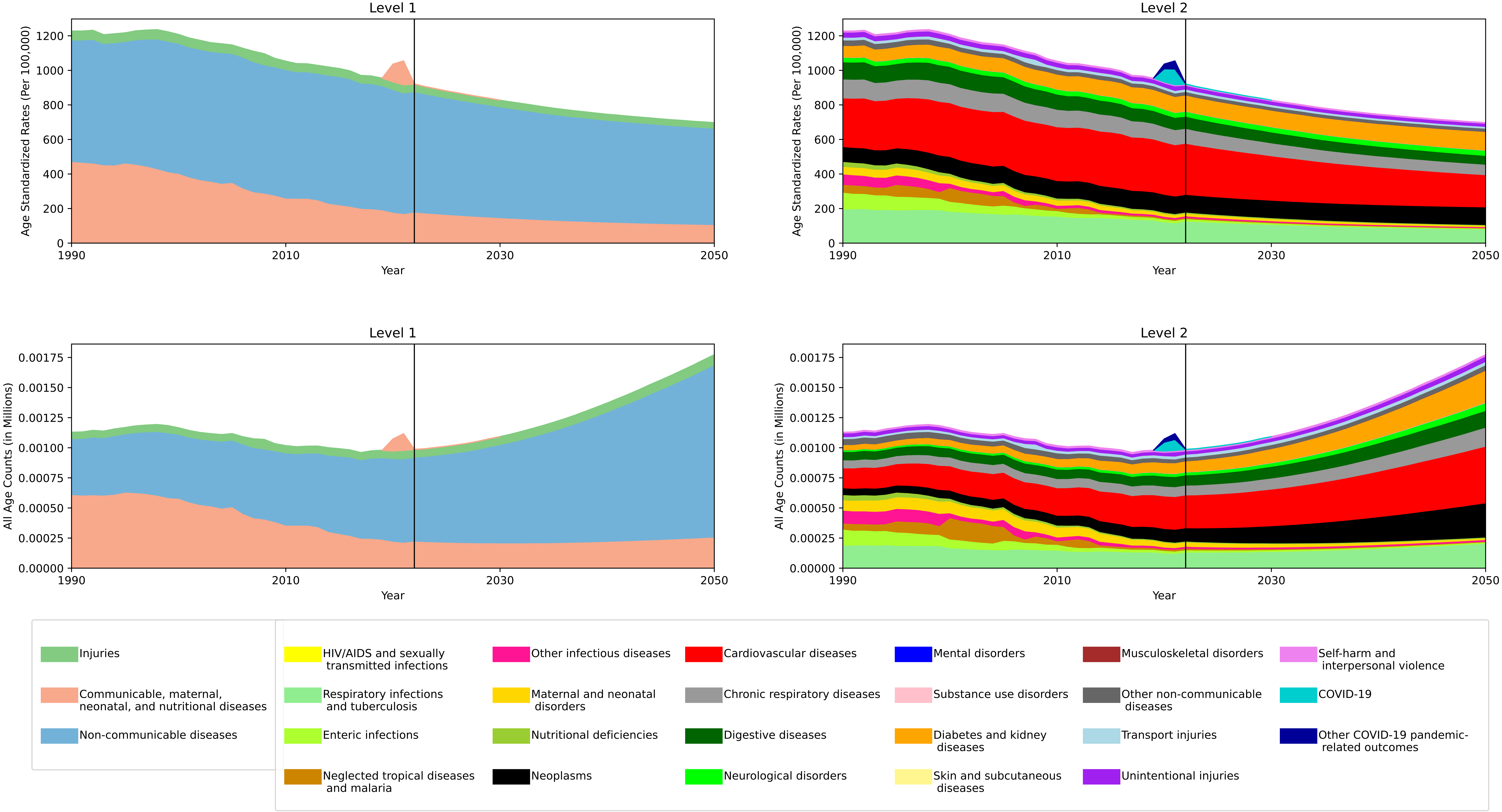

Senegal

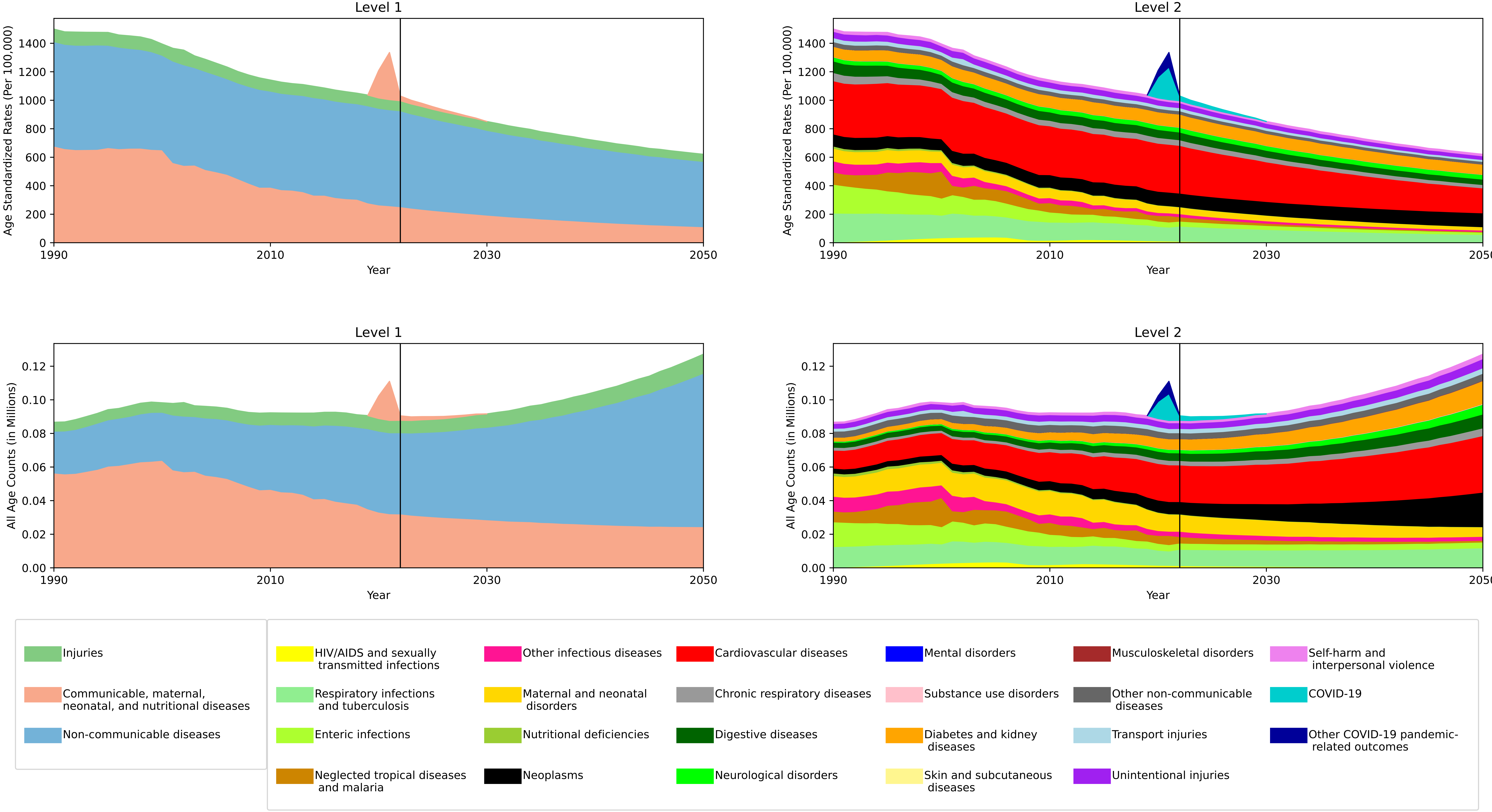
Level 1
Level 2
Age Standardized Rates (Per 100,000)
All Age Counts (in Millions)
Year
1990
2010
2030
2050
0
200
400
600
800
1000
1200
1400
0.00
0.02
0.04
0.06
0.08
0.10
0.12
Injuries
Communicable, maternal, neonatal, and nutritional diseases
Non-communicable diseases
HIV/AIDS and sexually transmitted infections
Respiratory infections and tuberculosis
Enteric infections
Neglected tropical diseases and malaria
Other infectious diseases
Maternal and neonatal disorders
Nutritional deficiencies
Neoplasms
Cardiovascular diseases
Chronic respiratory diseases
Digestive diseases
Neurological disorders
Mental disorders
Substance use disorders
Diabetes and kidney diseases
Skin and subcutaneous diseases
Musculoskeletal disorders
Other non-communicable diseases
Transport injuries
Unintentional injuries
Self-harm and interpersonal violence
COVID-19
Other COVID-19 pandemic-related outcomes

Sierra Leone

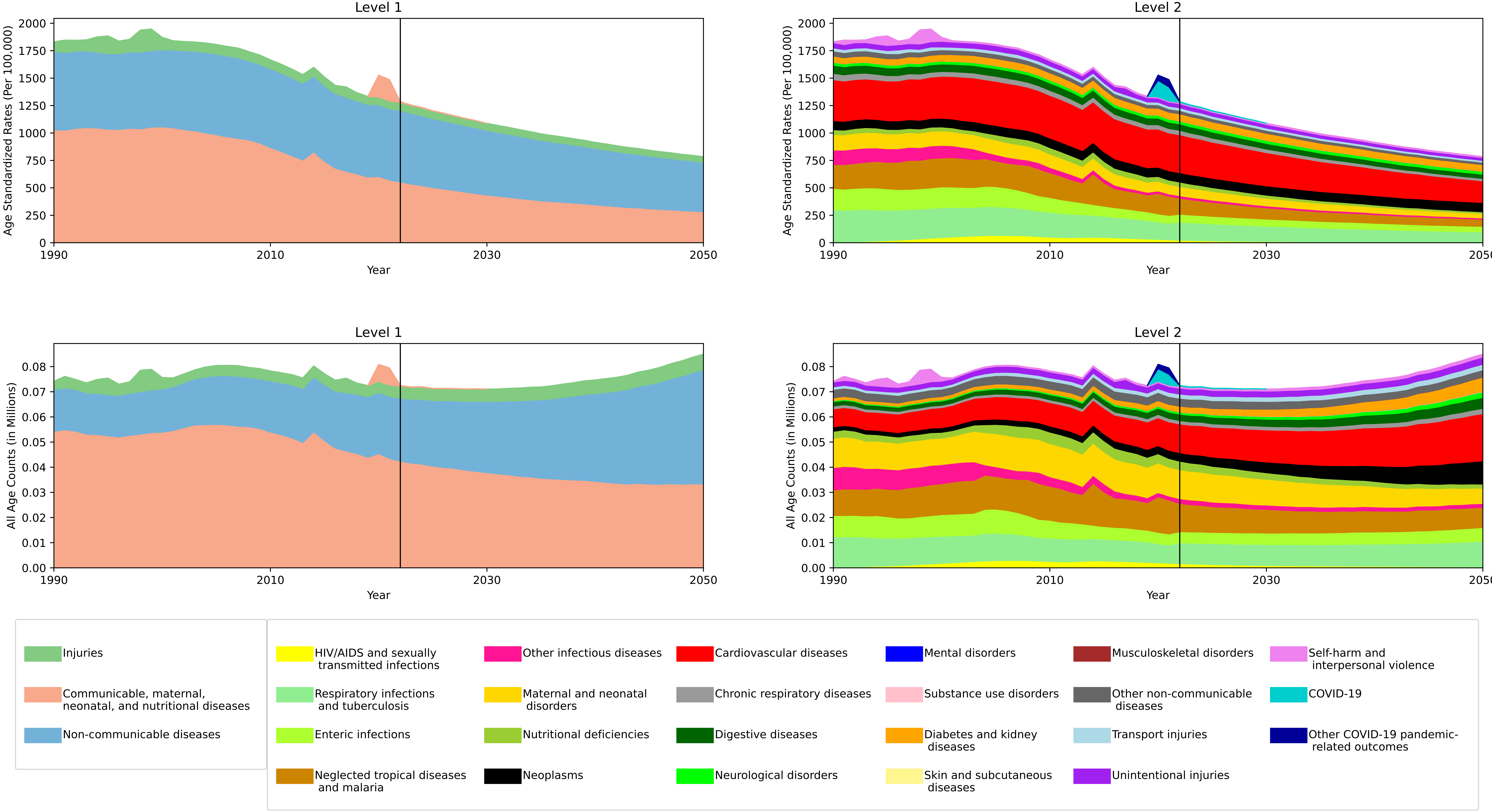

Togo

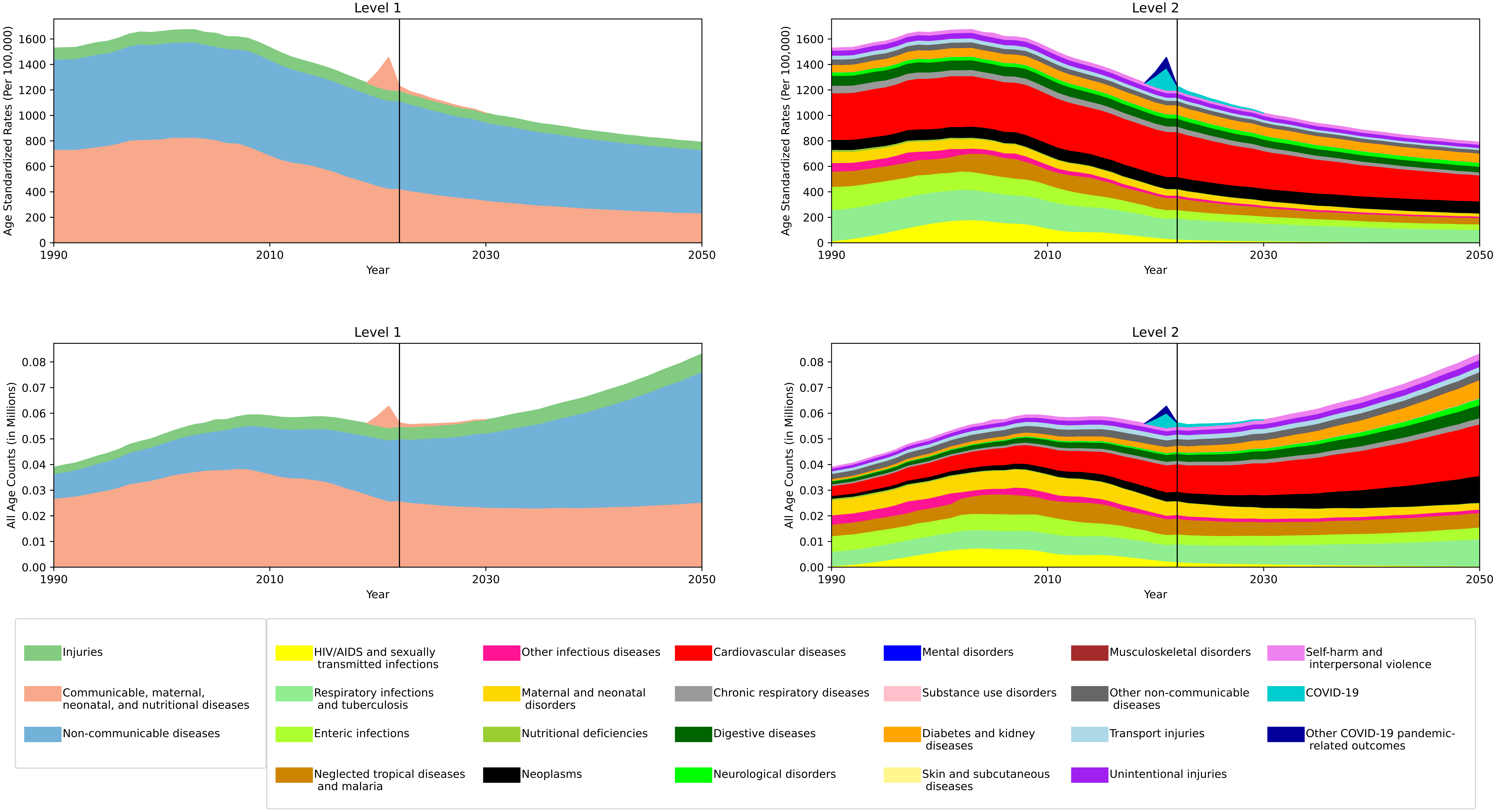
Level 1
Level 2
Age Standardized Rates (Per 100,000)
All Age Counts (in Millions)
Year
1990
2010
2030
2050
0
200
400
600
800
1000
1200
1400
1600
0.00
0.01
0.02
0.03
0.04
0.05
0.06
0.07
0.08
Injuries
Communicable, maternal, neonatal, and nutritional diseases
Non-communicable diseases
HIV/AIDS and sexually transmitted infections
Respiratory infections and tuberculosis
Enteric infections
Neglected tropical diseases and malaria
Other infectious diseases
Maternal and neonatal disorders
Nutritional deficiencies
Neoplasms
Cardiovascular diseases
Chronic respiratory diseases
Digestive diseases
Neurological disorders
Mental disorders
Substance use disorders
Diabetes and kidney diseases
Skin and subcutaneous diseases
Musculoskeletal disorders
Other non-communicable diseases
Transport injuries
Unintentional injuries
Self-harm and interpersonal violence
COVID-19
Other COVID-19 pandemic-related outcomes

Supplemental Results Figure S5. Age-standardised death rates and all age death counts and by Level 1 and Level 2 causes, 1990–2050

Global

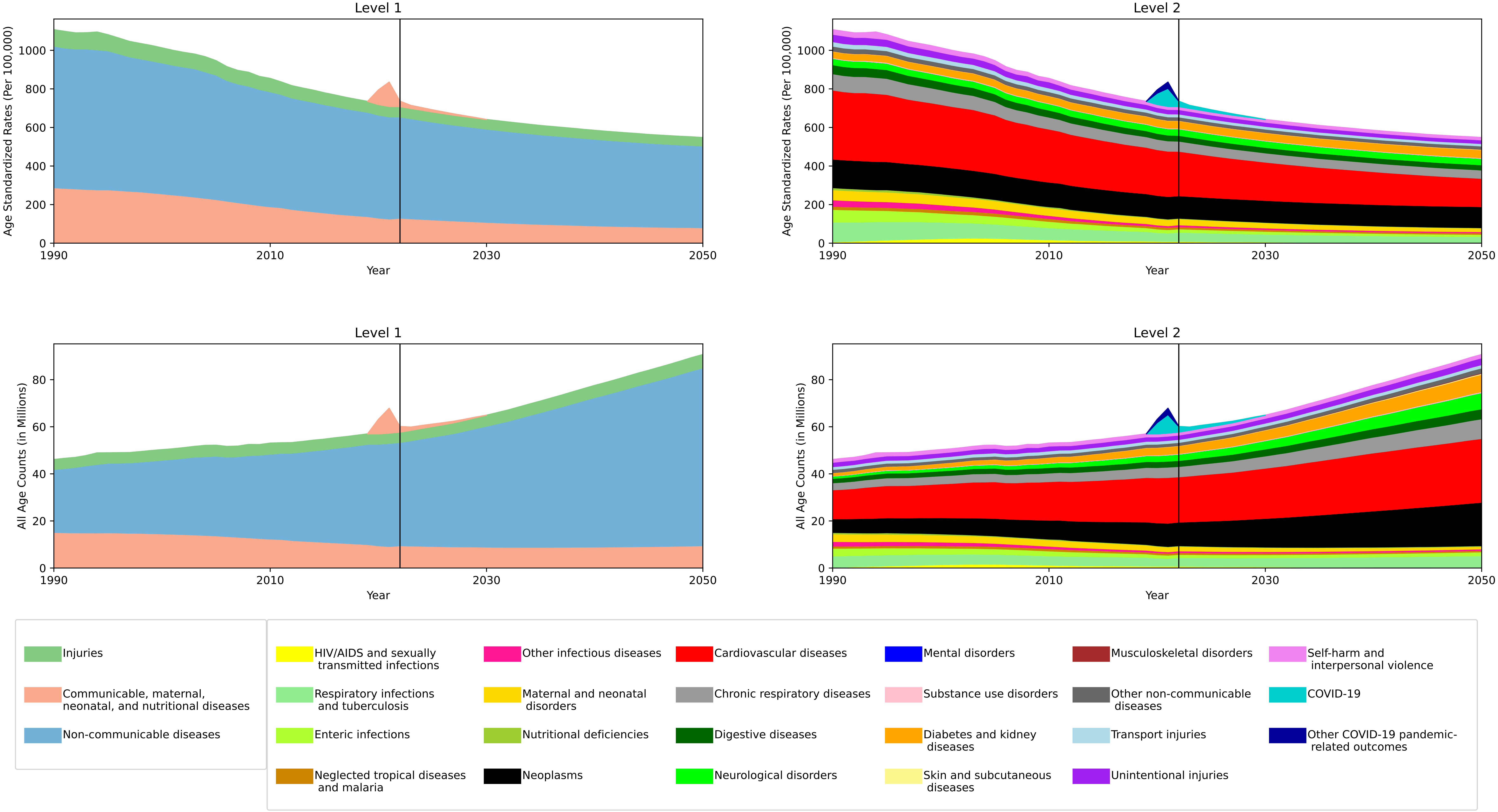

Central Europe, Eastern Europe, And Central Asia

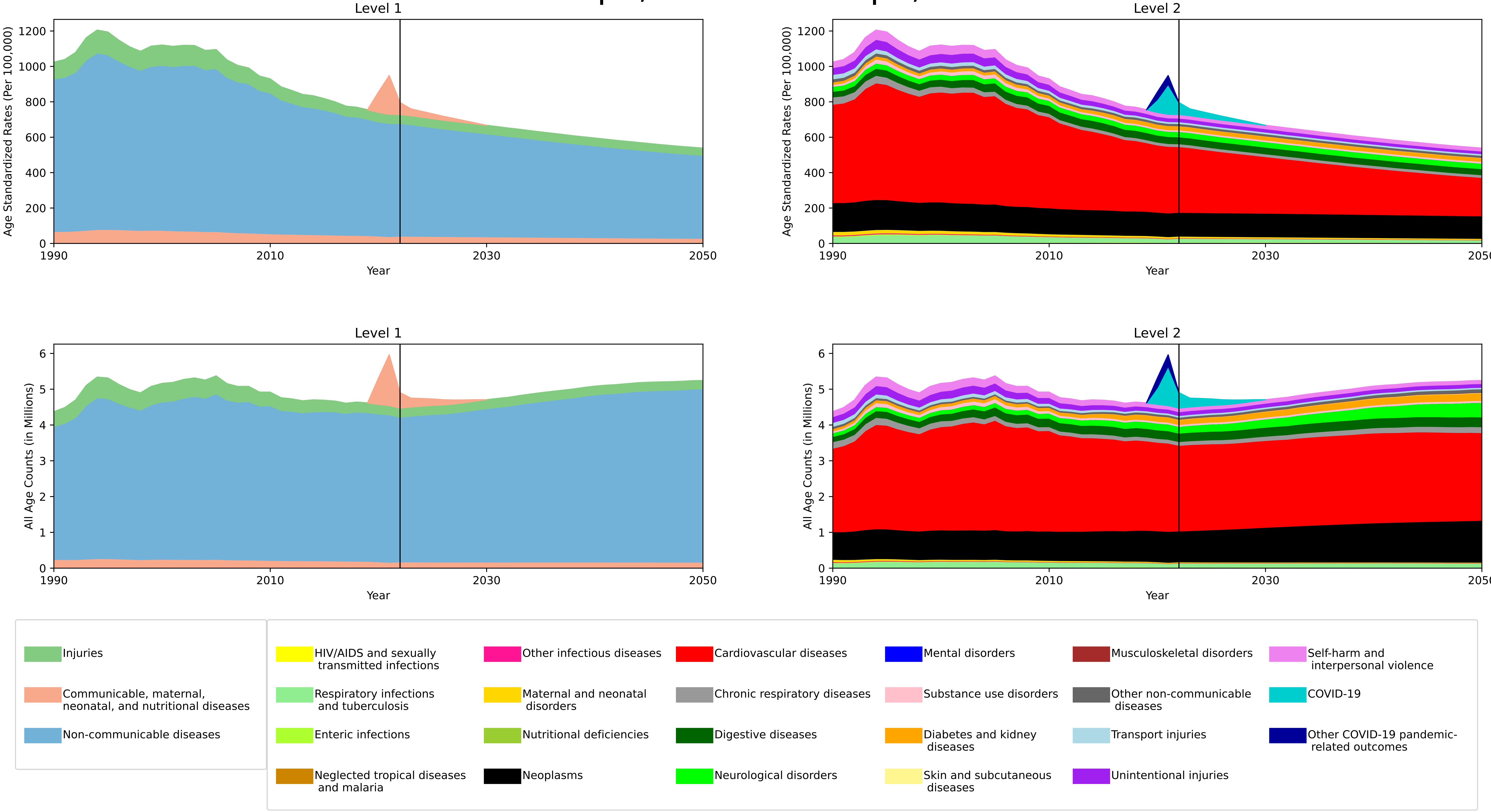

High Income

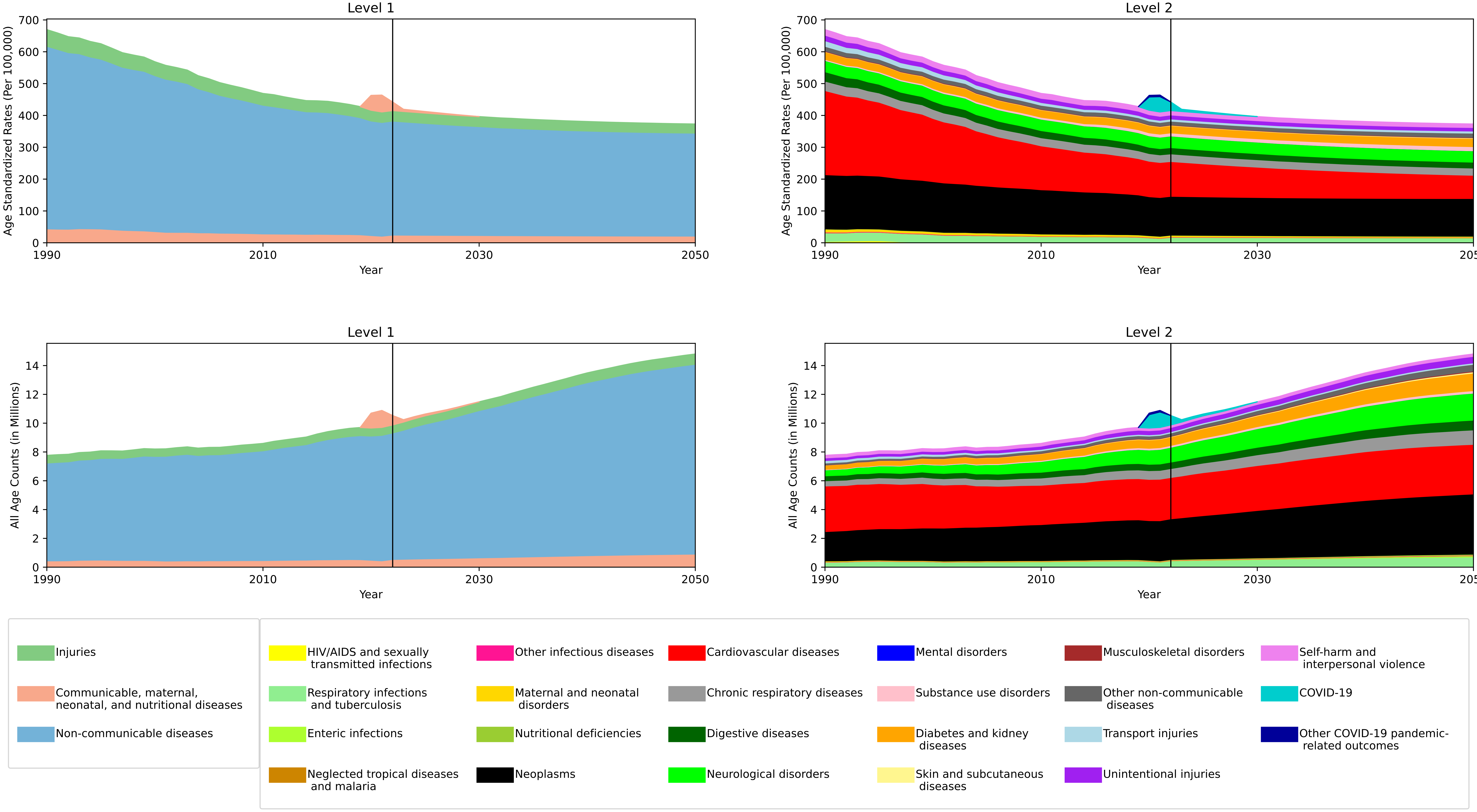
Level 1
Level 2
Age Standardized Rates (Per 100,000)
All Age Counts (in Millions)
Year
1990
2010
2030
2050
0
100
200
300
400
500
600
700
2
4
6
8
10
12
14
Injuries
Communicable, maternal, neonatal, and nutritional diseases
Non-communicable diseases
HIV/AIDS and sexually transmitted infections
Respiratory infections and tuberculosis
Enteric infections
Neglected tropical diseases and malaria
Other infectious diseases
Maternal and neonatal disorders
Nutritional deficiencies
Neoplasms
Cardiovascular diseases
Chronic respiratory diseases
Digestive diseases
Neurological disorders
Mental disorders
Substance use disorders
Diabetes and kidney diseases
Skin and subcutaneous diseases
Musculoskeletal disorders
Other non-communicable diseases
Transport injuries
Unintentional injuries
Self-harm and interpersonal violence
COVID-19
Other COVID-19 pandemic-related outcomes

Supplemental Results Figure S5. Age-standardised death rates and all age death counts and by Level 1 and Level 2 causes, 1990–2050

Latin America And Caribbean

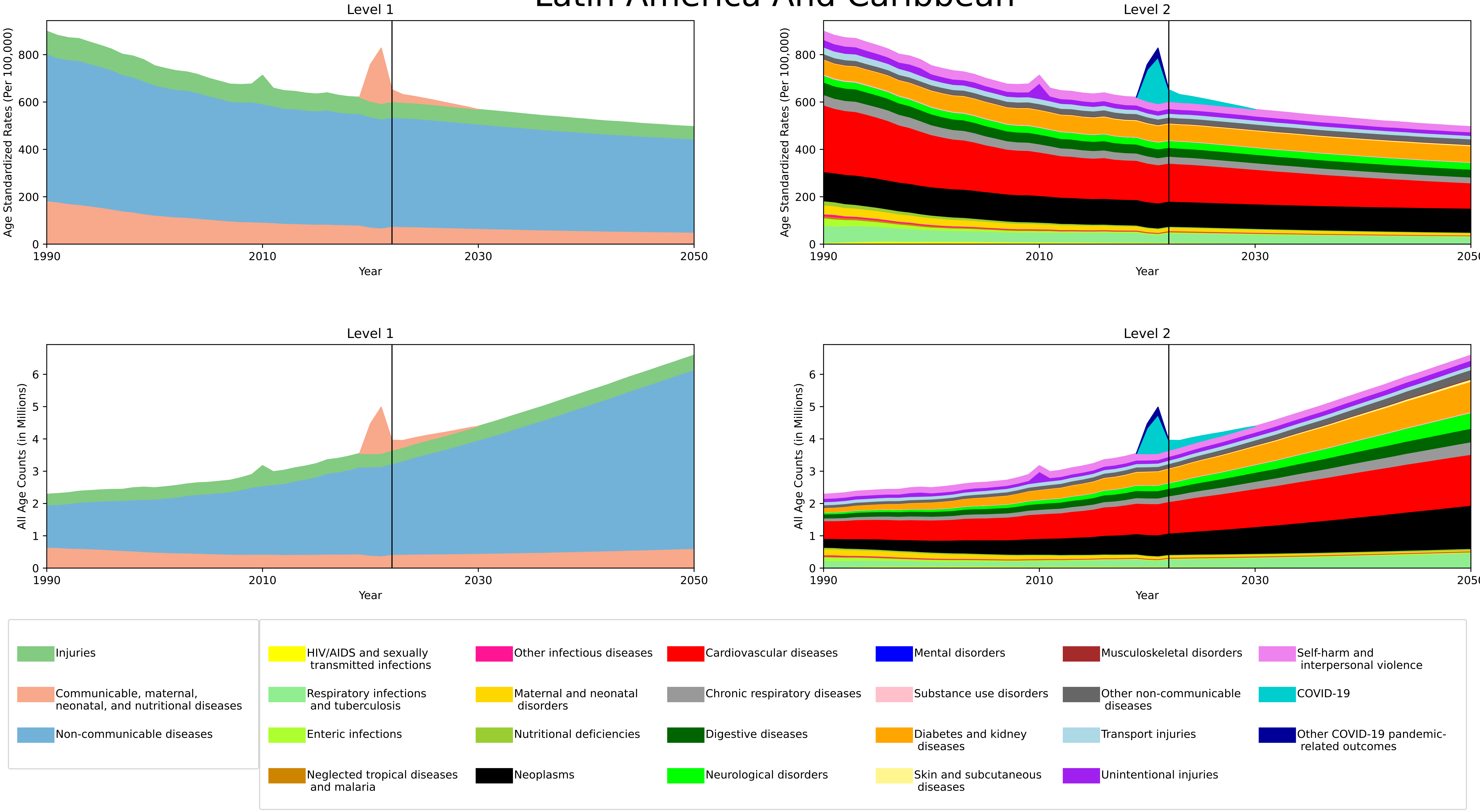

Supplemental Results Figure S5. Age-standardised death rates and all age death counts and by Level 1 and Level 2 causes, 1990–2050

North Africa And Middle East

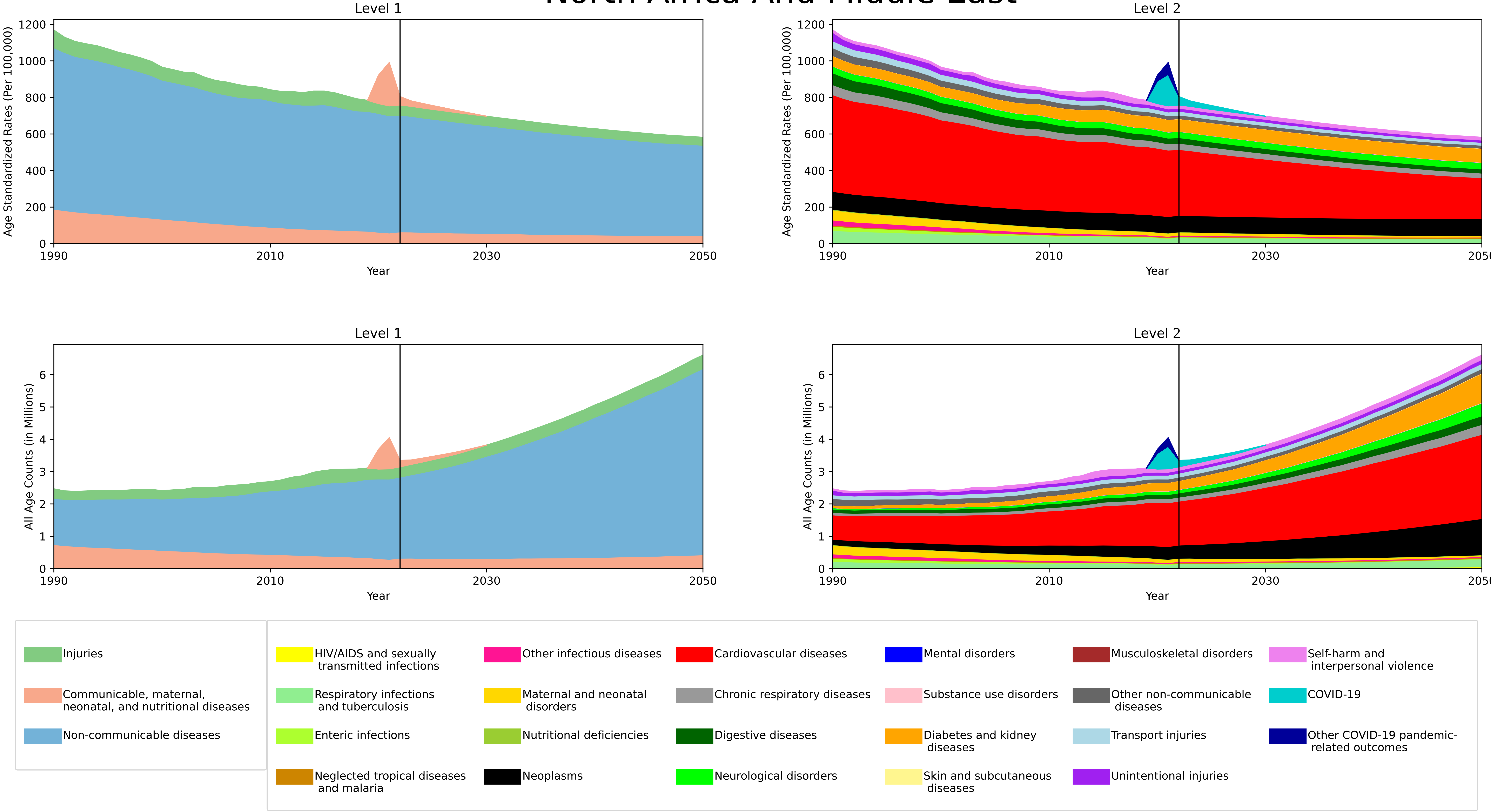

South Asia

Supplemental Results Figure S5. Age-standardised death rates and all age death counts and by Level 1 and Level 2 causes, 1990–2050

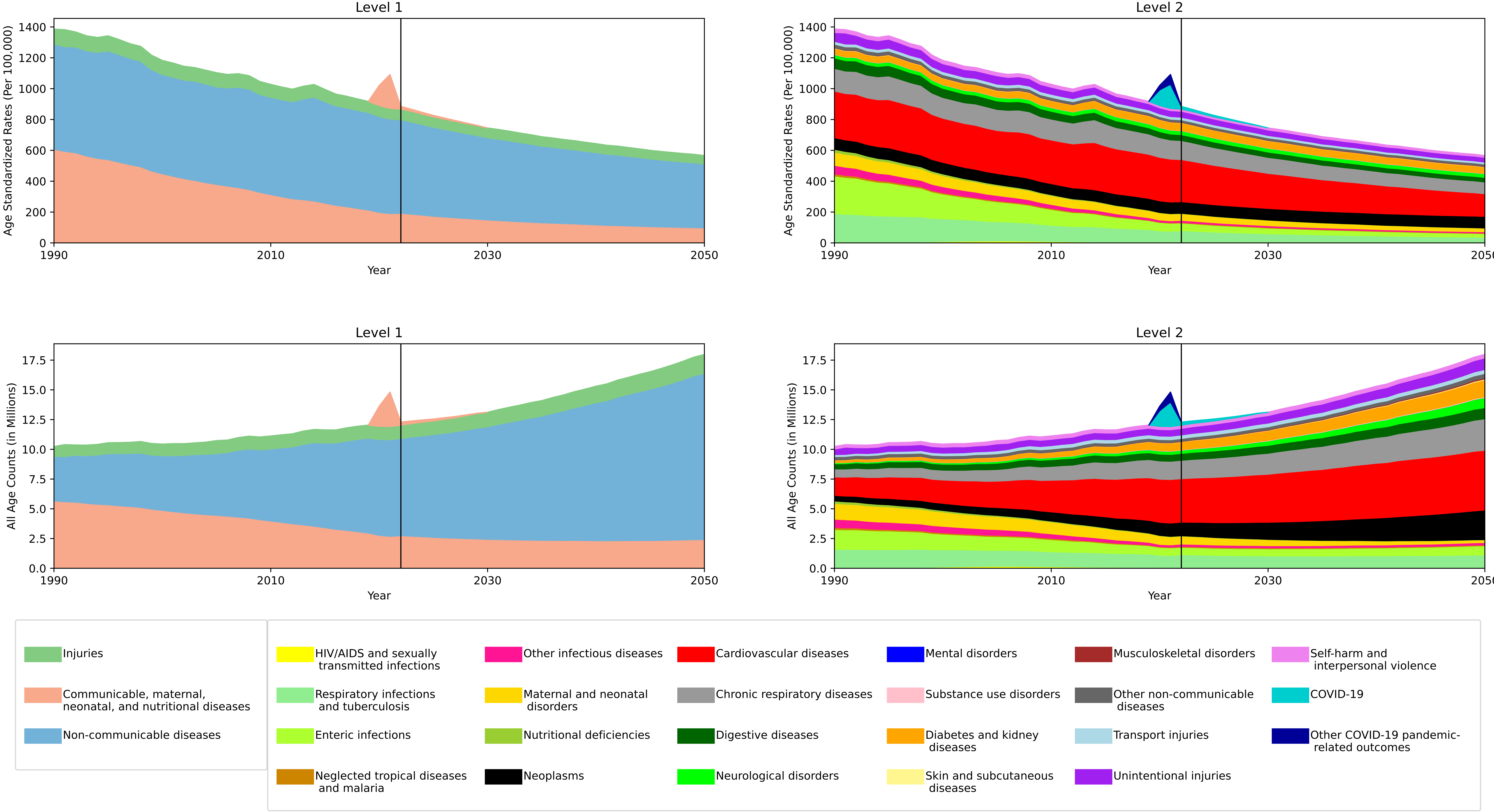

Supplemental Results Figure S5. Age-standardised death rates and all age death counts and by Level 1 and Level 2 causes, 1990–2050

Southeast Asia, East Asia, And Oceania

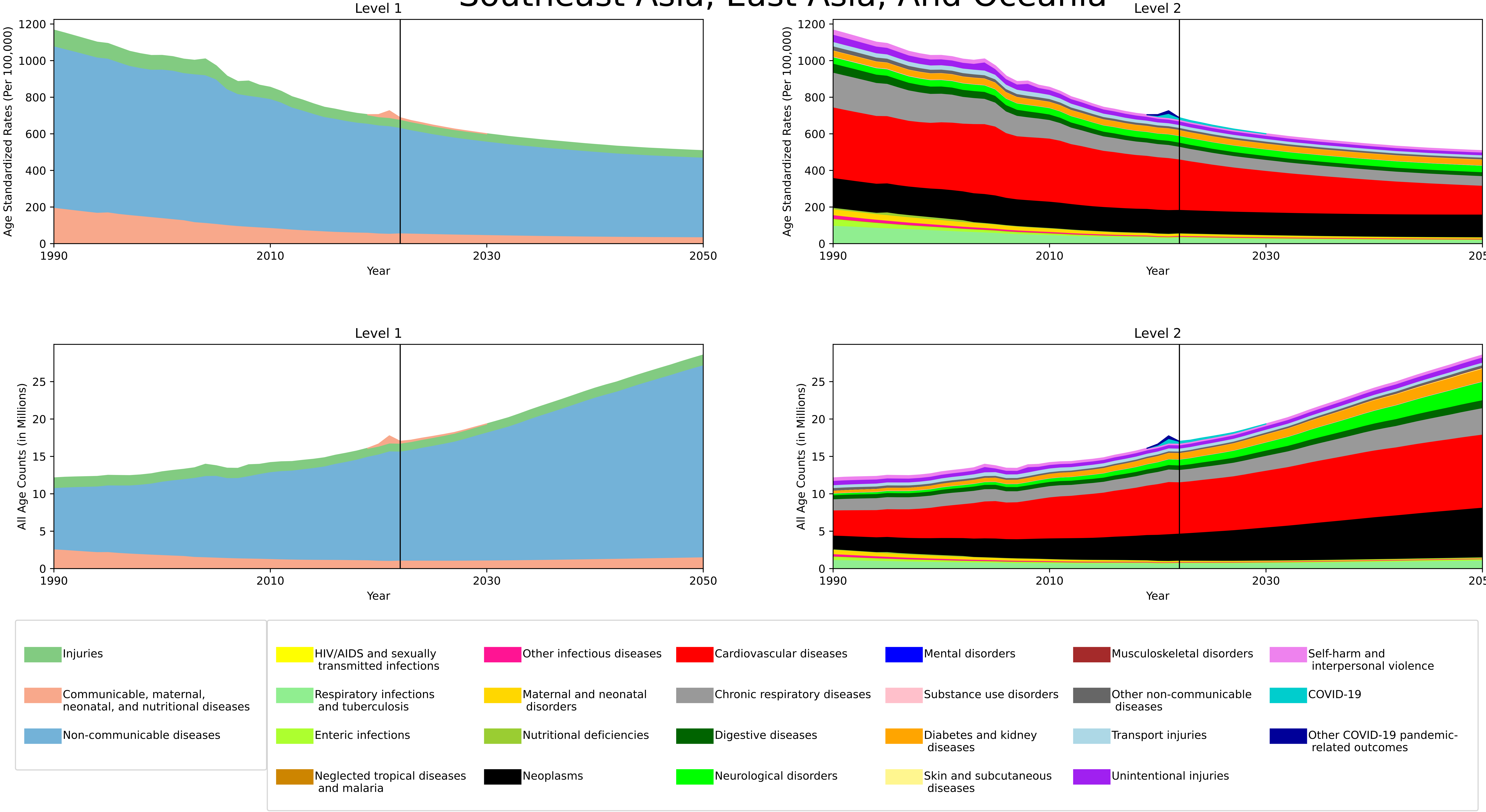

Sub-Saharan Africa

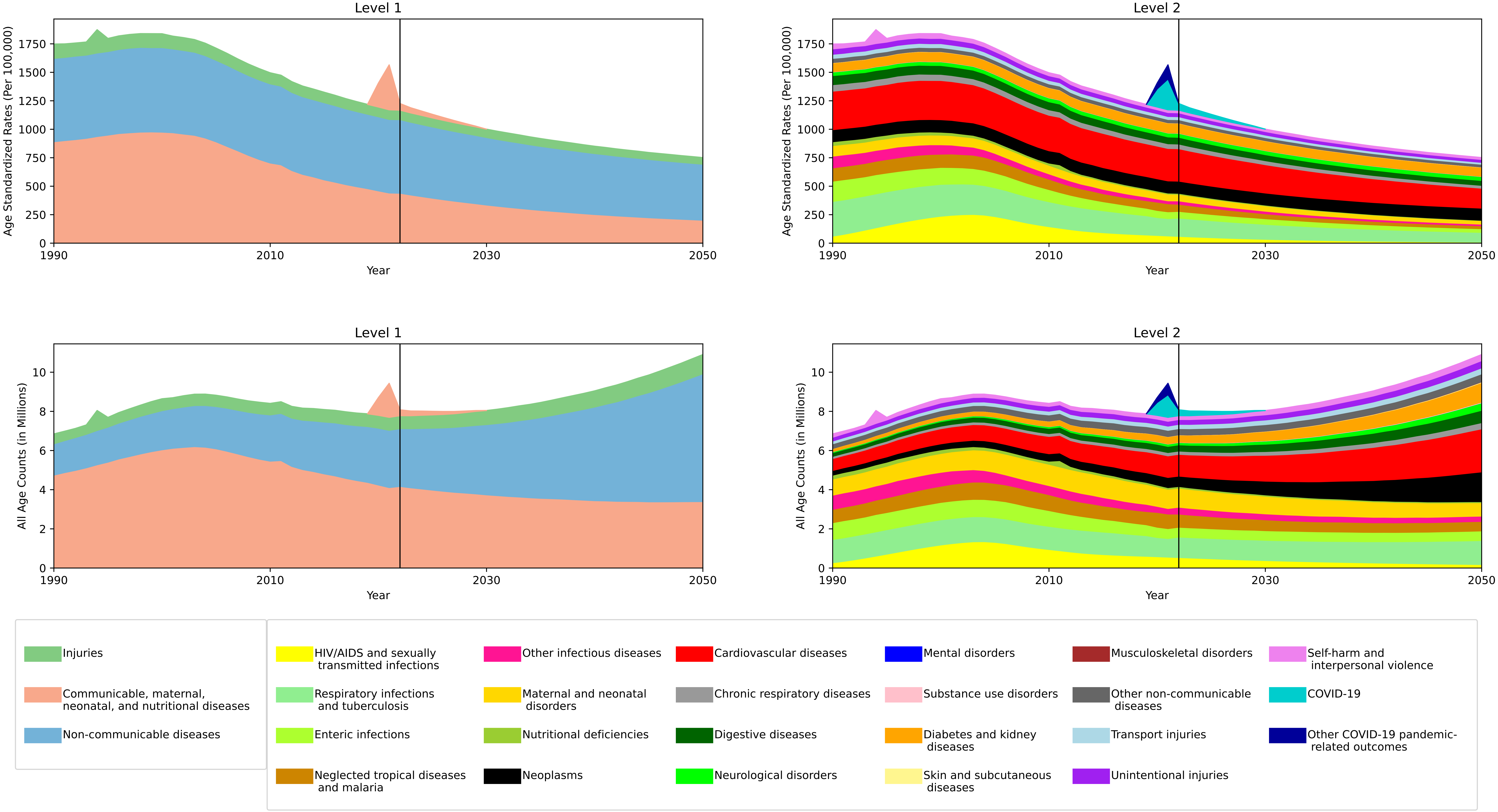

Central Asia

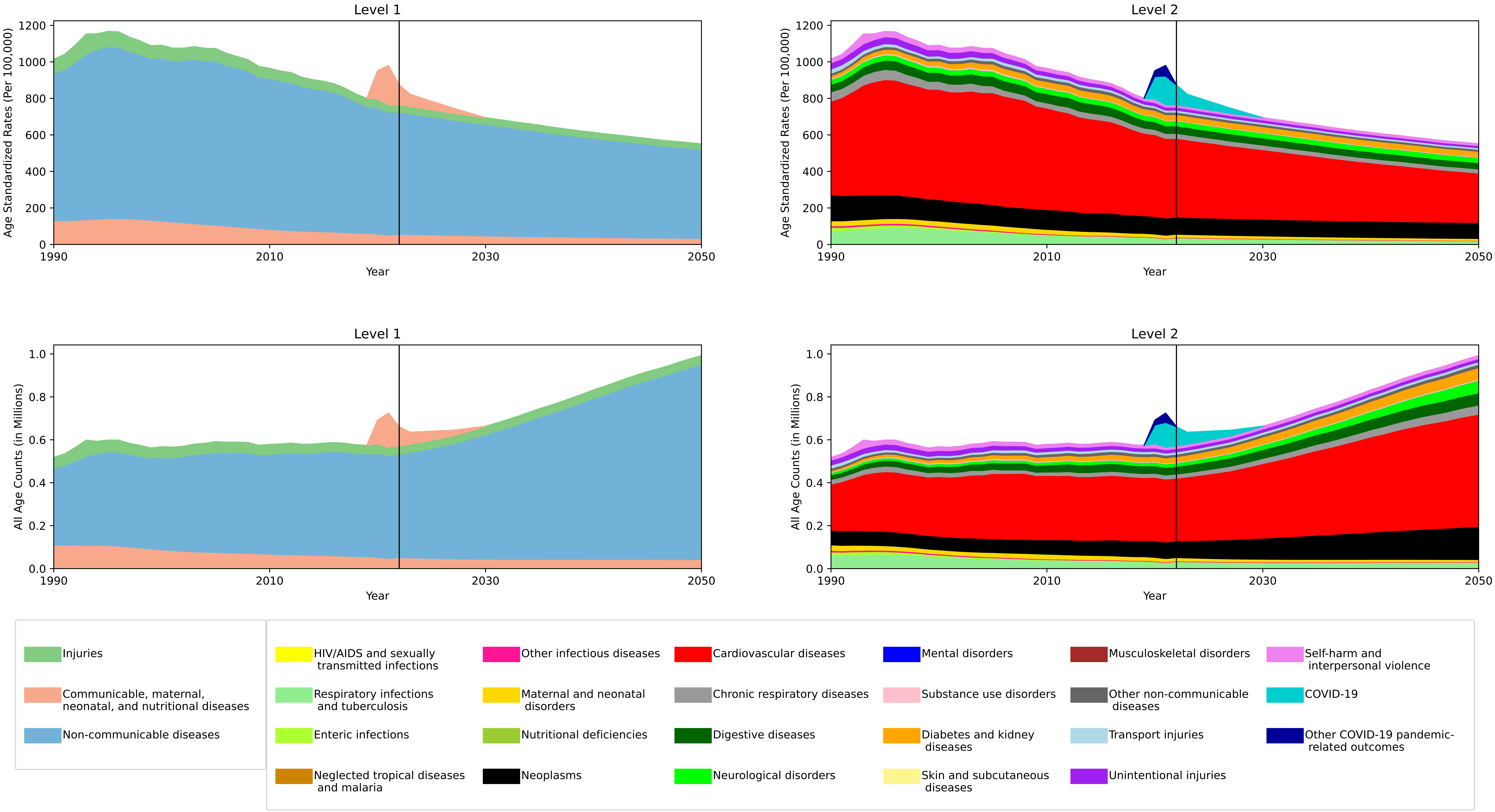

Central Europe

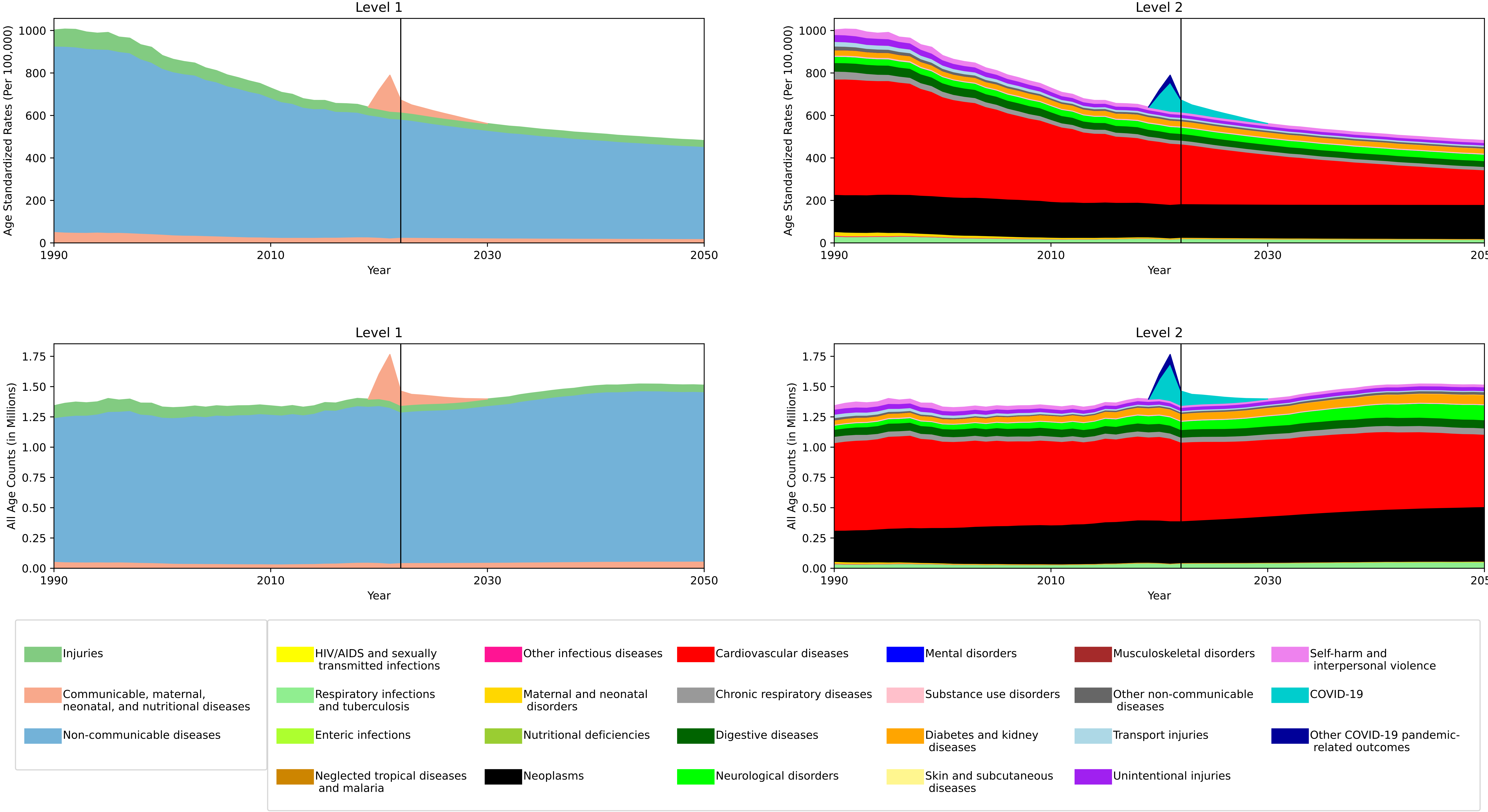

Eastern Europe

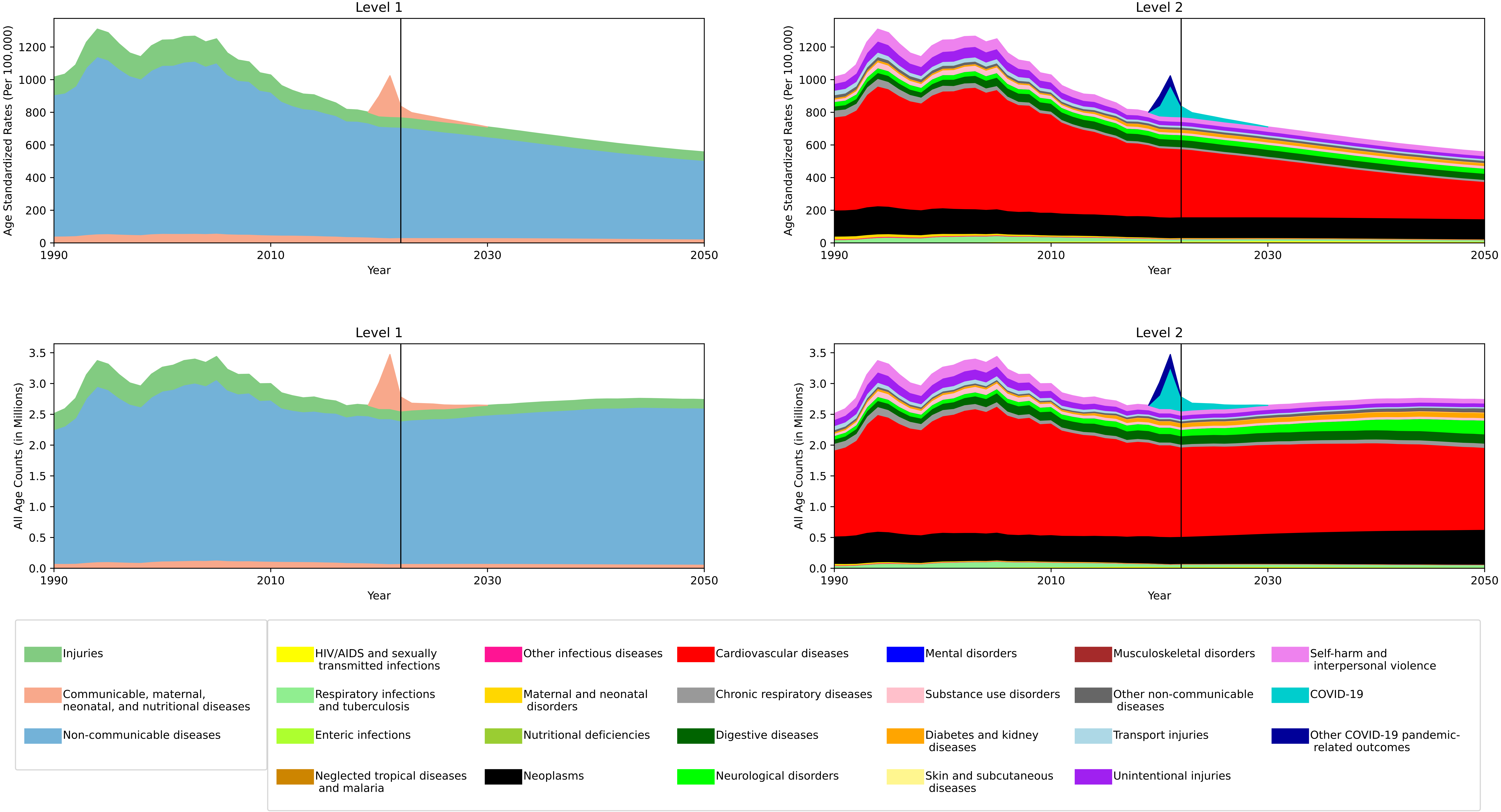

Australasia

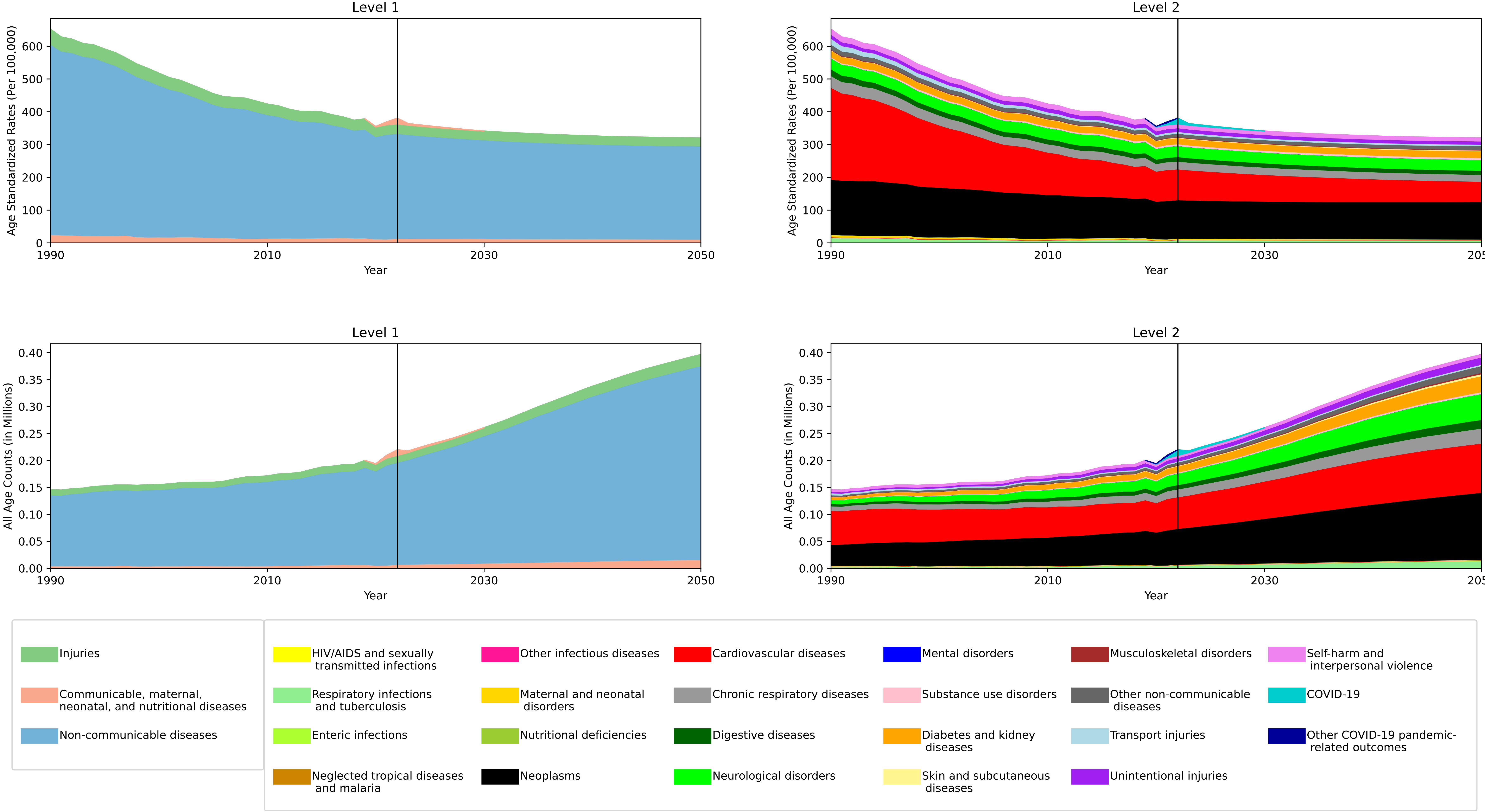

Supplemental Results Figure S5. Age-standardised death rates and all age death counts and by Level 1 and Level 2 causes, 1990–2050

High-Income Asia Pacific

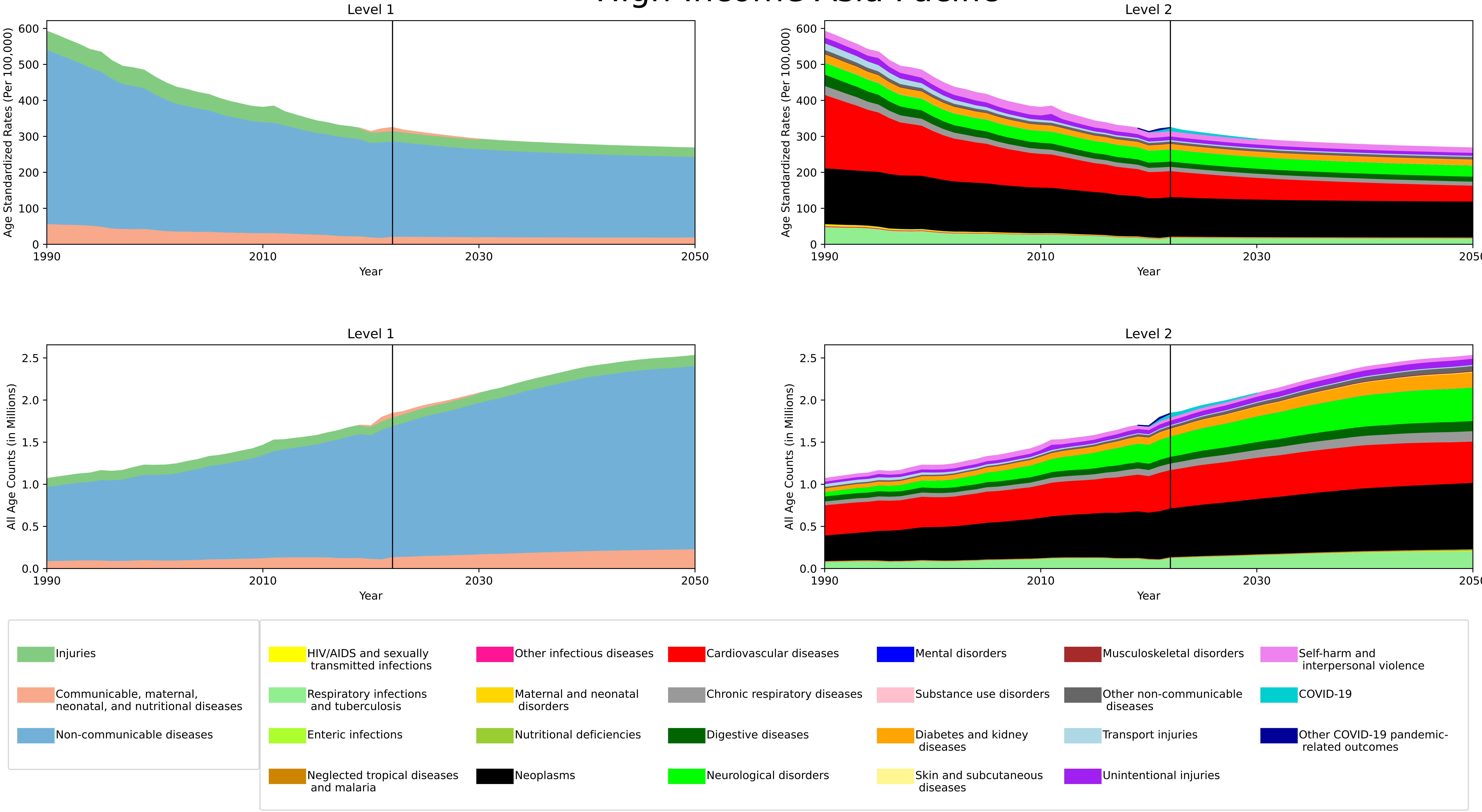

Supplemental Results Figure S5. Age-standardised death rates and all age death counts and by Level 1 and Level 2 causes, 1990–2050

High-Income North America

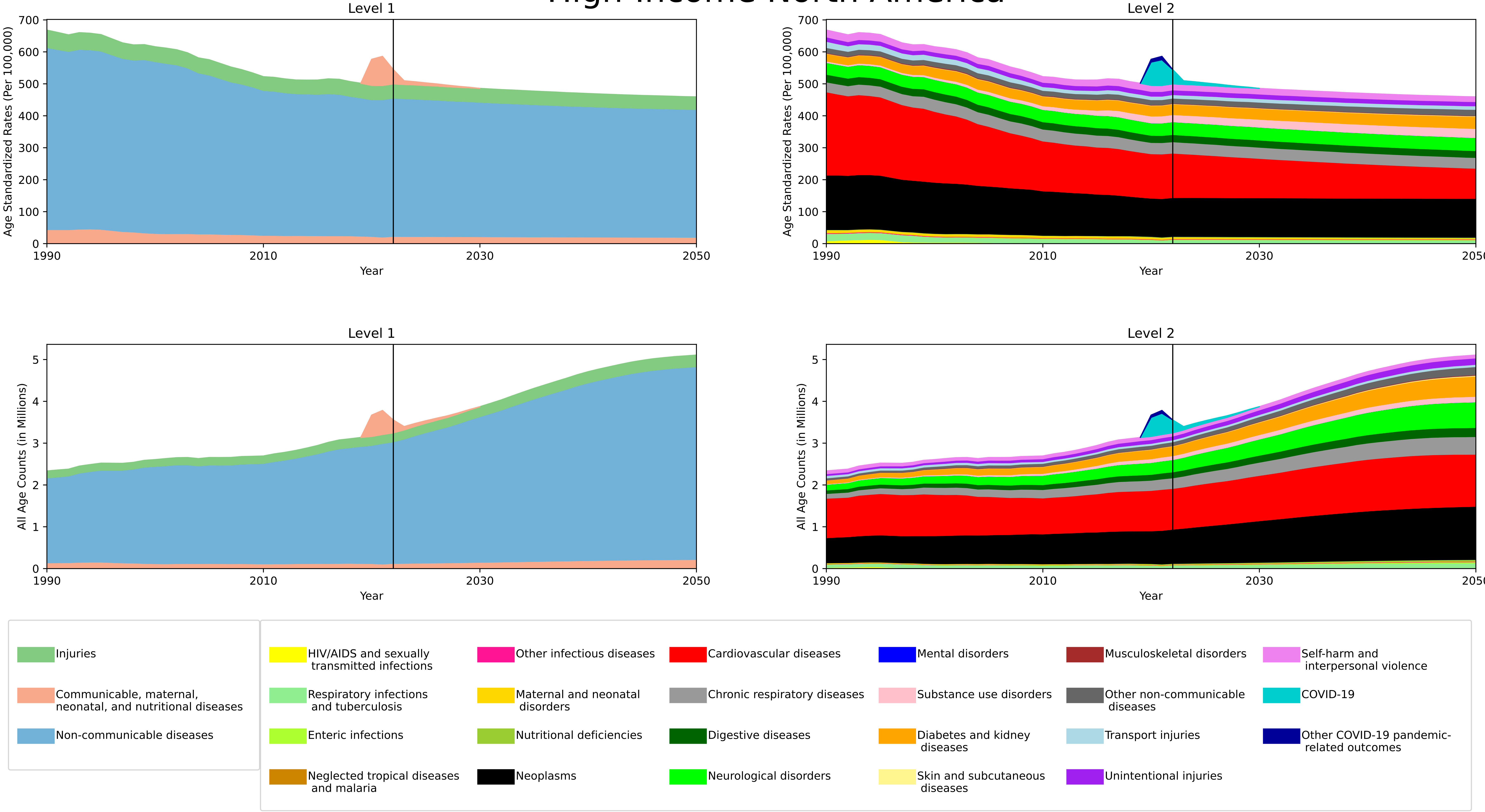

Supplemental Results Figure S5. Age-standardised death rates and all age death counts and by Level 1 and Level 2 causes, 1990–2050

Southern Latin America

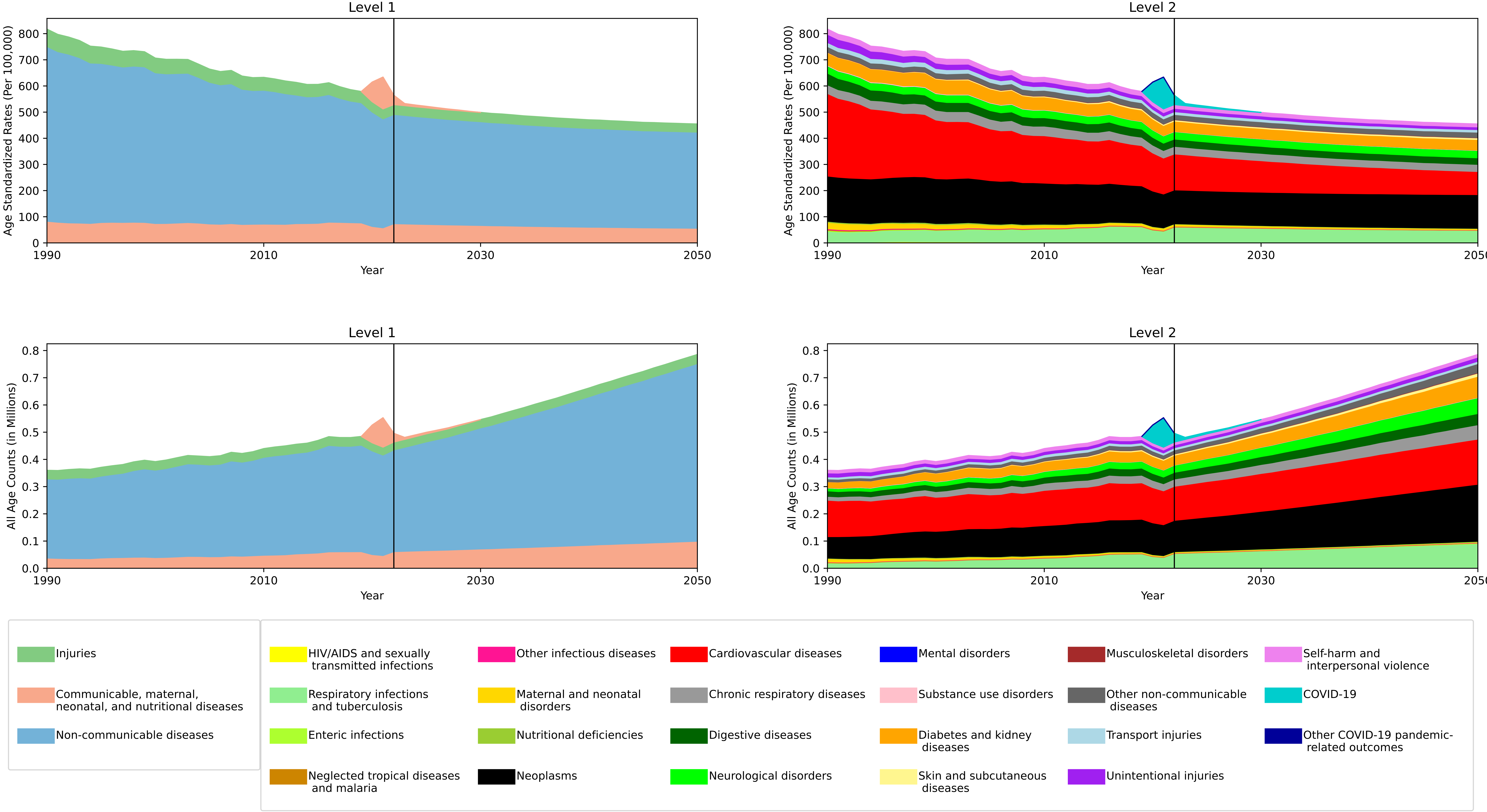

Western Europe

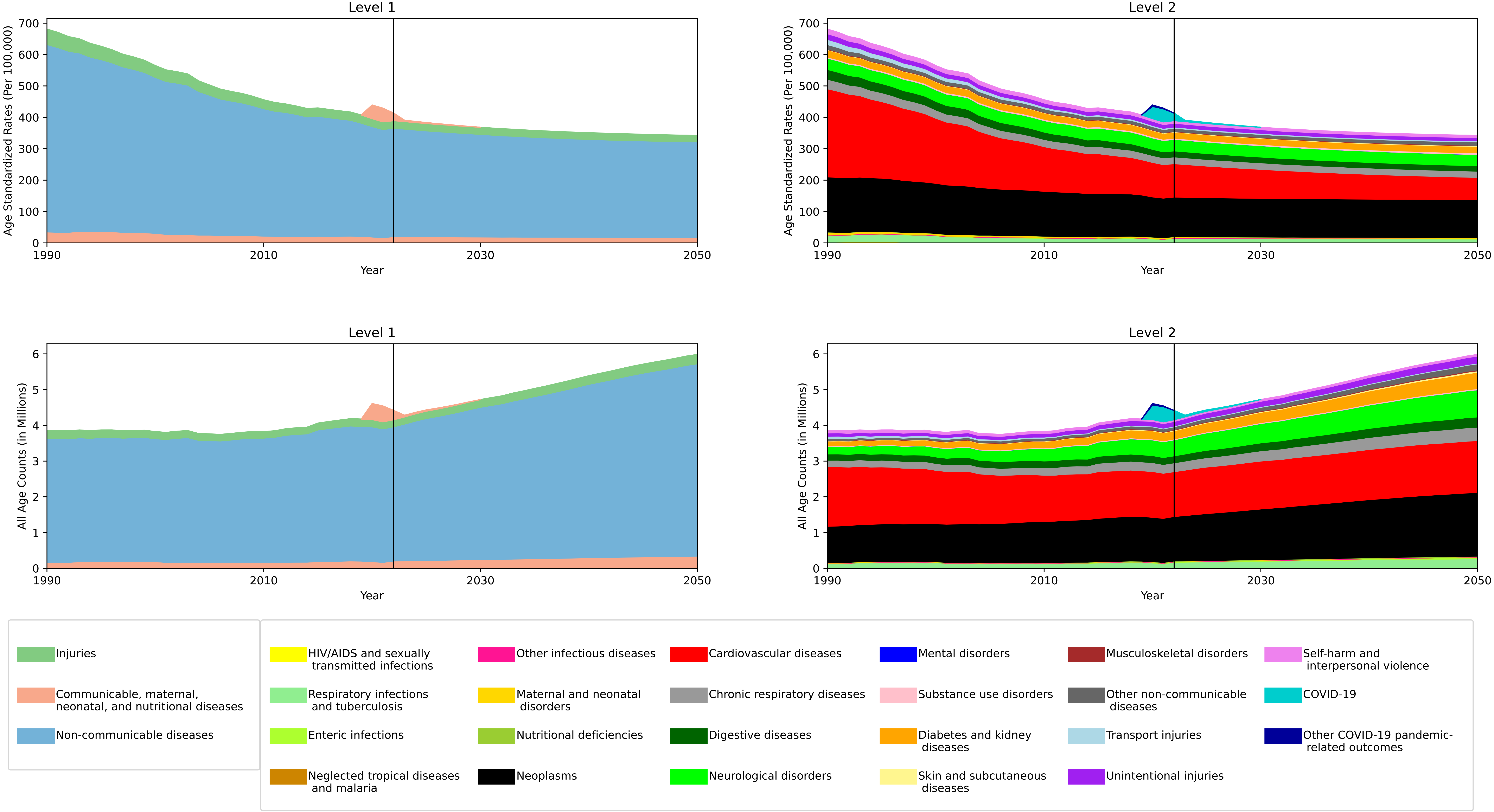

Andean Latin America

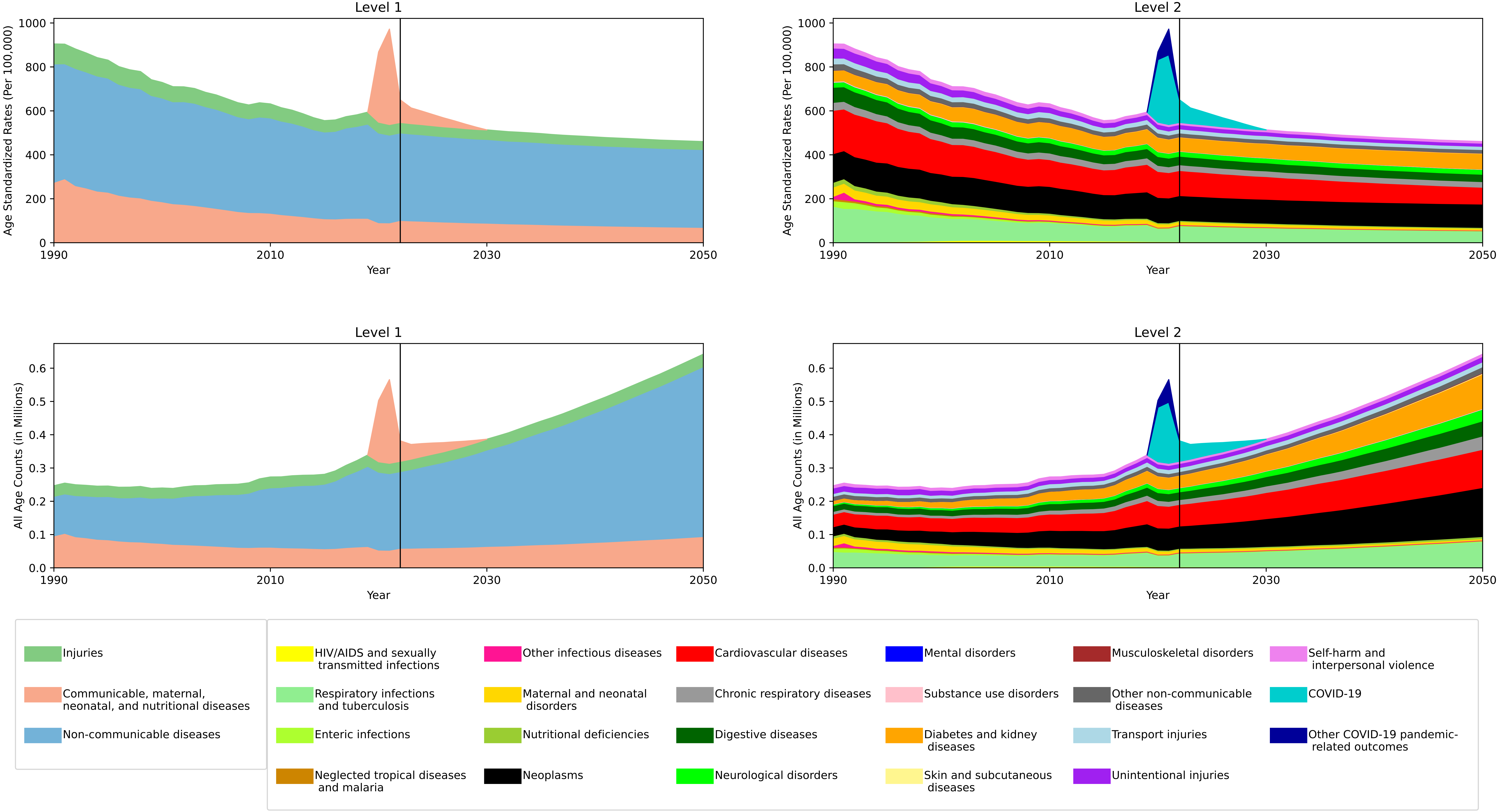

Caribbean

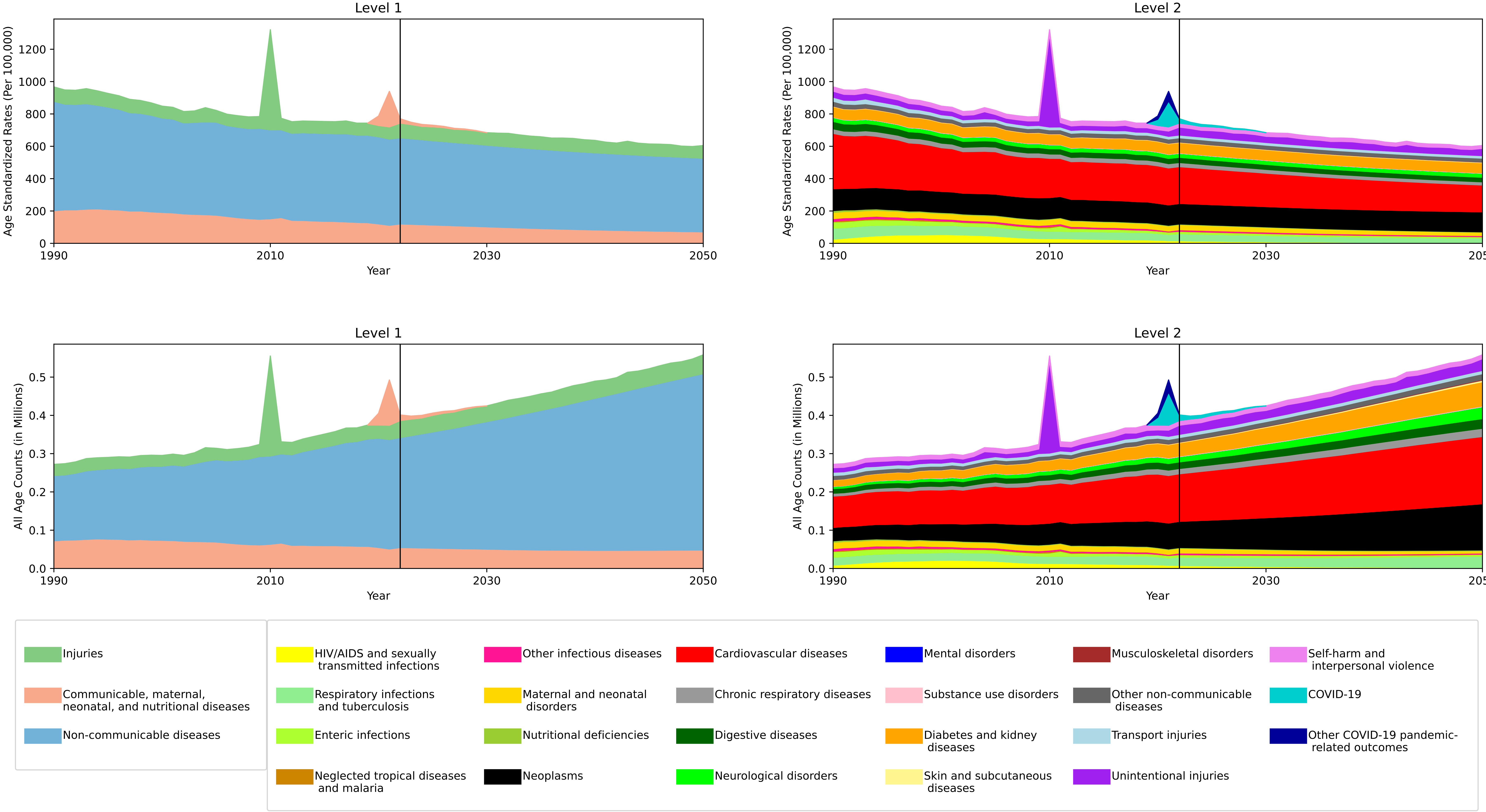
Level 1
Level 2
Age Standardized Rates (Per 100,000)
All Age Counts (in Millions)
Year
1990
2010
2030
2050
Injuries
Communicable, maternal, neonatal, and nutritional diseases
Non-communicable diseases
HIV/AIDS and sexually transmitted infections
Respiratory infections and tuberculosis
Enteric infections
Neglected tropical diseases and malaria
Other infectious diseases
Maternal and neonatal disorders
Nutritional deficiencies
Neoplasms
Cardiovascular diseases
Chronic respiratory diseases
Digestive diseases
Neurological disorders
Mental disorders
Substance use disorders
Diabetes and kidney diseases
Skin and subcutaneous diseases
Musculoskeletal disorders
Other non-communicable diseases
Transport injuries
Unintentional injuries
Self-harm and interpersonal violence
COVID-19
Other COVID-19 pandemic-related outcomes

Central Latin America

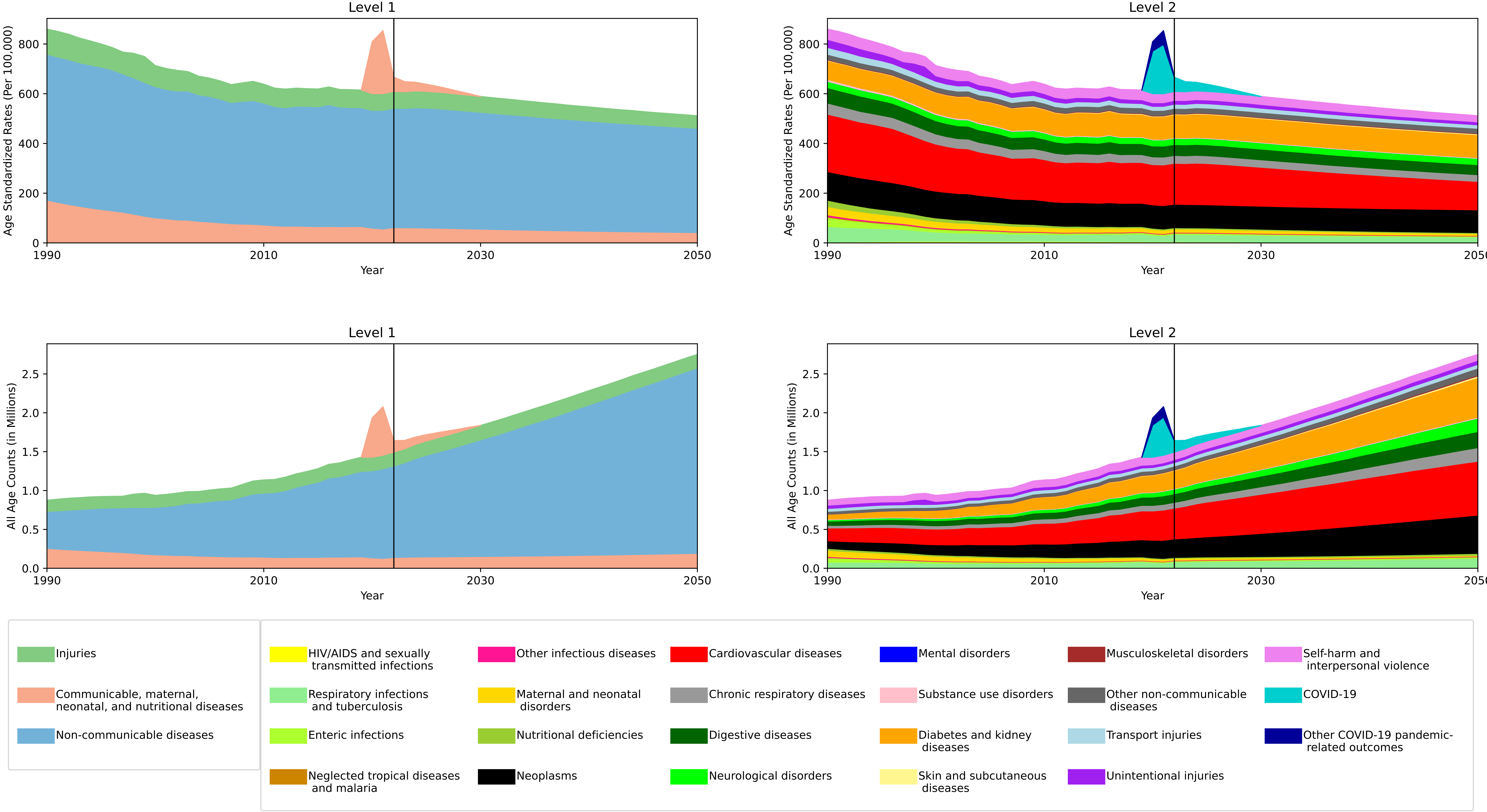

Tropical Latin America

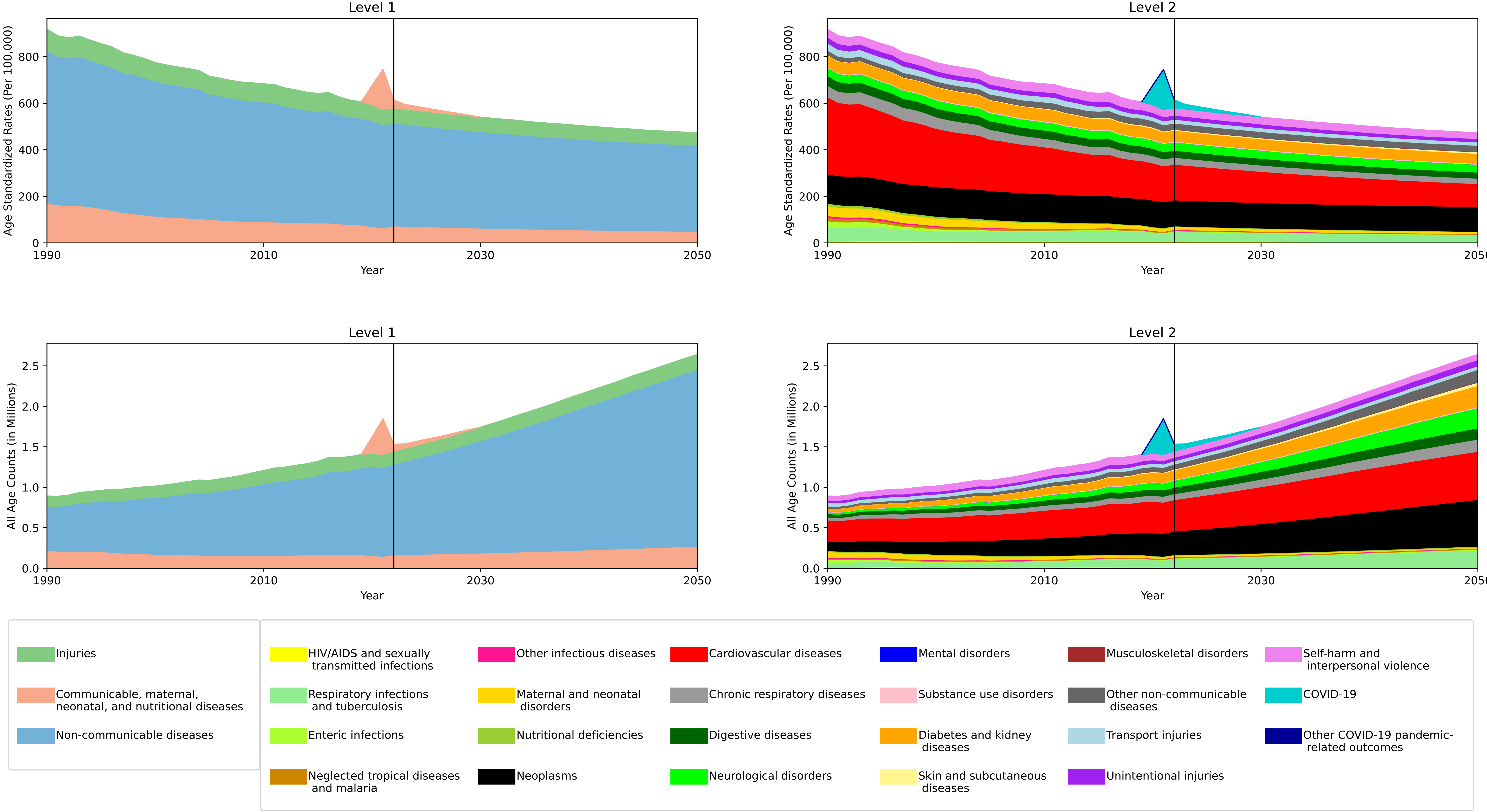

Supplemental Results Figure S5. Age-standardised death rates and all age death counts and by Level 1 and Level 2 causes, 1990–2050

North Africa And Middle East

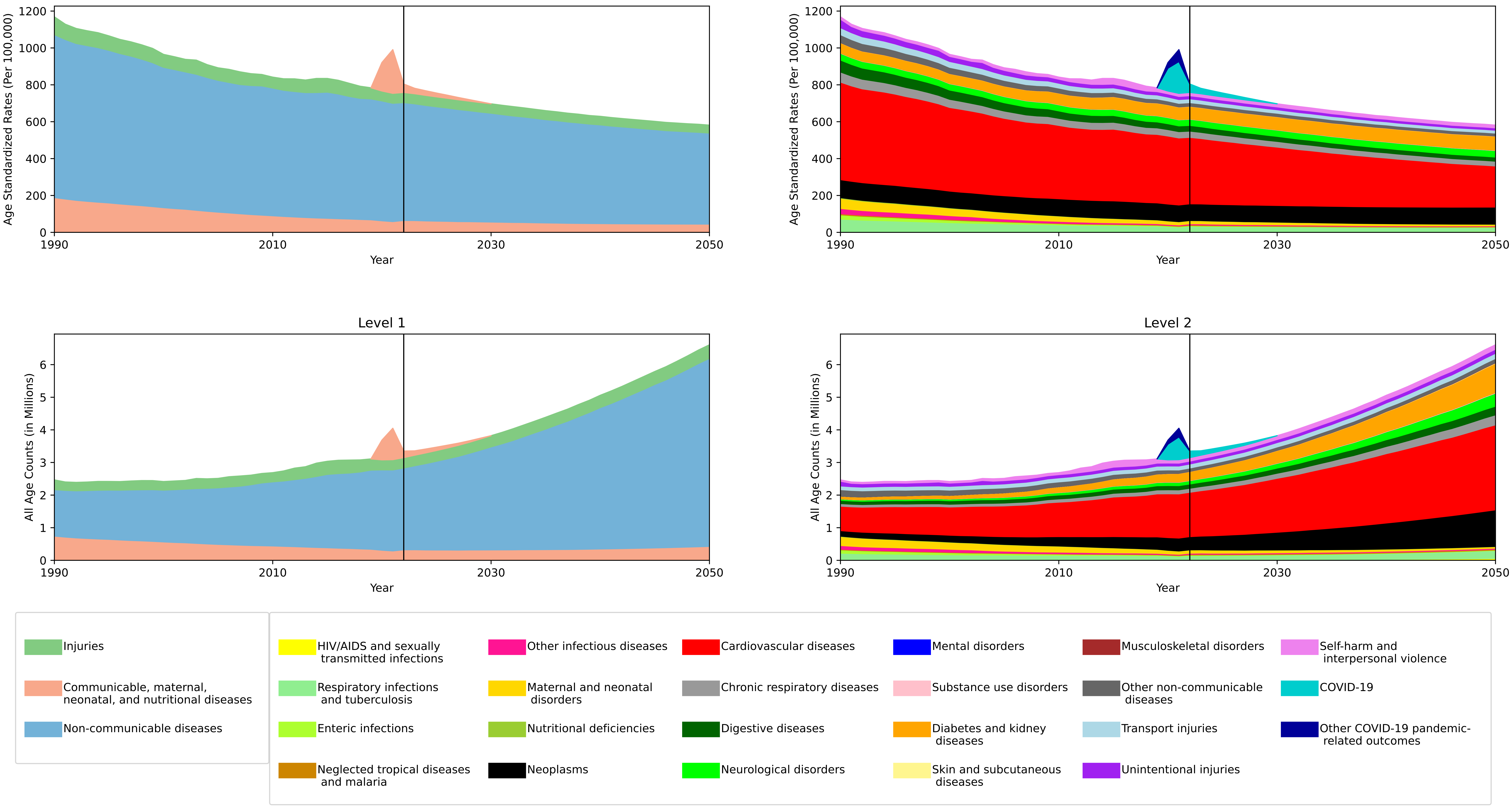

South Asia

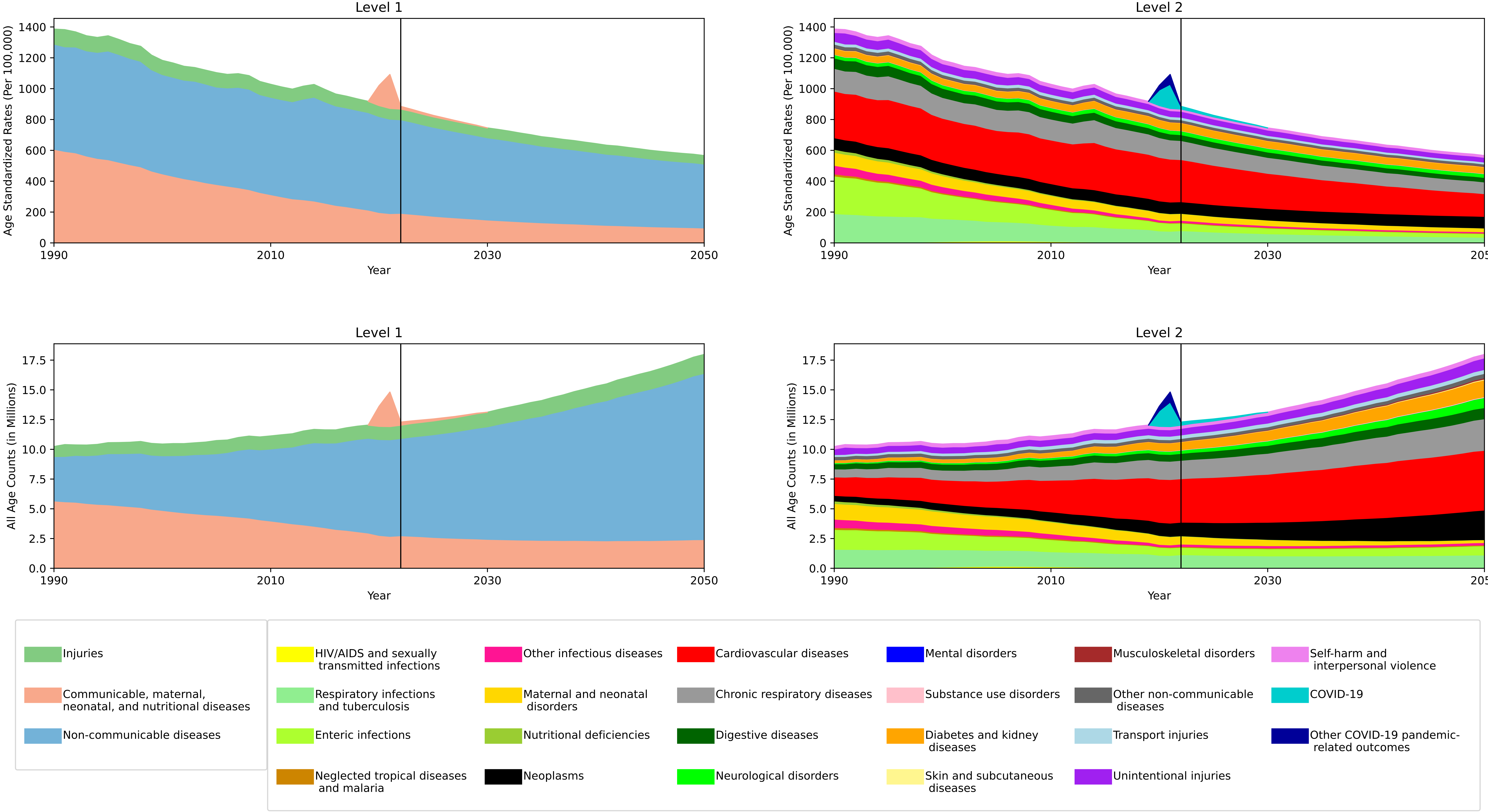
Level 1
Level 2
Age Standardized Rates (Per 100,000)
All Age Counts (in Millions)
Year
1990
2010
2030
2050
Injuries
Communicable, maternal, neonatal, and nutritional diseases
Non-communicable diseases
HIV/AIDS and sexually transmitted infections
Respiratory infections and tuberculosis
Enteric infections
Neglected tropical diseases and malaria
Other infectious diseases
Maternal and neonatal disorders
Nutritional deficiencies
Neoplasms
Cardiovascular diseases
Chronic respiratory diseases
Digestive diseases
Neurological disorders
Mental disorders
Substance use disorders
Diabetes and kidney diseases
Skin and subcutaneous diseases
Musculoskeletal disorders
Other non-communicable diseases
Transport injuries
Unintentional injuries
Self-harm and interpersonal violence
COVID-19
Other COVID-19 pandemic-related outcomes

East Asia

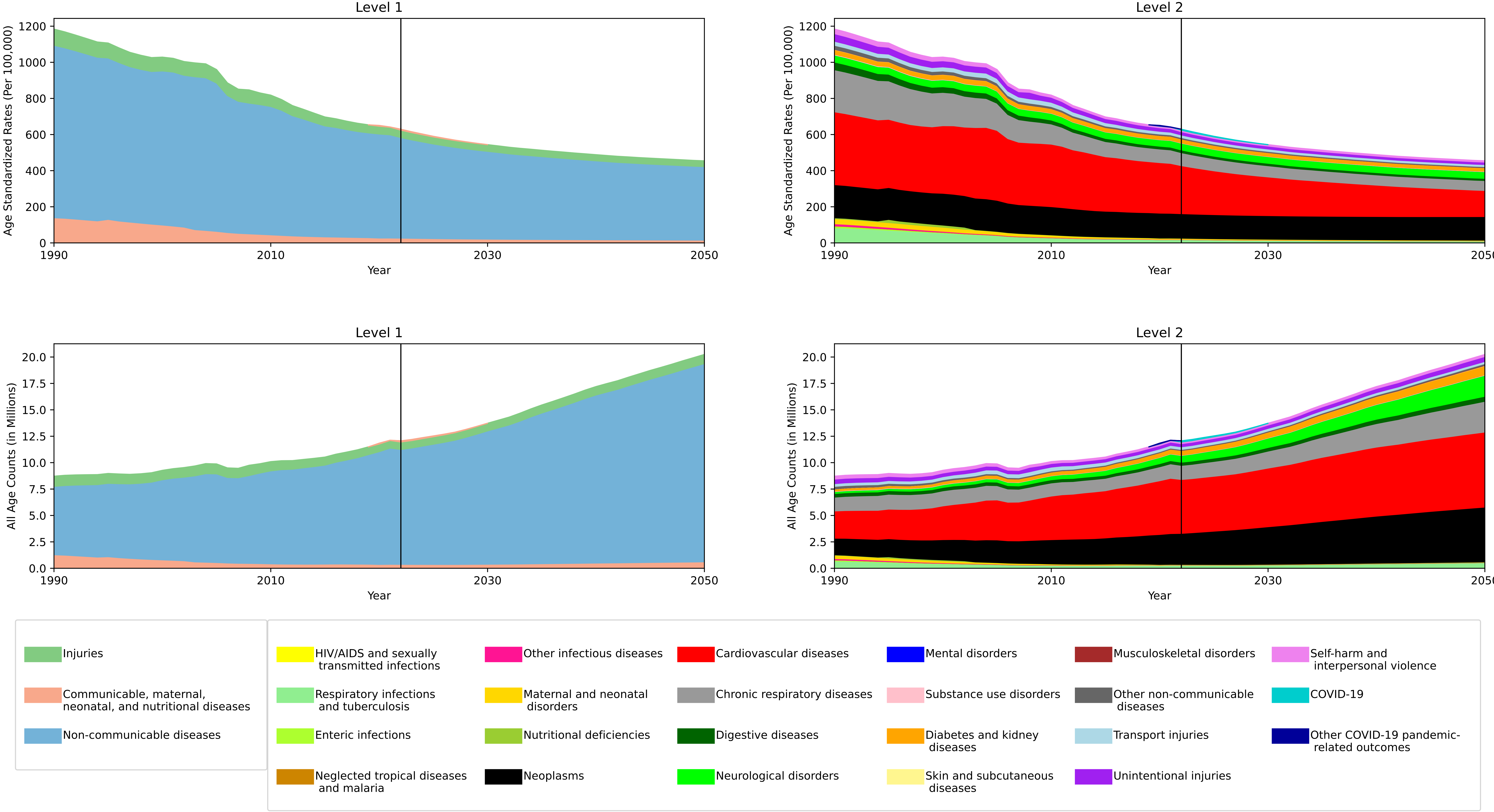
Level 1
Level 2
Age Standardized Rates (Per 100,000)
All Age Counts (in Millions)
Year
1990
2010
2030
2050
Injuries
Communicable, maternal, neonatal, and nutritional diseases
Non-communicable diseases
HIV/AIDS and sexually transmitted infections
Respiratory infections and tuberculosis
Enteric infections
Neglected tropical diseases and malaria
Other infectious diseases
Maternal and neonatal disorders
Nutritional deficiencies
Neoplasms
Cardiovascular diseases
Chronic respiratory diseases
Digestive diseases
Neurological disorders
Mental disorders
Substance use disorders
Diabetes and kidney diseases
Skin and subcutaneous diseases
Musculoskeletal disorders
Other non-communicable diseases
Transport injuries
Unintentional injuries
Self-harm and interpersonal violence
COVID-19
Other COVID-19 pandemic-related outcomes

Oceania

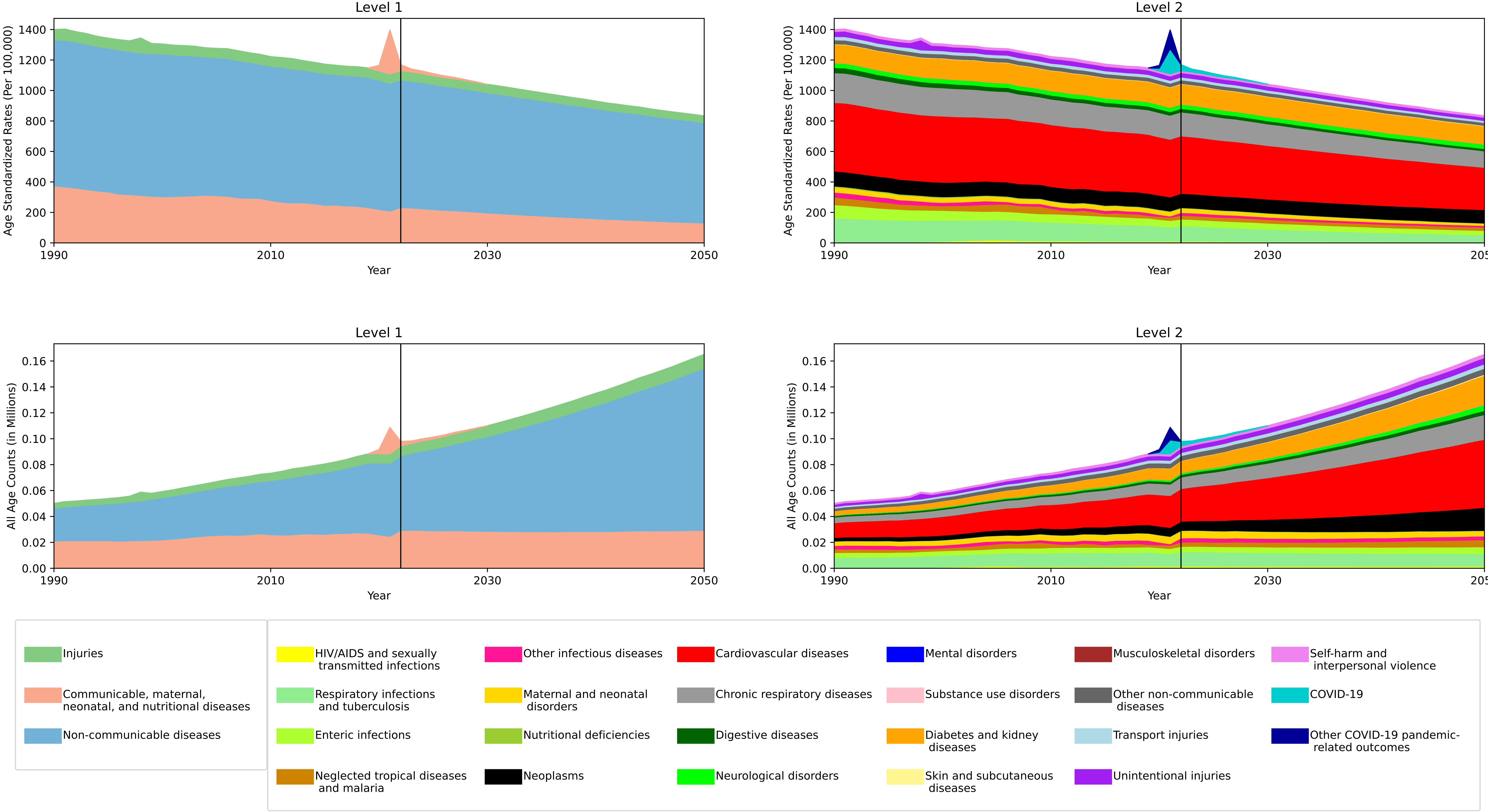
Level 1
Level 2
Age Standardized Rates (Per 100,000)
All Age Counts (in Millions)
Year
1990
2010
2030
2050
0
200
400
600
800
1000
1200
1400
0.00
0.02
0.04
0.06
0.08
0.10
0.12
0.14
0.16
Injuries
Communicable, maternal, neonatal, and nutritional diseases
Non-communicable diseases
HIV/AIDS and sexually transmitted infections
Respiratory infections and tuberculosis
Enteric infections
Neglected tropical diseases and malaria
Other infectious diseases
Maternal and neonatal disorders
Nutritional deficiencies
Neoplasms
Cardiovascular diseases
Chronic respiratory diseases
Digestive diseases
Neurological disorders
Mental disorders
Substance use disorders
Diabetes and kidney diseases
Skin and subcutaneous diseases
Musculoskeletal disorders
Other non-communicable diseases
Transport injuries
Unintentional injuries
Self-harm and interpersonal violence
COVID-19
Other COVID-19 pandemic-related outcomes

Southeast Asia

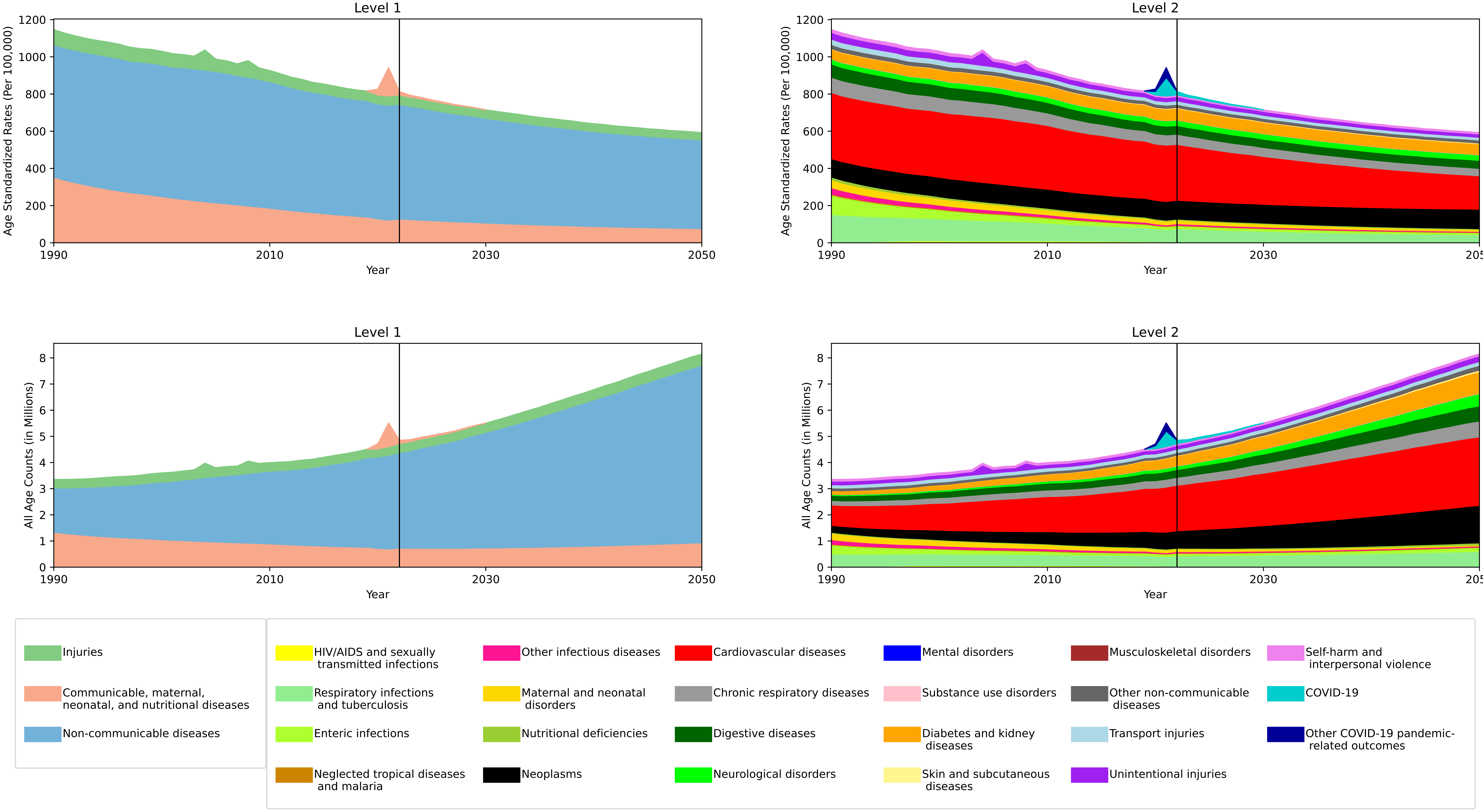

Supplemental Results Figure S5. Age-standardised death rates and all age death counts and by Level 1 and Level 2 causes, 1990–2050

Central Sub-Saharan Africa

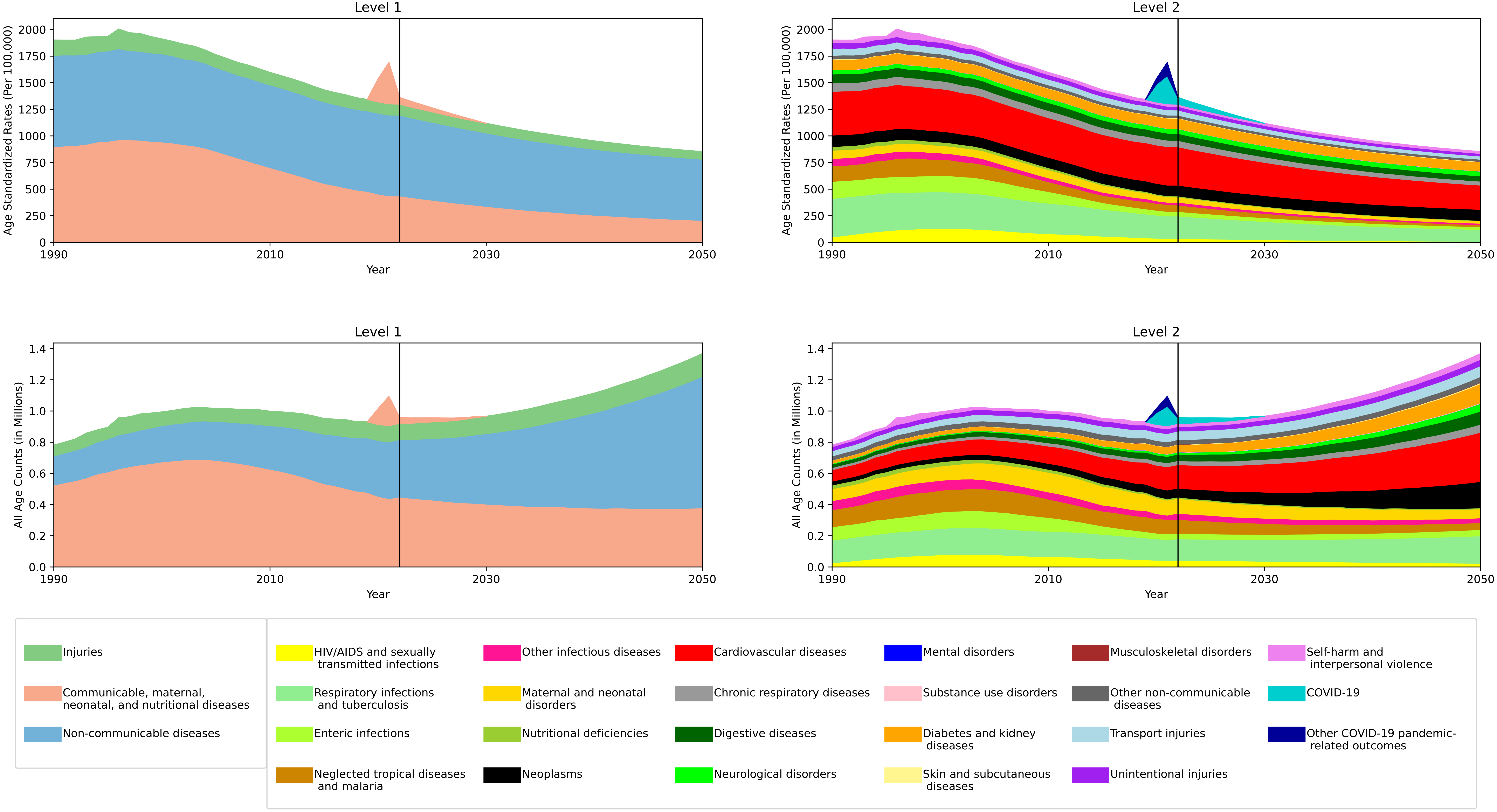

Supplemental Results Figure S5. Age-standardised death rates and all age death counts and by Level 1 and Level 2 causes, 1990–2050

Eastern Sub-Saharan Africa

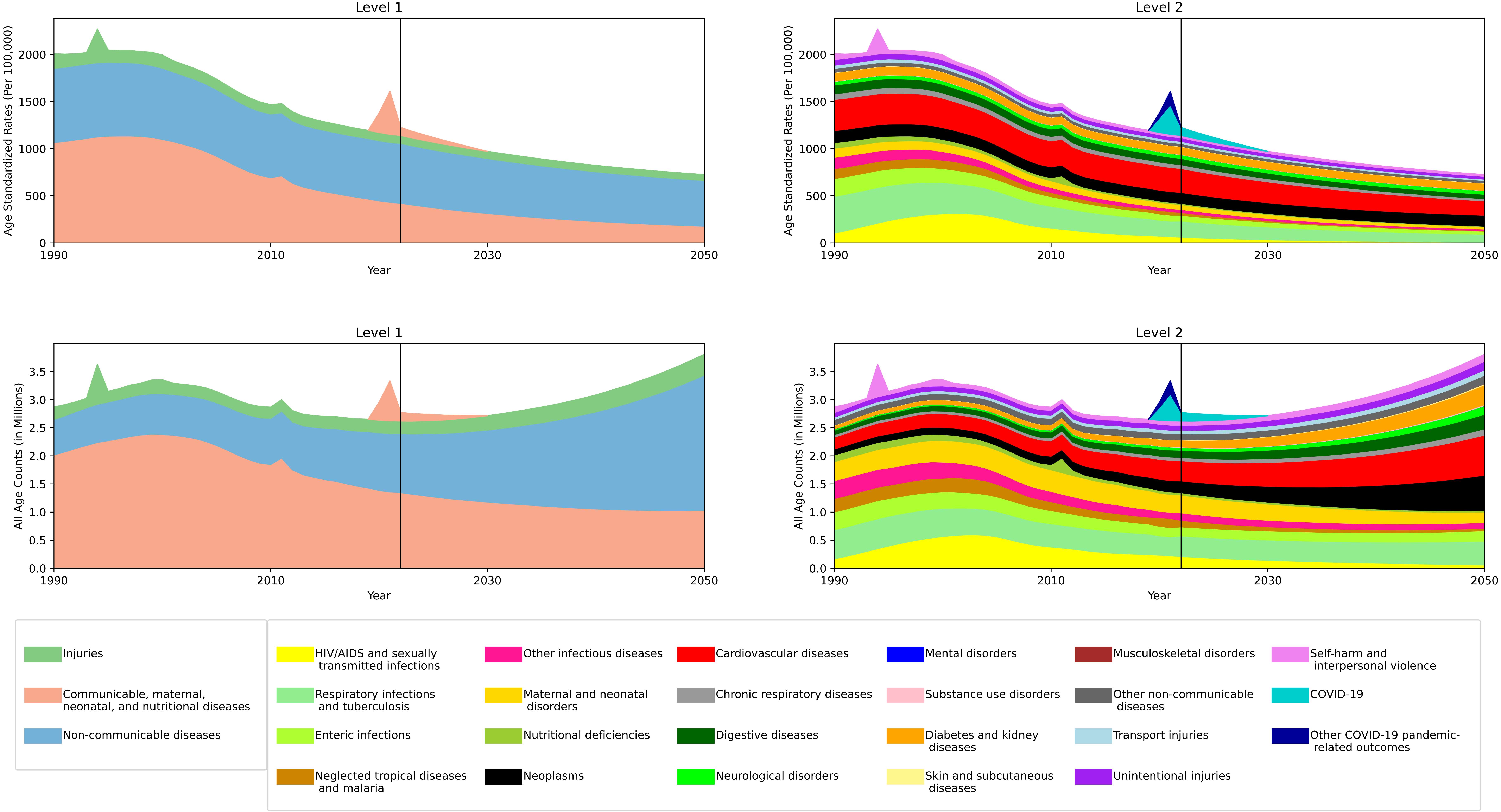

Supplemental Results Figure S5. Age-standardised death rates and all age death counts and by Level 1 and Level 2 causes, 1990–2050

Southern Sub-Saharan Africa

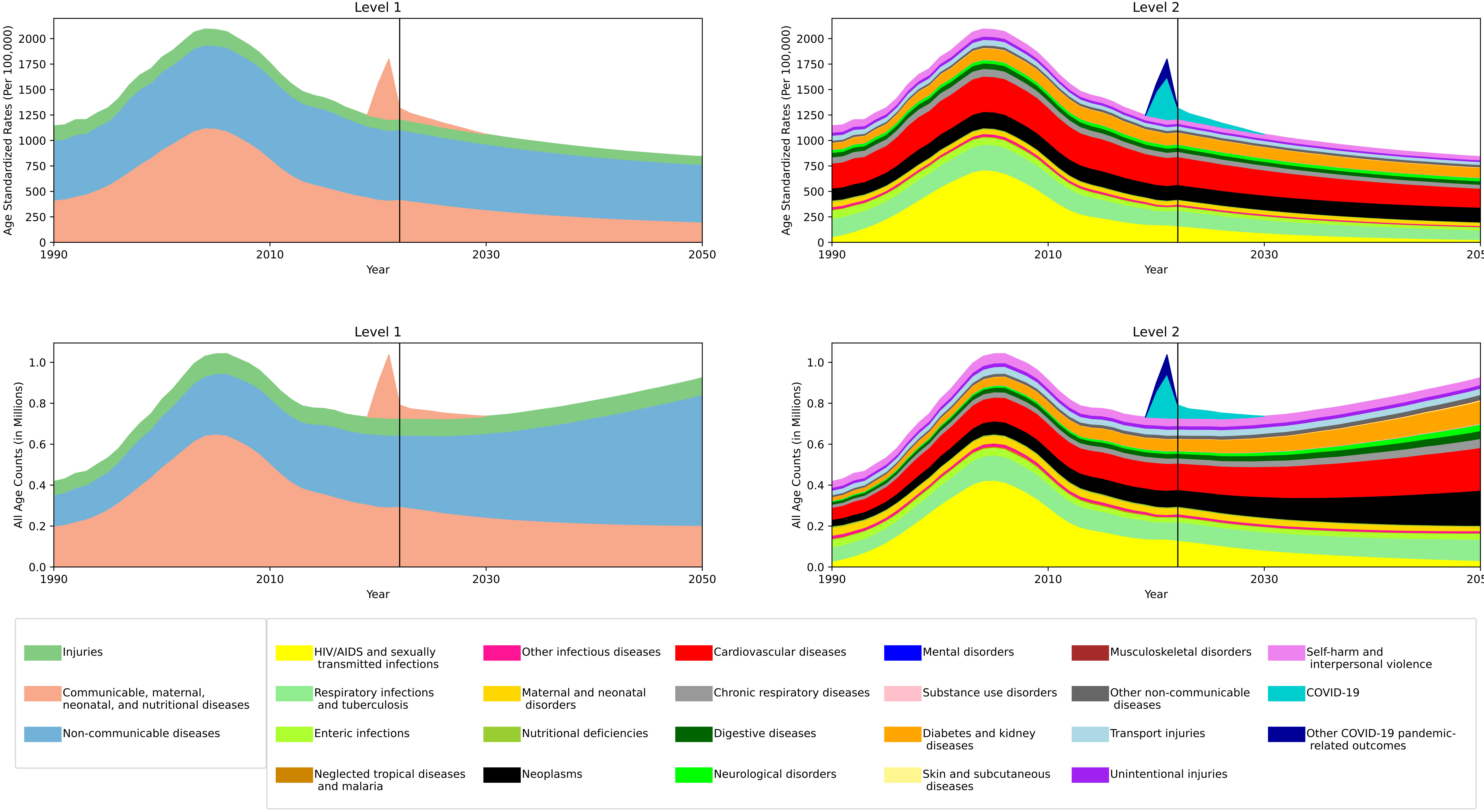

Supplemental Results Figure S5. Age-standardised death rates and all age death counts and by Level 1 and Level 2 causes, 1990–2050

Western Sub-Saharan Africa

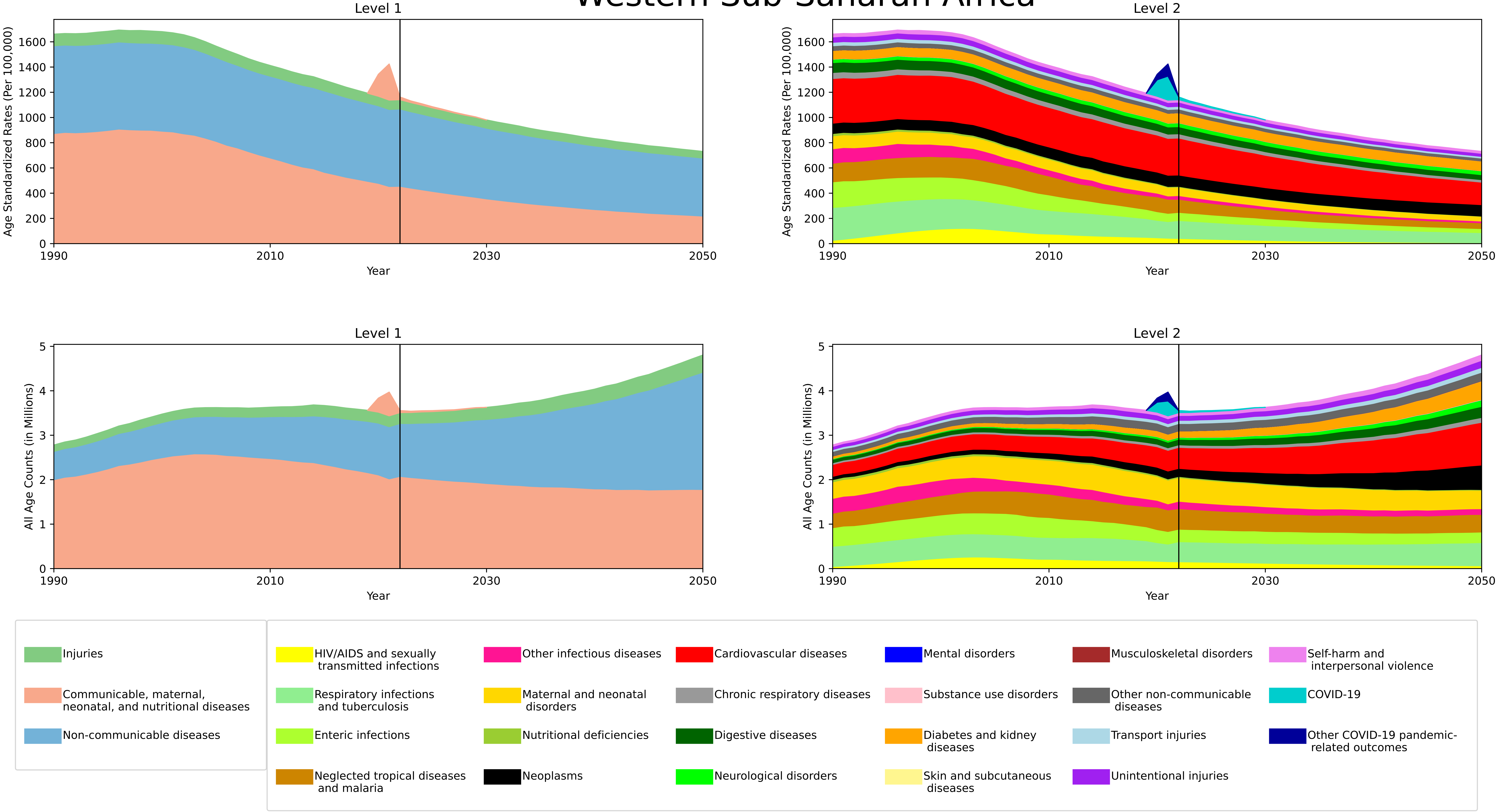

Armenia

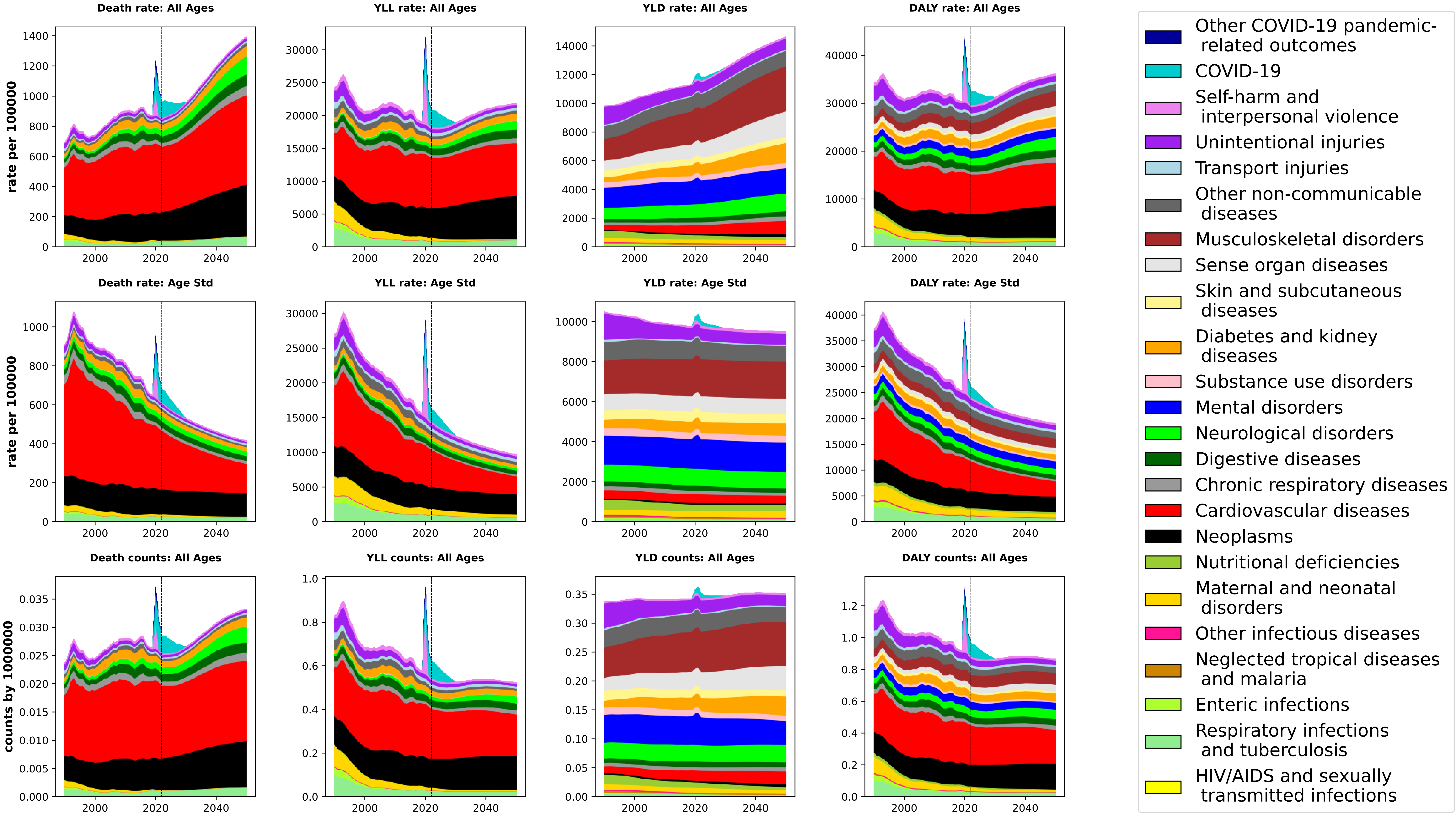
Death rate: All Ages
YLL rate: All Ages
YLD rate: All Ages
DALY rate: All Ages
Death rate: Age Std
YLL rate: Age Std
YLD rate: Age Std
DALY rate: Age Std
Death counts: All Ages
YLL counts: All Ages
YLD counts: All Ages
DALY counts: All Ages
rate per 100000
counts by 1000000
2000
2020
2040
Other COVID-19 pandemic-related outcomes
COVID-19
Self-harm and interpersonal violence
Unintentional injuries
Transport injuries
Other non-communicable diseases
Musculoskeletal disorders
Sense organ diseases
Skin and subcutaneous diseases
Diabetes and kidney diseases
Substance use disorders
Mental disorders
Neurological disorders
Digestive diseases
Chronic respiratory diseases
Cardiovascular diseases
Neoplasms
Nutritional deficiencies
Maternal and neonatal disorders
Other infectious diseases
Neglected tropical diseases and malaria
Enteric infections
Respiratory infections and tuberculosis
HIV/AIDS and sexually transmitted infections

Azerbaijan

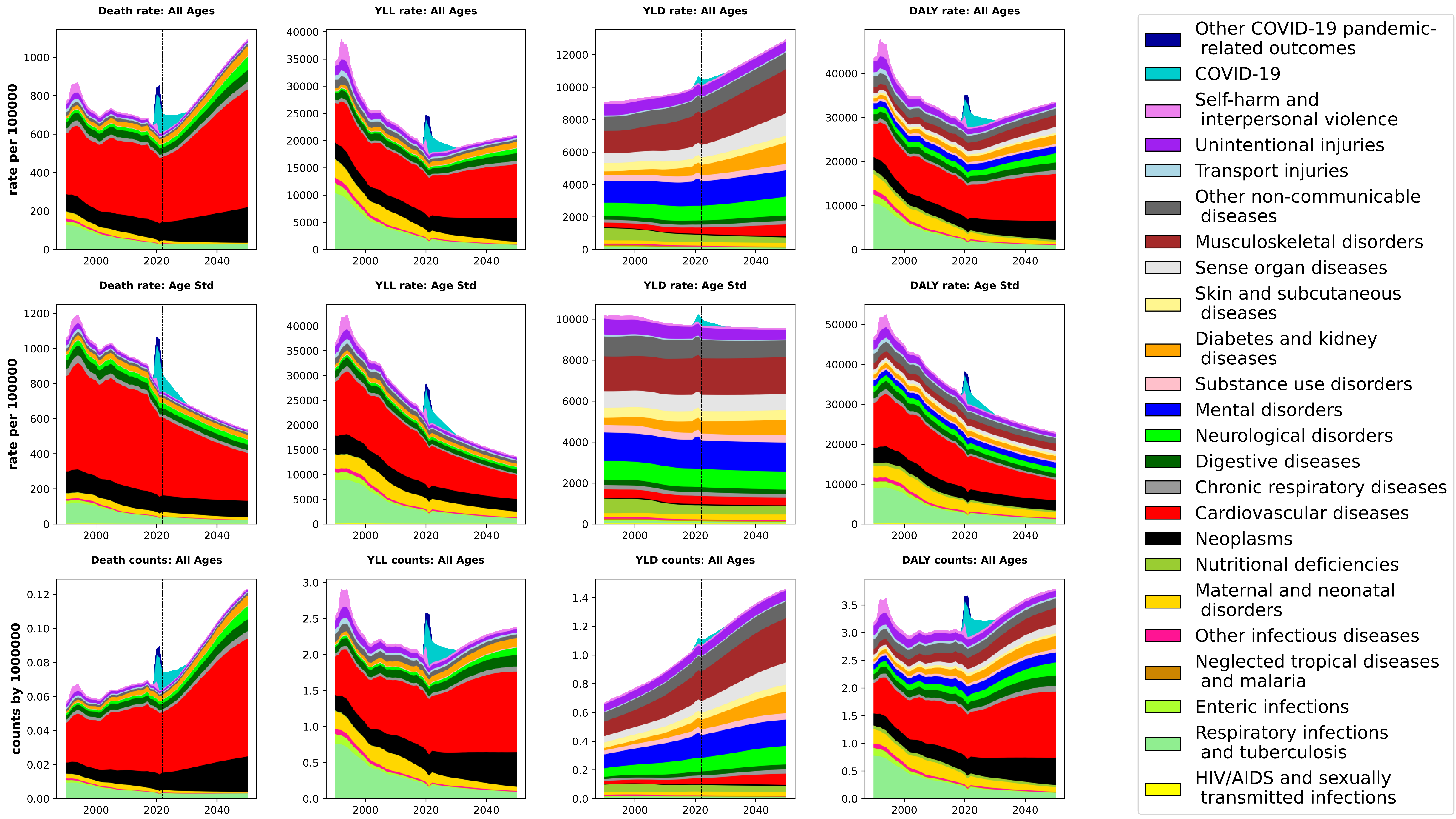
Death rate: All Ages
YLL rate: All Ages
YLD rate: All Ages
DALY rate: All Ages
Death rate: Age Std
YLL rate: Age Std
YLD rate: Age Std
DALY rate: Age Std
Death counts: All Ages
YLL counts: All Ages
YLD counts: All Ages
DALY counts: All Ages
rate per 100000
counts by 1000000
Other COVID-19 pandemic-related outcomes
COVID-19
Self-harm and interpersonal violence
Unintentional injuries
Transport injuries
Other non-communicable diseases
Musculoskeletal disorders
Sense organ diseases
Skin and subcutaneous diseases
Diabetes and kidney diseases
Substance use disorders
Mental disorders
Neurological disorders
Digestive diseases
Chronic respiratory diseases
Cardiovascular diseases
Neoplasms
Nutritional deficiencies
Maternal and neonatal disorders
Other infectious diseases
Neglected tropical diseases and malaria
Enteric infections
Respiratory infections and tuberculosis
HIV/AIDS and sexually transmitted infections

Georgia

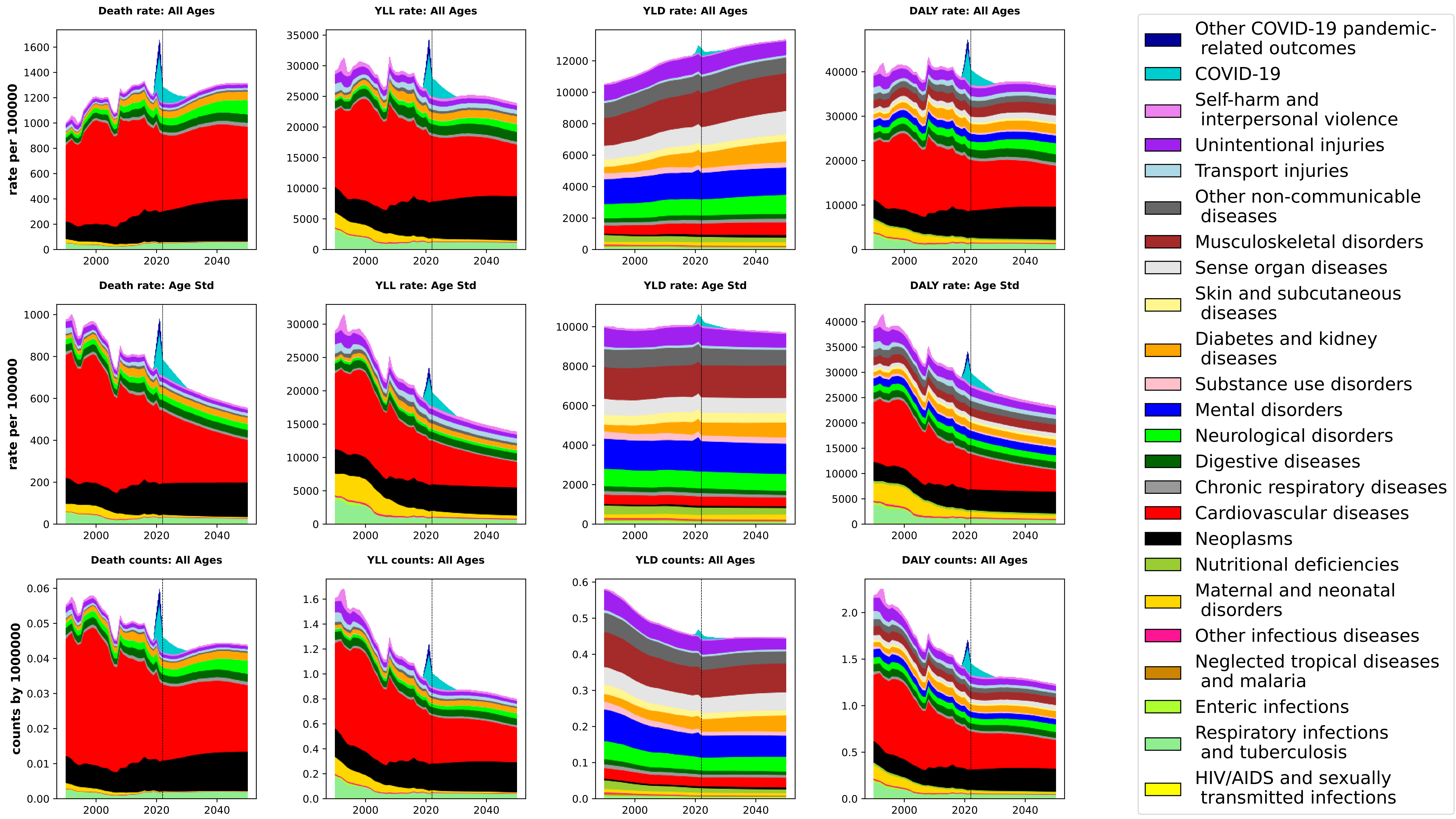
Death rate: All Ages
YLL rate: All Ages
YLD rate: All Ages
DALY rate: All Ages
Death rate: Age Std
YLL rate: Age Std
YLD rate: Age Std
DALY rate: Age Std
Death counts: All Ages
YLL counts: All Ages
YLD counts: All Ages
DALY counts: All Ages
rate per 100000
rate per 100000
counts by 1000000
Other COVID-19 pandemic-related outcomes
COVID-19
Self-harm and interpersonal violence
Unintentional injuries
Transport injuries
Other non-communicable diseases
Musculoskeletal disorders
Sense organ diseases
Skin and subcutaneous diseases
Diabetes and kidney diseases
Substance use disorders
Mental disorders
Neurological disorders
Digestive diseases
Chronic respiratory diseases
Cardiovascular diseases
Neoplasms
Nutritional deficiencies
Maternal and neonatal disorders
Other infectious diseases
Neglected tropical diseases and malaria
Enteric infections
Respiratory infections and tuberculosis
HIV/AIDS and sexually transmitted infections

Kazakhstan

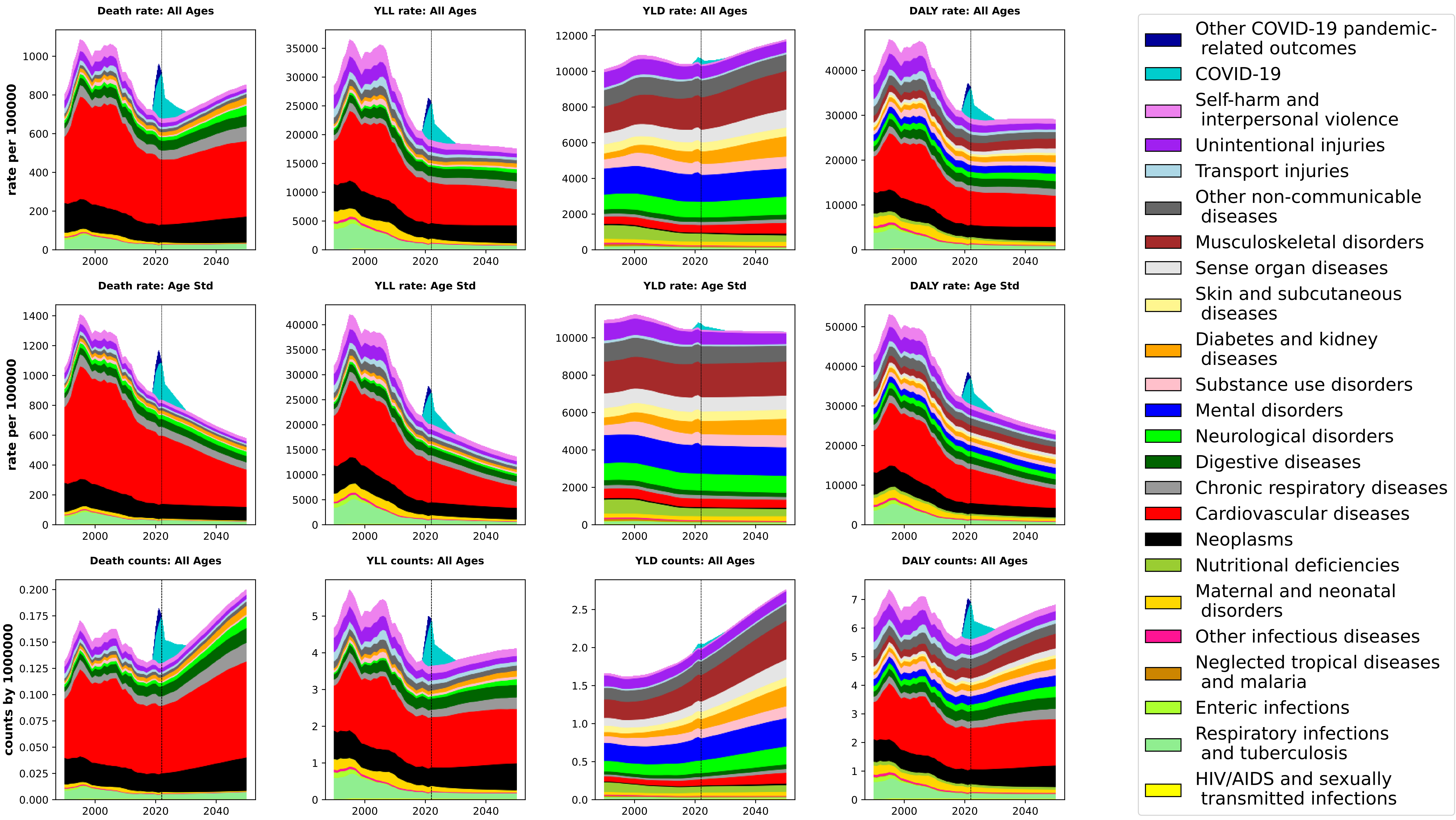
Death rate: All Ages
YLL rate: All Ages
YLD rate: All Ages
DALY rate: All Ages
Death rate: Age Std
YLL rate: Age Std
YLD rate: Age Std
DALY rate: Age Std
Death counts: All Ages
YLL counts: All Ages
YLD counts: All Ages
DALY counts: All Ages
rate per 100000
counts by 1000000
2000
2020
2040
Other COVID-19 pandemic-related outcomes
COVID-19
Self-harm and interpersonal violence
Unintentional injuries
Transport injuries
Other non-communicable diseases
Musculoskeletal disorders
Sense organ diseases
Skin and subcutaneous diseases
Diabetes and kidney diseases
Substance use disorders
Mental disorders
Neurological disorders
Digestive diseases
Chronic respiratory diseases
Cardiovascular diseases
Neoplasms
Nutritional deficiencies
Maternal and neonatal disorders
Other infectious diseases
Neglected tropical diseases and malaria
Enteric infections
Respiratory infections and tuberculosis
HIV/AIDS and sexually transmitted infections

Kyrgyzstan

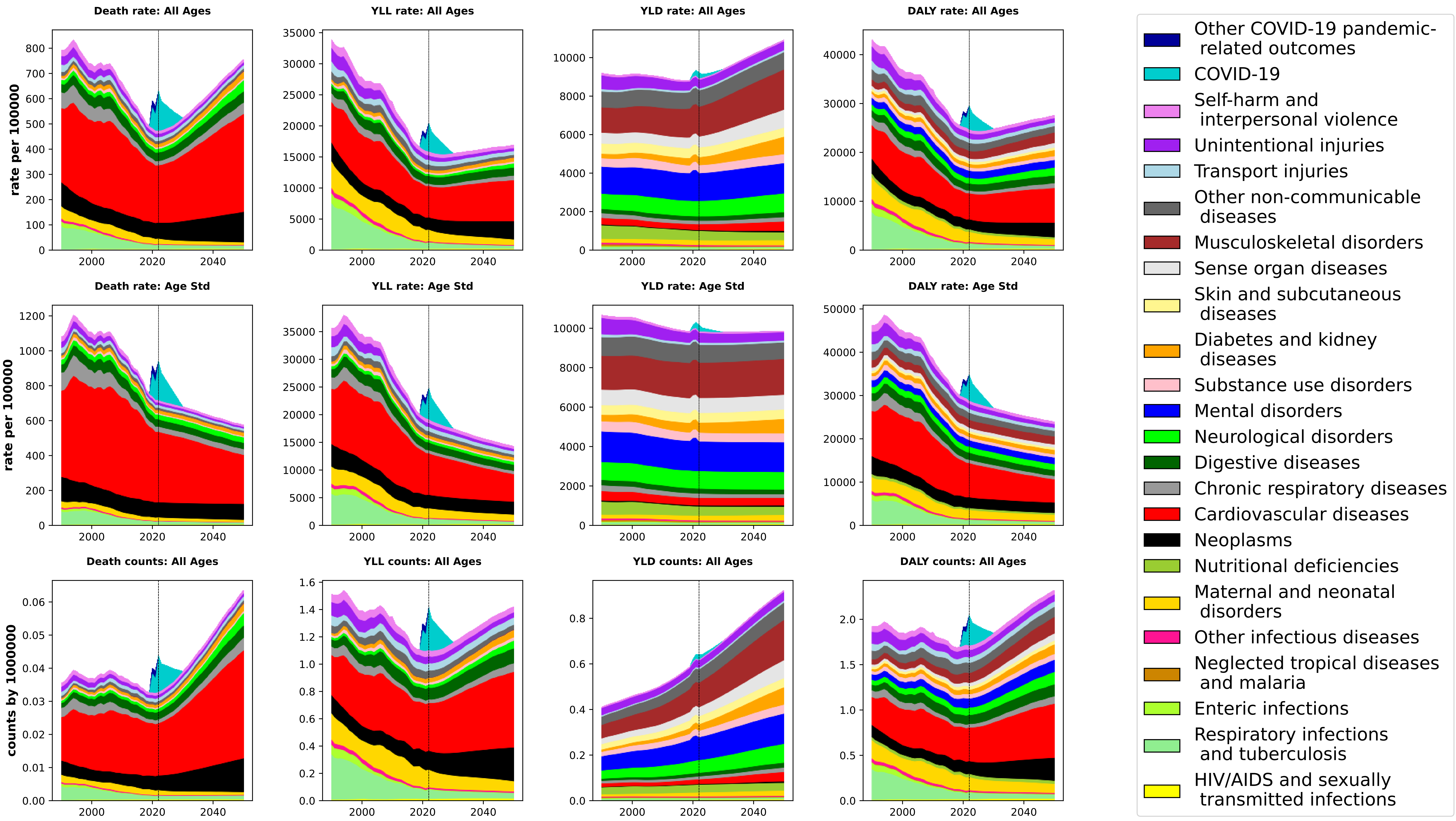
Death rate: All Ages
YLL rate: All Ages
YLD rate: All Ages
DALY rate: All Ages
Death rate: Age Std
YLL rate: Age Std
YLD rate: Age Std
DALY rate: Age Std
Death counts: All Ages
YLL counts: All Ages
YLD counts: All Ages
DALY counts: All Ages
rate per 100000
counts by 1000000
2000
2020
2040
Other COVID-19 pandemic-related outcomes
COVID-19
Self-harm and interpersonal violence
Unintentional injuries
Transport injuries
Other non-communicable diseases
Musculoskeletal disorders
Sense organ diseases
Skin and subcutaneous diseases
Diabetes and kidney diseases
Substance use disorders
Mental disorders
Neurological disorders
Digestive diseases
Chronic respiratory diseases
Cardiovascular diseases
Neoplasms
Nutritional deficiencies
Maternal and neonatal disorders
Other infectious diseases
Neglected tropical diseases and malaria
Enteric infections
Respiratory infections and tuberculosis
HIV/AIDS and sexually transmitted infections

Mongolia

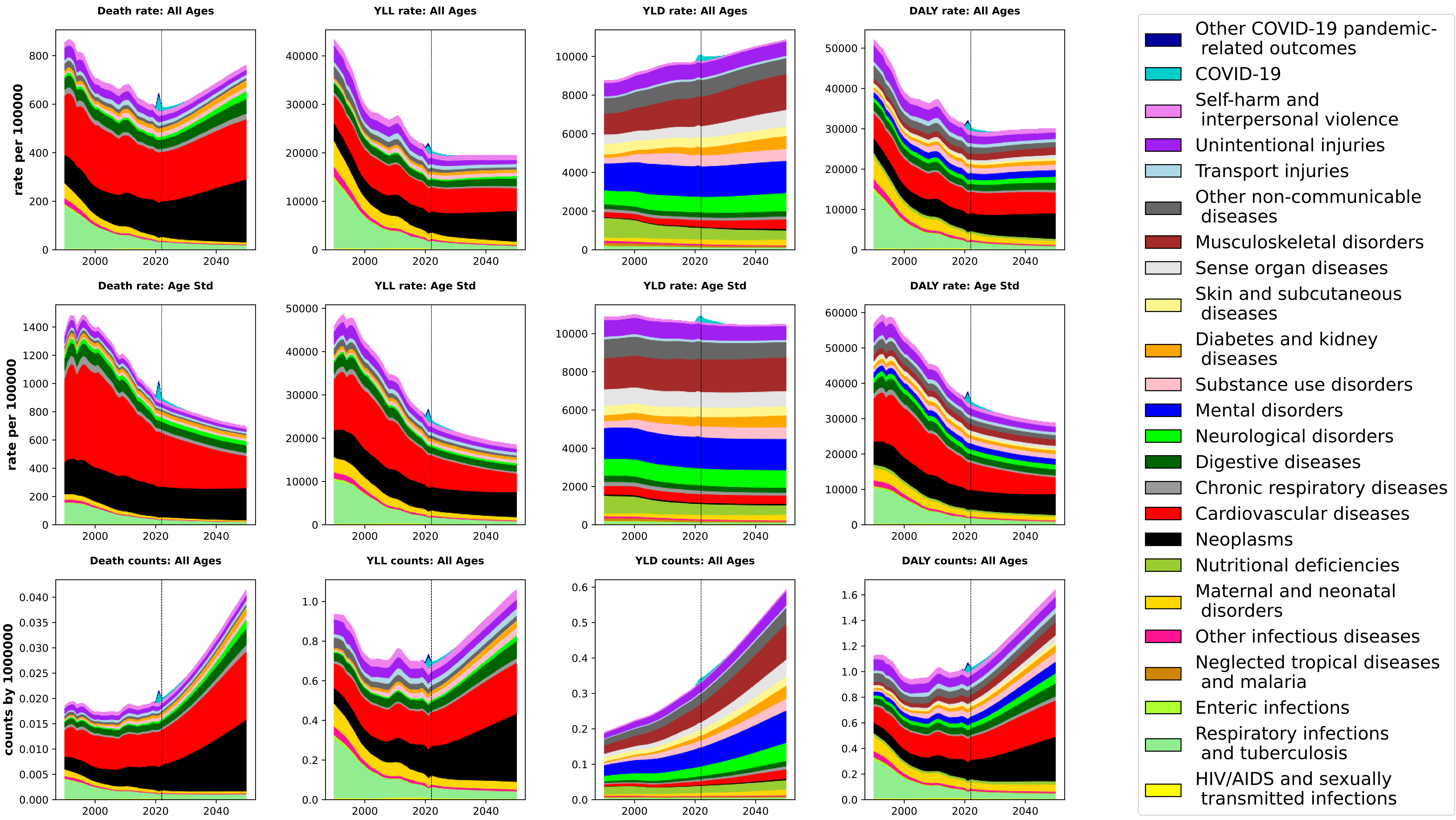
Death rate: All Ages
YLL rate: All Ages
YLD rate: All Ages
DALY rate: All Ages
Death rate: Age Std
YLL rate: Age Std
YLD rate: Age Std
DALY rate: Age Std
Death counts: All Ages
YLL counts: All Ages
YLD counts: All Ages
DALY counts: All Ages
rate per 100000
counts by 1000000
2000
2020
2040
Other COVID-19 pandemic-related outcomes
COVID-19
Self-harm and interpersonal violence
Unintentional injuries
Transport injuries
Other non-communicable diseases
Musculoskeletal disorders
Sense organ diseases
Skin and subcutaneous diseases
Diabetes and kidney diseases
Substance use disorders
Mental disorders
Neurological disorders
Digestive diseases
Chronic respiratory diseases
Cardiovascular diseases
Neoplasms
Nutritional deficiencies
Maternal and neonatal disorders
Other infectious diseases
Neglected tropical diseases and malaria
Enteric infections
Respiratory infections and tuberculosis
HIV/AIDS and sexually transmitted infections

Tajikistan

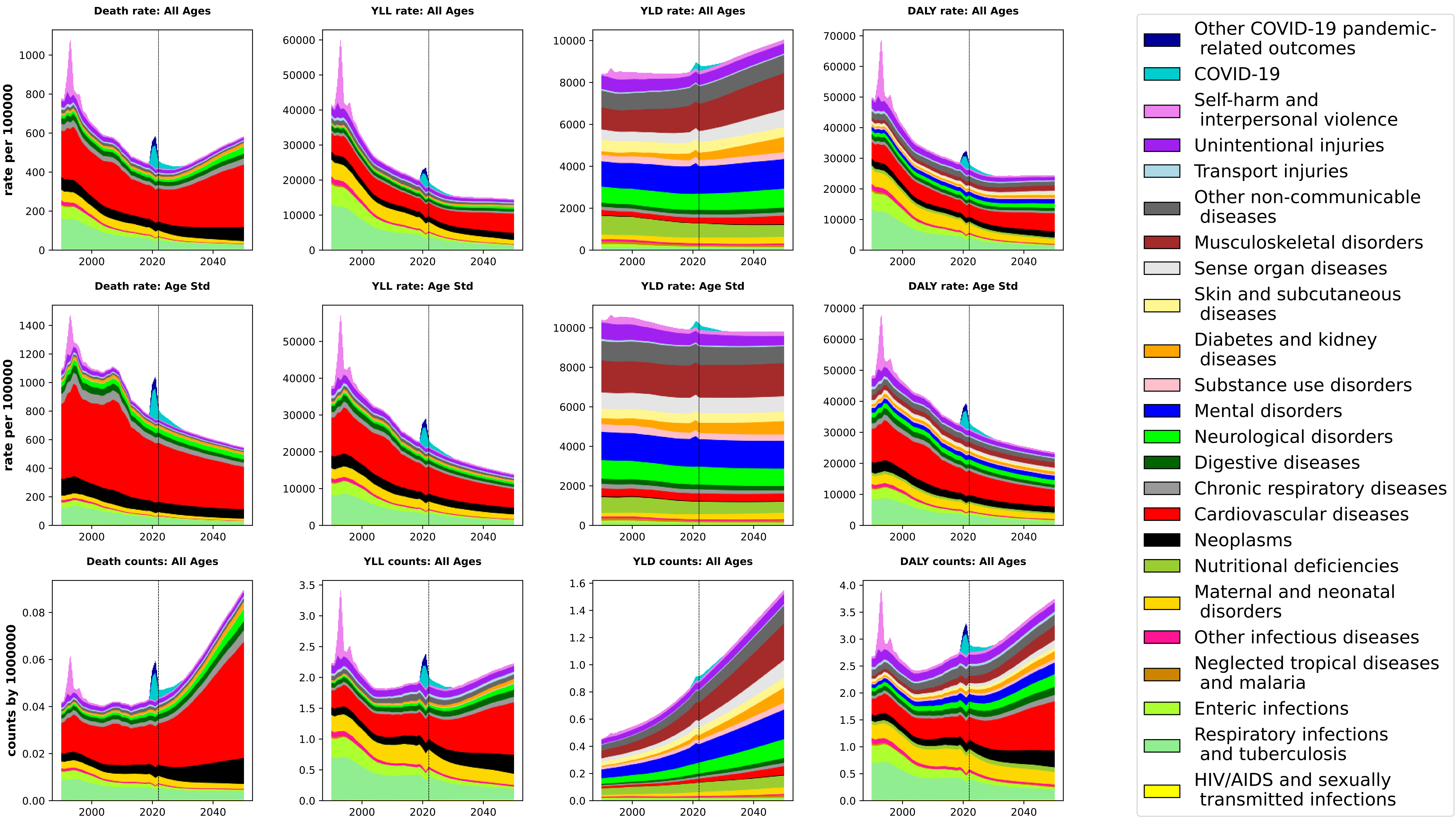
Death rate: All Ages
YLL rate: All Ages
YLD rate: All Ages
DALY rate: All Ages
Death rate: Age Std
YLL rate: Age Std
YLD rate: Age Std
DALY rate: Age Std
Death counts: All Ages
YLL counts: All Ages
YLD counts: All Ages
DALY counts: All Ages
rate per 100000
counts by 1000000
Other COVID-19 pandemic-related outcomes
COVID-19
Self-harm and interpersonal violence
Unintentional injuries
Transport injuries
Other non-communicable diseases
Musculoskeletal disorders
Sense organ diseases
Skin and subcutaneous diseases
Diabetes and kidney diseases
Substance use disorders
Mental disorders
Neurological disorders
Digestive diseases
Chronic respiratory diseases
Cardiovascular diseases
Neoplasms
Nutritional deficiencies
Maternal and neonatal disorders
Other infectious diseases
Neglected tropical diseases and malaria
Enteric infections
Respiratory infections and tuberculosis
HIV/AIDS and sexually transmitted infections

Turkmenistan

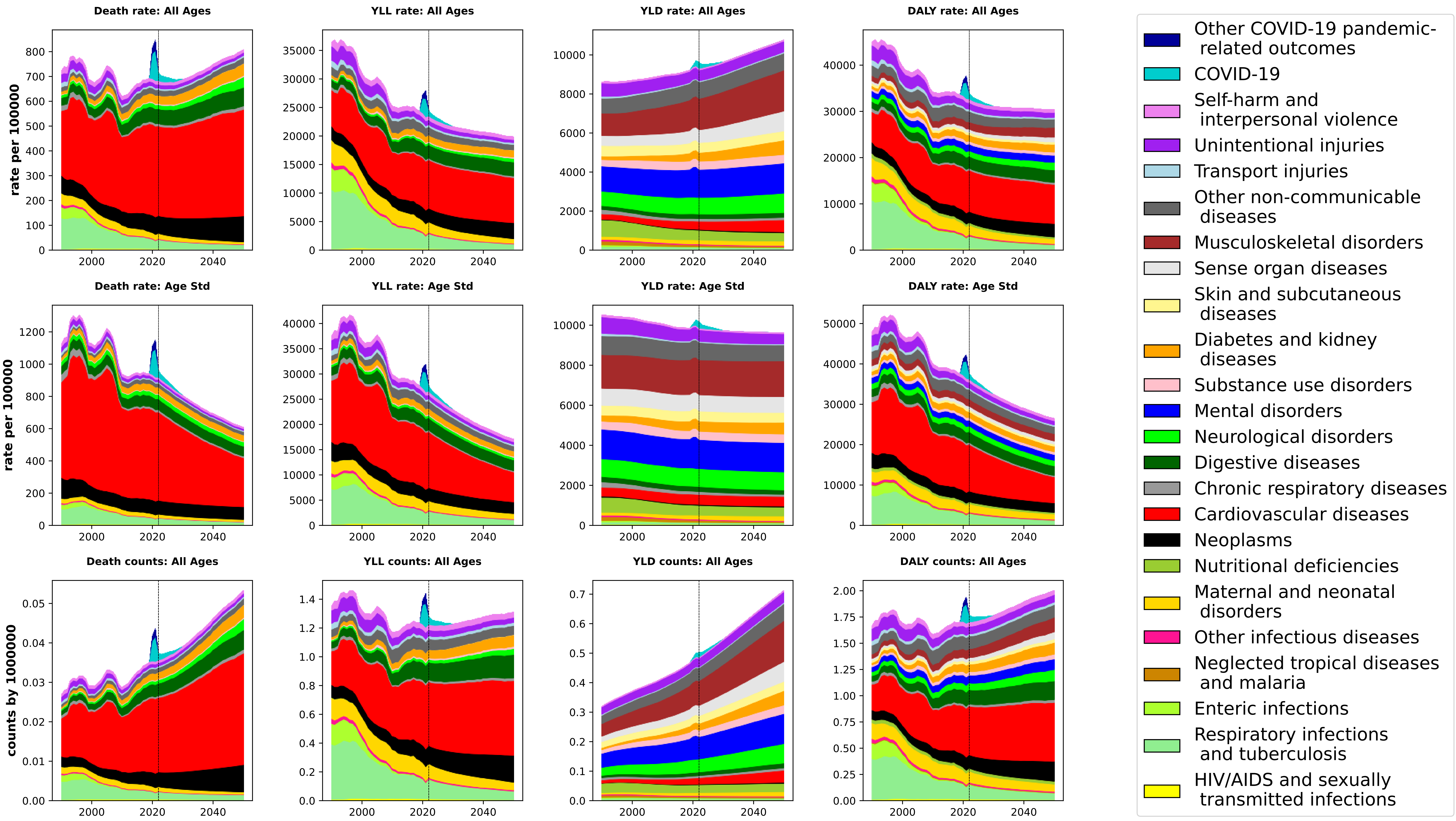

Uzbekistan

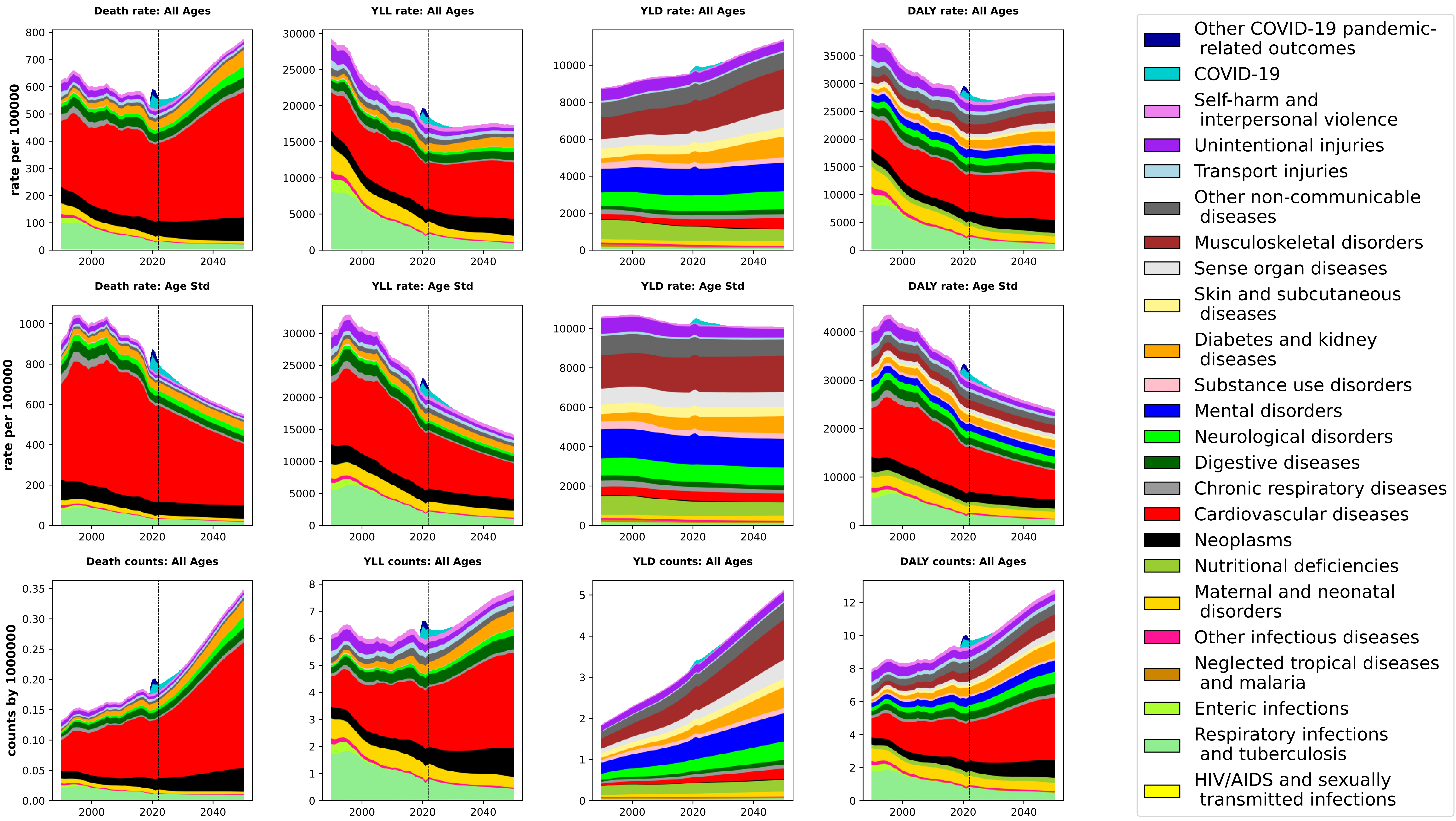
Death rate: All Ages
YLL rate: All Ages
YLD rate: All Ages
DALY rate: All Ages
Death rate: Age Std
YLL rate: Age Std
YLD rate: Age Std
DALY rate: Age Std
Death counts: All Ages
YLL counts: All Ages
YLD counts: All Ages
DALY counts: All Ages
rate per 100000
counts by 1000000
2000
2020
2040
Other COVID-19 pandemic-related outcomes
COVID-19
Self-harm and interpersonal violence
Unintentional injuries
Transport injuries
Other non-communicable diseases
Musculoskeletal disorders
Sense organ diseases
Skin and subcutaneous diseases
Diabetes and kidney diseases
Substance use disorders
Mental disorders
Neurological disorders
Digestive diseases
Chronic respiratory diseases
Cardiovascular diseases
Neoplasms
Nutritional deficiencies
Maternal and neonatal disorders
Other infectious diseases
Neglected tropical diseases and malaria
Enteric infections
Respiratory infections and tuberculosis
HIV/AIDS and sexually transmitted infections

Albania

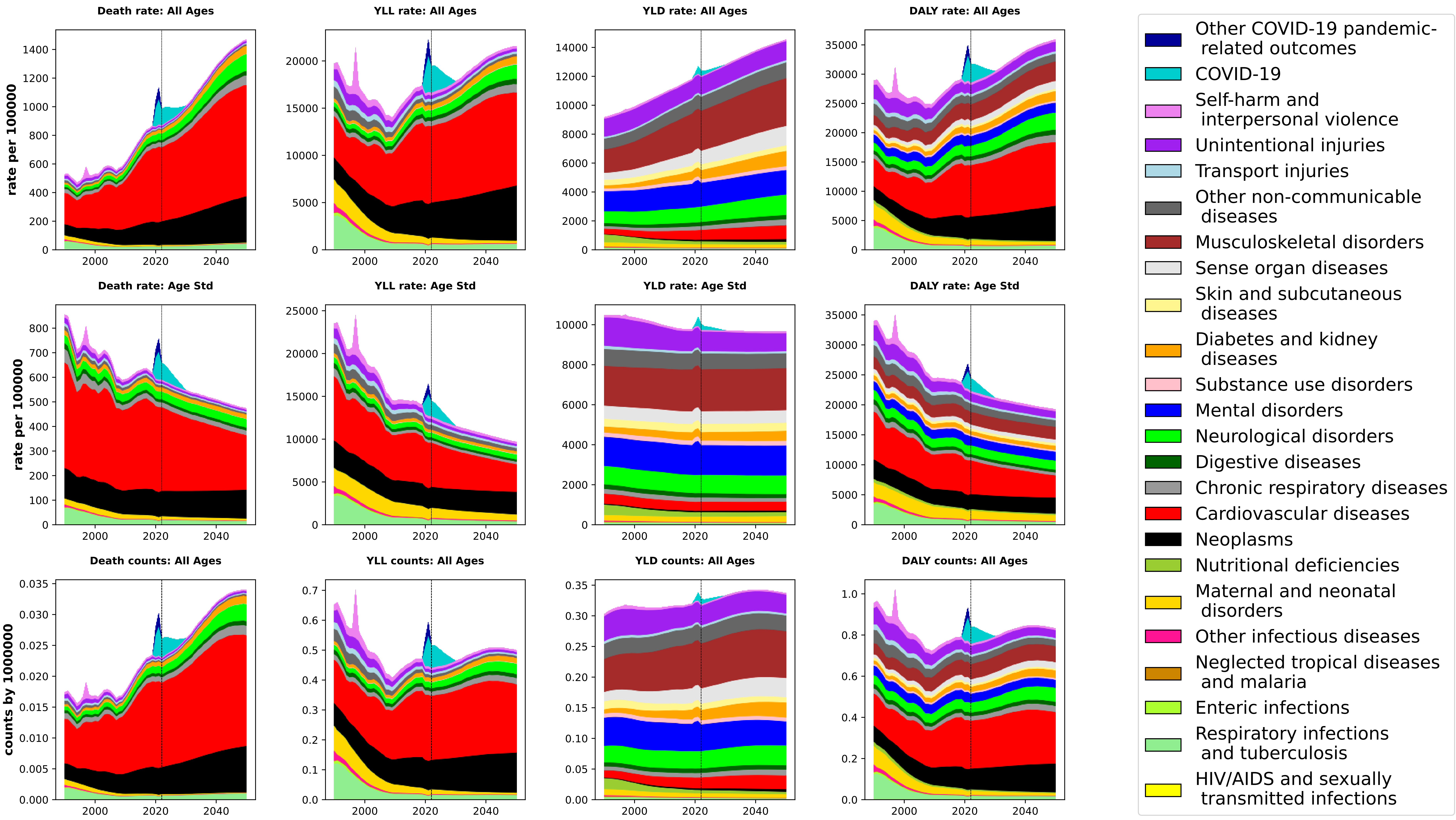

Death rate: All Ages
YLL rate: All Ages
YLD rate: All Ages
DALY rate: All Ages
Death rate: Age Std
YLL rate: Age Std
YLD rate: Age Std
DALY rate: Age Std
Death counts: All Ages
YLL counts: All Ages
YLD counts: All Ages
DALY counts: All Ages
rate per 100000
counts by 1000000
Other COVID-19 pandemic-related outcomes
COVID-19
Self-harm and interpersonal violence
Unintentional injuries
Transport injuries
Other non-communicable diseases
Musculoskeletal disorders
Sense organ diseases
Skin and subcutaneous diseases
Diabetes and kidney diseases
Substance use disorders
Mental disorders
Neurological disorders
Digestive diseases
Chronic respiratory diseases
Cardiovascular diseases
Neoplasms
Nutritional deficiencies
Maternal and neonatal disorders
Other infectious diseases
Neglected tropical diseases and malaria
Enteric infections
Respiratory infections and tuberculosis
HIV/AIDS and sexually transmitted infections

Bosnia and Herzegovina

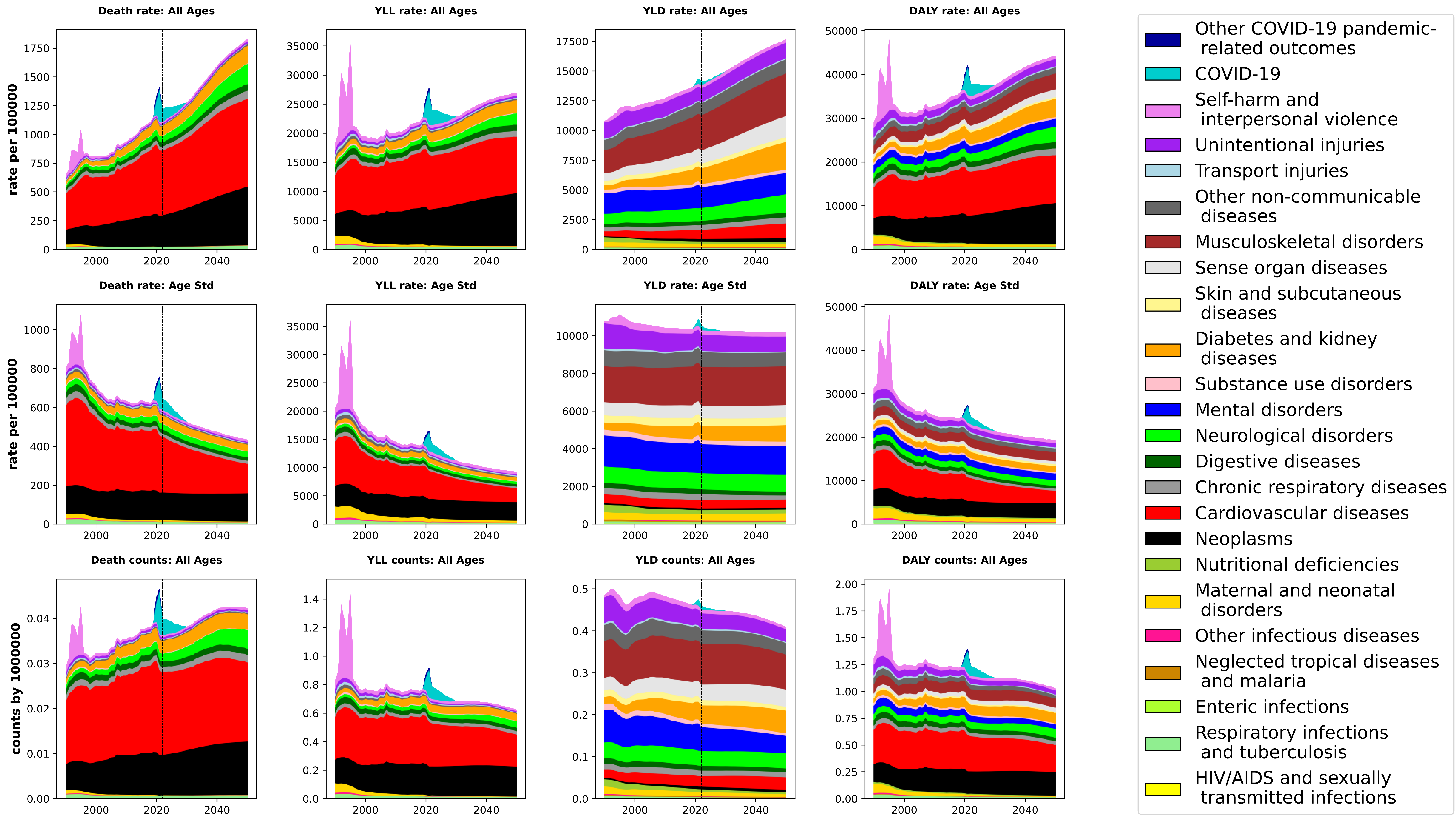

Bulgaria

Supplemental Results Figure S6. Cause level 2 area plots for 4 burden measures for reference scenario by location, 1990-2050

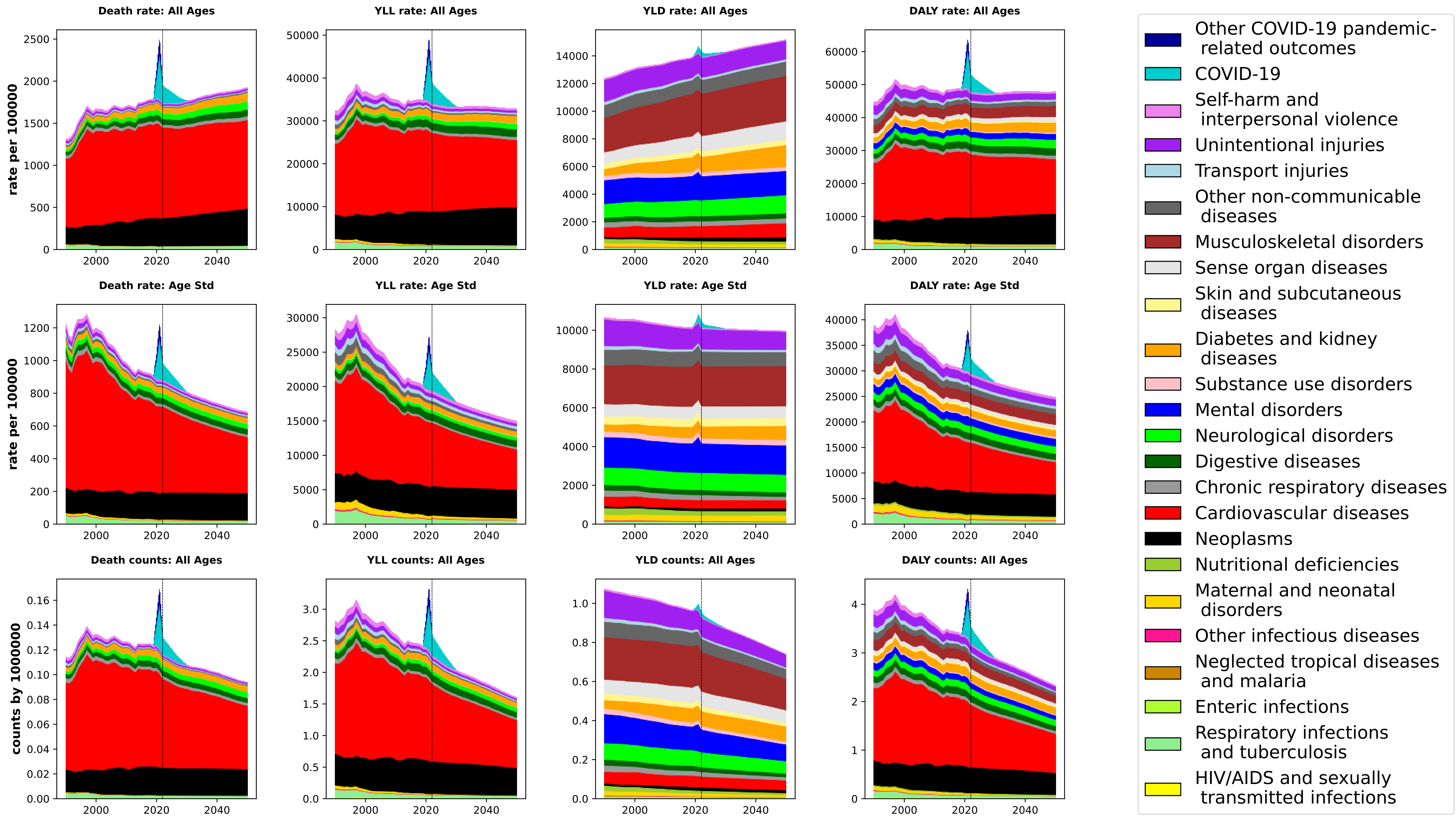

Croatia

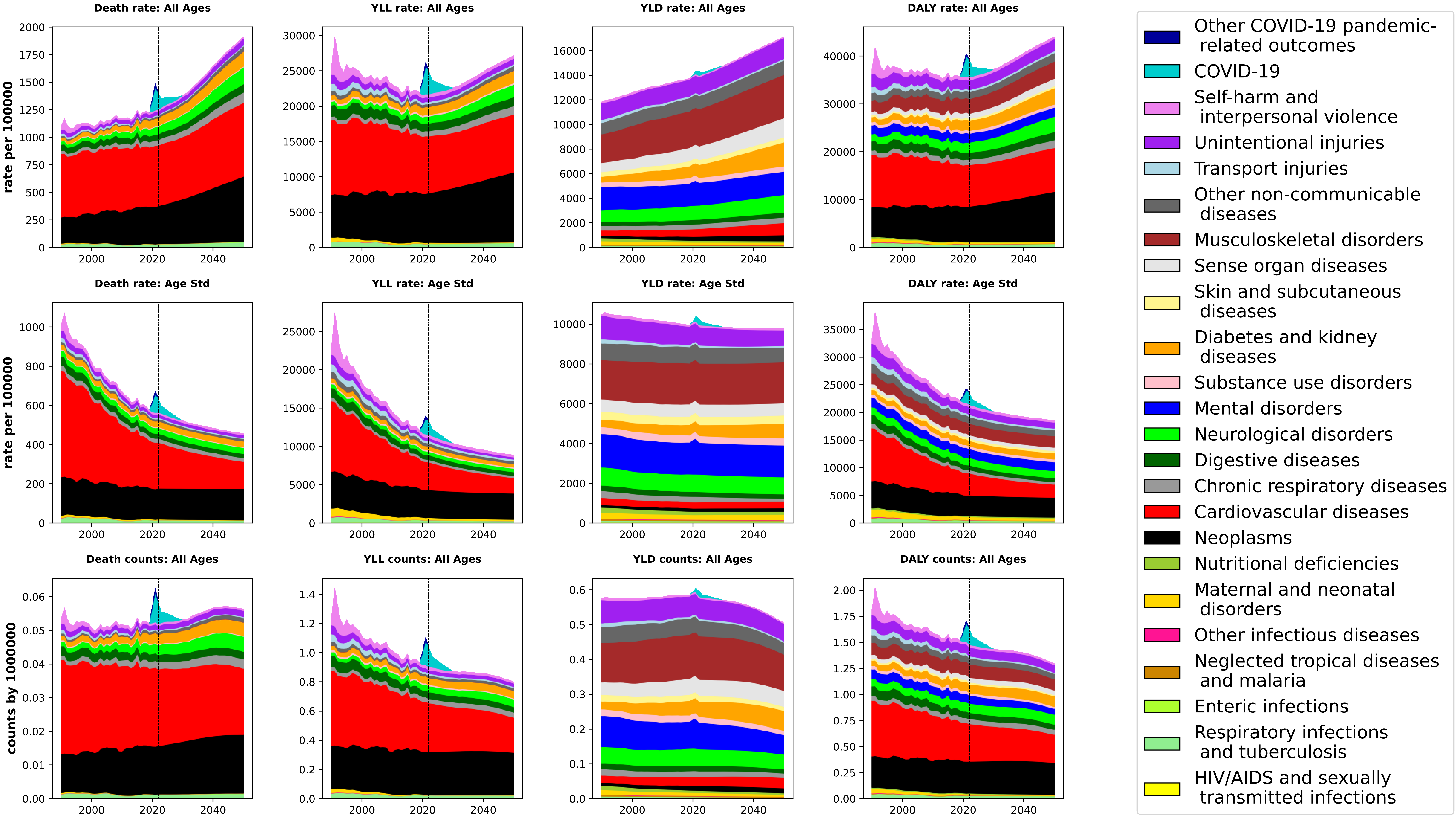
Death rate: All Ages
YLL rate: All Ages
YLD rate: All Ages
DALY rate: All Ages
Death rate: Age Std
YLL rate: Age Std
YLD rate: Age Std
DALY rate: Age Std
Death counts: All Ages
YLL counts: All Ages
YLD counts: All Ages
DALY counts: All Ages
rate per 100000
rate per 100000
counts by 1000000
2000
2020
2040
Other COVID-19 pandemic-related outcomes
COVID-19
Self-harm and interpersonal violence
Unintentional injuries
Transport injuries
Other non-communicable diseases
Musculoskeletal disorders
Sense organ diseases
Skin and subcutaneous diseases
Diabetes and kidney diseases
Substance use disorders
Mental disorders
Neurological disorders
Digestive diseases
Chronic respiratory diseases
Cardiovascular diseases
Neoplasms
Nutritional deficiencies
Maternal and neonatal disorders
Other infectious diseases
Neglected tropical diseases and malaria
Enteric infections
Respiratory infections and tuberculosis
HIV/AIDS and sexually transmitted infections

Czechia

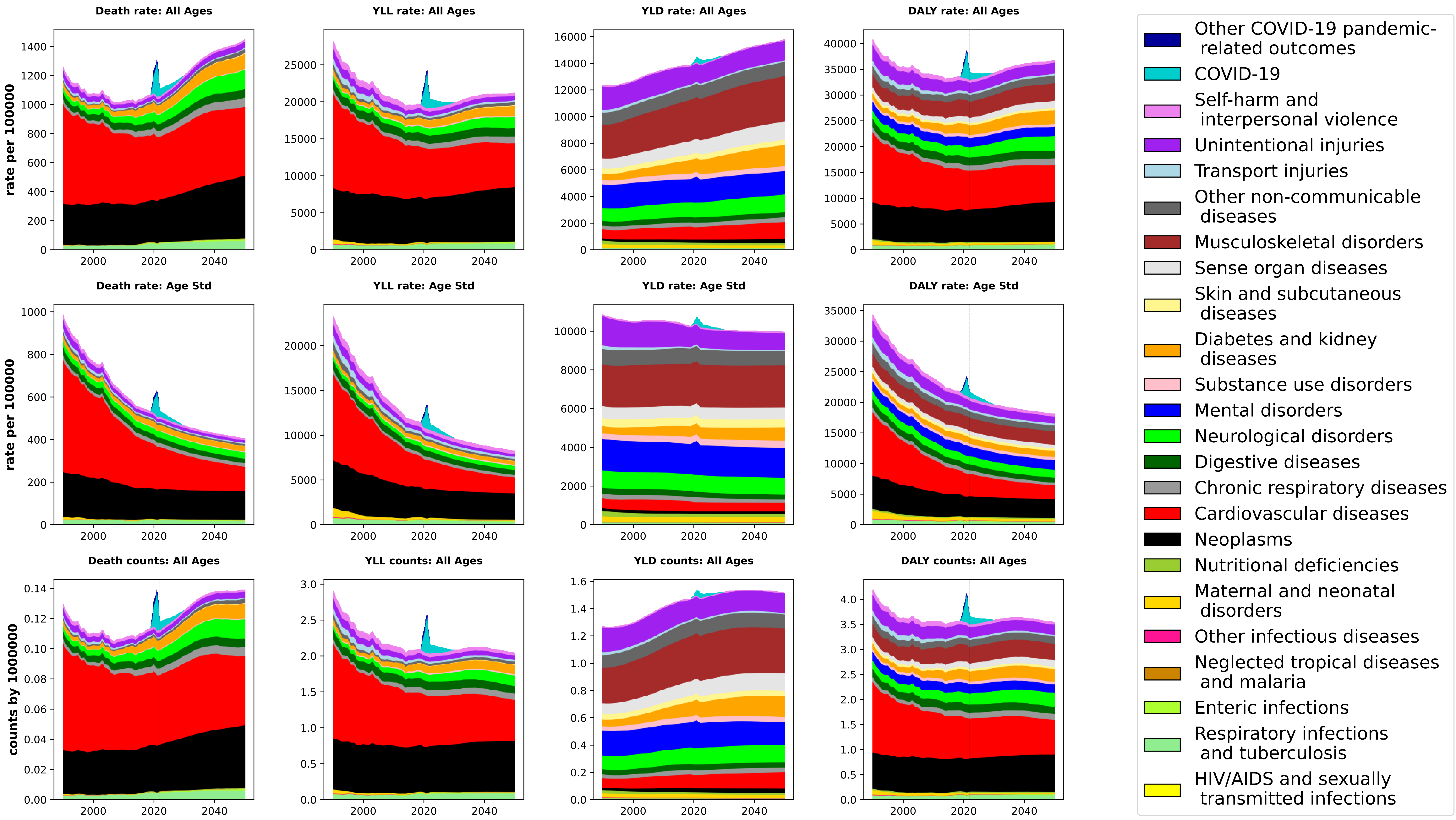
Death rate: All Ages
YLL rate: All Ages
YLD rate: All Ages
DALY rate: All Ages
Death rate: Age Std
YLL rate: Age Std
YLD rate: Age Std
DALY rate: Age Std
Death counts: All Ages
YLL counts: All Ages
YLD counts: All Ages
DALY counts: All Ages
rate per 100000
rate per 100000
counts by 1000000
Other COVID-19 pandemic-related outcomes
COVID-19
Self-harm and interpersonal violence
Unintentional injuries
Transport injuries
Other non-communicable diseases
Musculoskeletal disorders
Sense organ diseases
Skin and subcutaneous diseases
Diabetes and kidney diseases
Substance use disorders
Mental disorders
Neurological disorders
Digestive diseases
Chronic respiratory diseases
Cardiovascular diseases
Neoplasms
Nutritional deficiencies
Maternal and neonatal disorders
Other infectious diseases
Neglected tropical diseases and malaria
Enteric infections
Respiratory infections and tuberculosis
HIV/AIDS and sexually transmitted infections

Hungary

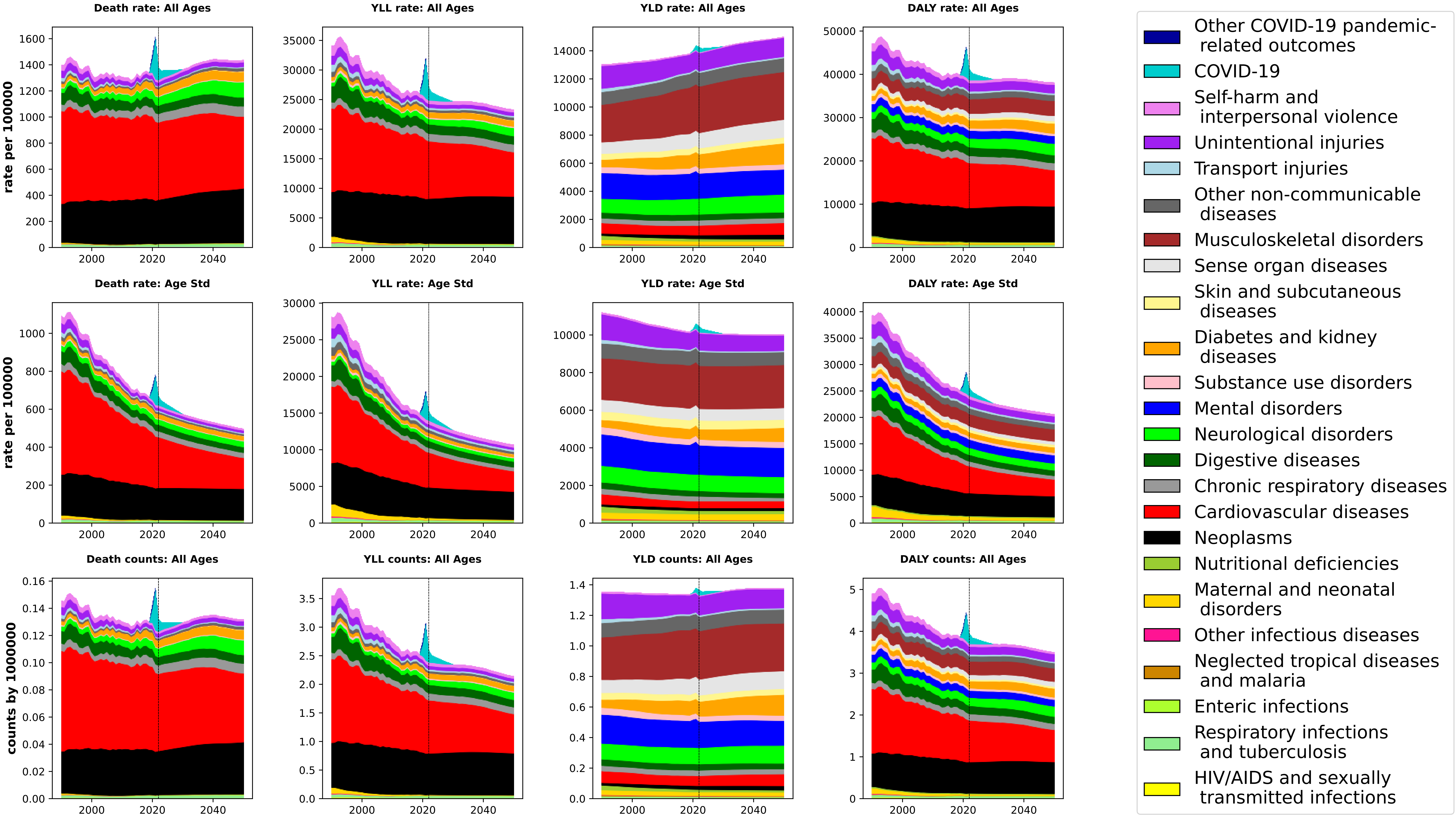
Death rate: All Ages
YLL rate: All Ages
YLD rate: All Ages
DALY rate: All Ages
Death rate: Age Std
YLL rate: Age Std
YLD rate: Age Std
DALY rate: Age Std
Death counts: All Ages
YLL counts: All Ages
YLD counts: All Ages
DALY counts: All Ages
rate per 100000
rate per 100000
counts by 1000000
Other COVID-19 pandemic-related outcomes
COVID-19
Self-harm and interpersonal violence
Unintentional injuries
Transport injuries
Other non-communicable diseases
Musculoskeletal disorders
Sense organ diseases
Skin and subcutaneous diseases
Diabetes and kidney diseases
Substance use disorders
Mental disorders
Neurological disorders
Digestive diseases
Chronic respiratory diseases
Cardiovascular diseases
Neoplasms
Nutritional deficiencies
Maternal and neonatal disorders
Other infectious diseases
Neglected tropical diseases and malaria
Enteric infections
Respiratory infections and tuberculosis
HIV/AIDS and sexually transmitted infections

Montenegro

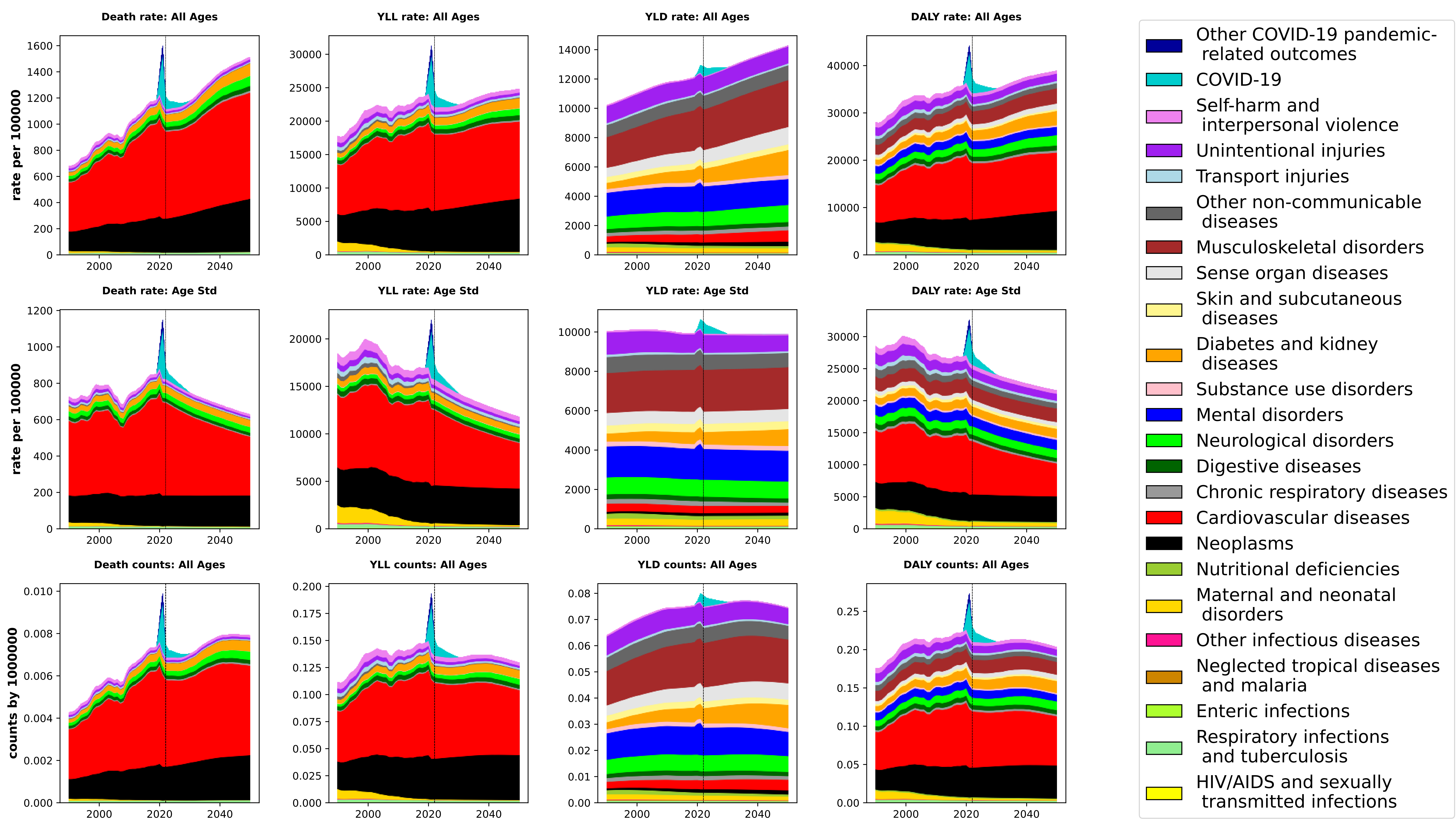
Death rate: All Ages
YLL rate: All Ages
YLD rate: All Ages
DALY rate: All Ages
Death rate: Age Std
YLL rate: Age Std
YLD rate: Age Std
DALY rate: Age Std
Death counts: All Ages
YLL counts: All Ages
YLD counts: All Ages
DALY counts: All Ages
rate per 100000
counts by 1000000
Other COVID-19 pandemic-related outcomes
COVID-19
Self-harm and interpersonal violence
Unintentional injuries
Transport injuries
Other non-communicable diseases
Musculoskeletal disorders
Sense organ diseases
Skin and subcutaneous diseases
Diabetes and kidney diseases
Substance use disorders
Mental disorders
Neurological disorders
Digestive diseases
Chronic respiratory diseases
Cardiovascular diseases
Neoplasms
Nutritional deficiencies
Maternal and neonatal disorders
Other infectious diseases
Neglected tropical diseases and malaria
Enteric infections
Respiratory infections and tuberculosis
HIV/AIDS and sexually transmitted infections

North Macedonia

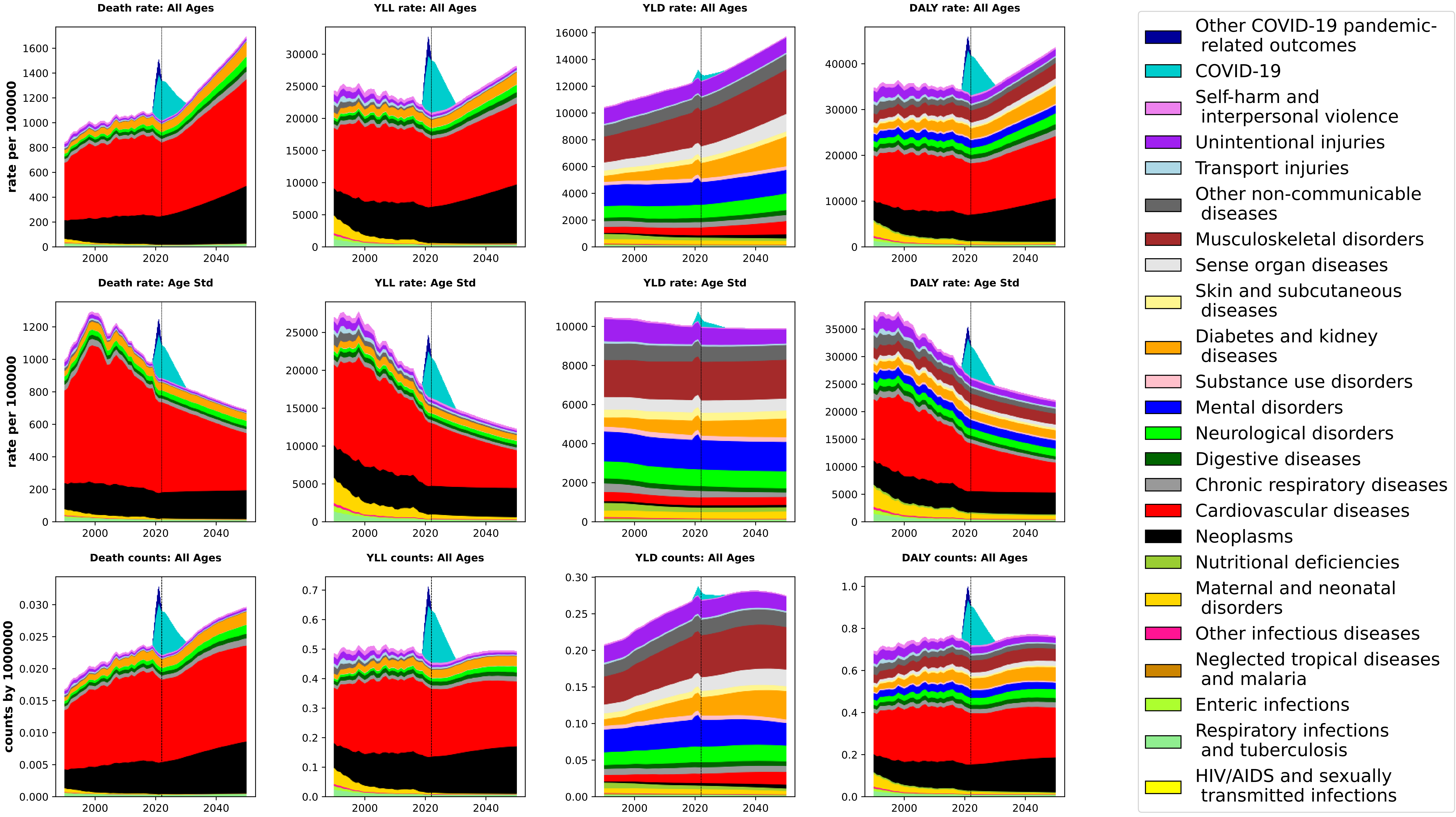

Poland

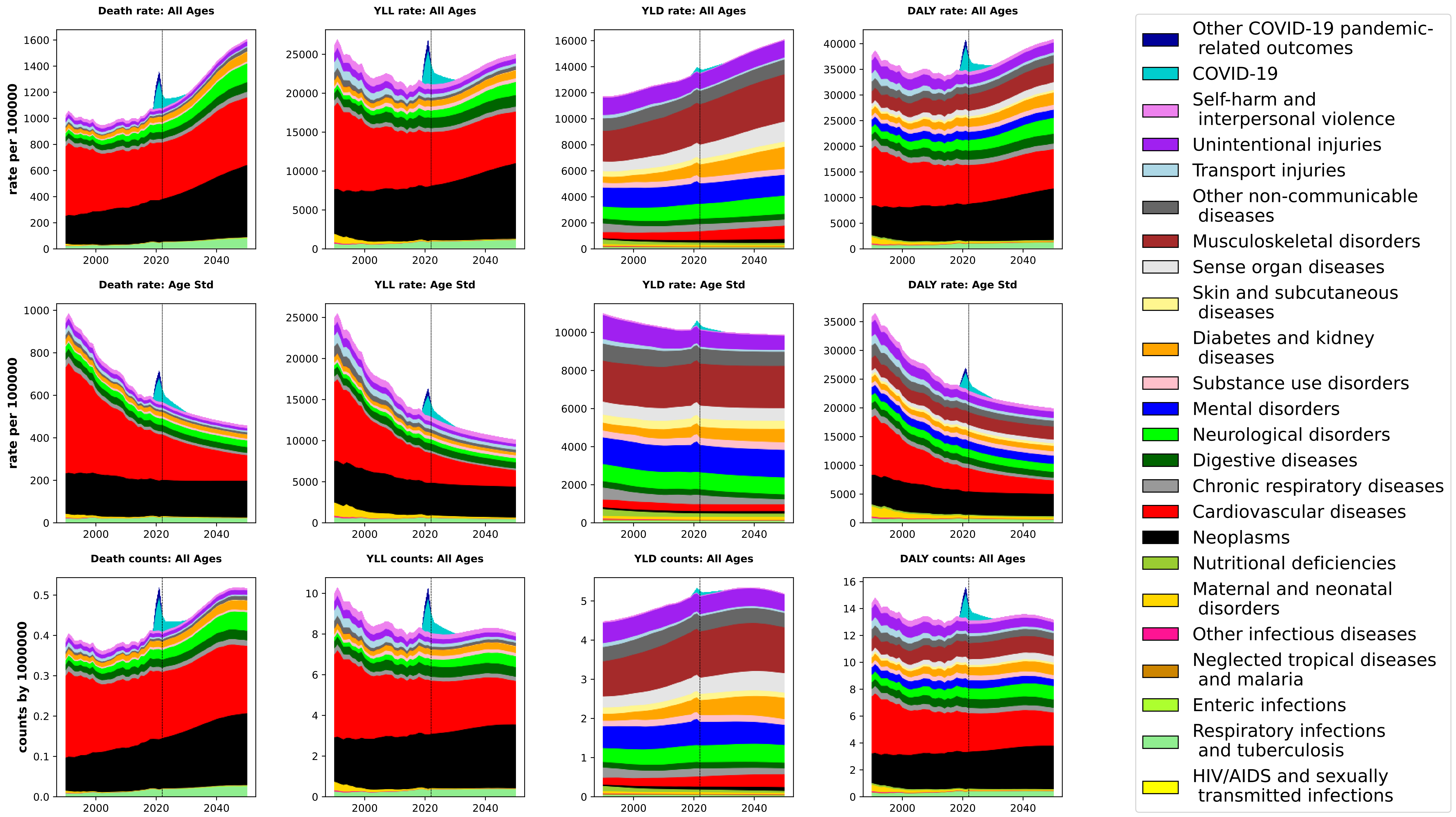
Death rate: All Ages
YLL rate: All Ages
YLD rate: All Ages
DALY rate: All Ages
Death rate: Age Std
YLL rate: Age Std
YLD rate: Age Std
DALY rate: Age Std
Death counts: All Ages
YLL counts: All Ages
YLD counts: All Ages
DALY counts: All Ages
rate per 100000
counts by 1000000
Other COVID-19 pandemic-related outcomes
COVID-19
Self-harm and interpersonal violence
Unintentional injuries
Transport injuries
Other non-communicable diseases
Musculoskeletal disorders
Sense organ diseases
Skin and subcutaneous diseases
Diabetes and kidney diseases
Substance use disorders
Mental disorders
Neurological disorders
Digestive diseases
Chronic respiratory diseases
Cardiovascular diseases
Neoplasms
Nutritional deficiencies
Maternal and neonatal disorders
Other infectious diseases
Neglected tropical diseases and malaria
Enteric infections
Respiratory infections and tuberculosis
HIV/AIDS and sexually transmitted infections

Romania

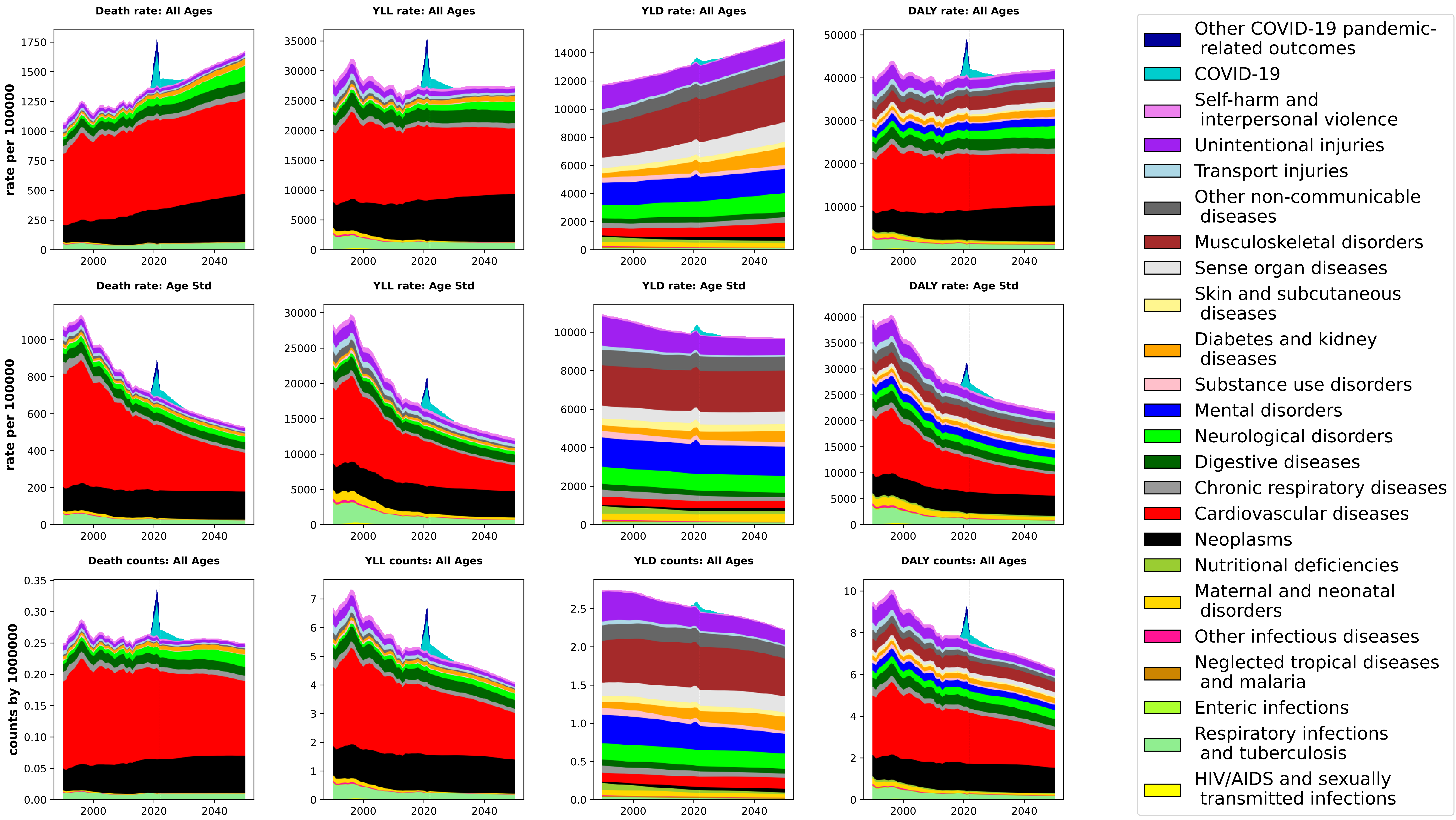
Death rate: All Ages
YLL rate: All Ages
YLD rate: All Ages
DALY rate: All Ages
Death rate: Age Std
YLL rate: Age Std
YLD rate: Age Std
DALY rate: Age Std
Death counts: All Ages
YLL counts: All Ages
YLD counts: All Ages
DALY counts: All Ages
rate per 100000
counts by 1000000
Other COVID-19 pandemic-related outcomes
COVID-19
Self-harm and interpersonal violence
Unintentional injuries
Transport injuries
Other non-communicable diseases
Musculoskeletal disorders
Sense organ diseases
Skin and subcutaneous diseases
Diabetes and kidney diseases
Substance use disorders
Mental disorders
Neurological disorders
Digestive diseases
Chronic respiratory diseases
Cardiovascular diseases
Neoplasms
Nutritional deficiencies
Maternal and neonatal disorders
Other infectious diseases
Neglected tropical diseases and malaria
Enteric infections
Respiratory infections and tuberculosis
HIV/AIDS and sexually transmitted infections

Serbia

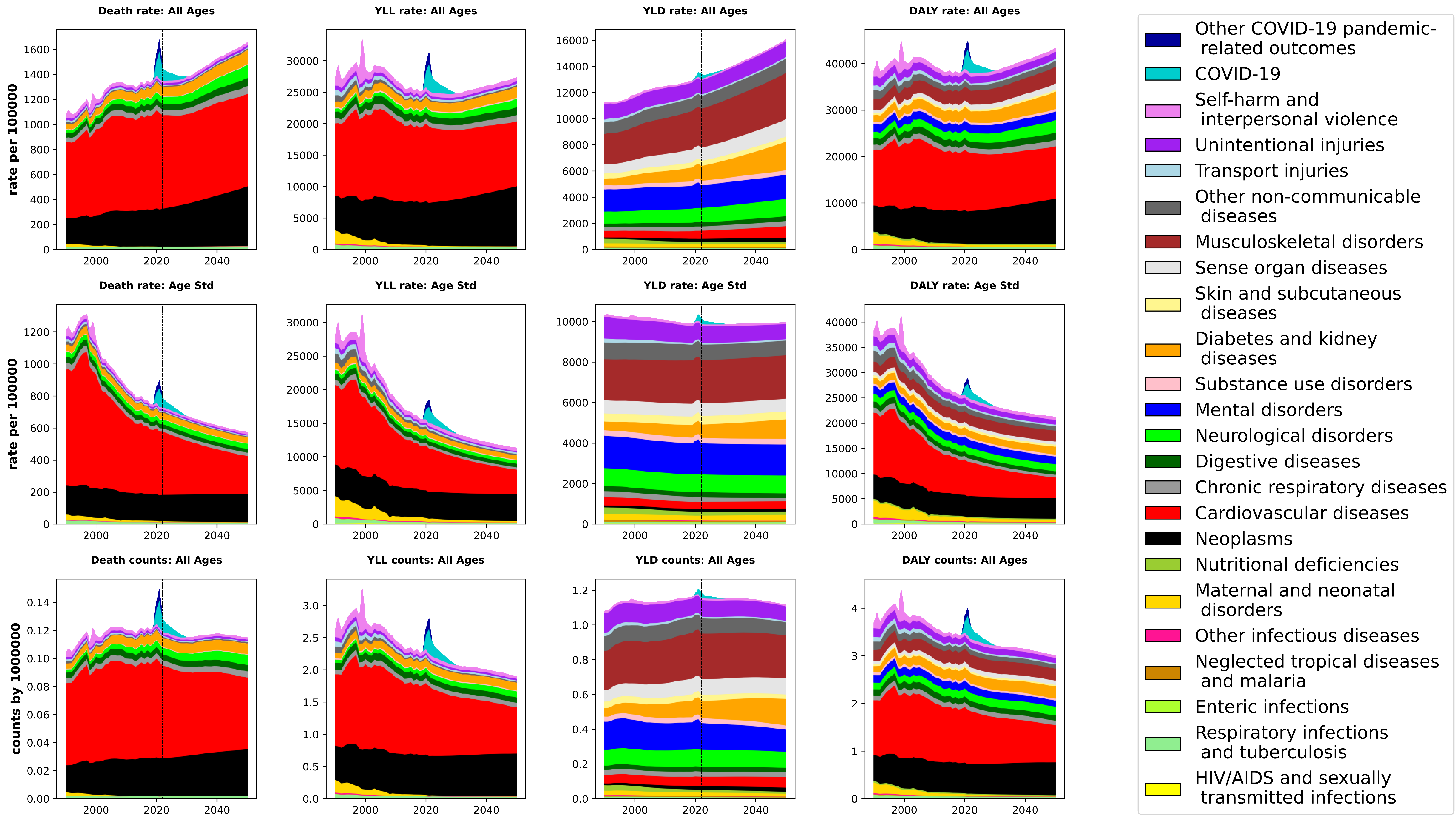
Death rate: All Ages
YLL rate: All Ages
YLD rate: All Ages
DALY rate: All Ages
Death rate: Age Std
YLL rate: Age Std
YLD rate: Age Std
DALY rate: Age Std
Death counts: All Ages
YLL counts: All Ages
YLD counts: All Ages
DALY counts: All Ages
rate per 100000
rate per 100000
counts by 1000000
2000
2020
2040
Other COVID-19 pandemic-related outcomes
COVID-19
Self-harm and interpersonal violence
Unintentional injuries
Transport injuries
Other non-communicable diseases
Musculoskeletal disorders
Sense organ diseases
Skin and subcutaneous diseases
Diabetes and kidney diseases
Substance use disorders
Mental disorders
Neurological disorders
Digestive diseases
Chronic respiratory diseases
Cardiovascular diseases
Neoplasms
Nutritional deficiencies
Maternal and neonatal disorders
Other infectious diseases
Neglected tropical diseases and malaria
Enteric infections
Respiratory infections and tuberculosis
HIV/AIDS and sexually transmitted infections

Slovakia

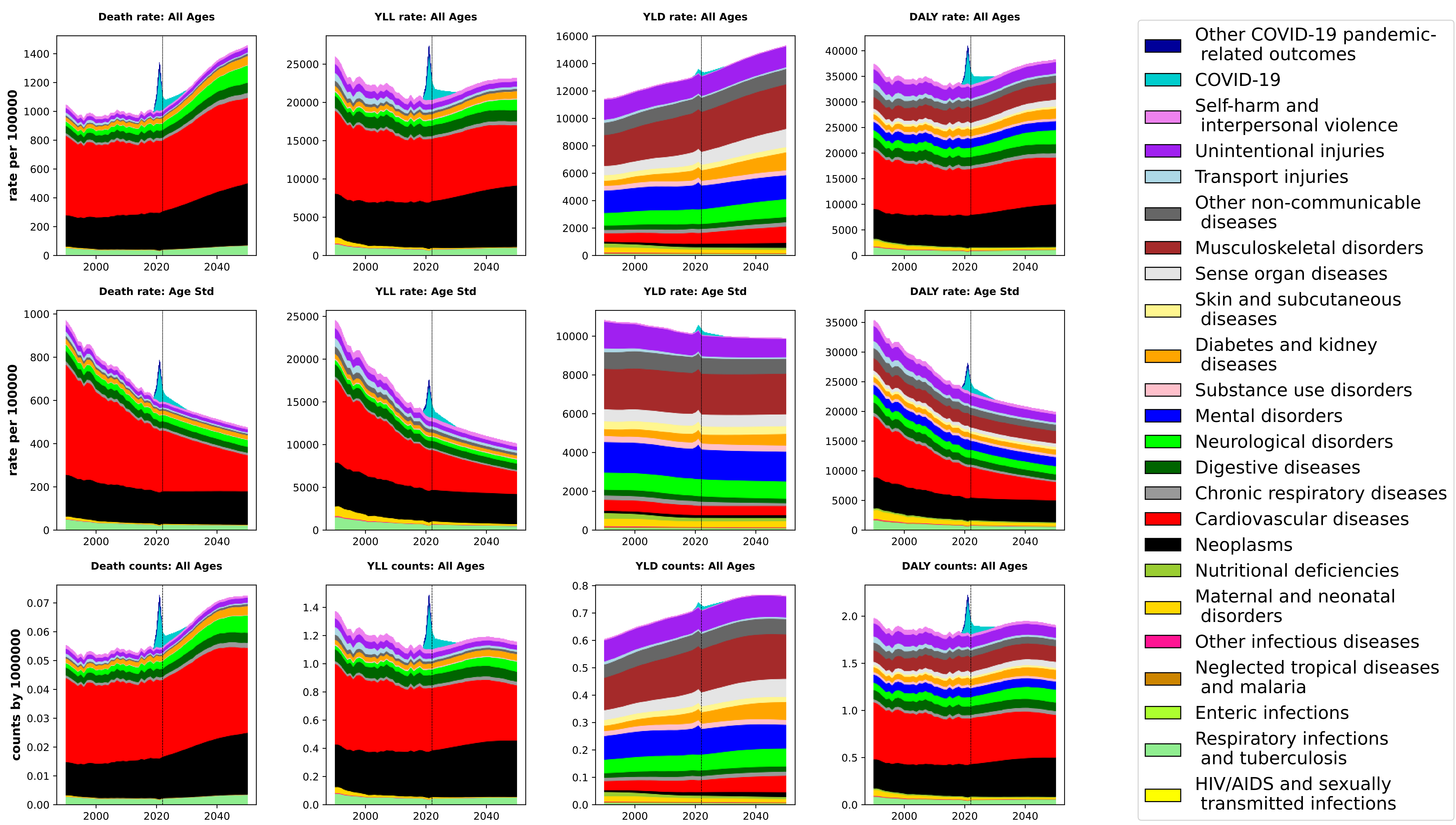
Death rate: All Ages
YLL rate: All Ages
YLD rate: All Ages
DALY rate: All Ages
Death rate: Age Std
YLL rate: Age Std
YLD rate: Age Std
DALY rate: Age Std
Death counts: All Ages
YLL counts: All Ages
YLD counts: All Ages
DALY counts: All Ages
rate per 100000
counts by 1000000
Other COVID-19 pandemic-related outcomes
COVID-19
Self-harm and interpersonal violence
Unintentional injuries
Transport injuries
Other non-communicable diseases
Musculoskeletal disorders
Sense organ diseases
Skin and subcutaneous diseases
Diabetes and kidney diseases
Substance use disorders
Mental disorders
Neurological disorders
Digestive diseases
Chronic respiratory diseases
Cardiovascular diseases
Neoplasms
Nutritional deficiencies
Maternal and neonatal disorders
Other infectious diseases
Neglected tropical diseases and malaria
Enteric infections
Respiratory infections and tuberculosis
HIV/AIDS and sexually transmitted infections

Slovenia

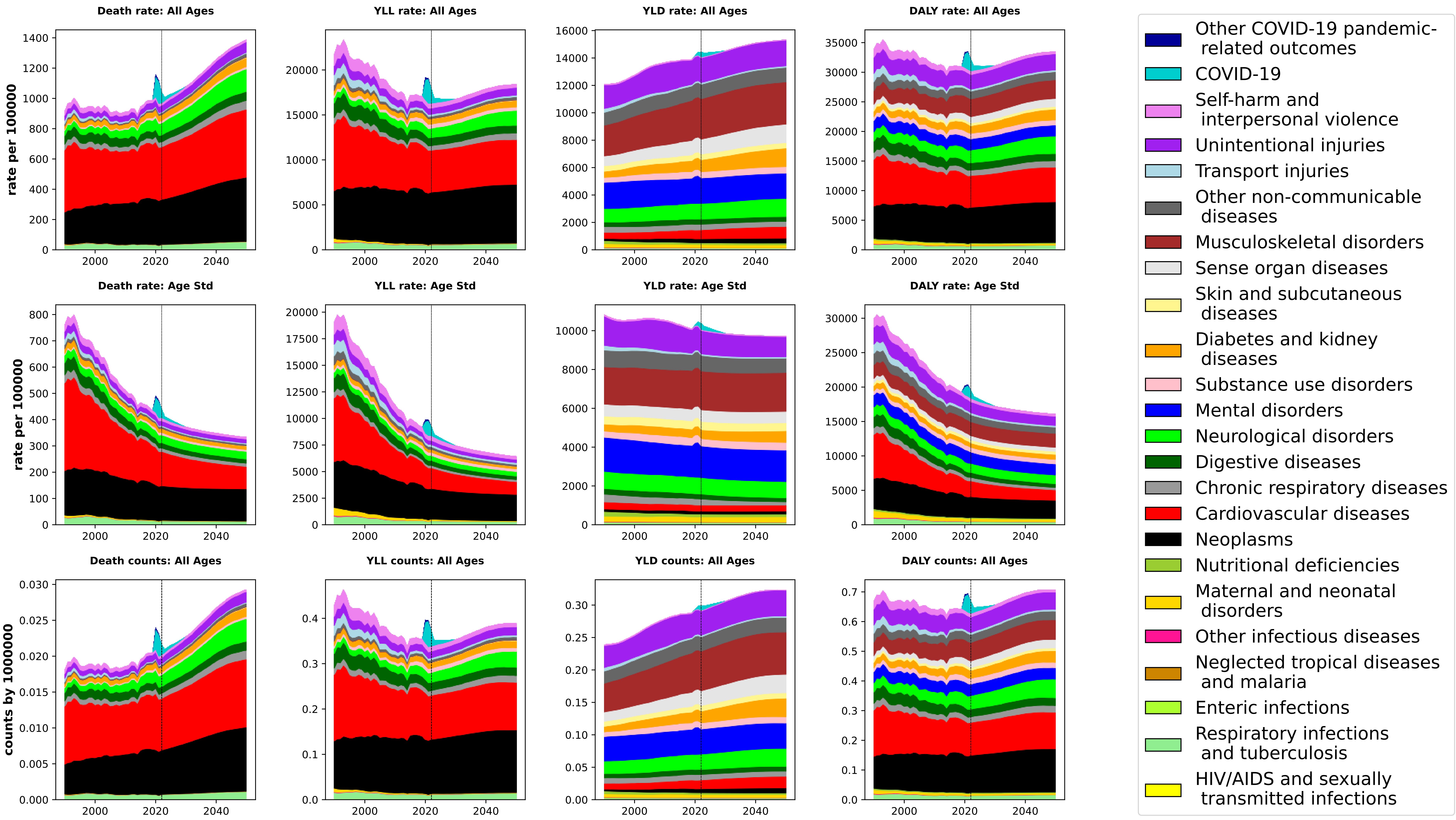
Death rate: All Ages
YLL rate: All Ages
YLD rate: All Ages
DALY rate: All Ages
Death rate: Age Std
YLL rate: Age Std
YLD rate: Age Std
DALY rate: Age Std
Death counts: All Ages
YLL counts: All Ages
YLD counts: All Ages
DALY counts: All Ages
rate per 100000
counts by 1000000
2000
2020
2040
Other COVID-19 pandemic-related outcomes
COVID-19
Self-harm and interpersonal violence
Unintentional injuries
Transport injuries
Other non-communicable diseases
Musculoskeletal disorders
Sense organ diseases
Skin and subcutaneous diseases
Diabetes and kidney diseases
Substance use disorders
Mental disorders
Neurological disorders
Digestive diseases
Chronic respiratory diseases
Cardiovascular diseases
Neoplasms
Nutritional deficiencies
Maternal and neonatal disorders
Other infectious diseases
Neglected tropical diseases and malaria
Enteric infections
Respiratory infections and tuberculosis
HIV/AIDS and sexually transmitted infections

Belarus

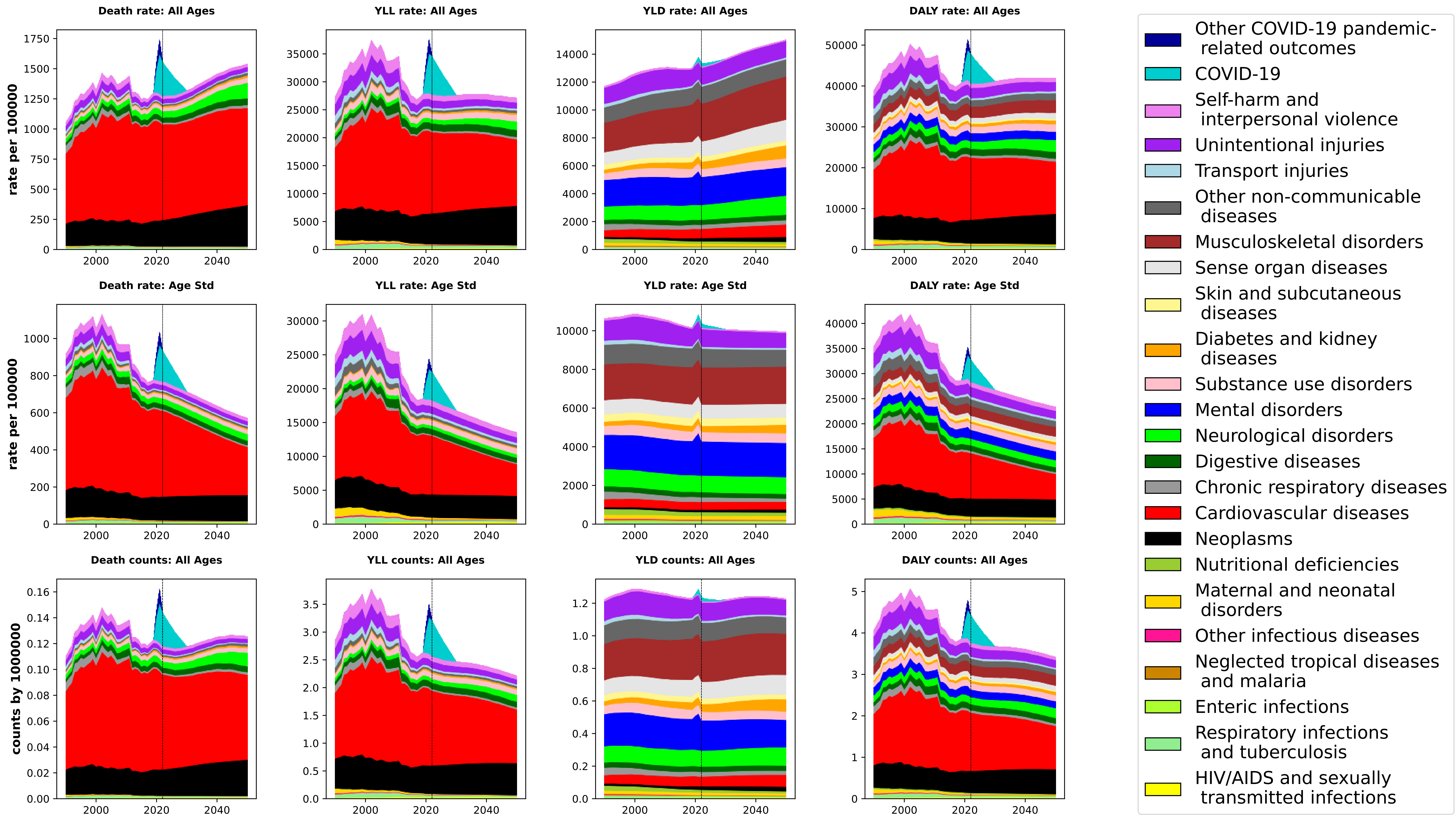
Death rate: All Ages
YLL rate: All Ages
YLD rate: All Ages
DALY rate: All Ages
Death rate: Age Std
YLL rate: Age Std
YLD rate: Age Std
DALY rate: Age Std
Death counts: All Ages
YLL counts: All Ages
YLD counts: All Ages
DALY counts: All Ages
rate per 100000
rate per 100000
counts by 1000000
2000
2020
2040
Other COVID-19 pandemic-related outcomes
COVID-19
Self-harm and interpersonal violence
Unintentional injuries
Transport injuries
Other non-communicable diseases
Musculoskeletal disorders
Sense organ diseases
Skin and subcutaneous diseases
Diabetes and kidney diseases
Substance use disorders
Mental disorders
Neurological disorders
Digestive diseases
Chronic respiratory diseases
Cardiovascular diseases
Neoplasms
Nutritional deficiencies
Maternal and neonatal disorders
Other infectious diseases
Neglected tropical diseases and malaria
Enteric infections
Respiratory infections and tuberculosis
HIV/AIDS and sexually transmitted infections

Estonia

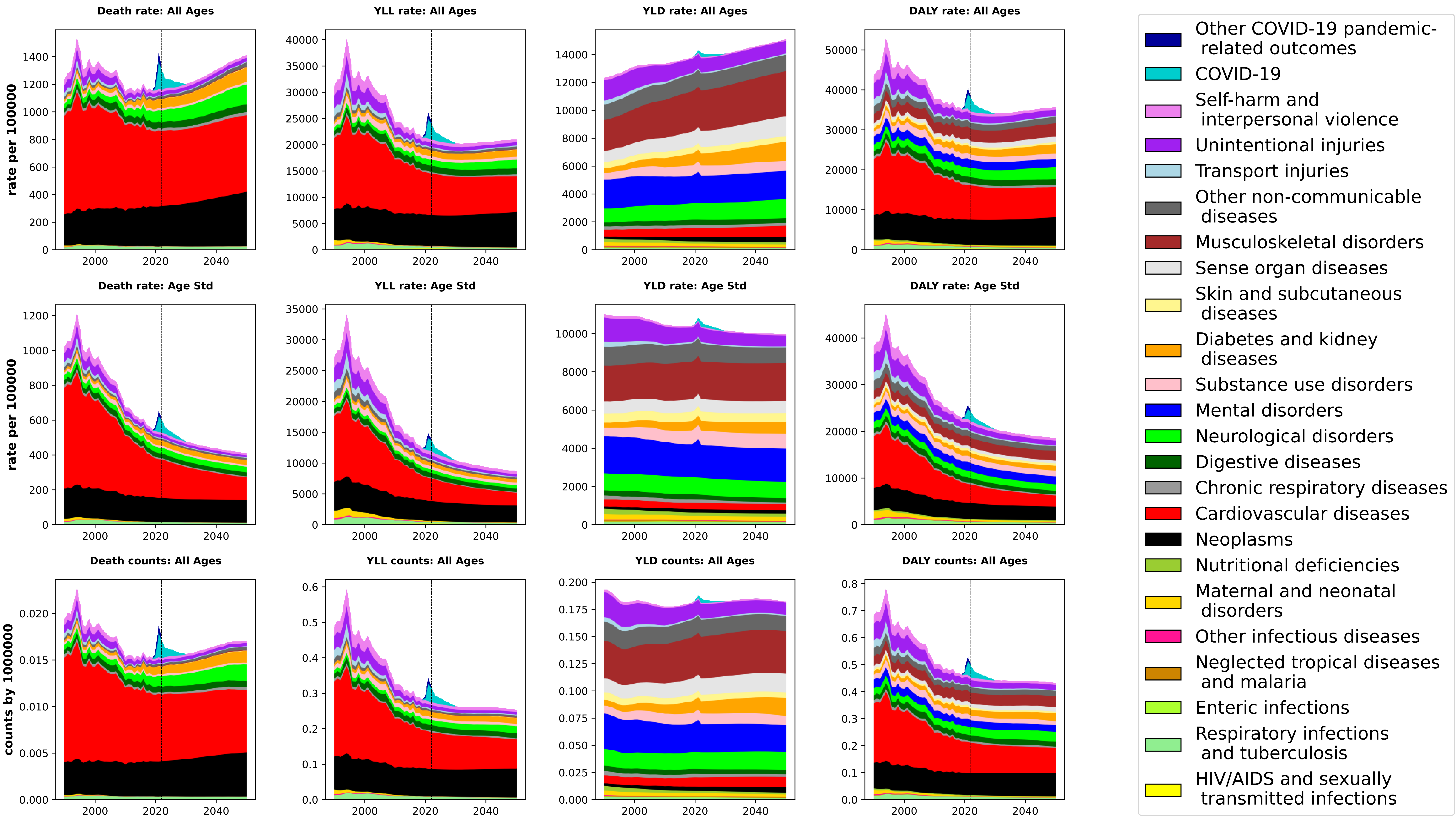
Death rate: All Ages
YLL rate: All Ages
YLD rate: All Ages
DALY rate: All Ages
Death rate: Age Std
YLL rate: Age Std
YLD rate: Age Std
DALY rate: Age Std
Death counts: All Ages
YLL counts: All Ages
YLD counts: All Ages
DALY counts: All Ages
rate per 100000
counts by 1000000
2000
2020
2040
Other COVID-19 pandemic-related outcomes
COVID-19
Self-harm and interpersonal violence
Unintentional injuries
Transport injuries
Other non-communicable diseases
Musculoskeletal disorders
Sense organ diseases
Skin and subcutaneous diseases
Diabetes and kidney diseases
Substance use disorders
Mental disorders
Neurological disorders
Digestive diseases
Chronic respiratory diseases
Cardiovascular diseases
Neoplasms
Nutritional deficiencies
Maternal and neonatal disorders
Other infectious diseases
Neglected tropical diseases and malaria
Enteric infections
Respiratory infections and tuberculosis
HIV/AIDS and sexually transmitted infections

Latvia

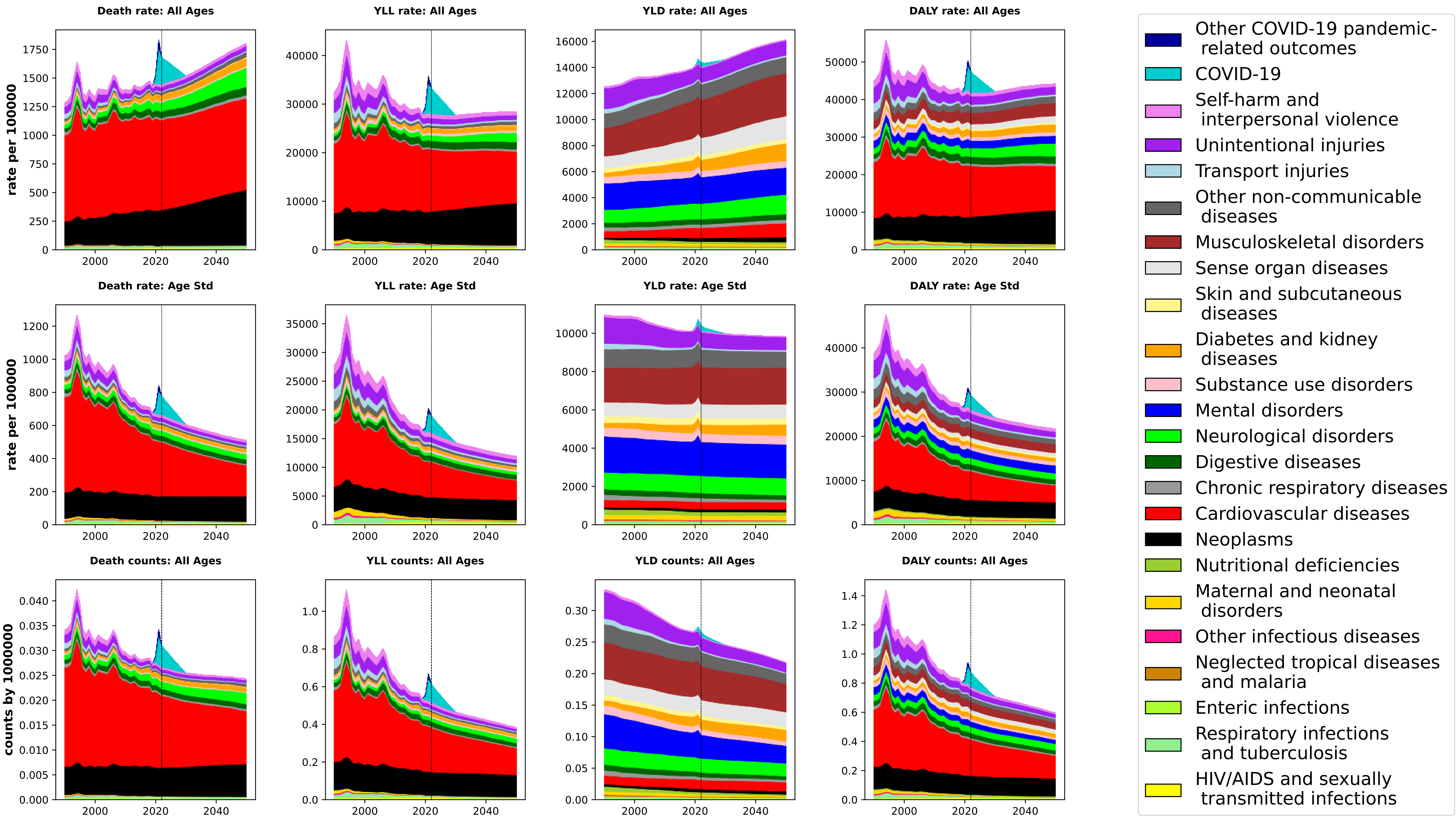
Death rate: All Ages
YLL rate: All Ages
YLD rate: All Ages
DALY rate: All Ages
Death rate: Age Std
YLL rate: Age Std
YLD rate: Age Std
DALY rate: Age Std
Death counts: All Ages
YLL counts: All Ages
YLD counts: All Ages
DALY counts: All Ages
rate per 100000
counts by 1000000
Other COVID-19 pandemic-related outcomes
COVID-19
Self-harm and interpersonal violence
Unintentional injuries
Transport injuries
Other non-communicable diseases
Musculoskeletal disorders
Sense organ diseases
Skin and subcutaneous diseases
Diabetes and kidney diseases
Substance use disorders
Mental disorders
Neurological disorders
Digestive diseases
Chronic respiratory diseases
Cardiovascular diseases
Neoplasms
Nutritional deficiencies
Maternal and neonatal disorders
Other infectious diseases
Neglected tropical diseases and malaria
Enteric infections
Respiratory infections and tuberculosis
HIV/AIDS and sexually transmitted infections

Lithuania

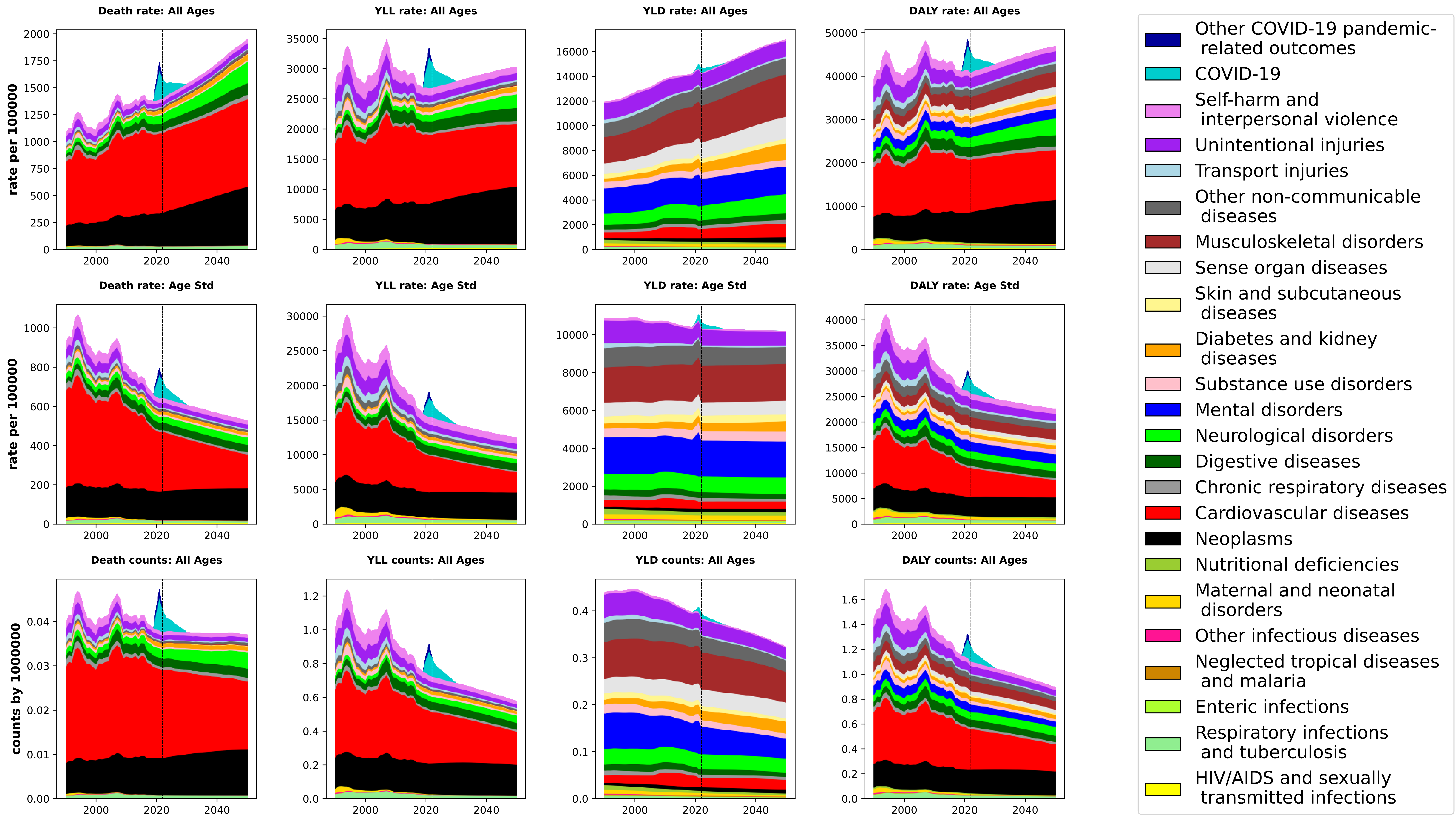
Death rate: All Ages
YLL rate: All Ages
YLD rate: All Ages
DALY rate: All Ages
Death rate: Age Std
YLL rate: Age Std
YLD rate: Age Std
DALY rate: Age Std
Death counts: All Ages
YLL counts: All Ages
YLD counts: All Ages
DALY counts: All Ages
rate per 100000
rate per 100000
counts by 1000000
2000
2020
2040
Other COVID-19 pandemic-related outcomes
COVID-19
Self-harm and interpersonal violence
Unintentional injuries
Transport injuries
Other non-communicable diseases
Musculoskeletal disorders
Sense organ diseases
Skin and subcutaneous diseases
Diabetes and kidney diseases
Substance use disorders
Mental disorders
Neurological disorders
Digestive diseases
Chronic respiratory diseases
Cardiovascular diseases
Neoplasms
Nutritional deficiencies
Maternal and neonatal disorders
Other infectious diseases
Neglected tropical diseases and malaria
Enteric infections
Respiratory infections and tuberculosis
HIV/AIDS and sexually transmitted infections

Moldova

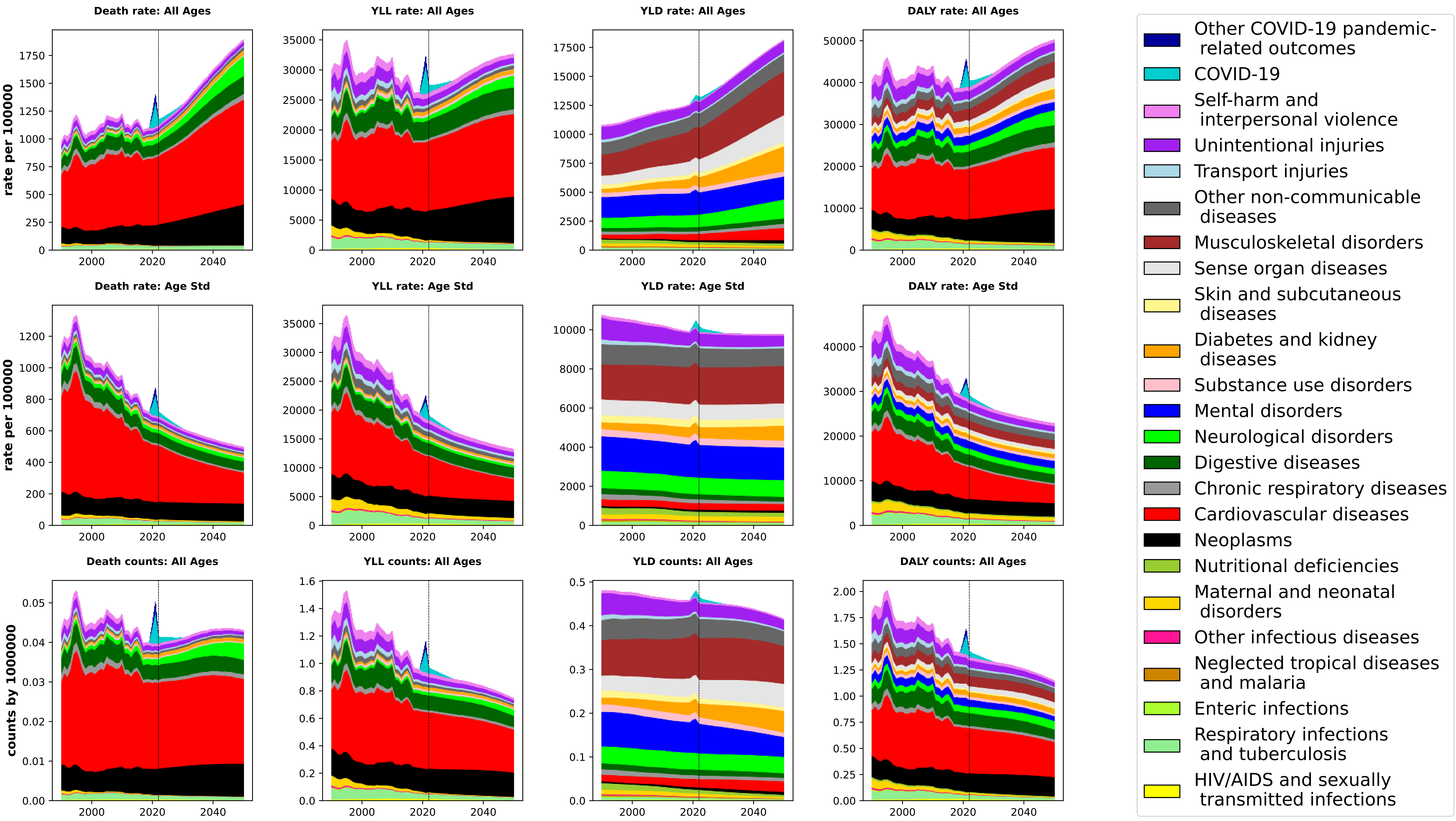
Death rate: All Ages
YLL rate: All Ages
YLD rate: All Ages
DALY rate: All Ages
Death rate: Age Std
YLL rate: Age Std
YLD rate: Age Std
DALY rate: Age Std
Death counts: All Ages
YLL counts: All Ages
YLD counts: All Ages
DALY counts: All Ages
rate per 100000
rate per 100000
counts by 1000000
2000
2020
2040
Other COVID-19 pandemic-related outcomes
COVID-19
Self-harm and interpersonal violence
Unintentional injuries
Transport injuries
Other non-communicable diseases
Musculoskeletal disorders
Sense organ diseases
Skin and subcutaneous diseases
Diabetes and kidney diseases
Substance use disorders
Mental disorders
Neurological disorders
Digestive diseases
Chronic respiratory diseases
Cardiovascular diseases
Neoplasms
Nutritional deficiencies
Maternal and neonatal disorders
Other infectious diseases
Neglected tropical diseases and malaria
Enteric infections
Respiratory infections and tuberculosis
HIV/AIDS and sexually transmitted infections

Russia

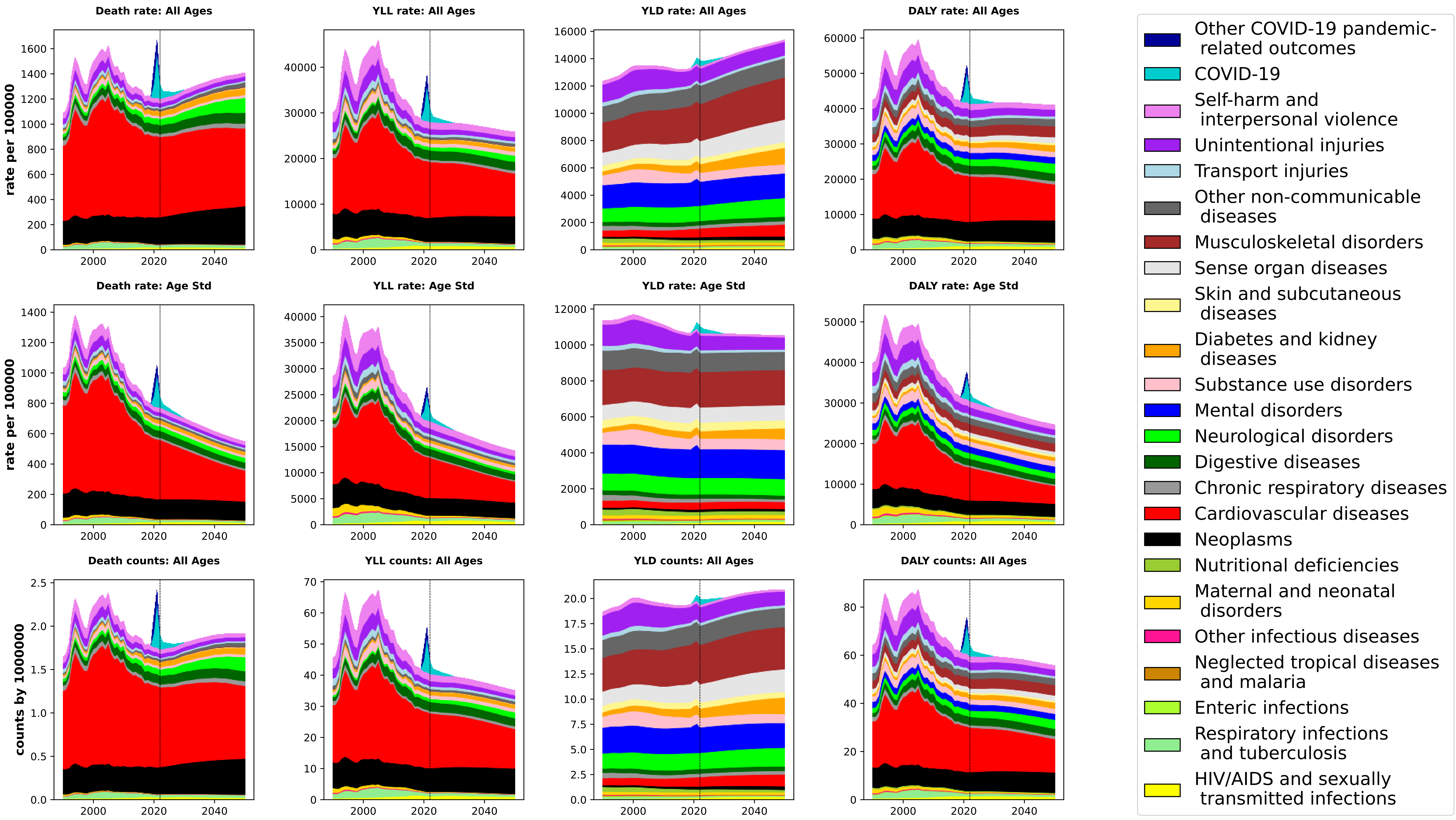
Death rate: All Ages
YLL rate: All Ages
YLD rate: All Ages
DALY rate: All Ages
Death rate: Age Std
YLL rate: Age Std
YLD rate: Age Std
DALY rate: Age Std
Death counts: All Ages
YLL counts: All Ages
YLD counts: All Ages
DALY counts: All Ages
rate per 100000
counts by 1000000
2000
2020
2040
Other COVID-19 pandemic-related outcomes
COVID-19
Self-harm and interpersonal violence
Unintentional injuries
Transport injuries
Other non-communicable diseases
Musculoskeletal disorders
Sense organ diseases
Skin and subcutaneous diseases
Diabetes and kidney diseases
Substance use disorders
Mental disorders
Neurological disorders
Digestive diseases
Chronic respiratory diseases
Cardiovascular diseases
Neoplasms
Nutritional deficiencies
Maternal and neonatal disorders
Other infectious diseases
Neglected tropical diseases and malaria
Enteric infections
Respiratory infections and tuberculosis
HIV/AIDS and sexually transmitted infections

Ukraine

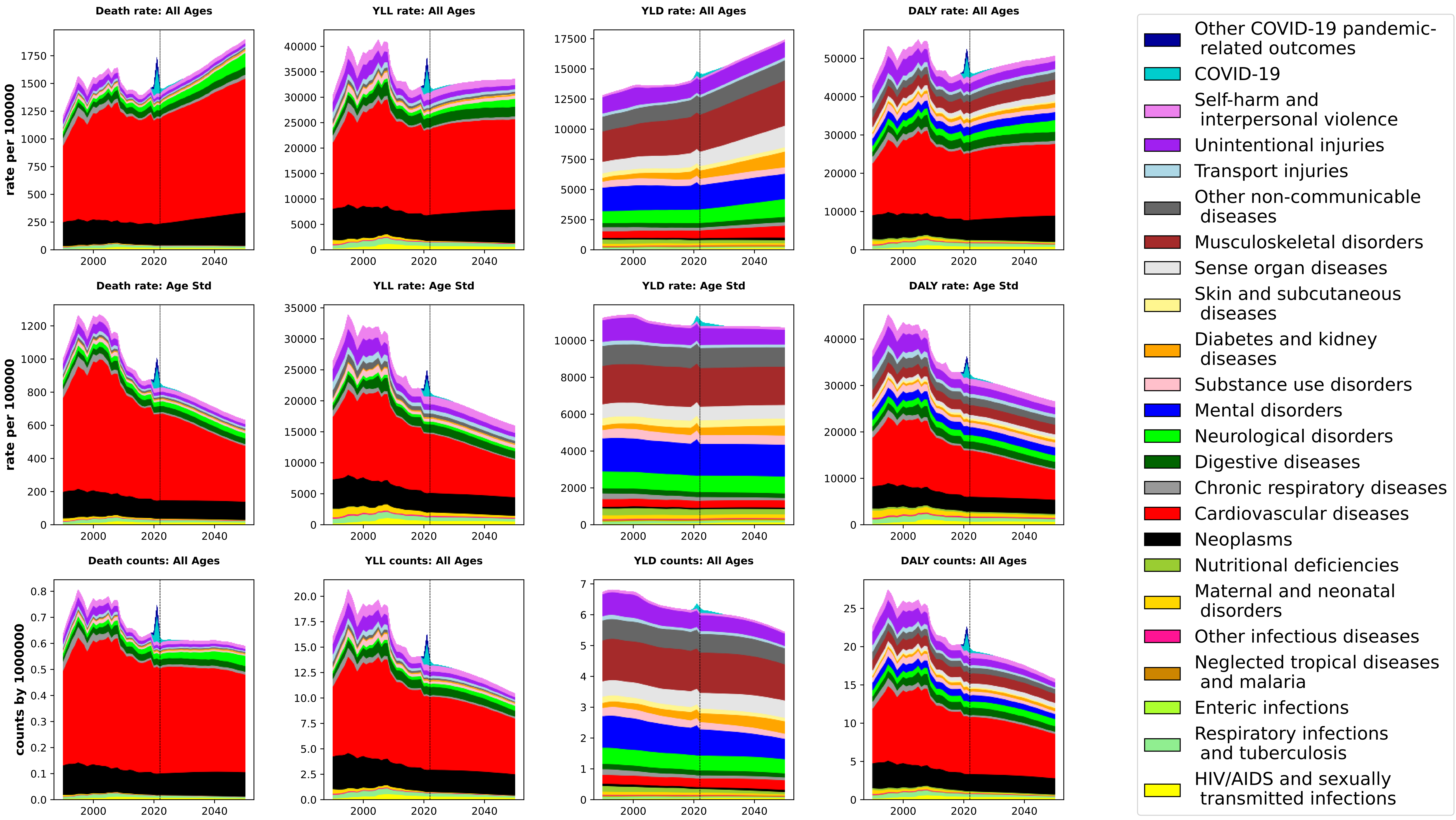
Death rate: All Ages
YLL rate: All Ages
YLD rate: All Ages
DALY rate: All Ages
Death rate: Age Std
YLL rate: Age Std
YLD rate: Age Std
DALY rate: Age Std
Death counts: All Ages
YLL counts: All Ages
YLD counts: All Ages
DALY counts: All Ages
rate per 100000
rate per 100000
counts by 1000000
2000
2020
2040
Other COVID-19 pandemic-related outcomes
COVID-19
Self-harm and interpersonal violence
Unintentional injuries
Transport injuries
Other non-communicable diseases
Musculoskeletal disorders
Sense organ diseases
Skin and subcutaneous diseases
Diabetes and kidney diseases
Substance use disorders
Mental disorders
Neurological disorders
Digestive diseases
Chronic respiratory diseases
Cardiovascular diseases
Neoplasms
Nutritional deficiencies
Maternal and neonatal disorders
Other infectious diseases
Neglected tropical diseases and malaria
Enteric infections
Respiratory infections and tuberculosis
HIV/AIDS and sexually transmitted infections

Australia

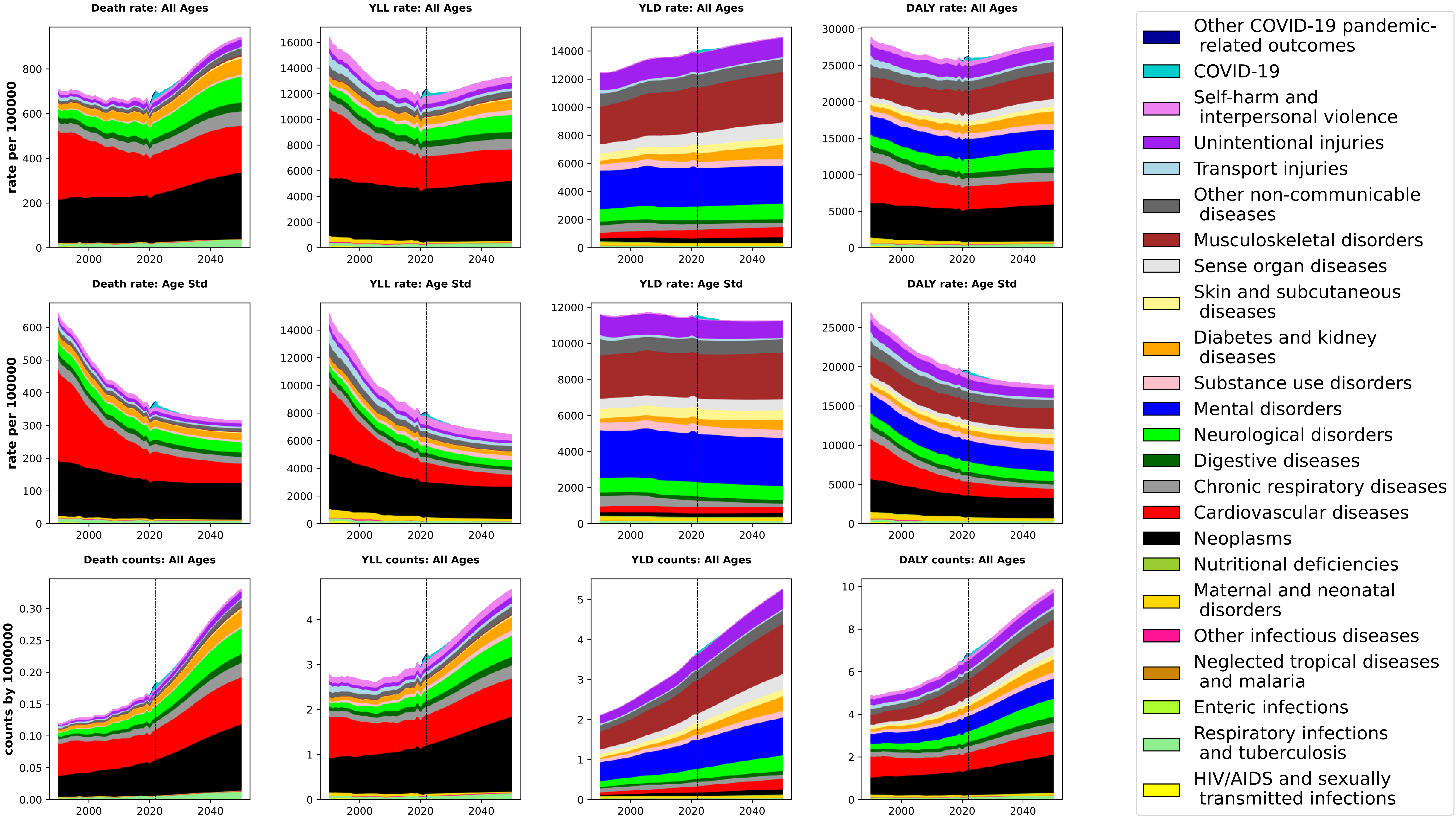
Death rate: All Ages
YLL rate: All Ages
YLD rate: All Ages
DALY rate: All Ages
Death rate: Age Std
YLL rate: Age Std
YLD rate: Age Std
DALY rate: Age Std
Death counts: All Ages
YLL counts: All Ages
YLD counts: All Ages
DALY counts: All Ages
rate per 100000
counts by 1000000
Other COVID-19 pandemic-related outcomes
COVID-19
Self-harm and interpersonal violence
Unintentional injuries
Transport injuries
Other non-communicable diseases
Musculoskeletal disorders
Sense organ diseases
Skin and subcutaneous diseases
Diabetes and kidney diseases
Substance use disorders
Mental disorders
Neurological disorders
Digestive diseases
Chronic respiratory diseases
Cardiovascular diseases
Neoplasms
Nutritional deficiencies
Maternal and neonatal disorders
Other infectious diseases
Neglected tropical diseases and malaria
Enteric infections
Respiratory infections and tuberculosis
HIV/AIDS and sexually transmitted infections

New Zealand

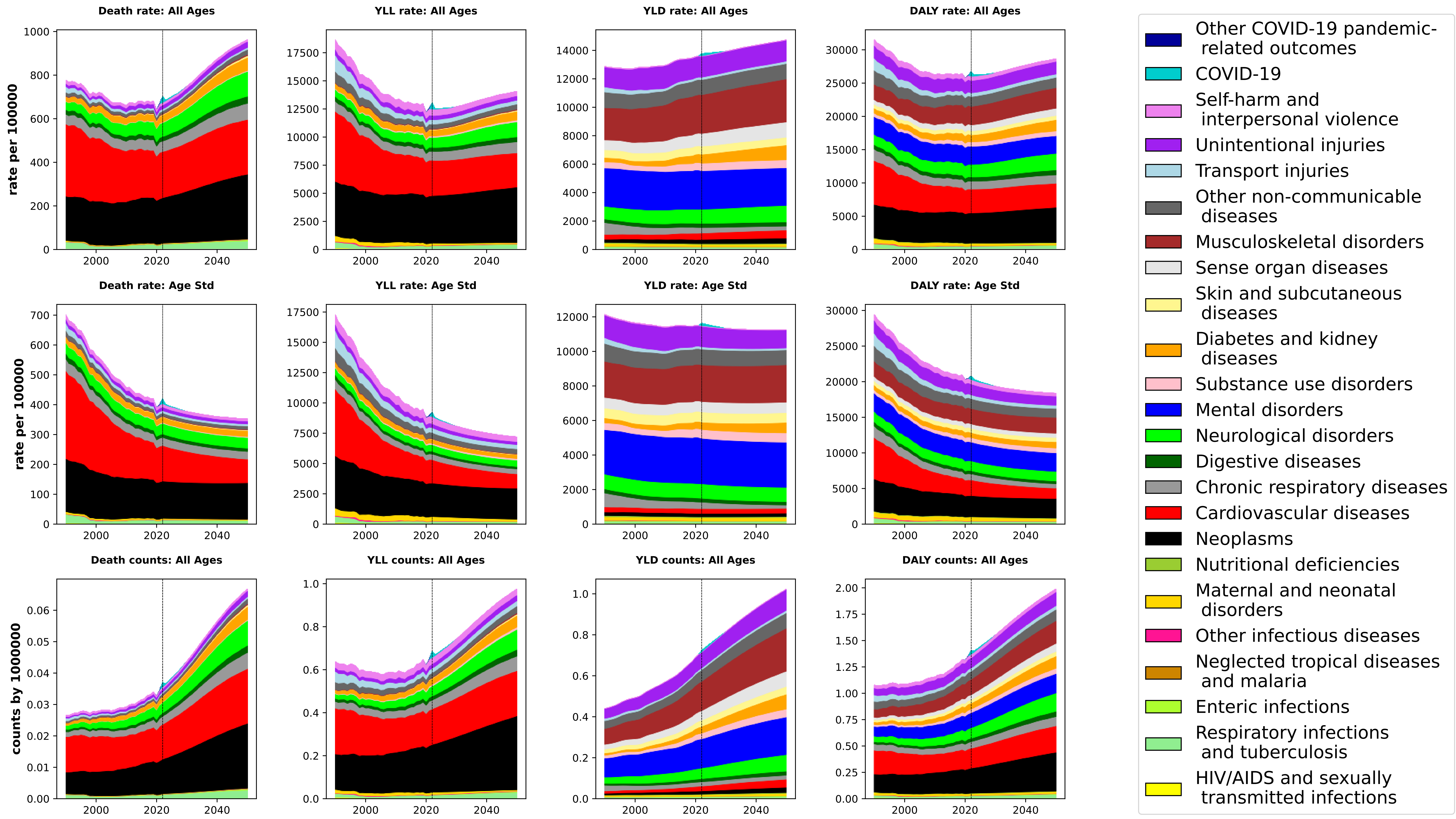
Death rate: All Ages
YLL rate: All Ages
YLD rate: All Ages
DALY rate: All Ages
Death rate: Age Std
YLL rate: Age Std
YLD rate: Age Std
DALY rate: Age Std
Death counts: All Ages
YLL counts: All Ages
YLD counts: All Ages
DALY counts: All Ages
rate per 100000
counts by 1000000
2000
2020
2040
Other COVID-19 pandemic-related outcomes
COVID-19
Self-harm and interpersonal violence
Unintentional injuries
Transport injuries
Other non-communicable diseases
Musculoskeletal disorders
Sense organ diseases
Skin and subcutaneous diseases
Diabetes and kidney diseases
Substance use disorders
Mental disorders
Neurological disorders
Digestive diseases
Chronic respiratory diseases
Cardiovascular diseases
Neoplasms
Nutritional deficiencies
Maternal and neonatal disorders
Other infectious diseases
Neglected tropical diseases and malaria
Enteric infections
Respiratory infections and tuberculosis
HIV/AIDS and sexually transmitted infections

Brunei

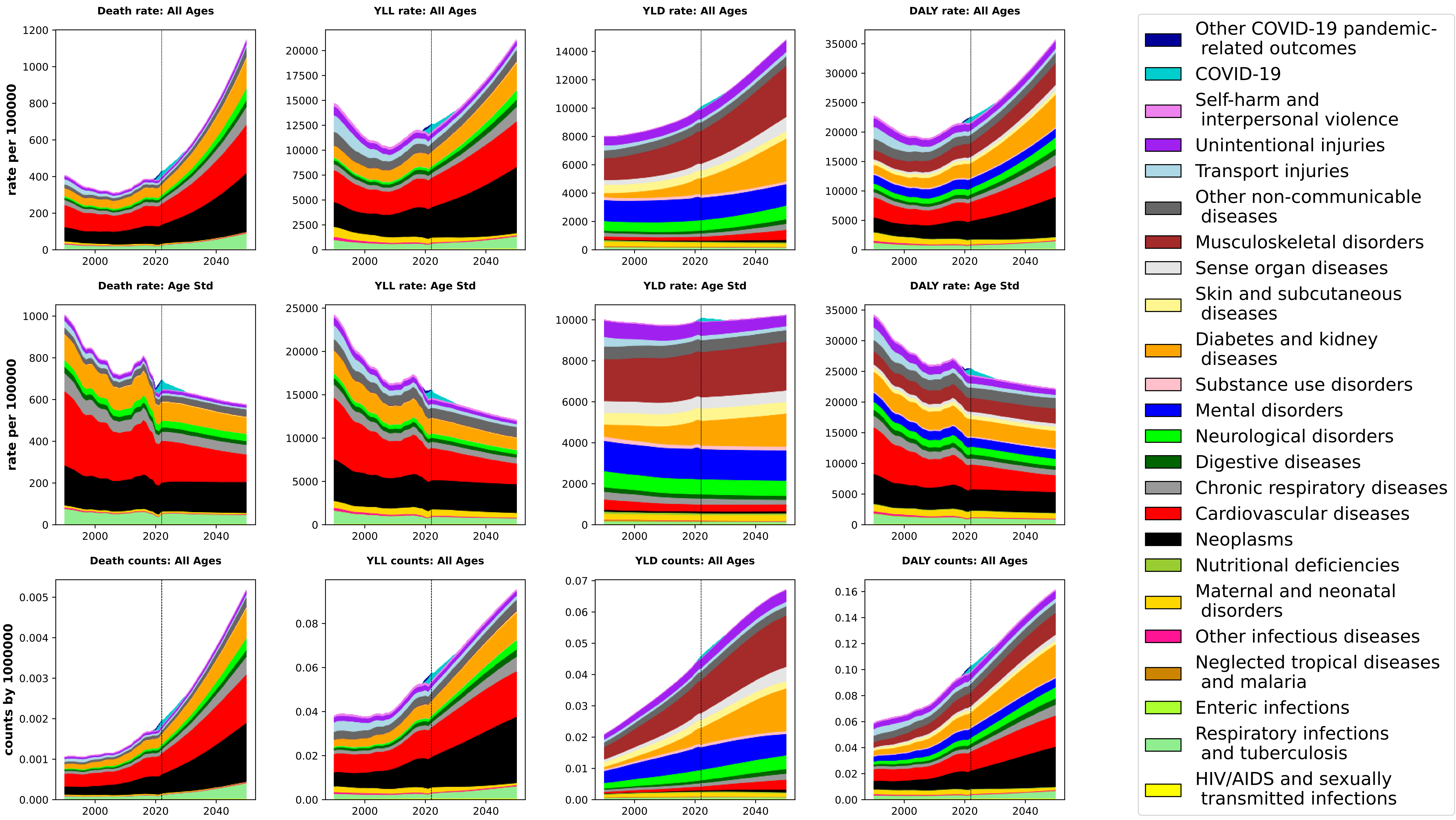
Death rate: All Ages
YLL rate: All Ages
YLD rate: All Ages
DALY rate: All Ages
Death rate: Age Std
YLL rate: Age Std
YLD rate: Age Std
DALY rate: Age Std
Death counts: All Ages
YLL counts: All Ages
YLD counts: All Ages
DALY counts: All Ages
rate per 100000
counts by 1000000
Other COVID-19 pandemic-related outcomes
COVID-19
Self-harm and interpersonal violence
Unintentional injuries
Transport injuries
Other non-communicable diseases
Musculoskeletal disorders
Sense organ diseases
Skin and subcutaneous diseases
Diabetes and kidney diseases
Substance use disorders
Mental disorders
Neurological disorders
Digestive diseases
Chronic respiratory diseases
Cardiovascular diseases
Neoplasms
Nutritional deficiencies
Maternal and neonatal disorders
Other infectious diseases
Neglected tropical diseases and malaria
Enteric infections
Respiratory infections and tuberculosis
HIV/AIDS and sexually transmitted infections

Japan

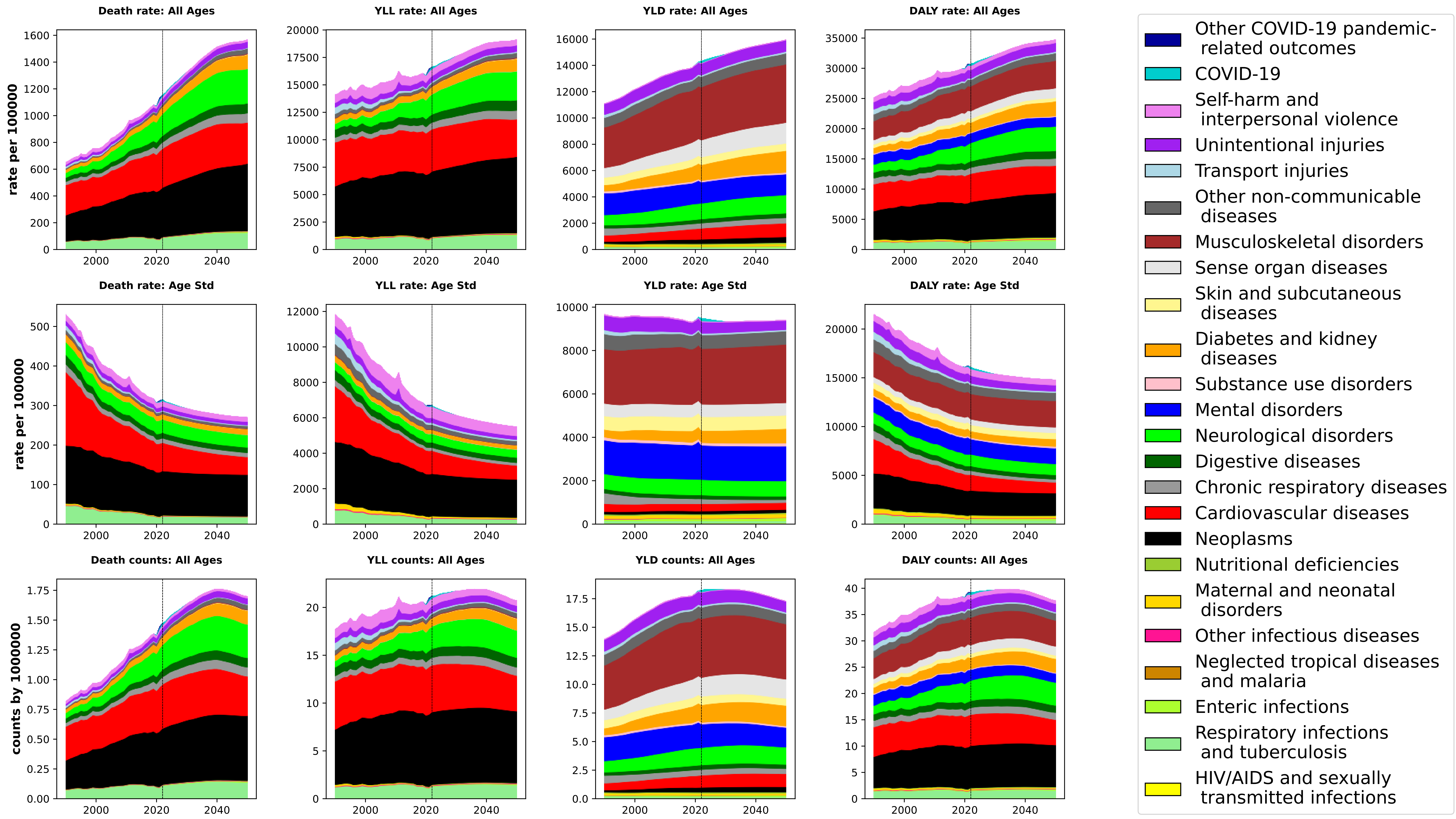
Death rate: All Ages
YLL rate: All Ages
YLD rate: All Ages
DALY rate: All Ages
Death rate: Age Std
YLL rate: Age Std
YLD rate: Age Std
DALY rate: Age Std
Death counts: All Ages
YLL counts: All Ages
YLD counts: All Ages
DALY counts: All Ages
rate per 100000
counts by 1000000
Other COVID-19 pandemic-related outcomes
COVID-19
Self-harm and interpersonal violence
Unintentional injuries
Transport injuries
Other non-communicable diseases
Musculoskeletal disorders
Sense organ diseases
Skin and subcutaneous diseases
Diabetes and kidney diseases
Substance use disorders
Mental disorders
Neurological disorders
Digestive diseases
Chronic respiratory diseases
Cardiovascular diseases
Neoplasms
Nutritional deficiencies
Maternal and neonatal disorders
Other infectious diseases
Neglected tropical diseases and malaria
Enteric infections
Respiratory infections and tuberculosis
HIV/AIDS and sexually transmitted infections

South Korea

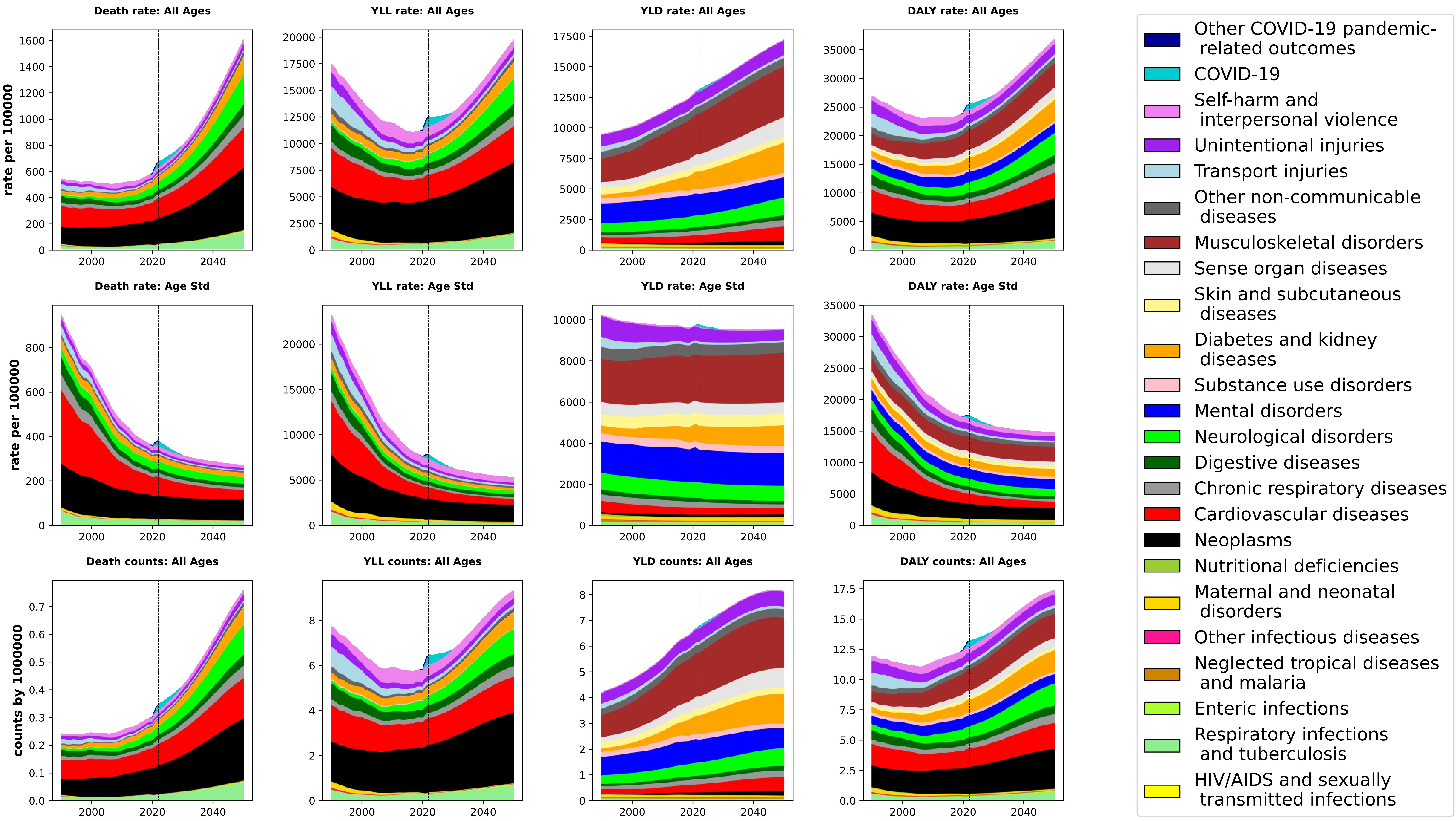
Death rate: All Ages
YLL rate: All Ages
YLD rate: All Ages
DALY rate: All Ages
Death rate: Age Std
YLL rate: Age Std
YLD rate: Age Std
DALY rate: Age Std
Death counts: All Ages
YLL counts: All Ages
YLD counts: All Ages
DALY counts: All Ages
rate per 100000
counts by 1000000
Other COVID-19 pandemic-related outcomes
COVID-19
Self-harm and interpersonal violence
Unintentional injuries
Transport injuries
Other non-communicable diseases
Musculoskeletal disorders
Sense organ diseases
Skin and subcutaneous diseases
Diabetes and kidney diseases
Substance use disorders
Mental disorders
Neurological disorders
Digestive diseases
Chronic respiratory diseases
Cardiovascular diseases
Neoplasms
Nutritional deficiencies
Maternal and neonatal disorders
Other infectious diseases
Neglected tropical diseases and malaria
Enteric infections
Respiratory infections and tuberculosis
HIV/AIDS and sexually transmitted infections

Singapore

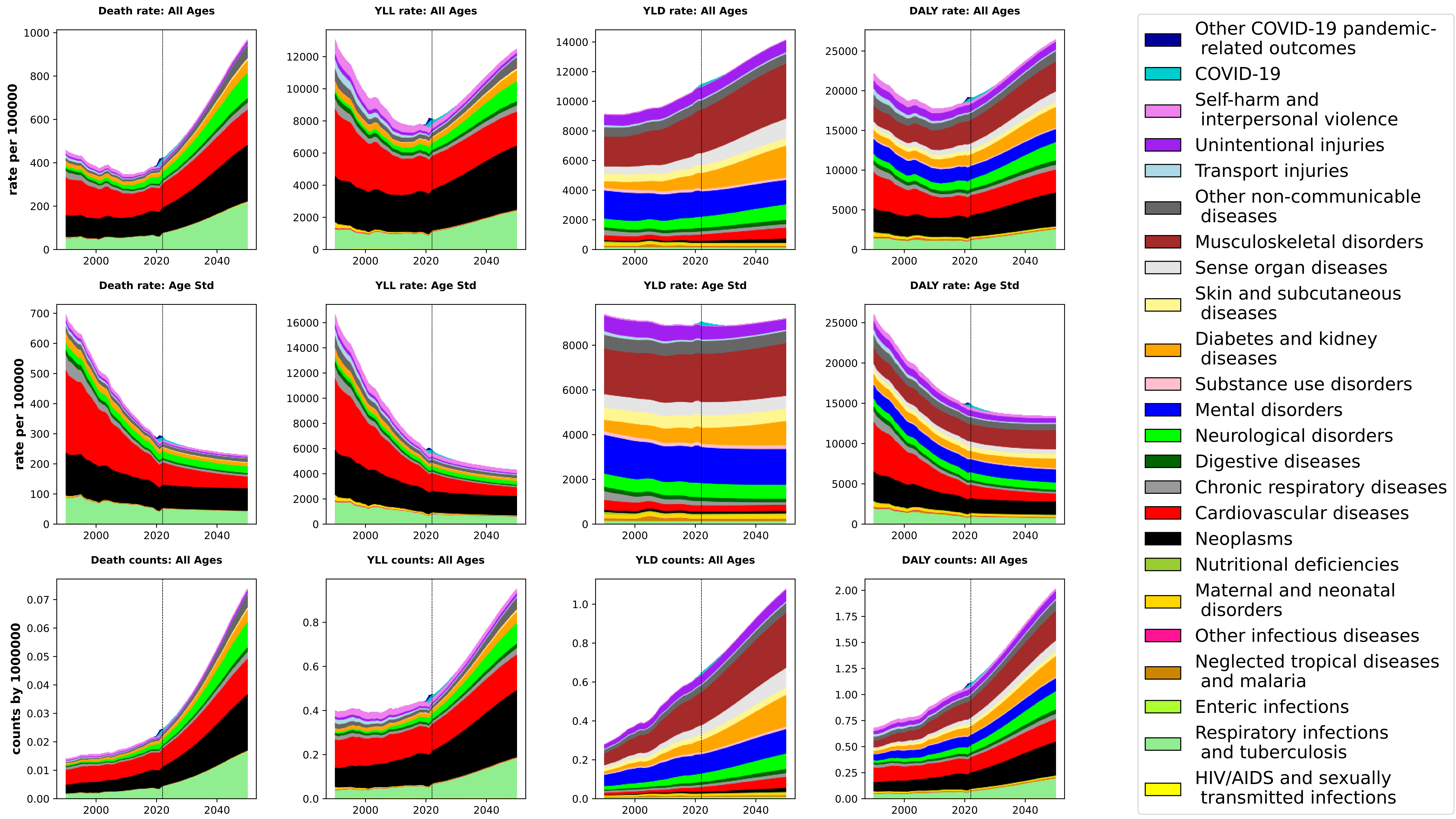
Death rate: All Ages
YLL rate: All Ages
YLD rate: All Ages
DALY rate: All Ages
Death rate: Age Std
YLL rate: Age Std
YLD rate: Age Std
DALY rate: Age Std
Death counts: All Ages
YLL counts: All Ages
YLD counts: All Ages
DALY counts: All Ages
rate per 100000
counts by 1000000
Other COVID-19 pandemic-related outcomes
COVID-19
Self-harm and interpersonal violence
Unintentional injuries
Transport injuries
Other non-communicable diseases
Musculoskeletal disorders
Sense organ diseases
Skin and subcutaneous diseases
Diabetes and kidney diseases
Substance use disorders
Mental disorders
Neurological disorders
Digestive diseases
Chronic respiratory diseases
Cardiovascular diseases
Neoplasms
Nutritional deficiencies
Maternal and neonatal disorders
Other infectious diseases
Neglected tropical diseases and malaria
Enteric infections
Respiratory infections and tuberculosis
HIV/AIDS and sexually transmitted infections

Canada

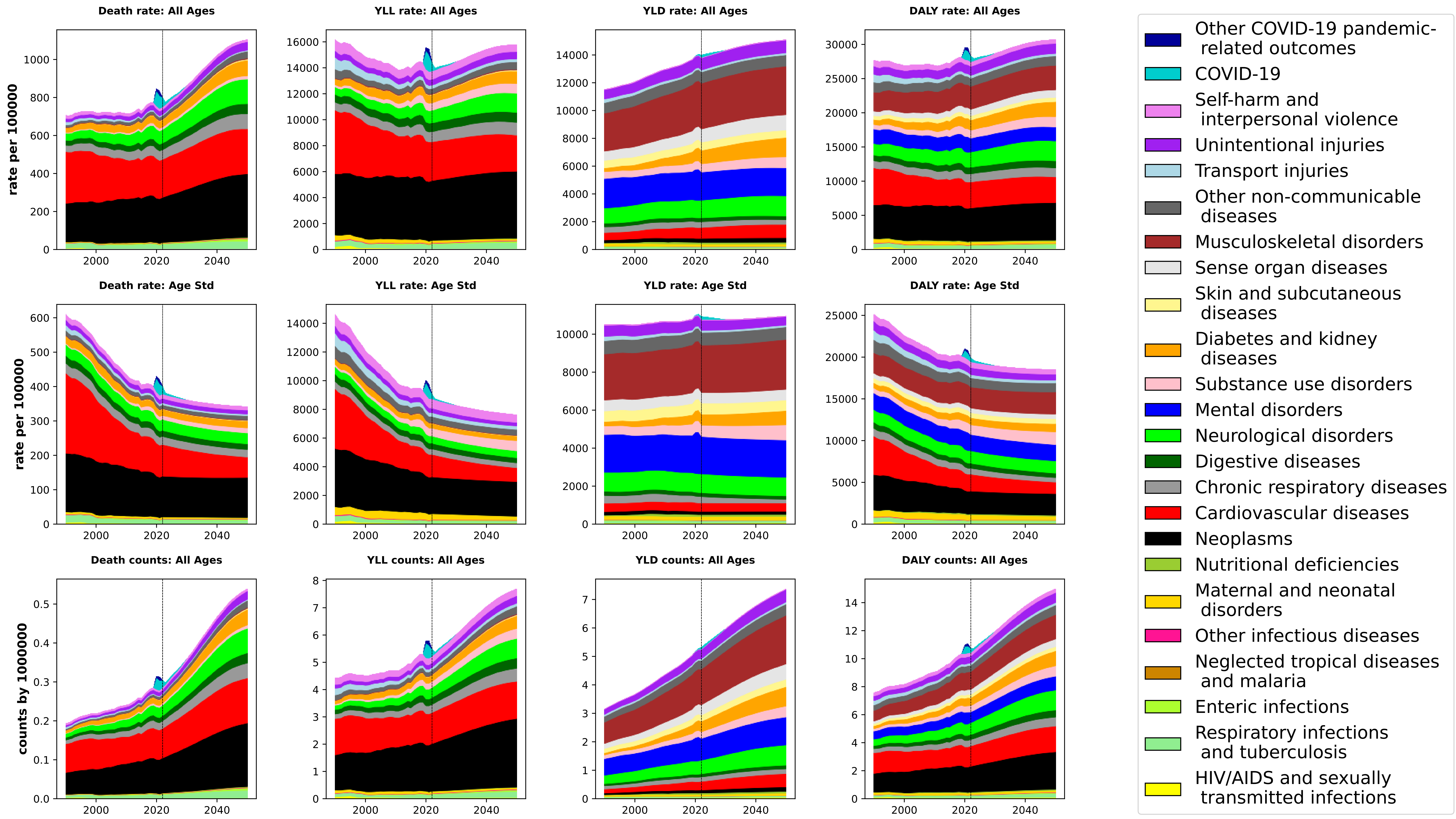
Death rate: All Ages
YLL rate: All Ages
YLD rate: All Ages
DALY rate: All Ages
Death rate: Age Std
YLL rate: Age Std
YLD rate: Age Std
DALY rate: Age Std
Death counts: All Ages
YLL counts: All Ages
YLD counts: All Ages
DALY counts: All Ages
rate per 100000
counts by 1000000
Other COVID-19 pandemic-related outcomes
COVID-19
Self-harm and interpersonal violence
Unintentional injuries
Transport injuries
Other non-communicable diseases
Musculoskeletal disorders
Sense organ diseases
Skin and subcutaneous diseases
Diabetes and kidney diseases
Substance use disorders
Mental disorders
Neurological disorders
Digestive diseases
Chronic respiratory diseases
Cardiovascular diseases
Neoplasms
Nutritional deficiencies
Maternal and neonatal disorders
Other infectious diseases
Neglected tropical diseases and malaria
Enteric infections
Respiratory infections and tuberculosis
HIV/AIDS and sexually transmitted infections

Greenland

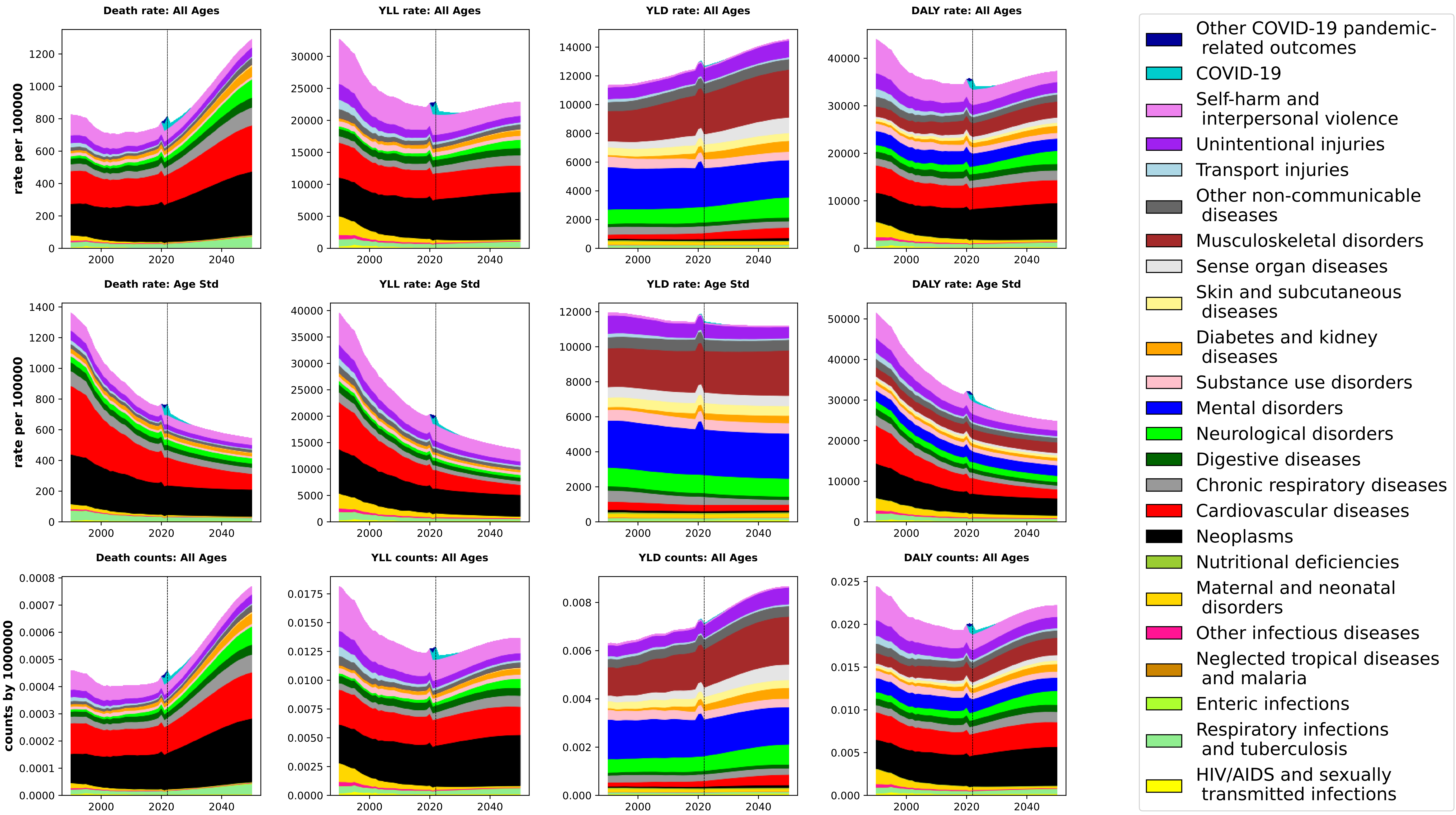
Death rate: All Ages
YLL rate: All Ages
YLD rate: All Ages
DALY rate: All Ages
Death rate: Age Std
YLL rate: Age Std
YLD rate: Age Std
DALY rate: Age Std
Death counts: All Ages
YLL counts: All Ages
YLD counts: All Ages
DALY counts: All Ages
rate per 100000
counts by 1000000
Other COVID-19 pandemic-related outcomes
COVID-19
Self-harm and interpersonal violence
Unintentional injuries
Transport injuries
Other non-communicable diseases
Musculoskeletal disorders
Sense organ diseases
Skin and subcutaneous diseases
Diabetes and kidney diseases
Substance use disorders
Mental disorders
Neurological disorders
Digestive diseases
Chronic respiratory diseases
Cardiovascular diseases
Neoplasms
Nutritional deficiencies
Maternal and neonatal disorders
Other infectious diseases
Neglected tropical diseases and malaria
Enteric infections
Respiratory infections and tuberculosis
HIV/AIDS and sexually transmitted infections

USA

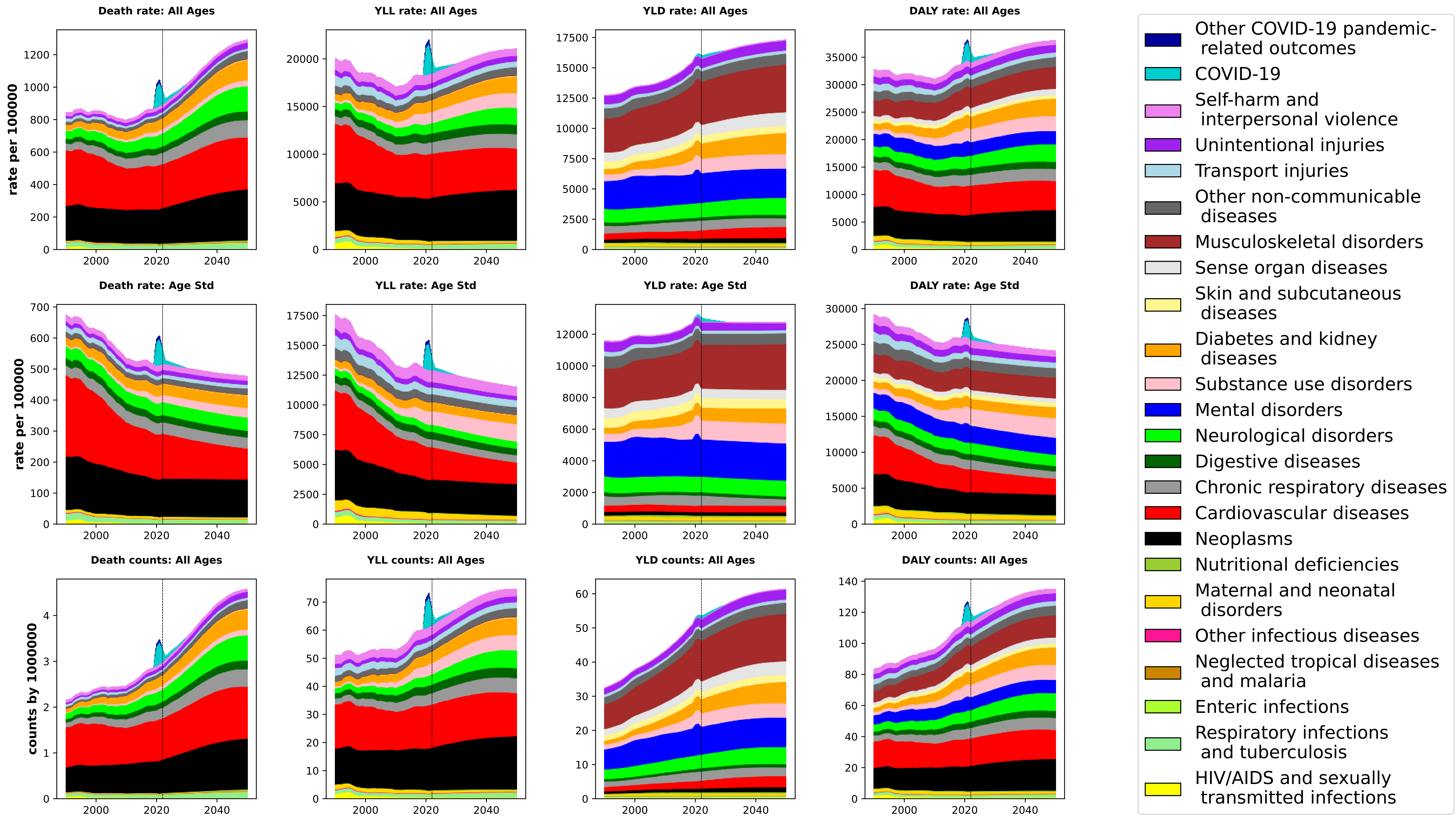
Death rate: All Ages
YLL rate: All Ages
YLD rate: All Ages
DALY rate: All Ages
Death rate: Age Std
YLL rate: Age Std
YLD rate: Age Std
DALY rate: Age Std
Death counts: All Ages
YLL counts: All Ages
YLD counts: All Ages
DALY counts: All Ages
rate per 100000
counts by 1000000
Other COVID-19 pandemic-related outcomes
COVID-19
Self-harm and interpersonal violence
Unintentional injuries
Transport injuries
Other non-communicable diseases
Musculoskeletal disorders
Sense organ diseases
Skin and subcutaneous diseases
Diabetes and kidney diseases
Substance use disorders
Mental disorders
Neurological disorders
Digestive diseases
Chronic respiratory diseases
Cardiovascular diseases
Neoplasms
Nutritional deficiencies
Maternal and neonatal disorders
Other infectious diseases
Neglected tropical diseases and malaria
Enteric infections
Respiratory infections and tuberculosis
HIV/AIDS and sexually transmitted infections

Argentina

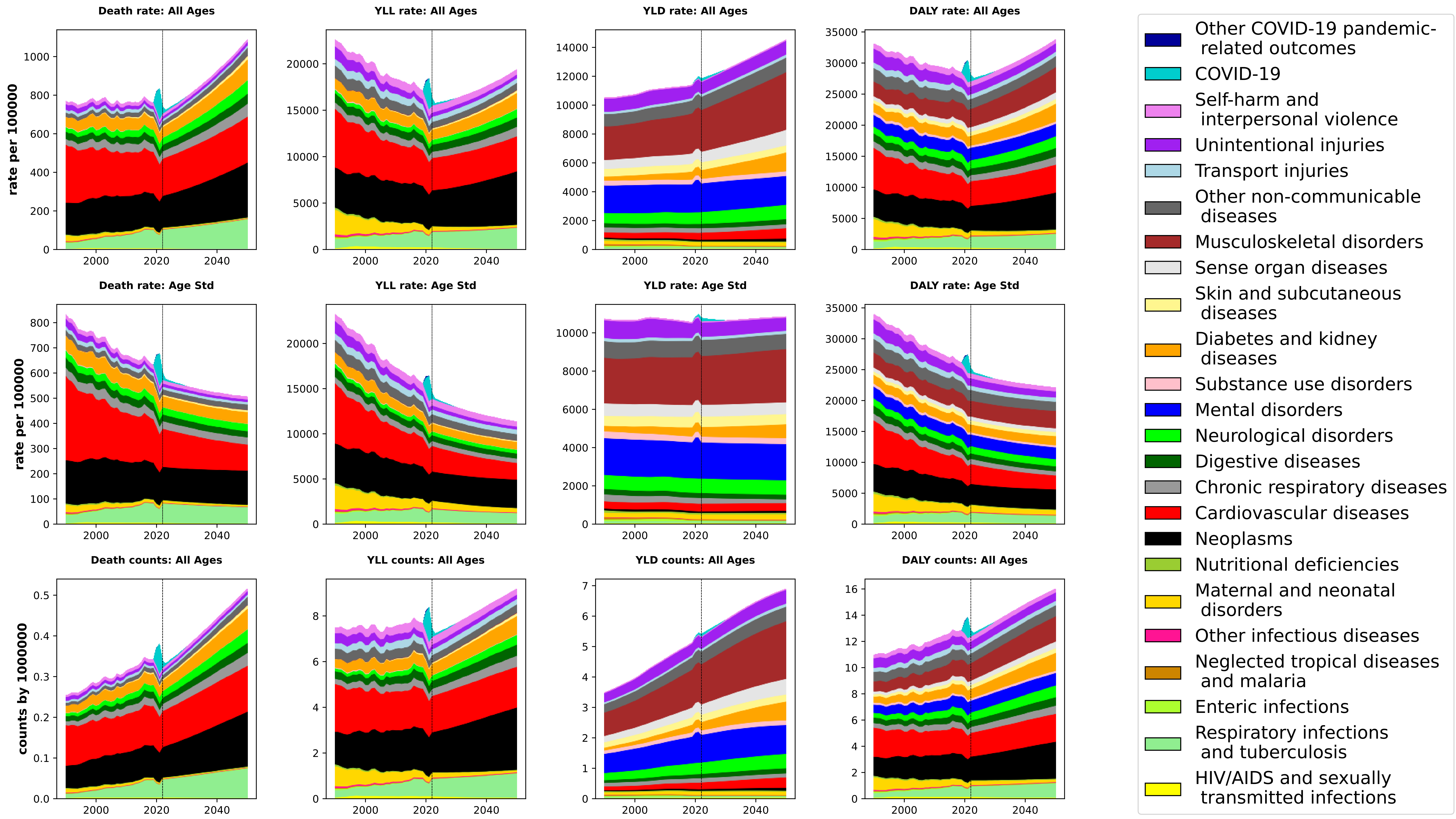
Death rate: All Ages
YLL rate: All Ages
YLD rate: All Ages
DALY rate: All Ages
Death rate: Age Std
YLL rate: Age Std
YLD rate: Age Std
DALY rate: Age Std
Death counts: All Ages
YLL counts: All Ages
YLD counts: All Ages
DALY counts: All Ages
rate per 100000
counts by 1000000
2000
2020
2040
Other COVID-19 pandemic-related outcomes
COVID-19
Self-harm and interpersonal violence
Unintentional injuries
Transport injuries
Other non-communicable diseases
Musculoskeletal disorders
Sense organ diseases
Skin and subcutaneous diseases
Diabetes and kidney diseases
Substance use disorders
Mental disorders
Neurological disorders
Digestive diseases
Chronic respiratory diseases
Cardiovascular diseases
Neoplasms
Nutritional deficiencies
Maternal and neonatal disorders
Other infectious diseases
Neglected tropical diseases and malaria
Enteric infections
Respiratory infections and tuberculosis
HIV/AIDS and sexually transmitted infections

Chile

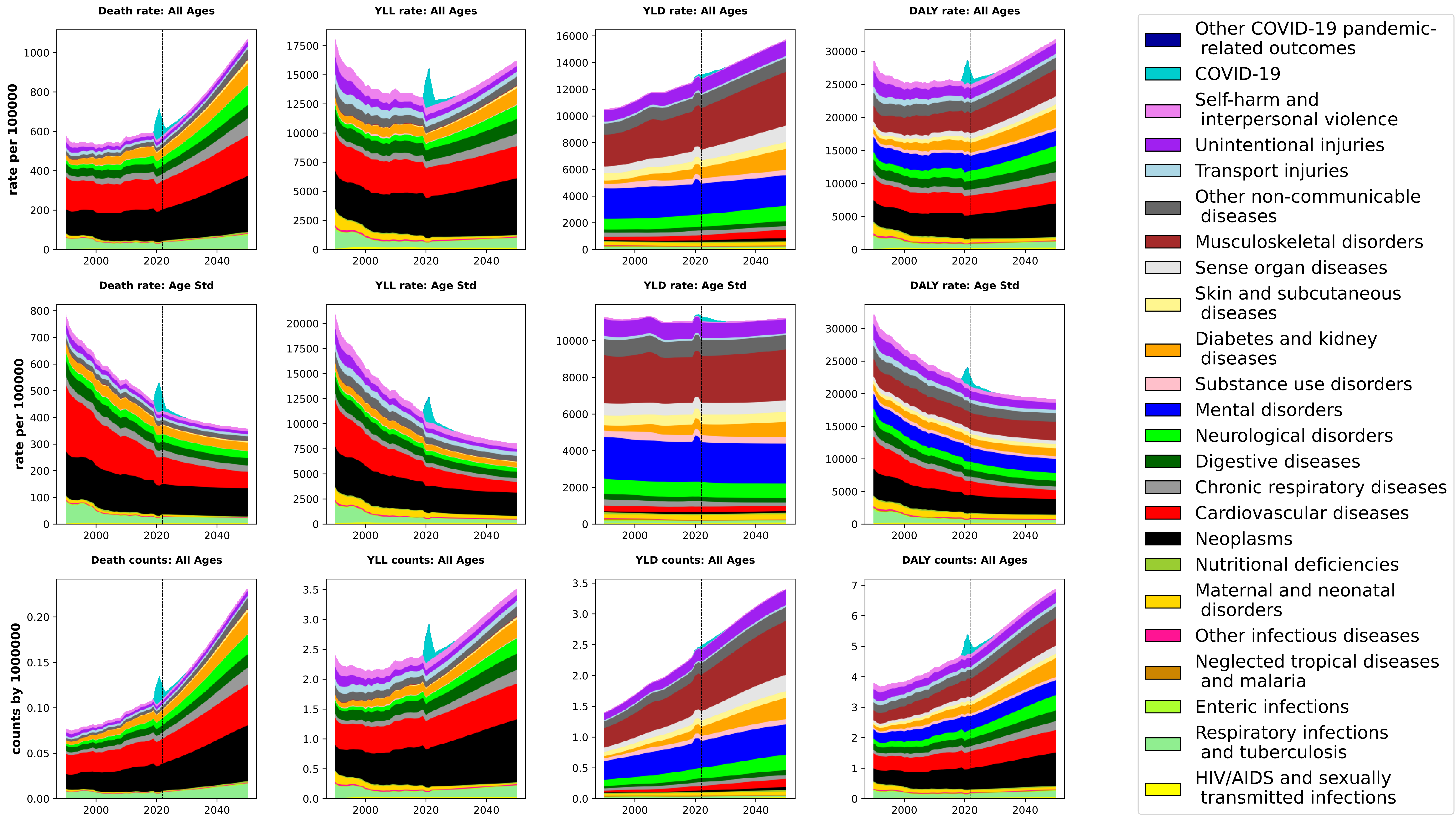
Death rate: All Ages
YLL rate: All Ages
YLD rate: All Ages
DALY rate: All Ages
Death rate: Age Std
YLL rate: Age Std
YLD rate: Age Std
DALY rate: Age Std
Death counts: All Ages
YLL counts: All Ages
YLD counts: All Ages
DALY counts: All Ages
rate per 100000
counts by 1000000
2000
2020
2040
Other COVID-19 pandemic-related outcomes
COVID-19
Self-harm and interpersonal violence
Unintentional injuries
Transport injuries
Other non-communicable diseases
Musculoskeletal disorders
Sense organ diseases
Skin and subcutaneous diseases
Diabetes and kidney diseases
Substance use disorders
Mental disorders
Neurological disorders
Digestive diseases
Chronic respiratory diseases
Cardiovascular diseases
Neoplasms
Nutritional deficiencies
Maternal and neonatal disorders
Other infectious diseases
Neglected tropical diseases and malaria
Enteric infections
Respiratory infections and tuberculosis
HIV/AIDS and sexually transmitted infections

Uruguay

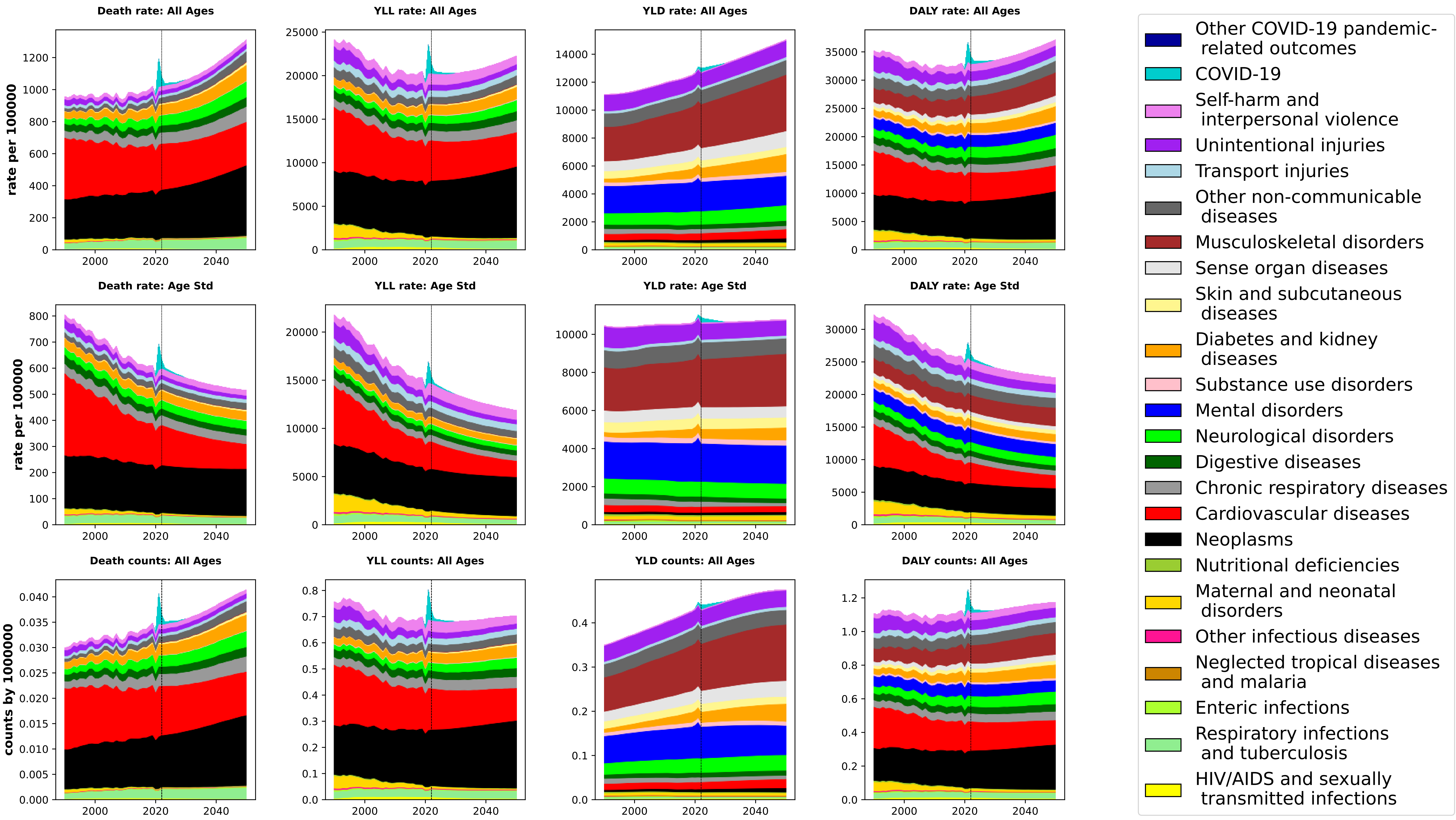
Death rate: All Ages
YLL rate: All Ages
YLD rate: All Ages
DALY rate: All Ages
Death rate: Age Std
YLL rate: Age Std
YLD rate: Age Std
DALY rate: Age Std
Death counts: All Ages
YLL counts: All Ages
YLD counts: All Ages
DALY counts: All Ages
rate per 100000
rate per 100000
counts by 1000000
Other COVID-19 pandemic-related outcomes
COVID-19
Self-harm and interpersonal violence
Unintentional injuries
Transport injuries
Other non-communicable diseases
Musculoskeletal disorders
Sense organ diseases
Skin and subcutaneous diseases
Diabetes and kidney diseases
Substance use disorders
Mental disorders
Neurological disorders
Digestive diseases
Chronic respiratory diseases
Cardiovascular diseases
Neoplasms
Nutritional deficiencies
Maternal and neonatal disorders
Other infectious diseases
Neglected tropical diseases and malaria
Enteric infections
Respiratory infections and tuberculosis
HIV/AIDS and sexually transmitted infections

Andorra

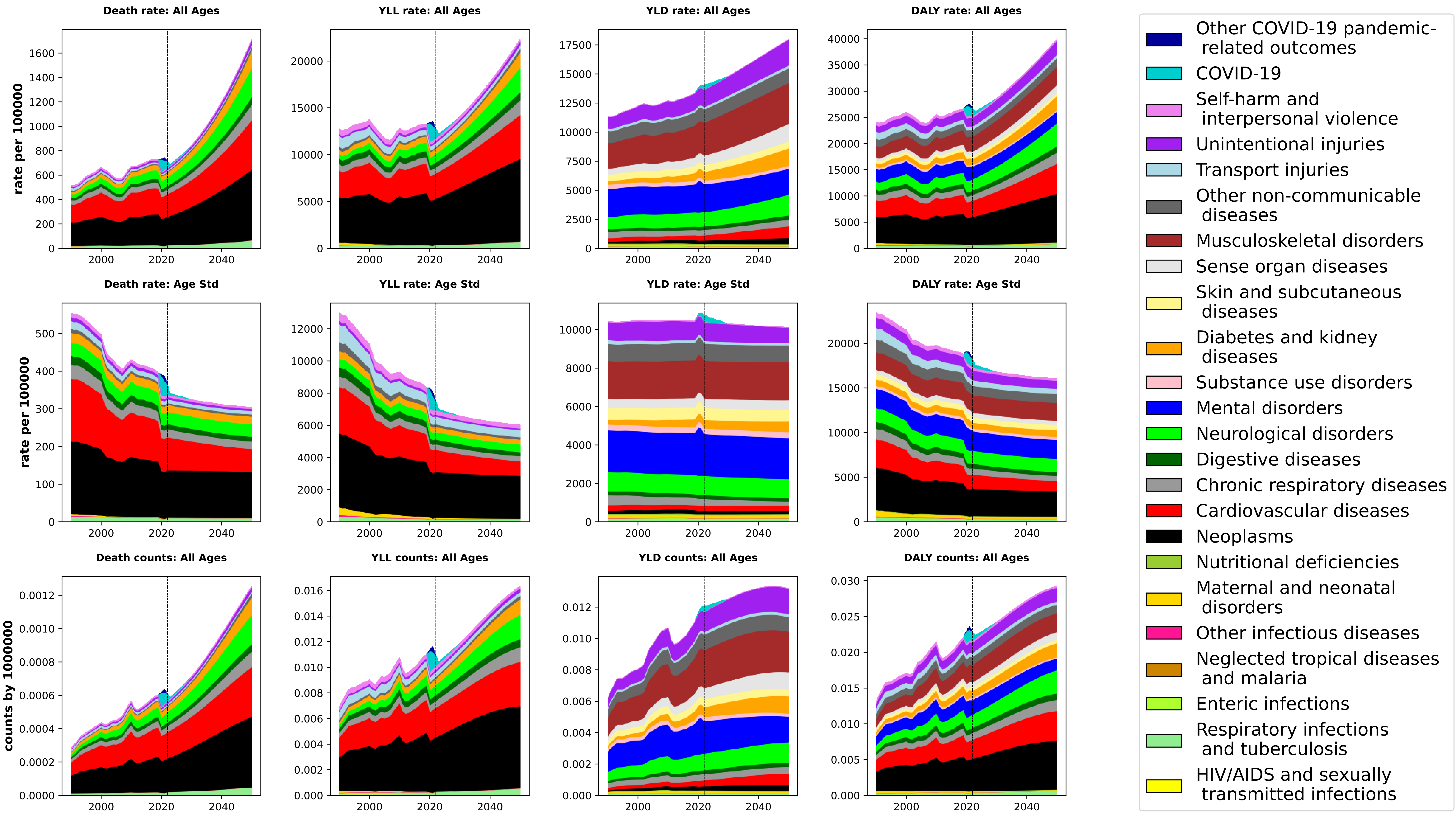
Death rate: All Ages
YLL rate: All Ages
YLD rate: All Ages
DALY rate: All Ages
Death rate: Age Std
YLL rate: Age Std
YLD rate: Age Std
DALY rate: Age Std
Death counts: All Ages
YLL counts: All Ages
YLD counts: All Ages
DALY counts: All Ages
rate per 100000
counts by 1000000
Other COVID-19 pandemic-related outcomes
COVID-19
Self-harm and interpersonal violence
Unintentional injuries
Transport injuries
Other non-communicable diseases
Musculoskeletal disorders
Sense organ diseases
Skin and subcutaneous diseases
Diabetes and kidney diseases
Substance use disorders
Mental disorders
Neurological disorders
Digestive diseases
Chronic respiratory diseases
Cardiovascular diseases
Neoplasms
Nutritional deficiencies
Maternal and neonatal disorders
Other infectious diseases
Neglected tropical diseases and malaria
Enteric infections
Respiratory infections and tuberculosis
HIV/AIDS and sexually transmitted infections

Austria

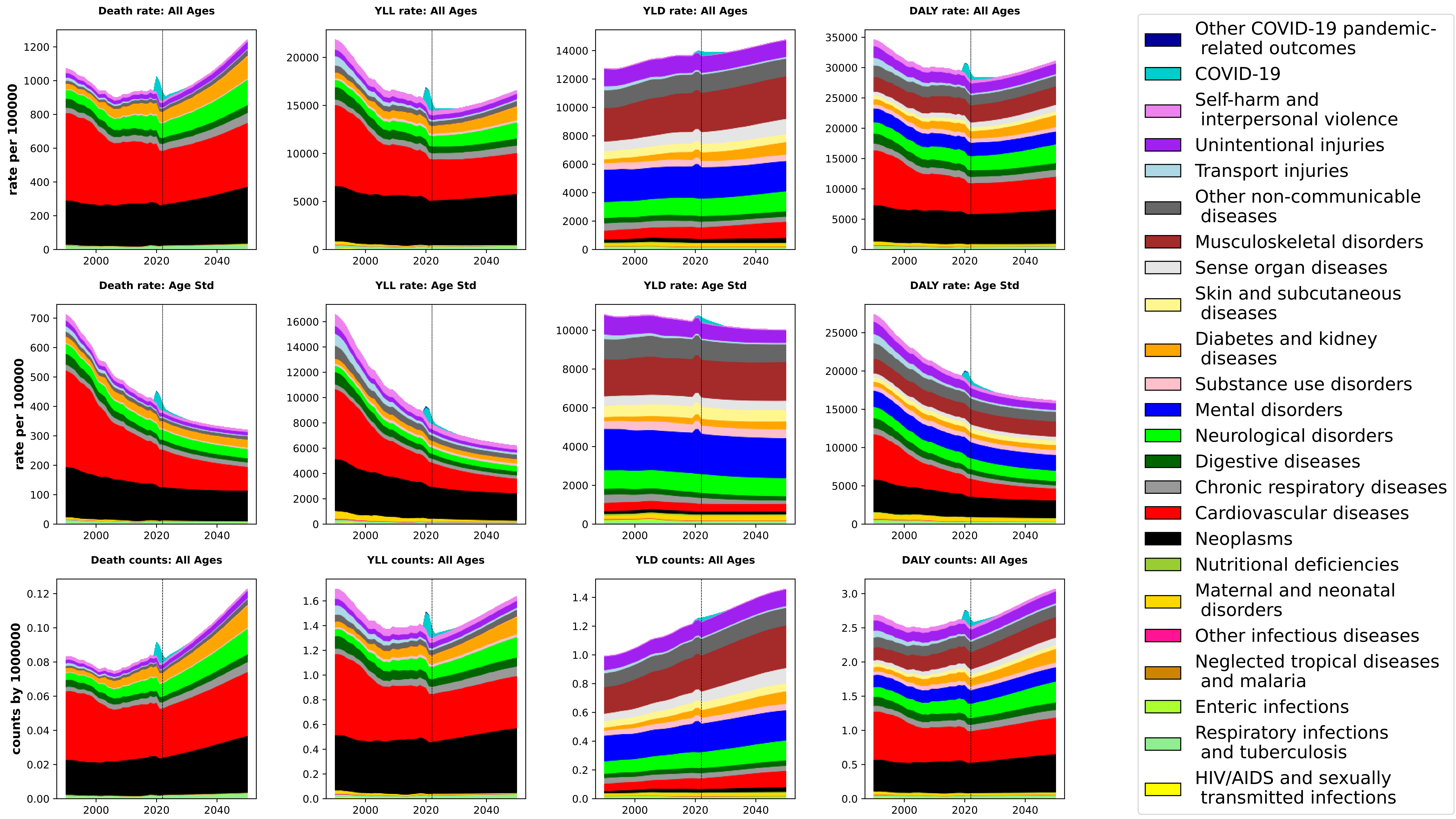
Death rate: All Ages
YLL rate: All Ages
YLD rate: All Ages
DALY rate: All Ages
Death rate: Age Std
YLL rate: Age Std
YLD rate: Age Std
DALY rate: Age Std
Death counts: All Ages
YLL counts: All Ages
YLD counts: All Ages
DALY counts: All Ages
rate per 100000
counts by 1000000
Other COVID-19 pandemic-related outcomes
COVID-19
Self-harm and interpersonal violence
Unintentional injuries
Transport injuries
Other non-communicable diseases
Musculoskeletal disorders
Sense organ diseases
Skin and subcutaneous diseases
Diabetes and kidney diseases
Substance use disorders
Mental disorders
Neurological disorders
Digestive diseases
Chronic respiratory diseases
Cardiovascular diseases
Neoplasms
Nutritional deficiencies
Maternal and neonatal disorders
Other infectious diseases
Neglected tropical diseases and malaria
Enteric infections
Respiratory infections and tuberculosis
HIV/AIDS and sexually transmitted infections

Belgium

Supplemental Results Figure S6. Cause level 2 area plots for 4 burden measures for reference scenario by location, 1990-2050

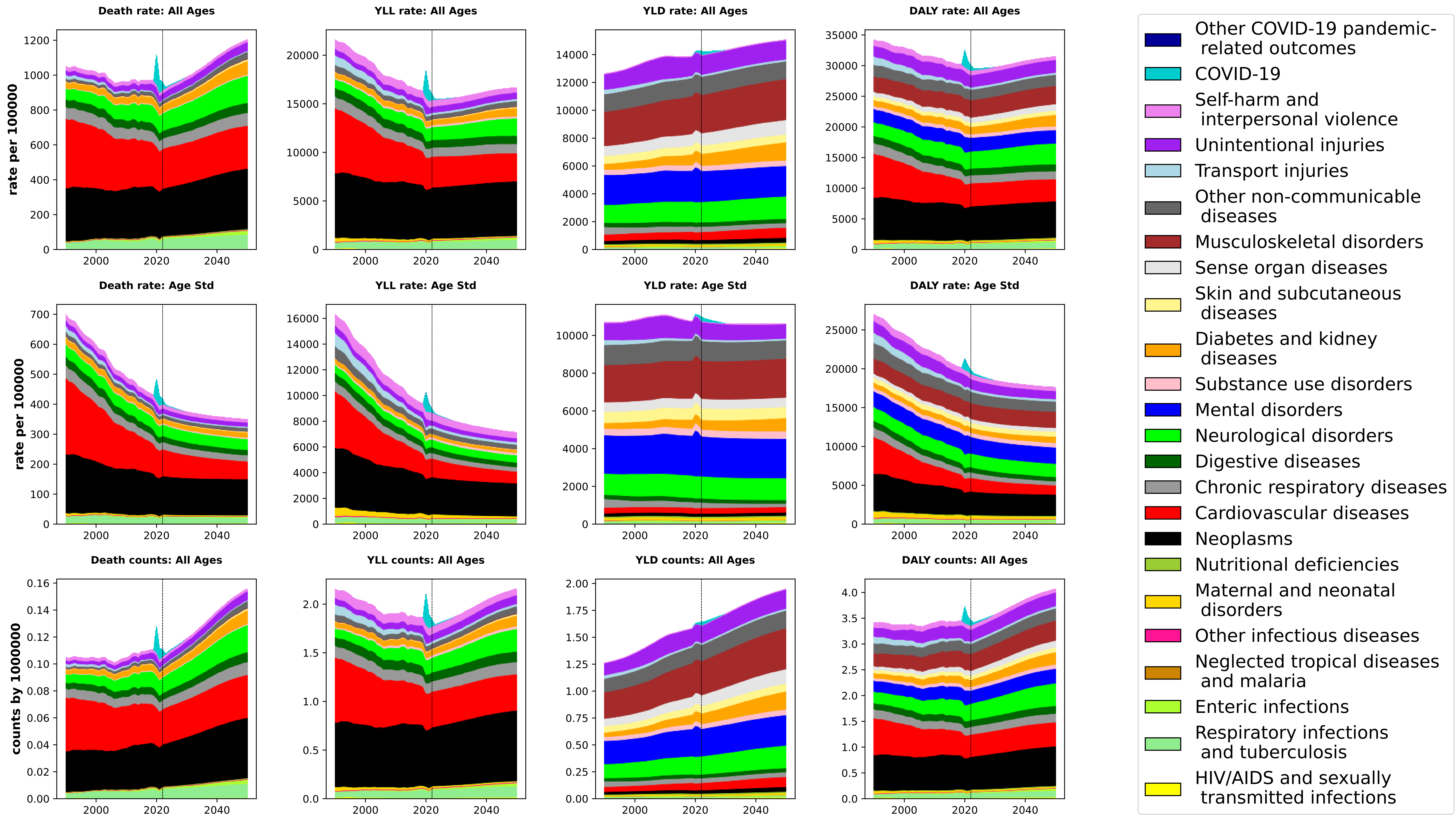

Cyprus

Supplemental Results Figure S6. Cause level 2 area plots for 4 burden measures for reference scenario by location, 1990-2050

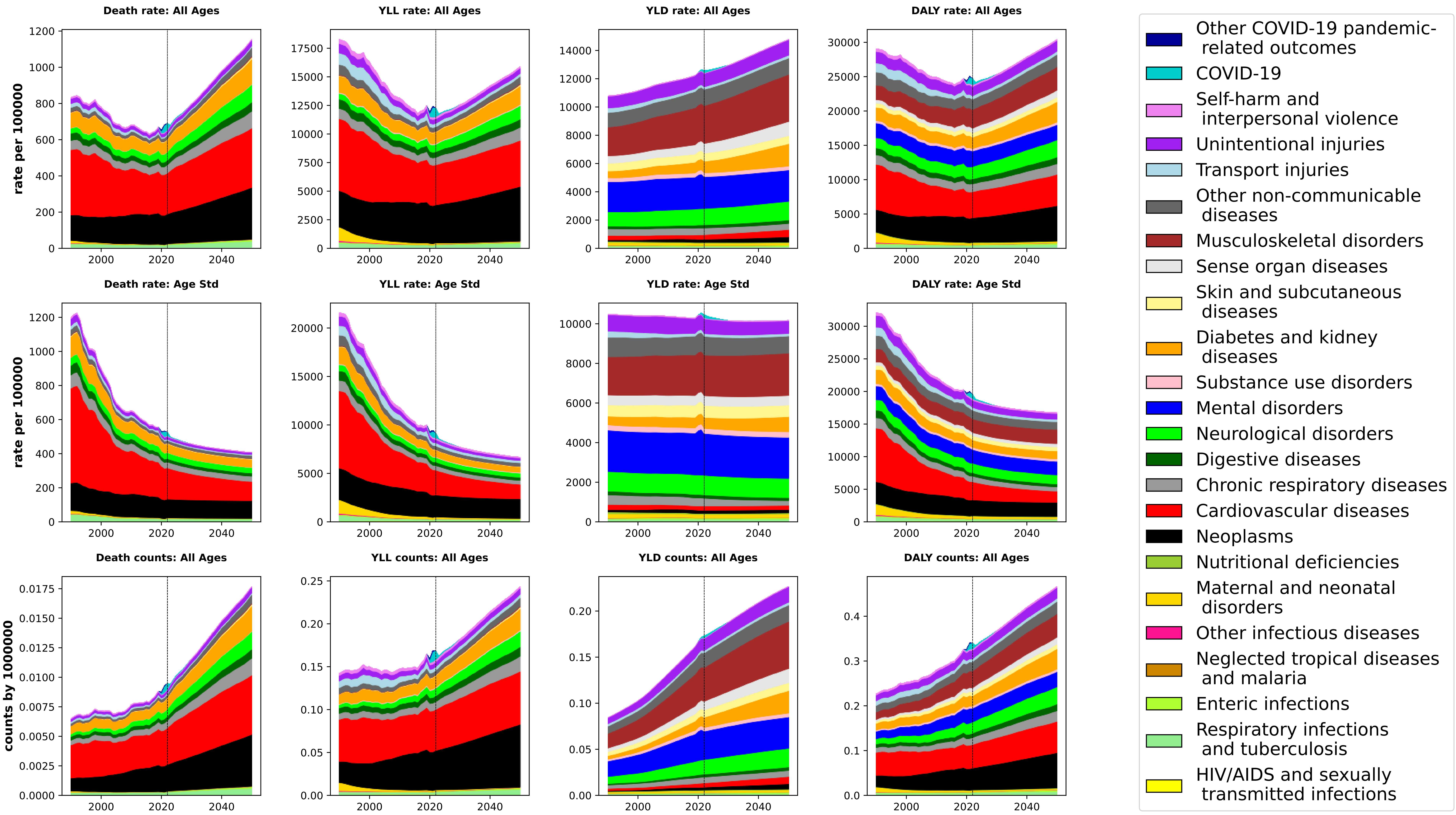

Denmark

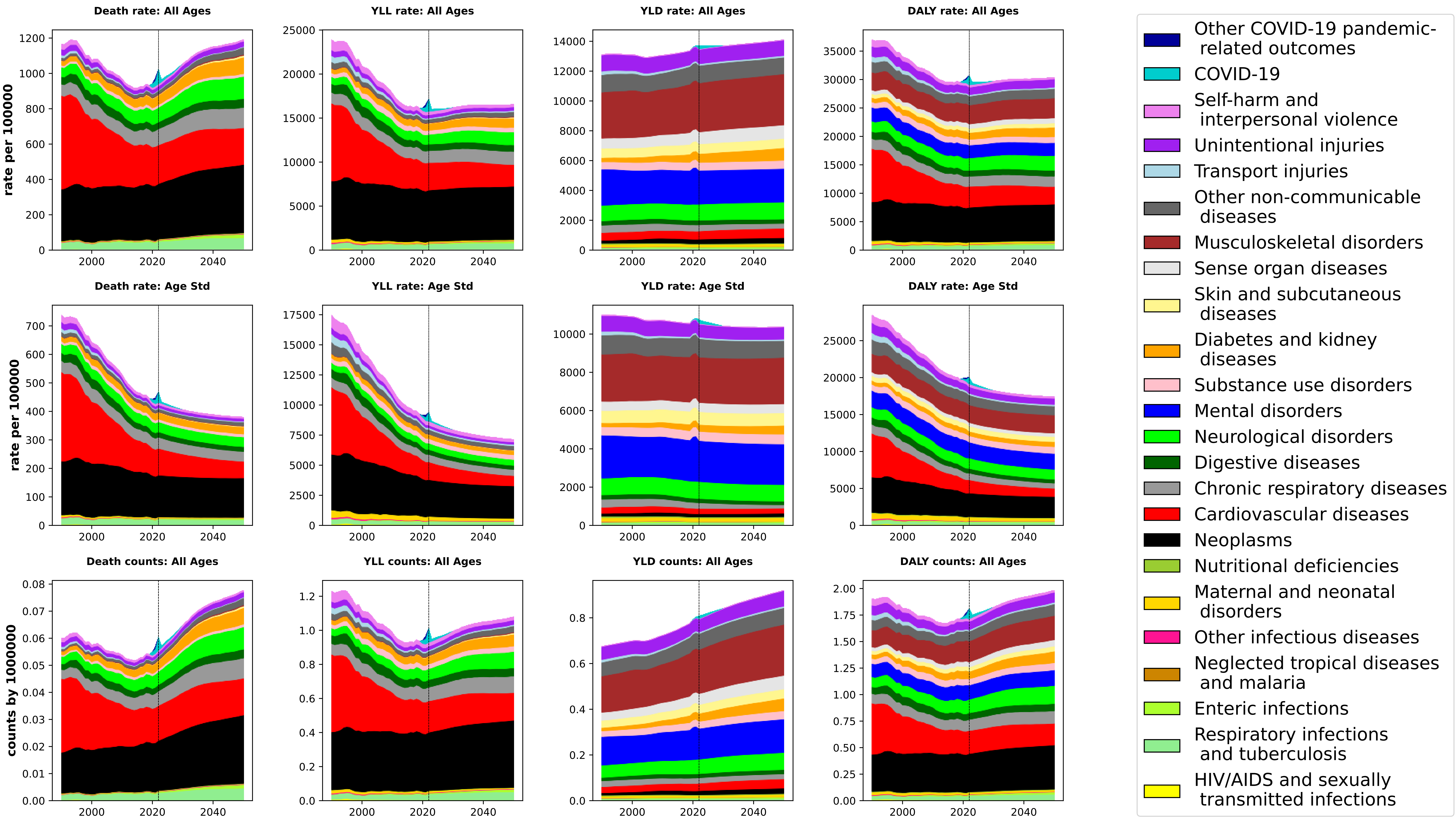
Death rate: All Ages
YLL rate: All Ages
YLD rate: All Ages
DALY rate: All Ages
Death rate: Age Std
YLL rate: Age Std
YLD rate: Age Std
DALY rate: Age Std
Death counts: All Ages
YLL counts: All Ages
YLD counts: All Ages
DALY counts: All Ages
rate per 100000
counts by 1000000
Other COVID-19 pandemic-related outcomes
COVID-19
Self-harm and interpersonal violence
Unintentional injuries
Transport injuries
Other non-communicable diseases
Musculoskeletal disorders
Sense organ diseases
Skin and subcutaneous diseases
Diabetes and kidney diseases
Substance use disorders
Mental disorders
Neurological disorders
Digestive diseases
Chronic respiratory diseases
Cardiovascular diseases
Neoplasms
Nutritional deficiencies
Maternal and neonatal disorders
Other infectious diseases
Neglected tropical diseases and malaria
Enteric infections
Respiratory infections and tuberculosis
HIV/AIDS and sexually transmitted infections

Finland

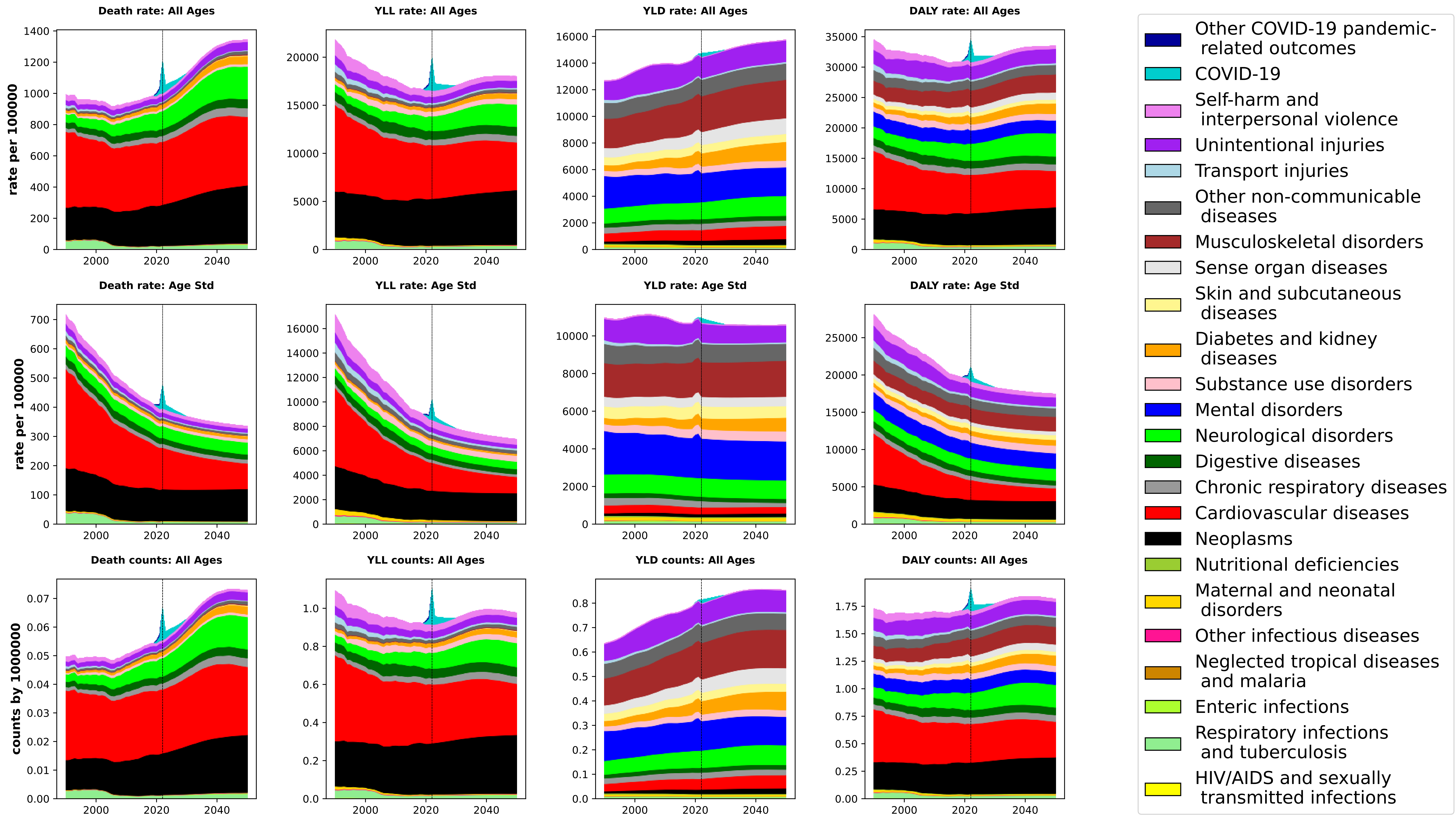
Death rate: All Ages
YLL rate: All Ages
YLD rate: All Ages
DALY rate: All Ages
Death rate: Age Std
YLL rate: Age Std
YLD rate: Age Std
DALY rate: Age Std
Death counts: All Ages
YLL counts: All Ages
YLD counts: All Ages
DALY counts: All Ages
rate per 100000
counts by 1000000
Other COVID-19 pandemic-related outcomes
COVID-19
Self-harm and interpersonal violence
Unintentional injuries
Transport injuries
Other non-communicable diseases
Musculoskeletal disorders
Sense organ diseases
Skin and subcutaneous diseases
Diabetes and kidney diseases
Substance use disorders
Mental disorders
Neurological disorders
Digestive diseases
Chronic respiratory diseases
Cardiovascular diseases
Neoplasms
Nutritional deficiencies
Maternal and neonatal disorders
Other infectious diseases
Neglected tropical diseases and malaria
Enteric infections
Respiratory infections and tuberculosis
HIV/AIDS and sexually transmitted infections

France

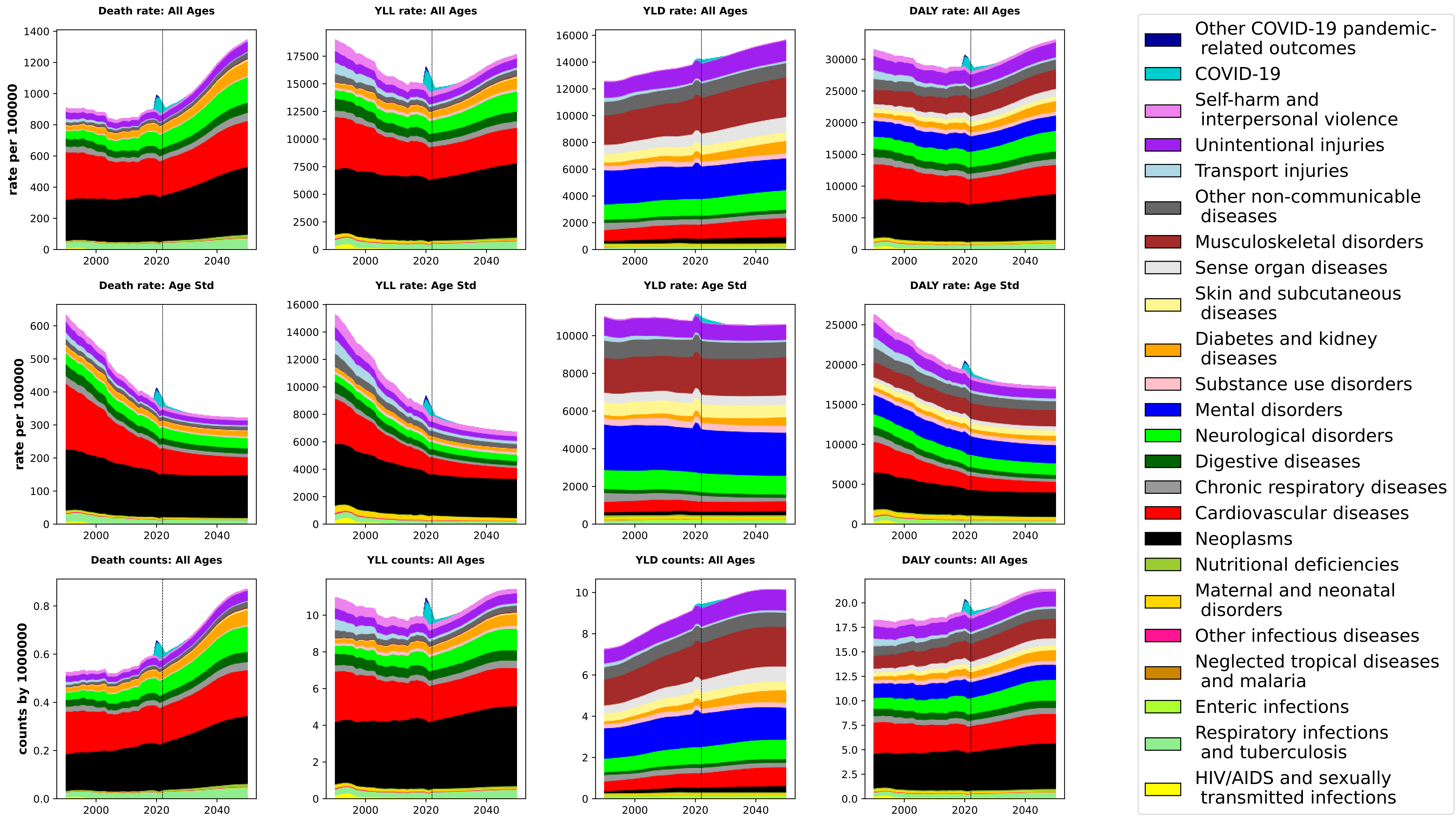
Death rate: All Ages
YLL rate: All Ages
YLD rate: All Ages
DALY rate: All Ages
Death rate: Age Std
YLL rate: Age Std
YLD rate: Age Std
DALY rate: Age Std
Death counts: All Ages
YLL counts: All Ages
YLD counts: All Ages
DALY counts: All Ages
rate per 100000
counts by 1000000
Other COVID-19 pandemic-related outcomes
COVID-19
Self-harm and interpersonal violence
Unintentional injuries
Transport injuries
Other non-communicable diseases
Musculoskeletal disorders
Sense organ diseases
Skin and subcutaneous diseases
Diabetes and kidney diseases
Substance use disorders
Mental disorders
Neurological disorders
Digestive diseases
Chronic respiratory diseases
Cardiovascular diseases
Neoplasms
Nutritional deficiencies
Maternal and neonatal disorders
Other infectious diseases
Neglected tropical diseases and malaria
Enteric infections
Respiratory infections and tuberculosis
HIV/AIDS and sexually transmitted infections

Germany

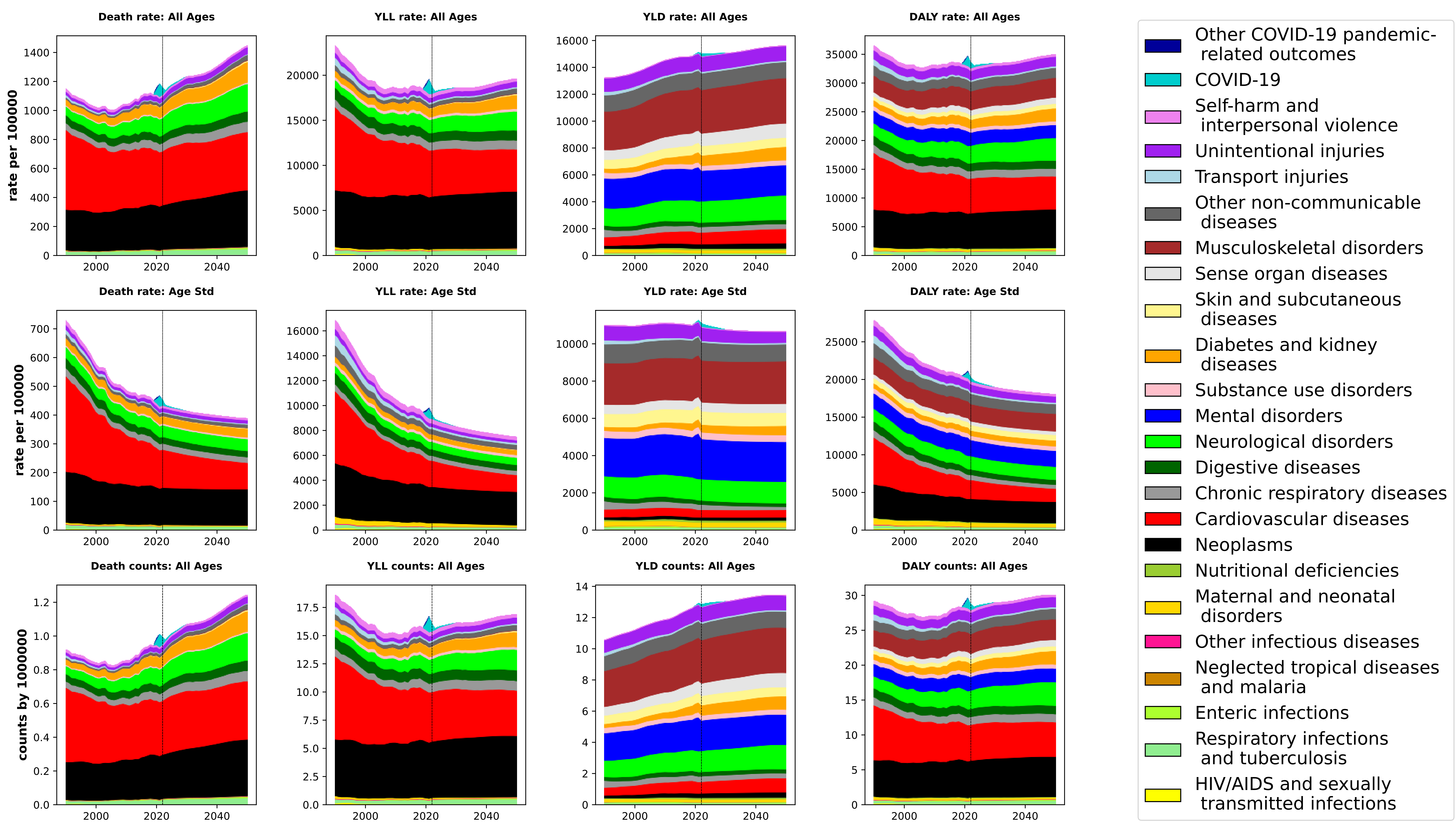
Death rate: All Ages
YLL rate: All Ages
YLD rate: All Ages
DALY rate: All Ages
Death rate: Age Std
YLL rate: Age Std
YLD rate: Age Std
DALY rate: Age Std
Death counts: All Ages
YLL counts: All Ages
YLD counts: All Ages
DALY counts: All Ages
rate per 100000
rate per 100000
counts by 1000000
Other COVID-19 pandemic-related outcomes
COVID-19
Self-harm and interpersonal violence
Unintentional injuries
Transport injuries
Other non-communicable diseases
Musculoskeletal disorders
Sense organ diseases
Skin and subcutaneous diseases
Diabetes and kidney diseases
Substance use disorders
Mental disorders
Neurological disorders
Digestive diseases
Chronic respiratory diseases
Cardiovascular diseases
Neoplasms
Nutritional deficiencies
Maternal and neonatal disorders
Other infectious diseases
Neglected tropical diseases and malaria
Enteric infections
Respiratory infections and tuberculosis
HIV/AIDS and sexually transmitted infections

Greece

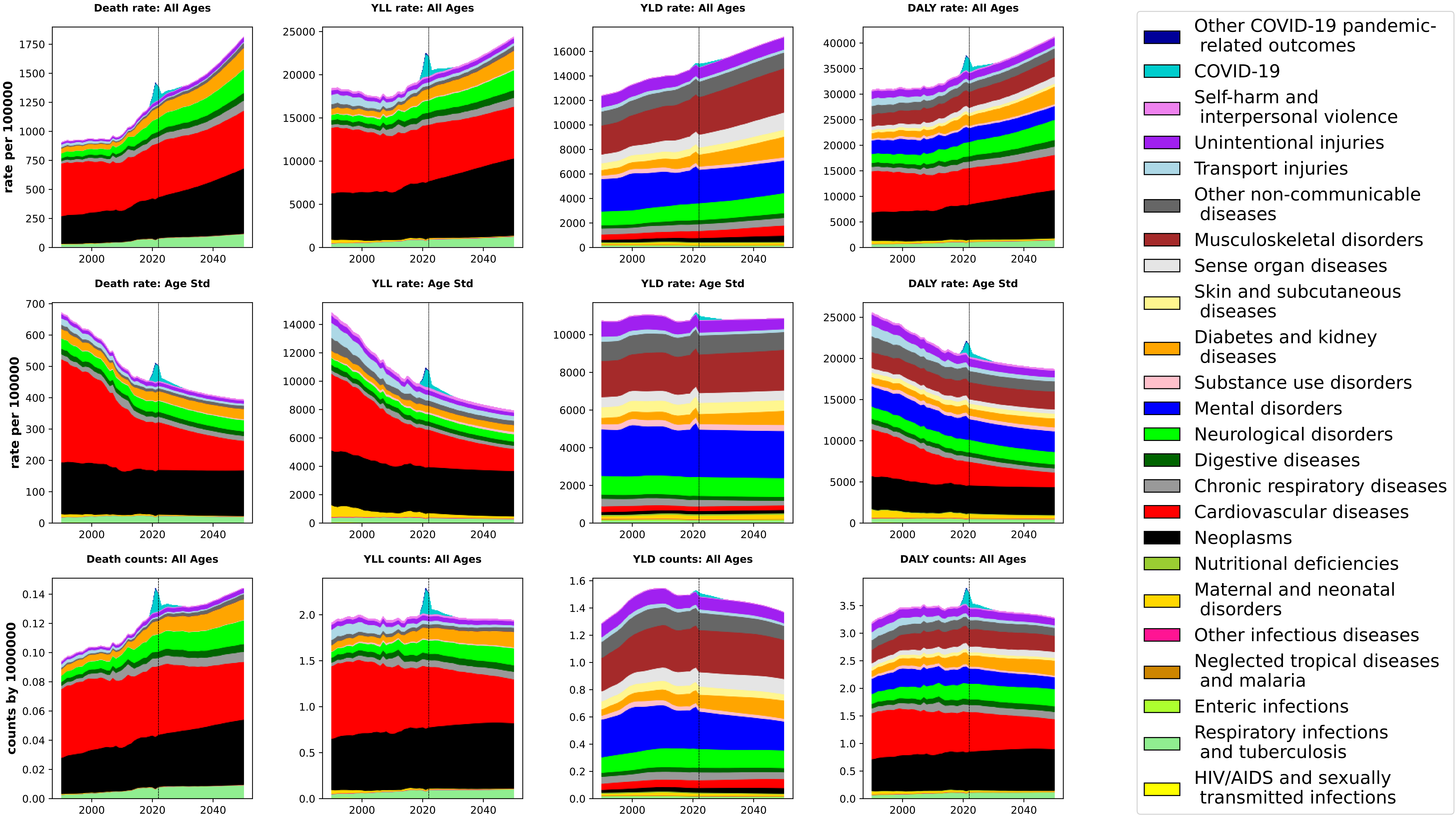
Death rate: All Ages
YLL rate: All Ages
YLD rate: All Ages
DALY rate: All Ages
Death rate: Age Std
YLL rate: Age Std
YLD rate: Age Std
DALY rate: Age Std
Death counts: All Ages
YLL counts: All Ages
YLD counts: All Ages
DALY counts: All Ages
rate per 100000
counts by 1000000
Other COVID-19 pandemic-related outcomes
COVID-19
Self-harm and interpersonal violence
Unintentional injuries
Transport injuries
Other non-communicable diseases
Musculoskeletal disorders
Sense organ diseases
Skin and subcutaneous diseases
Diabetes and kidney diseases
Substance use disorders
Mental disorders
Neurological disorders
Digestive diseases
Chronic respiratory diseases
Cardiovascular diseases
Neoplasms
Nutritional deficiencies
Maternal and neonatal disorders
Other infectious diseases
Neglected tropical diseases and malaria
Enteric infections
Respiratory infections and tuberculosis
HIV/AIDS and sexually transmitted infections

Iceland

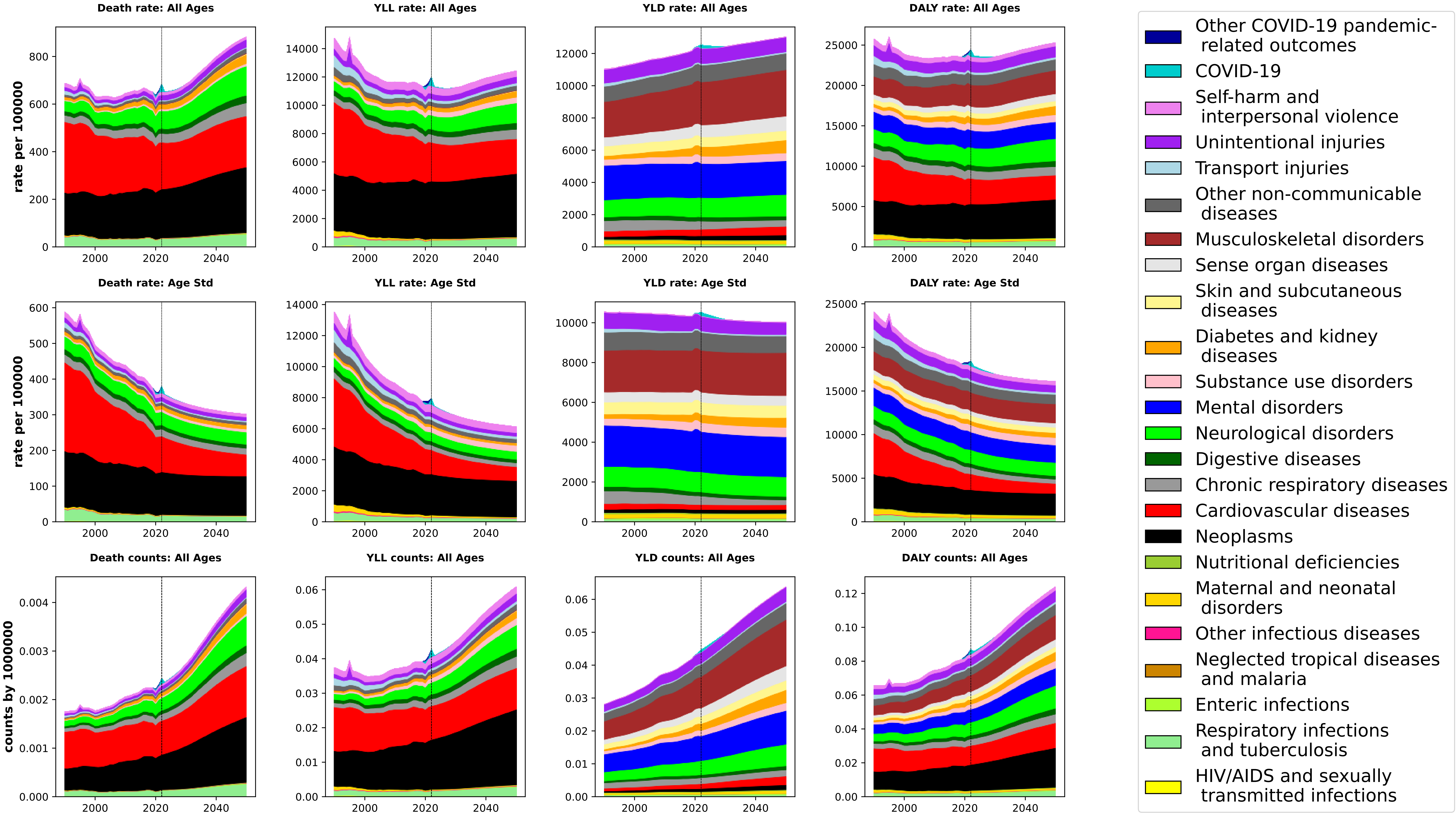
Death rate: All Ages
YLL rate: All Ages
YLD rate: All Ages
DALY rate: All Ages
Death rate: Age Std
YLL rate: Age Std
YLD rate: Age Std
DALY rate: Age Std
Death counts: All Ages
YLL counts: All Ages
YLD counts: All Ages
DALY counts: All Ages
rate per 100000
counts by 1000000
Other COVID-19 pandemic-related outcomes
COVID-19
Self-harm and interpersonal violence
Unintentional injuries
Transport injuries
Other non-communicable diseases
Musculoskeletal disorders
Sense organ diseases
Skin and subcutaneous diseases
Diabetes and kidney diseases
Substance use disorders
Mental disorders
Neurological disorders
Digestive diseases
Chronic respiratory diseases
Cardiovascular diseases
Neoplasms
Nutritional deficiencies
Maternal and neonatal disorders
Other infectious diseases
Neglected tropical diseases and malaria
Enteric infections
Respiratory infections and tuberculosis
HIV/AIDS and sexually transmitted infections

Ireland

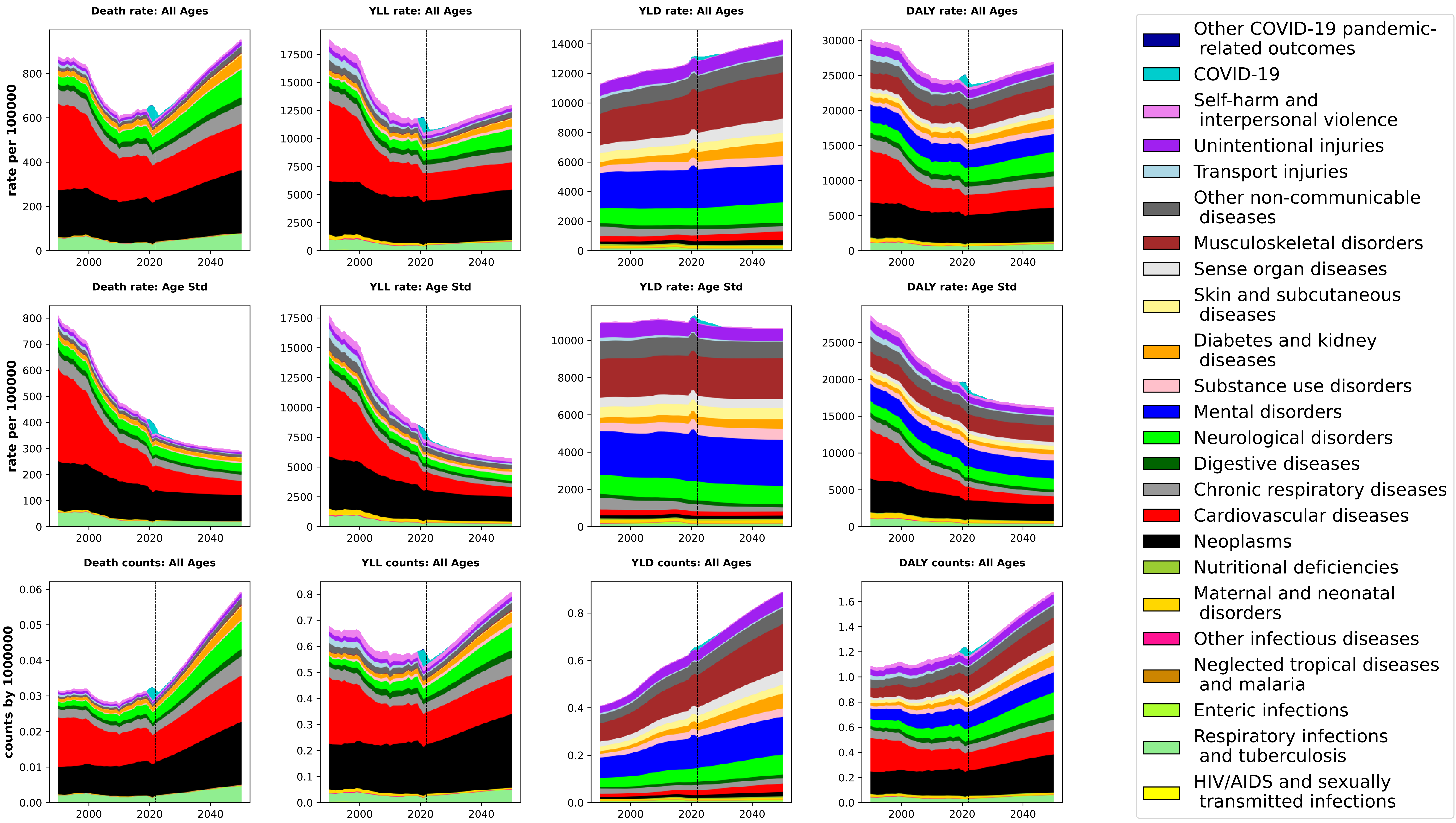
Death rate: All Ages
YLL rate: All Ages
YLD rate: All Ages
DALY rate: All Ages
Death rate: Age Std
YLL rate: Age Std
YLD rate: Age Std
DALY rate: Age Std
Death counts: All Ages
YLL counts: All Ages
YLD counts: All Ages
DALY counts: All Ages
rate per 100000
counts by 1000000
2000
2020
2040
Other COVID-19 pandemic-related outcomes
COVID-19
Self-harm and interpersonal violence
Unintentional injuries
Transport injuries
Other non-communicable diseases
Musculoskeletal disorders
Sense organ diseases
Skin and subcutaneous diseases
Diabetes and kidney diseases
Substance use disorders
Mental disorders
Neurological disorders
Digestive diseases
Chronic respiratory diseases
Cardiovascular diseases
Neoplasms
Nutritional deficiencies
Maternal and neonatal disorders
Other infectious diseases
Neglected tropical diseases and malaria
Enteric infections
Respiratory infections and tuberculosis
HIV/AIDS and sexually transmitted infections

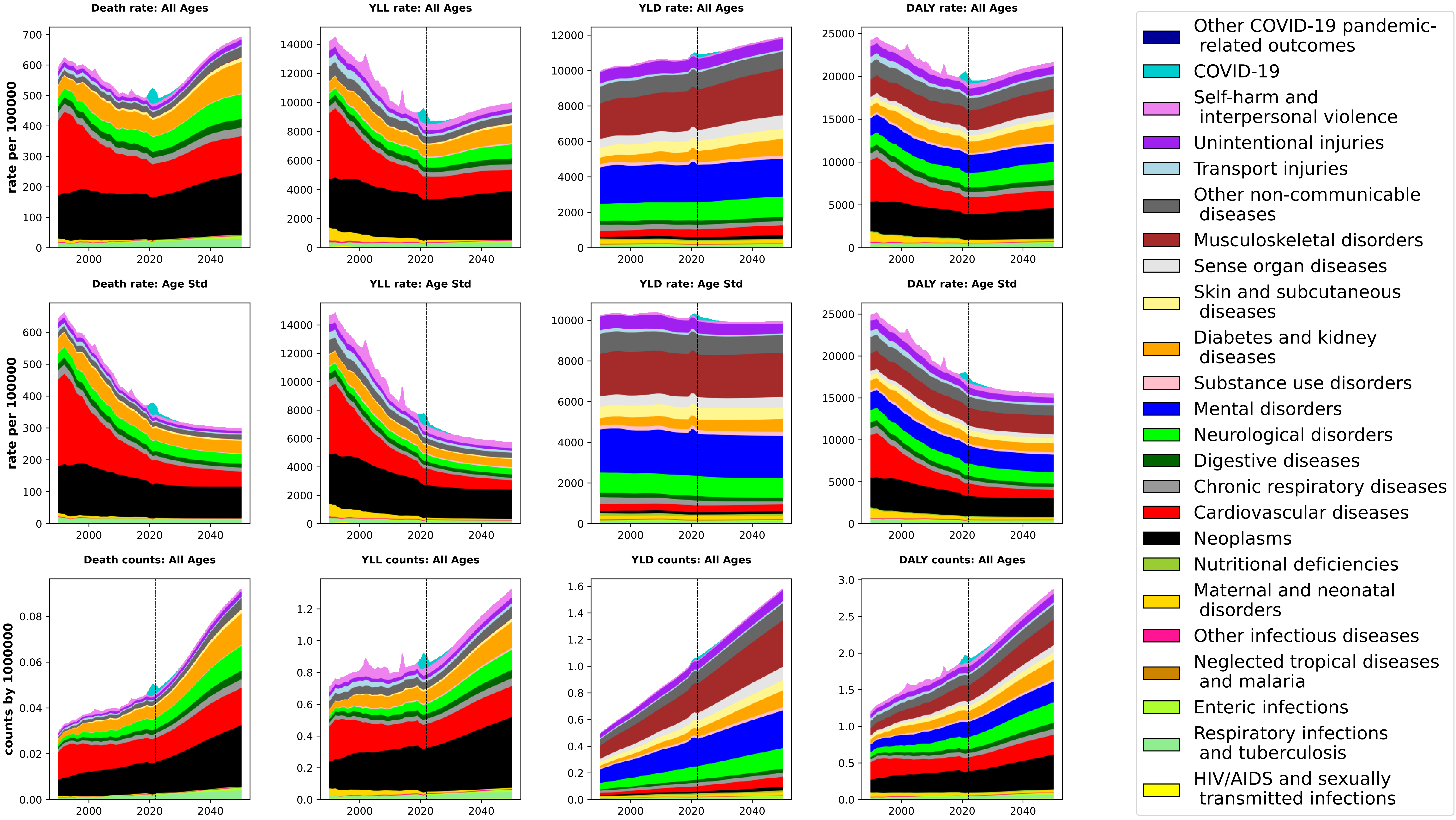
Death rate: All Ages
YLL rate: All Ages
YLD rate: All Ages
DALY rate: All Ages
Death rate: Age Std
YLL rate: Age Std
YLD rate: Age Std
DALY rate: Age Std
Death counts: All Ages
YLL counts: All Ages
YLD counts: All Ages
DALY counts: All Ages
rate per 100000
rate per 100000
counts by 1000000
Other COVID-19 pandemic-related outcomes
COVID-19
Self-harm and interpersonal violence
Unintentional injuries
Transport injuries
Other non-communicable diseases
Musculoskeletal disorders
Sense organ diseases
Skin and subcutaneous diseases
Diabetes and kidney diseases
Substance use disorders
Mental disorders
Neurological disorders
Digestive diseases
Chronic respiratory diseases
Cardiovascular diseases
Neoplasms
Nutritional deficiencies
Maternal and neonatal disorders
Other infectious diseases
Neglected tropical diseases and malaria
Enteric infections
Respiratory infections and tuberculosis
HIV/AIDS and sexually transmitted infections

Italy

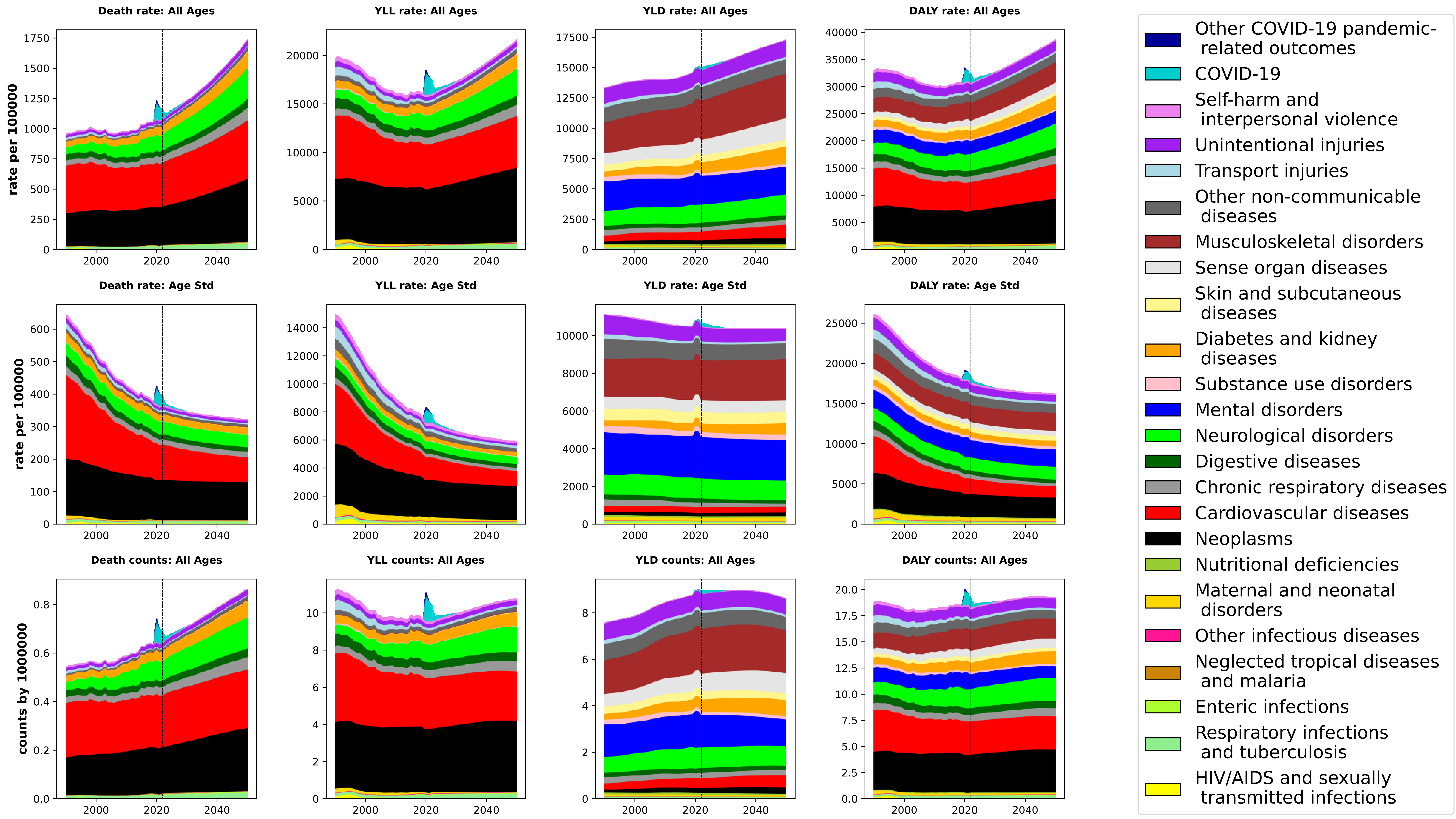
Death rate: All Ages
YLL rate: All Ages
YLD rate: All Ages
DALY rate: All Ages
Death rate: Age Std
YLL rate: Age Std
YLD rate: Age Std
DALY rate: Age Std
Death counts: All Ages
YLL counts: All Ages
YLD counts: All Ages
DALY counts: All Ages
rate per 100000
rate per 100000
counts by 1000000
Other COVID-19 pandemic-related outcomes
COVID-19
Self-harm and interpersonal violence
Unintentional injuries
Transport injuries
Other non-communicable diseases
Musculoskeletal disorders
Sense organ diseases
Skin and subcutaneous diseases
Diabetes and kidney diseases
Substance use disorders
Mental disorders
Neurological disorders
Digestive diseases
Chronic respiratory diseases
Cardiovascular diseases
Neoplasms
Nutritional deficiencies
Maternal and neonatal disorders
Other infectious diseases
Neglected tropical diseases and malaria
Enteric infections
Respiratory infections and tuberculosis
HIV/AIDS and sexually transmitted infections

Luxembourg

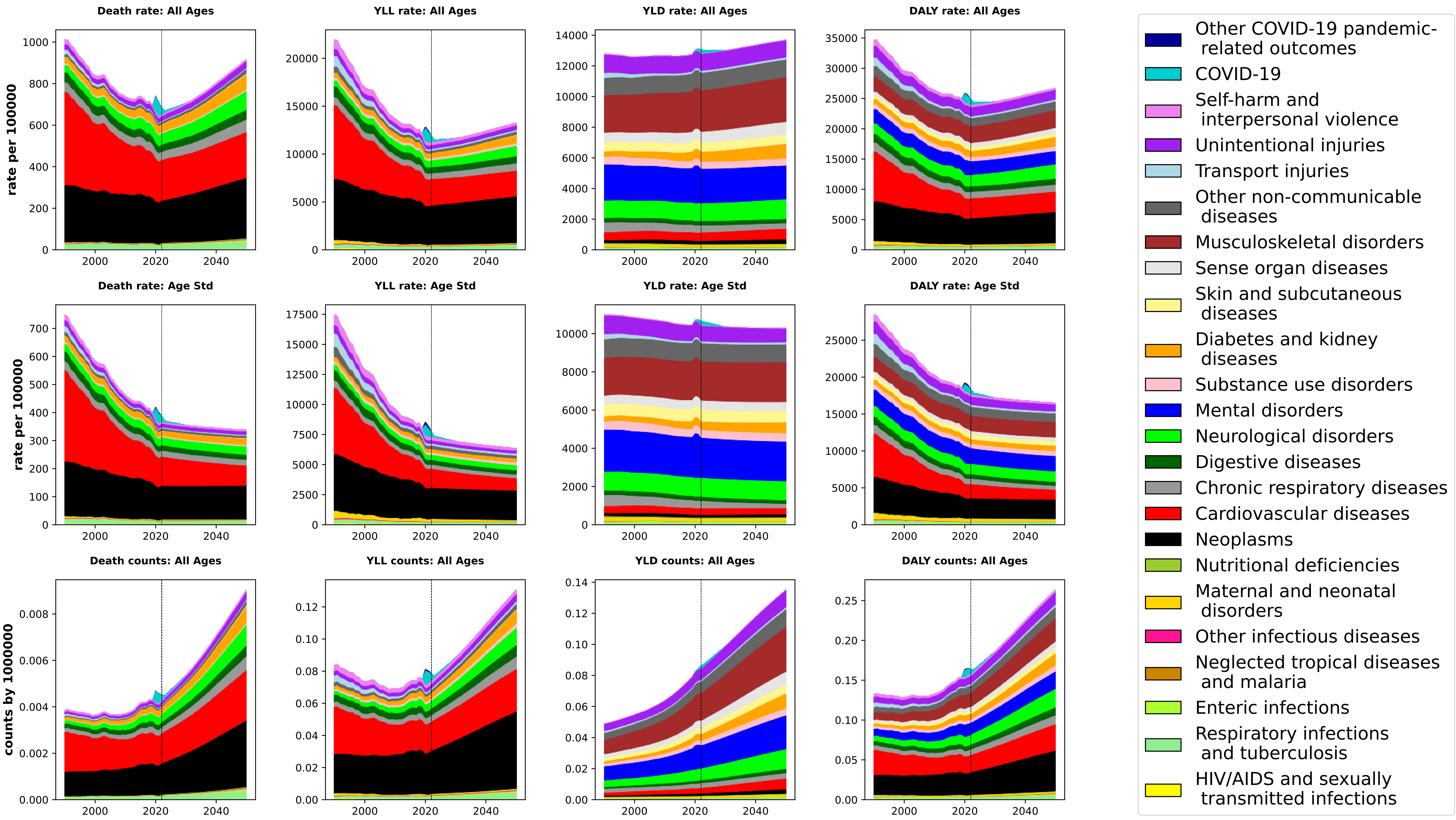
Death rate: All Ages
YLL rate: All Ages
YLD rate: All Ages
DALY rate: All Ages
Death rate: Age Std
YLL rate: Age Std
YLD rate: Age Std
DALY rate: Age Std
Death counts: All Ages
YLL counts: All Ages
YLD counts: All Ages
DALY counts: All Ages
rate per 100000
counts by 1000000
Other COVID-19 pandemic-related outcomes
COVID-19
Self-harm and interpersonal violence
Unintentional injuries
Transport injuries
Other non-communicable diseases
Musculoskeletal disorders
Sense organ diseases
Skin and subcutaneous diseases
Diabetes and kidney diseases
Substance use disorders
Mental disorders
Neurological disorders
Digestive diseases
Chronic respiratory diseases
Cardiovascular diseases
Neoplasms
Nutritional deficiencies
Maternal and neonatal disorders
Other infectious diseases
Neglected tropical diseases and malaria
Enteric infections
Respiratory infections and tuberculosis
HIV/AIDS and sexually transmitted infections

Malta

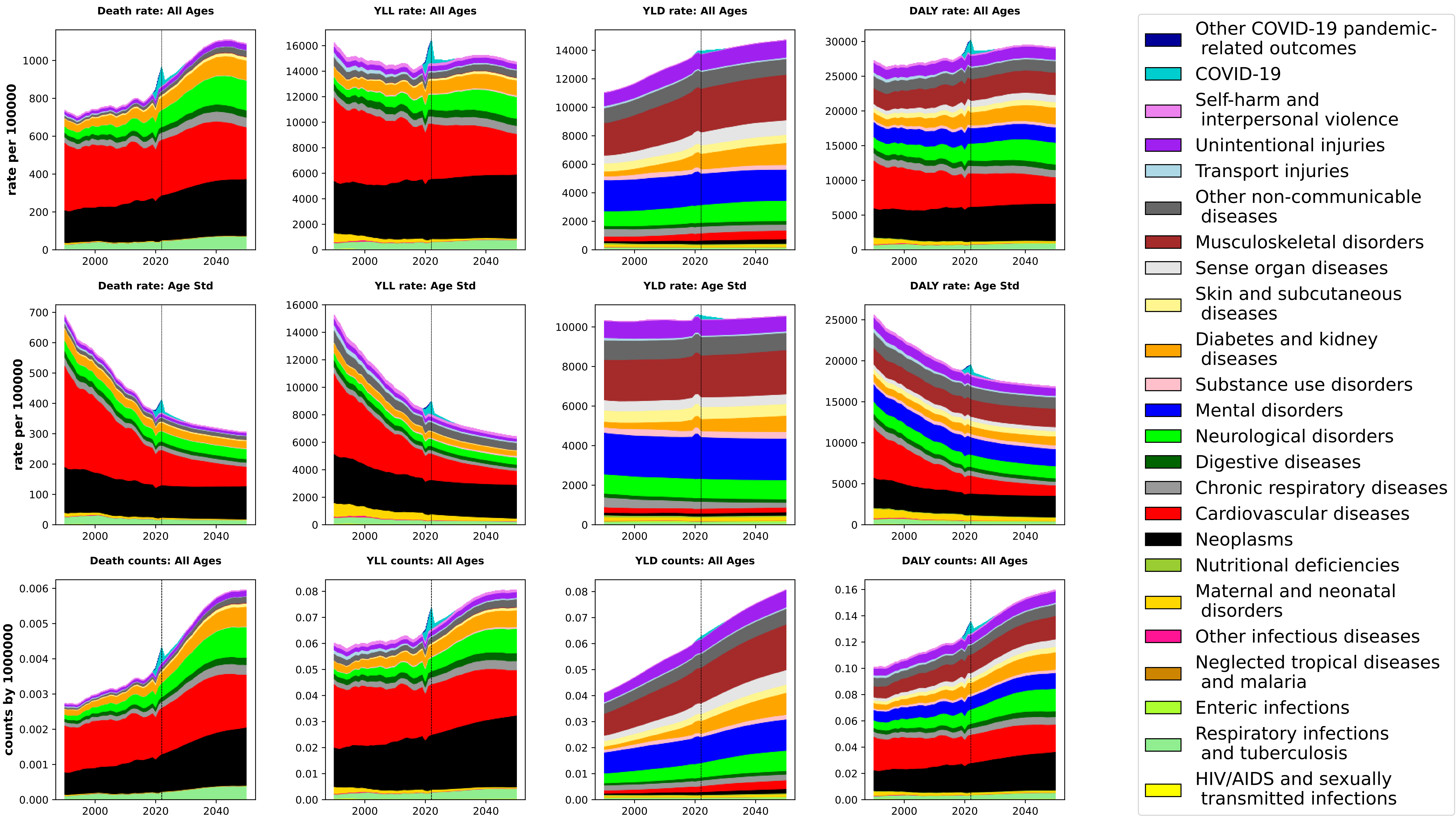
Death rate: All Ages
YLL rate: All Ages
YLD rate: All Ages
DALY rate: All Ages
Death rate: Age Std
YLL rate: Age Std
YLD rate: Age Std
DALY rate: Age Std
Death counts: All Ages
YLL counts: All Ages
YLD counts: All Ages
DALY counts: All Ages
rate per 100000
rate per 100000
counts by 1000000
Other COVID-19 pandemic-related outcomes
COVID-19
Self-harm and interpersonal violence
Unintentional injuries
Transport injuries
Other non-communicable diseases
Musculoskeletal disorders
Sense organ diseases
Skin and subcutaneous diseases
Diabetes and kidney diseases
Substance use disorders
Mental disorders
Neurological disorders
Digestive diseases
Chronic respiratory diseases
Cardiovascular diseases
Neoplasms
Nutritional deficiencies
Maternal and neonatal disorders
Other infectious diseases
Neglected tropical diseases and malaria
Enteric infections
Respiratory infections and tuberculosis
HIV/AIDS and sexually transmitted infections

Monaco

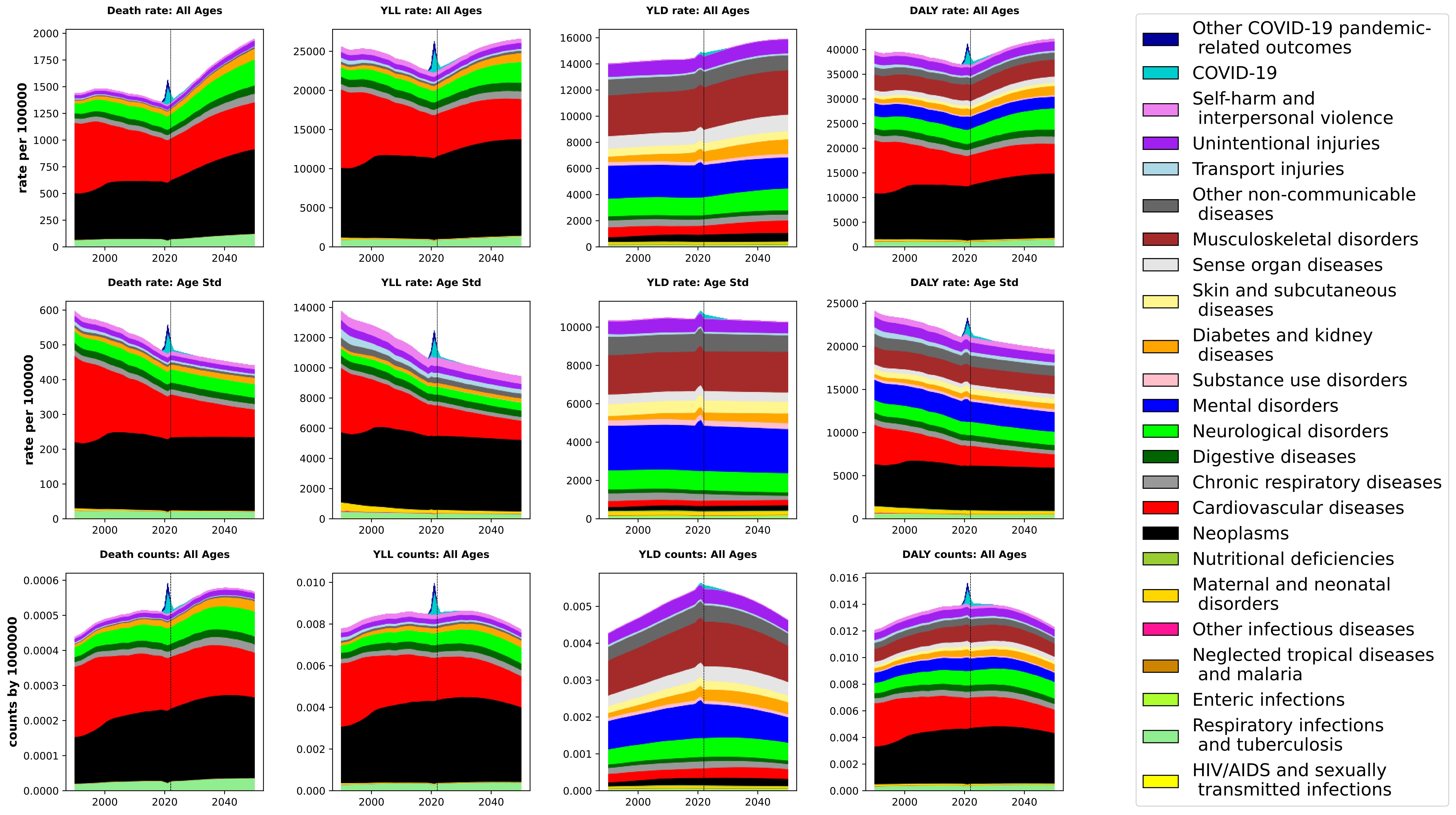
Death rate: All Ages
YLL rate: All Ages
YLD rate: All Ages
DALY rate: All Ages
Death rate: Age Std
YLL rate: Age Std
YLD rate: Age Std
DALY rate: Age Std
Death counts: All Ages
YLL counts: All Ages
YLD counts: All Ages
DALY counts: All Ages
rate per 100000
counts by 1000000
Other COVID-19 pandemic-related outcomes
COVID-19
Self-harm and interpersonal violence
Unintentional injuries
Transport injuries
Other non-communicable diseases
Musculoskeletal disorders
Sense organ diseases
Skin and subcutaneous diseases
Diabetes and kidney diseases
Substance use disorders
Mental disorders
Neurological disorders
Digestive diseases
Chronic respiratory diseases
Cardiovascular diseases
Neoplasms
Nutritional deficiencies
Maternal and neonatal disorders
Other infectious diseases
Neglected tropical diseases and malaria
Enteric infections
Respiratory infections and tuberculosis
HIV/AIDS and sexually transmitted infections

Netherlands

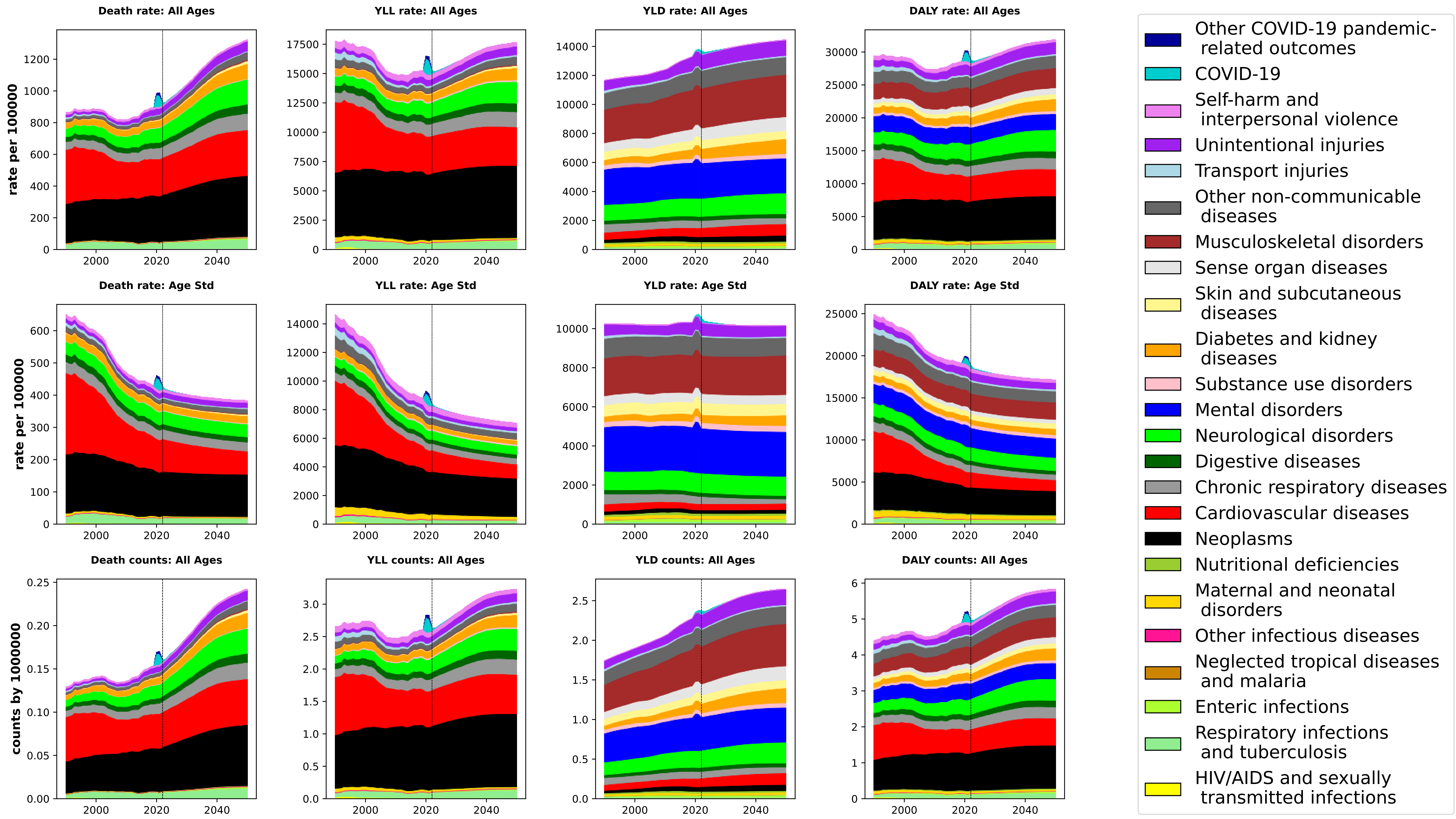

Norway

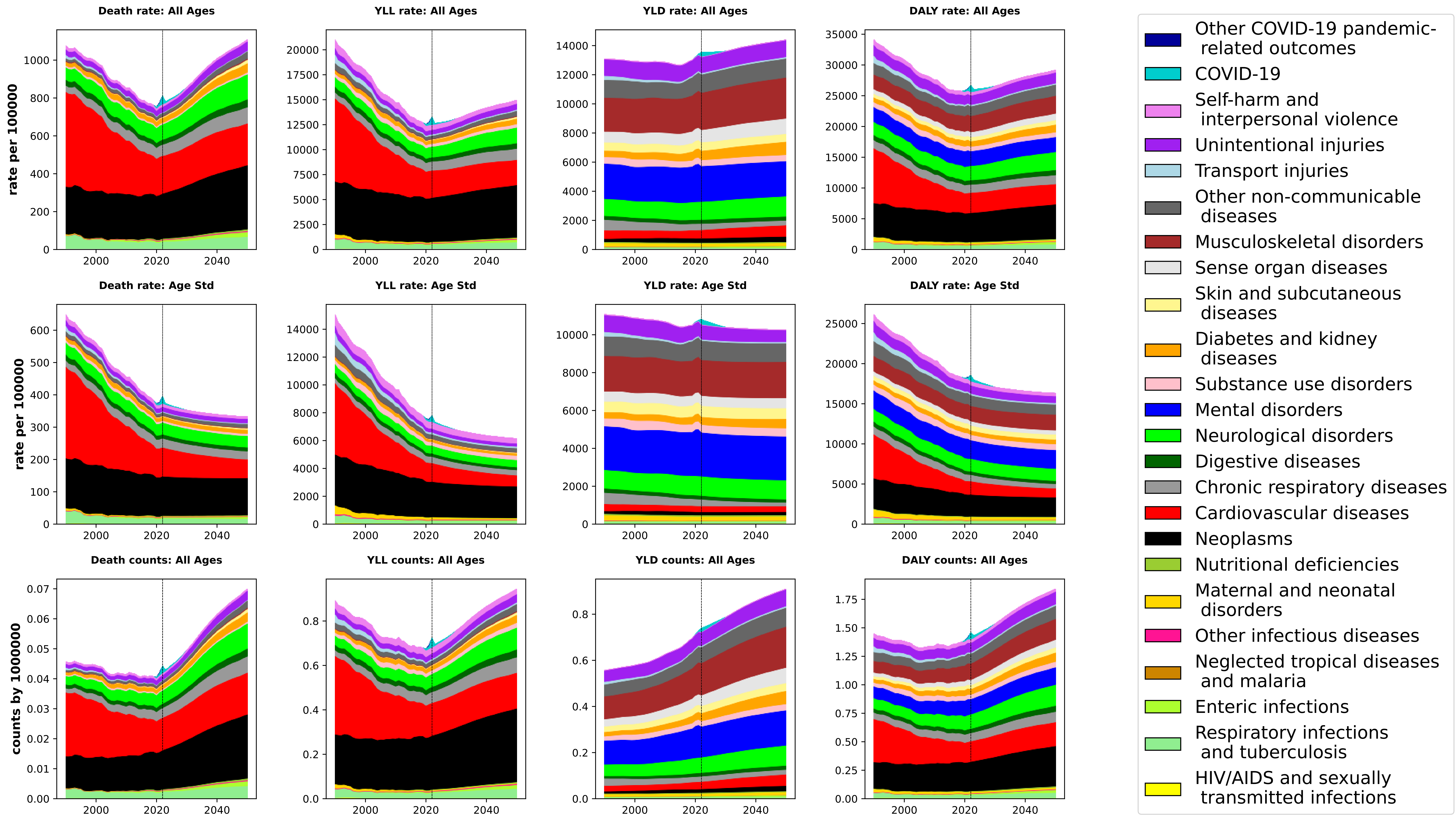
Death rate: All Ages
YLL rate: All Ages
YLD rate: All Ages
DALY rate: All Ages
Death rate: Age Std
YLL rate: Age Std
YLD rate: Age Std
DALY rate: Age Std
Death counts: All Ages
YLL counts: All Ages
YLD counts: All Ages
DALY counts: All Ages
rate per 100000
counts by 1000000
2000
2020
2040
Other COVID-19 pandemic-related outcomes
COVID-19
Self-harm and interpersonal violence
Unintentional injuries
Transport injuries
Other non-communicable diseases
Musculoskeletal disorders
Sense organ diseases
Skin and subcutaneous diseases
Diabetes and kidney diseases
Substance use disorders
Mental disorders
Neurological disorders
Digestive diseases
Chronic respiratory diseases
Cardiovascular diseases
Neoplasms
Nutritional deficiencies
Maternal and neonatal disorders
Other infectious diseases
Neglected tropical diseases and malaria
Enteric infections
Respiratory infections and tuberculosis
HIV/AIDS and sexually transmitted infections

Portugal

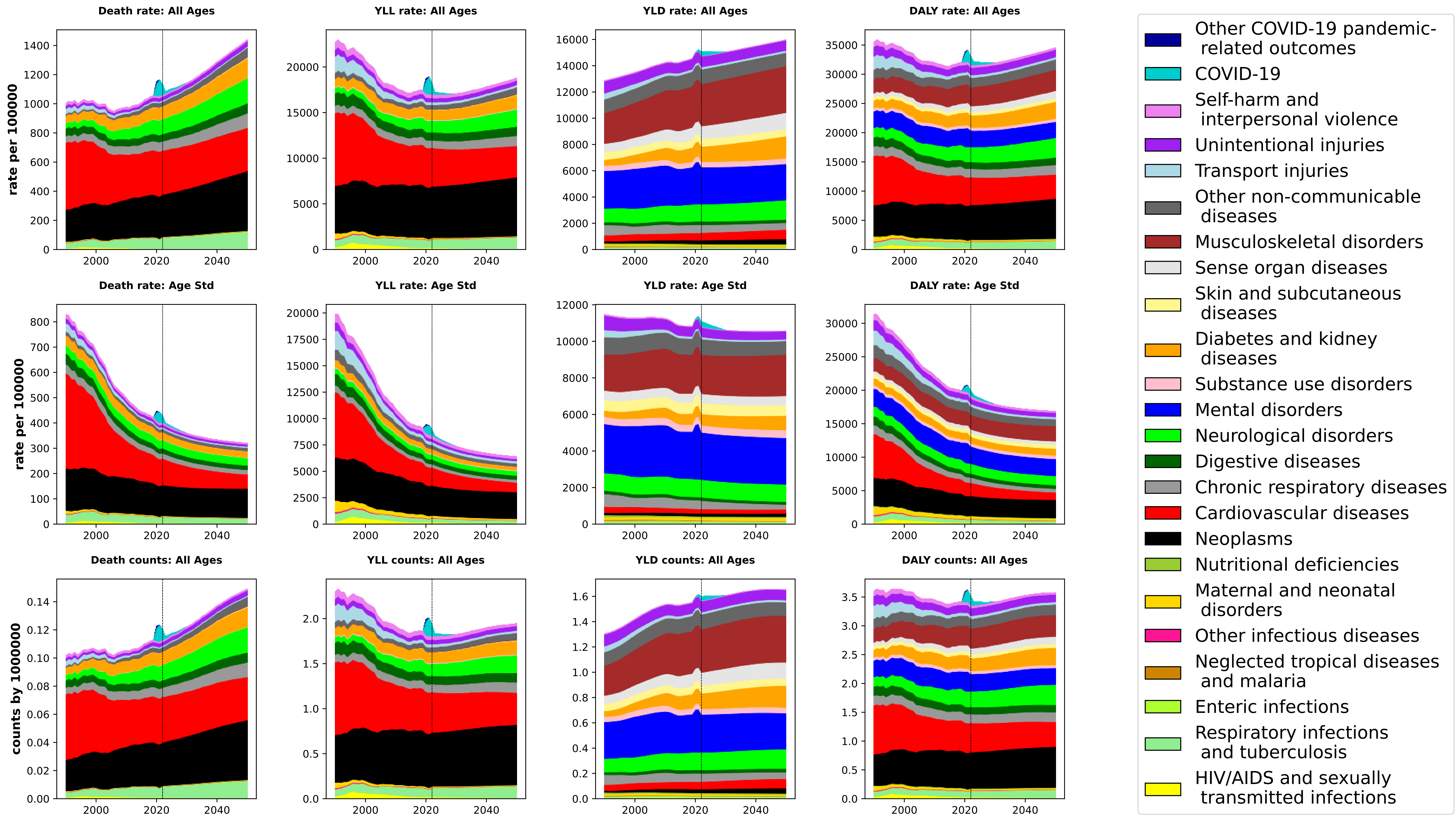
Death rate: All Ages
YLL rate: All Ages
YLD rate: All Ages
DALY rate: All Ages
Death rate: Age Std
YLL rate: Age Std
YLD rate: Age Std
DALY rate: Age Std
Death counts: All Ages
YLL counts: All Ages
YLD counts: All Ages
DALY counts: All Ages
rate per 100000
counts by 1000000
Other COVID-19 pandemic-related outcomes
COVID-19
Self-harm and interpersonal violence
Unintentional injuries
Transport injuries
Other non-communicable diseases
Musculoskeletal disorders
Sense organ diseases
Skin and subcutaneous diseases
Diabetes and kidney diseases
Substance use disorders
Mental disorders
Neurological disorders
Digestive diseases
Chronic respiratory diseases
Cardiovascular diseases
Neoplasms
Nutritional deficiencies
Maternal and neonatal disorders
Other infectious diseases
Neglected tropical diseases and malaria
Enteric infections
Respiratory infections and tuberculosis
HIV/AIDS and sexually transmitted infections

San Marino

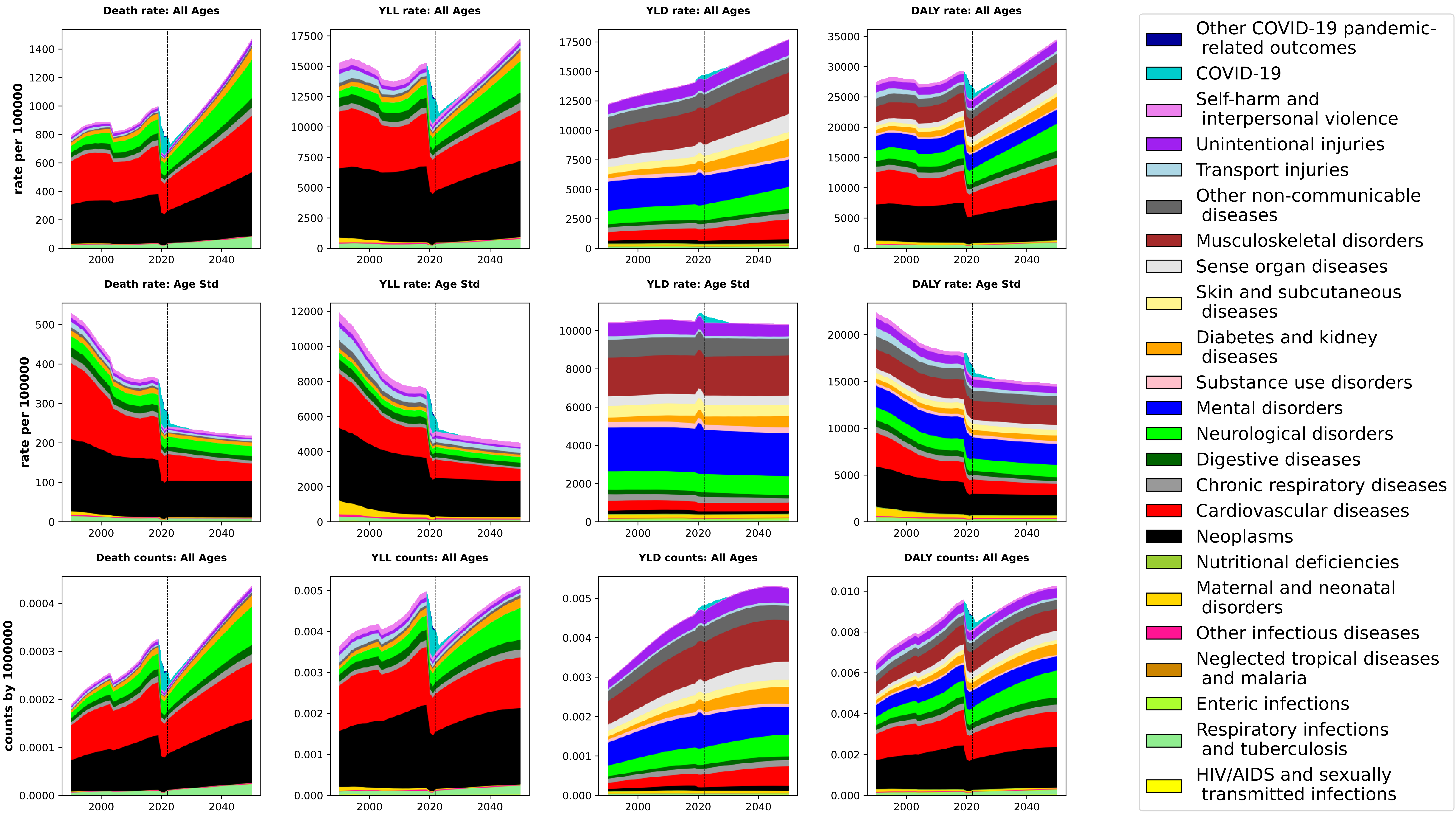
Death rate: All Ages
YLL rate: All Ages
YLD rate: All Ages
DALY rate: All Ages
Death rate: Age Std
YLL rate: Age Std
YLD rate: Age Std
DALY rate: Age Std
Death counts: All Ages
YLL counts: All Ages
YLD counts: All Ages
DALY counts: All Ages
rate per 100000
counts by 1000000
2000
2020
2040
Other COVID-19 pandemic-related outcomes
COVID-19
Self-harm and interpersonal violence
Unintentional injuries
Transport injuries
Other non-communicable diseases
Musculoskeletal disorders
Sense organ diseases
Skin and subcutaneous diseases
Diabetes and kidney diseases
Substance use disorders
Mental disorders
Neurological disorders
Digestive diseases
Chronic respiratory diseases
Cardiovascular diseases
Neoplasms
Nutritional deficiencies
Maternal and neonatal disorders
Other infectious diseases
Neglected tropical diseases and malaria
Enteric infections
Respiratory infections and tuberculosis
HIV/AIDS and sexually transmitted infections

Spain

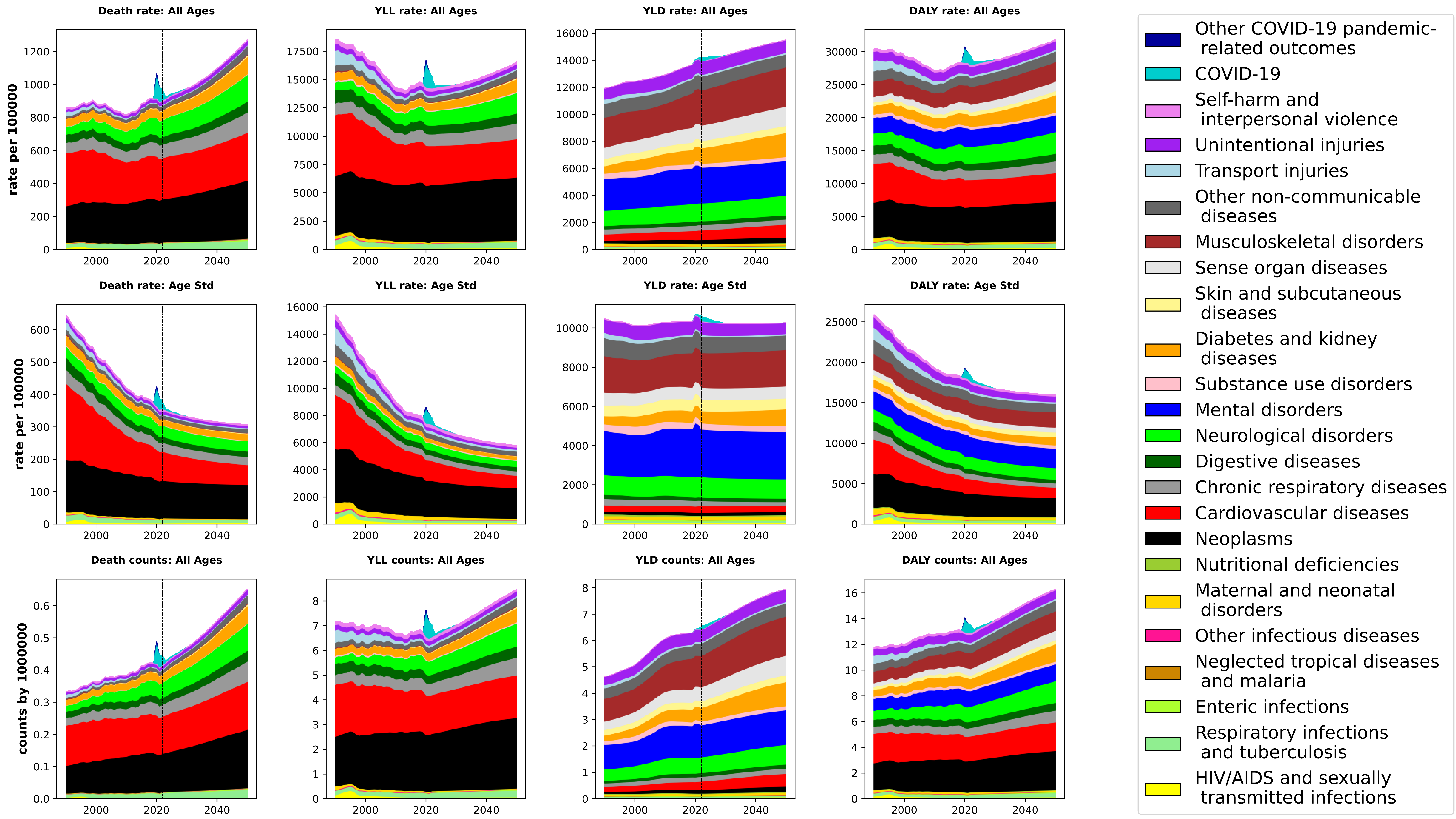
Death rate: All Ages
YLL rate: All Ages
YLD rate: All Ages
DALY rate: All Ages
Death rate: Age Std
YLL rate: Age Std
YLD rate: Age Std
DALY rate: Age Std
Death counts: All Ages
YLL counts: All Ages
YLD counts: All Ages
DALY counts: All Ages
rate per 100000
counts by 1000000
Other COVID-19 pandemic-related outcomes
COVID-19
Self-harm and interpersonal violence
Unintentional injuries
Transport injuries
Other non-communicable diseases
Musculoskeletal disorders
Sense organ diseases
Skin and subcutaneous diseases
Diabetes and kidney diseases
Substance use disorders
Mental disorders
Neurological disorders
Digestive diseases
Chronic respiratory diseases
Cardiovascular diseases
Neoplasms
Nutritional deficiencies
Maternal and neonatal disorders
Other infectious diseases
Neglected tropical diseases and malaria
Enteric infections
Respiratory infections and tuberculosis
HIV/AIDS and sexually transmitted infections

Sweden

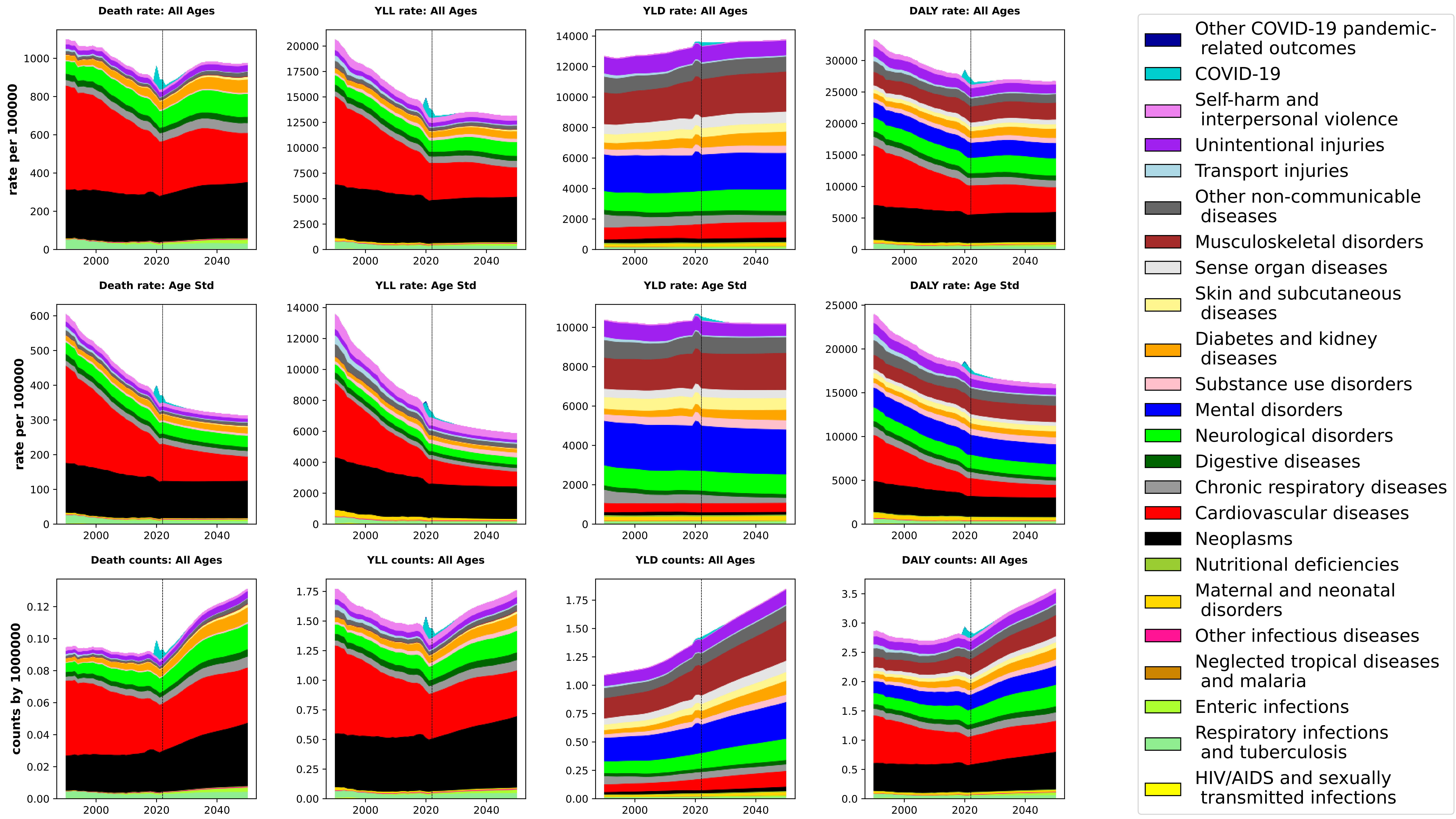
Death rate: All Ages
YLL rate: All Ages
YLD rate: All Ages
DALY rate: All Ages
Death rate: Age Std
YLL rate: Age Std
YLD rate: Age Std
DALY rate: Age Std
Death counts: All Ages
YLL counts: All Ages
YLD counts: All Ages
DALY counts: All Ages
rate per 100000
counts by 1000000
2000
2020
2040
Other COVID-19 pandemic-related outcomes
COVID-19
Self-harm and interpersonal violence
Unintentional injuries
Transport injuries
Other non-communicable diseases
Musculoskeletal disorders
Sense organ diseases
Skin and subcutaneous diseases
Diabetes and kidney diseases
Substance use disorders
Mental disorders
Neurological disorders
Digestive diseases
Chronic respiratory diseases
Cardiovascular diseases
Neoplasms
Nutritional deficiencies
Maternal and neonatal disorders
Other infectious diseases
Neglected tropical diseases and malaria
Enteric infections
Respiratory infections and tuberculosis
HIV/AIDS and sexually transmitted infections

Switzerland

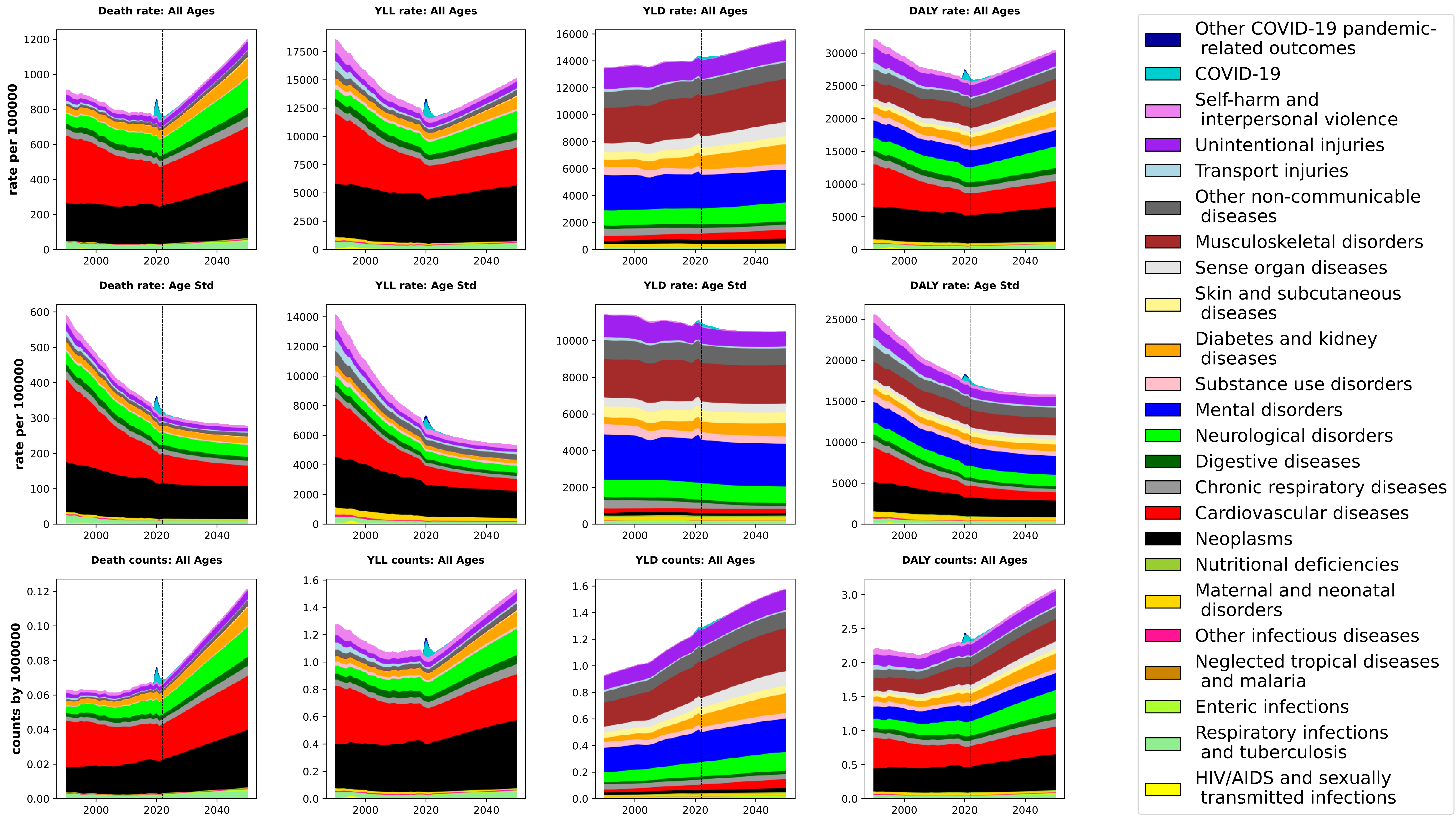

UK

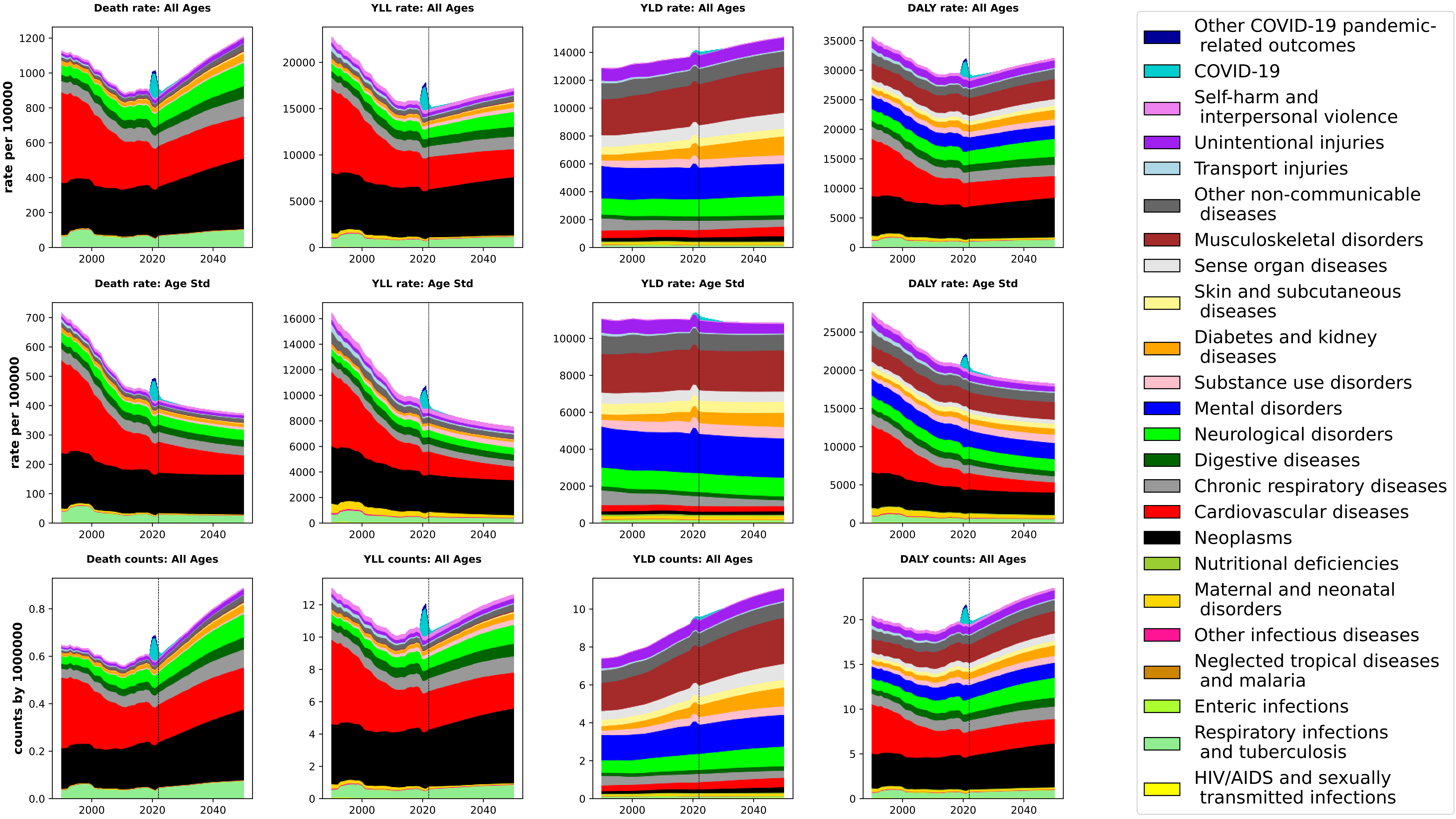
Death rate: All Ages
YLL rate: All Ages
YLD rate: All Ages
DALY rate: All Ages
Death rate: Age Std
YLL rate: Age Std
YLD rate: Age Std
DALY rate: Age Std
Death counts: All Ages
YLL counts: All Ages
YLD counts: All Ages
DALY counts: All Ages
rate per 100000
counts by 1000000
Other COVID-19 pandemic-related outcomes
COVID-19
Self-harm and interpersonal violence
Unintentional injuries
Transport injuries
Other non-communicable diseases
Musculoskeletal disorders
Sense organ diseases
Skin and subcutaneous diseases
Diabetes and kidney diseases
Substance use disorders
Mental disorders
Neurological disorders
Digestive diseases
Chronic respiratory diseases
Cardiovascular diseases
Neoplasms
Nutritional deficiencies
Maternal and neonatal disorders
Other infectious diseases
Neglected tropical diseases and malaria
Enteric infections
Respiratory infections and tuberculosis
HIV/AIDS and sexually transmitted infections

Bolivia

Supplemental Results Figure S6. Cause level 2 area plots for 4 burden measures for reference scenario by location, 1990-2050

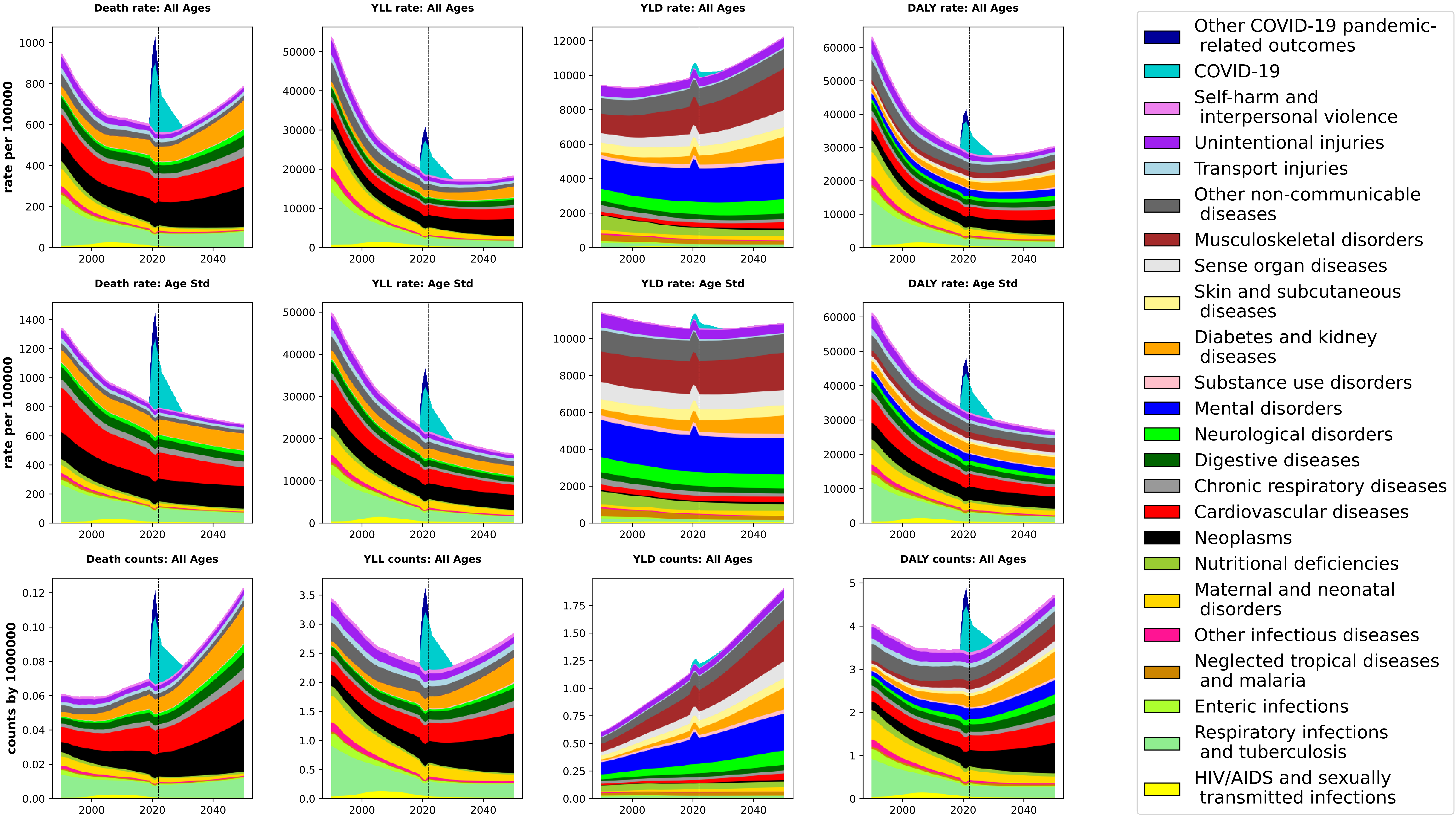

Ecuador

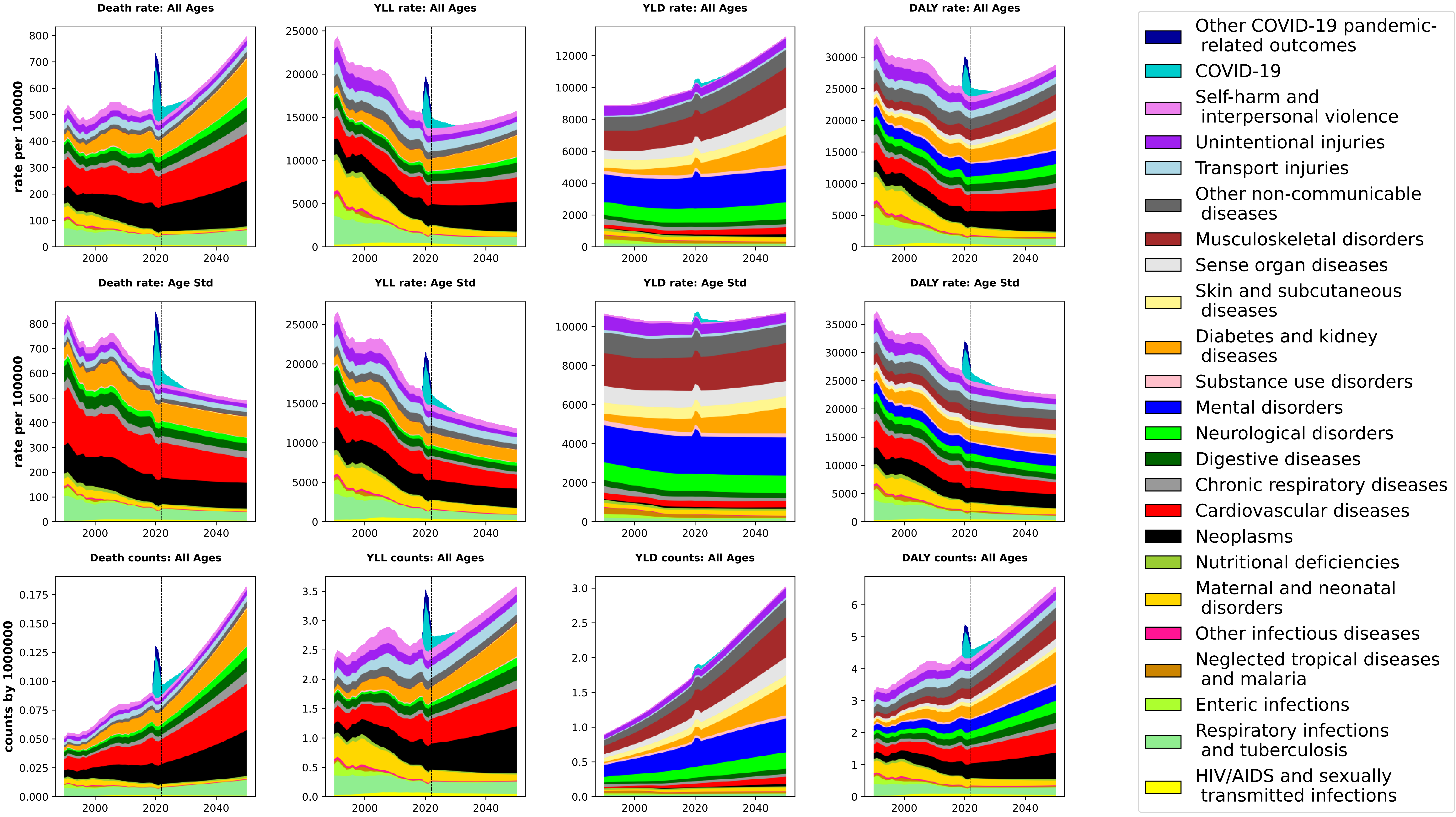
Death rate: All Ages
YLL rate: All Ages
YLD rate: All Ages
DALY rate: All Ages
Death rate: Age Std
YLL rate: Age Std
YLD rate: Age Std
DALY rate: Age Std
Death counts: All Ages
YLL counts: All Ages
YLD counts: All Ages
DALY counts: All Ages
rate per 100000
counts by 1000000
2000
2020
2040
Other COVID-19 pandemic-related outcomes
COVID-19
Self-harm and interpersonal violence
Unintentional injuries
Transport injuries
Other non-communicable diseases
Musculoskeletal disorders
Sense organ diseases
Skin and subcutaneous diseases
Diabetes and kidney diseases
Substance use disorders
Mental disorders
Neurological disorders
Digestive diseases
Chronic respiratory diseases
Cardiovascular diseases
Neoplasms
Nutritional deficiencies
Maternal and neonatal disorders
Other infectious diseases
Neglected tropical diseases and malaria
Enteric infections
Respiratory infections and tuberculosis
HIV/AIDS and sexually transmitted infections

Peru

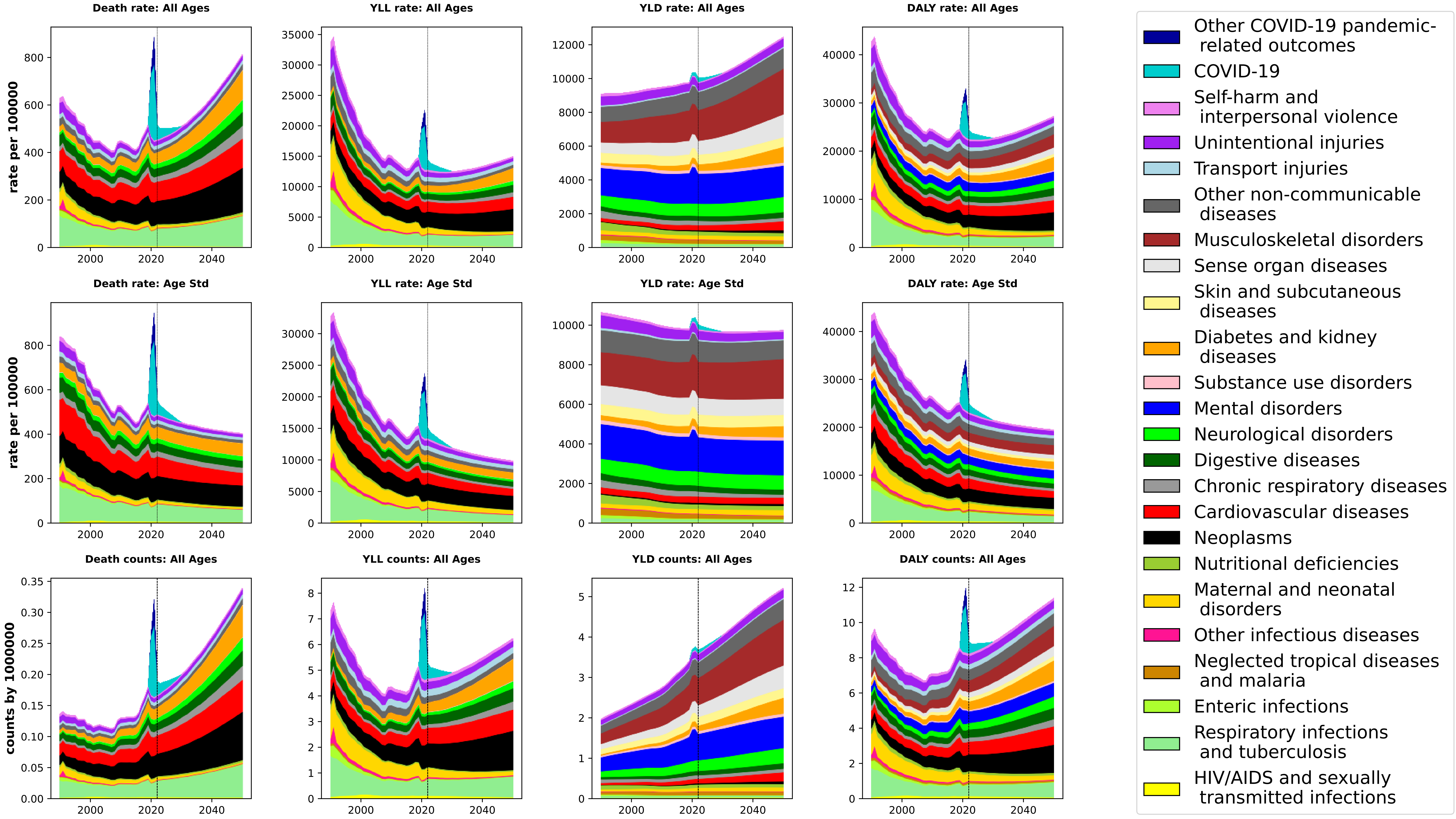
Death rate: All Ages
YLL rate: All Ages
YLD rate: All Ages
DALY rate: All Ages
Death rate: Age Std
YLL rate: Age Std
YLD rate: Age Std
DALY rate: Age Std
Death counts: All Ages
YLL counts: All Ages
YLD counts: All Ages
DALY counts: All Ages
rate per 100000
counts by 1000000
2000
2020
2040
Other COVID-19 pandemic-related outcomes
COVID-19
Self-harm and interpersonal violence
Unintentional injuries
Transport injuries
Other non-communicable diseases
Musculoskeletal disorders
Sense organ diseases
Skin and subcutaneous diseases
Diabetes and kidney diseases
Substance use disorders
Mental disorders
Neurological disorders
Digestive diseases
Chronic respiratory diseases
Cardiovascular diseases
Neoplasms
Nutritional deficiencies
Maternal and neonatal disorders
Other infectious diseases
Neglected tropical diseases and malaria
Enteric infections
Respiratory infections and tuberculosis
HIV/AIDS and sexually transmitted infections

Antigua and Barbuda

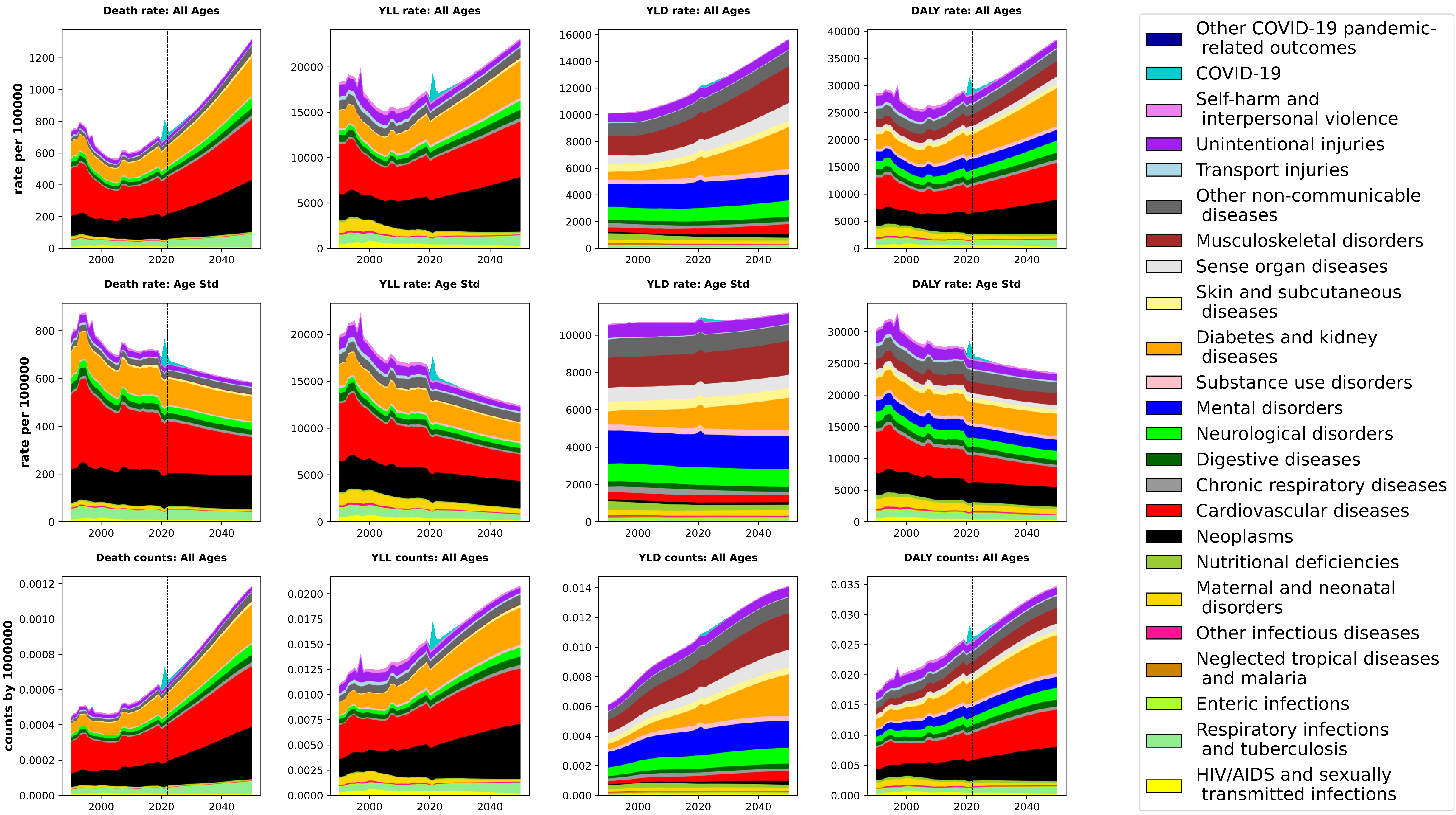

The Bahamas

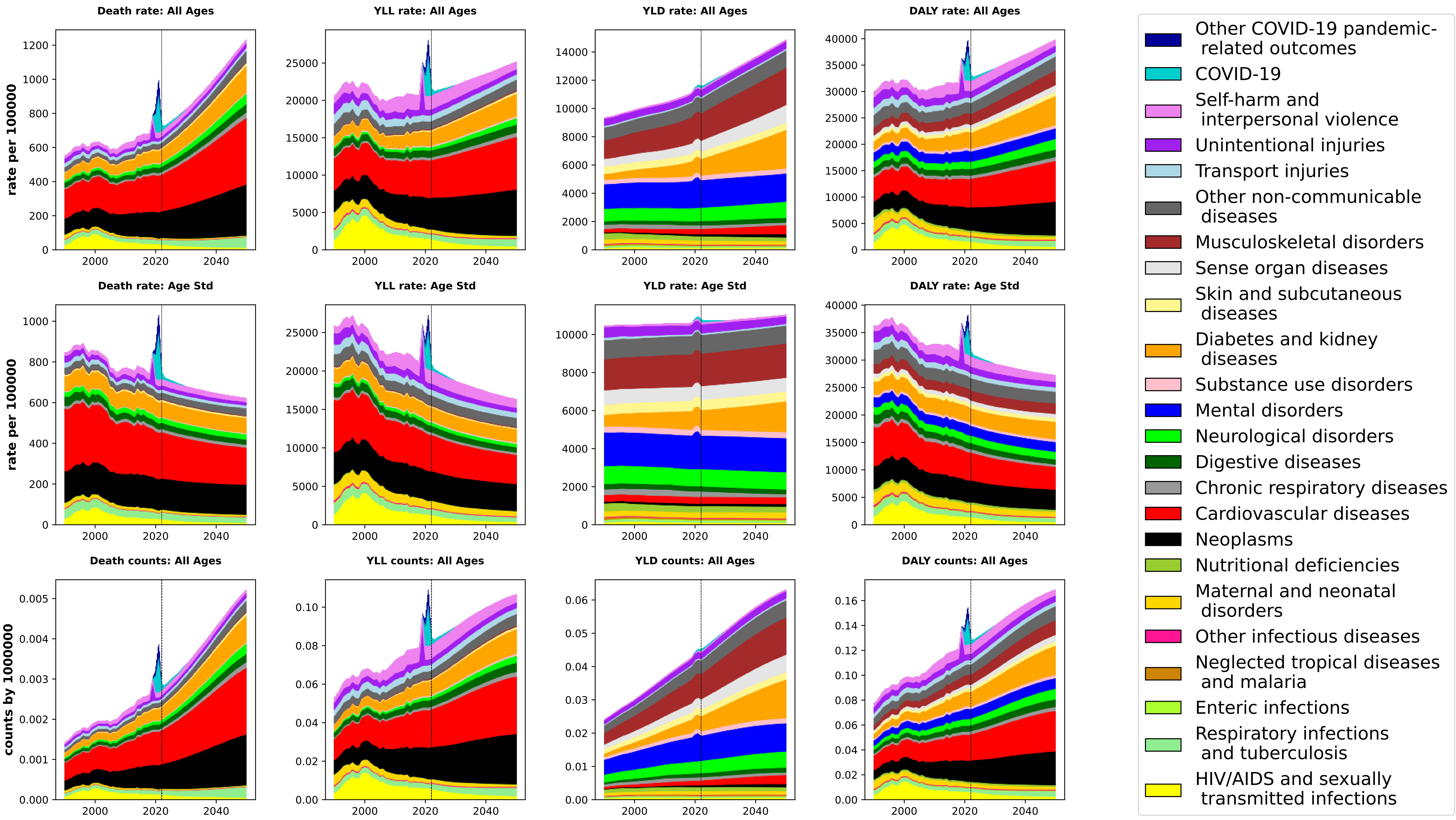
Death rate: All Ages
YLL rate: All Ages
YLD rate: All Ages
DALY rate: All Ages
Death rate: Age Std
YLL rate: Age Std
YLD rate: Age Std
DALY rate: Age Std
Death counts: All Ages
YLL counts: All Ages
YLD counts: All Ages
DALY counts: All Ages
rate per 100000
counts by 1000000
Other COVID-19 pandemic-related outcomes
COVID-19
Self-harm and interpersonal violence
Unintentional injuries
Transport injuries
Other non-communicable diseases
Musculoskeletal disorders
Sense organ diseases
Skin and subcutaneous diseases
Diabetes and kidney diseases
Substance use disorders
Mental disorders
Neurological disorders
Digestive diseases
Chronic respiratory diseases
Cardiovascular diseases
Neoplasms
Nutritional deficiencies
Maternal and neonatal disorders
Other infectious diseases
Neglected tropical diseases and malaria
Enteric infections
Respiratory infections and tuberculosis
HIV/AIDS and sexually transmitted infections

Barbados

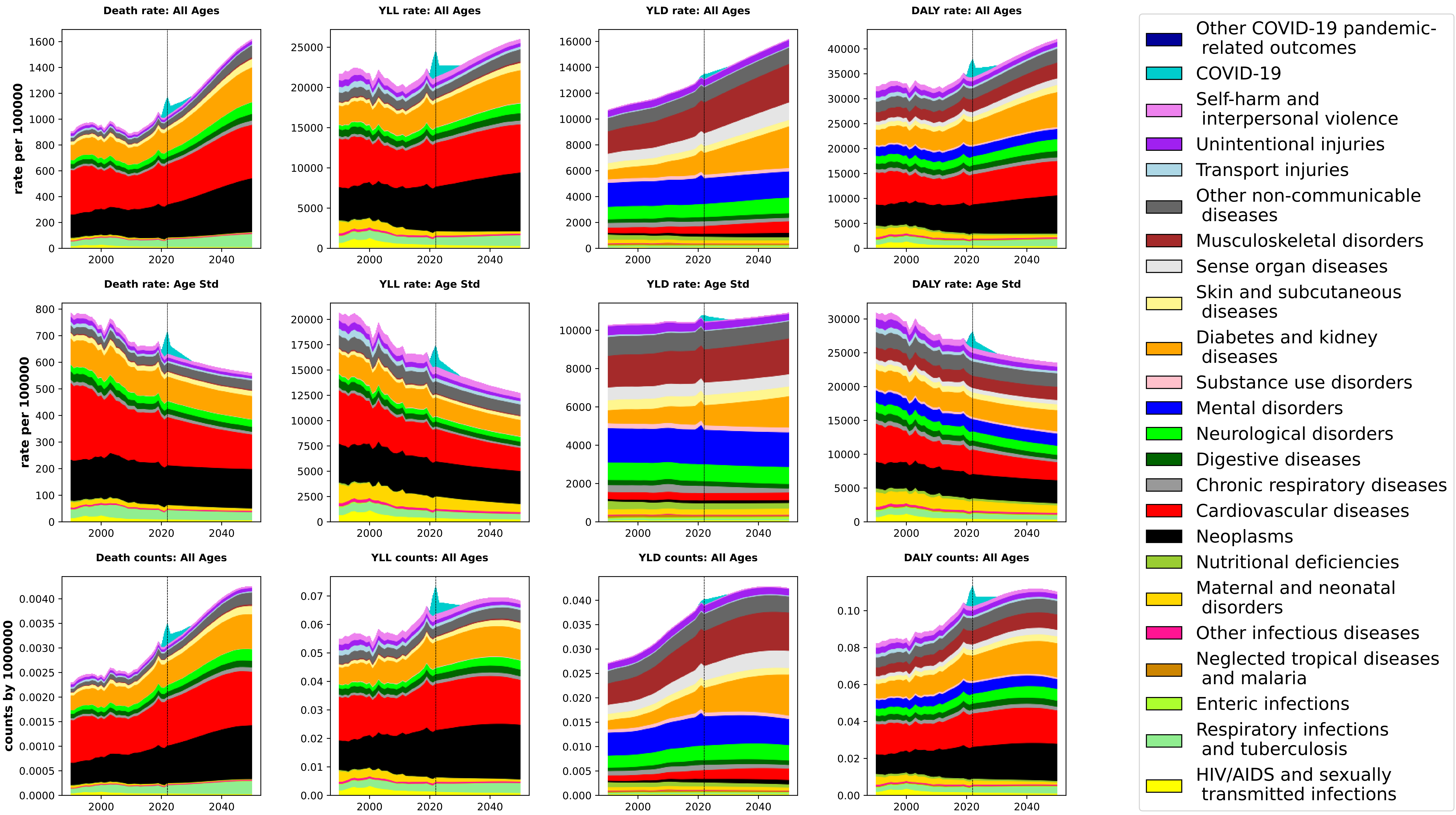
Death rate: All Ages
YLL rate: All Ages
YLD rate: All Ages
DALY rate: All Ages
Death rate: Age Std
YLL rate: Age Std
YLD rate: Age Std
DALY rate: Age Std
Death counts: All Ages
YLL counts: All Ages
YLD counts: All Ages
DALY counts: All Ages
rate per 100000
rate per 100000
counts by 1000000
2000
2020
2040
Other COVID-19 pandemic-related outcomes
COVID-19
Self-harm and interpersonal violence
Unintentional injuries
Transport injuries
Other non-communicable diseases
Musculoskeletal disorders
Sense organ diseases
Skin and subcutaneous diseases
Diabetes and kidney diseases
Substance use disorders
Mental disorders
Neurological disorders
Digestive diseases
Chronic respiratory diseases
Cardiovascular diseases
Neoplasms
Nutritional deficiencies
Maternal and neonatal disorders
Other infectious diseases
Neglected tropical diseases and malaria
Enteric infections
Respiratory infections and tuberculosis
HIV/AIDS and sexually transmitted infections

Belize

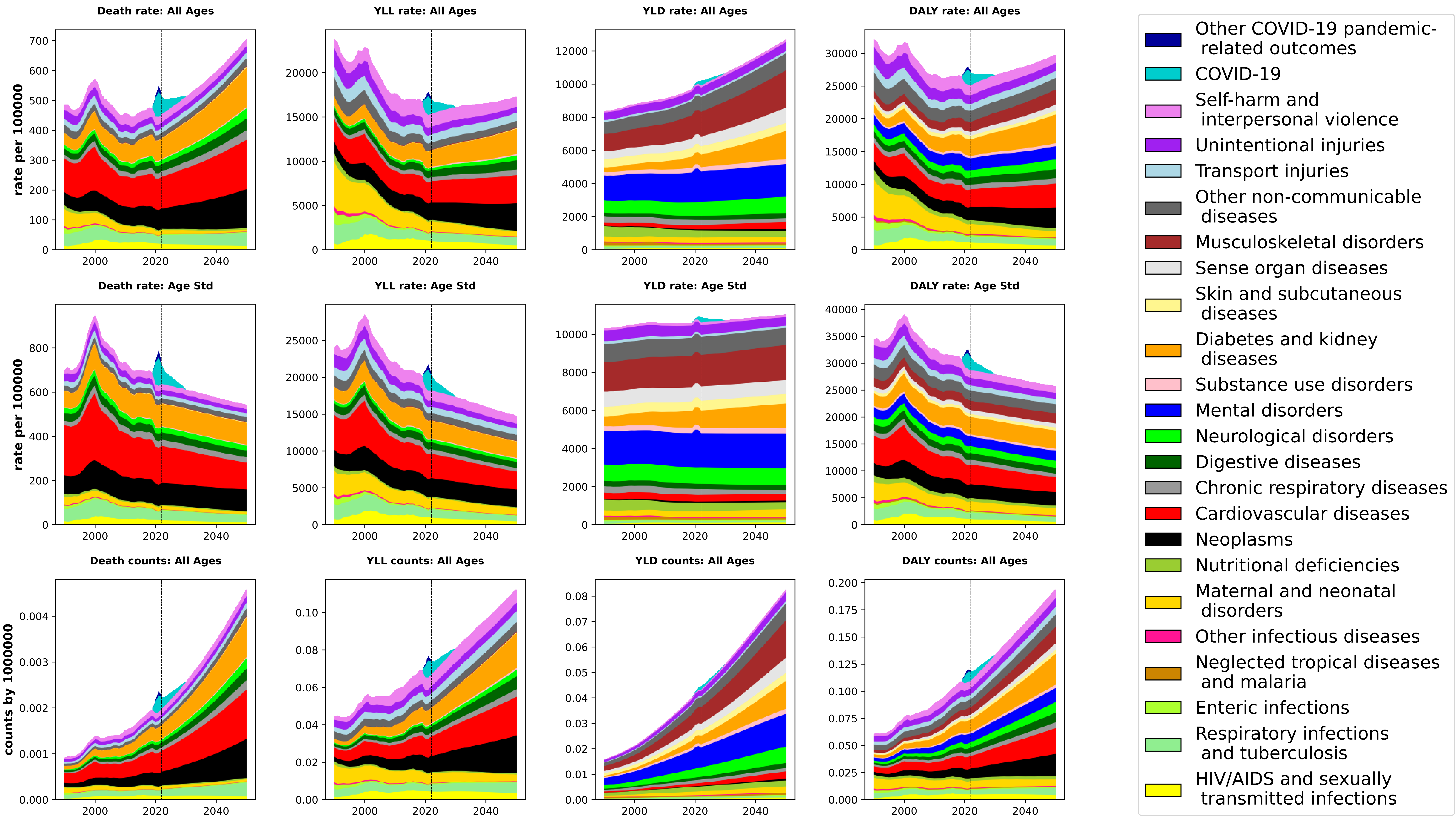
Death rate: All Ages
YLL rate: All Ages
YLD rate: All Ages
DALY rate: All Ages
Death rate: Age Std
YLL rate: Age Std
YLD rate: Age Std
DALY rate: Age Std
Death counts: All Ages
YLL counts: All Ages
YLD counts: All Ages
DALY counts: All Ages
rate per 100000
counts by 1000000
2000
2020
2040
Other COVID-19 pandemic-related outcomes
COVID-19
Self-harm and interpersonal violence
Unintentional injuries
Transport injuries
Other non-communicable diseases
Musculoskeletal disorders
Sense organ diseases
Skin and subcutaneous diseases
Diabetes and kidney diseases
Substance use disorders
Mental disorders
Neurological disorders
Digestive diseases
Chronic respiratory diseases
Cardiovascular diseases
Neoplasms
Nutritional deficiencies
Maternal and neonatal disorders
Other infectious diseases
Neglected tropical diseases and malaria
Enteric infections
Respiratory infections and tuberculosis
HIV/AIDS and sexually transmitted infections

Bermuda

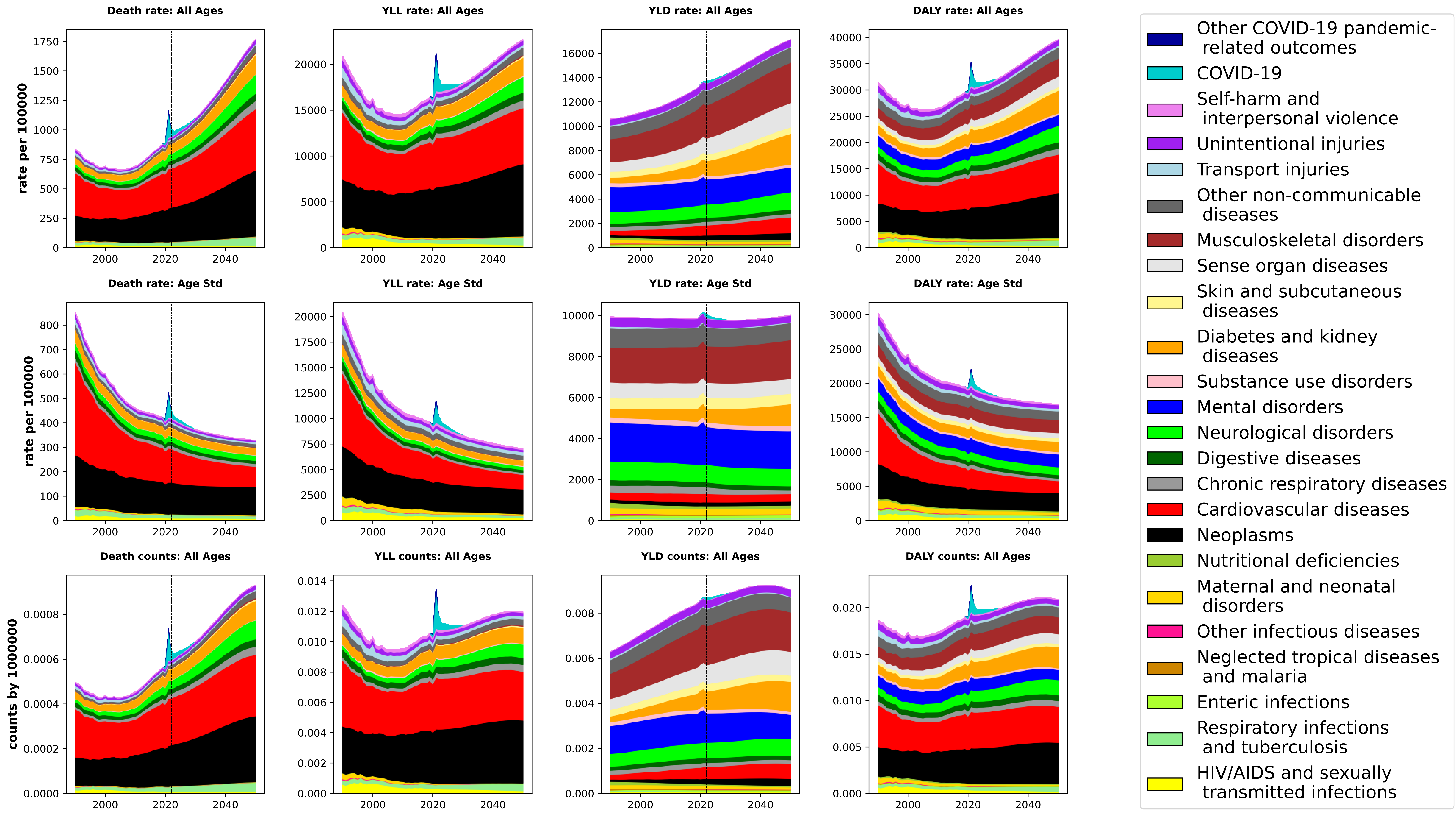
Death rate: All Ages
YLL rate: All Ages
YLD rate: All Ages
DALY rate: All Ages
Death rate: Age Std
YLL rate: Age Std
YLD rate: Age Std
DALY rate: Age Std
Death counts: All Ages
YLL counts: All Ages
YLD counts: All Ages
DALY counts: All Ages
rate per 100000
rate per 100000
counts by 1000000
2000
2020
2040
Other COVID-19 pandemic-related outcomes
COVID-19
Self-harm and interpersonal violence
Unintentional injuries
Transport injuries
Other non-communicable diseases
Musculoskeletal disorders
Sense organ diseases
Skin and subcutaneous diseases
Diabetes and kidney diseases
Substance use disorders
Mental disorders
Neurological disorders
Digestive diseases
Chronic respiratory diseases
Cardiovascular diseases
Neoplasms
Nutritional deficiencies
Maternal and neonatal disorders
Other infectious diseases
Neglected tropical diseases and malaria
Enteric infections
Respiratory infections and tuberculosis
HIV/AIDS and sexually transmitted infections

Cuba

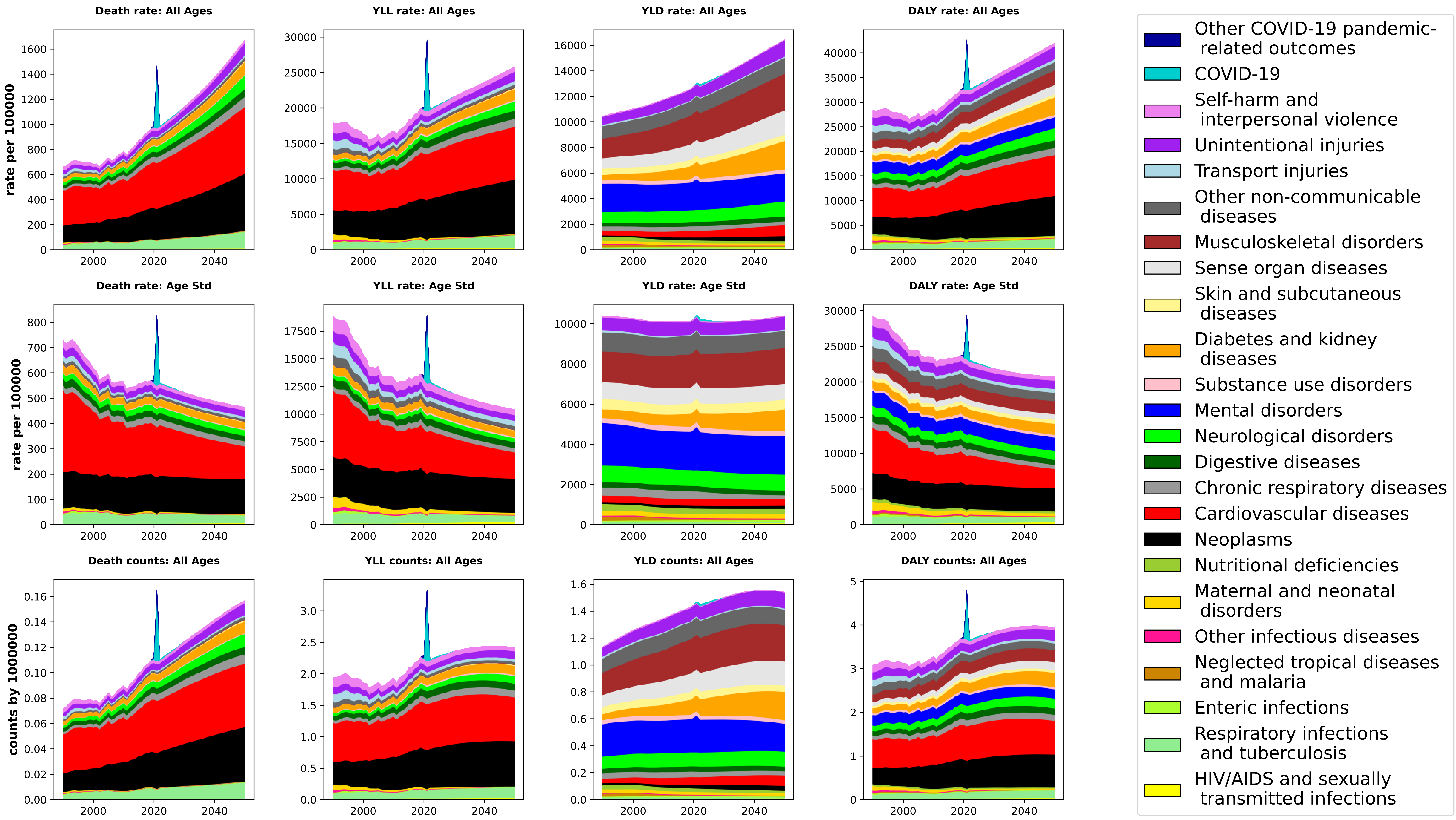
Death rate: All Ages
YLL rate: All Ages
YLD rate: All Ages
DALY rate: All Ages
Death rate: Age Std
YLL rate: Age Std
YLD rate: Age Std
DALY rate: Age Std
Death counts: All Ages
YLL counts: All Ages
YLD counts: All Ages
DALY counts: All Ages
rate per 100000
rate per 100000
counts by 1000000
2000
2020
2040
Other COVID-19 pandemic-related outcomes
COVID-19
Self-harm and interpersonal violence
Unintentional injuries
Transport injuries
Other non-communicable diseases
Musculoskeletal disorders
Sense organ diseases
Skin and subcutaneous diseases
Diabetes and kidney diseases
Substance use disorders
Mental disorders
Neurological disorders
Digestive diseases
Chronic respiratory diseases
Cardiovascular diseases
Neoplasms
Nutritional deficiencies
Maternal and neonatal disorders
Other infectious diseases
Neglected tropical diseases and malaria
Enteric infections
Respiratory infections and tuberculosis
HIV/AIDS and sexually transmitted infections

Dominica

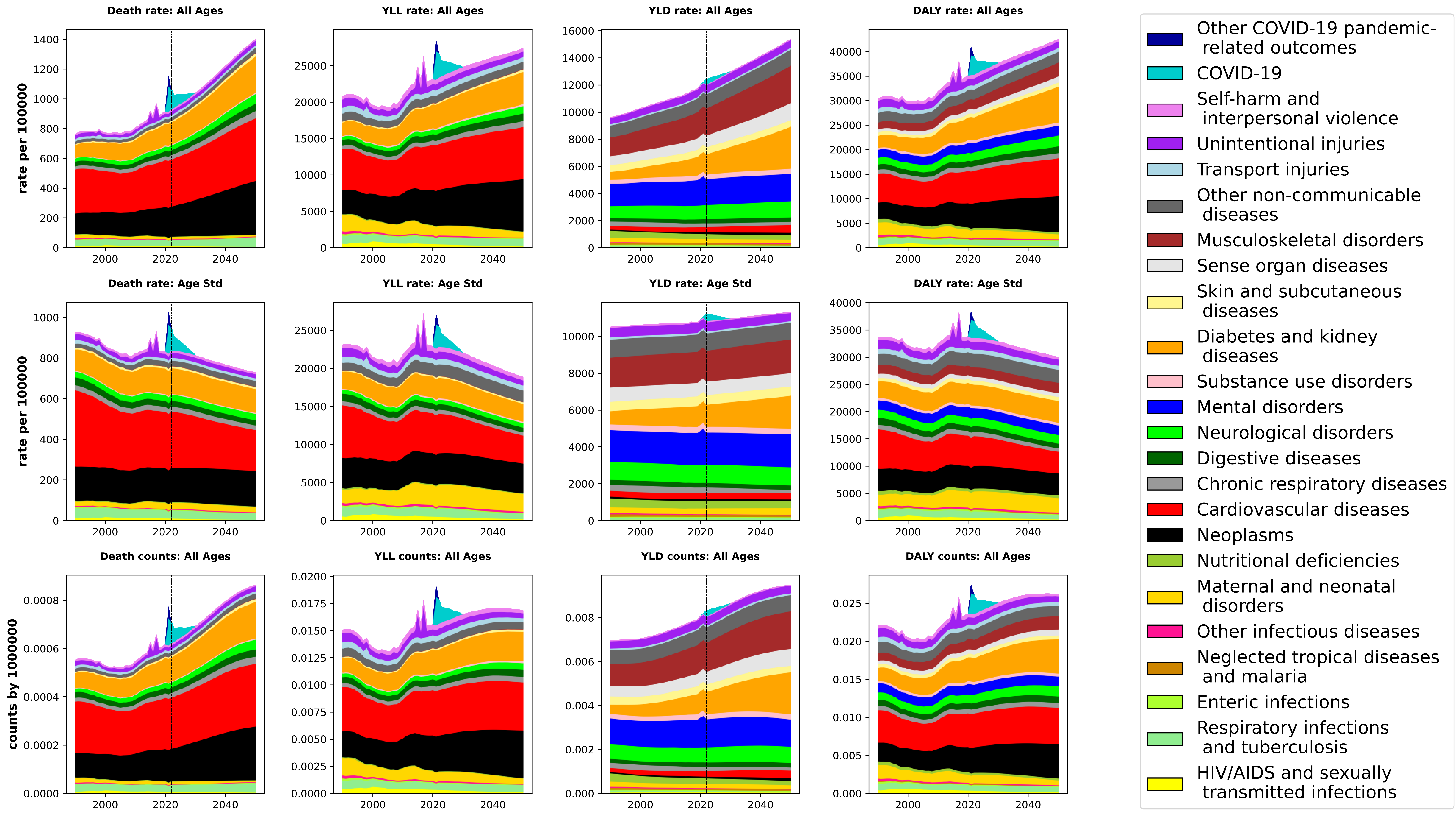
Death rate: All Ages
YLL rate: All Ages
YLD rate: All Ages
DALY rate: All Ages
Death rate: Age Std
YLL rate: Age Std
YLD rate: Age Std
DALY rate: Age Std
Death counts: All Ages
YLL counts: All Ages
YLD counts: All Ages
DALY counts: All Ages
rate per 100000
counts by 1000000
Other COVID-19 pandemic-related outcomes
COVID-19
Self-harm and interpersonal violence
Unintentional injuries
Transport injuries
Other non-communicable diseases
Musculoskeletal disorders
Sense organ diseases
Skin and subcutaneous diseases
Diabetes and kidney diseases
Substance use disorders
Mental disorders
Neurological disorders
Digestive diseases
Chronic respiratory diseases
Cardiovascular diseases
Neoplasms
Nutritional deficiencies
Maternal and neonatal disorders
Other infectious diseases
Neglected tropical diseases and malaria
Enteric infections
Respiratory infections and tuberculosis
HIV/AIDS and sexually transmitted infections

Dominican Republic

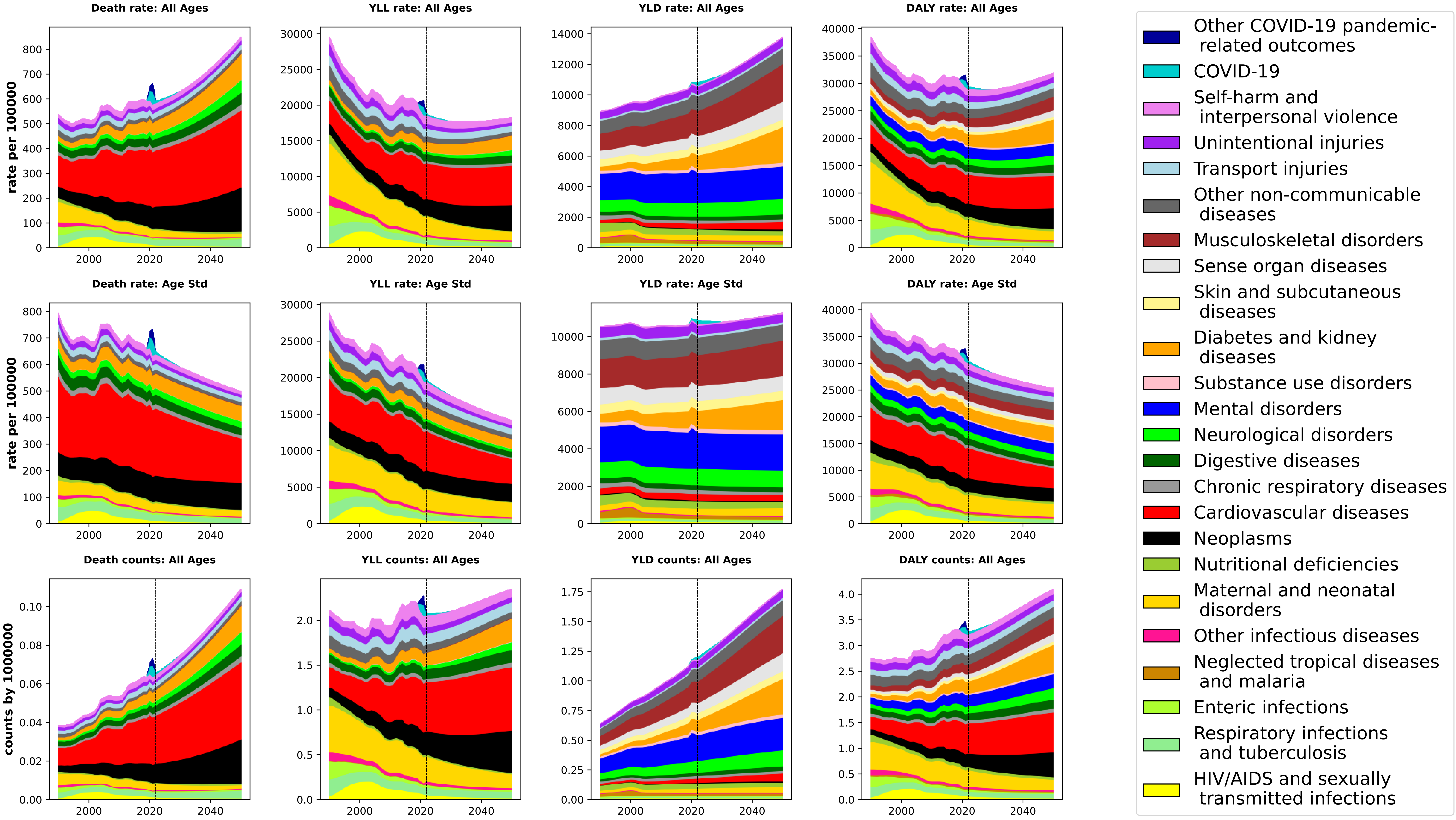

Grenada

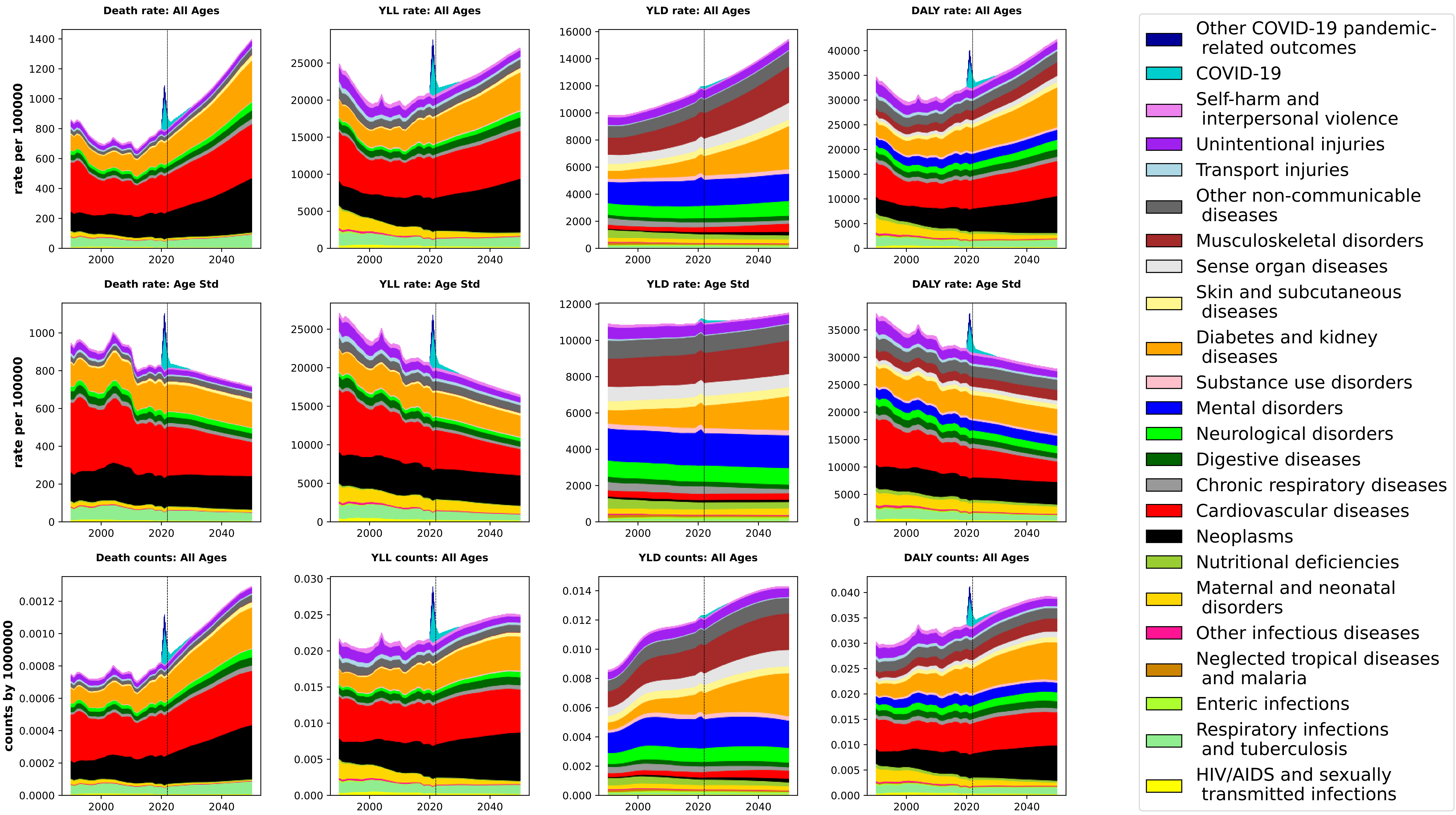
Death rate: All Ages
YLL rate: All Ages
YLD rate: All Ages
DALY rate: All Ages
Death rate: Age Std
YLL rate: Age Std
YLD rate: Age Std
DALY rate: Age Std
Death counts: All Ages
YLL counts: All Ages
YLD counts: All Ages
DALY counts: All Ages
rate per 100000
counts by 1000000
Other COVID-19 pandemic-related outcomes
COVID-19
Self-harm and interpersonal violence
Unintentional injuries
Transport injuries
Other non-communicable diseases
Musculoskeletal disorders
Sense organ diseases
Skin and subcutaneous diseases
Diabetes and kidney diseases
Substance use disorders
Mental disorders
Neurological disorders
Digestive diseases
Chronic respiratory diseases
Cardiovascular diseases
Neoplasms
Nutritional deficiencies
Maternal and neonatal disorders
Other infectious diseases
Neglected tropical diseases and malaria
Enteric infections
Respiratory infections and tuberculosis
HIV/AIDS and sexually transmitted infections

Guyana

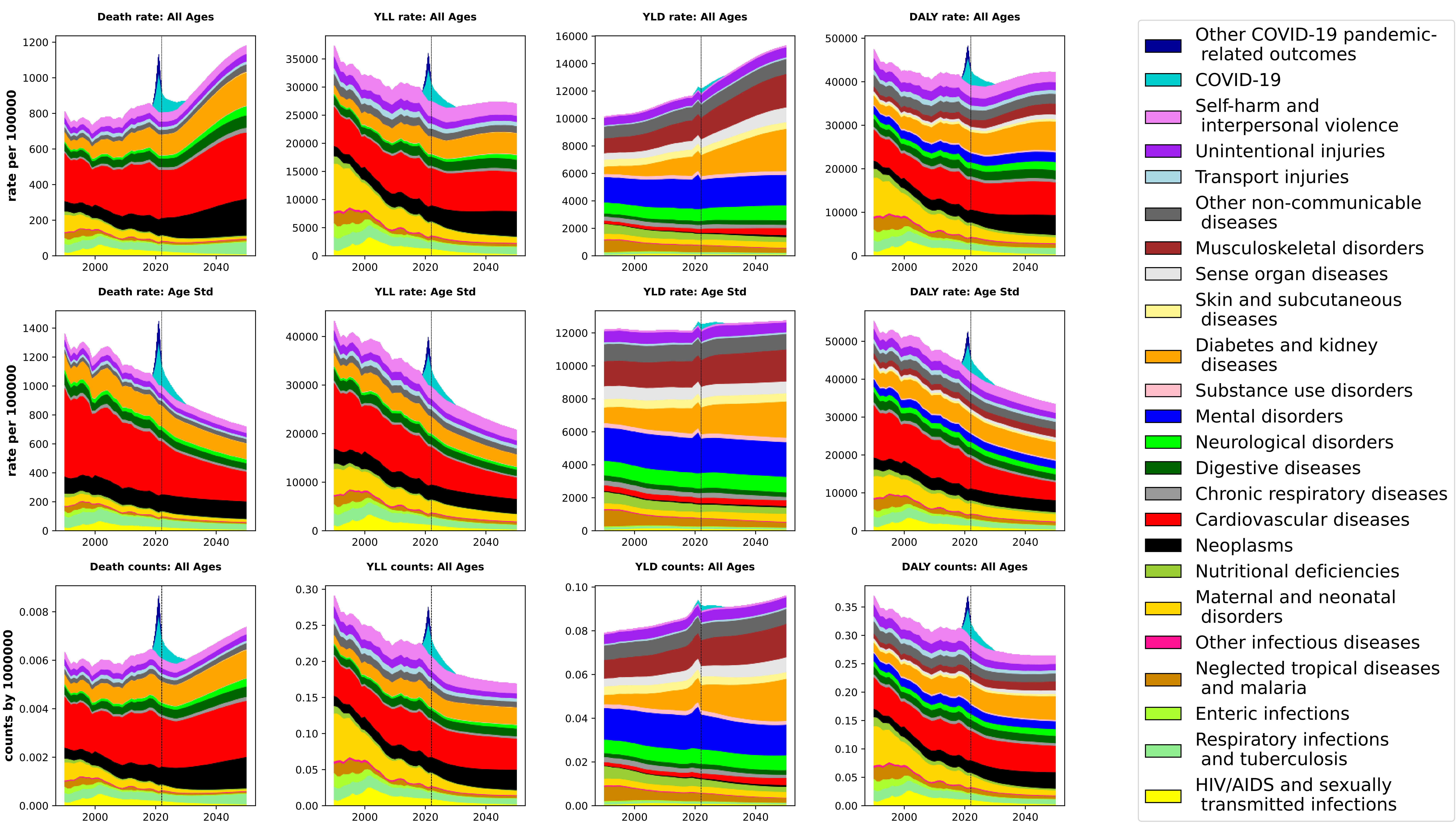
Death rate: All Ages
YLL rate: All Ages
YLD rate: All Ages
DALY rate: All Ages
Death rate: Age Std
YLL rate: Age Std
YLD rate: Age Std
DALY rate: Age Std
Death counts: All Ages
YLL counts: All Ages
YLD counts: All Ages
DALY counts: All Ages
rate per 100000
counts by 1000000
Other COVID-19 pandemic-related outcomes
COVID-19
Self-harm and interpersonal violence
Unintentional injuries
Transport injuries
Other non-communicable diseases
Musculoskeletal disorders
Sense organ diseases
Skin and subcutaneous diseases
Diabetes and kidney diseases
Substance use disorders
Mental disorders
Neurological disorders
Digestive diseases
Chronic respiratory diseases
Cardiovascular diseases
Neoplasms
Nutritional deficiencies
Maternal and neonatal disorders
Other infectious diseases
Neglected tropical diseases and malaria
Enteric infections
Respiratory infections and tuberculosis
HIV/AIDS and sexually transmitted infections

Haiti

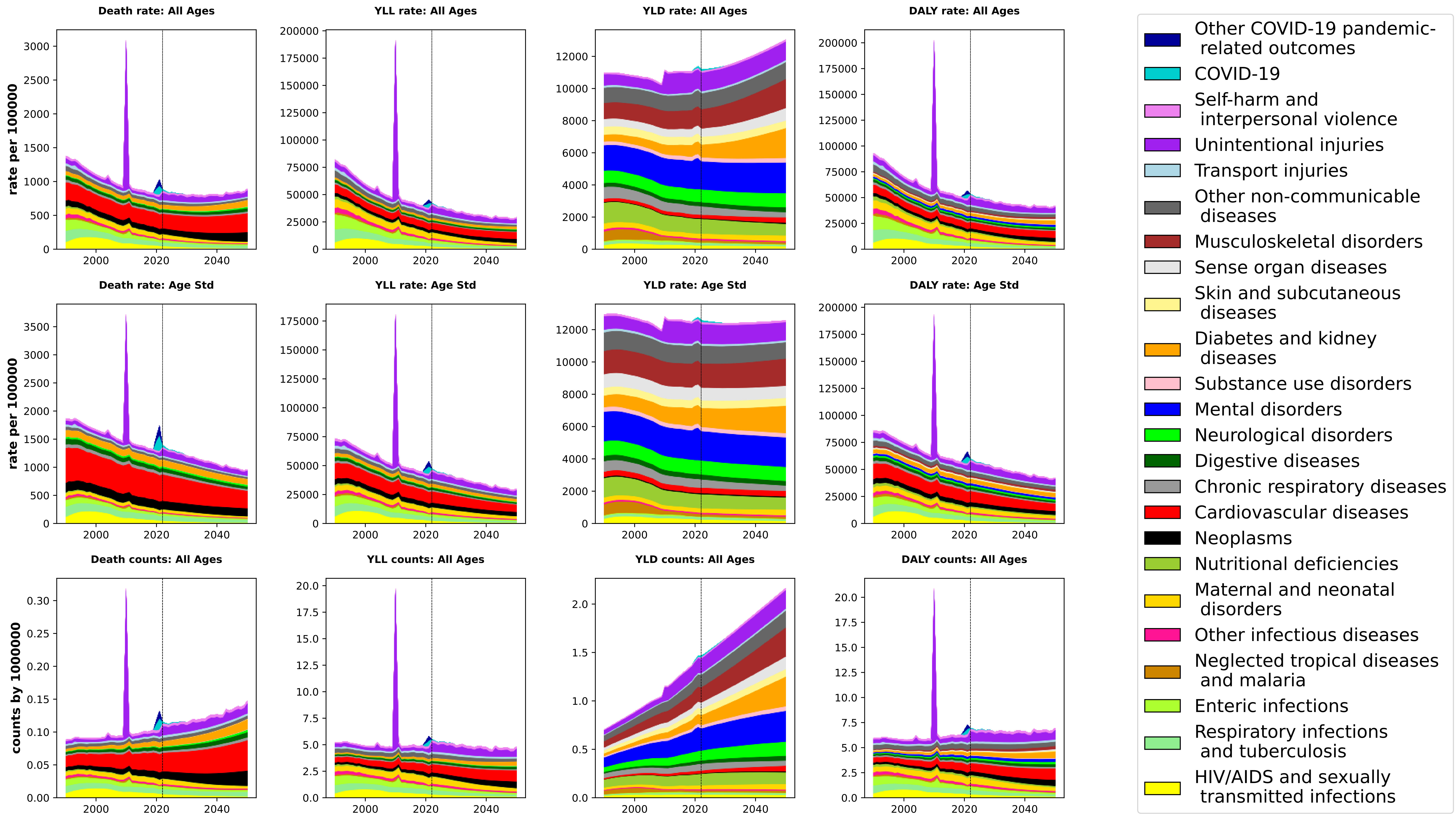
Death rate: All Ages
YLL rate: All Ages
YLD rate: All Ages
DALY rate: All Ages
Death rate: Age Std
YLL rate: Age Std
YLD rate: Age Std
DALY rate: Age Std
Death counts: All Ages
YLL counts: All Ages
YLD counts: All Ages
DALY counts: All Ages
rate per 100000
counts by 1000000
Other COVID-19 pandemic-related outcomes
COVID-19
Self-harm and interpersonal violence
Unintentional injuries
Transport injuries
Other non-communicable diseases
Musculoskeletal disorders
Sense organ diseases
Skin and subcutaneous diseases
Diabetes and kidney diseases
Substance use disorders
Mental disorders
Neurological disorders
Digestive diseases
Chronic respiratory diseases
Cardiovascular diseases
Neoplasms
Nutritional deficiencies
Maternal and neonatal disorders
Other infectious diseases
Neglected tropical diseases and malaria
Enteric infections
Respiratory infections and tuberculosis
HIV/AIDS and sexually transmitted infections

Jamaica

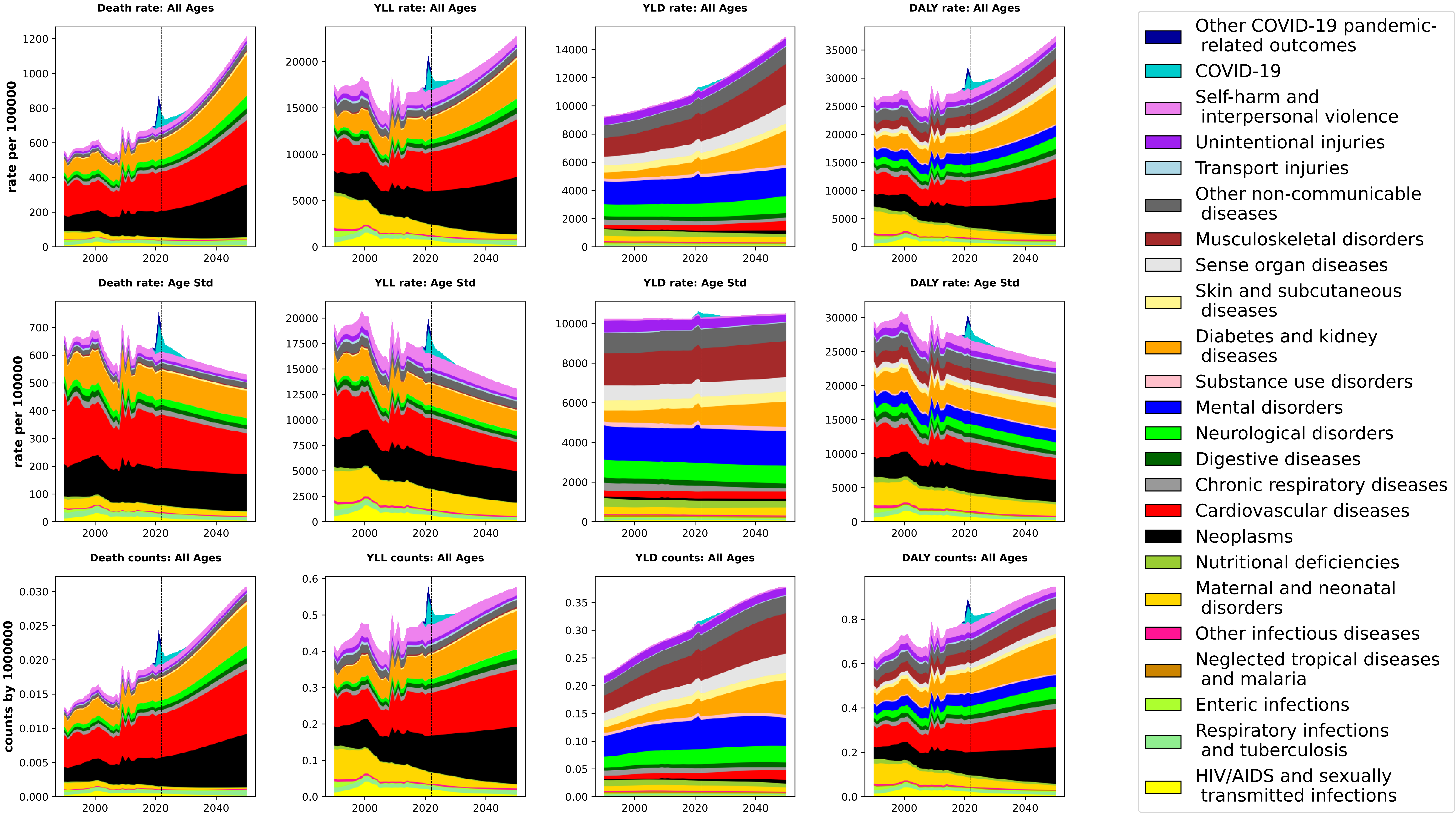
Death rate: All Ages
YLL rate: All Ages
YLD rate: All Ages
DALY rate: All Ages
Death rate: Age Std
YLL rate: Age Std
YLD rate: Age Std
DALY rate: Age Std
Death counts: All Ages
YLL counts: All Ages
YLD counts: All Ages
DALY counts: All Ages
rate per 100000
rate per 100000
counts by 1000000
Other COVID-19 pandemic-related outcomes
COVID-19
Self-harm and interpersonal violence
Unintentional injuries
Transport injuries
Other non-communicable diseases
Musculoskeletal disorders
Sense organ diseases
Skin and subcutaneous diseases
Diabetes and kidney diseases
Substance use disorders
Mental disorders
Neurological disorders
Digestive diseases
Chronic respiratory diseases
Cardiovascular diseases
Neoplasms
Nutritional deficiencies
Maternal and neonatal disorders
Other infectious diseases
Neglected tropical diseases and malaria
Enteric infections
Respiratory infections and tuberculosis
HIV/AIDS and sexually transmitted infections

Puerto Rico

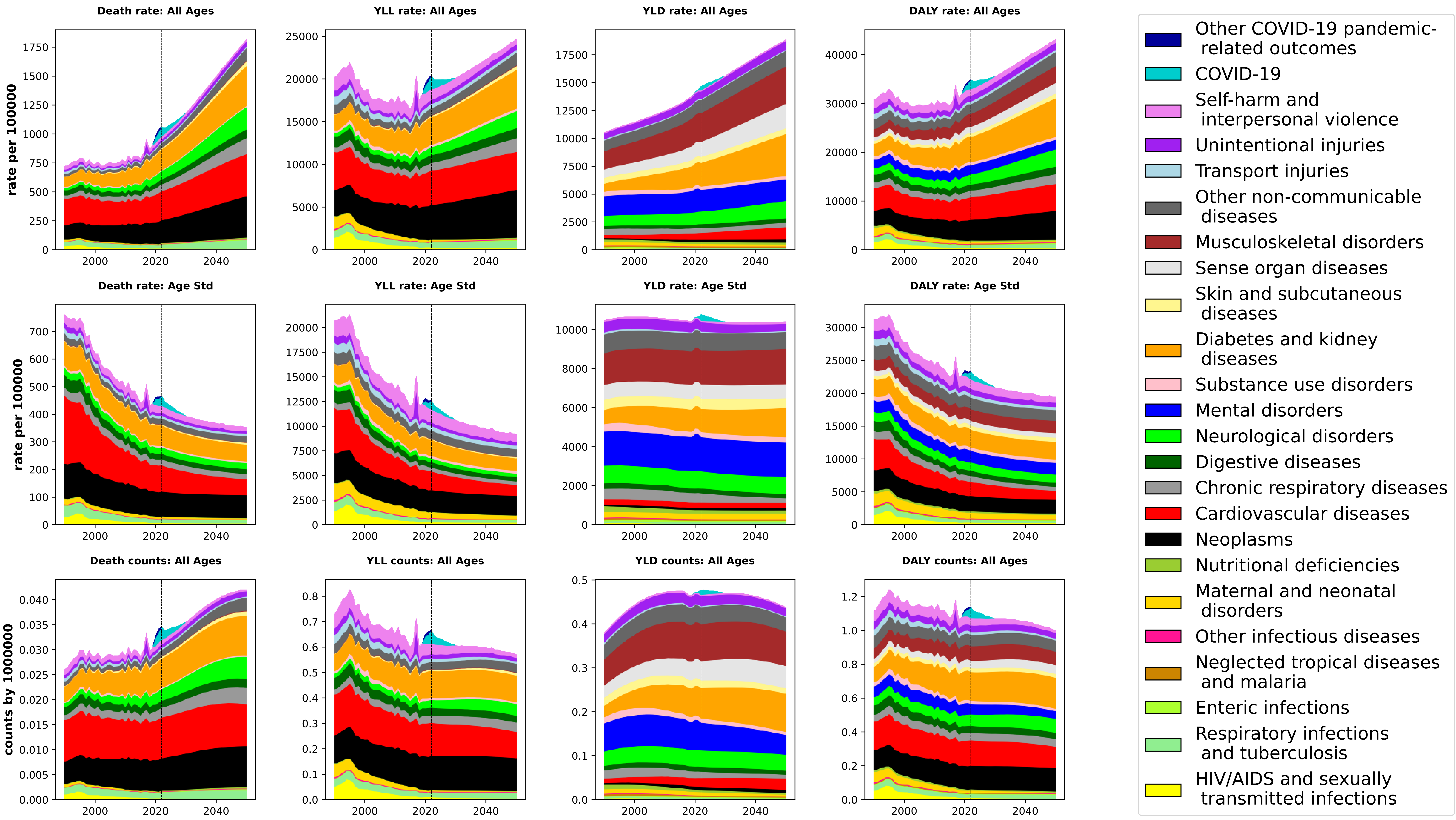
Death rate: All Ages
YLL rate: All Ages
YLD rate: All Ages
DALY rate: All Ages
Death rate: Age Std
YLL rate: Age Std
YLD rate: Age Std
DALY rate: Age Std
Death counts: All Ages
YLL counts: All Ages
YLD counts: All Ages
DALY counts: All Ages
rate per 100000
rate per 100000
counts by 1000000
Other COVID-19 pandemic-related outcomes
COVID-19
Self-harm and interpersonal violence
Unintentional injuries
Transport injuries
Other non-communicable diseases
Musculoskeletal disorders
Sense organ diseases
Skin and subcutaneous diseases
Diabetes and kidney diseases
Substance use disorders
Mental disorders
Neurological disorders
Digestive diseases
Chronic respiratory diseases
Cardiovascular diseases
Neoplasms
Nutritional deficiencies
Maternal and neonatal disorders
Other infectious diseases
Neglected tropical diseases and malaria
Enteric infections
Respiratory infections and tuberculosis
HIV/AIDS and sexually transmitted infections

Saint Kitts and Nevis

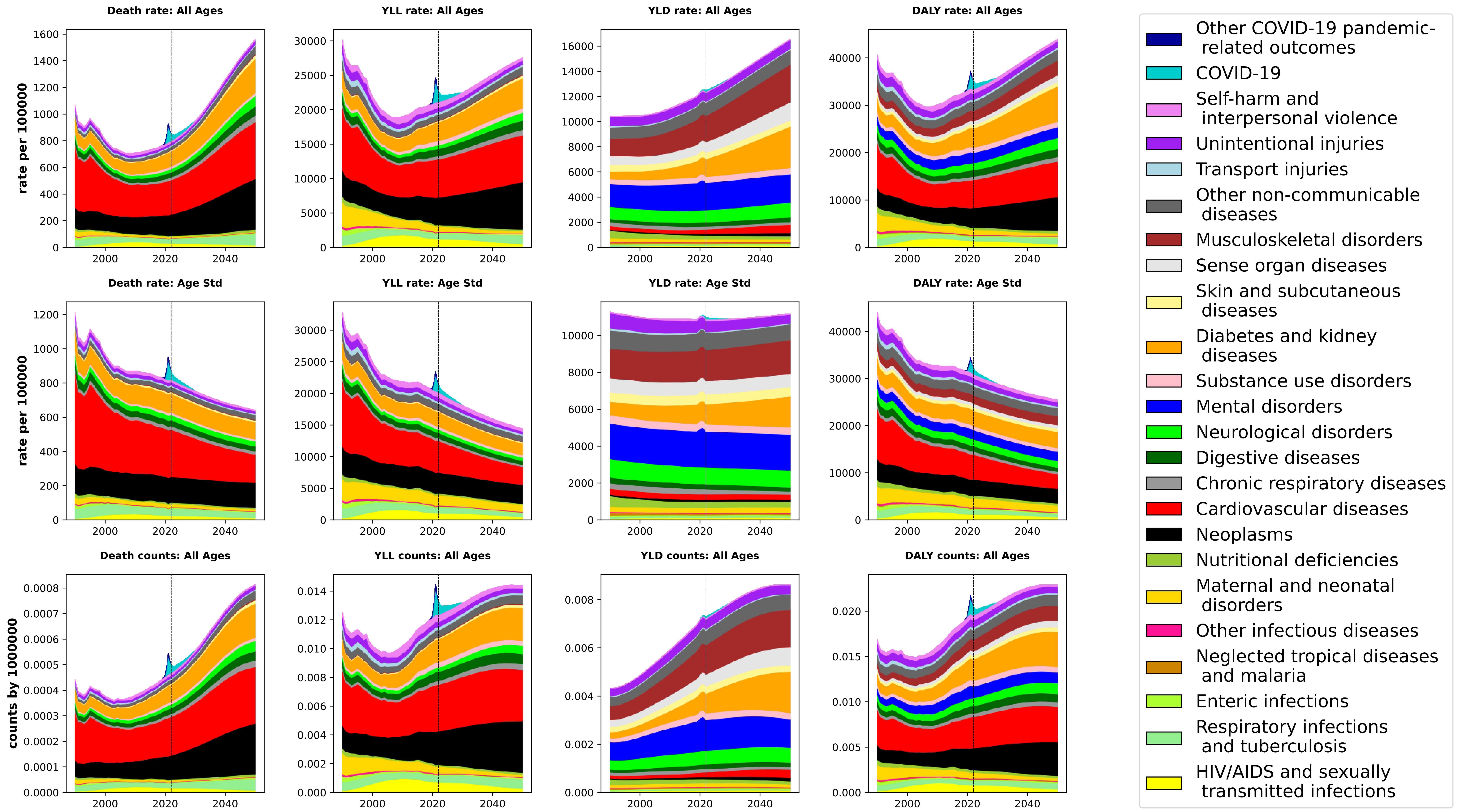

Saint Lucia

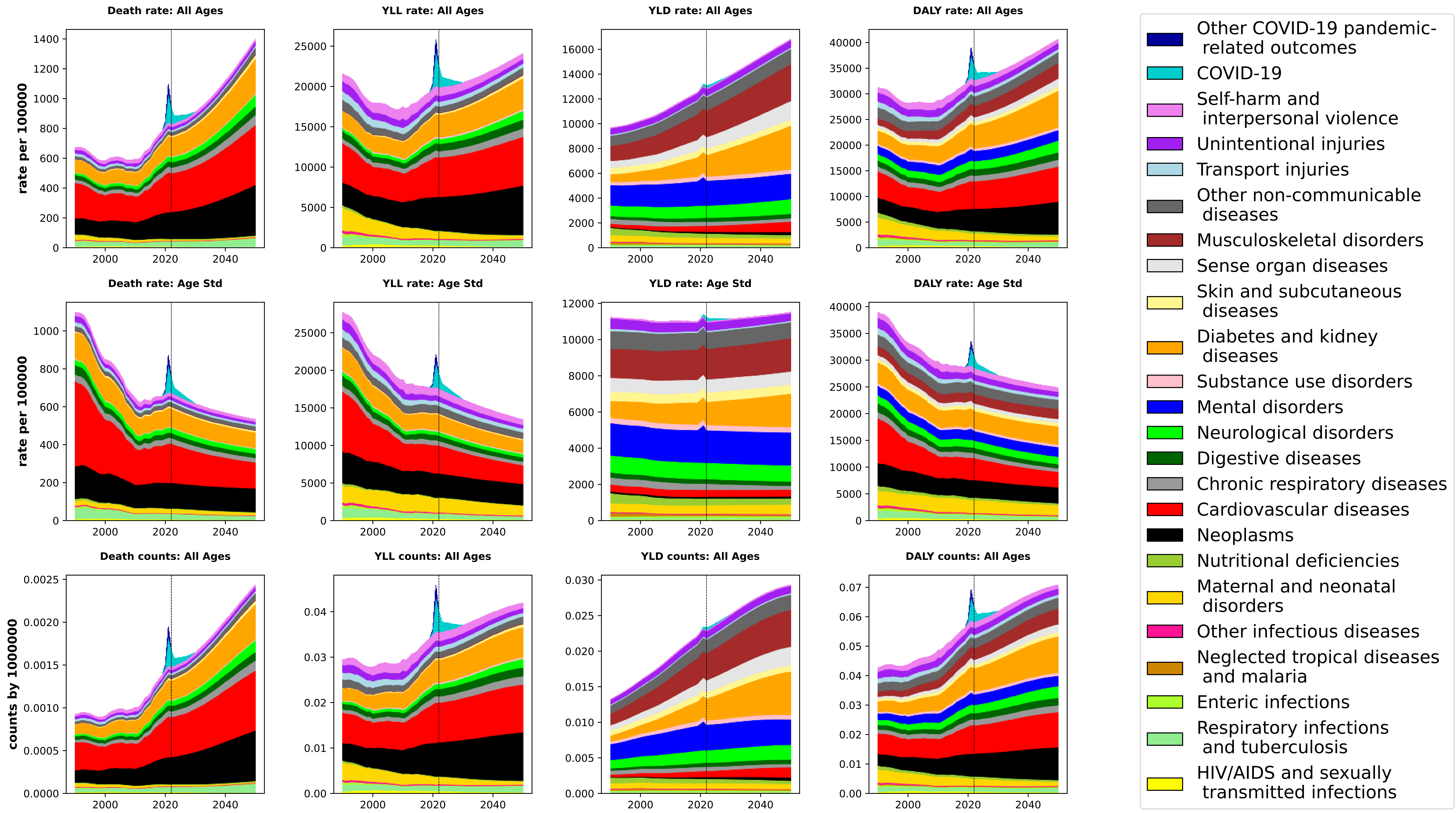
Death rate: All Ages
YLL rate: All Ages
YLD rate: All Ages
DALY rate: All Ages
Death rate: Age Std
YLL rate: Age Std
YLD rate: Age Std
DALY rate: Age Std
Death counts: All Ages
YLL counts: All Ages
YLD counts: All Ages
DALY counts: All Ages
rate per 100000
counts by 1000000
Other COVID-19 pandemic-related outcomes
COVID-19
Self-harm and interpersonal violence
Unintentional injuries
Transport injuries
Other non-communicable diseases
Musculoskeletal disorders
Sense organ diseases
Skin and subcutaneous diseases
Diabetes and kidney diseases
Substance use disorders
Mental disorders
Neurological disorders
Digestive diseases
Chronic respiratory diseases
Cardiovascular diseases
Neoplasms
Nutritional deficiencies
Maternal and neonatal disorders
Other infectious diseases
Neglected tropical diseases and malaria
Enteric infections
Respiratory infections and tuberculosis
HIV/AIDS and sexually transmitted infections

Saint Vincent and the Grenadines

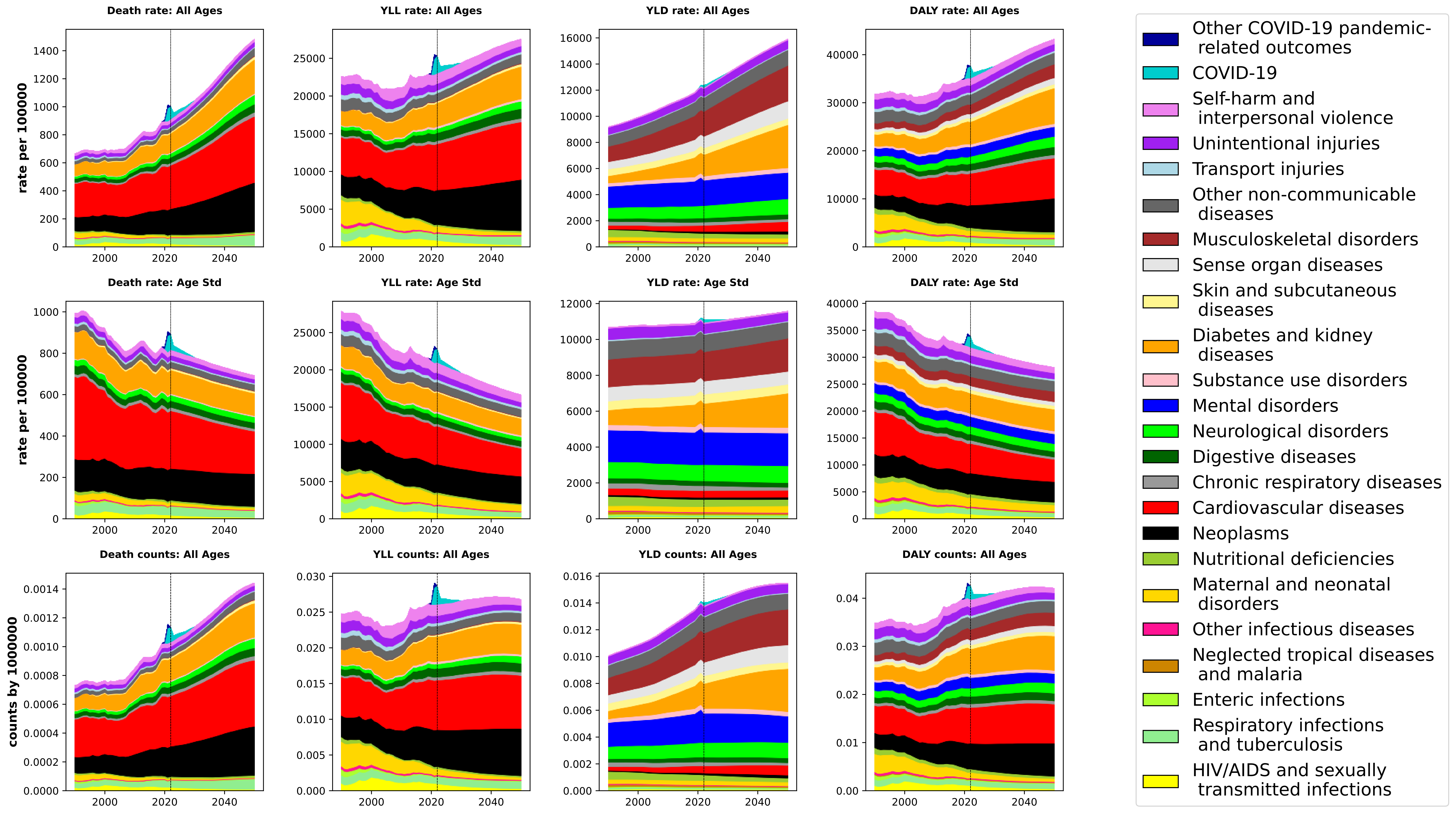

Suriname

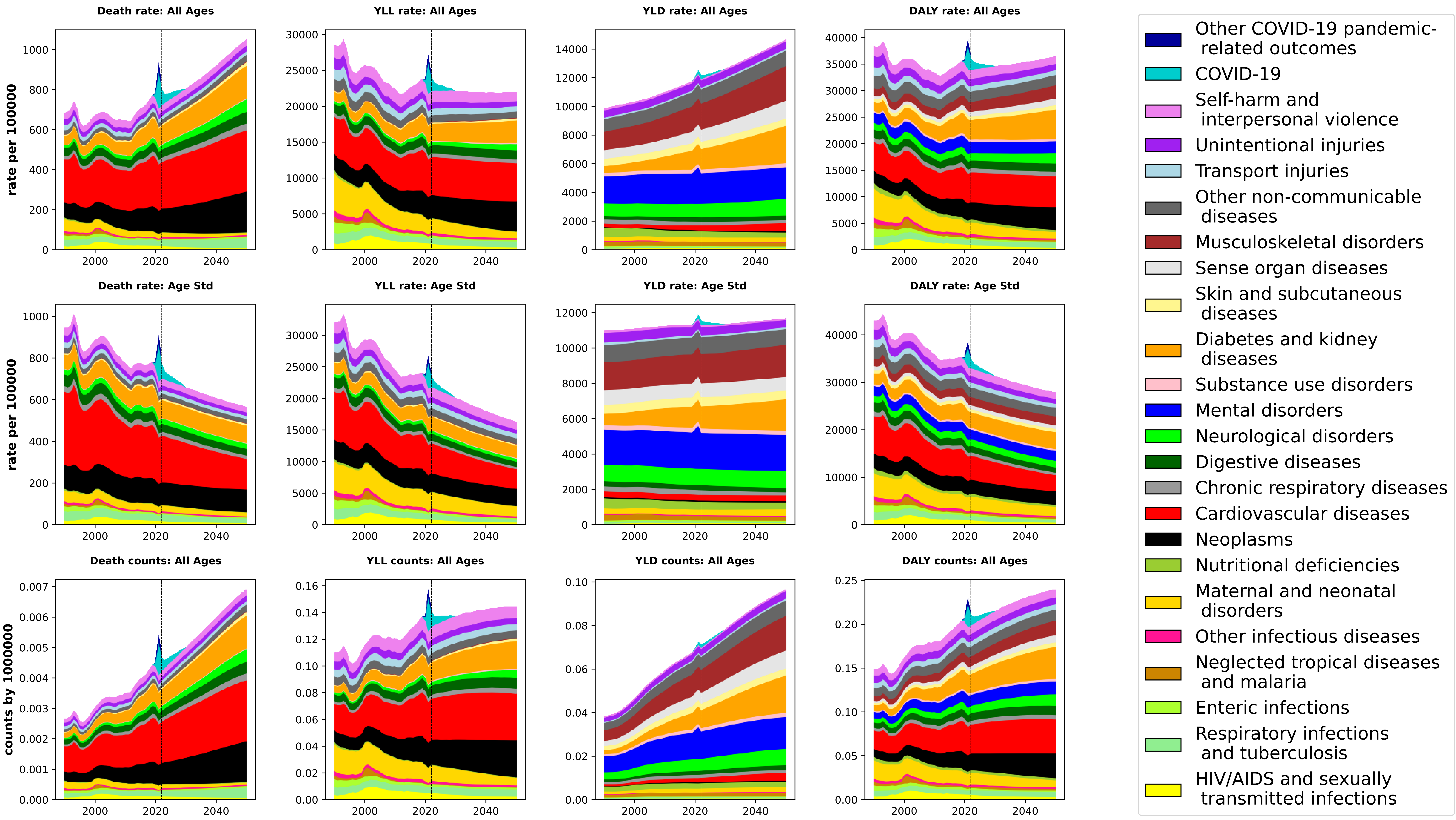
Death rate: All Ages
YLL rate: All Ages
YLD rate: All Ages
DALY rate: All Ages
Death rate: Age Std
YLL rate: Age Std
YLD rate: Age Std
DALY rate: Age Std
Death counts: All Ages
YLL counts: All Ages
YLD counts: All Ages
DALY counts: All Ages
rate per 100000
counts by 1000000
Other COVID-19 pandemic-related outcomes
COVID-19
Self-harm and interpersonal violence
Unintentional injuries
Transport injuries
Other non-communicable diseases
Musculoskeletal disorders
Sense organ diseases
Skin and subcutaneous diseases
Diabetes and kidney diseases
Substance use disorders
Mental disorders
Neurological disorders
Digestive diseases
Chronic respiratory diseases
Cardiovascular diseases
Neoplasms
Nutritional deficiencies
Maternal and neonatal disorders
Other infectious diseases
Neglected tropical diseases and malaria
Enteric infections
Respiratory infections and tuberculosis
HIV/AIDS and sexually transmitted infections

Trinidad and Tobago

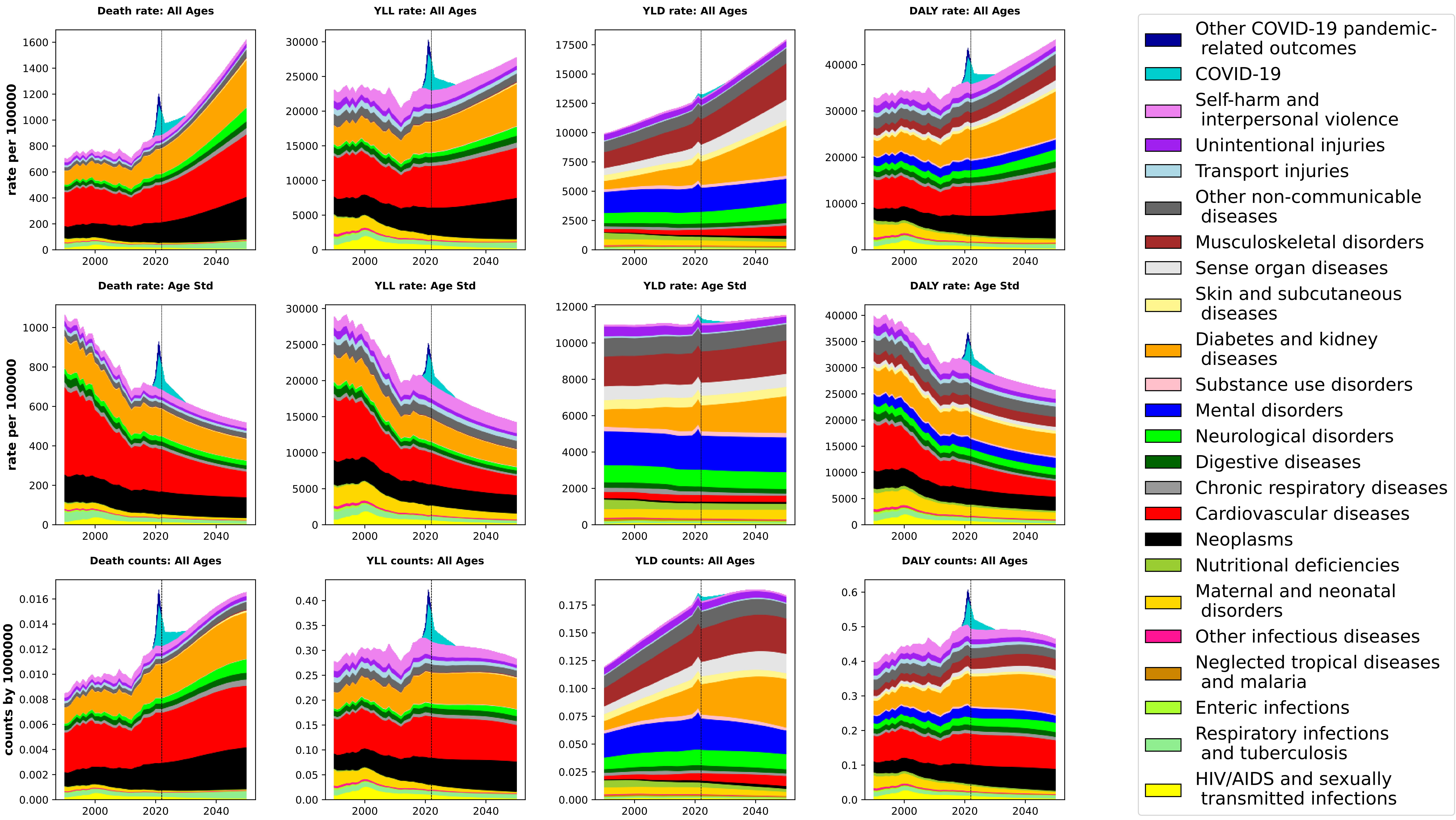

Virgin Islands

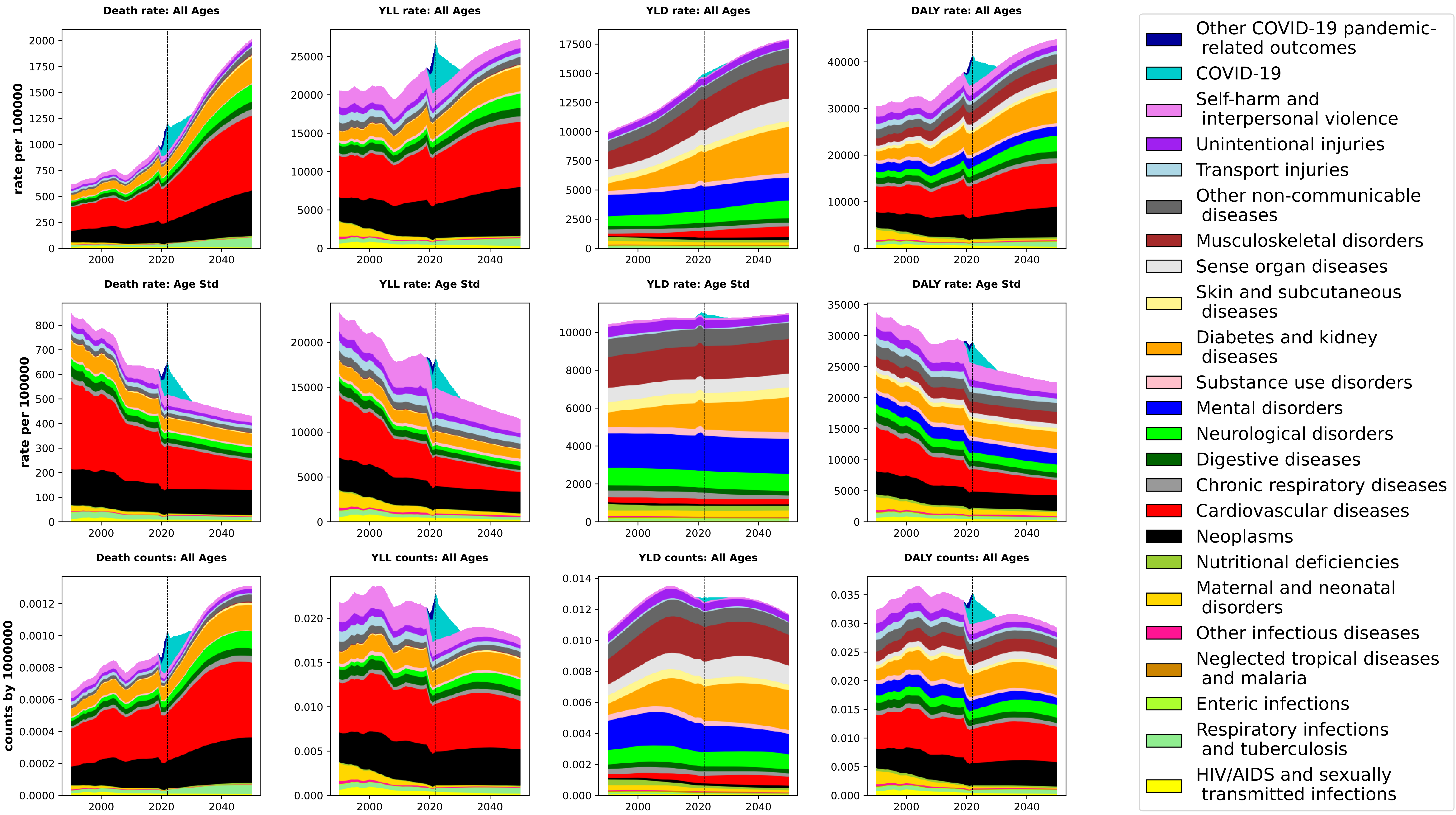

Colombia

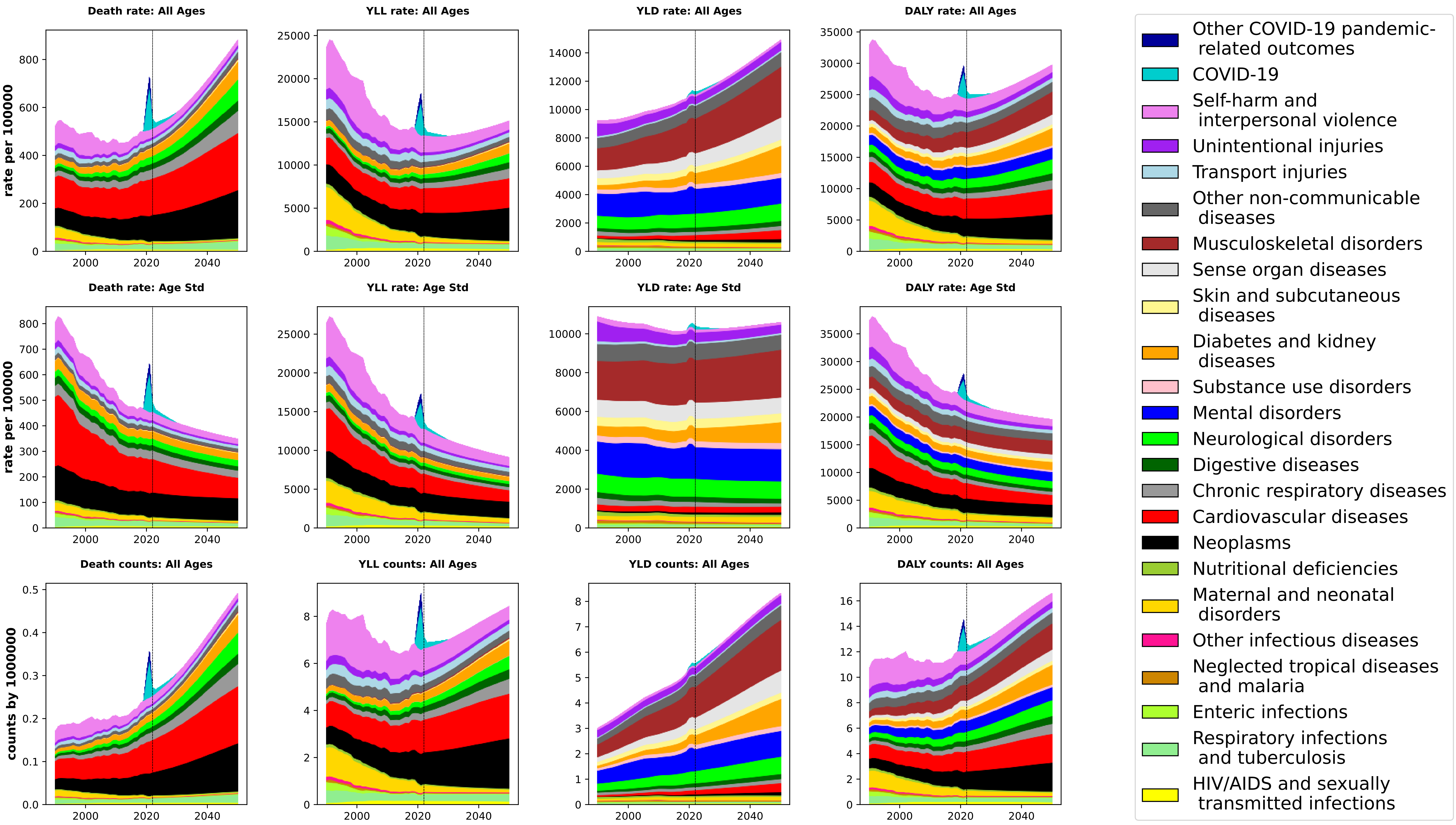
Death rate: All Ages
YLL rate: All Ages
YLD rate: All Ages
DALY rate: All Ages
Death rate: Age Std
YLL rate: Age Std
YLD rate: Age Std
DALY rate: Age Std
Death counts: All Ages
YLL counts: All Ages
YLD counts: All Ages
DALY counts: All Ages
rate per 100000
counts by 1000000
Other COVID-19 pandemic-related outcomes
COVID-19
Self-harm and interpersonal violence
Unintentional injuries
Transport injuries
Other non-communicable diseases
Musculoskeletal disorders
Sense organ diseases
Skin and subcutaneous diseases
Diabetes and kidney diseases
Substance use disorders
Mental disorders
Neurological disorders
Digestive diseases
Chronic respiratory diseases
Cardiovascular diseases
Neoplasms
Nutritional deficiencies
Maternal and neonatal disorders
Other infectious diseases
Neglected tropical diseases and malaria
Enteric infections
Respiratory infections and tuberculosis
HIV/AIDS and sexually transmitted infections

Costa Rica

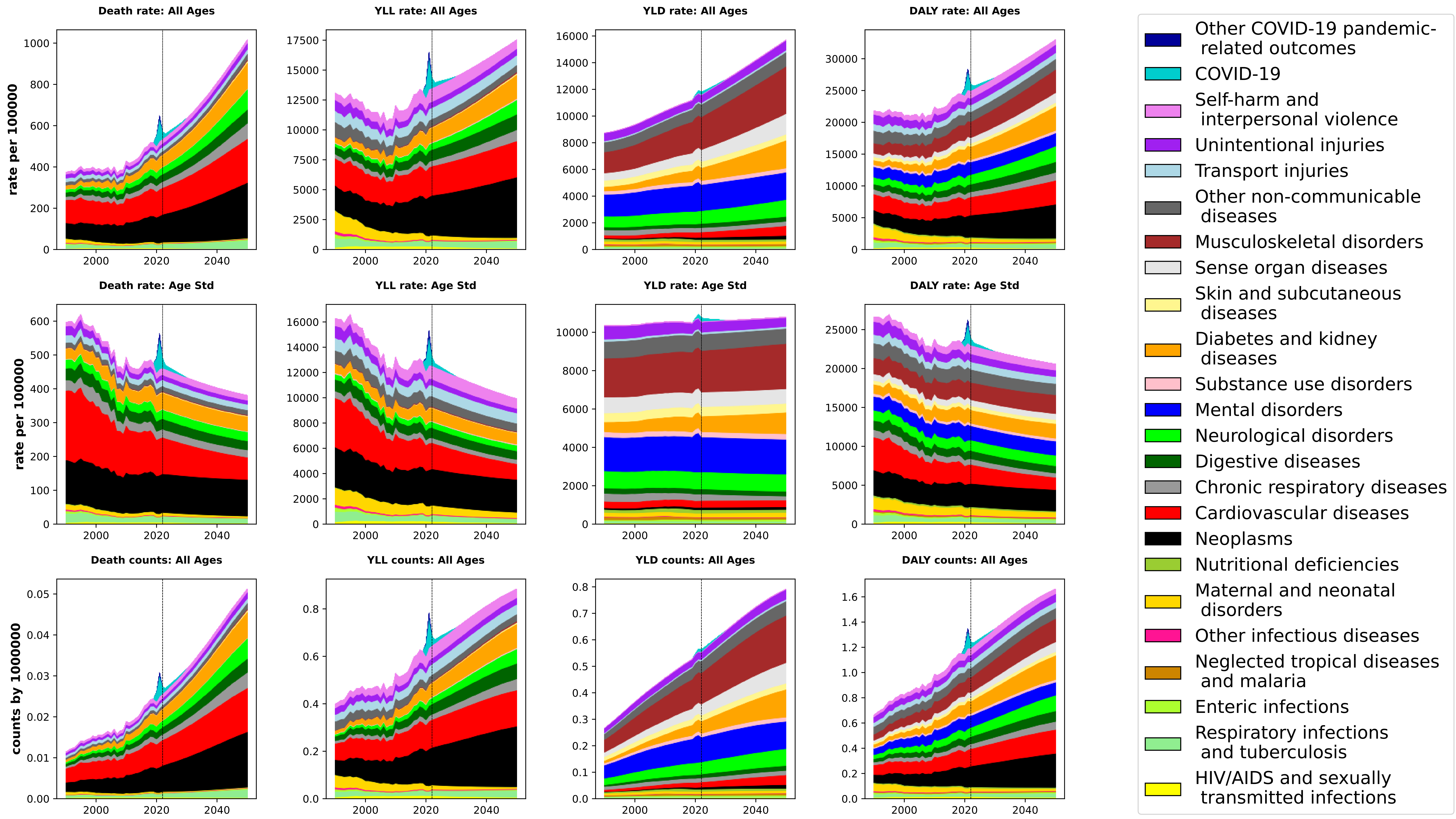
Death rate: All Ages
YLL rate: All Ages
YLD rate: All Ages
DALY rate: All Ages
Death rate: Age Std
YLL rate: Age Std
YLD rate: Age Std
DALY rate: Age Std
Death counts: All Ages
YLL counts: All Ages
YLD counts: All Ages
DALY counts: All Ages
rate per 100000
counts by 1000000
2000
2020
2040
Other COVID-19 pandemic-related outcomes
COVID-19
Self-harm and interpersonal violence
Unintentional injuries
Transport injuries
Other non-communicable diseases
Musculoskeletal disorders
Sense organ diseases
Skin and subcutaneous diseases
Diabetes and kidney diseases
Substance use disorders
Mental disorders
Neurological disorders
Digestive diseases
Chronic respiratory diseases
Cardiovascular diseases
Neoplasms
Nutritional deficiencies
Maternal and neonatal disorders
Other infectious diseases
Neglected tropical diseases and malaria
Enteric infections
Respiratory infections and tuberculosis
HIV/AIDS and sexually transmitted infections

El Salvador

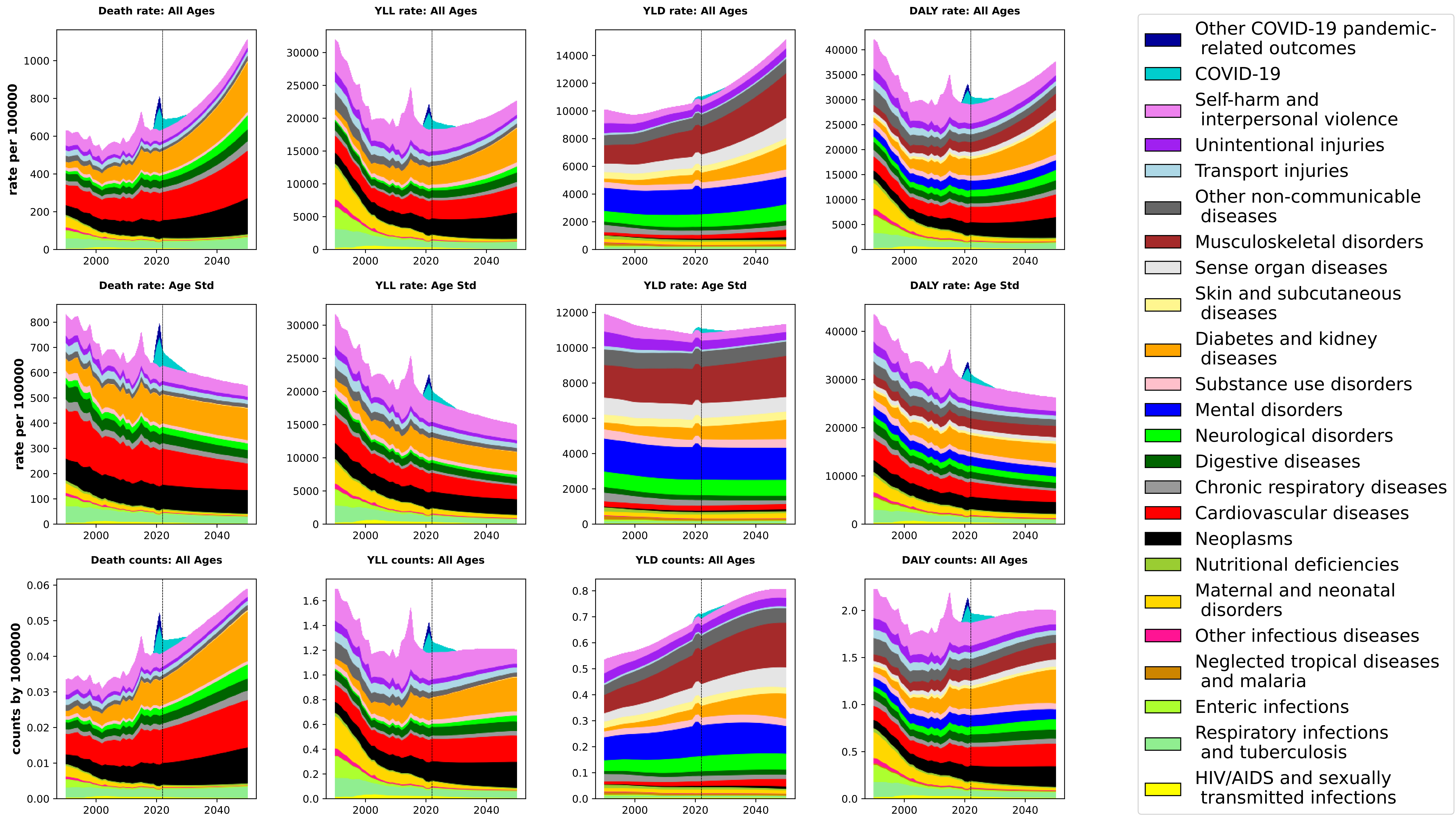
Death rate: All Ages
YLL rate: All Ages
YLD rate: All Ages
DALY rate: All Ages
Death rate: Age Std
YLL rate: Age Std
YLD rate: Age Std
DALY rate: Age Std
Death counts: All Ages
YLL counts: All Ages
YLD counts: All Ages
DALY counts: All Ages
rate per 100000
counts by 1000000
2000
2020
2040
Other COVID-19 pandemic-related outcomes
COVID-19
Self-harm and interpersonal violence
Unintentional injuries
Transport injuries
Other non-communicable diseases
Musculoskeletal disorders
Sense organ diseases
Skin and subcutaneous diseases
Diabetes and kidney diseases
Substance use disorders
Mental disorders
Neurological disorders
Digestive diseases
Chronic respiratory diseases
Cardiovascular diseases
Neoplasms
Nutritional deficiencies
Maternal and neonatal disorders
Other infectious diseases
Neglected tropical diseases and malaria
Enteric infections
Respiratory infections and tuberculosis
HIV/AIDS and sexually transmitted infections

Guatemala

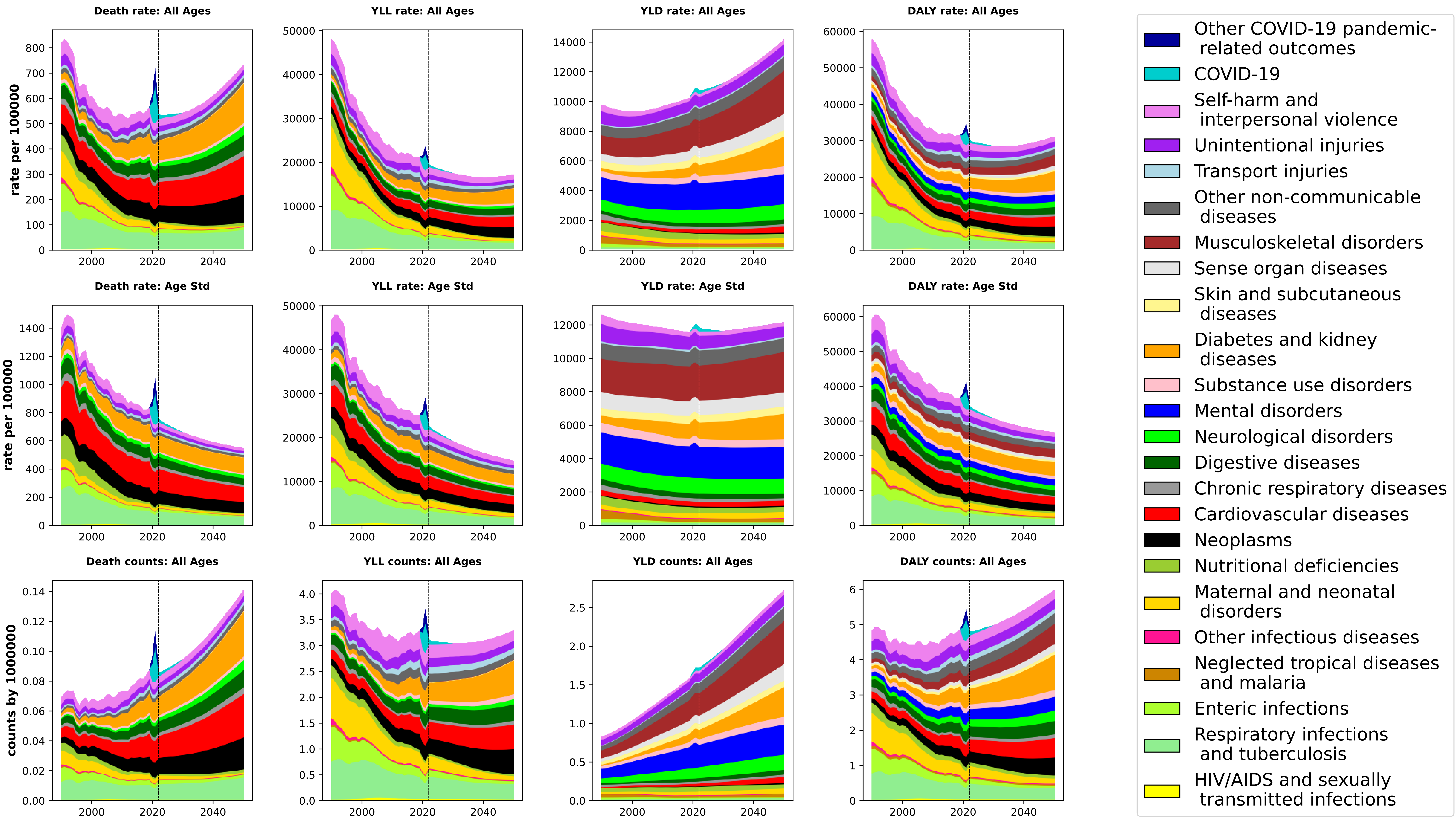
Death rate: All Ages
YLL rate: All Ages
YLD rate: All Ages
DALY rate: All Ages
Death rate: Age Std
YLL rate: Age Std
YLD rate: Age Std
DALY rate: Age Std
Death counts: All Ages
YLL counts: All Ages
YLD counts: All Ages
DALY counts: All Ages
rate per 100000
counts by 1000000
Other COVID-19 pandemic-related outcomes
COVID-19
Self-harm and interpersonal violence
Unintentional injuries
Transport injuries
Other non-communicable diseases
Musculoskeletal disorders
Sense organ diseases
Skin and subcutaneous diseases
Diabetes and kidney diseases
Substance use disorders
Mental disorders
Neurological disorders
Digestive diseases
Chronic respiratory diseases
Cardiovascular diseases
Neoplasms
Nutritional deficiencies
Maternal and neonatal disorders
Other infectious diseases
Neglected tropical diseases and malaria
Enteric infections
Respiratory infections and tuberculosis
HIV/AIDS and sexually transmitted infections

Honduras

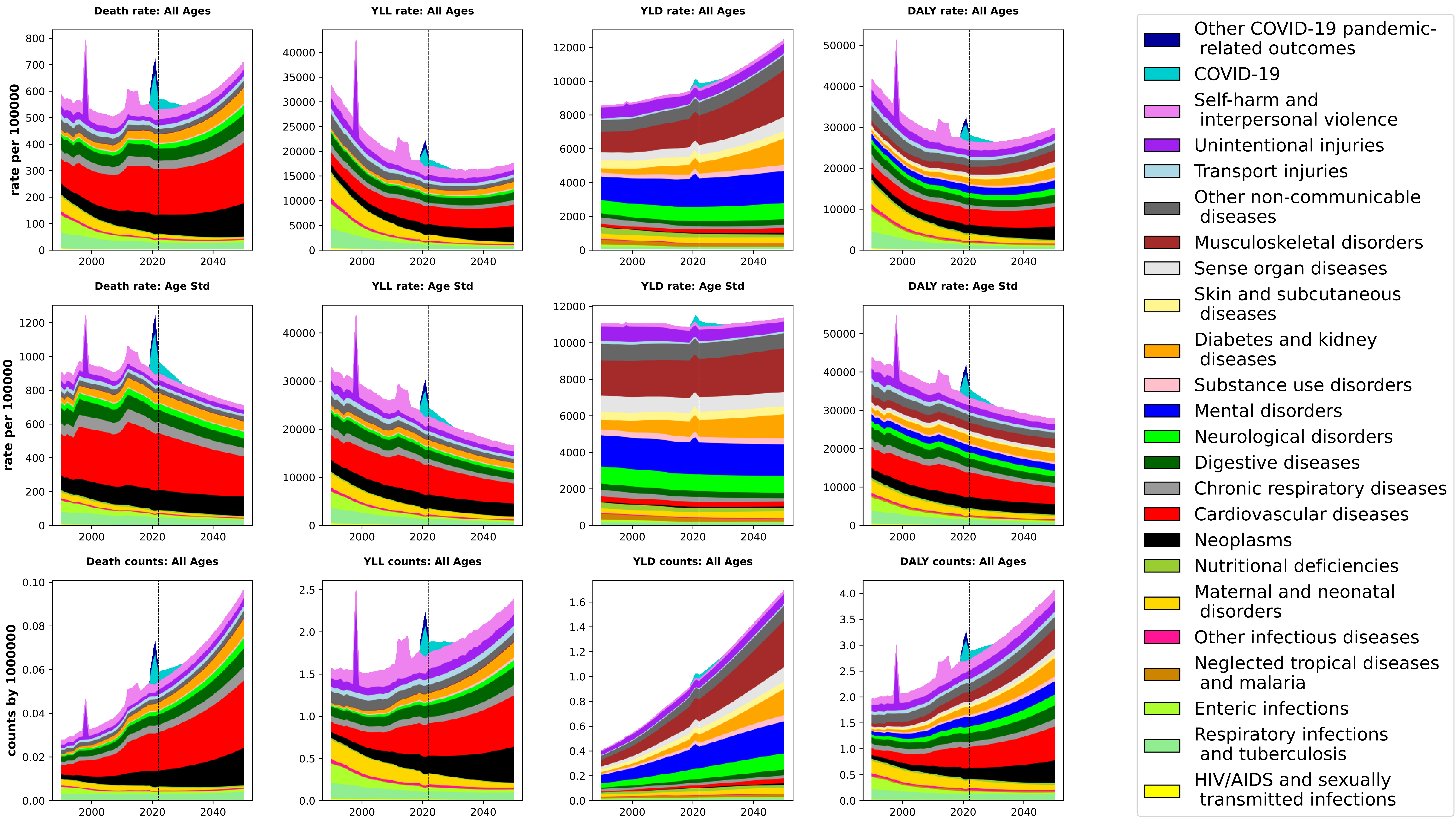
Death rate: All Ages
YLL rate: All Ages
YLD rate: All Ages
DALY rate: All Ages
Death rate: Age Std
YLL rate: Age Std
YLD rate: Age Std
DALY rate: Age Std
Death counts: All Ages
YLL counts: All Ages
YLD counts: All Ages
DALY counts: All Ages
rate per 100000
counts by 1000000
Other COVID-19 pandemic-related outcomes
COVID-19
Self-harm and interpersonal violence
Unintentional injuries
Transport injuries
Other non-communicable diseases
Musculoskeletal disorders
Sense organ diseases
Skin and subcutaneous diseases
Diabetes and kidney diseases
Substance use disorders
Mental disorders
Neurological disorders
Digestive diseases
Chronic respiratory diseases
Cardiovascular diseases
Neoplasms
Nutritional deficiencies
Maternal and neonatal disorders
Other infectious diseases
Neglected tropical diseases and malaria
Enteric infections
Respiratory infections and tuberculosis
HIV/AIDS and sexually transmitted infections

Mexico

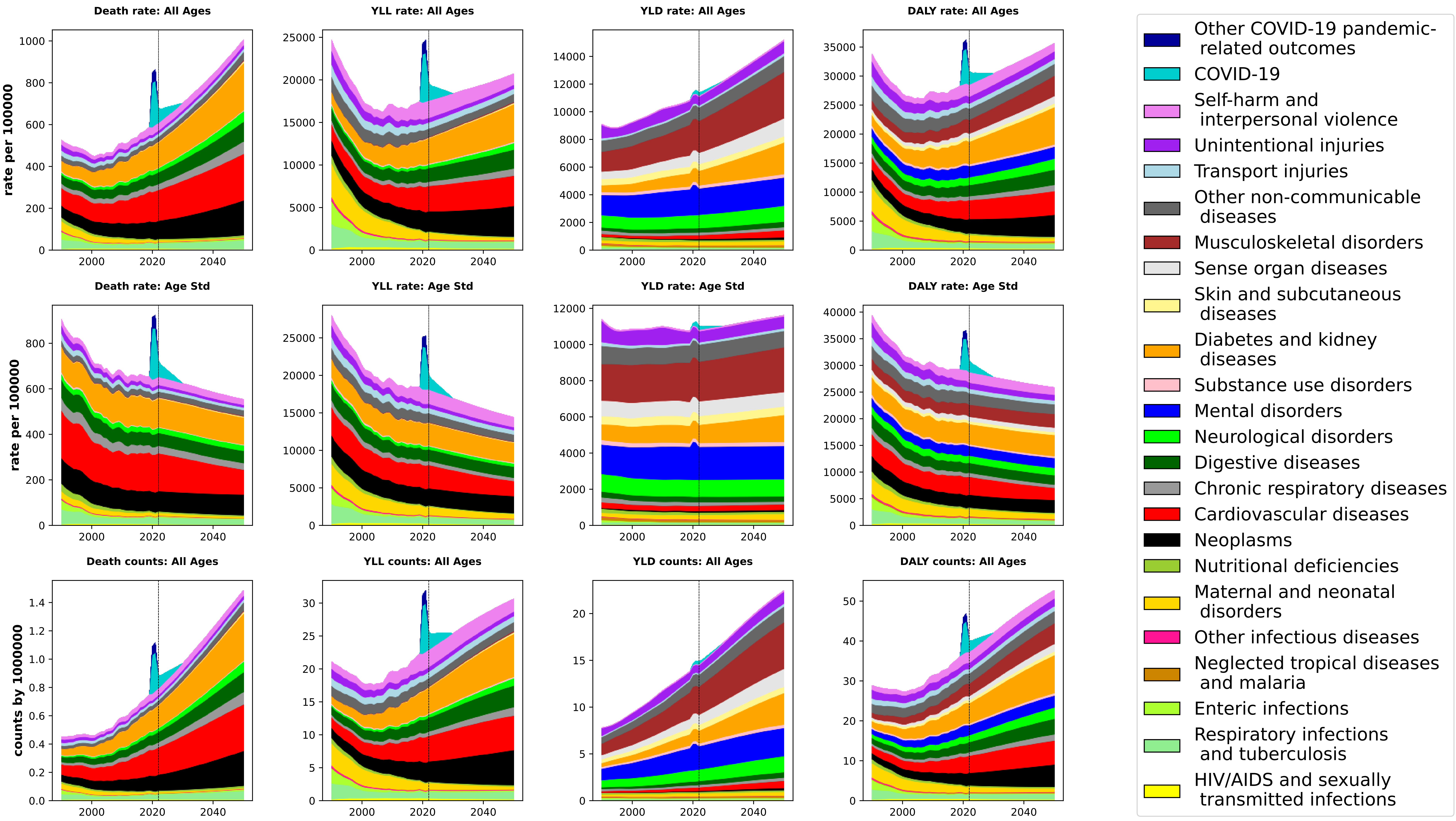
Death rate: All Ages
YLL rate: All Ages
YLD rate: All Ages
DALY rate: All Ages
Death rate: Age Std
YLL rate: Age Std
YLD rate: Age Std
DALY rate: Age Std
Death counts: All Ages
YLL counts: All Ages
YLD counts: All Ages
DALY counts: All Ages
rate per 100000
counts by 1000000
Other COVID-19 pandemic-related outcomes
COVID-19
Self-harm and interpersonal violence
Unintentional injuries
Transport injuries
Other non-communicable diseases
Musculoskeletal disorders
Sense organ diseases
Skin and subcutaneous diseases
Diabetes and kidney diseases
Substance use disorders
Mental disorders
Neurological disorders
Digestive diseases
Chronic respiratory diseases
Cardiovascular diseases
Neoplasms
Nutritional deficiencies
Maternal and neonatal disorders
Other infectious diseases
Neglected tropical diseases and malaria
Enteric infections
Respiratory infections and tuberculosis
HIV/AIDS and sexually transmitted infections

Nicaragua

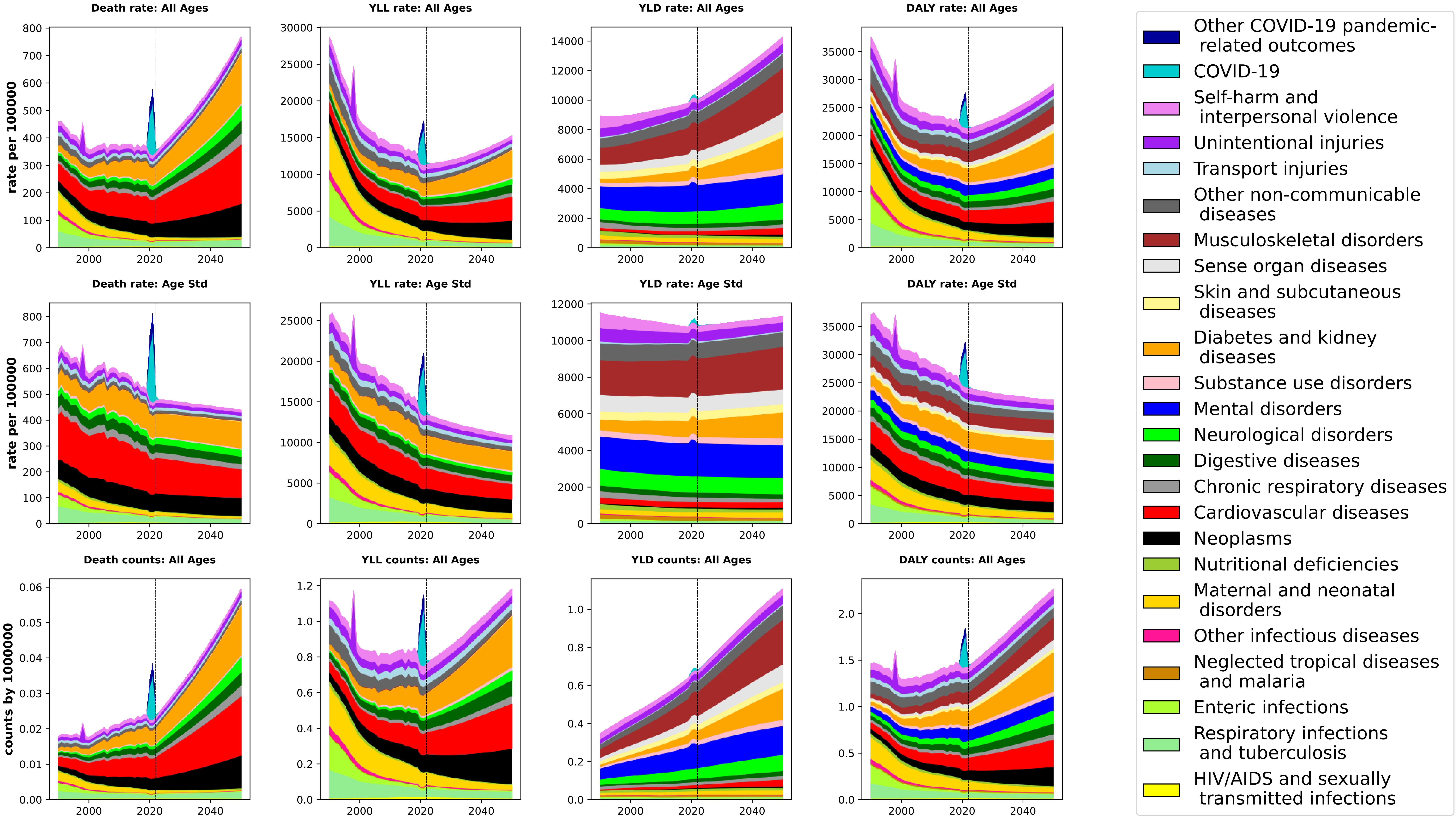
Death rate: All Ages
YLL rate: All Ages
YLD rate: All Ages
DALY rate: All Ages
Death rate: Age Std
YLL rate: Age Std
YLD rate: Age Std
DALY rate: Age Std
Death counts: All Ages
YLL counts: All Ages
YLD counts: All Ages
DALY counts: All Ages
rate per 100000
counts by 1000000
Other COVID-19 pandemic-related outcomes
COVID-19
Self-harm and interpersonal violence
Unintentional injuries
Transport injuries
Other non-communicable diseases
Musculoskeletal disorders
Sense organ diseases
Skin and subcutaneous diseases
Diabetes and kidney diseases
Substance use disorders
Mental disorders
Neurological disorders
Digestive diseases
Chronic respiratory diseases
Cardiovascular diseases
Neoplasms
Nutritional deficiencies
Maternal and neonatal disorders
Other infectious diseases
Neglected tropical diseases and malaria
Enteric infections
Respiratory infections and tuberculosis
HIV/AIDS and sexually transmitted infections

Panama

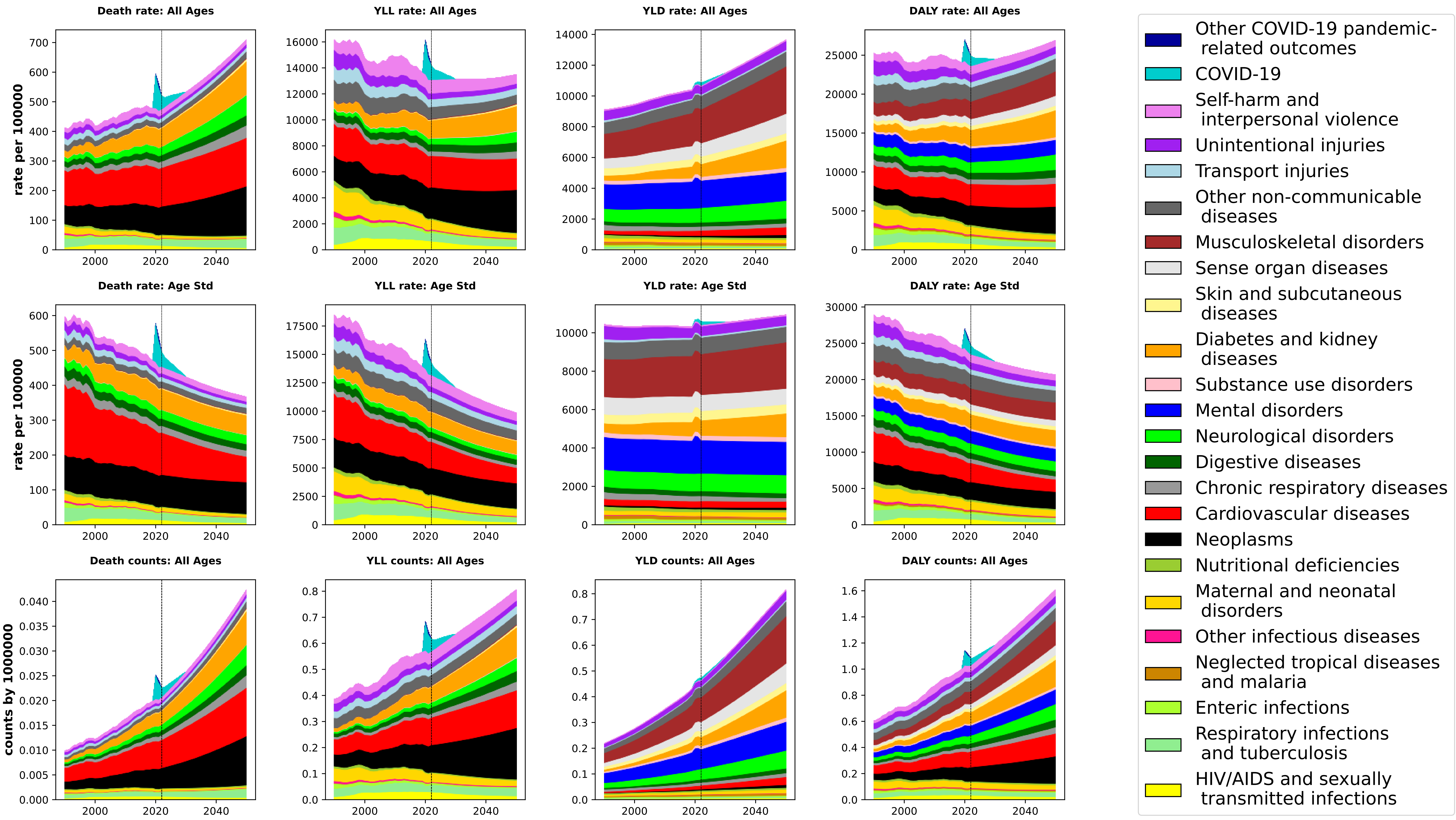
Death rate: All Ages
YLL rate: All Ages
YLD rate: All Ages
DALY rate: All Ages
Death rate: Age Std
YLL rate: Age Std
YLD rate: Age Std
DALY rate: Age Std
Death counts: All Ages
YLL counts: All Ages
YLD counts: All Ages
DALY counts: All Ages
rate per 100000
counts by 1000000
Other COVID-19 pandemic-related outcomes
COVID-19
Self-harm and interpersonal violence
Unintentional injuries
Transport injuries
Other non-communicable diseases
Musculoskeletal disorders
Sense organ diseases
Skin and subcutaneous diseases
Diabetes and kidney diseases
Substance use disorders
Mental disorders
Neurological disorders
Digestive diseases
Chronic respiratory diseases
Cardiovascular diseases
Neoplasms
Nutritional deficiencies
Maternal and neonatal disorders
Other infectious diseases
Neglected tropical diseases and malaria
Enteric infections
Respiratory infections and tuberculosis
HIV/AIDS and sexually transmitted infections

Venezuela

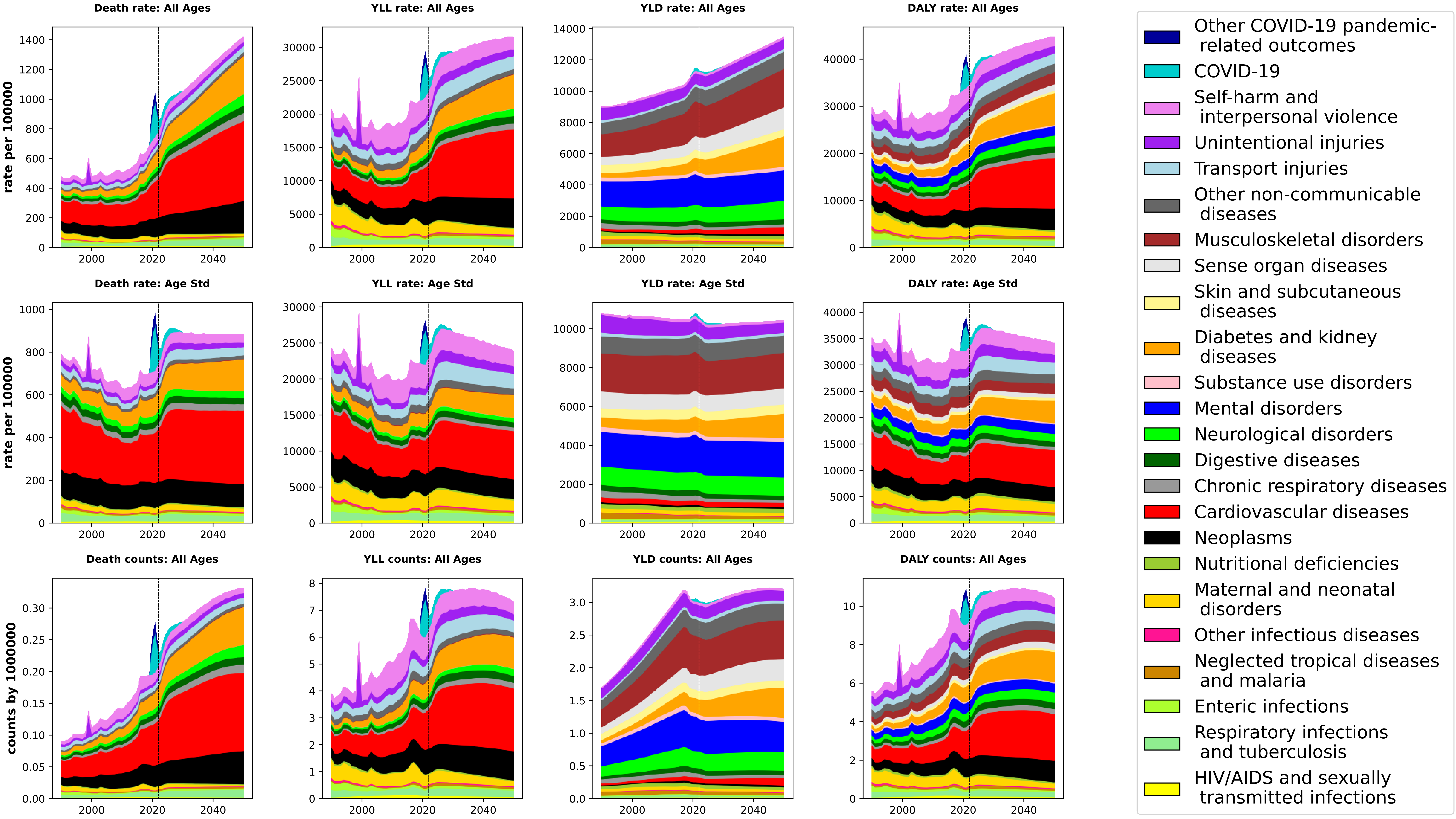
Death rate: All Ages
YLL rate: All Ages
YLD rate: All Ages
DALY rate: All Ages
Death rate: Age Std
YLL rate: Age Std
YLD rate: Age Std
DALY rate: Age Std
Death counts: All Ages
YLL counts: All Ages
YLD counts: All Ages
DALY counts: All Ages
rate per 100000
rate per 100000
counts by 1000000
2000
2020
2040
Other COVID-19 pandemic-related outcomes
COVID-19
Self-harm and interpersonal violence
Unintentional injuries
Transport injuries
Other non-communicable diseases
Musculoskeletal disorders
Sense organ diseases
Skin and subcutaneous diseases
Diabetes and kidney diseases
Substance use disorders
Mental disorders
Neurological disorders
Digestive diseases
Chronic respiratory diseases
Cardiovascular diseases
Neoplasms
Nutritional deficiencies
Maternal and neonatal disorders
Other infectious diseases
Neglected tropical diseases and malaria
Enteric infections
Respiratory infections and tuberculosis
HIV/AIDS and sexually transmitted infections

Brazil

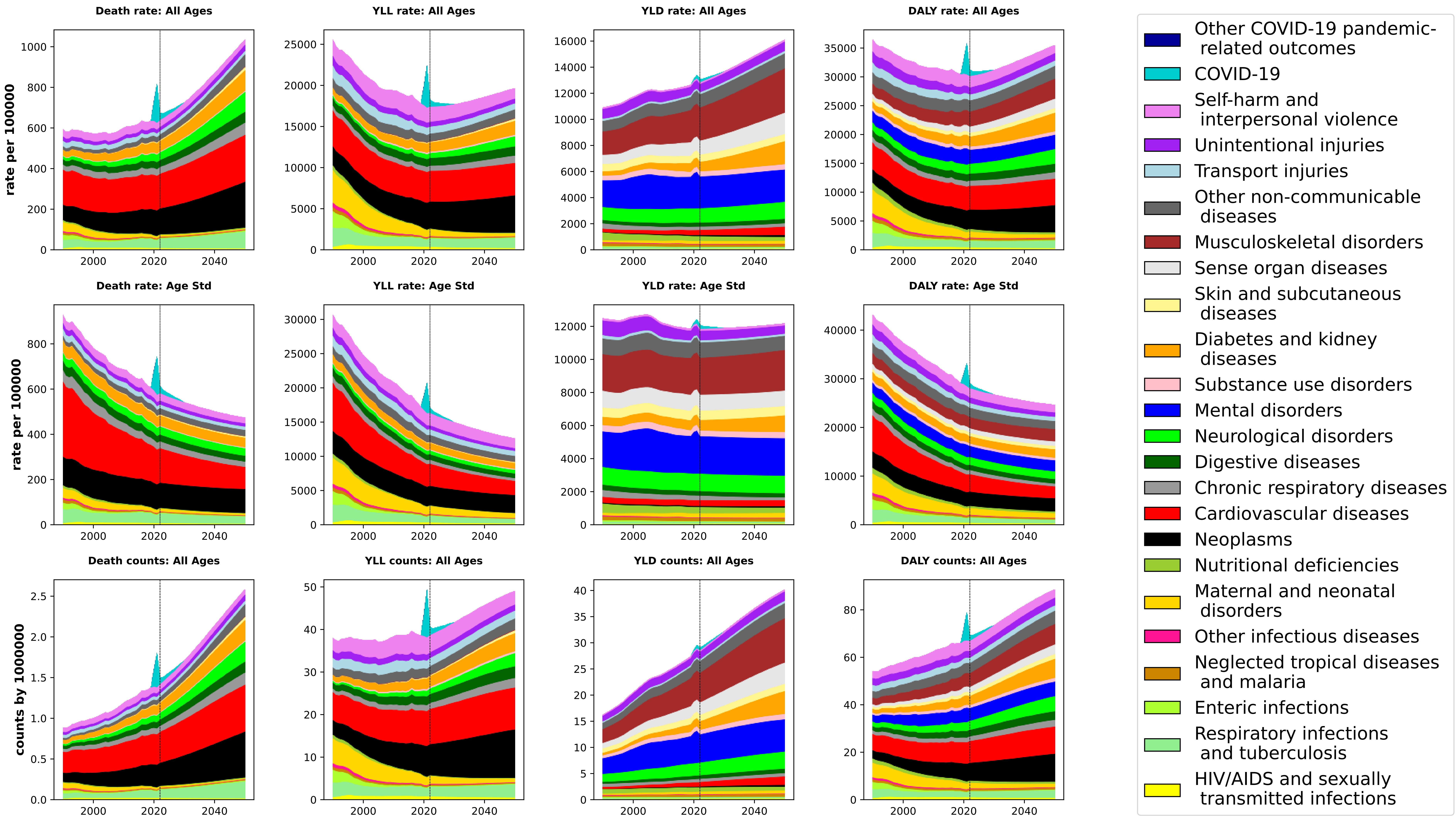
Death rate: All Ages
YLL rate: All Ages
YLD rate: All Ages
DALY rate: All Ages
Death rate: Age Std
YLL rate: Age Std
YLD rate: Age Std
DALY rate: Age Std
Death counts: All Ages
YLL counts: All Ages
YLD counts: All Ages
DALY counts: All Ages
rate per 100000
counts by 1000000
Other COVID-19 pandemic-related outcomes
COVID-19
Self-harm and interpersonal violence
Unintentional injuries
Transport injuries
Other non-communicable diseases
Musculoskeletal disorders
Sense organ diseases
Skin and subcutaneous diseases
Diabetes and kidney diseases
Substance use disorders
Mental disorders
Neurological disorders
Digestive diseases
Chronic respiratory diseases
Cardiovascular diseases
Neoplasms
Nutritional deficiencies
Maternal and neonatal disorders
Other infectious diseases
Neglected tropical diseases and malaria
Enteric infections
Respiratory infections and tuberculosis
HIV/AIDS and sexually transmitted infections

Paraguay

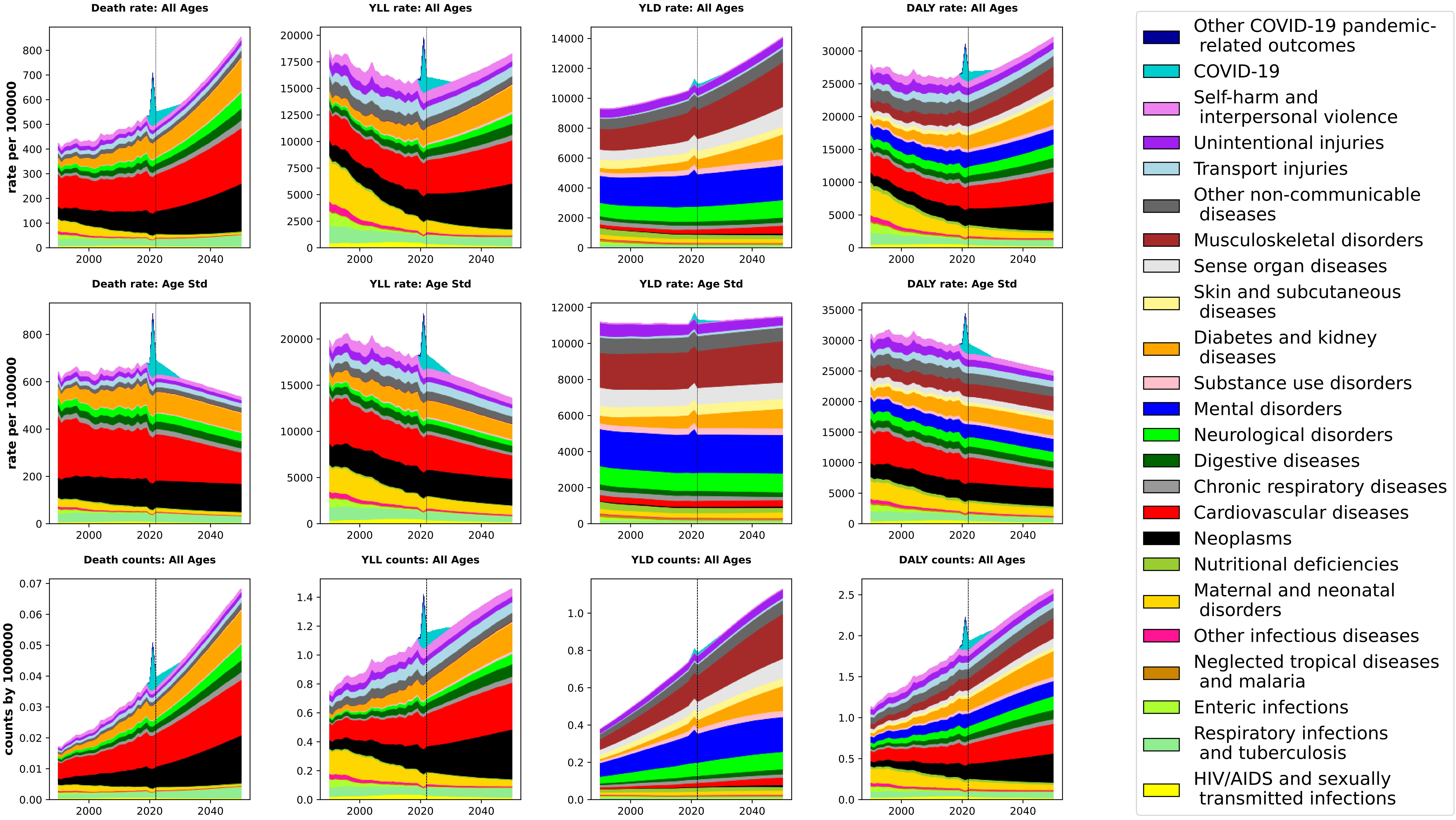
Death rate: All Ages
YLL rate: All Ages
YLD rate: All Ages
DALY rate: All Ages
Death rate: Age Std
YLL rate: Age Std
YLD rate: Age Std
DALY rate: Age Std
Death counts: All Ages
YLL counts: All Ages
YLD counts: All Ages
DALY counts: All Ages
rate per 100000
counts by 1000000
Other COVID-19 pandemic-related outcomes
COVID-19
Self-harm and interpersonal violence
Unintentional injuries
Transport injuries
Other non-communicable diseases
Musculoskeletal disorders
Sense organ diseases
Skin and subcutaneous diseases
Diabetes and kidney diseases
Substance use disorders
Mental disorders
Neurological disorders
Digestive diseases
Chronic respiratory diseases
Cardiovascular diseases
Neoplasms
Nutritional deficiencies
Maternal and neonatal disorders
Other infectious diseases
Neglected tropical diseases and malaria
Enteric infections
Respiratory infections and tuberculosis
HIV/AIDS and sexually transmitted infections

Afghanistan

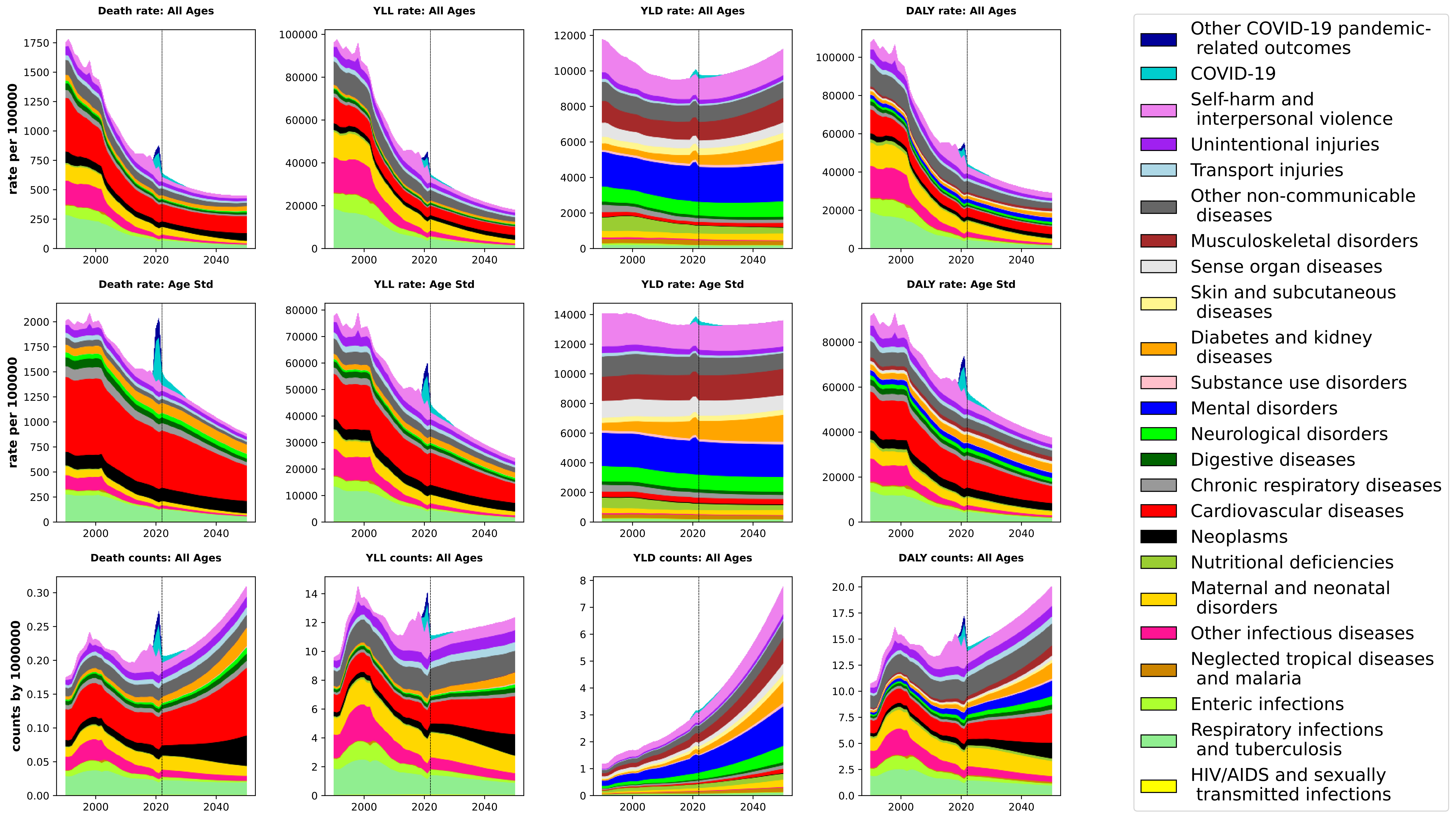

Algeria

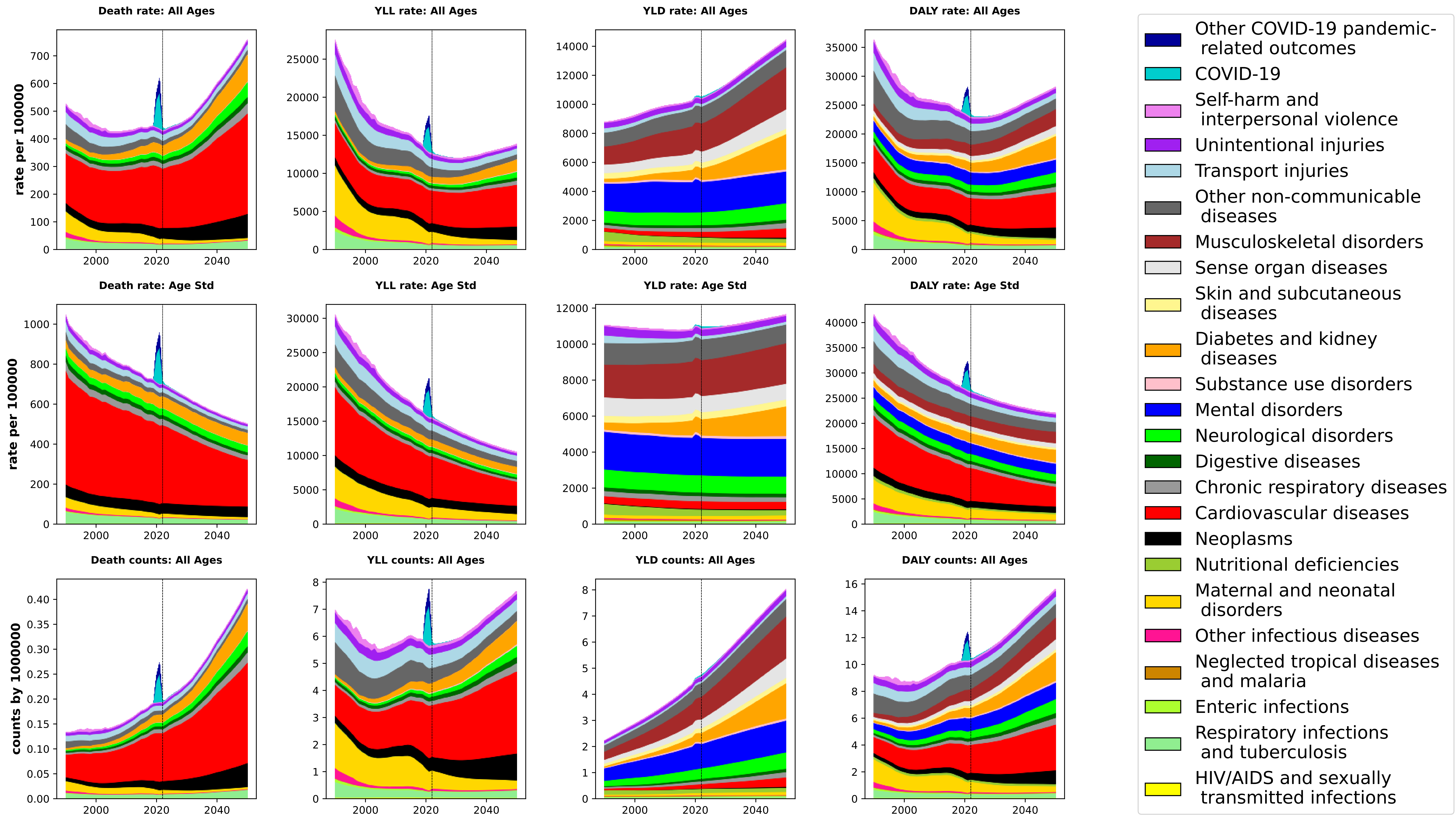
Death rate: All Ages
YLL rate: All Ages
YLD rate: All Ages
DALY rate: All Ages
Death rate: Age Std
YLL rate: Age Std
YLD rate: Age Std
DALY rate: Age Std
Death counts: All Ages
YLL counts: All Ages
YLD counts: All Ages
DALY counts: All Ages
rate per 100000
rate per 100000
counts by 1000000
2000
2020
2040
Other COVID-19 pandemic-related outcomes
COVID-19
Self-harm and interpersonal violence
Unintentional injuries
Transport injuries
Other non-communicable diseases
Musculoskeletal disorders
Sense organ diseases
Skin and subcutaneous diseases
Diabetes and kidney diseases
Substance use disorders
Mental disorders
Neurological disorders
Digestive diseases
Chronic respiratory diseases
Cardiovascular diseases
Neoplasms
Nutritional deficiencies
Maternal and neonatal disorders
Other infectious diseases
Neglected tropical diseases and malaria
Enteric infections
Respiratory infections and tuberculosis
HIV/AIDS and sexually transmitted infections

Bahrain

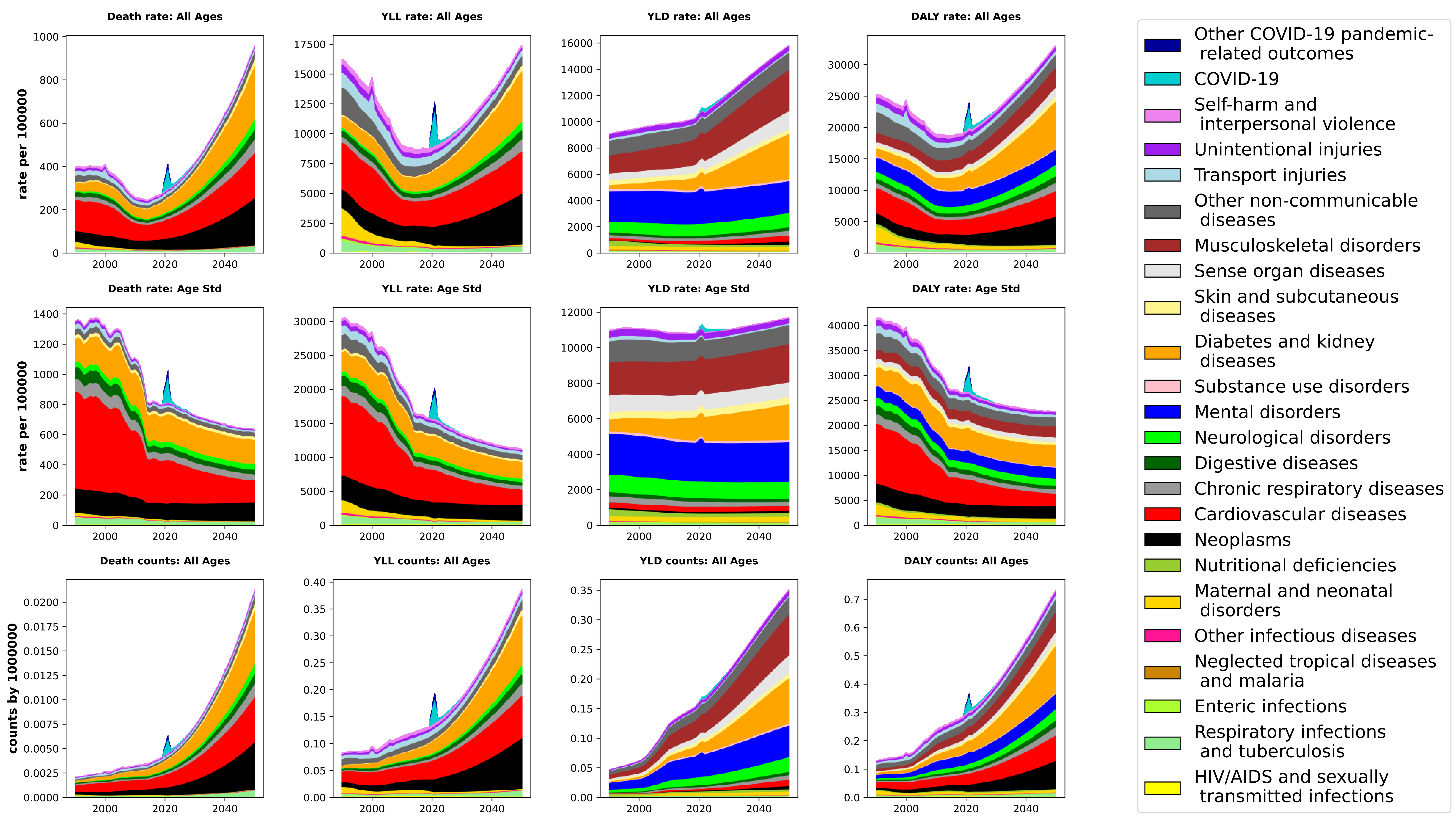
Death rate: All Ages
YLL rate: All Ages
YLD rate: All Ages
DALY rate: All Ages
Death rate: Age Std
YLL rate: Age Std
YLD rate: Age Std
DALY rate: Age Std
Death counts: All Ages
YLL counts: All Ages
YLD counts: All Ages
DALY counts: All Ages
rate per 100000
counts by 1000000
Other COVID-19 pandemic-related outcomes
COVID-19
Self-harm and interpersonal violence
Unintentional injuries
Transport injuries
Other non-communicable diseases
Musculoskeletal disorders
Sense organ diseases
Skin and subcutaneous diseases
Diabetes and kidney diseases
Substance use disorders
Mental disorders
Neurological disorders
Digestive diseases
Chronic respiratory diseases
Cardiovascular diseases
Neoplasms
Nutritional deficiencies
Maternal and neonatal disorders
Other infectious diseases
Neglected tropical diseases and malaria
Enteric infections
Respiratory infections and tuberculosis
HIV/AIDS and sexually transmitted infections

Egypt

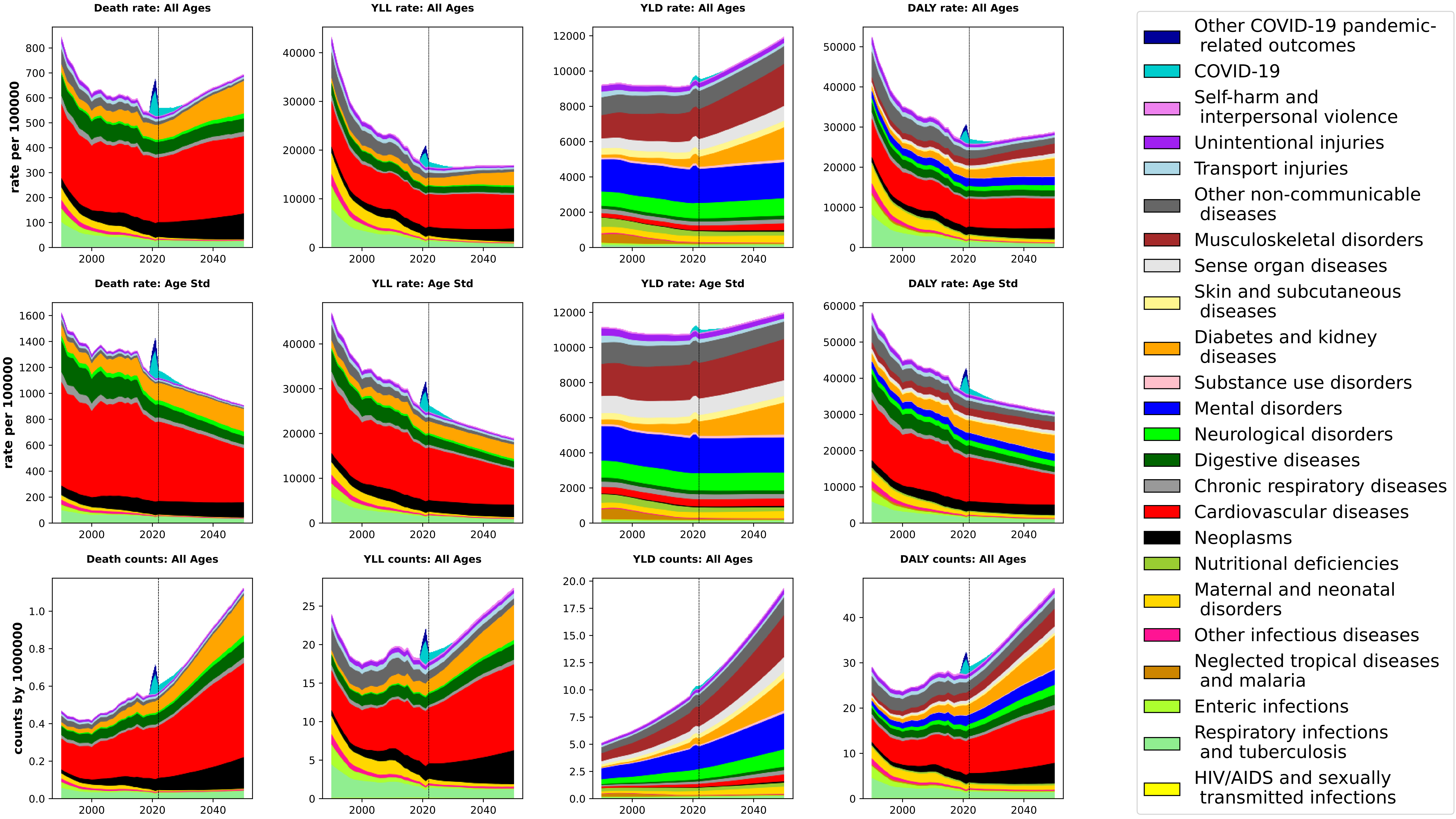
Death rate: All Ages
YLL rate: All Ages
YLD rate: All Ages
DALY rate: All Ages
Death rate: Age Std
YLL rate: Age Std
YLD rate: Age Std
DALY rate: Age Std
Death counts: All Ages
YLL counts: All Ages
YLD counts: All Ages
DALY counts: All Ages
rate per 100000
counts by 1000000
Other COVID-19 pandemic-related outcomes
COVID-19
Self-harm and interpersonal violence
Unintentional injuries
Transport injuries
Other non-communicable diseases
Musculoskeletal disorders
Sense organ diseases
Skin and subcutaneous diseases
Diabetes and kidney diseases
Substance use disorders
Mental disorders
Neurological disorders
Digestive diseases
Chronic respiratory diseases
Cardiovascular diseases
Neoplasms
Nutritional deficiencies
Maternal and neonatal disorders
Other infectious diseases
Neglected tropical diseases and malaria
Enteric infections
Respiratory infections and tuberculosis
HIV/AIDS and sexually transmitted infections

Iran

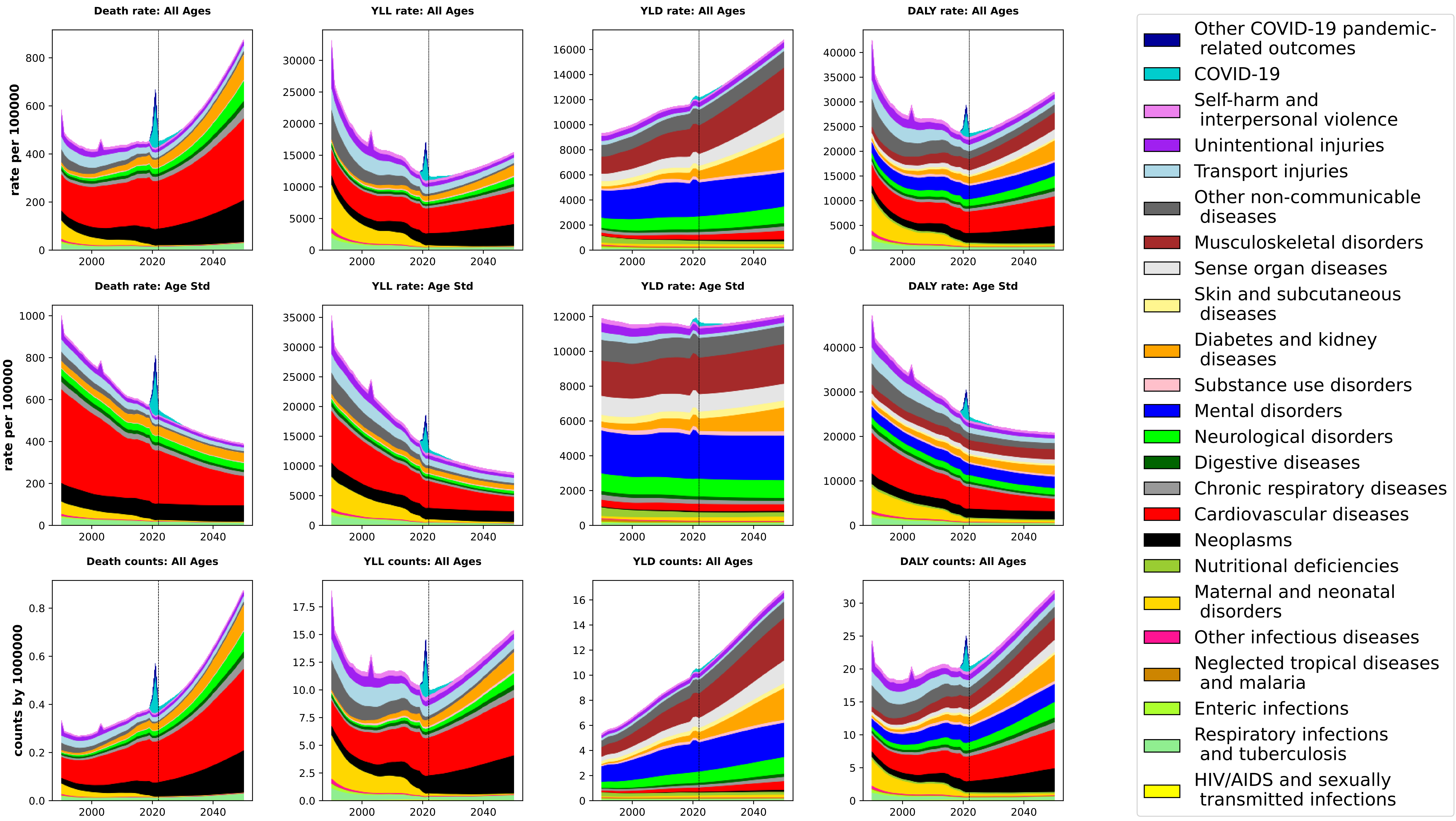
Death rate: All Ages
YLL rate: All Ages
YLD rate: All Ages
DALY rate: All Ages
Death rate: Age Std
YLL rate: Age Std
YLD rate: Age Std
DALY rate: Age Std
Death counts: All Ages
YLL counts: All Ages
YLD counts: All Ages
DALY counts: All Ages
rate per 100000
rate per 100000
counts by 1000000
2000
2020
2040
Other COVID-19 pandemic-related outcomes
COVID-19
Self-harm and interpersonal violence
Unintentional injuries
Transport injuries
Other non-communicable diseases
Musculoskeletal disorders
Sense organ diseases
Skin and subcutaneous diseases
Diabetes and kidney diseases
Substance use disorders
Mental disorders
Neurological disorders
Digestive diseases
Chronic respiratory diseases
Cardiovascular diseases
Neoplasms
Nutritional deficiencies
Maternal and neonatal disorders
Other infectious diseases
Neglected tropical diseases and malaria
Enteric infections
Respiratory infections and tuberculosis
HIV/AIDS and sexually transmitted infections

Iraq

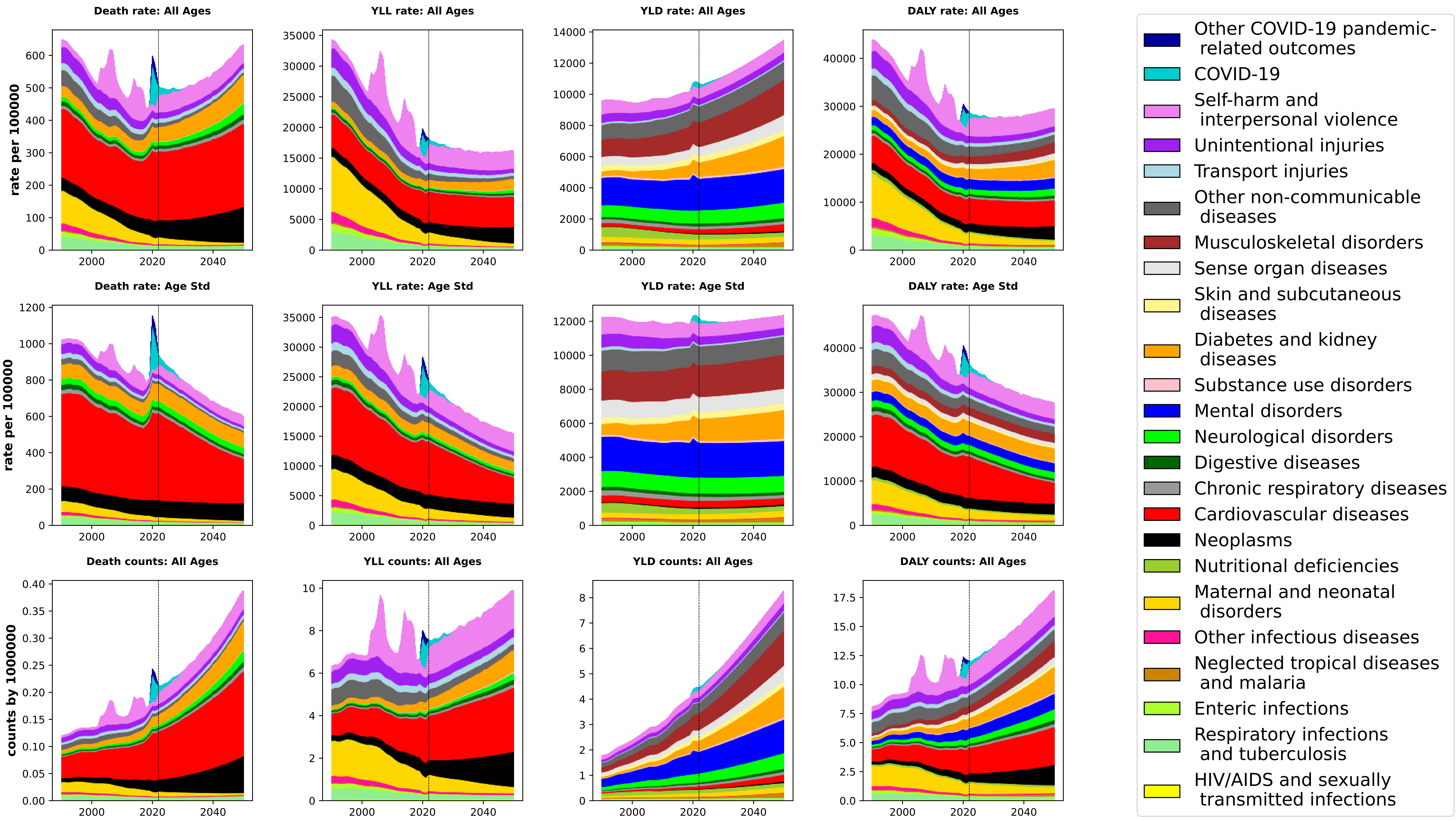
Death rate: All Ages
YLL rate: All Ages
YLD rate: All Ages
DALY rate: All Ages
Death rate: Age Std
YLL rate: Age Std
YLD rate: Age Std
DALY rate: Age Std
Death counts: All Ages
YLL counts: All Ages
YLD counts: All Ages
DALY counts: All Ages
rate per 100000
counts by 1000000
2000
2020
2040
Other COVID-19 pandemic-related outcomes
COVID-19
Self-harm and interpersonal violence
Unintentional injuries
Transport injuries
Other non-communicable diseases
Musculoskeletal disorders
Sense organ diseases
Skin and subcutaneous diseases
Diabetes and kidney diseases
Substance use disorders
Mental disorders
Neurological disorders
Digestive diseases
Chronic respiratory diseases
Cardiovascular diseases
Neoplasms
Nutritional deficiencies
Maternal and neonatal disorders
Other infectious diseases
Neglected tropical diseases and malaria
Enteric infections
Respiratory infections and tuberculosis
HIV/AIDS and sexually transmitted infections

Jordan

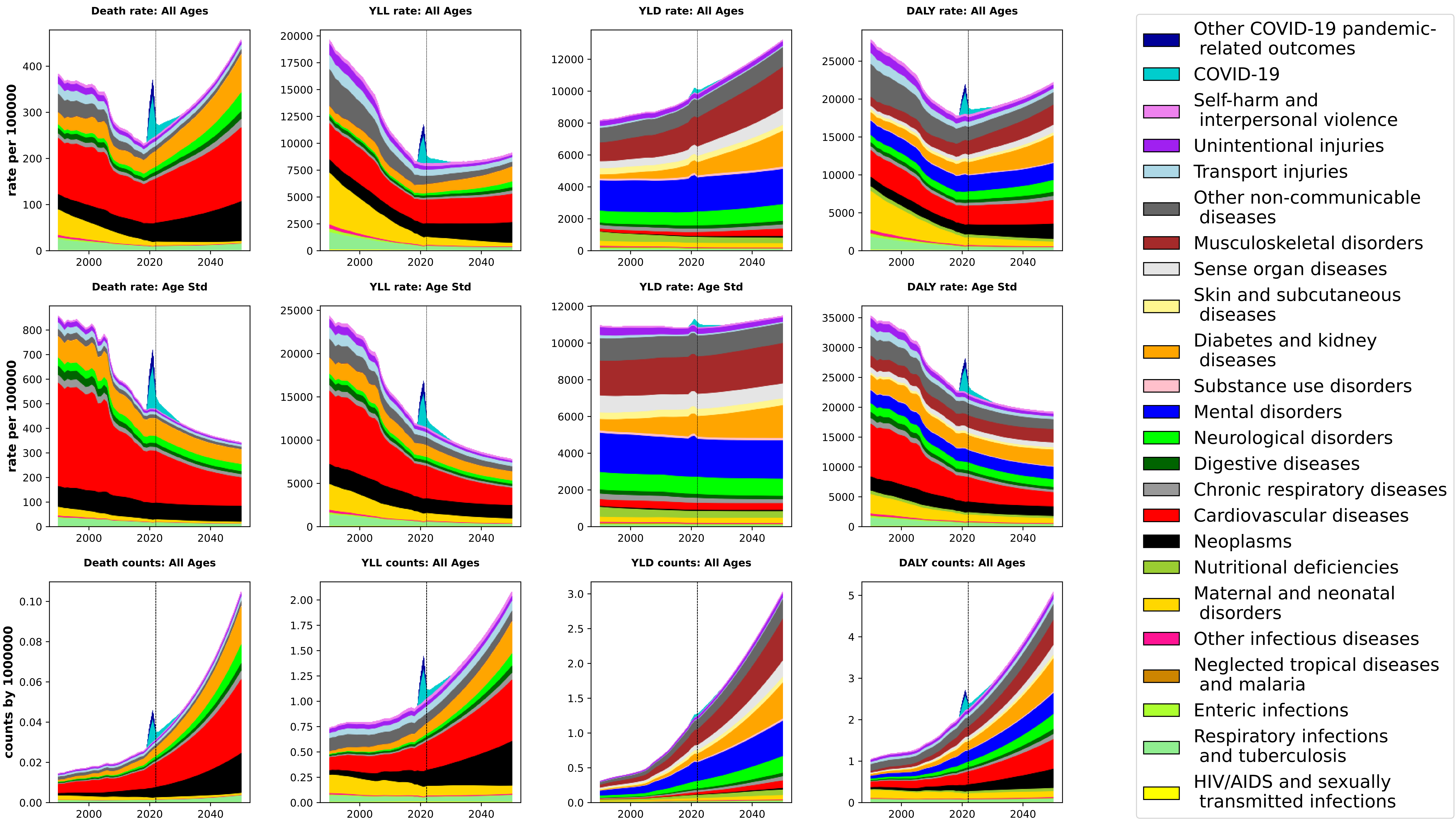
Death rate: All Ages
YLL rate: All Ages
YLD rate: All Ages
DALY rate: All Ages
Death rate: Age Std
YLL rate: Age Std
YLD rate: Age Std
DALY rate: Age Std
Death counts: All Ages
YLL counts: All Ages
YLD counts: All Ages
DALY counts: All Ages
rate per 100000
counts by 1000000
Other COVID-19 pandemic-related outcomes
COVID-19
Self-harm and interpersonal violence
Unintentional injuries
Transport injuries
Other non-communicable diseases
Musculoskeletal disorders
Sense organ diseases
Skin and subcutaneous diseases
Diabetes and kidney diseases
Substance use disorders
Mental disorders
Neurological disorders
Digestive diseases
Chronic respiratory diseases
Cardiovascular diseases
Neoplasms
Nutritional deficiencies
Maternal and neonatal disorders
Other infectious diseases
Neglected tropical diseases and malaria
Enteric infections
Respiratory infections and tuberculosis
HIV/AIDS and sexually transmitted infections

Kuwait

Supplemental Results Figure S6. Cause level 2 area plots for 4 burden measures for reference scenario by location, 1990-2050

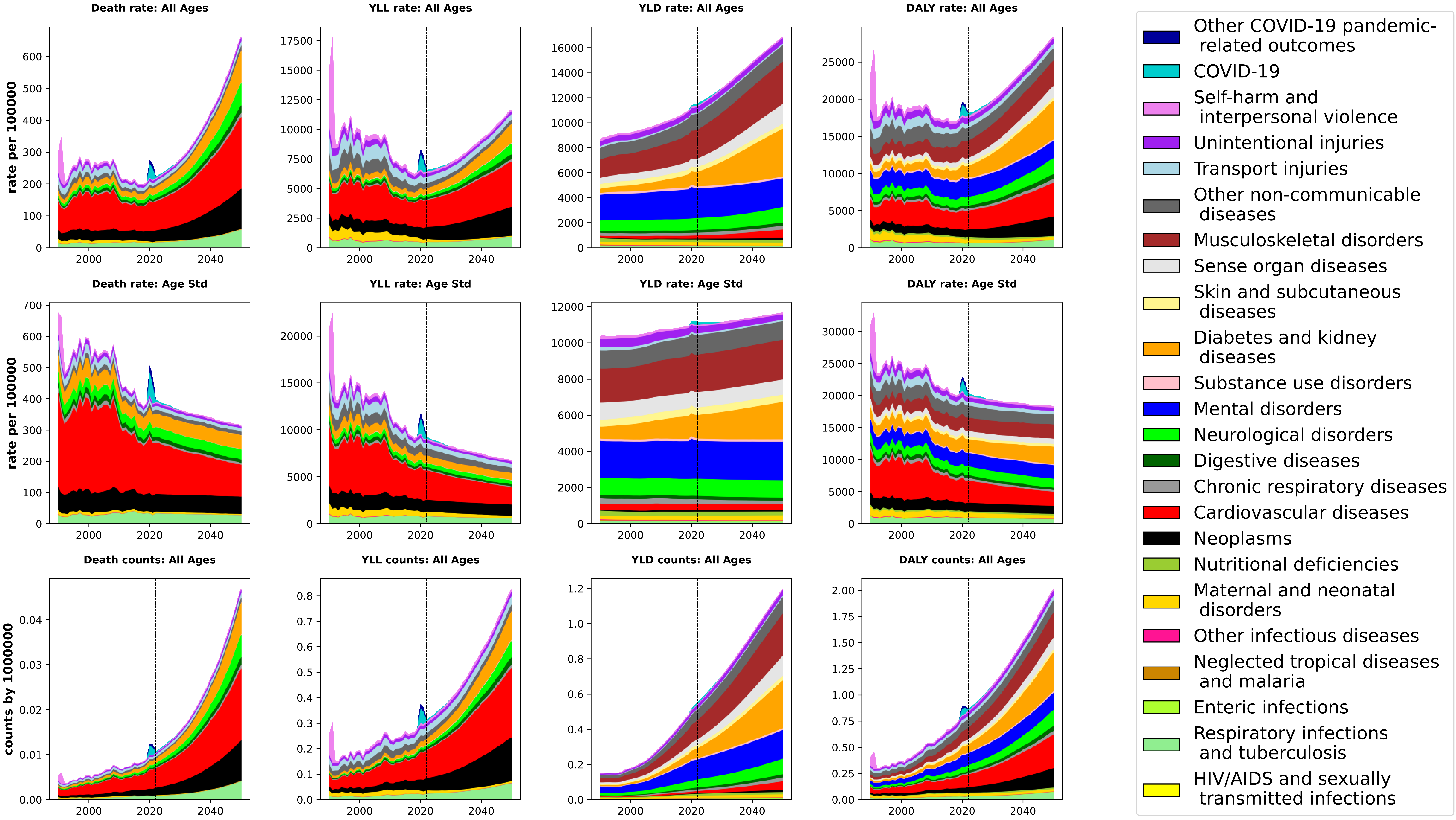

Lebanon

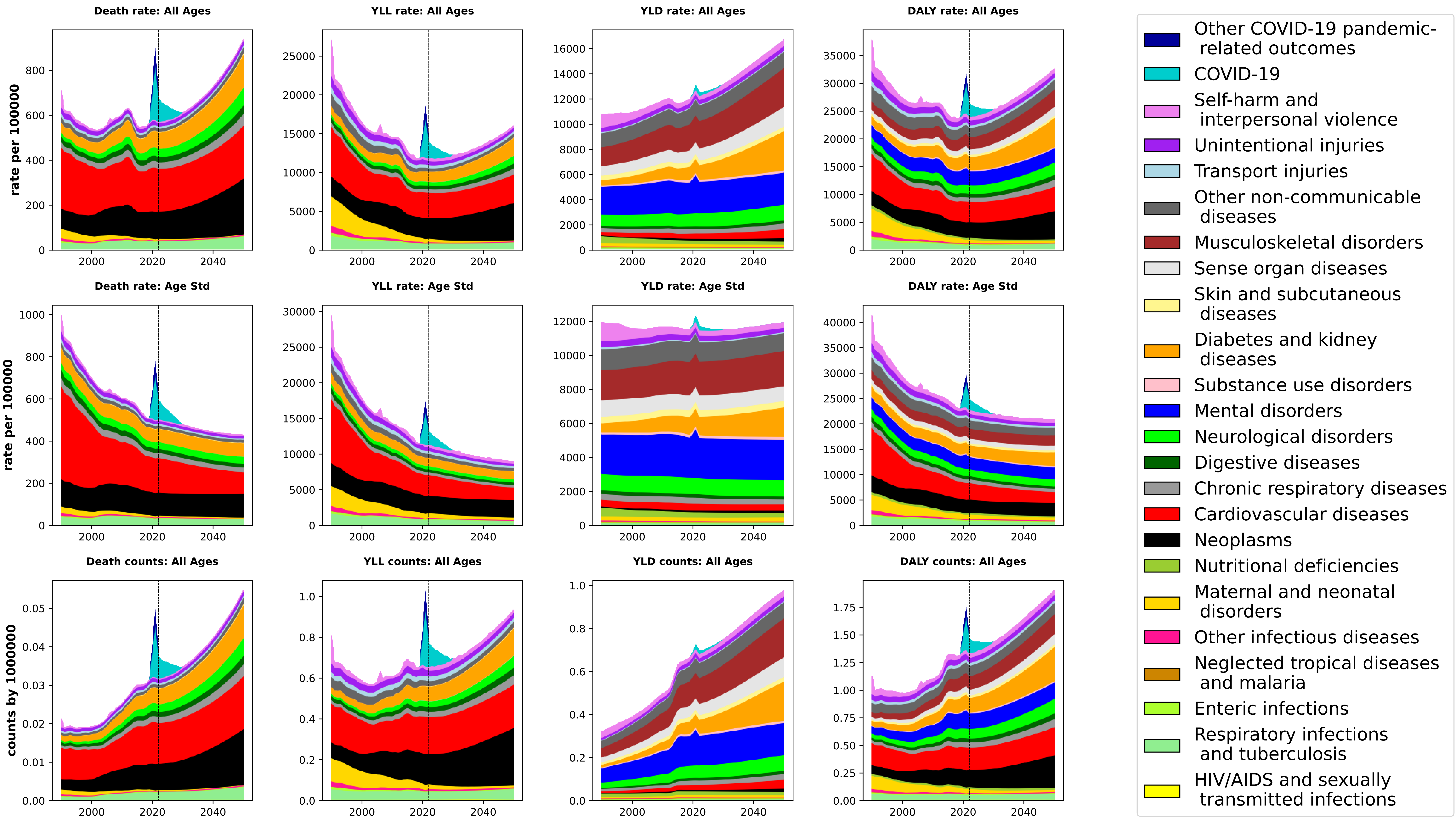
Death rate: All Ages
YLL rate: All Ages
YLD rate: All Ages
DALY rate: All Ages
Death rate: Age Std
YLL rate: Age Std
YLD rate: Age Std
DALY rate: Age Std
Death counts: All Ages
YLL counts: All Ages
YLD counts: All Ages
DALY counts: All Ages
rate per 100000
counts by 1000000
2000
2020
2040
Other COVID-19 pandemic-related outcomes
COVID-19
Self-harm and interpersonal violence
Unintentional injuries
Transport injuries
Other non-communicable diseases
Musculoskeletal disorders
Sense organ diseases
Skin and subcutaneous diseases
Diabetes and kidney diseases
Substance use disorders
Mental disorders
Neurological disorders
Digestive diseases
Chronic respiratory diseases
Cardiovascular diseases
Neoplasms
Nutritional deficiencies
Maternal and neonatal disorders
Other infectious diseases
Neglected tropical diseases and malaria
Enteric infections
Respiratory infections and tuberculosis
HIV/AIDS and sexually transmitted infections

Libya

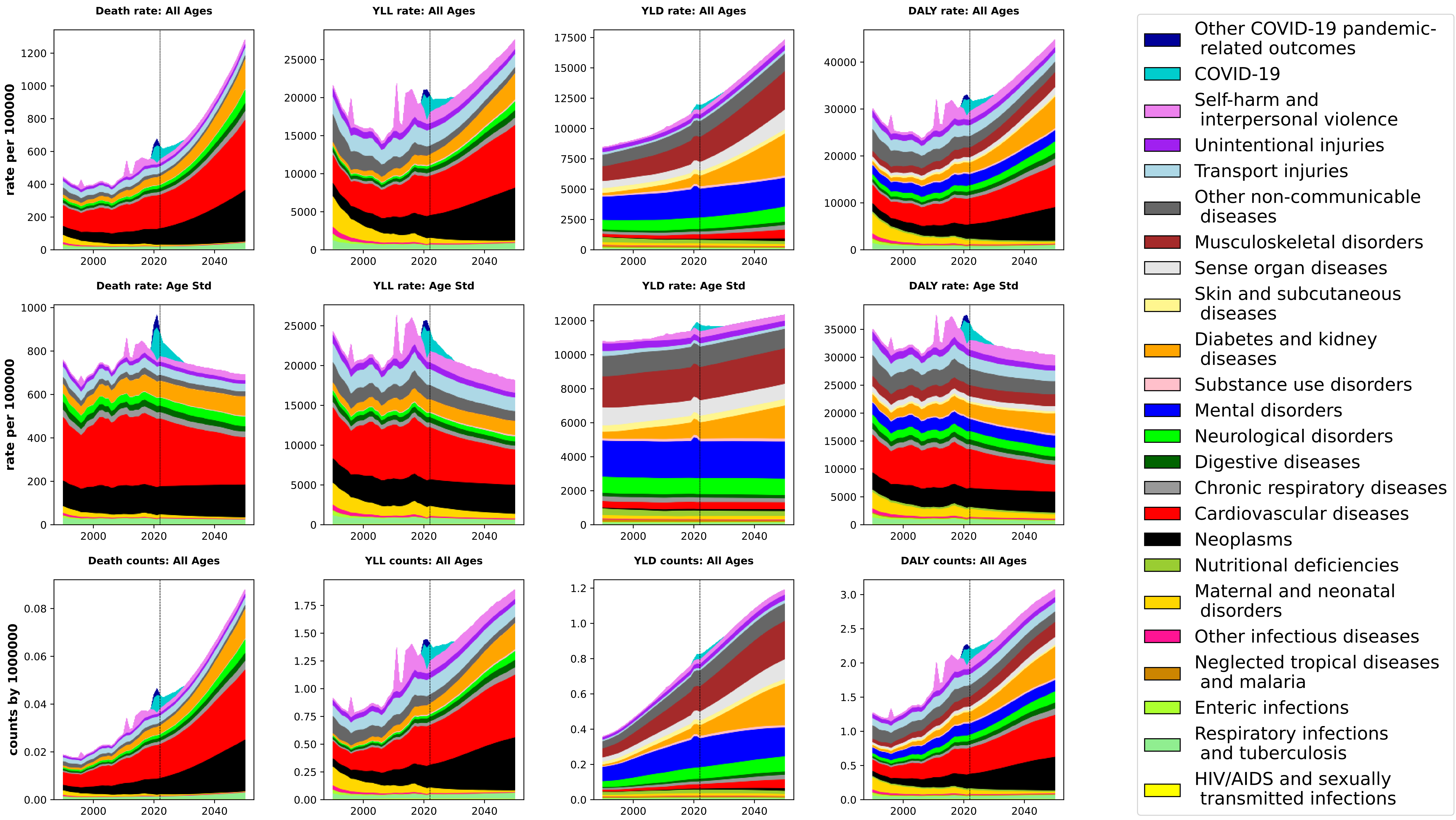
Death rate: All Ages
YLL rate: All Ages
YLD rate: All Ages
DALY rate: All Ages
Death rate: Age Std
YLL rate: Age Std
YLD rate: Age Std
DALY rate: Age Std
Death counts: All Ages
YLL counts: All Ages
YLD counts: All Ages
DALY counts: All Ages
rate per 100000
rate per 100000
counts by 1000000
2000
2020
2040
Other COVID-19 pandemic-related outcomes
COVID-19
Self-harm and interpersonal violence
Unintentional injuries
Transport injuries
Other non-communicable diseases
Musculoskeletal disorders
Sense organ diseases
Skin and subcutaneous diseases
Diabetes and kidney diseases
Substance use disorders
Mental disorders
Neurological disorders
Digestive diseases
Chronic respiratory diseases
Cardiovascular diseases
Neoplasms
Nutritional deficiencies
Maternal and neonatal disorders
Other infectious diseases
Neglected tropical diseases and malaria
Enteric infections
Respiratory infections and tuberculosis
HIV/AIDS and sexually transmitted infections

Morocco

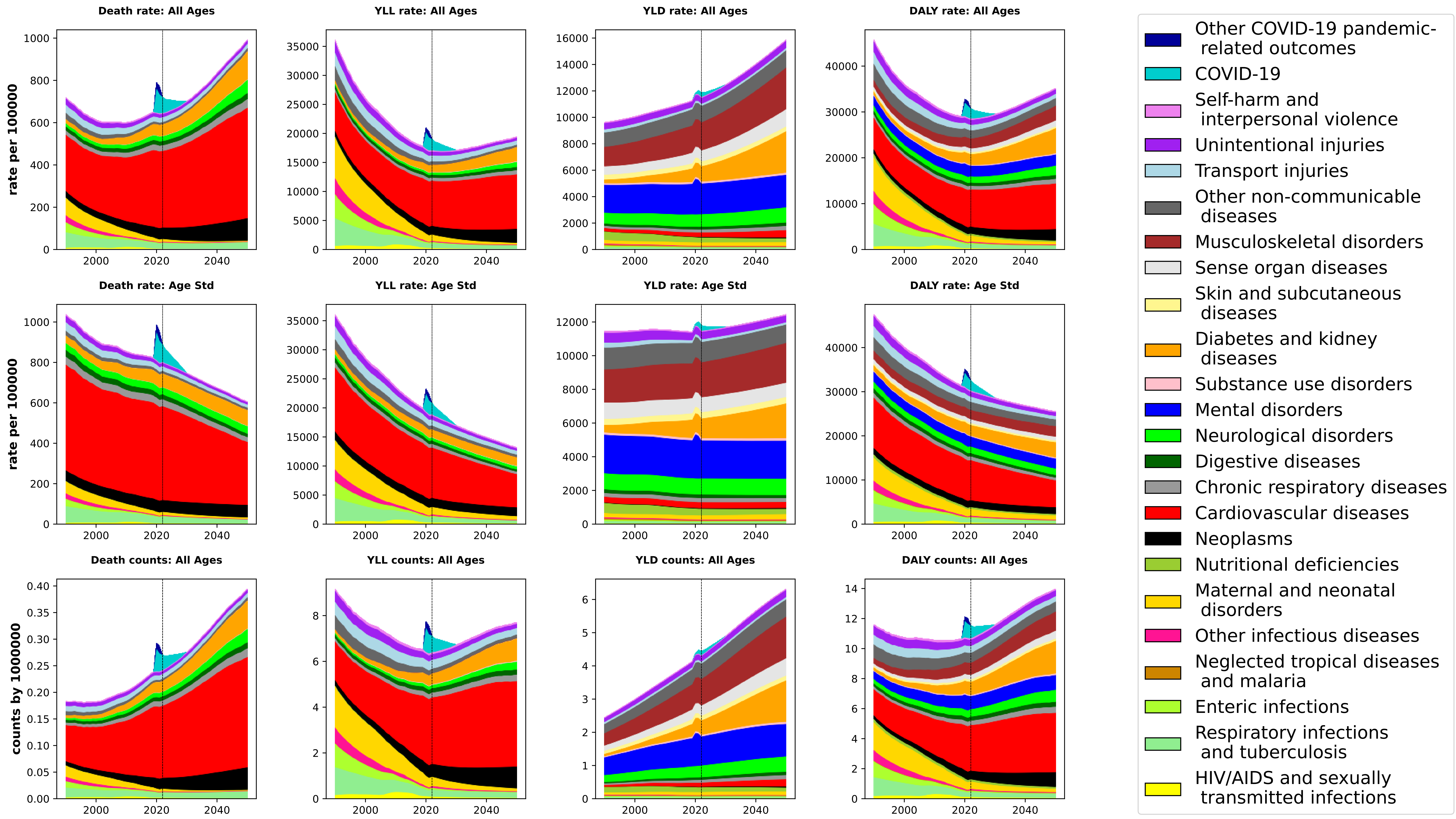
Death rate: All Ages
YLL rate: All Ages
YLD rate: All Ages
DALY rate: All Ages
Death rate: Age Std
YLL rate: Age Std
YLD rate: Age Std
DALY rate: Age Std
Death counts: All Ages
YLL counts: All Ages
YLD counts: All Ages
DALY counts: All Ages
rate per 100000
counts by 1000000
2000
2020
2040
Other COVID-19 pandemic-related outcomes
COVID-19
Self-harm and interpersonal violence
Unintentional injuries
Transport injuries
Other non-communicable diseases
Musculoskeletal disorders
Sense organ diseases
Skin and subcutaneous diseases
Diabetes and kidney diseases
Substance use disorders
Mental disorders
Neurological disorders
Digestive diseases
Chronic respiratory diseases
Cardiovascular diseases
Neoplasms
Nutritional deficiencies
Maternal and neonatal disorders
Other infectious diseases
Neglected tropical diseases and malaria
Enteric infections
Respiratory infections and tuberculosis
HIV/AIDS and sexually transmitted infections

Oman

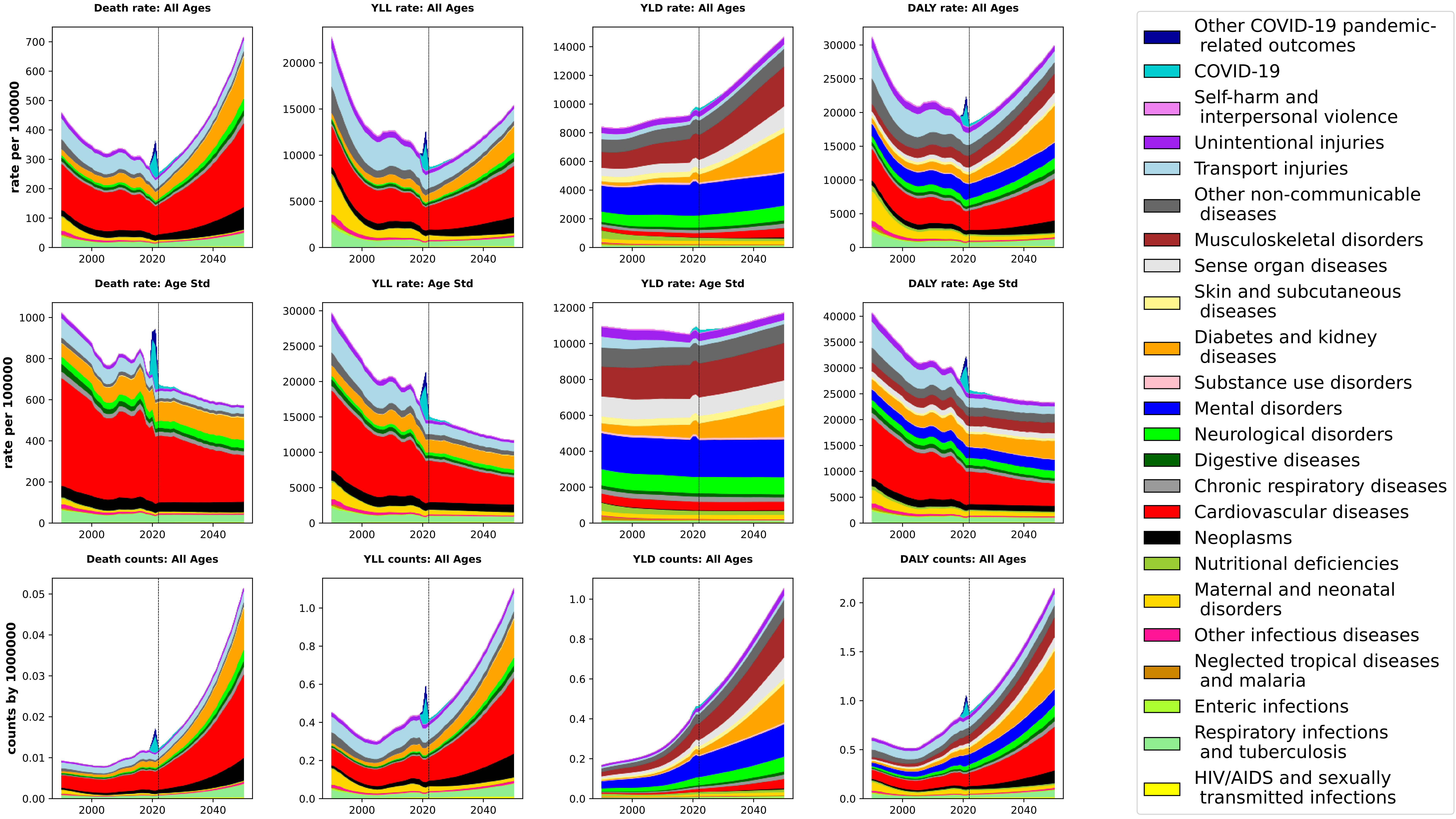
Death rate: All Ages
YLL rate: All Ages
YLD rate: All Ages
DALY rate: All Ages
Death rate: Age Std
YLL rate: Age Std
YLD rate: Age Std
DALY rate: Age Std
Death counts: All Ages
YLL counts: All Ages
YLD counts: All Ages
DALY counts: All Ages
rate per 100000
counts by 1000000
Other COVID-19 pandemic-related outcomes
COVID-19
Self-harm and interpersonal violence
Unintentional injuries
Transport injuries
Other non-communicable diseases
Musculoskeletal disorders
Sense organ diseases
Skin and subcutaneous diseases
Diabetes and kidney diseases
Substance use disorders
Mental disorders
Neurological disorders
Digestive diseases
Chronic respiratory diseases
Cardiovascular diseases
Neoplasms
Nutritional deficiencies
Maternal and neonatal disorders
Other infectious diseases
Neglected tropical diseases and malaria
Enteric infections
Respiratory infections and tuberculosis
HIV/AIDS and sexually transmitted infections

Palestine

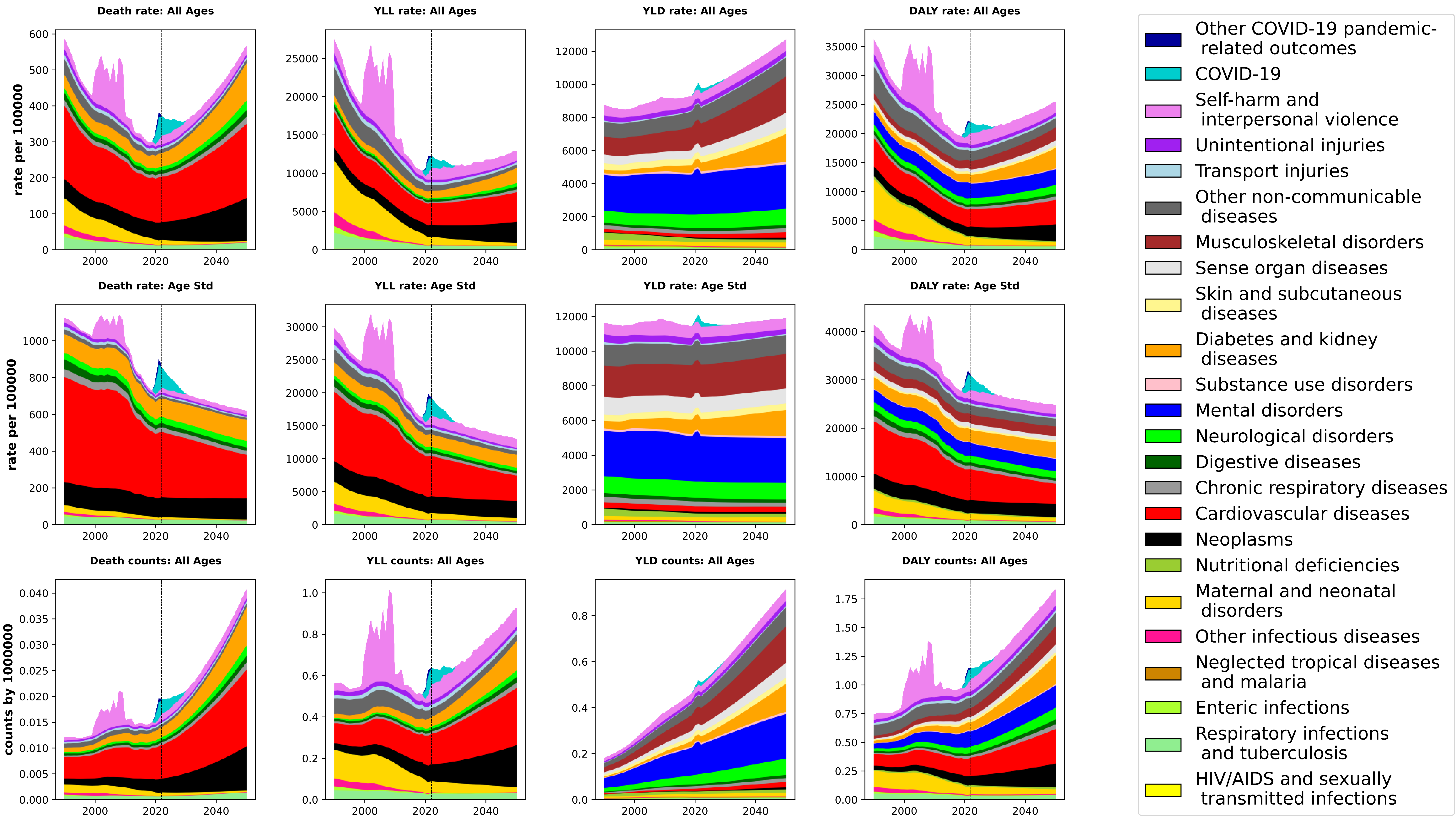

Death rate: All Ages
YLL rate: All Ages
YLD rate: All Ages
DALY rate: All Ages
Death rate: Age Std
YLL rate: Age Std
YLD rate: Age Std
DALY rate: Age Std
Death counts: All Ages
YLL counts: All Ages
YLD counts: All Ages
DALY counts: All Ages
rate per 100000
counts by 1000000
2000
2020
2040
Other COVID-19 pandemic-related outcomes
COVID-19
Self-harm and interpersonal violence
Unintentional injuries
Transport injuries
Other non-communicable diseases
Musculoskeletal disorders
Sense organ diseases
Skin and subcutaneous diseases
Diabetes and kidney diseases
Substance use disorders
Mental disorders
Neurological disorders
Digestive diseases
Chronic respiratory diseases
Cardiovascular diseases
Neoplasms
Nutritional deficiencies
Maternal and neonatal disorders
Other infectious diseases
Neglected tropical diseases and malaria
Enteric infections
Respiratory infections and tuberculosis
HIV/AIDS and sexually transmitted infections

Qatar

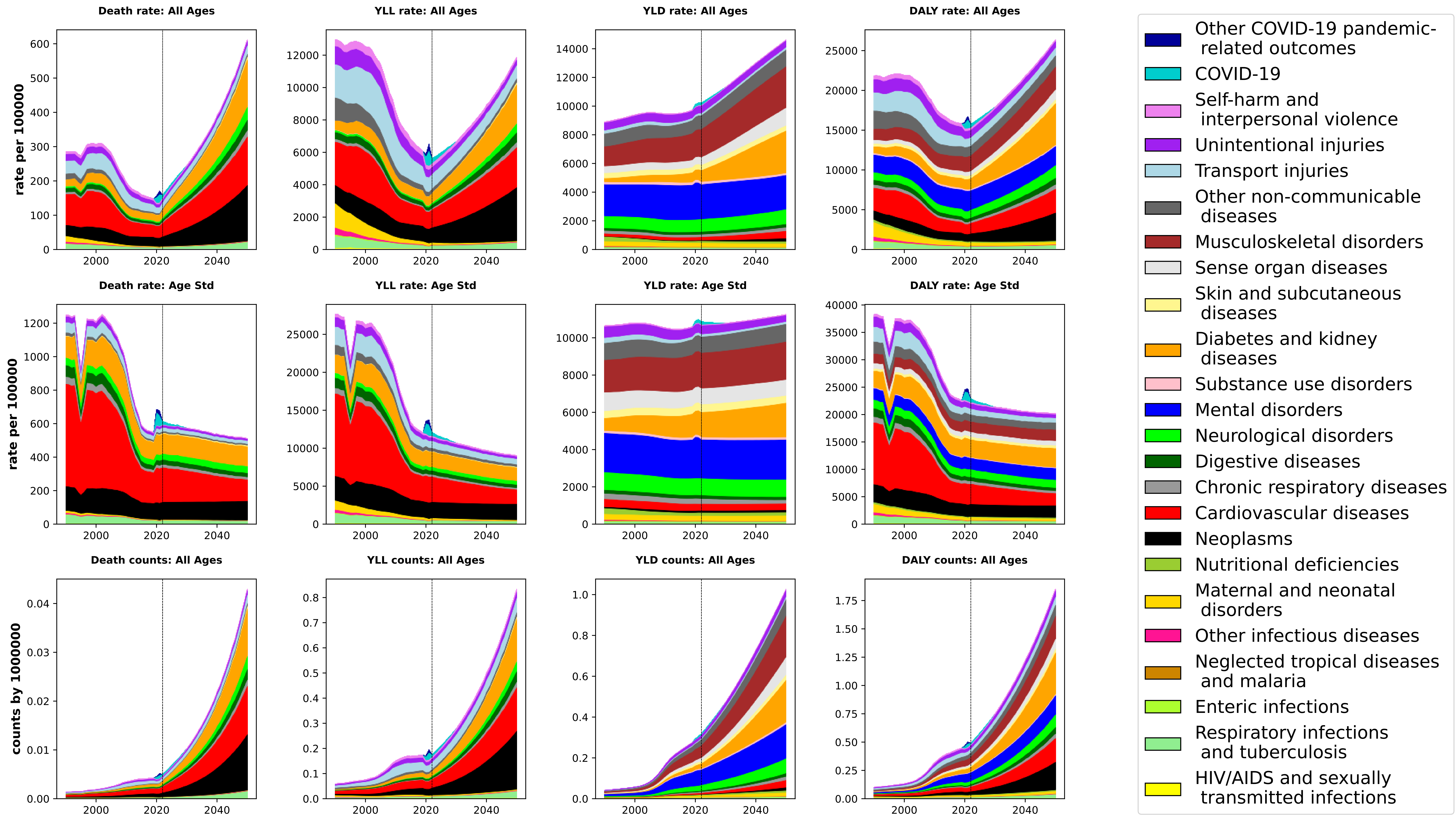
Death rate: All Ages
YLL rate: All Ages
YLD rate: All Ages
DALY rate: All Ages
Death rate: Age Std
YLL rate: Age Std
YLD rate: Age Std
DALY rate: Age Std
Death counts: All Ages
YLL counts: All Ages
YLD counts: All Ages
DALY counts: All Ages
rate per 100000
counts by 1000000
Other COVID-19 pandemic-related outcomes
COVID-19
Self-harm and interpersonal violence
Unintentional injuries
Transport injuries
Other non-communicable diseases
Musculoskeletal disorders
Sense organ diseases
Skin and subcutaneous diseases
Diabetes and kidney diseases
Substance use disorders
Mental disorders
Neurological disorders
Digestive diseases
Chronic respiratory diseases
Cardiovascular diseases
Neoplasms
Nutritional deficiencies
Maternal and neonatal disorders
Other infectious diseases
Neglected tropical diseases and malaria
Enteric infections
Respiratory infections and tuberculosis
HIV/AIDS and sexually transmitted infections

Saudi Arabia

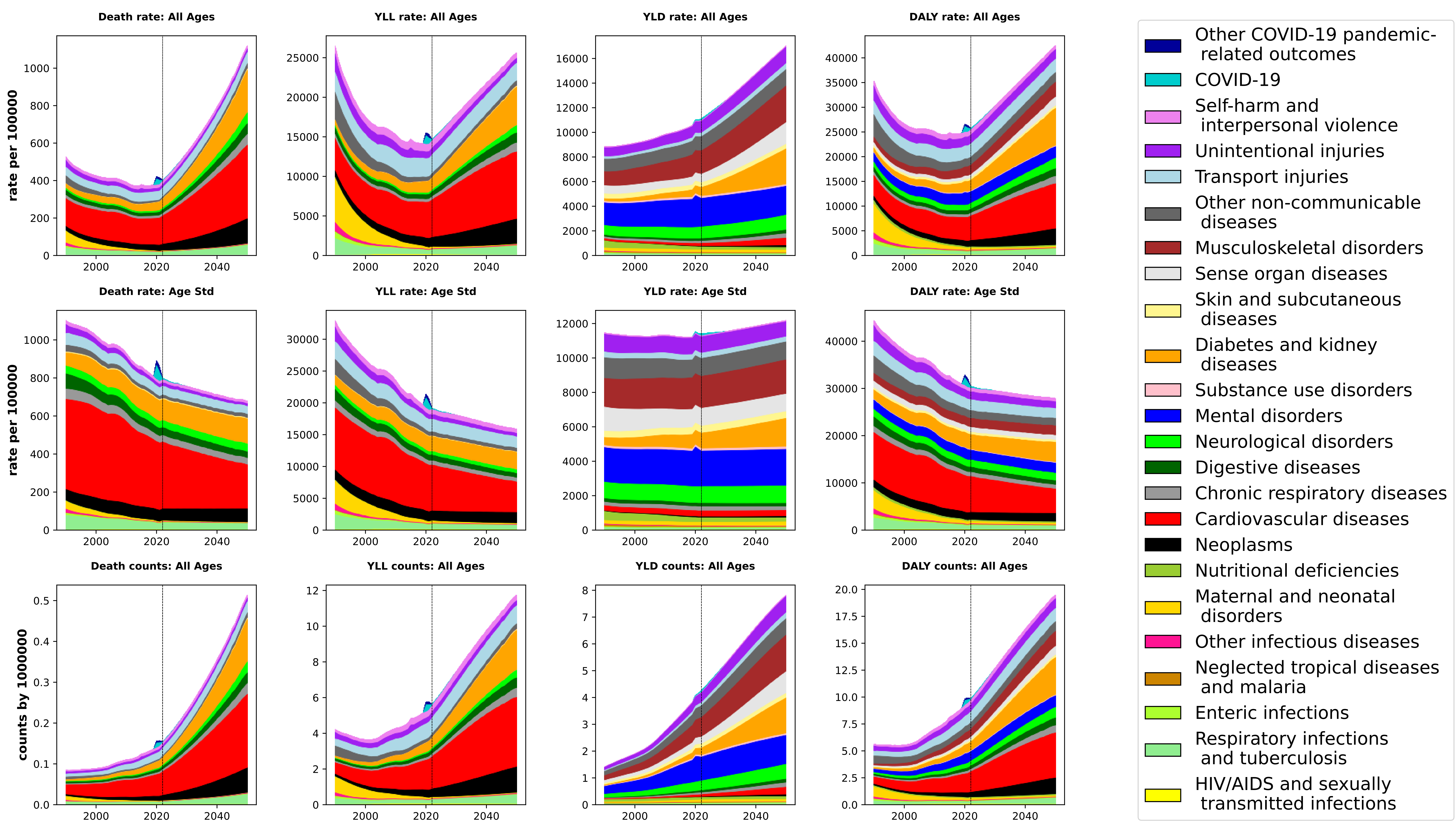

Sudan

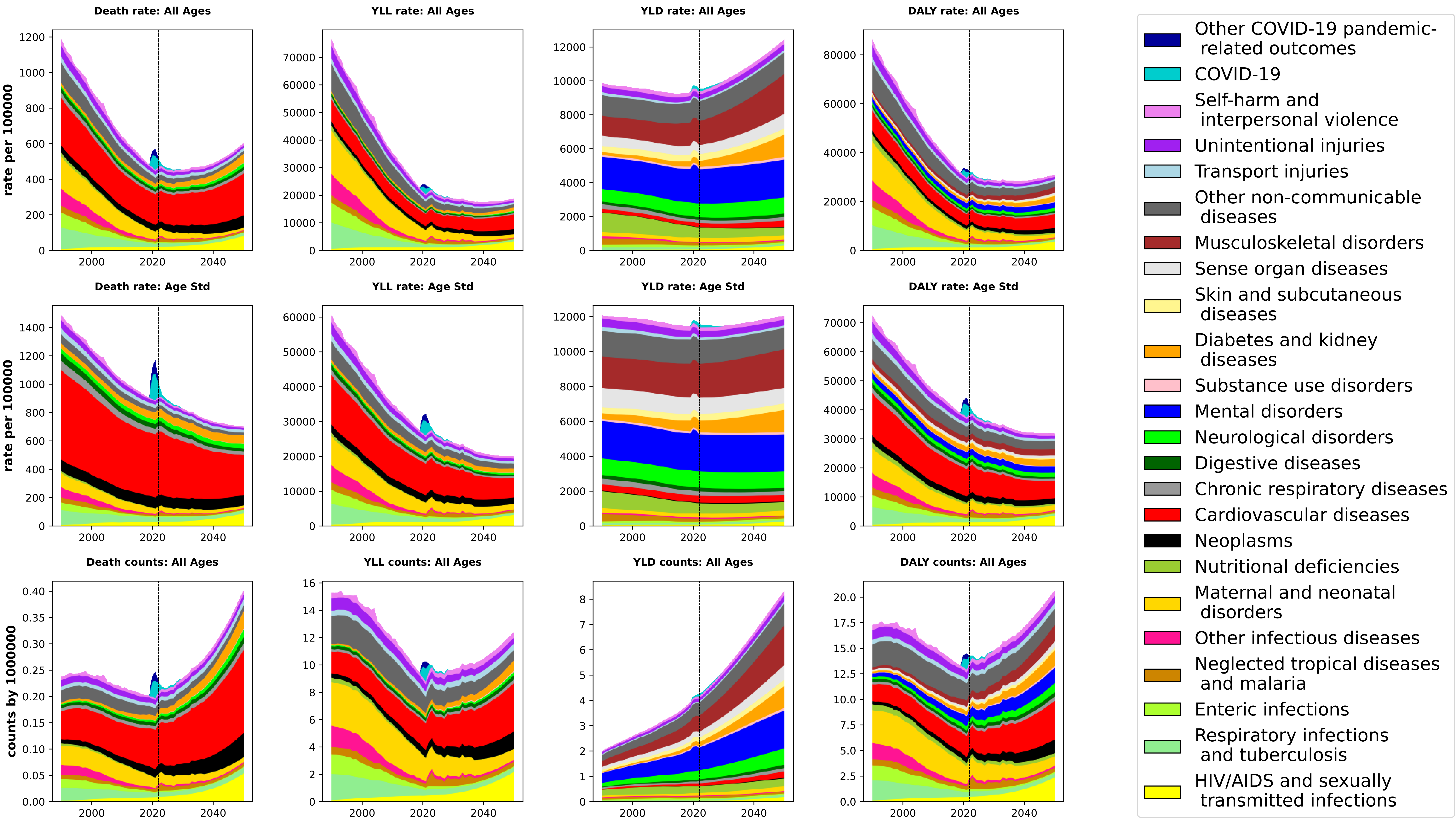
Death rate: All Ages
YLL rate: All Ages
YLD rate: All Ages
DALY rate: All Ages
Death rate: Age Std
YLL rate: Age Std
YLD rate: Age Std
DALY rate: Age Std
Death counts: All Ages
YLL counts: All Ages
YLD counts: All Ages
DALY counts: All Ages
rate per 100000
rate per 100000
counts by 1000000
2000
2020
2040
Other COVID-19 pandemic-related outcomes
COVID-19
Self-harm and interpersonal violence
Unintentional injuries
Transport injuries
Other non-communicable diseases
Musculoskeletal disorders
Sense organ diseases
Skin and subcutaneous diseases
Diabetes and kidney diseases
Substance use disorders
Mental disorders
Neurological disorders
Digestive diseases
Chronic respiratory diseases
Cardiovascular diseases
Neoplasms
Nutritional deficiencies
Maternal and neonatal disorders
Other infectious diseases
Neglected tropical diseases and malaria
Enteric infections
Respiratory infections and tuberculosis
HIV/AIDS and sexually transmitted infections

Syria

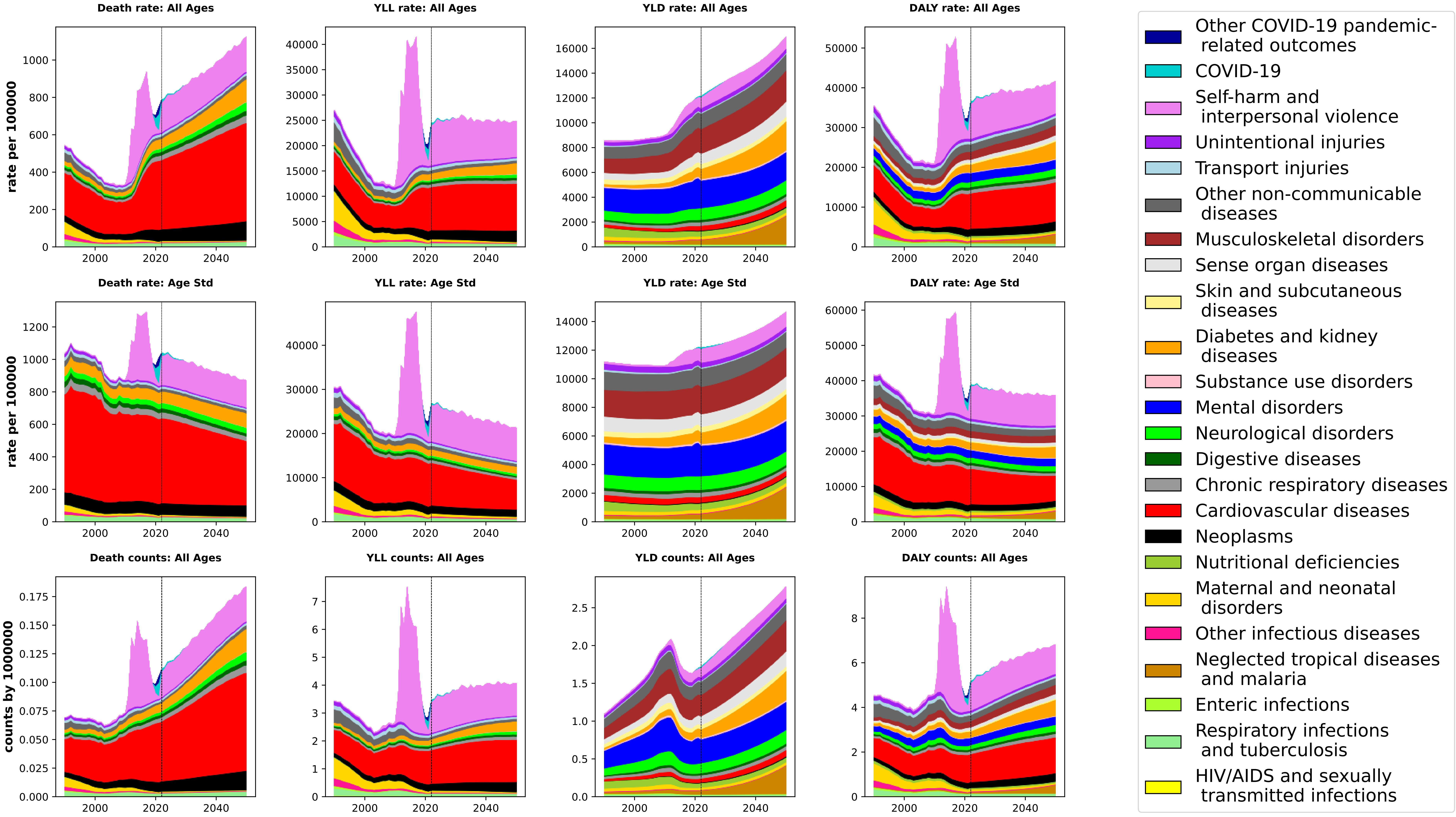
Death rate: All Ages
YLL rate: All Ages
YLD rate: All Ages
DALY rate: All Ages
Death rate: Age Std
YLL rate: Age Std
YLD rate: Age Std
DALY rate: Age Std
Death counts: All Ages
YLL counts: All Ages
YLD counts: All Ages
DALY counts: All Ages
rate per 100000
counts by 1000000
Other COVID-19 pandemic-related outcomes
COVID-19
Self-harm and interpersonal violence
Unintentional injuries
Transport injuries
Other non-communicable diseases
Musculoskeletal disorders
Sense organ diseases
Skin and subcutaneous diseases
Diabetes and kidney diseases
Substance use disorders
Mental disorders
Neurological disorders
Digestive diseases
Chronic respiratory diseases
Cardiovascular diseases
Neoplasms
Nutritional deficiencies
Maternal and neonatal disorders
Other infectious diseases
Neglected tropical diseases and malaria
Enteric infections
Respiratory infections and tuberculosis
HIV/AIDS and sexually transmitted infections

Tunisia

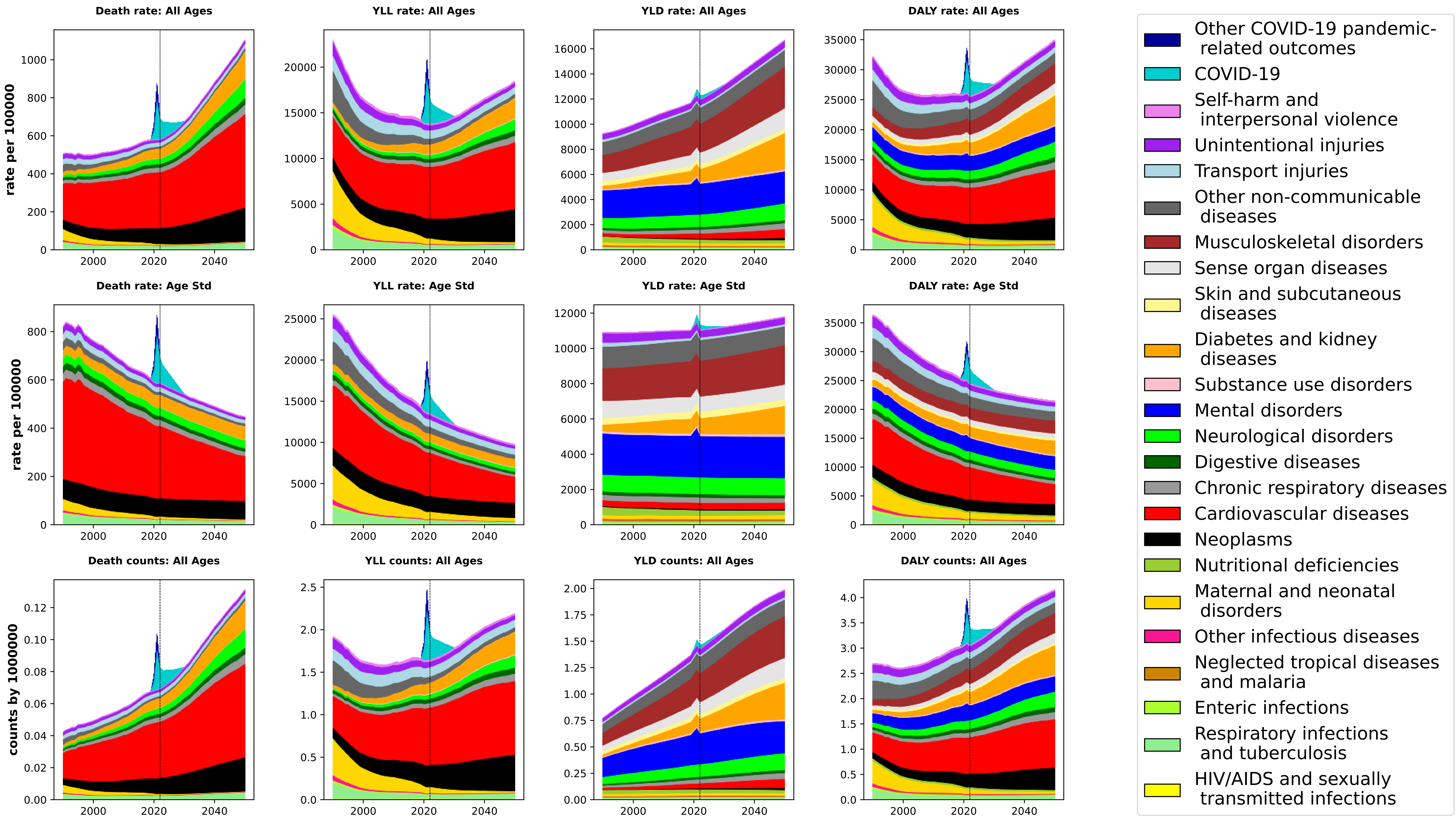
Death rate: All Ages
YLL rate: All Ages
YLD rate: All Ages
DALY rate: All Ages
Death rate: Age Std
YLL rate: Age Std
YLD rate: Age Std
DALY rate: Age Std
Death counts: All Ages
YLL counts: All Ages
YLD counts: All Ages
DALY counts: All Ages
rate per 100000
counts by 1000000
2000
2020
2040
Other COVID-19 pandemic-related outcomes
COVID-19
Self-harm and interpersonal violence
Unintentional injuries
Transport injuries
Other non-communicable diseases
Musculoskeletal disorders
Sense organ diseases
Skin and subcutaneous diseases
Diabetes and kidney diseases
Substance use disorders
Mental disorders
Neurological disorders
Digestive diseases
Chronic respiratory diseases
Cardiovascular diseases
Neoplasms
Nutritional deficiencies
Maternal and neonatal disorders
Other infectious diseases
Neglected tropical diseases and malaria
Enteric infections
Respiratory infections and tuberculosis
HIV/AIDS and sexually transmitted infections

Türkiye

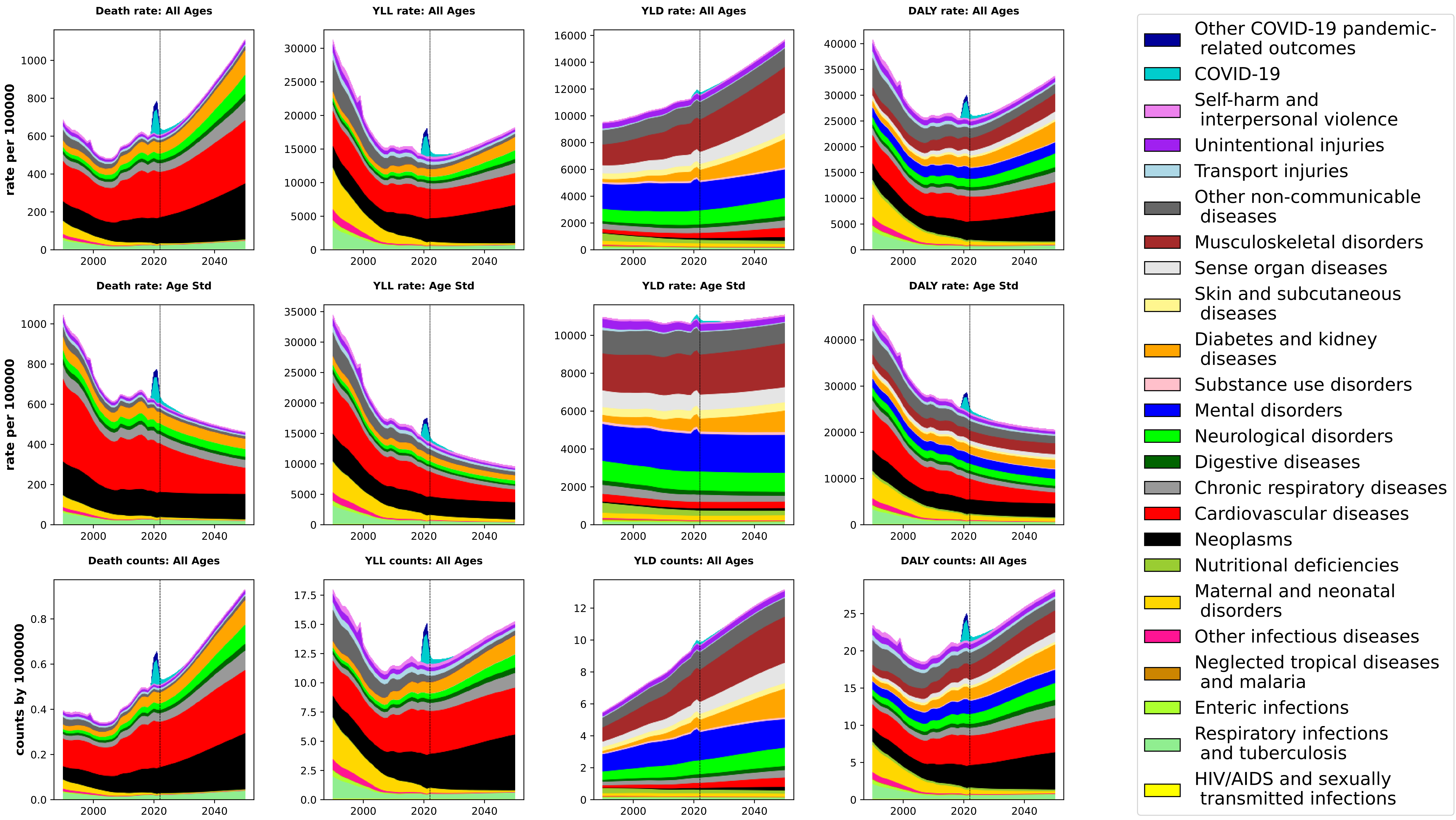

Death rate: All Ages
YLL rate: All Ages
YLD rate: All Ages
DALY rate: All Ages
Death rate: Age Std
YLL rate: Age Std
YLD rate: Age Std
DALY rate: Age Std
Death counts: All Ages
YLL counts: All Ages
YLD counts: All Ages
DALY counts: All Ages
rate per 100000
counts by 1000000
Other COVID-19 pandemic-related outcomes
COVID-19
Self-harm and interpersonal violence
Unintentional injuries
Transport injuries
Other non-communicable diseases
Musculoskeletal disorders
Sense organ diseases
Skin and subcutaneous diseases
Diabetes and kidney diseases
Substance use disorders
Mental disorders
Neurological disorders
Digestive diseases
Chronic respiratory diseases
Cardiovascular diseases
Neoplasms
Nutritional deficiencies
Maternal and neonatal disorders
Other infectious diseases
Neglected tropical diseases and malaria
Enteric infections
Respiratory infections and tuberculosis
HIV/AIDS and sexually transmitted infections

United Arab Emirates

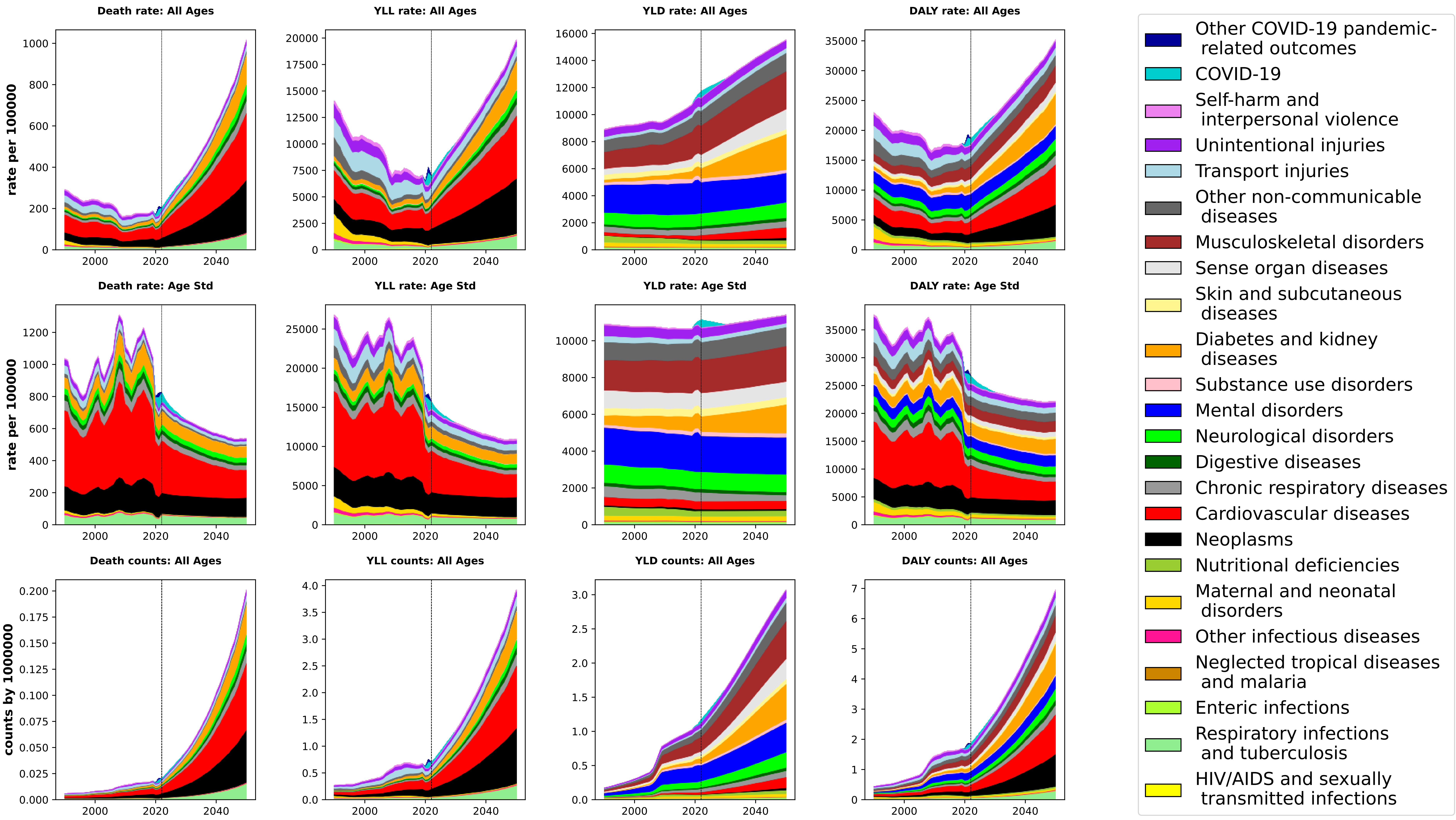

Yemen

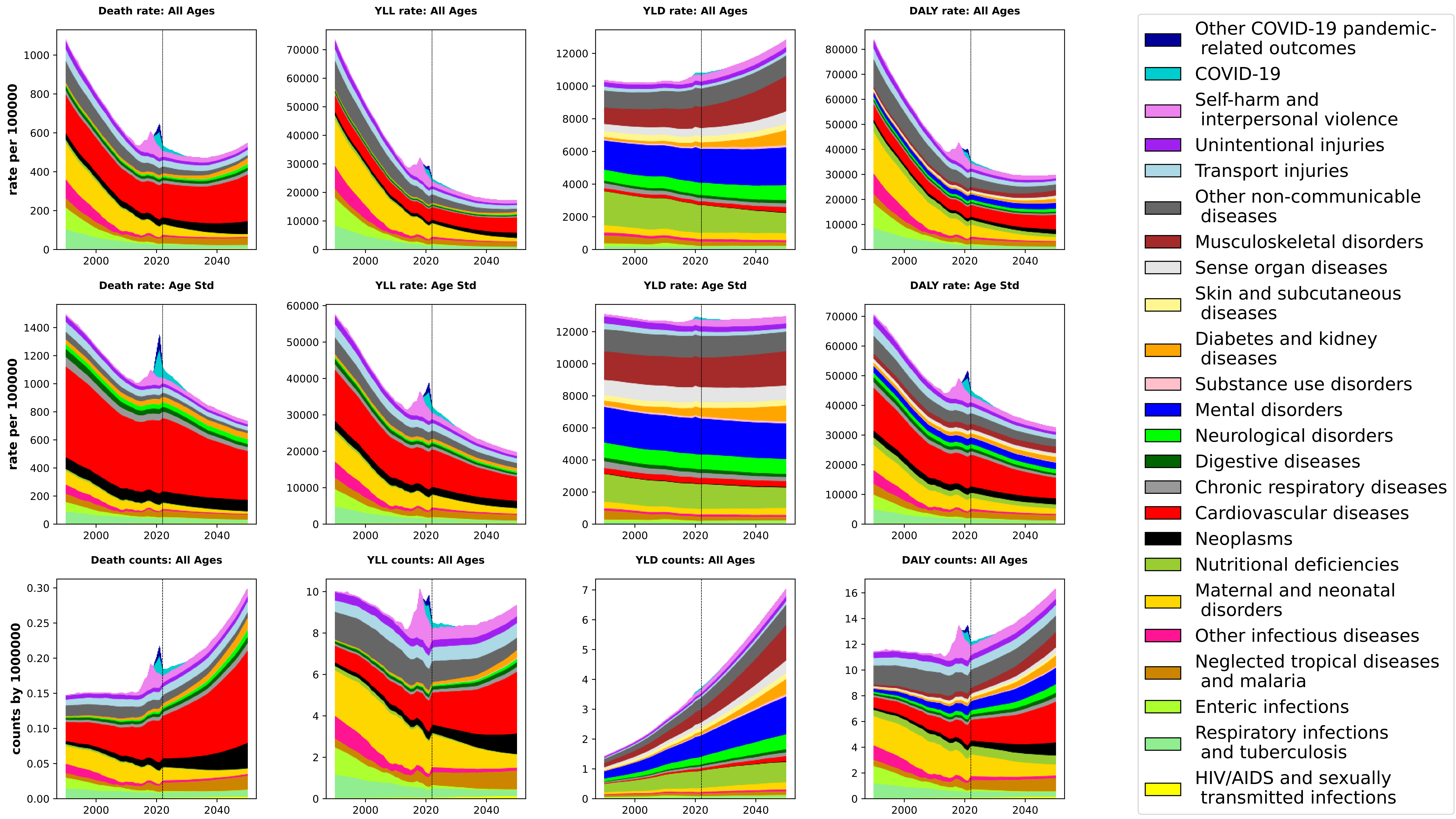
Death rate: All Ages
YLL rate: All Ages
YLD rate: All Ages
DALY rate: All Ages
Death rate: Age Std
YLL rate: Age Std
YLD rate: Age Std
DALY rate: Age Std
Death counts: All Ages
YLL counts: All Ages
YLD counts: All Ages
DALY counts: All Ages
rate per 100000
counts by 1000000
Other COVID-19 pandemic-related outcomes
COVID-19
Self-harm and interpersonal violence
Unintentional injuries
Transport injuries
Other non-communicable diseases
Musculoskeletal disorders
Sense organ diseases
Skin and subcutaneous diseases
Diabetes and kidney diseases
Substance use disorders
Mental disorders
Neurological disorders
Digestive diseases
Chronic respiratory diseases
Cardiovascular diseases
Neoplasms
Nutritional deficiencies
Maternal and neonatal disorders
Other infectious diseases
Neglected tropical diseases and malaria
Enteric infections
Respiratory infections and tuberculosis
HIV/AIDS and sexually transmitted infections

Bangladesh

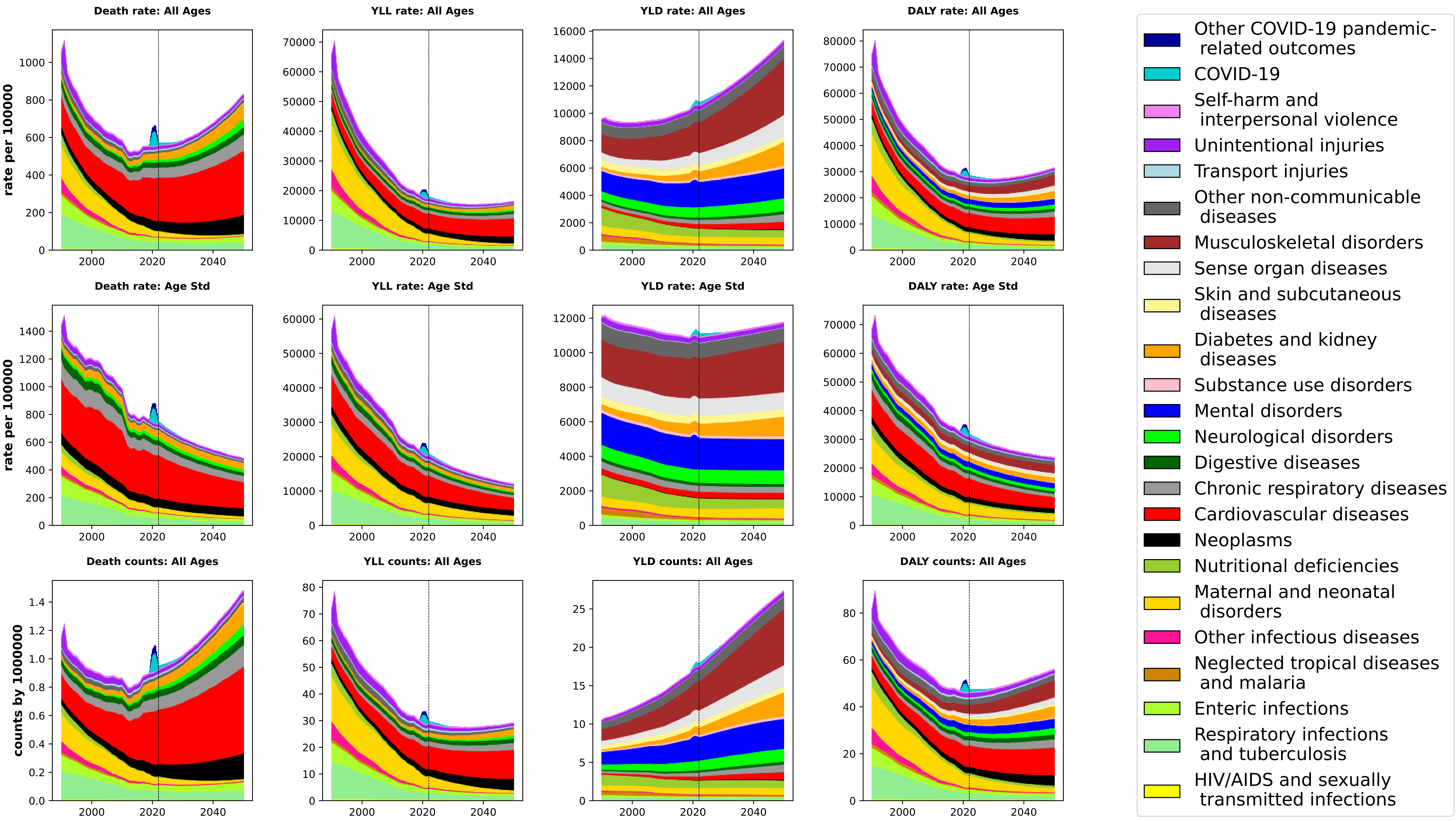
Death rate: All Ages
YLL rate: All Ages
YLD rate: All Ages
DALY rate: All Ages
Death rate: Age Std
YLL rate: Age Std
YLD rate: Age Std
DALY rate: Age Std
Death counts: All Ages
YLL counts: All Ages
YLD counts: All Ages
DALY counts: All Ages
rate per 100000
counts by 1000000
Other COVID-19 pandemic-related outcomes
COVID-19
Self-harm and interpersonal violence
Unintentional injuries
Transport injuries
Other non-communicable diseases
Musculoskeletal disorders
Sense organ diseases
Skin and subcutaneous diseases
Diabetes and kidney diseases
Substance use disorders
Mental disorders
Neurological disorders
Digestive diseases
Chronic respiratory diseases
Cardiovascular diseases
Neoplasms
Nutritional deficiencies
Maternal and neonatal disorders
Other infectious diseases
Neglected tropical diseases and malaria
Enteric infections
Respiratory infections and tuberculosis
HIV/AIDS and sexually transmitted infections

Bhutan

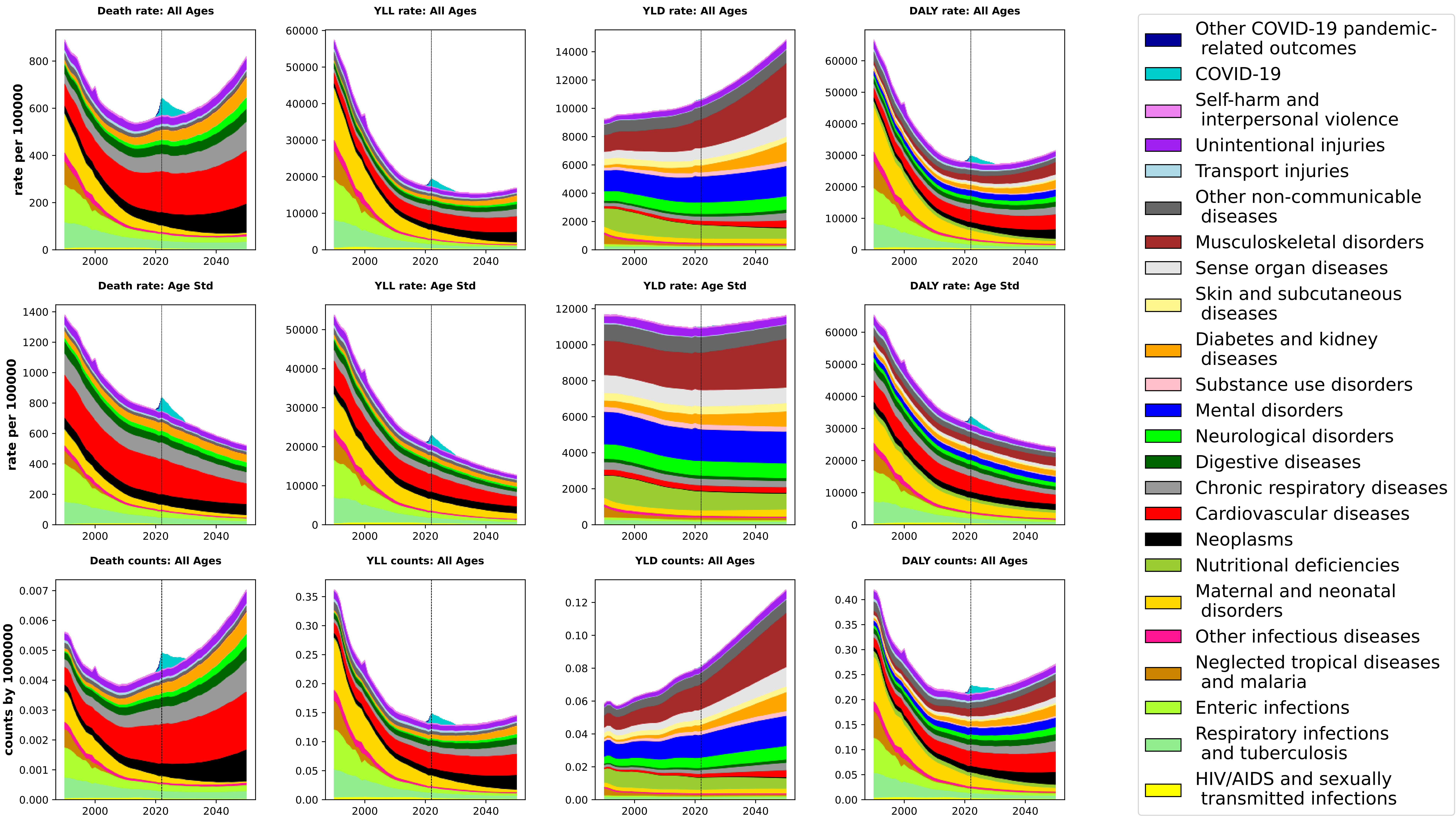
Death rate: All Ages
YLL rate: All Ages
YLD rate: All Ages
DALY rate: All Ages
Death rate: Age Std
YLL rate: Age Std
YLD rate: Age Std
DALY rate: Age Std
Death counts: All Ages
YLL counts: All Ages
YLD counts: All Ages
DALY counts: All Ages
rate per 100000
rate per 100000
counts by 1000000
2000
2020
2040
Other COVID-19 pandemic-related outcomes
COVID-19
Self-harm and interpersonal violence
Unintentional injuries
Transport injuries
Other non-communicable diseases
Musculoskeletal disorders
Sense organ diseases
Skin and subcutaneous diseases
Diabetes and kidney diseases
Substance use disorders
Mental disorders
Neurological disorders
Digestive diseases
Chronic respiratory diseases
Cardiovascular diseases
Neoplasms
Nutritional deficiencies
Maternal and neonatal disorders
Other infectious diseases
Neglected tropical diseases and malaria
Enteric infections
Respiratory infections and tuberculosis
HIV/AIDS and sexually transmitted infections

India

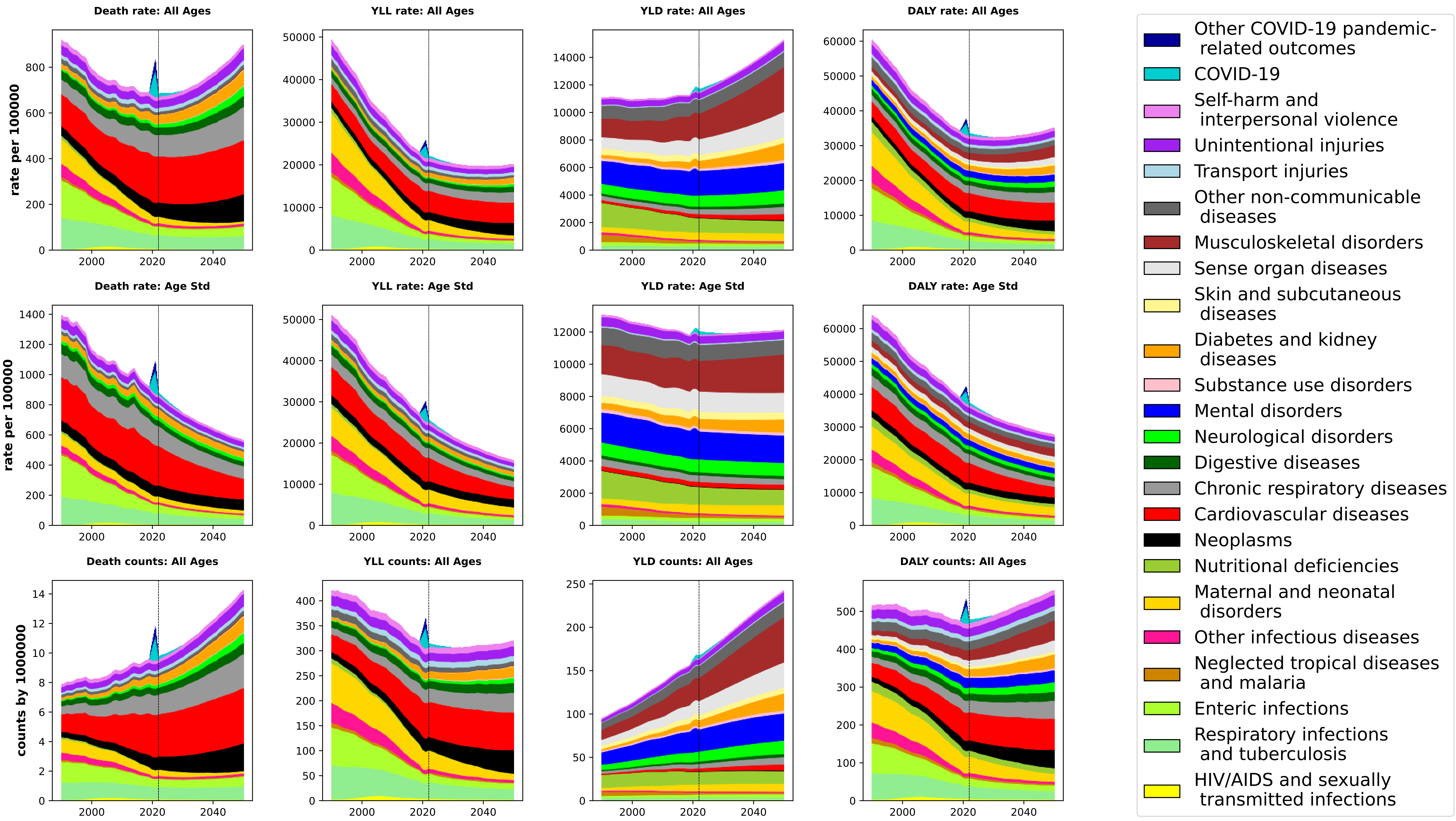
Death rate: All Ages
YLL rate: All Ages
YLD rate: All Ages
DALY rate: All Ages
Death rate: Age Std
YLL rate: Age Std
YLD rate: Age Std
DALY rate: Age Std
Death counts: All Ages
YLL counts: All Ages
YLD counts: All Ages
DALY counts: All Ages
rate per 100000
counts by 1000000
2000
2020
2040
Other COVID-19 pandemic-related outcomes
COVID-19
Self-harm and interpersonal violence
Unintentional injuries
Transport injuries
Other non-communicable diseases
Musculoskeletal disorders
Sense organ diseases
Skin and subcutaneous diseases
Diabetes and kidney diseases
Substance use disorders
Mental disorders
Neurological disorders
Digestive diseases
Chronic respiratory diseases
Cardiovascular diseases
Neoplasms
Nutritional deficiencies
Maternal and neonatal disorders
Other infectious diseases
Neglected tropical diseases and malaria
Enteric infections
Respiratory infections and tuberculosis
HIV/AIDS and sexually transmitted infections

Nepal

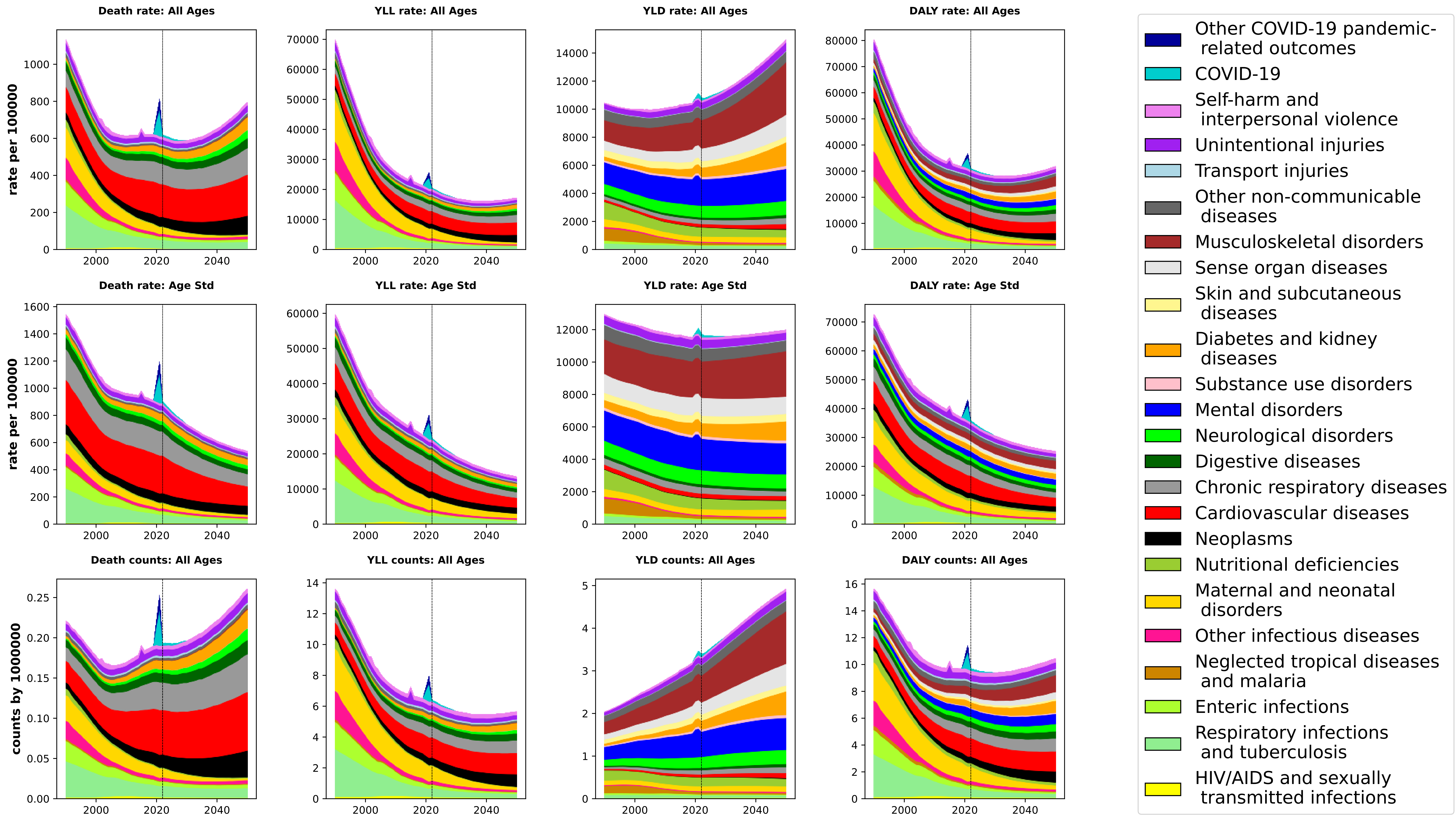
Death rate: All Ages
YLL rate: All Ages
YLD rate: All Ages
DALY rate: All Ages
Death rate: Age Std
YLL rate: Age Std
YLD rate: Age Std
DALY rate: Age Std
Death counts: All Ages
YLL counts: All Ages
YLD counts: All Ages
DALY counts: All Ages
rate per 100000
rate per 100000
counts by 1000000
Other COVID-19 pandemic-related outcomes
COVID-19
Self-harm and interpersonal violence
Unintentional injuries
Transport injuries
Other non-communicable diseases
Musculoskeletal disorders
Sense organ diseases
Skin and subcutaneous diseases
Diabetes and kidney diseases
Substance use disorders
Mental disorders
Neurological disorders
Digestive diseases
Chronic respiratory diseases
Cardiovascular diseases
Neoplasms
Nutritional deficiencies
Maternal and neonatal disorders
Other infectious diseases
Neglected tropical diseases and malaria
Enteric infections
Respiratory infections and tuberculosis
HIV/AIDS and sexually transmitted infections

Pakistan

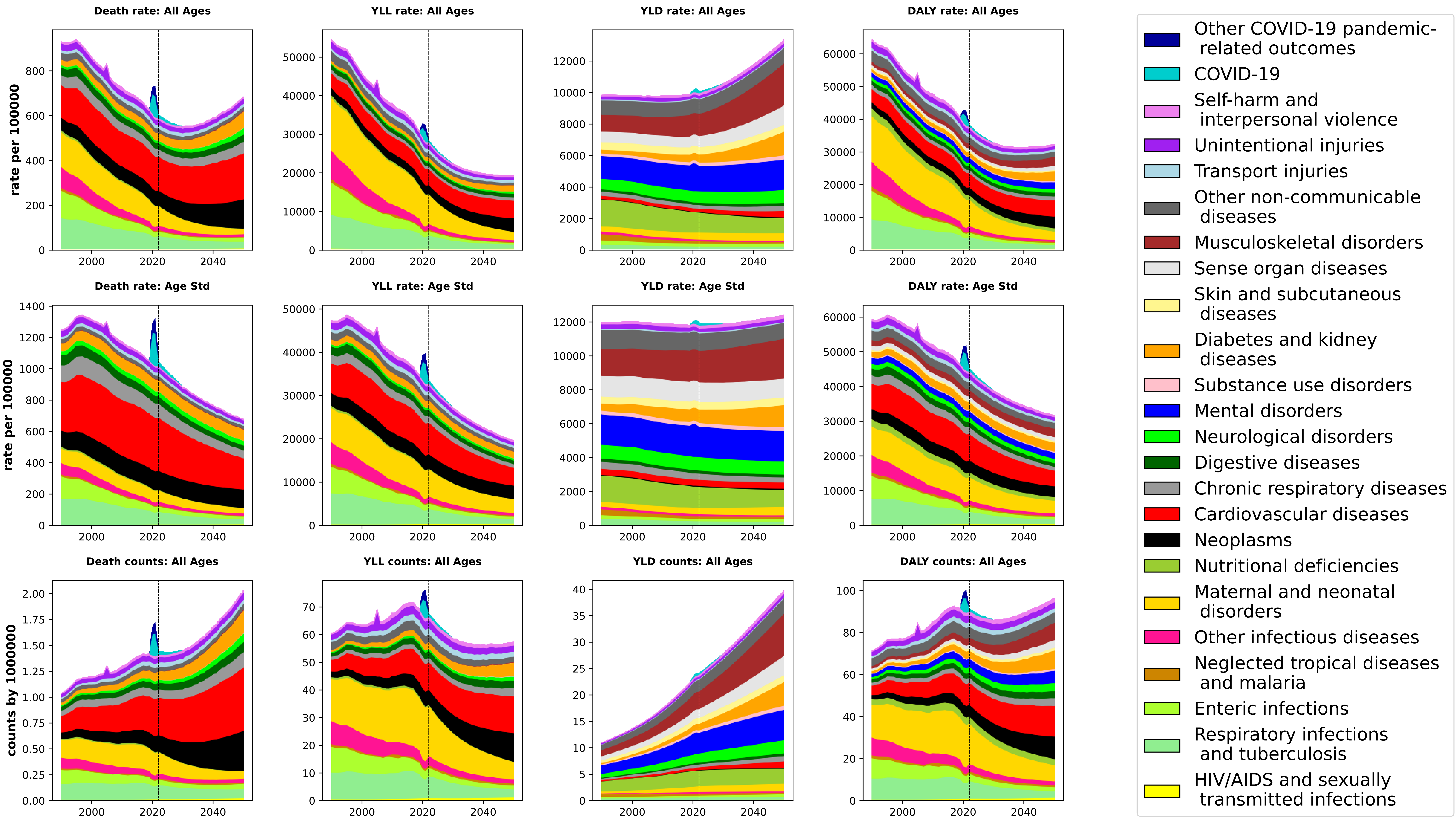
Death rate: All Ages
YLL rate: All Ages
YLD rate: All Ages
DALY rate: All Ages
Death rate: Age Std
YLL rate: Age Std
YLD rate: Age Std
DALY rate: Age Std
Death counts: All Ages
YLL counts: All Ages
YLD counts: All Ages
DALY counts: All Ages
rate per 100000
counts by 1000000
Other COVID-19 pandemic-related outcomes
COVID-19
Self-harm and interpersonal violence
Unintentional injuries
Transport injuries
Other non-communicable diseases
Musculoskeletal disorders
Sense organ diseases
Skin and subcutaneous diseases
Diabetes and kidney diseases
Substance use disorders
Mental disorders
Neurological disorders
Digestive diseases
Chronic respiratory diseases
Cardiovascular diseases
Neoplasms
Nutritional deficiencies
Maternal and neonatal disorders
Other infectious diseases
Neglected tropical diseases and malaria
Enteric infections
Respiratory infections and tuberculosis
HIV/AIDS and sexually transmitted infections

China

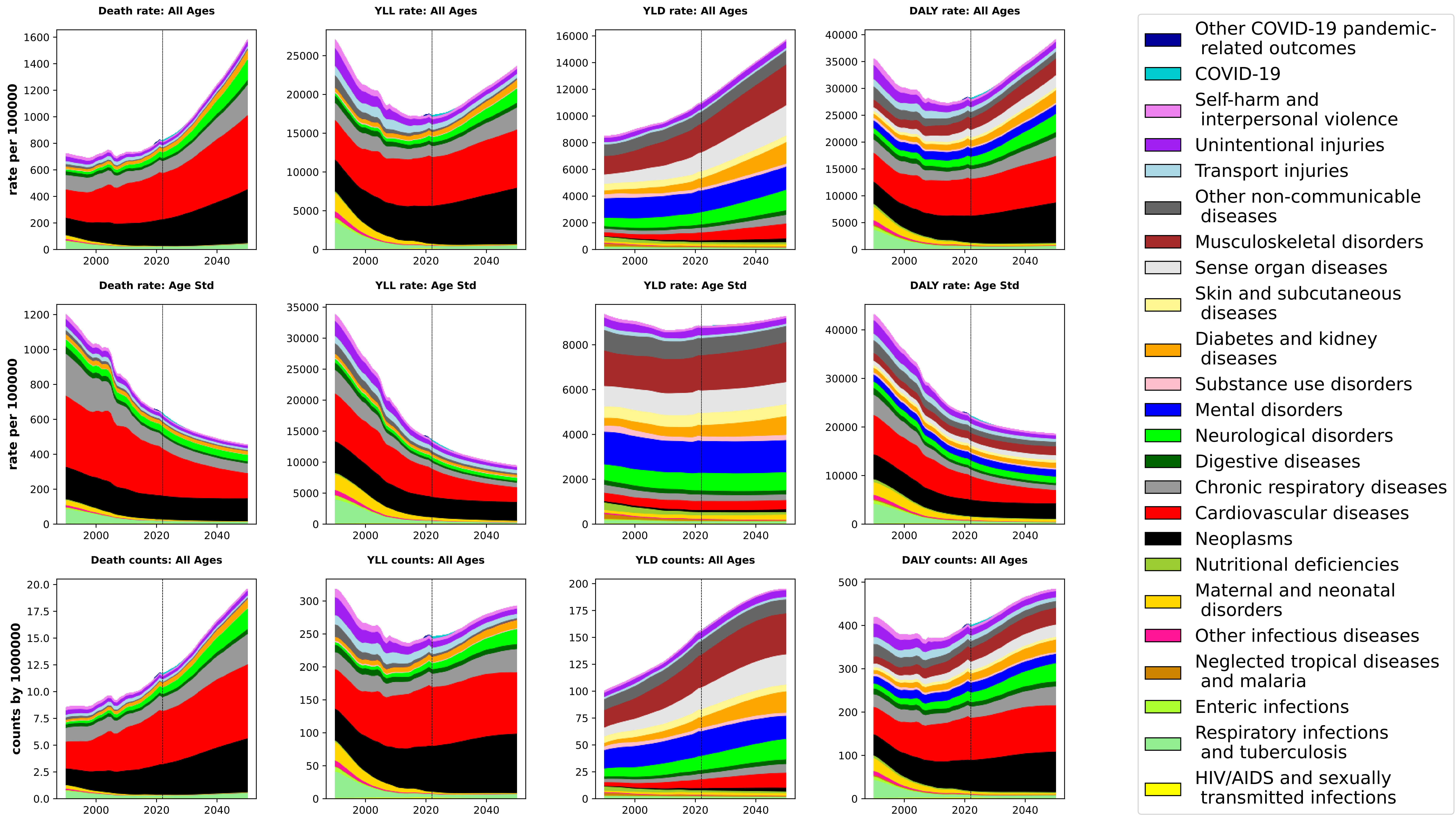
Death rate: All Ages
YLL rate: All Ages
YLD rate: All Ages
DALY rate: All Ages
Death rate: Age Std
YLL rate: Age Std
YLD rate: Age Std
DALY rate: Age Std
Death counts: All Ages
YLL counts: All Ages
YLD counts: All Ages
DALY counts: All Ages
rate per 100000
counts by 1000000
Other COVID-19 pandemic-related outcomes
COVID-19
Self-harm and interpersonal violence
Unintentional injuries
Transport injuries
Other non-communicable diseases
Musculoskeletal disorders
Sense organ diseases
Skin and subcutaneous diseases
Diabetes and kidney diseases
Substance use disorders
Mental disorders
Neurological disorders
Digestive diseases
Chronic respiratory diseases
Cardiovascular diseases
Neoplasms
Nutritional deficiencies
Maternal and neonatal disorders
Other infectious diseases
Neglected tropical diseases and malaria
Enteric infections
Respiratory infections and tuberculosis
HIV/AIDS and sexually transmitted infections

North Korea

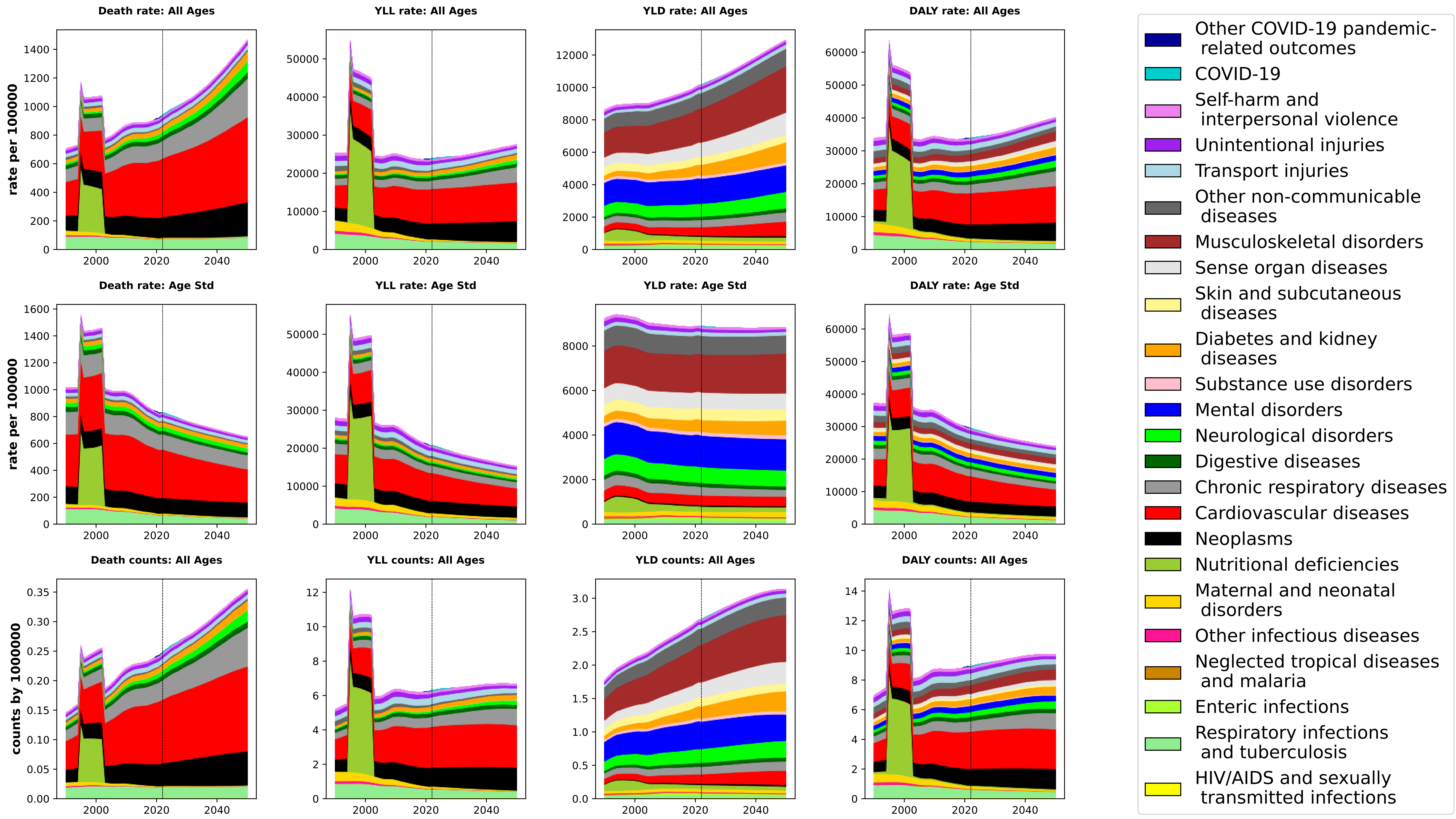
Death rate: All Ages
YLL rate: All Ages
YLD rate: All Ages
DALY rate: All Ages
Death rate: Age Std
YLL rate: Age Std
YLD rate: Age Std
DALY rate: Age Std
Death counts: All Ages
YLL counts: All Ages
YLD counts: All Ages
DALY counts: All Ages
rate per 100000
counts by 1000000
Other COVID-19 pandemic-related outcomes
COVID-19
Self-harm and interpersonal violence
Unintentional injuries
Transport injuries
Other non-communicable diseases
Musculoskeletal disorders
Sense organ diseases
Skin and subcutaneous diseases
Diabetes and kidney diseases
Substance use disorders
Mental disorders
Neurological disorders
Digestive diseases
Chronic respiratory diseases
Cardiovascular diseases
Neoplasms
Nutritional deficiencies
Maternal and neonatal disorders
Other infectious diseases
Neglected tropical diseases and malaria
Enteric infections
Respiratory infections and tuberculosis
HIV/AIDS and sexually transmitted infections

Taiwan (Province of China)

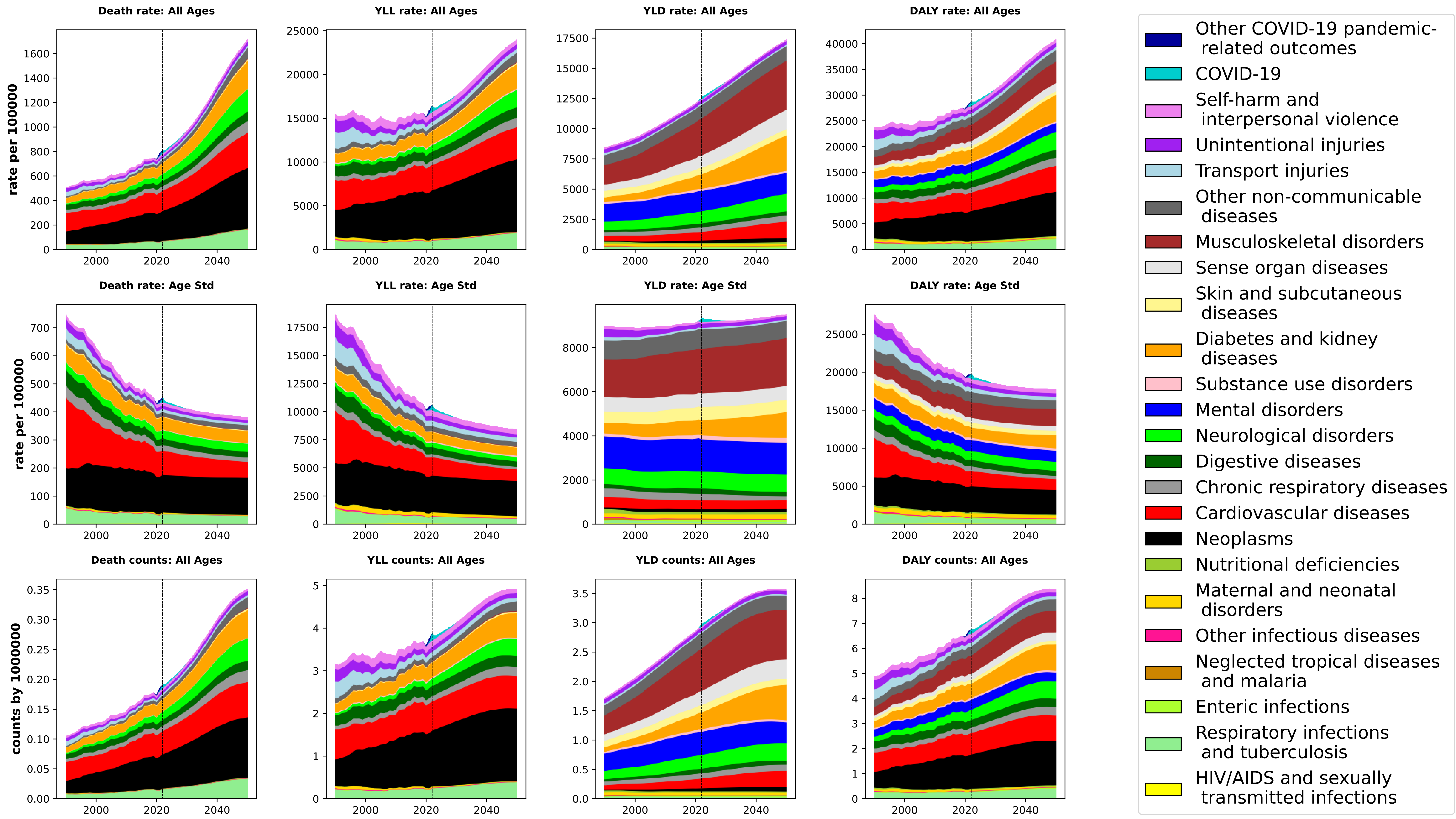

American Samoa

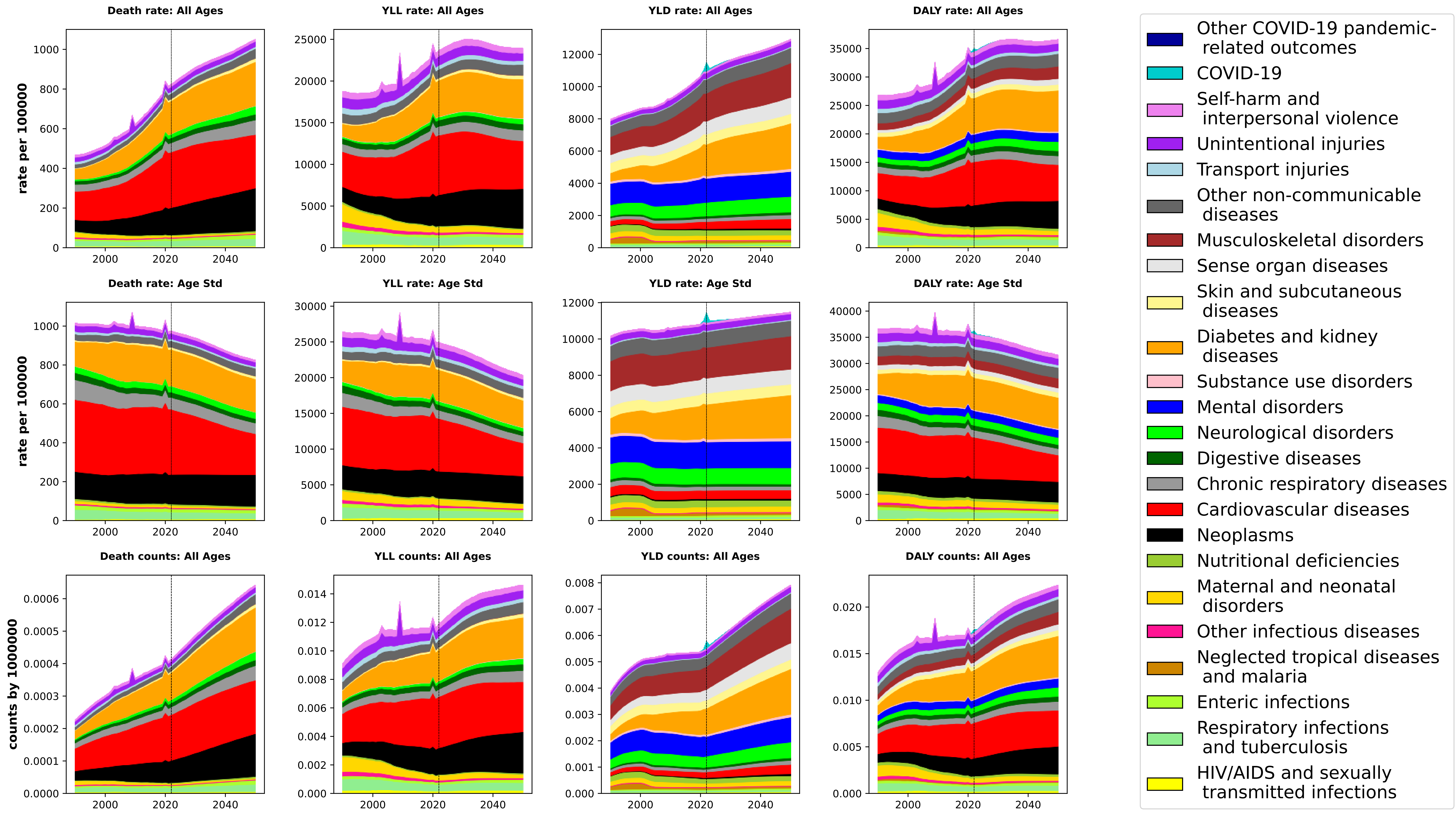

Cook Islands

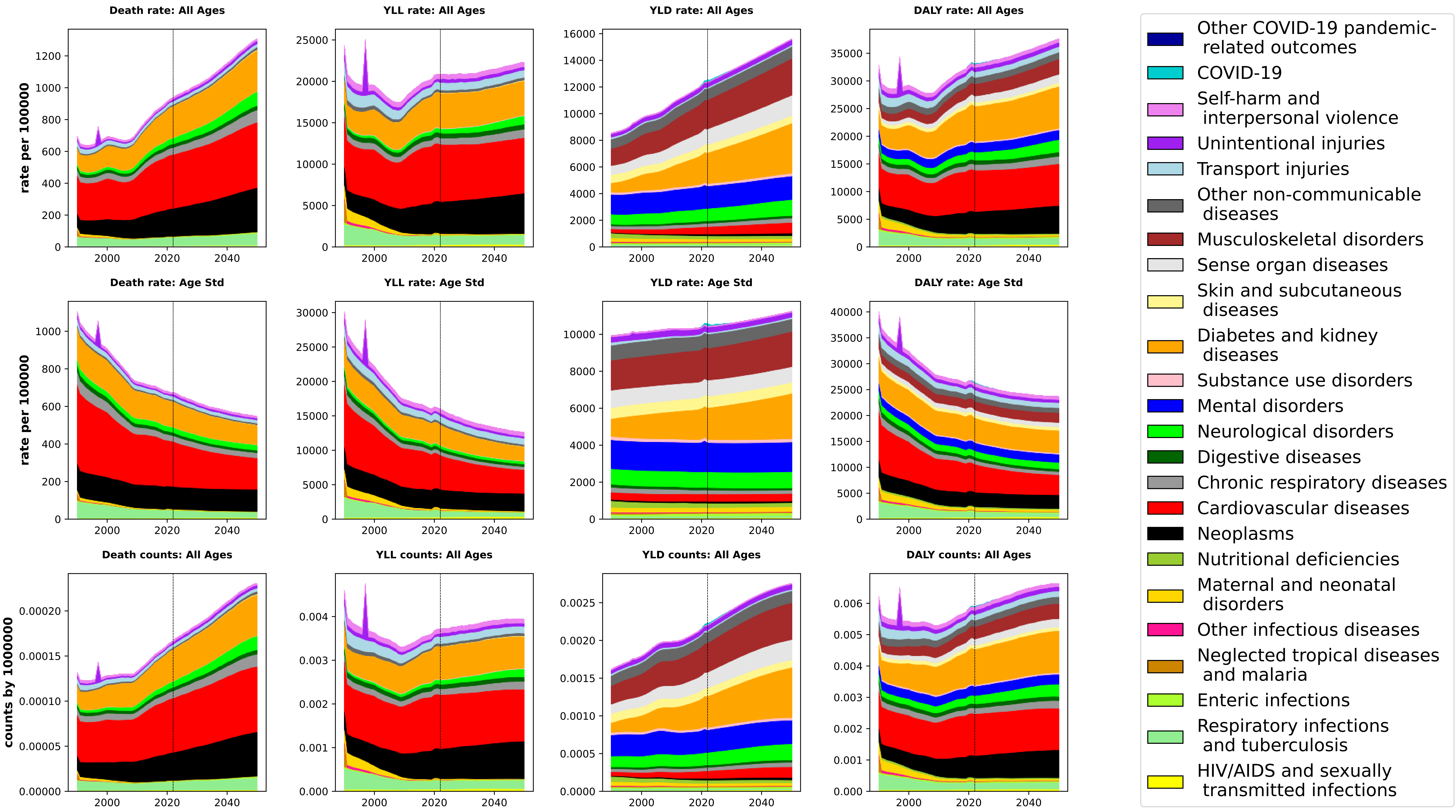

Fiji

Supplemental Results Figure S6. Cause level 2 area plots for 4 burden measures for reference scenario by location, 1990-2050

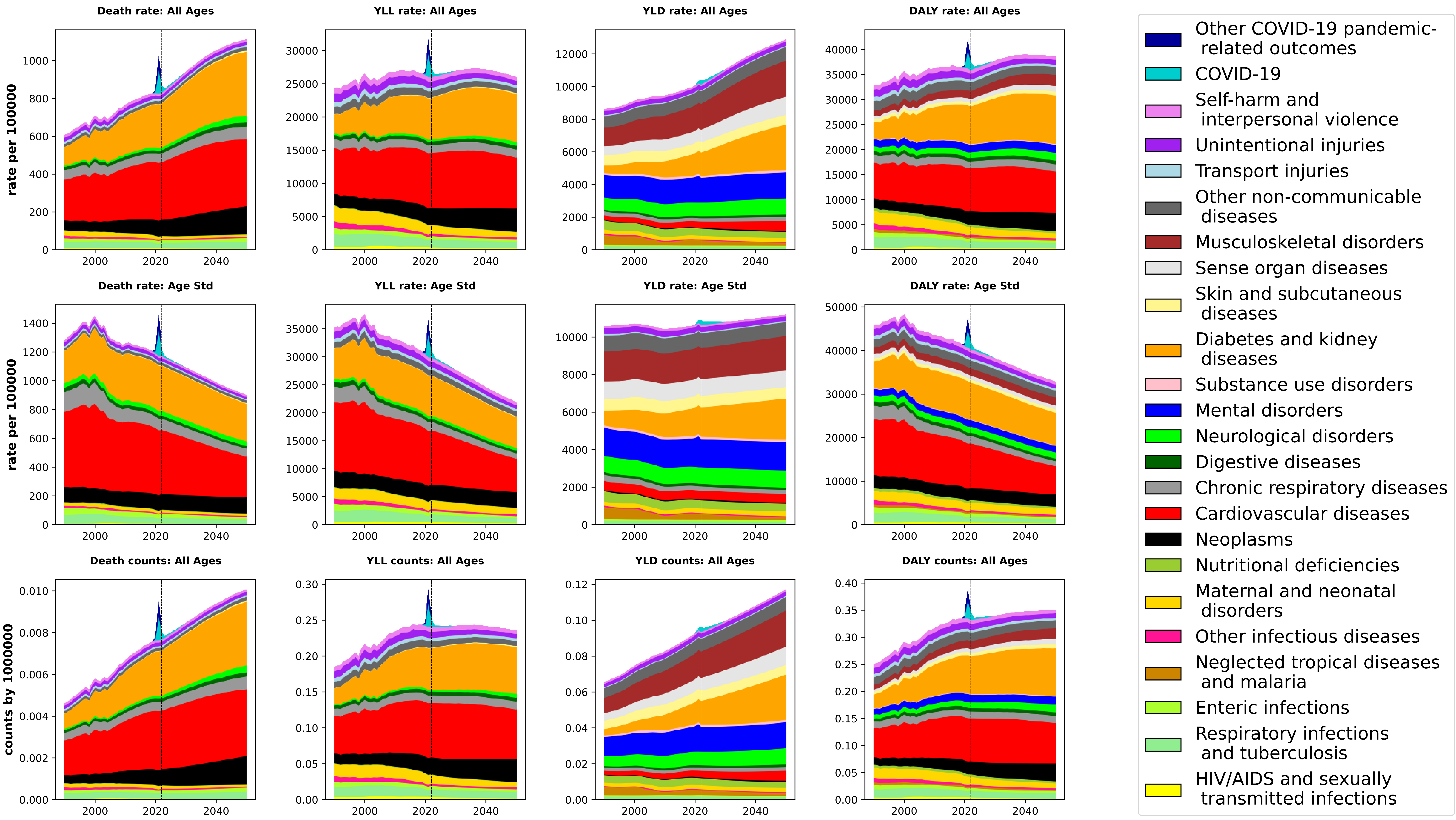

Guam

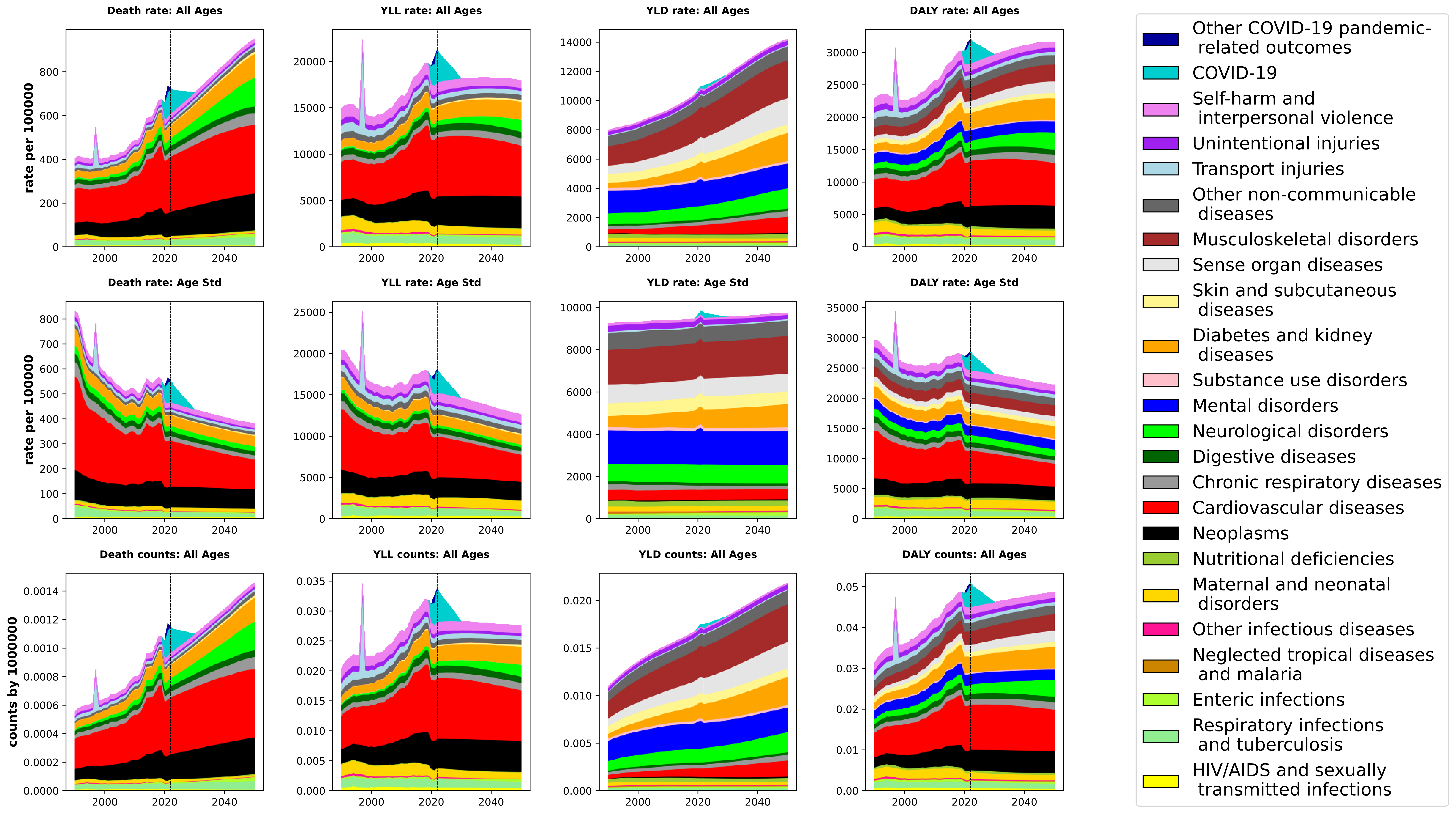
Death rate: All Ages
YLL rate: All Ages
YLD rate: All Ages
DALY rate: All Ages
Death rate: Age Std
YLL rate: Age Std
YLD rate: Age Std
DALY rate: Age Std
Death counts: All Ages
YLL counts: All Ages
YLD counts: All Ages
DALY counts: All Ages
rate per 100000
counts by 1000000
2000
2020
2040
Other COVID-19 pandemic-related outcomes
COVID-19
Self-harm and interpersonal violence
Unintentional injuries
Transport injuries
Other non-communicable diseases
Musculoskeletal disorders
Sense organ diseases
Skin and subcutaneous diseases
Diabetes and kidney diseases
Substance use disorders
Mental disorders
Neurological disorders
Digestive diseases
Chronic respiratory diseases
Cardiovascular diseases
Neoplasms
Nutritional deficiencies
Maternal and neonatal disorders
Other infectious diseases
Neglected tropical diseases and malaria
Enteric infections
Respiratory infections and tuberculosis
HIV/AIDS and sexually transmitted infections

Kiribati

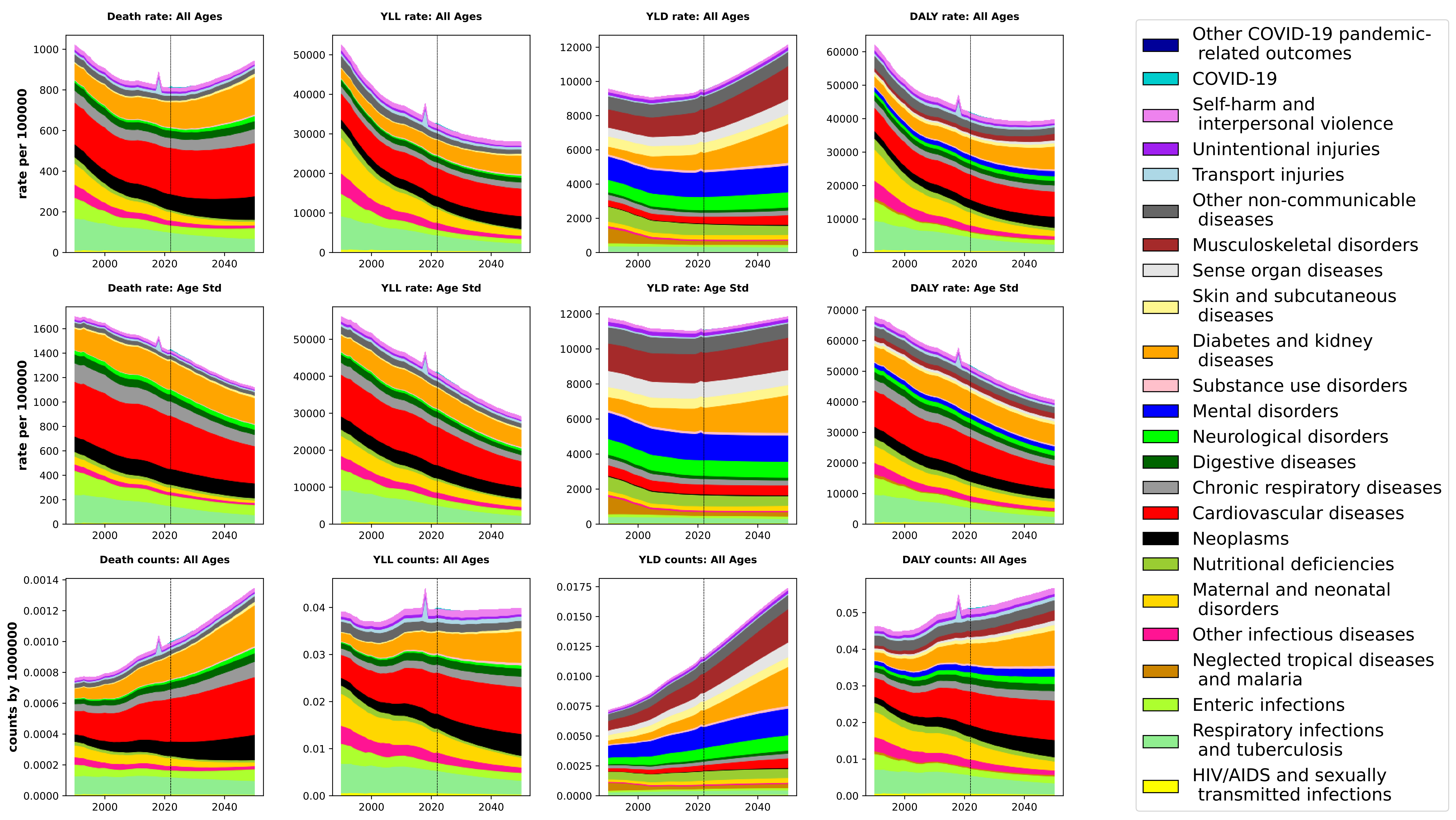
Death rate: All Ages
YLL rate: All Ages
YLD rate: All Ages
DALY rate: All Ages
Death rate: Age Std
YLL rate: Age Std
YLD rate: Age Std
DALY rate: Age Std
Death counts: All Ages
YLL counts: All Ages
YLD counts: All Ages
DALY counts: All Ages
rate per 100000
counts by 1000000
Other COVID-19 pandemic-related outcomes
COVID-19
Self-harm and interpersonal violence
Unintentional injuries
Transport injuries
Other non-communicable diseases
Musculoskeletal disorders
Sense organ diseases
Skin and subcutaneous diseases
Diabetes and kidney diseases
Substance use disorders
Mental disorders
Neurological disorders
Digestive diseases
Chronic respiratory diseases
Cardiovascular diseases
Neoplasms
Nutritional deficiencies
Maternal and neonatal disorders
Other infectious diseases
Neglected tropical diseases and malaria
Enteric infections
Respiratory infections and tuberculosis
HIV/AIDS and sexually transmitted infections

Marshall Islands

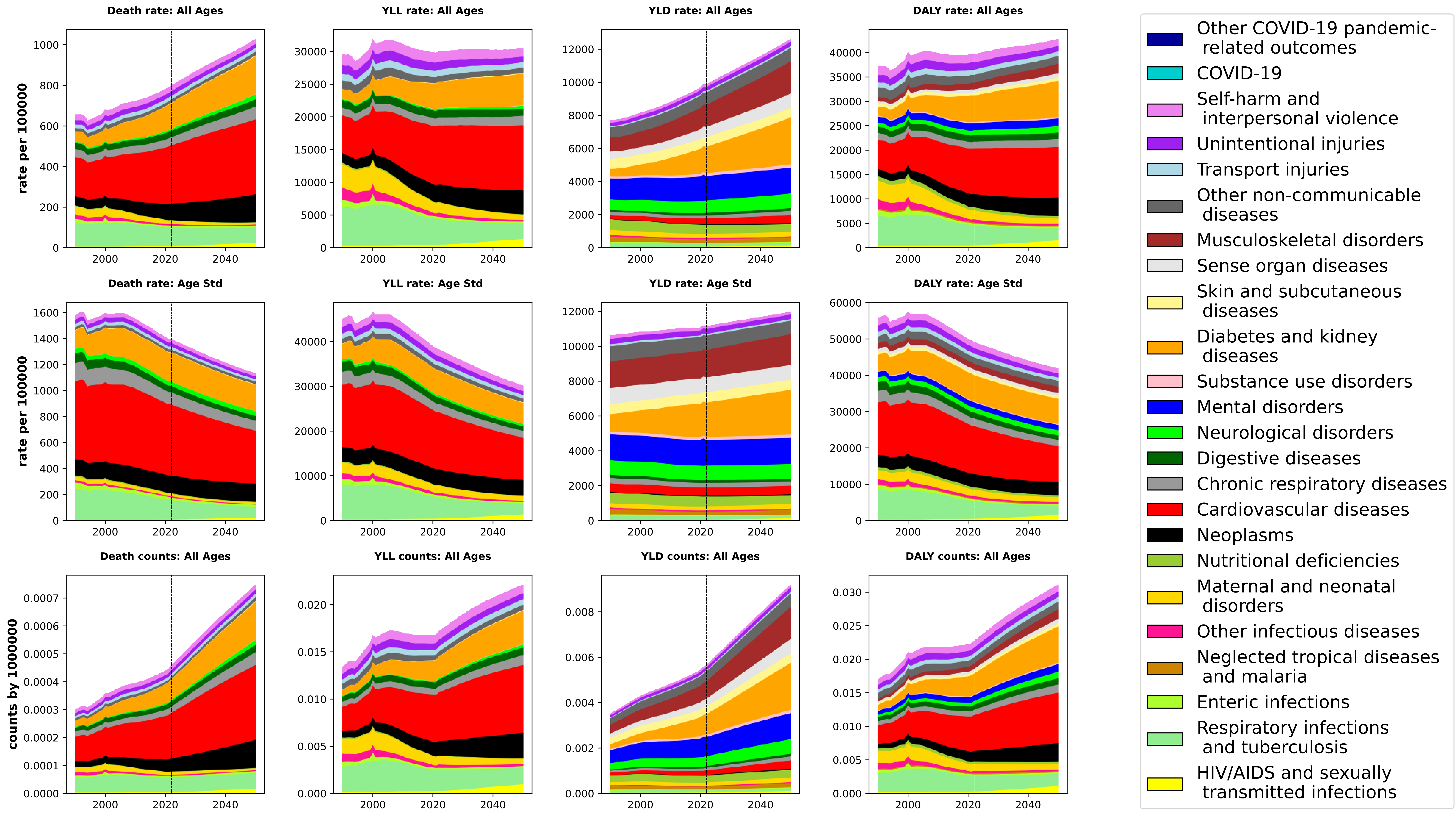

Federated States of Micronesia

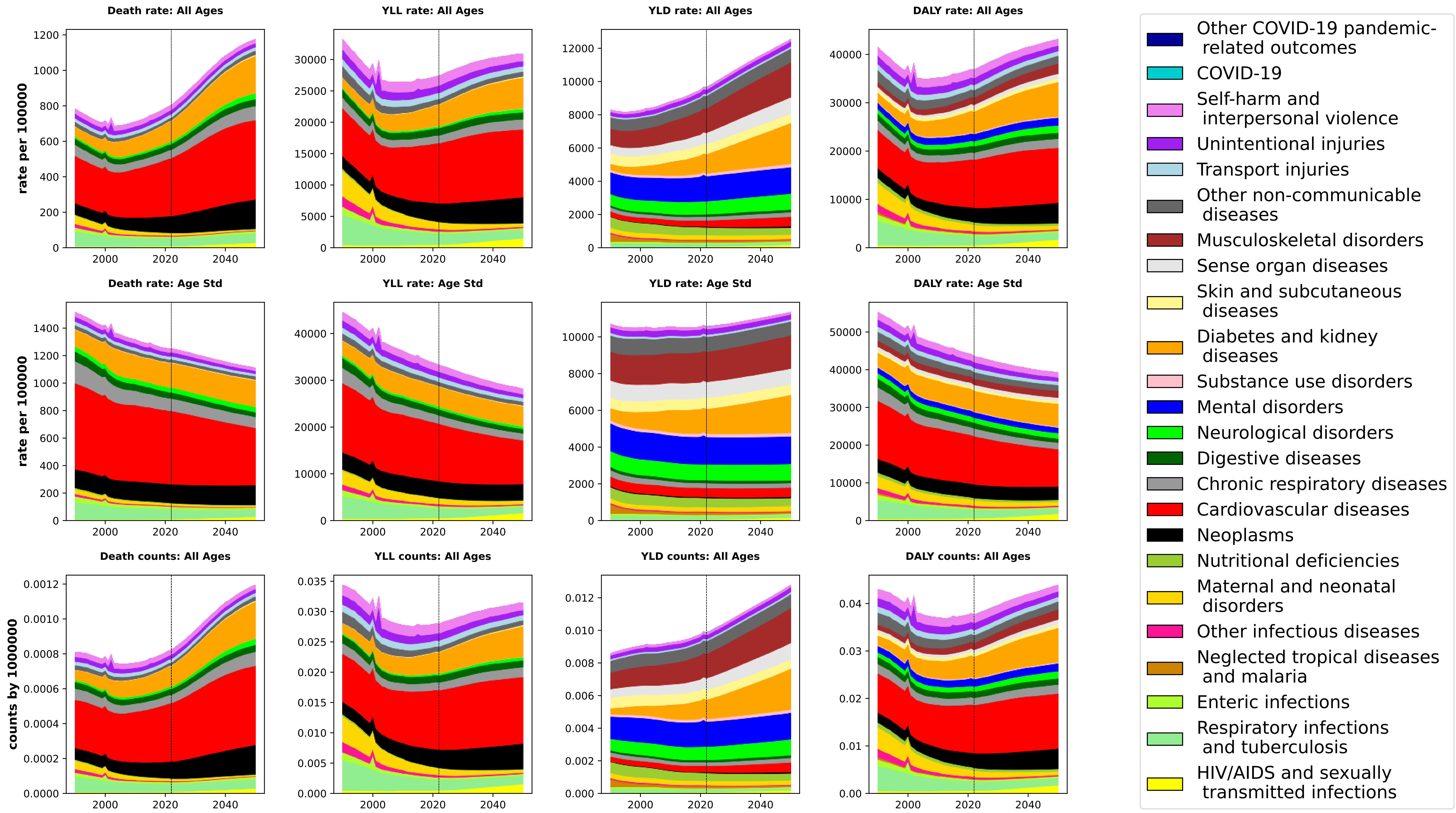

Nauru

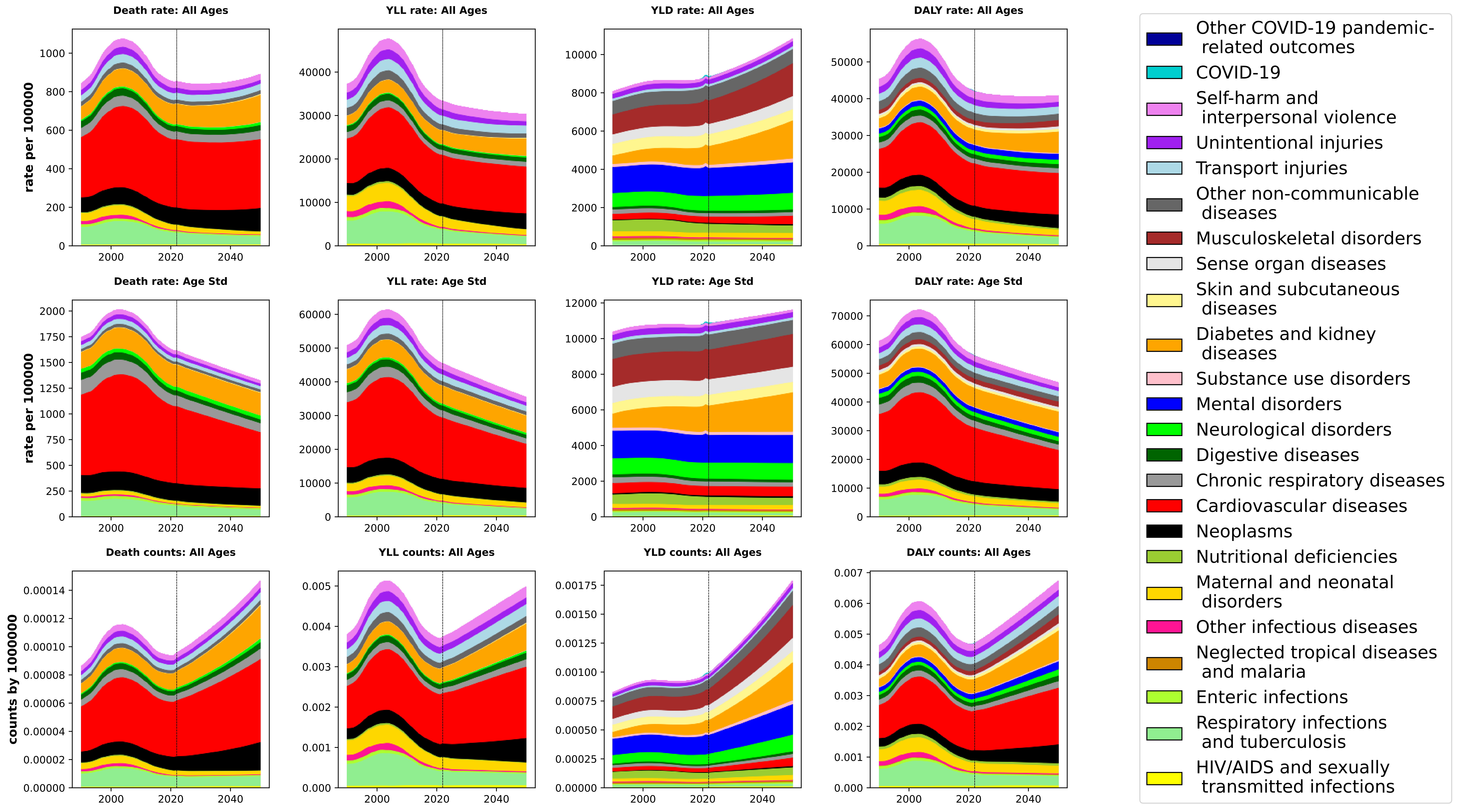
Death rate: All Ages
YLL rate: All Ages
YLD rate: All Ages
DALY rate: All Ages
Death rate: Age Std
YLL rate: Age Std
YLD rate: Age Std
DALY rate: Age Std
Death counts: All Ages
YLL counts: All Ages
YLD counts: All Ages
DALY counts: All Ages
rate per 100000
rate per 100000
counts by 1000000
Other COVID-19 pandemic-related outcomes
COVID-19
Self-harm and interpersonal violence
Unintentional injuries
Transport injuries
Other non-communicable diseases
Musculoskeletal disorders
Sense organ diseases
Skin and subcutaneous diseases
Diabetes and kidney diseases
Substance use disorders
Mental disorders
Neurological disorders
Digestive diseases
Chronic respiratory diseases
Cardiovascular diseases
Neoplasms
Nutritional deficiencies
Maternal and neonatal disorders
Other infectious diseases
Neglected tropical diseases and malaria
Enteric infections
Respiratory infections and tuberculosis
HIV/AIDS and sexually transmitted infections

Niue

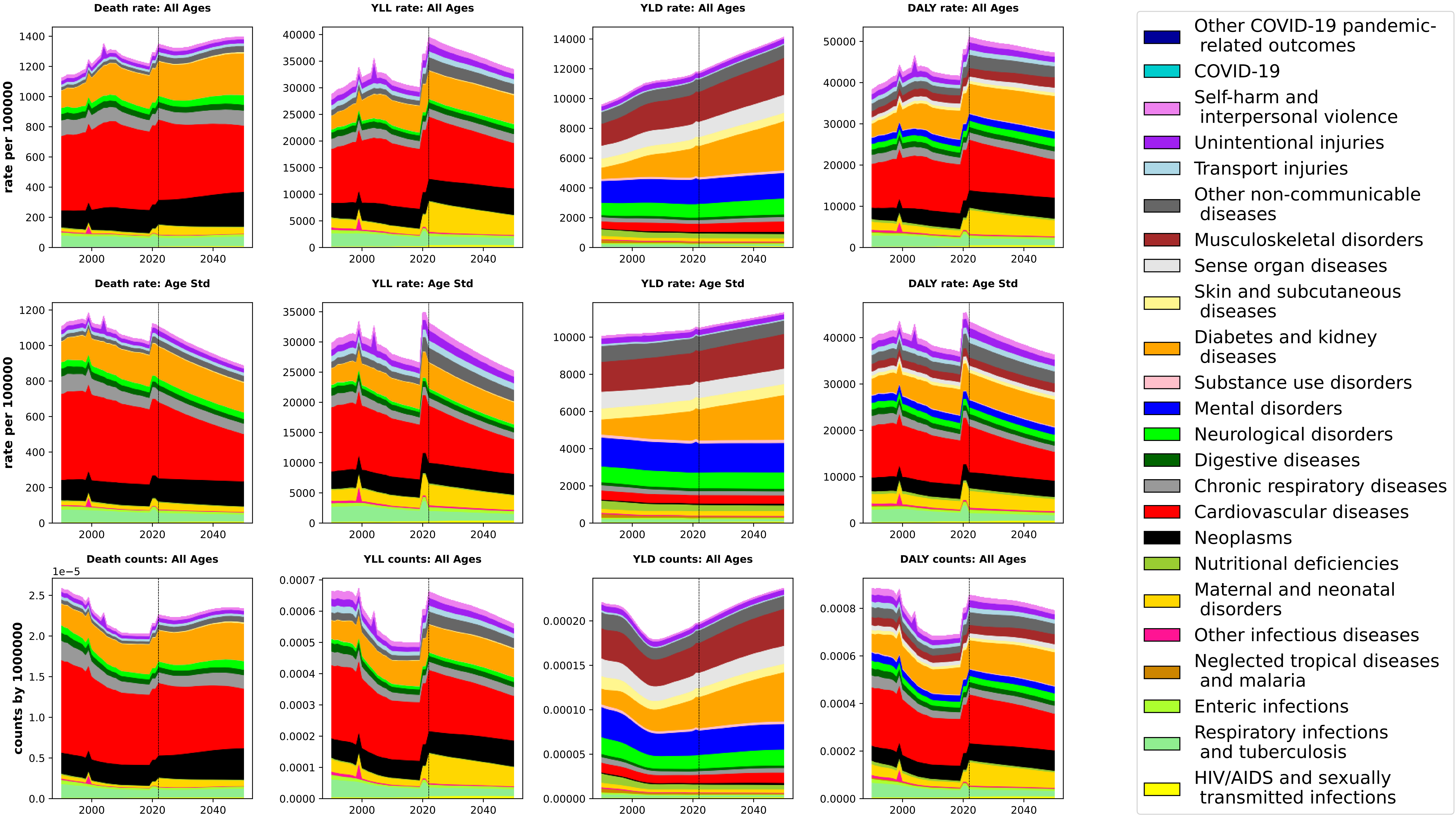
Death rate: All Ages
YLL rate: All Ages
YLD rate: All Ages
DALY rate: All Ages
Death rate: Age Std
YLL rate: Age Std
YLD rate: Age Std
DALY rate: Age Std
Death counts: All Ages
YLL counts: All Ages
YLD counts: All Ages
DALY counts: All Ages
rate per 100000
counts by 1000000
1e−5
Other COVID-19 pandemic-related outcomes
COVID-19
Self-harm and interpersonal violence
Unintentional injuries
Transport injuries
Other non-communicable diseases
Musculoskeletal disorders
Sense organ diseases
Skin and subcutaneous diseases
Diabetes and kidney diseases
Substance use disorders
Mental disorders
Neurological disorders
Digestive diseases
Chronic respiratory diseases
Cardiovascular diseases
Neoplasms
Nutritional deficiencies
Maternal and neonatal disorders
Other infectious diseases
Neglected tropical diseases and malaria
Enteric infections
Respiratory infections and tuberculosis
HIV/AIDS and sexually transmitted infections

Northern Mariana Islands

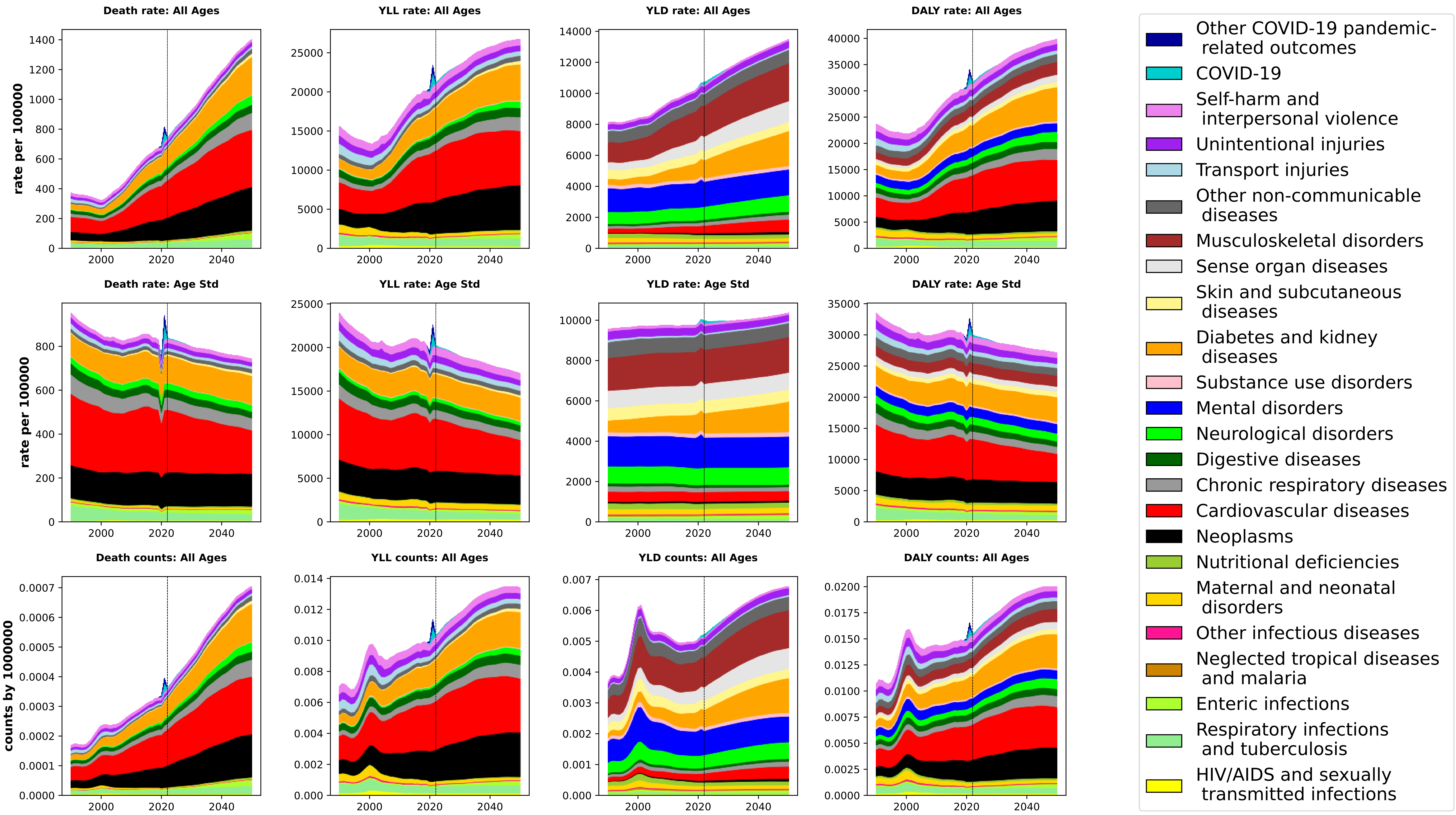

Palau

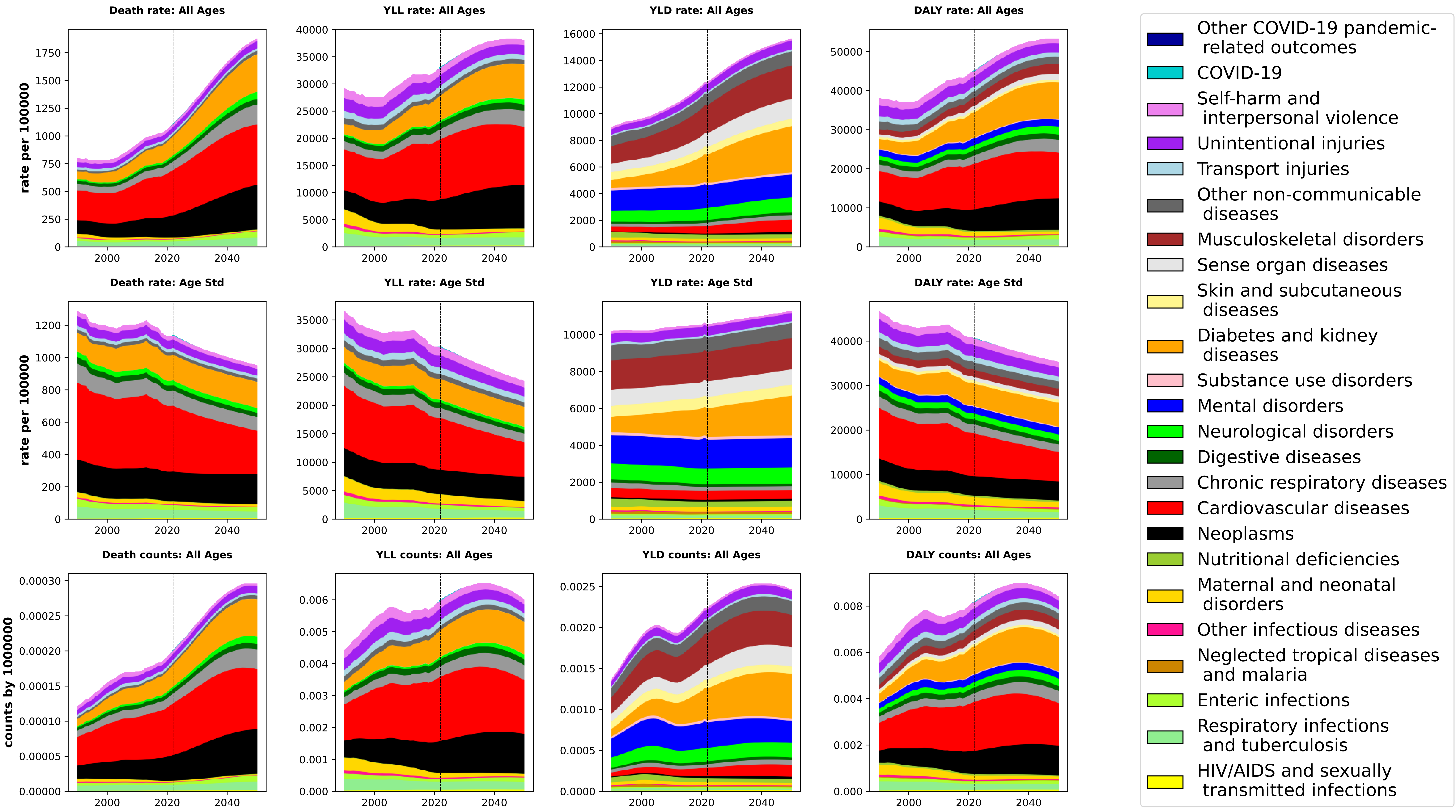
Death rate: All Ages
YLL rate: All Ages
YLD rate: All Ages
DALY rate: All Ages
Death rate: Age Std
YLL rate: Age Std
YLD rate: Age Std
DALY rate: Age Std
Death counts: All Ages
YLL counts: All Ages
YLD counts: All Ages
DALY counts: All Ages
rate per 100000
counts by 1000000
Other COVID-19 pandemic-related outcomes
COVID-19
Self-harm and interpersonal violence
Unintentional injuries
Transport injuries
Other non-communicable diseases
Musculoskeletal disorders
Sense organ diseases
Skin and subcutaneous diseases
Diabetes and kidney diseases
Substance use disorders
Mental disorders
Neurological disorders
Digestive diseases
Chronic respiratory diseases
Cardiovascular diseases
Neoplasms
Nutritional deficiencies
Maternal and neonatal disorders
Other infectious diseases
Neglected tropical diseases and malaria
Enteric infections
Respiratory infections and tuberculosis
HIV/AIDS and sexually transmitted infections

Papua New Guinea

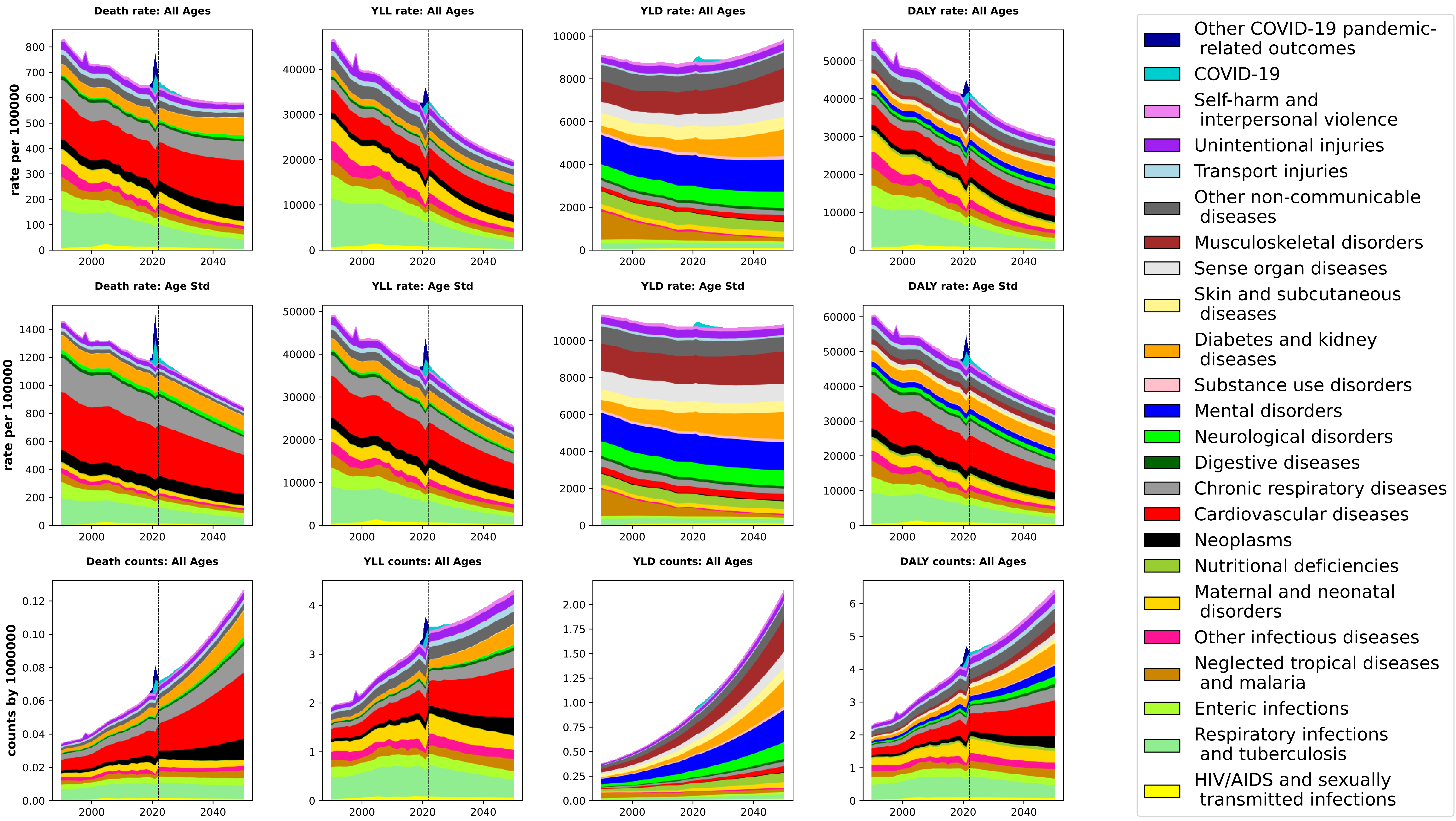

Samoa

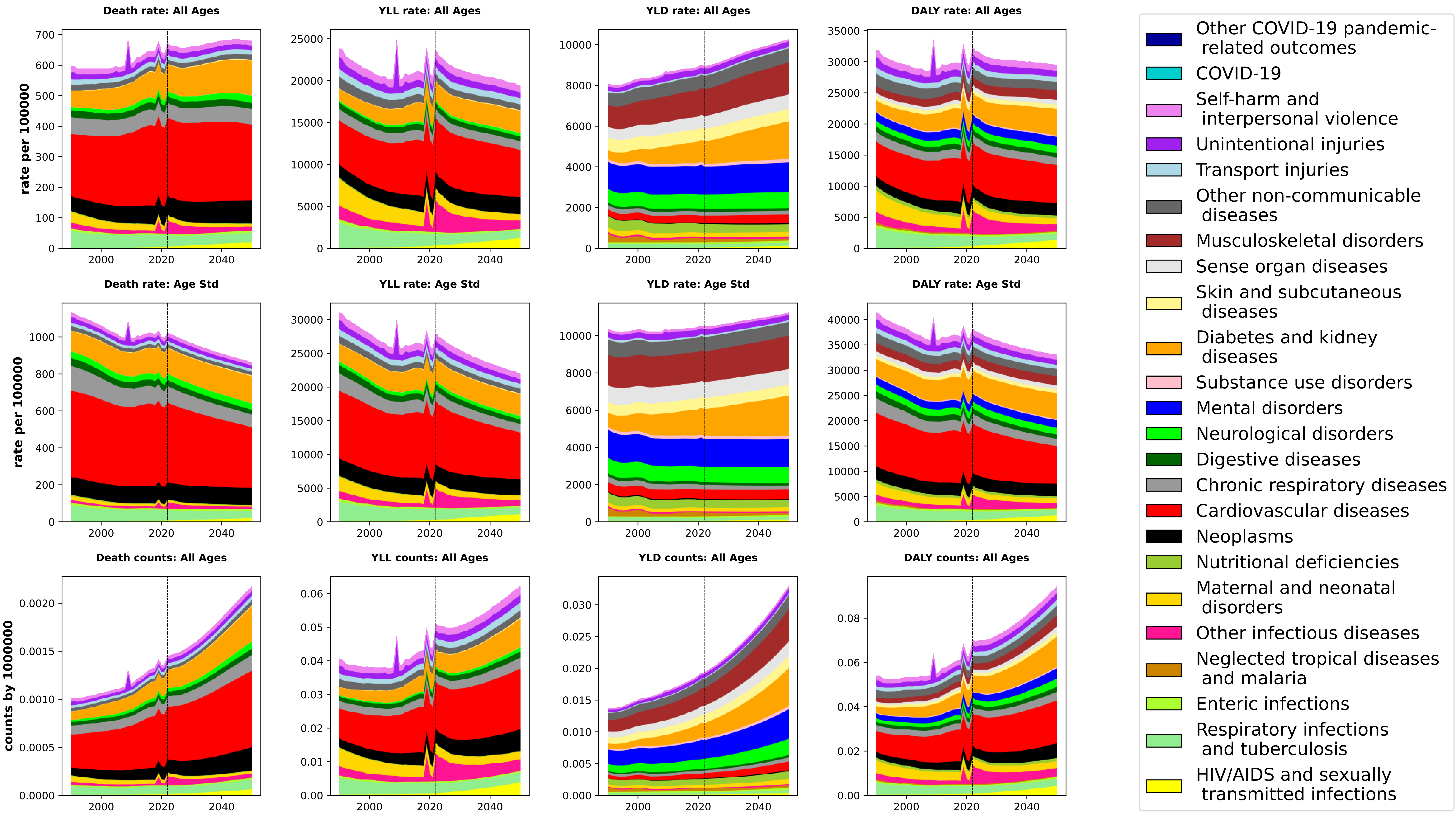
Death rate: All Ages
YLL rate: All Ages
YLD rate: All Ages
DALY rate: All Ages
Death rate: Age Std
YLL rate: Age Std
YLD rate: Age Std
DALY rate: Age Std
Death counts: All Ages
YLL counts: All Ages
YLD counts: All Ages
DALY counts: All Ages
rate per 100000
rate per 100000
counts by 1000000
2000
2020
2040
Other COVID-19 pandemic-related outcomes
COVID-19
Self-harm and interpersonal violence
Unintentional injuries
Transport injuries
Other non-communicable diseases
Musculoskeletal disorders
Sense organ diseases
Skin and subcutaneous diseases
Diabetes and kidney diseases
Substance use disorders
Mental disorders
Neurological disorders
Digestive diseases
Chronic respiratory diseases
Cardiovascular diseases
Neoplasms
Nutritional deficiencies
Maternal and neonatal disorders
Other infectious diseases
Neglected tropical diseases and malaria
Enteric infections
Respiratory infections and tuberculosis
HIV/AIDS and sexually transmitted infections

Solomon Islands

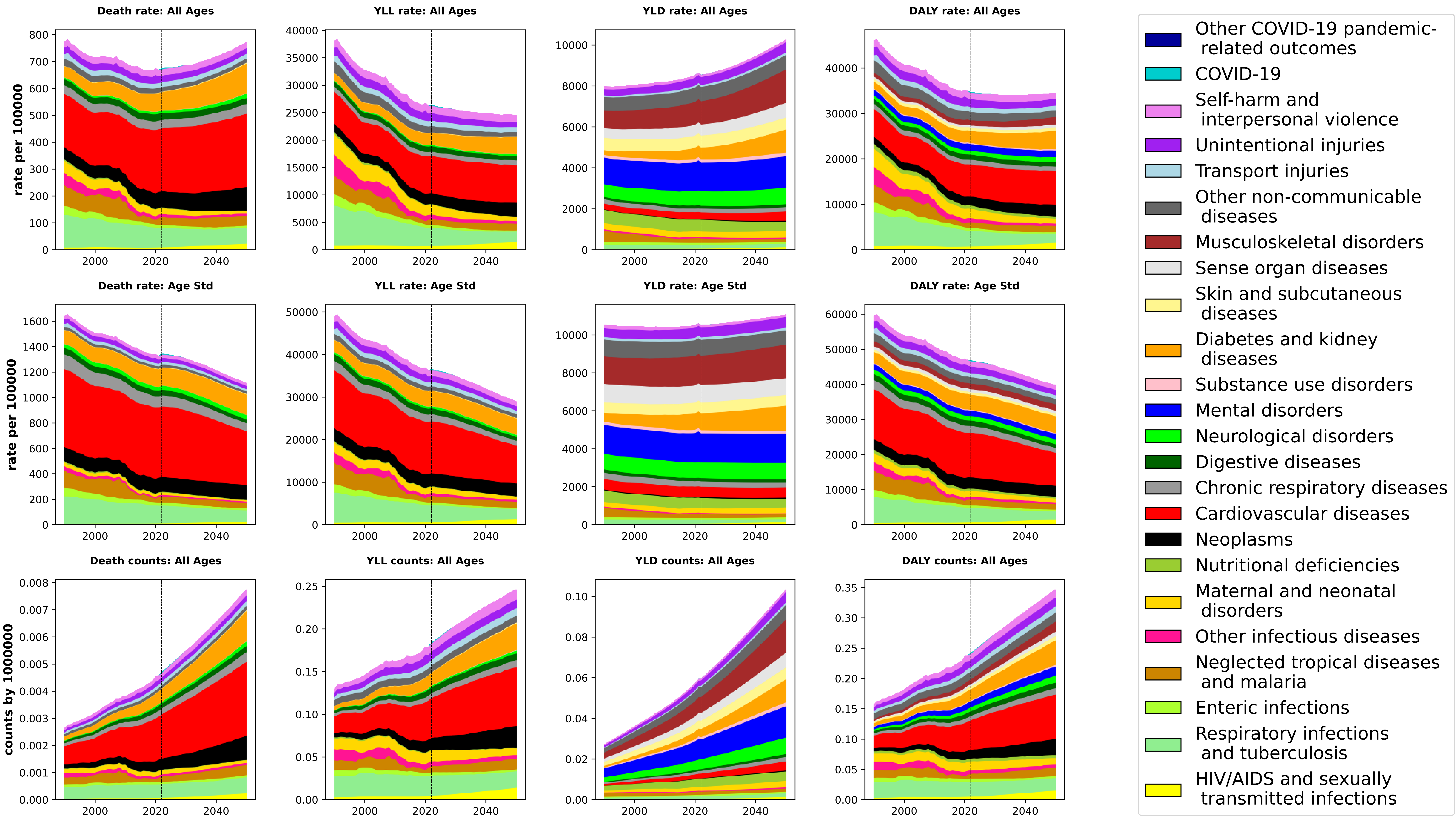

Tokelau

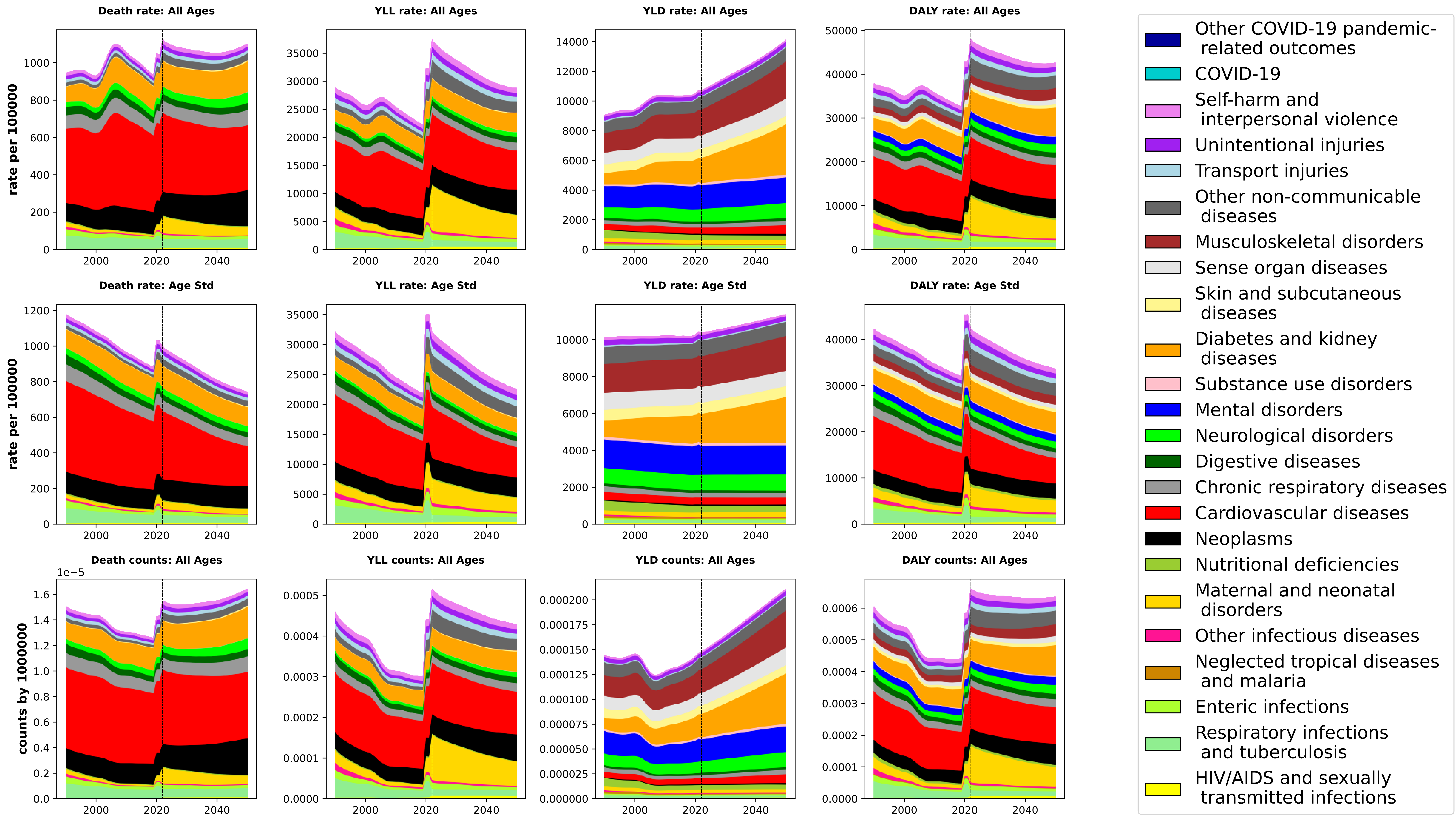
Death rate: All Ages
YLL rate: All Ages
YLD rate: All Ages
DALY rate: All Ages
Death rate: Age Std
YLL rate: Age Std
YLD rate: Age Std
DALY rate: Age Std
Death counts: All Ages
YLL counts: All Ages
YLD counts: All Ages
DALY counts: All Ages
rate per 100000
counts by 1000000
1e−5
2000
2020
2040
Other COVID-19 pandemic-related outcomes
COVID-19
Self-harm and interpersonal violence
Unintentional injuries
Transport injuries
Other non-communicable diseases
Musculoskeletal disorders
Sense organ diseases
Skin and subcutaneous diseases
Diabetes and kidney diseases
Substance use disorders
Mental disorders
Neurological disorders
Digestive diseases
Chronic respiratory diseases
Cardiovascular diseases
Neoplasms
Nutritional deficiencies
Maternal and neonatal disorders
Other infectious diseases
Neglected tropical diseases and malaria
Enteric infections
Respiratory infections and tuberculosis
HIV/AIDS and sexually transmitted infections

Tonga

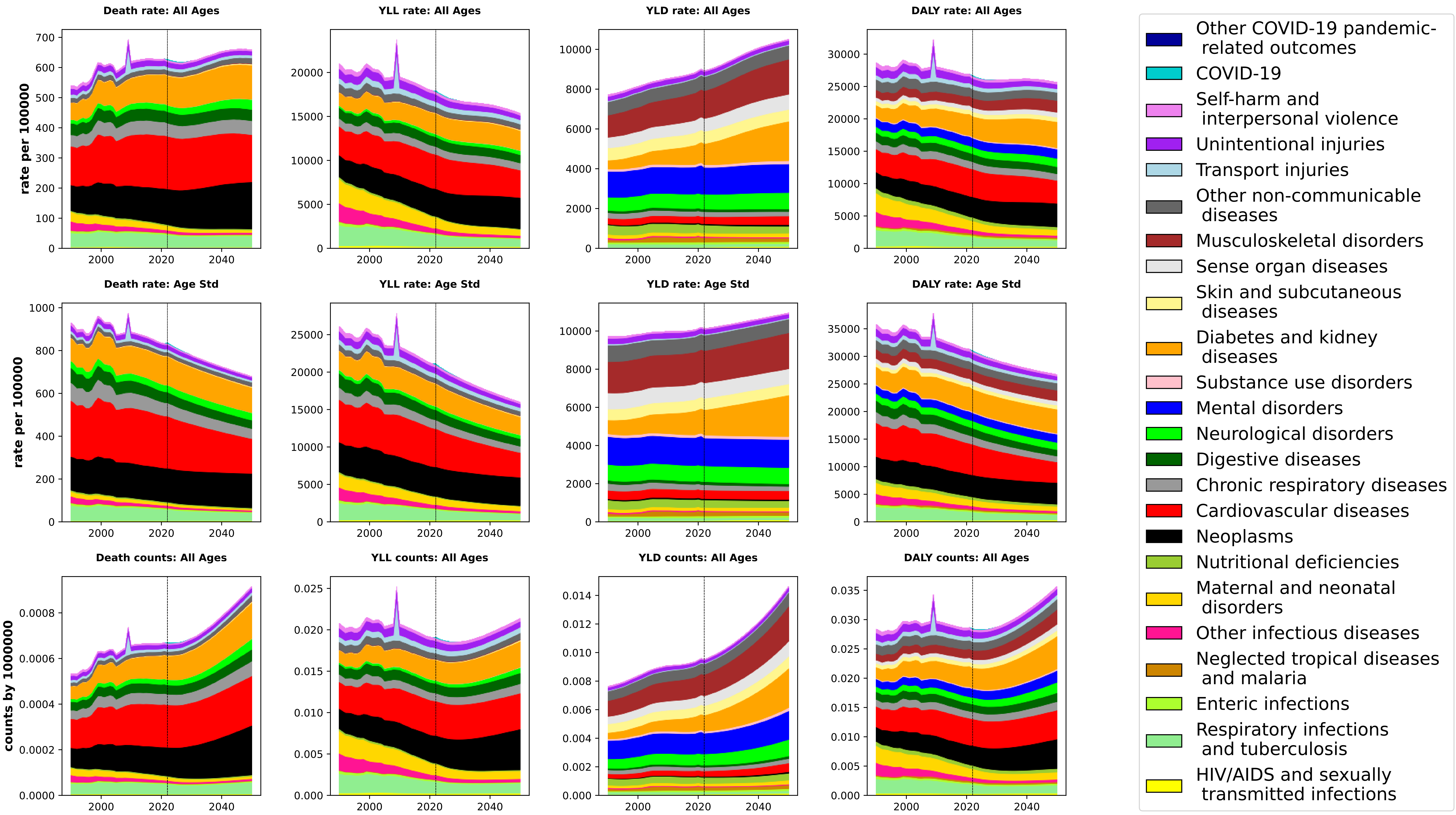
Death rate: All Ages
YLL rate: All Ages
YLD rate: All Ages
DALY rate: All Ages
Death rate: Age Std
YLL rate: Age Std
YLD rate: Age Std
DALY rate: Age Std
Death counts: All Ages
YLL counts: All Ages
YLD counts: All Ages
DALY counts: All Ages
rate per 100000
rate per 100000
counts by 1000000
2000
2020
2040
Other COVID-19 pandemic-related outcomes
COVID-19
Self-harm and interpersonal violence
Unintentional injuries
Transport injuries
Other non-communicable diseases
Musculoskeletal disorders
Sense organ diseases
Skin and subcutaneous diseases
Diabetes and kidney diseases
Substance use disorders
Mental disorders
Neurological disorders
Digestive diseases
Chronic respiratory diseases
Cardiovascular diseases
Neoplasms
Nutritional deficiencies
Maternal and neonatal disorders
Other infectious diseases
Neglected tropical diseases and malaria
Enteric infections
Respiratory infections and tuberculosis
HIV/AIDS and sexually transmitted infections

Tuvalu

Supplemental Results Figure S6. Cause level 2 area plots for 4 burden measures for reference scenario by location, 1990-2050

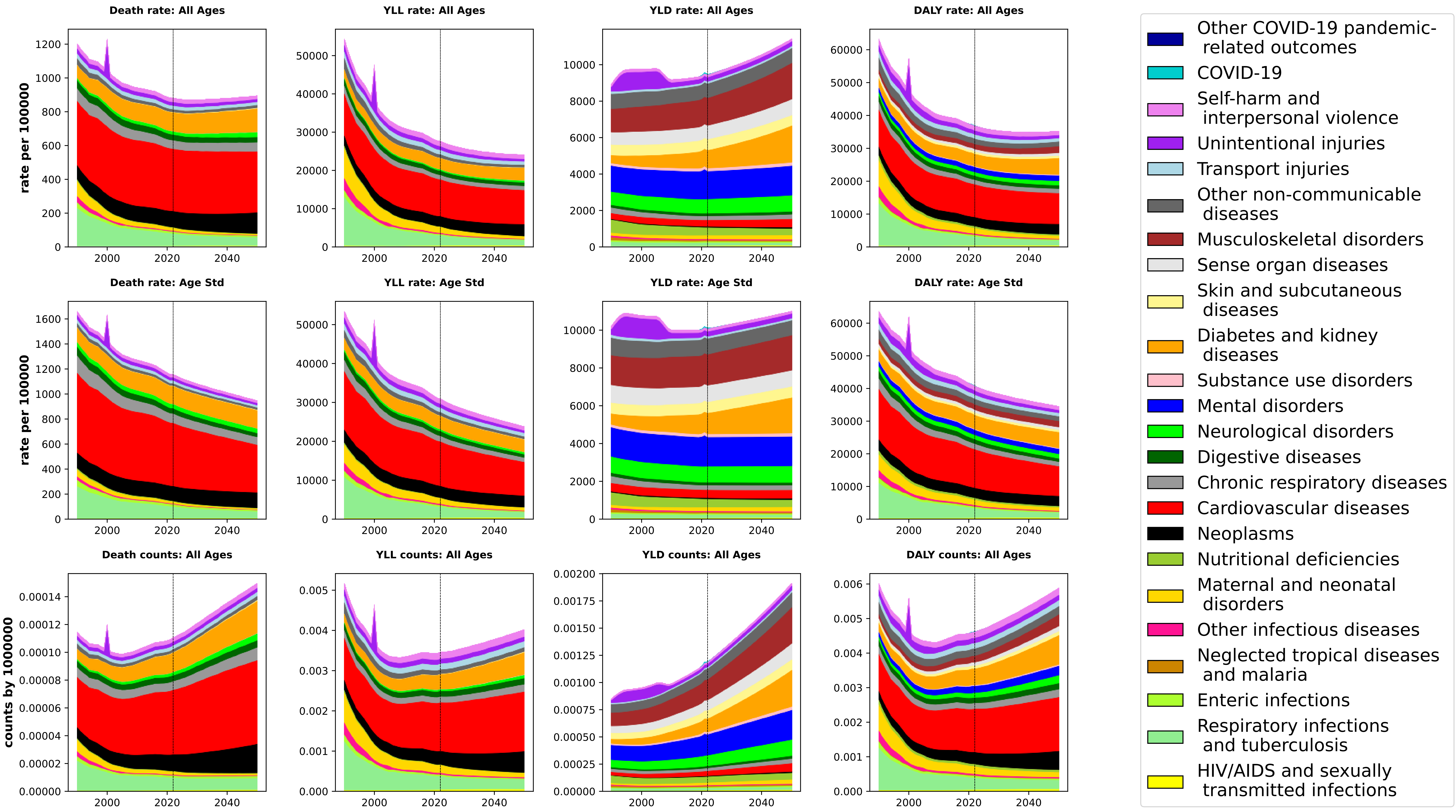

Vanuatu

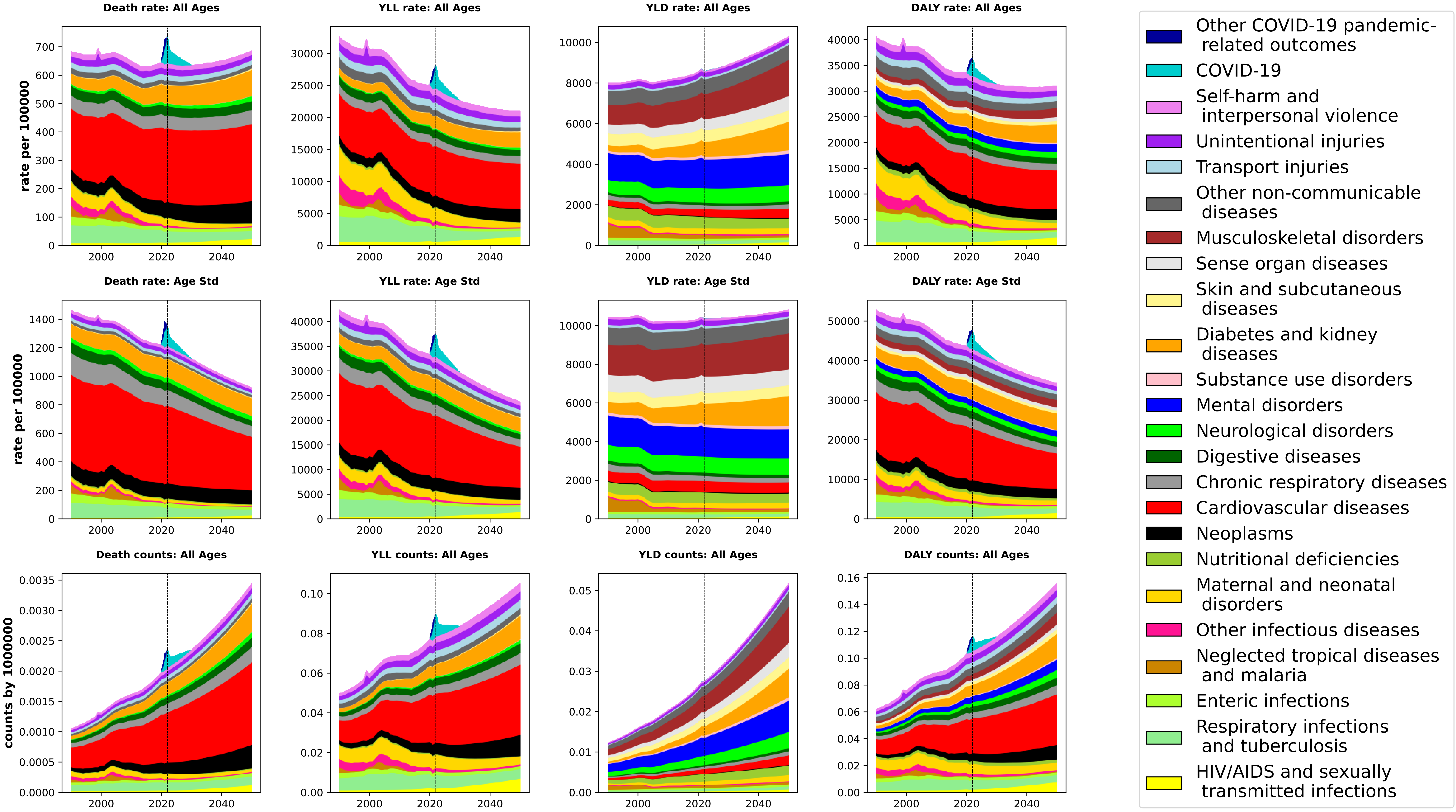
Death rate: All Ages
YLL rate: All Ages
YLD rate: All Ages
DALY rate: All Ages
Death rate: Age Std
YLL rate: Age Std
YLD rate: Age Std
DALY rate: Age Std
Death counts: All Ages
YLL counts: All Ages
YLD counts: All Ages
DALY counts: All Ages
rate per 100000
rate per 100000
counts by 1000000
Other COVID-19 pandemic-related outcomes
COVID-19
Self-harm and interpersonal violence
Unintentional injuries
Transport injuries
Other non-communicable diseases
Musculoskeletal disorders
Sense organ diseases
Skin and subcutaneous diseases
Diabetes and kidney diseases
Substance use disorders
Mental disorders
Neurological disorders
Digestive diseases
Chronic respiratory diseases
Cardiovascular diseases
Neoplasms
Nutritional deficiencies
Maternal and neonatal disorders
Other infectious diseases
Neglected tropical diseases and malaria
Enteric infections
Respiratory infections and tuberculosis
HIV/AIDS and sexually transmitted infections

Cambodia

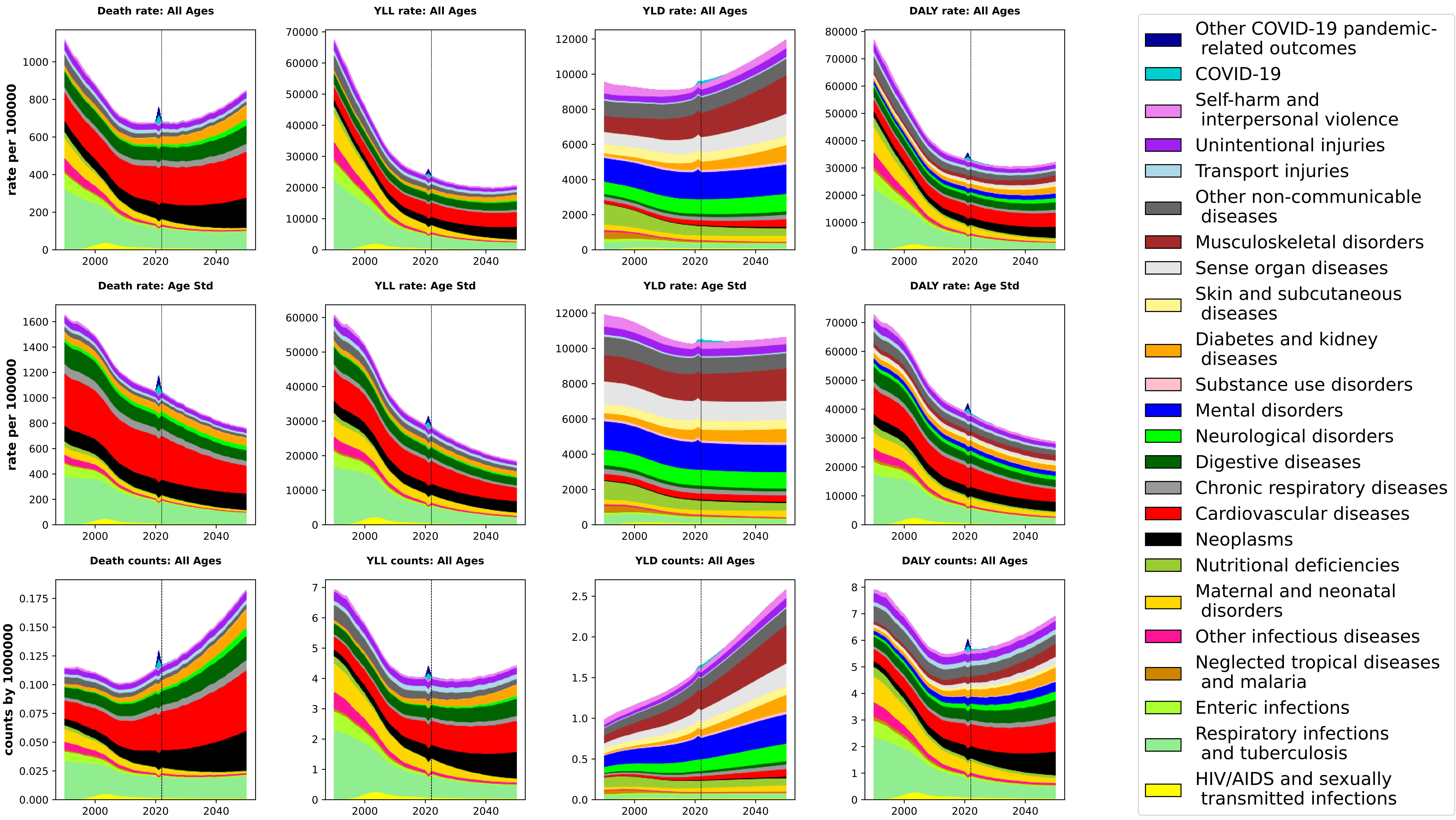

Death rate: All Ages
YLL rate: All Ages
YLD rate: All Ages
DALY rate: All Ages
Death rate: Age Std
YLL rate: Age Std
YLD rate: Age Std
DALY rate: Age Std
Death counts: All Ages
YLL counts: All Ages
YLD counts: All Ages
DALY counts: All Ages
rate per 100000
counts by 1000000
Other COVID-19 pandemic-related outcomes
COVID-19
Self-harm and interpersonal violence
Unintentional injuries
Transport injuries
Other non-communicable diseases
Musculoskeletal disorders
Sense organ diseases
Skin and subcutaneous diseases
Diabetes and kidney diseases
Substance use disorders
Mental disorders
Neurological disorders
Digestive diseases
Chronic respiratory diseases
Cardiovascular diseases
Neoplasms
Nutritional deficiencies
Maternal and neonatal disorders
Other infectious diseases
Neglected tropical diseases and malaria
Enteric infections
Respiratory infections and tuberculosis
HIV/AIDS and sexually transmitted infections

Indonesia

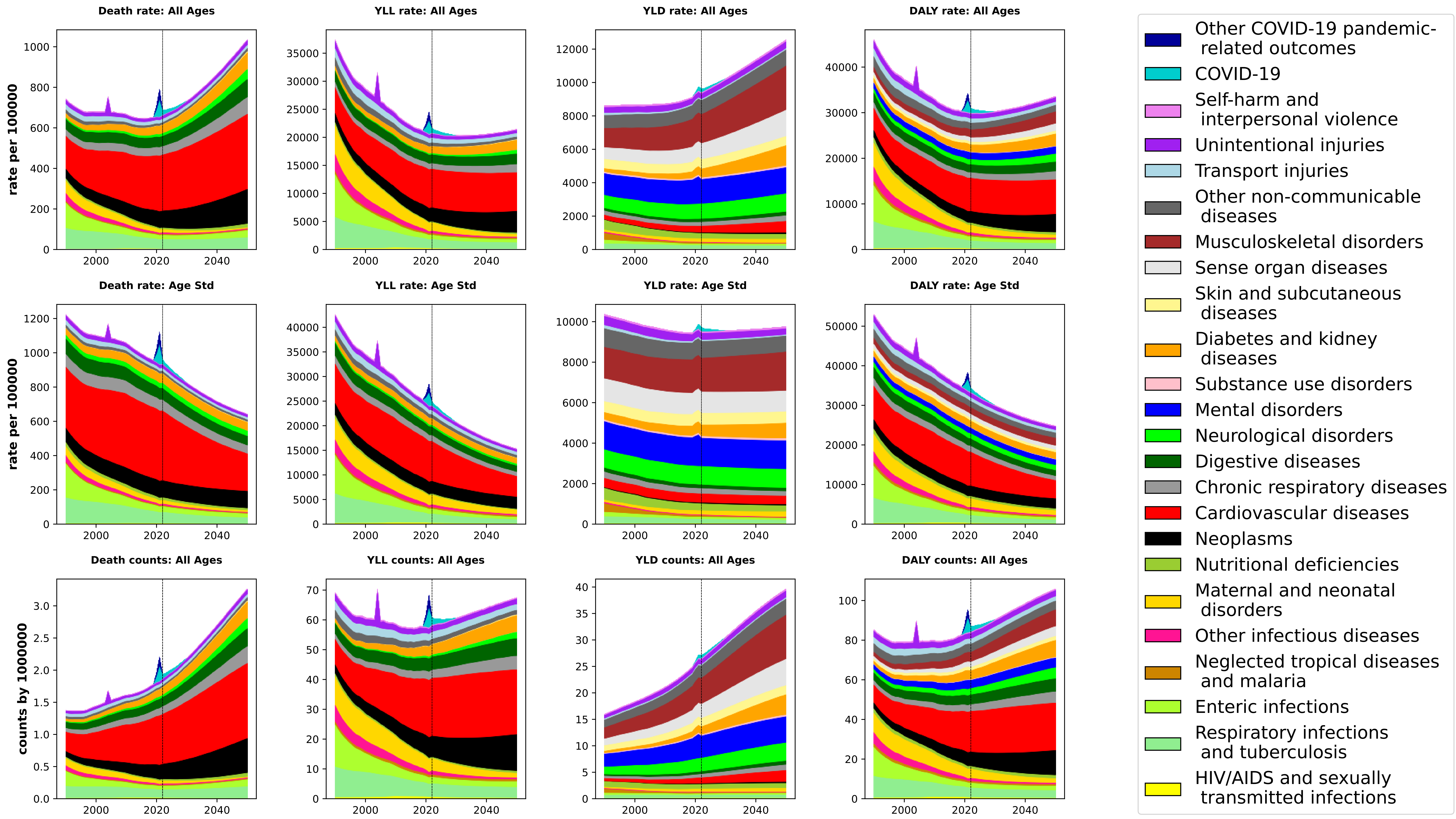
Death rate: All Ages
YLL rate: All Ages
YLD rate: All Ages
DALY rate: All Ages
Death rate: Age Std
YLL rate: Age Std
YLD rate: Age Std
DALY rate: Age Std
Death counts: All Ages
YLL counts: All Ages
YLD counts: All Ages
DALY counts: All Ages
rate per 100000
counts by 1000000
Other COVID-19 pandemic-related outcomes
COVID-19
Self-harm and interpersonal violence
Unintentional injuries
Transport injuries
Other non-communicable diseases
Musculoskeletal disorders
Sense organ diseases
Skin and subcutaneous diseases
Diabetes and kidney diseases
Substance use disorders
Mental disorders
Neurological disorders
Digestive diseases
Chronic respiratory diseases
Cardiovascular diseases
Neoplasms
Nutritional deficiencies
Maternal and neonatal disorders
Other infectious diseases
Neglected tropical diseases and malaria
Enteric infections
Respiratory infections and tuberculosis
HIV/AIDS and sexually transmitted infections

Laos

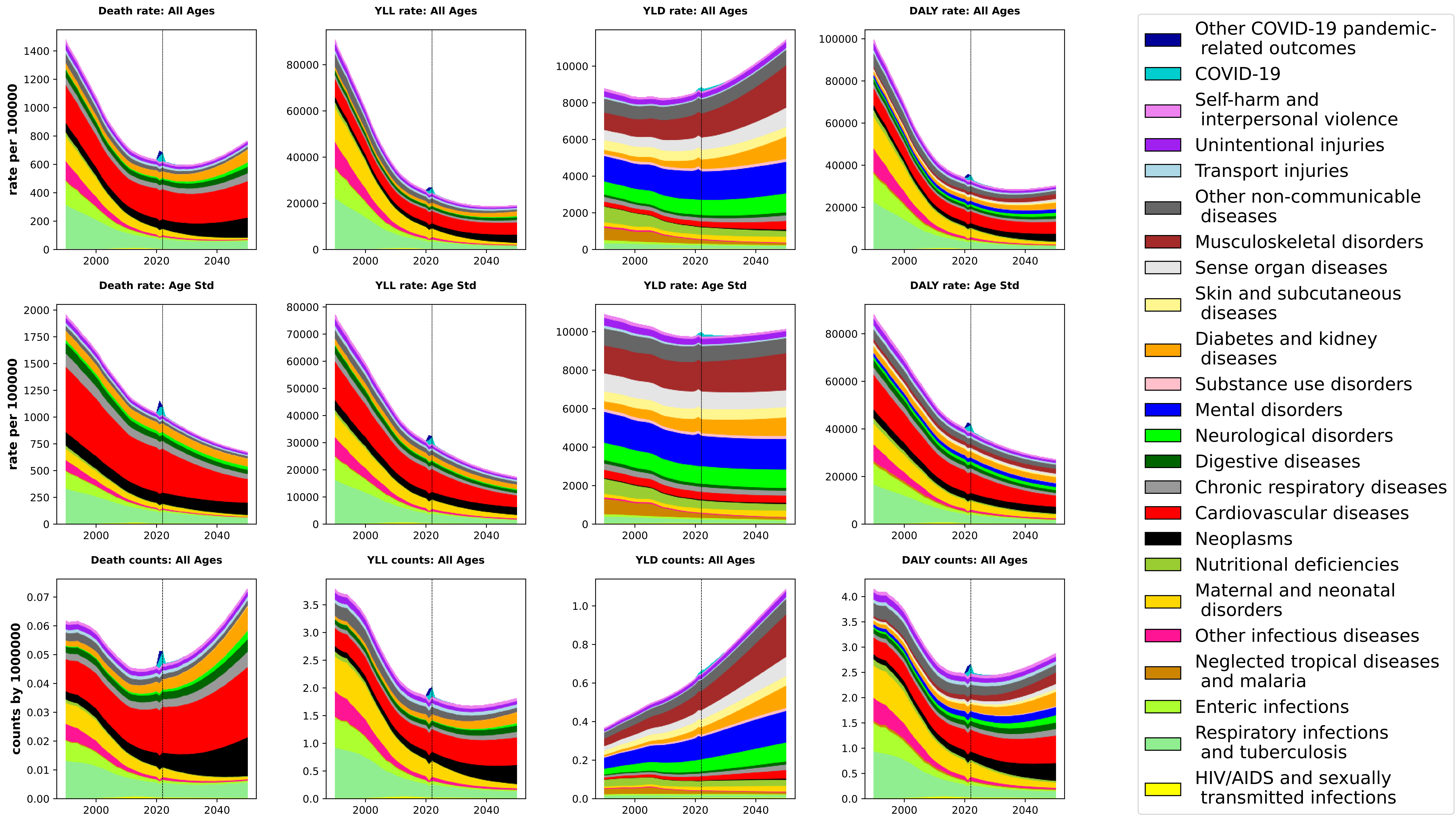
Death rate: All Ages
YLL rate: All Ages
YLD rate: All Ages
DALY rate: All Ages
Death rate: Age Std
YLL rate: Age Std
YLD rate: Age Std
DALY rate: Age Std
Death counts: All Ages
YLL counts: All Ages
YLD counts: All Ages
DALY counts: All Ages
rate per 100000
counts by 1000000
Other COVID-19 pandemic-related outcomes
COVID-19
Self-harm and interpersonal violence
Unintentional injuries
Transport injuries
Other non-communicable diseases
Musculoskeletal disorders
Sense organ diseases
Skin and subcutaneous diseases
Diabetes and kidney diseases
Substance use disorders
Mental disorders
Neurological disorders
Digestive diseases
Chronic respiratory diseases
Cardiovascular diseases
Neoplasms
Nutritional deficiencies
Maternal and neonatal disorders
Other infectious diseases
Neglected tropical diseases and malaria
Enteric infections
Respiratory infections and tuberculosis
HIV/AIDS and sexually transmitted infections

Malaysia

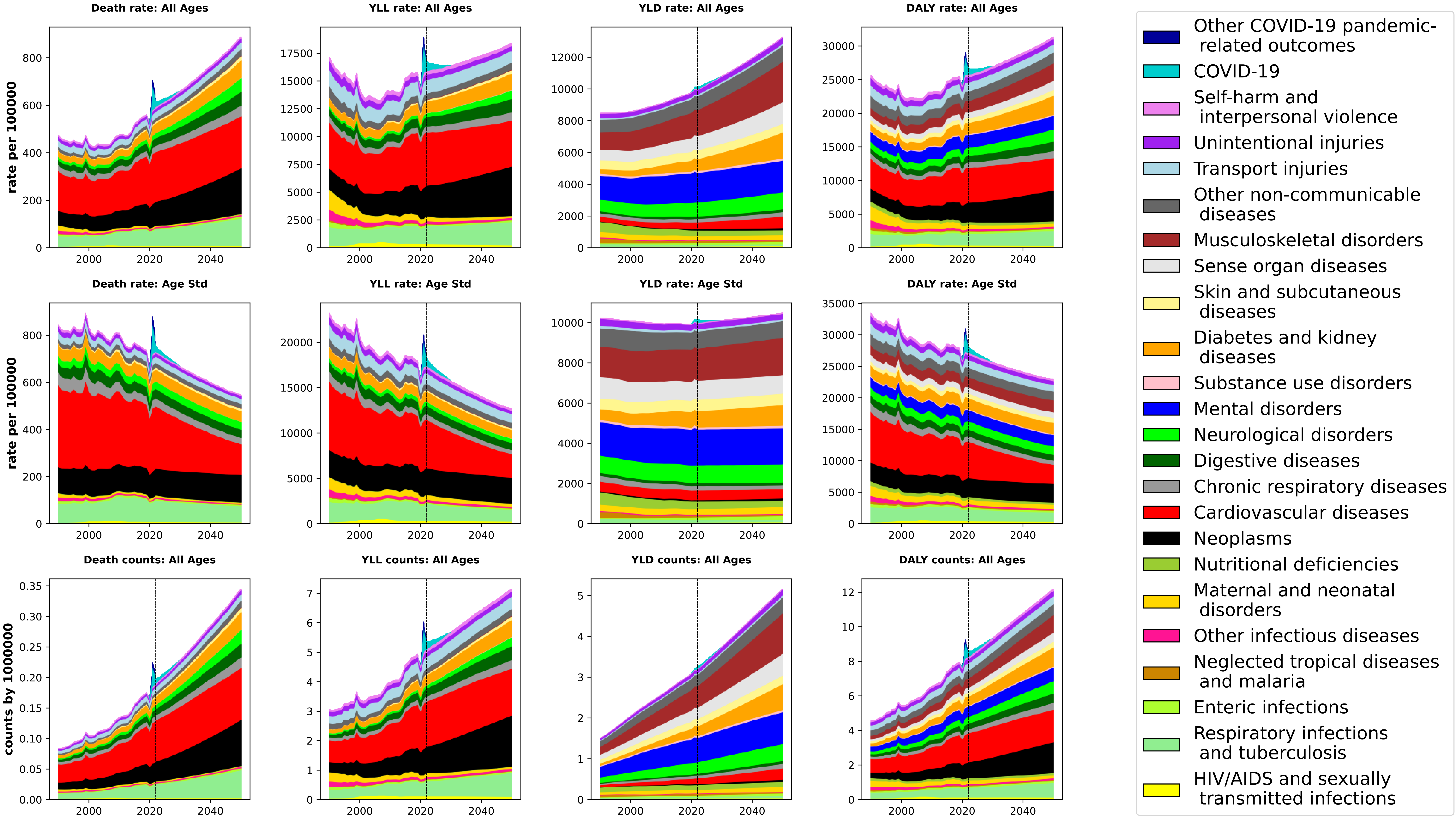
Death rate: All Ages
YLL rate: All Ages
YLD rate: All Ages
DALY rate: All Ages
Death rate: Age Std
YLL rate: Age Std
YLD rate: Age Std
DALY rate: Age Std
Death counts: All Ages
YLL counts: All Ages
YLD counts: All Ages
DALY counts: All Ages
rate per 100000
counts by 1000000
Other COVID-19 pandemic-related outcomes
COVID-19
Self-harm and interpersonal violence
Unintentional injuries
Transport injuries
Other non-communicable diseases
Musculoskeletal disorders
Sense organ diseases
Skin and subcutaneous diseases
Diabetes and kidney diseases
Substance use disorders
Mental disorders
Neurological disorders
Digestive diseases
Chronic respiratory diseases
Cardiovascular diseases
Neoplasms
Nutritional deficiencies
Maternal and neonatal disorders
Other infectious diseases
Neglected tropical diseases and malaria
Enteric infections
Respiratory infections and tuberculosis
HIV/AIDS and sexually transmitted infections

Maldives

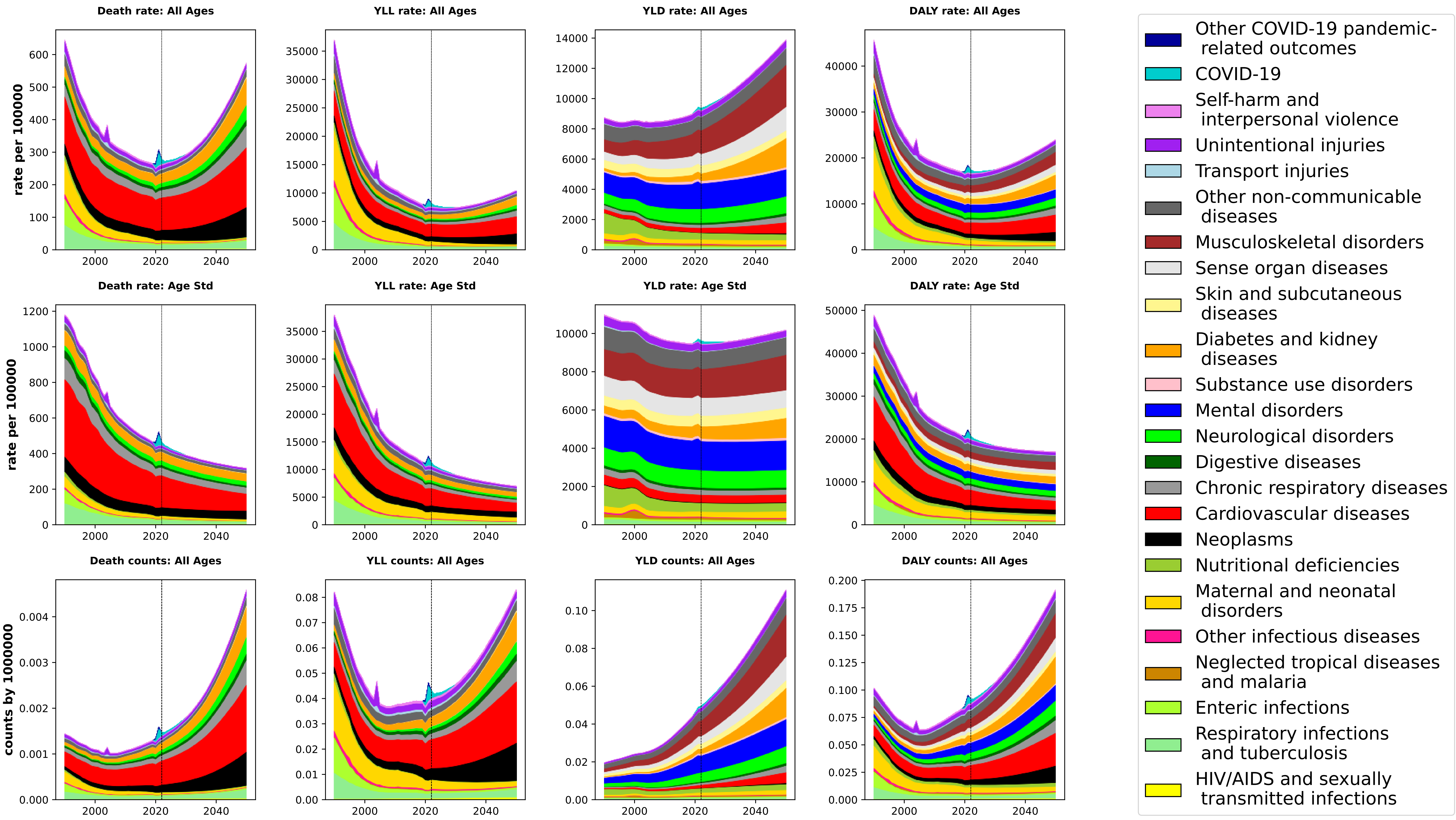
Death rate: All Ages
YLL rate: All Ages
YLD rate: All Ages
DALY rate: All Ages
Death rate: Age Std
YLL rate: Age Std
YLD rate: Age Std
DALY rate: Age Std
Death counts: All Ages
YLL counts: All Ages
YLD counts: All Ages
DALY counts: All Ages
rate per 100000
counts by 1000000
2000
2020
2040
Other COVID-19 pandemic-related outcomes
COVID-19
Self-harm and interpersonal violence
Unintentional injuries
Transport injuries
Other non-communicable diseases
Musculoskeletal disorders
Sense organ diseases
Skin and subcutaneous diseases
Diabetes and kidney diseases
Substance use disorders
Mental disorders
Neurological disorders
Digestive diseases
Chronic respiratory diseases
Cardiovascular diseases
Neoplasms
Nutritional deficiencies
Maternal and neonatal disorders
Other infectious diseases
Neglected tropical diseases and malaria
Enteric infections
Respiratory infections and tuberculosis
HIV/AIDS and sexually transmitted infections

Mauritius

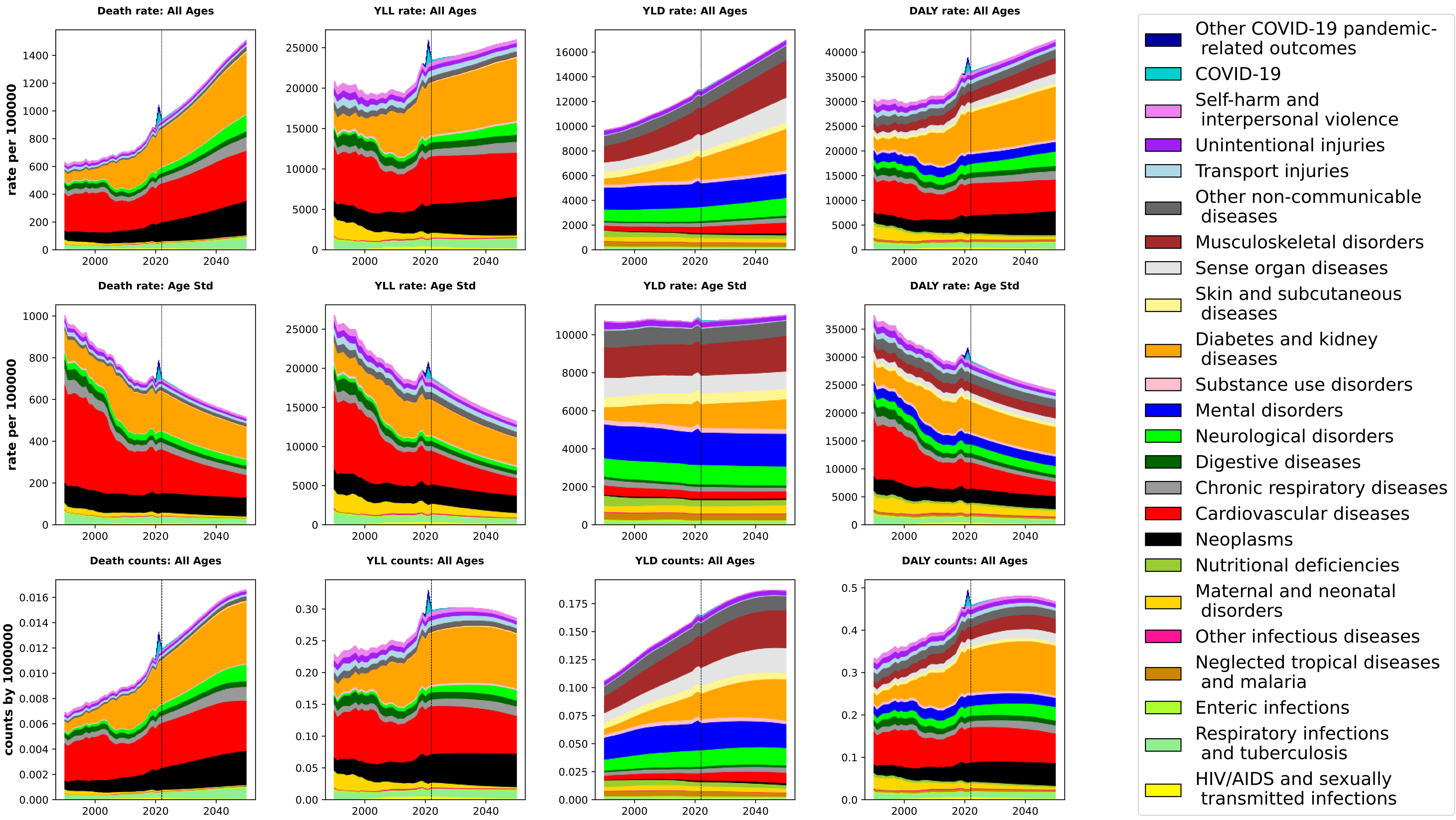
Death rate: All Ages
YLL rate: All Ages
YLD rate: All Ages
DALY rate: All Ages
Death rate: Age Std
YLL rate: Age Std
YLD rate: Age Std
DALY rate: Age Std
Death counts: All Ages
YLL counts: All Ages
YLD counts: All Ages
DALY counts: All Ages
rate per 100000
counts by 1000000
Other COVID-19 pandemic-related outcomes
COVID-19
Self-harm and interpersonal violence
Unintentional injuries
Transport injuries
Other non-communicable diseases
Musculoskeletal disorders
Sense organ diseases
Skin and subcutaneous diseases
Diabetes and kidney diseases
Substance use disorders
Mental disorders
Neurological disorders
Digestive diseases
Chronic respiratory diseases
Cardiovascular diseases
Neoplasms
Nutritional deficiencies
Maternal and neonatal disorders
Other infectious diseases
Neglected tropical diseases and malaria
Enteric infections
Respiratory infections and tuberculosis
HIV/AIDS and sexually transmitted infections

Myanmar

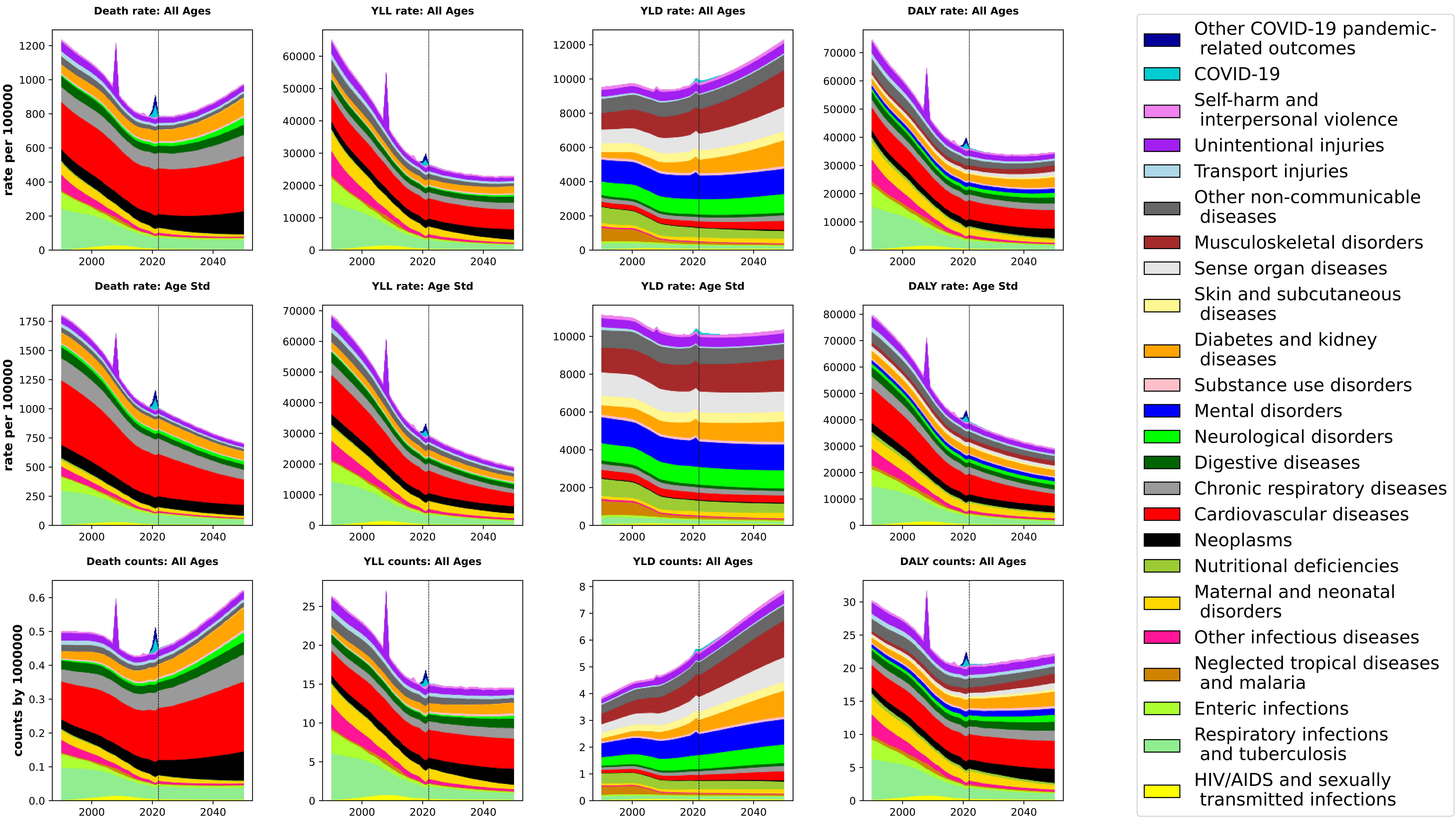
Death rate: All Ages
YLL rate: All Ages
YLD rate: All Ages
DALY rate: All Ages
Death rate: Age Std
YLL rate: Age Std
YLD rate: Age Std
DALY rate: Age Std
Death counts: All Ages
YLL counts: All Ages
YLD counts: All Ages
DALY counts: All Ages
rate per 100000
counts by 1000000
2000
2020
2040
Other COVID-19 pandemic-related outcomes
COVID-19
Self-harm and interpersonal violence
Unintentional injuries
Transport injuries
Other non-communicable diseases
Musculoskeletal disorders
Sense organ diseases
Skin and subcutaneous diseases
Diabetes and kidney diseases
Substance use disorders
Mental disorders
Neurological disorders
Digestive diseases
Chronic respiratory diseases
Cardiovascular diseases
Neoplasms
Nutritional deficiencies
Maternal and neonatal disorders
Other infectious diseases
Neglected tropical diseases and malaria
Enteric infections
Respiratory infections and tuberculosis
HIV/AIDS and sexually transmitted infections

Philippines

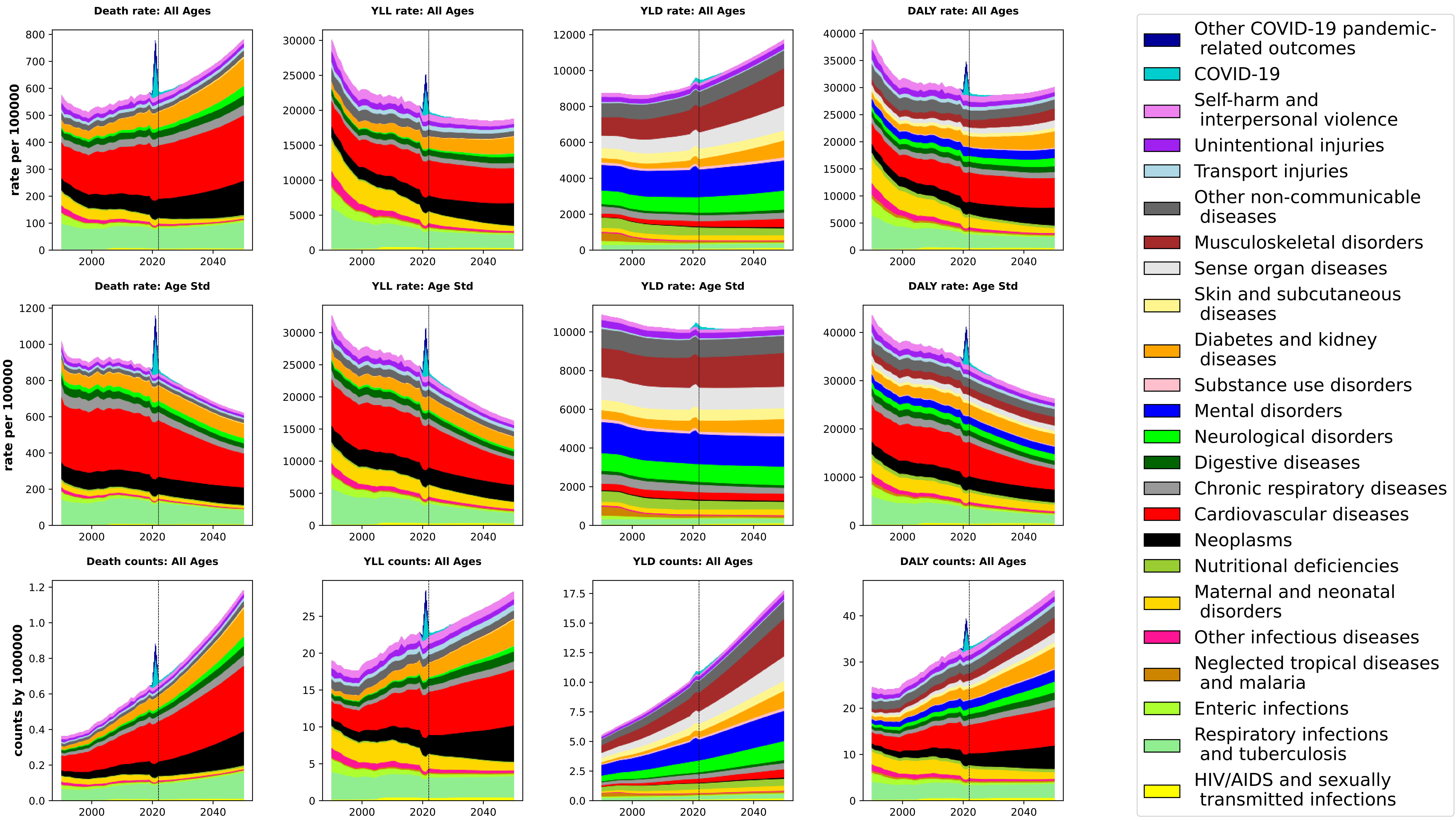

Seychelles

Supplemental Results Figure S6. Cause level 2 area plots for 4 burden measures for reference scenario by location, 1990-2050

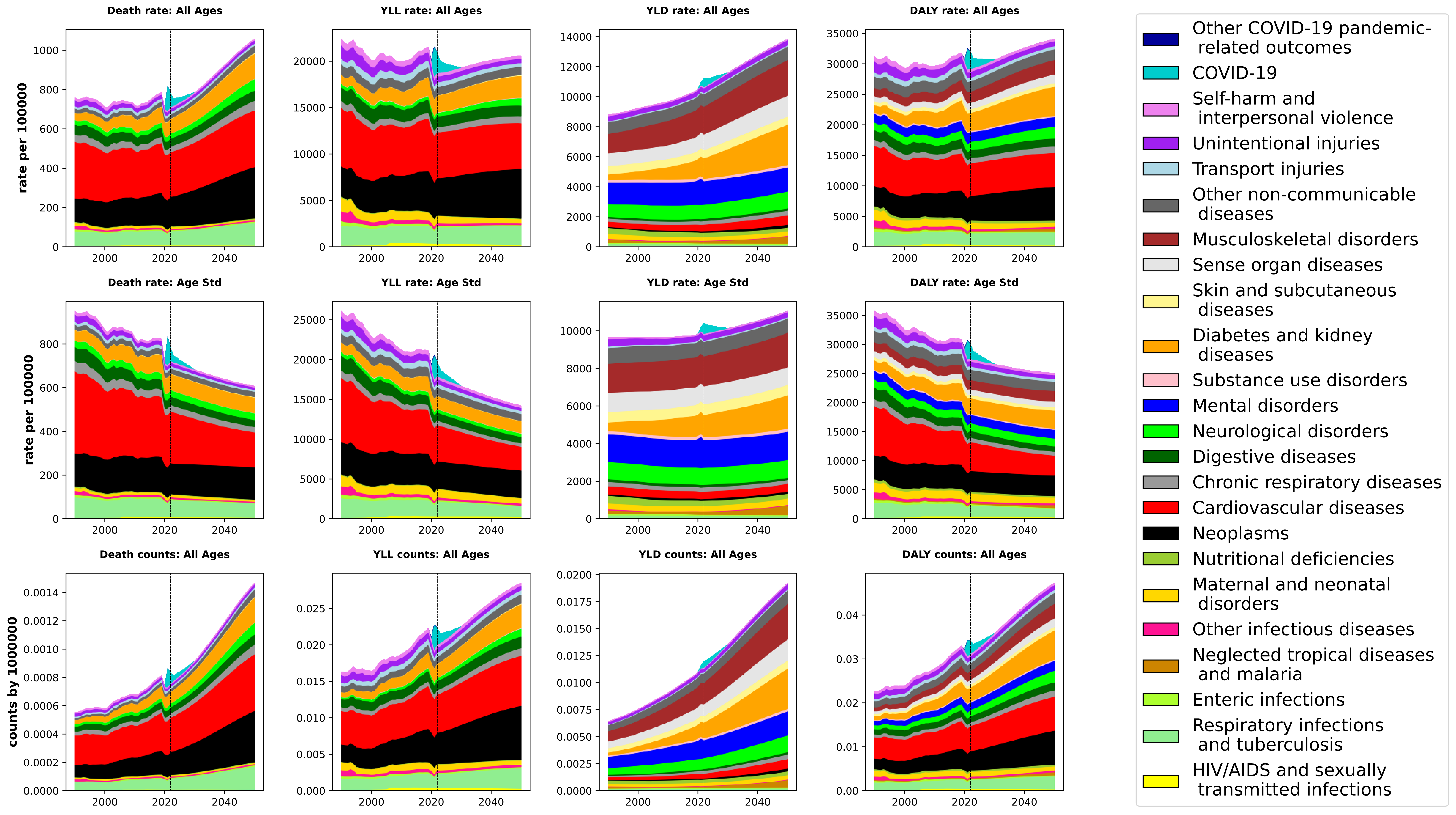

Sri Lanka

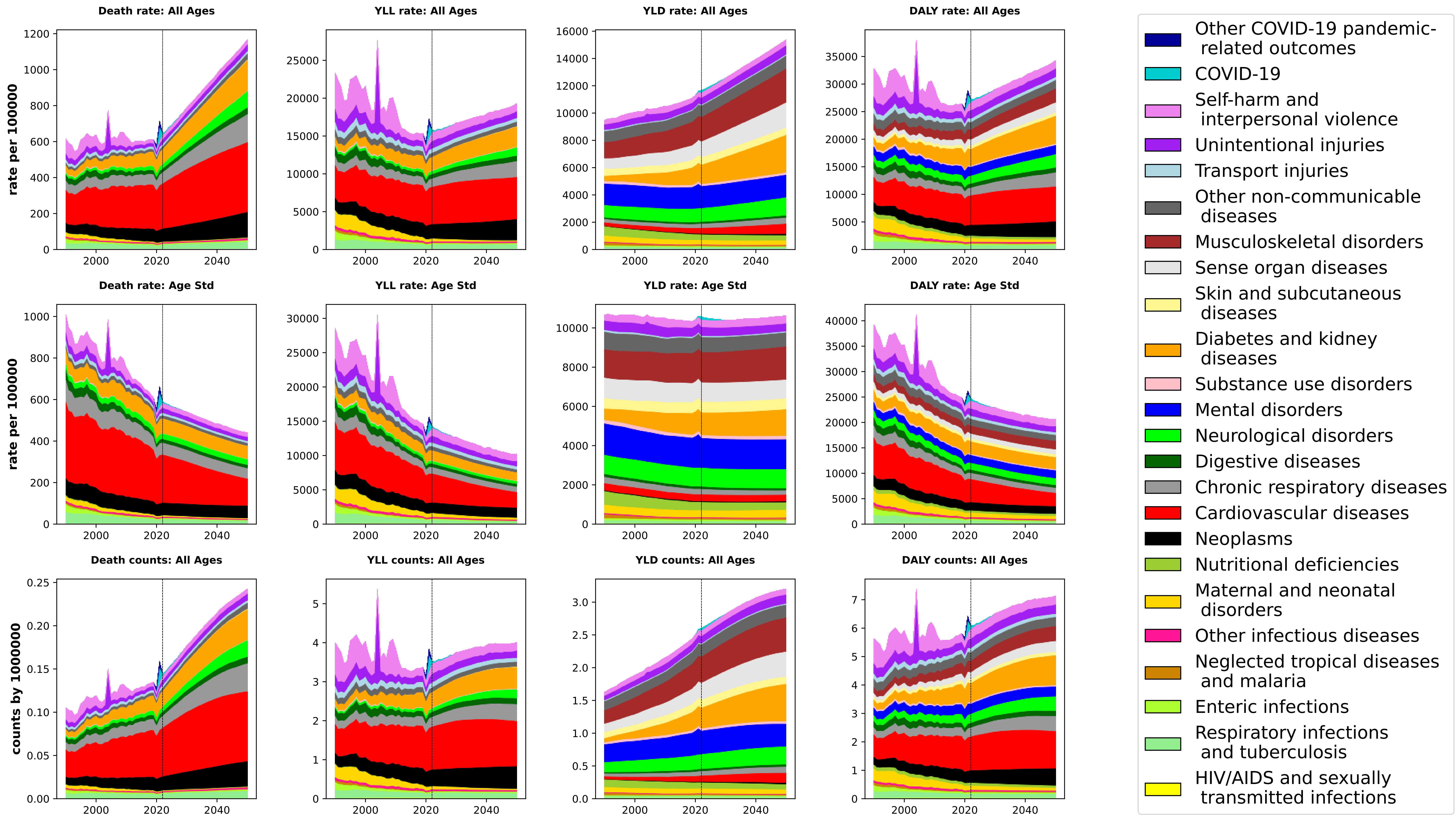
Death rate: All Ages
YLL rate: All Ages
YLD rate: All Ages
DALY rate: All Ages
Death rate: Age Std
YLL rate: Age Std
YLD rate: Age Std
DALY rate: Age Std
Death counts: All Ages
YLL counts: All Ages
YLD counts: All Ages
DALY counts: All Ages
rate per 100000
counts by 1000000
Other COVID-19 pandemic-related outcomes
COVID-19
Self-harm and interpersonal violence
Unintentional injuries
Transport injuries
Other non-communicable diseases
Musculoskeletal disorders
Sense organ diseases
Skin and subcutaneous diseases
Diabetes and kidney diseases
Substance use disorders
Mental disorders
Neurological disorders
Digestive diseases
Chronic respiratory diseases
Cardiovascular diseases
Neoplasms
Nutritional deficiencies
Maternal and neonatal disorders
Other infectious diseases
Neglected tropical diseases and malaria
Enteric infections
Respiratory infections and tuberculosis
HIV/AIDS and sexually transmitted infections

Thailand

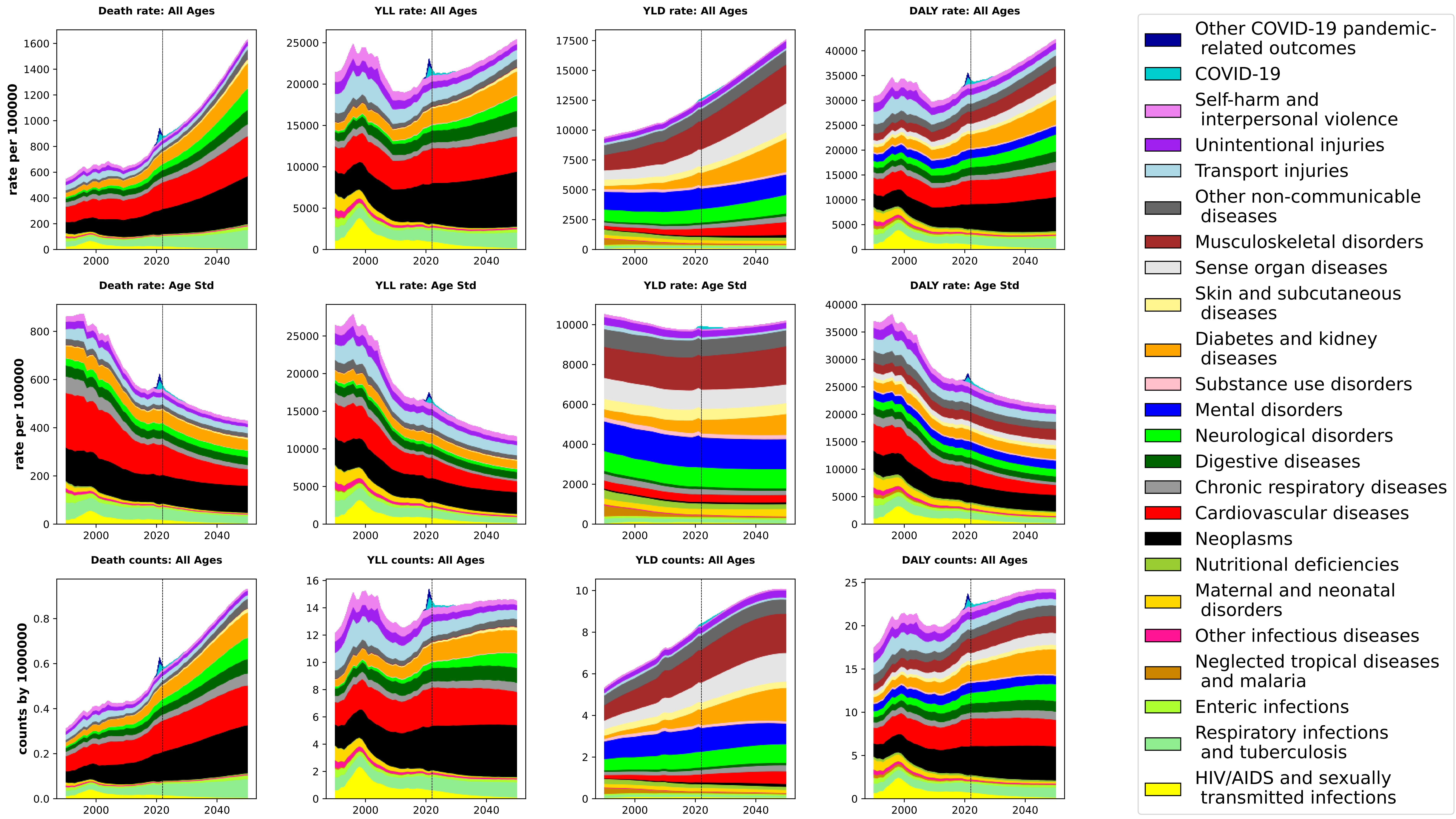
Death rate: All Ages
YLL rate: All Ages
YLD rate: All Ages
DALY rate: All Ages
Death rate: Age Std
YLL rate: Age Std
YLD rate: Age Std
DALY rate: Age Std
Death counts: All Ages
YLL counts: All Ages
YLD counts: All Ages
DALY counts: All Ages
rate per 100000
counts by 1000000
2000
2020
2040
Other COVID-19 pandemic-related outcomes
COVID-19
Self-harm and interpersonal violence
Unintentional injuries
Transport injuries
Other non-communicable diseases
Musculoskeletal disorders
Sense organ diseases
Skin and subcutaneous diseases
Diabetes and kidney diseases
Substance use disorders
Mental disorders
Neurological disorders
Digestive diseases
Chronic respiratory diseases
Cardiovascular diseases
Neoplasms
Nutritional deficiencies
Maternal and neonatal disorders
Other infectious diseases
Neglected tropical diseases and malaria
Enteric infections
Respiratory infections and tuberculosis
HIV/AIDS and sexually transmitted infections

Timor-Leste

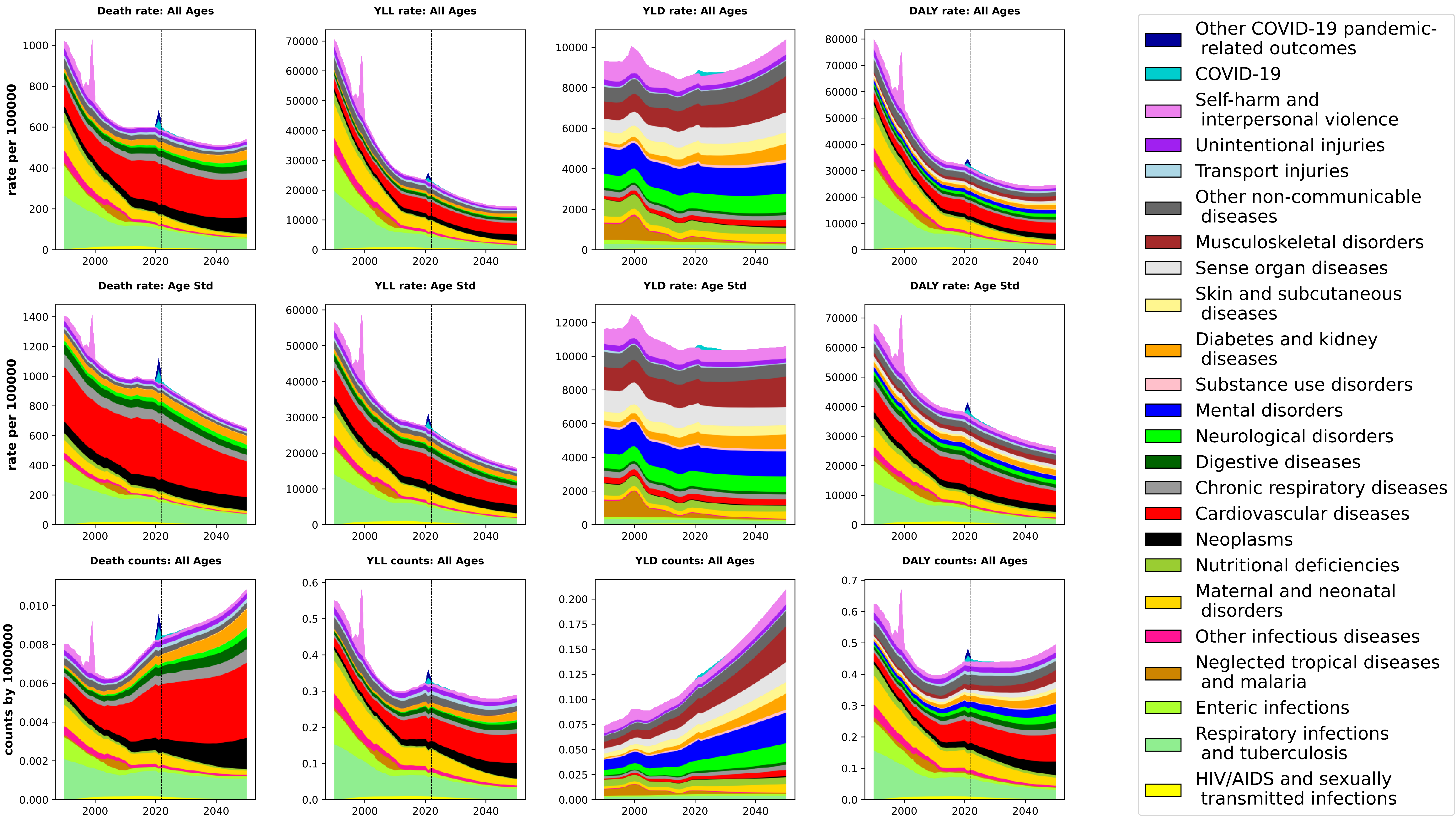
Death rate: All Ages
YLL rate: All Ages
YLD rate: All Ages
DALY rate: All Ages
Death rate: Age Std
YLL rate: Age Std
YLD rate: Age Std
DALY rate: Age Std
Death counts: All Ages
YLL counts: All Ages
YLD counts: All Ages
DALY counts: All Ages
rate per 100000
counts by 1000000
2000
2020
2040
Other COVID-19 pandemic-related outcomes
COVID-19
Self-harm and interpersonal violence
Unintentional injuries
Transport injuries
Other non-communicable diseases
Musculoskeletal disorders
Sense organ diseases
Skin and subcutaneous diseases
Diabetes and kidney diseases
Substance use disorders
Mental disorders
Neurological disorders
Digestive diseases
Chronic respiratory diseases
Cardiovascular diseases
Neoplasms
Nutritional deficiencies
Maternal and neonatal disorders
Other infectious diseases
Neglected tropical diseases and malaria
Enteric infections
Respiratory infections and tuberculosis
HIV/AIDS and sexually transmitted infections

Viet Nam

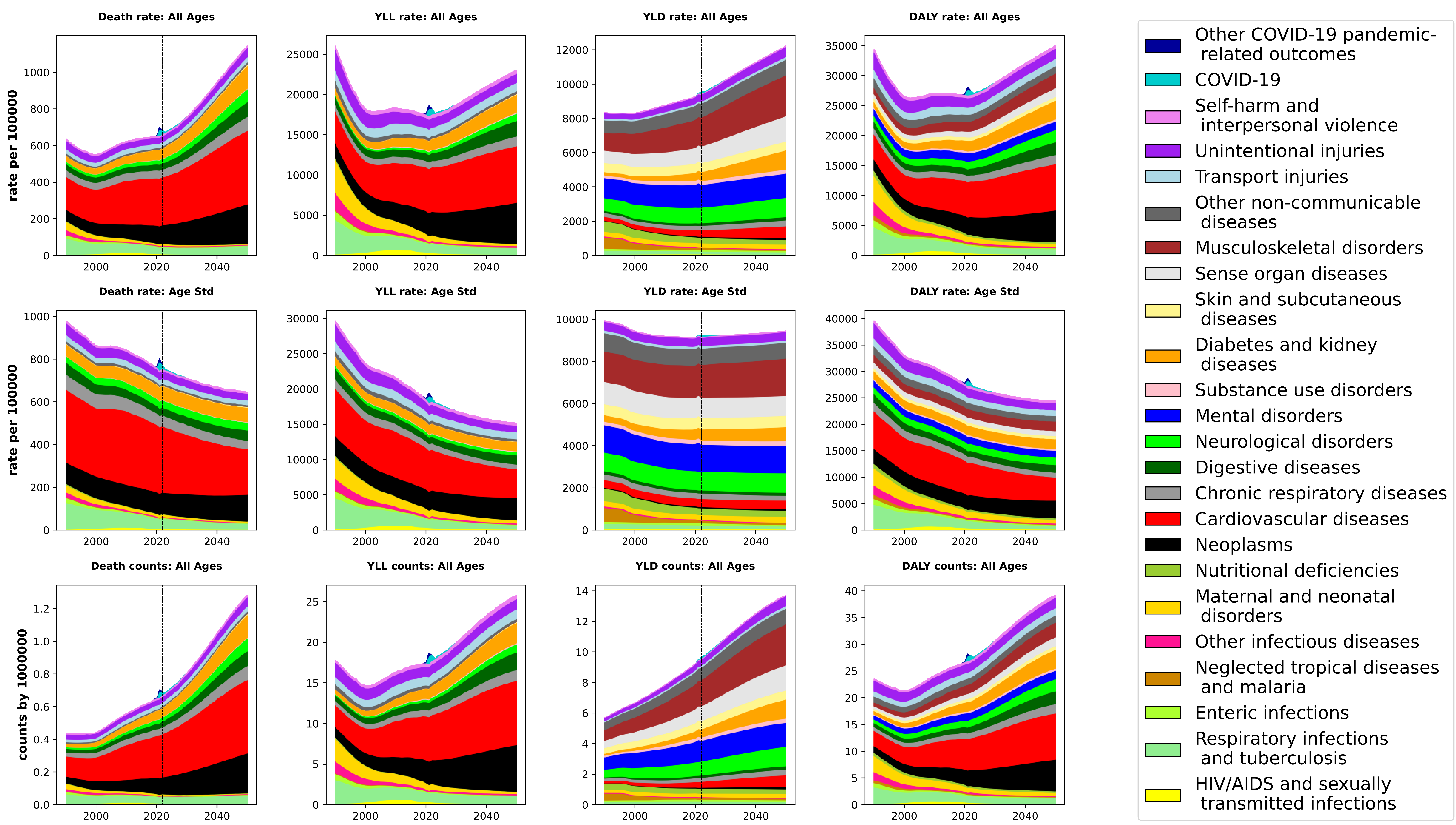
Death rate: All Ages
YLL rate: All Ages
YLD rate: All Ages
DALY rate: All Ages
Death rate: Age Std
YLL rate: Age Std
YLD rate: Age Std
DALY rate: Age Std
Death counts: All Ages
YLL counts: All Ages
YLD counts: All Ages
DALY counts: All Ages
rate per 100000
counts by 1000000
Other COVID-19 pandemic-related outcomes
COVID-19
Self-harm and interpersonal violence
Unintentional injuries
Transport injuries
Other non-communicable diseases
Musculoskeletal disorders
Sense organ diseases
Skin and subcutaneous diseases
Diabetes and kidney diseases
Substance use disorders
Mental disorders
Neurological disorders
Digestive diseases
Chronic respiratory diseases
Cardiovascular diseases
Neoplasms
Nutritional deficiencies
Maternal and neonatal disorders
Other infectious diseases
Neglected tropical diseases and malaria
Enteric infections
Respiratory infections and tuberculosis
HIV/AIDS and sexually transmitted infections

Angola

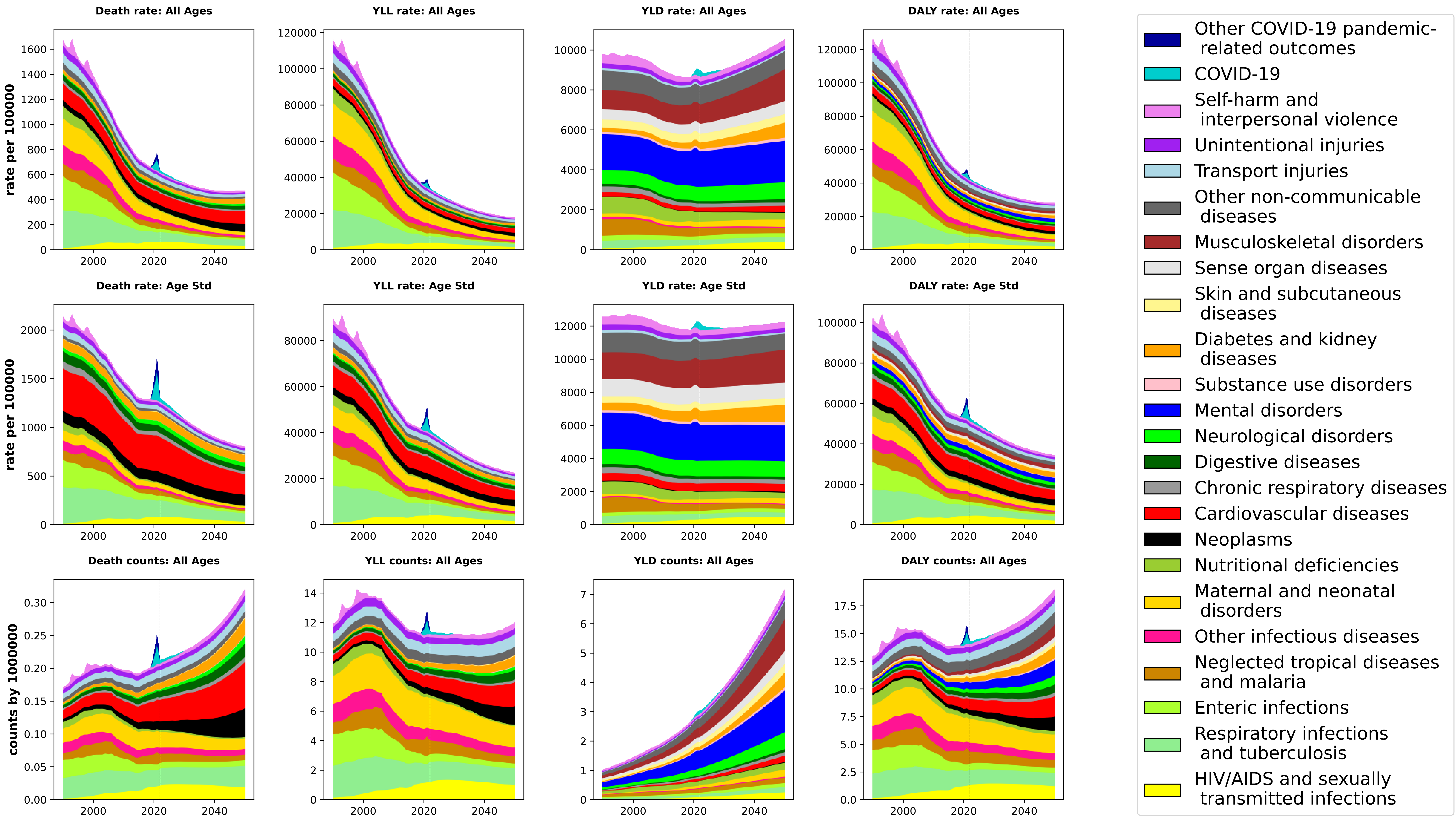
Death rate: All Ages
YLL rate: All Ages
YLD rate: All Ages
DALY rate: All Ages
Death rate: Age Std
YLL rate: Age Std
YLD rate: Age Std
DALY rate: Age Std
Death counts: All Ages
YLL counts: All Ages
YLD counts: All Ages
DALY counts: All Ages
rate per 100000
rate per 100000
counts by 1000000
2000
2020
2040
Other COVID-19 pandemic-related outcomes
COVID-19
Self-harm and interpersonal violence
Unintentional injuries
Transport injuries
Other non-communicable diseases
Musculoskeletal disorders
Sense organ diseases
Skin and subcutaneous diseases
Diabetes and kidney diseases
Substance use disorders
Mental disorders
Neurological disorders
Digestive diseases
Chronic respiratory diseases
Cardiovascular diseases
Neoplasms
Nutritional deficiencies
Maternal and neonatal disorders
Other infectious diseases
Neglected tropical diseases and malaria
Enteric infections
Respiratory infections and tuberculosis
HIV/AIDS and sexually transmitted infections

Central African Republic

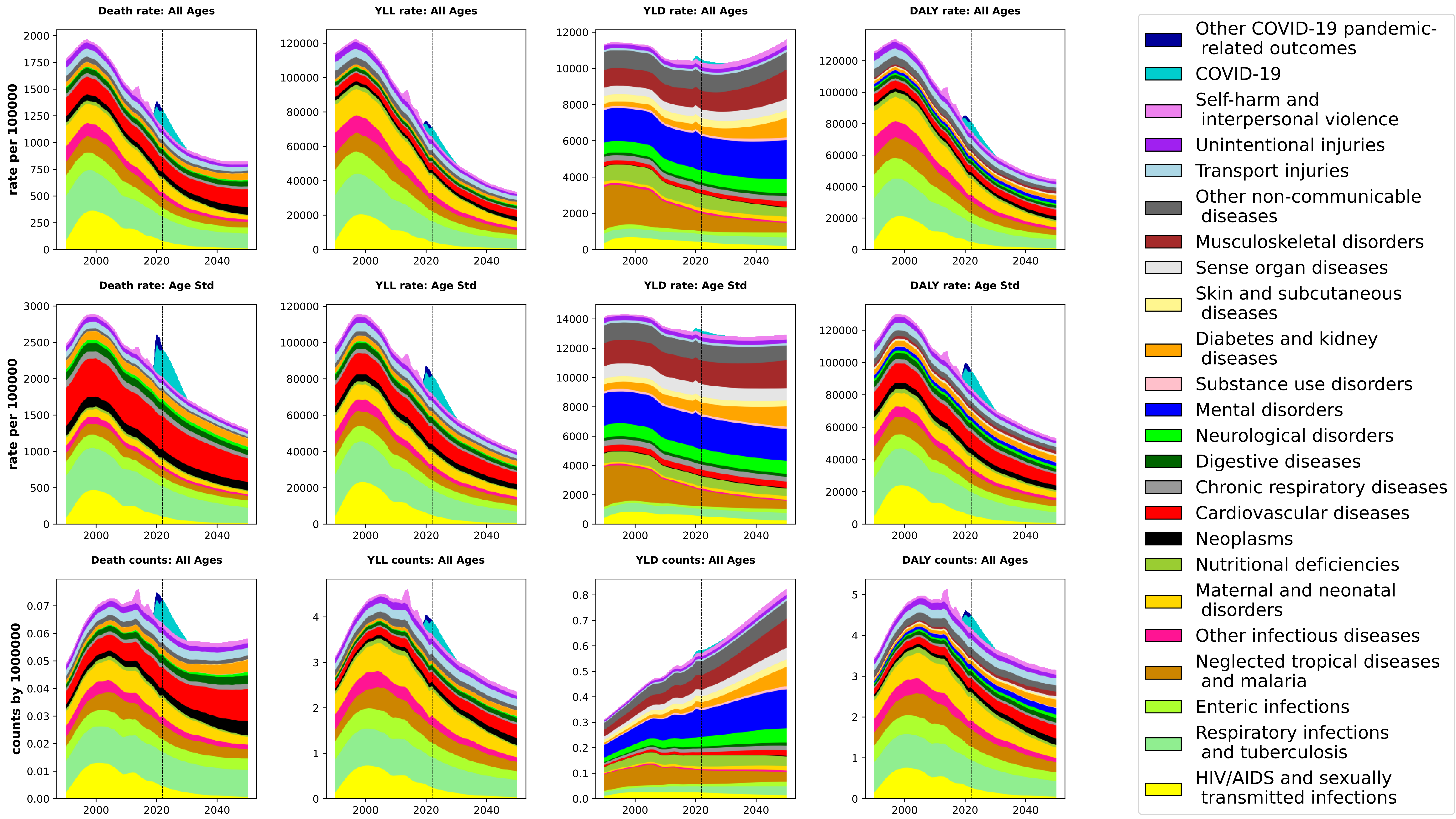

Congo (Brazzaville)

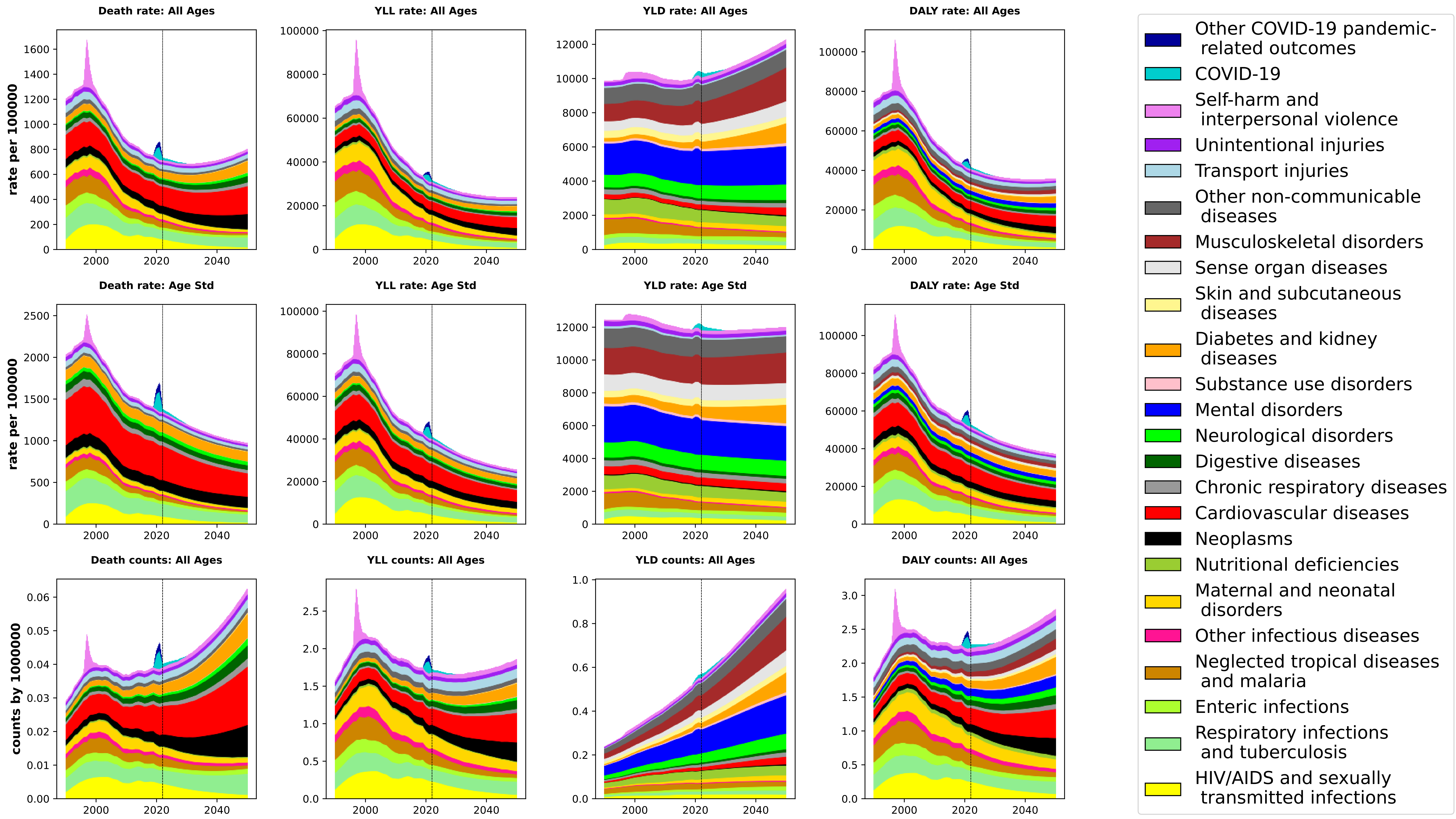

DR Congo

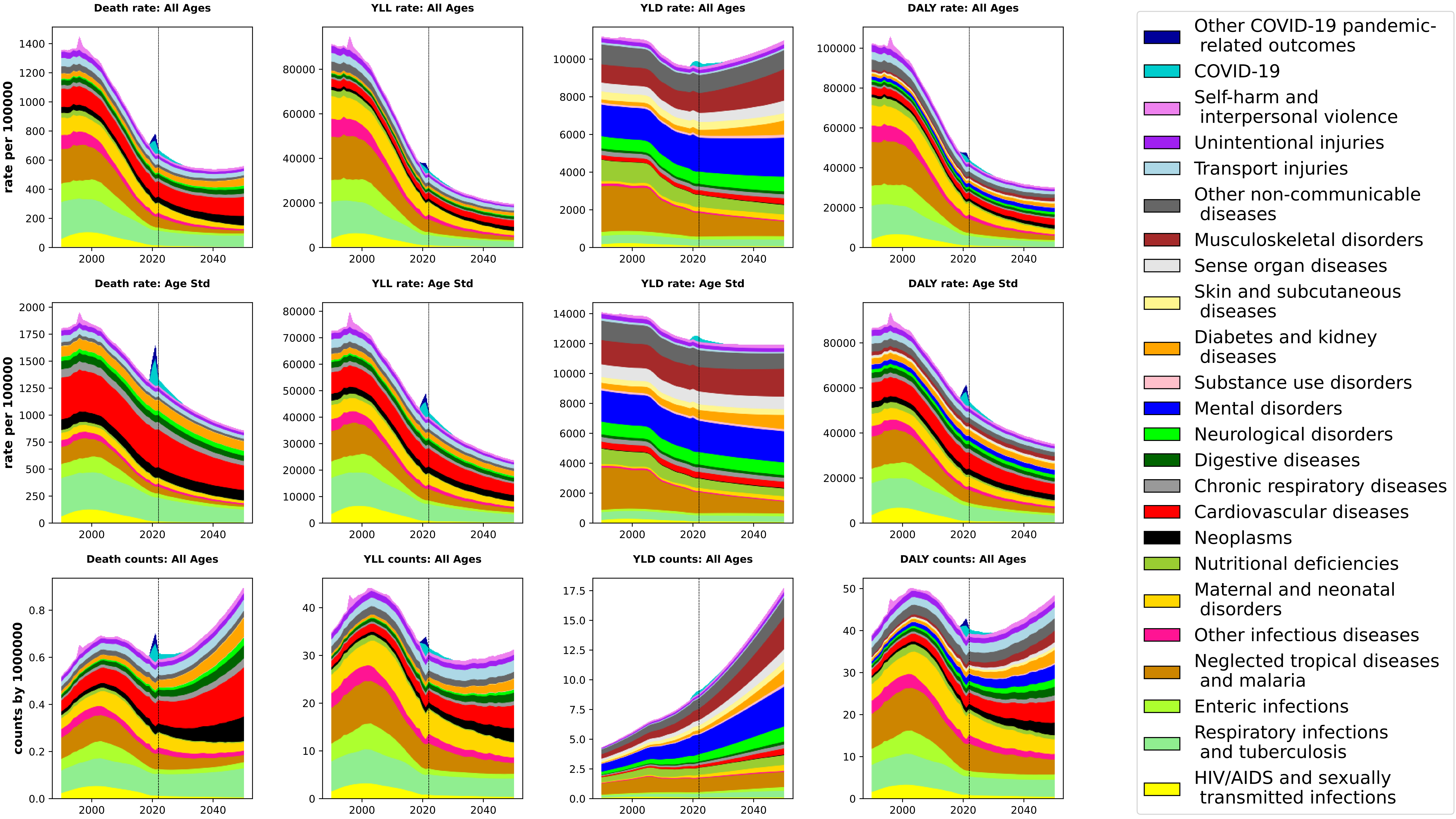
Death rate: All Ages
YLL rate: All Ages
YLD rate: All Ages
DALY rate: All Ages
Death rate: Age Std
YLL rate: Age Std
YLD rate: Age Std
DALY rate: Age Std
Death counts: All Ages
YLL counts: All Ages
YLD counts: All Ages
DALY counts: All Ages
rate per 100000
counts by 1000000
2000
2020
2040
Other COVID-19 pandemic-related outcomes
COVID-19
Self-harm and interpersonal violence
Unintentional injuries
Transport injuries
Other non-communicable diseases
Musculoskeletal disorders
Sense organ diseases
Skin and subcutaneous diseases
Diabetes and kidney diseases
Substance use disorders
Mental disorders
Neurological disorders
Digestive diseases
Chronic respiratory diseases
Cardiovascular diseases
Neoplasms
Nutritional deficiencies
Maternal and neonatal disorders
Other infectious diseases
Neglected tropical diseases and malaria
Enteric infections
Respiratory infections and tuberculosis
HIV/AIDS and sexually transmitted infections

Equatorial Guinea

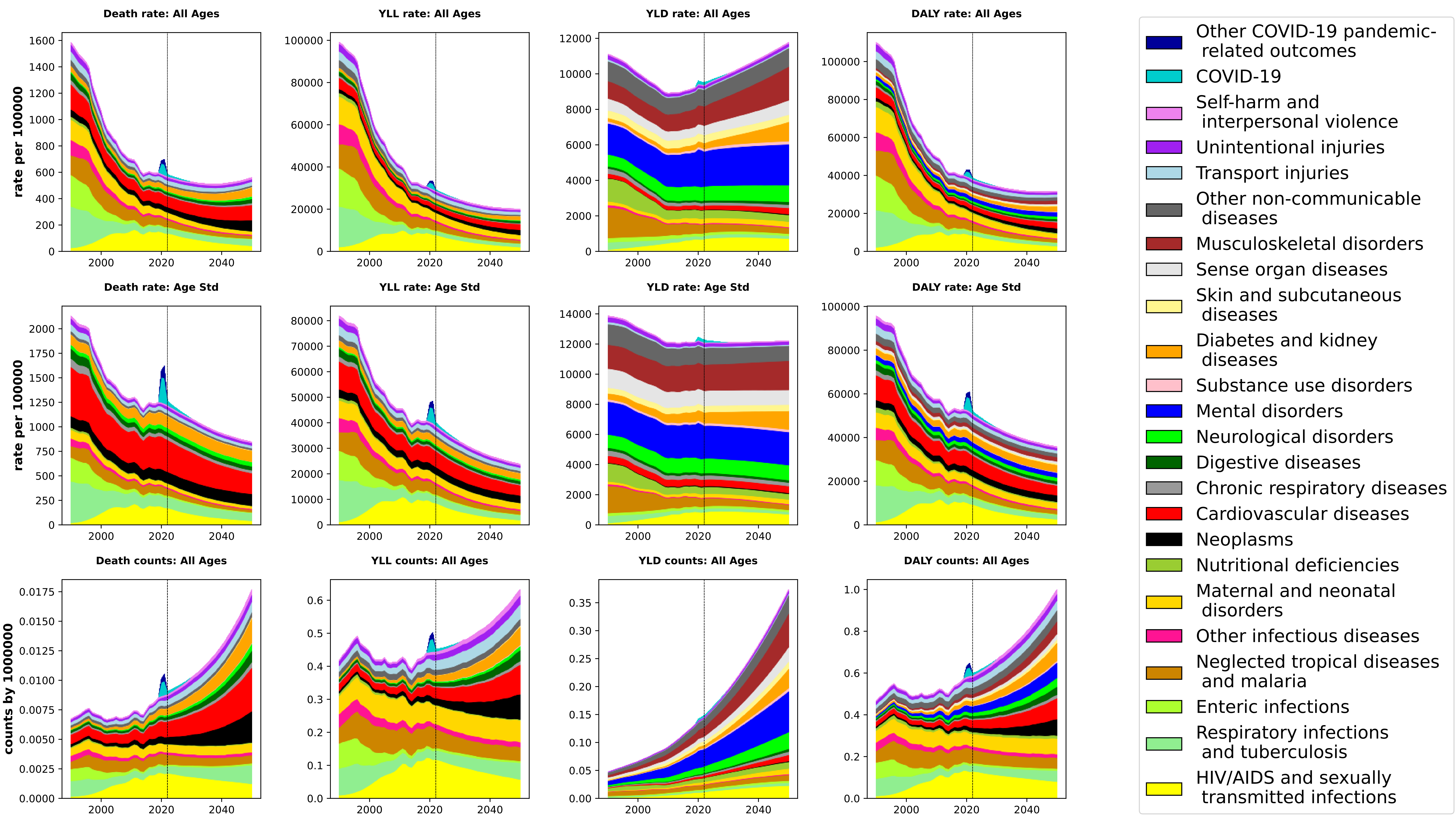

Gabon

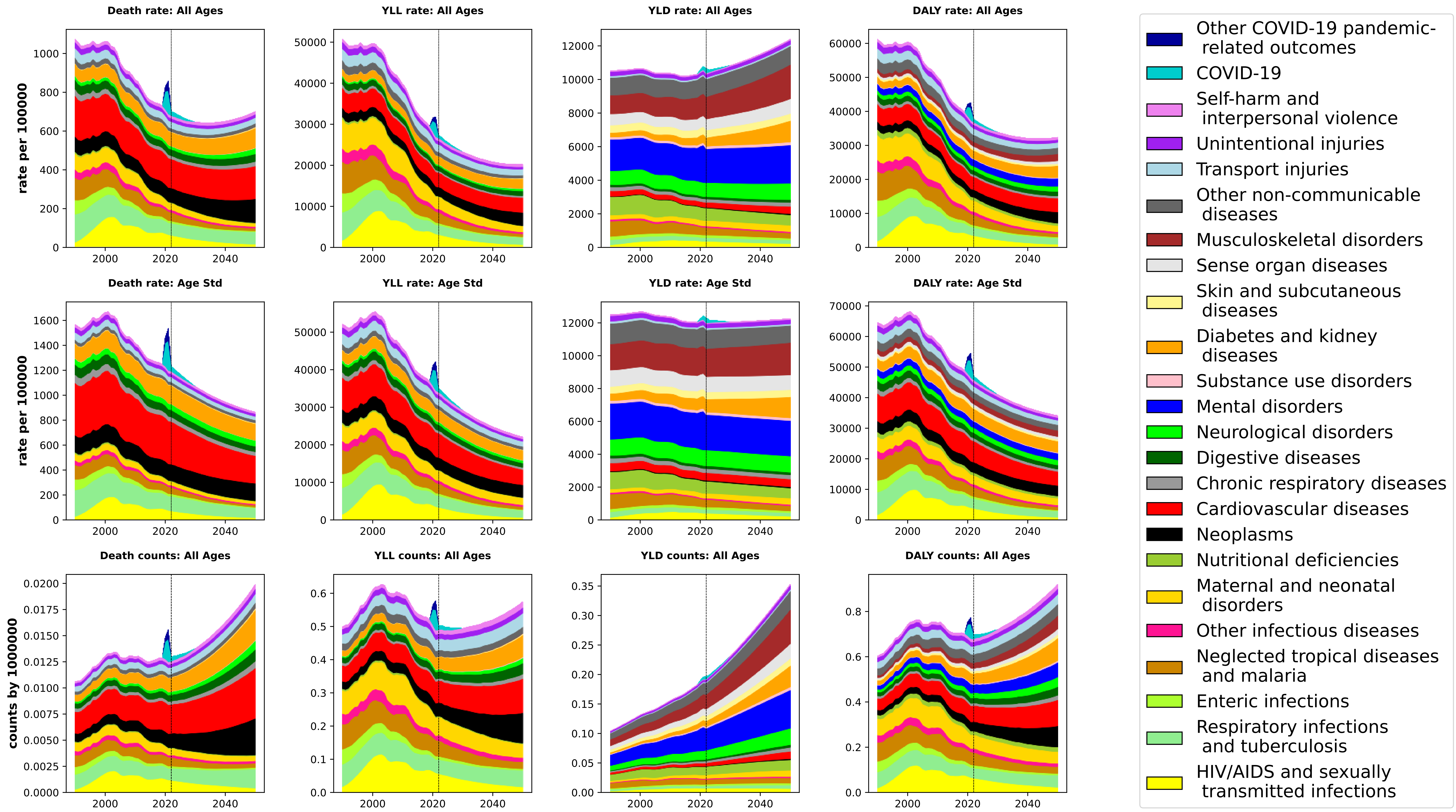
Death rate: All Ages
YLL rate: All Ages
YLD rate: All Ages
DALY rate: All Ages
Death rate: Age Std
YLL rate: Age Std
YLD rate: Age Std
DALY rate: Age Std
Death counts: All Ages
YLL counts: All Ages
YLD counts: All Ages
DALY counts: All Ages
rate per 100000
counts by 1000000
2000
2020
2040
Other COVID-19 pandemic-related outcomes
COVID-19
Self-harm and interpersonal violence
Unintentional injuries
Transport injuries
Other non-communicable diseases
Musculoskeletal disorders
Sense organ diseases
Skin and subcutaneous diseases
Diabetes and kidney diseases
Substance use disorders
Mental disorders
Neurological disorders
Digestive diseases
Chronic respiratory diseases
Cardiovascular diseases
Neoplasms
Nutritional deficiencies
Maternal and neonatal disorders
Other infectious diseases
Neglected tropical diseases and malaria
Enteric infections
Respiratory infections and tuberculosis
HIV/AIDS and sexually transmitted infections

Burundi

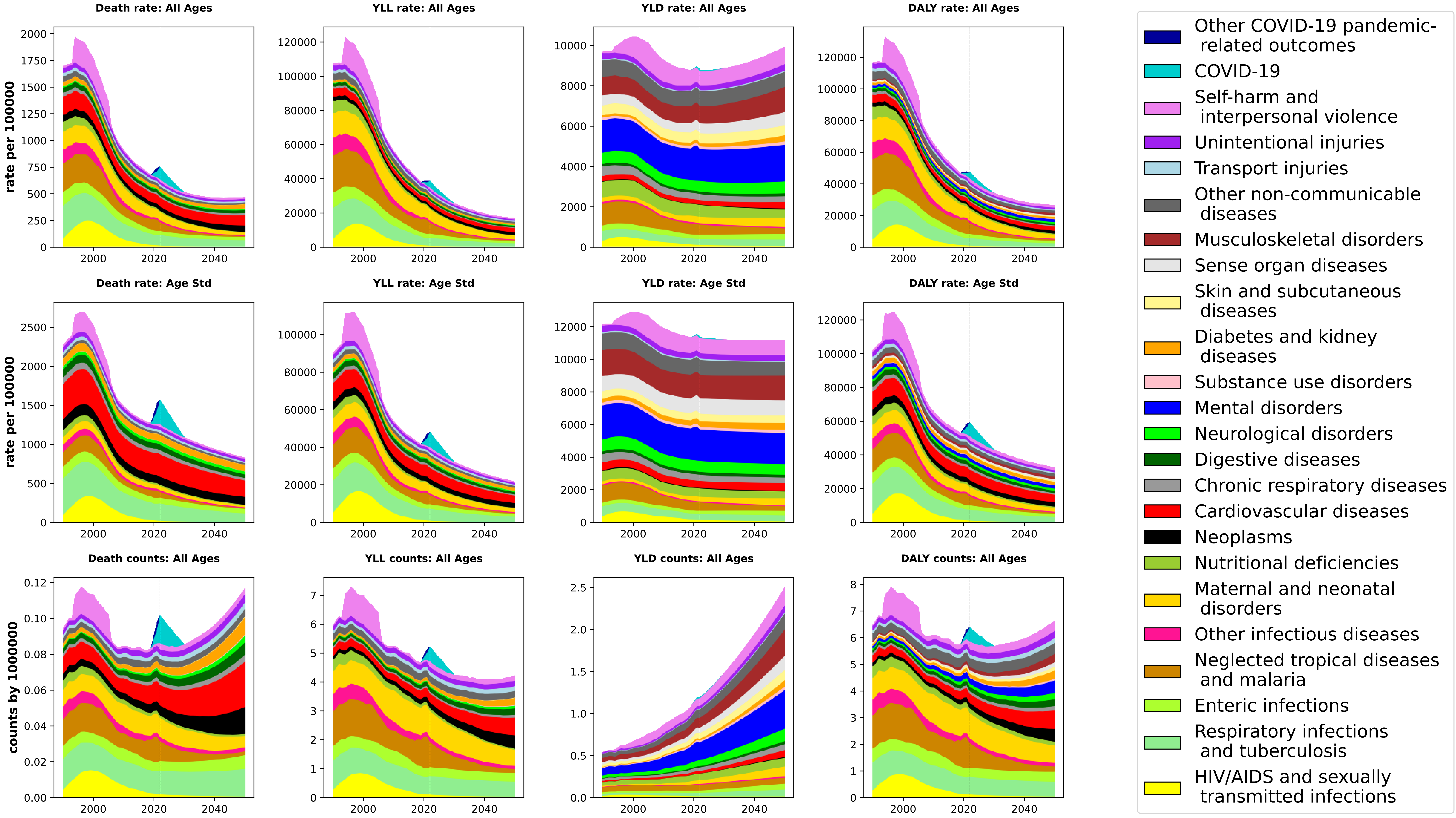
Death rate: All Ages
YLL rate: All Ages
YLD rate: All Ages
DALY rate: All Ages
Death rate: Age Std
YLL rate: Age Std
YLD rate: Age Std
DALY rate: Age Std
Death counts: All Ages
YLL counts: All Ages
YLD counts: All Ages
DALY counts: All Ages
rate per 100000
counts by 1000000
Other COVID-19 pandemic-related outcomes
COVID-19
Self-harm and interpersonal violence
Unintentional injuries
Transport injuries
Other non-communicable diseases
Musculoskeletal disorders
Sense organ diseases
Skin and subcutaneous diseases
Diabetes and kidney diseases
Substance use disorders
Mental disorders
Neurological disorders
Digestive diseases
Chronic respiratory diseases
Cardiovascular diseases
Neoplasms
Nutritional deficiencies
Maternal and neonatal disorders
Other infectious diseases
Neglected tropical diseases and malaria
Enteric infections
Respiratory infections and tuberculosis
HIV/AIDS and sexually transmitted infections

Comoros

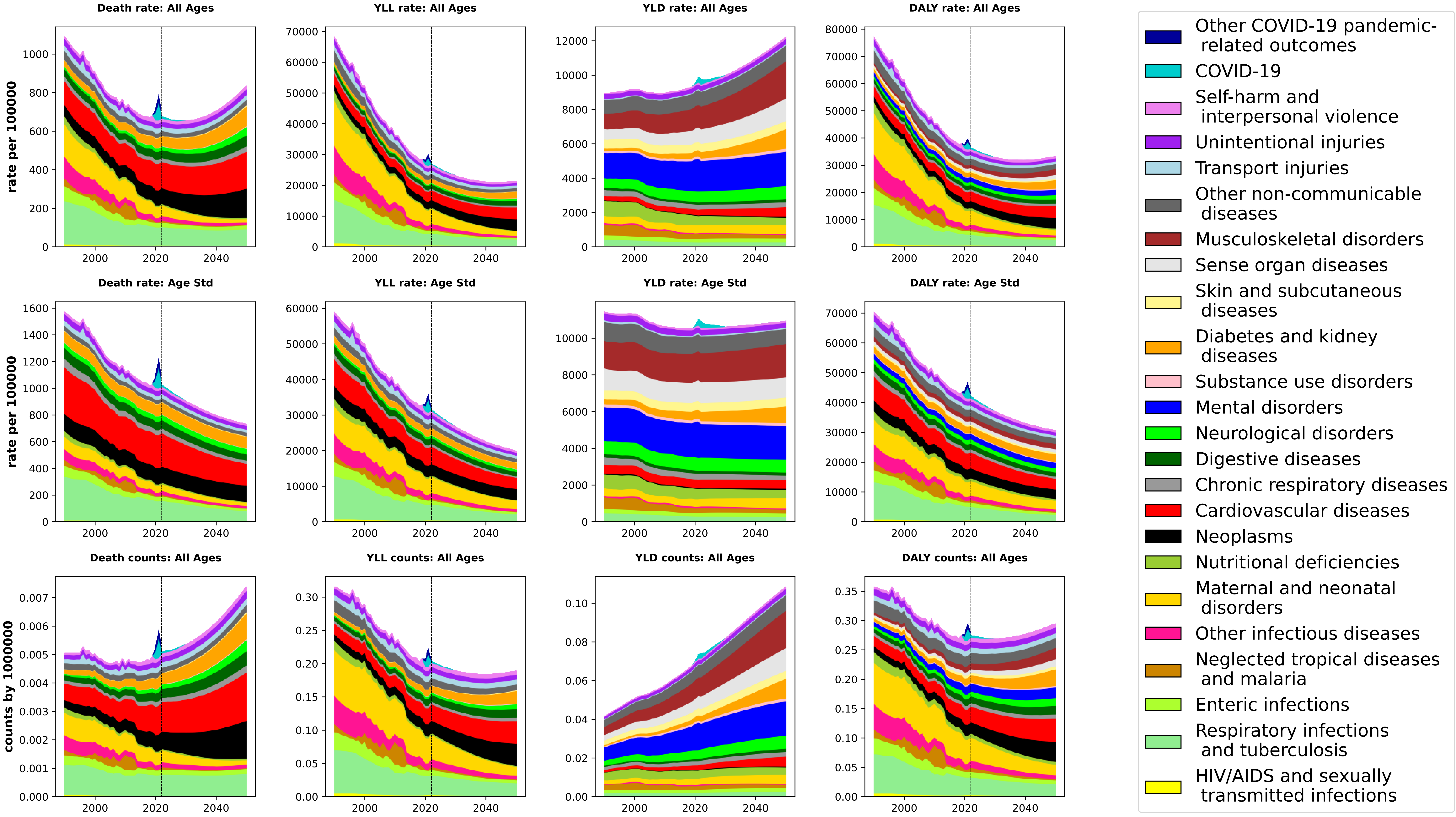
Death rate: All Ages
YLL rate: All Ages
YLD rate: All Ages
DALY rate: All Ages
Death rate: Age Std
YLL rate: Age Std
YLD rate: Age Std
DALY rate: Age Std
Death counts: All Ages
YLL counts: All Ages
YLD counts: All Ages
DALY counts: All Ages
rate per 100000
counts by 1000000
2000
2020
2040
Other COVID-19 pandemic-related outcomes
COVID-19
Self-harm and interpersonal violence
Unintentional injuries
Transport injuries
Other non-communicable diseases
Musculoskeletal disorders
Sense organ diseases
Skin and subcutaneous diseases
Diabetes and kidney diseases
Substance use disorders
Mental disorders
Neurological disorders
Digestive diseases
Chronic respiratory diseases
Cardiovascular diseases
Neoplasms
Nutritional deficiencies
Maternal and neonatal disorders
Other infectious diseases
Neglected tropical diseases and malaria
Enteric infections
Respiratory infections and tuberculosis
HIV/AIDS and sexually transmitted infections

Djibouti

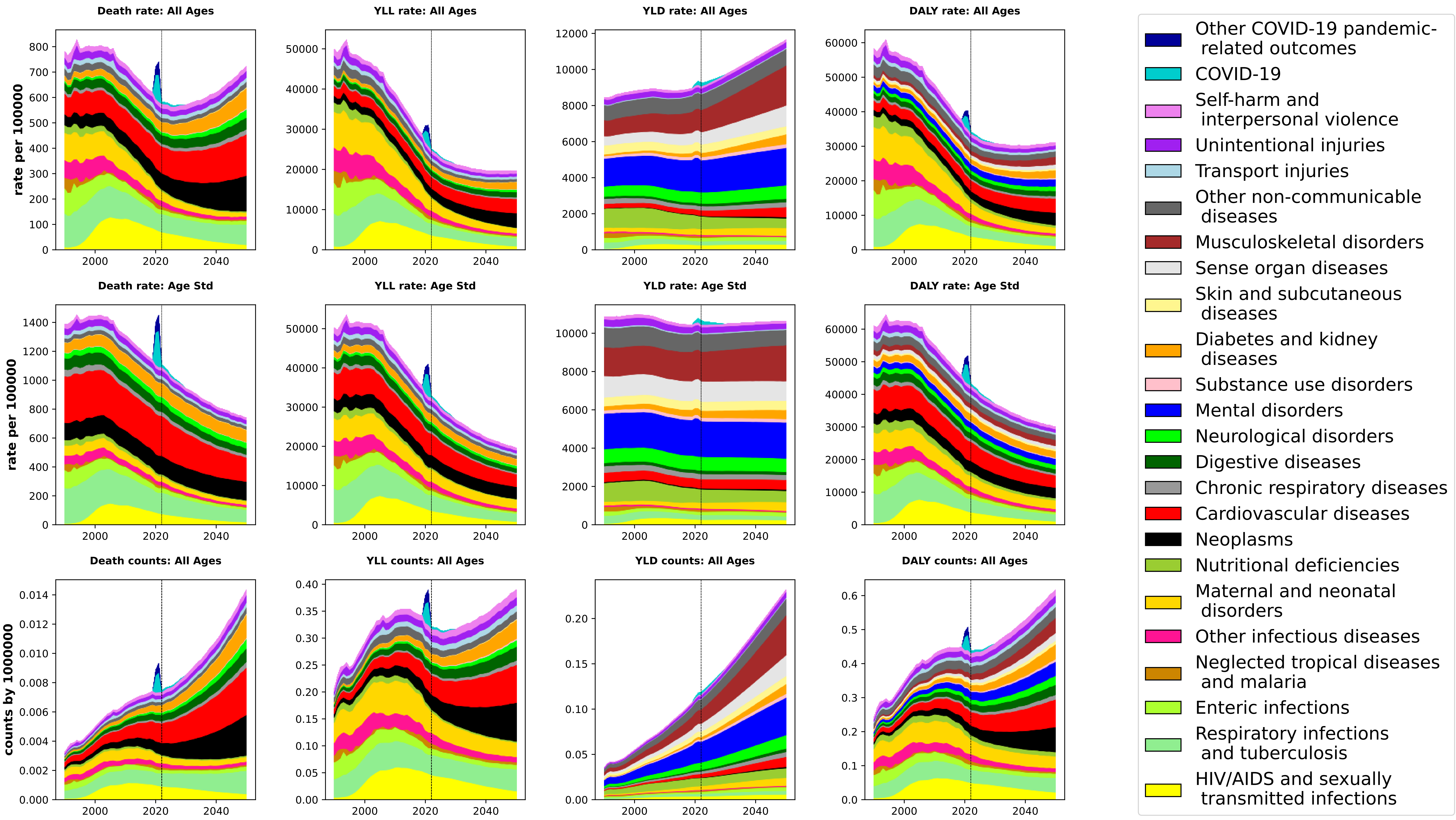
Death rate: All Ages
YLL rate: All Ages
YLD rate: All Ages
DALY rate: All Ages
Death rate: Age Std
YLL rate: Age Std
YLD rate: Age Std
DALY rate: Age Std
Death counts: All Ages
YLL counts: All Ages
YLD counts: All Ages
DALY counts: All Ages
rate per 100000
counts by 1000000
Other COVID-19 pandemic-related outcomes
COVID-19
Self-harm and interpersonal violence
Unintentional injuries
Transport injuries
Other non-communicable diseases
Musculoskeletal disorders
Sense organ diseases
Skin and subcutaneous diseases
Diabetes and kidney diseases
Substance use disorders
Mental disorders
Neurological disorders
Digestive diseases
Chronic respiratory diseases
Cardiovascular diseases
Neoplasms
Nutritional deficiencies
Maternal and neonatal disorders
Other infectious diseases
Neglected tropical diseases and malaria
Enteric infections
Respiratory infections and tuberculosis
HIV/AIDS and sexually transmitted infections

Eritrea

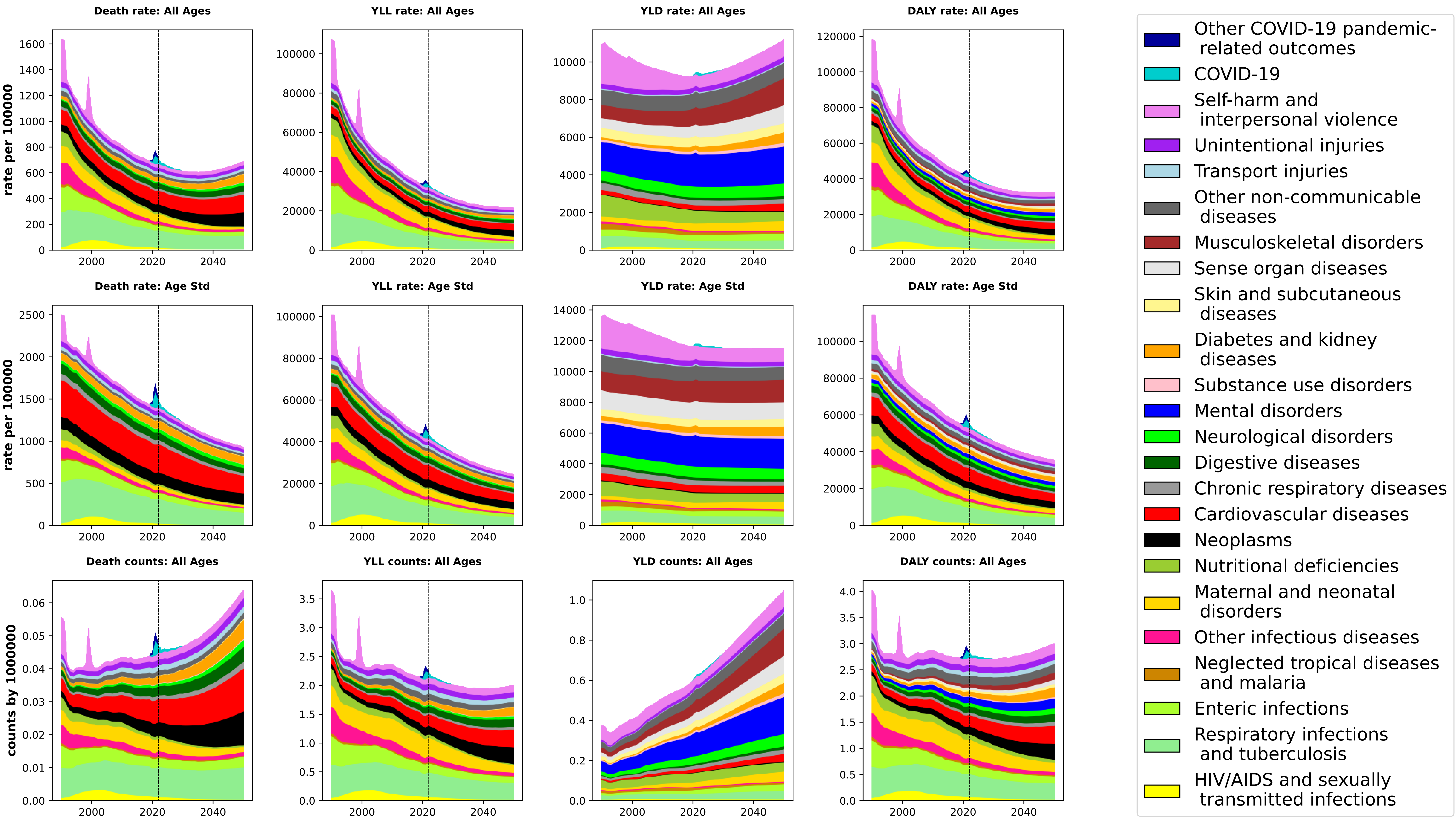
Death rate: All Ages
YLL rate: All Ages
YLD rate: All Ages
DALY rate: All Ages
Death rate: Age Std
YLL rate: Age Std
YLD rate: Age Std
DALY rate: Age Std
Death counts: All Ages
YLL counts: All Ages
YLD counts: All Ages
DALY counts: All Ages
rate per 100000
counts by 1000000
Other COVID-19 pandemic-related outcomes
COVID-19
Self-harm and interpersonal violence
Unintentional injuries
Transport injuries
Other non-communicable diseases
Musculoskeletal disorders
Sense organ diseases
Skin and subcutaneous diseases
Diabetes and kidney diseases
Substance use disorders
Mental disorders
Neurological disorders
Digestive diseases
Chronic respiratory diseases
Cardiovascular diseases
Neoplasms
Nutritional deficiencies
Maternal and neonatal disorders
Other infectious diseases
Neglected tropical diseases and malaria
Enteric infections
Respiratory infections and tuberculosis
HIV/AIDS and sexually transmitted infections

Ethiopia

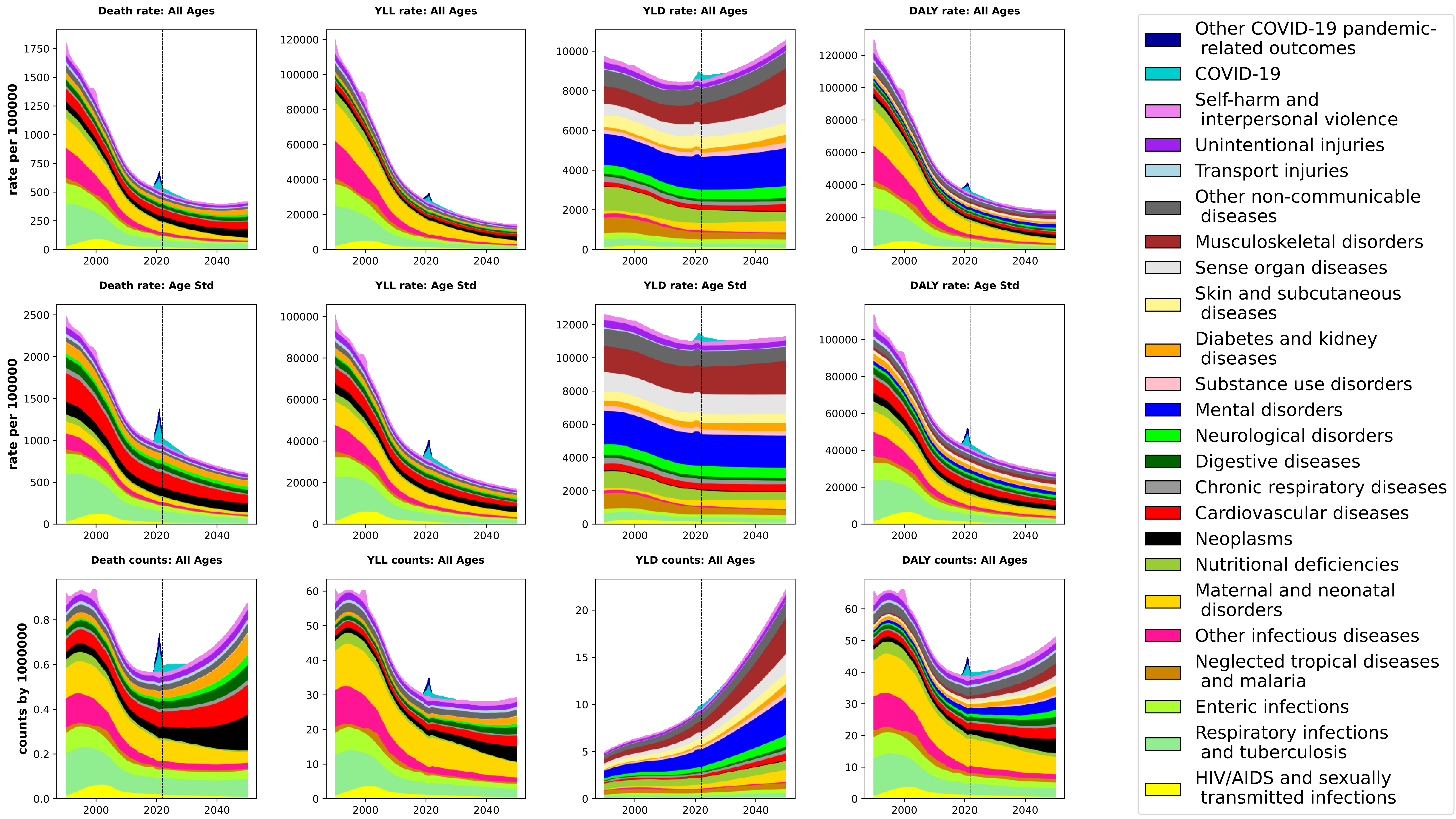
Death rate: All Ages
YLL rate: All Ages
YLD rate: All Ages
DALY rate: All Ages
Death rate: Age Std
YLL rate: Age Std
YLD rate: Age Std
DALY rate: Age Std
Death counts: All Ages
YLL counts: All Ages
YLD counts: All Ages
DALY counts: All Ages
rate per 100000
counts by 1000000
Other COVID-19 pandemic-related outcomes
COVID-19
Self-harm and interpersonal violence
Unintentional injuries
Transport injuries
Other non-communicable diseases
Musculoskeletal disorders
Sense organ diseases
Skin and subcutaneous diseases
Diabetes and kidney diseases
Substance use disorders
Mental disorders
Neurological disorders
Digestive diseases
Chronic respiratory diseases
Cardiovascular diseases
Neoplasms
Nutritional deficiencies
Maternal and neonatal disorders
Other infectious diseases
Neglected tropical diseases and malaria
Enteric infections
Respiratory infections and tuberculosis
HIV/AIDS and sexually transmitted infections

Kenya

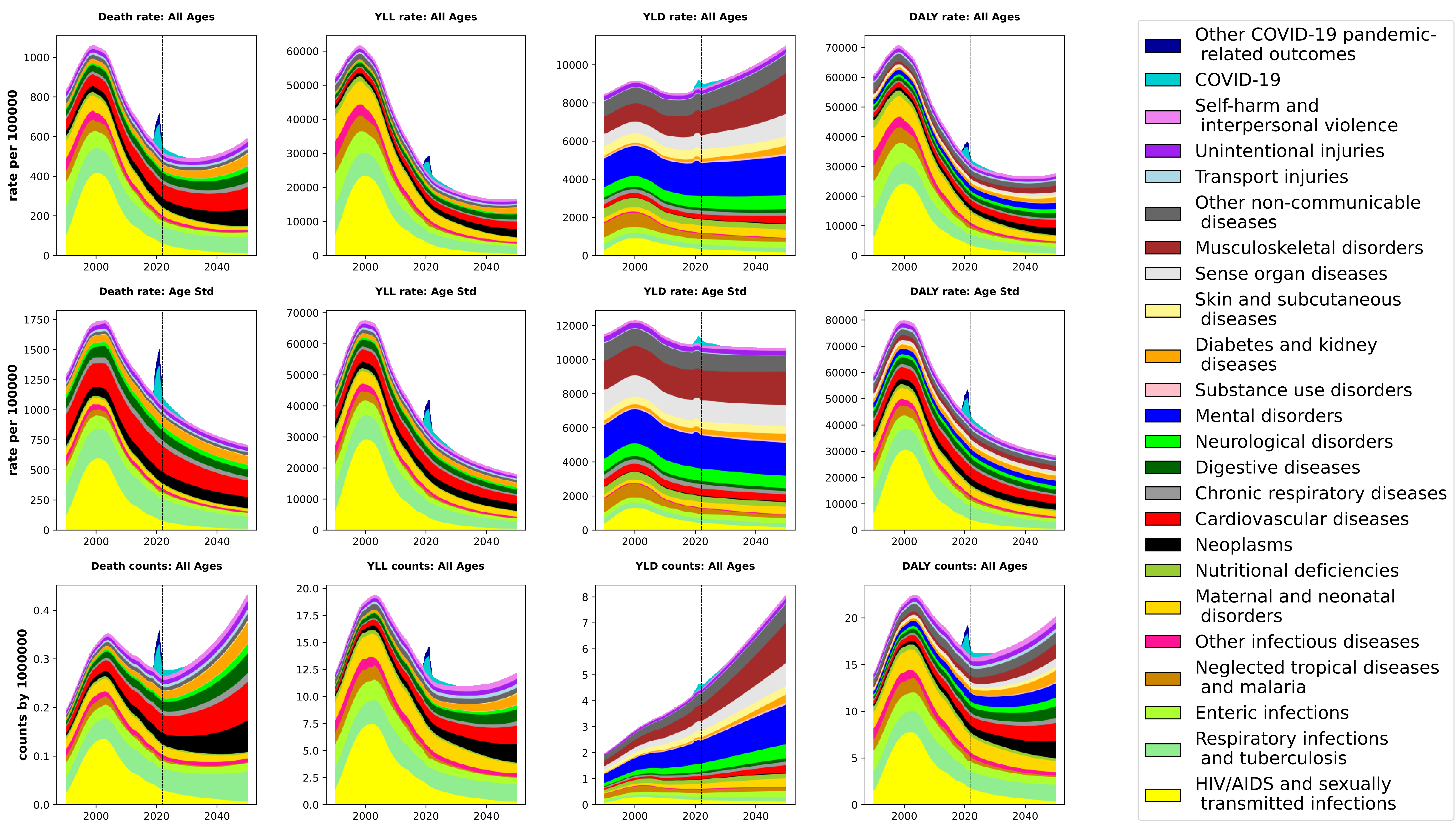
Death rate: All Ages
YLL rate: All Ages
YLD rate: All Ages
DALY rate: All Ages
Death rate: Age Std
YLL rate: Age Std
YLD rate: Age Std
DALY rate: Age Std
Death counts: All Ages
YLL counts: All Ages
YLD counts: All Ages
DALY counts: All Ages
rate per 100000
counts by 1000000
Other COVID-19 pandemic-related outcomes
COVID-19
Self-harm and interpersonal violence
Unintentional injuries
Transport injuries
Other non-communicable diseases
Musculoskeletal disorders
Sense organ diseases
Skin and subcutaneous diseases
Diabetes and kidney diseases
Substance use disorders
Mental disorders
Neurological disorders
Digestive diseases
Chronic respiratory diseases
Cardiovascular diseases
Neoplasms
Nutritional deficiencies
Maternal and neonatal disorders
Other infectious diseases
Neglected tropical diseases and malaria
Enteric infections
Respiratory infections and tuberculosis
HIV/AIDS and sexually transmitted infections

Madagascar

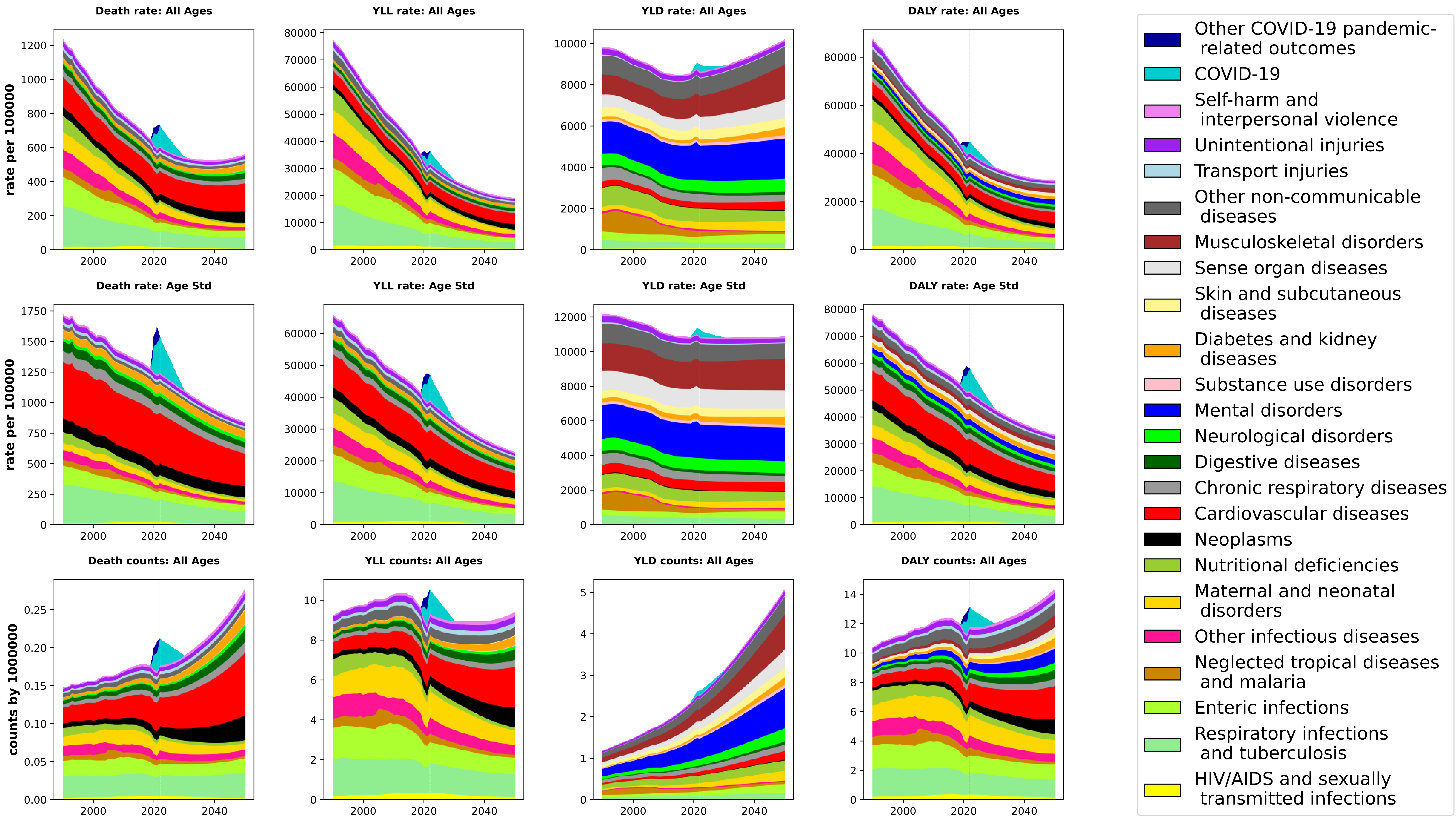
Death rate: All Ages
YLL rate: All Ages
YLD rate: All Ages
DALY rate: All Ages
Death rate: Age Std
YLL rate: Age Std
YLD rate: Age Std
DALY rate: Age Std
Death counts: All Ages
YLL counts: All Ages
YLD counts: All Ages
DALY counts: All Ages
rate per 100000
counts by 1000000
Other COVID-19 pandemic-related outcomes
COVID-19
Self-harm and interpersonal violence
Unintentional injuries
Transport injuries
Other non-communicable diseases
Musculoskeletal disorders
Sense organ diseases
Skin and subcutaneous diseases
Diabetes and kidney diseases
Substance use disorders
Mental disorders
Neurological disorders
Digestive diseases
Chronic respiratory diseases
Cardiovascular diseases
Neoplasms
Nutritional deficiencies
Maternal and neonatal disorders
Other infectious diseases
Neglected tropical diseases and malaria
Enteric infections
Respiratory infections and tuberculosis
HIV/AIDS and sexually transmitted infections

Malawi

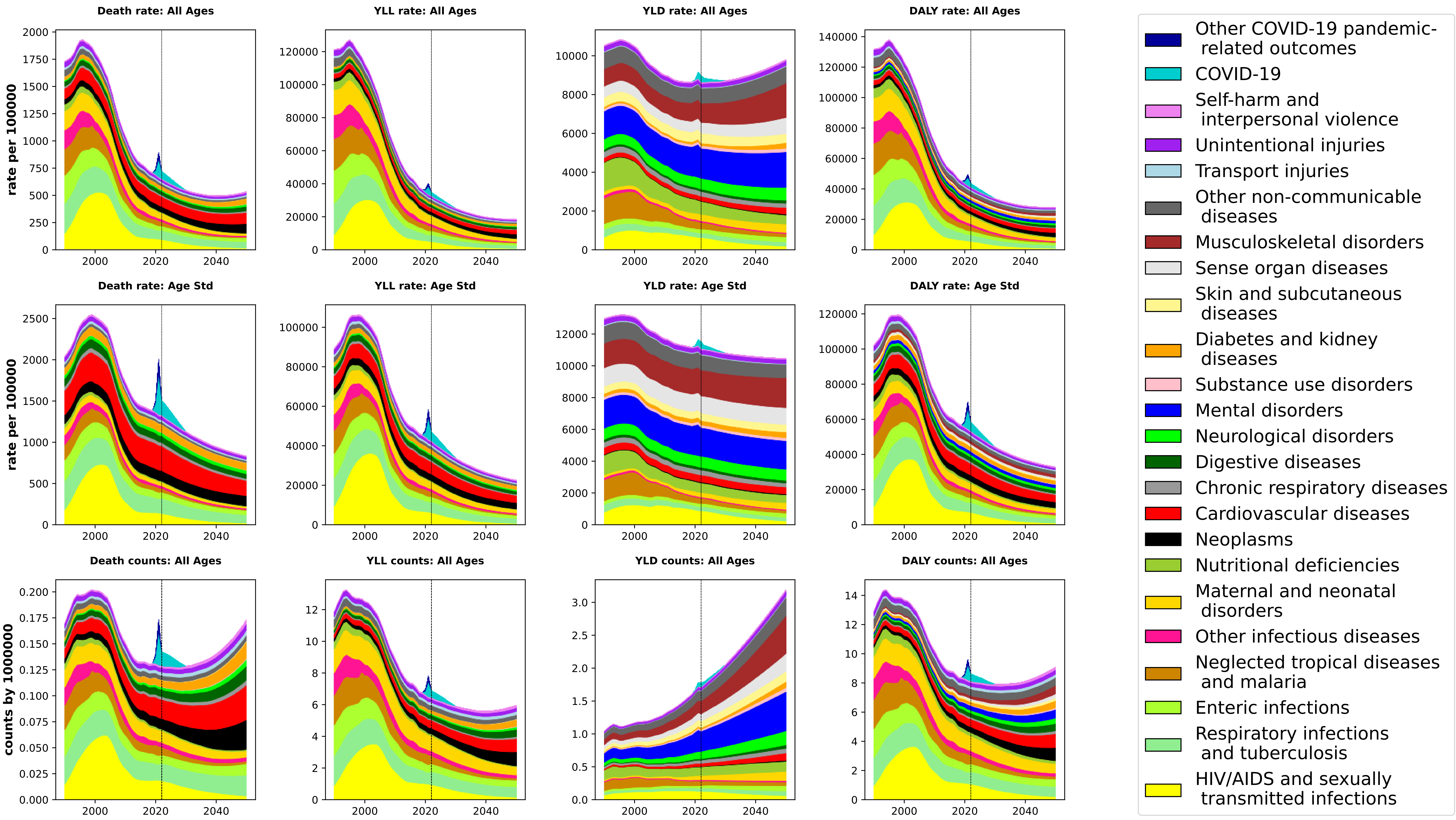

Death rate: All Ages
YLL rate: All Ages
YLD rate: All Ages
DALY rate: All Ages
Death rate: Age Std
YLL rate: Age Std
YLD rate: Age Std
DALY rate: Age Std
Death counts: All Ages
YLL counts: All Ages
YLD counts: All Ages
DALY counts: All Ages
rate per 100000
rate per 100000
counts by 1000000
Other COVID-19 pandemic-related outcomes
COVID-19
Self-harm and interpersonal violence
Unintentional injuries
Transport injuries
Other non-communicable diseases
Musculoskeletal disorders
Sense organ diseases
Skin and subcutaneous diseases
Diabetes and kidney diseases
Substance use disorders
Mental disorders
Neurological disorders
Digestive diseases
Chronic respiratory diseases
Cardiovascular diseases
Neoplasms
Nutritional deficiencies
Maternal and neonatal disorders
Other infectious diseases
Neglected tropical diseases and malaria
Enteric infections
Respiratory infections and tuberculosis
HIV/AIDS and sexually transmitted infections

Mozambique

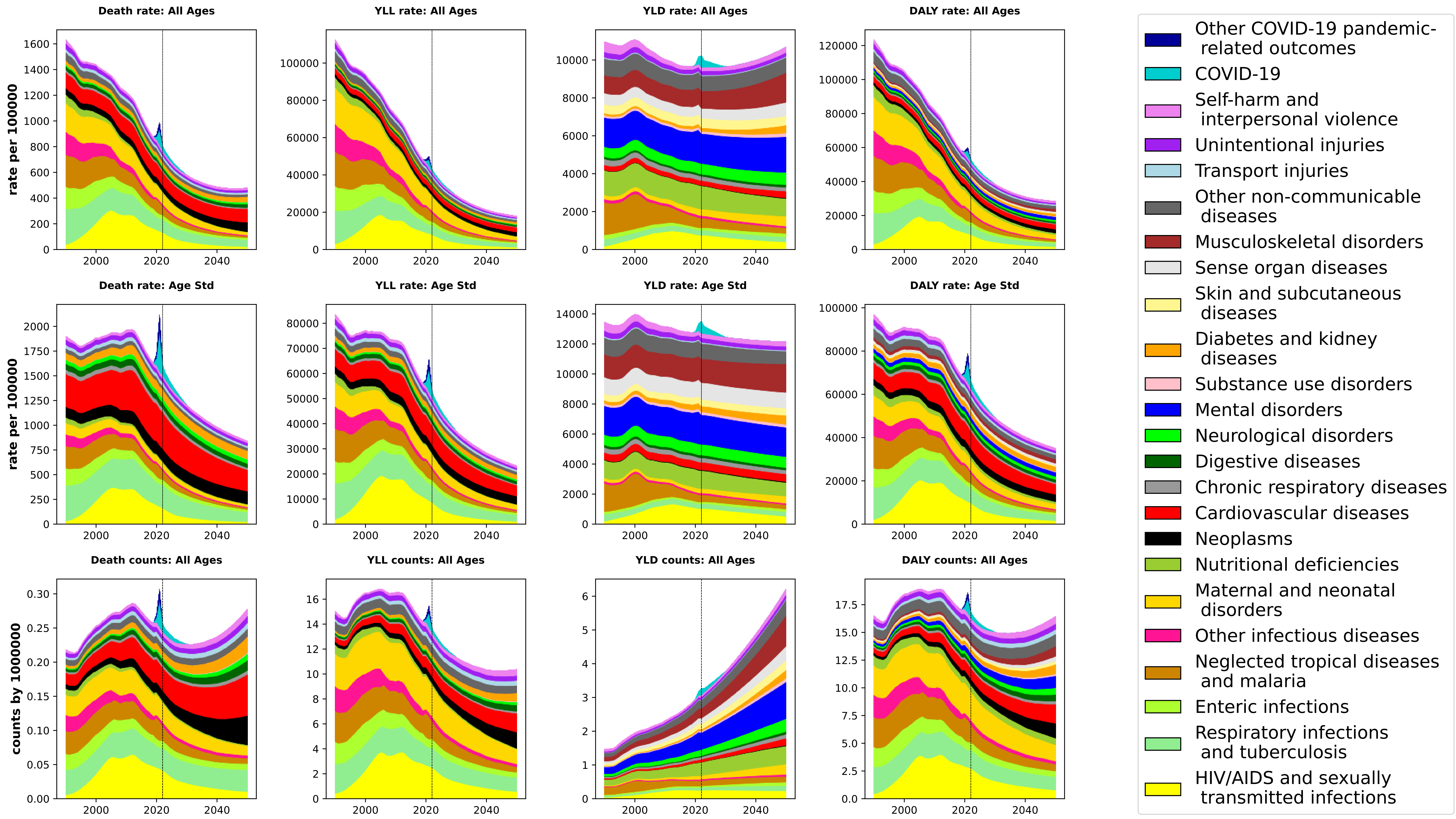
Death rate: All Ages
YLL rate: All Ages
YLD rate: All Ages
DALY rate: All Ages
Death rate: Age Std
YLL rate: Age Std
YLD rate: Age Std
DALY rate: Age Std
Death counts: All Ages
YLL counts: All Ages
YLD counts: All Ages
DALY counts: All Ages
rate per 100000
counts by 1000000
2000
2020
2040
Other COVID-19 pandemic-related outcomes
COVID-19
Self-harm and interpersonal violence
Unintentional injuries
Transport injuries
Other non-communicable diseases
Musculoskeletal disorders
Sense organ diseases
Skin and subcutaneous diseases
Diabetes and kidney diseases
Substance use disorders
Mental disorders
Neurological disorders
Digestive diseases
Chronic respiratory diseases
Cardiovascular diseases
Neoplasms
Nutritional deficiencies
Maternal and neonatal disorders
Other infectious diseases
Neglected tropical diseases and malaria
Enteric infections
Respiratory infections and tuberculosis
HIV/AIDS and sexually transmitted infections

Rwanda

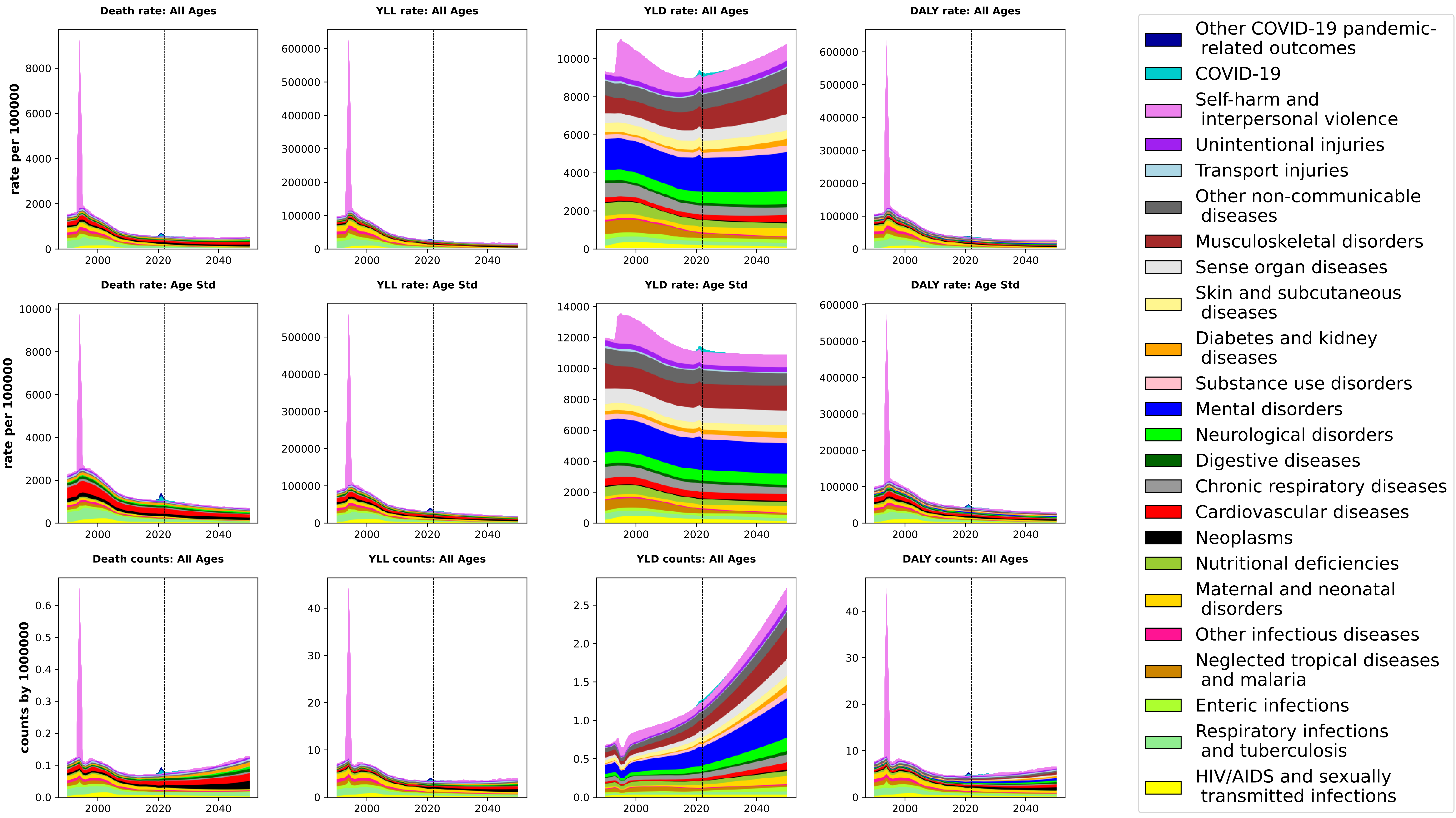
Death rate: All Ages
YLL rate: All Ages
YLD rate: All Ages
DALY rate: All Ages
Death rate: Age Std
YLL rate: Age Std
YLD rate: Age Std
DALY rate: Age Std
Death counts: All Ages
YLL counts: All Ages
YLD counts: All Ages
DALY counts: All Ages
rate per 100000
counts by 1000000
2000
2020
2040
Other COVID-19 pandemic-related outcomes
COVID-19
Self-harm and interpersonal violence
Unintentional injuries
Transport injuries
Other non-communicable diseases
Musculoskeletal disorders
Sense organ diseases
Skin and subcutaneous diseases
Diabetes and kidney diseases
Substance use disorders
Mental disorders
Neurological disorders
Digestive diseases
Chronic respiratory diseases
Cardiovascular diseases
Neoplasms
Nutritional deficiencies
Maternal and neonatal disorders
Other infectious diseases
Neglected tropical diseases and malaria
Enteric infections
Respiratory infections and tuberculosis
HIV/AIDS and sexually transmitted infections

Somalia

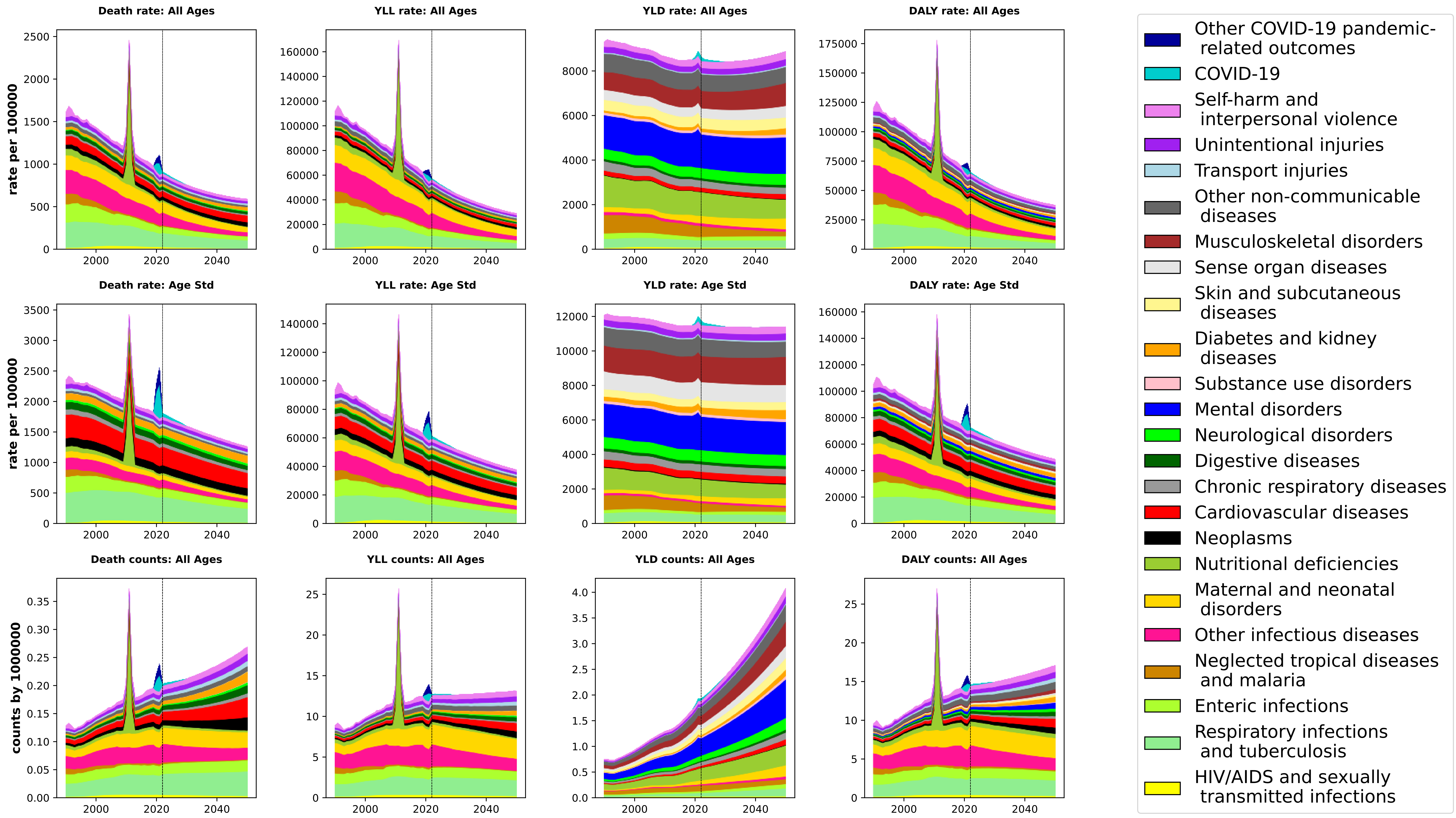
Death rate: All Ages
YLL rate: All Ages
YLD rate: All Ages
DALY rate: All Ages
Death rate: Age Std
YLL rate: Age Std
YLD rate: Age Std
DALY rate: Age Std
Death counts: All Ages
YLL counts: All Ages
YLD counts: All Ages
DALY counts: All Ages
rate per 100000
counts by 1000000
2000
2020
2040
Other COVID-19 pandemic-related outcomes
COVID-19
Self-harm and interpersonal violence
Unintentional injuries
Transport injuries
Other non-communicable diseases
Musculoskeletal disorders
Sense organ diseases
Skin and subcutaneous diseases
Diabetes and kidney diseases
Substance use disorders
Mental disorders
Neurological disorders
Digestive diseases
Chronic respiratory diseases
Cardiovascular diseases
Neoplasms
Nutritional deficiencies
Maternal and neonatal disorders
Other infectious diseases
Neglected tropical diseases and malaria
Enteric infections
Respiratory infections and tuberculosis
HIV/AIDS and sexually transmitted infections

South Sudan

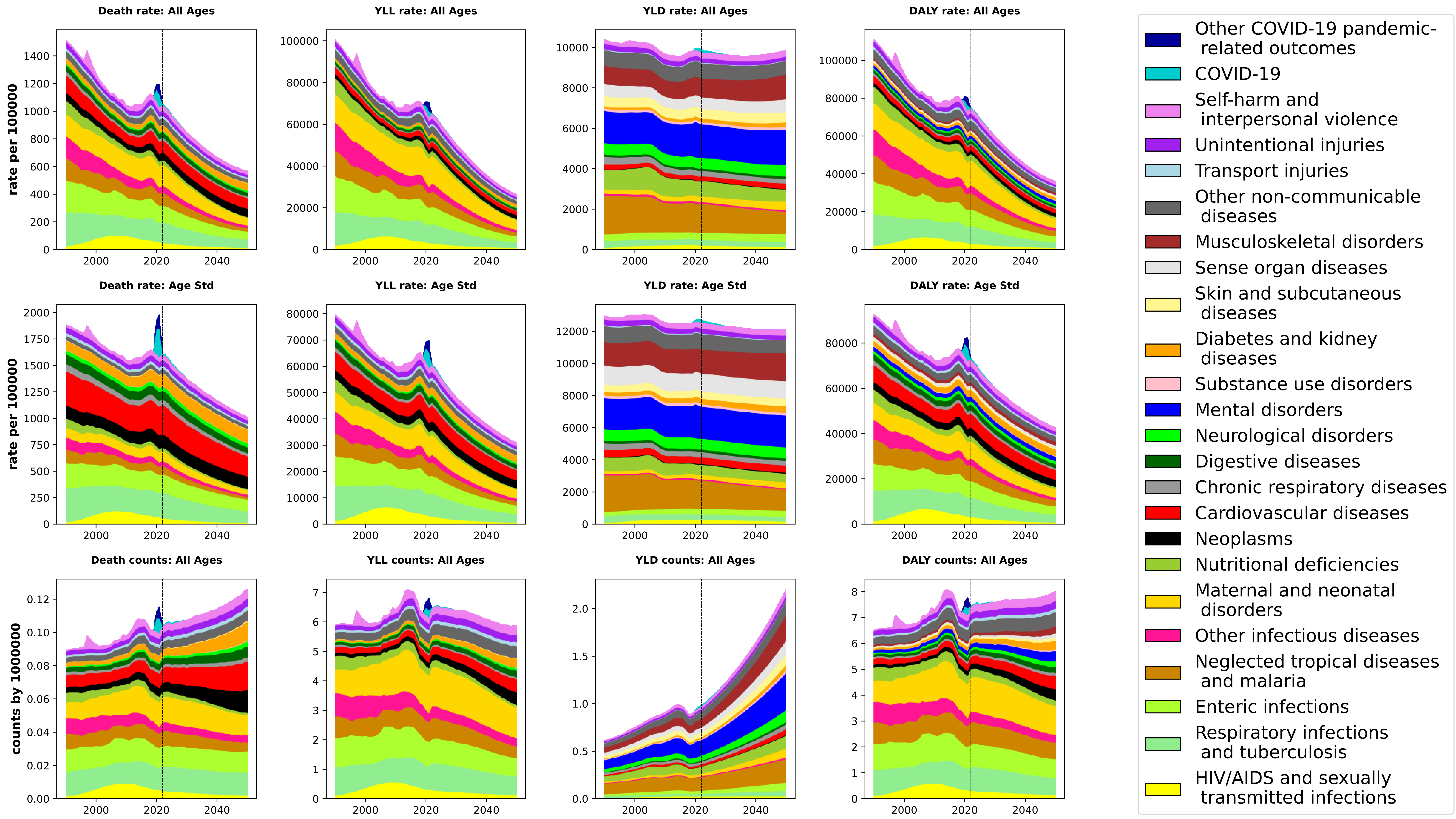
Death rate: All Ages
YLL rate: All Ages
YLD rate: All Ages
DALY rate: All Ages
Death rate: Age Std
YLL rate: Age Std
YLD rate: Age Std
DALY rate: Age Std
Death counts: All Ages
YLL counts: All Ages
YLD counts: All Ages
DALY counts: All Ages
rate per 100000
counts by 1000000
Other COVID-19 pandemic-related outcomes
COVID-19
Self-harm and interpersonal violence
Unintentional injuries
Transport injuries
Other non-communicable diseases
Musculoskeletal disorders
Sense organ diseases
Skin and subcutaneous diseases
Diabetes and kidney diseases
Substance use disorders
Mental disorders
Neurological disorders
Digestive diseases
Chronic respiratory diseases
Cardiovascular diseases
Neoplasms
Nutritional deficiencies
Maternal and neonatal disorders
Other infectious diseases
Neglected tropical diseases and malaria
Enteric infections
Respiratory infections and tuberculosis
HIV/AIDS and sexually transmitted infections

Uganda

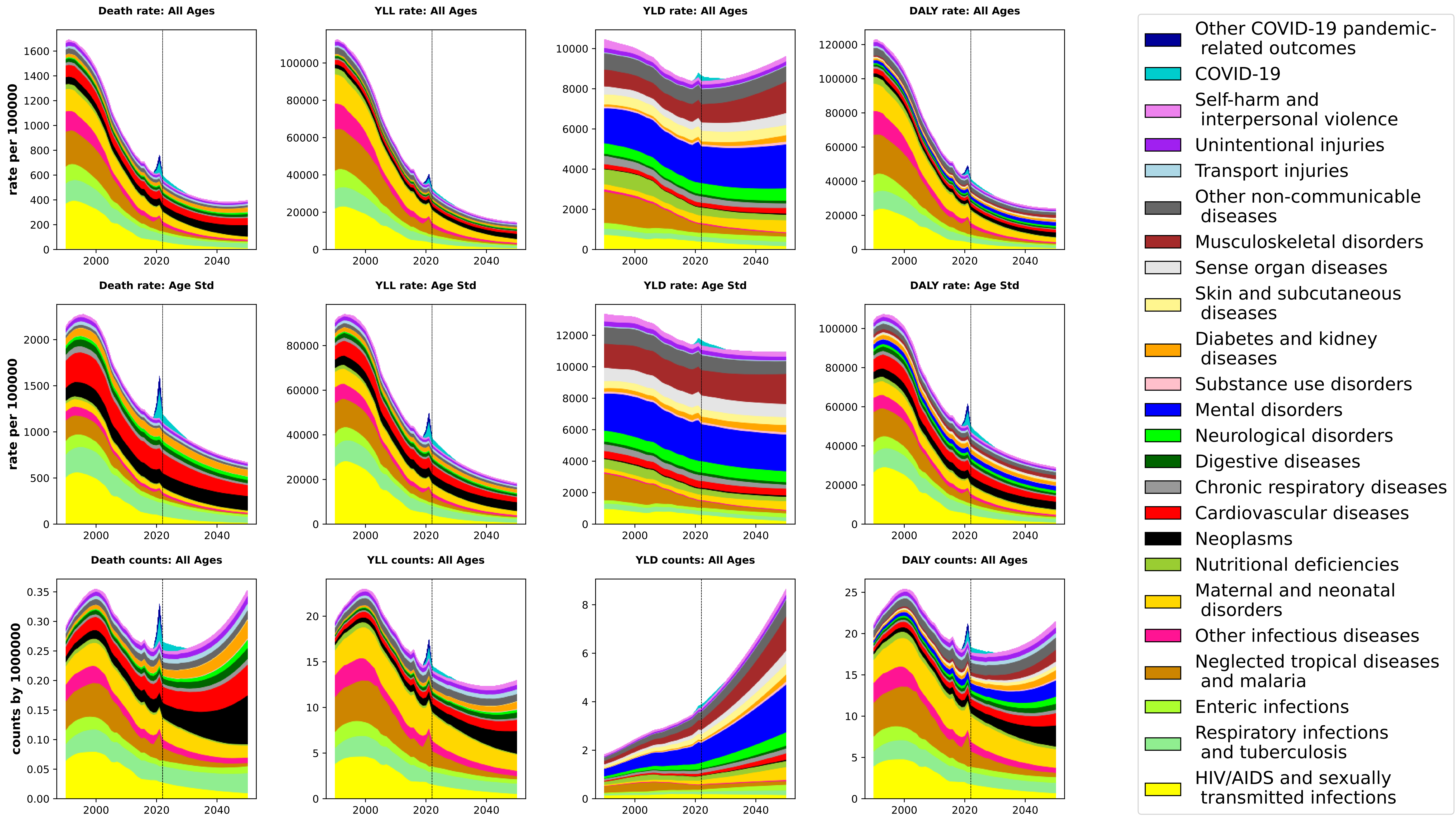
Death rate: All Ages
YLL rate: All Ages
YLD rate: All Ages
DALY rate: All Ages
Death rate: Age Std
YLL rate: Age Std
YLD rate: Age Std
DALY rate: Age Std
Death counts: All Ages
YLL counts: All Ages
YLD counts: All Ages
DALY counts: All Ages
rate per 100000
counts by 1000000
2000
2020
2040
Other COVID-19 pandemic-related outcomes
COVID-19
Self-harm and interpersonal violence
Unintentional injuries
Transport injuries
Other non-communicable diseases
Musculoskeletal disorders
Sense organ diseases
Skin and subcutaneous diseases
Diabetes and kidney diseases
Substance use disorders
Mental disorders
Neurological disorders
Digestive diseases
Chronic respiratory diseases
Cardiovascular diseases
Neoplasms
Nutritional deficiencies
Maternal and neonatal disorders
Other infectious diseases
Neglected tropical diseases and malaria
Enteric infections
Respiratory infections and tuberculosis
HIV/AIDS and sexually transmitted infections

Tanzania

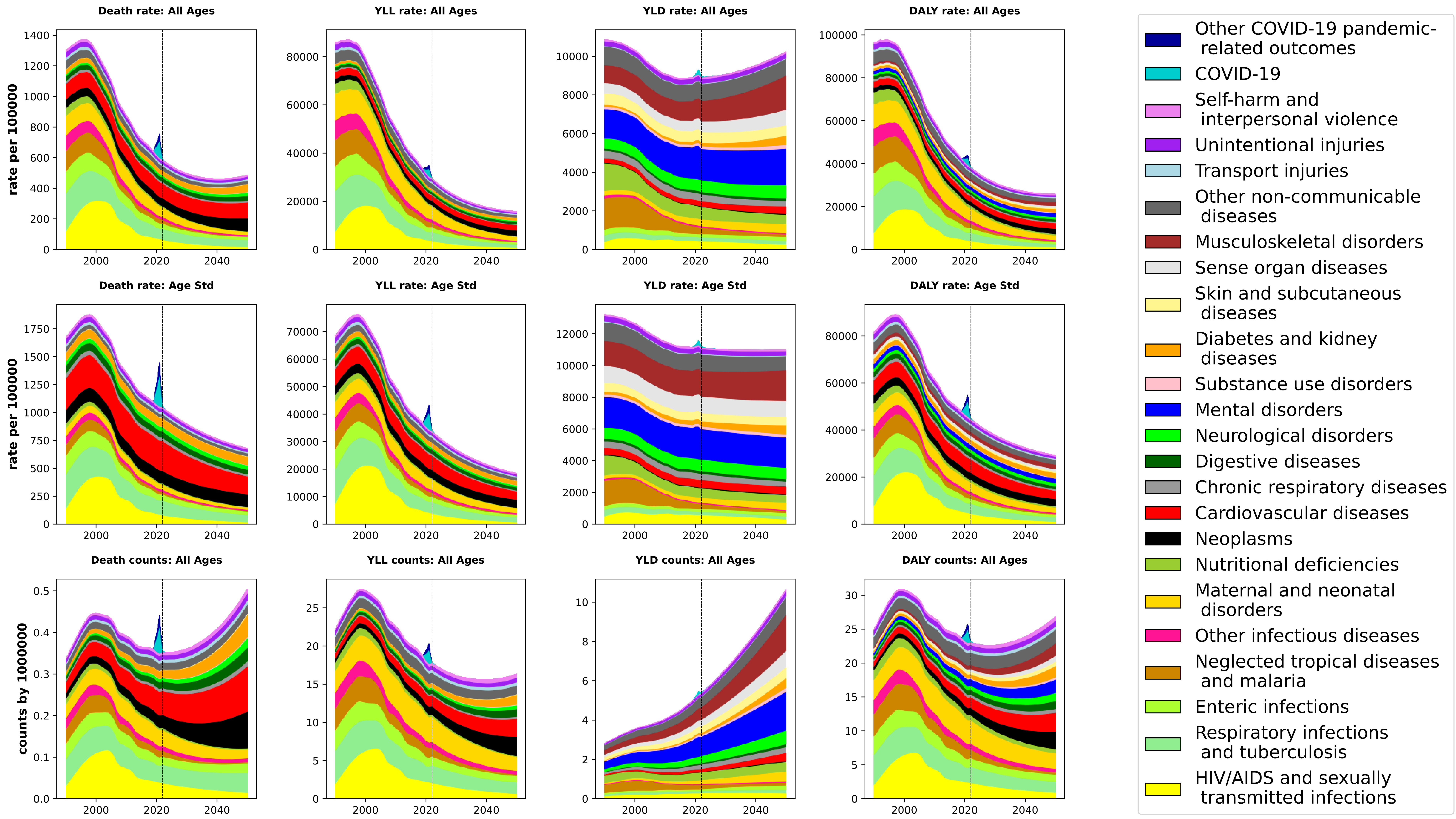
Death rate: All Ages
YLL rate: All Ages
YLD rate: All Ages
DALY rate: All Ages
Death rate: Age Std
YLL rate: Age Std
YLD rate: Age Std
DALY rate: Age Std
Death counts: All Ages
YLL counts: All Ages
YLD counts: All Ages
DALY counts: All Ages
rate per 100000
counts by 1000000
2000
2020
2040
Other COVID-19 pandemic-related outcomes
COVID-19
Self-harm and interpersonal violence
Unintentional injuries
Transport injuries
Other non-communicable diseases
Musculoskeletal disorders
Sense organ diseases
Skin and subcutaneous diseases
Diabetes and kidney diseases
Substance use disorders
Mental disorders
Neurological disorders
Digestive diseases
Chronic respiratory diseases
Cardiovascular diseases
Neoplasms
Nutritional deficiencies
Maternal and neonatal disorders
Other infectious diseases
Neglected tropical diseases and malaria
Enteric infections
Respiratory infections and tuberculosis
HIV/AIDS and sexually transmitted infections

Zambia

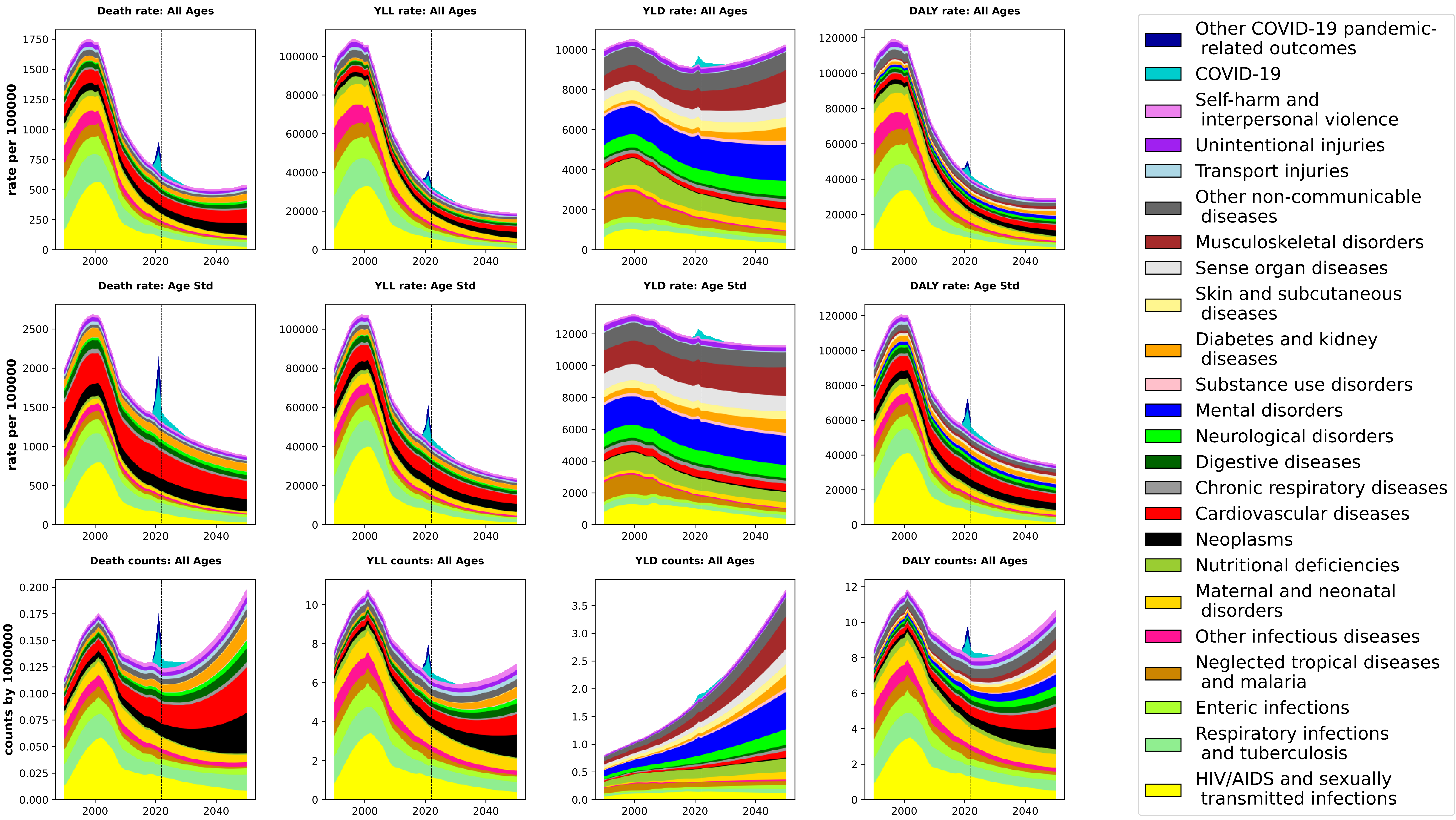
Death rate: All Ages
YLL rate: All Ages
YLD rate: All Ages
DALY rate: All Ages
Death rate: Age Std
YLL rate: Age Std
YLD rate: Age Std
DALY rate: Age Std
Death counts: All Ages
YLL counts: All Ages
YLD counts: All Ages
DALY counts: All Ages
rate per 100000
counts by 1000000
2000
2020
2040
Other COVID-19 pandemic-related outcomes
COVID-19
Self-harm and interpersonal violence
Unintentional injuries
Transport injuries
Other non-communicable diseases
Musculoskeletal disorders
Sense organ diseases
Skin and subcutaneous diseases
Diabetes and kidney diseases
Substance use disorders
Mental disorders
Neurological disorders
Digestive diseases
Chronic respiratory diseases
Cardiovascular diseases
Neoplasms
Nutritional deficiencies
Maternal and neonatal disorders
Other infectious diseases
Neglected tropical diseases and malaria
Enteric infections
Respiratory infections and tuberculosis
HIV/AIDS and sexually transmitted infections

Botswana

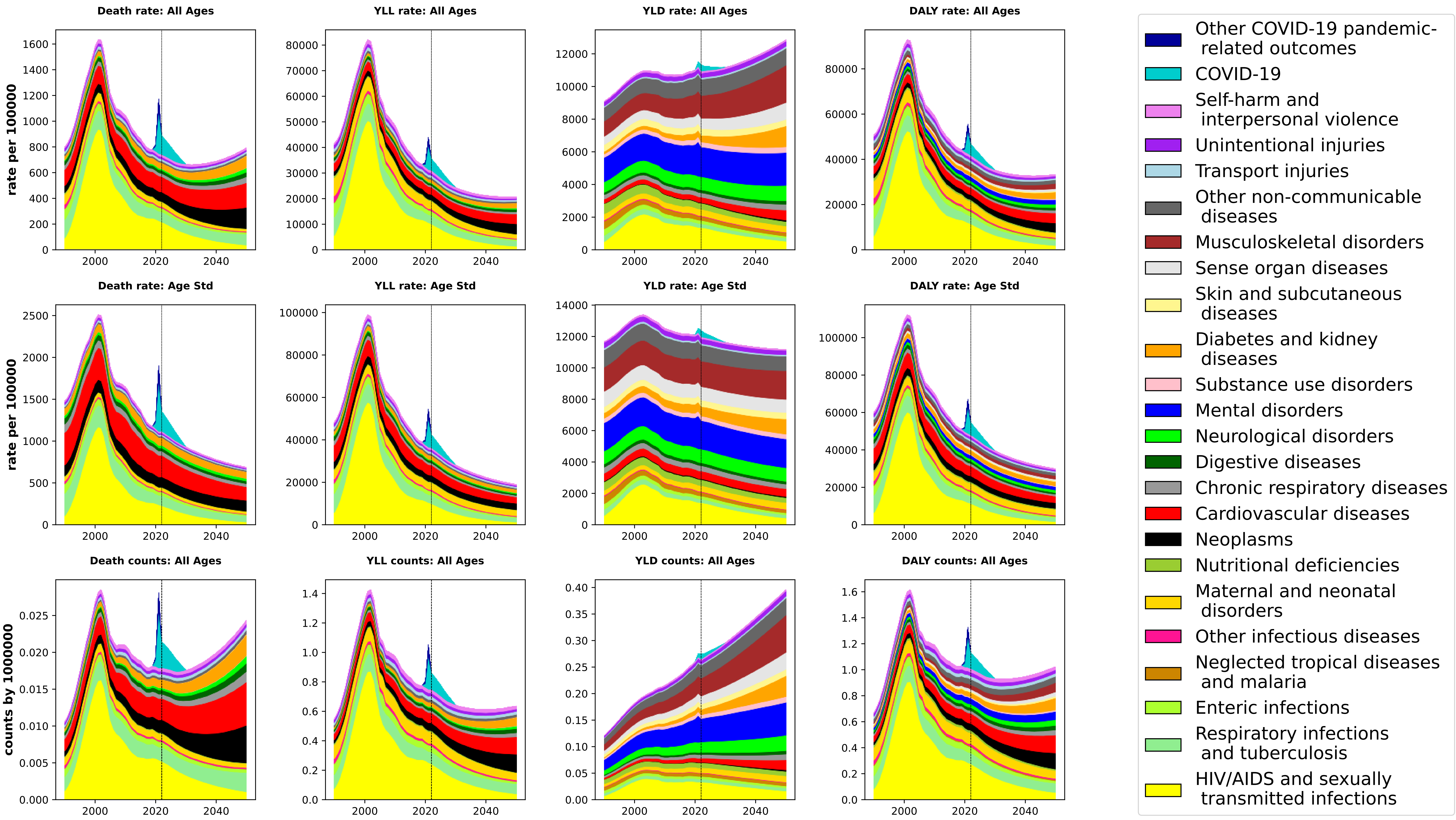

Death rate: All Ages
YLL rate: All Ages
YLD rate: All Ages
DALY rate: All Ages
Death rate: Age Std
YLL rate: Age Std
YLD rate: Age Std
DALY rate: Age Std
Death counts: All Ages
YLL counts: All Ages
YLD counts: All Ages
DALY counts: All Ages
rate per 100000
rate per 100000
counts by 1000000
2000
2020
2040
Other COVID-19 pandemic-related outcomes
COVID-19
Self-harm and interpersonal violence
Unintentional injuries
Transport injuries
Other non-communicable diseases
Musculoskeletal disorders
Sense organ diseases
Skin and subcutaneous diseases
Diabetes and kidney diseases
Substance use disorders
Mental disorders
Neurological disorders
Digestive diseases
Chronic respiratory diseases
Cardiovascular diseases
Neoplasms
Nutritional deficiencies
Maternal and neonatal disorders
Other infectious diseases
Neglected tropical diseases and malaria
Enteric infections
Respiratory infections and tuberculosis
HIV/AIDS and sexually transmitted infections

Eswatini

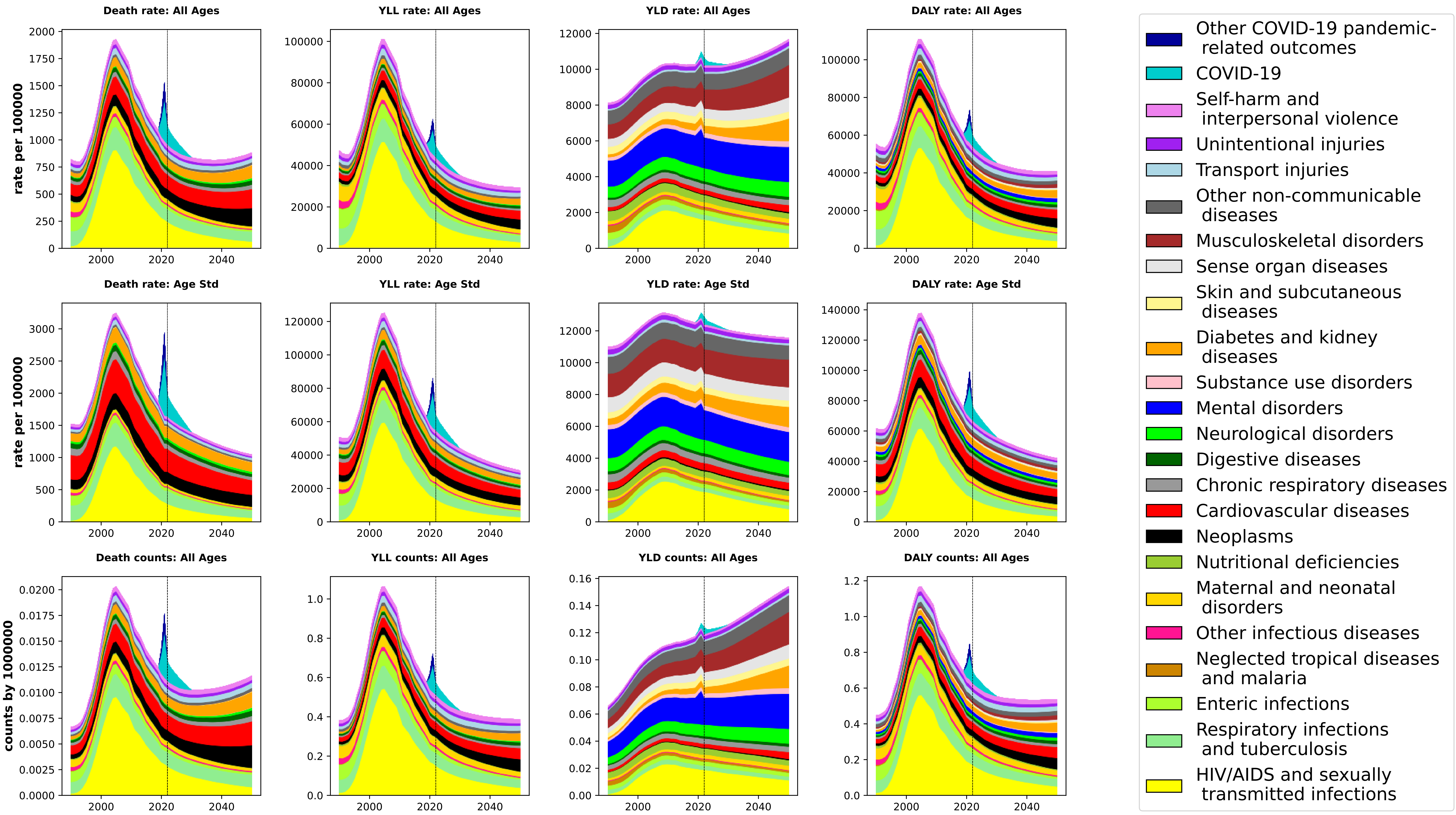
Death rate: All Ages
YLL rate: All Ages
YLD rate: All Ages
DALY rate: All Ages
Death rate: Age Std
YLL rate: Age Std
YLD rate: Age Std
DALY rate: Age Std
Death counts: All Ages
YLL counts: All Ages
YLD counts: All Ages
DALY counts: All Ages
rate per 100000
counts by 1000000
2000
2020
2040
Other COVID-19 pandemic-related outcomes
COVID-19
Self-harm and interpersonal violence
Unintentional injuries
Transport injuries
Other non-communicable diseases
Musculoskeletal disorders
Sense organ diseases
Skin and subcutaneous diseases
Diabetes and kidney diseases
Substance use disorders
Mental disorders
Neurological disorders
Digestive diseases
Chronic respiratory diseases
Cardiovascular diseases
Neoplasms
Nutritional deficiencies
Maternal and neonatal disorders
Other infectious diseases
Neglected tropical diseases and malaria
Enteric infections
Respiratory infections and tuberculosis
HIV/AIDS and sexually transmitted infections

Lesotho

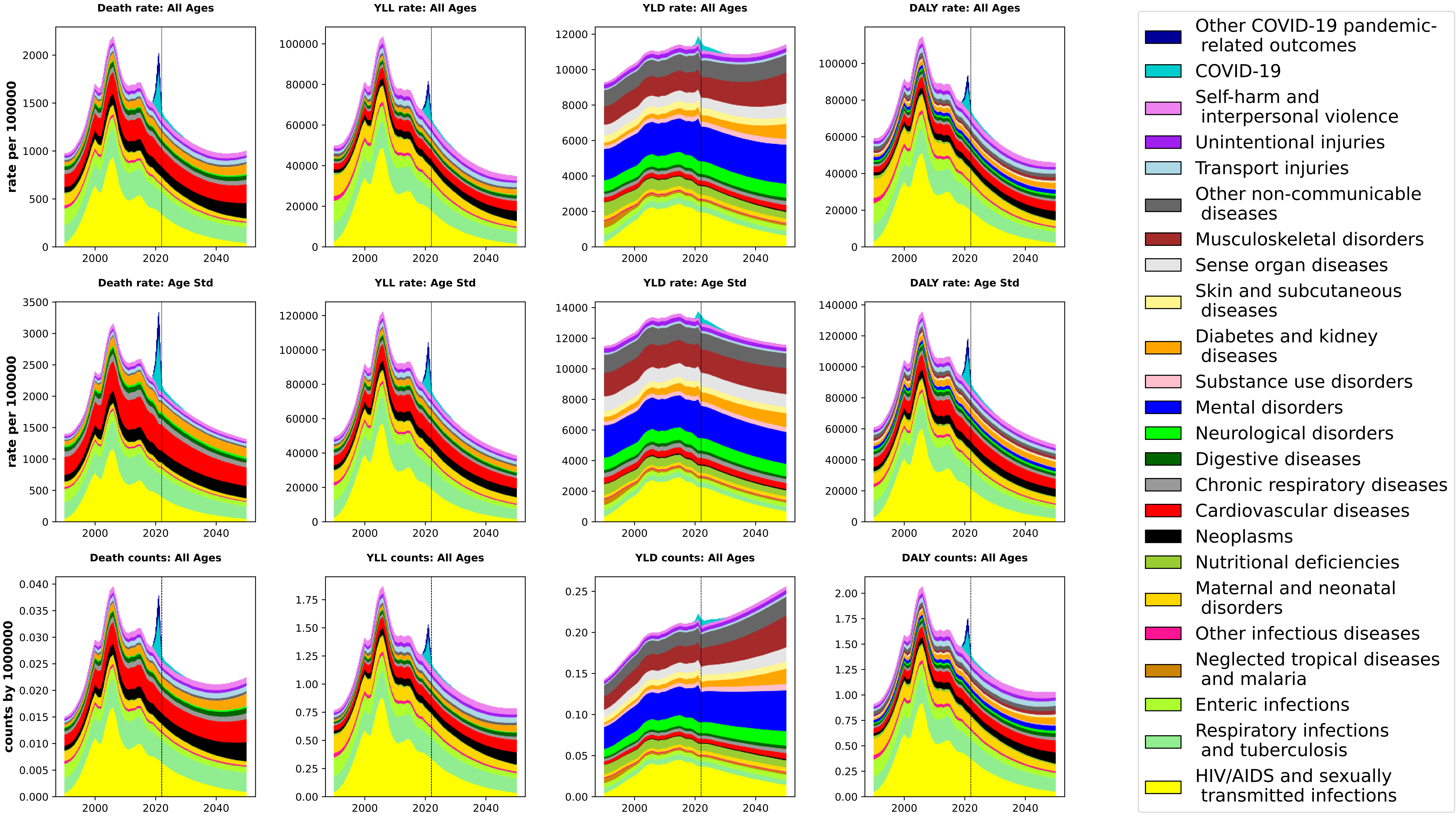
Death rate: All Ages
YLL rate: All Ages
YLD rate: All Ages
DALY rate: All Ages
Death rate: Age Std
YLL rate: Age Std
YLD rate: Age Std
DALY rate: Age Std
Death counts: All Ages
YLL counts: All Ages
YLD counts: All Ages
DALY counts: All Ages
rate per 100000
counts by 1000000
Other COVID-19 pandemic-related outcomes
COVID-19
Self-harm and interpersonal violence
Unintentional injuries
Transport injuries
Other non-communicable diseases
Musculoskeletal disorders
Sense organ diseases
Skin and subcutaneous diseases
Diabetes and kidney diseases
Substance use disorders
Mental disorders
Neurological disorders
Digestive diseases
Chronic respiratory diseases
Cardiovascular diseases
Neoplasms
Nutritional deficiencies
Maternal and neonatal disorders
Other infectious diseases
Neglected tropical diseases and malaria
Enteric infections
Respiratory infections and tuberculosis
HIV/AIDS and sexually transmitted infections

Namibia

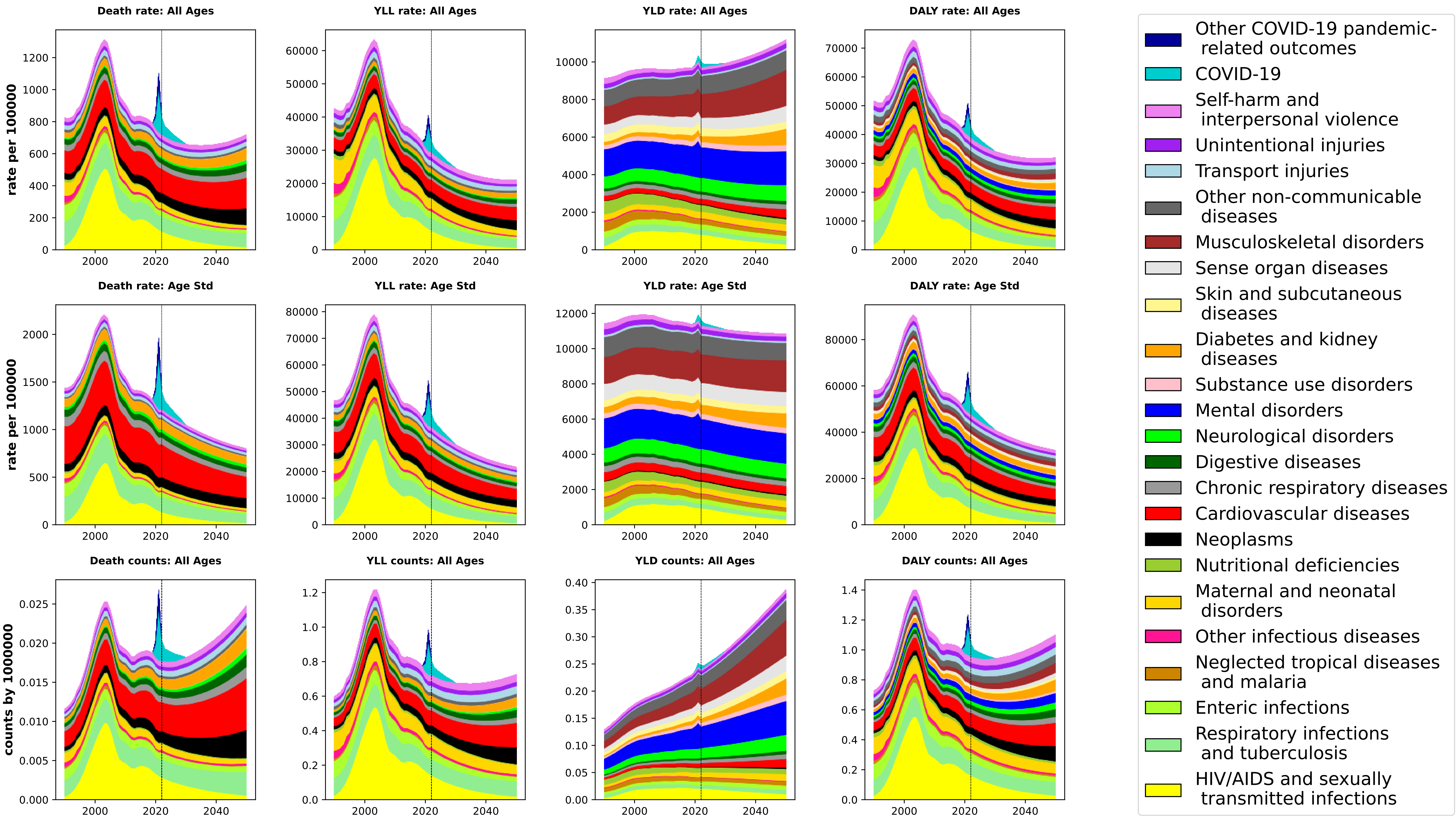
Death rate: All Ages
YLL rate: All Ages
YLD rate: All Ages
DALY rate: All Ages
Death rate: Age Std
YLL rate: Age Std
YLD rate: Age Std
DALY rate: Age Std
Death counts: All Ages
YLL counts: All Ages
YLD counts: All Ages
DALY counts: All Ages
rate per 100000
counts by 1000000
Other COVID-19 pandemic-related outcomes
COVID-19
Self-harm and interpersonal violence
Unintentional injuries
Transport injuries
Other non-communicable diseases
Musculoskeletal disorders
Sense organ diseases
Skin and subcutaneous diseases
Diabetes and kidney diseases
Substance use disorders
Mental disorders
Neurological disorders
Digestive diseases
Chronic respiratory diseases
Cardiovascular diseases
Neoplasms
Nutritional deficiencies
Maternal and neonatal disorders
Other infectious diseases
Neglected tropical diseases and malaria
Enteric infections
Respiratory infections and tuberculosis
HIV/AIDS and sexually transmitted infections

South Africa

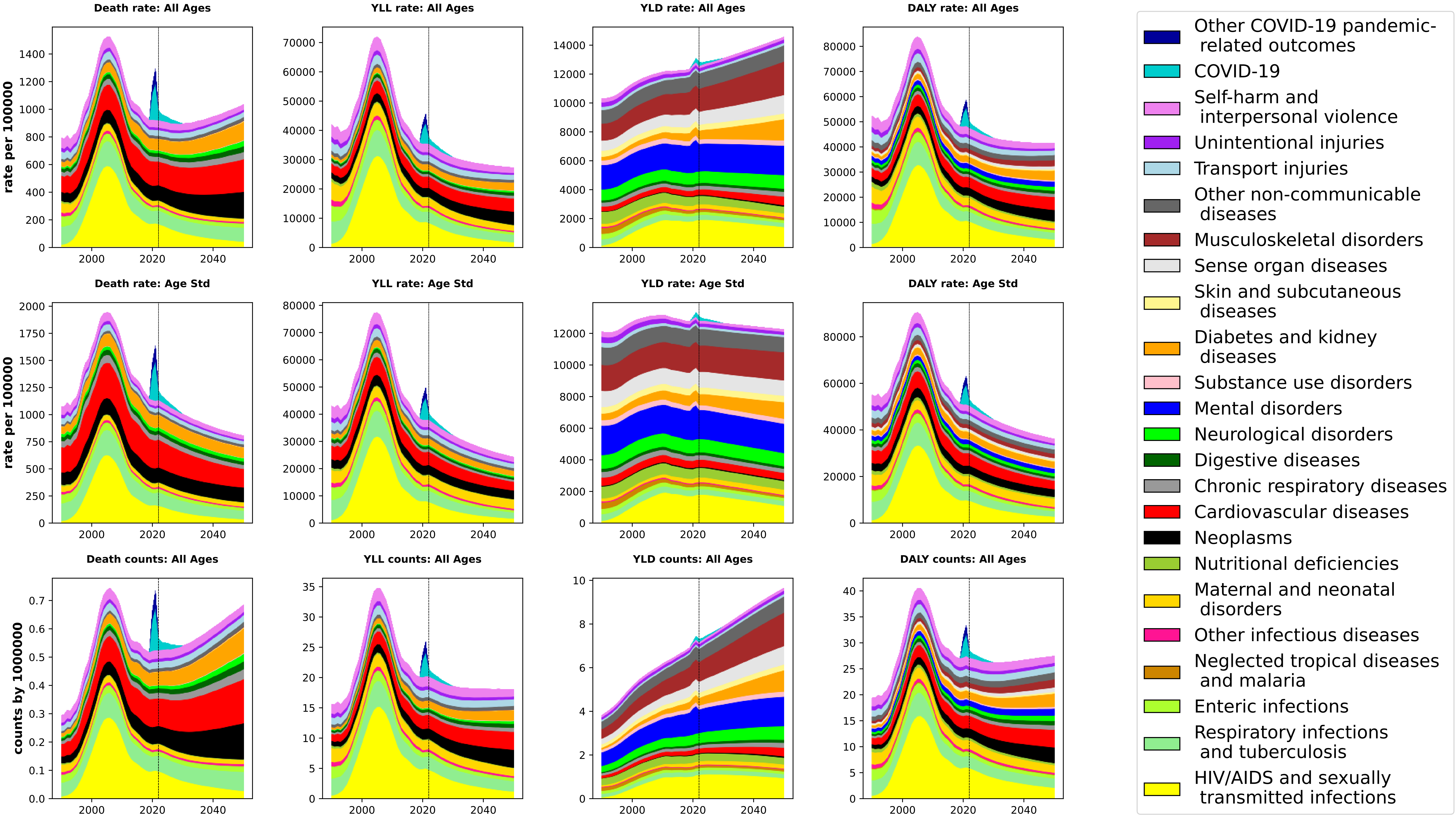

Zimbabwe

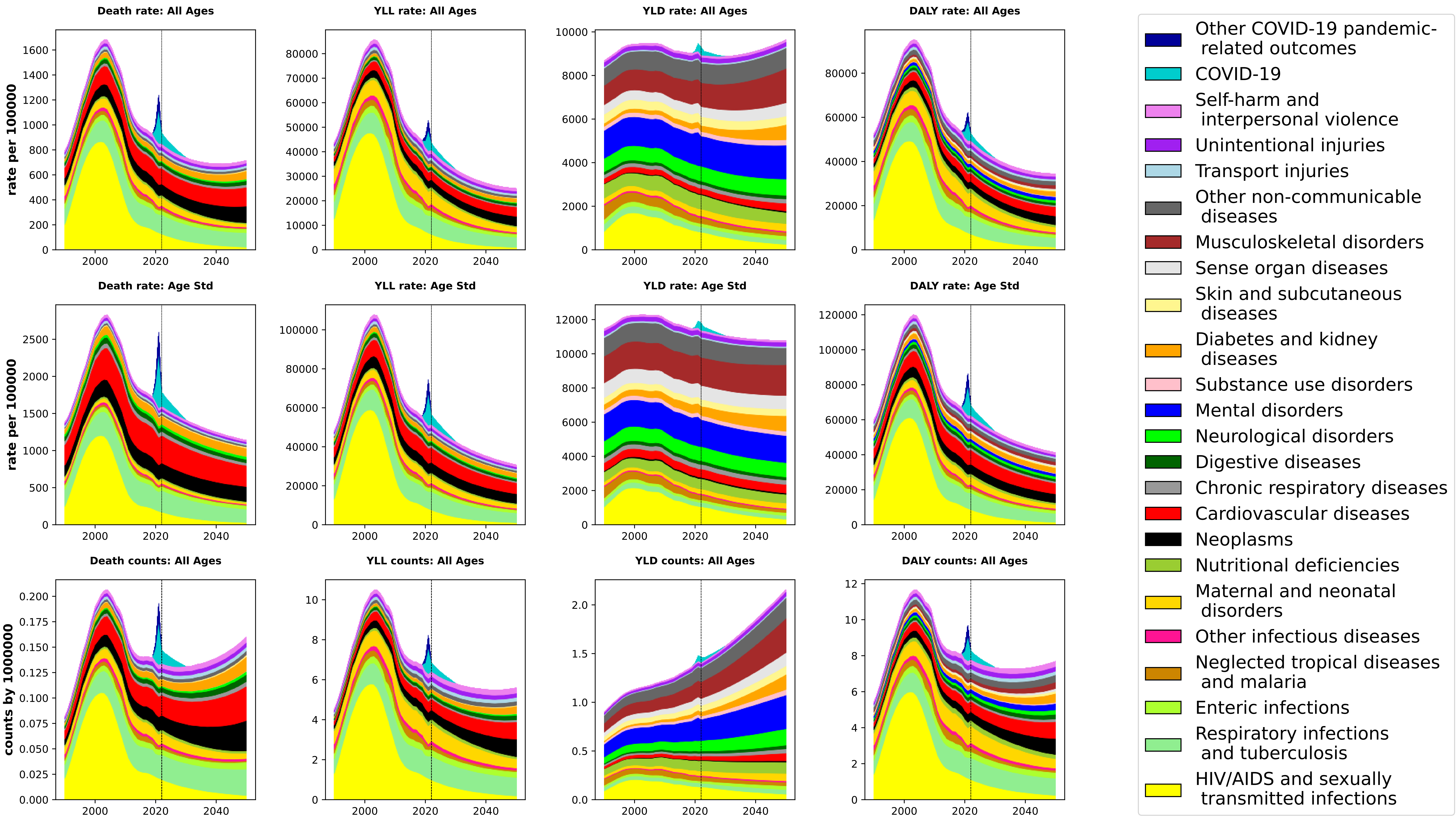
Death rate: All Ages
YLL rate: All Ages
YLD rate: All Ages
DALY rate: All Ages
Death rate: Age Std
YLL rate: Age Std
YLD rate: Age Std
DALY rate: Age Std
Death counts: All Ages
YLL counts: All Ages
YLD counts: All Ages
DALY counts: All Ages
rate per 100000
counts by 1000000
2000
2020
2040
Other COVID-19 pandemic-related outcomes
COVID-19
Self-harm and interpersonal violence
Unintentional injuries
Transport injuries
Other non-communicable diseases
Musculoskeletal disorders
Sense organ diseases
Skin and subcutaneous diseases
Diabetes and kidney diseases
Substance use disorders
Mental disorders
Neurological disorders
Digestive diseases
Chronic respiratory diseases
Cardiovascular diseases
Neoplasms
Nutritional deficiencies
Maternal and neonatal disorders
Other infectious diseases
Neglected tropical diseases and malaria
Enteric infections
Respiratory infections and tuberculosis
HIV/AIDS and sexually transmitted infections

Benin

Supplemental Results Figure S6. Cause level 2 area plots for 4 burden measures for reference scenario by location, 1990-2050

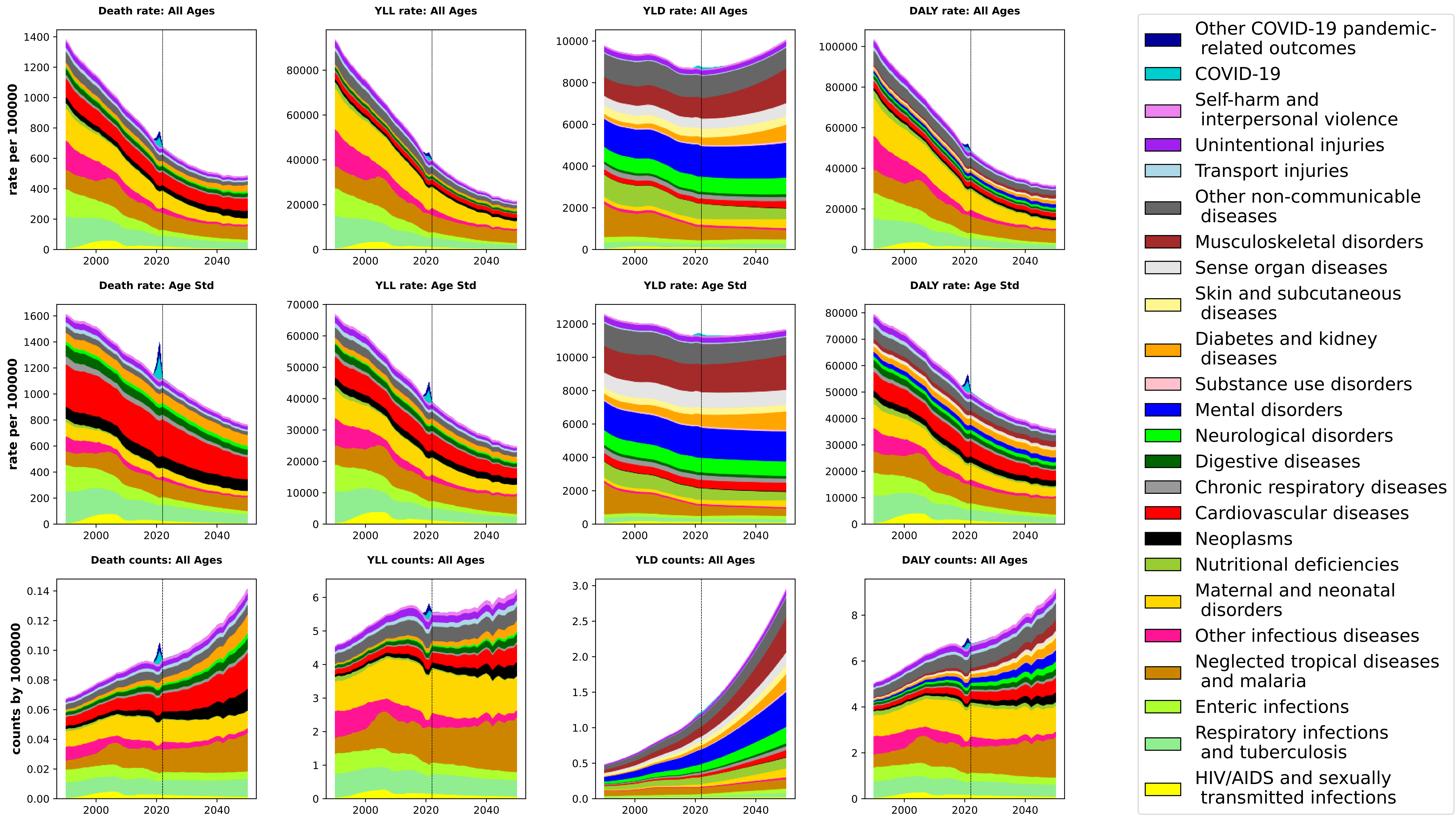

Burkina Faso

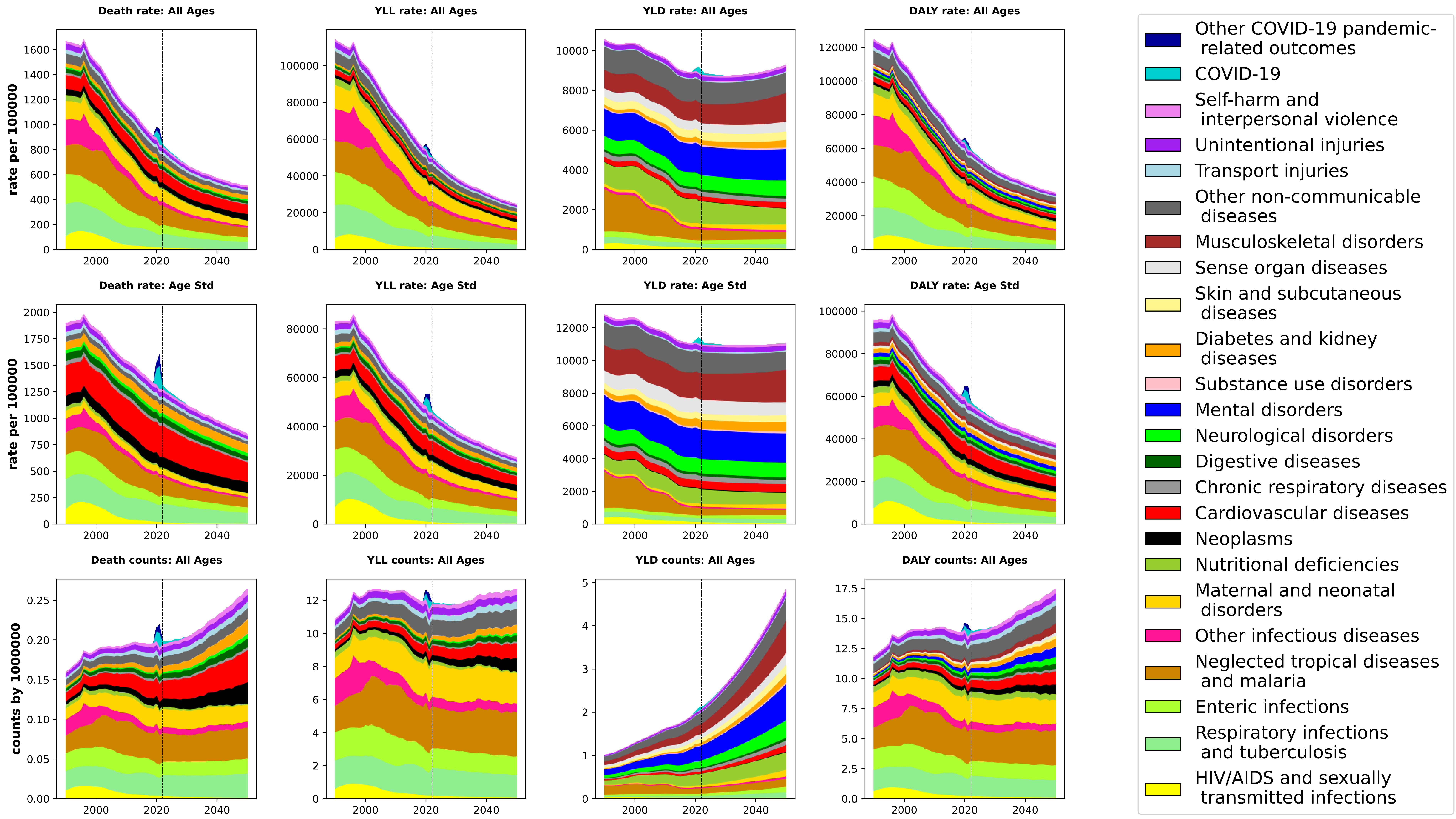

Cabo Verde

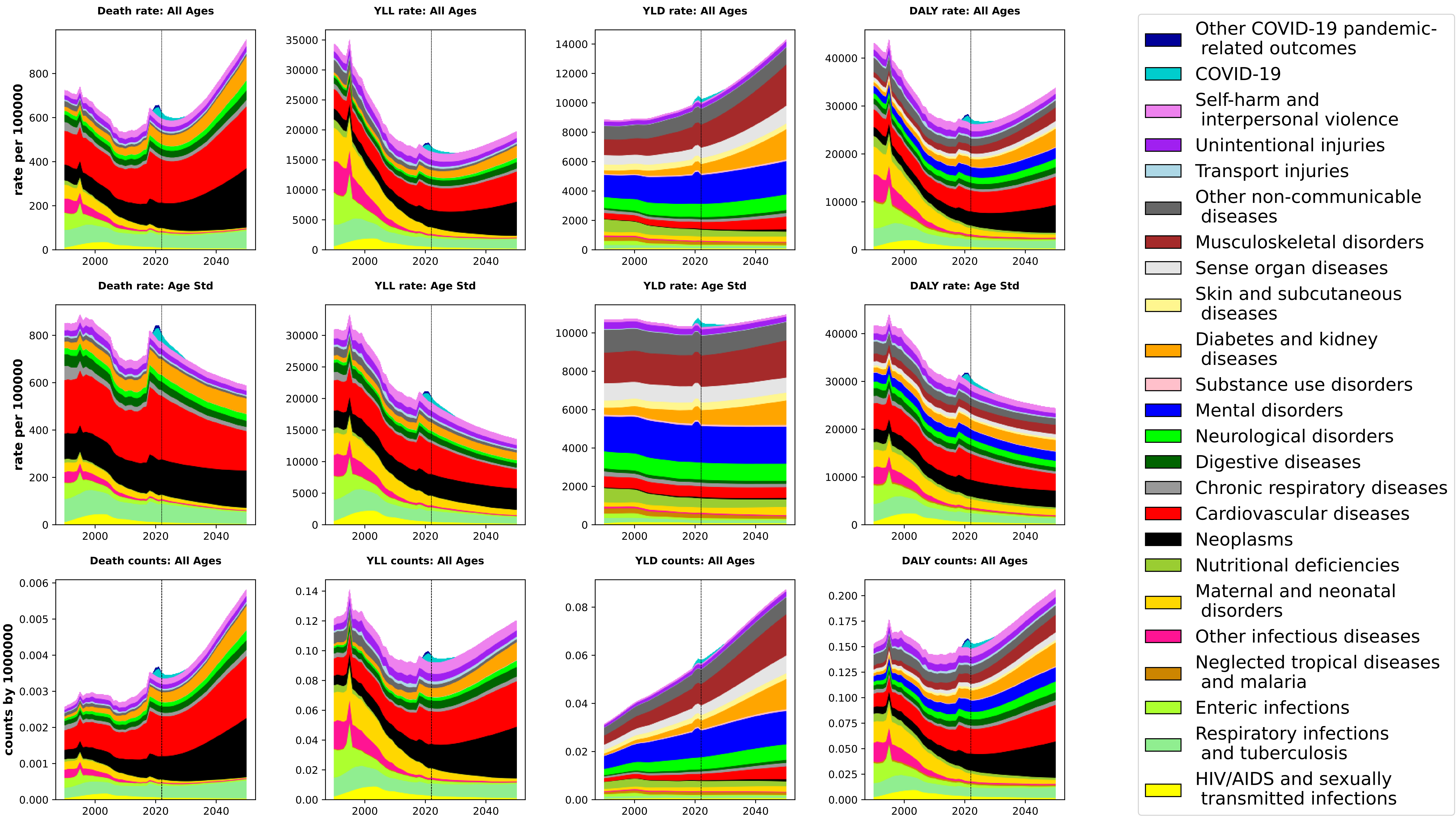
Death rate: All Ages
YLL rate: All Ages
YLD rate: All Ages
DALY rate: All Ages
Death rate: Age Std
YLL rate: Age Std
YLD rate: Age Std
DALY rate: Age Std
Death counts: All Ages
YLL counts: All Ages
YLD counts: All Ages
DALY counts: All Ages
rate per 100000
counts by 1000000
Other COVID-19 pandemic-related outcomes
COVID-19
Self-harm and interpersonal violence
Unintentional injuries
Transport injuries
Other non-communicable diseases
Musculoskeletal disorders
Sense organ diseases
Skin and subcutaneous diseases
Diabetes and kidney diseases
Substance use disorders
Mental disorders
Neurological disorders
Digestive diseases
Chronic respiratory diseases
Cardiovascular diseases
Neoplasms
Nutritional deficiencies
Maternal and neonatal disorders
Other infectious diseases
Neglected tropical diseases and malaria
Enteric infections
Respiratory infections and tuberculosis
HIV/AIDS and sexually transmitted infections

Cameroon

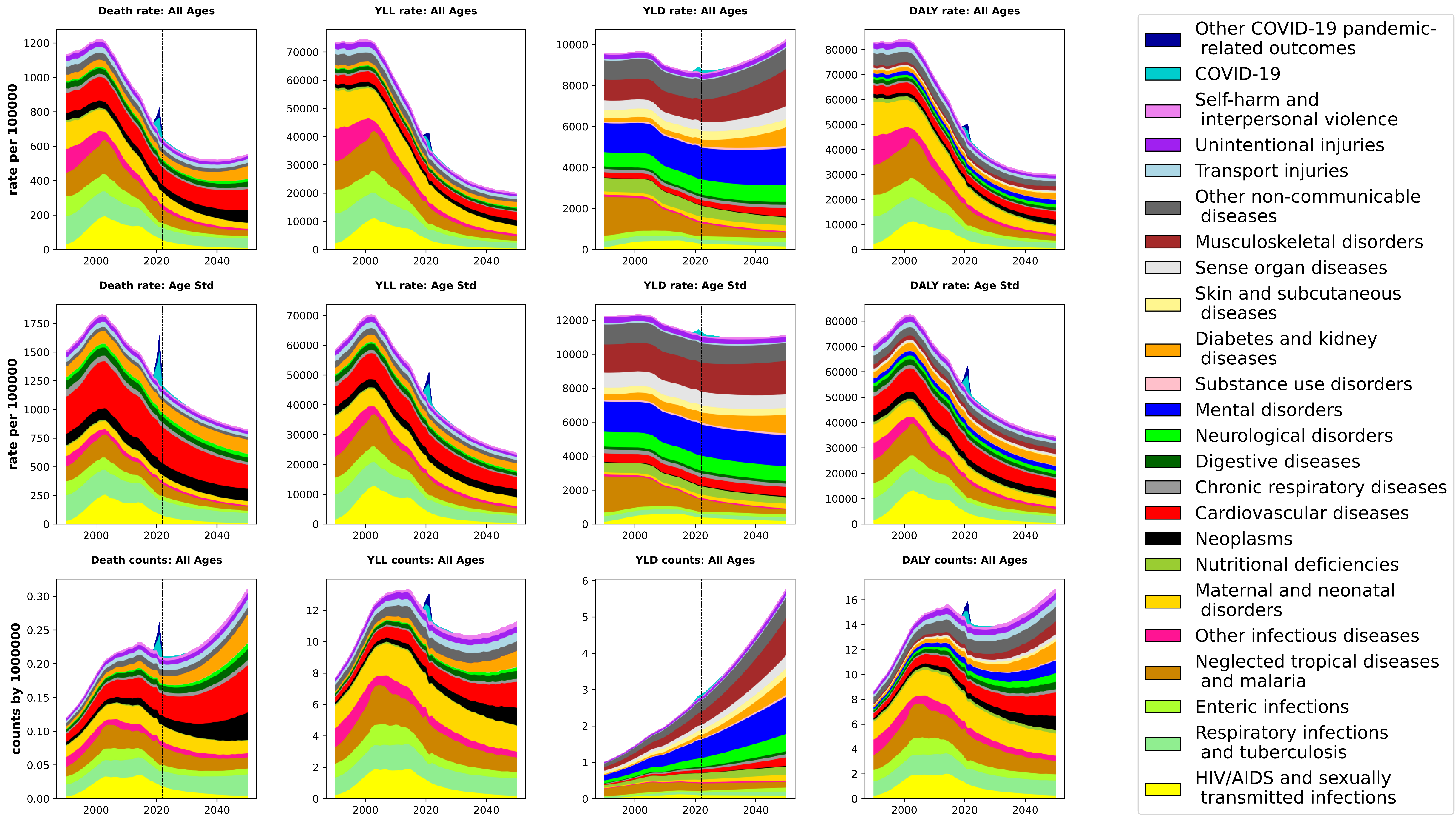

Death rate: All Ages
YLL rate: All Ages
YLD rate: All Ages
DALY rate: All Ages
Death rate: Age Std
YLL rate: Age Std
YLD rate: Age Std
DALY rate: Age Std
Death counts: All Ages
YLL counts: All Ages
YLD counts: All Ages
DALY counts: All Ages
rate per 100000
counts by 1000000
2000
2020
2040
Other COVID-19 pandemic-related outcomes
COVID-19
Self-harm and interpersonal violence
Unintentional injuries
Transport injuries
Other non-communicable diseases
Musculoskeletal disorders
Sense organ diseases
Skin and subcutaneous diseases
Diabetes and kidney diseases
Substance use disorders
Mental disorders
Neurological disorders
Digestive diseases
Chronic respiratory diseases
Cardiovascular diseases
Neoplasms
Nutritional deficiencies
Maternal and neonatal disorders
Other infectious diseases
Neglected tropical diseases and malaria
Enteric infections
Respiratory infections and tuberculosis
HIV/AIDS and sexually transmitted infections

Chad

Supplemental Results Figure S6. Cause level 2 area plots for 4 burden measures for reference scenario by location, 1990-2050

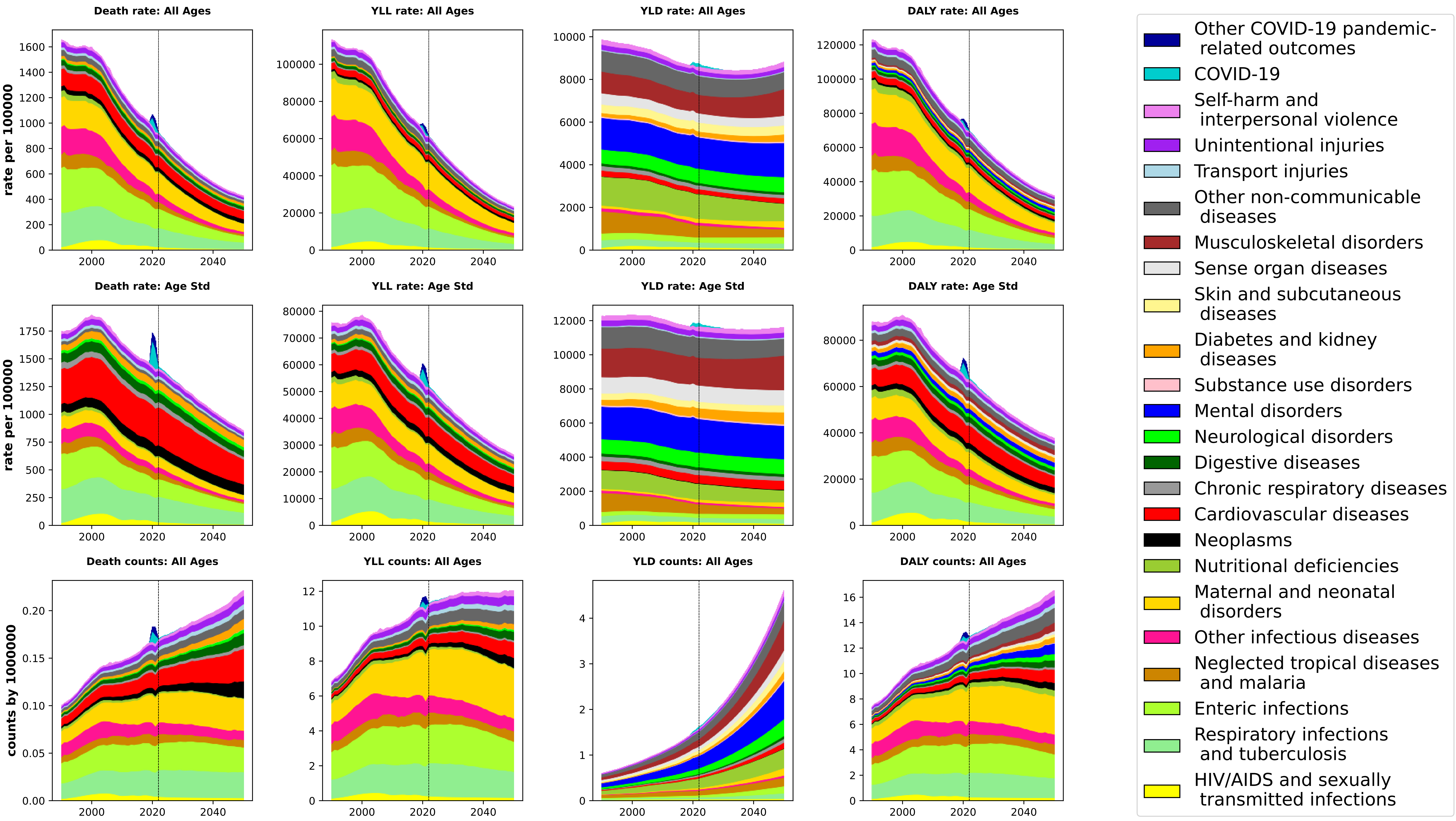

Côte d'Ivoire

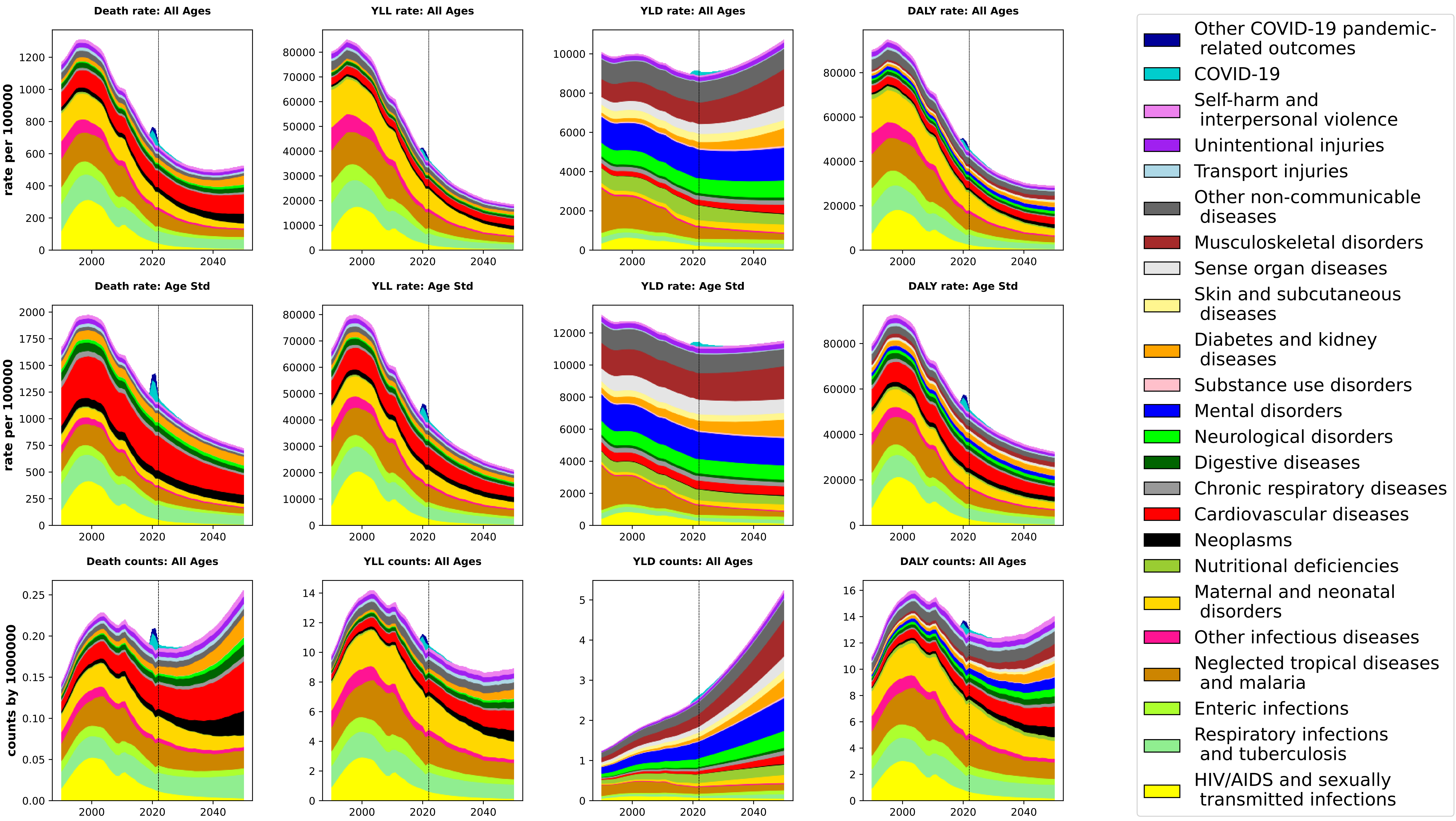

The Gambia

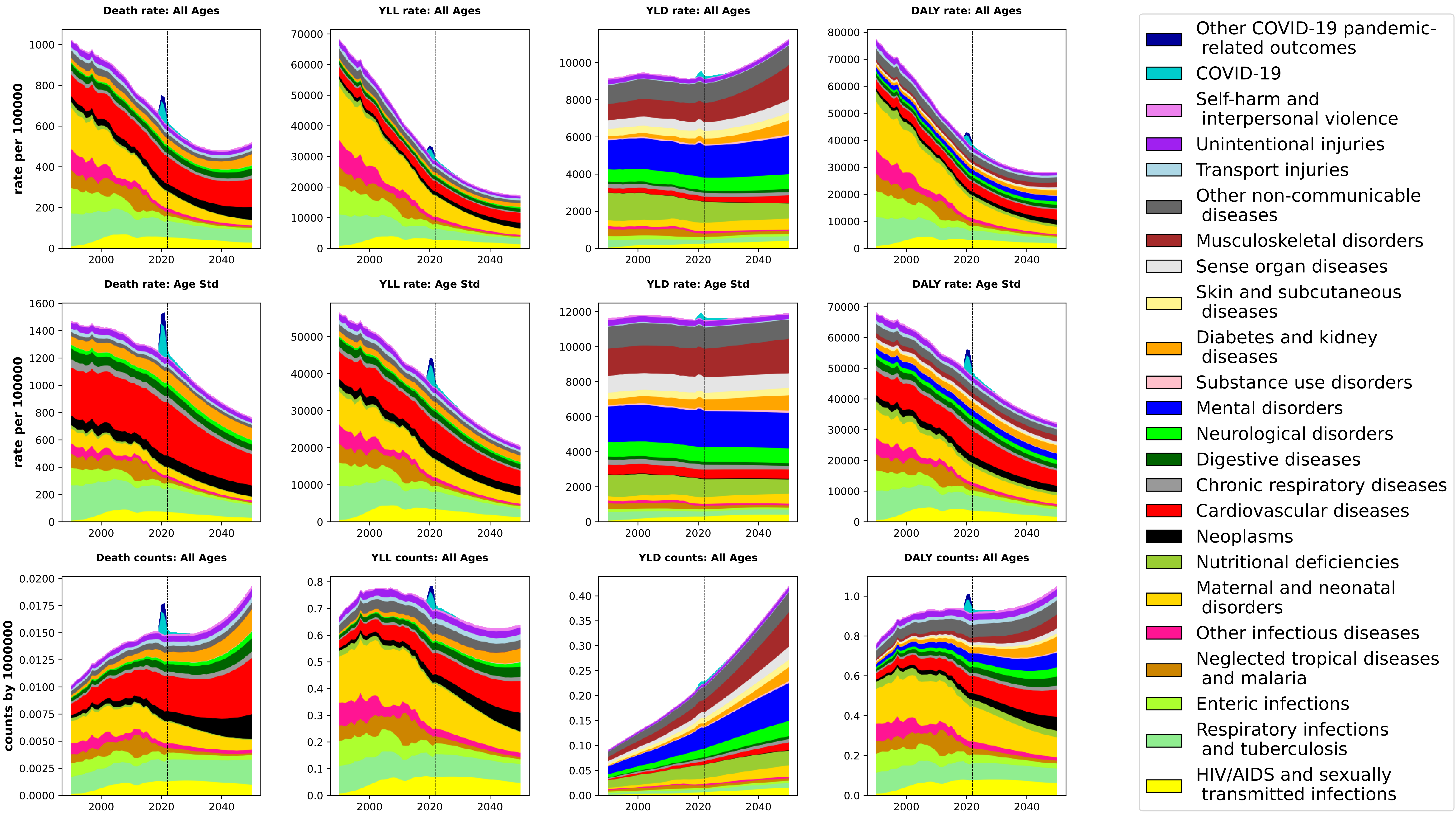

Death rate: All Ages
YLL rate: All Ages
YLD rate: All Ages
DALY rate: All Ages
Death rate: Age Std
YLL rate: Age Std
YLD rate: Age Std
DALY rate: Age Std
Death counts: All Ages
YLL counts: All Ages
YLD counts: All Ages
DALY counts: All Ages
rate per 100000
counts by 1000000
Other COVID-19 pandemic-related outcomes
COVID-19
Self-harm and interpersonal violence
Unintentional injuries
Transport injuries
Other non-communicable diseases
Musculoskeletal disorders
Sense organ diseases
Skin and subcutaneous diseases
Diabetes and kidney diseases
Substance use disorders
Mental disorders
Neurological disorders
Digestive diseases
Chronic respiratory diseases
Cardiovascular diseases
Neoplasms
Nutritional deficiencies
Maternal and neonatal disorders
Other infectious diseases
Neglected tropical diseases and malaria
Enteric infections
Respiratory infections and tuberculosis
HIV/AIDS and sexually transmitted infections

Ghana

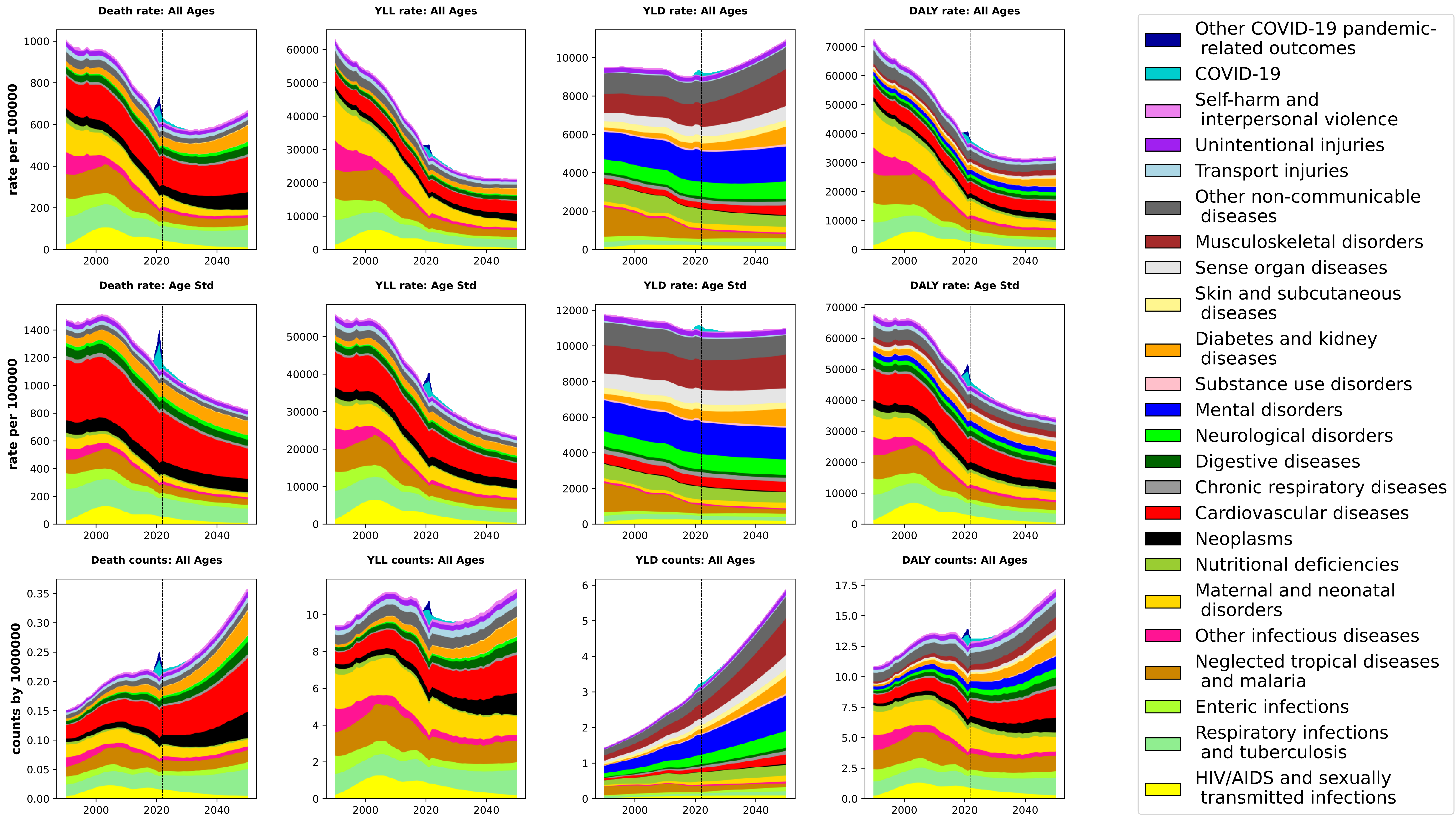
Death rate: All Ages
YLL rate: All Ages
YLD rate: All Ages
DALY rate: All Ages
Death rate: Age Std
YLL rate: Age Std
YLD rate: Age Std
DALY rate: Age Std
Death counts: All Ages
YLL counts: All Ages
YLD counts: All Ages
DALY counts: All Ages
rate per 100000
rate per 100000
counts by 1000000
2000
2020
2040
Other COVID-19 pandemic-related outcomes
COVID-19
Self-harm and interpersonal violence
Unintentional injuries
Transport injuries
Other non-communicable diseases
Musculoskeletal disorders
Sense organ diseases
Skin and subcutaneous diseases
Diabetes and kidney diseases
Substance use disorders
Mental disorders
Neurological disorders
Digestive diseases
Chronic respiratory diseases
Cardiovascular diseases
Neoplasms
Nutritional deficiencies
Maternal and neonatal disorders
Other infectious diseases
Neglected tropical diseases and malaria
Enteric infections
Respiratory infections and tuberculosis
HIV/AIDS and sexually transmitted infections

Guinea

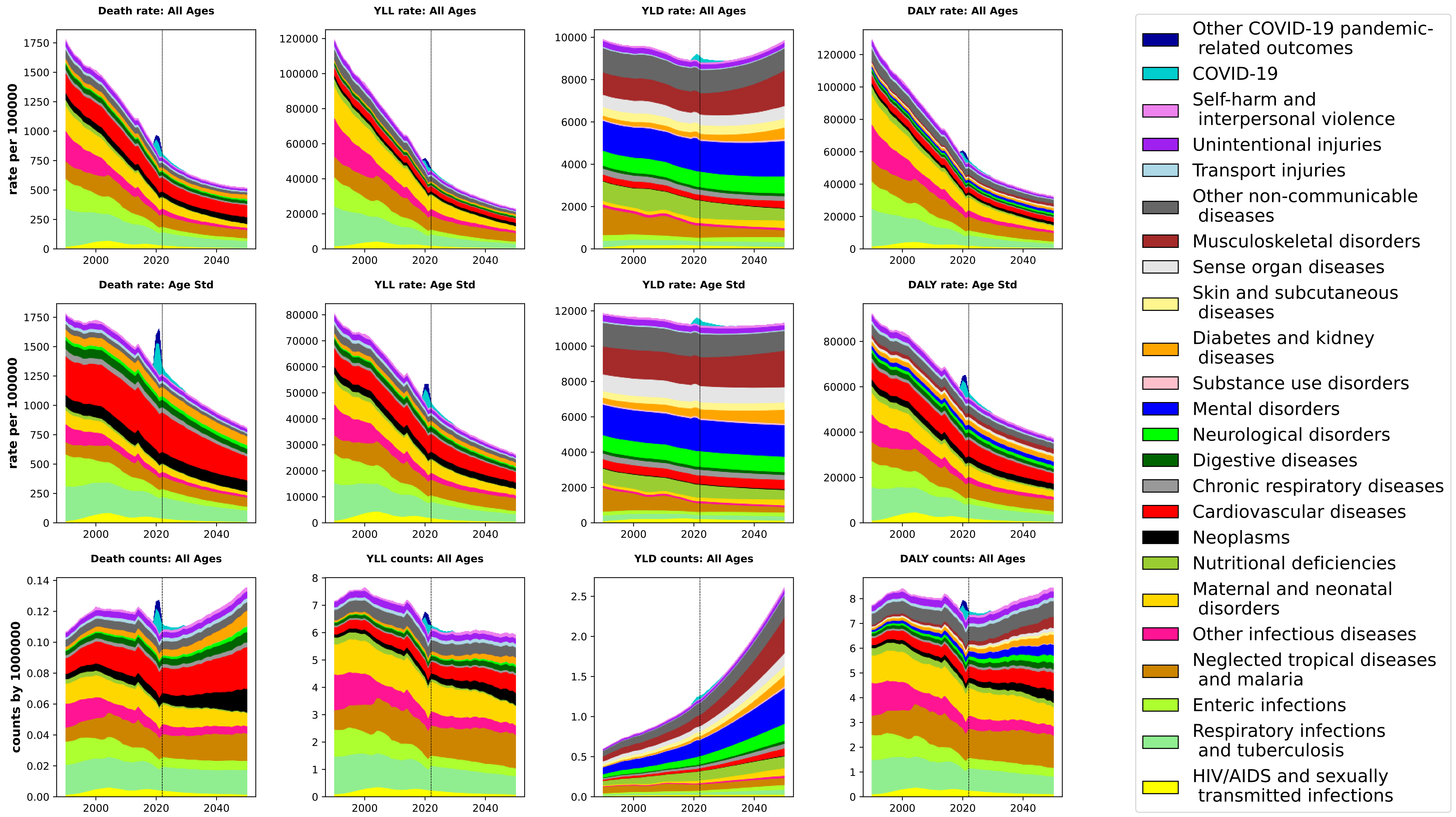
Death rate: All Ages
YLL rate: All Ages
YLD rate: All Ages
DALY rate: All Ages
Death rate: Age Std
YLL rate: Age Std
YLD rate: Age Std
DALY rate: Age Std
Death counts: All Ages
YLL counts: All Ages
YLD counts: All Ages
DALY counts: All Ages
rate per 100000
rate per 100000
counts by 1000000
Other COVID-19 pandemic-related outcomes
COVID-19
Self-harm and interpersonal violence
Unintentional injuries
Transport injuries
Other non-communicable diseases
Musculoskeletal disorders
Sense organ diseases
Skin and subcutaneous diseases
Diabetes and kidney diseases
Substance use disorders
Mental disorders
Neurological disorders
Digestive diseases
Chronic respiratory diseases
Cardiovascular diseases
Neoplasms
Nutritional deficiencies
Maternal and neonatal disorders
Other infectious diseases
Neglected tropical diseases and malaria
Enteric infections
Respiratory infections and tuberculosis
HIV/AIDS and sexually transmitted infections

Guinea-Bissau

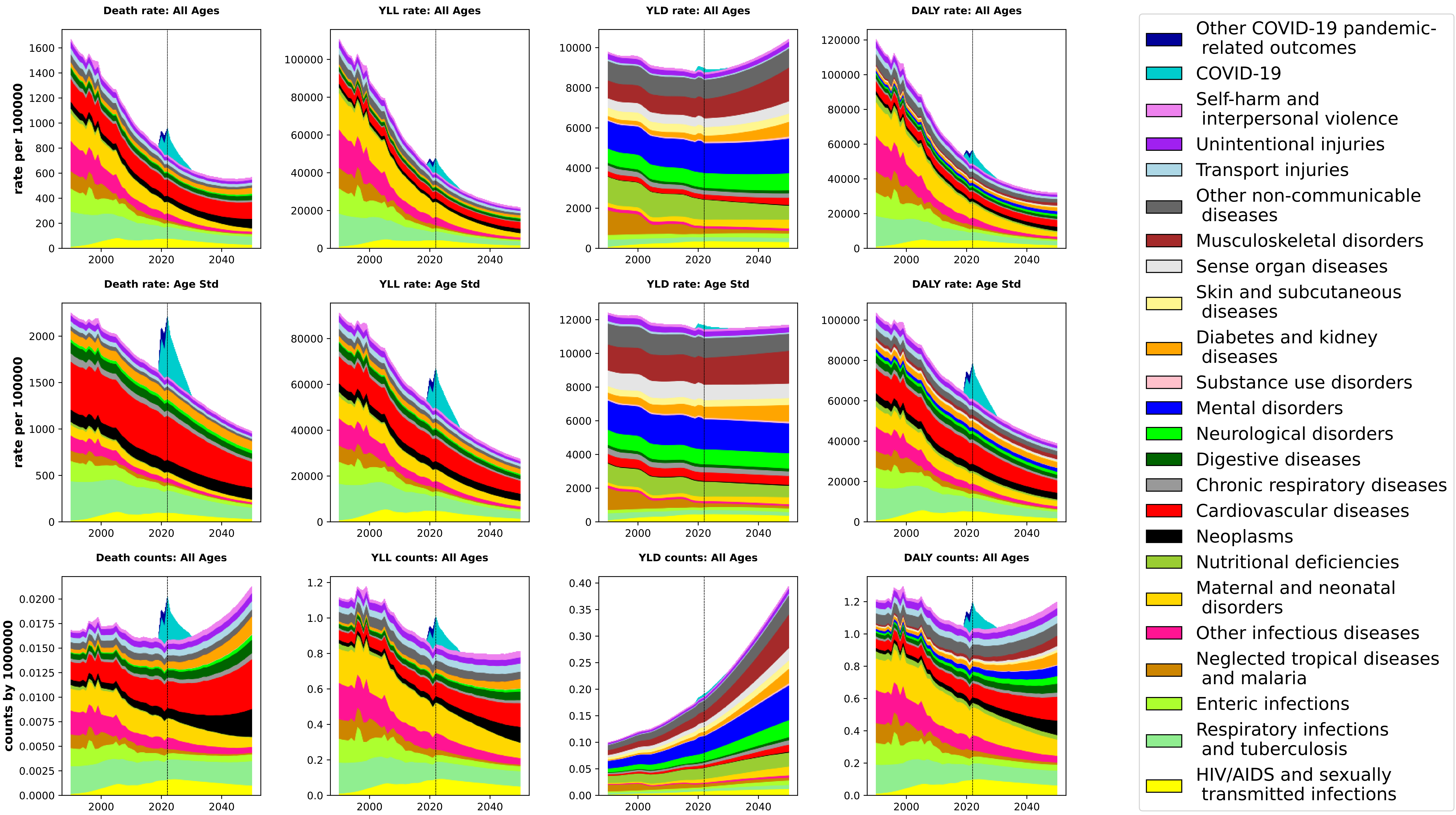

Liberia

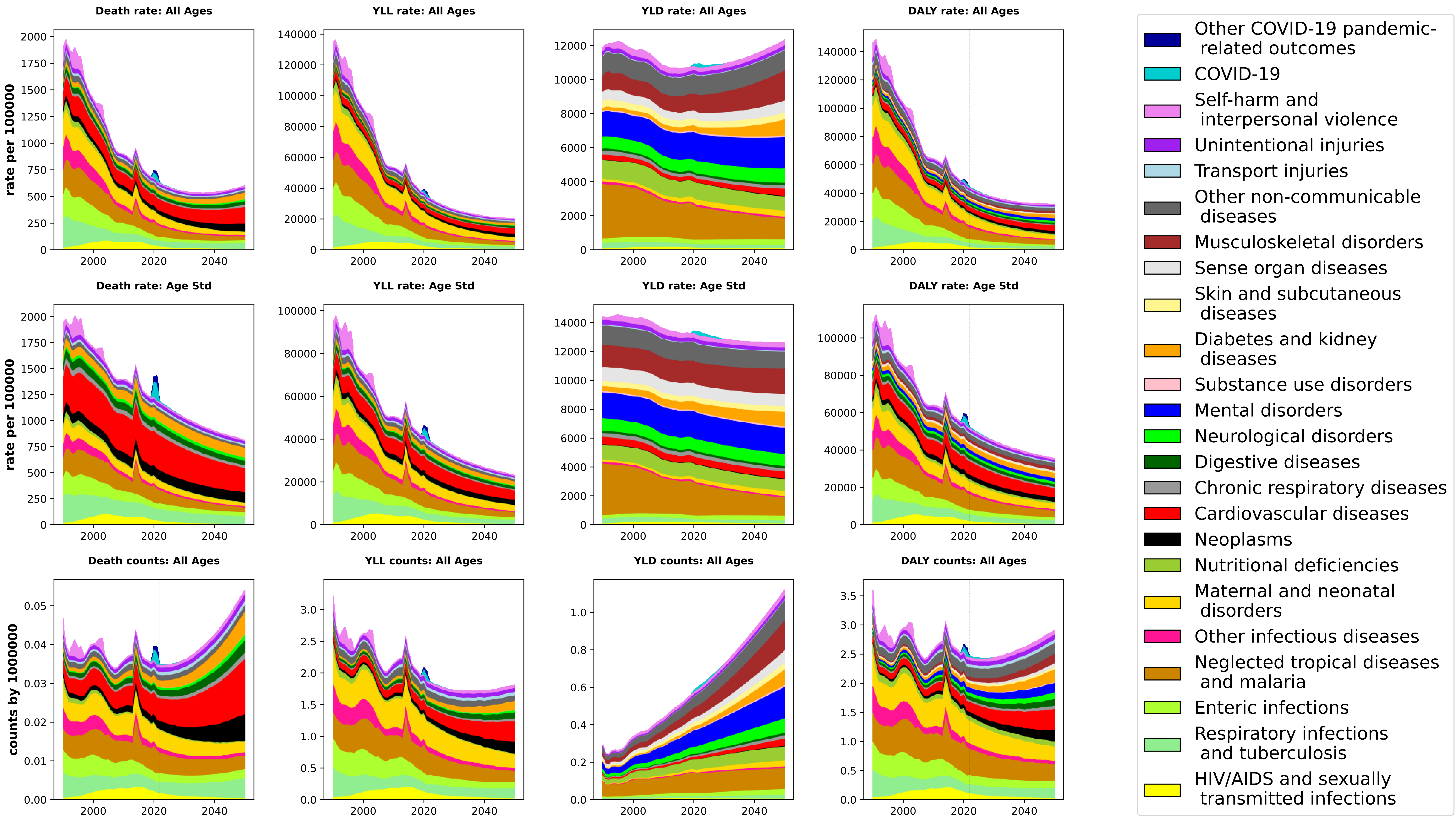
Death rate: All Ages
YLL rate: All Ages
YLD rate: All Ages
DALY rate: All Ages
Death rate: Age Std
YLL rate: Age Std
YLD rate: Age Std
DALY rate: Age Std
Death counts: All Ages
YLL counts: All Ages
YLD counts: All Ages
DALY counts: All Ages
rate per 100000
counts by 1000000
Other COVID-19 pandemic-related outcomes
COVID-19
Self-harm and interpersonal violence
Unintentional injuries
Transport injuries
Other non-communicable diseases
Musculoskeletal disorders
Sense organ diseases
Skin and subcutaneous diseases
Diabetes and kidney diseases
Substance use disorders
Mental disorders
Neurological disorders
Digestive diseases
Chronic respiratory diseases
Cardiovascular diseases
Neoplasms
Nutritional deficiencies
Maternal and neonatal disorders
Other infectious diseases
Neglected tropical diseases and malaria
Enteric infections
Respiratory infections and tuberculosis
HIV/AIDS and sexually transmitted infections

Mali

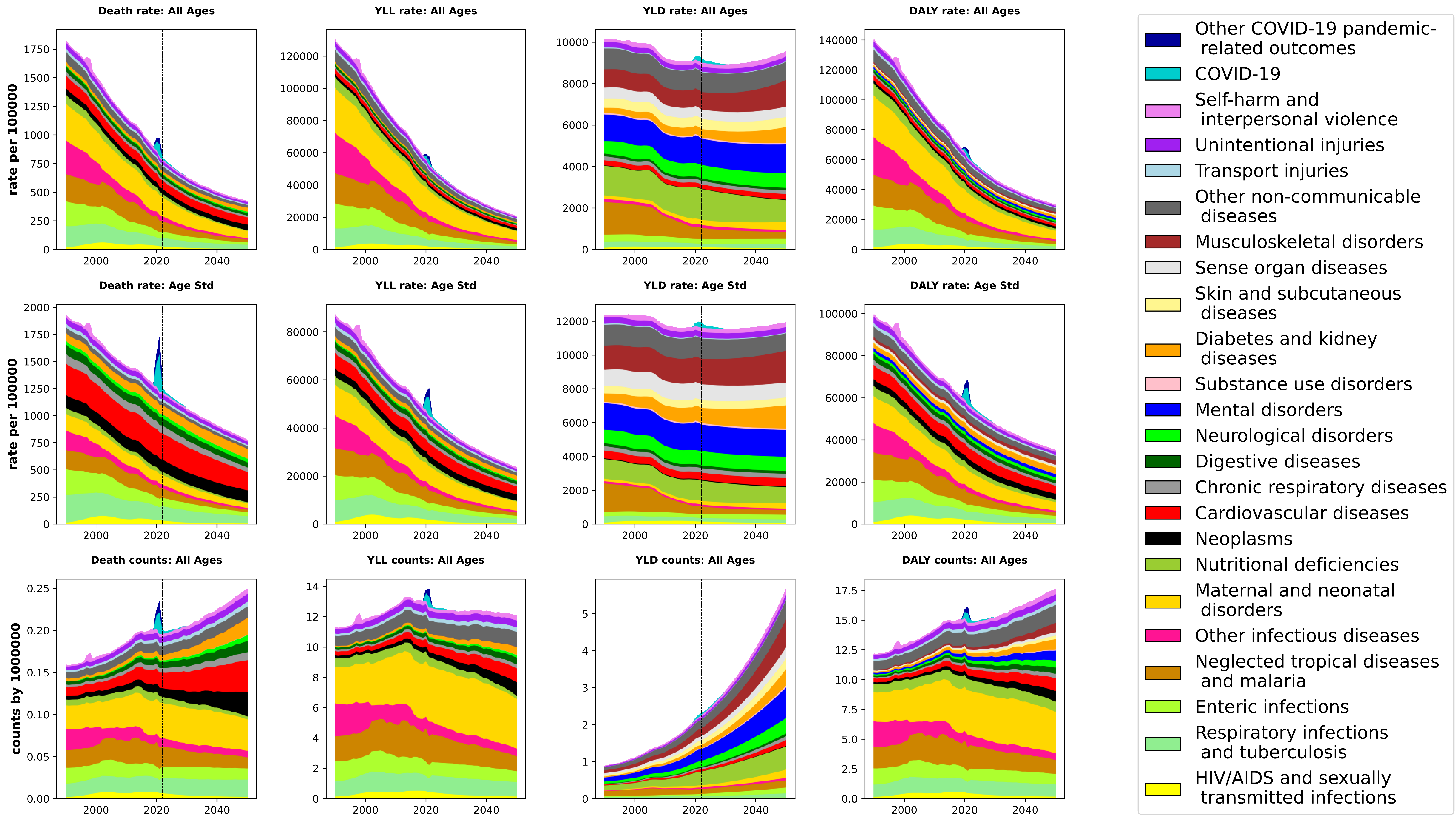

Death rate: All Ages
YLL rate: All Ages
YLD rate: All Ages
DALY rate: All Ages
Death rate: Age Std
YLL rate: Age Std
YLD rate: Age Std
DALY rate: Age Std
Death counts: All Ages
YLL counts: All Ages
YLD counts: All Ages
DALY counts: All Ages
rate per 100000
rate per 100000
counts by 1000000
Other COVID-19 pandemic-related outcomes
COVID-19
Self-harm and interpersonal violence
Unintentional injuries
Transport injuries
Other non-communicable diseases
Musculoskeletal disorders
Sense organ diseases
Skin and subcutaneous diseases
Diabetes and kidney diseases
Substance use disorders
Mental disorders
Neurological disorders
Digestive diseases
Chronic respiratory diseases
Cardiovascular diseases
Neoplasms
Nutritional deficiencies
Maternal and neonatal disorders
Other infectious diseases
Neglected tropical diseases and malaria
Enteric infections
Respiratory infections and tuberculosis
HIV/AIDS and sexually transmitted infections

Mauritania

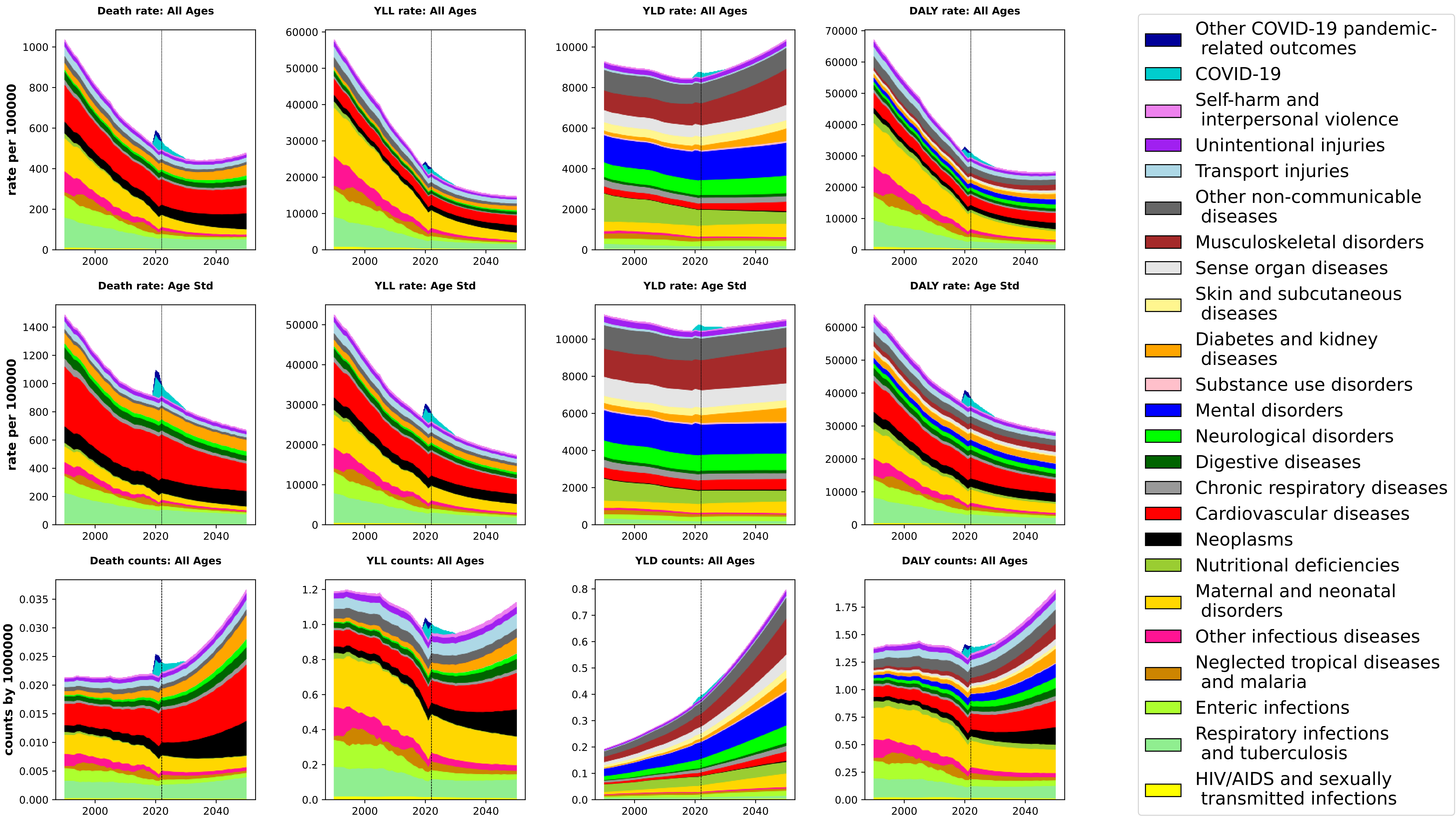
Death rate: All Ages
YLL rate: All Ages
YLD rate: All Ages
DALY rate: All Ages
Death rate: Age Std
YLL rate: Age Std
YLD rate: Age Std
DALY rate: Age Std
Death counts: All Ages
YLL counts: All Ages
YLD counts: All Ages
DALY counts: All Ages
rate per 100000
counts by 1000000
2000
2020
2040
Other COVID-19 pandemic-related outcomes
COVID-19
Self-harm and interpersonal violence
Unintentional injuries
Transport injuries
Other non-communicable diseases
Musculoskeletal disorders
Sense organ diseases
Skin and subcutaneous diseases
Diabetes and kidney diseases
Substance use disorders
Mental disorders
Neurological disorders
Digestive diseases
Chronic respiratory diseases
Cardiovascular diseases
Neoplasms
Nutritional deficiencies
Maternal and neonatal disorders
Other infectious diseases
Neglected tropical diseases and malaria
Enteric infections
Respiratory infections and tuberculosis
HIV/AIDS and sexually transmitted infections

Niger

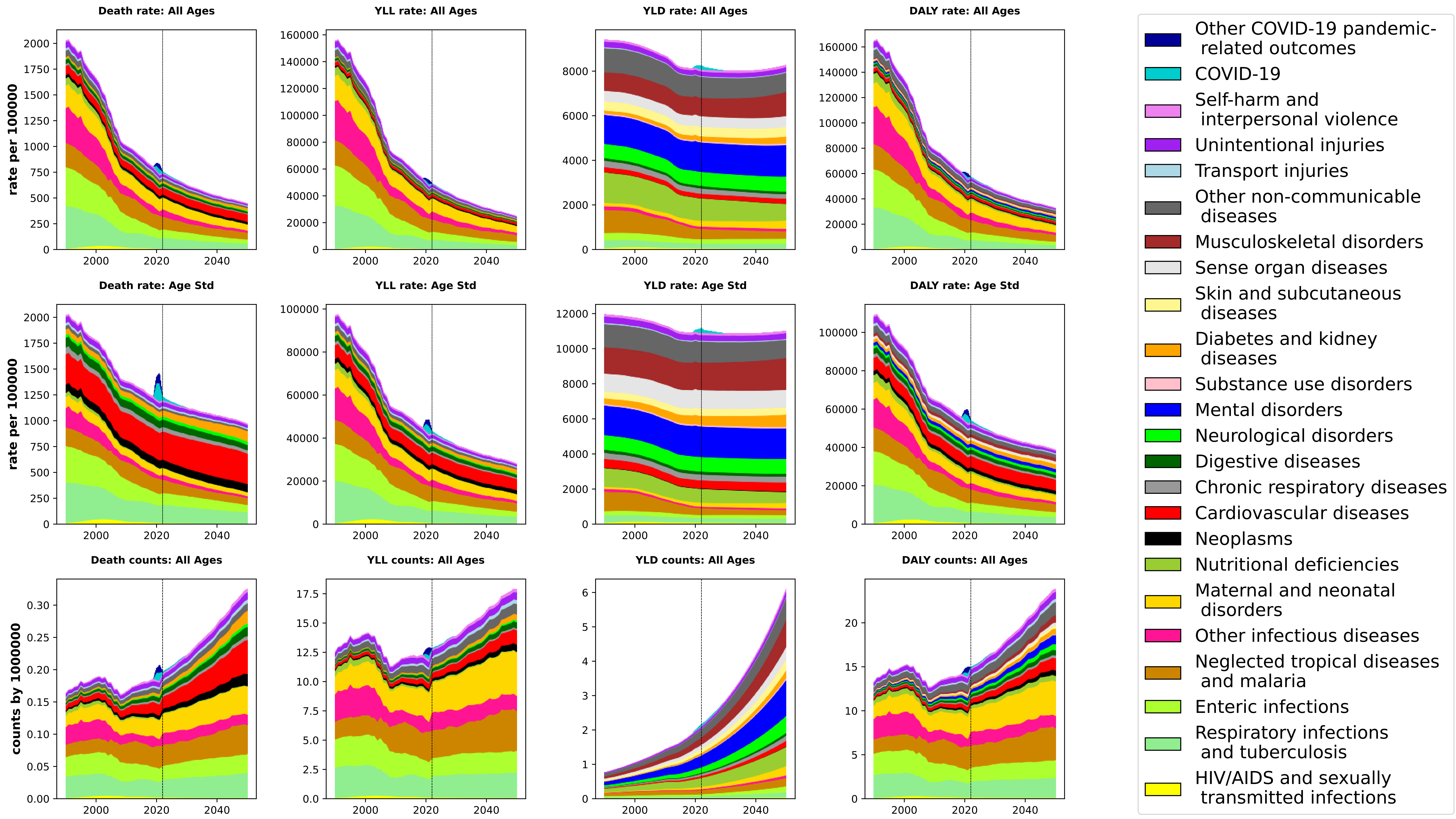
Death rate: All Ages
YLL rate: All Ages
YLD rate: All Ages
DALY rate: All Ages
Death rate: Age Std
YLL rate: Age Std
YLD rate: Age Std
DALY rate: Age Std
Death counts: All Ages
YLL counts: All Ages
YLD counts: All Ages
DALY counts: All Ages
rate per 100000
counts by 1000000
2000
2020
2040
Other COVID-19 pandemic-related outcomes
COVID-19
Self-harm and interpersonal violence
Unintentional injuries
Transport injuries
Other non-communicable diseases
Musculoskeletal disorders
Sense organ diseases
Skin and subcutaneous diseases
Diabetes and kidney diseases
Substance use disorders
Mental disorders
Neurological disorders
Digestive diseases
Chronic respiratory diseases
Cardiovascular diseases
Neoplasms
Nutritional deficiencies
Maternal and neonatal disorders
Other infectious diseases
Neglected tropical diseases and malaria
Enteric infections
Respiratory infections and tuberculosis
HIV/AIDS and sexually transmitted infections

Nigeria

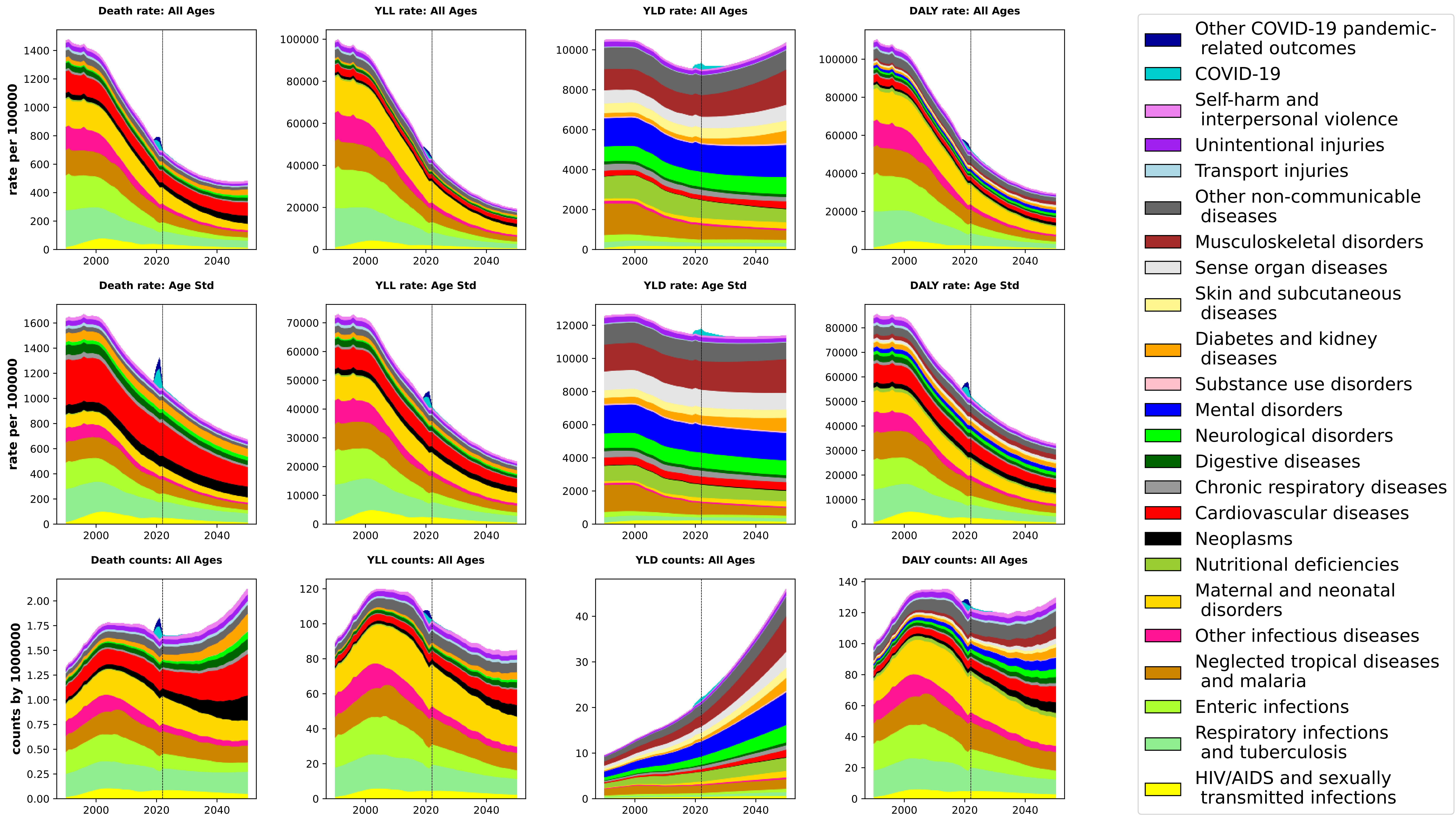
Death rate: All Ages
YLL rate: All Ages
YLD rate: All Ages
DALY rate: All Ages
Death rate: Age Std
YLL rate: Age Std
YLD rate: Age Std
DALY rate: Age Std
Death counts: All Ages
YLL counts: All Ages
YLD counts: All Ages
DALY counts: All Ages
rate per 100000
counts by 1000000
Other COVID-19 pandemic-related outcomes
COVID-19
Self-harm and interpersonal violence
Unintentional injuries
Transport injuries
Other non-communicable diseases
Musculoskeletal disorders
Sense organ diseases
Skin and subcutaneous diseases
Diabetes and kidney diseases
Substance use disorders
Mental disorders
Neurological disorders
Digestive diseases
Chronic respiratory diseases
Cardiovascular diseases
Neoplasms
Nutritional deficiencies
Maternal and neonatal disorders
Other infectious diseases
Neglected tropical diseases and malaria
Enteric infections
Respiratory infections and tuberculosis
HIV/AIDS and sexually transmitted infections

São Tomé and Príncipe

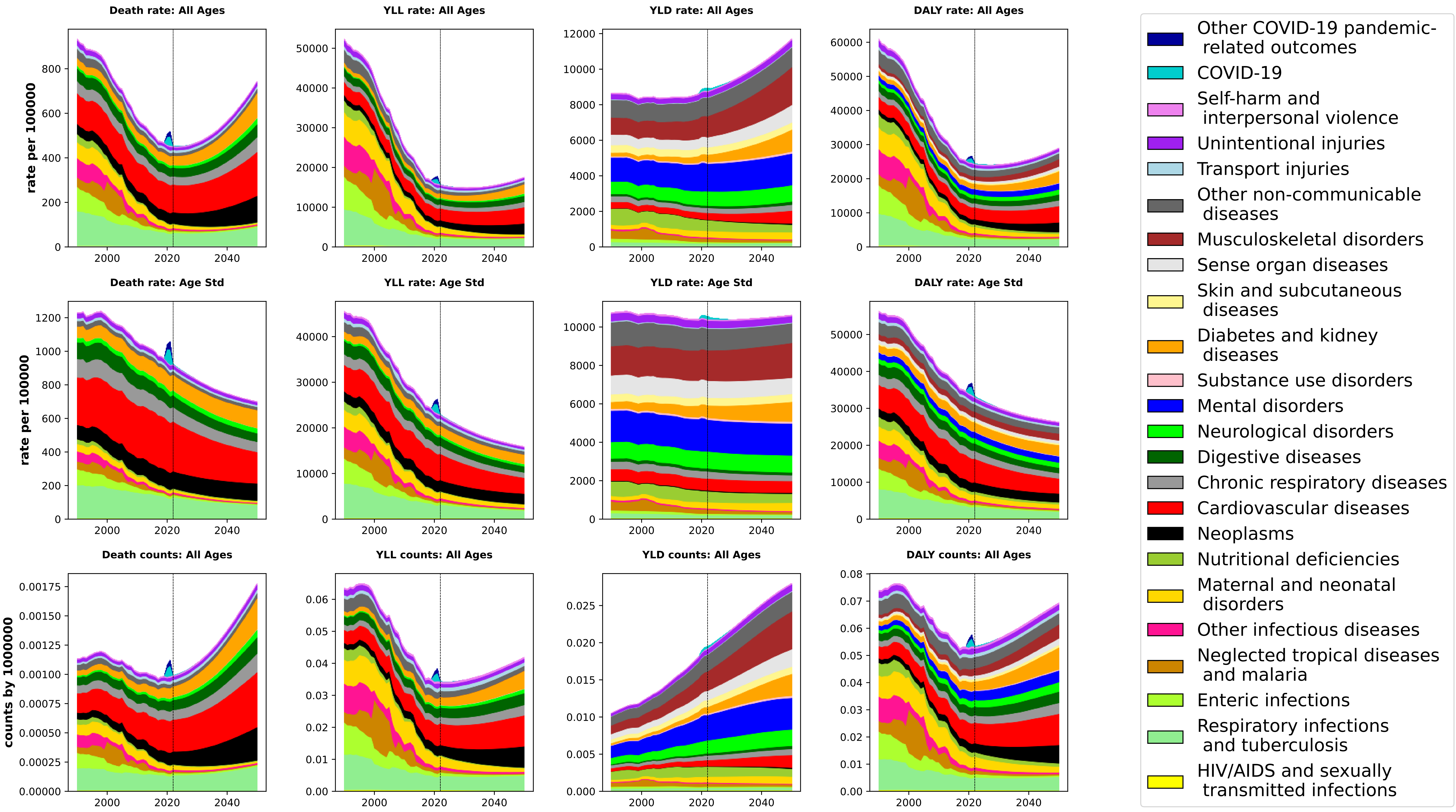

Senegal

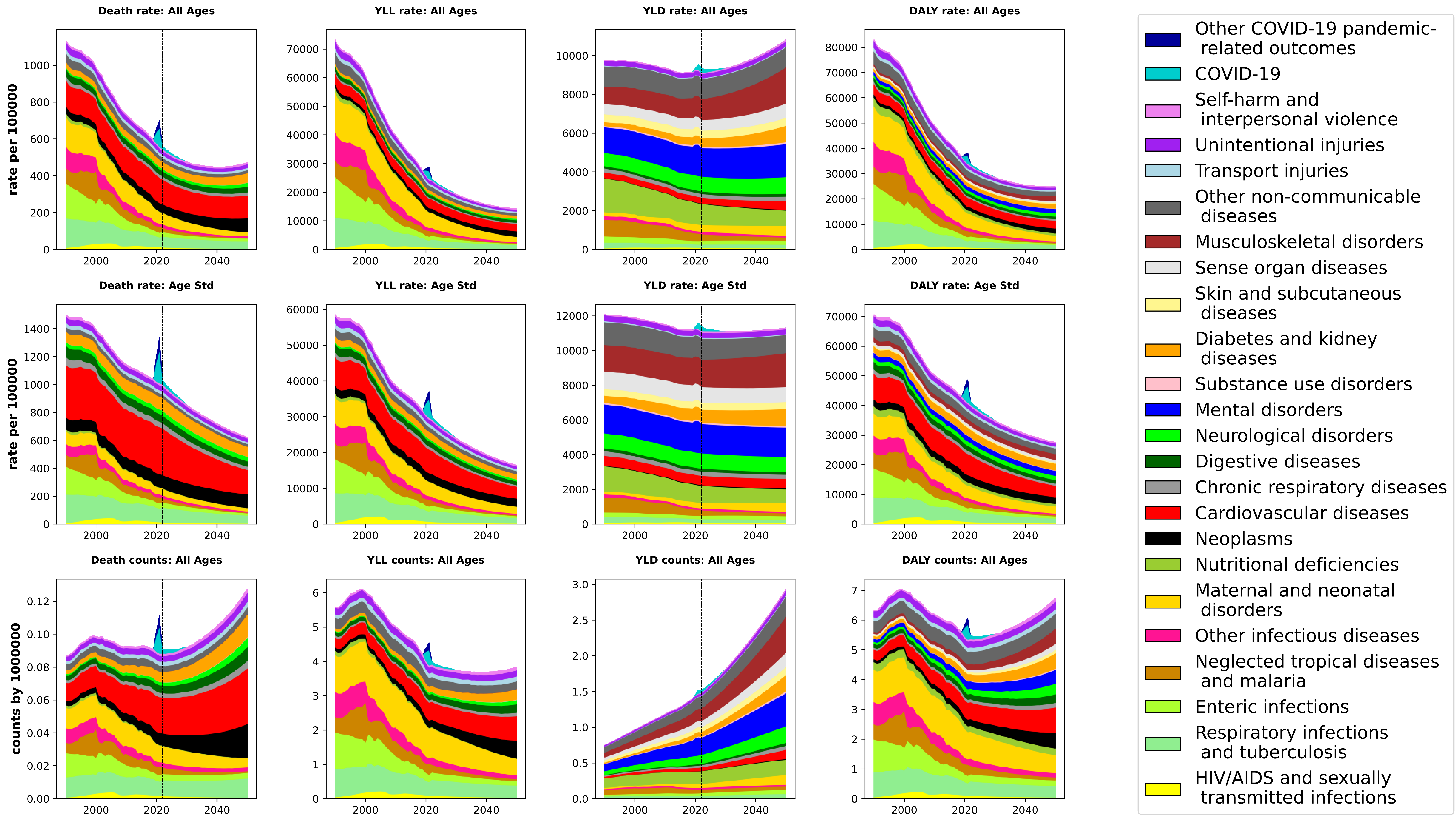
Death rate: All Ages
YLL rate: All Ages
YLD rate: All Ages
DALY rate: All Ages
Death rate: Age Std
YLL rate: Age Std
YLD rate: Age Std
DALY rate: Age Std
Death counts: All Ages
YLL counts: All Ages
YLD counts: All Ages
DALY counts: All Ages
rate per 100000
counts by 1000000
Other COVID-19 pandemic-related outcomes
COVID-19
Self-harm and interpersonal violence
Unintentional injuries
Transport injuries
Other non-communicable diseases
Musculoskeletal disorders
Sense organ diseases
Skin and subcutaneous diseases
Diabetes and kidney diseases
Substance use disorders
Mental disorders
Neurological disorders
Digestive diseases
Chronic respiratory diseases
Cardiovascular diseases
Neoplasms
Nutritional deficiencies
Maternal and neonatal disorders
Other infectious diseases
Neglected tropical diseases and malaria
Enteric infections
Respiratory infections and tuberculosis
HIV/AIDS and sexually transmitted infections

Sierra Leone

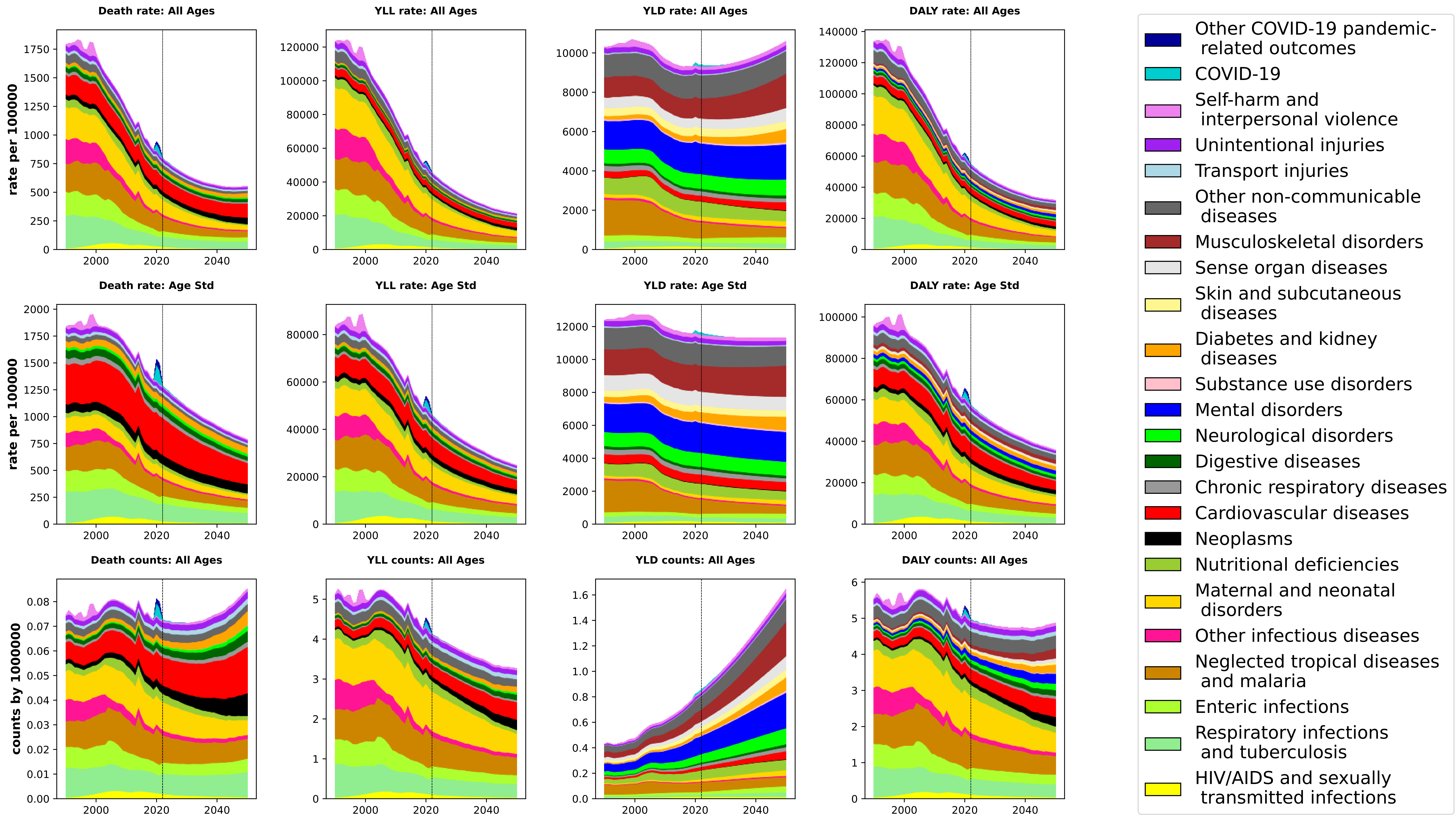

Togo

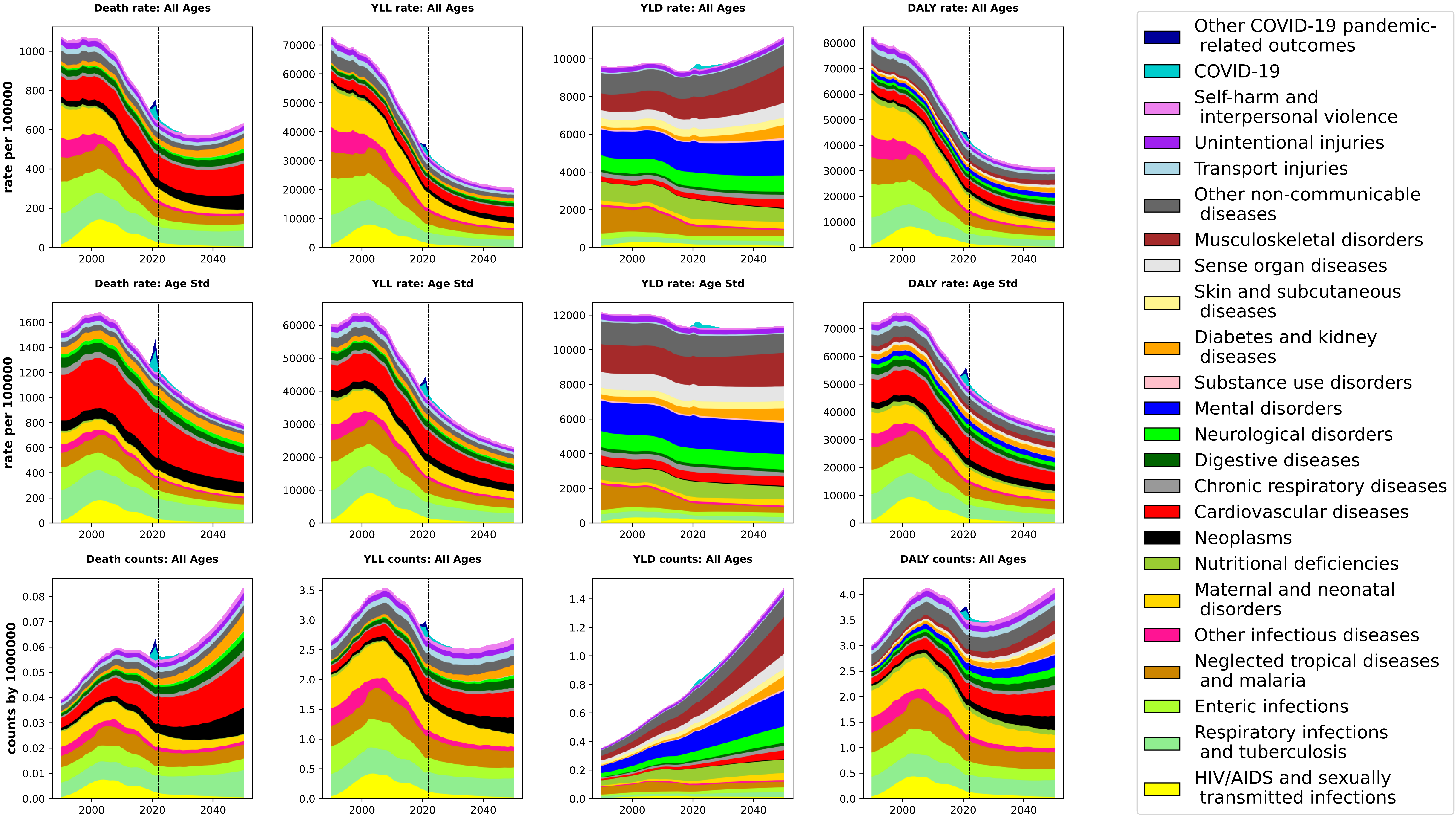
Death rate: All Ages
YLL rate: All Ages
YLD rate: All Ages
DALY rate: All Ages
Death rate: Age Std
YLL rate: Age Std
YLD rate: Age Std
DALY rate: Age Std
Death counts: All Ages
YLL counts: All Ages
YLD counts: All Ages
DALY counts: All Ages
rate per 100000
counts by 1000000
2000
2020
2040
Other COVID-19 pandemic-related outcomes
COVID-19
Self-harm and interpersonal violence
Unintentional injuries
Transport injuries
Other non-communicable diseases
Musculoskeletal disorders
Sense organ diseases
Skin and subcutaneous diseases
Diabetes and kidney diseases
Substance use disorders
Mental disorders
Neurological disorders
Digestive diseases
Chronic respiratory diseases
Cardiovascular diseases
Neoplasms
Nutritional deficiencies
Maternal and neonatal disorders
Other infectious diseases
Neglected tropical diseases and malaria
Enteric infections
Respiratory infections and tuberculosis
HIV/AIDS and sexually transmitted infections

Global

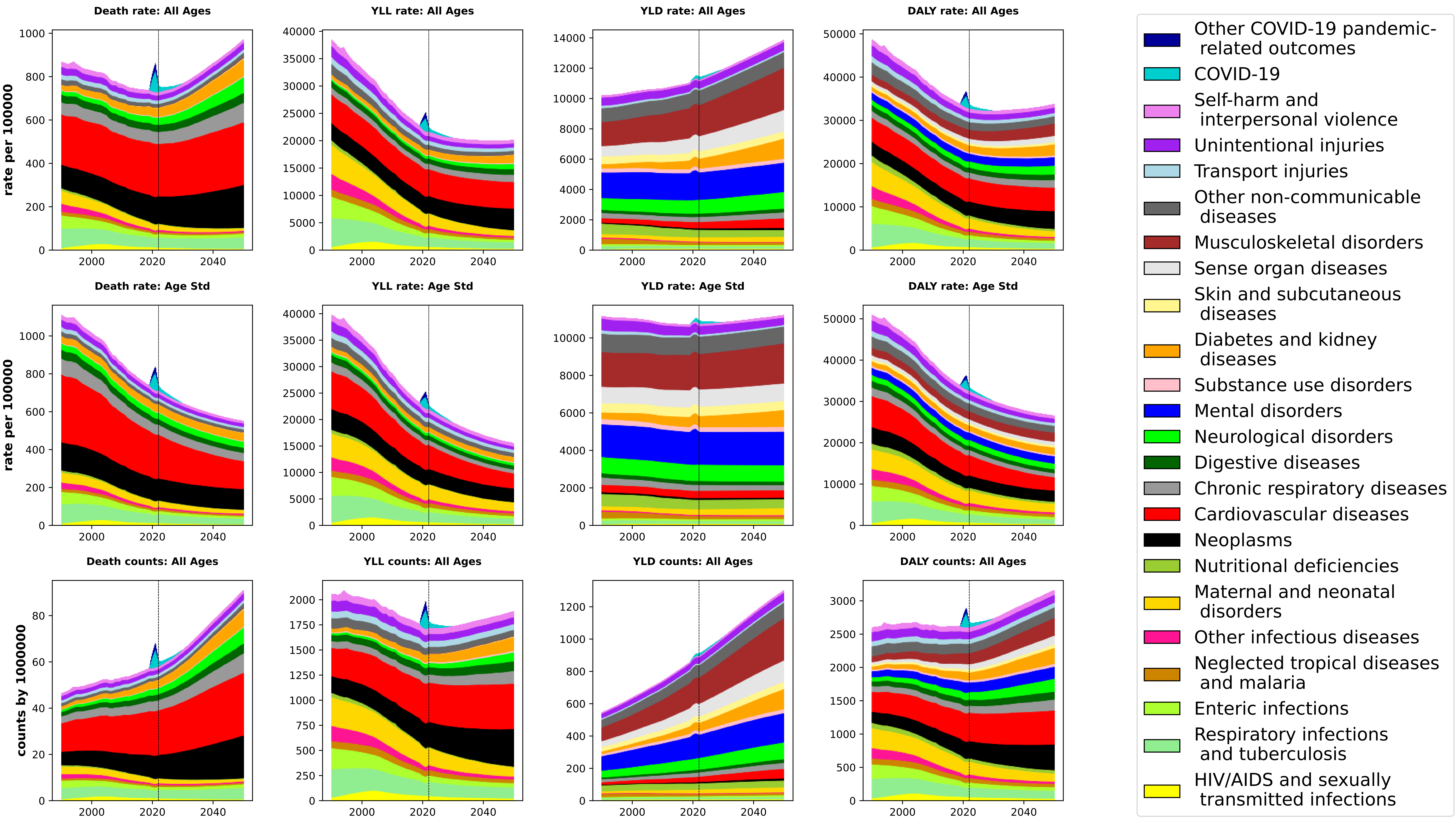
Death rate: All Ages
YLL rate: All Ages
YLD rate: All Ages
DALY rate: All Ages
Death rate: Age Std
YLL rate: Age Std
YLD rate: Age Std
DALY rate: Age Std
Death counts: All Ages
YLL counts: All Ages
YLD counts: All Ages
DALY counts: All Ages
rate per 100000
counts by 1000000
Other COVID-19 pandemic-related outcomes
COVID-19
Self-harm and interpersonal violence
Unintentional injuries
Transport injuries
Other non-communicable diseases
Musculoskeletal disorders
Sense organ diseases
Skin and subcutaneous diseases
Diabetes and kidney diseases
Substance use disorders
Mental disorders
Neurological disorders
Digestive diseases
Chronic respiratory diseases
Cardiovascular diseases
Neoplasms
Nutritional deficiencies
Maternal and neonatal disorders
Other infectious diseases
Neglected tropical diseases and malaria
Enteric infections
Respiratory infections and tuberculosis
HIV/AIDS and sexually transmitted infections

Central Europe, Eastern Europe, and Central Asia

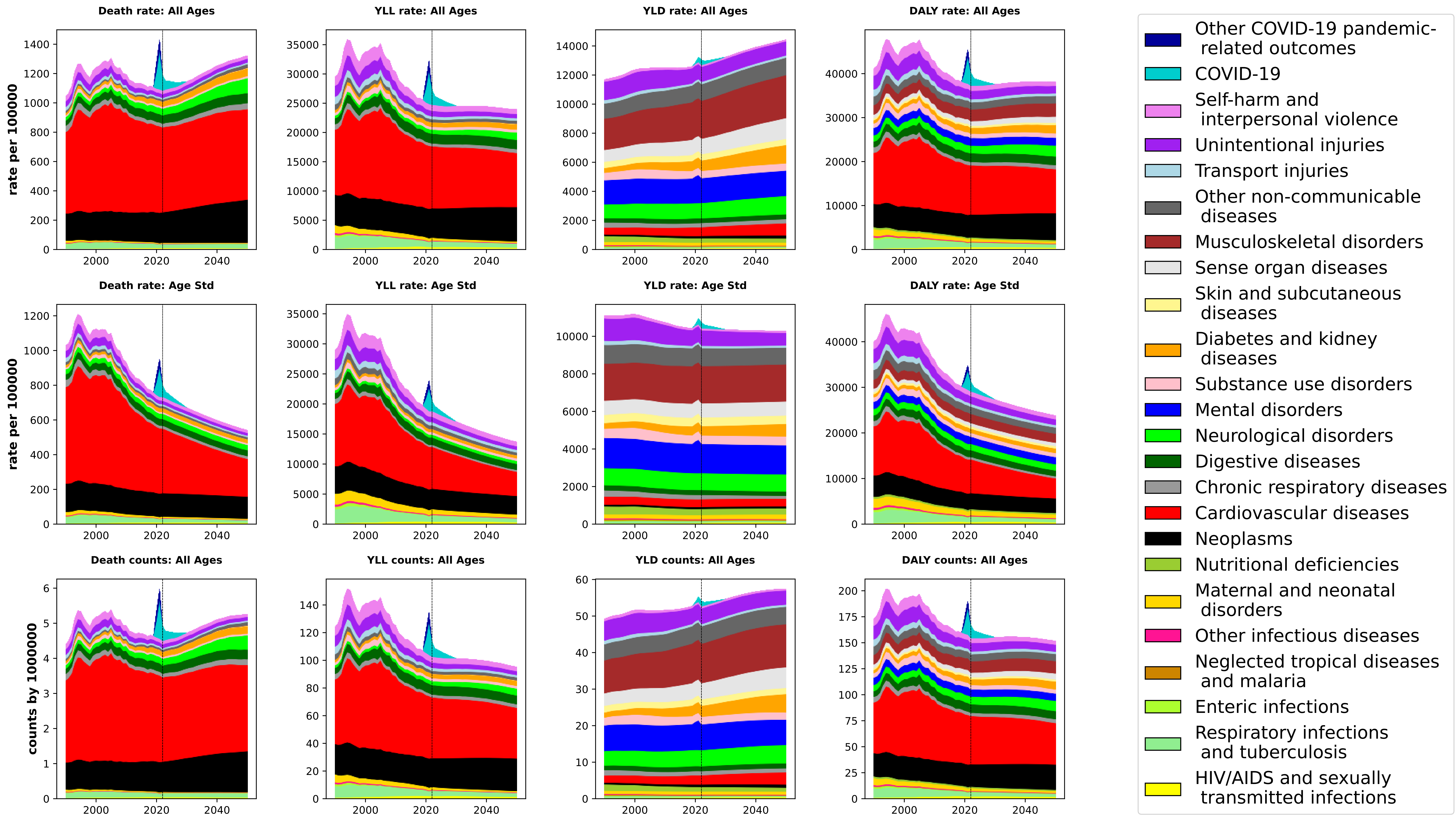

High income

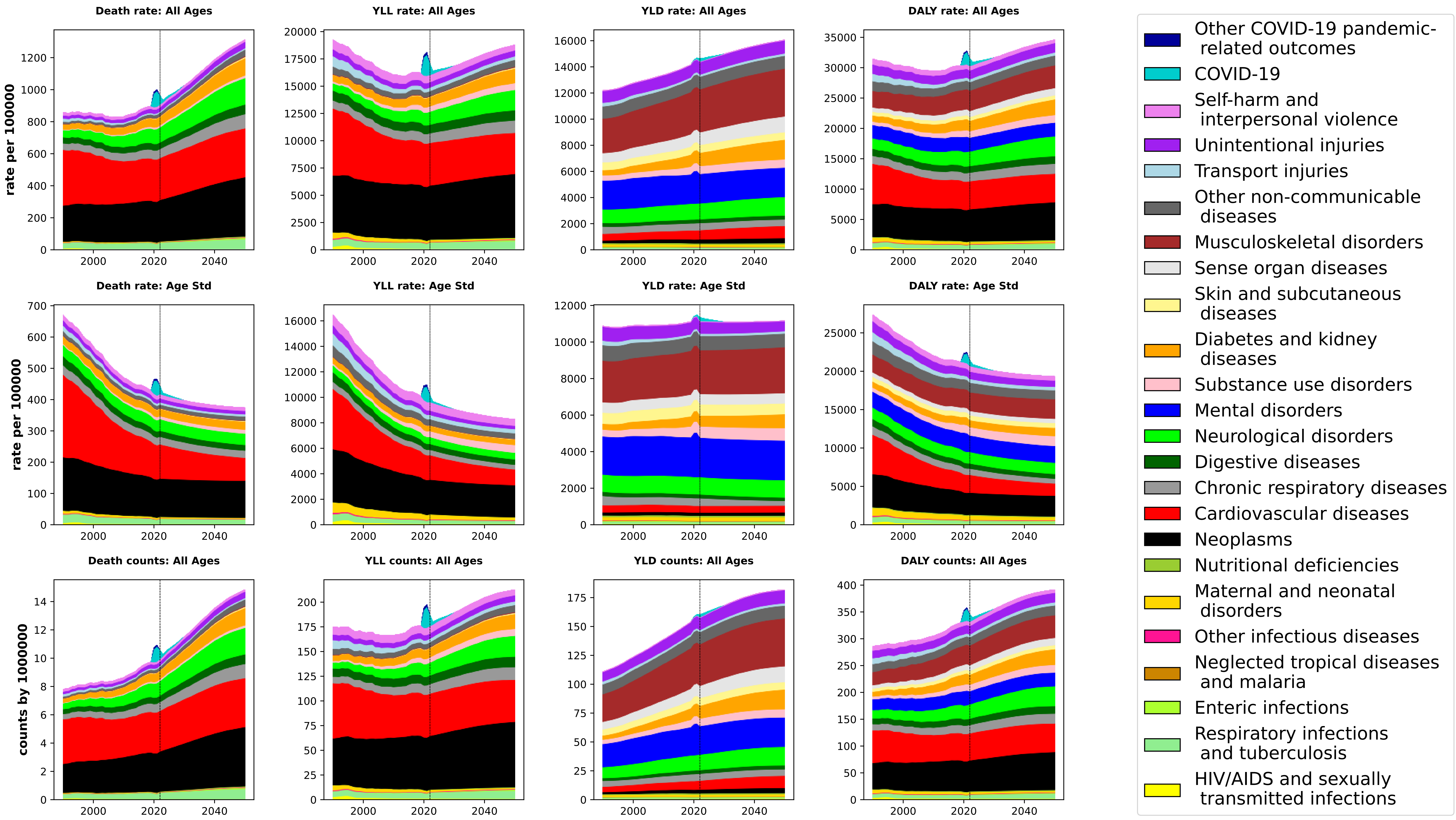
Death rate: All Ages
YLL rate: All Ages
YLD rate: All Ages
DALY rate: All Ages
Death rate: Age Std
YLL rate: Age Std
YLD rate: Age Std
DALY rate: Age Std
Death counts: All Ages
YLL counts: All Ages
YLD counts: All Ages
DALY counts: All Ages
rate per 100000
rate per 100000
counts by 1000000
Other COVID-19 pandemic-related outcomes
COVID-19
Self-harm and interpersonal violence
Unintentional injuries
Transport injuries
Other non-communicable diseases
Musculoskeletal disorders
Sense organ diseases
Skin and subcutaneous diseases
Diabetes and kidney diseases
Substance use disorders
Mental disorders
Neurological disorders
Digestive diseases
Chronic respiratory diseases
Cardiovascular diseases
Neoplasms
Nutritional deficiencies
Maternal and neonatal disorders
Other infectious diseases
Neglected tropical diseases and malaria
Enteric infections
Respiratory infections and tuberculosis
HIV/AIDS and sexually transmitted infections

Latin America and Caribbean

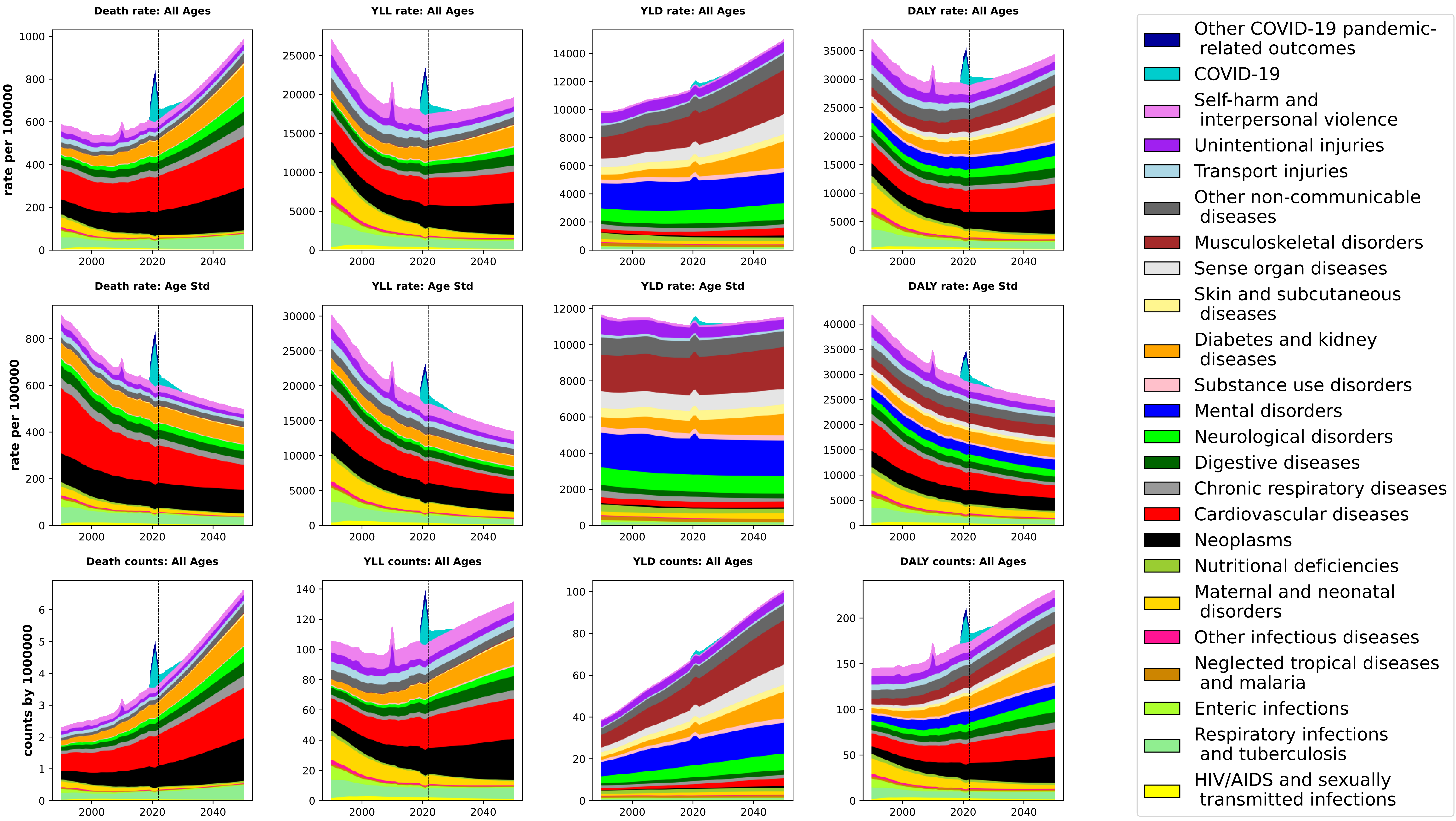

North Africa and Middle East

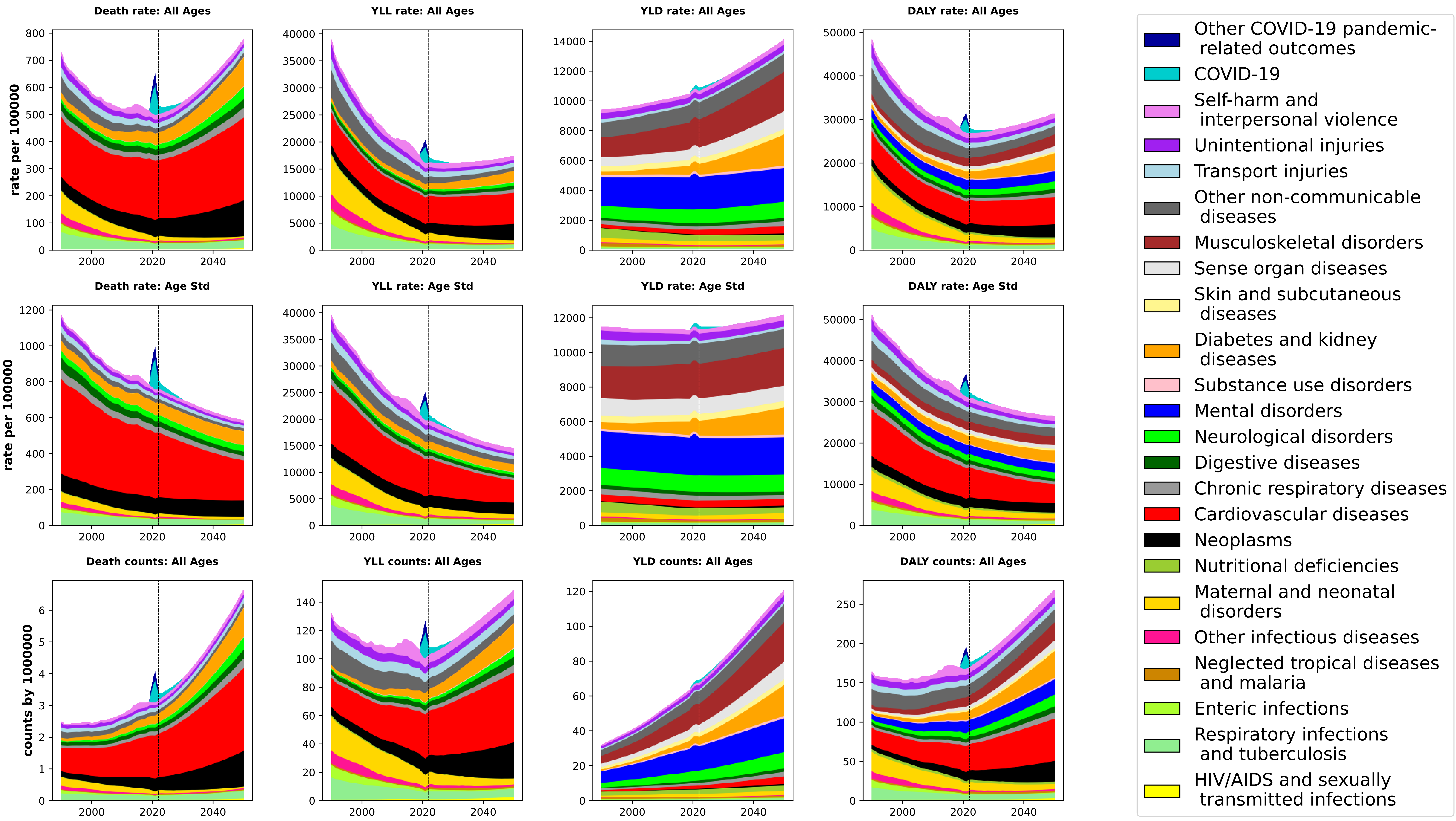

South Asia

Supplemental Results Figure S6. Cause level 2 area plots for 4 burden measures for reference scenario by location, 1990-2050

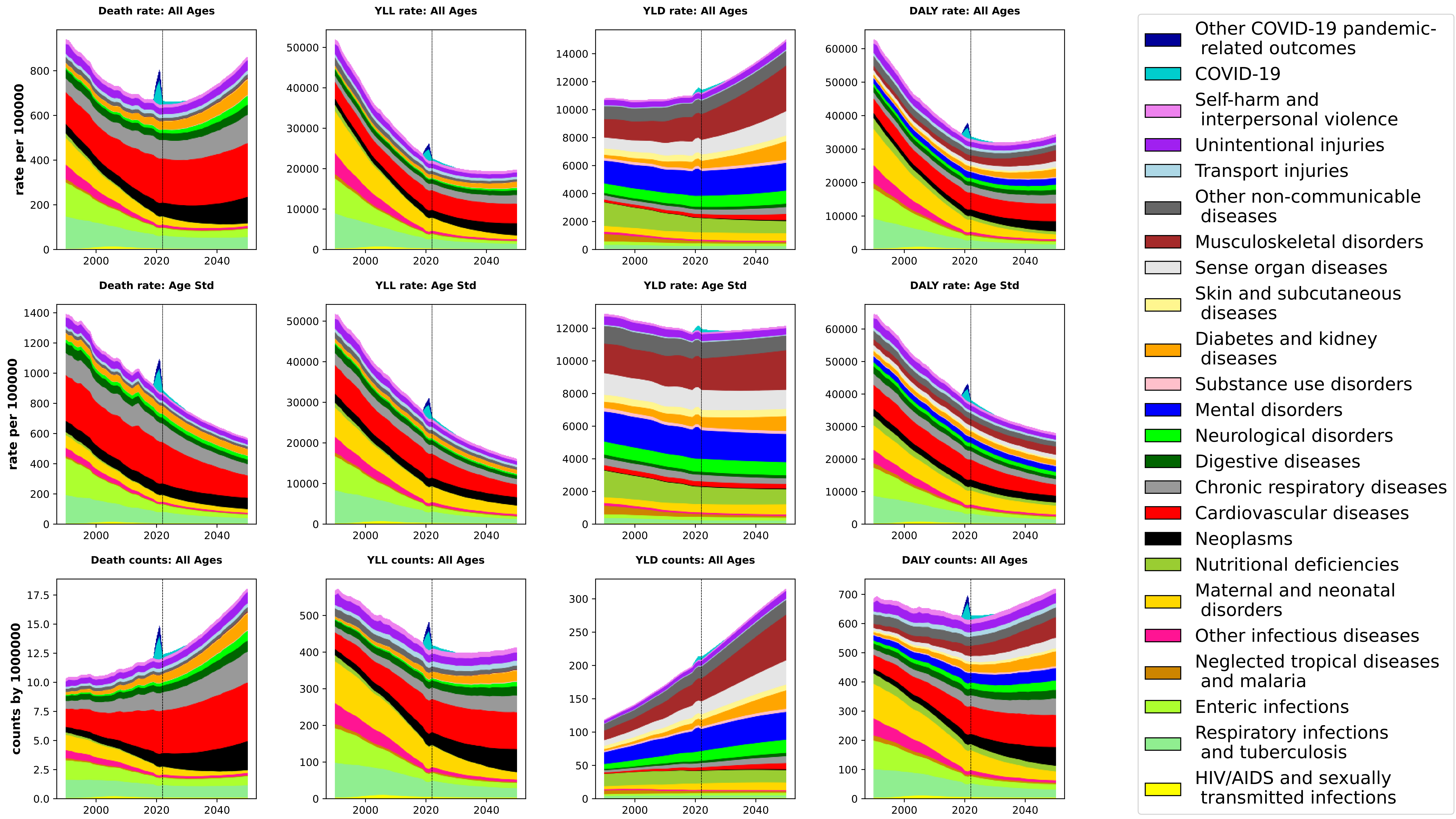

Southeast Asia, East Asia, and Oceania

Supplemental Results Figure S6. Cause level 2 area plots for 4 burden measures for reference scenario by location, 1990-2050

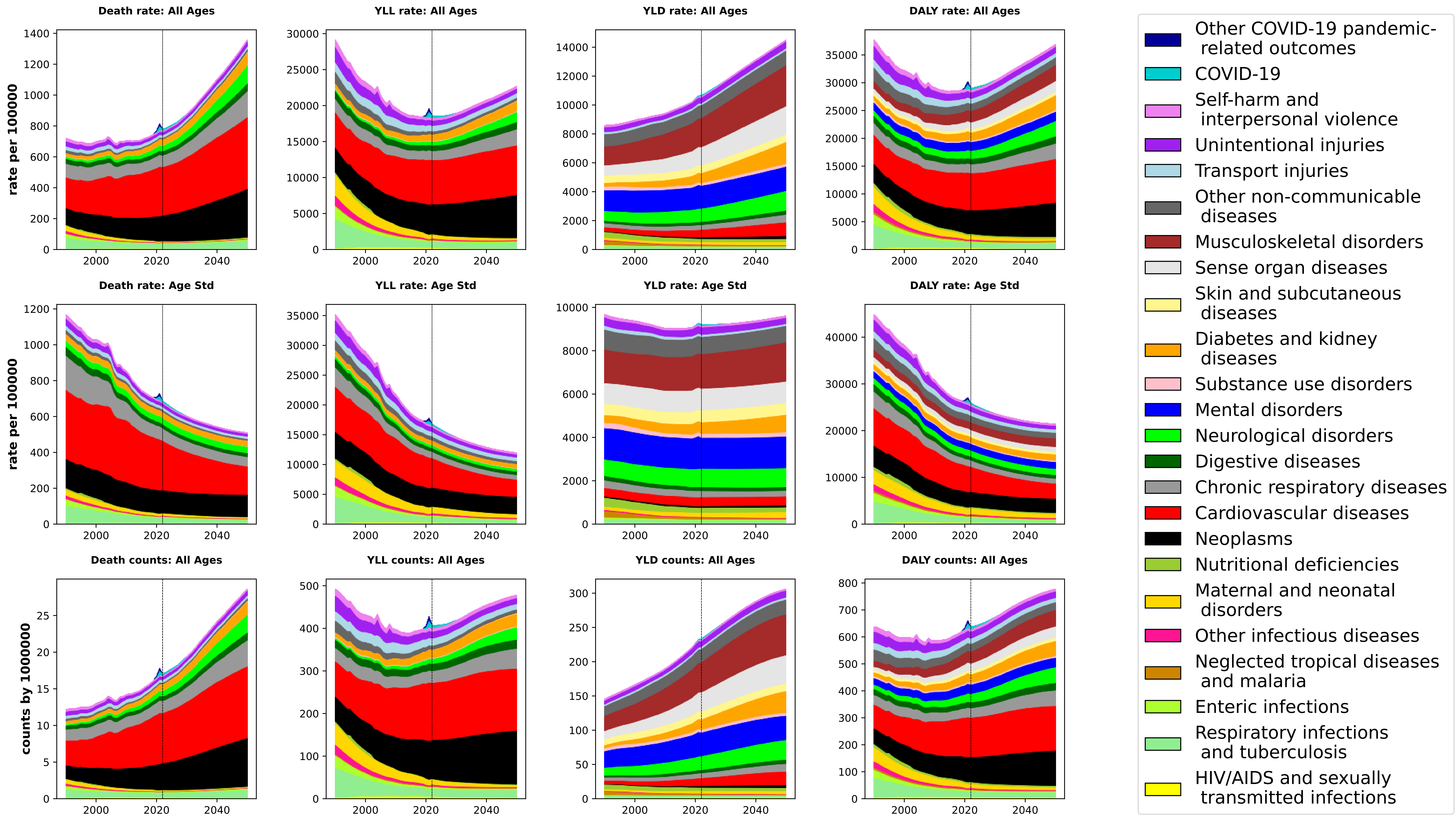

Sub-Saharan Africa

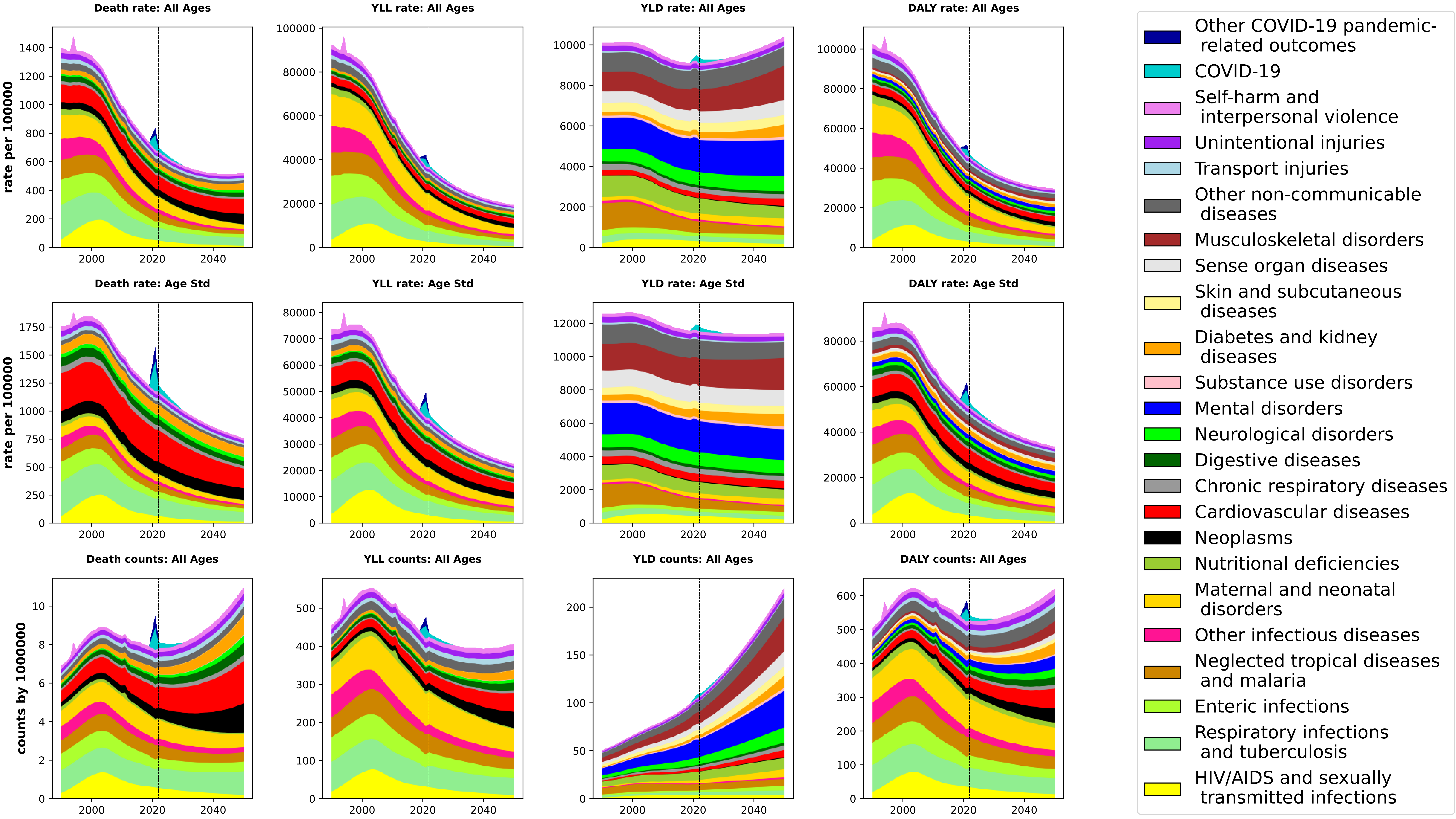

Central Asia

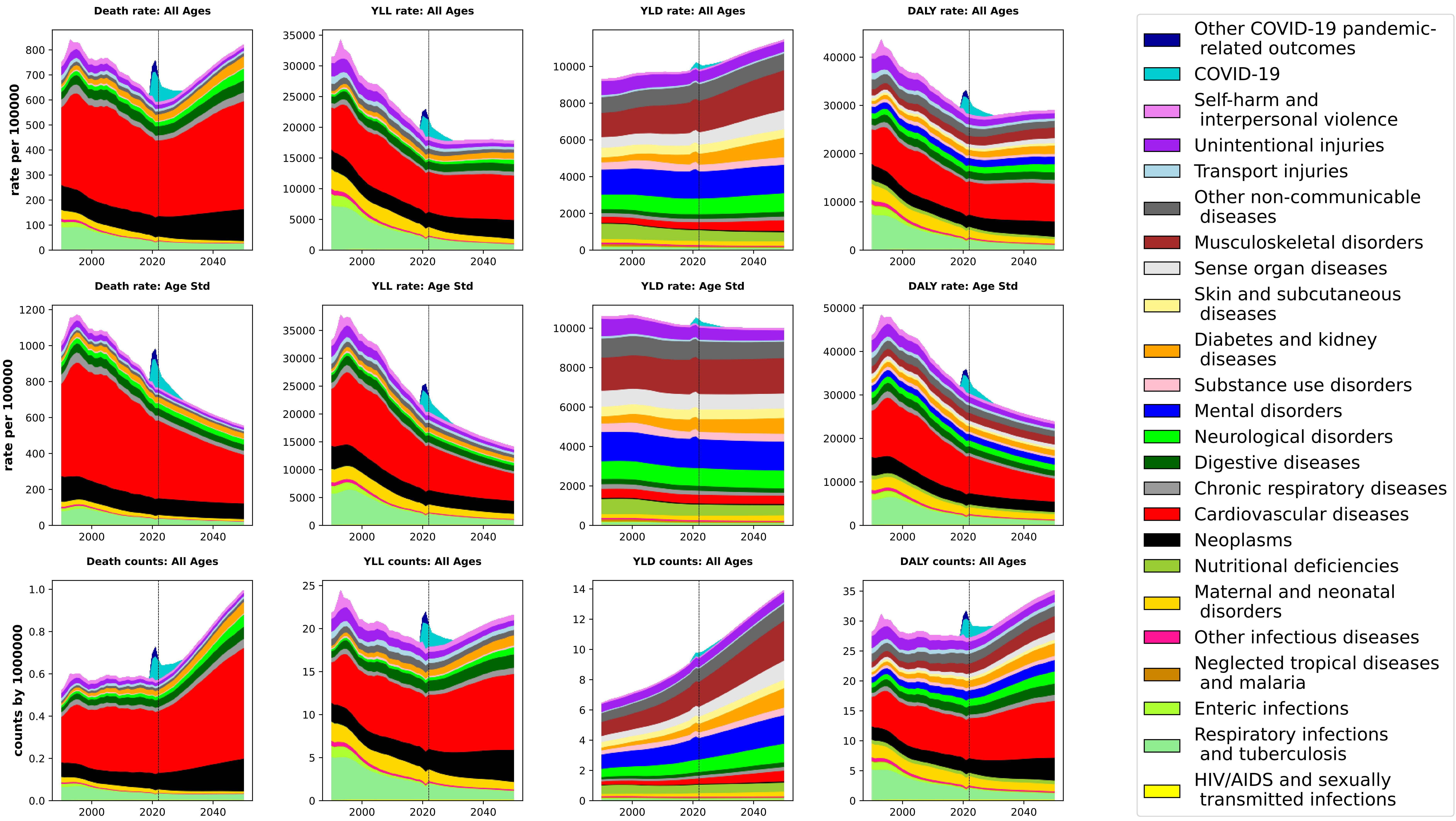

Central Europe

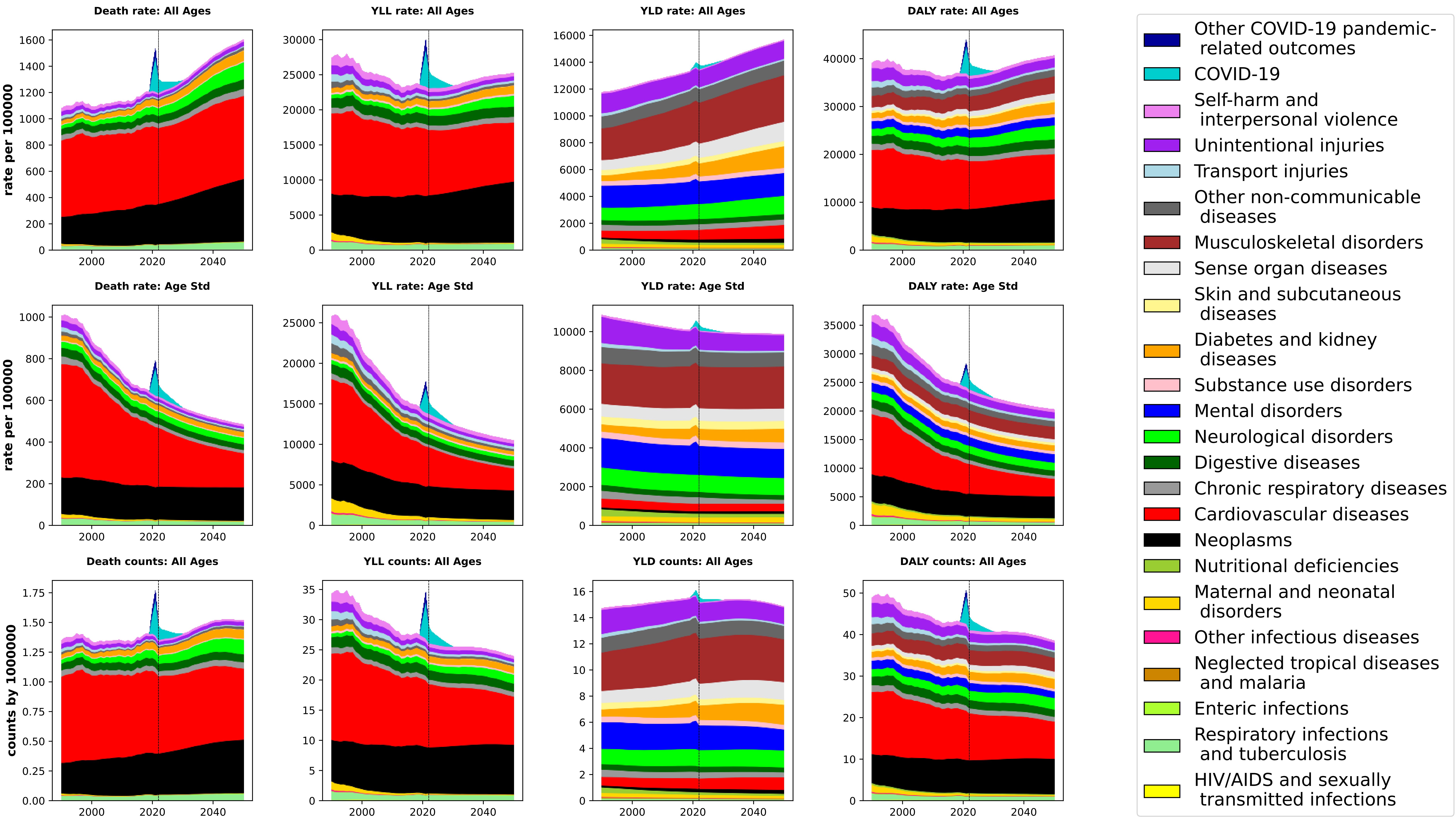

Eastern Europe

Supplemental Results Figure S6. Cause level 2 area plots for 4 burden measures for reference scenario by location, 1990-2050

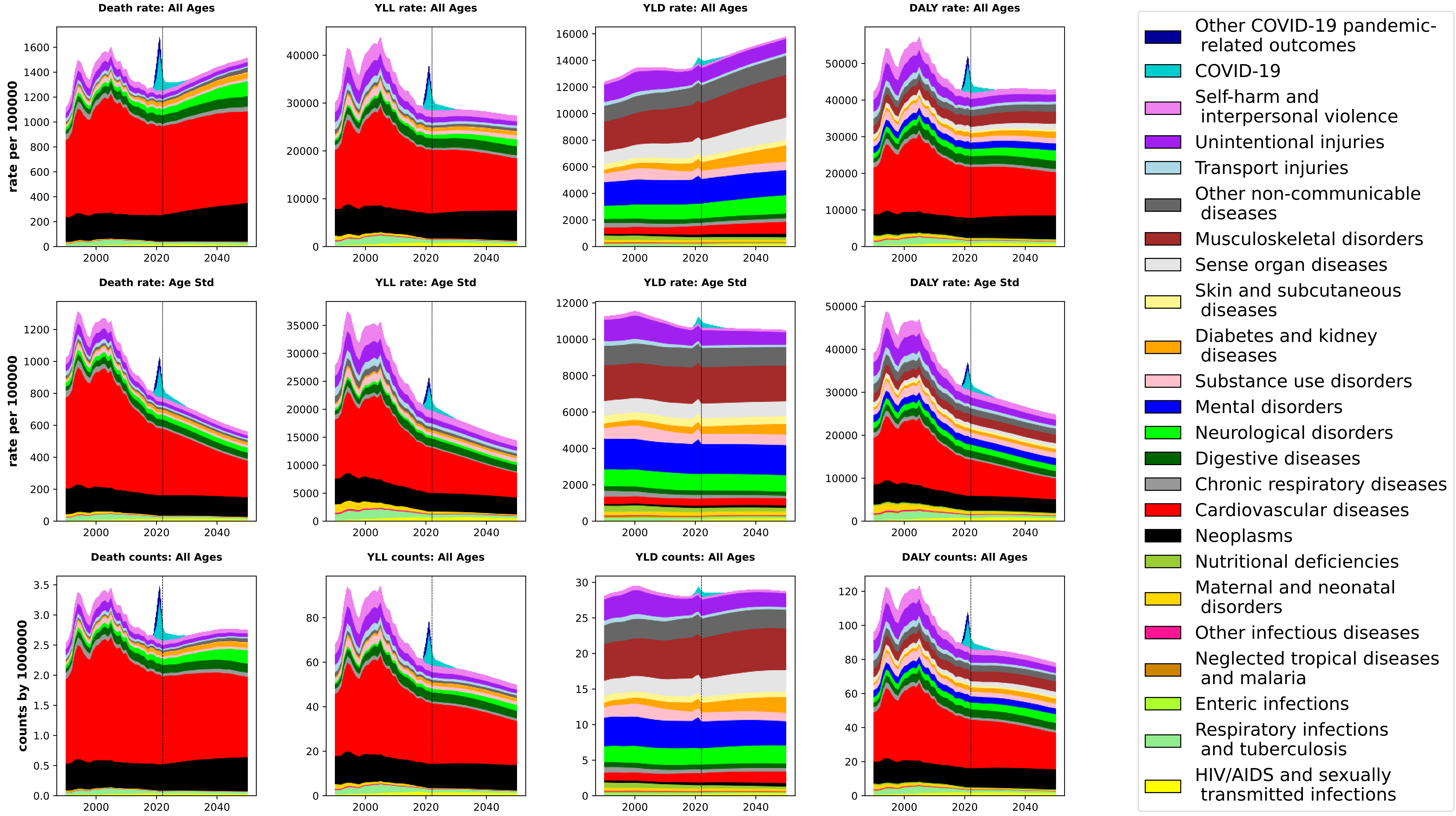

Australasia

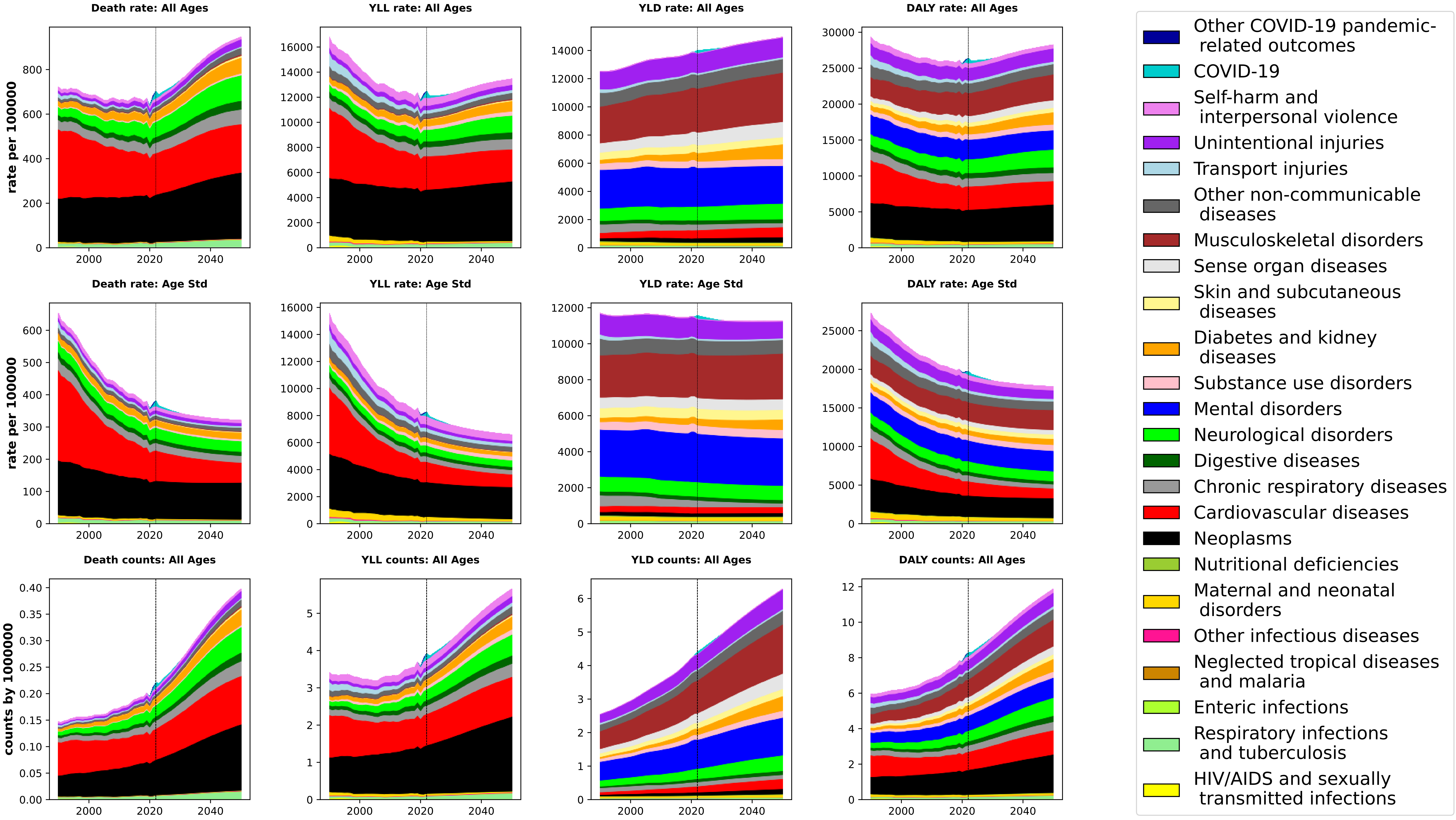

High-income Asia Pacific

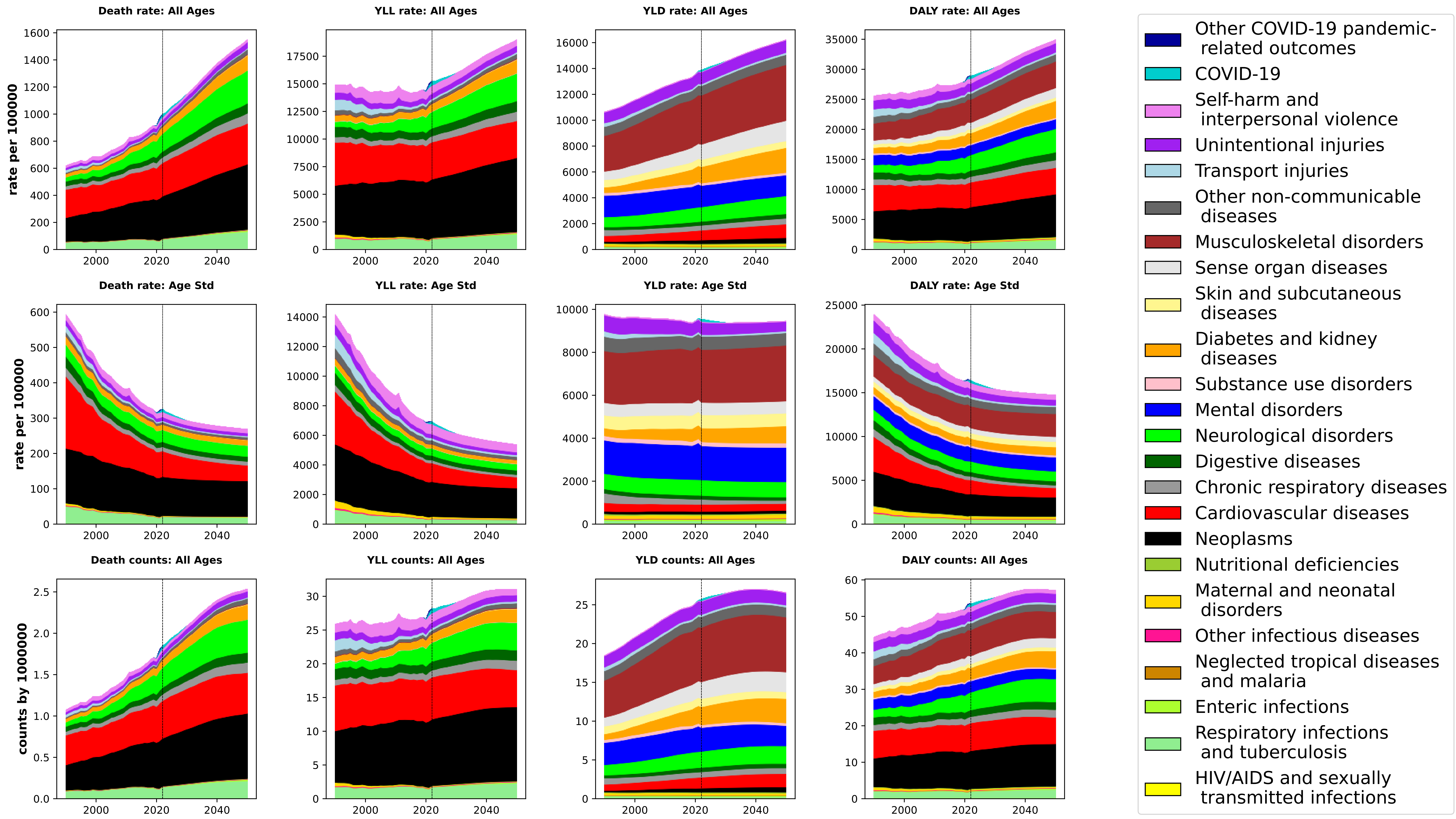

High-income North America

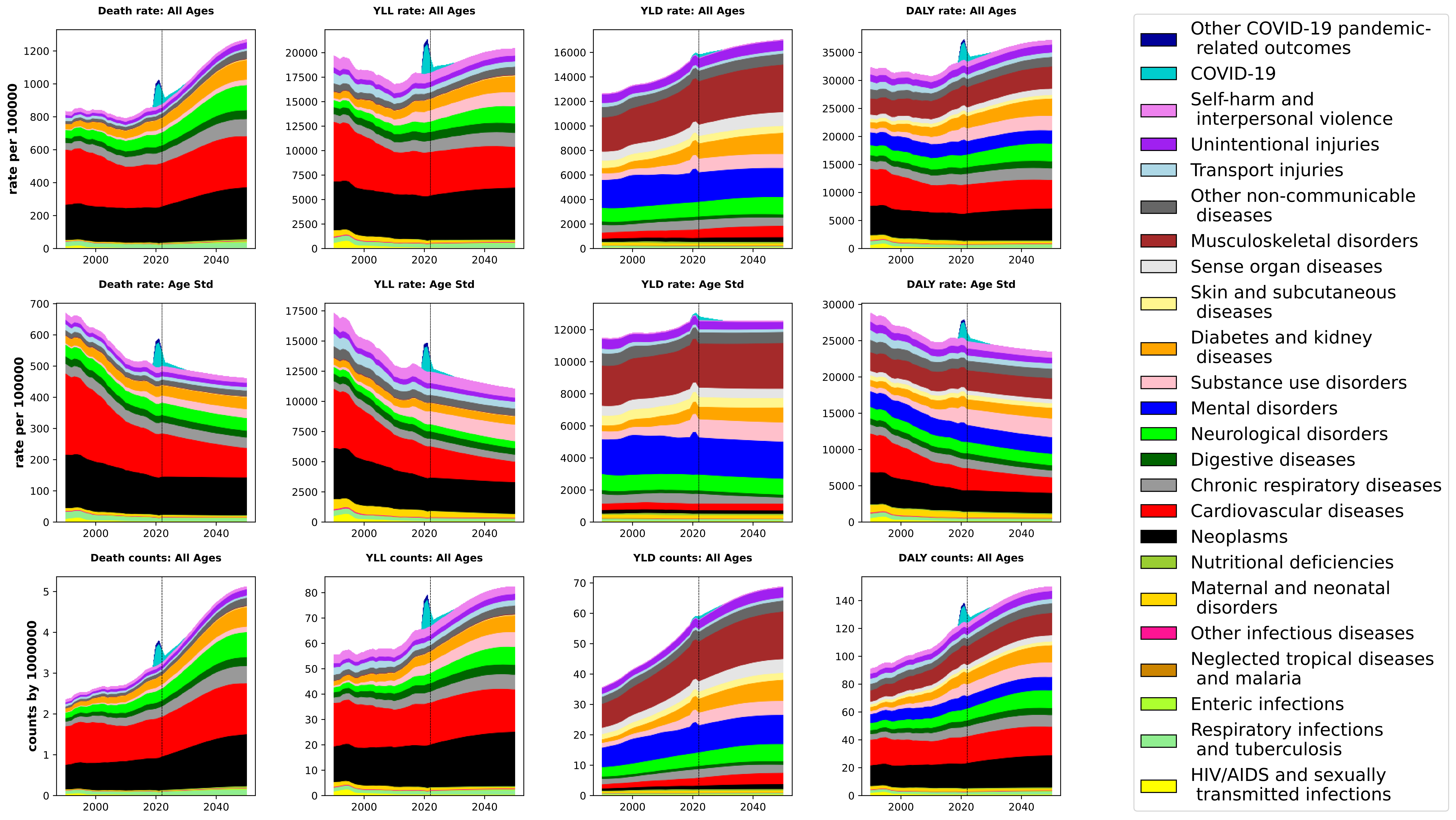

Southern Latin America

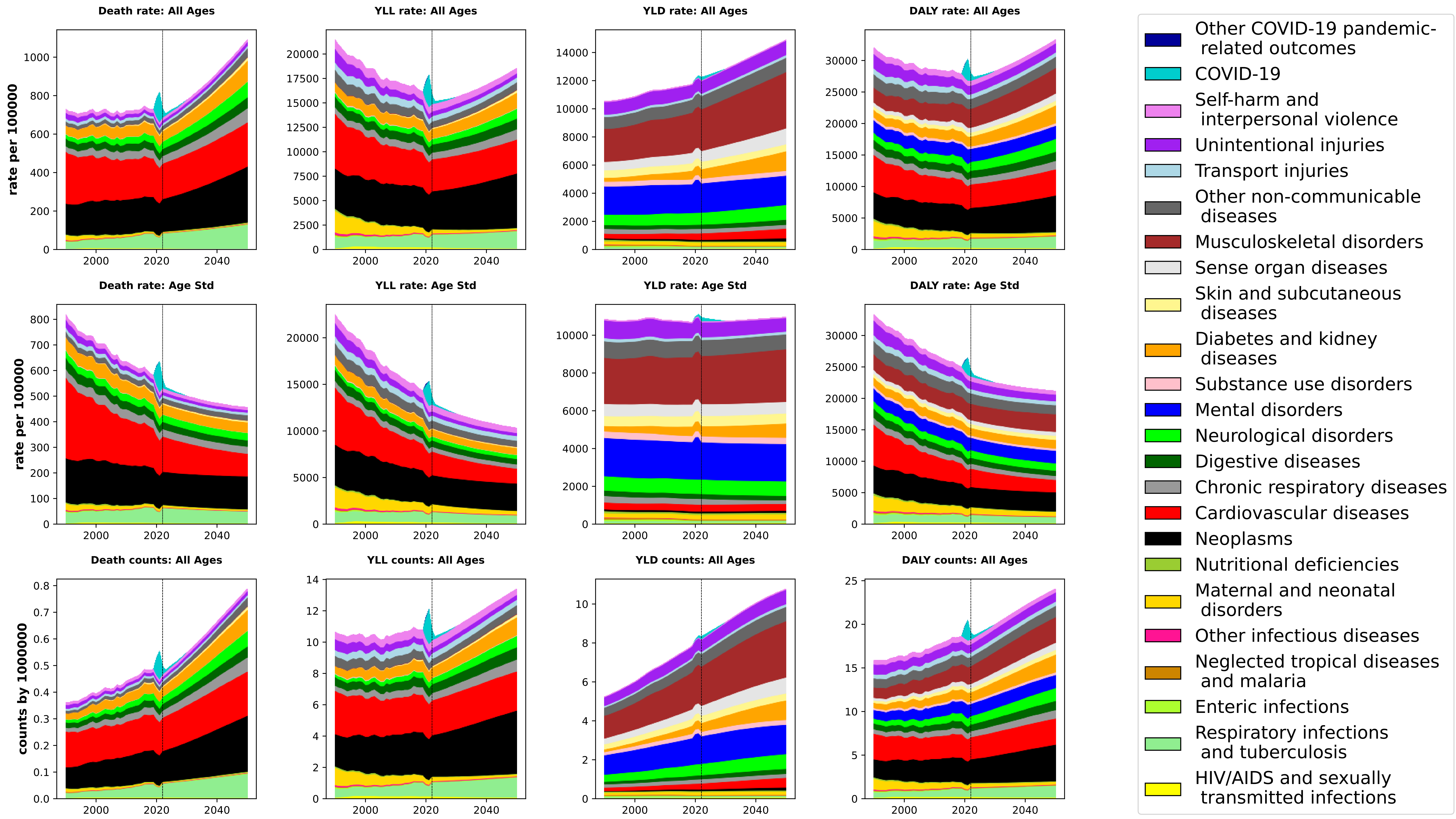

Western Europe

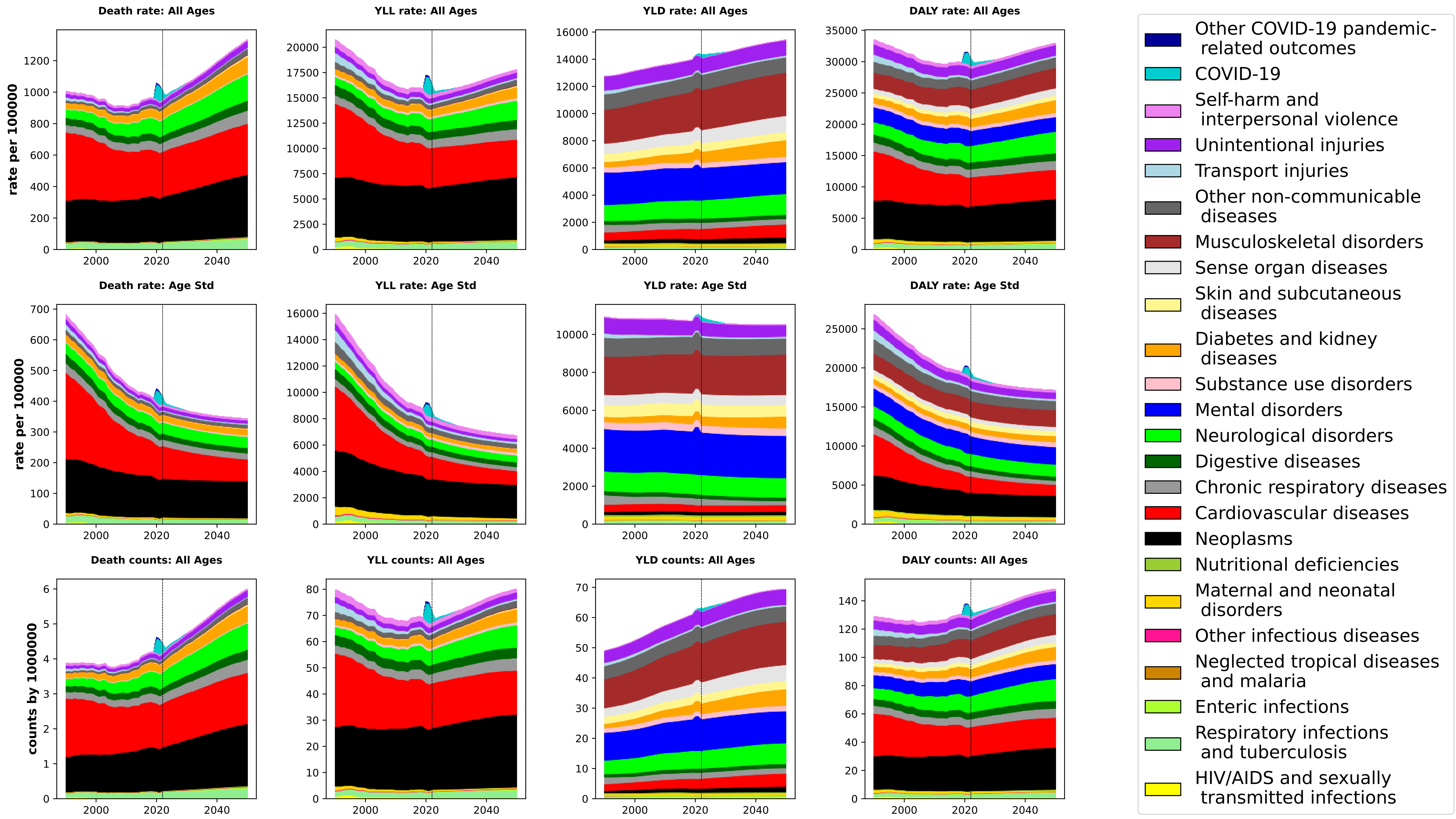

Andean Latin America

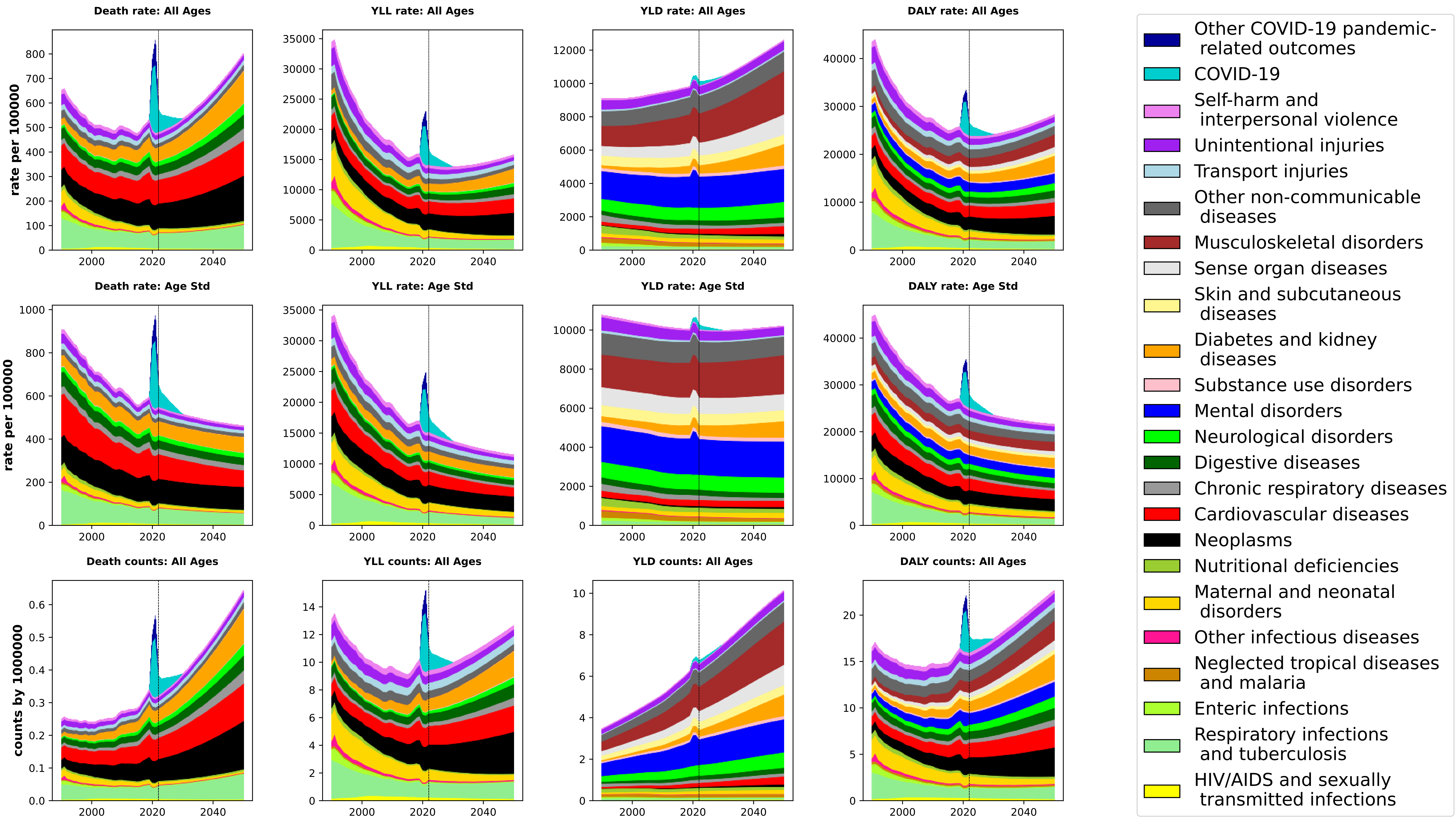

Caribbean

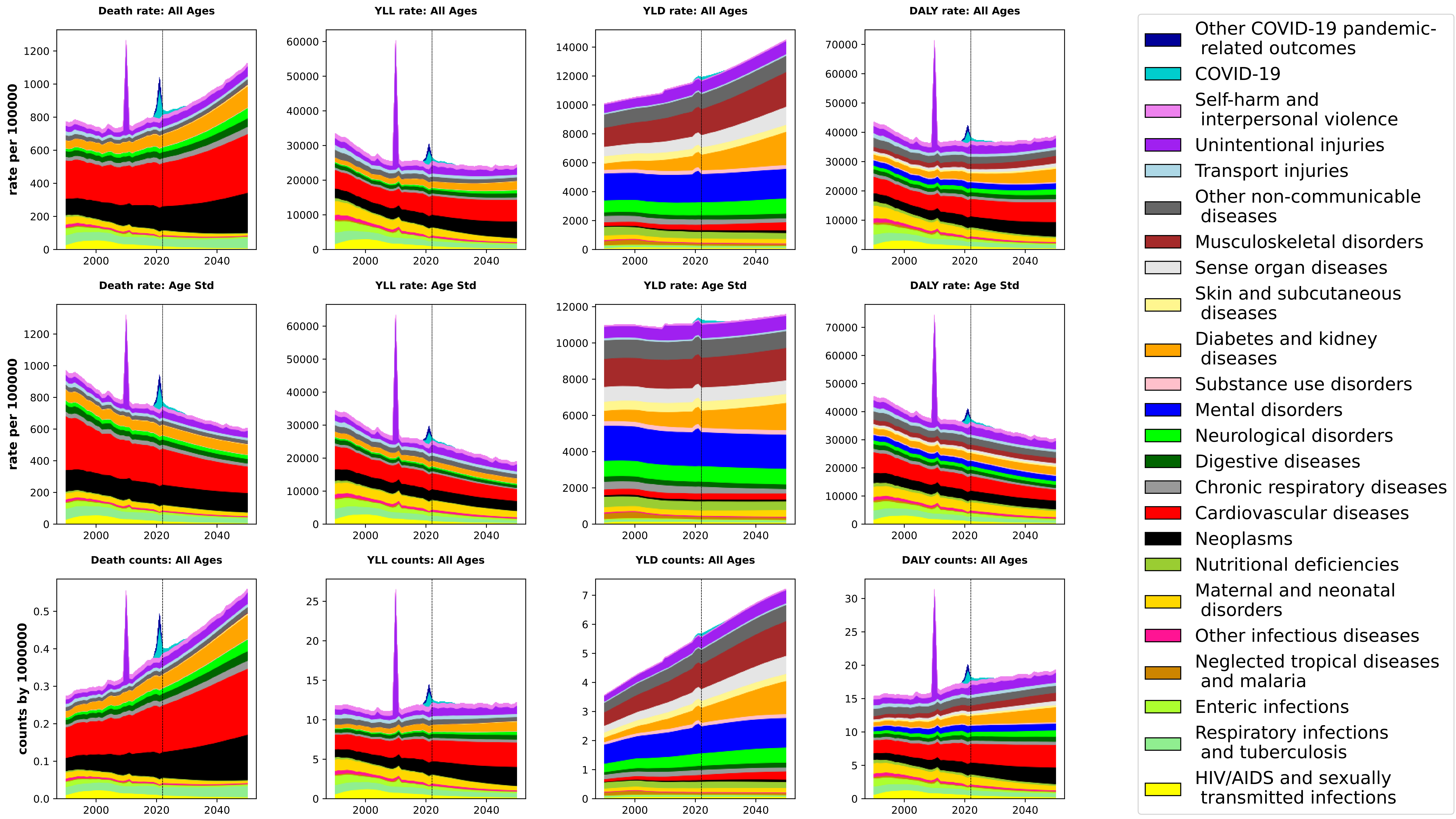
Death rate: All Ages
YLL rate: All Ages
YLD rate: All Ages
DALY rate: All Ages
Death rate: Age Std
YLL rate: Age Std
YLD rate: Age Std
DALY rate: Age Std
Death counts: All Ages
YLL counts: All Ages
YLD counts: All Ages
DALY counts: All Ages
rate per 100000
counts by 1000000
2000
2020
2040
Other COVID-19 pandemic-related outcomes
COVID-19
Self-harm and interpersonal violence
Unintentional injuries
Transport injuries
Other non-communicable diseases
Musculoskeletal disorders
Sense organ diseases
Skin and subcutaneous diseases
Diabetes and kidney diseases
Substance use disorders
Mental disorders
Neurological disorders
Digestive diseases
Chronic respiratory diseases
Cardiovascular diseases
Neoplasms
Nutritional deficiencies
Maternal and neonatal disorders
Other infectious diseases
Neglected tropical diseases and malaria
Enteric infections
Respiratory infections and tuberculosis
HIV/AIDS and sexually transmitted infections

Central Latin America

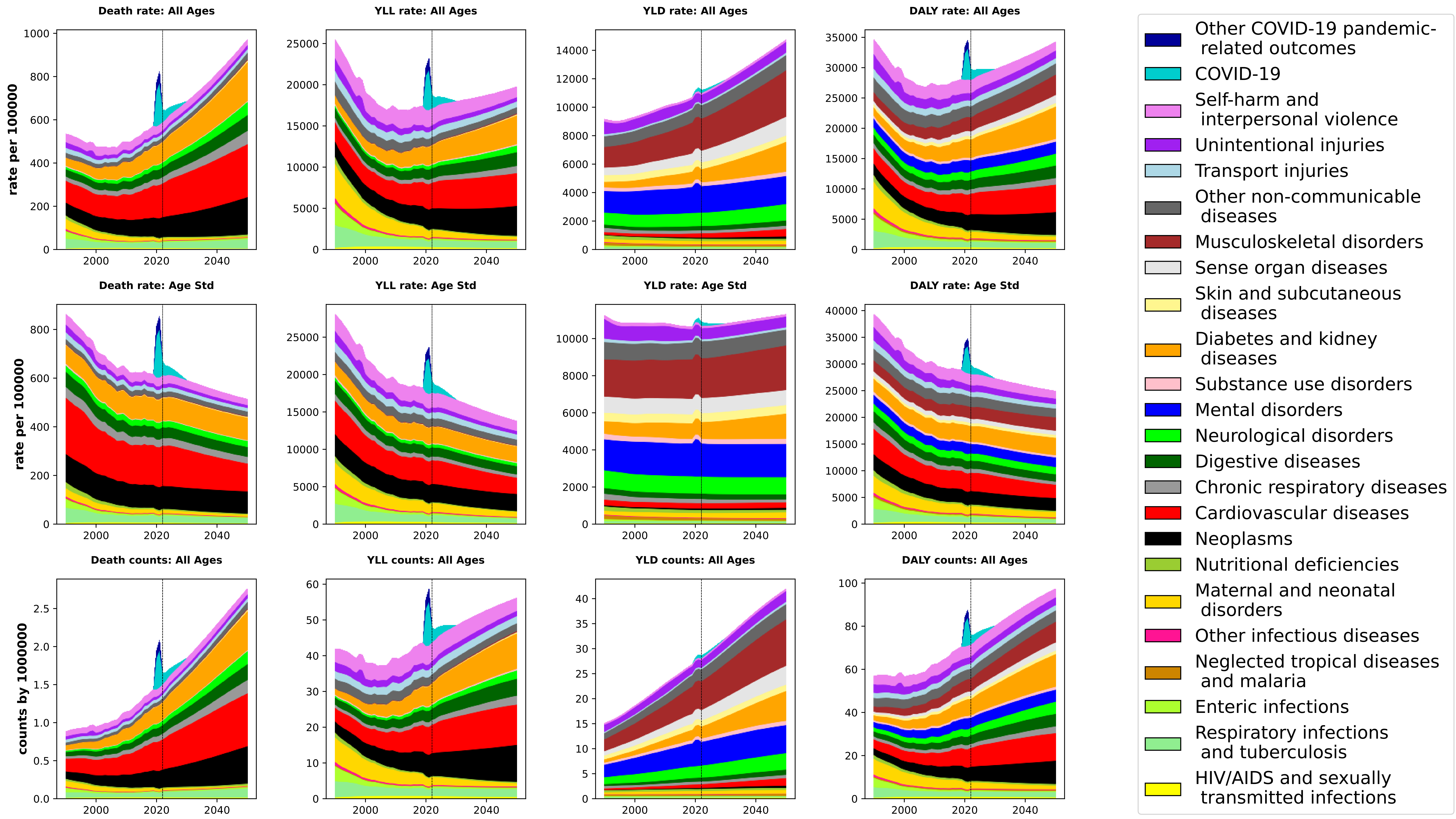

Tropical Latin America

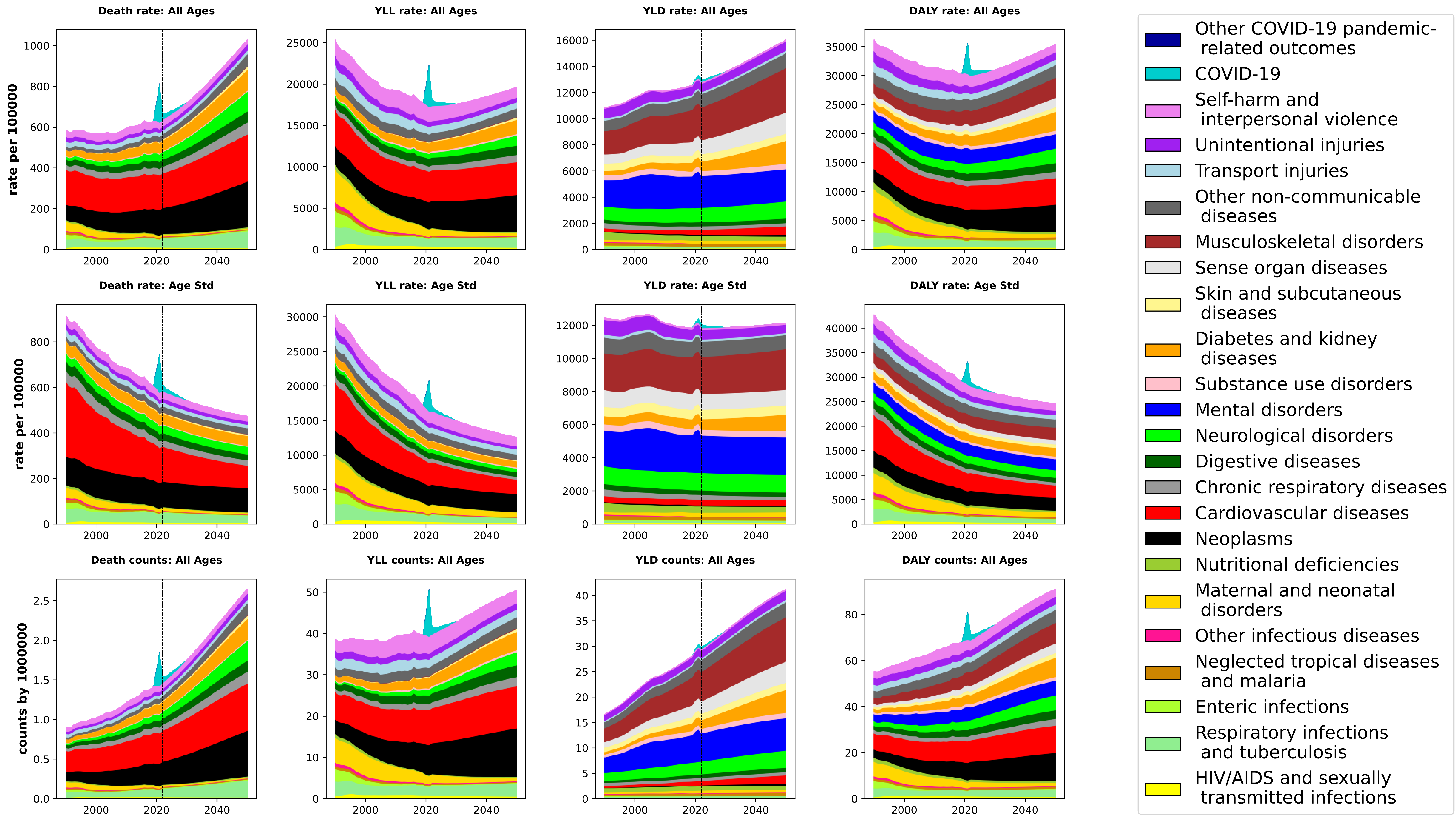

North Africa and Middle East

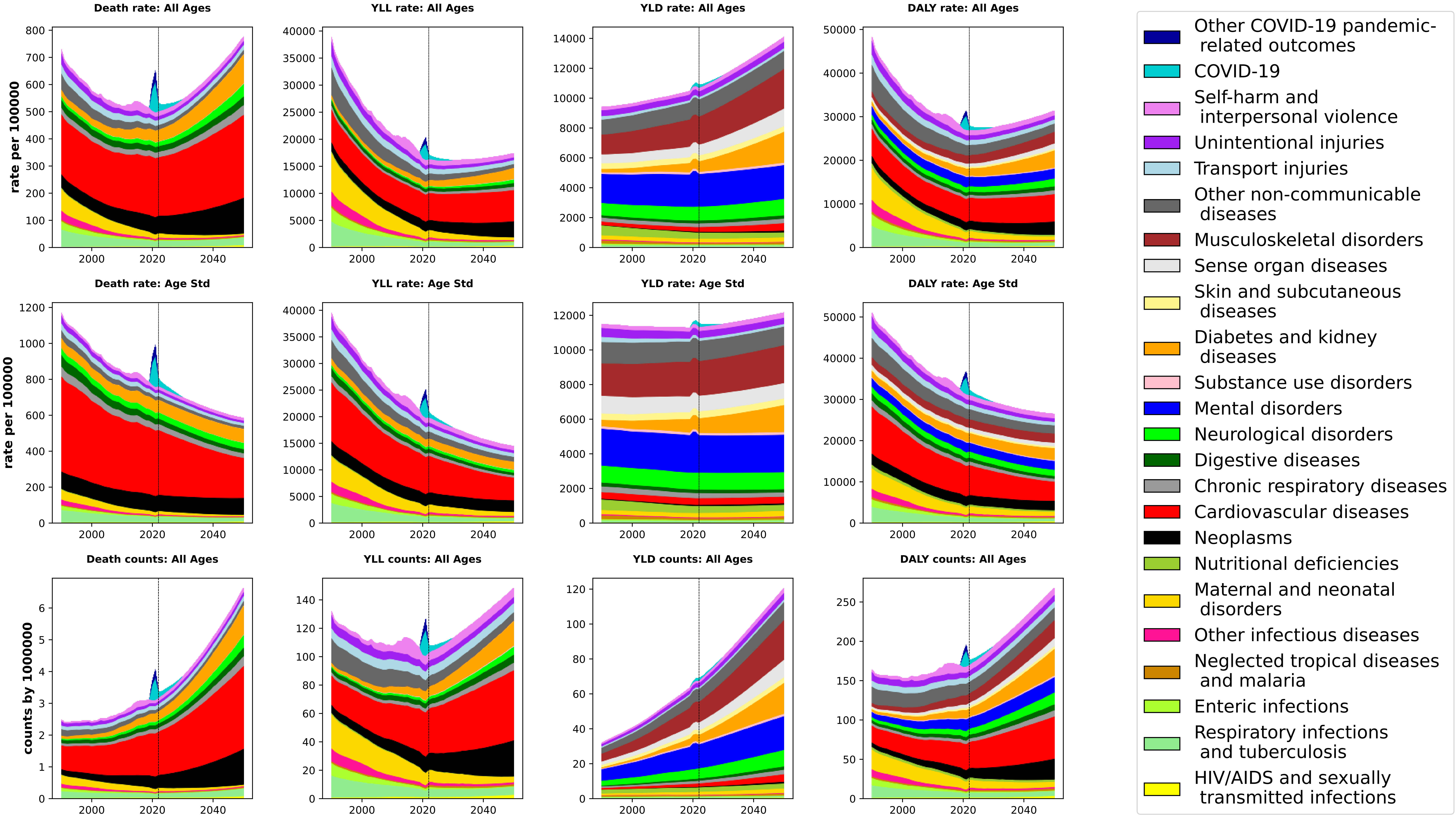

South Asia

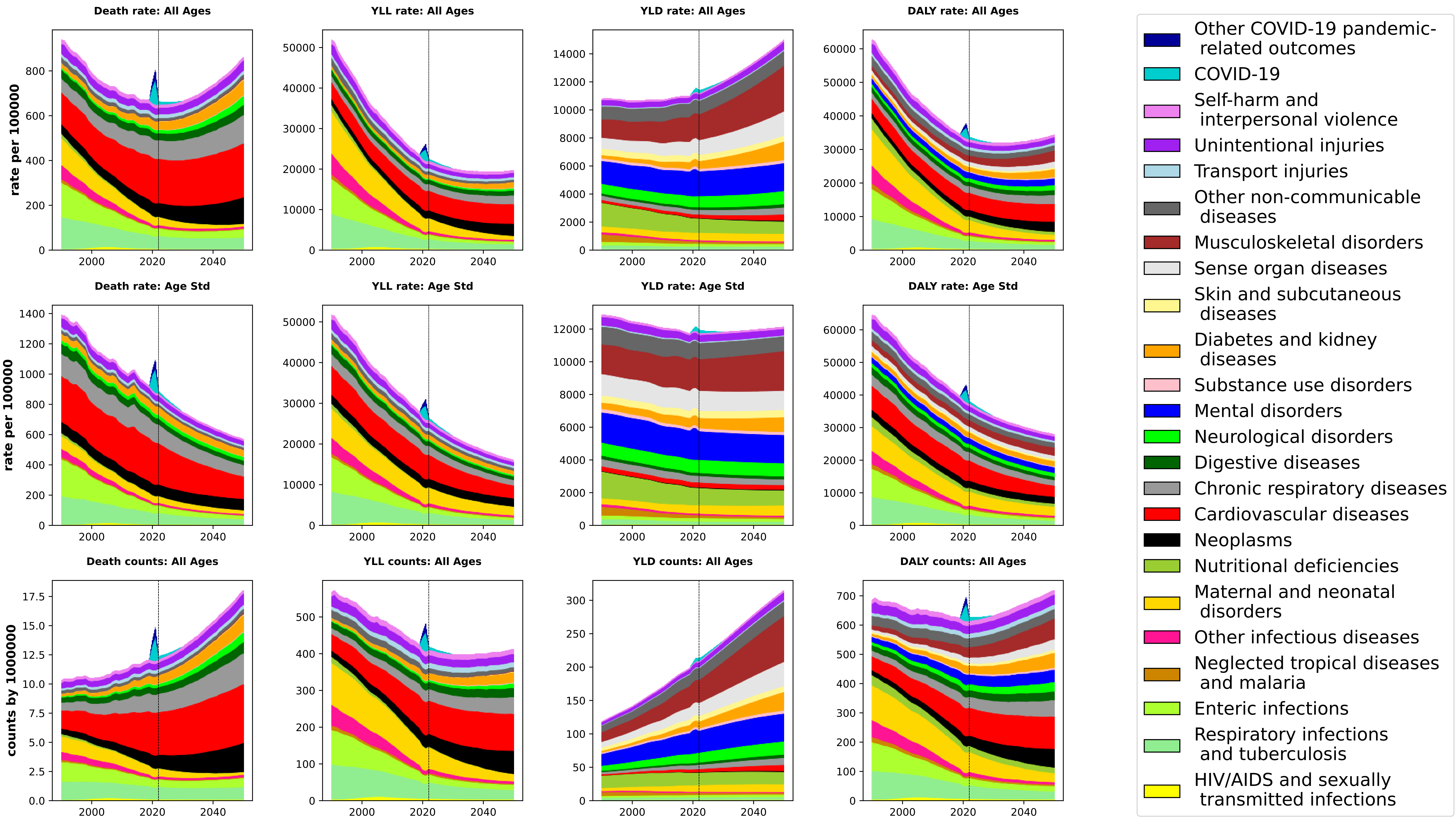
Death rate: All Ages
YLL rate: All Ages
YLD rate: All Ages
DALY rate: All Ages
Death rate: Age Std
YLL rate: Age Std
YLD rate: Age Std
DALY rate: Age Std
Death counts: All Ages
YLL counts: All Ages
YLD counts: All Ages
DALY counts: All Ages
rate per 100000
counts by 1000000
Other COVID-19 pandemic-related outcomes
COVID-19
Self-harm and interpersonal violence
Unintentional injuries
Transport injuries
Other non-communicable diseases
Musculoskeletal disorders
Sense organ diseases
Skin and subcutaneous diseases
Diabetes and kidney diseases
Substance use disorders
Mental disorders
Neurological disorders
Digestive diseases
Chronic respiratory diseases
Cardiovascular diseases
Neoplasms
Nutritional deficiencies
Maternal and neonatal disorders
Other infectious diseases
Neglected tropical diseases and malaria
Enteric infections
Respiratory infections and tuberculosis
HIV/AIDS and sexually transmitted infections

East Asia

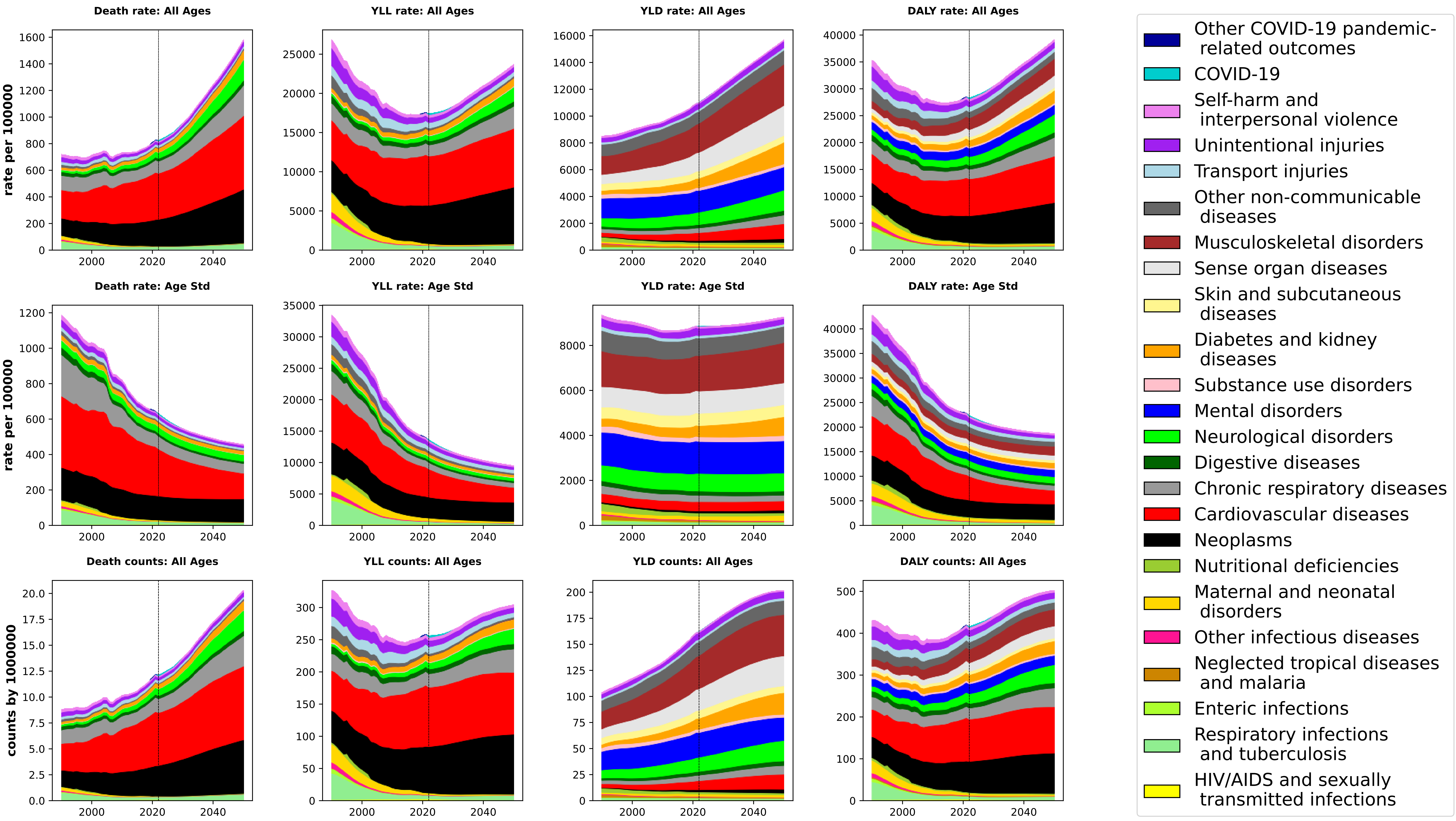
Death rate: All Ages
YLL rate: All Ages
YLD rate: All Ages
DALY rate: All Ages
Death rate: Age Std
YLL rate: Age Std
YLD rate: Age Std
DALY rate: Age Std
Death counts: All Ages
YLL counts: All Ages
YLD counts: All Ages
DALY counts: All Ages
rate per 100000
counts by 1000000
2000
2020
2040
Other COVID-19 pandemic-related outcomes
COVID-19
Self-harm and interpersonal violence
Unintentional injuries
Transport injuries
Other non-communicable diseases
Musculoskeletal disorders
Sense organ diseases
Skin and subcutaneous diseases
Diabetes and kidney diseases
Substance use disorders
Mental disorders
Neurological disorders
Digestive diseases
Chronic respiratory diseases
Cardiovascular diseases
Neoplasms
Nutritional deficiencies
Maternal and neonatal disorders
Other infectious diseases
Neglected tropical diseases and malaria
Enteric infections
Respiratory infections and tuberculosis
HIV/AIDS and sexually transmitted infections

Oceania

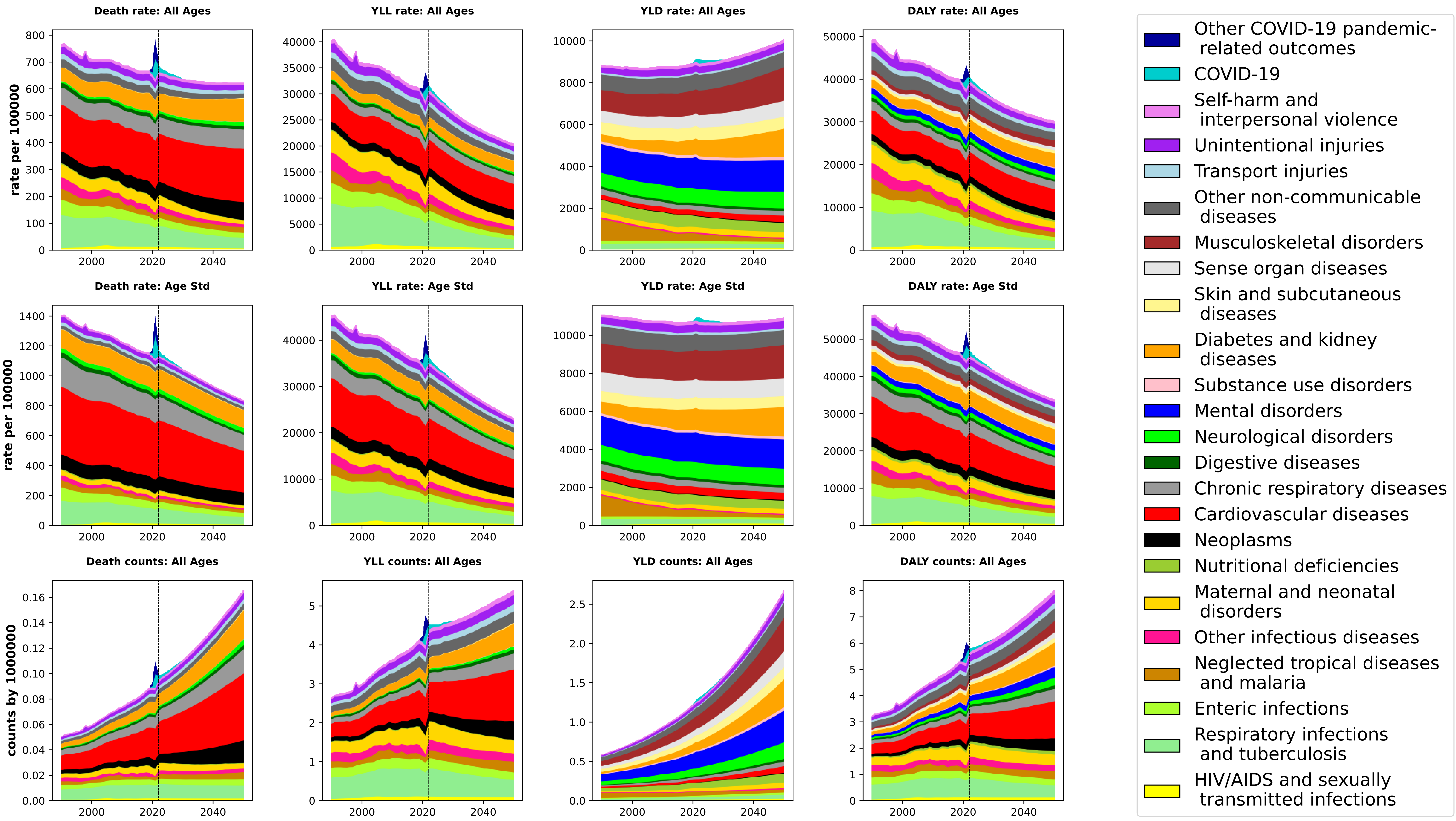
Death rate: All Ages
YLL rate: All Ages
YLD rate: All Ages
DALY rate: All Ages
Death rate: Age Std
YLL rate: Age Std
YLD rate: Age Std
DALY rate: Age Std
Death counts: All Ages
YLL counts: All Ages
YLD counts: All Ages
DALY counts: All Ages
rate per 100000
counts by 1000000
Other COVID-19 pandemic-related outcomes
COVID-19
Self-harm and interpersonal violence
Unintentional injuries
Transport injuries
Other non-communicable diseases
Musculoskeletal disorders
Sense organ diseases
Skin and subcutaneous diseases
Diabetes and kidney diseases
Substance use disorders
Mental disorders
Neurological disorders
Digestive diseases
Chronic respiratory diseases
Cardiovascular diseases
Neoplasms
Nutritional deficiencies
Maternal and neonatal disorders
Other infectious diseases
Neglected tropical diseases and malaria
Enteric infections
Respiratory infections and tuberculosis
HIV/AIDS and sexually transmitted infections

Southeast Asia

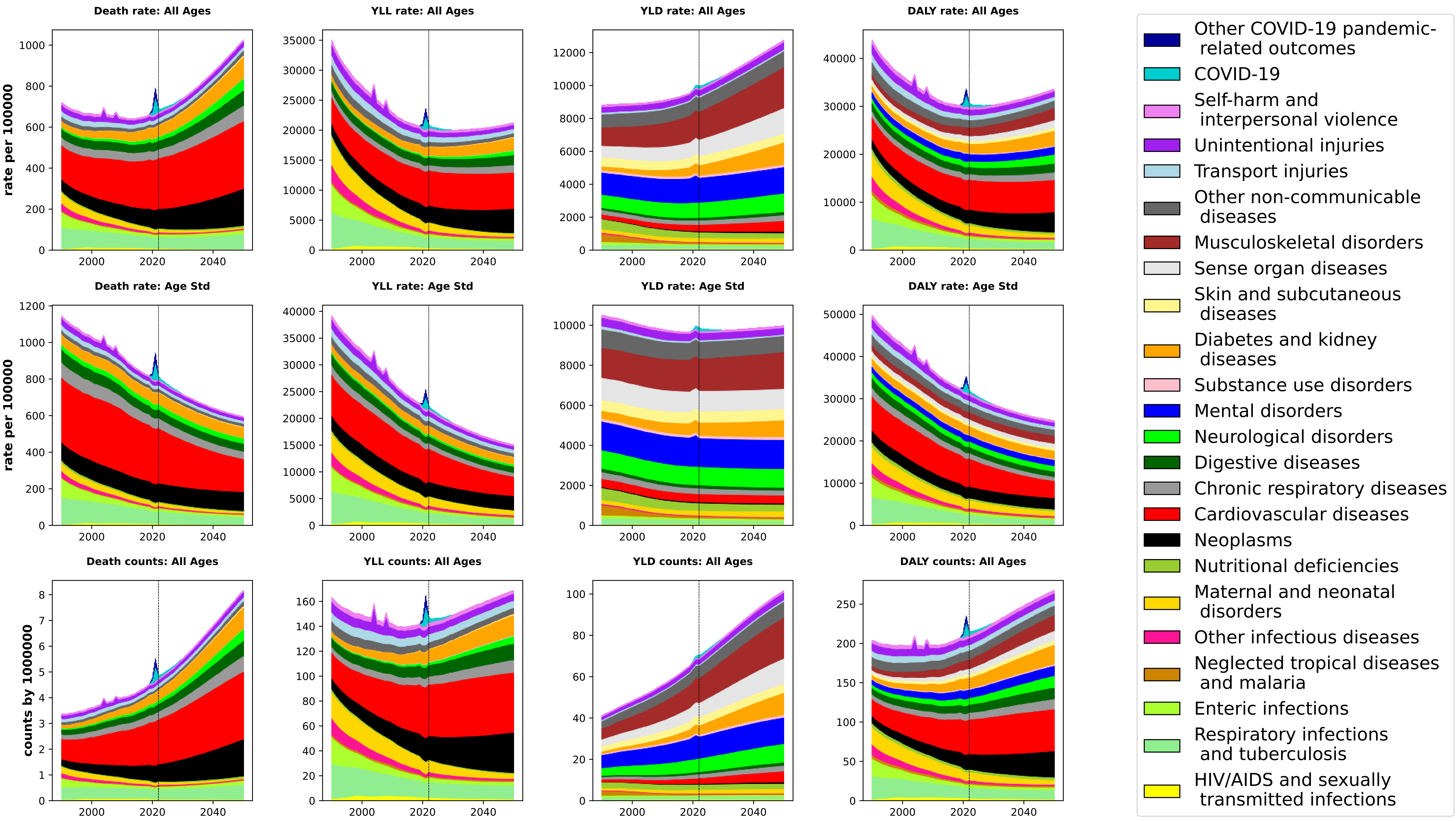

Central sub-Saharan Africa

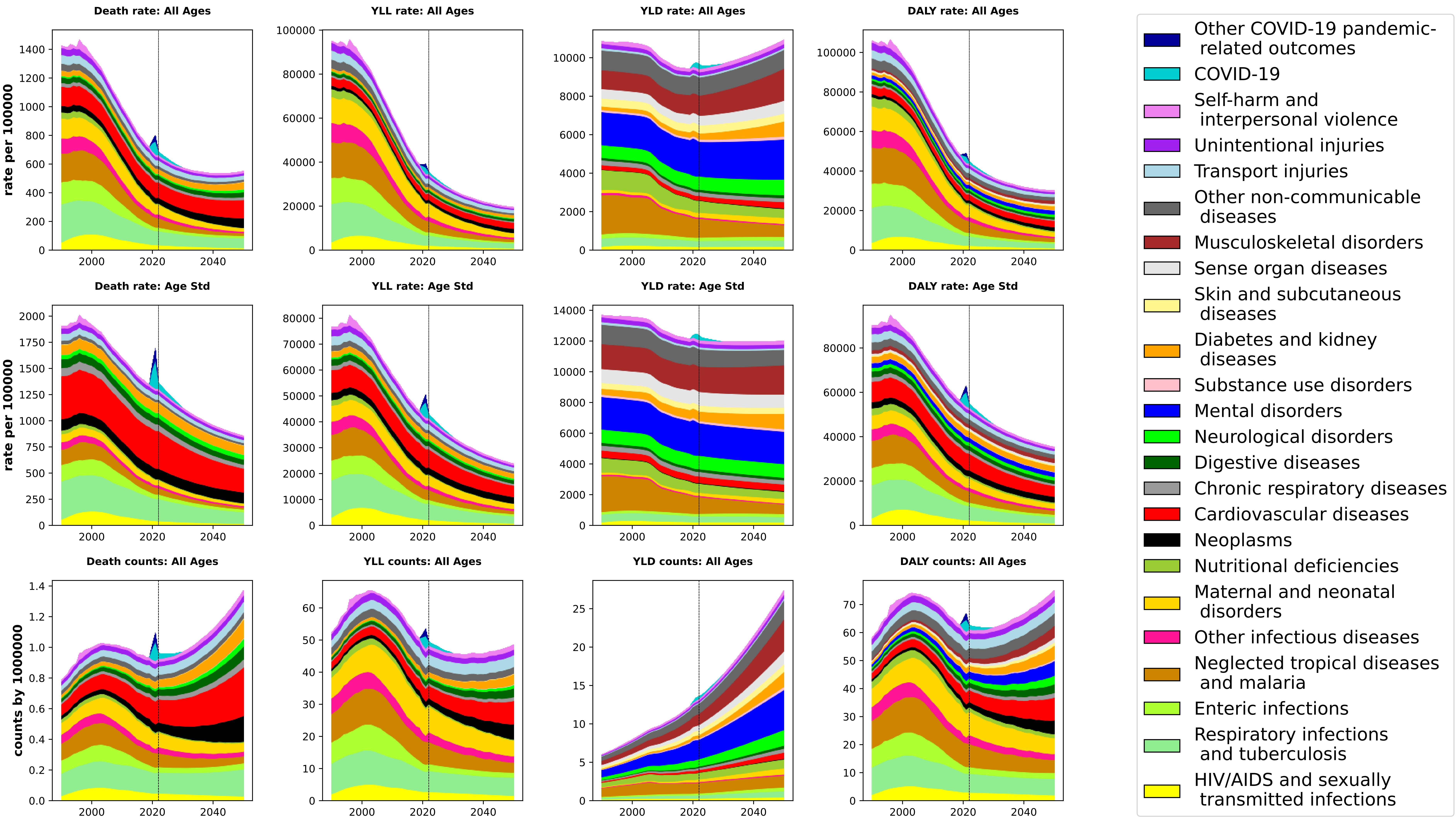

Eastern sub-Saharan Africa

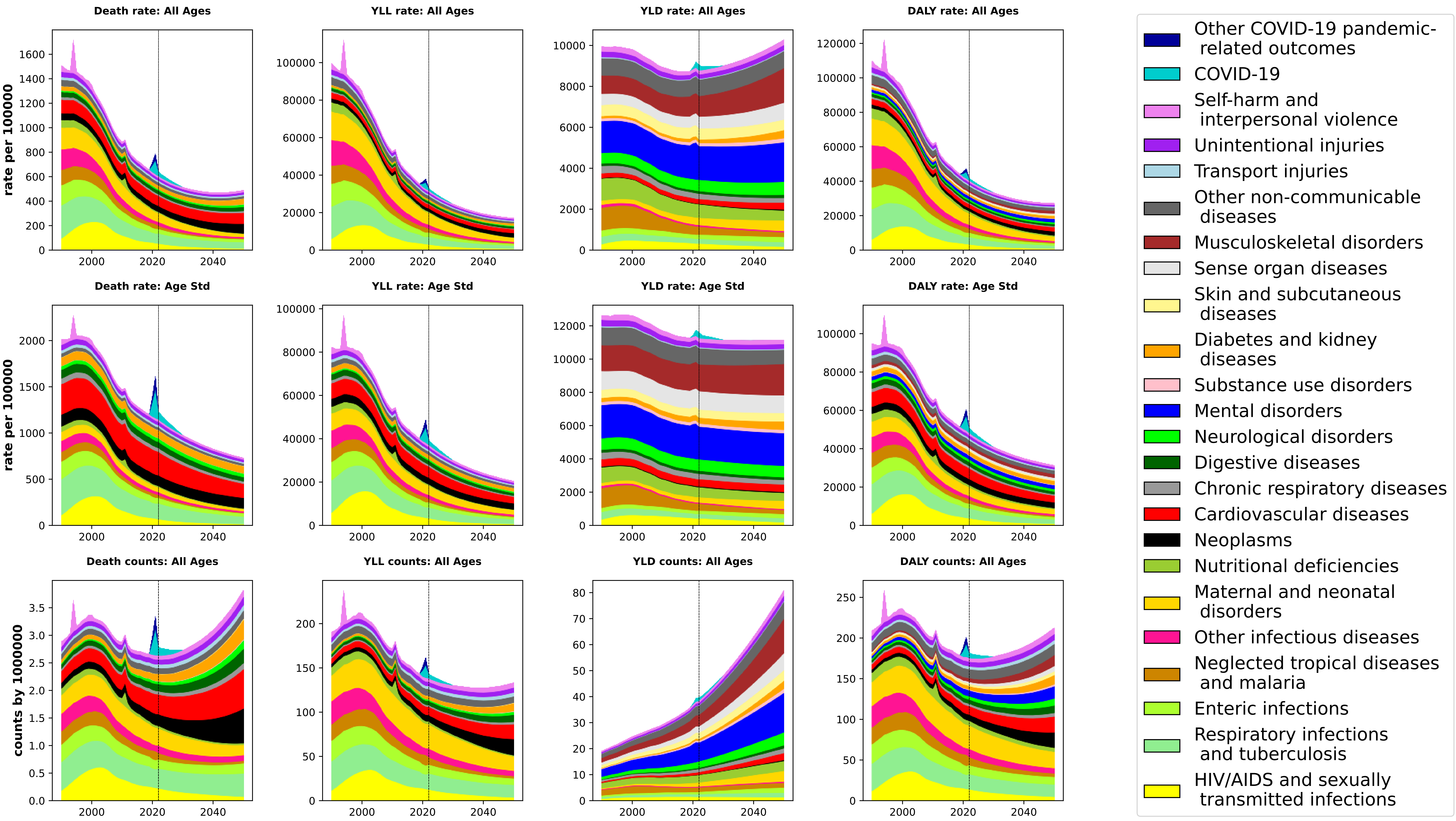

Southern sub-Saharan Africa

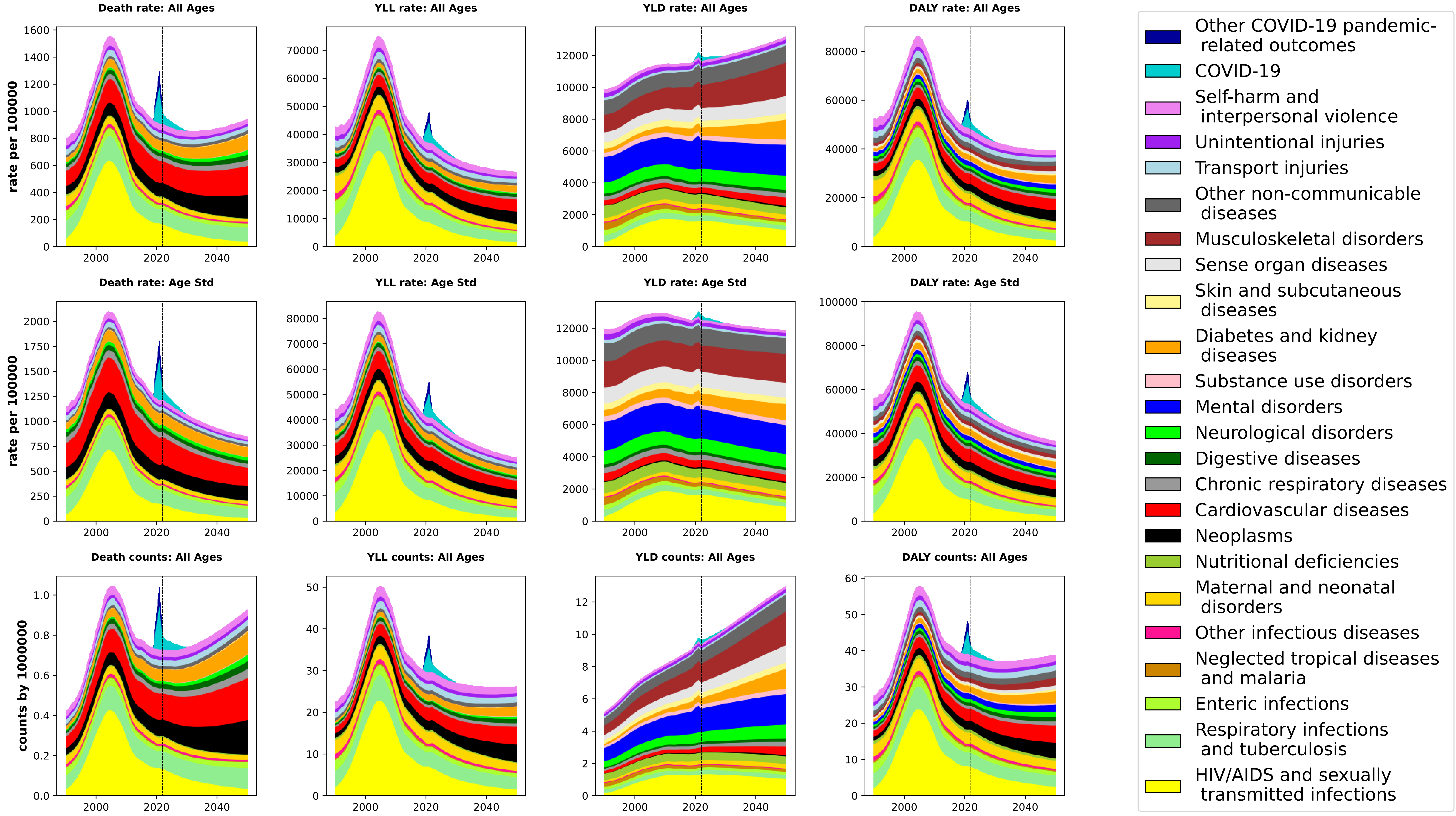

Western sub-Saharan Africa

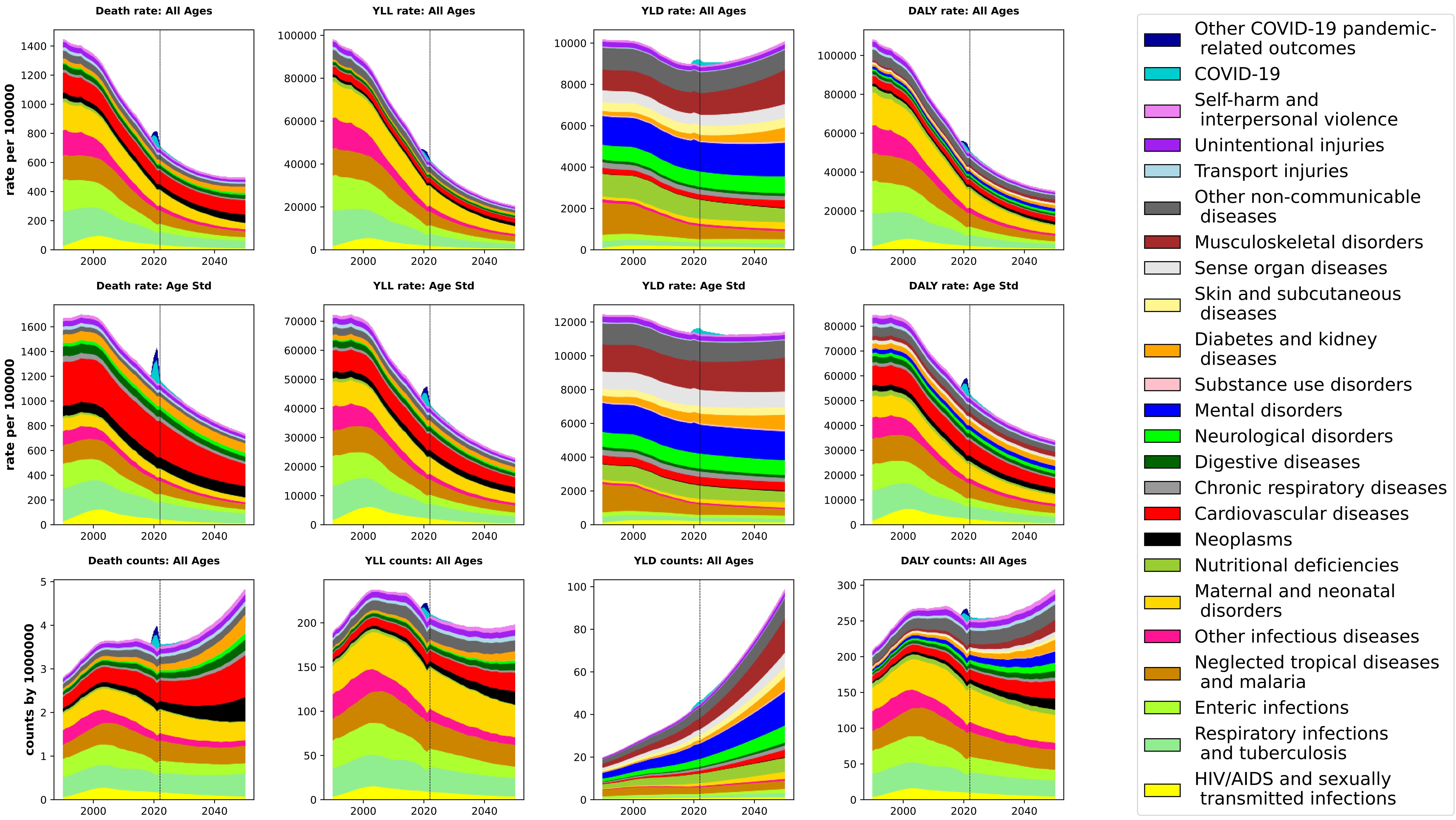

Supplemental Results Figure S7. Leading 20 level 3 causes of DALYs, and percentage change in number of DALYs and all-age and age-standardised DALY rates by location, 2022 and 2050

Armenia, Reference scenario

Legend:
Communicable, maternal, neonatal and nutritional
Non-communicable
Injuries

Leading Causes 2022	Leading Causes 2050	Mean % change number of DALY	Mean % change all-age DALY rate	Mean % change age-standardized DALY rate
1 Ischemic heart disease	1 Ischemic heart disease	-19.8 (-46.6 to 16.8)	0.872 (-33.2 to 42.1)	**-52.9 (-70.3 to -25.5)**
2 COVID-19	2 Stroke	**-21.3 (-38.9 to -0.445)**	-1.09 (-19.5 to 20.7)	**-50.7 (-60.4 to -38.3)**
3 Stroke	3 Low back pain	-0.0186 (-18.2 to 19.1)	**25.5 (18.4 to 33.6)**	**-3.42 (-3.62 to -3.19)**
4 Low back pain	4 Alzheimer's disease	**116 (70 to 164)**	**172 (121 to 231)**	**-9.3 (-11.1 to -7.46)**
5 Diabetes	5 Diabetes	11.2 (-10.5 to 32.9)	**39.8 (21 to 59.1)**	-10.7 (-20.7 to 0.386)
6 Lung cancer	6 Lung cancer	8.19 (-21.5 to 51.9)	36.1 (-3.39 to 83.4)	-16.3 (-39.3 to 12.2)
7 Lower respiratory infect	7 Age-related hearing loss	**37.1 (15.6 to 60.9)**	**72.4 (56 to 90.1)**	**-0.474 (-0.635 to -0.336)**
8 Neonatal disorders	8 COPD	**68.7 (20.5 to 130)**	**112 (59.3 to 179)**	-6.51 (-32.9 to 26.1)
9 Depressive disorders	9 Lower respiratory infect	-6.8 (-29.5 to 22.8)	17.2 (-10.6 to 44.8)	**-45.6 (-58.9 to -32.4)**
10 Headache disorders	10 Pancreatic cancer	**64.4 (7.1 to 138)**	**107 (41.1 to 202)**	23.1 (-15.6 to 73.5)
11 Age-related hearing loss	11 Depressive disorders	-4.82 (-21.1 to 12.1)	**19.5 (12.7 to 28.3)**	**-2.48 (-2.81 to -2.11)**
12 Alzheimer's disease	12 Hypertensive heart disease	**60.6 (3.02 to 139)**	**102 (31.2 to 199)**	-10.6 (-43.8 to 39.4)
13 Road injuries	13 Colorectal cancer	27.6 (-16 to 90.1)	**60.5 (7.77 to 138)**	-8.45 (-37.7 to 33.4)
14 Cirrhosis liver	14 Cirrhosis liver	0.373 (-18.7 to 23.3)	**26.1 (6.44 to 47.1)**	-11.2 (-27.6 to 6.72)
15 COPD	15 Blindness and vision loss	**38.8 (14.5 to 61.4)**	**74.5 (57.2 to 95.9)**	**-3.69 (-4.02 to -3.43)**
16 Breast cancer	16 Breast cancer	12.7 (-21 to 51.1)	**41.6 (4.22 to 89.3)**	-13.1 (-34.1 to 14.1)
17 Colorectal cancer	17 Osteoarthritis	**34.5 (13.8 to 55.4)**	**69.1 (55.4 to 84.3)**	0.171 (-0.383 to 0.73)
18 Oral disorders	18 Headache disorders	**-23 (-39.3 to -7.51)**	-3.5 (-9.31 to 6.83)	**-0.969 (-1.23 to -0.613)**
19 Congenital defects	19 Oral disorders	15.4 (-6.43 to 35.9)	**44.9 (33 to 58.5)**	**0.917 (0.694 to 1.16)**
20 Blindness and vision loss	20 Chronic kidney disease	37 (-10.5 to 97.6)	**72.1 (16.3 to 145)**	-3.26 (-38.2 to 50.7)
22 Pancreatic cancer	21 Road injuries			
23 Osteoarthritis	24 Neonatal disorders			
24 Hypertensive heart disease	43 Congenital defects			
26 Chronic kidney disease	150 COVID-19			

Supplemental Results Figure S7. Leading 20 level 3 causes of DALYs, and percentage change in number of DALYs and all-age and age-standardised DALY rates by location, 2022 and 2050

Azerbaijan, Reference scenario

Legend:
Communicable, maternal, neonatal and nutritional
Non-communicable
Injuries

Leading Causes 2022	Leading Causes 2050	Mean % change number of DALY	Mean % change all-age DALY rate	Mean % change age-standardized DALY rate
1 Ischemic heart disease	1 Ischemic heart disease	42.5 (-13.1 to 109)	33.9 (-14.4 to 90.6)	**-43.6 (-66.8 to -6.08)**
2 COVID-19	2 Stroke	**54.5 (17.1 to 96.7)**	**45 (16.7 to 78.2)**	**-36.8 (-50.9 to -16.9)**
3 Stroke	3 Diabetes	**75.5 (42 to 114)**	**64.7 (39.2 to 88.3)**	-0.265 (-14.7 to 15.4)
4 Neonatal disorders	4 Low back pain	**38.2 (17 to 59.8)**	**29.6 (22 to 37)**	**-3.29 (-3.48 to -3.1)**
5 Lower respiratory infect	5 Cirrhosis liver	**55.5 (23.5 to 91.3)**	**45.9 (23.1 to 73.5)**	-9.52 (-25 to 10.5)
6 Low back pain	6 Alzheimer's disease	**284 (179 to 401)**	**260 (178 to 350)**	**2.28 (0.45 to 4.48)**
7 Diabetes	7 Hypertensive heart disease	**166 (58.9 to 296)**	**150 (52.7 to 276)**	8.05 (-37.4 to 74.5)
8 Cirrhosis liver	8 Age-related hearing loss	**90.8 (59.2 to 126)**	**78.9 (61.7 to 98.5)**	**-0.477 (-0.621 to -0.324)**
9 Headache disorders	9 COPD	**144 (75.7 to 219)**	**129 (71.1 to 195)**	0.968 (-28.2 to 40.6)
10 Depressive disorders	10 Neonatal disorders	**-50.8 (-67.9 to -22.8)**	**-53.8 (-68.5 to -26.4)**	-36.5 (-60 to 1.9)
11 Cardiomyopathy	11 Lower respiratory infect	**-52.2 (-66.8 to -33.7)**	**-55.1 (-66.9 to -39.4)**	**-56.8 (-70.3 to -38.8)**
12 Congenital defects	12 Lung cancer	**60 (0.642 to 128)**	**49.8 (1.39 to 111)**	-9.19 (-37.8 to 26.8)
13 Age-related hearing loss	13 Headache disorders	7.09 (-8.48 to 23.2)	0.49 (-5.33 to 10.8)	**-0.679 (-0.862 to -0.437)**
14 Lung cancer	14 Depressive disorders	**27.5 (8.33 to 49)**	**19.5 (12.3 to 27.7)**	**-2.55 (-2.9 to -2.18)**
15 Dietary iron deficiency	15 Chronic kidney disease	**95.6 (20.6 to 190)**	**83.8 (16.7 to 172)**	14.9 (-32.1 to 88.2)
16 Stomach cancer	16 Blindness and vision loss	**94.4 (61.5 to 128)**	**82.3 (62.7 to 100)**	**-3.66 (-3.92 to -3.38)**
17 Gynecological diseases	17 Cardiomyopathy	13 (-12 to 40.3)	6.01 (-13.1 to 27.1)	**-23.3 (-34.8 to -9.2)**
18 Alcohol use disorders	18 Liver cancer	**125 (56.5 to 218)**	**112 (48.3 to 193)**	21.7 (-13.1 to 76)
19 COPD	19 Osteoarthritis	**93.8 (63.9 to 125)**	**81.8 (68.9 to 93.6)**	-0.048 (-0.632 to 0.546)
20 Hypertensive heart disease	20 Oral disorders	**57.4 (31.1 to 84.7)**	**47.6 (35.9 to 59.9)**	**0.966 (0.748 to 1.19)**
21 Oral disorders	21 Stomach cancer			
24 Chronic kidney disease	24 Alcohol use disorders			
26 Blindness and vision loss	26 Dietary iron deficiency			
28 Osteoarthritis	33 Gynecological diseases			
30 Alzheimer's disease	37 Congenital defects			
34 Liver cancer	155 COVID-19			

Supplemental Results Figure S7. Leading 20 level 3 causes of DALYs, and percentage change in number of DALYs and all-age and age-standardised DALY rates by location, 2022 and 2050

Georgia, Reference scenario

Legend:
Communicable, maternal, neonatal and nutritional
Non-communicable
Injuries

Leading Causes 2022
1 Stroke
2 Ischemic heart disease
3 COVID-19
4 Diabetes
5 Hypertensive heart disease
6 Low back pain
7 Lung cancer
8 Lower respiratory infect
9 Cirrhosis liver
10 Falls
11 Road injuries
12 Alzheimer's disease
13 Depressive disorders
14 Breast cancer
15 Neonatal disorders
16 Age-related hearing loss
17 Headache disorders
18 Colorectal cancer
19 COPD
20 Stomach cancer
21 Chronic kidney disease
22 Blindness and vision loss
23 Oral disorders

Leading Causes 2050	Mean % change number of DALY	Mean % change all-age DALY rate	Mean % change age-standardized DALY rate
1 Stroke	**-30.6 (-42.8 to -16.4)**	**-25.6 (-39.6 to -9.75)**	**-44.6 (-55.5 to -31.1)**
2 Ischemic heart disease	**-36.7 (-57.3 to -13.5)**	**-32.1 (-55 to -4.26)**	**-49 (-66.3 to -22.6)**
3 Hypertensive heart disease	27.2 (-16.9 to 80.6)	36.6 (-13.3 to 96.7)	-3.24 (-39.4 to 45.8)
4 Lung cancer	**45.1 (3.44 to 94.4)**	**55.1 (14.8 to 103)**	**38.4 (3.15 to 84.6)**
5 Diabetes	2.1 (-14.5 to 17.6)	9.33 (-3.35 to 24.5)	-4.49 (-14 to 7.24)
6 Alzheimer's disease	**76.1 (40.2 to 113)**	**88.6 (57.3 to 122)**	-0.834 (-2.95 to 1.18)
7 Low back pain	-1.08 (-15.1 to 13)	**5.79 (1.5 to 10.9)**	**-3.19 (-3.4 to -3.02)**
8 Cirrhosis liver	5.91 (-18.3 to 29.1)	13.3 (-5.34 to 35.3)	14.7 (-6.89 to 41.1)
9 Lower respiratory infect	-7.9 (-26 to 12.3)	-1.34 (-19.5 to 17.2)	**-26.3 (-40.9 to -10.8)**
10 Age-related hearing loss	**20 (2.33 to 35.4)**	**28.5 (17.5 to 40.8)**	**-0.572 (-0.669 to -0.45)**
11 Road injuries	-15 (-46.5 to 29.9)	-8.92 (-42.7 to 38.5)	-8.87 (-43.4 to 40.6)
12 Depressive disorders	-0.919 (-14.6 to 13.5)	**5.94 (1.97 to 9.65)**	**-1.92 (-2.2 to -1.65)**
13 Falls	**-18.5 (-33.5 to -0.631)**	**-12.9 (-23.6 to -2.21)**	**-21.8 (-32.3 to -10.4)**
14 Breast cancer	-2.27 (-29 to 29.7)	4.46 (-21.3 to 34.6)	-8.03 (-29.9 to 17.1)
15 Colorectal cancer	13 (-24.6 to 57)	20.8 (-19.4 to 69.2)	2.05 (-30.1 to 38.1)
16 Chronic kidney disease	29.6 (-14 to 81.7)	39 (-6.84 to 95.5)	12.2 (-28.1 to 70)
17 COPD	21.6 (-9.4 to 55.9)	30.4 (-2.49 to 66.2)	-4.55 (-28.9 to 25.7)
18 Headache disorders	-8.41 (-23.1 to 6.43)	**-2.16 (-4.22 to -0.162)**	-0.175 (-0.368 to 0.0135)
19 Blindness and vision loss	**19.2 (1.57 to 37.2)**	**27.6 (16.5 to 38.8)**	**-3.75 (-4.05 to -3.44)**
20 Oral disorders	7.7 (-7.3 to 23.1)	**15.2 (8.51 to 22.8)**	**0.685 (0.508 to 0.883)**
21 Neonatal disorders			
26 Stomach cancer			
158 COVID-19			

Supplemental Results Figure S7. Leading 20 level 3 causes of DALYs, and percentage change in number of DALYs and all-age and age-standardised DALY rates by location, 2022 and 2050

Kazakhstan, Reference scenario

Legend:
Communicable, maternal, neonatal and nutritional
Non-communicable
Injuries

Leading Causes 2022	Leading Causes 2050	Mean % change number of DALY	Mean % change all-age DALY rate	Mean % change age-standardized DALY rate
1 COVID-19	1 Ischemic heart disease	10.8 (-24.1 to 49.1)	-8.65 (-37.1 to 22.1)	**-51.2 (-69.1 to -26)**
2 Ischemic heart disease	2 Stroke	7.74 (-13.5 to 28.8)	-11.2 (-27.8 to 6.27)	**-48.8 (-59.8 to -33.6)**
3 Stroke	3 COPD	**78.2 (34.9 to 135)**	**46.9 (11.4 to 88.5)**	-11.5 (-37.6 to 19.6)
4 Cardiomyopathy	4 Low back pain	**35.4 (18.3 to 49.9)**	**11.5 (6.17 to 16.6)**	**-3.75 (-4.01 to -3.53)**
5 Cirrhosis liver	5 Cirrhosis liver	12.8 (-7.75 to 34.8)	-7.08 (-22.9 to 9.97)	**-23.2 (-36.9 to -6.8)**
6 Low back pain	6 Diabetes	**75.8 (54.6 to 96.3)**	**44.8 (33.6 to 56.5)**	**10.5 (2.7 to 18.8)**
7 Neonatal disorders	7 Cardiomyopathy	5.73 (-10.2 to 28.5)	-13 (-26 to 2.24)	**-25.3 (-36.8 to -9.96)**
8 COPD	8 Self-harm	1 (-24.9 to 31.4)	-16.8 (-33.8 to 6.05)	-15.2 (-33.2 to 8.64)
9 Self-harm	9 Depressive disorders	**31.3 (14.7 to 47.1)**	**8.06 (3.61 to 13.2)**	**-2.5 (-2.85 to -2.21)**
10 Lower respiratory infect	10 Alzheimer's disease	**200 (129 to 267)**	**147 (97.8 to 196)**	1.9 (-0.144 to 4.02)
11 Diabetes	11 Age-related hearing loss	**74.4 (51.6 to 95.4)**	**43.6 (31.7 to 55.5)**	**-0.589 (-0.843 to -0.348)**
12 Congenital defects	12 Lower respiratory infect	-8.44 (-27.2 to 12.2)	**-24.6 (-37.3 to -7.85)**	**-39.5 (-51.3 to -24.8)**
13 Road injuries	13 Alcohol use disorders	**19.6 (0.0183 to 37.9)**	-1.54 (-12.7 to 11.8)	1.24 (-9.4 to 15.9)
14 Depressive disorders	14 Headache disorders	**20.4 (5.4 to 33.7)**	-0.929 (-3.71 to 3.45)	**-0.347 (-0.487 to -0.189)**
15 Alcohol use disorders	15 Road injuries	3.24 (-38.3 to 68.2)	-14.8 (-48.8 to 36.8)	-14.4 (-48.7 to 38.6)
16 Headache disorders	16 Neonatal disorders	**-35 (-52.8 to -8.9)**	**-46.5 (-59.8 to -27.3)**	**-35.5 (-51.2 to -13.9)**
17 Falls	17 Chronic kidney disease	**102 (38.1 to 198)**	**66.2 (11.2 to 140)**	16 (-27.4 to 79.1)
18 Lung cancer	18 Lung cancer	30.1 (-12.4 to 78.4)	6.99 (-26.6 to 43.6)	-21.1 (-42.5 to 3.77)
19 Age-related hearing loss	19 Falls	10.4 (-7.77 to 29.2)	-9.15 (-21.1 to 3.69)	**-16.8 (-28.3 to -4.35)**
20 Dietary iron deficiency	20 Oral disorders	**51.3 (30.2 to 69.5)**	**24.5 (16.4 to 34)**	**0.605 (0.419 to 0.82)**
23 Oral disorders	25 Dietary iron deficiency			
25 Chronic kidney disease	26 Congenital defects			
31 Alzheimer's disease	153 COVID-19			

Supplemental Results Figure S7. Leading 20 level 3 causes of DALYs, and percentage change in number of DALYs and all-age and age-standardised DALY rates by location, 2022 and 2050

Kyrgyzstan, Reference scenario

Legend:
Communicable, maternal, neonatal and nutritional
Non-communicable
Injuries

Leading Causes 2022	Leading Causes 2050	Mean % change number of DALY	Mean % change all-age DALY rate	Mean % change age-standardized DALY rate
1 COVID-19	1 Ischemic heart disease	**72.3 (10.2 to 152)**	43.8 (-5.27 to 114)	-32.8 (-60.5 to 9.58)
2 Ischemic heart disease	2 Stroke	**32.6 (0.464 to 69.2)**	10.6 (-13.2 to 40)	**-37.9 (-53.3 to -16.9)**
3 Neonatal disorders	3 Neonatal disorders	-32.6 (-62.2 to 10.1)	**-43.9 (-65.9 to -10.3)**	-26.5 (-56.3 to 16.6)
4 Stroke	4 Low back pain	**51.1 (23.4 to 76)**	**25.8 (18.7 to 33.5)**	**-3.35 (-3.52 to -3.11)**
5 Cirrhosis liver	5 Cirrhosis liver	28.3 (-3.14 to 60.5)	6.91 (-12.6 to 30.4)	-16.2 (-33.4 to 3.85)
6 Low back pain	6 COPD	**166 (88.8 to 246)**	**122 (64.9 to 194)**	8.89 (-25.9 to 58.1)
7 Road injuries	7 Diabetes	**124 (82 to 165)**	**86.8 (66.1 to 109)**	**22.4 (10 to 35.2)**
8 Lower respiratory infect	8 Road injuries	2.16 (-45 to 81.5)	-14.8 (-51.9 to 48.9)	-17.4 (-53.3 to 43.8)
9 Congenital defects	9 Depressive disorders	**42.4 (16.3 to 66.6)**	**18.5 (12.3 to 25.3)**	**-2.17 (-2.5 to -1.85)**
10 Depressive disorders	10 Alzheimer's disease	**279 (176 to 387)**	**215 (146 to 299)**	**11.5 (8.53 to 14.3)**
11 Headache disorders	11 Headache disorders	**26.4 (3.75 to 50.2)**	**5.2 (0.366 to 13.7)**	-0.125 (-0.297 to 0.00915)
12 Alcohol use disorders	12 Age-related hearing loss	**96.4 (59.5 to 132)**	**63.5 (49 to 79)**	**-0.601 (-0.737 to -0.431)**
13 Dietary iron deficiency	13 Alcohol use disorders	**31.2 (1.54 to 62)**	9.24 (-6.35 to 28.2)	-1.76 (-14.9 to 15.6)
14 Self-harm	14 Hypertensive heart disease	**171 (59.7 to 304)**	**126 (38.2 to 235)**	12.6 (-34.9 to 82.4)
15 Diabetes	15 Lung cancer	**117 (35.5 to 205)**	**80.6 (24.2 to 157)**	8.96 (-21.9 to 50.7)
16 COPD	16 Chronic kidney disease	**94 (14.8 to 205)**	62 (-2 to 155)	16.2 (-33.3 to 93.6)
17 Age-related hearing loss	17 Dietary iron deficiency	1.32 (-17.9 to 22.1)	**-15.7 (-18.6 to -13)**	**-9.68 (-10 to -9.22)**
18 Falls	18 Self-harm	0.537 (-28.1 to 34.1)	-16.2 (-37.9 to 9.51)	-19.1 (-40.3 to 6.76)
19 Stomach cancer	19 Lower respiratory infect	**-40.5 (-56.9 to -21.5)**	**-50.4 (-63.3 to -35.5)**	**-48.8 (-62.2 to -31.5)**
20 Gynecological diseases	20 Oral disorders	**70.3 (39.6 to 103)**	**41.8 (31.1 to 53.4)**	**0.968 (0.745 to 1.2)**
23 Oral disorders	22 Stomach cancer			
24 Chronic kidney disease	24 Congenital defects			
27 Hypertensive heart disease	26 Falls			
28 Lung cancer	27 Gynecological diseases			
35 Alzheimer's disease	152 COVID-19			

Supplemental Results Figure S7. Leading 20 level 3 causes of DALYs, and percentage change in number of DALYs and all-age and age-standardised DALY rates by location, 2022 and 2050

Mongolia, Reference scenario

Legend:
Communicable, maternal, neonatal and nutritional
Non-communicable
Injuries

Leading Causes 2022	Leading Causes 2050	Mean % change number of DALY	Mean % change all-age DALY rate	Mean % change age-standardized DALY rate
1 Ischemic heart disease	1 Liver cancer	**192 (104 to 301)**	**81.3 (26 to 155)**	30.3 (-14.2 to 90.9)
2 Stroke	2 Ischemic heart disease	46.5 (-3.84 to 111)	-8.96 (-40.5 to 33.5)	**-47.6 (-69.2 to -14.2)**
3 Liver cancer	3 Stroke	**55.1 (24.3 to 90.8)**	-3.67 (-24.4 to 21.8)	**-36.3 (-51.7 to -18.2)**
4 Neonatal disorders	4 Alcohol use disorders	**50.4 (19.3 to 93)**	-6.67 (-26 to 16.7)	-3.88 (-23.7 to 22.4)
5 Alcohol use disorders	5 Cirrhosis liver	**124 (79.9 to 168)**	**39 (11.2 to 69.8)**	13.4 (-10.3 to 41)
6 Lower respiratory infect	6 Low back pain	**80.7 (65.7 to 97)**	**12 (5.57 to 20)**	**-3.88 (-4.12 to -3.55)**
7 Low back pain	7 Neonatal disorders	5.23 (-28 to 53.9)	**-34.7 (-54.3 to -4.9)**	-21 (-44.8 to 14.4)
8 Road injuries	8 Self-harm	**66.4 (26.2 to 127)**	3.24 (-22.2 to 38.9)	1.72 (-23 to 39)
9 Cirrhosis liver	9 Road injuries	45.1 (-10 to 127)	-9.88 (-43.6 to 44.3)	-9.94 (-43.4 to 44.7)
10 COVID-19	10 Depressive disorders	**75.3 (60.9 to 89.4)**	**8.71 (3.52 to 14.7)**	**-2.02 (-2.34 to -1.58)**
11 Self-harm	11 Stomach cancer	**42.5 (20.3 to 61.2)**	-11.6 (-25 to 1.52)	**-36.5 (-44 to -29.2)**
12 Stomach cancer	12 Diabetes	**109 (84.8 to 135)**	**29.5 (15.3 to 45.9)**	1.27 (-9.51 to 14.7)
13 Congenital defects	13 Lung cancer	**125 (43 to 223)**	39.6 (-8.15 to 99.4)	-2.7 (-32.4 to 33.3)
14 Depressive disorders	14 Falls	**61 (34.3 to 86.7)**	-0.174 (-17.7 to 12.7)	-6.85 (-21.3 to 5.76)
15 Tuberculosis	15 Headache disorders	**65.7 (53.3 to 80)**	2.71 (-0.653 to 6.77)	**0.241 (0.0535 to 0.486)**
16 Falls	16 Age-related hearing loss	**131 (104 to 157)**	**43.2 (29.8 to 56)**	**-0.959 (-1.05 to -0.861)**
17 Dietary iron deficiency	17 Esophageal cancer	**172 (134 to 217)**	**68.8 (44.8 to 96.8)**	8.35 (-6.3 to 23.1)
18 Headache disorders	18 Cardiomyopathy	**83.5 (49.3 to 131)**	13.8 (-6.67 to 48.2)	-0.394 (-18.8 to 25.2)
19 Diabetes	19 Dietary iron deficiency	**30.8 (16.8 to 45.3)**	**-19 (-21.5 to -16.9)**	**-12.9 (-13.1 to -12.5)**
20 Lung cancer	20 Alzheimer's disease	**338 (244 to 436)**	**171 (109 to 234)**	**3.95 (1.57 to 6.29)**
21 Cardiomyopathy	21 Lower respiratory infect			
23 Age-related hearing loss	24 Congenital defects			
27 Esophageal cancer	36 Tuberculosis			
41 Alzheimer's disease	157 COVID-19			

Supplemental Results Figure S7. Leading 20 level 3 causes of DALYs, and percentage change in number of DALYs and all-age and age-standardised DALY rates by location, 2022 and 2050

Tajikistan, Reference scenario

Legend:
Communicable, maternal, neonatal and nutritional
Non-communicable
Injuries

Leading Causes 2022	Leading Causes 2050	Mean % change number of DALY	Mean % change all-age DALY rate	Mean % change age-standardized DALY rate
1 Lower respiratory infect	1 Ischemic heart disease	**124 (37.9 to 215)**	50.5 (-6.58 to 134)	-29.4 (-59.8 to 18)
2 Neonatal disorders	2 Stroke	**102 (50 to 151)**	**35.4 (1.08 to 70.4)**	**-33.4 (-52.7 to -12.2)**
3 Ischemic heart disease	3 Neonatal disorders	-23.1 (-54.7 to 29.5)	**-48.4 (-69 to -9.69)**	-30.4 (-58.5 to 21.5)
4 Stroke	4 Lower respiratory infect	**-54.6 (-68.4 to -38.5)**	**-69.6 (-77.5 to -58.5)**	**-62.8 (-72.4 to -49.2)**
5 COVID-19	5 Low back pain	**85 (54.3 to 117)**	**23.7 (17.3 to 30.6)**	**-3.87 (-4.17 to -3.57)**
6 Diarrheal diseases	6 Diabetes	**146 (101 to 198)**	**64.5 (40 to 95.1)**	6.33 (-10.7 to 26.1)
7 Congenital defects	7 Hypertensive heart disease	**204 (82.9 to 355)**	**104 (19.7 to 224)**	-5.59 (-47.6 to 64.3)
8 Low back pain	8 Cirrhosis liver	**50.2 (13.4 to 89.5)**	0.597 (-22.2 to 25.6)	**-23.3 (-41.2 to -4.98)**
9 Dietary iron deficiency	9 Headache disorders	**57.9 (29.1 to 87)**	**5.55 (1.64 to 13.1)**	0.0824 (-0.0595 to 0.226)
10 Cirrhosis liver	10 COPD	**142 (67.7 to 218)**	**62.1 (13.9 to 117)**	-20.2 (-46.9 to 17.5)
11 Headache disorders	11 Dietary iron deficiency	**24.4 (2.97 to 49.4)**	**-16.9 (-19 to -14.7)**	**-11.9 (-12.2 to -11.7)**
12 Diabetes	12 Age-related hearing loss	**140 (97.4 to 183)**	**60.8 (47.2 to 75.4)**	**-0.865 (-0.96 to -0.765)**
13 Falls	13 Depressive disorders	**74.8 (45.9 to 109)**	**16.8 (11.5 to 23.2)**	**-2.13 (-2.63 to -1.66)**
14 Idiopathic epilepsy	14 Alzheimer's disease	**350 (228 to 476)**	**201 (135 to 269)**	0.847 (-1.89 to 3.15)
15 Depressive disorders	15 Congenital defects	**-27.8 (-45.8 to -7.7)**	**-51.7 (-61.3 to -41.3)**	**-36 (-47.2 to -22.2)**
16 Drowning	16 Falls	26.3 (-1.57 to 62.4)	-15.5 (-29.9 to 3.61)	**-19 (-30.4 to -4.17)**
17 Road injuries	17 Idiopathic epilepsy	28.5 (-27.6 to 105)	-13.7 (-51.7 to 41.5)	-9.95 (-49.9 to 45.4)
18 Tuberculosis	18 Oral disorders	**111 (73.9 to 149)**	**40.9 (30.8 to 51.2)**	**0.707 (0.393 to 1.06)**
19 COPD	19 Road injuries	22.8 (-32.6 to 100)	-17.5 (-54.7 to 42.3)	-18.2 (-55.6 to 44.9)
20 Age-related hearing loss	20 Blindness and vision loss	**144 (102 to 194)**	**63.3 (47.4 to 77.8)**	**-5.22 (-5.65 to -4.75)**
23 Hypertensive heart disease	22 Diarrheal diseases			
27 Oral disorders	32 Drowning			
31 Blindness and vision loss	44 Tuberculosis			
41 Alzheimer's disease	151 COVID-19			

Supplemental Results Figure S7. Leading 20 level 3 causes of DALYs, and percentage change in number of DALYs and all-age and age-standardised DALY rates by location, 2022 and 2050

Turkmenistan, Reference scenario

Legend:
Communicable, maternal, neonatal and nutritional
Non-communicable
Injuries

Leading Causes 2022	Leading Causes 2050	Mean % change number of DALY	Mean % change all-age DALY rate	Mean % change age-standardized DALY rate
1 Ischemic heart disease	1 Ischemic heart disease	21.4 (-28.1 to 83.5)	-3.52 (-40.8 to 48.7)	**-48.8 (-72.1 to -9.95)**
2 Stroke	2 Stroke	11.5 (-16.4 to 54.3)	-11.5 (-34.2 to 23.4)	**-45.7 (-61.9 to -19.3)**
3 Lower respiratory infect	3 Cirrhosis liver	**38.4 (6.3 to 80.1)**	9.87 (-16.7 to 47.5)	-5.55 (-27.7 to 24.8)
4 Cirrhosis liver	4 Low back pain	**52.6 (31.4 to 72)**	**20.8 (12.8 to 29.4)**	**-3.48 (-3.78 to -3.16)**
5 Neonatal disorders	5 Neonatal disorders	-35.3 (-63.2 to 11.9)	**-48.6 (-69.3 to -13.5)**	-34.8 (-60.7 to 6.73)
6 COVID-19	6 Diabetes	**49.2 (18.6 to 91.8)**	18.4 (-5.15 to 47.4)	-15.1 (-33.3 to 9.67)
7 Congenital defects	7 Lower respiratory infect	**-56.3 (-70.1 to -36.8)**	**-65.3 (-75.4 to -51.3)**	**-60.5 (-72 to -43.8)**
8 Low back pain	8 Chronic kidney disease	**76 (2.11 to 213)**	40 (-20.5 to 139)	9.57 (-42.5 to 98.6)
9 Diabetes	9 Age-related hearing loss	**95.5 (56.7 to 131)**	**54.7 (34.2 to 75.5)**	**-0.616 (-0.865 to -0.398)**
10 Headache disorders	10 Depressive disorders	**43.8 (23.9 to 64.1)**	**13.9 (7.84 to 21.3)**	**-2.5 (-3.17 to -1.96)**
11 Chronic kidney disease	11 Headache disorders	**30.6 (14 to 47)**	3.46 (-0.613 to 10.9)	**0.586 (0.169 to 0.896)**
12 Depressive disorders	12 Alzheimer's disease	**235 (121 to 364)**	**165 (82.4 to 244)**	-1.43 (-3.68 to 0.647)
13 Cardiomyopathy	13 Hypertensive heart disease	**126 (33.4 to 264)**	**79.4 (5.49 to 192)**	0.725 (-47 to 76.2)
14 Self-harm	14 Congenital defects	**-40.2 (-54.1 to -23.4)**	**-52.6 (-60.7 to -40.6)**	**-39.9 (-48.6 to -26.6)**
15 Dietary iron deficiency	15 Cardiomyopathy	14.6 (-6.16 to 42)	-9.25 (-23.4 to 8.4)	**-23.8 (-34.3 to -11.7)**
16 Alcohol use disorders	16 Self-harm	12.9 (-22.7 to 62.2)	-10.4 (-38 to 28)	-9.78 (-37.7 to 29)
17 Age-related hearing loss	17 Urinary diseases	**83.2 (31.9 to 154)**	**45.5 (6.42 to 102)**	7.85 (-25.9 to 58.9)
18 Road injuries	18 Alcohol use disorders	**26 (6.51 to 54)**	-0.116 (-12 to 19.2)	-4.62 (-15.9 to 11.3)
19 Tuberculosis	19 Dietary iron deficiency	6.45 (-9.25 to 22.4)	**-15.8 (-18.5 to -13)**	**-8.91 (-9.54 to -8.3)**
20 Hypertensive heart disease	20 Blindness and vision loss	**95.6 (56.4 to 133)**	**54.8 (34.1 to 75.4)**	**-4.39 (-4.73 to -4.08)**
23 Urinary diseases	25 Road injuries			
27 Blindness and vision loss	51 Tuberculosis			
38 Alzheimer's disease	150 COVID-19			

Supplemental Results Figure S7. Leading 20 level 3 causes of DALYs, and percentage change in number of DALYs and all-age and age-standardised DALY rates by location, 2022 and 2050

Uzbekistan, Reference scenario

Legend:
Communicable, maternal, neonatal and nutritional
Non-communicable
Injuries

Leading Causes 2022	Leading Causes 2050	Mean % change number of DALY	Mean % change all-age DALY rate	Mean % change age-standardized DALY rate
1 Ischemic heart disease	1 Ischemic heart disease	68.8 (-0.845 to 168)	29.6 (-17.9 to 89.2)	-36.3 (-63.1 to 5.88)
2 Lower respiratory infect	2 Stroke	**51 (3.53 to 113)**	16 (-8.47 to 45.5)	**-38.3 (-53.4 to -16.6)**
3 Stroke	3 Diabetes	**96.5 (39.4 to 172)**	**51.1 (26.4 to 86.6)**	0.37 (-16.4 to 25.9)
4 Neonatal disorders	4 Low back pain	**56.2 (15.3 to 100)**	**20 (13.6 to 26.7)**	**-3.72 (-3.9 to -3.52)**
5 COVID-19	5 Neonatal disorders	-12 (-49.3 to 55.6)	-32.6 (-56.3 to 5.28)	-11.4 (-42.2 to 33.2)
6 Diabetes	6 Cirrhosis liver	30.5 (-9.33 to 84.4)	0.213 (-18.6 to 28.2)	-16.7 (-34.3 to 7.45)
7 Low back pain	7 Lower respiratory infect	**-47.2 (-67.3 to -17.1)**	**-59.5 (-71.1 to -41.4)**	**-51.9 (-66.9 to -29.4)**
8 Cirrhosis liver	8 Chronic kidney disease	**141 (37.4 to 317)**	**85.1 (14.7 to 183)**	29.7 (-25.1 to 115)
9 Congenital defects	9 Dietary iron deficiency	8.58 (-22.3 to 40.6)	**-16.7 (-19 to -14.5)**	**-10 (-10.5 to -9.37)**
10 Dietary iron deficiency	10 Depressive disorders	**48.1 (8.93 to 89.2)**	**13.7 (8.42 to 20.9)**	**-2.26 (-2.53 to -1.96)**
11 Road injuries	11 Alzheimer's disease	**297 (148 to 445)**	**205 (124 to 289)**	**3.11 (0.98 to 5.32)**
12 Headache disorders	12 Age-related hearing loss	**109 (50.1 to 168)**	**60.8 (43.8 to 78.4)**	**-0.71 (-0.814 to -0.592)**
13 Depressive disorders	13 Headache disorders	31.6 (-1.81 to 68.5)	1.03 (-3 to 7.33)	**-0.282 (-0.43 to -0.149)**
14 Self-harm	14 Road injuries	7.67 (-49.1 to 117)	-17.4 (-56.9 to 59.1)	-17.2 (-57.6 to 61.8)
15 Age-related hearing loss	15 Hypertensive heart disease	**136 (34.9 to 273)**	**80.9 (11.2 to 178)**	-5.26 (-44.6 to 50.4)
16 Chronic kidney disease	16 Self-harm	5.01 (-30.2 to 57.7)	-19.5 (-42.1 to 12.9)	-17.4 (-41.2 to 15.9)
17 Gynecological diseases	17 Congenital defects	**-36.2 (-56.6 to -12.2)**	**-51.1 (-59.8 to -39.5)**	**-36.7 (-48.1 to -21.8)**
18 Falls	18 Blindness and vision loss	**112 (55.4 to 174)**	**63.1 (43.8 to 81.5)**	**-4.62 (-4.97 to -4.28)**
19 Drowning	19 Other musculoskeletal	**113 (52.3 to 171)**	**63.4 (52.1 to 78.7)**	**31.7 (27.9 to 36)**
20 Idiopathic epilepsy	20 Oral disorders	**75.4 (29.5 to 122)**	**34.7 (24.2 to 46.5)**	**1.29 (0.851 to 1.75)**
21 Hypertensive heart disease	23 Falls			
22 Oral disorders	24 Idiopathic epilepsy			
24 Blindness and vision loss	25 Gynecological diseases			
27 Other musculoskeletal	41 Drowning			
34 Alzheimer's disease	155 COVID-19			

Supplemental Results Figure S7. Leading 20 level 3 causes of DALYs, and percentage change in number of DALYs and all-age and age-standardised DALY rates by location, 2022 and 2050

Albania, Reference scenario

Legend:
Communicable, maternal, neonatal and nutritional
Non-communicable
Injuries

Leading Causes 2022	Leading Causes 2050	Mean % change number of DALY	Mean % change all-age DALY rate	Mean % change age-standardized DALY rate
1 Ischemic heart disease	1 Stroke	16.2 (-12.5 to 48.3)	**32.7 (5.73 to 55.3)**	**-33.5 (-48.8 to -15.7)**
2 Stroke	2 Ischemic heart disease	-9.22 (-36.8 to 24.7)	3.76 (-25.4 to 41.7)	**-44.3 (-62.8 to -15.3)**
3 COVID-19	3 Low back pain	0.104 (-15.5 to 17.3)	**14.3 (9.3 to 19.8)**	**-3.03 (-3.24 to -2.82)**
4 Low back pain	4 Alzheimer's disease	**119 (74.3 to 178)**	**151 (110 to 197)**	**13.9 (11.7 to 16)**
5 Lung cancer	5 Lung cancer	34.8 (-2.34 to 76.6)	**54.2 (14.6 to 97.3)**	8.19 (-17.2 to 37.1)
6 Neonatal disorders	6 COPD	**76 (32.5 to 131)**	**101 (52.5 to 154)**	11.5 (-15.1 to 47.7)
7 Age-related hearing loss	7 Diabetes	**36.6 (15 to 61)**	**56.1 (42.2 to 70.9)**	**11.6 (5.29 to 17.5)**
8 Diabetes	8 Age-related hearing loss	**25.9 (8.83 to 47.1)**	**43.8 (31.8 to 56.9)**	**-0.597 (-0.719 to -0.481)**
9 Mechanical forces	9 Mechanical forces	-4.13 (-21.9 to 12.8)	**9.42 (1.71 to 17.1)**	-3.84 (-10.2 to 3.25)
10 Headache disorders	10 Headache disorders	-12.5 (-27.1 to 5.11)	-0.22 (-4.51 to 7)	**-0.29 (-0.561 to -0.0496)**
11 Alzheimer's disease	11 Liver cancer	**74.9 (32 to 129)**	**99.8 (52.8 to 156)**	**40.9 (9.48 to 84.4)**
12 COPD	12 Oral disorders	14.2 (-1.84 to 32.9)	**30.5 (22.4 to 39.7)**	**0.421 (0.229 to 0.604)**
13 Lower respiratory infect	13 Chronic kidney disease	**67.1 (19.3 to 124)**	**90.9 (40.3 to 154)**	8.63 (-24.7 to 57.3)
14 Falls	14 Lower respiratory infect	-1.31 (-25.8 to 24.6)	12.8 (-12.8 to 38.9)	**-41.7 (-52.7 to -25.6)**
15 Depressive disorders	15 Falls	2.14 (-15.9 to 22.6)	**16.7 (2.28 to 36.8)**	**-12.2 (-23.8 to -1.55)**
16 Anxiety disorders	16 Depressive disorders	-4.77 (-19.9 to 11)	**8.7 (4.36 to 14)**	**-2.13 (-2.57 to -1.64)**
17 Oral disorders	17 Hypertensive heart disease	**81.5 (17.6 to 161)**	**108 (37.4 to 207)**	7.37 (-30.3 to 63.7)
18 Road injuries	18 Neonatal disorders	**-48.7 (-65.2 to -23.2)**	**-41.5 (-55.7 to -12.9)**	-30.4 (-52.3 to 12.4)
19 Stomach cancer	19 Anxiety disorders	-10.2 (-25.8 to 5.47)	2.43 (-2.47 to 7.56)	0.154 (-0.223 to 0.483)
20 Other cardiovascular	20 Osteoarthritis	**25 (8.21 to 43.6)**	**42.9 (33.5 to 54.1)**	**2.02 (1.65 to 2.4)**
21 Osteoarthritis	21 Other cardiovascular			
22 Liver cancer	26 Stomach cancer			
23 Chronic kidney disease	30 Road injuries			
30 Hypertensive heart disease	155 COVID-19			

Supplemental Results Figure S7. Leading 20 level 3 causes of DALYs, and percentage change in number of DALYs and all-age and age-standardised DALY rates by location, 2022 and 2050

Bosnia and Herzegovina, Reference scenario

Legend:
Communicable, maternal, neonatal and nutritional
Non-communicable
Injuries

Leading Causes 2022	Leading Causes 2050	Mean % change number of DALY	Mean % change all-age DALY rate	Mean % change age-standardized DALY rate
1 Ischemic heart disease	1 Ischemic heart disease	-30.4 (-53.6 to 4.07)	-2 (-35.7 to 48.5)	**-53.1 (-70.4 to -27.5)**
2 Stroke	2 Stroke	**-32.6 (-46.9 to -11.8)**	-5.17 (-26.2 to 22.1)	**-51.3 (-61.3 to -37.4)**
3 COVID-19	3 Diabetes	5.12 (-13.1 to 27.8)	**47.7 (25.6 to 74.2)**	-4.62 (-16.3 to 9.43)
4 Diabetes	4 Low back pain	**-18.2 (-32.1 to -4.89)**	**14.7 (9.42 to 21.2)**	**-3.35 (-3.55 to -3.2)**
5 Low back pain	5 Alzheimer's disease	**111 (60.1 to 168)**	**196 (137 to 266)**	**3.59 (1.42 to 5.87)**
6 Lung cancer	6 Lung cancer	-7.37 (-37.7 to 29.7)	29.9 (-8.05 to 66.9)	-10.8 (-35.3 to 17.2)
7 Colorectal cancer	7 COPD	28.5 (-7.45 to 78.3)	**80.7 (30.3 to 142)**	-5.65 (-32.4 to 30.4)
8 COPD	8 Colorectal cancer	16.4 (-26 to 73.9)	**63.2 (5.6 to 130)**	-2.06 (-34.7 to 35.2)
9 Age-related hearing loss	9 Age-related hearing loss	15.7 (-6.47 to 34.8)	**62.4 (46.8 to 82.6)**	**-0.712 (-0.87 to -0.555)**
10 Alzheimer's disease	10 Hypertensive heart disease	48 (-7.7 to 143)	**108 (24.9 to 240)**	-2.02 (-39.1 to 69)
11 Headache disorders	11 Pancreatic cancer	49.1 (-2.71 to 120)	**109 (39.2 to 192)**	33.2 (-9.43 to 86.6)
12 Depressive disorders	12 Cardiomyopathy	2.72 (-25.7 to 39.8)	**44.1 (11.4 to 83.6)**	**-23.4 (-33.7 to -11.1)**
13 Falls	13 Falls	-4.48 (-22.7 to 15.9)	**34 (17.9 to 53)**	-7.48 (-17.5 to 3.62)
14 Cardiomyopathy	14 Chronic kidney disease	37.3 (-7.11 to 108)	**93.2 (23.9 to 187)**	1.83 (-35.3 to 58.3)
15 Neonatal disorders	15 Oral disorders	-0.699 (-18.4 to 14.9)	**39.3 (28 to 51.1)**	0.0205 (-0.259 to 0.278)
16 Oral disorders	16 Depressive disorders	**-20 (-34 to -5.27)**	**12.2 (6.34 to 20.5)**	**-2.35 (-2.75 to -2)**
17 Cirrhosis liver	17 Osteoarthritis	8.96 (-7.67 to 24.9)	**53 (40.1 to 66.1)**	**1.65 (1.24 to 2.07)**
18 Hypertensive heart disease	18 Headache disorders	**-32.7 (-46.7 to -20.7)**	-5.78 (-11 to 1.59)	0.114 (-0.076 to 0.323)
19 Breast cancer	19 Breast cancer	-4.1 (-34.4 to 38.2)	34.4 (-2 to 80.1)	-9.88 (-32.8 to 19.4)
20 Anxiety disorders	20 Cirrhosis liver	-20.3 (-36.5 to 3.05)	12 (-8.71 to 39.5)	-8.42 (-24.5 to 15.2)
21 Osteoarthritis	27 Anxiety disorders			
23 Chronic kidney disease	30 Neonatal disorders			
25 Pancreatic cancer	155 COVID-19			

Supplemental Results Figure S7. Leading 20 level 3 causes of DALYs, and percentage change in number of DALYs and all-age and age-standardised DALY rates by location, 2022 and 2050

Bulgaria, Reference scenario

Legend:
Communicable, maternal, neonatal and nutritional
Non-communicable
Injuries

Leading Causes 2022	Leading Causes 2050	Mean % change number of DALY	Mean % change all-age DALY rate	Mean % change age-standardized DALY rate
1 Ischemic heart disease	1 Hypertensive heart disease	9.37 (-20.9 to 41.7)	**50.7 (7.09 to 95.4)**	13.6 (-22.5 to 58)
2 Stroke	2 Stroke	**-48.3 (-56.6 to -38.3)**	**-28.8 (-39.7 to -16.2)**	**-45.9 (-55.3 to -35.5)**
3 COVID-19	3 Ischemic heart disease	**-51 (-64.3 to -34)**	**-32.5 (-50.9 to -10.6)**	**-49.2 (-65.4 to -27.9)**
4 Hypertensive heart disease	4 Low back pain	**-24.8 (-33.7 to -16.3)**	**3.44 (0.233 to 6.92)**	**-2.04 (-2.16 to -1.91)**
5 Low back pain	5 Diabetes	**-15 (-25.8 to -3.44)**	**17 (6.88 to 26.4)**	-1.09 (-9.11 to 6.85)
6 Lung cancer	6 Lung cancer	**-28.2 (-47.4 to -6.58)**	-1.24 (-23.6 to 23.1)	-14.4 (-34.3 to 4.69)
7 Diabetes	7 Colorectal cancer	-11.1 (-37.1 to 22.1)	22.2 (-9.03 to 68.6)	-1.05 (-25.6 to 31.4)
8 Cirrhosis liver	8 Alzheimer's disease	14.5 (-7.21 to 40.1)	**57.5 (33.8 to 79.9)**	**2.14 (0.033 to 4.15)**
9 Colorectal cancer	9 Cirrhosis liver	**-27.4 (-40 to -14.3)**	0.0175 (-14.3 to 16.5)	-2.67 (-18 to 14.7)
10 Falls	10 Chronic kidney disease	5.6 (-24.3 to 33.1)	**45.5 (4.31 to 85.3)**	15.5 (-17.9 to 53.9)
11 Alzheimer's disease	11 Age-related hearing loss	**-11.7 (-24.1 to -0.706)**	**21.5 (13.4 to 28.7)**	**-0.461 (-0.568 to -0.333)**
12 Age-related hearing loss	12 COPD	-12.1 (-30.8 to 8.95)	21 (-3.56 to 46.7)	-7.32 (-28.9 to 15.4)
13 COPD	13 Falls	**-29 (-39.5 to -15.7)**	-2.21 (-13.1 to 10.8)	**-14 (-24.5 to -3.84)**
14 Chronic kidney disease	14 Pancreatic cancer	11.2 (-21.3 to 49.1)	**52.9 (13.9 to 106)**	29.3 (-5.07 to 69.7)
15 Breast cancer	15 Breast cancer	-18.6 (-37.8 to 3.49)	11.9 (-9.42 to 39.2)	-2.93 (-19 to 18.2)
16 Lower respiratory infect	16 Depressive disorders	**-24.3 (-33.5 to -15.8)**	**4.13 (1.06 to 7.45)**	**-1.34 (-1.69 to -1.02)**
17 Headache disorders	17 Lower respiratory infect	**-31.1 (-43.1 to -18.5)**	-5.05 (-18.8 to 11.7)	**-34 (-45.7 to -21.7)**
18 Depressive disorders	18 Headache disorders	**-29.6 (-37.5 to -22.3)**	**-3.1 (-5.48 to -0.233)**	**0.262 (0.117 to 0.377)**
19 Mechanical forces	19 Mechanical forces	**-28.3 (-36.7 to -19.6)**	-1.33 (-8 to 5.19)	-6.34 (-13.7 to 0.715)
20 Other cardiovascular	20 Osteoarthritis	**-13.9 (-24.3 to -3.87)**	**18.5 (12.9 to 23.9)**	**0.947 (0.604 to 1.27)**
22 Pancreatic cancer	22 Other cardiovascular			
24 Osteoarthritis	150 COVID-19			

Supplemental Results Figure S7. Leading 20 level 3 causes of DALYs, and percentage change in number of DALYs and all-age and age-standardised DALY rates by location, 2022 and 2050

Croatia, Reference scenario

Legend:
Communicable, maternal, neonatal and nutritional
Non-communicable
Injuries

Leading Causes 2022

1 Ischemic heart disease
2 COVID-19
3 Stroke
4 Low back pain
5 Lung cancer
6 Diabetes
7 Falls
8 Colorectal cancer
9 Alzheimer's disease
10 COPD
11 Age-related hearing loss
12 Depressive disorders
13 Cirrhosis liver
14 Headache disorders
15 Breast cancer
16 Oral disorders
17 Chronic kidney disease
18 Osteoarthritis
19 Self-harm
20 Anxiety disorders
21 Hypertensive heart disease
23 Pancreatic cancer
24 Prostate cancer

Leading Causes 2050

Leading Causes 2050	Mean % change number of DALY	Mean % change all-age DALY rate	Mean % change age-standardized DALY rate
1 Ischemic heart disease	**-35 (-53.4 to -13.3)**	-7.5 (-32 to 22.6)	**-49.8 (-63.6 to -31)**
2 Diabetes	10.7 (-6.36 to 28.1)	**57.5 (43.1 to 72.6)**	**12.7 (4.19 to 20.8)**
3 Stroke	**-36 (-47.4 to -20.6)**	-8.91 (-23 to 6.44)	**-45.4 (-53.4 to -35.7)**
4 Lung cancer	-7.9 (-29.9 to 16.6)	**31 (5.79 to 65.7)**	-6.73 (-24.3 to 16.6)
5 Low back pain	**-20.8 (-33.3 to -9.12)**	**12.5 (7.64 to 17.1)**	**-2.56 (-2.69 to -2.45)**
6 Alzheimer's disease	**43.6 (16.6 to 69.3)**	**104 (78.4 to 131)**	1.63 (-0.622 to 3.8)
7 Falls	-0.297 (-18.2 to 18.4)	**41.8 (26.1 to 60.8)**	**-13.4 (-21.2 to -5.84)**
8 Colorectal cancer	4.71 (-25.8 to 44.9)	**48.9 (5.08 to 95.2)**	-6.06 (-31.3 to 22.2)
9 COPD	18 (-9.53 to 46.8)	**67.9 (35.7 to 106)**	-4.17 (-23.5 to 17.2)
10 Age-related hearing loss	0.395 (-16.4 to 15.1)	**42.8 (34.3 to 52.6)**	**-0.518 (-0.653 to -0.374)**
11 Chronic kidney disease	**47.7 (5.22 to 99.2)**	**110 (54.7 to 175)**	16.4 (-13.3 to 54.7)
12 Hypertensive heart disease	37.5 (-5.87 to 101)	**95.7 (35.1 to 188)**	4 (-31.9 to 62.9)
13 Pancreatic cancer	30.5 (-6.62 to 75.5)	**85.5 (37.4 to 136)**	24 (-5.54 to 55.6)
14 Depressive disorders	**-22.2 (-34.4 to -10.6)**	**10.6 (6.37 to 15.5)**	**-1.84 (-2.15 to -1.56)**
15 Prostate cancer	30.4 (-0.569 to 64.9)	**85.4 (51.1 to 127)**	9.31 (-6.36 to 26.8)
16 Breast cancer	-10.4 (-32.8 to 14.4)	**27.4 (1.59 to 58.5)**	-14 (-29.9 to 2.79)
17 Osteoarthritis	-2.23 (-17.3 to 11)	**39 (32.6 to 46.4)**	**1.01 (0.701 to 1.31)**
18 Oral disorders	-10.5 (-25.6 to 3.65)	**27.2 (19.8 to 35.3)**	**0.257 (0.079 to 0.422)**
19 Cirrhosis liver	**-33.9 (-46.1 to -19)**	-6.05 (-18.4 to 8.23)	**-24.1 (-34.9 to -12.8)**
20 Headache disorders	**-32.4 (-44.5 to -20.8)**	-3.97 (-8.37 to 2.99)	0.113 (-0.0315 to 0.237)
25 Anxiety disorders			
28 Self-harm			
153 COVID-19			

Supplemental Results Figure S7. Leading 20 level 3 causes of DALYs, and percentage change in number of DALYs and all-age and age-standardised DALY rates by location, 2022 and 2050

Czechia, Reference scenario

Legend:
Communicable, maternal, neonatal and nutritional
Non-communicable
Injuries

Leading Causes 2022	Leading Causes 2050	Mean % change number of DALY	Mean % change all-age DALY rate	Mean % change age-standardized DALY rate
1 Ischemic heart disease	1 Ischemic heart disease	**-25.3 (-44.8 to -2.06)**	-17.8 (-38.7 to 10.1)	**-51 (-64.2 to -31.8)**
2 Low back pain	2 Low back pain	-4.59 (-12.9 to 7.29)	**4.99 (1 to 9.06)**	**-1.73 (-1.83 to -1.63)**
3 Stroke	3 Diabetes	**20.4 (6.76 to 36.5)**	**32.5 (21.7 to 45.1)**	1.14 (-5.9 to 9.63)
4 Diabetes	4 Alzheimer's disease	**71.2 (45.2 to 103)**	**88.3 (66.5 to 112)**	**-2.35 (-4.37 to -0.101)**
5 COVID-19	5 Stroke	**-21.4 (-34.3 to -8.81)**	**-13.5 (-26.5 to -1.07)**	**-41.9 (-50.8 to -32.9)**
6 Lung cancer	6 Lung cancer	0.604 (-20 to 28.4)	10.7 (-9.81 to 35.1)	-15.5 (-31.6 to 1.9)
7 Falls	7 Falls	12.8 (-4.75 to 30.2)	**24.2 (9.44 to 40.1)**	**-13.8 (-22.1 to -3.96)**
8 Colorectal cancer	8 COPD	19.7 (-6.78 to 46.8)	**31.8 (2.55 to 61.4)**	-10.4 (-30 to 11)
9 Alzheimer's disease	9 Colorectal cancer	13.7 (-19.9 to 56)	25.1 (-7.32 to 66.9)	-10.5 (-34.6 to 19.6)
10 COPD	10 Age-related hearing loss	**19.9 (7.91 to 35.6)**	**31.9 (24.5 to 39.3)**	**-0.336 (-0.489 to -0.178)**
11 Age-related hearing loss	11 Pancreatic cancer	**41.6 (3.56 to 85.5)**	**55.7 (16.9 to 103)**	18.5 (-10.7 to 54.6)
12 Lower respiratory infect	12 Lower respiratory infect	3.14 (-17.1 to 25.8)	13.5 (-4.4 to 35.9)	**-29.5 (-40.5 to -17.6)**
13 Cirrhosis liver	13 Depressive disorders	-5.61 (-14.2 to 5.4)	**3.88 (0.637 to 7.19)**	**-1.24 (-1.47 to -1.03)**
14 Headache disorders	14 Headache disorders	**-13.7 (-21.5 to -4.6)**	**-5 (-7.88 to -1.38)**	**0.199 (0.084 to 0.271)**
15 Depressive disorders	15 Osteoarthritis	**13 (2.45 to 25.5)**	**24.4 (19.3 to 29.4)**	**0.541 (0.311 to 0.794)**
16 Pancreatic cancer	16 Oral disorders	7.43 (-3.52 to 21.6)	**18.2 (12 to 24.7)**	0.0618 (-0.0766 to 0.196)
17 Oral disorders	17 Hypertensive heart disease	**53.8 (0.504 to 122)**	**69.3 (14.5 to 153)**	3.81 (-31.6 to 54.1)
18 Osteoarthritis	18 Cirrhosis liver	**-25 (-38.2 to -9.85)**	**-17.5 (-29.7 to -2.86)**	**-19.7 (-32.4 to -5.87)**
19 Self-harm	19 Atrial fibrillation	**62.2 (36.7 to 92.2)**	**78.5 (54.9 to 104)**	7.05 (-2.62 to 17.9)
20 Mechanical forces	20 Chronic kidney disease	**99.6 (46.9 to 160)**	**120 (59.8 to 187)**	33.9 (-4.33 to 80.1)
25 Hypertensive heart disease	23 Mechanical forces			
29 Atrial fibrillation	27 Self-harm			
34 Chronic kidney disease	151 COVID-19			

Supplemental Results Figure S7. Leading 20 level 3 causes of DALYs, and percentage change in number of DALYs and all-age and age-standardised DALY rates by location, 2022 and 2050

Hungary, Reference scenario

Legend:
Communicable, maternal, neonatal and nutritional
Non-communicable
Injuries

Leading Causes 2022	Leading Causes 2050	Mean % change number of DALY	Mean % change all-age DALY rate	Mean % change age-standardized DALY rate
1 Ischemic heart disease	1 Ischemic heart disease	**-39.5 (-55.9 to -16.1)**	**-36.7 (-53.6 to -15.3)**	**-54.1 (-67.8 to -34.2)**
2 COVID-19	2 Low back pain	-4.38 (-16.1 to 7.6)	0.0424 (-3.25 to 3.15)	**-2.4 (-2.53 to -2.28)**
3 Stroke	3 Lung cancer	-10.6 (-32.4 to 13.2)	-6.46 (-26.7 to 16)	-15.3 (-33.2 to 4.32)
4 Low back pain	4 Diabetes	**16.8 (0.601 to 33.7)**	**22.2 (12.3 to 31.9)**	**10.6 (1.96 to 19)**
5 Lung cancer	5 Hypertensive heart disease	**45.5 (3.52 to 104)**	**52.2 (7.97 to 118)**	15.8 (-20.3 to 69)
6 COPD	6 Stroke	**-35.8 (-47.2 to -21)**	**-32.8 (-42.9 to -21.3)**	**-46.6 (-54.6 to -36.2)**
7 Diabetes	7 COPD	-0.156 (-23.2 to 28.9)	4.54 (-17.9 to 32.5)	-15.1 (-34.2 to 10.1)
8 Colorectal cancer	8 Alzheimer's disease	**58.1 (28.8 to 91.5)**	**65.3 (43.1 to 88.5)**	**3.27 (1.14 to 5.33)**
9 Falls	9 Colorectal cancer	7.56 (-27.1 to 45.7)	12.4 (-19.5 to 45.4)	-5.44 (-30.8 to 23.9)
10 Hypertensive heart disease	10 Falls	11.4 (-5.34 to 30.5)	**16.5 (3.33 to 29.5)**	-7.11 (-16.5 to 1.26)
11 Cirrhosis liver	11 Age-related hearing loss	12.1 (-2.55 to 27.4)	**17.3 (10.5 to 24.8)**	**-0.432 (-0.613 to -0.238)**
12 Alzheimer's disease	12 Cirrhosis liver	**-21.7 (-35.4 to -5.48)**	**-18 (-29.4 to -2.68)**	**-18.5 (-30.4 to -4.36)**
13 Age-related hearing loss	13 Pancreatic cancer	35.9 (-2.77 to 85.4)	**42.1 (2.04 to 92.9)**	24.2 (-8.32 to 64.7)
14 Headache disorders	14 Depressive disorders	-4.65 (-15.8 to 6.4)	-0.243 (-2.72 to 2.31)	**-1.95 (-2.24 to -1.65)**
15 Depressive disorders	15 Headache disorders	-7.94 (-18.3 to 3.32)	**-3.67 (-5.9 to -1.63)**	**-0.115 (-0.226 to -0.00868)**
16 Self-harm	16 Chronic kidney disease	**57.8 (16.1 to 110)**	**65.2 (26 to 111)**	17.8 (-16.5 to 60.1)
17 Breast cancer	17 Breast cancer	-3.81 (-28.6 to 21.8)	0.622 (-22.5 to 28.1)	-12.9 (-31.2 to 9.02)
18 Cardiomyopathy	18 Osteoarthritis	7.07 (-5.76 to 21.4)	**12 (6.6 to 17.1)**	**0.62 (0.263 to 0.958)**
19 Pancreatic cancer	19 Oral disorders	5.57 (-7.97 to 20)	**10.4 (5.45 to 15.8)**	-0.0671 (-0.226 to 0.0947)
20 Osteoarthritis	20 Self-harm	**-23.5 (-37.6 to -5.41)**	**-20 (-31.8 to -4.31)**	**-23.2 (-35.3 to -7.71)**
22 Oral disorders	23 Cardiomyopathy			
27 Chronic kidney disease	151 COVID-19			

Supplemental Results Figure S7. Leading 20 level 3 causes of DALYs, and percentage change in number of DALYs and all-age and age-standardised DALY rates by location, 2022 and 2050

Montenegro, Reference scenario

Legend:
Communicable, maternal, neonatal and nutritional
Non-communicable
Injuries

Leading Causes 2022	Leading Causes 2050	Mean % change number of DALY	Mean % change all-age DALY rate	Mean % change age-standardized DALY rate
1 Ischemic heart disease	1 Stroke	-6.2 (-24.7 to 14.6)	10.6 (-7.48 to 32.1)	**-35.6 (-48.5 to -20.7)**
2 Stroke	2 Ischemic heart disease	**-31.9 (-52.8 to -5.02)**	-19.8 (-41.9 to 7.97)	**-50.3 (-65.5 to -29.4)**
3 COVID-19	3 Lung cancer	-3.62 (-29.2 to 23.9)	13.5 (-11.2 to 41.7)	-14.9 (-33 to 6.66)
4 Lung cancer	4 Diabetes	**24.9 (4.87 to 45.8)**	**47.2 (34 to 61.6)**	9.38 (-1.23 to 20)
5 Low back pain	5 Low back pain	-6.1 (-19 to 7.94)	**10.6 (7.49 to 14.1)**	**-2.49 (-2.64 to -2.35)**
6 Diabetes	6 Alzheimer's disease	**65.2 (34.2 to 98.2)**	**94.6 (69.7 to 123)**	**2.02 (0.0448 to 4.23)**
7 Falls	7 Chronic kidney disease	**56 (12.8 to 106)**	**83.9 (36.3 to 144)**	19.2 (-14.7 to 63.9)
8 Cardiomyopathy	8 Colorectal cancer	22.3 (-16.6 to 71.8)	**43.9 (0.902 to 90.8)**	-1.86 (-30.6 to 29.4)
9 Headache disorders	9 Falls	-3.03 (-20.5 to 18.7)	**14.2 (0.559 to 27.8)**	**-10.9 (-21.2 to -0.849)**
10 Age-related hearing loss	10 Age-related hearing loss	**16 (0.519 to 35.1)**	**36.7 (29.4 to 44.8)**	**-0.466 (-0.577 to -0.369)**
11 Colorectal cancer	11 Cardiomyopathy	-2.6 (-23.7 to 20.4)	14.7 (-3.76 to 36.1)	**-23.3 (-33.4 to -12.7)**
12 Breast cancer	12 Atrial fibrillation	**76.4 (41.7 to 118)**	**108 (76.1 to 147)**	**12.9 (0.18 to 29)**
13 Self-harm	13 Breast cancer	6.91 (-21.7 to 38.4)	25.9 (-1.52 to 59.6)	-6.4 (-26.6 to 18.5)
14 Depressive disorders	14 Pancreatic cancer	**54.2 (6.14 to 112)**	**81.6 (27.6 to 142)**	29.3 (-6.72 to 70)
15 Chronic kidney disease	15 Headache disorders	**-16.4 (-27.4 to -3.12)**	-1.54 (-4.5 to 3.73)	**-0.21 (-0.359 to -0.0748)**
16 Alzheimer's disease	16 Depressive disorders	-8.36 (-19.8 to 6.04)	**7.94 (4.23 to 12.1)**	**-1.96 (-2.28 to -1.65)**
17 Anxiety disorders	17 Prostate cancer	**47.3 (13.1 to 85.2)**	**73.6 (41.7 to 113)**	6.41 (-11.2 to 25.8)
18 Oral disorders	18 Brain cancer	**32.4 (12 to 56.3)**	**56 (43.3 to 72.9)**	**32.8 (22.9 to 44.6)**
19 Neonatal disorders	19 Oral disorders	5.96 (-7.81 to 23.6)	**24.8 (18.8 to 31.7)**	0.182 (-0.0015 to 0.34)
20 Road injuries	20 Hypertensive heart disease	**61.6 (9.48 to 128)**	**90.6 (31.7 to 176)**	10.6 (-27 to 65.9)
22 Pancreatic cancer	22 Self-harm			
24 Atrial fibrillation	23 Anxiety disorders			
25 Brain cancer	27 Road injuries			
26 Prostate cancer	28 Neonatal disorders			
30 Hypertensive heart disease	147 COVID-19			

Supplemental Results Figure S7. Leading 20 level 3 causes of DALYs, and percentage change in number of DALYs and all-age and age-standardised DALY rates by location, 2022 and 2050

North Macedonia, Reference scenario

Legend:
Communicable, maternal, neonatal and nutritional
Non-communicable
Injuries

Leading Causes 2022	Leading Causes 2050	Mean % change number of DALY	Mean % change all-age DALY rate	Mean % change age-standardized DALY rate
1 COVID-19	1 Stroke	-12 (-31.8 to 12.1)	9.47 (-10.2 to 28)	**-45.1 (-56.4 to -31.6)**
2 Stroke	2 Ischemic heart disease	-15.4 (-40.2 to 12.8)	5.41 (-24.6 to 38.1)	**-44.1 (-63.2 to -21.3)**
3 Ischemic heart disease	3 Diabetes	**30.1 (3.61 to 60.5)**	**61.8 (43.3 to 83.1)**	-0.0633 (-10.9 to 11.2)
4 Diabetes	4 Low back pain	-4.78 (-23.8 to 15.1)	**18.3 (14.1 to 23.1)**	**-2.52 (-2.66 to -2.39)**
5 Low back pain	5 Lung cancer	20.4 (-17 to 72.5)	**49.2 (18.5 to 95.3)**	-4.13 (-23.7 to 24.1)
6 Lung cancer	6 Hypertensive heart disease	**98 (31.3 to 182)**	**147 (63.1 to 241)**	14.3 (-30.1 to 69.3)
7 Hypertensive heart disease	7 COPD	**57.8 (13 to 109)**	**96.3 (49.3 to 144)**	4.58 (-21.3 to 34.6)
8 Headache disorders	8 Colorectal cancer	42.5 (-8.85 to 113)	**76.7 (25.4 to 145)**	2.54 (-24.3 to 42.2)
9 COPD	9 Alzheimer's disease	**106 (53.6 to 163)**	**155 (121 to 195)**	**12.7 (10 to 15.6)**
10 Colorectal cancer	10 Age-related hearing loss	24.4 (-1.56 to 50)	**54.5 (45.8 to 65)**	**-0.576 (-0.715 to -0.442)**
11 Age-related hearing loss	11 Breast cancer	16.9 (-17.7 to 61.1)	**44.9 (15.6 to 82.3)**	-5.75 (-25 to 18.5)
12 Cardiomyopathy	12 Cardiomyopathy	10.1 (-23.8 to 45.1)	**36.8 (9.31 to 77.1)**	**-23.6 (-34.5 to -10.9)**
13 Falls	13 Pancreatic cancer	**88.4 (22.7 to 168)**	**134 (70.9 to 227)**	**41.2 (2.99 to 95.3)**
14 Breast cancer	14 Chronic kidney disease	**68.4 (12.8 to 135)**	**110 (47.9 to 178)**	18.3 (-18.2 to 67.7)
15 Neonatal disorders	15 Liver cancer	**77.4 (32 to 138)**	**121 (70.6 to 175)**	**36.6 (5.29 to 76.6)**
16 Depressive disorders	16 Headache disorders	**-20.7 (-36.6 to -4.99)**	-1.44 (-7.09 to 7.38)	**0.296 (0.151 to 0.388)**
17 Oral disorders	17 Falls	-4.89 (-27.6 to 23.3)	**18 (2.62 to 35)**	**-18.7 (-27.2 to -8.52)**
18 Stomach cancer	18 Oral disorders	11.8 (-8.54 to 34.3)	**38.9 (31.3 to 46.9)**	**0.176 (0.00536 to 0.341)**
19 Anxiety disorders	19 Brain cancer	**43.3 (13.4 to 82.4)**	**77.9 (60.7 to 97.2)**	**28.6 (19.6 to 39.1)**
20 Mechanical forces	20 Depressive disorders	-8.44 (-27.1 to 10.2)	**13.7 (7.75 to 20.2)**	**-1.47 (-1.75 to -1.2)**
21 Alzheimer's disease	22 Stomach cancer			
22 Chronic kidney disease	23 Mechanical forces			
25 Brain cancer	24 Anxiety disorders			
27 Pancreatic cancer	28 Neonatal disorders			
28 Liver cancer	154 COVID-19			

Supplemental Results Figure S7. Leading 20 level 3 causes of DALYs, and percentage change in number of DALYs and all-age and age-standardised DALY rates by location, 2022 and 2050

Poland, Reference scenario

Legend:
Communicable, maternal, neonatal and nutritional
Non-communicable
Injuries

Leading Causes 2022	Leading Causes 2050	Mean % change number of DALY	Mean % change all-age DALY rate	Mean % change age-standardized DALY rate
1 Ischemic heart disease	1 Ischemic heart disease	**-30.3 (-48.9 to -9.57)**	-17.6 (-38.9 to 5.62)	**-55 (-67 to -41)**
2 COVID-19	2 Lung cancer	20.6 (-5.44 to 45.2)	**42.6 (14.1 to 73)**	-0.717 (-20.6 to 19.9)
3 Stroke	3 Low back pain	-5.74 (-15.8 to 3.89)	**11.4 (7.21 to 15.5)**	**-2.49 (-2.59 to -2.39)**
4 Low back pain	4 Diabetes	**21.1 (8.2 to 36.3)**	**43.2 (32.6 to 52)**	2.63 (-3.73 to 8.54)
5 Lung cancer	5 Stroke	**-27.4 (-38.4 to -14.2)**	-14.2 (-26.8 to 1.17)	**-48.2 (-55.2 to -39.7)**
6 Diabetes	6 Alzheimer's disease	**78.7 (57.3 to 104)**	**111 (92.6 to 134)**	**-2.03 (-3.8 to -0.0633)**
7 Colorectal cancer	7 Colorectal cancer	22.3 (-8.88 to 62.8)	**44.6 (7.48 to 90.1)**	-6.87 (-30.2 to 23.1)
8 Falls	8 Falls	3.64 (-12.5 to 21.3)	**22.5 (6.05 to 36.7)**	**-14.4 (-25.2 to -5.1)**
9 Alcohol use disorders	9 Age-related hearing loss	**20.9 (8.87 to 34.2)**	**42.9 (36.7 to 49.2)**	**-0.559 (-0.633 to -0.479)**
10 Lower respiratory infect	10 Lower respiratory infect	7.92 (-13.3 to 29.5)	**27.6 (3.85 to 50.8)**	**-25.1 (-37 to -12.4)**
11 Alzheimer's disease	11 Hypertensive heart disease	**59.3 (7.83 to 117)**	**88.3 (28.6 to 157)**	7.66 (-25.8 to 47.3)
12 Age-related hearing loss	12 COPD	18 (-9.33 to 51.1)	**39.4 (9.54 to 66.7)**	-14.3 (-31.7 to 3.58)
13 Cirrhosis liver	13 Alcohol use disorders	**-18.6 (-31.4 to -1)**	-3.77 (-15.8 to 11.2)	-4.16 (-15.6 to 10.2)
14 Headache disorders	14 Cirrhosis liver	**-15.2 (-27.8 to -0.902)**	0.285 (-12.5 to 14)	-7.45 (-20.5 to 5.1)
15 Self-harm	15 Oral disorders	8.69 (-4.02 to 19.9)	**28.5 (21.6 to 34.9)**	**0.245 (0.091 to 0.404)**
16 COPD	16 Pancreatic cancer	**49.4 (13.1 to 88.8)**	**76.7 (35 to 131)**	22.4 (-6.07 to 56.6)
17 Oral disorders	17 Breast cancer	7.75 (-18 to 35.4)	**27.4 (0.354 to 60.3)**	-12.3 (-29.9 to 6.49)
18 Breast cancer	18 Headache disorders	**-18.1 (-25.8 to -10.7)**	-3.21 (-6.68 to 2.64)	0.0714 (-0.0346 to 0.162)
19 Road injuries	19 Osteoarthritis	**19.5 (7.67 to 31.3)**	**41.3 (35.9 to 46.4)**	**1.13 (0.755 to 1.48)**
20 Hypertensive heart disease	20 Prostate cancer	**48 (20.3 to 81.4)**	**75 (45.5 to 110)**	5.79 (-8.94 to 22.8)
22 Osteoarthritis	23 Self-harm			
26 Pancreatic cancer	26 Road injuries			
29 Prostate cancer	151 COVID-19			

Supplemental Results Figure S7. Leading 20 level 3 causes of DALYs, and percentage change in number of DALYs and all-age and age-standardised DALY rates by location, 2022 and 2050

Romania, Reference scenario

Legend:
Communicable, maternal, neonatal and nutritional
Non-communicable
Injuries

Leading Causes 2022
1 Ischemic heart disease
2 Stroke
3 COVID-19
4 Low back pain
5 Cirrhosis liver
6 Lung cancer
7 Hypertensive heart disease
8 Cardiomyopathy
9 Lower respiratory infect
10 Colorectal cancer
11 Falls
12 Diabetes
13 COPD
14 Alzheimer's disease
15 Age-related hearing loss
16 Headache disorders
17 Road injuries
18 Depressive disorders
19 Breast cancer
20 Mechanical forces
23 Oral disorders
24 Pancreatic cancer
25 Osteoarthritis

Leading Causes 2050	Mean % change number of DALY	Mean % change all-age DALY rate	Mean % change age-standardized DALY rate
1 Ischemic heart disease	**-40 (-56.1 to -15.8)**	-23.6 (-43.2 to 2.68)	**-50.6 (-65.8 to -26.2)**
2 Stroke	**-37.6 (-49.8 to -23.7)**	**-20.6 (-32.6 to -7.81)**	**-46.5 (-55.5 to -34.3)**
3 Hypertensive heart disease	38.6 (-6.02 to 100)	**76.5 (25.7 to 154)**	11.5 (-24.2 to 75)
4 Low back pain	**-16.6 (-30 to -3.87)**	**6 (2.22 to 10.5)**	**-2.47 (-2.61 to -2.36)**
5 Alzheimer's disease	**53.2 (16.1 to 90.3)**	**94.6 (60.1 to 127)**	1.86 (-0.0664 to 3.77)
6 Lung cancer	-17.6 (-41.4 to 12.2)	4.67 (-20.1 to 37.3)	-12.6 (-32.4 to 12.8)
7 Cirrhosis liver	**-26.2 (-40.6 to -9.46)**	-6.2 (-19.8 to 10.6)	-12.1 (-26.4 to 5.76)
8 Colorectal cancer	4.61 (-33.2 to 48.2)	32.7 (-9.07 to 80.4)	0.00526 (-30 to 35)
9 Diabetes	12.4 (-6.29 to 31)	**42.9 (31.4 to 52.8)**	**16.1 (7.69 to 24.9)**
10 Cardiomyopathy	-13.3 (-29.6 to 6.38)	10.2 (-3.56 to 27.8)	**-15.9 (-26.9 to -2.85)**
11 COPD	4.15 (-18.5 to 34.1)	**32.5 (6.4 to 64.1)**	-7.17 (-28.1 to 19.6)
12 Age-related hearing loss	5.16 (-13.5 to 22.9)	**33.6 (25 to 42.9)**	**-0.582 (-0.71 to -0.456)**
13 Lower respiratory infect	**-24.1 (-38.3 to -4.33)**	-3.55 (-17.9 to 13.4)	**-35 (-46.1 to -22.2)**
14 Falls	**-22.5 (-36.9 to -7.28)**	-1.46 (-14.7 to 11)	**-17 (-28.1 to -5.58)**
15 Pancreatic cancer	28.6 (-13.2 to 74.5)	**63.3 (13.9 to 117)**	30.9 (-8.13 to 72.2)
16 Breast cancer	-9.22 (-35.9 to 19.3)	15.3 (-11.6 to 43.6)	-6.91 (-27.6 to 15.9)
17 Headache disorders	**-24.6 (-36.9 to -12)**	**-4.18 (-6.87 to -0.923)**	**0.298 (0.133 to 0.413)**
18 Depressive disorders	**-17.8 (-31.8 to -5.37)**	**4.41 (1.01 to 8.17)**	**-1.49 (-1.74 to -1.27)**
19 Osteoarthritis	0.702 (-16.5 to 15.3)	**28 (21.6 to 34.4)**	**1.4 (1.1 to 1.66)**
20 Oral disorders	-5.71 (-21.5 to 8.67)	**19.8 (12.9 to 27.2)**	**0.189 (0.00678 to 0.36)**
22 Road injuries			
23 Mechanical forces			
152 COVID-19			

Supplemental Results Figure S7. Leading 20 level 3 causes of DALYs, and percentage change in number of DALYs and all-age and age-standardised DALY rates by location, 2022 and 2050

Serbia, Reference scenario

Legend:
Communicable, maternal, neonatal and nutritional
Non-communicable
Injuries

Leading Causes 2022	Leading Causes 2050	Mean % change number of DALY	Mean % change all-age DALY rate	Mean % change age-standardized DALY rate
1 Ischemic heart disease	1 Ischemic heart disease	**-38.6 (-54.7 to -17.6)**	-21.1 (-41.3 to 4.85)	**-48.3 (-63.5 to -25.6)**
2 Stroke	2 Stroke	**-39.5 (-52.5 to -26.2)**	**-22.3 (-34.8 to -8.65)**	**-47.7 (-56.9 to -37)**
3 COVID-19	3 Diabetes	17.3 (-2 to 39.1)	**50.6 (35.4 to 64.9)**	**12.5 (2.56 to 22.5)**
4 Low back pain	4 Lung cancer	-0.383 (-24.8 to 28)	**27.9 (0.895 to 61.9)**	-5.85 (-25.8 to 18.9)
5 Lung cancer	5 Low back pain	-11.6 (-24.5 to 0.8)	**13.6 (10.1 to 16.6)**	**-3.03 (-3.2 to -2.86)**
6 Diabetes	6 Alzheimer's disease	**46.3 (16.1 to 77.8)**	**87.8 (57.4 to 118)**	**12.7 (10.2 to 15.5)**
7 Colorectal cancer	7 Hypertensive heart disease	40 (-7.08 to 89.9)	**79.9 (23 to 141)**	15.2 (-22.6 to 65.8)
8 COPD	8 Colorectal cancer	15.1 (-22.9 to 58.4)	**47.8 (5.98 to 90)**	3.77 (-24.1 to 32.7)
9 Hypertensive heart disease	9 COPD	13.2 (-12.7 to 43.9)	**45.4 (15.5 to 77.1)**	-1.67 (-24.6 to 24.7)
10 Alzheimer's disease	10 Chronic kidney disease	35.8 (-1.16 to 80.8)	**74.4 (30.6 to 128)**	16.8 (-14.2 to 59.5)
11 Age-related hearing loss	11 Age-related hearing loss	4.67 (-10.9 to 21.6)	**34.4 (27.7 to 41.2)**	**-0.614 (-0.72 to -0.495)**
12 Breast cancer	12 Breast cancer	-0.784 (-23.6 to 24.1)	**27.5 (4.73 to 53)**	-7.84 (-24.6 to 11.9)
13 Headache disorders	13 Falls	-3.23 (-21 to 17.6)	**24.3 (9.03 to 41.2)**	-6.26 (-17.1 to 7.26)
14 Falls	14 Pancreatic cancer	**53.3 (2.86 to 110)**	**96.7 (47.3 to 163)**	**40.6 (4.53 to 85.3)**
15 Depressive disorders	15 Depressive disorders	**-13.8 (-25.6 to -1.47)**	**10.7 (7.38 to 14.5)**	**-2.29 (-2.64 to -1.94)**
16 Cardiomyopathy	16 Headache disorders	**-21.5 (-33 to -9.82)**	0.859 (-3.96 to 9.2)	0.1 (-0.138 to 0.324)
17 Chronic kidney disease	17 Cardiomyopathy	-12.1 (-29 to 8.33)	12.9 (-5.36 to 35.9)	**-21.4 (-32.9 to -6.7)**
18 Self-harm	18 Oral disorders	-0.815 (-15 to 14.2)	**27.4 (22.6 to 32.5)**	**0.27 (0.0737 to 0.497)**
19 Oral disorders	19 Osteoarthritis	7.87 (-7.06 to 22.7)	**38.5 (34 to 42.8)**	**1.48 (1.09 to 1.89)**
20 Cirrhosis liver	20 Brain cancer	**30.6 (10.8 to 55.6)**	**67.7 (50 to 85.5)**	**26.7 (15.5 to 38.5)**
22 Osteoarthritis	21 Self-harm			
24 Pancreatic cancer	23 Cirrhosis liver			
29 Brain cancer	153 COVID-19			

Supplemental Results Figure S7. Leading 20 level 3 causes of DALYs, and percentage change in number of DALYs and all-age and age-standardised DALY rates by location, 2022 and 2050

Slovakia, Reference scenario

Legend:
Communicable, maternal, neonatal and nutritional
Non-communicable
Injuries

Leading Causes 2022	Leading Causes 2050	Mean % change number of DALY	Mean % change all-age DALY rate	Mean % change age-standardized DALY rate
1 Ischemic heart disease	1 Ischemic heart disease	-16.3 (-38.2 to 12.9)	-8.85 (-30 to 20)	**-48.5 (-61.7 to -27.9)**
2 COVID-19	2 Stroke	-14 (-26.5 to 1.51)	-6.34 (-18.3 to 8.16)	**-41.2 (-49 to -30.6)**
3 Stroke	3 Low back pain	-0.281 (-9.5 to 8.58)	**8.6 (4.88 to 12.7)**	**-1.91 (-2.08 to -1.77)**
4 Low back pain	4 Alzheimer's disease	**105 (70.1 to 143)**	**123 (91.5 to 154)**	**5.76 (3.54 to 8.68)**
5 Lung cancer	5 Colorectal cancer	30 (-8.2 to 71.6)	**41.4 (2.28 to 80.7)**	-3.87 (-29 to 22.3)
6 Falls	6 Diabetes	**32.1 (16.7 to 48.1)**	**43.8 (33 to 55)**	**8.66 (0.88 to 15.9)**
7 Colorectal cancer	7 Lung cancer	7.98 (-15.9 to 34.6)	17.5 (-7.49 to 41.4)	-12.9 (-31 to 5.9)
8 Diabetes	8 Falls	7.6 (-8.61 to 24.3)	**17.2 (3.36 to 30.5)**	**-14.2 (-23.1 to -5.78)**
9 Cirrhosis liver	9 Age-related hearing loss	**28.2 (14.2 to 42.6)**	**39.6 (31.3 to 47.2)**	**-0.32 (-0.511 to -0.132)**
10 Lower respiratory infect	10 Lower respiratory infect	**22.5 (0.822 to 51.7)**	**33.3 (11.2 to 61.1)**	**-24.5 (-36.3 to -11.7)**
11 Age-related hearing loss	11 Cirrhosis liver	-15.1 (-28.2 to 2.02)	-7.59 (-20.8 to 7.18)	**-15.8 (-28.8 to -0.561)**
12 Alzheimer's disease	12 COPD	**42.7 (11.7 to 77.1)**	**55.4 (25.8 to 89.9)**	-2.41 (-22.6 to 24.8)
13 Headache disorders	13 Pancreatic cancer	**58.3 (13.6 to 109)**	**72.2 (27.4 to 125)**	24.5 (-6.14 to 61.4)
14 Depressive disorders	14 Hypertensive heart disease	**77.4 (28.5 to 146)**	**93.4 (42.4 to 179)**	15 (-16.1 to 72)
15 Breast cancer	15 Headache disorders	**-12.5 (-20.6 to -3.95)**	**-4.67 (-7.74 to -0.626)**	0.0231 (-0.0847 to 0.103)
16 Mechanical forces	16 Depressive disorders	-2.62 (-11.5 to 7.29)	**6.04 (2.35 to 10)**	**-1.62 (-1.95 to -1.33)**
17 Self-harm	17 Breast cancer	10.9 (-13.4 to 41.7)	20.6 (-2.71 to 48.9)	-10.4 (-27.1 to 6.42)
18 COPD	18 Chronic kidney disease	**78 (33 to 137)**	**93.9 (50.3 to 156)**	22.3 (-8.52 to 67.7)
19 Oral disorders	19 Oral disorders	**14.2 (2.73 to 26.3)**	**24.4 (17.6 to 31.2)**	0.0373 (-0.124 to 0.183)
20 Anxiety disorders	20 Osteoarthritis	**22.6 (10.6 to 37.2)**	**33.5 (28.1 to 37.8)**	**0.288 (0.0162 to 0.564)**
21 Osteoarthritis	23 Mechanical forces			
23 Pancreatic cancer	24 Anxiety disorders			
26 Hypertensive heart disease	25 Self-harm			
29 Chronic kidney disease	154 COVID-19			

Supplemental Results Figure S7. Leading 20 level 3 causes of DALYs, and percentage change in number of DALYs and all-age and age-standardised DALY rates by location, 2022 and 2050

Slovenia, Reference scenario

Legend:
Communicable, maternal, neonatal and nutritional
Non-communicable
Injuries

Leading Causes 2022	Leading Causes 2050	Mean % change number of DALY	Mean % change all-age DALY rate	Mean % change age-standardized DALY rate
1 COVID-19	1 Low back pain	3.45 (-5.91 to 12.4)	1.68 (-1.52 to 5.27)	**-2.04 (-2.15 to -1.94)**
2 Low back pain	2 Falls	**26.4 (9.28 to 43.5)**	**24.2 (11.1 to 37.5)**	**-14.4 (-22.5 to -6.53)**
3 Ischemic heart disease	3 Alzheimer's disease	**76.2 (53 to 102)**	**73.1 (56.4 to 92.3)**	**-5.71 (-7.63 to -3.25)**
4 Falls	4 Ischemic heart disease	-21.2 (-42.7 to 3.16)	-22.5 (-43.2 to 0.212)	**-51.3 (-63 to -37)**
5 Stroke	5 Diabetes	**33.9 (19.5 to 47.7)**	**31.6 (22.1 to 41)**	**13.4 (5.39 to 20.7)**
6 Lung cancer	6 Lung cancer	2.8 (-19.2 to 29.7)	0.974 (-17.7 to 22.4)	**-18.3 (-33.1 to -0.21)**
7 Diabetes	7 Stroke	**-18.2 (-32.4 to -2.5)**	**-19.6 (-31.3 to -5.51)**	**-46.8 (-54.3 to -38.5)**
8 Alzheimer's disease	8 Hypertensive heart disease	**79.3 (12.9 to 168)**	**76.2 (12.3 to 159)**	-3.2 (-36.3 to 46.8)
9 Alcohol use disorders	9 Age-related hearing loss	**27.4 (14.7 to 40)**	**25.2 (18.9 to 31.2)**	**-0.519 (-0.669 to -0.366)**
10 Age-related hearing loss	10 COPD	**50.4 (17 to 91.6)**	**47.8 (16 to 87.7)**	-5.43 (-26.5 to 19.9)
11 Depressive disorders	11 Nonrheum valv diseases	**98.8 (53 to 154)**	**95.4 (54.2 to 145)**	7.62 (-10.9 to 31.3)
12 Colorectal cancer	12 Colorectal cancer	20.3 (-15.4 to 68.1)	18.1 (-14.6 to 60.3)	-18 (-40.1 to 8.98)
13 Headache disorders	13 Depressive disorders	3.61 (-5.72 to 13.3)	1.82 (-1.34 to 4.88)	**-1.26 (-1.48 to -1.04)**
14 COPD	14 Prostate cancer	**63 (29.3 to 106)**	**60.1 (30.3 to 99.2)**	3.98 (-13.2 to 22.9)
15 Hypertensive heart disease	15 Alcohol use disorders	-9.01 (-22.2 to 5.44)	-10.6 (-22 to 1.5)	-8.6 (-19.1 to 3.82)
16 Mechanical forces	16 Headache disorders	-3.64 (-11.8 to 3.69)	**-5.29 (-7.23 to -3.42)**	**0.396 (0.23 to 0.495)**
17 Self-harm	17 Oral disorders	**14.2 (2.36 to 24.7)**	**12.3 (6.99 to 17.2)**	0.129 (-0.0394 to 0.289)
18 Oral disorders	18 Osteoarthritis	**18.8 (7.2 to 31)**	**16.7 (12.5 to 20.5)**	**0.941 (0.689 to 1.18)**
19 Cirrhosis liver	19 Lower respiratory infect	**28.1 (3.37 to 55.4)**	**25.9 (3.79 to 52.8)**	**-24.3 (-33.2 to -10.3)**
20 Osteoarthritis	20 Pancreatic cancer	**48.3 (9.01 to 100)**	**45.6 (6.74 to 92.8)**	12.8 (-14.5 to 46.6)
21 Nonrheum valv diseases	21 Mechanical forces			
22 Lower respiratory infect	26 Self-harm			
24 Prostate cancer	28 Cirrhosis liver			
27 Pancreatic cancer	148 COVID-19			

Supplemental Results Figure S7. Leading 20 level 3 causes of DALYs, and percentage change in number of DALYs and all-age and age-standardised DALY rates by location, 2022 and 2050

Belarus, Reference scenario

Legend:
Communicable, maternal, neonatal and nutritional
Non-communicable
Injuries

Leading Causes 2022	Leading Causes 2050	Mean % change number of DALY	Mean % change all-age DALY rate	Mean % change age-standardized DALY rate
1 Ischemic heart disease	1 Ischemic heart disease	-27.1 (-51.7 to 3.4)	-17.3 (-46.1 to 16.9)	**-47.3 (-66.7 to -18.7)**
2 COVID-19	2 Stroke	**-29.9 (-47 to -11.8)**	**-20.6 (-37.5 to -1.83)**	**-44.6 (-55.8 to -27.2)**
3 Stroke	3 Low back pain	-1.91 (-18 to 12.4)	**11 (5.77 to 16)**	**-2.85 (-3.08 to -2.6)**
4 Low back pain	4 Alzheimer's disease	**101 (37.9 to 161)**	**127 (71.4 to 179)**	**13.5 (11.1 to 16)**
5 Alcohol use disorders	5 Alcohol use disorders	-2.82 (-26.1 to 26)	10.1 (-14.3 to 44.9)	3.42 (-19.7 to 34.9)
6 Falls	6 Falls	-9.59 (-26.4 to 9.11)	2.26 (-9.82 to 15.5)	-11.3 (-23.1 to 0.223)
7 Lung cancer	7 Lung cancer	1.16 (-36.9 to 40.9)	14.1 (-19.6 to 54.4)	-7.68 (-33 to 24.6)
8 Depressive disorders	8 Colorectal cancer	24.3 (-27.9 to 91.2)	40.2 (-11.5 to 110)	2.69 (-30.7 to 50.1)
9 Self-harm	9 Age-related hearing loss	20.5 (-4.32 to 43.1)	**36.2 (22.6 to 49.1)**	**-0.449 (-0.61 to -0.279)**
10 Colorectal cancer	10 Depressive disorders	-6.34 (-20.5 to 8.42)	**5.97 (2.03 to 10.4)**	**-1.71 (-1.99 to -1.47)**
11 Cirrhosis liver	11 Diabetes	**31.6 (9.36 to 58)**	**48.9 (34.8 to 63.6)**	**17.9 (8.43 to 27.4)**
12 Age-related hearing loss	12 Self-harm	-19.5 (-36.7 to 1.79)	-8.79 (-28.5 to 15.4)	-10.9 (-30.8 to 15.9)
13 Alzheimer's disease	13 Headache disorders	**-13.8 (-25.3 to -1.4)**	-2.47 (-5.53 to 1.05)	**-0.498 (-0.774 to -0.271)**
14 Headache disorders	14 COPD	36.3 (-1.51 to 79.5)	**54.5 (11.9 to 103)**	6.24 (-23.4 to 46.3)
15 Diabetes	15 Cirrhosis liver	**-21.6 (-37.6 to -3.52)**	-11.1 (-26.3 to 9.31)	-15.4 (-30.4 to 3.67)
16 Stomach cancer	16 Osteoarthritis	13.1 (-6.74 to 33.1)	**27.9 (18.8 to 35.3)**	**0.475 (0.0307 to 0.951)**
17 Cardiomyopathy	17 Pancreatic cancer	52.9 (-10.8 to 132)	**72.4 (9.84 to 151)**	35.5 (-12.8 to 94.8)
18 Gynecological diseases	18 Prostate cancer	**61 (9.84 to 117)**	**81.7 (31.8 to 138)**	26.1 (-2.19 to 57.2)
19 Road injuries	19 Oral disorders	7.09 (-11.1 to 23.9)	**21.1 (11.6 to 28.6)**	0.183 (-0.00877 to 0.36)
20 Osteoarthritis	20 Urinary diseases	**50.6 (17.7 to 86.4)**	**70.3 (41.5 to 105)**	**22.8 (2.12 to 47.1)**
23 COPD	23 Stomach cancer			
24 Oral disorders	24 Road injuries			
27 Pancreatic cancer	26 Gynecological diseases			
30 Urinary diseases	27 Cardiomyopathy			
32 Prostate cancer	149 COVID-19			

Supplemental Results Figure S7. Leading 20 level 3 causes of DALYs, and percentage change in number of DALYs and all-age and age-standardised DALY rates by location, 2022 and 2050

Estonia, Reference scenario

Legend:
Communicable, maternal, neonatal and nutritional
Non-communicable
Injuries

Leading Causes 2022	Leading Causes 2050	Mean % change number of DALY	Mean % change all-age DALY rate	Mean % change age-standardized DALY rate
1 COVID-19	1 Hypertensive heart disease	25.7 (-15.1 to 79.1)	36.3 (-8.23 to 86.6)	-8.09 (-39.8 to 36.7)
2 Ischemic heart disease	2 Ischemic heart disease	**-42.8 (-59.1 to -22.2)**	**-38 (-54.8 to -16.2)**	**-61.1 (-72.8 to -43.6)**
3 Hypertensive heart disease	3 Low back pain	-0.782 (-10.9 to 8.71)	**7.46 (4.67 to 11.4)**	**-2.71 (-2.95 to -2.47)**
4 Low back pain	4 Alzheimer's disease	**45.5 (19.5 to 72.9)**	**57.6 (34.2 to 82.5)**	**-6.36 (-8.57 to -3.88)**
5 Stroke	5 Diabetes	11.8 (-2.61 to 25.6)	**21.1 (9.52 to 32.6)**	1.48 (-7.48 to 10.2)
6 Diabetes	6 Chronic kidney disease	**52.4 (7.85 to 104)**	**65.1 (16.3 to 127)**	7.53 (-25.2 to 50.5)
7 Lung cancer	7 Lung cancer	-9.58 (-33 to 20)	-2.08 (-24.8 to 27.3)	**-25.5 (-42.9 to -4.55)**
8 Alzheimer's disease	8 Age-related hearing loss	**15.8 (2.59 to 26.7)**	**25.4 (18.6 to 32.3)**	**-0.394 (-0.654 to -0.05)**
9 Depressive disorders	9 Colorectal cancer	13.2 (-25.5 to 59.5)	22.5 (-17.8 to 71.1)	-11.7 (-38.4 to 19)
10 Falls	10 Stroke	**-42.4 (-53.2 to -30.8)**	**-37.6 (-47.3 to -25.9)**	**-52.9 (-60.2 to -44.5)**
11 Age-related hearing loss	11 Depressive disorders	-2.1 (-11.7 to 7.25)	**6.04 (3.05 to 9.23)**	**-1.68 (-2.09 to -1.32)**
12 Colorectal cancer	12 Falls	-9.75 (-23.5 to 9.13)	-2.25 (-16.2 to 13.4)	**-20.7 (-31.1 to -9.69)**
13 Cirrhosis liver	13 Prostate cancer	**42.8 (12.6 to 83.1)**	**54.7 (23.8 to 92.1)**	11.5 (-6.81 to 30.6)
14 Alcohol use disorders	14 Headache disorders	-8.95 (-17.5 to 0.126)	-1.39 (-3.67 to 1.77)	-0.174 (-0.464 to 0.00355)
15 Headache disorders	15 Drug use disorders	-9.06 (-21.6 to 5.61)	-1.54 (-10.2 to 7.98)	2.01 (-6.72 to 12.2)
16 Drug use disorders	16 Osteoarthritis	11.4 (-0.17 to 21.8)	**20.6 (15.8 to 26)**	-0.0807 (-0.492 to 0.336)
17 Chronic kidney disease	17 Pancreatic cancer	33.9 (-5.92 to 85)	**45 (4.33 to 99.7)**	10.5 (-18.7 to 45.4)
18 Self-harm	18 Alcohol use disorders	-15.4 (-30.2 to 1.91)	-8.32 (-23.4 to 10.8)	-11.6 (-24.9 to 4.12)
19 Osteoarthritis	19 Cirrhosis liver	**-23.2 (-37.1 to -8.59)**	**-16.8 (-29.6 to -1.85)**	**-23.5 (-36.9 to -8.63)**
20 Prostate cancer	20 Urinary diseases	**45.1 (19.6 to 71.2)**	**57.1 (34.9 to 84.5)**	12 (-3.33 to 29.6)
22 Pancreatic cancer	23 Self-harm			
30 Urinary diseases	152 COVID-19			

Supplemental Results Figure S7. Leading 20 level 3 causes of DALYs, and percentage change in number of DALYs and all-age and age-standardised DALY rates by location, 2022 and 2050

Latvia, Reference scenario

Legend:
Communicable, maternal, neonatal and nutritional
Non-communicable
Injuries

Leading Causes 2022	Leading Causes 2050	Mean % change number of DALY	Mean % change all-age DALY rate	Mean % change age-standardized DALY rate
1 Ischemic heart disease	1 Ischemic heart disease	**-44.3 (-60.7 to -26.9)**	-23.2 (-47.2 to 2.32)	**-53 (-68.3 to -33.2)**
2 COVID-19	2 Stroke	**-43.2 (-53.9 to -31.8)**	**-21.7 (-37.5 to -6.86)**	**-48.3 (-57.4 to -37.3)**
3 Stroke	3 Alzheimer's disease	**42 (13.4 to 66.7)**	**95.4 (65.3 to 126)**	**4.32 (1.77 to 7.1)**
4 Low back pain	4 Low back pain	**-19.8 (-30.5 to -10.3)**	**10.3 (5.62 to 16)**	**-2.54 (-2.79 to -2.33)**
5 Cardiomyopathy	5 Diabetes	**-14 (-26.3 to -0.762)**	**18.4 (4.85 to 31.7)**	-6.03 (-15 to 3.35)
6 Diabetes	6 Hypertensive heart disease	24.1 (-17.2 to 77.4)	**71.2 (13.7 to 147)**	13.8 (-23.2 to 68.5)
7 Lung cancer	7 Lung cancer	-15.3 (-35.9 to 8.77)	16.6 (-9.39 to 48.5)	-12 (-29.1 to 12)
8 Alzheimer's disease	8 Cardiomyopathy	**-39.4 (-52.7 to -24.6)**	-16.7 (-29.9 to 0.458)	**-23.9 (-38 to -5.24)**
9 Falls	9 Age-related hearing loss	-2.9 (-16.8 to 9.72)	**33.7 (23.7 to 45)**	**-0.332 (-0.593 to -0.116)**
10 Depressive disorders	10 Falls	**-20.5 (-35 to -6.63)**	9.43 (-4.27 to 24.9)	**-14.2 (-24.7 to -3.25)**
11 Age-related hearing loss	11 Colorectal cancer	-2.77 (-34.4 to 38)	33.7 (-6.74 to 84.6)	-5.76 (-30.4 to 27.1)
12 Colorectal cancer	12 Depressive disorders	**-22.6 (-33 to -13.8)**	**6.55 (2.45 to 11.1)**	**-1.39 (-1.72 to -1.14)**
13 Hypertensive heart disease	13 Prostate cancer	26.3 (-4.72 to 61.6)	**73.7 (40.8 to 117)**	**17.6 (1.21 to 40)**
14 Alcohol use disorders	14 Pancreatic cancer	19.6 (-18.1 to 67.4)	**64.6 (17 to 118)**	23 (-10.3 to 66.5)
15 Cirrhosis liver	15 Alcohol use disorders	**-31.6 (-44.1 to -16.6)**	-5.88 (-19.5 to 11)	-5.45 (-19.7 to 12.7)
16 Self-harm	16 Osteoarthritis	-8.86 (-20.1 to 1.26)	**25.5 (17.2 to 33.2)**	-0.129 (-0.493 to 0.288)
17 Headache disorders	17 Cirrhosis liver	**-32.5 (-44.2 to -21.4)**	-7.01 (-20.3 to 7.13)	-9.43 (-21.9 to 5.95)
18 Osteoarthritis	18 Urinary diseases	20 (-1.54 to 40.9)	**65.3 (39.9 to 92)**	13.2 (-1.98 to 27.7)
19 Breast cancer	19 Headache disorders	**-29.8 (-39.5 to -20.8)**	-3.41 (-6.38 to 0.549)	**-0.474 (-0.753 to -0.291)**
20 Pancreatic cancer	20 COPD	5.04 (-18.4 to 31.4)	**44.8 (10.2 to 83.9)**	-1.67 (-25.7 to 23.2)
22 Prostate cancer	21 Self-harm			
26 COPD	22 Breast cancer			
30 Urinary diseases	150 COVID-19			

Supplemental Results Figure S7. Leading 20 level 3 causes of DALYs, and percentage change in number of DALYs and all-age and age-standardised DALY rates by location, 2022 and 2050

Lithuania, Reference scenario

Legend:
Communicable, maternal, neonatal and nutritional
Non-communicable
Injuries

Leading Causes 2022	Leading Causes 2050	Mean % change number of DALY	Mean % change all-age DALY rate	Mean % change age-standardized DALY rate
1 Ischemic heart disease	1 Ischemic heart disease	**-40.9 (-60.3 to -18.8)**	-16.1 (-40.3 to 15.2)	**-52.2 (-67.9 to -30)**
2 COVID-19	2 Stroke	**-40.6 (-53.4 to -29.1)**	-15.6 (-30.2 to 1.46)	**-43.6 (-52.5 to -32.2)**
3 Stroke	3 Alzheimer's disease	**58.3 (27.4 to 91.4)**	**125 (91.2 to 162)**	**14.7 (11.8 to 18)**
4 Low back pain	4 Low back pain	**-20.5 (-29.9 to -9.77)**	**12.9 (8.47 to 18.2)**	**-2.66 (-2.84 to -2.48)**
5 Falls	5 Lung cancer	-10.9 (-34.9 to 17.8)	26.6 (-3.46 to 63.1)	-3.44 (-26.4 to 22.8)
6 Lung cancer	6 Falls	**-19 (-32.3 to -3.15)**	**15 (1.03 to 32.8)**	**-11.5 (-20.4 to -0.712)**
7 Self-harm	7 Diabetes	-5.9 (-20.9 to 8.78)	**33.7 (19.4 to 48.2)**	4.67 (-4.78 to 14.3)
8 Alzheimer's disease	8 Colorectal cancer	7.98 (-28.4 to 53.4)	**53.5 (6.33 to 111)**	3.44 (-28.4 to 36.7)
9 Diabetes	9 Age-related hearing loss	-2.59 (-15.1 to 10.8)	**38.4 (28.8 to 49.7)**	**-0.431 (-0.633 to -0.241)**
10 Depressive disorders	10 Depressive disorders	**-24.9 (-34.7 to -14.2)**	**6.63 (3.35 to 11.1)**	**-1.61 (-1.94 to -1.3)**
11 Cirrhosis liver	11 Self-harm	**-34.1 (-49 to -17.7)**	-6.49 (-23.5 to 11.5)	-9.39 (-27.2 to 11.4)
12 Age-related hearing loss	12 Cirrhosis liver	**-27.6 (-41.9 to -12.3)**	2.68 (-11.5 to 18.1)	-2.76 (-17.4 to 15.2)
13 Colorectal cancer	13 Pancreatic cancer	33.6 (-9.32 to 87.1)	**89.8 (33.8 to 157)**	39.6 (-2.12 to 85)
14 Alcohol use disorders	14 Prostate cancer	30.7 (-0.741 to 65.7)	**85.7 (45.9 to 135)**	**23.8 (4.06 to 45.1)**
15 Headache disorders	15 Hypertensive heart disease	41 (-5.25 to 103)	**100 (37.3 to 179)**	31.8 (-12 to 93.5)
16 Cardiomyopathy	16 COPD	16.5 (-13.6 to 50.7)	**65.6 (28.5 to 120)**	6.56 (-18.2 to 37.8)
17 Osteoarthritis	17 Osteoarthritis	-8.65 (-19 to 2.76)	**29.8 (22.8 to 37.3)**	0.316 (-0.0847 to 0.713)
18 Anxiety disorders	18 Breast cancer	-11.7 (-33.7 to 13.7)	25.4 (-2.79 to 55.3)	-7.86 (-26.3 to 11.5)
19 COPD	19 Alcohol use disorders	**-31.6 (-45.6 to -15.7)**	-2.91 (-15.9 to 15.4)	-3.93 (-16.3 to 13.7)
20 Breast cancer	20 Atrial fibrillation	**66.5 (34.4 to 108)**	**137 (95.3 to 183)**	**30 (17.5 to 45.9)**
23 Pancreatic cancer	21 Headache disorders			
24 Prostate cancer	24 Anxiety disorders			
25 Hypertensive heart disease	25 Cardiomyopathy			
38 Atrial fibrillation	152 COVID-19			

Supplemental Results Figure S7. Leading 20 level 3 causes of DALYs, and percentage change in number of DALYs and all-age and age-standardised DALY rates by location, 2022 and 2050

Moldova, Reference scenario

Legend:
Communicable, maternal, neonatal and nutritional
Non-communicable
Injuries

Leading Causes 2022	Leading Causes 2050	Mean % change number of DALY	Mean % change all-age DALY rate	Mean % change age-standardized DALY rate
1 Ischemic heart disease	1 Ischemic heart disease	-28.2 (-53.6 to 15.3)	12.3 (-29.7 to 70.5)	**-50.5 (-71 to -15.9)**
2 Stroke	2 Stroke	**-30.8 (-48.8 to -7.45)**	8.05 (-15.7 to 41.5)	**-44.6 (-56.7 to -25.1)**
3 Cirrhosis liver	3 Cirrhosis liver	**-26 (-43.4 to -4.53)**	15.3 (-4.61 to 38.8)	-11.5 (-26.6 to 10.8)
4 Low back pain	4 Alzheimer's disease	**129 (65.6 to 182)**	**257 (172 to 330)**	-1.28 (-3.34 to 0.718)
5 COVID-19	5 Hypertensive heart disease	52.4 (-8.62 to 149)	**138 (46.4 to 281)**	6.04 (-36.9 to 81.7)
6 Diabetes	6 Low back pain	-14.3 (-33.4 to 4.44)	**33.3 (23.4 to 44.4)**	**-2.58 (-2.81 to -2.28)**
7 Hypertensive heart disease	7 Diabetes	21.1 (-5.4 to 46.4)	**88.9 (66.8 to 114)**	**15.2 (6.33 to 27)**
8 Lower respiratory infect	8 Age-related hearing loss	**27.4 (1.57 to 56.4)**	**98.5 (73.5 to 129)**	**-0.426 (-0.633 to -0.27)**
9 Lung cancer	9 Colorectal cancer	8.79 (-34.4 to 62.7)	**69.2 (6.69 to 148)**	-5.83 (-41.6 to 34.5)
10 Colorectal cancer	10 Lung cancer	-3.16 (-38.1 to 42.3)	50.7 (-0.464 to 107)	-8.77 (-37.5 to 23.2)
11 Depressive disorders	11 COPD	36.3 (-1.18 to 91.9)	**113 (52.1 to 192)**	-1.13 (-28.7 to 45.7)
12 Headache disorders	12 Blindness and vision loss	**28.2 (1.1 to 55.5)**	**99.8 (72 to 131)**	**-2.63 (-2.81 to -2.43)**
13 Age-related hearing loss	13 Depressive disorders	**-23.4 (-42.2 to -4.34)**	**19 (10.7 to 29.1)**	**-1.38 (-1.75 to -0.955)**
14 Falls	14 Falls	-19.5 (-41.9 to 2.48)	**25.1 (7.14 to 48.9)**	-11 (-22.5 to 2.01)
15 Alzheimer's disease	15 Lower respiratory infect	**-40.2 (-57.3 to -14.5)**	-6.95 (-26.1 to 28.9)	**-33.9 (-48.9 to -8.24)**
16 Alcohol use disorders	16 Osteoarthritis	18.9 (-6.41 to 45.8)	**85.3 (67.3 to 106)**	**0.628 (0.237 to 0.952)**
17 Self-harm	17 Pancreatic cancer	38.3 (-17.4 to 105)	**115 (34 to 202)**	28.8 (-18.9 to 82.8)
18 COPD	18 Oral disorders	2.8 (-20.5 to 27.4)	**60 (43.7 to 81.5)**	**0.178 (0.00341 to 0.349)**
19 Gynecological diseases	19 Alcohol use disorders	**-33.9 (-52 to -10.4)**	2.59 (-15.8 to 29.8)	-0.717 (-16.9 to 21.3)
20 Road injuries	20 Headache disorders	**-39.7 (-56.8 to -23.6)**	-6.51 (-14.5 to 3.68)	-0.0657 (-0.366 to 0.376)
23 Blindness and vision loss	23 Self-harm			
25 Osteoarthritis	27 Road injuries			
26 Oral disorders	29 Gynecological diseases			
28 Pancreatic cancer	149 COVID-19			

Supplemental Results Figure S7. Leading 20 level 3 causes of DALYs, and percentage change in number of DALYs and all-age and age-standardised DALY rates by location, 2022 and 2050

Russia, Reference scenario

Legend:
Communicable, maternal, neonatal and nutritional
Non-communicable
Injuries

Leading Causes 2022	Leading Causes 2050	Mean % change number of DALY	Mean % change all-age DALY rate	Mean % change age-standardized DALY rate
1 Ischemic heart disease	1 Ischemic heart disease	**-30.2 (-52.2 to -5.78)**	**-25.7 (-47.9 to -1.89)**	**-52.7 (-68.6 to -34.9)**
2 Stroke	2 Stroke	**-28.3 (-43.3 to -14.4)**	**-23.7 (-36.3 to -11.5)**	**-49.3 (-57.7 to -39.3)**
3 COVID-19	3 Low back pain	4.4 (-7.44 to 18.3)	**11.1 (7.64 to 15.2)**	**-2.69 (-2.95 to -2.42)**
4 Low back pain	4 Diabetes	**27.7 (8.14 to 47.6)**	**35.9 (22.8 to 49.1)**	5.46 (-4.46 to 15.7)
5 Cardiomyopathy	5 Alzheimer's disease	**101 (62.4 to 151)**	**114 (79.3 to 150)**	-0.903 (-3.09 to 1.49)
6 Cirrhosis liver	6 Alcohol use disorders	-7.7 (-24.7 to 11.7)	-1.76 (-16.9 to 18.1)	0.707 (-13.4 to 19.4)
7 Diabetes	7 Age-related hearing loss	**29.1 (11.8 to 51.7)**	**37.3 (28.6 to 45.3)**	**-0.339 (-0.476 to -0.218)**
8 Self-harm	8 Cirrhosis liver	**-24.5 (-37.2 to -9.15)**	**-19.6 (-30.4 to -5.21)**	**-21.2 (-32.7 to -5.7)**
9 Alcohol use disorders	9 Lung cancer	-6.9 (-31 to 19.4)	-1.02 (-23.3 to 23.4)	**-21.5 (-39 to -3.01)**
10 Lung cancer	10 Cardiomyopathy	**-33.9 (-47 to -18.1)**	**-29.7 (-41.8 to -15)**	**-31.1 (-42.4 to -14.3)**
11 Falls	11 Falls	-8.09 (-21.9 to 9.88)	-2.26 (-12.6 to 9.53)	**-17.3 (-26.6 to -8.59)**
12 Headache disorders	12 Self-harm	**-22.1 (-37.6 to -2.43)**	-17.1 (-31.8 to 0.951)	-17.1 (-32.7 to 4.63)
13 HIV/AIDS	13 Colorectal cancer	9.68 (-25.8 to 51.1)	16.5 (-17.3 to 58.3)	-16 (-38.5 to 12.5)
14 Colorectal cancer	14 COPD	**33.4 (0.13 to 67.1)**	**41.8 (8.57 to 76.7)**	-5.66 (-28.6 to 22.3)
15 Road injuries	15 Headache disorders	-8.09 (-18.7 to 3.89)	-2.22 (-4.36 to 0.697)	**-0.581 (-0.876 to -0.358)**
16 Age-related hearing loss	16 Depressive disorders	-0.468 (-11.7 to 12.7)	**5.9 (2.72 to 8.7)**	**-1.12 (-1.23 to -1.03)**
17 Alzheimer's disease	17 Blindness and vision loss	**20.7 (4.93 to 39.7)**	**28.4 (20 to 37.7)**	**-2.55 (-2.83 to -2.27)**
18 Depressive disorders	18 Urinary diseases	**73.5 (39.2 to 110)**	**84.5 (55.6 to 116)**	**24.2 (3.81 to 43.7)**
19 Interpersonal violence	19 HIV/AIDS	**-21.8 (-37.4 to -8.01)**	**-16.8 (-29.3 to -7.46)**	-8.9 (-23 to 0.632)
20 COPD	20 Road injuries	-15.8 (-41.4 to 17.5)	-10.4 (-36.2 to 21.2)	-11.1 (-38.6 to 21)
24 Blindness and vision loss	31 Interpersonal violence			
32 Urinary diseases	153 COVID-19			

Supplemental Results Figure S7. Leading 20 level 3 causes of DALYs, and percentage change in number of DALYs and all-age and age-standardised DALY rates by location, 2022 and 2050

Ukraine, Reference scenario

Legend:
Communicable, maternal, neonatal and nutritional
Non-communicable
Injuries

Leading Causes 2022	Leading Causes 2050	Mean % change number of DALY	Mean % change all-age DALY rate	Mean % change age-standardized DALY rate
1 Ischemic heart disease	1 Ischemic heart disease	-21.3 (-47.2 to 13.6)	8.28 (-27.8 to 49.1)	**-39 (-62.8 to -0.031)**
2 Stroke	2 Stroke	**-29 (-47.7 to -8.81)**	-2.5 (-22 to 18)	**-38.8 (-52.7 to -21)**
3 Low back pain	3 Low back pain	-12.6 (-31.3 to 5.73)	**19.9 (14.3 to 25.4)**	**-1.9 (-2.86 to -1.01)**
4 COVID-19	4 Alzheimer's disease	**72 (12.2 to 155)**	**134 (63.6 to 208)**	**8.6 (2.52 to 12.3)**
5 Cirrhosis liver	5 Cirrhosis liver	**-33 (-48.5 to -12.4)**	-7.76 (-24.7 to 14.5)	-12.1 (-28.6 to 6.69)
6 Self-harm	6 Age-related hearing loss	10.2 (-17.1 to 39.5)	**50.8 (33.7 to 66.5)**	-0.129 (-0.324 to 0.101)
7 Cardiomyopathy	7 Diabetes	17.4 (-7.95 to 45.4)	**60.9 (48.1 to 73.8)**	**17.5 (9.54 to 25)**
8 Falls	8 Depressive disorders	-18.1 (-33.9 to 1.36)	**12.3 (7.13 to 17.5)**	**-1.09 (-1.64 to -0.596)**
9 Depressive disorders	9 Falls	-20.4 (-39.1 to 1.14)	9.06 (-2.87 to 23.2)	-9.56 (-19.9 to 0.276)
10 Alcohol use disorders	10 Colorectal cancer	2.44 (-37.7 to 66.2)	39.5 (-7.42 to 104)	-6.39 (-37.1 to 35.6)
11 Lung cancer	11 Cardiomyopathy	**-31.8 (-53.1 to -4.34)**	-6.6 (-26.5 to 18.9)	-17 (-33.6 to 3.42)
12 Age-related hearing loss	12 Lung cancer	-16.3 (-47.6 to 23.8)	14.2 (-20 to 57.6)	-18.7 (-42.9 to 11.9)
13 Alzheimer's disease	13 Self-harm	**-34.2 (-50 to -16.5)**	-9.41 (-27.5 to 9.44)	-5.98 (-28.5 to 20.2)
14 Road injuries	14 Alcohol use disorders	**-23.4 (-41.3 to -0.78)**	5.51 (-17.1 to 35.2)	8.11 (-13.1 to 38.4)
15 Headache disorders	15 COPD	23.7 (-10.3 to 68.7)	**69.9 (28.1 to 124)**	2.48 (-26.2 to 36.8)
16 HIV/AIDS	16 Headache disorders	**-28.7 (-44.4 to -13.4)**	-2.14 (-7.24 to 4.92)	**-0.603 (-1.18 to -0.124)**
17 Colorectal cancer	17 Road injuries	-30.2 (-55.3 to 7.51)	-3.78 (-38.8 to 44.8)	0.0798 (-39.4 to 54.6)
18 Diabetes	18 Osteoarthritis	6.7 (-16.7 to 34.7)	**46.2 (35.1 to 57.5)**	-0.319 (-1.29 to 0.442)
19 Lower respiratory infect	19 Oral disorders	-2.51 (-24.9 to 22.8)	**33.6 (24.6 to 44.1)**	-0.381 (-0.947 to 0.0967)
20 Gynecological diseases	20 HIV/AIDS	**-37.5 (-53.6 to -19.3)**	-14.1 (-34.9 to 5.01)	-5.01 (-24.1 to 10.6)
21 Oral disorders	25 Gynecological diseases			
23 COPD	26 Lower respiratory infect			
25 Osteoarthritis	155 COVID-19			

Supplemental Results Figure S7. Leading 20 level 3 causes of DALYs, and percentage change in number of DALYs and all-age and age-standardised DALY rates by location, 2022 and 2050

Australia, Reference scenario

Legend:
Communicable, maternal, neonatal and nutritional
Non-communicable
Injuries

Leading Causes 2022	Leading Causes 2050	Mean % change number of DALY	Mean % change all-age DALY rate	Mean % change age-standardized DALY rate
1 Low back pain	1 Low back pain	**37.4 (28.5 to 44.8)**	2.59 (-0.491 to 5.26)	**-2.75 (-2.87 to -2.62)**
2 Ischemic heart disease	2 Alzheimer's disease	**108 (87.9 to 130)**	**55.1 (42.9 to 69.6)**	**-5.1 (-8.7 to -0.695)**
3 Falls	3 Diabetes	**103 (85.5 to 122)**	**51.3 (40.7 to 63.2)**	**30.4 (21.5 to 38.9)**
4 Other musculoskeletal	4 Other musculoskeletal	**72.7 (61.2 to 83.8)**	**29 (24.8 to 33.1)**	**26.3 (24.9 to 27.7)**
5 Depressive disorders	5 Falls	**53 (38.3 to 67.9)**	**14.2 (5.9 to 23.5)**	**-14.9 (-21.1 to -8.88)**
6 Alzheimer's disease	6 Ischemic heart disease	-0.289 (-19.4 to 27)	**-25.5 (-40.4 to -7.36)**	**-51.4 (-61.7 to -37.3)**
7 Lung cancer	7 Depressive disorders	**33.1 (24.5 to 41.3)**	-0.66 (-1.95 to 0.727)	**-1.52 (-1.69 to -1.36)**
8 Stroke	8 COPD	**58.8 (35.1 to 87.5)**	**18.6 (0.687 to 40.2)**	-16 (-28.7 to 1.22)
9 Diabetes	9 Lung cancer	**33.9 (8.81 to 59.6)**	-0.0722 (-16.9 to 18.4)	**-19 (-32.2 to -6.25)**
10 COVID-19	10 Age-related hearing loss	**62.2 (50 to 73.8)**	**21.1 (15.9 to 26.9)**	**-0.792 (-0.898 to -0.698)**
11 Anxiety disorders	11 Stroke	**24.2 (10.7 to 38.7)**	-7.23 (-16.5 to 3.7)	**-33.4 (-40.7 to -25)**
12 COPD	12 Anxiety disorders	**33.2 (24.7 to 41.5)**	-0.595 (-1.86 to 0.929)	**0.244 (0.0807 to 0.419)**
13 Age-related hearing loss	13 Colorectal cancer	**58.9 (25.3 to 97.7)**	18.6 (-4.58 to 44.4)	-9.14 (-27.7 to 10.3)
14 Self-harm	14 Drug use disorders	**51.2 (35.9 to 66.9)**	**12.9 (2.21 to 23.5)**	**12.4 (2.35 to 22.4)**
15 Headache disorders	15 Headache disorders	**33.8 (25.8 to 41.7)**	-0.102 (-1.85 to 2.39)	-0.0447 (-0.196 to 0.112)
16 Colorectal cancer	16 Chronic kidney disease	**180 (129 to 251)**	**109 (73.8 to 160)**	**26 (2.32 to 64.8)**
17 Drug use disorders	17 Osteoarthritis	**55.3 (44.4 to 65.7)**	**16 (11.5 to 20.4)**	**2.33 (2.13 to 2.57)**
18 Osteoarthritis	18 Atrial fibrillation	**132 (105 to 159)**	**73.3 (55.8 to 90.5)**	**11.4 (3.32 to 18.4)**
19 Oral disorders	19 Self-harm	9.93 (-5.21 to 27.2)	**-17.9 (-29.7 to -4.45)**	**-18.4 (-30.9 to -3.48)**
20 Breast cancer	20 Oral disorders	**48.5 (38.4 to 58.3)**	**10.9 (6.47 to 14.4)**	**0.565 (0.427 to 0.722)**
27 Atrial fibrillation	23 Breast cancer			
30 Chronic kidney disease	155 COVID-19			

Supplemental Results Figure S7. Leading 20 level 3 causes of DALYs, and percentage change in number of DALYs and all-age and age-standardised DALY rates by location, 2022 and 2050

New Zealand, Reference scenario

Legend:
Communicable, maternal, neonatal and nutritional
Non-communicable
Injuries

Leading Causes 2022	Leading Causes 2050	Mean % change number of DALY	Mean % change all-age DALY rate	Mean % change age-standardized DALY rate
1 Ischemic heart disease	1 Low back pain	**40.2 (30.1 to 50.4)**	**5.11 (2.93 to 7.58)**	**-2.2 (-2.31 to -2.08)**
2 Low back pain	2 Ischemic heart disease	4.67 (-20.1 to 33)	**-21.5 (-38.2 to -0.701)**	**-48.8 (-60.4 to -35.1)**
3 Falls	3 Alzheimer's disease	**121 (95.6 to 146)**	**66 (53.3 to 78.3)**	-1.17 (-5.59 to 3.15)
4 Anxiety disorders	4 Diabetes	**102 (84.5 to 122)**	**51.1 (41.8 to 60.6)**	**27.6 (20.9 to 35.1)**
5 Stroke	5 Falls	**52.5 (36.2 to 66.8)**	**14.3 (5.63 to 23.9)**	**-12.6 (-18.2 to -7.35)**
6 Lung cancer	6 COPD	**75.5 (43.8 to 109)**	**31.6 (9.49 to 52.6)**	-7.48 (-23.3 to 10.7)
7 Alzheimer's disease	7 Anxiety disorders	**32.7 (22.5 to 42.9)**	-0.537 (-2.23 to 1.14)	**0.235 (0.118 to 0.362)**
8 Diabetes	8 Lung cancer	**40.7 (15.7 to 68.8)**	5.46 (-12.4 to 26)	-13.5 (-26 to 1.22)
9 COVID-19	9 Stroke	**22.8 (6.35 to 40.2)**	-7.94 (-19.1 to 3.78)	**-36.2 (-43.9 to -26.8)**
10 COPD	10 Colorectal cancer	**67.1 (29 to 115)**	25.3 (-1.85 to 58)	-8.25 (-25.8 to 14.3)
11 Depressive disorders	11 Age-related hearing loss	**64.9 (52.7 to 78.1)**	**23.6 (18.5 to 29.3)**	**-0.371 (-0.468 to -0.279)**
12 Colorectal cancer	12 Depressive disorders	**30.7 (20.4 to 40.7)**	**-1.99 (-3.69 to -0.494)**	**-0.936 (-1.02 to -0.864)**
13 Headache disorders	13 Headache disorders	**35 (24.8 to 45.7)**	1.19 (-0.748 to 4.65)	0.0238 (-0.129 to 0.139)
14 Age-related hearing loss	14 Other musculoskeletal	**67.8 (54 to 81)**	**25.8 (21.8 to 30)**	**19.3 (16.9 to 21.2)**
15 Self-harm	15 Chronic kidney disease	**132 (83.1 to 183)**	**74.2 (39.3 to 111)**	15.1 (-8.77 to 41.6)
16 Road injuries	16 Osteoarthritis	**58.4 (46.5 to 70.7)**	**18.8 (14.5 to 23.1)**	**1.44 (1.27 to 1.64)**
17 Oral disorders	17 Oral disorders	**50.2 (39.5 to 61.7)**	**12.6 (9.35 to 16)**	**0.333 (0.22 to 0.446)**
18 Osteoarthritis	18 Atrial fibrillation	**137 (110 to 166)**	**77.4 (58.5 to 93.9)**	**8.87 (1.59 to 16.7)**
19 Other musculoskeletal	19 Road injuries	20.9 (-10.2 to 56.7)	-9.37 (-32.3 to 16)	-11 (-35.8 to 15.9)
20 Mechanical forces	20 Mechanical forces	**37.8 (26.1 to 48.5)**	3.32 (-1.75 to 7.7)	-4.17 (-9.51 to 0.313)
27 Chronic kidney disease	23 Self-harm			
29 Atrial fibrillation	153 COVID-19			

Supplemental Results Figure S7. Leading 20 level 3 causes of DALYs, and percentage change in number of DALYs and all-age and age-standardised DALY rates by location, 2022 and 2050

Brunei, Reference scenario

Legend:
Communicable, maternal, neonatal and nutritional
Non-communicable
Injuries

Leading Causes 2022	Leading Causes 2050	Mean % change number of DALY	Mean % change all-age DALY rate	Mean % change age-standardized DALY rate
1 Diabetes	1 Diabetes	**109 (74.6 to 141)**	**110 (83.8 to 140)**	-2.21 (-12.8 to 10.2)
2 Ischemic heart disease	2 Stroke	**70.3 (40.8 to 105)**	**71 (48 to 101)**	**-34.4 (-45.2 to -19.3)**
3 Stroke	3 Ischemic heart disease	15.3 (-17.5 to 56.1)	15.8 (-15.9 to 61.1)	**-49.4 (-64.3 to -26.2)**
4 Low back pain	4 Chronic kidney disease	**199 (131 to 292)**	**200 (135 to 292)**	10.9 (-16.5 to 53.7)
5 COVID-19	5 COPD	**290 (218 to 366)**	**291 (226 to 374)**	8.65 (-12.2 to 38.2)
6 Neonatal disorders	6 Low back pain	**28.2 (10.8 to 49.4)**	**28.5 (18.6 to 40.1)**	**-2.93 (-3.16 to -2.69)**
7 Other musculoskeletal	7 Lung cancer	**172 (99.3 to 250)**	**172 (112 to 238)**	2.3 (-18.4 to 26)
8 Road injuries	8 Other musculoskeletal	**68.5 (37.7 to 98.5)**	**68.9 (53.6 to 90.3)**	**30.5 (28.9 to 32.2)**
9 Congenital defects	9 Lower respiratory infect	**164 (119 to 228)**	**165 (121 to 213)**	-16.2 (-30.4 to 1.94)
10 Headache disorders	10 Colorectal cancer	**129 (58.6 to 218)**	**129 (66.5 to 199)**	-2.92 (-27.8 to 26.5)
11 Falls	11 Alzheimer's disease	**366 (283 to 454)**	**367 (304 to 441)**	1.61 (-2.8 to 6.56)
12 Chronic kidney disease	12 Falls	**43.9 (20.6 to 70.4)**	**44.3 (30.2 to 60.7)**	**-16.9 (-23.8 to -8.6)**
13 Lung cancer	13 Osteoarthritis	**142 (110 to 179)**	**142 (126 to 160)**	**0.341 (0.0168 to 0.738)**
14 Colorectal cancer	14 Age-related hearing loss	**126 (94.1 to 166)**	**127 (107 to 148)**	**-0.308 (-0.527 to -0.104)**
15 Lower respiratory infect	15 Liver cancer	**184 (131 to 243)**	**185 (136 to 247)**	**24.2 (2.28 to 55.9)**
16 COPD	16 Road injuries	-3.81 (-29 to 34)	-3.41 (-27.5 to 35.5)	-21.1 (-44.1 to 17.3)
17 Anxiety disorders	17 Hypertensive heart disease	**268 (181 to 361)**	**269 (185 to 361)**	18.3 (-10.2 to 55.1)
18 Breast cancer	18 Headache disorders	-3.97 (-21.1 to 15.3)	-3.74 (-12 to 11.8)	**0.772 (0.329 to 1.07)**
19 Depressive disorders	19 Urinary diseases	**304 (233 to 388)**	**306 (241 to 380)**	**31.9 (13.4 to 60.1)**
20 Age-related hearing loss	20 Breast cancer	**40.4 (3.43 to 79)**	**40.7 (8.1 to 71.2)**	-16.2 (-34.1 to 4.83)
21 Osteoarthritis	22 Neonatal disorders			
26 Liver cancer	24 Anxiety disorders			
32 Alzheimer's disease	25 Congenital defects			
40 Hypertensive heart disease	29 Depressive disorders			
52 Urinary diseases	153 COVID-19			

Supplemental Results Figure S7. Leading 20 level 3 causes of DALYs, and percentage change in number of DALYs and all-age and age-standardised DALY rates by location, 2022 and 2050

Japan, Reference scenario

Legend:
Communicable, maternal, neonatal and nutritional
Non-communicable
Injuries

Leading Causes 2022	Leading Causes 2050	Mean % change number of DALY	Mean % change all-age DALY rate	Mean % change age-standardized DALY rate
1 Stroke	1 Alzheimer's disease	**23.3 (10.9 to 35.6)**	**44.5 (33.6 to 54.6)**	**-10.2 (-14.2 to -6.05)**
2 Alzheimer's disease	2 Low back pain	**-13.1 (-19.7 to -6.44)**	1.79 (-0.988 to 4.3)	**-2.02 (-2.07 to -1.97)**
3 Low back pain	3 Stroke	**-26.1 (-34.1 to -17.1)**	**-13.4 (-20.9 to -4.33)**	**-31 (-37.7 to -23.2)**
4 Ischemic heart disease	4 Diabetes	**10.1 (2.05 to 18.8)**	**29 (24.5 to 32.7)**	**14.6 (12.4 to 16.5)**
5 Lung cancer	5 Other musculoskeletal	4.44 (-3.34 to 13.2)	**22.4 (18.5 to 26.5)**	**19.9 (19.2 to 20.6)**
6 Other musculoskeletal	6 Lung cancer	-4.99 (-21.3 to 14.8)	11.3 (-6.21 to 30.8)	**-18.2 (-30.1 to -5.58)**
7 Diabetes	7 Lower respiratory infect	12.2 (-3.29 to 30.7)	**31.5 (14.1 to 47.4)**	**-18.6 (-28.6 to -8.42)**
8 Colorectal cancer	8 Colorectal cancer	2.19 (-17.7 to 25)	19.8 (-3.19 to 45.1)	-10.7 (-27.9 to 9.36)
9 Lower respiratory infect	9 Age-related hearing loss	5.42 (-2.27 to 13.9)	**23.5 (19.6 to 27.9)**	**-0.378 (-0.435 to -0.327)**
10 Age-related hearing loss	10 Chronic kidney disease	**66.2 (34.7 to 100)**	**94.9 (55.2 to 136)**	20.7 (-0.651 to 46.4)
11 Osteoarthritis	11 Ischemic heart disease	**-44.9 (-55.6 to -31.4)**	**-35.4 (-47.5 to -19.1)**	**-55.3 (-64.8 to -43.7)**
12 Stomach cancer	12 Osteoarthritis	-0.601 (-8.24 to 7)	**16.5 (13.1 to 19.4)**	**0.923 (0.786 to 1.09)**
13 Falls	13 Pancreatic cancer	**29.2 (4.4 to 58.2)**	**51.4 (24.1 to 79.1)**	19 (-1.96 to 39.6)
14 Self-harm	14 Falls	-2.89 (-14 to 9.15)	**13.8 (3.59 to 25.3)**	**-15.6 (-22.5 to -8.11)**
15 Pancreatic cancer	15 COPD	12.5 (-4.84 to 30.5)	**31.8 (14.1 to 51)**	-12.6 (-25.4 to 1.88)
16 COPD	16 Liver cancer	**29.9 (13.1 to 50.2)**	**52.3 (32.5 to 73)**	**28.1 (13.1 to 45.4)**
17 Chronic kidney disease	17 Stomach cancer	**-28.2 (-35.4 to -19.6)**	**-15.9 (-22.3 to -7.88)**	**-38.9 (-43.1 to -33.9)**
18 Headache disorders	18 Self-harm	**-30.5 (-38.7 to -22.2)**	**-18.5 (-27.4 to -9.09)**	**-14.9 (-26.5 to -1.25)**
19 Depressive disorders	19 Headache disorders	**-18.9 (-24.5 to -12.9)**	**-4.92 (-6.69 to -2.36)**	**0.108 (0.0241 to 0.177)**
20 Liver cancer	20 Depressive disorders	**-15 (-21.7 to -8.64)**	-0.401 (-1.75 to 1.2)	**-1.05 (-1.1 to -0.988)**

Supplemental Results Figure S7. Leading 20 level 3 causes of DALYs, and percentage change in number of DALYs and all-age and age-standardised DALY rates by location, 2022 and 2050

South Korea, Reference scenario

Legend:
Communicable, maternal, neonatal and nutritional
Non-communicable
Injuries

Leading Causes 2022
1 Diabetes
2 Stroke
3 Low back pain
4 COVID-19
5 Other musculoskeletal
6 Self-harm
7 Alzheimer's disease
8 Falls
9 Lung cancer
10 Ischemic heart disease
11 Liver cancer
12 Osteoarthritis
13 Age-related hearing loss
14 Headache disorders
15 COPD
16 Stomach cancer
17 Colorectal cancer
18 Cirrhosis liver
19 Road injuries
20 Lower respiratory infect
24 Chronic kidney disease
25 Pancreatic cancer
36 Atrial fibrillation

Leading Causes 2050	Mean % change number of DALY	Mean % change all-age DALY rate	Mean % change age-standardized DALY rate
1 Diabetes	**49.7 (37.3 to 63.3)**	**62.9 (53.9 to 73.5)**	**8.02 (2.51 to 12.3)**
2 Alzheimer's disease	**173 (144 to 206)**	**197 (172 to 225)**	**-18.1 (-21.5 to -14.2)**
3 Stroke	**24.3 (11.8 to 38.6)**	**35.3 (21.9 to 48.2)**	**-43.1 (-49.2 to -37.2)**
4 Low back pain	4.68 (-4.1 to 14.2)	**13.9 (7.98 to 20.9)**	**-2.4 (-2.54 to -2.26)**
5 Lung cancer	**47.6 (23.2 to 73.1)**	**60.6 (36.2 to 87)**	**-28.6 (-37.5 to -18.8)**
6 Falls	**35.4 (19.2 to 53.4)**	**47.3 (30.6 to 65.3)**	**-24.1 (-30.8 to -17)**
7 Lower respiratory infect	**152 (110 to 201)**	**175 (135 to 217)**	**-25.7 (-35.3 to -16)**
8 COPD	**114 (81 to 154)**	**133 (103 to 170)**	**-20.5 (-30.3 to -8.67)**
9 Other musculoskeletal	**15.1 (4.78 to 25.6)**	**25.2 (16.7 to 35.5)**	**23.6 (22.4 to 24.7)**
10 Liver cancer	**60.2 (39.4 to 82.8)**	**74.4 (52.5 to 97.1)**	5.36 (-7.86 to 19.5)
11 Age-related hearing loss	**74.3 (58.8 to 91.7)**	**89.7 (74.7 to 105)**	**-0.272 (-0.477 to -0.053)**
12 Chronic kidney disease	**194 (137 to 251)**	**219 (158 to 280)**	1.7 (-15.7 to 22.4)
13 Osteoarthritis	**45.8 (34.6 to 56.8)**	**58.7 (52.8 to 65.8)**	**0.326 (0.109 to 0.556)**
14 Colorectal cancer	**79.9 (44.7 to 120)**	**95.8 (61.4 to 138)**	-13.4 (-27.4 to 4.56)
15 Ischemic heart disease	2.08 (-17.2 to 22.3)	11.1 (-9.97 to 32.8)	**-61.1 (-67.8 to -53)**
16 Self-harm	**-27.4 (-36.4 to -18.8)**	**-21 (-29 to -12.8)**	**-24.2 (-32.9 to -14.1)**
17 Pancreatic cancer	**98 (59.4 to 139)**	**115 (77.3 to 159)**	6.43 (-10.9 to 25.2)
18 Stomach cancer	8.4 (-5.37 to 22.3)	**18 (5.66 to 29.1)**	**-44.8 (-48.3 to -41)**
19 Cirrhosis liver	7 (-7.34 to 23.4)	**16.4 (2.6 to 30.8)**	**-14.1 (-24.5 to -3)**
20 Atrial fibrillation	**157 (125 to 184)**	**179 (148 to 211)**	0.159 (-5.74 to 6.11)
21 Headache disorders			
24 Road injuries			
155 COVID-19			

Supplemental Results Figure S7. Leading 20 level 3 causes of DALYs, and percentage change in number of DALYs and all-age and age-standardised DALY rates by location, 2022 and 2050

Singapore, Reference scenario

Legend:
Communicable, maternal, neonatal and nutritional
Non-communicable
Injuries

Leading Causes 2022	Leading Causes 2050	Mean % change number of DALY	Mean % change all-age DALY rate	Mean % change age-standardized DALY rate
1 Ischemic heart disease	1 Lower respiratory infect	**203 (164 to 247)**	**128 (102 to 161)**	**-22.1 (-31 to -9.98)**
2 Low back pain	2 Diabetes	**164 (141 to 187)**	**99 (87 to 114)**	**53.1 (50.1 to 55.7)**
3 Lower respiratory infect	3 Alzheimer's disease	**294 (251 to 341)**	**197 (168 to 229)**	**-7.58 (-11.4 to -3.46)**
4 Diabetes	4 Other musculoskeletal	**87.7 (70.4 to 106)**	**41.4 (31.5 to 50.8)**	**30.4 (29.3 to 31.5)**
5 Other musculoskeletal	5 Low back pain	**44.4 (32.5 to 57)**	**8.8 (3.02 to 15.3)**	**-2.5 (-2.73 to -2.23)**
6 Stroke	6 Ischemic heart disease	-2.73 (-23.7 to 27.6)	**-26.7 (-41.8 to -5.1)**	**-63 (-70.9 to -51.7)**
7 Osteoarthritis	7 Age-related hearing loss	**127 (106 to 151)**	**71.2 (57 to 86.1)**	**-0.911 (-1.16 to -0.642)**
8 Age-related hearing loss	8 Osteoarthritis	**102 (87.2 to 118)**	**52.3 (44.4 to 61.2)**	**2.38 (2.12 to 2.62)**
9 Falls	9 Stroke	**62.7 (44 to 82.7)**	**22.6 (12.6 to 34.8)**	**-33.4 (-40.2 to -25.1)**
10 Lung cancer	10 Lung cancer	**104 (67.3 to 143)**	**54 (26.5 to 84.3)**	**-19.9 (-33.2 to -5.08)**
11 Headache disorders	11 Colorectal cancer	**132 (75.9 to 188)**	**74.9 (33.8 to 118)**	-7.9 (-27.4 to 12.3)
12 Alzheimer's disease	12 Urinary diseases	**372 (285 to 465)**	**256 (193 to 323)**	**24.4 (7.28 to 44.5)**
13 COVID-19	13 Falls	**63 (46.2 to 81.2)**	**22.9 (12.7 to 35.1)**	**-16.8 (-24.6 to -7.78)**
14 Colorectal cancer	14 Chronic kidney disease	**215 (145 to 307)**	**138 (86.8 to 205)**	-3.06 (-24.7 to 27.1)
15 Depressive disorders	15 COPD	**194 (141 to 252)**	**122 (86.3 to 159)**	-9.94 (-24.3 to 8.32)
16 Anxiety disorders	16 Headache disorders	**20.7 (11.6 to 30.9)**	**-9.04 (-12.7 to -3.67)**	**0.5 (0.195 to 0.766)**
17 Self-harm	17 Hypertensive heart disease	**201 (133 to 287)**	**126 (79.6 to 191)**	11.3 (-14.9 to 44.6)
18 Autism spectrum	18 Depressive disorders	**40.8 (30 to 52.5)**	**6.07 (1.21 to 10.7)**	**-1.48 (-1.73 to -1.19)**
19 Breast cancer	19 Pancreatic cancer	**186 (123 to 250)**	**116 (70.3 to 163)**	20.5 (-4.95 to 45.1)
20 Neck pain	20 Liver cancer	**134 (96.5 to 178)**	**76.1 (51.1 to 104)**	6.9 (-9.46 to 26.5)
21 Chronic kidney disease	22 Anxiety disorders			
25 COPD	24 Breast cancer			
28 Liver cancer	25 Neck pain			
29 Hypertensive heart disease	26 Autism spectrum			
30 Urinary diseases	27 Self-harm			
32 Pancreatic cancer	151 COVID-19			

Supplemental Results Figure S7. Leading 20 level 3 causes of DALYs, and percentage change in number of DALYs and all-age and age-standardised DALY rates by location, 2022 and 2050

Canada, Reference scenario

Legend:
Communicable, maternal, neonatal and nutritional
Non-communicable
Injuries

Leading Causes 2022
1 Ischemic heart disease
2 Low back pain
3 Other musculoskeletal
4 Diabetes
5 Lung cancer
6 Alzheimer's disease
7 Stroke
8 Drug use disorders
9 Falls
10 COVID-19
11 COPD
12 Headache disorders
13 Age-related hearing loss
14 Depressive disorders
15 Self-harm
16 Colorectal cancer
17 Anxiety disorders
18 Breast cancer
19 Osteoarthritis
20 Cirrhosis liver
23 Lower respiratory infect
25 Pancreatic cancer
27 Chronic kidney disease
29 Atrial fibrillation

Leading Causes 2050	Mean % change number of DALY	Mean % change all-age DALY rate	Mean % change age-standardized DALY rate
1 Diabetes	**67.2 (55.3 to 81.5)**	**29.7 (23 to 37)**	**16.4 (10.9 to 21.2)**
2 Alzheimer's disease	**108 (87.7 to 131)**	**61.3 (48 to 75.3)**	**-5.13 (-9.66 to -0.991)**
3 Other musculoskeletal	**45 (36.2 to 55.2)**	**12.5 (9.51 to 15.5)**	**14.9 (14.2 to 15.6)**
4 Low back pain	**30 (22.3 to 38.5)**	0.832 (-1.99 to 4.03)	**-1.61 (-1.69 to -1.52)**
5 Ischemic heart disease	-6.68 (-24.2 to 14.8)	**-27.6 (-42 to -10.7)**	**-51.6 (-61.6 to -39.5)**
6 Drug use disorders	**83.7 (64.6 to 105)**	**42.4 (29 to 57.2)**	**40.6 (27.4 to 55.3)**
7 Lung cancer	**25.3 (4.52 to 46.4)**	-2.86 (-16.9 to 14.9)	**-15.9 (-26.5 to -3.35)**
8 Falls	**63.7 (45.9 to 78.5)**	**27 (16.2 to 38.3)**	**-13.5 (-18.9 to -7.11)**
9 COPD	**60.4 (36.1 to 88.6)**	**24.4 (7.02 to 41.9)**	-9.42 (-23.1 to 4.05)
10 Stroke	**24 (11.6 to 36.1)**	-3.8 (-12 to 5.8)	**-27.8 (-34.8 to -20.2)**
11 Age-related hearing loss	**51.3 (41.2 to 62.4)**	**17.3 (11.4 to 23.2)**	**-0.421 (-0.495 to -0.338)**
12 Headache disorders	**27.6 (19.4 to 35.9)**	-1.07 (-2.51 to 0.428)	**-0.277 (-0.473 to -0.0522)**
13 Chronic kidney disease	**185 (139 to 242)**	**121 (82.5 to 160)**	**32.7 (9.39 to 61.5)**
14 Colorectal cancer	**48.2 (22.4 to 82.5)**	14.9 (-5.31 to 40.9)	-11.5 (-27.4 to 7.13)
15 Depressive disorders	**28.3 (20.5 to 37.5)**	-0.493 (-1.65 to 0.816)	**-0.989 (-1.1 to -0.876)**
16 Anxiety disorders	**28.9 (20.3 to 37.7)**	-0.0909 (-1.57 to 1.43)	0.0902 (-0.0765 to 0.248)
17 Self-harm	15.3 (-0.381 to 33.6)	-10.6 (-22 to 2.12)	-10.5 (-23.4 to 4.07)
18 Atrial fibrillation	**122 (96.6 to 147)**	**72.3 (56.3 to 90.1)**	**11.8 (4.94 to 17.9)**
19 Lower respiratory infect	**74.4 (53.7 to 99)**	**35.3 (17.8 to 53.3)**	**-18.2 (-27.1 to -7.01)**
20 Pancreatic cancer	**80.9 (50.1 to 120)**	**40.2 (17.8 to 71.4)**	**19.5 (1.91 to 43.3)**
21 Osteoarthritis			
22 Breast cancer			
28 Cirrhosis liver			
152 COVID-19			

Supplemental Results Figure S7. Leading 20 level 3 causes of DALYs, and percentage change in number of DALYs and all-age and age-standardised DALY rates by location, 2022 and 2050

Greenland, Reference scenario

Legend:
Communicable, maternal, neonatal and nutritional
Non-communicable
Injuries

Leading Causes 2022	Leading Causes 2050	Mean % change number of DALY	Mean % change all-age DALY rate	Mean % change age-standardized DALY rate
1 Self-harm	1 Lung cancer	17.8 (-4.93 to 46.5)	11.2 (-11 to 36.3)	**-17.5 (-32.9 to -0.279)**
2 Lung cancer	2 Self-harm	**-22.3 (-36.6 to -4.87)**	**-26.6 (-37.5 to -11.7)**	**-21.9 (-35 to -4.29)**
3 COVID-19	3 COPD	**73.7 (36.1 to 122)**	**63.9 (31.9 to 106)**	-14.6 (-31.3 to 7.21)
4 Ischemic heart disease	4 Stroke	17 (-6.02 to 43)	10.4 (-9.08 to 34.1)	**-42.4 (-52.8 to -29.8)**
5 Stroke	5 Ischemic heart disease	-17.3 (-40.5 to 12.3)	-22 (-43 to 7.55)	**-56.8 (-69.3 to -39.2)**
6 Depressive disorders	6 Falls	**58.5 (36.7 to 79.5)**	**49.6 (35 to 64.8)**	**-16.8 (-23 to -10.5)**
7 Low back pain	7 Other musculoskeletal	**33.9 (19.8 to 48.7)**	**26.3 (21.5 to 31.9)**	**25.9 (24.9 to 26.9)**
8 COPD	8 Alzheimer's disease	**260 (192 to 331)**	**240 (181 to 296)**	**-8.13 (-12.2 to -4.03)**
9 Other musculoskeletal	9 Low back pain	**15.6 (2.86 to 26.4)**	**9.09 (4.69 to 14.2)**	**-1.73 (-1.91 to -1.53)**
10 Falls	10 Depressive disorders	0.48 (-9.97 to 10.7)	**-5.18 (-6.89 to -3.21)**	**-0.938 (-1.17 to -0.679)**
11 Alcohol use disorders	11 Colorectal cancer	**36.6 (1.97 to 77.7)**	29 (-6.26 to 68.9)	-15.2 (-37.7 to 9.94)
12 Colorectal cancer	12 Diabetes	**45.6 (23.2 to 67.8)**	**37.4 (22.5 to 52.8)**	-3.76 (-12.5 to 6.27)
13 Headache disorders	13 Alcohol use disorders	-7.91 (-21.9 to 4.52)	**-13.1 (-21.8 to -1.65)**	-9.42 (-19.1 to 1.31)
14 Neonatal disorders	14 Pancreatic cancer	**70.7 (31.7 to 120)**	**61 (25.2 to 104)**	16.3 (-9.58 to 44.1)
15 Diabetes	15 Age-related hearing loss	**70.2 (47.9 to 90.9)**	**60.7 (46.9 to 75.9)**	**-1.45 (-1.73 to -1.12)**
16 Anxiety disorders	16 Lower respiratory infect	**87.3 (49.3 to 129)**	**76.8 (43.3 to 110)**	**-24.5 (-37.2 to -11.9)**
17 Pancreatic cancer	17 Headache disorders	3.15 (-7.99 to 13.7)	-2.65 (-5.12 to 2.4)	**1.04 (0.629 to 1.45)**
18 Age-related hearing loss	18 Chronic kidney disease	**130 (72 to 199)**	**117 (59.9 to 187)**	-0.351 (-25.1 to 33)
19 Interpersonal violence	19 Anxiety disorders	5.13 (-5.53 to 15.1)	-0.779 (-4.05 to 2.63)	**1.21 (0.814 to 1.59)**
20 Cirrhosis liver	20 Atrial fibrillation	**166 (118 to 214)**	**151 (113 to 187)**	-2.76 (-9.08 to 3.23)
21 Lower respiratory infect	24 Cirrhosis liver			
22 Alzheimer's disease	25 Neonatal disorders			
31 Chronic kidney disease	38 Interpersonal violence			
44 Atrial fibrillation	157 COVID-19			

Supplemental Results Figure S7. Leading 20 level 3 causes of DALYs, and percentage change in number of DALYs and all-age and age-standardised DALY rates by location, 2022 and 2050

USA, Reference scenario

Legend:
Communicable, maternal, neonatal and nutritional
Non-communicable
Injuries

Leading Causes 2022	Leading Causes 2050	Mean % change number of DALY	Mean % change all-age DALY rate	Mean % change age-standardized DALY rate
1 Ischemic heart disease	1 Drug use disorders	**25.5 (11 to 42.4)**	**18.2 (5.64 to 32)**	**19.8 (6.49 to 33.7)**
2 Drug use disorders	2 Diabetes	**25.3 (16 to 34.6)**	**18.1 (11.5 to 24)**	2.03 (-3.06 to 6.63)
3 COVID-19	3 Ischemic heart disease	**-25.6 (-38.8 to -10.2)**	**-29.9 (-40.8 to -16.6)**	**-51.9 (-60.7 to -41.1)**
4 Diabetes	4 Alzheimer's disease	**88.1 (69.8 to 107)**	**77.2 (64.1 to 90.1)**	1.33 (-2.13 to 4.97)
5 Low back pain	5 COPD	**32.4 (15.4 to 48.9)**	**24.7 (10.9 to 41)**	-8.78 (-20.4 to 3.44)
6 COPD	6 Low back pain	**11.7 (4.5 to 19.4)**	**5.26 (3.35 to 7.29)**	**-1.24 (-1.3 to -1.18)**
7 Other musculoskeletal	7 Chronic kidney disease	**103 (74 to 136)**	**91.4 (63.7 to 122)**	**31.5 (11.9 to 55.3)**
8 Stroke	8 Other musculoskeletal	**22.6 (15.3 to 31.3)**	**15.5 (13.6 to 17.6)**	**12.8 (12.2 to 13.3)**
9 Lung cancer	9 Stroke	1.86 (-6.59 to 11.6)	-4.03 (-10.3 to 3.05)	**-28.9 (-34.4 to -22.4)**
10 Alzheimer's disease	10 Lung cancer	7.48 (-7.48 to 24.4)	1.26 (-12.2 to 16.6)	**-17.5 (-27.9 to -6.64)**
11 Depressive disorders	11 Falls	**30.3 (18.5 to 44)**	**22.8 (13 to 33.5)**	**-10 (-15.9 to -3.39)**
12 Road injuries	12 Age-related hearing loss	**31.8 (23 to 40.7)**	**24.2 (20 to 28.9)**	**-0.118 (-0.206 to -0.0359)**
13 Falls	13 Depressive disorders	4.34 (-2.23 to 11.2)	**-1.7 (-2.63 to -0.661)**	**-0.853 (-0.95 to -0.759)**
14 Chronic kidney disease	14 Road injuries	10.6 (-11 to 33.9)	4.24 (-15.6 to 25.4)	4.09 (-18 to 29)
15 Anxiety disorders	15 Anxiety disorders	6.02 (-0.381 to 13)	-0.113 (-1.44 to 1.29)	-0.000416 (-0.0758 to 0.0837)
16 Headache disorders	16 Headache disorders	5.02 (-1.72 to 12.7)	-1.05 (-2.61 to 2.66)	**-0.228 (-0.378 to -0.028)**
17 Age-related hearing loss	17 Hypertensive heart disease	**72.5 (45.9 to 107)**	**62.6 (35.9 to 98.4)**	**25.8 (3.84 to 53.9)**
18 Self-harm	18 Osteoarthritis	**23 (14.9 to 31.7)**	**15.9 (13.1 to 18.9)**	**0.584 (0.436 to 0.725)**
19 Cirrhosis liver	19 Colorectal cancer	18.9 (-2.49 to 43.1)	12 (-7.92 to 32.5)	-11.9 (-26.9 to 3.32)
20 Osteoarthritis	20 Self-harm	-9.11 (-20.3 to 1.23)	**-14.3 (-22.8 to -4.89)**	**-13.3 (-22.9 to -2.28)**
21 Colorectal cancer	22 Cirrhosis liver			
24 Hypertensive heart disease	153 COVID-19			

Supplemental Results Figure S7. Leading 20 level 3 causes of DALYs, and percentage change in number of DALYs and all-age and age-standardised DALY rates by location, 2022 and 2050

Argentina, Reference scenario

Legend:
Communicable, maternal, neonatal and nutritional
Non-communicable
Injuries

Leading Causes 2022	Leading Causes 2050	Mean % change number of DALY	Mean % change all-age DALY rate	Mean % change age-standardized DALY rate
1 Lower respiratory infect	1 Lower respiratory infect	**42.5 (19.2 to 70.7)**	**38 (21.7 to 55)**	**-18.7 (-28.9 to -9.34)**
2 Ischemic heart disease	2 Other musculoskeletal	**64.6 (42 to 89.5)**	**59.4 (52.1 to 67.4)**	**30.6 (29.1 to 32.1)**
3 Low back pain	3 Diabetes	**77.2 (49.8 to 108)**	**71.6 (56.4 to 88.2)**	**11.8 (2.05 to 21)**
4 Stroke	4 Low back pain	**22.3 (6.06 to 41.2)**	**18.4 (13.3 to 23.9)**	**-3.78 (-3.88 to -3.67)**
5 Other musculoskeletal	5 Ischemic heart disease	-11.8 (-34.7 to 13.8)	-14.5 (-34.5 to 9.72)	**-48.4 (-61.9 to -30.8)**
6 COVID-19	6 Chronic kidney disease	**103 (60.4 to 153)**	**96.2 (58.2 to 135)**	17.8 (-7.81 to 44.1)
7 Diabetes	7 Stroke	4.16 (-12.4 to 24.3)	0.905 (-11.1 to 14.4)	**-35.3 (-44.2 to -25.8)**
8 Road injuries	8 Lung cancer	**67.1 (32 to 106)**	**61.7 (37.3 to 92.2)**	0.706 (-13.9 to 17.4)
9 Neonatal disorders	9 COPD	**62.9 (32.3 to 97.8)**	**57.9 (32.7 to 82.7)**	-7.16 (-22.7 to 8.37)
10 Chronic kidney disease	10 Alzheimer's disease	**94.3 (63.8 to 126)**	**88 (73.5 to 104)**	0.0238 (-4.52 to 5)
11 Anxiety disorders	11 Colorectal cancer	**58.9 (18.1 to 108)**	**53.7 (19 to 94.4)**	-4.08 (-23.9 to 20)
12 COPD	12 Age-related hearing loss	**57.8 (35.9 to 82.9)**	**52.8 (45.6 to 59.8)**	**-0.363 (-0.485 to -0.222)**
13 Lung cancer	13 Road injuries	0.358 (-22.8 to 26.6)	-2.74 (-23.9 to 21.8)	-2.69 (-25.9 to 23.4)
14 Colorectal cancer	14 Anxiety disorders	3.76 (-10.7 to 21.6)	0.43 (-2.77 to 3.7)	0.08 (-0.0312 to 0.183)
15 Headache disorders	15 Pancreatic cancer	**125 (74.2 to 192)**	**118 (79.9 to 167)**	**36 (12.2 to 64.7)**
16 Self-harm	16 Breast cancer	**45.1 (15.6 to 87.2)**	**40.4 (21.6 to 68.8)**	-7.29 (-20.2 to 9.44)
17 Depressive disorders	17 Hypertensive heart disease	**92.7 (48.4 to 138)**	**86.7 (52.1 to 121)**	7.09 (-14.7 to 30.3)
18 Alzheimer's disease	18 Other cardiovascular	**43.6 (22.2 to 69.9)**	**39.1 (27.5 to 53.8)**	**-13.1 (-21 to -4.32)**
19 Congenital defects	19 Headache disorders	6.55 (-9.16 to 25.2)	3.11 (-2.19 to 14.2)	-0.144 (-0.278 to 0.00201)
20 Age-related hearing loss	20 Osteoarthritis	**64.2 (42.5 to 88.1)**	**59 (52 to 65.3)**	**2.01 (1.75 to 2.3)**
21 Breast cancer	22 Depressive disorders			
22 Other cardiovascular	26 Neonatal disorders			
26 Osteoarthritis	27 Self-harm			
30 Hypertensive heart disease	36 Congenital defects			
32 Pancreatic cancer	159 COVID-19			

Supplemental Results Figure S7. Leading 20 level 3 causes of DALYs, and percentage change in number of DALYs and all-age and age-standardised DALY rates by location, 2022 and 2050

Chile, Reference scenario

Legend:
Communicable, maternal, neonatal and nutritional
Non-communicable
Injuries

Leading Causes 2022	Leading Causes 2050	Mean % change number of DALY	Mean % change all-age DALY rate	Mean % change age-standardized DALY rate
1 COVID-19	1 Diabetes	**106 (85.1 to 127)**	**80.1 (69.1 to 91.6)**	**20.4 (13.1 to 27.3)**
2 Low back pain	2 Other musculoskeletal	**65.2 (49.8 to 82.1)**	**44.8 (39.4 to 51.1)**	**28.4 (27.1 to 29.8)**
3 Other musculoskeletal	3 Low back pain	**30 (18.8 to 40.9)**	**13.9 (9.84 to 18.4)**	**-3.56 (-3.66 to -3.45)**
4 Ischemic heart disease	4 Alzheimer's disease	**166 (137 to 203)**	**133 (116 to 154)**	**-4.76 (-8.47 to -0.589)**
5 Stroke	5 Stroke	**15 (0.222 to 30.4)**	0.779 (-9.95 to 12.1)	**-42.7 (-48.7 to -35.2)**
6 Diabetes	6 Chronic kidney disease	**155 (108 to 214)**	**124 (84.3 to 170)**	7.67 (-11.2 to 29.8)
7 Depressive disorders	7 Falls	**53.6 (33 to 76.7)**	**34.5 (21.2 to 48.6)**	-8.05 (-17.4 to 0.891)
8 Anxiety disorders	8 Ischemic heart disease	-8.25 (-26.6 to 16.7)	-19.6 (-35 to 1.28)	**-54.1 (-63.2 to -41.2)**
9 Cirrhosis liver	9 Lower respiratory infect	**90.2 (61.9 to 124)**	**66.7 (44.2 to 89.1)**	**-23.6 (-32.4 to -16)**
10 Falls	10 Age-related hearing loss	**84.1 (64.9 to 107)**	**61.3 (53.2 to 69.7)**	**-0.286 (-0.413 to -0.15)**
11 Road injuries	11 COPD	**127 (89.9 to 176)**	**98.6 (70.7 to 136)**	-9.3 (-22.6 to 6.84)
12 Headache disorders	12 Depressive disorders	**13.3 (3.09 to 25.7)**	-0.716 (-4.26 to 3.46)	**-1.82 (-1.98 to -1.68)**
13 Alzheimer's disease	13 Cirrhosis liver	**27.1 (13.9 to 42.7)**	**11.4 (1.87 to 20.7)**	**-14.8 (-22 to -7.56)**
14 Age-related hearing loss	14 Anxiety disorders	**11.7 (1.37 to 25.2)**	-2.15 (-6.25 to 2.72)	-0.0132 (-0.277 to 0.195)
15 Self-harm	15 Lung cancer	**62.1 (33.4 to 89.2)**	**42.1 (21.5 to 65.5)**	-10.5 (-22.5 to 3.18)
16 Lower respiratory infect	16 Colorectal cancer	**77.5 (42.9 to 117)**	**55.6 (25.6 to 91.4)**	-6.79 (-24.2 to 12.2)
17 Chronic kidney disease	17 Hypertensive heart disease	**156 (102 to 214)**	**124 (82.2 to 167)**	2.5 (-17 to 23.1)
18 Stomach cancer	18 Osteoarthritis	**73 (57.2 to 89.5)**	**51.6 (45.8 to 57.3)**	**1.8 (1.54 to 2.06)**
19 Neonatal disorders	19 Road injuries	4.36 (-19.5 to 32.5)	-8.56 (-27 to 15.6)	-10.5 (-30.5 to 15.1)
20 Lung cancer	20 Urinary diseases	**184 (140 to 240)**	**149 (118 to 184)**	**18.8 (6.58 to 33.2)**
22 Colorectal cancer	21 Headache disorders			
23 COPD	24 Stomach cancer			
25 Osteoarthritis	27 Self-harm			
30 Hypertensive heart disease	31 Neonatal disorders			
37 Urinary diseases	153 COVID-19			

Supplemental Results Figure S7. Leading 20 level 3 causes of DALYs, and percentage change in number of DALYs and all-age and age-standardised DALY rates by location, 2022 and 2050

Uruguay, Reference scenario

Legend:
Communicable, maternal, neonatal and nutritional
Non-communicable
Injuries

Leading Causes 2022	Leading Causes 2050	Mean % change number of DALY	Mean % change all-age DALY rate	Mean % change age-standardized DALY rate
1 Ischemic heart disease	1 Diabetes	**36.6 (15.7 to 58.1)**	**46.7 (32.4 to 59.6)**	**9.12 (0.984 to 17.7)**
2 Stroke	2 Other musculoskeletal	**38.6 (18.7 to 60.1)**	**48.9 (43.1 to 54.7)**	**31 (29.5 to 32.6)**
3 COVID-19	3 Low back pain	3.83 (-11.5 to 19.6)	**11.5 (7.7 to 15.5)**	**-3.87 (-3.99 to -3.74)**
4 Low back pain	4 Lung cancer	14.1 (-8.45 to 40)	**22.5 (3.6 to 43.6)**	-11.8 (-25.3 to 1.53)
5 Lung cancer	5 Stroke	**-28.6 (-41 to -15.8)**	**-23.2 (-31.6 to -14)**	**-44.1 (-51.2 to -35.2)**
6 Diabetes	6 COPD	16.4 (-4.34 to 41.7)	**25.1 (6.56 to 44.7)**	**-15.1 (-28.2 to -0.156)**
7 Other musculoskeletal	7 Ischemic heart disease	**-36.8 (-51.6 to -17.5)**	**-32.1 (-46.1 to -13.3)**	**-52.8 (-63.2 to -38)**
8 COPD	8 Colorectal cancer	26.4 (-2.47 to 56.8)	**35.9 (7.7 to 65.1)**	-4.84 (-24.3 to 15)
9 Colorectal cancer	9 Alzheimer's disease	**40.8 (18.4 to 61.8)**	**51.2 (40.4 to 62)**	-2.82 (-7.13 to 1.56)
10 Self-harm	10 Lower respiratory infect	7.27 (-11.3 to 28.7)	**15.2 (1.8 to 30.6)**	**-24.1 (-32.2 to -13.9)**
11 Road injuries	11 Chronic kidney disease	**51 (21.5 to 88.5)**	**62.2 (33.6 to 94.6)**	9.49 (-11.6 to 37)
12 Lower respiratory infect	12 Road injuries	-8.22 (-29 to 23)	-1.45 (-20.7 to 24.4)	0.94 (-20.8 to 31.6)
13 Alzheimer's disease	13 Pancreatic cancer	**68.2 (32.5 to 111)**	**80.8 (47.5 to 117)**	**29.1 (7.04 to 55.7)**
14 Breast cancer	14 Breast cancer	14.5 (-6.01 to 37.4)	**23 (5.73 to 41.8)**	-9.51 (-23.1 to 3.39)
15 Anxiety disorders	15 Self-harm	**-20.3 (-34.1 to -5.89)**	**-14.4 (-24.7 to -2.41)**	-11.2 (-24.2 to 3.12)
16 Depressive disorders	16 Age-related hearing loss	**25.3 (6.32 to 43.2)**	**34.7 (28.6 to 40.2)**	**-0.469 (-0.567 to -0.368)**
17 Falls	17 Falls	16.7 (-1.44 to 36.3)	**25.4 (14.6 to 35.7)**	-1.81 (-11.2 to 6.3)
18 Age-related hearing loss	18 Urinary diseases	**78.2 (44.4 to 114)**	**91.4 (66.5 to 122)**	**24.6 (8.71 to 45.5)**
19 Chronic kidney disease	19 Anxiety disorders	-7.54 (-21.5 to 6.73)	-0.695 (-4.89 to 4.42)	**-0.256 (-0.446 to -0.0582)**
20 Headache disorders	20 Prostate cancer	**39.9 (15.9 to 67.9)**	**50.4 (31 to 72)**	1.87 (-10.3 to 14.5)
22 Pancreatic cancer	21 Depressive disorders			
24 Prostate cancer	23 Headache disorders			
32 Urinary diseases	153 COVID-19			

Supplemental Results Figure S7. Leading 20 level 3 causes of DALYs, and percentage change in number of DALYs and all-age and age-standardised DALY rates by location, 2022 and 2050

Andorra, Reference scenario

Legend:
Communicable, maternal, neonatal and nutritional
Non-communicable
Injuries

Leading Causes 2022
1 Low back pain
2 COVID-19
3 Ischemic heart disease
4 Falls
5 Lung cancer
6 Alzheimer's disease
7 Diabetes
8 Depressive disorders
9 Headache disorders
10 Stroke
11 COPD
12 Anxiety disorders
13 Colorectal cancer
14 Road injuries
15 Gynecological diseases
16 Age-related hearing loss
17 Breast cancer
18 Osteoarthritis
19 Pancreatic cancer
20 Liver cancer
25 Prostate cancer
28 Chronic kidney disease
36 Hypertensive heart disease
38 Atrial fibrillation

Leading Causes 2050	Mean % change number of DALY	Mean % change all-age DALY rate	Mean % change age-standardized DALY rate
1 Alzheimer's disease	**177 (123 to 241)**	**227 (184 to 273)**	1.24 (-3.96 to 6.42)
2 Falls	**47.3 (23.4 to 72)**	**74.1 (56.5 to 90)**	**-13.5 (-19.8 to -6.35)**
3 Ischemic heart disease	13 (-20.8 to 55.6)	33.6 (-5.2 to 84.6)	**-47.8 (-61.9 to -27.5)**
4 COPD	**109 (58.8 to 160)**	**147 (91.8 to 208)**	-4.86 (-22.6 to 17.7)
5 Low back pain	3.41 (-10.2 to 16)	**22.4 (15.1 to 30.8)**	**-0.648 (-0.785 to -0.524)**
6 Diabetes	**57.3 (29.9 to 86.9)**	**86.2 (64.2 to 111)**	9.67 (-0.578 to 18.6)
7 Lung cancer	18 (-13.6 to 54.1)	**39.3 (9.52 to 74.7)**	**-24.4 (-41.4 to -6.59)**
8 Colorectal cancer	**59.7 (10.2 to 120)**	**88.5 (35.5 to 145)**	-13.9 (-37.8 to 10.2)
9 Stroke	**32.8 (2.77 to 66.6)**	**57 (25.4 to 91.2)**	**-33.5 (-45.2 to -18.4)**
10 Chronic kidney disease	**240 (144 to 353)**	**302 (200 to 421)**	33.3 (-1.2 to 75.1)
11 Pancreatic cancer	**93.2 (36.9 to 170)**	**128 (71.3 to 194)**	18.5 (-8.14 to 50.2)
12 Age-related hearing loss	**68.6 (42.5 to 99.3)**	**99.4 (80.8 to 123)**	**-0.517 (-0.675 to -0.346)**
13 Liver cancer	**83.5 (46.1 to 128)**	**117 (78.8 to 166)**	**21.3 (0.0134 to 47.4)**
14 Prostate cancer	**129 (67.8 to 193)**	**170 (110 to 231)**	-1.65 (-17.5 to 14.4)
15 Osteoarthritis	**45.3 (27.4 to 64.8)**	**71.9 (64.9 to 79.8)**	**1.33 (1.1 to 1.56)**
16 Depressive disorders	**-16.3 (-25.8 to -2.95)**	-0.937 (-7.32 to 7.72)	**-0.179 (-0.28 to -0.0582)**
17 Hypertensive heart disease	**188 (115 to 268)**	**241 (156 to 341)**	17.5 (-12.7 to 58.1)
18 Breast cancer	19.3 (-9.18 to 52.3)	**41.1 (11.1 to 72.4)**	-10.7 (-28.9 to 6.65)
19 Anxiety disorders	**-20.4 (-31.3 to -7.95)**	-5.73 (-13.7 to 3.32)	**0.444 (0.321 to 0.585)**
20 Atrial fibrillation	**166 (124 to 211)**	**215 (177 to 257)**	**9.63 (2.66 to 16.9)**
22 Headache disorders			
25 Road injuries			
28 Gynecological diseases			
150 COVID-19			

Supplemental Results Figure S7. Leading 20 level 3 causes of DALYs, and percentage change in number of DALYs and all-age and age-standardised DALY rates by location, 2022 and 2050

Austria, Reference scenario

Legend:
Communicable, maternal, neonatal and nutritional
Non-communicable
Injuries

Leading Causes 2022	Leading Causes 2050	Mean % change number of DALY	Mean % change all-age DALY rate	Mean % change age-standardized DALY rate
1 Ischemic heart disease	1 Ischemic heart disease	-15 (-31.6 to 3.04)	**-22.1 (-37.6 to -5.7)**	**-51.7 (-62.3 to -40.3)**
2 Low back pain	2 Alzheimer's disease	**74.2 (52.8 to 96.1)**	**59.4 (48.3 to 70.9)**	**-9.94 (-14 to -6)**
3 Falls	3 Falls	**24.5 (8.77 to 40)**	**13.9 (4.72 to 24.6)**	**-15.7 (-22.2 to -8.8)**
4 Alzheimer's disease	4 Low back pain	**15.2 (5.3 to 25.3)**	**5.5 (2.61 to 8.92)**	**-1.91 (-1.99 to -1.83)**
5 COVID-19	5 Chronic kidney disease	**153 (105 to 202)**	**131 (87.7 to 171)**	**26.3 (3.21 to 50.7)**
6 Stroke	6 COPD	**37.5 (14.5 to 59.3)**	**26 (8.51 to 43.7)**	**-14.5 (-27.5 to -0.856)**
7 Lung cancer	7 Stroke	4.58 (-6.83 to 18.5)	-4.25 (-12.6 to 4.23)	**-34.5 (-40.6 to -27.7)**
8 COPD	8 Diabetes	**39.6 (23.7 to 58.3)**	**27.8 (15.9 to 40.4)**	2.6 (-5.2 to 9.98)
9 Diabetes	9 Lung cancer	3.51 (-13.6 to 21)	-5.26 (-18.5 to 10.8)	**-22.7 (-33.5 to -9.86)**
10 Anxiety disorders	10 Atrial fibrillation	**87.8 (64.2 to 112)**	**71.9 (57.7 to 86.3)**	3.45 (-2.13 to 10.9)
11 Headache disorders	11 Anxiety disorders	7.53 (-1.18 to 16.1)	-1.54 (-3.77 to 0.936)	**0.182 (0.0551 to 0.302)**
12 Depressive disorders	12 Headache disorders	3.52 (-5.06 to 11.9)	**-5.22 (-6.7 to -2.37)**	-0.0561 (-0.272 to 0.077)
13 Gynecological diseases	13 Hypertensive heart disease	**89.9 (56.7 to 127)**	**73.9 (45.7 to 108)**	0.606 (-17.7 to 23.6)
14 Age-related hearing loss	14 Age-related hearing loss	**43.7 (29.4 to 57.8)**	**31.5 (25.7 to 38.1)**	**-0.198 (-0.317 to -0.0457)**
15 Colorectal cancer	15 Pancreatic cancer	**58.4 (29.7 to 94.2)**	**45 (20 to 73.4)**	10.3 (-8.27 to 31.9)
16 Self-harm	16 Depressive disorders	8.43 (-0.406 to 17.1)	-0.729 (-2.51 to 1.32)	**-1.01 (-1.13 to -0.885)**
17 Neck pain	17 Colorectal cancer	**27.3 (0.0689 to 58.4)**	16.5 (-5.55 to 41.2)	**-18.5 (-33.1 to -1.29)**
18 Osteoarthritis	18 Osteoarthritis	**32.1 (20 to 43.7)**	**20.9 (17.2 to 24.6)**	**0.801 (0.641 to 0.971)**
19 Chronic kidney disease	19 Other musculoskeletal	**23.6 (12.2 to 35.8)**	**13.1 (9.65 to 17.2)**	**17.4 (16.3 to 18.1)**
20 Other musculoskeletal	20 Oral disorders	**27.4 (15.6 to 40.4)**	**16.6 (11.7 to 20.8)**	-0.0187 (-0.149 to 0.123)
23 Oral disorders	21 Neck pain			
25 Atrial fibrillation	22 Gynecological diseases			
26 Pancreatic cancer	28 Self-harm			
27 Hypertensive heart disease	153 COVID-19			

Supplemental Results Figure S7. Leading 20 level 3 causes of DALYs, and percentage change in number of DALYs and all-age and age-standardised DALY rates by location, 2022 and 2050

Belgium, Reference scenario

Legend:
Communicable, maternal, neonatal and nutritional
Non-communicable
Injuries

Leading Causes 2022	Leading Causes 2050	Mean % change number of DALY	Mean % change all-age DALY rate	Mean % change age-standardized DALY rate
1 Ischemic heart disease	1 Alzheimer's disease	**60.1 (41.4 to 78.2)**	**42.7 (30.8 to 52.9)**	**-8.52 (-12.7 to -4.57)**
2 Low back pain	2 Falls	**21.8 (9.17 to 35.6)**	**8.63 (1.04 to 16.9)**	**-13.3 (-19.2 to -6.96)**
3 Falls	3 Low back pain	**14.2 (4.01 to 25)**	1.82 (-0.721 to 4.57)	**-2.31 (-2.38 to -2.22)**
4 Lung cancer	4 Diabetes	**53.7 (38.8 to 67.9)**	**37 (30.4 to 43.7)**	**26 (20.4 to 31.1)**
5 Alzheimer's disease	5 Lung cancer	-1.85 (-18.1 to 17.7)	-12.5 (-24.6 to 2.92)	**-23.4 (-33.3 to -9.57)**
6 Stroke	6 COPD	**22 (3.59 to 42.7)**	8.77 (-5.81 to 24.1)	**-18.1 (-29.4 to -4.3)**
7 COPD	7 Ischemic heart disease	**-28 (-41.6 to -9.98)**	**-35.8 (-47.8 to -20.4)**	**-54.3 (-62.7 to -42.9)**
8 COVID-19	8 Stroke	-6.2 (-18.8 to 6.59)	**-16.4 (-24.5 to -7.53)**	**-35.7 (-42.7 to -27.7)**
9 Headache disorders	9 Lower respiratory infect	**38.5 (19.9 to 60.5)**	**23.5 (8.43 to 43.9)**	**-20.1 (-30 to -8.62)**
10 Diabetes	10 Headache disorders	**10.5 (1.47 to 20.2)**	-1.47 (-2.69 to 0.364)	-0.0114 (-0.141 to 0.139)
11 Depressive disorders	11 Depressive disorders	**12.2 (2.79 to 22.3)**	0.0576 (-1.51 to 1.77)	**-1.29 (-1.4 to -1.18)**
12 Lower respiratory infect	12 Chronic kidney disease	**147 (102 to 201)**	**120 (86.2 to 161)**	**36.7 (13.4 to 67.3)**
13 Self-harm	13 Colorectal cancer	**28.2 (0.964 to 60.2)**	14.3 (-8.68 to 39.8)	-11.3 (-28.6 to 7.65)
14 Anxiety disorders	14 Age-related hearing loss	**35.8 (23.6 to 48.9)**	**21.1 (16 to 26.8)**	**-0.548 (-0.693 to -0.396)**
15 Colorectal cancer	15 Anxiety disorders	**11.7 (1.91 to 20.9)**	-0.385 (-1.83 to 1.05)	**0.426 (0.35 to 0.498)**
16 Gynecological diseases	16 Pancreatic cancer	**56.9 (27.2 to 92.9)**	**39.8 (15.6 to 64.7)**	17.8 (-3.55 to 38.3)
17 Age-related hearing loss	17 Osteoarthritis	**26.5 (15 to 38)**	**12.8 (8.78 to 16.7)**	**1.8 (1.61 to 2)**
18 Breast cancer	18 Breast cancer	14.5 (-3.1 to 35.8)	2.02 (-11.6 to 18.3)	**-13.2 (-25.1 to -0.947)**
19 Oral disorders	19 Oral disorders	**22.9 (12.2 to 34.3)**	**9.55 (5.71 to 13.8)**	**0.392 (0.265 to 0.541)**
20 Osteoarthritis	20 Self-harm	**-12.1 (-25 to -0.257)**	**-21.6 (-30.5 to -12.2)**	**-22.6 (-32.1 to -12.3)**
23 Pancreatic cancer	22 Gynecological diseases			
27 Chronic kidney disease	149 COVID-19			

Supplemental Results Figure S7. Leading 20 level 3 causes of DALYs, and percentage change in number of DALYs and all-age and age-standardised DALY rates by location, 2022 and 2050

Cyprus, Reference scenario

Legend:
Communicable, maternal, neonatal and nutritional
Non-communicable
Injuries

Leading Causes 2022	Leading Causes 2050	Mean % change number of DALY	Mean % change all-age DALY rate	Mean % change age-standardized DALY rate
1 Ischemic heart disease	1 Diabetes	**77.1 (48.2 to 104)**	**60.4 (38.4 to 80.6)**	3.37 (-8.86 to 16.4)
2 Low back pain	2 Ischemic heart disease	-7.72 (-34.2 to 22.8)	-16.4 (-37.7 to 11.4)	**-54.2 (-66.7 to -35.2)**
3 Diabetes	3 Low back pain	**27.5 (13.2 to 44.7)**	**15.5 (9.81 to 20.6)**	**-2.43 (-2.62 to -2.2)**
4 COVID-19	4 Alzheimer's disease	**115 (82 to 150)**	**94.6 (76.9 to 115)**	**-7.47 (-11.7 to -3.77)**
5 Stroke	5 COPD	**100 (57 to 149)**	**81.5 (49.8 to 118)**	-8.1 (-26.4 to 14)
6 Falls	6 Falls	**48.2 (26.1 to 70.1)**	**34.2 (21.5 to 47.3)**	**-11 (-17.5 to -3.73)**
7 Headache disorders	7 Stroke	15.3 (-6.21 to 36.5)	4.41 (-12.4 to 23.1)	**-46.1 (-55 to -34.5)**
8 Anxiety disorders	8 Chronic kidney disease	**183 (114 to 270)**	**156 (95.9 to 225)**	18.5 (-10.2 to 56.1)
9 Lung cancer	9 Lung cancer	**32.6 (1.26 to 65.7)**	20 (-6.83 to 48.5)	**-23.5 (-39.9 to -7.84)**
10 COPD	10 Anxiety disorders	10.1 (-2.33 to 23.7)	-0.337 (-5.07 to 6.07)	0.139 (-0.177 to 0.466)
11 Alzheimer's disease	11 Headache disorders	3.48 (-8.83 to 18.1)	-6.32 (-10.3 to 1.33)	**-0.638 (-1.09 to -0.268)**
12 Depressive disorders	12 Other musculoskeletal	**53.2 (33.6 to 72.7)**	**38.7 (29.7 to 51.2)**	**27.3 (25.3 to 28.8)**
13 Gynecological diseases	13 Depressive disorders	12 (-1.95 to 26)	1.43 (-2.98 to 7.32)	**-1.86 (-2.22 to -1.49)**
14 Other musculoskeletal	14 Age-related hearing loss	**67.5 (46.6 to 89.2)**	**51.6 (42.5 to 60)**	**-0.791 (-0.979 to -0.596)**
15 Road injuries	15 Colorectal cancer	**69.7 (22.3 to 129)**	**53.6 (13.6 to 97.3)**	-11.6 (-32.5 to 11.2)
16 Breast cancer	16 Osteoarthritis	**69.6 (48.1 to 89.5)**	**53.5 (46.2 to 61)**	**2.31 (2.05 to 2.6)**
17 Age-related hearing loss	17 Breast cancer	**42.1 (13.5 to 73.4)**	**28.7 (6.87 to 53.3)**	**-17.2 (-30.2 to -2.49)**
18 Neck pain	18 Hypertensive heart disease	**161 (95.3 to 222)**	**136 (88.3 to 192)**	0.751 (-22.8 to 34.7)
19 Colorectal cancer	19 Oral disorders	**49.7 (30.6 to 68.2)**	**35.6 (27.5 to 45.2)**	**0.39 (0.223 to 0.586)**
20 Osteoarthritis	20 Prostate cancer	**92.2 (51.6 to 139)**	**74 (42 to 113)**	-3.89 (-19.1 to 12.1)
21 Chronic kidney disease	21 Gynecological diseases			
22 Oral disorders	26 Neck pain			
24 Prostate cancer	27 Road injuries			
27 Hypertensive heart disease	150 COVID-19			

Supplemental Results Figure S7. Leading 20 level 3 causes of DALYs, and percentage change in number of DALYs and all-age and age-standardised DALY rates by location, 2022 and 2050

Denmark, Reference scenario

Legend:
Communicable, maternal, neonatal and nutritional
Non-communicable
Injuries

Leading Causes 2022	Leading Causes 2050	Mean % change number of DALY	Mean % change all-age DALY rate	Mean % change age-standardized DALY rate
1 Low back pain	1 Low back pain	**11.6 (3 to 20.2)**	0.707 (-1.29 to 3)	**-1.62 (-1.67 to -1.56)**
2 COVID-19	2 COPD	**21.7 (4.44 to 42.1)**	9.82 (-4.28 to 25.6)	**-15.1 (-25.1 to -3.27)**
3 COPD	3 Lung cancer	1.96 (-12.2 to 17.7)	-8.03 (-19 to 3.79)	**-18.8 (-27.4 to -8.81)**
4 Lung cancer	4 Alzheimer's disease	**54.4 (38.1 to 73.2)**	**39.3 (28.8 to 50.7)**	**-6.55 (-10.5 to -2.04)**
5 Ischemic heart disease	5 Falls	**33.1 (20.3 to 47.8)**	**20.1 (11.7 to 30.2)**	-2.32 (-9.31 to 4.01)
6 Stroke	6 Diabetes	**27.5 (13 to 42.9)**	**15 (7.22 to 24.1)**	4.83 (-3.95 to 12.2)
7 Falls	7 Stroke	**-19.2 (-27.5 to -9.14)**	**-27.1 (-33.2 to -21.1)**	**-41.4 (-46 to -36.2)**
8 Alzheimer's disease	8 Ischemic heart disease	**-36.3 (-46.2 to -22.9)**	**-42.6 (-51.3 to -31.4)**	**-57.1 (-63.8 to -48.5)**
9 Diabetes	9 Colorectal cancer	15.3 (-7.52 to 42.3)	4.01 (-14 to 23.1)	**-15.2 (-29 to -0.454)**
10 Colorectal cancer	10 Chronic kidney disease	**114 (79.3 to 155)**	**93.4 (65.8 to 126)**	**31.2 (11.4 to 53.9)**
11 Depressive disorders	11 Lower respiratory infect	**40.4 (19.4 to 62.9)**	**26.6 (12.2 to 42.8)**	**-16.4 (-25 to -7.17)**
12 Headache disorders	12 Depressive disorders	**11 (2.54 to 19.6)**	0.12 (-1.12 to 1.49)	**-0.994 (-1.07 to -0.919)**
13 Anxiety disorders	13 Other musculoskeletal	**30.1 (19.7 to 41)**	**17.4 (14.8 to 20.9)**	**17.9 (16.8 to 18.8)**
14 Alcohol use disorders	14 Headache disorders	**9.8 (1.43 to 18.1)**	-0.937 (-1.85 to 0.468)	0.0282 (-0.0585 to 0.111)
15 Lower respiratory infect	15 Prostate cancer	**46.5 (23.3 to 70.2)**	**32.1 (14.9 to 50.1)**	2.62 (-7.02 to 14.3)
16 Other musculoskeletal	16 Anxiety disorders	**10.4 (1.81 to 18.6)**	-0.376 (-1.41 to 0.679)	**0.257 (0.208 to 0.308)**
17 Gynecological diseases	17 Alcohol use disorders	8.1 (-1.6 to 18)	-2.45 (-7.81 to 2.76)	-1.52 (-6.21 to 2.47)
18 Prostate cancer	18 Pancreatic cancer	**43.4 (20.4 to 71)**	**29.3 (9.62 to 47.9)**	14.3 (-1.44 to 30.4)
19 Breast cancer	19 Age-related hearing loss	**27.3 (15.8 to 38.2)**	**14.9 (10.5 to 18.8)**	**-0.378 (-0.479 to -0.249)**
20 Age-related hearing loss	20 Osteoarthritis	**19.6 (9.81 to 29.3)**	**7.89 (5.42 to 10.7)**	**1.24 (1.09 to 1.4)**
22 Osteoarthritis	22 Breast cancer			
24 Pancreatic cancer	26 Gynecological diseases			
25 Chronic kidney disease	148 COVID-19			

Supplemental Results Figure S7. Leading 20 level 3 causes of DALYs, and percentage change in number of DALYs and all-age and age-standardised DALY rates by location, 2022 and 2050

Finland, Reference scenario

Legend:
Communicable, maternal, neonatal and nutritional
Non-communicable
Injuries

Leading Causes 2022	Leading Causes 2050	Mean % change number of DALY	Mean % change all-age DALY rate	Mean % change age-standardized DALY rate
1 COVID-19	1 Alzheimer's disease	**63.8 (44 to 84.6)**	**67.7 (53.8 to 79.4)**	0.668 (-3.93 to 5.01)
2 Ischemic heart disease	2 Ischemic heart disease	**-33.9 (-47.3 to -19.1)**	**-32.2 (-45.5 to -18.8)**	**-56.1 (-65.2 to -44.6)**
3 Falls	3 Falls	13.5 (-0.00941 to 26)	**16.2 (7.67 to 25.1)**	**-7.99 (-14.2 to -1.71)**
4 Stroke	4 Low back pain	-0.564 (-10.1 to 9.29)	1.84 (-0.232 to 4.39)	**-2.31 (-2.38 to -2.25)**
5 Low back pain	5 Diabetes	**26.8 (14.1 to 39.1)**	**29.9 (25.6 to 35)**	**19.1 (15.6 to 22.5)**
6 Alzheimer's disease	6 Stroke	**-18.6 (-28.3 to -5.64)**	**-16.6 (-23.4 to -8.14)**	**-36.7 (-43.5 to -29.6)**
7 Diabetes	7 Lung cancer	-3.09 (-19.3 to 15.7)	-0.758 (-15.2 to 16)	**-14.3 (-26.6 to -2.44)**
8 Lung cancer	8 Depressive disorders	-2.78 (-11.4 to 6.16)	-0.414 (-1.77 to 0.962)	**-1.07 (-1.14 to -0.989)**
9 Depressive disorders	9 Hypertensive heart disease	**64.5 (32.2 to 104)**	**68.5 (38.5 to 103)**	8.65 (-10.3 to 32.5)
10 Headache disorders	10 COPD	**21.7 (4.99 to 42.5)**	**24.7 (11.1 to 42.6)**	-6.73 (-18.6 to 7.18)
11 Age-related hearing loss	11 Pancreatic cancer	**46.6 (15.5 to 77.2)**	**50.1 (24.4 to 77.3)**	**25.6 (6.04 to 45.2)**
12 COPD	12 Age-related hearing loss	**20 (7.44 to 32.5)**	**22.9 (17.7 to 28.1)**	**-0.6 (-0.78 to -0.415)**
13 Self-harm	13 Headache disorders	-3.64 (-12.7 to 5.49)	-1.29 (-2.84 to 2.03)	**0.296 (0.123 to 0.375)**
14 Colorectal cancer	14 Colorectal cancer	16.7 (-6.82 to 44)	19.5 (-3.11 to 44.4)	-6.93 (-23 to 11.5)
15 Cirrhosis liver	15 Prostate cancer	**43.6 (18.8 to 70.7)**	**47 (26.3 to 67.4)**	**12 (1.22 to 24.6)**
16 Pancreatic cancer	16 Osteoarthritis	9.61 (-1.66 to 20.1)	**12.3 (9.77 to 14.7)**	**1.37 (1.15 to 1.58)**
17 Osteoarthritis	17 Drug use disorders	**14.1 (0.26 to 28.4)**	**16.9 (6.22 to 27.8)**	**17.3 (6.02 to 29.9)**
18 Alcohol use disorders	18 Atrial fibrillation	**56.1 (34.6 to 77.8)**	**59.9 (43.5 to 75.8)**	**6.81 (0.497 to 13.3)**
19 Gynecological diseases	19 Oral disorders	6.31 (-4.1 to 16.5)	**8.89 (6.27 to 11.4)**	**0.301 (0.172 to 0.455)**
20 Hypertensive heart disease	20 Alcohol use disorders	-3.25 (-13.8 to 8.32)	-0.875 (-7.19 to 6.68)	-1.74 (-7.61 to 3.53)
22 Drug use disorders	25 Self-harm			
23 Oral disorders	26 Cirrhosis liver			
25 Prostate cancer	27 Gynecological diseases			
27 Atrial fibrillation	150 COVID-19			

Supplemental Results Figure S7. Leading 20 level 3 causes of DALYs, and percentage change in number of DALYs and all-age and age-standardised DALY rates by location, 2022 and 2050

France, Reference scenario

Legend:
Communicable, maternal, neonatal and nutritional
Non-communicable
Injuries

Leading Causes 2022	Leading Causes 2050	Mean % change number of DALY	Mean % change all-age DALY rate	Mean % change age-standardized DALY rate
1 Low back pain	1 Falls	**24.6 (11 to 39.2)**	**28.2 (17.4 to 38.3)**	-5.26 (-12.6 to 2.07)
2 Falls	2 Alzheimer's disease	**61.5 (42.5 to 80.7)**	**66.2 (56.1 to 77.2)**	**-3.76 (-7.63 to -0.146)**
3 Lung cancer	3 Low back pain	1.81 (-8.51 to 12.1)	**4.81 (1.41 to 8.04)**	**-2.68 (-2.77 to -2.6)**
4 Ischemic heart disease	4 Lung cancer	5.46 (-11.4 to 22.4)	8.54 (-5.18 to 23.9)	-7.11 (-18.8 to 5.72)
5 COVID-19	5 Diabetes	**51.1 (33.4 to 70.4)**	**55.6 (43.7 to 70.8)**	**28.3 (19.3 to 35.7)**
6 Alzheimer's disease	6 Ischemic heart disease	**-21.3 (-36 to -5.8)**	**-19 (-32.4 to -3.81)**	**-49.5 (-57.9 to -40.1)**
7 Stroke	7 Stroke	-8.46 (-20.4 to 3.98)	-5.76 (-14.1 to 3.48)	**-34.7 (-40.8 to -28.1)**
8 Depressive disorders	8 Other cardiovascular	**20.4 (5.97 to 34.6)**	**24 (14.9 to 35.6)**	**-10.6 (-17.4 to -3.49)**
9 Anxiety disorders	9 Colorectal cancer	**24.5 (0.834 to 53.5)**	**28.2 (5.99 to 56.1)**	-10 (-24.5 to 7.42)
10 Headache disorders	10 Depressive disorders	-2.09 (-13.1 to 8.21)	0.785 (-1.2 to 3.13)	**-1.68 (-1.84 to -1.52)**
11 Other cardiovascular	11 Anxiety disorders	-4.2 (-13.8 to 7.08)	-1.39 (-3.72 to 0.926)	0.0455 (-0.091 to 0.183)
12 Colorectal cancer	12 Age-related hearing loss	**27 (12.9 to 40.5)**	**30.8 (24.6 to 37.8)**	**-0.464 (-0.561 to -0.352)**
13 Diabetes	13 Headache disorders	-5.76 (-16.6 to 4.5)	-2.99 (-4.65 to 0.388)	**-0.273 (-0.613 to -0.0782)**
14 Age-related hearing loss	14 COPD	**39 (19 to 62.2)**	**43.1 (25.2 to 62.7)**	-7.87 (-18.9 to 4.41)
15 Breast cancer	15 Chronic kidney disease	**140 (98.8 to 197)**	**147 (106 to 195)**	**34.5 (13.7 to 58.4)**
16 Gynecological diseases	16 Pancreatic cancer	**55.1 (26.5 to 83.7)**	**59.7 (35.2 to 86.8)**	**24.5 (5.35 to 46.3)**
17 Self-harm	17 Lower respiratory infect	**49.3 (24.3 to 73)**	**53.7 (33.2 to 72.9)**	**-15.5 (-25.3 to -4.91)**
18 COPD	18 Osteoarthritis	**17.6 (5.78 to 29.6)**	**21.1 (16.4 to 25.2)**	**1.81 (1.6 to 2.07)**
19 Osteoarthritis	19 Breast cancer	4.93 (-11.5 to 22.8)	8.01 (-4.73 to 22.4)	**-15.2 (-24.8 to -6.29)**
20 Other musculoskeletal	20 Other musculoskeletal	**23.6 (10.9 to 36)**	**27.2 (22.8 to 32.4)**	**26.8 (25 to 28.2)**
21 Lower respiratory infect	24 Gynecological diseases			
22 Pancreatic cancer	27 Self-harm			
31 Chronic kidney disease	149 COVID-19			

Supplemental Results Figure S7. Leading 20 level 3 causes of DALYs, and percentage change in number of DALYs and all-age and age-standardised DALY rates by location, 2022 and 2050

Germany, Reference scenario

Legend:
Communicable, maternal, neonatal and nutritional
Non-communicable
Injuries

Leading Causes 2022	Leading Causes 2050	Mean % change number of DALY	Mean % change all-age DALY rate	Mean % change age-standardized DALY rate
1 Ischemic heart disease	1 Alzheimer's disease	**49.1 (30 to 66)**	**48.3 (39.5 to 57.3)**	-0.222 (-4.08 to 4.15)
2 Low back pain	2 Low back pain	0.878 (-9.68 to 9.42)	0.343 (-1.82 to 2.52)	**-1.55 (-1.65 to -1.46)**
3 Alzheimer's disease	3 Ischemic heart disease	**-34.3 (-47 to -21.6)**	**-34.7 (-46.3 to -23.9)**	**-52.5 (-60.9 to -43.8)**
4 Stroke	4 Falls	11.8 (-3 to 27.4)	**11.2 (1.05 to 21.9)**	**-12.3 (-19.4 to -4.81)**
5 Lung cancer	5 Chronic kidney disease	**113 (76.2 to 154)**	**111 (76.3 to 151)**	**36.3 (14.9 to 61.1)**
6 Falls	6 COPD	13.7 (-1.66 to 29.7)	13.1 (-0.0178 to 25.7)	**-10.8 (-21.1 to -0.385)**
7 COVID-19	7 Lung cancer	-9.43 (-23.6 to 5.27)	-9.91 (-19.6 to 1.83)	**-18.6 (-26.9 to -7.5)**
8 COPD	8 Stroke	**-18.9 (-28.4 to -8.82)**	**-19.4 (-24.7 to -13)**	**-34.4 (-40.1 to -28.9)**
9 Diabetes	9 Diabetes	12.3 (-0.929 to 26.7)	**11.7 (3.82 to 19.2)**	0.934 (-5.2 to 6.34)
10 Headache disorders	10 Anxiety disorders	0.0947 (-9.36 to 8.55)	-0.428 (-1.79 to 0.717)	**0.466 (0.234 to 0.681)**
11 Anxiety disorders	11 Hypertensive heart disease	**66.1 (38.4 to 98.8)**	**65.2 (39.7 to 90.7)**	11.3 (-6.89 to 29.7)
12 Depressive disorders	12 Headache disorders	-2.33 (-11.5 to 5.34)	**-2.84 (-3.8 to -1.87)**	0.329 (-0.0402 to 0.553)
13 Colorectal cancer	13 Colorectal cancer	9.67 (-9.95 to 33.6)	9.09 (-6.3 to 27.5)	-12.3 (-23.9 to 1.89)
14 Chronic kidney disease	14 Atrial fibrillation	**59.1 (40 to 80.5)**	**58.2 (44.5 to 73.4)**	**11.5 (4.17 to 18.1)**
15 Age-related hearing loss	15 Depressive disorders	0.185 (-9.55 to 8.29)	-0.341 (-1.77 to 0.969)	**-0.545 (-0.698 to -0.378)**
16 Breast cancer	16 Pancreatic cancer	**38.6 (16.9 to 63.7)**	**37.9 (19.9 to 59.5)**	**18.8 (3.98 to 35.9)**
17 Gynecological diseases	17 Age-related hearing loss	**18.2 (5.1 to 29.8)**	**17.6 (13.4 to 21.6)**	-0.0994 (-0.217 to 0.0214)
18 Cirrhosis liver	18 Other musculoskeletal	**12.8 (2.03 to 22.7)**	**12.3 (10.1 to 14.8)**	**15.8 (15.1 to 16.5)**
19 Other musculoskeletal	19 Osteoarthritis	9.66 (-1.7 to 20)	**9.07 (6.44 to 11.8)**	**0.632 (0.457 to 0.814)**
20 Osteoarthritis	20 Breast cancer	0.646 (-14.3 to 17.6)	0.0955 (-10.2 to 15.1)	**-14.6 (-23.4 to -4.78)**
21 Pancreatic cancer	24 Cirrhosis liver			
22 Atrial fibrillation	25 Gynecological diseases			
24 Hypertensive heart disease	147 COVID-19			

Supplemental Results Figure S7. Leading 20 level 3 causes of DALYs, and percentage change in number of DALYs and all-age and age-standardised DALY rates by location, 2022 and 2050

Greece, Reference scenario

Legend:
Communicable, maternal, neonatal and nutritional
Non-communicable
Injuries

Leading Causes 2022
1 Ischemic heart disease
2 COVID-19
3 Stroke
4 Lung cancer
5 Low back pain
6 Alzheimer's disease
7 Depressive disorders
8 Diabetes
9 COPD
10 Chronic kidney disease
11 Lower respiratory infect
12 Falls
13 Headache disorders
14 Anxiety disorders
15 Colorectal cancer
16 Age-related hearing loss
17 Breast cancer
18 Road injuries
19 Other musculoskeletal
20 Gynecological diseases
21 Osteoarthritis
22 Pancreatic cancer
25 Hypertensive heart disease

Leading Causes 2050	Mean % change number of DALY	Mean % change all-age DALY rate	Mean % change age-standardized DALY rate
1 Alzheimer's disease	**33.5 (14.1 to 55.2)**	**69.2 (55.1 to 83.5)**	0.733 (-3.67 to 5.69)
2 Ischemic heart disease	**-43.1 (-57.7 to -28.5)**	**-27.9 (-43 to -12.1)**	**-51.4 (-64.3 to -38.6)**
3 Stroke	**-29.2 (-41.6 to -16.1)**	-10.3 (-20.7 to 0.311)	**-39.5 (-47 to -33)**
4 Lung cancer	-8.31 (-26.6 to 13)	16.1 (-0.961 to 36.7)	**-14.5 (-26.8 to -0.269)**
5 Chronic kidney disease	**55.2 (23 to 92.6)**	**96.6 (58.3 to 137)**	16.6 (-3.93 to 40.2)
6 Diabetes	**26.2 (7.21 to 46.4)**	**59.9 (47.7 to 71.5)**	**31.8 (25.8 to 37)**
7 Low back pain	**-14.1 (-28.2 to -1.25)**	**8.73 (3.89 to 13.7)**	**-2.87 (-2.95 to -2.79)**
8 COPD	13 (-7.73 to 32.8)	**43.2 (22.2 to 64.9)**	-10.4 (-24 to 3.96)
9 Lower respiratory infect	11.1 (-9.69 to 32.4)	**40.8 (23.8 to 59.3)**	**-19.3 (-28.9 to -8.17)**
10 Depressive disorders	**-21.2 (-34.2 to -7.9)**	-0.334 (-4.21 to 3.75)	**-1.79 (-1.93 to -1.63)**
11 Colorectal cancer	16.9 (-8.85 to 46.7)	**48 (19.1 to 83.5)**	-0.436 (-19.3 to 25.2)
12 Falls	-2.36 (-17.9 to 14.1)	**23.7 (10.8 to 36)**	-5.24 (-13.7 to 3.42)
13 Pancreatic cancer	**49.7 (17.8 to 85.8)**	**89.5 (57.3 to 133)**	**36.2 (14.8 to 64.2)**
14 Age-related hearing loss	9.95 (-7.6 to 26.9)	**39.3 (30.7 to 47.2)**	**-0.726 (-0.79 to -0.657)**
15 Breast cancer	0.375 (-18.7 to 24)	**27.1 (8.35 to 50.2)**	-8.29 (-20.5 to 6.84)
16 Other musculoskeletal	11.3 (-8.93 to 30.3)	**40.9 (31.9 to 49.9)**	**33.4 (32.4 to 34.3)**
17 Hypertensive heart disease	**40 (9.24 to 71.6)**	**77.5 (42.2 to 111)**	8.74 (-13.5 to 32.8)
18 Headache disorders	**-27.8 (-40.5 to -15.4)**	**-8.63 (-12 to -3.49)**	-0.198 (-0.432 to 0.0322)
19 Anxiety disorders	**-22.6 (-36.1 to -8.99)**	-2.04 (-5.84 to 3.04)	**0.272 (0.143 to 0.381)**
20 Osteoarthritis	5.25 (-10.5 to 20.8)	**33.3 (25.8 to 40.7)**	**2.29 (2.03 to 2.55)**
25 Road injuries			
28 Gynecological diseases			
153 COVID-19			

Supplemental Results Figure S7. Leading 20 level 3 causes of DALYs, and percentage change in number of DALYs and all-age and age-standardised DALY rates by location, 2022 and 2050

Iceland, Reference scenario

Legend:
Communicable, maternal, neonatal and nutritional
Non-communicable
Injuries

Leading Causes 2022	Leading Causes 2050	Mean % change number of DALY	Mean % change all-age DALY rate	Mean % change age-standardized DALY rate
1 Ischemic heart disease	1 Low back pain	**44.1 (30.4 to 58.4)**	**3.69 (1.06 to 6.93)**	**-1.55 (-1.62 to -1.48)**
2 Low back pain	2 Alzheimer's disease	**117 (91.2 to 143)**	**56.5 (41.9 to 71)**	**-10.2 (-14.3 to -6.15)**
3 Lung cancer	3 Ischemic heart disease	-4.09 (-26.9 to 23.5)	**-31 (-45.1 to -12.5)**	**-54.8 (-64.7 to -41.6)**
4 Falls	4 Falls	**55.2 (35.7 to 75.2)**	**11.6 (1.61 to 23.2)**	**-13.6 (-20.9 to -5.24)**
5 Alzheimer's disease	5 Lung cancer	**29.6 (6.6 to 51.1)**	-6.73 (-22.1 to 8.5)	**-22.6 (-34.9 to -10.3)**
6 COVID-19	6 COPD	**72.4 (42.1 to 110)**	**24 (5.46 to 48.1)**	-16 (-30.2 to 2.64)
7 Headache disorders	7 Diabetes	**78.2 (59.7 to 97.8)**	**28.2 (21.5 to 35.9)**	**13 (8.58 to 17)**
8 Stroke	8 Headache disorders	**37 (24.9 to 50.2)**	-1.42 (-2.8 to 1.47)	**0.334 (0.207 to 0.436)**
9 COPD	9 Stroke	**25.9 (9.59 to 50.9)**	-9.46 (-19.7 to 2.23)	**-36 (-43.3 to -28.6)**
10 Anxiety disorders	10 Anxiety disorders	**38.6 (25.5 to 53.8)**	-0.313 (-1.74 to 1.25)	**0.394 (0.312 to 0.478)**
11 Diabetes	11 Drug use disorders	**59 (38.6 to 84)**	**14.4 (3.36 to 27.8)**	**16.8 (4.69 to 32.1)**
12 Depressive disorders	12 Depressive disorders	**40.1 (27.4 to 53.5)**	0.789 (-0.765 to 2.8)	**-0.734 (-0.846 to -0.615)**
13 Drug use disorders	13 Other musculoskeletal	**64.2 (50.6 to 78.1)**	**18.2 (14.9 to 22)**	**17.7 (16.8 to 18.5)**
14 Self-harm	14 Age-related hearing loss	**73.8 (57.7 to 91.9)**	**25 (18 to 32.2)**	**-0.417 (-0.506 to -0.329)**
15 Other musculoskeletal	15 Lower respiratory infect	**87.5 (58 to 124)**	**34.9 (16.1 to 58.8)**	**-21.3 (-31.1 to -10.6)**
16 Gynecological diseases	16 Atrial fibrillation	**149 (117 to 180)**	**78.9 (60.5 to 97.8)**	**10.2 (3.04 to 16.6)**
17 Age-related hearing loss	17 Osteoarthritis	**61.2 (46.3 to 76.9)**	**16 (10.5 to 21.2)**	**1.28 (1.13 to 1.44)**
18 Osteoarthritis	18 Prostate cancer	**98.2 (63.8 to 133)**	**42.7 (21 to 71.1)**	-2.8 (-14.6 to 11.6)
19 Lower respiratory infect	19 Pancreatic cancer	**90.5 (51.9 to 128)**	**37.1 (9.38 to 65.1)**	9.99 (-11.1 to 33.3)
20 Colorectal cancer	20 Colorectal cancer	**53 (21.2 to 92.8)**	10.2 (-11.9 to 37.4)	-17.2 (-33.4 to 1.78)
24 Prostate cancer	21 Self-harm			
25 Pancreatic cancer	22 Gynecological diseases			
28 Atrial fibrillation	149 COVID-19			

Supplemental Results Figure S7. Leading 20 level 3 causes of DALYs, and percentage change in number of DALYs and all-age and age-standardised DALY rates by location, 2022 and 2050

Ireland, Reference scenario

Legend:
Communicable, maternal, neonatal and nutritional
Non-communicable
Injuries

Leading Causes 2022	Leading Causes 2050	Mean % change number of DALY	Mean % change all-age DALY rate	Mean % change age-standardized DALY rate
1 Ischemic heart disease	1 Low back pain	**36.8 (24.3 to 48.8)**	**8.38 (4.83 to 12)**	**-1.9 (-2.02 to -1.78)**
2 Low back pain	2 Alzheimer's disease	**140 (115 to 170)**	**90.5 (74.7 to 106)**	**-10.1 (-13.7 to -6.06)**
3 COVID-19	3 Ischemic heart disease	-5.02 (-20.9 to 13.9)	**-24.8 (-36.1 to -11.7)**	**-57.9 (-63.2 to -50.8)**
4 Depressive disorders	4 COPD	**79.6 (55.1 to 109)**	**42.3 (23.7 to 63.7)**	**-20.4 (-29.5 to -10.8)**
5 Lung cancer	5 Falls	**53.1 (35.3 to 70.2)**	**21.3 (11 to 31.2)**	**-9.6 (-17.4 to -1.86)**
6 Anxiety disorders	6 Depressive disorders	**27.4 (16.2 to 37.8)**	0.943 (-0.814 to 3.08)	**-1.35 (-1.51 to -1.19)**
7 COPD	7 Diabetes	**85.8 (66.9 to 104)**	**47.2 (39.8 to 55)**	**14.9 (10.3 to 18.7)**
8 Falls	8 Lung cancer	**26.2 (8.61 to 47)**	-0.0158 (-11.6 to 13.1)	**-27.1 (-35.2 to -19.9)**
9 Headache disorders	9 Anxiety disorders	**24.3 (11.6 to 35.6)**	-1.53 (-4.71 to 0.943)	0.0184 (-0.169 to 0.193)
10 Alzheimer's disease	10 Lower respiratory infect	**106 (76 to 136)**	**63.4 (44.5 to 87.1)**	**-23.6 (-31.2 to -15.2)**
11 Stroke	11 Headache disorders	**21.6 (9.71 to 34.3)**	-3.66 (-5.73 to 0.614)	**-0.436 (-0.703 to -0.168)**
12 Diabetes	12 Other musculoskeletal	**59.8 (45.1 to 74.8)**	**26.6 (20.6 to 32.8)**	**21 (19.9 to 21.9)**
13 Gynecological diseases	13 Colorectal cancer	**60.7 (32.2 to 89.4)**	**27.3 (5.8 to 44.6)**	**-16.7 (-29.7 to -4.61)**
14 Other musculoskeletal	14 Stroke	**18.7 (4.18 to 36.2)**	-5.97 (-14.4 to 3.46)	**-43.8 (-48 to -38.6)**
15 Colorectal cancer	15 Age-related hearing loss	**78.5 (60.5 to 96.1)**	**41.4 (33.4 to 49.8)**	**-0.627 (-0.736 to -0.524)**
16 Lower respiratory infect	16 Osteoarthritis	**63.8 (47.8 to 77.8)**	**29.8 (24.7 to 35)**	**1.84 (1.68 to 2)**
17 Age-related hearing loss	17 Chronic kidney disease	**204 (156 to 260)**	**141 (107 to 183)**	**17.9 (2.36 to 40.2)**
18 Drug use disorders	18 Drug use disorders	**46.9 (29.9 to 64.5)**	**16.4 (5.63 to 27.3)**	**16.9 (6.38 to 28.6)**
19 Osteoarthritis	19 Oral disorders	**57.8 (42.3 to 73.2)**	**25 (19 to 32.1)**	**0.249 (0.145 to 0.365)**
20 Breast cancer	20 Gynecological diseases	6.08 (-15.9 to 37.9)	-16 (-32 to 7.64)	-16.1 (-30.8 to 5.99)
22 Oral disorders	21 Breast cancer			
32 Chronic kidney disease	150 COVID-19			

Supplemental Results Figure S7. Leading 20 level 3 causes of DALYs, and percentage change in number of DALYs and all-age and age-standardised DALY rates by location, 2022 and 2050

Israel, Reference scenario

Legend:
Communicable, maternal, neonatal and nutritional
Non-communicable
Injuries

Leading Causes 2022
1 Low back pain
2 COVID-19
3 Diabetes
4 Depressive disorders
5 Ischemic heart disease
6 Headache disorders
7 Alzheimer's disease
8 Stroke
9 Lung cancer
10 Falls
11 Gynecological diseases
12 Anxiety disorders
13 Other musculoskeletal
14 Chronic kidney disease
15 COPD
16 Age-related hearing loss
17 Colorectal cancer
18 Breast cancer
19 Neck pain
20 Osteoarthritis
21 Oral disorders
26 Pancreatic cancer

Leading Causes 2050	Mean % change number of DALY	Mean % change all-age DALY rate	Mean % change age-standardized DALY rate
1 Low back pain	**46.2 (25.1 to 68.6)**	**7.1 (4.42 to 9.79)**	**-2.65 (-2.74 to -2.54)**
2 Diabetes	**80.3 (52.8 to 109)**	**32.2 (20.1 to 44.6)**	2.64 (-5.28 to 10.8)
3 Alzheimer's disease	**112 (83.1 to 146)**	**55.3 (44.3 to 67.6)**	**-10.5 (-14.2 to -7.18)**
4 Depressive disorders	**41.7 (22 to 63.2)**	**3.76 (1.79 to 5.95)**	**-1.59 (-1.71 to -1.46)**
5 Chronic kidney disease	**176 (116 to 245)**	**102 (64.2 to 141)**	22.1 (-3.66 to 49.6)
6 Headache disorders	**37.3 (18.2 to 58.5)**	0.564 (-1.05 to 4.34)	**-0.21 (-0.473 to -0.0124)**
7 Ischemic heart disease	4.26 (-22.7 to 32.3)	**-23.6 (-39.7 to -5.84)**	**-50.3 (-61.4 to -37.3)**
8 Other musculoskeletal	**86.8 (62 to 115)**	**36.8 (33.2 to 41.2)**	**27.4 (26.2 to 28.4)**
9 Falls	**52.7 (27.9 to 82.3)**	**11.9 (2.53 to 20.4)**	**-10.8 (-18.4 to -2.81)**
10 Stroke	**27.5 (4.11 to 52.1)**	-6.61 (-15.5 to 3.93)	**-34.5 (-41.9 to -26.8)**
11 Lung cancer	**35.4 (9.04 to 69.2)**	-0.825 (-15.5 to 19.9)	**-24 (-35.8 to -7.95)**
12 COPD	**68.7 (34.7 to 102)**	**23.6 (5.12 to 45)**	**-17.2 (-30.3 to -2.59)**
13 Age-related hearing loss	**78 (53.3 to 105)**	**30.4 (25 to 36.4)**	**-0.452 (-0.577 to -0.33)**
14 Colorectal cancer	**83.1 (35.9 to 141)**	**34 (6.62 to 67.8)**	-5.88 (-24.3 to 17.2)
15 Anxiety disorders	**38.3 (19.2 to 58.8)**	1.29 (-0.737 to 3.51)	**0.282 (0.162 to 0.403)**
16 Gynecological diseases	15.3 (-20.8 to 53.5)	-15.5 (-36.2 to 10)	-17.5 (-37.2 to 6.3)
17 Osteoarthritis	**71 (46.9 to 96.2)**	**25.3 (20.9 to 29.7)**	**1.42 (1.19 to 1.66)**
18 Pancreatic cancer	**113 (63.8 to 165)**	**56.2 (27.3 to 90.1)**	17.6 (-3.59 to 41.7)
19 Oral disorders	**60.2 (38.9 to 84)**	**17.4 (13.7 to 21.8)**	0.0291 (-0.118 to 0.194)
20 Breast cancer	**49.1 (20.6 to 82.2)**	9.19 (-7.25 to 28)	**-15.3 (-27.5 to -2.35)**
22 Neck pain			
153 COVID-19			

Supplemental Results Figure S7. Leading 20 level 3 causes of DALYs, and percentage change in number of DALYs and all-age and age-standardised DALY rates by location, 2022 and 2050

Italy, Reference scenario

Legend:
Communicable, maternal, neonatal and nutritional
Non-communicable
Injuries

Leading Causes 2022	Leading Causes 2050	Mean % change number of DALY	Mean % change all-age DALY rate	Mean % change age-standardized DALY rate
1 Ischemic heart disease	1 Alzheimer's disease	**42.5 (24.1 to 58.2)**	**70.6 (55.8 to 83.7)**	**-8.78 (-14.8 to -3.13)**
2 Alzheimer's disease	2 Ischemic heart disease	**-25.9 (-40.9 to -8.22)**	-11.2 (-28.6 to 7.98)	**-48.7 (-59.7 to -36.3)**
3 COVID-19	3 Low back pain	**-10.6 (-18.1 to -2.97)**	**7.06 (2.89 to 11.1)**	**-2.67 (-2.73 to -2.61)**
4 Low back pain	4 Diabetes	**22.2 (9.97 to 35.2)**	**46.3 (35.7 to 57.6)**	**12.5 (6.01 to 19.3)**
5 Stroke	5 Falls	9.81 (-2.41 to 23.3)	**31.5 (19.8 to 45.1)**	**-9.27 (-16.1 to -1.36)**
6 Falls	6 Stroke	**-19.1 (-28.7 to -8.19)**	-3.05 (-12.6 to 8.62)	**-39.8 (-46.5 to -32.3)**
7 Lung cancer	7 Lung cancer	-2.23 (-17.6 to 16.3)	**17.1 (2.02 to 37.7)**	**-18.2 (-28.4 to -5.33)**
8 Diabetes	8 COPD	**38.4 (18.4 to 61.1)**	**65.8 (41.3 to 86.7)**	-6.87 (-21.5 to 5.09)
9 COPD	9 Hypertensive heart disease	**65.4 (31.7 to 94)**	**98.3 (58.6 to 136)**	9.53 (-13.6 to 33.4)
10 Depressive disorders	10 Age-related hearing loss	**19.9 (8.69 to 31.4)**	**43.5 (36.8 to 51.1)**	**-0.275 (-0.365 to -0.185)**
11 Headache disorders	11 Colorectal cancer	14.4 (-9.31 to 44.9)	**37 (11 to 70.5)**	-10.8 (-26.8 to 7.84)
12 Age-related hearing loss	12 Chronic kidney disease	**95.2 (62.4 to 136)**	**134 (93.9 to 177)**	20.1 (-1.97 to 42.6)
13 Colorectal cancer	13 Pancreatic cancer	**49.4 (25 to 80.8)**	**78.9 (48.3 to 114)**	**24.3 (2.25 to 46.6)**
14 Anxiety disorders	14 Depressive disorders	**-16.1 (-23.2 to -8.19)**	0.478 (-2.76 to 3.74)	**-1.64 (-1.74 to -1.52)**
15 Hypertensive heart disease	15 Osteoarthritis	8.65 (-0.171 to 18.1)	**30.1 (25.4 to 34.4)**	**1.28 (1.06 to 1.54)**
16 Other musculoskeletal	16 Headache disorders	**-23.7 (-30.3 to -17)**	**-8.68 (-10.9 to -3.9)**	**-0.191 (-0.576 to -0.00466)**
17 Osteoarthritis	17 Other musculoskeletal	3.97 (-6.95 to 14.5)	**24.5 (17.7 to 32)**	**27.6 (26.5 to 28.5)**
18 Breast cancer	18 Atrial fibrillation	**61.2 (43.1 to 80.8)**	**93.1 (76.7 to 111)**	**7.96 (1.46 to 14.4)**
19 Pancreatic cancer	19 Anxiety disorders	**-19 (-25.9 to -11.1)**	-3.01 (-5.85 to 0.00728)	**0.173 (0.0507 to 0.285)**
20 Neck pain	20 Breast cancer	-6.02 (-20.2 to 9.21)	12.5 (-0.804 to 30.8)	**-17.1 (-26.6 to -6.15)**
24 Chronic kidney disease	25 Neck pain			
26 Atrial fibrillation	152 COVID-19			

Supplemental Results Figure S7. Leading 20 level 3 causes of DALYs, and percentage change in number of DALYs and all-age and age-standardised DALY rates by location, 2022 and 2050

Luxembourg, Reference scenario

Legend:
Communicable, maternal, neonatal and nutritional
Non-communicable
Injuries

Leading Causes 2022	Leading Causes 2050	Mean % change number of DALY	Mean % change all-age DALY rate	Mean % change age-standardized DALY rate
1 Low back pain	1 Low back pain	**56.8 (45.9 to 68.4)**	**4.43 (2 to 7.25)**	**-1.17 (-1.29 to -1.04)**
2 Ischemic heart disease	2 Falls	**68.6 (49.6 to 87.9)**	**12.3 (2.46 to 23.7)**	**-9.77 (-16 to -2.56)**
3 Falls	3 Lung cancer	**58.7 (29.4 to 81.4)**	5.72 (-14.3 to 19.9)	**-14.2 (-29 to -4.08)**
4 Lung cancer	4 Ischemic heart disease	8.13 (-11.1 to 35.8)	**-28 (-40 to -7.51)**	**-50.1 (-57.9 to -35.1)**
5 COVID-19	5 Alzheimer's disease	**141 (113 to 170)**	**60.4 (47.2 to 77)**	0.455 (-3.5 to 4.75)
6 Stroke	6 Diabetes	**103 (85.6 to 121)**	**34.9 (27 to 42)**	**16.8 (11.9 to 22.2)**
7 Headache disorders	7 COPD	**100 (71.4 to 139)**	**33.4 (16.2 to 57)**	-7.58 (-17.8 to 8.56)
8 COPD	8 Headache disorders	**45.9 (35.3 to 56.4)**	**-2.81 (-4.23 to -0.489)**	**0.266 (0.138 to 0.413)**
9 Diabetes	9 Stroke	**29.4 (13.8 to 48.2)**	**-13.8 (-22.6 to -1.92)**	**-38 (-43 to -29.5)**
10 Anxiety disorders	10 Chronic kidney disease	**209 (158 to 285)**	**106 (74.8 to 150)**	**30.5 (11.6 to 62.8)**
11 Depressive disorders	11 Depressive disorders	**51 (40.7 to 60.9)**	0.588 (-1.17 to 2.26)	**-0.567 (-0.684 to -0.447)**
12 Alzheimer's disease	12 Anxiety disorders	**50 (39.3 to 60.1)**	-0.12 (-1.58 to 1.86)	**0.476 (0.378 to 0.602)**
13 Gynecological diseases	13 Colorectal cancer	**83 (41.5 to 117)**	21.9 (-5.16 to 43.2)	-9.71 (-29 to 6.78)
14 Colorectal cancer	14 Other musculoskeletal	**71 (59.1 to 82.5)**	**13.9 (11 to 17.6)**	**15.8 (15 to 16.5)**
15 Other musculoskeletal	15 Age-related hearing loss	**86.6 (72.2 to 102)**	**24.3 (18.5 to 31)**	**-0.557 (-0.738 to -0.377)**
16 Age-related hearing loss	16 Gynecological diseases	**37.1 (8.56 to 73.5)**	-8.65 (-27 to 16)	-6.34 (-24.7 to 16.8)
17 Osteoarthritis	17 Osteoarthritis	**77.9 (64.9 to 91.9)**	**18.5 (13.5 to 23.2)**	**1.48 (1.32 to 1.65)**
18 Alcohol use disorders	18 Pancreatic cancer	**135 (92.3 to 170)**	**56.4 (29.3 to 78.8)**	**22.8 (0.534 to 41.5)**
19 Breast cancer	19 Breast cancer	**64.9 (39.1 to 87.5)**	9.84 (-5.86 to 21.5)	**-9.67 (-22 to -0.017)**
20 Neck pain	20 Oral disorders	**70 (57.3 to 82.1)**	**13.2 (8.84 to 18.5)**	**0.324 (0.211 to 0.439)**
21 Oral disorders	22 Alcohol use disorders			
23 Chronic kidney disease	24 Neck pain			
26 Pancreatic cancer	146 COVID-19			

Supplemental Results Figure S7. Leading 20 level 3 causes of DALYs, and percentage change in number of DALYs and all-age and age-standardised DALY rates by location, 2022 and 2050

Malta, Reference scenario

Legend:
Communicable, maternal, neonatal and nutritional
Non-communicable
Injuries

Leading Causes 2022	Leading Causes 2050	Mean % change number of DALY	Mean % change all-age DALY rate	Mean % change age-standardized DALY rate
1 Ischemic heart disease	1 Alzheimer's disease	**93.4 (63.8 to 123)**	**56.8 (39.2 to 78.3)**	**-5.01 (-9.16 to -0.585)**
2 COVID-19	2 Diabetes	**43.8 (25.6 to 63.8)**	**16.5 (6.76 to 27.9)**	**14.2 (5.91 to 21.6)**
3 Low back pain	3 Ischemic heart disease	**-28.7 (-47 to -3.03)**	**-42.2 (-55.8 to -23.4)**	**-57.4 (-68.1 to -42.4)**
4 Diabetes	4 Low back pain	**19.1 (5.06 to 31.8)**	**-3.52 (-6.48 to -0.663)**	**-3.18 (-3.27 to -3.09)**
5 Alzheimer's disease	5 Falls	**44.2 (25.3 to 64.1)**	**16.8 (7.61 to 27.1)**	-0.899 (-7.02 to 6.27)
6 Falls	6 Lung cancer	18.8 (-8.75 to 45.3)	-3.75 (-22.6 to 16.8)	-8.16 (-25.7 to 9.9)
7 Stroke	7 Chronic kidney disease	**109 (57.5 to 175)**	**69.2 (32.6 to 114)**	10.8 (-11.8 to 45.2)
8 Lung cancer	8 Stroke	-10.1 (-24.7 to 7.1)	**-27.2 (-37.2 to -15.1)**	**-40.9 (-48.9 to -29.7)**
9 Headache disorders	9 Anxiety disorders	**23.9 (10.5 to 36.8)**	0.435 (-0.947 to 1.65)	**0.735 (0.655 to 0.79)**
10 Anxiety disorders	10 Headache disorders	**22.1 (8.98 to 34.7)**	-1.08 (-3.12 to 0.442)	**0.393 (0.223 to 0.476)**
11 Depressive disorders	11 Lower respiratory infect	**53.8 (26.6 to 83.3)**	**24.6 (5.95 to 46.7)**	**-25.3 (-34.6 to -12.1)**
12 Colorectal cancer	12 Colorectal cancer	36 (-0.913 to 72.7)	10.2 (-17.5 to 37.8)	-4.26 (-25.9 to 17.1)
13 COPD	13 Other musculoskeletal	**65.8 (47 to 82.4)**	**34.4 (30.5 to 38.1)**	**40 (38.6 to 41.2)**
14 Age-related hearing loss	14 COPD	**43.2 (15.7 to 77.6)**	16 (-4.49 to 38.7)	-10.9 (-24.8 to 6.94)
15 Lower respiratory infect	15 Age-related hearing loss	**41.2 (23.6 to 59.4)**	**14.4 (7.89 to 22.9)**	**-0.766 (-0.915 to -0.576)**
16 Gynecological diseases	16 Depressive disorders	**22.4 (9.07 to 35.8)**	-0.794 (-2.62 to 0.76)	**-1.59 (-1.71 to -1.46)**
17 Breast cancer	17 Pancreatic cancer	**71.7 (29.6 to 116)**	**39.1 (7.36 to 69.7)**	**29.3 (1.39 to 58.8)**
18 Osteoarthritis	18 Osteoarthritis	**30.3 (14.8 to 44.5)**	**5.6 (0.672 to 11.1)**	**2.49 (2.21 to 2.8)**
19 Other musculoskeletal	19 Breast cancer	19.8 (-4.11 to 46)	-2.93 (-19.5 to 13.8)	-10.4 (-25.2 to 5.78)
20 Chronic kidney disease	20 Gynecological diseases	5.84 (-28.4 to 46.6)	-14.2 (-38.6 to 16.3)	-11.8 (-35.9 to 17.4)
21 Pancreatic cancer	150 COVID-19			

Supplemental Results Figure S7. Leading 20 level 3 causes of DALYs, and percentage change in number of DALYs and all-age and age-standardised DALY rates by location, 2022 and 2050

Monaco, Reference scenario

Legend:
Communicable, maternal, neonatal and nutritional
Non-communicable
Injuries

Leading Causes 2022	Leading Causes 2050	Mean % change number of DALY	Mean % change all-age DALY rate	Mean % change age-standardized DALY rate
1 Lung cancer	1 Lung cancer	**-21.8 (-36.5 to -6.46)**	2.2 (-15 to 20.3)	-11.7 (-27.5 to 5.4)
2 Ischemic heart disease	2 Alzheimer's disease	**35.6 (15.2 to 60.1)**	**77.1 (56.9 to 98.1)**	2.6 (-2.01 to 7.93)
3 Stroke	3 Stroke	**-36.2 (-47.6 to -22.9)**	**-16.7 (-29.6 to -0.405)**	**-39.5 (-47.7 to -30.3)**
4 Low back pain	4 Ischemic heart disease	**-44.3 (-57.3 to -26.9)**	**-27.3 (-43.6 to -4.86)**	**-50.9 (-61.6 to -35.3)**
5 Alzheimer's disease	5 Colorectal cancer	-5.43 (-27.3 to 18.3)	23.5 (-2.5 to 49)	-7.07 (-27.4 to 12.3)
6 COVID-19	6 Low back pain	**-22.5 (-30.1 to -14.2)**	1.26 (-3.56 to 5.76)	**-1.58 (-1.7 to -1.45)**
7 Colorectal cancer	7 Lower respiratory infect	18.9 (-5.38 to 47.8)	**55.2 (30 to 81.9)**	-9.14 (-21.6 to 4.1)
8 Breast cancer	8 Breast cancer	**-17.1 (-31.4 to -0.441)**	8.33 (-8.16 to 25.4)	-8.57 (-21.3 to 4.61)
9 Depressive disorders	9 COPD	11.2 (-12 to 35.5)	**45.3 (17.7 to 74.1)**	-0.475 (-15.4 to 18.7)
10 Falls	10 Pancreatic cancer	19.6 (-2.56 to 44.4)	**56.2 (26.2 to 84)**	**27.7 (3.32 to 51)**
11 COPD	11 Falls	-8.94 (-22.3 to 4.17)	**18.9 (6.44 to 31)**	**-9.01 (-17.1 to -1.26)**
12 Lower respiratory infect	12 Diabetes	-1.44 (-13.4 to 11.2)	**28.8 (19.3 to 38.9)**	**17 (11.7 to 20.9)**
13 Diabetes	13 Chronic kidney disease	**70.2 (26.2 to 121)**	**122 (76.9 to 181)**	**31.6 (7.34 to 68.2)**
14 Headache disorders	14 Depressive disorders	**-24.2 (-31.6 to -15.9)**	-0.992 (-4.68 to 2.72)	**-1.38 (-1.58 to -1.21)**
15 Pancreatic cancer	15 Prostate cancer	14.1 (-6.66 to 40.3)	**49 (24.9 to 73.2)**	3.18 (-10.1 to 17.1)
16 Anxiety disorders	16 Age-related hearing loss	-3.44 (-14.3 to 8.79)	**26.1 (17.2 to 35.3)**	**-0.506 (-0.615 to -0.394)**
17 Age-related hearing loss	17 Osteoarthritis	**-14.5 (-23.4 to -5.05)**	**11.7 (5.17 to 17.7)**	**1.42 (1.27 to 1.58)**
18 Osteoarthritis	18 Headache disorders	**-27.3 (-33.9 to -20.3)**	**-5.01 (-7.3 to -2.76)**	**-0.769 (-1.09 to -0.406)**
19 Other musculoskeletal	19 Other musculoskeletal	**-14.3 (-23.1 to -5.08)**	**12 (6.66 to 18.3)**	**16.5 (15.8 to 17.1)**
20 Self-harm	20 Anxiety disorders	**-24.6 (-32.4 to -16.5)**	-1.52 (-4.23 to 0.992)	**-0.464 (-0.705 to -0.24)**
21 Prostate cancer	27 Self-harm			
26 Chronic kidney disease	150 COVID-19			

Supplemental Results Figure S7. Leading 20 level 3 causes of DALYs, and percentage change in number of DALYs and all-age and age-standardised DALY rates by location, 2022 and 2050

Netherlands, Reference scenario

Legend:
Communicable, maternal, neonatal and nutritional
Non-communicable
Injuries

Leading Causes 2022	Leading Causes 2050	Mean % change number of DALY	Mean % change all-age DALY rate	Mean % change age-standardized DALY rate
1 Lung cancer	1 Alzheimer's disease	**67 (47.9 to 88.5)**	**57.3 (46.8 to 68.9)**	-3.79 (-8.07 to 0.577)
2 Ischemic heart disease	2 Falls	**35.5 (19.4 to 54.1)**	**27.6 (17.2 to 38.9)**	**-11.5 (-17.5 to -4.35)**
3 Low back pain	3 COPD	**30.5 (12.5 to 50.2)**	**23 (7.11 to 41.9)**	-12.1 (-22.8 to 1.3)
4 COPD	4 Low back pain	7.11 (-2.45 to 16.8)	0.89 (-1.77 to 4.02)	**-1.97 (-2.03 to -1.9)**
5 Falls	5 Lung cancer	-4.8 (-18.1 to 9.48)	-10.3 (-20.4 to 0.59)	**-21.3 (-29.8 to -11.9)**
6 Stroke	6 Diabetes	**35.6 (21.5 to 48.1)**	**27.8 (19.5 to 36.7)**	**14.2 (7.68 to 20.3)**
7 Alzheimer's disease	7 Stroke	-8.32 (-19 to 3.45)	**-13.6 (-20.8 to -5.22)**	**-37.2 (-43 to -31.1)**
8 Colorectal cancer	8 Colorectal cancer	17.4 (-4.18 to 39.8)	10.6 (-6.88 to 28.3)	-11.5 (-26.3 to 1.4)
9 Diabetes	9 Ischemic heart disease	**-25.9 (-40.5 to -12.2)**	**-30.2 (-41.2 to -17.7)**	**-52.4 (-60.2 to -43.4)**
10 Anxiety disorders	10 Anxiety disorders	5.33 (-2.71 to 14.7)	-0.78 (-1.98 to 0.539)	**0.205 (0.133 to 0.274)**
11 Depressive disorders	11 Chronic kidney disease	**153 (109 to 204)**	**139 (101 to 181)**	**40.8 (17.8 to 68.2)**
12 Headache disorders	12 Depressive disorders	5.43 (-3.6 to 14.4)	-0.683 (-2.16 to 0.883)	**-1.16 (-1.27 to -1.07)**
13 COVID-19	13 Headache disorders	3.44 (-4.85 to 11.4)	**-2.55 (-3.51 to -1.02)**	-0.124 (-0.276 to 0.0406)
14 Other musculoskeletal	14 Lower respiratory infect	**47 (26.1 to 67.9)**	**38.5 (20.9 to 56.9)**	**-16.7 (-25.4 to -6.69)**
15 Breast cancer	15 Other musculoskeletal	**24.6 (14.2 to 34.9)**	**17.3 (14.2 to 21.3)**	**20.4 (19.4 to 21.3)**
16 Gynecological diseases	16 Age-related hearing loss	**28.2 (16 to 40.9)**	**20.7 (15.3 to 26)**	**-0.401 (-0.509 to -0.272)**
17 Age-related hearing loss	17 Breast cancer	11.4 (-4.77 to 28)	4.92 (-7 to 17.5)	**-11.2 (-21.6 to -0.153)**
18 Osteoarthritis	18 Atrial fibrillation	**92.9 (70.3 to 118)**	**81.7 (66.4 to 98.2)**	**17.6 (9.75 to 25.5)**
19 Oral disorders	19 Osteoarthritis	**17.4 (7.34 to 27.5)**	**10.6 (7.76 to 13.4)**	**1.41 (1.25 to 1.58)**
20 Lower respiratory infect	20 Oral disorders	**15.8 (6.24 to 25.6)**	**9.06 (5.56 to 12.2)**	**0.258 (0.166 to 0.361)**
25 Chronic kidney disease	24 Gynecological diseases			
27 Atrial fibrillation	150 COVID-19			

Supplemental Results Figure S7. Leading 20 level 3 causes of DALYs, and percentage change in number of DALYs and all-age and age-standardised DALY rates by location, 2022 and 2050

Norway, Reference scenario

Legend:
Communicable, maternal, neonatal and nutritional
Non-communicable
Injuries

Leading Causes 2022	Leading Causes 2050	Mean % change number of DALY	Mean % change all-age DALY rate	Mean % change age-standardized DALY rate
1 Ischemic heart disease	1 Alzheimer's disease	**77.8 (56.9 to 96.5)**	**53.8 (42.6 to 64.4)**	**-7.33 (-11.2 to -3.05)**
2 Low back pain	2 Low back pain	**23.1 (13.2 to 33.8)**	**6.56 (4.34 to 8.74)**	**-1.22 (-1.26 to -1.17)**
3 COVID-19	3 Falls	**47.1 (31.4 to 64.4)**	**27.4 (19.7 to 36.9)**	**-12.1 (-17.7 to -5.35)**
4 Falls	4 COPD	**42.7 (24.5 to 60)**	**23.5 (11.2 to 36.6)**	**-16.5 (-24.4 to -7.34)**
5 COPD	5 Ischemic heart disease	**-24 (-35 to -11)**	**-34.2 (-41.8 to -25.2)**	**-56.8 (-61.8 to -50.3)**
6 Alzheimer's disease	6 Lung cancer	10.5 (-3.78 to 24.4)	-4.36 (-13.5 to 6.22)	**-25.8 (-32.5 to -17.6)**
7 Stroke	7 Diabetes	**44.2 (31.5 to 59.1)**	**24.8 (19 to 30.8)**	**5.72 (1.34 to 9.81)**
8 Lung cancer	8 Stroke	4.81 (-6.01 to 16.8)	**-9.29 (-16 to -2.9)**	**-36.8 (-41.5 to -32.6)**
9 Anxiety disorders	9 Anxiety disorders	**14.5 (5.29 to 23.8)**	-0.919 (-2.4 to 0.649)	**0.285 (0.247 to 0.317)**
10 Depressive disorders	10 Colorectal cancer	**37.5 (17.3 to 59.5)**	**19 (2.94 to 34.7)**	**-15.3 (-26.5 to -5.44)**
11 Headache disorders	11 Depressive disorders	**15.4 (6.29 to 24.9)**	-0.139 (-1.5 to 1.42)	**-0.529 (-0.559 to -0.494)**
12 Diabetes	12 Headache disorders	**12.9 (3.55 to 21.9)**	-2.31 (-4.02 to 1.87)	**0.223 (0.148 to 0.278)**
13 Colorectal cancer	13 Lower respiratory infect	**75.1 (50.7 to 104)**	**51.5 (31.9 to 72.7)**	**-11.4 (-21.6 to -0.791)**
14 Age-related hearing loss	14 Age-related hearing loss	**47.6 (34.6 to 61.4)**	**27.7 (22.8 to 33.2)**	**-0.209 (-0.299 to -0.113)**
15 Self-harm	15 Prostate cancer	**84 (57.3 to 111)**	**59.3 (38.1 to 76.1)**	3.11 (-6.52 to 12.6)
16 Gynecological diseases	16 Osteoarthritis	**39.5 (28.1 to 51.9)**	**20.7 (17.7 to 24.2)**	**0.726 (0.618 to 0.851)**
17 Drug use disorders	17 Drug use disorders	**30.2 (15.9 to 48.5)**	**12.7 (4.67 to 25)**	**17.4 (8.22 to 31.8)**
18 Oral disorders	18 Oral disorders	**31.8 (21.6 to 43.8)**	**14.1 (10.6 to 17.7)**	**0.182 (0.103 to 0.265)**
19 Osteoarthritis	19 Pancreatic cancer	**66.5 (44.9 to 87.2)**	**44.2 (26.7 to 61.2)**	9.84 (-2.76 to 23.1)
20 Lower respiratory infect	20 Atrial fibrillation	**98 (74.7 to 122)**	**71.4 (57 to 86.8)**	**7.73 (0.424 to 15.6)**
22 Prostate cancer	24 Gynecological diseases			
23 Pancreatic cancer	27 Self-harm			
26 Atrial fibrillation	146 COVID-19			

Supplemental Results Figure S7. Leading 20 level 3 causes of DALYs, and percentage change in number of DALYs and all-age and age-standardised DALY rates by location, 2022 and 2050

Portugal, Reference scenario

Legend:
Communicable, maternal, neonatal and nutritional
Non-communicable
Injuries

Leading Causes 2022	Leading Causes 2050	Mean % change number of DALY	Mean % change all-age DALY rate	Mean % change age-standardized DALY rate
1 Stroke	1 Alzheimer's disease	**53.8 (33.5 to 76.2)**	**56.7 (45.1 to 67.9)**	**-10.4 (-14.3 to -6.71)**
2 Low back pain	2 Diabetes	**29.6 (13.6 to 48.3)**	**32 (23 to 41)**	**13.5 (7.25 to 19.7)**
3 Ischemic heart disease	3 Low back pain	0.134 (-10.7 to 11.2)	2.03 (-0.912 to 5.09)	**-3.57 (-3.69 to -3.46)**
4 COVID-19	4 Stroke	**-29.4 (-41.3 to -15.3)**	**-28 (-36.4 to -19.3)**	**-50.3 (-56.5 to -43.4)**
5 Diabetes	5 COPD	**37.5 (14.8 to 65.5)**	**40.1 (19.1 to 61.9)**	-14.1 (-27.5 to 0.36)
6 Alzheimer's disease	6 Lower respiratory infect	**33.6 (14.2 to 56.2)**	**36.1 (19.7 to 55.1)**	**-24.7 (-33.6 to -13.8)**
7 Depressive disorders	7 Colorectal cancer	23.5 (-3.28 to 58.2)	**25.9 (1.52 to 55.3)**	-6.88 (-24.2 to 14.8)
8 Lung cancer	8 Lung cancer	5.41 (-13.8 to 32)	7.38 (-10.1 to 28.6)	-10.2 (-23.7 to 6.5)
9 Anxiety disorders	9 Depressive disorders	-2.45 (-13 to 8.39)	-0.599 (-3.37 to 2.53)	**-2.56 (-2.76 to -2.34)**
10 COPD	10 Ischemic heart disease	**-38.5 (-52 to -23)**	**-37.4 (-49.6 to -23.3)**	**-56.9 (-65.7 to -46.2)**
11 Lower respiratory infect	11 Anxiety disorders	-3.63 (-14.2 to 6.75)	-1.8 (-4.17 to 0.649)	0.167 (-0.0227 to 0.357)
12 Colorectal cancer	12 Chronic kidney disease	**64.6 (30.1 to 101)**	**67.8 (37.5 to 99.4)**	-7.66 (-25.5 to 14.1)
13 Falls	13 Falls	9.28 (-5.79 to 24.6)	**11.4 (1.44 to 22.7)**	**-13.3 (-21.4 to -4.83)**
14 Headache disorders	14 Age-related hearing loss	**28 (12.4 to 42.4)**	**30.5 (24.8 to 36)**	**-0.843 (-0.925 to -0.753)**
15 Age-related hearing loss	15 Other musculoskeletal	**34.8 (18.9 to 51.7)**	**37.4 (31.8 to 43.5)**	**41.2 (39.8 to 42.3)**
16 Chronic kidney disease	16 Headache disorders	-8.46 (-18.4 to 1.47)	**-6.72 (-8.51 to -4.29)**	**-0.509 (-0.859 to -0.163)**
17 Other musculoskeletal	17 Osteoarthritis	**18.9 (5.38 to 32.2)**	**21.1 (17.5 to 24.7)**	**2.67 (2.35 to 3.01)**
18 Osteoarthritis	18 Pancreatic cancer	**54.2 (24 to 94)**	**57.1 (30 to 90.9)**	**25.4 (4.22 to 52.5)**
19 Stomach cancer	19 Urinary diseases	**97 (64.3 to 136)**	**101 (69 to 131)**	13 (-2.26 to 26.5)
20 Gynecological diseases	20 Prostate cancer	**44.2 (19.8 to 72.4)**	**46.9 (28.7 to 69.1)**	1.49 (-9.59 to 13.5)
29 Prostate cancer	28 Stomach cancer			
30 Pancreatic cancer	31 Gynecological diseases			
32 Urinary diseases	153 COVID-19			

Supplemental Results Figure S7. Leading 20 level 3 causes of DALYs, and percentage change in number of DALYs and all-age and age-standardised DALY rates by location, 2022 and 2050

San Marino, Reference scenario

Legend:
Communicable, maternal, neonatal and nutritional
Non-communicable
Injuries

Leading Causes 2022	Leading Causes 2050	Mean % change number of DALY	Mean % change all-age DALY rate	Mean % change age-standardized DALY rate
1 COVID-19	1 Alzheimer's disease	**135 (82.2 to 189)**	**161 (124 to 203)**	3.68 (-1.37 to 8.84)
2 Low back pain	2 Low back pain	0.0779 (-13.5 to 15.4)	**11.4 (6.37 to 17.4)**	**-2 (-2.2 to -1.76)**
3 Alzheimer's disease	3 Stroke	13 (-16.1 to 41.1)	25.7 (-0.0336 to 52.2)	**-34.8 (-45.3 to -20.3)**
4 Stroke	4 Diabetes	**52.9 (26.4 to 80.8)**	**70.3 (56.1 to 85.3)**	**27 (20.2 to 32.8)**
5 Ischemic heart disease	5 Falls	**34.8 (12.3 to 64.3)**	**50 (33.6 to 66)**	**-9.05 (-17.1 to -0.418)**
6 Depressive disorders	6 Ischemic heart disease	-1.95 (-34.1 to 36.5)	9.14 (-19.6 to 48.4)	**-49.3 (-61.2 to -29.5)**
7 Lung cancer	7 Other cardiovascular	**43.4 (14 to 71)**	**59.6 (39.1 to 83.4)**	**-10.7 (-19.7 to -0.426)**
8 Falls	8 Lung cancer	8.92 (-18.1 to 47)	21.1 (-7.38 to 54)	-16.7 (-34.2 to 0.0955)
9 Diabetes	9 Age-related hearing loss	**48.7 (22.9 to 75.1)**	**65.5 (52 to 81)**	**-0.588 (-0.775 to -0.42)**
10 Headache disorders	10 COPD	**83.4 (33.8 to 132)**	**104 (61.8 to 152)**	-0.987 (-19.9 to 27.4)
11 Other cardiovascular	11 Colorectal cancer	**52.1 (7.22 to 111)**	**69 (25.4 to 124)**	-6.44 (-29.9 to 19.8)
12 Anxiety disorders	12 Depressive disorders	-11.4 (-24.6 to 1.99)	-1.32 (-6.18 to 4.91)	**-2 (-2.33 to -1.71)**
13 Age-related hearing loss	13 Osteoarthritis	**26.3 (5.73 to 47.4)**	**40.7 (33.5 to 47.8)**	**1.74 (1.52 to 1.99)**
14 Colorectal cancer	14 Lower respiratory infect	**108 (51.9 to 166)**	**131 (79.9 to 187)**	-12.6 (-25.8 to 4.65)
15 Gynecological diseases	15 Atrial fibrillation	**130 (83 to 183)**	**156 (123 to 192)**	**12.5 (6.08 to 19.9)**
16 Osteoarthritis	16 Anxiety disorders	**-14.8 (-27.2 to -1.66)**	-5.13 (-10 to 0.155)	**-0.6 (-1.01 to -0.125)**
17 Other musculoskeletal	17 Headache disorders	**-21.6 (-32.6 to -9.11)**	**-12.7 (-16.1 to -6.68)**	**-1.49 (-2.13 to -0.826)**
18 COPD	18 Hypertensive heart disease	**151 (68.7 to 237)**	**179 (108 to 271)**	20.4 (-7.74 to 60.7)
19 Oral disorders	19 Other musculoskeletal	3.28 (-12.3 to 22.3)	**15 (6.31 to 24.2)**	**19.8 (19 to 20.6)**
20 Stomach cancer	20 Oral disorders	**18.8 (0.0678 to 38.9)**	**32.3 (22.6 to 42)**	**0.238 (0.0815 to 0.407)**
25 Lower respiratory infect	24 Stomach cancer			
31 Atrial fibrillation	28 Gynecological diseases			
34 Hypertensive heart disease	150 COVID-19			

Supplemental Results Figure S7. Leading 20 level 3 causes of DALYs, and percentage change in number of DALYs and all-age and age-standardised DALY rates by location, 2022 and 2050

Spain, Reference scenario

Legend:
Communicable, maternal, neonatal and nutritional
Non-communicable
Injuries

Leading Causes 2022	Leading Causes 2050	Mean % change number of DALY	Mean % change all-age DALY rate	Mean % change age-standardized DALY rate
1 Ischemic heart disease	1 Alzheimer's disease	**72.2 (53.5 to 92)**	**52.5 (40.2 to 63.7)**	**-6.93 (-11.1 to -2.79)**
2 COVID-19	2 Diabetes	**64.6 (45.8 to 79)**	**45.8 (37.5 to 53.7)**	**24.6 (20.2 to 28.5)**
3 Diabetes	3 COPD	**56.5 (30.2 to 81)**	**38.6 (19 to 57.6)**	**-13.7 (-25.6 to -0.525)**
4 Alzheimer's disease	4 Low back pain	**16.8 (5.2 to 26.8)**	3.44 (-0.335 to 7.41)	**-3 (-3.09 to -2.93)**
5 Low back pain	5 Depressive disorders	**11.7 (1.79 to 21.7)**	-1.11 (-2.89 to 0.87)	**-1.81 (-1.89 to -1.7)**
6 Stroke	6 Ischemic heart disease	-18.9 (-34.5 to 0.26)	**-28.2 (-41.8 to -13.8)**	**-52.2 (-62.3 to -40.8)**
7 Lung cancer	7 Stroke	5.11 (-8.6 to 20.1)	-6.92 (-16.4 to 4.24)	**-34.7 (-41.8 to -26.7)**
8 Depressive disorders	8 Lung cancer	-5.78 (-20.9 to 11.5)	**-16.5 (-29.6 to -1.71)**	**-31.2 (-40.8 to -20.4)**
9 COPD	9 Falls	**18.3 (4.89 to 33.4)**	4.73 (-4.38 to 13.9)	**-15.1 (-22.7 to -7.79)**
10 Falls	10 Colorectal cancer	**35.4 (9.1 to 67)**	20 (-1.45 to 46)	-13.8 (-29.1 to 3.57)
11 Headache disorders	11 Chronic kidney disease	**146 (93.8 to 201)**	**118 (80.3 to 164)**	**25.5 (1.23 to 56.2)**
12 Colorectal cancer	12 Age-related hearing loss	**47.8 (31.8 to 62.8)**	**30.8 (23.2 to 37)**	**-0.619 (-0.735 to -0.478)**
13 Age-related hearing loss	13 Headache disorders	6.22 (-2.96 to 15.6)	**-5.93 (-7.79 to -3.42)**	-0.12 (-0.378 to 0.0713)
14 Anxiety disorders	14 Other musculoskeletal	**46.2 (33.2 to 60.6)**	**29.5 (23.7 to 36)**	**31.7 (30.2 to 32.8)**
15 Gynecological diseases	15 Osteoarthritis	**36.3 (23.8 to 48.3)**	**20.7 (15.6 to 25.6)**	**2.11 (1.89 to 2.37)**
16 Other musculoskeletal	16 Anxiety disorders	**10.9 (0.508 to 20.4)**	-1.76 (-4.82 to 1.35)	**0.227 (0.109 to 0.333)**
17 Osteoarthritis	17 Atrial fibrillation	**97.7 (77.4 to 120)**	**75.1 (62 to 91)**	**10.7 (4.44 to 17.2)**
18 Blindness and vision loss	18 Blindness and vision loss	**36.8 (22.6 to 49.2)**	**21.1 (14.1 to 26.9)**	**-3.83 (-4.07 to -3.57)**
19 Lower respiratory infect	19 Lower respiratory infect	**49.8 (28.8 to 74.4)**	**32.7 (17.6 to 48.1)**	**-21.3 (-29.5 to -11.7)**
20 Chronic kidney disease	20 Pancreatic cancer	**70 (37.7 to 108)**	**50.6 (22.2 to 81.4)**	17.5 (-3.19 to 43.1)
22 Pancreatic cancer	23 Gynecological diseases			
26 Atrial fibrillation	153 COVID-19			

Supplemental Results Figure S7. Leading 20 level 3 causes of DALYs, and percentage change in number of DALYs and all-age and age-standardised DALY rates by location, 2022 and 2050

Sweden, Reference scenario

Legend:
Communicable, maternal, neonatal and nutritional
Non-communicable
Injuries

Leading Causes 2022	Leading Causes 2050	Mean % change number of DALY	Mean % change all-age DALY rate	Mean % change age-standardized DALY rate
1 Ischemic heart disease	1 Alzheimer's disease	**61.7 (40.7 to 81.2)**	**25.8 (15.2 to 38.4)**	-4.46 (-8.43 to 0.131)
2 Alzheimer's disease	2 Ischemic heart disease	**-25.3 (-38.2 to -10.5)**	**-41.9 (-51.3 to -31.1)**	**-53.2 (-60.3 to -44.1)**
3 Low back pain	3 Low back pain	**27.4 (16.4 to 37.8)**	-0.883 (-3.22 to 1.49)	**-1.83 (-1.93 to -1.74)**
4 Stroke	4 Falls	**35.7 (19.8 to 51.8)**	5.56 (-2.61 to 14.1)	**-11.2 (-17.5 to -3.17)**
5 Falls	5 Diabetes	**47.3 (33.5 to 62.9)**	**14.6 (6.02 to 22.2)**	**11.7 (5.17 to 17.2)**
6 COVID-19	6 Depressive disorders	**29.7 (19.2 to 39.5)**	0.884 (-0.147 to 1.87)	**-0.823 (-0.913 to -0.732)**
7 Depressive disorders	7 COPD	**35.4 (14.2 to 58.6)**	5.36 (-8.26 to 22.8)	-9.23 (-20.6 to 6.04)
8 Diabetes	8 Stroke	-5.64 (-16.8 to 6.61)	**-26.6 (-33.5 to -18.2)**	**-35.5 (-41.5 to -29)**
9 COPD	9 Atrial fibrillation	**80.2 (56.9 to 106)**	**40.2 (25.6 to 55.2)**	**9.92 (3.32 to 17)**
10 Headache disorders	10 Headache disorders	**30.3 (20.5 to 39.3)**	**1.36 (0.329 to 2.5)**	**0.312 (0.175 to 0.4)**
11 Lung cancer	11 Lung cancer	**20.1 (2.86 to 40.2)**	-6.53 (-18.3 to 5.27)	**-12.2 (-22.5 to -1.48)**
12 Anxiety disorders	12 Anxiety disorders	**29.2 (18.8 to 38.5)**	0.532 (-0.195 to 1.26)	**0.513 (0.453 to 0.575)**
13 Colorectal cancer	13 Other musculoskeletal	**54.7 (42.6 to 67.6)**	**20.4 (17.4 to 23.2)**	**20.7 (19.8 to 21.4)**
14 Atrial fibrillation	14 Colorectal cancer	**31.9 (6.66 to 58.2)**	2.6 (-15.1 to 22.1)	-10.9 (-25.1 to 4.96)
15 Other musculoskeletal	15 Chronic kidney disease	**131 (88.4 to 176)**	**79.4 (49.9 to 111)**	**30.8 (10.5 to 56.9)**
16 Self-harm	16 Prostate cancer	**57.7 (33.2 to 88.6)**	**22.7 (6.72 to 41.2)**	3.08 (-7.63 to 14.3)
17 Age-related hearing loss	17 Pancreatic cancer	**67.2 (37.1 to 99)**	**30 (9.16 to 51.6)**	**20.2 (2.41 to 40.1)**
18 Prostate cancer	18 Drug use disorders	**58.5 (40.3 to 80.5)**	**23.3 (11.8 to 37.1)**	**19.3 (6.95 to 34.1)**
19 Osteoarthritis	19 Age-related hearing loss	**35.6 (23.5 to 46.8)**	**5.5 (1.48 to 9.43)**	**-0.646 (-0.702 to -0.588)**
20 Drug use disorders	20 Osteoarthritis	**33.3 (21.5 to 45.6)**	**3.7 (0.0887 to 7.06)**	**1.5 (1.28 to 1.73)**
23 Pancreatic cancer	21 Self-harm			
29 Chronic kidney disease	148 COVID-19			

Supplemental Results Figure S7. Leading 20 level 3 causes of DALYs, and percentage change in number of DALYs and all-age and age-standardised DALY rates by location, 2022 and 2050

Switzerland, Reference scenario

Legend:
Communicable, maternal, neonatal and nutritional
Non-communicable
Injuries

Leading Causes 2022	Leading Causes 2050	Mean % change number of DALY	Mean % change all-age DALY rate	Mean % change age-standardized DALY rate
1 Low back pain	1 Alzheimer's disease	**93.2 (69.6 to 119)**	**71.4 (60.2 to 83.8)**	**-6.28 (-10.6 to -2.23)**
2 Ischemic heart disease	2 Falls	**43.9 (25.2 to 60.1)**	**27.6 (18.5 to 37)**	**-8.84 (-14.5 to -2.53)**
3 Falls	3 Low back pain	**19.7 (8.27 to 30.4)**	**6.21 (3.38 to 9.4)**	**-0.974 (-1.03 to -0.924)**
4 Alzheimer's disease	4 Diabetes	**54.9 (37.5 to 69.6)**	**37.5 (31.1 to 43.9)**	**13.9 (10.4 to 16.8)**
5 Diabetes	5 Ischemic heart disease	-9.07 (-21.9 to 5.14)	**-19.3 (-29.7 to -6.8)**	**-53.6 (-59.4 to -45.9)**
6 Anxiety disorders	6 COPD	**61.7 (40.5 to 84.9)**	**43.4 (29.4 to 62.7)**	-9.01 (-17 to 1.29)
7 Lung cancer	7 Chronic kidney disease	**184 (134 to 234)**	**152 (116 to 193)**	**29.3 (13 to 47.4)**
8 Depressive disorders	8 Anxiety disorders	**11.2 (1.8 to 20.6)**	-1.39 (-3.59 to 0.965)	**0.112 (0.0534 to 0.173)**
9 Stroke	9 Lung cancer	14 (-3.29 to 29.4)	1.06 (-10.2 to 12.1)	**-22.5 (-30.2 to -15.7)**
10 Headache disorders	10 Depressive disorders	**12.5 (3.15 to 21.7)**	-0.21 (-2.01 to 1.84)	**-0.507 (-0.572 to -0.447)**
11 COPD	11 Stroke	10.4 (-2.09 to 23.7)	-2.06 (-10.3 to 6.34)	**-35.7 (-40.7 to -30.2)**
12 Gynecological diseases	12 Age-related hearing loss	**52 (37.1 to 69)**	**34.8 (28 to 41.9)**	-0.0795 (-0.214 to 0.0603)
13 COVID-19	13 Headache disorders	6.5 (-3.09 to 17.1)	**-5.53 (-7.27 to -2.86)**	0.00939 (-0.149 to 0.0744)
14 Age-related hearing loss	14 Hypertensive heart disease	**131 (91.1 to 176)**	**104 (75.7 to 137)**	11.9 (-2.38 to 29.5)
15 Other musculoskeletal	15 Osteoarthritis	**38.6 (25.6 to 51.7)**	**22.9 (19.4 to 26.3)**	**0.36 (0.243 to 0.492)**
16 Osteoarthritis	16 Prostate cancer	**87.4 (61.3 to 118)**	**66.2 (46.7 to 87)**	-0.504 (-9.29 to 8.43)
17 Colorectal cancer	17 Colorectal cancer	**37.4 (13.7 to 61.4)**	**21.8 (1.96 to 38)**	**-18.3 (-30.4 to -7.62)**
18 Self-harm	18 Other musculoskeletal	**20.2 (8.07 to 32.6)**	**6.62 (3.02 to 11.2)**	**8.86 (8.15 to 9.38)**
19 Oral disorders	19 Pancreatic cancer	**71.5 (41.8 to 96.8)**	**52.1 (32 to 70.5)**	10.3 (-3.4 to 24.7)
20 Neck pain	20 Gynecological diseases	-3.89 (-18.8 to 14.6)	-14.7 (-28.6 to 0.0616)	-11.6 (-25.1 to 3.38)
22 Chronic kidney disease	21 Oral disorders			
24 Prostate cancer	24 Neck pain			
25 Pancreatic cancer	32 Self-harm			
27 Hypertensive heart disease	150 COVID-19			

Supplemental Results Figure S7. Leading 20 level 3 causes of DALYs, and percentage change in number of DALYs and all-age and age-standardised DALY rates by location, 2022 and 2050

UK, Reference scenario

Legend:
Communicable, maternal, neonatal and nutritional
Non-communicable
Injuries

Leading Causes 2022	Leading Causes 2050	Mean % change number of DALY	Mean % change all-age DALY rate	Mean % change age-standardized DALY rate
1 Ischemic heart disease	1 Low back pain	**14.7 (8.64 to 21.1)**	**6.17 (3.29 to 9.4)**	**-1.87 (-1.94 to -1.8)**
2 Low back pain	2 Alzheimer's disease	**73 (58.2 to 87.2)**	**60.1 (49 to 72.1)**	-2.69 (-6.47 to 1.89)
3 COPD	3 COPD	**28.9 (10.7 to 46.3)**	**19.4 (1.45 to 38.2)**	**-15.8 (-28.8 to -2.86)**
4 Lung cancer	4 Diabetes	**49.2 (41 to 57.9)**	**38 (32.1 to 44.1)**	**20.8 (17.3 to 24.2)**
5 Stroke	5 Lung cancer	19.6 (-0.241 to 40.7)	10.7 (-6.81 to 27.5)	**-15.8 (-26.5 to -3.83)**
6 Alzheimer's disease	6 Ischemic heart disease	**-30.5 (-43.3 to -14.8)**	**-35.6 (-47.9 to -20)**	**-55 (-63.9 to -43.4)**
7 Diabetes	7 Lower respiratory infect	**47.4 (29 to 65.8)**	**36.4 (20.5 to 53)**	**-16.9 (-26 to -5.29)**
8 Depressive disorders	8 Falls	**22.2 (10.7 to 34.8)**	**13.1 (3.35 to 24.7)**	**-14.7 (-21.6 to -6.44)**
9 Falls	9 Depressive disorders	**9.89 (3.83 to 15.9)**	1.67 (-0.25 to 4.11)	**-1.16 (-1.23 to -1.08)**
10 COVID-19	10 Stroke	**-9.64 (-17.8 to -1.39)**	**-16.4 (-24.1 to -8.48)**	**-39.3 (-44.7 to -32.6)**
11 Lower respiratory infect	11 Colorectal cancer	**33.3 (7.59 to 64.1)**	**23.3 (0.715 to 51.1)**	-10.2 (-25.8 to 9.22)
12 Headache disorders	12 Headache disorders	**6.54 (0.771 to 13.6)**	-1.43 (-3.17 to 2.73)	**-0.448 (-0.677 to -0.178)**
13 Colorectal cancer	13 Age-related hearing loss	**36.2 (28 to 44)**	**26.1 (20.8 to 30.9)**	**-0.596 (-0.647 to -0.552)**
14 Anxiety disorders	14 Drug use disorders	**34.6 (20.8 to 54.7)**	**24.5 (13.1 to 38.5)**	**26.4 (14.8 to 41.3)**
15 Age-related hearing loss	15 Other musculoskeletal	**35.8 (28.7 to 43.9)**	**25.6 (21.1 to 30.6)**	**20 (18.8 to 21)**
16 Drug use disorders	16 Osteoarthritis	**30.3 (22.9 to 37.5)**	**20.5 (16.1 to 25)**	**1.68 (1.53 to 1.84)**
17 Other musculoskeletal	17 Anxiety disorders	**7.89 (1.08 to 15.2)**	-0.193 (-1.88 to 1.62)	**0.1 (0.0167 to 0.185)**
18 Osteoarthritis	18 Prostate cancer	**59.7 (35.7 to 84.6)**	**47.8 (28.5 to 70.8)**	0.705 (-9.93 to 12.9)
19 Breast cancer	19 Breast cancer	**22.7 (5.41 to 42.5)**	13.5 (-0.699 to 29.5)	-11.7 (-22.6 to 2.02)
20 Gynecological diseases	20 Pancreatic cancer	**69.1 (36.2 to 104)**	**56.4 (28.5 to 86.9)**	**20.7 (0.657 to 43)**
27 Prostate cancer	24 Gynecological diseases			
28 Pancreatic cancer	150 COVID-19			

Supplemental Results Figure S7. Leading 20 level 3 causes of DALYs, and percentage change in number of DALYs and all-age and age-standardised DALY rates by location, 2022 and 2050

Bolivia, Reference scenario

Legend:
Communicable, maternal, neonatal and nutritional
Non-communicable
Injuries

Leading Causes 2022	Leading Causes 2050	Mean % change number of DALY	Mean % change all-age DALY rate	Mean % change age-standardized DALY rate
1 COVID-19	1 Diabetes	**123 (77.4 to 166)**	**70.4 (42 to 101)**	5.23 (-10.1 to 23.2)
2 Lower respiratory infect	2 Chronic kidney disease	**160 (65.9 to 270)**	**99.1 (37.5 to 176)**	25.1 (-16 to 79.1)
3 Neonatal disorders	3 Lower respiratory infect	0.266 (-22.9 to 24.3)	**-23.2 (-38.9 to -7.35)**	**-39.4 (-50.3 to -25.9)**
4 Congenital defects	4 Ischemic heart disease	40.8 (-13 to 117)	7.86 (-30.5 to 63)	**-35.8 (-60.3 to -0.463)**
5 Diabetes	5 Stroke	34.4 (-2.24 to 72.1)	2.92 (-20.3 to 31.1)	**-35.6 (-51.2 to -17.5)**
6 Ischemic heart disease	6 Other musculoskeletal	**142 (110 to 179)**	**85.4 (74.9 to 95.8)**	**47.1 (44.4 to 49.5)**
7 Stroke	7 Neonatal disorders	**-31.2 (-47.6 to -14.3)**	**-47.4 (-57.6 to -35.9)**	**-26.2 (-40.8 to -10.9)**
8 Chronic kidney disease	8 Cirrhosis liver	**72.1 (37.4 to 110)**	**31.8 (10.9 to 53.4)**	-13.1 (-26.3 to 0.0276)
9 Road injuries	9 Low back pain	**57.1 (36.8 to 81.5)**	**20.2 (13.9 to 26.2)**	**-4.42 (-4.55 to -4.3)**
10 Cirrhosis liver	10 Road injuries	14.8 (-30.8 to 80)	-12.1 (-45.4 to 33.3)	-20.9 (-51.4 to 22.8)
11 Anxiety disorders	11 Anxiety disorders	**39 (21.6 to 58.3)**	**6.4 (1.28 to 12.5)**	**0.564 (0.423 to 0.678)**
12 Low back pain	12 Depressive disorders	**52.3 (32.1 to 75.3)**	**16.5 (10.8 to 23.1)**	**-2.51 (-2.68 to -2.32)**
13 Depressive disorders	13 Stomach cancer	**38.3 (12.8 to 67.5)**	5.82 (-8.3 to 20.6)	**-34.8 (-41.4 to -27.8)**
14 Stomach cancer	14 Congenital defects	**-41.6 (-52 to -27.3)**	**-55.3 (-60.2 to -48.5)**	**-35.8 (-42.7 to -28.6)**
15 Other musculoskeletal	15 COPD	**139 (70.5 to 220)**	**83.3 (34.3 to 143)**	2.44 (-27.9 to 37.7)
16 Headache disorders	16 Oral disorders	**86.2 (60.1 to 115)**	**42.5 (32.6 to 52.1)**	**0.421 (0.23 to 0.603)**
17 Tuberculosis	17 Headache disorders	**37.9 (19.4 to 62.4)**	**5.57 (0.339 to 17)**	0.104 (-0.0263 to 0.217)
18 Dietary iron deficiency	18 Age-related hearing loss	**93.4 (64.2 to 126)**	**48 (36.4 to 59.2)**	**-0.617 (-0.759 to -0.442)**
19 Gynecological diseases	19 Falls	**57.9 (30.1 to 90.7)**	**20.8 (4.07 to 40.3)**	-7 (-17.9 to 5.14)
20 Oral disorders	20 Blindness and vision loss	**83.5 (55 to 114)**	**40.5 (29.4 to 50.6)**	**-5.33 (-5.73 to -4.91)**
21 Falls	26 Gynecological diseases			
23 Blindness and vision loss	30 Dietary iron deficiency			
24 Age-related hearing loss	47 Tuberculosis			
26 COPD	159 COVID-19			

Supplemental Results Figure S7. Leading 20 level 3 causes of DALYs, and percentage change in number of DALYs and all-age and age-standardised DALY rates by location, 2022 and 2050

Ecuador, Reference scenario

Legend:
Communicable, maternal, neonatal and nutritional
Non-communicable
Injuries

Leading Causes 2022	Leading Causes 2050	Mean % change number of DALY	Mean % change all-age DALY rate	Mean % change age-standardized DALY rate
1 COVID-19	1 Diabetes	**168 (122 to 218)**	**115 (87.3 to 144)**	**33.3 (19 to 48.6)**
2 Ischemic heart disease	2 Chronic kidney disease	**169 (81.5 to 263)**	**116 (53.5 to 199)**	26.6 (-11.5 to 80.5)
3 Road injuries	3 Ischemic heart disease	41.4 (-9.33 to 100)	13.5 (-23.2 to 62.4)	**-36.7 (-59.1 to -2.76)**
4 Diabetes	4 Road injuries	7.07 (-30.3 to 56.8)	-14 (-44.1 to 25.7)	-16.7 (-46.5 to 22.9)
5 Neonatal disorders	5 Other musculoskeletal	**120 (85.5 to 151)**	**76 (65.7 to 86.3)**	**38.8 (36.8 to 41.2)**
6 Lower respiratory infect	6 Stroke	**42.1 (10.2 to 77.1)**	14 (-8.25 to 40.2)	**-31.3 (-46.4 to -12.7)**
7 Chronic kidney disease	7 Lower respiratory infect	24.8 (-2.87 to 57.5)	0.114 (-18.6 to 22.2)	**-35.2 (-46.7 to -21.5)**
8 Stroke	8 Low back pain	**54.3 (32.6 to 77.3)**	**23.8 (17.5 to 31)**	**-3.62 (-3.77 to -3.46)**
9 Congenital defects	9 Depressive disorders	**43 (22.6 to 63)**	**14.6 (9.15 to 21.4)**	**-1.93 (-2.08 to -1.77)**
10 Anxiety disorders	10 Cirrhosis liver	**66.9 (38 to 95.1)**	**33.9 (18.6 to 51.7)**	**-12.9 (-22.3 to -0.619)**
11 Low back pain	11 Neonatal disorders	**-24.3 (-41 to -4.82)**	**-39.3 (-49.8 to -27.1)**	**-19.4 (-34.3 to -1.35)**
12 Depressive disorders	12 Anxiety disorders	**31.4 (11.9 to 51.4)**	5.27 (-0.347 to 11.5)	**0.545 (0.396 to 0.703)**
13 Other musculoskeletal	13 Age-related hearing loss	**92.2 (61.8 to 124)**	**54.1 (41.8 to 66.7)**	**-0.69 (-0.805 to -0.562)**
14 Headache disorders	14 Falls	**53.5 (22.3 to 89.1)**	**23 (9.02 to 39.8)**	-10.4 (-19.3 to 1.58)
15 Cirrhosis liver	15 Oral disorders	**79.5 (51.9 to 109)**	**43.9 (33.9 to 54.3)**	**0.461 (0.293 to 0.638)**
16 Interpersonal violence	16 Headache disorders	**29.5 (9.49 to 47.7)**	3.85 (-2.14 to 17.4)	0.115 (-0.0102 to 0.254)
17 Falls	17 Alzheimer's disease	**182 (127 to 253)**	**126 (92.5 to 160)**	**3.57 (0.558 to 6.27)**
18 Self-harm	18 COPD	**152 (80.5 to 232)**	**102 (51.8 to 154)**	-1.47 (-29.2 to 31.5)
19 Age-related hearing loss	19 Blindness and vision loss	**82.4 (52.7 to 113)**	**46.2 (35.9 to 57.4)**	**-4.61 (-4.95 to -4.27)**
20 Gynecological diseases	20 Osteoarthritis	**110 (79.5 to 140)**	**68.3 (57.4 to 79.4)**	**2.63 (2.3 to 3.04)**
22 Oral disorders	23 Self-harm			
25 Blindness and vision loss	26 Congenital defects			
26 COPD	27 Gynecological diseases			
27 Osteoarthritis	31 Interpersonal violence			
30 Alzheimer's disease	160 COVID-19			

Supplemental Results Figure S7. Leading 20 level 3 causes of DALYs, and percentage change in number of DALYs and all-age and age-standardised DALY rates by location, 2022 and 2050

Peru, Reference scenario

Legend:
Communicable, maternal, neonatal and nutritional
Non-communicable
Injuries

Leading Causes 2022	Leading Causes 2050	Mean % change number of DALY	Mean % change all-age DALY rate	Mean % change age-standardized DALY rate
1 COVID-19	1 Lower respiratory infect	**35.9 (6.04 to 67.5)**	20.1 (-2.1 to 49.4)	**-35.9 (-46.1 to -23.9)**
2 Lower respiratory infect	2 Chronic kidney disease	**180 (95.7 to 274)**	**148 (76.3 to 233)**	26.2 (-7.98 to 72.3)
3 Neonatal disorders	3 Diabetes	**116 (79.4 to 156)**	**91.3 (63.7 to 120)**	4.91 (-6.34 to 17.7)
4 Ischemic heart disease	4 Other musculoskeletal	**93.3 (69.5 to 118)**	**70.9 (59.7 to 82.9)**	**33 (31.5 to 34.3)**
5 Road injuries	5 Ischemic heart disease	35.1 (-13.2 to 97)	19.4 (-19.3 to 75.6)	**-40.7 (-59.7 to -10.4)**
6 Stroke	6 Low back pain	**43.1 (26.8 to 59.9)**	**26.5 (19.3 to 33.7)**	**-3.3 (-3.48 to -3.15)**
7 Diabetes	7 Stroke	**27.5 (0.389 to 59.8)**	12.7 (-8.41 to 37.8)	**-36.1 (-48.2 to -21.6)**
8 Low back pain	8 Cirrhosis liver	**56.2 (29.8 to 80.3)**	**38.1 (18.1 to 57.1)**	**-13.9 (-25.1 to -3.45)**
9 Anxiety disorders	9 Blindness and vision loss	**86.8 (64 to 113)**	**65.1 (50.5 to 77.8)**	**-3.84 (-4.07 to -3.6)**
10 Chronic kidney disease	10 Oral disorders	**75.6 (52.8 to 98.5)**	**55.2 (42.8 to 68.1)**	0.172 (-0.000845 to 0.359)
11 Other musculoskeletal	11 Anxiety disorders	**16.7 (1.77 to 33.3)**	3.12 (-2.7 to 11.1)	**0.276 (0.0133 to 0.567)**
12 Cirrhosis liver	12 Alzheimer's disease	**190 (139 to 245)**	**156 (121 to 190)**	-2.33 (-5.43 to 0.214)
13 Congenital defects	13 Road injuries	-4.07 (-36.1 to 45.2)	-15.2 (-42.7 to 24.9)	-20.2 (-46.7 to 19.9)
14 Oral disorders	14 Age-related hearing loss	**96.4 (71.7 to 124)**	**73.6 (60.6 to 86.1)**	**-0.555 (-0.659 to -0.457)**
15 Headache disorders	15 Neonatal disorders	**-41.6 (-53.3 to -27.2)**	**-48.4 (-57 to -38.6)**	**-29.6 (-40.5 to -16)**
16 Stomach cancer	16 Interstitial lung disease	**141 (105 to 184)**	**113 (83.2 to 145)**	-2.55 (-12.1 to 7.77)
17 Blindness and vision loss	17 Osteoarthritis	**110 (85.6 to 133)**	**85.4 (72.5 to 96.3)**	**1.94 (1.64 to 2.29)**
18 Gynecological diseases	18 Stomach cancer	**24 (4.64 to 44)**	9.59 (-5.29 to 23.5)	**-41.3 (-46.3 to -35.4)**
19 Depressive disorders	19 Headache disorders	14.9 (-1.45 to 30.2)	1.48 (-4.35 to 13.4)	-0.142 (-0.368 to 0.0883)
20 Foreign body	20 Lung cancer	**98.5 (50.9 to 156)**	**75.4 (34.2 to 121)**	-7.16 (-27 to 15.4)
21 Age-related hearing loss	22 Depressive disorders			
24 Osteoarthritis	26 Gynecological diseases			
25 Alzheimer's disease	32 Congenital defects			
26 Lung cancer	34 Foreign body			
28 Interstitial lung disease	162 COVID-19			

Supplemental Results Figure S7. Leading 20 level 3 causes of DALYs, and percentage change in number of DALYs and all-age and age-standardised DALY rates by location, 2022 and 2050

Antigua and Barbuda, Reference scenario

Legend:
Communicable, maternal, neonatal and nutritional
Non-communicable
Injuries

Leading Causes 2022
1 Diabetes
2 Stroke
3 Ischemic heart disease
4 Chronic kidney disease
5 COVID-19
6 Hypertensive heart disease
7 Low back pain
8 Lower respiratory infect
9 Neonatal disorders
10 Prostate cancer
11 Other musculoskeletal
12 Headache disorders
13 Breast cancer
14 Depressive disorders
15 Anxiety disorders
16 Alcohol use disorders
17 Mechanical forces
18 Age-related hearing loss
19 Endo/metab/blood/immune
20 HIV/AIDS
21 Cirrhosis liver
22 Colorectal cancer
27 Osteoarthritis
28 Alzheimer's disease

Leading Causes 2050	Mean % change number of DALY	Mean % change all-age DALY rate	Mean % change age-standardized DALY rate
1 Diabetes	**75.8 (47.8 to 105)**	**75.1 (56.1 to 96.5)**	9.89 (-3.15 to 26.3)
2 Chronic kidney disease	**89.3 (27.7 to 169)**	**88.6 (34.7 to 165)**	10.7 (-24.2 to 65.3)
3 Hypertensive heart disease	**123 (56.1 to 211)**	**122 (62.5 to 206)**	14.4 (-21.2 to 66.5)
4 Stroke	13.2 (-13.2 to 43.2)	12.8 (-9.48 to 40.8)	**-39.6 (-52.7 to -20.3)**
5 Ischemic heart disease	5.3 (-32 to 56.1)	4.95 (-32.3 to 57.3)	**-45.9 (-66.3 to -15.6)**
6 Prostate cancer	**109 (39.9 to 187)**	**108 (47 to 180)**	-4.43 (-27.8 to 20)
7 Lower respiratory infect	**53.3 (21.1 to 92.6)**	**52.7 (27.7 to 82.9)**	**-24.8 (-38.9 to -2.42)**
8 Other musculoskeletal	**55.6 (33.4 to 79.2)**	**54.9 (45.8 to 64.6)**	**28.3 (24.8 to 31.7)**
9 Low back pain	**19.7 (3.24 to 35.1)**	**19.2 (13.2 to 25.2)**	**-3.13 (-3.3 to -2.93)**
10 Breast cancer	**52.1 (7.94 to 106)**	**51.5 (9.35 to 99)**	-1.67 (-27.5 to 28.2)
11 Alzheimer's disease	**164 (116 to 207)**	**163 (119 to 197)**	**6.8 (3.76 to 9.71)**
12 Age-related hearing loss	**62.6 (41.1 to 86.3)**	**62 (52.1 to 76)**	**-0.533 (-0.63 to -0.449)**
13 Colorectal cancer	**82.7 (10.9 to 169)**	**82 (13.7 to 173)**	0.711 (-33.6 to 44.1)
14 Endo/metab/blood/immune	**54.8 (26 to 89.5)**	**54.2 (34.1 to 76.3)**	9.65 (-7.47 to 30.8)
15 Depressive disorders	9.84 (-6.12 to 26.8)	**9.37 (3.82 to 15.3)**	**-2.05 (-2.38 to -1.78)**
16 Alcohol use disorders	9.89 (-7.46 to 31.3)	9.45 (-3.25 to 25)	-2.95 (-11.8 to 9.05)
17 Headache disorders	-3.05 (-17.7 to 12.2)	-3.45 (-8.66 to 8.35)	**-0.33 (-0.526 to -0.0981)**
18 Anxiety disorders	0.866 (-14.2 to 18.9)	0.404 (-4.54 to 7.02)	0.0546 (-0.225 to 0.331)
19 Osteoarthritis	**63.5 (44.6 to 82.9)**	**62.9 (53.3 to 73.6)**	**1.95 (1.69 to 2.22)**
20 Cirrhosis liver	**27.3 (4.63 to 52.1)**	**26.9 (9.34 to 45.5)**	-13.1 (-24.9 to 1.8)
23 Mechanical forces			
25 Neonatal disorders			
41 HIV/AIDS			
153 COVID-19			

Supplemental Results Figure S7. Leading 20 level 3 causes of DALYs, and percentage change in number of DALYs and all-age and age-standardised DALY rates by location, 2022 and 2050

The Bahamas, Reference scenario

Legend:
Communicable, maternal, neonatal and nutritional
Non-communicable
Injuries

Leading Causes 2022	Leading Causes 2050	Mean % change number of DALY	Mean % change all-age DALY rate	Mean % change age-standardized DALY rate
1 Diabetes	1 Diabetes	**97.4 (62.4 to 136)**	**80.8 (60.4 to 104)**	**16.6 (4.67 to 31.5)**
2 Interpersonal violence	2 Hypertensive heart disease	**139 (65.2 to 230)**	**119 (57.9 to 208)**	21.2 (-14.8 to 77.1)
3 Ischemic heart disease	3 Chronic kidney disease	**114 (46.5 to 207)**	**96.3 (34.3 to 179)**	25.3 (-18 to 82.5)
4 HIV/AIDS	4 Ischemic heart disease	16.2 (-26.4 to 70.8)	6.51 (-28.6 to 62.4)	**-40.2 (-62.4 to -4.33)**
5 Hypertensive heart disease	5 Stroke	28 (-4.21 to 59)	17.3 (-6.89 to 47.7)	**-33.8 (-48.6 to -13.1)**
6 Stroke	6 Prostate cancer	**132 (55.6 to 240)**	**112 (51.8 to 200)**	-5.76 (-28 to 23.7)
7 COVID-19	7 Interpersonal violence	-41.8 (-68 to 15.9)	-46.7 (-70.4 to 2.29)	-37.1 (-66.4 to 27.8)
8 Chronic kidney disease	8 Other musculoskeletal	**55 (31.2 to 78.3)**	**42 (32.2 to 53.3)**	**15.2 (10.3 to 20.8)**
9 Road injuries	9 Lower respiratory infect	**68.8 (31 to 110)**	**54.6 (27.4 to 87.8)**	**-20.6 (-35.5 to -0.31)**
10 Low back pain	10 Low back pain	**34.3 (15.7 to 51.5)**	**23 (17.1 to 29.7)**	**-2.07 (-2.25 to -1.86)**
11 Breast cancer	11 Endo/metab/blood/immune	**58.9 (28.2 to 90.1)**	**45.6 (23.6 to 67.6)**	10.4 (-7.9 to 31.5)
12 Neonatal disorders	12 Breast cancer	37.4 (-2.97 to 94.4)	25.8 (-8.41 to 68.1)	-14.7 (-36.3 to 11.4)
13 Other musculoskeletal	13 Alzheimer's disease	**213 (144 to 294)**	**187 (139 to 234)**	**3.77 (0.954 to 6.5)**
14 Endo/metab/blood/immune	14 Age-related hearing loss	**83.2 (53.6 to 114)**	**67.7 (53.6 to 83)**	**-0.347 (-0.423 to -0.272)**
15 Headache disorders	15 Road injuries	2.93 (-38 to 63.6)	-5.62 (-42.8 to 56.8)	-0.844 (-39.7 to 70.1)
16 Lower respiratory infect	16 Colorectal cancer	**66.7 (2.66 to 156)**	52.4 (-4.79 to 131)	-10.3 (-41.3 to 29.8)
17 Depressive disorders	17 Depressive disorders	**22.3 (5.45 to 39.2)**	**12 (6.76 to 18.4)**	**-1.42 (-1.65 to -1.22)**
18 Anxiety disorders	18 Headache disorders	6.52 (-7.35 to 23)	-2.4 (-7.98 to 12.3)	**-0.345 (-0.525 to -0.137)**
19 Cirrhosis liver	19 Lung cancer	**62.8 (12 to 129)**	**48.9 (4.76 to 102)**	-7.68 (-33.9 to 19.7)
20 Prostate cancer	20 Anxiety disorders	10.2 (-5.3 to 26.1)	0.937 (-5.33 to 7.82)	-0.0974 (-0.305 to 0.125)
21 Colorectal cancer	21 Cirrhosis liver			
22 Age-related hearing loss	27 Neonatal disorders			
24 Lung cancer	28 HIV/AIDS			
36 Alzheimer's disease	156 COVID-19			

Supplemental Results Figure S7. Leading 20 level 3 causes of DALYs, and percentage change in number of DALYs and all-age and age-standardised DALY rates by location, 2022 and 2050

Barbados, Reference scenario

Legend:
Communicable, maternal, neonatal and nutritional
Non-communicable
Injuries

Leading Causes 2022	Leading Causes 2050	Mean % change number of DALY	Mean % change all-age DALY rate	Mean % change age-standardized DALY rate
1 COVID-19	1 Diabetes	**27.3 (3.2 to 58.3)**	**44.9 (21.7 to 76)**	8.54 (-7.11 to 27.8)
2 Diabetes	2 Stroke	-14.1 (-36.4 to 16.3)	-2.17 (-26.5 to 30.9)	**-35.6 (-51.1 to -11.8)**
3 Stroke	3 Chronic kidney disease	**63.7 (9.51 to 147)**	**86.5 (30.3 to 174)**	26.4 (-12.4 to 92.7)
4 Ischemic heart disease	4 Ischemic heart disease	-17.3 (-47.3 to 33.2)	-5.72 (-39.8 to 46.5)	**-40.4 (-61.9 to -5.96)**
5 Chronic kidney disease	5 Lower respiratory infect	27 (-1.85 to 65.2)	**44.6 (13.7 to 82.6)**	-22.2 (-37.3 to 0.0987)
6 Lower respiratory infect	6 Colorectal cancer	25.8 (-24 to 94.6)	42.7 (-13.5 to 107)	-3.59 (-37.7 to 33.3)
7 Low back pain	7 Prostate cancer	44.2 (-8.79 to 106)	**63.7 (3.13 to 125)**	-3.29 (-28.4 to 24.2)
8 Colorectal cancer	8 Other musculoskeletal	**25.1 (6.89 to 49.7)**	**42.3 (32.6 to 51.9)**	**25.4 (19.5 to 30.1)**
9 Breast cancer	9 Alzheimer's disease	**71.9 (29.6 to 115)**	**95.3 (58.1 to 128)**	1.36 (-1.85 to 4.13)
10 Other musculoskeletal	10 Breast cancer	8.25 (-26.6 to 50.3)	22.9 (-14.2 to 64)	-8.03 (-33.2 to 20.3)
11 Neonatal disorders	11 Low back pain	-2.98 (-16.2 to 13.3)	**10.4 (4.91 to 15.8)**	**-3.41 (-3.55 to -3.25)**
12 Prostate cancer	12 Hypertensive heart disease	**59.3 (11.3 to 133)**	**81.5 (24.7 to 159)**	16.4 (-21.8 to 70.4)
13 Age-related hearing loss	13 Urinary diseases	**76.3 (36.7 to 129)**	**101 (58.9 to 158)**	21.3 (-2.65 to 59.9)
14 Depressive disorders	14 Age-related hearing loss	**23 (2.55 to 46.5)**	**39.9 (26.5 to 51.7)**	**-0.382 (-0.491 to -0.27)**
15 Interpersonal violence	15 Depressive disorders	-7.43 (-19.3 to 7.96)	**5.29 (1.58 to 9.55)**	**-2.08 (-2.33 to -1.8)**
16 Headache disorders	16 Endo/metab/blood/immune	**25.2 (3.66 to 55.5)**	**42.5 (18.9 to 71.3)**	7.44 (-9.83 to 30.9)
17 Anxiety disorders	17 Osteoarthritis	16.6 (-0.264 to 37.9)	**32.6 (23.3 to 40.1)**	**1.49 (1.21 to 1.77)**
18 Alzheimer's disease	18 Headache disorders	**-15.2 (-25.5 to -2.03)**	-3.49 (-7.44 to 4.43)	**-0.517 (-0.718 to -0.277)**
19 Hypertensive heart disease	19 Anxiety disorders	-12.6 (-23.8 to 2.42)	-0.54 (-4.25 to 3.5)	-0.185 (-0.431 to 0.0384)
20 Urinary diseases	20 Oral disorders	7.74 (-6.96 to 25.7)	**22.5 (14.9 to 30.3)**	0.151 (-0.0848 to 0.359)
21 Osteoarthritis	22 Neonatal disorders			
22 Endo/metab/blood/immune	31 Interpersonal violence			
23 Oral disorders	153 COVID-19			

Supplemental Results Figure S7. Leading 20 level 3 causes of DALYs, and percentage change in number of DALYs and all-age and age-standardised DALY rates by location, 2022 and 2050

Belize, Reference scenario

Legend:
Communicable, maternal, neonatal and nutritional
Non-communicable
Injuries

Leading Causes 2022
1 COVID-19
2 Diabetes
3 Neonatal disorders
4 Interpersonal violence
5 HIV/AIDS
6 Ischemic heart disease
7 Road injuries
8 Chronic kidney disease
9 Lower respiratory infect
10 Stroke
11 Low back pain
12 Headache disorders
13 Depressive disorders
14 Cirrhosis liver
15 Anxiety disorders
16 Congenital defects
17 Other musculoskeletal
18 Dietary iron deficiency
19 Hypertensive heart disease
20 Gynecological diseases
21 Age-related hearing loss
22 Endo/metab/blood/immune
26 COPD
39 Alzheimer's disease

Leading Causes 2050	Mean % change number of DALY	Mean % change all-age DALY rate	Mean % change age-standardized DALY rate
1 Diabetes	**146 (104 to 197)**	**65.2 (42.6 to 95.8)**	-2.13 (-15.8 to 15.5)
2 Chronic kidney disease	**187 (94.2 to 313)**	**93.2 (30.8 to 187)**	21.5 (-20 to 86.8)
3 Ischemic heart disease	**84.3 (14.5 to 186)**	24 (-22.6 to 96.9)	-33.8 (-59.6 to 9.31)
4 Stroke	**81.9 (36 to 133)**	22.3 (-6.7 to 60.3)	**-33.3 (-50.4 to -11.8)**
5 Road injuries	55.5 (-5.16 to 155)	4.63 (-34.6 to 74.3)	2.19 (-37.2 to 73.3)
6 Interpersonal violence	4.33 (-39.8 to 84.3)	-30.2 (-59.3 to 18.5)	-26.8 (-57.3 to 25.2)
7 Neonatal disorders	-8.89 (-30 to 17.7)	**-38.8 (-50.4 to -23.6)**	-18.7 (-35 to 1.81)
8 Lower respiratory infect	**53.8 (21 to 94.9)**	3.37 (-16.6 to 29.8)	**-31.5 (-45.2 to -11.5)**
9 Other musculoskeletal	**176 (134 to 212)**	**85 (71.7 to 99.7)**	**40.8 (35.9 to 45.5)**
10 Low back pain	**88.2 (61.3 to 111)**	**26.4 (19.3 to 34.2)**	**-2.85 (-3.09 to -2.61)**
11 Hypertensive heart disease	**194 (104 to 311)**	**97.5 (37.8 to 185)**	5.5 (-28.3 to 55.3)
12 Cirrhosis liver	**105 (67.7 to 149)**	**38.1 (16.8 to 63.8)**	-8.16 (-21.7 to 7.49)
13 Depressive disorders	**73.7 (49.1 to 97.2)**	**16.6 (10.6 to 24.6)**	**-1.59 (-1.91 to -1.28)**
14 Headache disorders	**55.5 (33.5 to 82.1)**	4.37 (-2.71 to 21.8)	**0.483 (0.291 to 0.749)**
15 HIV/AIDS	-14.5 (-58.7 to 55.8)	-42.6 (-73 to 2.15)	**-49.5 (-76.3 to -9.37)**
16 Anxiety disorders	**58.5 (36.1 to 79.7)**	6.47 (-0.805 to 14.6)	**0.95 (0.632 to 1.29)**
17 Age-related hearing loss	**142 (106 to 178)**	**62.5 (48.9 to 74.1)**	**-0.991 (-1.24 to -0.741)**
18 COPD	**157 (87.8 to 239)**	**72.7 (28.3 to 132)**	-10.6 (-36.4 to 23.2)
19 Alzheimer's disease	**266 (191 to 330)**	**146 (105 to 183)**	1.07 (-1.65 to 3.82)
20 Endo/metab/blood/immune	**98.4 (59.1 to 147)**	**33.3 (12.6 to 67)**	6.51 (-12 to 34)
26 Dietary iron deficiency			
27 Gynecological diseases			
35 Congenital defects			
156 COVID-19			

Supplemental Results Figure S7. Leading 20 level 3 causes of DALYs, and percentage change in number of DALYs and all-age and age-standardised DALY rates by location, 2022 and 2050

Bermuda, Reference scenario

Legend:
Communicable, maternal, neonatal and nutritional
Non-communicable
Injuries

Leading Causes 2022	Leading Causes 2050	Mean % change number of DALY	Mean % change all-age DALY rate	Mean % change age-standardized DALY rate
1 Ischemic heart disease	1 Diabetes	**39.4 (20.1 to 60.9)**	**67.5 (51 to 84.6)**	**26.4 (15.3 to 37.2)**
2 Diabetes	2 Ischemic heart disease	-18.8 (-48.9 to 27)	-2.28 (-36.1 to 47.2)	**-50.8 (-65.3 to -24.7)**
3 COVID-19	3 Alzheimer's disease	**100 (60 to 140)**	**141 (106 to 175)**	**-8.86 (-11.5 to -6.13)**
4 Stroke	4 Prostate cancer	**54.7 (9.79 to 103)**	**85.8 (36.4 to 139)**	-5.66 (-26.2 to 15.2)
5 Lung cancer	5 Age-related hearing loss	**30.9 (14.2 to 50.8)**	**57.4 (45 to 70.2)**	**-0.402 (-0.496 to -0.305)**
6 Low back pain	6 Colorectal cancer	28 (-19.4 to 83.1)	53.7 (-1.98 to 115)	-15.4 (-43.1 to 15.7)
7 Colorectal cancer	7 Lung cancer	1.7 (-27.8 to 33.5)	22.2 (-12.5 to 58.6)	**-20.5 (-39.9 to -0.343)**
8 Age-related hearing loss	8 Chronic kidney disease	**62.7 (6.71 to 140)**	**95.6 (32.1 to 189)**	3.65 (-27.9 to 49.9)
9 Other musculoskeletal	9 Stroke	-19.2 (-40.3 to 5.9)	-2.84 (-25.6 to 26.4)	**-46.1 (-56 to -31.8)**
10 Alzheimer's disease	10 Low back pain	-7.98 (-18 to 3.91)	**10.7 (5.01 to 15.9)**	**-2.54 (-2.7 to -2.38)**
11 Prostate cancer	11 Other musculoskeletal	11.2 (-1.6 to 26)	**33.7 (26 to 44.8)**	**23.3 (21 to 25.2)**
12 Depressive disorders	12 Lower respiratory infect	**56.4 (16 to 98.8)**	**88 (45.7 to 134)**	**-25.3 (-38.4 to -9.43)**
13 Chronic kidney disease	13 Other cardiovascular	12.4 (-10.8 to 38.9)	**35.1 (11.9 to 61.6)**	**-25.3 (-34.9 to -13.2)**
14 Other cardiovascular	14 COPD	**52.4 (7.62 to 105)**	**83.1 (34 to 147)**	-11.5 (-32.1 to 19.5)
15 Headache disorders	15 Osteoarthritis	**14.6 (2.22 to 30.2)**	**37.7 (32.2 to 43.6)**	**1.64 (1.42 to 1.86)**
16 Osteoarthritis	16 Depressive disorders	**-12.1 (-20.8 to -0.536)**	**5.63 (0.621 to 11)**	**-1.48 (-1.68 to -1.3)**
17 Anxiety disorders	17 Hypertensive heart disease	**67.5 (6.64 to 148)**	**101 (32.7 to 189)**	0.799 (-30 to 41.9)
18 Breast cancer	18 Pancreatic cancer	54.5 (-11.7 to 136)	**85.5 (9.14 to 182)**	11.8 (-28.8 to 62.5)
19 Oral disorders	19 Breast cancer	0.756 (-29.4 to 37.7)	21 (-12.5 to 58)	**-23.6 (-41.7 to -4.6)**
20 Lower respiratory infect	20 Atrial fibrillation	**110 (64.2 to 162)**	**153 (109 to 207)**	2.11 (-9.28 to 14.2)
21 COPD	21 Oral disorders			
26 Pancreatic cancer	25 Anxiety disorders			
27 Hypertensive heart disease	26 Headache disorders			
35 Atrial fibrillation	152 COVID-19			

Supplemental Results Figure S7. Leading 20 level 3 causes of DALYs, and percentage change in number of DALYs and all-age and age-standardised DALY rates by location, 2022 and 2050

Cuba, Reference scenario

Legend:
Communicable, maternal, neonatal and nutritional
Non-communicable
Injuries

Leading Causes 2022	Leading Causes 2050	Mean % change number of DALY	Mean % change all-age DALY rate	Mean % change age-standardized DALY rate
1 Ischemic heart disease	1 Ischemic heart disease	-12.9 (-41 to 22.3)	4.44 (-28.4 to 45.1)	**-42.2 (-60.2 to -14.5)**
2 Stroke	2 Diabetes	**53.8 (32 to 74.7)**	**84.1 (69.9 to 95.6)**	**40.3 (30.8 to 48.9)**
3 Diabetes	3 Stroke	-14.4 (-32 to 5.55)	2.66 (-17.3 to 24.9)	**-41.2 (-52.1 to -28.6)**
4 Lung cancer	4 Falls	**64.7 (34.5 to 98.2)**	**97.2 (72.5 to 124)**	7.09 (-3.62 to 19.2)
5 Lower respiratory infect	5 Lower respiratory infect	21.4 (-1.69 to 50)	**45.3 (21.2 to 73.7)**	**-30.7 (-41.7 to -17.1)**
6 Falls	6 Lung cancer	7.53 (-23.4 to 44.1)	28.5 (-1.96 to 67.1)	-13.8 (-32.7 to 9.4)
7 COPD	7 COPD	27.4 (-3.58 to 63.8)	**52.7 (16.2 to 89.9)**	-14.5 (-35.1 to 11.8)
8 Low back pain	8 Alzheimer's disease	**95.5 (59.5 to 131)**	**134 (104 to 164)**	-2.59 (-5.89 to 0.375)
9 Depressive disorders	9 Prostate cancer	**59.6 (15.2 to 117)**	**90.8 (42.8 to 151)**	-0.393 (-19.7 to 22.2)
10 Age-related hearing loss	10 Chronic kidney disease	**52.2 (11 to 107)**	**82.5 (34.8 to 144)**	11.6 (-17.4 to 54.3)
11 Other musculoskeletal	11 Colorectal cancer	36.6 (-9.07 to 98.7)	**63.2 (14.6 to 133)**	-3.13 (-30 to 33.3)
12 Colorectal cancer	12 Age-related hearing loss	**32.6 (13.5 to 51.5)**	**58.8 (45.9 to 71.4)**	**-0.468 (-0.552 to -0.386)**
13 Chronic kidney disease	13 Hypertensive heart disease	**69 (22.6 to 128)**	**103 (46 to 175)**	16.3 (-15.9 to 59.4)
14 Headache disorders	14 Low back pain	-4.15 (-16.7 to 8.2)	**14.8 (9.9 to 20.8)**	**-2.94 (-3.09 to -2.76)**
15 Prostate cancer	15 Depressive disorders	-8.51 (-20.3 to 3.68)	**9.54 (5.24 to 15.9)**	**-1.58 (-1.75 to -1.42)**
16 Alzheimer's disease	16 Other musculoskeletal	8.67 (-6.16 to 22.4)	**30.1 (25.3 to 35.7)**	**27.7 (25.2 to 29.8)**
17 Cirrhosis liver	17 Blindness and vision loss	**23.5 (5.59 to 41.8)**	**47.9 (35.5 to 58.1)**	**-3.77 (-4 to -3.54)**
18 Anxiety disorders	18 Osteoarthritis	**20.5 (3.25 to 37.3)**	**44.3 (38.1 to 51.4)**	**1.8 (1.54 to 2.09)**
19 Self-harm	19 Oral disorders	10.3 (-5.9 to 26.2)	**32.1 (23.1 to 40.9)**	**0.268 (0.0897 to 0.452)**
20 Hypertensive heart disease	20 Cirrhosis liver	-14.2 (-28.9 to 2.43)	2.7 (-9 to 16.2)	-9.25 (-20.1 to 2.8)
21 Blindness and vision loss	22 Headache disorders			
22 Oral disorders	24 Anxiety disorders			
24 Osteoarthritis	26 Self-harm			

Supplemental Results Figure S7. Leading 20 level 3 causes of DALYs, and percentage change in number of DALYs and all-age and age-standardised DALY rates by location, 2022 and 2050

Dominica, Reference scenario

Legend:
Communicable, maternal, neonatal and nutritional
Non-communicable
Injuries

Leading Causes 2022	Leading Causes 2050	Mean % change number of DALY	Mean % change all-age DALY rate	Mean % change age-standardized DALY rate
1 COVID-19	1 Diabetes	**40.9 (13 to 70.8)**	**52.6 (31.7 to 77.2)**	3.28 (-11.2 to 19.5)
2 Diabetes	2 Chronic kidney disease	**54.7 (7.94 to 121)**	**67.7 (15.7 to 141)**	12.6 (-25 to 71.8)
3 Ischemic heart disease	3 Hypertensive heart disease	**77.7 (22.6 to 142)**	**92.6 (31.6 to 169)**	16.2 (-21.4 to 66.7)
4 Stroke	4 Stroke	-11.6 (-33.2 to 13.8)	-4.16 (-25.4 to 21.8)	**-41.4 (-55.7 to -18.7)**
5 Neonatal disorders	5 Ischemic heart disease	-15.5 (-44.7 to 29.5)	-8.4 (-41.6 to 38.7)	**-44.2 (-64.3 to -9.57)**
6 Chronic kidney disease	6 Prostate cancer	**75.1 (18.4 to 146)**	**89.6 (32.6 to 163)**	9.59 (-16 to 47.5)
7 Hypertensive heart disease	7 Neonatal disorders	**-38.1 (-53.6 to -20.3)**	**-33 (-45.7 to -14.8)**	-12.2 (-31.1 to 14.8)
8 Lower respiratory infect	8 Lower respiratory infect	11.5 (-14.1 to 41.1)	20.8 (-6.89 to 47.4)	**-28.5 (-42.8 to -9.19)**
9 Road injuries	9 Other musculoskeletal	**49.9 (25.2 to 75.5)**	**62.3 (52.9 to 71)**	**30.2 (27.4 to 32.9)**
10 Low back pain	10 Low back pain	11.8 (-7.06 to 29.2)	**21.1 (15.2 to 27.2)**	**-2.35 (-2.57 to -2.13)**
11 Headache disorders	11 Lung cancer	39.1 (-7.72 to 92.9)	**50.5 (4.57 to 99.5)**	-0.399 (-28.1 to 30.4)
12 Other musculoskeletal	12 Breast cancer	33.4 (-9.75 to 79.1)	**44.4 (3.62 to 90.4)**	-3.29 (-29.3 to 25.4)
13 Congenital defects	13 COPD	**58.5 (11.7 to 111)**	**71.7 (27.5 to 128)**	2.7 (-26.4 to 42.5)
14 Depressive disorders	14 Alzheimer's disease	**97.7 (53.1 to 138)**	**114 (80.5 to 145)**	**7.58 (4.65 to 10.9)**
15 Lung cancer	15 Age-related hearing loss	**36.8 (12.7 to 61.9)**	**48.1 (35.7 to 58.7)**	**-0.65 (-0.903 to -0.341)**
16 Cardiomyopathy	16 Road injuries	-14.8 (-48.8 to 34.3)	-7.67 (-39.8 to 45.8)	-4.22 (-39.7 to 55.2)
17 Stomach cancer	17 Colorectal cancer	48.6 (-12.3 to 122)	**60.7 (2.1 to 140)**	1.03 (-34 to 46.1)
18 Interpersonal violence	18 Depressive disorders	4.73 (-12.4 to 22.4)	**13.4 (8.13 to 19.4)**	**-0.88 (-1.17 to -0.655)**
19 Breast cancer	19 Cardiomyopathy	0.0857 (-19.8 to 19.3)	8.31 (-5.16 to 22)	**-21.7 (-28.3 to -14.6)**
20 Prostate cancer	20 Headache disorders	-6.38 (-21.7 to 10.6)	1.36 (-5.43 to 17)	**0.775 (0.532 to 0.935)**
22 Age-related hearing loss	23 Stomach cancer			
23 COPD	31 Congenital defects			
26 Colorectal cancer	37 Interpersonal violence			
32 Alzheimer's disease	156 COVID-19			

Supplemental Results Figure S7. Leading 20 level 3 causes of DALYs, and percentage change in number of DALYs and all-age and age-standardised DALY rates by location, 2022 and 2050

Dominican Republic, Reference scenario

Legend:
Communicable, maternal, neonatal and nutritional
Non-communicable
Injuries

Leading Causes 2022	Leading Causes 2050	Mean % change number of DALY	Mean % change all-age DALY rate	Mean % change age-standardized DALY rate
1 Ischemic heart disease	1 Ischemic heart disease	22.5 (-23.9 to 78.8)	6.44 (-31.4 to 59.8)	**-39.9 (-63 to -5.07)**
2 Neonatal disorders	2 Diabetes	**133 (87.7 to 174)**	**102 (79 to 128)**	**29.5 (15.3 to 44.2)**
3 Stroke	3 Stroke	22.5 (-10.4 to 56.9)	6.45 (-16 to 29.7)	**-37.8 (-51.2 to -21.9)**
4 Diabetes	4 Neonatal disorders	**-35.9 (-54.9 to -15.9)**	**-44.4 (-54.2 to -31.8)**	**-20.5 (-34.9 to -2.76)**
5 Road injuries	5 Chronic kidney disease	**118 (41.3 to 201)**	**89.2 (33.7 to 163)**	20.3 (-17.2 to 72.3)
6 Interpersonal violence	6 Road injuries	-5.53 (-42.6 to 42.8)	-18.1 (-45.8 to 25.1)	-17.8 (-47.7 to 26.8)
7 Cirrhosis liver	7 Other musculoskeletal	**124 (81.9 to 160)**	**94.2 (82.7 to 107)**	**56.5 (53.5 to 59.4)**
8 Low back pain	8 Cirrhosis liver	**37.2 (10 to 66)**	**19.2 (1.8 to 36.3)**	**-17.6 (-29.7 to -3.99)**
9 Depressive disorders	9 Low back pain	**38.7 (14.3 to 61.6)**	**20.4 (13.9 to 27.2)**	**-4.37 (-4.53 to -4.16)**
10 Chronic kidney disease	10 Depressive disorders	**32.6 (9.16 to 57.1)**	**15.2 (9.42 to 22.9)**	**-2.07 (-2.25 to -1.87)**
11 Headache disorders	11 Alzheimer's disease	**179 (119 to 239)**	**142 (99.7 to 186)**	2.44 (-0.265 to 5.53)
12 Anxiety disorders	12 Age-related hearing loss	**85.9 (52.3 to 118)**	**61.6 (48 to 77.2)**	**-0.961 (-1.1 to -0.841)**
13 Lower respiratory infect	13 Hypertensive heart disease	**120 (48.4 to 202)**	**91.4 (37.1 to 158)**	5.65 (-25.1 to 50.7)
14 Congenital defects	14 Headache disorders	16.9 (-5.46 to 39.6)	1.54 (-3.3 to 13.6)	**0.76 (0.486 to 0.946)**
15 COVID-19	15 Lung cancer	**96.3 (36.9 to 163)**	**70.8 (21.7 to 128)**	1.25 (-23.7 to 30.5)
16 Other musculoskeletal	16 Interpersonal violence	-35.7 (-62.2 to 7.48)	**-44 (-64.6 to -7.59)**	-38.6 (-61.6 to 3.84)
17 Age-related hearing loss	17 Anxiety disorders	20.6 (-1.12 to 42)	4.75 (-0.931 to 11.1)	**1.27 (1.14 to 1.41)**
18 HIV/AIDS	18 Lower respiratory infect	10.7 (-16.6 to 40.5)	-3.82 (-21.9 to 19.6)	**-34.5 (-44.4 to -21.1)**
19 Dietary iron deficiency	19 Prostate cancer	**149 (64.2 to 255)**	**116 (49.7 to 198)**	3.3 (-21.6 to 29.3)
20 Endo/metab/blood/immune	20 Oral disorders	**63.6 (33.2 to 89.9)**	**42.1 (30.8 to 53.9)**	**0.862 (0.575 to 1.16)**
21 Hypertensive heart disease	25 Endo/metab/blood/immune			
23 Lung cancer	30 Dietary iron deficiency			
25 Oral disorders	33 Congenital defects			
27 Alzheimer's disease	71 HIV/AIDS			
34 Prostate cancer	161 COVID-19			

Supplemental Results Figure S7. Leading 20 level 3 causes of DALYs, and percentage change in number of DALYs and all-age and age-standardised DALY rates by location, 2022 and 2050

Grenada, Reference scenario

Legend:
Communicable, maternal, neonatal and nutritional
Non-communicable
Injuries

Leading Causes 2022	Leading Causes 2050	Mean % change number of DALY	Mean % change all-age DALY rate	Mean % change age-standardized DALY rate
1 Diabetes	1 Diabetes	**50.8 (21.8 to 78)**	**66.9 (41.6 to 96.4)**	4.61 (-10.4 to 24)
2 COVID-19	2 Chronic kidney disease	**61.7 (7.12 to 127)**	**79.4 (18.4 to 158)**	11.5 (-28.9 to 68.1)
3 Ischemic heart disease	3 Ischemic heart disease	-9.11 (-43.3 to 38.6)	0.936 (-36.7 to 53)	**-42.9 (-65.9 to -5.07)**
4 Stroke	4 Stroke	-3.22 (-27.6 to 24)	7.24 (-16.9 to 38.1)	**-40.8 (-56.1 to -16.8)**
5 Chronic kidney disease	5 Prostate cancer	**101 (28.3 to 186)**	**122 (49.5 to 205)**	13.1 (-17.2 to 47.9)
6 Neonatal disorders	6 Hypertensive heart disease	**78.6 (27.8 to 148)**	**98.2 (37.3 to 180)**	8.03 (-26.1 to 62.5)
7 Lower respiratory infect	7 Lower respiratory infect	23 (-3.41 to 50)	**36.1 (10.4 to 64.1)**	**-27.7 (-43.1 to -7.82)**
8 Low back pain	8 Other musculoskeletal	**59.3 (32.7 to 85.3)**	**76 (64.3 to 89.5)**	**41.3 (35.9 to 46.7)**
9 Hypertensive heart disease	9 Low back pain	9.55 (-5.43 to 27.4)	**21.1 (14.3 to 27.4)**	**-3.8 (-3.99 to -3.58)**
10 Prostate cancer	10 Breast cancer	34.7 (-9.06 to 87.2)	**48.6 (2.87 to 99.4)**	-3.17 (-30.1 to 26.1)
11 Headache disorders	11 Colorectal cancer	60.8 (-4.36 to 151)	**77.2 (7.72 to 157)**	2.46 (-35.1 to 44.7)
12 Depressive disorders	12 Age-related hearing loss	**45 (21.6 to 71.7)**	**60.3 (45.3 to 73.9)**	**-0.778 (-1.05 to -0.466)**
13 Other musculoskeletal	13 Alzheimer's disease	**122 (72.4 to 175)**	**145 (103 to 189)**	-0.614 (-3.66 to 2.29)
14 Anxiety disorders	14 Depressive disorders	1.06 (-13.7 to 19.5)	**11.7 (6.09 to 17.7)**	**-1.67 (-1.91 to -1.43)**
15 Breast cancer	15 Neonatal disorders	**-44.9 (-58.7 to -28.2)**	**-39.2 (-50.4 to -23)**	-18.2 (-34.2 to 3.88)
16 Cirrhosis liver	16 Endo/metab/blood/immune	**29.7 (4.87 to 58.4)**	**43.6 (21 to 73)**	7.06 (-12.4 to 29.9)
17 Age-related hearing loss	17 Pancreatic cancer	**110 (16.9 to 248)**	**131 (30.7 to 275)**	38.3 (-18.2 to 116)
18 Endo/metab/blood/immune	18 Headache disorders	-9.21 (-23.9 to 8.15)	0.299 (-5.62 to 15.1)	**0.765 (0.411 to 0.956)**
19 Colorectal cancer	19 COPD	**59.8 (17.6 to 113)**	**77.2 (27.8 to 131)**	-4.67 (-35.6 to 29.7)
20 Congenital defects	20 Cirrhosis liver	9.19 (-10.7 to 31.6)	**20.9 (1.08 to 43.1)**	-15.1 (-29.8 to 1.05)
29 COPD	21 Anxiety disorders			
37 Alzheimer's disease	44 Congenital defects			
39 Pancreatic cancer	154 COVID-19			

Supplemental Results Figure S7. Leading 20 level 3 causes of DALYs, and percentage change in number of DALYs and all-age and age-standardised DALY rates by location, 2022 and 2050

Guyana, Reference scenario

Legend:
Communicable, maternal, neonatal and nutritional
Non-communicable
Injuries

Leading Causes 2022	Leading Causes 2050	Mean % change number of DALY	Mean % change all-age DALY rate	Mean % change age-standardized DALY rate
1 Diabetes	1 Diabetes	20.3 (-9.08 to 50.9)	**48.5 (25.6 to 76.6)**	-7.16 (-19.5 to 7.31)
2 COVID-19	2 Stroke	-22.5 (-43.4 to 1.83)	-4.07 (-29.2 to 28.2)	**-49.8 (-61.7 to -34.7)**
3 Ischemic heart disease	3 Ischemic heart disease	**-35.3 (-61 to -3.4)**	-19.7 (-49 to 22.7)	**-58.1 (-73.5 to -33.8)**
4 Neonatal disorders	4 Chronic kidney disease	19.9 (-22.7 to 72.2)	**48.5 (2.73 to 118)**	-6.95 (-37.1 to 36.4)
5 Stroke	5 Hypertensive heart disease	45 (-4.44 to 106)	**79.5 (23.5 to 153)**	-5.87 (-34.9 to 35.2)
6 Self-harm	6 Self-harm	-26.1 (-47.7 to 2.29)	-8.93 (-30.9 to 15)	-8.17 (-31.8 to 17.3)
7 Chronic kidney disease	7 Neonatal disorders	**-56 (-68.2 to -40)**	**-46 (-55.8 to -33.7)**	**-28.1 (-41.9 to -10.3)**
8 Interpersonal violence	8 Lower respiratory infect	-11.6 (-34.3 to 14.2)	9.34 (-13.4 to 42.9)	**-32.4 (-45.2 to -15.7)**
9 HIV/AIDS	9 Cirrhosis liver	-13.1 (-35.1 to 9.99)	7.16 (-8.38 to 26.4)	**-18.8 (-29.7 to -5.19)**
10 Lower respiratory infect	10 Other musculoskeletal	**67.6 (27.9 to 101)**	**106 (89.7 to 126)**	**70 (65.6 to 74.5)**
11 Hypertensive heart disease	11 Depressive disorders	-11.5 (-32 to 8.9)	**8.94 (2.46 to 16.3)**	**-2.62 (-2.95 to -2.2)**
12 Cirrhosis liver	12 Interpersonal violence	**-47 (-71.2 to -13.8)**	-35.1 (-58.1 to 0.582)	-30.2 (-55.1 to 9.79)
13 Road injuries	13 Prostate cancer	**96.1 (25.8 to 181)**	**142 (68.1 to 243)**	0.688 (-22.6 to 29.8)
14 Depressive disorders	14 Low back pain	-2.17 (-24.6 to 19.5)	**20.5 (11.1 to 30.2)**	**-4.96 (-5.36 to -4.45)**
15 Congenital defects	15 Road injuries	-31.5 (-58.8 to 5.84)	-15.5 (-44.6 to 31.7)	-14.1 (-43.8 to 33.7)
16 Low back pain	16 Falls	26.4 (-9.06 to 65.4)	**55.8 (28.7 to 92.2)**	-3.54 (-16.1 to 10.9)
17 Malaria	17 Endo/metab/blood/immune	5.21 (-21.6 to 36.5)	**29.7 (10.6 to 52.5)**	7.08 (-10.2 to 26.2)
18 Headache disorders	18 Age-related hearing loss	**43.4 (11.2 to 75.6)**	**77.1 (53.8 to 105)**	**-1.22 (-1.41 to -1.06)**
19 Endo/metab/blood/immune	19 Alzheimer's disease	**176 (99.5 to 250)**	**241 (159 to 325)**	1.91 (-1.09 to 4.53)
20 Anxiety disorders	20 Breast cancer	20.7 (-20.9 to 63.2)	**48.6 (9.65 to 100)**	3.67 (-24.6 to 40.2)
21 Other musculoskeletal	21 Headache disorders			
23 Falls	23 Anxiety disorders			
24 Breast cancer	24 Malaria			
26 Age-related hearing loss	31 Congenital defects			
31 Prostate cancer	45 HIV/AIDS			
49 Alzheimer's disease	161 COVID-19			

Supplemental Results Figure S7. Leading 20 level 3 causes of DALYs, and percentage change in number of DALYs and all-age and age-standardised DALY rates by location, 2022 and 2050

Haiti, Reference scenario

Legend:
Communicable, maternal, neonatal and nutritional
Non-communicable
Injuries

Leading Causes 2022	Leading Causes 2050	Mean % change number of DALY	Mean % change all-age DALY rate	Mean % change age-standardized DALY rate
1 Nature disaster	1 Nature disaster	848 (-99.3 to 15100)	620 (-99.4 to 12000)	640 (-99.4 to 12300)
2 Neonatal disorders	2 Diabetes	**107 (55 to 173)**	**65 (34 to 101)**	-2.48 (-20.4 to 23.7)
3 Lower respiratory infect	3 Ischemic heart disease	49.8 (-14.9 to 139)	19.5 (-28.8 to 96.5)	-34.6 (-63.1 to 13)
4 Diarrheal diseases	4 Stroke	25.3 (-14.6 to 74.9)	-0.0951 (-27.3 to 34.3)	**-41.3 (-59.1 to -15.3)**
5 Ischemic heart disease	5 Neonatal disorders	**-45.3 (-64.3 to -20.2)**	**-56.5 (-68.1 to -41.5)**	**-32.5 (-48.9 to -8.88)**
6 Congenital defects	6 Road injuries	-5.68 (-48.1 to 70.6)	-24.8 (-55.7 to 28)	-22.1 (-53.6 to 33)
7 Stroke	7 Lower respiratory infect	**-48.5 (-62.8 to -30.1)**	**-59 (-69.1 to -46.5)**	**-54.5 (-65 to -41.4)**
8 HIV/AIDS	8 Congenital defects	**-41 (-56.6 to -22.2)**	**-53.1 (-60.6 to -44)**	**-29 (-38.7 to -17.2)**
9 Diabetes	9 Chronic kidney disease	**108 (30.7 to 213)**	**66.2 (10.9 to 160)**	11.8 (-27.7 to 73.5)
10 Road injuries	10 Interpersonal violence	-24.6 (-56.7 to 24.7)	-40 (-62.9 to 0.11)	-37.7 (-61.4 to 3.56)
11 Interpersonal violence	11 Diarrheal diseases	**-62 (-80.6 to -29.7)**	**-69.8 (-83.1 to -47.2)**	**-58.7 (-76.7 to -27.6)**
12 Pertussis	12 Cirrhosis liver	32 (-1.94 to 70.4)	5.24 (-14.9 to 33.3)	**-29.7 (-42.7 to -12.7)**
13 Dietary iron deficiency	13 Hypertensive heart disease	**125 (46.2 to 220)**	**79.4 (25.3 to 152)**	-5.55 (-40.6 to 41.7)
14 Asthma	14 Other musculoskeletal	**146 (92.3 to 197)**	**95.6 (73.5 to 117)**	**54.6 (43.6 to 63.8)**
15 Drowning	15 Low back pain	**53 (21 to 84.3)**	**21.9 (13.7 to 30.6)**	**-5.21 (-5.48 to -4.92)**
16 Cirrhosis liver	16 Dietary iron deficiency	0.147 (-19.1 to 22.6)	**-20.4 (-24.5 to -16.7)**	**-13 (-13.7 to -12.3)**
17 COVID-19	17 Depressive disorders	**44.1 (14.8 to 73.5)**	**14.7 (8.51 to 22.5)**	**-2.98 (-3.28 to -2.62)**
18 Maternal disorders	18 Headache disorders	**33.2 (1.84 to 62.4)**	**6 (1.03 to 17.9)**	-0.0314 (-0.309 to 0.236)
19 Chronic kidney disease	19 Anxiety disorders	**36 (9.42 to 64.2)**	**8.21 (2.46 to 14.4)**	**0.579 (0.227 to 0.898)**
20 Headache disorders	20 COPD	**66.2 (11.8 to 135)**	32.5 (-2.67 to 80.6)	**-32.4 (-52.7 to -3.27)**
21 Low back pain	26 Asthma			
23 Depressive disorders	27 HIV/AIDS			
25 Anxiety disorders	31 Drowning			
29 Hypertensive heart disease	34 Pertussis			
31 COPD	46 Maternal disorders			
36 Other musculoskeletal	161 COVID-19			

Supplemental Results Figure S7. Leading 20 level 3 causes of DALYs, and percentage change in number of DALYs and all-age and age-standardised DALY rates by location, 2022 and 2050

Jamaica, Reference scenario

Legend:
Communicable, maternal, neonatal and nutritional
Non-communicable
Injuries

Leading Causes 2022	Leading Causes 2050	Mean % change number of DALY	Mean % change all-age DALY rate	Mean % change age-standardized DALY rate
1 Diabetes	1 Diabetes	**73.8 (39 to 118)**	**92.6 (61.3 to 132)**	3.51 (-12.5 to 24.3)
2 COVID-19	2 Stroke	17.8 (-9.77 to 54)	**30.7 (4.24 to 68)**	**-32.6 (-46.8 to -8.15)**
3 Stroke	3 Chronic kidney disease	**97 (34.5 to 186)**	**118 (50.8 to 217)**	25.4 (-13.9 to 87.4)
4 Interpersonal violence	4 Hypertensive heart disease	**107 (39.1 to 199)**	**130 (61 to 227)**	19.7 (-17.1 to 76.9)
5 Neonatal disorders	5 Ischemic heart disease	19.1 (-24.6 to 81)	32.2 (-12 to 109)	-33.6 (-57.2 to 8.71)
6 Ischemic heart disease	6 Prostate cancer	**93.2 (30.7 to 165)**	**114 (52.6 to 183)**	-1.67 (-24.9 to 24.1)
7 Chronic kidney disease	7 Other musculoskeletal	**50.2 (27.5 to 75.8)**	**66.3 (52 to 79.6)**	**25.3 (19.4 to 30.1)**
8 Low back pain	8 Low back pain	**18.7 (0.905 to 36.8)**	**31.5 (23 to 39.7)**	**-2.58 (-2.8 to -2.33)**
9 Hypertensive heart disease	9 Alzheimer's disease	**109 (56.6 to 163)**	**132 (88.6 to 174)**	**4.07 (0.84 to 7.25)**
10 HIV/AIDS	10 Interpersonal violence	**-48.2 (-66.3 to -17.5)**	**-42.7 (-61.1 to -13.2)**	**-39.2 (-59.9 to -6.03)**
11 Other musculoskeletal	11 Age-related hearing loss	**57.2 (30 to 83.7)**	**74.1 (59 to 89.3)**	**-0.558 (-0.691 to -0.461)**
12 Headache disorders	12 Breast cancer	**43.9 (3.2 to 87.5)**	**59.3 (16.2 to 105)**	-4.53 (-28.4 to 20.1)
13 Depressive disorders	13 Lung cancer	**58.9 (6.73 to 116)**	**75.8 (19.1 to 140)**	-7.11 (-35 to 20.6)
14 Anxiety disorders	14 Colorectal cancer	**70.9 (8.15 to 145)**	**89.1 (24.7 to 170)**	-0.433 (-33.5 to 37.9)
15 Prostate cancer	15 Neonatal disorders	**-54.3 (-65.8 to -40.8)**	**-49.4 (-58.2 to -38.3)**	**-25.6 (-39.4 to -6.47)**
16 Breast cancer	16 Depressive disorders	6.31 (-11.7 to 23.4)	**17.7 (9.73 to 27.3)**	**-1.45 (-1.75 to -1.18)**
17 Age-related hearing loss	17 COPD	**56.8 (13.4 to 119)**	**73.8 (32 to 135)**	-13.5 (-34.2 to 19.3)
18 Lung cancer	18 Headache disorders	-10.2 (-24.9 to 9.34)	-0.59 (-9.46 to 19.1)	0.0707 (-0.0813 to 0.247)
19 Alzheimer's disease	19 Anxiety disorders	-6.76 (-22.1 to 11.9)	3.21 (-5.04 to 14.2)	**0.399 (0.233 to 0.594)**
20 Colorectal cancer	20 Osteoarthritis	**72.2 (46.1 to 98.2)**	**90.8 (78.9 to 104)**	**2.19 (1.92 to 2.49)**
22 COPD	39 HIV/AIDS			
29 Osteoarthritis	154 COVID-19			

Supplemental Results Figure S7. Leading 20 level 3 causes of DALYs, and percentage change in number of DALYs and all-age and age-standardised DALY rates by location, 2022 and 2050

Puerto Rico, Reference scenario

Legend:
Communicable, maternal, neonatal and nutritional
Non-communicable
Injuries

Leading Causes 2022	Leading Causes 2050	Mean % change number of DALY	Mean % change all-age DALY rate	Mean % change age-standardized DALY rate
1 Diabetes	1 Diabetes	-1.95 (-14 to 13.5)	**37.6 (22.8 to 53.9)**	-5.19 (-13.1 to 3.55)
2 Ischemic heart disease	2 Chronic kidney disease	**39.6 (4.37 to 92.6)**	**96.2 (45.4 to 162)**	8.17 (-15.5 to 45.3)
3 COVID-19	3 Alzheimer's disease	**71.7 (39.4 to 102)**	**141 (108 to 178)**	**-6.16 (-8.61 to -3.64)**
4 Chronic kidney disease	4 Ischemic heart disease	**-35.8 (-54.3 to -13.2)**	-9.75 (-34 to 21.6)	**-51.4 (-63.1 to -33.7)**
5 Interpersonal violence	5 COPD	**40.5 (10.7 to 73.7)**	**97.4 (51.9 to 151)**	-6.72 (-24.5 to 15.8)
6 Low back pain	6 Age-related hearing loss	**18.8 (1.72 to 33.6)**	**66.7 (53.4 to 84.2)**	**-0.299 (-0.354 to -0.235)**
7 Alzheimer's disease	7 Low back pain	**-14.1 (-23.3 to -4.64)**	**20.5 (15.1 to 26.3)**	**-2.33 (-2.48 to -2.19)**
8 Stroke	8 Endo/metab/blood/immune	19 (-0.802 to 43.9)	**67 (43.5 to 94.6)**	4.64 (-5.91 to 17.3)
9 COPD	9 Hypertensive heart disease	**40.9 (1.25 to 83.8)**	**98 (41.5 to 162)**	5.79 (-19.4 to 39.9)
10 Age-related hearing loss	10 Other musculoskeletal	-3.49 (-15.5 to 7.96)	**35.3 (29.5 to 41.3)**	**18.5 (17.1 to 19.9)**
11 Other musculoskeletal	11 Stroke	**-25.9 (-37.5 to -11.9)**	4.06 (-11.4 to 22.8)	**-39.8 (-48.2 to -29.7)**
12 Colorectal cancer	12 Colorectal cancer	-0.457 (-31.4 to 33.2)	**39.6 (1.21 to 86.6)**	-14.9 (-38.4 to 14.8)
13 Cirrhosis liver	13 Osteoarthritis	8.94 (-3.23 to 20.8)	**52.9 (43.9 to 62.7)**	**1.2 (1.01 to 1.38)**
14 Endo/metab/blood/immune	14 Lower respiratory infect	11.7 (-13.4 to 39.3)	**56.9 (22.3 to 97.7)**	**-26.3 (-36.8 to -14.1)**
15 Headache disorders	15 Falls	3.27 (-15.9 to 23.7)	**45 (22.2 to 71.1)**	**-17.3 (-26.4 to -6.3)**
16 Depressive disorders	16 Oral disorders	-0.898 (-12.3 to 11.7)	**39 (29.5 to 50.2)**	**0.207 (0.0546 to 0.357)**
17 Anxiety disorders	17 Blindness and vision loss	12.4 (-2.84 to 26.7)	**57.8 (45.6 to 74)**	**-2.69 (-2.83 to -2.52)**
18 Osteoarthritis	18 Urinary diseases	**66.8 (34.1 to 110)**	**134 (88.2 to 192)**	12.5 (-1.74 to 27)
19 Falls	19 Prostate cancer	22.9 (-9.48 to 58.5)	**72.4 (32.6 to 124)**	-7.99 (-25 to 9.49)
20 Lower respiratory infect	20 Depressive disorders	**-20.7 (-30.9 to -12.1)**	**11.2 (6.02 to 18.3)**	**-1.23 (-1.46 to -1.05)**
22 Oral disorders	21 Cirrhosis liver			
23 Hypertensive heart disease	25 Anxiety disorders			
26 Blindness and vision loss	26 Headache disorders			
27 Prostate cancer	28 Interpersonal violence			
34 Urinary diseases	153 COVID-19			

Supplemental Results Figure S7. Leading 20 level 3 causes of DALYs, and percentage change in number of DALYs and all-age and age-standardised DALY rates by location, 2022 and 2050

Saint Kitts and Nevis, Reference scenario

Legend:
Communicable, maternal, neonatal and nutritional
Non-communicable
Injuries

Leading Causes 2022	Leading Causes 2050	Mean % change number of DALY	Mean % change all-age DALY rate	Mean % change age-standardized DALY rate
1 Diabetes	1 Diabetes	**63.6 (26.9 to 107)**	**85.3 (60.3 to 115)**	8.47 (-5.89 to 24.7)
2 Stroke	2 Chronic kidney disease	**70.3 (9.66 to 155)**	**93.1 (31.4 to 181)**	0.858 (-32.4 to 53.1)
3 Ischemic heart disease	3 Stroke	-4.53 (-32.5 to 32.2)	8.22 (-18.6 to 42.3)	**-50.5 (-63.5 to -29.7)**
4 COVID-19	4 Ischemic heart disease	-11.1 (-48.6 to 38.8)	0.788 (-38.9 to 55)	**-53.5 (-72.6 to -24.5)**
5 Chronic kidney disease	5 Prostate cancer	**129 (46.9 to 233)**	**159 (74.1 to 267)**	-0.541 (-29.6 to 29)
6 HIV/AIDS	6 Hypertensive heart disease	**106 (33.1 to 201)**	**133 (59.1 to 227)**	3.2 (-31.1 to 56.4)
7 Alcohol use disorders	7 Lower respiratory infect	**42.9 (6.3 to 92.7)**	**61.9 (28.6 to 100)**	**-32.4 (-45.9 to -13.2)**
8 Neonatal disorders	8 Other musculoskeletal	**41.7 (14.3 to 74.7)**	**60.5 (48.2 to 74.3)**	**29.2 (24.2 to 34.3)**
9 Interpersonal violence	9 Alcohol use disorders	-9.47 (-29.5 to 15.3)	2.59 (-11.6 to 21)	-12.9 (-24.4 to 1.29)
10 Depressive disorders	10 Low back pain	9.34 (-10.8 to 33.2)	**23.8 (17.2 to 30.4)**	**-2.79 (-2.99 to -2.61)**
11 Low back pain	11 Depressive disorders	-0.117 (-17.9 to 25.6)	**13.1 (5.53 to 21.5)**	**-1.26 (-1.46 to -1.08)**
12 Lower respiratory infect	12 Age-related hearing loss	**57.8 (28.1 to 92.8)**	**78.7 (63.4 to 93.6)**	**-0.607 (-0.733 to -0.45)**
13 Other musculoskeletal	13 Alzheimer's disease	**178 (109 to 259)**	**215 (161 to 268)**	0.561 (-2.08 to 3.37)
14 Prostate cancer	14 Colorectal cancer	61.7 (-0.97 to 148)	**82.8 (13.6 to 170)**	-9.57 (-40.6 to 31.3)
15 Headache disorders	15 Breast cancer	33.6 (-10.8 to 89.2)	**51.1 (7.9 to 99.7)**	-12.6 (-33.9 to 13.9)
16 Cirrhosis liver	16 COPD	**83.6 (26.8 to 165)**	**108 (53 to 182)**	-8.92 (-35.2 to 31.7)
17 Hypertensive heart disease	17 Urinary diseases	**119 (64 to 189)**	**148 (99 to 224)**	14.8 (-9.78 to 49.6)
18 Anxiety disorders	18 Cirrhosis liver	-6.03 (-26.4 to 18.7)	6.49 (-9.76 to 24.6)	**-30.7 (-40.4 to -17.6)**
19 Breast cancer	19 Osteoarthritis	**55.2 (26.9 to 89)**	**75.8 (66.3 to 84.7)**	**2.27 (1.99 to 2.53)**
20 Age-related hearing loss	20 Headache disorders	-15.1 (-30 to 5.26)	-3.83 (-9.75 to 9.26)	**0.583 (0.339 to 0.767)**
21 Colorectal cancer	21 Anxiety disorders			
23 COPD	25 Neonatal disorders			
29 Osteoarthritis	27 HIV/AIDS			
37 Urinary diseases	32 Interpersonal violence			
38 Alzheimer's disease	153 COVID-19			

Supplemental Results Figure S7. Leading 20 level 3 causes of DALYs, and percentage change in number of DALYs and all-age and age-standardised DALY rates by location, 2022 and 2050

Saint Lucia, Reference scenario

Legend:
Communicable, maternal, neonatal and nutritional
Non-communicable
Injuries

Leading Causes 2022	Leading Causes 2050	Mean % change number of DALY	Mean % change all-age DALY rate	Mean % change age-standardized DALY rate
1 Diabetes	1 Diabetes	**58.4 (27 to 92.9)**	**61.5 (38.9 to 89.2)**	5.85 (-7.56 to 23.2)
2 COVID-19	2 Stroke	3.4 (-25.4 to 43.7)	5.55 (-24.1 to 44.3)	**-43.6 (-58.6 to -20.5)**
3 Stroke	3 Chronic kidney disease	**63.4 (1.7 to 141)**	**66.9 (9.74 to 150)**	3.51 (-33.5 to 61.5)
4 Neonatal disorders	4 Hypertensive heart disease	**93.3 (29.8 to 184)**	**97.5 (35.1 to 189)**	3.47 (-32.1 to 58.4)
5 Chronic kidney disease	5 Prostate cancer	**102 (27.5 to 198)**	**105 (35.1 to 196)**	-6.74 (-31.6 to 22.3)
6 Ischemic heart disease	6 Ischemic heart disease	2.02 (-40.2 to 58.8)	4.25 (-34.2 to 66)	**-44.7 (-65.4 to -7.97)**
7 Interpersonal violence	7 Other musculoskeletal	**60.2 (34.8 to 83)**	**63.3 (51.9 to 74.5)**	**35.5 (31.5 to 39.1)**
8 Low back pain	8 COPD	**61 (16.6 to 115)**	**64.4 (21 to 128)**	-14.5 (-38.3 to 23.1)
9 Hypertensive heart disease	9 Low back pain	**17.4 (1.27 to 32.8)**	**19.7 (13.5 to 26.3)**	**-3.16 (-3.31 to -2.99)**
10 Prostate cancer	10 Alzheimer's disease	**133 (83.6 to 182)**	**137 (99.2 to 172)**	**-5.83 (-8.35 to -2.91)**
11 Depressive disorders	11 Age-related hearing loss	**63.4 (35.4 to 90.7)**	**66.5 (50.7 to 80.4)**	**-0.723 (-0.816 to -0.638)**
12 Other musculoskeletal	12 Lower respiratory infect	30.3 (-0.014 to 68.7)	**32.9 (5.35 to 65.6)**	**-34.6 (-48.6 to -13.2)**
13 Road injuries	13 Neonatal disorders	**-40.5 (-52.7 to -25.5)**	**-39.3 (-49.3 to -26.4)**	-21.4 (-39.1 to 1.94)
14 COPD	14 Depressive disorders	7.03 (-7.23 to 21.2)	**9.11 (4.1 to 15.1)**	**-1.54 (-1.69 to -1.4)**
15 Headache disorders	15 Cirrhosis liver	6.85 (-13.4 to 31.4)	9.03 (-7.68 to 31)	**-19.7 (-32.2 to -2.25)**
16 Lower respiratory infect	16 Breast cancer	23.7 (-18.8 to 70.6)	25.9 (-14.7 to 73.3)	-14 (-39.3 to 14.6)
17 Cirrhosis liver	17 Osteoarthritis	**60.6 (36.7 to 84)**	**63.7 (52.8 to 74.9)**	**2.73 (2.43 to 3)**
18 Anxiety disorders	18 Headache disorders	-5.59 (-19.4 to 8.62)	-3.73 (-9.12 to 8.73)	**0.447 (0.276 to 0.564)**
19 Age-related hearing loss	19 Anxiety disorders	-1.14 (-15 to 12.8)	0.799 (-4.75 to 7.51)	**0.84 (0.745 to 0.955)**
20 Breast cancer	20 Oral disorders	**37.3 (15.7 to 56.6)**	**40 (28.6 to 51.9)**	**0.681 (0.475 to 0.857)**
23 Alzheimer's disease	21 Road injuries			
24 Oral disorders	25 Interpersonal violence			
27 Osteoarthritis	154 COVID-19			

Supplemental Results Figure S7. Leading 20 level 3 causes of DALYs, and percentage change in number of DALYs and all-age and age-standardised DALY rates by location, 2022 and 2050

Saint Vincent and the Grenadines, Reference scenario

Legend: Communicable, maternal, neonatal and nutritional; Non-communicable; Injuries

Leading Causes 2022	Leading Causes 2050	Mean % change number of DALY	Mean % change all-age DALY rate	Mean % change age-standardized DALY rate
1 Diabetes	1 Diabetes	**33.5 (8.7 to 61.7)**	**57.1 (36.4 to 85.8)**	7.04 (-6.8 to 26)
2 COVID-19	2 Hypertensive heart disease	**65.5 (11.8 to 131)**	**95 (30.8 to 176)**	13.7 (-25.5 to 73.4)
3 Ischemic heart disease	3 Ischemic heart disease	-10.5 (-45.9 to 39.6)	5.45 (-34.6 to 68.5)	-40 (-65.4 to 2.4)
4 Stroke	4 Stroke	-9.95 (-32.1 to 20.8)	6.05 (-17.4 to 41.5)	**-36.9 (-53 to -11.2)**
5 Hypertensive heart disease	5 Chronic kidney disease	44.3 (-5.26 to 109)	**70 (13.7 to 148)**	12 (-29.1 to 72.6)
6 Interpersonal violence	6 Prostate cancer	**57.6 (1.07 to 126)**	**85.2 (20 to 160)**	-5.95 (-31 to 24.7)
7 Chronic kidney disease	7 Lower respiratory infect	11.5 (-15.3 to 36.2)	**31.2 (4.91 to 58.1)**	**-29.6 (-46.4 to -8.94)**
8 Neonatal disorders	8 Other musculoskeletal	**49.4 (24.2 to 71.9)**	**75.6 (63.3 to 89.4)**	**44.4 (38.9 to 49.8)**
9 Lower respiratory infect	9 Low back pain	1.4 (-12.7 to 17)	**19.2 (13.4 to 25.6)**	**-3.07 (-3.26 to -2.85)**
10 Prostate cancer	10 Alzheimer's disease	**103 (58.3 to 142)**	**139 (97.4 to 179)**	**6.32 (3.23 to 9.56)**
11 HIV/AIDS	11 Breast cancer	24.1 (-18.4 to 72.8)	**45.7 (1.07 to 105)**	3.44 (-26.4 to 41.6)
12 Low back pain	12 Age-related hearing loss	**32.2 (9.61 to 53)**	**55.5 (42.4 to 67.2)**	**-1.04 (-1.23 to -0.835)**
13 Depressive disorders	13 Interpersonal violence	**-47.4 (-70.7 to -7.36)**	-38.2 (-63 to 9.45)	-32.1 (-59.9 to 18.5)
14 Headache disorders	14 Depressive disorders	-5.72 (-19.7 to 8.16)	**10.8 (6.14 to 16.5)**	**-1.52 (-1.77 to -1.25)**
15 Other musculoskeletal	15 Endo/metab/blood/immune	16.7 (-9.33 to 42.6)	**37.3 (12.1 to 62.3)**	5.14 (-15.8 to 29.7)
16 Breast cancer	16 Cirrhosis liver	4.09 (-16.3 to 28.4)	**22.5 (0.884 to 50.4)**	-2.69 (-18.9 to 19.2)
17 Cirrhosis liver	17 Colorectal cancer	45.9 (-12.1 to 113)	**71.4 (7.86 to 154)**	6.23 (-30.1 to 55.7)
18 Anxiety disorders	18 Neonatal disorders	**-46.7 (-59 to -32.1)**	**-37.4 (-47.6 to -24.8)**	**-26 (-39.2 to -7.76)**
19 Alcohol use disorders	19 Headache disorders	**-14.7 (-28.6 to -0.283)**	0.268 (-4.99 to 12.5)	**1.06 (0.694 to 1.22)**
20 Age-related hearing loss	20 Falls	20.2 (-8.48 to 49)	**41.3 (14.3 to 66.7)**	-4.82 (-19.2 to 11.8)
21 Endo/metab/blood/immune	21 Alcohol use disorders			
23 Falls	22 Anxiety disorders			
24 Colorectal cancer	47 HIV/AIDS			
29 Alzheimer's disease	154 COVID-19			

Supplemental Results Figure S7. Leading 20 level 3 causes of DALYs, and percentage change in number of DALYs and all-age and age-standardised DALY rates by location, 2022 and 2050

Suriname, Reference scenario

Legend:
Communicable, maternal, neonatal and nutritional
Non-communicable
Injuries

Leading Causes 2022	Leading Causes 2050	Mean % change number of DALY	Mean % change all-age DALY rate	Mean % change age-standardized DALY rate
1 Diabetes	1 Diabetes	**68.6 (39.6 to 99.6)**	**48.7 (30.6 to 72.1)**	9.31 (-3.28 to 23.7)
2 COVID-19	2 Ischemic heart disease	7.28 (-36.4 to 64.4)	-5.16 (-44.3 to 49.8)	**-39.4 (-63.9 to -0.297)**
3 Ischemic heart disease	3 Stroke	6.27 (-20.1 to 43.1)	-6.19 (-28.3 to 24.2)	**-40 (-55 to -19.1)**
4 Stroke	4 Chronic kidney disease	**86.8 (23.1 to 160)**	**65 (10.8 to 137)**	16.1 (-22.4 to 73.9)
5 Neonatal disorders	5 Neonatal disorders	**-39.9 (-53.5 to -21)**	**-47.1 (-58.9 to -29.7)**	**-32.1 (-47.7 to -11.1)**
6 Chronic kidney disease	6 Self-harm	0.923 (-25.1 to 28)	-11 (-33 to 15.7)	-11.2 (-34.2 to 15.8)
7 Self-harm	7 Depressive disorders	**22.1 (3.58 to 39.3)**	**7.53 (3.76 to 12.4)**	**-1.98 (-2.26 to -1.76)**
8 Road injuries	8 Other musculoskeletal	**81.1 (54.3 to 110)**	**59.5 (49.6 to 71.3)**	**37.1 (31.9 to 42.4)**
9 Depressive disorders	9 Low back pain	**30.1 (11.8 to 47.8)**	**14.6 (8.51 to 21.5)**	**-3.62 (-3.85 to -3.32)**
10 HIV/AIDS	10 Road injuries	-0.526 (-41 to 58.8)	-12 (-47.7 to 44.1)	-9.94 (-46.7 to 49.8)
11 Lower respiratory infect	11 Hypertensive heart disease	**99.5 (35.9 to 179)**	**76.3 (17.1 to 152)**	8.46 (-26.9 to 59.6)
12 Low back pain	12 Alzheimer's disease	**192 (107 to 269)**	**157 (97.2 to 220)**	0.414 (-2.38 to 3.3)
13 Cirrhosis liver	13 Lower respiratory infect	15.4 (-12 to 45.7)	1.78 (-23.5 to 30.5)	**-38.7 (-51.8 to -22.3)**
14 Congenital defects	14 Age-related hearing loss	**76 (42.3 to 105)**	**55 (36.3 to 73)**	**-0.677 (-0.807 to -0.559)**
15 Headache disorders	15 Cirrhosis liver	13.8 (-6.46 to 38.1)	0.315 (-15.9 to 18.1)	**-18.4 (-31.5 to -4.19)**
16 Interpersonal violence	16 Headache disorders	13.1 (-3.46 to 32.4)	-0.448 (-4.16 to 8.72)	0.0415 (-0.131 to 0.228)
17 Other musculoskeletal	17 COPD	**74.1 (24 to 147)**	**53.7 (9.41 to 108)**	-10.9 (-35.6 to 27.5)
18 Anxiety disorders	18 Prostate cancer	**129 (37 to 234)**	**101 (24.4 to 196)**	3.93 (-26.2 to 36.9)
19 Hypertensive heart disease	19 Colorectal cancer	67.8 (-0.724 to 159)	47.4 (-8.77 to 120)	-2.01 (-35.6 to 40.2)
20 Age-related hearing loss	20 Anxiety disorders	16.2 (-1.13 to 32.1)	2.32 (-1.6 to 7.01)	**0.42 (0.202 to 0.661)**
23 COPD	29 HIV/AIDS			
24 Colorectal cancer	30 Congenital defects			
30 Alzheimer's disease	34 Interpersonal violence			
35 Prostate cancer	160 COVID-19			

Supplemental Results Figure S7. Leading 20 level 3 causes of DALYs, and percentage change in number of DALYs and all-age and age-standardised DALY rates by location, 2022 and 2050

Trinidad and Tobago, Reference scenario

Legend:
Communicable, maternal, neonatal and nutritional
Non-communicable
Injuries

Leading Causes 2022	Leading Causes 2050	Mean % change number of DALY	Mean % change all-age DALY rate	Mean % change age-standardized DALY rate
1 Diabetes	1 Diabetes	14 (-8.91 to 41.4)	**56 (31.8 to 85)**	-7.1 (-19.1 to 7.45)
2 COVID-19	2 Ischemic heart disease	-22.3 (-47.5 to 18.1)	6.35 (-28.6 to 58.6)	**-47.1 (-64.9 to -21.7)**
3 Ischemic heart disease	3 Chronic kidney disease	40.6 (-2.3 to 99.3)	**92.5 (39.2 to 166)**	8.78 (-21.6 to 53.9)
4 Stroke	4 Stroke	-16.3 (-35.9 to 7.37)	14.5 (-6.77 to 43.5)	**-43.1 (-53.6 to -29.6)**
5 Interpersonal violence	5 Alzheimer's disease	**133 (77.5 to 200)**	**218 (149 to 291)**	**-4.91 (-7.52 to -1.95)**
6 Chronic kidney disease	6 Prostate cancer	**59.9 (7.72 to 134)**	**118 (48 to 204)**	-8.92 (-31.1 to 16.6)
7 Neonatal disorders	7 Hypertensive heart disease	**66.4 (15.6 to 132)**	**128 (56.3 to 215)**	10.9 (-21.1 to 54.9)
8 Low back pain	8 Age-related hearing loss	**38 (11 to 67.9)**	**88.8 (67.5 to 110)**	**-0.533 (-0.62 to -0.441)**
9 Road injuries	9 Other musculoskeletal	12.5 (-7.5 to 35.4)	**53.8 (42.7 to 65.4)**	**23.4 (19.6 to 27.1)**
10 Depressive disorders	10 Low back pain	-7.68 (-22.9 to 9.25)	**26.3 (18.6 to 35.1)**	**-2.06 (-2.26 to -1.81)**
11 HIV/AIDS	11 Depressive disorders	**-19.1 (-33.5 to -2.63)**	**10.6 (3.54 to 19.1)**	**-0.917 (-1.11 to -0.734)**
12 Other musculoskeletal	12 Colorectal cancer	18.5 (-23.2 to 71.9)	**61.8 (6.48 to 127)**	-12.4 (-38.8 to 18)
13 Self-harm	13 Lower respiratory infect	13.8 (-13.9 to 50.2)	**55.6 (21.1 to 96.1)**	**-30.1 (-41.6 to -16.2)**
14 Headache disorders	14 Interpersonal violence	**-64.2 (-80 to -36.3)**	**-51.3 (-70.1 to -19.2)**	-36.8 (-64.7 to 15.4)
15 Prostate cancer	15 COPD	**47.7 (6.4 to 97.4)**	**102 (52 to 164)**	-8.95 (-29.4 to 15.1)
16 Age-related hearing loss	16 Breast cancer	-5.61 (-33.8 to 29.1)	28.9 (-5.29 to 61.3)	-16.5 (-37.1 to 2.15)
17 Breast cancer	17 Endo/metab/blood/immune	4.77 (-15.6 to 30.8)	**43.3 (23.2 to 66.3)**	3.33 (-11 to 19.9)
18 Hypertensive heart disease	18 Osteoarthritis	**31.7 (11.6 to 57.2)**	**80.2 (66 to 94.3)**	**1.87 (1.6 to 2.17)**
19 Congenital defects	19 Road injuries	**-40.2 (-61.9 to -6)**	-18.3 (-46.4 to 16.2)	-6.14 (-42 to 39.9)
20 Anxiety disorders	20 Neonatal disorders	**-57 (-66.5 to -45.7)**	**-41.4 (-49.1 to -31.9)**	**-23.4 (-34.5 to -9.52)**
21 Lower respiratory infect	22 Headache disorders			
22 Colorectal cancer	26 Anxiety disorders			
23 Endo/metab/blood/immune	27 Self-harm			
24 Alzheimer's disease	35 Congenital defects			
26 Osteoarthritis	43 HIV/AIDS			
30 COPD	156 COVID-19			

Supplemental Results Figure S7. Leading 20 level 3 causes of DALYs, and percentage change in number of DALYs and all-age and age-standardised DALY rates by location, 2022 and 2050

Virgin Islands, Reference scenario

Legend:
Communicable, maternal, neonatal and nutritional
Non-communicable
Injuries

Leading Causes 2022	Leading Causes 2050	Mean % change number of DALY	Mean % change all-age DALY rate	Mean % change age-standardized DALY rate
1 COVID-19	1 Diabetes	6.36 (-10.1 to 23.2)	**37.4 (21.9 to 55.6)**	**9.99 (1.19 to 18.8)**
2 Ischemic heart disease	2 Ischemic heart disease	-10.4 (-45 to 45.6)	15.8 (-28.6 to 87.3)	**-44.8 (-63.4 to -17.2)**
3 Diabetes	3 Alzheimer's disease	**124 (82.2 to 172)**	**189 (134 to 248)**	**4.85 (1.68 to 7.74)**
4 Interpersonal violence	4 Chronic kidney disease	**63.8 (14.7 to 144)**	**112 (43.5 to 224)**	19.1 (-15.9 to 77.2)
5 Stroke	5 Stroke	-11.7 (-32.8 to 14.5)	14.1 (-11.1 to 45.3)	**-34.5 (-47.4 to -19.4)**
6 Low back pain	6 Hypertensive heart disease	**74.5 (15.7 to 149)**	**126 (51.7 to 226)**	15.8 (-19.4 to 60.1)
7 Chronic kidney disease	7 Prostate cancer	**63.2 (2.98 to 143)**	**111 (40.1 to 203)**	1.69 (-22.4 to 27.2)
8 Age-related hearing loss	8 Age-related hearing loss	**16.7 (0.913 to 31.3)**	**50.8 (34 to 68.5)**	**-0.472 (-0.551 to -0.389)**
9 Other musculoskeletal	9 Low back pain	**-16.7 (-27.4 to -5.8)**	**7.54 (0.68 to 15.3)**	**-2.01 (-2.28 to -1.74)**
10 Depressive disorders	10 Lower respiratory infect	**68.1 (27.7 to 120)**	**117 (65.3 to 183)**	-16 (-30.5 to 0.804)
11 Road injuries	11 Other musculoskeletal	0.4 (-13.2 to 13.5)	**29.6 (20.6 to 40.9)**	**22.1 (19.3 to 24.2)**
12 Alzheimer's disease	12 Colorectal cancer	19.8 (-24.1 to 77.1)	54.6 (-2.45 to 127)	-7.45 (-38.8 to 26.7)
13 Lung cancer	13 Interpersonal violence	**-59.2 (-78.5 to -22.3)**	**-47.5 (-71.5 to -0.488)**	-44 (-72.5 to 8.74)
14 Colorectal cancer	14 Lung cancer	4.27 (-25.8 to 39.4)	34.5 (-2.2 to 77)	-3.71 (-28.9 to 22.5)
15 Hypertensive heart disease	15 COPD	**59.6 (11.7 to 130)**	**106 (49.3 to 185)**	1.22 (-23.9 to 35.4)
16 Prostate cancer	16 Atrial fibrillation	**149 (85.4 to 234)**	**221 (149 to 321)**	**12.4 (0.528 to 27.5)**
17 Breast cancer	17 Breast cancer	-0.933 (-30 to 31.3)	27.8 (-5.87 to 64)	-8.39 (-29.2 to 12.8)
18 Headache disorders	18 Osteoarthritis	-1.35 (-13.2 to 9.41)	**27.5 (18.1 to 39)**	**1.89 (1.66 to 2.15)**
19 Osteoarthritis	19 Depressive disorders	**-19.4 (-30.7 to -8.51)**	3.97 (-0.857 to 8.91)	**-1.16 (-1.44 to -0.874)**
20 Cirrhosis liver	20 Blindness and vision loss	9.05 (-6.01 to 22.7)	**40.9 (24.4 to 56.7)**	**-2.76 (-3.07 to -2.37)**
23 Lower respiratory infect	22 Road injuries			
25 Blindness and vision loss	25 Headache disorders			
28 COPD	26 Cirrhosis liver			
37 Atrial fibrillation	156 COVID-19			

Supplemental Results Figure S7. Leading 20 level 3 causes of DALYs, and percentage change in number of DALYs and all-age and age-standardised DALY rates by location, 2022 and 2050

Colombia, Reference scenario

Legend:
Communicable, maternal, neonatal and nutritional
Non-communicable
Injuries

Leading Causes 2022	Leading Causes 2050	Mean % change number of DALY	Mean % change all-age DALY rate	Mean % change age-standardized DALY rate
1 Ischemic heart disease	1 Diabetes	**137 (109 to 164)**	**109 (89.4 to 130)**	**32.5 (22 to 43)**
2 Interpersonal violence	2 Ischemic heart disease	30.1 (-15.8 to 92.8)	15.2 (-26.4 to 75.4)	**-44.8 (-65.2 to -13.6)**
3 COVID-19	3 Other musculoskeletal	**96.5 (76.3 to 116)**	**73.7 (62.8 to 85)**	**37.9 (34.6 to 41.2)**
4 Low back pain	4 COPD	**126 (71.4 to 187)**	**99.6 (50.3 to 157)**	-16.7 (-38.7 to 14.6)
5 Diabetes	5 Low back pain	**33.9 (20.2 to 47.3)**	**18.3 (12.1 to 25.1)**	**-4.52 (-4.7 to -4.33)**
6 Other musculoskeletal	6 Alzheimer's disease	**205 (154 to 263)**	**169 (128 to 209)**	**-4.53 (-7.67 to -1.46)**
7 Road injuries	7 Stroke	**25.2 (0.45 to 54.4)**	10.8 (-11.5 to 39.3)	**-40.9 (-53.3 to -24)**
8 Neonatal disorders	8 Age-related hearing loss	**96.8 (73.6 to 121)**	**73.9 (57.7 to 88.3)**	**-0.627 (-0.747 to -0.51)**
9 Stroke	9 Interpersonal violence	**-45.1 (-68.2 to -11)**	**-51.6 (-70.3 to -21)**	**-44.5 (-66.9 to -4.56)**
10 COPD	10 Chronic kidney disease	**132 (66.1 to 230)**	**105 (41.2 to 188)**	5.83 (-25.9 to 56.1)
11 Headache disorders	11 Blindness and vision loss	**84.1 (62.8 to 107)**	**62.7 (47.7 to 76.3)**	**-5 (-5.3 to -4.68)**
12 Anxiety disorders	12 Road injuries	-11.9 (-41.4 to 25.1)	-22.1 (-45.5 to 13.8)	-21.9 (-47.3 to 14.3)
13 Lower respiratory infect	13 Headache disorders	11.6 (-1.67 to 26.1)	-1.44 (-7.63 to 12.4)	**-0.355 (-0.591 to -0.0899)**
14 Age-related hearing loss	14 Anxiety disorders	**14.5 (0.578 to 29.8)**	1.1 (-5.25 to 8.48)	**0.412 (0.181 to 0.658)**
15 Congenital defects	15 Osteoarthritis	**98.9 (79.4 to 119)**	**75.8 (62.9 to 87.5)**	**2.36 (1.96 to 2.73)**
16 Alzheimer's disease	16 Lower respiratory infect	15.8 (-8.63 to 44)	2.49 (-19.8 to 29.1)	**-39.5 (-50.7 to -24.5)**
17 Depressive disorders	17 Oral disorders	**64 (47.2 to 81.6)**	**44.9 (33.9 to 55.9)**	**0.316 (0.0465 to 0.629)**
18 Blindness and vision loss	18 Colorectal cancer	**83.1 (15.6 to 174)**	**61.6 (7.31 to 128)**	-6.74 (-38.5 to 34)
19 Stomach cancer	19 Depressive disorders	**30.6 (16.5 to 44.4)**	**15.4 (9.96 to 21.8)**	**-1.86 (-2.08 to -1.62)**
20 Chronic kidney disease	20 Falls	**52.2 (30.1 to 75.5)**	**34.5 (18.4 to 52.6)**	-9.3 (-18.8 to 1.71)
21 Oral disorders	22 Neonatal disorders			
23 Falls	26 Stomach cancer			
26 Osteoarthritis	39 Congenital defects			
27 Colorectal cancer	158 COVID-19			

Supplemental Results Figure S7. Leading 20 level 3 causes of DALYs, and percentage change in number of DALYs and all-age and age-standardised DALY rates by location, 2022 and 2050

Costa Rica, Reference scenario

Legend:
Communicable, maternal, neonatal and nutritional
Non-communicable
Injuries

Leading Causes 2022	Leading Causes 2050	Mean % change number of DALY	Mean % change all-age DALY rate	Mean % change age-standardized DALY rate
1 Ischemic heart disease	1 Diabetes	**99.1 (69.4 to 125)**	**88.5 (74.3 to 105)**	**14.5 (5.46 to 23.1)**
2 Diabetes	2 Other musculoskeletal	**71.3 (49.3 to 92.3)**	**62.1 (55 to 71.1)**	**29.9 (27.4 to 32.4)**
3 COVID-19	3 Chronic kidney disease	**99.1 (41.3 to 176)**	**88.4 (34.3 to 160)**	0.722 (-29.7 to 42.2)
4 Road injuries	4 Ischemic heart disease	8.28 (-30.1 to 62)	2.5 (-32.6 to 50.2)	**-49.2 (-66.6 to -24.8)**
5 Low back pain	5 Alzheimer's disease	**198 (153 to 253)**	**182 (150 to 215)**	-1.76 (-4.39 to 0.755)
6 Other musculoskeletal	6 Low back pain	**29.5 (12.9 to 45.1)**	**22.6 (16.9 to 29)**	**-3.58 (-3.72 to -3.42)**
7 Chronic kidney disease	7 COPD	**133 (64.4 to 208)**	**121 (64.1 to 188)**	-11 (-35.4 to 17.4)
8 Interpersonal violence	8 Road injuries	-4.35 (-34.7 to 40.5)	-9.49 (-36.9 to 30.5)	-6.38 (-36.2 to 35.2)
9 Stroke	9 Age-related hearing loss	**88.7 (63.8 to 115)**	**78.6 (67 to 90)**	**-0.603 (-0.695 to -0.52)**
10 Depressive disorders	10 Stroke	26.7 (-1.77 to 56.7)	19.9 (-2.92 to 47.5)	**-40.3 (-52.3 to -26.1)**
11 Headache disorders	11 Depressive disorders	**22.7 (5.86 to 39.6)**	**16.1 (9.75 to 23.4)**	**-1.99 (-2.25 to -1.73)**
12 Neonatal disorders	12 Falls	**69.8 (41.6 to 101)**	**60.8 (39.9 to 84.8)**	-7.74 (-17.3 to 3.7)
13 Cirrhosis liver	13 Colorectal cancer	**73.9 (15.6 to 159)**	**64.6 (8 to 132)**	-8.02 (-37.7 to 29.4)
14 Anxiety disorders	14 Cirrhosis liver	**33.1 (11.7 to 55.2)**	**26 (11 to 41.2)**	**-15.6 (-26.4 to -4.9)**
15 Congenital defects	15 Blindness and vision loss	**78.9 (54.1 to 104)**	**69.4 (56.9 to 81.3)**	**-4.01 (-4.28 to -3.71)**
16 Age-related hearing loss	16 Headache disorders	3.93 (-9.14 to 22.7)	-1.63 (-8.39 to 13.4)	**-0.498 (-0.744 to -0.197)**
17 Self-harm	17 Lower respiratory infect	**43 (14.6 to 75.1)**	**35.4 (11 to 60.4)**	**-32.4 (-43.2 to -19)**
18 Falls	18 Osteoarthritis	**90.6 (66.5 to 114)**	**80.4 (74 to 87.4)**	**2.39 (2.14 to 2.64)**
19 Stomach cancer	19 Oral disorders	**56.8 (34.4 to 77.7)**	**48.4 (38.2 to 58.4)**	**0.548 (0.379 to 0.736)**
20 COPD	20 Anxiety disorders	6.25 (-8.47 to 22)	0.584 (-6.24 to 8.55)	0.056 (-0.184 to 0.295)
21 Colorectal cancer	21 Stomach cancer			
22 Alzheimer's disease	25 Self-harm			
23 Lower respiratory infect	26 Interpersonal violence			
24 Oral disorders	31 Neonatal disorders			
25 Blindness and vision loss	35 Congenital defects			
28 Osteoarthritis	158 COVID-19			

Supplemental Results Figure S7. Leading 20 level 3 causes of DALYs, and percentage change in number of DALYs and all-age and age-standardised DALY rates by location, 2022 and 2050

El Salvador, Reference scenario

Legend:
Communicable, maternal, neonatal and nutritional
Non-communicable
Injuries

Leading Causes 2022	Leading Causes 2050	Mean % change number of DALY	Mean % change all-age DALY rate	Mean % change age-standardized DALY rate
1 Interpersonal violence	1 Chronic kidney disease	**79.1 (20.6 to 145)**	**120 (47.5 to 204)**	21.7 (-20.5 to 74.3)
2 Ischemic heart disease	2 Diabetes	**54.9 (22.6 to 93.9)**	**90.2 (59.1 to 132)**	5.47 (-9.61 to 23.5)
3 Chronic kidney disease	3 Ischemic heart disease	15.9 (-27 to 77.1)	42.5 (-10.6 to 114)	-29.2 (-57.7 to 13.8)
4 COVID-19	4 Interpersonal violence	**-49.7 (-73.4 to -10.8)**	-38.9 (-64.1 to 5.89)	-32.8 (-62.9 to 17.9)
5 Diabetes	5 Other musculoskeletal	**62.3 (26.1 to 94.5)**	**98.7 (82.3 to 117)**	**45.8 (42.8 to 48.8)**
6 Road injuries	6 Alcohol use disorders	-1.96 (-30.9 to 26.9)	**19.8 (3.99 to 37.7)**	1 (-10.9 to 15.1)
7 Alcohol use disorders	7 Low back pain	4.44 (-19.3 to 25)	**27.9 (17.7 to 39.3)**	**-5.25 (-5.54 to -4.91)**
8 Low back pain	8 Alzheimer's disease	**115 (60 to 170)**	**164 (112 to 223)**	**8.36 (5.29 to 11.6)**
9 Neonatal disorders	9 Stroke	9.48 (-19 to 43.6)	**34.5 (4.3 to 71.4)**	**-30 (-45 to -8.82)**
10 Lower respiratory infect	10 Lower respiratory infect	-2.27 (-26.9 to 30.1)	20 (-5.89 to 54.7)	**-29.5 (-44.9 to -9.57)**
11 Stroke	11 Cirrhosis liver	6.26 (-18.9 to 31.5)	**30.3 (9.58 to 52.3)**	**-15.4 (-26.6 to -3.55)**
12 Cirrhosis liver	12 Road injuries	-27.1 (-56.2 to 17)	-10.8 (-44.2 to 44.1)	-21.8 (-54.1 to 31)
13 Other musculoskeletal	13 COPD	**74.1 (25.2 to 132)**	**114 (60 to 183)**	-3.09 (-30.7 to 31.1)
14 Depressive disorders	14 Age-related hearing loss	**47.7 (16.8 to 76.1)**	**81.2 (61.5 to 105)**	**-0.793 (-0.943 to -0.632)**
15 Headache disorders	15 Depressive disorders	-3.88 (-25.9 to 18.2)	**17.6 (9.63 to 28.2)**	**-3.27 (-3.67 to -2.84)**
16 Congenital defects	16 Blindness and vision loss	**36.1 (7.16 to 62.8)**	**66.9 (48.5 to 88.6)**	**-5.76 (-6.11 to -5.35)**
17 Anxiety disorders	17 Headache disorders	-16 (-39.5 to 4.38)	2.66 (-5.78 to 21.7)	**-1.13 (-1.71 to -0.585)**
18 Self-harm	18 Falls	19.9 (-8.36 to 49.1)	**47 (25.4 to 73.7)**	-6.63 (-16.8 to 5.29)
19 Age-related hearing loss	19 Anxiety disorders	-14.7 (-38 to 6)	4.28 (-3.57 to 13.8)	-0.226 (-0.701 to 0.222)
20 Stomach cancer	20 Oral disorders	**28.7 (1.06 to 56.1)**	**57.8 (42.8 to 74.3)**	**0.579 (0.229 to 0.86)**
21 Falls	22 Stomach cancer			
22 Alzheimer's disease	24 Neonatal disorders			
23 Blindness and vision loss	27 Self-harm			
24 COPD	38 Congenital defects			
25 Oral disorders	160 COVID-19			

Supplemental Results Figure S7. Leading 20 level 3 causes of DALYs, and percentage change in number of DALYs and all-age and age-standardised DALY rates by location, 2022 and 2050

Guatemala, Reference scenario

Legend:
Communicable, maternal, neonatal and nutritional
Non-communicable
Injuries

Leading Causes 2022	Leading Causes 2050	Mean % change number of DALY	Mean % change all-age DALY rate	Mean % change age-standardized DALY rate
1 Lower respiratory infect	1 Diabetes	**106 (75.9 to 141)**	**71.9 (46.2 to 99.4)**	-1.85 (-15.4 to 12.8)
2 Diabetes	2 Chronic kidney disease	**163 (84.8 to 272)**	**119 (55.3 to 208)**	28 (-12.4 to 84)
3 Neonatal disorders	3 Ischemic heart disease	**66.4 (6.91 to 149)**	38.6 (-8.91 to 110)	-31.6 (-58.5 to 12.6)
4 Interpersonal violence	4 Lower respiratory infect	**-29.3 (-42.7 to -15.3)**	**-41.1 (-51.4 to -27.6)**	**-50.8 (-59.5 to -40.6)**
5 Ischemic heart disease	5 Other musculoskeletal	**158 (129 to 188)**	**115 (101 to 131)**	**57.3 (53.9 to 60.3)**
6 Cirrhosis liver	6 Cirrhosis liver	**30.6 (9.7 to 51.2)**	8.68 (-4.94 to 24.3)	**-29.5 (-37.9 to -19.7)**
7 Chronic kidney disease	7 Low back pain	**50.4 (32.2 to 68.6)**	**25.1 (17.4 to 34.7)**	**-5.31 (-5.48 to -5.13)**
8 Diarrheal diseases	8 Interpersonal violence	-25.1 (-57.5 to 44.3)	-37.8 (-64.9 to 14.6)	-36.7 (-64.6 to 20.6)
9 Congenital defects	9 Alcohol use disorders	**39.8 (17.9 to 64.8)**	**16.3 (3.1 to 33.3)**	-6.3 (-16.1 to 5.66)
10 Road injuries	10 Depressive disorders	**48.4 (28.4 to 66.2)**	**23.5 (15.8 to 33.3)**	**-2.98 (-3.3 to -2.68)**
11 Low back pain	11 Stroke	**38.6 (11.6 to 73)**	15.3 (-8.93 to 42.1)	**-38.3 (-53 to -21.3)**
12 COVID-19	12 Neonatal disorders	**-49.9 (-60.7 to -35.8)**	**-58.4 (-65.9 to -48.9)**	**-37.6 (-48.5 to -23.9)**
13 Alcohol use disorders	13 Road injuries	-15.9 (-49.7 to 33.6)	-30 (-56.5 to 12.5)	-33.7 (-59.4 to 7.68)
14 Stroke	14 Headache disorders	**28.2 (9.64 to 51.3)**	6.57 (-0.903 to 25.7)	-0.163 (-0.379 to 0.101)
15 Depressive disorders	15 Falls	**47.9 (23.6 to 74.7)**	**23 (4.48 to 41.3)**	**-13.2 (-23.8 to -2.38)**
16 Other musculoskeletal	16 Age-related hearing loss	**104 (82.1 to 128)**	**69.8 (54.9 to 83.9)**	**-1.02 (-1.13 to -0.897)**
17 Headache disorders	17 Anxiety disorders	**27.6 (10.6 to 47.7)**	6.07 (-1.74 to 14.8)	**0.576 (0.361 to 0.817)**
18 Anxiety disorders	18 Alzheimer's disease	**189 (139 to 243)**	**140 (101 to 180)**	**-4.12 (-6.66 to -1.1)**
19 Mechanical forces	19 Blindness and vision loss	**89.3 (67.1 to 114)**	**57.6 (44.4 to 70.2)**	**-6.75 (-7.25 to -6.28)**
20 Falls	20 Stomach cancer	**25 (7.47 to 45.7)**	4.07 (-9.41 to 17.6)	**-42.3 (-48.2 to -36.8)**
21 Stomach cancer	22 Congenital defects			
23 Age-related hearing loss	23 Mechanical forces			
24 Blindness and vision loss	26 Diarrheal diseases			
34 Alzheimer's disease	158 COVID-19			

Supplemental Results Figure S7. Leading 20 level 3 causes of DALYs, and percentage change in number of DALYs and all-age and age-standardised DALY rates by location, 2022 and 2050

Honduras, Reference scenario

Legend:
Communicable, maternal, neonatal and nutritional
Non-communicable
Injuries

Leading Causes 2022	Leading Causes 2050	Mean % change number of DALY	Mean % change all-age DALY rate	Mean % change age-standardized DALY rate
1 Interpersonal violence	1 Ischemic heart disease	57.7 (-1.7 to 140)	20.4 (-23.2 to 79.6)	-33.8 (-62.5 to 7.27)
2 Ischemic heart disease	2 Diabetes	**149 (108 to 189)**	**90 (65.6 to 117)**	10.4 (-1.83 to 24.2)
3 COVID-19	3 Stroke	**42.6 (8.11 to 83.8)**	8.77 (-16.4 to 39.4)	**-38.3 (-54.9 to -17)**
4 Stroke	4 Interpersonal violence	-9.74 (-40.6 to 47.8)	-31.2 (-51.6 to 16)	-37.4 (-57 to 6.17)
5 Neonatal disorders	5 Other musculoskeletal	**169 (132 to 204)**	**105 (88.5 to 123)**	**49.4 (44.8 to 53.4)**
6 Diabetes	6 Low back pain	**62.2 (42.3 to 85)**	**23.7 (14.9 to 31)**	**-5.38 (-5.65 to -5.12)**
7 Low back pain	7 COPD	**86.7 (40.1 to 152)**	**42.5 (7.42 to 88.2)**	-22.8 (-46.7 to 8.05)
8 Road injuries	8 Chronic kidney disease	**173 (84.9 to 289)**	**108 (45.6 to 203)**	22.3 (-19.3 to 82)
9 Congenital defects	9 Cirrhosis liver	**53.6 (26.9 to 80.6)**	17.3 (-1.16 to 39.5)	**-28.2 (-38.2 to -17.4)**
10 Lower respiratory infect	10 Road injuries	28.5 (-22.2 to 113)	-1.9 (-41.6 to 63.3)	-22.4 (-53.3 to 27.9)
11 Cirrhosis liver	11 Neonatal disorders	**-34.8 (-51.1 to -18.5)**	**-50.4 (-59.2 to -40)**	**-31.8 (-43.7 to -17.4)**
12 Nature disaster	12 Depressive disorders	**63.8 (40.4 to 88.1)**	**24.9 (15.9 to 34.7)**	**-3.13 (-3.56 to -2.66)**
13 Other musculoskeletal	13 Headache disorders	**41.2 (20.9 to 67.9)**	**7.66 (0.592 to 23.8)**	**-0.733 (-1.14 to -0.281)**
14 COPD	14 Hypertensive heart disease	**135 (63.3 to 230)**	**79.7 (23.7 to 149)**	-4.37 (-36.6 to 37.8)
15 Headache disorders	15 Lung cancer	**131 (58.9 to 216)**	**76 (25.6 to 138)**	-2.06 (-28.1 to 32.3)
16 Diarrheal diseases	16 Lower respiratory infect	0.993 (-23 to 31.4)	**-23 (-38.2 to -0.084)**	**-39 (-51.1 to -24.6)**
17 Depressive disorders	17 Anxiety disorders	**40 (17.4 to 61.7)**	6.7 (-0.51 to 15.7)	0.102 (-0.261 to 0.463)
18 Anxiety disorders	18 Alcohol use disorders	**81 (50.2 to 108)**	**38.1 (23.3 to 55.8)**	0.866 (-8.38 to 10.3)
19 Chronic kidney disease	19 Age-related hearing loss	**106 (77.6 to 136)**	**56.8 (42.1 to 70.4)**	**-0.907 (-1.07 to -0.786)**
20 Alcohol use disorders	20 Nature disaster	780 (-99.2 to 15300)	573 (-99.4 to 11600)	617 (-99.4 to 12700)
21 Hypertensive heart disease	24 Congenital defects			
22 Lung cancer	28 Diarrheal diseases			
24 Age-related hearing loss	158 COVID-19			

Supplemental Results Figure S7. Leading 20 level 3 causes of DALYs, and percentage change in number of DALYs and all-age and age-standardised DALY rates by location, 2022 and 2050

Mexico, Reference scenario

Legend:
Communicable, maternal, neonatal and nutritional
Non-communicable
Injuries

Leading Causes 2022	Leading Causes 2050	Mean % change number of DALY	Mean % change all-age DALY rate	Mean % change age-standardized DALY rate
1 Diabetes	1 Diabetes	**76.3 (53.8 to 101)**	**55.3 (35.5 to 76.8)**	-1.29 (-13.6 to 11.9)
2 COVID-19	2 Chronic kidney disease	**111 (55.1 to 180)**	**86.4 (40.8 to 146)**	17.1 (-16.9 to 57.2)
3 Ischemic heart disease	3 Ischemic heart disease	29.2 (-8.34 to 79.1)	13.9 (-21.5 to 59.3)	**-39 (-60.9 to -8.08)**
4 Interpersonal violence	4 Cirrhosis liver	**54.3 (36.6 to 73.7)**	**35.9 (21.2 to 51)**	-1.22 (-12.5 to 11)
5 Chronic kidney disease	5 Other musculoskeletal	**96.2 (80.4 to 114)**	**72.8 (65.2 to 81.8)**	**37.5 (35 to 40.3)**
6 Cirrhosis liver	6 Interpersonal violence	-29.5 (-59.1 to 17.7)	-38.1 (-62.9 to 4.73)	-35 (-62.4 to 11.1)
7 Neonatal disorders	7 Low back pain	**35.3 (24.3 to 46.9)**	**19.1 (14.2 to 24.1)**	**-3.88 (-4.2 to -3.61)**
8 Other musculoskeletal	8 COPD	**107 (64 to 156)**	**82.1 (42.1 to 127)**	-13.8 (-37.6 to 17.1)
9 Low back pain	9 Stroke	**28.9 (9.2 to 52.9)**	13.5 (-3.1 to 34.7)	**-35 (-47.3 to -20.4)**
10 Road injuries	10 Road injuries	9.38 (-25.1 to 58.2)	-3.53 (-36.1 to 43.8)	-8.57 (-40.3 to 34.7)
11 Lower respiratory infect	11 Depressive disorders	**28.6 (17.9 to 39.7)**	**13.2 (9.27 to 17.9)**	**-2.27 (-2.46 to -2.04)**
12 Stroke	12 Lower respiratory infect	**20.5 (1.99 to 40.9)**	6.14 (-9.82 to 24.1)	**-33.9 (-46 to -22.1)**
13 Congenital defects	13 Falls	**75.7 (50 to 107)**	**54.6 (37.2 to 73.6)**	9.46 (-2.2 to 22.7)
14 Depressive disorders	14 Age-related hearing loss	**84.9 (66.3 to 105)**	**62.8 (52.2 to 75)**	**-0.797 (-0.934 to -0.641)**
15 Headache disorders	15 Alzheimer's disease	**188 (139 to 239)**	**153 (116 to 192)**	3.43 (-0.00453 to 6.3)
16 COPD	16 Headache disorders	**16.3 (5.03 to 31.4)**	2.37 (-2.64 to 16.2)	0.0513 (-0.165 to 0.275)
17 Falls	17 Neonatal disorders	**-35.6 (-46.1 to -24.6)**	**-43.3 (-52.3 to -33.5)**	**-27.9 (-39.7 to -15.8)**
18 Anxiety disorders	18 Oral disorders	**62.9 (47.9 to 77.4)**	**43.5 (34.1 to 52)**	**0.906 (0.572 to 1.28)**
19 Age-related hearing loss	19 Urinary diseases	**127 (96.2 to 165)**	**100 (73.5 to 129)**	15.1 (-1.79 to 34.7)
20 Self-harm	20 Blindness and vision loss	**73.7 (55 to 94.1)**	**52.9 (42.2 to 63.7)**	**-4.77 (-5.12 to -4.38)**
21 Oral disorders	22 Anxiety disorders			
23 Blindness and vision loss	27 Congenital defects			
27 Alzheimer's disease	28 Self-harm			
28 Urinary diseases	159 COVID-19			

Supplemental Results Figure S7. Leading 20 level 3 causes of DALYs, and percentage change in number of DALYs and all-age and age-standardised DALY rates by location, 2022 and 2050

Nicaragua, Reference scenario

Legend:
Communicable, maternal, neonatal and nutritional
Non-communicable
Injuries

Leading Causes 2022	Leading Causes 2050	Mean % change number of DALY	Mean % change all-age DALY rate	Mean % change age-standardized DALY rate
1 Neonatal disorders	1 Chronic kidney disease	**178 (81.8 to 312)**	**144 (62.5 to 247)**	29.7 (-11.9 to 91.6)
2 Diabetes	2 Diabetes	**157 (111 to 215)**	**125 (96.7 to 153)**	**17.7 (5.16 to 31.3)**
3 Chronic kidney disease	3 Ischemic heart disease	**109 (27.7 to 226)**	**83.1 (17.9 to 175)**	-24.8 (-54.3 to 22.5)
4 Ischemic heart disease	4 Other musculoskeletal	**125 (85.4 to 169)**	**97.5 (82.1 to 114)**	**44.5 (41.1 to 48.1)**
5 Low back pain	5 Low back pain	**45.6 (18.4 to 74.6)**	**27.5 (19 to 36.7)**	**-4.88 (-5.06 to -4.66)**
6 Other musculoskeletal	6 Stroke	**77.6 (33.4 to 138)**	**55.4 (23.7 to 93.4)**	**-30.1 (-44.9 to -10.4)**
7 Congenital defects	7 Cirrhosis liver	**59.6 (26.9 to 97.1)**	**39.7 (21.3 to 59.3)**	**-13.5 (-24.4 to -2.79)**
8 Lower respiratory infect	8 Alzheimer's disease	**312 (225 to 398)**	**261 (205 to 322)**	**15.2 (11.4 to 18.3)**
9 Cirrhosis liver	9 Depressive disorders	**36.2 (12.3 to 62.2)**	**19.2 (11.6 to 28.1)**	**-2.65 (-2.94 to -2.38)**
10 Headache disorders	10 COPD	**180 (101 to 278)**	**146 (85.3 to 212)**	-7.3 (-34.6 to 28.7)
11 Road injuries	11 Age-related hearing loss	**117 (77 to 154)**	**90.1 (71.8 to 109)**	**-0.678 (-0.801 to -0.553)**
12 Depressive disorders	12 Neonatal disorders	**-45.5 (-59.6 to -27.8)**	**-52.4 (-61 to -43.1)**	**-32.4 (-43.2 to -19.6)**
13 Stroke	13 Headache disorders	18.3 (-6.02 to 44.2)	3.51 (-3.68 to 22.7)	**-0.401 (-0.642 to -0.159)**
14 Anxiety disorders	14 Alcohol use disorders	**44.9 (16.2 to 79.1)**	**26.9 (12.8 to 41)**	1.98 (-7.2 to 11.7)
15 Alcohol use disorders	15 Anxiety disorders	19.5 (-5.04 to 45.4)	4.56 (-3.47 to 13.7)	**0.308 (0.0373 to 0.573)**
16 Interpersonal violence	16 Blindness and vision loss	**100 (62.2 to 134)**	**75.5 (60.6 to 91.3)**	**-5.48 (-5.87 to -5.13)**
17 Age-related hearing loss	17 Road injuries	0.346 (-40.5 to 64.2)	-12.4 (-43.1 to 33.7)	-18.6 (-48.2 to 26.7)
18 Self-harm	18 Falls	**79.4 (41 to 122)**	**57.2 (35 to 84.6)**	-4.52 (-13.5 to 7.97)
19 Falls	19 Oral disorders	**81.9 (48 to 113)**	**59.4 (45.6 to 72.1)**	0.139 (-0.193 to 0.468)
20 Blindness and vision loss	20 Lower respiratory infect	-20.3 (-40.7 to 3.67)	**-30.2 (-43.7 to -13.3)**	**-50.4 (-60 to -38.6)**
21 Oral disorders	25 Interpersonal violence			
24 COPD	26 Congenital defects			
27 Alzheimer's disease	27 Self-harm			

Supplemental Results Figure S7. Leading 20 level 3 causes of DALYs, and percentage change in number of DALYs and all-age and age-standardised DALY rates by location, 2022 and 2050

Panama, Reference scenario

Legend:
Communicable, maternal, neonatal and nutritional
Non-communicable
Injuries

Leading Causes 2022	Leading Causes 2050	Mean % change number of DALY	Mean % change all-age DALY rate	Mean % change age-standardized DALY rate
1 Diabetes	1 Diabetes	**135 (95 to 175)**	**70.6 (48.8 to 92.1)**	**18.7 (6.31 to 31.4)**
2 COVID-19	2 Other musculoskeletal	**132 (103 to 160)**	**68.3 (60.6 to 76.6)**	**38.7 (36.4 to 41.2)**
3 Ischemic heart disease	3 Chronic kidney disease	**131 (57.7 to 228)**	**68.1 (16.2 to 134)**	9.05 (-25 to 50.4)
4 Stroke	4 Low back pain	**58.7 (38.6 to 78.5)**	**15.2 (10.4 to 20.2)**	**-3.7 (-3.8 to -3.57)**
5 Neonatal disorders	5 Ischemic heart disease	19.5 (-24.1 to 77.5)	-13.2 (-43.3 to 31)	**-49.6 (-67.6 to -25.5)**
6 Low back pain	6 Stroke	**32.1 (1.29 to 71.3)**	-4.08 (-24.9 to 20)	**-42.1 (-54.6 to -26.6)**
7 Interpersonal violence	7 Alzheimer's disease	**219 (164 to 283)**	**132 (98.6 to 165)**	**4.16 (1.55 to 6.81)**
8 Other musculoskeletal	8 Age-related hearing loss	**107 (74.9 to 134)**	**49.9 (39.7 to 59.8)**	**-0.759 (-0.846 to -0.671)**
9 Congenital defects	9 Depressive disorders	**56.2 (36.5 to 77)**	**13.4 (9.37 to 18.6)**	**-1.87 (-2.02 to -1.7)**
10 Chronic kidney disease	10 Road injuries	24.8 (-23.1 to 93.3)	-9.36 (-44.6 to 39.3)	-12.7 (-47.1 to 36.4)
11 HIV/AIDS	11 COPD	**147 (82.8 to 220)**	**79.2 (31.8 to 131)**	-7.28 (-33.8 to 24.9)
12 Road injuries	12 Headache disorders	**41.3 (23.1 to 62.4)**	2.56 (-1.97 to 13.1)	**0.277 (0.157 to 0.384)**
13 Headache disorders	13 Neonatal disorders	-14.8 (-30.9 to 2.66)	**-38.2 (-47.6 to -28.2)**	**-24.8 (-36.7 to -12.2)**
14 Lower respiratory infect	14 Blindness and vision loss	**97.8 (69 to 126)**	**43.5 (31.8 to 54.6)**	**-4.78 (-5.14 to -4.41)**
15 Depressive disorders	15 Interpersonal violence	-13 (-47.5 to 65.9)	-36.9 (-60.1 to 18.8)	-34.9 (-60.2 to 21)
16 Anxiety disorders	16 Anxiety disorders	**42.2 (22.7 to 60.5)**	3.19 (-1.08 to 8)	**0.758 (0.674 to 0.846)**
17 Age-related hearing loss	17 Lower respiratory infect	18.2 (-11.8 to 47.9)	-14.2 (-32.8 to 7.49)	**-39.3 (-50.6 to -27.6)**
18 Alzheimer's disease	18 Congenital defects	**-22.9 (-36.6 to -9.12)**	**-44.1 (-49.4 to -37.8)**	**-27.9 (-33.8 to -21.5)**
19 Blindness and vision loss	19 Oral disorders	**81 (56.9 to 104)**	**31.4 (23.5 to 39.1)**	**0.661 (0.439 to 0.854)**
20 Oral disorders	20 Colorectal cancer	**114 (44.2 to 213)**	**55.2 (4.44 to 129)**	2.05 (-29.4 to 43.9)
21 COPD	32 HIV/AIDS			
24 Colorectal cancer	159 COVID-19			

Supplemental Results Figure S7. Leading 20 level 3 causes of DALYs, and percentage change in number of DALYs and all-age and age-standardised DALY rates by location, 2022 and 2050

Venezuela, Reference scenario

Legend:
Communicable, maternal, neonatal and nutritional
Non-communicable
Injuries

Leading Causes 2022	Leading Causes 2050	Mean % change number of DALY	Mean % change all-age DALY rate	Mean % change age-standardized DALY rate
1 Ischemic heart disease	1 Ischemic heart disease	57.6 (-54.1 to 302)	89.1 (-49 to 421)	39.7 (-73.3 to 318)
2 Interpersonal violence	2 Diabetes	**75.2 (23.7 to 141)**	**102 (42.1 to 203)**	44.3 (-7.52 to 149)
3 Diabetes	3 Chronic kidney disease	**121 (17.9 to 283)**	**158 (23.7 to 395)**	81.8 (-22.6 to 287)
4 COVID-19	4 Stroke	35.1 (-21.2 to 126)	57.2 (-13.5 to 192)	9.29 (-51.3 to 143)
5 Road injuries	5 Road injuries	2.09 (-65 to 142)	21.7 (-61.5 to 216)	24.7 (-58.7 to 219)
6 Neonatal disorders	6 Interpersonal violence	-50.7 (-85.6 to 18.9)	-46.1 (-81 to 28.8)	-41.2 (-81.5 to 45.6)
7 Stroke	7 Alzheimer's disease	129 (-27.4 to 284)	153 (-6.67 to 284)	**8.77 (6.01 to 11.8)**
8 Chronic kidney disease	8 Low back pain	6.84 (-19.7 to 33.9)	**20.7 (8.51 to 30.9)**	**3.33 (2.78 to 4.14)**
9 Low back pain	9 Hypertensive heart disease	**105 (13.1 to 242)**	**138 (23.2 to 331)**	49 (-42.8 to 247)
10 Lower respiratory infect	10 COPD	**57.3 (4.16 to 114)**	**80.8 (17.5 to 172)**	8.53 (-49.1 to 115)
11 Other musculoskeletal	11 Lower respiratory infect	-1.06 (-21.6 to 23.8)	12.5 (-9.92 to 38.2)	-20.3 (-44.3 to 23.4)
12 Congenital defects	12 Neonatal disorders	**-49.7 (-72.7 to -4.08)**	-41.3 (-68.5 to 20.1)	-25.2 (-57.7 to 39.1)
13 Depressive disorders	13 Age-related hearing loss	40.3 (-17.5 to 92)	**57.3 (8.01 to 93.3)**	**0.459 (0.137 to 0.64)**
14 Headache disorders	14 Depressive disorders	1.62 (-23.3 to 28.8)	**14.8 (6.21 to 24.2)**	**1.77 (1.29 to 2.31)**
15 Cirrhosis liver	15 Other musculoskeletal	-22.6 (-44.3 to 0.506)	**-12.7 (-24.7 to -1.27)**	**-24.5 (-29.1 to -18.7)**
16 COPD	16 Lung cancer	17.5 (-64.8 to 113)	29.6 (-53.3 to 121)	-18.4 (-61.1 to 33.1)
17 Anxiety disorders	17 Blindness and vision loss	42.3 (-17.4 to 99.3)	**59.6 (9.57 to 98.7)**	**4.07 (3.66 to 4.51)**
18 Lung cancer	18 Headache disorders	-13.3 (-29.9 to 7.04)	-1.71 (-7.53 to 7.47)	-0.334 (-0.908 to 0.302)
19 Mechanical forces	19 Cirrhosis liver	-6.82 (-26.5 to 12.6)	6.4 (-16.1 to 35)	-14 (-33.1 to 15.7)
20 Age-related hearing loss	20 Prostate cancer	63.2 (-57.1 to 215)	79.6 (-44.9 to 225)	-14.4 (-55.7 to 31.4)
21 Alzheimer's disease	21 Anxiety disorders			
23 Hypertensive heart disease	23 Congenital defects			
25 Blindness and vision loss	27 Mechanical forces			
36 Prostate cancer	160 COVID-19			

Supplemental Results Figure S7. Leading 20 level 3 causes of DALYs, and percentage change in number of DALYs and all-age and age-standardised DALY rates by location, 2022 and 2050

Brazil, Reference scenario

Legend:
Communicable, maternal, neonatal and nutritional
Non-communicable
Injuries

Leading Causes 2022	Leading Causes 2050	Mean % change number of DALY	Mean % change all-age DALY rate	Mean % change age-standardized DALY rate
1 Ischemic heart disease	1 Diabetes	**105 (71.7 to 140)**	**83.3 (62.9 to 108)**	**19.1 (4.4 to 35)**
2 Interpersonal violence	2 Ischemic heart disease	3.58 (-37.6 to 61)	-7.43 (-42.6 to 48.8)	**-42.9 (-65.7 to -6.28)**
3 Stroke	3 Low back pain	**29.1 (10.9 to 47.2)**	**15.1 (10.7 to 20.1)**	**-4.01 (-4.2 to -3.85)**
4 Diabetes	4 Alzheimer's disease	**165 (113 to 218)**	**136 (105 to 166)**	**-5.39 (-7.7 to -2.79)**
5 Low back pain	5 Stroke	10.2 (-19.5 to 44.8)	-1.68 (-25.5 to 29.5)	**-41.2 (-56.4 to -20.3)**
6 COVID-19	6 Other musculoskeletal	**79.8 (57 to 105)**	**60.2 (52.1 to 69.2)**	**38.8 (35.9 to 41.5)**
7 Neonatal disorders	7 Lower respiratory infect	**42.7 (10.9 to 75.3)**	**27.4 (0.283 to 56.3)**	**-32 (-47.8 to -11.3)**
8 Road injuries	8 Interpersonal violence	-26.8 (-61.5 to 29)	-34.9 (-64 to 22.2)	-26.2 (-61.4 to 43)
9 Lower respiratory infect	9 COPD	**72.2 (18.1 to 130)**	**53.7 (11.6 to 108)**	-20.5 (-44.4 to 13.9)
10 Anxiety disorders	10 Anxiety disorders	13.6 (-1.42 to 28.8)	1.22 (-1.88 to 5.15)	**0.425 (0.252 to 0.619)**
11 Other musculoskeletal	11 Chronic kidney disease	**118 (39.1 to 210)**	**94.4 (29.6 to 182)**	14.9 (-26.8 to 75.4)
12 Headache disorders	12 Road injuries	0.79 (-39.1 to 54.6)	-9.97 (-39.6 to 40.5)	-8.58 (-40.9 to 45.3)
13 Depressive disorders	13 Age-related hearing loss	**77 (49.9 to 103)**	**57.8 (47.4 to 67.7)**	**-0.923 (-1.02 to -0.814)**
14 Falls	14 Falls	**45.2 (19.8 to 78.2)**	**29.4 (13.2 to 48.6)**	**-11.8 (-20.2 to -0.663)**
15 COPD	15 Depressive disorders	**19.5 (2.13 to 36.5)**	**6.46 (3.27 to 10.7)**	**-2.87 (-3.1 to -2.67)**
16 Age-related hearing loss	16 Headache disorders	7.49 (-8.27 to 23.8)	-4.19 (-7.9 to 6.31)	-0.125 (-0.372 to 0.125)
17 Alzheimer's disease	17 Oral disorders	**61.5 (36.9 to 85.4)**	**44 (34.3 to 53.2)**	0.0901 (-0.267 to 0.456)
18 Congenital defects	18 Urinary diseases	**156 (103 to 226)**	**128 (83 to 179)**	15.5 (-8.2 to 50.1)
19 Chronic kidney disease	19 Blindness and vision loss	**64.4 (39.1 to 89.5)**	**46.6 (35.1 to 56.8)**	**-4.86 (-5.13 to -4.55)**
20 Oral disorders	20 Lung cancer	50.5 (-0.516 to 115)	33.9 (-8.21 to 82.7)	-17.2 (-41.3 to 10)
22 Blindness and vision loss	21 Neonatal disorders			
24 Lung cancer	37 Congenital defects			
33 Urinary diseases	161 COVID-19			

Supplemental Results Figure S7. Leading 20 level 3 causes of DALYs, and percentage change in number of DALYs and all-age and age-standardised DALY rates by location, 2022 and 2050

Paraguay, Reference scenario

Legend:
Communicable, maternal, neonatal and nutritional
Non-communicable
Injuries

Leading Causes 2022	Leading Causes 2050	Mean % change number of DALY	Mean % change all-age DALY rate	Mean % change age-standardized DALY rate
1 COVID-19	1 Diabetes	**100 (51.3 to 153)**	**82.6 (51.3 to 126)**	-1.45 (-17 to 21)
2 Diabetes	2 Ischemic heart disease	24.9 (-26.1 to 91.9)	14 (-25.9 to 82.6)	**-42 (-64 to -6.22)**
3 Ischemic heart disease	3 Stroke	30.4 (-9.4 to 74.9)	18.9 (-9.54 to 58.5)	**-38.7 (-53.1 to -15.7)**
4 Neonatal disorders	4 Other musculoskeletal	**105 (59.9 to 145)**	**87.1 (71.2 to 104)**	**41.9 (38.5 to 45.2)**
5 Stroke	5 Chronic kidney disease	**156 (60.7 to 279)**	**134 (52.1 to 244)**	27.1 (-14.8 to 89.1)
6 Road injuries	6 Road injuries	2.26 (-36.7 to 64.4)	-6.85 (-39.7 to 45.8)	-9.17 (-42 to 44.1)
7 Low back pain	7 Low back pain	**38.3 (8.31 to 65.3)**	**26 (16.6 to 35.6)**	**-3.71 (-3.92 to -3.46)**
8 Anxiety disorders	8 Anxiety disorders	18.4 (-8.47 to 43.3)	**7.77 (0.145 to 17.1)**	**0.957 (0.809 to 1.13)**
9 Interpersonal violence	9 Alzheimer's disease	**183 (108 to 258)**	**158 (108 to 208)**	**4.81 (2.43 to 7.38)**
10 Headache disorders	10 Lower respiratory infect	27.5 (-8.64 to 67.7)	16.4 (-9.33 to 48.7)	**-27.7 (-41.3 to -6.92)**
11 Other musculoskeletal	11 Neonatal disorders	**-34 (-55.2 to -9.36)**	**-40.1 (-52 to -22.9)**	-15.1 (-32.5 to 10.5)
12 Lower respiratory infect	12 Age-related hearing loss	**86 (44.4 to 122)**	**69.7 (52.6 to 86.4)**	**-0.782 (-0.877 to -0.683)**
13 Congenital defects	13 Headache disorders	9.37 (-18.1 to 37.3)	-0.483 (-7.43 to 21.8)	**0.499 (0.303 to 0.658)**
14 Depressive disorders	14 Hypertensive heart disease	**169 (68.7 to 280)**	**145 (67.5 to 255)**	17.6 (-20.8 to 70.9)
15 Chronic kidney disease	15 Depressive disorders	25.3 (-2.41 to 50.2)	**14.1 (7.67 to 23.7)**	**-1.76 (-2.03 to -1.44)**
16 Alcohol use disorders	16 Lung cancer	**95.7 (20.4 to 170)**	**78.6 (20.2 to 145)**	-6.54 (-34.7 to 25.3)
17 Age-related hearing loss	17 Alcohol use disorders	**38.1 (7.51 to 68.8)**	**25.9 (12 to 42.9)**	2.01 (-6.63 to 11.5)
18 Falls	18 COPD	**98.5 (37.9 to 172)**	**81.2 (34.4 to 145)**	-13.6 (-37.6 to 21.1)
19 Alzheimer's disease	19 Blindness and vision loss	**67.2 (32 to 99.2)**	**52.4 (37 to 68.4)**	**-4.84 (-5.23 to -4.42)**
20 Blindness and vision loss	20 Falls	**42.3 (7.92 to 76.2)**	**29.7 (10 to 51.1)**	-9.99 (-20.6 to 2.9)
22 Lung cancer	22 Interpersonal violence			
23 COPD	30 Congenital defects			
27 Hypertensive heart disease	160 COVID-19			

Supplemental Results Figure S7. Leading 20 level 3 causes of DALYs, and percentage change in number of DALYs and all-age and age-standardised DALY rates by location, 2022 and 2050

Afghanistan, Reference scenario

Legend:
Communicable, maternal, neonatal and nutritional
Non-communicable
Injuries

Leading Causes 2022
1 Neonatal disorders
2 Congenital defects
3 Lower respiratory infect
4 Conflict & terror
5 Ischemic heart disease
6 Stroke
7 Road injuries
8 Tuberculosis
9 COVID-19
10 Pertussis
11 Diarrheal diseases
12 Diabetes
13 Interpersonal violence
14 Depressive disorders
15 Maternal disorders
16 Low back pain
17 Headache disorders
18 Anxiety disorders
19 Chronic kidney disease
20 Meningitis
21 Falls
23 Cirrhosis liver
24 Hypertensive heart disease
26 Gynecological diseases

Leading Causes 2050	Mean % change number of DALY	Mean % change all-age DALY rate	Mean % change age-standardized DALY rate
1 Ischemic heart disease	**96.6 (9.33 to 242)**	-8.12 (-48.3 to 56.7)	-39.4 (-67.8 to 8.71)
2 Congenital defects	-7.26 (-39.9 to 27.3)	**-57 (-63.4 to -48.7)**	**-38.3 (-46 to -28.3)**
3 Neonatal disorders	-17.1 (-53 to 26.2)	**-61.4 (-73.7 to -43.3)**	**-42.4 (-59.5 to -19)**
4 Conflict & terror	80.3 (-36.7 to 375)	-16.3 (-70.2 to 96.2)	-19.8 (-71.1 to 80.1)
5 Diabetes	**266 (152 to 386)**	**70.3 (46 to 97.9)**	10.1 (-6.49 to 30.6)
6 Stroke	**67.3 (5.8 to 152)**	-22 (-45.1 to 8.61)	**-46.2 (-65.6 to -19.7)**
7 Road injuries	44.5 (-22.6 to 163)	-32.4 (-64.4 to 18.5)	-37.7 (-66.4 to 9.8)
8 Lower respiratory infect	**-41.3 (-63 to -18.4)**	**-72.7 (-78.9 to -63.9)**	**-59.6 (-69.2 to -47.2)**
9 Depressive disorders	**142 (72 to 215)**	**12.5 (7.74 to 18.2)**	**-4.03 (-4.45 to -3.7)**
10 Chronic kidney disease	**209 (93.7 to 362)**	44.4 (-6.17 to 113)	4.08 (-33.5 to 60.6)
11 Low back pain	**144 (72 to 219)**	**13.4 (7.49 to 19.5)**	**-7.96 (-8.4 to -7.62)**
12 Headache disorders	**143 (73.3 to 213)**	**12.8 (8.66 to 20.9)**	0.0705 (-0.294 to 0.334)
13 Interpersonal violence	67 (-7.69 to 152)	-22.6 (-46.7 to 13.3)	-28.4 (-49.3 to 1.26)
14 Hypertensive heart disease	**196 (76.2 to 342)**	38.2 (-10.7 to 103)	-18.1 (-50.1 to 30.5)
15 Anxiety disorders	**133 (65.4 to 204)**	**8.15 (4.37 to 13.2)**	**0.792 (0.351 to 1.19)**
16 Pertussis	-23.6 (-66.2 to 43.4)	**-64.7 (-81 to -39.9)**	**-50.8 (-73 to -17.7)**
17 Tuberculosis	-32.3 (-62.9 to 13.8)	**-68.3 (-81.2 to -48)**	**-72.5 (-84.2 to -54.2)**
18 Gynecological diseases	**105 (27.1 to 189)**	-4.68 (-31.1 to 22)	**-23.3 (-44.8 to -1.52)**
19 Falls	**60.9 (4.44 to 143)**	**-25.5 (-40 to -6.43)**	**-16.8 (-24.8 to -7.56)**
20 Cirrhosis liver	**66.2 (14.5 to 126)**	**-22.6 (-37.2 to -5.8)**	**-41.2 (-53.3 to -27.2)**
30 Maternal disorders			
37 Diarrheal diseases			
53 Meningitis			
157 COVID-19			

Supplemental Results Figure S7. Leading 20 level 3 causes of DALYs, and percentage change in number of DALYs and all-age and age-standardised DALY rates by location, 2022 and 2050

Algeria, Reference scenario

Legend:
Communicable, maternal, neonatal and nutritional
Non-communicable
Injuries

Leading Causes 2022	Leading Causes 2050	Mean % change number of DALY	Mean % change all-age DALY rate	Mean % change age-standardized DALY rate
1 Ischemic heart disease	1 Diabetes	**234 (168 to 293)**	**168 (141 to 196)**	**55.3 (41.9 to 68.7)**
2 Neonatal disorders	2 Ischemic heart disease	40.3 (-9.65 to 114)	13 (-23.7 to 72.4)	**-48.4 (-68.2 to -13.8)**
3 Stroke	3 Stroke	**52.6 (16.5 to 99.1)**	22.8 (-1.68 to 56.6)	**-45.7 (-57.8 to -22)**
4 Road injuries	4 Hypertensive heart disease	**203 (115 to 313)**	**144 (76 to 232)**	0.89 (-30.6 to 46.3)
5 Congenital defects	5 Chronic kidney disease	**186 (99.2 to 308)**	**131 (63.8 to 227)**	9.72 (-25.9 to 68.3)
6 Diabetes	6 Low back pain	**53.2 (24.5 to 77.1)**	**23.2 (16.2 to 31.2)**	**-5.12 (-5.21 to -5.03)**
7 Low back pain	7 Other musculoskeletal	**159 (110 to 203)**	**108 (92.7 to 126)**	**65.6 (63 to 68.1)**
8 Depressive disorders	8 Road injuries	-6.04 (-39.7 to 53.6)	-24.4 (-49.7 to 20.2)	-28.7 (-52.6 to 15.5)
9 Headache disorders	9 Alzheimer's disease	**282 (198 to 372)**	**207 (159 to 255)**	**4.68 (2 to 7.02)**
10 Anxiety disorders	10 Depressive disorders	**35.7 (13.5 to 60.2)**	**9.05 (3.07 to 16)**	**-2.65 (-2.81 to -2.48)**
11 Gynecological diseases	11 Neonatal disorders	**-41.7 (-60.3 to -14.4)**	**-53.2 (-65.3 to -32.3)**	**-33.1 (-51.1 to -4.65)**
12 Hypertensive heart disease	12 COPD	**165 (102 to 254)**	**114 (58 to 183)**	-5.76 (-32.7 to 34.4)
13 Chronic kidney disease	13 Age-related hearing loss	**120 (81.5 to 157)**	**76.6 (61.3 to 91.8)**	**-1.51 (-1.62 to -1.4)**
14 Other musculoskeletal	14 Headache disorders	**29.2 (4.86 to 50.4)**	3.77 (-0.92 to 12.2)	**0.406 (0.259 to 0.518)**
15 Lower respiratory infect	15 Anxiety disorders	**27.1 (3.6 to 50.4)**	2.06 (-3.02 to 8.65)	**1.03 (0.922 to 1.15)**
16 Age-related hearing loss	16 Blindness and vision loss	**113 (77.1 to 149)**	**71.5 (56.2 to 87)**	**-6.89 (-7.33 to -6.43)**
17 Neck pain	17 Congenital defects	**-42.9 (-57.5 to -26.7)**	**-54.2 (-62.1 to -45.8)**	**-33.2 (-41.8 to -21.5)**
18 COVID-19	18 Gynecological diseases	2.89 (-23.3 to 36.1)	-17.3 (-37.5 to 7.55)	-16.9 (-35.6 to 4.51)
19 COPD	19 Neck pain	**52.9 (28.1 to 79.6)**	**23 (12.8 to 34.8)**	**-0.387 (-0.528 to -0.231)**
20 Falls	20 Oral disorders	**84.2 (51.1 to 113)**	**48 (36.2 to 61.8)**	**0.831 (0.579 to 1.12)**
22 Blindness and vision loss	21 Lower respiratory infect			
23 Oral disorders	22 Falls			
24 Alzheimer's disease	162 COVID-19			

Supplemental Results Figure S7. Leading 20 level 3 causes of DALYs, and percentage change in number of DALYs and all-age and age-standardised DALY rates by location, 2022 and 2050

Bahrain, Reference scenario

Legend:
Communicable, maternal, neonatal and nutritional
Non-communicable
Injuries

Leading Causes 2022	Leading Causes 2050	Mean % change number of DALY	Mean % change all-age DALY rate	Mean % change age-standardized DALY rate
1 Diabetes	1 Diabetes	**303 (237 to 382)**	**181 (132 to 241)**	2.17 (-16.4 to 27.1)
2 Ischemic heart disease	2 Ischemic heart disease	**82.6 (19 to 171)**	27.3 (-15.8 to 88.4)	**-57.1 (-73.6 to -28.7)**
3 Depressive disorders	3 Chronic kidney disease	**371 (222 to 541)**	**228 (126 to 360)**	3.21 (-33.9 to 55.3)
4 Low back pain	4 Low back pain	**83.6 (66.5 to 101)**	**27.7 (18.8 to 38.4)**	**-2.77 (-3.36 to -2.14)**
5 Headache disorders	5 COPD	**432 (276 to 594)**	**271 (166 to 390)**	0.431 (-31.7 to 46.3)
6 Road injuries	6 Other musculoskeletal	**163 (130 to 192)**	**82.8 (67.1 to 98.9)**	**42.3 (38.7 to 45.3)**
7 COVID-19	7 Depressive disorders	**50.6 (35.7 to 68.6)**	4.72 (-1.46 to 13.2)	-0.365 (-1.35 to 0.665)
8 Stroke	8 Stroke	**116 (62 to 177)**	**50.7 (13.8 to 97.5)**	**-53.7 (-65.9 to -33.3)**
9 Anxiety disorders	9 Lung cancer	**370 (187 to 571)**	**227 (109 to 363)**	11 (-23.8 to 50.5)
10 Other musculoskeletal	10 Age-related hearing loss	**200 (168 to 238)**	**109 (90.5 to 131)**	**-0.81 (-1.26 to -0.334)**
11 Gynecological diseases	11 Alzheimer's disease	**638 (513 to 794)**	**413 (325 to 519)**	0.0599 (-2.23 to 2.63)
12 Chronic kidney disease	12 Headache disorders	**40.6 (25.9 to 60.5)**	-2.23 (-8.08 to 6.69)	**3.28 (1.89 to 4.44)**
13 Neonatal disorders	13 Hypertensive heart disease	**486 (303 to 691)**	**308 (183 to 469)**	-1.08 (-35.5 to 46.6)
14 Age-related hearing loss	14 Anxiety disorders	**39.2 (25.7 to 57.5)**	-3.18 (-9.59 to 5.63)	**3.31 (1.96 to 4.44)**
15 Neck pain	15 Road injuries	19.8 (-27 to 93)	-16.5 (-48 to 35.3)	-20.7 (-51.8 to 36)
16 Congenital defects	16 Blindness and vision loss	**203 (164 to 240)**	**111 (87.5 to 133)**	**-5.58 (-6.19 to -4.91)**
17 Oral disorders	17 Oral disorders	**129 (100 to 157)**	**59.4 (43.8 to 79.7)**	**1.87 (1.31 to 2.54)**
18 COPD	18 Cirrhosis liver	**155 (110 to 192)**	**77.2 (44.5 to 107)**	**-35.2 (-45.8 to -21.5)**
19 Lung cancer	19 Lower respiratory infect	**200 (127 to 280)**	**109 (57.7 to 171)**	-23.7 (-42.3 to 1.81)
20 Hemoglobinopathies	20 Neck pain	**83.6 (59 to 108)**	**27.7 (14.4 to 43)**	**2.91 (1.15 to 5.01)**
22 Cirrhosis liver	21 Gynecological diseases			
23 Blindness and vision loss	30 Neonatal disorders			
27 Lower respiratory infect	32 Hemoglobinopathies			
35 Hypertensive heart disease	36 Congenital defects			
40 Alzheimer's disease	150 COVID-19			

Supplemental Results Figure S7. Leading 20 level 3 causes of DALYs, and percentage change in number of DALYs and all-age and age-standardised DALY rates by location, 2022 and 2050

Egypt, Reference scenario

Legend:
Communicable, maternal, neonatal and nutritional
Non-communicable
Injuries

Leading Causes 2022	Leading Causes 2050	Mean % change number of DALY	Mean % change all-age DALY rate	Mean % change age-standardized DALY rate
1 Ischemic heart disease	1 Ischemic heart disease	**55.9 (3.72 to 125)**	3.93 (-31.5 to 49.7)	-31.6 (-58.8 to 7.56)
2 Stroke	2 Diabetes	**241 (193 to 298)**	**127 (96.4 to 162)**	**50.2 (27.4 to 75.2)**
3 Cirrhosis liver	3 Chronic kidney disease	**235 (143 to 343)**	**123 (68.9 to 200)**	**48 (0.842 to 112)**
4 Lower respiratory infect	4 Stroke	**48.7 (16.1 to 90.4)**	-0.882 (-22.5 to 25.6)	**-38.9 (-54.4 to -16)**
5 Diabetes	5 Cirrhosis liver	**41.5 (19.6 to 65.4)**	-5.78 (-19 to 8.52)	**-37.5 (-46.6 to -26.3)**
6 COVID-19	6 Hypertensive heart disease	**140 (75.1 to 228)**	**60.1 (19.5 to 116)**	-3.82 (-35.1 to 43.2)
7 Congenital defects	7 Low back pain	**69.2 (50.4 to 85.6)**	**12.7 (6.56 to 18.6)**	**-5 (-5.16 to -4.82)**
8 Chronic kidney disease	8 Other musculoskeletal	**218 (188 to 249)**	**112 (97.2 to 126)**	**75.5 (72.8 to 78.2)**
9 Neonatal disorders	9 Depressive disorders	**63.9 (47 to 82.1)**	**9.14 (4.68 to 14.4)**	**-2.05 (-2.3 to -1.76)**
10 Low back pain	10 Headache disorders	**60 (42.3 to 78)**	**6.51 (2.91 to 13.1)**	**1.1 (0.845 to 1.29)**
11 Road injuries	11 Lower respiratory infect	-16.1 (-33 to 5.21)	**-44.1 (-54.6 to -30.4)**	**-44.7 (-55.4 to -31.2)**
12 Headache disorders	12 Neonatal disorders	6.06 (-12.1 to 31.2)	**-29.4 (-40.8 to -15.8)**	**-16.9 (-29.8 to -0.78)**
13 Depressive disorders	13 Road injuries	14.4 (-29.8 to 84.5)	-23.7 (-52.9 to 27.1)	-29 (-56.5 to 19.4)
14 Hypertensive heart disease	14 Anxiety disorders	**56.9 (37.4 to 74.5)**	**4.45 (0.878 to 8.92)**	**1.81 (1.63 to 2)**
15 Anxiety disorders	15 Congenital defects	**-27.7 (-41.3 to -13.2)**	**-51.9 (-58.1 to -44.9)**	**-39.2 (-46 to -31.2)**
16 Gynecological diseases	16 Liver cancer	**124 (88.2 to 169)**	**49.4 (26.2 to 80.6)**	1.73 (-16.2 to 22.1)
17 Other musculoskeletal	17 COPD	**118 (62.3 to 187)**	**45.4 (8.98 to 90.8)**	-8.77 (-33.9 to 27.2)
18 Other cardiovascular	18 Gynecological diseases	28.4 (-9.01 to 67.8)	-14.4 (-39 to 11.7)	-21.7 (-43.6 to 1.76)
19 Liver cancer	19 Age-related hearing loss	**105 (77.8 to 134)**	**36.3 (23.2 to 50.1)**	**-1.92 (-2.1 to -1.72)**
20 COPD	20 Lung cancer	**142 (64.1 to 235)**	**61 (7.34 to 121)**	11.1 (-19.7 to 44.8)
21 Age-related hearing loss	27 Other cardiovascular			
27 Lung cancer	161 COVID-19			

Supplemental Results Figure S7. Leading 20 level 3 causes of DALYs, and percentage change in number of DALYs and all-age and age-standardised DALY rates by location, 2022 and 2050

Iran, Reference scenario

Legend:
Communicable, maternal, neonatal and nutritional
Non-communicable
Injuries

Leading Causes 2022	Leading Causes 2050	Mean % change number of DALY	Mean % change all-age DALY rate	Mean % change age-standardized DALY rate
1 Ischemic heart disease	1 Diabetes	**262 (216 to 304)**	**210 (182 to 244)**	**54.4 (40.3 to 70.7)**
2 Road injuries	2 Ischemic heart disease	34.4 (-11.4 to 101)	15.2 (-24 to 79.3)	**-52.1 (-70.1 to -23.3)**
3 Low back pain	3 Low back pain	**50.1 (34.6 to 71.8)**	**28.4 (20.7 to 37.6)**	**-3.95 (-4.04 to -3.85)**
4 Stroke	4 Stroke	**37.4 (8.64 to 76)**	17.7 (-8.07 to 53.1)	**-51.4 (-62.7 to -33.7)**
5 Depressive disorders	5 Alzheimer's disease	**309 (235 to 385)**	**250 (197 to 300)**	2.09 (-0.296 to 4.49)
6 Diabetes	6 Hypertensive heart disease	**232 (136 to 348)**	**184 (107 to 298)**	6.58 (-24.8 to 57.8)
7 Anxiety disorders	7 Road injuries	0.802 (-33.7 to 58.5)	-13.6 (-44.2 to 37.8)	-16.1 (-47.2 to 37.6)
8 COVID-19	8 Depressive disorders	**21.1 (7.1 to 36.9)**	3.57 (-2.5 to 10.3)	**-2.01 (-2.09 to -1.91)**
9 Headache disorders	9 Chronic kidney disease	**218 (125 to 334)**	**172 (93.9 to 290)**	13.9 (-21.2 to 71.2)
10 Gynecological diseases	10 Age-related hearing loss	**124 (97.4 to 152)**	**91.7 (76.5 to 109)**	**-1.22 (-1.29 to -1.14)**
11 Neck pain	11 Other musculoskeletal	**123 (93.4 to 154)**	**90.2 (72.9 to 109)**	**50.2 (48 to 52.3)**
12 Age-related hearing loss	12 COPD	**174 (102 to 257)**	**135 (73.9 to 220)**	-6.41 (-34.6 to 33.9)
13 Other musculoskeletal	13 Anxiety disorders	**15.5 (1.51 to 29.1)**	-1.29 (-6.24 to 5.14)	**0.974 (0.937 to 1.02)**
14 Neonatal disorders	14 Blindness and vision loss	**132 (102 to 163)**	**98.1 (80.7 to 115)**	**-5.86 (-6.16 to -5.53)**
15 Hypertensive heart disease	15 Headache disorders	**16.2 (1.96 to 34.7)**	-0.693 (-6.11 to 9.03)	**0.518 (0.349 to 0.608)**
16 Blindness and vision loss	16 Neck pain	**44.2 (22.9 to 64.1)**	**23.3 (10.8 to 38.3)**	-0.0187 (-0.113 to 0.0942)
17 Drug use disorders	17 Lung cancer	**156 (75 to 255)**	**118 (52.2 to 195)**	1.19 (-26.5 to 31.5)
18 COPD	18 Oral disorders	**85.3 (61.2 to 110)**	**58.4 (44.9 to 73.7)**	**0.749 (0.554 to 0.947)**
19 Alzheimer's disease	19 Gynecological diseases	-6.41 (-28.3 to 17.7)	-19.9 (-39.6 to 3.06)	-12.6 (-31.4 to 8.23)
20 Chronic kidney disease	20 Osteoarthritis	**150 (123 to 179)**	**114 (101 to 128)**	**3.33 (3.03 to 3.68)**
21 Oral disorders	21 Drug use disorders			
28 Lung cancer	29 Neonatal disorders			
30 Osteoarthritis	155 COVID-19			

Supplemental Results Figure S7. Leading 20 level 3 causes of DALYs, and percentage change in number of DALYs and all-age and age-standardised DALY rates by location, 2022 and 2050

Iraq, Reference scenario

Legend:
Communicable, maternal, neonatal and nutritional
Non-communicable
Injuries

Leading Causes 2022	Leading Causes 2050	Mean % change number of DALY	Mean % change all-age DALY rate	Mean % change age-standardized DALY rate
1 Ischemic heart disease	1 Conflict & terror	166 (-78.4 to 875)	82.5 (-84.2 to 638)	79.1 (-84.6 to 611)
2 Conflict & terror	2 Diabetes	**165 (76.7 to 246)**	**79.4 (54.5 to 110)**	1.15 (-12.2 to 18.4)
3 Neonatal disorders	3 Ischemic heart disease	39 (-21.2 to 123)	-5.52 (-42.7 to 47.7)	**-53.3 (-72.1 to -22)**
4 Stroke	4 Stroke	39 (-14.3 to 100)	-5.73 (-31.2 to 30.2)	**-53.1 (-66 to -31.2)**
5 Diabetes	5 Low back pain	**81.1 (27.1 to 133)**	**22.7 (15.9 to 29.7)**	**-4.31 (-4.48 to -4.15)**
6 COVID-19	6 Chronic kidney disease	**186 (67.7 to 323)**	**94.2 (29.8 to 180)**	2.26 (-32 to 60.3)
7 Congenital defects	7 Neonatal disorders	**-42.7 (-61.4 to -20.4)**	**-61.1 (-69.5 to -50.4)**	**-46.8 (-58.7 to -30.9)**
8 Low back pain	8 Depressive disorders	**62.9 (15.1 to 107)**	**10.3 (5.42 to 16)**	**-1.81 (-2.03 to -1.61)**
9 Road injuries	9 Headache disorders	**57.6 (8.61 to 104)**	**6.77 (1.51 to 16.9)**	**1.14 (0.776 to 1.39)**
10 Depressive disorders	10 Anxiety disorders	**51.2 (5.08 to 93.2)**	2.41 (-3.62 to 8.84)	**1.69 (1.44 to 1.92)**
11 Mechanical forces	11 Hypertensive heart disease	**217 (89.9 to 354)**	**115 (48.5 to 204)**	-3.77 (-38 to 48)
12 Headache disorders	12 Other musculoskeletal	**198 (93.5 to 280)**	**102 (79.1 to 122)**	**55.1 (40.7 to 62.2)**
13 Interpersonal violence	13 Road injuries	23.5 (-29.9 to 103)	-16.1 (-48.4 to 36.9)	-24.1 (-53.4 to 23)
14 Anxiety disorders	14 Mechanical forces	19.6 (-21.6 to 67.7)	-18.9 (-36 to 5.47)	**-26.6 (-41.8 to -3.33)**
15 Gynecological diseases	15 Age-related hearing loss	**142 (72.1 to 218)**	**63.9 (48.2 to 80.3)**	**-1.37 (-1.5 to -1.24)**
16 Chronic kidney disease	16 Gynecological diseases	47.1 (-4.21 to 101)	-0.0942 (-26.6 to 25.5)	-10.5 (-33.1 to 13)
17 Lower respiratory infect	17 Lung cancer	**179 (58 to 336)**	**88.4 (24.9 to 160)**	-3.81 (-30.6 to 29.3)
18 Age-related hearing loss	18 Alzheimer's disease	**324 (168 to 491)**	**186 (122 to 249)**	**2.89 (0.392 to 5.29)**
19 Neck pain	19 Congenital defects	**-34.9 (-54.7 to -14.2)**	**-55.9 (-62.2 to -49.7)**	**-37.8 (-44.7 to -29.3)**
20 Other musculoskeletal	20 Interpersonal violence	-5.16 (-47.5 to 51.9)	-35.9 (-57.8 to 1.58)	-37.9 (-59.7 to 0.195)
21 Hypertensive heart disease	21 Neck pain			
23 Lung cancer	30 Lower respiratory infect			
34 Alzheimer's disease	157 COVID-19			

Supplemental Results Figure S7. Leading 20 level 3 causes of DALYs, and percentage change in number of DALYs and all-age and age-standardised DALY rates by location, 2022 and 2050

Jordan, Reference scenario

Legend: Communicable, maternal, neonatal and nutritional; Non-communicable; Injuries

Leading Causes 2022	Leading Causes 2050	Mean % change number of DALY	Mean % change all-age DALY rate	Mean % change age-standardized DALY rate
1 Diabetes	1 Diabetes	**306 (253 to 363)**	**125 (99.7 to 150)**	**20.5 (7.31 to 32.7)**
2 Ischemic heart disease	2 Ischemic heart disease	**81.1 (20.2 to 164)**	0.642 (-34 to 49.8)	**-52.5 (-68.7 to -25.1)**
3 Neonatal disorders	3 Low back pain	**124 (103 to 145)**	**24.4 (18.8 to 32)**	**-2.32 (-2.55 to -2.11)**
4 COVID-19	4 Stroke	**99.4 (62.2 to 142)**	10.7 (-10.8 to 34.8)	**-50.4 (-60.6 to -38.2)**
5 Low back pain	5 Hypertensive heart disease	**344 (214 to 491)**	**147 (73 to 237)**	-3.43 (-35.2 to 38.3)
6 Stroke	6 Chronic kidney disease	**279 (168 to 411)**	**111 (48.2 to 196)**	-1.13 (-31.6 to 43.1)
7 Congenital defects	7 Other musculoskeletal	**216 (190 to 247)**	**75.4 (65.4 to 85.9)**	**34.2 (32.1 to 36)**
8 Depressive disorders	8 Depressive disorders	**98.7 (79.3 to 119)**	**10.2 (4.91 to 16.9)**	0.0128 (-0.398 to 0.425)
9 Headache disorders	9 Headache disorders	**88.1 (67.6 to 110)**	4.33 (-1.35 to 15.2)	**1.64 (1.1 to 2.05)**
10 Anxiety disorders	10 Alzheimer's disease	**527 (413 to 644)**	**248 (189 to 305)**	-1.26 (-3.59 to 0.834)
11 Road injuries	11 Anxiety disorders	**79.4 (63.9 to 102)**	-0.529 (-6.26 to 6.7)	**1.89 (1.46 to 2.37)**
12 Gynecological diseases	12 Neonatal disorders	10.1 (-12.6 to 36.4)	**-38.9 (-51.2 to -23.6)**	**-26.3 (-42.6 to -5.36)**
13 Other musculoskeletal	13 Age-related hearing loss	**212 (180 to 249)**	**73.3 (59 to 90.1)**	**-0.971 (-1.1 to -0.849)**
14 Chronic kidney disease	14 Gynecological diseases	**84.4 (49.6 to 127)**	2.38 (-20 to 23.1)	-6.98 (-26 to 10.5)
15 Dietary iron deficiency	15 Road injuries	41.9 (-7.71 to 118)	-21.2 (-48.5 to 21.5)	-23 (-50.4 to 22.7)
16 Lower respiratory infect	16 Neck pain	**137 (114 to 160)**	**31.4 (23.3 to 40.9)**	**2.02 (1.51 to 2.55)**
17 Age-related hearing loss	17 COPD	**295 (197 to 406)**	**119 (64.6 to 182)**	-2.5 (-29.1 to 33.9)
18 Hypertensive heart disease	18 Oral disorders	**166 (140 to 194)**	**47.8 (36.6 to 59)**	**0.485 (0.297 to 0.657)**
19 Neck pain	19 Congenital defects	-5.06 (-20.1 to 10.6)	**-47.4 (-53.8 to -41.2)**	**-33.5 (-40.4 to -25)**
20 Oral disorders	20 Blindness and vision loss	**210 (175 to 246)**	**71.7 (56 to 86.9)**	**-3.88 (-4.23 to -3.5)**
23 Blindness and vision loss	21 Dietary iron deficiency			
25 COPD	24 Lower respiratory infect			
26 Alzheimer's disease	155 COVID-19			

Supplemental Results Figure S7. Leading 20 level 3 causes of DALYs, and percentage change in number of DALYs and all-age and age-standardised DALY rates by location, 2022 and 2050

Kuwait, Reference scenario

Legend:
Communicable, maternal, neonatal and nutritional
Non-communicable
Injuries

Leading Causes 2022
1 Ischemic heart disease
2 Diabetes
3 Low back pain
4 Depressive disorders
5 Headache disorders
6 Gynecological diseases
7 Other musculoskeletal
8 Anxiety disorders
9 Road injuries
10 Stroke
11 Neonatal disorders
12 Congenital defects
13 Lower respiratory infect
14 Neck pain
15 Falls
16 Age-related hearing loss
17 Oral disorders
18 COVID-19
19 Blindness and vision loss
20 Schizophrenia
21 Alzheimer's disease
23 Osteoarthritis
24 Chronic kidney disease
26 Hypertensive heart disease

Leading Causes 2050	Mean % change number of DALY	Mean % change all-age DALY rate	Mean % change age-standardized DALY rate
1 Diabetes	369 (299 to 449)	216 (188 to 250)	35.4 (26.3 to 44.3)
2 Ischemic heart disease	111 (46.6 to 213)	41.9 (-3.22 to 110)	-46.6 (-63.3 to -17.7)
3 Low back pain	97.5 (67.9 to 125)	33 (21.3 to 45.4)	-0.538 (-0.937 to -0.112)
4 Alzheimer's disease	722 (596 to 856)	453 (395 to 510)	6.86 (4.3 to 9.33)
5 Other musculoskeletal	124 (89.1 to 166)	51 (33.5 to 70.1)	22.2 (21.3 to 23.1)
6 Depressive disorders	53.1 (31.2 to 76.1)	3.11 (-5.93 to 14)	0.194 (-0.289 to 0.755)
7 Hypertensive heart disease	716 (485 to 1060)	449 (295 to 648)	38.3 (-2.87 to 99.3)
8 Age-related hearing loss	251 (196 to 309)	136 (110 to 160)	-1.11 (-1.43 to -0.757)
9 Chronic kidney disease	573 (374 to 841)	353 (213 to 529)	42 (-0.689 to 107)
10 Lower respiratory infect	204 (134 to 292)	105 (62.1 to 158)	-27 (-40.9 to -7.62)
11 Stroke	146 (94 to 203)	65.5 (37 to 103)	-42.7 (-52.7 to -28.3)
12 Headache disorders	36.6 (21 to 59.9)	-8 (-14.3 to 3.75)	1.24 (0.857 to 1.63)
13 Blindness and vision loss	270 (212 to 346)	149 (117 to 180)	-1.35 (-1.74 to -1.01)
14 Anxiety disorders	39.9 (22.4 to 61.4)	-5.81 (-13.3 to 5.33)	1.42 (0.908 to 1.89)
15 Road injuries	43.2 (-4.77 to 122)	-3.51 (-34 to 49.9)	-11 (-42 to 45.4)
16 Gynecological diseases	8.48 (-19.1 to 42.5)	-26.9 (-46.4 to -5.44)	-8.49 (-30.6 to 13.6)
17 Osteoarthritis	281 (233 to 334)	157 (135 to 177)	3.7 (3.17 to 4.23)
18 Oral disorders	140 (103 to 188)	61.2 (42.6 to 83.4)	-0.849 (-1.31 to -0.381)
19 Neck pain	85.7 (54.6 to 134)	25.1 (5.49 to 46.1)	4.59 (3.51 to 5.67)
20 Falls	69.2 (44.4 to 98)	13.9 (1.52 to 27.5)	-20.8 (-28.5 to -11.3)
24 Neonatal disorders			
27 Congenital defects			
30 Schizophrenia			
153 COVID-19			

Supplemental Results Figure S7. Leading 20 level 3 causes of DALYs, and percentage change in number of DALYs and all-age and age-standardised DALY rates by location, 2022 and 2050

Lebanon, Reference scenario

Legend:
Communicable, maternal, neonatal and nutritional
Non-communicable
Injuries

Leading Causes 2022	Leading Causes 2050	Mean % change number of DALY	Mean % change all-age DALY rate	Mean % change age-standardized DALY rate
1 COVID-19	1 Diabetes	**141 (91.7 to 186)**	**129 (104 to 153)**	**33 (19.5 to 47)**
2 Ischemic heart disease	2 Ischemic heart disease	14.3 (-27.7 to 80.2)	8.92 (-28.3 to 67.5)	**-40.9 (-64.2 to -4.1)**
3 Diabetes	3 Chronic kidney disease	**131 (54.3 to 229)**	**120 (54.3 to 223)**	17.9 (-20.2 to 85.9)
4 Stroke	4 Low back pain	**30.7 (4.95 to 53.9)**	**24.5 (16.6 to 32.6)**	**-3.69 (-3.98 to -3.42)**
5 Low back pain	5 Alzheimer's disease	**132 (80.7 to 192)**	**121 (84.7 to 155)**	**8.22 (5.98 to 10.6)**
6 Depressive disorders	6 Lung cancer	**115 (43.1 to 197)**	**105 (44.7 to 173)**	3.75 (-24.5 to 35.4)
7 Anxiety disorders	7 Stroke	14.9 (-15.7 to 49.8)	9.47 (-13.1 to 40.5)	**-37.1 (-49.4 to -17.9)**
8 Headache disorders	8 COPD	**104 (39.8 to 179)**	**94 (45.4 to 157)**	1.26 (-31.3 to 46.7)
9 Lower respiratory infect	9 Depressive disorders	13.2 (-9.17 to 34.9)	7.73 (-0.0885 to 16.8)	**-2.34 (-2.8 to -1.91)**
10 Gynecological diseases	10 Age-related hearing loss	**75.9 (47 to 105)**	**67.6 (54.2 to 80.5)**	**-0.543 (-0.735 to -0.354)**
11 Chronic kidney disease	11 Lower respiratory infect	**33.1 (1.79 to 66.4)**	**26.8 (2.99 to 55.7)**	**-27.4 (-41.8 to -9.13)**
12 Neonatal disorders	12 Other musculoskeletal	**79.7 (46.8 to 115)**	**71.1 (53.6 to 86.2)**	**33.1 (28.8 to 35.4)**
13 Lung cancer	13 Anxiety disorders	2.45 (-17.2 to 19.5)	-2.46 (-9.11 to 6.33)	0.159 (-0.267 to 0.604)
14 Alzheimer's disease	14 Headache disorders	3.88 (-15.7 to 25.4)	-1.12 (-7.35 to 9.47)	**-0.523 (-0.87 to -0.111)**
15 COPD	15 Blindness and vision loss	**73.6 (43.9 to 103)**	**65.4 (51.3 to 77.7)**	**-4.23 (-4.5 to -3.98)**
16 Age-related hearing loss	16 Conflict & terror	99.5 (-47.4 to 519)	89.1 (-51.6 to 501)	67.5 (-67 to 518)
17 Other musculoskeletal	17 Breast cancer	**55.9 (9.33 to 112)**	**48.4 (6.54 to 99.4)**	-14.7 (-35.3 to 9.64)
18 Road injuries	18 Colorectal cancer	**108 (32.1 to 201)**	**98.2 (31.6 to 181)**	2.79 (-29.3 to 41.4)
19 Conflict & terror	19 Gynecological diseases	-17.5 (-42.6 to 9.64)	-21.4 (-42.9 to 3.06)	-16.9 (-38.2 to 5.34)
20 Neck pain	20 Oral disorders	**57.5 (30.7 to 88.4)**	**50 (38.5 to 62.2)**	**0.828 (0.58 to 1.07)**
21 Blindness and vision loss	21 Neck pain			
22 Breast cancer	26 Road injuries			
24 Oral disorders	27 Neonatal disorders			
28 Colorectal cancer	155 COVID-19			

Supplemental Results Figure S7. Leading 20 level 3 causes of DALYs, and percentage change in number of DALYs and all-age and age-standardised DALY rates by location, 2022 and 2050

Libya, Reference scenario

Legend:
Communicable, maternal, neonatal and nutritional
Non-communicable
Injuries

Leading Causes 2022	Leading Causes 2050	Mean % change number of DALY	Mean % change all-age DALY rate	Mean % change age-standardized DALY rate
1 Ischemic heart disease	1 Ischemic heart disease	46.1 (-13 to 123)	47.8 (-6.27 to 133)	-36.6 (-61.8 to 5.1)
2 COVID-19	2 Diabetes	**230 (161 to 300)**	**232 (188 to 274)**	**59.6 (38 to 81)**
3 Road injuries	3 Chronic kidney disease	**218 (109 to 347)**	**221 (126 to 376)**	34 (-8.46 to 102)
4 Stroke	4 Road injuries	-14 (-48.4 to 42)	-13.1 (-45.3 to 46.4)	-12.7 (-46.6 to 51)
5 Diabetes	5 Stroke	35.2 (-7.07 to 88.7)	**36.5 (1.47 to 95.7)**	**-41.3 (-56.7 to -13.4)**
6 Conflict & terror	6 Hypertensive heart disease	**267 (143 to 403)**	**270 (152 to 405)**	31.2 (-12 to 95.3)
7 Congenital defects	7 Lung cancer	**168 (63.4 to 268)**	**169 (83.6 to 271)**	7.74 (-23.4 to 42)
8 Low back pain	8 Low back pain	**33.3 (7.87 to 60.6)**	**34.1 (23.5 to 44.8)**	**-2.72 (-2.88 to -2.58)**
9 Depressive disorders	9 Alzheimer's disease	**343 (221 to 466)**	**345 (259 to 433)**	**21.2 (18.4 to 24.1)**
10 Neonatal disorders	10 COPD	**183 (104 to 284)**	**186 (106 to 288)**	8.52 (-23.7 to 58.3)
11 Chronic kidney disease	11 Conflict & terror	4.04e+07 (-100 to 35100)	4.24e+07 (-100 to 33900)	2.95e+07 (-100 to 49200)
12 Headache disorders	12 Depressive disorders	6.42 (-14 to 31.1)	7.02 (-1.56 to 18)	**-1.17 (-1.37 to -0.97)**
13 Anxiety disorders	13 Age-related hearing loss	**113 (67 to 158)**	**114 (88.9 to 140)**	**-0.881 (-0.984 to -0.775)**
14 Gynecological diseases	14 Other musculoskeletal	**73.9 (35.8 to 109)**	**75 (58.4 to 93.8)**	**35.5 (33.1 to 37.7)**
15 Lung cancer	15 Colorectal cancer	**163 (58 to 287)**	**164 (73.7 to 289)**	12.6 (-20.2 to 54.1)
16 Hypertensive heart disease	16 Lower respiratory infect	**49.1 (11.2 to 90.9)**	**50.4 (17.7 to 95.5)**	-25.3 (-41.5 to 1.92)
17 Other musculoskeletal	17 Blindness and vision loss	**120 (76.3 to 169)**	**121 (92.1 to 148)**	**-4.19 (-4.52 to -3.8)**
18 Lower respiratory infect	18 Headache disorders	-3.01 (-21 to 19.5)	-2.48 (-9.65 to 11.7)	**0.86 (0.565 to 1.08)**
19 Cirrhosis liver	19 Cirrhosis liver	**54.9 (18.8 to 90.4)**	**56.2 (26.9 to 90.8)**	**-25.2 (-36.5 to -7.52)**
20 Age-related hearing loss	20 Anxiety disorders	-5.57 (-23.3 to 12.8)	-5.03 (-13.5 to 4.98)	**1.08 (0.891 to 1.29)**
23 COPD	22 Gynecological diseases			
25 Blindness and vision loss	28 Congenital defects			
30 Colorectal cancer	32 Neonatal disorders			
31 Alzheimer's disease	156 COVID-19			

Supplemental Results Figure S7. Leading 20 level 3 causes of DALYs, and percentage change in number of DALYs and all-age and age-standardised DALY rates by location, 2022 and 2050

Morocco, Reference scenario

Legend: Communicable, maternal, neonatal and nutritional; Non-communicable; Injuries

Leading Causes 2022	Leading Causes 2050	Mean % change number of DALY	Mean % change all-age DALY rate	Mean % change age-standardized DALY rate
1 Ischemic heart disease	1 Ischemic heart disease	25.5 (-18.8 to 97.2)	18.3 (-22.6 to 84.6)	-35.1 (-62.6 to 11.8)
2 COVID-19	2 Diabetes	**149 (115 to 191)**	**134 (112 to 159)**	**47.7 (33.3 to 63.3)**
3 Stroke	3 Stroke	23.2 (-7.71 to 68.8)	15.9 (-11.6 to 52.9)	**-37.7 (-55.8 to -10.9)**
4 Diabetes	4 Chronic kidney disease	**154 (80.5 to 260)**	**139 (73.1 to 224)**	32.2 (-13.2 to 102)
5 Neonatal disorders	5 Hypertensive heart disease	**101 (42.4 to 174)**	**89.4 (36.9 to 157)**	-4.17 (-37 to 46.6)
6 Road injuries	6 Low back pain	**27.1 (8.99 to 45.1)**	**19.5 (13.2 to 26.4)**	**-5.89 (-6 to -5.77)**
7 Low back pain	7 Depressive disorders	**16.9 (1.4 to 34.1)**	**9.85 (5.04 to 16)**	**-3.05 (-3.2 to -2.89)**
8 Depressive disorders	8 Other musculoskeletal	**136 (102 to 172)**	**122 (108 to 139)**	**78.6 (72.6 to 83)**
9 Hypertensive heart disease	9 Alzheimer's disease	**185 (105 to 287)**	**168 (97.5 to 238)**	**9.04 (6.59 to 12)**
10 Chronic kidney disease	10 Road injuries	-20.1 (-51.3 to 32.5)	-24.7 (-53.1 to 21.1)	-32.9 (-59.1 to 10.9)
11 Headache disorders	11 COPD	**76 (30.4 to 134)**	**65.7 (24 to 112)**	-12.7 (-41 to 28.5)
12 Anxiety disorders	12 Age-related hearing loss	**72.3 (46.2 to 109)**	**61.9 (43.3 to 79.5)**	**-1.75 (-1.87 to -1.63)**
13 Lower respiratory infect	13 Headache disorders	11.2 (-4.41 to 26.9)	4.46 (-0.335 to 13.2)	**0.505 (0.314 to 0.64)**
14 Gynecological diseases	14 Anxiety disorders	8.15 (-6.78 to 23.7)	1.59 (-4.51 to 7.67)	**1.26 (1.16 to 1.37)**
15 COPD	15 Neonatal disorders	**-52.5 (-64.4 to -35.6)**	**-55.3 (-64.7 to -41.1)**	**-36.4 (-52.1 to -15.3)**
16 Other musculoskeletal	16 Lung cancer	**73.8 (11 to 154)**	**63 (6.39 to 128)**	-6.57 (-35 to 25.3)
17 Age-related hearing loss	17 Lower respiratory infect	-6.57 (-27 to 17.7)	-12.1 (-31.7 to 9.89)	**-42.2 (-55.4 to -23.1)**
18 Congenital defects	18 Gynecological diseases	-6.13 (-31.3 to 27.4)	-11.6 (-33.2 to 16.3)	-16.4 (-35.5 to 6.91)
19 Neck pain	19 Blindness and vision loss	**61.1 (36.9 to 95.7)**	**51.3 (34.8 to 68.3)**	**-7.61 (-8.1 to -7.1)**
20 Falls	20 Falls	**39.4 (16.1 to 68.6)**	**30.9 (13.8 to 47.7)**	-6.4 (-15.5 to 3.09)
22 Alzheimer's disease	22 Neck pain			
23 Blindness and vision loss	31 Congenital defects			
26 Lung cancer	160 COVID-19			

Supplemental Results Figure S7. Leading 20 level 3 causes of DALYs, and percentage change in number of DALYs and all-age and age-standardised DALY rates by location, 2022 and 2050

Oman, Reference scenario

Legend:
Communicable, maternal, neonatal and nutritional
Non-communicable
Injuries

Leading Causes 2022	Leading Causes 2050	Mean % change number of DALY	Mean % change all-age DALY rate	Mean % change age-standardized DALY rate
1 Road injuries	1 Diabetes	**524 (444 to 614)**	**325 (271 to 392)**	**48.8 (30.3 to 72.4)**
2 Ischemic heart disease	2 Ischemic heart disease	**175 (83.6 to 311)**	**87.6 (24.4 to 185)**	**-44.1 (-65.6 to -8.83)**
3 Diabetes	3 Road injuries	34.6 (-18.1 to 121)	-8.17 (-44.1 to 49.9)	-17.6 (-51.6 to 41.6)
4 Low back pain	4 Chronic kidney disease	**542 (371 to 782)**	**338 (217 to 513)**	34.2 (-6.33 to 101)
5 Depressive disorders	5 Hypertensive heart disease	**640 (425 to 869)**	**405 (258 to 589)**	27.6 (-15.6 to 86.6)
6 Neonatal disorders	6 Stroke	**162 (106 to 236)**	**78.8 (39.7 to 139)**	**-43.5 (-57.2 to -18.9)**
7 Headache disorders	7 Low back pain	**95.5 (72.3 to 117)**	**33 (22.1 to 46)**	**-2.75 (-3.57 to -1.97)**
8 Stroke	8 Other musculoskeletal	**177 (138 to 219)**	**88.8 (65.5 to 116)**	**45.6 (42.9 to 48.3)**
9 Anxiety disorders	9 Depressive disorders	**63 (45.5 to 84.4)**	**11 (1.72 to 23.5)**	-0.0548 (-1.4 to 1.32)
10 Congenital defects	10 Lower respiratory infect	**169 (106 to 233)**	**83.2 (40.3 to 135)**	-14.1 (-34.2 to 16.4)
11 Gynecological diseases	11 Blindness and vision loss	**243 (196 to 290)**	**134 (107 to 157)**	**-4.15 (-4.64 to -3.61)**
12 Other musculoskeletal	12 Age-related hearing loss	**237 (194 to 285)**	**129 (105 to 154)**	**-0.667 (-1.24 to -0.143)**
13 COVID-19	13 Headache disorders	**50 (33 to 68.9)**	2.09 (-4.78 to 12.5)	**3.67 (1.82 to 5.1)**
14 Lower respiratory infect	14 Alzheimer's disease	**694 (544 to 859)**	**440 (341 to 529)**	**12.3 (8.52 to 16.2)**
15 Falls	15 COPD	**455 (305 to 639)**	**278 (174 to 420)**	10.5 (-25 to 59.1)
16 Chronic kidney disease	16 Anxiety disorders	**48.6 (32.8 to 69.6)**	1.11 (-6.15 to 11.3)	**3.44 (1.94 to 4.88)**
17 Neck pain	17 Other cardiovascular	**163 (106 to 221)**	**79.4 (44 to 116)**	-19.9 (-36.3 to 4.82)
18 Blindness and vision loss	18 Falls	**92.8 (63.1 to 129)**	**31.1 (13.1 to 50.3)**	**-11.2 (-20.6 to -0.0519)**
19 Other cardiovascular	19 Gynecological diseases	**50.1 (14.2 to 87.5)**	2.3 (-21.8 to 30.6)	15.5 (-9.67 to 41.9)
20 Age-related hearing loss	20 Neonatal disorders	-10.3 (-28.5 to 16)	**-38.9 (-50.9 to -20.3)**	-19.4 (-37.7 to 7.38)
21 Hypertensive heart disease	22 Neck pain			
28 COPD	25 Congenital defects			
36 Alzheimer's disease	158 COVID-19			

Supplemental Results Figure S7. Leading 20 level 3 causes of DALYs, and percentage change in number of DALYs and all-age and age-standardised DALY rates by location, 2022 and 2050

Palestine, Reference scenario

Legend:
Communicable, maternal, neonatal and nutritional
Non-communicable
Injuries

Leading Causes 2022	Leading Causes 2050	Mean % change number of DALY	Mean % change all-age DALY rate	Mean % change age-standardized DALY rate
1 COVID-19	1 Diabetes	**251 (198 to 310)**	**155 (124 to 191)**	**22.3 (6.31 to 42.4)**
2 Ischemic heart disease	2 Ischemic heart disease	**79.6 (18.3 to 168)**	30.7 (-15.9 to 100)	**-38.8 (-63.1 to -0.557)**
3 Conflict & terror	3 Conflict & terror	223 (-89.4 to 1900)	133 (-92.2 to 1390)	109 (-92.3 to 1290)
4 Neonatal disorders	4 Depressive disorders	**60.1 (40.3 to 80)**	**16.3 (9.66 to 24.2)**	**-1.86 (-1.95 to -1.77)**
5 Depressive disorders	5 Stroke	**75.3 (37.8 to 121)**	**27.5 (0.761 to 60.7)**	**-41.1 (-56.1 to -18.6)**
6 Diabetes	6 Chronic kidney disease	**270 (165 to 404)**	**169 (94.3 to 275)**	35.2 (-9.52 to 105)
7 Stroke	7 Low back pain	**77.8 (56.5 to 99.7)**	**29.2 (22 to 36.1)**	**-3.79 (-3.92 to -3.68)**
8 Low back pain	8 Headache disorders	**50.8 (29.9 to 78)**	**9.45 (2.78 to 22.9)**	**0.534 (0.356 to 0.637)**
9 Congenital defects	9 Anxiety disorders	**41.5 (19.3 to 64.3)**	2.69 (-4.35 to 10.3)	**0.923 (0.842 to 1.01)**
10 Anxiety disorders	10 Hypertensive heart disease	**248 (156 to 376)**	**153 (83 to 258)**	2.61 (-33.3 to 57.4)
11 Headache disorders	11 Neonatal disorders	**-36.5 (-51.2 to -16.9)**	**-53.9 (-64 to -40.9)**	**-37.9 (-51.6 to -19.1)**
12 Gynecological diseases	12 Other musculoskeletal	**176 (137 to 220)**	**100 (77.5 to 123)**	**41.9 (30.6 to 48.9)**
13 Lower respiratory infect	13 Gynecological diseases	**50.3 (16.2 to 85.6)**	9.26 (-13.8 to 33.5)	-8.58 (-26.5 to 9.29)
14 Chronic kidney disease	14 Age-related hearing loss	**137 (104 to 172)**	**71.8 (56.8 to 90.1)**	**-1.13 (-1.28 to -0.975)**
15 Road injuries	15 Lung cancer	**160 (77.9 to 269)**	**88.8 (29 to 161)**	-7.09 (-32.3 to 23.5)
16 Interpersonal violence	16 Alzheimer's disease	**306 (228 to 399)**	**195 (144 to 246)**	**4.29 (2.15 to 6.85)**
17 Falls	17 Neck pain	**94.5 (70.2 to 121)**	**41.3 (29.8 to 53.5)**	-0.118 (-0.334 to 0.0836)
18 Age-related hearing loss	18 Congenital defects	**-24.3 (-38.1 to -8.18)**	**-45.1 (-52.1 to -36.4)**	**-22.8 (-31.8 to -11.9)**
19 Neck pain	19 Lower respiratory infect	15.8 (-8.82 to 45.4)	-15.8 (-32.2 to 5.19)	**-33.6 (-47.3 to -15.7)**
20 Other musculoskeletal	20 Colorectal cancer	**185 (85.5 to 323)**	**107 (33.6 to 192)**	3.33 (-28.9 to 42.8)
21 Hypertensive heart disease	26 Falls			
22 Lung cancer	27 Road injuries			
27 Colorectal cancer	30 Interpersonal violence			
35 Alzheimer's disease	155 COVID-19			

Supplemental Results Figure S7. Leading 20 level 3 causes of DALYs, and percentage change in number of DALYs and all-age and age-standardised DALY rates by location, 2022 and 2050

Qatar, Reference scenario

Legend:
Communicable, maternal, neonatal and nutritional
Non-communicable
Injuries

Leading Causes 2022	Leading Causes 2050	Mean % change number of DALY	Mean % change all-age DALY rate	Mean % change age-standardized DALY rate
1 Diabetes	1 Diabetes	738 (638 to 869)	265 (224 to 316)	4.31 (-9.86 to 21.9)
2 Road injuries	2 Ischemic heart disease	357 (214 to 543)	98.8 (36 to 182)	-49.3 (-65.8 to -21.8)
3 Low back pain	3 Low back pain	195 (165 to 233)	28.5 (16.5 to 41.4)	-0.523 (-1.19 to 0.226)
4 Depressive disorders	4 Chronic kidney disease	1110 (787 to 1600)	428 (289 to 641)	22.5 (-12.9 to 84.1)
5 Ischemic heart disease	5 Road injuries	120 (44.2 to 249)	-4.09 (-36.4 to 53.3)	-17.1 (-46.9 to 33.2)
6 Headache disorders	6 Depressive disorders	136 (111 to 165)	2.58 (-6.06 to 12.7)	1.4 (0.163 to 2.9)
7 Anxiety disorders	7 Other musculoskeletal	249 (197 to 297)	51.9 (32.7 to 73.3)	26.2 (24.6 to 27.8)
8 Other musculoskeletal	8 Stroke	389 (297 to 493)	113 (75.7 to 155)	-42.5 (-53.5 to -26)
9 COVID-19	9 Age-related hearing loss	421 (356 to 489)	126 (101 to 153)	-0.502 (-1.03 to 0.0533)
10 Falls	10 Headache disorders	123 (102 to 147)	-2.84 (-9.05 to 6.66)	4.29 (2.44 to 5.91)
11 Gynecological diseases	11 Liver cancer	1150 (921 to 1400)	442 (345 to 550)	23.3 (2.28 to 50.5)
12 Neonatal disorders	12 Anxiety disorders	127 (103 to 154)	-1.28 (-8.37 to 8.05)	4.1 (2.56 to 6.03)
13 Stroke	13 Alzheimer's disease	1740 (1450 to 2080)	698 (584 to 840)	5.84 (1.65 to 9.81)
14 Neck pain	14 Falls	143 (108 to 183)	5.81 (-8.27 to 22.2)	-20.7 (-29.6 to -8.79)
15 Congenital defects	15 Blindness and vision loss	473 (389 to 565)	149 (115 to 182)	-3.6 (-4.18 to -2.96)
16 Age-related hearing loss	16 Gynecological diseases	148 (107 to 202)	7.72 (-10.9 to 34)	30.4 (9.59 to 56)
17 Oral disorders	17 Lung cancer	746 (510 to 1070)	268 (170 to 405)	-5.87 (-30 to 22.1)
18 Schizophrenia	18 COPD	814 (590 to 1070)	298 (207 to 412)	-1.09 (-25.7 to 33.1)
19 Chronic kidney disease	19 Cirrhosis liver	488 (395 to 583)	156 (116 to 201)	-29.2 (-38.4 to -17.9)
20 Mechanical forces	20 Neck pain	202 (151 to 267)	31.3 (11.5 to 55.7)	5.66 (3.44 to 8.19)

Leading Causes 2022 (continued)	Leading Causes 2050 (continued)
22 Blindness and vision loss	22 Oral disorders
25 Cirrhosis liver	27 Neonatal disorders
31 Liver cancer	30 Schizophrenia
33 Lung cancer	31 Congenital defects
37 COPD	40 Mechanical forces
43 Alzheimer's disease	151 COVID-19

Supplemental Results Figure S7. Leading 20 level 3 causes of DALYs, and percentage change in number of DALYs and all-age and age-standardised DALY rates by location, 2022 and 2050

Saudi Arabia, Reference scenario

Legend:
Communicable, maternal, neonatal and nutritional
Non-communicable
Injuries

Leading Causes 2022	Leading Causes 2050	Mean % change number of DALY	Mean % change all-age DALY rate	Mean % change age-standardized DALY rate
1 Ischemic heart disease	1 Ischemic heart disease	**76.1 (11.1 to 171)**	46.9 (-2.09 to 120)	**-41.9 (-62.2 to -9.13)**
2 Road injuries	2 Chronic kidney disease	**334 (200 to 512)**	**262 (154 to 409)**	30 (-7.02 to 91.5)
3 Stroke	3 Diabetes	**308 (231 to 386)**	**240 (196 to 287)**	**41 (24 to 59.4)**
4 Diabetes	4 Road injuries	23.3 (-22.2 to 93.8)	2.92 (-33.4 to 60.4)	-6.44 (-40.8 to 54.6)
5 Chronic kidney disease	5 Hypertensive heart disease	**455 (278 to 671)**	**363 (226 to 532)**	27.1 (-11.1 to 82.8)
6 Low back pain	6 Stroke	**97.3 (45.2 to 169)**	**64.4 (24.5 to 120)**	**-42.7 (-56.1 to -20.8)**
7 Depressive disorders	7 Low back pain	**63.7 (39.4 to 90.3)**	**36.5 (23.7 to 51)**	**-1.95 (-2.23 to -1.64)**
8 Falls	8 Falls	**80 (49 to 108)**	**50 (36.1 to 64.1)**	-4.38 (-9.08 to 0.281)
9 Headache disorders	9 COPD	**344 (220 to 527)**	**271 (171 to 414)**	11.5 (-21.9 to 56.5)
10 Anxiety disorders	10 Lower respiratory infect	**163 (97.1 to 257)**	**119 (69.7 to 188)**	-7.28 (-27.1 to 20.3)
11 Gynecological diseases	11 Cirrhosis liver	**141 (95.8 to 206)**	**101 (68 to 136)**	**-23 (-32.4 to -9.68)**
12 Interpersonal violence	12 Depressive disorders	**29.2 (8.28 to 50.8)**	7.66 (-2.24 to 19.3)	-0.266 (-0.738 to 0.263)
13 Cirrhosis liver	13 Blindness and vision loss	**229 (166 to 286)**	**174 (138 to 206)**	**-4 (-4.28 to -3.69)**
14 Hypertensive heart disease	14 Alzheimer's disease	**781 (576 to 983)**	**634 (516 to 753)**	**13 (10.5 to 15.5)**
15 Lower respiratory infect	15 Age-related hearing loss	**190 (140 to 243)**	**142 (114 to 174)**	**-1.09 (-1.23 to -0.945)**
16 Other musculoskeletal	16 Other musculoskeletal	**89.6 (56.2 to 128)**	**58.1 (33 to 86.8)**	**38.3 (34.7 to 41.4)**
17 Self-harm	17 Headache disorders	15.9 (-3.02 to 35.6)	-3.38 (-11 to 9.1)	**1.83 (1.16 to 2.37)**
18 Neonatal disorders	18 Anxiety disorders	14.6 (-3.71 to 32.8)	-4.44 (-13.3 to 5.83)	**2.38 (1.76 to 2.96)**
19 Neck pain	19 Gynecological diseases	3.09 (-24.5 to 32.7)	-14 (-35.9 to 9.19)	0.23 (-23 to 23.6)
20 Mechanical forces	20 Neck pain	**63.6 (33.4 to 102)**	**36.4 (15.4 to 61.2)**	**2.99 (2.32 to 3.76)**
21 Age-related hearing loss	27 Self-harm			
22 Blindness and vision loss	28 Interpersonal violence			
24 COPD	29 Mechanical forces			
46 Alzheimer's disease	34 Neonatal disorders			

Supplemental Results Figure S7. Leading 20 level 3 causes of DALYs, and percentage change in number of DALYs and all-age and age-standardised DALY rates by location, 2022 and 2050

Sudan, Reference scenario

Legend:
Communicable, maternal, neonatal and nutritional
Non-communicable
Injuries

Leading Causes 2022	Leading Causes 2050	Mean % change number of DALY	Mean % change all-age DALY rate	Mean % change age-standardized DALY rate
1 Neonatal disorders	1 HIV/AIDS	458 (-36.3 to 3080)	274 (-56.7 to 1930)	199 (-64.5 to 1640)
2 Ischemic heart disease	2 Ischemic heart disease	80 (-0.336 to 189)	19.9 (-31.1 to 97.8)	-40.3 (-68 to 3.54)
3 Congenital defects	3 Diabetes	**322 (247 to 404)**	**180 (138 to 222)**	**44.2 (24.6 to 64.9)**
4 Malaria	4 Stroke	**63.7 (12 to 133)**	8.89 (-21 to 50.8)	**-42.9 (-60.2 to -16.3)**
5 COVID-19	5 Neonatal disorders	**-46.5 (-67.1 to -18)**	**-64.5 (-77.3 to -45)**	**-41.3 (-61.7 to -7.94)**
6 Stroke	6 Congenital defects	**-39.5 (-53.5 to -22.3)**	**-59.9 (-66.5 to -51.9)**	**-33.9 (-41.7 to -24.6)**
7 Lower respiratory infect	7 Low back pain	**94.4 (62.4 to 128)**	**29.1 (21.2 to 38)**	**-5.1 (-5.39 to -4.76)**
8 Road injuries	8 Hypertensive heart disease	**226 (109 to 365)**	**117 (43.7 to 212)**	-0.817 (-37.3 to 51.9)
9 HIV/AIDS	9 Chronic kidney disease	**233 (113 to 384)**	**121 (47.1 to 230)**	24.2 (-20.7 to 93.8)
10 Low back pain	10 Road injuries	36 (-19.5 to 126)	-9.49 (-45.9 to 47.3)	-26 (-55.5 to 19.9)
11 Depressive disorders	11 Depressive disorders	**76.9 (49.7 to 109)**	**17.5 (10.7 to 25.7)**	**-2.68 (-3.03 to -2.32)**
12 Headache disorders	12 Other musculoskeletal	**283 (218 to 350)**	**154 (137 to 177)**	**73.5 (69.7 to 77.1)**
13 Anxiety disorders	13 Headache disorders	**70.6 (41.5 to 106)**	**13.3 (5.56 to 28.9)**	**0.357 (0.0741 to 0.676)**
14 Diabetes	14 Anxiety disorders	**58.9 (30.2 to 91)**	5.48 (-2.19 to 14.7)	**1.33 (0.928 to 1.8)**
15 Gynecological diseases	15 Gynecological diseases	**59.3 (16.5 to 111)**	5.93 (-19.7 to 34.9)	-16 (-38 to 7.77)
16 Dietary iron deficiency	16 Lower respiratory infect	**-31.9 (-53.5 to -7.33)**	**-54.8 (-67.4 to -42)**	**-50.6 (-62.8 to -34.1)**
17 Diarrheal diseases	17 Age-related hearing loss	**151 (107 to 197)**	**67 (48.6 to 85.4)**	**-1.98 (-2.28 to -1.68)**
18 Hypertensive heart disease	18 Malaria	18.7 (-61.6 to 53.3)	**-21.5 (-72.7 to -6.53)**	-6.53 (-71.4 to 5.92)
19 Conflict & terror	19 COPD	**127 (53.3 to 214)**	**51 (4.43 to 112)**	-25.6 (-49.5 to 14.6)
20 Chronic kidney disease	20 Neck pain	**119 (86.2 to 154)**	**45.6 (32.2 to 59.6)**	0.492 (-0.437 to 1.36)
22 Other musculoskeletal	21 Dietary iron deficiency			
25 COPD	27 Conflict & terror			
26 Age-related hearing loss	43 Diarrheal diseases			
28 Neck pain	166 COVID-19			

Supplemental Results Figure S7. Leading 20 level 3 causes of DALYs, and percentage change in number of DALYs and all-age and age-standardised DALY rates by location, 2022 and 2050

Syria, Reference scenario

Legend:
Communicable, maternal, neonatal and nutritional
Non-communicable
Injuries

Leading Causes 2022	Leading Causes 2050	Mean % change number of DALY	Mean % change all-age DALY rate	Mean % change age-standardized DALY rate
1 Conflict & terror	1 Conflict & terror	26900 (-97.2 to 5280)	25300 (-97.4 to 4450)	4600 (-96.9 to 4380)
2 Ischemic heart disease	2 Ischemic heart disease	21.9 (-26 to 84.8)	6.26 (-34.1 to 58.2)	-30.6 (-61.6 to 18.7)
3 Stroke	3 Diabetes	**179 (123 to 241)**	**142 (113 to 178)**	**75.6 (53.9 to 99.8)**
4 Diabetes	4 Leishmaniasis	**709 (324 to 1150)**	**600 (307 to 914)**	**580 (303 to 872)**
5 Low back pain	5 Stroke	16.5 (-16.8 to 54.2)	1.29 (-25.4 to 35.2)	**-35.8 (-56 to -5.7)**
6 Chronic kidney disease	6 Chronic kidney disease	**133 (66.4 to 207)**	**103 (45.9 to 174)**	31.4 (-11.5 to 97.8)
7 Depressive disorders	7 Low back pain	**34.9 (10.7 to 61.4)**	**16.9 (9.16 to 26.2)**	**-2.74 (-3.37 to -2.11)**
8 Headache disorders	8 Hypertensive heart disease	**139 (70.9 to 218)**	**108 (52.1 to 181)**	15.5 (-23.3 to 77.6)
9 Anxiety disorders	9 Depressive disorders	22.1 (-2.96 to 48.2)	5.73 (-0.269 to 12.4)	**-1.77 (-3.04 to -0.574)**
10 Neonatal disorders	10 COPD	**69.8 (20.2 to 125)**	**47.7 (5.13 to 92.7)**	-10.9 (-41 to 28.5)
11 Road injuries	11 Headache disorders	18.8 (-4.29 to 45.3)	2.97 (-2.49 to 11.8)	-0.692 (-1.47 to 0.233)
12 Lower respiratory infect	12 Alzheimer's disease	**181 (99.2 to 298)**	**143 (79.5 to 221)**	**6.42 (4.17 to 9.42)**
13 Gynecological diseases	13 Anxiety disorders	13.5 (-9.57 to 37.5)	-1.72 (-7.44 to 3.95)	-0.742 (-1.93 to 0.604)
14 COVID-19	14 Age-related hearing loss	**69 (33.6 to 110)**	**46.5 (27.5 to 70.5)**	**-1.49 (-1.82 to -1.14)**
15 Congenital defects	15 Gynecological diseases	10.9 (-22.8 to 45.2)	-3.58 (-31.3 to 23.6)	-21.1 (-42.6 to 2.81)
16 COPD	16 Other musculoskeletal	**77.9 (42.8 to 113)**	**54.3 (39.8 to 68.4)**	**31.5 (26.2 to 35.5)**
17 Hypertensive heart disease	17 Blindness and vision loss	**67.1 (32.9 to 108)**	**44.8 (25.3 to 66.4)**	**-3.32 (-3.69 to -2.9)**
18 Age-related hearing loss	18 Road injuries	-10.2 (-47.5 to 48)	-21.8 (-51.5 to 27.4)	-23.5 (-53.7 to 28.4)
19 Neck pain	19 Lower respiratory infect	-5.85 (-30.2 to 20.1)	-18.3 (-34.9 to 0.873)	**-38.7 (-53.3 to -16.2)**
20 Cirrhosis liver	20 Neck pain	**38.5 (11.7 to 68.1)**	**20.1 (10.3 to 32.6)**	1.71 (-0.157 to 3.36)
21 Blindness and vision loss	22 Neonatal disorders			
22 Other musculoskeletal	23 Cirrhosis liver			
25 Leishmaniasis	30 Congenital defects			
29 Alzheimer's disease	163 COVID-19			

Supplemental Results Figure S7. Leading 20 level 3 causes of DALYs, and percentage change in number of DALYs and all-age and age-standardised DALY rates by location, 2022 and 2050

Tunisia, Reference scenario

Legend:
Communicable, maternal, neonatal and nutritional
Non-communicable
Injuries

Leading Causes 2022	Leading Causes 2050	Mean % change number of DALY	Mean % change all-age DALY rate	Mean % change age-standardized DALY rate
1 Ischemic heart disease	1 Ischemic heart disease	14.8 (-28.8 to 67.9)	14.7 (-24.8 to 70.4)	**-45.9 (-64.7 to -16.4)**
2 COVID-19	2 Diabetes	**155 (115 to 193)**	**155 (130 to 182)**	**56.2 (43.2 to 69.1)**
3 Stroke	3 Stroke	19.9 (-10.8 to 59)	19.7 (-6.52 to 56.6)	**-44.4 (-56 to -25.8)**
4 Diabetes	4 Hypertensive heart disease	**153 (73.2 to 254)**	**152 (78.6 to 245)**	9.01 (-23.8 to 55.5)
5 Depressive disorders	5 Chronic kidney disease	**146 (72.3 to 246)**	**146 (75.6 to 243)**	16.1 (-19.6 to 73.1)
6 Road injuries	6 Alzheimer's disease	**221 (141 to 306)**	**220 (153 to 284)**	**7.65 (5.33 to 9.95)**
7 Low back pain	7 Low back pain	**23.1 (6.68 to 41)**	**22.9 (15.4 to 30.4)**	**-4.69 (-4.87 to -4.47)**
8 Neonatal disorders	8 Depressive disorders	9.87 (-4.79 to 25.3)	**9.67 (3.96 to 16.7)**	**-2.41 (-2.69 to -2.15)**
9 Headache disorders	9 Other musculoskeletal	**88.6 (61.8 to 114)**	**88.3 (75.3 to 103)**	**54.6 (51.9 to 56.7)**
10 Hypertensive heart disease	10 COPD	**100 (44.6 to 170)**	**99.8 (49.7 to 164)**	-8.7 (-33.4 to 24.5)
11 Chronic kidney disease	11 Road injuries	-12.4 (-41.8 to 33.5)	-12.5 (-38.9 to 30.1)	-17.7 (-43.9 to 24)
12 Gynecological diseases	12 Age-related hearing loss	**78.3 (51.5 to 104)**	**78 (60.3 to 95.3)**	**-1.33 (-1.44 to -1.22)**
13 Anxiety disorders	13 Lung cancer	**51.9 (6.95 to 108)**	**51.6 (12 to 99.6)**	-13.1 (-32.9 to 11)
14 Other musculoskeletal	14 Headache disorders	-0.691 (-13 to 15.5)	-0.872 (-6.47 to 8.1)	-0.119 (-0.359 to 0.107)
15 Congenital defects	15 Blindness and vision loss	**74.2 (46.3 to 99.6)**	**73.9 (52.6 to 89.6)**	**-6.01 (-6.43 to -5.58)**
16 Lung cancer	16 Anxiety disorders	-1.46 (-16 to 12.9)	-1.65 (-7.12 to 5.51)	**0.441 (0.101 to 0.75)**
17 Age-related hearing loss	17 Oral disorders	**46.3 (25.9 to 67)**	**46 (33.9 to 58.5)**	**0.643 (0.372 to 0.909)**
18 COPD	18 Gynecological diseases	-19.6 (-40.9 to 5.82)	-19.7 (-38.2 to 2.63)	-16.6 (-35 to 2.9)
19 Alzheimer's disease	19 Lower respiratory infect	28.2 (-0.165 to 66.7)	**28 (2.53 to 61.7)**	**-37.7 (-48.2 to -21.9)**
20 Neck pain	20 Neck pain	**20.4 (2.01 to 38.3)**	**20.2 (8.78 to 32.1)**	**-0.809 (-1.22 to -0.36)**
21 Lower respiratory infect	26 Neonatal disorders			
22 Blindness and vision loss	30 Congenital defects			
24 Oral disorders	156 COVID-19			

Supplemental Results Figure S7. Leading 20 level 3 causes of DALYs, and percentage change in number of DALYs and all-age and age-standardised DALY rates by location, 2022 and 2050

Türkiye, Reference scenario

Legend:
Communicable, maternal, neonatal and nutritional
Non-communicable
Injuries

Leading Causes 2022	Leading Causes 2050	Mean % change number of DALY	Mean % change all-age DALY rate	Mean % change age-standardized DALY rate
1 Ischemic heart disease	1 Diabetes	**120 (88.3 to 151)**	**120 (94.8 to 144)**	**25.8 (12.3 to 41.7)**
2 Stroke	2 Ischemic heart disease	-15.7 (-41.7 to 20.3)	-15.5 (-41.7 to 21.4)	**-60.1 (-73.3 to -37.4)**
3 Diabetes	3 COPD	**99.9 (52.7 to 155)**	**100 (54.4 to 160)**	-9.21 (-32.2 to 22.2)
4 Low back pain	4 Stroke	5.65 (-13.1 to 26.8)	5.82 (-12.8 to 27.4)	**-49.6 (-58.7 to -35.4)**
5 COVID-19	5 Lung cancer	**45.4 (2.56 to 90.9)**	**45.3 (6.9 to 85.9)**	-18 (-38.7 to 2.28)
6 Lung cancer	6 Alzheimer's disease	**192 (131 to 252)**	**192 (146 to 240)**	**7.32 (4.77 to 10.1)**
7 COPD	7 Low back pain	**22.9 (11.1 to 35.9)**	**22.9 (17.1 to 28.8)**	**-3.95 (-4.05 to -3.87)**
8 Depressive disorders	8 Chronic kidney disease	**102 (50.8 to 174)**	**102 (50.2 to 188)**	-4.5 (-31.9 to 41.4)
9 Neonatal disorders	9 Hypertensive heart disease	**170 (99.8 to 262)**	**170 (99.3 to 273)**	9.77 (-22.6 to 62.6)
10 Headache disorders	10 Other musculoskeletal	**79.8 (62.3 to 95.9)**	**79.9 (68.9 to 90.9)**	**46.8 (44.6 to 48.9)**
11 Gynecological diseases	11 Age-related hearing loss	**72.6 (52.3 to 93.8)**	**72.7 (61.2 to 87.7)**	**-1.1 (-1.19 to -1)**
12 Other musculoskeletal	12 Depressive disorders	4.1 (-7.03 to 15.2)	4.17 (-0.618 to 9.68)	**-2.18 (-2.3 to -2.01)**
13 Chronic kidney disease	13 Headache disorders	-0.548 (-10.3 to 9.89)	-0.465 (-5.53 to 8.3)	**0.306 (0.16 to 0.432)**
14 Anxiety disorders	14 Colorectal cancer	**96.2 (27.1 to 171)**	**96 (34.7 to 165)**	4.65 (-24.8 to 37.6)
15 Age-related hearing loss	15 Lower respiratory infect	**38.6 (10.9 to 72.3)**	**38.7 (14.8 to 77)**	**-35.2 (-47.1 to -15.5)**
16 Congenital defects	16 Oral disorders	**47.3 (30.6 to 65.2)**	**47.4 (37.1 to 58.1)**	**0.827 (0.642 to 1.02)**
17 Road injuries	17 Pancreatic cancer	**162 (74.5 to 259)**	**162 (88.9 to 258)**	**41.3 (3.47 to 85.4)**
18 Alzheimer's disease	18 Blindness and vision loss	**62.8 (44.9 to 83.6)**	**62.8 (48.7 to 74.7)**	**-5.21 (-5.57 to -4.84)**
19 Lower respiratory infect	19 Anxiety disorders	-2.6 (-13.9 to 8.45)	-2.54 (-8.13 to 4.46)	**0.813 (0.73 to 0.894)**
20 Neck pain	20 Neck pain	**22.1 (8.21 to 36.4)**	**22.2 (11.8 to 32.2)**	**-0.577 (-0.774 to -0.394)**
21 Oral disorders	23 Gynecological diseases			
22 Hypertensive heart disease	25 Neonatal disorders			
24 Colorectal cancer	26 Road injuries			
25 Blindness and vision loss	34 Congenital defects			
32 Pancreatic cancer	156 COVID-19			

Supplemental Results Figure S7. Leading 20 level 3 causes of DALYs, and percentage change in number of DALYs and all-age and age-standardised DALY rates by location, 2022 and 2050

United Arab Emirates, Reference scenario

Legend:
Communicable, maternal, neonatal and nutritional
Non-communicable
Injuries

Leading Causes 2022	Leading Causes 2050	Mean % change number of DALY	Mean % change all-age DALY rate	Mean % change age-standardized DALY rate
1 Ischemic heart disease	1 Diabetes	**652 (519 to 801)**	**269 (220 to 333)**	**26 (8.17 to 43.1)**
2 Road injuries	2 Ischemic heart disease	**436 (256 to 735)**	**163 (73.2 to 288)**	**-52.2 (-68.6 to -23.1)**
3 Low back pain	3 Stroke	**490 (355 to 664)**	**190 (127 to 273)**	**-46.9 (-56.8 to -31.6)**
4 Diabetes	4 Chronic kidney disease	**1310 (829 to 1960)**	**592 (385 to 915)**	11.1 (-23.8 to 61.3)
5 COVID-19	5 Hypertensive heart disease	**1580 (996 to 2430)**	**727 (458 to 1120)**	17.3 (-17.4 to 79.9)
6 Depressive disorders	6 Lower respiratory infect	**981 (705 to 1290)**	**431 (304 to 581)**	**-23.6 (-39.7 to -4.98)**
7 Headache disorders	7 Road injuries	**157 (66 to 307)**	26 (-16.2 to 97.2)	-4.55 (-38.7 to 51.9)
8 Other musculoskeletal	8 Low back pain	**150 (110 to 195)**	**22.6 (9.33 to 37.8)**	0.743 (-0.227 to 1.72)
9 Stroke	9 COPD	**702 (485 to 1020)**	**294 (198 to 457)**	-10 (-32.8 to 22.4)
10 Anxiety disorders	10 Age-related hearing loss	**371 (285 to 471)**	**131 (97.1 to 172)**	**-0.827 (-1.21 to -0.424)**
11 Falls	11 Depressive disorders	**95.9 (65 to 126)**	-3.81 (-13.7 to 5.87)	**1.69 (0.308 to 3.18)**
12 Gynecological diseases	12 Alzheimer's disease	**1990 (1510 to 2540)**	**925 (740 to 1170)**	2.57 (-0.919 to 5.9)
13 Oral disorders	13 Other musculoskeletal	**160 (108 to 225)**	**27.5 (8.24 to 51.7)**	**19.4 (17.4 to 21.3)**
14 Neck pain	14 Headache disorders	**88.1 (63 to 113)**	-7.59 (-14.2 to 0.889)	**3.97 (2.39 to 5.25)**
15 Asthma	15 Liver cancer	**710 (526 to 896)**	**298 (225 to 398)**	16.4 (-0.819 to 38.5)
16 Neonatal disorders	16 Oral disorders	**213 (155 to 283)**	**53.7 (30.2 to 78.5)**	-0.129 (-0.523 to 0.323)
17 Age-related hearing loss	17 Pancreatic cancer	**961 (556 to 1480)**	**421 (234 to 642)**	14.8 (-22.5 to 55.1)
18 Congenital defects	18 Falls	**127 (86.1 to 174)**	11.4 (-5.45 to 27.5)	**-17.6 (-28.1 to -6.36)**
19 COPD	19 Blindness and vision loss	**414 (308 to 517)**	**152 (108 to 196)**	**-1.4 (-2.01 to -0.451)**
20 Schizophrenia	20 Lung cancer	**670 (409 to 978)**	**278 (160 to 411)**	-11.6 (-35.1 to 15.2)
22 Chronic kidney disease	21 Anxiety disorders			
23 Lower respiratory infect	24 Neck pain			
24 Blindness and vision loss	26 Gynecological diseases			
30 Hypertensive heart disease	30 Neonatal disorders			
32 Liver cancer	31 Asthma			
34 Lung cancer	38 Congenital defects			
42 Pancreatic cancer	39 Schizophrenia			

Supplemental Results Figure S7. Leading 20 level 3 causes of DALYs, and percentage change in number of DALYs and all-age and age-standardised DALY rates by location, 2022 and 2050

Yemen, Reference scenario

Legend:
Communicable, maternal, neonatal and nutritional
Non-communicable
Injuries

Leading Causes 2022	Leading Causes 2050	Mean % change number of DALY	Mean % change all-age DALY rate	Mean % change age-standardized DALY rate
1 Neonatal disorders	1 Ischemic heart disease	**99.6 (10.1 to 233)**	25.8 (-27.5 to 110)	-35.2 (-66.6 to 15.8)
2 Congenital defects	2 Malaria	76 (-49.2 to 119)	10.6 (-65.9 to 29.1)	-5.11 (-72.3 to 2.52)
3 Ischemic heart disease	3 Stroke	**80.7 (21.9 to 157)**	13.8 (-18.9 to 58)	**-39.4 (-61 to -9.26)**
4 Road injuries	4 Road injuries	17.3 (-38.2 to 105)	-26.1 (-58.5 to 29.7)	-33.4 (-62.9 to 15)
5 Malaria	5 Neonatal disorders	**-49.9 (-69.3 to -22.9)**	**-68.6 (-79.5 to -52.8)**	**-46.9 (-64.4 to -22.1)**
6 Conflict & terror	6 Dietary iron deficiency	17.6 (-3.76 to 47.7)	**-26.1 (-29.6 to -22.7)**	**-16.1 (-16.6 to -15.5)**
7 Dietary iron deficiency	7 Conflict & terror	356 (-95.7 to 3670)	186 (-97.2 to 2150)	221 (-97.2 to 2540)
8 Stroke	8 Diabetes	**327 (241 to 420)**	**169 (132 to 216)**	**44.1 (24.3 to 64)**
9 Lower respiratory infect	9 Congenital defects	**-42.1 (-59.5 to -22.4)**	**-63.7 (-71.9 to -54.7)**	**-37.3 (-45.9 to -26.9)**
10 COVID-19	10 Depressive disorders	**90.6 (61.3 to 124)**	**20.1 (11.8 to 29.6)**	**-3.37 (-3.59 to -3.13)**
11 Depressive disorders	11 Low back pain	**101 (69 to 139)**	**26.9 (17.6 to 37.7)**	**-6.46 (-6.62 to -6.27)**
12 Low back pain	12 Hypertensive heart disease	**196 (90.2 to 349)**	**86.9 (24.2 to 161)**	-11.5 (-46.9 to 41.8)
13 Headache disorders	13 Other musculoskeletal	**354 (285 to 433)**	**186 (160 to 215)**	**95.6 (91.4 to 99.5)**
14 Anxiety disorders	14 Headache disorders	**85.1 (54.3 to 123)**	**16.6 (8.76 to 31.2)**	**0.628 (0.38 to 0.809)**
15 Gynecological diseases	15 Anxiety disorders	**72.2 (41 to 109)**	**8.38 (1.23 to 17)**	**1.43 (1.23 to 1.63)**
16 Hypertensive heart disease	16 Gynecological diseases	**73.3 (21.6 to 129)**	9.19 (-21.5 to 39.2)	-15.9 (-39 to 5.63)
17 Diarrheal diseases	17 Lower respiratory infect	**-32.3 (-53.7 to -9.69)**	**-57.3 (-71 to -43)**	**-49.8 (-64.8 to -32.3)**
18 Diabetes	18 COPD	**141 (59.5 to 235)**	**52.1 (8.24 to 107)**	-22.4 (-49.8 to 14.7)
19 Pertussis	19 Chronic kidney disease	**247 (125 to 408)**	**119 (45 to 209)**	24 (-23 to 93.1)
20 Maternal disorders	20 Age-related hearing loss	**155 (109 to 206)**	**60.6 (40.5 to 82.4)**	**-1.95 (-2.12 to -1.78)**
22 COPD	48 Maternal disorders			
26 Age-related hearing loss	50 Pertussis			
28 Other musculoskeletal	55 Diarrheal diseases			
35 Chronic kidney disease	166 COVID-19			

Supplemental Results Figure S7. Leading 20 level 3 causes of DALYs, and percentage change in number of DALYs and all-age and age-standardised DALY rates by location, 2022 and 2050

Bangladesh, Reference scenario

Legend:
Communicable, maternal, neonatal and nutritional
Non-communicable
Injuries

Leading Causes 2022	Leading Causes 2050	Mean % change number of DALY	Mean % change all-age DALY rate	Mean % change age-standardized DALY rate
1 Neonatal disorders	1 Stroke	28.8 (-4.1 to 65.3)	20.3 (-9.29 to 56.3)	**-42.6 (-58.3 to -20.3)**
2 Stroke	2 Diabetes	**143 (96.4 to 193)**	**127 (90.7 to 160)**	**21.5 (2.36 to 44)**
3 Ischemic heart disease	3 Other musculoskeletal	**151 (110 to 187)**	**134 (118 to 149)**	**67.5 (63.7 to 71.2)**
4 Lower respiratory infect	4 Ischemic heart disease	15 (-28.6 to 83.3)	7.62 (-35.9 to 70.8)	**-45.5 (-69.3 to -7.32)**
5 COPD	5 COPD	**75.9 (28.1 to 142)**	**64.4 (17.9 to 120)**	-26 (-49.2 to 8.92)
6 Diabetes	6 Low back pain	**46.1 (22.1 to 69.6)**	**36.2 (28.6 to 45.4)**	**-5.47 (-5.82 to -5.16)**
7 Low back pain	7 Depressive disorders	**35.3 (11.8 to 56.5)**	**26.1 (18.6 to 35.5)**	**-3.3 (-3.64 to -3)**
8 Other musculoskeletal	8 Neonatal disorders	**-62.9 (-71.9 to -52.9)**	**-65.4 (-71.2 to -58.3)**	**-45.2 (-53.9 to -35.5)**
9 Depressive disorders	9 Age-related hearing loss	**84.5 (50.8 to 119)**	**71.9 (56.4 to 88.8)**	**-1.71 (-1.92 to -1.49)**
10 Cirrhosis liver	10 Blindness and vision loss	**94.7 (58.2 to 133)**	**81.5 (64.3 to 98.2)**	**-6.23 (-6.68 to -5.75)**
11 COVID-19	11 Cirrhosis liver	4.45 (-17.1 to 28.7)	-2.52 (-21.4 to 22.3)	**-37.1 (-50.7 to -20.1)**
12 Diarrheal diseases	12 Headache disorders	15.9 (-5.18 to 41.1)	**8 (0.222 to 26.4)**	**0.438 (0.164 to 0.718)**
13 Headache disorders	13 Alzheimer's disease	**211 (131 to 298)**	**189 (125 to 256)**	1.56 (-3.03 to 6.82)
14 Tuberculosis	14 Diarrheal diseases	3.11 (-33.4 to 45.9)	-3.61 (-41.6 to 32.3)	**-38.5 (-62.1 to -5.18)**
15 Congenital defects	15 Hypertensive heart disease	**143 (69.6 to 253)**	**127 (52.1 to 222)**	-3.77 (-39.6 to 46.3)
16 Dietary iron deficiency	16 Chronic kidney disease	**125 (54.4 to 219)**	**110 (41.6 to 204)**	17.4 (-25.3 to 83.1)
17 Age-related hearing loss	17 Lower respiratory infect	**-47.1 (-60.8 to -29.4)**	**-50.7 (-62.4 to -36.7)**	**-62.5 (-70.8 to -51)**
18 Rheumatic heart disease	18 Dietary iron deficiency	0.645 (-18.3 to 18.6)	-6.3 (-12.2 to 0.878)	**-18.4 (-19.1 to -17.6)**
19 Anxiety disorders	19 Anxiety disorders	**24.1 (1.87 to 46.9)**	**15.7 (7.71 to 25.6)**	**1.24 (0.882 to 1.52)**
20 Blindness and vision loss	20 Osteoarthritis	**121 (82 to 157)**	**106 (89.9 to 121)**	**6.5 (6.09 to 6.99)**
27 Chronic kidney disease	22 Rheumatic heart disease			
28 Hypertensive heart disease	26 Tuberculosis			
31 Alzheimer's disease	29 Congenital defects			
35 Osteoarthritis	161 COVID-19			

Supplemental Results Figure S7. Leading 20 level 3 causes of DALYs, and percentage change in number of DALYs and all-age and age-standardised DALY rates by location, 2022 and 2050

Bhutan, Reference scenario

Legend:
Communicable, maternal, neonatal and nutritional
Non-communicable
Injuries

Leading Causes 2022	Leading Causes 2050	Mean % change number of DALY	Mean % change all-age DALY rate	Mean % change age-standardized DALY rate
1 Neonatal disorders	1 COPD	**91.5 (37.8 to 155)**	**68.6 (17.8 to 131)**	-25 (-51.6 to 13.2)
2 COVID-19	2 Ischemic heart disease	22 (-29.2 to 99.1)	7.48 (-39.1 to 81)	**-47.8 (-72.1 to -3.59)**
3 Ischemic heart disease	3 Other musculoskeletal	**192 (153 to 235)**	**157 (139 to 177)**	**89.1 (83.9 to 94.2)**
4 COPD	4 Diabetes	**128 (86.5 to 175)**	**101 (64.1 to 141)**	4.53 (-14.7 to 31.1)
5 Stroke	5 Stroke	32.9 (-1.17 to 77.1)	17 (-14.6 to 60.9)	**-45.1 (-61.6 to -22)**
6 Dietary iron deficiency	6 Low back pain	**54.2 (34.4 to 73)**	**35.4 (26.9 to 45.4)**	**-6.27 (-6.49 to -6.05)**
7 Low back pain	7 Depressive disorders	**46.3 (27.8 to 65.3)**	**28.5 (19.2 to 39.9)**	**-3.03 (-3.27 to -2.76)**
8 Diabetes	8 Falls	**77.1 (43.6 to 115)**	**55.6 (29.4 to 80.1)**	**-11.7 (-21 to -0.998)**
9 Cirrhosis liver	9 Chronic kidney disease	**132 (52.2 to 228)**	**104 (29.2 to 201)**	8.82 (-34.2 to 72.7)
10 Lower respiratory infect	10 Age-related hearing loss	**94.5 (68.3 to 119)**	**70.8 (56.9 to 85.4)**	**-1.77 (-1.9 to -1.62)**
11 Diarrheal diseases	11 Cirrhosis liver	13.9 (-10.7 to 43.6)	0.109 (-21.5 to 30.4)	**-35.5 (-49.4 to -15.9)**
12 Other musculoskeletal	12 Neonatal disorders	**-64.2 (-73.4 to -55.4)**	**-68.6 (-75 to -61.3)**	**-44.6 (-54.3 to -31.8)**
13 Depressive disorders	13 Dietary iron deficiency	-11.7 (-25.3 to 0.149)	**-22.5 (-28.4 to -16.4)**	**-15.6 (-16.5 to -14.9)**
14 Headache disorders	14 Headache disorders	**20.9 (2.58 to 40.7)**	6.19 (-1.45 to 24.6)	**1.36 (0.827 to 1.59)**
15 Falls	15 Alzheimer's disease	**193 (128 to 254)**	**157 (107 to 214)**	4.36 (-1.44 to 10.6)
16 Congenital defects	16 Hypertensive heart disease	**121 (52.3 to 205)**	**94.4 (29.3 to 177)**	-13 (-46.2 to 34.2)
17 Road injuries	17 Lower respiratory infect	**-33.2 (-54.6 to -4.93)**	**-41.2 (-59.4 to -15.1)**	**-57.7 (-68.2 to -42.1)**
18 Age-related hearing loss	18 Blindness and vision loss	**98.1 (70.9 to 122)**	**74 (59.7 to 89)**	**-7.97 (-8.77 to -7.15)**
19 Tuberculosis	19 Anxiety disorders	**30.2 (11.8 to 51.2)**	**14.2 (4.87 to 26.3)**	**2.12 (1.88 to 2.38)**
20 Chronic kidney disease	20 Diarrheal diseases	**-35.9 (-58.9 to -8.53)**	**-43.6 (-65 to -15.3)**	**-61.9 (-75.9 to -41.5)**
22 Anxiety disorders	24 Road injuries			
28 Blindness and vision loss	34 Congenital defects			
32 Hypertensive heart disease	38 Tuberculosis			
33 Alzheimer's disease	164 COVID-19			

Supplemental Results Figure S7. Leading 20 level 3 causes of DALYs, and percentage change in number of DALYs and all-age and age-standardised DALY rates by location, 2022 and 2050

India, Reference scenario

Legend:
Communicable, maternal, neonatal and nutritional
Non-communicable
Injuries

Leading Causes 2022	Leading Causes 2050	Mean % change number of DALY	Mean % change all-age DALY rate	Mean % change age-standardized DALY rate
1 Neonatal disorders	1 COPD	**63.3 (18.1 to 112)**	**47.6 (9.91 to 98.4)**	-31.3 (-51.7 to 2.17)
2 Ischemic heart disease	2 Ischemic heart disease	-3.5 (-43.6 to 50.1)	-12.6 (-47.8 to 39.9)	**-53.1 (-73.9 to -18.3)**
3 COPD	3 Other musculoskeletal	**145 (110 to 179)**	**121 (107 to 135)**	**66 (62.4 to 69.7)**
4 Lower respiratory infect	4 Diabetes	**92.2 (60.8 to 126)**	**73.5 (51.8 to 101)**	-1.7 (-14.7 to 18.1)
5 Stroke	5 Stroke	12.7 (-11.6 to 44.2)	1.82 (-20.1 to 32.1)	**-47 (-60.6 to -30.3)**
6 Diarrheal diseases	6 Neonatal disorders	**-51 (-61.4 to -40.5)**	**-55.8 (-63.2 to -47)**	**-31.8 (-42.4 to -19)**
7 Diabetes	7 Falls	**76.3 (39.9 to 114)**	**59.1 (35.6 to 81.2)**	**-10.8 (-19.2 to -1.64)**
8 Tuberculosis	8 Low back pain	**47.1 (27 to 68.1)**	**32.7 (25.1 to 41.1)**	**-6.15 (-6.52 to -5.72)**
9 Dietary iron deficiency	9 Blindness and vision loss	**84 (53.5 to 116)**	**66 (50.7 to 81.3)**	**-6.54 (-7.04 to -6.05)**
10 Other musculoskeletal	10 Lower respiratory infect	**-32.6 (-46.2 to -15.7)**	**-39.2 (-50.5 to -26.4)**	**-55.3 (-63.7 to -43.3)**
11 Road injuries	11 Diarrheal diseases	-15.4 (-41.5 to 17.5)	-23.5 (-48.2 to 10.2)	**-52 (-70.3 to -24.8)**
12 Falls	12 Cirrhosis liver	**34.8 (6.95 to 68.8)**	21.7 (-2.95 to 50.5)	-15.9 (-32.5 to 5.77)
13 Cirrhosis liver	13 Age-related hearing loss	**80.5 (53.5 to 109)**	**62.8 (50 to 76.2)**	**-1.45 (-1.62 to -1.32)**
14 Self-harm	14 Depressive disorders	**35.1 (15.7 to 54.9)**	**21.8 (15.9 to 28.8)**	**-4.67 (-5.08 to -4.33)**
15 Low back pain	15 Dietary iron deficiency	0.877 (-13.4 to 16.5)	**-9.04 (-11.5 to -6.8)**	**-13.6 (-14.2 to -12.7)**
16 COVID-19	16 Road injuries	-0.251 (-38.4 to 49.1)	-9.78 (-42.7 to 42.8)	-20 (-51.5 to 29.4)
17 Depressive disorders	17 Headache disorders	**18.2 (0.589 to 41.3)**	**6.61 (0.42 to 22.7)**	**0.583 (0.292 to 0.775)**
18 Headache disorders	18 Chronic kidney disease	**89.4 (31.2 to 165)**	**71.2 (19.1 to 145)**	-2.81 (-35.1 to 44.5)
19 Congenital defects	19 Self-harm	-10 (-33.6 to 16.8)	-18.8 (-38.2 to 6.29)	-19.1 (-40 to 8.59)
20 Blindness and vision loss	20 Alzheimer's disease	**189 (120 to 275)**	**161 (115 to 209)**	**-6.92 (-11.4 to -2.69)**
21 Age-related hearing loss	21 Tuberculosis			
25 Chronic kidney disease	31 Congenital defects			
34 Alzheimer's disease	166 COVID-19			

Supplemental Results Figure S7. Leading 20 level 3 causes of DALYs, and percentage change in number of DALYs and all-age and age-standardised DALY rates by location, 2022 and 2050

Nepal, Reference scenario

Legend:
Communicable, maternal, neonatal and nutritional
Non-communicable
Injuries

Leading Causes 2022	Leading Causes 2050	Mean % change number of DALY	Mean % change all-age DALY rate	Mean % change age-standardized DALY rate
1 Neonatal disorders	1 COPD	27.8 (-10.4 to 72.9)	22.8 (-14.1 to 67.6)	**-45.8 (-62.8 to -19.8)**
2 Ischemic heart disease	2 Diabetes	**118 (82.2 to 165)**	**110 (77.4 to 142)**	13 (-3.98 to 31.8)
3 COPD	3 Ischemic heart disease	-5.35 (-44.7 to 54.6)	-8.98 (-44.6 to 51.1)	**-55.2 (-74.9 to -20)**
4 Stroke	4 Other musculoskeletal	**175 (137 to 218)**	**164 (146 to 187)**	**84.4 (80 to 89.4)**
5 Lower respiratory infect	5 Low back pain	**42.6 (23.1 to 64.5)**	**37.1 (27.8 to 47)**	**-7.15 (-7.55 to -6.68)**
6 Low back pain	6 Stroke	3.1 (-22.5 to 38.4)	-0.882 (-26.5 to 31.5)	**-52.6 (-64.9 to -33.1)**
7 Diabetes	7 Depressive disorders	**38.2 (19.7 to 56.9)**	**32.8 (24.5 to 43.4)**	**-3.92 (-4.34 to -3.51)**
8 Depressive disorders	8 Cirrhosis liver	20.9 (-9.82 to 59.2)	16.2 (-10.2 to 51.7)	**-25.1 (-41.5 to -1.3)**
9 Cirrhosis liver	9 Neonatal disorders	**-71.9 (-79.9 to -64.1)**	**-73.1 (-78.9 to -67.7)**	**-50.2 (-58.4 to -41.1)**
10 Other musculoskeletal	10 Falls	**37.1 (9.36 to 70.2)**	**31.9 (8.63 to 66.3)**	**-19.1 (-28.2 to -8.76)**
11 Self-harm	11 Age-related hearing loss	**79.5 (52.5 to 108)**	**72.6 (55.5 to 91.9)**	**-1.84 (-2.09 to -1.62)**
12 Tuberculosis	12 Blindness and vision loss	**79.5 (55.4 to 103)**	**72.6 (57.6 to 88)**	**-6.7 (-7.45 to -5.79)**
13 Falls	13 Headache disorders	15.7 (-1.91 to 41.1)	**11.2 (2.72 to 32.2)**	0.0832 (-0.29 to 0.593)
14 Headache disorders	14 Chronic kidney disease	**88.8 (24.5 to 175)**	**81.5 (24.6 to 168)**	-1.91 (-34.4 to 54.6)
15 Dietary iron deficiency	15 Self-harm	-2.39 (-28 to 36.4)	-6.33 (-27.3 to 22.7)	-12.3 (-33.8 to 17.1)
16 COVID-19	16 Hypertensive heart disease	**123 (53 to 211)**	**114 (45.8 to 200)**	-7.84 (-41.2 to 38.2)
17 Diarrheal diseases	17 Lower respiratory infect	**-55.9 (-69.9 to -34.4)**	**-57.7 (-70.1 to -39.1)**	**-64.3 (-72.3 to -52)**
18 Asthma	18 Dietary iron deficiency	-14.1 (-27.9 to 0.00295)	**-17.6 (-23.2 to -12)**	**-19.9 (-20.9 to -18.6)**
19 Age-related hearing loss	19 Alzheimer's disease	**186 (114 to 265)**	**175 (110 to 243)**	**-6.55 (-11.4 to -1.36)**
20 Rheumatic heart disease	20 Anxiety disorders	**23.4 (3.35 to 45.1)**	**18.6 (8.97 to 29.9)**	**0.958 (0.416 to 1.52)**
21 Blindness and vision loss	24 Asthma			
22 Anxiety disorders	25 Tuberculosis			
25 Chronic kidney disease	27 Rheumatic heart disease			
31 Hypertensive heart disease	28 Diarrheal diseases			
36 Alzheimer's disease	162 COVID-19			

Supplemental Results Figure S7. Leading 20 level 3 causes of DALYs, and percentage change in number of DALYs and all-age and age-standardised DALY rates by location, 2022 and 2050

Pakistan, Reference scenario

Legend:
Communicable, maternal, neonatal and nutritional
Non-communicable
Injuries

Leading Causes 2022
1 Neonatal disorders
2 Lower respiratory infect
3 Ischemic heart disease
4 Stroke
5 Tuberculosis
6 Dietary iron deficiency
7 Diarrheal diseases
8 Diabetes
9 Road injuries
10 Congenital defects
11 COPD
12 Cirrhosis liver
13 Low back pain
14 COVID-19
15 Depressive disorders
16 Headache disorders
17 Other musculoskeletal
18 Rheumatic heart disease
19 Chronic kidney disease
20 Meningitis
24 Anxiety disorders
25 Age-related hearing loss
29 Blindness and vision loss
30 Breast cancer
43 Hypertensive heart disease

Leading Causes 2050	Mean % change number of DALY	Mean % change all-age DALY rate	Mean % change age-standardized DALY rate
1 Neonatal disorders	**-60.4 (-73.6 to -44.9)**	**-68 (-75.7 to -58)**	**-43.4 (-54.3 to -28.4)**
2 Ischemic heart disease	31.4 (-25.3 to 111)	6.97 (-40.7 to 83.5)	**-45.9 (-71.3 to -4.75)**
3 Diabetes	**168 (125 to 218)**	**117 (80.9 to 161)**	11.2 (-7.19 to 35.4)
4 Stroke	**49.3 (10.9 to 99.3)**	21.5 (-9.83 to 74.4)	**-40.9 (-58.5 to -13.6)**
5 Other musculoskeletal	**241 (199 to 288)**	**177 (154 to 202)**	**86.7 (81.8 to 91.6)**
6 COPD	**82 (28 to 147)**	**48.1 (7.72 to 103)**	**-37.2 (-59.7 to -6.58)**
7 Chronic kidney disease	**166 (71.5 to 289)**	**116 (41.6 to 228)**	27 (-21 to 101)
8 Low back pain	**67.8 (47.6 to 89.4)**	**36.3 (25.3 to 46)**	**-6.51 (-7.34 to -5.52)**
9 Dietary iron deficiency	-6.5 (-20.4 to 10.9)	**-24.2 (-27.5 to -20.4)**	**-17.7 (-18.3 to -16.8)**
10 Depressive disorders	**60.3 (40.5 to 78.2)**	**30.2 (20.8 to 39.6)**	**-3.24 (-3.63 to -2.83)**
11 Road injuries	-16.9 (-52.2 to 37.6)	-32.4 (-59.1 to 17.5)	-37 (-62.7 to 9.24)
12 Headache disorders	**40 (20.3 to 64.7)**	**13.6 (7.16 to 29.9)**	**0.89 (0.497 to 1.13)**
13 Blindness and vision loss	**127 (99.8 to 161)**	**84.2 (61.3 to 105)**	**-8.65 (-9.35 to -8.04)**
14 Cirrhosis liver	5.24 (-17 to 32.1)	-14.4 (-32.3 to 9.4)	**-41.1 (-53.6 to -24.4)**
15 Lower respiratory infect	**-68.9 (-78.4 to -57.1)**	**-74.8 (-81.8 to -66.3)**	**-65.1 (-73.7 to -53.9)**
16 Age-related hearing loss	**108 (83.2 to 137)**	**69.3 (50.7 to 88.5)**	**-1.77 (-1.98 to -1.56)**
17 Breast cancer	**111 (32.9 to 196)**	**71.4 (9.89 to 144)**	3 (-31.9 to 40.4)
18 Diarrheal diseases	-33.6 (-62.7 to 11)	**-46 (-68.8 to -14.9)**	**-47.3 (-69 to -12.3)**
19 Anxiety disorders	**48.1 (30.7 to 67.8)**	**20.2 (12.8 to 27.7)**	**1.7 (1.52 to 1.93)**
20 Hypertensive heart disease	**162 (82.2 to 267)**	**113 (47.4 to 204)**	-8.47 (-41.8 to 37.6)
22 Tuberculosis			
25 Congenital defects			
33 Rheumatic heart disease			
59 Meningitis			
164 COVID-19			

Supplemental Results Figure S7. Leading 20 level 3 causes of DALYs, and percentage change in number of DALYs and all-age and age-standardised DALY rates by location, 2022 and 2050

China, Reference scenario

Legend:
Communicable, maternal, neonatal and nutritional
Non-communicable
Injuries

Leading Causes 2022	Leading Causes 2050	Mean % change number of DALY	Mean % change all-age DALY rate	Mean % change age-standardized DALY rate
1 Stroke	1 Stroke	3.1 (-16.5 to 24.4)	18.5 (-1.45 to 47.2)	**-44.8 (-54.2 to -30.9)**
2 Ischemic heart disease	2 COPD	**82.7 (40.3 to 126)**	**110 (66.8 to 165)**	-19.7 (-39.4 to 7.62)
3 COPD	3 Ischemic heart disease	-11.5 (-37.6 to 20.4)	1.79 (-27.8 to 42.1)	**-56.5 (-70.1 to -36.7)**
4 Lung cancer	4 Alzheimer's disease	**173 (127 to 231)**	**214 (172 to 263)**	-0.256 (-3.9 to 3.1)
5 Age-related hearing loss	5 Lung cancer	34.2 (-0.962 to 77.5)	**54 (16.4 to 99)**	-13.8 (-30.8 to 7.4)
6 Road injuries	6 Diabetes	**75.4 (54.4 to 95.3)**	**101 (84.8 to 117)**	**44.2 (33.1 to 54.7)**
7 Diabetes	7 Age-related hearing loss	**43.1 (27.9 to 60.4)**	**64.3 (53.1 to 75.9)**	**-0.821 (-0.969 to -0.67)**
8 Low back pain	8 Hypertensive heart disease	**140 (72.7 to 225)**	**176 (102 to 288)**	10.7 (-23.9 to 58.3)
9 Stomach cancer	9 Low back pain	8.16 (-2.97 to 20.7)	**24.3 (17.7 to 30.7)**	**-4.16 (-4.53 to -3.72)**
10 Alzheimer's disease	10 Other musculoskeletal	**50.1 (35.4 to 66)**	**72.5 (61.4 to 83.2)**	**55 (52.6 to 57.1)**
11 Falls	11 Colorectal cancer	**68.1 (16.7 to 127)**	**92.8 (40.4 to 158)**	11.8 (-17.4 to 44.5)
12 Depressive disorders	12 Esophageal cancer	**43 (22.9 to 65)**	**64.3 (46.4 to 86.3)**	-0.617 (-11.3 to 10.7)
13 Other musculoskeletal	13 Falls	**19.1 (2.13 to 39.5)**	**36.8 (19.4 to 56.9)**	**-22.7 (-29.5 to -14.6)**
14 Headache disorders	14 Stomach cancer	-11.4 (-22.6 to 3.5)	1.79 (-10.1 to 14.3)	**-40 (-44.8 to -35.2)**
15 Esophageal cancer	15 Blindness and vision loss	**32 (13.7 to 50.2)**	**51.6 (36.6 to 66.3)**	**-5.19 (-5.68 to -4.8)**
16 Colorectal cancer	16 Road injuries	-29.2 (-51.8 to 5.52)	-18.5 (-45.2 to 23.7)	-19.2 (-49 to 29.3)
17 Blindness and vision loss	17 Depressive disorders	4.64 (-4.31 to 14)	**20.2 (15.1 to 26.2)**	**-1.99 (-2.36 to -1.61)**
18 Hypertensive heart disease	18 Osteoarthritis	**38.4 (25.7 to 52.5)**	**59 (51.4 to 68)**	**3.54 (3.02 to 4.03)**
19 Anxiety disorders	19 Chronic kidney disease	**68.3 (26.7 to 117)**	**93.4 (46.8 to 154)**	1.13 (-26.7 to 37.6)
20 Osteoarthritis	20 Headache disorders	**-14.7 (-21.6 to -6.45)**	-1.98 (-6.54 to 6.31)	-0.025 (-0.417 to 0.49)
24 Chronic kidney disease	25 Anxiety disorders			

Supplemental Results Figure S7. Leading 20 level 3 causes of DALYs, and percentage change in number of DALYs and all-age and age-standardised DALY rates by location, 2022 and 2050

North Korea, Reference scenario

Legend:
Communicable, maternal, neonatal and nutritional
Non-communicable
Injuries

Leading Causes 2022	Leading Causes 2050	Mean % change number of DALY	Mean % change all-age DALY rate	Mean % change age-standardized DALY rate
1 Stroke	1 Stroke	-3.45 (-25.6 to 22.9)	5.27 (-16.1 to 33.2)	**-38.8 (-52.7 to -18.7)**
2 Ischemic heart disease	2 COPD	**71.7 (29.8 to 119)**	**87.2 (43 to 143)**	-8.47 (-35.1 to 29.7)
3 COPD	3 Ischemic heart disease	7 (-27.7 to 59.4)	16.8 (-20.4 to 69.7)	-32.3 (-56.8 to 7.58)
4 Road injuries	4 Diabetes	**36.1 (10.6 to 62.3)**	**48.3 (26.5 to 67.1)**	-2 (-13.9 to 12.5)
5 Tuberculosis	5 Road injuries	-22 (-51.6 to 27.5)	-14.8 (-45 to 39)	-13.1 (-45.7 to 45.2)
6 Diabetes	6 Lung cancer	27.9 (-10.3 to 72.7)	**39 (4.59 to 77.3)**	-9.98 (-29.1 to 10.5)
7 Low back pain	7 Low back pain	12.9 (-3.5 to 30.6)	**23 (16.5 to 30.1)**	**-3.72 (-4.11 to -3.38)**
8 Lung cancer	8 Hypertensive heart disease	**88.8 (25.8 to 169)**	**106 (41.3 to 185)**	5.21 (-31.2 to 57.8)
9 Stomach cancer	9 Alzheimer's disease	**120 (63.3 to 182)**	**140 (86.4 to 189)**	-3.43 (-6.74 to 0.145)
10 Lower respiratory infect	10 Age-related hearing loss	**46.5 (23.3 to 71)**	**59.5 (45 to 74.1)**	**-0.862 (-1.37 to -0.421)**
11 Cirrhosis liver	11 Other musculoskeletal	**37.5 (16.1 to 58.2)**	**49.7 (41.7 to 59.5)**	**26.3 (23.3 to 28.6)**
12 Neonatal disorders	12 Tuberculosis	**-45.6 (-63.8 to -20.7)**	**-40.6 (-59.1 to -14.2)**	**-56 (-70 to -33.5)**
13 Age-related hearing loss	13 Chronic kidney disease	**53.8 (12 to 105)**	**67.8 (26.1 to 123)**	10.9 (-18.7 to 56.9)
14 Hypertensive heart disease	14 Stomach cancer	-11.9 (-26.8 to 6.03)	-4.11 (-15 to 8.69)	**-35.2 (-41.4 to -28.9)**
15 Headache disorders	15 Lower respiratory infect	6.28 (-15.8 to 34.9)	15.8 (-3.67 to 38.3)	**-41.7 (-52.3 to -26.6)**
16 Other musculoskeletal	16 Cirrhosis liver	-9.95 (-30.9 to 20.7)	-1.77 (-23.1 to 26.6)	**-24.8 (-41.3 to -3.99)**
17 Depressive disorders	17 Depressive disorders	6.36 (-9.43 to 22.5)	**15.8 (10.2 to 21.5)**	**-1.8 (-2.27 to -1.41)**
18 Alzheimer's disease	18 Colorectal cancer	31.8 (-12.2 to 84.2)	**43.3 (1.07 to 99.6)**	-4.64 (-29.7 to 27)
19 Self-harm	19 Headache disorders	-8.98 (-22.1 to 6.48)	-0.883 (-5.49 to 7.26)	**0.34 (0.0286 to 0.606)**
20 Chronic kidney disease	20 Esophageal cancer	**43.7 (15.9 to 73)**	**56.5 (37.3 to 77.1)**	4.64 (-8.38 to 17)
22 Colorectal cancer	24 Self-harm			
25 Esophageal cancer	29 Neonatal disorders			

Supplemental Results Figure S7. Leading 20 level 3 causes of DALYs, and percentage change in number of DALYs and all-age and age-standardised DALY rates by location, 2022 and 2050

Taiwan (Province of China), Reference scenario

Legend:
Communicable, maternal, neonatal and nutritional
Non-communicable
Injuries

Leading Causes 2022	Leading Causes 2050	Mean % change number of DALY	Mean % change all-age DALY rate	Mean % change age-standardized DALY rate
1 Diabetes	1 Diabetes	**66.3 (48.6 to 85.2)**	**91.3 (76.2 to 107)**	**25 (13.6 to 36)**
2 Stroke	2 Alzheimer's disease	**155 (123 to 194)**	**194 (168 to 218)**	0.536 (-2.34 to 3.59)
3 Low back pain	3 Chronic kidney disease	**150 (108 to 198)**	**188 (141 to 241)**	**24.7 (2.69 to 53.6)**
4 Ischemic heart disease	4 Stroke	6.01 (-6.92 to 20.6)	**22 (11 to 34.7)**	**-34.8 (-42.3 to -26.7)**
5 Lung cancer	5 Lower respiratory infect	**72.1 (46.9 to 98.4)**	**98 (75.3 to 124)**	**-25.5 (-32.9 to -15.9)**
6 Lower respiratory infect	6 Lung cancer	**40.4 (15.3 to 64.8)**	**61.3 (35 to 83.1)**	-7.29 (-20.9 to 4.18)
7 Colorectal cancer	7 Low back pain	6.66 (-3.23 to 16.9)	**22.7 (19.9 to 26)**	**-2.72 (-2.79 to -2.64)**
8 COVID-19	8 Colorectal cancer	**51.8 (17.4 to 85.3)**	**74.5 (40.8 to 102)**	-1.55 (-19.5 to 15.1)
9 Alzheimer's disease	9 COPD	**87.7 (57 to 130)**	**116 (82.4 to 158)**	-15.6 (-30.4 to 1.48)
10 Other musculoskeletal	10 Age-related hearing loss	**43.3 (26.9 to 59.3)**	**64.8 (52.1 to 78.1)**	**-0.648 (-0.715 to -0.577)**
11 Cirrhosis liver	11 Other musculoskeletal	**27.7 (14.5 to 43.6)**	**46.9 (38.7 to 55.3)**	**30.7 (28.9 to 32.3)**
12 Age-related hearing loss	12 Ischemic heart disease	**-21.9 (-35.6 to -2.25)**	-10 (-25.7 to 13.3)	**-54.1 (-62.7 to -40.8)**
13 Chronic kidney disease	13 Urinary diseases	**161 (120 to 211)**	**200 (160 to 245)**	**21.2 (9.91 to 34.9)**
14 COPD	14 Hypertensive heart disease	**121 (75.2 to 178)**	**154 (98.7 to 224)**	18 (-7.6 to 55)
15 Road injuries	15 Osteoarthritis	**30.9 (18.1 to 43.3)**	**50.6 (45.4 to 56.3)**	**1.96 (1.76 to 2.17)**
16 Self-harm	16 Liver cancer	**38.4 (16.6 to 63.5)**	**59.3 (37.1 to 89.8)**	13.1 (-6.05 to 37)
17 Headache disorders	17 Cirrhosis liver	**-17.8 (-27.9 to -4.32)**	-5.43 (-15.2 to 6.4)	**-22.2 (-30.4 to -11.3)**
18 Depressive disorders	18 Pancreatic cancer	**78.6 (40.1 to 119)**	**105 (69.6 to 142)**	**30.5 (7.45 to 51.1)**
19 Osteoarthritis	19 Falls	**16.6 (0.852 to 34)**	**34.1 (20.7 to 46)**	**-18.6 (-27.1 to -10.5)**
20 Anxiety disorders	20 Depressive disorders	-3.61 (-13.9 to 6.67)	**10.9 (5.76 to 16.5)**	**-1.41 (-1.58 to -1.22)**
21 Liver cancer	21 Road injuries			
23 Falls	22 Headache disorders			
27 Hypertensive heart disease	24 Self-harm			
29 Urinary diseases	26 Anxiety disorders			
30 Pancreatic cancer	156 COVID-19			

Supplemental Results Figure S7. Leading 20 level 3 causes of DALYs, and percentage change in number of DALYs and all-age and age-standardised DALY rates by location, 2022 and 2050

American Samoa, Reference scenario

Legend:
Communicable, maternal, neonatal and nutritional
Non-communicable
Injuries

Leading Causes 2022	Leading Causes 2050	Mean % change number of DALY	Mean % change all-age DALY rate	Mean % change age-standardized DALY rate
1 Diabetes	1 Diabetes	**31.5 (12.8 to 50.4)**	5.8 (-7.77 to 21)	-11.7 (-22 to 2.36)
2 Ischemic heart disease	2 Ischemic heart disease	-13.9 (-39.7 to 24.8)	-30.6 (-52.8 to 1.74)	**-45 (-63 to -17.3)**
3 Stroke	3 Chronic kidney disease	**86.6 (41.8 to 150)**	**50.3 (13 to 105)**	20.7 (-12.3 to 66.8)
4 Chronic kidney disease	4 Stroke	10.2 (-10.5 to 32.6)	-11.3 (-27.8 to 9.73)	**-33.3 (-46.1 to -16.4)**
5 COPD	5 COPD	**85.9 (44.1 to 139)**	**49.7 (15 to 94.5)**	0.499 (-24.3 to 35.5)
6 Lower respiratory infect	6 Endo/metab/blood/immune	**77 (56.4 to 98.3)**	**42.4 (27.4 to 59.5)**	**22 (8.42 to 40.2)**
7 Neonatal disorders	7 Lung cancer	**74 (31.9 to 125)**	**39.7 (7.72 to 73.6)**	9.92 (-11.4 to 33.8)
8 Low back pain	8 Lower respiratory infect	**21.8 (6.22 to 39.5)**	-2.05 (-15.2 to 13.1)	**-22.2 (-31.9 to -9.33)**
9 Endo/metab/blood/immune	9 Low back pain	**37.8 (24.4 to 51.3)**	**10.8 (4.57 to 17.3)**	**-2.49 (-2.65 to -2.32)**
10 Lung cancer	10 Breast cancer	**51.7 (12.1 to 98.7)**	21.8 (-8.59 to 56.2)	4.01 (-19.2 to 31.5)
11 Breast cancer	11 Other musculoskeletal	**86.2 (69.8 to 107)**	**49.7 (40.7 to 60)**	**34.6 (31.6 to 37.1)**
12 Headache disorders	12 Neonatal disorders	-6.11 (-26.4 to 23.3)	**-24.5 (-39.6 to -2.22)**	-16.8 (-34.8 to 9.6)
13 COVID-19	13 Age-related hearing loss	**57.3 (40.7 to 75.9)**	**26.4 (15.7 to 35.9)**	**-0.776 (-0.847 to -0.71)**
14 Age-related hearing loss	14 Prostate cancer	**115 (64.5 to 172)**	**72.7 (31.8 to 114)**	3.94 (-11.7 to 20.1)
15 Road injuries	15 Alzheimer's disease	**158 (104 to 210)**	**107 (67 to 140)**	**7.84 (4.68 to 11.7)**
16 Other musculoskeletal	16 Headache disorders	**28.5 (16.7 to 40)**	**3.33 (0.207 to 8.73)**	**0.475 (0.33 to 0.592)**
17 Anxiety disorders	17 Colorectal cancer	**61.2 (16.1 to 114)**	29.4 (-5.11 to 68.1)	2.37 (-22.4 to 28)
18 Self-harm	18 Road injuries	21.2 (-26.3 to 100)	-2.34 (-40.4 to 62)	-5.43 (-43.8 to 59.5)
19 Cirrhosis liver	19 Anxiety disorders	**27.3 (15 to 38.6)**	2.31 (-1.26 to 6.2)	**0.816 (0.68 to 0.965)**
20 Stomach cancer	20 Diarrheal diseases	**56.1 (13.3 to 117)**	25.7 (-10 to 73.9)	-4.92 (-34.2 to 45.1)
24 Colorectal cancer	23 Self-harm			
26 Diarrheal diseases	25 Stomach cancer			
27 Prostate cancer	27 Cirrhosis liver			
31 Alzheimer's disease	162 COVID-19			

Supplemental Results Figure S7. Leading 20 level 3 causes of DALYs, and percentage change in number of DALYs and all-age and age-standardised DALY rates by location, 2022 and 2050

Cook Islands, Reference scenario

Legend:
Communicable, maternal, neonatal and nutritional
Non-communicable
Injuries

Leading Causes 2022	Leading Causes 2050	Mean % change number of DALY	Mean % change all-age DALY rate	Mean % change age-standardized DALY rate
1 Diabetes	1 Diabetes	**14.8 (0.191 to 35.6)**	**15.5 (0.765 to 33.9)**	-5.94 (-16.7 to 8.02)
2 Ischemic heart disease	2 Hypertensive heart disease	**59.3 (11.1 to 122)**	**60.4 (11.6 to 126)**	17.5 (-19.4 to 69.6)
3 Stroke	3 Ischemic heart disease	**-34.2 (-54.7 to -7.38)**	**-33.8 (-51.1 to -6.26)**	**-52.4 (-67.4 to -28.8)**
4 Hypertensive heart disease	4 Stroke	-4.77 (-20.5 to 16.1)	-4.2 (-19.1 to 15.7)	**-31 (-42.3 to -13.7)**
5 Lower respiratory infect	5 Alzheimer's disease	**111 (71.3 to 162)**	**112 (75.5 to 150)**	3.6 (-0.478 to 7.31)
6 Lung cancer	6 COPD	**55.3 (16.5 to 94.3)**	**56.3 (22.5 to 99.9)**	-5.92 (-30.9 to 30.2)
7 Road injuries	7 Lower respiratory infect	8.48 (-9.24 to 27.6)	9.09 (-4.75 to 24.1)	**-30.8 (-40.1 to -18.5)**
8 Low back pain	8 Lung cancer	9.64 (-16.5 to 42.8)	10.1 (-16.2 to 36.4)	-15.7 (-32 to 3.09)
9 Breast cancer	9 Breast cancer	**36.7 (1.73 to 79.7)**	**37.3 (3.45 to 76.1)**	1.38 (-21.7 to 23.8)
10 COPD	10 Chronic kidney disease	**53.1 (15.6 to 109)**	**54.1 (20 to 106)**	9.41 (-17.9 to 50)
11 Prostate cancer	11 Low back pain	**13.5 (0.254 to 26.3)**	**14.2 (8.4 to 20.2)**	**-1.98 (-2.38 to -1.47)**
12 Age-related hearing loss	12 Prostate cancer	**38.9 (5.56 to 77.5)**	**39.5 (7.81 to 71.9)**	**-16.2 (-31.7 to -3.13)**
13 Chronic kidney disease	13 Road injuries	-1.98 (-38.2 to 50.5)	-1.23 (-36.4 to 52.2)	-2.38 (-38.1 to 50.8)
14 Headache disorders	14 Age-related hearing loss	**29.6 (13.5 to 48.2)**	**30.3 (19.7 to 40.3)**	**-0.911 (-1.16 to -0.654)**
15 Alzheimer's disease	15 Other musculoskeletal	**57.5 (40.7 to 74.1)**	**58.4 (50.2 to 66.6)**	**41.2 (40 to 42.3)**
16 Depressive disorders	16 Headache disorders	2.31 (-9.61 to 15.4)	2.86 (-0.305 to 7.02)	-0.0864 (-0.464 to 0.43)
17 Self-harm	17 Depressive disorders	6.29 (-5.6 to 17.8)	**6.89 (2.99 to 11.2)**	**-1.2 (-1.56 to -0.828)**
18 Other musculoskeletal	18 Liver cancer	**44.6 (5.29 to 103)**	**45.6 (8.99 to 105)**	26 (-7.77 to 75.9)
19 Anxiety disorders	19 Self-harm	0.264 (-20.7 to 29.9)	0.83 (-17.8 to 27.6)	0.449 (-18.9 to 28.5)
20 Blindness and vision loss	20 Blindness and vision loss	**29.8 (12.8 to 49.9)**	**30.5 (18.3 to 41.1)**	**-3.32 (-3.65 to -3.05)**
21 Liver cancer	22 Anxiety disorders			

Supplemental Results Figure S7. Leading 20 level 3 causes of DALYs, and percentage change in number of DALYs and all-age and age-standardised DALY rates by location, 2022 and 2050

Fiji, Reference scenario

Legend:
Communicable, maternal, neonatal and nutritional
Non-communicable
Injuries

Leading Causes 2022	Leading Causes 2050	Mean % change number of DALY	Mean % change all-age DALY rate	Mean % change age-standardized DALY rate
1 Diabetes	1 Diabetes	19.7 (-6.97 to 54.8)	**23 (0.124 to 54.9)**	-16.9 (-33.5 to 5.96)
2 Ischemic heart disease	2 Ischemic heart disease	-18.6 (-44.8 to 21.4)	-16.2 (-43 to 22.9)	**-43.7 (-63.6 to -13.4)**
3 Stroke	3 Stroke	4.4 (-18.2 to 35.6)	7.32 (-15 to 36.3)	**-32.7 (-47.8 to -11.1)**
4 COVID-19	4 Chronic kidney disease	**74.2 (31.3 to 143)**	**79.1 (34.4 to 140)**	24.4 (-11.1 to 75.4)
5 Neonatal disorders	5 Neonatal disorders	**-33.4 (-51.4 to -5.02)**	**-31.6 (-50.3 to -4.81)**	-16.7 (-39.7 to 14.8)
6 Lower respiratory infect	6 COPD	**67.2 (25.9 to 131)**	**71.9 (29 to 128)**	-3.8 (-30.2 to 35.7)
7 Chronic kidney disease	7 Lower respiratory infect	-18.4 (-35.3 to 4.36)	-16.2 (-31.9 to 4.43)	**-29.3 (-44 to -8.44)**
8 Congenital defects	8 Low back pain	15.9 (-0.299 to 32.8)	**19.1 (10 to 27.2)**	**-3.79 (-4.13 to -3.42)**
9 Asthma	9 Hypertensive heart disease	**72.9 (20.9 to 148)**	**77.8 (27.4 to 154)**	10.8 (-24.6 to 60.3)
10 Low back pain	10 Other musculoskeletal	**72.5 (47.3 to 99.2)**	**77.2 (65.5 to 90.6)**	**47.2 (45.8 to 48.6)**
11 COPD	11 Breast cancer	28.7 (-7.22 to 72.3)	32.1 (-4.98 to 73.9)	-2.49 (-26.2 to 24.7)
12 Headache disorders	12 Age-related hearing loss	**40.5 (17.2 to 66.5)**	**44.3 (28.5 to 59.6)**	**-0.9 (-1.05 to -0.755)**
13 Breast cancer	13 Headache disorders	1.74 (-13.4 to 18.2)	**4.44 (0.672 to 12.8)**	**0.52 (0.29 to 0.709)**
14 Road injuries	14 Alzheimer's disease	**156 (80.2 to 241)**	**163 (91.6 to 232)**	**6.83 (3.21 to 10.8)**
15 Diarrheal diseases	15 Congenital defects	**-37.9 (-50.1 to -19.7)**	**-36.4 (-44.8 to -23.8)**	**-21 (-29.4 to -8.03)**
16 Self-harm	16 Depressive disorders	6.74 (-8.07 to 23.9)	**9.6 (4.95 to 15)**	**-1.64 (-1.87 to -1.38)**
17 Age-related hearing loss	17 Anxiety disorders	2.82 (-11.5 to 19.3)	**5.56 (1.1 to 10.5)**	**0.815 (0.605 to 1.04)**
18 Depressive disorders	18 Diarrheal diseases	-14.7 (-45.1 to 28.5)	-12.5 (-40.9 to 29.6)	-34.7 (-57.8 to 3.1)
19 Anxiety disorders	19 Road injuries	-16.6 (-49.6 to 50.1)	-14.3 (-49.8 to 46.1)	-13.6 (-49.5 to 47)
20 Hypertensive heart disease	20 Asthma	**-37.9 (-49.4 to -21.3)**	**-36.2 (-46.7 to -21.8)**	**-57.1 (-64.7 to -46.7)**
21 Other musculoskeletal	21 Self-harm			
39 Alzheimer's disease	160 COVID-19			

Supplemental Results Figure S7. Leading 20 level 3 causes of DALYs, and percentage change in number of DALYs and all-age and age-standardised DALY rates by location, 2022 and 2050

Guam, Reference scenario

Legend:
Communicable, maternal, neonatal and nutritional
Non-communicable
Injuries

Leading Causes 2022	Leading Causes 2050	Mean % change number of DALY	Mean % change all-age DALY rate	Mean % change age-standardized DALY rate
1 Ischemic heart disease	1 Ischemic heart disease	-22.3 (-44.2 to 5.36)	-19.3 (-42.7 to 13.4)	**-40.2 (-56.7 to -16.9)**
2 COVID-19	2 Diabetes	**21.7 (6.16 to 36.2)**	**26 (13.1 to 40.9)**	-0.0977 (-8.53 to 8.41)
3 Stroke	3 Alzheimer's disease	**207 (166 to 250)**	**219 (175 to 261)**	**-7.3 (-9.9 to -4.36)**
4 Diabetes	4 Stroke	1.89 (-14.7 to 17.2)	5.6 (-9.1 to 25.9)	**-24.3 (-35.1 to -10.8)**
5 Neonatal disorders	5 Chronic kidney disease	**57 (17.5 to 103)**	**62.8 (23.9 to 114)**	27.4 (-4.39 to 66)
6 Lung cancer	6 Age-related hearing loss	**61 (38.2 to 82.1)**	**66.8 (49.8 to 86.3)**	**-0.337 (-0.431 to -0.234)**
7 Self-harm	7 Lung cancer	7.8 (-16.9 to 30.5)	11.5 (-9.97 to 35.4)	-10.6 (-26.2 to 5.36)
8 Chronic kidney disease	8 COPD	**79.1 (38.9 to 123)**	**85.9 (46.9 to 141)**	-1.43 (-22.5 to 27.9)
9 Low back pain	9 Low back pain	14.4 (-1.12 to 29.1)	**18.4 (10.4 to 28)**	**-1.71 (-1.9 to -1.52)**
10 Lower respiratory infect	10 Neonatal disorders	-23.1 (-43.9 to 5.46)	-20.4 (-39.1 to 3.74)	2.88 (-21.1 to 35.1)
11 Age-related hearing loss	11 Self-harm	-19.6 (-39 to 3.43)	-16.9 (-32.2 to 1.26)	-7.95 (-25.4 to 12.1)
12 Cirrhosis liver	12 Other musculoskeletal	**22.1 (3.79 to 38.1)**	**26.3 (18.4 to 36.1)**	**15.8 (14.3 to 17.1)**
13 Headache disorders	13 Lower respiratory infect	-7.84 (-20.7 to 8.36)	-4.58 (-16.7 to 10.9)	**-26.6 (-35.8 to -15.5)**
14 Other musculoskeletal	14 Blindness and vision loss	**64.4 (44.4 to 84.6)**	**70.3 (52.9 to 92.3)**	**-2.08 (-2.29 to -1.88)**
15 Alzheimer's disease	15 Hypertensive heart disease	**47.8 (7.85 to 97.3)**	**53.4 (12.2 to 109)**	24 (-7.76 to 65)
16 Depressive disorders	16 Depressive disorders	1.98 (-13.4 to 15.9)	**5.44 (1.01 to 10.2)**	**-0.811 (-0.937 to -0.679)**
17 Road injuries	17 Osteoarthritis	**41.2 (24.3 to 55.2)**	**46.3 (34.3 to 59.6)**	**0.782 (0.595 to 0.986)**
18 COPD	18 Headache disorders	-8.61 (-24.7 to 4.23)	-5.58 (-8.91 to 0.216)	**0.264 (0.111 to 0.412)**
19 Anxiety disorders	19 Cirrhosis liver	**-24.2 (-37.6 to -10.1)**	**-21.5 (-32.8 to -6.46)**	**-21.4 (-30.9 to -7.85)**
20 Hypertensive heart disease	20 Road injuries	-12.1 (-42.8 to 30.9)	-8.95 (-38.4 to 35.1)	-0.296 (-32.7 to 49.9)
23 Osteoarthritis	21 Anxiety disorders			
24 Blindness and vision loss	157 COVID-19			

Supplemental Results Figure S7. Leading 20 level 3 causes of DALYs, and percentage change in number of DALYs and all-age and age-standardised DALY rates by location, 2022 and 2050

Kiribati, Reference scenario

Legend:
Communicable, maternal, neonatal and nutritional
Non-communicable
Injuries

Leading Causes 2022	Leading Causes 2050	Mean % change number of DALY	Mean % change all-age DALY rate	Mean % change age-standardized DALY rate
1 Diabetes	1 Diabetes	**63.3 (29.4 to 101)**	**40.9 (16.5 to 70.2)**	-7.91 (-23.4 to 13.5)
2 Neonatal disorders	2 Ischemic heart disease	19.5 (-18.9 to 73.8)	3.1 (-29.1 to 49.6)	-30.9 (-53.1 to 3.89)
3 Ischemic heart disease	3 Stroke	13.9 (-8.86 to 38.2)	-1.76 (-17.7 to 18.5)	**-34.2 (-46.2 to -17.6)**
4 Stroke	4 Neonatal disorders	**-47.2 (-64 to -22.3)**	**-54.6 (-67 to -35.2)**	**-36 (-53.6 to -11.5)**
5 Tuberculosis	5 Chronic kidney disease	**120 (64.8 to 203)**	**89.6 (48.1 to 160)**	36.4 (-0.619 to 87.8)
6 Diarrheal diseases	6 COPD	**71.1 (32 to 120)**	**47.6 (15.2 to 86.3)**	-11.4 (-33.6 to 23.4)
7 Lower respiratory infect	7 Tuberculosis	**-42.6 (-61.6 to -11.9)**	**-50.5 (-66.7 to -24.9)**	**-61 (-74.4 to -39.1)**
8 Congenital defects	8 Diarrheal diseases	4.85 (-31.4 to 48.8)	-9.71 (-39 to 27.8)	-20.7 (-49.2 to 19.5)
9 Self-harm	9 Self-harm	2.84 (-21.8 to 35.7)	-11.5 (-30.1 to 8.11)	-11.9 (-29.7 to 7.73)
10 COPD	10 Low back pain	**44.4 (24.6 to 66.3)**	**24.5 (15.5 to 35)**	**-4.32 (-4.62 to -3.92)**
11 Chronic kidney disease	11 Lower respiratory infect	**-29.2 (-43.5 to -13)**	**-39.1 (-50.2 to -26.2)**	**-40.7 (-51.3 to -29.6)**
12 Cirrhosis liver	12 Road injuries	9.57 (-28.3 to 76.8)	-5.64 (-38 to 43.8)	-8.75 (-40.1 to 38.5)
13 Road injuries	13 Other musculoskeletal	**123 (94 to 154)**	**92.5 (79.2 to 107)**	**50.3 (49.1 to 51.4)**
14 Pertussis	14 Cirrhosis liver	-5.06 (-22.9 to 17.5)	-18.1 (-32.2 to 0.851)	**-34.6 (-44.8 to -21.1)**
15 Low back pain	15 Cervical cancer	22.6 (-1.05 to 53.7)	5.79 (-11.5 to 29.5)	**-24.2 (-36.9 to -8.42)**
16 Protein-energy malnutrition	16 Headache disorders	**26.4 (6.78 to 47.4)**	**8.82 (3.91 to 17.7)**	-0.00546 (-0.229 to 0.246)
17 Dietary iron deficiency	17 Congenital defects	**-40.1 (-54.7 to -21.9)**	**-48.5 (-56.2 to -37.5)**	**-30.7 (-39.1 to -19.8)**
18 Cervical cancer	18 Breast cancer	**53.5 (11 to 109)**	32.3 (-4.24 to 74.6)	-6.66 (-27.8 to 17.6)
19 Asthma	19 Endo/metab/blood/immune	**64.8 (39 to 90.8)**	**42.1 (26.1 to 59.1)**	12 (-0.7 to 24.9)
20 Headache disorders	20 Age-related hearing loss	**68.1 (43.7 to 94.9)**	**44.9 (31 to 61.5)**	**-0.868 (-1.03 to -0.697)**
26 Breast cancer	21 Dietary iron deficiency			
27 Endo/metab/blood/immune	30 Asthma			
28 Age-related hearing loss	31 Pertussis			
30 Other musculoskeletal	33 Protein-energy malnutrition			

Supplemental Results Figure S7. Leading 20 level 3 causes of DALYs, and percentage change in number of DALYs and all-age and age-standardised DALY rates by location, 2022 and 2050

Marshall Islands, Reference scenario

Legend:
Communicable, maternal, neonatal and nutritional
Non-communicable
Injuries

Leading Causes 2022	Leading Causes 2050	Mean % change number of DALY	Mean % change all-age DALY rate	Mean % change age-standardized DALY rate
1 Ischemic heart disease	1 Diabetes	**64.8 (30 to 101)**	**28.5 (5.99 to 55.9)**	-11.5 (-27.9 to 10.9)
2 Diabetes	2 Ischemic heart disease	40.4 (-13.1 to 118)	9.71 (-28.4 to 71.8)	-26.3 (-56.4 to 17)
3 Stroke	3 Stroke	**43.5 (7.24 to 90.4)**	12 (-12.8 to 43.4)	-26.1 (-44.7 to 0.766)
4 Tuberculosis	4 Chronic kidney disease	**134 (64.2 to 243)**	**82.8 (32.2 to 160)**	33.5 (-10.3 to 99.4)
5 Neonatal disorders	5 HIV/AIDS	**614 (270 to 1060)**	**456 (189 to 795)**	**486 (203 to 841)**
6 Lower respiratory infect	6 Lower respiratory infect	2.04 (-20.1 to 26.7)	**-20.4 (-34.3 to -3.87)**	**-28.8 (-42.4 to -10.7)**
7 Self-harm	7 COPD	**102 (48.1 to 161)**	**57.8 (19.2 to 105)**	-6.61 (-32.7 to 26.8)
8 Chronic kidney disease	8 Tuberculosis	**-37 (-58.4 to -2.84)**	**-50.8 (-67.5 to -26.8)**	**-61.3 (-74.6 to -39.9)**
9 Road injuries	9 Self-harm	10.2 (-19.8 to 48.5)	-14 (-37.7 to 15.1)	-12.6 (-35.5 to 15.5)
10 COPD	10 Neonatal disorders	-25 (-46.8 to 7.84)	**-41.5 (-57.5 to -19.3)**	-24.4 (-46 to 5.62)
11 Cirrhosis liver	11 Road injuries	15.5 (-36.9 to 114)	-9.69 (-49.6 to 66.4)	-15.1 (-53.1 to 58.5)
12 Drowning	12 Lung cancer	**108 (43.4 to 192)**	**61.5 (17.1 to 121)**	3.65 (-19.6 to 28.1)
13 Congenital defects	13 Hypertensive heart disease	**107 (41.4 to 201)**	**62 (12.2 to 134)**	4.14 (-32.1 to 57.3)
14 Low back pain	14 Low back pain	**55.3 (31.1 to 81.2)**	**21.1 (13.3 to 28.3)**	**-3.47 (-3.85 to -3.01)**
15 Headache disorders	15 Other musculoskeletal	**136 (96.4 to 180)**	**84 (71.3 to 96.2)**	**51.2 (47.9 to 54.3)**
16 Lung cancer	16 Cirrhosis liver	13.7 (-10.8 to 43.2)	-11.2 (-27.6 to 10.8)	**-27.7 (-41.3 to -9.93)**
17 Hypertensive heart disease	17 Headache disorders	**36.2 (17.1 to 57.8)**	**6.1 (2.41 to 13.8)**	**0.629 (0.374 to 0.822)**
18 Interpersonal violence	18 Breast cancer	**78.7 (22.4 to 160)**	39 (-3.52 to 89.9)	4.89 (-23.3 to 38.9)
19 Dietary iron deficiency	19 Age-related hearing loss	**84.1 (50.6 to 119)**	**43.4 (28.9 to 58.9)**	**-1.03 (-1.16 to -0.902)**
20 Rheumatic heart disease	20 Depressive disorders	**41.7 (20 to 64.5)**	**10.4 (5.84 to 15.3)**	**-1.57 (-1.8 to -1.29)**
22 Depressive disorders	22 Drowning			
23 Breast cancer	24 Congenital defects			
26 Age-related hearing loss	25 Dietary iron deficiency			
27 Other musculoskeletal	26 Interpersonal violence			
34 HIV/AIDS	37 Rheumatic heart disease			

Supplemental Results Figure S7. Leading 20 level 3 causes of DALYs, and percentage change in number of DALYs and all-age and age-standardised DALY rates by location, 2022 and 2050

Federated States of Micronesia, Reference scenario

Legend:
Communicable, maternal, neonatal and nutritional
Non-communicable
Injuries

Leading Causes 2022	Leading Causes 2050	Mean % change number of DALY	Mean % change all-age DALY rate	Mean % change age-standardized DALY rate
1 Ischemic heart disease	1 Ischemic heart disease	11.5 (-29.5 to 62.4)	12.5 (-24 to 61.4)	-24.5 (-51.2 to 14.6)
2 Stroke	2 Diabetes	**44 (11.5 to 75.1)**	**45.2 (20.8 to 76.5)**	-0.638 (-18 to 20.2)
3 Diabetes	3 Stroke	14.2 (-15.8 to 43.4)	15.2 (-9.62 to 45.6)	**-24.4 (-40.6 to -2.53)**
4 Lower respiratory infect	4 Chronic kidney disease	**111 (45.6 to 187)**	**113 (59.7 to 182)**	**50.2 (6.95 to 110)**
5 Neonatal disorders	5 HIV/AIDS	**505 (270 to 910)**	**509 (266 to 855)**	**534 (282 to 908)**
6 Chronic kidney disease	6 COPD	**62.8 (16.9 to 109)**	**64.3 (23.4 to 114)**	-2.6 (-27.9 to 33.3)
7 Self-harm	7 Lower respiratory infect	-13.1 (-32.4 to 10.5)	-12.5 (-25.4 to 4.66)	**-29.9 (-41.3 to -13.9)**
8 Road injuries	8 Lung cancer	**58.8 (12 to 127)**	**59.9 (15.8 to 115)**	6.18 (-16.6 to 32.1)
9 COPD	9 Self-harm	-17.4 (-41.2 to 16.6)	-16.9 (-36.5 to 6.16)	-14.6 (-34.3 to 9.39)
10 Cirrhosis liver	10 Hypertensive heart disease	**65.1 (8.58 to 128)**	**66.6 (17.9 to 133)**	6.18 (-29.6 to 58.7)
11 Low back pain	11 Road injuries	-10 (-50.7 to 47.2)	-9.37 (-44.7 to 52.9)	-15.2 (-50.4 to 45.6)
12 Lung cancer	12 Low back pain	22.3 (-0.91 to 47.4)	**23 (13.8 to 34)**	**-3.63 (-3.91 to -3.27)**
13 Drowning	13 Neonatal disorders	**-42.2 (-62 to -18.1)**	**-42 (-55.9 to -20.1)**	**-27.6 (-45.1 to -0.588)**
14 Interpersonal violence	14 Other musculoskeletal	**78.3 (44.2 to 116)**	**79.4 (65.2 to 93.4)**	**45.3 (42 to 48.1)**
15 Hypertensive heart disease	15 Headache disorders	6.2 (-16 to 32.8)	**6.74 (2.2 to 15.1)**	**0.639 (0.338 to 0.855)**
16 Headache disorders	16 Cirrhosis liver	-12 (-34 to 12.5)	-11.3 (-26.6 to 7.06)	**-29.1 (-40.5 to -13)**
17 Congenital defects	17 Breast cancer	30 (-11.7 to 85)	30.8 (-6.39 to 80.6)	-6.63 (-30.1 to 19.5)
18 Breast cancer	18 Age-related hearing loss	**43.5 (14.4 to 75.2)**	**44.5 (25 to 65.9)**	**-0.921 (-1.06 to -0.751)**
19 Anxiety disorders	19 Endo/metab/blood/immune	**46.3 (15.3 to 79.8)**	**47.4 (31.2 to 68.9)**	**13.5 (0.698 to 30.5)**
20 Depressive disorders	20 Alzheimer's disease	**161 (88 to 265)**	**163 (90.9 to 247)**	**10.5 (6.71 to 14.1)**
23 Age-related hearing loss	21 Depressive disorders			
26 Other musculoskeletal	22 Anxiety disorders			
27 Endo/metab/blood/immune	23 Interpersonal violence			
33 HIV/AIDS	26 Drowning			
41 Alzheimer's disease	31 Congenital defects			

Supplemental Results Figure S7. Leading 20 level 3 causes of DALYs, and percentage change in number of DALYs and all-age and age-standardised DALY rates by location, 2022 and 2050

Nauru, Reference scenario

Legend:
Communicable, maternal, neonatal and nutritional
Non-communicable
Injuries

Leading Causes 2022	Leading Causes 2050	Mean % change number of DALY	Mean % change all-age DALY rate	Mean % change age-standardized DALY rate
1 Ischemic heart disease	1 Ischemic heart disease	42 (-16.4 to 119)	-3.83 (-39.3 to 53.9)	-30.1 (-58.3 to 19.3)
2 Stroke	2 Diabetes	**98.8 (55.4 to 147)**	**34.5 (9.27 to 66.1)**	-3.02 (-23.2 to 25.8)
3 Diabetes	3 Stroke	**49.1 (7.41 to 92.4)**	0.901 (-22.4 to 33.1)	-25.3 (-44.8 to 4.51)
4 Lower respiratory infect	4 Road injuries	37.4 (-29.3 to 142)	-7.04 (-49.7 to 73.6)	-10.9 (-51.2 to 64.6)
5 Neonatal disorders	5 Chronic kidney disease	**159 (78.2 to 264)**	**75 (25.2 to 150)**	37.7 (-6.23 to 108)
6 Road injuries	6 Neonatal disorders	-8.51 (-43.1 to 38.5)	**-38.3 (-57.4 to -8.18)**	-18.4 (-44 to 23)
7 Self-harm	7 Lower respiratory infect	-14.1 (-38.4 to 13.3)	**-42 (-53.4 to -27)**	**-36.5 (-50 to -18.1)**
8 Chronic kidney disease	8 Self-harm	38.7 (-1.76 to 89.5)	-6.21 (-30.3 to 23)	-8.98 (-31.2 to 17.8)
9 Congenital defects	9 COPD	**87.2 (33.1 to 142)**	26.7 (-4.15 to 65.8)	-7.96 (-36.1 to 31.9)
10 Drowning	10 Lung cancer	**108 (37.4 to 181)**	40.5 (-2.29 to 94.8)	-0.121 (-24 to 27.9)
11 Tuberculosis	11 Low back pain	**75.8 (51.7 to 102)**	**18.9 (10.3 to 28.6)**	**-3.29 (-3.74 to -2.72)**
12 COPD	12 Hypertensive heart disease	**120 (40.3 to 212)**	**48.7 (2.68 to 113)**	4.62 (-32.4 to 59.6)
13 Interpersonal violence	13 Cirrhosis liver	**45.8 (11.4 to 81.3)**	-1.36 (-19.7 to 24.4)	-21.2 (-36.2 to 0.719)
14 Cirrhosis liver	14 Congenital defects	-12.5 (-34.1 to 7.76)	**-41 (-49.7 to -29.7)**	**-25.2 (-34.6 to -12.5)**
15 Lung cancer	15 Other musculoskeletal	**161 (122 to 201)**	**76.2 (61.6 to 91.4)**	**45.4 (42 to 48.8)**
16 Low back pain	16 Headache disorders	**61.7 (38.4 to 89.5)**	**9.26 (4.82 to 16.5)**	**0.371 (0.173 to 0.583)**
17 Headache disorders	17 Breast cancer	**87.1 (24 to 159)**	26.6 (-12.7 to 80.1)	-2.93 (-27.5 to 29.5)
18 Hypertensive heart disease	18 Drowning	-7.56 (-41.6 to 39.6)	**-37.5 (-59.2 to -7.25)**	**-36.9 (-58 to -6.66)**
19 Depressive disorders	19 Interpersonal violence	12.6 (-11.5 to 41.9)	**-23.9 (-37.4 to -7.69)**	**-27.9 (-39.7 to -11.7)**
20 Breast cancer	20 Depressive disorders	**65.5 (41.9 to 90)**	**11.9 (5.26 to 19.1)**	**-1.57 (-1.79 to -1.33)**
27 Other musculoskeletal	25 Tuberculosis			

Supplemental Results Figure S7. Leading 20 level 3 causes of DALYs, and percentage change in number of DALYs and all-age and age-standardised DALY rates by location, 2022 and 2050

Niue, Reference scenario

Legend:
Communicable, maternal, neonatal and nutritional
Non-communicable
Injuries

Leading Causes 2022	Leading Causes 2050	Mean % change number of DALY	Mean % change all-age DALY rate	Mean % change age-standardized DALY rate
1 Ischemic heart disease	1 Diabetes	11.9 (-5.86 to 35.9)	12.9 (-4.91 to 32.9)	-1.41 (-15.4 to 16.5)
2 Neonatal disorders	2 Ischemic heart disease	**-35.2 (-53.6 to -9.5)**	**-34.5 (-53.9 to -5.97)**	**-45.7 (-63.2 to -20.1)**
3 Diabetes	3 Neonatal disorders	**-32.8 (-53.2 to -2.51)**	**-32.2 (-52.6 to -3.44)**	-26 (-44.7 to 1.76)
4 Stroke	4 Stroke	**-21.2 (-35.2 to -3.6)**	**-20.5 (-35.2 to -1.74)**	**-34.9 (-46.9 to -17.6)**
5 Congenital defects	5 Chronic kidney disease	**35.4 (1.39 to 80.5)**	**36.7 (3.42 to 82.4)**	15.3 (-17.3 to 60.1)
6 Chronic kidney disease	6 COPD	**31 (5.39 to 67.3)**	**32.1 (3.3 to 63.8)**	0.745 (-24.9 to 33.4)
7 Lower respiratory infect	7 Congenital defects	**-33.9 (-45.4 to -18.5)**	**-33.4 (-41.9 to -21.2)**	**-24.6 (-34.4 to -11.9)**
8 COPD	8 Lower respiratory infect	-18.3 (-32.7 to 0.945)	**-17.6 (-29.1 to -1.87)**	**-32.4 (-43.1 to -17.5)**
9 Road injuries	9 Lung cancer	31.8 (-5.69 to 68.2)	32.7 (-0.485 to 68.2)	9.67 (-11.5 to 32.8)
10 Lung cancer	10 Road injuries	-17.7 (-46.3 to 38.5)	-17 (-48.2 to 33.7)	-10.5 (-44.9 to 49.4)
11 Self-harm	11 Low back pain	7.35 (-6.45 to 22.2)	**8.2 (1.86 to 14.6)**	**-3.43 (-3.71 to -3.12)**
12 Low back pain	12 Alzheimer's disease	**61.8 (27.5 to 100)**	**63 (32.9 to 90)**	**12.4 (8.53 to 17.2)**
13 Drowning	13 Hypertensive heart disease	32.7 (-4.43 to 84.9)	34 (-6.37 to 90.5)	10 (-22.3 to 60.3)
14 Breast cancer	14 Other musculoskeletal	**53.3 (32.5 to 74.1)**	**54.5 (46.2 to 64.5)**	**40.3 (36.2 to 43.6)**
15 Hypertensive heart disease	15 Breast cancer	17 (-18.3 to 54.5)	17.8 (-11 to 49.9)	-2.01 (-24.8 to 23.3)
16 Age-related hearing loss	16 Age-related hearing loss	17.4 (-0.689 to 35.1)	**18.3 (8.07 to 27.6)**	**-0.867 (-1.02 to -0.708)**
17 Cirrhosis liver	17 Self-harm	-22.3 (-39.4 to 3.75)	-21.7 (-37.9 to 0.0515)	-17.8 (-35.3 to 5.86)
18 Headache disorders	18 Headache disorders	2.84 (-11.8 to 16.4)	**3.63 (0.637 to 8.41)**	**0.227 (6.65e-05 to 0.458)**
19 Depressive disorders	19 Depressive disorders	4.12 (-9.21 to 18.4)	**4.93 (1.15 to 9.4)**	**-1.58 (-1.76 to -1.4)**
20 Alzheimer's disease	20 Endo/metab/blood/immune	**22.1 (3.33 to 40.9)**	**23 (12.1 to 36.4)**	9.12 (-1.97 to 21.5)
21 Other musculoskeletal	21 Cirrhosis liver			
22 Endo/metab/blood/immune	28 Drowning			

Northern Mariana Islands, Reference scenario

Legend:
Communicable, maternal, neonatal and nutritional
Non-communicable
Injuries

Leading Causes 2022	Leading Causes 2050	Mean % change number of DALY	Mean % change all-age DALY rate	Mean % change age-standardized DALY rate
1 Ischemic heart disease	1 Diabetes	**46 (26.6 to 64.2)**	**40.9 (24.4 to 58.6)**	2.29 (-9.83 to 17.6)
2 Diabetes	2 Ischemic heart disease	-3 (-29.3 to 34.9)	-6.27 (-33.7 to 31.2)	**-38.1 (-56.9 to -7.83)**
3 Stroke	3 Chronic kidney disease	**114 (67.9 to 178)**	**107 (63 to 163)**	**34.6 (3.14 to 85.1)**
4 Chronic kidney disease	4 Stroke	**29.7 (10.1 to 58)**	**25.2 (4.84 to 49.7)**	**-26.5 (-39.6 to -8.36)**
5 Lung cancer	5 COPD	**116 (77.5 to 160)**	**109 (68.9 to 154)**	1.94 (-20 to 35.1)
6 COPD	6 Lung cancer	**45.3 (9.41 to 82)**	**40 (8.55 to 69.7)**	-9.16 (-25.5 to 9.7)
7 Low back pain	7 Low back pain	**22.6 (11.5 to 33.4)**	**18.3 (9.77 to 28.8)**	**-0.98 (-1.29 to -0.686)**
8 Cirrhosis liver	8 Alzheimer's disease	**238 (172 to 299)**	**226 (166 to 276)**	**4.06 (0.921 to 7.11)**
9 Self-harm	9 Lower respiratory infect	**42.1 (25.9 to 61.3)**	**37.2 (22.9 to 56)**	**-19.7 (-29 to -7.69)**
10 Road injuries	10 Age-related hearing loss	**55.7 (39.1 to 73.8)**	**50.2 (37.9 to 65.2)**	**-0.543 (-0.677 to -0.429)**
11 Lower respiratory infect	11 Diarrheal diseases	**157 (84.9 to 240)**	**149 (77.9 to 234)**	39.4 (-2.19 to 99.7)
12 Headache disorders	12 Road injuries	0.0381 (-37.7 to 66.6)	-3.31 (-40.5 to 63.9)	-4.74 (-42.8 to 65.1)
13 Age-related hearing loss	13 Cirrhosis liver	-12.9 (-26.1 to 3.51)	**-15.9 (-29.2 to -0.469)**	**-28.1 (-38.3 to -12.9)**
14 Neonatal disorders	14 Other musculoskeletal	**37.7 (26.2 to 51.9)**	**32.9 (24.3 to 42.1)**	**21.4 (20.4 to 22.5)**
15 Other musculoskeletal	15 Self-harm	-13.3 (-30.1 to 5.57)	-16.3 (-30.7 to 1.4)	-15.8 (-31.8 to 3.2)
16 Breast cancer	16 Headache disorders	3.11 (-5.25 to 13.1)	-0.549 (-3.85 to 4.77)	**0.74 (0.275 to 1.05)**
17 Depressive disorders	17 Colorectal cancer	**46.6 (0.0144 to 92.2)**	41.3 (-0.849 to 80.6)	-9.07 (-31.4 to 12.8)
18 Anxiety disorders	18 Breast cancer	21.8 (-7.97 to 56.2)	17.4 (-12.4 to 48.5)	-12.5 (-31.7 to 8.29)
19 Drowning	19 Falls	**46 (26.3 to 70.2)**	**40.8 (22.7 to 59.7)**	**-10 (-18.4 to -0.292)**
20 Colorectal cancer	20 Neonatal disorders	-5.73 (-25.2 to 25.9)	-9.08 (-26.8 to 19.3)	3.89 (-19.8 to 39.3)
21 Falls	21 Depressive disorders			
27 Diarrheal diseases	22 Anxiety disorders			
31 Alzheimer's disease	37 Drowning			

Supplemental Results Figure S7. Leading 20 level 3 causes of DALYs, and percentage change in number of DALYs and all-age and age-standardised DALY rates by location, 2022 and 2050

Palau, Reference scenario

Legend:
Communicable, maternal, neonatal and nutritional
Non-communicable
Injuries

Leading Causes 2022	Leading Causes 2050	Mean % change number of DALY	Mean % change all-age DALY rate	Mean % change age-standardized DALY rate
1 Ischemic heart disease	1 Diabetes	13.8 (-8.99 to 36.8)	**30.9 (10.5 to 51.9)**	-0.965 (-14.8 to 14.7)
2 Diabetes	2 Ischemic heart disease	-22.3 (-46 to 10.3)	-10.5 (-37.2 to 22.1)	**-36.9 (-56.5 to -9.64)**
3 Stroke	3 Stroke	-4.35 (-25.5 to 17.7)	10.1 (-9.48 to 32.3)	**-26.4 (-39.8 to -8.71)**
4 Lung cancer	4 Chronic kidney disease	**75.3 (29.1 to 131)**	**102 (52.7 to 157)**	36.8 (-0.0756 to 82.9)
5 Chronic kidney disease	5 COPD	**74.9 (33.7 to 123)**	**101 (59.8 to 152)**	2.11 (-23.1 to 36)
6 COPD	6 Lung cancer	**43.5 (2.3 to 90.4)**	**65 (23 to 107)**	6.04 (-14.6 to 27.3)
7 Lower respiratory infect	7 Lower respiratory infect	5.46 (-15 to 26.9)	**21.3 (2.13 to 40.7)**	**-27.8 (-39.3 to -12.3)**
8 Road injuries	8 Falls	14.3 (-6.59 to 37)	**31.4 (12.4 to 51.1)**	**-13.2 (-20.8 to -3.6)**
9 Low back pain	9 Low back pain	-4.56 (-16.9 to 9.36)	**9.83 (2.62 to 18.3)**	**-2.58 (-3.22 to -1.94)**
10 Neonatal disorders	10 Road injuries	-22.4 (-53.1 to 25)	-10.6 (-43.2 to 45)	-6.43 (-41.6 to 54.7)
11 Self-harm	11 Diarrheal diseases	**62.6 (15.1 to 116)**	**87.2 (36.6 to 149)**	-7.81 (-36.1 to 29.7)
12 Falls	12 Alzheimer's disease	**168 (107 to 242)**	**208 (147 to 276)**	**5.98 (2.32 to 10.3)**
13 Cirrhosis liver	13 Breast cancer	9.9 (-20 to 46.8)	26.4 (-7.94 to 61)	-9.92 (-30.9 to 12.9)
14 Breast cancer	14 Age-related hearing loss	**27.7 (7.13 to 46.2)**	**46.9 (29.9 to 64)**	**-0.457 (-0.785 to -0.0962)**
15 Interpersonal violence	15 Lip oral cavity cancer	**17.5 (0.291 to 40.3)**	**35.2 (19.6 to 53.3)**	6.62 (-3.46 to 16.1)
16 Age-related hearing loss	16 Prostate cancer	**125 (65.2 to 196)**	**159 (89.9 to 226)**	**18.2 (1.67 to 37.4)**
17 Headache disorders	17 Self-harm	**-31.1 (-46 to -10.1)**	-20.7 (-37.3 to 0.648)	-15.2 (-34 to 7.45)
18 Stomach cancer	18 Other musculoskeletal	10.5 (-3.23 to 25.8)	**27.2 (17.4 to 37.1)**	**25.4 (24.1 to 26.8)**
19 Depressive disorders	19 Pancreatic cancer	**103 (35.2 to 177)**	**133 (62.7 to 211)**	28.9 (-2.04 to 64)
20 Lip oral cavity cancer	20 Neonatal disorders	**-40.6 (-56.7 to -20.6)**	**-31.6 (-47.1 to -6.8)**	**-29.9 (-47.5 to -0.897)**
23 Diarrheal diseases	21 Depressive disorders			
24 Other musculoskeletal	23 Cirrhosis liver			
35 Alzheimer's disease	24 Headache disorders			
40 Prostate cancer	25 Stomach cancer			
42 Pancreatic cancer	32 Interpersonal violence			

Supplemental Results Figure S7. Leading 20 level 3 causes of DALYs, and percentage change in number of DALYs and all-age and age-standardised DALY rates by location, 2022 and 2050

Papua New Guinea, Reference scenario

Legend:
Communicable, maternal, neonatal and nutritional
Non-communicable
Injuries

Leading Causes 2022
1 Lower respiratory infect
2 Neonatal disorders
3 Malaria
4 Congenital defects
5 Stroke
6 Ischemic heart disease
7 Diarrheal diseases
8 Diabetes
9 COPD
10 COVID-19
11 Tuberculosis
12 Road injuries
13 Pertussis
14 Measles
15 Drowning
16 Interpersonal violence
17 Asthma
18 Low back pain
19 Headache disorders
20 Rheumatic heart disease
24 Depressive disorders
27 Anxiety disorders
29 Age-related hearing loss
31 Other musculoskeletal
33 Chronic kidney disease

Leading Causes 2050	Mean % change number of DALY	Mean % change all-age DALY rate	Mean % change age-standardized DALY rate
1 Diabetes	**176 (120 to 232)**	**36.7 (11.8 to 64.1)**	0.934 (-16.8 to 23.7)
2 Ischemic heart disease	**125 (40.2 to 247)**	11.2 (-28.7 to 68.9)	-19.8 (-50.9 to 24.6)
3 Stroke	**111 (57.6 to 169)**	4.42 (-19.3 to 32.6)	**-26.8 (-44.6 to -4.98)**
4 Neonatal disorders	-19.1 (-47.1 to 20.9)	**-60 (-72.7 to -42.3)**	**-44.3 (-60.4 to -21.4)**
5 COPD	**121 (60.9 to 191)**	9.45 (-18.1 to 48.1)	**-32.1 (-51.7 to -5.19)**
6 Lower respiratory infect	**-44.3 (-58.9 to -28.7)**	**-72.5 (-79.1 to -64.9)**	**-62.5 (-71 to -52.8)**
7 Malaria	118 (-87.3 to 420)	8.76 (-93.5 to 141)	7.92 (-92.2 to 158)
8 Congenital defects	-4.15 (-24.6 to 21.8)	**-52.7 (-60.6 to -41.8)**	**-36.3 (-45.2 to -25)**
9 Diarrheal diseases	-19.6 (-52.8 to 24.2)	**-60.2 (-75.3 to -38.6)**	**-50.7 (-70.6 to -23.1)**
10 Road injuries	48 (-18.1 to 152)	-26.7 (-57.7 to 30.7)	-28.5 (-58.2 to 24.7)
11 Low back pain	**128 (97.5 to 158)**	**13 (6.02 to 19.9)**	**-4.22 (-4.61 to -3.81)**
12 Other musculoskeletal	**315 (259 to 374)**	**105 (91.3 to 120)**	**79.6 (74.9 to 82.8)**
13 Headache disorders	**116 (88.9 to 143)**	**6.96 (3.53 to 11.7)**	**0.852 (0.504 to 1.1)**
14 Pertussis	15.1 (-42.2 to 113)	-43.2 (-71.5 to 0.0865)	-24.9 (-61.2 to 31.1)
15 Chronic kidney disease	**241 (125 to 378)**	**68.6 (13.2 to 137)**	45.5 (-2.58 to 108)
16 Depressive disorders	**117 (90.6 to 146)**	**7.51 (3.14 to 11.9)**	**-2.06 (-2.39 to -1.72)**
17 Anxiety disorders	**117 (88.9 to 146)**	**7.48 (3.86 to 11.5)**	**1.72 (1.38 to 2.04)**
18 Interpersonal violence	**48.2 (22.3 to 82.1)**	**-26.7 (-36.3 to -14.2)**	**-29.7 (-38.1 to -18.5)**
19 Age-related hearing loss	**164 (125 to 205)**	**30.4 (19.2 to 42.1)**	**-1.52 (-1.74 to -1.28)**
20 Tuberculosis	-28.1 (-54.8 to 8.3)	**-64.4 (-77.7 to -44)**	**-68.8 (-80.4 to -50.6)**
24 Drowning			
25 Asthma			
30 Rheumatic heart disease			
70 Measles			
163 COVID-19			

Supplemental Results Figure S7. Leading 20 level 3 causes of DALYs, and percentage change in number of DALYs and all-age and age-standardised DALY rates by location, 2022 and 2050

Samoa, Reference scenario

Legend:
Communicable, maternal, neonatal and nutritional
Non-communicable
Injuries

Leading Causes 2022	Leading Causes 2050	Mean % change number of DALY	Mean % change all-age DALY rate	Mean % change age-standardized DALY rate
1 Ischemic heart disease	1 Diabetes	**76.1 (43.5 to 107)**	**19.6 (3.53 to 35.7)**	3.58 (-10 to 20.4)
2 Measles	2 Ischemic heart disease	26.5 (-13.1 to 80.1)	-14.1 (-41 to 23.6)	-26.6 (-51.5 to 10.4)
3 Diabetes	3 Stroke	**30 (1.25 to 62.3)**	-11.7 (-26.6 to 10.1)	**-26.2 (-40.7 to -6.02)**
4 Stroke	4 Chronic kidney disease	**155 (85.1 to 235)**	**73.3 (28.5 to 133)**	**52.1 (9.49 to 109)**
5 Neonatal disorders	5 HIV/AIDS	**677 (290 to 1220)**	**427 (156 to 828)**	**406 (149 to 783)**
6 Lower respiratory infect	6 Neonatal disorders	-10.9 (-36.9 to 25.2)	**-39.6 (-56 to -16.5)**	**-28.8 (-47 to -5.39)**
7 COPD	7 COPD	**57.3 (22.2 to 98.1)**	6.86 (-16.6 to 36.8)	-15.4 (-38.5 to 15.2)
8 Road injuries	8 Lower respiratory infect	-5.22 (-21.6 to 15.7)	**-35.7 (-45.4 to -23.3)**	**-34.5 (-44.4 to -20.1)**
9 Chronic kidney disease	9 Road injuries	36 (-21.2 to 132)	-7.71 (-43.6 to 56.8)	-13.3 (-46.5 to 45.5)
10 Self-harm	10 Self-harm	**38 (5.77 to 79.4)**	-6.36 (-25.6 to 16.8)	-12.4 (-29.2 to 8.41)
11 Congenital defects	11 Low back pain	**57.2 (36.6 to 77.8)**	**6.67 (0.612 to 13.3)**	**-3.46 (-3.74 to -3.21)**
12 Low back pain	12 Measles	-74.1 (-95.1 to 43)	**-82.5 (-96.6 to -17.6)**	**-79.2 (-95.8 to -7.12)**
13 Headache disorders	13 Headache disorders	**55.8 (36 to 76.5)**	**5.68 (2.53 to 10)**	**0.386 (0.217 to 0.521)**
14 Anxiety disorders	14 Other musculoskeletal	**126 (98.2 to 157)**	**53.3 (43.8 to 64)**	**39.4 (36.6 to 41.6)**
15 Cirrhosis liver	15 Hypertensive heart disease	**84.5 (30.4 to 153)**	25.3 (-10.8 to 73)	5.42 (-28.4 to 50.9)
16 Hypertensive heart disease	16 Age-related hearing loss	**70.3 (48.9 to 93.4)**	**15.6 (7.11 to 24.3)**	**-0.754 (-0.948 to -0.546)**
17 Dietary iron deficiency	17 Anxiety disorders	**53.9 (33.2 to 75.5)**	**4.35 (1.71 to 7.78)**	**0.533 (0.301 to 0.765)**
18 Age-related hearing loss	18 Congenital defects	-5.44 (-23 to 13.5)	**-35.9 (-43.1 to -27)**	**-26.4 (-33.7 to -16.9)**
19 Depressive disorders	19 Depressive disorders	**56.4 (35.8 to 76.9)**	**6.12 (2.27 to 10.5)**	**-1.41 (-1.61 to -1.18)**
20 Drowning	20 Pertussis	54.2 (-20.4 to 172)	4.39 (-46.5 to 81.7)	22 (-35.6 to 99.4)
22 Pertussis	22 Dietary iron deficiency			
23 Other musculoskeletal	24 Cirrhosis liver			
29 HIV/AIDS	31 Drowning			

Supplemental Results Figure S7. Leading 20 level 3 causes of DALYs, and percentage change in number of DALYs and all-age and age-standardised DALY rates by location, 2022 and 2050

Solomon Islands, Reference scenario

Legend:
Communicable, maternal, neonatal and nutritional
Non-communicable
Injuries

Leading Causes 2022	Leading Causes 2050	Mean % change number of DALY	Mean % change all-age DALY rate	Mean % change age-standardized DALY rate
1 Ischemic heart disease	1 Ischemic heart disease	56 (-2.72 to 146)	8.69 (-28.3 to 61.2)	-23.2 (-51.7 to 19.1)
2 Lower respiratory infect	2 Diabetes	**112 (60.6 to 172)**	**47.3 (18.8 to 82.6)**	7.42 (-14.1 to 40.2)
3 Stroke	3 Stroke	**49.2 (11.6 to 92)**	3.9 (-16.1 to 27.6)	**-28.5 (-44.4 to -7.87)**
4 Diabetes	4 Lower respiratory infect	-14.6 (-33.4 to 8.03)	**-40.5 (-50.8 to -27)**	**-39.7 (-49.7 to -26.5)**
5 Neonatal disorders	5 HIV/AIDS	**628 (273 to 1040)**	**405 (167 to 641)**	**418 (171 to 665)**
6 Malaria	6 Malaria	128 (-59.2 to 276)	56 (-72.2 to 153)	25.6 (-80 to 95.9)
7 Self-harm	7 Self-harm	32.7 (-0.769 to 78.2)	-7.67 (-28.7 to 21.1)	-13.6 (-31.6 to 11.8)
8 Road injuries	8 Road injuries	37.7 (-25 to 145)	-4.05 (-44.2 to 70.2)	-13.5 (-48.1 to 51.2)
9 Congenital defects	9 Chronic kidney disease	**174 (86.8 to 296)**	**90.6 (37 to 167)**	**53.1 (6.93 to 118)**
10 Cirrhosis liver	10 Neonatal disorders	-22.2 (-46.5 to 7.34)	**-45.9 (-59.8 to -27.3)**	-25.5 (-44.1 to 1.51)
11 Drowning	11 COPD	**79.6 (28.7 to 141)**	25 (-4.85 to 60.6)	-20.4 (-42.8 to 12.2)
12 COPD	12 Low back pain	**69.1 (41.7 to 100)**	**17.7 (10.7 to 25)**	**-4.41 (-4.94 to -3.65)**
13 Low back pain	13 Headache disorders	**58.2 (29.3 to 88.4)**	**9.98 (6.42 to 16.3)**	**0.676 (0.382 to 0.894)**
14 Maternal disorders	14 Cirrhosis liver	9.7 (-17.3 to 37.2)	**-23.6 (-37.4 to -6.38)**	**-37 (-49 to -20.9)**
15 Headache disorders	15 Other musculoskeletal	**200 (147 to 259)**	**109 (94.8 to 124)**	**74.4 (69.6 to 78.3)**
16 Diarrheal diseases	16 Congenital defects	**-21.7 (-38.8 to -1.14)**	**-45.6 (-53.5 to -36.5)**	**-27.2 (-35 to -16.2)**
17 Chronic kidney disease	17 Lung cancer	**123 (58.1 to 201)**	**54.8 (12.6 to 107)**	9.72 (-14.5 to 36.4)
18 Dietary iron deficiency	18 Falls	**86.9 (51.9 to 124)**	**30 (13.5 to 44.5)**	5.6 (-4.15 to 15.8)
19 Tuberculosis	19 Drowning	-0.953 (-33.5 to 42.6)	**-31.1 (-53.1 to -0.0725)**	**-31.9 (-52.8 to -2.48)**
20 Anxiety disorders	20 Depressive disorders	**61.2 (34.7 to 92.7)**	**12.1 (7.81 to 17.4)**	**-2.07 (-2.39 to -1.71)**
21 Depressive disorders	21 Anxiety disorders			
24 Falls	23 Dietary iron deficiency			
27 Lung cancer	30 Maternal disorders			
34 Other musculoskeletal	32 Diarrheal diseases			
35 HIV/AIDS	41 Tuberculosis			

Supplemental Results Figure S7. Leading 20 level 3 causes of DALYs, and percentage change in number of DALYs and all-age and age-standardised DALY rates by location, 2022 and 2050

Tokelau, Reference scenario

Legend:
Communicable, maternal, neonatal and nutritional
Non-communicable
Injuries

Leading Causes 2022	Leading Causes 2050	Mean % change number of DALY	Mean % change all-age DALY rate	Mean % change age-standardized DALY rate
1 Neonatal disorders	1 Diabetes	**50 (23.1 to 77.7)**	**38.5 (17.8 to 64.2)**	4.93 (-9 to 23.5)
2 Ischemic heart disease	2 Neonatal disorders	**-43.6 (-62.8 to -18.3)**	**-47.9 (-65 to -21.6)**	**-37.8 (-56.3 to -13.7)**
3 Diabetes	3 Ischemic heart disease	-26.5 (-50.4 to 5.72)	-32.1 (-53.4 to 1.24)	**-50.3 (-67.3 to -24.2)**
4 Stroke	4 Stroke	-7.94 (-28.5 to 18.5)	-15 (-32.1 to 12.4)	**-35.9 (-50.6 to -12.5)**
5 Congenital defects	5 Chronic kidney disease	**49.4 (4.13 to 100)**	**38.1 (2.32 to 90.3)**	8.48 (-24.1 to 56.1)
6 Road injuries	6 Congenital defects	**-42.9 (-53.2 to -29.5)**	**-47.4 (-55 to -35.1)**	**-33.4 (-43 to -22)**
7 Lower respiratory infect	7 COPD	**44.6 (8.64 to 85)**	**33.6 (2.85 to 74.3)**	-5.07 (-28.8 to 29.7)
8 Chronic kidney disease	8 Lung cancer	**67.7 (20.5 to 119)**	**54.6 (14.6 to 97.7)**	11.1 (-12.4 to 36.9)
9 COPD	9 Other musculoskeletal	**109 (87 to 131)**	**92.5 (80.4 to 105)**	**56.3 (51.6 to 60.5)**
10 Diarrheal diseases	10 Lower respiratory infect	-8.23 (-25.7 to 9.39)	-15.3 (-30.8 to 4.21)	**-34.8 (-45.9 to -18.8)**
11 Self-harm	11 Low back pain	**29.7 (16.2 to 44.4)**	**19.7 (12.1 to 27.5)**	**-4.86 (-5.07 to -4.66)**
12 Low back pain	12 Road injuries	-22.3 (-54.2 to 33.5)	-28.2 (-56.1 to 21.7)	-17.7 (-47.8 to 46.1)
13 Lung cancer	13 Alzheimer's disease	**74.6 (39.7 to 117)**	**61 (30.7 to 90.2)**	**9.4 (5.12 to 13.9)**
14 Headache disorders	14 Age-related hearing loss	**41.5 (25.6 to 61.6)**	**30.5 (18.6 to 41)**	**-1.05 (-1.18 to -0.928)**
15 Age-related hearing loss	15 Self-harm	-16.6 (-38.1 to 13.3)	-23.1 (-42.2 to 3.38)	-14.2 (-34.1 to 16.8)
16 Cirrhosis liver	16 Breast cancer	**41.7 (1.81 to 81.7)**	30.7 (-3.61 to 69.1)	-2.98 (-27.7 to 22.8)
17 Breast cancer	17 Hypertensive heart disease	47.2 (-2.23 to 112)	36.1 (-5.96 to 97.8)	-0.733 (-33 to 47.2)
18 Depressive disorders	18 Headache disorders	**18.4 (5.06 to 35)**	**9.21 (3.68 to 21.5)**	**0.224 (0.0237 to 0.445)**
19 Alzheimer's disease	19 Depressive disorders	**22.1 (7.73 to 36.7)**	**12.6 (7.32 to 19.5)**	**-2.2 (-2.38 to -1.99)**
20 Hypertensive heart disease	20 Cirrhosis liver	2.13 (-18.4 to 24.2)	-5.67 (-22.7 to 17.1)	-19.8 (-34.7 to 1.05)
21 Other musculoskeletal	24 Diarrheal diseases			

Supplemental Results Figure S7. Leading 20 level 3 causes of DALYs, and percentage change in number of DALYs and all-age and age-standardised DALY rates by location, 2022 and 2050

Tonga, Reference scenario

Legend:
Communicable, maternal, neonatal and nutritional
Non-communicable
Injuries

Leading Causes 2022	Leading Causes 2050	Mean % change number of DALY	Mean % change all-age DALY rate	Mean % change age-standardized DALY rate
1 Diabetes	1 Diabetes	**55.7 (25 to 94.4)**	19.2 (-0.807 to 41.8)	0.445 (-15.1 to 18.3)
2 Ischemic heart disease	2 Ischemic heart disease	-1.09 (-30.7 to 42.2)	-24.2 (-48.5 to 11.3)	**-37.5 (-58.1 to -7.81)**
3 Stroke	3 Stroke	12.3 (-11.1 to 41)	-13.9 (-31 to 8.64)	**-31.8 (-44.8 to -13.9)**
4 Neonatal disorders	4 Neonatal disorders	-13.3 (-36.3 to 19.2)	**-33.7 (-49.3 to -11.1)**	-22.2 (-39.6 to 4.6)
5 Lower respiratory infect	5 Lower respiratory infect	-12.7 (-27.7 to 7.15)	**-33.1 (-43 to -20.3)**	**-36.2 (-46.2 to -22.1)**
6 COPD	6 COPD	**44.8 (7.92 to 88.4)**	11 (-14.3 to 43.7)	-16.4 (-37.2 to 9.91)
7 Low back pain	7 Low back pain	**41.6 (19.6 to 61.6)**	**8.31 (2.04 to 15)**	**-3.53 (-3.8 to -3.22)**
8 Cirrhosis liver	8 Chronic kidney disease	**124 (57.2 to 217)**	**71.5 (24.7 to 145)**	**44 (2.13 to 109)**
9 Road injuries	9 Other musculoskeletal	**109 (76.2 to 140)**	**59.9 (49 to 71.1)**	**42.9 (38.4 to 46.9)**
10 Lung cancer	10 Liver cancer	**68.7 (16.9 to 143)**	29.2 (-5.14 to 78.5)	15.6 (-16.2 to 64.1)
11 Headache disorders	11 Lung cancer	**50.6 (12.3 to 96)**	15.2 (-12.9 to 45.6)	-7.06 (-25 to 12.2)
12 Liver cancer	12 Headache disorders	**38.4 (16 to 59.8)**	**5.85 (2.36 to 10.4)**	-0.0682 (-0.384 to 0.341)
13 Breast cancer	13 Road injuries	11.2 (-32.6 to 94)	-14.8 (-46.4 to 48.7)	-19.4 (-50 to 40.4)
14 Congenital defects	14 Breast cancer	**41.3 (4.22 to 90.6)**	8.04 (-20 to 40.1)	-4.38 (-26.3 to 20.7)
15 Anxiety disorders	15 Cirrhosis liver	6.4 (-15.6 to 30.1)	**-18.6 (-32.1 to -2.49)**	**-24.6 (-36.5 to -9.26)**
16 Age-related hearing loss	16 Age-related hearing loss	**55.6 (32.3 to 78.3)**	**19.1 (9.67 to 29.2)**	**-0.965 (-1.11 to -0.837)**
17 Other musculoskeletal	17 Alzheimer's disease	**101 (53.7 to 150)**	**53.8 (20.9 to 85.3)**	**4.98 (1.85 to 8.24)**
18 Chronic kidney disease	18 Anxiety disorders	**36.8 (15.3 to 58.6)**	**4.6 (1.26 to 7.91)**	0.311 (-0.0441 to 0.641)
19 Dietary iron deficiency	19 Depressive disorders	**39.6 (18.2 to 61.8)**	**6.78 (2.89 to 11.1)**	**-1.77 (-2.12 to -1.42)**
20 Depressive disorders	20 Endo/metab/blood/immune	**60.2 (30.1 to 91)**	**22.6 (8.27 to 39.3)**	10.7 (-0.985 to 24.3)
22 Alzheimer's disease	21 Dietary iron deficiency			
25 Endo/metab/blood/immune	23 Congenital defects			

Supplemental Results Figure S7. Leading 20 level 3 causes of DALYs, and percentage change in number of DALYs and all-age and age-standardised DALY rates by location, 2022 and 2050

Tuvalu, Reference scenario

Legend:
Communicable, maternal, neonatal and nutritional
Non-communicable
Injuries

Leading Causes 2022	Leading Causes 2050	Mean % change number of DALY	Mean % change all-age DALY rate	Mean % change age-standardized DALY rate
1 Ischemic heart disease	1 Ischemic heart disease	23 (-17.1 to 78.6)	-8.14 (-38.3 to 38.1)	-24.8 (-51.2 to 15.1)
2 Stroke	2 Diabetes	**67.5 (36.3 to 103)**	**24.9 (4.74 to 50)**	4.5 (-13.1 to 25.8)
3 Diabetes	3 Stroke	26.9 (-2.37 to 58)	-5.28 (-25.1 to 18.9)	-22.3 (-39.8 to 0.366)
4 Neonatal disorders	4 Chronic kidney disease	**122 (63.1 to 196)**	**65.7 (25.7 to 124)**	**41.3 (2.49 to 103)**
5 Lower respiratory infect	5 COPD	**59.2 (23.3 to 98.2)**	18.8 (-5.01 to 50.5)	-4.3 (-28.9 to 27.9)
6 Road injuries	6 Lower respiratory infect	-15.5 (-32.3 to 0.533)	**-37 (-45.1 to -25.1)**	**-38.6 (-48.5 to -25.1)**
7 Tuberculosis	7 Road injuries	9.7 (-36.2 to 83.7)	-18.1 (-53.2 to 39.8)	-20.6 (-53.9 to 35.6)
8 Self-harm	8 Neonatal disorders	**-32.7 (-54.5 to -5.94)**	**-49.9 (-62.6 to -31.2)**	**-36.2 (-52.4 to -13.1)**
9 Chronic kidney disease	9 Self-harm	16.2 (-10.3 to 49.8)	-13.4 (-30.3 to 8.34)	-14.5 (-30.7 to 7.17)
10 COPD	10 Low back pain	**45.3 (25 to 65.5)**	**8.36 (0.544 to 15.8)**	**-5.07 (-5.91 to -4.29)**
11 Low back pain	11 Lung cancer	**76.1 (23.6 to 134)**	31.2 (-3.72 to 71.3)	10.5 (-13.4 to 34.1)
12 Congenital defects	12 Other musculoskeletal	**145 (111 to 176)**	**82.5 (69.1 to 95.4)**	**61.4 (58.2 to 64)**
13 Cirrhosis liver	13 Hypertensive heart disease	**65.8 (15.7 to 131)**	23.8 (-12.2 to 74.1)	-0.0169 (-32.5 to 55)
14 Headache disorders	14 Headache disorders	**43 (21.8 to 60.5)**	**6.51 (2.74 to 11)**	**0.801 (0.341 to 1.18)**
15 Lung cancer	15 Age-related hearing loss	**56.8 (33.5 to 82.2)**	**16.9 (5.14 to 27.5)**	**-1.06 (-1.32 to -0.745)**
16 Hypertensive heart disease	16 Cirrhosis liver	11.2 (-11.9 to 33.1)	-17 (-31.3 to 1.35)	**-28 (-39.6 to -13.6)**
17 Interpersonal violence	17 Depressive disorders	**42.6 (21.9 to 62)**	**6.24 (1.47 to 12.3)**	**-2.18 (-2.55 to -1.75)**
18 Depressive disorders	18 Tuberculosis	**-44.6 (-62 to -21.3)**	**-58.7 (-70.2 to -39.2)**	**-63.2 (-73.6 to -44.8)**
19 Drowning	19 Anxiety disorders	**40.8 (19.8 to 59.9)**	**4.87 (1.87 to 8.78)**	**1.23 (0.746 to 1.65)**
20 Age-related hearing loss	20 Breast cancer	**55.8 (7.17 to 122)**	16.1 (-16.7 to 56.4)	-3.17 (-27.3 to 27.9)
21 Anxiety disorders	23 Interpersonal violence			
23 Breast cancer	25 Congenital defects			
24 Other musculoskeletal	29 Drowning			

Supplemental Results Figure S7. Leading 20 level 3 causes of DALYs, and percentage change in number of DALYs and all-age and age-standardised DALY rates by location, 2022 and 2050

Vanuatu, Reference scenario

Legend:
Communicable, maternal, neonatal and nutritional
Non-communicable
Injuries

Leading Causes 2022	Leading Causes 2050	Mean % change number of DALY	Mean % change all-age DALY rate	Mean % change age-standardized DALY rate
1 Ischemic heart disease	1 Ischemic heart disease	49.1 (-1.45 to 118)	-4.68 (-36 to 42.3)	-31.3 (-56.2 to 7.75)
2 COVID-19	2 Diabetes	**114 (83.1 to 150)**	**36.9 (15.3 to 61.5)**	-1.23 (-17.8 to 20.1)
3 Stroke	3 Stroke	**40.3 (12.1 to 72.2)**	-10.4 (-27.8 to 12.4)	**-35.7 (-50.6 to -16)**
4 Neonatal disorders	4 HIV/AIDS	**751 (358 to 1250)**	**443 (190 to 778)**	**449 (193 to 785)**
5 Diabetes	5 Neonatal disorders	-27.1 (-47.1 to 0.124)	**-53.5 (-65.3 to -35.7)**	**-37.7 (-53.8 to -15.4)**
6 Lower respiratory infect	6 Chronic kidney disease	**162 (89.1 to 257)**	**67.2 (20.8 to 132)**	28 (-12.3 to 84.7)
7 Road injuries	7 COPD	**75.3 (37.4 to 126)**	12 (-10.5 to 47.2)	-25.4 (-44.6 to 2.59)
8 Self-harm	8 Road injuries	24.8 (-28.8 to 110)	-20.2 (-53.6 to 47)	-25.2 (-56.2 to 37.5)
9 COPD	9 Self-harm	**37.4 (4.85 to 79.1)**	-12.3 (-31.1 to 13.1)	-17.1 (-34.4 to 6.04)
10 Congenital defects	10 Lower respiratory infect	**-27.6 (-40.4 to -12.3)**	**-53.8 (-60.9 to -45.3)**	**-50.1 (-58.5 to -37.8)**
11 Cirrhosis liver	11 Low back pain	**86.1 (70.6 to 106)**	**18.8 (12.7 to 25.8)**	**-3.63 (-3.94 to -3.24)**
12 Low back pain	12 Other musculoskeletal	**213 (187 to 243)**	**100 (86.7 to 112)**	**63.6 (59.9 to 66.3)**
13 Chronic kidney disease	13 Headache disorders	**72.9 (57.5 to 92.1)**	**10.3 (6.17 to 15.5)**	**0.639 (0.4 to 0.861)**
14 Drowning	14 Cirrhosis liver	18.2 (-2.62 to 44)	**-24.5 (-38.4 to -5.3)**	**-37.8 (-48.8 to -22)**
15 Diarrheal diseases	15 Depressive disorders	**75.8 (61.5 to 93.8)**	**12.1 (7.53 to 17)**	**-1.75 (-2.04 to -1.46)**
16 Headache disorders	16 Anxiety disorders	**69.5 (53.8 to 88.1)**	**8.12 (3.65 to 13.2)**	**1.3 (0.973 to 1.6)**
17 Dietary iron deficiency	17 Age-related hearing loss	**108 (85.1 to 134)**	**32.7 (21.8 to 44.6)**	**-1.29 (-1.45 to -1.11)**
18 Rheumatic heart disease	18 Hypertensive heart disease	**118 (51.7 to 197)**	39.1 (-1.54 to 92.6)	-3.76 (-38.2 to 46.6)
19 Anxiety disorders	19 Dietary iron deficiency	**29.8 (16.1 to 45.8)**	**-17.3 (-20.1 to -14.6)**	**-14.1 (-14.9 to -13.2)**
20 Asthma	20 Congenital defects	-15.6 (-29.7 to 1.78)	**-46.2 (-52.6 to -37.3)**	**-30.7 (-37.8 to -21.6)**
21 Depressive disorders	22 Diarrheal diseases			
23 Age-related hearing loss	24 Drowning			
24 Hypertensive heart disease	28 Rheumatic heart disease			
26 Other musculoskeletal	32 Asthma			
33 HIV/AIDS	164 COVID-19			

Supplemental Results Figure S7. Leading 20 level 3 causes of DALYs, and percentage change in number of DALYs and all-age and age-standardised DALY rates by location, 2022 and 2050

Cambodia, Reference scenario

Legend:
Communicable, maternal, neonatal and nutritional
Non-communicable
Injuries

Leading Causes 2022	Leading Causes 2050	Mean % change number of DALY	Mean % change all-age DALY rate	Mean % change age-standardized DALY rate
1 Lower respiratory infect	1 Stroke	28.4 (-0.26 to 62.6)	2.35 (-15.3 to 24.9)	**-41.5 (-53.2 to -27)**
2 Stroke	2 Cirrhosis liver	**63.7 (29.4 to 98.6)**	**30.5 (10.1 to 53.1)**	-6.67 (-20.1 to 8.73)
3 Neonatal disorders	3 Lower respiratory infect	**-33.7 (-45.9 to -18.5)**	**-47.2 (-55.1 to -37.9)**	**-56.1 (-62.5 to -48.6)**
4 Ischemic heart disease	4 Ischemic heart disease	13.8 (-22.8 to 61)	-9.24 (-35.6 to 28)	**-46.5 (-63.4 to -20.2)**
5 Cirrhosis liver	5 Diabetes	**90.2 (54 to 134)**	**51.5 (29.5 to 73.6)**	-7.29 (-19.8 to 6.46)
6 Congenital defects	6 Other musculoskeletal	**172 (129 to 224)**	**117 (103 to 132)**	**76.6 (68.7 to 81.8)**
7 Tuberculosis	7 Chronic kidney disease	**121 (61.9 to 194)**	**76 (34.6 to 127)**	20.8 (-10.4 to 63.5)
8 Road injuries	8 Falls	**65.4 (31.4 to 104)**	**31.7 (14.5 to 52.1)**	-5.69 (-14.6 to 4.15)
9 Diabetes	9 Neonatal disorders	**-55.3 (-66 to -41.5)**	**-64.4 (-72 to -54.3)**	**-47.8 (-59.5 to -31.7)**
10 Falls	10 COPD	**75 (30.7 to 125)**	**39.5 (9.68 to 71.1)**	**-24 (-42.1 to -0.566)**
11 Headache disorders	11 Road injuries	1.95 (-31.2 to 45.1)	-18.7 (-44.1 to 21.6)	-23.4 (-47.9 to 15.7)
12 Low back pain	12 Low back pain	**53.2 (28.5 to 79.6)**	**22 (15.8 to 28.4)**	**-7.03 (-7.32 to -6.76)**
13 COPD	13 Hypertensive heart disease	**115 (47.7 to 206)**	**71.7 (26.1 to 134)**	-4.64 (-34.4 to 36.9)
14 Age-related hearing loss	14 Lung cancer	**98.3 (44.4 to 163)**	**57.9 (20.8 to 96.8)**	-4.66 (-23.7 to 16)
15 Chronic kidney disease	15 Age-related hearing loss	**80.7 (50.7 to 114)**	**44 (33.6 to 54.8)**	**-1.36 (-1.54 to -1.15)**
16 Upper digest diseases	16 Headache disorders	**35.1 (14.7 to 57.8)**	**7.59 (3.01 to 17.5)**	0.261 (-0.00811 to 0.607)
17 Lung cancer	17 Colorectal cancer	**141 (67.4 to 228)**	**92 (36.6 to 154)**	20 (-10 to 51.3)
18 Dietary iron deficiency	18 Alzheimer's disease	**190 (125 to 265)**	**131 (91.8 to 182)**	3.75 (-0.0403 to 7.56)
19 Anxiety disorders	19 Congenital defects	**-48.4 (-58.9 to -35.2)**	**-58.9 (-64.7 to -52.3)**	**-36.5 (-43.9 to -27.8)**
20 Depressive disorders	20 Depressive disorders	**42.1 (20.6 to 65.8)**	**13.2 (8.38 to 18.4)**	**-2.88 (-3.14 to -2.54)**
21 Hypertensive heart disease	21 Anxiety disorders			
22 Other musculoskeletal	22 Tuberculosis			
28 Colorectal cancer	25 Upper digest diseases			
35 Alzheimer's disease	27 Dietary iron deficiency			

Supplemental Results Figure S7. Leading 20 level 3 causes of DALYs, and percentage change in number of DALYs and all-age and age-standardised DALY rates by location, 2022 and 2050

Indonesia, Reference scenario

Legend:
Communicable, maternal, neonatal and nutritional
Non-communicable
Injuries

Leading Causes 2022	Leading Causes 2050	Mean % change number of DALY	Mean % change all-age DALY rate	Mean % change age-standardized DALY rate
1 Stroke	1 Stroke	14.3 (-5.33 to 32.7)	2.33 (-14.8 to 21.2)	**-48.2 (-56.8 to -38.1)**
2 Ischemic heart disease	2 Ischemic heart disease	-8.96 (-34.3 to 21.5)	-18.4 (-40.5 to 11.5)	**-56.1 (-69 to -37.7)**
3 Neonatal disorders	3 Diabetes	**88.4 (61.4 to 119)**	**68.6 (47.6 to 89.7)**	-4.69 (-17.4 to 8.72)
4 Cirrhosis liver	4 COPD	**112 (70.6 to 160)**	**89.7 (50.4 to 136)**	-13.5 (-30.9 to 13.5)
5 COVID-19	5 Cirrhosis liver	**19.2 (3.11 to 33.6)**	6.65 (-7.28 to 21.5)	**-30.2 (-38.1 to -20.1)**
6 Diabetes	6 Hypertensive heart disease	**127 (62.4 to 201)**	**103 (49.1 to 177)**	-3.44 (-32.9 to 40.4)
7 Tuberculosis	7 Other musculoskeletal	**112 (92.1 to 136)**	**89.8 (79 to 102)**	**56.2 (54.6 to 58)**
8 Road injuries	8 Chronic kidney disease	**87.6 (44.3 to 140)**	**68 (26.9 to 120)**	10.5 (-16.8 to 47.7)
9 COPD	9 Low back pain	**41.5 (27.4 to 56.4)**	**26.5 (20.3 to 34.2)**	**-4.51 (-5.28 to -3.78)**
10 Low back pain	10 Age-related hearing loss	**80.2 (60.1 to 106)**	**61.1 (49.5 to 74.4)**	**-1.1 (-1.19 to -1.02)**
11 Headache disorders	11 Lung cancer	**92.8 (45.6 to 145)**	**72.2 (34.2 to 111)**	-4.79 (-22.7 to 13.5)
12 Diarrheal diseases	12 Alzheimer's disease	**252 (184 to 331)**	**214 (166 to 276)**	1.08 (-2.48 to 5.31)
13 Lower respiratory infect	13 Headache disorders	**13.4 (1.99 to 28.2)**	1.41 (-2.9 to 12.3)	**0.502 (0.305 to 0.686)**
14 Chronic kidney disease	14 Diarrheal diseases	7.11 (-29.4 to 60.9)	-4 (-38.8 to 47.4)	**-45.1 (-62.3 to -16.5)**
15 Age-related hearing loss	15 Neonatal disorders	**-52.8 (-62.5 to -39.4)**	**-57.8 (-66.5 to -46.8)**	**-42.8 (-55.1 to -27)**
16 Other musculoskeletal	16 Road injuries	-13.6 (-39.2 to 23.6)	-22.7 (-45.4 to 7.32)	-21.1 (-46 to 9.4)
17 Lung cancer	17 Lower respiratory infect	7.84 (-13.2 to 29)	-3.55 (-19.4 to 14)	**-43.4 (-52 to -33.3)**
18 Hypertensive heart disease	18 Blindness and vision loss	**93.9 (68.8 to 121)**	**73.3 (56.7 to 91.2)**	**-5.58 (-6.04 to -5.11)**
19 Congenital defects	19 Tuberculosis	**-38.2 (-54.9 to -14.6)**	**-44.7 (-61 to -23.8)**	**-63.6 (-75.2 to -47.1)**
20 Anxiety disorders	20 Falls	**59.4 (35.4 to 87)**	**42.5 (24.6 to 60.9)**	**-13.4 (-19.9 to -5.85)**
23 Falls	22 Anxiety disorders			
24 Blindness and vision loss	32 Congenital defects			
31 Alzheimer's disease	164 COVID-19			

Supplemental Results Figure S7. Leading 20 level 3 causes of DALYs, and percentage change in number of DALYs and all-age and age-standardised DALY rates by location, 2022 and 2050

Laos, Reference scenario

Legend:
Communicable, maternal, neonatal and nutritional
Non-communicable
Injuries

Leading Causes 2022	Leading Causes 2050	Mean % change number of DALY	Mean % change all-age DALY rate	Mean % change age-standardized DALY rate
1 Neonatal disorders	1 Stroke	22.6 (-1.83 to 53.6)	-4.01 (-23.6 to 16.7)	**-49.8 (-60.5 to -37)**
2 Stroke	2 Ischemic heart disease	11.5 (-23.2 to 59.3)	-12.7 (-39.8 to 23.4)	**-53.8 (-68.8 to -32.3)**
3 Lower respiratory infect	3 Diabetes	**113 (76.1 to 150)**	**66.4 (36 to 90.1)**	-10.8 (-24.5 to 3.28)
4 Ischemic heart disease	4 Chronic kidney disease	**132 (69.3 to 213)**	**81.5 (33.8 to 139)**	9.87 (-18.5 to 47.9)
5 COVID-19	5 COPD	**115 (66.9 to 168)**	**68.4 (29.2 to 108)**	**-21.2 (-40.8 to -0.269)**
6 Congenital defects	6 Other musculoskeletal	**201 (165 to 237)**	**136 (120 to 154)**	**82.2 (75.9 to 87.3)**
7 Diabetes	7 Neonatal disorders	**-64.8 (-73.8 to -53.7)**	**-72.5 (-78.9 to -64.6)**	**-53.7 (-64.8 to -39.4)**
8 Road injuries	8 Lower respiratory infect	**-45.3 (-55.9 to -31.2)**	**-57.2 (-65 to -45.6)**	**-59.9 (-66.4 to -51.7)**
9 Tuberculosis	9 Hypertensive heart disease	**139 (64.3 to 244)**	**87.3 (30.3 to 160)**	-8.76 (-38.8 to 33.8)
10 Chronic kidney disease	10 Age-related hearing loss	**102 (75.2 to 126)**	**57.9 (45.3 to 70.8)**	**-1.57 (-1.67 to -1.47)**
11 Diarrheal diseases	11 Cirrhosis liver	**46.4 (23.1 to 78)**	14.6 (-3.5 to 37.2)	**-24.5 (-35.7 to -10.5)**
12 COPD	12 Low back pain	**66.7 (47.3 to 86.9)**	**30.5 (23.3 to 38.7)**	**-5.95 (-6.18 to -5.71)**
13 Cirrhosis liver	13 Road injuries	-0.337 (-34.7 to 51.6)	-21.9 (-48.7 to 20.4)	-25.2 (-51.1 to 16.1)
14 Headache disorders	14 Lung cancer	**140 (78.7 to 207)**	**87.8 (42 to 137)**	-2.25 (-23.5 to 18.2)
15 Low back pain	15 Headache disorders	**39.6 (22.5 to 58.9)**	**9.26 (4.02 to 20.7)**	**0.785 (0.551 to 0.916)**
16 Anxiety disorders	16 Anxiety disorders	**43.2 (24.3 to 61.5)**	**12 (5.63 to 19.8)**	**1.52 (1.42 to 1.61)**
17 Interpersonal violence	17 Congenital defects	**-54.1 (-63.5 to -44.7)**	**-64.1 (-70.6 to -57.6)**	**-39.1 (-47.8 to -29.8)**
18 Age-related hearing loss	18 Alzheimer's disease	**265 (187 to 343)**	**185 (136 to 236)**	**6.87 (4.05 to 10)**
19 Other musculoskeletal	19 Colorectal cancer	**166 (88 to 255)**	**108 (51.4 to 177)**	15.5 (-12.9 to 50.6)
20 Hypertensive heart disease	20 Depressive disorders	**46.3 (30.3 to 63.5)**	**14.5 (8.74 to 21.3)**	**-2.53 (-2.77 to -2.26)**
21 Depressive disorders	23 Interpersonal violence			
25 Lung cancer	24 Tuberculosis			
32 Colorectal cancer	39 Diarrheal diseases			
40 Alzheimer's disease	165 COVID-19			

Supplemental Results Figure S7. Leading 20 level 3 causes of DALYs, and percentage change in number of DALYs and all-age and age-standardised DALY rates by location, 2022 and 2050

Malaysia, Reference scenario

Legend:
Communicable, maternal, neonatal and nutritional
Non-communicable
Injuries

Leading Causes 2022	Leading Causes 2050	Mean % change number of DALY	Mean % change all-age DALY rate	Mean % change age-standardized DALY rate
1 Ischemic heart disease	1 Ischemic heart disease	-12.2 (-37 to 21.4)	**-27.7 (-47.6 to -0.00157)**	**-59.7 (-71.4 to -42.6)**
2 Stroke	2 Lower respiratory infect	**64.7 (37.5 to 94.4)**	**35.6 (18.3 to 53.5)**	**-25.3 (-36.2 to -12.5)**
3 Lower respiratory infect	3 Stroke	**23.3 (2.58 to 53.1)**	1.49 (-15 to 23.6)	**-43.6 (-53 to -30.3)**
4 Road injuries	4 Diabetes	**112 (83.1 to 141)**	**74.4 (58.1 to 90.8)**	2.44 (-6.43 to 11.7)
5 COVID-19	5 Chronic kidney disease	**113 (61.8 to 181)**	**75.3 (34.4 to 128)**	3.49 (-21.3 to 37.1)
6 Diabetes	6 Other musculoskeletal	**125 (92.5 to 152)**	**85 (74.7 to 94.9)**	**50.2 (47.4 to 53.3)**
7 Depressive disorders	7 Road injuries	7.1 (-24.7 to 62)	-11.8 (-37.6 to 30.2)	-17.3 (-42.5 to 25.4)
8 Low back pain	8 Age-related hearing loss	**84.3 (59.8 to 109)**	**51.7 (42.5 to 60.3)**	**-1.07 (-1.15 to -0.98)**
9 Chronic kidney disease	9 COPD	**104 (61.2 to 166)**	**67.8 (32.1 to 114)**	-13.7 (-34.4 to 13)
10 Headache disorders	10 Alzheimer's disease	**213 (162 to 271)**	**157 (125 to 185)**	**4.61 (1.38 to 7.95)**
11 Neonatal disorders	11 Low back pain	**54.8 (33.8 to 73.4)**	**27.4 (19.8 to 33.8)**	**-3.83 (-4.03 to -3.65)**
12 Age-related hearing loss	12 Lung cancer	**113 (65.6 to 173)**	**75.4 (38.3 to 121)**	0.653 (-17.2 to 22.4)
13 Other musculoskeletal	13 Depressive disorders	**33.4 (14.3 to 50.6)**	**9.75 (4.16 to 15.3)**	**-1.83 (-2 to -1.64)**
14 Anxiety disorders	14 Colorectal cancer	**118 (60 to 195)**	**79.5 (33.1 to 133)**	3.18 (-21.1 to 28.9)
15 COPD	15 Cirrhosis liver	**62.7 (34.1 to 97.6)**	**34 (11.1 to 58.1)**	-13 (-26.7 to 2.6)
16 Cirrhosis liver	16 Headache disorders	**25.6 (10.8 to 41.5)**	3.4 (-2.25 to 15.5)	**0.789 (0.437 to 0.995)**
17 Breast cancer	17 Breast cancer	**81.1 (38.2 to 136)**	**48.9 (16.2 to 89)**	-2.17 (-23 to 22.6)
18 Lung cancer	18 Anxiety disorders	**31.1 (13.2 to 50.9)**	**7.89 (1.17 to 15.6)**	**1.39 (1.24 to 1.54)**
19 Colorectal cancer	19 Neonatal disorders	-14.3 (-28.3 to 1.02)	**-29.5 (-38.1 to -20)**	-15.7 (-27.1 to 0.236)
20 Congenital defects	20 Urinary diseases	**132 (96.4 to 167)**	**90.6 (72.4 to 110)**	3.98 (-3.27 to 14.9)
21 Alzheimer's disease	34 Congenital defects			
31 Urinary diseases	159 COVID-19			

Supplemental Results Figure S7. Leading 20 level 3 causes of DALYs, and percentage change in number of DALYs and all-age and age-standardised DALY rates by location, 2022 and 2050

Maldives, Reference scenario

Legend:
Communicable, maternal, neonatal and nutritional
Non-communicable
Injuries

Leading Causes 2022	Leading Causes 2050	Mean % change number of DALY	Mean % change all-age DALY rate	Mean % change age-standardized DALY rate
1 Stroke	1 Diabetes	**381 (313 to 450)**	**220 (183 to 262)**	**25.6 (13 to 37.8)**
2 Ischemic heart disease	2 Stroke	**130 (82.5 to 179)**	**52.9 (23.6 to 84.8)**	**-44.1 (-54.8 to -27.5)**
3 Neonatal disorders	3 Ischemic heart disease	**94.6 (36.4 to 181)**	29.7 (-9.64 to 87.1)	**-51.3 (-67.5 to -25.8)**
4 COVID-19	4 COPD	**335 (231 to 441)**	**189 (117 to 267)**	-9.73 (-33.1 to 18.6)
5 Headache disorders	5 Other musculoskeletal	**220 (176 to 265)**	**113 (89.5 to 140)**	**72.7 (69.8 to 75.9)**
6 Diabetes	6 Age-related hearing loss	**197 (159 to 233)**	**97.5 (81.4 to 116)**	**-1.36 (-1.84 to -0.892)**
7 Low back pain	7 Chronic kidney disease	**251 (152 to 369)**	**134 (71.4 to 210)**	-1.53 (-27.2 to 34)
8 Other musculoskeletal	8 Low back pain	**105 (78.1 to 129)**	**36.3 (24.8 to 49)**	**-4.52 (-5.56 to -3.55)**
9 Age-related hearing loss	9 Alzheimer's disease	**440 (358 to 523)**	**259 (212 to 303)**	**-6.75 (-9.85 to -3.38)**
10 Depressive disorders	10 Headache disorders	**48.2 (30.5 to 70.9)**	-1.41 (-9.19 to 12.5)	**3.45 (0.671 to 5.43)**
11 Chronic kidney disease	11 Depressive disorders	**77.2 (54.3 to 105)**	**17.8 (8.59 to 30.2)**	-0.453 (-1.55 to 0.657)
12 Anxiety disorders	12 Neonatal disorders	-12 (-28.3 to 8.7)	**-41.5 (-52 to -28.2)**	**-26.9 (-44.5 to -1.61)**
13 COPD	13 Anxiety disorders	**64.3 (40.6 to 92.6)**	9.31 (-0.32 to 23.3)	**4.73 (3.33 to 6.35)**
14 Dietary iron deficiency	14 Falls	**118 (70.1 to 158)**	**45.1 (16.6 to 69.4)**	**-12.2 (-21.6 to -2.37)**
15 Congenital defects	15 Blindness and vision loss	**238 (193 to 285)**	**125 (101 to 145)**	**-7.49 (-8.15 to -6.7)**
16 Falls	16 Osteoarthritis	**287 (240 to 328)**	**157 (139 to 176)**	**3.03 (2.4 to 3.79)**
17 Lower respiratory infect	17 Oral disorders	**162 (127 to 200)**	**74.5 (57.3 to 93.3)**	**0.52 (0.000654 to 1.04)**
18 Schizophrenia	18 Endo/metab/blood/immune	**112 (73 to 152)**	**40.8 (17.4 to 63)**	-5.85 (-17.3 to 8.4)
19 Hemoglobinopathies	19 Lung cancer	**326 (213 to 453)**	**183 (115 to 261)**	-2.12 (-22.4 to 22.5)
20 Endo/metab/blood/immune	20 Lower respiratory infect	**80.8 (49.6 to 111)**	**20.4 (0.623 to 40.7)**	**-47 (-55.8 to -36)**
24 Oral disorders	21 Dietary iron deficiency			
25 Alzheimer's disease	24 Hemoglobinopathies			
26 Blindness and vision loss	25 Schizophrenia			
30 Osteoarthritis	31 Congenital defects			
35 Lung cancer	154 COVID-19			

Supplemental Results Figure S7. Leading 20 level 3 causes of DALYs, and percentage change in number of DALYs and all-age and age-standardised DALY rates by location, 2022 and 2050

Mauritius, Reference scenario

Legend:
Communicable, maternal, neonatal and nutritional
Non-communicable
Injuries

Leading Causes 2022	Leading Causes 2050	Mean % change number of DALY	Mean % change all-age DALY rate	Mean % change age-standardized DALY rate
1 Diabetes	1 Diabetes	8.36 (-9.25 to 28.6)	**25.9 (11.1 to 42.3)**	**-18.3 (-27.1 to -6.78)**
2 Ischemic heart disease	2 Chronic kidney disease	**30.2 (0.981 to 67)**	**51.4 (18.5 to 89.9)**	-0.0206 (-21.6 to 29.7)
3 Chronic kidney disease	3 Ischemic heart disease	**-35 (-51.2 to -11.8)**	-24.4 (-44.3 to 0.907)	**-58.1 (-70.3 to -40.9)**
4 Stroke	4 Stroke	**-26 (-37.4 to -11.1)**	-14 (-25.2 to 0.858)	**-47.8 (-55.2 to -37.8)**
5 Neonatal disorders	5 Alzheimer's disease	**140 (94 to 184)**	**178 (141 to 216)**	**-4.56 (-7.33 to -1.58)**
6 Low back pain	6 Age-related hearing loss	**31.8 (10.6 to 51.3)**	**53.2 (43.6 to 62.9)**	**-0.941 (-1.02 to -0.842)**
7 Age-related hearing loss	7 Hypertensive heart disease	**47.7 (8.72 to 107)**	**71.8 (26 to 146)**	-11.8 (-36.6 to 27.3)
8 Lower respiratory infect	8 Other musculoskeletal	**38.5 (18.1 to 59.5)**	**61 (52.5 to 69.7)**	**40.4 (36.3 to 43.7)**
9 Road injuries	9 Lower respiratory infect	**18.3 (1.24 to 40.3)**	**37.5 (21.5 to 54.2)**	**-31.3 (-39.3 to -22)**
10 Depressive disorders	10 COPD	**73 (44.2 to 114)**	**101 (69.3 to 138)**	-8.49 (-27.2 to 15.6)
11 Other musculoskeletal	11 Low back pain	5.06 (-9.88 to 20.5)	**22.1 (16.1 to 29.6)**	**-3.89 (-4.04 to -3.74)**
12 Hypertensive heart disease	12 Depressive disorders	-10.3 (-23.1 to 3.63)	4.21 (-0.0625 to 9.02)	**-1.74 (-1.88 to -1.58)**
13 Headache disorders	13 Colorectal cancer	27 (-5.46 to 65.1)	**47.4 (13.9 to 84.5)**	-6.17 (-26.1 to 16.7)
14 Cirrhosis liver	14 Blindness and vision loss	**48 (27.6 to 71.6)**	**72 (58.5 to 85.2)**	**-5.3 (-5.68 to -4.94)**
15 Breast cancer	15 Breast cancer	4.23 (-17.8 to 32.3)	21 (-0.417 to 48)	-10.4 (-27.7 to 8.23)
16 COPD	16 Headache disorders	**-17.4 (-29.7 to -3.98)**	-4.03 (-8.28 to 6.18)	**0.529 (0.319 to 0.629)**
17 Alzheimer's disease	17 Road injuries	**-34.2 (-52.9 to -11)**	-23.5 (-43.7 to 3.71)	-17.3 (-40.3 to 13)
18 Colorectal cancer	18 Lung cancer	**30.2 (2.44 to 64.7)**	**51.2 (20.4 to 83.2)**	-3.14 (-19.9 to 14.9)
19 Self-harm	19 Oral disorders	**24.8 (5.55 to 42.2)**	**45.1 (34.9 to 55)**	**0.401 (0.205 to 0.597)**
20 Anxiety disorders	20 Osteoarthritis	**34.1 (13.5 to 54.2)**	**55.8 (48.6 to 63.6)**	**2.62 (2.33 to 2.93)**
22 Blindness and vision loss	22 Cirrhosis liver			
23 Lung cancer	23 Neonatal disorders			
25 Oral disorders	24 Anxiety disorders			
29 Osteoarthritis	25 Self-harm			

Supplemental Results Figure S7. Leading 20 level 3 causes of DALYs, and percentage change in number of DALYs and all-age and age-standardised DALY rates by location, 2022 and 2050

Myanmar, Reference scenario

Legend:
Communicable, maternal, neonatal and nutritional
Non-communicable
Injuries

Leading Causes 2022	Leading Causes 2050	Mean % change number of DALY	Mean % change all-age DALY rate	Mean % change age-standardized DALY rate
1 Stroke	1 Stroke	5.17 (-17.7 to 31.5)	-6.1 (-24.9 to 14.9)	**-43.5 (-56 to -28.7)**
2 Neonatal disorders	2 Diabetes	**47.2 (18.2 to 79.4)**	**31.4 (8.53 to 52)**	**-17.6 (-30.3 to -4.43)**
3 Ischemic heart disease	3 COPD	**52.9 (12.6 to 96.4)**	**36.5 (4.14 to 70.5)**	**-28.5 (-46.3 to -5.83)**
4 Lower respiratory infect	4 Ischemic heart disease	-2.1 (-32.9 to 39.4)	-12.6 (-39.7 to 20.9)	**-49 (-65.7 to -23.8)**
5 Diabetes	5 Cirrhosis liver	**65.4 (32.1 to 102)**	**47.8 (22 to 72.2)**	**21.3 (1.76 to 42.4)**
6 COPD	6 Neonatal disorders	**-51.7 (-64.4 to -34.6)**	**-56.9 (-67.1 to -43)**	**-37.3 (-53.2 to -14.8)**
7 Congenital defects	7 Chronic kidney disease	**85.1 (30.9 to 151)**	**65.3 (20 to 117)**	14.2 (-19.2 to 58.3)
8 Cirrhosis liver	8 Lower respiratory infect	**-44.6 (-54.9 to -32.6)**	**-50.6 (-57.3 to -41.1)**	**-57 (-63.4 to -48.7)**
9 Tuberculosis	9 Other musculoskeletal	**111 (83.3 to 140)**	**88.6 (78.9 to 98.8)**	**58.4 (54.1 to 62.2)**
10 Road injuries	10 Age-related hearing loss	**59.9 (39 to 81.7)**	**42.8 (32.7 to 54.1)**	**-1.18 (-1.27 to -1.09)**
11 Headache disorders	11 Hypertensive heart disease	**97.3 (38.4 to 177)**	**76.3 (22.3 to 142)**	1.14 (-31.3 to 45.7)
12 Chronic kidney disease	12 Falls	**43.5 (18.6 to 71.6)**	**28.1 (12.5 to 44.8)**	-6.01 (-15.7 to 2.61)
13 Age-related hearing loss	13 Road injuries	-2.08 (-32.5 to 42.4)	-12.6 (-39.4 to 31.9)	-14 (-40.9 to 29)
14 Falls	14 Headache disorders	**17 (1.85 to 33.1)**	**4.44 (0.503 to 12.8)**	0.135 (-0.141 to 0.527)
15 Asthma	15 Low back pain	**36.7 (19 to 54.2)**	**22.1 (16.3 to 27.6)**	**-5.18 (-5.48 to -4.86)**
16 Low back pain	16 Alzheimer's disease	**164 (98.5 to 229)**	**136 (89.2 to 184)**	1.99 (-1.48 to 5.34)
17 Dietary iron deficiency	17 Congenital defects	**-52.5 (-62.5 to -41.4)**	**-57.6 (-64.1 to -50.6)**	**-38.2 (-46.8 to -28.9)**
18 Alcohol use disorders	18 Alcohol use disorders	22.3 (-3.32 to 52.9)	9.26 (-14 to 33)	-3.23 (-21.7 to 17.8)
19 Hypertensive heart disease	19 Lung cancer	**49.8 (9.7 to 98.6)**	**33.7 (1.62 to 74.1)**	-15.8 (-31.3 to 4.87)
20 Other musculoskeletal	20 Blindness and vision loss	**75 (47.6 to 103)**	**56.2 (41.8 to 71.1)**	**-6.23 (-6.54 to -5.92)**
24 Lung cancer	24 Dietary iron deficiency			
28 Blindness and vision loss	25 Tuberculosis			
33 Alzheimer's disease	28 Asthma			

Supplemental Results Figure S7. Leading 20 level 3 causes of DALYs, and percentage change in number of DALYs and all-age and age-standardised DALY rates by location, 2022 and 2050

Philippines, Reference scenario

Legend:
Communicable, maternal, neonatal and nutritional
Non-communicable
Injuries

Leading Causes 2022
1 Ischemic heart disease
2 Stroke
3 Neonatal disorders
4 Lower respiratory infect
5 Diabetes
6 Chronic kidney disease
7 Tuberculosis
8 Interpersonal violence
9 Congenital defects
10 Headache disorders
11 Road injuries
12 Low back pain
13 COVID-19
14 COPD
15 Hypertensive heart disease
16 Age-related hearing loss
17 Anxiety disorders
18 Cirrhosis liver
19 Asthma
20 Depressive disorders
21 Other musculoskeletal
24 Breast cancer
25 Blindness and vision loss
36 Alzheimer's disease

Leading Causes 2050	Mean % change number of DALY	Mean % change all-age DALY rate	Mean % change age-standardized DALY rate
1 Ischemic heart disease	7.93 (-20 to 42.1)	-18.3 (-38.1 to 8.03)	**-51.3 (-64.4 to -33.7)**
2 Stroke	**24.4 (5.15 to 48.5)**	-5.87 (-18.1 to 8.69)	**-43.9 (-51.5 to -34.2)**
3 Lower respiratory infect	19.4 (-0.306 to 41.6)	-9.7 (-22.7 to 3.72)	**-39.6 (-46.8 to -31.1)**
4 Chronic kidney disease	**128 (81.2 to 185)**	**72.4 (37.9 to 114)**	14.4 (-9.39 to 48)
5 Diabetes	**91.3 (64.3 to 125)**	**44.7 (25 to 62.4)**	**-12.7 (-23.5 to -2.08)**
6 Hypertensive heart disease	**166 (96.6 to 253)**	**101 (48 to 161)**	11.8 (-17.9 to 49.9)
7 Neonatal disorders	**-32.3 (-45.4 to -16.7)**	**-48.8 (-57.4 to -38.1)**	**-31.2 (-43.3 to -16)**
8 COPD	**93.7 (54.7 to 135)**	**46.5 (20.1 to 71.3)**	**-21.3 (-36.3 to -2.19)**
9 Low back pain	**68.4 (50.1 to 87.4)**	**27.4 (22.1 to 33.4)**	**-4.26 (-5.06 to -3.48)**
10 Age-related hearing loss	**94.9 (72.4 to 119)**	**47.4 (38.7 to 57.1)**	**-0.933 (-1.02 to -0.852)**
11 Other musculoskeletal	**142 (117 to 167)**	**82.8 (73.7 to 92.2)**	**45.1 (41.6 to 48.2)**
12 Headache disorders	**41 (25.5 to 62.6)**	**6.65 (2.1 to 18.2)**	**0.37 (0.172 to 0.514)**
13 Alzheimer's disease	**243 (183 to 299)**	**159 (125 to 195)**	3.87 (-1.64 to 10)
14 Road injuries	13 (-16.3 to 58.6)	-14.5 (-37.9 to 18.9)	-16.7 (-39.6 to 16.4)
15 Interpersonal violence	-7.38 (-33.3 to 33.6)	**-30 (-48.6 to -3.65)**	**-34.1 (-52.3 to -8.82)**
16 Blindness and vision loss	**102 (78.1 to 128)**	**52.9 (41.4 to 63.6)**	**-5.04 (-5.51 to -4.58)**
17 Cirrhosis liver	**52.6 (30.9 to 77.3)**	**15.4 (2.5 to 29)**	**-17.5 (-26.1 to -6.95)**
18 Depressive disorders	**52.3 (35.4 to 70.5)**	**15.2 (10.7 to 20.4)**	**-1.68 (-1.82 to -1.51)**
19 Breast cancer	**89.9 (43.7 to 137)**	**43.6 (16.5 to 77.5)**	-0.985 (-20.2 to 21.2)
20 Anxiety disorders	**44 (27.8 to 60.1)**	**8.88 (4.33 to 13.6)**	**0.681 (0.379 to 0.965)**
23 Tuberculosis			
24 Congenital defects			
35 Asthma			
165 COVID-19			

Supplemental Results Figure S7. Leading 20 level 3 causes of DALYs, and percentage change in number of DALYs and all-age and age-standardised DALY rates by location, 2022 and 2050

Seychelles, Reference scenario

Legend: Communicable, maternal, neonatal and nutritional; Non-communicable; Injuries

Leading Causes 2022	Leading Causes 2050	Mean % change number of DALY	Mean % change all-age DALY rate	Mean % change age-standardized DALY rate
1 COVID-19	1 Diabetes	**121 (89.9 to 154)**	**69 (56 to 84.2)**	**26.2 (17.4 to 36.3)**
2 Ischemic heart disease	2 Lower respiratory infect	**50.8 (25.3 to 74.7)**	15.1 (-0.179 to 29.5)	**-26.6 (-36.1 to -16.3)**
3 Stroke	3 Hypertensive heart disease	**141 (69.3 to 238)**	**84.2 (35.7 to 155)**	14.9 (-17.2 to 62.2)
4 Diabetes	4 Chronic kidney disease	**96.4 (49.4 to 155)**	**49.9 (18.6 to 92.8)**	8.21 (-16.9 to 45)
5 Lower respiratory infect	5 Stroke	13.8 (-9.33 to 39)	-13.1 (-25.4 to 3.02)	**-41.5 (-50.5 to -29)**
6 Chronic kidney disease	6 Ischemic heart disease	-10.6 (-37.7 to 28.4)	**-31.7 (-50.3 to -3.54)**	**-54.2 (-67.2 to -33.4)**
7 Hypertensive heart disease	7 Other musculoskeletal	**106 (82 to 129)**	**57.1 (49.7 to 65.3)**	**48.5 (47.1 to 50)**
8 Neonatal disorders	8 COPD	**118 (66.1 to 170)**	**66.6 (34.4 to 99.6)**	-1.28 (-22.2 to 23.5)
9 Cirrhosis liver	9 Age-related hearing loss	**72.2 (47.4 to 96.4)**	**31.4 (23.4 to 40.3)**	**-0.99 (-1.2 to -0.783)**
10 Age-related hearing loss	10 Alzheimer's disease	**192 (138 to 249)**	**123 (95.1 to 151)**	**4.52 (1.55 to 7.58)**
11 Low back pain	11 Prostate cancer	**165 (108 to 235)**	**102 (61.3 to 143)**	11 (-4.98 to 26.5)
12 Headache disorders	12 Colorectal cancer	**88.9 (40.7 to 157)**	**44 (9.67 to 84.2)**	2.89 (-19.9 to 30.1)
13 Other musculoskeletal	13 Cirrhosis liver	19.1 (-1.23 to 38.8)	-9.03 (-20.2 to 2.82)	**-18.1 (-28 to -5.59)**
14 Congenital defects	14 Low back pain	**43.2 (27 to 59.7)**	**9.3 (3.83 to 15.6)**	**-3.69 (-4.06 to -3.32)**
15 COPD	15 Neonatal disorders	-11.2 (-30.5 to 9.7)	**-32.2 (-45.4 to -18.6)**	**-25.4 (-39.7 to -9.26)**
16 Road injuries	16 Headache disorders	**29 (13.3 to 44.1)**	-1.51 (-3.68 to 1.46)	**1.34 (0.43 to 1.9)**
17 Colorectal cancer	17 Lung cancer	**81.2 (41.5 to 134)**	**38.2 (9.62 to 72.9)**	-2.31 (-20.5 to 18.3)
18 Depressive disorders	18 Dengue	**396 (307 to 493)**	**279 (213 to 320)**	**277 (211 to 318)**
19 Anxiety disorders	19 Urinary diseases	**132 (92.7 to 174)**	**77.4 (55.6 to 105)**	**15.4 (0.508 to 34.9)**
20 Breast cancer	20 Breast cancer	**52.7 (20.7 to 100)**	16.5 (-7.23 to 46.6)	-0.559 (-19.5 to 20.4)
21 Lung cancer	21 Depressive disorders			
22 Prostate cancer	22 Road injuries			
23 Alzheimer's disease	23 Anxiety disorders			
27 Urinary diseases	25 Congenital defects			
56 Dengue	153 COVID-19			

Supplemental Results Figure S7. Leading 20 level 3 causes of DALYs, and percentage change in number of DALYs and all-age and age-standardised DALY rates by location, 2022 and 2050

Sri Lanka, Reference scenario

Legend:
Communicable, maternal, neonatal and nutritional
Non-communicable
Injuries

Leading Causes 2022
1 Diabetes
2 Ischemic heart disease
3 Stroke
4 COPD
5 Self-harm
6 Age-related hearing loss
7 Low back pain
8 Neonatal disorders
9 Headache disorders
10 Conflict & terror
11 Chronic kidney disease
12 Road injuries
13 Other musculoskeletal
14 Falls
15 Lower respiratory infect
16 Depressive disorders
17 Cirrhosis liver
18 Nature disaster
19 COVID-19
20 Anxiety disorders
21 Alzheimer's disease
22 Blindness and vision loss
23 Hypertensive heart disease
27 Urinary diseases

Leading Causes 2050	Mean % change number of DALY	Mean % change all-age DALY rate	Mean % change age-standardized DALY rate
1 Diabetes	**49.2 (23.9 to 82.1)**	**61.2 (37.5 to 89.8)**	-6.63 (-16.2 to 4.63)
2 Stroke	14 (-10.8 to 48.3)	23.2 (-0.877 to 51.3)	**-45.2 (-53.9 to -33.2)**
3 COPD	**105 (52.5 to 174)**	**121 (71.7 to 184)**	-5.22 (-27.1 to 23.8)
4 Ischemic heart disease	-14 (-40 to 23.5)	-6.93 (-34 to 32)	**-52.1 (-66.7 to -30.6)**
5 Alzheimer's disease	**188 (112 to 281)**	**210 (141 to 287)**	**4.73 (1.72 to 7.82)**
6 Chronic kidney disease	**87.4 (37 to 155)**	**103 (50.4 to 167)**	13 (-15.3 to 53.5)
7 Age-related hearing loss	**44.6 (21 to 71.6)**	**56.1 (40.8 to 70)**	**-0.756 (-0.834 to -0.671)**
8 Low back pain	16.8 (-0.305 to 36.5)	**26.1 (17.8 to 34.2)**	**-3.62 (-3.97 to -3.24)**
9 Other musculoskeletal	**43.9 (21.7 to 65.7)**	**55.4 (46.6 to 64.1)**	**37.5 (36.3 to 38.5)**
10 Hypertensive heart disease	**97.9 (32.3 to 172)**	**114 (47.7 to 193)**	6.97 (-24.7 to 53.2)
11 Falls	**35.3 (9.69 to 67)**	**46 (21.5 to 72.5)**	**-11.5 (-18.9 to -1.54)**
12 Conflict & terror	3430 (-93.9 to 2180)	3240 (-93.3 to 2190)	3710 (-93.4 to 2780)
13 Headache disorders	-8.24 (-24 to 7.29)	-0.984 (-5.57 to 10.2)	0.0418 (-0.172 to 0.321)
14 Blindness and vision loss	**59 (31.1 to 94.4)**	**71.7 (50.6 to 91.1)**	**-4.24 (-4.54 to -3.98)**
15 Self-harm	**-21.7 (-38.7 to -4.72)**	**-15.4 (-27.6 to -0.614)**	**-18.5 (-31.7 to -1.94)**
16 Lower respiratory infect	11.2 (-11.4 to 41.4)	20.1 (-1.28 to 49.8)	**-38.1 (-45.5 to -26.2)**
17 Cirrhosis liver	3.12 (-13.2 to 25.5)	11.4 (-4.7 to 32.1)	**-19.2 (-30 to -6.01)**
18 Depressive disorders	-1.2 (-17.3 to 14.7)	**6.64 (2.41 to 12.1)**	**-1.41 (-1.56 to -1.27)**
19 Urinary diseases	**73.3 (37.1 to 122)**	**87.2 (56.9 to 130)**	0.797 (-9.78 to 12.9)
20 Road injuries	-19.1 (-42.2 to 14.2)	-12.5 (-38.1 to 26)	-16.2 (-41.3 to 21.7)
21 Anxiety disorders			
22 Neonatal disorders			
24 Nature disaster			
160 COVID-19			

Supplemental Results Figure S7. Leading 20 level 3 causes of DALYs, and percentage change in number of DALYs and all-age and age-standardised DALY rates by location, 2022 and 2050

Thailand, Reference scenario

Legend: Communicable, maternal, neonatal and nutritional; Non-communicable; Injuries

Leading Causes 2022	Leading Causes 2050	Mean % change number of DALY	Mean % change all-age DALY rate	Mean % change age-standardized DALY rate
1 Stroke	1 Diabetes	**57.4 (25.4 to 99.1)**	**82.9 (58.8 to 111)**	13.3 (-0.459 to 27.3)
2 Road injuries	2 Stroke	-11.2 (-30.5 to 12.4)	3.25 (-16.8 to 25.3)	**-42.4 (-53.1 to -30.1)**
3 Diabetes	3 Alzheimer's disease	**167 (111 to 239)**	**210 (163 to 261)**	-3.49 (-7.35 to 0.838)
4 Ischemic heart disease	4 Chronic kidney disease	**57.9 (15.1 to 117)**	**83.7 (38 to 142)**	0.667 (-24.2 to 32)
5 Lower respiratory infect	5 Lower respiratory infect	24.2 (-0.242 to 54.5)	**44.4 (23.4 to 72.7)**	**-41.3 (-48.3 to -32.6)**
6 Chronic kidney disease	6 Age-related hearing loss	**40.7 (17.5 to 68.1)**	**63.6 (51.6 to 75.8)**	**-0.907 (-0.978 to -0.83)**
7 Cirrhosis liver	7 COPD	**71.3 (30.7 to 125)**	**99.2 (55.8 to 151)**	-12.7 (-30.4 to 9.6)
8 HIV/AIDS	8 Ischemic heart disease	-25.3 (-48.9 to 4.4)	-13.1 (-36.6 to 18.6)	**-55.9 (-67.7 to -39.3)**
9 Lung cancer	9 Other musculoskeletal	**28.6 (8.96 to 52.7)**	**49.6 (40.5 to 59.1)**	**36.1 (32.5 to 39.3)**
10 Age-related hearing loss	10 Lung cancer	21 (-10.1 to 59.1)	**40.5 (13.4 to 69.4)**	**-15.9 (-30.3 to -0.339)**
11 Other musculoskeletal	11 Road injuries	**-37.5 (-55.6 to -11.7)**	**-27.2 (-45.2 to -2.32)**	-17.5 (-40.8 to 13.4)
12 Low back pain	12 Cirrhosis liver	-6.52 (-23.4 to 14.2)	8.72 (-6.78 to 23.5)	-3.8 (-18.6 to 9.95)
13 COVID-19	13 Low back pain	6.41 (-10.4 to 27.4)	**23.7 (16.7 to 32.6)**	**-3.73 (-4.04 to -3.36)**
14 Headache disorders	14 Colorectal cancer	30.3 (-7.46 to 74.9)	**51.3 (14.8 to 92.2)**	-5.64 (-27.6 to 20.4)
15 COPD	15 Urinary diseases	**115 (66.1 to 178)**	**150 (105 to 211)**	9.65 (-3.15 to 26.8)
16 Alzheimer's disease	16 Blindness and vision loss	**52.5 (26.2 to 82.3)**	**77.3 (62.1 to 91.6)**	**-4.93 (-5.27 to -4.62)**
17 Colorectal cancer	17 Liver cancer	**45.7 (7.6 to 95.7)**	**69.5 (29.8 to 124)**	24 (-5.32 to 64.7)
18 Depressive disorders	18 Falls	19.2 (-4.3 to 45.1)	**38.6 (21.4 to 58.8)**	**-13.5 (-22.8 to -2.69)**
19 Self-harm	19 Headache disorders	**-21.3 (-34.1 to -9.42)**	**-8.53 (-12.5 to -0.229)**	0.0186 (-0.247 to 0.398)
20 Falls	20 Depressive disorders	-8.48 (-23.3 to 7.24)	**6.43 (1.24 to 12.2)**	**-1.7 (-1.89 to -1.51)**
21 Liver cancer	27 Self-harm			
22 Blindness and vision loss	59 HIV/AIDS			
28 Urinary diseases	164 COVID-19			

Supplemental Results Figure S7. Leading 20 level 3 causes of DALYs, and percentage change in number of DALYs and all-age and age-standardised DALY rates by location, 2022 and 2050

Timor-Leste, Reference scenario

Legend:
Communicable, maternal, neonatal and nutritional
Non-communicable
Injuries

Leading Causes 2022	Leading Causes 2050	Mean % change number of DALY	Mean % change all-age DALY rate	Mean % change age-standardized DALY rate
1 Neonatal disorders	1 Stroke	18.3 (-5.79 to 51)	-15.5 (-32.3 to 6.33)	**-41.2 (-53.2 to -24.2)**
2 Lower respiratory infect	2 Ischemic heart disease	18.4 (-20.8 to 80.2)	-15.4 (-44.4 to 27)	**-41.9 (-63.5 to -11.2)**
3 Stroke	3 Neonatal disorders	**-53 (-67.4 to -34.7)**	**-66.6 (-74.6 to -56.9)**	**-49.1 (-60.3 to -32.7)**
4 Ischemic heart disease	4 Diabetes	**95.3 (64.4 to 137)**	**39.3 (18.7 to 63.9)**	-2.81 (-14.4 to 12.4)
5 Tuberculosis	5 Conflict & terror	168 (-70 to 493)	90.7 (-78.7 to 358)	29.1 (-79.6 to 207)
6 Congenital defects	6 Other musculoskeletal	**232 (182 to 289)**	**137 (109 to 161)**	**82.9 (70.6 to 90.2)**
7 Diarrheal diseases	7 Chronic kidney disease	**134 (74.6 to 216)**	**66.9 (22 to 128)**	24 (-8.6 to 76)
8 Road injuries	8 Lower respiratory infect	**-54.4 (-65.3 to -42.4)**	**-67.5 (-73.9 to -59.7)**	**-60.6 (-68.3 to -51.6)**
9 Diabetes	9 COPD	**61.7 (23.1 to 118)**	15.5 (-13.8 to 52.6)	-22.1 (-42 to 3.86)
10 Conflict & terror	10 Headache disorders	**59.7 (35.1 to 89.7)**	**13.7 (7.4 to 26.2)**	**0.684 (0.381 to 0.916)**
11 COPD	11 Age-related hearing loss	**80.8 (51.7 to 109)**	**28.9 (17.4 to 42.7)**	**-1.59 (-1.74 to -1.45)**
12 Headache disorders	12 Congenital defects	**-42.9 (-59.1 to -24.8)**	**-59.6 (-67.4 to -50.8)**	**-38.7 (-46 to -29.9)**
13 COVID-19	13 Tuberculosis	**-47.7 (-66.1 to -20.1)**	**-62.7 (-75.3 to -43)**	**-69.5 (-80.1 to -52.2)**
14 HIV/AIDS	14 Low back pain	**72.5 (49.5 to 98.6)**	**23 (13.2 to 34.8)**	**-5.62 (-5.95 to -5.24)**
15 Chronic kidney disease	15 Road injuries	4.58 (-35.1 to 61.7)	-25.5 (-51.7 to 17.6)	-28.9 (-53.9 to 13.1)
16 Low back pain	16 Hypertensive heart disease	**87.3 (31.1 to 165)**	33.8 (-12.1 to 91.9)	-10.6 (-40.8 to 37.7)
17 Age-related hearing loss	17 Cirrhosis liver	**45.9 (19.1 to 82.2)**	4.12 (-15 to 26.8)	**-25 (-36.4 to -8.44)**
18 Cirrhosis liver	18 Anxiety disorders	**57.9 (32.5 to 85.3)**	**12.4 (5.18 to 21.1)**	**1.47 (1.16 to 1.74)**
19 Drowning	19 Depressive disorders	**62.5 (36.9 to 89)**	**15.7 (9.14 to 24.2)**	**-2.52 (-2.8 to -2.2)**
20 Anxiety disorders	20 Blindness and vision loss	**78 (52 to 108)**	**27 (12.4 to 42.7)**	**-8.89 (-9.42 to -8.28)**
22 Hypertensive heart disease	32 Diarrheal diseases			
25 Depressive disorders	40 Drowning			
26 Other musculoskeletal	75 HIV/AIDS			
29 Blindness and vision loss	161 COVID-19			

Supplemental Results Figure S7. Leading 20 level 3 causes of DALYs, and percentage change in number of DALYs and all-age and age-standardised DALY rates by location, 2022 and 2050

Viet Nam, Reference scenario

Legend:
Communicable, maternal, neonatal and nutritional
Non-communicable
Injuries

Leading Causes 2022	Leading Causes 2050	Mean % change number of DALY	Mean % change all-age DALY rate	Mean % change age-standardized DALY rate
1 Stroke	1 Stroke	**40.7 (13.5 to 65.3)**	**26.8 (4.32 to 51)**	**-30.2 (-43 to -16.4)**
2 Ischemic heart disease	2 Diabetes	**81.7 (51.5 to 114)**	**63.7 (39.7 to 87.2)**	-6.72 (-19.9 to 8.32)
3 Diabetes	3 Cirrhosis liver	**138 (97.7 to 182)**	**114 (81.2 to 147)**	**62.4 (38.9 to 87.3)**
4 Road injuries	4 Ischemic heart disease	12.5 (-20.4 to 50.7)	1.33 (-27.4 to 35.9)	**-45 (-61.2 to -22)**
5 Neonatal disorders	5 COPD	**93.9 (53 to 145)**	**74.7 (41.1 to 121)**	-14 (-32.4 to 13.9)
6 COVID-19	6 Chronic kidney disease	**141 (80.6 to 207)**	**117 (67.3 to 180)**	31.3 (-0.954 to 77.6)
7 COPD	7 Road injuries	14 (-18.2 to 58.5)	2.76 (-24.5 to 44.3)	-3.59 (-31.1 to 35.6)
8 Cirrhosis liver	8 Liver cancer	**193 (107 to 309)**	**164 (88.1 to 279)**	**84.3 (34.9 to 164)**
9 Low back pain	9 Alzheimer's disease	**189 (139 to 245)**	**160 (118 to 207)**	**4.72 (1.43 to 8.01)**
10 Headache disorders	10 Falls	**83.8 (48.3 to 121)**	**65.5 (40.7 to 93.7)**	-0.00195 (-9.08 to 8.98)
11 Lower respiratory infect	11 Age-related hearing loss	**65.7 (49.6 to 85.1)**	**49.2 (38.8 to 60.8)**	**-1.08 (-1.15 to -1)**
12 Age-related hearing loss	12 Lung cancer	**63 (23.2 to 105)**	**46.7 (13.1 to 82.5)**	-4.55 (-23.6 to 15.5)
13 Lung cancer	13 Hypertensive heart disease	**153 (76.1 to 240)**	**128 (64.2 to 206)**	18.7 (-19 to 68.1)
14 Chronic kidney disease	14 Other musculoskeletal	**92.4 (74.3 to 114)**	**73.2 (62.8 to 84.3)**	**54.5 (51.6 to 56.8)**
15 Falls	15 Low back pain	**34.6 (21.1 to 48.8)**	**21.2 (14.8 to 26.9)**	**-4.48 (-4.81 to -4.14)**
16 Tuberculosis	16 Colorectal cancer	**137 (65.4 to 210)**	**113 (53.7 to 178)**	**35.3 (1.39 to 71.6)**
17 Other musculoskeletal	17 Headache disorders	**12.2 (2.14 to 25.1)**	1.05 (-2.26 to 8.91)	**0.4 (0.227 to 0.578)**
18 Drowning	18 Lower respiratory infect	0.306 (-16.2 to 18.1)	-9.6 (-22.9 to 4.59)	**-47.5 (-55.1 to -38.6)**
19 Liver cancer	19 Neonatal disorders	**-39.9 (-50.5 to -27.6)**	**-45.9 (-55.4 to -34.4)**	**-36.3 (-47.6 to -22.1)**
20 Hypertensive heart disease	20 Blindness and vision loss	**84.4 (63.8 to 107)**	**66 (51.6 to 80.6)**	**-6.22 (-6.55 to -5.87)**
22 Alzheimer's disease	27 Tuberculosis			
24 Colorectal cancer	30 Drowning			
26 Blindness and vision loss	166 COVID-19			

Supplemental Results Figure S7. Leading 20 level 3 causes of DALYs, and percentage change in number of DALYs and all-age and age-standardised DALY rates by location, 2022 and 2050

Angola, Reference scenario

Legend:
Communicable, maternal, neonatal and nutritional
Non-communicable
Injuries

Leading Causes 2022	Leading Causes 2050	Mean % change number of DALY	Mean % change all-age DALY rate	Mean % change age-standardized DALY rate
1 Neonatal disorders	1 Neonatal disorders	-23.5 (-44.3 to 3.86)	**-62.2 (-70.5 to -53.3)**	**-42.1 (-53.3 to -29)**
2 HIV/AIDS	2 HIV/AIDS	-0.962 (-38.7 to 68.9)	**-51 (-69.3 to -16.9)**	**-57.1 (-72.7 to -30.4)**
3 Malaria	3 Road injuries	24 (-39.5 to 143)	-38.6 (-70.3 to 15.6)	-41.3 (-70.1 to 11.9)
4 Lower respiratory infect	4 Lower respiratory infect	3.18 (-20.5 to 29.9)	**-48.9 (-58.9 to -36.5)**	**-39.1 (-48.1 to -26.3)**
5 Road injuries	5 Diabetes	**209 (149 to 273)**	**53 (26.3 to 79.2)**	-2.26 (-17.3 to 12.3)
6 Tuberculosis	6 Depressive disorders	**145 (116 to 174)**	**21.1 (14.5 to 29.1)**	**-4.26 (-4.51 to -4)**
7 Diarrheal diseases	7 Stroke	**75.5 (35.3 to 118)**	-13 (-31.1 to 5.8)	**-46.3 (-55.8 to -34.6)**
8 Congenital defects	8 Malaria	-37.7 (-89.7 to 195)	-68.7 (-94.8 to 55.5)	-63.4 (-93.8 to 90.1)
9 Pertussis	9 Cirrhosis liver	**93.9 (56.3 to 135)**	-3.92 (-22.5 to 15.2)	**-28.6 (-40.4 to -13.3)**
10 Stroke	10 Ischemic heart disease	**53.7 (7.65 to 113)**	-23.8 (-47.9 to 6.08)	**-54 (-68.3 to -34.1)**
11 Ischemic heart disease	11 Diarrheal diseases	-18.8 (-48.1 to 20.3)	**-59.8 (-72.5 to -42.7)**	**-54.2 (-67.1 to -34.9)**
12 COVID-19	12 Congenital defects	-13.4 (-34.2 to 10.5)	**-57.2 (-64.3 to -47.6)**	**-37.3 (-46.4 to -25.3)**
13 Depressive disorders	13 Low back pain	**147 (118 to 178)**	**22.3 (14.3 to 30.5)**	**-7.81 (-8.01 to -7.57)**
14 Cirrhosis liver	14 Other musculoskeletal	**498 (422 to 582)**	**196 (164 to 223)**	**117 (100 to 127)**
15 Diabetes	15 Hypertensive heart disease	**219 (142 to 311)**	**58.3 (21.4 to 102)**	-10.7 (-30.8 to 15.1)
16 Meningitis	16 Chronic kidney disease	**171 (101 to 255)**	34.3 (-2.11 to 72.7)	-1.11 (-24.8 to 30.7)
17 Protein-energy malnutrition	17 Tuberculosis	**-35.1 (-58.2 to -1.11)**	**-67.8 (-78.7 to -50.9)**	**-72.2 (-80.7 to -59.1)**
18 Low back pain	18 Headache disorders	**141 (107 to 173)**	**19.1 (13.4 to 30)**	**0.488 (0.222 to 0.765)**
19 Drowning	19 Pertussis	-16.7 (-52.7 to 34.5)	**-59 (-75.7 to -33.3)**	-39 (-62.7 to 2.29)
20 STIs	20 Anxiety disorders	**133 (102 to 167)**	**15.4 (9.21 to 22.3)**	**1.63 (1.32 to 1.88)**
21 Chronic kidney disease	29 Meningitis			
22 Headache disorders	38 Drowning			
26 Anxiety disorders	47 STIs			
27 Hypertensive heart disease	53 Protein-energy malnutrition			
41 Other musculoskeletal	168 COVID-19			

Supplemental Results Figure S7. Leading 20 level 3 causes of DALYs, and percentage change in number of DALYs and all-age and age-standardised DALY rates by location, 2022 and 2050

Central African Republic, Reference scenario

Legend:
Communicable, maternal, neonatal and nutritional
Non-communicable
Injuries

Leading Causes 2022	Leading Causes 2050	Mean % change number of DALY	Mean % change all-age DALY rate	Mean % change age-standardized DALY rate
1 Neonatal disorders	1 Neonatal disorders	**-57 (-71.9 to -39.8)**	**-66.2 (-74.8 to -55.1)**	**-42.7 (-55.9 to -26.7)**
2 Malaria	2 Malaria	-41.4 (-92 to 214)	-53 (-92.9 to 179)	-40 (-91.2 to 282)
3 Tuberculosis	3 Lower respiratory infect	**-32.5 (-53.5 to -10.5)**	**-46.7 (-56.8 to -34.3)**	**-33.7 (-45.1 to -20.5)**
4 Lower respiratory infect	4 Diarrheal diseases	**-34.6 (-60.1 to -1.77)**	**-48.3 (-66.8 to -24.1)**	-30.9 (-54 to 0.0975)
5 Diarrheal diseases	5 Road injuries	-21.7 (-65.3 to 45.5)	-37.9 (-69.6 to 16.3)	-39.3 (-69.1 to 11.2)
6 COVID-19	6 Tuberculosis	**-54 (-72.8 to -27.1)**	**-63.6 (-77.4 to -41.4)**	**-67.5 (-79.5 to -49.7)**
7 HIV/AIDS	7 Diabetes	**132 (67.4 to 205)**	**83 (54.1 to 116)**	**20.9 (2.34 to 41.5)**
8 Road injuries	8 Stroke	13.1 (-19.5 to 52.9)	-10.7 (-29.6 to 9.87)	**-42.4 (-55 to -27.6)**
9 Congenital defects	9 Ischemic heart disease	40.1 (-13.5 to 109)	10.9 (-26.5 to 57.3)	-28.5 (-54.1 to 8.19)
10 Stroke	10 Cirrhosis liver	2.67 (-26.5 to 31.8)	**-19.1 (-33.2 to -4.2)**	**-39.3 (-49.5 to -27.6)**
11 Protein-energy malnutrition	11 Depressive disorders	**49.2 (4.07 to 91.2)**	**17.4 (11.7 to 24.5)**	**-3.72 (-4.12 to -3.32)**
12 Pertussis	12 Congenital defects	**-50 (-65.2 to -30.8)**	**-60.7 (-67.6 to -51.9)**	**-38.1 (-47.1 to -25.9)**
13 Ischemic heart disease	13 Chronic kidney disease	**63.5 (8.87 to 131)**	29.2 (-4.38 to 72)	-2.97 (-29.3 to 29.2)
14 Cirrhosis liver	14 Hypertensive heart disease	**101 (39.1 to 177)**	**59.2 (19.3 to 105)**	-11.4 (-34.2 to 17.6)
15 Meningitis	15 Low back pain	**53.4 (8.16 to 99.6)**	**20.8 (13 to 30)**	**-7.01 (-7.42 to -6.28)**
16 Diabetes	16 Pertussis	-41.8 (-69.3 to 2.93)	**-54.4 (-74.8 to -21.1)**	-29 (-60.2 to 23.8)
17 Maternal disorders	17 COPD	**52 (6.82 to 100)**	20.1 (-5.09 to 48.9)	**-27.7 (-43.5 to -6.77)**
18 Drowning	18 Other musculoskeletal	**262 (154 to 371)**	**185 (156 to 214)**	**112 (103 to 121)**
19 Conflict & terror	19 Self-harm	26.4 (-11.5 to 69.3)	-0.331 (-21.9 to 23.1)	**-21 (-36.7 to -3.33)**
20 Depressive disorders	20 Headache disorders	**47.9 (5.15 to 87.8)**	**16.3 (11.3 to 24.7)**	**1.22 (0.674 to 1.88)**
23 Chronic kidney disease	21 Meningitis			
25 Low back pain	25 Protein-energy malnutrition			
27 Self-harm	27 Maternal disorders			
29 Hypertensive heart disease	28 HIV/AIDS			
30 COPD	30 Drowning			
31 Headache disorders	32 Conflict & terror			
48 Other musculoskeletal	170 COVID-19			

Supplemental Results Figure S7. Leading 20 level 3 causes of DALYs, and percentage change in number of DALYs and all-age and age-standardised DALY rates by location, 2022 and 2050

Congo (Brazzaville), Reference scenario

Legend:
Communicable, maternal, neonatal and nutritional
Non-communicable
Injuries

Leading Causes 2022	Leading Causes 2050	Mean % change number of DALY	Mean % change all-age DALY rate	Mean % change age-standardized DALY rate
1 HIV/AIDS	1 Diabetes	**146 (93.6 to 208)**	**72.8 (43.2 to 105)**	0.54 (-12.7 to 16.9)
2 Neonatal disorders	2 Stroke	**62 (26 to 105)**	13.7 (-5.27 to 38.2)	**-39.9 (-50 to -26.4)**
3 Malaria	3 Road injuries	15.5 (-39 to 97.9)	-18.8 (-56.9 to 49.2)	-23 (-59.4 to 43.1)
4 Road injuries	4 Ischemic heart disease	**49.5 (1.73 to 104)**	4.95 (-26.1 to 49.5)	**-44.1 (-61.6 to -19)**
5 Ischemic heart disease	5 Neonatal disorders	**-28.2 (-46.3 to -5.26)**	**-49.7 (-58.8 to -36.8)**	**-25.9 (-37.9 to -8.49)**
6 Lower respiratory infect	6 Lower respiratory infect	**46.7 (17.1 to 83.3)**	2.94 (-13.2 to 22.6)	**-23.3 (-34.2 to -10.4)**
7 Stroke	7 Hypertensive heart disease	**219 (141 to 316)**	**124 (74.8 to 187)**	10.6 (-13.9 to 42.2)
8 Tuberculosis	8 Chronic kidney disease	**148 (83.1 to 230)**	**74.3 (36.6 to 126)**	13.2 (-13.6 to 46.7)
9 Diarrheal diseases	9 Depressive disorders	**69.2 (40.8 to 100)**	**18.6 (12.5 to 25.6)**	**-2.42 (-2.64 to -2.18)**
10 Diabetes	10 Cirrhosis liver	**64.9 (33.1 to 103)**	15.7 (-2.29 to 37.9)	**-19.5 (-30.7 to -5)**
11 Cirrhosis liver	11 Diarrheal diseases	-3.4 (-36.1 to 36.1)	**-32.2 (-55.9 to -3)**	**-35.3 (-54.3 to -9.17)**
12 Depressive disorders	12 Low back pain	**84.2 (52.3 to 117)**	**29.1 (20.3 to 37.1)**	**-4.67 (-4.88 to -4.43)**
13 COVID-19	13 HIV/AIDS	**-74.7 (-83.8 to -60.3)**	**-82.3 (-88.1 to -72.9)**	**-84.1 (-89.2 to -75.7)**
14 Congenital defects	14 Tuberculosis	-29 (-51.3 to 0.881)	**-50.1 (-65.7 to -26.9)**	**-63 (-74.8 to -45.5)**
15 Chronic kidney disease	15 COPD	**157 (99.6 to 227)**	**80.6 (48.1 to 121)**	-6.41 (-23.4 to 17.3)
16 Low back pain	16 Other musculoskeletal	**212 (159 to 262)**	**119 (100 to 136)**	**60.9 (55.1 to 64.8)**
17 Hypertensive heart disease	17 Malaria	-66 (-92 to 85.4)	-76 (-94.5 to 42.7)	-74.5 (-94.3 to 65.7)
18 Dietary iron deficiency	18 Headache disorders	**60.3 (31.9 to 90)**	**12.3 (6.97 to 23.3)**	**0.587 (0.357 to 0.797)**
19 Headache disorders	19 Age-related hearing loss	**109 (71.2 to 144)**	**46.7 (33.6 to 59.5)**	**-1.22 (-1.42 to -0.998)**
20 Maternal disorders	20 Breast cancer	**125 (59 to 194)**	**57.6 (19.8 to 104)**	5.21 (-16.6 to 27.6)
26 COPD	24 Dietary iron deficiency			
30 Age-related hearing loss	25 Congenital defects			
34 Breast cancer	46 Maternal disorders			
36 Other musculoskeletal	168 COVID-19			

Supplemental Results Figure S7. Leading 20 level 3 causes of DALYs, and percentage change in number of DALYs and all-age and age-standardised DALY rates by location, 2022 and 2050

DR Congo, Reference scenario

Legend:
Communicable, maternal, neonatal and nutritional
Non-communicable
Injuries

Leading Causes 2022	Leading Causes 2050	Mean % change number of DALY	Mean % change all-age DALY rate	Mean % change age-standardized DALY rate
1 Malaria	1 Neonatal disorders	**-33.7 (-57.8 to -6.76)**	**-62.4 (-70.4 to -51.3)**	**-43.4 (-54.5 to -29.3)**
2 Neonatal disorders	2 Malaria	-55.4 (-94.4 to 240)	-75.2 (-96.6 to 91)	-66 (-95.4 to 150)
3 Lower respiratory infect	3 Road injuries	19.3 (-38.1 to 119)	-31.9 (-64.8 to 28.6)	-35.1 (-65 to 23.2)
4 Road injuries	4 Lower respiratory infect	7.05 (-27.2 to 43.8)	**-39.2 (-53.7 to -24.5)**	**-39.2 (-49.4 to -29.1)**
5 Tuberculosis	5 Diabetes	**198 (104 to 313)**	**69.3 (36.9 to 106)**	4.56 (-11.8 to 22.4)
6 Diarrheal diseases	6 Stroke	**57.6 (10.4 to 114)**	-10.3 (-28.1 to 11.1)	**-47 (-56.9 to -34.3)**
7 Congenital defects	7 Depressive disorders	**113 (51.3 to 177)**	**20.8 (14.5 to 28.8)**	**-2.9 (-3.07 to -2.68)**
8 Stroke	8 Tuberculosis	-26.5 (-52.6 to 11)	**-58.1 (-72.6 to -34.7)**	**-66.8 (-77.4 to -48.8)**
9 COVID-19	9 Ischemic heart disease	**70.1 (9.03 to 157)**	-3.03 (-34.3 to 48)	**-43.6 (-62.1 to -14.9)**
10 Ischemic heart disease	10 Cirrhosis liver	**81.3 (28.3 to 145)**	3.22 (-15.7 to 24.8)	**-27.7 (-38.6 to -15.9)**
11 Depressive disorders	11 Hypertensive heart disease	**220 (112 to 340)**	**81.9 (34.4 to 143)**	-3.47 (-26 to 28.5)
12 Cirrhosis liver	12 Low back pain	**124 (60.2 to 195)**	**27.2 (18 to 36.9)**	**-5.95 (-6.15 to -5.77)**
13 Maternal disorders	13 Chronic kidney disease	**143 (67.5 to 242)**	**38.3 (1.73 to 88.8)**	-4.14 (-27.7 to 30.2)
14 Diabetes	14 Diarrheal diseases	-0.257 (-35.6 to 56.9)	**-43.2 (-62.4 to -11.1)**	**-37.4 (-54.9 to -11.4)**
15 Measles	15 Other musculoskeletal	**347 (219 to 489)**	**154 (132 to 175)**	**82.6 (75.8 to 87.2)**
16 Onchocerciasis	16 Congenital defects	-17 (-45.9 to 14.8)	**-52.9 (-60.6 to -43.9)**	**-32.4 (-41.9 to -20.9)**
17 Pertussis	17 Headache disorders	**106 (46.1 to 170)**	**17.1 (10.7 to 28.2)**	**0.809 (0.456 to 1.08)**
18 Low back pain	18 COPD	**122 (55.3 to 211)**	26.6 (-1.66 to 59.1)	**-27.5 (-42.6 to -9.4)**
19 Meningitis	19 Onchocerciasis	12.8 (-19 to 46.7)	**-36 (-39.2 to -31.8)**	**-46 (-46.8 to -44.7)**
20 STIs	20 Anxiety disorders	**98.1 (38.8 to 153)**	**12.4 (5.58 to 19.5)**	**1.34 (1.12 to 1.63)**
21 Chronic kidney disease	26 Pertussis			
23 Headache disorders	28 Maternal disorders			
25 Hypertensive heart disease	33 Meningitis			
27 COPD	44 STIs			
29 Anxiety disorders	86 Measles			
40 Other musculoskeletal	169 COVID-19			

Supplemental Results Figure S7. Leading 20 level 3 causes of DALYs, and percentage change in number of DALYs and all-age and age-standardised DALY rates by location, 2022 and 2050

Equatorial Guinea, Reference scenario

Legend:
Communicable, maternal, neonatal and nutritional
Non-communicable
Injuries

Leading Causes 2022	Leading Causes 2050	Mean % change number of DALY	Mean % change all-age DALY rate	Mean % change age-standardized DALY rate
1 HIV/AIDS	1 HIV/AIDS	**-38.9 (-57.9 to -5.21)**	**-70 (-79.5 to -56.1)**	**-75.2 (-82 to -63.5)**
2 Malaria	2 Neonatal disorders	26.6 (-6.67 to 66.4)	**-38.1 (-49.5 to -21)**	-14.1 (-28.8 to 8.02)
3 Neonatal disorders	3 Diabetes	**349 (250 to 441)**	**120 (78.5 to 164)**	7.22 (-10.4 to 26.4)
4 Road injuries	4 Road injuries	59.3 (-13.4 to 210)	-21.7 (-58.8 to 55.6)	-24.3 (-59.2 to 51.1)
5 Lower respiratory infect	5 Malaria	-23.9 (-90.3 to 160)	-63.1 (-95.2 to 27.2)	-58.6 (-94.9 to 62.8)
6 Depressive disorders	6 Lower respiratory infect	**109 (61.4 to 155)**	2.32 (-19.1 to 23.9)	**-21.5 (-33.8 to -6.73)**
7 Ischemic heart disease	7 Depressive disorders	**153 (121 to 182)**	**23.9 (14.6 to 33.4)**	**-2.5 (-3.04 to -1.98)**
8 Diabetes	8 Chronic kidney disease	**252 (163 to 363)**	**72.6 (29.3 to 123)**	7.03 (-18.5 to 41.9)
9 Congenital defects	9 Ischemic heart disease	**118 (50.8 to 204)**	6.85 (-24.8 to 51.2)	**-50.9 (-65.8 to -31.3)**
10 COVID-19	10 Stroke	**155 (102 to 218)**	**25.1 (2.13 to 53.9)**	**-40.6 (-50.9 to -24.4)**
11 Stroke	11 Cirrhosis liver	**258 (196 to 326)**	**75.8 (42.1 to 111)**	0.879 (-14.3 to 20.7)
12 Chronic kidney disease	12 Low back pain	**188 (150 to 223)**	**41.2 (27.3 to 53.4)**	**-5.2 (-5.64 to -4.77)**
13 Diarrheal diseases	13 Hypertensive heart disease	**417 (292 to 556)**	**154 (95.9 to 219)**	13.9 (-10.6 to 44.6)
14 Tuberculosis	14 Other musculoskeletal	**384 (321 to 443)**	**137 (118 to 159)**	**56.7 (53.3 to 60)**
15 Low back pain	15 Headache disorders	**139 (108 to 177)**	**17 (8.15 to 35.7)**	0.32 (-0.453 to 0.927)
16 Mechanical forces	16 Gynecological diseases	**133 (90.6 to 180)**	14.2 (-5.97 to 36.4)	**-17.8 (-32.2 to -0.51)**
17 Headache disorders	17 Self-harm	**162 (106 to 231)**	**28.3 (4.06 to 60.1)**	-2.82 (-21.3 to 22.9)
18 STIs	18 Anxiety disorders	**125 (93 to 157)**	**10.1 (1.32 to 21.8)**	**1.35 (0.609 to 2.06)**
19 Cirrhosis liver	19 Age-related hearing loss	**206 (161 to 248)**	**50.2 (34 to 65)**	**-1.18 (-1.45 to -0.885)**
20 Pertussis	20 Idiopathic epilepsy	**137 (76.4 to 217)**	16.4 (-12.7 to 61)	13.3 (-14.6 to 54.9)
21 Anxiety disorders	21 Mechanical forces			
22 Gynecological diseases	22 Congenital defects			
25 Idiopathic epilepsy	25 Tuberculosis			
26 Self-harm	30 Diarrheal diseases			
30 Age-related hearing loss	46 Pertussis			
31 Hypertensive heart disease	50 STIs			
32 Other musculoskeletal	166 COVID-19			

Supplemental Results Figure S7. Leading 20 level 3 causes of DALYs, and percentage change in number of DALYs and all-age and age-standardised DALY rates by location, 2022 and 2050

Gabon, Reference scenario

Legend:
Communicable, maternal, neonatal and nutritional
Non-communicable
Injuries

Leading Causes 2022	Leading Causes 2050	Mean % change number of DALY	Mean % change all-age DALY rate	Mean % change age-standardized DALY rate
1 Neonatal disorders	1 Diabetes	**135 (93.2 to 179)**	**52.5 (27.9 to 78.2)**	-1.31 (-14 to 14)
2 HIV/AIDS	2 Neonatal disorders	-14.1 (-34.8 to 16.1)	**-44.4 (-54.6 to -32.1)**	**-19.7 (-32.1 to -4.29)**
3 Malaria	3 Road injuries	24.4 (-32.4 to 135)	-19.3 (-54.9 to 52.5)	-24.1 (-56.8 to 43.2)
4 Road injuries	4 Chronic kidney disease	**119 (66.1 to 181)**	**42.2 (10.8 to 83.3)**	-0.66 (-22.2 to 27.8)
5 Lower respiratory infect	5 Lower respiratory infect	**41.4 (15.3 to 68.1)**	-8.09 (-23.1 to 11.8)	**-25 (-35.6 to -12.6)**
6 Diabetes	6 Stroke	**46.3 (19.4 to 76)**	-4.91 (-20.3 to 13.3)	**-42.2 (-51 to -29.9)**
7 Ischemic heart disease	7 Depressive disorders	**80.1 (59.5 to 102)**	**17 (9.67 to 25.2)**	**-2.63 (-3.01 to -2.3)**
8 Stroke	8 Hypertensive heart disease	**176 (113 to 254)**	**79.3 (42 to 127)**	4.63 (-18.1 to 33)
9 COVID-19	9 Ischemic heart disease	16.1 (-17 to 57.1)	-24.6 (-44.7 to 1.16)	**-53.5 (-67.1 to -36.5)**
10 Chronic kidney disease	10 Cirrhosis liver	**57.4 (32.4 to 88.5)**	2.35 (-14.8 to 20.6)	**-26.4 (-36.3 to -14.2)**
11 Depressive disorders	11 Low back pain	**91.5 (73.7 to 113)**	**24.4 (15.3 to 34.7)**	**-4.68 (-4.96 to -4.33)**
12 Tuberculosis	12 Other musculoskeletal	**239 (208 to 280)**	**120 (101 to 140)**	**67.5 (63.8 to 71.8)**
13 Cirrhosis liver	13 HIV/AIDS	**-68.9 (-75.3 to -61.3)**	**-79.8 (-84.9 to -74.7)**	**-82.7 (-86.1 to -78.9)**
14 Congenital defects	14 Malaria	-63.5 (-90.6 to 107)	-76.6 (-94.1 to 41.6)	-74 (-94.1 to 55.6)
15 Dietary iron deficiency	15 Dietary iron deficiency	**29.5 (12.8 to 47.5)**	**-16 (-20.6 to -11.2)**	**-14.5 (-15.4 to -13.5)**
16 Low back pain	16 Headache disorders	**73.9 (54.3 to 98.7)**	**12.9 (6.7 to 23.2)**	**0.576 (0.269 to 0.909)**
17 Diarrheal diseases	17 COPD	**155 (102 to 213)**	**65.8 (36.1 to 104)**	2.1 (-17.2 to 25.7)
18 Hypertensive heart disease	18 Age-related hearing loss	**106 (83.1 to 130)**	**34.1 (22.7 to 48.5)**	**-1.66 (-1.88 to -1.44)**
19 Headache disorders	19 Gynecological diseases	**61.2 (29.9 to 93.6)**	4.75 (-12.9 to 27.3)	-14.4 (-29.2 to 3.19)
20 Gynecological diseases	20 Anxiety disorders	**68 (48.9 to 91.4)**	**9.02 (3.02 to 17.7)**	**1.18 (0.755 to 1.62)**
21 Anxiety disorders	23 Tuberculosis			
24 Age-related hearing loss	27 Congenital defects			
27 Other musculoskeletal	37 Diarrheal diseases			
28 COPD	166 COVID-19			

Supplemental Results Figure S7. Leading 20 level 3 causes of DALYs, and percentage change in number of DALYs and all-age and age-standardised DALY rates by location, 2022 and 2050

Burundi, Reference scenario

Legend:
Communicable, maternal, neonatal and nutritional
Non-communicable
Injuries

Leading Causes 2022	Leading Causes 2050	Mean % change number of DALY	Mean % change all-age DALY rate	Mean % change age-standardized DALY rate
1 Malaria	1 Neonatal disorders	**-31.6 (-49.2 to -10.8)**	**-62.9 (-70.2 to -54)**	**-42 (-51.4 to -30.3)**
2 Neonatal disorders	2 Diarrheal diseases	11.9 (-32.8 to 74.6)	**-39.2 (-61.7 to -7.82)**	**-30.6 (-53.4 to -1.57)**
3 COVID-19	3 Lower respiratory infect	-3.93 (-28.9 to 22.9)	**-47.8 (-58.9 to -34)**	**-39.6 (-49.5 to -28.1)**
4 Lower respiratory infect	4 Stroke	**85.2 (45 to 131)**	0.683 (-22 to 22.9)	**-36.5 (-49.3 to -21.1)**
5 Diarrheal diseases	5 Conflict & terror	134 (-88.2 to 772)	25.7 (-93.5 to 375)	0.394 (-92.6 to 254)
6 Tuberculosis	6 Ischemic heart disease	**119 (42.4 to 221)**	19.4 (-20.6 to 75.8)	-22.6 (-48.7 to 20.1)
7 Conflict & terror	7 Tuberculosis	-32.2 (-56.9 to 2.81)	**-63.1 (-76.8 to -43)**	**-70.7 (-80.7 to -56)**
8 Congenital defects	8 Depressive disorders	**128 (89.8 to 163)**	**23.9 (17.2 to 32)**	**-2.39 (-2.79 to -1.96)**
9 Stroke	9 Diabetes	**141 (86 to 198)**	**31 (4.54 to 57.6)**	-14.1 (-30.1 to 4.8)
10 Road injuries	10 Road injuries	32.8 (-24.6 to 141)	-27.8 (-58.9 to 23.6)	-37.1 (-62 to 3.03)
11 Ischemic heart disease	11 Congenital defects	**-21 (-38.9 to -1.4)**	**-57.2 (-64.2 to -48.6)**	**-36.5 (-45.1 to -26.8)**
12 Protein-energy malnutrition	12 Low back pain	**132 (94.6 to 167)**	**26 (17.3 to 34.7)**	**-5.63 (-5.94 to -5.34)**
13 Cirrhosis liver	13 Malaria	**-83.4 (-96.1 to -0.874)**	**-91 (-97.8 to -49.5)**	**-88 (-96.9 to -34.5)**
14 HIV/AIDS	14 Chronic kidney disease	**171 (97.4 to 259)**	**47.4 (10.9 to 97.4)**	6.43 (-20.3 to 44)
15 Depressive disorders	15 Cirrhosis liver	**47.5 (17.7 to 80.9)**	**-19.8 (-32.2 to -5.68)**	**-44.4 (-52.9 to -34.6)**
16 Maternal disorders	16 Age-related hearing loss	**156 (115 to 198)**	**38.8 (26.3 to 50.1)**	**-1.8 (-2.02 to -1.56)**
17 Meningitis	17 Anxiety disorders	**112 (77.3 to 145)**	**14.9 (9.18 to 21.9)**	**2.28 (1.89 to 2.67)**
18 Diabetes	18 COPD	**136 (81.7 to 203)**	**28.4 (0.371 to 68.5)**	**-24.3 (-39.5 to -0.903)**
19 Low back pain	19 Headache disorders	**119 (82.3 to 156)**	**18.8 (13 to 28.6)**	**1.74 (1.15 to 2.16)**
20 Dietary iron deficiency	20 Gynecological diseases	**84.3 (41.3 to 129)**	0.194 (-19.3 to 22.3)	-17.5 (-33.2 to 0.339)
23 Anxiety disorders	21 Dietary iron deficiency			
24 Chronic kidney disease	27 Meningitis			
25 Gynecological diseases	29 Maternal disorders			
26 Age-related hearing loss	39 Protein-energy malnutrition			
27 Headache disorders	77 HIV/AIDS			
29 COPD	167 COVID-19			

Supplemental Results Figure S7. Leading 20 level 3 causes of DALYs, and percentage change in number of DALYs and all-age and age-standardised DALY rates by location, 2022 and 2050

Comoros, Reference scenario

Legend: Communicable, maternal, neonatal and nutritional; Non-communicable; Injuries

Leading Causes 2022	Leading Causes 2050	Mean % change number of DALY	Mean % change all-age DALY rate	Mean % change age-standardized DALY rate
1 Neonatal disorders	1 Neonatal disorders	**-55.3 (-69 to -36.3)**	**-62 (-69.8 to -52.5)**	**-37.9 (-48.7 to -22)**
2 Lower respiratory infect	2 Diabetes	**131 (77.4 to 206)**	**98.4 (56.3 to 145)**	12.2 (-6.89 to 33.3)
3 Stroke	3 Stroke	28.6 (-5.49 to 77.7)	10.2 (-13.2 to 37.8)	**-42.3 (-54.1 to -26.3)**
4 Tuberculosis	4 Lower respiratory infect	**-30.8 (-49.1 to -7.22)**	**-40.7 (-54.2 to -24)**	**-50.9 (-60.3 to -37.5)**
5 Road injuries	5 Chronic kidney disease	**127 (60.1 to 221)**	**94.5 (46 to 153)**	14.4 (-15.2 to 54.7)
6 Diarrheal diseases	6 Ischemic heart disease	28.6 (-20 to 102)	10.1 (-27 to 62.8)	**-41.5 (-61 to -11.9)**
7 Congenital defects	7 Hypertensive heart disease	**127 (62.5 to 218)**	**94.7 (44 to 151)**	-4.22 (-28.3 to 26.1)
8 Ischemic heart disease	8 Road injuries	-7.19 (-47.9 to 58.2)	-20.7 (-51.9 to 31.8)	-30 (-59.5 to 20.1)
9 Diabetes	9 Low back pain	**53.8 (21.8 to 87.1)**	**31.8 (20.3 to 43.5)**	**-6.04 (-6.25 to -5.86)**
10 Cirrhosis liver	10 Depressive disorders	**45.6 (15.3 to 80)**	**24.8 (15.4 to 36.9)**	**-3.02 (-3.24 to -2.78)**
11 Low back pain	11 Age-related hearing loss	**84.2 (46 to 125)**	**58 (41 to 75.8)**	**-1.54 (-1.76 to -1.32)**
12 Depressive disorders	12 Cirrhosis liver	23.4 (-5.97 to 68)	5.72 (-15.4 to 29.4)	**-31.6 (-43.2 to -17.5)**
13 Chronic kidney disease	13 Other musculoskeletal	**179 (119 to 242)**	**139 (117 to 167)**	**74.9 (69.9 to 78.8)**
14 Meningitis	14 Tuberculosis	**-40.5 (-62.1 to -11.4)**	**-49.1 (-65.4 to -25.6)**	**-65.2 (-76.7 to -47.7)**
15 Interpersonal violence	15 Diarrheal diseases	-27.9 (-54.9 to 7.45)	**-38.2 (-58.7 to -10.1)**	**-44.5 (-63 to -20.4)**
16 Malaria	16 COPD	**76.7 (28.5 to 146)**	**51.5 (16.5 to 96.2)**	-23.3 (-40.2 to 0.439)
17 COVID-19	17 Alzheimer's disease	**181 (98.8 to 259)**	**141 (94.7 to 191)**	-1.79 (-5.59 to 1.65)
18 Age-related hearing loss	18 Congenital defects	**-51.2 (-67.2 to -28.8)**	**-58.4 (-67.4 to -48.4)**	**-33.8 (-43.8 to -18.8)**
19 Hypertensive heart disease	19 Breast cancer	**80.4 (28.1 to 137)**	**54.9 (17 to 98.8)**	-1.86 (-21.8 to 20.7)
20 Dietary iron deficiency	20 Interpersonal violence	-15.3 (-37.2 to 12)	**-27.5 (-40.7 to -11.8)**	**-32.5 (-44.4 to -18.7)**
25 COPD	30 Dietary iron deficiency			
30 Other musculoskeletal	34 Meningitis			
32 Breast cancer	67 Malaria			
44 Alzheimer's disease	163 COVID-19			

Supplemental Results Figure S7. Leading 20 level 3 causes of DALYs, and percentage change in number of DALYs and all-age and age-standardised DALY rates by location, 2022 and 2050

Djibouti, Reference scenario

Legend:
Communicable, maternal, neonatal and nutritional
Non-communicable
Injuries

Leading Causes 2022	Leading Causes 2050	Mean % change number of DALY	Mean % change all-age DALY rate	Mean % change age-standardized DALY rate
1 HIV/AIDS	1 Stroke	**103 (50.7 to 168)**	**31.8 (6.55 to 71.4)**	**-40 (-51.1 to -22.5)**
2 Neonatal disorders	2 Lower respiratory infect	**35.4 (0.809 to 73.5)**	-12 (-31.9 to 14.3)	**-35.6 (-47 to -20.6)**
3 Lower respiratory infect	3 Neonatal disorders	-13.6 (-35.8 to 11.7)	**-44 (-56 to -30.8)**	-12.3 (-26 to 7.05)
4 Stroke	4 Diabetes	**138 (78.5 to 209)**	**54.4 (23.2 to 92.1)**	**-21.8 (-37.1 to -1.88)**
5 Tuberculosis	5 Chronic kidney disease	**225 (134 to 345)**	**111 (60.9 to 177)**	12.4 (-14.3 to 47.7)
6 Diarrheal diseases	6 Ischemic heart disease	**60.4 (1.58 to 153)**	4.13 (-29.2 to 56.3)	**-50.8 (-67.4 to -26)**
7 Ischemic heart disease	7 HIV/AIDS	**-58.4 (-79.4 to -17.4)**	**-73 (-86.6 to -46.7)**	**-76.7 (-88.3 to -53.3)**
8 Congenital defects	8 Depressive disorders	**97.8 (66.8 to 126)**	**28.6 (20 to 40.4)**	**-2.34 (-2.73 to -1.96)**
9 Road injuries	9 Other musculoskeletal	**339 (274 to 405)**	**185 (165 to 211)**	**101 (94.6 to 105)**
10 Diabetes	10 Low back pain	**111 (81.3 to 143)**	**36.9 (26.9 to 47.9)**	**-5.66 (-6.02 to -5.24)**
11 Pertussis	11 Age-related hearing loss	**156 (115 to 199)**	**66.4 (50.8 to 83)**	**-1.84 (-2.15 to -1.51)**
12 Cirrhosis liver	12 Cirrhosis liver	**58.2 (21 to 104)**	2.81 (-15.7 to 26.2)	**-37.7 (-48.3 to -24.1)**
13 Interpersonal violence	13 Hypertensive heart disease	**205 (120 to 307)**	**97.9 (53.6 to 153)**	-18.3 (-36.9 to 6.12)
14 Depressive disorders	14 Road injuries	22.7 (-31.4 to 121)	-20.4 (-53.3 to 39.3)	-36 (-62.8 to 14.4)
15 Malaria	15 Diarrheal diseases	-9.35 (-43 to 31.4)	**-41.2 (-59.2 to -14.4)**	**-49.2 (-65.6 to -25.8)**
16 Low back pain	16 Interpersonal violence	18 (-8.33 to 48.4)	**-23.3 (-37.7 to -7.8)**	**-31.3 (-43.3 to -18.3)**
17 Maternal disorders	17 Tuberculosis	**-32.3 (-54.7 to -0.203)**	**-56 (-69.5 to -37.6)**	**-71.6 (-80.4 to -59.5)**
18 COVID-19	18 COPD	**213 (127 to 314)**	**103 (60.8 to 159)**	-9.67 (-29.5 to 16.2)
19 Chronic kidney disease	19 Headache disorders	**78.8 (54.4 to 108)**	**16.2 (9.71 to 29.5)**	**1.64 (1.02 to 2)**
20 Protein-energy malnutrition	20 Anxiety disorders	**72.5 (47.5 to 98.4)**	**12.1 (5.64 to 20.5)**	**2.52 (2.2 to 2.85)**
21 Age-related hearing loss	29 Congenital defects			
25 Anxiety disorders	42 Malaria			
26 Headache disorders	44 Protein-energy malnutrition			
28 Hypertensive heart disease	45 Maternal disorders			
30 Other musculoskeletal	51 Pertussis			
34 COPD	163 COVID-19			

Supplemental Results Figure S7. Leading 20 level 3 causes of DALYs, and percentage change in number of DALYs and all-age and age-standardised DALY rates by location, 2022 and 2050

Eritrea, Reference scenario

Legend:
Communicable, maternal, neonatal and nutritional
Non-communicable
Injuries

Leading Causes 2022	Leading Causes 2050	Mean % change number of DALY	Mean % change all-age DALY rate	Mean % change age-standardized DALY rate
1 Neonatal disorders	1 Lower respiratory infect	-18.1 (-50.8 to 16.3)	**-40 (-56 to -22.4)**	**-43.9 (-55.5 to -30)**
2 Lower respiratory infect	2 Neonatal disorders	**-49.7 (-70.1 to -27.9)**	**-63.5 (-70.5 to -54.8)**	**-42.4 (-51.7 to -29.4)**
3 Tuberculosis	3 Stroke	48.2 (-6.04 to 102)	8.55 (-14.1 to 35.7)	**-43 (-54.1 to -26.5)**
4 Diarrheal diseases	4 Diarrheal diseases	-22.3 (-56.1 to 20.1)	**-43.3 (-62.9 to -18.2)**	**-42.7 (-60.3 to -20.4)**
5 Stroke	5 Diabetes	**107 (29.8 to 184)**	**51.1 (24.5 to 83.2)**	-14.2 (-28.7 to 4.61)
6 Congenital defects	6 Tuberculosis	**-46.1 (-68.6 to -18.7)**	**-60.5 (-72.5 to -42.1)**	**-70.6 (-79.7 to -57.2)**
7 COVID-19	7 Conflict & terror	180 (-20.1 to 391)	105 (-38.9 to 294)	36.4 (-46.3 to 157)
8 Road injuries	8 Ischemic heart disease	41.2 (-17.8 to 109)	3.51 (-28.1 to 45.9)	**-44.3 (-62.2 to -18.8)**
9 Interpersonal violence	9 Cirrhosis liver	28.6 (-20.7 to 75.7)	-5.9 (-22.5 to 14.9)	**-37.9 (-47.9 to -24.7)**
10 Cirrhosis liver	10 Road injuries	0.8 (-51 to 80)	-26.3 (-56.8 to 30.3)	-36.4 (-62.7 to 9.69)
11 Ischemic heart disease	11 Chronic kidney disease	**152 (51.1 to 250)**	**84.2 (40 to 142)**	15.9 (-12.5 to 52.7)
12 Protein-energy malnutrition	12 Depressive disorders	**70.2 (10.7 to 133)**	**24.2 (15.3 to 34.9)**	**-4.02 (-4.48 to -3.62)**
13 Diabetes	13 Interpersonal violence	2.25 (-35.9 to 48.8)	**-25.5 (-39 to -7.5)**	**-31.4 (-42.6 to -17.1)**
14 Maternal disorders	14 Low back pain	**77 (14.6 to 144)**	**29.2 (18.6 to 41.9)**	**-8.1 (-8.46 to -7.79)**
15 HIV/AIDS	15 Hypertensive heart disease	**111 (29.4 to 193)**	**54.3 (17.8 to 94.7)**	**-28.7 (-46.2 to -6.34)**
16 Meningitis	16 Age-related hearing loss	**111 (38.7 to 188)**	**54 (39.1 to 72.4)**	**-2.01 (-2.35 to -1.61)**
17 Depressive disorders	17 Congenital defects	**-42.8 (-66.2 to -12.9)**	**-58.5 (-67.2 to -46.5)**	**-36.3 (-45.4 to -23.1)**
18 Conflict & terror	18 COPD	**98.3 (24.4 to 168)**	**45.2 (16.5 to 83.1)**	**-28 (-44 to -9.52)**
19 Dietary iron deficiency	19 Self-harm	42.9 (-11.3 to 105)	4.33 (-17.1 to 28.1)	-18 (-34.2 to 0.958)
20 Low back pain	20 Anxiety disorders	53.5 (-6.98 to 113)	**11.8 (5.71 to 20)**	**1.23 (0.839 to 1.6)**
21 Chronic kidney disease	22 Dietary iron deficiency			
22 Self-harm	31 Meningitis			
25 Hypertensive heart disease	36 Maternal disorders			
26 Anxiety disorders	38 Protein-energy malnutrition			
28 Age-related hearing loss	75 HIV/AIDS			
32 COPD	166 COVID-19			

Supplemental Results Figure S7. Leading 20 level 3 causes of DALYs, and percentage change in number of DALYs and all-age and age-standardised DALY rates by location, 2022 and 2050

Ethiopia, Reference scenario

Legend:
Communicable, maternal, neonatal and nutritional
Non-communicable
Injuries

Leading Causes 2022	Leading Causes 2050	Mean % change number of DALY	Mean % change all-age DALY rate	Mean % change age-standardized DALY rate
1 Neonatal disorders	1 Neonatal disorders	**-36.8 (-53.4 to -16.8)**	**-66.6 (-73.6 to -57.9)**	**-46 (-55.6 to -32.3)**
2 Lower respiratory infect	2 Lower respiratory infect	**-24 (-40.7 to -1.79)**	**-59.8 (-67.6 to -51.3)**	**-54.6 (-62.3 to -44.9)**
3 Diarrheal diseases	3 Diarrheal diseases	-21.2 (-50.3 to 19.7)	**-58.3 (-73.1 to -36.4)**	**-55.7 (-70.5 to -33.8)**
4 Congenital defects	4 Depressive disorders	**137 (102 to 174)**	**25.7 (19 to 33.7)**	**-3.92 (-4.1 to -3.69)**
5 COVID-19	5 Chronic kidney disease	**207 (122 to 309)**	**62.5 (23.8 to 115)**	7.93 (-19.3 to 42.9)
6 Tuberculosis	6 Other musculoskeletal	**554 (461 to 643)**	**246 (223 to 272)**	**139 (132 to 146)**
7 Stroke	7 Stroke	**80.5 (44.2 to 124)**	-4.36 (-21.5 to 16.5)	**-46 (-55.4 to -33.5)**
8 HIV/AIDS	8 Low back pain	**144 (105 to 182)**	**29.2 (21.4 to 38.6)**	**-7.84 (-8.13 to -7.47)**
9 Meningitis	9 Diabetes	**125 (83.4 to 169)**	**19 (0.279 to 42.3)**	**-30.7 (-40.9 to -18.5)**
10 Pertussis	10 Age-related hearing loss	**177 (133 to 218)**	**46.6 (35.5 to 56.6)**	**-2.21 (-2.4 to -2.02)**
11 Depressive disorders	11 Cirrhosis liver	**88.9 (51.8 to 140)**	0.0898 (-16.8 to 21.8)	**-35.9 (-46.4 to -22.3)**
12 Protein-energy malnutrition	12 Congenital defects	**-24.6 (-42.7 to -2.53)**	**-60.2 (-66.6 to -52.7)**	**-38.4 (-45.7 to -27.8)**
13 Road injuries	13 Anxiety disorders	**115 (81.2 to 147)**	**13.9 (8.65 to 20)**	**2.14 (1.87 to 2.42)**
14 Low back pain	14 Interpersonal violence	**64.4 (27.3 to 105)**	-13 (-25.7 to 2.36)	**-24.3 (-34.7 to -10.8)**
15 Cirrhosis liver	15 Road injuries	32.7 (-26.3 to 140)	-29.7 (-59.5 to 29.8)	-39.2 (-64.8 to 8.05)
16 Diabetes	16 Ischemic heart disease	50.7 (-2.28 to 124)	-20.1 (-46.4 to 19.6)	**-54.7 (-70.2 to -30)**
17 Interpersonal violence	17 Headache disorders	**124 (87.3 to 160)**	**18.5 (12.1 to 29.2)**	**1.1 (0.709 to 1.38)**
18 Ischemic heart disease	18 Gynecological diseases	**80.3 (41.4 to 130)**	-4.52 (-22.5 to 18)	**-24.3 (-38 to -6.41)**
19 Dietary iron deficiency	19 Tuberculosis	**-36.2 (-59.2 to -1.56)**	**-66.2 (-77.8 to -47.3)**	**-75.2 (-83.3 to -62.4)**
20 Chronic kidney disease	20 Idiopathic epilepsy	**76.1 (15.9 to 162)**	-6.76 (-35.4 to 31.4)	-9.44 (-36.1 to 27.6)
22 Age-related hearing loss	24 Dietary iron deficiency			
23 Anxiety disorders	32 Meningitis			
24 Gynecological diseases	36 Pertussis			
25 Idiopathic epilepsy	47 HIV/AIDS			
28 Headache disorders	48 Protein-energy malnutrition			
39 Other musculoskeletal	168 COVID-19			

Supplemental Results Figure S7. Leading 20 level 3 causes of DALYs, and percentage change in number of DALYs and all-age and age-standardised DALY rates by location, 2022 and 2050

Kenya, Reference scenario

Legend:
Communicable, maternal, neonatal and nutritional
Non-communicable
Injuries

Leading Causes 2022	Leading Causes 2050	Mean % change number of DALY	Mean % change all-age DALY rate	Mean % change age-standardized DALY rate
1 HIV/AIDS	1 Neonatal disorders	**-31.4 (-46.8 to -13.7)**	**-52.4 (-61 to -41.7)**	**-28.1 (-38.2 to -15.2)**
2 Neonatal disorders	2 Lower respiratory infect	9.46 (-12.4 to 31.5)	**-23.9 (-37.2 to -6.46)**	**-38.7 (-47.5 to -27.3)**
3 Lower respiratory infect	3 Stroke	**67.9 (41.3 to 94)**	16.8 (-2.27 to 37.6)	**-43.5 (-51.9 to -32.6)**
4 Tuberculosis	4 Cirrhosis liver	**73.9 (50.2 to 102)**	**20.9 (2.09 to 41.4)**	**-31.1 (-40 to -19.6)**
5 Diarrheal diseases	5 Diarrheal diseases	-10.5 (-38.7 to 30.7)	**-37.8 (-56.9 to -8.38)**	**-38.8 (-57.4 to -12)**
6 COVID-19	6 Low back pain	**104 (86.3 to 122)**	**41.5 (30.2 to 52.1)**	**-5.25 (-5.72 to -4.83)**
7 Stroke	7 Depressive disorders	**91.8 (74.1 to 112)**	**33.3 (24.8 to 42.9)**	**-2.57 (-2.76 to -2.38)**
8 Cirrhosis liver	8 Tuberculosis	-23.3 (-46.8 to 6.4)	**-46.6 (-62.6 to -25.3)**	**-65.6 (-76.1 to -51)**
9 Congenital defects	9 Diabetes	**157 (118 to 197)**	**78.4 (49.7 to 109)**	-8.79 (-22.2 to 5.9)
10 Depressive disorders	10 Chronic kidney disease	**183 (121 to 255)**	**96.8 (52.4 to 148)**	11.7 (-13.9 to 45)
11 Low back pain	11 Age-related hearing loss	**131 (108 to 151)**	**60.3 (44.7 to 75)**	**-1.24 (-1.38 to -1.1)**
12 Malaria	12 Other musculoskeletal	**276 (242 to 310)**	**162 (140 to 183)**	**71.5 (68.3 to 74.2)**
13 Ischemic heart disease	13 Ischemic heart disease	**57.9 (13.7 to 113)**	9.88 (-21.4 to 51)	**-46.6 (-61.4 to -26.7)**
14 Interpersonal violence	14 Hypertensive heart disease	**209 (143 to 285)**	**115 (65.9 to 169)**	-5.31 (-27.3 to 20.5)
15 Road injuries	15 COPD	**121 (76.8 to 162)**	**53.5 (24.2 to 84.6)**	**-26.6 (-40.7 to -11.4)**
16 Diabetes	16 Road injuries	30.4 (-12.2 to 98.8)	-9.31 (-38.7 to 38.6)	-29.3 (-53 to 8.43)
17 Age-related hearing loss	17 Headache disorders	**72.1 (55.1 to 97.1)**	**19.6 (11.8 to 32.9)**	**0.579 (0.347 to 0.834)**
18 Meningitis	18 Interpersonal violence	**23.7 (4.29 to 48.2)**	**-14.1 (-26.5 to -0.942)**	**-27.3 (-36.4 to -16)**
19 Chronic kidney disease	19 Gynecological diseases	**61.6 (33 to 91)**	12.3 (-6.18 to 34.4)	-13.8 (-27.6 to 1.92)
20 Protein-energy malnutrition	20 Anxiety disorders	**61.8 (44.1 to 79.5)**	**12.4 (6.01 to 20.4)**	**1.08 (0.894 to 1.31)**
21 Gynecological diseases	21 HIV/AIDS			
22 Anxiety disorders	25 Congenital defects			
23 Headache disorders	34 Meningitis			
26 COPD	42 Protein-energy malnutrition			
28 Hypertensive heart disease	97 Malaria			
29 Other musculoskeletal	167 COVID-19			

Supplemental Results Figure S7. Leading 20 level 3 causes of DALYs, and percentage change in number of DALYs and all-age and age-standardised DALY rates by location, 2022 and 2050

Madagascar, Reference scenario

Legend:
Communicable, maternal, neonatal and nutritional
Non-communicable
Injuries

Leading Causes 2022	Leading Causes 2050	Mean % change number of DALY	Mean % change all-age DALY rate	Mean % change age-standardized DALY rate
1 COVID-19	1 Diarrheal diseases	-7.88 (-38 to 45)	**-45.9 (-63.4 to -17.8)**	**-33 (-52.6 to -5.25)**
2 Neonatal disorders	2 Stroke	**64.2 (36.4 to 101)**	-3.51 (-21.8 to 18.3)	**-44 (-53.4 to -31.6)**
3 Diarrheal diseases	3 Neonatal disorders	**-34.9 (-47.5 to -18.8)**	**-61.8 (-68 to -54.2)**	**-42.6 (-50.8 to -32.2)**
4 Lower respiratory infect	4 Lower respiratory infect	**-19.6 (-34.2 to -0.474)**	**-52.7 (-61.8 to -41.3)**	**-46.3 (-54.6 to -35.7)**
5 Stroke	5 Ischemic heart disease	**72.4 (24 to 149)**	1.33 (-29.6 to 50.1)	**-41.8 (-59.5 to -15.7)**
6 Tuberculosis	6 Hypertensive heart disease	**181 (119 to 269)**	**65.2 (25.7 to 117)**	-13 (-33.1 to 13.9)
7 Malaria	7 Depressive disorders	**114 (92.2 to 137)**	**25.6 (18.5 to 34.1)**	**-2.85 (-3.05 to -2.62)**
8 Congenital defects	8 Low back pain	**123 (103 to 148)**	**31.1 (22.7 to 39.2)**	**-5.65 (-5.83 to -5.47)**
9 Protein-energy malnutrition	9 Diabetes	**141 (99.5 to 192)**	**41.7 (15.7 to 73.4)**	-14.2 (-26.7 to 2.31)
10 Ischemic heart disease	10 Tuberculosis	-24.4 (-48.7 to 13.9)	**-55.5 (-69.2 to -35)**	**-66.1 (-76.1 to -51)**
11 Measles	11 Age-related hearing loss	**147 (121 to 177)**	**45.2 (33.8 to 56.5)**	**-1.52 (-1.62 to -1.41)**
12 Road injuries	12 Congenital defects	**-22.7 (-37.8 to -5.81)**	**-54.7 (-61.6 to -46.3)**	**-34.2 (-42.4 to -23.3)**
13 Pertussis	13 Other musculoskeletal	**329 (290 to 376)**	**152 (135 to 167)**	**78.1 (73.4 to 81.7)**
14 Asthma	14 COPD	**142 (92.3 to 213)**	**42.4 (11.6 to 80.3)**	**-24.5 (-40.3 to -6.03)**
15 Depressive disorders	15 Cirrhosis liver	**66.8 (41.8 to 99.7)**	-1.93 (-17.4 to 17.7)	**-34.6 (-43.9 to -22.2)**
16 Meningitis	16 Chronic kidney disease	**174 (110 to 267)**	**61 (21.3 to 116)**	9.38 (-17.1 to 46.2)
17 Cirrhosis liver	17 Road injuries	18 (-27.8 to 103)	-30.7 (-56.9 to 15.1)	-37.6 (-59.8 to 1.32)
18 STIs	18 Anxiety disorders	**92.4 (72.4 to 112)**	**13 (5.95 to 20.8)**	**1.08 (0.876 to 1.29)**
19 Hypertensive heart disease	19 Dietary iron deficiency	**37.8 (20.3 to 55.6)**	**-19.1 (-24 to -15.1)**	**-17.3 (-17.8 to -16.5)**
20 Low back pain	20 Headache disorders	**100 (80.2 to 124)**	**17.6 (11.3 to 28.1)**	**0.394 (0.201 to 0.578)**
22 Dietary iron deficiency	23 Protein-energy malnutrition			
24 Diabetes	25 Asthma			
26 Age-related hearing loss	27 Pertussis			
27 COPD	28 Malaria			
28 Anxiety disorders	30 Meningitis			
30 Headache disorders	45 STIs			
32 Chronic kidney disease	84 Measles			

Supplemental Results Figure S7. Leading 20 level 3 causes of DALYs, and percentage change in number of DALYs and all-age and age-standardised DALY rates by location, 2022 and 2050

Malawi, Reference scenario

Legend:
Communicable, maternal, neonatal and nutritional
Non-communicable
Injuries

Leading Causes 2022	Leading Causes 2050	Mean % change number of DALY	Mean % change all-age DALY rate	Mean % change age-standardized DALY rate
1 HIV/AIDS	1 Neonatal disorders	**-35.4 (-52.1 to -14.3)**	**-60.8 (-68.3 to -50.4)**	**-37.3 (-48.3 to -23.8)**
2 Neonatal disorders	2 Lower respiratory infect	-15.9 (-31.6 to 3.46)	**-48.7 (-58.2 to -35.6)**	**-43.5 (-53 to -30.5)**
3 Lower respiratory infect	3 Diarrheal diseases	3.26 (-29.2 to 55.1)	**-37 (-57.5 to -5.54)**	**-34 (-55.1 to -0.788)**
4 COVID-19	4 Stroke	**60.8 (31.9 to 93.5)**	-1.82 (-21.8 to 22.4)	**-44.9 (-55.7 to -29.5)**
5 Malaria	5 Cirrhosis liver	**82.7 (53.2 to 115)**	11.5 (-8.06 to 35.5)	**-31.5 (-42.1 to -18.7)**
6 Diarrheal diseases	6 Chronic kidney disease	**144 (81.3 to 225)**	**48.9 (10 to 104)**	0.823 (-26 to 36.4)
7 Tuberculosis	7 Ischemic heart disease	**67.9 (12.9 to 152)**	2.64 (-33.4 to 60.2)	**-42.8 (-62.1 to -11.9)**
8 Congenital defects	8 Diabetes	**122 (80.9 to 170)**	**35.8 (9.49 to 70.4)**	**-20.6 (-36.1 to -0.118)**
9 Stroke	9 Low back pain	**125 (100 to 149)**	**37.4 (26.2 to 49.4)**	**-6.09 (-6.5 to -5.61)**
10 Meningitis	10 Depressive disorders	**114 (87.2 to 137)**	**30.3 (20.1 to 41.5)**	**-2.94 (-3.37 to -2.51)**
11 Road injuries	11 Other musculoskeletal	**369 (315 to 416)**	**186 (162 to 213)**	**88.3 (82.7 to 92.9)**
12 Cirrhosis liver	12 Road injuries	24.6 (-27.9 to 110)	-23.9 (-57 to 32.9)	-34.8 (-61.2 to 10.6)
13 Ischemic heart disease	13 Tuberculosis	-25.7 (-51.7 to 13.6)	**-54.6 (-71.7 to -28.8)**	**-67.7 (-79.1 to -50.1)**
14 Dietary iron deficiency	14 Age-related hearing loss	**145 (116 to 172)**	**49.5 (35.4 to 64.7)**	**-1.68 (-2.2 to -1.11)**
15 Protein-energy malnutrition	15 Congenital defects	**-32.8 (-50 to -15.1)**	**-59.2 (-66.7 to -50.8)**	**-36.8 (-45.6 to -24.9)**
16 Diabetes	16 Esophageal cancer	**187 (148 to 225)**	**75.2 (52.5 to 99.7)**	-7.89 (-18 to 1.1)
17 Low back pain	17 Self-harm	**89.8 (54.8 to 133)**	15.8 (-7.72 to 40.3)	-14.3 (-29.7 to 2.73)
18 Chronic kidney disease	18 Anxiety disorders	**90 (64.7 to 116)**	**15.7 (6.74 to 26)**	**1.36 (0.994 to 1.75)**
19 Depressive disorders	19 Hypertensive heart disease	**163 (101 to 241)**	**60.6 (19.1 to 117)**	-16.5 (-38.2 to 13.1)
20 Idiopathic epilepsy	20 Headache disorders	**100 (75.2 to 128)**	**22.1 (12.8 to 35.9)**	**0.691 (0.286 to 1.09)**
22 Self-harm	21 Idiopathic epilepsy			
23 Anxiety disorders	22 Dietary iron deficiency			
25 Age-related hearing loss	23 HIV/AIDS			
27 Headache disorders	27 Meningitis			
30 Esophageal cancer	30 Malaria			
33 Hypertensive heart disease	43 Protein-energy malnutrition			
38 Other musculoskeletal	167 COVID-19			

Supplemental Results Figure S7. Leading 20 level 3 causes of DALYs, and percentage change in number of DALYs and all-age and age-standardised DALY rates by location, 2022 and 2050

Mozambique, Reference scenario

Legend:
Communicable, maternal, neonatal and nutritional
Non-communicable
Injuries

Leading Causes 2022	Leading Causes 2050	Mean % change number of DALY	Mean % change all-age DALY rate	Mean % change age-standardized DALY rate
1 HIV/AIDS	1 Neonatal disorders	**-44.9 (-58.7 to -30.2)**	**-69.6 (-75.2 to -62.4)**	**-50.7 (-58.8 to -40.6)**
2 Neonatal disorders	2 Stroke	**49.3 (22.7 to 80.8)**	-17.4 (-34.9 to 0.212)	**-48.8 (-57.6 to -37.8)**
3 Malaria	3 Lower respiratory infect	**-19.1 (-33.6 to -0.537)**	**-55.3 (-63.7 to -45.3)**	**-47.5 (-55.7 to -38)**
4 Lower respiratory infect	4 HIV/AIDS	**-75.5 (-79.7 to -70.2)**	**-86.5 (-88.9 to -83.5)**	**-87.2 (-89.3 to -84.6)**
5 Tuberculosis	5 Diabetes	**165 (113 to 223)**	**46.5 (17.1 to 74.9)**	-5.19 (-21 to 13.6)
6 Stroke	6 Malaria	-62.1 (-93.4 to 69.8)	**-79 (-96.2 to -8.36)**	**-76.6 (-95.8 to -1.98)**
7 COVID-19	7 Dietary iron deficiency	**41.2 (21.7 to 59.3)**	**-22 (-24.8 to -18.8)**	**-23.5 (-24.3 to -22.7)**
8 Congenital defects	8 Tuberculosis	**-38.2 (-59.1 to -9.76)**	**-65.8 (-77.3 to -47.3)**	**-73.2 (-81.5 to -60)**
9 Diarrheal diseases	9 Depressive disorders	**130 (103 to 157)**	**27.1 (19.6 to 35.1)**	**-3.99 (-4.41 to -3.62)**
10 Dietary iron deficiency	10 Diarrheal diseases	-25.6 (-49.6 to 3.69)	**-58.8 (-72.3 to -40.6)**	**-51.8 (-65.3 to -33.7)**
11 Road injuries	11 Congenital defects	**-29.8 (-45.5 to -12.6)**	**-61.3 (-67.9 to -53)**	**-39.8 (-47.7 to -29.8)**
12 STIs	12 Low back pain	**130 (107 to 153)**	**27.2 (18.1 to 36.1)**	**-8.02 (-8.34 to -7.61)**
13 Diabetes	13 Road injuries	14.8 (-29.8 to 87.3)	-36.4 (-63.6 to 8.55)	**-45.7 (-66.9 to -9.41)**
14 Depressive disorders	14 Other musculoskeletal	**480 (396 to 558)**	**221 (184 to 251)**	**120 (101 to 136)**
15 Protein-energy malnutrition	15 Hypertensive heart disease	**160 (97 to 231)**	**43.9 (6.59 to 87.7)**	-19.4 (-39.3 to 3.53)
16 Low back pain	16 Chronic kidney disease	**137 (76.4 to 204)**	31.3 (-2.16 to 71.2)	2.01 (-22.8 to 34.4)
17 Meningitis	17 Self-harm	**114 (74.9 to 156)**	18.2 (-2.4 to 42.5)	-12.4 (-25.5 to 3.48)
18 Interpersonal violence	18 Age-related hearing loss	**151 (119 to 180)**	**38.5 (26.2 to 50)**	**-2.27 (-2.47 to -2.05)**
19 Maternal disorders	19 Anxiety disorders	**115 (88 to 145)**	**18.7 (11.7 to 27.4)**	**1.89 (1.49 to 2.27)**
20 Self-harm	20 Interpersonal violence	**62.7 (29.2 to 103)**	-10.1 (-26 to 6.74)	**-21.4 (-32.6 to -9)**
21 Chronic kidney disease	36 STIs			
22 Hypertensive heart disease	37 Meningitis			
26 Anxiety disorders	42 Maternal disorders			
29 Age-related hearing loss	52 Protein-energy malnutrition			
44 Other musculoskeletal	160 COVID-19			

Supplemental Results Figure S7. Leading 20 level 3 causes of DALYs, and percentage change in number of DALYs and all-age and age-standardised DALY rates by location, 2022 and 2050

Rwanda, Reference scenario

Legend:
Communicable, maternal, neonatal and nutritional
Non-communicable
Injuries

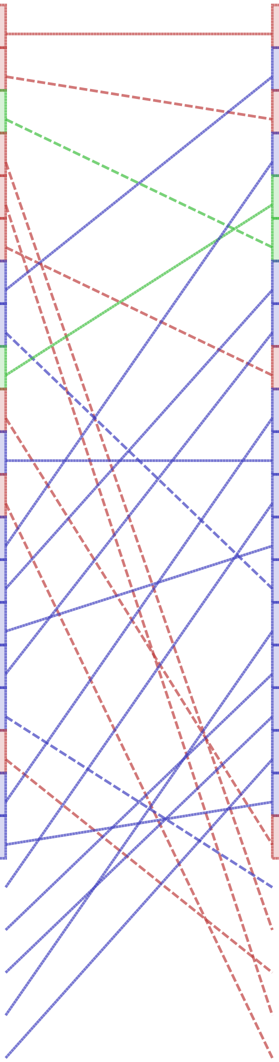

Leading Causes 2022	Leading Causes 2050	Mean % change number of DALY	Mean % change all-age DALY rate	Mean % change age-standardized DALY rate
1 Neonatal disorders	1 Neonatal disorders	**-27.1 (-43.6 to -11.3)**	**-61 (-68.4 to -53.2)**	**-37.6 (-48.2 to -25.9)**
2 Lower respiratory infect	2 Stroke	**60.1 (31 to 91.8)**	-14.2 (-29.1 to 1.39)	**-50.4 (-58.1 to -40.8)**
3 Conflict & terror	3 Lower respiratory infect	-9.46 (-28.7 to 13.2)	**-51.5 (-61.5 to -38.8)**	**-50.3 (-58.6 to -39)**
4 HIV/AIDS	4 Depressive disorders	**129 (96.1 to 161)**	**22.7 (16.1 to 30.4)**	**-4.34 (-4.68 to -3.88)**
5 Malaria	5 Road injuries	44.9 (-8.74 to 124)	-22.2 (-51.4 to 18.5)	-30.4 (-56.5 to 5.94)
6 Diarrheal diseases	6 Conflict & terror	159 (-31.4 to 425)	38.4 (-62.1 to 156)	1.91 (-65.6 to 90.6)
7 Stroke	7 Low back pain	**137 (106 to 166)**	**27.1 (20.3 to 35.4)**	**-7.29 (-7.53 to -6.96)**
8 Congenital defects	8 Diabetes	**133 (86.8 to 184)**	**24.7 (1.25 to 48.6)**	**-24.1 (-36.3 to -8.49)**
9 Road injuries	9 Diarrheal diseases	-4.64 (-40.3 to 39.9)	**-48.9 (-68.1 to -24.9)**	**-42.6 (-63 to -18.2)**
10 Tuberculosis	10 Chronic kidney disease	**173 (109 to 255)**	**46.2 (13.1 to 91.3)**	-2.09 (-25.2 to 30.6)
11 Cirrhosis liver	11 Cirrhosis liver	**57 (31.1 to 85)**	**-15.8 (-27.5 to -3.07)**	**-43.4 (-50.7 to -35)**
12 COVID-19	12 Age-related hearing loss	**171 (131 to 211)**	**45.4 (34.5 to 55.4)**	**-1.87 (-2.02 to -1.72)**
13 Depressive disorders	13 Ischemic heart disease	**52 (7.23 to 114)**	-18.5 (-42.3 to 17.6)	**-52.9 (-66.7 to -31.5)**
14 Low back pain	14 Congenital defects	**-25.5 (-40.7 to -8.08)**	**-60.2 (-66.6 to -52.1)**	**-38.5 (-47 to -28.4)**
15 Ischemic heart disease	15 COPD	**138 (84.7 to 201)**	27.5 (-0.0133 to 58.5)	**-28.5 (-43.3 to -11.5)**
16 Diabetes	16 Anxiety disorders	**109 (78.8 to 134)**	**12 (5.33 to 18.9)**	**1.02 (0.728 to 1.32)**
17 Asthma	17 Headache disorders	**117 (86.6 to 144)**	**16.2 (10.3 to 26.6)**	0.133 (-0.133 to 0.503)
18 Meningitis	18 Hypertensive heart disease	**164 (104 to 238)**	**41.6 (7.96 to 81.2)**	**-25.2 (-42.6 to -4.97)**
19 Chronic kidney disease	19 Gynecological diseases	**76.7 (42.5 to 116)**	-5.23 (-22.1 to 12.7)	**-23.8 (-37.7 to -9.73)**
20 Gynecological diseases	20 Tuberculosis	-27.8 (-50.1 to 4.97)	**-61.3 (-73.7 to -43.9)**	**-71.3 (-79.9 to -58.6)**
21 Age-related hearing loss	27 Asthma			
22 Anxiety disorders	28 HIV/AIDS			
26 Headache disorders	38 Meningitis			
27 COPD	78 Malaria			
30 Hypertensive heart disease	164 COVID-19			

Supplemental Results Figure S7. Leading 20 level 3 causes of DALYs, and percentage change in number of DALYs and all-age and age-standardised DALY rates by location, 2022 and 2050

Somalia, Reference scenario

Legend:
Communicable, maternal, neonatal and nutritional
Non-communicable
Injuries

Leading Causes 2022	Leading Causes 2050	Mean % change number of DALY	Mean % change all-age DALY rate	Mean % change age-standardized DALY rate
1 Neonatal disorders	1 Neonatal disorders	4.89 (-35.2 to 66.3)	**-48.6 (-62 to -25.3)**	-29.3 (-46.6 to 1.52)
2 Measles	2 Lower respiratory infect	5.94 (-34.2 to 56.5)	**-48.1 (-60.2 to -33.5)**	**-24.7 (-40 to -3.61)**
3 Diarrheal diseases	3 Diarrheal diseases	-6.07 (-52.6 to 97.2)	**-54 (-71.5 to -13.4)**	-37.9 (-60.4 to 18.1)
4 Lower respiratory infect	4 Tuberculosis	-22.9 (-60.8 to 50.7)	**-62.1 (-79 to -27.7)**	**-63.8 (-78.7 to -32.1)**
5 Tuberculosis	5 Pertussis	29.7 (-27.6 to 116)	-36.6 (-60.9 to 2.42)	-19.4 (-48.8 to 28.9)
6 Congenital defects	6 Stroke	**115 (36.2 to 230)**	5.39 (-20.1 to 44.4)	-20.8 (-44.3 to 20)
7 Pertussis	7 Congenital defects	3.94 (-33.1 to 47.9)	**-49.1 (-58 to -36.9)**	**-32.6 (-43.7 to -17.2)**
8 Protein-energy malnutrition	8 Road injuries	93.9 (-18.6 to 310)	-4.7 (-55 to 100)	-13.6 (-55.8 to 72.2)
9 Meningitis	9 Interpersonal violence	**71.9 (0.298 to 154)**	-15.9 (-37 to 8.4)	**-21.7 (-39.5 to -0.923)**
10 Maternal disorders	10 Diabetes	**188 (72.2 to 361)**	**41.4 (3.31 to 111)**	9.34 (-23 to 71.7)
11 Conflict & terror	11 Meningitis	-6.09 (-51.2 to 66.6)	**-53.9 (-71.7 to -22.4)**	**-46.1 (-64.4 to -15)**
12 Road injuries	12 Cirrhosis liver	**79 (15.6 to 167)**	-12.2 (-30.9 to 16.3)	**-29 (-44.2 to -5.77)**
13 Stroke	13 Chronic kidney disease	**181 (67.6 to 354)**	38 (-2.74 to 107)	14.8 (-19.7 to 75.4)
14 Tetanus	14 Conflict & terror	67.6 (-68.6 to 465)	-17.7 (-84.3 to 192)	-18.5 (-83.3 to 156)
15 Interpersonal violence	15 Ischemic heart disease	**162 (22.8 to 488)**	28.9 (-31.3 to 163)	-6.25 (-51.8 to 102)
16 COVID-19	16 Depressive disorders	**129 (52.7 to 197)**	**12.3 (7.99 to 17.3)**	**-3.67 (-4.13 to -3.19)**
17 Cirrhosis liver	17 Measles	-80.5 (-98.2 to 8.38)	**-90.8 (-98.9 to -49.1)**	**-88.3 (-98.5 to -35.4)**
18 Malaria	18 Maternal disorders	-4.22 (-51.6 to 78.9)	**-53 (-73.1 to -17.6)**	**-57.9 (-74.6 to -27.6)**
19 HIV/AIDS	19 Low back pain	**128 (50.7 to 196)**	**11.5 (6.21 to 17.7)**	**-6.95 (-7.42 to -6.58)**
20 STIs	20 Protein-energy malnutrition	-38.4 (-72.9 to 60.5)	**-69.7 (-85.4 to -24.3)**	-59.2 (-79.4 to 0.0628)
22 Diabetes	41 Tetanus			
23 Depressive disorders	43 STIs			
24 Ischemic heart disease	58 HIV/AIDS			
25 Chronic kidney disease	92 Malaria			
27 Low back pain	165 COVID-19			

Supplemental Results Figure S7. Leading 20 level 3 causes of DALYs, and percentage change in number of DALYs and all-age and age-standardised DALY rates by location, 2022 and 2050

South Sudan, Reference scenario

Legend:
Communicable, maternal, neonatal and nutritional
Non-communicable
Injuries

Leading Causes 2022	Leading Causes 2050	Mean % change number of DALY	Mean % change all-age DALY rate	Mean % change age-standardized DALY rate
1 Neonatal disorders	1 Neonatal disorders	-16.8 (-46.6 to 31)	**-62.8 (-74.5 to -42.3)**	**-50.4 (-65.4 to -22.9)**
2 Diarrheal diseases	2 Diarrheal diseases	-14 (-58.1 to 60.2)	**-61.5 (-81 to -24.5)**	**-48.2 (-74.2 to -0.559)**
3 Malaria	3 Lower respiratory infect	-25.5 (-50.4 to 1.75)	**-66.7 (-76.1 to -55.1)**	**-54 (-65.8 to -38.1)**
4 Lower respiratory infect	4 Malaria	-51.9 (-90.7 to 159)	-77.7 (-95.6 to 16.6)	-73.6 (-94.1 to 25)
5 Congenital defects	5 Congenital defects	5.91 (-23.8 to 37.9)	**-52.7 (-62.2 to -39.5)**	**-40.5 (-51.2 to -24.1)**
6 Tuberculosis	6 Stroke	**75.6 (24.4 to 144)**	-21.5 (-42 to 9.02)	-32.9 (-52.9 to 3.17)
7 Protein-energy malnutrition	7 Chronic kidney disease	**190 (74.4 to 353)**	29.7 (-17.6 to 103)	24.7 (-25.1 to 105)
8 HIV/AIDS	8 Interpersonal violence	**72.3 (30.9 to 118)**	**-23.1 (-35.4 to -6.55)**	**-26.8 (-38.1 to -11)**
9 Meningitis	9 Onchocerciasis	**75.8 (46.6 to 110)**	**-21.5 (-25.7 to -17.3)**	**-27.6 (-29.4 to -25.1)**
10 Conflict & terror	10 Diabetes	**123 (55.9 to 212)**	-0.468 (-25.8 to 42.2)	-13.1 (-38.9 to 34.4)
11 Stroke	11 Tuberculosis	-42.4 (-75 to 20.6)	**-74.2 (-88.3 to -42)**	**-72.4 (-86.9 to -40.4)**
12 Interpersonal violence	12 Depressive disorders	**139 (97.1 to 184)**	**6.64 (1.21 to 12.3)**	**-2.99 (-3.74 to -2.13)**
13 STIs	13 Ischemic heart disease	82.6 (-15.1 to 276)	-18.3 (-60.5 to 76.5)	-31.4 (-68.8 to 51.5)
14 Maternal disorders	14 Protein-energy malnutrition	-49.1 (-75.7 to 22.6)	**-77.2 (-89.1 to -45.2)**	**-71.5 (-86.4 to -29.4)**
15 Road injuries	15 Cirrhosis liver	**59.4 (17.2 to 111)**	**-28.7 (-44.2 to -6.57)**	**-37.1 (-50.6 to -17.1)**
16 Onchocerciasis	16 Road injuries	21.3 (-46.4 to 153)	-45.7 (-74.6 to 25.4)	-46.6 (-73.5 to 17.6)
17 Pertussis	17 Low back pain	**133 (92.6 to 181)**	4.16 (-2.07 to 11)	**-6.26 (-6.86 to -5.62)**
18 Cirrhosis liver	18 Maternal disorders	5.4 (-42.4 to 80.2)	**-52.9 (-72.9 to -18.1)**	**-58.3 (-75.6 to -28.7)**
19 Ischemic heart disease	19 Meningitis	-32 (-61.4 to 24.6)	**-69.6 (-81.6 to -42.3)**	**-61.6 (-75.5 to -32.1)**
20 Diabetes	20 Anxiety disorders	**141 (97.9 to 188)**	**7.82 (4.25 to 12)**	**2.07 (1.34 to 2.81)**
21 Chronic kidney disease	21 Conflict & terror			
23 Depressive disorders	24 Pertussis			
26 Low back pain	26 STIs			
31 Anxiety disorders	37 HIV/AIDS			

Supplemental Results Figure S7. Leading 20 level 3 causes of DALYs, and percentage change in number of DALYs and all-age and age-standardised DALY rates by location, 2022 and 2050

Uganda, Reference scenario

Legend:
Communicable, maternal, neonatal and nutritional
Non-communicable
Injuries

Leading Causes 2022	Leading Causes 2050	Mean % change number of DALY	Mean % change all-age DALY rate	Mean % change age-standardized DALY rate
1 Neonatal disorders	1 Neonatal disorders	**-27 (-45.2 to -5.26)**	**-63.8 (-70.3 to -55.5)**	**-43.6 (-52.3 to -32.5)**
2 Malaria	2 Depressive disorders	**157 (123 to 197)**	**27.7 (19.6 to 36.5)**	**-3.79 (-4.05 to -3.51)**
3 HIV/AIDS	3 Lower respiratory infect	-12 (-34.2 to 11.9)	**-56.3 (-66 to -44.4)**	**-49.3 (-57.9 to -39.3)**
4 Lower respiratory infect	4 Diarrheal diseases	10.3 (-32.3 to 57.4)	**-45.2 (-64.5 to -19.1)**	**-38.2 (-57.3 to -12.1)**
5 Congenital defects	5 Low back pain	**162 (127 to 197)**	**29.9 (21.5 to 37.3)**	**-7.44 (-7.67 to -7.18)**
6 Diarrheal diseases	6 Diabetes	**163 (108 to 230)**	**30.7 (7.82 to 56.4)**	**-21.5 (-34.4 to -7.08)**
7 COVID-19	7 Congenital defects	-17.4 (-34.7 to 3.7)	**-59.1 (-64.8 to -50.7)**	**-38.4 (-46 to -27.5)**
8 Tuberculosis	8 Stroke	**60 (23.3 to 94.6)**	**-20.6 (-34.8 to -6.04)**	**-52.3 (-59.7 to -43.6)**
9 Measles	9 Other musculoskeletal	**505 (423 to 592)**	**200 (178 to 224)**	**107 (96.7 to 113)**
10 Depressive disorders	10 Road injuries	38.8 (-20.3 to 138)	-31.1 (-58 to 10)	**-37.6 (-60.4 to -0.914)**
11 Road injuries	11 HIV/AIDS	**-67.7 (-74.6 to -59.8)**	**-84 (-86.8 to -80.7)**	**-87.1 (-89.3 to -84.2)**
12 Stroke	12 Chronic kidney disease	**158 (83.7 to 245)**	27.9 (-4.24 to 72.5)	-10.1 (-31.1 to 18.4)
13 Meningitis	13 Malaria	**-77.3 (-95 to -7.78)**	**-88.6 (-97.5 to -50.2)**	**-84.4 (-96.5 to -31.1)**
14 STIs	14 Anxiety disorders	**135 (105 to 166)**	**16.5 (9.7 to 25.5)**	**1.48 (1.21 to 1.75)**
15 Ischemic heart disease	15 Cirrhosis liver	**71.4 (39.4 to 107)**	-14.9 (-28.7 to 2)	**-43.5 (-51.5 to -33.5)**
16 Protein-energy malnutrition	16 Tuberculosis	-27.4 (-51.4 to 6.87)	**-63.9 (-76 to -47.4)**	**-71.8 (-80.1 to -59.1)**
17 Low back pain	17 Ischemic heart disease	**56.4 (6.81 to 123)**	-22.4 (-46.7 to 13)	**-54.5 (-68 to -36.2)**
18 Diabetes	18 Age-related hearing loss	**191 (154 to 227)**	**44.7 (33.2 to 54.2)**	**-2.14 (-2.27 to -2.03)**
19 Cirrhosis liver	19 Headache disorders	**144 (113 to 180)**	**21.2 (14.8 to 31.9)**	0.267 (-0.0415 to 0.531)
20 Pertussis	20 Gynecological diseases	**102 (57.9 to 149)**	0.394 (-18 to 22.1)	**-25.6 (-39.9 to -10.8)**
22 Chronic kidney disease	29 Meningitis			
23 Anxiety disorders	36 Pertussis			
28 Gynecological diseases	42 STIs			
29 Headache disorders	55 Protein-energy malnutrition			
31 Age-related hearing loss	95 Measles			
38 Other musculoskeletal	169 COVID-19			

Supplemental Results Figure S7. Leading 20 level 3 causes of DALYs, and percentage change in number of DALYs and all-age and age-standardised DALY rates by location, 2022 and 2050

Tanzania, Reference scenario

Legend:
Communicable, maternal, neonatal and nutritional
Non-communicable
Injuries

Leading Causes 2022	Leading Causes 2050	Mean % change number of DALY	Mean % change all-age DALY rate	Mean % change age-standardized DALY rate
1 Neonatal disorders	1 Neonatal disorders	**-32.8 (-47.8 to -15.2)**	**-61.5 (-67.8 to -53.9)**	**-40.9 (-49.4 to -28.5)**
2 HIV/AIDS	2 Lower respiratory infect	**-31.1 (-47.9 to -13.2)**	**-60.5 (-68.8 to -50.5)**	**-52.1 (-60.7 to -41)**
3 Lower respiratory infect	3 Diarrheal diseases	-9.97 (-40.9 to 30)	**-48.4 (-65.6 to -25.2)**	**-44.1 (-61.1 to -21)**
4 Malaria	4 Stroke	**60.1 (28.9 to 98.9)**	-8.23 (-24.1 to 10.2)	**-46 (-54.7 to -35.1)**
5 Diarrheal diseases	5 Diabetes	**183 (131 to 254)**	**62.1 (32.5 to 92.6)**	2.3 (-13.4 to 19.6)
6 Congenital defects	6 Ischemic heart disease	**55.5 (4.62 to 117)**	-10.8 (-37.6 to 26)	**-47.2 (-63.8 to -24.3)**
7 Tuberculosis	7 Depressive disorders	**116 (85.3 to 146)**	**23.6 (16.8 to 30.5)**	**-3.38 (-3.75 to -3.05)**
8 Stroke	8 Chronic kidney disease	**183 (108 to 271)**	**62 (24.1 to 116)**	12.1 (-13.1 to 45)
9 Ischemic heart disease	9 Congenital defects	**-27.8 (-43.1 to -8)**	**-58.7 (-65 to -49.7)**	**-37.8 (-45.8 to -24.9)**
10 STIs	10 Low back pain	**122 (90.3 to 153)**	**26.8 (18.7 to 34.5)**	**-6.73 (-6.96 to -6.49)**
11 Road injuries	11 Other musculoskeletal	**388 (319 to 457)**	**179 (161 to 198)**	**102 (95.7 to 106)**
12 Depressive disorders	12 HIV/AIDS	**-67.1 (-76 to -55.8)**	**-81.1 (-86.1 to -75.7)**	**-82.9 (-87.2 to -78.2)**
13 Dietary iron deficiency	13 Cirrhosis liver	**80.7 (50.3 to 117)**	3.55 (-11.3 to 23.9)	**-30.1 (-39.8 to -16.5)**
14 Maternal disorders	14 Age-related hearing loss	**152 (115 to 191)**	**44.5 (33 to 56.3)**	**-1.89 (-2.05 to -1.71)**
15 Cirrhosis liver	15 Dietary iron deficiency	**30.3 (7.6 to 51.5)**	**-25.5 (-30.2 to -21.5)**	**-19.3 (-20.4 to -17.9)**
16 Low back pain	16 Anxiety disorders	**98.9 (71 to 127)**	**13.8 (7.94 to 20.9)**	**1.38 (1.08 to 1.69)**
17 Diabetes	17 Hypertensive heart disease	**181 (109 to 264)**	**61.2 (22.3 to 108)**	-11.9 (-32.2 to 14.7)
18 Protein-energy malnutrition	18 Road injuries	19 (-26.3 to 90.9)	-31.7 (-56.1 to 17.5)	-39 (-60 to 4.34)
19 Meningitis	19 Headache disorders	**105 (76.7 to 137)**	**17.5 (11.9 to 26.9)**	**0.457 (0.174 to 0.721)**
20 Chronic kidney disease	20 Gynecological diseases	**69.6 (30.9 to 106)**	-2.84 (-22.2 to 18.1)	**-22.4 (-37.2 to -5.47)**
24 Anxiety disorders	21 Tuberculosis			
25 Gynecological diseases	33 Maternal disorders			
26 Headache disorders	40 Meningitis			
27 Age-related hearing loss	43 STIs			
30 Hypertensive heart disease	53 Malaria			
35 Other musculoskeletal	59 Protein-energy malnutrition			

Supplemental Results Figure S7. Leading 20 level 3 causes of DALYs, and percentage change in number of DALYs and all-age and age-standardised DALY rates by location, 2022 and 2050

Zambia, Reference scenario

Legend:
Communicable, maternal, neonatal and nutritional
Non-communicable
Injuries

Leading Causes 2022
1 HIV/AIDS
2 Neonatal disorders
3 Lower respiratory infect
4 Malaria
5 COVID-19
6 Diarrheal diseases
7 Congenital defects
8 Stroke
9 Dietary iron deficiency
10 Road injuries
11 Cirrhosis liver
12 Ischemic heart disease
13 Idiopathic epilepsy
14 Meningitis
15 Diabetes
16 Interpersonal violence
17 Tuberculosis
18 Chronic kidney disease
19 Depressive disorders
20 STIs
21 Low back pain
25 Headache disorders
27 Hypertensive heart disease
28 Age-related hearing loss
37 Other musculoskeletal

Leading Causes 2050	Mean % change number of DALY	Mean % change all-age DALY rate	Mean % change age-standardized DALY rate
1 Neonatal disorders	-20.7 (-38.3 to 1.45)	**-56.8 (-63.9 to -47.6)**	**-35.9 (-44.9 to -23.9)**
2 Stroke	**86.3 (49.6 to 127)**	1.75 (-14.1 to 20.6)	**-43.8 (-51.8 to -33)**
3 Lower respiratory infect	-6.9 (-27.7 to 16.7)	**-49.2 (-58.3 to -38.4)**	**-44 (-51.9 to -33.5)**
4 HIV/AIDS	**-64.1 (-71.4 to -55.2)**	**-80.4 (-83.5 to -76.6)**	**-83.4 (-85.9 to -80.3)**
5 Diabetes	**212 (154 to 286)**	**70.5 (44.2 to 101)**	-0.972 (-13.9 to 14.3)
6 Chronic kidney disease	**212 (133 to 309)**	**70.6 (29.3 to 116)**	16.8 (-8.82 to 51.1)
7 Diarrheal diseases	-14.2 (-45.7 to 33.4)	**-53.2 (-69.5 to -30.8)**	**-47.6 (-64.8 to -25.3)**
8 Cirrhosis liver	**93.6 (56.6 to 133)**	5.81 (-11.9 to 25.3)	**-31.8 (-40.9 to -19.8)**
9 Depressive disorders	**134 (97.7 to 169)**	**28 (19.9 to 36.5)**	**-2.37 (-2.64 to -2.09)**
10 Ischemic heart disease	**79.5 (23.6 to 154)**	-1.91 (-30.5 to 35.1)	**-45.9 (-61.6 to -23.4)**
11 Idiopathic epilepsy	**77.7 (25 to 149)**	-3.02 (-29.9 to 36.4)	-7.6 (-32.5 to 29.5)
12 Hypertensive heart disease	**244 (155 to 348)**	**87.7 (45.4 to 137)**	-3.43 (-25.7 to 26.4)
13 Congenital defects	-17.7 (-37.8 to 6.53)	**-55.2 (-62.6 to -48.5)**	**-35.1 (-43.8 to -25.8)**
14 Low back pain	**145 (107 to 180)**	**34 (24.8 to 44.1)**	**-5.01 (-5.26 to -4.73)**
15 Road injuries	46.8 (-10 to 149)	-19.8 (-51.1 to 36)	-31 (-57 to 15.8)
16 Other musculoskeletal	**361 (284 to 429)**	**152 (132 to 173)**	**75.2 (72.2 to 78.5)**
17 Dietary iron deficiency	**39.8 (13.8 to 68.4)**	**-23.8 (-29 to -18.6)**	**-13.7 (-14.8 to -12.6)**
18 Age-related hearing loss	**169 (128 to 209)**	**47 (33.2 to 60.4)**	**-1.46 (-1.62 to -1.3)**
19 Interpersonal violence	**45.5 (14.7 to 74.6)**	**-20.6 (-34 to -8.95)**	**-31.2 (-41.7 to -21.9)**
20 Headache disorders	**120 (85.5 to 157)**	**20 (12.8 to 31.9)**	**0.984 (0.633 to 1.25)**
30 Meningitis			
33 Malaria			
34 Tuberculosis			
47 STIs			
167 COVID-19			

Supplemental Results Figure S7. Leading 20 level 3 causes of DALYs, and percentage change in number of DALYs and all-age and age-standardised DALY rates by location, 2022 and 2050

Botswana, Reference scenario

Legend:
Communicable, maternal, neonatal and nutritional
Non-communicable
Injuries

Leading Causes 2022
1 HIV/AIDS
2 COVID-19
3 Neonatal disorders
4 Lower respiratory infect
5 Diarrheal diseases
6 Tuberculosis
7 Stroke
8 Diabetes
9 Road injuries
10 Ischemic heart disease
11 Depressive disorders
12 Interpersonal violence
13 Low back pain
14 Congenital defects
15 Gynecological diseases
16 Headache disorders
17 Self-harm
18 COPD
19 Hypertensive heart disease
20 Cirrhosis liver
22 Chronic kidney disease
25 Other musculoskeletal
28 Age-related hearing loss
29 Blindness and vision loss
31 Lung cancer

Leading Causes 2050	Mean % change number of DALY	Mean % change all-age DALY rate	Mean % change age-standardized DALY rate
1 Diabetes	**163 (117 to 218)**	**106 (74.7 to 141)**	-3.5 (-15.5 to 10.9)
2 Neonatal disorders	**-28.6 (-45 to -9.88)**	**-44 (-53.7 to -32.5)**	-13.2 (-26.5 to 1.64)
3 Lower respiratory infect	4.01 (-18.8 to 27.6)	**-18.3 (-32.7 to -2.37)**	**-39 (-48.1 to -27.7)**
4 Stroke	**58.8 (26.8 to 91.9)**	**24.7 (5.24 to 45)**	**-44.9 (-52.2 to -35.9)**
5 HIV/AIDS	**-82 (-85.9 to -77.8)**	**-85.8 (-88.4 to -82.9)**	**-87.9 (-89.9 to -85.7)**
6 Hypertensive heart disease	**201 (125 to 277)**	**136 (81.1 to 204)**	0.903 (-22 to 29.1)
7 Ischemic heart disease	43.7 (-0.813 to 88.4)	12.9 (-19 to 53.9)	**-48.2 (-62.3 to -28.9)**
8 COPD	**162 (101 to 217)**	**105 (64.4 to 149)**	-9.6 (-25.2 to 9.18)
9 Depressive disorders	**52.7 (32.9 to 74.4)**	**19.8 (11.6 to 28.3)**	**-2.65 (-2.88 to -2.45)**
10 Low back pain	**75.8 (53.5 to 99.7)**	**38 (27.2 to 47.6)**	**-5.32 (-5.53 to -5.14)**
11 Chronic kidney disease	**159 (96.6 to 227)**	**104 (59 to 155)**	8.37 (-13.4 to 37.7)
12 Other musculoskeletal	**180 (138 to 221)**	**120 (102 to 138)**	**61.7 (58.3 to 64.3)**
13 Road injuries	-5.98 (-43.8 to 53.2)	-26 (-54.4 to 27.3)	-27.3 (-57.4 to 29.9)
14 Diarrheal diseases	**-60.4 (-74.5 to -42.5)**	**-68.9 (-79.2 to -53.4)**	**-69 (-79 to -53.2)**
15 Headache disorders	**36.3 (16.6 to 58.9)**	**6.96 (0.689 to 19.4)**	**0.316 (0.119 to 0.455)**
16 Tuberculosis	**-47.3 (-62.7 to -25.8)**	**-58.5 (-71.3 to -41.2)**	**-69.7 (-79.3 to -56.2)**
17 Lung cancer	**146 (82.5 to 205)**	**92.8 (48.2 to 133)**	-5.84 (-22.8 to 10.6)
18 Age-related hearing loss	**106 (78.5 to 136)**	**61.6 (46.8 to 76.6)**	**-1.18 (-1.31 to -1.04)**
19 Blindness and vision loss	**108 (79 to 142)**	**63.6 (45 to 79.4)**	**-6.01 (-6.74 to -5.24)**
20 Cirrhosis liver	**43 (19.2 to 65.3)**	12.4 (-3.65 to 29.7)	**-28.8 (-37.5 to -18.5)**
22 Gynecological diseases			
25 Self-harm			
27 Interpersonal violence			
36 Congenital defects			
161 COVID-19			

Supplemental Results Figure S7. Leading 20 level 3 causes of DALYs, and percentage change in number of DALYs and all-age and age-standardised DALY rates by location, 2022 and 2050

Eswatini, Reference scenario

Legend:
Communicable, maternal, neonatal and nutritional
Non-communicable
Injuries

Leading Causes 2022	Leading Causes 2050	Mean % change number of DALY	Mean % change all-age DALY rate	Mean % change age-standardized DALY rate
1 HIV/AIDS	1 HIV/AIDS	**-71.9 (-77.4 to -65.3)**	**-75.4 (-78.5 to -70.9)**	**-78 (-80.7 to -74.3)**
2 COVID-19	2 Diabetes	**81.3 (41.6 to 128)**	**59.2 (30.9 to 98.5)**	**-17.7 (-29.4 to -2.18)**
3 Neonatal disorders	3 Lower respiratory infect	**-24.9 (-38.3 to -8.19)**	**-34 (-44.3 to -20.9)**	**-41 (-49.7 to -31.8)**
4 Lower respiratory infect	4 Neonatal disorders	**-32.4 (-48.5 to -13.1)**	**-40.7 (-51.9 to -27.1)**	-8.9 (-23.6 to 9.05)
5 Road injuries	5 Road injuries	-9.67 (-45.2 to 53.4)	-20.5 (-52.7 to 35.2)	-27.3 (-57.1 to 24)
6 Tuberculosis	6 Stroke	19.6 (-3.48 to 46.2)	5.06 (-12.2 to 25.5)	**-45.2 (-53 to -35.7)**
7 Diarrheal diseases	7 Chronic kidney disease	**120 (73.6 to 183)**	**93.8 (52.8 to 147)**	19.5 (-4.87 to 50.6)
8 Diabetes	8 Ischemic heart disease	20.2 (-12.7 to 65.7)	5.74 (-24.5 to 45.1)	**-44.5 (-60 to -22.8)**
9 Stroke	9 Hypertensive heart disease	**124 (69.3 to 189)**	**97.2 (52.4 to 155)**	-1.5 (-23.9 to 24.7)
10 Interpersonal violence	10 Tuberculosis	**-52.6 (-66.6 to -33.3)**	**-58.3 (-71.1 to -43.4)**	**-68.6 (-77.9 to -55.4)**
11 Ischemic heart disease	11 Self-harm	6.36 (-16.1 to 30.8)	-6.64 (-23.2 to 11.4)	**-18.1 (-32.8 to -1.01)**
12 Self-harm	12 Diarrheal diseases	**-57.6 (-70.4 to -38.7)**	**-62.7 (-75.4 to -46.4)**	**-58.6 (-71.1 to -39.8)**
13 Chronic kidney disease	13 COPD	**67.7 (32.9 to 102)**	**47.4 (18.9 to 79.2)**	**-23 (-37 to -5.84)**
14 Congenital defects	14 Depressive disorders	**39.4 (17.3 to 63.1)**	**22.3 (15.7 to 30.8)**	**-2.64 (-2.87 to -2.4)**
15 Cirrhosis liver	15 Interpersonal violence	**-25.6 (-40.7 to -7.88)**	**-34.8 (-43.3 to -25.3)**	**-35 (-42.6 to -25.6)**
16 Depressive disorders	16 Cirrhosis liver	**21.5 (1.94 to 48)**	6.71 (-7.93 to 22.9)	**-30.4 (-38.1 to -21.4)**
17 Hypertensive heart disease	17 Other musculoskeletal	**153 (116 to 206)**	**122 (102 to 142)**	**59.7 (54.1 to 64)**
18 COPD	18 Liver cancer	**92.6 (46.9 to 153)**	**69.3 (31.6 to 121)**	3.66 (-17.4 to 32)
19 Asthma	19 Low back pain	**56 (33.4 to 78.6)**	**36.9 (26.9 to 47.2)**	**-5.36 (-5.68 to -5.02)**
20 Headache disorders	20 Headache disorders	**28.4 (7.96 to 50)**	**12.7 (6.51 to 25.7)**	**0.352 (0.105 to 0.587)**
22 Low back pain	28 Congenital defects			
29 Liver cancer	32 Asthma			
35 Other musculoskeletal	162 COVID-19			

Supplemental Results Figure S7. Leading 20 level 3 causes of DALYs, and percentage change in number of DALYs and all-age and age-standardised DALY rates by location, 2022 and 2050

Lesotho, Reference scenario

Legend: Communicable, maternal, neonatal and nutritional; Non-communicable; Injuries

Leading Causes 2022	Leading Causes 2050	Mean % change number of DALY	Mean % change all-age DALY rate	Mean % change age-standardized DALY rate
1 HIV/AIDS	1 Lower respiratory infect	**-18.1 (-31.1 to -1.54)**	**-30.8 (-42.3 to -16)**	**-36 (-44.6 to -24.2)**
2 Tuberculosis	2 Tuberculosis	**-43.1 (-59.6 to -20.5)**	**-51.9 (-66.4 to -33)**	**-63.8 (-74.3 to -50.3)**
3 Neonatal disorders	3 Neonatal disorders	**-41.6 (-55.4 to -25.9)**	**-50.9 (-60.6 to -39.3)**	**-28.3 (-40.2 to -14.1)**
4 Lower respiratory infect	4 Diarrheal diseases	**-37.5 (-57.7 to -10.9)**	**-47.2 (-64.9 to -23.6)**	**-43.1 (-60.6 to -18.4)**
5 Diarrheal diseases	5 Stroke	10.4 (-11.5 to 30.2)	-6.78 (-24.2 to 9.82)	**-37 (-46.2 to -26.4)**
6 Road injuries	6 Road injuries	-1.28 (-38 to 61.7)	-16.5 (-50 to 40.1)	-25.4 (-55.3 to 23.8)
7 Stroke	7 Diabetes	**64.1 (32 to 102)**	**38.5 (11.5 to 66.8)**	-6.81 (-21 to 11)
8 Interpersonal violence	8 HIV/AIDS	**-88.5 (-91.1 to -85.6)**	**-90.3 (-92.3 to -88.5)**	**-90.7 (-92.4 to -89.1)**
9 COVID-19	9 Interpersonal violence	**-23.1 (-36.5 to -2.72)**	**-35.2 (-42.6 to -26.4)**	**-36.4 (-43.2 to -27.1)**
10 Diabetes	10 Hypertensive heart disease	**89.3 (43.4 to 143)**	**60 (20.6 to 108)**	1.17 (-21.8 to 28.9)
11 Ischemic heart disease	11 Chronic kidney disease	**104 (59.4 to 158)**	**72.2 (30.8 to 117)**	25.4 (-1.68 to 58.7)
12 Self-harm	12 COPD	**53.4 (21.6 to 88.3)**	**29.5 (2.8 to 58.5)**	-10.1 (-25.6 to 10.5)
13 Depressive disorders	13 Ischemic heart disease	24 (-11.4 to 68.2)	4.83 (-27.1 to 44.8)	**-31.6 (-50.5 to -5.37)**
14 COPD	14 Depressive disorders	**40.9 (22.5 to 62.7)**	**18.7 (10.7 to 27.2)**	**-2.36 (-2.64 to -2.06)**
15 Congenital defects	15 Self-harm	10.3 (-8.85 to 32.9)	-6.95 (-22 to 9.07)	**-20.7 (-31.9 to -7.85)**
16 Cirrhosis liver	16 Cirrhosis liver	**22.1 (3.38 to 41.5)**	3.01 (-12.8 to 17.6)	**-29.4 (-37.2 to -21)**
17 Hypertensive heart disease	17 Low back pain	**51 (32 to 73)**	**27.3 (15.4 to 37.6)**	**-4.64 (-4.91 to -4.39)**
18 Chronic kidney disease	18 Lung cancer	**95.5 (47.6 to 150)**	**64.6 (27.4 to 103)**	5.56 (-13.5 to 25.2)
19 Protein-energy malnutrition	19 Cervical cancer	**34.8 (12.6 to 55.1)**	13.8 (-4.92 to 32.9)	**-26.2 (-35 to -16.4)**
20 Low back pain	20 Other musculoskeletal	**137 (103 to 175)**	**99.5 (81.3 to 119)**	**51.2 (46.6 to 54.5)**
21 Cervical cancer	24 Congenital defects			
30 Lung cancer	50 Protein-energy malnutrition			
38 Other musculoskeletal	160 COVID-19			

Supplemental Results Figure S7. Leading 20 level 3 causes of DALYs, and percentage change in number of DALYs and all-age and age-standardised DALY rates by location, 2022 and 2050

Namibia, Reference scenario

Legend:
Communicable, maternal, neonatal and nutritional
Non-communicable
Injuries

Leading Causes 2022
1 HIV/AIDS
2 COVID-19
3 Neonatal disorders
4 Lower respiratory infect
5 Diarrheal diseases
6 Stroke
7 Tuberculosis
8 Road injuries
9 Ischemic heart disease
10 Diabetes
11 Interpersonal violence
12 COPD
13 Congenital defects
14 Cirrhosis liver
15 Self-harm
16 Low back pain
17 Depressive disorders
18 Hypertensive heart disease
19 Headache disorders
20 Gynecological diseases
23 Breast cancer
24 Chronic kidney disease
27 Other musculoskeletal

Leading Causes 2050	Mean % change number of DALY	Mean % change all-age DALY rate	Mean % change age-standardized DALY rate
1 Lower respiratory infect	15.4 (-7.69 to 41.9)	**-17.6 (-29.3 to -4.46)**	**-33.9 (-42.2 to -24.4)**
2 Neonatal disorders	-18 (-36.6 to 2.41)	**-41.5 (-51.6 to -29.6)**	**-16.7 (-29.6 to -0.0988)**
3 Stroke	**45.2 (18.9 to 77.5)**	3.76 (-11 to 22.4)	**-43.6 (-50.2 to -34.9)**
4 Diabetes	**110 (70.2 to 156)**	**50 (26.4 to 84.1)**	**-15.7 (-26.9 to -2.08)**
5 Road injuries	22.9 (-25.3 to 99.3)	-12.2 (-44.3 to 36.9)	-19.6 (-50.3 to 26.7)
6 Hypertensive heart disease	**200 (129 to 284)**	**114 (69.5 to 171)**	12.4 (-13 to 43)
7 Ischemic heart disease	27.5 (-8.34 to 74.2)	-8.95 (-30.8 to 21.3)	**-49 (-61.8 to -30)**
8 Diarrheal diseases	**-37.6 (-58.7 to -8.02)**	**-55.5 (-70 to -30)**	**-53.6 (-69.5 to -31)**
9 COPD	**101 (61.6 to 150)**	**43.9 (18.4 to 69.9)**	**-21 (-34.2 to -5.74)**
10 Cirrhosis liver	**91.4 (56.2 to 129)**	**36.7 (16.9 to 58.2)**	-8.35 (-19.2 to 5.22)
11 Low back pain	**83.2 (54.8 to 109)**	**30.8 (21.8 to 39.3)**	**-4.51 (-4.68 to -4.3)**
12 Interpersonal violence	-1.11 (-19.2 to 22.2)	**-29.4 (-39.2 to -18.7)**	**-30 (-39 to -19.5)**
13 Tuberculosis	**-36.6 (-56.1 to -11.7)**	**-54.7 (-66.7 to -36.4)**	**-65.8 (-75.1 to -51.5)**
14 Depressive disorders	**66.3 (42 to 90.6)**	**18.7 (12.7 to 25)**	**-2.14 (-2.31 to -1.96)**
15 Other musculoskeletal	**186 (143 to 228)**	**104 (89 to 120)**	**53.2 (50.2 to 55.5)**
16 Self-harm	**48.2 (17.3 to 82.6)**	5.79 (-9 to 25.5)	-7.08 (-19.3 to 12.3)
17 Chronic kidney disease	**143 (85.8 to 207)**	**73.3 (40.9 to 117)**	8.06 (-12.6 to 35.2)
18 HIV/AIDS	**-86 (-89.8 to -80.2)**	**-90 (-92.3 to -86.2)**	**-91.2 (-92.9 to -88.4)**
19 Breast cancer	**120 (70.2 to 175)**	**57.2 (25.9 to 95)**	-0.154 (-16.2 to 17.8)
20 Headache disorders	**54.3 (31.3 to 78.6)**	**10.2 (5.05 to 20.6)**	**0.259 (0.0757 to 0.417)**
21 Gynecological diseases			
26 Congenital defects			
162 COVID-19			

Supplemental Results Figure S7. Leading 20 level 3 causes of DALYs, and percentage change in number of DALYs and all-age and age-standardised DALY rates by location, 2022 and 2050

South Africa, Reference scenario

Legend:
Communicable, maternal, neonatal and nutritional
Non-communicable
Injuries

Leading Causes 2022	Leading Causes 2050	Mean % change number of DALY	Mean % change all-age DALY rate	Mean % change age-standardized DALY rate
1 HIV/AIDS	1 HIV/AIDS	**-66 (-71 to -59.9)**	**-70.5 (-74.3 to -67.1)**	**-73.1 (-75.9 to -70)**
2 Neonatal disorders	2 Diabetes	**76.7 (54.3 to 105)**	**53 (38.3 to 70.6)**	**-11.7 (-21 to -1.42)**
3 COVID-19	3 Neonatal disorders	-12.1 (-27.9 to 7.08)	**-23.9 (-35.1 to -11.7)**	6.65 (-9.32 to 22.6)
4 Lower respiratory infect	4 Lower respiratory infect	-4.27 (-19.1 to 10.7)	**-17.1 (-25.5 to -8.16)**	**-36.4 (-42.5 to -29.6)**
5 Road injuries	5 Road injuries	-6.63 (-35.9 to 39.4)	-19.1 (-43.5 to 21.6)	-17.8 (-43.5 to 26.4)
6 Interpersonal violence	6 Stroke	16.4 (-0.281 to 33.5)	0.834 (-9.89 to 12.2)	**-44 (-49.3 to -38.3)**
7 Diabetes	7 Interpersonal violence	**-30.1 (-41.9 to -18.5)**	**-39.5 (-45.4 to -32.7)**	**-35.9 (-43 to -27.8)**
8 Stroke	8 Hypertensive heart disease	**133 (88.9 to 180)**	**102 (71.2 to 145)**	9.46 (-9.77 to 30.9)
9 Tuberculosis	9 Ischemic heart disease	5.61 (-18.1 to 32.5)	-8.45 (-27.3 to 15.4)	**-47.8 (-59.3 to -32.3)**
10 Diarrheal diseases	10 Chronic kidney disease	**99 (64.1 to 140)**	**72.4 (44.1 to 103)**	8.9 (-8.7 to 31.2)
11 Ischemic heart disease	11 COPD	**82.9 (55.4 to 115)**	**58.4 (40.9 to 80.2)**	-12.7 (-23.4 to 0.095)
12 Self-harm	12 Diarrheal diseases	-21.9 (-43.6 to 5.89)	**-32.3 (-49.6 to -9.89)**	**-39.5 (-56.9 to -15.8)**
13 Depressive disorders	13 Depressive disorders	**31.7 (15.9 to 47.7)**	**14 (10 to 19)**	**-1.84 (-2.01 to -1.67)**
14 COPD	14 Low back pain	**49.3 (30.6 to 68.3)**	**29.3 (23.6 to 35.8)**	**-3.69 (-3.87 to -3.54)**
15 Low back pain	15 Tuberculosis	**-47.1 (-60.1 to -30.6)**	**-54.2 (-64.8 to -39.9)**	**-63.4 (-72.2 to -51.2)**
16 Hypertensive heart disease	16 Other musculoskeletal	**98.6 (71.4 to 125)**	**71.9 (62 to 82.4)**	**33.3 (30.7 to 35.4)**
17 Chronic kidney disease	17 Self-harm	-3.47 (-19.6 to 13.9)	**-16.4 (-26.9 to -2.05)**	-15.4 (-27 to 0.755)
18 Dietary iron deficiency	18 Blindness and vision loss	**61.2 (38.7 to 83.4)**	**39.5 (28.9 to 51.5)**	**-3.57 (-4.03 to -3.12)**
19 Headache disorders	19 Endo/metab/blood/immune	**52.9 (32.1 to 76.5)**	**32.4 (20.7 to 44)**	6.04 (-3.22 to 13.8)
20 Gynecological diseases	20 Lung cancer	**55 (24.9 to 84.4)**	**34.2 (12.9 to 54.4)**	**-19.6 (-30.8 to -7.82)**
22 Endo/metab/blood/immune	21 Headache disorders			
24 Lung cancer	25 Gynecological diseases			
25 Blindness and vision loss	29 Dietary iron deficiency			
27 Other musculoskeletal	162 COVID-19			

Supplemental Results Figure S7. Leading 20 level 3 causes of DALYs, and percentage change in number of DALYs and all-age and age-standardised DALY rates by location, 2022 and 2050

Zimbabwe, Reference scenario

Legend:
Communicable, maternal, neonatal and nutritional
Non-communicable
Injuries

Leading Causes 2022	Leading Causes 2050	Mean % change number of DALY	Mean % change all-age DALY rate	Mean % change age-standardized DALY rate
1 HIV/AIDS	1 Lower respiratory infect	-12.7 (-27.4 to 5.97)	**-38.1 (-47.3 to -23.5)**	**-33.9 (-43.1 to -20.5)**
2 Neonatal disorders	2 Neonatal disorders	**-45.3 (-59.2 to -26.6)**	**-61.3 (-68.3 to -49.9)**	**-46.8 (-56.1 to -33.7)**
3 Lower respiratory infect	3 Stroke	**44.7 (12 to 79.3)**	2.7 (-16.8 to 26.3)	**-33.5 (-46.1 to -15.5)**
4 COVID-19	4 Diarrheal diseases	-2.05 (-41.7 to 48.1)	-30.6 (-54.6 to 5.56)	-22.6 (-45.6 to 11)
5 Tuberculosis	5 Diabetes	**116 (71.8 to 166)**	**53.5 (26.6 to 88)**	-1.46 (-17.2 to 20.1)
6 Diarrheal diseases	6 Tuberculosis	**-39.6 (-61.4 to -12.2)**	**-57.1 (-71.2 to -34.8)**	**-63.8 (-75.1 to -45.5)**
7 Malaria	7 Ischemic heart disease	**66.9 (13 to 140)**	18.6 (-17.5 to 72.8)	-24.1 (-48.4 to 10.7)
8 Stroke	8 Chronic kidney disease	**147 (82.4 to 224)**	**75.3 (35.4 to 128)**	29.3 (-0.314 to 72.3)
9 Protein-energy malnutrition	9 Road injuries	13.4 (-37.2 to 101)	-19.5 (-54 to 46.5)	-27.3 (-56.5 to 28.7)
10 Road injuries	10 Self-harm	29.2 (-1.42 to 65.4)	-8.36 (-25.2 to 15.1)	**-20.1 (-34.2 to -0.948)**
11 Ischemic heart disease	11 HIV/AIDS	**-82.3 (-85.9 to -78.1)**	**-87.4 (-89.5 to -85)**	**-88.3 (-90.3 to -86)**
12 Congenital defects	12 Low back pain	**74.9 (48 to 101)**	**24 (15.3 to 32.2)**	**-4.47 (-4.66 to -4.3)**
13 Self-harm	13 Hypertensive heart disease	**137 (77.4 to 203)**	**68.4 (30.8 to 121)**	3.6 (-20.5 to 35)
14 Diabetes	14 Congenital defects	**-22.2 (-38.7 to -5.04)**	**-44.9 (-51.2 to -36.4)**	**-27.5 (-34.6 to -17.9)**
15 Interpersonal violence	15 Headache disorders	**57.6 (32.8 to 82.3)**	**11.6 (6.46 to 19.7)**	0.0874 (-0.138 to 0.286)
16 Dietary iron deficiency	16 Depressive disorders	**68.1 (40.1 to 94.5)**	**19.1 (12.5 to 26.8)**	**-2.16 (-2.39 to -1.96)**
17 Cirrhosis liver	17 Protein-energy malnutrition	**-44.9 (-73.3 to -0.224)**	**-60.9 (-80.3 to -29.6)**	**-51.1 (-75 to -9.81)**
18 Meningitis	18 Other musculoskeletal	**176 (132 to 219)**	**95.4 (80.6 to 110)**	**51.1 (48.2 to 53.8)**
19 Low back pain	19 Cirrhosis liver	21.9 (-1.4 to 46.4)	-13.5 (-25.9 to 3.04)	**-36.1 (-44.4 to -25)**
20 Chronic kidney disease	20 Interpersonal violence	0.0189 (-20.7 to 25.7)	**-29.2 (-38.8 to -18.4)**	**-32.1 (-41.8 to -22.3)**
22 Headache disorders	21 Dietary iron deficiency			
25 Depressive disorders	34 Malaria			
27 Hypertensive heart disease	37 Meningitis			
36 Other musculoskeletal	164 COVID-19			

Supplemental Results Figure S7. Leading 20 level 3 causes of DALYs, and percentage change in number of DALYs and all-age and age-standardised DALY rates by location, 2022 and 2050

Benin, Reference scenario

Legend:
Communicable, maternal, neonatal and nutritional
Non-communicable
Injuries

Leading Causes 2022	Leading Causes 2050	Mean % change number of DALY	Mean % change all-age DALY rate	Mean % change age-standardized DALY rate
1 Neonatal disorders	1 Malaria	50.2 (-79.7 to 705)	-26.8 (-90 to 297)	-13.1 (-87.3 to 345)
2 Malaria	2 Neonatal disorders	-17 (-37.8 to 5.94)	**-60.6 (-68.6 to -50.8)**	**-39 (-49.3 to -24.5)**
3 Lower respiratory infect	3 Lower respiratory infect	-18.1 (-39.7 to 7.99)	**-61.1 (-69.3 to -49.2)**	**-47.3 (-56.8 to -32.7)**
4 Congenital defects	4 Stroke	**76 (41.3 to 117)**	-16.2 (-31.8 to 0.966)	**-43.7 (-52.8 to -30.8)**
5 Diarrheal diseases	5 Diabetes	**232 (172 to 298)**	**58 (35.5 to 80.1)**	6.03 (-7.18 to 19.9)
6 Measles	6 Congenital defects	-11.5 (-29.8 to 10.8)	**-58 (-64 to -49.2)**	**-38.9 (-46.1 to -28.9)**
7 Hemoglobinopathies	7 Hemoglobinopathies	**31.8 (1.72 to 62.8)**	**-37.5 (-47.2 to -26.6)**	**-26.4 (-35 to -17.9)**
8 Stroke	8 Chronic kidney disease	**214 (141 to 329)**	**49.4 (15.5 to 97.2)**	24.5 (-1.67 to 63.4)
9 Road injuries	9 Diarrheal diseases	-19.2 (-49.4 to 23.6)	**-61.6 (-75.4 to -43.1)**	**-55.8 (-68.4 to -39.5)**
10 Meningitis	10 Other musculoskeletal	**560 (464 to 661)**	**214 (192 to 236)**	**139 (132 to 146)**
11 HIV/AIDS	11 Depressive disorders	**150 (118 to 185)**	**19.1 (13.3 to 25.7)**	**-4.15 (-4.8 to -3.36)**
12 Pertussis	12 Road injuries	39.1 (-22.8 to 145)	-33.8 (-62.6 to 13.7)	-35.1 (-60.9 to 8.29)
13 Diabetes	13 Headache disorders	**143 (110 to 180)**	**15.4 (10.9 to 24.4)**	**0.805 (0.184 to 1.29)**
14 Dietary iron deficiency	14 Low back pain	**149 (112 to 182)**	**18.2 (11 to 25)**	**-8.28 (-8.75 to -7.47)**
15 Ischemic heart disease	15 Ischemic heart disease	**75.4 (25.6 to 145)**	-16.5 (-39.8 to 13.4)	**-45.5 (-61 to -25.5)**
16 Depressive disorders	16 Dietary iron deficiency	**56.6 (30.8 to 83.6)**	**-25.6 (-28.8 to -21.9)**	**-24.1 (-25.1 to -23.1)**
17 Cirrhosis liver	17 Cirrhosis liver	**67.1 (36 to 105)**	**-20.5 (-34.1 to -2.29)**	**-38.7 (-47.3 to -25.5)**
18 iNTS	18 Anxiety disorders	**134 (103 to 173)**	**11.1 (6.4 to 16.4)**	**2.01 (1.45 to 2.65)**
19 Tuberculosis	19 Gynecological diseases	**89.7 (49.4 to 140)**	-9.73 (-24.6 to 11)	**-26.9 (-40.1 to -11.3)**
20 Maternal disorders	20 Interpersonal violence	**69.6 (32.5 to 112)**	**-19.4 (-32.5 to -3.88)**	**-23.8 (-34.7 to -9.38)**
21 Headache disorders	22 Meningitis			
22 Chronic kidney disease	24 iNTS			
23 Low back pain	29 Pertussis			
25 Interpersonal violence	33 Maternal disorders			
26 Gynecological diseases	35 Tuberculosis			
27 Anxiety disorders	48 HIV/AIDS			
39 Other musculoskeletal	89 Measles			

Burkina Faso, Reference scenario

Legend:
Communicable, maternal, neonatal and nutritional
Non-communicable
Injuries

Leading Causes 2022	Leading Causes 2050	Mean % change number of DALY	Mean % change all-age DALY rate	Mean % change age-standardized DALY rate
1 Malaria	1 Malaria	14.4 (-92.4 to 600)	-47.1 (-96.4 to 186)	-41.4 (-95.5 to 208)
2 Neonatal disorders	2 Neonatal disorders	-2.91 (-25.2 to 23.7)	**-56.5 (-65.5 to -45.8)**	**-37.5 (-48.2 to -24.6)**
3 Lower respiratory infect	3 Lower respiratory infect	-15.9 (-35.3 to 9.08)	**-62.3 (-70 to -52.9)**	**-44.6 (-54.3 to -33.2)**
4 Diarrheal diseases	4 Diarrheal diseases	-8.29 (-44.6 to 40.3)	**-58.9 (-74.1 to -37.7)**	**-43.6 (-62.6 to -18)**
5 Congenital defects	5 Congenital defects	-0.311 (-19.7 to 22.3)	**-55.4 (-61.5 to -47.2)**	**-39.1 (-46.9 to -29.2)**
6 Road injuries	6 Road injuries	44.4 (-17.5 to 146)	-35.2 (-63.2 to 13.8)	-36.4 (-61.3 to 7.23)
7 Hemoglobinopathies	7 Hemoglobinopathies	**41.8 (2.2 to 75)**	**-36.5 (-52.5 to -24)**	**-26.3 (-41.1 to -15.5)**
8 Meningitis	8 Stroke	**51.2 (22.8 to 80.8)**	**-32.2 (-45.1 to -18.2)**	**-43.5 (-53 to -31.7)**
9 iNTS	9 Dietary iron deficiency	**58 (38.1 to 80)**	**-29.3 (-32 to -26.6)**	**-22 (-23.5 to -20.8)**
10 Tuberculosis	10 Diabetes	**159 (117 to 204)**	16.1 (-0.76 to 35.2)	-6.04 (-19.2 to 9.32)
11 Stroke	11 iNTS	24.7 (-33.2 to 103)	**-44.1 (-68.9 to -10.8)**	-31.7 (-61.4 to 9.17)
12 Protein-energy malnutrition	12 Depressive disorders	**149 (127 to 173)**	**11.7 (6.72 to 17.5)**	**-3.98 (-4.53 to -3.33)**
13 Dietary iron deficiency	13 Other musculoskeletal	**507 (450 to 565)**	**172 (153 to 190)**	**127 (119 to 133)**
14 Ischemic heart disease	14 Chronic kidney disease	**123 (62.7 to 196)**	-0.0612 (-27.8 to 34.4)	-5.84 (-29.7 to 22)
15 Measles	15 Headache disorders	**149 (127 to 174)**	**11.5 (7.85 to 17)**	**0.707 (0.048 to 1.22)**
16 COVID-19	16 Low back pain	**143 (121 to 165)**	**9.16 (3.43 to 15.5)**	**-7.63 (-8.4 to -6.97)**
17 Interpersonal violence	17 Ischemic heart disease	**61.4 (8.19 to 124)**	-27.6 (-51 to 1.85)	**-42.8 (-60 to -20.1)**
18 Chronic kidney disease	18 Interpersonal violence	**80.1 (46.3 to 122)**	**-19.3 (-32.8 to -4.42)**	**-22.9 (-33.4 to -10.3)**
19 Typhoid & paratyph	19 Meningitis	-9.16 (-45 to 42.9)	**-59.3 (-75 to -35.2)**	**-49.9 (-67.1 to -25.9)**
20 Diabetes	20 Cirrhosis liver	**66 (35.9 to 102)**	**-25.6 (-39.1 to -11.1)**	**-36.2 (-46.2 to -25.1)**
21 Depressive disorders	22 Tuberculosis			
24 Headache disorders	25 Typhoid & paratyph			
25 Low back pain	36 Protein-energy malnutrition			
26 Cirrhosis liver	75 Measles			
45 Other musculoskeletal	166 COVID-19			

Supplemental Results Figure S7. Leading 20 level 3 causes of DALYs, and percentage change in number of DALYs and all-age and age-standardised DALY rates by location, 2022 and 2050

Cabo Verde, Reference scenario

Legend: Communicable, maternal, neonatal and nutritional; Non-communicable; Injuries

Leading Causes 2022	Leading Causes 2050	Mean % change number of DALY	Mean % change all-age DALY rate	Mean % change age-standardized DALY rate
1 Ischemic heart disease	1 Diabetes	**161 (114 to 203)**	**143 (114 to 179)**	**24 (10.5 to 39.9)**
2 Stroke	2 Ischemic heart disease	30.6 (-6.68 to 80.1)	21.8 (-9.97 to 69.6)	**-44.5 (-60.9 to -22.7)**
3 Neonatal disorders	3 Stroke	**32.3 (6.83 to 60.4)**	**23.3 (3.05 to 51)**	**-41.3 (-50 to -28.4)**
4 Lower respiratory infect	4 Lower respiratory infect	**29.7 (4.02 to 53.6)**	**20.9 (3.61 to 47.3)**	**-35.5 (-43.5 to -25.1)**
5 Diabetes	5 Depressive disorders	**37.2 (16.7 to 58.2)**	**27.8 (17.9 to 39.5)**	**-3.27 (-3.63 to -2.9)**
6 COVID-19	6 Chronic kidney disease	**143 (86 to 210)**	**127 (81.3 to 185)**	18.1 (-8.15 to 49.8)
7 Depressive disorders	7 Other musculoskeletal	**194 (143 to 240)**	**174 (151 to 199)**	**93.1 (89.8 to 96.3)**
8 Interpersonal violence	8 Low back pain	**48.6 (28.7 to 69.3)**	**38.5 (28.2 to 49.7)**	**-6.57 (-6.87 to -6.3)**
9 Low back pain	9 Lung cancer	**142 (91.6 to 208)**	**126 (78.2 to 174)**	4.77 (-13 to 23.8)
10 Self-harm	10 Prostate cancer	**204 (129 to 301)**	**183 (117 to 260)**	17.7 (-3.64 to 45.9)
11 Cirrhosis liver	11 Cirrhosis liver	16.3 (-5.97 to 41)	8.38 (-11.1 to 30.3)	**-35.7 (-45.4 to -23.5)**
12 Headache disorders	12 Hypertensive heart disease	**116 (63 to 175)**	**101 (59 to 156)**	-5.67 (-25.9 to 19.1)
13 Gynecological diseases	13 Age-related hearing loss	**79 (50.4 to 105)**	**66.8 (52.2 to 83.7)**	**-1.68 (-1.92 to -1.48)**
14 Chronic kidney disease	14 Headache disorders	16.2 (-3.27 to 38.7)	8.24 (-0.404 to 27.9)	**1.04 (0.603 to 1.28)**
15 Drowning	15 Self-harm	7.44 (-12.6 to 33)	0.0551 (-16.7 to 18.6)	**-20.9 (-33.3 to -7.09)**
16 Dietary iron deficiency	16 Esophageal cancer	**108 (72.3 to 146)**	**93.9 (66.2 to 126)**	-6.02 (-16.1 to 4.49)
17 HIV/AIDS	17 Neonatal disorders	**-46.7 (-60.1 to -33.5)**	**-50.4 (-58.8 to -41.5)**	**-27.2 (-36.9 to -15.6)**
18 Diarrheal diseases	18 Pancreatic cancer	**275 (173 to 388)**	**250 (163 to 344)**	**64.4 (24.6 to 105)**
19 Age-related hearing loss	19 Stomach cancer	**49.3 (22.5 to 77.1)**	**39.1 (21.3 to 56.1)**	**-35 (-39.6 to -29.6)**
20 Stomach cancer	20 COPD	**101 (57.5 to 146)**	**87.3 (51.7 to 132)**	-11.3 (-27.9 to 11)
22 Other musculoskeletal	21 Interpersonal violence			
25 Hypertensive heart disease	25 Gynecological diseases			
26 Esophageal cancer	35 Dietary iron deficiency			
27 Lung cancer	37 Drowning			
29 COPD	42 Diarrheal diseases			
36 Prostate cancer	45 HIV/AIDS			
43 Pancreatic cancer	158 COVID-19			

Cameroon, Reference scenario

Legend:
Communicable, maternal, neonatal and nutritional
Non-communicable
Injuries

Leading Causes 2022	Leading Causes 2050	Mean % change number of DALY	Mean % change all-age DALY rate	Mean % change age-standardized DALY rate
1 Neonatal disorders	1 Neonatal disorders	-14.1 (-41.1 to 16.8)	**-50.4 (-60.4 to -38.2)**	**-22 (-34.8 to -7.29)**
2 Malaria	2 Lower respiratory infect	-12.3 (-35.2 to 16.4)	**-49.2 (-58.8 to -36.5)**	**-39.7 (-49.9 to -27.4)**
3 Lower respiratory infect	3 Malaria	-47.5 (-95 to 193)	-69.4 (-96.8 to 68.3)	-65.2 (-96.5 to 111)
4 HIV/AIDS	4 Stroke	**75.4 (32.4 to 123)**	1.71 (-17.2 to 22.6)	**-40.3 (-50 to -26.9)**
5 Diarrheal diseases	5 Diabetes	**232 (147 to 308)**	**92.3 (61.8 to 129)**	13.1 (-2.77 to 30.5)
6 Congenital defects	6 Chronic kidney disease	**225 (130 to 336)**	**88.4 (43 to 145)**	**35.4 (4.71 to 77.3)**
7 Stroke	7 Road injuries	38.1 (-21 to 131)	-20 (-50 to 39.5)	-26.3 (-52.4 to 27)
8 Road injuries	8 Ischemic heart disease	**89.7 (25 to 175)**	10 (-23.2 to 51.7)	**-39.2 (-57.6 to -11.6)**
9 Ischemic heart disease	9 Depressive disorders	**119 (81.8 to 163)**	**27 (18.9 to 36.1)**	**-2.9 (-3.27 to -2.51)**
10 Diabetes	10 Congenital defects	-24.8 (-45.8 to 1.12)	**-56.5 (-63.8 to -47.9)**	**-36 (-44 to -25.1)**
11 Pertussis	11 Diarrheal diseases	-25.4 (-58.6 to 14.3)	**-56.8 (-74.3 to -33.2)**	**-48.3 (-66.9 to -25.2)**
12 Meningitis	12 Low back pain	**125 (88.4 to 168)**	**30.6 (21.5 to 40.4)**	**-5.9 (-6.25 to -5.59)**
13 Chronic kidney disease	13 Other musculoskeletal	**351 (268 to 431)**	**161 (141 to 182)**	**86.6 (81.9 to 91.2)**
14 Tuberculosis	14 Headache disorders	**101 (62.4 to 145)**	**16.4 (10.2 to 29.4)**	**0.678 (0.351 to 0.995)**
15 Depressive disorders	15 Cirrhosis liver	**60.8 (23.2 to 98.8)**	-6.73 (-22.4 to 11.8)	**-35.7 (-44.7 to -24.1)**
16 Maternal disorders	16 Hypertensive heart disease	**196 (109 to 304)**	**71.7 (28.6 to 120)**	-5.35 (-28.1 to 21.4)
17 Cirrhosis liver	17 Gynecological diseases	**79.2 (36.2 to 129)**	3.92 (-14.3 to 22.4)	**-18.7 (-32.3 to -3.63)**
18 Low back pain	18 Anxiety disorders	**89.5 (54.5 to 129)**	**9.78 (3.11 to 18.6)**	**1.53 (1.27 to 1.82)**
19 Headache disorders	19 Age-related hearing loss	**146 (100 to 193)**	**42.6 (29.5 to 57.1)**	**-1.87 (-2.09 to -1.7)**
20 Measles	20 Dietary iron deficiency	**28.6 (2.36 to 57.1)**	**-25.6 (-30.4 to -20.9)**	**-19.1 (-19.7 to -18.3)**
21 Dietary iron deficiency	24 HIV/AIDS			
24 Gynecological diseases	29 Tuberculosis			
27 Anxiety disorders	31 Meningitis			
31 Hypertensive heart disease	34 Pertussis			
32 Age-related hearing loss	35 Maternal disorders			
34 Other musculoskeletal	117 Measles			

Supplemental Results Figure S7. Leading 20 level 3 causes of DALYs, and percentage change in number of DALYs and all-age and age-standardised DALY rates by location, 2022 and 2050

Chad, Reference scenario

Legend:
Communicable, maternal, neonatal and nutritional
Non-communicable
Injuries

Leading Causes 2022	Leading Causes 2050	Mean % change number of DALY	Mean % change all-age DALY rate	Mean % change age-standardized DALY rate
1 Neonatal disorders	1 Neonatal disorders	14.8 (-11.4 to 51.7)	**-59.6 (-67.7 to -49.6)**	**-44.6 (-55.3 to -30.6)**
2 Diarrheal diseases	2 Diarrheal diseases	-16.2 (-50 to 33.1)	**-70.5 (-80.9 to -52.9)**	**-58 (-71.6 to -36.3)**
3 Lower respiratory infect	3 Lower respiratory infect	-19 (-39.1 to 6.98)	**-71.5 (-77.7 to -63.8)**	**-55.7 (-63.5 to -46.2)**
4 Malaria	4 Congenital defects	**34 (5.53 to 70.7)**	**-52.9 (-60.4 to -44.3)**	**-38.5 (-47.7 to -28.2)**
5 Congenital defects	5 Malaria	-26.8 (-89.8 to 62.3)	**-72.7 (-96.3 to -41.1)**	**-69.2 (-95.2 to -23.4)**
6 Tuberculosis	6 Stroke	**82.4 (44.5 to 129)**	**-35.8 (-48.4 to -21)**	**-38.6 (-51.4 to -21.2)**
7 Measles	7 Road injuries	69.7 (-8.81 to 209)	-40.3 (-68.7 to 8.27)	-40.2 (-66.3 to 3.08)
8 Meningitis	8 Dietary iron deficiency	**107 (77.8 to 134)**	**-27.1 (-28.9 to -25.2)**	**-23.6 (-24.8 to -22.3)**
9 Pertussis	9 Depressive disorders	**211 (165 to 252)**	**9.39 (6.02 to 12.8)**	**-3.58 (-4.16 to -2.99)**
10 Stroke	10 Pertussis	12.5 (-33.3 to 84.8)	**-60.5 (-76.1 to -36.8)**	**-50.7 (-70.1 to -22.2)**
11 Maternal disorders	11 Cirrhosis liver	**129 (82.6 to 194)**	**-19.3 (-34.7 to -0.311)**	**-26.8 (-40 to -10.3)**
12 Road injuries	12 Maternal disorders	17 (-28.5 to 81.3)	**-58.9 (-73.7 to -35.3)**	**-64.5 (-76.8 to -45.8)**
13 Dietary iron deficiency	13 Headache disorders	**219 (174 to 260)**	**12.2 (9.57 to 15.8)**	**1.19 (0.728 to 1.57)**
14 Protein-energy malnutrition	14 Diabetes	**193 (138 to 257)**	3.21 (-12.6 to 22)	-7.79 (-21.6 to 8.89)
15 HIV/AIDS	15 Low back pain	**198 (155 to 235)**	**4.89 (1.48 to 8.06)**	**-7.57 (-8.11 to -7.1)**
16 Cirrhosis liver	16 Other musculoskeletal	**728 (611 to 843)**	**191 (175 to 206)**	**149 (139 to 156)**
17 Drowning	17 Ischemic heart disease	**97 (27.6 to 195)**	-30.7 (-55.1 to 5.26)	**-38.2 (-61 to -5.81)**
18 Ischemic heart disease	18 Interpersonal violence	**136 (85 to 202)**	-16.9 (-29.7 to 2.62)	**-19.8 (-31.9 to -3.43)**
19 Depressive disorders	19 Meningitis	-25.2 (-54.2 to 25.8)	**-73.7 (-84.1 to -57.5)**	**-65 (-77.7 to -46.6)**
20 iNTS	20 Tuberculosis	-36.3 (-62.9 to 7.88)	**-77.6 (-86.7 to -62.3)**	**-74.8 (-84.4 to -60.4)**
21 Interpersonal violence	28 Drowning			
23 Diabetes	31 iNTS			
24 Low back pain	35 Protein-energy malnutrition			
25 Headache disorders	41 HIV/AIDS			
59 Other musculoskeletal	71 Measles			

Supplemental Results Figure S7. Leading 20 level 3 causes of DALYs, and percentage change in number of DALYs and all-age and age-standardised DALY rates by location, 2022 and 2050

Côte d'Ivoire, Reference scenario

Legend:
Communicable, maternal, neonatal and nutritional
Non-communicable
Injuries

Leading Causes 2022	Leading Causes 2050	Mean % change number of DALY	Mean % change all-age DALY rate	Mean % change age-standardized DALY rate
1 Neonatal disorders	1 Neonatal disorders	**-42.2 (-56.8 to -25.9)**	**-66.3 (-72.8 to -57.8)**	**-45.9 (-55.1 to -33.7)**
2 Malaria	2 Malaria	-46 (-95.3 to 172)	-67.7 (-97.2 to 64.6)	-61.3 (-96.7 to 111)
3 Lower respiratory infect	3 Lower respiratory infect	**-25.9 (-41.6 to -4.79)**	**-56.6 (-65.9 to -44.6)**	**-47.5 (-56.8 to -34.5)**
4 HIV/AIDS	4 Diabetes	**231 (170 to 297)**	**93.6 (66.5 to 124)**	10.7 (-4.03 to 26.2)
5 Congenital defects	5 Stroke	**53.8 (22.1 to 92.4)**	-10 (-27 to 11.3)	**-49 (-58.1 to -36.7)**
6 Diarrheal diseases	6 Ischemic heart disease	**65 (9.12 to 132)**	-3.42 (-32 to 37.7)	**-49.3 (-64.5 to -25.6)**
7 Stroke	7 Chronic kidney disease	**168 (95.3 to 254)**	**56.7 (21.3 to 107)**	7.19 (-18.3 to 43.6)
8 Ischemic heart disease	8 Other musculoskeletal	**412 (342 to 476)**	**200 (178 to 220)**	**123 (118 to 128)**
9 Road injuries	9 Congenital defects	**-27.9 (-44.3 to -11.9)**	**-57.9 (-64.4 to -48.7)**	**-37 (-44.9 to -26.3)**
10 COVID-19	10 Low back pain	**114 (87.7 to 140)**	**25.3 (17.4 to 33.2)**	**-7 (-7.4 to -6.6)**
11 Tuberculosis	11 Depressive disorders	**112 (84.6 to 140)**	**24.2 (17.4 to 31.4)**	**-2.95 (-3.45 to -2.4)**
12 Dietary iron deficiency	12 Diarrheal diseases	-28.6 (-56.7 to 7.4)	**-58.2 (-73.3 to -37.9)**	**-51.1 (-66.8 to -30.2)**
13 Diabetes	13 Headache disorders	**96 (67.1 to 121)**	**14.5 (9.43 to 23.1)**	**1.57 (1.04 to 1.97)**
14 iNTS	14 Road injuries	20.3 (-31.6 to 107)	-29.5 (-57.4 to 26.9)	-36.2 (-60.6 to 12.1)
15 Meningitis	15 Cirrhosis liver	**47.2 (16.7 to 78.8)**	-13.9 (-27.6 to 3.54)	**-39.4 (-48.7 to -26.6)**
16 Cirrhosis liver	16 Dietary iron deficiency	**22.9 (2.36 to 40.3)**	**-28.2 (-31.2 to -25)**	**-21 (-22 to -20)**
17 Low back pain	17 Hypertensive heart disease	**172 (101 to 261)**	**59.3 (23.2 to 113)**	-15.7 (-36.4 to 13.7)
18 Maternal disorders	18 Interpersonal violence	**41.2 (12.2 to 74.8)**	-17.5 (-32.6 to 1.11)	**-27 (-39.1 to -12.9)**
19 Headache disorders	19 Age-related hearing loss	**145 (113 to 185)**	**43.5 (31.3 to 55)**	**-2.41 (-2.63 to -2.18)**
20 Interpersonal violence	20 Anxiety disorders	**89.3 (64.5 to 114)**	**10.6 (5.45 to 16.1)**	**2.32 (1.98 to 2.67)**
21 Depressive disorders	29 Tuberculosis			
22 Chronic kidney disease	33 iNTS			
26 Anxiety disorders	35 Maternal disorders			
30 Hypertensive heart disease	36 Meningitis			
33 Age-related hearing loss	42 HIV/AIDS			
37 Other musculoskeletal	169 COVID-19			

Supplemental Results Figure S7. Leading 20 level 3 causes of DALYs, and percentage change in number of DALYs and all-age and age-standardised DALY rates by location, 2022 and 2050

The Gambia, Reference scenario

Legend:
Communicable, maternal, neonatal and nutritional
Non-communicable
Injuries

Leading Causes 2022	Leading Causes 2050	Mean % change number of DALY	Mean % change all-age DALY rate	Mean % change age-standardized DALY rate
1 Neonatal disorders	1 Neonatal disorders	**-42.3 (-55.3 to -25)**	**-62.1 (-69 to -55.2)**	**-36.7 (-46.6 to -25.3)**
2 HIV/AIDS	2 Lower respiratory infect	-12.4 (-29.6 to 8.52)	**-42.4 (-51.2 to -30.1)**	**-45.8 (-53.7 to -36.2)**
3 Lower respiratory infect	3 HIV/AIDS	-13.5 (-41.8 to 44.9)	-43 (-62.7 to 1.23)	**-55 (-70 to -20.5)**
4 Stroke	4 Stroke	**35.7 (8.75 to 65.8)**	-10.7 (-25.3 to 8.19)	**-48.9 (-57.5 to -37.1)**
5 Diarrheal diseases	5 Diabetes	**158 (107 to 212)**	**69.6 (40.5 to 98.3)**	-3.17 (-16.8 to 13.5)
6 Congenital defects	6 Ischemic heart disease	**43 (1.52 to 88.6)**	-5.86 (-32.9 to 31.7)	**-48.3 (-63.8 to -26.5)**
7 Ischemic heart disease	7 Depressive disorders	**96.4 (67.7 to 125)**	**29.2 (20.9 to 37.6)**	**-3.08 (-3.7 to -2.41)**
8 Dietary iron deficiency	8 Chronic kidney disease	**150 (88.2 to 228)**	**64.7 (26.2 to 113)**	13.7 (-13.1 to 48)
9 Tuberculosis	9 Liver cancer	**131 (67.8 to 210)**	**51.9 (14.4 to 100)**	-7.25 (-29.5 to 21.9)
10 Malaria	10 Other musculoskeletal	**364 (301 to 427)**	**205 (185 to 228)**	**113 (107 to 118)**
11 Road injuries	11 Dietary iron deficiency	11.5 (-5.31 to 31.1)	**-26.7 (-30 to -22.8)**	**-18.8 (-19.7 to -17.7)**
12 Maternal disorders	12 Low back pain	**102 (75.7 to 127)**	**32.7 (23.4 to 40.5)**	**-6.95 (-7.25 to -6.68)**
13 Depressive disorders	13 Headache disorders	**78.5 (51.2 to 106)**	**17.4 (10.6 to 30.6)**	**0.875 (0.573 to 1.14)**
14 COVID-19	14 Road injuries	9.36 (-32.3 to 71.2)	-28.1 (-54.4 to 19.4)	-38.4 (-60 to 0.178)
15 Diabetes	15 Hypertensive heart disease	**142 (80.7 to 204)**	**59 (20.3 to 103)**	-14.2 (-35.2 to 11.5)
16 Hemoglobinopathies	16 Congenital defects	**-32.6 (-45.9 to -16.2)**	**-55.7 (-61.6 to -48.7)**	**-33 (-40.3 to -23)**
17 Meningitis	17 Cirrhosis liver	**44.3 (17.7 to 80.4)**	-4.99 (-20.9 to 14.9)	**-37.8 (-47.8 to -25.3)**
18 Chronic kidney disease	18 Diarrheal diseases	**-46 (-64.8 to -20.5)**	**-64.5 (-76.3 to -46.4)**	**-59.8 (-72.4 to -41.2)**
19 Liver cancer	19 Gynecological diseases	**54.8 (22.8 to 93.3)**	1.87 (-15.5 to 23)	**-20.7 (-34.3 to -5.8)**
20 Headache disorders	20 Hemoglobinopathies	0.895 (-16.7 to 20.4)	**-33.7 (-41.6 to -27.8)**	**-26.3 (-34 to -20.6)**
21 Cirrhosis liver	23 Tuberculosis			
22 Low back pain	31 Maternal disorders			
23 Gynecological diseases	33 Meningitis			
24 Hypertensive heart disease	47 Malaria			
38 Other musculoskeletal	165 COVID-19			

Supplemental Results Figure S7. Leading 20 level 3 causes of DALYs, and percentage change in number of DALYs and all-age and age-standardised DALY rates by location, 2022 and 2050

Ghana, Reference scenario

Legend:
Communicable, maternal, neonatal and nutritional
Non-communicable
Injuries

Leading Causes 2022	Leading Causes 2050	Mean % change number of DALY	Mean % change all-age DALY rate	Mean % change age-standardized DALY rate
1 Neonatal disorders	1 Malaria	0.719 (-93.1 to 533)	-34.6 (-95.7 to 300)	-20.5 (-94.9 to 454)
2 Malaria	2 Neonatal disorders	**-33 (-49.8 to -14.1)**	**-56.6 (-64.6 to -46.9)**	**-32.1 (-42.8 to -18.6)**
3 HIV/AIDS	3 Lower respiratory infect	**51.4 (18.5 to 89.3)**	-1.46 (-20.9 to 21.8)	-15 (-29.5 to 1.5)
4 Lower respiratory infect	4 Stroke	**50.7 (21.6 to 85.3)**	-1.91 (-21.4 to 21.2)	**-44.4 (-54.7 to -32)**
5 Stroke	5 Diabetes	**188 (133 to 245)**	**87.1 (54 to 119)**	9.03 (-7.01 to 27.7)
6 Congenital defects	6 Chronic kidney disease	**163 (93.2 to 250)**	**71.1 (27.5 to 124)**	16.1 (-12.7 to 52.2)
7 Road injuries	7 Ischemic heart disease	**55.1 (4.57 to 121)**	1.02 (-30.5 to 44.6)	**-44.5 (-61.9 to -20.5)**
8 Ischemic heart disease	8 Road injuries	22 (-31 to 126)	-20.7 (-54.1 to 42)	-25.1 (-56.4 to 31.7)
9 Diarrheal diseases	9 Diarrheal diseases	34.1 (-11.1 to 95.6)	-12.8 (-40 to 25.9)	-18.1 (-43.9 to 18.1)
10 Tuberculosis	10 Depressive disorders	**91.7 (64.7 to 121)**	**24.6 (15.6 to 34.5)**	**-3.04 (-3.42 to -2.65)**
11 Diabetes	11 Other musculoskeletal	**262 (211 to 316)**	**135 (117 to 157)**	**70.6 (67 to 73.6)**
12 Hemoglobinopathies	12 Low back pain	**99.8 (73.9 to 129)**	**29.9 (19.4 to 40.5)**	**-5.48 (-5.66 to -5.29)**
13 COVID-19	13 Hypertensive heart disease	**185 (117 to 274)**	**85.5 (37.7 to 146)**	1.76 (-23.6 to 37.2)
14 Chronic kidney disease	14 Headache disorders	**74.2 (44.7 to 99.7)**	**13.2 (7.38 to 28.4)**	0.0462 (-0.237 to 0.324)
15 Dietary iron deficiency	15 Cirrhosis liver	**57.1 (28.1 to 94.9)**	2.24 (-16.2 to 24.2)	**-32.2 (-42 to -18.5)**
16 Depressive disorders	16 Hemoglobinopathies	5.74 (-14.9 to 28.1)	**-31.4 (-39.4 to -24.1)**	**-24 (-30.8 to -17.4)**
17 Cirrhosis liver	17 Tuberculosis	-4.56 (-36.2 to 42.2)	**-37.9 (-57.1 to -10.5)**	**-57.3 (-71.2 to -38.2)**
18 Meningitis	18 Congenital defects	**-26.2 (-42.4 to -6.52)**	**-52.2 (-58.6 to -44.1)**	**-31 (-38.1 to -21.8)**
19 Headache disorders	19 Dietary iron deficiency	19.9 (-2.63 to 43)	**-22.3 (-28.4 to -17.3)**	**-15.5 (-16.4 to -14.2)**
20 Low back pain	20 Gynecological diseases	**52 (20.4 to 87.9)**	-1.07 (-19.7 to 21.7)	**-18.9 (-33.7 to -2.94)**
21 Gynecological diseases	21 HIV/AIDS			
22 Hypertensive heart disease	24 Meningitis			
25 Other musculoskeletal	165 COVID-19			

Supplemental Results Figure S7. Leading 20 level 3 causes of DALYs, and percentage change in number of DALYs and all-age and age-standardised DALY rates by location, 2022 and 2050

Guinea, Reference scenario

Legend: Communicable, maternal, neonatal and nutritional; Non-communicable; Injuries

Leading Causes 2022	Leading Causes 2050	Mean % change number of DALY	Mean % change all-age DALY rate	Mean % change age-standardized DALY rate
1 Malaria	1 Malaria	15.4 (-88.5 to 614)	-37 (-94.4 to 287)	-14.2 (-92 to 423)
2 Neonatal disorders	2 Neonatal disorders	**-31.8 (-46.9 to -13.5)**	**-64.6 (-71.5 to -56.4)**	**-46.1 (-55.2 to -33)**
3 Lower respiratory infect	3 Lower respiratory infect	**-23.7 (-41.5 to -3.57)**	**-60.4 (-69 to -50.1)**	**-47.4 (-56.8 to -35.9)**
4 Congenital defects	4 Stroke	**44.8 (13.8 to 74.9)**	**-24.7 (-37.8 to -8.16)**	**-44.2 (-53.5 to -30)**
5 Diarrheal diseases	5 Congenital defects	-17.8 (-33.2 to 3.01)	**-57.3 (-62.8 to -50.4)**	**-38.7 (-45.9 to -29.4)**
6 Stroke	6 Diabetes	**167 (112 to 219)**	**38.6 (16.4 to 63.6)**	-0.878 (-14.6 to 15.9)
7 Pertussis	7 Diarrheal diseases	-13.9 (-44.4 to 23.5)	**-55.3 (-71.1 to -35.9)**	**-52.2 (-66.2 to -33.3)**
8 Meningitis	8 Other musculoskeletal	**513 (433 to 591)**	**218 (198 to 240)**	**145 (137 to 152)**
9 HIV/AIDS	9 Ischemic heart disease	**51.9 (2.68 to 110)**	-21 (-45.4 to 13.9)	**-44.8 (-62.3 to -19.6)**
10 COVID-19	10 Depressive disorders	**127 (100 to 156)**	**18 (12.1 to 25.2)**	**-4.18 (-5 to -3.41)**
11 iNTS	11 Pertussis	-11.4 (-50.6 to 42.6)	**-54.1 (-73 to -28.5)**	-34.3 (-60.8 to 1.88)
12 Road injuries	12 Low back pain	**125 (96.7 to 155)**	**17 (11 to 24.6)**	**-8.2 (-8.68 to -7.7)**
13 Ischemic heart disease	13 Headache disorders	**122 (93.3 to 154)**	**15.3 (10.8 to 24.4)**	**0.579 (0.0693 to 1.25)**
14 Tuberculosis	14 Chronic kidney disease	**145 (81.5 to 223)**	27.5 (-3.42 to 67.5)	8.04 (-18.2 to 42)
15 Maternal disorders	15 Hemoglobinopathies	**29.7 (3.8 to 58.3)**	**-32.7 (-43.1 to -23)**	**-23.1 (-33.2 to -13.9)**
16 Measles	16 Road injuries	9.02 (-36.6 to 87.2)	**-43.3 (-67.5 to -3.23)**	**-45.1 (-67.4 to -9.06)**
17 Hemoglobinopathies	17 iNTS	-5.85 (-53.7 to 59.7)	**-51.1 (-74.5 to -15.6)**	-36.4 (-64.6 to 9.81)
18 Protein-energy malnutrition	18 Cirrhosis liver	**47.8 (18.9 to 82.5)**	**-23.2 (-36.5 to -5.59)**	**-40.6 (-51 to -27.8)**
19 Dietary iron deficiency	19 Dietary iron deficiency	**32 (12.5 to 52.2)**	**-31.5 (-34.2 to -28.5)**	**-25.3 (-26.4 to -24.1)**
20 Cirrhosis liver	20 Meningitis	-34.3 (-59.4 to 9.19)	**-65.8 (-78.4 to -42.9)**	**-56.6 (-71.6 to -31)**
21 Diabetes	21 Maternal disorders			
22 Depressive disorders	28 Tuberculosis			
23 Low back pain	42 Protein-energy malnutrition			
24 Headache disorders	48 HIV/AIDS			
27 Chronic kidney disease	103 Measles			
44 Other musculoskeletal	166 COVID-19			

Supplemental Results Figure S7. Leading 20 level 3 causes of DALYs, and percentage change in number of DALYs and all-age and age-standardised DALY rates by location, 2022 and 2050

Guinea-Bissau, Reference scenario

Legend: Communicable, maternal, neonatal and nutritional; Non-communicable; Injuries

Leading Causes 2022	Leading Causes 2050	Mean % change number of DALY	Mean % change all-age DALY rate	Mean % change age-standardized DALY rate
1 Neonatal disorders	1 Neonatal disorders	**-42.1 (-57.2 to -24.8)**	**-67.4 (-74.2 to -60.2)**	**-45.3 (-54.5 to -33.1)**
2 COVID-19	2 Lower respiratory infect	0.833 (-22.5 to 26.3)	**-43.1 (-54.9 to -29.5)**	**-42.3 (-52.2 to -30.5)**
3 HIV/AIDS	3 Stroke	**49.9 (17.5 to 89.7)**	-15.4 (-32.5 to 4.33)	**-48.1 (-59 to -34.7)**
4 Lower respiratory infect	4 HIV/AIDS	**-38.2 (-59 to -4.84)**	**-65.2 (-76.2 to -48.2)**	**-68.6 (-77.5 to -53.8)**
5 Measles	5 Road injuries	26.9 (-34.1 to 133)	-28.4 (-61.8 to 30.8)	-33.1 (-63.7 to 18.8)
6 Stroke	6 Diabetes	**183 (132 to 244)**	**59.8 (31.1 to 89.2)**	-2.96 (-18.5 to 13.6)
7 Diarrheal diseases	7 Ischemic heart disease	**67.4 (8.36 to 145)**	-5.48 (-36.8 to 37.4)	**-45.5 (-64.3 to -19.8)**
8 Road injuries	8 Diarrheal diseases	-9.23 (-42.3 to 30.4)	**-48.8 (-65.4 to -28.7)**	**-44.4 (-61.7 to -22.6)**
9 Congenital defects	9 Chronic kidney disease	**156 (83.9 to 249)**	**44.3 (5.3 to 92.3)**	5.1 (-21.6 to 37.3)
10 Tuberculosis	10 Congenital defects	-20.8 (-38.4 to 1.96)	**-55.5 (-61.8 to -47.8)**	**-32.8 (-40.7 to -22.5)**
11 Ischemic heart disease	11 Cirrhosis liver	**53.3 (21.4 to 97.1)**	-13.5 (-30.8 to 5.73)	**-40 (-50.8 to -27.4)**
12 Malaria	12 Depressive disorders	**121 (89 to 153)**	**24.7 (17.2 to 33.6)**	**-3.9 (-4.4 to -3.4)**
13 Dietary iron deficiency	13 Other musculoskeletal	**426 (347 to 511)**	**196 (173 to 222)**	**115 (107 to 121)**
14 Cirrhosis liver	14 Dietary iron deficiency	**28 (5.04 to 53.8)**	**-28 (-31.1 to -24.5)**	**-21.2 (-22.1 to -20.1)**
15 Pertussis	15 Headache disorders	**109 (73.8 to 142)**	**17.6 (11.9 to 31.6)**	**0.536 (0.211 to 0.846)**
16 Meningitis	16 Low back pain	**125 (92.3 to 158)**	**27.1 (18.5 to 37)**	**-7.42 (-7.69 to -7.07)**
17 Diabetes	17 Hypertensive heart disease	**156 (84.2 to 248)**	**44.8 (8.11 to 95.1)**	-18 (-38.3 to 11.6)
18 Interpersonal violence	18 Tuberculosis	**-35.4 (-59.1 to -1.35)**	**-63.5 (-75.5 to -47.9)**	**-72.2 (-81.3 to -59.1)**
19 Chronic kidney disease	19 Interpersonal violence	**47.1 (11.5 to 83.4)**	-17.2 (-31 to 0.137)	**-26.5 (-37.5 to -11.2)**
20 Depressive disorders	20 Self-harm	**85.3 (45 to 131)**	4.58 (-16.5 to 28.4)	**-19.7 (-34.3 to -2.63)**
21 Headache disorders	25 Meningitis			
23 Low back pain	29 Pertussis			
25 Self-harm	45 Measles			
28 Hypertensive heart disease	75 Malaria			
40 Other musculoskeletal	166 COVID-19			

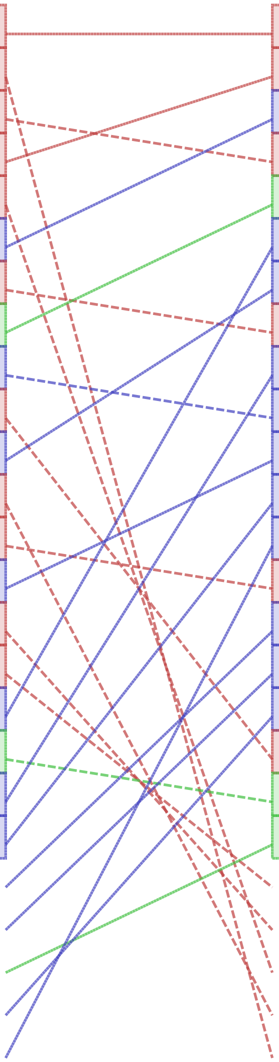

Supplemental Results Figure S7. Leading 20 level 3 causes of DALYs, and percentage change in number of DALYs and all-age and age-standardised DALY rates by location, 2022 and 2050

Liberia, Reference scenario

Legend:
Communicable, maternal, neonatal and nutritional
Non-communicable
Injuries

Leading Causes 2022	Leading Causes 2050	Mean % change number of DALY	Mean % change all-age DALY rate	Mean % change age-standardized DALY rate
1 Malaria	1 Neonatal disorders	**-37.3 (-56.8 to -12.2)**	**-61.5 (-71 to -49.3)**	**-35.6 (-48.3 to -18.4)**
2 Neonatal disorders	2 Malaria	-42.3 (-90.1 to 175)	-64.2 (-93.8 to 80.6)	-59.4 (-93.1 to 97)
3 Diarrheal diseases	3 Stroke	**83.9 (39.8 to 139)**	13.6 (-10.1 to 45.3)	**-40.7 (-53.5 to -20.9)**
4 Lower respiratory infect	4 Diabetes	**238 (166 to 316)**	**109 (75.8 to 149)**	11.5 (-4.71 to 33.8)
5 Congenital defects	5 Lower respiratory infect	7.69 (-17.1 to 33.6)	**-33.5 (-45.9 to -18.2)**	**-35.2 (-46.7 to -19)**
6 Stroke	6 Diarrheal diseases	-11.4 (-48.8 to 45)	**-45.3 (-67.1 to -10.5)**	-36.8 (-61.2 to 1.52)
7 HIV/AIDS	7 Ischemic heart disease	**128 (49.3 to 257)**	41 (-7.7 to 116)	-32.2 (-56.8 to 9.34)
8 Onchocerciasis	8 Chronic kidney disease	**178 (103 to 291)**	**72 (26.4 to 134)**	10.8 (-21.1 to 57)
9 Maternal disorders	9 Depressive disorders	**112 (73 to 154)**	**30.8 (21.3 to 42.9)**	**-1.94 (-3.23 to -0.561)**
10 Dietary iron deficiency	10 Dietary iron deficiency	**37.9 (13.6 to 66.3)**	**-15.1 (-19.2 to -11)**	**-13.9 (-15 to -12.1)**
11 Ischemic heart disease	11 Low back pain	**121 (80.3 to 158)**	**36.3 (25.6 to 48.7)**	**-5.18 (-5.64 to -4.73)**
12 Road injuries	12 Hypertensive heart disease	**213 (130 to 333)**	**93.6 (42.6 to 163)**	-5.79 (-32.2 to 32.1)
13 Diabetes	13 Onchocerciasis	7.98 (-12.2 to 31)	**-33.4 (-40.6 to -24.7)**	**-45.7 (-47.9 to -42.8)**
14 Cirrhosis liver	14 Cirrhosis liver	**65 (25.7 to 108)**	1.93 (-19.8 to 27.8)	**-34.2 (-46.1 to -18)**
15 Depressive disorders	15 Congenital defects	**-31.2 (-49.7 to -7.35)**	**-57.8 (-65 to -47.7)**	**-34.8 (-43.2 to -23.1)**
16 STIs	16 Headache disorders	**87.4 (56.3 to 121)**	**15.5 (8.1 to 29.1)**	**0.918 (0.433 to 1.4)**
17 Chronic kidney disease	17 Road injuries	23.2 (-30.6 to 135)	-23.9 (-57.4 to 38.8)	-30.9 (-61.5 to 23.4)
18 Tuberculosis	18 Other musculoskeletal	**317 (239 to 393)**	**157 (134 to 183)**	**77.2 (68.8 to 82.4)**
19 Meningitis	19 Liver cancer	**179 (103 to 288)**	**72.6 (27.8 to 131)**	1.6 (-25.3 to 41.7)
20 Hemoglobinopathies	20 Gynecological diseases	**71.7 (34.4 to 122)**	6.02 (-16.4 to 31.6)	-15.3 (-31.5 to 3.97)
22 Headache disorders	21 Hemoglobinopathies			
23 Low back pain	25 Maternal disorders			
26 Gynecological diseases	27 Tuberculosis			
29 Hypertensive heart disease	34 Meningitis			
33 Liver cancer	44 STIs			
41 Other musculoskeletal	71 HIV/AIDS			

Supplemental Results Figure S7. Leading 20 level 3 causes of DALYs, and percentage change in number of DALYs and all-age and age-standardised DALY rates by location, 2022 and 2050

Mali, Reference scenario

Legend:
Communicable, maternal, neonatal and nutritional
Non-communicable
Injuries

Leading Causes 2022	Leading Causes 2050	Mean % change number of DALY	Mean % change all-age DALY rate	Mean % change age-standardized DALY rate
1 Neonatal disorders	1 Neonatal disorders	-6.16 (-29.3 to 24.5)	**-60.4 (-69.2 to -47.8)**	**-45 (-57.2 to -28.1)**
2 Malaria	2 Malaria	-46.6 (-93.9 to 198)	-76 (-97.4 to 26.4)	-71.6 (-96.3 to 64.1)
3 Lower respiratory infect	3 Congenital defects	9.91 (-14.7 to 36.2)	**-53.6 (-60.7 to -45)**	**-38.7 (-47.6 to -30)**
4 Congenital defects	4 Lower respiratory infect	-14.6 (-36.7 to 10.6)	**-63.9 (-71.6 to -54)**	**-47.3 (-56.5 to -35)**
5 Protein-energy malnutrition	5 Diabetes	**241 (174 to 312)**	**44.2 (24 to 64.1)**	**16.9 (2.51 to 33.1)**
6 Diarrheal diseases	6 Dietary iron deficiency	**74.2 (49.9 to 105)**	**-26.4 (-28.2 to -24.9)**	**-21.1 (-22.1 to -20.1)**
7 Dietary iron deficiency	7 Diarrheal diseases	-10.5 (-47.2 to 41)	**-62.2 (-76.5 to -42.6)**	**-52.6 (-68.4 to -31.9)**
8 Measles	8 Stroke	**73.8 (32.1 to 122)**	**-26.6 (-41.4 to -10.7)**	**-36.2 (-49 to -20.7)**
9 iNTS	9 iNTS	4.4 (-48.3 to 94.3)	**-56 (-78.1 to -19.7)**	**-48.2 (-72.6 to -8.81)**
10 HIV/AIDS	10 Headache disorders	**165 (125 to 214)**	**12 (9.03 to 16.2)**	**0.896 (0.464 to 1.28)**
11 Stroke	11 Other musculoskeletal	**576 (461 to 701)**	**186 (168 to 202)**	**138 (129 to 145)**
12 Meningitis	12 Road injuries	37.2 (-21.2 to 133)	**-42.1 (-67 to -2.19)**	**-42.9 (-65.7 to -7.23)**
13 Road injuries	13 Chronic kidney disease	**178 (88.4 to 274)**	17.5 (-16.7 to 59.2)	13 (-15.4 to 51.9)
14 COVID-19	14 Depressive disorders	**163 (123 to 206)**	**10.9 (6.84 to 15.4)**	**-3.9 (-4.43 to -3.3)**
15 Tuberculosis	15 Low back pain	**155 (116 to 198)**	**7.74 (2.57 to 12.1)**	**-8.22 (-8.54 to -7.88)**
16 Diabetes	16 Ischemic heart disease	**100 (25.3 to 194)**	-15.5 (-45.4 to 23.4)	-31.7 (-56.1 to 1.22)
17 Maternal disorders	17 Hemoglobinopathies	**60.2 (25.5 to 92.9)**	**-32.4 (-39.8 to -25.3)**	**-23.5 (-29.7 to -17.3)**
18 Pertussis	18 Interpersonal violence	**88.6 (49.6 to 142)**	**-20.4 (-32.8 to -1.41)**	**-23.2 (-34.3 to -6.46)**
19 STIs	19 Protein-energy malnutrition	**-59.1 (-81.2 to -17.2)**	**-82.7 (-92.1 to -64.8)**	**-75.8 (-88.1 to -54.1)**
20 Hemoglobinopathies	20 Liver cancer	**144 (80.1 to 213)**	2.89 (-19.8 to 29.8)	-12.7 (-33 to 11.4)
21 Interpersonal violence	23 Meningitis			
22 Ischemic heart disease	26 Maternal disorders			
25 Headache disorders	28 Pertussis			
26 Chronic kidney disease	35 STIs			
27 Low back pain	36 Tuberculosis			
28 Depressive disorders	71 Measles			
30 Liver cancer	84 HIV/AIDS			

Supplemental Results Figure S7. Leading 20 level 3 causes of DALYs, and percentage change in number of DALYs and all-age and age-standardised DALY rates by location, 2022 and 2050

Mauritania, Reference scenario

Legend:
Communicable, maternal, neonatal and nutritional
Non-communicable
Injuries

Leading Causes 2022	Leading Causes 2050	Mean % change number of DALY	Mean % change all-age DALY rate	Mean % change age-standardized DALY rate
1 Neonatal disorders	1 Neonatal disorders	-9.5 (-30.8 to 16.6)	**-47 (-55.6 to -35.9)**	**-23 (-33.7 to -9.17)**
2 Lower respiratory infect	2 Lower respiratory infect	8.31 (-16.3 to 36.1)	**-36.4 (-48 to -20.8)**	**-32.5 (-43.8 to -17.2)**
3 Road injuries	3 Road injuries	32.2 (-31.4 to 143)	-22.4 (-57.1 to 42.2)	-25.3 (-57 to 33.7)
4 COVID-19	4 Stroke	**51.1 (19.8 to 86.3)**	-11.2 (-28 to 9.97)	**-42.4 (-53.2 to -26.6)**
5 Stroke	5 Diabetes	**162 (117 to 218)**	**54.2 (30.2 to 84.6)**	0.947 (-15.3 to 20.3)
6 Diarrheal diseases	6 Ischemic heart disease	43.4 (-0.336 to 105)	-15.7 (-40.1 to 17.2)	**-46.3 (-63.3 to -21.2)**
7 Malaria	7 Chronic kidney disease	**192 (119 to 287)**	**71.3 (34.9 to 122)**	24.3 (-4.83 to 65.3)
8 Congenital defects	8 Other musculoskeletal	**348 (288 to 408)**	**163 (145 to 184)**	**94 (90 to 97.7)**
9 Ischemic heart disease	9 Low back pain	**112 (81.7 to 140)**	**24.7 (16.9 to 33.7)**	**-6.43 (-6.65 to -6.21)**
10 Maternal disorders	10 Headache disorders	**97.5 (69.5 to 127)**	**15.9 (10.3 to 26.9)**	**0.411 (0.196 to 0.606)**
11 Dietary iron deficiency	11 Depressive disorders	**110 (77.8 to 139)**	**23.1 (15.5 to 31)**	**-3.2 (-3.5 to -2.89)**
12 Diabetes	12 Hypertensive heart disease	**153 (90.7 to 229)**	**48.9 (15 to 89.8)**	-5.59 (-28.7 to 23.8)
13 Headache disorders	13 Diarrheal diseases	-20 (-50.7 to 14.4)	**-53.1 (-69.6 to -34.3)**	**-48.9 (-64.8 to -29.4)**
14 Low back pain	14 Congenital defects	-14.6 (-35.3 to 7.44)	**-50 (-57.2 to -42.7)**	**-29.7 (-37.8 to -19.2)**
15 Depressive disorders	15 Dietary iron deficiency	**25.4 (3.13 to 47.3)**	**-26.5 (-31.4 to -22.7)**	**-18.7 (-19.5 to -17.6)**
16 Chronic kidney disease	16 Liver cancer	**150 (89.4 to 238)**	**46.9 (15.2 to 94.2)**	1.12 (-22 to 35.6)
17 Pertussis	17 Gynecological diseases	**75.8 (39.5 to 116)**	3.22 (-15.4 to 25.7)	**-20.2 (-35.1 to -2.84)**
18 Cirrhosis liver	18 Cirrhosis liver	**55.1 (24.2 to 92.3)**	-8.9 (-25.2 to 11.5)	**-34.2 (-45.1 to -19.6)**
19 Gynecological diseases	19 Age-related hearing loss	**124 (90.9 to 158)**	**31.5 (19.9 to 43.3)**	**-1.98 (-2.13 to -1.85)**
20 Hypertensive heart disease	20 Anxiety disorders	**86.8 (58.2 to 119)**	**9.57 (3.6 to 17.7)**	**1.5 (1.23 to 1.79)**
23 Anxiety disorders	26 Maternal disorders			
24 Liver cancer	32 Malaria			
27 Age-related hearing loss	45 Pertussis			
29 Other musculoskeletal	162 COVID-19			

Supplemental Results Figure S7. Leading 20 level 3 causes of DALYs, and percentage change in number of DALYs and all-age and age-standardised DALY rates by location, 2022 and 2050

Niger, Reference scenario

Legend:
Communicable, maternal, neonatal and nutritional
Non-communicable
Injuries

Leading Causes 2022	Leading Causes 2050	Mean % change number of DALY	Mean % change all-age DALY rate	Mean % change age-standardized DALY rate
1 Neonatal disorders	1 Neonatal disorders	**53.3 (19.6 to 91.8)**	**-45.6 (-56.4 to -33)**	**-31.6 (-43.8 to -17.1)**
2 Malaria	2 Malaria	30.9 (-92.4 to 659)	-52.8 (-97.3 to 172)	-49.5 (-96 to 192)
3 Lower respiratory infect	3 Lower respiratory infect	24.9 (-4.38 to 65.5)	**-55.7 (-65.3 to -40.3)**	**-36.2 (-47.6 to -21.8)**
4 Diarrheal diseases	4 Diarrheal diseases	17.4 (-24.4 to 70.1)	**-58.3 (-72.5 to -39.4)**	**-44 (-61.7 to -21.9)**
5 Measles	5 Congenital defects	**43.7 (14.7 to 77)**	**-49 (-56.7 to -38.8)**	**-37.5 (-45.8 to -27)**
6 Congenital defects	6 Stroke	**141 (87.9 to 205)**	-14.4 (-28.9 to 3.81)	-7.32 (-23.8 to 14.2)
7 Meningitis	7 iNTS	76 (-7.02 to 192)	**-37.5 (-66.9 to -0.529)**	-30.9 (-60.7 to 6.79)
8 Tuberculosis	8 Pertussis	**90.4 (5.75 to 194)**	-32.5 (-60 to 2.54)	-20.5 (-51.2 to 20.4)
9 iNTS	9 Meningitis	22.1 (-26.7 to 100)	**-56.6 (-73.7 to -27.6)**	**-47.8 (-65.6 to -18.5)**
10 Pertussis	10 Diabetes	**307 (235 to 388)**	**44.5 (26.4 to 67.4)**	**48.1 (25.9 to 74.6)**
11 Stroke	11 Dietary iron deficiency	**119 (84.1 to 153)**	**-22.5 (-24.7 to -20.3)**	**-19.2 (-20.3 to -18)**
12 Maternal disorders	12 Ischemic heart disease	**235 (130 to 359)**	19.1 (-16.1 to 62.8)	23.1 (-14.3 to 77.8)
13 Dietary iron deficiency	13 Maternal disorders	**53 (1.2 to 122)**	**-45.6 (-63.1 to -20.2)**	**-53.9 (-68.1 to -33.8)**
14 Road injuries	14 Headache disorders	**210 (169 to 257)**	**9.97 (6.91 to 14.3)**	**0.429 (0.043 to 0.881)**
15 Protein-energy malnutrition	15 Depressive disorders	**204 (163 to 249)**	**7.74 (3.48 to 13.1)**	**-3.53 (-4.25 to -2.73)**
16 Cirrhosis liver	16 Road injuries	**102 (14 to 235)**	-28 (-58.1 to 23.4)	-28.4 (-55 to 15.1)
17 COVID-19	17 Low back pain	**195 (152 to 241)**	**4.57 (0.251 to 9.93)**	**-6.65 (-7.03 to -6.24)**
18 Drowning	18 Cirrhosis liver	**90 (53.8 to 131)**	**-32.5 (-42.8 to -18.3)**	**-35.4 (-44.9 to -23.3)**
19 Hemoglobinopathies	19 Tuberculosis	-10.7 (-46.2 to 46.2)	**-68.2 (-80.7 to -48.8)**	**-66.3 (-78 to -49.2)**
20 Headache disorders	20 Hemoglobinopathies	**101 (64.3 to 140)**	**-28.7 (-36.2 to -20.9)**	**-19.8 (-25.9 to -13.7)**
21 Depressive disorders	26 Measles			
22 Ischemic heart disease	34 Drowning			
23 Low back pain	36 Protein-energy malnutrition			
24 Diabetes	167 COVID-19			

Supplemental Results Figure S7. Leading 20 level 3 causes of DALYs, and percentage change in number of DALYs and all-age and age-standardised DALY rates by location, 2022 and 2050

Nigeria, Reference scenario

Legend:
Communicable, maternal, neonatal and nutritional
Non-communicable
Injuries

Leading Causes 2022	Leading Causes 2050	Mean % change number of DALY	Mean % change all-age DALY rate	Mean % change age-standardized DALY rate
1 Neonatal disorders	1 Neonatal disorders	**-24.7 (-45 to -3.92)**	**-60 (-67.6 to -51.7)**	**-36.4 (-46.6 to -23.4)**
2 Malaria	2 Malaria	-24.4 (-92.1 to 400)	-58.6 (-95.6 to 173)	-50.3 (-94.7 to 253)
3 Lower respiratory infect	3 Lower respiratory infect	**-42.2 (-55.3 to -26.1)**	**-69.2 (-75.1 to -61.9)**	**-54.8 (-62.6 to -44.7)**
4 Diarrheal diseases	4 Diarrheal diseases	**-57.9 (-73 to -34.4)**	**-77.5 (-85.4 to -65.4)**	**-67.7 (-78.4 to -51.7)**
5 Congenital defects	5 Congenital defects	-21.2 (-41.6 to 0.654)	**-58.1 (-65.7 to -49.2)**	**-37.2 (-45.6 to -25.7)**
6 HIV/AIDS	6 Diabetes	**210 (156 to 271)**	**65.4 (40.1 to 92.9)**	-1.15 (-14 to 12.2)
7 Meningitis	7 Stroke	**74.4 (45.2 to 110)**	-6.9 (-21.3 to 9.67)	**-45.6 (-53.3 to -36.3)**
8 Tuberculosis	8 Ischemic heart disease	**76.5 (26.7 to 146)**	-5.76 (-33.8 to 30.4)	**-48.2 (-63 to -28.3)**
9 Stroke	9 Other musculoskeletal	**410 (344 to 473)**	**172 (150 to 192)**	**93.5 (88.7 to 97.7)**
10 Pertussis	10 Low back pain	**138 (108 to 168)**	**27.1 (19.1 to 36.5)**	**-6.72 (-7.2 to -6.22)**
11 Dietary iron deficiency	11 Headache disorders	**118 (87.2 to 151)**	**16.3 (10.1 to 27.3)**	0.116 (-0.241 to 0.467)
12 Ischemic heart disease	12 Dietary iron deficiency	**37.3 (15.8 to 61.5)**	**-26.9 (-29.7 to -24.1)**	**-19.1 (-19.9 to -18.1)**
13 iNTS	13 Chronic kidney disease	**199 (129 to 294)**	**59.5 (18.9 to 112)**	14.3 (-11.7 to 50.4)
14 Hemoglobinopathies	14 Depressive disorders	**134 (105 to 166)**	**25 (17.8 to 33.7)**	**-3.5 (-4 to -3.04)**
15 Maternal disorders	15 HIV/AIDS	**-45.1 (-61.4 to -36.3)**	**-70.8 (-78.3 to -65.4)**	**-75.5 (-81.5 to -71.8)**
16 Headache disorders	16 Hemoglobinopathies	**23.8 (2.04 to 52.2)**	**-34.1 (-42.5 to -26)**	**-23.5 (-30.6 to -16.5)**
17 Road injuries	17 Cirrhosis liver	**51.2 (25.2 to 83.5)**	**-19.3 (-33.1 to -1.92)**	**-39.8 (-48.2 to -27.9)**
18 Cirrhosis liver	18 Gynecological diseases	**94.8 (59.6 to 138)**	3.99 (-13.8 to 23.3)	**-20 (-32.6 to -6.95)**
19 Low back pain	19 Road injuries	39.9 (-11.4 to 129)	-25.3 (-53.6 to 20.6)	-32.6 (-56.9 to 8.82)
20 Diabetes	20 Age-related hearing loss	**155 (122 to 190)**	**35.9 (25.4 to 47)**	**-2.05 (-2.28 to -1.8)**
22 Depressive disorders	24 Tuberculosis			
24 Gynecological diseases	26 Meningitis			
25 Chronic kidney disease	27 iNTS			
28 Age-related hearing loss	30 Pertussis			
34 Other musculoskeletal	31 Maternal disorders			

Supplemental Results Figure S7. Leading 20 level 3 causes of DALYs, and percentage change in number of DALYs and all-age and age-standardised DALY rates by location, 2022 and 2050

São Tomé and Príncipe, Reference scenario

Legend:
Communicable, maternal, neonatal and nutritional
Non-communicable
Injuries

Leading Causes 2022	Leading Causes 2050	Mean % change number of DALY	Mean % change all-age DALY rate	Mean % change age-standardized DALY rate
1 Lower respiratory infect	1 Chronic kidney disease	**141 (73.9 to 216)**	**124 (70.8 to 198)**	17.1 (-10.4 to 52.5)
2 Neonatal disorders	2 Stroke	**33.5 (2.95 to 66.7)**	23.8 (-0.41 to 53.8)	**-43.8 (-52.9 to -31.5)**
3 Stroke	3 Lower respiratory infect	11.2 (-14 to 37.1)	3.1 (-16.5 to 27.8)	**-37.3 (-45.9 to -26.7)**
4 Cirrhosis liver	4 Diabetes	**171 (128 to 222)**	**152 (121 to 190)**	**25.1 (13.1 to 36.4)**
5 Ischemic heart disease	5 Ischemic heart disease	**48 (1.04 to 103)**	37.2 (-1.56 to 94.5)	**-40.8 (-58.3 to -17.8)**
6 Chronic kidney disease	6 COPD	**103 (56.5 to 153)**	**88.2 (47.2 to 136)**	**-20.4 (-34.8 to -1.22)**
7 COPD	7 Cirrhosis liver	22.1 (-8.02 to 48.9)	13.2 (-8.6 to 40)	**-34.6 (-44.2 to -23)**
8 Congenital defects	8 Neonatal disorders	**-45.4 (-60 to -26.8)**	**-49.7 (-58.1 to -41.5)**	**-26.3 (-36.5 to -14.2)**
9 Headache disorders	9 Low back pain	**54.9 (28.5 to 79.2)**	**43.4 (30.7 to 57.4)**	**-5.37 (-5.52 to -5.21)**
10 Dietary iron deficiency	10 Other musculoskeletal	**169 (128 to 219)**	**149 (127 to 178)**	**70.4 (65.9 to 73.9)**
11 Diabetes	11 Depressive disorders	**39.3 (15.6 to 64.8)**	**28.8 (18.7 to 41.1)**	**-2.49 (-2.77 to -2.2)**
12 Depressive disorders	12 Headache disorders	21.7 (-0.173 to 47.3)	**12.5 (4.5 to 33.6)**	**0.751 (0.461 to 0.926)**
13 Road injuries	13 Falls	**54.4 (21.2 to 90.8)**	**43.1 (17.7 to 75.5)**	**-11.9 (-21 to -2.08)**
14 Low back pain	14 Other cardiovascular	**76.1 (43.4 to 114)**	**63.4 (33.4 to 99.8)**	**-19.6 (-29.5 to -8.14)**
15 Asthma	15 Age-related hearing loss	**82.9 (55.3 to 115)**	**69.4 (52.5 to 87.3)**	**-1.4 (-1.54 to -1.24)**
16 Gynecological diseases	16 Lung cancer	**141 (88.2 to 211)**	**123 (73.2 to 181)**	-2.9 (-19.7 to 17.2)
17 Diarrheal diseases	17 Road injuries	-4.68 (-46.4 to 55)	-11.8 (-43.8 to 40.5)	-28.6 (-56.2 to 13.5)
18 Falls	18 Gynecological diseases	10.8 (-14.8 to 39.9)	2.6 (-16.8 to 24.3)	-16.4 (-30.4 to 0.507)
19 Anxiety disorders	19 Hypertensive heart disease	**139 (73.3 to 213)**	**121 (69.8 to 192)**	-2.74 (-25.1 to 25.8)
20 Hemoglobinopathies	20 Blindness and vision loss	**97.9 (66.1 to 128)**	**83.4 (62.3 to 106)**	**-7.41 (-8.1 to -6.74)**
21 Other cardiovascular	21 Dietary iron deficiency			
22 Age-related hearing loss	22 Anxiety disorders			
24 Other musculoskeletal	27 Asthma			
31 Lung cancer	30 Congenital defects			
32 Blindness and vision loss	34 Hemoglobinopathies			
34 Hypertensive heart disease	42 Diarrheal diseases			

Supplemental Results Figure S7. Leading 20 level 3 causes of DALYs, and percentage change in number of DALYs and all-age and age-standardised DALY rates by location, 2022 and 2050

Senegal, Reference scenario

Legend:
Communicable, maternal, neonatal and nutritional
Non-communicable
Injuries

Leading Causes 2022	Leading Causes 2050	Mean % change number of DALY	Mean % change all-age DALY rate	Mean % change age-standardized DALY rate
1 Neonatal disorders	1 Neonatal disorders	**-32.6 (-48.5 to -14.9)**	**-59.7 (-66.3 to -52)**	**-36.9 (-45.2 to -25.7)**
2 Lower respiratory infect	2 Diabetes	**146 (106 to 190)**	**47.4 (28 to 71.9)**	-8.16 (-19.1 to 5.11)
3 Stroke	3 Stroke	**26.2 (2.44 to 52.4)**	**-24.5 (-36.8 to -11)**	**-53.5 (-60.4 to -44.8)**
4 Congenital defects	4 Lower respiratory infect	**-20 (-38.1 to -0.0297)**	**-52.2 (-61.2 to -41.4)**	**-46.7 (-55.3 to -36.9)**
5 Malaria	5 Ischemic heart disease	23.9 (-12.2 to 65.4)	-25.9 (-45.7 to 0.752)	**-56 (-68.4 to -38.6)**
6 Diarrheal diseases	6 Other musculoskeletal	**392 (341 to 456)**	**194 (176 to 216)**	**116 (111 to 121)**
7 Ischemic heart disease	7 Dietary iron deficiency	**21.8 (3.55 to 39.3)**	**-27.2 (-30.9 to -23.7)**	**-19 (-19.7 to -18.1)**
8 Dietary iron deficiency	8 Depressive disorders	**106 (81.9 to 135)**	**23.5 (16.6 to 32)**	**-3.87 (-4.19 to -3.54)**
9 Diabetes	9 Chronic kidney disease	**98.8 (45.5 to 158)**	18.9 (-7.42 to 53.2)	-17.1 (-35 to 8.15)
10 Road injuries	10 Low back pain	**109 (85.9 to 134)**	**25 (17.4 to 33.1)**	**-7.71 (-7.95 to -7.51)**
11 COVID-19	11 Headache disorders	**92.2 (68 to 117)**	**15 (9.62 to 26.2)**	**0.585 (0.261 to 0.836)**
12 Tuberculosis	12 Congenital defects	**-25.6 (-40.3 to -6.9)**	**-55.6 (-61.9 to -47.8)**	**-34.4 (-41.6 to -24.3)**
13 Chronic kidney disease	13 Diarrheal diseases	-18.3 (-44.4 to 15.7)	**-51.1 (-66.7 to -31.5)**	**-49.8 (-63.9 to -31.6)**
14 Maternal disorders	14 Road injuries	13.7 (-33.4 to 96)	-32 (-57.4 to 13.8)	**-38.7 (-61.3 to -0.505)**
15 Depressive disorders	15 Hypertensive heart disease	**129 (73.4 to 195)**	**36.8 (6.88 to 77.4)**	-19.2 (-36.4 to 7.16)
16 Headache disorders	16 Cirrhosis liver	**45.6 (18.4 to 75.8)**	-12.9 (-27.3 to 5.09)	**-38.7 (-47.5 to -27.3)**
17 Meningitis	17 Age-related hearing loss	**128 (102 to 157)**	**36.6 (26.3 to 48.6)**	**-2.18 (-2.31 to -2.04)**
18 Cirrhosis liver	18 Gynecological diseases	**61 (28.8 to 96.9)**	-3.63 (-20.8 to 15)	**-24.2 (-36.3 to -9.72)**
19 Low back pain	19 COPD	**102 (59.4 to 142)**	21 (-0.558 to 48)	**-26.9 (-41 to -10.3)**
20 Hemoglobinopathies	20 Anxiety disorders	**83.3 (60.4 to 108)**	**9.61 (3.48 to 17.7)**	**2.03 (1.79 to 2.29)**
22 Gynecological diseases	23 Hemoglobinopathies			
26 Hypertensive heart disease	30 Tuberculosis			
27 Anxiety disorders	31 Meningitis			
29 COPD	36 Maternal disorders			
30 Age-related hearing loss	46 Malaria			
32 Other musculoskeletal	164 COVID-19			

Supplemental Results Figure S7. Leading 20 level 3 causes of DALYs, and percentage change in number of DALYs and all-age and age-standardised DALY rates by location, 2022 and 2050

Sierra Leone, Reference scenario

Legend:
Communicable, maternal, neonatal and nutritional
Non-communicable
Injuries

Leading Causes 2022	Leading Causes 2050	Mean % change number of DALY	Mean % change all-age DALY rate	Mean % change age-standardized DALY rate
1 Neonatal disorders	1 Neonatal disorders	**-47.2 (-60.6 to -29)**	**-69 (-76 to -58.5)**	**-47.6 (-58.9 to -30.4)**
2 Malaria	2 Malaria	-37.8 (-93.3 to 228)	-61.5 (-96.2 to 113)	-50.4 (-95.2 to 131)
3 Lower respiratory infect	3 Lower respiratory infect	-16.2 (-31.5 to 1.85)	**-50.7 (-61 to -39.9)**	**-42 (-52.3 to -31.1)**
4 Protein-energy malnutrition	4 Diarrheal diseases	-1.62 (-38.4 to 43.1)	**-42.2 (-62.2 to -16.6)**	**-34 (-53.3 to -10)**
5 Diarrheal diseases	5 Stroke	**32.2 (6.04 to 62.6)**	**-22.3 (-37.9 to -1.8)**	**-49.9 (-59.4 to -37.3)**
6 Congenital defects	6 Diabetes	**183 (135 to 236)**	**66.2 (37.6 to 96.8)**	3.34 (-11 to 19.4)
7 Stroke	7 Ischemic heart disease	**50.9 (1.81 to 112)**	-11.2 (-41.9 to 27.9)	**-47.8 (-65.4 to -23.8)**
8 Tuberculosis	8 Congenital defects	**-33.1 (-49 to -15.2)**	**-60.8 (-68 to -51.7)**	**-38.6 (-47.1 to -26.7)**
9 Ischemic heart disease	9 Depressive disorders	**110 (82.6 to 136)**	**23.1 (16 to 31.6)**	**-3.09 (-3.83 to -2.32)**
10 Road injuries	10 Road injuries	18 (-30.1 to 93.9)	-30.6 (-58.1 to 10.8)	-32.7 (-58.1 to 9.83)
11 HIV/AIDS	11 Low back pain	**116 (88.7 to 142)**	**26.9 (19.6 to 37.1)**	**-6.63 (-7.16 to -6.18)**
12 Maternal disorders	12 Other musculoskeletal	**387 (324 to 452)**	**186 (166 to 209)**	**108 (101 to 113)**
13 iNTS	13 Headache disorders	**95.5 (69.3 to 125)**	**14.8 (9.39 to 26.5)**	**0.967 (0.369 to 1.51)**
14 Hemoglobinopathies	14 Protein-energy malnutrition	**-65.6 (-82.6 to -38.7)**	**-79.7 (-89.3 to -62.3)**	**-69.3 (-83.4 to -42.4)**
15 Meningitis	15 Chronic kidney disease	**127 (67.3 to 203)**	33.6 (-0.122 to 76.1)	0.346 (-25.9 to 32.3)
16 Dietary iron deficiency	16 Hemoglobinopathies	12.2 (-11.3 to 37.9)	**-34.2 (-42.7 to -24.1)**	**-22.8 (-30.5 to -13.2)**
17 Depressive disorders	17 Cirrhosis liver	**38.5 (11.9 to 64)**	**-18.6 (-32.7 to -0.873)**	**-42.6 (-52.3 to -30.9)**
18 Diabetes	18 Dietary iron deficiency	**23.1 (3.67 to 41)**	**-27.8 (-31.6 to -24.1)**	**-20.4 (-21.2 to -19.2)**
19 Cirrhosis liver	19 Tuberculosis	**-36.2 (-58.6 to -3.35)**	**-62.4 (-76.5 to -43.1)**	**-67.8 (-79.1 to -51.7)**
20 Headache disorders	20 Maternal disorders	-26.5 (-53 to 9.81)	**-56.9 (-71.1 to -37.6)**	**-59.1 (-72.7 to -40.9)**
21 Low back pain	25 iNTS			
24 Chronic kidney disease	29 Meningitis			
40 Other musculoskeletal	63 HIV/AIDS			

Supplemental Results Figure S7. Leading 20 level 3 causes of DALYs, and percentage change in number of DALYs and all-age and age-standardised DALY rates by location, 2022 and 2050

Togo, Reference scenario

Legend:
Communicable, maternal, neonatal and nutritional
Non-communicable
Injuries

Leading Causes 2022	Leading Causes 2050	Mean % change number of DALY	Mean % change all-age DALY rate	Mean % change age-standardized DALY rate
1 Neonatal disorders	1 Lower respiratory infect	1.47 (-22.5 to 24.4)	**-34.4 (-46.5 to -19.8)**	**-37.5 (-47.4 to -24.1)**
2 Malaria	2 Neonatal disorders	**-46 (-60.2 to -30.5)**	**-65.1 (-71.5 to -56.4)**	**-42.6 (-51.8 to -29.5)**
3 Lower respiratory infect	3 Malaria	-37.2 (-92.3 to 154)	-59.7 (-95.1 to 72.7)	-55.9 (-94.7 to 93)
4 Diarrheal diseases	4 Stroke	**41.1 (9.88 to 74.7)**	-8.71 (-26 to 15.2)	**-49.7 (-59.1 to -35.3)**
5 Stroke	5 Diarrheal diseases	-12.7 (-42.9 to 25.7)	**-43.5 (-62.6 to -16.3)**	**-38 (-58.1 to -12.1)**
6 HIV/AIDS	6 Diabetes	**187 (125 to 251)**	**85.7 (55 to 122)**	6.16 (-9.74 to 25)
7 Congenital defects	7 Ischemic heart disease	48.4 (-0.487 to 120)	-3.81 (-34.5 to 45.3)	**-49.4 (-66.8 to -22.2)**
8 Road injuries	8 Road injuries	4.22 (-39.1 to 82.6)	-32.4 (-61.3 to 20.5)	-34.3 (-61.4 to 15.7)
9 Hemoglobinopathies	9 Depressive disorders	**96.9 (63.9 to 133)**	**27.1 (20.9 to 34.6)**	**-3.93 (-4.41 to -3.41)**
10 Ischemic heart disease	10 Chronic kidney disease	**162 (95.1 to 251)**	**69.5 (30.4 to 129)**	9.81 (-17.9 to 49.5)
11 Dietary iron deficiency	11 Other musculoskeletal	**336 (264 to 406)**	**181 (163 to 200)**	**101 (96.2 to 105)**
12 Tuberculosis	12 Hemoglobinopathies	2.44 (-19 to 24.2)	**-33.9 (-40.1 to -27.3)**	**-26.7 (-33.5 to -20)**
13 COVID-19	13 Low back pain	**101 (69.6 to 134)**	**29.7 (22.4 to 37.3)**	**-7.22 (-7.71 to -6.78)**
14 iNTS	14 Dietary iron deficiency	6.48 (-15.2 to 28.2)	**-31.4 (-36.2 to -27.3)**	**-17.5 (-18.7 to -16.5)**
15 Depressive disorders	15 Headache disorders	**79.2 (45.9 to 111)**	**15.7 (10.6 to 28)**	0.295 (-0.102 to 0.683)
16 Cirrhosis liver	16 Hypertensive heart disease	**170 (100 to 266)**	**74.8 (31 to 133)**	-8.89 (-32.2 to 21.8)
17 Diabetes	17 Cirrhosis liver	**43.4 (13.7 to 75.6)**	-7.25 (-23.1 to 11.8)	**-37.2 (-47.6 to -24.4)**
18 Low back pain	18 Congenital defects	**-30.8 (-46.4 to -15.4)**	**-55.3 (-61.3 to -47.7)**	**-32.4 (-40.1 to -22.9)**
19 Headache disorders	19 Tuberculosis	-22.5 (-47.7 to 16.3)	**-49.8 (-65.3 to -24.7)**	**-63.9 (-75.5 to -46)**
20 Meningitis	20 Gynecological diseases	**47.5 (17.3 to 83.4)**	-4.6 (-22.8 to 18.2)	**-24.5 (-38.9 to -6.36)**
21 Chronic kidney disease	26 iNTS			
22 Gynecological diseases	34 Meningitis			
27 Hypertensive heart disease	50 HIV/AIDS			
34 Other musculoskeletal	166 COVID-19			

Supplemental Results Figure S7. Leading 20 level 3 causes of DALYs, and percentage change in number of DALYs and all-age and age-standardised DALY rates by location, 2022 and 2050

Global, Reference scenario

Legend:
Communicable, maternal, neonatal and nutritional
Non-communicable
Injuries

Leading Causes 2022
1 Ischemic heart disease
2 Neonatal disorders
3 Stroke
4 Lower respiratory infect
5 Diabetes
6 COPD
7 COVID-19
8 Low back pain
9 Road injuries
10 Diarrheal diseases
11 Congenital defects
12 Malaria
13 Depressive disorders
14 Headache disorders
15 Cirrhosis liver
16 Lung cancer
17 Other musculoskeletal
18 Age-related hearing loss
19 Falls
20 Tuberculosis
22 Alzheimer's disease
23 Chronic kidney disease
27 Blindness and vision loss
30 Hypertensive heart disease

Leading Causes 2050	Mean % change number of DALY	Mean % change all-age DALY rate	Mean % change age-standardized DALY rate
1 Ischemic heart disease	1.61 (-28.3 to 44.6)	-13.5 (-40.7 to 27.6)	**-44.4 (-63.9 to -13.7)**
2 Stroke	12.9 (-4.67 to 34)	-3.94 (-18.4 to 16)	**-38.8 (-49.7 to -24.1)**
3 Diabetes	**97.5 (82.1 to 114)**	**68 (51.7 to 86.5)**	**22.4 (10.4 to 36.6)**
4 COPD	**72.8 (39.6 to 113)**	**47 (19 to 86.1)**	-13.8 (-34.6 to 15.6)
5 Neonatal disorders	**-37.2 (-48.4 to -23)**	**-46.6 (-55.8 to -34.4)**	**-32.7 (-43.2 to -17.5)**
6 Other musculoskeletal	**109 (97.2 to 119)**	**77.5 (70.8 to 84.9)**	**54.8 (52.3 to 57.6)**
7 Low back pain	**34.1 (28.1 to 40.4)**	**13.9 (10.3 to 18)**	**-5.23 (-5.58 to -4.9)**
8 Alzheimer's disease	**142 (106 to 173)**	**106 (80.6 to 129)**	**-2.91 (-4.6 to -1.47)**
9 Lower respiratory infect	**-14.6 (-25 to -4.23)**	**-27.4 (-36.3 to -16.1)**	**-44.1 (-53.2 to -32.1)**
10 Chronic kidney disease	**122 (71.7 to 183)**	**88.8 (43.8 to 151)**	28.6 (-6.61 to 77.7)
11 Age-related hearing loss	**66.7 (56 to 78.8)**	**41.7 (33.4 to 50.1)**	0.387 (-0.408 to 1.17)
12 Depressive disorders	**35.4 (28.9 to 41.6)**	**15.1 (11.8 to 18.9)**	**3.8 (3.02 to 4.51)**
13 Road injuries	-1.47 (-34.3 to 50.4)	-16.1 (-44.8 to 31.3)	-18.7 (-48.2 to 30.6)
14 Lung cancer	**36.9 (3.35 to 74.6)**	16.3 (-9.81 to 44.2)	**-23.5 (-37.7 to -6.91)**
15 Falls	**45.1 (32.2 to 59.1)**	**23.3 (13.3 to 32.7)**	**-14.9 (-18.6 to -11.5)**
16 Hypertensive heart disease	**135 (78.8 to 203)**	**99.8 (49.4 to 167)**	22.6 (-12.9 to 68.4)
17 Cirrhosis liver	**27.3 (11 to 45.7)**	8.27 (-6.41 to 27)	-12 (-24.5 to 4.22)
18 Headache disorders	**19.8 (13.8 to 28.9)**	1.84 (-1.1 to 8.87)	-0.299 (-0.866 to 0.375)
19 Blindness and vision loss	**71.9 (57.2 to 87.2)**	**46.1 (34.5 to 56.6)**	**3.69 (1.76 to 5.39)**
20 Diarrheal diseases	-20.2 (-44.3 to 15.3)	**-32.1 (-53.2 to -0.585)**	**-40.9 (-61.1 to -8.07)**
25 Congenital defects			
27 Malaria			
32 Tuberculosis			
172 COVID-19			

Supplemental Results Figure S7. Leading 20 level 3 causes of DALYs, and percentage change in number of DALYs and all-age and age-standardised DALY rates by location, 2022 and 2050

Central Europe, Eastern Europe, and Central Asia, Reference scenario

Legend:
Communicable, maternal, neonatal and nutritional
Non-communicable
Injuries

Leading Causes 2022	Leading Causes 2050	Mean % change number of DALY	Mean % change all-age DALY rate	Mean % change age-standardized DALY rate
1 Ischemic heart disease	1 Ischemic heart disease	-18.3 (-41.9 to 14.3)	-14.2 (-40 to 19.3)	**-45.4 (-64.3 to -19.6)**
2 Stroke	2 Stroke	**-21.3 (-33.1 to -8.77)**	**-17.4 (-29.4 to -3.69)**	**-44.3 (-54.3 to -32.1)**
3 COVID-19	3 Low back pain	2.86 (-3.04 to 10.3)	**7.92 (4.53 to 11.4)**	**-5.11 (-5.43 to -4.75)**
4 Low back pain	4 Diabetes	**32.5 (22.1 to 44)**	**39.1 (27.4 to 51.6)**	8.92 (-0.909 to 20.2)
5 Cirrhosis liver	5 Alzheimer's disease	**95.7 (60.9 to 128)**	**105 (72.3 to 136)**	1.28 (-0.0805 to 2.48)
6 Diabetes	6 Lung cancer	1.18 (-22 to 28.5)	6.08 (-16.5 to 31.8)	-18.5 (-34.8 to 0.694)
7 Lung cancer	7 Cirrhosis liver	-13.1 (-24.6 to 2.69)	-8.74 (-20.5 to 7.56)	-14.2 (-26.2 to 2.02)
8 Lower respiratory infect	8 Age-related hearing loss	**29.7 (19.4 to 40.9)**	**36.1 (27.6 to 44.6)**	**-0.537 (-0.769 to -0.351)**
9 Cardiomyopathy	9 COPD	**36.5 (9.45 to 66.2)**	**43.3 (14.7 to 76.3)**	-4.35 (-26.7 to 23.7)
10 Falls	10 Falls	-5.12 (-13.2 to 3.47)	-0.468 (-7.37 to 6.23)	**-18 (-22.6 to -13.2)**
11 Self-harm	11 Colorectal cancer	14.2 (-19.7 to 54.9)	19.7 (-16.2 to 61.9)	-14.6 (-38.6 to 15.1)
12 Alcohol use disorders	12 Alcohol use disorders	-8.01 (-18.5 to 5.01)	-3.45 (-15.2 to 12.5)	-1.74 (-13.5 to 12.8)
13 Headache disorders	13 Hypertensive heart disease	**58.5 (9.37 to 126)**	**66.5 (15.1 to 132)**	7.46 (-29.8 to 59.4)
14 Colorectal cancer	14 Depressive disorders	1.29 (-4.68 to 7.58)	**6.26 (2.84 to 9.53)**	**-1.08 (-1.65 to -0.46)**
15 Age-related hearing loss	15 Headache disorders	**-7.75 (-12.5 to -1.93)**	**-3.21 (-5.41 to -0.781)**	**-1.13 (-3.08 to -0.289)**
16 Neonatal disorders	16 Cardiomyopathy	**-24 (-32.2 to -14.2)**	**-20.3 (-28.2 to -12.4)**	**-28.7 (-36.5 to -20)**
17 Depressive disorders	17 Lower respiratory infect	**-27.8 (-38.8 to -11.7)**	**-24.2 (-35.4 to -8.61)**	**-42.8 (-55.1 to -23.8)**
18 Alzheimer's disease	18 Self-harm	**-21.5 (-32.1 to -8.09)**	**-17.6 (-29.9 to -4.59)**	-16.2 (-29.2 to 0.0409)
19 Road injuries	19 Road injuries	-15.6 (-43.8 to 27.5)	-11.3 (-39.7 to 31.9)	-11.2 (-42.1 to 36)
20 COPD	20 Osteoarthritis	**25.1 (16.3 to 34.3)**	**31.3 (25.6 to 36.6)**	-0.0285 (-0.382 to 0.336)
23 Hypertensive heart disease	25 Neonatal disorders			
25 Osteoarthritis	157 COVID-19			

Supplemental Results Figure S7. Leading 20 level 3 causes of DALYs, and percentage change in number of DALYs and all-age and age-standardised DALY rates by location, 2022 and 2050

High income, Reference scenario

Legend:
Communicable, maternal, neonatal and nutritional
Non-communicable
Injuries

Leading Causes 2022	Leading Causes 2050	Mean % change number of DALY	Mean % change all-age DALY rate	Mean % change age-standardized DALY rate
1 Ischemic heart disease	1 Alzheimer's disease	**67.8 (56.5 to 76.4)**	**62.3 (53.7 to 70.9)**	**-5.02 (-6.72 to -3.41)**
2 Low back pain	2 Diabetes	**36 (30.2 to 41.8)**	**31.6 (25.3 to 37.4)**	**11.9 (7.25 to 16.5)**
3 COVID-19	3 Low back pain	**7.76 (4.79 to 10.8)**	**4.22 (1.96 to 6.48)**	**-2.07 (-2.3 to -1.83)**
4 Stroke	4 Ischemic heart disease	**-25.8 (-37.8 to -10.8)**	**-28.2 (-39.7 to -13.8)**	**-51.7 (-60.6 to -40.6)**
5 Diabetes	5 COPD	**36.3 (22 to 51.3)**	**31.8 (17.8 to 45.8)**	-8.72 (-19.5 to 2.36)
6 Alzheimer's disease	6 Stroke	-6.08 (-12.1 to 0.603)	**-9.16 (-15.3 to -2)**	**-33.5 (-39.4 to -27.3)**
7 Lung cancer	7 Lung cancer	8.27 (-5.73 to 23.1)	4.71 (-8.48 to 20)	**-17.6 (-27.2 to -8)**
8 COPD	8 Other musculoskeletal	**26.2 (22 to 30.4)**	**22.1 (19.6 to 24.7)**	**19.7 (18.9 to 20.6)**
9 Falls	9 Falls	**24.8 (19.1 to 31.3)**	**20.7 (15.4 to 26.5)**	**-11.5 (-13.8 to -8.89)**
10 Other musculoskeletal	10 Chronic kidney disease	**110 (81.6 to 139)**	**103 (75.4 to 132)**	**29.2 (9.75 to 52.8)**
11 Drug use disorders	11 Drug use disorders	**27.7 (15.8 to 39.2)**	**23.5 (13 to 34.8)**	**28 (16.8 to 39.8)**
12 Depressive disorders	12 Age-related hearing loss	**32.2 (27.8 to 37.5)**	**27.8 (23.5 to 32.6)**	0.0685 (-0.243 to 0.367)
13 Headache disorders	13 Colorectal cancer	**24.7 (3.2 to 49.5)**	**20.5 (0.203 to 44.2)**	-10.6 (-25.3 to 5.74)
14 Age-related hearing loss	14 Depressive disorders	**4.1 (0.86 to 7.79)**	0.674 (-0.403 to 2.23)	**0.705 (0.435 to 1.01)**
15 Anxiety disorders	15 Lower respiratory infect	**41.1 (30.1 to 51.1)**	**36.4 (26.3 to 46.1)**	**-16.1 (-22.8 to -9.15)**
16 Colorectal cancer	16 Headache disorders	0.895 (-2.36 to 4.54)	-2.42 (-3.74 to 0.492)	0.555 (-0.193 to 1.08)
17 Self-harm	17 Anxiety disorders	**3.63 (0.295 to 7.53)**	0.217 (-1.26 to 1.75)	**1.87 (1.57 to 2.2)**
18 Lower respiratory infect	18 Osteoarthritis	**23 (19.6 to 26.9)**	**19 (16.1 to 21.9)**	**1.01 (0.801 to 1.27)**
19 Osteoarthritis	19 Pancreatic cancer	**57 (31.9 to 85.2)**	**51.9 (28.8 to 79.2)**	**19.5 (2.83 to 38)**
20 Chronic kidney disease	20 Hypertensive heart disease	**75.8 (50.2 to 105)**	**70.1 (43.7 to 98.4)**	**21 (1.43 to 45.3)**
24 Pancreatic cancer	24 Self-harm			
30 Hypertensive heart disease	156 COVID-19			

Supplemental Results Figure S7. Leading 20 level 3 causes of DALYs, and percentage change in number of DALYs and all-age and age-standardised DALY rates by location, 2022 and 2050

Latin America and Caribbean, Reference scenario

Legend:
Communicable, maternal, neonatal and nutritional
Non-communicable
Injuries

Leading Causes 2022	Leading Causes 2050	Mean % change number of DALY	Mean % change all-age DALY rate	Mean % change age-standardized DALY rate
1 Ischemic heart disease	1 Diabetes	**94.3 (72.4 to 116)**	**73.6 (52.9 to 97.4)**	12.4 (-0.846 to 28.6)
2 COVID-19	2 Ischemic heart disease	22.8 (-24.7 to 91)	9.95 (-32.9 to 78.5)	-37.4 (-63.2 to 5.63)
3 Diabetes	3 Chronic kidney disease	**121 (54.5 to 199)**	**97.5 (39 to 174)**	21.7 (-18.5 to 78.4)
4 Interpersonal violence	4 Other musculoskeletal	**86.1 (72.7 to 100)**	**66.2 (58.5 to 75)**	**37.2 (34.6 to 40)**
5 Neonatal disorders	5 Stroke	19 (-5.99 to 47.6)	6.41 (-17.2 to 38.3)	**-37.4 (-53.2 to -16.3)**
6 Stroke	6 Low back pain	**31.9 (22.1 to 40.9)**	**17.8 (13.2 to 22.6)**	**-4.12 (-4.37 to -3.86)**
7 Lower respiratory infect	7 Lower respiratory infect	19.7 (-0.897 to 38.9)	6.98 (-10.5 to 26.9)	**-37.2 (-48.7 to -22.3)**
8 Low back pain	8 Alzheimer's disease	**170 (126 to 209)**	**141 (107 to 172)**	**-2.23 (-3.94 to -0.544)**
9 Road injuries	9 Interpersonal violence	-32 (-61.4 to 16.3)	-39.4 (-63.2 to 2.84)	-33.2 (-61.6 to 14.9)
10 Chronic kidney disease	10 COPD	**87.5 (43.7 to 135)**	**67.6 (27.6 to 118)**	-17 (-40.7 to 15.1)
11 Other musculoskeletal	11 Road injuries	1.18 (-34.6 to 56.5)	-9.44 (-42.4 to 44.4)	-10.1 (-43.2 to 45)
12 Anxiety disorders	12 Cirrhosis liver	**36.6 (21.8 to 51.7)**	**22 (9.72 to 37.7)**	-8.63 (-19.5 to 3.71)
13 Headache disorders	13 Age-related hearing loss	**79.7 (61.7 to 96.1)**	**60.4 (50.2 to 70.8)**	**-1.04 (-1.18 to -0.89)**
14 Congenital defects	14 Depressive disorders	**24.2 (15.1 to 33.6)**	**10.9 (7.41 to 15.5)**	**-2.43 (-2.73 to -2.13)**
15 Cirrhosis liver	15 Anxiety disorders	**14.7 (6.29 to 23.2)**	2.38 (-1.32 to 7.13)	-0.319 (-0.865 to 0.279)
16 Depressive disorders	16 Falls	**52.1 (35.5 to 68.2)**	**35.8 (23.4 to 47)**	**-6.84 (-12.4 to -1.1)**
17 COPD	17 Neonatal disorders	**-39.2 (-50.5 to -26.3)**	**-45.6 (-55.6 to -33.1)**	**-27.9 (-41.7 to -10.1)**
18 Falls	18 Headache disorders	**10.9 (1.47 to 23.8)**	-0.985 (-5.43 to 11.6)	-0.0452 (-0.499 to 0.511)
19 Age-related hearing loss	19 Blindness and vision loss	**69.1 (52.5 to 83.3)**	**50.9 (39.2 to 61.1)**	**-4.34 (-4.61 to -4.07)**
20 Oral disorders	20 Oral disorders	**61.2 (46.6 to 73.3)**	**43.9 (35.4 to 52)**	**0.614 (0.253 to 0.973)**
21 Alzheimer's disease	30 Congenital defects			
22 Blindness and vision loss	165 COVID-19			

Supplemental Results Figure S7. Leading 20 level 3 causes of DALYs, and percentage change in number of DALYs and all-age and age-standardised DALY rates by location, 2022 and 2050

North Africa and Middle East, Reference scenario

Legend:
Communicable, maternal, neonatal and nutritional
Non-communicable
Injuries

Leading Causes 2022	Leading Causes 2050	Mean % change number of DALY	Mean % change all-age DALY rate	Mean % change age-standardized DALY rate
1 Ischemic heart disease	1 Ischemic heart disease	47 (-1.31 to 124)	8.8 (-29.3 to 67.4)	**-41.5 (-63.3 to -6.12)**
2 Neonatal disorders	2 Diabetes	**227 (199 to 256)**	**142 (119 to 169)**	**41.7 (27.9 to 58.6)**
3 Stroke	3 Stroke	**47.6 (16.5 to 86.6)**	9.19 (-13.9 to 41.2)	**-43.1 (-56.4 to -22.1)**
4 Congenital defects	4 Chronic kidney disease	**219 (134 to 327)**	**136 (71.6 to 223)**	28.1 (-9.1 to 87.5)
5 Road injuries	5 Low back pain	**61.1 (50.4 to 71.2)**	**19 (13.4 to 24.9)**	**-5.17 (-5.46 to -4.91)**
6 Diabetes	6 Hypertensive heart disease	**207 (126 to 312)**	**127 (66.5 to 209)**	8.27 (-26.5 to 58.5)
7 COVID-19	7 Road injuries	12.5 (-27.9 to 77.1)	-16.8 (-47.4 to 34.4)	-22.2 (-51.8 to 26)
8 Low back pain	8 Depressive disorders	**44.8 (35.6 to 55.5)**	**6.93 (2.67 to 12.3)**	**-2.32 (-2.96 to -1.65)**
9 Depressive disorders	9 Other musculoskeletal	**157 (138 to 176)**	**90 (77.5 to 102)**	**54.2 (51 to 57.2)**
10 Lower respiratory infect	10 Neonatal disorders	**-34 (-50.4 to -12.1)**	**-51.2 (-62.4 to -35.4)**	**-37.3 (-52.4 to -15.4)**
11 Conflict & terror	11 COPD	**140 (84.2 to 209)**	**77.9 (38 to 135)**	-12.7 (-37.5 to 23.5)
12 Headache disorders	12 Alzheimer's disease	**281 (211 to 345)**	**181 (136 to 227)**	**5.76 (4.12 to 7.55)**
13 Anxiety disorders	13 Headache disorders	**39.6 (30 to 51.5)**	3.15 (-0.804 to 10.3)	0.69 (-0.0384 to 1.21)
14 Chronic kidney disease	14 Conflict & terror	60.1 (-59.4 to 350)	18.5 (-69.8 to 235)	21.5 (-68.9 to 251)
15 Gynecological diseases	15 Age-related hearing loss	**122 (99.9 to 142)**	**63.8 (51.2 to 77.2)**	**-1.77 (-2.03 to -1.51)**
16 Hypertensive heart disease	16 Anxiety disorders	**36 (26.2 to 45.5)**	0.448 (-3.83 to 5.36)	0.213 (-0.49 to 0.826)
17 Cirrhosis liver	17 Lower respiratory infect	2.15 (-14.2 to 21.9)	**-24.5 (-36.6 to -7.31)**	**-42 (-53.8 to -25.3)**
18 Other musculoskeletal	18 Congenital defects	**-31.3 (-43.5 to -20.5)**	**-49.3 (-56.3 to -41.5)**	**-31.7 (-38.6 to -22.9)**
19 COPD	19 Cirrhosis liver	**57.3 (38.3 to 77.2)**	**16.3 (0.994 to 34.2)**	**-30.7 (-39.8 to -17.9)**
20 Age-related hearing loss	20 Blindness and vision loss	**124 (101 to 145)**	**65.2 (49.6 to 79.4)**	**-3.32 (-3.95 to -2.67)**
25 Blindness and vision loss	22 Gynecological diseases			
27 Alzheimer's disease	167 COVID-19			

Supplemental Results Figure S7. Leading 20 level 3 causes of DALYs, and percentage change in number of DALYs and all-age and age-standardised DALY rates by location, 2022 and 2050

South Asia, Reference scenario

Legend:
Communicable, maternal, neonatal and nutritional
Non-communicable
Injuries

Leading Causes 2022	Leading Causes 2050	Mean % change number of DALY	Mean % change all-age DALY rate	Mean % change age-standardized DALY rate
1 Neonatal disorders	1 Ischemic heart disease	1.4 (-39.9 to 57.8)	-9.19 (-46.1 to 46.7)	**-51.5 (-73.2 to -15.8)**
2 Ischemic heart disease	2 COPD	**64.3 (19.9 to 112)**	**47 (9.5 to 99.4)**	-32.2 (-52.7 to 0.761)
3 COPD	3 Diabetes	**107 (80 to 135)**	**84.5 (62.3 to 113)**	3.49 (-10.3 to 24.5)
4 Lower respiratory infect	4 Other musculoskeletal	**154 (126 to 182)**	**127 (114 to 140)**	**67.5 (64 to 71.3)**
5 Stroke	5 Stroke	18.7 (-7.69 to 48.6)	6.13 (-16.5 to 38.6)	**-45.4 (-59.5 to -27)**
6 Diarrheal diseases	6 Neonatal disorders	**-54.8 (-63.6 to -44.5)**	**-59.6 (-66.4 to -50.8)**	**-35.5 (-45.6 to -22.3)**
7 Diabetes	7 Falls	**72.3 (39 to 106)**	**53.7 (32.2 to 73.5)**	**-12.7 (-20.2 to -4.75)**
8 Tuberculosis	8 Low back pain	**49.3 (32.9 to 66.3)**	**33.3 (25.9 to 40.9)**	**-5.8 (-6.14 to -5.44)**
9 Dietary iron deficiency	9 Blindness and vision loss	**88.2 (62.7 to 117)**	**68 (53.6 to 82.9)**	**-6.79 (-7.1 to -6.45)**
10 Other musculoskeletal	10 Depressive disorders	**38.1 (22.4 to 55.2)**	**23.3 (17.8 to 30.6)**	**-4.05 (-4.45 to -3.61)**
11 Road injuries	11 Age-related hearing loss	**83.3 (59 to 109)**	**63.6 (51.3 to 76.7)**	**-1.74 (-1.91 to -1.57)**
12 Low back pain	12 Cirrhosis liver	**28.2 (3.31 to 57.4)**	14.6 (-7.56 to 42.9)	**-21.1 (-37.1 to -1.04)**
13 Cirrhosis liver	13 Lower respiratory infect	**-41 (-52.5 to -27.2)**	**-47.3 (-56.6 to -35.9)**	**-57.5 (-65.7 to -46.2)**
14 Depressive disorders	14 Diarrheal diseases	-16.8 (-41.6 to 16.7)	-25.6 (-49.3 to 8.23)	**-51.2 (-69.9 to -22.2)**
15 COVID-19	15 Dietary iron deficiency	-0.56 (-12.1 to 12.3)	**-11.3 (-13.4 to -9.18)**	**-13.7 (-14.8 to -12.6)**
16 Falls	16 Road injuries	-4.34 (-40.9 to 45.2)	-14.4 (-45.8 to 36.8)	-23.1 (-52.9 to 25.6)
17 Self-harm	17 Headache disorders	**20.4 (6.48 to 41.7)**	**7.43 (1.56 to 23.8)**	0.422 (-0.117 to 0.668)
18 Headache disorders	18 Chronic kidney disease	**104 (41.2 to 185)**	**82.4 (26.2 to 164)**	4.93 (-32 to 59)
19 Congenital defects	19 Self-harm	-9.2 (-31.1 to 16.1)	-18.9 (-37.9 to 6.93)	-20.3 (-40.2 to 6.49)
20 Blindness and vision loss	20 Alzheimer's disease	**196 (127 to 272)**	**164 (114 to 214)**	**-5.84 (-9.4 to -2.54)**
21 Age-related hearing loss	22 Tuberculosis			
25 Chronic kidney disease	30 Congenital defects			
38 Alzheimer's disease	166 COVID-19			

Supplemental Results Figure S7. Leading 20 level 3 causes of DALYs, and percentage change in number of DALYs and all-age and age-standardised DALY rates by location, 2022 and 2050

Southeast Asia, East Asia, and Oceania, Reference scenario

Legend:
Communicable, maternal, neonatal and nutritional
Non-communicable
Injuries

Leading Causes 2022	Leading Causes 2050	Mean % change number of DALY	Mean % change all-age DALY rate	Mean % change age-standardized DALY rate
1 Stroke	1 Stroke	7.42 (-9.3 to 28.7)	11.9 (-3.77 to 35.6)	**-41.5 (-50.7 to -28.6)**
2 Ischemic heart disease	2 COPD	**84.5 (47.2 to 122)**	**92.2 (54 to 138)**	-18 (-35.7 to 8.95)
3 COPD	3 Ischemic heart disease	-8.65 (-33.7 to 23.2)	-4.81 (-29.6 to 29.7)	**-52 (-66 to -31.3)**
4 Lung cancer	4 Diabetes	**75.9 (58.4 to 92.6)**	**83.1 (66.4 to 99.8)**	**25.2 (13.7 to 36.3)**
5 Diabetes	5 Alzheimer's disease	**179 (136 to 230)**	**190 (153 to 234)**	-0.379 (-3.61 to 2.41)
6 Road injuries	6 Lung cancer	**40.2 (4.26 to 81.4)**	**45.7 (10.8 to 85.8)**	-14.8 (-31.8 to 4.86)
7 Age-related hearing loss	7 Age-related hearing loss	**50.5 (37.5 to 65.2)**	**56.6 (46.3 to 66.4)**	**-0.87 (-1.23 to -0.558)**
8 Low back pain	8 Hypertensive heart disease	**137 (74.2 to 206)**	**146 (80 to 238)**	17.8 (-18 to 62.9)
9 Neonatal disorders	9 Other musculoskeletal	**66.3 (54.6 to 79.5)**	**73.1 (63.8 to 81.9)**	**54.4 (52 to 56.6)**
10 Headache disorders	10 Low back pain	**17.9 (10 to 27.1)**	**22.7 (17.2 to 28.1)**	**-3.43 (-3.73 to -3.06)**
11 Alzheimer's disease	11 Chronic kidney disease	**85.5 (43.6 to 135)**	**93.2 (51.7 to 150)**	20.6 (-8.45 to 61.1)
12 Lower respiratory infect	12 Colorectal cancer	**72.9 (23 to 131)**	**79.7 (31.4 to 137)**	8.97 (-18.8 to 39.8)
13 Stomach cancer	13 Road injuries	-21.2 (-43.4 to 15.3)	-17.8 (-42.2 to 21)	-17.6 (-43.5 to 23.2)
14 Other musculoskeletal	14 Falls	**29.2 (14.5 to 46.7)**	**34.5 (21.4 to 49.9)**	**-18.4 (-23.1 to -13.3)**
15 Falls	15 Lower respiratory infect	**15.2 (0.676 to 30)**	**19.9 (7.02 to 33)**	**-42.8 (-50.2 to -33.3)**
16 Depressive disorders	16 Cirrhosis liver	**19.3 (1.9 to 39)**	**24.2 (6.8 to 48)**	-2.29 (-16 to 16.3)
17 Cirrhosis liver	17 Blindness and vision loss	**46.7 (30.8 to 63)**	**52.6 (37.9 to 64.6)**	**-4.18 (-5.18 to -3.39)**
18 Chronic kidney disease	18 Depressive disorders	**10.4 (3.6 to 18.5)**	**14.9 (10.7 to 19.6)**	**-2.22 (-2.78 to -1.61)**
19 Colorectal cancer	19 Headache disorders	-3.16 (-9.49 to 3.72)	0.793 (-3.3 to 6.89)	**2.66 (0.607 to 4.22)**
20 Hypertensive heart disease	20 Stomach cancer	-8.88 (-19.2 to 3.15)	-5.19 (-15.3 to 5.44)	**-42.9 (-47 to -38.5)**
22 Blindness and vision loss	26 Neonatal disorders			

Supplemental Results Figure S7. Leading 20 level 3 causes of DALYs, and percentage change in number of DALYs and all-age and age-standardised DALY rates by location, 2022 and 2050

Sub-Saharan Africa, Reference scenario

Legend:
Communicable, maternal, neonatal and nutritional
Non-communicable
Injuries

Leading Causes 2022	Leading Causes 2050	Mean % change number of DALY	Mean % change all-age DALY rate	Mean % change age-standardized DALY rate
1 Neonatal disorders	1 Neonatal disorders	**-23.3 (-38.8 to -5.55)**	**-57.9 (-65.8 to -47.8)**	**-37.8 (-48.4 to -24)**
2 Malaria	2 Lower respiratory infect	**-19.3 (-31.6 to -5.55)**	**-55.7 (-62.6 to -48)**	**-45.8 (-53.4 to -36.2)**
3 Lower respiratory infect	3 Malaria	-33.3 (-79.8 to 145)	-63.4 (-88.8 to 36.7)	-56.4 (-86.4 to 62)
4 Diarrheal diseases	4 Diarrheal diseases	-26.1 (-49.5 to 10.2)	**-59.4 (-72.4 to -40.2)**	**-50.3 (-65.3 to -26.3)**
5 HIV/AIDS	5 Stroke	**62 (38.3 to 89.9)**	-11 (-25.4 to 6.5)	**-44.6 (-53.3 to -33.1)**
6 Congenital defects	6 Diabetes	**167 (135 to 202)**	**46.8 (25.7 to 71.1)**	-7.28 (-19.6 to 6.91)
7 Tuberculosis	7 Congenital defects	**-16.8 (-30.8 to -1.18)**	**-54.3 (-60.6 to -46.8)**	**-35.5 (-42 to -25)**
8 Stroke	8 Depressive disorders	**120 (109 to 133)**	**20.8 (15.7 to 26.3)**	**-2.85 (-3.19 to -2.53)**
9 COVID-19	9 Road injuries	25.5 (-25.5 to 116)	-31 (-59.8 to 17.8)	-36 (-61.6 to 7.38)
10 Road injuries	10 Ischemic heart disease	**63.8 (16.5 to 128)**	-9.92 (-37 to 27.8)	**-45.5 (-62 to -22.4)**
11 Meningitis	11 Chronic kidney disease	**170 (107 to 248)**	**48.5 (14.2 to 92.9)**	8.5 (-17.5 to 42.1)
12 Ischemic heart disease	12 Low back pain	**126 (114 to 138)**	**24 (18.1 to 29.8)**	**-6.13 (-6.33 to -5.95)**
13 Diabetes	13 Other musculoskeletal	**379 (352 to 407)**	**163 (147 to 178)**	**94.6 (90.1 to 98.4)**
14 Dietary iron deficiency	14 Cirrhosis liver	**67.5 (44.8 to 94.4)**	-7.93 (-22.5 to 10.1)	**-33.3 (-42.8 to -21.1)**
15 Pertussis	15 Tuberculosis	-31 (-54.1 to 4.03)	**-62 (-74.5 to -42.1)**	**-68.6 (-78.4 to -53.5)**
16 Depressive disorders	16 Headache disorders	**109 (94.8 to 126)**	**15 (10.3 to 23.4)**	**0.779 (0.0505 to 1.37)**
17 Cirrhosis liver	17 Dietary iron deficiency	**37.3 (25.5 to 48.8)**	**-24.7 (-26.1 to -23.2)**	**-18.1 (-18.8 to -17.4)**
18 Measles	18 HIV/AIDS	**-65.6 (-70.5 to -59.9)**	**-81.1 (-83.8 to -78)**	**-83.2 (-85.3 to -81.1)**
19 Maternal disorders	19 Hypertensive heart disease	**174 (118 to 244)**	**50.8 (17.9 to 91.9)**	-12 (-32.2 to 12.8)
20 Protein-energy malnutrition	20 Age-related hearing loss	**148 (132 to 165)**	**36.3 (27.2 to 44)**	**-1.03 (-1.37 to -0.631)**
21 Low back pain	27 Meningitis			
23 Chronic kidney disease	31 Pertussis			
24 Headache disorders	34 Maternal disorders			
32 Age-related hearing loss	48 Protein-energy malnutrition			
35 Hypertensive heart disease	100 Measles			
37 Other musculoskeletal	170 COVID-19			

Supplemental Results Figure S7. Leading 20 level 3 causes of DALYs, and percentage change in number of DALYs and all-age and age-standardised DALY rates by location, 2022 and 2050

Central Asia, Reference scenario

Legend:
Communicable, maternal, neonatal and nutritional
Non-communicable
Injuries

Leading Causes 2022	Leading Causes 2050	Mean % change number of DALY	Mean % change all-age DALY rate	Mean % change age-standardized DALY rate
1 Ischemic heart disease	1 Ischemic heart disease	47.4 (-3 to 120)	17.8 (-23.6 to 72)	**-38 (-63.2 to -1.44)**
2 COVID-19	2 Stroke	**30.8 (4.58 to 62.3)**	4.45 (-14.8 to 28.5)	**-41.6 (-55.1 to -23.2)**
3 Stroke	3 Diabetes	**83.2 (56.9 to 119)**	**46.3 (27 to 68.7)**	2.68 (-10.9 to 22.4)
4 Lower respiratory infect	4 Low back pain	**47.2 (31.6 to 62.9)**	**17.5 (12.8 to 22.8)**	**-3.9 (-4.14 to -3.66)**
5 Neonatal disorders	5 Cirrhosis liver	**31.7 (6.67 to 64.4)**	5.23 (-13.9 to 28.5)	-14 (-30.3 to 7.97)
6 Low back pain	6 Neonatal disorders	-24.5 (-51.1 to 19.9)	**-39.7 (-58.6 to -6.56)**	-23.9 (-47.9 to 15.1)
7 Cirrhosis liver	7 Lower respiratory infect	**-44.1 (-57.9 to -26.5)**	**-55.4 (-65.2 to -39.3)**	**-54.1 (-65.6 to -35)**
8 Diabetes	8 COPD	**108 (55.7 to 174)**	**65.9 (26.3 to 115)**	-9.26 (-35.8 to 28.2)
9 Congenital defects	9 Alzheimer's disease	**236 (157 to 321)**	**168 (111 to 228)**	**1.39 (0.219 to 2.61)**
10 Road injuries	10 Depressive disorders	**40.7 (25.4 to 56.1)**	**12.3 (8.19 to 17.4)**	**-2.26 (-2.76 to -1.84)**
11 Headache disorders	11 Age-related hearing loss	**92.4 (67.8 to 118)**	**53.5 (41.1 to 67.3)**	**-0.569 (-0.812 to -0.333)**
12 Dietary iron deficiency	12 Headache disorders	**26.5 (12.5 to 42)**	0.945 (-2.25 to 6.4)	**-0.238 (-0.433 to -0.0437)**
13 Depressive disorders	13 Hypertensive heart disease	**123 (39.9 to 239)**	**78.6 (12.6 to 163)**	-4.15 (-44.2 to 54.4)
14 Self-harm	14 Chronic kidney disease	**110 (30.6 to 232)**	**67.5 (3.79 to 157)**	19.2 (-30.3 to 100)
15 COPD	15 Road injuries	6.49 (-39.2 to 92.4)	-14.9 (-51.2 to 50.1)	-14.9 (-51.8 to 51.8)
16 Cardiomyopathy	16 Dietary iron deficiency	6.55 (-4.83 to 19.6)	**-15 (-16.4 to -13.4)**	**-8.59 (-9.67 to -7.54)**
17 Age-related hearing loss	17 Self-harm	6.54 (-17.5 to 38.8)	-14.9 (-33.6 to 8.71)	-14.1 (-33.5 to 9.82)
18 Falls	18 Lung cancer	**56.9 (6.49 to 117)**	25.1 (-12.6 to 67.1)	-16.3 (-40.7 to 13)
19 Alcohol use disorders	19 Alcohol use disorders	**27.8 (11.2 to 50.8)**	2.05 (-7.64 to 15.6)	-0.351 (-9.77 to 13.2)
20 Gynecological diseases	20 Blindness and vision loss	**94.2 (66.6 to 120)**	**55 (40.7 to 68.8)**	**-3.9 (-4.15 to -3.67)**
21 Lung cancer	22 Falls			
22 Chronic kidney disease	23 Congenital defects			
23 Hypertensive heart disease	25 Cardiomyopathy			
27 Blindness and vision loss	29 Gynecological diseases			
30 Alzheimer's disease	157 COVID-19			

Supplemental Results Figure S7. Leading 20 level 3 causes of DALYs, and percentage change in number of DALYs and all-age and age-standardised DALY rates by location, 2022 and 2050

Central Europe, Reference scenario

Legend:
Communicable, maternal, neonatal and nutritional
Non-communicable
Injuries

Leading Causes 2022	Leading Causes 2050	Mean % change number of DALY	Mean % change all-age DALY rate	Mean % change age-standardized DALY rate
1 Ischemic heart disease	1 Ischemic heart disease	**-35 (-50.4 to -14.4)**	-21.1 (-40.7 to 2.19)	**-52.3 (-65.2 to -33.9)**
2 Stroke	2 Stroke	**-33.3 (-41.3 to -23.9)**	**-19.1 (-28.7 to -7.98)**	**-47.5 (-54.6 to -38.7)**
3 COVID-19	3 Low back pain	**-10.5 (-15.1 to -5.05)**	**8.52 (5.15 to 11.8)**	**-2.19 (-2.35 to -2.06)**
4 Low back pain	4 Lung cancer	1.24 (-18.3 to 22.1)	22.8 (-0.581 to 48.1)	-7.01 (-24 to 11.1)
5 Lung cancer	5 Diabetes	**14.7 (7.58 to 23.6)**	**39.2 (30.4 to 48.3)**	6.23 (-1.18 to 13.6)
6 Diabetes	6 Alzheimer's disease	**64.3 (43.4 to 82.1)**	**99.2 (76.6 to 121)**	**1.31 (0.259 to 2.43)**
7 Colorectal cancer	7 Hypertensive heart disease	37.4 (-0.801 to 87.7)	**66.7 (20.9 to 127)**	3.84 (-27.4 to 46.8)
8 Falls	8 Colorectal cancer	12.3 (-17.9 to 49)	36.1 (-1.6 to 78.3)	-4.63 (-30 to 22.8)
9 Cirrhosis liver	9 Falls	-2.02 (-10.3 to 5.86)	**18.8 (10.1 to 26.8)**	**-13.3 (-17.7 to -8.56)**
10 Hypertensive heart disease	10 COPD	12 (-10.3 to 34.4)	**35.8 (10.3 to 61.3)**	-10.2 (-28.6 to 10.2)
11 Alzheimer's disease	11 Age-related hearing loss	**12.2 (3.89 to 19.9)**	**36.1 (29.8 to 42.1)**	**-0.446 (-0.547 to -0.33)**
12 COPD	12 Cirrhosis liver	**-22.3 (-31.7 to -11.6)**	-5.7 (-16.8 to 6.71)	**-14.1 (-24.5 to -2.15)**
13 Age-related hearing loss	13 Lower respiratory infect	-4.69 (-16.3 to 6.69)	15.6 (-0.149 to 30.7)	**-29.7 (-39.3 to -19.6)**
14 Lower respiratory infect	14 Pancreatic cancer	**39.6 (4.26 to 80.9)**	**69.3 (28.1 to 118)**	25.5 (-4.15 to 60.8)
15 Headache disorders	15 Breast cancer	-1.56 (-19.8 to 19.6)	19.4 (-2.72 to 42.9)	-10.6 (-26.8 to 5.98)
16 Depressive disorders	16 Headache disorders	**-20.3 (-25.1 to -15.5)**	-3.37 (-6.16 to 0.954)	0.114 (-0.0423 to 0.259)
17 Alcohol use disorders	17 Oral disorders	2.05 (-4.29 to 8.66)	**23.8 (18 to 29.3)**	**0.473 (0.293 to 0.633)**
18 Breast cancer	18 Chronic kidney disease	**46.3 (10.8 to 85.3)**	**77.5 (33.9 to 125)**	12.7 (-17 to 49.4)
19 Self-harm	19 Osteoarthritis	**9.32 (2.65 to 15)**	**32.6 (28.1 to 37.3)**	**1.23 (0.964 to 1.51)**
20 Cardiomyopathy	20 Depressive disorders	**-13.2 (-19.3 to -8.4)**	**5.24 (2.4 to 8.27)**	**-1.75 (-2.18 to -1.35)**
21 Oral disorders	21 Cardiomyopathy			
23 Osteoarthritis	23 Alcohol use disorders			
26 Pancreatic cancer	26 Self-harm			
29 Chronic kidney disease	153 COVID-19			

Supplemental Results Figure S7. Leading 20 level 3 causes of DALYs, and percentage change in number of DALYs and all-age and age-standardised DALY rates by location, 2022 and 2050

Eastern Europe, Reference scenario

Legend:
Communicable, maternal, neonatal and nutritional
Non-communicable
Injuries

Leading Causes 2022	Leading Causes 2050	Mean % change number of DALY	Mean % change all-age DALY rate	Mean % change age-standardized DALY rate
1 Ischemic heart disease	1 Ischemic heart disease	**-27.7 (-49.5 to -2.46)**	-18 (-42.9 to 12.3)	**-49.4 (-66.9 to -26.9)**
2 Stroke	2 Stroke	**-29 (-42.8 to -17.2)**	**-19.5 (-32.1 to -7.49)**	**-46.7 (-56 to -36.1)**
3 COVID-19	3 Low back pain	-0.828 (-10.8 to 10.3)	**12.3 (8.73 to 16.3)**	**-3.06 (-3.39 to -2.77)**
4 Low back pain	4 Alzheimer's disease	**92.8 (54.3 to 137)**	**118 (77.8 to 154)**	1.63 (-0.806 to 3.57)
5 Cirrhosis liver	5 Diabetes	**25.1 (9.88 to 39.3)**	**41.7 (30.1 to 53.6)**	8.65 (-0.441 to 18.8)
6 Cardiomyopathy	6 Age-related hearing loss	**23.6 (9.38 to 41.9)**	**39.9 (30.2 to 48.9)**	**-0.259 (-0.427 to -0.128)**
7 Self-harm	7 Cirrhosis liver	**-26.5 (-37.6 to -12.6)**	**-16.7 (-27.7 to -2.03)**	**-19.3 (-31.2 to -4.23)**
8 Alcohol use disorders	8 Alcohol use disorders	-10.9 (-26 to 7.64)	0.872 (-15 to 22.8)	2.68 (-11.9 to 22.2)
9 Diabetes	9 Lung cancer	-8.54 (-32.5 to 21.9)	3.38 (-20.7 to 30.9)	-19.9 (-37.7 to 1.58)
10 Lung cancer	10 Falls	-11.3 (-22.8 to 3.17)	0.343 (-8.63 to 10.1)	**-15.5 (-23 to -7.97)**
11 Falls	11 Colorectal cancer	8.58 (-27 to 54.3)	22.7 (-15 to 67.4)	-12.6 (-37.2 to 18.9)
12 Headache disorders	12 Cardiomyopathy	**-33.7 (-45.4 to -21)**	**-24.9 (-35.8 to -13.3)**	**-27.6 (-38.3 to -14.6)**
13 Depressive disorders	13 Self-harm	**-25.1 (-38.1 to -9.17)**	-15.1 (-27.9 to 1.03)	-14.7 (-29 to 4.12)
14 Colorectal cancer	14 COPD	31.1 (-0.168 to 63.1)	**48.4 (14.8 to 84.5)**	-3.07 (-26.4 to 25.5)
15 Age-related hearing loss	15 Depressive disorders	-6.51 (-15.3 to 4.15)	**5.83 (2.41 to 9.23)**	**-2.27 (-2.56 to -1.98)**
16 HIV/AIDS	16 Headache disorders	**-13.4 (-22 to -4.26)**	-2.01 (-4.75 to 1.47)	-0.0797 (-0.479 to 0.297)
17 Alzheimer's disease	17 Road injuries	-19.7 (-44 to 10.7)	-8.95 (-36.3 to 27.1)	-9.09 (-39.3 to 26.7)
18 Road injuries	18 Osteoarthritis	**17.2 (4.54 to 32.6)**	**32.7 (26 to 38.8)**	0.204 (-0.263 to 0.576)
19 Interpersonal violence	19 Blindness and vision loss	**18.2 (4.07 to 34.6)**	**33.8 (24.4 to 43.9)**	**-1.22 (-1.79 to -0.629)**
20 COPD	20 Oral disorders	9.24 (-3.03 to 22.9)	**23.6 (17.3 to 30.3)**	-0.0124 (-0.234 to 0.228)
24 Oral disorders	21 HIV/AIDS			
26 Osteoarthritis	33 Interpersonal violence			
27 Blindness and vision loss	154 COVID-19			

Supplemental Results Figure S7. Leading 20 level 3 causes of DALYs, and percentage change in number of DALYs and all-age and age-standardised DALY rates by location, 2022 and 2050

Australasia, Reference scenario

Legend:
Communicable, maternal, neonatal and nutritional
Non-communicable
Injuries

Leading Causes 2022	Leading Causes 2050	Mean % change number of DALY	Mean % change all-age DALY rate	Mean % change age-standardized DALY rate
1 Low back pain	1 Low back pain	37.9 (30.5 to 44.6)	3.01 (0.0809 to 5.53)	-2.64 (-2.77 to -2.53)
2 Ischemic heart disease	2 Alzheimer's disease	110 (92.1 to 131)	56.9 (45.8 to 70)	-4.38 (-7.71 to -0.688)
3 Falls	3 Diabetes	102 (87.3 to 118)	51.3 (41.1 to 62.4)	29.9 (21.6 to 37.7)
4 Depressive disorders	4 Falls	52.9 (40 to 67.1)	14.3 (6.96 to 22.9)	-14.6 (-20 to -9.44)
5 Alzheimer's disease	5 Ischemic heart disease	0.653 (-18.3 to 27)	-24.8 (-38.8 to -5.77)	-50.9 (-60.9 to -37.3)
6 Other musculoskeletal	6 Other musculoskeletal	72.5 (63.3 to 82.8)	28.9 (24.8 to 32.9)	25.7 (24.2 to 27.2)
7 Lung cancer	7 COPD	61.8 (38.5 to 88.6)	20.9 (2.66 to 41.6)	-14.4 (-27.3 to 3.51)
8 Stroke	8 Depressive disorders	32.8 (26 to 39.9)	-0.787 (-1.97 to 0.548)	-1.47 (-1.7 to -1.22)
9 Diabetes	9 Lung cancer	35 (12.4 to 60.2)	0.856 (-15.4 to 18.9)	-18 (-31.4 to -5.46)
10 Anxiety disorders	10 Age-related hearing loss	62.6 (51.6 to 73.3)	21.5 (16.5 to 27.3)	-0.734 (-0.832 to -0.654)
11 COVID-19	11 Stroke	23.9 (11.2 to 38.2)	-7.38 (-16 to 3.54)	-33.8 (-41.1 to -25.3)
12 COPD	12 Anxiety disorders	33 (26 to 40.5)	-0.645 (-2 to 0.762)	0.151 (-0.151 to 0.396)
13 Age-related hearing loss	13 Colorectal cancer	60.4 (27.6 to 99.8)	19.8 (-4.67 to 45.8)	-8.91 (-27.1 to 10.9)
14 Headache disorders	14 Headache disorders	34 (27.4 to 40.9)	0.107 (-1.53 to 2.71)	-0.0329 (-0.202 to 0.134)
15 Self-harm	15 Chronic kidney disease	171 (123 to 235)	102 (67.8 to 150)	23.8 (-0.203 to 58.9)
16 Colorectal cancer	16 Osteoarthritis	55.8 (46.9 to 64.5)	16.4 (12.2 to 20.5)	2.17 (1.98 to 2.41)
17 Drug use disorders	17 Drug use disorders	51.6 (37 to 65.7)	13.3 (3.03 to 23.5)	12.9 (3.1 to 22.2)
18 Osteoarthritis	18 Atrial fibrillation	133 (109 to 155)	74 (58.3 to 88.5)	11 (4.07 to 17)
19 Oral disorders	19 Oral disorders	48.8 (40.8 to 56.9)	11.2 (7.09 to 14.5)	0.58 (0.416 to 0.729)
20 Road injuries	20 Self-harm	8.75 (-4.86 to 23.9)	-18.7 (-29.3 to -6.54)	-19.2 (-30.4 to -5.62)
27 Atrial fibrillation	26 Road injuries			
31 Chronic kidney disease	155 COVID-19			

Supplemental Results Figure S7. Leading 20 level 3 causes of DALYs, and percentage change in number of DALYs and all-age and age-standardised DALY rates by location, 2022 and 2050

High-income Asia Pacific, Reference scenario

Legend:
Communicable, maternal, neonatal and nutritional
Non-communicable
Injuries

Leading Causes 2022	Leading Causes 2050	Mean % change number of DALY	Mean % change all-age DALY rate	Mean % change age-standardized DALY rate
1 Stroke	1 Alzheimer's disease	**48.9 (37.6 to 61.5)**	**67.9 (56.9 to 77.9)**	**-12.2 (-15.2 to -9.13)**
2 Alzheimer's disease	2 Diabetes	**29 (21.4 to 36.9)**	**45.5 (39.7 to 50.7)**	**17.6 (14.1 to 20.4)**
3 Low back pain	3 Stroke	**-13.6 (-21.4 to -4.09)**	-2.57 (-10.4 to 6.84)	**-33.6 (-39.5 to -26.5)**
4 Diabetes	4 Low back pain	**-7.56 (-13.2 to -2.01)**	**4.24 (1.31 to 7.64)**	**-2.5 (-2.74 to -2.29)**
5 Ischemic heart disease	5 Other musculoskeletal	**9.6 (3.11 to 17.2)**	**23.6 (18.9 to 28.2)**	**20.5 (19.5 to 21.4)**
6 Other musculoskeletal	6 Lung cancer	8.74 (-7.81 to 27.4)	**22.6 (4.55 to 41.3)**	**-21.2 (-31.8 to -9.89)**
7 Lung cancer	7 Lower respiratory infect	**42.5 (26.9 to 59.3)**	**60.7 (44.4 to 76.9)**	**-17.2 (-25.5 to -8.66)**
8 Lower respiratory infect	8 Age-related hearing loss	**22.2 (14.9 to 30.1)**	**37.9 (32.2 to 44.2)**	**-0.606 (-1.06 to -0.155)**
9 Colorectal cancer	9 Colorectal cancer	17.7 (-4.16 to 44.6)	**32.8 (8.7 to 60.7)**	-12.5 (-29.2 to 5.66)
10 Age-related hearing loss	10 Chronic kidney disease	**93.1 (58.1 to 129)**	**118 (78.9 to 160)**	15.4 (-3.92 to 40.4)
11 Falls	11 Falls	**11.3 (1.08 to 21.1)**	**25.5 (16 to 34.7)**	**-17 (-22.2 to -11.5)**
12 Self-harm	12 Ischemic heart disease	**-34.6 (-46.7 to -20.1)**	**-26.3 (-39.6 to -9.59)**	**-55.9 (-64.8 to -44.9)**
13 Osteoarthritis	13 Osteoarthritis	**13.2 (7.25 to 19.7)**	**27.7 (24.3 to 31)**	**1 (0.748 to 1.29)**
14 Stomach cancer	14 COPD	**43.5 (24.6 to 63.4)**	**61.9 (42.8 to 83.3)**	-9.33 (-21.2 to 2.58)
15 COVID-19	15 Pancreatic cancer	**42.8 (15.8 to 72.7)**	**61 (32.4 to 89.8)**	12.6 (-6.25 to 31.8)
16 COPD	16 Liver cancer	**43.1 (26.5 to 62.4)**	**61.4 (43.8 to 79.8)**	**18.3 (5.75 to 32)**
17 Headache disorders	17 Stomach cancer	**-19.6 (-26.6 to -12.6)**	**-9.31 (-15.8 to -2.39)**	**-41.8 (-45 to -38.4)**
18 Pancreatic cancer	18 Self-harm	**-28.9 (-36 to -21.7)**	**-19.8 (-26.9 to -12.2)**	**-18.3 (-27.3 to -8.8)**
19 Liver cancer	19 Headache disorders	**-17.7 (-22.4 to -12.5)**	**-7.19 (-9.82 to -2.66)**	-0.207 (-0.605 to 0.228)
20 Chronic kidney disease	20 Depressive disorders	**-10 (-15.4 to -4.47)**	1.45 (-0.968 to 4.02)	**-0.597 (-0.86 to -0.311)**
21 Depressive disorders	154 COVID-19			

Supplemental Results Figure S7. Leading 20 level 3 causes of DALYs, and percentage change in number of DALYs and all-age and age-standardised DALY rates by location, 2022 and 2050

High-income North America, Reference scenario

Legend:
Communicable, maternal, neonatal and nutritional
Non-communicable
Injuries

Leading Causes 2022	Leading Causes 2050	Mean % change number of DALY	Mean % change all-age DALY rate	Mean % change age-standardized DALY rate
1 Ischemic heart disease	1 Drug use disorders	**28.3 (13.7 to 44.2)**	**18.3 (5.9 to 31.4)**	**19.5 (6.98 to 32.7)**
2 Drug use disorders	2 Diabetes	**28.7 (19.9 to 37.5)**	**18.6 (12.2 to 24.5)**	2.92 (-2.22 to 7.48)
3 COVID-19	3 Ischemic heart disease	**-24.3 (-37.2 to -9.26)**	**-30.2 (-41 to -17)**	**-52.2 (-61 to -41.4)**
4 Diabetes	4 Alzheimer's disease	**90 (72.4 to 107)**	**75.2 (63.2 to 87.2)**	0.359 (-2.98 to 3.78)
5 Low back pain	5 COPD	**34.1 (17.2 to 50.3)**	**23.7 (10.1 to 39.8)**	-9.3 (-20.7 to 2.73)
6 COPD	6 Low back pain	**13.4 (6.93 to 20.4)**	**4.52 (2.52 to 6.65)**	**-1.55 (-1.71 to -1.41)**
7 Other musculoskeletal	7 Other musculoskeletal	**25 (18.2 to 33)**	**15.3 (13.2 to 17.5)**	**13.1 (12.4 to 13.6)**
8 Stroke	8 Chronic kidney disease	**107 (77.2 to 140)**	**90.8 (63.1 to 121)**	**30.5 (10.8 to 53.9)**
9 Lung cancer	9 Lung cancer	9.36 (-6.02 to 25.7)	0.826 (-12.1 to 16.4)	**-17.3 (-27.4 to -6.57)**
10 Alzheimer's disease	10 Stroke	3.65 (-4.48 to 12.5)	-4.42 (-10.5 to 1.97)	**-29.1 (-34.7 to -22.6)**
11 Depressive disorders	11 Falls	**34.1 (23.3 to 46.6)**	**23.7 (14.8 to 33.2)**	**-10.3 (-16 to -3.95)**
12 Falls	12 Age-related hearing loss	**33.8 (26 to 42.2)**	**23.4 (19.2 to 28.3)**	**-0.231 (-0.322 to -0.139)**
13 Road injuries	13 Depressive disorders	**6.13 (0.228 to 12.5)**	**-2.15 (-3.06 to -1.14)**	**-1.48 (-1.65 to -1.31)**
14 Headache disorders	14 Road injuries	11.1 (-10.4 to 34.5)	2.47 (-16.6 to 22.9)	2.13 (-19.1 to 25.7)
15 Anxiety disorders	15 Headache disorders	**7.28 (1.4 to 14)**	-1.08 (-2.51 to 2.23)	**-0.274 (-0.481 to -0.0552)**
16 Chronic kidney disease	16 Anxiety disorders	**7.82 (2.02 to 14.4)**	-0.588 (-1.94 to 0.836)	**-0.502 (-0.759 to -0.261)**
17 Age-related hearing loss	17 Hypertensive heart disease	**73.4 (46.3 to 107)**	**59.9 (34 to 94.8)**	**24.2 (2.64 to 51.8)**
18 Self-harm	18 Colorectal cancer	**22.2 (0.675 to 46.8)**	12.7 (-7.21 to 33.4)	-11.8 (-26.4 to 3.43)
19 Cirrhosis liver	19 Osteoarthritis	**24.3 (17 to 32.5)**	**14.6 (11.8 to 17.6)**	**0.343 (0.2 to 0.483)**
20 Colorectal cancer	20 Self-harm	-6.95 (-17.5 to 2.45)	**-14.2 (-22.6 to -5.04)**	**-13.1 (-22.4 to -2.98)**
21 Osteoarthritis	24 Cirrhosis liver			
26 Hypertensive heart disease	153 COVID-19			

Supplemental Results Figure S7. Leading 20 level 3 causes of DALYs, and percentage change in number of DALYs and all-age and age-standardised DALY rates by location, 2022 and 2050

Southern Latin America, Reference scenario

Legend:
Communicable, maternal, neonatal and nutritional
Non-communicable
Injuries

Leading Causes 2022	Leading Causes 2050	Mean % change number of DALY	Mean % change all-age DALY rate	Mean % change age-standardized DALY rate
1 Ischemic heart disease	1 Lower respiratory infect	**46.6 (27.5 to 66.5)**	**38.7 (22.9 to 55)**	**-21.5 (-30.5 to -13)**
2 Low back pain	2 Diabetes	**82.8 (62.1 to 105)**	**72.9 (59.4 to 87.2)**	**14.2 (5.13 to 22.1)**
3 Lower respiratory infect	3 Other musculoskeletal	**63.5 (46.9 to 80.9)**	**54.5 (48.4 to 61.7)**	**29.8 (28.5 to 31.3)**
4 COVID-19	4 Low back pain	**23.5 (11.8 to 37.5)**	**16.8 (12.2 to 21.5)**	**-3.73 (-3.85 to -3.61)**
5 Stroke	5 Chronic kidney disease	**112 (71.4 to 155)**	**101 (63.3 to 139)**	15.1 (-8.22 to 40.4)
6 Other musculoskeletal	6 Ischemic heart disease	-12.8 (-31.7 to 10.4)	-17.5 (-35.4 to 4.99)	**-50.7 (-62.7 to -35.5)**
7 Diabetes	7 Stroke	4.4 (-8.83 to 20.2)	-1.28 (-11.8 to 10.4)	**-38 (-45.6 to -30.1)**
8 Road injuries	8 Alzheimer's disease	**111 (87.6 to 135)**	**99.4 (85.2 to 115)**	-2.18 (-5.45 to 1.1)
9 Anxiety disorders	9 COPD	**70.8 (44.2 to 96.7)**	**61.5 (39.5 to 86.2)**	-8.98 (-22.8 to 5.9)
10 Neonatal disorders	10 Lung cancer	**60.7 (33.6 to 93.5)**	**51.8 (30.4 to 77.8)**	-3.2 (-16.7 to 11.4)
11 Lung cancer	11 Colorectal cancer	**59.9 (23.2 to 101)**	**51.1 (18.7 to 87.2)**	-5.67 (-25 to 17.6)
12 COPD	12 Age-related hearing loss	**63.7 (48.1 to 81.5)**	**54.7 (47.3 to 61.6)**	**-0.271 (-0.462 to -0.0965)**
13 Chronic kidney disease	13 Road injuries	0.816 (-20.8 to 26.5)	-4.64 (-24.4 to 18.3)	-5.13 (-26.2 to 18.5)
14 Depressive disorders	14 Anxiety disorders	5.52 (-4.83 to 17)	-0.272 (-3.42 to 3.85)	0.126 (-0.248 to 0.507)
15 Headache disorders	15 Falls	**44.7 (29.1 to 63.2)**	**36.8 (27.3 to 47)**	-1.77 (-7.84 to 4.92)
16 Self-harm	16 Depressive disorders	7.62 (-3.79 to 19.3)	1.71 (-1.22 to 5.5)	**-1.37 (-1.81 to -0.808)**
17 Colorectal cancer	17 Hypertensive heart disease	**105 (66.6 to 143)**	**94.1 (59.9 to 130)**	5.42 (-15.5 to 27.5)
18 Alzheimer's disease	18 Pancreatic cancer	**121 (72.4 to 177)**	**109 (71.6 to 156)**	**32.1 (9.68 to 59.9)**
19 Falls	19 Osteoarthritis	**64.7 (49.1 to 81.5)**	**55.7 (49.1 to 61.2)**	**1.93 (1.67 to 2.22)**
20 Age-related hearing loss	20 Cirrhosis liver	**24.5 (11.1 to 41.1)**	**17.7 (7.61 to 27.5)**	**-15.5 (-22.5 to -9.04)**
21 Cirrhosis liver	21 Headache disorders			
25 Osteoarthritis	26 Self-harm			
30 Hypertensive heart disease	28 Neonatal disorders			
32 Pancreatic cancer	157 COVID-19			

Supplemental Results Figure S7. Leading 20 level 3 causes of DALYs, and percentage change in number of DALYs and all-age and age-standardised DALY rates by location, 2022 and 2050

Western Europe, Reference scenario

Legend:
Communicable, maternal, neonatal and nutritional
Non-communicable
Injuries

Leading Causes 2022	Leading Causes 2050	Mean % change number of DALY	Mean % change all-age DALY rate	Mean % change age-standardized DALY rate
1 Ischemic heart disease	1 Alzheimer's disease	**58.8 (48.6 to 67.9)**	**54.5 (47.2 to 62.2)**	**-5.52 (-7.21 to -3.75)**
2 Low back pain	2 Low back pain	**5.84 (2.21 to 9.44)**	**3 (0.554 to 5.53)**	**-2.56 (-2.76 to -2.4)**
3 Alzheimer's disease	3 Ischemic heart disease	**-28 (-39.4 to -15.3)**	**-29.9 (-41.4 to -17.2)**	**-52.9 (-61.2 to -42.8)**
4 COVID-19	4 Falls	**20.1 (13.4 to 26.6)**	**16.9 (11.6 to 22)**	**-11.6 (-14.5 to -8.51)**
5 Stroke	5 Diabetes	**38.4 (32 to 44.9)**	**34.7 (27.8 to 41.1)**	**16.4 (11.1 to 21.5)**
6 Lung cancer	6 Lung cancer	1.81 (-10.8 to 14.9)	-0.935 (-12.6 to 12.1)	**-18.6 (-27.9 to -9.11)**
7 Falls	7 COPD	**32.3 (17.9 to 46.2)**	**28.8 (15.1 to 43.3)**	**-11.6 (-21.9 to -1.31)**
8 Diabetes	8 Stroke	**-12 (-18.4 to -5.28)**	**-14.4 (-20.4 to -7.45)**	**-37.8 (-43.5 to -32.2)**
9 COPD	9 Depressive disorders	3.17 (-0.334 to 6.95)	0.39 (-1.14 to 2.11)	**-0.923 (-1.19 to -0.614)**
10 Depressive disorders	10 Colorectal cancer	**23.5 (2.6 to 45.5)**	**20.2 (0.804 to 43.5)**	-11.4 (-24.9 to 4.85)
11 Headache disorders	11 Chronic kidney disease	**118 (89.5 to 149)**	**112 (84 to 146)**	**25.5 (6.65 to 47.9)**
12 Anxiety disorders	12 Age-related hearing loss	**29.9 (24.4 to 35.3)**	**26.4 (21.8 to 30.8)**	**-0.551 (-0.677 to -0.416)**
13 Colorectal cancer	13 Headache disorders	-1.24 (-4.86 to 2.16)	**-3.9 (-5.21 to -1.13)**	-0.311 (-0.65 to 0.106)
14 Age-related hearing loss	14 Anxiety disorders	1.04 (-2.56 to 5.24)	**-1.68 (-3.34 to -0.0235)**	-0.374 (-0.685 to 0.041)
15 Other musculoskeletal	15 Lower respiratory infect	**40.8 (30.8 to 51.2)**	**37.1 (27.5 to 47.4)**	**-16.8 (-24 to -9.85)**
16 Osteoarthritis	16 Other musculoskeletal	**25.8 (19.8 to 30.9)**	**22.4 (19.1 to 26.5)**	**23.1 (22.1 to 24)**
17 Breast cancer	17 Pancreatic cancer	**54.8 (31.6 to 79.8)**	**50.6 (28 to 75.4)**	**19.4 (1.58 to 37.8)**
18 Gynecological diseases	18 Osteoarthritis	**21 (16.8 to 25)**	**17.7 (14.8 to 20.8)**	**1.53 (1.31 to 1.74)**
19 Lower respiratory infect	19 Atrial fibrillation	**72.5 (63 to 82.5)**	**67.8 (59.2 to 76.5)**	**7.7 (4.58 to 10.6)**
20 Oral disorders	20 Breast cancer	7.99 (-3.73 to 19.8)	5.08 (-5.83 to 17.3)	**-15.4 (-23.3 to -6.73)**
21 Pancreatic cancer	23 Oral disorders			
23 Chronic kidney disease	24 Gynecological diseases			
27 Atrial fibrillation	152 COVID-19			

Supplemental Results Figure S7. Leading 20 level 3 causes of DALYs, and percentage change in number of DALYs and all-age and age-standardised DALY rates by location, 2022 and 2050

Andean Latin America, Reference scenario

Legend:
Communicable, maternal, neonatal and nutritional
Non-communicable
Injuries

Leading Causes 2022	Leading Causes 2050	Mean % change number of DALY	Mean % change all-age DALY rate	Mean % change age-standardized DALY rate
1 COVID-19	1 Diabetes	**136 (104 to 169)**	**97.4 (73 to 122)**	**15.9 (4.17 to 29.9)**
2 Lower respiratory infect	2 Chronic kidney disease	**171 (88.8 to 261)**	**127 (62.8 to 206)**	25.6 (-11.9 to 75.4)
3 Neonatal disorders	3 Lower respiratory infect	**26 (1.38 to 52.9)**	5.46 (-12.3 to 26.5)	**-36.9 (-47.1 to -24.7)**
4 Ischemic heart disease	4 Ischemic heart disease	38 (-8.68 to 100)	15.6 (-22.4 to 68.5)	**-38.5 (-59.7 to -6.12)**
5 Diabetes	5 Other musculoskeletal	**108 (90.2 to 130)**	**74.5 (65.4 to 84.1)**	**37.3 (35.7 to 39.2)**
6 Road injuries	6 Stroke	**32.9 (6.35 to 63)**	11.3 (-11 to 36.3)	**-34.6 (-48.1 to -18.5)**
7 Stroke	7 Low back pain	**48.5 (36.1 to 61.4)**	**24.3 (18.4 to 30.3)**	**-3.59 (-3.8 to -3.36)**
8 Chronic kidney disease	8 Cirrhosis liver	**61.8 (38.8 to 83.7)**	**35.5 (18.1 to 52.5)**	**-13.6 (-23.3 to -3.1)**
9 Congenital defects	9 Road injuries	3.21 (-31.2 to 54.2)	-13.5 (-43.3 to 28.9)	-18.2 (-47.4 to 23.3)
10 Anxiety disorders	10 Anxiety disorders	**24.6 (12.6 to 37.6)**	4.28 (-1.06 to 10.6)	**0.399 (0.0447 to 0.737)**
11 Low back pain	11 Neonatal disorders	**-34.7 (-46.7 to -22.8)**	**-45.4 (-53.4 to -36.1)**	**-25.4 (-36.1 to -12.7)**
12 Other musculoskeletal	12 Oral disorders	**78.4 (62.5 to 97.7)**	**49.4 (39.1 to 60)**	0.296 (-0.0648 to 0.671)
13 Cirrhosis liver	13 Age-related hearing loss	**94.6 (77.6 to 117)**	**62.9 (53.1 to 72.7)**	**-0.515 (-0.743 to -0.287)**
14 Headache disorders	14 Blindness and vision loss	**85.3 (68.2 to 107)**	**55.2 (43.9 to 65.3)**	**-4.36 (-4.82 to -3.97)**
15 Depressive disorders	15 Alzheimer's disease	**186 (143 to 232)**	**140 (108 to 171)**	0.335 (-1.69 to 2.17)
16 Stomach cancer	16 Depressive disorders	**40.2 (27.6 to 54.4)**	**17.4 (12.1 to 23.6)**	-0.0892 (-0.765 to 0.63)
17 Oral disorders	17 Falls	**48.1 (29.5 to 71.1)**	**23.9 (12.2 to 35.5)**	**-10.2 (-17.7 to -1.84)**
18 Gynecological diseases	18 Stomach cancer	**27.7 (11.7 to 41.3)**	6.95 (-2.44 to 16.3)	**-39.1 (-42.9 to -35)**
19 Falls	19 Headache disorders	**23.4 (12 to 38.1)**	3.32 (-2.18 to 15.1)	0.511 (-0.179 to 1.13)
20 Blindness and vision loss	20 COPD	**147 (86.9 to 215)**	**107 (60 to 161)**	-2.24 (-27.3 to 29.1)
21 Age-related hearing loss	25 Congenital defects			
30 Alzheimer's disease	27 Gynecological diseases			
32 COPD	162 COVID-19			

Supplemental Results Figure S7. Leading 20 level 3 causes of DALYs, and percentage change in number of DALYs and all-age and age-standardised DALY rates by location, 2022 and 2050

Caribbean, Reference scenario

Legend:
Communicable, maternal, neonatal and nutritional
Non-communicable
Injuries

Leading Causes 2022	Leading Causes 2050	Mean % change number of DALY	Mean % change all-age DALY rate	Mean % change age-standardized DALY rate
1 Ischemic heart disease	1 Diabetes	**73.4 (50.3 to 97.3)**	**67.2 (47.9 to 89.9)**	**19.7 (6.19 to 36)**
2 Neonatal disorders	2 Ischemic heart disease	11.2 (-29 to 58.1)	7.41 (-31.9 to 61.9)	-32.2 (-58 to 7.6)
3 Diabetes	3 Stroke	9.58 (-14.4 to 35.3)	5.75 (-17.5 to 31)	**-30.4 (-47.1 to -8.75)**
4 Stroke	4 Nature disaster	768 (-99.3 to 13300)	721 (-99.3 to 13000)	835 (-99.3 to 15000)
5 Nature disaster	5 Chronic kidney disease	**80.8 (29.8 to 150)**	**74.5 (23.8 to 145)**	19.1 (-18 to 73.3)
6 Lower respiratory infect	6 Neonatal disorders	**-42.5 (-56.3 to -27)**	**-44.6 (-55.9 to -29.3)**	**-25.1 (-39.9 to -5.8)**
7 Interpersonal violence	7 Lower respiratory infect	**-21.2 (-35.8 to -3.03)**	**-23.9 (-37 to -6.11)**	**-47.8 (-56.7 to -36.1)**
8 COVID-19	8 Hypertensive heart disease	**94.3 (37.4 to 154)**	**87.6 (32.2 to 152)**	17.7 (-18.9 to 64.4)
9 Road injuries	9 Road injuries	-6.72 (-39.3 to 42.2)	-9.95 (-41.6 to 42)	-6.01 (-40.4 to 49.8)
10 Congenital defects	10 Other musculoskeletal	**66 (48.9 to 85.8)**	**60 (50.2 to 69)**	**38.8 (33.8 to 44)**
11 Diarrheal diseases	11 Low back pain	**20.2 (9.49 to 31.8)**	**15.9 (11.2 to 20.4)**	**-3.7 (-4.39 to -3.01)**
12 HIV/AIDS	12 Alzheimer's disease	**121 (83 to 153)**	**113 (82.7 to 139)**	-0.801 (-2.71 to 1.19)
13 Chronic kidney disease	13 Depressive disorders	**14.7 (4.47 to 25.3)**	**10.6 (6.71 to 15)**	**-1.91 (-3 to -1.01)**
14 Low back pain	14 Age-related hearing loss	**56.3 (38.3 to 72.6)**	**50.7 (38.8 to 61.2)**	**-0.904 (-1.24 to -0.542)**
15 Depressive disorders	15 COPD	**46.8 (13.9 to 82.7)**	**41.7 (10.4 to 78.5)**	-17.7 (-39.4 to 8.33)
16 Cirrhosis liver	16 Cirrhosis liver	16.8 (-0.617 to 33.9)	12.8 (-3.02 to 30.7)	-12 (-24.4 to 3.31)
17 Headache disorders	17 Lung cancer	33.5 (-6.48 to 78.1)	28.8 (-7.25 to 70.9)	-16.4 (-37.5 to 9.29)
18 Anxiety disorders	18 Prostate cancer	**94.2 (35.9 to 156)**	**87.2 (37.5 to 141)**	3.19 (-20 to 28.1)
19 Lung cancer	19 Falls	**55.8 (33.1 to 75.4)**	**50.3 (32.9 to 66.7)**	**-7.31 (-13.5 to -1.56)**
20 Other musculoskeletal	20 Interpersonal violence	**-37.2 (-59.2 to -1.9)**	**-39.5 (-59.4 to -4.61)**	-34.3 (-56.9 to 5.97)
21 COPD	21 Headache disorders			
22 Age-related hearing loss	23 Anxiety disorders			
23 Hypertensive heart disease	24 Congenital defects			
26 Falls	31 Diarrheal diseases			
29 Alzheimer's disease	40 HIV/AIDS			
30 Prostate cancer	164 COVID-19			

Supplemental Results Figure S7. Leading 20 level 3 causes of DALYs, and percentage change in number of DALYs and all-age and age-standardised DALY rates by location, 2022 and 2050

Central Latin America, Reference scenario

Legend:
Communicable, maternal, neonatal and nutritional
Non-communicable
Injuries

Leading Causes 2022	Leading Causes 2050	Mean % change number of DALY	Mean % change all-age DALY rate	Mean % change age-standardized DALY rate
1 Ischemic heart disease	1 Diabetes	**87 (66.7 to 112)**	**67.9 (46.4 to 94.2)**	6.81 (-7.34 to 22.9)
2 Diabetes	2 Ischemic heart disease	37.8 (-17.3 to 123)	24.1 (-26.7 to 105)	-33.1 (-63.7 to 20.4)
3 COVID-19	3 Chronic kidney disease	**118 (55.6 to 193)**	**96.1 (39.2 to 168)**	21.4 (-18.4 to 75)
4 Interpersonal violence	4 Other musculoskeletal	**89.3 (77.3 to 103)**	**69.8 (61.5 to 79.7)**	**35.2 (32.6 to 38.1)**
5 Chronic kidney disease	5 Low back pain	**33.6 (23.9 to 42.3)**	**19.9 (14.4 to 25.3)**	**-3.49 (-3.78 to -3.22)**
6 Neonatal disorders	6 Cirrhosis liver	**46.1 (32.8 to 59.9)**	**31.1 (17.7 to 48.3)**	-5.15 (-16.2 to 6.72)
7 Road injuries	7 Interpersonal violence	-35.4 (-62.2 to 10.4)	**-42.3 (-64.6 to -3.95)**	-38 (-64 to 3.98)
8 Low back pain	8 Stroke	**31.2 (5.08 to 64.6)**	18 (-8.15 to 54.7)	**-33 (-50.2 to -8.02)**
9 Stroke	9 COPD	**105 (62.6 to 150)**	**84.6 (40.3 to 131)**	-13.5 (-38.8 to 21)
10 Other musculoskeletal	10 Road injuries	2.55 (-35.9 to 66.8)	-7.69 (-43.4 to 52.4)	-9.66 (-45.4 to 52.6)
11 Cirrhosis liver	11 Alzheimer's disease	**184 (129 to 236)**	**155 (109 to 200)**	1.1 (-2.03 to 3.29)
12 Lower respiratory infect	12 Lower respiratory infect	5.61 (-10.9 to 20.9)	-5.17 (-20.4 to 11.5)	**-38.7 (-49.6 to -25.8)**
13 Congenital defects	13 Age-related hearing loss	**84 (64.9 to 101)**	**65.1 (51.4 to 78.2)**	**-0.628 (-0.753 to -0.483)**
14 Depressive disorders	14 Depressive disorders	**28 (18.4 to 38.3)**	**14.8 (10.2 to 20.3)**	**-1.6 (-1.94 to -1.29)**
15 Headache disorders	15 Falls	**61.8 (43.2 to 82.3)**	**45.1 (31.4 to 60.2)**	2.33 (-5.45 to 10.8)
16 COPD	16 Headache disorders	**13.4 (4.21 to 28)**	1.7 (-3.4 to 14.9)	-0.157 (-0.405 to 0.117)
17 Anxiety disorders	17 Neonatal disorders	**-39.2 (-51.1 to -26)**	**-45.4 (-56.1 to -32.4)**	**-28 (-41.9 to -8.15)**
18 Age-related hearing loss	18 Blindness and vision loss	**73.9 (55.1 to 90.5)**	**56 (42.1 to 68.7)**	**-4.22 (-4.64 to -3.89)**
19 Falls	19 Anxiety disorders	**14.4 (5.42 to 24.1)**	2.6 (-2.5 to 8.16)	0.131 (-0.267 to 0.495)
20 Self-harm	20 Oral disorders	**59.6 (46.7 to 72.4)**	**43.2 (33.5 to 52.9)**	**0.729 (0.45 to 1.11)**
21 Oral disorders	28 Congenital defects			
22 Blindness and vision loss	31 Self-harm			
24 Alzheimer's disease	162 COVID-19			

Supplemental Results Figure S7. Leading 20 level 3 causes of DALYs, and percentage change in number of DALYs and all-age and age-standardised DALY rates by location, 2022 and 2050

Tropical Latin America, Reference scenario

Legend:
Communicable, maternal, neonatal and nutritional
Non-communicable
Injuries

Leading Causes 2022	Leading Causes 2050	Mean % change number of DALY	Mean % change all-age DALY rate	Mean % change age-standardized DALY rate
1 Ischemic heart disease	1 Diabetes	**105 (72.8 to 138)**	**83.2 (62.8 to 108)**	**18.5 (3.89 to 34.7)**
2 Interpersonal violence	2 Ischemic heart disease	4.15 (-37.1 to 61.1)	-6.86 (-42.3 to 49.6)	**-42.9 (-65.6 to -6.22)**
3 Stroke	3 Low back pain	**29.3 (11 to 46.7)**	**15.4 (11.1 to 20.2)**	**-4.05 (-4.23 to -3.88)**
4 Diabetes	4 Alzheimer's disease	**165 (114 to 218)**	**136 (105 to 167)**	**-5.19 (-7.45 to -2.68)**
5 Low back pain	5 Stroke	10.7 (-18.3 to 45.2)	-1.12 (-25.2 to 30.4)	**-41.1 (-56.2 to -20.2)**
6 COVID-19	6 Other musculoskeletal	**80.5 (58.4 to 106)**	**61 (52.6 to 69.9)**	**38.9 (36 to 41.6)**
7 Neonatal disorders	7 Lower respiratory infect	**42.4 (10.9 to 73.7)**	**27.1 (0.16 to 55.6)**	**-31.9 (-47.6 to -11.2)**
8 Road injuries	8 Interpersonal violence	-26.9 (-61.7 to 28.8)	-34.9 (-64 to 22.1)	-26.2 (-61.4 to 42.5)
9 Lower respiratory infect	9 COPD	**72.6 (18.9 to 132)**	**54.1 (12.2 to 108)**	-20.5 (-44.3 to 13.8)
10 Anxiety disorders	10 Anxiety disorders	13.7 (-0.921 to 28.7)	1.4 (-1.64 to 5.39)	**0.421 (0.228 to 0.629)**
11 Other musculoskeletal	11 Chronic kidney disease	**119 (40.3 to 210)**	**95.6 (30.7 to 184)**	15.4 (-26.1 to 76)
12 Headache disorders	12 Road injuries	0.846 (-37.8 to 54.1)	-9.86 (-39.6 to 40.2)	-8.61 (-40.5 to 45.5)
13 Depressive disorders	13 Age-related hearing loss	**77.2 (50.1 to 102)**	**58.1 (47.7 to 67.8)**	**-0.925 (-1.03 to -0.818)**
14 Falls	14 Falls	**45.1 (20.5 to 77)**	**29.4 (13.3 to 48.4)**	**-11.8 (-20.1 to -1.01)**
15 COPD	15 Depressive disorders	**19.6 (2.5 to 36.1)**	**6.65 (3.45 to 11)**	**-2.92 (-3.15 to -2.71)**
16 Age-related hearing loss	16 Headache disorders	7.54 (-8.07 to 24.2)	-4.09 (-7.87 to 6.83)	-0.119 (-0.344 to 0.169)
17 Alzheimer's disease	17 Oral disorders	**61.6 (37.2 to 85.5)**	**44.1 (34.6 to 53.2)**	-0.0468 (-0.417 to 0.336)
18 Congenital defects	18 Urinary diseases	**155 (104 to 224)**	**128 (82.9 to 179)**	15.3 (-8.08 to 49.9)
19 Chronic kidney disease	19 Blindness and vision loss	**64.5 (39.2 to 88.5)**	**46.7 (35.6 to 56.7)**	**-4.87 (-5.15 to -4.57)**
20 Cirrhosis liver	20 Lung cancer	**51.5 (0.579 to 116)**	35 (-7.54 to 83.7)	-16.9 (-41.2 to 10.3)
21 Oral disorders	21 Neonatal disorders			
22 Blindness and vision loss	25 Cirrhosis liver			
24 Lung cancer	36 Congenital defects			
33 Urinary diseases	162 COVID-19			

Supplemental Results Figure S7. Leading 20 level 3 causes of DALYs, and percentage change in number of DALYs and all-age and age-standardised DALY rates by location, 2022 and 2050

North Africa and Middle East, Reference scenario

Legend:
Communicable, maternal, neonatal and nutritional
Non-communicable
Injuries

Leading Causes 2022	Leading Causes 2050	Mean % change number of DALY	Mean % change all-age DALY rate	Mean % change age-standardized DALY rate
1 Ischemic heart disease	1 Ischemic heart disease	47 (-1.31 to 124)	8.8 (-29.3 to 67.4)	**-41.5 (-63.3 to -6.12)**
2 Neonatal disorders	2 Diabetes	**227 (199 to 256)**	**142 (119 to 169)**	**41.7 (27.9 to 58.6)**
3 Stroke	3 Stroke	**47.6 (16.5 to 86.6)**	9.19 (-13.9 to 41.2)	**-43.1 (-56.4 to -22.1)**
4 Congenital defects	4 Chronic kidney disease	**219 (134 to 327)**	**136 (71.6 to 223)**	28.1 (-9.1 to 87.5)
5 Road injuries	5 Low back pain	**61.1 (50.4 to 71.2)**	**19 (13.4 to 24.9)**	**-5.17 (-5.46 to -4.91)**
6 Diabetes	6 Hypertensive heart disease	**207 (126 to 312)**	**127 (66.5 to 209)**	8.27 (-26.5 to 58.5)
7 COVID-19	7 Road injuries	12.5 (-27.9 to 77.1)	-16.8 (-47.4 to 34.4)	-22.2 (-51.8 to 26)
8 Low back pain	8 Depressive disorders	**44.8 (35.6 to 55.5)**	**6.93 (2.67 to 12.3)**	**-2.32 (-2.96 to -1.65)**
9 Depressive disorders	9 Other musculoskeletal	**157 (138 to 176)**	**90 (77.5 to 102)**	**54.2 (51 to 57.2)**
10 Lower respiratory infect	10 Neonatal disorders	**-34 (-50.4 to -12.1)**	**-51.2 (-62.4 to -35.4)**	**-37.3 (-52.4 to -15.4)**
11 Conflict & terror	11 COPD	**140 (84.2 to 209)**	**77.9 (38 to 135)**	-12.7 (-37.5 to 23.5)
12 Headache disorders	12 Alzheimer's disease	**281 (211 to 345)**	**181 (136 to 227)**	**5.76 (4.12 to 7.55)**
13 Anxiety disorders	13 Headache disorders	**39.6 (30 to 51.5)**	3.15 (-0.804 to 10.3)	0.69 (-0.0384 to 1.21)
14 Chronic kidney disease	14 Conflict & terror	60.1 (-59.4 to 350)	18.5 (-69.8 to 235)	21.5 (-68.9 to 251)
15 Gynecological diseases	15 Age-related hearing loss	**122 (99.9 to 142)**	**63.8 (51.2 to 77.2)**	**-1.77 (-2.03 to -1.51)**
16 Hypertensive heart disease	16 Anxiety disorders	**36 (26.2 to 45.5)**	0.448 (-3.83 to 5.36)	0.213 (-0.49 to 0.826)
17 Cirrhosis liver	17 Lower respiratory infect	2.15 (-14.2 to 21.9)	**-24.5 (-36.6 to -7.31)**	**-42 (-53.8 to -25.3)**
18 Other musculoskeletal	18 Congenital defects	**-31.3 (-43.5 to -20.5)**	**-49.3 (-56.3 to -41.5)**	**-31.7 (-38.6 to -22.9)**
19 COPD	19 Cirrhosis liver	**57.3 (38.3 to 77.2)**	**16.3 (0.994 to 34.2)**	**-30.7 (-39.8 to -17.9)**
20 Age-related hearing loss	20 Blindness and vision loss	**124 (101 to 145)**	**65.2 (49.6 to 79.4)**	**-3.32 (-3.95 to -2.67)**
25 Blindness and vision loss	22 Gynecological diseases			
27 Alzheimer's disease	167 COVID-19			

Supplemental Results Figure S7. Leading 20 level 3 causes of DALYs, and percentage change in number of DALYs and all-age and age-standardised DALY rates by location, 2022 and 2050

South Asia, Reference scenario

Legend:
Communicable, maternal, neonatal and nutritional
Non-communicable
Injuries

Leading Causes 2022
1 Neonatal disorders
2 Ischemic heart disease
3 COPD
4 Lower respiratory infect
5 Stroke
6 Diarrheal diseases
7 Diabetes
8 Tuberculosis
9 Dietary iron deficiency
10 Other musculoskeletal
11 Road injuries
12 Low back pain
13 Cirrhosis liver
14 Depressive disorders
15 COVID-19
16 Falls
17 Self-harm
18 Headache disorders
19 Congenital defects
20 Blindness and vision loss
21 Age-related hearing loss
25 Chronic kidney disease
38 Alzheimer's disease

Leading Causes 2050	Mean % change number of DALY	Mean % change all-age DALY rate	Mean % change age-standardized DALY rate
1 Ischemic heart disease	1.4 (-39.9 to 57.8)	-9.19 (-46.1 to 46.7)	**-51.5 (-73.2 to -15.8)**
2 COPD	**64.3 (19.9 to 112)**	**47 (9.5 to 99.4)**	-32.2 (-52.7 to 0.761)
3 Diabetes	**107 (80 to 135)**	**84.5 (62.3 to 113)**	3.49 (-10.3 to 24.5)
4 Other musculoskeletal	**154 (126 to 182)**	**127 (114 to 140)**	**67.5 (64 to 71.3)**
5 Stroke	18.7 (-7.69 to 48.6)	6.13 (-16.5 to 38.6)	**-45.4 (-59.5 to -27)**
6 Neonatal disorders	**-54.8 (-63.6 to -44.5)**	**-59.6 (-66.4 to -50.8)**	**-35.5 (-45.6 to -22.3)**
7 Falls	**72.3 (39 to 106)**	**53.7 (32.2 to 73.5)**	**-12.7 (-20.2 to -4.75)**
8 Low back pain	**49.3 (32.9 to 66.3)**	**33.3 (25.9 to 40.9)**	**-5.8 (-6.14 to -5.44)**
9 Blindness and vision loss	**88.2 (62.7 to 117)**	**68 (53.6 to 82.9)**	**-6.79 (-7.1 to -6.45)**
10 Depressive disorders	**38.1 (22.4 to 55.2)**	**23.3 (17.8 to 30.6)**	**-4.05 (-4.45 to -3.61)**
11 Age-related hearing loss	**83.3 (59 to 109)**	**63.6 (51.3 to 76.7)**	**-1.74 (-1.91 to -1.57)**
12 Cirrhosis liver	**28.2 (3.31 to 57.4)**	14.6 (-7.56 to 42.9)	**-21.1 (-37.1 to -1.04)**
13 Lower respiratory infect	**-41 (-52.5 to -27.2)**	**-47.3 (-56.6 to -35.9)**	**-57.5 (-65.7 to -46.2)**
14 Diarrheal diseases	-16.8 (-41.6 to 16.7)	-25.6 (-49.3 to 8.23)	**-51.2 (-69.9 to -22.2)**
15 Dietary iron deficiency	-0.56 (-12.1 to 12.3)	**-11.3 (-13.4 to -9.18)**	**-13.7 (-14.8 to -12.6)**
16 Road injuries	-4.34 (-40.9 to 45.2)	-14.4 (-45.8 to 36.8)	-23.1 (-52.9 to 25.6)
17 Headache disorders	**20.4 (6.48 to 41.7)**	**7.43 (1.56 to 23.8)**	0.422 (-0.117 to 0.668)
18 Chronic kidney disease	**104 (41.2 to 185)**	**82.4 (26.2 to 164)**	4.93 (-32 to 59)
19 Self-harm	-9.2 (-31.1 to 16.1)	-18.9 (-37.9 to 6.93)	-20.3 (-40.2 to 6.49)
20 Alzheimer's disease	**196 (127 to 272)**	**164 (114 to 214)**	**-5.84 (-9.4 to -2.54)**
22 Tuberculosis			
30 Congenital defects			
166 COVID-19			

Supplemental Results Figure S7. Leading 20 level 3 causes of DALYs, and percentage change in number of DALYs and all-age and age-standardised DALY rates by location, 2022 and 2050

East Asia, Reference scenario

Legend:
Communicable, maternal, neonatal and nutritional
Non-communicable
Injuries

Leading Causes 2022	Leading Causes 2050	Mean % change number of DALY	Mean % change all-age DALY rate	Mean % change age-standardized DALY rate
1 Stroke	1 Stroke	2.92 (-16.4 to 24)	18.2 (-1.53 to 46.7)	**-44.4 (-53.8 to -30.3)**
2 Ischemic heart disease	2 COPD	**82.4 (40.9 to 125)**	**109 (66.3 to 163)**	-19.1 (-38.9 to 8.26)
3 COPD	3 Ischemic heart disease	-11.1 (-37 to 21.2)	2.07 (-27.4 to 42.5)	**-55.7 (-69.4 to -35.7)**
4 Lung cancer	4 Alzheimer's disease	**172 (127 to 229)**	**212 (171 to 261)**	0.00387 (-3.54 to 3.35)
5 Age-related hearing loss	5 Lung cancer	34.2 (-0.635 to 76.7)	**53.8 (16.5 to 98.3)**	-13.6 (-30.6 to 7.45)
6 Diabetes	6 Diabetes	**74.3 (53.8 to 93.5)**	**100 (83.8 to 115)**	**42.3 (31.2 to 52.7)**
7 Road injuries	7 Age-related hearing loss	**43.1 (27.9 to 60.1)**	**64.2 (53 to 75.8)**	**-0.819 (-0.983 to -0.657)**
8 Low back pain	8 Hypertensive heart disease	**139 (72.4 to 221)**	**174 (100 to 283)**	11 (-23.4 to 58.2)
9 Stomach cancer	9 Low back pain	8.21 (-2.51 to 20.2)	**24.2 (17.8 to 30.4)**	**-4.13 (-4.51 to -3.67)**
10 Alzheimer's disease	10 Other musculoskeletal	**49.4 (35.3 to 64.9)**	**71.5 (60.6 to 82)**	**53.8 (51.4 to 55.9)**
11 Falls	11 Colorectal cancer	**67.1 (16.7 to 125)**	**91.5 (39.7 to 154)**	11.1 (-17.7 to 42.9)
12 Depressive disorders	12 Esophageal cancer	**42.4 (23.3 to 63.9)**	**63.5 (45.9 to 85)**	-0.463 (-10.9 to 10.5)
13 Other musculoskeletal	13 Falls	**19 (2.53 to 38.7)**	**36.5 (19.4 to 56.1)**	**-22.5 (-29.3 to -14.6)**
14 Headache disorders	14 Stomach cancer	-11.3 (-22.3 to 3.31)	1.72 (-10 to 13.9)	**-39.8 (-44.4 to -35.2)**
15 Colorectal cancer	15 Road injuries	-28.9 (-51.4 to 6.08)	-18.3 (-44.8 to 23.8)	-18.7 (-48.1 to 29.5)
16 Esophageal cancer	16 Depressive disorders	4.55 (-4.17 to 13.9)	**20 (14.9 to 25.9)**	**-1.97 (-2.32 to -1.61)**
17 Blindness and vision loss	17 Blindness and vision loss	**32 (13.7 to 50.1)**	**51.4 (36.5 to 66.1)**	**-5.12 (-5.59 to -4.75)**
18 Hypertensive heart disease	18 Chronic kidney disease	**70.4 (29.4 to 119)**	**95.7 (49.3 to 154)**	2.14 (-25.4 to 38.3)
19 Anxiety disorders	19 Osteoarthritis	**38.3 (25.8 to 51.7)**	**58.7 (51.3 to 67.5)**	**3.42 (2.92 to 3.9)**
20 Osteoarthritis	20 Headache disorders	**-14.7 (-21.2 to -6.44)**	-2.04 (-6.59 to 6.29)	-0.023 (-0.398 to 0.474)
24 Chronic kidney disease	25 Anxiety disorders			

Supplemental Results Figure S7. Leading 20 level 3 causes of DALYs, and percentage change in number of DALYs and all-age and age-standardised DALY rates by location, 2022 and 2050

Oceania, Reference scenario

Legend:
Communicable, maternal, neonatal and nutritional
Non-communicable
Injuries

Leading Causes 2022
1 Lower respiratory infect
2 Neonatal disorders
3 Ischemic heart disease
4 Diabetes
5 Stroke
6 Congenital defects
7 Malaria
8 Diarrheal diseases
9 COVID-19
10 COPD
11 Road injuries
12 Tuberculosis
13 Pertussis
14 Measles
15 Drowning
16 Low back pain
17 Asthma
18 Interpersonal violence
19 Headache disorders
20 Rheumatic heart disease
21 Dietary iron deficiency
22 Depressive disorders
23 Anxiety disorders
24 Chronic kidney disease
29 Age-related hearing loss
31 Other musculoskeletal

Leading Causes 2050	Mean % change number of DALY	Mean % change all-age DALY rate	Mean % change age-standardized DALY rate
1 Diabetes	**130 (88.3 to 174)**	**23.7 (3.44 to 47.8)**	-7.23 (-22.6 to 14.6)
2 Ischemic heart disease	**82 (16.4 to 170)**	-2.04 (-37.5 to 47.8)	-27.6 (-55.1 to 12.4)
3 Stroke	**89.4 (43.8 to 137)**	1.89 (-18.8 to 27)	**-26 (-43.9 to -5.05)**
4 Neonatal disorders	-19.8 (-46.2 to 17.7)	**-56.9 (-70.3 to -38.5)**	**-42 (-58.7 to -18.5)**
5 Lower respiratory infect	**-41.8 (-56 to -26.6)**	**-68.7 (-75.7 to -60.4)**	**-59.7 (-68.1 to -49.4)**
6 COPD	**115 (58.7 to 181)**	15.8 (-13.6 to 54.1)	-24.3 (-45.2 to 5.52)
7 Malaria	117 (-86.9 to 449)	17.4 (-92.7 to 178)	16.2 (-91.7 to 200)
8 Congenital defects	-6.08 (-23.9 to 17.5)	**-49.6 (-57.7 to -39)**	**-34 (-42.9 to -23.8)**
9 Road injuries	42.6 (-19.2 to 142)	-23.2 (-55.6 to 37.5)	-24.8 (-56 to 30.9)
10 Diarrheal diseases	-18.5 (-50.9 to 24.8)	**-56.2 (-72.9 to -32.4)**	**-48 (-68.6 to -19.1)**
11 Low back pain	**108 (83.2 to 132)**	**11.6 (5.33 to 17.8)**	**-4.41 (-4.84 to -3.96)**
12 Chronic kidney disease	**183 (98.4 to 285)**	**52.2 (5.37 to 116)**	24.8 (-13.6 to 81.5)
13 Other musculoskeletal	**263 (219 to 311)**	**95.2 (83 to 107)**	**70.7 (65.9 to 74.2)**
14 Headache disorders	**97.9 (77.4 to 120)**	**6.38 (3.09 to 10.4)**	**0.741 (0.403 to 1.02)**
15 Depressive disorders	**100 (78.8 to 123)**	**7.58 (3.59 to 11.7)**	**-1.59 (-2.01 to -1.19)**
16 Anxiety disorders	**98.7 (76.6 to 122)**	**6.79 (3.51 to 10.7)**	**1.49 (1.08 to 2.01)**
17 Age-related hearing loss	**139 (108 to 172)**	**28.4 (17.8 to 38.9)**	**-1.24 (-1.52 to -0.958)**
18 Pertussis	11.5 (-41.2 to 103)	-40.1 (-68.8 to 5.75)	-22.4 (-57.9 to 35.5)
19 Interpersonal violence	**42.1 (19.4 to 71.5)**	**-23.7 (-32.8 to -12.1)**	**-26.3 (-34.5 to -15.7)**
20 Dietary iron deficiency	**50.5 (31.8 to 70.1)**	**-19.2 (-21.8 to -16.8)**	**-15.7 (-17.2 to -13.7)**
23 Tuberculosis			
25 Drowning			
27 Asthma			
32 Rheumatic heart disease			
73 Measles			
163 COVID-19			

Supplemental Results Figure S7. Leading 20 level 3 causes of DALYs, and percentage change in number of DALYs and all-age and age-standardised DALY rates by location, 2022 and 2050

Southeast Asia, Reference scenario

Legend:
Communicable, maternal, neonatal and nutritional
Non-communicable
Injuries

Leading Causes 2022	Leading Causes 2050	Mean % change number of DALY	Mean % change all-age DALY rate	Mean % change age-standardized DALY rate
1 Stroke	1 Stroke	**17.4 (1.6 to 34.4)**	3.96 (-11.6 to 21.1)	**-41.6 (-51.2 to -30.9)**
2 Ischemic heart disease	2 Ischemic heart disease	-4.67 (-29.1 to 26)	-15.5 (-37.8 to 13.5)	**-51.2 (-64.9 to -30.6)**
3 Neonatal disorders	3 Diabetes	**76.6 (58.1 to 96.2)**	**56.4 (38.6 to 74.6)**	-6.82 (-17.6 to 5.01)
4 Diabetes	4 COPD	**92.8 (59.9 to 127)**	**70.8 (40.5 to 109)**	-14.9 (-31.1 to 9.49)
5 Lower respiratory infect	5 Chronic kidney disease	**99.4 (58.1 to 150)**	**76.7 (36.1 to 126)**	13.8 (-13.1 to 54.4)
6 Road injuries	6 Cirrhosis liver	**39.9 (23.8 to 57.9)**	**23.9 (7.99 to 41.5)**	-9.6 (-20.9 to 5.12)
7 Cirrhosis liver	7 Lower respiratory infect	3.43 (-8.61 to 14.9)	-8.43 (-18.8 to 2.86)	**-45.1 (-51.5 to -36.5)**
8 COPD	8 Other musculoskeletal	**100 (87.5 to 114)**	**77.2 (69.2 to 86.4)**	**49.3 (46.6 to 51.7)**
9 COVID-19	9 Age-related hearing loss	**71.7 (58.5 to 86)**	**52 (42.8 to 61.7)**	**-1.02 (-1.2 to -0.806)**
10 Tuberculosis	10 Hypertensive heart disease	**133 (74.8 to 203)**	**106 (51.9 to 174)**	12.4 (-20.7 to 57.3)
11 Chronic kidney disease	11 Low back pain	**40.3 (30.1 to 50.1)**	**24.2 (19.2 to 30)**	**-3.78 (-4.16 to -3.4)**
12 Low back pain	12 Alzheimer's disease	**209 (167 to 251)**	**173 (137 to 210)**	1.16 (-1.15 to 3.31)
13 Headache disorders	13 Lung cancer	**70.9 (30.7 to 110)**	**51.1 (21.3 to 82.8)**	-8.35 (-24.6 to 7.93)
14 Age-related hearing loss	14 Road injuries	-7.71 (-31.2 to 28.2)	-18.2 (-40.3 to 13.8)	-19.3 (-42.1 to 13.5)
15 Congenital defects	15 Headache disorders	**14.8 (7.39 to 26.3)**	1.6 (-2.27 to 11.6)	0.202 (-0.128 to 0.762)
16 Other musculoskeletal	16 Neonatal disorders	**-46.4 (-55.4 to -35.3)**	**-52.5 (-60 to -42.1)**	**-37.8 (-48.7 to -22.3)**
17 Lung cancer	17 Falls	**57 (39.6 to 72.7)**	**38.9 (26.1 to 50.7)**	**-9.99 (-14.1 to -5.96)**
18 Diarrheal diseases	18 Blindness and vision loss	**86.3 (69.3 to 103)**	**64.9 (52.1 to 77.1)**	**-3.55 (-4.36 to -2.78)**
19 Hypertensive heart disease	19 Colorectal cancer	**91.7 (39.2 to 146)**	**69.5 (26.8 to 115)**	4.87 (-19.2 to 31.7)
20 Depressive disorders	20 Depressive disorders	**24.8 (17.1 to 32.7)**	**10.4 (6.85 to 14.2)**	**-1.7 (-2.23 to -1.2)**
21 Falls	23 Tuberculosis			
24 Blindness and vision loss	24 Diarrheal diseases			
26 Colorectal cancer	30 Congenital defects			
27 Alzheimer's disease	167 COVID-19			

Supplemental Results Figure S7. Leading 20 level 3 causes of DALYs, and percentage change in number of DALYs and all-age and age-standardised DALY rates by location, 2022 and 2050

Central sub-Saharan Africa, Reference scenario

Legend:
Communicable, maternal, neonatal and nutritional
Non-communicable
Injuries

Leading Causes 2022	Leading Causes 2050	Mean % change number of DALY	Mean % change all-age DALY rate	Mean % change age-standardized DALY rate
1 Neonatal disorders	1 Neonatal disorders	**-31.7 (-49.4 to -12.7)**	**-61.9 (-69.6 to -52)**	**-42.7 (-53.3 to -29.2)**
2 Malaria	2 Road injuries	18 (-37.9 to 108)	-33.9 (-65.6 to 22.9)	-36.8 (-65.8 to 14.2)
3 Lower respiratory infect	3 Lower respiratory infect	4.4 (-20.7 to 30.5)	**-41.7 (-53.2 to -30.3)**	**-39.1 (-48.2 to -29.4)**
4 Road injuries	4 Malaria	-51.8 (-91.1 to 174)	-73.4 (-94.1 to 53.7)	-65.3 (-92.4 to 101)
5 Tuberculosis	5 Diabetes	**194 (126 to 279)**	**63.9 (36.9 to 94.4)**	2.48 (-13.2 to 18.4)
6 HIV/AIDS	6 Stroke	**59.3 (24.3 to 102)**	-11 (-27.1 to 8.91)	**-46.6 (-56.2 to -34.2)**
7 Diarrheal diseases	7 Depressive disorders	**116 (73.5 to 157)**	**20.3 (14.6 to 27)**	**-3.18 (-3.42 to -2.9)**
8 Congenital defects	8 Ischemic heart disease	**62.7 (12.9 to 132)**	-8.99 (-37.5 to 33.9)	**-46.3 (-63.6 to -20.3)**
9 Stroke	9 Tuberculosis	-31.2 (-54.3 to 0.185)	**-61.5 (-74.5 to -39.8)**	**-68.6 (-78.5 to -52.2)**
10 COVID-19	10 Cirrhosis liver	**78.9 (43.5 to 123)**	0.0134 (-16.8 to 19)	**-28.5 (-39.2 to -16.1)**
11 Ischemic heart disease	11 Hypertensive heart disease	**214 (138 to 311)**	**75.6 (35 to 130)**	-4.88 (-27.8 to 23.9)
12 Depressive disorders	12 Diarrheal diseases	-11.3 (-38.6 to 30.8)	**-50.4 (-65.5 to -25)**	**-43.8 (-59.8 to -19.8)**
13 Cirrhosis liver	13 Chronic kidney disease	**145 (82 to 226)**	**37.2 (2.54 to 83.7)**	-3.27 (-26.9 to 29.3)
14 Pertussis	14 Low back pain	**125 (81.2 to 171)**	**25.4 (18.4 to 32.9)**	**-6.34 (-6.57 to -6.13)**
15 Diabetes	15 Congenital defects	-18.4 (-37.9 to 1.52)	**-54.5 (-61.4 to -46.3)**	**-34.6 (-43.4 to -23.4)**
16 Maternal disorders	16 Other musculoskeletal	**368 (286 to 460)**	**161 (142 to 180)**	**89.9 (82.2 to 95.5)**
17 Meningitis	17 HIV/AIDS	-35.1 (-57.3 to 6.38)	**-63.8 (-75.3 to -44.3)**	**-68.4 (-77.9 to -52.5)**
18 Measles	18 Headache disorders	**110 (71.1 to 150)**	**17.1 (11.5 to 28)**	**0.745 (0.401 to 1.03)**
19 Low back pain	19 COPD	**126 (75.3 to 193)**	**26.2 (0.233 to 58)**	**-26.9 (-42.1 to -8.72)**
20 Chronic kidney disease	20 Anxiety disorders	**102 (63.5 to 144)**	**12.8 (6.59 to 19.2)**	**1.43 (1.13 to 1.74)**
25 Headache disorders	24 Pertussis			
26 Hypertensive heart disease	32 Meningitis			
30 Anxiety disorders	33 Maternal disorders			
32 COPD	97 Measles			
40 Other musculoskeletal	169 COVID-19			

Supplemental Results Figure S7. Leading 20 level 3 causes of DALYs, and percentage change in number of DALYs and all-age and age-standardised DALY rates by location, 2022 and 2050

Eastern sub-Saharan Africa, Reference scenario

Legend:
Communicable, maternal, neonatal and nutritional
Non-communicable
Injuries

Leading Causes 2022	Leading Causes 2050	Mean % change number of DALY	Mean % change all-age DALY rate	Mean % change age-standardized DALY rate
1 Neonatal disorders	1 Neonatal disorders	**-30.3 (-46.6 to -11.2)**	**-61.3 (-68.8 to -51.6)**	**-40.8 (-51.1 to -25.7)**
2 Lower respiratory infect	2 Lower respiratory infect	**-15.4 (-31.9 to -0.297)**	**-53 (-60.7 to -43.3)**	**-46.7 (-54.8 to -36.1)**
3 HIV/AIDS	3 Diarrheal diseases	-11.1 (-40.3 to 35)	**-50.6 (-66.9 to -25.3)**	**-44.4 (-62.2 to -16.7)**
4 Diarrheal diseases	4 Stroke	**68.5 (42.8 to 99.2)**	-6.38 (-22 to 13.7)	**-45.3 (-54.5 to -33.1)**
5 Malaria	5 Depressive disorders	**126 (111 to 142)**	**25.6 (19 to 32.9)**	**-2.81 (-3.19 to -2.46)**
6 Tuberculosis	6 Diabetes	**152 (115 to 195)**	**39.9 (17.4 to 66.7)**	-15.2 (-28 to 0.132)
7 Congenital defects	7 Chronic kidney disease	**181 (113 to 271)**	**56.3 (18.4 to 108)**	7.46 (-19.1 to 43.1)
8 COVID-19	8 Low back pain	**131 (114 to 146)**	**28.2 (20.2 to 35.9)**	**-6.92 (-7.13 to -6.71)**
9 Stroke	9 Congenital defects	**-21.9 (-36.3 to -5.13)**	**-56.7 (-62.7 to -48.6)**	**-36.1 (-43.6 to -25.2)**
10 Road injuries	10 Other musculoskeletal	**417 (377 to 452)**	**187 (168 to 208)**	**103 (97.2 to 107)**
11 Meningitis	11 Cirrhosis liver	**76.8 (52.2 to 107)**	-1.76 (-16.3 to 18.8)	**-35 (-44.2 to -22.7)**
12 Protein-energy malnutrition	12 Ischemic heart disease	**64.5 (11.1 to 141)**	-8.54 (-38.2 to 37.3)	**-47 (-64.1 to -20.9)**
13 Depressive disorders	13 Tuberculosis	-30.9 (-54.5 to 6.66)	**-61.6 (-74.7 to -40)**	**-70.5 (-80.3 to -55.2)**
14 Measles	14 Road injuries	33.1 (-23 to 129)	-26 (-56.1 to 28)	-35.3 (-60.7 to 8.62)
15 Ischemic heart disease	15 Age-related hearing loss	**158 (139 to 175)**	**43.4 (32.5 to 53.1)**	**-2.23 (-2.44 to -1.99)**
16 Cirrhosis liver	16 Anxiety disorders	**105 (90.5 to 119)**	**13.8 (8.31 to 20.8)**	**1.74 (1.42 to 2.08)**
17 Diabetes	17 Hypertensive heart disease	**171 (109 to 245)**	**50.6 (15.7 to 97.5)**	-18.5 (-38.4 to 6.02)
18 Low back pain	18 Interpersonal violence	**51.6 (25.8 to 79.2)**	**-15.9 (-28.4 to -0.0947)**	**-24 (-33.6 to -11)**
19 Dietary iron deficiency	19 Headache disorders	**112 (95.4 to 132)**	**17.6 (12.1 to 27.6)**	**0.401 (0.00315 to 0.688)**
20 Pertussis	20 HIV/AIDS	**-72.5 (-76.3 to -68.3)**	**-84.7 (-86.8 to -82.5)**	**-86.3 (-87.8 to -84.6)**
22 Interpersonal violence	21 Dietary iron deficiency			
23 Chronic kidney disease	27 Malaria			
26 Anxiety disorders	29 Meningitis			
27 Age-related hearing loss	34 Pertussis			
29 Headache disorders	44 Protein-energy malnutrition			
32 Hypertensive heart disease	94 Measles			
39 Other musculoskeletal	170 COVID-19			

Supplemental Results Figure S7. Leading 20 level 3 causes of DALYs, and percentage change in number of DALYs and all-age and age-standardised DALY rates by location, 2022 and 2050

Southern sub-Saharan Africa, Reference scenario

Legend:
Communicable, maternal, neonatal and nutritional
Non-communicable
Injuries

Leading Causes 2022	Leading Causes 2050	Mean % change number of DALY	Mean % change all-age DALY rate	Mean % change age-standardized DALY rate
1 HIV/AIDS	1 Diabetes	**82.9 (63.5 to 107)**	**50.4 (34.4 to 69.6)**	-11 (-20.5 to 0.0522)
2 Neonatal disorders	2 HIV/AIDS	**-70.3 (-74.2 to -65.9)**	**-75.6 (-78.7 to -72.6)**	**-78 (-80.4 to -75.5)**
3 Lower respiratory infect	3 Lower respiratory infect	-7.15 (-20 to 6.2)	**-23.7 (-32.1 to -13.6)**	**-36.3 (-42.4 to -28)**
4 COVID-19	4 Neonatal disorders	**-24.3 (-38.1 to -7.59)**	**-37.8 (-47.6 to -25.7)**	-15.1 (-27.1 to 0.329)
5 Road injuries	5 Stroke	**23.1 (8.55 to 38.7)**	1.23 (-10.2 to 15.5)	**-41.6 (-47.7 to -33.5)**
6 Tuberculosis	6 Road injuries	-3.73 (-35.4 to 49.8)	-20.7 (-45.6 to 23.6)	-22.2 (-46.9 to 22.5)
7 Interpersonal violence	7 Hypertensive heart disease	**135 (92.5 to 180)**	**93.5 (63.3 to 135)**	8.14 (-11.8 to 30.4)
8 Diarrheal diseases	8 Ischemic heart disease	17.8 (-10.5 to 52.9)	-3 (-25.4 to 27.8)	**-43.1 (-57.4 to -24.4)**
9 Stroke	9 Diarrheal diseases	-20.7 (-43.6 to 11.1)	**-34.8 (-52.6 to -10.1)**	**-37.9 (-54.9 to -12.1)**
10 Diabetes	10 Interpersonal violence	**-27.2 (-36.8 to -17.4)**	**-40.1 (-45.5 to -34.1)**	**-38.5 (-44.3 to -32)**
11 Ischemic heart disease	11 Chronic kidney disease	**109 (72 to 151)**	**72.2 (41.7 to 106)**	12.9 (-6.97 to 39)
12 Self-harm	12 COPD	**86.5 (62.9 to 115)**	**53.4 (34.4 to 77.8)**	-12.5 (-23.8 to 1.31)
13 Depressive disorders	13 Tuberculosis	**-44.5 (-58.9 to -25.8)**	**-54.3 (-66.4 to -37.7)**	**-62.6 (-73 to -48.3)**
14 Low back pain	14 Low back pain	**55.5 (41 to 71.1)**	**27.8 (22.4 to 34.3)**	**-3.34 (-3.59 to -3.05)**
15 Congenital defects	15 Depressive disorders	**38 (25.3 to 50.9)**	**13.4 (8.74 to 18.6)**	**-3.39 (-3.94 to -2.82)**
16 Hypertensive heart disease	16 Self-harm	5.71 (-11.1 to 22.6)	-13.1 (-23.9 to 0.0608)	**-15.4 (-26.4 to -3.2)**
17 Chronic kidney disease	17 Other musculoskeletal	**115 (92.9 to 136)**	**76.9 (67.5 to 87.7)**	**38.1 (35.7 to 40.3)**
18 COPD	18 Headache disorders	**29.3 (16.5 to 44)**	**6.27 (2.07 to 15.2)**	-0.0536 (-0.568 to 0.447)
19 Dietary iron deficiency	19 Cirrhosis liver	**30.7 (15.3 to 47.1)**	7.46 (-2.38 to 19.1)	**-21.4 (-28.2 to -13.1)**
20 Headache disorders	20 Blindness and vision loss	**65.8 (47 to 83.6)**	**36.2 (25.4 to 47.4)**	**-5.38 (-6.02 to -4.9)**
22 Cirrhosis liver	28 Dietary iron deficiency			
26 Blindness and vision loss	31 Congenital defects			
29 Other musculoskeletal	164 COVID-19			

Supplemental Results Figure S7. Leading 20 level 3 causes of DALYs, and percentage change in number of DALYs and all-age and age-standardised DALY rates by location, 2022 and 2050

Western sub-Saharan Africa, Reference scenario

Legend:
Communicable, maternal, neonatal and nutritional
Non-communicable
Injuries

Leading Causes 2022	Leading Causes 2050	Mean % change number of DALY	Mean % change all-age DALY rate	Mean % change age-standardized DALY rate
1 Neonatal disorders	1 Neonatal disorders	-17.6 (-33.9 to 1.62)	**-57.7 (-65.4 to -47.2)**	**-37.7 (-47.5 to -24.4)**
2 Malaria	2 Malaria	-18.7 (-81.3 to 207)	-58.1 (-90.5 to 54.3)	-51.9 (-88 to 81.8)
3 Lower respiratory infect	3 Lower respiratory infect	**-25.9 (-38.5 to -12.3)**	**-61.9 (-68.3 to -54)**	**-48.1 (-55.8 to -38.2)**
4 Diarrheal diseases	4 Diarrheal diseases	**-36.8 (-57.3 to -6.48)**	**-67.5 (-78.1 to -50.8)**	**-56.9 (-70.1 to -36.8)**
5 Congenital defects	5 Stroke	**66.5 (43.6 to 95)**	-14.3 (-27.9 to 2.63)	**-44.4 (-53 to -32.8)**
6 HIV/AIDS	6 Congenital defects	-12.6 (-26.4 to 4.77)	**-55.1 (-61.2 to -46.3)**	**-37.1 (-44.4 to -27.1)**
7 Stroke	7 Diabetes	**208 (175 to 247)**	**58.4 (38.6 to 82)**	3.34 (-9.13 to 17.8)
8 Meningitis	8 Ischemic heart disease	**75.1 (25.9 to 141)**	-9.78 (-36 to 25.2)	**-44.7 (-61.3 to -22.7)**
9 Tuberculosis	9 Chronic kidney disease	**182 (118 to 262)**	**45.2 (11.3 to 90.6)**	12.1 (-14.2 to 46.9)
10 Dietary iron deficiency	10 Other musculoskeletal	**418 (381 to 454)**	**166 (152 to 180)**	**101 (96.9 to 105)**
11 Road injuries	11 Depressive disorders	**134 (118 to 151)**	**20.1 (15.3 to 25.6)**	**-3.42 (-3.83 to -2.99)**
12 Ischemic heart disease	12 Low back pain	**136 (120 to 152)**	**21.3 (15.9 to 27.1)**	**-6.6 (-6.9 to -6.28)**
13 Pertussis	13 Headache disorders	**121 (103 to 141)**	**13.8 (9.57 to 23.5)**	**0.469 (0.11 to 0.737)**
14 Hemoglobinopathies	14 Dietary iron deficiency	**44.6 (29.9 to 59.1)**	**-25.7 (-27.4 to -23.8)**	**-19.1 (-20 to -18.1)**
15 iNTS	15 Road injuries	37.3 (-17.3 to 130)	-29.3 (-58.3 to 20.7)	-33.4 (-58.9 to 11.8)
16 Maternal disorders	16 Hemoglobinopathies	**27.8 (14.1 to 45.4)**	**-34.3 (-39.6 to -29.3)**	**-24.8 (-29 to -20.6)**
17 Measles	17 Cirrhosis liver	**59.1 (35.6 to 85)**	**-18.1 (-31.1 to -1.19)**	**-37.4 (-46.1 to -25.5)**
18 Diabetes	18 Gynecological diseases	**87.6 (57.1 to 123)**	-3.42 (-20.9 to 13.6)	**-21.7 (-34.4 to -7.05)**
19 Cirrhosis liver	19 Age-related hearing loss	**153 (133 to 177)**	**30.1 (22.2 to 37.5)**	**-2.09 (-2.31 to -1.9)**
20 COVID-19	20 Anxiety disorders	**113 (95.5 to 131)**	**9.4 (5.31 to 13.8)**	**1.56 (1.18 to 1.96)**
21 Headache disorders	21 Meningitis			
22 Depressive disorders	22 iNTS			
23 Low back pain	23 Tuberculosis			
25 Chronic kidney disease	26 HIV/AIDS			
27 Gynecological diseases	27 Pertussis			
29 Anxiety disorders	28 Maternal disorders			
32 Age-related hearing loss	90 Measles			

Supplemental Results Figure S8. Leading 20 level 3 causes of deaths, and percentage change in number of deaths and all-age and age-standardised death rates by location, 2022 and 2050

Armenia, Reference scenario

Legend:
Communicable, maternal, neonatal and nutritional
Non-communicable
Injuries

Leading Causes 2022	Leading Causes 2050	Mean % change number of DEATH	Mean % change all-age DEATH rate	Mean % change age-standardized DEATH rate
1 Ischemic heart disease	1 Ischemic heart disease	3.62 (-31.6 to 48.6)	30.3 (-14.7 to 78.7)	**-52.8 (-71.1 to -25.9)**
2 COVID-19	2 Stroke	-9.61 (-31 to 19.1)	13.7 (-12.9 to 45.8)	**-58.2 (-69.2 to -43.6)**
3 Stroke	3 Alzheimer's disease	**175 (99.9 to 262)**	**246 (162 to 339)**	**-8.53 (-11.1 to -6.35)**
4 Lung cancer	4 Lung cancer	28.3 (-8.21 to 80.8)	**61.5 (12.7 to 121)**	-12.8 (-36.7 to 16.5)
5 Lower respiratory infect	5 Lower respiratory infect	**66.2 (30.6 to 113)**	**109 (65.2 to 155)**	**-27.6 (-42.8 to -7.39)**
6 Alzheimer's disease	6 COPD	**118 (53.9 to 213)**	**175 (100 to 277)**	-6.33 (-34.5 to 30.7)
7 Cirrhosis liver	7 Hypertensive heart disease	**92.5 (24.1 to 179)**	**142 (54.5 to 252)**	-13.4 (-45.5 to 36.8)
8 Hypertensive heart disease	8 Pancreatic cancer	**103 (27.7 to 201)**	**156 (66.4 to 290)**	27.2 (-10.8 to 78.4)
9 Diabetes	9 Colorectal cancer	**58.3 (1.19 to 139)**	**99.2 (29.1 to 210)**	-5.24 (-34.5 to 37.1)
10 COPD	10 Cirrhosis liver	**27.4 (5.52 to 53.7)**	**60.2 (35.3 to 86.3)**	-14.1 (-28.1 to 2.68)
11 Colorectal cancer	11 Chronic kidney disease	**89.8 (29.8 to 163)**	**139 (65.5 to 231)**	3.38 (-31.2 to 51.9)
12 Breast cancer	12 Breast cancer	45.1 (-2.94 to 108)	**82.6 (23.7 to 161)**	-7.94 (-31.1 to 20.8)
13 Pancreatic cancer	13 Diabetes	7.99 (-23.5 to 49.6)	35.9 (-1.54 to 81.9)	**-38.5 (-56.1 to -16.2)**
14 Stomach cancer	14 Aortic aneurysm	35.2 (-0.794 to 82.7)	**70.3 (32.1 to 133)**	**-24.7 (-35.2 to -9.01)**
15 Chronic kidney disease	15 Liver cancer	**70.9 (23.2 to 129)**	**115 (59.3 to 179)**	11.8 (-19.5 to 53)
16 Aortic aneurysm	16 Prostate cancer	**77.6 (27.1 to 158)**	**124 (61.9 to 213)**	-4.19 (-22.1 to 18.6)
17 Road injuries	17 Stomach cancer	-4.53 (-22.3 to 14.2)	**20.2 (1.24 to 45.7)**	**-39.7 (-46.9 to -31.1)**
18 Liver cancer	18 Urinary diseases	**109 (60.1 to 176)**	**162 (103 to 238)**	5.09 (-18.3 to 35.6)
19 Prostate cancer	19 Vascular intestinal	**84.3 (36.1 to 134)**	**132 (72.2 to 213)**	-10 (-24.4 to 4)
20 Upper digest diseases	20 Brain cancer	**63.9 (33.4 to 101)**	**106 (79.2 to 133)**	**29.2 (18.4 to 40.1)**
21 Brain cancer	23 Road injuries			
23 Vascular intestinal	25 Upper digest diseases			
24 Urinary diseases	130 COVID-19			

Supplemental Results Figure S8. Leading 20 level 3 causes of deaths, and percentage change in number of deaths and all-age and age-standardised death rates by location, 2022 and 2050

Azerbaijan, Reference scenario

Legend:
Communicable, maternal, neonatal and nutritional
Non-communicable
Injuries

Leading Causes 2022	Leading Causes 2050	Mean % change number of DEATH	Mean % change all-age DEATH rate	Mean % change age-standardized DEATH rate
1 Ischemic heart disease	1 Ischemic heart disease	**88 (19.6 to 161)**	**76.6 (15.7 to 140)**	**-41.7 (-67 to -2.21)**
2 COVID-19	2 Stroke	**96.3 (48.2 to 152)**	**84.2 (45.9 to 129)**	**-38.4 (-55.2 to -16.5)**
3 Stroke	3 Alzheimer's disease	**388 (221 to 596)**	**357 (211 to 528)**	**3.88 (1.54 to 6.62)**
4 Cirrhosis liver	4 Cirrhosis liver	**100 (58.6 to 143)**	**88 (58.9 to 117)**	-9.13 (-23.3 to 9.8)
5 Lower respiratory infect	5 Hypertensive heart disease	**231 (103 to 390)**	**211 (97.1 to 352)**	6.43 (-39 to 70)
6 Diabetes	6 COPD	**216 (131 to 311)**	**197 (119 to 279)**	0.401 (-30.7 to 43.1)
7 Hypertensive heart disease	7 Lung cancer	**97.1 (22.4 to 181)**	**84.5 (22.6 to 170)**	-4.19 (-33.7 to 32.3)
8 Lung cancer	8 Diabetes	**65.5 (12.2 to 130)**	**55.5 (9.09 to 110)**	-27.1 (-51.3 to 6.47)
9 Neonatal disorders	9 Chronic kidney disease	**182 (85.5 to 295)**	**165 (73.4 to 267)**	23.2 (-24.7 to 89.7)
10 Cardiomyopathy	10 Lower respiratory infect	4.05 (-24.3 to 40.1)	-2.34 (-27.5 to 27.1)	**-46.6 (-60.5 to -28.6)**
11 Stomach cancer	11 Liver cancer	**176 (94.6 to 269)**	**159 (85.5 to 245)**	24 (-10.2 to 75.2)
12 Alzheimer's disease	12 Cardiomyopathy	**50.9 (12.1 to 111)**	**41.5 (11.8 to 90.7)**	**-23.3 (-35.3 to -11.2)**
13 COPD	13 Stomach cancer	**52.7 (22 to 85.9)**	**43.2 (20 to 65.6)**	**-34.2 (-41.5 to -26.6)**
14 Chronic kidney disease	14 Colorectal cancer	**135 (37.9 to 265)**	**120 (25.7 to 236)**	3.44 (-33.9 to 50.6)
15 Breast cancer	15 Breast cancer	**79.1 (18.1 to 157)**	**67.7 (14.7 to 135)**	-4.19 (-31.5 to 28.9)
16 Liver cancer	16 Pancreatic cancer	**218 (88.1 to 380)**	**198 (72.6 to 364)**	39.3 (-10 to 101)
17 Colorectal cancer	17 Esophageal cancer	**139 (101 to 192)**	**124 (93.3 to 160)**	1.62 (-11.2 to 17.5)
18 Road injuries	18 Parkinson's disease	**297 (207 to 417)**	**273 (191 to 359)**	0.76 (-12.3 to 18)
19 Esophageal cancer	19 Brain cancer	**99.9 (67.7 to 135)**	**87.5 (69.8 to 110)**	**33 (23.6 to 44.3)**
20 Brain cancer	20 Prostate cancer	**204 (104 to 324)**	**185 (102 to 278)**	7.49 (-16.6 to 32.2)
22 Pancreatic cancer	23 Road injuries			
28 Prostate cancer	24 Neonatal disorders			
33 Parkinson's disease	130 COVID-19			

Supplemental Results Figure S8. Leading 20 level 3 causes of deaths, and percentage change in number of deaths and all-age and age-standardised death rates by location, 2022 and 2050

Georgia, Reference scenario

Legend:
Communicable, maternal, neonatal and nutritional
Non-communicable
Injuries

Leading Causes 2022	Leading Causes 2050	Mean % change number of DEATH	Mean % change all-age DEATH rate	Mean % change age-standardized DEATH rate
1 Stroke	1 Stroke	**-23 (-38.9 to -6.52)**	-17.5 (-35.7 to 1.93)	**-48.8 (-60.6 to -34.5)**
2 Ischemic heart disease	2 Ischemic heart disease	-24.3 (-50 to 5.81)	-18.7 (-47 to 11)	**-49.3 (-67.6 to -25.2)**
3 COVID-19	3 Hypertensive heart disease	43.5 (-5.98 to 102)	54.2 (-0.432 to 121)	-6.52 (-42 to 45.4)
4 Hypertensive heart disease	4 Alzheimer's disease	**113 (57.7 to 166)**	**128 (74.6 to 187)**	-1.52 (-3.95 to 1.08)
5 Lower respiratory infect	5 Lung cancer	**62.5 (14.9 to 117)**	**73.8 (27.1 to 124)**	**43.8 (9 to 89.7)**
6 Lung cancer	6 Lower respiratory infect	15 (-7.25 to 38.2)	23.3 (-0.226 to 49.1)	-16.5 (-32 to 0.218)
7 Alzheimer's disease	7 Cirrhosis liver	13 (-8.1 to 34.1)	**21 (4.4 to 40.8)**	12.3 (-7.28 to 35.6)
8 Cirrhosis liver	8 Chronic kidney disease	**62.4 (12.4 to 118)**	**74.3 (16.4 to 135)**	20 (-19.5 to 73.5)
9 Diabetes	9 Breast cancer	15.7 (-16.6 to 56.1)	23.7 (-11.5 to 66.7)	-3.19 (-25.7 to 22.4)
10 Breast cancer	10 Colorectal cancer	29.7 (-16.3 to 83.8)	38.7 (-10 to 98)	5.61 (-26.5 to 46.1)
11 Colorectal cancer	11 Diabetes	-3.27 (-31.6 to 25.4)	3.76 (-24.7 to 40.6)	**-25.6 (-46.1 to -1.75)**
12 Chronic kidney disease	12 COPD	34.9 (-0.713 to 75)	**44.7 (5.44 to 94.5)**	-5.85 (-31.1 to 24.7)
13 Stomach cancer	13 Prostate cancer	**38.5 (0.0289 to 84.9)**	**48.2 (7.4 to 95.4)**	4.57 (-16.6 to 26.9)
14 COPD	14 Pancreatic cancer	**67.2 (11.4 to 144)**	**78.7 (19.5 to 144)**	38.9 (-2.16 to 92.3)
15 Prostate cancer	15 Atrial fibrillation	**67.1 (30.2 to 115)**	**79 (44.2 to 128)**	-2.21 (-12.8 to 10.9)
16 Road injuries	16 Stomach cancer	**-22.1 (-35.6 to -7.12)**	**-16.6 (-27.9 to -2.3)**	**-35.7 (-42.7 to -27.3)**
17 Falls	17 Road injuries	-12.2 (-42.6 to 32.5)	-5.87 (-39.8 to 42)	-9.44 (-43.8 to 39.1)
18 Pancreatic cancer	18 Brain cancer	**42.6 (19.3 to 64.8)**	**52.6 (39 to 70)**	**33.8 (24.4 to 46.1)**
19 Cardiomyopathy	19 Falls	9.72 (-10.8 to 31.5)	17.4 (-0.64 to 37.8)	-13.2 (-25.6 to 0.327)
20 Brain cancer	20 Skin cancer	**82.3 (29.9 to 156)**	**95.3 (41.7 to 171)**	18.1 (-2.08 to 47.2)
21 Atrial fibrillation	25 Cardiomyopathy			
35 Skin cancer	130 COVID-19			

Supplemental Results Figure S8. Leading 20 level 3 causes of deaths, and percentage change in number of deaths and all-age and age-standardised death rates by location, 2022 and 2050

Kazakhstan, Reference scenario

Legend:
Communicable, maternal, neonatal and nutritional
Non-communicable
Injuries

Leading Causes 2022	Leading Causes 2050	Mean % change number of DEATH	Mean % change all-age DEATH rate	Mean % change age-standardized DEATH rate
1 COVID-19	1 Ischemic heart disease	42.5 (-1.63 to 88.8)	17.5 (-17.3 to 54.2)	**-48.1 (-68.7 to -19.5)**
2 Ischemic heart disease	2 Stroke	**33.1 (5.1 to 61.5)**	9.73 (-11.7 to 30.5)	**-50.2 (-63 to -32.8)**
3 Stroke	3 COPD	**122 (69.3 to 186)**	**83.1 (40.9 to 131)**	-7.53 (-35.6 to 25.7)
4 Cardiomyopathy	4 Cirrhosis liver	**41.9 (18.3 to 65.9)**	16.9 (-0.983 to 32.9)	**-19.8 (-32.9 to -3.7)**
5 COPD	5 Cardiomyopathy	**38.9 (12.1 to 73.7)**	14.3 (-6.46 to 39.1)	**-24 (-34.1 to -10.6)**
6 Cirrhosis liver	6 Alzheimer's disease	**272 (157 to 394)**	**206 (124 to 295)**	**4.26 (1.93 to 7.1)**
7 Lower respiratory infect	7 Lower respiratory infect	**54.7 (27.1 to 85.8)**	**27.5 (8.09 to 53.7)**	**-24.9 (-38.6 to -5.57)**
8 Self-harm	8 Chronic kidney disease	**175 (96 to 281)**	**127 (58.5 to 208)**	27 (-17.7 to 88.4)
9 Lung cancer	9 Lung cancer	**55.8 (1.39 to 119)**	28.1 (-13.6 to 72.5)	-16.2 (-39.1 to 9.69)
10 Road injuries	10 Self-harm	13.2 (-12.1 to 40.3)	-6.78 (-22.8 to 15.3)	-13.5 (-29.1 to 10.7)
11 Alzheimer's disease	11 Colorectal cancer	**105 (27.2 to 208)**	**68.8 (2.98 to 144)**	-0.317 (-32.5 to 37.1)
12 Chronic kidney disease	12 Hypertensive heart disease	**142 (64.1 to 236)**	**99.7 (32.9 to 180)**	-4.68 (-43.2 to 53.8)
13 Stomach cancer	13 Pancreatic cancer	**162 (63.9 to 290)**	**115 (35.2 to 210)**	35.3 (-8.71 to 85)
14 Colorectal cancer	14 Urinary diseases	**130 (84.8 to 179)**	**89.2 (56 to 127)**	11.5 (-11.5 to 40.6)
15 Neonatal disorders	15 Diabetes	**55.6 (13.9 to 105)**	28.3 (-5.88 to 69.6)	-27.7 (-49 to 0.207)
16 Diabetes	16 Stomach cancer	**21.2 (0.914 to 42.7)**	-0.18 (-12.3 to 14.5)	**-36.8 (-43.4 to -28.9)**
17 Breast cancer	17 Breast cancer	**62.8 (8.43 to 124)**	33.8 (-7.42 to 82.5)	-9.42 (-33.3 to 17.4)
18 Asthma	18 Road injuries	9.38 (-32.4 to 74.7)	-9.78 (-43.7 to 41.7)	-14.6 (-48.3 to 37.5)
19 Congenital defects	19 Parkinson's disease	**218 (156 to 285)**	**162 (114 to 211)**	4.84 (-10.7 to 19.1)
20 Hypertensive heart disease	20 Liver cancer	**130 (71.7 to 200)**	**89.2 (42.8 to 143)**	16.6 (-15.4 to 61.6)
21 Urinary diseases	26 Asthma			
23 Pancreatic cancer	37 Congenital defects			
25 Liver cancer	39 Neonatal disorders			
30 Parkinson's disease	130 COVID-19			

Supplemental Results Figure S8. Leading 20 level 3 causes of deaths, and percentage change in number of deaths and all-age and age-standardised death rates by location, 2022 and 2050

Kyrgyzstan, Reference scenario

Legend:
Communicable, maternal, neonatal and nutritional
Non-communicable
Injuries

Leading Causes 2022	Leading Causes 2050	Mean % change number of DEATH	Mean % change all-age DEATH rate	Mean % change age-standardized DEATH rate
1 COVID-19	1 Ischemic heart disease	**120 (45.3 to 211)**	**83.2 (25.4 to 152)**	-29.1 (-59.3 to 19.9)
2 Ischemic heart disease	2 Stroke	**60 (19.8 to 103)**	**33.5 (4.14 to 71.6)**	**-39.2 (-56.7 to -16.3)**
3 Stroke	3 COPD	**244 (149 to 344)**	**187 (117 to 275)**	13.4 (-24.3 to 70.8)
4 Cirrhosis liver	4 Cirrhosis liver	**55.7 (21.3 to 89.9)**	**29.8 (7.4 to 54.4)**	-12.6 (-28.8 to 6.36)
5 Neonatal disorders	5 Alzheimer's disease	**347 (191 to 515)**	**272 (164 to 398)**	**14.7 (11.2 to 18)**
6 COPD	6 Hypertensive heart disease	**223 (103 to 373)**	**169 (69.6 to 295)**	12.4 (-37.2 to 88.7)
7 Road injuries	7 Lung cancer	**165 (62.3 to 281)**	**120 (48 to 215)**	17.5 (-15.8 to 60.3)
8 Lower respiratory infect	8 Chronic kidney disease	**177 (77.6 to 294)**	**131 (47.1 to 237)**	29.9 (-20.7 to 107)
9 Stomach cancer	9 Stomach cancer	**56.1 (22.3 to 92.6)**	**30 (11.6 to 51.1)**	**-30.1 (-38.7 to -21.4)**
10 Hypertensive heart disease	10 Road injuries	11.1 (-39.5 to 95.7)	-7.31 (-46.2 to 61.1)	-17 (-52.6 to 44.5)
11 Alzheimer's disease	11 Lower respiratory infect	9.4 (-16.2 to 37.5)	-8.77 (-28.8 to 11.3)	**-31.4 (-48.6 to -13.2)**
12 Self-harm	12 Pancreatic cancer	**249 (102 to 463)**	**190 (83.2 to 332)**	49.1 (-0.677 to 114)
13 Lung cancer	13 Neonatal disorders	-41.3 (-72.3 to 7)	**-51.1 (-76.3 to -12.8)**	-33.1 (-67.7 to 16.1)
14 Cardiomyopathy	14 Colorectal cancer	**143 (39.6 to 281)**	**102 (23.7 to 201)**	1.28 (-34 to 45.5)
15 Chronic kidney disease	15 Self-harm	17.2 (-14.3 to 50.8)	-2.35 (-24.1 to 23.2)	-16.6 (-35.9 to 7.74)
16 Congenital defects	16 Cardiomyopathy	33.1 (-2.41 to 89.2)	10.8 (-14.5 to 45.2)	**-26.8 (-42.5 to -6.25)**
17 Diabetes	17 Diabetes	**78.8 (22.5 to 149)**	**49 (2.09 to 98.3)**	-20.3 (-47.9 to 11.2)
18 Breast cancer	18 Breast cancer	**88.2 (23.5 to 170)**	**56.6 (8.18 to 114)**	-7.36 (-31.9 to 23)
19 Colorectal cancer	19 Liver cancer	**223 (120 to 343)**	**170 (87.5 to 279)**	39.4 (-5.92 to 103)
20 Alcohol use disorders	20 Urinary diseases	**193 (105 to 279)**	**144 (80.8 to 224)**	31.2 (-9.3 to 88.1)
22 Pancreatic cancer	21 Alcohol use disorders			
29 Liver cancer	34 Congenital defects			
31 Urinary diseases	130 COVID-19			

Supplemental Results Figure S8. Leading 20 level 3 causes of deaths, and percentage change in number of deaths and all-age and age-standardised death rates by location, 2022 and 2050

Mongolia, Reference scenario

Legend:
Communicable, maternal, neonatal and nutritional
Non-communicable
Injuries

Leading Causes 2022	Leading Causes 2050	Mean % change number of DEATH	Mean % change all-age DEATH rate	Mean % change age-standardized DEATH rate
1 Ischemic heart disease	1 Ischemic heart disease	**90.4 (27.3 to 160)**	18.3 (-20 to 64.8)	**-46.7 (-70 to -9.42)**
2 Stroke	2 Liver cancer	**247 (148 to 360)**	**116 (52.5 to 197)**	29.2 (-14.7 to 85.4)
3 Liver cancer	3 Stroke	**92.2 (57.7 to 132)**	19.3 (-5.26 to 46.5)	**-37.9 (-53.9 to -19.2)**
4 Cirrhosis liver	4 Cirrhosis liver	**174 (126 to 220)**	**69.8 (38.4 to 105)**	10.6 (-12.1 to 36)
5 Stomach cancer	5 Stomach cancer	**75.7 (45.6 to 102)**	9.01 (-10.1 to 26.2)	**-35.2 (-42.4 to -27.5)**
6 Alcohol use disorders	6 Lung cancer	**176 (72.7 to 304)**	**71.2 (10.1 to 149)**	1.4 (-29.5 to 36.8)
7 Lower respiratory infect	7 Alzheimer's disease	**433 (271 to 606)**	**230 (128 to 339)**	**4.18 (1.51 to 7.06)**
8 Lung cancer	8 Esophageal cancer	**221 (175 to 271)**	**98.9 (69.3 to 132)**	5.83 (-7.93 to 20.4)
9 COVID-19	9 COPD	**235 (154 to 333)**	**108 (53.4 to 167)**	-8.16 (-39.2 to 31.6)
10 Road injuries	10 Chronic kidney disease	**211 (108 to 332)**	**93.3 (30.2 to 174)**	15.2 (-29.3 to 80.4)
11 Self-harm	11 Alcohol use disorders	46.8 (-1.57 to 106)	-8.86 (-40.3 to 32.9)	-9.25 (-41.1 to 35.8)
12 Neonatal disorders	12 Self-harm	**74.6 (35.7 to 128)**	8.36 (-15.9 to 41.8)	1.63 (-20.7 to 34.9)
13 Tuberculosis	13 Cardiomyopathy	**115 (74.2 to 168)**	**33.5 (7.57 to 70.6)**	-6.72 (-21.3 to 15.1)
14 Cardiomyopathy	14 Road injuries	50.7 (-3.63 to 128)	-6.39 (-39.9 to 46.7)	-10.6 (-42.6 to 41.8)
15 Esophageal cancer	15 Pancreatic cancer	**289 (128 to 500)**	**141 (40.5 to 265)**	45.2 (-5.1 to 111)
16 COPD	16 Lower respiratory infect	17.9 (-5.33 to 50.2)	**-26.8 (-42.4 to -8.22)**	**-36.8 (-50.1 to -18.5)**
17 Chronic kidney disease	17 Hypertensive heart disease	**230 (107 to 372)**	**105 (24.5 to 204)**	-5.56 (-51.2 to 58.1)
18 Alzheimer's disease	18 Colorectal cancer	**171 (58.5 to 303)**	67.9 (-2.86 to 151)	-0.546 (-35.3 to 41.3)
19 Congenital defects	19 Neonatal disorders	-12.3 (-50.5 to 44.5)	**-45.6 (-70.2 to -11.7)**	-31.7 (-61 to 11)
20 Diabetes	20 Diabetes	**70.4 (18.6 to 123)**	5.81 (-25.4 to 42.7)	**-33.4 (-56.6 to -6.19)**
22 Pancreatic cancer	22 Tuberculosis			
24 Colorectal cancer	35 Congenital defects			
26 Hypertensive heart disease	129 COVID-19			

Supplemental Results Figure S8. Leading 20 level 3 causes of deaths, and percentage change in number of deaths and all-age and age-standardised death rates by location, 2022 and 2050

Tajikistan, Reference scenario

Legend:
Communicable, maternal, neonatal and nutritional
Non-communicable
Injuries

Leading Causes 2022	Leading Causes 2050	Mean % change number of DEATH	Mean % change all-age DEATH rate	Mean % change age-standardized DEATH rate
1 Ischemic heart disease	1 Ischemic heart disease	**191 (90 to 298)**	**95.4 (25.4 to 182)**	-25.6 (-58.2 to 25.5)
2 Stroke	2 Stroke	**153 (88 to 216)**	**69.4 (28.4 to 107)**	**-33.7 (-54.8 to -8.28)**
3 Lower respiratory infect	3 Hypertensive heart disease	**269 (126 to 436)**	**147 (47.8 to 273)**	-7.62 (-49.8 to 63.2)
4 COVID-19	4 COPD	**204 (117 to 300)**	**104 (46.8 to 160)**	-21 (-47.4 to 16.1)
5 Neonatal disorders	5 Alzheimer's disease	**439 (262 to 642)**	**260 (151 to 375)**	1.84 (-1.92 to 4.79)
6 Cirrhosis liver	6 Lower respiratory infect	**-25.4 (-45.1 to -4.83)**	**-50.1 (-61 to -34.6)**	**-51.7 (-63.1 to -36.3)**
7 Hypertensive heart disease	7 Cirrhosis liver	**88.6 (45.3 to 131)**	26.4 (-1.03 to 51.4)	**-21.5 (-38 to -4.42)**
8 Diarrheal diseases	8 Diabetes	**139 (60 to 244)**	**60.8 (5.25 to 131)**	-14.4 (-46.6 to 26.5)
9 COPD	9 Neonatal disorders	-33.4 (-68.7 to 25.5)	**-55.3 (-78.8 to -12.6)**	-37.8 (-70.5 to 20.5)
10 Diabetes	10 Stomach cancer	**81.9 (43.3 to 121)**	**21.6 (3.66 to 41.6)**	**-38.1 (-44 to -31.4)**
11 Stomach cancer	11 Lung cancer	**146 (47.3 to 274)**	**64.2 (6.37 to 140)**	-12.4 (-37.5 to 23.5)
12 Congenital defects	12 Parkinson's disease	**375 (256 to 523)**	**217 (158 to 290)**	-4.48 (-17.3 to 10.9)
13 Tuberculosis	13 Brain cancer	**141 (99.5 to 192)**	**61.4 (45.5 to 81.1)**	**31.2 (20.5 to 44.1)**
14 Alzheimer's disease	14 Esophageal cancer	**169 (119 to 219)**	**79.7 (57.5 to 108)**	**-17.2 (-27.3 to -5.45)**
15 Road injuries	15 Breast cancer	**129 (48.1 to 263)**	**53 (3.15 to 127)**	-3.33 (-31.4 to 34.6)
16 Drowning	16 Liver cancer	**238 (133 to 349)**	**127 (59.9 to 215)**	13.3 (-22.4 to 59.2)
17 Lung cancer	17 Road injuries	31.3 (-27.5 to 109)	-11.8 (-50.4 to 49.4)	-19.6 (-56 to 41.8)
18 Idiopathic epilepsy	18 Urinary diseases	**205 (119 to 286)**	**104 (47.2 to 156)**	10.3 (-25.7 to 47.3)
19 Falls	19 Colorectal cancer	**188 (55.7 to 379)**	**91.7 (15.4 to 202)**	-2.25 (-36.5 to 43.7)
20 Brain cancer	20 Idiopathic epilepsy	30.5 (-25.5 to 112)	-12.4 (-50.4 to 46.5)	-11.5 (-50.9 to 49.7)
21 Breast cancer	21 Falls			
22 Esophageal cancer	24 Diarrheal diseases			
25 Colorectal cancer	26 Congenital defects			
27 Urinary diseases	27 Tuberculosis			
28 Liver cancer	28 Drowning			
30 Parkinson's disease	130 COVID-19			

Supplemental Results Figure S8. Leading 20 level 3 causes of deaths, and percentage change in number of deaths and all-age and age-standardised death rates by location, 2022 and 2050

Turkmenistan, Reference scenario

Legend:
Communicable, maternal, neonatal and nutritional
Non-communicable
Injuries

Leading Causes 2022	Leading Causes 2050	Mean % change number of DEATH	Mean % change all-age DEATH rate	Mean % change age-standardized DEATH rate
1 Ischemic heart disease	1 Ischemic heart disease	55.8 (-2.3 to 129)	23.7 (-22 to 80.2)	**-46 (-71.2 to -3.81)**
2 Stroke	2 Stroke	30.2 (-2.45 to 79.2)	3.29 (-22.9 to 38.5)	**-48.1 (-65.8 to -19.8)**
3 Cirrhosis liver	3 Cirrhosis liver	**61.8 (27.8 to 106)**	**28.4 (1.65 to 62.7)**	-3.94 (-25.6 to 24.6)
4 COVID-19	4 Alzheimer's disease	**287 (125 to 479)**	**206 (81.6 to 342)**	-1.06 (-4.04 to 1.7)
5 Lower respiratory infect	5 Hypertensive heart disease	**168 (64.5 to 304)**	**113 (29.5 to 227)**	-0.954 (-49 to 75.7)
6 Neonatal disorders	6 Chronic kidney disease	**143 (54.3 to 282)**	**93.3 (22.6 to 202)**	18.7 (-32.7 to 104)
7 Diabetes	7 Diabetes	50.1 (-1.17 to 122)	19.3 (-21.4 to 74.5)	-27.3 (-56.1 to 18.3)
8 Chronic kidney disease	8 Urinary diseases	**164 (87.5 to 252)**	**109 (53.7 to 180)**	20.1 (-19.7 to 83.1)
9 Hypertensive heart disease	9 Lower respiratory infect	**-32.8 (-48.9 to -11.6)**	**-46.7 (-60.4 to -30)**	**-51.7 (-65.3 to -33.5)**
10 Cardiomyopathy	10 Cardiomyopathy	**42.1 (11.1 to 84.8)**	12.4 (-8.21 to 37.3)	**-24.3 (-34.4 to -12.1)**
11 Congenital defects	11 COPD	**120 (59.4 to 192)**	**74.4 (27 to 130)**	-17.5 (-50.3 to 30.7)
12 Alzheimer's disease	12 Esophageal cancer	**107 (66.8 to 148)**	**64.2 (41.5 to 90.4)**	-8.35 (-21.3 to 9.01)
13 Self-harm	13 Self-harm	27 (-7.85 to 74.1)	0.825 (-26.5 to 37.8)	-8.76 (-34.4 to 26.7)
14 Urinary diseases	14 Lung cancer	63.8 (-5.45 to 155)	29.3 (-22.4 to 95.4)	-19.9 (-46.8 to 13.4)
15 Stomach cancer	15 Liver cancer	**154 (54.1 to 280)**	**102 (25.2 to 206)**	23.7 (-26.7 to 106)
16 COPD	16 Neonatal disorders	-43.9 (-74.5 to 8.66)	**-55.4 (-79.1 to -16.2)**	-41.2 (-70.4 to 5.09)
17 Lung cancer	17 Brain cancer	**94.5 (63.3 to 124)**	**54 (38.6 to 70.8)**	**26.3 (16.5 to 36.2)**
18 Tuberculosis	18 Breast cancer	**65.6 (1.85 to 155)**	30.8 (-15.6 to 91.5)	-10.7 (-38.4 to 26)
19 Esophageal cancer	19 Stomach cancer	**29.4 (1.8 to 61.1)**	2.5 (-15.8 to 22.4)	**-39.4 (-47 to -31.8)**
20 Road injuries	20 Colorectal cancer	**114 (15.6 to 264)**	69.1 (-5.47 to 176)	-4.02 (-40.2 to 45.3)
21 Breast cancer	21 Road injuries			
22 Brain cancer	23 Congenital defects			
23 Liver cancer	30 Tuberculosis			
24 Colorectal cancer	130 COVID-19			

Supplemental Results Figure S8. Leading 20 level 3 causes of deaths, and percentage change in number of deaths and all-age and age-standardised death rates by location, 2022 and 2050

Uzbekistan, Reference scenario

Legend:
Communicable, maternal, neonatal and nutritional
Non-communicable
Injuries

Leading Causes 2022
1 Ischemic heart disease
2 Stroke
3 COVID-19
4 Lower respiratory infect
5 Cirrhosis liver
6 Diabetes
7 Neonatal disorders
8 Hypertensive heart disease
9 Road injuries
10 Chronic kidney disease
11 Self-harm
12 Alzheimer's disease
13 Congenital defects
14 Stomach cancer
15 Lung cancer
16 Breast cancer
17 COPD
18 Drowning
19 Brain cancer
20 Rheumatic heart disease
22 Colorectal cancer
23 Liver cancer
25 Esophageal cancer
33 Pancreatic cancer

Leading Causes 2050	Mean % change number of DEATH	Mean % change all-age DEATH rate	Mean % change age-standardized DEATH rate
1 Ischemic heart disease	**117 (33.3 to 231)**	**66.8 (8.88 to 124)**	-34.1 (-62.6 to 10.8)
2 Stroke	**82.8 (27.4 to 151)**	**40.4 (9.8 to 71.6)**	**-41.2 (-58.3 to -17.2)**
3 Alzheimer's disease	**385 (184 to 595)**	**273 (136 to 421)**	**4.19 (1.63 to 6.98)**
4 Diabetes	**99 (28.4 to 211)**	**53 (6.18 to 122)**	-15.8 (-44.6 to 33.1)
5 Cirrhosis liver	**58 (10.4 to 120)**	**21.4 (2.72 to 51.6)**	-15.4 (-31.9 to 8.11)
6 Chronic kidney disease	**249 (113 to 438)**	**168 (79 to 268)**	42.2 (-15.4 to 128)
7 Hypertensive heart disease	**183 (63.4 to 336)**	**118 (39.3 to 220)**	-7.52 (-47.1 to 49.3)
8 Lower respiratory infect	-19.6 (-45.3 to 18.7)	**-38.3 (-51.3 to -18.2)**	**-43.6 (-58 to -18.7)**
9 COPD	**220 (119 to 357)**	**146 (82.3 to 210)**	-3.72 (-37.2 to 42)
10 Lung cancer	**126 (32.4 to 232)**	**73.9 (7.42 to 153)**	-2.23 (-34.3 to 34)
11 Neonatal disorders	-21 (-62.1 to 57.4)	-39.5 (-67.6 to 3.75)	-17.3 (-55.1 to 34.1)
12 Road injuries	15.8 (-43.2 to 131)	-11.2 (-52.3 to 67.9)	-18.3 (-57 to 56.4)
13 Self-harm	16.9 (-20.7 to 67.7)	-10.3 (-31.2 to 19.4)	-16.5 (-38.6 to 15)
14 Breast cancer	**85.9 (8.17 to 186)**	43 (-6.17 to 102)	-5.48 (-33.3 to 27.9)
15 Liver cancer	**172 (68.4 to 338)**	**109 (39.1 to 204)**	20.9 (-23.4 to 84.5)
16 Colorectal cancer	**149 (38.5 to 293)**	**91.3 (14 to 199)**	4.15 (-32.7 to 52.4)
17 Stomach cancer	**44.9 (3.33 to 89.5)**	11.4 (-3.97 to 29.7)	**-38 (-44.3 to -31)**
18 Brain cancer	**110 (53.2 to 172)**	**61.1 (45.7 to 78.6)**	**28.1 (18.5 to 38.4)**
19 Esophageal cancer	**134 (71.3 to 210)**	**80.1 (53 to 104)**	-6.91 (-21.4 to 6.71)
20 Pancreatic cancer	**246 (91 to 446)**	**166 (55.8 to 296)**	42.1 (-7.67 to 106)
25 Congenital defects			
26 Rheumatic heart disease			
33 Drowning			
130 COVID-19			

Supplemental Results Figure S8. Leading 20 level 3 causes of deaths, and percentage change in number of deaths and all-age and age-standardised death rates by location, 2022 and 2050

Albania, Reference scenario

Legend:
Communicable, maternal, neonatal and nutritional
Non-communicable
Injuries

Leading Causes 2022	Leading Causes 2050	Mean % change number of DEATH	Mean % change all-age DEATH rate	Mean % change age-standardized DEATH rate
1 Ischemic heart disease	1 Stroke	**39.7 (4.25 to 82.6)**	**59.6 (24.9 to 94.2)**	**-32.4 (-49.8 to -12.4)**
2 Stroke	2 Ischemic heart disease	11.1 (-23.4 to 51.8)	27 (-8.56 to 72.3)	**-43.6 (-63.9 to -11.2)**
3 COVID-19	3 Alzheimer's disease	**165 (103 to 250)**	**203 (135 to 275)**	**14.3 (11.6 to 17.2)**
4 Lung cancer	4 Lung cancer	**63.9 (15.2 to 115)**	**87.4 (34.7 to 145)**	15.4 (-10.1 to 43.7)
5 Alzheimer's disease	5 COPD	**111 (48.3 to 179)**	**142 (79.6 to 218)**	8.84 (-21.5 to 48.8)
6 COPD	6 Chronic kidney disease	**125 (65.1 to 208)**	**157 (90.9 to 238)**	20.5 (-12.5 to 71.2)
7 Lower respiratory infect	7 Lower respiratory infect	**66.5 (25.5 to 111)**	**90.3 (45.3 to 138)**	**-21.4 (-37.8 to -4.12)**
8 Stomach cancer	8 Hypertensive heart disease	**113 (38.8 to 211)**	**144 (60.4 to 253)**	5.91 (-32.5 to 64.1)
9 Hypertensive heart disease	9 Liver cancer	**109 (58.6 to 171)**	**138 (86 to 197)**	**45.2 (13.6 to 89.4)**
10 Chronic kidney disease	10 Colorectal cancer	**99.8 (21.6 to 199)**	**128 (42.3 to 250)**	19.4 (-15.4 to 68.2)
11 Liver cancer	11 Pancreatic cancer	**136 (46 to 232)**	**170 (68 to 270)**	**55.4 (5.62 to 101)**
12 Cardiomyopathy	12 Prostate cancer	**88 (29.9 to 175)**	**115 (56.6 to 197)**	3.79 (-16.9 to 31.4)
13 Colorectal cancer	13 Cardiomyopathy	**56.2 (12.3 to 115)**	**78.4 (30 to 146)**	**-18.2 (-31.3 to -2.93)**
14 Prostate cancer	14 Atrial fibrillation	**191 (107 to 304)**	**233 (145 to 346)**	**25.1 (5.87 to 49.4)**
15 Other cardiovascular	15 Stomach cancer	16.6 (-6.55 to 54.7)	**33.2 (9.21 to 64.3)**	**-26.3 (-33.9 to -16.7)**
16 Cirrhosis liver	16 Parkinson's disease	**98.7 (55.7 to 157)**	**127 (79.3 to 181)**	-0.55 (-15.9 to 16.1)
17 Pancreatic cancer	17 Other cardiovascular	**53.8 (15.5 to 96.8)**	**75.8 (35.9 to 120)**	-15.6 (-31.4 to 5.32)
18 Parkinson's disease	18 Breast cancer	**72.1 (15.8 to 150)**	**96.7 (36.7 to 187)**	10.2 (-16 to 41.3)
19 Breast cancer	19 Brain cancer	**78.3 (48.7 to 117)**	**104 (82.1 to 127)**	**43 (33.3 to 54.7)**
20 Diabetes	20 Cirrhosis liver	**26.5 (3.14 to 52.4)**	**44.5 (22.3 to 66.6)**	-3.02 (-16.2 to 13.6)
22 Brain cancer	21 Diabetes			
23 Atrial fibrillation	129 COVID-19			

Supplemental Results Figure S8. Leading 20 level 3 causes of deaths, and percentage change in number of deaths and all-age and age-standardised death rates by location, 2022 and 2050

Bosnia and Herzegovina, Reference scenario

Legend:
Communicable, maternal, neonatal and nutritional
Non-communicable
Injuries

Leading Causes 2022	Leading Causes 2050	Mean % change number of DEATH	Mean % change all-age DEATH rate	Mean % change age-standardized DEATH rate
1 Ischemic heart disease	1 Ischemic heart disease	-9.71 (-42.3 to 36.4)	27.1 (-18.7 to 90.9)	**-51.6 (-71.2 to -23.8)**
2 Stroke	2 Stroke	-21.3 (-39.4 to 3.38)	10.8 (-17.8 to 47.2)	**-56.2 (-67.9 to -39.5)**
3 COVID-19	3 Alzheimer's disease	**176 (90.8 to 272)**	**288 (183 to 416)**	**5.73 (2.81 to 8.64)**
4 Lung cancer	4 Lung cancer	14.9 (-24.5 to 64)	**61.1 (11 to 109)**	-4.74 (-31 to 25.3)
5 Diabetes	5 Diabetes	11 (-21.3 to 54.1)	**56.1 (11.4 to 116)**	-28.7 (-49.8 to 1.46)
6 Alzheimer's disease	6 Colorectal cancer	47.7 (-10.2 to 130)	**107 (29.4 to 204)**	3.86 (-31.4 to 43.5)
7 Colorectal cancer	7 Hypertensive heart disease	**78.7 (9.85 to 186)**	**152 (52.5 to 295)**	-2.22 (-43.1 to 69.5)
8 COPD	8 COPD	**54.2 (7.89 to 113)**	**117 (52.4 to 195)**	-5.98 (-35.3 to 34.5)
9 Cardiomyopathy	9 Cardiomyopathy	38.1 (-5.06 to 110)	**93.7 (44.6 to 172)**	**-23.2 (-35.6 to -6.74)**
10 Hypertensive heart disease	10 Chronic kidney disease	**84.1 (25.7 to 170)**	**159 (66.8 to 276)**	10.6 (-30 to 71)
11 Chronic kidney disease	11 Pancreatic cancer	**88 (17.3 to 180)**	**164 (69.2 to 275)**	41.2 (-2.74 to 96.4)
12 Cirrhosis liver	12 Prostate cancer	**73.8 (18.7 to 152)**	**144 (77.3 to 244)**	8.48 (-14 to 32.3)
13 Breast cancer	13 Breast cancer	27.6 (-18.7 to 92.5)	**78.8 (20.6 to 160)**	-4.2 (-28.9 to 29.1)
14 Pancreatic cancer	14 Liver cancer	**69.9 (24.3 to 139)**	**139 (72.8 to 249)**	36.7 (-6.47 to 99.7)
15 Stomach cancer	15 Atrial fibrillation	**139 (77 to 224)**	**236 (158 to 349)**	11.5 (-2.91 to 29.3)
16 Lower respiratory infect	16 Lower respiratory infect	**37.4 (6.22 to 78.2)**	**93 (49.9 to 150)**	**-23.5 (-38 to -1.73)**
17 Prostate cancer	17 Brain cancer	**45.1 (16.9 to 72.4)**	**104 (72.3 to 143)**	**29.9 (19.2 to 41.5)**
18 Brain cancer	18 Parkinson's disease	**69.4 (33.1 to 108)**	**138 (94.6 to 187)**	-2.51 (-14.9 to 13)
19 Liver cancer	19 Cirrhosis liver	-6.16 (-24.1 to 20)	**31.9 (8.73 to 64.5)**	-7.63 (-22.7 to 15.4)
20 Self-harm	20 Stomach cancer	-11.8 (-30.8 to 7.32)	**23.9 (3.38 to 49.6)**	**-35.7 (-42.2 to -28)**
21 Parkinson's disease	25 Self-harm			
24 Atrial fibrillation	130 COVID-19			

Supplemental Results Figure S8. Leading 20 level 3 causes of deaths, and percentage change in number of deaths and all-age and age-standardised death rates by location, 2022 and 2050

Bulgaria, Reference scenario

Legend:
Communicable, maternal, neonatal and nutritional
Non-communicable
Injuries

Leading Causes 2022	Leading Causes 2050	Mean % change number of DEATH	Mean % change all-age DEATH rate	Mean % change age-standardized DEATH rate
1 Ischemic heart disease	1 Hypertensive heart disease	19.3 (-16.7 to 56.7)	**64.4 (15.3 to 115)**	9.8 (-27.4 to 55.2)
2 Stroke	2 Ischemic heart disease	**-42.4 (-57.2 to -24.1)**	-20.7 (-42 to 4.85)	**-47.3 (-64.7 to -21.9)**
3 Hypertensive heart disease	3 Stroke	**-43.9 (-53.7 to -33)**	**-22.7 (-35.7 to -7.54)**	**-48.6 (-60.1 to -35.5)**
4 COVID-19	4 Colorectal cancer	1.51 (-30.9 to 44.4)	**39.5 (0.143 to 101)**	3.62 (-22 to 40.2)
5 Lung cancer	5 Lung cancer	-19.4 (-40.7 to 3.9)	10.9 (-14.3 to 38.2)	-10.2 (-30.6 to 10.4)
6 Colorectal cancer	6 Alzheimer's disease	28.4 (-5.3 to 63.1)	**76.6 (39.1 to 116)**	1.97 (-0.569 to 4.43)
7 Cirrhosis liver	7 Chronic kidney disease	25 (-7.93 to 59.9)	**72.1 (28.8 to 119)**	23.1 (-13 to 62.1)
8 Alzheimer's disease	8 Cirrhosis liver	**-21.3 (-34.7 to -7.95)**	8.42 (-6.2 to 23.3)	-0.945 (-14.9 to 14.9)
9 Chronic kidney disease	9 COPD	-1.74 (-21.7 to 24)	**35.3 (9.69 to 66.8)**	-6.55 (-28.6 to 19.8)
10 Diabetes	10 Pancreatic cancer	24.9 (-15 to 71.9)	**71.6 (24.5 to 136)**	33.9 (-0.593 to 76.9)
11 COPD	11 Diabetes	**-26.3 (-42.3 to -7.15)**	1.52 (-18.3 to 24.5)	**-28.2 (-44 to -6.92)**
12 Lower respiratory infect	12 Breast cancer	-3.81 (-29.1 to 29.5)	**32.2 (0.892 to 71.8)**	1.72 (-17.1 to 26.3)
13 Breast cancer	13 Lower respiratory infect	-8.49 (-24.7 to 11.7)	**26 (7.9 to 48.7)**	**-19.7 (-33.5 to -6.33)**
14 Stomach cancer	14 Prostate cancer	10.7 (-16.3 to 45.9)	**52.3 (19.4 to 96.2)**	7.42 (-9.12 to 28.2)
15 Pancreatic cancer	15 Atrial fibrillation	29.1 (-1.7 to 70.3)	**77.4 (41.9 to 124)**	10.7 (-2.78 to 27.6)
16 Other cardiovascular	16 Brain cancer	**25.1 (7.31 to 43.5)**	**72.2 (56 to 90.5)**	**42 (30.6 to 53.2)**
17 Prostate cancer	17 Other cardiovascular	**-20.5 (-35.9 to -4.53)**	9.43 (-8.56 to 29.1)	**-19.8 (-31.7 to -6.49)**
18 Parkinson's disease	18 Parkinson's disease	10.9 (-9.8 to 36.2)	**52.6 (25.4 to 82)**	1.29 (-13.5 to 16.3)
19 Brain cancer	19 Stomach cancer	**-36.4 (-47.1 to -23.6)**	**-12.5 (-23.8 to -0.0669)**	**-33.3 (-39.8 to -26.4)**
20 Self-harm	20 Bladder cancer	-4.98 (-24.3 to 19.3)	**30.7 (8.69 to 55.9)**	-4.61 (-16.8 to 7.06)
21 Atrial fibrillation	21 Self-harm			
22 Bladder cancer	129 COVID-19			

Supplemental Results Figure S8. Leading 20 level 3 causes of deaths, and percentage change in number of deaths and all-age and age-standardised death rates by location, 2022 and 2050

Croatia, Reference scenario

Legend:
Communicable, maternal, neonatal and nutritional
Non-communicable
Injuries

Leading Causes 2022	Leading Causes 2050	Mean % change number of DEATH	Mean % change all-age DEATH rate	Mean % change age-standardized DEATH rate
1 Ischemic heart disease	1 Ischemic heart disease	-22.5 (-46.3 to 3.89)	10.3 (-21.2 to 48.2)	**-48.1 (-64.8 to -25.3)**
2 Stroke	2 Stroke	**-33.1 (-46.5 to -14.4)**	-4.84 (-23.3 to 18.3)	**-52 (-61.5 to -39.7)**
3 COVID-19	3 Alzheimer's disease	**68.5 (31.6 to 105)**	**140 (99.2 to 181)**	**4.88 (2.33 to 7.75)**
4 Lung cancer	4 Lung cancer	8.13 (-17.6 to 38.3)	**53.7 (23.5 to 96.5)**	-0.9 (-18.8 to 23.9)
5 Colorectal cancer	5 Colorectal cancer	27.8 (-12.3 to 79.7)	**81.8 (24.5 to 150)**	1.62 (-26.5 to 33.9)
6 Alzheimer's disease	6 COPD	**40 (4.53 to 79.3)**	**99.4 (55.3 to 154)**	0.832 (-22.7 to 28.7)
7 COPD	7 Chronic kidney disease	**78.4 (25.3 to 138)**	**154 (85 to 234)**	27.5 (-6.71 to 73.1)
8 Diabetes	8 Hypertensive heart disease	**58.1 (8.44 to 135)**	**125 (52.7 to 230)**	4.51 (-35.2 to 62.5)
9 Hypertensive heart disease	9 Falls	**38 (1.63 to 79.2)**	**96.2 (57.4 to 150)**	-7.7 (-22.4 to 10.1)
10 Chronic kidney disease	10 Diabetes	-0.733 (-27.2 to 29.2)	**41.3 (7.38 to 80)**	**-26.8 (-44 to -5.51)**
11 Falls	11 Prostate cancer	**48.8 (9.35 to 92.8)**	**112 (62.5 to 171)**	12.9 (-4.87 to 35)
12 Cirrhosis liver	12 Pancreatic cancer	**55.4 (8.61 to 110)**	**121 (62.7 to 188)**	**31.9 (0.405 to 67.5)**
13 Breast cancer	13 Urinary diseases	**104 (49.8 to 176)**	**190 (116 to 282)**	**41.3 (7.46 to 85.4)**
14 Prostate cancer	14 Lower respiratory infect	24.7 (-1.03 to 54.7)	**77.5 (45.1 to 117)**	**-16.4 (-29.5 to -0.445)**
15 Lower respiratory infect	15 Breast cancer	13.1 (-20.6 to 52.3)	**60.7 (19.9 to 111)**	-7.87 (-26 to 14.5)
16 Pancreatic cancer	16 Brain cancer	**55.1 (28 to 86.4)**	**121 (93.7 to 155)**	**34 (23.3 to 45.5)**
17 Cardiomyopathy	17 Cardiomyopathy	10.7 (-14.4 to 41.9)	**57.5 (28.1 to 91.4)**	**-17.9 (-29.7 to -3.39)**
18 Stomach cancer	18 Cirrhosis liver	**-23.4 (-37.9 to -8.27)**	9.01 (-4.54 to 23.7)	**-21.9 (-31.9 to -11.1)**
19 Self-harm	19 Nonrheum valv diseases	**58.3 (17.6 to 107)**	**125 (75.2 to 185)**	10.7 (-10 to 31.8)
20 Urinary diseases	20 Parkinson's disease	**38.3 (10.2 to 69.7)**	**96.8 (64.6 to 133)**	1.41 (-12.5 to 17.3)
21 Brain cancer	23 Stomach cancer			
23 Parkinson's disease	27 Self-harm			
24 Nonrheum valv diseases	130 COVID-19			

Supplemental Results Figure S8. Leading 20 level 3 causes of deaths, and percentage change in number of deaths and all-age and age-standardised death rates by location, 2022 and 2050

Czechia, Reference scenario

Legend:
Communicable, maternal, neonatal and nutritional
Non-communicable
Injuries

Leading Causes 2022	Leading Causes 2050	Mean % change number of DEATH	Mean % change all-age DEATH rate	Mean % change age-standardized DEATH rate
1 Ischemic heart disease	1 Ischemic heart disease	-9.68 (-34.8 to 19.7)	-0.545 (-26.8 to 33.3)	**-50 (-65 to -28.1)**
2 Stroke	2 Alzheimer's disease	**108 (68.6 to 156)**	**128 (93.3 to 169)**	0.0795 (-2.54 to 3.01)
3 Lung cancer	3 Stroke	**-20.3 (-38.2 to -3.39)**	-12.3 (-30 to 5.57)	**-53.4 (-64.5 to -42.1)**
4 COVID-19	4 Lung cancer	16.7 (-8.14 to 48.6)	**28.4 (3.77 to 60.2)**	-10.8 (-27.1 to 7.59)
5 Alzheimer's disease	5 Colorectal cancer	39 (-4.45 to 96)	**52.8 (10.8 to 113)**	-4.52 (-29 to 26.4)
6 Colorectal cancer	6 Diabetes	**33.3 (2.57 to 70.3)**	**46.8 (15.9 to 84.7)**	-18.6 (-37.6 to 4.53)
7 Diabetes	7 Lower respiratory infect	**31.7 (4.42 to 63)**	**44.9 (21.7 to 73.9)**	**-22.7 (-36.7 to -7.23)**
8 Lower respiratory infect	8 COPD	**39.6 (7.59 to 77)**	**53.7 (17.8 to 91.5)**	-7.46 (-30.6 to 16)
9 COPD	9 Pancreatic cancer	**67.3 (19.8 to 127)**	**84.1 (34.1 to 141)**	24.2 (-7.23 to 62.3)
10 Pancreatic cancer	10 Hypertensive heart disease	**78.9 (14.5 to 164)**	**96.9 (29.9 to 189)**	2.91 (-35.1 to 57.6)
11 Cirrhosis liver	11 Chronic kidney disease	**150 (82.3 to 233)**	**175 (109 to 264)**	**46.4 (5.17 to 98.2)**
12 Hypertensive heart disease	12 Prostate cancer	**67.9 (25.8 to 124)**	**84.7 (41.2 to 141)**	9.29 (-9.52 to 34.4)
13 Falls	13 Falls	**55.7 (20.5 to 94.6)**	**71.2 (36.7 to 109)**	-10.5 (-23.1 to 2.61)
14 Breast cancer	14 Breast cancer	26.8 (-4.9 to 68.6)	**39.5 (6.5 to 80.2)**	-12 (-27 to 6.9)
15 Prostate cancer	15 Atrial fibrillation	**114 (68 to 170)**	**136 (91.7 to 190)**	13.5 (-2.22 to 32.3)
16 Chronic kidney disease	16 Cirrhosis liver	-14.7 (-29.2 to 1.12)	-6.14 (-19.3 to 9.01)	**-18.2 (-30.3 to -4.6)**
17 Kidney cancer	17 Urinary diseases	**147 (81.4 to 231)**	**172 (107 to 262)**	**40.4 (8.45 to 90.3)**
18 Self-harm	18 Kidney cancer	39.5 (-1.44 to 96.2)	**53.5 (9.08 to 120)**	2.92 (-24.4 to 44.5)
19 Stomach cancer	19 Parkinson's disease	**60.5 (29.9 to 92.5)**	**76.5 (48.7 to 109)**	0.048 (-12.9 to 19.2)
20 Parkinson's disease	20 Nonrheum valv diseases	**78.5 (38.5 to 134)**	**96.3 (58.2 to 156)**	5.08 (-12.2 to 29.9)
21 Atrial fibrillation	25 Self-harm			
24 Nonrheum valv diseases	27 Stomach cancer			
27 Urinary diseases	128 COVID-19			

Supplemental Results Figure S8. Leading 20 level 3 causes of deaths, and percentage change in number of deaths and all-age and age-standardised death rates by location, 2022 and 2050

Hungary, Reference scenario

Legend:
Communicable, maternal, neonatal and nutritional
Non-communicable
Injuries

Leading Causes 2022	Leading Causes 2050	Mean % change number of DEATH	Mean % change all-age DEATH rate	Mean % change age-standardized DEATH rate
1 Ischemic heart disease	1 Ischemic heart disease	**-27.2 (-48.1 to -1.88)**	**-23.8 (-45 to -1.14)**	**-51.5 (-66.5 to -28.5)**
2 Stroke	2 Hypertensive heart disease	**63.7 (16.7 to 127)**	**71.3 (23.1 to 140)**	14.4 (-23.3 to 71)
3 COVID-19	3 Lung cancer	1.71 (-23.4 to 31.1)	6.34 (-18 to 32)	-10.4 (-28.5 to 10.7)
4 Lung cancer	4 Alzheimer's disease	**89.4 (46.1 to 134)**	**98 (60.2 to 138)**	**5.72 (3.07 to 8.22)**
5 Hypertensive heart disease	5 Stroke	**-33 (-47.3 to -13.1)**	**-29.9 (-43.1 to -14.8)**	**-52.9 (-62.2 to -41.3)**
6 COPD	6 COPD	16 (-12.3 to 48.8)	21.5 (-7.2 to 54.7)	-10.6 (-32 to 18)
7 Colorectal cancer	7 Colorectal cancer	27 (-14.7 to 78.6)	32.6 (-8.39 to 77.5)	1.64 (-26.1 to 31.5)
8 Alzheimer's disease	8 Chronic kidney disease	**89.3 (41.8 to 149)**	**98.1 (50.4 to 153)**	28.2 (-6.37 to 77.2)
9 Cirrhosis liver	9 Pancreatic cancer	**56.6 (8.5 to 115)**	**63.8 (15.2 to 125)**	31.4 (-2.63 to 75.4)
10 Diabetes	10 Cirrhosis liver	-14.3 (-28.4 to 3.38)	-10.4 (-22.7 to 6.45)	**-15.7 (-27.1 to -2.27)**
11 Breast cancer	11 Breast cancer	15.6 (-18.1 to 52.2)	20.8 (-12.6 to 59.9)	-6.91 (-28.6 to 20.4)
12 Pancreatic cancer	12 Diabetes	-7.33 (-26.8 to 17.2)	-2.94 (-21.5 to 23.6)	**-30.5 (-45.5 to -11.4)**
13 Chronic kidney disease	13 Falls	**34.8 (6.53 to 70.9)**	**41 (14.1 to 68.3)**	-6.72 (-17.5 to 8.33)
14 Cardiomyopathy	14 Nonrheum valv diseases	**67.2 (24.7 to 124)**	**74.9 (34.9 to 130)**	17.2 (-2.75 to 47.6)
15 Falls	15 Prostate cancer	**54.8 (12.8 to 101)**	**61.7 (28 to 108)**	18.5 (-1.97 to 40.5)
16 Self-harm	16 Cardiomyopathy	-4.87 (-26.5 to 21.9)	-0.519 (-20 to 26.7)	**-22.6 (-38.5 to -3.88)**
17 Lower respiratory infect	17 Lower respiratory infect	19.6 (-4.5 to 49.2)	**25.1 (2.94 to 51.2)**	**-21.3 (-34.9 to -4.15)**
18 Prostate cancer	18 Atrial fibrillation	**93.8 (47.5 to 153)**	**103 (60.8 to 154)**	**20 (2.04 to 39.1)**
19 Nonrheum valv diseases	19 Parkinson's disease	**49.3 (23.8 to 79)**	**56.2 (36.2 to 78.9)**	9.43 (-4.35 to 25.2)
20 Stomach cancer	20 Self-harm	-11.3 (-27.4 to 8.05)	-7.17 (-19.9 to 8.59)	**-18.6 (-31.4 to -2.15)**
22 Parkinson's disease	24 Stomach cancer			
25 Atrial fibrillation	129 COVID-19			

Supplemental Results Figure S8. Leading 20 level 3 causes of deaths, and percentage change in number of deaths and all-age and age-standardised death rates by location, 2022 and 2050

Montenegro, Reference scenario

Legend:
Communicable, maternal, neonatal and nutritional
Non-communicable
Injuries

Leading Causes 2022

1 Stroke
2 Ischemic heart disease
3 COVID-19
4 Lung cancer
5 Cardiomyopathy
6 Diabetes
7 Colorectal cancer
8 Chronic kidney disease
9 Alzheimer's disease
10 Breast cancer
11 Atrial fibrillation
12 Prostate cancer
13 Pancreatic cancer
14 Hypertensive heart disease
15 Self-harm
16 Aortic aneurysm
17 Stomach cancer
18 Brain cancer
19 Lower respiratory infect
20 Liver cancer
22 Parkinson's disease
24 COPD

Leading Causes 2050	Mean % change number of DEATH	Mean % change all-age DEATH rate	Mean % change age-standardized DEATH rate
1 Stroke	9.28 (-13.5 to 35.1)	**28.8 (6.43 to 55.2)**	**-34 (-49.5 to -15.9)**
2 Ischemic heart disease	-19.1 (-44.7 to 13.3)	-4.71 (-30.9 to 28.7)	**-48.6 (-66 to -24.9)**
3 Lung cancer	13.9 (-17.4 to 47.6)	**34.1 (3.12 to 68.6)**	-9.46 (-28.7 to 12.5)
4 Chronic kidney disease	**95.4 (45.6 to 153)**	**130 (75 to 197)**	28.8 (-6.98 to 74.8)
5 Atrial fibrillation	**114 (62.5 to 170)**	**152 (102 to 216)**	**18.5 (0.0693 to 40.2)**
6 Alzheimer's disease	**90.8 (45.9 to 142)**	**125 (84.3 to 173)**	**3.58 (0.946 to 6.69)**
7 Cardiomyopathy	18.6 (-9.8 to 56.3)	**39.7 (9.25 to 78.9)**	**-23.1 (-36.5 to -6.24)**
8 Colorectal cancer	47 (-3.33 to 111)	**73 (16.9 to 137)**	3.92 (-27.6 to 37)
9 Pancreatic cancer	**83.2 (24 to 150)**	**116 (47.4 to 189)**	36 (-1.37 to 77.6)
10 Hypertensive heart disease	**82.5 (25.5 to 155)**	**115 (46.5 to 217)**	9.83 (-29.6 to 71.2)
11 Breast cancer	33.1 (-8.18 to 80.6)	**56.7 (14.6 to 109)**	0.279 (-22.7 to 30)
12 Prostate cancer	**67.3 (22.8 to 118)**	**97 (50.9 to 151)**	8.28 (-11.3 to 30.7)
13 Diabetes	2.37 (-22.3 to 32.3)	20.6 (-9.02 to 54.4)	**-30 (-47.8 to -9.35)**
14 Brain cancer	**59.1 (34.4 to 91.1)**	**87.4 (70.2 to 110)**	**40.9 (29.9 to 53.4)**
15 Liver cancer	**68.5 (26.8 to 117)**	**98.6 (56.3 to 149)**	**29.4 (0.127 to 65.7)**
16 Parkinson's disease	**75.9 (41.1 to 126)**	**107 (70.9 to 151)**	2.46 (-14.6 to 20.6)
17 Aortic aneurysm	20.7 (-9.74 to 60.4)	**42 (13.1 to 78.5)**	**-15 (-28.6 to -0.162)**
18 Lower respiratory infect	25.7 (-1.66 to 58.6)	**48.1 (19.3 to 79.7)**	**-20.3 (-34.6 to -2.7)**
19 Self-harm	-12.6 (-27.7 to 5.79)	2.98 (-11.3 to 19.1)	-15.4 (-28.3 to 1.13)
20 COPD	**47.8 (6.53 to 93.1)**	**74.3 (32 to 123)**	-3.94 (-30.3 to 28.1)
22 Stomach cancer			
130 COVID-19			

Supplemental Results Figure S8. Leading 20 level 3 causes of deaths, and percentage change in number of deaths and all-age and age-standardised death rates by location, 2022 and 2050

North Macedonia, Reference scenario

Legend:
Communicable, maternal, neonatal and nutritional
Non-communicable
Injuries

Leading Causes 2022	Leading Causes 2050	Mean % change number of DEATH	Mean % change all-age DEATH rate	Mean % change age-standardized DEATH rate
1 COVID-19	1 Stroke	0.902 (-22.7 to 26.5)	**25.5 (1.74 to 50.9)**	**-45.8 (-58.2 to -31.5)**
2 Stroke	2 Ischemic heart disease	4.09 (-25.9 to 40.6)	29.6 (-8.2 to 69.4)	**-40.6 (-61.3 to -12)**
3 Ischemic heart disease	3 Hypertensive heart disease	**129 (51.3 to 223)**	**184 (88.9 to 292)**	15.3 (-30.7 to 72.1)
4 Lung cancer	4 Lung cancer	43.2 (-2.94 to 107)	**77.3 (36.5 to 135)**	3.96 (-16.9 to 34.9)
5 Hypertensive heart disease	5 Diabetes	27 (-13.6 to 71.1)	**58 (12.9 to 104)**	**-22 (-43.3 to -0.0753)**
6 Diabetes	6 Colorectal cancer	**74.3 (6.35 to 162)**	**116 (47.8 to 209)**	11.4 (-19.5 to 53.3)
7 Colorectal cancer	7 COPD	**89.2 (35.7 to 157)**	**135 (78.1 to 200)**	7.95 (-21.2 to 42.6)
8 Cardiomyopathy	8 Alzheimer's disease	**146 (70.5 to 225)**	**206 (145 to 284)**	**16.5 (13 to 19.8)**
9 COPD	9 Cardiomyopathy	38.1 (-6.74 to 89)	**71.5 (31.7 to 128)**	**-21.3 (-35.9 to -1.27)**
10 Stomach cancer	10 Chronic kidney disease	**114 (45.2 to 193)**	**167 (93.1 to 245)**	30.6 (-8.1 to 82.8)
11 Breast cancer	11 Pancreatic cancer	**126 (43.9 to 231)**	**180 (99.1 to 297)**	**51.2 (9.9 to 107)**
12 Chronic kidney disease	12 Breast cancer	48.5 (-1.54 to 113)	**84 (37.8 to 149)**	1.19 (-21.3 to 34.2)
13 Alzheimer's disease	13 Liver cancer	**115 (62.3 to 177)**	**167 (109 to 225)**	**49.5 (13.8 to 100)**
14 Pancreatic cancer	14 Stomach cancer	13.2 (-14.2 to 47.4)	**40.7 (19.1 to 71.5)**	**-25.6 (-34.9 to -13.8)**
15 Liver cancer	15 Prostate cancer	**107 (40.2 to 184)**	**156 (96.9 to 227)**	**23 (1.35 to 45)**
16 Cirrhosis liver	16 Brain cancer	**82.9 (42.8 to 135)**	**127 (103 to 157)**	**42.2 (31.8 to 55.8)**
17 Brain cancer	17 Atrial fibrillation	**171 (80.6 to 272)**	**235 (155 to 331)**	**27.9 (3.2 to 56.5)**
18 Prostate cancer	18 Parkinson's disease	**120 (56.5 to 198)**	**173 (124 to 240)**	11.8 (-5.03 to 32.5)
19 Self-harm	19 Lower respiratory infect	**71.5 (28.9 to 122)**	**113 (71.1 to 169)**	-8.59 (-27.3 to 12.9)
20 Lower respiratory infect	20 Bladder cancer	**78.8 (31.6 to 135)**	**122 (79.5 to 176)**	11.7 (-2.66 to 27.4)
21 Parkinson's disease	21 Cirrhosis liver			
22 Bladder cancer	26 Self-harm			
23 Atrial fibrillation	130 COVID-19			

Supplemental Results Figure S8. Leading 20 level 3 causes of deaths, and percentage change in number of deaths and all-age and age-standardised death rates by location, 2022 and 2050

Poland, Reference scenario

Legend:
Communicable, maternal, neonatal and nutritional
Non-communicable
Injuries

Leading Causes 2022	Leading Causes 2050	Mean % change number of DEATH	Mean % change all-age DEATH rate	Mean % change age-standardized DEATH rate
1 Ischemic heart disease	1 Ischemic heart disease	-15 (-40.1 to 13.3)	0.454 (-27.9 to 32.1)	**-54.6 (-68.6 to -38.8)**
2 Stroke	2 Lung cancer	**41.6 (11 to 74.6)**	**67.4 (35.7 to 107)**	6.12 (-14.8 to 28.2)
3 COVID-19	3 Alzheimer's disease	**114 (82.6 to 151)**	**153 (120 to 185)**	-0.313 (-2.92 to 2.47)
4 Lung cancer	4 Stroke	-19.4 (-34.9 to 0.482)	-4.74 (-22.5 to 18.1)	**-54.9 (-63.3 to -44.4)**
5 Colorectal cancer	5 Colorectal cancer	**51.6 (10.2 to 105)**	**79.3 (30.9 to 139)**	-0.715 (-24.9 to 29.5)
6 Lower respiratory infect	6 Lower respiratory infect	**42.8 (12.4 to 76.1)**	**68.9 (34.7 to 103)**	**-18.2 (-31.9 to -2.85)**
7 Alzheimer's disease	7 Hypertensive heart disease	**88.7 (23.9 to 160)**	**123 (47.9 to 203)**	4.76 (-30.4 to 43.6)
8 Hypertensive heart disease	8 Prostate cancer	**72.1 (33.3 to 122)**	**103 (59.3 to 154)**	8.62 (-7.02 to 28.7)
9 Diabetes	9 Cardiomyopathy	**36.1 (3.42 to 75.2)**	**60.8 (24.2 to 106)**	**-19.3 (-32.4 to -3.4)**
10 Cirrhosis liver	10 Pancreatic cancer	**79.7 (35.7 to 129)**	**113 (59.3 to 177)**	28.5 (-0.167 to 67.6)
11 Cardiomyopathy	11 COPD	**35.9 (1.85 to 77.5)**	**60.5 (22.6 to 101)**	-14.2 (-33.4 to 6.52)
12 COPD	12 Breast cancer	39.3 (-0.554 to 86.3)	**64.7 (20.6 to 119)**	-7.74 (-27 to 17.2)
13 Breast cancer	13 Diabetes	19.9 (-5.98 to 57.7)	**41.8 (13.2 to 75.2)**	**-27.7 (-42.5 to -10.8)**
14 Prostate cancer	14 Chronic kidney disease	**120 (61.4 to 186)**	**160 (91.4 to 246)**	25.5 (-6.71 to 59.3)
15 Pancreatic cancer	15 Atrial fibrillation	**131 (85.5 to 187)**	**173 (122 to 238)**	12.3 (-5.06 to 32.7)
16 Stomach cancer	16 Cirrhosis liver	-4.83 (-18.4 to 9.64)	12.5 (-0.262 to 27.3)	-6.03 (-17.8 to 6.21)
17 Falls	17 Falls	**51.8 (14.6 to 96.3)**	**79.3 (43.4 to 129)**	-11.4 (-24.3 to 1.88)
18 Self-harm	18 Urinary diseases	**155 (87.3 to 238)**	**201 (119 to 292)**	**37.2 (5.9 to 72)**
19 Bladder cancer	19 Bladder cancer	**44.6 (19.7 to 74.4)**	**70.9 (48.4 to 104)**	-6.89 (-17.8 to 6.84)
20 Chronic kidney disease	20 Parkinson's disease	**67.6 (38 to 105)**	**98.1 (68.6 to 134)**	-0.462 (-14.4 to 13.9)
22 Atrial fibrillation	24 Stomach cancer			
24 Parkinson's disease	27 Self-harm			
29 Urinary diseases	129 COVID-19			

Supplemental Results Figure S8. Leading 20 level 3 causes of deaths, and percentage change in number of deaths and all-age and age-standardised death rates by location, 2022 and 2050

Romania, Reference scenario

Legend:
Communicable, maternal, neonatal and nutritional
Non-communicable
Injuries

Leading Causes 2022
1 Ischemic heart disease
2 Stroke
3 Hypertensive heart disease
4 COVID-19
5 Lung cancer
6 Cirrhosis liver
7 Cardiomyopathy
8 Alzheimer's disease
9 Colorectal cancer
10 Lower respiratory infect
11 COPD
12 Breast cancer
13 Stomach cancer
14 Pancreatic cancer
15 Chronic kidney disease
16 Prostate cancer
17 Diabetes
18 Parkinson's disease
19 Road injuries
20 Falls
22 Bladder cancer
24 Brain cancer
26 Atrial fibrillation

Leading Causes 2050	Mean % change number of DEATH	Mean % change all-age DEATH rate	Mean % change age-standardized DEATH rate
1 Ischemic heart disease	**-27.9 (-47 to -2)**	-8.24 (-32.6 to 22.7)	**-49.7 (-65.9 to -23.1)**
2 Stroke	**-32 (-47 to -17.2)**	-13.5 (-28.5 to 1.57)	**-51.2 (-61.8 to -36.3)**
3 Hypertensive heart disease	**55.5 (6.75 to 125)**	**97.9 (43.5 to 184)**	9.18 (-27.2 to 79.2)
4 Alzheimer's disease	**80.6 (29.3 to 133)**	**129 (73 to 184)**	1.78 (-0.93 to 4.4)
5 Lung cancer	-3.74 (-32.6 to 31.3)	22.2 (-8.44 to 60.6)	-8.05 (-29.8 to 18.7)
6 Cardiomyopathy	7.67 (-16.1 to 39.7)	**36.8 (14 to 65.9)**	**-15.3 (-27.4 to -1.57)**
7 Colorectal cancer	24 (-24.1 to 77.4)	**57.3 (3.84 to 122)**	4.79 (-26.6 to 42.2)
8 Cirrhosis liver	-16.6 (-32.8 to 1.5)	6.03 (-7.48 to 23.5)	-10.3 (-23.8 to 6.58)
9 COPD	19.7 (-8.82 to 54.5)	**52.3 (19.5 to 91.7)**	-7.36 (-29.4 to 20.6)
10 Lower respiratory infect	3.06 (-17.3 to 26.7)	**31 (10.9 to 53.1)**	**-20.9 (-34.2 to -4.1)**
11 Pancreatic cancer	**50.7 (0.652 to 107)**	**91.2 (30.5 to 157)**	36 (-4.42 to 75.4)
12 Chronic kidney disease	**53.3 (15.4 to 103)**	**95.1 (46.3 to 160)**	17.7 (-15.1 to 66.5)
13 Breast cancer	11.5 (-25.7 to 53)	**41.6 (3.86 to 90)**	-2.08 (-26.4 to 21.2)
14 Prostate cancer	39.4 (-2.27 to 84)	**76.9 (33.7 to 122)**	9.35 (-10.9 to 30.3)
15 Parkinson's disease	**38.4 (9.99 to 68.6)**	**75.9 (48.1 to 104)**	0.669 (-12.8 to 15.9)
16 Stomach cancer	**-25.8 (-43.2 to -10.6)**	-5.75 (-18.3 to 9.25)	**-34.9 (-41.5 to -26.9)**
17 Atrial fibrillation	**87.1 (31.7 to 151)**	**138 (84.1 to 203)**	15.8 (-1.18 to 34.8)
18 Diabetes	-5.98 (-29.6 to 26.6)	19.6 (-7.09 to 55.3)	**-26.1 (-44.2 to -0.349)**
19 Brain cancer	**36 (12.1 to 62.5)**	**72.9 (56.2 to 93.3)**	**34.1 (24 to 45.2)**
20 Bladder cancer	17 (-13.3 to 47.5)	**48.6 (23.6 to 77.8)**	-4.79 (-17 to 8.54)
21 Falls			
25 Road injuries			
130 COVID-19			

Supplemental Results Figure S8. Leading 20 level 3 causes of deaths, and percentage change in number of deaths and all-age and age-standardised death rates by location, 2022 and 2050

Serbia, Reference scenario

Legend:
Communicable, maternal, neonatal and nutritional
Non-communicable
Injuries

Leading Causes 2022	Leading Causes 2050	Mean % change number of DEATH	Mean % change all-age DEATH rate	Mean % change age-standardized DEATH rate
1 Ischemic heart disease	1 Ischemic heart disease	**-30.7 (-50.7 to -8.07)**	-10.9 (-35.1 to 18.2)	**-45.9 (-63.5 to -18.7)**
2 Stroke	2 Stroke	**-36.7 (-51.1 to -20.2)**	**-18.8 (-32.7 to -0.651)**	**-50.1 (-60.9 to -36.8)**
3 COVID-19	3 Lung cancer	10 (-19.1 to 39.9)	**41.3 (9.3 to 79.5)**	-0.51 (-20.9 to 25.5)
4 Lung cancer	4 Hypertensive heart disease	51.6 (-0.95 to 109)	**94.7 (33.9 to 161)**	16.7 (-24.9 to 73)
5 Hypertensive heart disease	5 Alzheimer's disease	**63.7 (21.5 to 113)**	**110 (60.9 to 163)**	**16.8 (13.6 to 20.5)**
6 Diabetes	6 Colorectal cancer	30.6 (-14.7 to 83.7)	**67.7 (17.2 to 124)**	11.1 (-18.2 to 44.5)
7 Colorectal cancer	7 Chronic kidney disease	**59.6 (16 to 107)**	**105 (57 to 164)**	29 (-8.23 to 74.5)
8 COPD	8 COPD	23 (-6.34 to 57.4)	**58 (21.8 to 96.1)**	0.381 (-24.6 to 29.1)
9 Alzheimer's disease	9 Diabetes	-15.7 (-36.1 to 13.7)	8.2 (-15 to 36.7)	**-30.2 (-46.5 to -5.62)**
10 Cardiomyopathy	10 Breast cancer	15.8 (-13.1 to 53.3)	**48.8 (17 to 86.7)**	-0.0366 (-18.7 to 23)
11 Chronic kidney disease	11 Pancreatic cancer	**71.4 (13.7 to 138)**	**120 (61.5 to 196)**	**49.1 (12.4 to 96.9)**
12 Breast cancer	12 Cardiomyopathy	-1.54 (-22.6 to 24.7)	**26.5 (3.17 to 56.9)**	**-19.7 (-32.5 to -5.07)**
13 Pancreatic cancer	13 Prostate cancer	**38.5 (3.96 to 73.1)**	**78 (40.8 to 119)**	14.6 (-4.01 to 34.4)
14 Self-harm	14 Brain cancer	**53.2 (28.9 to 80.2)**	**96.8 (77.3 to 118)**	**38.8 (26.4 to 52.7)**
15 Prostate cancer	15 Lower respiratory infect	11.6 (-11.3 to 42.2)	**43.2 (20.5 to 72.3)**	-12.4 (-25.5 to 5.88)
16 Cirrhosis liver	16 Atrial fibrillation	**76.3 (34 to 133)**	**126 (82.1 to 184)**	**30.6 (12.2 to 52.9)**
17 Lower respiratory infect	17 Nonrheum valv diseases	**38.3 (5.84 to 80.4)**	**77.6 (46.5 to 122)**	9.16 (-7.58 to 28.7)
18 Stomach cancer	18 Liver cancer	**46.8 (15.4 to 84.8)**	**88.6 (50.5 to 131)**	**31.4 (4.79 to 65.8)**
19 Nonrheum valv diseases	19 Cirrhosis liver	-7.4 (-24.3 to 13)	**18.9 (3.57 to 34.6)**	-11.1 (-22.6 to 1.33)
20 Brain cancer	20 Self-harm	-11.6 (-28.3 to 6.44)	13.6 (-1.77 to 32.3)	-13.8 (-26.3 to 3.08)
21 Liver cancer	23 Stomach cancer			
24 Atrial fibrillation	130 COVID-19			

Supplemental Results Figure S8. Leading 20 level 3 causes of deaths, and percentage change in number of deaths and all-age and age-standardised death rates by location, 2022 and 2050

Slovakia, Reference scenario

Legend:
Communicable, maternal, neonatal and nutritional
Non-communicable
Injuries

Leading Causes 2022	Leading Causes 2050	Mean % change number of DEATH	Mean % change all-age DEATH rate	Mean % change age-standardized DEATH rate
1 Ischemic heart disease	1 Ischemic heart disease	3.61 (-24.2 to 37.7)	12.8 (-14 to 46.6)	**-45.9 (-61.6 to -20.9)**
2 Stroke	2 Stroke	-7.89 (-25.9 to 11.4)	0.317 (-17.2 to 19.9)	**-48.1 (-57.6 to -36.2)**
3 COVID-19	3 Alzheimer's disease	**151 (96.9 to 211)**	**173 (117 to 232)**	**9.26 (6.39 to 12.4)**
4 Lung cancer	4 Colorectal cancer	**60.4 (8.74 to 121)**	**74.4 (21.9 to 131)**	3.7 (-23.4 to 32.5)
5 Colorectal cancer	5 Lung cancer	29.2 (-2.44 to 61.7)	**40.6 (9.04 to 70)**	-6.28 (-25.8 to 13.2)
6 Alzheimer's disease	6 Lower respiratory infect	**73.5 (37.2 to 117)**	**88.9 (52.7 to 136)**	-8.05 (-23.5 to 11.3)
7 Lower respiratory infect	7 Hypertensive heart disease	**111 (49.9 to 201)**	**130 (68 to 233)**	16.4 (-17.3 to 77.4)
8 Cirrhosis liver	8 Chronic kidney disease	**133 (76.8 to 210)**	**154 (95.1 to 239)**	**35.5 (0.571 to 85.2)**
9 Hypertensive heart disease	9 Pancreatic cancer	**91.5 (34.1 to 153)**	**108 (50.5 to 176)**	**32 (0.257 to 69.5)**
10 Breast cancer	10 COPD	**72.6 (33.6 to 115)**	**88 (46.4 to 135)**	2.09 (-20.3 to 32.9)
11 Pancreatic cancer	11 Prostate cancer	**100 (58.1 to 155)**	**118 (72.9 to 170)**	**20.7 (0.426 to 41.6)**
12 COPD	12 Cirrhosis liver	-2.11 (-16.7 to 15.2)	6.59 (-7.99 to 21)	-13.1 (-26 to 0.398)
13 Diabetes	13 Breast cancer	**40 (4.05 to 86)**	**52.3 (17.3 to 96.4)**	-4.85 (-22.8 to 17.8)
14 Chronic kidney disease	14 Atrial fibrillation	**159 (94.4 to 229)**	**182 (116 to 248)**	**23.6 (6.39 to 41.8)**
15 Falls	15 Falls	**58.4 (20.1 to 97.7)**	**72.4 (37.4 to 115)**	-7.74 (-19.8 to 6.2)
16 Stomach cancer	16 Diabetes	16.4 (-12.4 to 49.7)	26.7 (-2.77 to 58.8)	**-28.3 (-44.2 to -7.02)**
17 Prostate cancer	17 Stomach cancer	6.51 (-13.8 to 30.9)	15.9 (-2.57 to 37.1)	**-30.2 (-38.6 to -20.3)**
18 Self-harm	18 Parkinson's disease	**97.4 (59.6 to 139)**	**115 (79.2 to 151)**	8.35 (-6.16 to 24.1)
19 Atrial fibrillation	19 Kidney cancer	**53.9 (4.35 to 115)**	**67.4 (16.9 to 122)**	9.44 (-19.8 to 42.5)
20 Kidney cancer	20 Brain cancer	**73 (49.8 to 97.7)**	**88.4 (69.6 to 108)**	**37.4 (26.1 to 48.4)**
23 Brain cancer	23 Self-harm			
25 Parkinson's disease	129 COVID-19			

Supplemental Results Figure S8. Leading 20 level 3 causes of deaths, and percentage change in number of deaths and all-age and age-standardised death rates by location, 2022 and 2050

Slovenia, Reference scenario

Legend:
Communicable, maternal, neonatal and nutritional
Non-communicable
Injuries

Leading Causes 2022	Leading Causes 2050	Mean % change number of DEATH	Mean % change all-age DEATH rate	Mean % change age-standardized DEATH rate
1 Ischemic heart disease	1 Alzheimer's disease	**106 (74.2 to 143)**	**102 (77.9 to 131)**	**-3.34 (-5.69 to -0.0539)**
2 Stroke	2 Ischemic heart disease	-7.26 (-37.8 to 28.3)	-8.82 (-38.1 to 25.2)	**-52.8 (-66.8 to -35.5)**
3 COVID-19	3 Hypertensive heart disease	**99.7 (24.5 to 200)**	**96.3 (22.2 to 191)**	-3.31 (-38.7 to 51.2)
4 Lung cancer	4 Nonrheum valv diseases	**139 (69.4 to 225)**	**135 (68.6 to 220)**	15.5 (-11.3 to 51.6)
5 Alzheimer's disease	5 Lung cancer	26.5 (-3.38 to 63.3)	24.3 (-1.24 to 54)	-12.1 (-27.8 to 6.76)
6 Hypertensive heart disease	6 Stroke	-15.8 (-33.2 to 5.07)	-17.3 (-34.2 to 2.91)	**-56.5 (-65.2 to -45.5)**
7 Colorectal cancer	7 Colorectal cancer	**52.5 (2.77 to 117)**	**49.8 (3.64 to 110)**	-10.6 (-33.9 to 18.7)
8 Falls	8 Falls	**79.7 (37.1 to 128)**	**76.4 (42 to 115)**	-10.7 (-24 to 3.69)
9 Nonrheum valv diseases	9 COPD	**83 (35.1 to 147)**	**79.9 (33.8 to 141)**	-1.4 (-25.2 to 31)
10 Cardiomyopathy	10 Prostate cancer	**95 (49.8 to 155)**	**91.5 (48.8 to 145)**	9.69 (-10.2 to 31.7)
11 COPD	11 Lower respiratory infect	**72.1 (34.1 to 113)**	**69.2 (34.5 to 108)**	-14.3 (-28 to 5.89)
12 Lower respiratory infect	12 Cardiomyopathy	**48.6 (6.43 to 100)**	**46 (7.87 to 93.9)**	**-25.8 (-38.7 to -7.1)**
13 Prostate cancer	13 Chronic kidney disease	**140 (73.1 to 217)**	**136 (72.9 to 215)**	21 (-10.4 to 68)
14 Breast cancer	14 Pancreatic cancer	**83 (29.8 to 153)**	**79.7 (28.1 to 141)**	20.2 (-9.37 to 56.3)
15 Diabetes	15 Breast cancer	31.8 (-5.68 to 90)	29.5 (-7.39 to 79.6)	-20.1 (-36.4 to 0.647)
16 Cirrhosis liver	16 Atrial fibrillation	**136 (84.3 to 205)**	**131 (86.9 to 193)**	12.9 (-4.68 to 34.9)
17 Pancreatic cancer	17 Diabetes	8.27 (-17.1 to 39.1)	6.48 (-19.7 to 37.3)	**-38.7 (-52.8 to -21)**
18 Self-harm	18 Parkinson's disease	**71.2 (41.2 to 109)**	**68.2 (43.6 to 99.6)**	-3.62 (-16.6 to 11.8)
19 Chronic kidney disease	19 Cirrhosis liver	-8.12 (-21.2 to 5.89)	-9.7 (-21.1 to 2.2)	**-28.5 (-37.5 to -18.9)**
20 Stomach cancer	20 Bladder cancer	**57.3 (24.3 to 96.7)**	**54.5 (28.6 to 90.3)**	-8.42 (-20.3 to 6.89)
22 Parkinson's disease	23 Self-harm			
23 Bladder cancer	24 Stomach cancer			
24 Atrial fibrillation	129 COVID-19			

Supplemental Results Figure S8. Leading 20 level 3 causes of deaths, and percentage change in number of deaths and all-age and age-standardised death rates by location, 2022 and 2050

Belarus, Reference scenario

Legend:
Communicable, maternal, neonatal and nutritional
Non-communicable
Injuries

Leading Causes 2022	Leading Causes 2050	Mean % change number of DEATH	Mean % change all-age DEATH rate	Mean % change age-standardized DEATH rate
1 Ischemic heart disease	1 Ischemic heart disease	-10.2 (-39.1 to 24.5)	1.74 (-31.4 to 37.6)	**-45 (-67 to -11.8)**
2 COVID-19	2 Stroke	-20.7 (-41.4 to 1.47)	-10.2 (-31.4 to 12)	**-49.4 (-63 to -28.4)**
3 Stroke	3 Alzheimer's disease	**140 (46.8 to 237)**	**170 (82.9 to 260)**	**16.3 (13.3 to 19.8)**
4 Lung cancer	4 Colorectal cancer	53.3 (-16.4 to 147)	**72.9 (0.335 to 165)**	10.1 (-25.7 to 61.1)
5 Alzheimer's disease	5 Lung cancer	20.2 (-26.5 to 71.9)	35.6 (-8.15 to 84.9)	-0.375 (-26.8 to 32.9)
6 Colorectal cancer	6 Alcohol use disorders	22.2 (-20.3 to 70.1)	38.5 (-7.58 to 97.9)	7.47 (-28 to 59.1)
7 Alcohol use disorders	7 Pancreatic cancer	**86.4 (4.28 to 186)**	**110 (29.2 to 213)**	45 (-4.87 to 107)
8 Stomach cancer	8 Prostate cancer	**84.6 (16.5 to 166)**	**108 (41.3 to 188)**	28 (-1.81 to 62.5)
9 Cirrhosis liver	9 COPD	**57.8 (13.2 to 110)**	**78.7 (27.7 to 137)**	9.49 (-21.7 to 50.9)
10 Self-harm	10 Stomach cancer	-4.66 (-27.6 to 16.8)	7.83 (-10.7 to 25.2)	**-26.7 (-34.6 to -19.7)**
11 Breast cancer	11 Self-harm	-5.84 (-25 to 16)	6.63 (-12.5 to 26.6)	-7.3 (-26.3 to 14.9)
12 Cardiomyopathy	12 Breast cancer	28.2 (-21.6 to 90.4)	44.6 (-4.83 to 101)	-1.08 (-27.2 to 28.4)
13 COPD	13 Cirrhosis liver	-9.49 (-26 to 11.7)	2.53 (-14.4 to 21.2)	-11.5 (-26.3 to 7.76)
14 Falls	14 Atrial fibrillation	**156 (67.2 to 267)**	**188 (96.9 to 286)**	**30.1 (11.3 to 53)**
15 Pancreatic cancer	15 Urinary diseases	**121 (57.5 to 197)**	**150 (93.9 to 219)**	**47.9 (12.2 to 95.4)**
16 Prostate cancer	16 Parkinson's disease	**104 (45.9 to 156)**	**131 (81.6 to 185)**	**19.7 (0.994 to 39.9)**
17 Lower respiratory infect	17 Falls	12.2 (-16.4 to 42.9)	**26.9 (1.48 to 53.5)**	-6.62 (-24.8 to 13)
18 Road injuries	18 Peripheral artery	**94.5 (19.9 to 190)**	**119 (39.5 to 211)**	21.2 (-5.29 to 52.5)
19 Parkinson's disease	19 Vascular intestinal	**87.9 (18.1 to 159)**	**112 (43 to 172)**	12.7 (-5.94 to 32.3)
20 Urinary diseases	20 Kidney cancer	51.9 (-14.3 to 134)	**71.3 (0.763 to 159)**	23.7 (-22.4 to 76.5)
22 Peripheral artery	21 Cardiomyopathy			
23 Atrial fibrillation	23 Lower respiratory infect			
24 Kidney cancer	28 Road injuries			
28 Vascular intestinal	129 COVID-19			

Estonia, Reference scenario

Legend:
Communicable, maternal, neonatal and nutritional
Non-communicable
Injuries

Leading Causes 2022	Leading Causes 2050	Mean % change number of DEATH	Mean % change all-age DEATH rate	Mean % change age-standardized DEATH rate
1 Ischemic heart disease	1 Hypertensive heart disease	39.6 (-7.14 to 90.6)	**51.4 (1.45 to 109)**	-9.28 (-42.6 to 36.7)
2 Hypertensive heart disease	2 Ischemic heart disease	**-32.8 (-53.8 to -8.18)**	-27.2 (-48.3 to 0.93)	**-60.2 (-73.7 to -38.7)**
3 COVID-19	3 Alzheimer's disease	**70 (34.8 to 111)**	**84.1 (47.7 to 125)**	-2.48 (-5.08 to 0.542)
4 Stroke	4 Chronic kidney disease	**91.6 (35.9 to 154)**	**108 (47.9 to 186)**	22.4 (-16 to 72.1)
5 Alzheimer's disease	5 Colorectal cancer	34.3 (-15.6 to 96.7)	45.3 (-5.02 to 111)	-3.77 (-32 to 29.7)
6 Lung cancer	6 Lung cancer	7.03 (-22.9 to 42.3)	15.9 (-15.2 to 51.1)	-17.5 (-37.5 to 5.38)
7 Chronic kidney disease	7 Stroke	**-43.7 (-57.5 to -28.4)**	**-39 (-52.1 to -23.3)**	**-63.6 (-71.5 to -52.6)**
8 Colorectal cancer	8 Prostate cancer	**62.8 (20 to 119)**	**76.4 (33.4 to 133)**	15.8 (-6.13 to 39.9)
9 Prostate cancer	9 Pancreatic cancer	**56.9 (9.11 to 126)**	**69.9 (19.1 to 139)**	18.8 (-13.1 to 58.4)
10 Diabetes	10 Atrial fibrillation	**74.2 (30.3 to 137)**	**88.6 (44.1 to 147)**	0.631 (-14.9 to 19.6)
11 Pancreatic cancer	11 Urinary diseases	**101 (54.8 to 153)**	**118 (71.8 to 173)**	**29.9 (2.02 to 65.1)**
12 Cirrhosis liver	12 Breast cancer	11.6 (-21.6 to 63)	20.9 (-13.4 to 69.5)	-17 (-36.2 to 7.87)
13 Stomach cancer	13 COPD	35.5 (-1.75 to 76.6)	**46.8 (8.57 to 91.4)**	-6.39 (-30.5 to 26.4)
14 Breast cancer	14 Cirrhosis liver	-13.6 (-27 to 1.24)	-6.34 (-19.4 to 9.01)	**-19.7 (-32.4 to -5.82)**
15 Cardiomyopathy	15 Diabetes	-21.1 (-39.5 to 1.36)	-14.4 (-33.2 to 11)	**-44.6 (-57 to -28.3)**
16 Lower respiratory infect	16 Lower respiratory infect	10.7 (-9.24 to 34.7)	**19.9 (0.195 to 44)**	**-24.4 (-37.8 to -9.35)**
17 Self-harm	17 Parkinson's disease	**47 (19 to 82.7)**	**59.2 (35.6 to 91.3)**	1.94 (-13.2 to 19.5)
18 Atrial fibrillation	18 Stomach cancer	**-18.7 (-32.6 to -4.54)**	-12 (-23.8 to 1.26)	**-38.7 (-45.2 to -32.1)**
19 COPD	19 Cardiomyopathy	-12.1 (-29 to 9.35)	-4.79 (-20.5 to 16.7)	**-31.9 (-43.9 to -17.1)**
20 Alcohol use disorders	20 Self-harm	-8.6 (-24.8 to 9.26)	-0.994 (-15.6 to 15.7)	**-20.4 (-33.8 to -5.79)**
21 Parkinson's disease	28 Alcohol use disorders			
22 Urinary diseases	129 COVID-19			

Supplemental Results Figure S8. Leading 20 level 3 causes of deaths, and percentage change in number of deaths and all-age and age-standardised death rates by location, 2022 and 2050

Latvia, Reference scenario

Legend:
Communicable, maternal, neonatal and nutritional
Non-communicable
Injuries

Leading Causes 2022	Leading Causes 2050	Mean % change number of DEATH	Mean % change all-age DEATH rate	Mean % change age-standardized DEATH rate
1 Ischemic heart disease	1 Ischemic heart disease	**-31.9 (-52.9 to -9.59)**	-6.1 (-36 to 25.1)	**-50.4 (-67.6 to -27.9)**
2 Stroke	2 Stroke	**-38 (-51.9 to -22.2)**	-14.5 (-35.9 to 7.23)	**-53.5 (-64.6 to -41)**
3 COVID-19	3 Alzheimer's disease	**68.7 (29 to 109)**	**132 (85.1 to 190)**	**7.59 (4.22 to 10.7)**
4 Alzheimer's disease	4 Hypertensive heart disease	40.8 (-7.11 to 99.8)	**94.2 (25.5 to 181)**	12.5 (-27.1 to 73.6)
5 Hypertensive heart disease	5 Lung cancer	0.224 (-25.2 to 31.1)	**37.9 (6.13 to 75.9)**	-4.33 (-23.2 to 20.4)
6 Lung cancer	6 Colorectal cancer	17.6 (-24.4 to 73.6)	**61.7 (6.3 to 128)**	2.12 (-25.2 to 39.7)
7 Cardiomyopathy	7 Prostate cancer	**46.2 (5.49 to 91.9)**	**101 (54.1 to 155)**	**24 (3.18 to 48.7)**
8 Colorectal cancer	8 Cardiomyopathy	**-25 (-41.7 to -7.13)**	3.23 (-12.6 to 25.6)	**-21.7 (-35.1 to -4.96)**
9 Diabetes	9 Pancreatic cancer	44.1 (-5.35 to 108)	**98.2 (38.1 to 172)**	31.5 (-3.47 to 77.3)
10 Prostate cancer	10 Chronic kidney disease	**69.8 (25.7 to 129)**	**134 (70.6 to 217)**	28.8 (-9.1 to 78.9)
11 Pancreatic cancer	11 Urinary diseases	**66.1 (30.5 to 112)**	**129 (84.7 to 185)**	**29.8 (5.02 to 56.9)**
12 Breast cancer	12 Breast cancer	1.87 (-28.6 to 39.7)	**40.2 (3.02 to 92.7)**	-8.6 (-27.1 to 14.9)
13 Stomach cancer	13 Atrial fibrillation	**88.4 (38.1 to 146)**	**159 (100 to 236)**	**24.6 (5.91 to 48.3)**
14 Cirrhosis liver	14 Diabetes	**-25.2 (-44.2 to -0.411)**	2.98 (-21.5 to 30.7)	**-38.7 (-52 to -20.8)**
15 Self-harm	15 COPD	18.5 (-8.95 to 51)	**63.4 (22 to 106)**	2.45 (-23.3 to 27.5)
16 Lower respiratory infect	16 Lower respiratory infect	-2.1 (-23 to 20.7)	**34.9 (8.69 to 59.5)**	**-16.1 (-31.7 to -0.279)**
17 Chronic kidney disease	17 Cirrhosis liver	**-22.4 (-34.8 to -10.6)**	6.87 (-8.74 to 21.7)	-5.74 (-18.1 to 8.28)
18 COPD	18 Stomach cancer	**-26.1 (-38.8 to -11.4)**	1.73 (-12.9 to 19.5)	**-32.3 (-39.2 to -23.2)**
19 Urinary diseases	19 Parkinson's disease	**36.1 (6.11 to 71.9)**	**87.4 (54.3 to 132)**	8.36 (-6.79 to 26)
20 Falls	20 Falls	7.41 (-14.5 to 37.4)	**47.9 (18.8 to 80.7)**	-10.1 (-20.9 to 4.01)
21 Atrial fibrillation	22 Self-harm			
22 Parkinson's disease	129 COVID-19			

Supplemental Results Figure S8. Leading 20 level 3 causes of deaths, and percentage change in number of deaths and all-age and age-standardised death rates by location, 2022 and 2050

Lithuania, Reference scenario

Legend:
Communicable, maternal, neonatal and nutritional
Non-communicable
Injuries

Leading Causes 2022	Leading Causes 2050	Mean % change number of DEATH	Mean % change all-age DEATH rate	Mean % change age-standardized DEATH rate
1 Ischemic heart disease	1 Ischemic heart disease	**-28.4 (-53.2 to -0.63)**	1.76 (-29.3 to 35.5)	**-50 (-67.4 to -24)**
2 COVID-19	2 Stroke	**-34.8 (-51 to -19)**	-7.37 (-27 to 16.1)	**-51.1 (-62.1 to -37.6)**
3 Stroke	3 Alzheimer's disease	**87.1 (45.8 to 138)**	**166 (112 to 217)**	**19.4 (16.2 to 22.7)**
4 Alzheimer's disease	4 Lung cancer	6.02 (-24 to 42.8)	**50.7 (10.3 to 101)**	3.36 (-21.1 to 31.6)
5 Lung cancer	5 Colorectal cancer	33.4 (-16.1 to 93.6)	**89.6 (25.6 to 165)**	11.7 (-22 to 47.8)
6 Colorectal cancer	6 Hypertensive heart disease	**65 (9.9 to 136)**	**134 (57.4 to 224)**	30.6 (-15.5 to 94.1)
7 Cirrhosis liver	7 Pancreatic cancer	**63.7 (7.67 to 131)**	**133 (57.7 to 228)**	**48.7 (5.47 to 95)**
8 Self-harm	8 Prostate cancer	**54.7 (9.59 to 113)**	**120 (60.9 to 195)**	**27.8 (3.13 to 53.6)**
9 Hypertensive heart disease	9 COPD	**37 (0.071 to 76)**	**94.8 (49 to 167)**	9.98 (-16.3 to 43.8)
10 Prostate cancer	10 Atrial fibrillation	**121 (64.6 to 193)**	**214 (140 to 298)**	**42.1 (19.6 to 69.5)**
11 Pancreatic cancer	11 Breast cancer	14.5 (-18.5 to 56.2)	**62.8 (20.5 to 116)**	0.289 (-20.8 to 24)
12 Stomach cancer	12 Cirrhosis liver	**-16.9 (-31.7 to -1.04)**	**17.9 (3.97 to 33)**	1.1 (-13.2 to 17)
13 Breast cancer	13 Self-harm	**-20.4 (-36.7 to -3.25)**	13 (-4.6 to 32.6)	-5.82 (-23.7 to 13.7)
14 COPD	14 Chronic kidney disease	**76.1 (23.7 to 135)**	**150 (81.5 to 246)**	37.4 (-2.63 to 90.9)
15 Diabetes	15 Falls	14.5 (-12.1 to 48.2)	**62.5 (30.7 to 102)**	-4.7 (-18.5 to 10.2)
16 Cardiomyopathy	16 Stomach cancer	-17 (-31.2 to 0.731)	**17.9 (1.21 to 37.5)**	**-25.6 (-33.1 to -17.3)**
17 Falls	17 Urinary diseases	**85.1 (42.2 to 140)**	**163 (110 to 244)**	**42.1 (12.8 to 82)**
18 Lower respiratory infect	18 Parkinson's disease	**57.6 (21.6 to 92.2)**	**124 (82.3 to 171)**	**18.3 (2.04 to 37.1)**
19 Chronic kidney disease	19 Vascular intestinal	**50 (12.2 to 91.1)**	**113 (64.3 to 172)**	12.3 (-2.87 to 30.8)
20 Atrial fibrillation	20 Lower respiratory infect	2.12 (-17.2 to 26.9)	**45 (21.2 to 72)**	-12.1 (-26.6 to 8.23)
21 Vascular intestinal	21 Diabetes			
22 Parkinson's disease	22 Cardiomyopathy			
27 Urinary diseases	129 COVID-19			

Supplemental Results Figure S8. Leading 20 level 3 causes of deaths, and percentage change in number of deaths and all-age and age-standardised death rates by location, 2022 and 2050

Moldova, Reference scenario

Legend:
Communicable, maternal, neonatal and nutritional
Non-communicable
Injuries

Leading Causes 2022	Leading Causes 2050	Mean % change number of DEATH	Mean % change all-age DEATH rate	Mean % change age-standardized DEATH rate
1 Ischemic heart disease	1 Ischemic heart disease	-6.64 (-39.5 to 45.9)	46.1 (-10.2 to 120)	**-50.5 (-70.7 to -15.5)**
2 Stroke	2 Stroke	-18.2 (-40.7 to 7.9)	27.8 (-4.01 to 69.5)	**-49.9 (-63.3 to -29.1)**
3 Cirrhosis liver	3 Alzheimer's disease	**194 (103 to 278)**	**359 (226 to 470)**	**-2.68 (-5.3 to -0.0639)**
4 Hypertensive heart disease	4 Hypertensive heart disease	**75.9 (6.75 to 183)**	**175 (68 to 337)**	3.33 (-38.3 to 81.7)
5 COVID-19	5 Cirrhosis liver	-9.61 (-29.2 to 13.9)	**41 (17.2 to 69.2)**	-10.1 (-25.2 to 10.8)
6 Alzheimer's disease	6 Colorectal cancer	30.4 (-24.4 to 94.7)	**103 (21.3 to 202)**	-3.11 (-38.4 to 37.8)
7 Colorectal cancer	7 Lung cancer	12.9 (-29.8 to 64.3)	**75.9 (14.6 to 143)**	-5 (-34.1 to 27.7)
8 Lung cancer	8 COPD	**67.7 (21.6 to 129)**	**162 (84.2 to 257)**	-0.74 (-29.7 to 48.2)
9 Lower respiratory infect	9 Pancreatic cancer	66.4 (-3.73 to 153)	**159 (55.8 to 270)**	32.3 (-15.4 to 86.4)
10 COPD	10 Lower respiratory infect	-13.2 (-34.7 to 19.1)	**35.3 (8.39 to 72.7)**	-22.2 (-38.6 to 5.37)
11 Breast cancer	11 Breast cancer	6.53 (-32.1 to 52.5)	**65.7 (9.95 to 126)**	-12.6 (-36.1 to 15.9)
12 Diabetes	12 Urinary diseases	**81.3 (32.6 to 133)**	**183 (117 to 268)**	20.8 (-6.39 to 67.9)
13 Self-harm	13 Atrial fibrillation	**149 (84.2 to 230)**	**289 (184 to 380)**	3.74 (-9.79 to 19.7)
14 Stomach cancer	14 Prostate cancer	**58.2 (4.63 to 120)**	**147 (70 to 227)**	8.13 (-17.9 to 32.3)
15 Pancreatic cancer	15 Diabetes	-9.76 (-36.5 to 31.5)	41.1 (-0.0934 to 102)	**-37.5 (-55.4 to -6.6)**
16 Cardiomyopathy	16 Stomach cancer	-19.1 (-36.9 to 3.86)	**26.1 (7.1 to 50.7)**	**-34.4 (-42.2 to -25.4)**
17 Prostate cancer	17 Vascular intestinal	**67.6 (14.7 to 127)**	**161 (88.4 to 240)**	-6.49 (-21.2 to 8.33)
18 Alcohol use disorders	18 Self-harm	**-31.4 (-51.2 to -10.2)**	6.59 (-12.7 to 32.7)	-10.3 (-27.7 to 14.3)
19 Road injuries	19 Liver cancer	**81.3 (25.4 to 153)**	**183 (101 to 298)**	37.2 (-2.46 to 110)
20 Pancreatitis	20 Parkinson's disease	**80.9 (39.4 to 125)**	**182 (131 to 244)**	-1.14 (-14.1 to 11.1)
21 Urinary diseases	21 Pancreatitis			
24 Vascular intestinal	25 Cardiomyopathy			
25 Atrial fibrillation	27 Alcohol use disorders			
32 Parkinson's disease	28 Road injuries			
33 Liver cancer	129 COVID-19			

Supplemental Results Figure S8. Leading 20 level 3 causes of deaths, and percentage change in number of deaths and all-age and age-standardised death rates by location, 2022 and 2050

Russia, Reference scenario

Legend:
Communicable, maternal, neonatal and nutritional
Non-communicable
Injuries

Leading Causes 2022	Leading Causes 2050	Mean % change number of DEATH	Mean % change all-age DEATH rate	Mean % change age-standardized DEATH rate
1 Ischemic heart disease	1 Ischemic heart disease	-11.5 (-39.3 to 17.6)	-5.81 (-33.1 to 19.9)	**-51.3 (-67.5 to -30.9)**
2 Stroke	2 Stroke	-16.2 (-33.5 to 2.46)	-10.8 (-27.9 to 5.99)	**-54.1 (-63.7 to -41.8)**
3 COVID-19	3 Alzheimer's disease	**148 (85.3 to 225)**	**163 (103 to 227)**	-0.117 (-3.21 to 3.01)
4 Lung cancer	4 Colorectal cancer	35.8 (-11.7 to 93.1)	44.2 (-2.07 to 97)	-11.3 (-34.2 to 18.7)
5 Cardiomyopathy	5 Lung cancer	12 (-18.2 to 48.4)	19 (-8.85 to 51.2)	-15.4 (-32.7 to 3.94)
6 Alzheimer's disease	6 COPD	**63.9 (21.9 to 105)**	**74.2 (32.4 to 111)**	-2.04 (-26.4 to 26.8)
7 Cirrhosis liver	7 Urinary diseases	**154 (88.4 to 231)**	**170 (107 to 250)**	**42.9 (10.4 to 79.5)**
8 Colorectal cancer	8 Cardiomyopathy	**-20.3 (-34.9 to -1.45)**	-15.2 (-28.7 to 2.01)	**-30.9 (-42 to -16.6)**
9 Diabetes	9 Cirrhosis liver	-13.7 (-27.4 to 1.7)	-8.18 (-18.1 to 5.17)	**-19.1 (-30.4 to -5.68)**
10 Self-harm	10 Diabetes	4.03 (-23.4 to 38.1)	10.7 (-16.6 to 37.7)	**-35.9 (-51.7 to -14.1)**
11 COPD	11 Pancreatic cancer	**78.8 (19 to 156)**	**89.9 (32.3 to 160)**	27.4 (-5.58 to 68.9)
12 Stomach cancer	12 Chronic kidney disease	**122 (51.6 to 202)**	**136 (65.6 to 204)**	**41.8 (1.48 to 95.7)**
13 Breast cancer	13 Self-harm	-10.8 (-27.5 to 9.01)	-5.12 (-19.3 to 10.4)	-15 (-29.6 to 3.45)
14 Lower respiratory infect	14 Hypertensive heart disease	**102 (33.7 to 176)**	**115 (43.5 to 194)**	18.8 (-22.7 to 76.4)
15 Pancreatic cancer	15 Lower respiratory infect	20.8 (-2.97 to 49.3)	**28.5 (9.17 to 49.8)**	**-17 (-31.1 to -1.22)**
16 Alcohol use disorders	16 Breast cancer	18.1 (-15.1 to 66.1)	25.5 (-6.62 to 70.1)	-14.3 (-31.5 to 9.31)
17 HIV/AIDS	17 Stomach cancer	-3.68 (-21.3 to 18.1)	2.41 (-13.1 to 18.9)	**-32.8 (-40.9 to -24.7)**
18 Urinary diseases	18 Prostate cancer	**91.4 (42.5 to 152)**	**103 (56.1 to 157)**	19.8 (-1.09 to 44.8)
19 Interpersonal violence	19 Atrial fibrillation	**157 (89.7 to 242)**	**173 (110 to 250)**	13.3 (-0.548 to 34.8)
20 Road injuries	20 Vascular intestinal	**79.3 (34.3 to 135)**	**90.6 (48.6 to 146)**	-0.579 (-16.2 to 16.8)
21 Prostate cancer	21 Alcohol use disorders			
22 Hypertensive heart disease	27 Road injuries			
23 Chronic kidney disease	29 HIV/AIDS			
24 Vascular intestinal	35 Interpersonal violence			
29 Atrial fibrillation	129 COVID-19			

Supplemental Results Figure S8. Leading 20 level 3 causes of deaths, and percentage change in number of deaths and all-age and age-standardised death rates by location, 2022 and 2050

Ukraine, Reference scenario

Legend:
Communicable, maternal, neonatal and nutritional
Non-communicable
Injuries

Leading Causes 2022	Leading Causes 2050	Mean % change number of DEATH	Mean % change all-age DEATH rate	Mean % change age-standardized DEATH rate
1 Ischemic heart disease	1 Ischemic heart disease	-5.98 (-37.2 to 36.4)	29.1 (-11.5 to 82.1)	-36.3 (-61.7 to 7.19)
2 Stroke	2 Stroke	-19.2 (-44 to 7.6)	10.7 (-14.1 to 38.6)	**-43.2 (-58.7 to -21.8)**
3 COVID-19	3 Alzheimer's disease	**102 (15.4 to 235)**	**175 (63 to 309)**	9.93 (-0.446 to 14.2)
4 Alzheimer's disease	4 Colorectal cancer	21.4 (-27.9 to 108)	**65.1 (3.52 to 154)**	-1 (-32.1 to 42.5)
5 Cirrhosis liver	5 Cardiomyopathy	-6.73 (-40.3 to 41.6)	27.2 (-10.7 to 72.7)	-18.2 (-33 to 1.11)
6 Cardiomyopathy	6 Lung cancer	-1.23 (-40.2 to 50)	34.6 (-8.99 to 89.9)	-12.9 (-38.4 to 18.4)
7 Lung cancer	7 Cirrhosis liver	**-23.4 (-39.8 to -1.3)**	5.35 (-12.2 to 26.1)	-9.77 (-26 to 8.44)
8 Colorectal cancer	8 COPD	**46.8 (1.6 to 110)**	**101 (48.4 to 173)**	6.25 (-24.5 to 44.2)
9 Self-harm	9 Hypertensive heart disease	**72.7 (16.6 to 148)**	**138 (59.1 to 234)**	30 (-21.6 to 105)
10 Stomach cancer	10 Breast cancer	7.69 (-33.1 to 66)	**46.9 (4.56 to 105)**	-5.91 (-28.2 to 24.7)
11 Breast cancer	11 Self-harm	-19.8 (-40.2 to 2.46)	10.1 (-10.8 to 29.6)	-3.7 (-23.4 to 19.4)
12 COPD	12 Pancreatic cancer	47.7 (-10.2 to 142)	**101 (31.9 to 196)**	28.8 (-12.8 to 84.5)
13 Lower respiratory infect	13 Atrial fibrillation	**124 (28.6 to 253)**	**205 (89.4 to 326)**	**27 (7.06 to 50.7)**
14 HIV/AIDS	14 Prostate cancer	59.4 (-2.41 to 145)	**117 (42.8 to 212)**	23.2 (-5.39 to 61.8)
15 Hypertensive heart disease	15 Stomach cancer	-18.9 (-41.9 to 4.87)	11 (-6.92 to 27.9)	**-29.3 (-37.8 to -22)**
16 Pancreatic cancer	16 Peripheral artery	88.1 (-1.61 to 237)	**155 (38.9 to 305)**	16.3 (-10.5 to 48.2)
17 Alcohol use disorders	17 Parkinson's disease	**79.1 (21.9 to 148)**	**145 (86.8 to 215)**	**20.3 (5.01 to 40.7)**
18 Road injuries	18 Lower respiratory infect	-23.1 (-42.7 to 0.569)	5.65 (-14.5 to 27.3)	-19.1 (-37.1 to 0.0935)
19 Prostate cancer	19 Alcohol use disorders	-14.5 (-39.6 to 25.1)	17.9 (-21.2 to 69.1)	14.4 (-23.5 to 63.1)
20 Interpersonal violence	20 Vascular intestinal	52.5 (-0.211 to 130)	**108 (57.6 to 179)**	9.13 (-6.04 to 26.1)
21 Atrial fibrillation	21 Road injuries			
22 Parkinson's disease	22 HIV/AIDS			
23 Peripheral artery	37 Interpersonal violence			
26 Vascular intestinal	129 COVID-19			

Supplemental Results Figure S8. Leading 20 level 3 causes of deaths, and percentage change in number of deaths and all-age and age-standardised death rates by location, 2022 and 2050

Australia, Reference scenario

Legend:
Communicable, maternal, neonatal and nutritional
Non-communicable
Injuries

Leading Causes 2022	Leading Causes 2050	Mean % change number of DEATH	Mean % change all-age DEATH rate	Mean % change age-standardized DEATH rate
1 Ischemic heart disease	1 Alzheimer's disease	**138 (115 to 167)**	**78.1 (61.8 to 96.5)**	-2.76 (-6.87 to 2.02)
2 Alzheimer's disease	2 Ischemic heart disease	20.3 (-4.42 to 53.4)	-10.1 (-29.2 to 15)	**-49.8 (-62 to -33.6)**
3 Stroke	3 COPD	**90.2 (60.2 to 126)**	**42 (18.6 to 68.8)**	-12.4 (-26.5 to 5.97)
4 Lung cancer	4 Chronic kidney disease	**242 (178 to 328)**	**155 (112 to 216)**	**39.6 (13.2 to 82.4)**
5 COPD	5 Lung cancer	**61.8 (29.3 to 97.5)**	20.8 (-0.863 to 45.2)	-12.4 (-26.2 to 2.86)
6 COVID-19	6 Stroke	**25.4 (8.69 to 44.6)**	-6.34 (-18.3 to 6.87)	**-45.7 (-53.3 to -36.2)**
7 Colorectal cancer	7 Colorectal cancer	**97.6 (52.1 to 150)**	**47.5 (15.6 to 84.9)**	-2.77 (-22.6 to 16.9)
8 Chronic kidney disease	8 Atrial fibrillation	**199 (159 to 238)**	**123 (95.3 to 148)**	**20.9 (10.1 to 31.9)**
9 Lower respiratory infect	9 Lower respiratory infect	**126 (94.2 to 166)**	**68.8 (46.5 to 97.4)**	-12.6 (-25.1 to 0.836)
10 Prostate cancer	10 Falls	**124 (92.8 to 160)**	**67.5 (44.5 to 93.4)**	-8.44 (-18.8 to 3.29)
11 Falls	11 Prostate cancer	**110 (70.5 to 159)**	**57 (29.9 to 88.4)**	-3.63 (-16 to 10.4)
12 Diabetes	12 Pancreatic cancer	**137 (87.6 to 190)**	**76.7 (39.6 to 113)**	**26 (2.1 to 48.4)**
13 Pancreatic cancer	13 Diabetes	**75.3 (37.6 to 114)**	**30.9 (3.27 to 60.5)**	**-22.7 (-39.3 to -4.66)**
14 Atrial fibrillation	14 Breast cancer	**74.5 (43.1 to 113)**	**30.2 (8.15 to 57)**	-7.8 (-20.8 to 8.19)
15 Breast cancer	15 Urinary diseases	**260 (206 to 329)**	**169 (128 to 218)**	**42 (21.4 to 70.4)**
16 Self-harm	16 Nonrheum valv diseases	**204 (163 to 251)**	**127 (98.8 to 158)**	**21 (9.21 to 33.1)**
17 Parkinson's disease	17 Parkinson's disease	**100 (75.9 to 124)**	**49.3 (34.1 to 66.2)**	-5.02 (-12.2 to 4.72)
18 Liver cancer	18 Liver cancer	**91.3 (65.1 to 118)**	**42.8 (24.6 to 62.5)**	13.5 (-0.305 to 30.3)
19 Leukemia	19 Endo/metab/blood/immune	**145 (105 to 188)**	**82.8 (50.4 to 115)**	**17 (2.44 to 32.3)**
20 Lymphoma	20 Leukemia	**79.7 (49 to 110)**	**34.1 (12.9 to 52.7)**	-8.19 (-19.2 to 2.12)
22 Nonrheum valv diseases	21 Lymphoma			
25 Endo/metab/blood/immune	22 Self-harm			
29 Urinary diseases	129 COVID-19			

Supplemental Results Figure S8. Leading 20 level 3 causes of deaths, and percentage change in number of deaths and all-age and age-standardised death rates by location, 2022 and 2050

New Zealand, Reference scenario

Legend:
Communicable, maternal, neonatal and nutritional
Non-communicable
Injuries

Leading Causes 2022	Leading Causes 2050	Mean % change number of DEATH	Mean % change all-age DEATH rate	Mean % change age-standardized DEATH rate
1 Ischemic heart disease	1 Ischemic heart disease	27.9 (-4.44 to 61)	-4.13 (-25.7 to 20.5)	**-46.1 (-59.6 to -29.8)**
2 Stroke	2 Alzheimer's disease	**153 (123 to 183)**	**89.6 (70.6 to 107)**	0.356 (-4.64 to 5.38)
3 Alzheimer's disease	3 COPD	**110 (71.2 to 149)**	**57.6 (29.6 to 85.7)**	-3.45 (-20.2 to 17.7)
4 COPD	4 Colorectal cancer	**108 (56.3 to 172)**	**56.2 (18.6 to 103)**	-0.461 (-21.3 to 23.7)
5 Lung cancer	5 Stroke	**26.3 (6.74 to 46.8)**	-5.32 (-18.4 to 7.93)	**-46.1 (-54.1 to -36.2)**
6 Colorectal cancer	6 Lung cancer	**65.7 (34.5 to 100)**	**24.2 (3.1 to 51.3)**	-8.04 (-21.9 to 8.2)
7 COVID-19	7 Chronic kidney disease	**197 (137 to 260)**	**123 (78.9 to 164)**	**28.2 (1.17 to 58)**
8 Lower respiratory infect	8 Lower respiratory infect	**136 (103 to 170)**	**76.9 (54.4 to 104)**	**-13.4 (-25.1 to -0.563)**
9 Chronic kidney disease	9 Atrial fibrillation	**203 (165 to 242)**	**127 (100 to 153)**	**17.8 (7.34 to 29.3)**
10 Prostate cancer	10 Prostate cancer	**119 (74.2 to 170)**	**64.3 (33.3 to 102)**	-1.46 (-14.7 to 15.4)
11 Breast cancer	11 Nonrheum valv diseases	**221 (170 to 277)**	**141 (104 to 178)**	**25.9 (12.5 to 45)**
12 Atrial fibrillation	12 Falls	**132 (93.7 to 170)**	**74.1 (48.2 to 102)**	-8.48 (-18.4 to 3.34)
13 Falls	13 Pancreatic cancer	**137 (88.9 to 199)**	**77.5 (40.7 to 117)**	**26.2 (2.28 to 52.3)**
14 Diabetes	14 Breast cancer	**68.8 (37.1 to 104)**	**26.5 (4.5 to 49.9)**	-11.4 (-24.5 to 3.48)
15 Pancreatic cancer	15 Diabetes	**93.3 (51.7 to 150)**	**44.9 (13.8 to 82.6)**	-13.9 (-32.8 to 8.82)
16 Self-harm	16 Parkinson's disease	**123 (96.2 to 153)**	**67 (49.5 to 86.2)**	0.189 (-7.99 to 9.29)
17 Nonrheum valv diseases	17 Urinary diseases	**275 (205 to 357)**	**181 (139 to 237)**	**47.1 (26.1 to 76.8)**
18 Parkinson's disease	18 Melanoma	**105 (83.1 to 127)**	**53.8 (39.4 to 70)**	6.51 (-1.47 to 15.9)
19 Lymphoma	19 Lymphoma	**81.4 (48.1 to 116)**	**36 (12.9 to 58.5)**	-6.17 (-19.5 to 8.3)
20 Stomach cancer	20 Leukemia	**81.1 (56.1 to 116)**	**35.8 (16.8 to 57)**	-8.71 (-18.5 to 3.66)
23 Leukemia	24 Self-harm			
24 Melanoma	34 Stomach cancer			
35 Urinary diseases	127 COVID-19			

Supplemental Results Figure S8. Leading 20 level 3 causes of deaths, and percentage change in number of deaths and all-age and age-standardised death rates by location, 2022 and 2050

Brunei, Reference scenario

Legend:
Communicable, maternal, neonatal and nutritional
Non-communicable
Injuries

Leading Causes 2022	Leading Causes 2050	Mean % change number of DEATH	Mean % change all-age DEATH rate	Mean % change age-standardized DEATH rate
1 Ischemic heart disease	1 Chronic kidney disease	**355 (263 to 481)**	**356 (265 to 477)**	22.9 (-6.02 to 65.9)
2 Stroke	2 Ischemic heart disease	**67.3 (24 to 120)**	**68.1 (24.7 to 127)**	**-48.2 (-62.5 to -25.4)**
3 Diabetes	3 COPD	**413 (317 to 519)**	**415 (327 to 535)**	15.3 (-8.9 to 48.2)
4 COVID-19	4 Stroke	**111 (73 to 159)**	**112 (74.4 to 152)**	**-42.6 (-54 to -27.3)**
5 Chronic kidney disease	5 Lower respiratory infect	**327 (253 to 429)**	**329 (261 to 407)**	-5.75 (-24.2 to 14.7)
6 Lung cancer	6 Diabetes	**106 (48.5 to 175)**	**107 (49.2 to 180)**	**-36.1 (-54.9 to -9.48)**
7 Lower respiratory infect	7 Lung cancer	**266 (164 to 383)**	**267 (182 to 363)**	11.5 (-10.7 to 37.2)
8 COPD	8 Colorectal cancer	**222 (119 to 346)**	**222 (129 to 324)**	5.29 (-21.4 to 37.9)
9 Colorectal cancer	9 Alzheimer's disease	**490 (372 to 627)**	**492 (389 to 602)**	3.16 (-2.2 to 9.06)
10 Road injuries	10 Hypertensive heart disease	**420 (307 to 551)**	**422 (321 to 537)**	16.9 (-10.5 to 52.3)
11 Breast cancer	11 Urinary diseases	**557 (426 to 690)**	**559 (443 to 684)**	**50.4 (27 to 87.4)**
12 Liver cancer	12 Liver cancer	**258 (196 to 334)**	**259 (201 to 336)**	**25.4 (3.71 to 57.9)**
13 Alzheimer's disease	13 Pancreatic cancer	**363 (237 to 517)**	**364 (260 to 503)**	**46.8 (17.3 to 85.7)**
14 Stomach cancer	14 Endo/metab/blood/immune	**218 (157 to 285)**	**219 (169 to 275)**	**27.3 (10.3 to 47.4)**
15 Cirrhosis liver	15 Atrial fibrillation	**511 (394 to 649)**	**513 (420 to 625)**	**29.2 (16.2 to 45.1)**
16 Hypertensive heart disease	16 Prostate cancer	**437 (291 to 594)**	**439 (318 to 584)**	**29 (6.79 to 52.9)**
17 Endo/metab/blood/immune	17 Breast cancer	**86 (36.3 to 143)**	**86.4 (44.3 to 126)**	-12 (-30.7 to 8.12)
18 Congenital defects	18 Stomach cancer	**113 (78.9 to 155)**	**114 (86.4 to 146)**	**-28.4 (-33.9 to -21.6)**
19 Neonatal disorders	19 Lymphoma	**196 (111 to 285)**	**197 (116 to 267)**	3.87 (-19.3 to 26.2)
20 Cardiomyopathy	20 Parkinson's disease	**415 (321 to 517)**	**416 (351 to 484)**	**14.1 (2.89 to 25.8)**
21 Lymphoma	21 Cardiomyopathy			
22 Urinary diseases	22 Cirrhosis liver			
24 Pancreatic cancer	24 Road injuries			
32 Prostate cancer	53 Congenital defects			
34 Atrial fibrillation	65 Neonatal disorders			
37 Parkinson's disease	130 COVID-19			

Supplemental Results Figure S8. Leading 20 level 3 causes of deaths, and percentage change in number of deaths and all-age and age-standardised death rates by location, 2022 and 2050

Japan, Reference scenario

Legend:
Communicable, maternal, neonatal and nutritional
Non-communicable
Injuries

Leading Causes 2022	Leading Causes 2050	Mean % change number of DEATH	Mean % change all-age DEATH rate	Mean % change age-standardized DEATH rate
1 Alzheimer's disease	1 Alzheimer's disease	**38.6 (22.7 to 54.1)**	**62.4 (48.1 to 74.8)**	**-9.31 (-13.7 to -4.39)**
2 Stroke	2 Lower respiratory infect	**26.3 (8.34 to 47.3)**	**48 (27.6 to 66.4)**	**-12.4 (-23.6 to -1.47)**
3 Ischemic heart disease	3 Chronic kidney disease	**94 (53.8 to 135)**	**127 (79.6 to 172)**	**29.6 (5.5 to 57.1)**
4 Lower respiratory infect	4 Lung cancer	10.7 (-10.1 to 35.9)	**29.7 (6.61 to 57.4)**	-12.7 (-25.5 to 1.08)
5 Lung cancer	5 Stroke	**-29.9 (-40.7 to -19.3)**	**-17.8 (-28.8 to -6.39)**	**-45.6 (-52.9 to -37.4)**
6 Colorectal cancer	6 Colorectal cancer	22.5 (-2.96 to 50.6)	**43.5 (14.2 to 74.2)**	-6.81 (-24.7 to 13)
7 Stomach cancer	7 Ischemic heart disease	**-36.3 (-49.9 to -19.4)**	**-25.3 (-40.6 to -5.47)**	**-55.8 (-65.7 to -43.9)**
8 Chronic kidney disease	8 Pancreatic cancer	**50 (20.2 to 83.8)**	**75.8 (43.4 to 109)**	**23.5 (1.46 to 44.9)**
9 Pancreatic cancer	9 Stomach cancer	**-15.3 (-24.4 to -4.44)**	-0.788 (-9.69 to 9.46)	**-35.8 (-40.2 to -30.1)**
10 COPD	10 Liver cancer	**41.8 (21.8 to 65)**	**66.2 (43.5 to 92.4)**	**25.5 (11.1 to 41.1)**
11 Liver cancer	11 Nonrheum valv diseases	**97.3 (66.4 to 134)**	**131 (95.4 to 179)**	**23 (8.7 to 38.4)**
12 Interstitial lung disease	12 COPD	**24.8 (1.79 to 47.2)**	**46.2 (23.5 to 72.6)**	-12.7 (-26.2 to 1.68)
13 Aortic aneurysm	13 Interstitial lung disease	**34.2 (17 to 54.4)**	**57.2 (40 to 75.6)**	8.17 (-2.02 to 19.1)
14 Nonrheum valv diseases	14 Urinary diseases	**126 (85.6 to 181)**	**164 (123 to 230)**	**41.7 (22.5 to 69.2)**
15 Cirrhosis liver	15 Aortic aneurysm	8.77 (-6.63 to 25.1)	**27.5 (11.2 to 45.8)**	**-18.5 (-27.4 to -9.25)**
16 Gallbladder cancer	16 Hypertensive heart disease	**47.9 (12 to 94.9)**	**73.4 (31.9 to 126)**	-0.202 (-21.3 to 26.8)
17 Self-harm	17 Parkinson's disease	**21.7 (9.97 to 36.1)**	**42.6 (31 to 57.2)**	-0.0456 (-7.51 to 8.69)
18 Parkinson's disease	18 Atrial fibrillation	**59.6 (40.2 to 82.4)**	**87.1 (67.1 to 108)**	**13.7 (4.35 to 23.8)**
19 COVID-19	19 Cirrhosis liver	0.764 (-9.53 to 11.5)	**18.1 (7.68 to 29.2)**	-2.53 (-10.4 to 7.36)
20 Breast cancer	20 Prostate cancer	**36.8 (9.02 to 69.5)**	**60.4 (28 to 98.5)**	5.08 (-9.16 to 20.4)
22 Prostate cancer	22 Gallbladder cancer			
23 Hypertensive heart disease	25 Self-harm			
26 Atrial fibrillation	26 Breast cancer			
27 Urinary diseases	128 COVID-19			

Supplemental Results Figure S8. Leading 20 level 3 causes of deaths, and percentage change in number of deaths and all-age and age-standardised death rates by location, 2022 and 2050

South Korea, Reference scenario

Legend:
Communicable, maternal, neonatal and nutritional
Non-communicable
Injuries

Leading Causes 2022	Leading Causes 2050	Mean % change number of DEATH	Mean % change all-age DEATH rate	Mean % change age-standardized DEATH rate
1 Stroke	1 Alzheimer's disease	**246 (206 to 290)**	**277 (242 to 316)**	**-17.3 (-21.7 to -12.5)**
2 Alzheimer's disease	2 Lower respiratory infect	**227 (173 to 291)**	**256 (204 to 311)**	**-19.1 (-30.8 to -6.88)**
3 Ischemic heart disease	3 Stroke	**50.2 (29.6 to 72)**	**63.5 (41.5 to 84.6)**	**-55.7 (-61.8 to -49.9)**
4 Lung cancer	4 Lung cancer	**105 (67.3 to 144)**	**123 (86.1 to 164)**	**-22.4 (-32.2 to -11.1)**
5 COVID-19	5 Chronic kidney disease	**319 (243 to 398)**	**355 (271 to 440)**	13.4 (-5.49 to 35.6)
6 Lower respiratory infect	6 Ischemic heart disease	**37.8 (8.98 to 66.9)**	**50 (19.2 to 81.1)**	**-62.6 (-70.4 to -53.7)**
7 Liver cancer	7 COPD	**211 (154 to 279)**	**238 (181 to 304)**	**-20.5 (-33.1 to -5.71)**
8 Self-harm	8 Liver cancer	**132 (97.5 to 167)**	**152 (120 to 192)**	11.9 (-1.48 to 25.6)
9 Stomach cancer	9 Colorectal cancer	**166 (109 to 231)**	**189 (131 to 256)**	-6.42 (-22.2 to 12.7)
10 Colorectal cancer	10 Pancreatic cancer	**182 (123 to 242)**	**206 (148 to 267)**	14 (-4.61 to 33)
11 COPD	11 Hypertensive heart disease	**312 (232 to 415)**	**348 (258 to 448)**	-7.08 (-25.3 to 11.6)
12 Diabetes	12 Stomach cancer	**62.2 (40.9 to 84.8)**	**76.4 (56.4 to 97.2)**	**-40.1 (-44.6 to -35.1)**
13 Chronic kidney disease	13 Diabetes	**70 (34.1 to 113)**	**85 (47.2 to 131)**	**-46 (-56.1 to -34.1)**
14 Cirrhosis liver	14 Cirrhosis liver	**59.1 (36.1 to 86.8)**	**73.1 (50.8 to 101)**	**-12.9 (-23.1 to -2.9)**
15 Pancreatic cancer	15 Self-harm	8.77 (-5.87 to 22.4)	**18.4 (5.25 to 31.2)**	**-21.9 (-29.6 to -13.2)**
16 Gallbladder cancer	16 Atrial fibrillation	**333 (279 to 394)**	**371 (321 to 428)**	**9.65 (0.24 to 19.3)**
17 Falls	17 Falls	**173 (130 to 225)**	**198 (148 to 250)**	**-18 (-26.6 to -8.96)**
18 Hypertensive heart disease	18 Parkinson's disease	**204 (165 to 240)**	**231 (198 to 268)**	-5.39 (-14.2 to 2.28)
19 Road injuries	19 Urinary diseases	**423 (317 to 553)**	**470 (357 to 606)**	**23.9 (5.5 to 48.9)**
20 Parkinson's disease	20 Gallbladder cancer	**82.2 (58.3 to 114)**	**98.3 (71.2 to 124)**	**-35.8 (-41.1 to -29.2)**
21 Atrial fibrillation	24 Road injuries			
31 Urinary diseases	132 COVID-19			

Supplemental Results Figure S8. Leading 20 level 3 causes of deaths, and percentage change in number of deaths and all-age and age-standardised death rates by location, 2022 and 2050

Singapore, Reference scenario

Legend:
Communicable, maternal, neonatal and nutritional
Non-communicable
Injuries

Leading Causes 2022	Leading Causes 2050	Mean % change number of DEATH	Mean % change all-age DEATH rate	Mean % change age-standardized DEATH rate
1 Lower respiratory infect	1 Lower respiratory infect	**299 (247 to 354)**	**201 (163 to 245)**	**-16 (-26.3 to -1.96)**
2 Ischemic heart disease	2 Alzheimer's disease	**399 (335 to 470)**	**276 (232 to 316)**	**-6.04 (-10.7 to -1.14)**
3 Alzheimer's disease	3 Ischemic heart disease	**35.6 (3.05 to 78.9)**	2.13 (-20.5 to 31.5)	**-63 (-71.1 to -50.4)**
4 Lung cancer	4 Urinary diseases	**619 (483 to 767)**	**442 (341 to 542)**	**42.4 (15.7 to 71.7)**
5 Stroke	5 Chronic kidney disease	**348 (253 to 478)**	**238 (168 to 330)**	7.15 (-14.6 to 38.3)
6 Colorectal cancer	6 Lung cancer	**179 (121 to 240)**	**110 (68.5 to 159)**	-14.2 (-28.1 to 1.88)
7 Chronic kidney disease	7 Colorectal cancer	**232 (141 to 324)**	**150 (85.7 to 216)**	-1.95 (-23.7 to 21.5)
8 COVID-19	8 Hypertensive heart disease	**316 (215 to 436)**	**214 (144 to 299)**	10.9 (-15.9 to 43.8)
9 Urinary diseases	9 COPD	**305 (225 to 392)**	**205 (151 to 265)**	-8.64 (-23.4 to 10.9)
10 Hypertensive heart disease	10 Stroke	**70.7 (43.8 to 99.9)**	**28.7 (11.7 to 48)**	**-53.3 (-59.7 to -44.2)**
11 COPD	11 Pancreatic cancer	**286 (194 to 379)**	**191 (123 to 256)**	**27.6 (0.459 to 52.8)**
12 Liver cancer	12 Liver cancer	**205 (155 to 264)**	**130 (97.7 to 168)**	12.2 (-4.94 to 31.9)
13 Breast cancer	13 Prostate cancer	**337 (248 to 433)**	**229 (163 to 303)**	10.4 (-6.74 to 26.5)
14 Pancreatic cancer	14 Breast cancer	**111 (63.2 to 157)**	**59.3 (28.1 to 92.7)**	-10 (-26 to 7.24)
15 Self-harm	15 Parkinson's disease	**306 (260 to 357)**	**206 (176 to 242)**	1.15 (-6.69 to 10.6)
16 Stomach cancer	16 Stomach cancer	**126 (96.6 to 155)**	**70.3 (51.9 to 88.7)**	**-34.7 (-40.1 to -29.7)**
17 Prostate cancer	17 Falls	**234 (183 to 291)**	**152 (115 to 189)**	**-14 (-22.6 to -5.97)**
18 Parkinson's disease	18 Lymphoma	**189 (132 to 243)**	**118 (78.6 to 154)**	-6 (-22 to 8.54)
19 Lymphoma	19 Self-harm	**30.1 (11.4 to 49.5)**	-1.95 (-14 to 11.8)	**-25.3 (-36.6 to -13.7)**
20 Falls	20 Aortic aneurysm	**213 (161 to 278)**	**136 (99 to 174)**	**-13.5 (-24.6 to -0.618)**
23 Aortic aneurysm	127 COVID-19			

Supplemental Results Figure S8. Leading 20 level 3 causes of deaths, and percentage change in number of deaths and all-age and age-standardised death rates by location, 2022 and 2050

Canada, Reference scenario

Legend:
Communicable, maternal, neonatal and nutritional
Non-communicable
Injuries

Leading Causes 2022	Leading Causes 2050	Mean % change number of DEATH	Mean % change all-age DEATH rate	Mean % change age-standardized DEATH rate
1 Ischemic heart disease	1 Ischemic heart disease	17 (-8.01 to 44.1)	-9.26 (-28.4 to 12.1)	**-50.4 (-61.7 to -36.3)**
2 Lung cancer	2 Alzheimer's disease	**153 (127 to 180)**	**96.2 (77.4 to 115)**	-3.11 (-7.76 to 1.18)
3 COVID-19	3 Lung cancer	**53.9 (27.1 to 82.9)**	**19.3 (0.297 to 42)**	-10.9 (-22.6 to 2.99)
4 Alzheimer's disease	4 COPD	**101 (66.5 to 136)**	**55.7 (31.3 to 81.4)**	-7.09 (-22 to 8.51)
5 Stroke	5 Chronic kidney disease	**261 (202 to 328)**	**180 (133 to 230)**	**44.7 (19.7 to 76.4)**
6 COPD	6 Stroke	**27.8 (10.6 to 45.7)**	-0.874 (-12.9 to 11.7)	**-44 (-51.4 to -35.5)**
7 Colorectal cancer	7 Colorectal cancer	**92.8 (54.3 to 138)**	**49.5 (20.3 to 84.3)**	-5.63 (-22.6 to 13.7)
8 Lower respiratory infect	8 Lower respiratory infect	**116 (89.1 to 149)**	**67.8 (46.1 to 93)**	**-13.4 (-23.7 to -0.536)**
9 Chronic kidney disease	9 Falls	**134 (98.1 to 169)**	**81.7 (55.1 to 108)**	-7.68 (-18.1 to 1.67)
10 Falls	10 Pancreatic cancer	**125 (82.3 to 180)**	**74.1 (44.4 to 117)**	**25.3 (6.16 to 51.2)**
11 Diabetes	11 Prostate cancer	**129 (89.1 to 183)**	**77.7 (46.8 to 123)**	2.39 (-9.55 to 17.4)
12 Breast cancer	12 Atrial fibrillation	**214 (171 to 252)**	**144 (115 to 171)**	**18.7 (8.88 to 28.6)**
13 Pancreatic cancer	13 Nonrheum valv diseases	**241 (197 to 291)**	**164 (131 to 210)**	**26.5 (12.6 to 40.9)**
14 Prostate cancer	14 Diabetes	**73.1 (38.6 to 114)**	**34.2 (6.21 to 62.9)**	**-23.3 (-37.8 to -8.3)**
15 Cirrhosis liver	15 Breast cancer	**72.3 (44.3 to 105)**	**33.6 (13.4 to 55.1)**	-7.36 (-17.8 to 6.62)
16 Self-harm	16 Urinary diseases	**290 (217 to 366)**	**202 (148 to 254)**	**48.7 (25 to 73.2)**
17 Parkinson's disease	17 Parkinson's disease	**119 (95.7 to 147)**	**69.9 (53.5 to 91.1)**	1.25 (-7.38 to 9.72)
18 Atrial fibrillation	18 Liver cancer	**110 (81.9 to 134)**	**62.5 (43.7 to 82.9)**	**30.8 (16.7 to 45.8)**
19 Drug use disorders	19 Interstitial lung disease	**115 (86.8 to 144)**	**66.7 (44.9 to 87.8)**	5.43 (-5.11 to 15.4)
20 Lymphoma	20 Drug use disorders	**85.9 (57.4 to 119)**	**44.2 (22.6 to 68.4)**	**38.7 (16.2 to 64.3)**
21 Nonrheum valv diseases	22 Lymphoma			
22 Liver cancer	24 Cirrhosis liver			
24 Interstitial lung disease	28 Self-harm			
29 Urinary diseases	128 COVID-19			

Supplemental Results Figure S8. Leading 20 level 3 causes of deaths, and percentage change in number of deaths and all-age and age-standardised death rates by location, 2022 and 2050

Greenland, Reference scenario

Legend:
Communicable, maternal, neonatal and nutritional
Non-communicable
Injuries

Leading Causes 2022	Leading Causes 2050	Mean % change number of DEATH	Mean % change all-age DEATH rate	Mean % change age-standardized DEATH rate
1 Ischemic heart disease	1 Lung cancer	**56.9 (23.3 to 96.9)**	**48.1 (17.3 to 82.3)**	-13.8 (-29.8 to 4.54)
2 Lung cancer	2 COPD	**147 (89.9 to 222)**	**133 (77.4 to 201)**	-13.8 (-30.8 to 9.68)
3 COVID-19	3 Ischemic heart disease	28.9 (-9.38 to 75.9)	21.6 (-12.1 to 64.9)	**-54.7 (-67.9 to -36.2)**
4 Stroke	4 Stroke	**64.6 (24.6 to 106)**	**55.3 (21.3 to 92.8)**	**-49.2 (-59.2 to -37.1)**
5 Self-harm	5 Alzheimer's disease	**423 (305 to 537)**	**394 (285 to 490)**	**-8.16 (-12.4 to -2.85)**
6 COPD	6 Lower respiratory infect	**226 (150 to 296)**	**208 (142 to 269)**	**-15.9 (-30.9 to -2.32)**
7 Colorectal cancer	7 Colorectal cancer	**102 (46.1 to 165)**	**90.8 (36.7 to 146)**	-10.6 (-34 to 14.5)
8 Pancreatic cancer	8 Chronic kidney disease	**272 (172 to 387)**	**251 (160 to 355)**	8.41 (-19.4 to 44.2)
9 Lower respiratory infect	9 Self-harm	-14.7 (-29.3 to 1.21)	**-19.5 (-29.8 to -4.56)**	**-22.3 (-33.9 to -6.25)**
10 Alzheimer's disease	10 Pancreatic cancer	**136 (77.6 to 203)**	**122 (69.9 to 184)**	20.8 (-5.64 to 49.5)
11 Falls	11 Falls	**187 (128 to 248)**	**170 (121 to 221)**	**-15.3 (-23.1 to -7.47)**
12 Alcohol use disorders	12 Atrial fibrillation	**365 (259 to 466)**	**339 (252 to 411)**	4.35 (-3.46 to 15)
13 Esophageal cancer	13 Urinary diseases	**467 (304 to 637)**	**435 (290 to 580)**	**36 (9.68 to 70.2)**
14 Chronic kidney disease	14 Parkinson's disease	**270 (195 to 343)**	**249 (191 to 312)**	-4.59 (-12.4 to 4.22)
15 Cirrhosis liver	15 Esophageal cancer	**47.7 (22.6 to 70)**	**39.4 (22.5 to 56.5)**	**-14.4 (-22.6 to -6)**
16 Cardiomyopathy	16 Nonrheum valv diseases	**354 (249 to 463)**	**328 (234 to 431)**	6.36 (-5.78 to 19.9)
17 Stomach cancer	17 Diabetes	**76.8 (21 to 133)**	**66.9 (16.5 to 118)**	**-28.2 (-47.5 to -6.04)**
18 Breast cancer	18 Prostate cancer	**199 (118 to 292)**	**182 (118 to 258)**	-0.677 (-16.3 to 13.6)
19 Diabetes	19 Liver cancer	**122 (79.7 to 166)**	**110 (75.9 to 148)**	16.3 (-1.84 to 40.5)
20 Interpersonal violence	20 Other digestive	**269 (186 to 366)**	**248 (172 to 328)**	**24.9 (1.97 to 53.5)**
21 Liver cancer	21 Breast cancer			
26 Atrial fibrillation	23 Cardiomyopathy			
27 Parkinson's disease	25 Alcohol use disorders			
29 Prostate cancer	26 Stomach cancer			
36 Urinary diseases	27 Cirrhosis liver			
38 Nonrheum valv diseases	50 Interpersonal violence			
39 Other digestive	127 COVID-19			

Supplemental Results Figure S8. Leading 20 level 3 causes of deaths, and percentage change in number of deaths and all-age and age-standardised death rates by location, 2022 and 2050

USA, Reference scenario

Legend:
Communicable, maternal, neonatal and nutritional
Non-communicable
Injuries

Leading Causes 2022	Leading Causes 2050	Mean % change number of DEATH	Mean % change all-age DEATH rate	Mean % change age-standardized DEATH rate
1 Ischemic heart disease	1 Ischemic heart disease	-5.57 (-22.1 to 12.6)	-11 (-24.5 to 5.79)	**-49 (-57.9 to -37.2)**
2 COVID-19	2 Alzheimer's disease	**118 (94.7 to 142)**	**106 (87.2 to 124)**	2.5 (-1.69 to 6.91)
3 COPD	3 Chronic kidney disease	**160 (123 to 200)**	**145 (113 to 182)**	**42.6 (21.5 to 67.8)**
4 Alzheimer's disease	4 COPD	**63 (40.7 to 88.1)**	**53.6 (33.5 to 77.6)**	-5.11 (-17.6 to 8.88)
5 Stroke	5 Lung cancer	**30 (10.6 to 52.3)**	**22.4 (4.99 to 43.9)**	**-12.1 (-22.8 to -0.169)**
6 Lung cancer	6 Stroke	6.73 (-5.38 to 19.6)	0.569 (-9.34 to 9.23)	**-40.3 (-46.8 to -33.5)**
7 Chronic kidney disease	7 Hypertensive heart disease	**107 (76.5 to 145)**	**95.4 (65.4 to 134)**	**22.8 (2.39 to 50.3)**
8 Colorectal cancer	8 Colorectal cancer	**49.5 (21.5 to 84.3)**	**40.9 (14.4 to 71.8)**	-6.22 (-22.1 to 10.2)
9 Diabetes	9 Pancreatic cancer	**87 (57.1 to 122)**	**76.1 (48.5 to 113)**	**26.5 (10.2 to 45.4)**
10 Drug use disorders	10 Lower respiratory infect	**63.8 (42.1 to 84.7)**	**54.3 (35.8 to 74.1)**	**-10.4 (-20.7 to -0.465)**
11 Hypertensive heart disease	11 Drug use disorders	**47.1 (22.6 to 75.7)**	**38.6 (18 to 62.2)**	**34.8 (12.3 to 61.1)**
12 Cirrhosis liver	12 Atrial fibrillation	**166 (132 to 208)**	**150 (123 to 178)**	**27.7 (16.6 to 37.8)**
13 Lower respiratory infect	13 Falls	**83.9 (58.9 to 111)**	**73.3 (52.3 to 97.2)**	-3.11 (-11.5 to 6.24)
14 Pancreatic cancer	14 Endo/metab/blood/immune	**113 (82 to 147)**	**101 (74.5 to 128)**	**30.2 (17.7 to 43.3)**
15 Breast cancer	15 Prostate cancer	**85.5 (57.2 to 119)**	**74.8 (50.5 to 103)**	5.48 (-5.32 to 17.4)
16 Falls	16 Nonrheum valv diseases	**188 (152 to 235)**	**172 (138 to 209)**	**33.9 (21.1 to 49.5)**
17 Self-harm	17 Diabetes	6.07 (-12.3 to 25.1)	-0.0285 (-16.7 to 18.6)	**-35.7 (-46.1 to -24.9)**
18 Prostate cancer	18 Breast cancer	**41.4 (19.6 to 62.9)**	**33.2 (16.1 to 51.9)**	-6.21 (-16.8 to 4.89)
19 Endo/metab/blood/immune	19 Parkinson's disease	**97.9 (76.1 to 121)**	**86.5 (68.9 to 104)**	7.11 (-1.83 to 15.9)
20 Road injuries	20 Cirrhosis liver	6.1 (-4.88 to 16.3)	-0.0417 (-7.25 to 7.11)	**-12.1 (-19.1 to -6.11)**
21 Parkinson's disease	23 Self-harm			
22 Atrial fibrillation	24 Road injuries			
25 Nonrheum valv diseases	129 COVID-19			

Supplemental Results Figure S8. Leading 20 level 3 causes of deaths, and percentage change in number of deaths and all-age and age-standardised death rates by location, 2022 and 2050

Argentina, Reference scenario

Legend:
Communicable, maternal, neonatal and nutritional
Non-communicable
Injuries

Leading Causes 2022	Leading Causes 2050	Mean % change number of DEATH	Mean % change all-age DEATH rate	Mean % change age-standardized DEATH rate
1 Lower respiratory infect	1 Lower respiratory infect	**67 (41.6 to 98.1)**	**61.7 (43.4 to 82.5)**	**-12.9 (-24.9 to -0.822)**
2 Ischemic heart disease	2 Chronic kidney disease	**138 (91.2 to 196)**	**131 (87.7 to 170)**	**26.9 (0.888 to 53.7)**
3 Stroke	3 Ischemic heart disease	-1.24 (-24.9 to 24.9)	-4.27 (-26.1 to 20.3)	**-47 (-60.5 to -29.2)**
4 COVID-19	4 COPD	**81.5 (48.3 to 119)**	**75.9 (48.4 to 101)**	-3.98 (-20.9 to 12.3)
5 Chronic kidney disease	5 Alzheimer's disease	**108 (73.5 to 148)**	**101 (80.5 to 124)**	1.22 (-3.92 to 6.46)
6 COPD	6 Stroke	2.49 (-15.9 to 22.8)	-0.718 (-13.8 to 13.2)	**-42.9 (-52.2 to -33.6)**
7 Alzheimer's disease	7 Lung cancer	**87.6 (47.6 to 131)**	**81.5 (53.8 to 117)**	7.32 (-7.76 to 26.2)
8 Lung cancer	8 Colorectal cancer	**81.6 (33.2 to 137)**	**75.6 (37 to 123)**	1.38 (-18.2 to 26.2)
9 Colorectal cancer	9 Hypertensive heart disease	**103 (57.9 to 151)**	**96.5 (64.1 to 131)**	4.94 (-16.3 to 27.5)
10 Diabetes	10 Urinary diseases	**175 (125 to 239)**	**166 (129 to 213)**	**41.3 (19.1 to 64)**
11 Hypertensive heart disease	11 Pancreatic cancer	**150 (89.9 to 223)**	**142 (97.4 to 199)**	**41.6 (17.9 to 70)**
12 Breast cancer	12 Diabetes	**34.6 (3.32 to 69.4)**	**30.4 (3.32 to 62)**	**-25.9 (-43.1 to -7.76)**
13 Cardiomyopathy	13 Breast cancer	**68.5 (33.4 to 120)**	**63 (37.3 to 97.3)**	-1.9 (-15.7 to 16.3)
14 Cirrhosis liver	14 Prostate cancer	**101 (59.5 to 155)**	**94.9 (65.6 to 130)**	7.97 (-6.91 to 24.2)
15 Other cardiovascular	15 Cardiomyopathy	**44.4 (20.1 to 74.1)**	**39.8 (24.1 to 60)**	**-20.9 (-28.3 to -12.4)**
16 Pancreatic cancer	16 Other cardiovascular	**54 (29.9 to 78.8)**	**49.1 (36.2 to 65.2)**	**-17.6 (-24.7 to -9.2)**
17 Urinary diseases	17 Cirrhosis liver	**33.9 (14.8 to 60.3)**	**29.7 (17 to 41.7)**	**-16.5 (-24.6 to -9.32)**
18 Prostate cancer	18 Bacterial skin	**227 (138 to 331)**	**217 (129 to 299)**	**77.8 (25.4 to 130)**
19 Road injuries	19 Ileus & obstruction	**91.8 (59.5 to 128)**	**85.7 (66.4 to 104)**	2.26 (-11.1 to 13.9)
20 Self-harm	20 Parkinson's disease	**99.5 (69.5 to 137)**	**93.2 (72.2 to 113)**	4.5 (-4.2 to 13.7)
22 Ileus & obstruction	23 Road injuries			
23 Parkinson's disease	26 Self-harm			
35 Bacterial skin	133 COVID-19			

Supplemental Results Figure S8. Leading 20 level 3 causes of deaths, and percentage change in number of deaths and all-age and age-standardised death rates by location, 2022 and 2050

Chile, Reference scenario

Legend:
Communicable, maternal, neonatal and nutritional
Non-communicable
Injuries

Leading Causes 2022	Leading Causes 2050	Mean % change number of DEATH	Mean % change all-age DEATH rate	Mean % change age-standardized DEATH rate
1 COVID-19	1 Chronic kidney disease	**240 (181 to 319)**	**198 (147 to 259)**	16.5 (-3.26 to 41.9)
2 Ischemic heart disease	2 Alzheimer's disease	**213 (175 to 261)**	**174 (149 to 202)**	-4.43 (-8.65 to 0.285)
3 Stroke	3 Lower respiratory infect	**162 (122 to 211)**	**130 (95.6 to 164)**	**-16.5 (-28.4 to -4.44)**
4 Lower respiratory infect	4 Stroke	**28.7 (8.99 to 50)**	12.7 (-2.09 to 27.8)	**-51.7 (-58 to -44.5)**
5 Chronic kidney disease	5 COPD	**189 (139 to 259)**	**153 (115 to 206)**	-5.57 (-20.8 to 14.1)
6 Alzheimer's disease	6 Ischemic heart disease	16.2 (-9.53 to 49.8)	1.83 (-19 to 29.2)	**-54.7 (-64.3 to -42.9)**
7 Cirrhosis liver	7 Hypertensive heart disease	**204 (137 to 278)**	**167 (110 to 219)**	-0.5 (-20.3 to 20.6)
8 COPD	8 Urinary diseases	**316 (248 to 401)**	**264 (212 to 325)**	**35.7 (18 to 57.5)**
9 Stomach cancer	9 Colorectal cancer	**128 (81.6 to 182)**	**100 (60.1 to 147)**	-1.74 (-19.6 to 17.3)
10 Lung cancer	10 Lung cancer	**99.9 (63.8 to 134)**	**75.2 (48 to 105)**	-4.91 (-18.1 to 9.07)
11 Colorectal cancer	11 Prostate cancer	**169 (125 to 224)**	**136 (101 to 178)**	2.24 (-9.7 to 16.3)
12 Hypertensive heart disease	12 Cirrhosis liver	**45.9 (31.9 to 63.3)**	**27.9 (17.8 to 38.2)**	**-14.8 (-21.3 to -8.23)**
13 Diabetes	13 Interstitial lung disease	**156 (122 to 197)**	**124 (100 to 149)**	-0.138 (-8.03 to 8.33)
14 Prostate cancer	14 Diabetes	**89.8 (46.7 to 142)**	**66.3 (31.5 to 105)**	**-28.8 (-42.7 to -12.5)**
15 Interstitial lung disease	15 Stomach cancer	**41.8 (23.6 to 63.5)**	**24.3 (14.1 to 37.5)**	**-36.8 (-41.3 to -31.2)**
16 Urinary diseases	16 Pancreatic cancer	**182 (125 to 240)**	**148 (99.5 to 199)**	**31 (8.77 to 56)**
17 Road injuries	17 Breast cancer	**99.8 (62.8 to 137)**	**75.1 (43.7 to 107)**	-6.67 (-20.8 to 7.96)
18 Self-harm	18 Falls	**153 (112 to 199)**	**121 (91.2 to 156)**	**-11.5 (-20.5 to -0.956)**
19 Gallbladder cancer	19 Atrial fibrillation	**249 (203 to 293)**	**205 (176 to 239)**	**8.29 (0.575 to 17.2)**
20 Pancreatic cancer	20 Parkinson's disease	**167 (130 to 201)**	**134 (109 to 160)**	-3.05 (-11 to 6.54)
21 Breast cancer	21 Gallbladder cancer			
22 Falls	24 Road injuries			
23 Parkinson's disease	25 Self-harm			
24 Atrial fibrillation	131 COVID-19			

Supplemental Results Figure S8. Leading 20 level 3 causes of deaths, and percentage change in number of deaths and all-age and age-standardised death rates by location, 2022 and 2050

Uruguay, Reference scenario

Legend:
Communicable, maternal, neonatal and nutritional
Non-communicable
Injuries

Leading Causes 2022	Leading Causes 2050	Mean % change number of DEATH	Mean % change all-age DEATH rate	Mean % change age-standardized DEATH rate
1 Ischemic heart disease	1 COPD	**29.7 (6.03 to 57.8)**	**39.3 (17.7 to 61.9)**	-10.6 (-24 to 5.39)
2 Stroke	2 Ischemic heart disease	**-30.7 (-47 to -10.6)**	**-25.5 (-41.8 to -5.82)**	**-51.6 (-62.8 to -35.7)**
3 COPD	3 Stroke	**-31 (-43.8 to -18.2)**	**-25.9 (-35.7 to -15.7)**	**-50.8 (-58.1 to -42.5)**
4 COVID-19	4 Colorectal cancer	**42.3 (9.81 to 77.2)**	**53 (21.6 to 89.7)**	1.07 (-18.8 to 22.1)
5 Lung cancer	5 Lung cancer	**29.2 (3.98 to 59.4)**	**38.8 (17.7 to 63.7)**	-5.07 (-18.8 to 9.29)
6 Lower respiratory infect	6 Alzheimer's disease	**47.5 (23.6 to 73.4)**	**58.5 (43.8 to 73.2)**	-1.29 (-6.6 to 3.84)
7 Colorectal cancer	7 Lower respiratory infect	**22.9 (1.65 to 49.3)**	**32 (15.4 to 50.2)**	**-16.2 (-26.8 to -3.12)**
8 Alzheimer's disease	8 Chronic kidney disease	**71.4 (39 to 111)**	**84.1 (53.5 to 117)**	17.5 (-4.45 to 43.9)
9 Chronic kidney disease	9 Urinary diseases	**107 (64.7 to 150)**	**122 (87.7 to 162)**	**39.3 (17.3 to 67.6)**
10 Diabetes	10 Pancreatic cancer	**86.6 (45.2 to 134)**	**101 (61.3 to 142)**	**35.8 (12.1 to 64)**
11 Breast cancer	11 Prostate cancer	**46.2 (18.5 to 76.8)**	**57.1 (35.2 to 82.6)**	2.98 (-9.98 to 16.3)
12 Prostate cancer	12 Breast cancer	**31.2 (5.33 to 59.8)**	**41 (17.3 to 66.6)**	-3.84 (-18.6 to 11.4)
13 Hypertensive heart disease	13 Hypertensive heart disease	**41.2 (12 to 78.5)**	**51.7 (25.4 to 85.9)**	-1.12 (-20.8 to 23.6)
14 Pancreatic cancer	14 Diabetes	16.8 (-11.3 to 51.7)	25.5 (-4.05 to 57.1)	-19.3 (-38.5 to 3.36)
15 Urinary diseases	15 Nonrheum valv diseases	**75.1 (42.9 to 108)**	**88.2 (65.9 to 114)**	**17.9 (6.24 to 31)**
16 Self-harm	16 Self-harm	-11.8 (-26.1 to 1.95)	-5.19 (-14.2 to 5.42)	**-12.4 (-23.2 to -0.0293)**
17 Stomach cancer	17 Parkinson's disease	**42.9 (18.2 to 70.7)**	**53.5 (39.3 to 70.2)**	0.136 (-8.47 to 9.06)
18 Road injuries	18 Stomach cancer	-12.5 (-26.6 to 1.78)	-5.95 (-14.1 to 1.74)	**-36.9 (-41.1 to -32.5)**
19 Nonrheum valv diseases	19 Road injuries	-5.01 (-26.4 to 25.2)	2.02 (-17.5 to 28)	-2.58 (-22.9 to 25.8)
20 Cirrhosis liver	20 Kidney cancer	**42.1 (12.1 to 77.6)**	**52.8 (27.5 to 85.3)**	8.34 (-8.68 to 30.6)
21 Parkinson's disease	23 Cirrhosis liver			
22 Kidney cancer	129 COVID-19			

Supplemental Results Figure S8. Leading 20 level 3 causes of deaths, and percentage change in number of deaths and all-age and age-standardised death rates by location, 2022 and 2050

Andorra, Reference scenario

Legend:
Communicable, maternal, neonatal and nutritional
Non-communicable
Injuries

Leading Causes 2022	Leading Causes 2050	Mean % change number of DEATH	Mean % change all-age DEATH rate	Mean % change age-standardized DEATH rate
1 Ischemic heart disease	1 Alzheimer's disease	**205 (136 to 283)**	**260 (198 to 330)**	1.62 (-3.81 to 7.08)
2 Alzheimer's disease	2 Ischemic heart disease	40.9 (-5.61 to 97.4)	**66.6 (14.1 to 131)**	**-47.9 (-64 to -26.5)**
3 COVID-19	3 COPD	**164 (94.3 to 242)**	**212 (136 to 297)**	-3.08 (-22.5 to 25)
4 Lung cancer	4 Chronic kidney disease	**312 (195 to 445)**	**387 (264 to 529)**	**42.5 (5.9 to 84.2)**
5 Stroke	5 Lung cancer	**60.9 (15.5 to 118)**	**90 (46.9 to 141)**	**-19.6 (-36 to -2.37)**
6 COPD	6 Colorectal cancer	**119 (44.7 to 218)**	**158 (77.4 to 245)**	-8.17 (-32.9 to 17)
7 Colorectal cancer	7 Stroke	**42.7 (4.25 to 88)**	**68.7 (27 to 113)**	**-45.8 (-57.4 to -29.5)**
8 Chronic kidney disease	8 Hypertensive heart disease	**219 (135 to 309)**	**277 (182 to 392)**	14.1 (-15.2 to 52.3)
9 Pancreatic cancer	9 Prostate cancer	**178 (97.3 to 273)**	**229 (146 to 325)**	1.58 (-16.8 to 20.8)
10 Liver cancer	10 Pancreatic cancer	**163 (83.1 to 266)**	**210 (128 to 310)**	23.9 (-3.57 to 55.5)
11 Prostate cancer	11 Lower respiratory infect	**180 (104 to 261)**	**231 (155 to 320)**	-3.23 (-20.6 to 17.8)
12 Lower respiratory infect	12 Liver cancer	**142 (86.8 to 205)**	**186 (131 to 252)**	**23.6 (2.37 to 47.9)**
13 Diabetes	13 Diabetes	**100 (38.2 to 176)**	**137 (63.8 to 223)**	-25.4 (-45.3 to 0.81)
14 Hypertensive heart disease	14 Falls	**151 (81.3 to 220)**	**196 (126 to 267)**	-10 (-20.1 to 1.38)
15 Breast cancer	15 Breast cancer	**76.7 (26.4 to 141)**	**109 (53 to 170)**	-6.63 (-26.9 to 13.2)
16 Cirrhosis liver	16 Atrial fibrillation	**251 (179 to 338)**	**315 (245 to 398)**	**18.6 (9.58 to 29)**
17 Stomach cancer	17 Urinary diseases	**342 (213 to 485)**	**422 (298 to 595)**	**50.7 (18.6 to 90.9)**
18 Falls	18 Cardiomyopathy	**108 (57.7 to 173)**	**146 (102 to 204)**	**-21.3 (-29.3 to -13.2)**
19 Cardiomyopathy	19 Parkinson's disease	**193 (136 to 257)**	**246 (198 to 305)**	3.67 (-5.32 to 13.4)
20 Brain cancer	20 Brain cancer	**104 (69.5 to 148)**	**142 (113 to 176)**	**41.7 (31 to 51.7)**
23 Atrial fibrillation	21 Stomach cancer			
27 Parkinson's disease	26 Cirrhosis liver			
30 Urinary diseases	126 COVID-19			

Supplemental Results Figure S8. Leading 20 level 3 causes of deaths, and percentage change in number of deaths and all-age and age-standardised death rates by location, 2022 and 2050

Austria, Reference scenario

Legend:
Communicable, maternal, neonatal and nutritional
Non-communicable
Injuries

Leading Causes 2022	Leading Causes 2050	Mean % change number of DEATH	Mean % change all-age DEATH rate	Mean % change age-standardized DEATH rate
1 Ischemic heart disease	1 Ischemic heart disease	-1.67 (-21.8 to 21)	-9.94 (-28.9 to 8.84)	**-51.1 (-62.6 to -38.1)**
2 Alzheimer's disease	2 Alzheimer's disease	**93 (67.7 to 117)**	**76.6 (63 to 91.9)**	**-7.99 (-12.2 to -3.78)**
3 Stroke	3 Chronic kidney disease	**190 (135 to 244)**	**165 (116 to 215)**	**36.6 (10.1 to 64.5)**
4 COVID-19	4 Hypertensive heart disease	**106 (69.8 to 148)**	**89.1 (58 to 125)**	-0.131 (-18.8 to 23.1)
5 Lung cancer	5 COPD	**60.8 (32.9 to 88.6)**	**47.3 (24.5 to 69.6)**	-11.8 (-25.4 to 3.64)
6 Chronic kidney disease	6 Lung cancer	**22.2 (0.686 to 44.7)**	11.8 (-4.82 to 32)	**-19.1 (-30.3 to -5.69)**
7 COPD	7 Stroke	-6.22 (-19.6 to 8.66)	**-14.1 (-25.5 to -3.11)**	**-51.3 (-58 to -44.3)**
8 Hypertensive heart disease	8 Atrial fibrillation	**129 (95.4 to 164)**	**109 (85.8 to 134)**	8.41 (-0.5 to 18.3)
9 Colorectal cancer	9 Colorectal cancer	**58.2 (22.4 to 102)**	**44.7 (16 to 77.3)**	-12.5 (-27.8 to 4.86)
10 Diabetes	10 Pancreatic cancer	**89.5 (56 to 136)**	**73.4 (44.2 to 110)**	15.8 (-3.3 to 38.6)
11 Pancreatic cancer	11 Falls	**77.6 (48.7 to 109)**	**62.6 (42.2 to 86.8)**	**-11.1 (-19.6 to -1.94)**
12 Falls	12 Nonrheum valv diseases	**148 (112 to 195)**	**127 (94.9 to 157)**	**16.2 (3.74 to 29.2)**
13 Breast cancer	13 Prostate cancer	**93.5 (57 to 140)**	**77.2 (46.9 to 114)**	1.06 (-12.4 to 15)
14 Atrial fibrillation	14 Diabetes	**40.6 (9.69 to 78.2)**	**28.8 (2.43 to 62)**	**-29.1 (-45.6 to -13.8)**
15 Cardiomyopathy	15 Lower respiratory infect	**66.1 (45 to 97.5)**	**52.1 (35 to 77.2)**	**-17.5 (-28 to -5.6)**
16 Cirrhosis liver	16 Breast cancer	**39.7 (16.4 to 67.6)**	**27.8 (7.5 to 53.5)**	**-17.7 (-28.4 to -4.56)**
17 Prostate cancer	17 Cardiomyopathy	**44 (20.6 to 73.2)**	**31.8 (14 to 54.3)**	**-26.6 (-33.3 to -18.2)**
18 Lower respiratory infect	18 Parkinson's disease	**86.3 (60.7 to 111)**	**70.5 (52.4 to 86.2)**	-2.3 (-11.1 to 6.16)
19 Nonrheum valv diseases	19 Cirrhosis liver	**12.3 (1.01 to 24.8)**	2.81 (-4.7 to 10.9)	**-18 (-24.6 to -11.9)**
20 Self-harm	20 Liver cancer	**91 (66.1 to 123)**	**74.9 (52 to 101)**	**24.5 (9.56 to 39.5)**
21 Parkinson's disease	23 Self-harm			
22 Liver cancer	129 COVID-19			

Supplemental Results Figure S8. Leading 20 level 3 causes of deaths, and percentage change in number of deaths and all-age and age-standardised death rates by location, 2022 and 2050

Belgium, Reference scenario

Legend:
Communicable, maternal, neonatal and nutritional
Non-communicable
Injuries

Leading Causes 2022	Leading Causes 2050	Mean % change number of DEATH	Mean % change all-age DEATH rate	Mean % change age-standardized DEATH rate
1 Ischemic heart disease	1 Alzheimer's disease	**75.9 (54.2 to 96.7)**	**56.8 (42 to 69.2)**	**-7.24 (-11.7 to -3)**
2 Alzheimer's disease	2 Lower respiratory infect	**57 (34.3 to 83.1)**	**40 (21 to 63.9)**	**-14.9 (-26.5 to -0.945)**
3 Stroke	3 Ischemic heart disease	-16.8 (-33.7 to 5.8)	**-25.8 (-40.6 to -7.13)**	**-53.7 (-63.8 to -40.9)**
4 Lung cancer	4 Chronic kidney disease	**187 (131 to 250)**	**156 (115 to 206)**	**49.4 (22 to 81.9)**
5 Lower respiratory infect	5 COPD	**40.6 (18.4 to 66.4)**	**25.3 (7.02 to 43.9)**	**-16.6 (-28.4 to -2.43)**
6 COPD	6 Lung cancer	15.2 (-4.97 to 38.2)	2.66 (-13.1 to 21.7)	**-19.4 (-29.6 to -6.14)**
7 COVID-19	7 Stroke	-7.03 (-21.7 to 8.08)	**-17.1 (-27.6 to -6.72)**	**-45.6 (-52.9 to -37.9)**
8 Colorectal cancer	8 Colorectal cancer	**54.2 (20.5 to 93.3)**	**37.4 (8.36 to 66.6)**	-6.19 (-23.9 to 13.7)
9 Chronic kidney disease	9 Nonrheum valv diseases	**141 (106 to 182)**	**115 (88.1 to 145)**	**24.4 (12.5 to 39.6)**
10 Falls	10 Falls	**57.9 (35.3 to 83.2)**	**40.8 (22.5 to 60.4)**	**-11.8 (-20 to -3.44)**
11 Breast cancer	11 Pancreatic cancer	**84.3 (47.7 to 127)**	**64.3 (34.6 to 96.2)**	**23 (1.75 to 43)**
12 Pancreatic cancer	12 Prostate cancer	**80.9 (49.1 to 119)**	**61.2 (35.1 to 94.9)**	4.48 (-7.73 to 19)
13 Prostate cancer	13 Breast cancer	**35.8 (11.8 to 65.5)**	**21.1 (1.43 to 44.7)**	-10.2 (-22.6 to 4.45)
14 Nonrheum valv diseases	14 Urinary diseases	**166 (121 to 225)**	**137 (99.8 to 181)**	**39.5 (18.9 to 65.7)**
15 Self-harm	15 Atrial fibrillation	**136 (102 to 174)**	**110 (85.8 to 136)**	**23.5 (13.6 to 34.5)**
16 Cirrhosis liver	16 Diarrheal diseases	**206 (78 to 395)**	**173 (58.2 to 341)**	56.8 (-10.3 to 158)
17 Diabetes	17 Parkinson's disease	**69.8 (49 to 89.3)**	**51.4 (36.8 to 66.3)**	0.658 (-7.58 to 9.79)
18 Atrial fibrillation	18 Self-harm	-4.81 (-17.5 to 7.89)	**-15.1 (-23.1 to -5.54)**	**-21.4 (-30 to -12.7)**
19 Parkinson's disease	19 Endocarditis	**141 (86.7 to 214)**	**115 (62.1 to 176)**	**36.4 (4.7 to 73.7)**
20 Urinary diseases	20 Diabetes	17.4 (-9.86 to 48.2)	4.67 (-17.4 to 27.3)	**-34.2 (-48.2 to -17.8)**
24 Diarrheal diseases	23 Cirrhosis liver			
32 Endocarditis	128 COVID-19			

Supplemental Results Figure S8. Leading 20 level 3 causes of deaths, and percentage change in number of deaths and all-age and age-standardised death rates by location, 2022 and 2050

Cyprus, Reference scenario

Legend:
Communicable, maternal, neonatal and nutritional
Non-communicable
Injuries

Leading Causes 2022	Leading Causes 2050	Mean % change number of DEATH	Mean % change all-age DEATH rate	Mean % change age-standardized DEATH rate
1 Ischemic heart disease	1 Ischemic heart disease	13.4 (-20 to 49.3)	2.68 (-24 to 37.1)	**-53.3 (-67.5 to -35)**
2 Stroke	2 Chronic kidney disease	**251 (166 to 352)**	**218 (143 to 298)**	28.6 (-2.05 to 69.4)
3 Diabetes	3 COPD	**147 (87 to 217)**	**124 (81.5 to 179)**	-5.01 (-25.9 to 21.1)
4 COVID-19	4 Alzheimer's disease	**145 (104 to 191)**	**122 (93.2 to 151)**	**-7.93 (-12.1 to -3.98)**
5 COPD	5 Stroke	**27 (1.88 to 53.7)**	15 (-4.85 to 37.9)	**-52.3 (-61.2 to -40.5)**
6 Alzheimer's disease	6 Diabetes	**59 (14.1 to 114)**	**44 (5.61 to 90.2)**	**-35.1 (-53.2 to -10.8)**
7 Lung cancer	7 Hypertensive heart disease	**201 (126 to 271)**	**173 (117 to 236)**	-0.798 (-25.2 to 35.4)
8 Chronic kidney disease	8 Lung cancer	**51.4 (13.5 to 91.3)**	**37 (4.79 to 70.6)**	**-20.4 (-36.8 to -4.14)**
9 Colorectal cancer	9 Colorectal cancer	**107 (42.1 to 188)**	**86.9 (33.8 to 148)**	-6.37 (-28.3 to 17.9)
10 Hypertensive heart disease	10 Nonrheum valv diseases	**234 (167 to 309)**	**202 (152 to 260)**	**20.9 (6.84 to 37.2)**
11 Breast cancer	11 Lower respiratory infect	**148 (101 to 207)**	**125 (87.4 to 172)**	-14.3 (-29.2 to 3.78)
12 Lower respiratory infect	12 Urinary diseases	**289 (199 to 375)**	**252 (179 to 315)**	**37.8 (8.68 to 66.1)**
13 Prostate cancer	13 Prostate cancer	**115 (63.1 to 178)**	**94.4 (51.3 to 149)**	-2.66 (-18.7 to 17)
14 Nonrheum valv diseases	14 Atrial fibrillation	**230 (157 to 311)**	**199 (147 to 270)**	**10.6 (0.185 to 24.2)**
15 Falls	15 Breast cancer	**79.5 (33.7 to 129)**	**62.5 (26.8 to 102)**	-12.9 (-29.3 to 4.07)
16 Pancreatic cancer	16 Pancreatic cancer	**158 (85.9 to 243)**	**133 (73.2 to 200)**	26.5 (-1.69 to 54.4)
17 Atrial fibrillation	17 Falls	**128 (82.1 to 184)**	**106 (68.8 to 150)**	**-13.2 (-22.7 to -2.11)**
18 Urinary diseases	18 Parkinson's disease	**118 (82.2 to 160)**	**97.5 (70.2 to 127)**	-6.59 (-14.9 to 3.79)
19 Stomach cancer	19 Interstitial lung disease	**110 (77.3 to 150)**	**90.5 (60.9 to 126)**	-4.63 (-13.7 to 6.74)
20 Cirrhosis liver	20 Leukemia	**90.4 (36.9 to 143)**	**72.4 (34.3 to 121)**	-7.46 (-24.5 to 11.1)
21 Leukemia	25 Stomach cancer			
22 Parkinson's disease	26 Cirrhosis liver			
23 Interstitial lung disease	129 COVID-19			

Supplemental Results Figure S8. Leading 20 level 3 causes of deaths, and percentage change in number of deaths and all-age and age-standardised death rates by location, 2022 and 2050

Denmark, Reference scenario

Legend:
Communicable, maternal, neonatal and nutritional
Non-communicable
Injuries

Leading Causes 2022
1 Ischemic heart disease
2 COVID-19
3 COPD
4 Lung cancer
5 Stroke
6 Alzheimer's disease
7 Lower respiratory infect
8 Colorectal cancer
9 Prostate cancer
10 Chronic kidney disease
11 Diabetes
12 Breast cancer
13 Pancreatic cancer
14 Falls
15 Atrial fibrillation
16 Cirrhosis liver
17 Nonrheum valv diseases
18 Parkinson's disease
19 Urinary diseases
20 Self-harm
21 Leukemia
27 Brain cancer
31 Diarrheal diseases

Leading Causes 2050	Mean % change number of DEATH	Mean % change all-age DEATH rate	Mean % change age-standardized DEATH rate
1 COPD	**42.2 (20.4 to 70.6)**	**28.3 (9.51 to 50.6)**	-11 (-24.2 to 3.98)
2 Alzheimer's disease	**74.3 (54 to 96)**	**57.2 (45.3 to 70.6)**	-4.47 (-8.77 to 0.836)
3 Lung cancer	**18.8 (1.27 to 37.5)**	7.15 (-6.16 to 21.6)	**-13.6 (-22.7 to -3.44)**
4 Chronic kidney disease	**150 (109 to 199)**	**126 (93.8 to 167)**	**41.5 (19.6 to 68.4)**
5 Lower respiratory infect	**61.3 (35.9 to 88.8)**	**45.5 (27.8 to 66.2)**	-11 (-21.6 to 0.4)
6 Ischemic heart disease	**-26.8 (-39.2 to -9.96)**	**-33.9 (-45.1 to -19.4)**	**-57 (-65 to -47)**
7 Colorectal cancer	**37.7 (9.47 to 71.7)**	**24.2 (1.81 to 49.8)**	-9.5 (-24.2 to 5.84)
8 Stroke	**-19.6 (-29.3 to -8.26)**	**-27.4 (-35.6 to -20)**	**-51 (-56.2 to -44.7)**
9 Prostate cancer	**68.6 (39.1 to 98.8)**	**52.1 (28.2 to 79.4)**	4.96 (-6.47 to 18.9)
10 Pancreatic cancer	**66.5 (38.6 to 101)**	**50.1 (25.6 to 72.9)**	**20.3 (3.21 to 37)**
11 Atrial fibrillation	**108 (82.1 to 135)**	**87.8 (69.2 to 106)**	**14.2 (4.76 to 23)**
12 Urinary diseases	**179 (131 to 243)**	**151 (113 to 197)**	**50.6 (26.7 to 76.2)**
13 Breast cancer	**34.3 (12.1 to 62)**	**21.1 (3.49 to 42.1)**	-10.5 (-20.6 to 2.09)
14 Diabetes	19.7 (-1.98 to 48.9)	7.91 (-9.81 to 29.5)	**-28.9 (-40.8 to -14.8)**
15 Falls	**63.2 (40 to 90.7)**	**47.2 (29.4 to 67.7)**	-6.25 (-14.7 to 3.23)
16 Nonrheum valv diseases	**123 (90.7 to 161)**	**101 (77.2 to 128)**	**22.9 (11.3 to 39.4)**
17 Diarrheal diseases	**234 (118 to 427)**	**201 (94.1 to 368)**	**81.1 (13.7 to 187)**
18 Parkinson's disease	**67 (45.3 to 90.4)**	**50.6 (34.3 to 67)**	4.42 (-6.09 to 14.2)
19 Brain cancer	**71 (54 to 90.3)**	**54.3 (44.7 to 64.2)**	**33.4 (26.4 to 40.3)**
20 Leukemia	**33.4 (15.6 to 56.1)**	**20.3 (7.83 to 34.8)**	**-10.8 (-18.3 to -2.69)**
24 Cirrhosis liver			
26 Self-harm			
128 COVID-19			

Supplemental Results Figure S8. Leading 20 level 3 causes of deaths, and percentage change in number of deaths and all-age and age-standardised death rates by location, 2022 and 2050

Finland, Reference scenario

Legend:
Communicable, maternal, neonatal and nutritional
Non-communicable
Injuries

Leading Causes 2022	Leading Causes 2050	Mean % change number of DEATH	Mean % change all-age DEATH rate	Mean % change age-standardized DEATH rate
1 Ischemic heart disease	1 Ischemic heart disease	**-20.3 (-37.3 to -1.79)**	**-18.3 (-35.9 to -0.332)**	**-54.7 (-65.4 to -42.6)**
2 COVID-19	2 Alzheimer's disease	**92.8 (66.4 to 118)**	**97.5 (79.4 to 115)**	3.79 (-1.38 to 8.38)
3 Stroke	3 Hypertensive heart disease	**88.1 (50.4 to 134)**	**92.7 (56.7 to 133)**	7.62 (-12 to 32.1)
4 Alzheimer's disease	4 Stroke	**-19.8 (-30.6 to -5.71)**	**-17.9 (-26.9 to -7.25)**	**-49.9 (-56.1 to -42)**
5 Lung cancer	5 Lung cancer	14.6 (-7.19 to 36.7)	17.3 (-1.86 to 38.8)	-8.91 (-22.4 to 5.05)
6 Hypertensive heart disease	6 Pancreatic cancer	**75.1 (34.8 to 115)**	**79.3 (45.2 to 117)**	**32.9 (11.5 to 54.4)**
7 Colorectal cancer	7 Falls	**59.6 (33 to 92.6)**	**63.5 (38.3 to 87.7)**	-6.04 (-16 to 4.12)
8 Pancreatic cancer	8 Nonrheum valv diseases	**127 (90.6 to 169)**	**133 (103 to 165)**	**24.3 (11.5 to 38.5)**
9 COPD	9 Colorectal cancer	**42.2 (11.9 to 77.4)**	**45.6 (18.2 to 79.3)**	-1.35 (-18.3 to 18.1)
10 Falls	10 COPD	**41.7 (18.4 to 68)**	**45.2 (25.7 to 68)**	-4.86 (-17.2 to 9.92)
11 Prostate cancer	11 Chronic kidney disease	**149 (99.4 to 211)**	**156 (105 to 211)**	**34.3 (7.92 to 63.9)**
12 Breast cancer	12 Prostate cancer	**70.6 (35.8 to 111)**	**74.7 (43.4 to 111)**	11.1 (-2.45 to 26.3)
13 Cirrhosis liver	13 Atrial fibrillation	**113 (78.1 to 146)**	**118 (92.5 to 144)**	**17.3 (8.94 to 27.3)**
14 Nonrheum valv diseases	14 Breast cancer	**31 (5 to 62.9)**	**34.1 (13.5 to 56.9)**	-6.7 (-19.1 to 7.82)
15 Self-harm	15 Lower respiratory infect	**66.5 (39.1 to 102)**	**70.5 (46 to 96.6)**	-8.04 (-19.5 to 4.26)
16 Chronic kidney disease	16 Parkinson's disease	**52.5 (29.1 to 74.1)**	**56.1 (40.7 to 71)**	3.92 (-3.99 to 12.7)
17 Parkinson's disease	17 Cirrhosis liver	**-12.9 (-23.8 to -0.914)**	**-10.8 (-17.4 to -3.47)**	**-18.6 (-25.5 to -11.4)**
18 Lower respiratory infect	18 Liver cancer	**48.9 (27.2 to 74.9)**	**52.5 (35.8 to 72)**	**20.1 (7.31 to 35.3)**
19 Atrial fibrillation	19 Urinary diseases	**176 (125 to 233)**	**183 (135 to 241)**	**46.1 (28.6 to 68.9)**
20 Diabetes	20 Self-harm	**-13.1 (-23.9 to -3.24)**	**-11 (-17.5 to -3.24)**	**-21.6 (-29.6 to -13.5)**
22 Liver cancer	25 Diabetes			
39 Urinary diseases	128 COVID-19			

Supplemental Results Figure S8. Leading 20 level 3 causes of deaths, and percentage change in number of deaths and all-age and age-standardised death rates by location, 2022 and 2050

France, Reference scenario

Legend:
- Communicable, maternal, neonatal and nutritional
- Non-communicable
- Injuries

Leading Causes 2022	Leading Causes 2050	Mean % change number of DEATH	Mean % change all-age DEATH rate	Mean % change age-standardized DEATH rate
1 Ischemic heart disease	1 Alzheimer's disease	**75.2 (53 to 96.9)**	**80.4 (67.4 to 94.4)**	**-4.67 (-9.27 to -0.597)**
2 Alzheimer's disease	2 Ischemic heart disease	-11.4 (-31 to 8.53)	-8.77 (-27.1 to 11.7)	**-51.4 (-61.3 to -40.1)**
3 Stroke	3 Lung cancer	**25.3 (3.85 to 47.3)**	**29 (11.3 to 49.1)**	-3.62 (-16.3 to 9.29)
4 COVID-19	4 Chronic kidney disease	**175 (125 to 246)**	**183 (136 to 239)**	**45.2 (22.4 to 75.4)**
5 Lung cancer	5 Lower respiratory infect	**69.9 (40.9 to 98.5)**	**75 (49.5 to 99.7)**	-10.1 (-22.7 to 2.46)
6 Colorectal cancer	6 Stroke	-8.79 (-21.8 to 5.5)	-6.1 (-16.4 to 6.1)	**-46.9 (-52.7 to -39.7)**
7 Lower respiratory infect	7 Colorectal cancer	**52.2 (20.5 to 90.4)**	**56.7 (29.3 to 95.7)**	-5.99 (-20.4 to 11.8)
8 Falls	8 Falls	**58.9 (32 to 89.9)**	**63.6 (41.8 to 88.5)**	**-9.56 (-18.6 to -0.122)**
9 COPD	9 Pancreatic cancer	**87.2 (52.2 to 127)**	**92.7 (62 to 127)**	**29.7 (9.8 to 50.1)**
10 Breast cancer	10 COPD	**63 (37.1 to 99.3)**	**67.8 (41 to 96.5)**	-7.06 (-20.3 to 6.72)
11 Chronic kidney disease	11 Nonrheum valv diseases	**144 (103 to 189)**	**151 (114 to 192)**	**27.1 (11.1 to 44.3)**
12 Pancreatic cancer	12 Prostate cancer	**76.5 (42.5 to 111)**	**81.8 (51.8 to 116)**	3.42 (-9.47 to 16.8)
13 Prostate cancer	13 Atrial fibrillation	**115 (85.7 to 142)**	**121 (97.9 to 145)**	**15.4 (4.05 to 25.5)**
14 Diabetes	14 Breast cancer	**30.4 (6.98 to 55.7)**	**34.3 (14.4 to 58.9)**	**-12.1 (-22.8 to -0.381)**
15 Atrial fibrillation	15 Diabetes	**44.2 (14.7 to 83.8)**	**48.4 (21.8 to 89)**	**-20.6 (-33.5 to -0.159)**
16 Nonrheum valv diseases	16 Hypertensive heart disease	**94.7 (55.7 to 136)**	**100 (63.1 to 142)**	4.64 (-14.2 to 25.4)
17 Hypertensive heart disease	17 Liver cancer	**62.4 (39.9 to 85.5)**	**67.2 (47.7 to 87.6)**	**23 (8.52 to 36.5)**
18 Self-harm	18 Parkinson's disease	**60.8 (41.8 to 84)**	**65.6 (51.1 to 81.7)**	-2.85 (-9.85 to 4.31)
19 Cirrhosis liver	19 Other cardiovascular	**48.7 (29.9 to 69.6)**	**53.1 (37.5 to 69.9)**	**-17.3 (-25.2 to -8.73)**
20 Liver cancer	20 Protein-energy malnutrition	**140 (46 to 246)**	**148 (51.9 to 268)**	16.7 (-28.2 to 71.2)
21 Other cardiovascular	28 Cirrhosis liver			
22 Parkinson's disease	31 Self-harm			
31 Protein-energy malnutrition	130 COVID-19			

Supplemental Results Figure S8. Leading 20 level 3 causes of deaths, and percentage change in number of deaths and all-age and age-standardised death rates by location, 2022 and 2050

Germany, Reference scenario

Legend:
Communicable, maternal, neonatal and nutritional
Non-communicable
Injuries

Leading Causes 2022	Leading Causes 2050	Mean % change number of DEATH	Mean % change all-age DEATH rate	Mean % change age-standardized DEATH rate
1 Ischemic heart disease	1 Alzheimer's disease	**61.9 (40.1 to 82.5)**	**61 (49.3 to 72.6)**	-0.675 (-4.72 to 4.44)
2 Alzheimer's disease	2 Ischemic heart disease	**-24.5 (-39.8 to -8.32)**	**-24.9 (-39.7 to -12)**	**-51.2 (-60.7 to -41)**
3 Stroke	3 Chronic kidney disease	**139 (97 to 186)**	**138 (96.6 to 182)**	**46 (21.3 to 73.9)**
4 Lung cancer	4 Hypertensive heart disease	**78.5 (47.7 to 115)**	**77.5 (48.9 to 106)**	9.89 (-9.75 to 29.8)
5 COVID-19	5 Lung cancer	4.97 (-10.6 to 22.7)	4.41 (-7.91 to 19.1)	**-14.4 (-23.6 to -3.48)**
6 Chronic kidney disease	6 COPD	**31.4 (12.4 to 51.8)**	**30.7 (14.9 to 46.6)**	-7.26 (-18.5 to 4.73)
7 COPD	7 Stroke	**-25.8 (-35.6 to -13.9)**	**-26.2 (-34.3 to -17.9)**	**-48.5 (-54.5 to -42.5)**
8 Hypertensive heart disease	8 Atrial fibrillation	**95.6 (67.5 to 124)**	**94.5 (75.3 to 119)**	**19.5 (9.81 to 29.8)**
9 Colorectal cancer	9 Colorectal cancer	**32.4 (8.52 to 62)**	**31.7 (11.2 to 55.3)**	-5.92 (-18.2 to 9.74)
10 Lower respiratory infect	10 Nonrheum valv diseases	**110 (77 to 148)**	**109 (83.7 to 140)**	**27.4 (13.1 to 44)**
11 Falls	11 Pancreatic cancer	**61.6 (34.7 to 93.4)**	**60.8 (38.9 to 86.7)**	**24.7 (9.27 to 42.6)**
12 Pancreatic cancer	12 Falls	**49.4 (24 to 75.8)**	**48.6 (27.9 to 71.7)**	-5.31 (-14.7 to 5.32)
13 Diabetes	13 Lower respiratory infect	**39.8 (15.4 to 65)**	**39 (21.4 to 58)**	-9.85 (-20.1 to 1.27)
14 Atrial fibrillation	14 Prostate cancer	**54.4 (28.1 to 83.9)**	**53.6 (31.4 to 78.8)**	6.85 (-5.23 to 19.7)
15 Breast cancer	15 Breast cancer	**20.8 (0.938 to 45.9)**	**20.1 (4.51 to 44.3)**	-9.9 (-19 to 2.99)
16 Prostate cancer	16 Diabetes	8.86 (-13 to 36.3)	8.2 (-13.1 to 30.9)	**-30.1 (-42.6 to -16.6)**
17 Nonrheum valv diseases	17 Parkinson's disease	**50.5 (30.7 to 71.2)**	**49.7 (37.2 to 64.1)**	4.72 (-2.76 to 13.2)
18 Cirrhosis liver	18 Urinary diseases	**139 (95.3 to 182)**	**138 (102 to 178)**	**49.9 (28.4 to 71.5)**
19 Parkinson's disease	19 Cirrhosis liver	1.08 (-11 to 14.2)	0.533 (-6.2 to 7.41)	**-8.71 (-14.8 to -1.58)**
20 Stomach cancer	20 Liver cancer	**67.9 (46.7 to 89.9)**	**67 (49.9 to 83.8)**	**37.3 (22.2 to 49.5)**
24 Liver cancer	25 Stomach cancer			
27 Urinary diseases	128 COVID-19			

Supplemental Results Figure S8. Leading 20 level 3 causes of deaths, and percentage change in number of deaths and all-age and age-standardised death rates by location, 2022 and 2050

Greece, Reference scenario

Legend:
Communicable, maternal, neonatal and nutritional
Non-communicable
Injuries

Leading Causes 2022	Leading Causes 2050	Mean % change number of DEATH	Mean % change all-age DEATH rate	Mean % change age-standardized DEATH rate
1 Ischemic heart disease	1 Ischemic heart disease	**-33.7 (-50.7 to -16.3)**	-15.9 (-35 to 0.472)	**-50.5 (-63.7 to -37.9)**
2 Stroke	2 Alzheimer's disease	**42.9 (20.2 to 68.7)**	**81 (62.2 to 101)**	2.9 (-1.69 to 8.47)
3 COVID-19	3 Chronic kidney disease	**73.4 (37.7 to 116)**	**120 (78.3 to 164)**	**25.1 (0.929 to 50.7)**
4 Alzheimer's disease	4 Stroke	**-28.7 (-40.8 to -14.8)**	-9.62 (-21.6 to 3.2)	**-46.1 (-53.5 to -39.3)**
5 Lung cancer	5 Lung cancer	4.82 (-16 to 29.2)	**32.8 (12.6 to 57.5)**	-11.7 (-24.7 to 2.82)
6 Lower respiratory infect	6 Lower respiratory infect	21 (-1.01 to 44.5)	**53.4 (34.5 to 74.1)**	**-13.9 (-24.9 to -0.905)**
7 Chronic kidney disease	7 COPD	**26.3 (1.09 to 51)**	**60.1 (33.5 to 89.7)**	-8.94 (-26.1 to 6.8)
8 COPD	8 Colorectal cancer	**34.8 (3.05 to 70.5)**	**70.7 (35.8 to 114)**	4.1 (-15.5 to 27.2)
9 Colorectal cancer	9 Hypertensive heart disease	**48.5 (16.9 to 83.8)**	**88.4 (50.4 to 124)**	7.52 (-14.8 to 30.6)
10 Hypertensive heart disease	10 Pancreatic cancer	**73.2 (35.7 to 116)**	**119 (79.1 to 172)**	**41.2 (19.5 to 69)**
11 Breast cancer	11 Breast cancer	26.4 (-1.34 to 58.6)	**60.1 (33.2 to 92.7)**	-0.847 (-14.7 to 15.3)
12 Pancreatic cancer	12 Prostate cancer	**33 (6.63 to 65.8)**	**68.5 (41.5 to 102)**	2.13 (-10.3 to 16.4)
13 Prostate cancer	13 Nonrheum valv diseases	**82.1 (47 to 122)**	**131 (97.2 to 170)**	**28.2 (14.4 to 44.9)**
14 Stomach cancer	14 Urinary diseases	**123 (69.9 to 182)**	**182 (126 to 246)**	**55.6 (27.3 to 88.7)**
15 Parkinson's disease	15 Atrial fibrillation	**57.4 (29.5 to 88.1)**	**99.5 (74.9 to 128)**	**11.9 (3.69 to 22.1)**
16 Bladder cancer	16 Parkinson's disease	**32.2 (9.1 to 56.6)**	**67.5 (49.4 to 87.2)**	2.12 (-6.17 to 11.8)
17 Diabetes	17 Brain cancer	**60 (34.2 to 84.7)**	**103 (85 to 117)**	**44.7 (37.6 to 50.3)**
18 Leukemia	18 Bladder cancer	20.4 (-0.462 to 43.1)	**52.5 (34.1 to 74.6)**	-6.81 (-15.1 to 2.69)
19 Atrial fibrillation	19 Leukemia	21.1 (-0.452 to 45.9)	**53.3 (34.7 to 75.1)**	-2.7 (-11.7 to 7.42)
20 Nonrheum valv diseases	20 Diabetes	4.81 (-21.1 to 35.6)	**32.9 (4.58 to 66)**	**-22.7 (-39.2 to -3.77)**
21 Brain cancer	22 Stomach cancer			
24 Urinary diseases	130 COVID-19			

Supplemental Results Figure S8. Leading 20 level 3 causes of deaths, and percentage change in number of deaths and all-age and age-standardised death rates by location, 2022 and 2050

Iceland, Reference scenario

Legend:
Communicable, maternal, neonatal and nutritional
Non-communicable
Injuries

Leading Causes 2022	Leading Causes 2050	Mean % change number of DEATH	Mean % change all-age DEATH rate	Mean % change age-standardized DEATH rate
1 Ischemic heart disease	1 Ischemic heart disease	15.5 (-13.1 to 48.5)	-16.9 (-35.3 to 6.72)	**-53.7 (-64.2 to -38.7)**
2 Alzheimer's disease	2 Alzheimer's disease	**145 (112 to 177)**	**76.5 (57.3 to 95.7)**	**-8.34 (-12.8 to -3.57)**
3 Lung cancer	3 Lower respiratory infect	**119 (83.9 to 165)**	**57.6 (34.7 to 87.6)**	**-16 (-28 to -2.59)**
4 Stroke	4 COPD	**109 (70.8 to 157)**	**50.7 (23.1 to 82.5)**	-12.6 (-28.1 to 7.55)
5 COVID-19	5 Lung cancer	**56.1 (27.7 to 83.1)**	12.3 (-7.35 to 33.6)	**-18.1 (-30.5 to -5.43)**
6 COPD	6 Stroke	**22.6 (3.3 to 50.1)**	-11.8 (-24 to 1.36)	**-49.5 (-56.4 to -41.5)**
7 Lower respiratory infect	7 Prostate cancer	**137 (90.3 to 191)**	**70.7 (38.6 to 115)**	0.468 (-12.9 to 15.7)
8 Colorectal cancer	8 Atrial fibrillation	**206 (160 to 253)**	**120 (92.8 to 150)**	**15.2 (6.18 to 26)**
9 Prostate cancer	9 Chronic kidney disease	**239 (173 to 312)**	**144 (100 to 200)**	**30.7 (8.07 to 59)**
10 Pancreatic cancer	10 Colorectal cancer	**89.8 (49.4 to 137)**	**36.6 (8.6 to 72)**	-12 (-29 to 6.94)
11 Falls	11 Pancreatic cancer	**131 (81.9 to 180)**	**66.1 (30.1 to 102)**	15.3 (-6.59 to 37.2)
12 Breast cancer	12 Falls	**128 (91.1 to 175)**	**64 (43.2 to 96.7)**	-9.19 (-19.1 to 2.34)
13 Atrial fibrillation	13 Nonrheum valv diseases	**205 (162 to 253)**	**119 (89.4 to 151)**	**17.4 (4.48 to 31.1)**
14 Self-harm	14 Parkinson's disease	**129 (100 to 161)**	**64.5 (46.5 to 85.1)**	-2.98 (-11.1 to 6.52)
15 Parkinson's disease	15 Breast cancer	**64.8 (34.2 to 95.6)**	18.6 (-2.35 to 40.9)	**-16.7 (-28.7 to -3.52)**
16 Chronic kidney disease	16 Urinary diseases	**249 (191 to 323)**	**151 (113 to 193)**	**32.4 (14 to 53.6)**
17 Nonrheum valv diseases	17 Hypertensive heart disease	**167 (112 to 234)**	**92.4 (54.6 to 138)**	5.23 (-16.2 to 28)
18 Brain cancer	18 Brain cancer	**115 (93.9 to 139)**	**54.9 (44.9 to 66.8)**	**27.8 (22.7 to 34.1)**
19 Kidney cancer	19 Self-harm	**29.5 (11.6 to 50)**	-6.87 (-16.8 to 3.89)	**-16.8 (-26.9 to -5.16)**
20 Diabetes	20 Liver cancer	**127 (90.1 to 167)**	**63.1 (40 to 87.7)**	**24.3 (10.3 to 40.9)**
22 Liver cancer	21 Kidney cancer			
23 Hypertensive heart disease	23 Diabetes			
29 Urinary diseases	127 COVID-19			

Supplemental Results Figure S8. Leading 20 level 3 causes of deaths, and percentage change in number of deaths and all-age and age-standardised death rates by location, 2022 and 2050

Ireland, Reference scenario

Legend:
Communicable, maternal, neonatal and nutritional
Non-communicable
Injuries

Leading Causes 2022	Leading Causes 2050	Mean % change number of DEATH	Mean % change all-age DEATH rate	Mean % change age-standardized DEATH rate
1 Ischemic heart disease	1 Alzheimer's disease	**182 (149 to 221)**	**124 (102 to 143)**	**-8.27 (-12.3 to -3.78)**
2 Alzheimer's disease	2 Ischemic heart disease	18.7 (-3.76 to 41.6)	-5.99 (-21.5 to 12.3)	**-57.1 (-63.1 to -48.5)**
3 COPD	3 COPD	**118 (84.8 to 161)**	**72.8 (46.9 to 102)**	**-18.4 (-29.5 to -7.38)**
4 Lung cancer	4 Lower respiratory infect	**154 (116 to 197)**	**101 (75.8 to 135)**	**-19.5 (-29.1 to -8.57)**
5 Stroke	5 Lung cancer	**54.1 (30.2 to 81.5)**	**22.1 (6.15 to 40.7)**	**-23.4 (-31.5 to -15.4)**
6 COVID-19	6 Chronic kidney disease	**274 (215 to 339)**	**197 (152 to 249)**	**28.5 (9.2 to 52.3)**
7 Lower respiratory infect	7 Colorectal cancer	**106 (68 to 143)**	**63.4 (34 to 88.9)**	-11 (-25 to 0.697)
8 Colorectal cancer	8 Stroke	**26.2 (8.93 to 45.1)**	-0.0177 (-11.8 to 12.9)	**-54.1 (-58.6 to -48.5)**
9 Chronic kidney disease	9 Prostate cancer	**153 (106 to 197)**	**100 (69.5 to 132)**	-1.48 (-13.3 to 9.84)
10 Breast cancer	10 Pancreatic cancer	**151 (111 to 198)**	**98.5 (65.9 to 129)**	**17.5 (1.35 to 31.9)**
11 Prostate cancer	11 Atrial fibrillation	**238 (194 to 294)**	**168 (138 to 207)**	9.7 (-0.14 to 19.3)
12 Pancreatic cancer	12 Breast cancer	**77 (48.5 to 106)**	**40.2 (20.7 to 59.6)**	**-13 (-23.1 to -3.22)**
13 Atrial fibrillation	13 Urinary diseases	**328 (254 to 410)**	**239 (185 to 302)**	**42.2 (22 to 64.1)**
14 Esophageal cancer	14 Parkinson's disease	**146 (117 to 183)**	**94.8 (76 to 117)**	-2.85 (-10.6 to 7.91)
15 Diabetes	15 Interstitial lung disease	**127 (97 to 155)**	**80.1 (62.5 to 98.2)**	-2.66 (-10.7 to 4.85)
16 Self-harm	16 Nonrheum valv diseases	**239 (187 to 301)**	**168 (134 to 211)**	**15.7 (4.32 to 29.4)**
17 Interstitial lung disease	17 Diabetes	**103 (61.3 to 144)**	**61.2 (33.4 to 92.1)**	**-25.9 (-37.5 to -11)**
18 Parkinson's disease	18 Falls	**141 (110 to 174)**	**90.7 (66 to 113)**	**-11.8 (-20.5 to -3.55)**
19 Stomach cancer	19 Brain cancer	**116 (89.5 to 139)**	**71.1 (59.9 to 82.2)**	**26.4 (20.5 to 33.2)**
20 Falls	20 Cardiomyopathy	**95.6 (68.5 to 123)**	**55 (35.4 to 78.3)**	**-27.3 (-33.3 to -19.7)**
21 Cardiomyopathy	21 Esophageal cancer			
22 Brain cancer	29 Stomach cancer			
26 Urinary diseases	32 Self-harm			
28 Nonrheum valv diseases	128 COVID-19			

Supplemental Results Figure S8. Leading 20 level 3 causes of deaths, and percentage change in number of deaths and all-age and age-standardised death rates by location, 2022 and 2050

Israel, Reference scenario

Legend:
Communicable, maternal, neonatal and nutritional
Non-communicable
Injuries

Leading Causes 2022	Leading Causes 2050	Mean % change number of DEATH	Mean % change all-age DEATH rate	Mean % change age-standardized DEATH rate
1 Ischemic heart disease	1 Chronic kidney disease	**230 (164 to 307)**	**142 (99.1 to 187)**	**31.7 (4.82 to 60.7)**
2 Alzheimer's disease	2 Alzheimer's disease	**136 (101 to 173)**	**73 (58.1 to 88.5)**	**-9.67 (-13.7 to -5.57)**
3 COVID-19	3 Ischemic heart disease	17.1 (-17.2 to 51.1)	-14.2 (-35.6 to 9.57)	**-51.8 (-64.4 to -37.2)**
4 Chronic kidney disease	4 Diabetes	**79.5 (37.5 to 132)**	**31.6 (5.08 to 58.6)**	**-25.4 (-41.5 to -7.6)**
5 Stroke	5 Colorectal cancer	**118 (59.8 to 191)**	**59.9 (26.2 to 101)**	-1.26 (-19.7 to 22.4)
6 Diabetes	6 Lung cancer	**59.4 (27.5 to 98.7)**	16.8 (-2.25 to 42)	**-19.3 (-31.1 to -3.41)**
7 Lung cancer	7 Lower respiratory infect	**103 (64 to 146)**	**48.7 (28.5 to 69.2)**	**-19 (-29.9 to -5.39)**
8 Lower respiratory infect	8 Stroke	22.7 (-0.995 to 49.7)	-10.1 (-21.2 to 2.77)	**-47.3 (-54.8 to -38.5)**
9 Colorectal cancer	9 COPD	**95 (52.2 to 136)**	**42.9 (18.8 to 67.1)**	**-16.7 (-31.1 to -1.87)**
10 COPD	10 Pancreatic cancer	**150 (91.1 to 216)**	**83 (47 to 122)**	**23.1 (1.66 to 47.1)**
11 Breast cancer	11 Urinary diseases	**245 (175 to 327)**	**153 (114 to 204)**	**34 (12.9 to 63.2)**
12 Pancreatic cancer	12 Breast cancer	**83 (43.9 to 125)**	**34 (11.5 to 59)**	-11.3 (-24 to 2.83)
13 Urinary diseases	13 Atrial fibrillation	**182 (134 to 234)**	**107 (83 to 132)**	8.94 (-0.517 to 18.5)
14 Atrial fibrillation	14 Nonrheum valv diseases	**190 (138 to 245)**	**113 (88 to 145)**	**13.4 (1.84 to 27.1)**
15 Lymphoma	15 Endo/metab/blood/immune	**144 (95.6 to 197)**	**79 (54.5 to 113)**	11.7 (-1.49 to 27.5)
16 Nonrheum valv diseases	16 Prostate cancer	**128 (76.9 to 196)**	**66.7 (36.6 to 107)**	-1.22 (-14.4 to 13.3)
17 Leukemia	17 Parkinson's disease	**118 (85.8 to 155)**	**59.5 (45.8 to 74.9)**	-4.58 (-12.1 to 4.27)
18 Stomach cancer	18 Lymphoma	**85.7 (47.4 to 127)**	**36.1 (14.7 to 59)**	-9.65 (-21.8 to 4.25)
19 Prostate cancer	19 Diarrheal diseases	**272 (93.1 to 514)**	**172 (44.8 to 361)**	44.4 (-24 to 144)
20 Parkinson's disease	20 Leukemia	**83.7 (44.4 to 131)**	**34.6 (10.9 to 63)**	-11.4 (-23.7 to 4.31)
21 Endo/metab/blood/immune	26 Stomach cancer			
33 Diarrheal diseases	129 COVID-19			

Supplemental Results Figure S8. Leading 20 level 3 causes of deaths, and percentage change in number of deaths and all-age and age-standardised death rates by location, 2022 and 2050

Italy, Reference scenario

Legend:
Communicable, maternal, neonatal and nutritional
Non-communicable
Injuries

Leading Causes 2022	Leading Causes 2050	Mean % change number of DEATH	Mean % change all-age DEATH rate	Mean % change age-standardized DEATH rate
1 Ischemic heart disease	1 Alzheimer's disease	**60.9 (41.6 to 81.2)**	**92.6 (76.8 to 108)**	-1.89 (-6.66 to 3.25)
2 Alzheimer's disease	2 Ischemic heart disease	-14.9 (-33.3 to 5.62)	2.03 (-18 to 24.6)	**-47.5 (-59 to -33.5)**
3 Stroke	3 Hypertensive heart disease	**76.5 (42.2 to 108)**	**112 (66.5 to 151)**	8.7 (-14.9 to 32.4)
4 COVID-19	4 Stroke	**-17.2 (-27.6 to -4.4)**	-0.796 (-12.9 to 12.9)	**-47.1 (-54.5 to -39.7)**
5 Lung cancer	5 COPD	**58.2 (32.4 to 84.3)**	**89.5 (59.9 to 120)**	-2.74 (-20.5 to 12.4)
6 Hypertensive heart disease	6 Chronic kidney disease	**122 (87.3 to 169)**	**166 (122 to 215)**	**32.5 (7.66 to 59.3)**
7 COPD	7 Lung cancer	17.9 (-1.6 to 42)	**41.2 (21.2 to 68.4)**	**-13.6 (-24.5 to -0.393)**
8 Colorectal cancer	8 Colorectal cancer	**38.9 (8.5 to 79.3)**	**66.2 (32.5 to 110)**	-5.51 (-22.5 to 16.8)
9 Chronic kidney disease	9 Pancreatic cancer	**79.7 (48 to 117)**	**115 (77.1 to 159)**	**30.5 (8.29 to 52.8)**
10 Diabetes	10 Diabetes	19.4 (-4.77 to 48.3)	**43 (14 to 75.8)**	**-26 (-40.6 to -8.65)**
11 Pancreatic cancer	11 Lower respiratory infect	**46.4 (22.5 to 72.9)**	**75.5 (51.9 to 104)**	-10.8 (-21.7 to 0.435)
12 Breast cancer	12 Atrial fibrillation	**94.1 (69.2 to 124)**	**132 (110 to 159)**	**17.4 (7.96 to 27.3)**
13 Lower respiratory infect	13 Falls	**52.3 (27.9 to 78.3)**	**82.4 (58.2 to 110)**	-6.28 (-15.2 to 3.64)
14 Falls	14 Prostate cancer	**69.8 (43.4 to 111)**	**103 (74 to 147)**	9.14 (-3.68 to 23.6)
15 Stomach cancer	15 Breast cancer	19.6 (-1.62 to 42.7)	**43.2 (21.7 to 70.7)**	-12.8 (-22.9 to 1.11)
16 Prostate cancer	16 Parkinson's disease	**57.6 (38.2 to 78.4)**	**88.7 (70.5 to 107)**	2.59 (-5.72 to 11.4)
17 Atrial fibrillation	17 Nonrheum valv diseases	**99 (68.3 to 132)**	**138 (105 to 171)**	**21.4 (8.79 to 36.4)**
18 Cirrhosis liver	18 Bladder cancer	**48 (28.6 to 73.8)**	**77.2 (56.9 to 101)**	-3.65 (-12.2 to 5.7)
19 Parkinson's disease	19 Stomach cancer	-3.08 (-13.8 to 10.6)	**16.1 (5.41 to 27.7)**	**-34.3 (-39.4 to -28.8)**
20 Bladder cancer	20 Liver cancer	**50.6 (28.6 to 75.6)**	**80.4 (58.2 to 102)**	**18.9 (4.58 to 33.8)**
22 Nonrheum valv diseases	22 Cirrhosis liver			
23 Liver cancer	128 COVID-19			

Supplemental Results Figure S8. Leading 20 level 3 causes of deaths, and percentage change in number of deaths and all-age and age-standardised death rates by location, 2022 and 2050

Luxembourg, Reference scenario

Legend:
Communicable, maternal, neonatal and nutritional
Non-communicable
Injuries

Leading Causes 2022	Leading Causes 2050	Mean % change number of DEATH	Mean % change all-age DEATH rate	Mean % change age-standardized DEATH rate
1 Ischemic heart disease	1 Ischemic heart disease	22.3 (-1.06 to 56.8)	-18.6 (-33 to 4.46)	**-49.5 (-58.3 to -32.9)**
2 Stroke	2 Alzheimer's disease	**161 (128 to 195)**	**73.6 (55.3 to 93.9)**	2.29 (-2.17 to 7.03)
3 Lung cancer	3 Chronic kidney disease	**250 (192 to 338)**	**133 (97.4 to 182)**	**39.6 (19.8 to 72.9)**
4 Alzheimer's disease	4 Lung cancer	**88.9 (52.9 to 119)**	**25.9 (1.02 to 44.1)**	-8.29 (-24.2 to 2.23)
5 COVID-19	5 COPD	**133 (94.5 to 180)**	**55.4 (32.5 to 82.6)**	-3.17 (-15.8 to 15.4)
6 COPD	6 Stroke	**29.2 (10.6 to 50.7)**	**-13.9 (-23.9 to -0.571)**	**-46 (-51.9 to -37.6)**
7 Colorectal cancer	7 Colorectal cancer	**120 (65 to 166)**	**46.2 (11.8 to 72.6)**	-3.54 (-23.3 to 15.2)
8 Chronic kidney disease	8 Lower respiratory infect	**140 (104 to 182)**	**59.6 (37.6 to 85.5)**	-4.25 (-15.4 to 11.7)
9 Lower respiratory infect	9 Pancreatic cancer	**178 (125 to 222)**	**85.2 (51.3 to 114)**	**29.7 (7.63 to 48.1)**
10 Breast cancer	10 Nonrheum valv diseases	**202 (156 to 255)**	**101 (70.5 to 133)**	**20.4 (4.76 to 35.4)**
11 Falls	11 Atrial fibrillation	**191 (149 to 230)**	**93.9 (69.4 to 118)**	**15 (3.96 to 26.3)**
12 Pancreatic cancer	12 Falls	**132 (98.2 to 170)**	**54.3 (30.8 to 77.8)**	-4.67 (-14.1 to 5.43)
13 Atrial fibrillation	13 Prostate cancer	**165 (113 to 221)**	**76.7 (45.9 to 110)**	9.47 (-4.51 to 23.4)
14 Nonrheum valv diseases	14 Breast cancer	**96.3 (58.6 to 129)**	**30.7 (6.74 to 47.7)**	-6.14 (-20.4 to 4.55)
15 Prostate cancer	15 Hypertensive heart disease	**161 (113 to 228)**	**74 (44.6 to 115)**	6.09 (-11.1 to 36.1)
16 Cirrhosis liver	16 Parkinson's disease	**154 (122 to 194)**	**69.5 (50.9 to 94.7)**	5.22 (-3.02 to 15.4)
17 Diabetes	17 Diabetes	**91.5 (52.9 to 134)**	**27.6 (3.32 to 54.9)**	**-21.5 (-34.8 to -4.53)**
18 Hypertensive heart disease	18 Cirrhosis liver	**50.6 (35.4 to 68.3)**	0.283 (-7.93 to 9.6)	**-17.2 (-23.1 to -10.5)**
19 Parkinson's disease	19 Leukemia	**110 (79.2 to 140)**	**39.8 (20.6 to 56.6)**	-4.72 (-15.4 to 3.57)
20 Self-harm	20 Endocarditis	**254 (174 to 361)**	**136 (81.1 to 207)**	**48.2 (17 to 95.2)**
21 Leukemia	28 Self-harm			
31 Endocarditis	127 COVID-19			

Supplemental Results Figure S8. Leading 20 level 3 causes of deaths, and percentage change in number of deaths and all-age and age-standardised death rates by location, 2022 and 2050

Malta, Reference scenario

Legend:
Communicable, maternal, neonatal and nutritional
Non-communicable
Injuries

Leading Causes 2022	Leading Causes 2050	Mean % change number of DEATH	Mean % change all-age DEATH rate	Mean % change age-standardized DEATH rate
1 Ischemic heart disease	1 Alzheimer's disease	**138 (97.2 to 186)**	**93.1 (64.5 to 127)**	-3.83 (-9.23 to 0.98)
2 COVID-19	2 Ischemic heart disease	-12.9 (-36.7 to 17.7)	**-29.4 (-47.6 to -7.57)**	**-57.4 (-68.5 to -41.7)**
3 Alzheimer's disease	3 Chronic kidney disease	**167 (103 to 240)**	**116 (70.7 to 172)**	20.7 (-3.34 to 55.5)
4 Stroke	4 Lower respiratory infect	**89.5 (54.7 to 126)**	**53.6 (28.6 to 81.8)**	**-19.4 (-30.4 to -5.03)**
5 Lower respiratory infect	5 Lung cancer	**37.7 (4.17 to 71.4)**	11.6 (-11.3 to 36.8)	-2.3 (-20.9 to 17.7)
6 Lung cancer	6 Stroke	-5.02 (-23.8 to 14.4)	**-23 (-35.3 to -8.81)**	**-51.6 (-59.5 to -40.4)**
7 Diabetes	7 Colorectal cancer	**64.5 (18.1 to 114)**	33.2 (-1.16 to 70.5)	-0.429 (-22.7 to 21)
8 Chronic kidney disease	8 Pancreatic cancer	**103 (51.9 to 156)**	**64.3 (24.6 to 104)**	**34.8 (6.17 to 65.8)**
9 Colorectal cancer	9 COPD	**70.8 (32.8 to 109)**	**38.3 (10.9 to 70.3)**	-11.1 (-26.6 to 8.1)
10 COPD	10 Diabetes	19 (-11.3 to 55.2)	-3.62 (-25.8 to 24.8)	**-37.7 (-51.4 to -19.1)**
11 Breast cancer	11 Atrial fibrillation	**163 (118 to 216)**	**113 (83.2 to 144)**	**13.5 (4.88 to 24.3)**
12 Pancreatic cancer	12 Hypertensive heart disease	**126 (67.3 to 191)**	**82.8 (38.3 to 138)**	2.67 (-19.6 to 32.3)
13 Falls	13 Breast cancer	**42.9 (11.6 to 80.6)**	15.8 (-6.91 to 40)	-7.83 (-24 to 9.34)
14 Hypertensive heart disease	14 Urinary diseases	**210 (147 to 292)**	**151 (103 to 209)**	**36.2 (12.6 to 65.8)**
15 Atrial fibrillation	15 Falls	**92 (57.7 to 133)**	**55.6 (29.3 to 86.1)**	**-11.2 (-20 to -1.67)**
16 Parkinson's disease	16 Prostate cancer	**97.2 (48.6 to 154)**	**59.8 (23.2 to 103)**	10.4 (-7.54 to 27.2)
17 Prostate cancer	17 Parkinson's disease	**75.5 (46.7 to 105)**	**42.2 (25.9 to 61.5)**	-0.177 (-8.86 to 8.7)
18 Interstitial lung disease	18 Interstitial lung disease	**69.9 (38.6 to 98.5)**	**37.6 (20.1 to 57.4)**	1.51 (-8.21 to 11.5)
19 Stomach cancer	19 Nonrheum valv diseases	**145 (99.7 to 208)**	**98.2 (67.3 to 136)**	**15.7 (3.85 to 31.8)**
20 Bladder cancer	20 Bladder cancer	**64.2 (35.2 to 95.8)**	**33.1 (14.3 to 54.8)**	-6.41 (-15.8 to 2.39)
21 Urinary diseases	26 Stomach cancer			
27 Nonrheum valv diseases	129 COVID-19			

Supplemental Results Figure S8. Leading 20 level 3 causes of deaths, and percentage change in number of deaths and all-age and age-standardised death rates by location, 2022 and 2050

Monaco, Reference scenario

Legend:
Communicable, maternal, neonatal and nutritional
Non-communicable
Injuries

Leading Causes 2022	Leading Causes 2050	Mean % change number of DEATH	Mean % change all-age DEATH rate	Mean % change age-standardized DEATH rate
1 Lung cancer	1 Lung cancer	-3.94 (-22.5 to 16.6)	**25.5 (4.18 to 49.4)**	-6.8 (-23 to 10.2)
2 Ischemic heart disease	2 Alzheimer's disease	**51.1 (26.9 to 79.6)**	**97.2 (70.2 to 127)**	3.83 (-1.36 to 9.55)
3 Stroke	3 Ischemic heart disease	**-34.3 (-50.9 to -12.2)**	-14.2 (-34.5 to 13.9)	**-50.7 (-62.2 to -34.9)**
4 Alzheimer's disease	4 Stroke	**-34.9 (-48.4 to -18.9)**	-15.1 (-30.6 to 2.15)	**-49.7 (-57.5 to -39.9)**
5 COVID-19	5 Lower respiratory infect	**34.2 (6.12 to 67.7)**	**75.2 (45.1 to 108)**	-4.91 (-18.5 to 11.7)
6 Colorectal cancer	6 Colorectal cancer	17.7 (-11.4 to 50)	**53.6 (18.7 to 88.3)**	-1.48 (-22.6 to 20.1)
7 Lower respiratory infect	7 Chronic kidney disease	**98.8 (46.5 to 156)**	**160 (108 to 231)**	**41.4 (15.7 to 81)**
8 Breast cancer	8 Pancreatic cancer	**46.4 (17.7 to 80.5)**	**91.3 (53.5 to 128)**	**33.6 (9.14 to 55.7)**
9 COPD	9 COPD	**33.2 (1.51 to 67.5)**	**74.1 (34.8 to 113)**	1.69 (-16.7 to 22.6)
10 Pancreatic cancer	10 Breast cancer	5.2 (-16.6 to 33.5)	**37.4 (11.5 to 68.6)**	-3.07 (-17.5 to 11.7)
11 Prostate cancer	11 Prostate cancer	**33.6 (7.44 to 68)**	**74.5 (42.6 to 108)**	4.88 (-10 to 20.2)
12 Chronic kidney disease	12 Hypertensive heart disease	**59.4 (14.1 to 114)**	**108 (60.2 to 171)**	16.9 (-5.93 to 50.6)
13 Hypertensive heart disease	13 Parkinson's disease	**35.8 (14.3 to 58.9)**	**77.4 (56.2 to 101)**	5.23 (-4.61 to 13.8)
14 Leukemia	14 Atrial fibrillation	**75.1 (43.5 to 114)**	**129 (96.5 to 161)**	**22.6 (12.1 to 32.9)**
15 Cardiomyopathy	15 Liver cancer	**31.8 (8.82 to 57.8)**	**72.2 (49.4 to 100)**	**33.2 (17.6 to 51.8)**
16 Parkinson's disease	16 Leukemia	8.63 (-9.9 to 29.2)	**41.9 (18.4 to 64.6)**	-2.98 (-14.4 to 7.98)
17 Liver cancer	17 Cardiomyopathy	3.49 (-13.3 to 25.3)	**35.1 (16.6 to 57.5)**	**-20.2 (-26.8 to -11.4)**
18 Cirrhosis liver	18 Bladder cancer	**17.4 (0.0935 to 39)**	**53.4 (33.2 to 74.2)**	-5.64 (-15 to 4.87)
19 Stomach cancer	19 Adverse medical treatment	26.8 (-10.4 to 66.6)	**65.6 (21 to 123)**	7.04 (-16.7 to 38.2)
20 Bladder cancer	20 Aortic aneurysm	8.61 (-10.7 to 30.5)	**41.9 (18.3 to 66.1)**	**-11.5 (-22 to -1.55)**
21 Atrial fibrillation	21 Stomach cancer			
22 Aortic aneurysm	23 Cirrhosis liver			
25 Adverse medical treatment	127 COVID-19			

Netherlands, Reference scenario

Legend:
- Communicable, maternal, neonatal and nutritional
- Non-communicable
- Injuries

Leading Causes 2022

Leading Causes 2022
1 Ischemic heart disease
2 Stroke
3 Lung cancer
4 Alzheimer's disease
5 COPD
6 Colorectal cancer
7 Lower respiratory infect
8 Falls
9 Chronic kidney disease
10 Breast cancer
11 Prostate cancer
12 Nonrheum valv diseases
13 Pancreatic cancer
14 Diabetes
15 COVID-19
16 Atrial fibrillation
17 Urinary diseases
18 Esophageal cancer
19 Parkinson's disease
20 Self-harm
26 Endocarditis
45 Bacterial skin

Leading Causes 2050

Leading Causes 2050	Mean % change number of DEATH	Mean % change all-age DEATH rate	Mean % change age-standardized DEATH rate
1 Alzheimer's disease	**85.3 (63.6 to 111)**	**74.5 (62.2 to 88.7)**	-2.99 (-7.4 to 2.35)
2 COPD	**56.5 (33.7 to 83.3)**	**47.4 (26.7 to 72.8)**	-8.8 (-21.5 to 6.63)
3 Chronic kidney disease	**194 (143 to 252)**	**177 (133 to 227)**	**52.8 (26.3 to 83.8)**
4 Lung cancer	13 (-3.79 to 31)	6.38 (-5.87 to 21.6)	**-17.3 (-26.4 to -7.14)**
5 Ischemic heart disease	-13.8 (-32 to 2.7)	**-18.7 (-33.2 to -2.92)**	**-52 (-61.4 to -42)**
6 Stroke	-7.2 (-19.8 to 6.92)	**-12.6 (-22 to -2.37)**	**-47.7 (-53.8 to -40.9)**
7 Lower respiratory infect	**68.6 (43 to 96.2)**	**58.9 (36.9 to 81.7)**	-11.4 (-23.2 to 0.608)
8 Colorectal cancer	**43 (14.7 to 73.8)**	**34.6 (12.1 to 59.3)**	-6.76 (-21.6 to 8.01)
9 Falls	**82.2 (54.7 to 112)**	**71.6 (50.7 to 94.3)**	-4.45 (-13.9 to 6.05)
10 Nonrheum valv diseases	**145 (115 to 189)**	**131 (105 to 160)**	**25.8 (14.5 to 40.2)**
11 Urinary diseases	**197 (146 to 252)**	**179 (135 to 236)**	**53.4 (29.1 to 82.5)**
12 Atrial fibrillation	**142 (109 to 172)**	**128 (104 to 150)**	**25.8 (15.4 to 36.4)**
13 Prostate cancer	**66.5 (38.4 to 97.1)**	**56.8 (35.2 to 78.8)**	2.1 (-10.3 to 14.8)
14 Pancreatic cancer	**73.5 (42.4 to 107)**	**63.4 (36.8 to 90)**	**22.5 (4.31 to 40.9)**
15 Breast cancer	**37.8 (14.8 to 58.5)**	**29.8 (10.3 to 47.6)**	-6.5 (-17.4 to 5.58)
16 Diabetes	26.1 (-0.653 to 55.3)	18.8 (-3.54 to 45.2)	**-30.5 (-42.5 to -15.3)**
17 Parkinson's disease	**84.5 (62.2 to 116)**	**73.8 (55.5 to 92.4)**	5.34 (-2.97 to 14.2)
18 Endocarditis	**173 (112 to 247)**	**157 (104 to 217)**	**49.9 (19 to 84.4)**
19 Bacterial skin	**272 (187 to 389)**	**250 (167 to 369)**	**100 (55.5 to 164)**
20 Esophageal cancer	**17.2 (4.19 to 31.9)**	**10.4 (0.221 to 21.6)**	**-14 (-20.5 to -6.49)**
31 Self-harm			
128 COVID-19			

Supplemental Results Figure S8. Leading 20 level 3 causes of deaths, and percentage change in number of deaths and all-age and age-standardised death rates by location, 2022 and 2050

Norway, Reference scenario

Legend:
Communicable, maternal, neonatal and nutritional
Non-communicable
Injuries

Leading Causes 2022	Leading Causes 2050	Mean % change number of DEATH	Mean % change all-age DEATH rate	Mean % change age-standardized DEATH rate
1 Ischemic heart disease	1 Alzheimer's disease	**94.5 (70.8 to 116)**	**68.4 (56.1 to 78.7)**	-4.59 (-9.36 to 0.349)
2 Alzheimer's disease	2 COPD	**67.5 (44.7 to 89.3)**	**45 (27.6 to 63)**	-11.1 (-21.1 to 0.0418)
3 COPD	3 Ischemic heart disease	-14.2 (-27.8 to 2.4)	**-25.8 (-35.8 to -12.7)**	**-56.5 (-62.7 to -48.7)**
4 Stroke	4 Lower respiratory infect	**90.9 (62.2 to 123)**	**65.2 (42.9 to 90.1)**	-6.77 (-19 to 6.92)
5 Lung cancer	5 Colorectal cancer	**65.3 (39.1 to 91.2)**	**43.1 (22.8 to 62.1)**	-8.97 (-20.7 to 2.18)
6 COVID-19	6 Lung cancer	**29.4 (12.4 to 46.6)**	12 (-0.249 to 24.6)	**-21.3 (-28.7 to -12.9)**
7 Lower respiratory infect	7 Falls	**95.3 (70 to 129)**	**69 (48.1 to 91.8)**	-3.62 (-13.6 to 7.79)
8 Colorectal cancer	8 Prostate cancer	**115 (77.1 to 151)**	**85.8 (56.3 to 111)**	9.25 (-4.25 to 20.8)
9 Falls	9 Stroke	-6.7 (-17.9 to 4.9)	**-19.3 (-26.9 to -11.9)**	**-51.3 (-55.8 to -46.5)**
10 Prostate cancer	10 Nonrheum valv diseases	**172 (134 to 213)**	**135 (107 to 167)**	**31.4 (16.9 to 46.4)**
11 Pancreatic cancer	11 Chronic kidney disease	**176 (135 to 227)**	**139 (110 to 178)**	**35.1 (18.1 to 58.9)**
12 Nonrheum valv diseases	12 Pancreatic cancer	**96.4 (71.8 to 122)**	**70 (48.5 to 90.8)**	**16.5 (2.5 to 29.7)**
13 Chronic kidney disease	13 Atrial fibrillation	**140 (111 to 174)**	**108 (89.3 to 131)**	**17.2 (6.82 to 27.5)**
14 Atrial fibrillation	14 Urinary diseases	**231 (176 to 294)**	**187 (144 to 235)**	**60.2 (36.6 to 84.4)**
15 Breast cancer	15 Diarrheal diseases	**301 (172 to 494)**	**247 (133 to 404)**	**94 (28.1 to 187)**
16 Parkinson's disease	16 Parkinson's disease	**93.1 (72.7 to 118)**	**67.1 (54.8 to 82.9)**	2.55 (-4.79 to 11.6)
17 Diabetes	17 Bacterial skin	**364 (250 to 505)**	**301 (211 to 413)**	**126 (79.9 to 186)**
18 Self-harm	18 Breast cancer	**48.6 (27.4 to 72)**	**28.6 (12.9 to 47.2)**	**-11.7 (-20.8 to -2.54)**
19 Urinary diseases	19 Aortic aneurysm	**50.9 (31.6 to 73.1)**	**30.6 (16.1 to 47.7)**	**-19.9 (-27.6 to -10.5)**
20 Aortic aneurysm	20 Diabetes	**25.1 (3.08 to 48.2)**	8.22 (-11 to 26.9)	**-36.6 (-46.6 to -26.7)**
24 Diarrheal diseases	28 Self-harm			
38 Bacterial skin	128 COVID-19			

Supplemental Results Figure S8. Leading 20 level 3 causes of deaths, and percentage change in number of deaths and all-age and age-standardised death rates by location, 2022 and 2050

Portugal, Reference scenario

Legend:
Communicable, maternal, neonatal and nutritional
Non-communicable
Injuries

Leading Causes 2022	Leading Causes 2050	Mean % change number of DEATH	Mean % change all-age DEATH rate	Mean % change age-standardized DEATH rate
1 Stroke	1 Alzheimer's disease	**72.5 (47 to 101)**	**75.7 (60.5 to 92)**	**-9.95 (-14.3 to -5.85)**
2 Ischemic heart disease	2 Lower respiratory infect	**55.1 (31.5 to 84.5)**	**58.1 (39 to 82.1)**	**-19.1 (-30.3 to -7.79)**
3 Alzheimer's disease	3 Stroke	**-24.7 (-37.7 to -8.05)**	**-23.3 (-33.9 to -12)**	**-57.1 (-63.3 to -50.1)**
4 COVID-19	4 Chronic kidney disease	**93.3 (55.3 to 134)**	**97 (61.2 to 132)**	-0.0999 (-19.5 to 23.1)
5 Lower respiratory infect	5 COPD	**60.2 (31.5 to 93.6)**	**63.2 (35.7 to 92.1)**	-14.5 (-29.6 to 2.5)
6 COPD	6 Ischemic heart disease	**-28.9 (-46.4 to -9.11)**	**-27.5 (-42.8 to -10.6)**	**-58.9 (-68.4 to -47.5)**
7 Chronic kidney disease	7 Colorectal cancer	**51.2 (15 to 94.3)**	**54.1 (21.4 to 90.6)**	-3.21 (-21.6 to 18.7)
8 Colorectal cancer	8 Lung cancer	**28.4 (5.28 to 58.3)**	**30.9 (8.66 to 59.4)**	-4.87 (-19.6 to 12.5)
9 Lung cancer	9 Urinary diseases	**146 (101 to 200)**	**151 (109 to 198)**	**27.4 (4.23 to 50.1)**
10 Diabetes	10 Diabetes	21.4 (-5.18 to 59.4)	23.6 (-2.07 to 55.7)	**-32 (-46.7 to -14.9)**
11 Stomach cancer	11 Prostate cancer	**60.1 (28.6 to 101)**	**63.1 (38.4 to 97.9)**	-3.82 (-14.7 to 10.2)
12 Prostate cancer	12 Pancreatic cancer	**85.4 (46.1 to 135)**	**88.8 (54.7 to 132)**	**30.1 (8.28 to 57.8)**
13 Hypertensive heart disease	13 Hypertensive heart disease	**64.1 (28.2 to 105)**	**67.2 (31.5 to 102)**	-14.7 (-34 to 4.09)
14 Breast cancer	14 Breast cancer	**33.3 (8.39 to 63.5)**	**35.8 (12.1 to 60.2)**	-11.3 (-23.7 to 3.45)
15 Urinary diseases	15 Nonrheum valv diseases	**93 (62.1 to 132)**	**96.6 (70.3 to 125)**	4.03 (-6.39 to 15.9)
16 Pancreatic cancer	16 Stomach cancer	-10 (-22.4 to 3.68)	-8.32 (-17 to 1.18)	**-40.4 (-44.3 to -36)**
17 Cirrhosis liver	17 Falls	**53.4 (28.8 to 80)**	**56.4 (36.6 to 78.3)**	**-12.6 (-20.3 to -3.38)**
18 Falls	18 Atrial fibrillation	**86.6 (58.1 to 118)**	**90.1 (69 to 116)**	-2.96 (-11 to 6.22)
19 Nonrheum valv diseases	19 Parkinson's disease	**47.9 (27 to 71.8)**	**50.6 (37 to 66.4)**	**-9.83 (-17.4 to -2.54)**
20 Parkinson's disease	20 Liver cancer	**67.2 (41.1 to 94.5)**	**70.4 (47.4 to 92.1)**	**36 (19.4 to 53.6)**
23 Atrial fibrillation	21 Cirrhosis liver			
24 Liver cancer	129 COVID-19			

Supplemental Results Figure S8. Leading 20 level 3 causes of deaths, and percentage change in number of deaths and all-age and age-standardised death rates by location, 2022 and 2050

San Marino, Reference scenario

Legend:
Communicable, maternal, neonatal and nutritional
Non-communicable
Injuries

Leading Causes 2022	Leading Causes 2050	Mean % change number of DEATH	Mean % change all-age DEATH rate	Mean % change age-standardized DEATH rate
1 COVID-19	1 Alzheimer's disease	**170 (104 to 243)**	**200 (148 to 262)**	4.06 (-1.33 to 9.33)
2 Alzheimer's disease	2 Ischemic heart disease	19.6 (-22.2 to 69.5)	33.2 (-5.96 to 85.9)	**-51.7 (-65.6 to -29.9)**
3 Ischemic heart disease	3 Stroke	19.4 (-14.9 to 56.5)	**32.8 (0.621 to 71.9)**	**-48.2 (-58.6 to -34.9)**
4 Stroke	4 Lower respiratory infect	**141 (77.4 to 214)**	**169 (104 to 238)**	-7.06 (-22.4 to 12)
5 Lung cancer	5 Colorectal cancer	**102 (38.5 to 185)**	**124 (60.8 to 200)**	-1.6 (-25.5 to 24.5)
6 Colorectal cancer	6 Lung cancer	**43.1 (5.48 to 98.6)**	**58.9 (19.3 to 107)**	-13 (-31.7 to 5.36)
7 Lower respiratory infect	7 Hypertensive heart disease	**189 (97 to 301)**	**221 (135 to 335)**	17.3 (-9.11 to 56.1)
8 Stomach cancer	8 Chronic kidney disease	**261 (143 to 391)**	**302 (193 to 436)**	**39.9 (6.64 to 82.6)**
9 COPD	9 COPD	**133 (62.3 to 212)**	**160 (93.3 to 249)**	-0.916 (-21.9 to 31.1)
10 Hypertensive heart disease	10 Atrial fibrillation	**217 (140 to 315)**	**252 (186 to 337)**	**21.1 (11.2 to 32.1)**
11 Other cardiovascular	11 Prostate cancer	**112 (47.6 to 189)**	**136 (73.8 to 220)**	-3.24 (-20.2 to 11.3)
12 Cirrhosis liver	12 Other cardiovascular	**105 (53.2 to 167)**	**128 (82.3 to 179)**	-12.2 (-22.5 to 1.81)
13 Prostate cancer	13 Stomach cancer	35.2 (-0.313 to 73.6)	**50.2 (26.9 to 80)**	**-33.4 (-38.9 to -28.1)**
14 Pancreatic cancer	14 Pancreatic cancer	**136 (64.4 to 233)**	**162 (95.9 to 241)**	**32.1 (4.03 to 62.4)**
15 Chronic kidney disease	15 Cirrhosis liver	**47.7 (11 to 88.1)**	**64.2 (36.9 to 95)**	-9.29 (-19.9 to 3.66)
16 Breast cancer	16 Breast cancer	**75 (20.2 to 147)**	**94.3 (41.2 to 164)**	-0.81 (-22.4 to 24.5)
17 Atrial fibrillation	17 Bladder cancer	**96.7 (43.2 to 161)**	**119 (73.9 to 173)**	-8.43 (-18.1 to 3.52)
18 Diabetes	18 Cardiomyopathy	**92.2 (44.9 to 162)**	**114 (67.5 to 172)**	**-18.2 (-26.6 to -10.2)**
19 Lymphoma	19 Diabetes	**77 (15.3 to 146)**	**97 (38 to 174)**	**-27.2 (-47.5 to -0.643)**
20 Bladder cancer	20 Brain cancer	**87.5 (52.7 to 132)**	**109 (83.4 to 138)**	**41.6 (34.1 to 48.6)**
21 Cardiomyopathy	21 Lymphoma			
22 Brain cancer	128 COVID-19			

Supplemental Results Figure S8. Leading 20 level 3 causes of deaths, and percentage change in number of deaths and all-age and age-standardised death rates by location, 2022 and 2050

Spain, Reference scenario

Legend:
Communicable, maternal, neonatal and nutritional
Non-communicable
Injuries

Leading Causes 2022	Leading Causes 2050	Mean % change number of DEATH	Mean % change all-age DEATH rate	Mean % change age-standardized DEATH rate
1 Ischemic heart disease	1 Alzheimer's disease	**81.4 (61.8 to 103)**	**60.7 (45.9 to 74.8)**	**-6.05 (-11.4 to -1.47)**
2 Alzheimer's disease	2 COPD	**76.8 (45.5 to 109)**	**56.5 (33.1 to 82.3)**	-9.42 (-23.6 to 7.03)
3 COVID-19	3 Chronic kidney disease	**175 (118 to 235)**	**143 (103 to 191)**	**38.1 (11.2 to 71.4)**
4 Stroke	4 Ischemic heart disease	-5.46 (-25.6 to 16.6)	-16.3 (-33.4 to 2.62)	**-50.4 (-62.2 to -37)**
5 COPD	5 Stroke	3.23 (-12.9 to 20.1)	-8.59 (-20 to 3.68)	**-44.1 (-52.5 to -34.8)**
6 Lung cancer	6 Colorectal cancer	**64.4 (29.8 to 108)**	**45.6 (15.9 to 82.5)**	-7.76 (-23.8 to 11.4)
7 Colorectal cancer	7 Lung cancer	14.3 (-4.43 to 36.1)	1.2 (-15.5 to 19.6)	**-27.4 (-37.8 to -15.8)**
8 Chronic kidney disease	8 Lower respiratory infect	**68.1 (43.3 to 97.2)**	**48.9 (29.7 to 67.7)**	**-13.8 (-23.9 to -2.81)**
9 Lower respiratory infect	9 Urinary diseases	**183 (137 to 236)**	**150 (114 to 193)**	**43.6 (23 to 68.9)**
10 Hypertensive heart disease	10 Hypertensive heart disease	**88.2 (45.6 to 129)**	**66.7 (31.9 to 97.7)**	-1.59 (-21.5 to 22.1)
11 Diabetes	11 Pancreatic cancer	**106 (65 to 149)**	**82.3 (47.5 to 122)**	**24.5 (3.12 to 48.9)**
12 Pancreatic cancer	12 Atrial fibrillation	**132 (103 to 167)**	**106 (83.6 to 133)**	**19.3 (8.94 to 29.2)**
13 Breast cancer	13 Nonrheum valv diseases	**130 (98.8 to 169)**	**104 (81 to 133)**	**18.6 (6.2 to 31.5)**
14 Prostate cancer	14 Prostate cancer	**96.6 (64.3 to 137)**	**74.2 (47 to 106)**	5.78 (-7.31 to 20)
15 Urinary diseases	15 Diabetes	**42.6 (9.23 to 78.4)**	26.3 (-0.539 to 54.2)	**-27 (-44.2 to -8.67)**
16 Atrial fibrillation	16 Parkinson's disease	**86.8 (60 to 113)**	**65.4 (47.7 to 83.6)**	0.977 (-7.47 to 10.7)
17 Nonrheum valv diseases	17 Breast cancer	**40.5 (18.2 to 67.7)**	**24.5 (4.12 to 46.9)**	-13.8 (-25.2 to 0.474)
18 Stomach cancer	18 Interstitial lung disease	**84.9 (59.3 to 112)**	**63.8 (45 to 83.7)**	1.63 (-6.78 to 11.3)
19 Cirrhosis liver	19 Bladder cancer	**62.9 (39.3 to 88.3)**	**44.3 (26.2 to 64.9)**	**-11.1 (-20.1 to -2.01)**
20 Parkinson's disease	20 Liver cancer	**65.1 (40.1 to 88.9)**	**46.2 (27.6 to 65.3)**	5.07 (-8.07 to 18.5)
21 Bladder cancer	23 Stomach cancer			
22 Interstitial lung disease	24 Cirrhosis liver			
24 Liver cancer	131 COVID-19			

Supplemental Results Figure S8. Leading 20 level 3 causes of deaths, and percentage change in number of deaths and all-age and age-standardised death rates by location, 2022 and 2050

Sweden, Reference scenario

Legend:
Communicable, maternal, neonatal and nutritional
Non-communicable
Injuries

Leading Causes 2022	Leading Causes 2050	Mean % change number of DEATH	Mean % change all-age DEATH rate	Mean % change age-standardized DEATH rate
1 Ischemic heart disease	1 Alzheimer's disease	**79.8 (54 to 102)**	**39.9 (25.3 to 55.6)**	-2.83 (-7.55 to 2.41)
2 Alzheimer's disease	2 Ischemic heart disease	**-19.1 (-33.8 to -0.815)**	**-37.1 (-48.6 to -23.7)**	**-54.7 (-63.1 to -43.6)**
3 Stroke	3 Chronic kidney disease	**162 (114 to 215)**	**103 (69.8 to 143)**	**41 (18 to 68.5)**
4 COVID-19	4 Atrial fibrillation	**126 (91.2 to 165)**	**75.6 (53.8 to 101)**	**20.2 (10.2 to 32.5)**
5 Lung cancer	5 Stroke	**-15.2 (-26.5 to -1.93)**	**-34.1 (-41.3 to -24.7)**	**-50 (-56.3 to -42.1)**
6 COPD	6 COPD	**52.8 (27.2 to 79.4)**	**18.9 (1.96 to 40.6)**	-6.51 (-19.5 to 11.9)
7 Colorectal cancer	7 Lung cancer	**34.5 (13.3 to 60.1)**	4.66 (-9.17 to 19.9)	-7.66 (-18.7 to 3.77)
8 Atrial fibrillation	8 Colorectal cancer	**53 (21.3 to 85.6)**	19 (-3.81 to 43.5)	-4.89 (-20.1 to 11.8)
9 Prostate cancer	9 Prostate cancer	**80 (47.7 to 123)**	**40 (18.4 to 67.8)**	7.03 (-5.97 to 20.7)
10 Chronic kidney disease	10 Pancreatic cancer	**88.7 (52.5 to 128)**	**46.8 (20.5 to 72.6)**	**26.1 (8.19 to 45.1)**
11 Lower respiratory infect	11 Lower respiratory infect	**54.3 (26.2 to 83.4)**	**20 (3.37 to 38.4)**	**-14.3 (-26.7 to -1.17)**
12 Pancreatic cancer	12 Hypertensive heart disease	**96.5 (53.7 to 148)**	**52.8 (23.3 to 89.6)**	6.9 (-13.8 to 32.2)
13 Falls	13 Falls	**71.9 (42.4 to 102)**	**33.8 (14.9 to 53.6)**	-5.27 (-14.2 to 4.95)
14 Diabetes	14 Nonrheum valv diseases	**143 (103 to 188)**	**89.1 (63.1 to 122)**	**29.5 (16 to 45.8)**
15 Hypertensive heart disease	15 Diarrheal diseases	**216 (93.2 to 390)**	**146 (48.8 to 275)**	**70.4 (1.57 to 169)**
16 Breast cancer	16 Diabetes	19.4 (-8.24 to 48.8)	-7.12 (-29.2 to 14.6)	**-32.2 (-45.8 to -17)**
17 Nonrheum valv diseases	17 Breast cancer	**44.3 (18.2 to 73.2)**	12.2 (-6.09 to 31.6)	-7.43 (-19 to 5.28)
18 Self-harm	18 Parkinson's disease	**66.7 (43.5 to 90.8)**	**29.7 (17.3 to 45.6)**	3.11 (-4.33 to 12.5)
19 Parkinson's disease	19 Bacterial skin	**310 (204 to 420)**	**219 (142 to 304)**	**128 (75.5 to 196)**
20 Aortic aneurysm	20 Urinary diseases	**187 (136 to 245)**	**124 (86.1 to 168)**	**54.8 (29.6 to 79.6)**
22 Diarrheal diseases	21 Self-harm			
33 Urinary diseases	23 Aortic aneurysm			
40 Bacterial skin	128 COVID-19			

Supplemental Results Figure S8. Leading 20 level 3 causes of deaths, and percentage change in number of deaths and all-age and age-standardised death rates by location, 2022 and 2050

Switzerland, Reference scenario

Legend:
Communicable, maternal, neonatal and nutritional
Non-communicable
Injuries

Leading Causes 2022
1 Ischemic heart disease
2 Alzheimer's disease
3 Stroke
4 Lung cancer
5 Chronic kidney disease
6 COPD
7 Hypertensive heart disease
8 Falls
9 Colorectal cancer
10 COVID-19
11 Lower respiratory infect
12 Prostate cancer
13 Pancreatic cancer
14 Breast cancer
15 Diabetes
16 Self-harm
17 Parkinson's disease
18 Nonrheum valv diseases
19 Endocarditis
20 Atrial fibrillation
28 Urinary diseases
30 Other digestive

Leading Causes 2050	Mean % change number of DEATH	Mean % change all-age DEATH rate	Mean % change age-standardized DEATH rate
1 Alzheimer's disease	**113 (86.3 to 141)**	**89.2 (75.6 to 103)**	-4.49 (-9 to 0.0862)
2 Ischemic heart disease	4.61 (-11.7 to 23.6)	-7.16 (-20.5 to 6.98)	**-53 (-59.6 to -44.6)**
3 Chronic kidney disease	**221 (161 to 278)**	**185 (141 to 228)**	**39.4 (19.7 to 61.6)**
4 Hypertensive heart disease	**149 (104 to 196)**	**121 (87.3 to 157)**	11.7 (-4.77 to 29.3)
5 Lung cancer	**37.4 (17.2 to 56.7)**	**21.8 (6.94 to 36.1)**	**-18.2 (-26.5 to -10)**
6 COPD	**94.4 (67 to 126)**	**72.5 (51.1 to 98.6)**	-4.71 (-13.7 to 7.93)
7 Falls	**104 (73.3 to 137)**	**81.3 (57.1 to 103)**	-4.66 (-15 to 6.37)
8 Stroke	6.84 (-7.64 to 21.6)	-5.24 (-15.8 to 5.95)	**-48.8 (-53.9 to -42.2)**
9 Prostate cancer	**124 (89.3 to 166)**	**99.1 (70.4 to 131)**	6.14 (-6.07 to 18.3)
10 Lower respiratory infect	**98.5 (68.6 to 132)**	**76.1 (53.7 to 99.4)**	-10.9 (-21.8 to 0.991)
11 Colorectal cancer	**71 (38.9 to 103)**	**51.7 (26.9 to 74.7)**	**-11.9 (-24.2 to -0.88)**
12 Pancreatic cancer	**106 (68.2 to 139)**	**82.4 (56.1 to 105)**	**15.9 (1.45 to 30.6)**
13 Endocarditis	**244 (180 to 334)**	**205 (154 to 281)**	**57.1 (32.6 to 91.1)**
14 Nonrheum valv diseases	**186 (140 to 233)**	**154 (118 to 191)**	**25.5 (10.3 to 41.3)**
15 Breast cancer	**48.6 (22.6 to 77.5)**	**31.8 (13.3 to 50.4)**	**-15.9 (-25.5 to -6.11)**
16 Atrial fibrillation	**165 (131 to 207)**	**135 (112 to 162)**	**17.1 (8.37 to 27.4)**
17 Parkinson's disease	**111 (82.6 to 141)**	**86.9 (69.4 to 107)**	3.63 (-4.18 to 12.7)
18 Urinary diseases	**244 (195 to 310)**	**205 (161 to 257)**	**51.2 (29.6 to 72.4)**
19 Diabetes	**50.2 (19 to 85.1)**	**33.3 (6.18 to 61.5)**	**-31.6 (-42.6 to -18.7)**
20 Other digestive	**219 (162 to 273)**	**183 (143 to 225)**	**46.1 (29.1 to 70.5)**
26 Self-harm			
128 COVID-19			

Supplemental Results Figure S8. Leading 20 level 3 causes of deaths, and percentage change in number of deaths and all-age and age-standardised death rates by location, 2022 and 2050

UK, Reference scenario

Legend:
Communicable, maternal, neonatal and nutritional
Non-communicable
Injuries

Leading Causes 2022	Leading Causes 2050	Mean % change number of DEATH	Mean % change all-age DEATH rate	Mean % change age-standardized DEATH rate
1 Ischemic heart disease	1 Alzheimer's disease	**90.8 (74.1 to 107)**	**76.6 (63.4 to 91.7)**	-0.762 (-5.29 to 4.26)
2 Lower respiratory infect	2 Lower respiratory infect	**69.2 (47.4 to 91.6)**	**56.6 (35.9 to 77.6)**	-11.5 (-22.7 to 1.18)
3 Stroke	3 Ischemic heart disease	**-18.5 (-34.3 to -0.182)**	**-24.5 (-39.4 to -6.11)**	**-53.8 (-63.4 to -40.6)**
4 COPD	4 COPD	**49.2 (26.6 to 73.1)**	**38.1 (15.9 to 61.1)**	-12.7 (-27.3 to 1.59)
5 Lung cancer	5 Lung cancer	**41.6 (16 to 70.2)**	**31 (8.74 to 51.9)**	-9.6 (-22.7 to 4.29)
6 Alzheimer's disease	6 Colorectal cancer	**61.2 (26.9 to 102)**	**49 (19.9 to 83.9)**	-4.03 (-20.6 to 15.3)
7 COVID-19	7 Stroke	**-12 (-22.6 to -2.56)**	**-18.6 (-29.8 to -9.8)**	**-50.2 (-56.8 to -43.7)**
8 Colorectal cancer	8 Prostate cancer	**81.4 (50.2 to 120)**	**67.8 (41.3 to 103)**	2.77 (-10.6 to 18.6)
9 Prostate cancer	9 Pancreatic cancer	**99.3 (58.1 to 144)**	**84.3 (51.8 to 120)**	**27.2 (6.81 to 50.5)**
10 Breast cancer	10 Chronic kidney disease	**133 (93.1 to 171)**	**116 (77 to 150)**	22.5 (-0.172 to 46)
11 Pancreatic cancer	11 Urinary diseases	**189 (146 to 240)**	**167 (126 to 219)**	**49.8 (25.6 to 76.1)**
12 Falls	12 Breast cancer	**50.4 (25 to 79)**	**39.1 (19.3 to 62.9)**	-6.34 (-18.3 to 7.43)
13 Chronic kidney disease	13 Atrial fibrillation	**109 (86 to 136)**	**93.4 (74.7 to 117)**	**8.61 (0.0549 to 18.7)**
14 Esophageal cancer	14 Falls	**69.7 (46.6 to 94.2)**	**57 (37.3 to 79.7)**	-7.91 (-15.6 to 2.36)
15 Atrial fibrillation	15 Nonrheum valv diseases	**143 (107 to 176)**	**125 (97.6 to 152)**	**26.6 (13.9 to 40.6)**
16 Cirrhosis liver	16 Interstitial lung disease	**77.9 (56.3 to 102)**	**64.6 (46.4 to 83.8)**	6.3 (-2.89 to 17)
17 Interstitial lung disease	17 Parkinson's disease	**77 (57.4 to 97.5)**	**63.8 (48.8 to 81)**	2.65 (-6.49 to 11.8)
18 Parkinson's disease	18 Other digestive	**163 (125 to 208)**	**144 (111 to 185)**	**43.6 (24.1 to 68.4)**
19 Urinary diseases	19 Esophageal cancer	**27.1 (12.5 to 39.8)**	**17.6 (6.67 to 29.8)**	**-16.2 (-23.3 to -9.3)**
20 Liver cancer	20 Liver cancer	**67.6 (47.6 to 87.8)**	**55.1 (37.3 to 72.7)**	**12.2 (0.308 to 23.9)**
21 Nonrheum valv diseases	26 Cirrhosis liver			
30 Other digestive	128 COVID-19			

Supplemental Results Figure S8. Leading 20 level 3 causes of deaths, and percentage change in number of deaths and all-age and age-standardised death rates by location, 2022 and 2050

Bolivia, Reference scenario

Legend:
Communicable, maternal, neonatal and nutritional
Non-communicable
Injuries

Leading Causes 2022
1 COVID-19
2 Ischemic heart disease
3 Lower respiratory infect
4 Stroke
5 Chronic kidney disease
6 Diabetes
7 Cirrhosis liver
8 Stomach cancer
9 Neonatal disorders
10 Road injuries
11 COPD
12 Congenital defects
13 Tuberculosis
14 Lung cancer
15 Colorectal cancer
16 Cervical cancer
17 Hypertensive heart disease
18 Prostate cancer
19 Alzheimer's disease
20 Breast cancer
21 Interstitial lung disease
22 Falls
27 Pancreatic cancer
36 Urinary diseases

Leading Causes 2050	Mean % change number of DEATH	Mean % change all-age DEATH rate	Mean % change age-standardized DEATH rate
1 Chronic kidney disease	**217 (103 to 349)**	**142 (64.9 to 232)**	38.1 (-8.15 to 101)
2 Lower respiratory infect	**77.1 (32.4 to 120)**	**35.5 (5.76 to 66.6)**	**-21.3 (-37.9 to -1.38)**
3 Ischemic heart disease	58.4 (-4.03 to 147)	21.3 (-25.2 to 84.2)	-34.1 (-61.5 to 4.51)
4 Stroke	**49.7 (9.9 to 91.9)**	14.6 (-11.8 to 43.6)	**-36.2 (-52.2 to -17.3)**
5 Diabetes	**77.4 (23 to 141)**	35.8 (-2.35 to 86.8)	-22 (-46.3 to 6.54)
6 Cirrhosis liver	**93.6 (58.2 to 137)**	**48.2 (27.4 to 71.2)**	-10.5 (-24.1 to 3.97)
7 COPD	**167 (91.7 to 258)**	**104 (47.5 to 174)**	3.9 (-31 to 40.2)
8 Stomach cancer	**59.4 (24.7 to 97.6)**	**21.9 (2.3 to 45.4)**	**-30.2 (-38.7 to -20.5)**
9 Lung cancer	**158 (70.8 to 268)**	**96.9 (39.8 to 171)**	12.3 (-17.6 to 49.7)
10 Colorectal cancer	**151 (53.2 to 279)**	**92 (24.2 to 184)**	8.41 (-27.4 to 53.1)
11 Prostate cancer	**196 (87.7 to 347)**	**126 (48.4 to 229)**	16.5 (-12.9 to 55.9)
12 Hypertensive heart disease	**161 (72.6 to 264)**	**99.8 (39.1 to 174)**	2.55 (-34 to 45.8)
13 Alzheimer's disease	**212 (115 to 312)**	**139 (77.4 to 202)**	**8.08 (4.17 to 11.8)**
14 Road injuries	33.1 (-18.8 to 105)	1.99 (-35.8 to 51.2)	-20.8 (-50.8 to 21.6)
15 Pancreatic cancer	**252 (104 to 478)**	**169 (64.4 to 326)**	52.2 (-7.91 to 132)
16 Interstitial lung disease	**172 (109 to 242)**	**108 (66.3 to 149)**	10.3 (-6.69 to 32)
17 Breast cancer	**112 (46.3 to 201)**	**62.4 (16.2 to 127)**	0.987 (-24.7 to 37.5)
18 Cervical cancer	**65.2 (27.8 to 103)**	**26.5 (2.36 to 52.8)**	**-20.7 (-36.3 to -4.67)**
19 Urinary diseases	**233 (151 to 330)**	**155 (95.7 to 226)**	**37.3 (2.06 to 80.3)**
20 Falls	**91.3 (50.3 to 145)**	**46.3 (20.2 to 80.6)**	-7.45 (-18.7 to 4.97)
24 Neonatal disorders			
27 Tuberculosis			
29 Congenital defects			
134 COVID-19			

Supplemental Results Figure S8. Leading 20 level 3 causes of deaths, and percentage change in number of deaths and all-age and age-standardised death rates by location, 2022 and 2050

Ecuador, Reference scenario

Legend:
Communicable, maternal, neonatal and nutritional
Non-communicable
Injuries

Leading Causes 2022	Leading Causes 2050	Mean % change number of DEATH	Mean % change all-age DEATH rate	Mean % change age-standardized DEATH rate
1 Ischemic heart disease	1 Chronic kidney disease	**240 (134 to 374)**	**173 (91.1 to 267)**	36.6 (-5.97 to 100)
2 COVID-19	2 Ischemic heart disease	**71.7 (8.45 to 143)**	37.8 (-10.1 to 94.3)	-35.5 (-60.1 to 1.01)
3 Chronic kidney disease	3 Lower respiratory infect	**115 (63.8 to 178)**	**71.9 (33.9 to 113)**	-20.5 (-37.4 to 0.653)
4 Stroke	4 Stroke	**60.3 (19.8 to 103)**	**28.5 (1.3 to 60.8)**	**-36 (-52 to -16.4)**
5 Lower respiratory infect	5 Diabetes	**109 (52.3 to 167)**	**67.5 (24.8 to 115)**	-15.8 (-39.4 to 14.5)
6 Diabetes	6 COPD	**201 (111 to 311)**	**142 (75.5 to 214)**	0.108 (-30.2 to 39.5)
7 Road injuries	7 Cirrhosis liver	**90.8 (55.8 to 123)**	**53 (35.1 to 72.8)**	**-12.4 (-21.8 to -0.753)**
8 Cirrhosis liver	8 Alzheimer's disease	**215 (139 to 305)**	**152 (103 to 207)**	3.58 (-0.129 to 6.91)
9 Stomach cancer	9 Hypertensive heart disease	**211 (112 to 330)**	**149 (72.2 to 236)**	7.53 (-30.2 to 61.9)
10 COPD	10 Road injuries	20.9 (-19.7 to 73.8)	-2.9 (-35.4 to 41.8)	-18.2 (-47.3 to 19.7)
11 Alzheimer's disease	11 Stomach cancer	**56.4 (21.9 to 97.3)**	**25.4 (7.26 to 49.3)**	**-34.4 (-41.1 to -25.7)**
12 Hypertensive heart disease	12 Prostate cancer	**184 (80.1 to 322)**	**128 (51.6 to 226)**	3.01 (-23 to 33.1)
13 Neonatal disorders	13 Colorectal cancer	**170 (65 to 320)**	**116 (42.2 to 221)**	11.8 (-23.8 to 59.3)
14 Prostate cancer	14 Lung cancer	**172 (83.9 to 292)**	**117 (54.7 to 199)**	12.4 (-12.9 to 46.9)
15 Colorectal cancer	15 Interstitial lung disease	**185 (120 to 265)**	**128 (83.2 to 177)**	4.73 (-10.3 to 21.6)
16 Self-harm	16 Falls	**112 (61.8 to 170)**	**69.9 (40.3 to 105)**	-10.5 (-21.2 to 1.79)
17 Interpersonal violence	17 Pancreatic cancer	**242 (97.9 to 461)**	**173 (60.6 to 325)**	43 (-11.1 to 117)
18 Lung cancer	18 Breast cancer	**118 (49.9 to 204)**	**74.1 (23.9 to 134)**	0.759 (-25.5 to 33)
19 Falls	19 Urinary diseases	**279 (172 to 399)**	**204 (133 to 285)**	**36.1 (6.72 to 71.3)**
20 Congenital defects	20 Atrial fibrillation	**272 (172 to 398)**	**198 (132 to 290)**	20 (-2.01 to 42.5)
21 Interstitial lung disease	22 Self-harm			
22 Breast cancer	31 Interpersonal violence			
26 Pancreatic cancer	33 Neonatal disorders			
31 Urinary diseases	41 Congenital defects			
32 Atrial fibrillation	134 COVID-19			

Supplemental Results Figure S8. Leading 20 level 3 causes of deaths, and percentage change in number of deaths and all-age and age-standardised death rates by location, 2022 and 2050

Peru, Reference scenario

Legend:
Communicable, maternal, neonatal and nutritional
Non-communicable
Injuries

Leading Causes 2022	Leading Causes 2050	Mean % change number of DEATH	Mean % change all-age DEATH rate	Mean % change age-standardized DEATH rate
1 Lower respiratory infect	1 Lower respiratory infect	**114 (66.3 to 164)**	**88.7 (50.3 to 133)**	**-24.2 (-38.2 to -5.43)**
2 COVID-19	2 Chronic kidney disease	**275 (156 to 415)**	**231 (134 to 350)**	**39.3 (1.79 to 91.3)**
3 Ischemic heart disease	3 Ischemic heart disease	**66.1 (0.662 to 156)**	46.8 (-7.38 to 124)	**-40.4 (-61.6 to -5.51)**
4 Chronic kidney disease	4 Stroke	**50 (14 to 91.5)**	**32.6 (4.28 to 66.5)**	**-40.3 (-53.6 to -24.5)**
5 Stroke	5 Alzheimer's disease	**224 (152 to 301)**	**187 (133 to 247)**	-1.99 (-5.49 to 1.32)
6 Cirrhosis liver	6 Cirrhosis liver	**86 (54.3 to 117)**	**64.4 (40.7 to 85.7)**	**-13.8 (-25.2 to -1.71)**
7 Stomach cancer	7 Interstitial lung disease	**192 (139 to 260)**	**158 (115 to 213)**	1.48 (-12.8 to 17.7)
8 Diabetes	8 Diabetes	**95.7 (45.4 to 157)**	**73 (28 to 120)**	**-24 (-42.3 to -0.629)**
9 Road injuries	9 Stomach cancer	**56.3 (30.2 to 85.5)**	**38.1 (16.5 to 60.8)**	**-37.7 (-44.1 to -30.2)**
10 Alzheimer's disease	10 Lung cancer	**145 (82.2 to 223)**	**116 (60.9 to 175)**	-1.08 (-23.2 to 23.7)
11 Interstitial lung disease	11 COPD	**191 (108 to 309)**	**157 (89.3 to 250)**	-5.31 (-31 to 29.4)
12 Lung cancer	12 Prostate cancer	**188 (102 to 309)**	**154 (81 to 256)**	-1.4 (-25.5 to 27.4)
13 Neonatal disorders	13 Colorectal cancer	**145 (60.5 to 262)**	**116 (41 to 214)**	-4.39 (-34.4 to 31.1)
14 Colorectal cancer	14 Urinary diseases	**274 (179 to 384)**	**230 (159 to 312)**	26.5 (-1.03 to 55.6)
15 COPD	15 Pancreatic cancer	**223 (103 to 383)**	**185 (72.4 to 320)**	30.2 (-17.4 to 90)
16 Prostate cancer	16 Road injuries	15 (-23.6 to 70.5)	1.69 (-31.2 to 48.6)	-20.8 (-46.8 to 18)
17 Cervical cancer	17 Hypertensive heart disease	**176 (92.9 to 296)**	**144 (72.2 to 243)**	-2.54 (-31.3 to 38.7)
18 Tuberculosis	18 Parkinson's disease	**173 (116 to 239)**	**141 (98.7 to 188)**	-8.65 (-21.5 to 6.21)
19 Foreign body	19 Atrial fibrillation	**262 (179 to 370)**	**220 (156 to 309)**	14.3 (-3.23 to 37)
20 Breast cancer	20 Breast cancer	**89.6 (39.9 to 154)**	**67.5 (26.2 to 121)**	-11 (-30.3 to 11.3)
22 Pancreatic cancer	21 Cervical cancer			
24 Urinary diseases	28 Foreign body			
25 Hypertensive heart disease	34 Tuberculosis			
26 Parkinson's disease	36 Neonatal disorders			
35 Atrial fibrillation	134 COVID-19			

Supplemental Results Figure S8. Leading 20 level 3 causes of deaths, and percentage change in number of deaths and all-age and age-standardised death rates by location, 2022 and 2050

Antigua and Barbuda, Reference scenario

Legend:
Communicable, maternal, neonatal and nutritional
Non-communicable
Injuries

Leading Causes 2022	Leading Causes 2050	Mean % change number of DEATH	Mean % change all-age DEATH rate	Mean % change age-standardized DEATH rate
1 Stroke	1 Chronic kidney disease	**148 (68.7 to 243)**	**147 (80.9 to 243)**	19.7 (-18.3 to 81.5)
2 Ischemic heart disease	2 Hypertensive heart disease	**164 (87.4 to 262)**	**163 (90.6 to 248)**	13.8 (-23.8 to 69.6)
3 Diabetes	3 Diabetes	**65.7 (17.2 to 122)**	**65.1 (24 to 111)**	-23.4 (-44.3 to 6.19)
4 Chronic kidney disease	4 Stroke	31.2 (-0.0512 to 66.9)	**30.7 (2.81 to 61.9)**	**-41.8 (-57.3 to -20.8)**
5 Hypertensive heart disease	5 Ischemic heart disease	25 (-23.4 to 86.4)	24.6 (-22.3 to 85.2)	**-45 (-68.4 to -9.18)**
6 Lower respiratory infect	6 Prostate cancer	**148 (53.5 to 257)**	**147 (62.1 to 249)**	0.784 (-26.6 to 34)
7 COVID-19	7 Lower respiratory infect	**116 (71.6 to 169)**	**115 (80.2 to 154)**	-13.8 (-32.9 to 14.9)
8 Prostate cancer	8 Alzheimer's disease	**204 (134 to 266)**	**203 (137 to 263)**	**8.02 (4.11 to 11.9)**
9 Breast cancer	9 Breast cancer	**97.5 (27.2 to 180)**	**96.7 (34.5 to 170)**	6.71 (-22.4 to 38.9)
10 Colorectal cancer	10 Colorectal cancer	**128 (31.2 to 246)**	**127 (33.3 to 255)**	6.71 (-31.1 to 54.3)
11 Cirrhosis liver	11 Endo/metab/blood/immune	**148 (104 to 206)**	**148 (110 to 189)**	**24.5 (3.57 to 49.7)**
12 Alzheimer's disease	12 Lung cancer	**108 (34.1 to 184)**	**107 (42 to 177)**	6.33 (-24.5 to 36.8)
13 Stomach cancer	13 Cirrhosis liver	**51.7 (26.8 to 79.6)**	**51.1 (32.9 to 71.4)**	-12.2 (-24.1 to 3.18)
14 Lung cancer	14 Urinary diseases	**228 (151 to 306)**	**227 (171 to 302)**	**39.7 (7.03 to 96.1)**
15 Endo/metab/blood/immune	15 COPD	**143 (77 to 213)**	**142 (83.8 to 204)**	4.16 (-27.9 to 46)
16 Other cardiovascular	16 Atrial fibrillation	**235 (153 to 324)**	**234 (162 to 325)**	18.2 (-0.226 to 44.2)
17 HIV/AIDS	17 Pancreatic cancer	**182 (55 to 349)**	**181 (53.5 to 344)**	38.3 (-16.5 to 110)
18 COPD	18 Other cardiovascular	**74.1 (41.7 to 109)**	**73.4 (48 to 106)**	**-19.3 (-34.3 to -2.61)**
19 Alcohol use disorders	19 Parkinson's disease	**162 (103 to 232)**	**161 (114 to 213)**	0.212 (-14.8 to 17.3)
20 Cardiomyopathy	20 Stomach cancer	**36.1 (8.11 to 63.4)**	**35.6 (15.3 to 57.9)**	**-33.9 (-42.8 to -24.3)**
21 Pancreatic cancer	22 Cardiomyopathy			
24 Parkinson's disease	23 Alcohol use disorders			
25 Urinary diseases	41 HIV/AIDS			
26 Atrial fibrillation	130 COVID-19			

Supplemental Results Figure S8. Leading 20 level 3 causes of deaths, and percentage change in number of deaths and all-age and age-standardised death rates by location, 2022 and 2050

The Bahamas, Reference scenario

Legend:
Communicable, maternal, neonatal and nutritional
Non-communicable
Injuries

Leading Causes 2022	Leading Causes 2050	Mean % change number of DEATH	Mean % change all-age DEATH rate	Mean % change age-standardized DEATH rate
1 Ischemic heart disease	1 Hypertensive heart disease	**197 (104 to 309)**	**172 (93.5 to 274)**	19.2 (-19.3 to 78.6)
2 Hypertensive heart disease	2 Chronic kidney disease	**193 (100 to 315)**	**168 (92 to 272)**	31.5 (-11.2 to 96)
3 Stroke	3 Ischemic heart disease	45 (-9.21 to 120)	32.9 (-15.6 to 104)	**-41.1 (-65.2 to -1.37)**
4 Chronic kidney disease	4 Stroke	**53.5 (12.2 to 96.3)**	**40.6 (7.62 to 77.3)**	**-38.7 (-54.1 to -17.2)**
5 Diabetes	5 Prostate cancer	**176 (79.5 to 319)**	**152 (68.8 to 287)**	-4.62 (-28.6 to 29.6)
6 COVID-19	6 Lower respiratory infect	**153 (90.2 to 223)**	**131 (87.9 to 180)**	-10.7 (-30.1 to 13.5)
7 Interpersonal violence	7 Diabetes	**69 (22.8 to 123)**	**54.8 (17.2 to 104)**	**-28.8 (-48.1 to -2.27)**
8 HIV/AIDS	8 Alzheimer's disease	**267 (175 to 377)**	**236 (163 to 308)**	**3.57 (0.278 to 6.97)**
9 Lower respiratory infect	9 Breast cancer	**81.9 (22.1 to 171)**	**66.4 (14.3 to 135)**	-8.17 (-33.5 to 22)
10 Prostate cancer	10 Colorectal cancer	**119 (27 to 254)**	**100 (18.7 to 214)**	-5.48 (-38.4 to 38.8)
11 Breast cancer	11 Endo/metab/blood/immune	**141 (88.8 to 197)**	**121 (85 to 164)**	20.1 (-0.106 to 42.6)
12 Colorectal cancer	12 Urinary diseases	**273 (173 to 383)**	**241 (164 to 322)**	**37.8 (6.25 to 78.6)**
13 Cirrhosis liver	13 Lung cancer	**106 (38.6 to 199)**	**88.7 (29.1 to 168)**	-2.07 (-30.1 to 28.6)
14 Lung cancer	14 COPD	**179 (99.8 to 275)**	**155 (92.6 to 232)**	3.38 (-25.5 to 40.8)
15 Road injuries	15 Cirrhosis liver	**36.6 (11.2 to 61.1)**	**25.1 (9.02 to 43.2)**	**-22.5 (-32.1 to -9.91)**
16 Endo/metab/blood/immune	16 Atrial fibrillation	**301 (195 to 430)**	**267 (183 to 360)**	17 (-2.71 to 37.7)
17 Alzheimer's disease	17 Interpersonal violence	-41.1 (-69.1 to 18.6)	-46 (-71.1 to 5.81)	-39 (-68.6 to 26.2)
18 Cardiomyopathy	18 Road injuries	12 (-32.2 to 77.9)	2.66 (-37.3 to 68.6)	-3.32 (-41.9 to 64.3)
19 COPD	19 Other cardiovascular	**87.3 (47.8 to 129)**	**71.5 (41.7 to 104)**	-17.5 (-32.1 to 0.516)
20 Stomach cancer	20 Cardiomyopathy	**42.7 (13.3 to 72.9)**	**30.6 (13.2 to 50.1)**	**-26.8 (-33.4 to -19.4)**
21 Other cardiovascular	25 Stomach cancer			
22 Urinary diseases	29 HIV/AIDS			
29 Atrial fibrillation	130 COVID-19			

Supplemental Results Figure S8. Leading 20 level 3 causes of deaths, and percentage change in number of deaths and all-age and age-standardised death rates by location, 2022 and 2050

Barbados, Reference scenario

Legend:
Communicable, maternal, neonatal and nutritional
Non-communicable
Injuries

Leading Causes 2022	Leading Causes 2050	Mean % change number of DEATH	Mean % change all-age DEATH rate	Mean % change age-standardized DEATH rate
1 COVID-19	1 Diabetes	24.1 (-14.8 to 83.8)	41.3 (-0.848 to 107)	-23.6 (-47.3 to 14.3)
2 Stroke	2 Stroke	-3.2 (-30.5 to 33.3)	10.1 (-20.2 to 51.8)	**-39.8 (-56.2 to -15.4)**
3 Ischemic heart disease	3 Chronic kidney disease	**112 (38.3 to 221)**	**141 (61 to 264)**	37 (-7.72 to 116)
4 Diabetes	4 Ischemic heart disease	-1.91 (-40.8 to 60.7)	11.8 (-32.2 to 76.6)	-40.1 (-64.7 to 1.26)
5 Lower respiratory infect	5 Lower respiratory infect	**58 (20.5 to 111)**	**79.8 (37.9 to 130)**	-13.2 (-32.4 to 15)
6 Chronic kidney disease	6 Prostate cancer	68 (-1.94 to 152)	**90.6 (11.8 to 177)**	-1.59 (-31.5 to 31.3)
7 Colorectal cancer	7 Colorectal cancer	54.1 (-13.3 to 148)	74.7 (-1.14 to 166)	0.73 (-36.5 to 45.4)
8 Prostate cancer	8 Urinary diseases	**136 (77.5 to 230)**	**168 (103 to 250)**	**36.6 (1.64 to 90.1)**
9 Breast cancer	9 Hypertensive heart disease	**83.4 (23.6 to 166)**	**109 (39.8 to 206)**	13.5 (-25.8 to 69.2)
10 Hypertensive heart disease	10 Alzheimer's disease	**96.8 (40.4 to 152)**	**124 (69 to 175)**	2.03 (-2.22 to 5.3)
11 Alzheimer's disease	11 Breast cancer	36 (-14.2 to 96)	54.3 (-1.6 to 121)	-1.85 (-31.7 to 33.7)
12 Urinary diseases	12 Decubitus ulcer	48.8 (-17.1 to 137)	68.9 (-3.88 to 148)	-20 (-44.4 to 6.79)
13 Decubitus ulcer	13 Pancreatic cancer	**95.1 (3.54 to 227)**	**121 (17.9 to 256)**	35.2 (-22.3 to 109)
14 Stomach cancer	14 Bacterial skin	**211 (87.2 to 447)**	**254 (110 to 500)**	**92.1 (7.24 to 239)**
15 Lung cancer	15 Atrial fibrillation	**147 (67.3 to 240)**	**181 (103 to 269)**	**24.3 (2.78 to 47.7)**
16 Pancreatic cancer	16 Endo/metab/blood/immune	**85 (46.5 to 141)**	**111 (69.7 to 170)**	20.3 (-0.345 to 47.3)
17 COPD	17 Peripheral artery	**131 (27.9 to 257)**	**162 (52.7 to 279)**	24.9 (-10.9 to 63.4)
18 Endo/metab/blood/immune	18 Lung cancer	35.5 (-15 to 95.1)	53.8 (-0.4 to 108)	-2.42 (-31.9 to 28.4)
19 Cirrhosis liver	19 COPD	**57.9 (12.3 to 129)**	**79.6 (26.3 to 148)**	-5.19 (-35.4 to 40.6)
20 Cardiomyopathy	20 Parkinson's disease	**74.2 (33.2 to 131)**	**98.2 (56.2 to 154)**	-2.48 (-18.4 to 12.7)
23 Atrial fibrillation	21 Stomach cancer			
24 Peripheral artery	22 Cardiomyopathy			
25 Parkinson's disease	23 Cirrhosis liver			
31 Bacterial skin	130 COVID-19			

Supplemental Results Figure S8. Leading 20 level 3 causes of deaths, and percentage change in number of deaths and all-age and age-standardised death rates by location, 2022 and 2050

Belize, Reference scenario

Legend:
Communicable, maternal, neonatal and nutritional
Non-communicable
Injuries

Leading Causes 2022	Leading Causes 2050	Mean % change number of DEATH	Mean % change all-age DEATH rate	Mean % change age-standardized DEATH rate
1 COVID-19	1 Chronic kidney disease	**265 (152 to 403)**	**146 (71.5 to 255)**	28.1 (-14.5 to 93.3)
2 Ischemic heart disease	2 Ischemic heart disease	**112 (29.9 to 224)**	42.5 (-13.1 to 121)	-34.3 (-62.5 to 13.4)
3 Diabetes	3 Diabetes	**119 (55.6 to 200)**	**47.4 (7.38 to 100)**	-26.6 (-48.9 to 5.04)
4 Stroke	4 Stroke	**104 (49.2 to 169)**	**37.4 (3.9 to 77.7)**	**-36.6 (-55.7 to -12.6)**
5 Chronic kidney disease	5 Lower respiratory infect	**143 (87.6 to 206)**	**63.1 (30.2 to 103)**	**-22.8 (-40.8 to -0.161)**
6 Lower respiratory infect	6 Hypertensive heart disease	**224 (132 to 347)**	**118 (52.1 to 209)**	0.814 (-31.2 to 50.9)
7 Interpersonal violence	7 COPD	**192 (113 to 283)**	**96.1 (46.5 to 166)**	-12.6 (-39 to 21.2)
8 HIV/AIDS	8 Cirrhosis liver	**127 (88.3 to 169)**	**52.7 (31.9 to 76.2)**	-10.2 (-22.8 to 6.94)
9 Hypertensive heart disease	9 Alzheimer's disease	**302 (197 to 398)**	**170 (109 to 224)**	0.738 (-2.44 to 4.08)
10 Cirrhosis liver	10 Prostate cancer	**229 (90.2 to 396)**	**120 (40.2 to 226)**	-7.94 (-35 to 26.4)
11 Road injuries	11 Road injuries	**64.8 (1.08 to 171)**	10.9 (-30.5 to 83.1)	-0.79 (-39.3 to 68.2)
12 COPD	12 Lung cancer	**191 (87.1 to 308)**	**95 (29.2 to 166)**	1.48 (-28.8 to 34.2)
13 Neonatal disorders	13 Interpersonal violence	7.34 (-40 to 93.4)	-28.2 (-59.5 to 26.3)	-28 (-60 to 27.6)
14 Prostate cancer	14 Colorectal cancer	**213 (77.1 to 373)**	**110 (21.9 to 225)**	4.24 (-31.3 to 52.1)
15 Alzheimer's disease	15 Urinary diseases	**299 (208 to 398)**	**168 (118 to 224)**	26.2 (-2.45 to 65.5)
16 Lung cancer	16 Pancreatic cancer	**336 (132 to 589)**	**192 (58.6 to 380)**	48.3 (-14.5 to 130)
17 Cervical cancer	17 HIV/AIDS	-15.2 (-60.1 to 59)	-43.1 (-72.7 to 2.45)	**-53 (-77.6 to -13.9)**
18 Stomach cancer	18 Endo/metab/blood/immune	**182 (129 to 240)**	**89.5 (63.1 to 125)**	16.8 (-2.57 to 43.3)
19 Colorectal cancer	19 Cervical cancer	**105 (62.9 to 165)**	**37.8 (10.7 to 69.5)**	-15.6 (-31.9 to 6.94)
20 Endo/metab/blood/immune	20 Breast cancer	**169 (81.2 to 280)**	**80.2 (25.8 to 146)**	3.62 (-24.4 to 37.1)
21 Breast cancer	21 Stomach cancer			
26 Urinary diseases	28 Neonatal disorders			
27 Pancreatic cancer	132 COVID-19			

Supplemental Results Figure S8. Leading 20 level 3 causes of deaths, and percentage change in number of deaths and all-age and age-standardised death rates by location, 2022 and 2050

Bermuda, Reference scenario

Legend:
Communicable, maternal, neonatal and nutritional
Non-communicable
Injuries

Leading Causes 2022	Leading Causes 2050	Mean % change number of DEATH	Mean % change all-age DEATH rate	Mean % change age-standardized DEATH rate
1 Ischemic heart disease	1 Ischemic heart disease	7.26 (-38.7 to 69.7)	29 (-23.1 to 97.6)	**-51.3 (-68.8 to -19.2)**
2 Stroke	2 Alzheimer's disease	**150 (90.2 to 209)**	**200 (142 to 256)**	**-7.02 (-10.3 to -3.19)**
3 COVID-19	3 Chronic kidney disease	**132 (45 to 252)**	**179 (86.7 to 314)**	12.3 (-21.7 to 66.1)
4 Lung cancer	4 Prostate cancer	**87.5 (19.8 to 156)**	**125 (50.9 to 210)**	-5.43 (-29.8 to 21.1)
5 Prostate cancer	5 Colorectal cancer	**73.8 (2.76 to 161)**	**109 (28.1 to 204)**	-10.6 (-39.8 to 24.5)
6 Alzheimer's disease	6 Stroke	-5.28 (-35 to 31.2)	13.8 (-19.3 to 54.5)	**-53.8 (-64.1 to -37.5)**
7 Colorectal cancer	7 Lung cancer	38 (-3.51 to 87.5)	**65.8 (16.7 to 121)**	-15 (-37.1 to 7.89)
8 Diabetes	8 Lower respiratory infect	**103 (46.6 to 164)**	**143 (85.5 to 208)**	-18.3 (-34.7 to 1.98)
9 Chronic kidney disease	9 Diabetes	27.9 (-11.2 to 72.8)	**53.7 (6.38 to 107)**	**-36.8 (-53.6 to -15.3)**
10 Lower respiratory infect	10 Hypertensive heart disease	**115 (34.8 to 222)**	**159 (63.8 to 273)**	0.625 (-33.5 to 48.4)
11 Breast cancer	11 COPD	**95.3 (34.4 to 170)**	**135 (66.7 to 225)**	-10.3 (-31.8 to 24)
12 Hypertensive heart disease	12 Pancreatic cancer	**112 (11.2 to 237)**	**154 (38.2 to 303)**	19.1 (-26.1 to 77.2)
13 COPD	13 Breast cancer	51.3 (-4.72 to 124)	**81.7 (18 to 165)**	-19 (-40.3 to 8.62)
14 Pancreatic cancer	14 Atrial fibrillation	**195 (116 to 286)**	**254 (173 to 355)**	6.7 (-10.2 to 27)
15 Other cardiovascular	15 Endo/metab/blood/immune	**133 (72.6 to 208)**	**180 (112 to 256)**	9.91 (-6.77 to 32.5)
16 Cardiomyopathy	16 Other cardiovascular	**65 (23.7 to 122)**	**98.4 (54.9 to 158)**	**-25.8 (-37.9 to -9.54)**
17 Cirrhosis liver	17 Nonrheum valv diseases	**119 (71.2 to 176)**	**163 (109 to 221)**	2.56 (-11 to 17.1)
18 Endo/metab/blood/immune	18 Peripheral artery	**175 (78.2 to 284)**	**230 (117 to 339)**	6.1 (-17.4 to 32.6)
19 Atrial fibrillation	19 Parkinson's disease	**92 (43.1 to 138)**	**131 (79.7 to 178)**	-11.1 (-27.1 to 4.06)
20 Stomach cancer	20 Urinary diseases	**210 (120 to 308)**	**273 (174 to 398)**	**26.9 (0.132 to 61.7)**
23 Parkinson's disease	22 Cardiomyopathy			
24 Nonrheum valv diseases	27 Cirrhosis liver			
30 Peripheral artery	28 Stomach cancer			
35 Urinary diseases	128 COVID-19			

Cuba, Reference scenario

Legend:
Communicable, maternal, neonatal and nutritional
Non-communicable
Injuries

Leading Causes 2022	Leading Causes 2050	Mean % change number of DEATH	Mean % change all-age DEATH rate	Mean % change age-standardized DEATH rate
1 Ischemic heart disease	1 Ischemic heart disease	10.1 (-29.6 to 56.8)	32.1 (-12 to 87.4)	**-41.2 (-61.7 to -8.79)**
2 Stroke	2 Lower respiratory infect	**55.3 (22.7 to 94.5)**	**85.9 (53.9 to 127)**	**-23.3 (-38 to -2.73)**
3 Lower respiratory infect	3 Stroke	3.55 (-21.2 to 30.4)	24.2 (-5.21 to 53.6)	**-43.9 (-56.6 to -27.9)**
4 Lung cancer	4 Lung cancer	35 (-6.68 to 84.1)	**61.3 (18.2 to 110)**	-10.2 (-30.2 to 13.4)
5 COPD	5 Falls	**104 (52.1 to 157)**	**145 (94.8 to 203)**	-2.42 (-16.6 to 14.3)
6 Falls	6 Alzheimer's disease	**124 (73.4 to 173)**	**168 (120 to 212)**	-2.55 (-6.19 to 0.604)
7 Prostate cancer	7 COPD	**55.7 (16.2 to 99.8)**	**86.6 (38.5 to 138)**	-13.9 (-36.3 to 14)
8 Colorectal cancer	8 Chronic kidney disease	**105 (50 to 178)**	**146 (82.2 to 225)**	19.6 (-12.1 to 67)
9 Alzheimer's disease	9 Prostate cancer	**83.4 (22.6 to 160)**	**119 (49.1 to 199)**	-3.64 (-26.4 to 21.9)
10 Chronic kidney disease	10 Hypertensive heart disease	**101 (42.5 to 168)**	**141 (69.2 to 233)**	12.3 (-19.5 to 58.2)
11 Hypertensive heart disease	11 Colorectal cancer	**75.7 (11.5 to 169)**	**110 (42.7 to 207)**	0.688 (-26.4 to 39.8)
12 Cirrhosis liver	12 Breast cancer	**53.1 (2.12 to 106)**	**83 (26.6 to 143)**	-2.25 (-25.6 to 22.6)
13 Diabetes	13 Atrial fibrillation	**161 (87.6 to 241)**	**212 (138 to 295)**	13 (-8.87 to 35)
14 Breast cancer	14 Diabetes	31.9 (-1.51 to 70.6)	**58.1 (19.4 to 98.2)**	**-25.9 (-43.5 to -3.35)**
15 Self-harm	15 Cirrhosis liver	1.13 (-15.2 to 20.2)	**21.1 (8 to 37.5)**	-8.43 (-18.4 to 2.88)
16 Cardiomyopathy	16 Pancreatic cancer	**96.4 (19.3 to 200)**	**135 (45.1 to 259)**	28.3 (-16.6 to 83.2)
17 Atrial fibrillation	17 Parkinson's disease	**96.3 (54 to 147)**	**135 (91.3 to 185)**	-3.99 (-18.8 to 11.5)
18 Pancreatic cancer	18 Self-harm	2.17 (-17.7 to 24.7)	**22.4 (2.57 to 44.3)**	-11.2 (-26.1 to 5.88)
19 Road injuries	19 Peripheral artery	**123 (55.7 to 199)**	**166 (95.5 to 240)**	8.35 (-10.6 to 28.7)
20 Stomach cancer	20 Cardiomyopathy	15.5 (-8.72 to 44.8)	**38.1 (14.2 to 67.4)**	**-20 (-31.8 to -4.93)**
22 Parkinson's disease	22 Road injuries			
32 Peripheral artery	27 Stomach cancer			

Supplemental Results Figure S8. Leading 20 level 3 causes of deaths, and percentage change in number of deaths and all-age and age-standardised death rates by location, 2022 and 2050

Dominica, Reference scenario

Legend:
Communicable, maternal, neonatal and nutritional
Non-communicable
Injuries

Leading Causes 2022	Leading Causes 2050	Mean % change number of DEATH	Mean % change all-age DEATH rate	Mean % change age-standardized DEATH rate
1 COVID-19	1 Chronic kidney disease	**93.6 (36.9 to 172)**	**110 (48.4 to 188)**	21.9 (-17.9 to 85.4)
2 Stroke	2 Hypertensive heart disease	**97.8 (39.4 to 168)**	**114 (49.4 to 191)**	15.1 (-24.3 to 68.3)
3 Ischemic heart disease	3 Diabetes	24.7 (-11.3 to 62.9)	35.1 (-0.86 to 76.1)	-24.9 (-47.6 to 5.01)
4 Diabetes	4 Stroke	-3.21 (-27.3 to 25.9)	4.87 (-18.4 to 32.9)	**-43.7 (-58.8 to -18.8)**
5 Chronic kidney disease	5 Ischemic heart disease	-3.47 (-38.3 to 48)	4.62 (-34.4 to 59.3)	**-43.1 (-65.9 to -3.47)**
6 Hypertensive heart disease	6 Prostate cancer	**116 (37.8 to 223)**	**134 (52.3 to 243)**	23.6 (-6.07 to 71.4)
7 Lower respiratory infect	7 Lower respiratory infect	**55.3 (16.9 to 97)**	**68.2 (30.3 to 106)**	-14.7 (-35.1 to 11.3)
8 Prostate cancer	8 COPD	**85.6 (30.5 to 148)**	**101 (48.5 to 164)**	8.03 (-24.5 to 53)
9 Stomach cancer	9 Lung cancer	**66 (7.16 to 134)**	**79.5 (21.4 to 143)**	8.46 (-21.1 to 41.9)
10 Lung cancer	10 Alzheimer's disease	**118 (58.2 to 168)**	**136 (82.9 to 182)**	**8.46 (4.93 to 12.5)**
11 COPD	11 Colorectal cancer	77.8 (-1.77 to 177)	**92.3 (16.2 to 197)**	7.53 (-30.2 to 55.3)
12 Cardiomyopathy	12 Breast cancer	**57.6 (0.136 to 123)**	**70.5 (16.5 to 140)**	1.34 (-26.5 to 34.9)
13 Breast cancer	13 Stomach cancer	10.6 (-13.2 to 43.1)	19.7 (-1.32 to 40.5)	**-30.9 (-39.7 to -21.8)**
14 Colorectal cancer	14 Cardiomyopathy	19.3 (-7.46 to 46.3)	**29 (8.17 to 53)**	**-20.6 (-28.8 to -11.8)**
15 Alzheimer's disease	15 Pancreatic cancer	**127 (19.7 to 281)**	**145 (37.1 to 289)**	43.5 (-14.1 to 124)
16 Cirrhosis liver	16 Atrial fibrillation	**148 (77 to 229)**	**169 (96.4 to 237)**	**24.1 (3.81 to 49.6)**
17 Road injuries	17 Cirrhosis liver	15.2 (-6.43 to 40.6)	**24.8 (8.75 to 42.1)**	**-18.2 (-29.3 to -3.77)**
18 Neonatal disorders	18 Endo/metab/blood/immune	**87 (40.3 to 139)**	**102 (71.4 to 144)**	**25 (3.23 to 49.8)**
19 Pancreatic cancer	19 Parkinson's disease	**103 (58.3 to 150)**	**120 (79.2 to 164)**	6.36 (-9.77 to 24.6)
20 Cervical cancer	20 Road injuries	-6 (-42.4 to 44.2)	1.85 (-33.5 to 60.1)	-7.3 (-41.4 to 49)
22 Endo/metab/blood/immune	22 Cervical cancer			
23 Atrial fibrillation	31 Neonatal disorders			
27 Parkinson's disease	129 COVID-19			

Supplemental Results Figure S8. Leading 20 level 3 causes of deaths, and percentage change in number of deaths and all-age and age-standardised death rates by location, 2022 and 2050

Dominican Republic, Reference scenario

Legend:
Communicable, maternal, neonatal and nutritional
Non-communicable
Injuries

Leading Causes 2022	Leading Causes 2050	Mean % change number of DEATH	Mean % change all-age DEATH rate	Mean % change age-standardized DEATH rate
1 Ischemic heart disease	1 Ischemic heart disease	49.6 (-8.52 to 124)	30 (-17.4 to 93.1)	-38 (-63.7 to 0.0948)
2 Stroke	2 Stroke	**42.2 (5.53 to 81.4)**	23.5 (-3.59 to 52)	**-39.2 (-53.6 to -22.4)**
3 Neonatal disorders	3 Chronic kidney disease	**193 (96.6 to 307)**	**154 (81.7 to 242)**	30.7 (-8.78 to 86.6)
4 Diabetes	4 Diabetes	**99.2 (41 to 164)**	**73.1 (28.3 to 123)**	-12.8 (-38.2 to 18.5)
5 Cirrhosis liver	5 Alzheimer's disease	**214 (128 to 297)**	**173 (106 to 244)**	**3.58 (0.307 to 7.52)**
6 Chronic kidney disease	6 Hypertensive heart disease	**148 (63.5 to 250)**	**116 (55 to 199)**	1.88 (-29.5 to 49)
7 Road injuries	7 Cirrhosis liver	**68.5 (36.7 to 100)**	**46.4 (29.6 to 67.5)**	**-16 (-27.1 to -2.44)**
8 Lower respiratory infect	8 Prostate cancer	**174 (69.1 to 321)**	**138 (52.2 to 261)**	2.57 (-25.3 to 33.9)
9 Hypertensive heart disease	9 Lower respiratory infect	**89.7 (44 to 150)**	**64.8 (29.1 to 108)**	**-24.3 (-37.7 to -6.84)**
10 Interpersonal violence	10 Lung cancer	**141 (61.4 to 230)**	**109 (42.4 to 189)**	8.73 (-18.9 to 41.4)
11 Alzheimer's disease	11 COPD	**106 (40 to 175)**	**79.1 (30.2 to 143)**	-20.4 (-45 to 13.7)
12 Lung cancer	12 Road injuries	4.59 (-35.5 to 56.1)	-9.28 (-40 to 35.3)	-19.5 (-48.3 to 23.9)
13 Prostate cancer	13 Colorectal cancer	**164 (51.9 to 308)**	**130 (41.4 to 253)**	13.6 (-23.5 to 59.8)
14 COPD	14 Pancreatic cancer	**236 (85.5 to 428)**	**192 (66.1 to 357)**	50.7 (-4.52 to 126)
15 COVID-19	15 Breast cancer	**129 (50.7 to 224)**	**99.1 (36.2 to 175)**	10.4 (-18 to 45.8)
16 Colorectal cancer	16 Atrial fibrillation	**259 (142 to 372)**	**212 (124 to 303)**	**20.3 (0.192 to 44.1)**
17 Breast cancer	17 Neonatal disorders	**-44.7 (-63.3 to -23.6)**	**-52.1 (-62.6 to -37.3)**	**-26.4 (-41.9 to -7.29)**
18 HIV/AIDS	18 Interpersonal violence	-34.2 (-63.1 to 11)	**-42.7 (-65 to -1.44)**	-40 (-63.8 to 3.87)
19 Stomach cancer	19 Parkinson's disease	**167 (101 to 237)**	**132 (86.3 to 186)**	-1.48 (-15.3 to 13.4)
20 Tuberculosis	20 Endo/metab/blood/immune	**92.4 (45.2 to 145)**	**67.3 (33.5 to 109)**	3.41 (-13.7 to 21.9)
23 Pancreatic cancer	21 Stomach cancer			
24 Endo/metab/blood/immune	39 Tuberculosis			
28 Atrial fibrillation	58 HIV/AIDS			
29 Parkinson's disease	132 COVID-19			

Supplemental Results Figure S8. Leading 20 level 3 causes of deaths, and percentage change in number of deaths and all-age and age-standardised death rates by location, 2022 and 2050

Grenada, Reference scenario

Legend:
Communicable, maternal, neonatal and nutritional
Non-communicable
Injuries

Leading Causes 2022	Leading Causes 2050	Mean % change number of DEATH	Mean % change all-age DEATH rate	Mean % change age-standardized DEATH rate
1 Ischemic heart disease	1 Chronic kidney disease	**110 (40.6 to 189)**	**133 (57.3 to 226)**	20.7 (-20.9 to 78.9)
2 Diabetes	2 Diabetes	**45.4 (5.05 to 95.6)**	**61.2 (16.5 to 118)**	-21.9 (-48 to 11.9)
3 Stroke	3 Ischemic heart disease	8.06 (-34.4 to 64.9)	20 (-25.5 to 83.4)	-41.7 (-67 to 1.45)
4 COVID-19	4 Stroke	11.4 (-17.4 to 44.2)	23.4 (-6.52 to 56.9)	**-43.3 (-60.4 to -17.6)**
5 Chronic kidney disease	5 Lower respiratory infect	**77.3 (39.8 to 117)**	**96.2 (58.9 to 133)**	-17 (-36.7 to 9.09)
6 Lower respiratory infect	6 Prostate cancer	**149 (49.4 to 270)**	**174 (72.6 to 304)**	29.5 (-8.57 to 77.3)
7 Hypertensive heart disease	7 Hypertensive heart disease	**107 (48.7 to 179)**	**129 (62.2 to 217)**	5.53 (-30.3 to 62.9)
8 Prostate cancer	8 Colorectal cancer	**99.9 (13.9 to 227)**	**120 (27.6 to 232)**	8.54 (-31.8 to 53.8)
9 Breast cancer	9 Breast cancer	**65.9 (6.41 to 139)**	**82.9 (19.4 to 165)**	0.401 (-28.6 to 35.3)
10 Colorectal cancer	10 Alzheimer's disease	**155 (87.9 to 231)**	**182 (117 to 253)**	-0.237 (-4.31 to 4.22)
11 Cirrhosis liver	11 Pancreatic cancer	**156 (34.1 to 334)**	**181 (53.3 to 372)**	46.1 (-13.5 to 129)
12 Cardiomyopathy	12 COPD	**97.2 (47.7 to 161)**	**119 (61.5 to 185)**	2.62 (-33 to 43.5)
13 COPD	13 Lung cancer	**79.3 (11.6 to 170)**	**97.5 (23.6 to 180)**	7.1 (-27.2 to 46.4)
14 Lung cancer	14 Bacterial skin	**243 (91 to 466)**	**281 (109 to 538)**	84.1 (-6.52 to 256)
15 Alzheimer's disease	15 Cirrhosis liver	**26.9 (3.76 to 53.4)**	**40.5 (19.3 to 65)**	-13.1 (-27.2 to 3.41)
16 Lymphoma	16 Endo/metab/blood/immune	**93.9 (58.7 to 133)**	**115 (79.5 to 157)**	18.1 (-3.84 to 44.4)
17 Pancreatic cancer	17 Cardiomyopathy	**28.5 (1.18 to 63.4)**	**42 (22 to 69.9)**	**-23.3 (-30.7 to -14.1)**
18 Endo/metab/blood/immune	18 Lymphoma	**50 (0.107 to 110)**	**65.3 (16.2 to 124)**	-1.81 (-27.6 to 27.1)
19 Cervical cancer	19 Atrial fibrillation	**195 (115 to 293)**	**225 (145 to 317)**	**18.4 (1.87 to 39.5)**
20 Neonatal disorders	20 Falls	**103 (45.1 to 186)**	**124 (69.4 to 186)**	0.856 (-14 to 17.5)
23 Falls	25 Cervical cancer			
27 Bacterial skin	50 Neonatal disorders			
30 Atrial fibrillation	130 COVID-19			

Supplemental Results Figure S8. Leading 20 level 3 causes of deaths, and percentage change in number of deaths and all-age and age-standardised death rates by location, 2022 and 2050

Guyana, Reference scenario

Legend:
Communicable, maternal, neonatal and nutritional
Non-communicable
Injuries

Leading Causes 2022	Leading Causes 2050	Mean % change number of DEATH	Mean % change all-age DEATH rate	Mean % change age-standardized DEATH rate
1 COVID-19	1 Stroke	-3.41 (-30.5 to 30.8)	19.6 (-13.3 to 62.7)	**-52.8 (-64.3 to -36.3)**
2 Ischemic heart disease	2 Ischemic heart disease	-16.1 (-49.4 to 30.8)	4.17 (-33.9 to 60.8)	**-58.1 (-74.7 to -31.7)**
3 Stroke	3 Hypertensive heart disease	**87.4 (26 to 166)**	**132 (59 to 233)**	-8.93 (-36.9 to 32)
4 Diabetes	4 Diabetes	15.1 (-21.5 to 64.5)	**42.5 (1.65 to 101)**	**-37.5 (-53.9 to -14.9)**
5 Chronic kidney disease	5 Chronic kidney disease	**63 (7.66 to 135)**	**102 (38.1 to 196)**	-3.99 (-33.8 to 39)
6 Hypertensive heart disease	6 Lower respiratory infect	**67.6 (21.2 to 126)**	**107 (54.4 to 180)**	**-22 (-37.4 to -1.06)**
7 Lower respiratory infect	7 Prostate cancer	**141 (47.8 to 260)**	**198 (93 to 343)**	1.35 (-24.7 to 31.1)
8 Self-harm	8 Cirrhosis liver	4.66 (-20.1 to 30.2)	**29.2 (9.09 to 53.1)**	**-18.4 (-29.6 to -5.33)**
9 Cirrhosis liver	9 Alzheimer's disease	**251 (139 to 378)**	**334 (204 to 459)**	1.2 (-2.38 to 4.37)
10 Neonatal disorders	10 Self-harm	-17 (-39.6 to 13.1)	2.25 (-21.6 to 27.3)	-10.3 (-31.2 to 11.8)
11 HIV/AIDS	11 Colorectal cancer	**94.3 (8.12 to 197)**	**139 (47.5 to 255)**	7.43 (-28.3 to 51.9)
12 Interpersonal violence	12 Breast cancer	**56.7 (0.958 to 118)**	**93.2 (37.1 to 164)**	8.26 (-20.7 to 46.6)
13 Prostate cancer	13 COPD	**77.5 (25.2 to 147)**	**120 (54 to 201)**	-15.7 (-39.2 to 13.3)
14 Road injuries	14 Falls	**99.7 (35.2 to 182)**	**146 (80.7 to 246)**	-10.4 (-23.1 to 4.85)
15 Breast cancer	15 Endo/metab/blood/immune	**64.9 (24.3 to 115)**	**104 (65.8 to 149)**	15.8 (-3.02 to 35.6)
16 Colorectal cancer	16 Pancreatic cancer	**174 (47.9 to 340)**	**237 (93 to 443)**	**61.6 (0.12 to 149)**
17 Cardiomyopathy	17 Cardiomyopathy	18.3 (-9.78 to 48.1)	**46 (22.1 to 75.6)**	**-22.4 (-29.1 to -15.5)**
18 Malaria	18 Lung cancer	**82.9 (14.5 to 159)**	**126 (54.7 to 204)**	14.1 (-15.6 to 45.9)
19 COPD	19 Interpersonal violence	**-45.8 (-70.5 to -11.6)**	-33.5 (-57.6 to 3.28)	-31.5 (-56.7 to 10.9)
20 Endo/metab/blood/immune	20 Urinary diseases	**148 (78.8 to 234)**	**207 (126 to 324)**	18.8 (-8.13 to 51)
21 Falls	21 Road injuries			
22 Alzheimer's disease	24 Neonatal disorders			
27 Lung cancer	26 Malaria			
33 Urinary diseases	38 HIV/AIDS			
34 Pancreatic cancer	132 COVID-19			

Supplemental Results Figure S8. Leading 20 level 3 causes of deaths, and percentage change in number of deaths and all-age and age-standardised death rates by location, 2022 and 2050

Haiti, Reference scenario

Legend:
Communicable, maternal, neonatal and nutritional
Non-communicable
Injuries

Leading Causes 2022	Leading Causes 2050	Mean % change number of DEATH	Mean % change all-age DEATH rate	Mean % change age-standardized DEATH rate
1 Ischemic heart disease	1 Ischemic heart disease	69.7 (-2.5 to 162)	35.3 (-17.7 to 111)	-32.8 (-63.3 to 18.5)
2 Stroke	2 Stroke	**49.9 (0.643 to 107)**	19.4 (-11 to 58.6)	**-39.5 (-59.5 to -9.16)**
3 Nature disaster	3 Nature disaster	inf (-100 to nan)	inf (-100 to nan)	inf (-100 to nan)
4 Lower respiratory infect	4 Diabetes	**76.2 (15.2 to 164)**	40.4 (-5.83 to 97)	-26.3 (-53.9 to 13.3)
5 Neonatal disorders	5 Lower respiratory infect	2.39 (-27.3 to 39.2)	-18.5 (-36.3 to 4.55)	**-38.2 (-53.8 to -20.9)**
6 Diabetes	6 Chronic kidney disease	**163 (68.7 to 280)**	**110 (44.4 to 211)**	18.5 (-24.7 to 83.8)
7 HIV/AIDS	7 Hypertensive heart disease	**146 (62.2 to 247)**	**96.2 (39.6 to 172)**	-8.65 (-43.2 to 36.9)
8 Diarrheal diseases	8 Cirrhosis liver	**54.2 (16.2 to 97.4)**	**22.9 (2.35 to 51.2)**	**-28.2 (-42 to -10.8)**
9 Congenital defects	9 Prostate cancer	**210 (68.1 to 397)**	**146 (44.1 to 284)**	1.42 (-26.1 to 38.3)
10 Road injuries	10 COPD	**81.5 (23.2 to 156)**	**44.6 (5.83 to 96)**	**-33.7 (-54.9 to -4.69)**
11 Interpersonal violence	11 Road injuries	1.04 (-44.3 to 78.8)	-19.5 (-52.3 to 36.1)	-24.9 (-55.3 to 27)
12 Cirrhosis liver	12 Neonatal disorders	**-51.8 (-70.1 to -24.6)**	**-61.7 (-73.8 to -45.4)**	**-37.8 (-54.9 to -12.8)**
13 COVID-19	13 Diarrheal diseases	-39.1 (-69.9 to 9.04)	**-51.5 (-74 to -14.9)**	**-45.3 (-70.2 to -1.87)**
14 Chronic kidney disease	14 Colorectal cancer	**160 (35.4 to 336)**	**107 (19.3 to 248)**	4.31 (-33.8 to 58.3)
15 Hypertensive heart disease	15 Breast cancer	**112 (26.1 to 224)**	**68.4 (8.16 to 149)**	0.237 (-32.2 to 38.7)
16 COPD	16 Interpersonal violence	-22.4 (-57 to 34.3)	-38.3 (-63.1 to 6.5)	-39.3 (-63.6 to 5.11)
17 Tuberculosis	17 Congenital defects	**-41.4 (-57.8 to -20.6)**	**-53.4 (-62.5 to -42.7)**	**-30 (-40 to -17.2)**
18 Drowning	18 Cervical cancer	**46.8 (8.05 to 96.8)**	17.1 (-7.41 to 48.3)	**-25 (-41.6 to -5.14)**
19 Maternal disorders	19 Stomach cancer	**54.4 (16.2 to 99.5)**	**23 (2.61 to 49.7)**	**-36.2 (-43.6 to -28.2)**
20 Pertussis	20 Alzheimer's disease	**237 (103 to 404)**	**168 (78.3 to 280)**	2.14 (-1.18 to 5.07)
21 Cervical cancer	26 HIV/AIDS			
22 Stomach cancer	28 Tuberculosis			
23 Prostate cancer	30 Drowning			
24 Breast cancer	39 Maternal disorders			
27 Colorectal cancer	46 Pertussis			
36 Alzheimer's disease	131 COVID-19			

Supplemental Results Figure S8. Leading 20 level 3 causes of deaths, and percentage change in number of deaths and all-age and age-standardised death rates by location, 2022 and 2050

Jamaica, Reference scenario

Legend:
Communicable, maternal, neonatal and nutritional
Non-communicable
Injuries

Leading Causes 2022	Leading Causes 2050	Mean % change number of DEATH	Mean % change all-age DEATH rate	Mean % change age-standardized DEATH rate
1 Stroke	1 Diabetes	**71.2 (20.4 to 140)**	**89.8 (36.3 to 157)**	-13.8 (-38.5 to 20.7)
2 Diabetes	2 Stroke	24.2 (-5.89 to 67.2)	**37.7 (5.63 to 82.2)**	**-35.7 (-51 to -9.19)**
3 COVID-19	3 Chronic kidney disease	**143 (63.1 to 252)**	**170 (86.7 to 288)**	34 (-7.6 to 100)
4 Ischemic heart disease	4 Hypertensive heart disease	**118 (47.6 to 217)**	**142 (66 to 238)**	16 (-21.1 to 73.1)
5 Hypertensive heart disease	5 Ischemic heart disease	30 (-23.8 to 111)	44.2 (-10.7 to 138)	-33.3 (-58.8 to 12.7)
6 Chronic kidney disease	6 Prostate cancer	**108 (33.3 to 193)**	**130 (52.4 to 222)**	0.678 (-25.9 to 29.2)
7 Prostate cancer	7 Alzheimer's disease	**110 (45.5 to 184)**	**132 (73.7 to 192)**	3.35 (-0.209 to 6.82)
8 Interpersonal violence	8 Colorectal cancer	**97.5 (19.7 to 196)**	**118 (35.7 to 222)**	3.93 (-32.2 to 43.3)
9 Alzheimer's disease	9 Lung cancer	**85.4 (18.8 to 160)**	**105 (35.6 to 189)**	-0.354 (-29.5 to 29.3)
10 Lung cancer	10 Breast cancer	**72.2 (18.2 to 132)**	**90.6 (33.2 to 159)**	0.707 (-26 to 30.1)
11 Colorectal cancer	11 COPD	**70.6 (21.8 to 152)**	**89.1 (41.2 to 167)**	-13.9 (-35.2 to 24.2)
12 COPD	12 Lower respiratory infect	**47.8 (9.71 to 101)**	**63.8 (25.1 to 109)**	**-24.6 (-40.1 to -1.11)**
13 Breast cancer	13 Urinary diseases	**171 (95.2 to 274)**	**200 (127 to 294)**	**35.4 (5.09 to 83.5)**
14 Lower respiratory infect	14 Interpersonal violence	**-39 (-60.8 to -3.03)**	-32.6 (-54.7 to 5.69)	**-39.7 (-59.8 to -6.55)**
15 HIV/AIDS	15 Atrial fibrillation	**142 (65.6 to 228)**	**167 (101 to 247)**	**16.8 (0.678 to 40)**
16 Neonatal disorders	16 Pancreatic cancer	**159 (51.4 to 315)**	**186 (68.7 to 345)**	37.9 (-16.1 to 109)
17 Stomach cancer	17 Stomach cancer	23.7 (-1.39 to 59.2)	**37.1 (15.2 to 68)**	**-34.3 (-42.2 to -25)**
18 Cervical cancer	18 Parkinson's disease	**112 (62.7 to 184)**	**134 (87.6 to 195)**	0.683 (-13.9 to 16.5)
19 Urinary diseases	19 Cervical cancer	**30.6 (2.41 to 64.2)**	**44.7 (20.3 to 75.4)**	-18.3 (-33.6 to 0.916)
20 Atrial fibrillation	20 Decubitus ulcer	61.8 (-4.04 to 141)	**78.8 (7.29 to 160)**	-20.4 (-40 to 1.64)
22 Decubitus ulcer	37 HIV/AIDS			
25 Parkinson's disease	44 Neonatal disorders			
26 Pancreatic cancer	130 COVID-19			

Supplemental Results Figure S8. Leading 20 level 3 causes of deaths, and percentage change in number of deaths and all-age and age-standardised death rates by location, 2022 and 2050

Puerto Rico, Reference scenario

Legend:
Communicable, maternal, neonatal and nutritional
Non-communicable
Injuries

Leading Causes 2022	Leading Causes 2050	Mean % change number of DEATH	Mean % change all-age DEATH rate	Mean % change age-standardized DEATH rate
1 Ischemic heart disease	1 Chronic kidney disease	**91.6 (40.8 to 163)**	**169 (95.8 to 269)**	14.4 (-13.6 to 53.2)
2 Diabetes	2 Alzheimer's disease	**109 (66.2 to 158)**	**193 (140 to 257)**	**-6.23 (-8.89 to -3.4)**
3 Chronic kidney disease	3 Ischemic heart disease	-19.9 (-45.6 to 15.4)	12.6 (-23.6 to 59.2)	**-53.5 (-67.3 to -33.7)**
4 COVID-19	4 Diabetes	-5.05 (-25 to 21.8)	**33.4 (1.18 to 68.6)**	**-40.8 (-53.1 to -25.2)**
5 Alzheimer's disease	5 COPD	**79.8 (36 to 127)**	**153 (79 to 224)**	-3.97 (-26 to 23.1)
6 Stroke	6 Hypertensive heart disease	**74.6 (19.9 to 136)**	**145 (71.1 to 235)**	2.86 (-23.4 to 39.2)
7 COPD	7 Lower respiratory infect	**46.4 (8.69 to 87.4)**	**106 (54.4 to 169)**	**-19.3 (-33.8 to -1.39)**
8 Lower respiratory infect	8 Colorectal cancer	29.6 (-15.1 to 84)	**81.7 (27 to 152)**	-11.2 (-35.5 to 17.4)
9 Colorectal cancer	9 Endo/metab/blood/immune	**86.5 (48.8 to 136)**	**162 (109 to 224)**	**14.2 (1.63 to 30)**
10 Hypertensive heart disease	10 Stroke	**-18.6 (-34.7 to -0.187)**	14.3 (-7.41 to 41.1)	**-49 (-57.9 to -37.6)**
11 Cirrhosis liver	11 Urinary diseases	**157 (90.4 to 234)**	**261 (165 to 379)**	**31.1 (4.4 to 60.1)**
12 Prostate cancer	12 Prostate cancer	**50.1 (1.67 to 107)**	**110 (49.2 to 198)**	-8.02 (-26.4 to 12)
13 Lung cancer	13 Lung cancer	30.1 (-4.86 to 70.4)	**82.5 (44.1 to 140)**	-5.16 (-22.1 to 15.8)
14 Endo/metab/blood/immune	14 Atrial fibrillation	**115 (61.9 to 176)**	**201 (132 to 278)**	5.22 (-10.5 to 26.2)
15 Interpersonal violence	15 Bacterial skin	**207 (102 to 362)**	**332 (176 to 546)**	**75.2 (17.7 to 166)**
16 Breast cancer	16 Pancreatic cancer	**60.8 (3.8 to 129)**	**125 (51.8 to 223)**	18.9 (-14.7 to 67)
17 Pancreatic cancer	17 Cirrhosis liver	-8.94 (-21.4 to 5.72)	**27.8 (12 to 44.5)**	**-20.7 (-28.6 to -13.3)**
18 Urinary diseases	18 Breast cancer	15.8 (-16.9 to 56.8)	**62.3 (22.9 to 115)**	-13.3 (-28.1 to 5.67)
19 Atrial fibrillation	19 Parkinson's disease	**48.1 (17.1 to 80.9)**	**108 (67.1 to 160)**	-8.95 (-21.6 to 6.22)
20 Parkinson's disease	20 Falls	**52.1 (11.9 to 97.7)**	**113 (65.1 to 180)**	**-16.1 (-27 to -2.3)**
21 Falls	38 Interpersonal violence			
26 Bacterial skin	129 COVID-19			

Supplemental Results Figure S8. Leading 20 level 3 causes of deaths, and percentage change in number of deaths and all-age and age-standardised death rates by location, 2022 and 2050

Saint Kitts and Nevis, Reference scenario

Legend:
Communicable, maternal, neonatal and nutritional
Non-communicable
Injuries

Leading Causes 2022	Leading Causes 2050	Mean % change number of DEATH	Mean % change all-age DEATH rate	Mean % change age-standardized DEATH rate
1 Stroke	1 Chronic kidney disease	**142 (55.3 to 257)**	**174 (94.2 to 294)**	11.2 (-24.9 to 71.6)
2 Ischemic heart disease	2 Stroke	21.3 (-14.9 to 66.6)	**37.5 (1.11 to 82.1)**	**-51.3 (-66.3 to -27.6)**
3 Chronic kidney disease	3 Ischemic heart disease	15.9 (-34.6 to 80.1)	31.5 (-22.3 to 99.1)	**-51.8 (-73.5 to -19.6)**
4 COVID-19	4 Prostate cancer	**192 (74.2 to 359)**	**231 (106 to 382)**	5.3 (-28.8 to 41.6)
5 Diabetes	5 Diabetes	**58.6 (4.43 to 126)**	**79.7 (25.9 to 147)**	-30.6 (-52.4 to 2.15)
6 Lower respiratory infect	6 Hypertensive heart disease	**165 (74.6 to 285)**	**201 (103 to 309)**	4.62 (-33 to 62.7)
7 Prostate cancer	7 Lower respiratory infect	**120 (64.7 to 197)**	**149 (102 to 212)**	-20.2 (-39.1 to 6.86)
8 Hypertensive heart disease	8 Colorectal cancer	**117 (22.3 to 245)**	**146 (43.9 to 268)**	-2.33 (-37 to 41.9)
9 HIV/AIDS	9 COPD	**140 (61.5 to 240)**	**172 (101 to 268)**	-4.43 (-33 to 42.2)
10 Cirrhosis liver	10 Alzheimer's disease	**237 (141 to 352)**	**281 (199 to 366)**	2.43 (-1.13 to 6.24)
11 Breast cancer	11 Breast cancer	**83.3 (15.8 to 172)**	**107 (37.9 to 187)**	-5.29 (-30.9 to 25)
12 Alcohol use disorders	12 Urinary diseases	**233 (143 to 358)**	**277 (192 to 420)**	32.9 (-3.89 to 91.2)
13 Colorectal cancer	13 Cirrhosis liver	19.3 (-6.35 to 51)	**35.2 (14.5 to 57.1)**	**-26.8 (-37.6 to -14.5)**
14 COPD	14 Pancreatic cancer	**185 (46.1 to 396)**	**222 (72.8 to 446)**	34.1 (-24.2 to 113)
15 Interpersonal violence	15 Parkinson's disease	**211 (130 to 290)**	**252 (185 to 320)**	-1.66 (-17.3 to 15.3)
16 Cardiomyopathy	16 Lung cancer	**84.7 (20.4 to 178)**	**109 (36.6 to 196)**	-5.59 (-33.3 to 27.4)
17 Lung cancer	17 Atrial fibrillation	**278 (155 to 413)**	**328 (219 to 444)**	10.4 (-8.13 to 33.1)
18 Alzheimer's disease	18 Alcohol use disorders	5.48 (-21.7 to 46)	19.5 (-6.4 to 58.1)	-18.9 (-35.9 to 5.81)
19 Urinary diseases	19 Cardiomyopathy	**43.4 (9.84 to 84.7)**	**62.3 (34.8 to 97.6)**	**-29 (-36.1 to -22.2)**
20 Stomach cancer	20 Endo/metab/blood/immune	**136 (70.5 to 218)**	**167 (120 to 230)**	13.9 (-6.61 to 40.9)
21 Pancreatic cancer	23 Stomach cancer			
25 Parkinson's disease	32 HIV/AIDS			
26 Endo/metab/blood/immune	41 Interpersonal violence			
30 Atrial fibrillation	128 COVID-19			

Supplemental Results Figure S8. Leading 20 level 3 causes of deaths, and percentage change in number of deaths and all-age and age-standardised death rates by location, 2022 and 2050

Saint Lucia, Reference scenario

Legend:
Communicable, maternal, neonatal and nutritional
Non-communicable
Injuries

Leading Causes 2022	Leading Causes 2050	Mean % change number of DEATH	Mean % change all-age DEATH rate	Mean % change age-standardized DEATH rate
1 COVID-19	1 Stroke	20.7 (-16.7 to 69.5)	23.2 (-14.3 to 70.4)	**-47.5 (-64 to -21.8)**
2 Stroke	2 Chronic kidney disease	**124 (40.9 to 219)**	**129 (49 to 228)**	8.65 (-30.4 to 74.5)
3 Diabetes	3 Diabetes	53 (-2.18 to 115)	**56.2 (6.95 to 121)**	-30.3 (-54 to 10.5)
4 Ischemic heart disease	4 Hypertensive heart disease	**127 (52 to 225)**	**132 (58.1 to 233)**	-1.61 (-37.3 to 54.4)
5 Chronic kidney disease	5 Prostate cancer	**130 (32.5 to 254)**	**133 (43.2 to 262)**	-7.56 (-34.6 to 22.9)
6 Hypertensive heart disease	6 Ischemic heart disease	22.4 (-33.3 to 97.9)	25 (-25.9 to 107)	**-46 (-70.6 to -5.25)**
7 Prostate cancer	7 COPD	**95.4 (40.4 to 160)**	**99.5 (47.8 to 176)**	-14.8 (-40.5 to 27.6)
8 Lower respiratory infect	8 Lower respiratory infect	**81.2 (37.2 to 136)**	**84.7 (46.3 to 126)**	-26.9 (-45.4 to 0.562)
9 COPD	9 Alzheimer's disease	**159 (93.4 to 233)**	**164 (108 to 221)**	**-6.83 (-9.79 to -3.52)**
10 Cirrhosis liver	10 Colorectal cancer	**106 (7.06 to 246)**	**110 (18.3 to 242)**	-4.09 (-40.2 to 41.8)
11 Alzheimer's disease	11 Breast cancer	**67.7 (1.73 to 149)**	**70.6 (8.71 to 147)**	-8.83 (-36.3 to 25.5)
12 Breast cancer	12 Lung cancer	**84 (9.88 to 167)**	**87.1 (20.4 to 164)**	-4.81 (-33.6 to 28.3)
13 Cardiomyopathy	13 Cirrhosis liver	**32.7 (7.79 to 62)**	**35.4 (13.7 to 59.5)**	**-19.5 (-31.3 to -2.44)**
14 Interpersonal violence	14 Atrial fibrillation	**198 (113 to 297)**	**203 (132 to 286)**	4.09 (-15 to 25.9)
15 Lung cancer	15 Pancreatic cancer	**168 (31.2 to 354)**	**172 (42.3 to 358)**	32.6 (-24.5 to 109)
16 Colorectal cancer	16 Urinary diseases	**191 (101 to 298)**	**196 (124 to 282)**	21.8 (-10.2 to 63.3)
17 Stomach cancer	17 Cardiomyopathy	**36.2 (5.12 to 70.9)**	**38.7 (16.8 to 65.9)**	**-28.1 (-34.9 to -20.9)**
18 Road injuries	18 Stomach cancer	**27.5 (0.318 to 60.5)**	**30 (8.08 to 54)**	**-37.8 (-44.7 to -30.2)**
19 Other cardiovascular	19 Parkinson's disease	**136 (79.6 to 199)**	**140 (95 to 193)**	-10.3 (-25.8 to 4.36)
20 Neonatal disorders	20 Other cardiovascular	**62.5 (21.5 to 107)**	**65.6 (31.9 to 107)**	**-25.6 (-41 to -4.44)**
21 Pancreatic cancer	28 Road injuries			
22 Atrial fibrillation	35 Interpersonal violence			
24 Urinary diseases	53 Neonatal disorders			
25 Parkinson's disease	131 COVID-19			

Supplemental Results Figure S8. Leading 20 level 3 causes of deaths, and percentage change in number of deaths and all-age and age-standardised death rates by location, 2022 and 2050

Saint Vincent and the Grenadines, Reference scenario

Legend:
Communicable, maternal, neonatal and nutritional
Non-communicable
Injuries

Leading Causes 2022	Leading Causes 2050	Mean % change number of DEATH	Mean % change all-age DEATH rate	Mean % change age-standardized DEATH rate
1 Ischemic heart disease	1 Hypertensive heart disease	**93.3 (29.7 to 159)**	**128 (54 to 206)**	9.46 (-31 to 69.9)
2 Stroke	2 Ischemic heart disease	9.18 (-34.7 to 64.9)	28.6 (-22.2 to 96)	-38.7 (-67.6 to 9.38)
3 COVID-19	3 Diabetes	35.2 (-4.14 to 89.3)	**59.2 (14.7 to 112)**	-18 (-45.1 to 21.5)
4 Diabetes	4 Stroke	4.6 (-20.7 to 39.8)	23.2 (-3.92 to 58.6)	**-39.7 (-57.2 to -12.4)**
5 Hypertensive heart disease	5 Chronic kidney disease	**91.3 (30.7 to 164)**	**125 (57.3 to 206)**	20.6 (-23.6 to 83.3)
6 Chronic kidney disease	6 Prostate cancer	**81.7 (8.58 to 176)**	**113 (29 to 224)**	-4.44 (-31.7 to 31.1)
7 Prostate cancer	7 Lower respiratory infect	**53.4 (19.8 to 86.3)**	**80.5 (43 to 113)**	-20.7 (-39.7 to 4.48)
8 Lower respiratory infect	8 Alzheimer's disease	**127 (67.8 to 178)**	**167 (107 to 230)**	**5.6 (2.56 to 9.1)**
9 Interpersonal violence	9 Breast cancer	**62.3 (0.923 to 136)**	**90.6 (22.9 to 189)**	12 (-22.2 to 53.6)
10 Breast cancer	10 Colorectal cancer	**84.9 (4.18 to 183)**	**117 (26.5 to 233)**	11.9 (-27.5 to 63.3)
11 Cirrhosis liver	11 Urinary diseases	**140 (85.9 to 204)**	**182 (123 to 264)**	32.2 (-3.48 to 78)
12 HIV/AIDS	12 Cirrhosis liver	18.1 (-3.61 to 43.1)	**39 (18.4 to 64.7)**	-3.99 (-19 to 16.4)
13 Colorectal cancer	13 COPD	**60.4 (16.2 to 118)**	**88.7 (40 to 148)**	-10.9 (-38.5 to 29.7)
14 Alzheimer's disease	14 Endo/metab/blood/immune	**78.4 (38.4 to 118)**	**110 (72.5 to 148)**	19 (-6.57 to 43.8)
15 Other cardiovascular	15 Atrial fibrillation	**163 (87.7 to 250)**	**209 (133 to 307)**	19.2 (-2.43 to 44.1)
16 COPD	16 Lung cancer	**60.9 (0.563 to 133)**	**89 (21.3 to 170)**	6.81 (-25.5 to 44.1)
17 Stomach cancer	17 Pancreatic cancer	**128 (14.6 to 267)**	**168 (43.7 to 336)**	47.7 (-13.8 to 134)
18 Lung cancer	18 Other cardiovascular	27.9 (-0.282 to 57.5)	**50.4 (22.3 to 84.6)**	-21.8 (-37.9 to 0.556)
19 Endo/metab/blood/immune	19 Falls	**66.8 (10.3 to 126)**	**96 (41 to 156)**	-7.62 (-25.2 to 8.98)
20 Cervical cancer	20 Stomach cancer	4.31 (-16.3 to 27.2)	**22.7 (4.2 to 44.3)**	**-34.1 (-42.1 to -25.4)**
21 Urinary diseases	22 Cervical cancer			
22 Falls	24 Interpersonal violence			
27 Pancreatic cancer	53 HIV/AIDS			
28 Atrial fibrillation	129 COVID-19			

Supplemental Results Figure S8. Leading 20 level 3 causes of deaths, and percentage change in number of deaths and all-age and age-standardised death rates by location, 2022 and 2050

Suriname, Reference scenario

Legend:
Communicable, maternal, neonatal and nutritional
Non-communicable
Injuries

Leading Causes 2022	Leading Causes 2050	Mean % change number of DEATH	Mean % change all-age DEATH rate	Mean % change age-standardized DEATH rate
1 Stroke	1 Ischemic heart disease	39.3 (-21.9 to 113)	23.1 (-29.5 to 89.4)	-38.7 (-64.5 to 5.04)
2 Ischemic heart disease	2 Stroke	32.2 (-0.18 to 75.8)	16.7 (-12.8 to 54.3)	**-41.4 (-57.6 to -19)**
3 COVID-19	3 Chronic kidney disease	**156 (67.9 to 263)**	**126 (48.7 to 216)**	22.8 (-18.3 to 78.7)
4 Diabetes	4 Diabetes	**61.4 (16.9 to 117)**	**42.4 (6.15 to 97.2)**	-23.6 (-46.3 to 8.86)
5 Chronic kidney disease	5 Lower respiratory infect	**94.3 (44.7 to 149)**	**71.3 (26.7 to 120)**	**-26.4 (-42.3 to -7.25)**
6 Lower respiratory infect	6 Alzheimer's disease	**257 (131 to 386)**	**214 (118 to 317)**	0.0721 (-3.27 to 3.64)
7 Self-harm	7 Hypertensive heart disease	**144 (60.1 to 250)**	**116 (40.7 to 205)**	4.72 (-30.8 to 57.9)
8 Hypertensive heart disease	8 Prostate cancer	**180 (55.6 to 333)**	**146 (41 to 281)**	6.13 (-27.3 to 44.7)
9 Neonatal disorders	9 COPD	**118 (55.6 to 200)**	**92.6 (37.5 to 164)**	-10.9 (-36.9 to 29)
10 Cirrhosis liver	10 Colorectal cancer	**118 (23.6 to 247)**	**91.5 (11.7 to 195)**	2.51 (-33.3 to 47.6)
11 Road injuries	11 Lung cancer	**88 (18.9 to 177)**	**65.2 (11 to 136)**	-2.17 (-28.6 to 32.2)
12 HIV/AIDS	12 Cirrhosis liver	**35.3 (11 to 62.5)**	**19.3 (2.24 to 39.3)**	**-17.5 (-30.4 to -4.29)**
13 COPD	13 Self-harm	13.5 (-12.2 to 45.1)	0.0655 (-19.9 to 25.2)	-11.5 (-31.7 to 14)
14 Prostate cancer	14 Urinary diseases	**223 (134 to 350)**	**184 (109 to 269)**	28.1 (-2.69 to 66.4)
15 Alzheimer's disease	15 Breast cancer	**81.7 (13.3 to 168)**	**59.6 (6.49 to 127)**	-1.95 (-29.9 to 34.1)
16 Lung cancer	16 Atrial fibrillation	**268 (137 to 410)**	**224 (125 to 329)**	10.9 (-4.43 to 34.7)
17 Colorectal cancer	17 Pancreatic cancer	**177 (43.2 to 371)**	**143 (32 to 313)**	39.1 (-19 to 116)
18 Breast cancer	18 Road injuries	5.16 (-38 to 66.9)	-7 (-42.8 to 51.2)	-11.8 (-47.4 to 45.9)
19 Interpersonal violence	19 Falls	**148 (75.6 to 228)**	**118 (61.5 to 182)**	-8.83 (-20.5 to 4.46)
20 Cervical cancer	20 Ileus & obstruction	**124 (68.7 to 196)**	**97.2 (49.7 to 156)**	-3.4 (-27.5 to 28.9)
22 Urinary diseases	23 Cervical cancer			
23 Pancreatic cancer	24 Neonatal disorders			
26 Ileus & obstruction	31 HIV/AIDS			
28 Falls	36 Interpersonal violence			
29 Atrial fibrillation	131 COVID-19			

Supplemental Results Figure S8. Leading 20 level 3 causes of deaths, and percentage change in number of deaths and all-age and age-standardised death rates by location, 2022 and 2050

Trinidad and Tobago, Reference scenario

Legend:
Communicable, maternal, neonatal and nutritional
Non-communicable
Injuries

Leading Causes 2022	Leading Causes 2050	Mean % change number of DEATH	Mean % change all-age DEATH rate	Mean % change age-standardized DEATH rate
1 COVID-19	1 Diabetes	18.8 (-18.4 to 58.6)	**62.6 (21.1 to 111)**	**-35.2 (-51.9 to -15.5)**
2 Ischemic heart disease	2 Ischemic heart disease	3.66 (-31.9 to 58.4)	41.9 (-7.75 to 120)	**-48.1 (-66.4 to -18.6)**
3 Diabetes	3 Chronic kidney disease	**101 (37.5 to 186)**	**175 (97.6 to 279)**	14.2 (-17.3 to 61.1)
4 Stroke	4 Stroke	4.75 (-23.4 to 36.4)	**43.3 (13.7 to 82)**	**-48.3 (-59.4 to -34.7)**
5 Chronic kidney disease	5 Prostate cancer	**95.6 (21.7 to 206)**	**167 (68.4 to 290)**	-8.12 (-31.1 to 18.1)
6 Prostate cancer	6 Alzheimer's disease	**195 (110 to 305)**	**303 (190 to 424)**	**-5.53 (-8.66 to -2.25)**
7 Interpersonal violence	7 Hypertensive heart disease	**124 (54.2 to 217)**	**206 (110 to 335)**	7.37 (-25.5 to 51.4)
8 Hypertensive heart disease	8 Lower respiratory infect	**90.1 (39.2 to 164)**	**160 (97.5 to 235)**	-19.9 (-35.6 to 0.499)
9 Lower respiratory infect	9 Colorectal cancer	60.5 (-1.34 to 148)	**119 (36.1 to 225)**	-8.29 (-36.8 to 25.9)
10 Colorectal cancer	10 COPD	**89.6 (30 to 163)**	**159 (87.5 to 246)**	-8.92 (-30.7 to 18.9)
11 Alzheimer's disease	11 Breast cancer	33 (-12.2 to 91.6)	**81.5 (25.9 to 147)**	-12.3 (-34 to 8.56)
12 Breast cancer	12 Atrial fibrillation	**234 (128 to 358)**	**356 (228 to 505)**	8.83 (-9.41 to 30)
13 COPD	13 Urinary diseases	**162 (85.3 to 265)**	**258 (175 to 359)**	20.8 (-2.57 to 47.7)
14 Road injuries	14 Lung cancer	37.2 (-5.52 to 90.1)	**87.4 (33.7 to 149)**	-10.9 (-31.5 to 13.8)
15 HIV/AIDS	15 Endo/metab/blood/immune	**80.1 (36.5 to 134)**	**146 (100 to 198)**	10.4 (-5.32 to 28.8)
16 Lung cancer	16 Pancreatic cancer	**102 (15.2 to 229)**	**175 (61 to 333)**	22.3 (-22.8 to 76.1)
17 Self-harm	17 Falls	**103 (36.6 to 179)**	**176 (99.1 to 269)**	**-14.5 (-25.9 to -1.75)**
18 Cirrhosis liver	18 Cirrhosis liver	-3.69 (-21.1 to 16.4)	**31.8 (15.5 to 50.6)**	**-20 (-27.9 to -8.21)**
19 Endo/metab/blood/immune	19 Parkinson's disease	**111 (60.9 to 174)**	**189 (131 to 251)**	-8.23 (-21.5 to 6.71)
20 Cervical cancer	20 Self-harm	**-21.5 (-38 to -3.39)**	7.44 (-9.21 to 27.6)	-8.65 (-24 to 12.1)

Leading Causes 2022 (below rank 20)	Leading Causes 2050 (below rank 20)
21 Urinary diseases	21 Road injuries
22 Pancreatic cancer	23 Interpersonal violence
23 Atrial fibrillation	26 Cervical cancer
27 Falls	45 HIV/AIDS
30 Parkinson's disease	131 COVID-19

Supplemental Results Figure S8. Leading 20 level 3 causes of deaths, and percentage change in number of deaths and all-age and age-standardised death rates by location, 2022 and 2050

Virgin Islands, Reference scenario

Legend:
Communicable, maternal, neonatal and nutritional
Non-communicable
Injuries

Leading Causes 2022	Leading Causes 2050	Mean % change number of DEATH	Mean % change all-age DEATH rate	Mean % change age-standardized DEATH rate
1 COVID-19	1 Ischemic heart disease	37 (-20.6 to 133)	**77.1 (3.35 to 193)**	**-42.9 (-65.6 to -9.77)**
2 Ischemic heart disease	2 Chronic kidney disease	**168 (84.9 to 315)**	**246 (136 to 445)**	30.2 (-8.75 to 94.8)
3 Stroke	3 Alzheimer's disease	**213 (141 to 289)**	**305 (212 to 411)**	**5.42 (1.33 to 9.01)**
4 Diabetes	4 Stroke	24 (-10.2 to 67)	**60.2 (18.6 to 115)**	**-40.4 (-53.4 to -24.8)**
5 Chronic kidney disease	5 Diabetes	40.3 (-1.85 to 94.4)	**81.3 (25.3 to 147)**	**-29.1 (-47.2 to -6.57)**
6 Prostate cancer	6 Hypertensive heart disease	**155 (64.4 to 280)**	**230 (110 to 388)**	15.1 (-21 to 65.6)
7 Hypertensive heart disease	7 Prostate cancer	**122 (27.5 to 247)**	**187 (74.4 to 331)**	5.71 (-23.2 to 38.1)
8 Alzheimer's disease	8 Lower respiratory infect	**156 (87.5 to 254)**	**231 (140 to 351)**	-9.07 (-28.6 to 10.6)
9 Interpersonal violence	9 Colorectal cancer	**82.1 (4.41 to 185)**	**135 (36.4 to 258)**	0.328 (-35 to 41.5)
10 Colorectal cancer	10 Atrial fibrillation	**356 (213 to 541)**	**489 (316 to 705)**	**22.2 (1.32 to 47.4)**
11 Lung cancer	11 Lung cancer	50 (-0.453 to 110)	**93.5 (35.7 to 162)**	4.52 (-22.7 to 33.4)
12 Lower respiratory infect	12 COPD	**139 (59.9 to 257)**	**209 (113 to 359)**	3.55 (-25.1 to 43.7)
13 Breast cancer	13 Breast cancer	59.9 (-0.252 to 125)	**106 (30.7 to 185)**	-1.68 (-25.1 to 23.1)
14 Cirrhosis liver	14 Urinary diseases	**310 (180 to 516)**	**429 (262 to 678)**	**45.9 (15 to 93.4)**
15 COPD	15 Parkinson's disease	**120 (61.9 to 203)**	**184 (121 to 276)**	1.53 (-14.3 to 20.4)
16 Road injuries	16 Pancreatic cancer	**110 (14.1 to 242)**	**171 (49.4 to 321)**	35.7 (-17.4 to 99.7)
17 Stomach cancer	17 Cirrhosis liver	1.93 (-16.2 to 22.3)	**31.6 (10.2 to 54.3)**	**-15.3 (-24.4 to -4.5)**
18 Parkinson's disease	18 Endo/metab/blood/immune	**147 (77 to 239)**	**219 (133 to 324)**	**20 (0.16 to 40)**
19 Cardiomyopathy	19 Peripheral artery	**323 (159 to 512)**	**447 (245 to 701)**	**29.9 (1.11 to 66)**
20 Pancreatic cancer	20 Falls	**170 (92.4 to 270)**	**249 (156 to 368)**	-8.64 (-20.4 to 5.44)
21 Atrial fibrillation	21 Cardiomyopathy			
23 Endo/metab/blood/immune	23 Stomach cancer			
24 Urinary diseases	24 Interpersonal violence			
27 Falls	27 Road injuries			
39 Peripheral artery	127 COVID-19			

Supplemental Results Figure S8. Leading 20 level 3 causes of deaths, and percentage change in number of deaths and all-age and age-standardised death rates by location, 2022 and 2050

Colombia, Reference scenario

Legend:
Communicable, maternal, neonatal and nutritional
Non-communicable
Injuries

Leading Causes 2022
1 Ischemic heart disease
2 COVID-19
3 COPD
4 Stroke
5 Interpersonal violence
6 Alzheimer's disease
7 Lower respiratory infect
8 Chronic kidney disease
9 Stomach cancer
10 Road injuries
11 Diabetes
12 Lung cancer
13 Colorectal cancer
14 Hypertensive heart disease
15 Breast cancer
16 Prostate cancer
17 Cirrhosis liver
18 Self-harm
19 Urinary diseases
20 Neonatal disorders
22 Pancreatic cancer
23 Atrial fibrillation
27 Parkinson's disease

Leading Causes 2050	Mean % change number of DEATH	Mean % change all-age DEATH rate	Mean % change age-standardized DEATH rate
1 Ischemic heart disease	**62.7 (2.4 to 149)**	44.1 (-13.4 to 128)	**-44.4 (-67 to -8.54)**
2 COPD	**175 (103 to 252)**	**144 (75.7 to 213)**	-15.3 (-40.1 to 19.8)
3 Alzheimer's disease	**263 (187 to 346)**	**221 (152 to 281)**	**-3.46 (-6.96 to -0.0157)**
4 Chronic kidney disease	**214 (120 to 332)**	**178 (93.7 to 301)**	13.6 (-21.1 to 67.5)
5 Stroke	**45.1 (9.85 to 83.5)**	28.4 (-2.23 to 64.4)	**-45.8 (-59.4 to -27.2)**
6 Lower respiratory infect	**100 (59 to 150)**	**77.1 (40.6 to 119)**	**-28 (-42.7 to -8.55)**
7 Lung cancer	**127 (58.2 to 218)**	**101 (40.5 to 167)**	-5.47 (-28.9 to 24.5)
8 Colorectal cancer	**133 (40.9 to 260)**	**106 (29.7 to 194)**	-3.19 (-36.5 to 39.8)
9 Diabetes	**82.5 (37.4 to 136)**	**61.5 (23 to 107)**	**-30.3 (-47.4 to -8.28)**
10 Hypertensive heart disease	**197 (105 to 309)**	**163 (79.7 to 278)**	-2.96 (-34.5 to 41.4)
11 Prostate cancer	**168 (76 to 273)**	**137 (61.7 to 228)**	-4.07 (-28.3 to 23.7)
12 Urinary diseases	**270 (195 to 370)**	**227 (157 to 315)**	22.6 (-4.52 to 51.3)
13 Stomach cancer	**42.1 (19.1 to 73.7)**	**25.6 (7.52 to 49.2)**	**-40.2 (-47 to -32.5)**
14 Atrial fibrillation	**284 (196 to 374)**	**239 (162 to 323)**	11.5 (-8.88 to 33.4)
15 Breast cancer	**89 (28.8 to 154)**	**66.9 (14.4 to 123)**	-9.78 (-32.7 to 16.4)
16 Interpersonal violence	-41.7 (-68.6 to 0.427)	**-48.6 (-70.8 to -13.6)**	**-45.8 (-70.1 to -4.57)**
17 Pancreatic cancer	**207 (71 to 377)**	**171 (50.9 to 315)**	30.4 (-21.5 to 92.3)
18 Road injuries	1.56 (-29.3 to 45)	-10.1 (-36.4 to 31.7)	-23.8 (-47.3 to 13.1)
19 Parkinson's disease	**174 (129 to 234)**	**142 (103 to 189)**	-8.58 (-22.2 to 5.1)
20 Cirrhosis liver	**64.7 (43.2 to 86.6)**	**45.6 (26.4 to 65.2)**	**-20.9 (-29 to -11.8)**
33 Self-harm			
56 Neonatal disorders			
135 COVID-19			

Supplemental Results Figure S8. Leading 20 level 3 causes of deaths, and percentage change in number of deaths and all-age and age-standardised death rates by location, 2022 and 2050

Costa Rica, Reference scenario

Legend:
Communicable, maternal, neonatal and nutritional
Non-communicable
Injuries

Leading Causes 2022	Leading Causes 2050	Mean % change number of DEATH	Mean % change all-age DEATH rate	Mean % change age-standardized DEATH rate
1 Ischemic heart disease	1 Chronic kidney disease	**169 (89.5 to 273)**	**155 (80.6 to 245)**	5.64 (-26 to 50.1)
2 COVID-19	2 Ischemic heart disease	37.8 (-15.4 to 116)	30.4 (-19.9 to 99.8)	**-50 (-69.4 to -22.4)**
3 Chronic kidney disease	3 Alzheimer's disease	**250 (187 to 329)**	**231 (182 to 277)**	-1.72 (-5.06 to 1.14)
4 Stroke	4 COPD	**186 (98.8 to 287)**	**171 (92 to 264)**	-11 (-37.6 to 26.8)
5 COPD	5 Stroke	**45.1 (7.54 to 86.9)**	**37.4 (5.45 to 73.7)**	**-47.6 (-60.9 to -32.3)**
6 Alzheimer's disease	6 Lower respiratory infect	**123 (74 to 173)**	**111 (67.9 to 153)**	**-22.3 (-37.4 to -4.42)**
7 Diabetes	7 Colorectal cancer	**124 (42.3 to 242)**	**112 (33.4 to 217)**	-4.67 (-35.5 to 35.8)
8 Stomach cancer	8 Diabetes	**82.3 (30.3 to 142)**	**72.6 (30.7 to 121)**	**-30.4 (-47 to -8.91)**
9 Road injuries	9 Hypertensive heart disease	**215 (107 to 338)**	**198 (103 to 306)**	2.94 (-30.1 to 52.1)
10 Cirrhosis liver	10 Prostate cancer	**171 (86.5 to 288)**	**156 (77.7 to 262)**	-3.96 (-26.4 to 27.5)
11 Lower respiratory infect	11 Cirrhosis liver	**55.5 (31.7 to 80.5)**	**47.2 (30 to 64.4)**	**-16.3 (-26.3 to -5.86)**
12 Colorectal cancer	12 Stomach cancer	**46.4 (21.5 to 74.9)**	**38.6 (18.6 to 63)**	**-37.7 (-44.2 to -29.1)**
13 Interpersonal violence	13 Falls	**152 (99 to 221)**	**138 (96.2 to 190)**	-10.8 (-22.1 to 2.94)
14 Hypertensive heart disease	14 Breast cancer	**98 (41.4 to 179)**	**87.4 (35 to 154)**	-6.08 (-29 to 23.6)
15 Prostate cancer	15 Road injuries	8.5 (-25.2 to 58.7)	2.66 (-28.6 to 48.3)	-9.85 (-37.8 to 31.7)
16 Breast cancer	16 Pancreatic cancer	**202 (81.5 to 370)**	**186 (68.3 to 342)**	32.1 (-17.1 to 96.8)
17 Self-harm	17 Urinary diseases	**282 (182 to 395)**	**261 (179 to 362)**	30.2 (-1.68 to 71.1)
18 Lung cancer	18 Atrial fibrillation	**280 (197 to 389)**	**259 (192 to 338)**	8.55 (-7.5 to 27.6)
19 Falls	19 Lung cancer	**118 (54.2 to 208)**	**106 (49.2 to 177)**	-6.02 (-30.1 to 22.9)
20 Liver cancer	20 Liver cancer	**150 (88.3 to 210)**	**136 (88.7 to 187)**	12.3 (-10.2 to 38.7)
21 Pancreatic cancer	22 Self-harm			
23 Atrial fibrillation	30 Interpersonal violence			
24 Urinary diseases	134 COVID-19			

Supplemental Results Figure S8. Leading 20 level 3 causes of deaths, and percentage change in number of deaths and all-age and age-standardised death rates by location, 2022 and 2050

El Salvador, Reference scenario

Legend:
Communicable, maternal, neonatal and nutritional
Non-communicable
Injuries

Leading Causes 2022	Leading Causes 2050	Mean % change number of DEATH	Mean % change all-age DEATH rate	Mean % change age-standardized DEATH rate
1 Ischemic heart disease	1 Chronic kidney disease	**125 (55.4 to 203)**	**176 (84.6 to 282)**	30.5 (-15.5 to 85.9)
2 Chronic kidney disease	2 Ischemic heart disease	33.2 (-18.4 to 106)	**63.7 (2.11 to 149)**	-28.1 (-59.1 to 18.5)
3 COVID-19	3 Diabetes	41.4 (-1.11 to 94.5)	**73.9 (28.2 to 137)**	-21.3 (-43.8 to 7.99)
4 Interpersonal violence	4 Alzheimer's disease	**128 (60.3 to 203)**	**179 (104 to 263)**	**9.82 (5.74 to 13.7)**
5 Diabetes	5 Lower respiratory infect	**44.8 (12 to 87.1)**	**78.1 (41.7 to 129)**	-18.2 (-35.4 to 3.66)
6 Stroke	6 Stroke	22.7 (-9.44 to 64)	**50.9 (15.3 to 92.1)**	**-32.4 (-49.1 to -10.1)**
7 Lower respiratory infect	7 COPD	**87.9 (33.1 to 154)**	**131 (70.4 to 207)**	-4.46 (-32.4 to 33.2)
8 Cirrhosis liver	8 Interpersonal violence	**-47.2 (-73.9 to -3.48)**	-35.8 (-65.8 to 16)	-34.4 (-66.2 to 22.7)
9 Alzheimer's disease	9 Cirrhosis liver	22.6 (-6.05 to 49.9)	**50.5 (27.7 to 76.7)**	**-16 (-26.2 to -4.88)**
10 COPD	10 Stomach cancer	17.9 (-8.43 to 46.7)	**44.8 (20.4 to 75.1)**	**-33 (-40.3 to -25)**
11 Road injuries	11 Lung cancer	**106 (37.3 to 203)**	**152 (72.3 to 257)**	15.7 (-10.7 to 51.9)
12 Stomach cancer	12 Prostate cancer	**90.9 (17.8 to 201)**	**134 (49.6 to 264)**	-2.81 (-28.6 to 32.7)
13 Alcohol use disorders	13 Atrial fibrillation	**164 (73.5 to 258)**	**224 (130 to 341)**	**28.5 (9.51 to 58.7)**
14 Prostate cancer	14 Road injuries	-15.2 (-48.8 to 32.7)	3.99 (-33.4 to 62.9)	-23.1 (-54.1 to 27.2)
15 Lung cancer	15 Colorectal cancer	**88.6 (12.5 to 198)**	**132 (39 to 263)**	6.94 (-28.1 to 54.6)
16 Cervical cancer	16 Alcohol use disorders	10.2 (-22.2 to 48.3)	**35 (6.63 to 67.3)**	-5.09 (-25.2 to 17.8)
17 Self-harm	17 Pancreatic cancer	**175 (49.2 to 366)**	**237 (84.8 to 445)**	55.2 (-9.87 to 135)
18 Colorectal cancer	18 Urinary diseases	**133 (77.4 to 197)**	**187 (128 to 256)**	26.4 (-0.301 to 61.1)
19 Breast cancer	19 Breast cancer	**64.4 (7.96 to 142)**	**102 (38.8 to 189)**	1.11 (-25.6 to 34.4)
20 Neonatal disorders	20 Hypertensive heart disease	**90.3 (32.2 to 171)**	**134 (62.9 to 220)**	0.788 (-32.4 to 51.6)
21 Atrial fibrillation	21 Cervical cancer			
23 Hypertensive heart disease	24 Self-harm			
24 Urinary diseases	54 Neonatal disorders			
30 Pancreatic cancer	132 COVID-19			

Supplemental Results Figure S8. Leading 20 level 3 causes of deaths, and percentage change in number of deaths and all-age and age-standardised death rates by location, 2022 and 2050

Guatemala, Reference scenario

Legend:
Communicable, maternal, neonatal and nutritional
Non-communicable
Injuries

Leading Causes 2022	Leading Causes 2050	Mean % change number of DEATH	Mean % change all-age DEATH rate	Mean % change age-standardized DEATH rate
1 Lower respiratory infect	1 Chronic kidney disease	**237 (143 to 364)**	**180 (106 to 289)**	37.9 (-3.63 to 103)
2 Ischemic heart disease	2 Ischemic heart disease	**107 (36.2 to 195)**	**72.3 (16.2 to 152)**	-30.1 (-59.9 to 19.1)
3 Diabetes	3 Lower respiratory infect	**49.7 (22.3 to 82)**	**24.7 (0.48 to 56.3)**	**-37.7 (-49.4 to -23.4)**
4 Chronic kidney disease	4 Diabetes	**64.2 (21.4 to 111)**	**36.8 (3.13 to 72.8)**	**-33 (-52.4 to -8.47)**
5 Cirrhosis liver	5 Cirrhosis liver	**47.5 (27.9 to 68.7)**	**22.8 (8.08 to 39.3)**	**-29.3 (-37.2 to -20.3)**
6 Stroke	6 Stroke	**67.4 (34.6 to 105)**	**39.3 (12.2 to 72.8)**	**-39.4 (-54.9 to -19.1)**
7 Interpersonal violence	7 Alzheimer's disease	**227 (153 to 307)**	**172 (111 to 232)**	**-4.34 (-7.29 to -0.269)**
8 COVID-19	8 Stomach cancer	**50.9 (27.2 to 79.5)**	**25.7 (5.12 to 47)**	**-39.5 (-46.3 to -33.2)**
9 Stomach cancer	9 COPD	**114 (66.1 to 186)**	**78 (36.5 to 137)**	-30.6 (-50.8 to 0.104)
10 Diarrheal diseases	10 Interpersonal violence	-22.6 (-60.3 to 48.3)	-35.7 (-66.8 to 24.6)	-39 (-68.2 to 20.6)
11 Neonatal disorders	11 Prostate cancer	**176 (80 to 315)**	**130 (48.8 to 243)**	-6.59 (-29.8 to 24.6)
12 Road injuries	12 Road injuries	-9.63 (-46 to 43.1)	-24.8 (-53.3 to 19.8)	-35.8 (-61.1 to 4.68)
13 COPD	13 Diarrheal diseases	-21.6 (-52 to 12)	**-34.7 (-59.1 to -3.73)**	**-59.3 (-76 to -35.7)**
14 Alzheimer's disease	14 Alcohol use disorders	**51.7 (19.8 to 93.4)**	**26.3 (1.47 to 60.3)**	-14.6 (-31.9 to 7.31)
15 Congenital defects	15 Protein-energy malnutrition	**78.6 (9.17 to 166)**	48.9 (-10 to 123)	-33 (-65.7 to 19.7)
16 Alcohol use disorders	16 Colorectal cancer	**150 (58.2 to 277)**	**108 (29.8 to 209)**	-0.613 (-34.8 to 41.3)
17 Protein-energy malnutrition	17 Falls	**85 (49.1 to 126)**	**54 (25.2 to 83.6)**	**-18.6 (-28.1 to -6.61)**
18 Prostate cancer	18 Atrial fibrillation	**316 (214 to 435)**	**246 (171 to 355)**	**20.2 (1.85 to 42.9)**
19 Falls	19 Lung cancer	**136 (70.1 to 216)**	**96.1 (37.6 to 163)**	-5.75 (-29.5 to 20.8)
20 Cervical cancer	20 Cervical cancer	**55 (29.3 to 86.3)**	**29.1 (8.34 to 54.8)**	**-28.8 (-41.3 to -14.7)**
23 Colorectal cancer	31 Neonatal disorders			
25 Lung cancer	34 Congenital defects			
39 Atrial fibrillation	133 COVID-19			

Supplemental Results Figure S8. Leading 20 level 3 causes of deaths, and percentage change in number of deaths and all-age and age-standardised death rates by location, 2022 and 2050

Honduras, Reference scenario

Legend:
Communicable, maternal, neonatal and nutritional
Non-communicable
Injuries

Leading Causes 2022
1 Ischemic heart disease
2 Stroke
3 COVID-19
4 Interpersonal violence
5 COPD
6 Cirrhosis liver
7 Lower respiratory infect
8 Diabetes
9 Hypertensive heart disease
10 Road injuries
11 Chronic kidney disease
12 Lung cancer
13 Stomach cancer
14 Neonatal disorders
15 Diarrheal diseases
16 Nature disaster
17 Alzheimer's disease
18 Congenital defects
19 Upper digest diseases
20 Other digestive
22 Urinary diseases
25 Breast cancer
26 Parkinson's disease
27 Prostate cancer
33 Pancreatic cancer

Leading Causes 2050	Mean % change number of DEATH	Mean % change all-age DEATH rate	Mean % change age-standardized DEATH rate
1 Ischemic heart disease	**70.9 (9.07 to 150)**	30.5 (-15.5 to 86.6)	-30.8 (-62.7 to 17.9)
2 Stroke	**55.2 (20.2 to 90.3)**	18.4 (-8.01 to 46.2)	**-36.7 (-54.6 to -13.4)**
3 COPD	**100 (55.6 to 159)**	**52.7 (16.8 to 92.8)**	-21.7 (-46.6 to 13.5)
4 Chronic kidney disease	**211 (119 to 328)**	**137 (66.2 to 233)**	31.8 (-12.8 to 99)
5 Hypertensive heart disease	**144 (76.6 to 229)**	**85.9 (33.4 to 146)**	-6.57 (-39.2 to 37)
6 Cirrhosis liver	**69 (39.4 to 96.7)**	**29 (9.81 to 50)**	**-26.1 (-35.8 to -14.8)**
7 Lung cancer	**148 (64.3 to 245)**	**89.3 (33.5 to 165)**	1.98 (-25.9 to 37.9)
8 Interpersonal violence	-5.14 (-42.1 to 60.3)	-27.7 (-52.8 to 26.6)	-39.9 (-60.6 to 6.05)
9 Lower respiratory infect	**61.5 (27.8 to 98.4)**	**23.2 (1.28 to 49.3)**	**-27.1 (-44.6 to -6.13)**
10 Diabetes	**70.9 (22.6 to 129)**	30.4 (-4.04 to 71.9)	-27.6 (-49.3 to 3.39)
11 Alzheimer's disease	**203 (125 to 292)**	**131 (77.2 to 196)**	**6.2 (3.07 to 9.76)**
12 Road injuries	43.2 (-12.2 to 130)	9.34 (-33.7 to 74.9)	-25.3 (-54.1 to 20.3)
13 Stomach cancer	**64.5 (36.4 to 94)**	**25.5 (6.92 to 47.1)**	**-31.6 (-38.8 to -24.1)**
14 Other digestive	**162 (91.4 to 236)**	**99.9 (44.9 to 163)**	15.7 (-16.9 to 60.2)
15 Urinary diseases	**194 (135 to 259)**	**124 (80.5 to 167)**	21.4 (-7.75 to 53.6)
16 Diarrheal diseases	14.9 (-30.7 to 75.2)	-12.3 (-45.3 to 34.8)	**-40.4 (-65 to -0.318)**
17 Pancreatic cancer	**277 (111 to 516)**	**187 (71.7 to 370)**	54.1 (-6.19 to 136)
18 Prostate cancer	**178 (75.5 to 315)**	**112 (35.9 to 213)**	4.62 (-25.8 to 43.2)
19 Parkinson's disease	**161 (108 to 218)**	**99 (63 to 136)**	-1.84 (-19 to 16.6)
20 Breast cancer	**140 (69.2 to 237)**	**83.3 (25.3 to 155)**	4.48 (-22.8 to 37.6)
22 Nature disaster			
25 Upper digest diseases			
41 Neonatal disorders			
43 Congenital defects			
133 COVID-19			

Supplemental Results Figure S8. Leading 20 level 3 causes of deaths, and percentage change in number of deaths and all-age and age-standardised death rates by location, 2022 and 2050

Mexico, Reference scenario

Legend:
Communicable, maternal, neonatal and nutritional
Non-communicable
Injuries

Leading Causes 2022	Leading Causes 2050	Mean % change number of DEATH	Mean % change all-age DEATH rate	Mean % change age-standardized DEATH rate
1 Ischemic heart disease	1 Ischemic heart disease	**60.2 (13.6 to 118)**	41.2 (-1.64 to 92)	**-38.5 (-62.8 to -5.55)**
2 COVID-19	2 Chronic kidney disease	**170 (109 to 256)**	**138 (82.2 to 211)**	23.7 (-11.2 to 69.9)
3 Diabetes	3 Diabetes	**68.8 (33.3 to 109)**	**48.7 (17.4 to 85.6)**	**-26 (-44.3 to -4.93)**
4 Chronic kidney disease	4 Cirrhosis liver	**74.6 (55.1 to 94.9)**	**53.8 (40 to 68.6)**	-2.65 (-12.9 to 8.63)
5 Cirrhosis liver	5 COPD	**143 (89.8 to 202)**	**114 (66.4 to 166)**	-14.5 (-39.9 to 20.9)
6 Interpersonal violence	6 Lower respiratory infect	**89.2 (59.6 to 130)**	**66.6 (42.3 to 99.3)**	**-22.4 (-36.9 to -6.91)**
7 Stroke	7 Stroke	**46.9 (20.1 to 75.8)**	**29.4 (7.33 to 54.9)**	**-40.4 (-53.9 to -24.7)**
8 Lower respiratory infect	8 Alzheimer's disease	**226 (154 to 306)**	**186 (128 to 249)**	2.78 (-1.23 to 6.34)
9 COPD	9 Urinary diseases	**217 (163 to 279)**	**179 (134 to 234)**	**30.3 (5.51 to 59.1)**
10 Road injuries	10 Interpersonal violence	-26.1 (-59.1 to 30.5)	-35.1 (-62.2 to 13.2)	-35.8 (-63.4 to 12.3)
11 Alzheimer's disease	11 Hypertensive heart disease	**202 (128 to 285)**	**166 (102 to 249)**	7.93 (-25.9 to 55.9)
12 Stomach cancer	12 Colorectal cancer	**135 (49.1 to 247)**	**107 (35.6 to 202)**	8.19 (-26.9 to 48.8)
13 Colorectal cancer	13 Prostate cancer	**172 (74.5 to 286)**	**139 (59.7 to 230)**	0.164 (-25.7 to 30.2)
14 Neonatal disorders	14 Lung cancer	**133 (61.2 to 225)**	**105 (47.3 to 181)**	2.63 (-22.1 to 33.3)
15 Urinary diseases	15 Pancreatic cancer	**227 (87.6 to 408)**	**187 (70 to 342)**	48.4 (-6.84 to 121)
16 Breast cancer	16 Road injuries	11.3 (-24.5 to 62.2)	-1.87 (-36.2 to 42.8)	-15.5 (-45 to 24.7)
17 Lung cancer	17 Atrial fibrillation	**273 (191 to 380)**	**228 (165 to 320)**	**20.2 (2.25 to 40)**
18 Prostate cancer	18 Breast cancer	**100 (47.1 to 164)**	**76.1 (31.8 to 140)**	3.85 (-18.5 to 32.9)
19 Hypertensive heart disease	19 Stomach cancer	**48.8 (25.4 to 73.5)**	**31 (12.3 to 51.4)**	**-33 (-40.6 to -24.5)**
20 Congenital defects	20 Endo/metab/blood/immune	**161 (121 to 200)**	**130 (102 to 162)**	**23.8 (5.8 to 41.5)**
23 Pancreatic cancer	44 Congenital defects			
25 Endo/metab/blood/immune	45 Neonatal disorders			
29 Atrial fibrillation	133 COVID-19			

Supplemental Results Figure S8. Leading 20 level 3 causes of deaths, and percentage change in number of deaths and all-age and age-standardised death rates by location, 2022 and 2050

Nicaragua, Reference scenario

Legend:
Communicable, maternal, neonatal and nutritional
Non-communicable
Injuries

Leading Causes 2022	Leading Causes 2050	Mean % change number of DEATH	Mean % change all-age DEATH rate	Mean % change age-standardized DEATH rate
1 Ischemic heart disease	1 Chronic kidney disease	**277 (148 to 459)**	**230 (126 to 358)**	40.6 (-7.8 to 103)
2 Chronic kidney disease	2 Ischemic heart disease	**182 (67.1 to 333)**	**147 (56.5 to 258)**	-20.6 (-54.7 to 31.9)
3 Stroke	3 Diabetes	**137 (64 to 241)**	**107 (59 to 172)**	-17.7 (-41.4 to 15.3)
4 Diabetes	4 Stroke	**130 (71 to 209)**	**101 (56.6 to 159)**	**-29.6 (-48.2 to -3.59)**
5 Cirrhosis liver	5 Alzheimer's disease	**392 (275 to 516)**	**332 (242 to 431)**	**17.5 (12.9 to 21.7)**
6 Lower respiratory infect	6 COPD	**258 (153 to 390)**	**214 (131 to 301)**	-4.9 (-36.1 to 36.1)
7 COPD	7 Cirrhosis liver	**93.8 (56.6 to 138)**	**69.7 (49.1 to 93.4)**	**-11.7 (-21.9 to -0.0669)**
8 Neonatal disorders	8 Hypertensive heart disease	**292 (164 to 453)**	**244 (133 to 359)**	10.1 (-29.2 to 63.9)
9 Alzheimer's disease	9 Lower respiratory infect	**93.7 (52.2 to 148)**	**69.8 (37.9 to 111)**	**-31 (-44.9 to -14.8)**
10 Road injuries	10 Prostate cancer	**288 (161 to 479)**	**240 (116 to 427)**	16.2 (-14.8 to 58)
11 COVID-19	11 Stomach cancer	**99.3 (56.1 to 149)**	**74.7 (45.5 to 110)**	**-28.8 (-36.5 to -19.4)**
12 Interpersonal violence	12 Colorectal cancer	**210 (95.4 to 377)**	**172 (56.5 to 329)**	10.3 (-27.1 to 58.1)
13 Stomach cancer	13 Atrial fibrillation	**501 (327 to 685)**	**427 (295 to 574)**	**37.6 (14.4 to 69.9)**
14 Hypertensive heart disease	14 Lung cancer	**219 (117 to 367)**	**180 (91.4 to 309)**	14.5 (-15.6 to 56.7)
15 Congenital defects	15 Road injuries	17.6 (-29.9 to 90.4)	2.73 (-34.3 to 54.6)	-19.3 (-48.5 to 26)
16 Self-harm	16 Pancreatic cancer	**350 (157 to 655)**	**295 (113 to 564)**	62.2 (-2.05 to 152)
17 Cervical cancer	17 Falls	**185 (123 to 278)**	**150 (91.7 to 222)**	-4.28 (-15.6 to 10.5)
18 Nature disaster	18 Cervical cancer	**86 (40.8 to 142)**	**63 (30.9 to 102)**	**-19.8 (-33.7 to -0.0227)**
19 Alcohol use disorders	19 Breast cancer	**144 (64.5 to 247)**	**114 (53.9 to 202)**	2.09 (-24.3 to 37.2)
20 Colorectal cancer	20 Parkinson's disease	**289 (207 to 389)**	**241 (184 to 308)**	9.52 (-5.14 to 23.7)
21 Prostate cancer	21 Alcohol use disorders			
22 Breast cancer	22 Nature disaster			
23 Lung cancer	24 Self-harm			
24 Falls	26 Interpersonal violence			
30 Pancreatic cancer	31 Neonatal disorders			
31 Atrial fibrillation	46 Congenital defects			
32 Parkinson's disease	133 COVID-19			

Supplemental Results Figure S8. Leading 20 level 3 causes of deaths, and percentage change in number of deaths and all-age and age-standardised death rates by location, 2022 and 2050

Panama, Reference scenario

Legend:
Communicable, maternal, neonatal and nutritional
Non-communicable
Injuries

Leading Causes 2022	Leading Causes 2050	Mean % change number of DEATH	Mean % change all-age DEATH rate	Mean % change age-standardized DEATH rate
1 Ischemic heart disease	1 Chronic kidney disease	**196 (100 to 315)**	**115 (46.2 to 196)**	13.6 (-22.5 to 59.2)
2 Stroke	2 Ischemic heart disease	45.5 (-12.3 to 127)	5.65 (-33.7 to 66)	**-49.9 (-70.4 to -21.4)**
3 COVID-19	3 Alzheimer's disease	**272 (194 to 363)**	**170 (119 to 225)**	**5.48 (2.55 to 8.83)**
4 Chronic kidney disease	4 Stroke	**47.8 (10.6 to 93.5)**	7.33 (-17.7 to 38.2)	**-47.2 (-59.9 to -30)**
5 Diabetes	5 Diabetes	**104 (45.8 to 170)**	**48.1 (5.31 to 93.8)**	-23.7 (-45.5 to 1.48)
6 Alzheimer's disease	6 COPD	**190 (105 to 299)**	**111 (50.8 to 191)**	-7.91 (-36.2 to 28.7)
7 Lower respiratory infect	7 Lower respiratory infect	**116 (62.3 to 181)**	**57 (20.8 to 98.2)**	**-24.4 (-39.3 to -5.62)**
8 COPD	8 Colorectal cancer	**166 (70.6 to 312)**	**93 (24.3 to 192)**	6.09 (-28.5 to 48.2)
9 Interpersonal violence	9 Prostate cancer	**194 (92.4 to 330)**	**113 (41.8 to 213)**	1.06 (-24.7 to 34.1)
10 HIV/AIDS	10 Hypertensive heart disease	**211 (108 to 361)**	**126 (48.9 to 224)**	6.5 (-30.4 to 50.5)
11 Road injuries	11 Lung cancer	**154 (82.3 to 262)**	**84.2 (35.3 to 157)**	3.03 (-20.6 to 35.5)
12 Colorectal cancer	12 Atrial fibrillation	**289 (187 to 413)**	**182 (120 to 265)**	11.6 (-6.56 to 33.2)
13 Stomach cancer	13 Cirrhosis liver	**88.1 (54.2 to 124)**	**36.5 (18.4 to 54.6)**	-9.81 (-20.5 to 2.59)
14 Cirrhosis liver	14 Breast cancer	**130 (63.9 to 222)**	**67.1 (23.5 to 135)**	4.12 (-21 to 40.9)
15 Prostate cancer	15 Stomach cancer	**61.7 (27.8 to 98.6)**	17.4 (-2.25 to 41.7)	**-35.7 (-43.3 to -28.5)**
16 Lung cancer	16 Road injuries	35.1 (-16.1 to 113)	-1.83 (-39.8 to 49.6)	-15.8 (-48.6 to 30.9)
17 Hypertensive heart disease	17 Pancreatic cancer	**255 (114 to 478)**	**158 (60 to 329)**	44 (-4.48 to 124)
18 Congenital defects	18 Urinary diseases	**282 (182 to 402)**	**177 (109 to 254)**	29 (-1.46 to 59.8)
19 Breast cancer	19 Parkinson's disease	**193 (119 to 267)**	**112 (72.4 to 152)**	-2.53 (-18 to 14)
20 Neonatal disorders	20 Interpersonal violence	-9.97 (-46.1 to 74.1)	-34.7 (-60.2 to 23.7)	-36.3 (-62.8 to 23.4)
22 Atrial fibrillation	35 Congenital defects			
25 Parkinson's disease	37 HIV/AIDS			
27 Pancreatic cancer	41 Neonatal disorders			
28 Urinary diseases	134 COVID-19			

Supplemental Results Figure S8. Leading 20 level 3 causes of deaths, and percentage change in number of deaths and all-age and age-standardised death rates by location, 2022 and 2050

Venezuela, Reference scenario

Legend:
Communicable, maternal, neonatal and nutritional
Non-communicable
Injuries

Leading Causes 2022	Leading Causes 2050	Mean % change number of DEATH	Mean % change all-age DEATH rate	Mean % change age-standardized DEATH rate
1 Ischemic heart disease	1 Ischemic heart disease	72.7 (-42.7 to 311)	105 (-36 to 438)	43.8 (-74.5 to 340)
2 COVID-19	2 Chronic kidney disease	**167 (59.5 to 309)**	**209 (72.1 to 423)**	91.1 (-19.5 to 305)
3 Stroke	3 Stroke	54.5 (-8.59 to 146)	**78.8 (1.96 to 213)**	12.8 (-57.5 to 173)
4 Diabetes	4 Diabetes	**88.9 (4.77 to 214)**	**120 (16 to 298)**	37.2 (-50.8 to 230)
5 Chronic kidney disease	5 Alzheimer's disease	161 (-49 to 393)	186 (-33.2 to 393)	**9.94 (5.99 to 13.5)**
6 Interpersonal violence	6 Hypertensive heart disease	**120 (37.6 to 230)**	**153 (50.7 to 288)**	45.9 (-45.5 to 245)
7 Road injuries	7 COPD	**87.6 (31 to 168)**	**113 (48.7 to 190)**	17 (-46.9 to 141)
8 Lower respiratory infect	8 Lower respiratory infect	67.7 (-6.44 to 141)	**88.3 (20.2 to 143)**	0.921 (-31.7 to 59.3)
9 COPD	9 Road injuries	8.33 (-62.2 to 156)	29 (-57.2 to 228)	23.3 (-60.5 to 220)
10 Hypertensive heart disease	10 Lung cancer	47.5 (-63.4 to 183)	62.1 (-52.3 to 193)	-14.7 (-59.6 to 40.5)
11 Alzheimer's disease	11 Prostate cancer	97.9 (-60 to 332)	117 (-49.5 to 324)	-10.3 (-56.5 to 40)
12 Lung cancer	12 Interpersonal violence	-50.7 (-87 to 22.9)	-46.2 (-82.7 to 33.9)	-42.7 (-83.1 to 46.3)
13 Cirrhosis liver	13 Cirrhosis liver	10.3 (-13.1 to 36.5)	**25.3 (5.19 to 52.2)**	-13.2 (-31.8 to 14.4)
14 Neonatal disorders	14 Breast cancer	31.7 (-60.4 to 153)	45.1 (-46.1 to 159)	-10.7 (-52.7 to 41.2)
15 Prostate cancer	15 Colorectal cancer	42.2 (-73.5 to 209)	55.5 (-64.8 to 209)	-21.5 (-69.8 to 39.1)
16 Stomach cancer	16 Stomach cancer	14.5 (-42.7 to 69.6)	28.1 (-25.9 to 68.6)	**-28.9 (-36.8 to -18.1)**
17 Breast cancer	17 Atrial fibrillation	200 (-25.2 to 424)	231 (-3.92 to 449)	**33.5 (8.4 to 60.3)**
18 Colorectal cancer	18 Cervical cancer	15.2 (-11 to 49)	**31.7 (2.26 to 71.9)**	-3.28 (-34.5 to 47.2)
19 Cervical cancer	19 Pancreatic cancer	77.4 (-72.9 to 321)	93.5 (-64 to 326)	-1.82 (-71.4 to 99.1)
20 Self-harm	20 Diarrheal diseases	57.2 (-35.1 to 241)	85.5 (-25.7 to 346)	33.9 (-67.1 to 313)
23 Diarrheal diseases	24 Self-harm			
25 Pancreatic cancer	25 Neonatal disorders			
28 Atrial fibrillation	136 COVID-19			

Supplemental Results Figure S8. Leading 20 level 3 causes of deaths, and percentage change in number of deaths and all-age and age-standardised death rates by location, 2022 and 2050

Brazil, Reference scenario

Legend:
Communicable, maternal, neonatal and nutritional
Non-communicable
Injuries

Leading Causes 2022	Leading Causes 2050	Mean % change number of DEATH	Mean % change all-age DEATH rate	Mean % change age-standardized DEATH rate
1 Ischemic heart disease	1 Lower respiratory infect	**111 (63.6 to 156)**	**88.1 (51.5 to 126)**	-23.6 (-44.5 to 0.193)
2 Stroke	2 Ischemic heart disease	25 (-27 to 94.2)	11.7 (-34.4 to 81.6)	**-44.4 (-68.1 to -5.07)**
3 Lower respiratory infect	3 Alzheimer's disease	**207 (137 to 283)**	**174 (126 to 225)**	**-7.3 (-9.94 to -4.85)**
4 COVID-19	4 Stroke	29 (-10.2 to 72.6)	15.1 (-16.1 to 54.3)	**-45.4 (-62 to -22)**
5 Diabetes	5 COPD	**108 (40.1 to 177)**	**85.9 (30.4 to 154)**	-20.4 (-45.6 to 18.1)
6 COPD	6 Chronic kidney disease	**185 (82.9 to 300)**	**154 (69.8 to 265)**	19.6 (-24.1 to 83.1)
7 Alzheimer's disease	7 Diabetes	**79 (24.3 to 138)**	**59.8 (15.7 to 115)**	-23.9 (-47.5 to 8.7)
8 Interpersonal violence	8 Urinary diseases	**268 (185 to 371)**	**229 (156 to 302)**	28.5 (-3.53 to 74.3)
9 Chronic kidney disease	9 Hypertensive heart disease	**166 (74.2 to 265)**	**138 (58.7 to 229)**	4.56 (-34.2 to 60.5)
10 Road injuries	10 Lung cancer	**83.3 (16.4 to 166)**	**63.2 (7.52 to 129)**	-13.4 (-39.4 to 15.3)
11 Lung cancer	11 Colorectal cancer	**105 (13.6 to 231)**	**82.6 (6.45 to 183)**	-5.12 (-40.3 to 38.3)
12 Cirrhosis liver	12 Prostate cancer	**171 (65.5 to 308)**	**142 (51.6 to 261)**	3.53 (-26.9 to 39.3)
13 Urinary diseases	13 Falls	**130 (78.8 to 197)**	**105 (62.1 to 151)**	-12 (-23 to 2.2)
14 Hypertensive heart disease	14 Pancreatic cancer	**181 (42.8 to 378)**	**150 (32.1 to 313)**	30.3 (-25.3 to 101)
15 Colorectal cancer	15 Breast cancer	**77 (14.1 to 154)**	**57.5 (6.02 to 128)**	-6.63 (-32.8 to 26.9)
16 Stomach cancer	16 Cirrhosis liver	**33.7 (8.94 to 58.3)**	**19.3 (0.729 to 38.9)**	**-17.7 (-30.8 to -1.44)**
17 Breast cancer	17 Interpersonal violence	-25.9 (-61.4 to 34)	-34.1 (-64.9 to 24.7)	-27.5 (-62.6 to 42.1)
18 Falls	18 Atrial fibrillation	**256 (172 to 363)**	**217 (157 to 297)**	9.51 (-5.36 to 31.5)
19 Prostate cancer	19 Road injuries	5.59 (-34.9 to 62.5)	-5.67 (-37.3 to 45.2)	-10.9 (-42.2 to 42.6)
20 Neonatal disorders	20 Bacterial skin	**338 (129 to 603)**	**292 (111 to 556)**	81.6 (-8.21 to 225)
23 Pancreatic cancer	21 Stomach cancer			
28 Atrial fibrillation	54 Neonatal disorders			
37 Bacterial skin	136 COVID-19			

Supplemental Results Figure S8. Leading 20 level 3 causes of deaths, and percentage change in number of deaths and all-age and age-standardised death rates by location, 2022 and 2050

Paraguay, Reference scenario

Legend:
Communicable, maternal, neonatal and nutritional
Non-communicable
Injuries

Leading Causes 2022	Leading Causes 2050	Mean % change number of DEATH	Mean % change all-age DEATH rate	Mean % change age-standardized DEATH rate
1 Ischemic heart disease	1 Ischemic heart disease	41.2 (-19.1 to 117)	28.9 (-18.6 to 111)	**-42 (-65.9 to -1.93)**
2 Stroke	2 Diabetes	**80.9 (19.2 to 158)**	**65.2 (18.9 to 134)**	-23.4 (-45.6 to 13.7)
3 COVID-19	3 Stroke	42.2 (-1.46 to 94.6)	29.7 (-4.42 to 74.3)	**-41.9 (-57.8 to -16.2)**
4 Diabetes	4 Chronic kidney disease	**216 (94.5 to 367)**	**188 (92.7 to 313)**	33.8 (-11.5 to 104)
5 Lower respiratory infect	5 Alzheimer's disease	**206 (110 to 303)**	**179 (105 to 250)**	**3.75 (0.764 to 6.98)**
6 Chronic kidney disease	6 Lower respiratory infect	**95.8 (40.8 to 167)**	**78.7 (39.3 to 130)**	-19.6 (-36 to 7.45)
7 Road injuries	7 Hypertensive heart disease	**190 (80.9 to 322)**	**164 (74.7 to 274)**	13.1 (-26.5 to 70.1)
8 Alzheimer's disease	8 COPD	**117 (47.9 to 202)**	**98 (44.7 to 169)**	-15.9 (-39.3 to 20.2)
9 Hypertensive heart disease	9 Lung cancer	**120 (32.1 to 211)**	**101 (31.9 to 179)**	-3.1 (-31.8 to 27.5)
10 COPD	10 Prostate cancer	**192 (63.2 to 336)**	**166 (61.7 to 295)**	11.9 (-22.9 to 48.3)
11 Lung cancer	11 Road injuries	15.6 (-26.2 to 81.8)	5.4 (-29.8 to 63.2)	-10.3 (-43 to 41.9)
12 Interpersonal violence	12 Colorectal cancer	**158 (44.7 to 285)**	**135 (40.6 to 254)**	10.9 (-28.6 to 56.7)
13 Neonatal disorders	13 Breast cancer	**122 (29.7 to 210)**	**102 (37 to 184)**	5.69 (-25.9 to 42.6)
14 Cirrhosis liver	14 Cirrhosis liver	**75.9 (31.8 to 126)**	**60.4 (30.5 to 98.1)**	-8.55 (-23.2 to 12.9)
15 Colorectal cancer	15 Pancreatic cancer	**255 (75.3 to 469)**	**224 (73.1 to 415)**	52.4 (-9.76 to 137)
16 Breast cancer	16 Atrial fibrillation	**261 (152 to 385)**	**229 (137 to 320)**	**22.2 (2.8 to 44)**
17 Prostate cancer	17 Endo/metab/blood/immune	**168 (102 to 244)**	**144 (95.8 to 204)**	**28.1 (9.03 to 59.6)**
18 Cervical cancer	18 Urinary diseases	**206 (135 to 295)**	**179 (122 to 244)**	19.6 (-0.172 to 44.6)
19 Stomach cancer	19 Cervical cancer	**58.4 (13.2 to 102)**	**44.4 (14.7 to 82.1)**	-18.5 (-33.5 to 3.86)
20 Congenital defects	20 Stomach cancer	**60.9 (24.3 to 106)**	**46.8 (22.5 to 76.5)**	**-30 (-38.2 to -19.4)**
22 Pancreatic cancer	23 Interpersonal violence			
23 Endo/metab/blood/immune	33 Neonatal disorders			
25 Atrial fibrillation	49 Congenital defects			
28 Urinary diseases	133 COVID-19			

Supplemental Results Figure S8. Leading 20 level 3 causes of deaths, and percentage change in number of deaths and all-age and age-standardised death rates by location, 2022 and 2050

Afghanistan, Reference scenario

Legend:
Communicable, maternal, neonatal and nutritional
Non-communicable
Injuries

Leading Causes 2022	Leading Causes 2050	Mean % change number of DEATH	Mean % change all-age DEATH rate	Mean % change age-standardized DEATH rate
1 Ischemic heart disease	1 Ischemic heart disease	**113 (20.9 to 262)**	-0.603 (-43.4 to 66.6)	-37.4 (-67.9 to 15.3)
2 Neonatal disorders	2 Stroke	**85.8 (16.6 to 181)**	-13.4 (-41.7 to 20)	**-44.4 (-66.2 to -12.9)**
3 Congenital defects	3 Chronic kidney disease	**266 (133 to 428)**	**70.9 (13.9 to 141)**	10.2 (-31 to 68.7)
4 Stroke	4 Hypertensive heart disease	**202 (81.2 to 342)**	40.8 (-7.63 to 104)	-20.2 (-52.5 to 30)
5 Lower respiratory infect	5 Congenital defects	-9 (-41.1 to 25.4)	**-57.8 (-64.2 to -49.4)**	**-39.6 (-47.2 to -29)**
6 COVID-19	6 Lower respiratory infect	-8.92 (-38.6 to 29.2)	**-57.6 (-65.9 to -47.4)**	**-46.8 (-60.3 to -30.9)**
7 Tuberculosis	7 Neonatal disorders	-29.5 (-62.6 to 14.3)	**-67.2 (-79.8 to -48.1)**	**-50.4 (-68.7 to -24.5)**
8 Conflict & terror	8 Road injuries	50.6 (-19.3 to 170)	-29.6 (-62.4 to 22.9)	-39.4 (-66.9 to 6.25)
9 Road injuries	9 Diabetes	**140 (36.7 to 266)**	12.1 (-31.6 to 70.8)	-28.4 (-59 to 21.6)
10 Hypertensive heart disease	10 Cirrhosis liver	**96.8 (37.4 to 162)**	-8.38 (-23.9 to 10.2)	**-39 (-51 to -23.5)**
11 Chronic kidney disease	11 COPD	**99.9 (23.7 to 189)**	-6.82 (-37.3 to 27.6)	**-45.1 (-66.1 to -15.3)**
12 Pertussis	12 Stomach cancer	**116 (43.4 to 201)**	0.168 (-15 to 15)	**-36.8 (-43.5 to -28.5)**
13 Diarrheal diseases	13 Tuberculosis	-20.9 (-55.4 to 30.5)	**-63 (-78.3 to -38.4)**	**-71.3 (-83.9 to -50.8)**
14 Cirrhosis liver	14 Conflict & terror	111 (-94.6 to 1330)	-1.9 (-97.4 to 471)	10.6 (-98.4 to 653)
15 COPD	15 Alzheimer's disease	**285 (125 to 500)**	**78.7 (23.1 to 133)**	**7.08 (1.73 to 12)**
16 Maternal disorders	16 Interpersonal violence	61.4 (-16.1 to 154)	-25.2 (-50.7 to 14.7)	**-33.5 (-54.9 to -2.22)**
17 Diabetes	17 Colorectal cancer	**293 (99.6 to 566)**	**81.9 (9.44 to 183)**	15.2 (-22.3 to 71.2)
18 Interpersonal violence	18 Leukemia	**156 (55 to 297)**	18.5 (-10.7 to 61)	-0.648 (-21.7 to 29)
19 Stomach cancer	19 Falls	**84.3 (18.4 to 174)**	-14.6 (-32.4 to 10)	-10.7 (-21.4 to 3.99)
20 Falls	20 Lung cancer	**284 (107 to 553)**	**77.9 (14.4 to 166)**	9.87 (-23.1 to 52.3)
24 Leukemia	21 Pertussis			
29 Alzheimer's disease	23 Maternal disorders			
31 Colorectal cancer	43 Diarrheal diseases			
34 Lung cancer	132 COVID-19			

Supplemental Results Figure S8. Leading 20 level 3 causes of deaths, and percentage change in number of deaths and all-age and age-standardised death rates by location, 2022 and 2050

Algeria, Reference scenario

Legend:
Communicable, maternal, neonatal and nutritional
Non-communicable
Injuries

Leading Causes 2022	Leading Causes 2050	Mean % change number of DEATH	Mean % change all-age DEATH rate	Mean % change age-standardized DEATH rate
1 Ischemic heart disease	1 Ischemic heart disease	**82.3 (20.1 to 170)**	**46.8 (0.333 to 115)**	**-47 (-68.4 to -7.9)**
2 Stroke	2 Stroke	**84.5 (42 to 140)**	**48.6 (16.1 to 86.7)**	**-49.3 (-63.8 to -20.8)**
3 Hypertensive heart disease	3 Hypertensive heart disease	**265 (161 to 381)**	**194 (109 to 283)**	-0.999 (-35.1 to 46.3)
4 Chronic kidney disease	4 Chronic kidney disease	**295 (179 to 444)**	**218 (132 to 333)**	16.4 (-23.3 to 83.4)
5 Road injuries	5 Alzheimer's disease	**338 (225 to 476)**	**252 (177 to 334)**	**5.04 (1.73 to 8.25)**
6 Neonatal disorders	6 COPD	**230 (153 to 319)**	**166 (103 to 238)**	-11.4 (-37.9 to 25.2)
7 Lower respiratory infect	7 Lower respiratory infect	**162 (96.5 to 235)**	**111 (69 to 166)**	-20.7 (-39.6 to 4.81)
8 Alzheimer's disease	8 Diabetes	**169 (86.6 to 262)**	**116 (58.1 to 198)**	-14.5 (-43 to 30.3)
9 Diabetes	9 Road injuries	9.67 (-29.1 to 73)	-11.7 (-40.2 to 35.4)	-30.8 (-54 to 10.8)
10 Congenital defects	10 Cirrhosis liver	**108 (63.1 to 154)**	**67.2 (43.2 to 92.6)**	**-28.1 (-39.3 to -12.1)**
11 COPD	11 Lung cancer	**200 (90.1 to 336)**	**141 (58.3 to 241)**	5.62 (-23.2 to 41.2)
12 COVID-19	12 Atrial fibrillation	**446 (255 to 683)**	**339 (193 to 488)**	20 (-3.54 to 46.5)
13 Cirrhosis liver	13 Colorectal cancer	**270 (113 to 473)**	**197 (76.2 to 354)**	19.8 (-19.5 to 65.7)
14 Lung cancer	14 Other cardiovascular	**140 (79.8 to 208)**	**93.4 (55.4 to 151)**	**-23.7 (-40.2 to -2.17)**
15 Other cardiovascular	15 Parkinson's disease	**275 (181 to 386)**	**202 (135 to 284)**	-6.66 (-24.6 to 10.8)
16 Asthma	16 Breast cancer	**153 (56.9 to 267)**	**103 (31.8 to 182)**	7.43 (-20.9 to 43.3)
17 Breast cancer	17 Falls	**137 (64.6 to 226)**	**90.3 (41.9 to 157)**	-12.3 (-23.6 to 0.791)
18 Falls	18 Neonatal disorders	**-52.4 (-71.2 to -22.3)**	**-61.8 (-74.7 to -38.3)**	**-40.5 (-60.4 to -8.31)**
19 Tuberculosis	19 Urinary diseases	**350 (226 to 499)**	**261 (180 to 383)**	20 (-12.3 to 68.1)
20 Colorectal cancer	20 Pancreatic cancer	**399 (210 to 664)**	**301 (149 to 480)**	**65.5 (15.7 to 123)**
22 Parkinson's disease	21 Asthma			
24 Atrial fibrillation	22 Congenital defects			
30 Urinary diseases	32 Tuberculosis			
39 Pancreatic cancer	131 COVID-19			

Supplemental Results Figure S8. Leading 20 level 3 causes of deaths, and percentage change in number of deaths and all-age and age-standardised death rates by location, 2022 and 2050

Bahrain, Reference scenario

Legend:
Communicable, maternal, neonatal and nutritional
Non-communicable
Injuries

Leading Causes 2022	Leading Causes 2050	Mean % change number of DEATH	Mean % change all-age DEATH rate	Mean % change age-standardized DEATH rate
1 Ischemic heart disease	1 Diabetes	**424 (270 to 631)**	**265 (152 to 410)**	-18.3 (-48 to 25.3)
2 Diabetes	2 Ischemic heart disease	**162 (69.4 to 280)**	**82.9 (16.6 to 171)**	**-56 (-75.1 to -22.1)**
3 Stroke	3 Chronic kidney disease	**597 (386 to 835)**	**386 (241 to 571)**	10 (-31.7 to 70.8)
4 Chronic kidney disease	4 COPD	**700 (471 to 912)**	**457 (300 to 625)**	2.9 (-32.9 to 55.7)
5 COVID-19	5 Lung cancer	**592 (302 to 907)**	**381 (188 to 591)**	24.3 (-16.3 to 70.3)
6 Road injuries	6 Stroke	**201 (116 to 304)**	**110 (52.4 to 177)**	**-57.1 (-71.6 to -33.1)**
7 Lung cancer	7 Hypertensive heart disease	**688 (446 to 961)**	**449 (281 to 642)**	0.219 (-36.2 to 50.8)
8 COPD	8 Alzheimer's disease	**827 (600 to 1060)**	**544 (396 to 729)**	3.21 (-0.123 to 6.98)
9 Cirrhosis liver	9 Lower respiratory infect	**476 (338 to 635)**	**301 (208 to 412)**	-12.4 (-35.8 to 20.2)
10 Hypertensive heart disease	10 Cirrhosis liver	**282 (215 to 340)**	**166 (117 to 210)**	**-30.4 (-42.6 to -14.8)**
11 Lower respiratory infect	11 Bacterial skin	**848 (561 to 1270)**	**560 (365 to 836)**	36.4 (-20.8 to 117)
12 Breast cancer	12 Colorectal cancer	**526 (247 to 825)**	**335 (156 to 545)**	14.1 (-24.1 to 62.3)
13 Colorectal cancer	13 Prostate cancer	**958 (530 to 1530)**	**635 (349 to 1010)**	28.7 (-12 to 80.8)
14 Alzheimer's disease	14 Pancreatic cancer	**727 (392 to 1100)**	**475 (270 to 711)**	**49.7 (5.5 to 111)**
15 Self-harm	15 Urinary diseases	**629 (437 to 872)**	**407 (287 to 566)**	-5.67 (-23.8 to 17.7)
16 Endo/metab/blood/immune	16 Breast cancer	**210 (87.6 to 347)**	**116 (39.6 to 207)**	-21 (-44.2 to 10.7)
17 Bacterial skin	17 Road injuries	**69.6 (9.77 to 154)**	18.2 (-21.7 to 83.6)	-21.2 (-50.7 to 29.7)
18 Pancreatic cancer	18 Endo/metab/blood/immune	**379 (272 to 511)**	**233 (159 to 326)**	10 (-12.3 to 43.8)
19 Urinary diseases	19 Liver cancer	**560 (416 to 721)**	**359 (269 to 481)**	21.8 (-5.98 to 61.6)
20 Stomach cancer	20 Parkinson's disease	**898 (641 to 1210)**	**593 (427 to 805)**	0.902 (-21.3 to 22.8)
23 Prostate cancer	23 Stomach cancer			
24 Liver cancer	42 Self-harm			
37 Parkinson's disease	129 COVID-19			

Supplemental Results Figure S8. Leading 20 level 3 causes of deaths, and percentage change in number of deaths and all-age and age-standardised death rates by location, 2022 and 2050

Egypt, Reference scenario

Legend:
Communicable, maternal, neonatal and nutritional
Non-communicable
Injuries

Leading Causes 2022	Leading Causes 2050	Mean % change number of DEATH	Mean % change all-age DEATH rate	Mean % change age-standardized DEATH rate
1 Ischemic heart disease	1 Ischemic heart disease	**77 (20.4 to 149)**	18 (-19.2 to 68.6)	-30.9 (-59.4 to 11.8)
2 Stroke	2 Chronic kidney disease	**300 (200 to 421)**	**167 (103 to 253)**	**55.9 (4.16 to 128)**
3 Cirrhosis liver	3 Stroke	**63.8 (27.8 to 110)**	9.14 (-13.4 to 37.5)	**-40.6 (-57.2 to -15.6)**
4 Chronic kidney disease	4 Cirrhosis liver	**60.6 (34.9 to 86.1)**	6.96 (-7.7 to 22)	**-35.9 (-46.1 to -23.9)**
5 COVID-19	5 Diabetes	**156 (92.7 to 229)**	**70.5 (29.3 to 121)**	5.89 (-29.3 to 48.1)
6 Diabetes	6 Hypertensive heart disease	**168 (96.2 to 255)**	**78.9 (37.7 to 140)**	-4.69 (-36.1 to 42.1)
7 Hypertensive heart disease	7 Lower respiratory infect	**42.1 (13.3 to 75.2)**	-5.39 (-21 to 14.9)	**-31.5 (-44.4 to -13.8)**
8 Lower respiratory infect	8 Liver cancer	**147 (109 to 194)**	**64.3 (41.2 to 96.1)**	2.87 (-16.4 to 23.5)
9 Liver cancer	9 Lung cancer	**181 (85.6 to 296)**	**86.8 (20.5 to 163)**	18.6 (-14.7 to 53.4)
10 Congenital defects	10 COPD	**136 (81.1 to 208)**	**57.5 (23.8 to 106)**	-13.9 (-40.6 to 24.5)
11 Road injuries	11 Alzheimer's disease	**276 (159 to 404)**	**150 (77.1 to 229)**	**14.7 (10.8 to 18.1)**
12 COPD	12 Breast cancer	**159 (66.3 to 261)**	**72.1 (13.5 to 143)**	19.1 (-18.2 to 58.4)
13 Lung cancer	13 Colorectal cancer	**199 (84.4 to 342)**	**98.9 (23.1 to 199)**	25.4 (-14.7 to 72.9)
14 Other cardiovascular	14 Pancreatic cancer	**334 (183 to 531)**	**189 (78 to 311)**	**78.5 (24.5 to 139)**
15 Alzheimer's disease	15 Brain cancer	**182 (143 to 226)**	**88.1 (67.3 to 111)**	**38.4 (30.6 to 47.1)**
16 Breast cancer	16 Road injuries	13.7 (-32.2 to 88.8)	-24.2 (-53.8 to 27.1)	-33.1 (-59.8 to 14.2)
17 Neonatal disorders	17 Other cardiovascular	**53.5 (27.2 to 87.7)**	2.24 (-13.9 to 20.1)	**-28.3 (-42.5 to -14.5)**
18 Colorectal cancer	18 Leukemia	**118 (67 to 184)**	**45.4 (12.7 to 90.8)**	5.23 (-13.4 to 28.6)
19 Brain cancer	19 Congenital defects	**-33.2 (-46.4 to -18.5)**	**-55.6 (-61.8 to -48.5)**	**-43.3 (-50.7 to -35.4)**
20 Leukemia	20 Stomach cancer	**61.2 (28.8 to 93.4)**	7.34 (-12.3 to 27.9)	**-32.9 (-40.6 to -23.7)**
21 Stomach cancer	27 Neonatal disorders			
22 Pancreatic cancer	132 COVID-19			

Supplemental Results Figure S8. Leading 20 level 3 causes of deaths, and percentage change in number of deaths and all-age and age-standardised death rates by location, 2022 and 2050

Iran, Reference scenario

Legend:
Communicable, maternal, neonatal and nutritional
Non-communicable
Injuries

Leading Causes 2022	Leading Causes 2050	Mean % change number of DEATH	Mean % change all-age DEATH rate	Mean % change age-standardized DEATH rate
1 Ischemic heart disease	1 Ischemic heart disease	**70.6 (11.6 to 150)**	46.2 (-4.17 to 122)	**-51.7 (-71.8 to -19.9)**
2 Stroke	2 Hypertensive heart disease	**292 (181 to 412)**	**236 (144 to 360)**	4.68 (-29.8 to 57.2)
3 Hypertensive heart disease	3 Alzheimer's disease	**373 (257 to 492)**	**304 (215 to 394)**	**4.14 (1.21 to 6.9)**
4 COVID-19	4 Stroke	**59.8 (21 to 108)**	**36.9 (1.74 to 82.1)**	**-56.6 (-69.9 to -36)**
5 Road injuries	5 Chronic kidney disease	**326 (202 to 463)**	**265 (162 to 400)**	20.1 (-17.9 to 80.9)
6 Alzheimer's disease	6 Diabetes	**196 (113 to 303)**	**154 (80.6 to 248)**	-14.4 (-42.4 to 27.8)
7 Diabetes	7 COPD	**245 (155 to 338)**	**196 (119 to 291)**	-7.28 (-37.6 to 35.6)
8 Chronic kidney disease	8 Lung cancer	**237 (122 to 384)**	**188 (92.3 to 309)**	8.63 (-20.8 to 45.1)
9 COPD	9 Lower respiratory infect	**165 (115 to 217)**	**127 (82.9 to 168)**	**-24.8 (-41.3 to -3.76)**
10 Lower respiratory infect	10 Road injuries	18 (-22.2 to 81.7)	1.13 (-34.5 to 56.4)	-19.6 (-49.1 to 30.2)
11 Stomach cancer	11 Stomach cancer	**105 (73.1 to 144)**	**75.7 (51.8 to 106)**	**-35.7 (-42.7 to -27.2)**
12 Lung cancer	12 Colorectal cancer	**231 (100 to 387)**	**183 (76.8 to 313)**	6.04 (-27.5 to 46.1)
13 Cirrhosis liver	13 Cirrhosis liver	**135 (101 to 166)**	**101 (76 to 130)**	**-20.3 (-31.8 to -3.64)**
14 Colorectal cancer	14 Prostate cancer	**288 (137 to 467)**	**231 (106 to 380)**	-3.07 (-30.7 to 32.3)
15 Other cardiovascular	15 Pancreatic cancer	**369 (197 to 570)**	**300 (161 to 456)**	**49.2 (3.65 to 98.2)**
16 Breast cancer	16 Brain cancer	**201 (160 to 246)**	**157 (131 to 182)**	**29.9 (22.8 to 36.3)**
17 Brain cancer	17 Other cardiovascular	**153 (108 to 203)**	**117 (82.2 to 157)**	**-25 (-40.3 to -6.9)**
18 Leukemia	18 Atrial fibrillation	**415 (273 to 567)**	**341 (224 to 471)**	12.7 (-7.12 to 33.2)
19 Self-harm	19 Parkinson's disease	**272 (204 to 352)**	**218 (163 to 292)**	-8.84 (-23.8 to 8.64)
20 Prostate cancer	20 Leukemia	**141 (79.6 to 222)**	**106 (55.7 to 165)**	-3.94 (-22 to 16.1)
24 Pancreatic cancer	21 Breast cancer			
26 Parkinson's disease	31 Self-harm			
32 Atrial fibrillation	132 COVID-19			

Supplemental Results Figure S8. Leading 20 level 3 causes of deaths, and percentage change in number of deaths and all-age and age-standardised death rates by location, 2022 and 2050

Iraq, Reference scenario

Legend:
Communicable, maternal, neonatal and nutritional
Non-communicable
Injuries

Leading Causes 2022	Leading Causes 2050	Mean % change number of DEATH	Mean % change all-age DEATH rate	Mean % change age-standardized DEATH rate
1 Ischemic heart disease	1 Ischemic heart disease	65.2 (-6.65 to 166)	12.1 (-30.8 to 73.5)	**-52.1 (-73.3 to -16.4)**
2 Stroke	2 Stroke	60.3 (-0.583 to 134)	8.74 (-22.2 to 50.8)	**-54.9 (-70.3 to -29.8)**
3 Conflict & terror	3 Chronic kidney disease	**269 (116 to 444)**	**151 (77.1 to 253)**	9.16 (-28.7 to 73.9)
4 COVID-19	4 Conflict & terror	618 (-96.2 to 4310)	400 (-97.7 to 2830)	413 (-97.7 to 2900)
5 Diabetes	5 Diabetes	**130 (40.6 to 244)**	**56.2 (11.6 to 126)**	-29.8 (-53.8 to 12.8)
6 Chronic kidney disease	6 Hypertensive heart disease	**266 (124 to 417)**	**148 (73.9 to 243)**	-4.56 (-39.6 to 49.1)
7 Neonatal disorders	7 Alzheimer's disease	**378 (183 to 597)**	**223 (127 to 318)**	**4.06 (1.28 to 7.01)**
8 Hypertensive heart disease	8 Lung cancer	**231 (78.5 to 433)**	**124 (40.6 to 217)**	1.13 (-28.6 to 36.8)
9 Road injuries	9 Road injuries	38.4 (-19.8 to 120)	-5.92 (-40.5 to 49.4)	-25.6 (-54.1 to 19)
10 Lung cancer	10 Cirrhosis liver	**129 (56.6 to 198)**	**55 (29.3 to 83.5)**	**-24.1 (-36.2 to -6.57)**
11 Congenital defects	11 COPD	**217 (100 to 357)**	**115 (58.3 to 200)**	-14.2 (-42.6 to 27.8)
12 Interpersonal violence	12 Breast cancer	**156 (51.5 to 290)**	**73.1 (15.1 to 137)**	0.0411 (-29.3 to 32.2)
13 Mechanical forces	13 Pancreatic cancer	**396 (172 to 692)**	**235 (111 to 384)**	**56.2 (9.34 to 116)**
14 Lower respiratory infect	14 Lower respiratory infect	**58.6 (6.52 to 114)**	7.65 (-14 to 36.1)	**-30.4 (-45.9 to -8)**
15 Alzheimer's disease	15 Brain cancer	**219 (118 to 306)**	**116 (91.3 to 139)**	**36.9 (30.2 to 44.6)**
16 Cirrhosis liver	16 Mechanical forces	20.8 (-19.9 to 70.2)	-18 (-35.6 to 8.92)	**-30.4 (-44.7 to -5.69)**
17 Breast cancer	17 Colorectal cancer	**239 (76.7 to 436)**	**128 (38.1 to 235)**	8.33 (-27 to 49.5)
18 COPD	18 Interpersonal violence	-0.927 (-49 to 72.6)	-33.1 (-59.6 to 12.5)	-39.9 (-64.1 to 1.87)
19 Self-harm	19 Liver cancer	**247 (122 to 366)**	**135 (86.3 to 192)**	12.4 (-13.7 to 46.2)
20 Brain cancer	20 Parkinson's disease	**322 (168 to 522)**	**185 (118 to 263)**	-2.33 (-19.1 to 17)
22 Colorectal cancer	24 Neonatal disorders			
26 Pancreatic cancer	26 Self-harm			
27 Liver cancer	28 Congenital defects			
31 Parkinson's disease	131 COVID-19			

Supplemental Results Figure S8. Leading 20 level 3 causes of deaths, and percentage change in number of deaths and all-age and age-standardised death rates by location, 2022 and 2050

Jordan, Reference scenario

Legend:
Communicable, maternal, neonatal and nutritional
Non-communicable
Injuries

Leading Causes 2022	Leading Causes 2050	Mean % change number of DEATH	Mean % change all-age DEATH rate	Mean % change age-standardized DEATH rate
1 Ischemic heart disease	1 Ischemic heart disease	**142 (57.4 to 252)**	34.2 (-14.2 to 102)	**-52.9 (-71.6 to -21.2)**
2 Stroke	2 Hypertensive heart disease	**477 (304 to 678)**	**220 (122 to 331)**	-3.57 (-36.4 to 42)
3 COVID-19	3 Chronic kidney disease	**457 (294 to 652)**	**209 (118 to 331)**	5.64 (-28.7 to 54.6)
4 Diabetes	4 Stroke	**138 (76.3 to 206)**	32.1 (-3.51 to 70.5)	**-58.6 (-70.8 to -45.2)**
5 Chronic kidney disease	5 Diabetes	**233 (143 to 342)**	**84.6 (30.6 to 144)**	**-34.3 (-55.1 to -8.97)**
6 Hypertensive heart disease	6 Alzheimer's disease	**703 (520 to 927)**	**345 (247 to 450)**	-0.723 (-3.28 to 1.77)
7 Road injuries	7 COPD	**431 (284 to 582)**	**195 (116 to 285)**	-7.62 (-34.1 to 30.2)
8 Neonatal disorders	8 Lung cancer	**283 (157 to 432)**	**112 (47.3 to 194)**	-5.59 (-31 to 24.8)
9 Lower respiratory infect	9 Lower respiratory infect	**213 (146 to 298)**	**73.7 (36 to 129)**	**-31.1 (-44.8 to -10.6)**
10 Alzheimer's disease	10 Colorectal cancer	**325 (153 to 537)**	**135 (45.3 to 249)**	-3.16 (-33.2 to 34.2)
11 Congenital defects	11 Road injuries	**65.4 (8.62 to 151)**	-8.14 (-39.7 to 39.9)	-24.5 (-51.7 to 19.1)
12 Lung cancer	12 Breast cancer	**194 (99.3 to 300)**	**62.9 (11.1 to 118)**	-15.9 (-38.9 to 12.1)
13 Breast cancer	13 Cirrhosis liver	**219 (174 to 275)**	**77 (50.6 to 105)**	**-28 (-39.2 to -14.1)**
14 Colorectal cancer	14 Leukemia	**222 (159 to 294)**	**78.7 (51 to 115)**	-3.92 (-13.6 to 7.8)
15 COPD	15 Pancreatic cancer	**469 (265 to 713)**	**216 (104 to 345)**	32.4 (-3.47 to 81.3)
16 Cirrhosis liver	16 Prostate cancer	**519 (274 to 851)**	**243 (113 to 419)**	-1.72 (-28.7 to 36.7)
17 Leukemia	17 Parkinson's disease	**503 (362 to 656)**	**234 (164 to 318)**	-12.1 (-27.6 to 6.93)
18 Interpersonal violence	18 Brain cancer	**270 (230 to 316)**	**105 (85.7 to 124)**	**23.4 (15.8 to 30.2)**
19 Lymphoma	19 Lymphoma	**218 (116 to 338)**	**76.3 (23.6 to 141)**	-12.1 (-34.2 to 14.2)
20 Brain cancer	20 Atrial fibrillation	**779 (526 to 1040)**	**387 (255 to 536)**	6.21 (-9.26 to 26.6)
22 Pancreatic cancer	21 Neonatal disorders			
24 Prostate cancer	24 Congenital defects			
26 Parkinson's disease	35 Interpersonal violence			
39 Atrial fibrillation	132 COVID-19			

Supplemental Results Figure S8. Leading 20 level 3 causes of deaths, and percentage change in number of deaths and all-age and age-standardised death rates by location, 2022 and 2050

Kuwait, Reference scenario

Legend:
Communicable, maternal, neonatal and nutritional
Non-communicable
Injuries

Leading Causes 2022	Leading Causes 2050	Mean % change number of DEATH	Mean % change all-age DEATH rate	Mean % change age-standardized DEATH rate
1 Ischemic heart disease	1 Ischemic heart disease	**193 (97.2 to 342)**	**97.3 (31.4 to 196)**	**-47.2 (-65.1 to -16.2)**
2 Stroke	2 Alzheimer's disease	**744 (594 to 909)**	**468 (388 to 542)**	**8.67 (5.43 to 11.6)**
3 Lower respiratory infect	3 Chronic kidney disease	**877 (575 to 1280)**	**558 (352 to 810)**	**53 (3.8 to 128)**
4 Diabetes	4 Hypertensive heart disease	**892 (615 to 1310)**	**568 (381 to 829)**	38.6 (-4.66 to 108)
5 Alzheimer's disease	5 Lower respiratory infect	**445 (313 to 610)**	**267 (190 to 366)**	-15.1 (-34.1 to 10.7)
6 Road injuries	6 Diabetes	**430 (290 to 634)**	**257 (162 to 390)**	-23.4 (-43.8 to 10.6)
7 Chronic kidney disease	7 Stroke	**207 (133 to 305)**	**107 (62.7 to 170)**	**-53.4 (-64.3 to -35.1)**
8 Hypertensive heart disease	8 Colorectal cancer	**445 (233 to 685)**	**266 (133 to 416)**	-5.62 (-35.2 to 24.4)
9 Colorectal cancer	9 Lung cancer	**494 (289 to 761)**	**300 (169 to 459)**	-4.59 (-31.7 to 26.1)
10 Lung cancer	10 Pancreatic cancer	**657 (401 to 958)**	**409 (245 to 577)**	30 (-7.23 to 68.2)
11 COVID-19	11 Road injuries	**94.3 (34.3 to 193)**	30.9 (-9.64 to 98.1)	-13.6 (-42.8 to 38.7)
12 Congenital defects	12 Prostate cancer	**621 (343 to 975)**	**385 (208 to 591)**	-12.2 (-40 to 19.5)
13 Cirrhosis liver	13 Cirrhosis liver	**325 (245 to 416)**	**186 (147 to 232)**	**-27.1 (-36.4 to -13.5)**
14 Breast cancer	14 Breast cancer	**297 (152 to 451)**	**167 (78.4 to 262)**	3.19 (-25.2 to 32.4)
15 Falls	15 Urinary diseases	**887 (593 to 1210)**	**565 (382 to 786)**	41.7 (-1.56 to 103)
16 Pancreatic cancer	16 Atrial fibrillation	**822 (615 to 1060)**	**520 (402 to 657)**	**26.1 (4.47 to 53.6)**
17 Neonatal disorders	17 Parkinson's disease	**677 (515 to 877)**	**422 (325 to 539)**	-7.51 (-23.7 to 12.9)
18 Prostate cancer	18 Falls	**256 (173 to 353)**	**139 (87.1 to 189)**	**-18.1 (-29.8 to -4.83)**
19 Self-harm	19 Bladder cancer	**510 (375 to 692)**	**310 (224 to 405)**	-11.8 (-25.4 to 4.12)
20 Lymphoma	20 COPD	**617 (410 to 921)**	**383 (251 to 556)**	-3.25 (-34.4 to 37.9)
22 Parkinson's disease	21 Lymphoma			
23 Bladder cancer	40 Self-harm			
25 Atrial fibrillation	42 Congenital defects			
27 Urinary diseases	54 Neonatal disorders			
28 COPD	129 COVID-19			

Supplemental Results Figure S8. Leading 20 level 3 causes of deaths, and percentage change in number of deaths and all-age and age-standardised death rates by location, 2022 and 2050

Lebanon, Reference scenario

Legend:
Communicable, maternal, neonatal and nutritional
Non-communicable
Injuries

Leading Causes 2022	Leading Causes 2050	Mean % change number of DEATH	Mean % change all-age DEATH rate	Mean % change age-standardized DEATH rate
1 Ischemic heart disease	1 Ischemic heart disease	19.1 (-25.6 to 84.4)	13.5 (-26.1 to 74.6)	-41.6 (-67.4 to 0.608)
2 COVID-19	2 Chronic kidney disease	**158 (75.8 to 253)**	**146 (74.7 to 255)**	24.9 (-19.1 to 104)
3 Stroke	3 Alzheimer's disease	**137 (71.6 to 208)**	**126 (75.2 to 176)**	**10 (7.01 to 13.4)**
4 Chronic kidney disease	4 Lung cancer	**135 (51 to 233)**	**123 (52.3 to 206)**	9.44 (-20.1 to 43.8)
5 Lower respiratory infect	5 Lower respiratory infect	**69.2 (25.9 to 115)**	**61.1 (31.1 to 96)**	-17.2 (-36.9 to 6.77)
6 Diabetes	6 Diabetes	**69.8 (14.1 to 125)**	**61.7 (17.3 to 111)**	-19.3 (-43.9 to 17.7)
7 Alzheimer's disease	7 Stroke	11.1 (-21.2 to 51.7)	5.86 (-21.1 to 40.6)	**-45.3 (-62.1 to -18.7)**
8 Lung cancer	8 COPD	**110 (44.5 to 188)**	**100 (46.8 to 168)**	0.547 (-34.5 to 52.4)
9 COPD	9 Hypertensive heart disease	**111 (39.5 to 192)**	**101 (42.7 to 169)**	2.03 (-34.5 to 55.6)
10 Hypertensive heart disease	10 Colorectal cancer	**129 (38.2 to 246)**	**118 (40.4 to 218)**	8.06 (-25.9 to 49.7)
11 Cirrhosis liver	11 Breast cancer	**95 (19.4 to 190)**	**85.5 (21.2 to 171)**	-4.34 (-33 to 29)
12 Colorectal cancer	12 Cirrhosis liver	**60.2 (29.3 to 91)**	**52.7 (32.1 to 73.2)**	**-20.6 (-33.7 to -4.41)**
13 Breast cancer	13 Prostate cancer	**92.3 (19 to 191)**	**83.1 (17.7 to 172)**	-9.95 (-35.2 to 22.8)
14 Prostate cancer	14 Bladder cancer	**102 (49.6 to 173)**	**92.6 (49.4 to 148)**	-4.7 (-19.6 to 13.6)
15 Bladder cancer	15 Urinary diseases	**169 (104 to 253)**	**156 (95.7 to 225)**	28.7 (-7.27 to 76.6)
16 Road injuries	16 Pancreatic cancer	**216 (94.6 to 366)**	**200 (91.7 to 330)**	47.5 (-0.363 to 104)
17 Falls	17 Lymphoma	**91.3 (28.9 to 166)**	**82.2 (30 to 149)**	-4.82 (-27.8 to 20.5)
18 Lymphoma	18 Falls	**75 (28.2 to 130)**	**66.5 (27.8 to 112)**	-9.55 (-21.9 to 0.873)
19 Urinary diseases	19 Atrial fibrillation	**150 (80.1 to 233)**	**138 (75.2 to 201)**	16.8 (-3.84 to 43.2)
20 Stomach cancer	20 Leukemia	**88.8 (33.3 to 159)**	**79.8 (31.4 to 140)**	-1.85 (-22.5 to 22)
21 Leukemia	22 Road injuries			
22 Pancreatic cancer	24 Stomach cancer			
26 Atrial fibrillation	132 COVID-19			

Supplemental Results Figure S8. Leading 20 level 3 causes of deaths, and percentage change in number of deaths and all-age and age-standardised death rates by location, 2022 and 2050

Libya, Reference scenario

Legend:
Communicable, maternal, neonatal and nutritional
Non-communicable
Injuries

Leading Causes 2022	Leading Causes 2050	Mean % change number of DEATH	Mean % change all-age DEATH rate	Mean % change age-standardized DEATH rate
1 Ischemic heart disease	1 Ischemic heart disease	**82.4 (6.91 to 170)**	**84.5 (13.9 to 183)**	-37.1 (-63.9 to 8.24)
2 COVID-19	2 Chronic kidney disease	**319 (181 to 467)**	**323 (201 to 501)**	40.7 (-4.6 to 118)
3 Stroke	3 Hypertensive heart disease	**332 (187 to 479)**	**336 (203 to 483)**	29.9 (-16 to 95.9)
4 Road injuries	4 Stroke	**67.9 (13.7 to 140)**	**69.5 (22.2 to 140)**	**-44.9 (-62.9 to -12.9)**
5 Chronic kidney disease	5 Alzheimer's disease	**355 (199 to 509)**	**357 (234 to 479)**	**23.7 (20.3 to 27.1)**
6 Hypertensive heart disease	6 Lung cancer	**241 (100 to 379)**	**242 (123 to 387)**	14.5 (-19.7 to 53.1)
7 Lung cancer	7 COPD	**255 (152 to 379)**	**258 (161 to 388)**	9.41 (-24.6 to 66.8)
8 Diabetes	8 Diabetes	**153 (65.8 to 267)**	**156 (75.9 to 284)**	-14.3 (-43.3 to 38.4)
9 Conflict & terror	9 Road injuries	7.08 (-34 to 73.1)	8.26 (-31.6 to 81.4)	-14 (-46.6 to 47.6)
10 Cirrhosis liver	10 Lower respiratory infect	**154 (93 to 214)**	**156 (107 to 216)**	-9.97 (-31 to 27)
11 Alzheimer's disease	11 Colorectal cancer	**248 (95.3 to 429)**	**249 (118 to 433)**	19.6 (-16.8 to 67.9)
12 Lower respiratory infect	12 Cirrhosis liver	**109 (59.9 to 158)**	**111 (73.8 to 152)**	**-22.4 (-34.9 to -2.9)**
13 COPD	13 Pancreatic cancer	**380 (182 to 613)**	**382 (213 to 612)**	**63 (13.4 to 120)**
14 Congenital defects	14 Liver cancer	**242 (157 to 333)**	**245 (176 to 334)**	**26.2 (0.187 to 66.2)**
15 Colorectal cancer	15 Conflict & terror	inf (-100 to nan)	inf (-100 to nan)	inf (-100 to nan)
16 Neonatal disorders	16 Prostate cancer	**320 (131 to 548)**	**320 (152 to 528)**	8.01 (-25.1 to 47.2)
17 Other cardiovascular	17 Bladder cancer	**265 (141 to 417)**	**267 (167 to 378)**	7.45 (-12.6 to 30.5)
18 Breast cancer	18 Lymphoma	**175 (71.9 to 292)**	**176 (82.7 to 290)**	7.54 (-23.6 to 37.5)
19 Liver cancer	19 Breast cancer	**144 (42.5 to 270)**	**145 (56.7 to 261)**	11 (-23.7 to 51.7)
20 Pancreatic cancer	20 Parkinson's disease	**298 (192 to 412)**	**301 (225 to 397)**	3.72 (-12.9 to 26.8)
23 Lymphoma	22 Other cardiovascular			
28 Bladder cancer	38 Congenital defects			
29 Prostate cancer	57 Neonatal disorders			
30 Parkinson's disease	131 COVID-19			

Supplemental Results Figure S8. Leading 20 level 3 causes of deaths, and percentage change in number of deaths and all-age and age-standardised death rates by location, 2022 and 2050

Morocco, Reference scenario

Legend:
Communicable, maternal, neonatal and nutritional
Non-communicable
Injuries

Leading Causes 2022	Leading Causes 2050	Mean % change number of DEATH	Mean % change all-age DEATH rate	Mean % change age-standardized DEATH rate
1 Ischemic heart disease	1 Ischemic heart disease	46.6 (-3.43 to 117)	38.2 (-7.38 to 101)	-34.2 (-62.8 to 18.7)
2 Stroke	2 Stroke	**40.2 (6.69 to 86.8)**	**32 (1.79 to 72.2)**	**-39.8 (-60.3 to -8.18)**
3 COVID-19	3 Chronic kidney disease	**210 (125 to 308)**	**192 (121 to 277)**	38.6 (-10.3 to 113)
4 Hypertensive heart disease	4 Hypertensive heart disease	**125 (65.7 to 192)**	**112 (56.4 to 176)**	-6.51 (-40.7 to 45.5)
5 Chronic kidney disease	5 Alzheimer's disease	**223 (106 to 371)**	**203 (97.9 to 320)**	**10.1 (7.05 to 13.3)**
6 Road injuries	6 Diabetes	**90.5 (36.2 to 157)**	**79.3 (30.3 to 144)**	-10.5 (-41.3 to 33.8)
7 Diabetes	7 COPD	**96.8 (48.6 to 151)**	**85.2 (43 to 131)**	-17.2 (-45.7 to 24.5)
8 Lower respiratory infect	8 Lower respiratory infect	**59.4 (27.8 to 92.2)**	**49.9 (24.1 to 75.7)**	**-28.2 (-46 to -2.98)**
9 COPD	9 Lung cancer	**101 (21.5 to 203)**	**88.3 (16.9 to 178)**	-2.25 (-33 to 35)
10 Alzheimer's disease	10 Road injuries	-8.42 (-42.6 to 46.5)	-13.7 (-43.6 to 34.6)	-34.5 (-59.6 to 6.8)
11 Cirrhosis liver	11 Cirrhosis liver	**45 (22.7 to 71.3)**	**36.3 (19.1 to 56.1)**	**-30.4 (-42 to -15.7)**
12 Lung cancer	12 Colorectal cancer	**153 (45 to 314)**	**137 (36.9 to 281)**	20.5 (-19.3 to 70.2)
13 Neonatal disorders	13 Parkinson's disease	**160 (92.2 to 251)**	**144 (90.3 to 202)**	-1.02 (-17.3 to 19.2)
14 Asthma	14 Other cardiovascular	**64.8 (32.5 to 104)**	**54.8 (32.9 to 82)**	**-25.2 (-41.5 to -4.95)**
15 Tuberculosis	15 Atrial fibrillation	**260 (134 to 456)**	**237 (121 to 382)**	**23.3 (2.98 to 45.1)**
16 Colorectal cancer	16 Breast cancer	**93.6 (26.3 to 198)**	**81.2 (20 to 163)**	11.9 (-18.6 to 51.6)
17 Other cardiovascular	17 Falls	**75.5 (26.3 to 139)**	**64.7 (23.1 to 108)**	**-13.2 (-22.4 to -3.4)**
18 Breast cancer	18 Lymphoma	**119 (37.6 to 223)**	**106 (34.7 to 195)**	9.23 (-19.3 to 43.3)
19 Falls	19 Asthma	-11.7 (-29.6 to 10.5)	-17.1 (-29.7 to 0.793)	**-61.4 (-69.8 to -49.4)**
20 Parkinson's disease	20 Urinary diseases	**174 (111 to 253)**	**158 (112 to 229)**	13.7 (-17.8 to 57.6)

Leading Causes 2022	Leading Causes 2050
23 Lymphoma	24 Tuberculosis
25 Atrial fibrillation	29 Neonatal disorders
27 Urinary diseases	131 COVID-19

Supplemental Results Figure S8. Leading 20 level 3 causes of deaths, and percentage change in number of deaths and all-age and age-standardised death rates by location, 2022 and 2050

Oman, Reference scenario

Legend:
Communicable, maternal, neonatal and nutritional
Non-communicable
Injuries

Leading Causes 2022	Leading Causes 2050	Mean % change number of DEATH	Mean % change all-age DEATH rate	Mean % change age-standardized DEATH rate
1 Ischemic heart disease	1 Ischemic heart disease	**246 (132 to 410)**	**136 (56.8 to 253)**	-41 (-65.7 to 0.337)
2 Road injuries	2 Chronic kidney disease	**751 (549 to 1010)**	**480 (327 to 681)**	45 (-2.96 to 119)
3 Stroke	3 Hypertensive heart disease	**779 (551 to 1040)**	**499 (337 to 692)**	31.1 (-15.6 to 93.4)
4 Diabetes	4 Diabetes	**472 (323 to 657)**	**289 (178 to 413)**	0.142 (-32 to 59.1)
5 Chronic kidney disease	5 Stroke	**224 (144 to 329)**	**121 (67.9 to 206)**	**-46.7 (-63.9 to -16.5)**
6 Hypertensive heart disease	6 Lower respiratory infect	**412 (309 to 538)**	**249 (176 to 340)**	2.53 (-22.8 to 41.7)
7 Lower respiratory infect	7 Road injuries	**78 (13.1 to 178)**	21.4 (-24.2 to 91.6)	-17.3 (-50.7 to 40.5)
8 COVID-19	8 Alzheimer's disease	**774 (547 to 1030)**	**494 (341 to 656)**	**17 (13.6 to 21)**
9 Neonatal disorders	9 COPD	**641 (446 to 849)**	**405 (267 to 564)**	12.3 (-28 to 62.9)
10 Other cardiovascular	10 Other cardiovascular	**339 (261 to 431)**	**199 (150 to 265)**	-14.5 (-34.2 to 13)
11 Cirrhosis liver	11 Cirrhosis liver	**320 (248 to 399)**	**186 (136 to 233)**	**-21.5 (-34.5 to -2.94)**
12 Alzheimer's disease	12 Lung cancer	**608 (337 to 893)**	**381 (209 to 580)**	19.8 (-17.9 to 63.6)
13 Congenital defects	13 Liver cancer	**625 (459 to 798)**	**394 (285 to 513)**	**34.4 (5.35 to 74.2)**
14 COPD	14 Parkinson's disease	**769 (543 to 1060)**	**491 (345 to 662)**	21.3 (-2.64 to 52.7)
15 Falls	15 Cardiomyopathy	**351 (275 to 447)**	**206 (161 to 264)**	**-11.9 (-21.2 to -0.693)**
16 Cardiomyopathy	16 Urinary diseases	**748 (545 to 1050)**	**478 (334 to 670)**	44.9 (-3.02 to 131)
17 Stomach cancer	17 Colorectal cancer	**644 (329 to 1020)**	**406 (208 to 656)**	30.3 (-17.2 to 83.8)
18 Upper respiratory infect	18 Upper respiratory infect	**417 (307 to 539)**	**252 (186 to 332)**	5.94 (-3.13 to 13.2)
19 Lung cancer	19 Falls	**232 (146 to 325)**	**125 (72.1 to 189)**	-4.01 (-17.3 to 12.1)
20 Lymphoma	20 Lymphoma	**417 (216 to 621)**	**251 (122 to 393)**	9.76 (-24.8 to 47.7)
21 Liver cancer	22 Stomach cancer			
26 Colorectal cancer	34 Neonatal disorders			
27 Parkinson's disease	37 Congenital defects			
30 Urinary diseases	133 COVID-19			

Supplemental Results Figure S8. Leading 20 level 3 causes of deaths, and percentage change in number of deaths and all-age and age-standardised death rates by location, 2022 and 2050

Palestine, Reference scenario

Legend:
Communicable, maternal, neonatal and nutritional
Non-communicable
Injuries

Leading Causes 2022	Leading Causes 2050	Mean % change number of DEATH	Mean % change all-age DEATH rate	Mean % change age-standardized DEATH rate
1 Ischemic heart disease	1 Ischemic heart disease	**110 (39.1 to 204)**	**52.7 (1.67 to 126)**	-36.8 (-63.8 to 7.06)
2 COVID-19	2 Chronic kidney disease	**375 (257 to 527)**	**245 (158 to 368)**	45.6 (-2.77 to 120)
3 Stroke	3 Stroke	**105 (60.6 to 158)**	**49.1 (15.1 to 87.6)**	**-42.4 (-60.5 to -17.4)**
4 Diabetes	4 Diabetes	**204 (126 to 299)**	**121 (62.2 to 193)**	-8.92 (-38.2 to 27.4)
5 Conflict & terror	5 Hypertensive heart disease	**286 (197 to 425)**	**181 (109 to 284)**	1.51 (-35.5 to 59.8)
6 Chronic kidney disease	6 Lung cancer	**214 (108 to 360)**	**128 (49.8 to 226)**	1.06 (-27.9 to 34.3)
7 Hypertensive heart disease	7 Alzheimer's disease	**337 (225 to 469)**	**217 (142 to 300)**	**4.85 (1.66 to 8.14)**
8 Neonatal disorders	8 Conflict & terror	2350 (-98.6 to 20300)	1660 (-98.9 to 13500)	1680 (-98.9 to 13600)
9 Lower respiratory infect	9 Lower respiratory infect	**118 (78.7 to 171)**	**58.4 (31.1 to 92)**	-20.7 (-38.2 to 3.3)
10 Lung cancer	10 Colorectal cancer	**252 (116 to 446)**	**155 (53.9 to 282)**	11 (-25.5 to 56.1)
11 Congenital defects	11 COPD	**233 (154 to 309)**	**142 (84.6 to 200)**	-6.55 (-36.5 to 34.1)
12 Colorectal cancer	12 Breast cancer	**181 (85.1 to 301)**	**104 (36.4 to 185)**	0.476 (-29.3 to 36.4)
13 Alzheimer's disease	13 Brain cancer	**245 (196 to 297)**	**150 (124 to 180)**	**42 (34.6 to 50.7)**
14 Road injuries	14 Cirrhosis liver	**145 (112 to 186)**	**78.2 (55.5 to 106)**	**-23.8 (-36.1 to -6.17)**
15 Breast cancer	15 Pancreatic cancer	**388 (204 to 627)**	**255 (124 to 414)**	**53 (5.17 to 111)**
16 Cirrhosis liver	16 Prostate cancer	**341 (171 to 565)**	**220 (102 to 382)**	16.2 (-17.4 to 58.8)
17 COPD	17 Liver cancer	**262 (205 to 334)**	**163 (124 to 218)**	16.7 (-6.8 to 48.6)
18 Interpersonal violence	18 Road injuries	31.6 (-18.4 to 105)	-4.34 (-40.9 to 51.5)	-25.5 (-55.6 to 22.7)
19 Brain cancer	19 Leukemia	**121 (88.8 to 160)**	**60.7 (43.3 to 82.2)**	**-9.24 (-15.1 to -1.23)**
20 Leukemia	20 Atrial fibrillation	**384 (247 to 565)**	**251 (150 to 366)**	19.8 (-0.704 to 45.5)
21 Liver cancer	25 Neonatal disorders			
24 Prostate cancer	26 Congenital defects			
25 Pancreatic cancer	29 Interpersonal violence			
34 Atrial fibrillation	131 COVID-19			

Supplemental Results Figure S8. Leading 20 level 3 causes of deaths, and percentage change in number of deaths and all-age and age-standardised death rates by location, 2022 and 2050

Qatar, Reference scenario

Legend:
Communicable, maternal, neonatal and nutritional
Non-communicable
Injuries

Leading Causes 2022	Leading Causes 2050	Mean % change number of DEATH	Mean % change all-age DEATH rate	Mean % change age-standardized DEATH rate
1 Ischemic heart disease	1 Diabetes	**1090 (765 to 1560)**	**417 (267 to 640)**	-18.6 (-42.9 to 19.7)
2 Road injuries	2 Ischemic heart disease	**591 (342 to 886)**	**201 (98.2 to 334)**	**-46.3 (-66.4 to -12.3)**
3 Diabetes	3 Chronic kidney disease	**1790 (1260 to 2530)**	**721 (498 to 1020)**	35 (-7.42 to 110)
4 Stroke	4 Stroke	**678 (496 to 889)**	**239 (160 to 332)**	**-46.6 (-59.4 to -28.1)**
5 COVID-19	5 Liver cancer	**1700 (1370 to 2060)**	**683 (535 to 848)**	**36 (10.2 to 69.1)**
6 Chronic kidney disease	6 Alzheimer's disease	**2040 (1580 to 2550)**	**832 (642 to 1030)**	**12.1 (8.05 to 16.1)**
7 Cirrhosis liver	7 Road injuries	**228 (114 to 396)**	42.6 (-5.55 to 120)	-13.6 (-44.3 to 37.6)
8 Liver cancer	8 Lung cancer	**1180 (815 to 1700)**	**456 (303 to 684)**	6.27 (-22.9 to 39.8)
9 Lung cancer	9 Hypertensive heart disease	**2050 (1410 to 2780)**	**836 (554 to 1150)**	29.5 (-15.4 to 89.4)
10 Lower respiratory infect	10 Cirrhosis liver	**822 (660 to 977)**	**301 (230 to 366)**	**-22.8 (-33.3 to -10.8)**
11 Self-harm	11 Lower respiratory infect	**972 (738 to 1230)**	**366 (271 to 476)**	-5.94 (-26.4 to 21.8)
12 Falls	12 Prostate cancer	**2420 (1590 to 3480)**	**997 (643 to 1410)**	28.9 (-5.44 to 76.9)
13 Breast cancer	13 Colorectal cancer	**1140 (692 to 1670)**	**441 (243 to 653)**	1.52 (-31.2 to 42)
14 Colorectal cancer	14 COPD	**1690 (1210 to 2230)**	**677 (473 to 933)**	6.9 (-22.3 to 49.7)
15 Alzheimer's disease	15 Pancreatic cancer	**1630 (1040 to 2210)**	**652 (401 to 880)**	35.9 (-5.65 to 74.9)
16 Hypertensive heart disease	16 Breast cancer	**672 (402 to 968)**	**235 (122 to 376)**	-19.8 (-42.1 to 11.6)
17 Congenital defects	17 Parkinson's disease	**2230 (1740 to 2810)**	**914 (715 to 1150)**	10.6 (-9.66 to 37.6)
18 Mechanical forces	18 Falls	**452 (300 to 654)**	**140 (75.5 to 223)**	-4.41 (-19.6 to 18.9)
19 COPD	19 Brain cancer	**958 (741 to 1270)**	**360 (274 to 487)**	**42.7 (29.2 to 61.2)**
20 Pancreatic cancer	20 Bacterial skin	**3140 (2080 to 4590)**	**1310 (847 to 1880)**	**115 (36.4 to 251)**
21 Prostate cancer	32 Self-harm			
25 Brain cancer	39 Mechanical forces			
32 Parkinson's disease	51 Congenital defects			
44 Bacterial skin	129 COVID-19			

Supplemental Results Figure S8. Leading 20 level 3 causes of deaths, and percentage change in number of deaths and all-age and age-standardised death rates by location, 2022 and 2050

Saudi Arabia, Reference scenario

Legend:
Communicable, maternal, neonatal and nutritional
Non-communicable
Injuries

Leading Causes 2022	Leading Causes 2050	Mean % change number of DEATH	Mean % change all-age DEATH rate	Mean % change age-standardized DEATH rate
1 Ischemic heart disease	1 Chronic kidney disease	**537 (349 to 781)**	**431 (281 to 634)**	32.4 (-6.9 to 100)
2 Road injuries	2 Ischemic heart disease	**141 (52.2 to 261)**	**101 (31.6 to 199)**	**-43.5 (-65.2 to -8.43)**
3 Stroke	3 Hypertensive heart disease	**655 (425 to 924)**	**529 (347 to 741)**	25 (-17 to 82.7)
4 Chronic kidney disease	4 Stroke	**172 (96.5 to 279)**	**127 (69.5 to 205)**	**-48 (-62.9 to -22.1)**
5 Hypertensive heart disease	5 Road injuries	50.1 (-5.57 to 130)	25.3 (-17.8 to 90.5)	-8.4 (-41.8 to 50)
6 Cirrhosis liver	6 Lower respiratory infect	**333 (231 to 482)**	**261 (181 to 360)**	-5.27 (-28 to 26.8)
7 Diabetes	7 Diabetes	**294 (179 to 440)**	**229 (140 to 337)**	-11.8 (-38.5 to 24.9)
8 Lower respiratory infect	8 Cirrhosis liver	**245 (177 to 326)**	**187 (139 to 238)**	**-22.9 (-33.2 to -8.69)**
9 Interpersonal violence	9 COPD	**540 (356 to 774)**	**434 (295 to 623)**	9.9 (-26.8 to 58.6)
10 Self-harm	10 Alzheimer's disease	**910 (613 to 1210)**	**740 (552 to 946)**	**13.1 (9.87 to 15.8)**
11 COPD	11 Colorectal cancer	**416 (202 to 653)**	**329 (171 to 510)**	20.9 (-17.1 to 61.8)
12 Alzheimer's disease	12 Liver cancer	**576 (432 to 776)**	**463 (360 to 586)**	**28.2 (2.94 to 61.4)**
13 Colorectal cancer	13 Lung cancer	**452 (239 to 667)**	**359 (204 to 519)**	14.1 (-19.5 to 50.3)
14 COVID-19	14 Parkinson's disease	**731 (533 to 995)**	**592 (451 to 745)**	0.511 (-19.7 to 24.4)
15 Fire & heat	15 Urinary diseases	**725 (473 to 1040)**	**588 (390 to 796)**	43.2 (-1.16 to 116)
16 Mechanical forces	16 Interstitial lung disease	**473 (342 to 639)**	**378 (281 to 488)**	12.2 (-1.76 to 29.7)
17 Breast cancer	17 Pancreatic cancer	**642 (363 to 937)**	**517 (299 to 764)**	**57 (9.44 to 109)**
18 Lung cancer	18 Endo/metab/blood/immune	**314 (212 to 444)**	**245 (174 to 332)**	**26.8 (4.41 to 54.1)**
19 Congenital defects	19 Other cardiovascular	**263 (180 to 364)**	**202 (143 to 268)**	-15.4 (-32 to 2.57)
20 Asthma	20 Lymphoma	**289 (146 to 447)**	**224 (117 to 349)**	5.24 (-23.1 to 34.8)

Leading Causes 2022	Leading Causes 2050
21 Liver cancer	21 Breast cancer
23 Other cardiovascular	22 Self-harm
24 Endo/metab/blood/immune	26 Interpersonal violence
26 Lymphoma	31 Asthma
31 Interstitial lung disease	41 Fire & heat
33 Parkinson's disease	42 Mechanical forces
34 Urinary diseases	50 Congenital defects

Supplemental Results Figure S8. Leading 20 level 3 causes of deaths, and percentage change in number of deaths and all-age and age-standardised death rates by location, 2022 and 2050

Sudan, Reference scenario

Legend:
Communicable, maternal, neonatal and nutritional
Non-communicable
Injuries

Leading Causes 2022	Leading Causes 2050	Mean % change number of DEATH	Mean % change all-age DEATH rate	Mean % change age-standardized DEATH rate
1 Ischemic heart disease	1 Ischemic heart disease	**98.7 (9.52 to 213)**	32.3 (-24.6 to 112)	-39.5 (-69.8 to 9.04)
2 Stroke	2 HIV/AIDS	514 (-31.5 to 3590)	312 (-56.4 to 2300)	186 (-67.8 to 1650)
3 COVID-19	3 Stroke	**85.1 (24 to 155)**	23.2 (-15.4 to 70.5)	**-44 (-64.1 to -13.3)**
4 Neonatal disorders	4 Hypertensive heart disease	**245 (121 to 374)**	**129 (49.7 to 212)**	-3.26 (-40.7 to 50.1)
5 Congenital defects	5 Chronic kidney disease	**313 (159 to 488)**	**174 (83.5 to 294)**	29.7 (-20.2 to 106)
6 Malaria	6 Road injuries	54.8 (-7.43 to 155)	3.09 (-38 to 68.6)	-30.7 (-58.2 to 12.6)
7 Hypertensive heart disease	7 Alzheimer's disease	**311 (167 to 481)**	**173 (79.5 to 274)**	**5.34 (2 to 8.53)**
8 Lower respiratory infect	8 Lower respiratory infect	32.1 (-7.76 to 71.2)	-12.2 (-36.6 to 9.56)	**-35.3 (-52.3 to -13.6)**
9 Road injuries	9 COPD	**139 (64.8 to 224)**	**58.9 (10.2 to 117)**	-30.4 (-54.7 to 7.97)
10 HIV/AIDS	10 Diabetes	**148 (60.6 to 267)**	**65.5 (8.27 to 144)**	-22.1 (-51.2 to 30.1)
11 Chronic kidney disease	11 Cirrhosis liver	**93.1 (54.2 to 132)**	**28.4 (5.88 to 52.1)**	**-36 (-47.7 to -18.1)**
12 Cirrhosis liver	12 Neonatal disorders	**-54.4 (-75 to -24.1)**	**-69.7 (-82.8 to -49.2)**	**-46.6 (-68 to -10.5)**
13 COPD	13 Congenital defects	**-41.4 (-55.9 to -23.8)**	**-61.2 (-68.1 to -52.7)**	**-35.4 (-44.3 to -25.3)**
14 Diabetes	14 Stomach cancer	**99.3 (59.3 to 151)**	**32.4 (9.52 to 56.5)**	**-38 (-45.6 to -29.2)**
15 Stomach cancer	15 Malaria	49.3 (-48.1 to 89.4)	-1.21 (-66.5 to 13.5)	-2.6 (-79.3 to 9.4)
16 Alzheimer's disease	16 Lung cancer	**189 (85.1 to 335)**	**91.4 (21.6 to 186)**	-12.2 (-37.9 to 19)
17 Maternal disorders	17 Colorectal cancer	**251 (110 to 466)**	**133 (43.5 to 263)**	9.77 (-26.7 to 52.3)
18 Diarrheal diseases	18 Leukemia	**115 (50.9 to 201)**	**42.6 (5.07 to 95.2)**	-1.18 (-23.9 to 27.2)
19 Asthma	19 Brain cancer	**216 (155 to 277)**	**110 (87.8 to 138)**	**38.7 (30.1 to 47.5)**
20 Conflict & terror	20 Other cardiovascular	**71.7 (26 to 114)**	14.1 (-11.6 to 40.9)	**-31.5 (-47.5 to -8.9)**
22 Other cardiovascular	26 Asthma			
23 Lung cancer	29 Conflict & terror			
24 Leukemia	41 Diarrheal diseases			
27 Colorectal cancer	44 Maternal disorders			
33 Brain cancer	134 COVID-19			

Supplemental Results Figure S8. Leading 20 level 3 causes of deaths, and percentage change in number of deaths and all-age and age-standardised death rates by location, 2022 and 2050

Syria, Reference scenario

Legend:
Communicable, maternal, neonatal and nutritional
Non-communicable
Injuries

Leading Causes 2022	Leading Causes 2050	Mean % change number of DEATH	Mean % change all-age DEATH rate	Mean % change age-standardized DEATH rate
1 Ischemic heart disease	1 Ischemic heart disease	56.6 (-2.54 to 123)	36.5 (-12.8 to 91)	-26 (-60.7 to 29.7)
2 Conflict & terror	2 Conflict & terror	inf (-100 to 1.51e+06)	inf (-100 to 1.19e+06)	inf (-100 to 1.11e+06)
3 Stroke	3 Chronic kidney disease	**215 (132 to 311)**	**174 (104 to 257)**	44.6 (-7.27 to 118)
4 Chronic kidney disease	4 Stroke	**47.2 (2.97 to 93)**	27.9 (-7.07 to 66.7)	-34.8 (-58.6 to 1.68)
5 Hypertensive heart disease	5 Hypertensive heart disease	**192 (114 to 279)**	**154 (89.6 to 228)**	18.2 (-22.7 to 82.5)
6 COPD	6 Alzheimer's disease	**229 (113 to 411)**	**184 (91.7 to 322)**	**9.55 (6.62 to 12.9)**
7 COVID-19	7 COPD	**113 (51.1 to 181)**	**84.7 (35.4 to 142)**	-9.83 (-42.1 to 31.6)
8 Lower respiratory infect	8 Diabetes	**99.1 (42.3 to 184)**	**73.3 (25.1 to 139)**	-3.8 (-37.8 to 51.4)
9 Diabetes	9 Lower respiratory infect	**58.6 (19.3 to 104)**	**37.7 (6.72 to 77.1)**	-24.8 (-43.4 to 5.91)
10 Cirrhosis liver	10 Cirrhosis liver	**43 (9.6 to 75.1)**	**24.1 (2.65 to 46.4)**	**-29.6 (-42.3 to -11.1)**
11 Road injuries	11 Lung cancer	70 (-2.61 to 194)	46.7 (-13.4 to 141)	-12.2 (-39 to 21.4)
12 Alzheimer's disease	12 Urinary diseases	**236 (143 to 348)**	**192 (115 to 287)**	40.5 (-6.11 to 101)
13 Lung cancer	13 Brain cancer	**129 (77.5 to 199)**	**98.7 (65.3 to 136)**	**35.9 (27.1 to 44.7)**
14 Other cardiovascular	14 Road injuries	3.53 (-36.6 to 61.8)	-9.81 (-42.6 to 40)	-25.5 (-54.5 to 25.5)
15 Asthma	15 Other cardiovascular	**79 (32.3 to 136)**	**55.3 (21.2 to 93.3)**	-17.8 (-35.2 to 8.79)
16 Brain cancer	16 Colorectal cancer	**110 (10.6 to 254)**	**81.6 (1.94 to 213)**	2.55 (-30.6 to 45.4)
17 Leukemia	17 Parkinson's disease	**184 (92.8 to 306)**	**146 (72.1 to 231)**	-4.09 (-22.4 to 15.4)
18 Breast cancer	18 Liver cancer	**107 (57.2 to 158)**	**79.7 (43.4 to 122)**	10.7 (-14.4 to 48.7)
19 Colorectal cancer	19 Breast cancer	**72.7 (1.41 to 177)**	49 (-10 to 138)	5.44 (-27.7 to 49.5)
20 Congenital defects	20 Leukemia	**68.3 (7.48 to 157)**	45.3 (-1.99 to 111)	-7.45 (-28.9 to 19.6)
22 Liver cancer	26 Asthma			
24 Urinary diseases	32 Congenital defects			
27 Parkinson's disease	132 COVID-19			

Supplemental Results Figure S8. Leading 20 level 3 causes of deaths, and percentage change in number of deaths and all-age and age-standardised death rates by location, 2022 and 2050

Tunisia, Reference scenario

Legend: Communicable, maternal, neonatal and nutritional; Non-communicable; Injuries

Leading Causes 2022	Leading Causes 2050	Mean % change number of DEATH	Mean % change all-age DEATH rate	Mean % change age-standardized DEATH rate
1 Ischemic heart disease	1 Ischemic heart disease	47 (-9.52 to 115)	46.8 (-3.6 to 116)	**-45 (-65.6 to -12.7)**
2 COVID-19	2 Stroke	**43.3 (5.82 to 95.6)**	**43.1 (8.56 to 93.3)**	**-48.5 (-62.9 to -27.2)**
3 Stroke	3 Hypertensive heart disease	**199 (105 to 318)**	**198 (106 to 301)**	5.45 (-28.2 to 53.6)
4 Hypertensive heart disease	4 Chronic kidney disease	**223 (122 to 354)**	**222 (124 to 337)**	20.5 (-17.6 to 85.1)
5 Chronic kidney disease	5 Alzheimer's disease	**278 (155 to 408)**	**277 (172 to 394)**	**7.9 (5.02 to 10.8)**
6 Alzheimer's disease	6 COPD	**145 (71.6 to 245)**	**145 (77.2 to 232)**	-13.8 (-39.3 to 23.4)
7 Road injuries	7 Diabetes	**120 (54 to 203)**	**120 (62.8 to 197)**	-11.3 (-34.5 to 24)
8 Lung cancer	8 Lung cancer	**86.5 (28 to 165)**	**86.1 (32.9 to 147)**	-8.42 (-30.1 to 17.5)
9 Diabetes	9 Lower respiratory infect	**113 (61.9 to 178)**	**112 (68.1 to 169)**	**-23.8 (-39.5 to -2.43)**
10 COPD	10 Cirrhosis liver	**81 (46.5 to 117)**	**80.6 (55.5 to 107)**	**-21.1 (-30.9 to -9.35)**
11 Lower respiratory infect	11 Colorectal cancer	**170 (60.7 to 312)**	**169 (70.7 to 292)**	16.2 (-19.5 to 56.9)
12 Cirrhosis liver	12 Road injuries	3.38 (-30.6 to 51.4)	3.35 (-28.4 to 56.1)	-20.3 (-44.7 to 19.7)
13 Colorectal cancer	13 Atrial fibrillation	**327 (180 to 498)**	**326 (198 to 498)**	16.6 (-1.61 to 39.5)
14 Breast cancer	14 Breast cancer	**105 (36.5 to 180)**	**104 (42.3 to 176)**	8.18 (-18.6 to 35.2)
15 Other cardiovascular	15 Other cardiovascular	**107 (58.1 to 166)**	**107 (61.4 to 160)**	**-21.8 (-35.2 to -6.03)**
16 Asthma	16 Parkinson's disease	**191 (112 to 281)**	**190 (123 to 267)**	-4.52 (-20.5 to 15.7)
17 Neonatal disorders	17 Pancreatic cancer	**251 (117 to 413)**	**250 (131 to 406)**	**57.4 (12.1 to 110)**
18 Congenital defects	18 Prostate cancer	**190 (75 to 327)**	**189 (88.1 to 315)**	0.55 (-25.3 to 34.5)
19 Stomach cancer	19 Lymphoma	**118 (44.6 to 199)**	**117 (56.7 to 192)**	3.67 (-19.2 to 29.8)
20 Lymphoma	20 Falls	**117 (57 to 192)**	**116 (62.2 to 187)**	-10.6 (-19.6 to 0.086)
21 Falls	23 Asthma			
22 Parkinson's disease	24 Stomach cancer			
23 Atrial fibrillation	46 Congenital defects			
26 Prostate cancer	49 Neonatal disorders			
27 Pancreatic cancer	131 COVID-19			

Supplemental Results Figure S8. Leading 20 level 3 causes of deaths, and percentage change in number of deaths and all-age and age-standardised death rates by location, 2022 and 2050

Türkiye, Reference scenario

Legend:
Communicable, maternal, neonatal and nutritional
Non-communicable
Injuries

Leading Causes 2022	Leading Causes 2050	Mean % change number of DEATH	Mean % change all-age DEATH rate	Mean % change age-standardized DEATH rate
1 Ischemic heart disease	1 Ischemic heart disease	5.13 (-30.3 to 53)	5.36 (-29.4 to 53.3)	**-58.4 (-74.9 to -30.8)**
2 Stroke	2 COPD	**140 (80.3 to 215)**	**140 (80.8 to 215)**	-8.01 (-34.5 to 29.2)
3 COPD	3 Stroke	20.7 (-4.68 to 51)	20.9 (-4.44 to 47.9)	**-53.8 (-65.2 to -35.6)**
4 Lung cancer	4 Chronic kidney disease	**162 (100 to 254)**	**163 (96.5 to 265)**	3.87 (-27.4 to 58.7)
5 COVID-19	5 Alzheimer's disease	**236 (143 to 335)**	**235 (160 to 318)**	**9.57 (6.5 to 13)**
6 Chronic kidney disease	6 Hypertensive heart disease	**207 (123 to 312)**	**208 (131 to 323)**	9.42 (-24.9 to 67.5)
7 Diabetes	7 Lung cancer	**80 (21.2 to 143)**	**79.9 (28.9 to 134)**	-11.5 (-35.1 to 10.5)
8 Hypertensive heart disease	8 Diabetes	**78.8 (35 to 135)**	**79.1 (35 to 138)**	-27.5 (-46.1 to 6.13)
9 Alzheimer's disease	9 Lower respiratory infect	**108 (68.5 to 160)**	**108 (73.7 to 162)**	-21.4 (-36.8 to 4.55)
10 Lower respiratory infect	10 Colorectal cancer	**154 (54.8 to 269)**	**154 (62.2 to 254)**	13.1 (-20.6 to 50.5)
11 Colorectal cancer	11 Pancreatic cancer	**229 (108 to 357)**	**229 (125 to 359)**	**51.9 (8.94 to 100)**
12 Stomach cancer	12 Prostate cancer	**197 (85.4 to 324)**	**196 (93.5 to 333)**	10.9 (-18.2 to 46.3)
13 Road injuries	13 Stomach cancer	**45.2 (19.6 to 81)**	**45.2 (21.9 to 72.8)**	**-33.6 (-41.3 to -23.9)**
14 Cirrhosis liver	14 Brain cancer	**154 (111 to 191)**	**154 (126 to 184)**	**37.4 (28.8 to 46.5)**
15 Pancreatic cancer	15 Cirrhosis liver	**63.5 (35 to 99.1)**	**63.7 (39 to 94.6)**	**-25.1 (-38.3 to -7.06)**
16 Breast cancer	16 Falls	**121 (62.8 to 182)**	**121 (66.6 to 168)**	**-15.5 (-26.7 to -2.09)**
17 Prostate cancer	17 Parkinson's disease	**193 (124 to 271)**	**193 (129 to 264)**	1.72 (-16.5 to 21.9)
18 Falls	18 Breast cancer	**90 (22.1 to 176)**	**89.7 (28.5 to 164)**	-1.86 (-27.8 to 28.1)
19 Brain cancer	19 Atrial fibrillation	**268 (164 to 412)**	**267 (181 to 396)**	**18.8 (1.95 to 39.2)**
20 Leukemia	20 Leukemia	**97.9 (41.7 to 165)**	**97.8 (52.6 to 157)**	-0.472 (-19.1 to 17.9)
23 Parkinson's disease	23 Road injuries			
28 Atrial fibrillation	131 COVID-19			

Supplemental Results Figure S8. Leading 20 level 3 causes of deaths, and percentage change in number of deaths and all-age and age-standardised death rates by location, 2022 and 2050

United Arab Emirates, Reference scenario

Legend:
Communicable, maternal, neonatal and nutritional
Non-communicable
Injuries

Leading Causes 2022	Leading Causes 2050	Mean % change number of DEATH	Mean % change all-age DEATH rate	Mean % change age-standardized DEATH rate
1 Ischemic heart disease	1 Ischemic heart disease	**747 (455 to 1200)**	**316 (171 to 523)**	**-55.2 (-71.5 to -24)**
2 Stroke	2 Chronic kidney disease	**2050 (1310 to 2950)**	**958 (621 to 1380)**	2.76 (-31.5 to 47)
3 Road injuries	3 Stroke	**890 (638 to 1260)**	**386 (264 to 547)**	**-57.6 (-67.8 to -41.8)**
4 COVID-19	4 Hypertensive heart disease	**2510 (1580 to 3770)**	**1180 (769 to 1770)**	9.96 (-23.8 to 71.8)
5 Diabetes	5 Lower respiratory infect	**1830 (1350 to 2360)**	**847 (634 to 1100)**	-23.5 (-41.4 to 0.105)
6 Chronic kidney disease	6 Diabetes	**1050 (708 to 1500)**	**463 (314 to 684)**	**-36 (-53.5 to -14.1)**
7 Lower respiratory infect	7 COPD	**1620 (1090 to 2300)**	**744 (510 to 1070)**	-14.8 (-38.9 to 19.6)
8 Hypertensive heart disease	8 Alzheimer's disease	**3060 (2210 to 3990)**	**1450 (1090 to 1930)**	2.33 (-2.59 to 7.56)
9 COPD	9 Pancreatic cancer	**1540 (897 to 2360)**	**707 (410 to 1060)**	13.6 (-25.3 to 55.3)
10 Liver cancer	10 Liver cancer	**1130 (841 to 1430)**	**502 (382 to 665)**	16.3 (-2.6 to 38.8)
11 Lung cancer	11 Lung cancer	**1130 (693 to 1610)**	**502 (314 to 722)**	-9.9 (-36 to 17.1)
12 Cirrhosis liver	12 Road injuries	**277 (145 to 507)**	**84.9 (24.4 to 185)**	-10.3 (-41.9 to 43.1)
13 Colorectal cancer	13 Colorectal cancer	**1100 (592 to 1640)**	**488 (267 to 761)**	-19.8 (-48.5 to 15.4)
14 Breast cancer	14 Prostate cancer	**2660 (1650 to 3790)**	**1250 (812 to 1780)**	10.2 (-22.9 to 48.2)
15 Pancreatic cancer	15 Cirrhosis liver	**838 (638 to 1080)**	**361 (277 to 463)**	**-38.5 (-46.9 to -27.4)**
16 Brain cancer	16 Breast cancer	**750 (411 to 1180)**	**317 (166 to 509)**	-24.8 (-48.4 to 3.1)
17 Falls	17 Brain cancer	**852 (600 to 1150)**	**367 (260 to 493)**	**33.2 (19 to 46.2)**
18 Leukemia	18 Cardiomyopathy	**919 (661 to 1190)**	**400 (292 to 523)**	**-31.2 (-41.5 to -20.6)**
19 Self-harm	19 Stomach cancer	**788 (568 to 1040)**	**336 (247 to 445)**	**-42 (-50.7 to -30)**
20 Congenital defects	20 Ovarian cancer	**1260 (827 to 1790)**	**566 (355 to 810)**	-11 (-34.1 to 12)
21 Alzheimer's disease	21 Falls			
22 Stomach cancer	23 Leukemia			
23 Cardiomyopathy	43 Self-harm			
28 Prostate cancer	55 Congenital defects			
29 Ovarian cancer	128 COVID-19			

Supplemental Results Figure S8. Leading 20 level 3 causes of deaths, and percentage change in number of deaths and all-age and age-standardised death rates by location, 2022 and 2050

Yemen, Reference scenario

Legend:
Communicable, maternal, neonatal and nutritional
Non-communicable
Injuries

Leading Causes 2022	Leading Causes 2050	Mean % change number of DEATH	Mean % change all-age DEATH rate	Mean % change age-standardized DEATH rate
1 Ischemic heart disease	1 Ischemic heart disease	**116 (22.5 to 257)**	36.4 (-20.2 to 115)	-33.7 (-67.8 to 22.4)
2 Stroke	2 Stroke	**101 (34.8 to 178)**	26.9 (-9.56 to 70.6)	**-38.4 (-62.6 to -4.95)**
3 Neonatal disorders	3 Hypertensive heart disease	**206 (105 to 331)**	**92.7 (32.6 to 160)**	-14.1 (-49.8 to 41.3)
4 Malaria	4 Malaria	104 (-42.3 to 156)	28.3 (-60.4 to 52.5)	-4.65 (-74.2 to 3.57)
5 Road injuries	5 Road injuries	30.2 (-29.3 to 125)	-17.9 (-52.7 to 38.6)	-36.1 (-64 to 9.63)
6 Congenital defects	6 Chronic kidney disease	**314 (172 to 490)**	**161 (81.5 to 260)**	30.4 (-21.1 to 109)
7 COVID-19	7 Lower respiratory infect	28.8 (-12.5 to 69)	-18.8 (-42.2 to 2.87)	**-34.8 (-52.8 to -9.35)**
8 Conflict & terror	8 Conflict & terror	inf (-100 to 185000)	inf (-100 to 102000)	inf (-100 to 149000)
9 Hypertensive heart disease	9 COPD	**149 (73.8 to 238)**	**56.6 (14.3 to 109)**	-26.7 (-54 to 15.7)
10 Lower respiratory infect	10 Alzheimer's disease	**325 (164 to 557)**	**168 (65.2 to 294)**	**8.38 (5.76 to 11.2)**
11 COPD	11 Neonatal disorders	**-61.1 (-80.6 to -32.6)**	**-75.6 (-87 to -57.8)**	**-54.7 (-73.5 to -27.2)**
12 Cirrhosis liver	12 Cirrhosis liver	**95.1 (52.9 to 142)**	**22.9 (2.06 to 47)**	**-36.7 (-49.1 to -20.2)**
13 Stomach cancer	13 Congenital defects	**-44.1 (-61.4 to -24.6)**	**-65 (-72.9 to -55.6)**	**-38.9 (-47.7 to -28.1)**
14 Chronic kidney disease	14 Stomach cancer	**114 (64.6 to 170)**	**34.6 (9.37 to 65.5)**	**-33.5 (-42.2 to -24.6)**
15 Diabetes	15 Diabetes	**176 (78.5 to 312)**	**74.1 (19.8 to 147)**	-11 (-45.7 to 43)
16 Maternal disorders	16 Lung cancer	**253 (108 to 457)**	**122 (35.5 to 251)**	7.56 (-23.6 to 50.4)
17 Alzheimer's disease	17 Colorectal cancer	**296 (121 to 530)**	**150 (45.1 to 310)**	23 (-20.8 to 76.9)
18 Asthma	18 Leukemia	**152 (83.4 to 253)**	**58.5 (15.6 to 120)**	5.87 (-16.1 to 38)
19 Diarrheal diseases	19 Other cardiovascular	**90.9 (45.8 to 141)**	20.3 (-0.913 to 48.4)	**-29 (-44.5 to -9.44)**
20 Pertussis	20 Brain cancer	**254 (192 to 329)**	**123 (92.3 to 154)**	**44.4 (37 to 53.6)**
21 Lung cancer	24 Asthma			
22 Other cardiovascular	34 Maternal disorders			
24 Leukemia	47 Pertussis			
29 Colorectal cancer	54 Diarrheal diseases			
33 Brain cancer	133 COVID-19			

Supplemental Results Figure S8. Leading 20 level 3 causes of deaths, and percentage change in number of deaths and all-age and age-standardised death rates by location, 2022 and 2050

Bangladesh, Reference scenario

Legend:
Communicable, maternal, neonatal and nutritional
Non-communicable
Injuries

Leading Causes 2022	Leading Causes 2050	Mean % change number of DEATH	Mean % change all-age DEATH rate	Mean % change age-standardized DEATH rate
1 Stroke	1 Stroke	**53.8 (10.3 to 101)**	**43.5 (5.97 to 82.5)**	**-43.5 (-61.8 to -17.3)**
2 Ischemic heart disease	2 Ischemic heart disease	35.7 (-16.5 to 112)	27 (-24.1 to 102)	**-45.5 (-70.9 to -4.77)**
3 COPD	3 COPD	**84.5 (31.2 to 155)**	**72.4 (22.9 to 138)**	**-33.2 (-56.5 to -0.0404)**
4 Neonatal disorders	4 Diabetes	**130 (61.4 to 214)**	**115 (51.1 to 188)**	-21.3 (-49.9 to 23.7)
5 Diabetes	5 Hypertensive heart disease	**176 (91.8 to 276)**	**158 (76.9 to 247)**	-8.42 (-44.2 to 42.2)
6 Lower respiratory infect	6 Alzheimer's disease	**260 (132 to 390)**	**235 (131 to 342)**	-0.32 (-5.46 to 6.51)
7 Cirrhosis liver	7 Diarrheal diseases	**85.5 (31.3 to 154)**	**73.3 (24.3 to 131)**	-33.2 (-57.4 to 0.801)
8 Diarrheal diseases	8 Chronic kidney disease	**244 (145 to 371)**	**221 (123 to 330)**	36.8 (-14.2 to 110)
9 Tuberculosis	9 Lower respiratory infect	23.2 (-7.27 to 60.5)	14.8 (-11.9 to 46.2)	**-47.3 (-59.1 to -31.9)**
10 Hypertensive heart disease	10 Cirrhosis liver	**37.8 (8.21 to 68.9)**	**28.6 (4.92 to 52.7)**	**-35.8 (-48.3 to -19.1)**
11 Rheumatic heart disease	11 Rheumatic heart disease	12.1 (-18.4 to 56)	4.76 (-25.4 to 39.6)	**-52.1 (-67.9 to -29.2)**
12 COVID-19	12 Atrial fibrillation	**373 (191 to 615)**	**340 (203 to 506)**	**25 (3.35 to 54.8)**
13 Alzheimer's disease	13 Urinary diseases	**201 (140 to 284)**	**181 (130 to 247)**	14.9 (-11 to 48.6)
14 Chronic kidney disease	14 Tuberculosis	**-32.8 (-58 to -0.717)**	**-37.2 (-62.1 to -4.56)**	**-69.6 (-83 to -49.1)**
15 Asthma	15 Lung cancer	**116 (37.5 to 220)**	**101 (29.2 to 190)**	-6.1 (-33.5 to 23.8)
16 Road injuries	16 Cardiomyopathy	**102 (54.4 to 160)**	**88.3 (50.4 to 135)**	**-19.9 (-26 to -12.7)**
17 Congenital defects	17 Parkinson's disease	**183 (101 to 297)**	**164 (106 to 253)**	-8.7 (-26.9 to 16.2)
18 Cardiomyopathy	18 Colorectal cancer	**158 (47 to 303)**	**139 (40.2 to 253)**	7.87 (-28.7 to 51.4)
19 Drowning	19 Lip oral cavity cancer	**73.8 (40.6 to 108)**	**62.1 (36.6 to 88.6)**	**-21.8 (-31.8 to -11.3)**
20 Lung cancer	20 Falls	**110 (45.2 to 204)**	**95.4 (39.4 to 169)**	**-19.1 (-30 to -6.01)**
24 Lip oral cavity cancer	21 Asthma			
25 Urinary diseases	25 Neonatal disorders			
29 Falls	26 Road injuries			
30 Parkinson's disease	52 Congenital defects			
31 Colorectal cancer	54 Drowning			
33 Atrial fibrillation	133 COVID-19			

Supplemental Results Figure S8. Leading 20 level 3 causes of deaths, and percentage change in number of deaths and all-age and age-standardised death rates by location, 2022 and 2050

Bhutan, Reference scenario

Legend:
Communicable, maternal, neonatal and nutritional
Non-communicable
Injuries

Leading Causes 2022	Leading Causes 2050	Mean % change number of DEATH	Mean % change all-age DEATH rate	Mean % change age-standardized DEATH rate
1 Ischemic heart disease	1 COPD	**97.2 (40.5 to 157)**	**73.5 (20.9 to 131)**	-26.6 (-53.3 to 16)
2 COVID-19	2 Ischemic heart disease	34.5 (-20.5 to 110)	18.5 (-31.7 to 94.3)	-46.8 (-72.7 to 1.14)
3 COPD	3 Stroke	38.7 (-1.39 to 86.4)	22.1 (-12.9 to 65.1)	**-46.8 (-66.7 to -18.6)**
4 Stroke	4 Chronic kidney disease	**190 (96.5 to 293)**	**155 (64.9 to 258)**	19.1 (-27.2 to 92.2)
5 Neonatal disorders	5 Diabetes	**85.4 (29.6 to 165)**	**63.2 (10.2 to 133)**	-27.4 (-54.5 to 17)
6 Diabetes	6 Alzheimer's disease	**215 (117 to 305)**	**177 (100 to 261)**	**7.55 (0.739 to 13.6)**
7 Cirrhosis liver	7 Falls	**131 (71.3 to 193)**	**103 (56.9 to 153)**	-8.71 (-19.1 to 4.25)
8 Diarrheal diseases	8 Cirrhosis liver	**43.7 (18.8 to 76.6)**	**26.4 (2.59 to 57.5)**	**-32.6 (-46.6 to -13.5)**
9 Lower respiratory infect	9 Hypertensive heart disease	**128 (60.5 to 199)**	**101 (36.6 to 166)**	-15.2 (-48.7 to 32.2)
10 Chronic kidney disease	10 Lower respiratory infect	**34.6 (0.841 to 72)**	18.4 (-10.3 to 54)	**-41.5 (-57.6 to -20.6)**
11 Falls	11 Diarrheal diseases	0.915 (-26.4 to 34.3)	-11.3 (-35.8 to 15.8)	**-58.3 (-73.1 to -36.2)**
12 Tuberculosis	12 Urinary diseases	**203 (145 to 275)**	**166 (116 to 235)**	22 (-9.75 to 63.3)
13 Hypertensive heart disease	13 Lung cancer	**187 (76.8 to 318)**	**152 (63.7 to 260)**	13 (-19.1 to 55.7)
14 Alzheimer's disease	14 Parkinson's disease	**184 (118 to 259)**	**149 (101 to 213)**	3.43 (-15.3 to 30.4)
15 Rheumatic heart disease	15 Colorectal cancer	**205 (63.7 to 367)**	**167 (50.9 to 319)**	20.6 (-24.6 to 72.4)
16 Road injuries	16 Atrial fibrillation	**266 (161 to 379)**	**221 (136 to 313)**	**27.2 (7.94 to 46.6)**
17 Asthma	17 Rheumatic heart disease	-5.54 (-36.4 to 32.6)	-16.9 (-45.4 to 19.3)	**-58 (-75 to -30.7)**
18 Urinary diseases	18 Cardiomyopathy	**109 (65.8 to 163)**	**83.3 (52.3 to 121)**	**-12.9 (-19.1 to -4.13)**
19 Mechanical forces	19 Breast cancer	**149 (51.1 to 257)**	**118 (40.1 to 214)**	11.2 (-22.2 to 53.3)
20 Self-harm	20 Liver cancer	**169 (119 to 227)**	**136 (88 to 183)**	10.3 (-10.7 to 37.7)
24 Cardiomyopathy	21 Tuberculosis			
26 Lung cancer	22 Asthma			
27 Parkinson's disease	24 Road injuries			
31 Breast cancer	27 Self-harm			
32 Colorectal cancer	36 Neonatal disorders			
33 Liver cancer	37 Mechanical forces			
36 Atrial fibrillation	133 COVID-19			

Supplemental Results Figure S8. Leading 20 level 3 causes of deaths, and percentage change in number of deaths and all-age and age-standardised death rates by location, 2022 and 2050

India, Reference scenario

Legend:
Communicable, maternal, neonatal and nutritional
Non-communicable
Injuries

Leading Causes 2022	Leading Causes 2050	Mean % change number of DEATH	Mean % change all-age DEATH rate	Mean % change age-standardized DEATH rate
1 Ischemic heart disease	1 COPD	**82.3 (35.4 to 134)**	**64.8 (23.5 to 117)**	-34.4 (-55.7 to 1.02)
2 COPD	2 Ischemic heart disease	14.9 (-31.6 to 73.1)	3.97 (-35.2 to 58.7)	**-53 (-74.3 to -16.4)**
3 Stroke	3 Stroke	25.7 (-1.31 to 59.4)	13.6 (-11.6 to 45.1)	**-49.7 (-64.5 to -31.6)**
4 Diarrheal diseases	4 Diarrheal diseases	**34 (2.57 to 68.3)**	21.1 (-8.35 to 57.4)	**-52.1 (-68.8 to -25.4)**
5 Lower respiratory infect	5 Lower respiratory infect	**33.5 (8.81 to 61.9)**	20.5 (-1.03 to 44.9)	**-43 (-53.3 to -28.3)**
6 Tuberculosis	6 Diabetes	**76.6 (30.7 to 124)**	**59.5 (18 to 109)**	-30 (-53.5 to 5.24)
7 Neonatal disorders	7 Falls	**138 (76.7 to 212)**	**114 (74.2 to 165)**	**-12.6 (-21.5 to -0.904)**
8 Diabetes	8 Alzheimer's disease	**257 (145 to 386)**	**222 (143 to 322)**	**-6.21 (-11.9 to -1.45)**
9 Cirrhosis liver	9 Cirrhosis liver	**61.5 (30.3 to 94.2)**	**45.9 (21 to 73.6)**	-15.3 (-30.5 to 5.51)
10 Falls	10 Chronic kidney disease	**143 (73.1 to 227)**	**119 (58.9 to 202)**	2.45 (-31.3 to 52.1)
11 Road injuries	11 Hypertensive heart disease	**144 (74.9 to 219)**	**121 (64.3 to 199)**	-12 (-42.8 to 35.4)
12 COVID-19	12 Tuberculosis	-29.6 (-57.6 to 5.44)	**-36.3 (-60.5 to -1.91)**	**-64.5 (-79.2 to -40.1)**
13 Asthma	13 Road injuries	13.6 (-27.9 to 65.1)	2.74 (-31.4 to 59)	-21.7 (-50.5 to 24.7)
14 Self-harm	14 Urinary diseases	**191 (143 to 247)**	**163 (128 to 209)**	17 (-4.27 to 44.9)
15 Chronic kidney disease	15 Self-harm	6.61 (-18.9 to 35.4)	-3.76 (-24.2 to 23)	-18 (-37 to 7.85)
16 Rheumatic heart disease	16 Asthma	-8.64 (-26.8 to 11.4)	**-17.5 (-31 to -2.35)**	**-63.6 (-71.4 to -51.9)**
17 Hypertensive heart disease	17 Breast cancer	**114 (31.6 to 219)**	**92.6 (26.4 to 168)**	5.35 (-27.9 to 41.6)
18 Alzheimer's disease	18 Rheumatic heart disease	-0.898 (-33.5 to 37.7)	-10.4 (-39 to 28.4)	**-54.5 (-71.9 to -29.1)**
19 Breast cancer	19 Lip oral cavity cancer	**121 (83.1 to 160)**	**99.3 (77.6 to 122)**	3.07 (-6.59 to 15.2)
20 Urinary diseases	20 Lung cancer	**101 (28.3 to 190)**	**81.4 (22.4 to 152)**	-7.56 (-33.6 to 20.8)
21 Lung cancer	23 Neonatal disorders			
23 Lip oral cavity cancer	133 COVID-19			

Supplemental Results Figure S8. Leading 20 level 3 causes of deaths, and percentage change in number of deaths and all-age and age-standardised death rates by location, 2022 and 2050

Nepal, Reference scenario

Legend:
Communicable, maternal, neonatal and nutritional
Non-communicable
Injuries

Leading Causes 2022	Leading Causes 2050	Mean % change number of DEATH	Mean % change all-age DEATH rate	Mean % change age-standardized DEATH rate
1 Ischemic heart disease	1 COPD	42.8 (-1.12 to 94.1)	37.2 (-3.31 to 81.5)	**-47.8 (-65.3 to -21.1)**
2 COPD	2 Ischemic heart disease	13.2 (-33.9 to 80.9)	8.9 (-34.3 to 71.6)	**-54.6 (-74.9 to -18.1)**
3 Stroke	3 Stroke	19.3 (-12.5 to 57.1)	14.7 (-16.6 to 50.2)	**-53.4 (-68.6 to -31.3)**
4 Neonatal disorders	4 Diabetes	**90.3 (32.1 to 167)**	**82.8 (29 to 146)**	-24.8 (-49.5 to 18.2)
5 Lower respiratory infect	5 Chronic kidney disease	**143 (65.9 to 245)**	**134 (58.7 to 220)**	2.62 (-31.1 to 62.9)
6 Tuberculosis	6 Hypertensive heart disease	**150 (75.8 to 237)**	**140 (67.4 to 218)**	-13.1 (-45.6 to 34.6)
7 Diabetes	7 Cirrhosis liver	**46.4 (11.4 to 84.6)**	**40.8 (9.51 to 73.4)**	**-25 (-41.4 to -2.02)**
8 Cirrhosis liver	8 Alzheimer's disease	**261 (147 to 395)**	**247 (134 to 361)**	-2.66 (-8.58 to 4.18)
9 Asthma	9 Lower respiratory infect	1.93 (-25.7 to 35.7)	-1.99 (-27.9 to 29.1)	**-51.4 (-62.4 to -35.6)**
10 Chronic kidney disease	10 Falls	**109 (45.2 to 190)**	**101 (44.6 to 182)**	**-18 (-27.7 to -3.5)**
11 Self-harm	11 Self-harm	20.2 (-5.88 to 57.7)	15.5 (-6.84 to 45.2)	-13.4 (-32.8 to 15.1)
12 Diarrheal diseases	12 Asthma	-13.6 (-32.3 to 9.99)	-16.9 (-33.9 to 2.98)	**-64.4 (-73.3 to -53.1)**
13 Hypertensive heart disease	13 Urinary diseases	**191 (131 to 277)**	**180 (129 to 242)**	15.9 (-12.7 to 54.9)
14 Rheumatic heart disease	14 Diarrheal diseases	3.38 (-28 to 36.6)	-0.566 (-31.5 to 33.8)	**-59.7 (-73.8 to -40.1)**
15 COVID-19	15 Tuberculosis	**-40.5 (-64.7 to -8.96)**	**-42.8 (-66.5 to -10.9)**	**-71.8 (-83.8 to -51.1)**
16 Falls	16 Rheumatic heart disease	-6.08 (-38.8 to 34)	-9.74 (-41.6 to 27.9)	**-57.1 (-73.4 to -30.7)**
17 Alzheimer's disease	17 Interstitial lung disease	**169 (116 to 225)**	**159 (117 to 204)**	7.67 (-5.36 to 20.2)
18 Upper digest diseases	18 Breast cancer	**146 (56.5 to 266)**	**136 (52.5 to 246)**	19.6 (-16.6 to 68.1)
19 Road injuries	19 Upper digest diseases	25.3 (-3.78 to 57.7)	20.4 (-3.08 to 47.3)	**-46.6 (-58.6 to -27.5)**
20 Drowning	20 Parkinson's disease	**201 (115 to 310)**	**189 (120 to 283)**	-1.38 (-19.6 to 21)
21 Urinary diseases	27 Road injuries			
27 Breast cancer	31 Neonatal disorders			
30 Interstitial lung disease	37 Drowning			
39 Parkinson's disease	133 COVID-19			

Supplemental Results Figure S8. Leading 20 level 3 causes of deaths, and percentage change in number of deaths and all-age and age-standardised death rates by location, 2022 and 2050

Pakistan, Reference scenario

Legend:
Communicable, maternal, neonatal and nutritional
Non-communicable
Injuries

Leading Causes 2022	Leading Causes 2050	Mean % change number of DEATH	Mean % change all-age DEATH rate	Mean % change age-standardized DEATH rate
1 Ischemic heart disease	1 Ischemic heart disease	56.1 (-10.2 to 140)	27.1 (-27.8 to 106)	**-44.9 (-71.9 to -2.93)**
2 Neonatal disorders	2 Stroke	**70.2 (25.4 to 124)**	**38.5 (3.48 to 92.4)**	**-43 (-61.9 to -15)**
3 Stroke	3 COPD	**96.5 (43.4 to 161)**	**59.9 (17.4 to 117)**	**-41 (-62.6 to -8.15)**
4 Lower respiratory infect	4 Chronic kidney disease	**258 (144 to 405)**	**191 (98.5 to 318)**	38.6 (-14 to 123)
5 COPD	5 Diabetes	**115 (50 to 196)**	**74.8 (21.3 to 140)**	-25.8 (-55.5 to 16.9)
6 Tuberculosis	6 Neonatal disorders	**-66.1 (-79.1 to -49.8)**	**-72.6 (-80.6 to -63)**	**-48.1 (-59.4 to -31.6)**
7 Diabetes	7 Hypertensive heart disease	**194 (110 to 292)**	**139 (75 to 224)**	-11.6 (-45.4 to 34.5)
8 Cirrhosis liver	8 Cirrhosis liver	**34.9 (12.3 to 62.3)**	9.7 (-10.1 to 33.8)	**-39.1 (-50.4 to -23.3)**
9 Diarrheal diseases	9 Breast cancer	**163 (58.1 to 271)**	**113 (30.5 to 219)**	10.4 (-26.3 to 53.9)
10 Road injuries	10 Alzheimer's disease	**296 (167 to 453)**	**222 (127 to 333)**	-2.33 (-11.2 to 5.55)
11 COVID-19	11 Diarrheal diseases	25.1 (-22.1 to 74.9)	1.74 (-33.9 to 42.9)	**-41 (-63.7 to -6.04)**
12 Chronic kidney disease	12 Lower respiratory infect	**-37 (-54.5 to -13.4)**	**-48.8 (-62.1 to -29.7)**	**-52.4 (-65.1 to -35.8)**
13 Rheumatic heart disease	13 Lip oral cavity cancer	**127 (93.1 to 165)**	**84.2 (58.9 to 110)**	-10.1 (-20.5 to 1.25)
14 Hypertensive heart disease	14 Tuberculosis	**-37.8 (-62.9 to -2.41)**	**-49.4 (-69.7 to -16.3)**	**-71.1 (-83.9 to -49.7)**
15 Breast cancer	15 Lung cancer	**128 (43.8 to 238)**	**84.7 (16.2 to 166)**	-18.5 (-43.7 to 12.8)
16 Congenital defects	16 Road injuries	-4.18 (-40.8 to 49.1)	-22 (-50.9 to 30.4)	-38.2 (-61.4 to 3.26)
17 Lip oral cavity cancer	17 Urinary diseases	**195 (138 to 273)**	**140 (93 to 191)**	14.4 (-13.8 to 57.7)
18 Lung cancer	18 Falls	**121 (58.7 to 205)**	**79.8 (30.1 to 151)**	-15.8 (-30.3 to 2.77)
19 Drowning	19 Colorectal cancer	**217 (84.6 to 401)**	**157 (52.1 to 300)**	13.5 (-24.8 to 66.6)
20 Self-harm	20 Rheumatic heart disease	-10.3 (-39.9 to 33.4)	-27 (-53 to 11.1)	**-59.3 (-75.3 to -33.2)**
22 Falls	29 Self-harm			
24 Alzheimer's disease	44 Congenital defects			
25 Urinary diseases	47 Drowning			
35 Colorectal cancer	132 COVID-19			

Supplemental Results Figure S8. Leading 20 level 3 causes of deaths, and percentage change in number of deaths and all-age and age-standardised death rates by location, 2022 and 2050

China, Reference scenario

Legend:
Communicable, maternal, neonatal and nutritional
Non-communicable
Injuries

Leading Causes 2022	Leading Causes 2050	Mean % change number of DEATH	Mean % change all-age DEATH rate	Mean % change age-standardized DEATH rate
1 Stroke	1 Stroke	**27.9 (2.71 to 57.8)**	**47 (20.1 to 84.5)**	**-48 (-58.5 to -32.3)**
2 Ischemic heart disease	2 COPD	**124 (74 to 179)**	**157 (103 to 219)**	-22.2 (-41.5 to 4.72)
3 COPD	3 Ischemic heart disease	17.3 (-16.1 to 57.2)	34.8 (-3.19 to 85.9)	**-56.4 (-70.9 to -36.6)**
4 Lung cancer	4 Alzheimer's disease	**232 (161 to 320)**	**280 (213 to 362)**	-0.968 (-4.91 to 3.13)
5 Alzheimer's disease	5 Lung cancer	**79.6 (27.4 to 145)**	**106 (50.9 to 173)**	-7.48 (-26.4 to 15.8)
6 Stomach cancer	6 Hypertensive heart disease	**194 (115 to 297)**	**238 (150 to 371)**	6.19 (-26.6 to 54.7)
7 Hypertensive heart disease	7 Colorectal cancer	**130 (51.8 to 223)**	**163 (84.6 to 264)**	16.1 (-13.4 to 49.5)
8 Esophageal cancer	8 Esophageal cancer	**79.8 (51.8 to 110)**	**107 (80.6 to 136)**	-0.499 (-10.9 to 10.6)
9 Colorectal cancer	9 Stomach cancer	18.6 (-1.36 to 41.6)	**36.1 (17.2 to 59)**	**-37.4 (-42.5 to -32.5)**
10 Road injuries	10 Chronic kidney disease	**142 (83 to 203)**	**178 (115 to 260)**	6.86 (-21.8 to 45.2)
11 Chronic kidney disease	11 Lower respiratory infect	**123 (85 to 165)**	**156 (114 to 196)**	**-29.9 (-39.5 to -17.5)**
12 Lower respiratory infect	12 Falls	**118 (64.9 to 176)**	**150 (99.4 to 209)**	**-13.4 (-21.4 to -4.6)**
13 Diabetes	13 Pancreatic cancer	**151 (72.9 to 235)**	**187 (108 to 286)**	**38.5 (4.52 to 76.1)**
14 Liver cancer	14 Liver cancer	**71.8 (26.7 to 127)**	**97.5 (47 to 161)**	10.8 (-20.4 to 56.1)
15 Cirrhosis liver	15 Atrial fibrillation	**327 (231 to 449)**	**390 (289 to 519)**	**20.3 (7.28 to 33.9)**
16 Falls	16 Diabetes	**57.5 (22.2 to 102)**	**81.1 (42.5 to 144)**	**-28.7 (-46.8 to -1.77)**
17 Self-harm	17 Parkinson's disease	**171 (124 to 226)**	**211 (168 to 261)**	-2.04 (-10.2 to 7.72)
18 Pancreatic cancer	18 Cirrhosis liver	23.2 (-2.78 to 55.9)	**41.6 (13.9 to 80.2)**	-20.6 (-37.3 to 2.17)
19 COVID-19	19 Road injuries	-19.5 (-44.2 to 14.6)	-7.31 (-36.6 to 34.1)	-21.8 (-50.1 to 23.3)
20 Parkinson's disease	20 Self-harm	**27.8 (3.24 to 53.4)**	**46.9 (23.4 to 74.7)**	-14.8 (-29.8 to 4.32)
24 Atrial fibrillation	132 COVID-19			

Supplemental Results Figure S8. Leading 20 level 3 causes of deaths, and percentage change in number of deaths and all-age and age-standardised death rates by location, 2022 and 2050

North Korea, Reference scenario

Legend:
Communicable, maternal, neonatal and nutritional
Non-communicable
Injuries

Leading Causes 2022	Leading Causes 2050	Mean % change number of DEATH	Mean % change all-age DEATH rate	Mean % change age-standardized DEATH rate
1 Stroke	1 Stroke	16.9 (-8.56 to 50.1)	**27.4 (1.53 to 59.7)**	**-38 (-52.9 to -15.3)**
2 Ischemic heart disease	2 COPD	**106 (55.1 to 168)**	**124 (75.5 to 183)**	-5.34 (-33.4 to 38.4)
3 COPD	3 Ischemic heart disease	38.7 (-4.22 to 97.3)	**51.3 (5.89 to 112)**	-28.5 (-54 to 14.1)
4 Tuberculosis	4 Hypertensive heart disease	**118 (49.7 to 210)**	**137 (62.2 to 226)**	2.44 (-34.6 to 57.5)
5 Lung cancer	5 Alzheimer's disease	**148 (69.5 to 231)**	**170 (90.2 to 246)**	-3.79 (-8.53 to 1.85)
6 Road injuries	6 Lung cancer	**56.2 (6.48 to 117)**	**69.8 (23.5 to 121)**	-4.25 (-24.4 to 16.8)
7 Hypertensive heart disease	7 Lower respiratory infect	**65.6 (29.6 to 109)**	**80.3 (49.5 to 116)**	**-26.4 (-40.2 to -9.38)**
8 Stomach cancer	8 Chronic kidney disease	**106 (53 to 170)**	**125 (73.3 to 190)**	18.9 (-11.9 to 65.1)
9 Lower respiratory infect	9 Tuberculosis	**-28.7 (-50.7 to -1.93)**	-22.2 (-44.7 to 8.67)	**-53.3 (-68 to -31.4)**
10 Alzheimer's disease	10 Stomach cancer	7.92 (-11.1 to 31.6)	**17.5 (2.8 to 33.8)**	**-32 (-37.6 to -25.7)**
11 Cirrhosis liver	11 Road injuries	-14.5 (-46 to 37.1)	-6.63 (-37.7 to 50.3)	-13.6 (-45.4 to 42.4)
12 Diabetes	12 Diabetes	34.5 (-5.49 to 81.1)	**46.7 (6.28 to 93.1)**	-24.2 (-46.2 to 8.62)
13 Chronic kidney disease	13 Colorectal cancer	**65.7 (6.46 to 137)**	**80.1 (21.5 to 159)**	0.115 (-24.5 to 32.2)
14 Colorectal cancer	14 Esophageal cancer	**68.4 (35.8 to 103)**	**83.3 (61.6 to 108)**	7.23 (-6.13 to 20.3)
15 Esophageal cancer	15 Cirrhosis liver	8.28 (-16.2 to 42)	18.1 (-7.06 to 51.9)	**-23.5 (-40.6 to -2.12)**
16 Self-harm	16 Liver cancer	**67.8 (17.4 to 143)**	**83.1 (30 to 163)**	16.2 (-19.4 to 75.8)
17 Liver cancer	17 Atrial fibrillation	**190 (91.1 to 303)**	**215 (114 to 325)**	11.7 (-3.16 to 32.8)
18 Falls	18 Parkinson's disease	**137 (84.9 to 193)**	**158 (118 to 205)**	8.97 (-3.16 to 20.1)
19 Breast cancer	19 Falls	**64 (19.2 to 121)**	**78.4 (37.1 to 133)**	**-9.77 (-18.6 to -0.535)**
20 Upper digest diseases	20 Self-harm	6.69 (-16.1 to 35.1)	16.3 (-5.52 to 43.9)	-12.1 (-29.2 to 10.3)
23 Parkinson's disease	22 Breast cancer			
27 Atrial fibrillation	23 Upper digest diseases			

Supplemental Results Figure S8. Leading 20 level 3 causes of deaths, and percentage change in number of deaths and all-age and age-standardised death rates by location, 2022 and 2050

Taiwan (Province of China), Reference scenario

Legend:
Communicable, maternal, neonatal and nutritional
Non-communicable
Injuries

Leading Causes 2022	Leading Causes 2050	Mean % change number of DEATH	Mean % change all-age DEATH rate	Mean % change age-standardized DEATH rate
1 Lower respiratory infect	1 Lower respiratory infect	**119 (86.9 to 154)**	**152 (122 to 185)**	**-18.1 (-27.2 to -6.38)**
2 Ischemic heart disease	2 Chronic kidney disease	**239 (181 to 308)**	**291 (228 to 366)**	**34.2 (9 to 64)**
3 Stroke	3 Alzheimer's disease	**201 (159 to 248)**	**246 (209 to 281)**	2.23 (-1.35 to 6.16)
4 Lung cancer	4 Lung cancer	**88.9 (51.2 to 122)**	**117 (79.1 to 151)**	-0.962 (-15.4 to 11.2)
5 Diabetes	5 Colorectal cancer	**111 (62.7 to 164)**	**142 (92.4 to 185)**	3.86 (-15.4 to 21.2)
6 Alzheimer's disease	6 COPD	**132 (92.9 to 184)**	**168 (123 to 222)**	-14.9 (-30.6 to 2.87)
7 Colorectal cancer	7 Stroke	13 (-2.09 to 33.1)	**30 (14.5 to 50)**	**-47.6 (-54.6 to -39.2)**
8 Chronic kidney disease	8 Diabetes	**44.9 (17.9 to 75.5)**	**66.8 (37 to 97.8)**	**-36.1 (-46.6 to -23.3)**
9 COPD	9 Urinary diseases	**291 (223 to 374)**	**350 (282 to 432)**	**41.7 (21.8 to 67.1)**
10 Cirrhosis liver	10 Ischemic heart disease	-1.61 (-22.3 to 25.6)	13.3 (-10.9 to 45.2)	**-57 (-66.7 to -42.3)**
11 COVID-19	11 Hypertensive heart disease	**176 (117 to 256)**	**218 (147 to 314)**	13.7 (-11.6 to 52.1)
12 Hypertensive heart disease	12 Pancreatic cancer	**136 (84.7 to 194)**	**171 (121 to 220)**	**35.1 (12 to 56.7)**
13 Liver cancer	13 Liver cancer	**75.8 (49.1 to 108)**	**102 (74.6 to 140)**	13.8 (-3.29 to 36.3)
14 Urinary diseases	14 Atrial fibrillation	**252 (200 to 324)**	**305 (247 to 368)**	**18.3 (6.79 to 31)**
15 Stomach cancer	15 Cirrhosis liver	9.35 (-4.81 to 26.1)	**25.8 (10.9 to 40.2)**	**-23.1 (-30.9 to -12.3)**
16 Self-harm	16 Parkinson's disease	**140 (106 to 176)**	**176 (149 to 206)**	-2.82 (-11.8 to 7.65)
17 Road injuries	17 Stomach cancer	**33.6 (15 to 55.6)**	**53.7 (35 to 74.8)**	**-35.3 (-41.5 to -28.6)**
18 Pancreatic cancer	18 Prostate cancer	**131 (86.6 to 179)**	**166 (125 to 207)**	0.199 (-11.3 to 10.5)
19 Esophageal cancer	19 Falls	**84.7 (52.9 to 114)**	**112 (83.6 to 140)**	**-13.2 (-20.7 to -5.42)**
20 Breast cancer	20 Esophageal cancer	**22 (7.64 to 38.5)**	**40.3 (25.7 to 55.8)**	-4.9 (-14.5 to 6.95)
21 Falls	21 Breast cancer			
23 Parkinson's disease	22 Self-harm			
24 Prostate cancer	23 Road injuries			
25 Atrial fibrillation	131 COVID-19			

Supplemental Results Figure S8. Leading 20 level 3 causes of deaths, and percentage change in number of deaths and all-age and age-standardised death rates by location, 2022 and 2050

American Samoa, Reference scenario

Legend:
Communicable, maternal, neonatal and nutritional
Non-communicable
Injuries

Leading Causes 2022	Leading Causes 2050	Mean % change number of DEATH	Mean % change all-age DEATH rate	Mean % change age-standardized DEATH rate
1 Ischemic heart disease	1 Ischemic heart disease	1.63 (-28.7 to 41.3)	-18.1 (-42.8 to 15.6)	**-45.4 (-63.3 to -18.7)**
2 Diabetes	2 Chronic kidney disease	**130 (77.6 to 194)**	**85.1 (42.5 to 140)**	24.4 (-8.37 to 68.3)
3 Stroke	3 Diabetes	27.6 (-2.05 to 59.1)	2.72 (-20.5 to 29.5)	**-30.4 (-47.7 to -6.92)**
4 Chronic kidney disease	4 Stroke	24.1 (-0.0677 to 49.1)	-0.118 (-19 to 22.5)	**-37.8 (-51.5 to -21.1)**
5 COPD	5 COPD	**122 (79.3 to 172)**	**78.8 (38.8 to 123)**	-0.268 (-24.8 to 34.5)
6 Lung cancer	6 Lung cancer	**112 (55.3 to 180)**	**69.9 (26.8 to 116)**	15.8 (-7.06 to 39.8)
7 Lower respiratory infect	7 Prostate cancer	**152 (85.4 to 225)**	**102 (48.1 to 161)**	6.09 (-10.2 to 24.4)
8 Breast cancer	8 Lower respiratory infect	**74.3 (47.4 to 102)**	**40.2 (19.8 to 61.4)**	**-14.1 (-24.6 to -0.965)**
9 Prostate cancer	9 Alzheimer's disease	**188 (113 to 263)**	**131 (76 to 177)**	**5.6 (1.67 to 9.89)**
10 Endo/metab/blood/immune	10 Breast cancer	**81.4 (28.9 to 145)**	**45.7 (4.43 to 90.8)**	8.14 (-16.4 to 35.1)
11 Stomach cancer	11 Endo/metab/blood/immune	**124 (92.8 to 156)**	**80 (59.9 to 105)**	**29.5 (15.8 to 47.2)**
12 Colorectal cancer	12 Colorectal cancer	**99.4 (40 to 169)**	**60.1 (14.1 to 113)**	6.75 (-17.6 to 32)
13 Alzheimer's disease	13 Diarrheal diseases	**136 (73 to 210)**	**90.1 (36.8 to 155)**	2.56 (-30.2 to 54.9)
14 Cirrhosis liver	14 Hypertensive heart disease	**106 (51.8 to 179)**	**66 (19.1 to 130)**	8.97 (-25.6 to 56.3)
15 Hypertensive heart disease	15 Stomach cancer	**28 (7.02 to 50.7)**	2.96 (-11.9 to 19.9)	**-30.6 (-35.2 to -25.1)**
16 Diarrheal diseases	16 Urinary diseases	**198 (142 to 263)**	**140 (93.8 to 184)**	**40.3 (13.8 to 72.8)**
17 Road injuries	17 Bacterial skin	**183 (91.9 to 304)**	**128 (52.3 to 237)**	**67 (11.9 to 147)**
18 Self-harm	18 Cirrhosis liver	10.9 (-5.92 to 30.6)	-10.7 (-24.6 to 6.64)	**-24.3 (-35.9 to -10.4)**
19 Drowning	19 Pancreatic cancer	**167 (92.1 to 265)**	**115 (55.4 to 179)**	**47.6 (14.1 to 84.9)**
20 Asthma	20 Liver cancer	**92.2 (47.9 to 156)**	**54.8 (17.9 to 108)**	19.6 (-10.9 to 63.4)
22 Liver cancer	23 Road injuries			
24 Urinary diseases	25 Self-harm			
26 Bacterial skin	30 Asthma			
29 Pancreatic cancer	31 Drowning			

Supplemental Results Figure S8. Leading 20 level 3 causes of deaths, and percentage change in number of deaths and all-age and age-standardised death rates by location, 2022 and 2050

Cook Islands, Reference scenario

Legend:
Communicable, maternal, neonatal and nutritional
Non-communicable
Injuries

Leading Causes 2022	Leading Causes 2050	Mean % change number of DEATH	Mean % change all-age DEATH rate	Mean % change age-standardized DEATH rate
1 Diabetes	1 Diabetes	17.3 (-7.53 to 47.6)	18.1 (-7.16 to 46.2)	**-31.2 (-46 to -7.44)**
2 Ischemic heart disease	2 Hypertensive heart disease	**90.6 (35.8 to 166)**	**91.9 (36.2 to 164)**	14.1 (-22.3 to 65.5)
3 Stroke	3 Ischemic heart disease	-17.3 (-41.9 to 13.5)	-16.8 (-38 to 13.5)	**-52.4 (-68 to -29.3)**
4 Hypertensive heart disease	4 Stroke	7.13 (-13 to 32.2)	7.77 (-10.1 to 30.3)	**-38.2 (-50.5 to -20.7)**
5 Lower respiratory infect	5 Lower respiratory infect	**47 (24 to 78.8)**	**47.7 (27.1 to 68.8)**	**-25.7 (-35.6 to -12.7)**
6 Lung cancer	6 Alzheimer's disease	**148 (91.5 to 224)**	**149 (95.9 to 215)**	3.06 (-1.22 to 7.08)
7 Prostate cancer	7 COPD	**96.6 (51.6 to 152)**	**97.8 (56.4 to 152)**	-4.26 (-31.3 to 37.8)
8 COPD	8 Prostate cancer	**66.3 (21.9 to 122)**	**67 (22.7 to 112)**	-15.1 (-31.4 to 1.06)
9 Chronic kidney disease	9 Chronic kidney disease	**102 (54.1 to 162)**	**104 (60.4 to 166)**	15.8 (-13.7 to 61.3)
10 Alzheimer's disease	10 Lung cancer	**36.5 (0.566 to 82.6)**	**37 (0.949 to 74.8)**	-10.7 (-28.3 to 10.1)
11 Breast cancer	11 Breast cancer	**81.8 (27.5 to 148)**	**82.5 (30.3 to 146)**	12.4 (-12.1 to 38.5)
12 Road injuries	12 Liver cancer	**66 (24.4 to 128)**	**67.1 (28.2 to 129)**	27.2 (-5.68 to 77)
13 Liver cancer	13 Atrial fibrillation	**183 (115 to 282)**	**185 (120 to 254)**	**20.9 (10.2 to 33.1)**
14 Self-harm	14 Road injuries	1.38 (-32.8 to 50.1)	2.14 (-33.3 to 55.1)	-5.63 (-39.5 to 45.9)
15 Stomach cancer	15 Colorectal cancer	**83.3 (25.9 to 162)**	**83.9 (32 to 153)**	7.63 (-17.8 to 36.8)
16 Colorectal cancer	16 Endo/metab/blood/immune	**93.9 (60.2 to 134)**	**95 (63.9 to 128)**	**17.7 (3.81 to 33)**
17 Atrial fibrillation	17 Self-harm	9.48 (-11 to 34.6)	10.1 (-6.38 to 30.5)	-1.02 (-18.7 to 24)
18 Endo/metab/blood/immune	18 Pancreatic cancer	**86.2 (35.6 to 148)**	**87 (36.5 to 144)**	26.2 (-3.94 to 60.9)
19 Asthma	19 Parkinson's disease	**82.1 (51.2 to 118)**	**83 (57.4 to 109)**	-7.1 (-16.4 to 1.63)
20 Parkinson's disease	20 Falls	**72 (34.6 to 121)**	**72.9 (37.6 to 111)**	-10 (-19.4 to 0.66)
21 Pancreatic cancer	21 Stomach cancer			
23 Falls	23 Asthma			

Supplemental Results Figure S8. Leading 20 level 3 causes of deaths, and percentage change in number of deaths and all-age and age-standardised death rates by location, 2022 and 2050

Fiji, Reference scenario

Legend:
Communicable, maternal, neonatal and nutritional
Non-communicable
Injuries

Leading Causes 2022	Leading Causes 2050	Mean % change number of DEATH	Mean % change all-age DEATH rate	Mean % change age-standardized DEATH rate
1 Diabetes	1 Diabetes	28.5 (-6.64 to 74.7)	32.1 (-0.613 to 76.3)	-24.6 (-45.3 to 5.24)
2 Ischemic heart disease	2 Ischemic heart disease	-0.785 (-31.6 to 46.8)	1.98 (-27.6 to 45.6)	**-43 (-61.9 to -13.1)**
3 Stroke	3 Stroke	23.2 (-4.45 to 64.3)	26.6 (-0.619 to 63.1)	**-35.8 (-51.4 to -13.1)**
4 COVID-19	4 Chronic kidney disease	**126 (71.5 to 207)**	**132 (75 to 207)**	32 (-4.69 to 81.3)
5 Chronic kidney disease	5 COPD	**106 (53.5 to 187)**	**111 (60.5 to 183)**	-2.8 (-29.3 to 33.4)
6 Lower respiratory infect	6 Hypertensive heart disease	**104 (45.6 to 192)**	**110 (49.2 to 194)**	7.58 (-27.8 to 55.3)
7 Asthma	7 Lower respiratory infect	**38.3 (9.16 to 77)**	**42.1 (14.7 to 72.7)**	-18 (-31.5 to 1.22)
8 COPD	8 Breast cancer	**61.5 (9.61 to 127)**	**65.6 (15.5 to 120)**	2.71 (-20.6 to 29.5)
9 Breast cancer	9 Alzheimer's disease	**203 (90.1 to 336)**	**211 (105 to 318)**	3.68 (-0.572 to 7.93)
10 Hypertensive heart disease	10 Asthma	-14.7 (-33.4 to 13)	-12.4 (-31.5 to 11.1)	**-55.5 (-63.4 to -44.6)**
11 Diarrheal diseases	11 Diarrheal diseases	43.4 (-0.37 to 107)	**47.3 (1.44 to 107)**	-29.7 (-55.9 to 9.3)
12 Neonatal disorders	12 Lung cancer	**109 (39.1 to 197)**	**114 (46.4 to 186)**	20.8 (-6.37 to 49.1)
13 Cervical cancer	13 Colorectal cancer	**95.2 (21.9 to 187)**	**100 (31.1 to 179)**	10.7 (-16.6 to 42.1)
14 Road injuries	14 Prostate cancer	**157 (57.4 to 288)**	**164 (59.6 to 262)**	17.8 (-6.14 to 42.5)
15 Self-harm	15 Cervical cancer	19.7 (-6.16 to 52.9)	23 (-0.811 to 55)	-18.3 (-35.2 to 2.89)
16 Congenital defects	16 Atrial fibrillation	**205 (105 to 331)**	**213 (120 to 319)**	**18.7 (7.67 to 31.8)**
17 Alzheimer's disease	17 Self-harm	-7.87 (-30.7 to 23.8)	-5.4 (-26.1 to 16.2)	-13.9 (-33.3 to 8.17)
18 Lung cancer	18 Neonatal disorders	**-40.4 (-62.1 to -8.68)**	**-38.9 (-61.2 to -7.59)**	-22.3 (-48.7 to 14.1)
19 Cirrhosis liver	19 Road injuries	-10.3 (-44.8 to 54)	-7.8 (-44.1 to 51.9)	-15.5 (-49.6 to 41.2)
20 Colorectal cancer	20 Liver cancer	**88.8 (31.2 to 165)**	**94.1 (39 to 170)**	23.8 (-13.8 to 80.4)
24 Prostate cancer	21 Cirrhosis liver			
27 Liver cancer	29 Congenital defects			
35 Atrial fibrillation	131 COVID-19			

Supplemental Results Figure S8. Leading 20 level 3 causes of deaths, and percentage change in number of deaths and all-age and age-standardised death rates by location, 2022 and 2050

Guam, Reference scenario

Legend: Communicable, maternal, neonatal and nutritional; Non-communicable; Injuries

Leading Causes 2022	Leading Causes 2050	Mean % change number of DEATH	Mean % change all-age DEATH rate	Mean % change age-standardized DEATH rate
1 Ischemic heart disease	1 Ischemic heart disease	11.1 (-22 to 53.3)	15.4 (-18.9 to 64.5)	**-41.8 (-58.7 to -18.4)**
2 COVID-19	2 Alzheimer's disease	**287 (231 to 341)**	**301 (243 to 359)**	**-9.51 (-12.6 to -6.25)**
3 Stroke	3 Chronic kidney disease	**136 (79 to 201)**	**145 (84.2 to 221)**	28.1 (-1.45 to 65.1)
4 Lung cancer	4 Stroke	16.3 (-6.53 to 40.2)	20.6 (-4.99 to 52.8)	**-33.7 (-44.3 to -20)**
5 Chronic kidney disease	5 Lung cancer	**42.9 (7.74 to 78.4)**	**47.8 (15.5 to 82.9)**	-7.86 (-23.5 to 8.35)
6 Alzheimer's disease	6 COPD	**141 (83.6 to 200)**	**150 (90.7 to 223)**	-4.43 (-23.4 to 22.4)
7 Diabetes	7 Lower respiratory infect	**72.9 (41.8 to 107)**	**79.2 (46.6 to 111)**	**-21 (-30 to -11.2)**
8 Lower respiratory infect	8 Hypertensive heart disease	**96.2 (41.4 to 158)**	**104 (50.4 to 184)**	20.1 (-11.3 to 60.1)
9 COPD	9 Diabetes	8.85 (-15.9 to 37.1)	13 (-12.3 to 45.5)	**-37.9 (-49.7 to -21)**
10 Cirrhosis liver	10 Colorectal cancer	**56.4 (10.5 to 101)**	**61.7 (19.8 to 110)**	-10.6 (-31.4 to 11.1)
11 Self-harm	11 Prostate cancer	**141 (84.2 to 210)**	**149 (91.9 to 212)**	-3.29 (-16.5 to 10.7)
12 Hypertensive heart disease	12 Cirrhosis liver	-7.81 (-23.6 to 8.19)	-4.49 (-19.3 to 15.7)	**-21.9 (-31 to -8.5)**
13 Colorectal cancer	13 Diarrheal diseases	**283 (149 to 508)**	**298 (147 to 545)**	25.1 (-13.8 to 81.6)
14 Road injuries	14 Self-harm	-14 (-32.6 to 4.94)	-11.1 (-25.4 to 4.25)	-8.34 (-24.5 to 9.41)
15 Neonatal disorders	15 Liver cancer	**57.6 (19.5 to 94.9)**	**63.6 (23.9 to 106)**	18.1 (-11 to 53.9)
16 Breast cancer	16 Atrial fibrillation	**244 (182 to 304)**	**256 (194 to 319)**	**9.31 (1.95 to 17.1)**
17 Prostate cancer	17 Bacterial skin	**197 (108 to 324)**	**209 (109 to 346)**	**79.8 (31.2 to 147)**
18 Liver cancer	18 Breast cancer	7.16 (-19.7 to 33.5)	10.7 (-13.4 to 37.6)	-13.5 (-32 to 5.08)
19 Stomach cancer	19 Urinary diseases	**217 (136 to 319)**	**229 (151 to 331)**	**25.1 (5.94 to 48.2)**
20 Tuberculosis	20 Pancreatic cancer	**75.2 (33 to 119)**	**81.2 (40 to 125)**	22.1 (-1.78 to 50.2)
21 Pancreatic cancer	21 Road injuries			
22 Diarrheal diseases	22 Neonatal disorders			
25 Bacterial skin	23 Stomach cancer			
28 Atrial fibrillation	33 Tuberculosis			
33 Urinary diseases	130 COVID-19			

Supplemental Results Figure S8. Leading 20 level 3 causes of deaths, and percentage change in number of deaths and all-age and age-standardised death rates by location, 2022 and 2050

Kiribati, Reference scenario

Legend:
Communicable, maternal, neonatal and nutritional
Non-communicable
Injuries

Leading Causes 2022

1 Ischemic heart disease
2 Diabetes
3 Stroke
4 Tuberculosis
5 Neonatal disorders
6 Diarrheal diseases
7 COPD
8 Chronic kidney disease
9 Lower respiratory infect
10 Cirrhosis liver
11 Self-harm
12 Cervical cancer
13 Asthma
14 Congenital defects
15 Stomach cancer
16 Road injuries
17 Breast cancer
18 Protein-energy malnutrition
19 Lung cancer
20 Upper digest diseases
23 Hypertensive heart disease
25 Alzheimer's disease
28 Bacterial skin

Leading Causes 2050

Leading Causes 2050	Mean % change number of DEATH	Mean % change all-age DEATH rate	Mean % change age-standardized DEATH rate
1 Diabetes	**53.2 (15.9 to 95.8)**	**32.2 (2.79 to 69.6)**	-19.9 (-39.6 to 9.42)
2 Ischemic heart disease	35.3 (-3.89 to 85.8)	16.7 (-16.5 to 59.7)	-29 (-53 to 4.38)
3 Stroke	**29.4 (2.78 to 55.2)**	11.6 (-6.7 to 35.1)	**-34.2 (-47.9 to -14.9)**
4 COPD	**98.9 (55.1 to 153)**	**71.6 (35.9 to 113)**	-10.1 (-32.5 to 26)
5 Chronic kidney disease	**171 (108 to 249)**	**133 (88.4 to 203)**	**47.3 (10.7 to 99.6)**
6 Diarrheal diseases	**78.9 (27.3 to 138)**	**54.3 (14.1 to 104)**	-4.94 (-39.6 to 43.6)
7 Tuberculosis	-29.8 (-51.3 to 3.17)	**-39.5 (-57.1 to -11.9)**	**-59 (-72.4 to -37.7)**
8 Lower respiratory infect	15.3 (-6.42 to 38.2)	-0.681 (-15 to 16.5)	**-27.5 (-38.2 to -14.3)**
9 Cervical cancer	**39.6 (13.1 to 67.5)**	**20.4 (0.342 to 42.9)**	**-22.2 (-34.7 to -8.83)**
10 Breast cancer	**81.5 (28.2 to 150)**	**56.4 (12 to 110)**	-1.28 (-22.4 to 24.9)
11 Cirrhosis liver	7.67 (-11 to 31.3)	-7.11 (-22.9 to 12.4)	**-32.7 (-43.2 to -18.7)**
12 Lung cancer	**101 (39 to 174)**	**73.1 (24.7 to 133)**	2.28 (-18.1 to 24.6)
13 Self-harm	7.85 (-16.2 to 39.6)	-7.15 (-25.2 to 12)	-12.1 (-28.9 to 6.15)
14 Alzheimer's disease	**192 (100 to 292)**	**151 (75.9 to 237)**	**5.92 (0.763 to 10.2)**
15 Neonatal disorders	**-52.7 (-70.4 to -26.4)**	**-59.4 (-73.2 to -38.4)**	**-40.5 (-59.7 to -13.7)**
16 Stomach cancer	**28.9 (5.34 to 55.9)**	11.1 (-7.71 to 28.3)	**-30.7 (-35.9 to -25.1)**
17 Hypertensive heart disease	**116 (57.2 to 190)**	**86.7 (32.1 to 150)**	3.49 (-30.4 to 47.8)
18 Bacterial skin	**207 (109 to 333)**	**165 (82.4 to 280)**	**83.2 (21.3 to 182)**
19 Road injuries	17.8 (-19.6 to 84.3)	1.49 (-31.6 to 50.4)	-9.94 (-40 to 36.8)
20 Protein-energy malnutrition	15.2 (-31.3 to 81.6)	-0.719 (-38.5 to 55.9)	-19.9 (-56.1 to 41.1)
22 Asthma			
24 Upper digest diseases			
27 Congenital defects			

Supplemental Results Figure S8. Leading 20 level 3 causes of deaths, and percentage change in number of deaths and all-age and age-standardised death rates by location, 2022 and 2050

Marshall Islands, Reference scenario

Legend:
Communicable, maternal, neonatal and nutritional
Non-communicable
Injuries

Leading Causes 2022	Leading Causes 2050	Mean % change number of DEATH	Mean % change all-age DEATH rate	Mean % change age-standardized DEATH rate
1 Ischemic heart disease	1 Ischemic heart disease	**60.6 (5.59 to 139)**	25.4 (-14.9 to 81.4)	-27.2 (-56.5 to 16.5)
2 Stroke	2 Stroke	**66.7 (25.5 to 120)**	**30 (3.6 to 62.8)**	**-28 (-48.1 to -1.32)**
3 Diabetes	3 Diabetes	**58.1 (12.1 to 114)**	23.3 (-7.9 to 63.9)	-25 (-48.5 to 9.18)
4 Tuberculosis	4 Chronic kidney disease	**189 (109 to 300)**	**125 (69.5 to 202)**	40.6 (-3.51 to 104)
5 Lower respiratory infect	5 COPD	**144 (85 to 206)**	**90.4 (48.6 to 138)**	-7.36 (-34.2 to 25.8)
6 Chronic kidney disease	6 Lower respiratory infect	**58.2 (23.5 to 95.4)**	**23.2 (3.18 to 46.6)**	**-19.1 (-34.2 to -1.71)**
7 COPD	7 Tuberculosis	-25 (-50 to 9.2)	**-41.5 (-59.4 to -16.5)**	**-60.9 (-74.3 to -41)**
8 Self-harm	8 Lung cancer	**144 (62.7 to 252)**	**89.5 (31.7 to 166)**	8.34 (-15 to 34.3)
9 Cirrhosis liver	9 Hypertensive heart disease	**138 (64.2 to 230)**	**85.5 (34.2 to 155)**	1.52 (-35.3 to 53)
10 Road injuries	10 HIV/AIDS	**604 (257 to 1020)**	**448 (184 to 778)**	**441 (182 to 765)**
11 Lung cancer	11 Self-harm	16.9 (-13.3 to 53.1)	-8.78 (-31.9 to 20.6)	-12.8 (-34.9 to 15.3)
12 Hypertensive heart disease	12 Cirrhosis liver	26.4 (-1.09 to 57.1)	-1.4 (-18.3 to 22.1)	**-27.6 (-41.1 to -9.76)**
13 Neonatal disorders	13 Breast cancer	**115 (41.5 to 226)**	**67 (13 to 134)**	12.3 (-18.2 to 48.6)
14 Drowning	14 Road injuries	27.3 (-24.2 to 119)	-0.528 (-40.3 to 70.2)	-17.7 (-53.1 to 46.9)
15 Breast cancer	15 Alzheimer's disease	**321 (165 to 524)**	**227 (119 to 356)**	**10.7 (6.2 to 14.5)**
16 Stomach cancer	16 Colorectal cancer	**145 (57.8 to 273)**	**90.2 (24.6 to 184)**	11.1 (-19.1 to 45.4)
17 Asthma	17 Prostate cancer	**213 (103 to 352)**	**143 (62.5 to 242)**	2.41 (-12.8 to 20.6)
18 Rheumatic heart disease	18 Stomach cancer	**43.2 (10.1 to 82.6)**	11.5 (-6.21 to 33.3)	**-31.5 (-37.1 to -25.1)**
19 Diarrheal diseases	19 Diarrheal diseases	**55.5 (5.45 to 115)**	21.2 (-17.2 to 63.9)	-32.4 (-59.2 to 13)
20 Congenital defects	20 Cervical cancer	**59.1 (24.5 to 97.2)**	24.2 (-0.443 to 52.7)	-11 (-31.6 to 12.5)
21 Colorectal cancer	21 Neonatal disorders			
22 Cervical cancer	22 Drowning			
25 Prostate cancer	24 Asthma			
26 Alzheimer's disease	30 Rheumatic heart disease			
27 HIV/AIDS	32 Congenital defects			

Supplemental Results Figure S8. Leading 20 level 3 causes of deaths, and percentage change in number of deaths and all-age and age-standardised death rates by location, 2022 and 2050

Federated States of Micronesia, Reference scenario

Legend:
Communicable, maternal, neonatal and nutritional
Non-communicable
Injuries

Leading Causes 2022
1 Ischemic heart disease
2 Stroke
3 Diabetes
4 Chronic kidney disease
5 COPD
6 Lower respiratory infect
7 Lung cancer
8 Hypertensive heart disease
9 Self-harm
10 Road injuries
11 Cirrhosis liver
12 Breast cancer
13 Tuberculosis
14 Stomach cancer
15 Asthma
16 Neonatal disorders
17 Drowning
18 Interpersonal violence
19 Diarrheal diseases
20 Colorectal cancer
21 Alzheimer's disease
25 Endo/metab/blood/immune
26 Prostate cancer
29 HIV/AIDS
33 Atrial fibrillation

Leading Causes 2050	Mean % change number of DEATH	Mean % change all-age DEATH rate	Mean % change age-standardized DEATH rate
1 Ischemic heart disease	31.8 (-12.7 to 86.3)	33.1 (-7.74 to 84.4)	-23 (-50.3 to 16.3)
2 Stroke	**35.7 (1.86 to 72.3)**	**36.9 (5.88 to 71.6)**	**-24.6 (-42.8 to -1.99)**
3 Diabetes	37.5 (-1.94 to 75.1)	**38.8 (4.93 to 83.1)**	-17.3 (-40.4 to 13.9)
4 Chronic kidney disease	**170 (89 to 254)**	**173 (105 to 254)**	**63.8 (17.7 to 126)**
5 COPD	**102 (45.7 to 159)**	**104 (49.1 to 164)**	0.171 (-25.9 to 34.8)
6 Lower respiratory infect	**37.5 (5.01 to 74.4)**	**38.6 (11.5 to 69)**	**-18.5 (-31.9 to -2.1)**
7 Lung cancer	**91.8 (29.8 to 180)**	**93.3 (34.6 to 171)**	14.6 (-8.88 to 41.3)
8 Hypertensive heart disease	**91.5 (32.1 to 165)**	**93.4 (37.7 to 165)**	4.21 (-30.8 to 52.2)
9 HIV/AIDS	**492 (261 to 878)**	**496 (261 to 837)**	**480 (254 to 809)**
10 Alzheimer's disease	**195 (97.4 to 343)**	**197 (97.2 to 311)**	**9.16 (4.89 to 13.3)**
11 Breast cancer	**55.8 (2.99 to 128)**	**57 (6.91 to 118)**	-1.12 (-24.7 to 25.8)
12 Road injuries	2.72 (-39.8 to 61.5)	3.53 (-34.2 to 64.6)	-16.4 (-49.4 to 38.9)
13 Self-harm	-11 (-35.1 to 19)	-10.4 (-28.3 to 13.5)	-14.6 (-32.7 to 7.98)
14 Cirrhosis liver	-1.18 (-24.5 to 25.2)	-0.405 (-16.4 to 19.4)	**-27 (-38.1 to -10.7)**
15 Prostate cancer	**175 (77.1 to 328)**	**177 (81.4 to 300)**	**25.4 (8.27 to 46.6)**
16 Colorectal cancer	**87.3 (19.5 to 184)**	**88.7 (22 to 184)**	8.66 (-17.2 to 38.6)
17 Stomach cancer	14.7 (-12.8 to 44.6)	15.7 (-8.84 to 42.4)	**-30.3 (-35.5 to -24.2)**
18 Diarrheal diseases	**55.8 (5.64 to 109)**	**57.3 (8.77 to 112)**	-22.1 (-50.8 to 24.9)
19 Endo/metab/blood/immune	**97 (56.1 to 150)**	**98.5 (65.9 to 134)**	**24.6 (10.4 to 43.5)**
20 Atrial fibrillation	**203 (111 to 324)**	**206 (113 to 323)**	**26.2 (15.7 to 37.4)**
21 Asthma			
26 Drowning			
29 Tuberculosis			
31 Interpersonal violence			
32 Neonatal disorders			

Supplemental Results Figure S8. Leading 20 level 3 causes of deaths, and percentage change in number of deaths and all-age and age-standardised death rates by location, 2022 and 2050

Nauru, Reference scenario

Legend:
Communicable, maternal, neonatal and nutritional
Non-communicable
Injuries

Leading Causes 2022	Leading Causes 2050	Mean % change number of DEATH	Mean % change all-age DEATH rate	Mean % change age-standardized DEATH rate
1 Ischemic heart disease	1 Ischemic heart disease	45.5 (-9.54 to 110)	-1.51 (-34.9 to 50.9)	-29.5 (-57.5 to 20.9)
2 Stroke	2 Stroke	**52.4 (12.3 to 95.7)**	3.08 (-19.9 to 31.4)	-27.3 (-47.9 to 3.39)
3 Diabetes	3 Diabetes	**67.1 (18.1 to 124)**	13.1 (-15.8 to 57)	-14.7 (-41.7 to 28.9)
4 Lower respiratory infect	4 Chronic kidney disease	**185 (107 to 276)**	**92.6 (46.9 to 151)**	45.9 (-0.196 to 117)
5 Road injuries	5 COPD	**92.4 (40 to 147)**	30.1 (-0.435 to 64.9)	-7.33 (-35.4 to 33.2)
6 Chronic kidney disease	6 Lower respiratory infect	18.1 (-7.14 to 45.7)	**-20.2 (-32.3 to -4.14)**	**-25.1 (-40 to -6.99)**
7 COPD	7 Road injuries	41.3 (-25.1 to 142)	-4.38 (-47.2 to 75)	-12.7 (-51.5 to 56.7)
8 Self-harm	8 Lung cancer	**123 (42.5 to 207)**	**50.5 (0.529 to 115)**	4.9 (-20 to 33.5)
9 Neonatal disorders	9 Self-harm	**43.9 (3.91 to 91.8)**	-2.68 (-25.8 to 25.9)	-9.57 (-30.5 to 16.1)
10 Lung cancer	10 Hypertensive heart disease	**123 (47.8 to 205)**	**50.6 (8.15 to 113)**	2.75 (-33.9 to 59)
11 Tuberculosis	11 Breast cancer	**106 (32.6 to 190)**	39.4 (-6.4 to 104)	5.29 (-21.9 to 38.1)
12 Cirrhosis liver	12 Cirrhosis liver	**54.8 (21.6 to 90.6)**	4.78 (-13.8 to 27.4)	**-21.4 (-36.2 to -0.411)**
13 Hypertensive heart disease	13 Neonatal disorders	-16.4 (-53.5 to 34.4)	**-43.6 (-66.6 to -9.96)**	-23 (-53.5 to 23.9)
14 Drowning	14 Colorectal cancer	**122 (36.1 to 217)**	50.3 (-5.56 to 123)	7.13 (-21.9 to 38.4)
15 Breast cancer	15 Drowning	0.548 (-34.7 to 48.9)	**-32 (-54.4 to -1.79)**	**-35.1 (-55.6 to -4.93)**
16 Congenital defects	16 Stomach cancer	**32.8 (4.6 to 60.7)**	-10.2 (-23.8 to 5.38)	**-34.1 (-38.9 to -27.6)**
17 Stomach cancer	17 Alzheimer's disease	**137 (52.9 to 223)**	**60 (9.74 to 124)**	**9.1 (4.92 to 13.3)**
18 Interpersonal violence	18 Tuberculosis	-26.4 (-53.1 to 8.87)	**-50.1 (-66.3 to -24.2)**	**-61 (-74.4 to -39.8)**
19 Asthma	19 Endo/metab/blood/immune	**136 (94 to 180)**	**59.3 (38.7 to 81)**	**24.6 (6.39 to 46.4)**
20 Colorectal cancer	20 Interpersonal violence	10.4 (-15 to 43.2)	**-25.3 (-40.9 to -5.22)**	**-31.5 (-44.5 to -13)**
24 Alzheimer's disease	22 Congenital defects			
26 Endo/metab/blood/immune	26 Asthma			

Supplemental Results Figure S8. Leading 20 level 3 causes of deaths, and percentage change in number of deaths and all-age and age-standardised death rates by location, 2022 and 2050

Niue, Reference scenario

Legend:
Communicable, maternal, neonatal and nutritional
Non-communicable
Injuries

Leading Causes 2022	Leading Causes 2050	Mean % change number of DEATH	Mean % change all-age DEATH rate	Mean % change age-standardized DEATH rate
1 Ischemic heart disease	1 Ischemic heart disease	**-28.2 (-47.4 to -1.93)**	-27.5 (-47.1 to 0.338)	**-44.9 (-62.6 to -18.6)**
2 Stroke	2 Diabetes	3.24 (-20 to 30)	4.2 (-19.5 to 31.3)	-21.3 (-40.8 to 5.02)
3 Diabetes	3 Stroke	-16.7 (-32.5 to 3.04)	-16 (-30.5 to 2.85)	**-38.1 (-50.4 to -21.2)**
4 Chronic kidney disease	4 Chronic kidney disease	**60.1 (24.3 to 104)**	**61.5 (26.4 to 108)**	23.2 (-10.7 to 67.9)
5 Neonatal disorders	5 COPD	**43.6 (17 to 84.9)**	**44.8 (16.5 to 79.5)**	2.04 (-22.3 to 37.1)
6 COPD	6 Lower respiratory infect	14.4 (-3.98 to 40.6)	15.3 (-1.45 to 34.7)	**-17.5 (-29.9 to -3.18)**
7 Lower respiratory infect	7 Lung cancer	**52.5 (6.77 to 99.2)**	**53.5 (12 to 98.7)**	17.2 (-5.91 to 41.8)
8 Lung cancer	8 Alzheimer's disease	**62.3 (24.2 to 105)**	**63.5 (27.4 to 100)**	**9.22 (5.73 to 13.3)**
9 Hypertensive heart disease	9 Hypertensive heart disease	**43.6 (6.09 to 94.5)**	**45 (1.03 to 98.9)**	8.9 (-23.9 to 59.5)
10 Alzheimer's disease	10 Neonatal disorders	**-34.3 (-55.3 to -3.18)**	**-33.7 (-55.4 to -4.08)**	-28.8 (-49.7 to 0.805)
11 Breast cancer	11 Breast cancer	36.8 (-7.89 to 84.1)	37.7 (-0.273 to 80.1)	5.27 (-19.5 to 32.9)
12 Congenital defects	12 Prostate cancer	**68.9 (18.1 to 120)**	**70 (23.8 to 121)**	15.9 (-2.61 to 33.6)
13 Road injuries	13 Colorectal cancer	48.9 (-1.28 to 110)	**49.9 (4.43 to 100)**	12.2 (-14.5 to 41.7)
14 Cirrhosis liver	14 Road injuries	-14.5 (-42.8 to 39.4)	-13.7 (-43.8 to 34.3)	-12.5 (-45.7 to 42.6)
15 Colorectal cancer	15 Cirrhosis liver	-15.1 (-28.8 to 2.12)	-14.3 (-26.5 to 1.11)	**-24.8 (-35.8 to -10.9)**
16 Self-harm	16 Atrial fibrillation	**68 (31.4 to 108)**	**69.2 (39.4 to 108)**	**14.8 (4.1 to 26.2)**
17 Prostate cancer	17 Endo/metab/blood/immune	**54.3 (28.8 to 87)**	**55.5 (38.6 to 77.3)**	**22 (8.27 to 37.2)**
18 Stomach cancer	18 Congenital defects	**-34.4 (-45.8 to -18.8)**	**-33.9 (-42.6 to -21.4)**	**-25.2 (-35.6 to -12.2)**
19 Asthma	19 Diarrheal diseases	8.8 (-21.2 to 50)	9.78 (-20.9 to 51.7)	-22.8 (-49.2 to 22.6)
20 Diarrheal diseases	20 Pancreatic cancer	**107 (42 to 186)**	**108 (48.1 to 175)**	**58.2 (23.1 to 100)**
22 Endo/metab/blood/immune	21 Stomach cancer			
24 Atrial fibrillation	22 Self-harm			
29 Pancreatic cancer	26 Asthma			

Supplemental Results Figure S8. Leading 20 level 3 causes of deaths, and percentage change in number of deaths and all-age and age-standardised death rates by location, 2022 and 2050

Northern Mariana Islands, Reference scenario

Legend: Communicable, maternal, neonatal and nutritional; Non-communicable; Injuries

Leading Causes 2022	Leading Causes 2050	Mean % change number of DEATH	Mean % change all-age DEATH rate	Mean % change age-standardized DEATH rate
1 Ischemic heart disease	1 Ischemic heart disease	**35.2 (0.64 to 82.3)**	30.6 (-6.03 to 78)	**-37.7 (-58.1 to -7.6)**
2 Stroke	2 Chronic kidney disease	**214 (151 to 287)**	**203 (145 to 275)**	**43.7 (10.5 to 93.8)**
3 Diabetes	3 Stroke	**74.4 (44.1 to 110)**	**68.4 (40 to 101)**	**-30.3 (-43.7 to -11.7)**
4 Chronic kidney disease	4 COPD	**206 (154 to 271)**	**196 (145 to 254)**	3.91 (-18.9 to 36)
5 Lung cancer	5 Diabetes	**63 (31.5 to 101)**	**57.4 (24.1 to 94.1)**	**-23.7 (-42.2 to -1.11)**
6 COPD	6 Lung cancer	**95.6 (44.4 to 153)**	**88.5 (43 to 133)**	-4.22 (-21.6 to 15)
7 Cirrhosis liver	7 Alzheimer's disease	**336 (240 to 438)**	**320 (226 to 411)**	3.52 (-0.219 to 6.83)
8 Lower respiratory infect	8 Lower respiratory infect	**135 (106 to 172)**	**127 (100 to 159)**	**-12.6 (-22 to -1.24)**
9 Self-harm	9 Diarrheal diseases	**423 (284 to 584)**	**405 (271 to 570)**	**55.1 (4.82 to 129)**
10 Breast cancer	10 Colorectal cancer	**110 (39.1 to 187)**	**102 (37.4 to 169)**	-4.17 (-26.7 to 18.2)
11 Colorectal cancer	11 Prostate cancer	**214 (128 to 304)**	**202 (125 to 289)**	-1.86 (-16.7 to 16.5)
12 Road injuries	12 Cirrhosis liver	11.5 (-5.34 to 28.6)	7.7 (-9.26 to 25.1)	**-26.2 (-36.2 to -10.6)**
13 Stomach cancer	13 Breast cancer	**75.6 (26.5 to 136)**	**69.3 (20.8 to 125)**	-6.29 (-25.5 to 16.7)
14 Alzheimer's disease	14 Pancreatic cancer	**191 (95.6 to 292)**	**181 (93.1 to 263)**	**36.1 (4.59 to 71.6)**
15 Tuberculosis	15 Bacterial skin	**307 (172 to 480)**	**293 (162 to 476)**	**84.8 (21.2 to 180)**
16 Prostate cancer	16 Urinary diseases	**309 (240 to 384)**	**295 (226 to 369)**	**47.1 (21 to 79.2)**
17 Drowning	17 Atrial fibrillation	**331 (232 to 423)**	**315 (229 to 398)**	**19.5 (9.16 to 29.6)**
18 Diarrheal diseases	18 Stomach cancer	**45.5 (20.1 to 69)**	**40.4 (19.4 to 58.4)**	**-32.6 (-38.1 to -26.9)**
19 Cervical cancer	19 Hypertensive heart disease	**177 (101 to 280)**	**167 (90.3 to 272)**	17 (-21.7 to 70.2)
20 Pancreatic cancer	20 Self-harm	-0.114 (-17.1 to 18.1)	-3.59 (-19.2 to 13.5)	-15.6 (-30.1 to 1.28)
23 Hypertensive heart disease	22 Road injuries			
25 Bacterial skin	27 Cervical cancer			
26 Urinary diseases	30 Tuberculosis			
29 Atrial fibrillation	40 Drowning			

Supplemental Results Figure S8. Leading 20 level 3 causes of deaths, and percentage change in number of deaths and all-age and age-standardised death rates by location, 2022 and 2050

Palau, Reference scenario

Legend:
Communicable, maternal, neonatal and nutritional
Non-communicable
Injuries

Leading Causes 2022	Leading Causes 2050	Mean % change number of DEATH	Mean % change all-age DEATH rate	Mean % change age-standardized DEATH rate
1 Ischemic heart disease	1 Ischemic heart disease	6.36 (-25.7 to 48.4)	22.4 (-13.4 to 66.2)	**-37.8 (-57.5 to -10.4)**
2 Stroke	2 Stroke	24.4 (-5.71 to 59.2)	**43 (14.9 to 77.6)**	**-31.3 (-45.5 to -14.2)**
3 Diabetes	3 Diabetes	**37.7 (1.82 to 79.8)**	**58.5 (22.7 to 102)**	-19.3 (-38.6 to 4.22)
4 COPD	4 COPD	**145 (84.5 to 206)**	**182 (118 to 245)**	2.83 (-20.4 to 35.8)
5 Lung cancer	5 Chronic kidney disease	**154 (88.4 to 236)**	**193 (124 to 276)**	**44.8 (8.45 to 94.1)**
6 Chronic kidney disease	6 Lung cancer	**93.7 (35.5 to 163)**	**123 (58.2 to 187)**	12.7 (-10.1 to 36.6)
7 Lower respiratory infect	7 Lower respiratory infect	**76.7 (39.7 to 122)**	**103 (66 to 145)**	**-17.6 (-29.4 to -3.82)**
8 Breast cancer	8 Diarrheal diseases	**167 (90.1 to 252)**	**207 (126 to 307)**	-1.34 (-32 to 40.5)
9 Stomach cancer	9 Alzheimer's disease	**238 (147 to 341)**	**288 (192 to 398)**	3.67 (-0.905 to 8.67)
10 Cirrhosis liver	10 Prostate cancer	**199 (108 to 308)**	**244 (143 to 342)**	**23.3 (5.35 to 42.2)**
11 Road injuries	11 Breast cancer	47.9 (-0.233 to 106)	**70.1 (17.7 to 128)**	-11.1 (-32.2 to 11.6)
12 Diarrheal diseases	12 Lip oral cavity cancer	**60.8 (33.8 to 100)**	**85 (60.1 to 114)**	7.07 (-2.18 to 15.9)
13 Asthma	13 Pancreatic cancer	**166 (69 to 281)**	**205 (102 to 324)**	28.8 (-2.84 to 64.3)
14 Lip oral cavity cancer	14 Colorectal cancer	**71.2 (9.51 to 144)**	**96.8 (30.6 to 163)**	-11.9 (-32.7 to 12.5)
15 Cervical cancer	15 Falls	**86.4 (36.3 to 142)**	**114 (64.3 to 169)**	**-19.1 (-27.6 to -9.58)**
16 Self-harm	16 Stomach cancer	8.5 (-12.2 to 34.4)	**24.7 (2.85 to 46.8)**	**-34 (-39.6 to -27.6)**
17 Colorectal cancer	17 Cervical cancer	14 (-9.33 to 39.7)	**31.2 (7.45 to 58.8)**	**-25.2 (-36.5 to -9.93)**
18 Prostate cancer	18 Hypertensive heart disease	**91 (34.8 to 158)**	**120 (55.4 to 205)**	6.55 (-26.3 to 53.4)
19 Falls	19 Cirrhosis liver	-16.5 (-32.6 to 2.56)	-3.9 (-18.4 to 14.6)	**-20.7 (-32.7 to -5.05)**
20 Alzheimer's disease	20 Liver cancer	**56.5 (9.27 to 118)**	**80.2 (32.2 to 150)**	37.3 (-1.65 to 92.4)
21 Pancreatic cancer	21 Road injuries			
22 Liver cancer	22 Asthma			
25 Hypertensive heart disease	24 Self-harm			

Supplemental Results Figure S8. Leading 20 level 3 causes of deaths, and percentage change in number of deaths and all-age and age-standardised death rates by location, 2022 and 2050

Papua New Guinea, Reference scenario

Legend:
Communicable, maternal, neonatal and nutritional
Non-communicable
Injuries

Leading Causes 2022	Leading Causes 2050	Mean % change number of DEATH	Mean % change all-age DEATH rate	Mean % change age-standardized DEATH rate
1 Stroke	1 Stroke	**149 (91.4 to 214)**	23.4 (-4.68 to 49.5)	**-25.5 (-44.1 to -1.99)**
2 Lower respiratory infect	2 Ischemic heart disease	**157 (65.2 to 281)**	27.4 (-17 to 86)	-18.4 (-49.7 to 25.3)
3 Ischemic heart disease	3 COPD	**153 (85.3 to 230)**	25.3 (-5.52 to 65)	**-31.6 (-51.7 to -4.29)**
4 COPD	4 Diabetes	**182 (101 to 264)**	**39.7 (4.28 to 80.1)**	-6.63 (-33.4 to 28.4)
5 Diabetes	5 Lower respiratory infect	-17.5 (-34.5 to 0.724)	**-59.2 (-66.7 to -49.9)**	**-49.9 (-59.6 to -38.5)**
6 Neonatal disorders	6 Malaria	142 (-83.9 to 482)	20.9 (-91.3 to 191)	9.43 (-87.6 to 163)
7 Malaria	7 Diarrheal diseases	33.1 (-13 to 89.9)	**-34.1 (-56.5 to -3.68)**	-36.8 (-62.1 to 0.314)
8 Diarrheal diseases	8 Chronic kidney disease	**338 (203 to 486)**	**117 (56.8 to 187)**	**59.6 (8.59 to 126)**
9 Tuberculosis	9 Lung cancer	**216 (113 to 333)**	**56 (9.85 to 111)**	-2.65 (-23.2 to 21.6)
10 Congenital defects	10 Neonatal disorders	-30.5 (-60 to 11.3)	**-65.7 (-79.4 to -46.9)**	**-51.4 (-69.5 to -26.8)**
11 COVID-19	11 Road injuries	51.7 (-13.4 to 153)	-24.9 (-56 to 29.6)	-31.8 (-59.6 to 17.1)
12 Road injuries	12 Congenital defects	-6.14 (-26.3 to 19.6)	**-53.6 (-61.7 to -42.7)**	**-37.6 (-46.6 to -26.4)**
13 Asthma	13 Hypertensive heart disease	**189 (94.7 to 302)**	43 (-0.639 to 103)	-12.4 (-44.7 to 30.9)
14 Pertussis	14 Tuberculosis	-10.8 (-41.1 to 24.8)	**-55.8 (-70.2 to -35.8)**	**-67.2 (-79 to -49.9)**
15 Rheumatic heart disease	15 Alzheimer's disease	**399 (252 to 611)**	**146 (76.8 to 223)**	**7.78 (3.92 to 11.4)**
16 Measles	16 Asthma	**29.9 (3.26 to 61.2)**	**-35.8 (-48 to -21.9)**	**-60.2 (-68.7 to -50.3)**
17 Drowning	17 Breast cancer	**177 (89.7 to 294)**	36.8 (-5.2 to 88.2)	9.26 (-19.1 to 42.9)
18 Lung cancer	18 Prostate cancer	**312 (175 to 469)**	**104 (40.1 to 171)**	3.72 (-13.2 to 21.3)
19 Interpersonal violence	19 Stomach cancer	**97.1 (57.5 to 143)**	-2.55 (-16.7 to 14.3)	**-35.2 (-40.3 to -29.5)**
20 HIV/AIDS	20 Interpersonal violence	**44.4 (16.7 to 81.8)**	**-28.6 (-39.4 to -14.7)**	**-33 (-42.3 to -19.4)**
21 Hypertensive heart disease	21 Pertussis			
23 Chronic kidney disease	22 Drowning			
24 Stomach cancer	24 Rheumatic heart disease			
25 Breast cancer	29 HIV/AIDS			
29 Alzheimer's disease	65 Measles			
34 Prostate cancer	132 COVID-19			

Supplemental Results Figure S8. Leading 20 level 3 causes of deaths, and percentage change in number of deaths and all-age and age-standardised death rates by location, 2022 and 2050

Samoa, Reference scenario

Legend:
Communicable, maternal, neonatal and nutritional
Non-communicable
Injuries

Leading Causes 2022	Leading Causes 2050	Mean % change number of DEATH	Mean % change all-age DEATH rate	Mean % change age-standardized DEATH rate
1 Ischemic heart disease	1 Ischemic heart disease	**40 (1.2 to 90.8)**	-4.87 (-31.4 to 29.2)	-24.8 (-50.8 to 10.7)
2 Stroke	2 Stroke	**38.8 (10.7 to 69.7)**	-5.73 (-19.9 to 13.4)	**-28 (-43.7 to -6.7)**
3 Diabetes	3 Diabetes	**48.6 (17.1 to 84.2)**	0.962 (-17.6 to 24.7)	-18.2 (-36.7 to 7.76)
4 Measles	4 Chronic kidney disease	**194 (123 to 271)**	**99.9 (55.4 to 153)**	**61.4 (16.9 to 119)**
5 COPD	5 COPD	**72 (36.7 to 111)**	16.8 (-4.58 to 41.9)	-15.1 (-38.2 to 16)
6 Lower respiratory infect	6 Lower respiratory infect	**32.3 (10.6 to 57.4)**	-10.2 (-22.9 to 1.18)	**-25.3 (-36.1 to -12.2)**
7 Chronic kidney disease	7 Hypertensive heart disease	**96.6 (41.7 to 163)**	33.5 (-0.939 to 80.1)	2.42 (-31.8 to 51.4)
8 Hypertensive heart disease	8 HIV/AIDS	**619 (271 to 1140)**	**387 (147 to 754)**	**358 (138 to 703)**
9 Neonatal disorders	9 Alzheimer's disease	**129 (67.2 to 211)**	**55.6 (17.9 to 101)**	0.486 (-4.27 to 4.55)
10 Road injuries	10 Road injuries	36.6 (-18 to 128)	-7.3 (-41.6 to 54.2)	-15.3 (-46.5 to 39.8)
11 Self-harm	11 Self-harm	**40.5 (9.3 to 77.2)**	-4.67 (-22.9 to 16.4)	-12.7 (-28.5 to 6.88)
12 Alzheimer's disease	12 Breast cancer	**71.4 (21.6 to 134)**	16.3 (-16 to 49.9)	-1.57 (-25.5 to 22.1)
13 Cirrhosis liver	13 Cirrhosis liver	17.4 (-6.02 to 42.3)	**-20.3 (-33.9 to -5.37)**	**-28.8 (-40.8 to -14.1)**
14 Asthma	14 Neonatal disorders	-23.6 (-51.3 to 15.5)	**-48.2 (-67 to -22.1)**	**-38.5 (-59.5 to -11)**
15 Breast cancer	15 Colorectal cancer	**90.6 (32 to 175)**	29.4 (-9.65 to 79.5)	1.87 (-21.9 to 28.6)
16 Stomach cancer	16 Endo/metab/blood/immune	**118 (82.2 to 149)**	**48 (31.5 to 65.3)**	**23.1 (8.59 to 36.7)**
17 Congenital defects	17 Measles	-73.7 (-94.6 to 39.5)	**-82.3 (-96.2 to -20)**	**-78.9 (-95.4 to -9.96)**
18 Tuberculosis	18 Lung cancer	**69.2 (26.5 to 126)**	14.9 (-15.1 to 49.9)	-6.36 (-25.1 to 13)
19 Drowning	19 Diarrheal diseases	**109 (54.8 to 173)**	**42.3 (2.69 to 91.5)**	8.04 (-30.5 to 64.2)
20 Colorectal cancer	20 Prostate cancer	**149 (83.1 to 245)**	**69.5 (25.2 to 127)**	**19.6 (0.92 to 38.2)**
21 Lung cancer	22 Stomach cancer			
22 Endo/metab/blood/immune	24 Asthma			
26 Diarrheal diseases	29 Drowning			
29 HIV/AIDS	30 Congenital defects			
32 Prostate cancer	37 Tuberculosis			

Supplemental Results Figure S8. Leading 20 level 3 causes of deaths, and percentage change in number of deaths and all-age and age-standardised death rates by location, 2022 and 2050

Solomon Islands, Reference scenario

Legend:
Communicable, maternal, neonatal and nutritional
Non-communicable
Injuries

Leading Causes 2022	Leading Causes 2050	Mean % change number of DEATH	Mean % change all-age DEATH rate	Mean % change age-standardized DEATH rate
1 Ischemic heart disease	1 Ischemic heart disease	**73.8 (13 to 158)**	21.1 (-16.3 to 73)	-20.7 (-51.2 to 21.7)
2 Stroke	2 Stroke	**64.7 (23.1 to 109)**	14.7 (-8.71 to 40)	**-27.3 (-45.3 to -4.44)**
3 Lower respiratory infect	3 Diabetes	**120 (54.7 to 190)**	**53.3 (16.6 to 98.2)**	6.23 (-23.9 to 49.4)
4 Diabetes	4 Lower respiratory infect	**26.8 (1.27 to 57.4)**	-11.8 (-25.6 to 2.13)	**-31.4 (-41.6 to -17.8)**
5 Malaria	5 Malaria	164 (-55 to 369)	80.8 (-71.8 to 219)	24.2 (-73.8 to 125)
6 COPD	6 COPD	**91.8 (39.6 to 152)**	**33.5 (4.03 to 66)**	-22 (-43.1 to 8.54)
7 Self-harm	7 Chronic kidney disease	**220 (124 to 337)**	**123 (65.7 to 197)**	**63.5 (15.8 to 129)**
8 Cirrhosis liver	8 HIV/AIDS	**623 (280 to 1040)**	**401 (176 to 640)**	**388 (167 to 617)**
9 Road injuries	9 Lung cancer	**152 (75.6 to 254)**	**75 (24.8 to 141)**	14.4 (-10.2 to 42.6)
10 Neonatal disorders	10 Self-harm	**36.8 (2.39 to 81.1)**	-4.77 (-23.8 to 22.1)	-14.1 (-31.4 to 9.5)
11 Chronic kidney disease	11 Road injuries	37.6 (-23.2 to 143)	-4.11 (-43.2 to 68.1)	-17.7 (-50.1 to 42.3)
12 Drowning	12 Cirrhosis liver	19.5 (-7.79 to 49.9)	-16.8 (-31 to 0.182)	**-36.3 (-48.2 to -21.2)**
13 Tuberculosis	13 Hypertensive heart disease	**113 (42.8 to 195)**	**48.4 (6.67 to 101)**	-7.97 (-40.9 to 36.4)
14 Lung cancer	14 Alzheimer's disease	**207 (110 to 326)**	**113 (49 to 182)**	**14.8 (9.88 to 19.3)**
15 Congenital defects	15 Breast cancer	**122 (53.2 to 230)**	**54.6 (7.84 to 121)**	15.8 (-12.6 to 50.6)
16 Diarrheal diseases	16 Stomach cancer	**41.3 (14 to 71.7)**	-1.71 (-16.9 to 12.5)	**-32.5 (-37.6 to -26.4)**
17 Stomach cancer	17 Colorectal cancer	**162 (73.2 to 292)**	**82.2 (20.6 to 172)**	20 (-10.4 to 56.5)
18 Maternal disorders	18 Drowning	6.29 (-27.4 to 52.7)	-26 (-47.4 to 5.75)	**-30.4 (-50.6 to -1.6)**
19 Hypertensive heart disease	19 Prostate cancer	**178 (80.7 to 304)**	**93.3 (29.4 to 168)**	4.64 (-13.3 to 25.2)
20 Asthma	20 Diarrheal diseases	3.01 (-29.4 to 48.2)	**-28.3 (-49.3 to -1.21)**	**-42 (-64.9 to -8.66)**
21 Breast cancer	24 Neonatal disorders			
26 Alzheimer's disease	26 Congenital defects			
27 Colorectal cancer	27 Tuberculosis			
30 HIV/AIDS	28 Asthma			
31 Prostate cancer	29 Maternal disorders			

Supplemental Results Figure S8. Leading 20 level 3 causes of deaths, and percentage change in number of deaths and all-age and age-standardised death rates by location, 2022 and 2050

Tokelau, Reference scenario

Legend:
Communicable, maternal, neonatal and nutritional
Non-communicable
Injuries

Leading Causes 2022	Leading Causes 2050	Mean % change number of DEATH	Mean % change all-age DEATH rate	Mean % change age-standardized DEATH rate
1 Ischemic heart disease	1 Ischemic heart disease	-23.3 (-47.2 to 8.97)	-29.2 (-50.6 to 3.82)	**-49.9 (-66.9 to -22.8)**
2 Stroke	2 Stroke	-7.36 (-27 to 16)	-14.4 (-32.3 to 9.54)	**-39.8 (-54.1 to -16.6)**
3 Diabetes	3 Diabetes	13.9 (-18 to 52)	5.23 (-22.5 to 42.7)	-26.6 (-48 to 4.02)
4 Neonatal disorders	4 COPD	**48.8 (13.5 to 88.3)**	**37.4 (8.88 to 78.5)**	-5.72 (-31.3 to 30.3)
5 COPD	5 Chronic kidney disease	**72.2 (26.9 to 124)**	**59.1 (23.5 to 112)**	14.9 (-18 to 59.9)
6 Lower respiratory infect	6 Lung cancer	**85.5 (29.5 to 148)**	**71 (22.5 to 120)**	18.5 (-6.08 to 43.6)
7 Chronic kidney disease	7 Lower respiratory infect	16.1 (-4.18 to 39.4)	7.18 (-9.44 to 29.4)	**-24.7 (-37 to -8.75)**
8 Congenital defects	8 Alzheimer's disease	**70.4 (31 to 122)**	**57.1 (23.2 to 93)**	**7.8 (3.67 to 11.8)**
9 Alzheimer's disease	9 Neonatal disorders	**-45.4 (-65.4 to -19.2)**	**-49.5 (-66.9 to -22.6)**	**-41.4 (-61.1 to -15.6)**
10 Lung cancer	10 Hypertensive heart disease	**49.9 (0.606 to 108)**	38.6 (-3.54 to 102)	-2.42 (-35.4 to 46.6)
11 Hypertensive heart disease	11 Breast cancer	**59.3 (12 to 113)**	**46.8 (3.97 to 92.8)**	3.74 (-20.3 to 30)
12 Diarrheal diseases	12 Prostate cancer	**84.2 (28.9 to 151)**	**69.8 (22 to 117)**	12.3 (-8.42 to 32.3)
13 Road injuries	13 Colorectal cancer	**77.5 (19.4 to 144)**	**63.6 (13.2 to 119)**	13.8 (-13.4 to 43.4)
14 Breast cancer	14 Congenital defects	**-43.2 (-53.4 to -29.6)**	**-47.6 (-55.2 to -35.1)**	**-33.9 (-43.5 to -22.2)**
15 Self-harm	15 Cirrhosis liver	11.7 (-11.4 to 35.9)	3.19 (-16.3 to 26.1)	-19.3 (-33.9 to 0.523)
16 Prostate cancer	16 Road injuries	-16.8 (-49.4 to 41.2)	-23.2 (-51.5 to 27.9)	-19 (-49.4 to 43.1)
17 Cirrhosis liver	17 Atrial fibrillation	**84.2 (44.9 to 139)**	**69.8 (35.4 to 111)**	**16.5 (4.9 to 28.2)**
18 Asthma	18 Diarrheal diseases	-25.1 (-47.1 to 2.58)	**-30.9 (-50.2 to -6.53)**	**-50.2 (-65.2 to -25.4)**
19 Stomach cancer	19 Self-harm	-8.42 (-28.9 to 20.4)	-15.5 (-34.3 to 9.66)	-13.7 (-32.9 to 14.8)
20 Colorectal cancer	20 Endo/metab/blood/immune	**68.2 (44.1 to 95.7)**	**55.2 (35.7 to 78.4)**	15.4 (-0.187 to 32.2)
22 Atrial fibrillation	21 Stomach cancer			
24 Endo/metab/blood/immune	26 Asthma			

Supplemental Results Figure S8. Leading 20 level 3 causes of deaths, and percentage change in number of deaths and all-age and age-standardised death rates by location, 2022 and 2050

Tonga, Reference scenario

Legend:
Communicable, maternal, neonatal and nutritional
Non-communicable
Injuries

Leading Causes 2022	Leading Causes 2050	Mean % change number of DEATH	Mean % change all-age DEATH rate	Mean % change age-standardized DEATH rate
1 Ischemic heart disease	1 Ischemic heart disease	9.49 (-22.9 to 57)	-16.1 (-41.9 to 18)	**-36.4 (-57.1 to -6.03)**
2 Diabetes	2 Diabetes	**32.4 (0.324 to 83.9)**	1.51 (-23.7 to 34.1)	-23.5 (-44.6 to 3.45)
3 Stroke	3 Stroke	16.9 (-7.41 to 47.7)	-10.4 (-30.1 to 11.5)	**-35.2 (-50.1 to -15.8)**
4 Lower respiratory infect	4 COPD	**55 (18.8 to 104)**	18.8 (-6.62 to 50.3)	-16.6 (-37.9 to 10.3)
5 COPD	5 Lower respiratory infect	**23.8 (0.473 to 54)**	-5.17 (-20.5 to 13.8)	**-26.6 (-36.9 to -11.2)**
6 Lung cancer	6 Chronic kidney disease	**168 (95.2 to 264)**	**105 (52.9 to 178)**	**56.3 (13 to 119)**
7 Cirrhosis liver	7 Lung cancer	**73.2 (27 to 129)**	32.5 (-2.7 to 71.5)	-1.18 (-20.6 to 20.1)
8 Alzheimer's disease	8 Alzheimer's disease	**105 (48.2 to 166)**	**57.3 (15.2 to 102)**	**4.74 (0.864 to 9.07)**
9 Liver cancer	9 Liver cancer	**86.5 (34.6 to 162)**	**42.9 (5.23 to 100)**	18.4 (-12.2 to 65.7)
10 Breast cancer	10 Breast cancer	**65.7 (16.9 to 124)**	26.8 (-10.3 to 67.7)	2.7 (-19.1 to 29.3)
11 Chronic kidney disease	11 Prostate cancer	**108 (50.4 to 171)**	**59.5 (15.7 to 110)**	8.06 (-9.56 to 25.6)
12 Neonatal disorders	12 Cirrhosis liver	14.2 (-7.34 to 38.2)	-12.5 (-26 to 4.86)	**-24.1 (-35.8 to -9.07)**
13 Stomach cancer	13 Stomach cancer	13.7 (-8.9 to 38)	-13 (-26.7 to 1.96)	**-34.1 (-39.3 to -28.7)**
14 Prostate cancer	14 Road injuries	12.4 (-30.1 to 91)	-13.8 (-44.3 to 46.8)	-21.5 (-50 to 32.9)
15 Road injuries	15 Pancreatic cancer	**160 (83.7 to 256)**	**98.9 (41.5 to 159)**	**49.3 (14.1 to 88.8)**
16 Upper digest diseases	16 Endo/metab/blood/immune	**101 (64.9 to 145)**	**53.8 (34.4 to 79.2)**	**22.9 (8 to 39.1)**
17 Asthma	17 Colorectal cancer	**88 (29.7 to 158)**	43.8 (-1.18 to 94.5)	6.76 (-16.4 to 33.8)
18 Cervical cancer	18 Cervical cancer	**30.6 (2.45 to 66.3)**	0.0338 (-19.9 to 22.5)	-18.6 (-32.9 to 1.45)
19 Endo/metab/blood/immune	19 Hypertensive heart disease	**78.2 (24.3 to 153)**	36.7 (-3.63 to 97.3)	2.15 (-30.9 to 49.9)
20 Hypertensive heart disease	20 Neonatal disorders	-24.2 (-49.1 to 12.4)	**-42 (-58.7 to -16.6)**	-31.3 (-51.5 to 0.0261)
22 Colorectal cancer	23 Upper digest diseases			
25 Pancreatic cancer	28 Asthma			

Supplemental Results Figure S8. Leading 20 level 3 causes of deaths, and percentage change in number of deaths and all-age and age-standardised death rates by location, 2022 and 2050

Tuvalu, Reference scenario

Legend:
Communicable, maternal, neonatal and nutritional
Non-communicable
Injuries

Leading Causes 2022	Leading Causes 2050	Mean % change number of DEATH	Mean % change all-age DEATH rate	Mean % change age-standardized DEATH rate
1 Ischemic heart disease	1 Ischemic heart disease	27.1 (-12.7 to 76.2)	-5.05 (-32.3 to 36.5)	-25.9 (-52.5 to 13.1)
2 Stroke	2 Stroke	**29.4 (0.252 to 57.6)**	-3.42 (-21.6 to 17)	**-25.5 (-43.5 to -2.41)**
3 Diabetes	3 Diabetes	**35.1 (1.88 to 71.1)**	0.786 (-20.9 to 27.7)	-17 (-40.1 to 15.4)
4 Lower respiratory infect	4 Chronic kidney disease	**149 (89.5 to 216)**	**85.6 (43.9 to 143)**	**50.2 (11 to 112)**
5 COPD	5 COPD	**71 (34.6 to 109)**	**27.6 (4.29 to 51.8)**	-4.78 (-29.8 to 29.9)
6 Tuberculosis	6 Lower respiratory infect	**22.7 (1.84 to 43.8)**	-8.51 (-21.1 to 5.29)	**-25.2 (-36.7 to -10.7)**
7 Chronic kidney disease	7 Lung cancer	**93.6 (32.1 to 161)**	**44.3 (2.58 to 92.1)**	18 (-6.59 to 42.6)
8 Lung cancer	8 Hypertensive heart disease	**70.5 (22.6 to 125)**	27.3 (-6.98 to 73.3)	-2.87 (-34 to 51.8)
9 Hypertensive heart disease	9 Alzheimer's disease	**127 (59.1 to 208)**	**69.1 (18.1 to 124)**	**8.1 (2.96 to 13.1)**
10 Road injuries	10 Road injuries	10.5 (-34.3 to 79.7)	-17.5 (-51.7 to 38.2)	-22.6 (-54.6 to 30.7)
11 Self-harm	11 Breast cancer	**69.2 (11.1 to 146)**	26.1 (-13.3 to 74.1)	1.87 (-22.4 to 32.9)
12 Cirrhosis liver	12 Tuberculosis	**-41.1 (-58.4 to -19.8)**	**-56 (-68 to -38)**	**-62.8 (-73.7 to -45.1)**
13 Neonatal disorders	13 Self-harm	20.1 (-4.59 to 50.6)	-10.5 (-26.3 to 10.9)	-14.4 (-29.5 to 5.98)
14 Alzheimer's disease	14 Cirrhosis liver	15.1 (-8.54 to 37.2)	-14.1 (-27.9 to 3.47)	**-27.5 (-39 to -13.3)**
15 Breast cancer	15 Prostate cancer	**118 (37.8 to 206)**	**62.5 (8.81 to 122)**	16.5 (-4.34 to 42.1)
16 Stomach cancer	16 Colorectal cancer	**90.9 (22.1 to 178)**	42.2 (-7.87 to 94.9)	13.1 (-14.5 to 44.5)
17 Asthma	17 Stomach cancer	11.4 (-9.37 to 35.7)	**-17 (-30.9 to -4.49)**	**-32.6 (-37.9 to -27.1)**
18 Diarrheal diseases	18 Diarrheal diseases	16.3 (-14.7 to 52.4)	-13.2 (-35.5 to 12)	-33.2 (-57.6 to 7.85)
19 Colorectal cancer	19 Endo/metab/blood/immune	**96.9 (69.8 to 130)**	**46.8 (28.8 to 67.1)**	**19.8 (6.66 to 37.9)**
20 Drowning	20 Atrial fibrillation	**151 (82.4 to 228)**	**86.8 (39.8 to 141)**	**25.9 (13.6 to 39.1)**
21 Prostate cancer	22 Neonatal disorders			
28 Endo/metab/blood/immune	24 Asthma			
30 Atrial fibrillation	28 Drowning			

Supplemental Results Figure S8. Leading 20 level 3 causes of deaths, and percentage change in number of deaths and all-age and age-standardised death rates by location, 2022 and 2050

Vanuatu, Reference scenario

Legend:
Communicable, maternal, neonatal and nutritional
Non-communicable
Injuries

Leading Causes 2022	Leading Causes 2050	Mean % change number of DEATH	Mean % change all-age DEATH rate	Mean % change age-standardized DEATH rate
1 Ischemic heart disease	1 Ischemic heart disease	**67 (17.7 to 132)**	6.69 (-23.8 to 53)	-29.6 (-55.4 to 10.2)
2 COVID-19	2 Stroke	**55.5 (25.8 to 85.9)**	-0.679 (-19.3 to 22)	**-36.4 (-53 to -15.2)**
3 Stroke	3 Diabetes	**96.3 (53.1 to 148)**	25.4 (-3.89 to 59.9)	-14.8 (-42 to 21.5)
4 Diabetes	4 COPD	**98.1 (61.8 to 147)**	**26.5 (3.23 to 60)**	-25.3 (-45.1 to 1.82)
5 Lower respiratory infect	5 Chronic kidney disease	**207 (132 to 295)**	**95.8 (51.5 to 154)**	34.5 (-8 to 89.2)
6 COPD	6 Lower respiratory infect	**15.8 (0.702 to 32.1)**	**-26.1 (-35.1 to -17.5)**	**-38.3 (-47.8 to -24.4)**
7 Neonatal disorders	7 HIV/AIDS	**735 (356 to 1220)**	**433 (188 to 754)**	**409 (172 to 714)**
8 Road injuries	8 Hypertensive heart disease	**137 (70.7 to 210)**	**51.2 (12.2 to 104)**	-6.49 (-40.3 to 43.3)
9 Chronic kidney disease	9 Road injuries	26.6 (-25.7 to 112)	-19.1 (-52.1 to 46.3)	-27.7 (-57.3 to 30.8)
10 Cirrhosis liver	10 Self-harm	**42.5 (12.4 to 80.2)**	-9.02 (-27.3 to 15.5)	-17.6 (-33.7 to 3.85)
11 Self-harm	11 Cirrhosis liver	**27 (5.14 to 52.5)**	**-18.9 (-33.1 to -0.23)**	**-37.4 (-48.7 to -21.5)**
12 Hypertensive heart disease	12 Lung cancer	**122 (56.1 to 205)**	**41.4 (1.42 to 91.7)**	-7.9 (-28.4 to 13.5)
13 Diarrheal diseases	13 Alzheimer's disease	**262 (167 to 379)**	**130 (70.4 to 204)**	**6.55 (2.9 to 10.4)**
14 Tuberculosis	14 Diarrheal diseases	**58.2 (10.8 to 106)**	0.979 (-27.3 to 34)	-26.5 (-54 to 16.3)
15 Asthma	15 Breast cancer	**139 (62.4 to 233)**	**52.6 (7.87 to 108)**	10.1 (-18.1 to 39.5)
16 Lung cancer	16 Colorectal cancer	**162 (71.2 to 270)**	**66.9 (11.2 to 132)**	9.83 (-18 to 41)
17 Drowning	17 Neonatal disorders	**-42 (-64.2 to -9.59)**	**-63 (-77.1 to -44.5)**	**-48.1 (-66.8 to -21.8)**
18 Rheumatic heart disease	18 Stomach cancer	**48 (23.9 to 73.7)**	-5.63 (-18.6 to 7.8)	**-35.8 (-41.4 to -30.6)**
19 Stomach cancer	19 Prostate cancer	**190 (98.1 to 307)**	**85 (31.2 to 156)**	-1.41 (-15.3 to 16.1)
20 Congenital defects	20 Asthma	-3.43 (-19.9 to 20.4)	**-38.4 (-49.4 to -23.5)**	**-59 (-66.8 to -47.2)**
22 Breast cancer	21 Drowning			
23 Alzheimer's disease	25 Tuberculosis			
24 Colorectal cancer	27 Rheumatic heart disease			
26 Prostate cancer	28 Congenital defects			
27 HIV/AIDS	130 COVID-19			

Supplemental Results Figure S8. Leading 20 level 3 causes of deaths, and percentage change in number of deaths and all-age and age-standardised death rates by location, 2022 and 2050

Cambodia, Reference scenario

Legend:
Communicable, maternal, neonatal and nutritional
Non-communicable
Injuries

Leading Causes 2022	Leading Causes 2050	Mean % change number of DEATH	Mean % change all-age DEATH rate	Mean % change age-standardized DEATH rate
1 Stroke	1 Stroke	**49.6 (15.4 to 92.4)**	19.2 (-2.31 to 43.5)	**-39.9 (-53.7 to -23.8)**
2 Lower respiratory infect	2 Lower respiratory infect	**28.7 (2.21 to 62.6)**	2.49 (-14.8 to 21.1)	**-41.3 (-49.8 to -30.7)**
3 Ischemic heart disease	3 Ischemic heart disease	37.9 (-6.42 to 94.2)	9.98 (-21.6 to 48.2)	**-42.7 (-62.1 to -13.3)**
4 Cirrhosis liver	4 Cirrhosis liver	**87.8 (51.4 to 126)**	**49.7 (26 to 76.1)**	-5.72 (-19.2 to 11.1)
5 Tuberculosis	5 Chronic kidney disease	**188 (115 to 283)**	**129 (79.6 to 190)**	33.8 (-0.0973 to 81.4)
6 COPD	6 COPD	**92.8 (44 to 151)**	**53.6 (20.6 to 89.7)**	**-27.5 (-45.6 to -5.5)**
7 Neonatal disorders	7 Hypertensive heart disease	**134 (61.9 to 228)**	**86.7 (36.8 to 147)**	-7.89 (-36.4 to 33.5)
8 Diabetes	8 Diabetes	**80.2 (32 to 137)**	**43.6 (9.22 to 78.3)**	-19.9 (-40.7 to 3.85)
9 Hypertensive heart disease	9 Lung cancer	**126 (60 to 203)**	**79.6 (34.5 to 131)**	0.888 (-19.5 to 23.2)
10 Lung cancer	10 Alzheimer's disease	**251 (152 to 364)**	**179 (114 to 263)**	**8.86 (4.72 to 12.8)**
11 Road injuries	11 Falls	**113 (62 to 181)**	**69.7 (37.7 to 108)**	-6.18 (-16.1 to 4.76)
12 Chronic kidney disease	12 Colorectal cancer	**180 (89.7 to 290)**	**123 (55.4 to 204)**	25.5 (-4.75 to 57.4)
13 Upper digest diseases	13 Tuberculosis	**-36.4 (-55.7 to -10.1)**	**-49.3 (-63.1 to -28.9)**	**-68.9 (-79.3 to -53.5)**
14 Falls	14 Breast cancer	**132 (59.5 to 206)**	**84.4 (36.6 to 132)**	15 (-10.8 to 43.1)
15 Congenital defects	15 Road injuries	13.6 (-21 to 59.2)	-9.4 (-36 to 32.1)	-24 (-47.6 to 12.9)
16 Colorectal cancer	16 Upper digest diseases	18.6 (-7.14 to 54.1)	-5.48 (-23.3 to 17)	**-49.1 (-59.6 to -35.3)**
17 Alzheimer's disease	17 Liver cancer	**167 (93.7 to 259)**	**113 (62.6 to 183)**	22.2 (-9.32 to 67)
18 Breast cancer	18 Stomach cancer	**49.1 (22.6 to 84.7)**	**18.8 (2.64 to 37.7)**	**-32.5 (-37.5 to -26.3)**
19 Stomach cancer	19 Pancreatic cancer	**299 (171 to 453)**	**218 (128 to 327)**	**79.2 (36.6 to 129)**
20 Asthma	20 Ileus & obstruction	**78.6 (34.9 to 129)**	**42.3 (10.1 to 75.8)**	-13.3 (-29.4 to 8.09)
24 Ileus & obstruction	25 Neonatal disorders			
25 Liver cancer	28 Congenital defects			
38 Pancreatic cancer	30 Asthma			

Supplemental Results Figure S8. Leading 20 level 3 causes of deaths, and percentage change in number of deaths and all-age and age-standardised death rates by location, 2022 and 2050

Indonesia, Reference scenario

Legend:
Communicable, maternal, neonatal and nutritional
Non-communicable
Injuries

Leading Causes 2022	Leading Causes 2050	Mean % change number of DEATH	Mean % change all-age DEATH rate	Mean % change age-standardized DEATH rate
1 Stroke	1 Stroke	**43 (19.2 to 72.4)**	**28 (6.95 to 55.3)**	**-49.7 (-58.9 to -38)**
2 Ischemic heart disease	2 Ischemic heart disease	21.6 (-12.4 to 55.4)	8.93 (-20.7 to 44.1)	**-54.4 (-68.4 to -34.9)**
3 Cirrhosis liver	3 COPD	**167 (114 to 233)**	**139 (93.9 to 199)**	-14 (-32.6 to 10.8)
4 COPD	4 Hypertensive heart disease	**178 (102 to 264)**	**149 (82.1 to 232)**	-6.01 (-35.4 to 33.3)
5 Tuberculosis	5 Cirrhosis liver	**61.5 (36.8 to 84.7)**	**44.5 (28.5 to 63.4)**	**-27.4 (-35.3 to -17.5)**
6 COVID-19	6 Chronic kidney disease	**163 (105 to 225)**	**136 (88.3 to 200)**	18.1 (-10 to 56.6)
7 Diabetes	7 Lung cancer	**141 (78.5 to 216)**	**115 (63.9 to 171)**	0.671 (-18.1 to 20.5)
8 Hypertensive heart disease	8 Diabetes	**85.3 (45.8 to 130)**	**65.9 (34.8 to 107)**	**-25.8 (-42.7 to -4.99)**
9 Lung cancer	9 Alzheimer's disease	**349 (248 to 493)**	**301 (219 to 415)**	2.9 (-1.75 to 7.7)
10 Chronic kidney disease	10 Diarrheal diseases	**109 (41.6 to 205)**	**87.5 (25.7 to 175)**	-30.6 (-53.5 to 5.22)
11 Diarrheal diseases	11 Lower respiratory infect	**118 (85.4 to 162)**	**95 (69.4 to 127)**	**-24.6 (-34.4 to -13.7)**
12 Lower respiratory infect	12 Tuberculosis	-9.28 (-34.4 to 17.4)	-18.7 (-39.4 to 6.45)	**-60.6 (-72.2 to -44.3)**
13 Road injuries	13 Colorectal cancer	**151 (74.5 to 241)**	**124 (61.5 to 200)**	2.72 (-20.9 to 27.9)
14 Neonatal disorders	14 Protein-energy malnutrition	**196 (93.4 to 340)**	**165 (78.7 to 290)**	-18.7 (-53.3 to 43.9)
15 Colorectal cancer	15 Breast cancer	**94.4 (42.1 to 160)**	**73.6 (29 to 127)**	1.3 (-20.5 to 24.3)
16 Asthma	16 Falls	**140 (86.2 to 206)**	**115 (69.8 to 160)**	**-12.7 (-20.6 to -2.28)**
17 Breast cancer	17 Road injuries	3.75 (-23.8 to 43.9)	-7.06 (-32.4 to 28.3)	-23.1 (-46.3 to 5.68)
18 Alzheimer's disease	18 Ileus & obstruction	**165 (118 to 238)**	**138 (95.5 to 204)**	-3.43 (-21.7 to 18.4)
19 Falls	19 Atrial fibrillation	**404 (288 to 591)**	**350 (246 to 477)**	**12.9 (0.84 to 24)**
20 Protein-energy malnutrition	20 Prostate cancer	**265 (168 to 380)**	**226 (145 to 317)**	10.7 (-6.59 to 28.6)
22 Ileus & obstruction	25 Asthma			
27 Prostate cancer	31 Neonatal disorders			
34 Atrial fibrillation	133 COVID-19			

Supplemental Results Figure S8. Leading 20 level 3 causes of deaths, and percentage change in number of deaths and all-age and age-standardised death rates by location, 2022 and 2050

Laos, Reference scenario

Legend:
Communicable, maternal, neonatal and nutritional
Non-communicable
Injuries

Leading Causes 2022	Leading Causes 2050	Mean % change number of DEATH	Mean % change all-age DEATH rate	Mean % change age-standardized DEATH rate
1 Stroke	1 Stroke	**45.1 (15.4 to 84.8)**	13.6 (-9.89 to 37.7)	**-49.3 (-61.5 to -34.7)**
2 Ischemic heart disease	2 Ischemic heart disease	38.5 (-2.65 to 90.6)	8.45 (-23.7 to 46.5)	**-51.2 (-67.4 to -27)**
3 COVID-19	3 Chronic kidney disease	**207 (134 to 306)**	**141 (79.7 to 209)**	20 (-11 to 59.2)
4 Lower respiratory infect	4 COPD	**137 (88.8 to 193)**	**85.8 (45.3 to 126)**	**-24.9 (-43.5 to -4.6)**
5 Neonatal disorders	5 Lower respiratory infect	**24.6 (0.822 to 61.8)**	-2.49 (-19.4 to 23)	**-43.6 (-51.7 to -33.8)**
6 COPD	6 Hypertensive heart disease	**167 (86.9 to 273)**	**109 (47.2 to 184)**	-12 (-41.8 to 29.4)
7 Chronic kidney disease	7 Lung cancer	**180 (100 to 264)**	**119 (62.6 to 182)**	3.32 (-17.9 to 25.1)
8 Tuberculosis	8 Diabetes	**70.1 (24.1 to 120)**	33.3 (-2.4 to 73)	**-36.9 (-54.8 to -15.5)**
9 Diabetes	9 Cirrhosis liver	**73.9 (46 to 109)**	**36.2 (14.1 to 61)**	**-23.6 (-35 to -9.7)**
10 Cirrhosis liver	10 Alzheimer's disease	**314 (199 to 429)**	**224 (148 to 300)**	**8.12 (4.66 to 11.9)**
11 Hypertensive heart disease	11 Colorectal cancer	**221 (118 to 339)**	**151 (76.1 to 240)**	21 (-7.98 to 55.8)
12 Congenital defects	12 Breast cancer	**162 (86.9 to 240)**	**105 (51.1 to 159)**	15.2 (-11.5 to 45.2)
13 Road injuries	13 Road injuries	11 (-27 to 67.6)	-13.1 (-42 to 28.9)	-27.4 (-51.9 to 11.2)
14 Lung cancer	14 Tuberculosis	**-31.4 (-53.1 to -1.91)**	**-46.2 (-63.3 to -25.4)**	**-70 (-79.7 to -56)**
15 Diarrheal diseases	15 Falls	**119 (76.7 to 170)**	**71.4 (40.8 to 102)**	-8.34 (-16.5 to 0.0707)
16 Asthma	16 Liver cancer	**175 (97.7 to 270)**	**116 (52.8 to 184)**	10.2 (-24.6 to 50.5)
17 Upper digest diseases	17 Upper digest diseases	**41.7 (9.36 to 81.9)**	10.9 (-13.8 to 37.5)	**-48.8 (-60.1 to -35.2)**
18 Colorectal cancer	18 Neonatal disorders	**-74 (-82.6 to -61.4)**	**-79.7 (-85.9 to -70.6)**	**-61.4 (-72.8 to -46.6)**
19 Interpersonal violence	19 Pancreatic cancer	**386 (237 to 552)**	**280 (166 to 398)**	**82 (41.3 to 133)**
20 Alzheimer's disease	20 Stomach cancer	**77.9 (46.8 to 112)**	**39.2 (19 to 60.4)**	**-32.2 (-37 to -26.7)**
21 Breast cancer	21 Asthma			
22 Falls	27 Congenital defects			
25 Stomach cancer	28 Diarrheal diseases			
26 Liver cancer	29 Interpersonal violence			
42 Pancreatic cancer	133 COVID-19			

Supplemental Results Figure S8. Leading 20 level 3 causes of deaths, and percentage change in number of deaths and all-age and age-standardised death rates by location, 2022 and 2050

Malaysia, Reference scenario

Legend:
Communicable, maternal, neonatal and nutritional
Non-communicable
Injuries

Leading Causes 2022	Leading Causes 2050	Mean % change number of DEATH	Mean % change all-age DEATH rate	Mean % change age-standardized DEATH rate
1 Ischemic heart disease	1 Lower respiratory infect	**114 (82.3 to 145)**	**75.9 (55.4 to 97.5)**	**-21.8 (-34 to -8.48)**
2 Stroke	2 Ischemic heart disease	3.38 (-25.9 to 41.7)	-14.9 (-38 to 15.3)	**-59.3 (-71.6 to -41.2)**
3 Lower respiratory infect	3 Stroke	**33 (8.47 to 64.5)**	9.55 (-10.9 to 33.3)	**-49 (-59.7 to -35.1)**
4 COVID-19	4 Chronic kidney disease	**168 (105 to 246)**	**120 (71.6 to 179)**	9.12 (-16.5 to 43.1)
5 Chronic kidney disease	5 Alzheimer's disease	**270 (200 to 356)**	**205 (153 to 255)**	**5.15 (1.37 to 9.03)**
6 Road injuries	6 COPD	**130 (83.7 to 195)**	**89.2 (49.2 to 134)**	-17.4 (-36.9 to 8.84)
7 COPD	7 Colorectal cancer	**160 (86 to 256)**	**114 (56.6 to 183)**	7.49 (-16 to 34.2)
8 Diabetes	8 Lung cancer	**147 (87.5 to 219)**	**103 (58.1 to 159)**	6.35 (-13.1 to 29.9)
9 Lung cancer	9 Road injuries	24.9 (-10.8 to 80.5)	2.86 (-26.3 to 47.6)	-18.6 (-42 to 20.3)
10 Cirrhosis liver	10 Cirrhosis liver	**81.5 (53.2 to 119)**	**49.5 (26.1 to 74.2)**	-15.1 (-27.7 to 0.483)
11 Colorectal cancer	11 Breast cancer	**115 (61.7 to 181)**	**77.1 (34.8 to 123)**	3.55 (-18.8 to 27.9)
12 Alzheimer's disease	12 Diabetes	**51.5 (18.2 to 94.3)**	24.8 (-1.15 to 54.3)	**-37.3 (-51.8 to -18.4)**
13 Breast cancer	13 Urinary diseases	**202 (147 to 256)**	**148 (112 to 188)**	10.3 (-0.956 to 25.7)
14 Urinary diseases	14 Liver cancer	**161 (97.8 to 256)**	**115 (61.8 to 190)**	17.8 (-14.1 to 63.2)
15 Tuberculosis	15 Atrial fibrillation	**316 (244 to 405)**	**243 (191 to 313)**	**15.1 (3.51 to 28.2)**
16 HIV/AIDS	16 Hypertensive heart disease	**152 (87 to 247)**	**107 (50.9 to 190)**	-3.8 (-31.4 to 41.3)
17 Stomach cancer	17 Prostate cancer	**195 (128 to 275)**	**143 (93.8 to 203)**	5.96 (-9.63 to 20.6)
18 Self-harm	18 Falls	**117 (79.9 to 157)**	**78.7 (55.6 to 105)**	**-10.6 (-18 to -2.38)**
19 Liver cancer	19 Decubitus ulcer	**167 (61.2 to 303)**	**119 (29.6 to 220)**	-9.2 (-38.8 to 23.3)
20 Falls	20 Parkinson's disease	**210 (161 to 260)**	**155 (126 to 183)**	3.87 (-5.7 to 14)
21 Hypertensive heart disease	26 Stomach cancer			
25 Decubitus ulcer	32 Self-harm			
27 Prostate cancer	38 HIV/AIDS			
31 Parkinson's disease	39 Tuberculosis			
37 Atrial fibrillation	131 COVID-19			

Supplemental Results Figure S8. Leading 20 level 3 causes of deaths, and percentage change in number of deaths and all-age and age-standardised death rates by location, 2022 and 2050

Maldives, Reference scenario

Legend:
Communicable, maternal, neonatal and nutritional
Non-communicable
Injuries

Leading Causes 2022	Leading Causes 2050	Mean % change number of DEATH	Mean % change all-age DEATH rate	Mean % change age-standardized DEATH rate
1 Ischemic heart disease	1 Stroke	**170 (113 to 235)**	**79.7 (41.7 to 125)**	**-48.3 (-60.8 to -30.2)**
2 Stroke	2 Ischemic heart disease	**147 (70.5 to 252)**	**64.9 (12.7 to 135)**	**-51 (-68 to -24.5)**
3 COPD	3 Chronic kidney disease	**408 (275 to 543)**	**238 (153 to 332)**	6.96 (-20.1 to 45.4)
4 Chronic kidney disease	4 COPD	**390 (274 to 502)**	**226 (149 to 311)**	-14.7 (-39.1 to 15.4)
5 COVID-19	5 Alzheimer's disease	**474 (360 to 597)**	**282 (210 to 347)**	**-5.81 (-9.23 to -2.26)**
6 Diabetes	6 Diabetes	**230 (149 to 320)**	**120 (66.9 to 180)**	**-33.7 (-50.5 to -11.4)**
7 Lower respiratory infect	7 Lower respiratory infect	**224 (169 to 276)**	**116 (85.7 to 143)**	**-35.7 (-45.3 to -22.4)**
8 Alzheimer's disease	8 Lung cancer	**437 (284 to 620)**	**256 (163 to 361)**	4.48 (-16.8 to 31.6)
9 Neonatal disorders	9 Hypertensive heart disease	**332 (198 to 504)**	**188 (98.2 to 304)**	-20 (-46.4 to 22.7)
10 Cirrhosis liver	10 Liver cancer	**457 (319 to 656)**	**271 (169 to 407)**	13.2 (-19.4 to 58)
11 Hypertensive heart disease	11 Endo/metab/blood/immune	**332 (205 to 426)**	**187 (103 to 241)**	7.79 (-5.56 to 26.4)
12 Lung cancer	12 Cirrhosis liver	**126 (76.4 to 179)**	**50.7 (22.4 to 82.9)**	**-38.5 (-48.5 to -25.4)**
13 Tuberculosis	13 Colorectal cancer	**398 (234 to 610)**	**231 (121 to 361)**	2.37 (-24.9 to 33.7)
14 Endo/metab/blood/immune	14 Atrial fibrillation	**560 (409 to 718)**	**339 (251 to 425)**	9.5 (-3.18 to 21.9)
15 Falls	15 Prostate cancer	**570 (387 to 760)**	**345 (241 to 447)**	13.3 (-7.25 to 32.5)
16 Congenital defects	16 Urinary diseases	**510 (383 to 630)**	**306 (227 to 377)**	18.4 (-6.21 to 54)
17 Drowning	17 Parkinson's disease	**439 (358 to 532)**	**259 (217 to 313)**	-8.59 (-18.3 to 1.66)
18 Liver cancer	18 Falls	**202 (94.2 to 276)**	**101 (31.4 to 147)**	**-21.6 (-31.5 to -14)**
19 Breast cancer	19 Interstitial lung disease	**349 (259 to 444)**	**199 (140 to 253)**	-5.66 (-15.7 to 6.14)
20 Road injuries	20 Pancreatic cancer	**644 (410 to 903)**	**394 (239 to 542)**	**50.1 (9.39 to 89.7)**
21 Colorectal cancer	21 Breast cancer			
25 Interstitial lung disease	28 Tuberculosis			
26 Parkinson's disease	35 Neonatal disorders			
27 Atrial fibrillation	38 Road injuries			
28 Urinary diseases	43 Drowning			
31 Prostate cancer	48 Congenital defects			
39 Pancreatic cancer	129 COVID-19			

Supplemental Results Figure S8. Leading 20 level 3 causes of deaths, and percentage change in number of deaths and all-age and age-standardised death rates by location, 2022 and 2050

Mauritius, Reference scenario

Legend:
Communicable, maternal, neonatal and nutritional
Non-communicable
Injuries

Leading Causes 2022	Leading Causes 2050	Mean % change number of DEATH	Mean % change all-age DEATH rate	Mean % change age-standardized DEATH rate
1 Diabetes	1 Chronic kidney disease	**80.6 (42.8 to 132)**	**110 (70.1 to 151)**	1.87 (-20.4 to 28.6)
2 Ischemic heart disease	2 Diabetes	15.8 (-6.11 to 44.2)	**34.6 (8.8 to 60)**	**-34.5 (-47.8 to -18.2)**
3 Chronic kidney disease	3 Ischemic heart disease	-9.8 (-31.4 to 19.3)	4.93 (-20.9 to 36)	**-58.5 (-71.3 to -40.8)**
4 Stroke	4 Stroke	-13.1 (-27.6 to 4.72)	1.06 (-15.1 to 19.6)	**-55.7 (-63 to -46.2)**
5 Hypertensive heart disease	5 Alzheimer's disease	**204 (139 to 265)**	**253 (194 to 308)**	**-4.43 (-7.6 to -0.998)**
6 Lower respiratory infect	6 Hypertensive heart disease	**90.2 (42.2 to 165)**	**121 (64.2 to 205)**	-14.7 (-39.5 to 25.7)
7 Alzheimer's disease	7 Lower respiratory infect	**91.7 (62.5 to 125)**	**123 (95.7 to 150)**	**-19.1 (-28.7 to -7.75)**
8 COPD	8 COPD	**130 (90.7 to 183)**	**168 (124 to 216)**	-9.6 (-27.9 to 15.1)
9 Colorectal cancer	9 Colorectal cancer	**71.9 (24.5 to 128)**	**99.5 (50.4 to 155)**	-1.54 (-22 to 22.5)
10 Breast cancer	10 Interstitial lung disease	**104 (69.3 to 144)**	**137 (108 to 166)**	-4.72 (-11.9 to 4.33)
11 Cirrhosis liver	11 Lung cancer	**68.7 (30.6 to 114)**	**96 (53.1 to 142)**	2.85 (-14.6 to 21.2)
12 Road injuries	12 Breast cancer	**35.4 (4.19 to 72)**	**57.2 (28.2 to 96.5)**	-7.26 (-23.6 to 12.3)
13 Lung cancer	13 Prostate cancer	**121 (73.2 to 181)**	**157 (109 to 213)**	2.12 (-9.73 to 16.7)
14 Stomach cancer	14 Atrial fibrillation	**215 (159 to 278)**	**266 (213 to 332)**	-3.07 (-12.2 to 6.31)
15 Interstitial lung disease	15 Pancreatic cancer	**123 (62.8 to 189)**	**159 (96 to 229)**	**38.1 (8.3 to 68.7)**
16 Self-harm	16 Cirrhosis liver	-6.7 (-20.3 to 11)	8.49 (-4.73 to 21)	**-26.7 (-36.4 to -17)**
17 Prostate cancer	17 Stomach cancer	2.57 (-14.3 to 20.3)	**19.2 (5.74 to 31.4)**	**-38.9 (-43.3 to -34.5)**
18 Asthma	18 Urinary diseases	**169 (120 to 222)**	**213 (169 to 260)**	**20.3 (2.08 to 43)**
19 COVID-19	19 Parkinson's disease	**128 (88.4 to 168)**	**165 (135 to 200)**	-8.56 (-15.7 to 0.656)
20 Pancreatic cancer	20 Road injuries	-23.8 (-44.5 to 3.64)	-11.3 (-32.4 to 18.8)	-19.1 (-40.5 to 8.91)
23 Atrial fibrillation	22 Self-harm			
25 Parkinson's disease	25 Asthma			
28 Urinary diseases	132 COVID-19			

Supplemental Results Figure S8. Leading 20 level 3 causes of deaths, and percentage change in number of deaths and all-age and age-standardised death rates by location, 2022 and 2050

Myanmar, Reference scenario

Legend:
Communicable, maternal, neonatal and nutritional
Non-communicable
Injuries

Leading Causes 2022	Leading Causes 2050	Mean % change number of DEATH	Mean % change all-age DEATH rate	Mean % change age-standardized DEATH rate
1 Stroke	1 Stroke	25.2 (-3.01 to 60.1)	11.8 (-10.3 to 35.3)	**-43.3 (-57.2 to -27)**
2 Ischemic heart disease	2 COPD	**74.4 (28.4 to 123)**	**55.7 (17.6 to 91.2)**	**-30 (-48.9 to -6)**
3 COPD	3 Ischemic heart disease	22.6 (-16.3 to 69.4)	9.45 (-20.9 to 46.5)	**-45.9 (-64.8 to -18.7)**
4 Diabetes	4 Diabetes	**38.2 (1.72 to 82.1)**	23.3 (-5.76 to 53.2)	**-34.3 (-51.7 to -13.4)**
5 Lower respiratory infect	5 Chronic kidney disease	**156 (87.9 to 234)**	**129 (73.6 to 189)**	27.6 (-8.63 to 71.7)
6 Tuberculosis	6 Hypertensive heart disease	**122 (55.7 to 209)**	**98.6 (39 to 167)**	-2.77 (-35.1 to 40.2)
7 Neonatal disorders	7 Lower respiratory infect	12.3 (-9.6 to 37.6)	0.24 (-15.2 to 17)	**-41.7 (-49.9 to -32)**
8 Cirrhosis liver	8 Cirrhosis liver	**80.1 (45.6 to 120)**	**60.9 (34.7 to 85.8)**	**20.2 (0.658 to 40.5)**
9 Hypertensive heart disease	9 Alzheimer's disease	**206 (115 to 309)**	**173 (101 to 260)**	3.73 (-0.489 to 8.09)
10 Chronic kidney disease	10 Lung cancer	**74.8 (23.9 to 132)**	**56 (15.2 to 108)**	-12.9 (-28 to 8.18)
11 Asthma	11 Colorectal cancer	**120 (47.5 to 207)**	**96 (34.4 to 170)**	8.53 (-19.3 to 35.9)
12 Lung cancer	12 Falls	**90.1 (49.6 to 140)**	**69.6 (36.5 to 107)**	-6.88 (-15.1 to 1.64)
13 Congenital defects	13 Tuberculosis	**-35.1 (-54.6 to -9.66)**	**-42 (-58.3 to -20.3)**	**-64.9 (-76.2 to -48.2)**
14 Road injuries	14 Breast cancer	**75.6 (29.7 to 136)**	**56.8 (14.5 to 105)**	2.95 (-19.7 to 29.9)
15 Alzheimer's disease	15 Asthma	-14.1 (-32.3 to 9.5)	**-23.3 (-36.6 to -9.67)**	**-61.6 (-68.5 to -53)**
16 Falls	16 Road injuries	7.1 (-25 to 52.2)	-4.36 (-32.4 to 39.6)	-16.1 (-40.9 to 24.1)
17 Alcohol use disorders	17 Alcohol use disorders	30.1 (-3.26 to 65.6)	16.2 (-12.8 to 45.1)	-4.45 (-27.4 to 20.6)
18 Colorectal cancer	18 Atrial fibrillation	**276 (164 to 410)**	**235 (142 to 334)**	**26.8 (14.6 to 40.4)**
19 Breast cancer	19 Neonatal disorders	**-60.5 (-72.5 to -42)**	**-64.8 (-75.5 to -49.3)**	**-43.9 (-61 to -20)**
20 Diarrheal diseases	20 Urinary diseases	**175 (114 to 240)**	**145 (99.4 to 199)**	**26.4 (1.59 to 52)**
31 Urinary diseases	21 Diarrheal diseases			
37 Atrial fibrillation	29 Congenital defects			

Supplemental Results Figure S8. Leading 20 level 3 causes of deaths, and percentage change in number of deaths and all-age and age-standardised death rates by location, 2022 and 2050

Philippines, Reference scenario

Legend:
Communicable, maternal, neonatal and nutritional
Non-communicable
Injuries

Leading Causes 2022	Leading Causes 2050	Mean % change number of DEATH	Mean % change all-age DEATH rate	Mean % change age-standardized DEATH rate
1 Ischemic heart disease	1 Ischemic heart disease	35.6 (-1.42 to 78.4)	2.59 (-25 to 31.3)	**-50 (-63.5 to -31.4)**
2 Stroke	2 Lower respiratory infect	**107 (72.4 to 145)**	**56.7 (36.4 to 79.1)**	**-28.4 (-37.3 to -18.2)**
3 Lower respiratory infect	3 Stroke	**43 (20.5 to 70.7)**	8.13 (-6.68 to 23.5)	**-46.4 (-54.5 to -37)**
4 Chronic kidney disease	4 Chronic kidney disease	**195 (135 to 268)**	**123 (80.7 to 170)**	21.1 (-3.73 to 55.6)
5 Diabetes	5 Hypertensive heart disease	**202 (127 to 297)**	**128 (69.8 to 191)**	7.56 (-22.8 to 46.6)
6 Tuberculosis	6 Diabetes	**76.7 (41.4 to 111)**	**33.7 (8.76 to 60.2)**	**-30 (-44.2 to -13.5)**
7 Hypertensive heart disease	7 COPD	**119 (72.5 to 170)**	**65.8 (32.5 to 95.6)**	**-25.9 (-40.3 to -7.93)**
8 COPD	8 Alzheimer's disease	**286 (209 to 367)**	**192 (142 to 243)**	-0.459 (-5.03 to 4.15)
9 Neonatal disorders	9 Lung cancer	**113 (67.3 to 166)**	**61 (29.6 to 92.4)**	-12.3 (-26.4 to 1.96)
10 Interpersonal violence	10 Colorectal cancer	**157 (92.4 to 231)**	**94.2 (46.6 to 144)**	6.2 (-14.6 to 28)
11 Cirrhosis liver	11 Cirrhosis liver	**77 (51.3 to 107)**	**33.9 (18.9 to 48.5)**	**-16.7 (-25.1 to -6.87)**
12 COVID-19	12 Breast cancer	**123 (67.3 to 183)**	**68.5 (34.9 to 109)**	2.96 (-16.1 to 25.8)
13 Lung cancer	13 Tuberculosis	-16.9 (-38.3 to 11.7)	**-37.1 (-54.3 to -17.4)**	**-62.5 (-73.4 to -47.9)**
14 Road injuries	14 Urinary diseases	**238 (179 to 312)**	**156 (123 to 201)**	**29.5 (13 to 48.1)**
15 Breast cancer	15 Road injuries	24.5 (-7.52 to 70.6)	-5.75 (-30 to 29)	-18.2 (-40.6 to 13.4)
16 Alzheimer's disease	16 Prostate cancer	**248 (164 to 343)**	**163 (106 to 226)**	14.6 (-2.98 to 33.6)
17 Colorectal cancer	17 Liver cancer	**167 (111 to 228)**	**102 (62.8 to 145)**	17.7 (-5.82 to 47)
18 Asthma	18 Interpersonal violence	-6.5 (-37.3 to 45.4)	-29.4 (-51.4 to 6.35)	**-36.9 (-56.7 to -6.23)**
19 Congenital defects	19 Atrial fibrillation	**354 (262 to 452)**	**243 (182 to 305)**	**16.4 (5.48 to 30)**
20 Upper digest diseases	20 Neonatal disorders	**-44.6 (-58.5 to -27.8)**	**-58.1 (-67.3 to -46.4)**	**-38.7 (-51.9 to -21.9)**
22 Liver cancer	23 Upper digest diseases			
24 Urinary diseases	24 Asthma			
26 Prostate cancer	37 Congenital defects			
37 Atrial fibrillation	133 COVID-19			

Supplemental Results Figure S8. Leading 20 level 3 causes of deaths, and percentage change in number of deaths and all-age and age-standardised death rates by location, 2022 and 2050

Seychelles, Reference scenario

Legend:
Communicable, maternal, neonatal and nutritional
Non-communicable
Injuries

Leading Causes 2022	Leading Causes 2050	Mean % change number of DEATH	Mean % change all-age DEATH rate	Mean % change age-standardized DEATH rate
1 Ischemic heart disease	1 Lower respiratory infect	**117 (82.4 to 151)**	**65.9 (45.3 to 86.3)**	**-16.9 (-27.8 to -4.52)**
2 Stroke	2 Hypertensive heart disease	**182 (100 to 285)**	**115 (58.9 to 190)**	10.1 (-21.8 to 56.4)
3 Lower respiratory infect	3 Chronic kidney disease	**157 (99 to 228)**	**96.3 (58.3 to 142)**	12 (-12.9 to 47.5)
4 COVID-19	4 Stroke	**29 (3.47 to 58.6)**	-1.49 (-17.2 to 18.2)	**-47.6 (-57.8 to -34.8)**
5 Hypertensive heart disease	5 Ischemic heart disease	13.3 (-21.6 to 60.8)	-13.5 (-36.9 to 19.5)	**-53.4 (-67.9 to -31.9)**
6 Chronic kidney disease	6 Prostate cancer	**209 (134 to 298)**	**136 (83 to 189)**	13.9 (-4.62 to 30.7)
7 Cirrhosis liver	7 COPD	**162 (98.6 to 224)**	**99.9 (59.4 to 141)**	-3.36 (-24.9 to 23)
8 Diabetes	8 Alzheimer's disease	**229 (165 to 307)**	**151 (110 to 192)**	**3.67 (0.091 to 7.31)**
9 COPD	9 Colorectal cancer	**143 (75.8 to 234)**	**85.5 (37.8 to 141)**	8.12 (-15.1 to 34.7)
10 Colorectal cancer	10 Diabetes	**70.1 (30.4 to 109)**	**29.9 (6.72 to 59.3)**	**-27.3 (-43.1 to -7.88)**
11 Prostate cancer	11 Cirrhosis liver	**44.5 (19.6 to 67.1)**	10.3 (-2.99 to 23.5)	**-17.1 (-26.4 to -5.07)**
12 Alzheimer's disease	12 Lung cancer	**130 (74 to 204)**	**75.4 (36.2 to 125)**	4.96 (-14.7 to 26.8)
13 Lung cancer	13 Urinary diseases	**229 (167 to 289)**	**151 (114 to 193)**	**28.8 (8.06 to 59.5)**
14 Breast cancer	14 Breast cancer	**91.8 (48 to 156)**	**46.4 (14.4 to 83.7)**	2.2 (-17.2 to 25.6)
15 Road injuries	15 Pancreatic cancer	**222 (138 to 326)**	**145 (84.5 to 216)**	**50.5 (19.8 to 85.3)**
16 Urinary diseases	16 Parkinson's disease	**209 (157 to 265)**	**136 (108 to 164)**	2.97 (-6.66 to 13.1)
17 Neonatal disorders	17 Atrial fibrillation	**275 (190 to 373)**	**186 (133 to 232)**	**15 (1.64 to 26.8)**
18 Drowning	18 Lip oral cavity cancer	**121 (81.8 to 166)**	**68.8 (43.5 to 90.6)**	8.25 (-4.47 to 21.3)
19 Cervical cancer	19 Cardiomyopathy	**110 (71.1 to 153)**	**59.9 (38.1 to 88.7)**	**-13.3 (-20.1 to -5.69)**
20 Lip oral cavity cancer	20 Road injuries	17.4 (-20.9 to 69.8)	-10.4 (-38.2 to 26.4)	-19.5 (-45.6 to 16.6)
21 Cardiomyopathy	24 Cervical cancer			
23 Pancreatic cancer	33 Drowning			
27 Parkinson's disease	40 Neonatal disorders			
36 Atrial fibrillation	130 COVID-19			

Supplemental Results Figure S8. Leading 20 level 3 causes of deaths, and percentage change in number of deaths and all-age and age-standardised death rates by location, 2022 and 2050

Sri Lanka, Reference scenario

Legend:
Communicable, maternal, neonatal and nutritional
Non-communicable
Injuries

Leading Causes 2022
1 Stroke
2 Ischemic heart disease
3 Diabetes
4 COPD
5 Chronic kidney disease
6 Lower respiratory infect
7 Hypertensive heart disease
8 Alzheimer's disease
9 Self-harm
10 Cirrhosis liver
11 Road injuries
12 Lung cancer
13 Urinary diseases
14 COVID-19
15 Asthma
16 Nature disaster
17 Breast cancer
18 Falls
19 Conflict & terror
20 Colorectal cancer
21 Lip oral cavity cancer
23 Parkinson's disease
28 Atrial fibrillation

Leading Causes 2050	Mean % change number of DEATH	Mean % change all-age DEATH rate	Mean % change age-standardized DEATH rate
1 Stroke	**45.3 (8.74 to 95.4)**	**56.9 (22.2 to 106)**	**-48.5 (-58.3 to -36.2)**
2 COPD	**167 (92 to 269)**	**188 (115 to 289)**	-4.12 (-26.1 to 25.8)
3 Ischemic heart disease	16.7 (-20.7 to 72.8)	26.2 (-14 to 78.3)	**-50.4 (-66.8 to -27.2)**
4 Diabetes	**65.1 (19.5 to 124)**	**78.5 (32.2 to 134)**	-27.1 (-44.5 to 0.427)
5 Chronic kidney disease	**173 (98.7 to 278)**	**195 (119 to 295)**	21.9 (-8.1 to 66.5)
6 Alzheimer's disease	**278 (151 to 441)**	**307 (187 to 475)**	**4.95 (1.52 to 8.81)**
7 Hypertensive heart disease	**146 (65.2 to 241)**	**166 (83 to 264)**	4.75 (-26.5 to 53.4)
8 Lower respiratory infect	**75.6 (33.8 to 138)**	**89.6 (49 to 141)**	**-28.8 (-37.1 to -15.5)**
9 Urinary diseases	**156 (89.3 to 265)**	**177 (113 to 281)**	8.19 (-6 to 26.5)
10 Cirrhosis liver	**35.2 (10.5 to 70.2)**	**46.1 (20.7 to 75.6)**	**-16.4 (-27.3 to -2.94)**
11 Self-harm	4.32 (-15.6 to 31)	12.7 (-4.17 to 32.5)	**-15 (-28 to -0.0403)**
12 Atrial fibrillation	**353 (207 to 567)**	**388 (242 to 586)**	**14.8 (4.8 to 26.9)**
13 Lung cancer	**65.9 (17.9 to 126)**	**78.8 (32.6 to 132)**	-5.62 (-23.7 to 14.3)
14 Falls	**98.7 (49.4 to 169)**	**114 (65.8 to 176)**	**-10.2 (-18.2 to -1.74)**
15 Breast cancer	**64.7 (19.5 to 129)**	**77.5 (29.2 to 135)**	-4.22 (-24.6 to 14.8)
16 Parkinson's disease	**185 (118 to 272)**	**208 (146 to 288)**	2.81 (-7.4 to 13.5)
17 Lip oral cavity cancer	**126 (79.8 to 187)**	**143 (99.2 to 205)**	**18.5 (5.28 to 31.8)**
18 Road injuries	-3.5 (-29.3 to 33)	4.41 (-25.6 to 45.6)	-16.2 (-41.4 to 22.4)
19 Colorectal cancer	**103 (33.3 to 188)**	**118 (47.6 to 194)**	1.2 (-21.5 to 26.2)
20 Asthma	4.89 (-23.9 to 47)	13.3 (-13.1 to 53.7)	**-59.1 (-65.2 to -49.9)**
22 Nature disaster			
24 Conflict & terror			
131 COVID-19			

Supplemental Results Figure S8. Leading 20 level 3 causes of deaths, and percentage change in number of deaths and all-age and age-standardised death rates by location, 2022 and 2050

Thailand, Reference scenario

Legend:
Communicable, maternal, neonatal and nutritional
Non-communicable
Injuries

Leading Causes 2022	Leading Causes 2050	Mean % change number of DEATH	Mean % change all-age DEATH rate	Mean % change age-standardized DEATH rate
1 Stroke	1 Stroke	15.1 (-13.1 to 50.3)	**33.7 (4.1 to 69.4)**	**-49 (-59.8 to -36.8)**
2 Ischemic heart disease	2 Chronic kidney disease	**134 (70 to 223)**	**172 (106 to 260)**	2.3 (-22.3 to 32.2)
3 Lower respiratory infect	3 Alzheimer's disease	**223 (145 to 327)**	**276 (204 to 356)**	**-7.67 (-11 to -4.23)**
4 Chronic kidney disease	4 Lower respiratory infect	**84.2 (44.4 to 133)**	**114 (79.5 to 157)**	**-33.1 (-41.6 to -22.3)**
5 Lung cancer	5 Ischemic heart disease	2.85 (-33.4 to 47.5)	19.6 (-17.9 to 63.3)	**-58 (-71.2 to -41.8)**
6 Diabetes	6 COPD	**121 (63.4 to 195)**	**157 (98.4 to 230)**	-19.7 (-35.8 to 1.69)
7 Alzheimer's disease	7 Lung cancer	**67.3 (21.9 to 129)**	**94.3 (53.7 to 143)**	-12.1 (-27.1 to 3.38)
8 COPD	8 Urinary diseases	**253 (164 to 375)**	**310 (220 to 433)**	**21.8 (3.44 to 49.7)**
9 Road injuries	9 Diabetes	32.1 (-3.86 to 76.9)	**53.5 (16.2 to 104)**	**-36.1 (-49.1 to -17)**
10 Cirrhosis liver	10 Colorectal cancer	**84.6 (28.3 to 155)**	**114 (58.1 to 178)**	-3.34 (-24.5 to 22.6)
11 Colorectal cancer	11 Cirrhosis liver	**25.4 (2.21 to 55.3)**	**45.8 (24.6 to 68.1)**	-3.92 (-17.1 to 9.21)
12 HIV/AIDS	12 Liver cancer	**100 (50.9 to 169)**	**133 (80.6 to 203)**	27.5 (-0.989 to 68.3)
13 COVID-19	13 Atrial fibrillation	**277 (181 to 407)**	**338 (249 to 439)**	3.07 (-6.86 to 13.3)
14 Liver cancer	14 Diarrheal diseases	**144 (57 to 274)**	**184 (89.1 to 318)**	-17.5 (-42.8 to 19)
15 Urinary diseases	15 Road injuries	-27.6 (-47.3 to 0.773)	-15.8 (-35.5 to 12.2)	-17.7 (-39.7 to 12.1)
16 Tuberculosis	16 Parkinson's disease	**160 (109 to 234)**	**202 (158 to 251)**	-7.3 (-14.9 to 2.42)
17 Breast cancer	17 Bacterial skin	**304 (159 to 483)**	**370 (215 to 576)**	**84.6 (32.5 to 159)**
18 Self-harm	18 Pancreatic cancer	**133 (69.5 to 218)**	**170 (108 to 245)**	**32.4 (4.33 to 65.2)**
19 Stomach cancer	19 Breast cancer	33.8 (-1.68 to 75.7)	**55.4 (23.4 to 92.1)**	-8.92 (-27.1 to 12.9)
20 Gallbladder cancer	20 Prostate cancer	**143 (83.6 to 228)**	**182 (119 to 252)**	-1.8 (-15.3 to 11.8)
21 Diarrheal diseases	26 Gallbladder cancer			
23 Parkinson's disease	27 Stomach cancer			
24 Pancreatic cancer	28 Tuberculosis			
25 Atrial fibrillation	32 Self-harm			
27 Prostate cancer	55 HIV/AIDS			
37 Bacterial skin	133 COVID-19			

Supplemental Results Figure S8. Leading 20 level 3 causes of deaths, and percentage change in number of deaths and all-age and age-standardised death rates by location, 2022 and 2050

Timor-Leste, Reference scenario

Legend:
Communicable, maternal, neonatal and nutritional
Non-communicable
Injuries

Leading Causes 2022	Leading Causes 2050	Mean % change number of DEATH	Mean % change all-age DEATH rate	Mean % change age-standardized DEATH rate
1 Stroke	1 Stroke	**27.8 (1.56 to 62.7)**	-8.79 (-27.6 to 14.4)	**-40.4 (-54.1 to -20.8)**
2 Ischemic heart disease	2 Ischemic heart disease	32.2 (-8.79 to 92.1)	-5.59 (-36.6 to 34.4)	**-39.4 (-62.5 to -5.06)**
3 Lower respiratory infect	3 Chronic kidney disease	**173 (108 to 271)**	**95 (45.5 to 157)**	32.7 (-1.5 to 87.7)
4 Tuberculosis	4 Lower respiratory infect	-4.99 (-23.5 to 19.3)	**-32.2 (-44.9 to -16.3)**	**-42.9 (-52.2 to -32.7)**
5 Neonatal disorders	5 COPD	**64.3 (25.8 to 113)**	17.3 (-12.4 to 51.4)	**-26.9 (-45.1 to -1.35)**
6 COPD	6 Hypertensive heart disease	**92.9 (37.1 to 162)**	37.8 (-5.71 to 90.6)	-13.1 (-42.9 to 36.5)
7 Chronic kidney disease	7 Tuberculosis	**-37.7 (-56.5 to -10.7)**	**-55.5 (-70.7 to -35.3)**	**-67.5 (-77.9 to -50.5)**
8 Hypertensive heart disease	8 Alzheimer's disease	**223 (115 to 344)**	**130 (58.3 to 212)**	**14.8 (10.6 to 19.2)**
9 Congenital defects	9 Lung cancer	**138 (61.6 to 218)**	**69.4 (15.9 to 130)**	16.6 (-10.2 to 45.1)
10 Diabetes	10 Cirrhosis liver	**54.1 (28.2 to 91.9)**	10 (-9.51 to 33.2)	**-24.2 (-35.5 to -7.79)**
11 Cirrhosis liver	11 Diabetes	**51.6 (13.7 to 104)**	8.24 (-21.4 to 44.6)	-25.7 (-47.2 to 5.28)
12 Road injuries	12 Colorectal cancer	**169 (71.7 to 283)**	**91.6 (25.9 to 180)**	29.4 (-5.45 to 68.1)
13 Diarrheal diseases	13 Road injuries	11.4 (-31 to 70.9)	-20.6 (-47.4 to 23.2)	-30.3 (-54.7 to 10.8)
14 Upper digest diseases	14 Neonatal disorders	**-67 (-79.7 to -50.3)**	**-76.6 (-84.4 to -66)**	**-61.1 (-73 to -43.8)**
15 Lung cancer	15 Breast cancer	**145 (68.8 to 247)**	**74.4 (23.9 to 133)**	18.5 (-10.2 to 51.5)
16 HIV/AIDS	16 Falls	**83.1 (42.6 to 131)**	**30.5 (6.36 to 60.8)**	-7.06 (-15.4 to 1.08)
17 Asthma	17 Upper digest diseases	15 (-10.3 to 44.5)	-17.9 (-36.4 to 0.669)	**-45.7 (-58.1 to -28.1)**
18 COVID-19	18 Congenital defects	**-46.2 (-63.5 to -27.1)**	**-61.9 (-69.8 to -53.3)**	**-41.4 (-49.2 to -31.6)**
19 Alzheimer's disease	19 Stomach cancer	**49.6 (22 to 81.5)**	6.74 (-9.32 to 25.7)	**-27.6 (-33.3 to -21.2)**
20 Drowning	20 Asthma	**-22.7 (-37.6 to -3.67)**	**-44.9 (-53.6 to -32.6)**	**-61.6 (-68 to -53)**
21 Falls	22 Diarrheal diseases			
22 Colorectal cancer	34 Drowning			
25 Stomach cancer	55 HIV/AIDS			
26 Breast cancer	132 COVID-19			

Supplemental Results Figure S8. Leading 20 level 3 causes of deaths, and percentage change in number of deaths and all-age and age-standardised death rates by location, 2022 and 2050

Viet Nam, Reference scenario

Legend:
Communicable, maternal, neonatal and nutritional
Non-communicable
Injuries

Leading Causes 2022	Leading Causes 2050	Mean % change number of DEATH	Mean % change all-age DEATH rate	Mean % change age-standardized DEATH rate
1 Stroke	1 Stroke	**61.8 (31.7 to 94.8)**	**45.8 (20.4 to 72.9)**	**-33.7 (-47.4 to -19.6)**
2 Ischemic heart disease	2 Ischemic heart disease	36.7 (-3.13 to 81.6)	23.1 (-11 to 64.4)	**-44.8 (-62.1 to -20.6)**
3 COPD	3 Chronic kidney disease	**221 (141 to 305)**	**189 (127 to 258)**	**39.5 (6.14 to 85.2)**
4 Diabetes	4 COPD	**122 (77.1 to 179)**	**100 (61.4 to 149)**	-16.9 (-34.5 to 12.3)
5 Cirrhosis liver	5 Cirrhosis liver	**179 (135 to 232)**	**151 (116 to 188)**	**56.2 (33 to 79.4)**
6 Chronic kidney disease	6 Alzheimer's disease	**231 (153 to 311)**	**198 (137 to 268)**	**6.38 (2.55 to 10.2)**
7 Road injuries	7 Diabetes	**85.6 (42.1 to 137)**	**67.2 (27.7 to 109)**	-22.5 (-41.9 to 2.94)
8 Lung cancer	8 Hypertensive heart disease	**182 (99.3 to 277)**	**154 (86.5 to 235)**	10.6 (-24.5 to 57.4)
9 Lower respiratory infect	9 Liver cancer	**246 (151 to 373)**	**212 (130 to 334)**	**91 (38.9 to 171)**
10 COVID-19	10 Lung cancer	**93.4 (42.7 to 146)**	**74.1 (31.1 to 119)**	-1.06 (-20.4 to 19.3)
11 Tuberculosis	11 Falls	**141 (77.5 to 210)**	**117 (65.3 to 175)**	-1.21 (-11 to 11.6)
12 Hypertensive heart disease	12 Lower respiratory infect	**70 (41.5 to 101)**	**53.1 (32.7 to 79.5)**	**-33 (-42.4 to -21.5)**
13 Alzheimer's disease	13 Colorectal cancer	**184 (93.4 to 279)**	**156 (79.8 to 235)**	**37.4 (4.33 to 73.6)**
14 Falls	14 Road injuries	29.7 (-7.05 to 79.6)	16.9 (-13.6 to 61.4)	-5.51 (-31 to 32.3)
15 Liver cancer	15 Atrial fibrillation	**332 (229 to 450)**	**289 (200 to 391)**	**35.8 (22.8 to 51.3)**
16 Colorectal cancer	16 Tuberculosis	-11.9 (-36.3 to 19.2)	-20.6 (-41 to 6.67)	**-59.8 (-71.3 to -41.7)**
17 Self-harm	17 Breast cancer	**119 (63.5 to 190)**	**97.1 (51.5 to 153)**	21.5 (-2.3 to 51.2)
18 Drowning	18 Parkinson's disease	**208 (153 to 269)**	**178 (136 to 220)**	5.24 (-4.55 to 14.5)
19 Stomach cancer	19 Self-harm	**56.3 (29 to 82.8)**	**40.8 (19.6 to 63.6)**	9.73 (-7.36 to 30.1)
20 Neonatal disorders	20 Stomach cancer	**34.2 (12.3 to 58.6)**	**20.9 (2.94 to 38.4)**	**-32.2 (-37.4 to -26.6)**
22 Breast cancer	28 Drowning			
24 Parkinson's disease	48 Neonatal disorders			
26 Atrial fibrillation	132 COVID-19			

Supplemental Results Figure S8. Leading 20 level 3 causes of deaths, and percentage change in number of deaths and all-age and age-standardised death rates by location, 2022 and 2050

Angola, Reference scenario

Legend:
Communicable, maternal, neonatal and nutritional
Non-communicable
Injuries

Leading Causes 2022	Leading Causes 2050	Mean % change number of DEATH	Mean % change all-age DEATH rate	Mean % change age-standardized DEATH rate
1 Neonatal disorders	1 Lower respiratory infect	**74.3 (38 to 115)**	-13.6 (-29.2 to 4.57)	**-30 (-38.8 to -19.4)**
2 HIV/AIDS	2 Stroke	**90.3 (42.9 to 139)**	-5.7 (-26.3 to 17)	**-49 (-58.8 to -37)**
3 Lower respiratory infect	3 Ischemic heart disease	**69.9 (19.3 to 131)**	-15.8 (-40 to 14.3)	**-54.4 (-68.7 to -35.1)**
4 Malaria	4 Hypertensive heart disease	**250 (168 to 340)**	**73.3 (36.1 to 115)**	-14.1 (-32.2 to 8.61)
5 Stroke	5 HIV/AIDS	-10.9 (-45.7 to 57.4)	**-55.8 (-73.2 to -23.5)**	**-63.6 (-77.7 to -39.7)**
6 Tuberculosis	6 Neonatal disorders	**-32.4 (-52.4 to -5.63)**	**-66.6 (-75 to -57)**	**-48.3 (-60.3 to -35.2)**
7 Road injuries	7 Road injuries	32.4 (-31 to 147)	-34.5 (-66 to 19.4)	-42.6 (-67.9 to 3.87)
8 Ischemic heart disease	8 Chronic kidney disease	**247 (162 to 347)**	**71.8 (30 to 117)**	5.79 (-17 to 34.4)
9 Diarrheal diseases	9 Cirrhosis liver	**117 (77.3 to 163)**	7.64 (-12.2 to 27.3)	**-26.4 (-38.1 to -10.9)**
10 Cirrhosis liver	10 Diabetes	**130 (70.3 to 198)**	14.2 (-11.7 to 45.9)	**-32.3 (-47.6 to -11.9)**
11 Congenital defects	11 Tuberculosis	-19.7 (-44.1 to 12.6)	**-60.2 (-72.8 to -43.7)**	**-71.1 (-78.9 to -60.4)**
12 COVID-19	12 Diarrheal diseases	2.38 (-29.4 to 38.5)	**-49.3 (-64.3 to -30.7)**	**-54.4 (-65.2 to -41)**
13 Hypertensive heart disease	13 Alzheimer's disease	**431 (282 to 583)**	**163 (93.7 to 244)**	2.46 (-0.876 to 5.52)
14 Diabetes	14 Malaria	-39.2 (-95.8 to 286)	-69.4 (-97.9 to 94.2)	-68.9 (-97.6 to 139)
15 Chronic kidney disease	15 COPD	**157 (101 to 217)**	**27.3 (0.393 to 55)**	**-33.8 (-46 to -17.4)**
16 Pertussis	16 Self-harm	**141 (93.2 to 201)**	19.3 (-3.28 to 44.2)	**-16.7 (-29.9 to -2.64)**
17 Meningitis	17 Breast cancer	**273 (186 to 382)**	**84.6 (43.7 to 139)**	**23.8 (2.48 to 49.8)**
18 Protein-energy malnutrition	18 Lung cancer	**240 (155 to 331)**	**68.6 (28.7 to 114)**	4.88 (-14.3 to 25.2)
19 Self-harm	19 Cardiomyopathy	**150 (106 to 199)**	**24.1 (7.19 to 43.4)**	**-20.5 (-23.7 to -17.1)**
20 COPD	20 Congenital defects	-18.6 (-38.8 to 6.04)	**-59.8 (-67.6 to -49.3)**	**-40.5 (-50.4 to -27)**
25 Cardiomyopathy	24 Pertussis			
26 Alzheimer's disease	28 Meningitis			
27 Breast cancer	34 Protein-energy malnutrition			
28 Lung cancer	136 COVID-19			

Supplemental Results Figure S8. Leading 20 level 3 causes of deaths, and percentage change in number of deaths and all-age and age-standardised death rates by location, 2022 and 2050

Central African Republic, Reference scenario

Legend:
Communicable, maternal, neonatal and nutritional
Non-communicable
Injuries

Leading Causes 2022	Leading Causes 2050	Mean % change number of DEATH	Mean % change all-age DEATH rate	Mean % change age-standardized DEATH rate
1 Tuberculosis	1 Lower respiratory infect	8.62 (-21 to 43.7)	-14.2 (-30.8 to 6.49)	**-22.1 (-33.5 to -9.84)**
2 COVID-19	2 Tuberculosis	**-42.8 (-63.7 to -14.7)**	**-54.8 (-69 to -33.8)**	**-65.7 (-76.7 to -49.7)**
3 Neonatal disorders	3 Ischemic heart disease	**62.2 (2.27 to 136)**	28.4 (-13.5 to 76)	-23.5 (-50.4 to 14.5)
4 Malaria	4 Stroke	29 (-6.72 to 75)	1.92 (-19.5 to 25.2)	**-41.1 (-54.9 to -25.1)**
5 Lower respiratory infect	5 Diarrheal diseases	-5.34 (-40.5 to 36.9)	-25.2 (-48.4 to 5.31)	-17 (-40.4 to 17.1)
6 Diarrheal diseases	6 Malaria	-35.7 (-95.3 to 180)	-48.5 (-96.1 to 143)	-43.6 (-95 to 164)
7 HIV/AIDS	7 Road injuries	-17.4 (-60.9 to 48.1)	-34.6 (-66.1 to 18.4)	-41.4 (-68.3 to 2.01)
8 Road injuries	8 Diabetes	**97.1 (37 to 167)**	**55.8 (19 to 98.6)**	-3.67 (-28.5 to 29.2)
9 Stroke	9 Neonatal disorders	**-59.4 (-74.3 to -41.9)**	**-68.1 (-76.8 to -56.6)**	**-45.1 (-58.3 to -28.9)**
10 Ischemic heart disease	10 Hypertensive heart disease	**118 (55.1 to 196)**	**71.9 (34.2 to 117)**	-15.4 (-36.1 to 10.3)
11 Cirrhosis liver	11 Cirrhosis liver	12.6 (-19.3 to 44.6)	-11.2 (-24.8 to 3.5)	**-38.5 (-47.9 to -27.8)**
12 Congenital defects	12 Chronic kidney disease	**100 (40.6 to 172)**	**58.3 (21.2 to 98.5)**	3.6 (-22.2 to 34.6)
13 Diabetes	13 COPD	**60.6 (16.5 to 110)**	**26.9 (1.44 to 56.7)**	**-31.2 (-45.4 to -12.4)**
14 Hypertensive heart disease	14 Self-harm	36.6 (-1.42 to 77.2)	7.68 (-12.4 to 27.4)	**-21.4 (-34.8 to -6.66)**
15 Protein-energy malnutrition	15 Cervical cancer	**46.5 (3.74 to 90.6)**	15.6 (-6.12 to 38.6)	**-20.9 (-35.6 to -3.84)**
16 Chronic kidney disease	16 Alzheimer's disease	**222 (106 to 376)**	**153 (76.5 to 234)**	2.97 (-2.55 to 7.98)
17 Maternal disorders	17 Congenital defects	**-52 (-66.5 to -33.1)**	**-62.3 (-69.4 to -53)**	**-40.1 (-49.3 to -27)**
18 Meningitis	18 Meningitis	**-34.8 (-59.2 to -3.2)**	**-48.4 (-65.6 to -22.5)**	**-46.1 (-62.3 to -21.5)**
19 Pertussis	19 Breast cancer	**128 (44 to 225)**	**79.5 (32 to 135)**	20.2 (-0.13 to 45.9)
20 COPD	20 Cardiomyopathy	**50.1 (3.08 to 101)**	18.1 (-0.216 to 40.6)	**-21.2 (-25.1 to -17)**
22 Self-harm	22 Pertussis			
25 Cervical cancer	23 Protein-energy malnutrition			
29 Cardiomyopathy	24 Maternal disorders			
34 Breast cancer	29 HIV/AIDS			
40 Alzheimer's disease	136 COVID-19			

Supplemental Results Figure S8. Leading 20 level 3 causes of deaths, and percentage change in number of deaths and all-age and age-standardised death rates by location, 2022 and 2050

Congo (Brazzaville), Reference scenario

Legend:
Communicable, maternal, neonatal and nutritional
Non-communicable
Injuries

Leading Causes 2022	Leading Causes 2050	Mean % change number of DEATH	Mean % change all-age DEATH rate	Mean % change age-standardized DEATH rate
1 HIV/AIDS	1 Ischemic heart disease	**67.9 (18.5 to 123)**	17.8 (-15.2 to 61.9)	**-43.8 (-61.2 to -18.1)**
2 Ischemic heart disease	2 Stroke	**76.2 (35.4 to 123)**	**23.6 (2.25 to 51)**	**-42.6 (-53.4 to -28.5)**
3 Stroke	3 Hypertensive heart disease	**247 (165 to 350)**	**143 (97.4 to 203)**	5.71 (-16.1 to 32.8)
4 Malaria	4 Lower respiratory infect	**117 (75.9 to 170)**	**52.1 (30.3 to 76.7)**	**-17.1 (-26.6 to -4.05)**
5 Lower respiratory infect	5 Chronic kidney disease	**217 (138 to 311)**	**123 (77.7 to 174)**	21.1 (-3.7 to 51.5)
6 Road injuries	6 Diabetes	**105 (54.1 to 163)**	**43.7 (13 to 80.9)**	**-24.1 (-39.7 to -3.91)**
7 Tuberculosis	7 Cirrhosis liver	**89.8 (54 to 135)**	**33.2 (12.5 to 58)**	**-17.8 (-28.9 to -3.44)**
8 Neonatal disorders	8 Road injuries	28.1 (-27.3 to 108)	-10.1 (-48.5 to 55.7)	-23.9 (-56.4 to 34.3)
9 Diabetes	9 COPD	**185 (124 to 260)**	**100 (65.5 to 144)**	-9.21 (-25.3 to 12.4)
10 Cirrhosis liver	10 Tuberculosis	-13.1 (-39 to 17)	**-39 (-55.2 to -16.7)**	**-61.4 (-72.3 to -46.3)**
11 Hypertensive heart disease	11 Diarrheal diseases	37.1 (-5.05 to 84.6)	-3.71 (-33.8 to 29.5)	**-31.2 (-48.5 to -5.65)**
12 Chronic kidney disease	12 Alzheimer's disease	**291 (184 to 412)**	**174 (116 to 239)**	2.67 (-1.03 to 6.74)
13 Diarrheal diseases	13 Breast cancer	**161 (82 to 244)**	**82.7 (38.5 to 137)**	9.32 (-11.5 to 30.7)
14 COVID-19	14 Neonatal disorders	**-36.6 (-54.3 to -14.2)**	**-55.6 (-65.4 to -42.2)**	**-31.3 (-44.9 to -12.4)**
15 COPD	15 Lung cancer	**190 (100 to 290)**	**103 (53 to 163)**	7.1 (-11.2 to 28.4)
16 Self-harm	16 Self-harm	**89.9 (51.5 to 136)**	**33.2 (11.9 to 57.9)**	-9.03 (-23.1 to 8.7)
17 Cervical cancer	17 HIV/AIDS	**-76.7 (-86.4 to -63.4)**	**-83.7 (-90.2 to -74.5)**	**-86.3 (-91.7 to -79)**
18 Breast cancer	18 Prostate cancer	**299 (157 to 453)**	**179 (93.1 to 290)**	19.2 (-3.55 to 45.4)
19 Congenital defects	19 Cervical cancer	**78.2 (40.2 to 121)**	**25 (3.97 to 48.1)**	**-21.6 (-33.3 to -7.44)**
20 Maternal disorders	20 Esophageal cancer	**188 (133 to 253)**	**102 (70.3 to 135)**	5.86 (-5.64 to 18.2)
22 Alzheimer's disease	22 Malaria			
23 Lung cancer	35 Congenital defects			
26 Esophageal cancer	43 Maternal disorders			
31 Prostate cancer	135 COVID-19			

Supplemental Results Figure S8. Leading 20 level 3 causes of deaths, and percentage change in number of deaths and all-age and age-standardised death rates by location, 2022 and 2050

DR Congo, Reference scenario

Legend:
Communicable, maternal, neonatal and nutritional
Non-communicable
Injuries

Leading Causes 2022	Leading Causes 2050	Mean % change number of DEATH	Mean % change all-age DEATH rate	Mean % change age-standardized DEATH rate
1 Malaria	1 Lower respiratory infect	**74.6 (21.8 to 132)**	-0.81 (-20.3 to 16.9)	**-29.8 (-39.4 to -20.6)**
2 Neonatal disorders	2 Stroke	**72.4 (17.2 to 134)**	-1.93 (-23 to 20.2)	**-47.2 (-58 to -33.4)**
3 Tuberculosis	3 Ischemic heart disease	**87.1 (19.8 to 182)**	6.62 (-27.8 to 56.2)	**-42.6 (-61 to -13.5)**
4 Lower respiratory infect	4 Hypertensive heart disease	**234 (121 to 371)**	**90.1 (45.2 to 141)**	-8.62 (-29.1 to 20.3)
5 Stroke	5 Road injuries	30.8 (-28.2 to 125)	-25.4 (-58.7 to 36)	-35.4 (-62.3 to 15.3)
6 Road injuries	6 Tuberculosis	-9.78 (-40.9 to 30.2)	**-48.6 (-63.8 to -24.7)**	**-64.8 (-74.6 to -48.3)**
7 Ischemic heart disease	7 Chronic kidney disease	**199 (104 to 316)**	**70.1 (31.6 to 123)**	1.76 (-21.4 to 34.2)
8 COVID-19	8 Diabetes	**145 (58.3 to 249)**	**39.1 (4.08 to 82.3)**	-19.4 (-38.7 to 4.34)
9 Cirrhosis liver	9 Cirrhosis liver	**99.7 (41.4 to 168)**	13.7 (-5.73 to 35.7)	**-26.4 (-37.3 to -14.6)**
10 Hypertensive heart disease	10 Malaria	-58.9 (-98.3 to 220)	-77.2 (-98.9 to 80.6)	-71.2 (-98.6 to 118)
11 Diarrheal diseases	11 COPD	**136 (62.6 to 231)**	**34.4 (5.95 to 66.8)**	**-30.4 (-43.9 to -14)**
12 Diabetes	12 Neonatal disorders	**-41.7 (-63.7 to -16.1)**	**-66.9 (-75.1 to -54.9)**	**-49 (-60.5 to -34.8)**
13 Chronic kidney disease	13 Alzheimer's disease	**322 (156 to 512)**	**140 (71.5 to 206)**	0.079 (-3.39 to 3.99)
14 COPD	14 Diarrheal diseases	38.6 (-13.9 to 109)	-21 (-49.3 to 15.7)	**-30 (-48 to -2.86)**
15 Maternal disorders	15 Self-harm	**122 (50.9 to 197)**	**26.1 (4.23 to 50.1)**	-11.8 (-25.2 to 2.41)
16 Congenital defects	16 Cervical cancer	**110 (40.4 to 182)**	19.7 (-0.397 to 49.5)	**-25.1 (-36.2 to -8.94)**
17 Measles	17 Breast cancer	**206 (88.4 to 348)**	**73.2 (27.3 to 117)**	6.26 (-13 to 26.7)
18 Self-harm	18 Cardiomyopathy	**135 (66.5 to 211)**	**33.7 (13.8 to 52.8)**	**-19.5 (-24.7 to -14.2)**
19 Meningitis	19 Prostate cancer	**348 (163 to 605)**	**154 (75.6 to 244)**	**27.8 (1.29 to 53.5)**
20 Pertussis	20 Esophageal cancer	**196 (104 to 302)**	**68 (43.3 to 98)**	-3.61 (-14.8 to 7.54)
21 Cervical cancer	22 Congenital defects			
24 Alzheimer's disease	26 Maternal disorders			
25 Cardiomyopathy	28 Meningitis			
30 Breast cancer	31 Pertussis			
34 Esophageal cancer	81 Measles			
41 Prostate cancer	136 COVID-19			

Supplemental Results Figure S8. Leading 20 level 3 causes of deaths, and percentage change in number of deaths and all-age and age-standardised death rates by location, 2022 and 2050

Equatorial Guinea, Reference scenario

Legend:
Communicable, maternal, neonatal and nutritional
Non-communicable
Injuries

Leading Causes 2022	Leading Causes 2050	Mean % change number of DEATH	Mean % change all-age DEATH rate	Mean % change age-standardized DEATH rate
1 HIV/AIDS	1 Lower respiratory infect	**203 (149 to 257)**	**48.5 (24.3 to 77.2)**	**-18.4 (-29 to -4.49)**
2 Malaria	2 Ischemic heart disease	**118 (50.7 to 200)**	7.07 (-25.4 to 48.3)	**-51.6 (-66.4 to -31.8)**
3 Neonatal disorders	3 HIV/AIDS	**-42.5 (-61.2 to -13.4)**	**-71.8 (-80.7 to -58.3)**	**-78.6 (-85.1 to -68.8)**
4 Ischemic heart disease	4 Chronic kidney disease	**334 (226 to 449)**	**113 (67.1 to 164)**	14.1 (-10.1 to 45.6)
5 Road injuries	5 Stroke	**148 (93.5 to 210)**	21.9 (-2.05 to 49.7)	**-45.3 (-55.9 to -29.3)**
6 Lower respiratory infect	6 Hypertensive heart disease	**416 (307 to 549)**	**153 (102 to 208)**	10.8 (-11.5 to 39.2)
7 Stroke	7 Diabetes	**231 (146 to 329)**	**62.6 (23.5 to 107)**	**-21.7 (-39.2 to -0.0243)**
8 Diabetes	8 Road injuries	**84.3 (7.99 to 239)**	-9.48 (-48.2 to 71.3)	-23.2 (-54.5 to 43.4)
9 COVID-19	9 Cirrhosis liver	**302 (235 to 374)**	**97.5 (61.4 to 137)**	1.47 (-13.4 to 20.5)
10 Chronic kidney disease	10 Neonatal disorders	18 (-14.3 to 58.1)	**-42.3 (-54.3 to -24.2)**	-17.9 (-34.5 to 5.87)
11 Tuberculosis	11 Malaria	-24.5 (-95.6 to 299)	-63.3 (-97.9 to 97.6)	-67.3 (-97.7 to 69.9)
12 Hypertensive heart disease	12 Alzheimer's disease	**407 (271 to 578)**	**148 (82.4 to 224)**	**6 (2.6 to 10.1)**
13 Cirrhosis liver	13 COPD	**315 (222 to 412)**	**103 (61.9 to 148)**	-7.27 (-25.7 to 14.6)
14 Congenital defects	14 Breast cancer	**324 (224 to 453)**	**108 (61 to 161)**	8.52 (-9.02 to 28.1)
15 Mechanical forces	15 Lung cancer	**386 (266 to 533)**	**138 (77.7 to 199)**	10.1 (-9.63 to 31.7)
16 Diarrheal diseases	16 Self-harm	**208 (144 to 275)**	**51.2 (25 to 84.1)**	-3.3 (-20 to 18.1)
17 Self-harm	17 Tuberculosis	**61 (16.3 to 123)**	-21 (-43.3 to 8.74)	**-56.1 (-67.1 to -40.2)**
18 COPD	18 Colorectal cancer	**417 (282 to 594)**	**153 (85.1 to 237)**	20.1 (-2.17 to 45.2)
19 Maternal disorders	19 Mechanical forces	**97.4 (52.3 to 150)**	-3.17 (-22.8 to 24.1)	**-22.6 (-36.3 to -1.45)**
20 Meningitis	20 Esophageal cancer	**418 (330 to 512)**	**154 (113 to 200)**	**11.6 (1.25 to 23.2)**
21 Breast cancer	27 Diarrheal diseases			
22 Alzheimer's disease	28 Congenital defects			
26 Lung cancer	29 Meningitis			
30 Colorectal cancer	34 Maternal disorders			
31 Esophageal cancer	134 COVID-19			

Supplemental Results Figure S8. Leading 20 level 3 causes of deaths, and percentage change in number of deaths and all-age and age-standardised death rates by location, 2022 and 2050

Gabon, Reference scenario

Legend:
Communicable, maternal, neonatal and nutritional
Non-communicable
Injuries

Leading Causes 2022	Leading Causes 2050	Mean % change number of DEATH	Mean % change all-age DEATH rate	Mean % change age-standardized DEATH rate
1 Ischemic heart disease	1 Chronic kidney disease	**165 (108 to 232)**	**72.2 (39.7 to 111)**	6.59 (-14 to 32.4)
2 HIV/AIDS	2 Lower respiratory infect	**95.6 (64.1 to 132)**	**27.2 (9.15 to 52.2)**	**-19.8 (-29.6 to -8.79)**
3 Stroke	3 Hypertensive heart disease	**192 (129 to 266)**	**89.7 (53.2 to 136)**	1.41 (-19.5 to 26.9)
4 Lower respiratory infect	4 Stroke	**47.8 (19.8 to 78.3)**	-3.95 (-20.4 to 15.8)	**-46.3 (-55.6 to -33.8)**
5 Malaria	5 Ischemic heart disease	25 (-9.77 to 67)	-18.7 (-39.8 to 7.21)	**-53.2 (-67.1 to -36.4)**
6 Diabetes	6 Diabetes	**83.3 (43.4 to 127)**	19.1 (-4.19 to 44.4)	**-26 (-39.7 to -8.32)**
7 Chronic kidney disease	7 Road injuries	34 (-22.7 to 136)	-13 (-48.6 to 57.4)	-26.1 (-55.1 to 32.7)
8 Road injuries	8 Cirrhosis liver	**71.7 (44.2 to 103)**	11.7 (-7.15 to 30.9)	**-24.8 (-34.3 to -12.5)**
9 Neonatal disorders	9 Alzheimer's disease	**234 (159 to 323)**	**117 (71.2 to 182)**	**3.95 (0.434 to 7.7)**
10 Hypertensive heart disease	10 COPD	**168 (113 to 226)**	**74.4 (44.3 to 117)**	-1.19 (-19 to 21.3)
11 COVID-19	11 Lung cancer	**167 (102 to 238)**	**73.7 (31.1 to 118)**	9.59 (-8.58 to 29.3)
12 Cirrhosis liver	12 Breast cancer	**170 (106 to 247)**	**75.4 (35.2 to 124)**	13.4 (-5.83 to 33.4)
13 Tuberculosis	13 Neonatal disorders	-25.6 (-47.6 to 3.21)	**-51.9 (-63 to -37.5)**	**-26.2 (-40.2 to -9.37)**
14 COPD	14 Tuberculosis	-11.1 (-35.2 to 18.3)	**-42.2 (-56.7 to -22.1)**	**-61.1 (-71.1 to -47.2)**
15 Alzheimer's disease	15 Colorectal cancer	**183 (112 to 269)**	**83.8 (35.1 to 146)**	13.5 (-7.35 to 36.2)
16 Diarrheal diseases	16 Self-harm	**85.4 (52.5 to 120)**	**20.5 (3.79 to 43)**	-13.2 (-24.7 to 0.501)
17 Self-harm	17 HIV/AIDS	**-68.1 (-78.6 to -57.6)**	**-79.3 (-86 to -72.5)**	**-82.7 (-88.6 to -77.7)**
18 Lung cancer	18 Prostate cancer	**198 (110 to 304)**	**93.5 (37.7 to 168)**	8.68 (-13.1 to 32.5)
19 Breast cancer	19 Liver cancer	**148 (90.8 to 213)**	**61.2 (26.1 to 102)**	2.09 (-17.6 to 28.9)
20 Colorectal cancer	20 Esophageal cancer	**140 (106 to 176)**	**55.9 (31.8 to 82.8)**	-3.45 (-11.3 to 5.52)
24 Liver cancer	21 Malaria			
25 Esophageal cancer	28 Diarrheal diseases			
27 Prostate cancer	135 COVID-19			

Supplemental Results Figure S8. Leading 20 level 3 causes of deaths, and percentage change in number of deaths and all-age and age-standardised death rates by location, 2022 and 2050

Burundi, Reference scenario

Legend:
Communicable, maternal, neonatal and nutritional
Non-communicable
Injuries

Leading Causes 2022	Leading Causes 2050	Mean % change number of DEATH	Mean % change all-age DEATH rate	Mean % change age-standardized DEATH rate
1 COVID-19	1 Stroke	**99.2 (51.2 to 154)**	8.26 (-17.2 to 31.2)	**-34.8 (-48.2 to -17.8)**
2 Malaria	2 Lower respiratory infect	**54.9 (19.6 to 96.5)**	-15.9 (-31.9 to 0.613)	**-28.6 (-38.4 to -16.8)**
3 Neonatal disorders	3 Ischemic heart disease	**148 (63.1 to 252)**	34.8 (-8.99 to 99.1)	-14.6 (-44 to 31.5)
4 Tuberculosis	4 Diarrheal diseases	51.3 (-0.72 to 116)	-17.7 (-44.5 to 18.8)	-21.4 (-44 to 14.9)
5 Lower respiratory infect	5 Tuberculosis	-17.9 (-44 to 17.3)	**-55.4 (-68.6 to -35.9)**	**-68.6 (-77.6 to -55.2)**
6 Stroke	6 Neonatal disorders	**-41.5 (-58.9 to -19.5)**	**-68.3 (-75.6 to -58.8)**	**-49.3 (-59.1 to -36.5)**
7 Diarrheal diseases	7 Chronic kidney disease	**230 (147 to 315)**	**79.3 (37.9 to 130)**	15.6 (-10.8 to 51.4)
8 Ischemic heart disease	8 Diabetes	**139 (71.9 to 202)**	29.9 (-2.2 to 65.3)	-17.6 (-38.1 to 7.63)
9 Road injuries	9 Cirrhosis liver	**64 (31.8 to 98.4)**	-10.8 (-24.9 to 4.72)	**-42.7 (-51.3 to -32.4)**
10 Cirrhosis liver	10 Road injuries	46.7 (-11.3 to 147)	-20.2 (-51.6 to 28.6)	**-39.1 (-60.2 to -5.43)**
11 Conflict & terror	11 Hypertensive heart disease	**145 (80.5 to 210)**	**33.4 (1.02 to 69.4)**	**-27.2 (-44.1 to -3.7)**
12 Congenital defects	12 COPD	**126 (74 to 179)**	23 (-2.23 to 51.1)	**-30.8 (-43.4 to -12.3)**
13 Diabetes	13 Alzheimer's disease	**260 (146 to 385)**	**95.7 (37.5 to 150)**	1.23 (-1.81 to 4.53)
14 Chronic kidney disease	14 Self-harm	**97.7 (59.3 to 142)**	7.4 (-11.5 to 26.6)	**-24.4 (-36.1 to -12)**
15 HIV/AIDS	15 Congenital defects	**-24.8 (-42.8 to -4.52)**	**-59.2 (-66.5 to -49.7)**	**-39 (-48.3 to -28.1)**
16 Protein-energy malnutrition	16 Falls	**162 (96.3 to 235)**	**42.1 (14.8 to 73.1)**	**-5.82 (-10.5 to -0.274)**
17 COPD	17 Urinary diseases	**208 (155 to 275)**	**67.1 (44.4 to 93.8)**	8.25 (-3.85 to 23.1)
18 Maternal disorders	18 Cervical cancer	**124 (73.2 to 173)**	22.1 (-1.42 to 48.2)	**-20.5 (-33.5 to -3.95)**
19 Hypertensive heart disease	19 Breast cancer	**217 (127 to 316)**	**72 (29.8 to 119)**	10.7 (-6.22 to 34)
20 Meningitis	20 Ileus & obstruction	**143 (87.3 to 206)**	**32.2 (5.49 to 58.9)**	-9.66 (-26.9 to 10.6)
21 Self-harm	21 Malaria			
22 Cervical cancer	26 Meningitis			
24 Falls	27 Protein-energy malnutrition			
27 Ileus & obstruction	28 Maternal disorders			
28 Alzheimer's disease	29 Conflict & terror			
30 Urinary diseases	55 HIV/AIDS			
33 Breast cancer	134 COVID-19			

Supplemental Results Figure S8. Leading 20 level 3 causes of deaths, and percentage change in number of deaths and all-age and age-standardised death rates by location, 2022 and 2050

Comoros, Reference scenario

Legend:
Communicable, maternal, neonatal and nutritional
Non-communicable
Injuries

Leading Causes 2022	Leading Causes 2050	Mean % change number of DEATH	Mean % change all-age DEATH rate	Mean % change age-standardized DEATH rate
1 Stroke	1 Stroke	**40.9 (1.56 to 93.8)**	20.8 (-8.38 to 54.4)	**-44.4 (-56.9 to -27.5)**
2 Lower respiratory infect	2 Chronic kidney disease	**193 (112 to 302)**	**151 (88.2 to 223)**	22.7 (-6.04 to 60.5)
3 Neonatal disorders	3 Lower respiratory infect	26.7 (-3.18 to 64.8)	8.69 (-13.2 to 31.6)	**-38.7 (-48.2 to -26.1)**
4 Tuberculosis	4 Ischemic heart disease	45.6 (-7.61 to 125)	24.7 (-16.7 to 79.7)	**-41.6 (-62.1 to -11.2)**
5 Ischemic heart disease	5 Hypertensive heart disease	**147 (76.6 to 240)**	**111 (60.9 to 164)**	-8.44 (-29.8 to 17.6)
6 Diabetes	6 Diabetes	**92.3 (37.8 to 174)**	**64.8 (20.1 to 117)**	-20.2 (-40.7 to 7.64)
7 Chronic kidney disease	7 Alzheimer's disease	**218 (109 to 321)**	**173 (104 to 242)**	1.48 (-1.61 to 4.37)
8 Road injuries	8 Tuberculosis	-21 (-45 to 12.9)	**-32.4 (-51.2 to -8.92)**	**-62.8 (-73.4 to -47.9)**
9 Cirrhosis liver	9 Cirrhosis liver	**45.3 (9.27 to 92.2)**	**24.6 (0.616 to 52.1)**	**-30.7 (-42 to -15.7)**
10 Hypertensive heart disease	10 Road injuries	18.6 (-25.1 to 83.4)	1.47 (-32.6 to 51.7)	-29.3 (-54.8 to 10.4)
11 Diarrheal diseases	11 COPD	**92.4 (37.5 to 160)**	**65 (26.9 to 106)**	**-28 (-42.5 to -8.76)**
12 COPD	12 Diarrheal diseases	12 (-25.2 to 65.6)	-3.97 (-31.3 to 30.7)	**-40.6 (-57 to -16.5)**
13 Congenital defects	13 Urinary diseases	**173 (103 to 248)**	**134 (87.5 to 182)**	**16.4 (1.51 to 33.9)**
14 Meningitis	14 Breast cancer	**118 (54 to 185)**	**87.4 (40.6 to 141)**	3.73 (-14.6 to 24.3)
15 Alzheimer's disease	15 Prostate cancer	**216 (104 to 326)**	**171 (82.4 to 261)**	15.2 (-9.35 to 41.1)
16 Interpersonal violence	16 Neonatal disorders	**-63.5 (-76.6 to -45.9)**	**-69 (-77.8 to -58.5)**	**-44.5 (-55.8 to -28.2)**
17 Malaria	17 Esophageal cancer	**101 (57.7 to 153)**	**72.3 (42.9 to 98.6)**	**-13.6 (-24.7 to -1.31)**
18 Cervical cancer	18 Colorectal cancer	**178 (85.5 to 281)**	**139 (70.3 to 218)**	16.8 (-6.14 to 42.2)
19 Breast cancer	19 Falls	**135 (69.2 to 202)**	**102 (62.2 to 145)**	**-5.66 (-10.2 to -0.781)**
20 Esophageal cancer	20 Ileus & obstruction	**105 (47.7 to 175)**	**75.8 (36.6 to 118)**	-1.77 (-21.2 to 25.2)
22 Urinary diseases	21 Cervical cancer			
23 Falls	23 Interpersonal violence			
25 Ileus & obstruction	26 Meningitis			
27 Prostate cancer	35 Congenital defects			
28 Colorectal cancer	70 Malaria			

Supplemental Results Figure S8. Leading 20 level 3 causes of deaths, and percentage change in number of deaths and all-age and age-standardised death rates by location, 2022 and 2050

Djibouti, Reference scenario

Legend:
Communicable, maternal, neonatal and nutritional
Non-communicable
Injuries

Leading Causes 2022	Leading Causes 2050	Mean % change number of DEATH	Mean % change all-age DEATH rate	Mean % change age-standardized DEATH rate
1 HIV/AIDS	1 Stroke	**131 (65.8 to 209)**	**49.8 (17.4 to 97.4)**	**-43.5 (-55.7 to -25.3)**
2 Stroke	2 Lower respiratory infect	**145 (83.2 to 209)**	**59.1 (28.1 to 94.9)**	**-24.8 (-35.4 to -10.5)**
3 Lower respiratory infect	3 Chronic kidney disease	**349 (223 to 497)**	**191 (127 to 269)**	21.5 (-4.93 to 56.8)
4 Ischemic heart disease	4 Ischemic heart disease	**91.3 (18.8 to 192)**	24.1 (-15.7 to 82.7)	**-51.1 (-68.1 to -25.1)**
5 Tuberculosis	5 Hypertensive heart disease	**262 (161 to 369)**	**135 (85.5 to 190)**	-21.7 (-38.6 to 0.077)
6 Neonatal disorders	6 Diabetes	**151 (70 to 254)**	**62.6 (16.4 to 117)**	**-32.3 (-49.8 to -5.72)**
7 Cirrhosis liver	7 Cirrhosis liver	**92.4 (44.9 to 147)**	**25 (1.84 to 54.3)**	**-36.6 (-46.9 to -23.1)**
8 Diabetes	8 Alzheimer's disease	**520 (342 to 749)**	**302 (198 to 419)**	0.509 (-2.76 to 3.83)
9 Chronic kidney disease	9 Tuberculosis	-7.43 (-37.2 to 28.6)	**-39.9 (-55.8 to -20.7)**	**-69.6 (-77.8 to -59.2)**
10 Diarrheal diseases	10 HIV/AIDS	**-60.5 (-84.1 to -17.8)**	**-74.3 (-88.7 to -45.5)**	**-79.5 (-91.1 to -55.9)**
11 Road injuries	11 COPD	**256 (159 to 367)**	**131 (81.5 to 190)**	-18.4 (-34.1 to 2.89)
12 Hypertensive heart disease	12 Breast cancer	**261 (169 to 362)**	**135 (86 to 196)**	16.3 (-1.41 to 34.7)
13 Interpersonal violence	13 Colorectal cancer	**321 (195 to 471)**	**174 (99.6 to 267)**	16.3 (-7.17 to 39.1)
14 Malaria	14 Road injuries	53 (-5.58 to 152)	-0.677 (-34.6 to 59.9)	-37.4 (-59.7 to 1.4)
15 Maternal disorders	15 Prostate cancer	**358 (210 to 553)**	**197 (107 to 313)**	4.89 (-17.8 to 29.4)
16 COVID-19	16 Diarrheal diseases	35.7 (-7.85 to 92)	-12 (-36.2 to 21.7)	**-50.1 (-62.4 to -31.6)**
17 Pertussis	17 Urinary diseases	**292 (201 to 397)**	**154 (107 to 209)**	9.01 (-6.7 to 26.5)
18 Meningitis	18 Neonatal disorders	**-29.1 (-52.8 to -1.99)**	**-54.2 (-66.1 to -40)**	-20.1 (-34.4 to 1.68)
19 Congenital defects	19 Esophageal cancer	**204 (137 to 275)**	**97.6 (66.2 to 137)**	**-13.7 (-23.2 to -3.01)**
20 COPD	20 Lung cancer	**275 (172 to 402)**	**143 (83.2 to 211)**	7.2 (-13.8 to 30.3)
21 Breast cancer	22 Interpersonal violence			
24 Esophageal cancer	29 Meningitis			
26 Colorectal cancer	35 Malaria			
27 Urinary diseases	41 Maternal disorders			
28 Alzheimer's disease	43 Congenital defects			
30 Lung cancer	60 Pertussis			
31 Prostate cancer	133 COVID-19			

Supplemental Results Figure S8. Leading 20 level 3 causes of deaths, and percentage change in number of deaths and all-age and age-standardised death rates by location, 2022 and 2050

Eritrea, Reference scenario

Legend:
Communicable, maternal, neonatal and nutritional
Non-communicable
Injuries

Leading Causes 2022	Leading Causes 2050	Mean % change number of DEATH	Mean % change all-age DEATH rate	Mean % change age-standardized DEATH rate
1 Tuberculosis	1 Lower respiratory infect	44.4 (-7.99 to 96.8)	5.91 (-18.4 to 32.5)	**-32.9 (-43 to -19.3)**
2 Lower respiratory infect	2 Stroke	**70.1 (8.99 to 134)**	24.7 (-1.41 to 54.4)	**-42.8 (-54.6 to -25.3)**
3 Stroke	3 Ischemic heart disease	65.1 (-3.94 to 138)	21.1 (-13.9 to 67)	**-43.1 (-61.5 to -17.5)**
4 Neonatal disorders	4 Tuberculosis	-29.4 (-58.2 to 1.09)	**-48.3 (-62.2 to -29.2)**	**-68.1 (-76.9 to -56.2)**
5 Diarrheal diseases	5 Chronic kidney disease	**239 (110 to 365)**	**149 (94.8 to 213)**	**28.4 (0.562 to 65.3)**
6 COVID-19	6 Diarrheal diseases	13.3 (-42 to 71.2)	-17.2 (-49 to 19)	**-36.7 (-54.6 to -11.1)**
7 Ischemic heart disease	7 Diabetes	**103 (23.6 to 194)**	**48.7 (13.5 to 88.2)**	**-26.3 (-45.8 to -1.16)**
8 Cirrhosis liver	8 Hypertensive heart disease	**134 (48 to 224)**	**71.7 (36.4 to 112)**	**-31.2 (-46.5 to -12.4)**
9 Road injuries	9 Cirrhosis liver	53.7 (-1.95 to 106)	12.5 (-8.83 to 36.6)	**-35.9 (-45.7 to -23)**
10 Diabetes	10 Road injuries	20.3 (-36.7 to 99.9)	-12 (-44.1 to 43)	-36.4 (-59.5 to 0.344)
11 Hypertensive heart disease	11 COPD	**123 (44 to 200)**	**63.4 (31.4 to 104)**	**-30.4 (-44.3 to -13.2)**
12 Interpersonal violence	12 Alzheimer's disease	**297 (147 to 494)**	**190 (121 to 274)**	-2.52 (-6.74 to 1.37)
13 Congenital defects	13 Neonatal disorders	**-62.1 (-78.7 to -42.8)**	**-72.5 (-80.1 to -63.4)**	**-53.1 (-62.6 to -39.9)**
14 Chronic kidney disease	14 Interpersonal violence	9.85 (-31 to 57.7)	-19.9 (-35.6 to 0.893)	**-32.1 (-43.4 to -18.5)**
15 HIV/AIDS	15 Self-harm	**70.5 (8.9 to 140)**	**24.6 (1.69 to 49.7)**	-16 (-30.6 to 0.0395)
16 Maternal disorders	16 Breast cancer	**150 (50.9 to 268)**	**82.5 (39.1 to 137)**	1.41 (-14 to 19.2)
17 Protein-energy malnutrition	17 Cervical cancer	59.9 (-0.924 to 123)	16.9 (-4.93 to 41.9)	**-32 (-43 to -16.9)**
18 Meningitis	18 Colorectal cancer	**238 (99.3 to 409)**	**147 (71.3 to 241)**	21.3 (-2.63 to 48.4)
19 COPD	19 Esophageal cancer	**136 (56.6 to 218)**	**72.4 (45.8 to 104)**	**-17.1 (-26.6 to -6.68)**
20 Self-harm	20 Urinary diseases	**198 (97.9 to 303)**	**118 (83.2 to 169)**	**17.6 (3.79 to 33.2)**
21 Cervical cancer	25 Meningitis			
22 Breast cancer	28 Congenital defects			
24 Esophageal cancer	29 Protein-energy malnutrition			
30 Urinary diseases	33 Maternal disorders			
32 Colorectal cancer	69 HIV/AIDS			
33 Alzheimer's disease	134 COVID-19			

Supplemental Results Figure S8. Leading 20 level 3 causes of deaths, and percentage change in number of deaths and all-age and age-standardised death rates by location, 2022 and 2050

Ethiopia, Reference scenario

Legend:
Communicable, maternal, neonatal and nutritional
Non-communicable
Injuries

Leading Causes 2022	Leading Causes 2050	Mean % change number of DEATH	Mean % change all-age DEATH rate	Mean % change age-standardized DEATH rate
1 Neonatal disorders	1 Chronic kidney disease	**290 (196 to 392)**	**107 (62.9 to 157)**	19.3 (-8.63 to 53.7)
2 Lower respiratory infect	2 Lower respiratory infect	**31.1 (7.74 to 59.8)**	**-30.6 (-40.7 to -19.7)**	**-46.4 (-54.1 to -37.6)**
3 Diarrheal diseases	3 Stroke	**85.5 (45.3 to 129)**	-1.73 (-19.8 to 18.9)	**-47.7 (-58.2 to -33.7)**
4 COVID-19	4 Neonatal disorders	**-49.1 (-65.2 to -30.5)**	**-73.1 (-80.2 to -65.4)**	**-54.5 (-64.6 to -41.2)**
5 Stroke	5 Diarrheal diseases	3.55 (-28.8 to 46.6)	**-45.1 (-61.5 to -26.2)**	**-56 (-68.3 to -37.1)**
6 Tuberculosis	6 Cirrhosis liver	**113 (72.9 to 161)**	12.8 (-3.81 to 34.2)	**-34.7 (-44.9 to -21.7)**
7 Ischemic heart disease	7 Ischemic heart disease	**61.1 (4.23 to 134)**	-14.6 (-42.9 to 23.2)	**-54.2 (-70.7 to -29)**
8 Chronic kidney disease	8 Alzheimer's disease	**317 (230 to 406)**	**121 (80.1 to 168)**	0.391 (-3.7 to 4.92)
9 Cirrhosis liver	9 Hypertensive heart disease	**149 (91.1 to 216)**	**31.8 (3.04 to 66)**	**-33.3 (-49.2 to -10.6)**
10 Congenital defects	10 Colorectal cancer	**323 (192 to 474)**	**124 (65.3 to 199)**	20.7 (-6.22 to 50.1)
11 Diabetes	11 Diabetes	**74.1 (31.4 to 127)**	-7.77 (-27.6 to 16.9)	**-48.9 (-61.3 to -32.3)**
12 HIV/AIDS	12 Urinary diseases	**250 (195 to 314)**	**85.5 (64.1 to 107)**	6.85 (-5.87 to 20.4)
13 Meningitis	13 Tuberculosis	-20.9 (-44.8 to 11.7)	**-58.1 (-70.2 to -42)**	**-74 (-81.4 to -63.3)**
14 Road injuries	14 Breast cancer	**286 (190 to 412)**	**105 (59.9 to 160)**	19.4 (-1.5 to 39.9)
15 Hypertensive heart disease	15 Falls	**209 (151 to 271)**	**63.5 (38.9 to 88)**	**-5.64 (-11.2 to -0.206)**
16 Protein-energy malnutrition	16 Road injuries	51.7 (-8.89 to 152)	-19.6 (-49.4 to 34.7)	**-42 (-63.3 to -5.93)**
17 Interpersonal violence	17 COPD	**87.8 (46.6 to 129)**	-0.501 (-19 to 18.9)	**-49 (-59.8 to -37.3)**
18 COPD	18 Self-harm	**127 (81.9 to 177)**	**20.3 (0.999 to 42)**	**-20.1 (-33.5 to -5.26)**
19 Pertussis	19 Interpersonal violence	**74.3 (35.2 to 118)**	-7.75 (-22.8 to 10.1)	**-28 (-38.4 to -14.5)**
20 Maternal disorders	20 Meningitis	-2.1 (-27.6 to 34.6)	**-48.1 (-61.9 to -30.1)**	**-54.1 (-65.6 to -36.8)**
21 Alzheimer's disease	26 Congenital defects			
23 Self-harm	33 Protein-energy malnutrition			
24 Urinary diseases	35 HIV/AIDS			
25 Colorectal cancer	36 Pertussis			
26 Falls	38 Maternal disorders			
28 Breast cancer	135 COVID-19			

Supplemental Results Figure S8. Leading 20 level 3 causes of deaths, and percentage change in number of deaths and all-age and age-standardised death rates by location, 2022 and 2050

Kenya, Reference scenario

Legend:
Communicable, maternal, neonatal and nutritional
Non-communicable
Injuries

Leading Causes 2022	Leading Causes 2050	Mean % change number of DEATH	Mean % change all-age DEATH rate	Mean % change age-standardized DEATH rate
1 HIV/AIDS	1 Lower respiratory infect	**88.8 (61.4 to 115)**	**31.4 (10.4 to 51.6)**	**-28.9 (-37.2 to -18.9)**
2 Tuberculosis	2 Stroke	**80.3 (51.3 to 109)**	**25.4 (4.46 to 49.4)**	**-45.7 (-55.4 to -33.9)**
3 Lower respiratory infect	3 Chronic kidney disease	**259 (187 to 334)**	**150 (97.8 to 206)**	19.1 (-5.77 to 49.9)
4 Stroke	4 Cirrhosis liver	**100 (73.1 to 129)**	**39.1 (18.5 to 61.3)**	**-30 (-38.8 to -18.6)**
5 Neonatal disorders	5 Tuberculosis	-3.11 (-29.3 to 25.2)	**-32.6 (-50 to -10.7)**	**-63.3 (-73 to -50.5)**
6 COVID-19	6 Hypertensive heart disease	**231 (163 to 304)**	**130 (82 to 184)**	-10.3 (-29.7 to 13.3)
7 Cirrhosis liver	7 Ischemic heart disease	**72.8 (25.1 to 127)**	20.2 (-14.7 to 61.7)	**-47.2 (-63.4 to -27)**
8 Diarrheal diseases	8 Diabetes	**125 (82.8 to 172)**	**56.8 (26.6 to 90.9)**	**-27 (-41.9 to -9.62)**
9 Ischemic heart disease	9 Diarrheal diseases	31.8 (-6.56 to 88)	-8.33 (-34.4 to 28.8)	**-35.9 (-51.3 to -11.1)**
10 Chronic kidney disease	10 COPD	**146 (103 to 190)**	**71.2 (40.3 to 102)**	**-27.5 (-40.3 to -14.6)**
11 Diabetes	11 Alzheimer's disease	**311 (218 to 408)**	**186 (124 to 260)**	-3.24 (-8.38 to 1.63)
12 Hypertensive heart disease	12 Urinary diseases	**253 (206 to 302)**	**145 (111 to 183)**	**20.5 (8.45 to 34.7)**
13 COPD	13 Road injuries	**61.4 (15.3 to 123)**	12.3 (-18.8 to 57.5)	-28.9 (-49.2 to 2.09)
14 Road injuries	14 Neonatal disorders	**-44.8 (-60.2 to -27.5)**	**-61.8 (-71 to -51.1)**	**-36.5 (-47.7 to -22.6)**
15 Interpersonal violence	15 Breast cancer	**196 (132 to 264)**	**106 (60.7 to 154)**	4.45 (-10.5 to 22.2)
16 Meningitis	16 Falls	**192 (141 to 249)**	**103 (65.5 to 147)**	**-6.57 (-10.9 to -1.35)**
17 Malaria	17 Ileus & obstruction	**169 (118 to 211)**	**87.2 (53.8 to 126)**	-1.67 (-18.9 to 17.6)
18 Congenital defects	18 Esophageal cancer	**163 (128 to 195)**	**82.9 (60.5 to 106)**	**-15.8 (-24.7 to -6.83)**
19 Self-harm	19 Self-harm	**105 (74.3 to 136)**	**42.5 (21.5 to 66.9)**	**-14.7 (-25.2 to -2.28)**
20 Maternal disorders	20 Interpersonal violence	**35 (13.6 to 61.9)**	-6.18 (-22 to 9.47)	**-30.3 (-39.2 to -19.9)**
22 Alzheimer's disease	21 HIV/AIDS			
23 Falls	23 Meningitis			
24 Breast cancer	39 Congenital defects			
25 Ileus & obstruction	47 Maternal disorders			
26 Esophageal cancer	93 Malaria			
27 Urinary diseases	135 COVID-19			

Supplemental Results Figure S8. Leading 20 level 3 causes of deaths, and percentage change in number of deaths and all-age and age-standardised death rates by location, 2022 and 2050

Madagascar, Reference scenario

Legend:
Communicable, maternal, neonatal and nutritional
Non-communicable
Injuries

Leading Causes 2022	Leading Causes 2050	Mean % change number of DEATH	Mean % change all-age DEATH rate	Mean % change age-standardized DEATH rate
1 COVID-19	1 Stroke	**88.9 (55 to 133)**	11 (-8.93 to 38.1)	**-43.5 (-53.4 to -30.8)**
2 Stroke	2 Lower respiratory infect	**40.9 (18.1 to 71.3)**	**-17.1 (-30.2 to -1.67)**	**-32.3 (-40.6 to -21.7)**
3 Lower respiratory infect	3 Hypertensive heart disease	**219 (150 to 310)**	**87.2 (47.2 to 142)**	-15.5 (-32.9 to 7.98)
4 Diarrheal diseases	4 Diarrheal diseases	29.8 (-15.5 to 102)	-23.7 (-50.1 to 14.3)	-19 (-40.1 to 11.4)
5 Neonatal disorders	5 Ischemic heart disease	**99.3 (40.5 to 177)**	17.1 (-19.5 to 70.1)	**-40.1 (-57.8 to -14.5)**
6 Tuberculosis	6 COPD	**177 (120 to 250)**	**62.6 (28.6 to 102)**	**-26.7 (-40.7 to -9.57)**
7 Ischemic heart disease	7 Tuberculosis	-2.54 (-28.5 to 36)	**-42.7 (-58.4 to -20.3)**	**-63.4 (-72.5 to -49.9)**
8 Hypertensive heart disease	8 Chronic kidney disease	**263 (181 to 368)**	**113 (60.3 to 173)**	18.7 (-8.1 to 52.3)
9 Malaria	9 Cirrhosis liver	**90.9 (61.7 to 126)**	12.2 (-4.76 to 33)	**-33.7 (-43 to -21.5)**
10 Protein-energy malnutrition	10 Diabetes	**134 (85.2 to 206)**	**37.6 (6.45 to 81.5)**	**-25.5 (-42.1 to -3.37)**
11 Cirrhosis liver	11 Neonatal disorders	**-48 (-60.6 to -32.6)**	**-69.5 (-75.5 to -61.8)**	**-51.9 (-61.4 to -40.4)**
12 Congenital defects	12 Road injuries	39.8 (-7.83 to 130)	-17.8 (-46.6 to 30.6)	**-37.2 (-57 to -6.06)**
13 COPD	13 Alzheimer's disease	**376 (243 to 548)**	**179 (104 to 262)**	-0.445 (-4.09 to 2.81)
14 Road injuries	14 Protein-energy malnutrition	-15 (-45.7 to 27.3)	**-50 (-68 to -23.6)**	**-45.4 (-64 to -16.6)**
15 Diabetes	15 Breast cancer	**195 (124 to 275)**	**73.5 (33.3 to 115)**	3.57 (-13.1 to 22.8)
16 Measles	16 Self-harm	**102 (71 to 140)**	18.6 (-0.631 to 38.7)	**-18.6 (-29.2 to -4.17)**
17 HIV/AIDS	17 Cervical cancer	**105 (73.6 to 150)**	20.3 (-0.466 to 48)	**-25.1 (-35.7 to -10.1)**
18 Meningitis	18 Esophageal cancer	**162 (125 to 208)**	**54.2 (34.2 to 77.2)**	**-17.9 (-27.5 to -7.93)**
19 Chronic kidney disease	19 Ileus & obstruction	**158 (110 to 223)**	**51.8 (26 to 85.2)**	-4.67 (-21.2 to 16.5)
20 Maternal disorders	20 Other unspecified infectious	**71.6 (19 to 149)**	0.842 (-31.3 to 44.5)	-3.81 (-33.1 to 38.2)
23 Self-harm	22 Congenital defects			
25 Cervical cancer	24 Meningitis			
26 Other unspecified infectious	30 Malaria			
27 Breast cancer	33 Maternal disorders			
28 Esophageal cancer	55 HIV/AIDS			
29 Ileus & obstruction	82 Measles			
36 Alzheimer's disease	133 COVID-19			

Supplemental Results Figure S8. Leading 20 level 3 causes of deaths, and percentage change in number of deaths and all-age and age-standardised death rates by location, 2022 and 2050

Malawi, Reference scenario

Legend:
Communicable, maternal, neonatal and nutritional
Non-communicable
Injuries

Leading Causes 2022	Leading Causes 2050	Mean % change number of DEATH	Mean % change all-age DEATH rate	Mean % change age-standardized DEATH rate
1 HIV/AIDS	1 Stroke	**64.2 (34 to 97.2)**	0.315 (-19.6 to 25.2)	**-45 (-57.3 to -27)**
2 COVID-19	2 Lower respiratory infect	**41.7 (19.9 to 67.7)**	-13.5 (-28.3 to 2.21)	**-30.7 (-41.1 to -18.4)**
3 Lower respiratory infect	3 Diarrheal diseases	**45.4 (3.6 to 102)**	-11.2 (-37.4 to 25.7)	-25.6 (-47.2 to 8.38)
4 Neonatal disorders	4 Chronic kidney disease	**193 (132 to 261)**	**79.2 (35.9 to 127)**	8.95 (-16.9 to 44)
5 Stroke	5 Ischemic heart disease	**73.4 (18.8 to 157)**	6.01 (-31.1 to 61.2)	**-42.2 (-63 to -10.3)**
6 Diarrheal diseases	6 Cirrhosis liver	**98.9 (69 to 133)**	**21.4 (0.787 to 45.6)**	**-30.8 (-41.6 to -17.3)**
7 Malaria	7 Diabetes	**110 (61.6 to 165)**	28.1 (-4.02 to 64)	**-26.6 (-44.5 to -2.49)**
8 Tuberculosis	8 Esophageal cancer	**193 (156 to 233)**	**79.1 (56.1 to 105)**	-6.66 (-16.3 to 1.78)
9 Ischemic heart disease	9 Tuberculosis	-9.97 (-37 to 28.4)	**-45 (-63.3 to -19.1)**	**-65.3 (-76.2 to -48.6)**
10 Cirrhosis liver	10 Hypertensive heart disease	**162 (108 to 225)**	**59.9 (25.8 to 106)**	-19.7 (-39.5 to 7.2)
11 Diabetes	11 Neonatal disorders	**-46.3 (-63.1 to -24)**	**-67.4 (-75.5 to -55.7)**	**-45 (-56.3 to -30.8)**
12 Chronic kidney disease	12 Road injuries	44.2 (-10.1 to 130)	-11.9 (-45.6 to 43.8)	-35.3 (-58.8 to 2.13)
13 Road injuries	13 COPD	**119 (78 to 163)**	**33.5 (7.32 to 64.3)**	**-28.8 (-43.5 to -10.6)**
14 Congenital defects	14 Self-harm	**112 (77.1 to 153)**	**29.5 (6.15 to 54.7)**	**-14.8 (-28 to -1.14)**
15 Meningitis	15 Lymphoma	**152 (80 to 226)**	**53.5 (12.1 to 101)**	6.43 (-13.3 to 28.4)
16 Hypertensive heart disease	16 Alzheimer's disease	**247 (162 to 333)**	**112 (53.2 to 167)**	0.298 (-4.52 to 4.63)
17 Esophageal cancer	17 Cervical cancer	**120 (83 to 160)**	**34.2 (7.99 to 67.5)**	**-22.9 (-35.8 to -5.62)**
18 COPD	18 Breast cancer	**224 (149 to 314)**	**97.7 (51.9 to 147)**	11.6 (-6.74 to 32)
19 Self-harm	19 Falls	**147 (101 to 194)**	**50.6 (21.6 to 79.4)**	**-6.31 (-11.2 to -0.774)**
20 Protein-energy malnutrition	20 HIV/AIDS	**-83.8 (-88.9 to -78)**	**-90.1 (-93.1 to -86.8)**	**-90.7 (-93.8 to -87.4)**
21 Cervical cancer	21 Meningitis			
23 Lymphoma	25 Congenital defects			
24 Falls	29 Protein-energy malnutrition			
27 Alzheimer's disease	30 Malaria			
28 Breast cancer	134 COVID-19			

Supplemental Results Figure S8. Leading 20 level 3 causes of deaths, and percentage change in number of deaths and all-age and age-standardised death rates by location, 2022 and 2050

Mozambique, Reference scenario

Legend:
Communicable, maternal, neonatal and nutritional
Non-communicable
Injuries

Leading Causes 2022	Leading Causes 2050	Mean % change number of DEATH	Mean % change all-age DEATH rate	Mean % change age-standardized DEATH rate
1 HIV/AIDS	1 Stroke	**54.3 (25.3 to 88.8)**	-14.6 (-33.6 to 3.39)	**-48.9 (-58.1 to -37.9)**
2 Neonatal disorders	2 Lower respiratory infect	**30.4 (10.4 to 51.7)**	**-27.9 (-39.8 to -15.9)**	**-39 (-46.7 to -30.2)**
3 Stroke	3 Hypertensive heart disease	**164 (102 to 240)**	**46.2 (9.05 to 82.1)**	**-23.8 (-41.4 to -2.74)**
4 Malaria	4 Tuberculosis	-26.3 (-46.6 to 1.1)	**-59.2 (-70.9 to -42)**	**-72.1 (-79.5 to -61.4)**
5 Tuberculosis	5 Diabetes	**133 (80.2 to 189)**	28.7 (-2.73 to 62.5)	-18.6 (-37.1 to 4.5)
6 Lower respiratory infect	6 Neonatal disorders	**-54.7 (-67.8 to -39.3)**	**-75 (-80.4 to -67.4)**	**-58.3 (-66.5 to -47.9)**
7 COVID-19	7 Chronic kidney disease	**202 (134 to 279)**	**67.4 (26.3 to 109)**	12.6 (-12.5 to 44)
8 Diarrheal diseases	8 HIV/AIDS	**-79.2 (-84.2 to -74.2)**	**-88.5 (-91.2 to -85.8)**	**-90.1 (-92.8 to -87.5)**
9 Congenital defects	9 Diarrheal diseases	-10.1 (-38.6 to 22.9)	**-50.2 (-65.7 to -30.6)**	**-51.1 (-62.4 to -33.3)**
10 Road injuries	10 Liver cancer	**199 (135 to 272)**	**65.6 (27.3 to 100)**	-1.16 (-20.5 to 23.5)
11 Hypertensive heart disease	11 Road injuries	27.4 (-17.6 to 99.1)	-29.4 (-56.5 to 14.1)	**-46.7 (-64.8 to -16.3)**
12 Diabetes	12 Malaria	-64.3 (-98 to 69.2)	**-80.2 (-98.9 to -3.06)**	-81.4 (-98.6 to 1.8)
13 Ischemic heart disease	13 Self-harm	**128 (90.3 to 173)**	**26.3 (6.67 to 49.1)**	**-14 (-24.8 to -1.78)**
14 Chronic kidney disease	14 Ischemic heart disease	**54.5 (4.91 to 109)**	-14.4 (-42.8 to 18.2)	**-49.2 (-66.1 to -28.2)**
15 Cirrhosis liver	15 Cirrhosis liver	**106 (74.6 to 149)**	14.3 (-4.17 to 34.3)	**-28 (-38 to -15.8)**
16 Self-harm	16 Breast cancer	**266 (166 to 364)**	**102 (52.5 to 151)**	**28.4 (8.46 to 51.1)**
17 Protein-energy malnutrition	17 Alzheimer's disease	**299 (186 to 473)**	**120 (61.1 to 207)**	3.52 (-0.082 to 6.73)
18 Liver cancer	18 Cervical cancer	**113 (77.6 to 153)**	18.1 (-0.721 to 41.1)	**-23.9 (-34.6 to -9.51)**
19 Maternal disorders	19 Urinary diseases	**187 (144 to 242)**	**58.6 (37.8 to 86.5)**	5.46 (-5.43 to 19.3)
20 Cervical cancer	20 Ileus & obstruction	**128 (83.8 to 179)**	**26.1 (2.53 to 56.3)**	-12.7 (-27.9 to 5.03)
25 Ileus & obstruction	21 Congenital defects			
26 Urinary diseases	30 Maternal disorders			
28 Breast cancer	33 Protein-energy malnutrition			
31 Alzheimer's disease	133 COVID-19			

Supplemental Results Figure S8. Leading 20 level 3 causes of deaths, and percentage change in number of deaths and all-age and age-standardised death rates by location, 2022 and 2050

Rwanda, Reference scenario

Legend:
Communicable, maternal, neonatal and nutritional
Non-communicable
Injuries

Leading Causes 2022	Leading Causes 2050	Mean % change number of DEATH	Mean % change all-age DEATH rate	Mean % change age-standardized DEATH rate
1 Stroke	1 Stroke	**74.2 (40.3 to 111)**	-6.61 (-23 to 11.1)	**-50.8 (-59.3 to -40.6)**
2 Neonatal disorders	2 Lower respiratory infect	**62.2 (34.4 to 95.3)**	-13 (-27.2 to 5.28)	**-38.9 (-46.6 to -29.7)**
3 Lower respiratory infect	3 Chronic kidney disease	**253 (169 to 348)**	**89.2 (48.9 to 137)**	7.15 (-16.3 to 39)
4 HIV/AIDS	4 Ischemic heart disease	**70.2 (19.8 to 136)**	-8.69 (-36 to 26.5)	**-52 (-66.8 to -29.3)**
5 Tuberculosis	5 Cirrhosis liver	**81.7 (53.5 to 116)**	-2.6 (-16.5 to 12.9)	**-40.9 (-48.7 to -32.1)**
6 Ischemic heart disease	6 Hypertensive heart disease	**187 (124 to 264)**	**54 (21.4 to 89.5)**	**-27.8 (-43 to -8.39)**
7 Road injuries	7 Diabetes	**117 (69.3 to 173)**	16.6 (-8.87 to 45.8)	**-35.3 (-48.6 to -18.9)**
8 Cirrhosis liver	8 Road injuries	**70 (15.4 to 150)**	-8.79 (-38.6 to 31.4)	**-31.9 (-53.7 to -1.22)**
9 Malaria	9 Alzheimer's disease	**366 (229 to 509)**	**149 (86.3 to 219)**	-2.3 (-5.25 to 0.673)
10 Conflict & terror	10 COPD	**137 (84.4 to 194)**	27 (-1.26 to 51.4)	**-36.1 (-48.4 to -22.1)**
11 Diarrheal diseases	11 Diarrheal diseases	31.4 (-19.3 to 94.1)	-29.6 (-55.4 to 1.87)	**-38.4 (-57 to -13.4)**
12 Diabetes	12 Neonatal disorders	**-42.2 (-57.7 to -24.8)**	**-69.1 (-76 to -60.6)**	**-46.6 (-57.4 to -34.2)**
13 COVID-19	13 Tuberculosis	-7.71 (-32.6 to 24.6)	**-50.5 (-64.2 to -33)**	**-68.8 (-77.2 to -57.9)**
14 Chronic kidney disease	14 Breast cancer	**223 (136 to 318)**	**72.7 (36.4 to 115)**	2.41 (-12.7 to 22)
15 Hypertensive heart disease	15 Urinary diseases	**267 (206 to 344)**	**96.6 (64.4 to 126)**	**13.9 (3.21 to 27.3)**
16 Congenital defects	16 Falls	**223 (149 to 301)**	**72.7 (40 to 107)**	**-6.36 (-10.7 to -1.77)**
17 COPD	17 Prostate cancer	**336 (198 to 474)**	**133 (72.8 to 203)**	17.5 (-4.93 to 42.2)
18 Meningitis	18 Self-harm	**114 (79.9 to 158)**	14.9 (-2.86 to 33.9)	**-21.2 (-32.2 to -8.87)**
19 Self-harm	19 Colorectal cancer	**284 (162 to 417)**	**105 (50.1 to 162)**	13.7 (-8.66 to 37.5)
20 Breast cancer	20 Esophageal cancer	**164 (124 to 214)**	**41.6 (24.2 to 59.4)**	**-21.8 (-29.3 to -13.5)**
23 Alzheimer's disease	24 HIV/AIDS			
25 Esophageal cancer	27 Meningitis			
26 Falls	28 Congenital defects			
27 Urinary diseases	68 Conflict & terror			
32 Colorectal cancer	71 Malaria			
33 Prostate cancer	134 COVID-19			

Supplemental Results Figure S8. Leading 20 level 3 causes of deaths, and percentage change in number of deaths and all-age and age-standardised death rates by location, 2022 and 2050

Somalia, Reference scenario

Legend:
Communicable, maternal, neonatal and nutritional
Non-communicable
Injuries

Leading Causes 2022	Leading Causes 2050	Mean % change number of DEATH	Mean % change all-age DEATH rate	Mean % change age-standardized DEATH rate
1 Neonatal disorders	1 Lower respiratory infect	50.3 (-3.85 to 114)	**-26.4 (-41.3 to -7.82)**	-9.79 (-24.8 to 12.3)
2 Tuberculosis	2 Neonatal disorders	-1.14 (-39.1 to 60.7)	**-51.6 (-65.1 to -27.7)**	**-33.8 (-52 to -1.83)**
3 Measles	3 Diarrheal diseases	18 (-37.6 to 132)	-42.2 (-64.7 to 7.35)	-27 (-52.2 to 40.8)
4 Lower respiratory infect	4 Tuberculosis	-8.23 (-48.6 to 67.4)	**-54.9 (-72 to -21)**	**-61.8 (-75.8 to -30)**
5 Diarrheal diseases	5 Stroke	**129 (45.7 to 246)**	12.3 (-13.9 to 51)	-19.2 (-44.9 to 28.6)
6 Stroke	6 Ischemic heart disease	**172 (31.6 to 491)**	33.7 (-26.6 to 168)	-6.59 (-52.4 to 103)
7 Congenital defects	7 Chronic kidney disease	**220 (106 to 383)**	**57.2 (18.8 to 119)**	20.6 (-13.3 to 81)
8 Meningitis	8 Cirrhosis liver	**92.2 (27.5 to 180)**	-5.67 (-23.4 to 20.4)	**-28.8 (-43.7 to -6.38)**
9 Pertussis	9 Diabetes	**186 (65.2 to 395)**	40.2 (-6.47 to 121)	6.92 (-33.7 to 85.7)
10 Cirrhosis liver	10 Road injuries	103 (-8.84 to 315)	-0.278 (-48.1 to 99.1)	-18.7 (-54.7 to 53.9)
11 Protein-energy malnutrition	11 Pertussis	29.8 (-27.3 to 117)	-36.6 (-60.4 to 2.37)	-19.8 (-48.7 to 27.3)
12 Maternal disorders	12 Interpersonal violence	**74.2 (2.1 to 160)**	-14.7 (-37.4 to 10.5)	**-23.6 (-41.1 to -3.31)**
13 Road injuries	13 Hypertensive heart disease	**162 (63.9 to 303)**	28.4 (-0.311 to 80.5)	-18.7 (-43.2 to 30.9)
14 COVID-19	14 COPD	**190 (91.7 to 311)**	**42.8 (13.4 to 80.3)**	-5.35 (-28.8 to 36.8)
15 Ischemic heart disease	15 Meningitis	6.58 (-42 to 76.9)	**-47.6 (-66.6 to -17.3)**	**-43.4 (-60.9 to -14.7)**
16 Interpersonal violence	16 Congenital defects	-0.116 (-36.5 to 44.6)	**-51.1 (-60.7 to -37.9)**	**-34.7 (-47.5 to -18.2)**
17 Tetanus	17 Maternal disorders	-5.62 (-51.8 to 72.6)	**-53.7 (-73 to -19.7)**	**-58.6 (-74.5 to -29.5)**
18 Diabetes	18 Cervical cancer	**99.7 (26.1 to 186)**	-2.09 (-22.3 to 23.8)	**-26 (-41.7 to -6.17)**
19 Conflict & terror	19 Conflict & terror	82.3 (-91 to 829)	-10.2 (-95.9 to 362)	-11.8 (-96 to 351)
20 Chronic kidney disease	20 Protein-energy malnutrition	-34.4 (-74.5 to 82.4)	**-67.7 (-86 to -13.6)**	-53.2 (-78 to 20.1)
22 Hypertensive heart disease	21 Measles			
25 COPD	36 Tetanus			
26 Cervical cancer	134 COVID-19			

Supplemental Results Figure S8. Leading 20 level 3 causes of deaths, and percentage change in number of deaths and all-age and age-standardised death rates by location, 2022 and 2050

South Sudan, Reference scenario

Legend:
Communicable, maternal, neonatal and nutritional
Non-communicable
Injuries

Leading Causes 2022	Leading Causes 2050	Mean % change number of DEATH	Mean % change all-age DEATH rate	Mean % change age-standardized DEATH rate
1 Neonatal disorders	1 Diarrheal diseases	10.4 (-47.8 to 100)	**-50.5 (-74.7 to -9.03)**	-33.7 (-67.3 to 32.5)
2 Diarrheal diseases	2 Neonatal disorders	-22.3 (-52.4 to 25.2)	**-65.3 (-77.4 to -44.3)**	**-54.1 (-69.9 to -26.1)**
3 Lower respiratory infect	3 Lower respiratory infect	7.21 (-21.3 to 32.6)	**-52.1 (-61.9 to -40.8)**	**-39.4 (-51.1 to -23.7)**
4 Malaria	4 Stroke	**91.9 (35.1 to 164)**	-14.2 (-37 to 16)	-32 (-55.7 to 10.1)
5 Tuberculosis	5 Chronic kidney disease	**256 (135 to 410)**	**59.2 (10.3 to 124)**	36.3 (-16.3 to 119)
6 Stroke	6 Ischemic heart disease	95.5 (-7.13 to 280)	-12.5 (-56.3 to 76.7)	-29.8 (-69.9 to 54.6)
7 HIV/AIDS	7 Diabetes	**132 (52.3 to 237)**	3.9 (-28.4 to 53.5)	-11.6 (-44.8 to 49.9)
8 Congenital defects	8 Malaria	-54.4 (-95.6 to 147)	-78.8 (-97.9 to 12.1)	-75.9 (-97 to 44.4)
9 Protein-energy malnutrition	9 Tuberculosis	-30.1 (-64.7 to 31.4)	**-68.7 (-83.8 to -39.6)**	**-70.1 (-85.1 to -39.9)**
10 Ischemic heart disease	10 Cirrhosis liver	**68.7 (29.9 to 113)**	**-24.6 (-39.4 to -5.83)**	**-36.6 (-50.5 to -16.7)**
11 Meningitis	11 Congenital defects	2.43 (-26.3 to 33.2)	**-54.3 (-64.3 to -40.8)**	**-42.3 (-53.7 to -25)**
12 Cirrhosis liver	12 Interpersonal violence	**72.4 (31.1 to 118)**	**-23 (-36.2 to -6.58)**	**-29.1 (-40.1 to -14.5)**
13 Chronic kidney disease	13 Hypertensive heart disease	**122 (43.2 to 225)**	-0.718 (-32.5 to 45.4)	-26.8 (-55.1 to 22.6)
14 Diabetes	14 Road injuries	26.5 (-38.4 to 149)	-43.4 (-70.8 to 22.1)	-47.8 (-72.3 to 7.23)
15 Maternal disorders	15 Maternal disorders	7.05 (-39.9 to 82)	**-52.1 (-71.8 to -17.7)**	**-57.9 (-75 to -30.3)**
16 Road injuries	16 COPD	**76.5 (25.8 to 134)**	-21.2 (-39.9 to 0.473)	**-42.6 (-61.1 to -14.6)**
17 Interpersonal violence	17 Meningitis	-20.1 (-51.8 to 36.4)	**-64.3 (-77.1 to -37.1)**	**-54.9 (-69.9 to -24.9)**
18 Conflict & terror	18 Self-harm	**107 (58.7 to 165)**	-7.4 (-25.4 to 17.2)	**-22.5 (-37.6 to -1.78)**
19 STIs	19 Urinary diseases	**170 (119 to 231)**	**20.6 (3.93 to 40.1)**	1.81 (-16 to 24.9)
20 Hypertensive heart disease	20 Alzheimer's disease	**264 (102 to 446)**	62.3 (-5.81 to 133)	**8.35 (4.16 to 13.3)**
21 COPD	22 Protein-energy malnutrition			
24 Self-harm	31 STIs			
29 Urinary diseases	32 HIV/AIDS			
37 Alzheimer's disease	35 Conflict & terror			

Supplemental Results Figure S8. Leading 20 level 3 causes of deaths, and percentage change in number of deaths and all-age and age-standardised death rates by location, 2022 and 2050

Uganda, Reference scenario

Legend:
Communicable, maternal, neonatal and nutritional
Non-communicable
Injuries

Leading Causes 2022	Leading Causes 2050	Mean % change number of DEATH	Mean % change all-age DEATH rate	Mean % change age-standardized DEATH rate
1 Neonatal disorders	1 Lower respiratory infect	**46.6 (16 to 77.1)**	**-27.2 (-40.6 to -13.6)**	**-40.7 (-48.8 to -32.2)**
2 HIV/AIDS	2 Stroke	**63.8 (24.9 to 104)**	**-18.7 (-33.9 to -2.3)**	**-54.3 (-63 to -44.9)**
3 Malaria	3 Neonatal disorders	**-40 (-56.7 to -19.4)**	**-70.3 (-76.7 to -61.7)**	**-52.8 (-62.5 to -41.4)**
4 Lower respiratory infect	4 Chronic kidney disease	**220 (136 to 315)**	**59 (24 to 103)**	-3.35 (-23.5 to 23.4)
5 COVID-19	5 Ischemic heart disease	**65.8 (12.6 to 130)**	-17.7 (-43 to 19.5)	**-54.1 (-67.8 to -35.9)**
6 Stroke	6 Diabetes	**123 (65.1 to 178)**	10.5 (-16.3 to 39.1)	**-36.4 (-51.4 to -19.4)**
7 Tuberculosis	7 Diarrheal diseases	32.8 (-14.9 to 85.6)	**-34 (-56.6 to -6.52)**	**-38.7 (-54.1 to -16)**
8 Ischemic heart disease	8 Cirrhosis liver	**93 (56.8 to 133)**	-4.11 (-19.3 to 13.6)	**-41 (-49.2 to -31.3)**
9 Diarrheal diseases	9 Road injuries	**63 (3.44 to 160)**	-19.1 (-46.2 to 19.4)	**-37.4 (-56.4 to -8.01)**
10 Congenital defects	10 Tuberculosis	-9.36 (-36.5 to 25.8)	**-55 (-68.6 to -38.5)**	**-69.9 (-77.6 to -58.7)**
11 Road injuries	11 Hypertensive heart disease	**200 (123 to 275)**	**49.1 (16.7 to 86.3)**	**-24.2 (-40.8 to -3.97)**
12 Diabetes	12 Prostate cancer	**328 (214 to 463)**	**113 (56.6 to 169)**	13.9 (-9.45 to 41.1)
13 Cirrhosis liver	13 Breast cancer	**280 (201 to 373)**	**88.7 (50.3 to 133)**	12.5 (-5.5 to 31.1)
14 Measles	14 Alzheimer's disease	**339 (220 to 450)**	**118 (62.8 to 162)**	0.476 (-2.98 to 4.05)
15 Chronic kidney disease	15 HIV/AIDS	**-70.2 (-77.5 to -61.4)**	**-85.2 (-88.4 to -81.1)**	**-88.8 (-91.3 to -84.9)**
16 Meningitis	16 COPD	**114 (66.3 to 165)**	6.34 (-14.9 to 28.4)	**-42.8 (-53.2 to -28.5)**
17 Hypertensive heart disease	17 Esophageal cancer	**189 (141 to 237)**	**43.5 (25.1 to 59.2)**	**-19.5 (-27.2 to -12)**
18 COPD	18 Colorectal cancer	**320 (207 to 445)**	**109 (57.9 to 165)**	19.5 (-4.38 to 45.8)
19 Protein-energy malnutrition	19 Self-harm	**138 (93.2 to 189)**	18.3 (-0.673 to 39.9)	**-19.8 (-30.9 to -7.61)**
20 STIs	20 Urinary diseases	**259 (197 to 329)**	**78.4 (52.6 to 103)**	**12.6 (0.933 to 25.1)**
22 Self-harm	22 Congenital defects			
23 Breast cancer	25 Malaria			
25 Prostate cancer	26 Meningitis			
26 Esophageal cancer	35 Protein-energy malnutrition			
29 Alzheimer's disease	48 STIs			
31 Urinary diseases	88 Measles			
36 Colorectal cancer	136 COVID-19			

Supplemental Results Figure S8. Leading 20 level 3 causes of deaths, and percentage change in number of deaths and all-age and age-standardised death rates by location, 2022 and 2050

Tanzania, Reference scenario

Legend:
Communicable, maternal, neonatal and nutritional
Non-communicable
Injuries

Leading Causes 2022	Leading Causes 2050	Mean % change number of DEATH	Mean % change all-age DEATH rate	Mean % change age-standardized DEATH rate
1 HIV/AIDS	1 Stroke	**66.2 (31.6 to 107)**	-4.81 (-24.1 to 14.1)	**-48.3 (-57.4 to -36.1)**
2 Neonatal disorders	2 Lower respiratory infect	**26 (2.37 to 51.2)**	**-27.8 (-40.7 to -14.9)**	**-38.4 (-46.2 to -28.3)**
3 Lower respiratory infect	3 Ischemic heart disease	**66.5 (13.8 to 129)**	-4.54 (-32.8 to 34.9)	**-46.9 (-63.9 to -24.8)**
4 Stroke	4 Chronic kidney disease	**251 (163 to 348)**	**101 (55.3 to 152)**	19.2 (-5.15 to 49.8)
5 Ischemic heart disease	5 Hypertensive heart disease	**191 (118 to 266)**	**66.6 (29 to 106)**	-16.2 (-34 to 6.2)
6 Diarrheal diseases	6 Diarrheal diseases	25.6 (-9.48 to 72.1)	**-28.1 (-49 to -2.39)**	**-39.6 (-55.8 to -18)**
7 Tuberculosis	7 Diabetes	**139 (85.3 to 203)**	**37 (6.26 to 69.6)**	-19.9 (-35.9 to 1.81)
8 Malaria	8 Cirrhosis liver	**98.1 (64.9 to 136)**	13.5 (-2.84 to 34.3)	**-30.8 (-40.3 to -18.6)**
9 Congenital defects	9 Neonatal disorders	**-44.8 (-58.2 to -29.4)**	**-68.5 (-74.9 to -60.6)**	**-49.7 (-58.9 to -37.1)**
10 Cirrhosis liver	10 Alzheimer's disease	**280 (179 to 389)**	**117 (65.5 to 164)**	-0.0245 (-3.06 to 3.3)
11 Chronic kidney disease	11 Tuberculosis	-17.6 (-43.2 to 11.3)	**-52.8 (-65.6 to -34.6)**	**-68.3 (-77 to -55.5)**
12 Diabetes	12 Breast cancer	**231 (144 to 338)**	**89.3 (48.5 to 136)**	15.9 (-2.6 to 36.9)
13 Hypertensive heart disease	13 HIV/AIDS	**-66.4 (-78.2 to -52)**	**-80.7 (-87 to -72.8)**	**-83.4 (-88.9 to -76.7)**
14 Road injuries	14 Road injuries	37.2 (-10 to 107)	-21.3 (-46.5 to 26.8)	**-39.5 (-58 to -4.24)**
15 Maternal disorders	15 COPD	**110 (68.1 to 161)**	20 (-3.71 to 46.1)	**-36.6 (-48.5 to -22.2)**
16 Meningitis	16 Urinary diseases	**217 (161 to 280)**	**81.3 (54.8 to 106)**	11.9 (-0.524 to 25.2)
17 COPD	17 Colorectal cancer	**272 (160 to 389)**	**112 (56.8 to 179)**	21.7 (-4.08 to 48.7)
18 Protein-energy malnutrition	18 Falls	**158 (104 to 222)**	**47.9 (22.3 to 74.2)**	**-8.77 (-13.9 to -3.36)**
19 STIs	19 Prostate cancer	**264 (155 to 382)**	**108 (50.4 to 164)**	10.5 (-9.15 to 31.6)
20 Alzheimer's disease	20 Congenital defects	**-30.7 (-46.7 to -11.8)**	**-60.4 (-66.5 to -50.5)**	**-39.9 (-48.4 to -26.3)**
21 Breast cancer	28 Meningitis			
25 Falls	33 Maternal disorders			
29 Urinary diseases	36 Protein-energy malnutrition			
31 Colorectal cancer	51 STIs			
33 Prostate cancer	62 Malaria			

Supplemental Results Figure S8. Leading 20 level 3 causes of deaths, and percentage change in number of deaths and all-age and age-standardised death rates by location, 2022 and 2050

Zambia, Reference scenario

Legend:
Communicable, maternal, neonatal and nutritional
Non-communicable
Injuries

Leading Causes 2022	Leading Causes 2050	Mean % change number of DEATH	Mean % change all-age DEATH rate	Mean % change age-standardized DEATH rate
1 HIV/AIDS	1 Stroke	**93.2 (55.1 to 137)**	5.53 (-11.4 to 27)	**-44.5 (-53.2 to -32.4)**
2 Neonatal disorders	2 Chronic kidney disease	**287 (191 to 401)**	**111 (66.3 to 167)**	**27.6 (1.39 to 60.4)**
3 Stroke	3 Lower respiratory infect	**51.4 (20.4 to 83.7)**	**-17.3 (-30.2 to -1.99)**	**-33.5 (-40.8 to -24.7)**
4 COVID-19	4 Hypertensive heart disease	**251 (168 to 349)**	**91.9 (52 to 141)**	-6.08 (-26.4 to 20.1)
5 Lower respiratory infect	5 Ischemic heart disease	**90.4 (32.6 to 162)**	4.01 (-26.7 to 38.5)	**-45 (-61.4 to -23.1)**
6 Malaria	6 Cirrhosis liver	**119 (75.5 to 160)**	**19.4 (0.2 to 38.7)**	**-29.8 (-38.9 to -17.9)**
7 Ischemic heart disease	7 HIV/AIDS	**-63.8 (-72.3 to -53.1)**	**-80.2 (-84.1 to -74.9)**	**-83.5 (-87 to -78.1)**
8 Diarrheal diseases	8 Diabetes	**134 (82.8 to 198)**	**27.7 (1.88 to 55.1)**	**-28.3 (-42.9 to -9.63)**
9 Cirrhosis liver	9 Neonatal disorders	**-31 (-48.1 to -9.52)**	**-62.4 (-69.9 to -52.5)**	**-42.4 (-53 to -30.1)**
10 Chronic kidney disease	10 Diarrheal diseases	13.5 (-22.8 to 58.9)	**-38.1 (-56.6 to -15.9)**	**-42.6 (-57.7 to -22.5)**
11 Congenital defects	11 Breast cancer	**239 (162 to 346)**	**84.9 (44.3 to 133)**	11 (-6.63 to 29.4)
12 Hypertensive heart disease	12 Road injuries	**69.1 (11.5 to 167)**	-7.64 (-39 to 46.4)	-31.6 (-53.2 to 4.07)
13 Diabetes	13 Prostate cancer	**288 (186 to 411)**	**112 (63.4 to 167)**	4.57 (-14.4 to 23.5)
14 Tuberculosis	14 COPD	**151 (99.4 to 218)**	**37.1 (11.9 to 69.2)**	**-30.3 (-42.6 to -15.7)**
15 Road injuries	15 Idiopathic epilepsy	**116 (60 to 188)**	17.8 (-9.93 to 56.9)	-3.87 (-25.3 to 27.7)
16 Meningitis	16 Esophageal cancer	**215 (160 to 275)**	**72.3 (47.4 to 103)**	**-9.87 (-20.4 to -1.06)**
17 Interpersonal violence	17 Self-harm	**125 (82.6 to 174)**	**23.1 (5.57 to 46.5)**	**-16.7 (-27.9 to -3.65)**
18 Idiopathic epilepsy	18 Colorectal cancer	**291 (175 to 416)**	**114 (55.2 to 175)**	17 (-7.47 to 42.7)
19 Self-harm	19 Urinary diseases	**241 (181 to 309)**	**86.1 (62.4 to 117)**	**11.3 (0.74 to 22.5)**
20 COPD	20 Cervical cancer	**145 (98.1 to 196)**	**33.7 (13 to 59.1)**	**-21.4 (-31.1 to -8.09)**
21 Breast cancer	22 Interpersonal violence			
22 Cervical cancer	24 Tuberculosis			
24 Esophageal cancer	25 Congenital defects			
28 Urinary diseases	28 Meningitis			
29 Prostate cancer	38 Malaria			
30 Colorectal cancer	136 COVID-19			

Supplemental Results Figure S8. Leading 20 level 3 causes of deaths, and percentage change in number of deaths and all-age and age-standardised death rates by location, 2022 and 2050

Botswana, Reference scenario

Legend:
Communicable, maternal, neonatal and nutritional
Non-communicable
Injuries

Leading Causes 2022	Leading Causes 2050	Mean % change number of DEATH	Mean % change all-age DEATH rate	Mean % change age-standardized DEATH rate
1 HIV/AIDS	1 Lower respiratory infect	**80.5 (47.9 to 121)**	**41.7 (21.8 to 65.3)**	**-27.8 (-36.1 to -18.6)**
2 COVID-19	2 Stroke	**69.4 (32.2 to 107)**	**33 (10.7 to 54.7)**	**-49.2 (-56.9 to -40.2)**
3 Stroke	3 Hypertensive heart disease	**237 (154 to 321)**	**165 (106 to 230)**	-2.54 (-23.5 to 22.5)
4 Lower respiratory infect	4 Diabetes	**121 (70.6 to 179)**	**73.7 (39.6 to 109)**	**-29 (-42.6 to -12.6)**
5 Ischemic heart disease	5 Ischemic heart disease	**64.6 (13.5 to 114)**	29.3 (-7.96 to 70.9)	**-48.5 (-63.3 to -29.6)**
6 Neonatal disorders	6 Chronic kidney disease	**245 (163 to 330)**	**171 (114 to 234)**	16.8 (-4.24 to 46.4)
7 Tuberculosis	7 COPD	**202 (131 to 264)**	**137 (93.4 to 184)**	-10.7 (-25.3 to 7.71)
8 Diabetes	8 HIV/AIDS	**-80.9 (-86.2 to -73.6)**	**-85 (-88.6 to -79.5)**	**-88.1 (-90.8 to -84.5)**
9 Diarrheal diseases	9 Alzheimer's disease	**347 (234 to 464)**	**250 (173 to 314)**	3.32 (-0.279 to 7.26)
10 Hypertensive heart disease	10 Prostate cancer	**299 (192 to 428)**	**212 (139 to 293)**	17.8 (-3.65 to 41.8)
11 Road injuries	11 Lung cancer	**189 (113 to 266)**	**127 (72.3 to 177)**	0.476 (-16.5 to 17.7)
12 COPD	12 Tuberculosis	**-28.9 (-48.1 to -5.83)**	**-44.1 (-59.3 to -25.3)**	**-67.3 (-76.2 to -55.3)**
13 Chronic kidney disease	13 Neonatal disorders	**-35 (-51.7 to -16)**	**-49.1 (-59.5 to -36.1)**	**-16.8 (-31.5 to -0.748)**
14 Cirrhosis liver	14 Cirrhosis liver	**79.7 (48.5 to 113)**	**41.1 (20.4 to 62.3)**	**-26 (-34.5 to -15.7)**
15 Self-harm	15 Breast cancer	**175 (103 to 256)**	**116 (68.3 to 174)**	4.73 (-11 to 22.3)
16 Interpersonal violence	16 Colorectal cancer	**248 (148 to 354)**	**173 (108 to 246)**	17.1 (-2.92 to 38.1)
17 Lung cancer	17 Diarrheal diseases	**-36.6 (-56.9 to -12.1)**	**-50.2 (-64.7 to -31.1)**	**-66.1 (-74.8 to -52.7)**
18 Breast cancer	18 Road injuries	8.47 (-30.6 to 64.3)	-14.7 (-44.9 to 36.4)	-28.8 (-56 to 21.5)
19 Asthma	19 Esophageal cancer	**180 (130 to 238)**	**120 (93.5 to 152)**	-3.4 (-12.2 to 5.6)
20 Prostate cancer	20 Cardiomyopathy	**144 (97.4 to 192)**	**91.7 (60.5 to 118)**	**-18.9 (-23.5 to -14)**
21 Alzheimer's disease	22 Self-harm			
23 Cardiomyopathy	27 Asthma			
25 Colorectal cancer	28 Interpersonal violence			
26 Esophageal cancer	132 COVID-19			

Supplemental Results Figure S8. Leading 20 level 3 causes of deaths, and percentage change in number of deaths and all-age and age-standardised death rates by location, 2022 and 2050

Eswatini, Reference scenario

Legend:
Communicable, maternal, neonatal and nutritional
Non-communicable
Injuries

Leading Causes 2022	Leading Causes 2050	Mean % change number of DEATH	Mean % change all-age DEATH rate	Mean % change age-standardized DEATH rate
1 HIV/AIDS	1 Lower respiratory infect	16.1 (-5.39 to 43.7)	1.98 (-13.8 to 20.9)	**-32 (-39.7 to -23)**
2 COVID-19	2 Stroke	25.3 (-1.92 to 54.9)	10 (-10.3 to 34)	**-47 (-55.6 to -37.6)**
3 Lower respiratory infect	3 Diabetes	**41.3 (7.47 to 77.2)**	24.2 (-3.51 to 57.9)	**-37.5 (-49.9 to -22.8)**
4 Stroke	4 HIV/AIDS	**-73.1 (-78.7 to -65.4)**	**-76.4 (-80.2 to -70.4)**	**-79.8 (-82.9 to -75.8)**
5 Tuberculosis	5 Chronic kidney disease	**168 (110 to 242)**	**136 (86.5 to 196)**	**28.6 (4.6 to 58.6)**
6 Diabetes	6 Hypertensive heart disease	**139 (83.1 to 205)**	**110 (64.1 to 168)**	-3.9 (-25.4 to 20)
7 Road injuries	7 Ischemic heart disease	28.4 (-7.32 to 73.8)	12.9 (-17.7 to 52.1)	**-45 (-60.3 to -24.4)**
8 Ischemic heart disease	8 Road injuries	-1.98 (-37.8 to 58.3)	-13.7 (-46.8 to 40.1)	-28.6 (-56.3 to 17.2)
9 Neonatal disorders	9 COPD	**79.5 (38.7 to 117)**	**57.7 (25.1 to 93.1)**	**-23.1 (-36.6 to -7.41)**
10 Diarrheal diseases	10 Tuberculosis	**-40.8 (-56.6 to -20.5)**	**-48 (-62.2 to -31.4)**	**-66 (-74.9 to -54.2)**
11 Chronic kidney disease	11 Cirrhosis liver	**40 (15.8 to 70)**	**23 (5.51 to 42.5)**	**-28 (-35.2 to -19.5)**
12 Hypertensive heart disease	12 Liver cancer	**123 (71.1 to 185)**	**96.1 (55.7 to 148)**	8.95 (-12.1 to 35.2)
13 COPD	13 Neonatal disorders	**-36.2 (-53.2 to -15.6)**	**-44.1 (-55.8 to -29)**	-11.6 (-27.8 to 7.42)
14 Interpersonal violence	14 Self-harm	17.8 (-5.37 to 41.6)	3.39 (-12.3 to 20)	**-18 (-30.4 to -2.62)**
15 Cirrhosis liver	15 Lung cancer	**97.1 (49.8 to 157)**	**72.8 (36.5 to 117)**	-10.2 (-24.7 to 5.48)
16 Self-harm	16 Diarrheal diseases	**-42.9 (-61.1 to -19.6)**	**-49.8 (-65.8 to -30.4)**	**-54.9 (-66.6 to -37.9)**
17 Cervical cancer	17 Esophageal cancer	**107 (63.3 to 151)**	**81.9 (50.2 to 117)**	-7.77 (-18 to 1.38)
18 Liver cancer	18 Breast cancer	**111 (58.1 to 182)**	**84.9 (44.2 to 135)**	1.28 (-13.4 to 15.8)
19 Lung cancer	19 Cervical cancer	**50.9 (19.2 to 83.7)**	**32.5 (11.5 to 58.6)**	**-26.2 (-35 to -15)**
20 Asthma	20 Colorectal cancer	**141 (70.5 to 239)**	**111 (58.7 to 183)**	9.93 (-10.5 to 33.7)
21 Esophageal cancer	21 Interpersonal violence			
22 Breast cancer	27 Asthma			
25 Colorectal cancer	132 COVID-19			

Supplemental Results Figure S8. Leading 20 level 3 causes of deaths, and percentage change in number of deaths and all-age and age-standardised death rates by location, 2022 and 2050

Lesotho, Reference scenario

Legend:
Communicable, maternal, neonatal and nutritional
Non-communicable
Injuries

Leading Causes 2022	Leading Causes 2050	Mean % change number of DEATH	Mean % change all-age DEATH rate	Mean % change age-standardized DEATH rate
1 HIV/AIDS	1 Lower respiratory infect	16.2 (-1.38 to 35.7)	-1.95 (-17.9 to 14.1)	**-20.3 (-28.4 to -10.4)**
2 Tuberculosis	2 Stroke	6.47 (-16.7 to 28.7)	-10.1 (-28.4 to 6.85)	**-37.2 (-46.8 to -25.7)**
3 Stroke	3 Tuberculosis	**-36.6 (-53.6 to -15.2)**	**-46.4 (-60.6 to -27.9)**	**-61.2 (-71.2 to -48.1)**
4 Lower respiratory infect	4 Diabetes	**34 (3.77 to 68)**	13.2 (-12.6 to 42)	-17.6 (-33.1 to 2.34)
5 Diarrheal diseases	5 Diarrheal diseases	-13.7 (-39.8 to 18)	-27 (-50.4 to 3.85)	-27.3 (-47.3 to 0.642)
6 Neonatal disorders	6 Hypertensive heart disease	**78.7 (35.3 to 126)**	**51 (13.9 to 91)**	-2.62 (-23 to 21.2)
7 Diabetes	7 Road injuries	6.6 (-30.8 to 66.7)	-9.85 (-43.2 to 43.2)	-26 (-53.3 to 17.5)
8 COVID-19	8 Chronic kidney disease	**119 (71.9 to 174)**	**84.9 (43.5 to 131)**	**36 (8.69 to 69.4)**
9 Road injuries	9 Ischemic heart disease	19.9 (-14.9 to 62.9)	1.33 (-29.1 to 37.1)	**-31.1 (-51.4 to -5.13)**
10 Ischemic heart disease	10 COPD	**50.3 (18.3 to 83.7)**	26.9 (-0.00231 to 54)	-9 (-24.8 to 10.7)
11 Interpersonal violence	11 HIV/AIDS	**-87.8 (-92.1 to -83.7)**	**-89.7 (-92.9 to -86.4)**	**-89.9 (-93.2 to -86.7)**
12 Hypertensive heart disease	12 Neonatal disorders	**-44.2 (-58.2 to -27.9)**	**-53 (-63.5 to -40.8)**	**-30.2 (-42.5 to -15.9)**
13 COPD	13 Cirrhosis liver	**27.9 (8.1 to 49.6)**	7.96 (-9.12 to 24.5)	**-27.8 (-35.4 to -19.2)**
14 Cirrhosis liver	14 Interpersonal violence	-20.3 (-34.1 to 2.01)	**-32.9 (-40.3 to -23.7)**	**-37.5 (-44.2 to -27.9)**
15 Chronic kidney disease	15 Lung cancer	**98.1 (49.3 to 156)**	**66.8 (27.9 to 108)**	12.2 (-6.61 to 32.1)
16 Self-harm	16 Self-harm	**20.3 (1.36 to 43.3)**	1.54 (-14.7 to 17.6)	**-20.1 (-30.2 to -8.72)**
17 Cervical cancer	17 Cervical cancer	**37 (15.1 to 59.1)**	15.6 (-1.78 to 32.5)	**-23.3 (-32 to -13.7)**
18 Asthma	18 Liver cancer	**91.6 (49.2 to 140)**	**61.8 (24.9 to 103)**	9.17 (-11.9 to 35.7)
19 Lung cancer	19 Esophageal cancer	**91 (56.7 to 136)**	**61.1 (36 to 90.8)**	2.63 (-7.43 to 13.5)
20 Liver cancer	20 Breast cancer	**81 (38.8 to 130)**	**52.5 (17.3 to 93)**	4.09 (-12.3 to 20.2)
21 Esophageal cancer	26 Asthma			
23 Breast cancer	130 COVID-19			

Supplemental Results Figure S8. Leading 20 level 3 causes of deaths, and percentage change in number of deaths and all-age and age-standardised death rates by location, 2022 and 2050

Namibia, Reference scenario

Legend:
Communicable, maternal, neonatal and nutritional
Non-communicable
Injuries

Leading Causes 2022	Leading Causes 2050	Mean % change number of DEATH	Mean % change all-age DEATH rate	Mean % change age-standardized DEATH rate
1 HIV/AIDS	1 Stroke	**50 (22 to 86.2)**	7.16 (-9.56 to 26.9)	**-46.5 (-53.9 to -37.7)**
2 COVID-19	2 Lower respiratory infect	**67.9 (37 to 106)**	**19.9 (1.2 to 39)**	**-26.6 (-33.5 to -18.4)**
3 Stroke	3 Hypertensive heart disease	**216 (146 to 302)**	**126 (81.3 to 178)**	7.53 (-15.6 to 35.4)
4 Lower respiratory infect	4 Ischemic heart disease	**39.1 (2.29 to 88.1)**	-0.624 (-24.8 to 30.8)	**-48.8 (-61.9 to -30.3)**
5 Ischemic heart disease	5 Diabetes	**68.4 (32.3 to 115)**	20.3 (-2.55 to 50.1)	**-36 (-46.9 to -22.2)**
6 Tuberculosis	6 COPD	**114 (72.7 to 166)**	**53.3 (25.2 to 82.4)**	**-22.8 (-35.1 to -7.86)**
7 Diabetes	7 Road injuries	34.3 (-14.3 to 108)	-4.04 (-36.2 to 45)	-20.8 (-48.8 to 21.2)
8 Diarrheal diseases	8 Chronic kidney disease	**195 (126 to 270)**	**111 (69.5 to 160)**	15.7 (-6.05 to 41.5)
9 Neonatal disorders	9 Cirrhosis liver	**114 (73.9 to 155)**	**52.9 (32.7 to 76.2)**	-8.13 (-18.3 to 4.74)
10 Road injuries	10 Tuberculosis	-21.8 (-43.6 to 2.8)	**-44.2 (-57.5 to -24.9)**	**-63.5 (-72.4 to -50.8)**
11 Hypertensive heart disease	11 Diarrheal diseases	-12.7 (-40.5 to 23.5)	**-37.6 (-56.3 to -9.87)**	**-49.5 (-63.3 to -29.3)**
12 COPD	12 Breast cancer	**148 (90.4 to 214)**	**76.9 (41.4 to 121)**	4.35 (-9.35 to 20.4)
13 Cirrhosis liver	13 Neonatal disorders	**-25.9 (-44.5 to -5.74)**	**-47.1 (-58.4 to -33.5)**	**-21.3 (-35.3 to -3.21)**
14 Interpersonal violence	14 Alzheimer's disease	**242 (157 to 358)**	**144 (92.3 to 219)**	-0.681 (-4.02 to 2.22)
15 Chronic kidney disease	15 Self-harm	**64.7 (31.4 to 100)**	**17.6 (1.81 to 35.5)**	-7.07 (-17.6 to 8.95)
16 Self-harm	16 Prostate cancer	**198 (120 to 289)**	**113 (61.1 to 178)**	6.95 (-12.5 to 26.9)
17 Breast cancer	17 HIV/AIDS	**-84 (-89.3 to -74.6)**	**-88.6 (-92 to -81.6)**	**-90.2 (-92.8 to -85.1)**
18 Asthma	18 Interpersonal violence	0.666 (-17.7 to 24.7)	**-28.1 (-38.1 to -17.5)**	**-31.6 (-40.6 to -22)**
19 Alzheimer's disease	19 Cardiomyopathy	**113 (75.5 to 161)**	**52.5 (31.2 to 78.2)**	**-18.6 (-23.3 to -13.8)**
20 Cardiomyopathy	20 Ileus & obstruction	**131 (85.4 to 186)**	**64.7 (36.5 to 95.6)**	-2.32 (-17.1 to 13.8)
21 Prostate cancer	24 Asthma			
26 Ileus & obstruction	132 COVID-19			

Supplemental Results Figure S8. Leading 20 level 3 causes of deaths, and percentage change in number of deaths and all-age and age-standardised death rates by location, 2022 and 2050

South Africa, Reference scenario

Legend:
Communicable, maternal, neonatal and nutritional
Non-communicable
Injuries

Leading Causes 2050	Mean % change number of DEATH	Mean % change all-age DEATH rate	Mean % change age-standardized DEATH rate
1 Diabetes	**57.7 (34.1 to 88)**	**36.6 (20 to 57.4)**	**-29.9 (-39.3 to -18.1)**
2 Lower respiratory infect	**53 (31.2 to 73.2)**	**32.5 (20.5 to 43.1)**	**-23.4 (-29.4 to -17.1)**
3 Stroke	**25.4 (8.55 to 43.5)**	8.61 (-2.28 to 20)	**-47.6 (-53.3 to -41.3)**
4 Hypertensive heart disease	**162 (114 to 211)**	**127 (95 to 164)**	6.44 (-11.2 to 26.6)
5 Ischemic heart disease	23.1 (-3.48 to 53)	6.67 (-15.3 to 32.1)	**-47.1 (-59 to -31.1)**
6 Chronic kidney disease	**163 (117 to 211)**	**128 (95.2 to 161)**	**19.9 (1.99 to 41.1)**
7 COPD	**109 (78.2 to 142)**	**81.2 (60.8 to 105)**	**-12.2 (-22.5 to -1.25)**
8 HIV/AIDS	**-73.5 (-78.4 to -68.8)**	**-77.1 (-80.1 to -74.2)**	**-79.6 (-82.1 to -77.4)**
9 Road injuries	1.18 (-28.6 to 45.2)	-12.3 (-37 to 28.7)	-18.2 (-42 to 22.8)
10 Diarrheal diseases	**32.8 (1.09 to 68.4)**	15.1 (-10.6 to 43.1)	**-27.4 (-44.4 to -4.48)**
11 Alzheimer's disease	**189 (141 to 230)**	**150 (125 to 175)**	-1.01 (-4.07 to 1.98)
12 Lung cancer	**77.9 (43.7 to 112)**	**54 (29.7 to 76.3)**	**-15 (-26.4 to -3.55)**
13 Prostate cancer	**184 (130 to 247)**	**146 (102 to 193)**	**17.1 (0.771 to 34.7)**
14 Tuberculosis	**-30.4 (-46.1 to -11.8)**	**-39.7 (-51.8 to -23.9)**	**-59.8 (-68.3 to -48.2)**
15 Interpersonal violence	**-27.2 (-39.3 to -15.6)**	**-37 (-43.2 to -30.2)**	**-36.4 (-43.2 to -29.2)**
16 Neonatal disorders	-15 (-31.5 to 5.55)	**-26.5 (-39 to -12.9)**	5.64 (-11.8 to 23)
17 Breast cancer	**90.3 (53.8 to 125)**	**64.7 (37.6 to 88.9)**	-5.83 (-18.4 to 6.44)
18 Cirrhosis liver	**56.2 (35.6 to 76.7)**	**35.3 (24.5 to 45.9)**	**-15.4 (-22.3 to -9.13)**
19 Colorectal cancer	**127 (73.7 to 176)**	**96.1 (61.1 to 131)**	4.33 (-12 to 21)
20 Endo/metab/blood/immune	**97.3 (70.8 to 127)**	**70.8 (54.6 to 84.5)**	**15.4 (6.64 to 23.9)**
22 Self-harm			
26 Cervical cancer			
132 COVID-19			

Leading Causes 2022
1 HIV/AIDS
2 COVID-19
3 Stroke
4 Lower respiratory infect
5 Diabetes
6 Ischemic heart disease
7 Road injuries
8 Tuberculosis
9 Interpersonal violence
10 Hypertensive heart disease
11 Neonatal disorders
12 Diarrheal diseases
13 Chronic kidney disease
14 COPD
15 Lung cancer
16 Self-harm
17 Cirrhosis liver
18 Breast cancer
19 Alzheimer's disease
20 Cervical cancer
21 Prostate cancer
22 Endo/metab/blood/immune
26 Colorectal cancer

Supplemental Results Figure S8. Leading 20 level 3 causes of deaths, and percentage change in number of deaths and all-age and age-standardised death rates by location, 2022 and 2050

Zimbabwe, Reference scenario

Legend:
Communicable, maternal, neonatal and nutritional
Non-communicable
Injuries

Leading Causes 2022	Leading Causes 2050	Mean % change number of DEATH	Mean % change all-age DEATH rate	Mean % change age-standardized DEATH rate
1 HIV/AIDS	1 Lower respiratory infect	**32.3 (10.9 to 60)**	-6.16 (-18.5 to 12.6)	**-20.7 (-30 to -8.39)**
2 Lower respiratory infect	2 Stroke	**50.2 (15.6 to 84.6)**	6.62 (-14.1 to 30.4)	**-33.4 (-46.5 to -14.2)**
3 COVID-19	3 Ischemic heart disease	**75.2 (21 to 145)**	24.4 (-12.4 to 75.8)	-22.5 (-47.1 to 13.5)
4 Tuberculosis	4 Tuberculosis	**-27.9 (-50.2 to -0.229)**	**-48.8 (-63.2 to -27)**	**-61.1 (-71.7 to -44.5)**
5 Neonatal disorders	5 Chronic kidney disease	**188 (118 to 263)**	**104 (61.9 to 162)**	**39.6 (10.5 to 81.5)**
6 Stroke	6 Diabetes	**79.5 (34.1 to 130)**	27.5 (-1.34 to 65.5)	-16 (-36.3 to 11.2)
7 Ischemic heart disease	7 Diarrheal diseases	34.1 (-20.7 to 92.1)	-4.95 (-40 to 38)	-6.66 (-31.2 to 28.2)
8 Diarrheal diseases	8 Hypertensive heart disease	**145 (88.6 to 207)**	**73.8 (38.6 to 120)**	2.54 (-20.1 to 30.7)
9 Diabetes	9 Neonatal disorders	**-50.8 (-64.6 to -31.7)**	**-65.2 (-72.8 to -53.3)**	**-51.4 (-60.5 to -37.7)**
10 Malaria	10 Self-harm	**38.9 (8.49 to 73.5)**	-1.51 (-17.1 to 21.3)	**-19.8 (-31.8 to -3.51)**
11 Road injuries	11 Road injuries	22.6 (-28.9 to 106)	-12.9 (-46.7 to 51.1)	-28.6 (-54.2 to 17.7)
12 Protein-energy malnutrition	12 Cirrhosis liver	**36.5 (11.2 to 61.9)**	-3.15 (-17.6 to 14.5)	**-33.7 (-42.2 to -22.3)**
13 Self-harm	13 HIV/AIDS	**-82.3 (-86.4 to -77.1)**	**-87.4 (-89.8 to -83.8)**	**-87.8 (-90.4 to -84.1)**
14 Chronic kidney disease	14 COPD	**115 (71.1 to 163)**	**52.9 (24.6 to 86.7)**	-9 (-25.5 to 11.9)
15 Hypertensive heart disease	15 Prostate cancer	**170 (86.5 to 266)**	**91.5 (32.7 to 158)**	6.82 (-16.1 to 29.6)
16 Cirrhosis liver	16 Cervical cancer	**53.4 (20.9 to 86.8)**	8.93 (-9.92 to 33.2)	**-25 (-35.6 to -11.3)**
17 Cervical cancer	17 Breast cancer	**107 (49.1 to 175)**	**46.7 (8.72 to 88.4)**	1.1 (-18.8 to 20.8)
18 Interpersonal violence	18 Liver cancer	**123 (69.5 to 183)**	**58 (24.9 to 103)**	4.22 (-16.9 to 36.8)
19 Congenital defects	19 Esophageal cancer	**110 (66.9 to 149)**	**48.9 (28.2 to 72.9)**	-7.38 (-15.3 to 3.35)
20 Asthma	20 Lung cancer	**138 (74.5 to 211)**	**68.9 (25.5 to 117)**	7.96 (-12.1 to 27.8)
22 COPD	21 Protein-energy malnutrition			
24 Breast cancer	28 Interpersonal violence			
25 Esophageal cancer	29 Asthma			
26 Liver cancer	31 Congenital defects			
27 Prostate cancer	46 Malaria			
29 Lung cancer	133 COVID-19			

Supplemental Results Figure S8. Leading 20 level 3 causes of deaths, and percentage change in number of deaths and all-age and age-standardised death rates by location, 2022 and 2050

Benin, Reference scenario

Legend:
Communicable, maternal, neonatal and nutritional
Non-communicable
Injuries

Leading Causes 2022	Leading Causes 2050	Mean % change number of DEATH	Mean % change all-age DEATH rate	Mean % change age-standardized DEATH rate
1 Malaria	1 Malaria	58 (-85.4 to 677)	-23.4 (-92.7 to 288)	-22.7 (-91.7 to 283)
2 Neonatal disorders	2 Stroke	**83 (41.7 to 126)**	-12.9 (-30 to 5.48)	**-44.4 (-54.2 to -30.5)**
3 Lower respiratory infect	3 Neonatal disorders	**-23.9 (-43.7 to -1.63)**	**-63.9 (-71.5 to -53.3)**	**-43.9 (-54.9 to -28.2)**
4 Stroke	4 Lower respiratory infect	**28 (3.81 to 56.9)**	**-39.1 (-49.6 to -26.4)**	**-38.6 (-47.3 to -26.4)**
5 Diarrheal diseases	5 Chronic kidney disease	**304 (217 to 410)**	**92.2 (48 to 142)**	**36.6 (9.85 to 74.2)**
6 Ischemic heart disease	6 Ischemic heart disease	**84.1 (32.9 to 154)**	-12.4 (-37.7 to 15.9)	**-44.3 (-60.5 to -24.3)**
7 Congenital defects	7 Diabetes	**130 (78.7 to 189)**	9.53 (-17.5 to 36.4)	**-26.7 (-43.7 to -7.86)**
8 Measles	8 Diarrheal diseases	-7.73 (-39.1 to 36.4)	**-56.1 (-70.7 to -38.3)**	**-58.6 (-68.3 to -45.4)**
9 Road injuries	9 Cirrhosis liver	**86.6 (55.8 to 127)**	-11.2 (-25.7 to 6.81)	**-37.5 (-45.9 to -25.5)**
10 Chronic kidney disease	10 Road injuries	50.1 (-8.08 to 155)	-28.5 (-56.5 to 16)	**-38.2 (-60.1 to -2.48)**
11 HIV/AIDS	11 Hypertensive heart disease	**159 (103 to 231)**	23.5 (-5.49 to 56.9)	**-24.8 (-40.4 to -4.47)**
12 Cirrhosis liver	12 Congenital defects	-14.6 (-32.9 to 10)	**-59.5 (-65.7 to -50.1)**	**-40.6 (-48.3 to -30.6)**
13 Tuberculosis	13 Hemoglobinopathies	**38.7 (7.35 to 74.5)**	**-34.2 (-46.5 to -21.1)**	**-27.1 (-35.8 to -18.5)**
14 Diabetes	14 Liver cancer	**185 (117 to 261)**	**35.6 (6.05 to 70.8)**	-7.55 (-27.6 to 18.2)
15 Hemoglobinopathies	15 Alzheimer's disease	**282 (190 to 373)**	**81.9 (40.3 to 119)**	3.25 (-0.587 to 6.81)
16 Meningitis	16 Prostate cancer	**291 (170 to 420)**	**86.1 (25.6 to 144)**	14.6 (-10.5 to 43.9)
17 Maternal disorders	17 Falls	**165 (112 to 221)**	**25.8 (4.6 to 49)**	**-7.04 (-12.9 to -0.948)**
18 Hypertensive heart disease	18 Self-harm	**127 (86.2 to 180)**	8.28 (-10 to 31.2)	**-19.6 (-32 to -5.18)**
19 COPD	19 COPD	**69.7 (31.9 to 109)**	**-19.3 (-37.6 to -1.32)**	**-49.2 (-59 to -37.7)**
20 Pertussis	20 Breast cancer	**280 (181 to 383)**	**80.5 (38.3 to 125)**	**24 (1.68 to 50.7)**
24 Liver cancer	21 Tuberculosis			
25 Self-harm	22 Meningitis			
26 Falls	27 Maternal disorders			
30 Alzheimer's disease	33 Pertussis			
31 Prostate cancer	38 HIV/AIDS			
37 Breast cancer	83 Measles			

Supplemental Results Figure S8. Leading 20 level 3 causes of deaths, and percentage change in number of deaths and all-age and age-standardised death rates by location, 2022 and 2050

Burkina Faso, Reference scenario

Legend:
Communicable, maternal, neonatal and nutritional
Non-communicable
Injuries

Leading Causes 2022	Leading Causes 2050	Mean % change number of DEATH	Mean % change all-age DEATH rate	Mean % change age-standardized DEATH rate
1 Malaria	1 Malaria	10.9 (-94.8 to 523)	-48.9 (-97.7 to 193)	-50.2 (-96.6 to 221)
2 Lower respiratory infect	2 Lower respiratory infect	**23.3 (1.2 to 48.5)**	**-44.7 (-53.2 to -34.1)**	**-30.6 (-40.2 to -19.1)**
3 Neonatal disorders	3 Neonatal disorders	-8.53 (-31 to 19)	**-59 (-68.5 to -47.4)**	**-41.5 (-52.8 to -27.9)**
4 Diarrheal diseases	4 Stroke	**60 (27.4 to 92.9)**	**-28.2 (-44.2 to -14)**	**-41.3 (-52.7 to -28.5)**
5 Stroke	5 Diarrheal diseases	11.7 (-27.9 to 68)	**-49.9 (-67.3 to -24.5)**	**-35.5 (-54.5 to -8.5)**
6 Ischemic heart disease	6 Ischemic heart disease	**68 (14.4 to 131)**	-24.6 (-49 to 4.86)	**-40.3 (-58.7 to -17.4)**
7 Congenital defects	7 Chronic kidney disease	**169 (99.9 to 238)**	20.7 (-8.2 to 49.6)	3.97 (-20.2 to 31.6)
8 Tuberculosis	8 Road injuries	49.2 (-8.05 to 143)	-33.1 (-59.5 to 10.9)	**-39.8 (-60.6 to -4.53)**
9 Road injuries	9 Hypertensive heart disease	**115 (57.6 to 174)**	-3.47 (-27.3 to 21.8)	**-27.9 (-44.3 to -6.96)**
10 Chronic kidney disease	10 Congenital defects	-2.76 (-21.1 to 19.1)	**-56.5 (-62.8 to -48.6)**	**-40.2 (-47.9 to -30.5)**
11 Meningitis	11 Diabetes	**139 (84.6 to 199)**	6.98 (-18.8 to 33.7)	-10.5 (-30.2 to 13.9)
12 COVID-19	12 Cirrhosis liver	**79.9 (45.5 to 112)**	**-19.3 (-33.3 to -5.22)**	**-35 (-44.6 to -24.5)**
13 Hypertensive heart disease	13 Liver cancer	**159 (111 to 214)**	16.4 (-5.53 to 41.2)	-6.62 (-23.8 to 17.2)
14 iNTS	14 Tuberculosis	-15.8 (-42.9 to 20.1)	**-62.2 (-74.9 to -45.3)**	**-67.4 (-77.1 to -54.1)**
15 Cirrhosis liver	15 iNTS	27.7 (-29.9 to 105)	**-42.8 (-67.2 to -10.6)**	-34 (-60.6 to 2.96)
16 Hemoglobinopathies	16 Hemoglobinopathies	22.8 (-10 to 62.1)	**-45 (-59.2 to -27.5)**	**-34.4 (-48.4 to -17.9)**
17 Diabetes	17 Breast cancer	**236 (152 to 341)**	**50.4 (12.4 to 94.6)**	**24.8 (2.84 to 53.3)**
18 Protein-energy malnutrition	18 Interpersonal violence	**81.1 (48.9 to 124)**	**-18.9 (-31.3 to -3.76)**	**-27 (-36.7 to -15.8)**
19 HIV/AIDS	19 Meningitis	4.1 (-29.2 to 53.8)	**-53.3 (-69.2 to -31.1)**	**-46.7 (-61 to -26.6)**
20 Interpersonal violence	20 Falls	**135 (89.6 to 181)**	5.42 (-12.4 to 22.2)	**-6.99 (-12 to -1.98)**
22 Liver cancer	36 Protein-energy malnutrition			
26 Falls	62 HIV/AIDS			
29 Breast cancer	134 COVID-19			

Supplemental Results Figure S8. Leading 20 level 3 causes of deaths, and percentage change in number of deaths and all-age and age-standardised death rates by location, 2022 and 2050

Cabo Verde, Reference scenario

Legend:
Communicable, maternal, neonatal and nutritional
Non-communicable
Injuries

Leading Causes 2022	Leading Causes 2050	Mean % change number of DEATH	Mean % change all-age DEATH rate	Mean % change age-standardized DEATH rate
1 Ischemic heart disease	1 Ischemic heart disease	40 (0.921 to 91.2)	30.6 (-3.3 to 76.6)	-44 (-60.7 to -23.2)
2 Stroke	2 Stroke	37.3 (9.9 to 68)	28 (5.37 to 56.4)	-44.6 (-54.2 to -29.9)
3 Lower respiratory infect	3 Lower respiratory infect	64.3 (33.9 to 93.4)	53.2 (32.9 to 85.4)	-31 (-38.5 to -21.5)
4 Diabetes	4 Chronic kidney disease	202 (136 to 273)	182 (130 to 249)	28 (1.88 to 58.8)
5 COVID-19	5 Diabetes	89.9 (47 to 138)	77.1 (41.9 to 122)	-23 (-40.1 to -1.14)
6 Chronic kidney disease	6 Prostate cancer	205 (127 to 301)	184 (109 to 262)	14.7 (-5.23 to 41.8)
7 Cirrhosis liver	7 Lung cancer	180 (120 to 259)	161 (102 to 218)	12.2 (-5.92 to 31.3)
8 Stomach cancer	8 Hypertensive heart disease	124 (72.1 to 177)	109 (68 to 161)	-9.63 (-27.7 to 14.2)
9 Hypertensive heart disease	9 Alzheimer's disease	159 (109 to 224)	142 (98.4 to 185)	3.97 (1.19 to 7.62)
10 Self-harm	10 Pancreatic cancer	328 (213 to 466)	299 (199 to 416)	72.4 (33.1 to 113)
11 Interpersonal violence	11 Stomach cancer	70.2 (40.4 to 102)	58.6 (35.3 to 79.8)	-31.3 (-36.3 to -26.2)
12 Lung cancer	12 Esophageal cancer	132 (92.7 to 177)	116 (83.3 to 153)	-3.24 (-13.4 to 7.63)
13 Prostate cancer	13 Cirrhosis liver	40.3 (14.5 to 68.4)	30.7 (9.45 to 56.9)	-33.3 (-42.6 to -20.8)
14 Esophageal cancer	14 Liver cancer	133 (78.1 to 200)	117 (69.1 to 176)	2.42 (-20.7 to 32.1)
15 Alzheimer's disease	15 COPD	109 (66.6 to 150)	94.4 (59.5 to 136)	-17.9 (-31.8 to -0.254)
16 Neonatal disorders	16 Colorectal cancer	216 (133 to 317)	194 (123 to 275)	26.3 (4.24 to 51)
17 COPD	17 Self-harm	32 (7.05 to 58.4)	23 (2.82 to 43.7)	-19.6 (-30.5 to -6.33)
18 Liver cancer	18 Breast cancer	147 (92.3 to 219)	130 (80 to 187)	11.4 (-6.07 to 30.4)
19 Tuberculosis	19 Atrial fibrillation	214 (152 to 296)	193 (136 to 254)	25.9 (19.2 to 34.4)
20 Drowning	20 Interpersonal violence	-23.9 (-42.1 to -4.1)	-29.2 (-42.4 to -14)	-36.8 (-49.6 to -22.7)
24 Pancreatic cancer	26 Drowning			
25 Breast cancer	27 Tuberculosis			
26 Colorectal cancer	44 Neonatal disorders			
31 Atrial fibrillation	131 COVID-19			

Supplemental Results Figure S8. Leading 20 level 3 causes of deaths, and percentage change in number of deaths and all-age and age-standardised death rates by location, 2022 and 2050

Cameroon, Reference scenario

Legend:
Communicable, maternal, neonatal and nutritional
Non-communicable
Injuries

Leading Causes 2022	Leading Causes 2050	Mean % change number of DEATH	Mean % change all-age DEATH rate	Mean % change age-standardized DEATH rate
1 Malaria	1 Stroke	**91.1 (41.1 to 146)**	10.8 (-10.5 to 36.6)	**-40.9 (-51.8 to -26.9)**
2 Neonatal disorders	2 Lower respiratory infect	**49.7 (13.3 to 87.5)**	-13.1 (-27.8 to 4.73)	**-27.5 (-37.1 to -16.2)**
3 Lower respiratory infect	3 Chronic kidney disease	**328 (201 to 459)**	**148 (96.2 to 214)**	**49.3 (18.4 to 89.2)**
4 HIV/AIDS	4 Ischemic heart disease	**105 (38.2 to 187)**	19 (-14.7 to 58.6)	**-37.8 (-56.1 to -10.9)**
5 Stroke	5 Neonatal disorders	-19.3 (-44.6 to 12.3)	**-53.4 (-63.9 to -40.7)**	**-25.4 (-38.5 to -10.2)**
6 Ischemic heart disease	6 Diabetes	**176 (92.4 to 259)**	**60.3 (22.8 to 99.7)**	-9.18 (-28.6 to 14.7)
7 Road injuries	7 Malaria	-41.2 (-97 to 225)	-65.8 (-98.3 to 83.3)	-68.4 (-97.7 to 73)
8 Diarrheal diseases	8 Road injuries	57.3 (-1.48 to 150)	-8.81 (-38.3 to 48.6)	-28.1 (-50 to 15)
9 Chronic kidney disease	9 Hypertensive heart disease	**210 (118 to 310)**	**79.6 (37.7 to 122)**	-9.29 (-29.5 to 14.3)
10 Congenital defects	10 Cirrhosis liver	**85.6 (39.4 to 128)**	7.67 (-9.52 to 27)	**-33.8 (-42.7 to -22.3)**
11 Diabetes	11 Diarrheal diseases	-0.828 (-40.9 to 47.3)	**-42.5 (-65.2 to -14.8)**	**-44.8 (-61.1 to -22.9)**
12 Tuberculosis	12 Liver cancer	**207 (131 to 286)**	**78.3 (48.3 to 111)**	4.14 (-12.3 to 26.1)
13 Cirrhosis liver	13 COPD	**128 (61.8 to 185)**	**31.9 (3.97 to 62.5)**	**-31.4 (-43.5 to -15.8)**
14 Hypertensive heart disease	14 Prostate cancer	**278 (160 to 416)**	**119 (55.3 to 199)**	8.49 (-12.8 to 32.1)
15 Maternal disorders	15 Tuberculosis	-1.46 (-33.6 to 38.7)	**-42.9 (-61.1 to -22.5)**	**-63.3 (-73.8 to -50.4)**
16 Meningitis	16 Self-harm	**105 (54.5 to 158)**	**19 (0.439 to 40.1)**	**-18.5 (-29.3 to -4.84)**
17 Pertussis	17 Alzheimer's disease	**313 (193 to 466)**	**140 (78.8 to 207)**	**6.03 (2.48 to 10.2)**
18 COPD	18 Breast cancer	**238 (144 to 346)**	**96.2 (45.6 to 153)**	16.6 (-3.57 to 37.4)
19 Self-harm	19 Congenital defects	-25.4 (-45.6 to 2.06)	**-56.9 (-64.2 to -47.8)**	**-37.2 (-45.3 to -25.4)**
20 Measles	20 Falls	**165 (102 to 237)**	**53.5 (27.9 to 90.2)**	**-7.33 (-12.5 to -2.61)**
21 Liver cancer	24 HIV/AIDS			
26 Falls	27 Meningitis			
28 Breast cancer	32 Maternal disorders			
30 Prostate cancer	37 Pertussis			
35 Alzheimer's disease	93 Measles			

Supplemental Results Figure S8. Leading 20 level 3 causes of deaths, and percentage change in number of deaths and all-age and age-standardised death rates by location, 2022 and 2050

Chad, Reference scenario

Legend:
Communicable, maternal, neonatal and nutritional
Non-communicable
Injuries

Leading Causes 2022	Leading Causes 2050	Mean % change number of DEATH	Mean % change all-age DEATH rate	Mean % change age-standardized DEATH rate
1 Diarrheal diseases	1 Neonatal disorders	9.87 (-16.5 to 45.9)	**-61.4 (-70.1 to -50.9)**	**-47.7 (-59.3 to -33)**
2 Neonatal disorders	2 Diarrheal diseases	-7.16 (-41.5 to 46.8)	**-67.4 (-79.3 to -50.8)**	**-49.3 (-64.7 to -25.6)**
3 Lower respiratory infect	3 Lower respiratory infect	5.05 (-16.8 to 33.6)	**-63.1 (-69 to -55.1)**	**-42.5 (-50.4 to -32.4)**
4 Malaria	4 Stroke	**90.4 (47.7 to 138)**	**-33 (-46 to -18.2)**	**-36.9 (-52.2 to -17.4)**
5 Stroke	5 Ischemic heart disease	**98.9 (30.5 to 189)**	-30.1 (-53.4 to 0.404)	**-36.3 (-60.5 to -2.8)**
6 Tuberculosis	6 Cirrhosis liver	**135 (93.1 to 193)**	**-17.3 (-30.5 to -1.4)**	**-27.2 (-39.2 to -11.6)**
7 Congenital defects	7 Malaria	-39.4 (-97 to 79.9)	**-77.3 (-98.9 to -36)**	**-77.8 (-97.8 to -7.25)**
8 Ischemic heart disease	8 Congenital defects	**29.3 (1.78 to 64.3)**	**-54.5 (-62.2 to -45.8)**	**-39.9 (-49.6 to -29)**
9 Maternal disorders	9 Chronic kidney disease	**216 (134 to 322)**	11.2 (-15.8 to 43.4)	10.2 (-18.1 to 46.1)
10 Meningitis	10 Road injuries	71.3 (-3.41 to 200)	-39.7 (-66.8 to 5.69)	**-43.1 (-65.3 to -8.42)**
11 Measles	11 Tuberculosis	-24.5 (-51.4 to 18.9)	**-73.4 (-82.7 to -59.1)**	**-72.7 (-82 to -60)**
12 Cirrhosis liver	12 Maternal disorders	17.4 (-27.2 to 77.8)	**-58.7 (-72.9 to -35.6)**	**-64.7 (-76.6 to -47.3)**
13 Pertussis	13 Diabetes	**139 (77.5 to 218)**	-15.8 (-35.8 to 8.19)	-18.5 (-39.6 to 10.9)
14 Road injuries	14 Hypertensive heart disease	**148 (84.2 to 237)**	-12.8 (-33.6 to 14.8)	-26 (-46.7 to 0.523)
15 HIV/AIDS	15 Pertussis	12.5 (-32.5 to 83.9)	**-60.5 (-76.1 to -37.1)**	**-50.8 (-70.2 to -22.8)**
16 Diabetes	16 Meningitis	-16.2 (-47.1 to 34.1)	**-70.5 (-81.7 to -54.7)**	**-59.6 (-72.2 to -41.4)**
17 Chronic kidney disease	17 Interpersonal violence	**132 (81.7 to 200)**	-18.4 (-33.1 to 1.33)	**-24.2 (-36.7 to -8.72)**
18 Protein-energy malnutrition	18 Self-harm	**175 (123 to 236)**	-3.21 (-18 to 15.2)	**-17.1 (-29.3 to -3.48)**
19 Hypertensive heart disease	19 Liver cancer	**187 (118 to 273)**	1.11 (-19.5 to 28.8)	-9.29 (-31.2 to 19.4)
20 Drowning	20 COPD	**70.5 (32.5 to 112)**	**-40.1 (-50.9 to -28.6)**	**-46.5 (-57.3 to -33.1)**
21 COPD	25 Drowning			
22 Interpersonal violence	33 HIV/AIDS			
24 Self-harm	43 Protein-energy malnutrition			
30 Liver cancer	62 Measles			

Supplemental Results Figure S8. Leading 20 level 3 causes of deaths, and percentage change in number of deaths and all-age and age-standardised death rates by location, 2022 and 2050

Côte d'Ivoire, Reference scenario

Legend:
Communicable, maternal, neonatal and nutritional
Non-communicable
Injuries

Leading Causes 2022	Leading Causes 2050	Mean % change number of DEATH	Mean % change all-age DEATH rate	Mean % change age-standardized DEATH rate
1 Malaria	1 Lower respiratory infect	**39.4 (12.7 to 68.9)**	**-18.5 (-32.7 to -0.808)**	**-34.8 (-44.4 to -21.6)**
2 Neonatal disorders	2 Stroke	**77 (36.5 to 124)**	3.52 (-17.3 to 27.4)	**-50.1 (-59.6 to -36.8)**
3 Lower respiratory infect	3 Ischemic heart disease	**90.9 (29.2 to 162)**	11.7 (-21.3 to 52.8)	**-47.8 (-63.5 to -24.2)**
4 Stroke	4 Malaria	-30.5 (-97.2 to 286)	-58.2 (-98.3 to 144)	-60.2 (-98.1 to 122)
5 Ischemic heart disease	5 Chronic kidney disease	**271 (181 to 369)**	**117 (70.7 to 169)**	17.1 (-7.39 to 50.7)
6 HIV/AIDS	6 Neonatal disorders	**-48.9 (-62.7 to -31.9)**	**-70.2 (-77.2 to -61.5)**	**-51.2 (-61.3 to -38.3)**
7 Diarrheal diseases	7 Diabetes	**164 (103 to 234)**	**54.5 (21.2 to 95)**	-22.3 (-38.5 to 2.02)
8 Congenital defects	8 Hypertensive heart disease	**203 (128 to 292)**	**77.4 (38.3 to 127)**	-19.1 (-37.5 to 6.59)
9 Tuberculosis	9 Prostate cancer	**358 (207 to 529)**	**167 (82.7 to 262)**	12 (-10.7 to 39.4)
10 COVID-19	10 Cirrhosis liver	**77.7 (43.1 to 117)**	3.96 (-12.7 to 24.4)	**-37.5 (-47 to -25.4)**
11 Chronic kidney disease	11 Road injuries	39.8 (-13.6 to 122)	-18.1 (-46.3 to 36.9)	-38.3 (-58.8 to 0.666)
12 Cirrhosis liver	12 Diarrheal diseases	-5.9 (-40.6 to 35.2)	**-45 (-65.1 to -20.8)**	**-51.5 (-64.6 to -31.3)**
13 Road injuries	13 Breast cancer	**276 (164 to 379)**	**120 (63.9 to 182)**	24.1 (-1.72 to 52)
14 Diabetes	14 Alzheimer's disease	**390 (237 to 529)**	**186 (121 to 251)**	3.38 (-0.743 to 7.01)
15 Hypertensive heart disease	15 COPD	**119 (68.7 to 173)**	**27.8 (2.45 to 56.2)**	**-42.4 (-53.9 to -28.5)**
16 Maternal disorders	16 Tuberculosis	-7.77 (-34.1 to 24.9)	**-46 (-60.9 to -27.1)**	**-66.9 (-76 to -54.3)**
17 Meningitis	17 Self-harm	**106 (67.1 to 151)**	**20.2 (0.643 to 42.4)**	**-20 (-32.1 to -4.9)**
18 Interpersonal violence	18 Falls	**194 (133 to 268)**	**71.6 (42.6 to 103)**	-6.01 (-11.2 to 0.287)
19 iNTS	19 Congenital defects	**-29 (-44 to -11.6)**	**-58.6 (-65.2 to -49)**	**-38.4 (-46.9 to -26.6)**
20 Self-harm	20 Interpersonal violence	**45.1 (15.4 to 80.6)**	-15.2 (-31.2 to 5.35)	**-31.6 (-43.4 to -17.9)**
21 COPD	22 Meningitis			
22 Prostate cancer	25 Maternal disorders			
24 Breast cancer	28 iNTS			
25 Falls	31 HIV/AIDS			
30 Alzheimer's disease	136 COVID-19			

Supplemental Results Figure S8. Leading 20 level 3 causes of deaths, and percentage change in number of deaths and all-age and age-standardised death rates by location, 2022 and 2050

The Gambia, Reference scenario

Legend:
Communicable, maternal, neonatal and nutritional
Non-communicable
Injuries

Leading Causes 2022	Leading Causes 2050	Mean % change number of DEATH	Mean % change all-age DEATH rate	Mean % change age-standardized DEATH rate
1 Neonatal disorders	1 Stroke	**38.9 (10.8 to 72.5)**	-8.58 (-24.6 to 10.2)	**-48.8 (-58.4 to -36.1)**
2 Lower respiratory infect	2 Lower respiratory infect	**32 (8.71 to 56.8)**	**-13.1 (-24.3 to -0.169)**	**-39.7 (-47.3 to -31)**
3 Stroke	3 Ischemic heart disease	**47.4 (5.77 to 96.9)**	-3.01 (-30.3 to 33.9)	**-46.5 (-62.7 to -24.9)**
4 HIV/AIDS	4 Chronic kidney disease	**206 (140 to 285)**	**101 (58.7 to 152)**	24.3 (-3.22 to 56.9)
5 Ischemic heart disease	5 Liver cancer	**156 (90.7 to 234)**	**68.7 (30.8 to 115)**	-3 (-26.6 to 27)
6 Tuberculosis	6 HIV/AIDS	-21.8 (-51.5 to 45.6)	-48.4 (-66 to 2.45)	**-62.1 (-74.9 to -24.6)**
7 Diarrheal diseases	7 Hypertensive heart disease	**142 (86.1 to 205)**	**59.5 (25.1 to 98.2)**	-16.9 (-35.9 to 5.08)
8 COVID-19	8 Neonatal disorders	**-53.1 (-66.4 to -37.5)**	**-69.2 (-76.3 to -61.9)**	**-45.2 (-55.8 to -33.1)**
9 Chronic kidney disease	9 Diabetes	**88.5 (48.1 to 138)**	24.1 (-3.04 to 53.5)	**-28.2 (-44.1 to -7.44)**
10 Diabetes	10 Cirrhosis liver	**63.5 (35.4 to 98.3)**	7.66 (-10.1 to 30.3)	**-36.1 (-45.8 to -24.5)**
11 Liver cancer	11 Road injuries	26.5 (-17.6 to 84.5)	-16.8 (-43.2 to 28)	**-39.2 (-57.9 to -8.04)**
12 Road injuries	12 Tuberculosis	**-29.5 (-48.9 to -8.14)**	**-53.6 (-65.8 to -37.5)**	**-70.6 (-78.8 to -61)**
13 Malaria	13 COPD	**60.7 (29.7 to 97.8)**	5.78 (-13.2 to 26.4)	**-42.4 (-52.5 to -29.7)**
14 Hypertensive heart disease	14 Falls	**121 (79.2 to 168)**	**45.8 (18.1 to 75.6)**	**-6.7 (-11.5 to -1.19)**
15 Cirrhosis liver	15 Diarrheal diseases	**-32.4 (-53.7 to -6.09)**	**-55.5 (-69.1 to -37.7)**	**-59.2 (-69.5 to -43.7)**
16 Maternal disorders	16 Alzheimer's disease	**196 (117 to 293)**	**95.1 (46.9 to 155)**	**4.01 (1.07 to 7.79)**
17 Congenital defects	17 Self-harm	**73.3 (41 to 109)**	14 (-3.43 to 33.9)	**-22.8 (-33.5 to -9.86)**
18 COPD	18 Ileus & obstruction	**117 (71.6 to 163)**	**42.6 (16.7 to 71.3)**	-8.05 (-24.1 to 10.6)
19 Meningitis	19 Cervical cancer	**99.9 (64.7 to 141)**	**31.5 (12.2 to 57.3)**	**-21.9 (-33.5 to -6.25)**
20 Falls	20 Congenital defects	**-32.2 (-46.3 to -14.2)**	**-55.5 (-61.7 to -47.2)**	**-34.2 (-41.8 to -23.5)**
22 Self-harm	22 Meningitis			
25 Alzheimer's disease	26 Maternal disorders			
28 Cervical cancer	37 Malaria			
29 Ileus & obstruction	135 COVID-19			

Supplemental Results Figure S8. Leading 20 level 3 causes of deaths, and percentage change in number of deaths and all-age and age-standardised death rates by location, 2022 and 2050

Ghana, Reference scenario

Legend:
Communicable, maternal, neonatal and nutritional
Non-communicable
Injuries

Leading Causes 2022
1 Stroke
2 Neonatal disorders
3 Malaria
4 Lower respiratory infect
5 HIV/AIDS
6 Ischemic heart disease
7 Tuberculosis
8 Chronic kidney disease
9 Diabetes
10 Road injuries
11 Cirrhosis liver
12 Diarrheal diseases
13 COVID-19
14 Hypertensive heart disease
15 Congenital defects
16 Meningitis
17 Hemoglobinopathies
18 Cardiomyopathy
19 Breast cancer
20 Falls
23 COPD
25 Prostate cancer
28 Alzheimer's disease
30 Liver cancer

Leading Causes 2050	Mean % change number of DEATH	Mean % change all-age DEATH rate	Mean % change age-standardized DEATH rate
1 Stroke	**59.5 (26.1 to 96.1)**	3.81 (-19.2 to 27.2)	**-46.7 (-57.8 to -33.6)**
2 Lower respiratory infect	**125 (89 to 175)**	**46.7 (22.1 to 72.5)**	-9.71 (-20.5 to 3.5)
3 Chronic kidney disease	**238 (162 to 330)**	**120 (65.4 to 172)**	24.7 (-3.47 to 58.8)
4 Ischemic heart disease	**68.4 (14.3 to 130)**	9.64 (-25 to 52.6)	**-44.3 (-62.1 to -20.7)**
5 Malaria	7.23 (-95.7 to 484)	-30.4 (-97.3 to 287)	-32.6 (-97.2 to 274)
6 Diabetes	**134 (78.6 to 194)**	**52.5 (16.3 to 92.2)**	-17.8 (-35.3 to 4.79)
7 Hypertensive heart disease	**205 (131 to 283)**	**98.8 (52.4 to 155)**	-1.38 (-24.5 to 29.3)
8 Cirrhosis liver	**84.4 (48 to 126)**	**20 (0.488 to 45)**	**-29.6 (-39.4 to -16.3)**
9 Neonatal disorders	**-39.7 (-57 to -20.6)**	**-61 (-69.4 to -50.4)**	**-36.7 (-48.1 to -23.1)**
10 Diarrheal diseases	**80.5 (28.2 to 143)**	17.5 (-16.6 to 61.2)	-19.8 (-42.4 to 10.5)
11 Tuberculosis	13.5 (-19.4 to 58.3)	-26.2 (-47.2 to 2.68)	**-57.2 (-69.8 to -40.9)**
12 Road injuries	36.8 (-18.1 to 134)	-11 (-47 to 50.6)	-26.8 (-54.9 to 22.1)
13 Alzheimer's disease	**302 (187 to 441)**	**162 (93.8 to 240)**	**8.03 (4.54 to 12.1)**
14 Breast cancer	**189 (115 to 273)**	**88 (41.1 to 140)**	12.7 (-7.58 to 37.1)
15 Prostate cancer	**239 (130 to 366)**	**120 (52.3 to 194)**	3.61 (-18.4 to 24)
16 Cardiomyopathy	**115 (77.7 to 159)**	**39.9 (17.5 to 59.7)**	**-20.3 (-25.3 to -16.2)**
17 Falls	**163 (106 to 229)**	**70.9 (39 to 106)**	**-6.07 (-9.9 to -1.24)**
18 COPD	**151 (93.4 to 209)**	**63.6 (29.7 to 99.2)**	**-18.3 (-34.7 to -0.259)**
19 Liver cancer	**193 (118 to 281)**	**90.7 (45.1 to 145)**	10.4 (-12.6 to 46.2)
20 Meningitis	21.7 (-12.4 to 81.1)	-20.8 (-42.4 to 13)	**-36.8 (-53.9 to -5)**
21 Hemoglobinopathies			
25 HIV/AIDS			
31 Congenital defects			
134 COVID-19			

Supplemental Results Figure S8. Leading 20 level 3 causes of deaths, and percentage change in number of deaths and all-age and age-standardised death rates by location, 2022 and 2050

Guinea, Reference scenario

Legend:
Communicable, maternal, neonatal and nutritional
Non-communicable
Injuries

Leading Causes 2022	Leading Causes 2050	Mean % change number of DEATH	Mean % change all-age DEATH rate	Mean % change age-standardized DEATH rate
1 Malaria	1 Malaria	24.5 (-90.6 to 527)	-32.5 (-95.2 to 239)	-19.5 (-94.7 to 261)
2 Lower respiratory infect	2 Lower respiratory infect	11 (-10.6 to 34.2)	**-42.3 (-52.7 to -31.2)**	**-38.9 (-47.5 to -28.5)**
3 Neonatal disorders	3 Stroke	**49.9 (16.1 to 80.7)**	**-22.1 (-36.1 to -4.34)**	**-44.2 (-54.2 to -29.3)**
4 Stroke	4 Ischemic heart disease	**55.1 (8.2 to 110)**	-19.4 (-43.2 to 10.7)	**-43.3 (-61.4 to -18.4)**
5 Ischemic heart disease	5 Neonatal disorders	**-38.8 (-52.7 to -20.8)**	**-68.2 (-74.9 to -59.8)**	**-51.4 (-61.2 to -37.9)**
6 Diarrheal diseases	6 Chronic kidney disease	**197 (126 to 269)**	**54.5 (22.5 to 95.3)**	18.8 (-6.18 to 53.3)
7 COVID-19	7 Diabetes	**110 (58.2 to 164)**	9.06 (-13.8 to 39.3)	-19.7 (-37.3 to 6.92)
8 Congenital defects	8 Diarrheal diseases	-7.74 (-35.2 to 24.7)	**-52.1 (-66.6 to -35.7)**	**-55.8 (-67.5 to -39.1)**
9 Tuberculosis	9 Cirrhosis liver	**59.9 (30.5 to 90.1)**	**-16.9 (-29.4 to -0.868)**	**-39.4 (-49.5 to -27.8)**
10 HIV/AIDS	10 Hypertensive heart disease	**116 (66.1 to 167)**	12.5 (-10.6 to 41.7)	**-24.7 (-41.3 to -2.64)**
11 Cirrhosis liver	11 Liver cancer	**135 (78.2 to 204)**	22.4 (-4.33 to 61.1)	-11.9 (-32.1 to 18.1)
12 Meningitis	12 Congenital defects	-19.5 (-34.9 to 2.05)	**-58.2 (-64 to -51.1)**	**-39.9 (-47.4 to -30.1)**
13 Pertussis	13 Road injuries	16.3 (-28.5 to 88.7)	**-39.5 (-62.9 to -3.1)**	**-47.2 (-65.9 to -18.1)**
14 Road injuries	14 Tuberculosis	-25.4 (-48.2 to 2.25)	**-61.2 (-72.4 to -45.4)**	**-70.5 (-79.1 to -58.9)**
15 Diabetes	15 COPD	**57.9 (25.3 to 90.8)**	**-17.9 (-32.8 to -0.85)**	**-42.7 (-53.3 to -28.1)**
16 Maternal disorders	16 Cervical cancer	**104 (63.4 to 147)**	6.02 (-12.3 to 26.5)	**-21.9 (-33.6 to -9.81)**
17 Chronic kidney disease	17 Pertussis	-11.4 (-50.4 to 42.4)	**-54.1 (-72.9 to -28.4)**	-34.6 (-60.7 to 1.94)
18 iNTS	18 Breast cancer	**250 (157 to 351)**	**81.7 (35.8 to 135)**	**30.8 (5.53 to 58)**
19 Hypertensive heart disease	19 Alzheimer's disease	**203 (118 to 292)**	**57.1 (15.5 to 102)**	**3.9 (0.357 to 7.88)**
20 Measles	20 iNTS	-1.43 (-49.9 to 64.1)	**-48.8 (-70.9 to -13.7)**	-38.4 (-64 to 3.65)
21 COPD	21 Maternal disorders			
23 Liver cancer	22 Meningitis			
25 Cervical cancer	39 HIV/AIDS			
32 Alzheimer's disease	92 Measles			
35 Breast cancer	135 COVID-19			

Supplemental Results Figure S8. Leading 20 level 3 causes of deaths, and percentage change in number of deaths and all-age and age-standardised death rates by location, 2022 and 2050

Guinea-Bissau, Reference scenario

Legend:
Communicable, maternal, neonatal and nutritional
Non-communicable
Injuries

Leading Causes 2022	Leading Causes 2050	Mean % change number of DEATH	Mean % change all-age DEATH rate	Mean % change age-standardized DEATH rate
1 COVID-19	1 Stroke	**61.9 (28.1 to 107)**	-8.67 (-26.4 to 11.9)	**-47 (-59 to -32.6)**
2 Neonatal disorders	2 Lower respiratory infect	**52.2 (23.7 to 89.4)**	-14.2 (-28.2 to 0.806)	**-35.9 (-44.6 to -26)**
3 HIV/AIDS	3 Ischemic heart disease	**78.3 (20.2 to 156)**	0.666 (-30.7 to 41.2)	**-44.1 (-62.6 to -18.3)**
4 Stroke	4 Chronic kidney disease	**213 (136 to 309)**	**76.4 (39.2 to 119)**	13.9 (-14.2 to 43)
5 Lower respiratory infect	5 HIV/AIDS	**-39 (-59.6 to -10)**	**-65.7 (-76.7 to -51)**	**-71.3 (-80.6 to -57.5)**
6 Ischemic heart disease	6 Neonatal disorders	**-49.9 (-64.3 to -32.8)**	**-71.8 (-78.3 to -64.4)**	**-51.2 (-61 to -39.7)**
7 Tuberculosis	7 Diabetes	**130 (69.4 to 205)**	30 (-0.0185 to 64)	-21.8 (-41.7 to 2.04)
8 Measles	8 Hypertensive heart disease	**168 (104 to 247)**	**51.1 (18.2 to 93.2)**	-20.8 (-39.4 to 3.88)
9 Road injuries	9 Road injuries	34.9 (-27.4 to 138)	-23.8 (-56.8 to 35)	-35.4 (-62.8 to 11.4)
10 Diarrheal diseases	10 Cirrhosis liver	**71.3 (37.2 to 113)**	-3.34 (-21 to 15.6)	**-38.9 (-49.3 to -26.5)**
11 Cirrhosis liver	11 Diarrheal diseases	15.1 (-21.2 to 63.6)	**-35 (-53 to -14.1)**	**-41.2 (-56.9 to -17.4)**
12 Congenital defects	12 Tuberculosis	-22.7 (-46.3 to 10.5)	**-56.4 (-68.9 to -40.2)**	**-70.5 (-79 to -58.6)**
13 Diabetes	13 Liver cancer	**181 (107 to 278)**	**58.7 (20.4 to 104)**	-4.32 (-28.1 to 25.3)
14 Chronic kidney disease	14 COPD	**105 (61.5 to 155)**	15.8 (-7.07 to 40.6)	**-37.1 (-49.4 to -21.1)**
15 Malaria	15 Self-harm	**102 (62 to 148)**	13.9 (-7.26 to 36)	**-20.7 (-34 to -5.78)**
16 Hypertensive heart disease	16 Cervical cancer	**115 (72.1 to 166)**	21.5 (-2.75 to 44.8)	**-22.5 (-34.9 to -8.52)**
17 Meningitis	17 Interpersonal violence	**49.1 (12.8 to 87.8)**	-16.1 (-30.5 to 3.31)	**-30 (-41.1 to -15.5)**
18 Interpersonal violence	18 Breast cancer	**227 (129 to 325)**	**84.3 (35.6 to 138)**	17.3 (-5.4 to 41.3)
19 COPD	19 Congenital defects	-19.3 (-37 to 3.56)	**-54.6 (-61.3 to -46.3)**	**-33 (-40.8 to -22.5)**
20 Pertussis	20 Falls	**153 (97.7 to 208)**	**42.6 (18 to 68)**	**-7.03 (-11 to -2.1)**
21 Self-harm	21 Meningitis			
22 Cervical cancer	35 Pertussis			
24 Liver cancer	47 Measles			
30 Falls	69 Malaria			
31 Breast cancer	134 COVID-19			

Supplemental Results Figure S8. Leading 20 level 3 causes of deaths, and percentage change in number of deaths and all-age and age-standardised death rates by location, 2022 and 2050

Liberia, Reference scenario

Legend:
Communicable, maternal, neonatal and nutritional
Non-communicable
Injuries

Leading Causes 2022	Leading Causes 2050	Mean % change number of DEATH	Mean % change all-age DEATH rate	Mean % change age-standardized DEATH rate
1 Malaria	1 Stroke	**111 (58.1 to 173)**	**30.3 (2.83 to 65.9)**	**-39.5 (-54.2 to -17.7)**
2 Neonatal disorders	2 Ischemic heart disease	**152 (66.1 to 275)**	**55.8 (3.23 to 131)**	-30.3 (-55.7 to 11.5)
3 Stroke	3 Lower respiratory infect	**91 (44 to 135)**	17.8 (-3.15 to 43.3)	**-24.5 (-35 to -9.87)**
4 Lower respiratory infect	4 Chronic kidney disease	**269 (172 to 388)**	**128 (76.8 to 193)**	19.5 (-11.4 to 63)
5 Ischemic heart disease	5 Malaria	-36.3 (-96.8 to 274)	-60 (-98.1 to 131)	-64.5 (-97.7 to 101)
6 Diarrheal diseases	6 Hypertensive heart disease	**241 (153 to 348)**	**110 (61.4 to 178)**	-8.67 (-32.7 to 25.3)
7 HIV/AIDS	7 Diabetes	**188 (111 to 285)**	**77.6 (34.9 to 135)**	-13 (-36 to 21)
8 Chronic kidney disease	8 Diarrheal diseases	28.3 (-25.9 to 109)	-20.7 (-53.9 to 21.9)	-30.3 (-54.9 to 6.65)
9 Maternal disorders	9 Neonatal disorders	**-44 (-63.5 to -17)**	**-65.6 (-75.5 to -52.7)**	**-40.2 (-53.3 to -23)**
10 Cirrhosis liver	10 Cirrhosis liver	**100 (55.5 to 145)**	**23.7 (0.735 to 48.7)**	**-32.5 (-43.8 to -17.5)**
11 Congenital defects	11 Liver cancer	**236 (144 to 360)**	**108 (53.1 to 180)**	6.42 (-22.3 to 46.8)
12 Diabetes	12 Road injuries	47.4 (-8.97 to 159)	-8.87 (-43.6 to 55.5)	-31.6 (-58.7 to 13.7)
13 Tuberculosis	13 COPD	**158 (90.8 to 229)**	**59.2 (29.8 to 95.6)**	**-31.4 (-46.1 to -11.9)**
14 Road injuries	14 Tuberculosis	15.9 (-20.8 to 70)	-28.3 (-50.3 to 3.93)	**-59.6 (-72.9 to -40)**
15 Hypertensive heart disease	15 Prostate cancer	**356 (184 to 591)**	**181 (80.1 to 288)**	12.3 (-11.5 to 38.4)
16 Meningitis	16 Alzheimer's disease	**305 (170 to 451)**	**149 (76.6 to 226)**	**3.99 (0.857 to 8.1)**
17 Liver cancer	17 Self-harm	**107 (62.6 to 154)**	**27.6 (5.63 to 55.1)**	-18 (-31 to 2.18)
18 COPD	18 Falls	**178 (97.7 to 259)**	**71.4 (37.8 to 114)**	**-6.03 (-10.7 to -0.449)**
19 Self-harm	19 Breast cancer	**236 (135 to 366)**	**107 (51.3 to 174)**	10.4 (-14 to 37.4)
20 STIs	20 Cervical cancer	**133 (81.1 to 193)**	**44.2 (16.6 to 75.7)**	**-20.5 (-34.6 to -2.87)**
24 Cervical cancer	21 Congenital defects			
25 Falls	22 Maternal disorders			
30 Breast cancer	23 Meningitis			
34 Alzheimer's disease	48 HIV/AIDS			
36 Prostate cancer	52 STIs			

Supplemental Results Figure S8. Leading 20 level 3 causes of deaths, and percentage change in number of deaths and all-age and age-standardised death rates by location, 2022 and 2050

Mali, Reference scenario

Legend:
Communicable, maternal, neonatal and nutritional
Non-communicable
Injuries

Leading Causes 2022	Leading Causes 2050	Mean % change number of DEATH	Mean % change all-age DEATH rate	Mean % change age-standardized DEATH rate
1 Neonatal disorders	1 Neonatal disorders	-10.8 (-33.8 to 20.1)	**-62.4 (-71.5 to -49.7)**	**-48.2 (-60.7 to -31.3)**
2 Malaria	2 Stroke	**93.5 (45 to 148)**	**-18.3 (-35.7 to -2.39)**	**-34 (-48.4 to -16.8)**
3 Lower respiratory infect	3 Lower respiratory infect	21.4 (-3.76 to 53.9)	**-48.7 (-57.2 to -39.4)**	**-34.3 (-42.8 to -23.3)**
4 Stroke	4 Malaria	-48.9 (-97 to 229)	-77.1 (-98.6 to 39.5)	-74.8 (-97.3 to 45.8)
5 Diarrheal diseases	5 Ischemic heart disease	**114 (37.4 to 205)**	-9.61 (-39 to 26.7)	-30 (-54.6 to 4.22)
6 Congenital defects	6 Chronic kidney disease	**250 (156 to 352)**	**47.9 (13.3 to 84.4)**	24.8 (-4.34 to 62.8)
7 Protein-energy malnutrition	7 Diabetes	**179 (104 to 264)**	17.6 (-11.1 to 46)	-2.92 (-26.5 to 26.7)
8 Ischemic heart disease	8 Congenital defects	7.8 (-18 to 37.9)	**-54.5 (-61.9 to -44.2)**	**-39.4 (-48.7 to -28.5)**
9 Tuberculosis	9 Diarrheal diseases	-2.95 (-40.8 to 48.6)	**-59 (-73.7 to -40.8)**	**-52.5 (-67.5 to -33.1)**
10 HIV/AIDS	10 Liver cancer	**160 (90.6 to 243)**	9.71 (-14.1 to 37.5)	-9.56 (-31.1 to 16)
11 COVID-19	11 COPD	**98.3 (51.7 to 152)**	-16.2 (-33.4 to 0.442)	**-35.8 (-50 to -19.2)**
12 iNTS	12 Cirrhosis liver	**74.7 (36.9 to 117)**	**-26.2 (-37.6 to -12.2)**	**-38.8 (-48.1 to -26.9)**
13 Road injuries	13 Road injuries	43.5 (-13.1 to 132)	**-39.4 (-62.9 to -2.77)**	**-44.1 (-63.6 to -15.2)**
14 Measles	14 Hypertensive heart disease	**141 (75.9 to 214)**	1.86 (-23.5 to 30.2)	**-24 (-42.5 to -0.873)**
15 Cirrhosis liver	15 iNTS	8.1 (-45 to 94.1)	**-54.4 (-76.7 to -19.4)**	**-48.9 (-71.1 to -13.1)**
16 Meningitis	16 Tuberculosis	-20 (-46.9 to 11.8)	**-66.2 (-76 to -53.4)**	**-71.4 (-79.5 to -59.8)**
17 Diabetes	17 Alzheimer's disease	**308 (194 to 442)**	**72.1 (25.1 to 118)**	**4.63 (1.46 to 8.48)**
18 COPD	18 Protein-energy malnutrition	**-51.8 (-77.8 to -6.67)**	**-79.7 (-90.3 to -60.8)**	**-68.9 (-83.5 to -44.8)**
19 Chronic kidney disease	19 Meningitis	0.329 (-32.9 to 51.7)	**-57.6 (-72 to -37.4)**	**-52.8 (-65.4 to -34.7)**
20 Maternal disorders	20 Interpersonal violence	**79.9 (40.4 to 134)**	**-24 (-37.2 to -5.06)**	**-29.5 (-39.8 to -14)**
21 Liver cancer	23 Maternal disorders			
22 Hypertensive heart disease	62 Measles			
25 Interpersonal violence	63 HIV/AIDS			
36 Alzheimer's disease	134 COVID-19			

Supplemental Results Figure S8. Leading 20 level 3 causes of deaths, and percentage change in number of deaths and all-age and age-standardised death rates by location, 2022 and 2050

Mauritania, Reference scenario

Legend:
Communicable, maternal, neonatal and nutritional
Non-communicable
Injuries

Leading Causes 2022	Leading Causes 2050	Mean % change number of DEATH	Mean % change all-age DEATH rate	Mean % change age-standardized DEATH rate
1 Stroke	1 Stroke	**51.8 (17.8 to 85.5)**	-10.8 (-28.4 to 10.1)	**-45.1 (-57.1 to -28.3)**
2 Neonatal disorders	2 Lower respiratory infect	**72.5 (42.1 to 103)**	1.31 (-13.6 to 21.1)	**-22.9 (-33.5 to -9.26)**
3 Ischemic heart disease	3 Ischemic heart disease	**50.2 (5.74 to 105)**	-11.7 (-37 to 21.4)	**-45.7 (-63.5 to -20.9)**
4 Lower respiratory infect	4 Chronic kidney disease	**251 (176 to 340)**	**106 (62.8 to 155)**	**33.3 (5.23 to 70.4)**
5 COVID-19	5 Hypertensive heart disease	**158 (100 to 227)**	**51.5 (19.4 to 84.6)**	-8.36 (-29.1 to 16.4)
6 Road injuries	6 Road injuries	42.8 (-19.3 to 148)	-16.2 (-50.1 to 46.2)	-28.1 (-56 to 21.5)
7 Chronic kidney disease	7 Neonatal disorders	-25.1 (-44.2 to 2.78)	**-56.2 (-65.1 to -44.1)**	**-32.8 (-44.9 to -18.1)**
8 Malaria	8 Diabetes	**89.6 (43 to 143)**	11.4 (-12.7 to 41.6)	**-28.6 (-45.8 to -3.16)**
9 Diarrheal diseases	9 Liver cancer	**171 (109 to 259)**	**59.3 (26.7 to 107)**	4.35 (-19.8 to 40.9)
10 Diabetes	10 Cirrhosis liver	**70.6 (40 to 106)**	0.242 (-15.6 to 19.7)	**-32.8 (-43.2 to -19.2)**
11 Hypertensive heart disease	11 Alzheimer's disease	**228 (140 to 341)**	**92.8 (43.9 to 154)**	**5.16 (2.33 to 8.89)**
12 Cirrhosis liver	12 Prostate cancer	**206 (113 to 340)**	**80 (26.2 to 155)**	6.55 (-14.9 to 30.4)
13 Maternal disorders	13 COPD	**114 (69.5 to 160)**	**25.6 (2 to 52.7)**	**-24.1 (-37.8 to -4.75)**
14 Congenital defects	14 Breast cancer	**196 (121 to 290)**	**73.6 (31.5 to 129)**	15.2 (-4.2 to 40.1)
15 Liver cancer	15 Diarrheal diseases	-13.9 (-41.7 to 19.7)	**-49.5 (-65.8 to -29.1)**	**-54.9 (-67.3 to -38.4)**
16 Tuberculosis	16 Falls	**150 (95.3 to 213)**	**46.7 (18.5 to 80.1)**	-4.49 (-9.64 to 1.09)
17 COPD	17 Colorectal cancer	**234 (143 to 362)**	**96.1 (44.5 to 165)**	**23.2 (1.27 to 50)**
18 Prostate cancer	18 Cervical cancer	**104 (67.2 to 145)**	**20.2 (0.228 to 45.9)**	**-19.5 (-31.7 to -4.38)**
19 Meningitis	19 Lung cancer	**175 (102 to 271)**	**61.2 (19.2 to 112)**	2.25 (-16.6 to 22.9)
20 Alzheimer's disease	20 Pancreatic cancer	**390 (251 to 588)**	**188 (107 to 290)**	**82.1 (38.3 to 129)**
22 Cervical cancer	21 Tuberculosis			
23 Falls	22 Congenital defects			
24 Breast cancer	24 Maternal disorders			
30 Lung cancer	32 Malaria			
33 Colorectal cancer	33 Meningitis			
41 Pancreatic cancer	134 COVID-19			

Supplemental Results Figure S8. Leading 20 level 3 causes of deaths, and percentage change in number of deaths and all-age and age-standardised death rates by location, 2022 and 2050

Niger, Reference scenario

Legend:
Communicable, maternal, neonatal and nutritional
Non-communicable
Injuries

Leading Causes 2022	Leading Causes 2050	Mean % change number of DEATH	Mean % change all-age DEATH rate	Mean % change age-standardized DEATH rate
1 Malaria	1 Neonatal disorders	**46.2 (13.3 to 86.1)**	**-48.1 (-59.1 to -35.1)**	**-35.7 (-48.3 to -20.5)**
2 Neonatal disorders	2 Malaria	26.5 (-96.5 to 653)	-54.4 (-98.7 to 164)	-52.5 (-97.5 to 164)
3 Lower respiratory infect	3 Lower respiratory infect	**53.5 (20.3 to 93.4)**	**-45.5 (-54.5 to -32.3)**	**-22.7 (-32.9 to -10.3)**
4 Diarrheal diseases	4 Stroke	**174 (111 to 244)**	-2.78 (-21.3 to 19.6)	3.22 (-16.7 to 26.8)
5 Measles	5 Diarrheal diseases	30.6 (-12.7 to 81.5)	**-53.7 (-68.8 to -34.9)**	**-36.2 (-54.2 to -11.3)**
6 Stroke	6 Ischemic heart disease	**267 (158 to 388)**	30.3 (-5.01 to 70.5)	38.2 (-2.54 to 96.6)
7 Tuberculosis	7 Diabetes	**399 (286 to 517)**	**77.2 (42.7 to 117)**	**94.3 (51.4 to 142)**
8 Congenital defects	8 Chronic kidney disease	**327 (229 to 446)**	**51.5 (17.5 to 86.8)**	**64 (25.5 to 109)**
9 Ischemic heart disease	9 Congenital defects	**37.5 (9.01 to 72.2)**	**-51.2 (-58.8 to -40.7)**	**-39.4 (-48.2 to -27.7)**
10 Meningitis	10 Cirrhosis liver	**95.9 (58.1 to 136)**	**-30.5 (-40.1 to -19.1)**	**-33.5 (-43 to -21.9)**
11 Maternal disorders	11 iNTS	77.3 (-2.8 to 191)	**-37 (-64.7 to -0.776)**	-33.1 (-61.2 to 2.78)
12 Cirrhosis liver	12 Tuberculosis	-1.82 (-34.7 to 45.4)	**-65.1 (-76.2 to -48.8)**	**-65.1 (-75.6 to -50.5)**
13 iNTS	13 Maternal disorders	**51.8 (2.26 to 119)**	**-46.1 (-63.4 to -22.1)**	**-55.1 (-68.6 to -36.5)**
14 COVID-19	14 Meningitis	27.4 (-21.4 to 102)	**-54.7 (-71.5 to -26.9)**	**-45.4 (-61.2 to -20.1)**
15 Pertussis	15 Pertussis	**90.7 (6.91 to 194)**	-32.5 (-59.5 to 2.7)	-20.6 (-50.5 to 19.9)
16 Road injuries	16 Road injuries	**103 (19.9 to 229)**	-27.8 (-55.5 to 17.3)	-30.9 (-53.7 to 4.96)
17 Chronic kidney disease	17 Hypertensive heart disease	**148 (89 to 211)**	-11.8 (-30.5 to 9.6)	-18.3 (-37.4 to 3.87)
18 Diabetes	18 COPD	**141 (92.2 to 194)**	-14.6 (-30 to 2.81)	-17.6 (-32.9 to 1.46)
19 Hypertensive heart disease	19 Interpersonal violence	**103 (61.9 to 152)**	**-27.9 (-40.1 to -13.3)**	**-30.3 (-40 to -18.3)**
20 COPD	20 Falls	**150 (92.1 to 215)**	-11.3 (-26.3 to 5.75)	**-6.22 (-12 to -0.598)**
23 Interpersonal violence	26 Measles			
24 Falls	135 COVID-19			

Supplemental Results Figure S8. Leading 20 level 3 causes of deaths, and percentage change in number of deaths and all-age and age-standardised death rates by location, 2022 and 2050

Nigeria, Reference scenario

Legend:
Communicable, maternal, neonatal and nutritional
Non-communicable
Injuries

Leading Causes 2022	Leading Causes 2050	Mean % change number of DEATH	Mean % change all-age DEATH rate	Mean % change age-standardized DEATH rate
1 Neonatal disorders	1 Malaria	-19.5 (-96.1 to 442)	-56 (-97.9 to 198)	-56.3 (-97.4 to 194)
2 Malaria	2 Neonatal disorders	**-28.7 (-48.4 to -8.03)**	**-62.1 (-70 to -53.3)**	**-39.2 (-49.7 to -25.7)**
3 Lower respiratory infect	3 Lower respiratory infect	0.809 (-18.9 to 25.1)	**-46.2 (-55.8 to -35.1)**	**-38 (-45.5 to -29)**
4 Diarrheal diseases	4 Ischemic heart disease	**95.2 (40.1 to 162)**	4.21 (-27.2 to 39.8)	**-48.2 (-62.9 to -28.2)**
5 HIV/AIDS	5 Stroke	**88.3 (53.8 to 126)**	0.509 (-17.7 to 18.5)	**-49.2 (-57.9 to -39.1)**
6 Ischemic heart disease	6 Chronic kidney disease	**303 (219 to 410)**	**115 (70.6 to 167)**	20 (-5.19 to 51.2)
7 Stroke	7 Diarrheal diseases	**-40.4 (-61.6 to -10.4)**	**-68.2 (-80 to -53.4)**	**-60.1 (-72 to -45)**
8 Congenital defects	8 Diabetes	**140 (89.4 to 198)**	**28.2 (0.149 to 56.1)**	**-32.1 (-44.8 to -15.9)**
9 Tuberculosis	9 Hypertensive heart disease	**232 (159 to 318)**	**77.2 (37.2 to 120)**	-11.6 (-28.9 to 12.7)
10 Meningitis	10 Prostate cancer	**305 (184 to 443)**	**116 (52.1 to 187)**	-2.63 (-25.7 to 22.3)
11 Cirrhosis liver	11 Cirrhosis liver	**86.3 (54.5 to 123)**	-0.601 (-17 to 17.6)	**-37 (-45 to -26.3)**
12 Diabetes	12 Breast cancer	**246 (150 to 345)**	**84.7 (42.1 to 134)**	12.7 (-8.68 to 36.5)
13 Chronic kidney disease	13 Tuberculosis	2.54 (-26.8 to 40.8)	**-45.2 (-61.1 to -25.7)**	**-63.4 (-72.4 to -49.5)**
14 Maternal disorders	14 Alzheimer's disease	**376 (266 to 491)**	**153 (103 to 206)**	**5.53 (0.329 to 10.4)**
15 Road injuries	15 HIV/AIDS	**-44 (-60.4 to -33.1)**	**-70.2 (-78 to -65)**	**-76.3 (-82.3 to -72.3)**
16 Pertussis	16 Congenital defects	**-23.4 (-43.1 to -0.897)**	**-59.3 (-66.8 to -49.9)**	**-38.7 (-47.2 to -26.2)**
17 iNTS	17 Road injuries	**63.9 (12.5 to 142)**	-12.5 (-41.1 to 30.8)	**-34.5 (-54.3 to -1.25)**
18 Hypertensive heart disease	18 COPD	**155 (103 to 209)**	**36.3 (11.9 to 63.1)**	**-33.9 (-46 to -19.2)**
19 Interpersonal violence	19 Falls	**197 (137 to 268)**	**58.5 (31.8 to 88.6)**	**-8.15 (-13.3 to -3.13)**
20 Breast cancer	20 Interpersonal violence	**58.9 (29 to 94.1)**	-15.4 (-27.3 to 0.063)	**-28.7 (-37.8 to -18)**
21 Prostate cancer	21 Meningitis			
23 COPD	23 Maternal disorders			
26 Falls	26 iNTS			
27 Alzheimer's disease	30 Pertussis			

Supplemental Results Figure S8. Leading 20 level 3 causes of deaths, and percentage change in number of deaths and all-age and age-standardised death rates by location, 2022 and 2050

São Tomé and Príncipe, Reference scenario

Legend:
Communicable, maternal, neonatal and nutritional
Non-communicable
Injuries

Leading Causes 2022	Leading Causes 2050	Mean % change number of DEATH	Mean % change all-age DEATH rate	Mean % change age-standardized DEATH rate
1 Stroke	1 Chronic kidney disease	**222 (148 to 298)**	**198 (137 to 277)**	**27.5 (0.753 to 62.5)**
2 Lower respiratory infect	2 Lower respiratory infect	**68.9 (35.2 to 99.7)**	**56.6 (33.4 to 89.3)**	**-31.1 (-39.4 to -21.5)**
3 Ischemic heart disease	3 Stroke	**48.4 (16.7 to 80.7)**	**37.7 (10.3 to 70.2)**	**-45.6 (-55.7 to -31.3)**
4 Chronic kidney disease	4 Ischemic heart disease	**67.6 (18.8 to 119)**	**55.5 (14.7 to 115)**	**-39.5 (-57.2 to -16.1)**
5 Cirrhosis liver	5 COPD	**131 (84.5 to 185)**	**115 (73.3 to 159)**	**-21.3 (-35.2 to -4.78)**
6 COPD	6 Cirrhosis liver	**52.4 (16.9 to 84.9)**	**41.3 (13.7 to 73.5)**	**-32.1 (-42.1 to -20.8)**
7 Neonatal disorders	7 Lung cancer	**185 (123 to 265)**	**164 (104 to 235)**	3.54 (-13.2 to 22.8)
8 Asthma	8 Hypertensive heart disease	**162 (98.3 to 232)**	**143 (91.7 to 211)**	-6.71 (-26.4 to 17.5)
9 Road injuries	9 Colorectal cancer	**238 (155 to 350)**	**214 (130 to 308)**	22.1 (-2.15 to 48.4)
10 Lung cancer	10 Diabetes	**99.9 (55.6 to 155)**	**85.4 (45.7 to 138)**	**-24.6 (-40.1 to -4.25)**
11 Other cardiovascular	11 Alzheimer's disease	**224 (173 to 287)**	**200 (148 to 255)**	**3.85 (0.573 to 7.24)**
12 Tuberculosis	12 Other cardiovascular	**96.7 (61.6 to 136)**	**82.6 (50.8 to 114)**	**-23.3 (-33 to -11.6)**
13 Diabetes	13 Falls	**118 (75.8 to 169)**	**102 (62.7 to 158)**	**-7.55 (-13.8 to -1.12)**
14 Hypertensive heart disease	14 Prostate cancer	**275 (180 to 392)**	**248 (155 to 350)**	19.3 (-3.57 to 43.5)
15 Cervical cancer	15 Stomach cancer	**89.7 (56.7 to 122)**	**75.9 (50.8 to 107)**	**-30.5 (-35.2 to -25.9)**
16 Stomach cancer	16 Breast cancer	**135 (82.6 to 205)**	**118 (67.6 to 175)**	5.23 (-13 to 26.3)
17 Falls	17 Road injuries	17 (-28 to 77.7)	8.38 (-28.6 to 64.4)	-28.9 (-52.4 to 7.16)
18 Diarrheal diseases	18 Cervical cancer	**66 (31.6 to 102)**	**53.9 (26.8 to 83.6)**	**-22.9 (-32.7 to -11.6)**
19 Congenital defects	19 Asthma	-5.73 (-24.9 to 15.9)	-12.6 (-26.8 to 2.81)	**-59.9 (-65.3 to -53.3)**
20 Breast cancer	20 Urinary diseases	**170 (113 to 222)**	**151 (113 to 193)**	**13.2 (2.68 to 24.3)**
21 Alzheimer's disease	23 Tuberculosis			
22 Colorectal cancer	28 Neonatal disorders			
25 Prostate cancer	30 Diarrheal diseases			
28 Urinary diseases	37 Congenital defects			

Supplemental Results Figure S8. Leading 20 level 3 causes of deaths, and percentage change in number of deaths and all-age and age-standardised death rates by location, 2022 and 2050

Senegal, Reference scenario

Legend:
Communicable, maternal, neonatal and nutritional
Non-communicable
Injuries

Leading Causes 2022
1 Stroke
2 Neonatal disorders
3 Ischemic heart disease
4 Lower respiratory infect
5 Malaria
6 Diabetes
7 Chronic kidney disease
8 Diarrheal diseases
9 Tuberculosis
10 COVID-19
11 Road injuries
12 Cirrhosis liver
13 Congenital defects
14 Hypertensive heart disease
15 COPD
16 Maternal disorders
17 Meningitis
18 Self-harm
19 HIV/AIDS
20 Falls
21 Alzheimer's disease
22 Prostate cancer
23 Cervical cancer
25 Liver cancer
27 Breast cancer
32 Lung cancer

Leading Causes 2050	Mean % change number of DEATH	Mean % change all-age DEATH rate	Mean % change age-standardized DEATH rate
1 Stroke	**34.8 (7.75 to 66.8)**	**-19.4 (-33.4 to -2.66)**	**-54.3 (-61.6 to -44.7)**
2 Ischemic heart disease	35 (-3.07 to 78.8)	-19.2 (-41.2 to 5.97)	**-55 (-68.1 to -37.4)**
3 Lower respiratory infect	**37.6 (12.1 to 64)**	**-17.7 (-29.5 to -4.57)**	**-35.5 (-44.1 to -25.5)**
4 Chronic kidney disease	**147 (86.3 to 208)**	**48 (17.6 to 84.5)**	-11.6 (-28.2 to 12.3)
5 Diabetes	**96.2 (51.5 to 145)**	17.4 (-5.88 to 49.8)	**-31.6 (-45.5 to -13.7)**
6 Hypertensive heart disease	**138 (83.1 to 195)**	**42.6 (14.1 to 75.8)**	-22.2 (-38 to 1.08)
7 Neonatal disorders	**-45.4 (-59.8 to -28.2)**	**-67.4 (-74 to -59.2)**	**-46 (-54.9 to -34.6)**
8 Cirrhosis liver	**63.8 (35.2 to 94.2)**	-2 (-16.6 to 16.3)	**-37.6 (-46.6 to -27)**
9 Alzheimer's disease	**267 (178 to 384)**	**119 (66.5 to 183)**	1.91 (-1.4 to 4.82)
10 COPD	**104 (58.7 to 151)**	**22.2 (1.05 to 45.6)**	**-33.1 (-45.3 to -18.5)**
11 Road injuries	28.9 (-18.3 to 105)	-22.9 (-48.3 to 21.1)	**-40 (-59.3 to -7.88)**
12 Prostate cancer	**240 (143 to 359)**	**104 (48.6 to 168)**	7.91 (-11.9 to 27.3)
13 Diarrheal diseases	-1.38 (-28 to 29.6)	**-41 (-57 to -22.1)**	**-53.5 (-65 to -37.9)**
14 Falls	**158 (109 to 215)**	**54.6 (26.1 to 82.3)**	**-6.95 (-11.6 to -2.02)**
15 Self-harm	**92.6 (57.9 to 125)**	15.2 (-1.59 to 33.4)	**-20.6 (-31.4 to -9.53)**
16 Breast cancer	**212 (140 to 295)**	**86.7 (45.7 to 138)**	16.3 (-1.02 to 38.6)
17 Tuberculosis	**-23.6 (-45.4 to -0.204)**	**-54.3 (-65.8 to -39.7)**	**-70.9 (-78.5 to -59.9)**
18 Liver cancer	**165 (110 to 221)**	**58.3 (28.6 to 94.7)**	-3.14 (-21.4 to 22.9)
19 Lung cancer	**191 (120 to 267)**	**74 (31.9 to 120)**	2.15 (-16 to 20.7)
20 Cervical cancer	**102 (69.8 to 143)**	**21.1 (5.71 to 42.8)**	**-22.8 (-33.1 to -11.9)**
22 Congenital defects			
27 Meningitis			
33 Maternal disorders			
42 Malaria			
64 HIV/AIDS			
135 COVID-19			

Supplemental Results Figure S8. Leading 20 level 3 causes of deaths, and percentage change in number of deaths and all-age and age-standardised death rates by location, 2022 and 2050

Sierra Leone, Reference scenario

Legend:
Communicable, maternal, neonatal and nutritional
Non-communicable
Injuries

Leading Causes 2022	Leading Causes 2050	Mean % change number of DEATH	Mean % change all-age DEATH rate	Mean % change age-standardized DEATH rate
1 Malaria	1 Malaria	-30.1 (-96.6 to 235)	-57.1 (-98 to 106)	-55.7 (-97.3 to 101)
2 Neonatal disorders	2 Lower respiratory infect	**40.8 (15.6 to 65.8)**	**-17.2 (-33.5 to -1.79)**	**-30.4 (-40 to -19.6)**
3 Lower respiratory infect	3 Stroke	**43.8 (12.2 to 77.4)**	-15.5 (-33.7 to 5.5)	**-49.2 (-59.8 to -35.6)**
4 Stroke	4 Ischemic heart disease	**58.7 (7.8 to 119)**	-6.62 (-37.7 to 29.5)	**-46 (-64.1 to -21.7)**
5 Ischemic heart disease	5 Neonatal disorders	**-50.5 (-64.1 to -31.7)**	**-70.9 (-78.2 to -60.1)**	**-50.2 (-61.7 to -32.7)**
6 Protein-energy malnutrition	6 Diarrheal diseases	38.5 (-6.95 to 95.8)	-18.6 (-44.9 to 16.4)	-26.6 (-47.4 to 2.14)
7 Diarrheal diseases	7 Chronic kidney disease	**188 (119 to 261)**	**69.1 (26.8 to 112)**	9.4 (-15.5 to 40.6)
8 Tuberculosis	8 Diabetes	**124 (68.5 to 184)**	31.9 (-1.05 to 67.1)	-19.2 (-39.5 to 3.05)
9 Congenital defects	9 Hypertensive heart disease	**147 (90.8 to 210)**	**45.1 (11.6 to 81.6)**	-20.3 (-38.7 to 3.97)
10 HIV/AIDS	10 Cirrhosis liver	**58 (30 to 83.1)**	-7.13 (-22.4 to 11.6)	**-41.2 (-50.2 to -30)**
11 Road injuries	11 Tuberculosis	-16.9 (-42.2 to 15.9)	**-51.1 (-66.3 to -31.4)**	**-65.6 (-76.2 to -52.4)**
12 Maternal disorders	12 Road injuries	32.9 (-14.5 to 106)	-21.8 (-49.7 to 18.6)	-34.7 (-55.8 to 0.493)
13 Cirrhosis liver	13 Protein-energy malnutrition	**-50.9 (-72.8 to -18.9)**	**-71.1 (-83.8 to -51)**	**-60.5 (-76.9 to -34.2)**
14 Diabetes	14 Congenital defects	**-34.1 (-50.2 to -17.6)**	**-61.3 (-68.1 to -52.5)**	**-39.8 (-48.7 to -27.5)**
15 Chronic kidney disease	15 COPD	**78.5 (40.5 to 117)**	4.87 (-17.9 to 25.8)	**-40.5 (-52 to -26.2)**
16 Meningitis	16 Prostate cancer	**250 (136 to 372)**	**105 (45.3 to 174)**	15.4 (-6.3 to 40.9)
17 Hypertensive heart disease	17 Alzheimer's disease	**232 (146 to 341)**	**94.5 (49 to 150)**	1.05 (-2.99 to 5.56)
18 iNTS	18 Falls	**135 (85.3 to 201)**	**37.9 (15 to 64)**	**-7.84 (-12.9 to -1.35)**
19 COPD	19 Liver cancer	**161 (102 to 225)**	**53.5 (21.5 to 93)**	-5.69 (-26.3 to 20.6)
20 Hemoglobinopathies	20 Maternal disorders	-24.7 (-50.7 to 11.1)	**-55.9 (-70.1 to -37)**	**-59 (-72.4 to -41.6)**
25 Falls	24 Meningitis			
26 Liver cancer	26 iNTS			
30 Prostate cancer	31 Hemoglobinopathies			
31 Alzheimer's disease	45 HIV/AIDS			

Supplemental Results Figure S8. Leading 20 level 3 causes of deaths, and percentage change in number of deaths and all-age and age-standardised death rates by location, 2022 and 2050

Togo, Reference scenario

Legend:
Communicable, maternal, neonatal and nutritional
Non-communicable
Injuries

Leading Causes 2022	Leading Causes 2050	Mean % change number of DEATH	Mean % change all-age DEATH rate	Mean % change age-standardized DEATH rate
1 Malaria	1 Lower respiratory infect	**76.1 (40.7 to 113)**	13.8 (-3.81 to 33.8)	**-28.8 (-38.6 to -16.1)**
2 Stroke	2 Stroke	**62.5 (23.4 to 103)**	5.12 (-16.5 to 33)	**-50.1 (-60.3 to -34.7)**
3 Lower respiratory infect	3 Ischemic heart disease	**72.5 (17.8 to 148)**	11.7 (-24.7 to 62.3)	**-48 (-66.1 to -19.6)**
4 Neonatal disorders	4 Chronic kidney disease	**254 (173 to 352)**	**129 (79.5 to 195)**	18.8 (-7.78 to 57.4)
5 Ischemic heart disease	5 Malaria	-28.2 (-95.8 to 233)	-53.8 (-97.4 to 116)	-60.2 (-97.4 to 83.4)
6 Diarrheal diseases	6 Diarrheal diseases	31.2 (-12.4 to 88.2)	-15.1 (-43.5 to 20.8)	**-34.3 (-52.6 to -7.38)**
7 Tuberculosis	7 Hypertensive heart disease	**200 (129 to 295)**	**94.3 (49.4 to 151)**	-13 (-34.3 to 13.3)
8 HIV/AIDS	8 Diabetes	**148 (85 to 218)**	**60.5 (23.5 to 105)**	-19.6 (-39.1 to 7.19)
9 Road injuries	9 Cirrhosis liver	**67.3 (34.5 to 105)**	8.22 (-10 to 30)	**-36.1 (-46.4 to -23.2)**
10 Cirrhosis liver	10 Neonatal disorders	**-54.1 (-66.8 to -38.6)**	**-70.4 (-76.9 to -61.5)**	**-48.7 (-58.4 to -34.8)**
11 COVID-19	11 Road injuries	15.4 (-27.9 to 94.4)	-25.1 (-54.9 to 29.6)	-35.7 (-60.3 to 7.9)
12 Chronic kidney disease	12 Tuberculosis	-1.82 (-29.6 to 38.7)	**-36.4 (-53.4 to -10.1)**	**-63.2 (-73.7 to -47.3)**
13 Diabetes	13 COPD	**102 (54.3 to 150)**	**30.9 (4.75 to 64.1)**	**-40.9 (-52.9 to -25.3)**
14 Congenital defects	14 Alzheimer's disease	**381 (243 to 554)**	**210 (121 to 307)**	1.72 (-1.61 to 5.55)
15 Hypertensive heart disease	15 Prostate cancer	**352 (183 to 584)**	**191 (87.9 to 313)**	22.6 (-7.07 to 49.3)
16 Hemoglobinopathies	16 Hemoglobinopathies	**25.8 (1.5 to 55.9)**	**-18.8 (-28.1 to -7.9)**	**-26.5 (-32.9 to -19.8)**
17 COPD	17 Self-harm	**97.3 (61.6 to 144)**	**27.6 (8.1 to 54.4)**	-15.2 (-28.7 to 1.03)
18 iNTS	18 Breast cancer	**201 (116 to 307)**	**93.9 (44.6 to 152)**	10.8 (-11.1 to 31.8)
19 Self-harm	19 Falls	**186 (118 to 275)**	**84.4 (44.9 to 126)**	**-6.85 (-12.5 to -1.29)**
20 Meningitis	20 Cervical cancer	**93.3 (57 to 132)**	**25.1 (6.02 to 49.2)**	**-26.4 (-37.3 to -12.2)**
22 Cervical cancer	26 Congenital defects			
25 Breast cancer	30 Meningitis			
26 Falls	31 iNTS			
35 Alzheimer's disease	41 HIV/AIDS			
36 Prostate cancer	134 COVID-19			

Supplemental Results Figure S8. Leading 20 level 3 causes of deaths, and percentage change in number of deaths and all-age and age-standardised death rates by location, 2022 and 2050

Global, Reference scenario

Legend:
Communicable, maternal, neonatal and nutritional
Non-communicable
Injuries

Leading Causes 2022	Leading Causes 2050	Mean % change number of DEATH	Mean % change all-age DEATH rate	Mean % change age-standardized DEATH rate
1 Ischemic heart disease	1 Ischemic heart disease	20.6 (-12.7 to 64)	2.69 (-27 to 44.4)	**-44.8 (-64.3 to -15.4)**
2 Stroke	2 Stroke	**28.5 (7.89 to 51.1)**	9.27 (-7.5 to 30.9)	**-41.3 (-53.4 to -25.9)**
3 COPD	3 COPD	**102 (66.1 to 141)**	**71.8 (40.1 to 113)**	-13.7 (-34.3 to 15.6)
4 Lower respiratory infect	4 Alzheimer's disease	**173 (124 to 222)**	**132 (95 to 168)**	**-3.48 (-5.38 to -1.07)**
5 COVID-19	5 Chronic kidney disease	**182 (126 to 245)**	**140 (90.5 to 202)**	33.1 (-0.751 to 79)
6 Lung cancer	6 Lower respiratory infect	**56.7 (42.3 to 69.5)**	**33.3 (20.1 to 46.5)**	**-26.6 (-36.8 to -13.3)**
7 Alzheimer's disease	7 Hypertensive heart disease	**165 (105 to 232)**	**126 (74.3 to 192)**	17.1 (-16.5 to 61)
8 Neonatal disorders	8 Lung cancer	**68.1 (24.5 to 117)**	**42.7 (8.87 to 80.4)**	-17 (-31.9 to 0.776)
9 Diabetes	9 Diabetes	**76.2 (43.2 to 115)**	**49.9 (18.6 to 89.6)**	-13.9 (-33.8 to 14.9)
10 Chronic kidney disease	10 Cirrhosis liver	**50.7 (34.5 to 67)**	**28.2 (12.6 to 45.3)**	-9.06 (-20.9 to 6.14)
11 Cirrhosis liver	11 Colorectal cancer	**103 (41.1 to 177)**	**72.1 (23.7 to 131)**	-3.02 (-24.8 to 23.1)
12 Hypertensive heart disease	12 Falls	**113 (82.1 to 146)**	**81.3 (58.4 to 107)**	**-5.34 (-10.6 to -0.215)**
13 Road injuries	13 Diarrheal diseases	30.9 (-0.247 to 68.6)	11.4 (-16.5 to 47.9)	-34 (-54.1 to 0.13)
14 Tuberculosis	14 Road injuries	9.8 (-24.3 to 61.4)	-6.52 (-36.1 to 41.6)	-18.1 (-46.7 to 28.6)
15 Diarrheal diseases	15 Pancreatic cancer	**140 (72.1 to 219)**	**104 (50.3 to 169)**	18.1 (-6.89 to 46.9)
16 Colorectal cancer	16 Breast cancer	**81.8 (36.1 to 137)**	**54.4 (17.9 to 96.1)**	1.42 (-18.9 to 24.8)
17 Stomach cancer	17 Stomach cancer	**24.7 (12.6 to 37)**	5.99 (-3.94 to 16.1)	**-38.1 (-41.6 to -34.4)**
18 Falls	18 Atrial fibrillation	**229 (177 to 278)**	**179 (143 to 216)**	**17.3 (11.9 to 22.5)**
19 Self-harm	19 Urinary diseases	**203 (176 to 225)**	**158 (133 to 182)**	**33.5 (11.4 to 60)**
20 Malaria	20 Prostate cancer	**145 (86 to 209)**	**108 (60.6 to 158)**	5.51 (-8.93 to 23)
22 Breast cancer	24 Neonatal disorders			
25 Pancreatic cancer	25 Tuberculosis			
27 Prostate cancer	26 Self-harm			
32 Urinary diseases	30 Malaria			
34 Atrial fibrillation	137 COVID-19			

Supplemental Results Figure S8. Leading 20 level 3 causes of deaths, and percentage change in number of deaths and all-age and age-standardised death rates by location, 2022 and 2050

Central Europe, Eastern Europe, and Central Asia, Reference scenario

Legend:
Communicable, maternal, neonatal and nutritional
Non-communicable
Injuries

Leading Causes 2022	Leading Causes 2050	Mean % change number of DEATH	Mean % change all-age DEATH rate	Mean % change age-standardized DEATH rate
1 Ischemic heart disease	1 Ischemic heart disease	-0.47 (-27 to 32.5)	4.5 (-25.2 to 38.7)	**-43.9 (-63.5 to -17.4)**
2 Stroke	2 Stroke	-11.9 (-26.5 to 0.831)	-7.48 (-21.9 to 7.03)	**-49.3 (-59.9 to -35.9)**
3 COVID-19	3 Alzheimer's disease	**134 (78.5 to 192)**	**146 (92 to 204)**	**2.34 (0.461 to 4.02)**
4 Lung cancer	4 Lung cancer	19.5 (-9.5 to 52.5)	25.3 (-2.74 to 57.3)	-13 (-30.2 to 7)
5 Alzheimer's disease	5 Hypertensive heart disease	**78.8 (26.8 to 142)**	**87.8 (32.4 to 153)**	2.02 (-34.8 to 53.8)
6 Cirrhosis liver	6 Colorectal cancer	38.7 (-5.66 to 93.2)	45.4 (-1.37 to 102)	-9.89 (-34 to 20.2)
7 Colorectal cancer	7 COPD	**62.8 (31.2 to 94.1)**	**70.9 (37 to 105)**	-1.32 (-24.1 to 26.8)
8 Cardiomyopathy	8 Cirrhosis liver	1.03 (-10.2 to 14.8)	6.05 (-5.39 to 19.4)	-10.9 (-22.6 to 4.5)
9 Hypertensive heart disease	9 Lower respiratory infect	**15.5 (2.77 to 29.4)**	**21.2 (6.77 to 36.2)**	**-29.6 (-42.1 to -11.5)**
10 Lower respiratory infect	10 Cardiomyopathy	-2.54 (-15.5 to 11.7)	2.23 (-10.1 to 14.6)	**-28.1 (-34.8 to -21.2)**
11 COPD	11 Chronic kidney disease	**115 (56.4 to 176)**	**125 (64.2 to 189)**	35.3 (-8.93 to 96.2)
12 Diabetes	12 Diabetes	15.2 (-9.51 to 43.8)	20.9 (-6.07 to 50.1)	**-26.1 (-44.2 to -0.425)**
13 Self-harm	13 Pancreatic cancer	**77 (25.8 to 142)**	**85.5 (32.1 to 150)**	23.4 (-7.68 to 61.1)
14 Stomach cancer	14 Breast cancer	23.9 (-8.05 to 65.8)	29.9 (-2.28 to 69.9)	-12.7 (-30.1 to 8.68)
15 Breast cancer	15 Prostate cancer	**74.8 (36.3 to 116)**	**83.3 (44.2 to 125)**	7.14 (-7.15 to 22.7)
16 Pancreatic cancer	16 Urinary diseases	**139 (99.7 to 184)**	**151 (107 to 197)**	**38.6 (8.69 to 72.3)**
17 Chronic kidney disease	17 Stomach cancer	-3.46 (-13.3 to 6.53)	1.27 (-8.48 to 10.5)	**-32.3 (-36.3 to -28.6)**
18 Prostate cancer	18 Atrial fibrillation	**136 (94.6 to 181)**	**147 (106 to 194)**	**10.2 (2.04 to 20.6)**
19 Road injuries	19 Self-harm	-10.3 (-21.4 to 1.76)	-5.89 (-17.7 to 6.39)	**-14.8 (-27.5 to -1.11)**
20 Alcohol use disorders	20 Parkinson's disease	**87.4 (64.2 to 109)**	**96.5 (76.1 to 117)**	4.11 (-3.6 to 11.2)
22 Urinary diseases	24 Alcohol use disorders			
23 Parkinson's disease	26 Road injuries			
24 Atrial fibrillation	131 COVID-19			

Supplemental Results Figure S8. Leading 20 level 3 causes of deaths, and percentage change in number of deaths and all-age and age-standardised death rates by location, 2022 and 2050

High income, Reference scenario

Legend:
Communicable, maternal, neonatal and nutritional
Non-communicable
Injuries

Leading Causes 2022	Leading Causes 2050	Mean % change number of DEATH	Mean % change all-age DEATH rate	Mean % change age-standardized DEATH rate
1 Ischemic heart disease	1 Alzheimer's disease	**88 (73.7 to 99.9)**	**81.8 (69.2 to 93.5)**	**-3.91 (-5.84 to -1.82)**
2 Alzheimer's disease	2 Ischemic heart disease	-10.6 (-25.7 to 5.95)	-13.5 (-28.1 to 3.08)	**-50.3 (-60.1 to -37.9)**
3 Stroke	3 Chronic kidney disease	**155 (122 to 187)**	**146 (115 to 180)**	**38.5 (18.2 to 62.4)**
4 COVID-19	4 COPD	**63.8 (45.5 to 82.7)**	**58.4 (40.8 to 76)**	-4.94 (-17 to 7.66)
5 Lung cancer	5 Lung cancer	**29.6 (10.8 to 49.6)**	**25.4 (7.73 to 46)**	**-12.7 (-23.1 to -2)**
6 COPD	6 Stroke	-6 (-14.5 to 2.42)	**-9.08 (-17 to -0.913)**	**-45.4 (-51.5 to -38.8)**
7 Lower respiratory infect	7 Lower respiratory infect	**66.5 (55.1 to 78.2)**	**61.1 (50 to 72.3)**	**-10.3 (-17.9 to -2.67)**
8 Chronic kidney disease	8 Colorectal cancer	**53.1 (24.3 to 86.6)**	**48 (21.6 to 79.4)**	-5.62 (-20.8 to 12.1)
9 Colorectal cancer	9 Hypertensive heart disease	**97.9 (71.3 to 127)**	**91.4 (64.5 to 121)**	13.9 (-3.56 to 34.6)
10 Pancreatic cancer	10 Pancreatic cancer	**86.4 (55 to 120)**	**80.3 (51.5 to 113)**	**24.5 (7.51 to 43.4)**
11 Hypertensive heart disease	11 Atrial fibrillation	**132 (119 to 147)**	**124 (112 to 135)**	**20.2 (16.3 to 24.1)**
12 Diabetes	12 Falls	**73.8 (61.4 to 85.1)**	**68.1 (56.8 to 79)**	**-5.57 (-9.13 to -2.05)**
13 Breast cancer	13 Prostate cancer	**82.1 (60.4 to 107)**	**76.1 (55.2 to 100)**	6.3 (-2.49 to 15.6)
14 Falls	14 Nonrheum valv diseases	**143 (129 to 163)**	**135 (121 to 152)**	**23.3 (17.8 to 29)**
15 Cirrhosis liver	15 Urinary diseases	**186 (162 to 205)**	**177 (156 to 194)**	**50.1 (37.5 to 63.1)**
16 Prostate cancer	16 Diabetes	**24.3 (7.37 to 41.2)**	**20.2 (3.88 to 38.4)**	**-28.6 (-38.8 to -17)**
17 Stomach cancer	17 Breast cancer	**36.9 (18.5 to 55.3)**	**32.4 (15.7 to 50.8)**	-8.64 (-18.4 to 1.99)
18 Self-harm	18 Parkinson's disease	**77.8 (68.7 to 86.3)**	**71.9 (63.2 to 80.4)**	4.07 (-0.2 to 7.73)
19 Atrial fibrillation	19 Liver cancer	**69.5 (54.2 to 84.5)**	**63.9 (49.3 to 79)**	**19.2 (8.02 to 30.3)**
20 Parkinson's disease	20 Cirrhosis liver	**9.12 (1.99 to 15.7)**	5.54 (-1.15 to 11.6)	**-12 (-17.5 to -7.01)**
21 Nonrheum valv diseases	23 Stomach cancer			
22 Liver cancer	26 Self-harm			
25 Urinary diseases	135 COVID-19			

Supplemental Results Figure S8. Leading 20 level 3 causes of deaths, and percentage change in number of deaths and all-age and age-standardised death rates by location, 2022 and 2050

Latin America and Caribbean, Reference scenario

Legend:
Communicable, maternal, neonatal and nutritional
Non-communicable
Injuries

Leading Causes 2022	Leading Causes 2050	Mean % change number of DEATH	Mean % change all-age DEATH rate	Mean % change age-standardized DEATH rate
1 Ischemic heart disease	1 Ischemic heart disease	48.3 (-8.2 to 122)	32.8 (-18.2 to 106)	-38.3 (-65.4 to 5.11)
2 COVID-19	2 Chronic kidney disease	**185 (105 to 267)**	**154 (84.6 to 243)**	27.4 (-13.6 to 85.9)
3 Stroke	3 Lower respiratory infect	**95.5 (67.8 to 123)**	**74.6 (52.1 to 101)**	**-24.4 (-39.5 to -5.34)**
4 Diabetes	4 Diabetes	**73.6 (30.2 to 115)**	**55.2 (17.7 to 98.3)**	-23 (-44 to 6.77)
5 Lower respiratory infect	5 Stroke	**37.3 (5.05 to 69.1)**	22.7 (-5.51 to 57.9)	**-41.5 (-57.9 to -19.6)**
6 Chronic kidney disease	6 Alzheimer's disease	**210 (146 to 272)**	**176 (122 to 228)**	**-2.97 (-5.08 to -0.975)**
7 COPD	7 COPD	**125 (71.9 to 179)**	**101 (51.8 to 155)**	-16.3 (-41.6 to 17.7)
8 Interpersonal violence	8 Cirrhosis liver	**57.6 (42.1 to 71)**	**40.8 (28.2 to 55.5)**	-9.17 (-19.1 to 2.86)
9 Alzheimer's disease	9 Hypertensive heart disease	**164 (83.9 to 239)**	**136 (66.6 to 217)**	3.37 (-33.2 to 55.4)
10 Cirrhosis liver	10 Urinary diseases	**249 (190 to 310)**	**211 (162 to 263)**	**30.4 (2.79 to 70.4)**
11 Road injuries	11 Lung cancer	**95.7 (27.8 to 174)**	**74.5 (18 to 140)**	-9.82 (-34.6 to 18.6)
12 Lung cancer	12 Prostate cancer	**159 (66.3 to 264)**	**131 (53.4 to 226)**	-0.786 (-27.1 to 27.6)
13 Stomach cancer	13 Colorectal cancer	**115 (28.3 to 227)**	**91.3 (18.1 to 190)**	-2.56 (-35.4 to 37.3)
14 Hypertensive heart disease	14 Road injuries	8.42 (-30.7 to 66.9)	-2.96 (-38.1 to 54.3)	-13.2 (-45.3 to 40)
15 Colorectal cancer	15 Pancreatic cancer	**193 (59.3 to 378)**	**161 (46.1 to 324)**	34.7 (-19.5 to 105)
16 Neonatal disorders	16 Interpersonal violence	-29.9 (-62 to 22.6)	-37.6 (-64.1 to 9.88)	-34.5 (-64 to 16.3)
17 Prostate cancer	17 Breast cancer	**82.8 (24.6 to 157)**	**63 (13.1 to 128)**	-5.13 (-29.6 to 26.3)
18 Breast cancer	18 Stomach cancer	**41.6 (26.2 to 54.9)**	**26.4 (16.3 to 38.2)**	**-34.5 (-37.8 to -30.7)**
19 Urinary diseases	19 Falls	**118 (80.3 to 157)**	**94.4 (62.4 to 129)**	**-12 (-18.9 to -3.27)**
20 Falls	20 Atrial fibrillation	**253 (187 to 317)**	**215 (165 to 262)**	**14.3 (5.18 to 25.2)**
24 Pancreatic cancer	43 Neonatal disorders			
26 Atrial fibrillation	136 COVID-19			

Supplemental Results Figure S8. Leading 20 level 3 causes of deaths, and percentage change in number of deaths and all-age and age-standardised death rates by location, 2022 and 2050

North Africa and Middle East, Reference scenario

Legend:
Communicable, maternal, neonatal and nutritional
Non-communicable
Injuries

Leading Causes 2022	Leading Causes 2050	Mean % change number of DEATH	Mean % change all-age DEATH rate	Mean % change age-standardized DEATH rate
1 Ischemic heart disease	1 Ischemic heart disease	**72 (18.4 to 151)**	27.3 (-15.2 to 90.5)	**-43.2 (-65.1 to -5.62)**
2 Stroke	2 Stroke	**67.4 (31.6 to 108)**	23.8 (-3.03 to 58.2)	**-47.5 (-62.4 to -25)**
3 COVID-19	3 Chronic kidney disease	**301 (204 to 415)**	**197 (124 to 292)**	32.2 (-7.37 to 95)
4 Chronic kidney disease	4 Hypertensive heart disease	**252 (166 to 354)**	**160 (96.8 to 242)**	5.42 (-29.7 to 57)
5 Hypertensive heart disease	5 Alzheimer's disease	**326 (224 to 422)**	**215 (145 to 288)**	**7.14 (4.55 to 9.29)**
6 Diabetes	6 Diabetes	**160 (101 to 235)**	**92.5 (48.8 to 152)**	-12.9 (-37.9 to 26.9)
7 Road injuries	7 COPD	**181 (122 to 248)**	**108 (64 to 166)**	-15.3 (-40.7 to 21.2)
8 Lower respiratory infect	8 Lower respiratory infect	**98.2 (73.4 to 125)**	**46.5 (27.6 to 71.3)**	**-25.2 (-39.2 to -3.57)**
9 Cirrhosis liver	9 Lung cancer	**154 (72 to 255)**	**87.4 (30.5 to 160)**	-9.99 (-33.1 to 17.9)
10 COPD	10 Cirrhosis liver	**91 (69.5 to 112)**	**41.1 (25 to 58.8)**	**-30.3 (-39.4 to -17.7)**
11 Neonatal disorders	11 Road injuries	29.1 (-14.4 to 99.4)	-4.42 (-38.4 to 50.6)	-22.3 (-51.4 to 25.6)
12 Lung cancer	12 Colorectal cancer	**220 (105 to 375)**	**136 (51.6 to 248)**	11.5 (-22.4 to 53.7)
13 Alzheimer's disease	13 Pancreatic cancer	**342 (184 to 537)**	**226 (116 to 368)**	**52.8 (8.53 to 105)**
14 Congenital defects	14 Liver cancer	**219 (172 to 272)**	**136 (101 to 181)**	19.7 (-1.22 to 48.6)
15 Conflict & terror	15 Conflict & terror	96.3 (-79.9 to 677)	45.4 (-85.4 to 487)	30.8 (-87 to 379)
16 Stomach cancer	16 Breast cancer	**145 (65.9 to 245)**	**80.9 (22.8 to 151)**	4.77 (-25 to 39.5)
17 Colorectal cancer	17 Stomach cancer	**88.8 (66.3 to 114)**	**39.4 (24.3 to 52.7)**	**-32.9 (-37 to -28.7)**
18 Breast cancer	18 Brain cancer	**200 (175 to 229)**	**122 (103 to 139)**	**34.9 (29.9 to 40.3)**
19 Other cardiovascular	19 Prostate cancer	**275 (139 to 446)**	**176 (77.3 to 302)**	3.23 (-25.2 to 33.9)
20 Leukemia	20 Other cardiovascular	**110 (87.5 to 136)**	**55.5 (36.9 to 72.9)**	**-24.2 (-37 to -9.45)**
22 Liver cancer	22 Leukemia			
24 Brain cancer	26 Congenital defects			
27 Pancreatic cancer	27 Neonatal disorders			
30 Prostate cancer	134 COVID-19			

Supplemental Results Figure S8. Leading 20 level 3 causes of deaths, and percentage change in number of deaths and all-age and age-standardised death rates by location, 2022 and 2050

South Asia, Reference scenario

Legend:
Communicable, maternal, neonatal and nutritional
Non-communicable
Injuries

Leading Causes 2022	Leading Causes 2050	Mean % change number of DEATH	Mean % change all-age DEATH rate	Mean % change age-standardized DEATH rate
1 Ischemic heart disease	1 Ischemic heart disease	20.3 (-28.1 to 82.5)	7.66 (-33.7 to 67.1)	**-51.4 (-73.7 to -13.5)**
2 COPD	2 COPD	**82.2 (35.4 to 132)**	**62.9 (22.4 to 115)**	**-35.4 (-56.4 to -0.966)**
3 Stroke	3 Stroke	**34.3 (5.35 to 67.3)**	20 (-5.93 to 54.8)	**-47.9 (-63.4 to -27.9)**
4 Neonatal disorders	4 Diabetes	**85.9 (41.1 to 138)**	**66.2 (21.8 to 120)**	-28.3 (-52.4 to 8.8)
5 Lower respiratory infect	5 Diarrheal diseases	**36 (5.18 to 69.7)**	21.6 (-7.11 to 57.1)	**-50.8 (-68 to -22.7)**
6 Diarrheal diseases	6 Lower respiratory infect	**23.8 (1.89 to 49.3)**	10.5 (-7.04 to 31.3)	**-44.8 (-54.6 to -30.8)**
7 Tuberculosis	7 Falls	**136 (80.8 to 203)**	**110 (71.5 to 158)**	**-13.6 (-22.2 to -3.72)**
8 Diabetes	8 Chronic kidney disease	**165 (91.5 to 254)**	**137 (72.7 to 227)**	11.1 (-26.9 to 66.6)
9 Cirrhosis liver	9 Alzheimer's disease	**260 (150 to 374)**	**221 (136 to 322)**	**-5.4 (-9.86 to -1.27)**
10 Road injuries	10 Cirrhosis liver	**55.9 (29.7 to 82.9)**	**39.3 (16.6 to 66.9)**	**-20.4 (-35.2 to -1.77)**
11 Falls	11 Hypertensive heart disease	**153 (83.8 to 229)**	**126 (68.2 to 206)**	-11.3 (-42.6 to 35.8)
12 COVID-19	12 Tuberculosis	-31.1 (-57.8 to 2.2)	**-38.3 (-61.9 to -3.83)**	**-65.7 (-80 to -42.6)**
13 Chronic kidney disease	13 Road injuries	9.76 (-29.8 to 61.5)	-1.8 (-35.7 to 53.2)	-24.7 (-52.8 to 20.1)
14 Asthma	14 Urinary diseases	**192 (152 to 239)**	**161 (129 to 199)**	16.8 (-5.24 to 46)
15 Self-harm	15 Breast cancer	**121 (35.5 to 230)**	**97 (32.2 to 177)**	8.62 (-25 to 44.1)
16 Rheumatic heart disease	16 Self-harm	7.1 (-15.4 to 35.2)	-4.32 (-24.5 to 20.9)	-19.3 (-37.8 to 6.07)
17 Hypertensive heart disease	17 Lip oral cavity cancer	**118 (87.7 to 149)**	**94.9 (77.3 to 112)**	0.811 (-8.34 to 11.4)
18 Alzheimer's disease	18 Rheumatic heart disease	-0.941 (-32.4 to 36.6)	-11.4 (-39.6 to 26.3)	**-54.9 (-71.8 to -29.7)**
19 Breast cancer	19 Asthma	-8.57 (-24 to 9.39)	**-18.3 (-31.7 to -3.89)**	**-63.9 (-71.4 to -52.6)**
20 Congenital defects	20 Lung cancer	**106 (30.4 to 198)**	**84 (22.2 to 155)**	-7.66 (-33.7 to 22.5)
21 Lung cancer	21 Neonatal disorders			
22 Lip oral cavity cancer	52 Congenital defects			
23 Urinary diseases	133 COVID-19			

Supplemental Results Figure S8. Leading 20 level 3 causes of deaths, and percentage change in number of deaths and all-age and age-standardised death rates by location, 2022 and 2050

Southeast Asia, East Asia, and Oceania, Reference scenario

Legend:
Communicable, maternal, neonatal and nutritional
Non-communicable
Injuries

Leading Causes 2022	Leading Causes 2050	Mean % change number of DEATH	Mean % change all-age DEATH rate	Mean % change age-standardized DEATH rate
1 Stroke	1 Stroke	**31.4 (10 to 56.1)**	**36.8 (15.4 to 68)**	**-45.2 (-55.5 to -30.7)**
2 Ischemic heart disease	2 COPD	**124 (77.6 to 172)**	**134 (87.9 to 185)**	-21.1 (-38.7 to 4.9)
3 COPD	3 Ischemic heart disease	19.1 (-12.9 to 58.8)	24.1 (-9.33 to 66.3)	**-53.1 (-68.2 to -33.2)**
4 Lung cancer	4 Alzheimer's disease	**236 (172 to 312)**	**249 (189 to 323)**	-0.986 (-4.87 to 2.62)
5 Alzheimer's disease	5 Lung cancer	**83.9 (31.3 to 145)**	**91.1 (41.7 to 149)**	-8.7 (-27.1 to 12.8)
6 Stomach cancer	6 Hypertensive heart disease	**186 (115 to 275)**	**198 (121 to 298)**	10.5 (-23.1 to 55.9)
7 Hypertensive heart disease	7 Chronic kidney disease	**157 (104 to 217)**	**167 (112 to 237)**	21.8 (-6.94 to 60.7)
8 Lower respiratory infect	8 Lower respiratory infect	**101 (77.5 to 124)**	**110 (85.4 to 130)**	**-27.6 (-36 to -16.9)**
9 Diabetes	9 Colorectal cancer	**131 (57.7 to 218)**	**140 (69.4 to 228)**	13.6 (-14.5 to 44.7)
10 Chronic kidney disease	10 Diabetes	**63.1 (32.5 to 99.4)**	**69.8 (35.4 to 111)**	-21.1 (-38.7 to 4.25)
11 Colorectal cancer	11 Stomach cancer	**20.7 (2.54 to 40.3)**	**25.5 (8.31 to 44.7)**	**-39.7 (-44.5 to -34.1)**
12 Road injuries	12 Esophageal cancer	**79.9 (55.9 to 106)**	**87.2 (64.4 to 113)**	-7.23 (-16.4 to 2.76)
13 Cirrhosis liver	13 Cirrhosis liver	**50.8 (31.3 to 73.5)**	**57 (35.9 to 83.3)**	-2.19 (-15.4 to 16.1)
14 Esophageal cancer	14 Falls	**117 (75.5 to 164)**	**126 (88.4 to 168)**	**-10.8 (-16.9 to -4.52)**
15 COVID-19	15 Liver cancer	**89.8 (47 to 149)**	**97.8 (50.2 to 162)**	14.3 (-15.8 to 57.4)
16 Liver cancer	16 Pancreatic cancer	**162 (84.1 to 250)**	**172 (97.8 to 262)**	**37.4 (5.12 to 72.7)**
17 Tuberculosis	17 Atrial fibrillation	**326 (244 to 426)**	**343 (265 to 436)**	**20.5 (11.2 to 31)**
18 Falls	18 Parkinson's disease	**182 (145 to 226)**	**193 (160 to 230)**	-0.573 (-7.68 to 6.95)
19 Self-harm	19 Road injuries	-10.2 (-34.3 to 26.4)	-6.37 (-32.6 to 32.1)	-19.5 (-44.3 to 19.1)
20 Breast cancer	20 Breast cancer	**66.2 (23.1 to 113)**	**72.8 (30.8 to 119)**	8.33 (-14.2 to 33.9)
21 Pancreatic cancer	21 Self-harm			
23 Parkinson's disease	24 Tuberculosis			
25 Atrial fibrillation	134 COVID-19			

Supplemental Results Figure S8. Leading 20 level 3 causes of deaths, and percentage change in number of deaths and all-age and age-standardised death rates by location, 2022 and 2050

Sub-Saharan Africa, Reference scenario

Legend:
Communicable, maternal, neonatal and nutritional
Non-communicable
Injuries

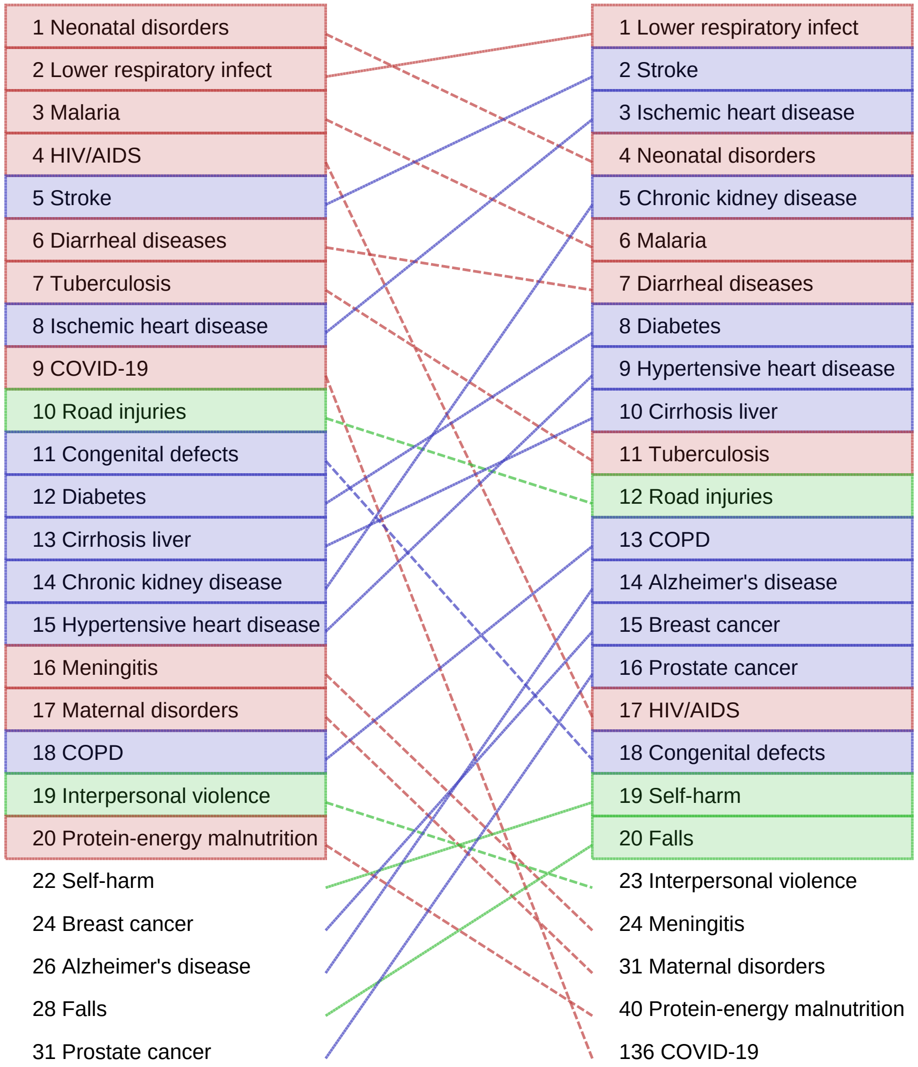

Leading Causes 2022	Leading Causes 2050	Mean % change number of DEATH	Mean % change all-age DEATH rate	Mean % change age-standardized DEATH rate
1 Neonatal disorders	1 Lower respiratory infect	**35 (19.2 to 52.1)**	**-25.8 (-35.4 to -14.7)**	**-33.6 (-40.8 to -24.7)**
2 Lower respiratory infect	2 Stroke	**73 (48.6 to 99.7)**	-4.9 (-20.5 to 12.2)	**-46.2 (-55.7 to -34.2)**
3 Malaria	3 Ischemic heart disease	**78.7 (30.6 to 141)**	-1.74 (-29.7 to 34.5)	**-44.8 (-61.5 to -22)**
4 HIV/AIDS	4 Neonatal disorders	**-30.4 (-46.1 to -11.2)**	**-61.8 (-69.3 to -51.4)**	**-42.7 (-54 to -28.4)**
5 Stroke	5 Chronic kidney disease	**244 (180 to 318)**	**88.9 (51.3 to 131)**	17 (-7.92 to 48)
6 Diarrheal diseases	6 Malaria	-30.1 (-80.4 to 149)	-61.7 (-89.3 to 34.8)	-61.1 (-88.7 to 32.5)
7 Tuberculosis	7 Diarrheal diseases	0.153 (-28.6 to 40.7)	**-44.9 (-61.3 to -24)**	**-44.6 (-58.7 to -21.9)**
8 Ischemic heart disease	8 Diabetes	**119 (80.5 to 161)**	20.5 (-2.67 to 47.6)	**-28.5 (-42.9 to -10.6)**
9 COVID-19	9 Hypertensive heart disease	**191 (141 to 253)**	**60 (30.5 to 95.3)**	-14.7 (-33.3 to 7.57)
10 Road injuries	10 Cirrhosis liver	**90 (68.2 to 115)**	4.41 (-10.2 to 21.6)	**-31.9 (-40.8 to -20.5)**
11 Congenital defects	11 Tuberculosis	-13.2 (-37.1 to 20.7)	**-52.3 (-65.2 to -33)**	**-66.6 (-75.5 to -53.6)**
12 Diabetes	12 Road injuries	39.8 (-11.3 to 126)	-23.1 (-52 to 24.4)	**-36.8 (-58.9 to -0.937)**
13 Cirrhosis liver	13 COPD	**123 (95.1 to 156)**	**22.7 (5.38 to 43.2)**	**-32.7 (-44.1 to -18.3)**
14 Chronic kidney disease	14 Alzheimer's disease	**302 (227 to 377)**	**121 (80.9 to 159)**	2.07 (-0.615 to 4.16)
15 Hypertensive heart disease	15 Breast cancer	**218 (148 to 283)**	**74.6 (37.2 to 112)**	11.6 (-6.23 to 30.7)
16 Meningitis	16 Prostate cancer	**274 (174 to 364)**	**105 (51.4 to 157)**	7.56 (-11.3 to 26.8)
17 Maternal disorders	17 HIV/AIDS	**-67.4 (-72.4 to -62.5)**	**-82.1 (-85 to -79.2)**	**-84.8 (-87 to -82.5)**
18 COPD	18 Congenital defects	**-19.6 (-33.6 to -1.63)**	**-55.9 (-61.9 to -47.2)**	**-37.4 (-44.8 to -26.4)**
19 Interpersonal violence	19 Self-harm	**101 (79.4 to 129)**	10.7 (-3.99 to 27.5)	**-19.3 (-29.1 to -7.47)**
20 Protein-energy malnutrition	20 Falls	**173 (131 to 212)**	**49.6 (27.6 to 68.9)**	**-5.52 (-8.63 to -2.79)**
22 Self-harm	23 Interpersonal violence			
24 Breast cancer	24 Meningitis			
26 Alzheimer's disease	31 Maternal disorders			
28 Falls	40 Protein-energy malnutrition			
31 Prostate cancer	136 COVID-19			

Supplemental Results Figure S8. Leading 20 level 3 causes of deaths, and percentage change in number of deaths and all-age and age-standardised death rates by location, 2022 and 2050

Central Asia, Reference scenario

Legend:
Communicable, maternal, neonatal and nutritional
Non-communicable
Injuries

Leading Causes 2022	Leading Causes 2050	Mean % change number of DEATH	Mean % change all-age DEATH rate	Mean % change age-standardized DEATH rate
1 Ischemic heart disease	1 Ischemic heart disease	**87.9 (27.6 to 160)**	**50.1 (1.61 to 104)**	-35.3 (-62.1 to 2.98)
2 COVID-19	2 Stroke	**55.5 (24.8 to 87.9)**	**24.2 (0.947 to 49.3)**	**-44.6 (-60.1 to -23.9)**
3 Stroke	3 Cirrhosis liver	**61 (34.4 to 91.4)**	**28.6 (9.26 to 49.9)**	-12.4 (-28.2 to 7.75)
4 Cirrhosis liver	4 Alzheimer's disease	**305 (179 to 439)**	**223 (128 to 330)**	**2.38 (0.77 to 3.98)**
5 Lower respiratory infect	5 COPD	**157 (97.2 to 224)**	**105 (59.6 to 151)**	-8.04 (-35.7 to 29.9)
6 Neonatal disorders	6 Hypertensive heart disease	**161 (69 to 272)**	**109 (36.6 to 201)**	-8.64 (-48 to 49.7)
7 COPD	7 Chronic kidney disease	**192 (98.3 to 309)**	**133 (57.4 to 224)**	29.1 (-20.5 to 104)
8 Diabetes	8 Diabetes	**76.6 (20.6 to 148)**	**41.1 (0.99 to 96.5)**	-19 (-44.9 to 23.7)
9 Hypertensive heart disease	9 Lower respiratory infect	-0.231 (-17.7 to 21.2)	**-20.3 (-32.5 to -5.18)**	**-42.9 (-55.5 to -23.7)**
10 Cardiomyopathy	10 Lung cancer	**87.1 (24.4 to 161)**	**49.3 (1.66 to 101)**	-12.4 (-36.8 to 16.2)
11 Lung cancer	11 Liver cancer	**182 (104 to 293)**	**126 (62.8 to 206)**	32.7 (-9.67 to 90)
12 Alzheimer's disease	12 Cardiomyopathy	**43 (20.2 to 72.4)**	14.1 (-1.83 to 30.3)	**-28.4 (-34.5 to -20.7)**
13 Road injuries	13 Colorectal cancer	**111 (28.9 to 211)**	**68.6 (2.99 to 148)**	-8.09 (-37.3 to 30.6)
14 Chronic kidney disease	14 Stomach cancer	**39.8 (21.1 to 58.3)**	**11.6 (1.75 to 21.6)**	**-35.7 (-39 to -31.9)**
15 Stomach cancer	15 Breast cancer	**69 (14.5 to 133)**	34.8 (-6.29 to 82.3)	-14 (-37.4 to 14.5)
16 Self-harm	16 Road injuries	13.9 (-33.8 to 103)	-8.91 (-46.2 to 57.9)	-16.1 (-51.9 to 47.5)
17 Congenital defects	17 Self-harm	18.8 (-5.23 to 49.2)	-5.12 (-24.1 to 17.5)	-13.9 (-31.6 to 8.39)
18 Breast cancer	18 Pancreatic cancer	**184 (75.1 to 318)**	**126 (42.4 to 224)**	25.7 (-13.5 to 72.9)
19 Colorectal cancer	19 Neonatal disorders	-35.1 (-65.8 to 15.7)	**-48.2 (-72 to -10.3)**	-31.6 (-62.2 to 13.6)
20 Liver cancer	20 Esophageal cancer	**128 (101 to 157)**	**82 (68.2 to 96)**	0.875 (-8.8 to 12.8)
23 Esophageal cancer	30 Congenital defects			
25 Pancreatic cancer	131 COVID-19			

Supplemental Results Figure S8. Leading 20 level 3 causes of deaths, and percentage change in number of deaths and all-age and age-standardised death rates by location, 2022 and 2050

Central Europe, Reference scenario

Legend:
Communicable, maternal, neonatal and nutritional
Non-communicable
Injuries

Leading Causes 2022
1 Ischemic heart disease
2 Stroke
3 COVID-19
4 Lung cancer
5 Hypertensive heart disease
6 Colorectal cancer
7 Alzheimer's disease
8 Lower respiratory infect
9 COPD
10 Cirrhosis liver
11 Diabetes
12 Cardiomyopathy
13 Breast cancer
14 Pancreatic cancer
15 Chronic kidney disease
16 Prostate cancer
17 Stomach cancer
18 Falls
19 Self-harm
20 Bladder cancer
21 Parkinson's disease
22 Atrial fibrillation
23 Brain cancer

Leading Causes 2050	Mean % change number of DEATH	Mean % change all-age DEATH rate	Mean % change age-standardized DEATH rate
1 Ischemic heart disease	-21.5 (-40.3 to 2.82)	-4.73 (-28.7 to 22.9)	**-51.1 (-65.7 to -30.5)**
2 Stroke	**-27.4 (-36.7 to -16.1)**	-11.9 (-23.2 to 0.614)	**-52.5 (-60.6 to -42.9)**
3 Lung cancer	18.7 (-6.01 to 45.3)	**43.9 (16.4 to 77.8)**	-0.765 (-19 to 18.4)
4 Alzheimer's disease	**96 (63.3 to 126)**	**138 (100 to 173)**	**3 (1.35 to 4.73)**
5 Hypertensive heart disease	**56 (13.4 to 108)**	**89.3 (38.7 to 153)**	1.05 (-30.6 to 45.4)
6 Colorectal cancer	36.3 (-3.51 to 84.6)	**65.2 (16.8 to 122)**	1.64 (-25.3 to 32.4)
7 Lower respiratory infect	**29.2 (13.1 to 45.4)**	**56.7 (36.6 to 76.1)**	**-17.5 (-29.7 to -5.38)**
8 COPD	**29.3 (3.58 to 53.9)**	**56.9 (27.4 to 87.1)**	-8.94 (-29 to 12.1)
9 Chronic kidney disease	**84.5 (43.1 to 127)**	**124 (72.2 to 179)**	22.9 (-7.38 to 63.5)
10 Pancreatic cancer	**65.2 (21.5 to 117)**	**100 (48.6 to 156)**	**32 (1.71 to 69.8)**
11 Diabetes	6.4 (-11.2 to 28.4)	**29.1 (7.7 to 53.9)**	**-26.6 (-40.6 to -10.3)**
12 Cardiomyopathy	16.2 (-0.284 to 34.6)	**40.9 (22.6 to 63.2)**	**-19.6 (-25.8 to -13.1)**
13 Prostate cancer	**57.9 (27.5 to 89.7)**	**91.4 (55.4 to 127)**	11.6 (-3.37 to 28)
14 Breast cancer	23.5 (-5.19 to 55.2)	**49.7 (14.8 to 88.9)**	-5.47 (-22.5 to 13)
15 Cirrhosis liver	**-13 (-23 to -1.78)**	5.56 (-5.34 to 17.5)	**-12.5 (-22.6 to -0.762)**
16 Atrial fibrillation	**107 (73.1 to 137)**	**151 (114 to 182)**	**17.1 (8.87 to 26.8)**
17 Falls	**39.3 (18 to 58.9)**	**68.9 (45.1 to 92.2)**	**-9.34 (-16.2 to -2.23)**
18 Parkinson's disease	**52.2 (38.4 to 67.6)**	**84.6 (69.5 to 103)**	1.48 (-5.39 to 8.78)
19 Bladder cancer	**32.2 (15.7 to 49.1)**	**60.3 (43.6 to 79.8)**	-4.1 (-11.2 to 3.25)
20 Brain cancer	**52.9 (42.8 to 63.6)**	**85.5 (76.3 to 96.1)**	**34.4 (29.6 to 39.7)**
22 Stomach cancer			
25 Self-harm			
130 COVID-19			

Supplemental Results Figure S8. Leading 20 level 3 causes of deaths, and percentage change in number of deaths and all-age and age-standardised death rates by location, 2022 and 2050

Eastern Europe, Reference scenario

Legend:
Communicable, maternal, neonatal and nutritional
Non-communicable
Injuries

Leading Causes 2022	Leading Causes 2050	Mean % change number of DEATH	Mean % change all-age DEATH rate	Mean % change age-standardized DEATH rate
1 Ischemic heart disease	1 Ischemic heart disease	-10.3 (-36.1 to 15.3)	1.64 (-27.2 to 32.7)	**-47.9 (-66.1 to -24)**
2 Stroke	2 Stroke	**-17.7 (-34.6 to -2.91)**	-6.78 (-23.5 to 9.3)	**-51.6 (-62.3 to -38.3)**
3 COVID-19	3 Alzheimer's disease	**135 (71.2 to 207)**	**165 (98.1 to 234)**	2.42 (-0.721 to 5.23)
4 Lung cancer	4 Colorectal cancer	33.2 (-13.7 to 94.9)	**50.5 (0.731 to 110)**	-7.5 (-32.9 to 25.8)
5 Alzheimer's disease	5 Lung cancer	9.52 (-20.3 to 47.6)	23.8 (-7.26 to 60.1)	-13.6 (-31.8 to 8.53)
6 Cardiomyopathy	6 COPD	**59.5 (22.5 to 95)**	**80.6 (39.9 to 117)**	0.698 (-23.2 to 30.5)
7 Cirrhosis liver	7 Cirrhosis liver	**-15.7 (-27.3 to -2.37)**	-4.48 (-15.2 to 7.58)	**-16.7 (-27.7 to -2.98)**
8 Colorectal cancer	8 Cardiomyopathy	**-17.9 (-31.7 to -0.0851)**	-7.06 (-21 to 8.58)	**-27.8 (-37.8 to -17.3)**
9 Self-harm	9 Pancreatic cancer	**72.3 (17.3 to 139)**	**94.7 (35.8 to 166)**	29.1 (-4.17 to 73.2)
10 Diabetes	10 Urinary diseases	**141 (84.9 to 201)**	**173 (117 to 245)**	**46.1 (14.1 to 83.6)**
11 Stomach cancer	11 Hypertensive heart disease	**85.1 (26.3 to 145)**	**110 (45.5 to 180)**	14.3 (-26.3 to 72.9)
12 COPD	12 Diabetes	1.2 (-24.1 to 31.9)	14.6 (-13.5 to 44)	**-34 (-50.9 to -10.3)**
13 Breast cancer	13 Self-harm	-13.1 (-26 to 1.54)	-1.57 (-15 to 13.3)	-12.4 (-25.7 to 4.07)
14 Lower respiratory infect	14 Breast cancer	15.5 (-16.3 to 61.8)	30.6 (-3.38 to 74.6)	-11.8 (-30.4 to 12.5)
15 Alcohol use disorders	15 Prostate cancer	**81.1 (35.8 to 140)**	**105 (57.1 to 159)**	**20.1 (0.3 to 41.8)**
16 Pancreatic cancer	16 Stomach cancer	-7.37 (-22.1 to 8.9)	4.79 (-8.79 to 17.2)	**-31.5 (-37.6 to -25.7)**
17 Hypertensive heart disease	17 Atrial fibrillation	**147 (88 to 224)**	**179 (117 to 259)**	**16.4 (4.16 to 33.7)**
18 HIV/AIDS	18 Chronic kidney disease	**109 (46.8 to 177)**	**136 (68.3 to 205)**	40.2 (-1.02 to 95.9)
19 Road injuries	19 Lower respiratory infect	10.9 (-6.93 to 33.3)	**25.5 (7.55 to 45.3)**	**-17.3 (-30.8 to -1.24)**
20 Prostate cancer	20 Vascular intestinal	**73.8 (30.9 to 122)**	**96.5 (54.1 to 146)**	2.64 (-10.3 to 18.5)
22 Urinary diseases	21 Alcohol use disorders			
23 Chronic kidney disease	26 Road injuries			
24 Vascular intestinal	30 HIV/AIDS			
27 Atrial fibrillation	129 COVID-19			

Supplemental Results Figure S8. Leading 20 level 3 causes of deaths, and percentage change in number of deaths and all-age and age-standardised death rates by location, 2022 and 2050

Australasia, Reference scenario

Legend:
Communicable, maternal, neonatal and nutritional
Non-communicable
Injuries

Leading Causes 2022	Leading Causes 2050	Mean % change number of DEATH	Mean % change all-age DEATH rate	Mean % change age-standardized DEATH rate
1 Ischemic heart disease	1 Alzheimer's disease	**141 (118 to 166)**	**79.9 (63.1 to 96.4)**	-2.21 (-5.86 to 1.92)
2 Alzheimer's disease	2 Ischemic heart disease	21.7 (-2.98 to 55.3)	-9.02 (-28.5 to 13.8)	**-48.9 (-61 to -33.5)**
3 Stroke	3 COPD	**93.7 (62.8 to 128)**	**44.7 (21.6 to 70.2)**	-10.7 (-24.7 to 7.46)
4 Lung cancer	4 Chronic kidney disease	**235 (177 to 308)**	**150 (108 to 205)**	**37.6 (11.9 to 77)**
5 COPD	5 Lung cancer	**62.5 (32.2 to 97)**	**21.4 (0.776 to 44.5)**	-11.7 (-25.6 to 2.21)
6 COVID-19	6 Stroke	**25.5 (10 to 43.8)**	-6.18 (-17.5 to 6.36)	**-45.7 (-53.4 to -36.1)**
7 Colorectal cancer	7 Colorectal cancer	**99.6 (55.8 to 152)**	**49.1 (16.7 to 88.1)**	-2.2 (-21.9 to 18.1)
8 Chronic kidney disease	8 Lower respiratory infect	**128 (99.2 to 164)**	**70.2 (50.6 to 93.1)**	**-12.7 (-24 to -0.982)**
9 Lower respiratory infect	9 Atrial fibrillation	**200 (165 to 234)**	**124 (98.8 to 145)**	**20.5 (10.9 to 29.2)**
10 Prostate cancer	10 Falls	**125 (95.7 to 158)**	**68.5 (49.1 to 90.7)**	-8.49 (-17.3 to 1.94)
11 Falls	11 Prostate cancer	**112 (72.4 to 156)**	**58.2 (30.9 to 87.9)**	-3.26 (-15.1 to 10.5)
12 Diabetes	12 Pancreatic cancer	**137 (88.9 to 191)**	**76.9 (40.5 to 113)**	**26 (3.19 to 48.3)**
13 Atrial fibrillation	13 Diabetes	**77.7 (41.8 to 115)**	**32.8 (6.54 to 62.4)**	**-21.5 (-36.4 to -3.42)**
14 Pancreatic cancer	14 Breast cancer	**73.5 (42.6 to 111)**	**29.6 (8.32 to 55.8)**	-8.41 (-21.1 to 6.62)
15 Breast cancer	15 Nonrheum valv diseases	**208 (172 to 248)**	**130 (105 to 156)**	**22.6 (13 to 32.2)**
16 Self-harm	16 Urinary diseases	**262 (213 to 327)**	**170 (133 to 216)**	**42.6 (23 to 69.2)**
17 Parkinson's disease	17 Parkinson's disease	**104 (85.5 to 126)**	**52.1 (39.7 to 67.4)**	-4.15 (-10.6 to 4.11)
18 Leukemia	18 Liver cancer	**91.6 (69.9 to 117)**	**43.2 (26 to 62.2)**	13.6 (-0.269 to 30)
19 Liver cancer	19 Endo/metab/blood/immune	**142 (106 to 180)**	**80.5 (52 to 108)**	**16.8 (4.54 to 29.7)**
20 Lymphoma	20 Leukemia	**80 (51.5 to 110)**	**34.4 (14.5 to 53.5)**	-8.34 (-19 to 2.77)
21 Nonrheum valv diseases	21 Lymphoma			
26 Endo/metab/blood/immune	22 Self-harm			
29 Urinary diseases	129 COVID-19			

Supplemental Results Figure S8. Leading 20 level 3 causes of deaths, and percentage change in number of deaths and all-age and age-standardised death rates by location, 2022 and 2050

High-income Asia Pacific, Reference scenario

Legend:
Communicable, maternal, neonatal and nutritional
Non-communicable
Injuries

Leading Causes 2022	Leading Causes 2050	Mean % change number of DEATH	Mean % change all-age DEATH rate	Mean % change age-standardized DEATH rate
1 Alzheimer's disease	1 Alzheimer's disease	**68.2 (53.2 to 85.2)**	**89.7 (75.9 to 104)**	**-11.2 (-14.4 to -7.48)**
2 Stroke	2 Lower respiratory infect	**64.6 (47 to 84.6)**	**85.6 (66.2 to 108)**	-9.57 (-19.1 to 0.408)
3 Ischemic heart disease	3 Stroke	**-13 (-23 to -1.17)**	-1.88 (-13.5 to 10.7)	**-46.6 (-52.8 to -39.4)**
4 Lower respiratory infect	4 Lung cancer	**31.5 (8.64 to 57.1)**	**48.3 (22.5 to 73.8)**	**-15.1 (-26.6 to -2.02)**
5 Lung cancer	5 Chronic kidney disease	**133 (89.5 to 175)**	**162 (115 to 211)**	**26 (3.39 to 52.3)**
6 Colorectal cancer	6 Colorectal cancer	**46.3 (15.5 to 82.4)**	**65.1 (32.4 to 103)**	-8.08 (-25.3 to 10.3)
7 Stomach cancer	7 Ischemic heart disease	**-21.9 (-37.7 to -3.88)**	-11.9 (-29.6 to 9.57)	**-56 (-65.5 to -44.5)**
8 Chronic kidney disease	8 Pancreatic cancer	**71.5 (38.9 to 107)**	**93.3 (60.2 to 129)**	17 (-3.35 to 36)
9 Pancreatic cancer	9 Liver cancer	**70.6 (50 to 93.5)**	**92.5 (69.1 to 117)**	**22.5 (8.92 to 36.1)**
10 Liver cancer	10 COPD	**74.7 (49.4 to 101)**	**97.1 (71.6 to 129)**	-3.46 (-16.7 to 11.7)
11 COPD	11 Stomach cancer	-0.953 (-10.6 to 8.7)	**11.7 (2.54 to 21.3)**	**-38.3 (-42.2 to -34.1)**
12 COVID-19	12 Nonrheum valv diseases	**113 (86.7 to 150)**	**140 (108 to 182)**	6.73 (-4.23 to 18)
13 Self-harm	13 Hypertensive heart disease	**116 (69.8 to 165)**	**144 (94.5 to 200)**	18.1 (-5.08 to 46.7)
14 Cirrhosis liver	14 Urinary diseases	**184 (143 to 238)**	**221 (174 to 278)**	**45.7 (26.5 to 70.6)**
15 Gallbladder cancer	15 Interstitial lung disease	**45 (27.6 to 62.1)**	**63.5 (47.3 to 80.2)**	-5.44 (-14 to 3.14)
16 Interstitial lung disease	16 Atrial fibrillation	**113 (95.3 to 135)**	**140 (121 to 163)**	**20.2 (12.4 to 29.8)**
17 Aortic aneurysm	17 Cirrhosis liver	**17.8 (6.54 to 31.5)**	**32.9 (20.3 to 45.8)**	-6.26 (-14.2 to 2.56)
18 Nonrheum valv diseases	18 Falls	**65.5 (44.2 to 86.1)**	**86.7 (66.1 to 107)**	**-8.38 (-15.2 to -0.809)**
19 Parkinson's disease	19 Parkinson's disease	**55.1 (43.4 to 69.2)**	**75 (62.8 to 87.8)**	-0.313 (-5.9 to 7.03)
20 Falls	20 Prostate cancer	**71.7 (39.7 to 107)**	**93.7 (57.1 to 130)**	4.92 (-8.83 to 19.6)
21 Hypertensive heart disease	21 Self-harm			
24 Prostate cancer	22 Aortic aneurysm			
26 Atrial fibrillation	23 Gallbladder cancer			
28 Urinary diseases	132 COVID-19			

Supplemental Results Figure S8. Leading 20 level 3 causes of deaths, and percentage change in number of deaths and all-age and age-standardised death rates by location, 2022 and 2050

High-income North America, Reference scenario

Legend:
Communicable, maternal, neonatal and nutritional
Non-communicable
Injuries

Leading Causes 2022	Leading Causes 2050	Mean % change number of DEATH	Mean % change all-age DEATH rate	Mean % change age-standardized DEATH rate
1 Ischemic heart disease	1 Ischemic heart disease	-3.8 (-20.1 to 14.6)	-11.3 (-24.8 to 5.89)	**-49.4 (-58.3 to -37.5)**
2 COVID-19	2 Alzheimer's disease	**121 (98.7 to 142)**	**104 (87 to 122)**	1.41 (-2.5 to 5.34)
3 COPD	3 Chronic kidney disease	**166 (128 to 208)**	**145 (113 to 181)**	**41.6 (21.1 to 65.4)**
4 Alzheimer's disease	4 COPD	**65.7 (43.2 to 89.9)**	**52.8 (33.4 to 76.4)**	-5.71 (-17.7 to 7.89)
5 Stroke	5 Lung cancer	**32.6 (13.6 to 53.8)**	**22.3 (5.06 to 43.8)**	**-12 (-22.5 to -0.336)**
6 Lung cancer	6 Stroke	8.47 (-3.32 to 20)	0.0221 (-9.48 to 8.55)	**-40.8 (-47 to -33.8)**
7 Chronic kidney disease	7 Hypertensive heart disease	**109 (77.4 to 147)**	**92.4 (63.1 to 130)**	**21 (1.2 to 48.1)**
8 Colorectal cancer	8 Colorectal cancer	**54.9 (26.5 to 90.3)**	**42.9 (16.4 to 73.6)**	-5.91 (-21.1 to 10.6)
9 Diabetes	9 Lower respiratory infect	**70.2 (49 to 90.3)**	**56.9 (39.6 to 73.8)**	**-10.6 (-20.3 to -1.24)**
10 Drug use disorders	10 Pancreatic cancer	**90.5 (60.2 to 125)**	**75.6 (47.9 to 112)**	**26.3 (10.1 to 45.4)**
11 Lower respiratory infect	11 Drug use disorders	**49 (24.8 to 76.2)**	**37.3 (17.9 to 60)**	**33.5 (12.6 to 58.5)**
12 Cirrhosis liver	12 Falls	**90.5 (68 to 114)**	**75.7 (56.5 to 95.2)**	-3.3 (-10.7 to 4.85)
13 Hypertensive heart disease	13 Atrial fibrillation	**170 (140 to 209)**	**149 (124 to 176)**	**26.4 (16.7 to 34.7)**
14 Pancreatic cancer	14 Endo/metab/blood/immune	**116 (84.1 to 147)**	**98.8 (73 to 125)**	**28.7 (17.3 to 41)**
15 Breast cancer	15 Prostate cancer	**90.4 (64.2 to 122)**	**75.6 (52.6 to 103)**	5.21 (-4.77 to 16.6)
16 Falls	16 Nonrheum valv diseases	**194 (160 to 239)**	**171 (138 to 206)**	**32.8 (20.7 to 46.5)**
17 Self-harm	17 Diabetes	11.5 (-6.21 to 30.6)	2.81 (-13.6 to 20.5)	**-34.8 (-44.8 to -24.3)**
18 Prostate cancer	18 Breast cancer	**44.6 (23.6 to 66.8)**	**33.3 (15.6 to 52.1)**	-6.38 (-16.7 to 4.66)
19 Endo/metab/blood/immune	19 Parkinson's disease	**100 (79.7 to 121)**	**84.4 (67.3 to 101)**	6.3 (-2.27 to 14.3)
20 Road injuries	20 Urinary diseases	**200 (163 to 241)**	**176 (140 to 210)**	**50.6 (33.9 to 68.9)**
21 Parkinson's disease	21 Cirrhosis liver			
22 Atrial fibrillation	23 Self-harm			
24 Nonrheum valv diseases	26 Road injuries			
29 Urinary diseases	129 COVID-19			

Supplemental Results Figure S8. Leading 20 level 3 causes of deaths, and percentage change in number of deaths and all-age and age-standardised death rates by location, 2022 and 2050

Southern Latin America, Reference scenario

Legend:
Communicable, maternal, neonatal and nutritional
Non-communicable
Injuries

Leading Causes 2022	Leading Causes 2050	Mean % change number of DEATH	Mean % change all-age DEATH rate	Mean % change age-standardized DEATH rate
1 Lower respiratory infect	1 Lower respiratory infect	**76.1 (54.8 to 101)**	**66.5 (48.8 to 86.3)**	**-17.5 (-27.5 to -7)**
2 Ischemic heart disease	2 Chronic kidney disease	**160 (116 to 205)**	**145 (104 to 188)**	**23.4 (0.722 to 47.7)**
3 Stroke	3 Ischemic heart disease	0.157 (-20.7 to 27)	-5.25 (-25.3 to 19.7)	**-50.6 (-62.5 to -35.2)**
4 COVID-19	4 Alzheimer's disease	**132 (104 to 164)**	**120 (100 to 140)**	-1.66 (-4.78 to 2.15)
5 Chronic kidney disease	5 COPD	**98.7 (67.3 to 128)**	**87.9 (61.7 to 118)**	-6.22 (-20.8 to 9.11)
6 COPD	6 Stroke	6.47 (-8.34 to 22.1)	0.685 (-11.4 to 13.9)	**-46.2 (-54.1 to -38.3)**
7 Alzheimer's disease	7 Lung cancer	**84.4 (51.6 to 122)**	**74.2 (48.7 to 105)**	2.63 (-11.7 to 18.5)
8 Lung cancer	8 Colorectal cancer	**87.7 (45.5 to 139)**	**77.3 (38.8 to 120)**	-1.26 (-20.2 to 21.8)
9 Colorectal cancer	9 Hypertensive heart disease	**125 (82.2 to 165)**	**112 (77.7 to 149)**	2.71 (-17.2 to 24)
10 Diabetes	10 Urinary diseases	**204 (164 to 249)**	**188 (156 to 228)**	**37.5 (20.2 to 57)**
11 Hypertensive heart disease	11 Pancreatic cancer	**151 (93.7 to 214)**	**137 (94.7 to 193)**	**36.9 (14.5 to 64.6)**
12 Cirrhosis liver	12 Diabetes	**45.5 (19.3 to 76.3)**	**37.7 (12.6 to 69.2)**	**-26.3 (-41.4 to -9.73)**
13 Breast cancer	13 Prostate cancer	**117 (79.1 to 159)**	**105 (77.6 to 139)**	6.16 (-4.95 to 18.3)
14 Stomach cancer	14 Breast cancer	**70.7 (42 to 108)**	**61.3 (37.4 to 92.4)**	-4.67 (-17.7 to 11.5)
15 Prostate cancer	15 Cirrhosis liver	**38 (23.3 to 55)**	**30.5 (20.1 to 40.8)**	**-14.7 (-21.1 to -8.72)**
16 Urinary diseases	16 Stomach cancer	**24.9 (11.9 to 40.7)**	**18.1 (9.77 to 27.4)**	**-33.8 (-37.6 to -29.6)**
17 Pancreatic cancer	17 Cardiomyopathy	**44.8 (25.9 to 67.9)**	**36.8 (23.1 to 54)**	**-25.1 (-30.9 to -18.8)**
18 Road injuries	18 Other cardiovascular	**56.4 (39 to 77.5)**	**47.9 (36.1 to 61.2)**	**-22.4 (-28 to -15.8)**
19 Cardiomyopathy	19 Interstitial lung disease	**123 (100 to 149)**	**111 (94.8 to 130)**	**9.47 (2.88 to 16.8)**
20 Self-harm	20 Parkinson's disease	**114 (90.1 to 139)**	**102 (84.8 to 120)**	1.58 (-4.42 to 7.97)
21 Other cardiovascular	24 Road injuries			
22 Parkinson's disease	27 Self-harm			
23 Interstitial lung disease	134 COVID-19			

Supplemental Results Figure S8. Leading 20 level 3 causes of deaths, and percentage change in number of deaths and all-age and age-standardised death rates by location, 2022 and 2050

Western Europe, Reference scenario

Legend:
Communicable, maternal, neonatal and nutritional
Non-communicable
Injuries

Leading Causes 2022	Leading Causes 2050	Mean % change number of DEATH	Mean % change all-age DEATH rate	Mean % change age-standardized DEATH rate
1 Ischemic heart disease	1 Alzheimer's disease	**74.3 (62.7 to 84.9)**	**69.6 (59.5 to 79.3)**	**-3.72 (-5.72 to -1.7)**
2 Alzheimer's disease	2 Ischemic heart disease	**-16.9 (-30.6 to -2.24)**	**-19.1 (-32.6 to -4.75)**	**-52.2 (-61.6 to -41)**
3 Stroke	3 Chronic kidney disease	**149 (118 to 181)**	**142 (112 to 177)**	**35.9 (16.3 to 59.7)**
4 COVID-19	4 COPD	**54 (36.1 to 70.1)**	**49.9 (33.2 to 67.2)**	-8.62 (-20.3 to 2.44)
5 Lung cancer	5 Lung cancer	**20.9 (5.04 to 36.8)**	**17.6 (2.56 to 34.2)**	**-14.1 (-23.7 to -4.23)**
6 COPD	6 Stroke	**-14.1 (-21.6 to -6.74)**	**-16.4 (-23.9 to -8.67)**	**-48.8 (-54.6 to -43.2)**
7 Lower respiratory infect	7 Lower respiratory infect	**61.2 (50.3 to 72.4)**	**56.9 (45.7 to 67.8)**	**-10.7 (-18.9 to -2.49)**
8 Colorectal cancer	8 Colorectal cancer	**49.7 (22.5 to 78.1)**	**45.6 (21.2 to 75.4)**	-5.72 (-19.8 to 10.5)
9 Chronic kidney disease	9 Hypertensive heart disease	**83.1 (58.7 to 107)**	**78.2 (54.5 to 102)**	2.36 (-14.5 to 19.8)
10 Hypertensive heart disease	10 Pancreatic cancer	**83.7 (54.4 to 115)**	**78.7 (50.4 to 109)**	**25.4 (7.57 to 44.4)**
11 Breast cancer	11 Atrial fibrillation	**110 (98.3 to 124)**	**105 (93.9 to 119)**	**15.3 (11.6 to 19)**
12 Pancreatic cancer	12 Prostate cancer	**74.4 (54.5 to 98.7)**	**69.7 (50.2 to 93.7)**	4.64 (-4.15 to 14.1)
13 Falls	13 Falls	**61.1 (49 to 72.1)**	**56.7 (45.7 to 66.9)**	**-8.34 (-12.3 to -4.54)**
14 Diabetes	14 Nonrheum valv diseases	**127 (113 to 143)**	**121 (107 to 135)**	**24.1 (18.2 to 30.6)**
15 Prostate cancer	15 Breast cancer	**33 (15.6 to 50.1)**	**29.4 (12.9 to 48.2)**	**-11.1 (-20.5 to -0.967)**
16 Atrial fibrillation	16 Diabetes	**28 (9.8 to 46)**	**24.6 (8.05 to 43)**	**-27.1 (-38 to -14.8)**
17 Nonrheum valv diseases	17 Urinary diseases	**171 (150 to 192)**	**164 (144 to 185)**	**47.9 (35.2 to 63.8)**
18 Cirrhosis liver	18 Parkinson's disease	**66.2 (57.6 to 74.5)**	**61.8 (54.8 to 69)**	1.54 (-2.14 to 5)
19 Parkinson's disease	19 Bladder cancer	**52.1 (41.3 to 64.8)**	**48 (37.6 to 59.7)**	**-7.19 (-11.1 to -2.24)**
20 Stomach cancer	20 Liver cancer	**64.6 (49.4 to 77.5)**	**60.2 (45.8 to 72.4)**	**19.6 (7.68 to 30)**
21 Bladder cancer	21 Cirrhosis liver			
24 Liver cancer	24 Stomach cancer			
25 Urinary diseases	131 COVID-19			

Supplemental Results Figure S8. Leading 20 level 3 causes of deaths, and percentage change in number of deaths and all-age and age-standardised death rates by location, 2022 and 2050

Andean Latin America, Reference scenario

Legend:
Communicable, maternal, neonatal and nutritional
Non-communicable
Injuries

Leading Causes 2022	Leading Causes 2050	Mean % change number of DEATH	Mean % change all-age DEATH rate	Mean % change age-standardized DEATH rate
1 COVID-19	1 Chronic kidney disease	**250 (146 to 362)**	**193 (114 to 285)**	36.9 (-4.83 to 95)
2 Lower respiratory infect	2 Lower respiratory infect	**108 (67 to 150)**	**73.8 (42.7 to 108)**	**-23.3 (-36.7 to -6.66)**
3 Ischemic heart disease	3 Ischemic heart disease	**66 (4.47 to 145)**	39.1 (-8.39 to 103)	**-37.9 (-61 to -1.69)**
4 Chronic kidney disease	4 Stroke	**52.4 (19.8 to 89.5)**	**27.6 (0.997 to 55.2)**	**-38.4 (-51.6 to -22.1)**
5 Stroke	5 Diabetes	**94.6 (44.2 to 147)**	**63 (23.2 to 105)**	-21.7 (-42.1 to 2.34)
6 Diabetes	6 Cirrhosis liver	**88.3 (62.4 to 113)**	**57.7 (39.6 to 75.7)**	**-13 (-23.3 to -1.93)**
7 Cirrhosis liver	7 Alzheimer's disease	**220 (155 to 282)**	**168 (117 to 214)**	0.402 (-2.42 to 2.86)
8 Stomach cancer	8 COPD	**187 (117 to 272)**	**141 (85.4 to 210)**	-2.54 (-30.4 to 30.3)
9 Road injuries	9 Stomach cancer	**56.5 (36.2 to 77.8)**	**31 (18.1 to 46.2)**	**-35.5 (-40.3 to -30.5)**
10 Alzheimer's disease	10 Interstitial lung disease	**188 (145 to 239)**	**141 (108 to 179)**	3.7 (-7.3 to 17.3)
11 Neonatal disorders	11 Lung cancer	**153 (83.9 to 247)**	**111 (55.7 to 177)**	4.27 (-20.3 to 31.8)
12 COPD	12 Prostate cancer	**187 (102 to 301)**	**140 (74.2 to 226)**	2.31 (-20.9 to 29.2)
13 Interstitial lung disease	13 Colorectal cancer	**152 (68 to 271)**	**111 (44 to 203)**	2.15 (-29.1 to 41.8)
14 Lung cancer	14 Hypertensive heart disease	**186 (105 to 275)**	**140 (72.2 to 223)**	2.73 (-31.7 to 46.5)
15 Colorectal cancer	15 Road injuries	20.1 (-19.3 to 77.1)	0.645 (-32 to 47.5)	-19.5 (-47 to 21.1)
16 Prostate cancer	16 Pancreatic cancer	**232 (104 to 415)**	**178 (69.4 to 322)**	37.3 (-14.1 to 105)
17 Congenital defects	17 Urinary diseases	**268 (190 to 352)**	**208 (155 to 267)**	**29.9 (5.13 to 59.3)**
18 Hypertensive heart disease	18 Breast cancer	**102 (49.3 to 170)**	**68.7 (25.5 to 128)**	-4.9 (-26.3 to 24.3)
19 Cervical cancer	19 Parkinson's disease	**172 (127 to 230)**	**127 (95.2 to 165)**	-5.75 (-16.2 to 5.33)
20 Tuberculosis	20 Falls	**99.3 (68.3 to 140)**	**66.8 (46.3 to 92.2)**	**-12.1 (-18.5 to -4.99)**
21 Breast cancer	22 Cervical cancer			
22 Falls	34 Neonatal disorders			
24 Pancreatic cancer	37 Tuberculosis			
29 Parkinson's disease	40 Congenital defects			
30 Urinary diseases	135 COVID-19			

Supplemental Results Figure S8. Leading 20 level 3 causes of deaths, and percentage change in number of deaths and all-age and age-standardised death rates by location, 2022 and 2050

Caribbean, Reference scenario

Legend:
Communicable, maternal, neonatal and nutritional
Non-communicable
Injuries

Leading Causes 2022	Leading Causes 2050	Mean % change number of DEATH	Mean % change all-age DEATH rate	Mean % change age-standardized DEATH rate
1 Ischemic heart disease	1 Ischemic heart disease	30 (-17.2 to 84.7)	25.5 (-20.4 to 86.3)	-32.9 (-59 to 6.6)
2 Stroke	2 Stroke	25.7 (-1.76 to 52.9)	21.3 (-4.83 to 50.2)	**-32.7 (-49.2 to -13.5)**
3 Diabetes	3 Chronic kidney disease	**132 (71.5 to 209)**	**124 (65.1 to 212)**	25.1 (-12.1 to 80.3)
4 Lower respiratory infect	4 Diabetes	**51.8 (13.6 to 90.1)**	**46.5 (9.46 to 92.7)**	-17.5 (-42.1 to 15.8)
5 COVID-19	5 Lower respiratory infect	**43.5 (20.8 to 71.7)**	**38.5 (18.5 to 65.2)**	**-30.6 (-43.1 to -14.8)**
6 Chronic kidney disease	6 Hypertensive heart disease	**120 (56.4 to 186)**	**112 (50.6 to 184)**	13.7 (-21.6 to 60.4)
7 Nature disaster	7 Alzheimer's disease	**149 (97.9 to 197)**	**140 (93.5 to 177)**	-0.891 (-3.54 to 1.29)
8 Hypertensive heart disease	8 Prostate cancer	**117 (42.9 to 199)**	**110 (46.2 to 177)**	3.21 (-22 to 29.6)
9 COPD	9 COPD	**73 (34.4 to 115)**	**67 (29.5 to 111)**	-16.6 (-38.7 to 11.7)
10 Lung cancer	10 Lung cancer	**60.6 (10.1 to 116)**	**54.9 (8.11 to 108)**	-11.5 (-34.1 to 14.2)
11 Neonatal disorders	11 Colorectal cancer	**91.6 (16.4 to 186)**	**84.7 (17.7 to 176)**	-0.302 (-31.6 to 40.7)
12 Cirrhosis liver	12 Nature disaster	16800 (-100 to 166000)	15600 (-100 to 162000)	16200 (-100 to 164000)
13 Prostate cancer	13 Cirrhosis liver	**37.4 (18.4 to 55.2)**	**32.6 (16.6 to 51.3)**	-9.58 (-21.2 to 5.89)
14 Alzheimer's disease	14 Falls	**104 (60.2 to 147)**	**96.5 (59.4 to 136)**	-9.85 (-19.9 to 1.2)
15 Colorectal cancer	15 Breast cancer	**72.6 (16.2 to 130)**	**66.3 (15.8 to 121)**	2.08 (-25.3 to 32.6)
16 Interpersonal violence	16 Road injuries	1.95 (-32.6 to 53.7)	-1.56 (-34.8 to 51.9)	-6.9 (-40 to 45.9)
17 Road injuries	17 Atrial fibrillation	**185 (127 to 240)**	**175 (123 to 223)**	**16.6 (6.29 to 27.8)**
18 HIV/AIDS	18 Pancreatic cancer	**143 (37.5 to 280)**	**135 (40.1 to 263)**	34.3 (-14.8 to 99.8)
19 Breast cancer	19 Parkinson's disease	**116 (86.1 to 151)**	**109 (83.6 to 135)**	-1.61 (-9.72 to 8.31)
20 Diarrheal diseases	20 Stomach cancer	**26.8 (11.8 to 46.6)**	**22.3 (9.6 to 34.7)**	**-27.2 (-32.1 to -21.5)**
21 Falls	23 Neonatal disorders			
24 Stomach cancer	24 Interpersonal violence			
27 Pancreatic cancer	27 Diarrheal diseases			
28 Atrial fibrillation	41 HIV/AIDS			
30 Parkinson's disease	136 COVID-19			

Supplemental Results Figure S8. Leading 20 level 3 causes of deaths, and percentage change in number of deaths and all-age and age-standardised death rates by location, 2022 and 2050

Central Latin America, Reference scenario

Legend:
Communicable, maternal, neonatal and nutritional
Non-communicable
Injuries

Leading Causes 2022	Leading Causes 2050	Mean % change number of DEATH	Mean % change all-age DEATH rate	Mean % change age-standardized DEATH rate
1 Ischemic heart disease	1 Ischemic heart disease	**65.6 (2.12 to 151)**	49 (-8.9 to 132)	-34.2 (-65.2 to 15.3)
2 COVID-19	2 Chronic kidney disease	**178 (108 to 265)**	**150 (84.7 to 237)**	27.2 (-14 to 81)
3 Diabetes	3 Diabetes	**72 (34.8 to 113)**	**54.5 (19.6 to 98.9)**	-23.5 (-44.8 to 4.54)
4 Chronic kidney disease	4 COPD	**145 (92.9 to 202)**	**120 (69.8 to 168)**	-13.1 (-38.8 to 24.8)
5 Stroke	5 Stroke	**50.2 (19.1 to 84.5)**	**34.9 (5.62 to 73)**	**-37.7 (-55.8 to -13.6)**
6 Interpersonal violence	6 Alzheimer's disease	**227 (143 to 311)**	**193 (122 to 264)**	1.05 (-3.28 to 3.87)
7 Lower respiratory infect	7 Lower respiratory infect	**81.1 (56 to 104)**	**62.5 (42.3 to 86)**	**-25.1 (-38.3 to -11.2)**
8 Cirrhosis liver	8 Cirrhosis liver	**65.9 (51.9 to 81.2)**	**49 (35.6 to 64.1)**	-6.29 (-15.9 to 5.43)
9 COPD	9 Hypertensive heart disease	**175 (99.6 to 253)**	**148 (76.6 to 232)**	3.03 (-34.1 to 54.1)
10 Road injuries	10 Urinary diseases	**221 (178 to 270)**	**188 (144 to 231)**	**29.6 (4.68 to 61.3)**
11 Alzheimer's disease	11 Lung cancer	**115 (40.4 to 210)**	**92.3 (27.1 to 176)**	-3.88 (-29.9 to 29.6)
12 Stomach cancer	12 Interpersonal violence	-32.4 (-61.6 to 20.9)	-39.6 (-63.9 to 4.7)	-39 (-65.2 to 5.26)
13 Lung cancer	13 Colorectal cancer	**124 (37.1 to 238)**	**100 (25 to 203)**	1.24 (-31.1 to 42.2)
14 Colorectal cancer	14 Prostate cancer	**157 (62.3 to 268)**	**130 (47.9 to 227)**	-3.41 (-29.6 to 26.6)
15 Hypertensive heart disease	15 Road injuries	8.88 (-32.8 to 76)	-1.98 (-41.1 to 63.4)	-14.6 (-48.7 to 45.3)
16 Neonatal disorders	16 Stomach cancer	**43.7 (24.9 to 63.2)**	**28.9 (14.5 to 43.9)**	**-34.9 (-39.4 to -30.2)**
17 Prostate cancer	17 Atrial fibrillation	**267 (189 to 352)**	**229 (165 to 302)**	**18.6 (8.29 to 32.3)**
18 Breast cancer	18 Pancreatic cancer	**207 (72.3 to 396)**	**175 (58.5 to 341)**	40.3 (-13.1 to 112)
19 Self-harm	19 Breast cancer	**88.1 (28.2 to 160)**	**68.4 (18.6 to 135)**	-3.23 (-27.3 to 27)
20 Urinary diseases	20 Falls	**108 (64.7 to 148)**	**86.6 (50.4 to 122)**	**-9.87 (-16.4 to -0.743)**
22 Falls	29 Self-harm			
24 Pancreatic cancer	43 Neonatal disorders			
29 Atrial fibrillation	136 COVID-19			

Supplemental Results Figure S8. Leading 20 level 3 causes of deaths, and percentage change in number of deaths and all-age and age-standardised death rates by location, 2022 and 2050

Tropical Latin America, Reference scenario

Legend:
Communicable, maternal, neonatal and nutritional
Non-communicable
Injuries

Leading Causes 2022	Leading Causes 2050	Mean % change number of DEATH	Mean % change all-age DEATH rate	Mean % change age-standardized DEATH rate
1 Ischemic heart disease	1 Lower respiratory infect	**111 (63.8 to 156)**	**87.9 (51.4 to 125)**	-23.6 (-44.3 to 0.175)
2 Stroke	2 Ischemic heart disease	25.5 (-26.7 to 94.5)	12.2 (-34.1 to 82.2)	**-44.3 (-67.9 to -4.94)**
3 Lower respiratory infect	3 Alzheimer's disease	**207 (136 to 281)**	**174 (126 to 224)**	**-7.1 (-9.72 to -4.65)**
4 COVID-19	4 Stroke	29.4 (-9.71 to 72)	15.5 (-15.4 to 54.7)	**-45.2 (-61.8 to -21.9)**
5 Diabetes	5 Chronic kidney disease	**186 (85.3 to 300)**	**155 (70.5 to 266)**	20.1 (-23.7 to 83.6)
6 COPD	6 COPD	**108 (40 to 177)**	**86 (31 to 153)**	-20.4 (-45.5 to 18)
7 Alzheimer's disease	7 Diabetes	**79.1 (26.2 to 139)**	**60 (16.2 to 114)**	-23.7 (-47.3 to 9.39)
8 Interpersonal violence	8 Urinary diseases	**268 (185 to 367)**	**228 (156 to 301)**	28.4 (-3.47 to 74.4)
9 Chronic kidney disease	9 Hypertensive heart disease	**167 (74.8 to 264)**	**138 (59.7 to 231)**	4.88 (-33.6 to 60.9)
10 Road injuries	10 Lung cancer	**84.2 (17.7 to 167)**	**64 (8.12 to 130)**	-13.1 (-39.3 to 15.7)
11 Lung cancer	11 Colorectal cancer	**106 (14.5 to 232)**	**83.6 (7.15 to 184)**	-4.81 (-40.1 to 38.4)
12 Cirrhosis liver	12 Prostate cancer	**172 (65.5 to 306)**	**142 (51.9 to 261)**	3.78 (-26.8 to 39.5)
13 Hypertensive heart disease	13 Falls	**130 (78.8 to 196)**	**106 (62.9 to 151)**	-12 (-22.8 to 1.99)
14 Urinary diseases	14 Pancreatic cancer	**183 (44.6 to 379)**	**152 (33.4 to 317)**	30.7 (-25.2 to 102)
15 Colorectal cancer	15 Breast cancer	**78 (14.4 to 155)**	**58.5 (6.8 to 130)**	-6.3 (-32.7 to 27.5)
16 Stomach cancer	16 Cirrhosis liver	**34.6 (9.93 to 59.7)**	**20.2 (1.76 to 39.8)**	**-17.4 (-30.6 to -1.07)**
17 Breast cancer	17 Road injuries	5.95 (-34.3 to 62.5)	-5.3 (-36.9 to 45.4)	-10.8 (-41.6 to 42.3)
18 Prostate cancer	18 Atrial fibrillation	**256 (173 to 359)**	**218 (156 to 297)**	9.76 (-4.88 to 31.2)
19 Falls	19 Interpersonal violence	-25.9 (-61.6 to 33.8)	-34.1 (-64.6 to 24.5)	-27.6 (-62.5 to 42)
20 Neonatal disorders	20 Bacterial skin	**339 (129 to 603)**	**292 (111 to 556)**	81.6 (-8.22 to 224)
23 Pancreatic cancer	21 Stomach cancer			
28 Atrial fibrillation	54 Neonatal disorders			
37 Bacterial skin	136 COVID-19			

Supplemental Results Figure S8. Leading 20 level 3 causes of deaths, and percentage change in number of deaths and all-age and age-standardised death rates by location, 2022 and 2050

North Africa and Middle East, Reference scenario

Legend:
Communicable, maternal, neonatal and nutritional
Non-communicable
Injuries

Leading Causes 2022	Leading Causes 2050	Mean % change number of DEATH	Mean % change all-age DEATH rate	Mean % change age-standardized DEATH rate
1 Ischemic heart disease	1 Ischemic heart disease	**72 (18.4 to 151)**	27.3 (-15.2 to 90.5)	**-43.2 (-65.1 to -5.62)**
2 Stroke	2 Stroke	**67.4 (31.6 to 108)**	23.8 (-3.03 to 58.2)	**-47.5 (-62.4 to -25)**
3 COVID-19	3 Chronic kidney disease	**301 (204 to 415)**	**197 (124 to 292)**	32.2 (-7.37 to 95)
4 Chronic kidney disease	4 Hypertensive heart disease	**252 (166 to 354)**	**160 (96.8 to 242)**	5.42 (-29.7 to 57)
5 Hypertensive heart disease	5 Alzheimer's disease	**326 (224 to 422)**	**215 (145 to 288)**	**7.14 (4.55 to 9.29)**
6 Diabetes	6 Diabetes	**160 (101 to 235)**	**92.5 (48.8 to 152)**	-12.9 (-37.9 to 26.9)
7 Road injuries	7 COPD	**181 (122 to 248)**	**108 (64 to 166)**	-15.3 (-40.7 to 21.2)
8 Lower respiratory infect	8 Lower respiratory infect	**98.2 (73.4 to 125)**	**46.5 (27.6 to 71.3)**	**-25.2 (-39.2 to -3.57)**
9 Cirrhosis liver	9 Lung cancer	**154 (72 to 255)**	**87.4 (30.5 to 160)**	-9.99 (-33.1 to 17.9)
10 COPD	10 Cirrhosis liver	**91 (69.5 to 112)**	**41.1 (25 to 58.8)**	**-30.3 (-39.4 to -17.7)**
11 Neonatal disorders	11 Road injuries	29.1 (-14.4 to 99.4)	-4.42 (-38.4 to 50.6)	-22.3 (-51.4 to 25.6)
12 Lung cancer	12 Colorectal cancer	**220 (105 to 375)**	**136 (51.6 to 248)**	11.5 (-22.4 to 53.7)
13 Alzheimer's disease	13 Pancreatic cancer	**342 (184 to 537)**	**226 (116 to 368)**	**52.8 (8.53 to 105)**
14 Congenital defects	14 Liver cancer	**219 (172 to 272)**	**136 (101 to 181)**	19.7 (-1.22 to 48.6)
15 Conflict & terror	15 Conflict & terror	96.3 (-79.9 to 677)	45.4 (-85.4 to 487)	30.8 (-87 to 379)
16 Stomach cancer	16 Breast cancer	**145 (65.9 to 245)**	**80.9 (22.8 to 151)**	4.77 (-25 to 39.5)
17 Colorectal cancer	17 Stomach cancer	**88.8 (66.3 to 114)**	**39.4 (24.3 to 52.7)**	**-32.9 (-37 to -28.7)**
18 Breast cancer	18 Brain cancer	**200 (175 to 229)**	**122 (103 to 139)**	**34.9 (29.9 to 40.3)**
19 Other cardiovascular	19 Prostate cancer	**275 (139 to 446)**	**176 (77.3 to 302)**	3.23 (-25.2 to 33.9)
20 Leukemia	20 Other cardiovascular	**110 (87.5 to 136)**	**55.5 (36.9 to 72.9)**	**-24.2 (-37 to -9.45)**
22 Liver cancer	22 Leukemia			
24 Brain cancer	26 Congenital defects			
27 Pancreatic cancer	27 Neonatal disorders			
30 Prostate cancer	134 COVID-19			

Supplemental Results Figure S8. Leading 20 level 3 causes of deaths, and percentage change in number of deaths and all-age and age-standardised death rates by location, 2022 and 2050

South Asia, Reference scenario

Legend:
Communicable, maternal, neonatal and nutritional
Non-communicable
Injuries

Leading Causes 2022	Leading Causes 2050	Mean % change number of DEATH	Mean % change all-age DEATH rate	Mean % change age-standardized DEATH rate
1 Ischemic heart disease	1 Ischemic heart disease	20.3 (-28.1 to 82.5)	7.66 (-33.7 to 67.1)	**-51.4 (-73.7 to -13.5)**
2 COPD	2 COPD	**82.2 (35.4 to 132)**	**62.9 (22.4 to 115)**	**-35.4 (-56.4 to -0.966)**
3 Stroke	3 Stroke	**34.3 (5.35 to 67.3)**	20 (-5.93 to 54.8)	**-47.9 (-63.4 to -27.9)**
4 Neonatal disorders	4 Diabetes	**85.9 (41.1 to 138)**	**66.2 (21.8 to 120)**	-28.3 (-52.4 to 8.8)
5 Lower respiratory infect	5 Diarrheal diseases	**36 (5.18 to 69.7)**	21.6 (-7.11 to 57.1)	**-50.8 (-68 to -22.7)**
6 Diarrheal diseases	6 Lower respiratory infect	**23.8 (1.89 to 49.3)**	10.5 (-7.04 to 31.3)	**-44.8 (-54.6 to -30.8)**
7 Tuberculosis	7 Falls	**136 (80.8 to 203)**	**110 (71.5 to 158)**	**-13.6 (-22.2 to -3.72)**
8 Diabetes	8 Chronic kidney disease	**165 (91.5 to 254)**	**137 (72.7 to 227)**	11.1 (-26.9 to 66.6)
9 Cirrhosis liver	9 Alzheimer's disease	**260 (150 to 374)**	**221 (136 to 322)**	**-5.4 (-9.86 to -1.27)**
10 Road injuries	10 Cirrhosis liver	**55.9 (29.7 to 82.9)**	**39.3 (16.6 to 66.9)**	**-20.4 (-35.2 to -1.77)**
11 Falls	11 Hypertensive heart disease	**153 (83.8 to 229)**	**126 (68.2 to 206)**	-11.3 (-42.6 to 35.8)
12 COVID-19	12 Tuberculosis	-31.1 (-57.8 to 2.2)	**-38.3 (-61.9 to -3.83)**	**-65.7 (-80 to -42.6)**
13 Chronic kidney disease	13 Road injuries	9.76 (-29.8 to 61.5)	-1.8 (-35.7 to 53.2)	-24.7 (-52.8 to 20.1)
14 Asthma	14 Urinary diseases	**192 (152 to 239)**	**161 (129 to 199)**	16.8 (-5.24 to 46)
15 Self-harm	15 Breast cancer	**121 (35.5 to 230)**	**97 (32.2 to 177)**	8.62 (-25 to 44.1)
16 Rheumatic heart disease	16 Self-harm	7.1 (-15.4 to 35.2)	-4.32 (-24.5 to 20.9)	-19.3 (-37.8 to 6.07)
17 Hypertensive heart disease	17 Lip oral cavity cancer	**118 (87.7 to 149)**	**94.9 (77.3 to 112)**	0.811 (-8.34 to 11.4)
18 Alzheimer's disease	18 Rheumatic heart disease	-0.941 (-32.4 to 36.6)	-11.4 (-39.6 to 26.3)	**-54.9 (-71.8 to -29.7)**
19 Breast cancer	19 Asthma	-8.57 (-24 to 9.39)	**-18.3 (-31.7 to -3.89)**	**-63.9 (-71.4 to -52.6)**
20 Congenital defects	20 Lung cancer	**106 (30.4 to 198)**	**84 (22.2 to 155)**	-7.66 (-33.7 to 22.5)
21 Lung cancer	21 Neonatal disorders			
22 Lip oral cavity cancer	52 Congenital defects			
23 Urinary diseases	133 COVID-19			

Supplemental Results Figure S8. Leading 20 level 3 causes of deaths, and percentage change in number of deaths and all-age and age-standardised death rates by location, 2022 and 2050

East Asia, Reference scenario

Legend:
Communicable, maternal, neonatal and nutritional
Non-communicable
Injuries

Leading Causes 2022	Leading Causes 2050	Mean % change number of DEATH	Mean % change all-age DEATH rate	Mean % change age-standardized DEATH rate
1 Stroke	1 Stroke	**27.5 (2.84 to 56.9)**	**46.4 (19.9 to 83.5)**	**-47.6 (-58.1 to -32)**
2 Ischemic heart disease	2 COPD	**123 (74.8 to 177)**	**156 (103 to 218)**	-21.6 (-40.8 to 5.22)
3 COPD	3 Ischemic heart disease	17.5 (-15.7 to 57.8)	35 (-2.83 to 86.1)	**-55.7 (-70.3 to -35.8)**
4 Lung cancer	4 Alzheimer's disease	**230 (161 to 316)**	**278 (212 to 360)**	-0.575 (-4.32 to 3.49)
5 Alzheimer's disease	5 Lung cancer	**79.5 (27.7 to 144)**	**106 (50.8 to 171)**	-7.25 (-26.2 to 15.8)
6 Stomach cancer	6 Hypertensive heart disease	**192 (114 to 293)**	**235 (148 to 366)**	6.55 (-26.1 to 54.8)
7 Hypertensive heart disease	7 Colorectal cancer	**128 (51.5 to 219)**	**161 (84.2 to 260)**	15.4 (-13.6 to 47.7)
8 Esophageal cancer	8 Esophageal cancer	**79.1 (52.1 to 109)**	**105 (80.1 to 135)**	-0.257 (-10.6 to 10.6)
9 Colorectal cancer	9 Chronic kidney disease	**145 (86.1 to 203)**	**182 (120 to 262)**	7.97 (-20.7 to 46)
10 Road injuries	10 Stomach cancer	18.5 (-1.2 to 40.7)	**35.9 (17.3 to 57.9)**	**-37.2 (-42.2 to -32.2)**
11 Lower respiratory infect	11 Lower respiratory infect	**121 (85 to 160)**	**153 (113 to 191)**	**-29.7 (-39.2 to -18.1)**
12 Chronic kidney disease	12 Pancreatic cancer	**150 (73.3 to 234)**	**186 (108 to 283)**	**38.4 (4.77 to 75.8)**
13 Diabetes	13 Falls	**116 (65 to 173)**	**148 (99.1 to 206)**	**-13 (-20.9 to -4.4)**
14 Liver cancer	14 Liver cancer	**71.8 (27.4 to 126)**	**97.3 (47.8 to 161)**	10.9 (-19.8 to 55.6)
15 Cirrhosis liver	15 Diabetes	**56.2 (22.6 to 99.3)**	**79.3 (41.3 to 140)**	**-29.2 (-47 to -2.96)**
16 Falls	16 Atrial fibrillation	**322 (231 to 442)**	**384 (288 to 507)**	**20.4 (8.12 to 33.7)**
17 Self-harm	17 Parkinson's disease	**170 (124 to 223)**	**210 (168 to 257)**	-1.8 (-9.83 to 7.67)
18 Pancreatic cancer	18 Cirrhosis liver	22.2 (-3.18 to 53.5)	**40.3 (13.8 to 78.2)**	-20.6 (-37.2 to 1.83)
19 COVID-19	19 Road injuries	-19 (-43.6 to 15.5)	-6.9 (-36.1 to 34.2)	-21.3 (-49.1 to 24.1)
20 Parkinson's disease	20 Self-harm	**26.5 (2.98 to 51.1)**	**45.2 (22.4 to 72.3)**	-14.8 (-29.5 to 4.11)
24 Atrial fibrillation	133 COVID-19			

Supplemental Results Figure S8. Leading 20 level 3 causes of deaths, and percentage change in number of deaths and all-age and age-standardised death rates by location, 2022 and 2050

Oceania, Reference scenario

Legend:
Communicable, maternal, neonatal and nutritional
Non-communicable
Injuries

Leading Causes 2022	Leading Causes 2050	Mean % change number of DEATH	Mean % change all-age DEATH rate	Mean % change age-standardized DEATH rate
1 Ischemic heart disease	1 Ischemic heart disease	**106 (38.8 to 197)**	10.9 (-25.2 to 59.2)	-27.5 (-55.1 to 11.8)
2 Stroke	2 Stroke	**122 (71.6 to 170)**	19.2 (-5.08 to 44.3)	**-25.3 (-43.7 to -3.67)**
3 Lower respiratory infect	3 Diabetes	**126 (69.8 to 184)**	21.5 (-5.8 to 53.6)	-17.4 (-40.3 to 13.1)
4 Diabetes	4 COPD	**147 (87.5 to 213)**	**32.8 (2.51 to 73.4)**	-23.8 (-44.7 to 3.62)
5 COPD	5 Lower respiratory infect	-10.7 (-26.5 to 5.76)	**-51.9 (-59.8 to -42.3)**	**-47 (-56.8 to -35.9)**
6 Neonatal disorders	6 Chronic kidney disease	**248 (155 to 352)**	**87 (39.1 to 148)**	31.7 (-6.09 to 87.9)
7 Malaria	7 Malaria	141 (-82.9 to 534)	30.3 (-90.1 to 221)	18.1 (-86.5 to 201)
8 COVID-19	8 Diarrheal diseases	35.6 (-9.14 to 89.2)	-27 (-51.1 to 5.36)	-33.2 (-58.7 to 1.46)
9 Diarrheal diseases	9 Lung cancer	**181 (92.5 to 287)**	**51.2 (8.21 to 105)**	-0.674 (-21.4 to 25.9)
10 Tuberculosis	10 Hypertensive heart disease	**162 (79.2 to 259)**	**40.9 (0.535 to 94.5)**	-10.8 (-42.8 to 36.6)
11 Congenital defects	11 Road injuries	45.9 (-15.4 to 143)	-21.4 (-53.8 to 36.3)	-27.6 (-57 to 24.2)
12 Road injuries	12 Neonatal disorders	-31.3 (-60 to 8.3)	**-63.1 (-77.8 to -43.2)**	**-49.3 (-68 to -23.9)**
13 Asthma	13 Alzheimer's disease	**327 (210 to 461)**	**130 (72.4 to 191)**	3.59 (-0.84 to 7.87)
14 Chronic kidney disease	14 Tuberculosis	-12.9 (-41.1 to 21.7)	**-53.1 (-68.2 to -32.4)**	**-64 (-76.4 to -46.1)**
15 Lung cancer	15 Congenital defects	-7.97 (-25.9 to 15.1)	**-50.6 (-59.1 to -40)**	**-35.4 (-44.3 to -25.1)**
16 Hypertensive heart disease	16 Asthma	21.5 (-1.08 to 48.6)	**-34.6 (-45.1 to -21.5)**	**-58.1 (-66.5 to -49.4)**
17 Drowning	17 Breast cancer	**140 (66.7 to 232)**	29.1 (-8.39 to 81.2)	-0.663 (-25 to 28.2)
18 Rheumatic heart disease	18 Prostate cancer	**263 (148 to 408)**	**94.9 (36.2 to 163)**	3.93 (-13 to 23.2)
19 Pertussis	19 Stomach cancer	**79.4 (50.1 to 114)**	-3.6 (-15.8 to 9.85)	**-33.8 (-38.1 to -28.7)**
20 Measles	20 Cirrhosis liver	**45.4 (19.4 to 75.5)**	**-21.8 (-34.3 to -4.23)**	**-37.6 (-49.1 to -21.9)**
22 Breast cancer	22 Drowning			
23 Stomach cancer	24 Pertussis			
24 Cirrhosis liver	27 Rheumatic heart disease			
27 Alzheimer's disease	67 Measles			
33 Prostate cancer	132 COVID-19			

Supplemental Results Figure S8. Leading 20 level 3 causes of deaths, and percentage change in number of deaths and all-age and age-standardised death rates by location, 2022 and 2050

Southeast Asia, Reference scenario

Legend:
Communicable, maternal, neonatal and nutritional
Non-communicable
Injuries

Leading Causes 2022	Leading Causes 2050	Mean % change number of DEATH	Mean % change all-age DEATH rate	Mean % change age-standardized DEATH rate
1 Stroke	1 Stroke	**42.1 (23 to 62.4)**	**25.9 (7 to 45.3)**	**-43.6 (-53.3 to -32.6)**
2 Ischemic heart disease	2 Ischemic heart disease	22.7 (-7.77 to 56.7)	8.75 (-18.8 to 40.5)	**-50.2 (-64.9 to -30.3)**
3 Lower respiratory infect	3 COPD	**131 (92.3 to 168)**	**105 (71.7 to 141)**	-17.6 (-34.8 to 3.99)
4 COPD	4 Chronic kidney disease	**171 (121 to 229)**	**140 (92.1 to 200)**	18.3 (-8.38 to 58)
5 Diabetes	5 Lower respiratory infect	**87 (69.9 to 106)**	**65.5 (50.4 to 81.2)**	**-30.8 (-38.4 to -21.2)**
6 Cirrhosis liver	6 Hypertensive heart disease	**174 (108 to 244)**	**142 (82.3 to 211)**	7.82 (-23.6 to 47.6)
7 Chronic kidney disease	7 Alzheimer's disease	**268 (201 to 339)**	**225 (172 to 283)**	-0.0147 (-3.01 to 2.64)
8 Tuberculosis	8 Cirrhosis liver	**75.7 (55.8 to 95)**	**55.5 (37.4 to 73.1)**	-7.36 (-18.4 to 6.24)
9 Lung cancer	9 Diabetes	**67.7 (38.3 to 101)**	**48.6 (20.9 to 77.9)**	**-28.9 (-44.4 to -9.82)**
10 Hypertensive heart disease	10 Lung cancer	**110 (56.4 to 161)**	**85.4 (46 to 129)**	-3.87 (-20.9 to 12.9)
11 COVID-19	11 Colorectal cancer	**141 (71.4 to 213)**	**113 (55.9 to 174)**	8.37 (-15.8 to 34.3)
12 Road injuries	12 Diarrheal diseases	**95.8 (35.8 to 174)**	**73.5 (19.6 to 145)**	-26.8 (-49.4 to 10)
13 Alzheimer's disease	13 Tuberculosis	-15.1 (-36.9 to 8.27)	**-24.8 (-44.3 to -2.54)**	**-59.5 (-70.9 to -43.3)**
14 Neonatal disorders	14 Road injuries	6.69 (-18.9 to 43.2)	-5.47 (-29.3 to 30.6)	-20.3 (-42.1 to 10.9)
15 Colorectal cancer	15 Breast cancer	**93.2 (47 to 144)**	**70.8 (29.6 to 112)**	5.62 (-14.8 to 29.1)
16 Diarrheal diseases	16 Falls	**121 (80.2 to 157)**	**95.4 (62.2 to 124)**	**-10.4 (-15.1 to -5.75)**
17 Breast cancer	17 Liver cancer	**161 (110 to 232)**	**132 (80.6 to 198)**	**31.2 (0.553 to 76.3)**
18 Asthma	18 Urinary diseases	**223 (188 to 262)**	**186 (154 to 222)**	**21.2 (3.44 to 44.3)**
19 Falls	19 Atrial fibrillation	**338 (259 to 416)**	**287 (223 to 350)**	**17 (10.7 to 23.3)**
20 Liver cancer	20 Parkinson's disease	**226 (186 to 268)**	**189 (159 to 217)**	1.94 (-3.38 to 8.31)
24 Urinary diseases	25 Asthma			
32 Parkinson's disease	35 Neonatal disorders			
35 Atrial fibrillation	134 COVID-19			

Supplemental Results Figure S8. Leading 20 level 3 causes of deaths, and percentage change in number of deaths and all-age and age-standardised death rates by location, 2022 and 2050

Central sub-Saharan Africa, Reference scenario

Legend:
Communicable, maternal, neonatal and nutritional
Non-communicable
Injuries

Leading Causes 2022	Leading Causes 2050	Mean % change number of DEATH	Mean % change all-age DEATH rate	Mean % change age-standardized DEATH rate
1 Malaria	1 Lower respiratory infect	**72.1 (35.2 to 115)**	-3.93 (-19.3 to 11.6)	**-29.8 (-38.3 to -19.9)**
2 Neonatal disorders	2 Stroke	**73.9 (33 to 123)**	-2.85 (-20.9 to 18.2)	**-47.6 (-57.8 to -34.3)**
3 Tuberculosis	3 Ischemic heart disease	**79.6 (25.3 to 157)**	0.42 (-29.2 to 42.3)	**-45.7 (-62.8 to -20.5)**
4 Lower respiratory infect	4 Hypertensive heart disease	**233 (153 to 332)**	**86.1 (47.3 to 131)**	-9.45 (-29.3 to 16.8)
5 Stroke	5 Road injuries	28.4 (-27.7 to 120)	-28 (-59.6 to 28.9)	-37.3 (-63.1 to 6.73)
6 Road injuries	6 Chronic kidney disease	**205 (134 to 302)**	**70.5 (32.4 to 118)**	2.9 (-19.5 to 32.7)
7 Ischemic heart disease	7 Tuberculosis	-15 (-40 to 18.1)	**-52.5 (-66.3 to -32)**	**-66.7 (-75.9 to -52.1)**
8 COVID-19	8 Diabetes	**136 (76.3 to 215)**	**31.8 (2.13 to 68.5)**	-23.1 (-40.5 to 0.0808)
9 HIV/AIDS	9 Cirrhosis liver	**98.3 (58.3 to 149)**	10.8 (-5.91 to 29.7)	**-26.8 (-36.8 to -14.4)**
10 Diarrheal diseases	10 Neonatal disorders	**-39.6 (-57.4 to -19.7)**	**-66.3 (-74.5 to -55.7)**	**-48.3 (-59.6 to -34.9)**
11 Cirrhosis liver	11 Malaria	-53.7 (-94.8 to 176)	-74.4 (-97.3 to 56.1)	-70 (-96.4 to 78.5)
12 Hypertensive heart disease	12 COPD	**139 (82.2 to 207)**	**33.3 (7.17 to 62.7)**	**-30.7 (-43.9 to -14.9)**
13 Diabetes	13 Diarrheal diseases	21.9 (-15.4 to 75.5)	**-31.9 (-52.2 to -4.59)**	**-37.9 (-52.8 to -15.4)**
14 Chronic kidney disease	14 Alzheimer's disease	**338 (207 to 488)**	**144 (82.2 to 202)**	0.369 (-2.54 to 3.87)
15 Congenital defects	15 Self-harm	**120 (72.1 to 173)**	**22.6 (3.01 to 43.2)**	-13.7 (-26.9 to 0.115)
16 COPD	16 HIV/AIDS	**-41.2 (-62.6 to -1.47)**	**-67.2 (-78.1 to -47)**	**-73.1 (-81.5 to -57.7)**
17 Maternal disorders	17 Breast cancer	**214 (117 to 332)**	**75.2 (30.4 to 119)**	10.6 (-9.2 to 31.2)
18 Pertussis	18 Cervical cancer	**107 (62.1 to 159)**	15.7 (-1.87 to 41)	**-25.5 (-36.3 to -9.66)**
19 Meningitis	19 Cardiomyopathy	**133 (83.2 to 186)**	**30.2 (14.1 to 46.4)**	**-19.9 (-23.7 to -16)**
20 Self-harm	20 Prostate cancer	**337 (181 to 554)**	**143 (68.1 to 226)**	21.3 (-2.92 to 47.8)
22 Cervical cancer	23 Congenital defects			
26 Alzheimer's disease	27 Meningitis			
27 Cardiomyopathy	28 Pertussis			
29 Breast cancer	29 Maternal disorders			
41 Prostate cancer	136 COVID-19			

Supplemental Results Figure S8. Leading 20 level 3 causes of deaths, and percentage change in number of deaths and all-age and age-standardised death rates by location, 2022 and 2050

Eastern sub-Saharan Africa, Reference scenario

Legend:
Communicable, maternal, neonatal and nutritional
Non-communicable
Injuries

Leading Causes 2022	Leading Causes 2050	Mean % change number of DEATH	Mean % change all-age DEATH rate	Mean % change age-standardized DEATH rate
1 Neonatal disorders	1 Stroke	**77.8 (49.7 to 109)**	-1.18 (-17.6 to 18.8)	**-46.4 (-56.3 to -33.3)**
2 Lower respiratory infect	2 Lower respiratory infect	**42.3 (23.1 to 60.5)**	**-20.9 (-32.3 to -8.87)**	**-36.8 (-44.4 to -27.6)**
3 HIV/AIDS	3 Chronic kidney disease	**254 (185 to 332)**	**96.6 (55.1 to 147)**	16.1 (-10.1 to 49.3)
4 Stroke	4 Ischemic heart disease	**76.7 (21.2 to 149)**	-1.75 (-32.3 to 43.3)	**-46.7 (-64.4 to -20.8)**
5 Tuberculosis	5 Diarrheal diseases	18.8 (-16.7 to 70.5)	**-34 (-54.1 to -7.03)**	**-41.8 (-57.9 to -16.1)**
6 COVID-19	6 Neonatal disorders	**-41.4 (-58 to -23.8)**	**-67.5 (-74.9 to -57.8)**	**-48.7 (-59.8 to -33.7)**
7 Diarrheal diseases	7 Hypertensive heart disease	**184 (131 to 246)**	**57.8 (27.2 to 97.4)**	-21.3 (-39.2 to 0.797)
8 Malaria	8 Cirrhosis liver	**97.9 (73.3 to 128)**	10 (-5.75 to 30.1)	**-34 (-42.8 to -22)**
9 Ischemic heart disease	9 Diabetes	**118 (74.4 to 169)**	21.1 (-4.19 to 53.5)	**-31.4 (-46.9 to -12.3)**
10 Cirrhosis liver	10 Tuberculosis	-14.3 (-38.5 to 20.6)	**-52.4 (-65.8 to -31.2)**	**-68.9 (-77.6 to -55.8)**
11 Congenital defects	11 Alzheimer's disease	**306 (227 to 370)**	**125 (85.5 to 165)**	-0.573 (-2.69 to 1.25)
12 Diabetes	12 Road injuries	52.1 (-2.67 to 143)	-15.4 (-46.4 to 36.2)	**-37 (-58.6 to -1.86)**
13 Chronic kidney disease	13 COPD	**122 (90.5 to 155)**	**23.5 (3.26 to 44.5)**	**-35.9 (-47.3 to -21.5)**
14 Road injuries	14 Breast cancer	**240 (168 to 311)**	**88.6 (48.1 to 133)**	13.1 (-4.05 to 33)
15 Hypertensive heart disease	15 Urinary diseases	**232 (204 to 266)**	**84.5 (65.9 to 103)**	10.6 (-1.58 to 23.3)
16 Meningitis	16 Self-harm	**117 (88.1 to 148)**	**20.5 (4.17 to 40.3)**	**-18.1 (-28.2 to -5.44)**
17 Protein-energy malnutrition	17 Colorectal cancer	**289 (175 to 386)**	**116 (56.3 to 183)**	19.3 (-5.41 to 45)
18 COPD	18 Falls	**182 (137 to 220)**	**56.5 (32.5 to 78.7)**	**-7.11 (-10.2 to -4)**
19 Maternal disorders	19 Esophageal cancer	**175 (151 to 196)**	**52.7 (36.5 to 68.8)**	**-16.3 (-22.9 to -9.24)**
20 Measles	20 Cervical cancer	**121 (90.3 to 157)**	**22.6 (4.55 to 47.5)**	**-24.8 (-35.6 to -9.74)**
22 Self-harm	21 HIV/AIDS			
24 Cervical cancer	22 Congenital defects			
25 Alzheimer's disease	25 Meningitis			
26 Breast cancer	31 Protein-energy malnutrition			
27 Urinary diseases	32 Malaria			
28 Falls	34 Maternal disorders			
31 Esophageal cancer	89 Measles			

Supplemental Results Figure S8. Leading 20 level 3 causes of deaths, and percentage change in number of deaths and all-age and age-standardised death rates by location, 2022 and 2050

Southern sub-Saharan Africa, Reference scenario

Legend:
Communicable, maternal, neonatal and nutritional
Non-communicable
Injuries

Leading Causes 2022	Leading Causes 2050	Mean % change number of DEATH	Mean % change all-age DEATH rate	Mean % change age-standardized DEATH rate
1 HIV/AIDS	1 Lower respiratory infect	**46.8 (29.1 to 64.2)**	**20.7 (9.74 to 32.1)**	**-23.2 (-29.6 to -15.6)**
2 COVID-19	2 Stroke	**30.3 (15 to 47)**	7.17 (-5.26 to 20.9)	**-45.5 (-51.8 to -37.5)**
3 Stroke	3 Diabetes	**60.3 (37.5 to 85.9)**	**31.9 (15.4 to 52.4)**	**-28.9 (-38.8 to -15.5)**
4 Lower respiratory infect	4 Hypertensive heart disease	**160 (119 to 204)**	**114 (83.6 to 150)**	5.21 (-13.2 to 26.2)
5 Diabetes	5 Ischemic heart disease	**32.3 (2.72 to 66.4)**	8.91 (-14.4 to 39.9)	**-43.6 (-57.4 to -25.8)**
6 Ischemic heart disease	6 Chronic kidney disease	**168 (125 to 210)**	**120 (87.7 to 157)**	**22.3 (2.63 to 45.4)**
7 Tuberculosis	7 COPD	**109 (83.6 to 137)**	**72.1 (53.7 to 94.8)**	**-12.5 (-23.2 to -0.344)**
8 Neonatal disorders	8 HIV/AIDS	**-76 (-79.8 to -72.4)**	**-80.3 (-82.7 to -78)**	**-82.8 (-84.7 to -81.1)**
9 Road injuries	9 Diarrheal diseases	26.1 (-7.15 to 64.2)	3.74 (-22.7 to 33.3)	**-26.2 (-43.5 to -2.19)**
10 Interpersonal violence	10 Road injuries	4.4 (-27.6 to 56.1)	-14.1 (-39.6 to 29.6)	-22.5 (-45.3 to 18.4)
11 Diarrheal diseases	11 Tuberculosis	**-30.1 (-45.6 to -11.5)**	**-42.5 (-55.6 to -25.1)**	**-59.9 (-69.4 to -47.5)**
12 Hypertensive heart disease	12 Alzheimer's disease	**191 (149 to 226)**	**139 (114 to 166)**	-0.438 (-3.19 to 2.32)
13 Chronic kidney disease	13 Prostate cancer	**183 (128 to 242)**	**133 (90.1 to 180)**	16.3 (-1.38 to 33.9)
14 COPD	14 Lung cancer	**86.2 (52.8 to 119)**	**53 (27.2 to 76.4)**	**-12.8 (-24.5 to -0.838)**
15 Self-harm	15 Neonatal disorders	**-28.7 (-43.4 to -11.4)**	**-41.4 (-52.1 to -28.9)**	**-17.8 (-30.9 to -1.07)**
16 Cirrhosis liver	16 Interpersonal violence	**-24.9 (-35.3 to -15.4)**	**-38.3 (-43.9 to -32.4)**	**-39.3 (-44.9 to -33.1)**
17 Lung cancer	17 Cirrhosis liver	**53 (35.8 to 71.3)**	**25.8 (14.7 to 37.4)**	**-19 (-25.7 to -11.6)**
18 Breast cancer	18 Breast cancer	**96.1 (55.2 to 132)**	**61.1 (34.3 to 86.7)**	-3.65 (-15.8 to 9.05)
19 Cervical cancer	19 Self-harm	**17 (1.2 to 32.5)**	-3.78 (-14.7 to 8.39)	**-14.8 (-24.2 to -3.91)**
20 Alzheimer's disease	20 Colorectal cancer	**130 (78.1 to 181)**	**89 (51.5 to 128)**	5.37 (-11.9 to 23.6)
22 Prostate cancer	24 Cervical cancer			
26 Colorectal cancer	133 COVID-19			

Supplemental Results Figure S8. Leading 20 level 3 causes of deaths, and percentage change in number of deaths and all-age and age-standardised death rates by location, 2022 and 2050

Western sub-Saharan Africa, Reference scenario

Legend:
Communicable, maternal, neonatal and nutritional
Non-communicable
Injuries

Leading Causes 2022	Leading Causes 2050	Mean % change number of DEATH	Mean % change all-age DEATH rate	Mean % change age-standardized DEATH rate
1 Neonatal disorders	1 Lower respiratory infect	**21.6 (5.36 to 39.6)**	**-37.4 (-45.6 to -27.3)**	**-34.1 (-42 to -24.6)**
2 Malaria	2 Malaria	-14.4 (-81.1 to 221)	-55.9 (-89.7 to 62.5)	-57.3 (-89.6 to 56.1)
3 Lower respiratory infect	3 Stroke	**80 (54.7 to 109)**	-7.31 (-22.2 to 8.77)	**-46.1 (-55.7 to -34.2)**
4 Diarrheal diseases	4 Neonatal disorders	**-22.6 (-38.3 to -3.08)**	**-60.2 (-67.7 to -49.2)**	**-41.2 (-52.2 to -27.2)**
5 Stroke	5 Ischemic heart disease	**91 (40.9 to 152)**	-1.63 (-28.3 to 32.4)	**-44 (-60.5 to -21.8)**
6 Ischemic heart disease	6 Chronic kidney disease	**267 (203 to 341)**	**89.1 (52.2 to 132)**	20.3 (-4.57 to 52.7)
7 HIV/AIDS	7 Diarrheal diseases	-17.5 (-42.7 to 18.3)	**-57.5 (-70.9 to -41)**	**-51.4 (-64.4 to -32.2)**
8 Congenital defects	8 Diabetes	**149 (107 to 196)**	**28.1 (3.03 to 52.9)**	**-22.4 (-38.1 to -3.55)**
9 Tuberculosis	9 Hypertensive heart disease	**192 (135 to 260)**	**50.2 (22.1 to 86.5)**	-15.6 (-33.9 to 7.46)
10 Cirrhosis liver	10 Cirrhosis liver	**84.4 (60.6 to 113)**	-5.04 (-18.5 to 10.7)	**-35.7 (-44.1 to -24.7)**
11 Road injuries	11 Road injuries	52.5 (-1.5 to 141)	-21.4 (-49.5 to 26)	-35.5 (-56.9 to 1.1)
12 Diabetes	12 Tuberculosis	-3.99 (-29.5 to 32.4)	**-50.5 (-64.6 to -31.4)**	**-64.8 (-74.1 to -51)**
13 Chronic kidney disease	13 Prostate cancer	**286 (181 to 397)**	**98.7 (43.6 to 151)**	4.06 (-15.2 to 23.8)
14 Meningitis	14 Congenital defects	-14.6 (-29.1 to 3.84)	**-56.1 (-62.4 to -47.7)**	**-38.4 (-45.7 to -27.6)**
15 Maternal disorders	15 Breast cancer	**241 (155 to 324)**	**75.4 (35.8 to 121)**	16.9 (-4.65 to 39.3)
16 Hypertensive heart disease	16 Alzheimer's disease	**327 (248 to 414)**	**119 (79.6 to 163)**	**4.89 (1.75 to 7.74)**
17 COVID-19	17 COPD	**124 (92.5 to 159)**	15.4 (-3.15 to 35.2)	**-35.3 (-47.1 to -20.8)**
18 iNTS	18 Falls	**173 (131 to 221)**	**40.6 (19.7 to 61.3)**	**-8.51 (-11.7 to -4.99)**
19 Pertussis	19 HIV/AIDS	**-59 (-68.7 to -52.1)**	**-78.9 (-83.6 to -75.6)**	**-82.1 (-86.1 to -79.5)**
20 COPD	20 Liver cancer	**185 (138 to 242)**	**47 (20.3 to 80.8)**	-5.88 (-23.6 to 18.2)
25 Breast cancer	21 Meningitis			
27 Prostate cancer	24 Maternal disorders			
29 Falls	26 iNTS			
30 Liver cancer	31 Pertussis			
31 Alzheimer's disease	136 COVID-19			

Supplemental Results Figure S9. Population aging by global and GBD super region, 1990-2050

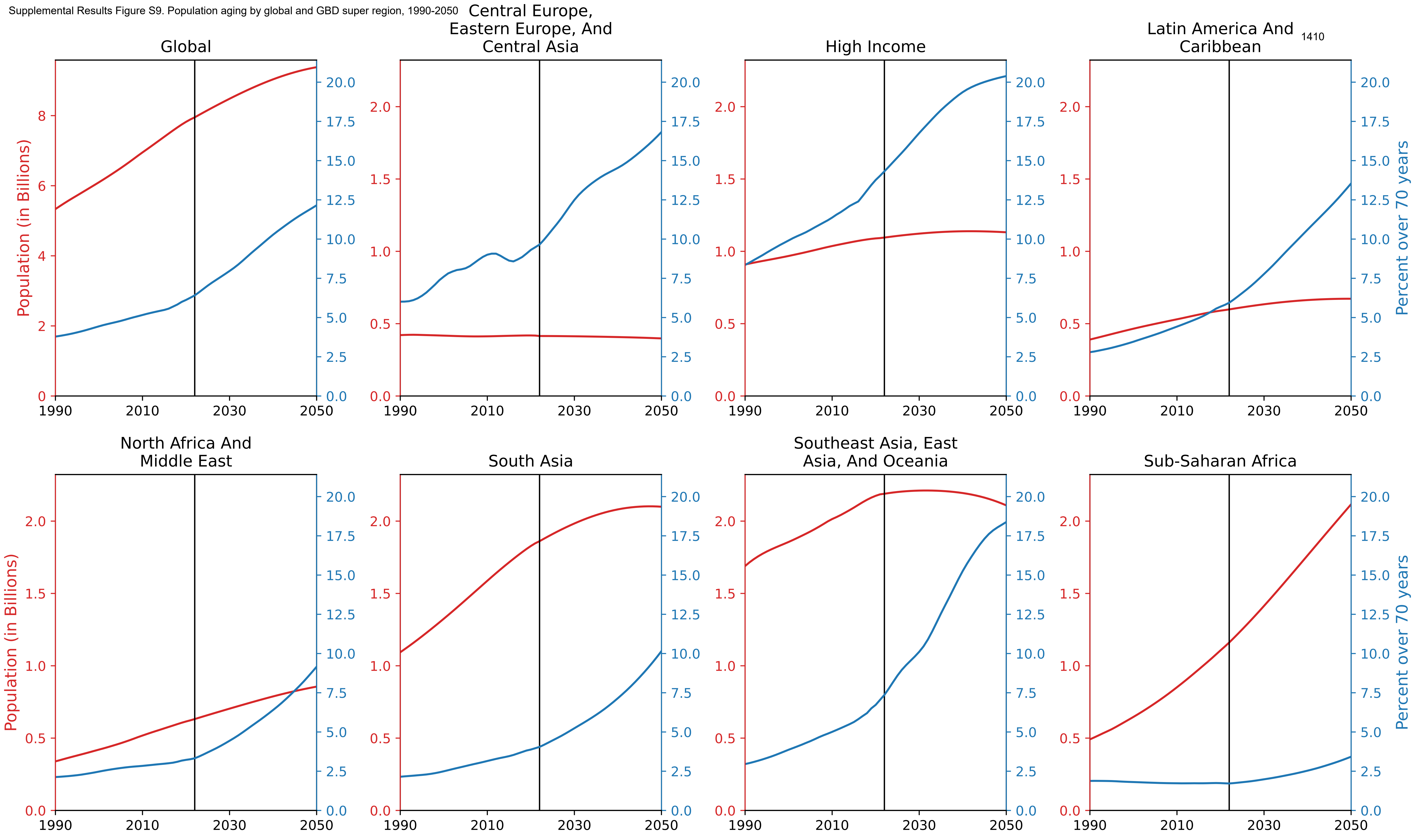

Armenia

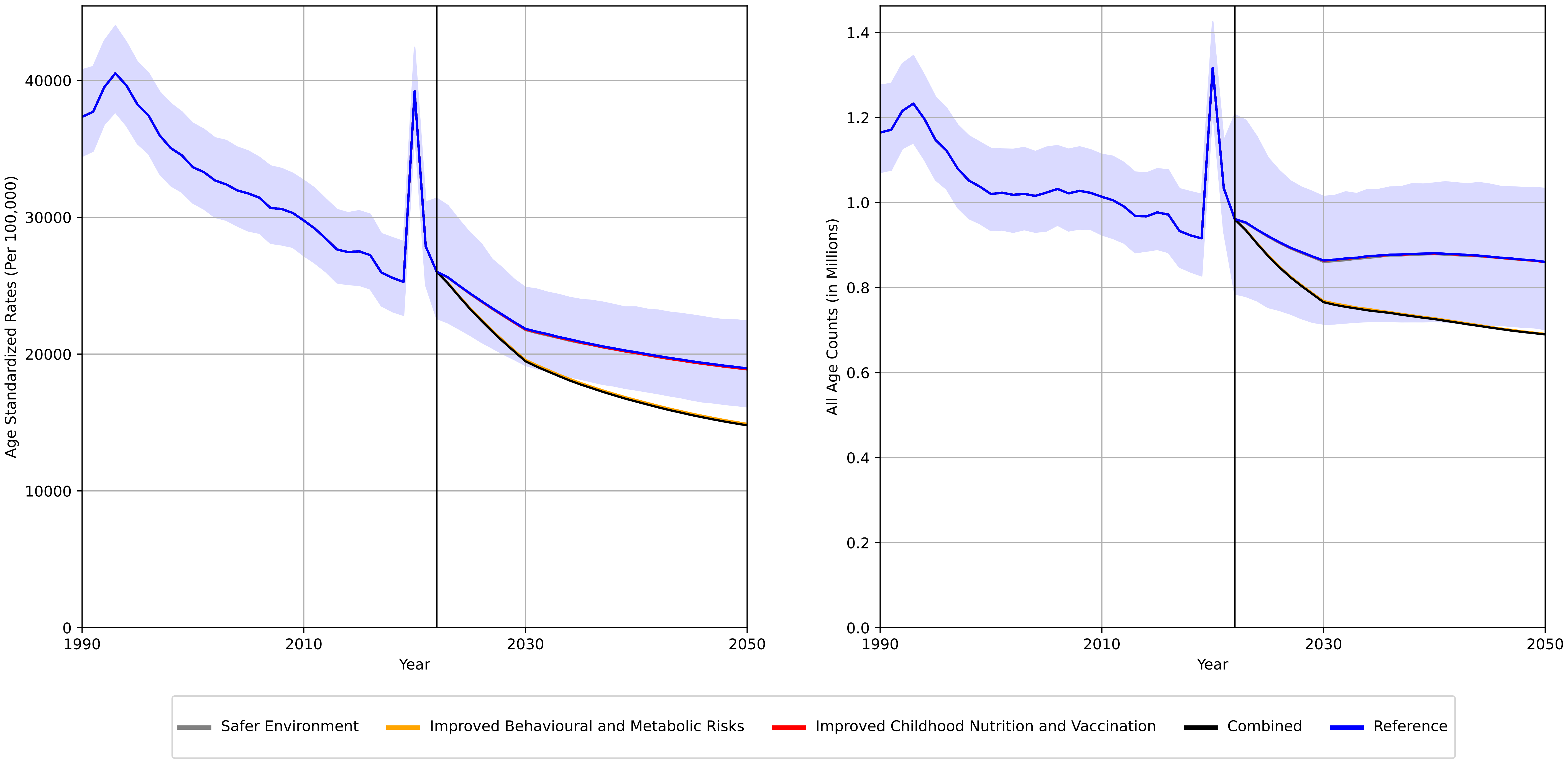
Age Standardized Rates (Per 100,000)
40000
30000
20000
10000
0
1990
2010
2030
2050
Year
All Age Counts (in Millions)
1.4
1.2
1.0
0.8
0.6
0.4
0.2
0.0
Safer Environment
Improved Behavioural and Metabolic Risks
Improved Childhood Nutrition and Vaccination
Combined
Reference

Azerbaijan

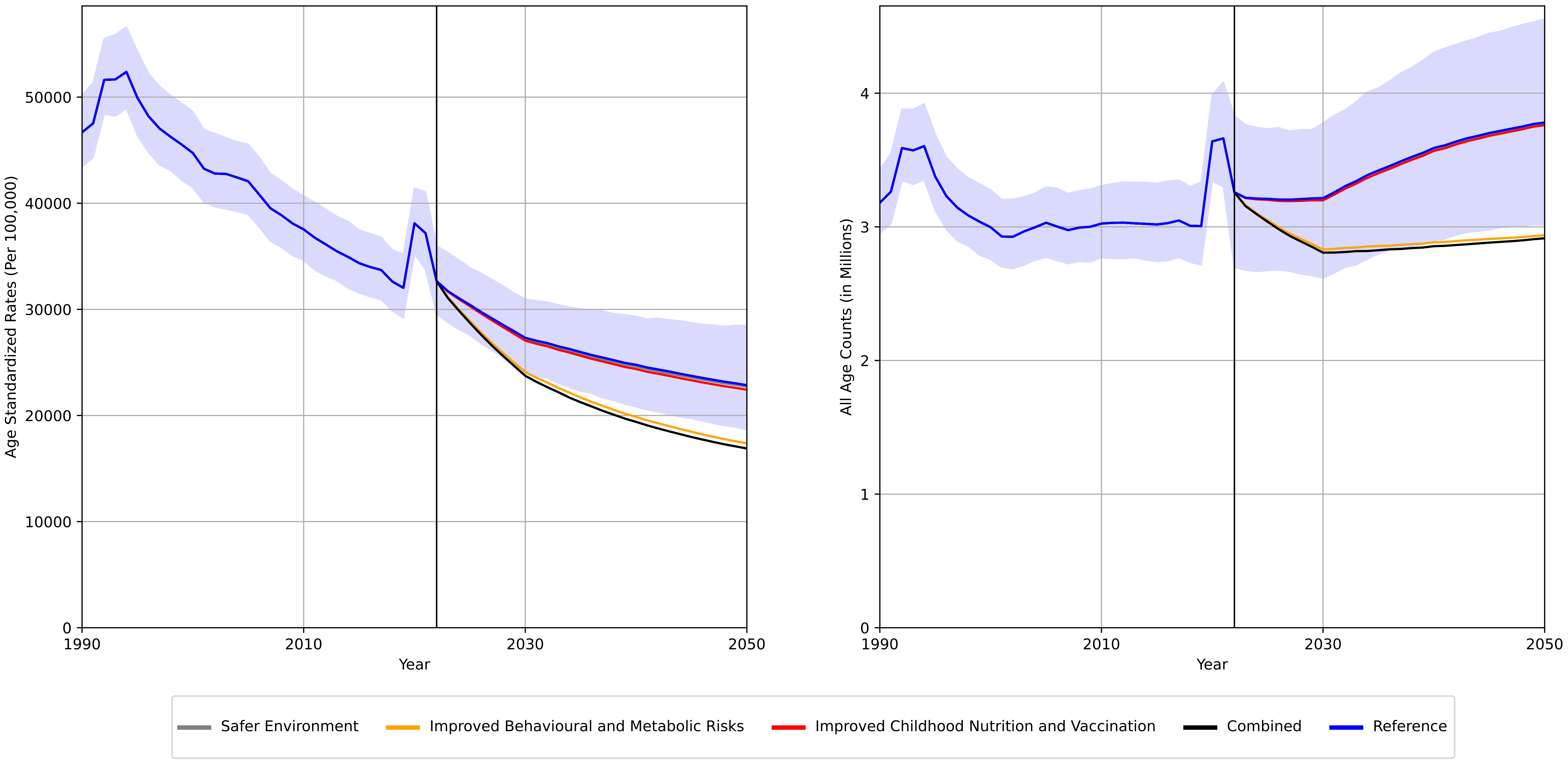

Age Standardized Rates (Per 100,000)
0
10000
20000
30000
40000
50000
1990
2010
2030
2050
Year
All Age Counts (in Millions)
0
1
2
3
4
1990
2010
2030
2050
Year
Safer Environment
Improved Behavioural and Metabolic Risks
Improved Childhood Nutrition and Vaccination
Combined
Reference

Georgia

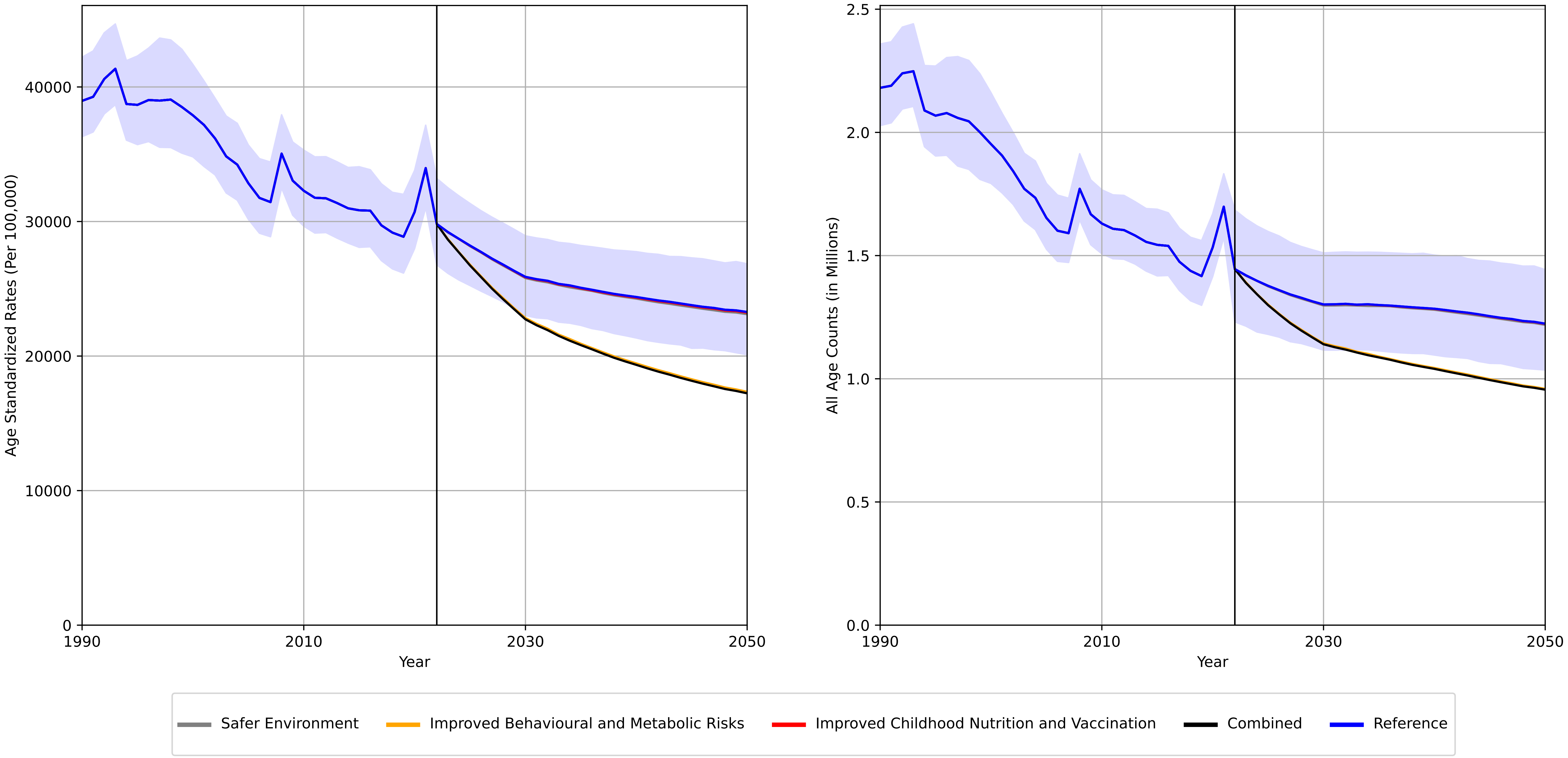
Age Standardized Rates (Per 100,000)
40000
30000
20000
10000
0
1990
2010
2030
2050
Year
All Age Counts (in Millions)
2.5
2.0
1.5
1.0
0.5
0.0
1990
2010
2030
2050
Year
Safer Environment
Improved Behavioural and Metabolic Risks
Improved Childhood Nutrition and Vaccination
Combined
Reference

Kazakhstan

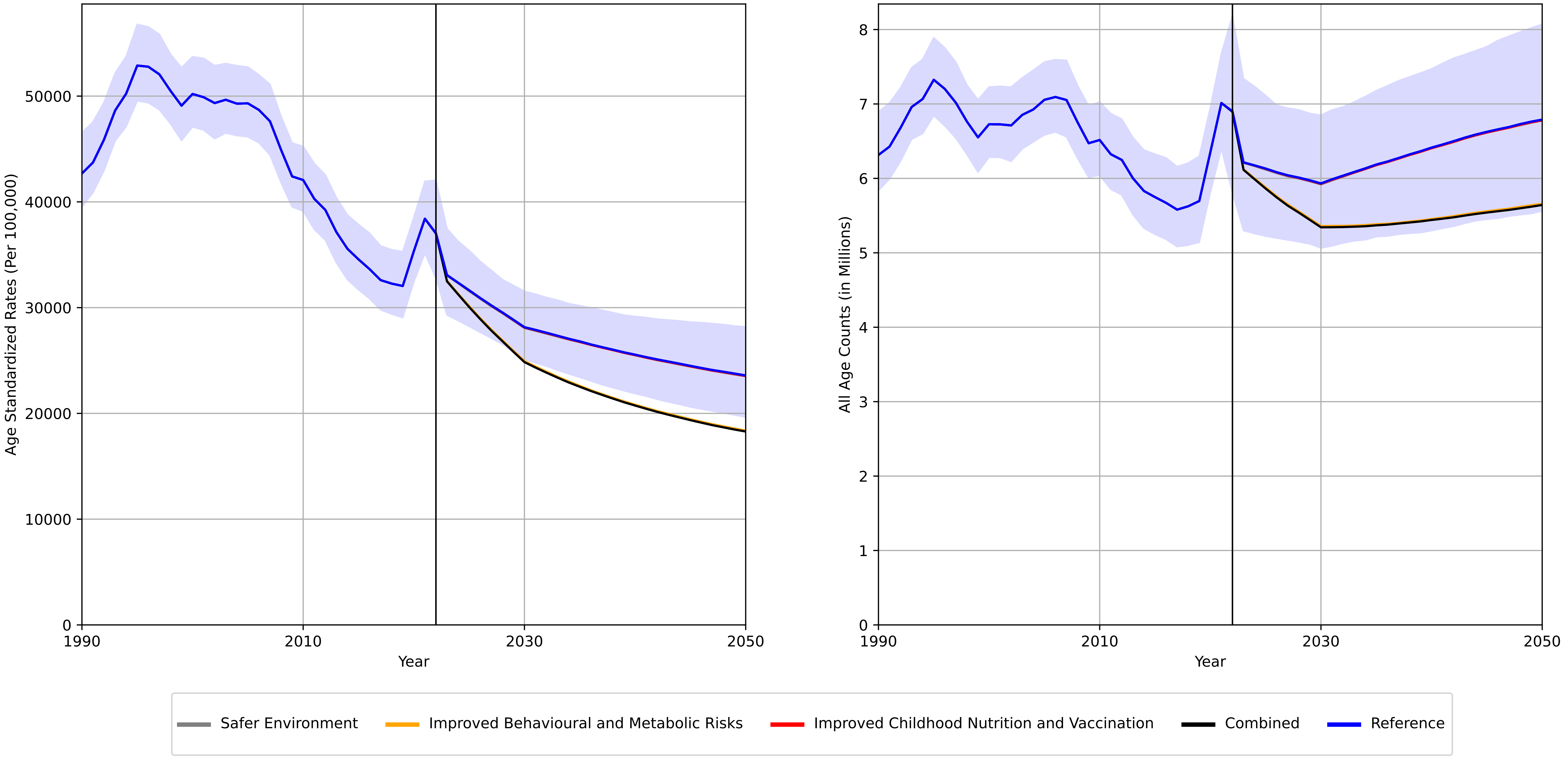
Age Standardized Rates (Per 100,000)
0
10000
20000
30000
40000
50000
1990
2010
2030
2050
Year
All Age Counts (in Millions)
0
1
2
3
4
5
6
7
8
Safer Environment
Improved Behavioural and Metabolic Risks
Improved Childhood Nutrition and Vaccination
Combined
Reference

Kyrgyzstan

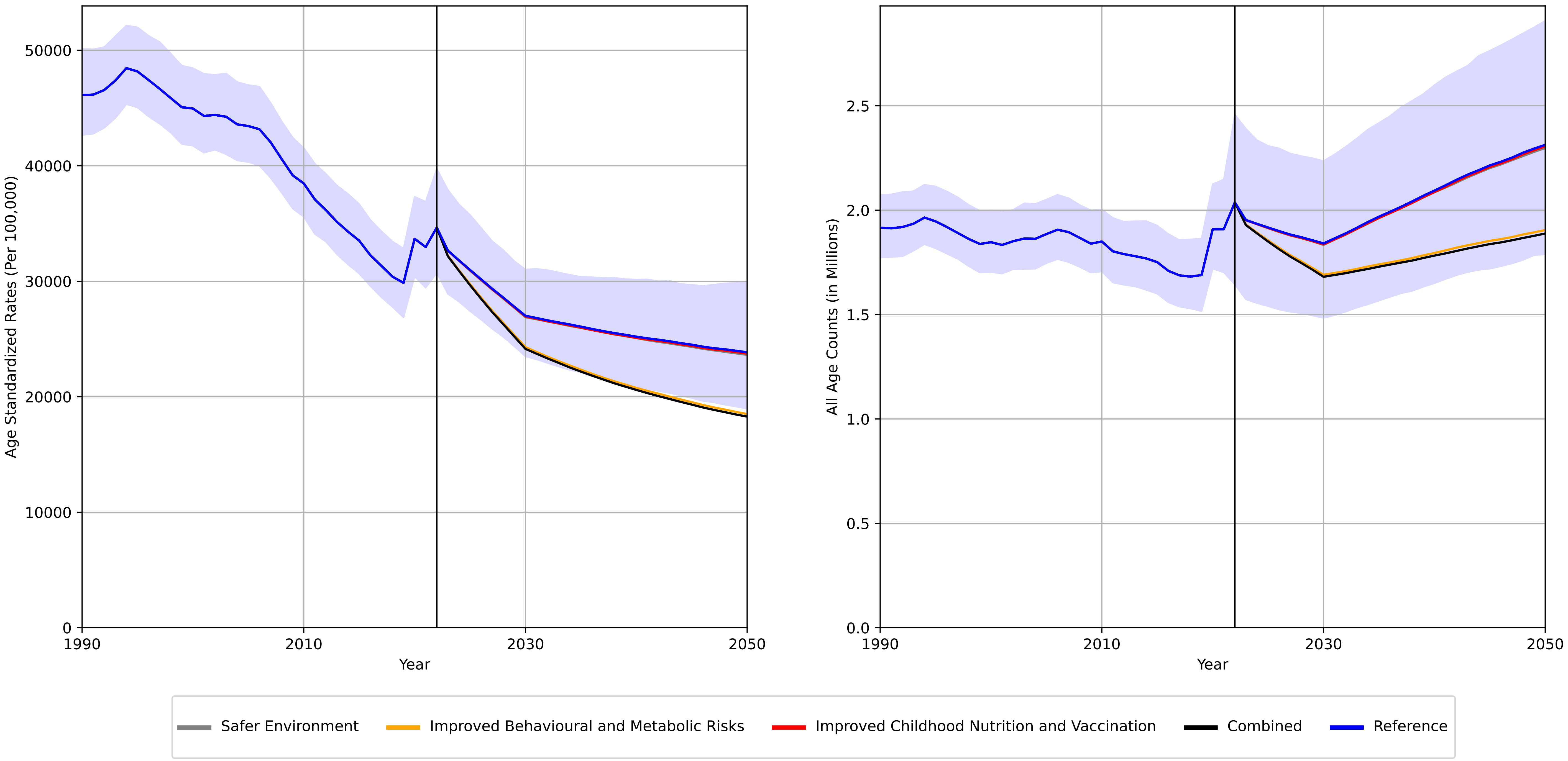

Age Standardized Rates (Per 100,000)
50000
40000
30000
20000
10000
0
1990
2010
2030
2050
Year
All Age Counts (in Millions)
2.5
2.0
1.5
1.0
0.5
0.0
1990
2010
2030
2050
Year
Safer Environment
Improved Behavioural and Metabolic Risks
Improved Childhood Nutrition and Vaccination
Combined
Reference

Mongolia

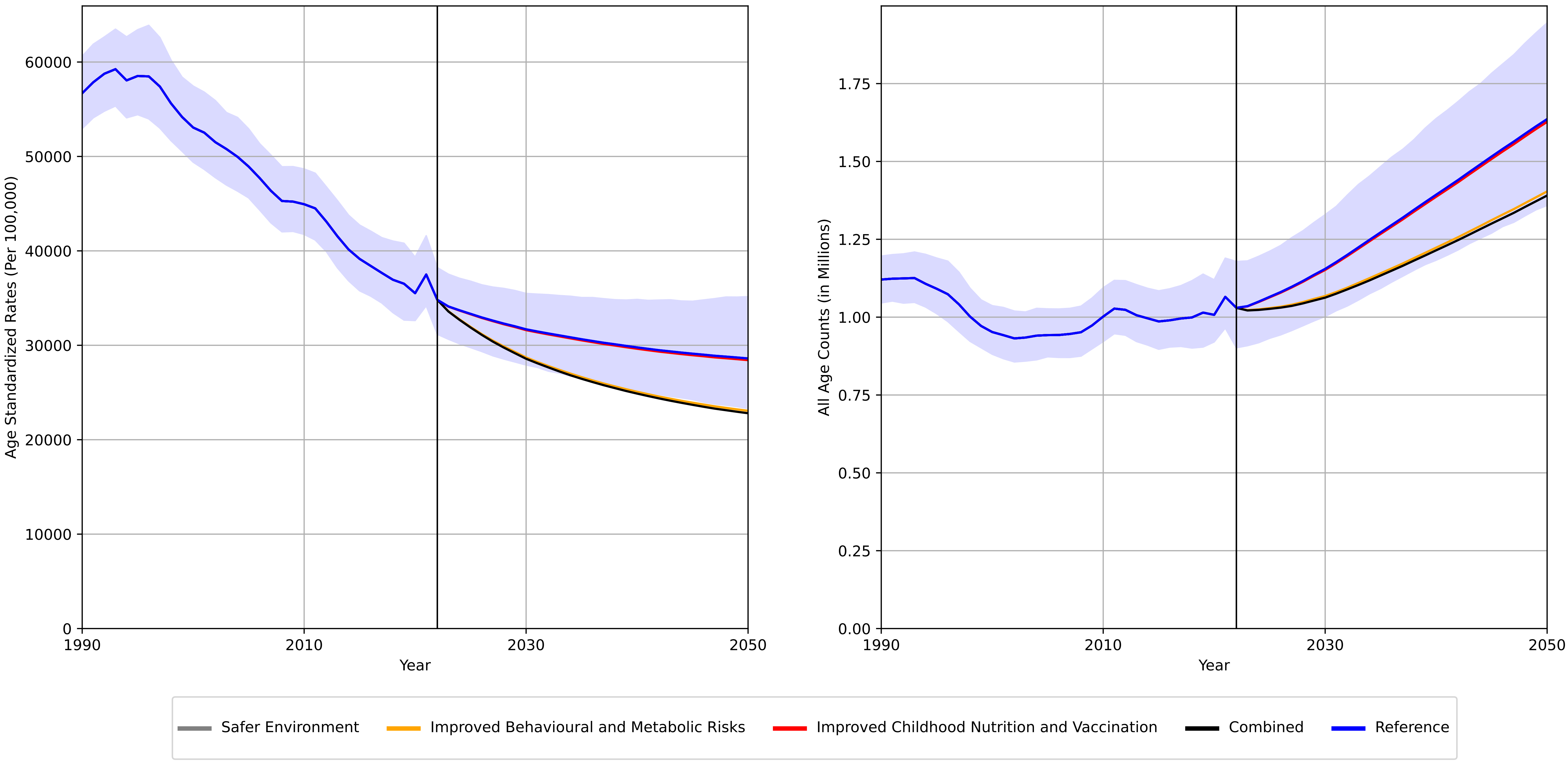

Age Standardized Rates (Per 100,000)
60000
50000
40000
30000
20000
10000
0
1990
2010
2030
2050
Year
All Age Counts (in Millions)
1.75
1.50
1.25
1.00
0.75
0.50
0.25
0.00
Safer Environment
Improved Behavioural and Metabolic Risks
Improved Childhood Nutrition and Vaccination
Combined
Reference

Tajikistan

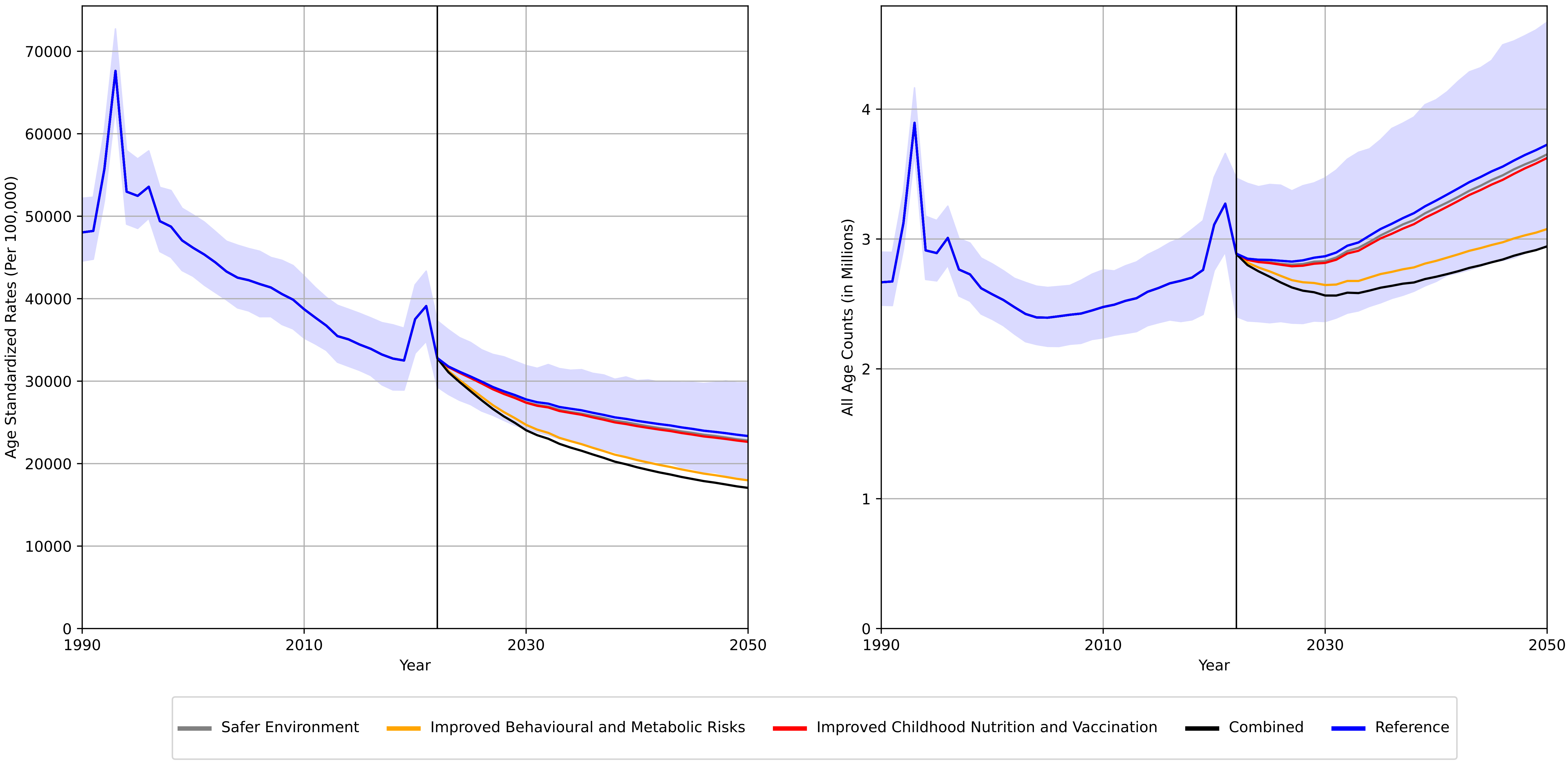

Age Standardized Rates (Per 100,000)
0
10000
20000
30000
40000
50000
60000
70000
1990
2010
2030
2050
Year
All Age Counts (in Millions)
0
1
2
3
4
1990
2010
2030
2050
Year
Safer Environment
Improved Behavioural and Metabolic Risks
Improved Childhood Nutrition and Vaccination
Combined
Reference

Turkmenistan

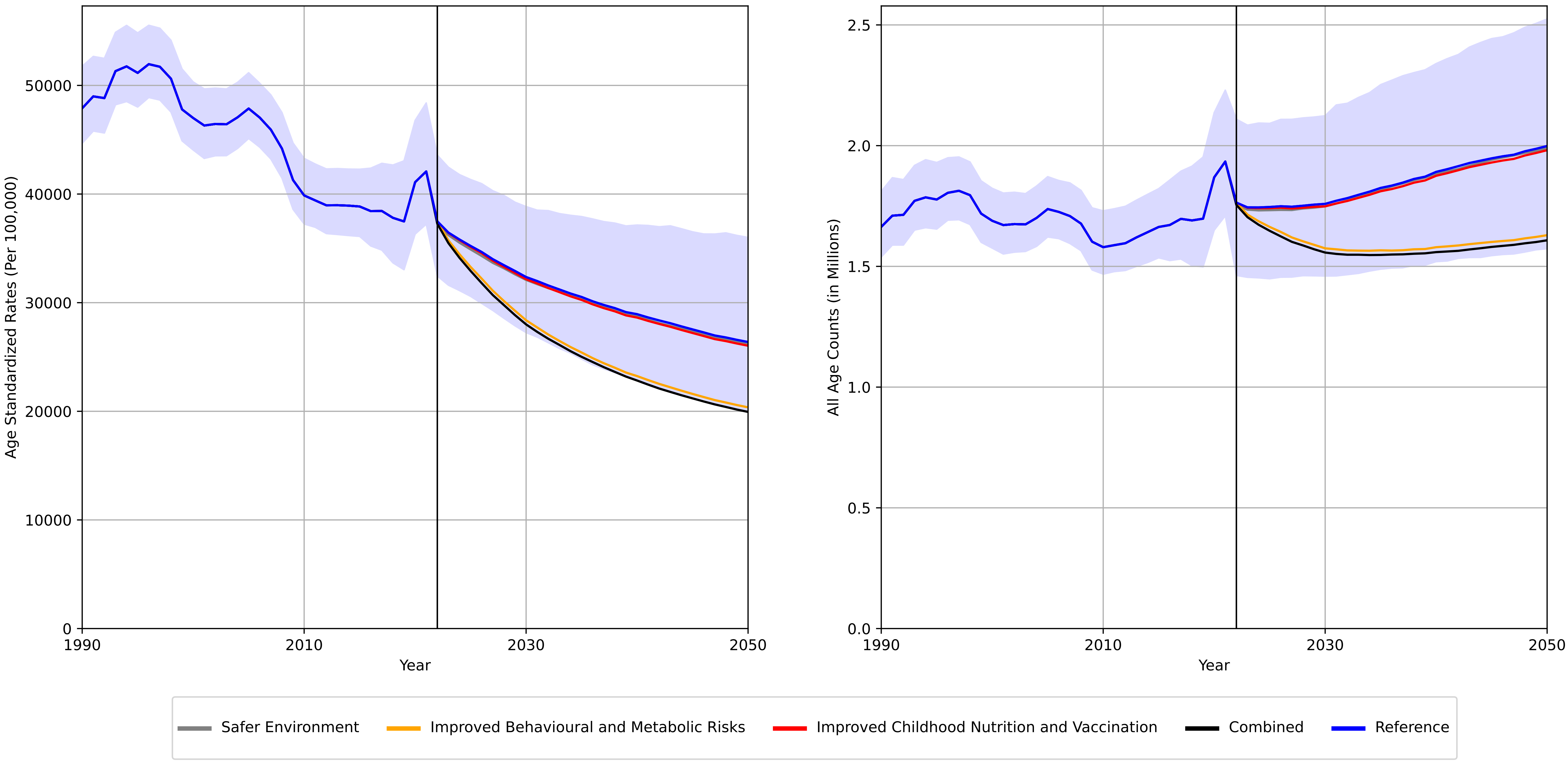

Uzbekistan

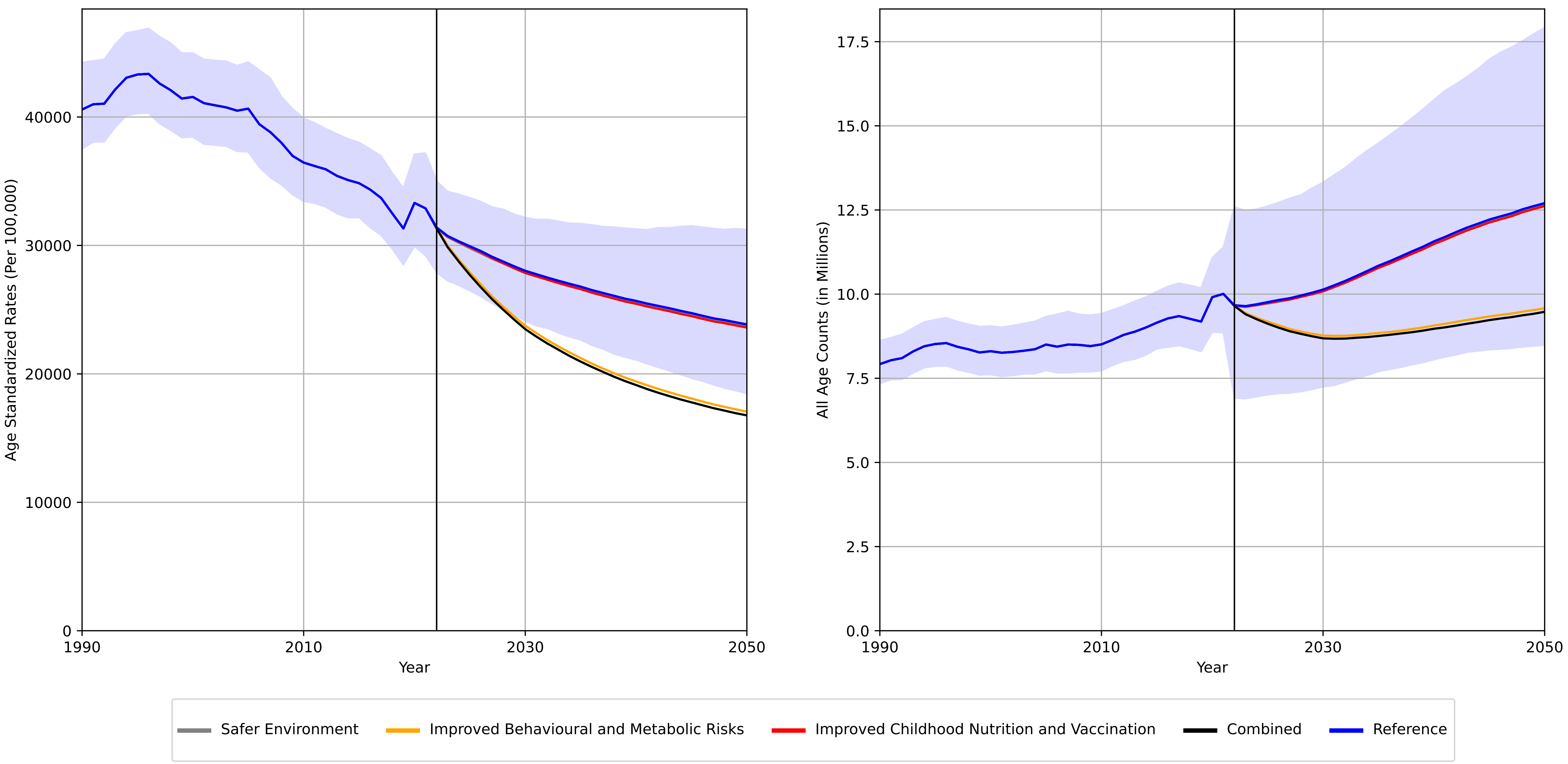
Age Standardized Rates (Per 100,000)
0
10000
20000
30000
40000
1990
2010
2030
2050
Year
All Age Counts (in Millions)
0.0
2.5
5.0
7.5
10.0
12.5
15.0
17.5
1990
2010
2030
2050
Year
Safer Environment
Improved Behavioural and Metabolic Risks
Improved Childhood Nutrition and Vaccination
Combined
Reference

Albania

Supplemental Results Figure S10. All-cause age standardised DALY rates (A) and all age DALY counts (B), for the past and for five future scenarios, 1990–2050

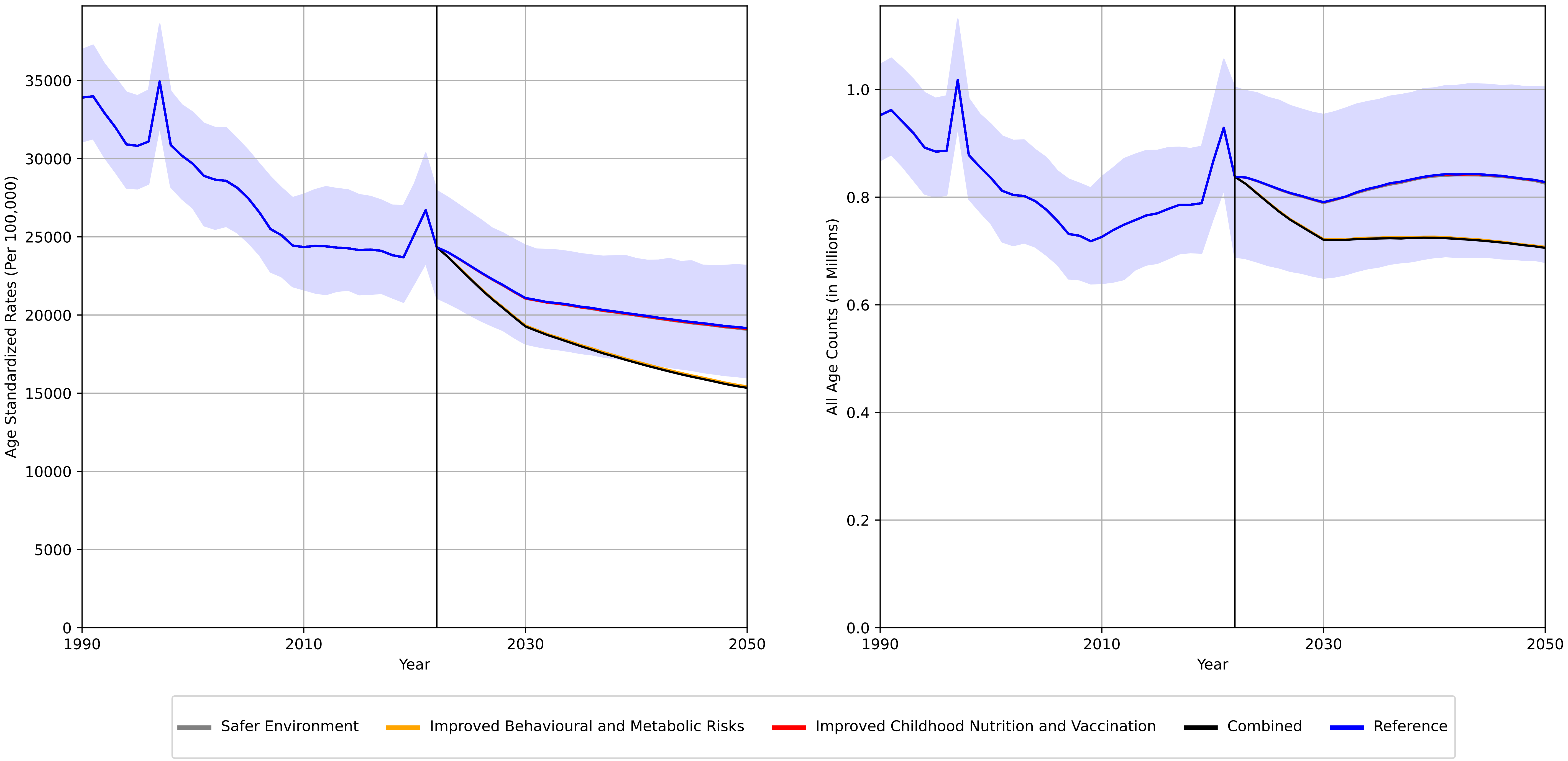

Bosnia And Herzegovina

Supplemental Results Figure S10. All-cause age standardised DALY rates (A) and all age DALY counts (B), for the past and for five future scenarios, 1990–2050

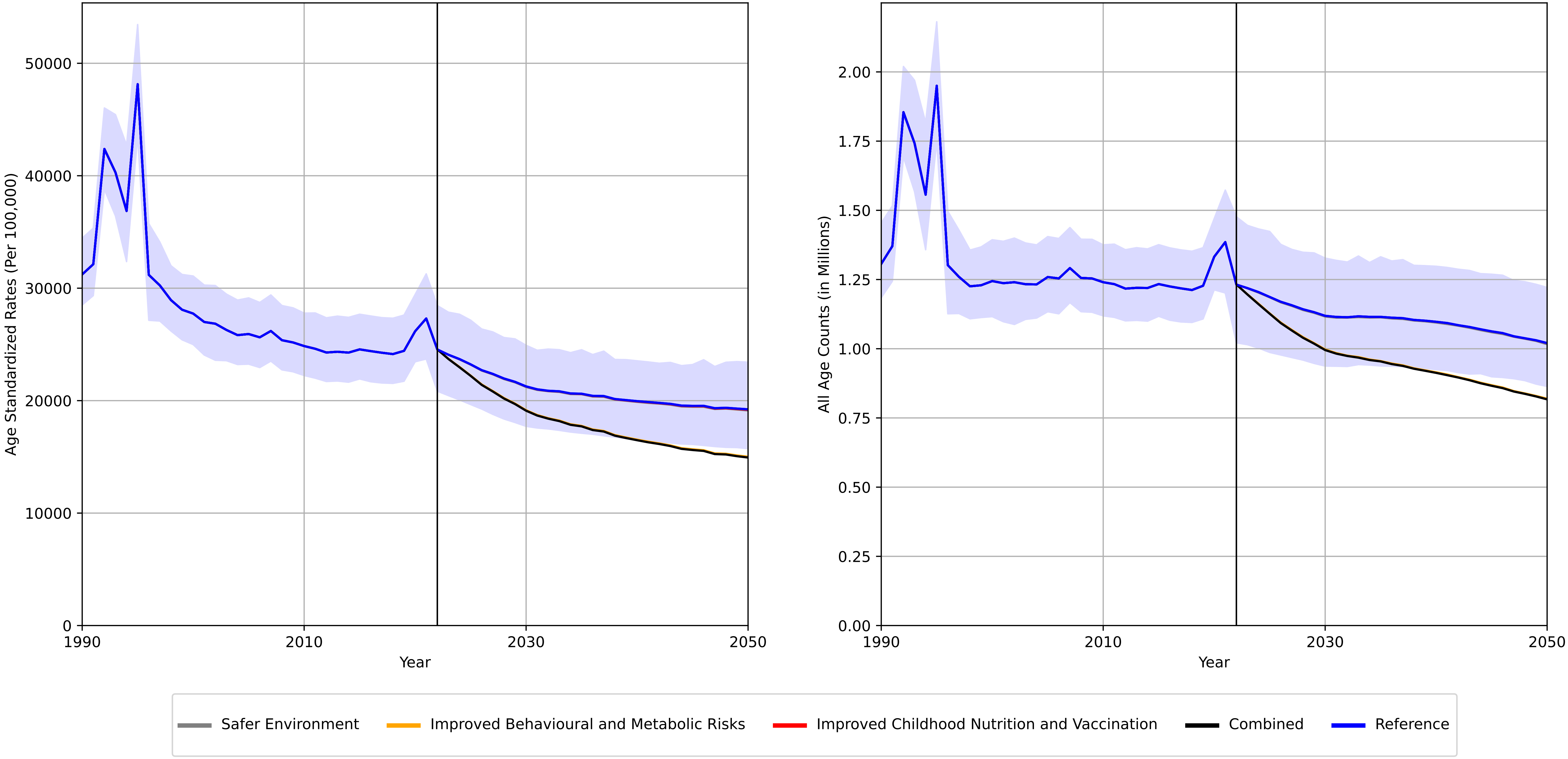

Bulgaria

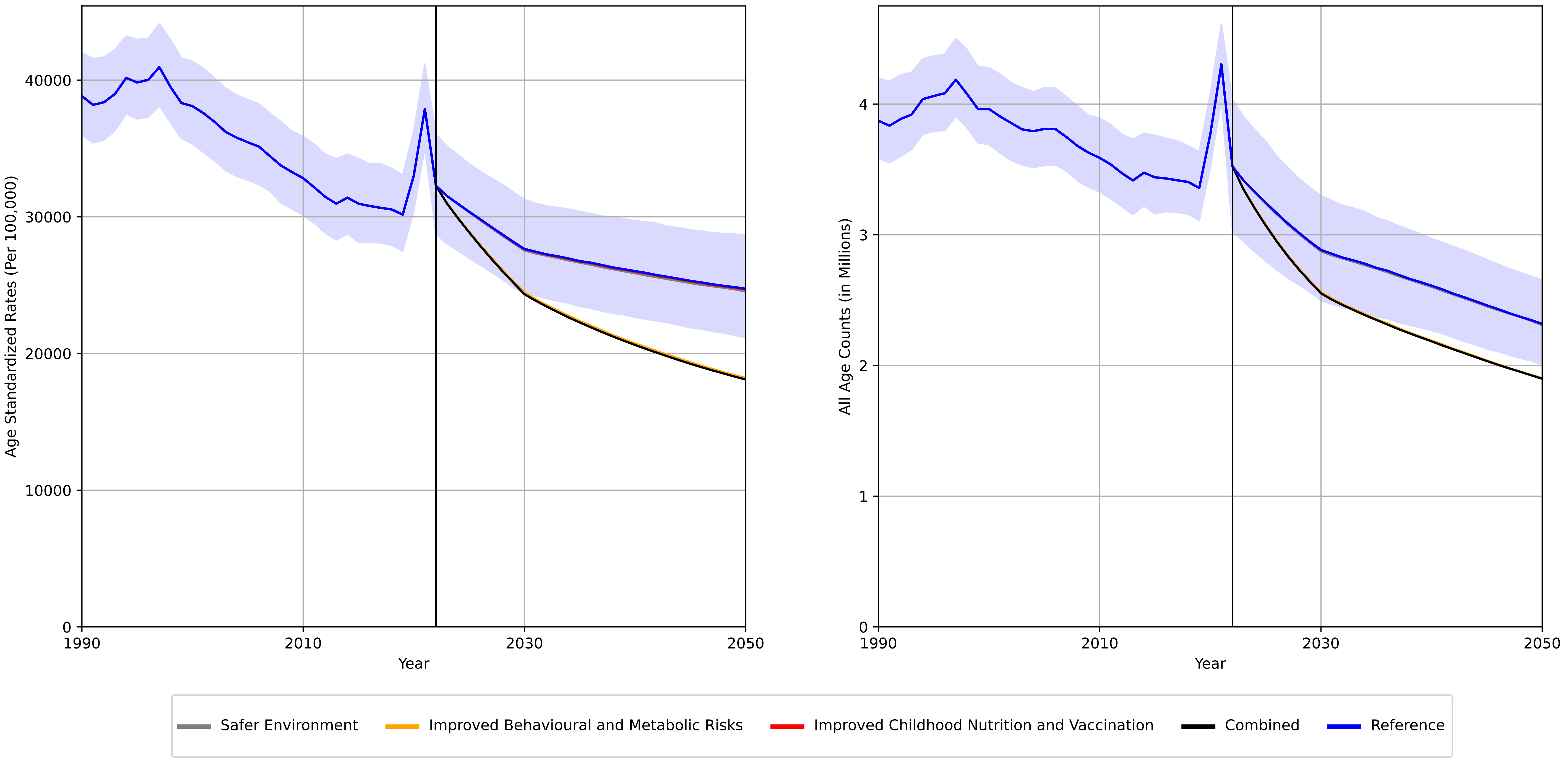
Age Standardized Rates (Per 100,000)
Year
All Age Counts (in Millions)
Year
Safer Environment
Improved Behavioural and Metabolic Risks
Improved Childhood Nutrition and Vaccination
Combined
Reference

Croatia

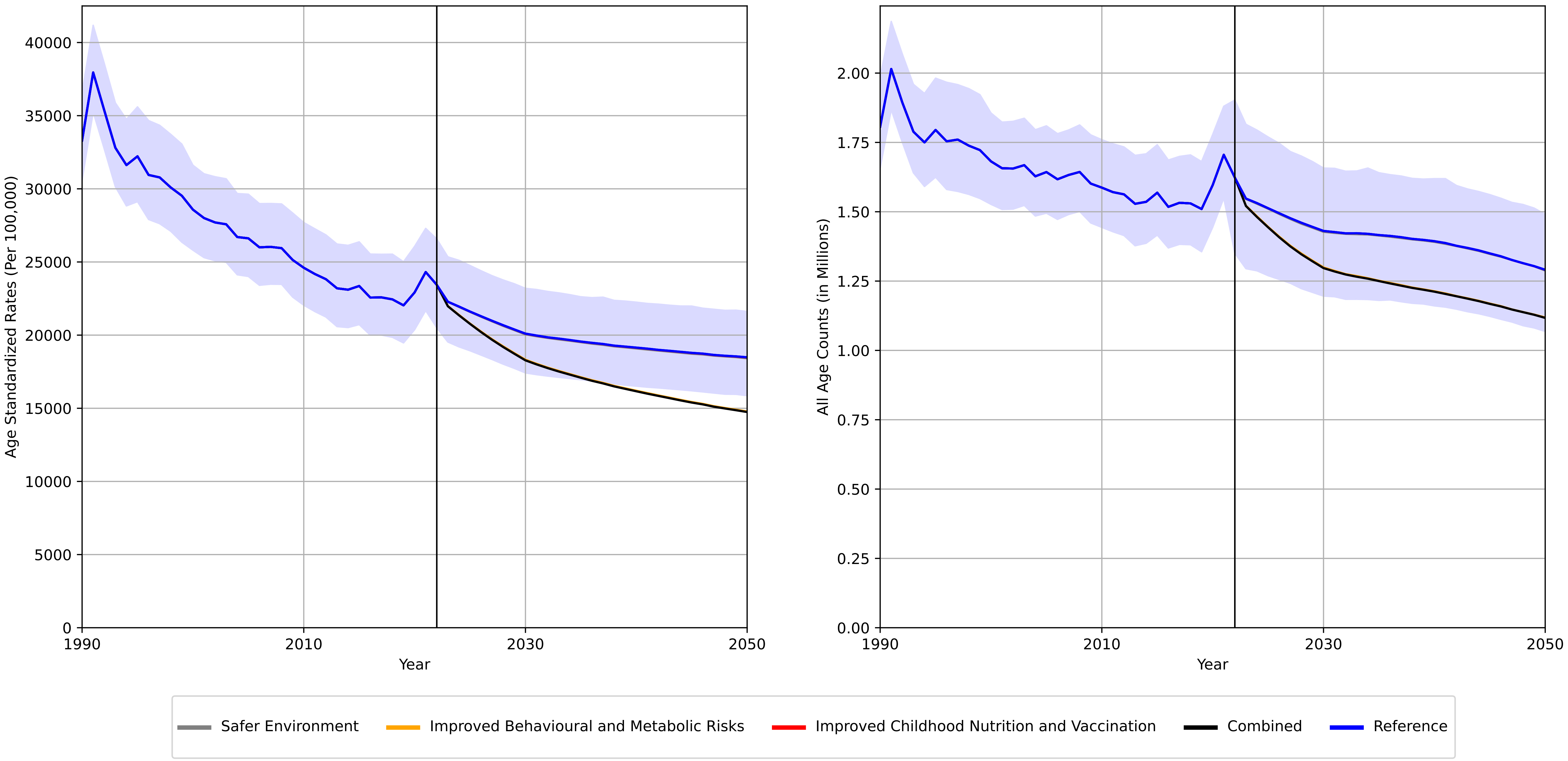
Age Standardized Rates (Per 100,000)
40000
35000
30000
25000
20000
15000
10000
5000
0
1990
2010
2030
2050
Year
All Age Counts (in Millions)
2.00
1.75
1.50
1.25
1.00
0.75
0.50
0.25
0.00
1990
2010
2030
2050
Year
Safer Environment
Improved Behavioural and Metabolic Risks
Improved Childhood Nutrition and Vaccination
Combined
Reference

Czechia

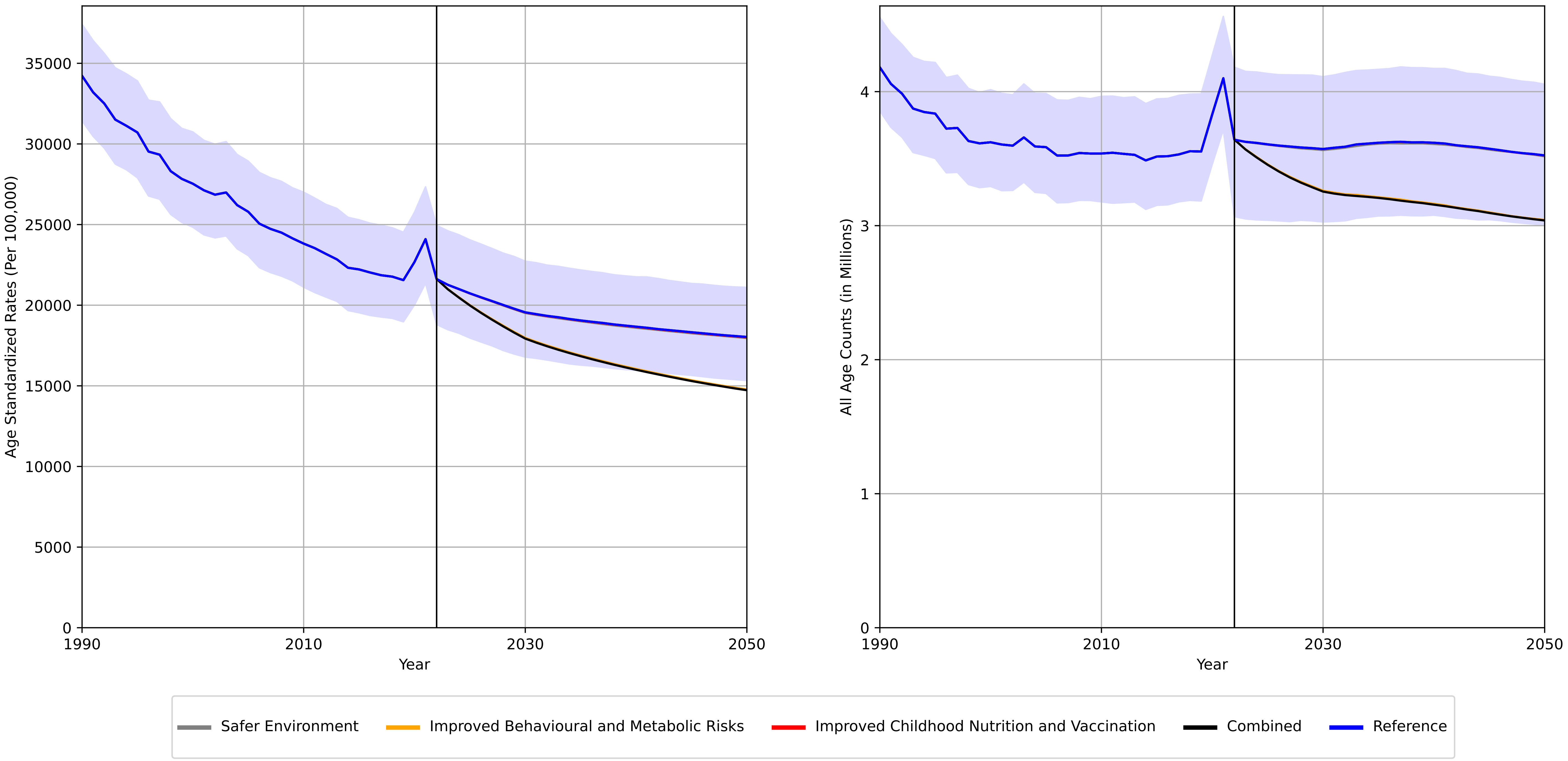

Age Standardized Rates (Per 100,000)
35000
30000
25000
20000
15000
10000
5000
0
1990
2010
2030
2050
Year
All Age Counts (in Millions)
4
3
2
1
0
1990
2010
2030
2050
Year
Safer Environment
Improved Behavioural and Metabolic Risks
Improved Childhood Nutrition and Vaccination
Combined
Reference

Hungary

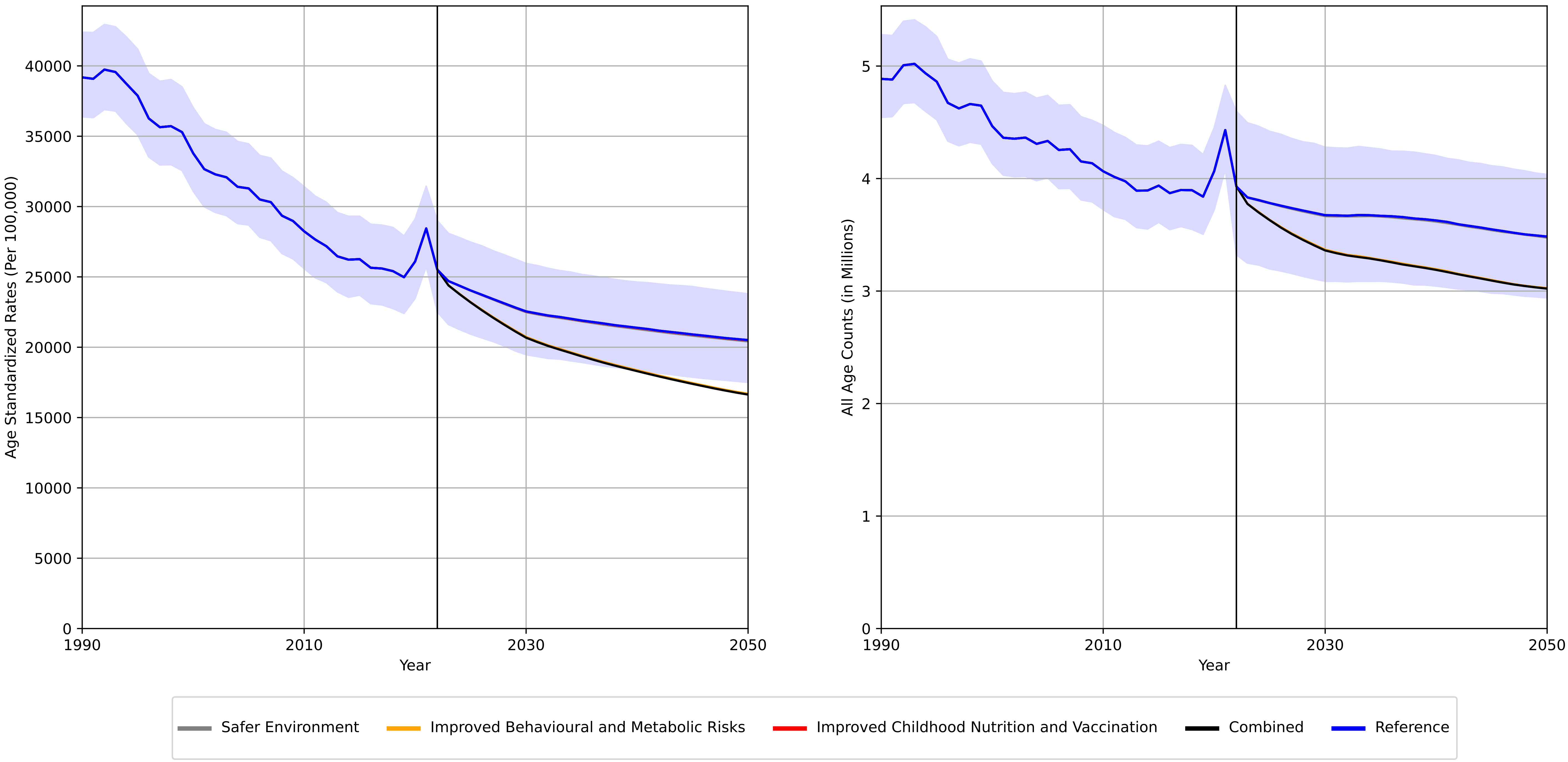
Age Standardized Rates (Per 100,000)
40000
35000
30000
25000
20000
15000
10000
5000
0
1990
2010
2030
2050
Year
All Age Counts (in Millions)
5
4
3
2
1
0
1990
2010
2030
2050
Year
Safer Environment
Improved Behavioural and Metabolic Risks
Improved Childhood Nutrition and Vaccination
Combined
Reference

Montenegro

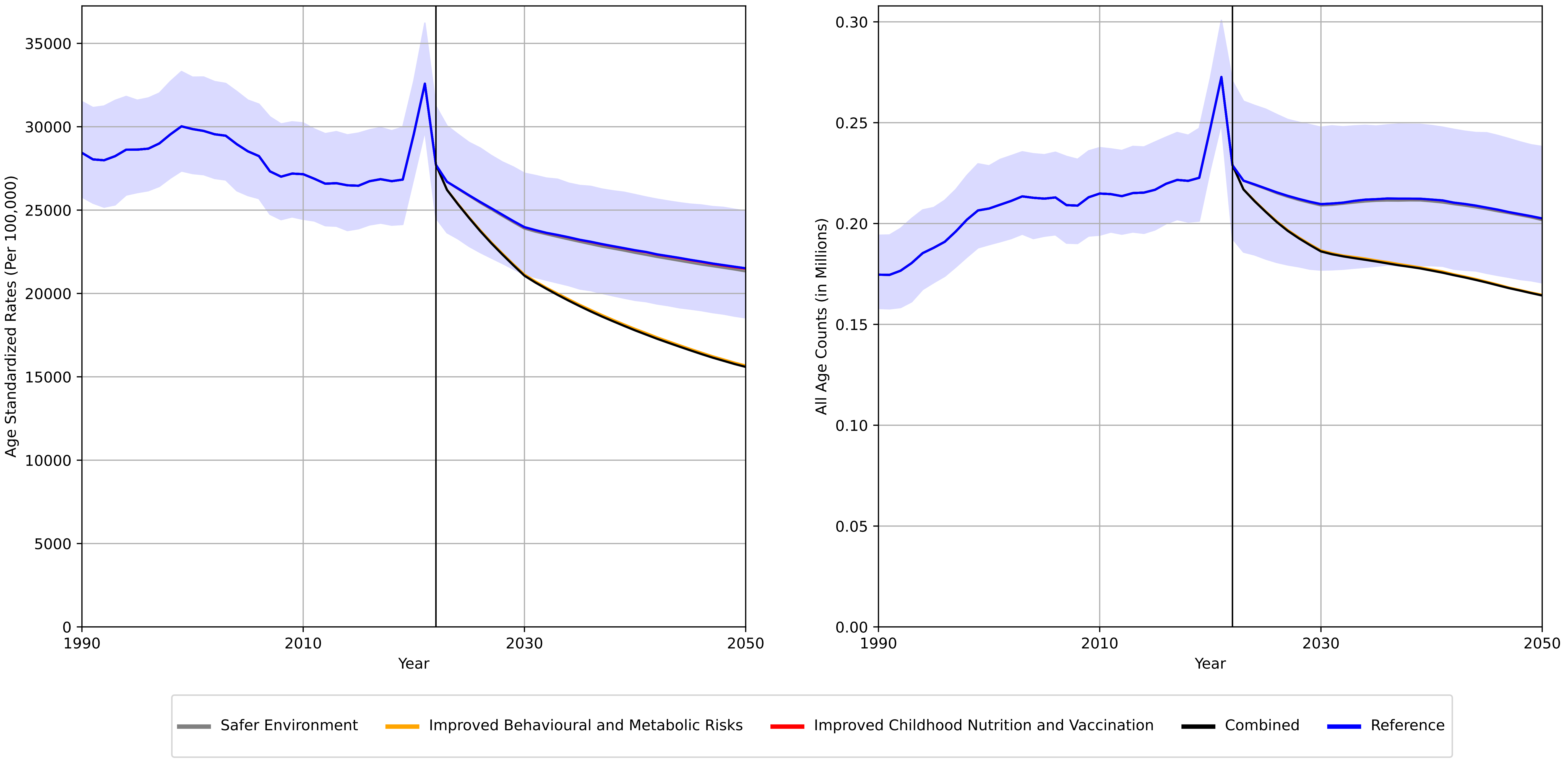
Age Standardized Rates (Per 100,000)
35000
30000
25000
20000
15000
10000
5000
0
1990
2010
2030
2050
Year
All Age Counts (in Millions)
0.30
0.25
0.20
0.15
0.10
0.05
0.00
1990
2010
2030
2050
Year
Safer Environment
Improved Behavioural and Metabolic Risks
Improved Childhood Nutrition and Vaccination
Combined
Reference

North Macedonia

Supplemental Results Figure S10. All-cause age standardised DALY rates (A) and all age DALY counts (B), for the past and for five future scenarios, 1990–2050

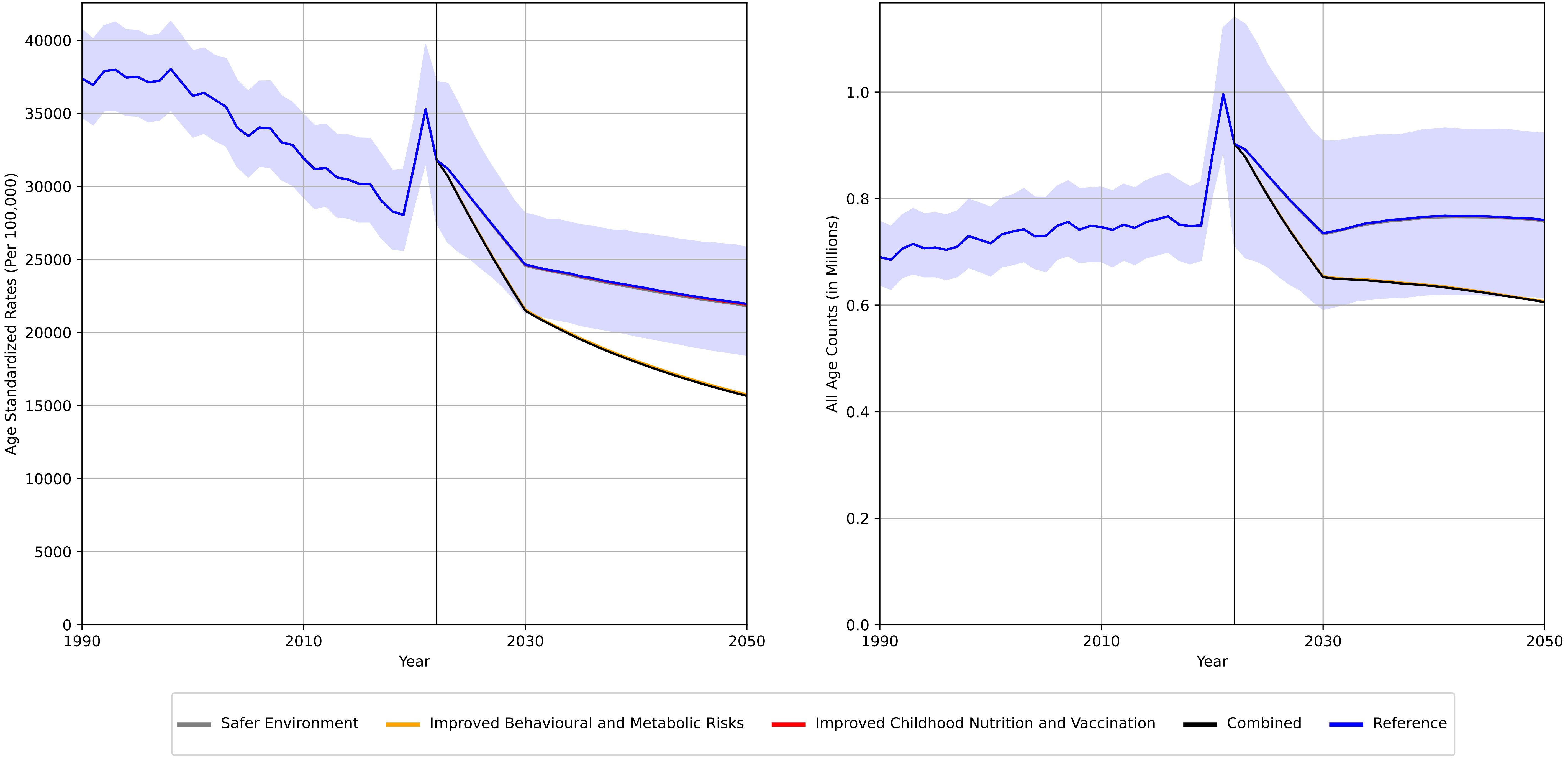

Poland

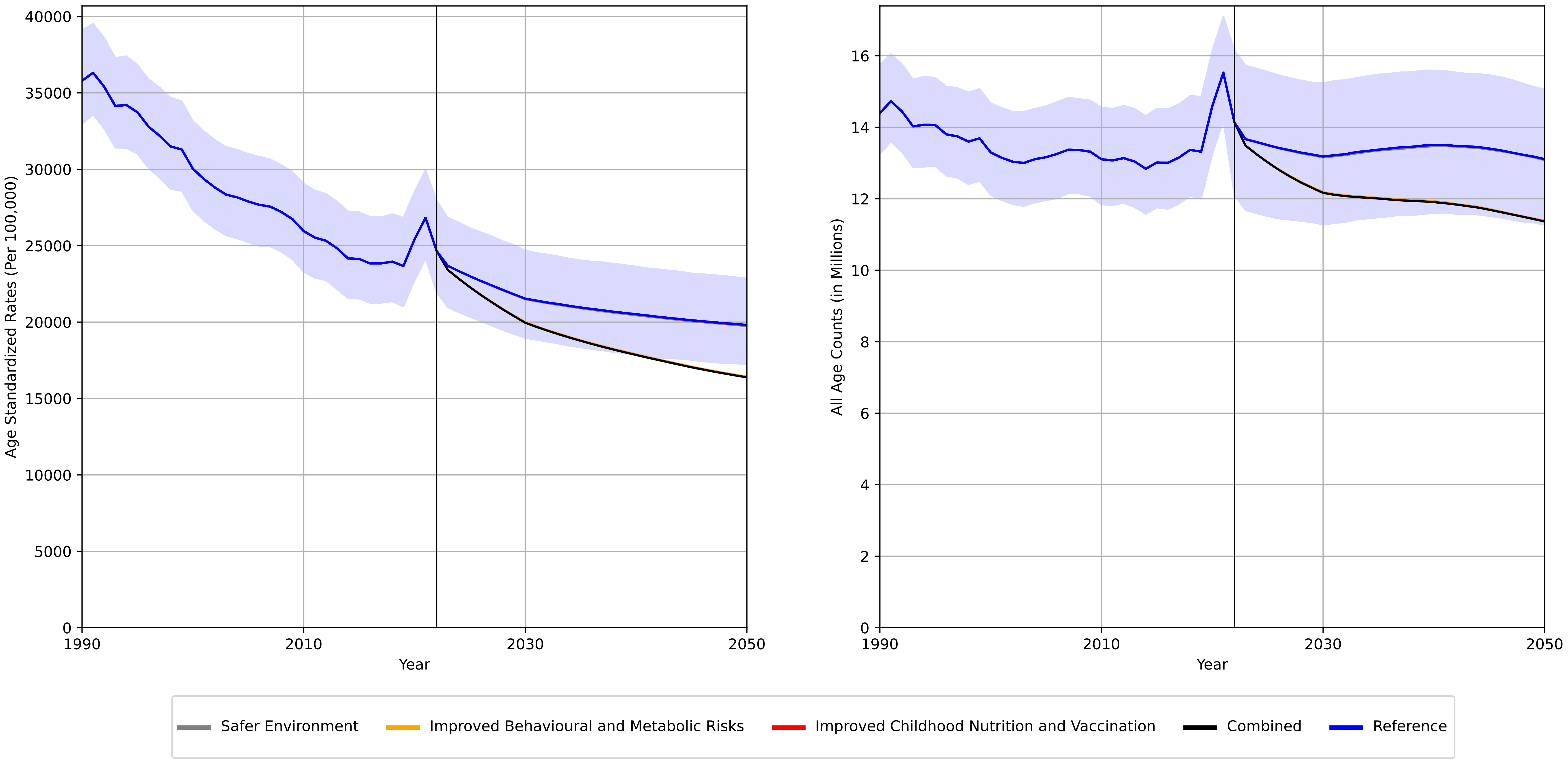

Age Standardized Rates (Per 100,000)
40000
35000
30000
25000
20000
15000
10000
5000
0
1990
2010
2030
2050
Year
All Age Counts (in Millions)
16
14
12
10
8
6
4
2
0
1990
2010
2030
2050
Year
Safer Environment
Improved Behavioural and Metabolic Risks
Improved Childhood Nutrition and Vaccination
Combined
Reference

Romania

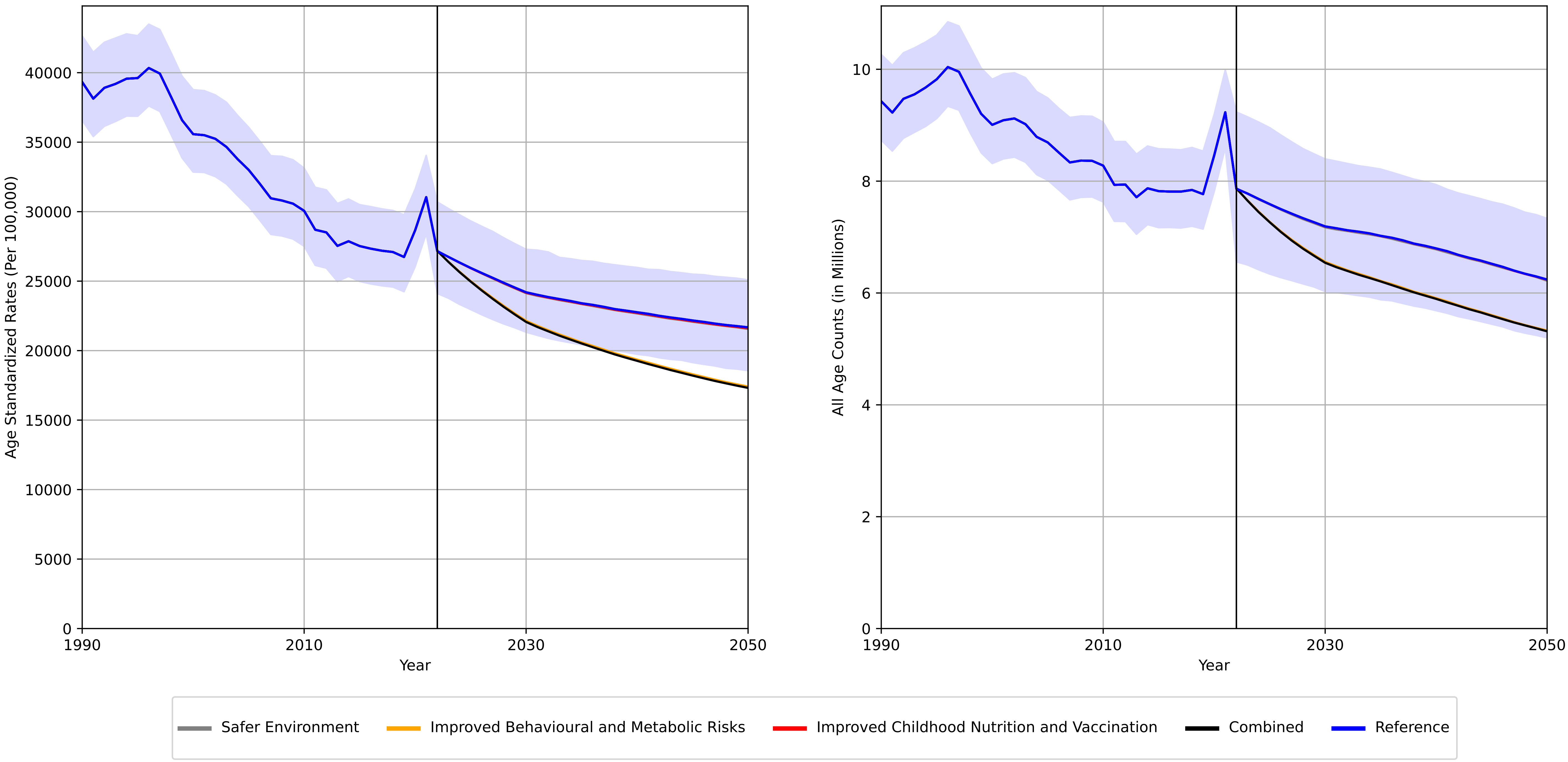
Age Standardized Rates (Per 100,000)
0
5000
10000
15000
20000
25000
30000
35000
40000
1990
2010
2030
2050
Year
All Age Counts (in Millions)
0
2
4
6
8
10
1990
2010
2030
2050
Year
Safer Environment
Improved Behavioural and Metabolic Risks
Improved Childhood Nutrition and Vaccination
Combined
Reference

Serbia

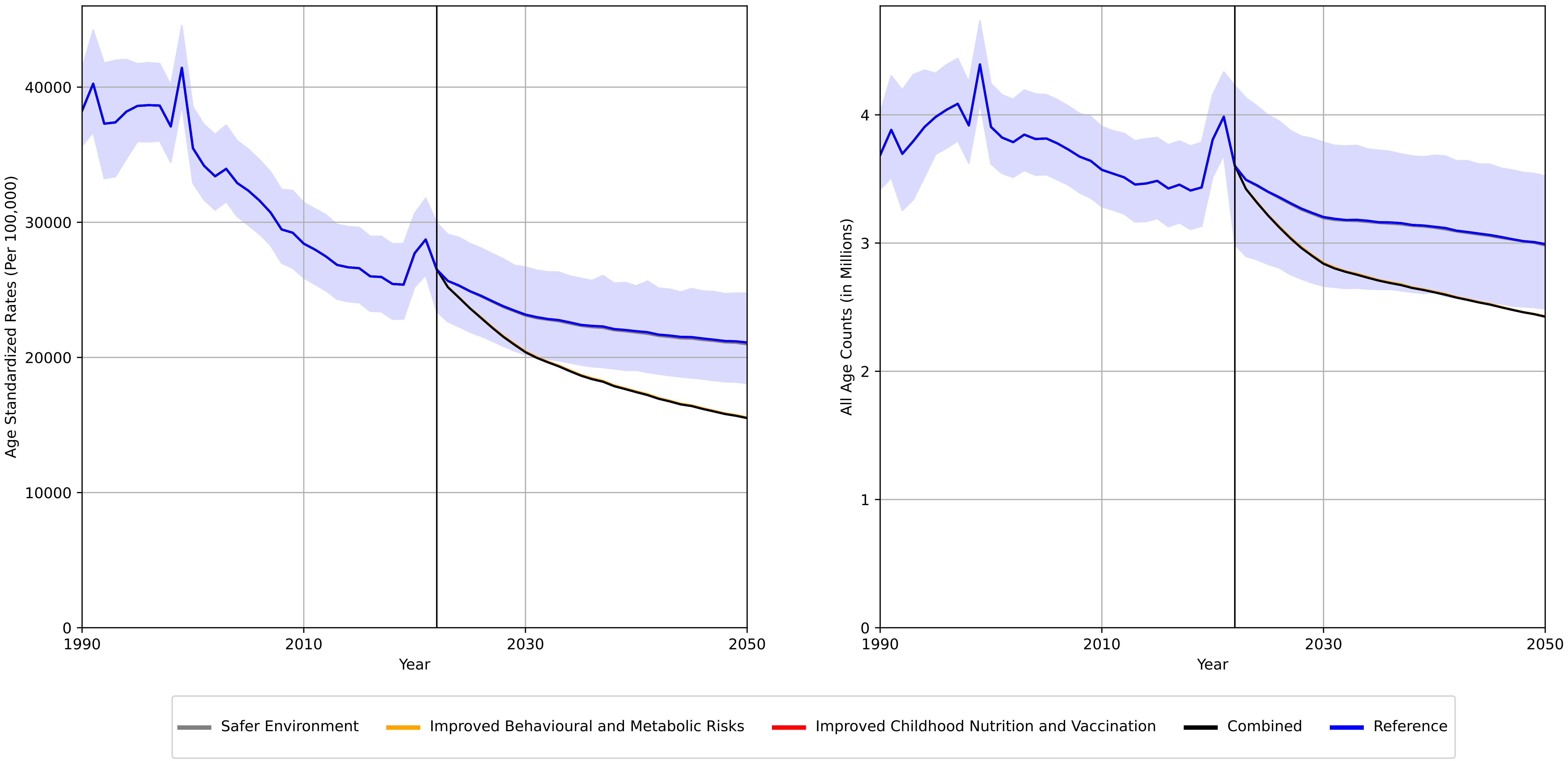
Age Standardized Rates (Per 100,000)
0
10000
20000
30000
40000
1990
2010
2030
2050
Year
All Age Counts (in Millions)
0
1
2
3
4
1990
2010
2030
2050
Year
Safer Environment
Improved Behavioural and Metabolic Risks
Improved Childhood Nutrition and Vaccination
Combined
Reference

Slovakia

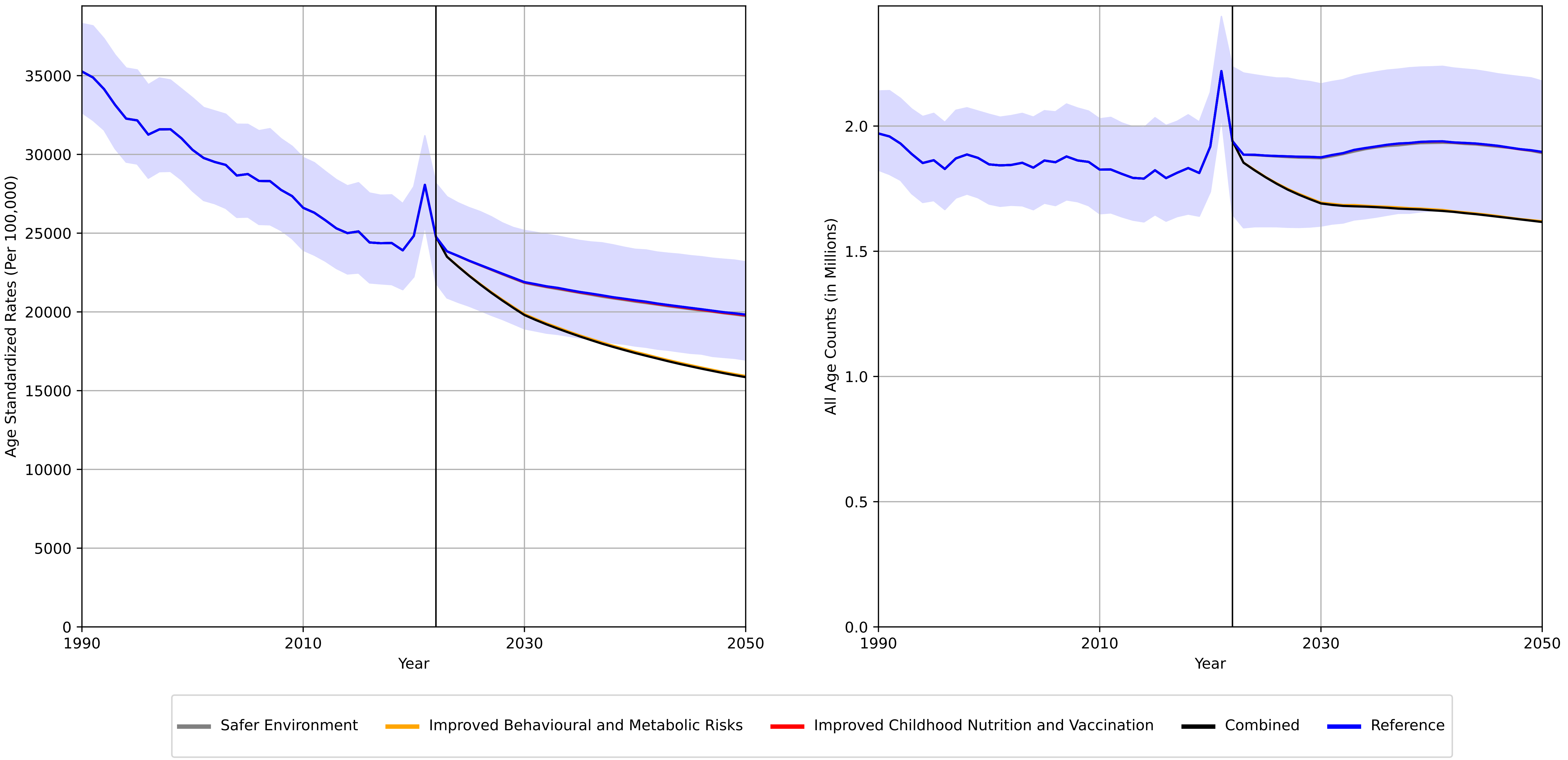

Age Standardized Rates (Per 100,000)
35000
30000
25000
20000
15000
10000
5000
0
1990
2010
2030
2050
Year
All Age Counts (in Millions)
2.0
1.5
1.0
0.5
0.0
1990
2010
2030
2050
Year
Safer Environment
Improved Behavioural and Metabolic Risks
Improved Childhood Nutrition and Vaccination
Combined
Reference

Slovenia

Supplemental Results Figure S10. All-cause age standardised DALY rates (A) and all age DALY counts (B), for the past and for five future scenarios, 1990–2050

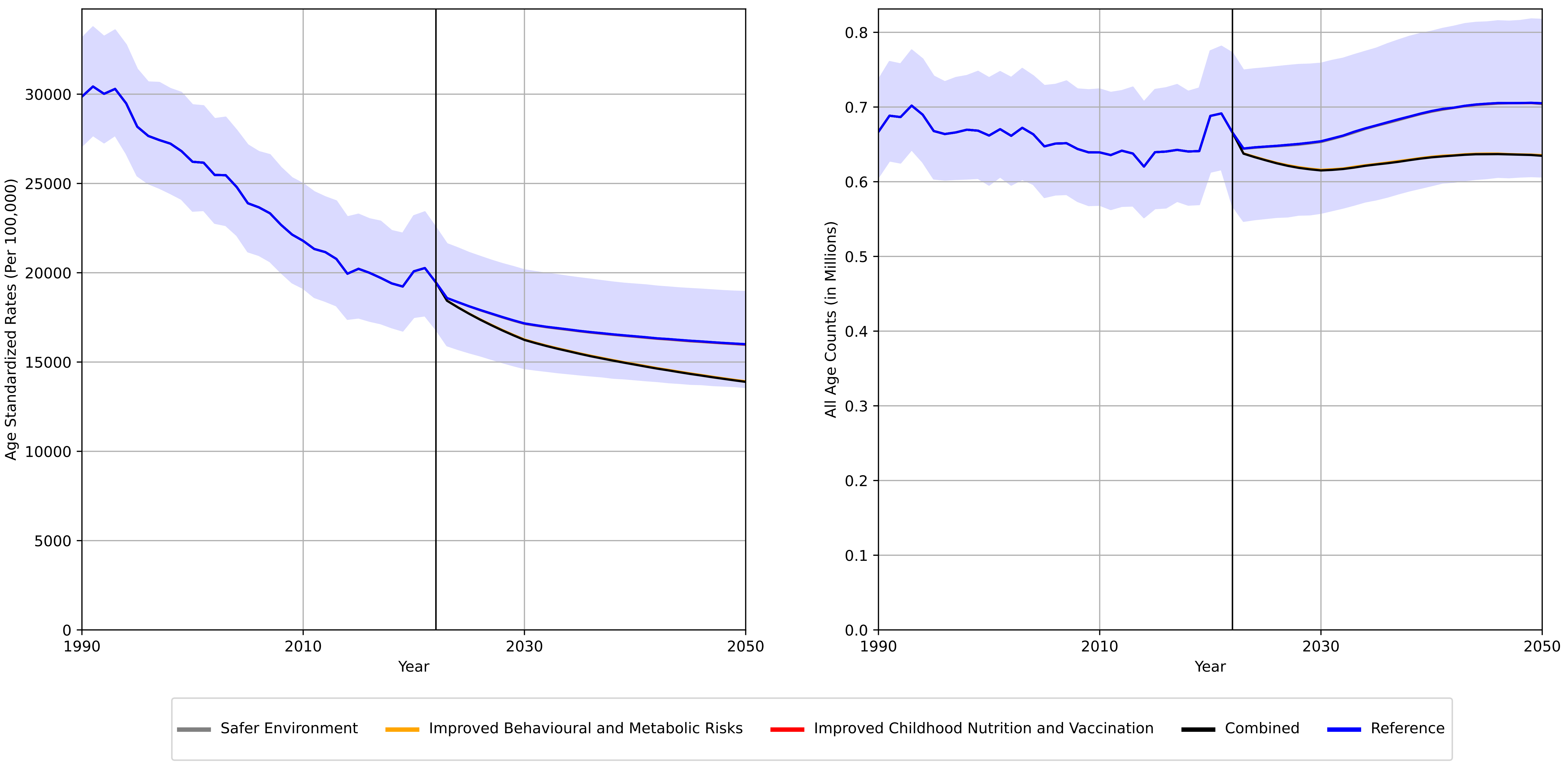

Belarus

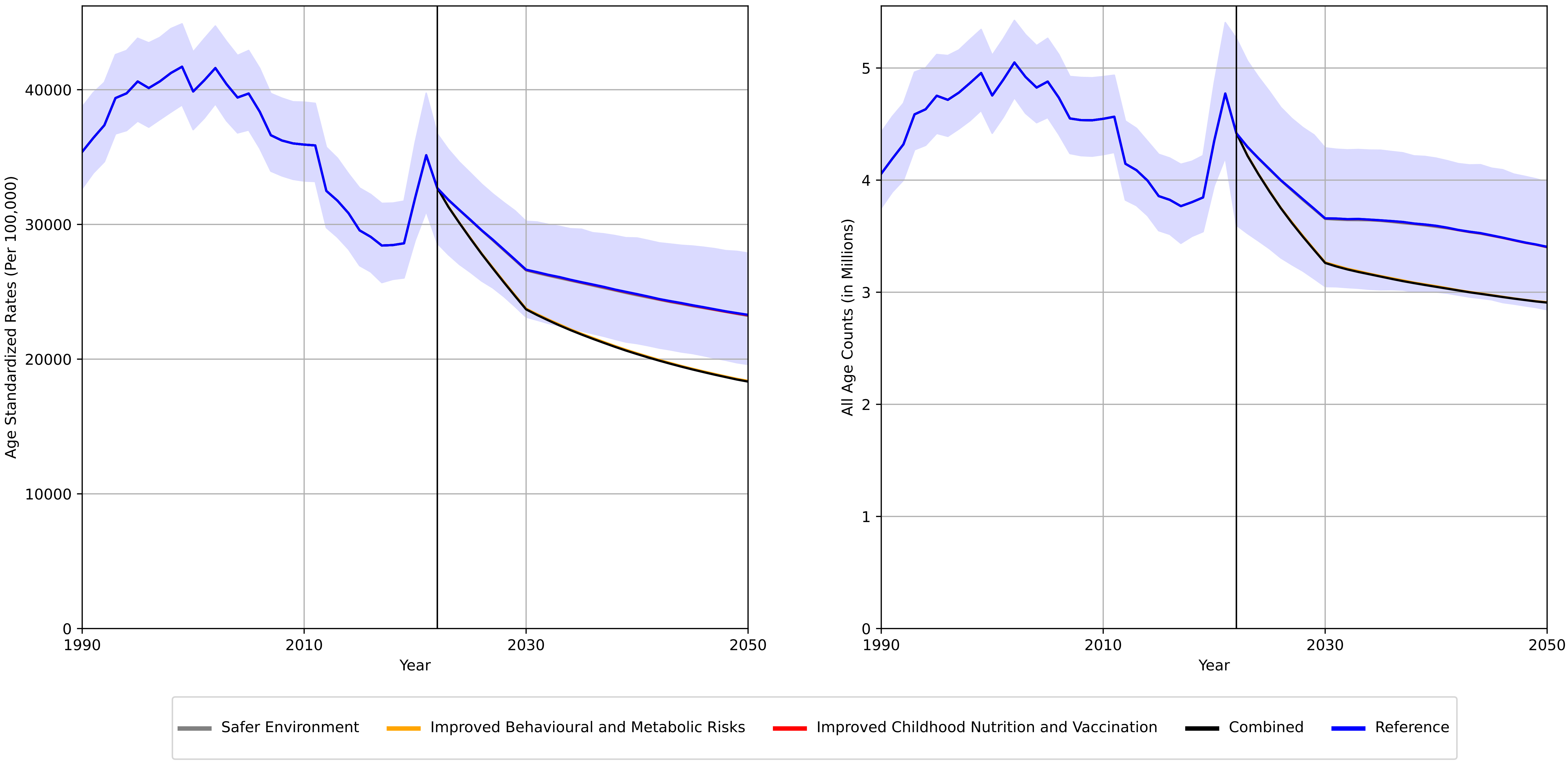
Age Standardized Rates (Per 100,000)
0
10000
20000
30000
40000
1990
2010
2030
2050
Year
All Age Counts (in Millions)
0
1
2
3
4
5
1990
2010
2030
2050
Year
Safer Environment
Improved Behavioural and Metabolic Risks
Improved Childhood Nutrition and Vaccination
Combined
Reference

Estonia

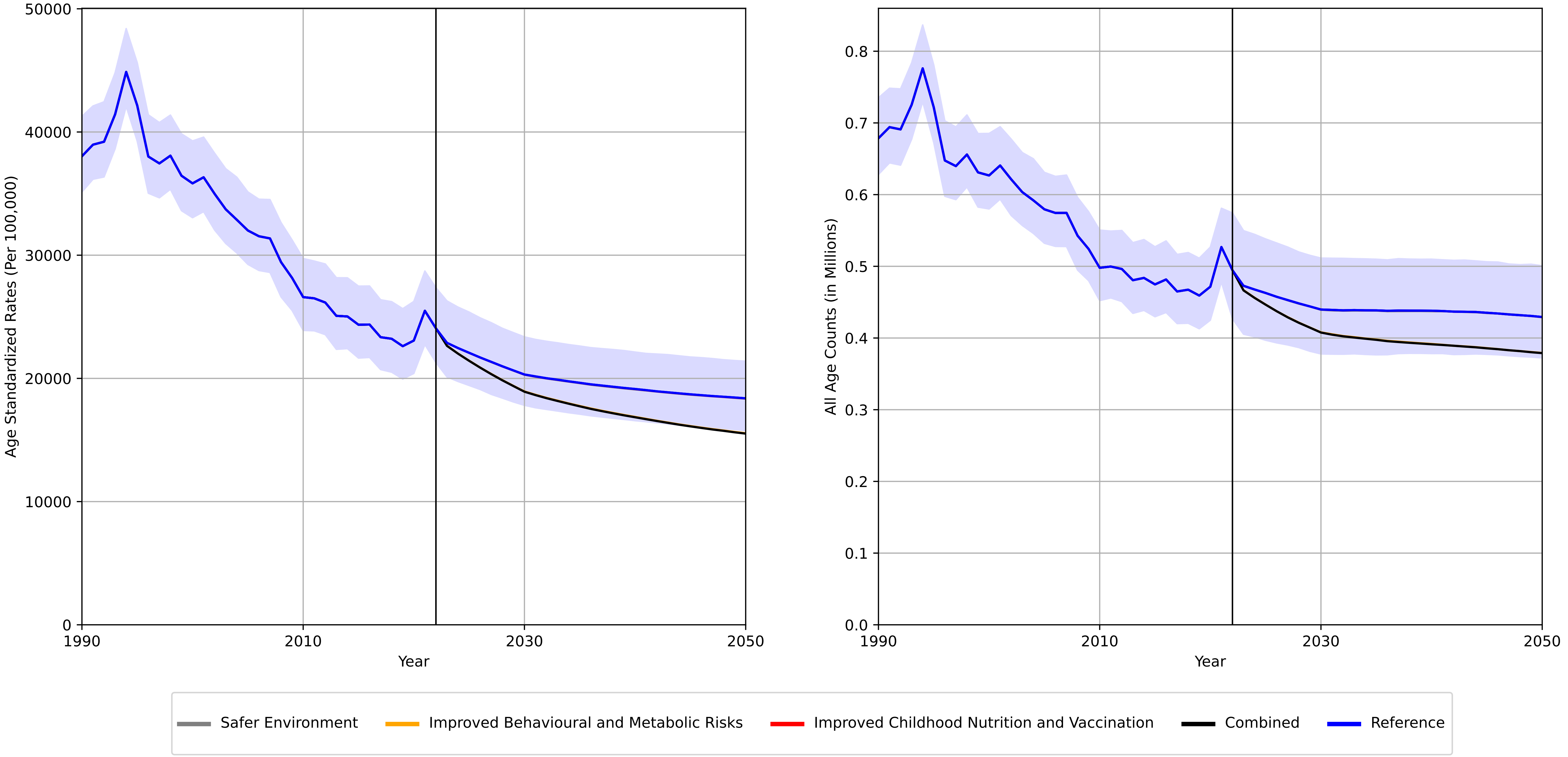

Age Standardized Rates (Per 100,000)
50000
40000
30000
20000
10000
0
1990
2010
2030
2050
Year
All Age Counts (in Millions)
0.8
0.7
0.6
0.5
0.4
0.3
0.2
0.1
0.0
1990
2010
2030
2050
Year
Safer Environment
Improved Behavioural and Metabolic Risks
Improved Childhood Nutrition and Vaccination
Combined
Reference

Latvia

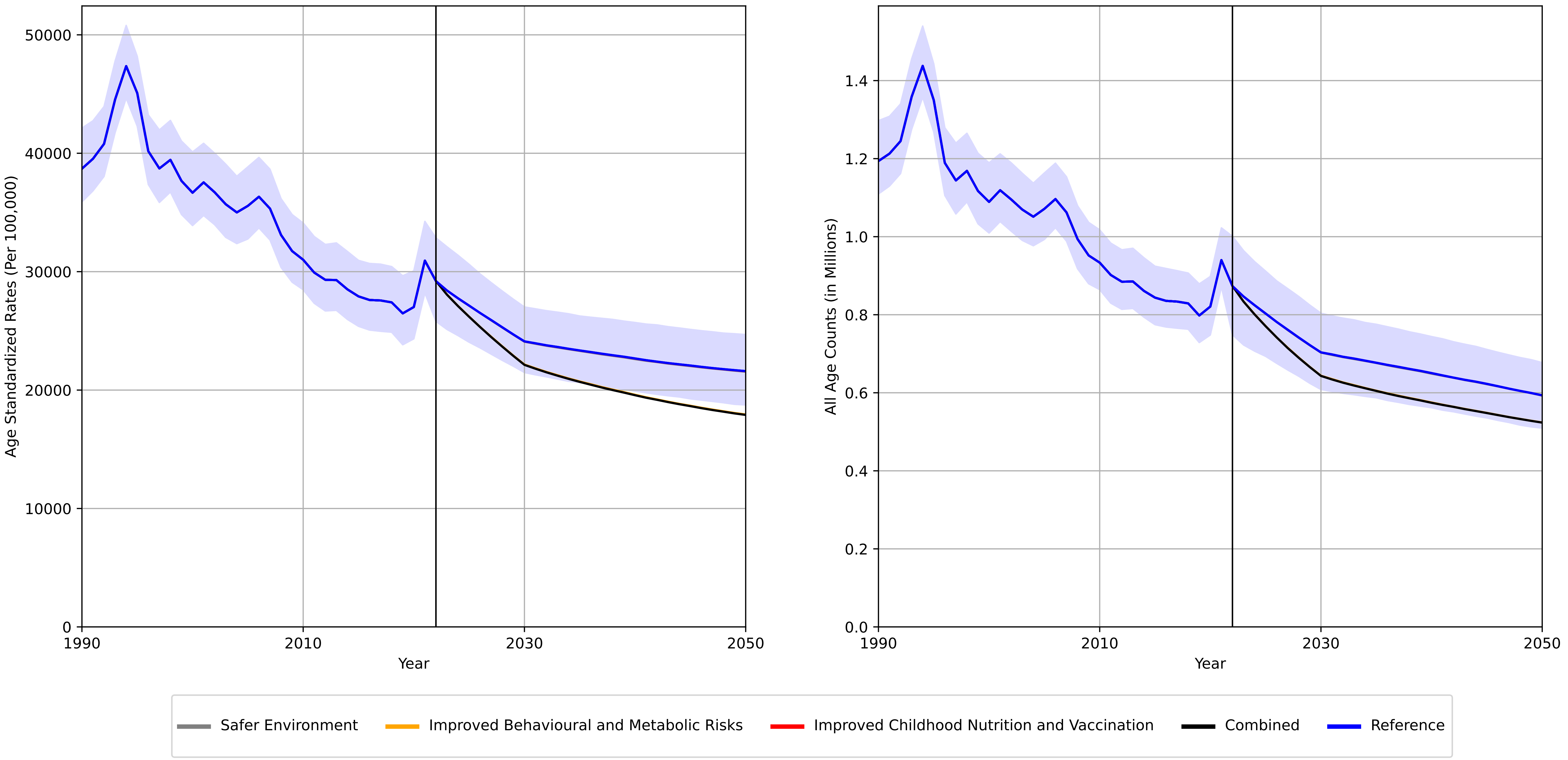
Age Standardized Rates (Per 100,000)
50000
40000
30000
20000
10000
0
1990
2010
2030
2050
Year
All Age Counts (in Millions)
1.4
1.2
1.0
0.8
0.6
0.4
0.2
0.0
1990
2010
2030
2050
Year
Safer Environment
Improved Behavioural and Metabolic Risks
Improved Childhood Nutrition and Vaccination
Combined
Reference

Supplemental Results Figure S10. All-cause age standardised DALY rates (A) and all age DALY counts (B), for the past and for five future scenarios, 1990–2050

Lithuania

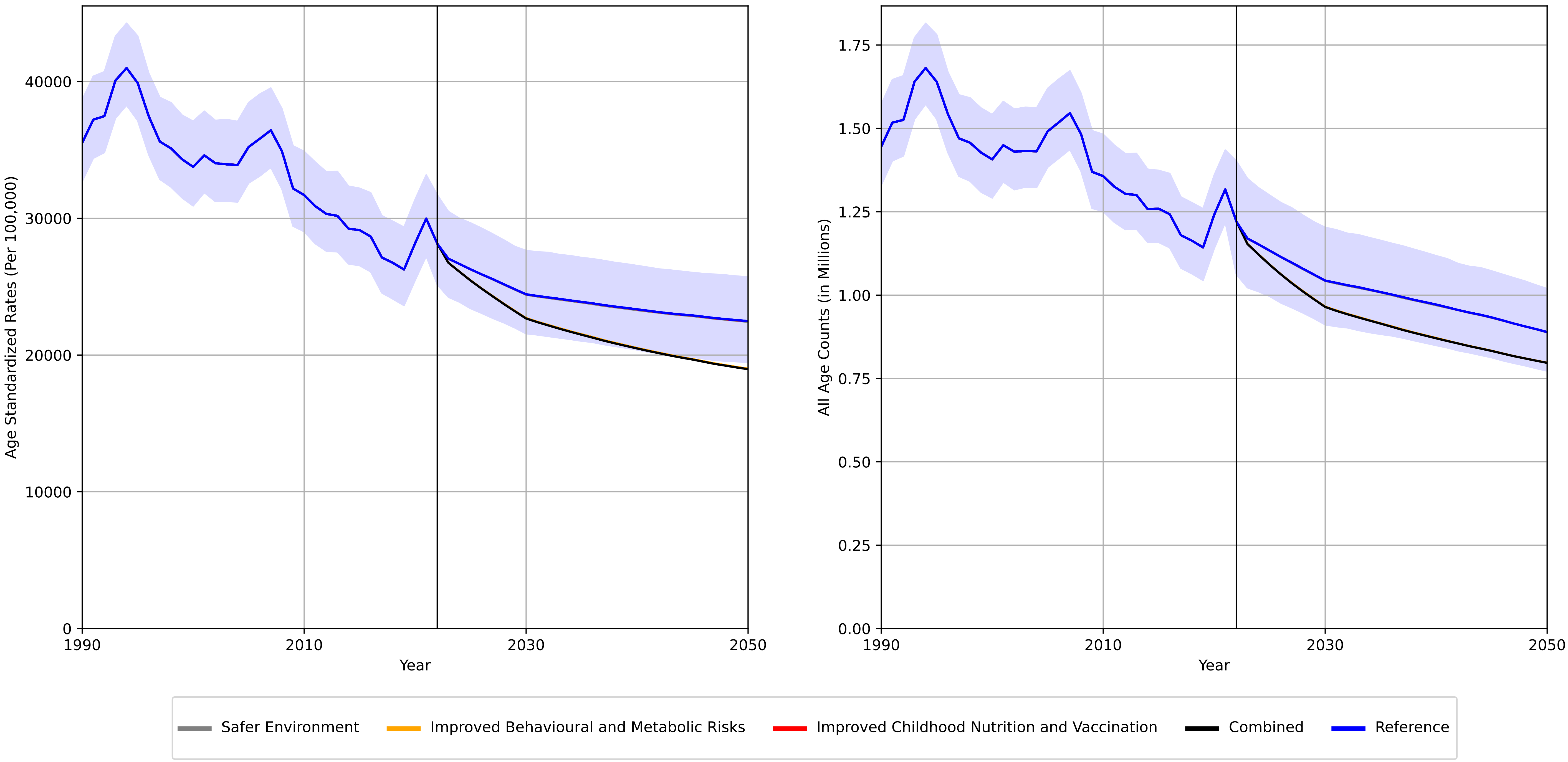

Moldova

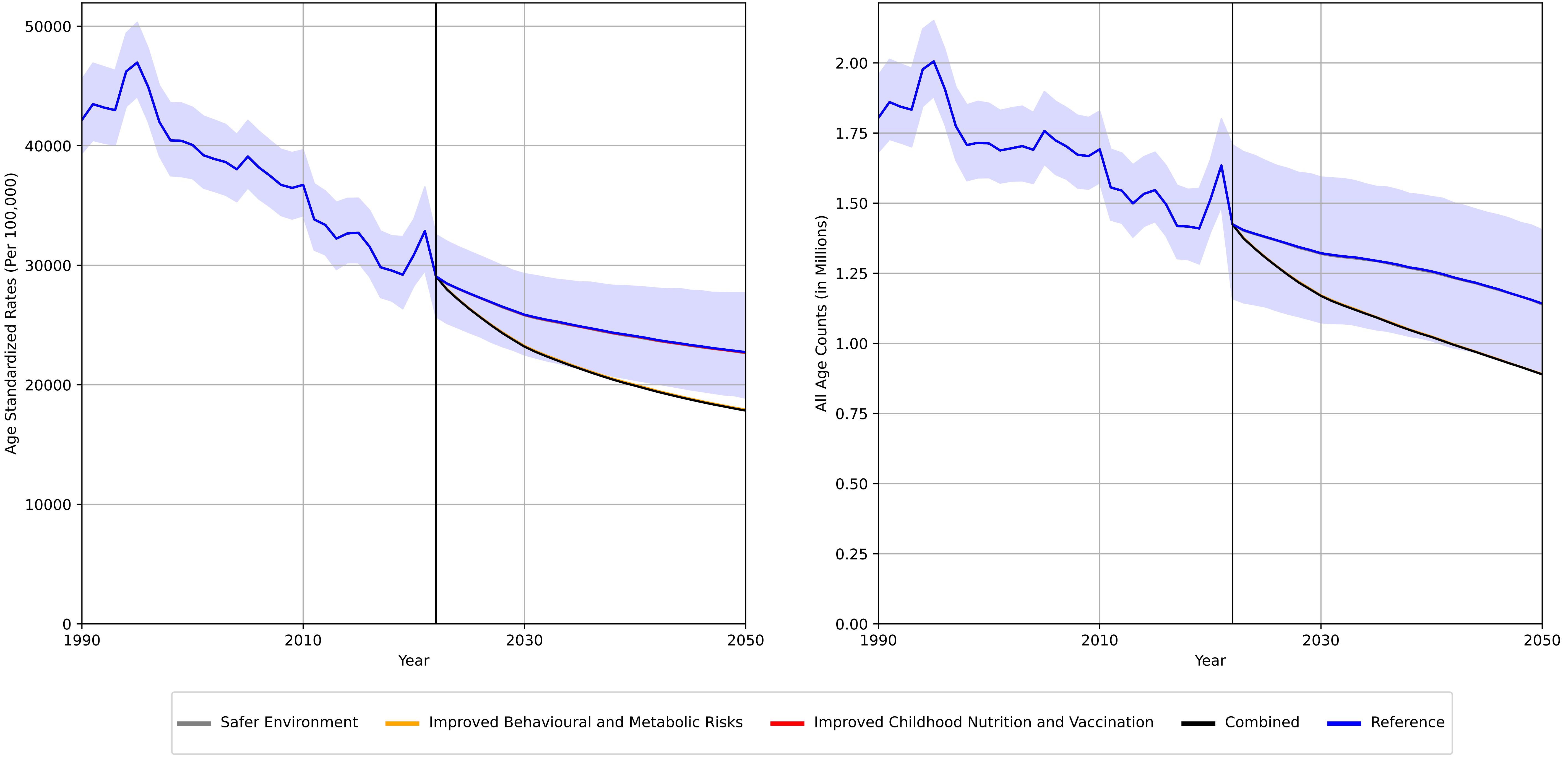
Age Standardized Rates (Per 100,000)
50000
40000
30000
20000
10000
0
1990
2010
2030
2050
Year
All Age Counts (in Millions)
2.00
1.75
1.50
1.25
1.00
0.75
0.50
0.25
0.00
1990
2010
2030
2050
Year
Safer Environment
Improved Behavioural and Metabolic Risks
Improved Childhood Nutrition and Vaccination
Combined
Reference

Russia

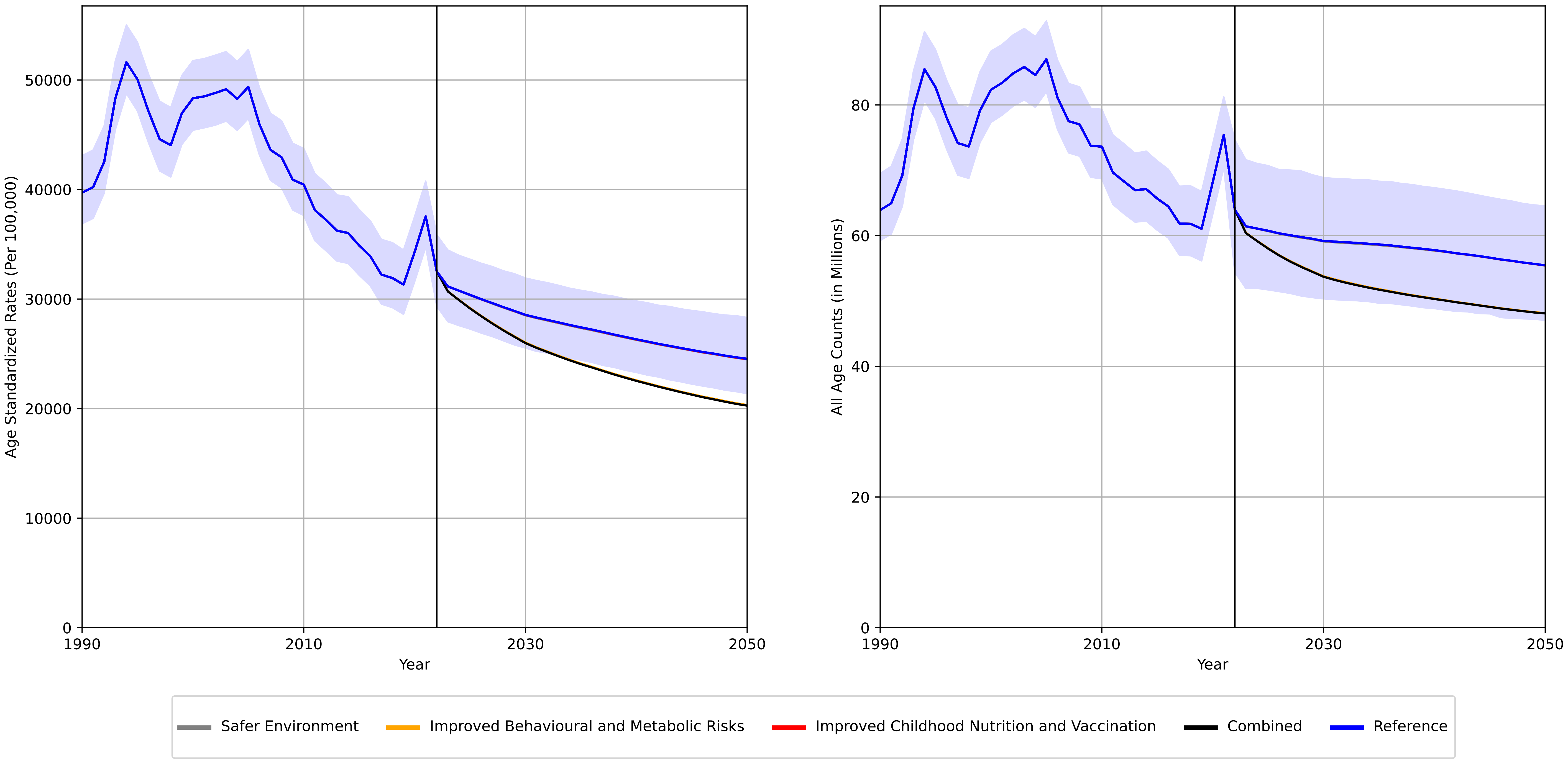

Age Standardized Rates (Per 100,000)
0
10000
20000
30000
40000
50000
1990
2010
2030
2050
Year
All Age Counts (in Millions)
0
20
40
60
80
1990
2010
2030
2050
Year
Safer Environment
Improved Behavioural and Metabolic Risks
Improved Childhood Nutrition and Vaccination
Combined
Reference

Ukraine

Supplemental Results Figure S10. All-cause age standardised DALY rates (A) and all age DALY counts (B), for the past and for five future scenarios, 1990–2050

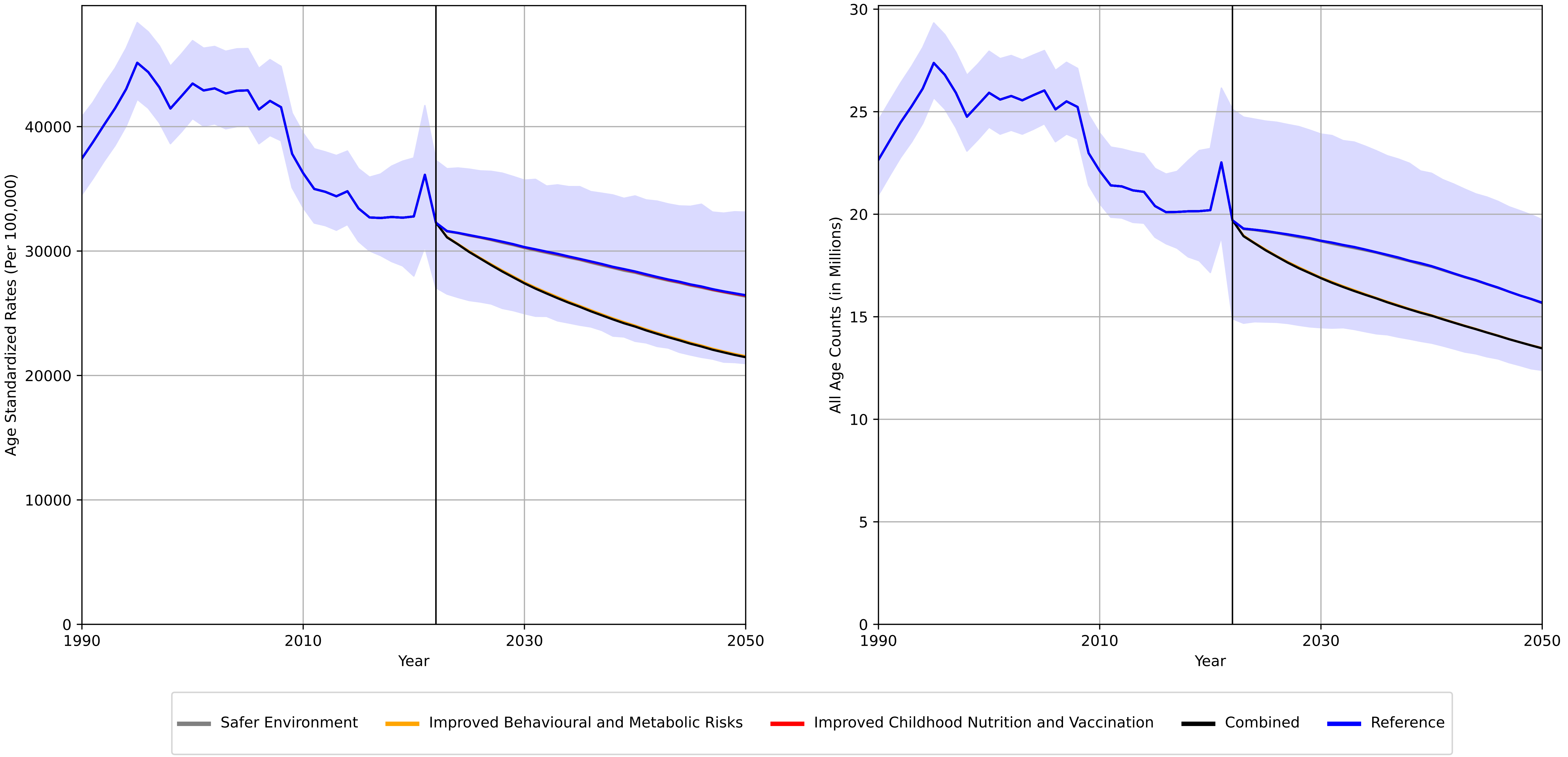

Australia

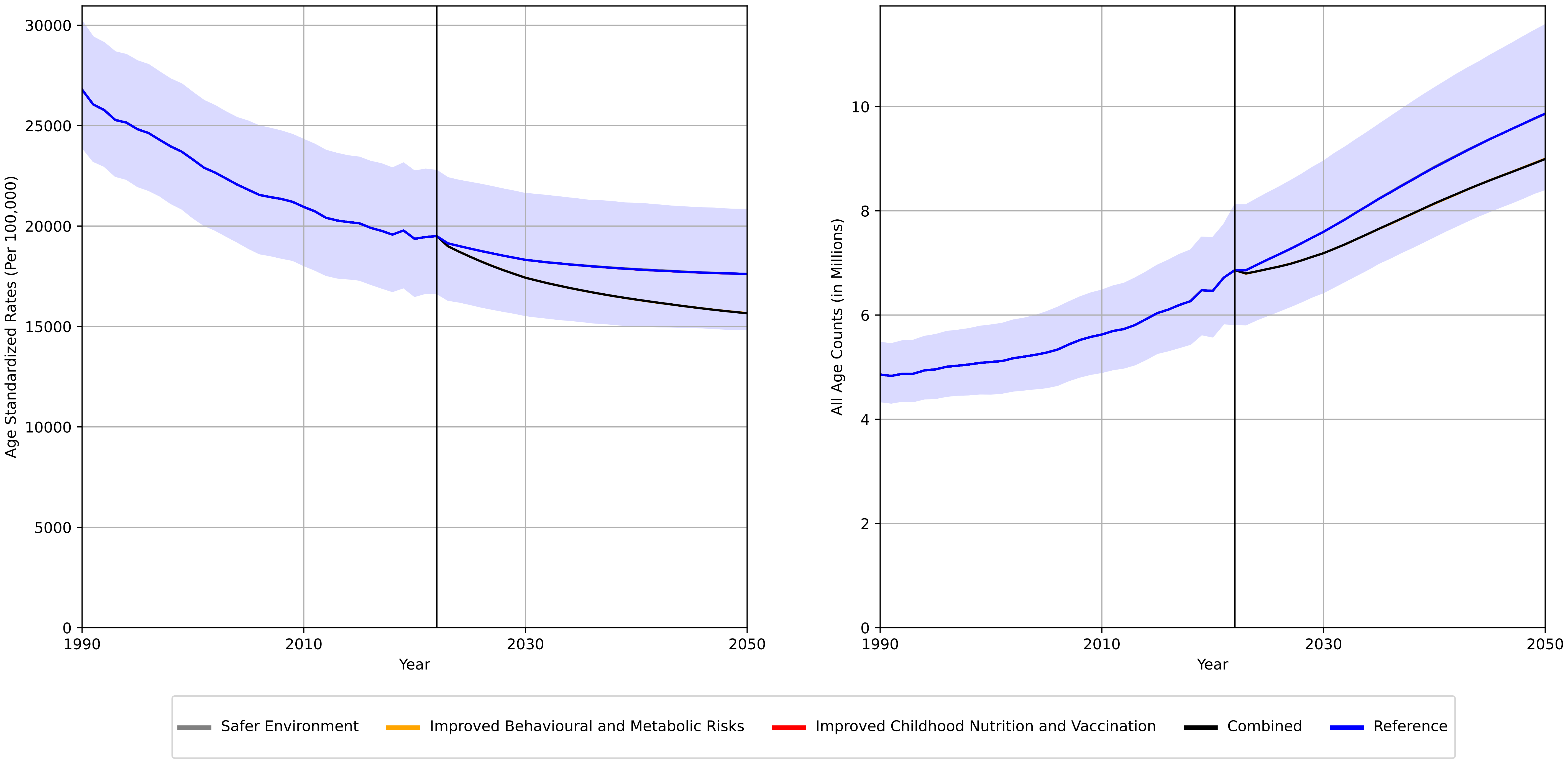
Age Standardized Rates (Per 100,000)
30000
25000
20000
15000
10000
5000
0
1990
2010
2030
2050
Year
All Age Counts (in Millions)
10
8
6
4
2
0
1990
2010
2030
2050
Year
Safer Environment
Improved Behavioural and Metabolic Risks
Improved Childhood Nutrition and Vaccination
Combined
Reference

New Zealand

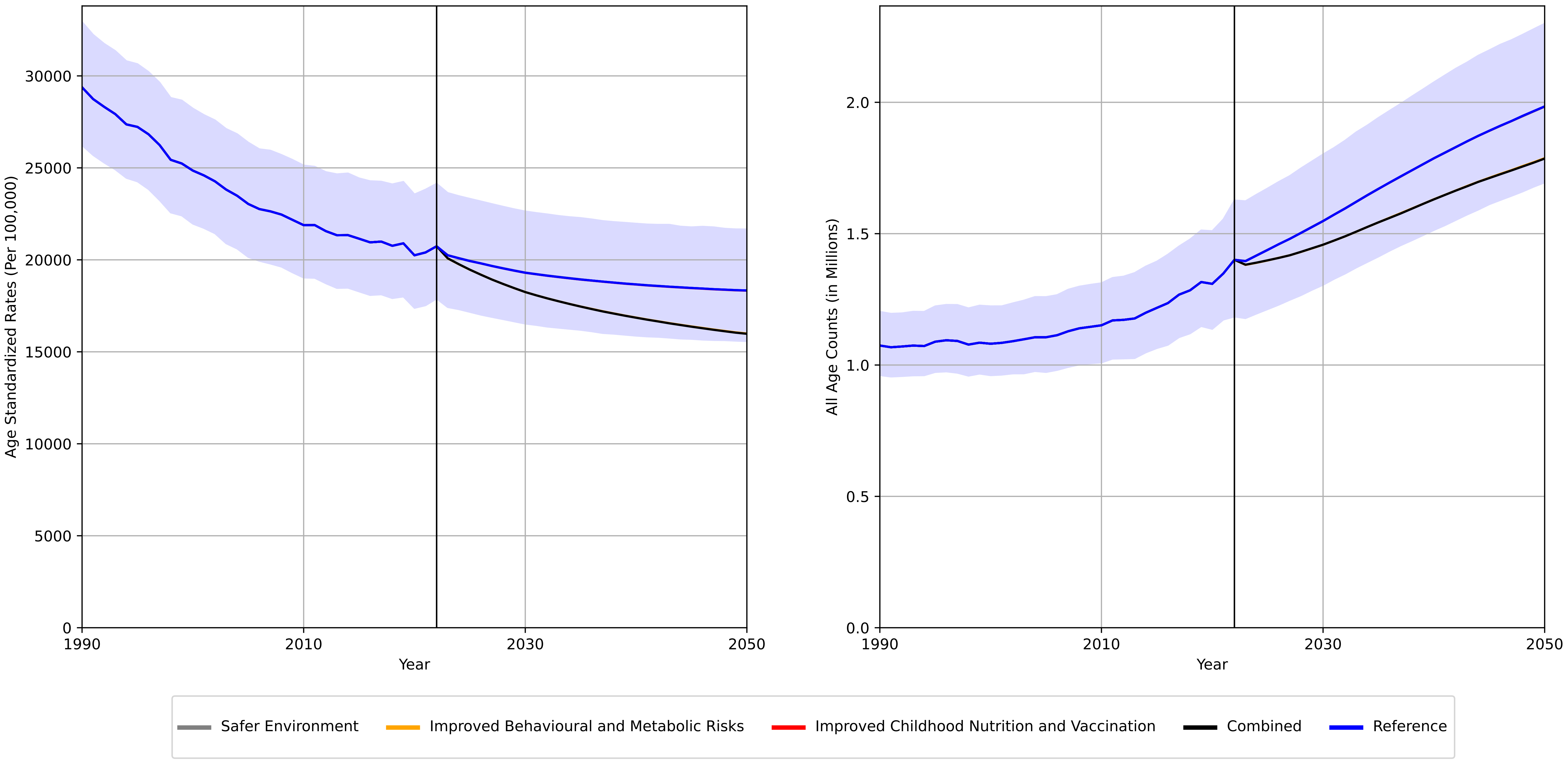

Age Standardized Rates (Per 100,000)
30000
25000
20000
15000
10000
5000
0
1990
2010
2030
2050
Year
All Age Counts (in Millions)
2.0
1.5
1.0
0.5
0.0
1990
2010
2030
2050
Year
Safer Environment
Improved Behavioural and Metabolic Risks
Improved Childhood Nutrition and Vaccination
Combined
Reference

Brunei

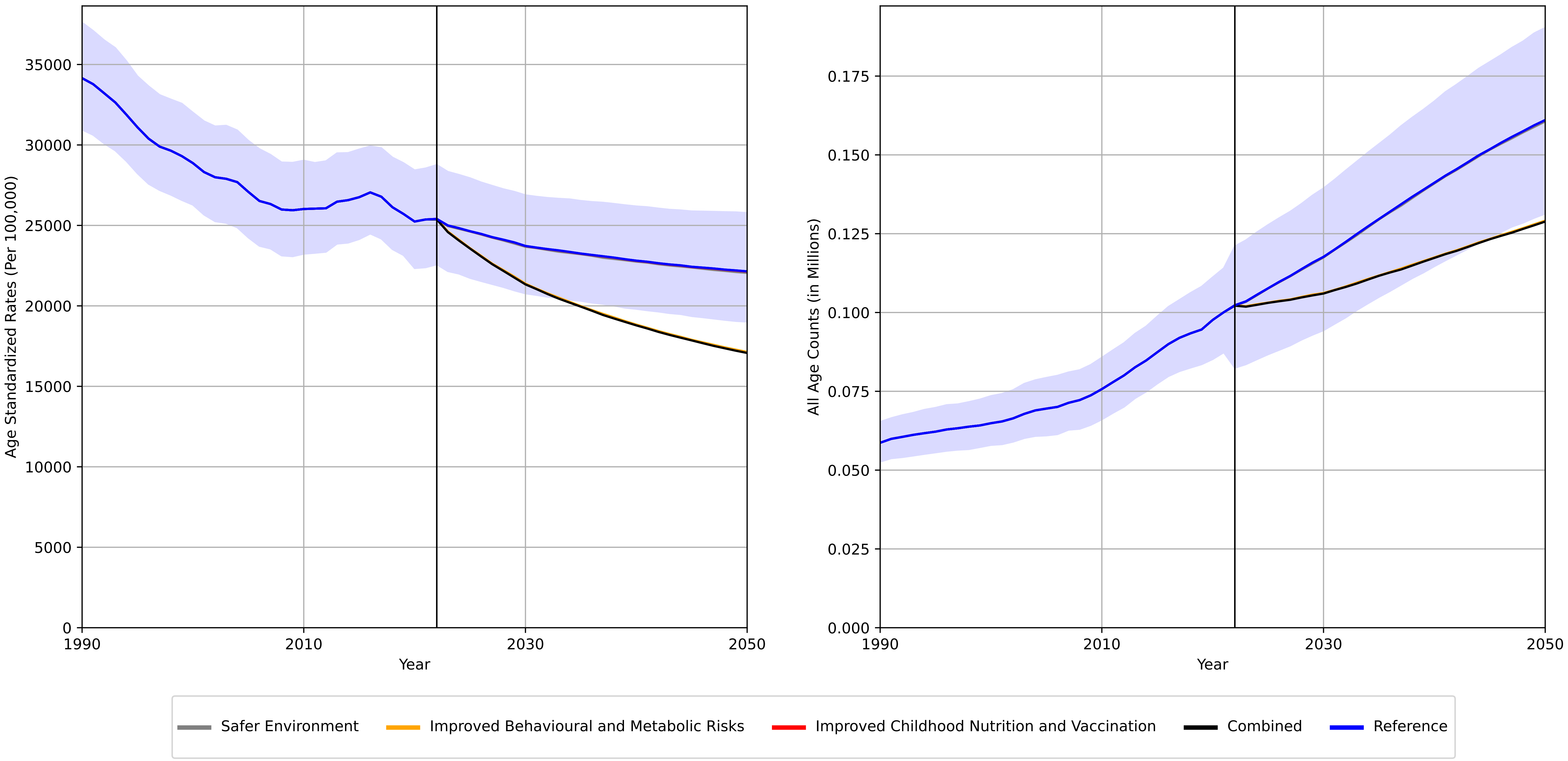

Age Standardized Rates (Per 100,000)
35000
30000
25000
20000
15000
10000
5000
0
1990
2010
2030
2050
Year
All Age Counts (in Millions)
0.175
0.150
0.125
0.100
0.075
0.050
0.025
0.000
1990
2010
2030
2050
Year
Safer Environment
Improved Behavioural and Metabolic Risks
Improved Childhood Nutrition and Vaccination
Combined
Reference

Japan

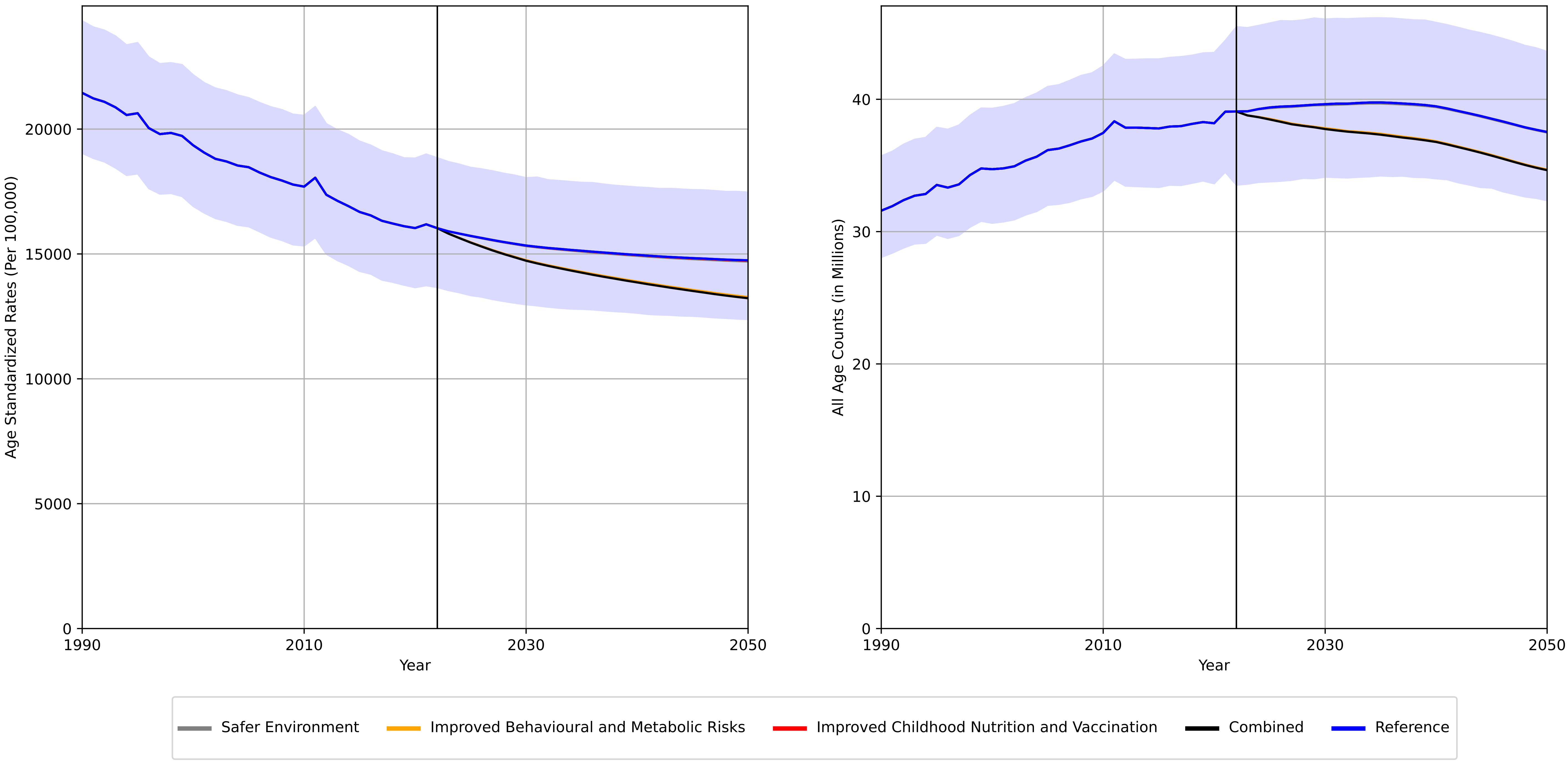

Age Standardized Rates (Per 100,000)
0
5000
10000
15000
20000
1990
2010
2030
2050
Year
All Age Counts (in Millions)
0
10
20
30
40
1990
2010
2030
2050
Year
Safer Environment
Improved Behavioural and Metabolic Risks
Improved Childhood Nutrition and Vaccination
Combined
Reference

South Korea

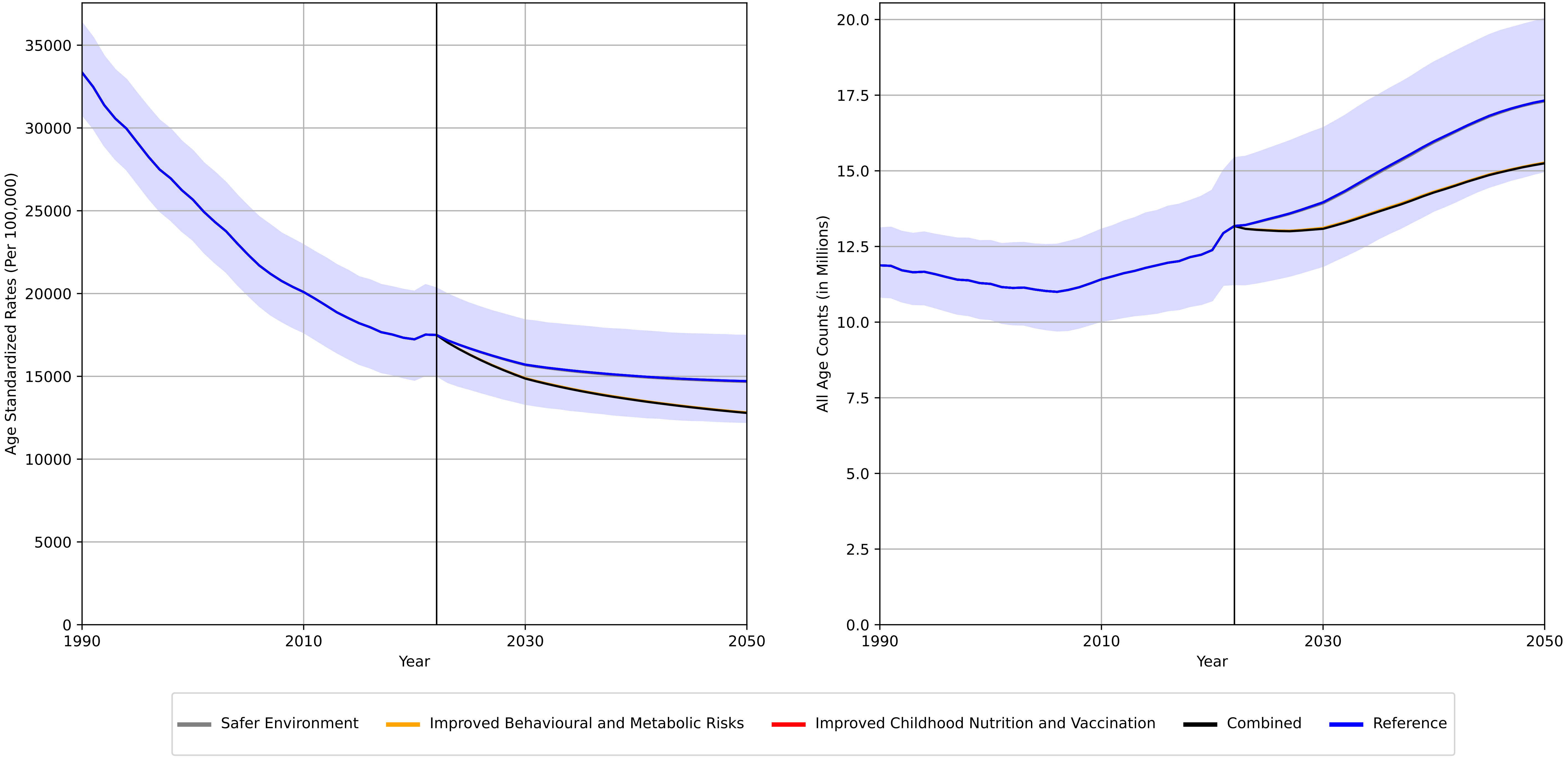
Age Standardized Rates (Per 100,000)
35000
30000
25000
20000
15000
10000
5000
0
1990
2010
2030
2050
Year
All Age Counts (in Millions)
20.0
17.5
15.0
12.5
10.0
7.5
5.0
2.5
0.0
1990
2010
2030
2050
Year
Safer Environment
Improved Behavioural and Metabolic Risks
Improved Childhood Nutrition and Vaccination
Combined
Reference

Singapore

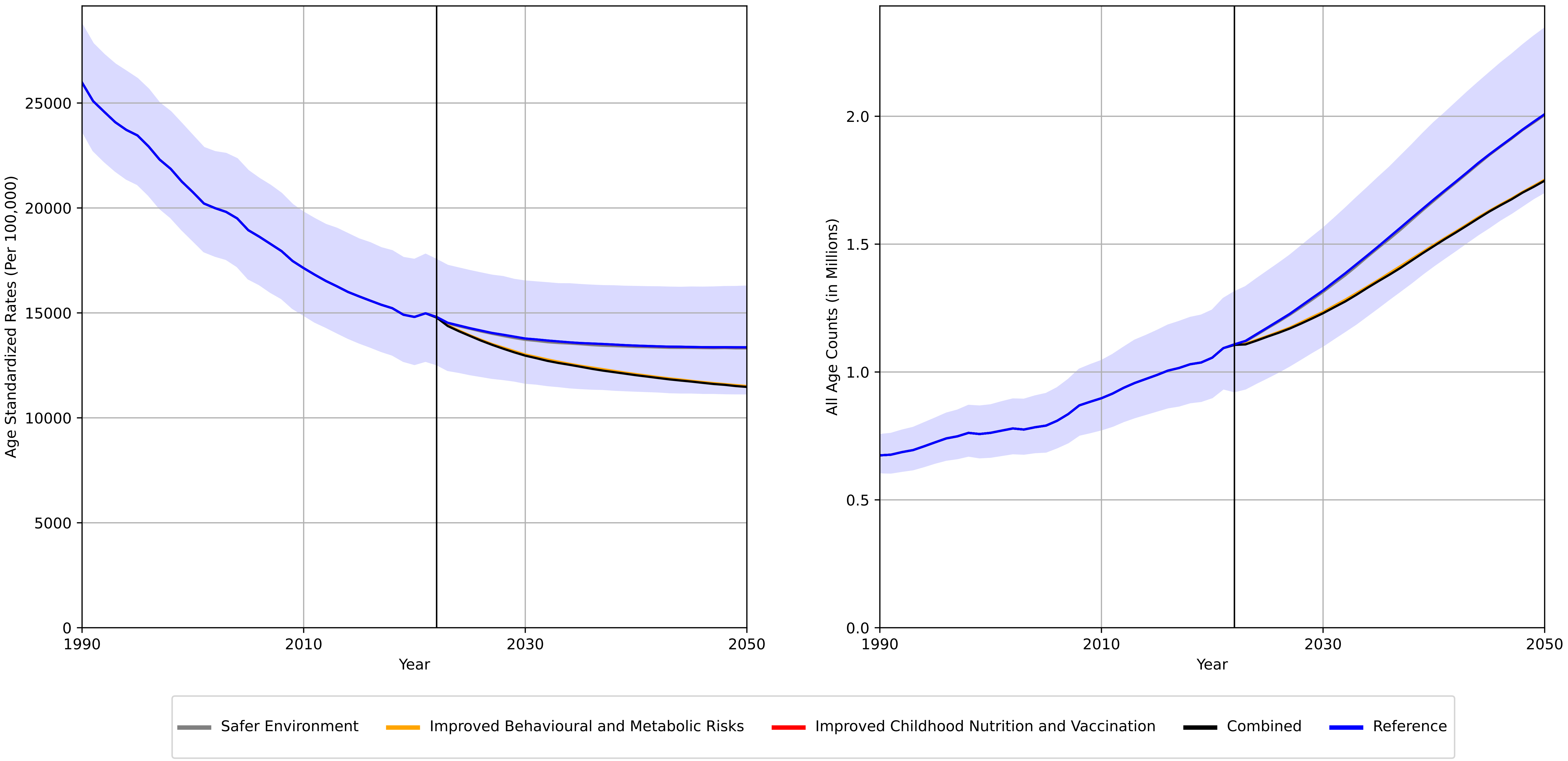
Age Standardized Rates (Per 100,000)
0
5000
10000
15000
20000
25000
1990
2010
2030
2050
Year
All Age Counts (in Millions)
0.0
0.5
1.0
1.5
2.0
1990
2010
2030
2050
Year
Safer Environment
Improved Behavioural and Metabolic Risks
Improved Childhood Nutrition and Vaccination
Combined
Reference

Canada

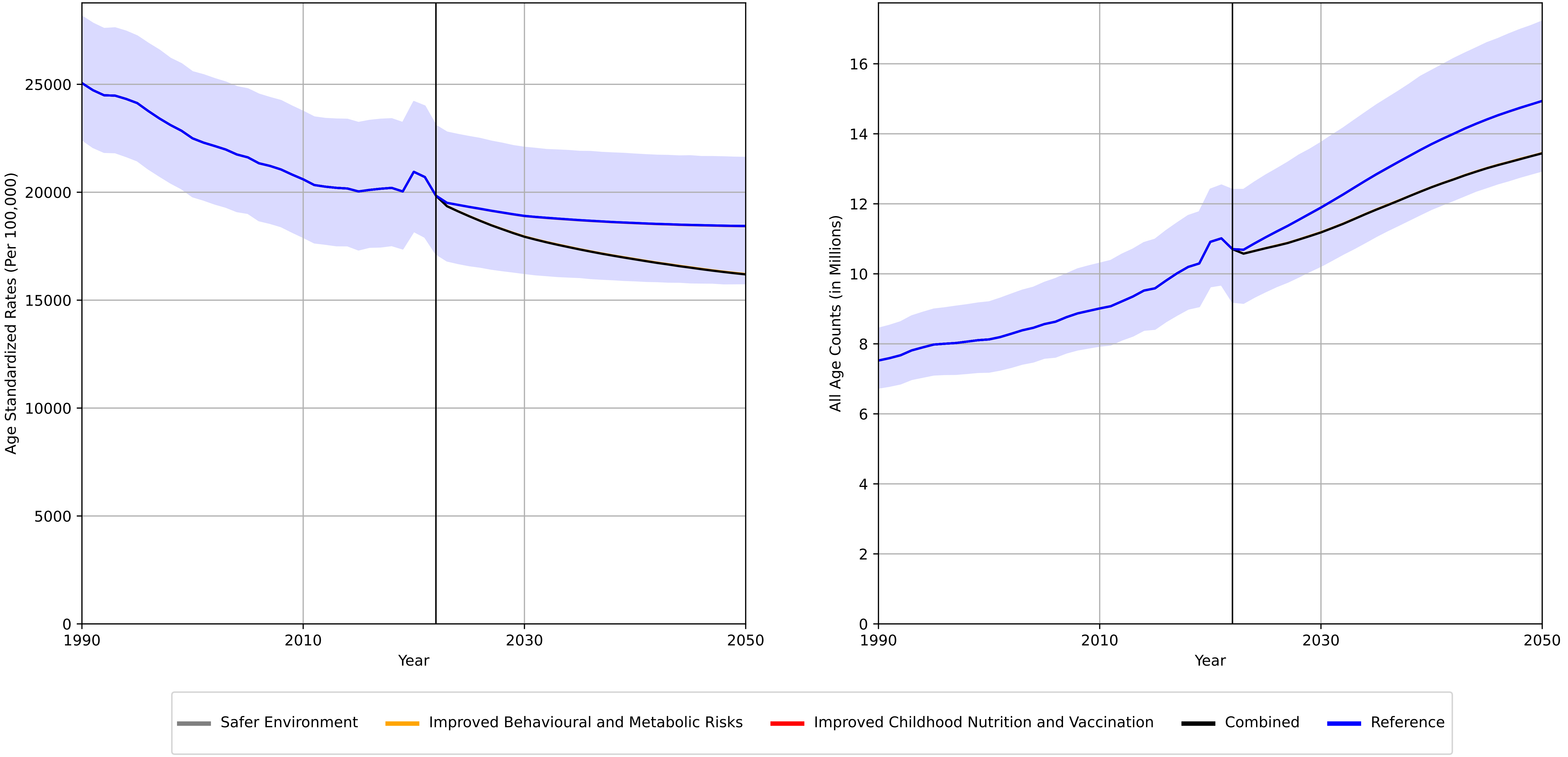
Age Standardized Rates (Per 100,000)
0
5000
10000
15000
20000
25000
1990
2010
2030
2050
Year
All Age Counts (in Millions)
0
2
4
6
8
10
12
14
16
1990
2010
2030
2050
Year
Safer Environment
Improved Behavioural and Metabolic Risks
Improved Childhood Nutrition and Vaccination
Combined
Reference

Greenland

Supplemental Results Figure S10. All-cause age standardised DALY rates (A) and all age DALY counts (B), for the past and for five future scenarios, 1990–2050

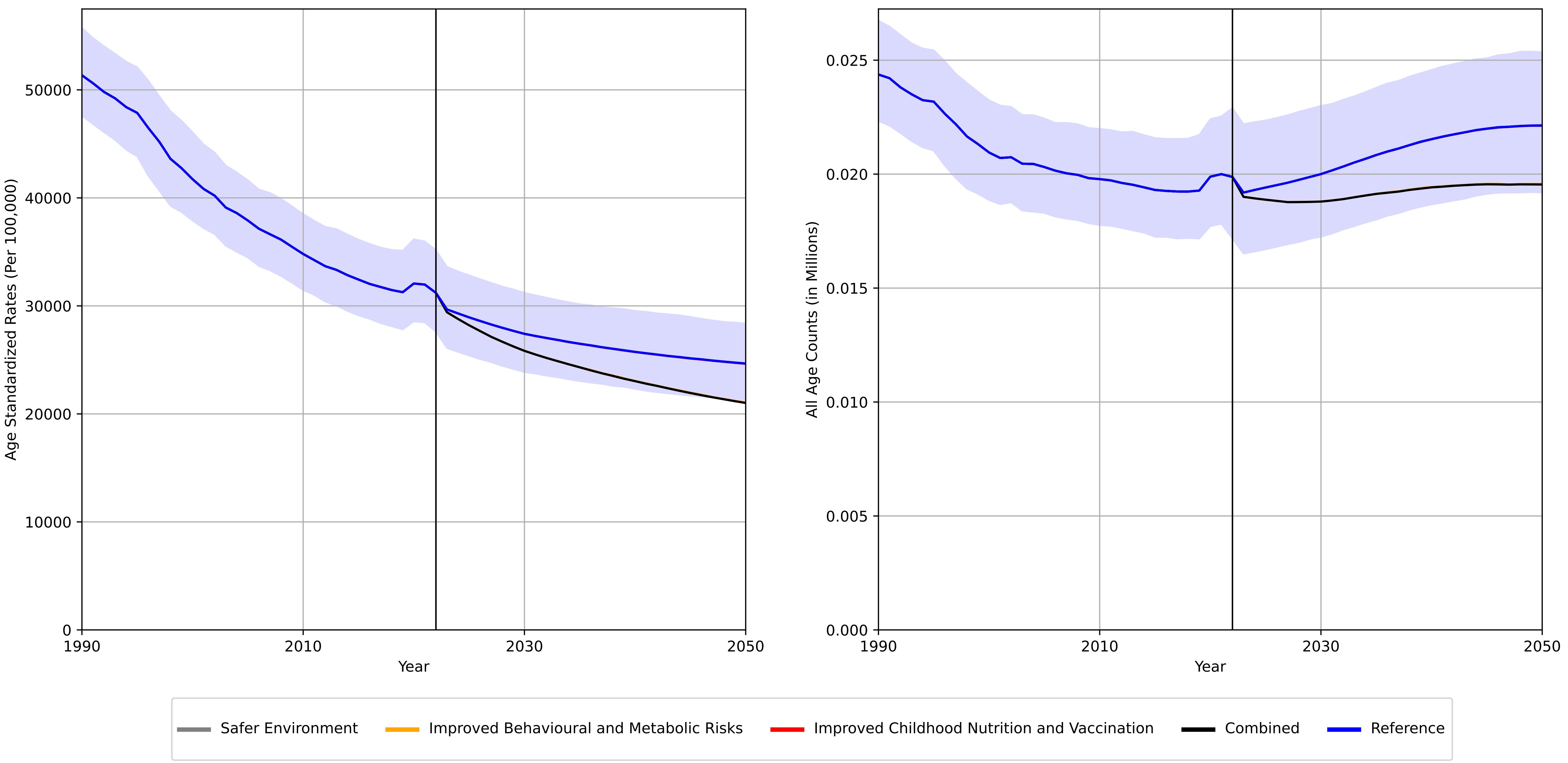

USA

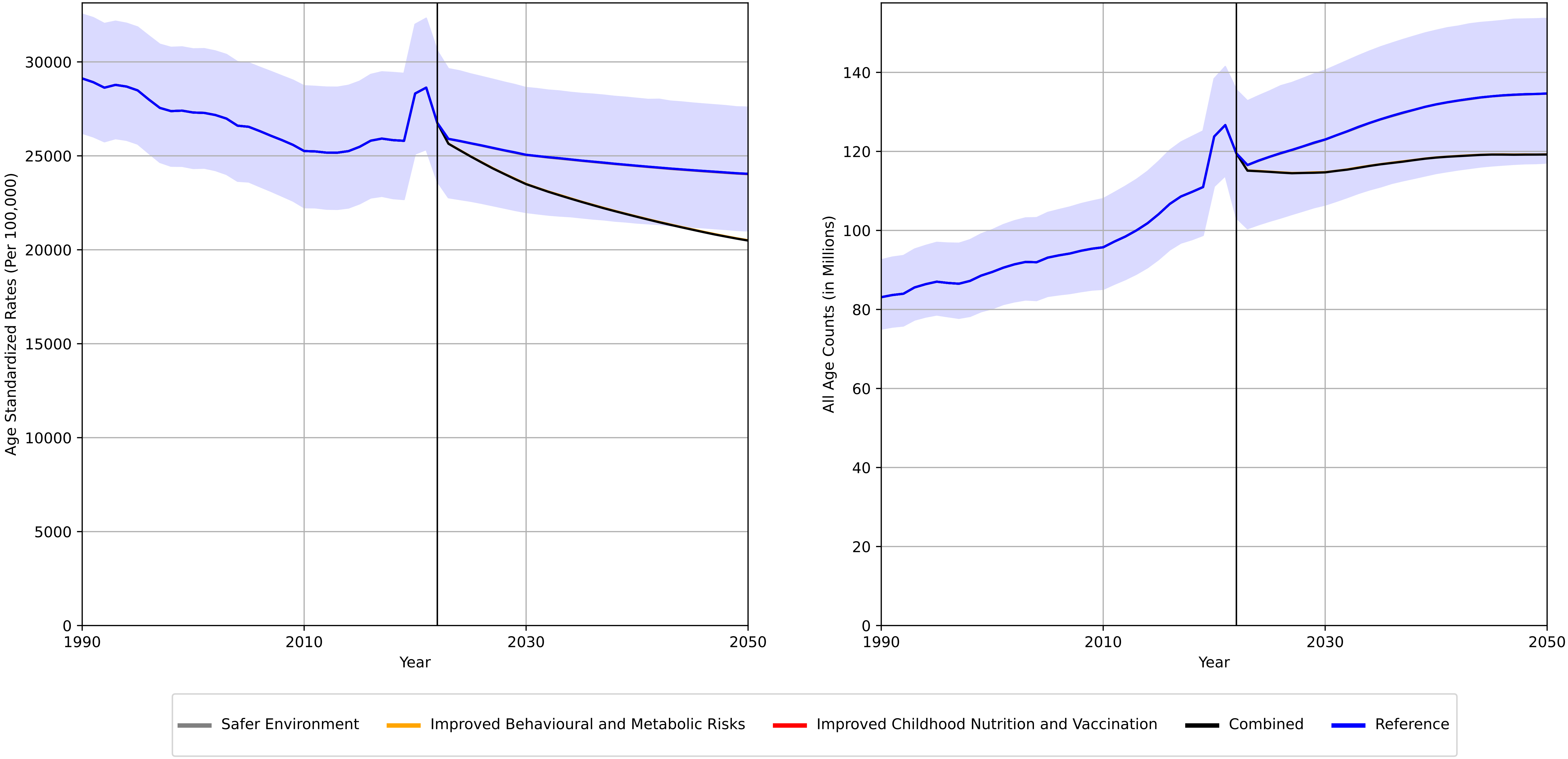

Age Standardized Rates (Per 100,000)
30000
25000
20000
15000
10000
5000
0
1990
2010
2030
2050
Year
All Age Counts (in Millions)
140
120
100
80
60
40
20
0
1990
2010
2030
2050
Year
Safer Environment
Improved Behavioural and Metabolic Risks
Improved Childhood Nutrition and Vaccination
Combined
Reference

Argentina

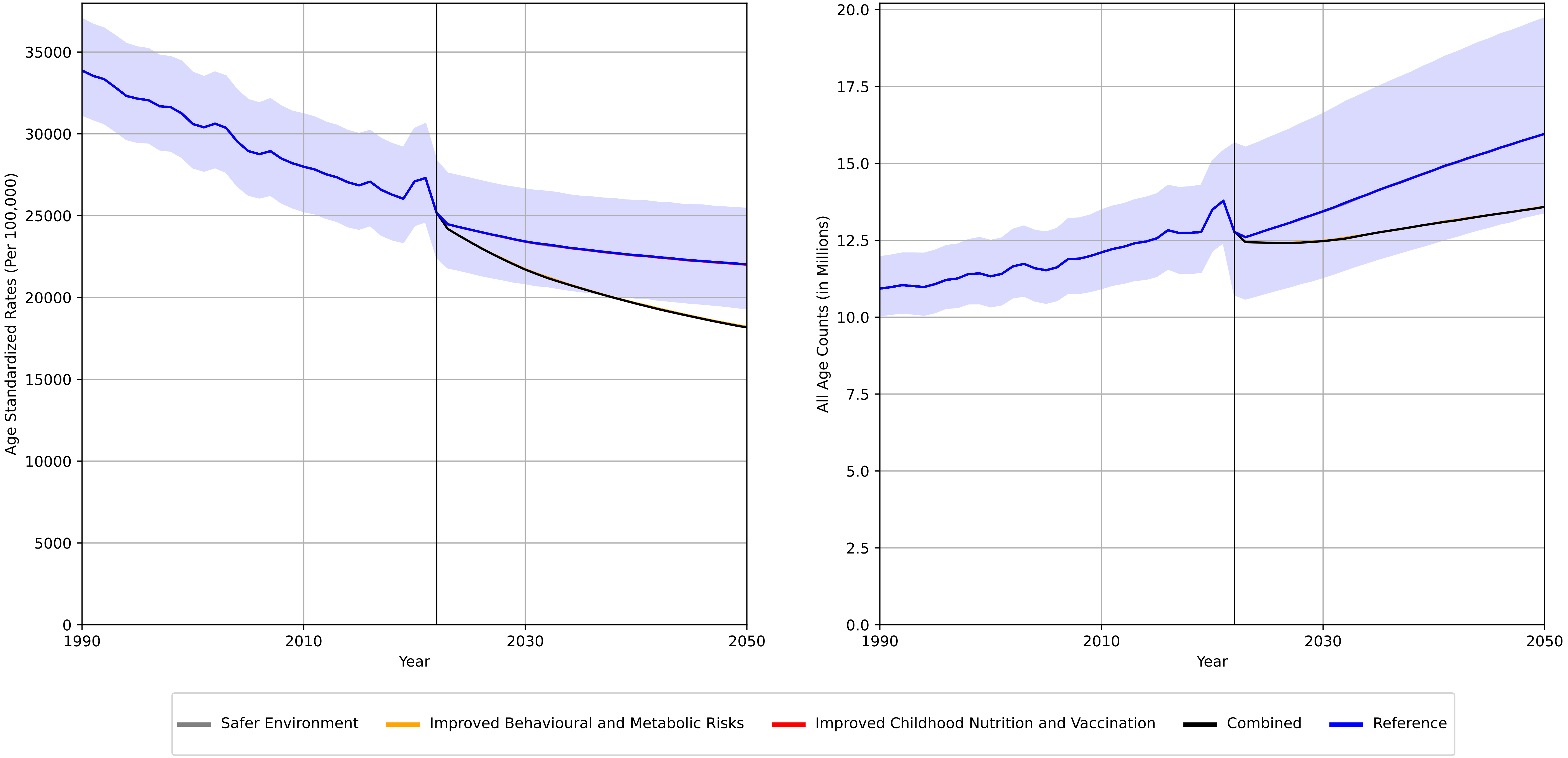
Age Standardized Rates (Per 100,000)
0
5000
10000
15000
20000
25000
30000
35000
1990
2010
2030
2050
Year
All Age Counts (in Millions)
0.0
2.5
5.0
7.5
10.0
12.5
15.0
17.5
20.0
1990
2010
2030
2050
Year
Safer Environment
Improved Behavioural and Metabolic Risks
Improved Childhood Nutrition and Vaccination
Combined
Reference

Chile

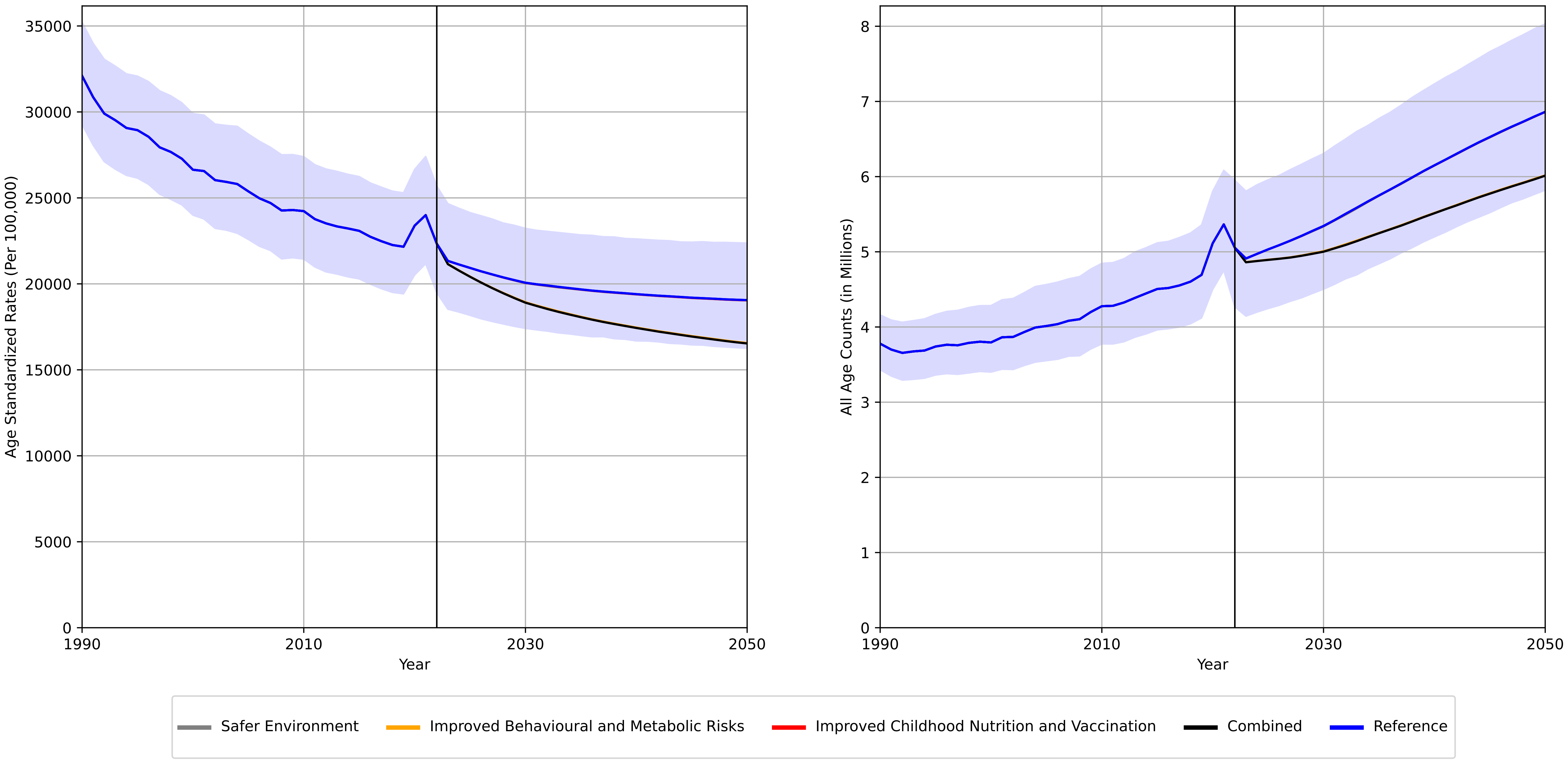
Age Standardized Rates (Per 100,000)
35000
30000
25000
20000
15000
10000
5000
0
1990
2010
2030
2050
Year
All Age Counts (in Millions)
8
7
6
5
4
3
2
1
0
Safer Environment
Improved Behavioural and Metabolic Risks
Improved Childhood Nutrition and Vaccination
Combined
Reference

Uruguay

Supplemental Results Figure S10. All-cause age standardised DALY rates (A) and all age DALY counts (B), for the past and for five future scenarios, 1990–2050

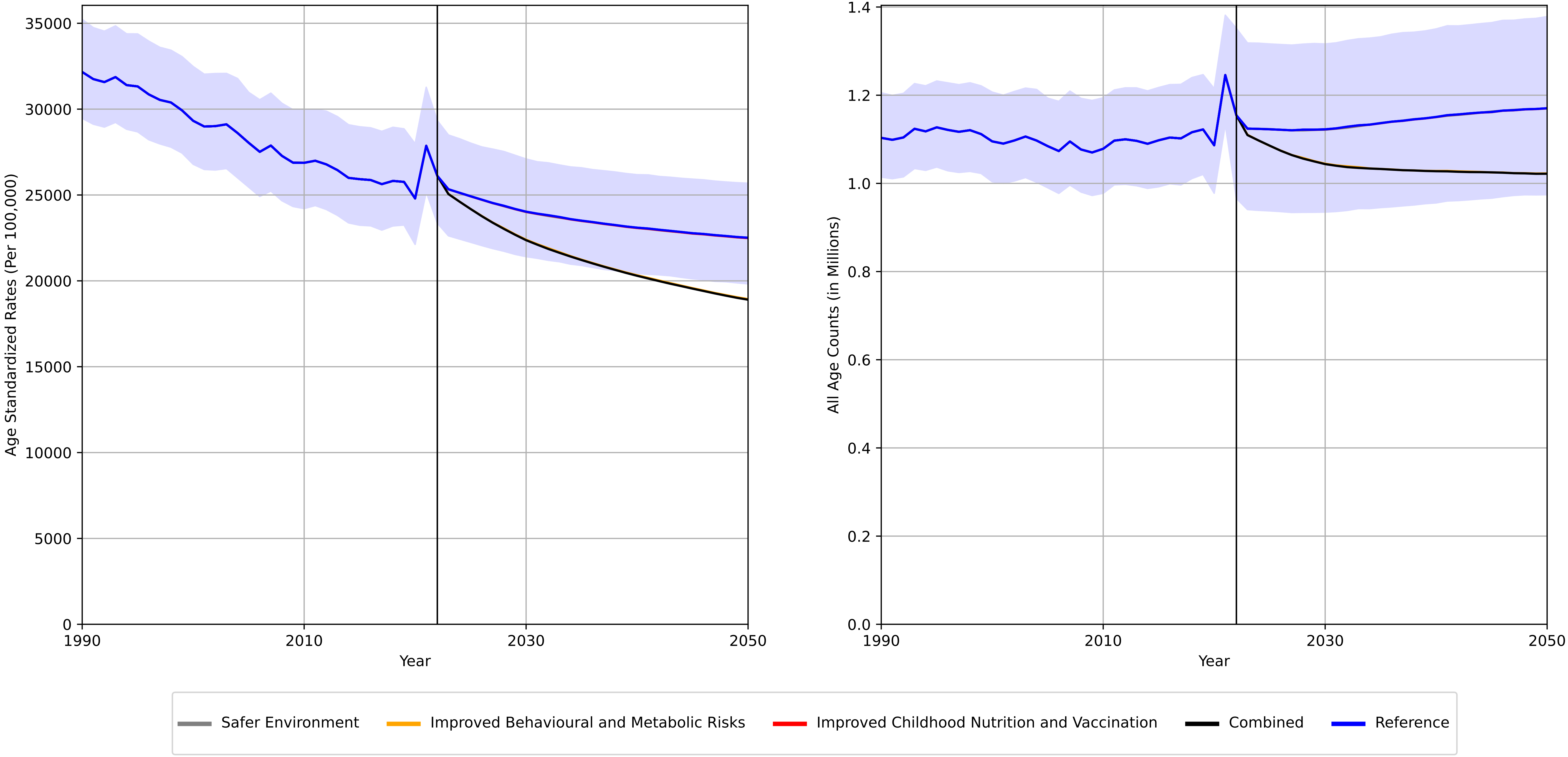

Andorra

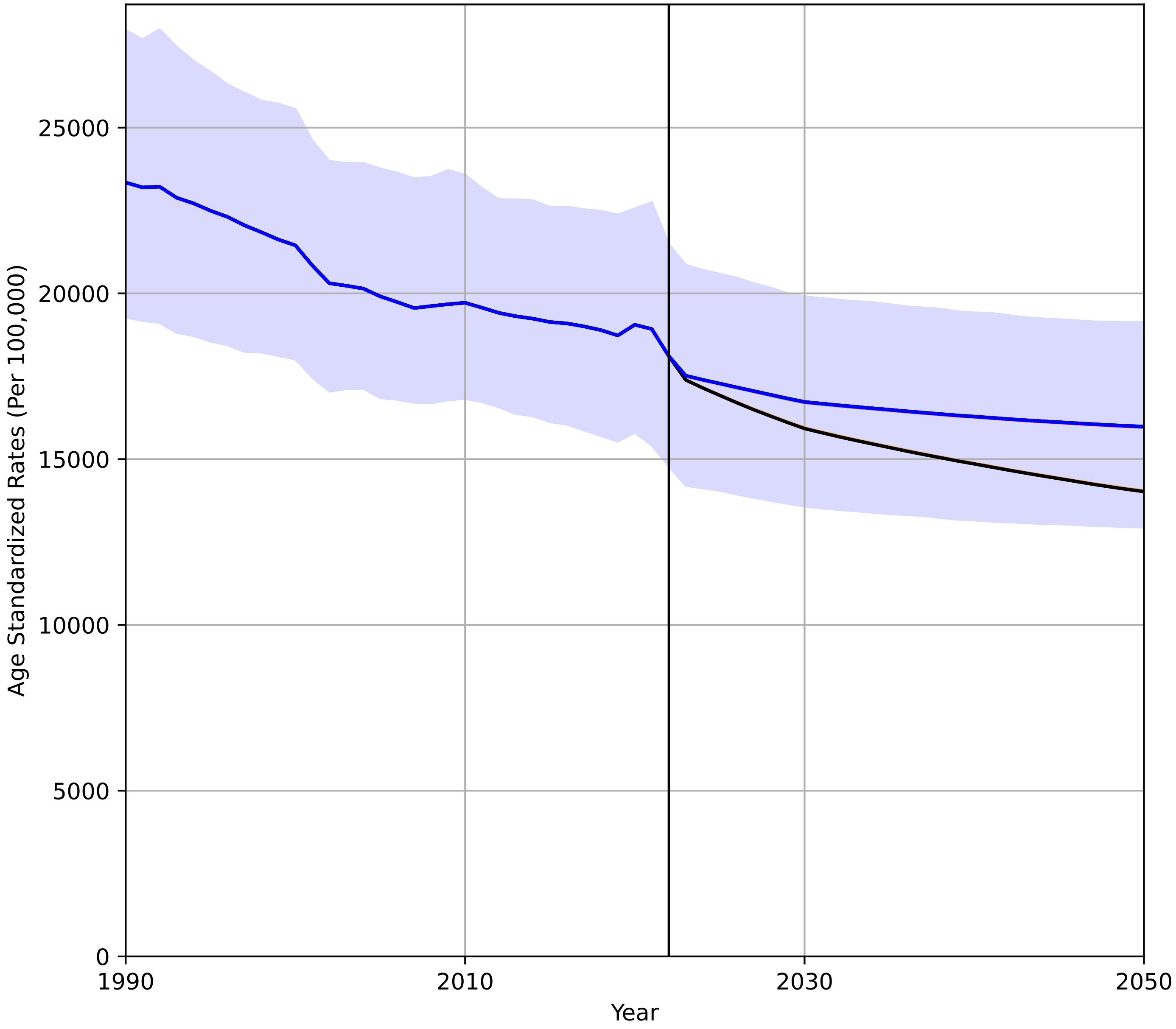
Age Standardized Rates (Per 100,000)
25000
20000
15000
10000
5000
0
1990
2010
2030
2050
Year

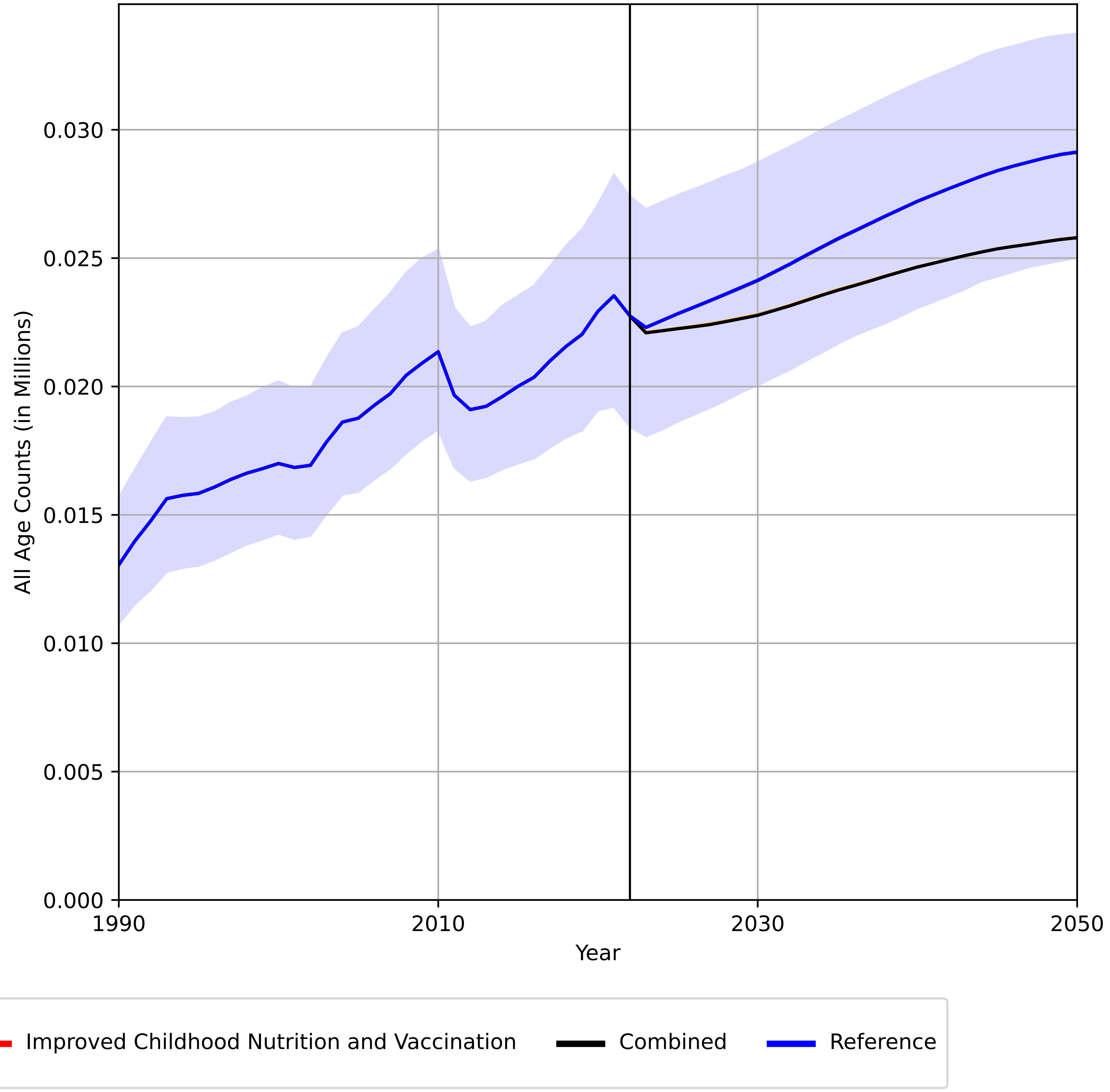
All Age Counts (in Millions)
0.030
0.025
0.020
0.015
0.010
0.005
0.000
1990
2010
2030
2050
Year
Safer Environment
Improved Behavioural and Metabolic Risks
Improved Childhood Nutrition and Vaccination
Combined
Reference

Austria

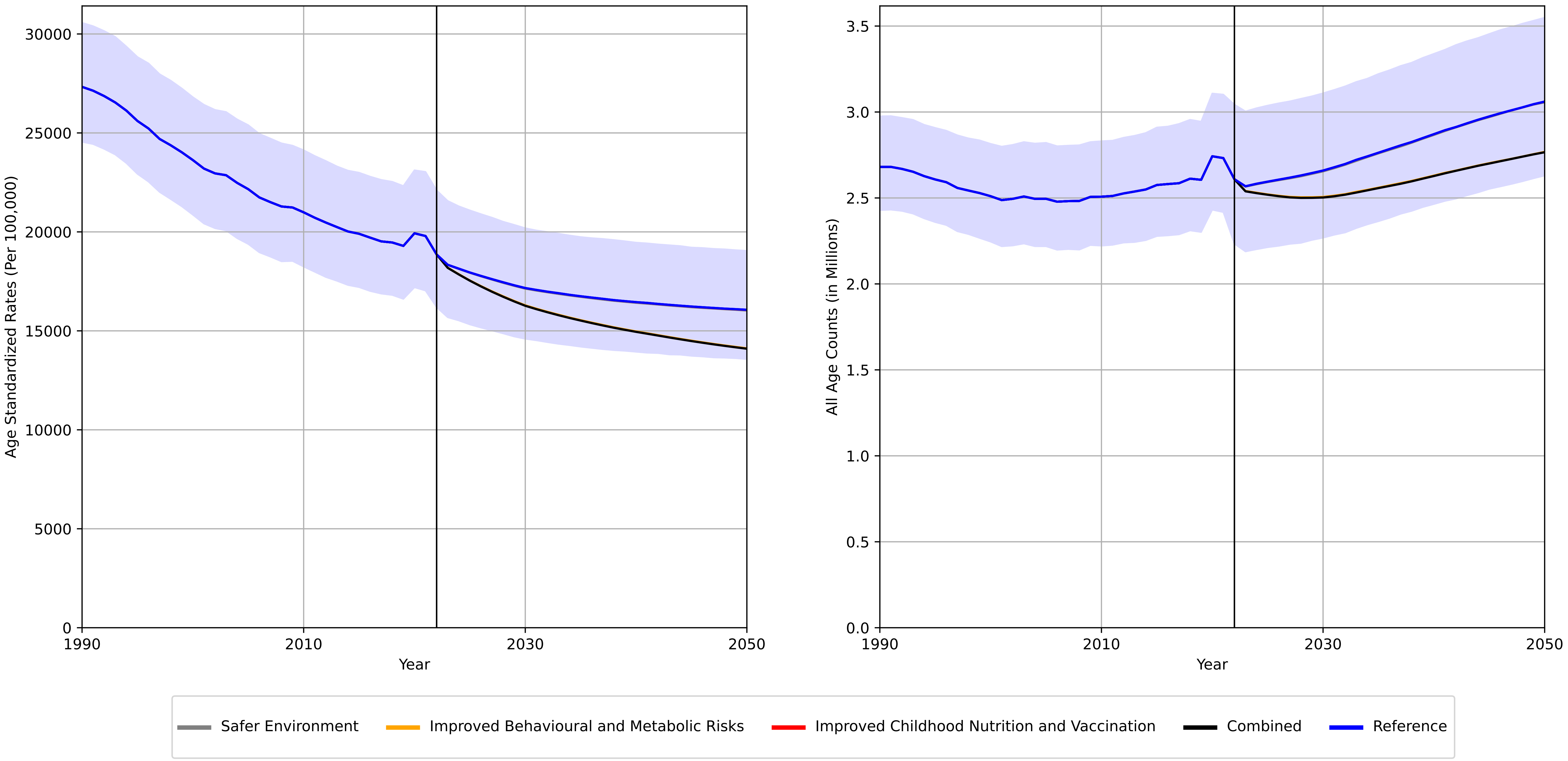

Age Standardized Rates (Per 100,000)
30000
25000
20000
15000
10000
5000
0
1990
2010
2030
2050
Year
All Age Counts (in Millions)
3.5
3.0
2.5
2.0
1.5
1.0
0.5
0.0
Safer Environment
Improved Behavioural and Metabolic Risks
Improved Childhood Nutrition and Vaccination
Combined
Reference

Belgium

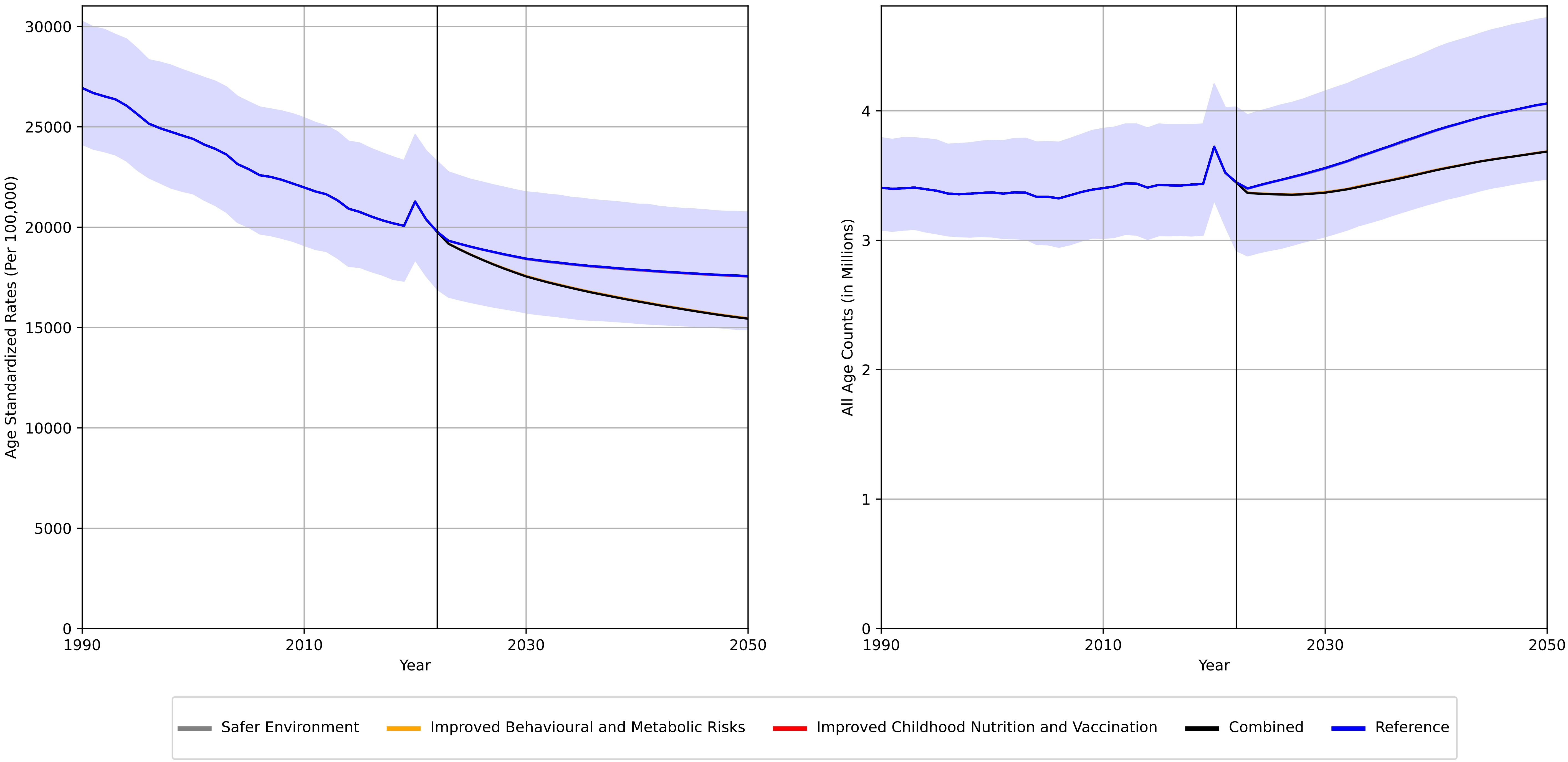
Age Standardized Rates (Per 100,000)
30000
25000
20000
15000
10000
5000
0
1990
2010
2030
2050
Year
All Age Counts (in Millions)
4
3
2
1
0
1990
2010
2030
2050
Year
Safer Environment
Improved Behavioural and Metabolic Risks
Improved Childhood Nutrition and Vaccination
Combined
Reference

Cyprus

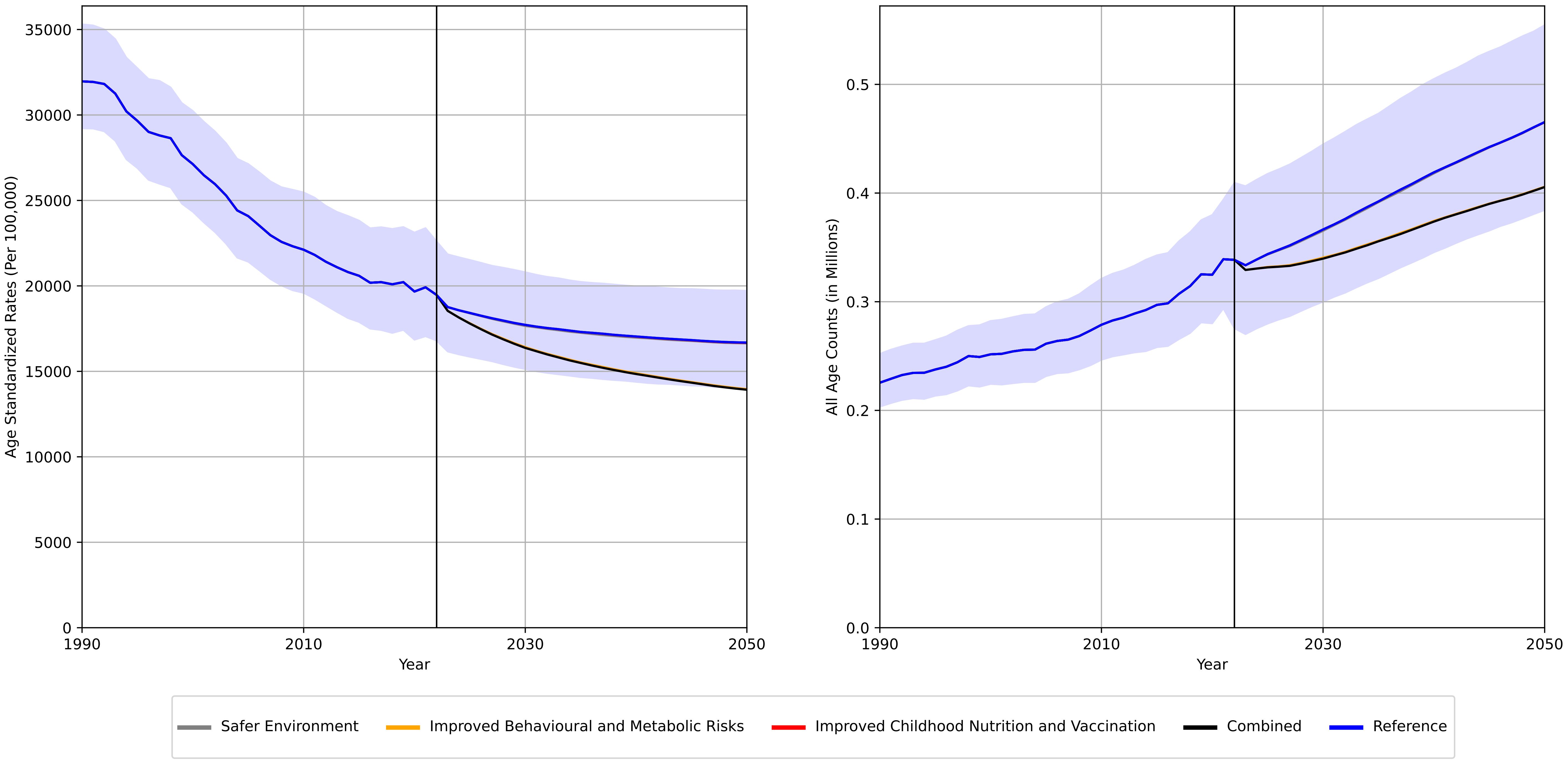
Age Standardized Rates (Per 100,000)
35000
30000
25000
20000
15000
10000
5000
0
1990
2010
2030
2050
Year
All Age Counts (in Millions)
0.5
0.4
0.3
0.2
0.1
0.0
1990
2010
2030
2050
Year
Safer Environment
Improved Behavioural and Metabolic Risks
Improved Childhood Nutrition and Vaccination
Combined
Reference

Denmark

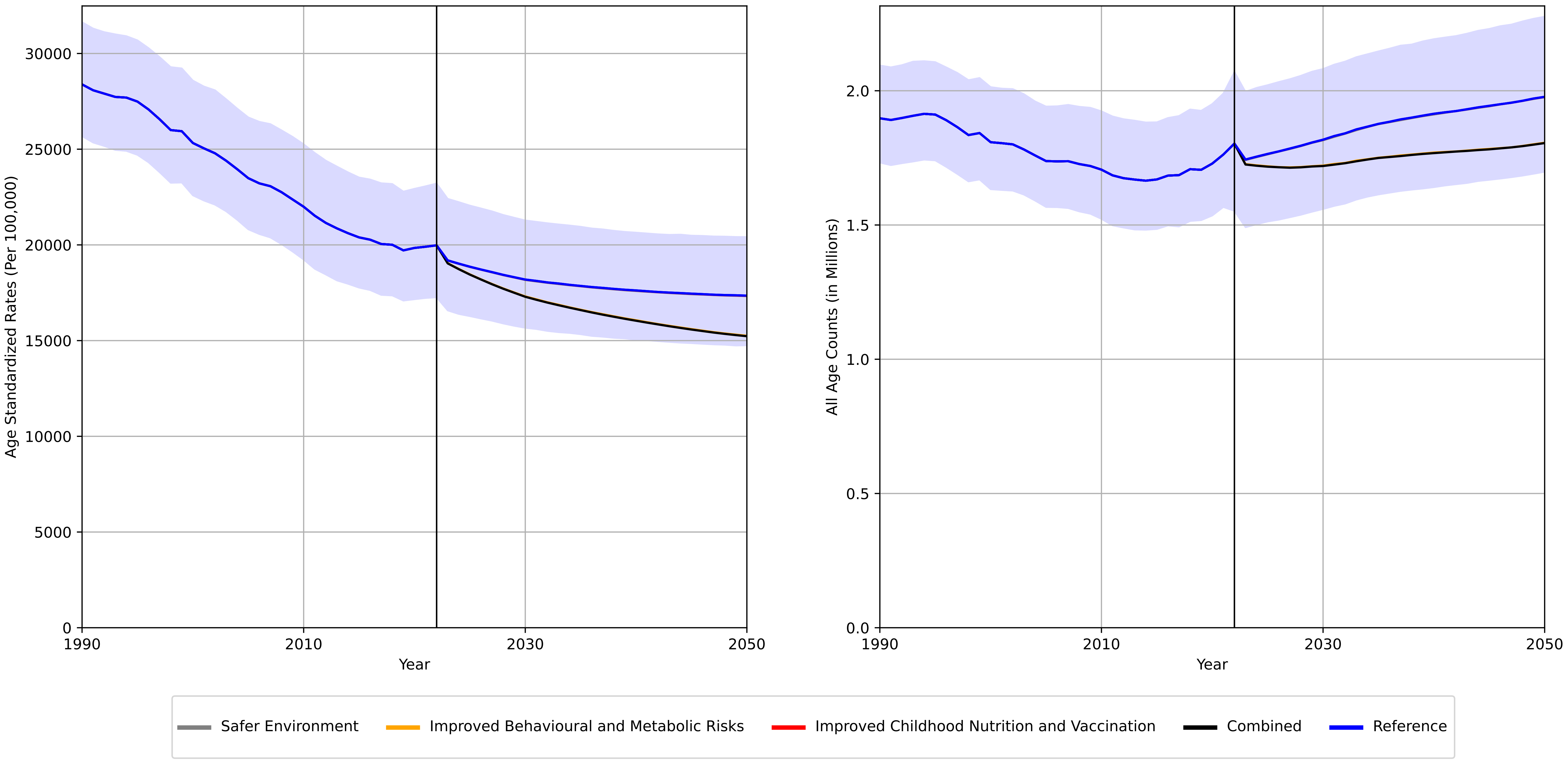

Age Standardized Rates (Per 100,000)
30000
25000
20000
15000
10000
5000
0
1990
2010
2030
2050
Year
All Age Counts (in Millions)
2.0
1.5
1.0
0.5
0.0
1990
2010
2030
2050
Year
Safer Environment
Improved Behavioural and Metabolic Risks
Improved Childhood Nutrition and Vaccination
Combined
Reference

Finland

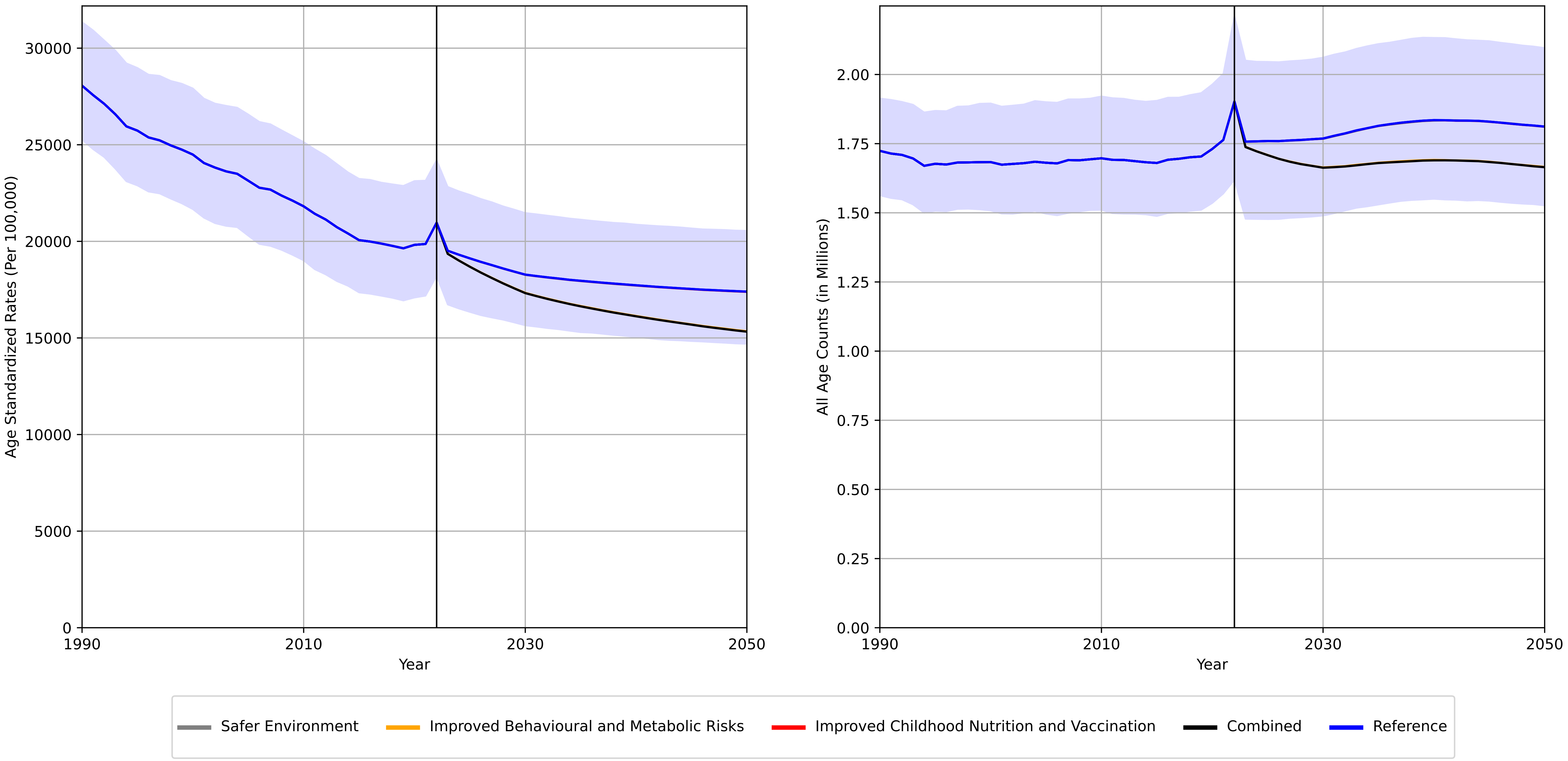

Age Standardized Rates (Per 100,000)
30000
25000
20000
15000
10000
5000
0
1990
2010
2030
2050
Year
All Age Counts (in Millions)
2.00
1.75
1.50
1.25
1.00
0.75
0.50
0.25
0.00
Safer Environment
Improved Behavioural and Metabolic Risks
Improved Childhood Nutrition and Vaccination
Combined
Reference

France

Supplemental Results Figure S10. All-cause age standardised DALY rates (A) and all age DALY counts (B), for the past and for five future scenarios, 1990–2050

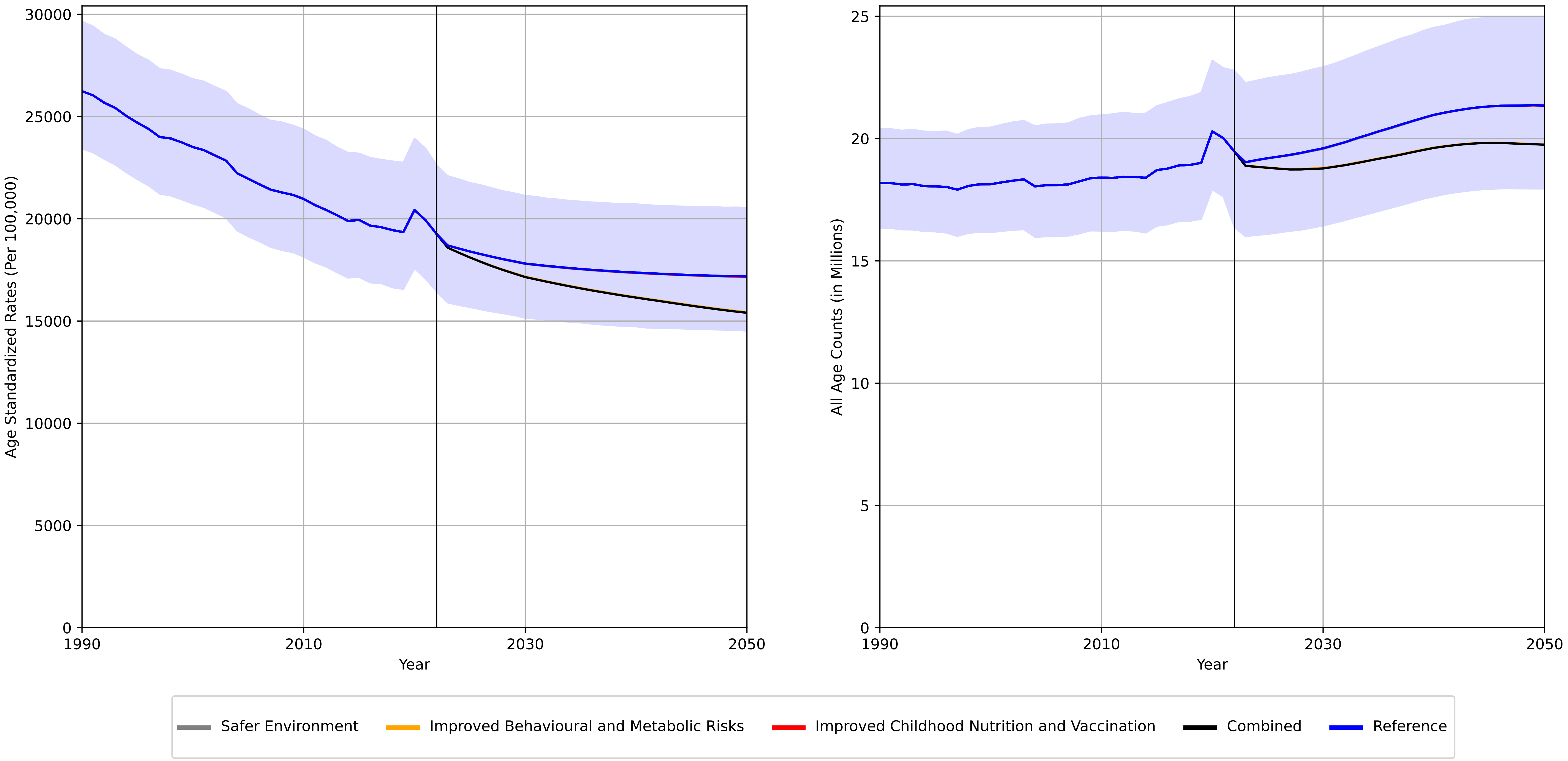

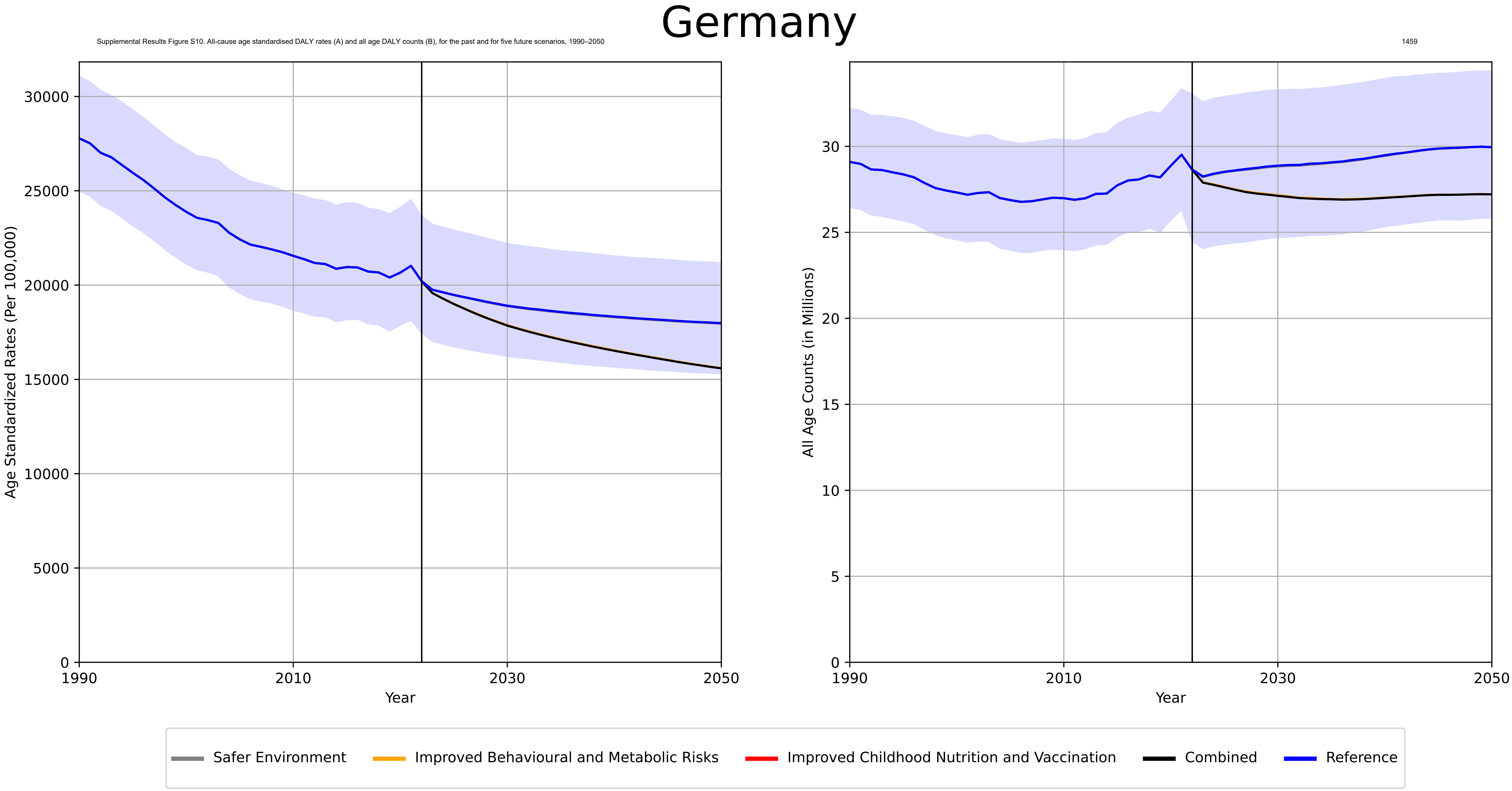
Germany
Age Standardized Rates (Per 100,000)
30000
25000
20000
15000
10000
5000
0
1990
2010
2030
2050
Year
All Age Counts (in Millions)
30
25
20
15
10
5
0
1990
2010
2030
2050
Year
Safer Environment
Improved Behavioural and Metabolic Risks
Improved Childhood Nutrition and Vaccination
Combined
Reference

Greece

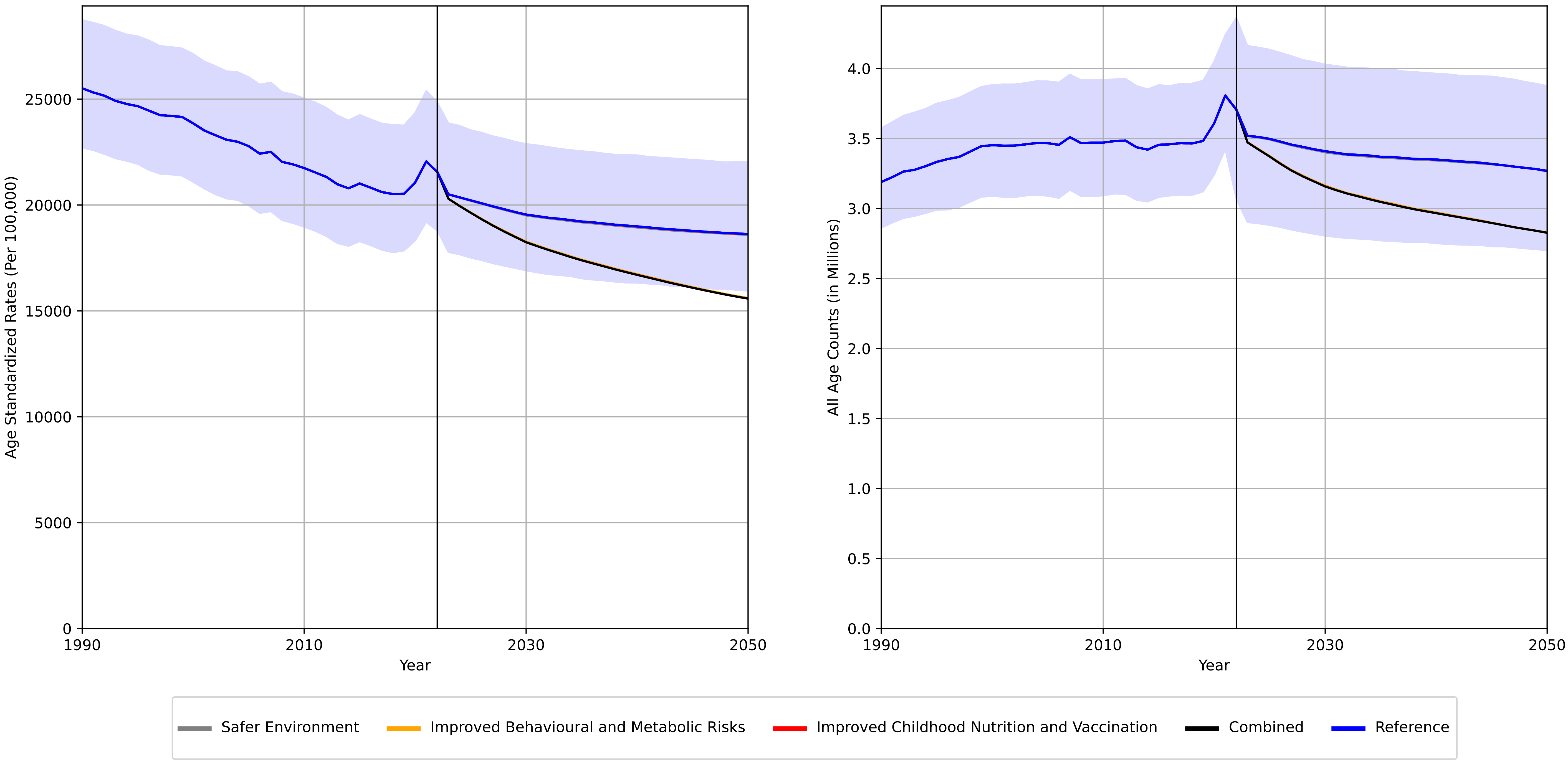

Age Standardized Rates (Per 100,000)
0
5000
10000
15000
20000
25000
1990
2010
2030
2050
Year
All Age Counts (in Millions)
0.0
0.5
1.0
1.5
2.0
2.5
3.0
3.5
4.0
1990
2010
2030
2050
Year
Safer Environment
Improved Behavioural and Metabolic Risks
Improved Childhood Nutrition and Vaccination
Combined
Reference

Iceland

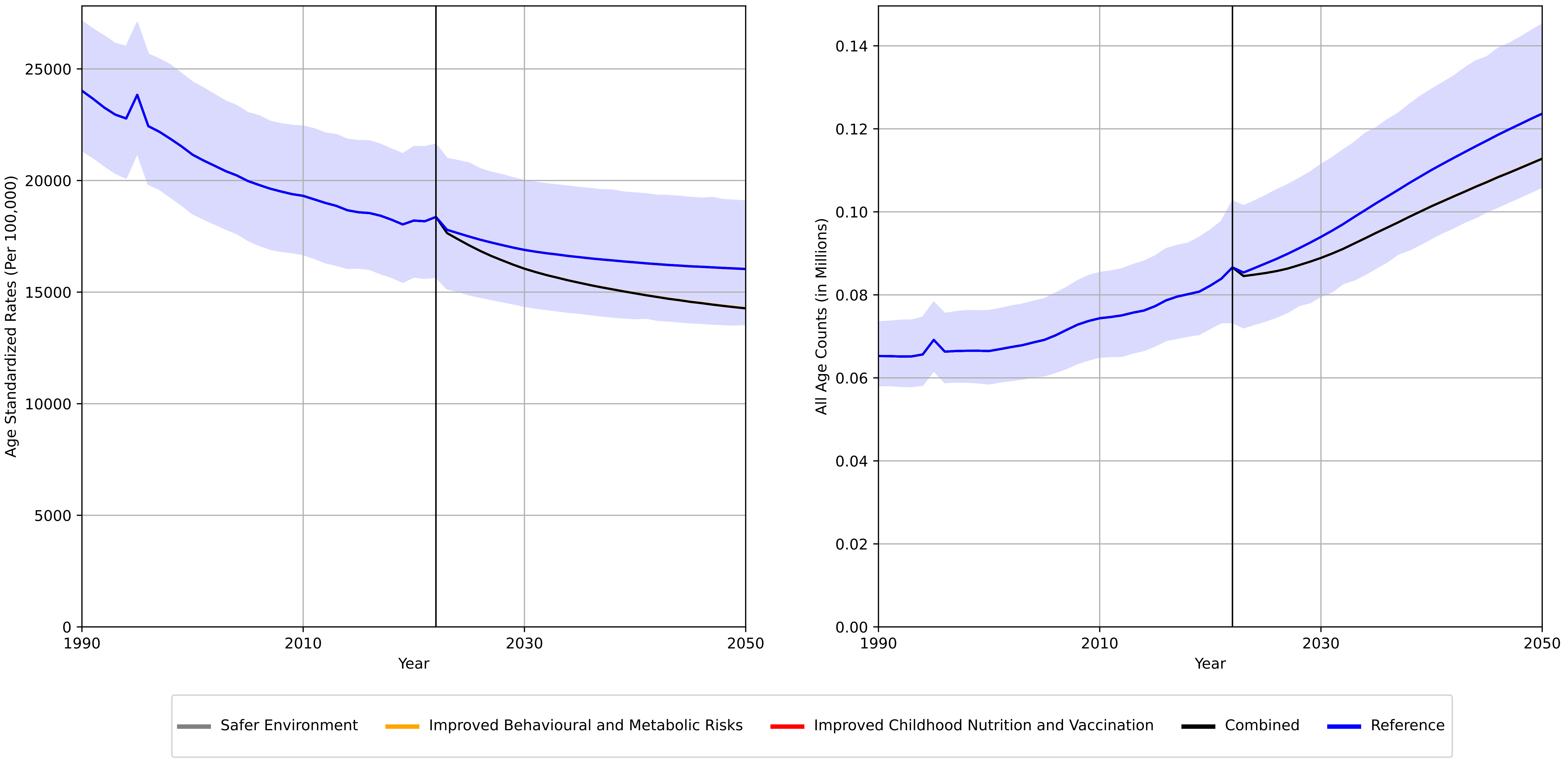

Age Standardized Rates (Per 100,000)
25000
20000
15000
10000
5000
0
1990
2010
2030
2050
Year
All Age Counts (in Millions)
0.14
0.12
0.10
0.08
0.06
0.04
0.02
0.00
1990
2010
2030
2050
Year
Safer Environment
Improved Behavioural and Metabolic Risks
Improved Childhood Nutrition and Vaccination
Combined
Reference

Ireland

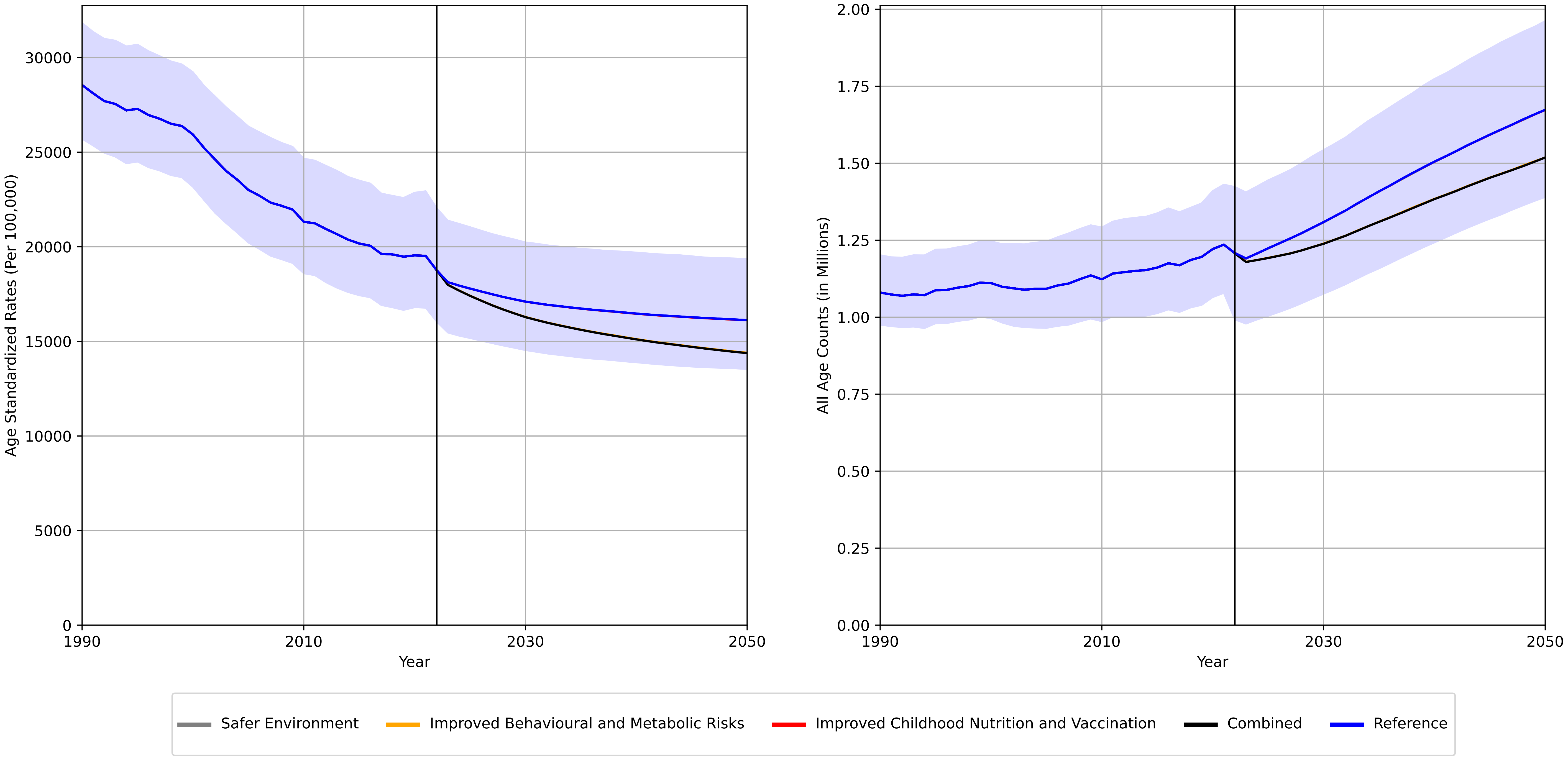

Age Standardized Rates (Per 100,000)
0
5000
10000
15000
20000
25000
30000
1990
2010
2030
2050
Year
All Age Counts (in Millions)
0.00
0.25
0.50
0.75
1.00
1.25
1.50
1.75
2.00
1990
2010
2030
2050
Year
Safer Environment
Improved Behavioural and Metabolic Risks
Improved Childhood Nutrition and Vaccination
Combined
Reference

Israel

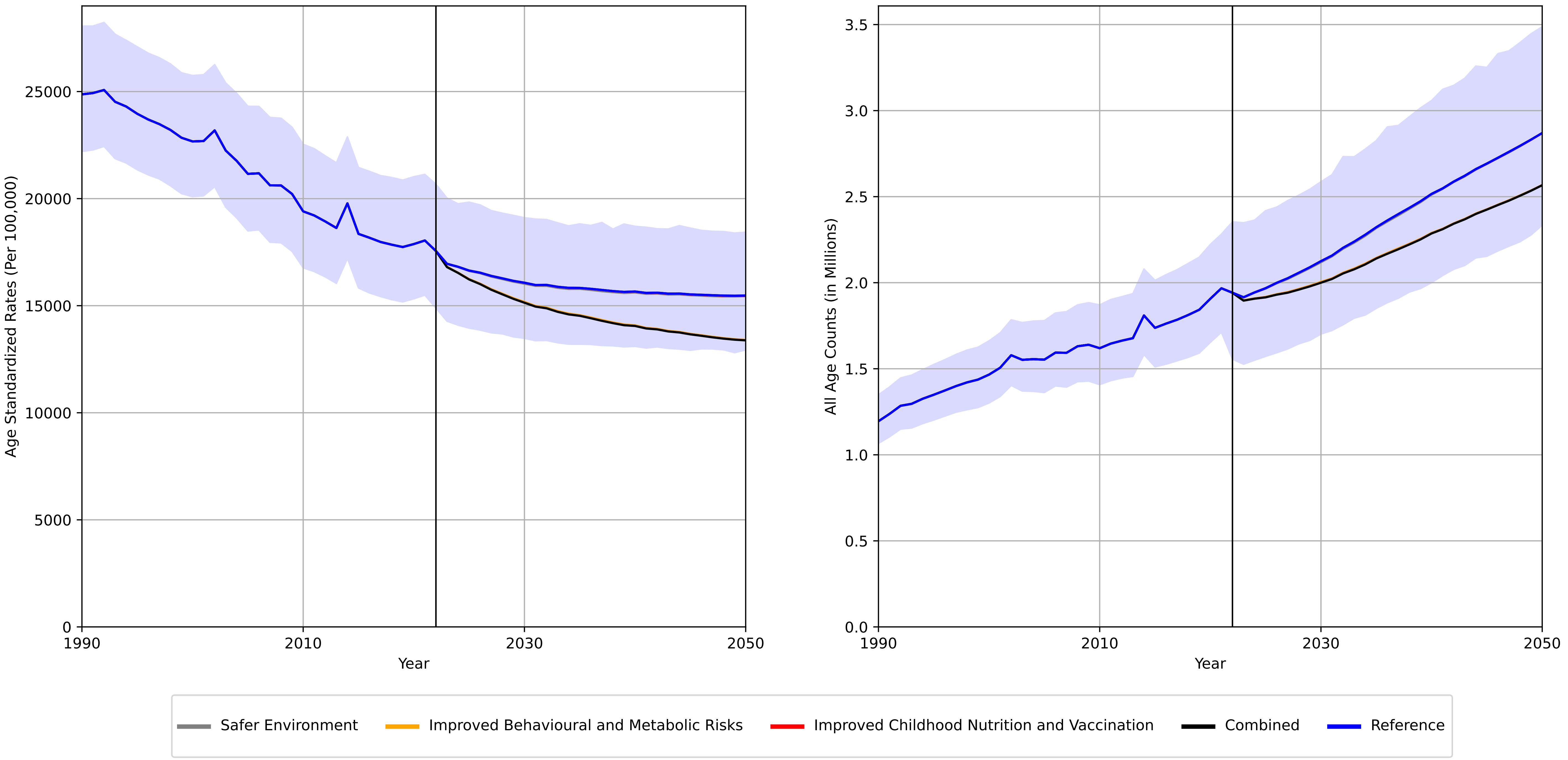

Age Standardized Rates (Per 100,000)
0
5000
10000
15000
20000
25000
1990
2010
2030
2050
Year
All Age Counts (in Millions)
0.0
0.5
1.0
1.5
2.0
2.5
3.0
3.5
1990
2010
2030
2050
Year
Safer Environment
Improved Behavioural and Metabolic Risks
Improved Childhood Nutrition and Vaccination
Combined
Reference

Italy

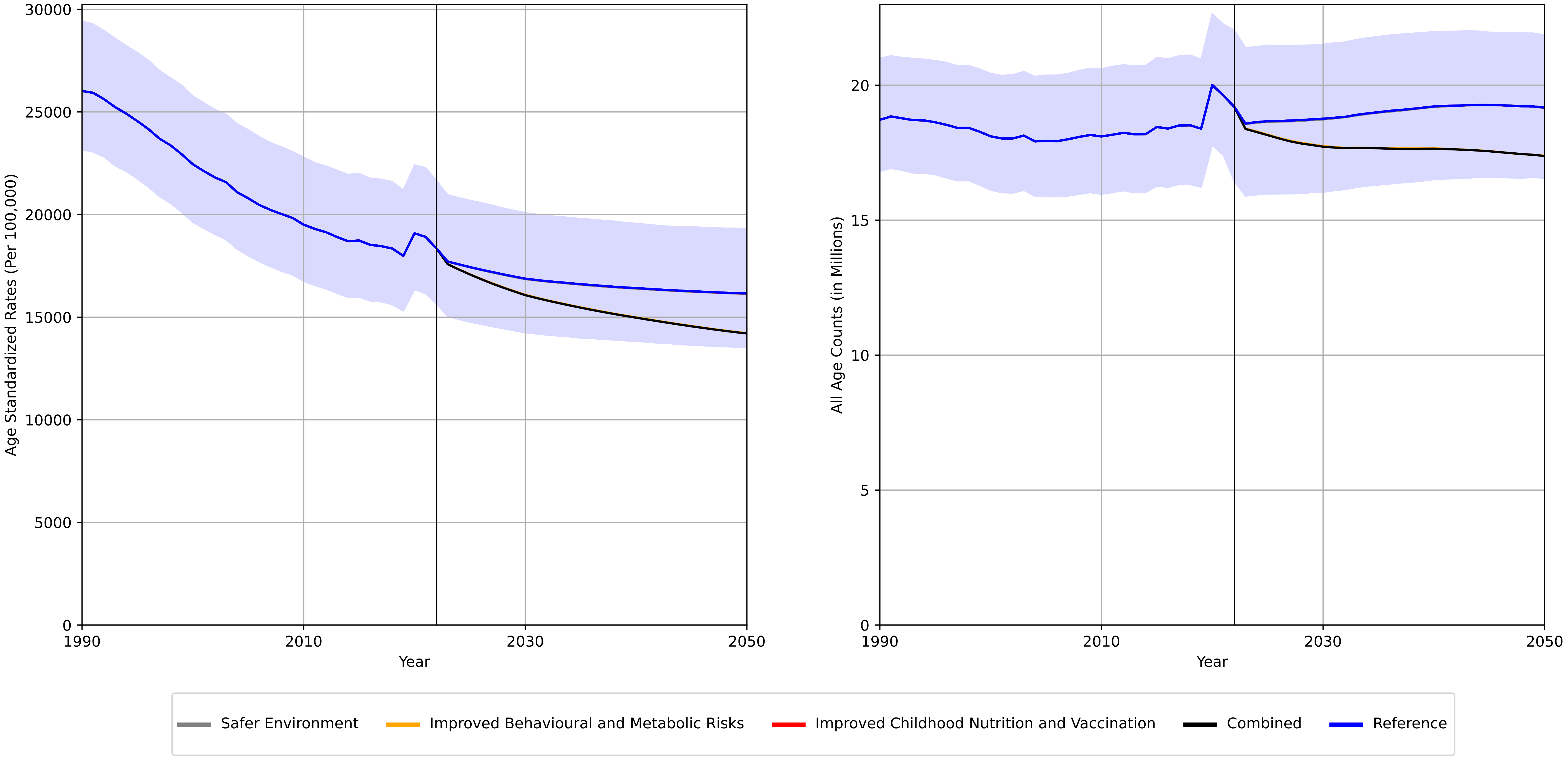
Age Standardized Rates (Per 100,000)
30000
25000
20000
15000
10000
5000
0
1990
2010
2030
2050
Year
All Age Counts (in Millions)
20
15
10
5
0
1990
2010
2030
2050
Year
Safer Environment
Improved Behavioural and Metabolic Risks
Improved Childhood Nutrition and Vaccination
Combined
Reference

Luxembourg

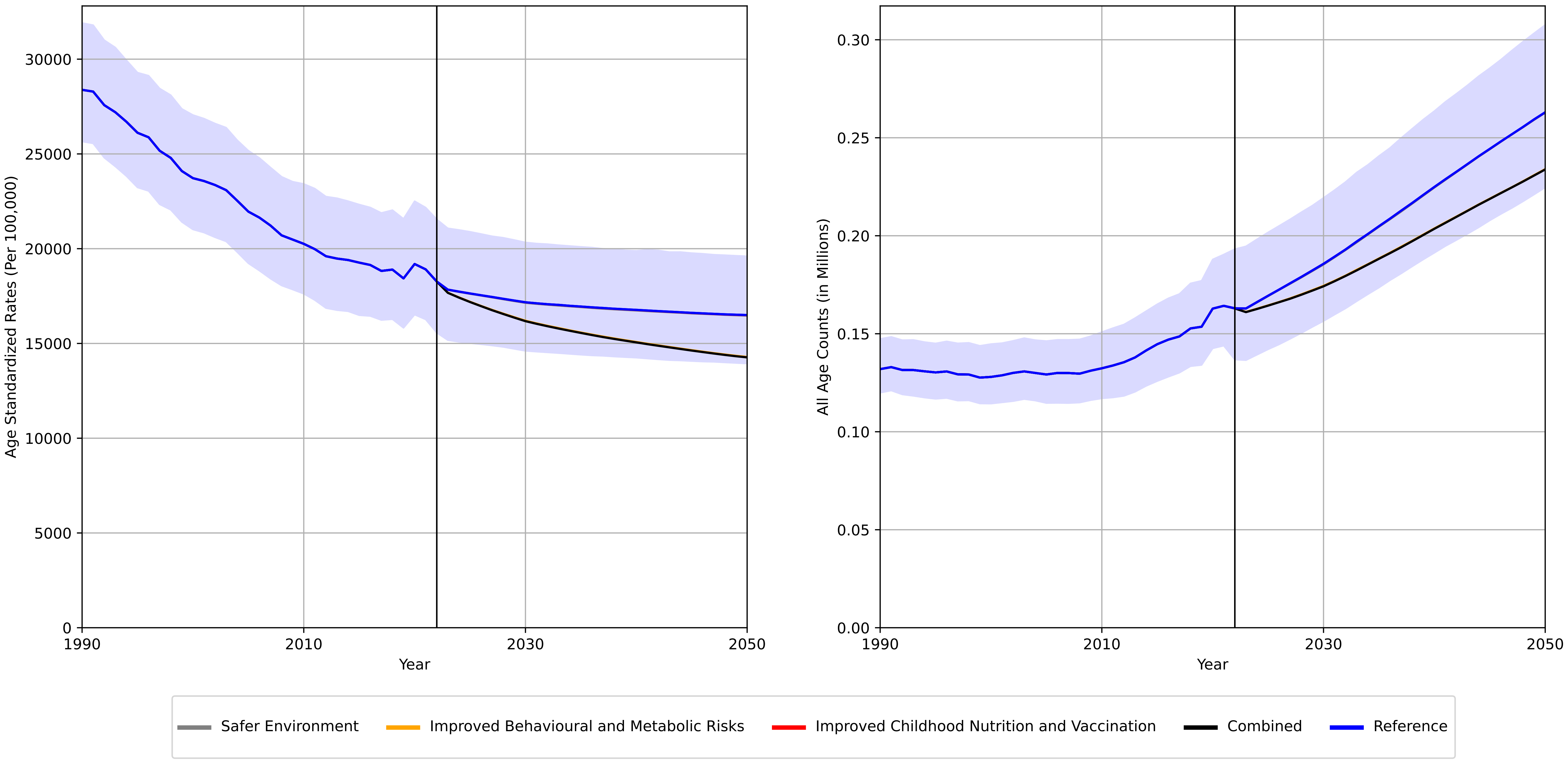
Age Standardized Rates (Per 100,000)
0
5000
10000
15000
20000
25000
30000
1990
2010
2030
2050
Year
All Age Counts (in Millions)
0.00
0.05
0.10
0.15
0.20
0.25
0.30
1990
2010
2030
2050
Year
Safer Environment
Improved Behavioural and Metabolic Risks
Improved Childhood Nutrition and Vaccination
Combined
Reference

Malta

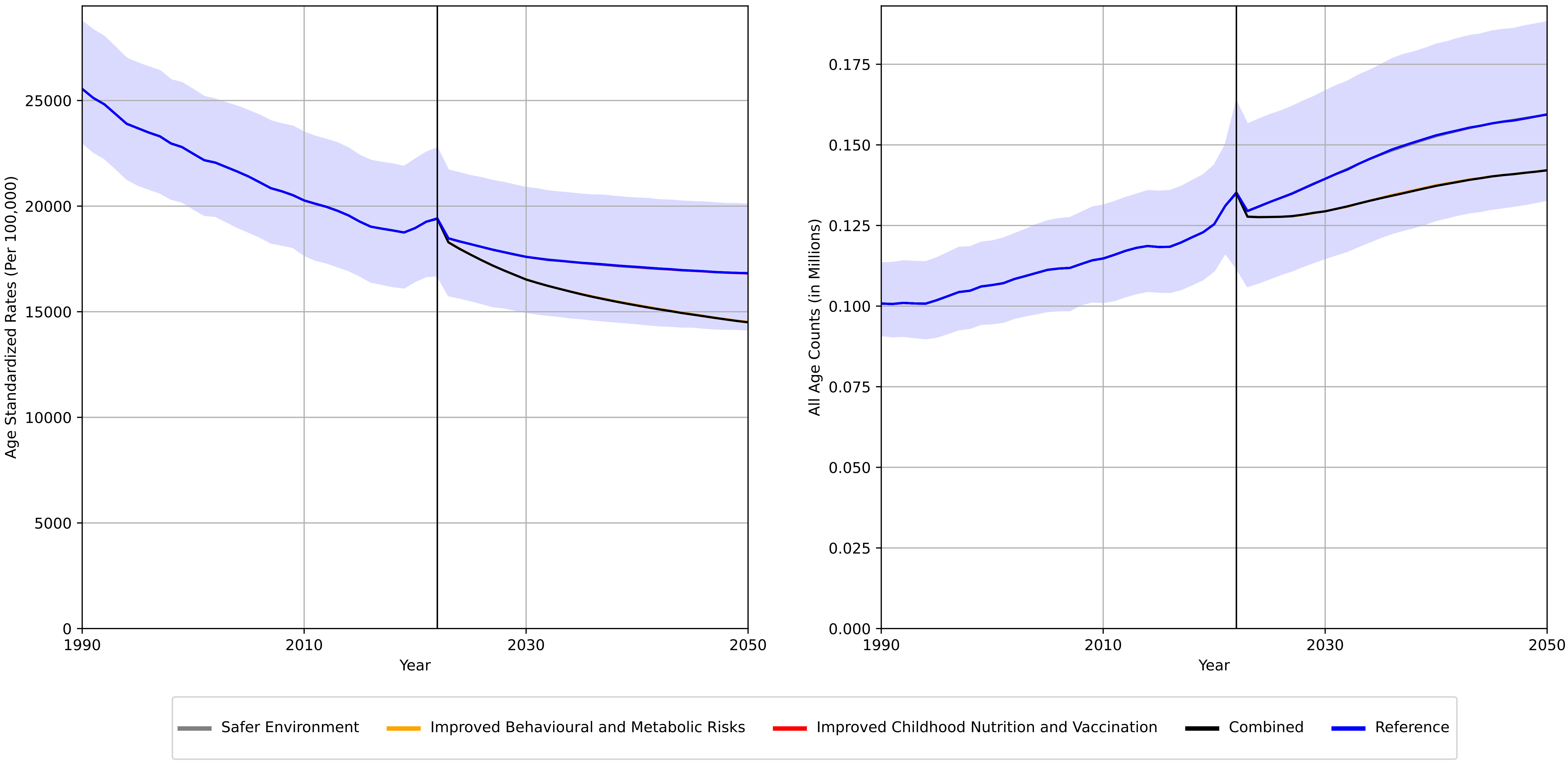
Age Standardized Rates (Per 100,000)
0
5000
10000
15000
20000
25000
1990
2010
2030
2050
Year
All Age Counts (in Millions)
0.000
0.025
0.050
0.075
0.100
0.125
0.150
0.175
1990
2010
2030
2050
Year
Safer Environment
Improved Behavioural and Metabolic Risks
Improved Childhood Nutrition and Vaccination
Combined
Reference

Monaco

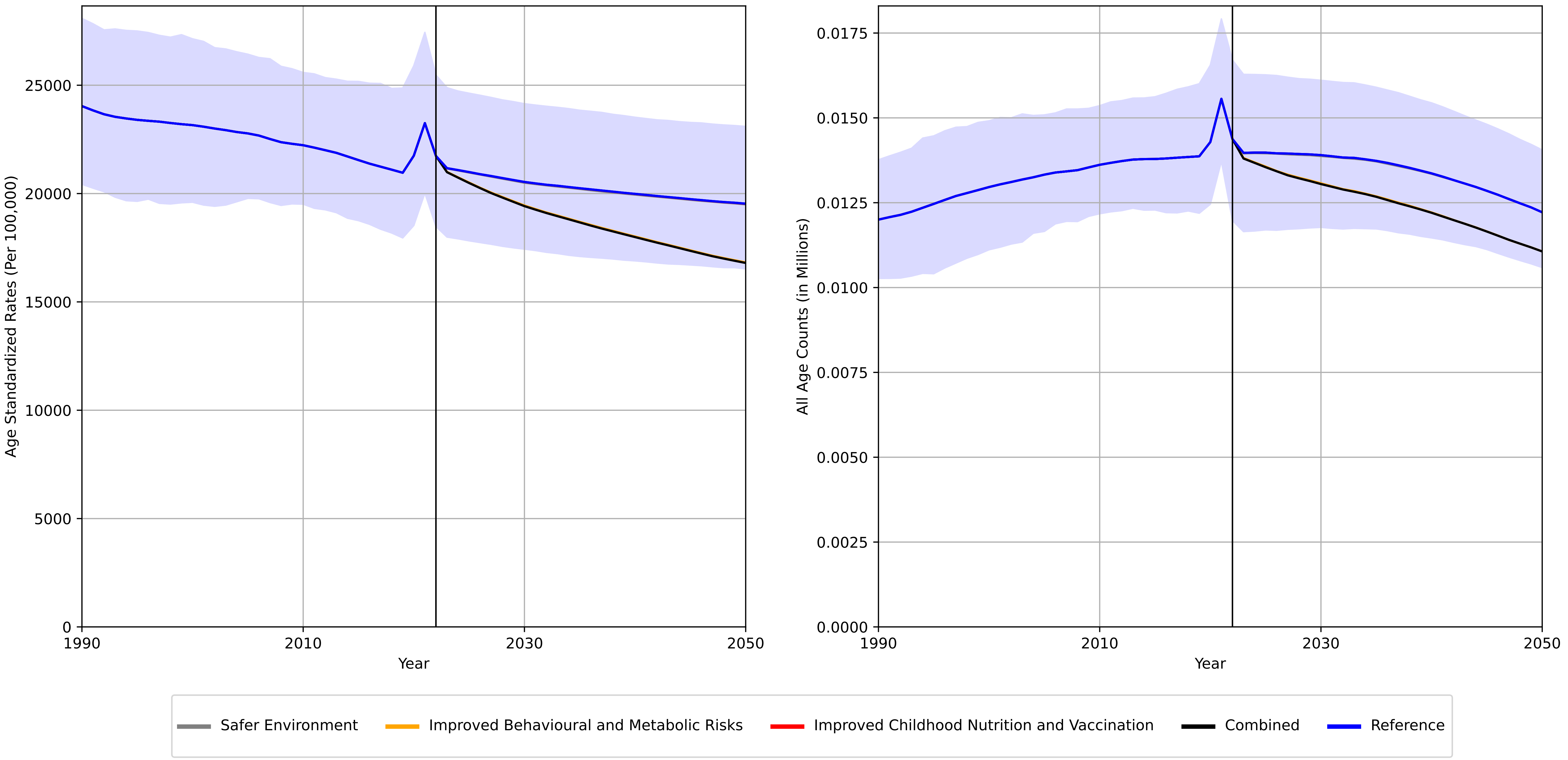

Age Standardized Rates (Per 100,000)
25000
20000
15000
10000
5000
0
1990
2010
2030
2050
Year
All Age Counts (in Millions)
0.0175
0.0150
0.0125
0.0100
0.0075
0.0050
0.0025
0.0000
1990
2010
2030
2050
Year
Safer Environment
Improved Behavioural and Metabolic Risks
Improved Childhood Nutrition and Vaccination
Combined
Reference

Netherlands

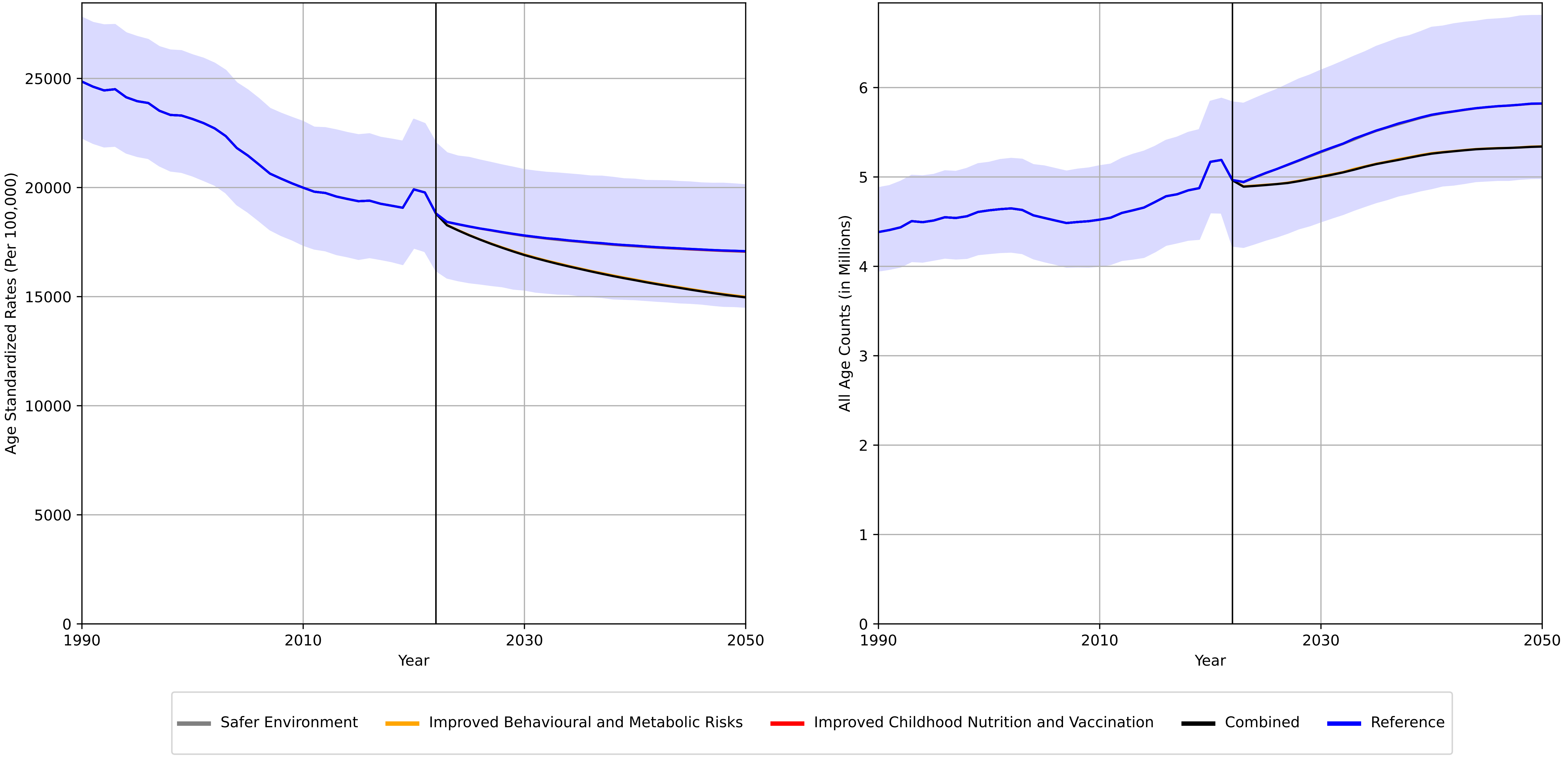

Norway

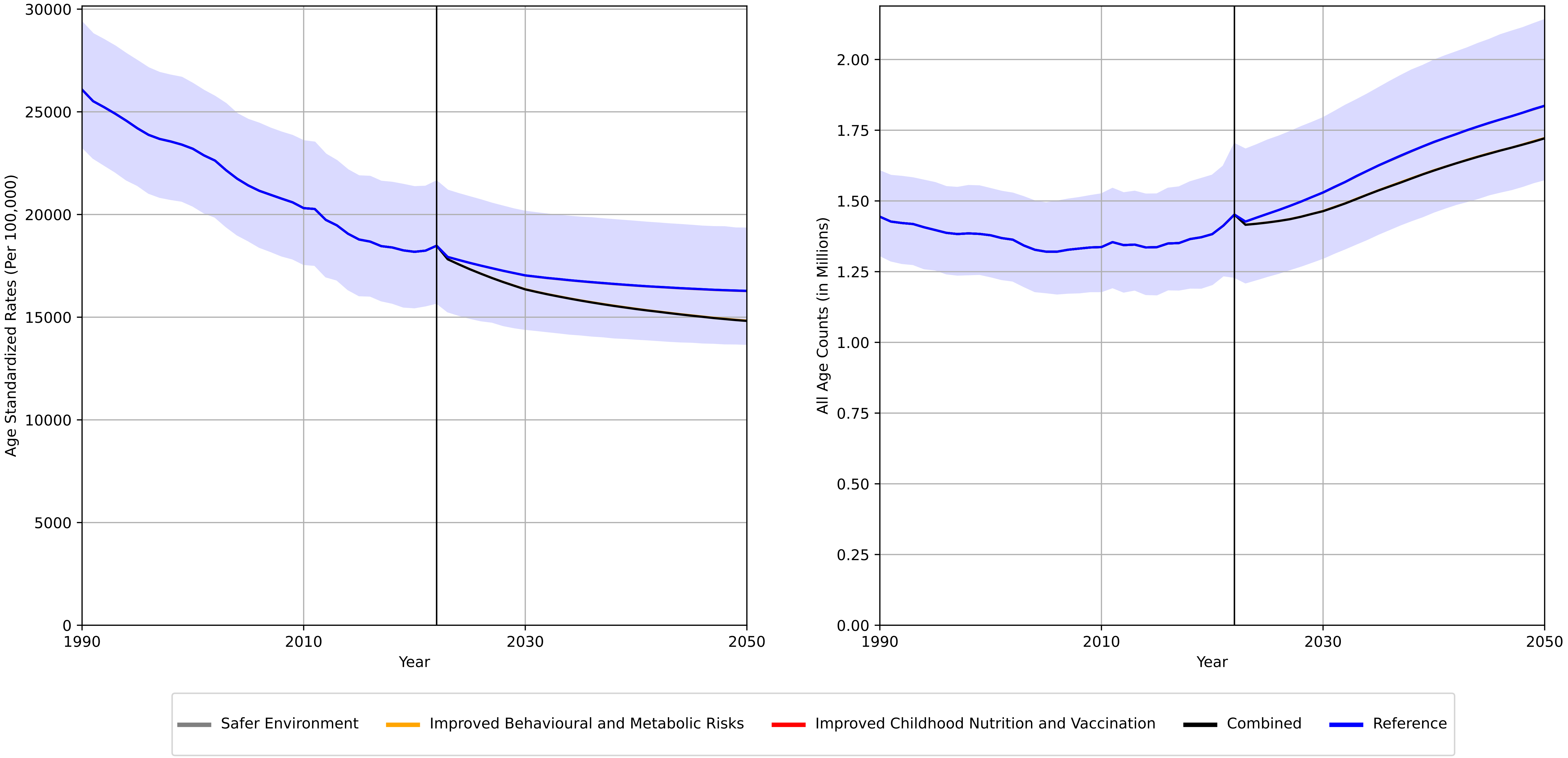

Age Standardized Rates (Per 100,000)
30000
25000
20000
15000
10000
5000
0
1990
2010
2030
2050
Year
All Age Counts (in Millions)
2.00
1.75
1.50
1.25
1.00
0.75
0.50
0.25
0.00
1990
2010
2030
2050
Year
Safer Environment
Improved Behavioural and Metabolic Risks
Improved Childhood Nutrition and Vaccination
Combined
Reference

Portugal

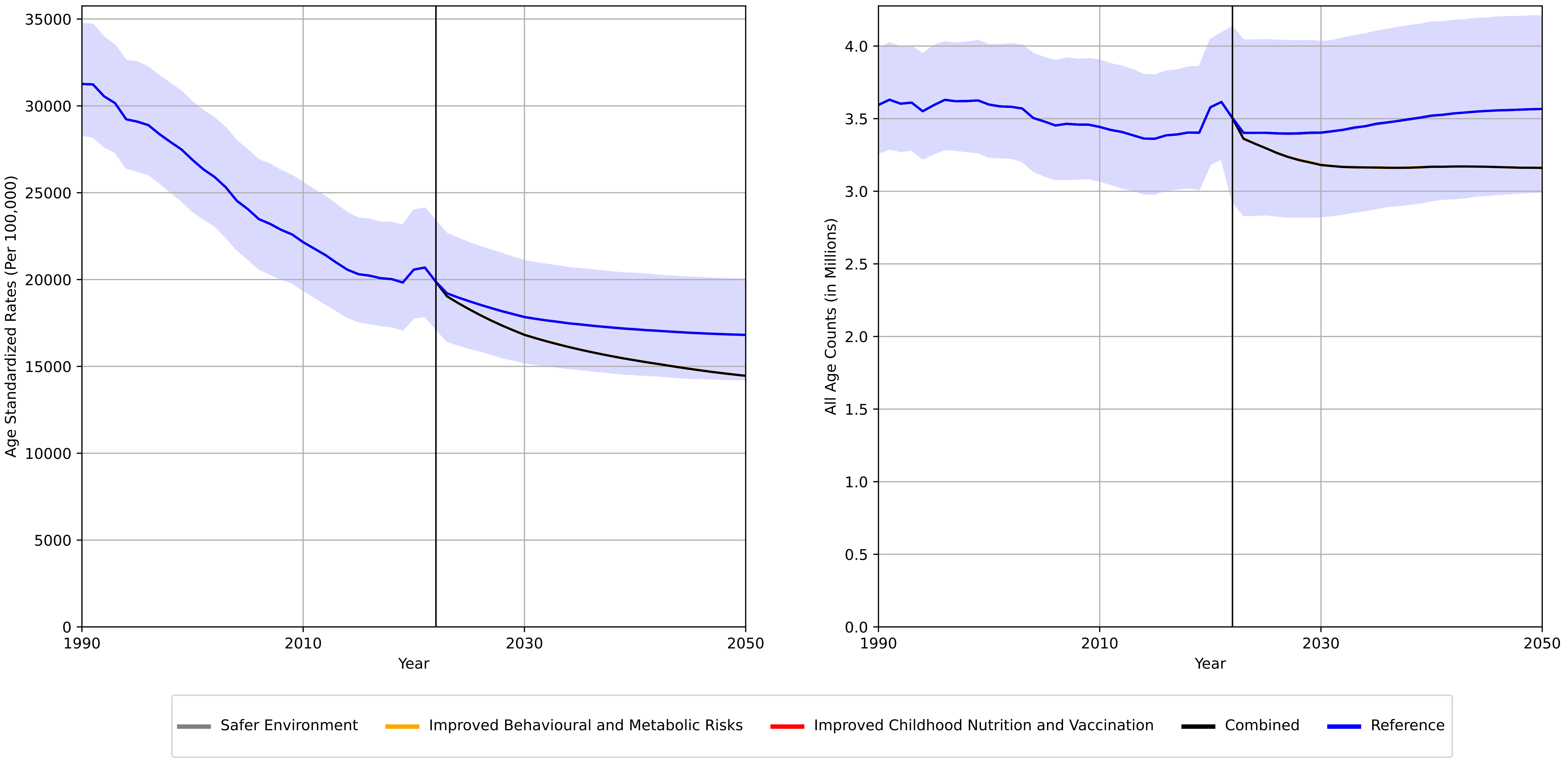

Age Standardized Rates (Per 100,000)
35000
30000
25000
20000
15000
10000
5000
0
1990
2010
2030
2050
Year
All Age Counts (in Millions)
4.0
3.5
3.0
2.5
2.0
1.5
1.0
0.5
0.0
1990
2010
2030
2050
Year
Safer Environment
Improved Behavioural and Metabolic Risks
Improved Childhood Nutrition and Vaccination
Combined
Reference

San Marino

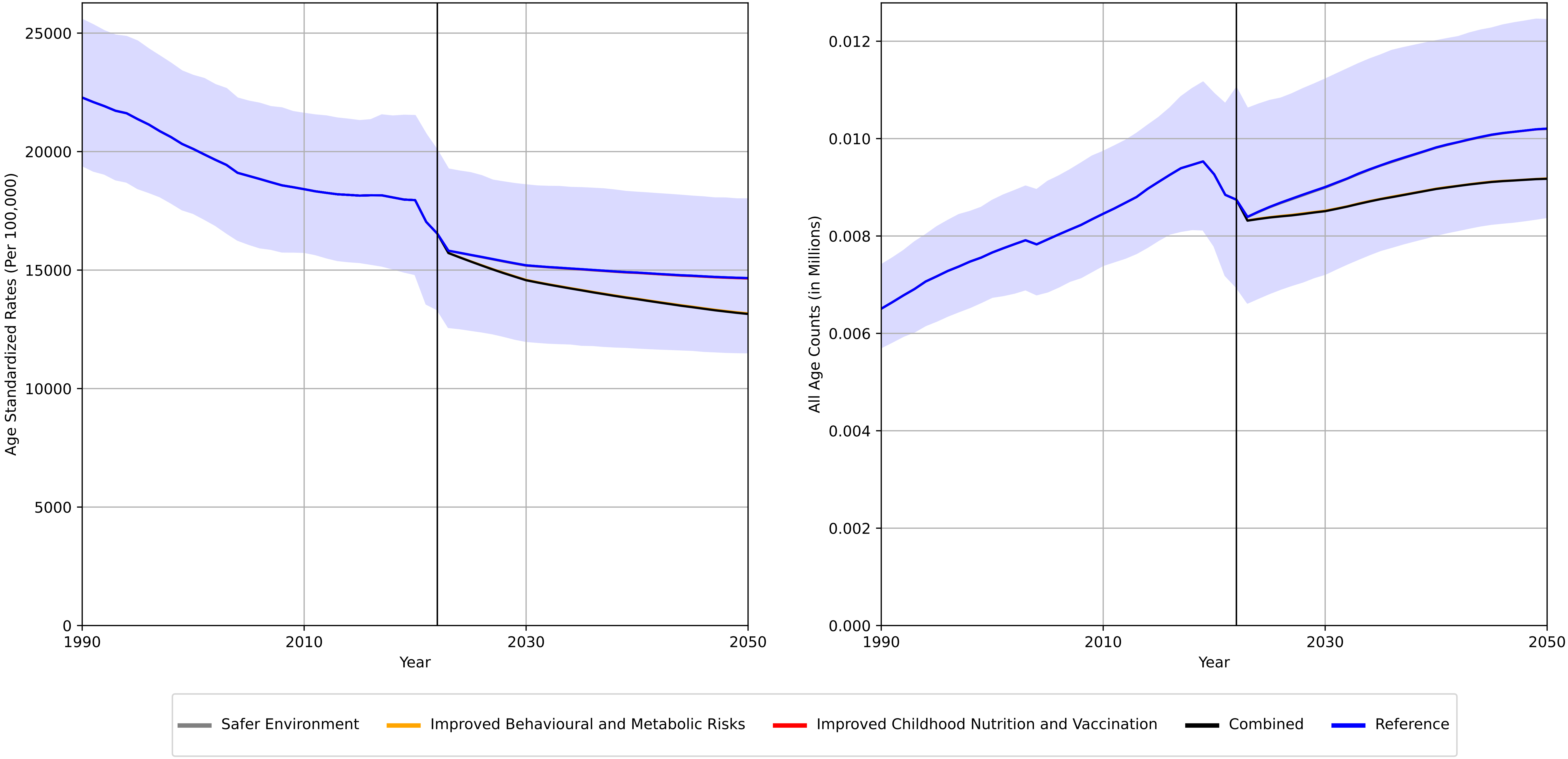

Age Standardized Rates (Per 100,000)
25000
20000
15000
10000
5000
0
1990
2010
2030
2050
Year
All Age Counts (in Millions)
0.012
0.010
0.008
0.006
0.004
0.002
0.000
1990
2010
2030
2050
Year
Safer Environment
Improved Behavioural and Metabolic Risks
Improved Childhood Nutrition and Vaccination
Combined
Reference

Spain

Supplemental Results Figure S10. All-cause age standardised DALY rates (A) and all age DALY counts (B), for the past and for five future scenarios, 1990–2050

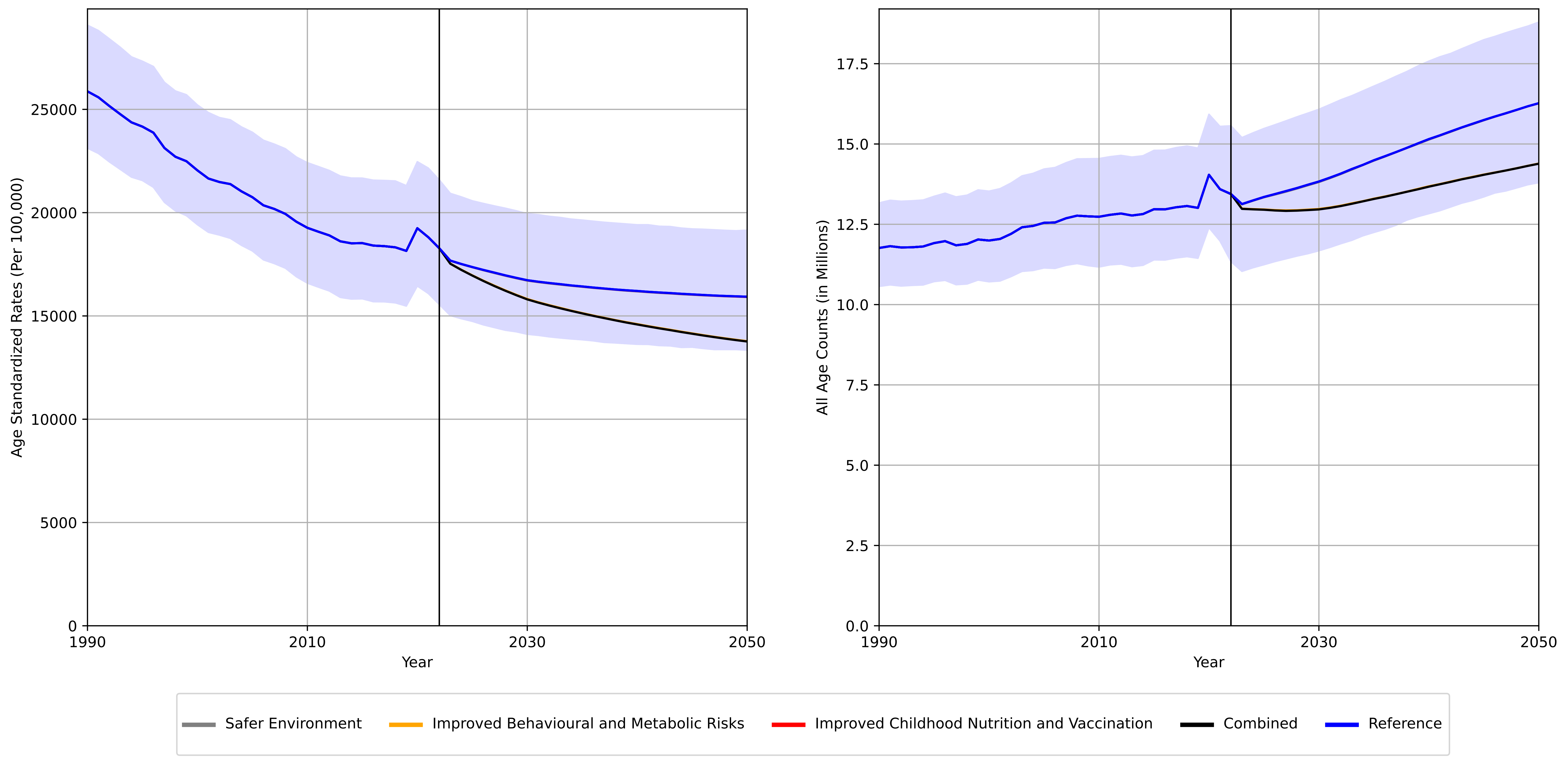

Sweden

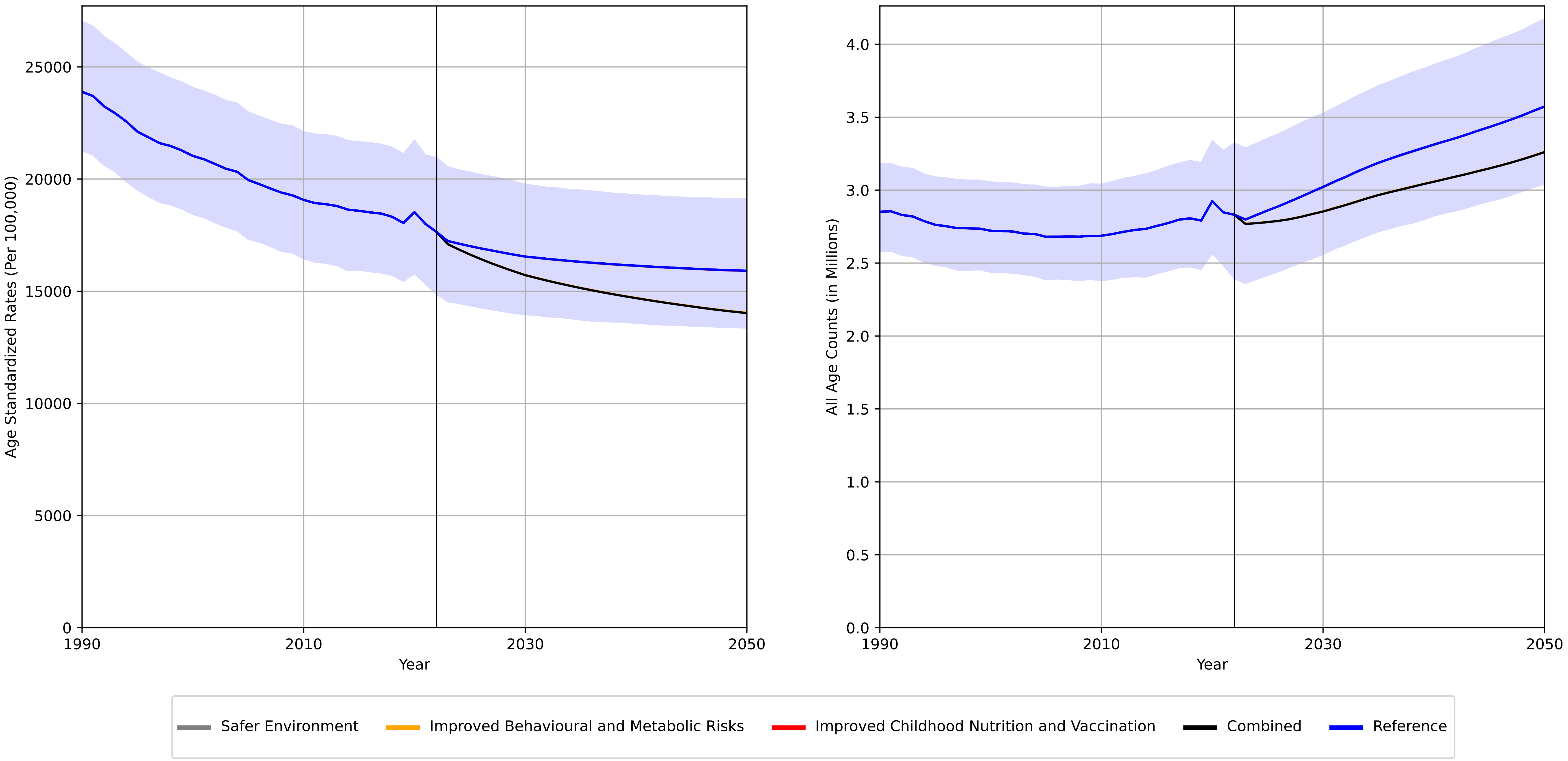

Age Standardized Rates (Per 100,000)
25000
20000
15000
10000
5000
0
1990
2010
2030
2050
Year
All Age Counts (in Millions)
4.0
3.5
3.0
2.5
2.0
1.5
1.0
0.5
0.0
1990
2010
2030
2050
Year
Safer Environment
Improved Behavioural and Metabolic Risks
Improved Childhood Nutrition and Vaccination
Combined
Reference

Switzerland

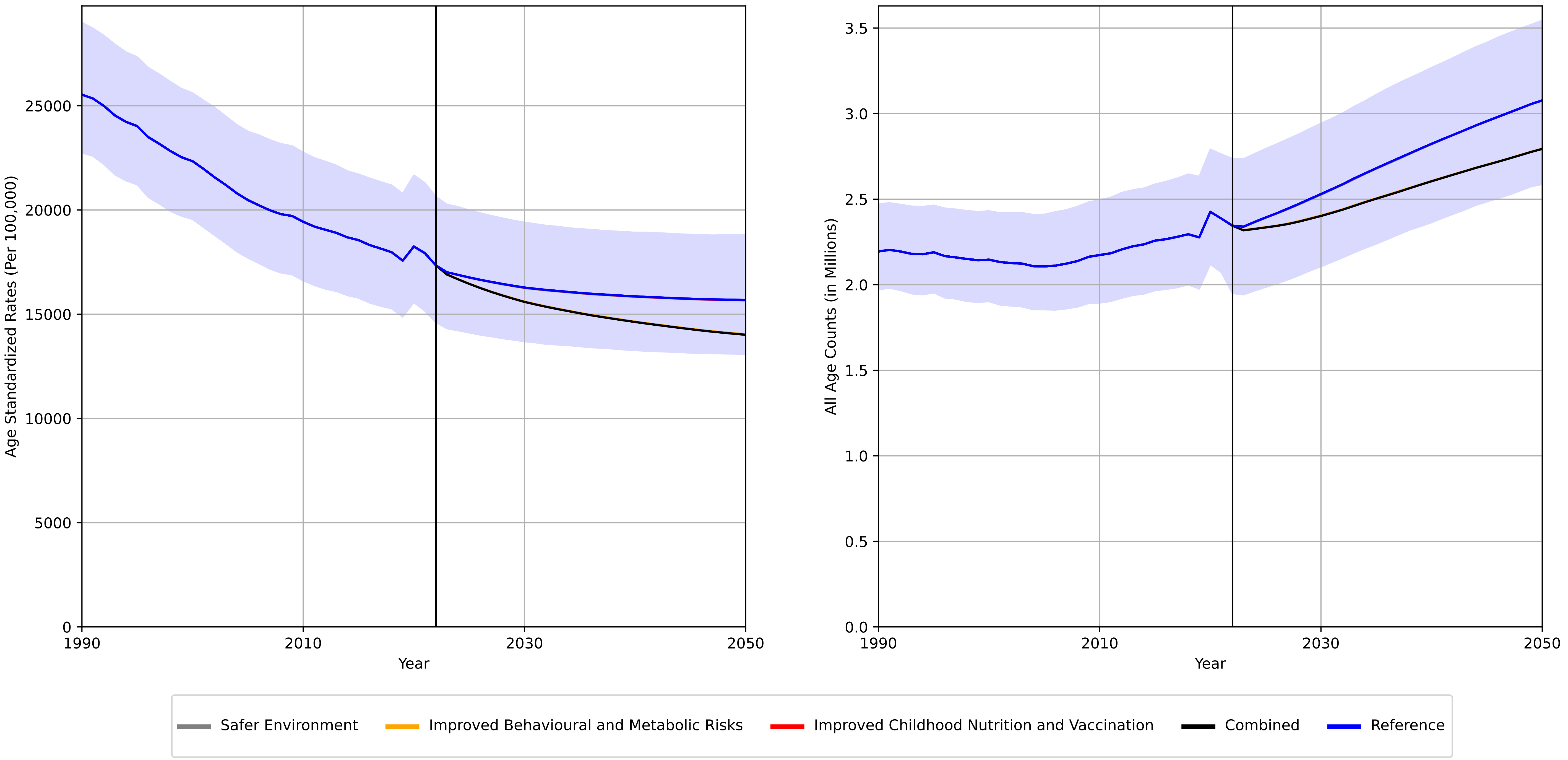

UK

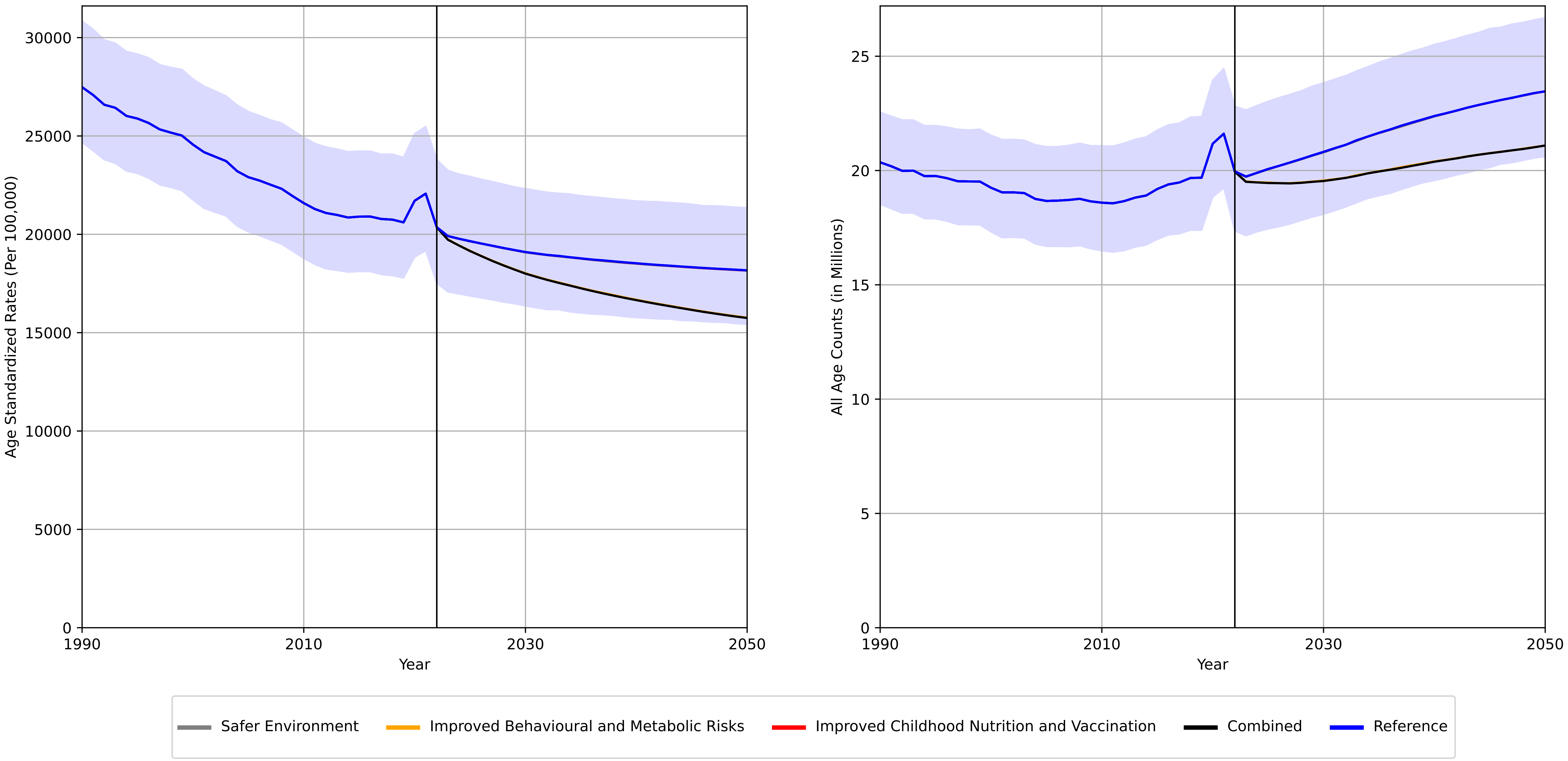
Age Standardized Rates (Per 100,000)
30000
25000
20000
15000
10000
5000
0
1990
2010
2030
2050
Year
All Age Counts (in Millions)
25
20
15
10
5
0
Safer Environment
Improved Behavioural and Metabolic Risks
Improved Childhood Nutrition and Vaccination
Combined
Reference

Bolivia

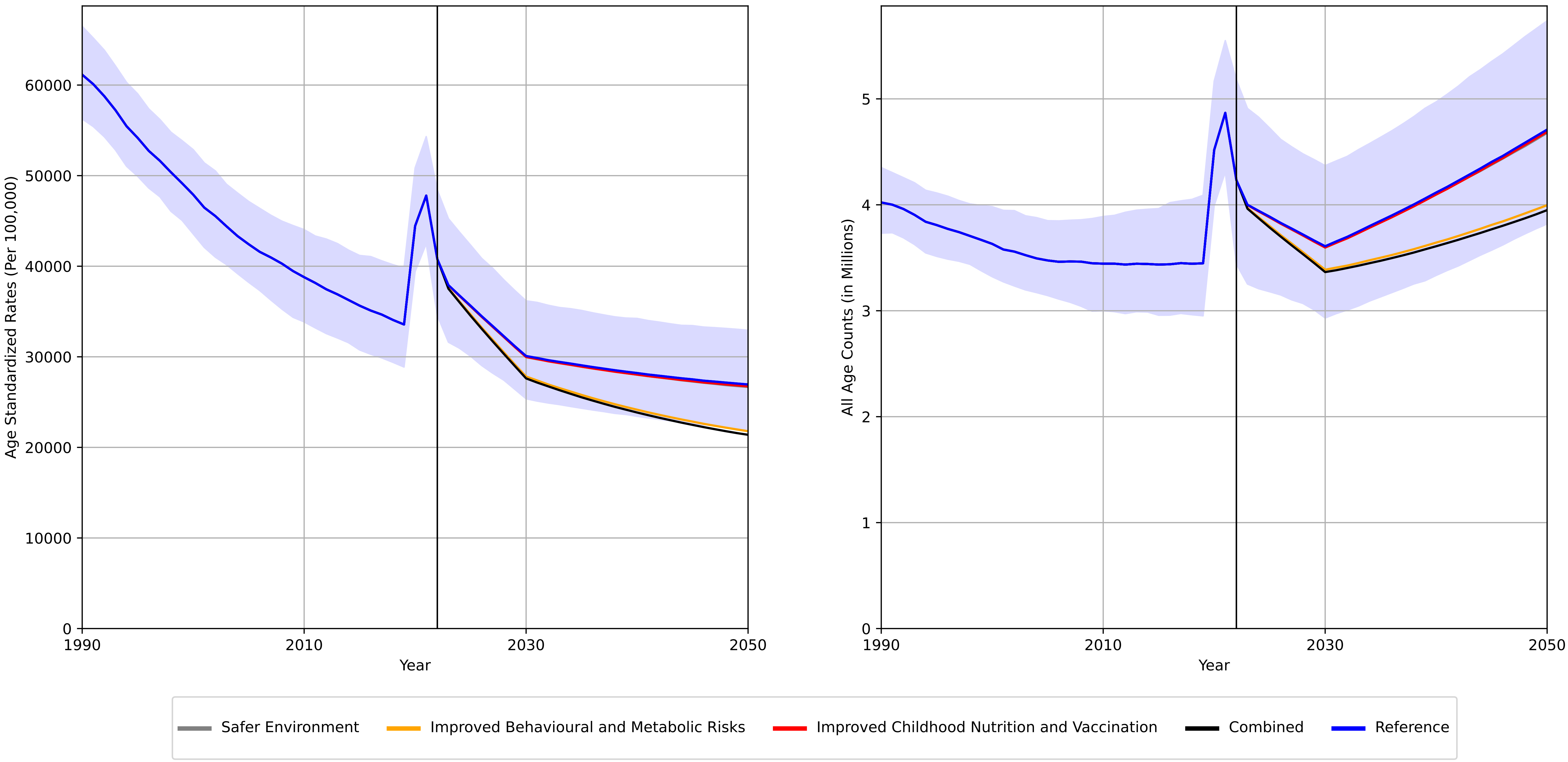

Age Standardized Rates (Per 100,000)
60000
50000
40000
30000
20000
10000
0
1990
2010
2030
2050
Year
All Age Counts (in Millions)
5
4
3
2
1
0
1990
2010
2030
2050
Year
Safer Environment
Improved Behavioural and Metabolic Risks
Improved Childhood Nutrition and Vaccination
Combined
Reference

Ecuador

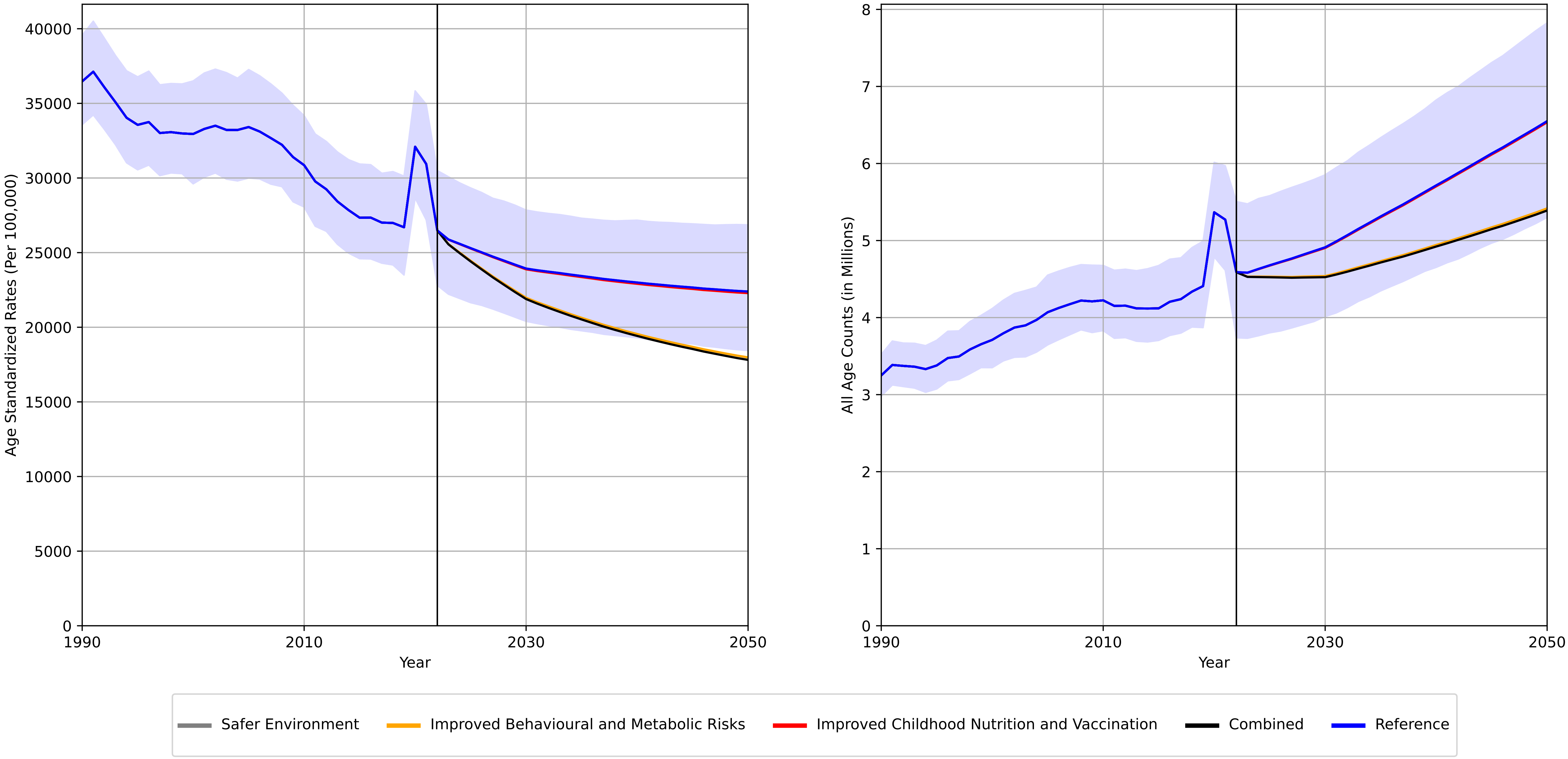

Age Standardized Rates (Per 100,000)
40000
35000
30000
25000
20000
15000
10000
5000
0
1990
2010
2030
2050
Year
All Age Counts (in Millions)
8
7
6
5
4
3
2
1
0
1990
2010
2030
2050
Year
Safer Environment
Improved Behavioural and Metabolic Risks
Improved Childhood Nutrition and Vaccination
Combined
Reference

Peru

Supplemental Results Figure S10. All-cause age standardised DALY rates (A) and all age DALY counts (B), for the past and for five future scenarios, 1990–2050

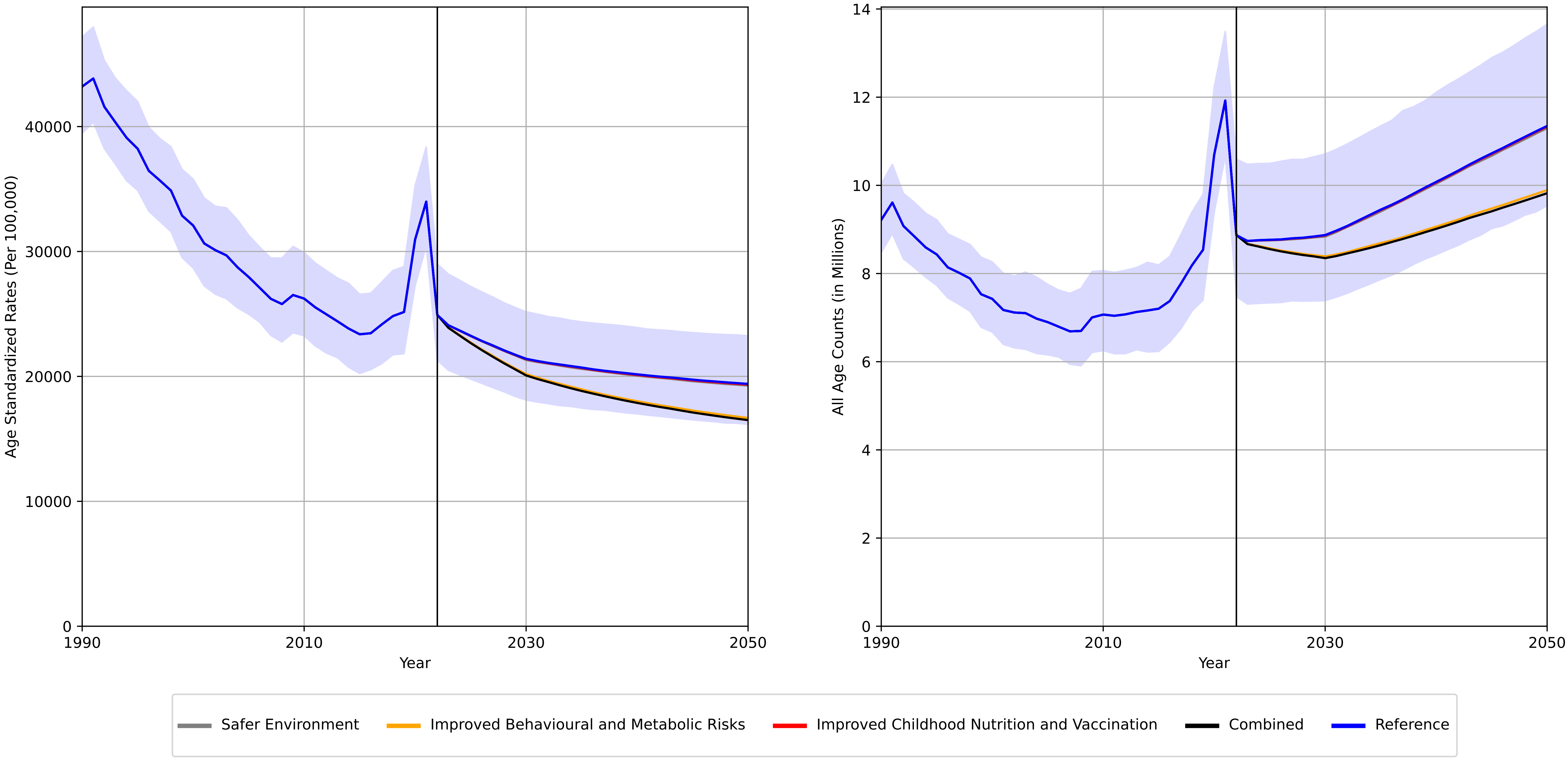

Antigua And Barbuda

Supplemental Results Figure S10. All-cause age standardised DALY rates (A) and all age DALY counts (B), for the past and for five future scenarios, 1990–2050

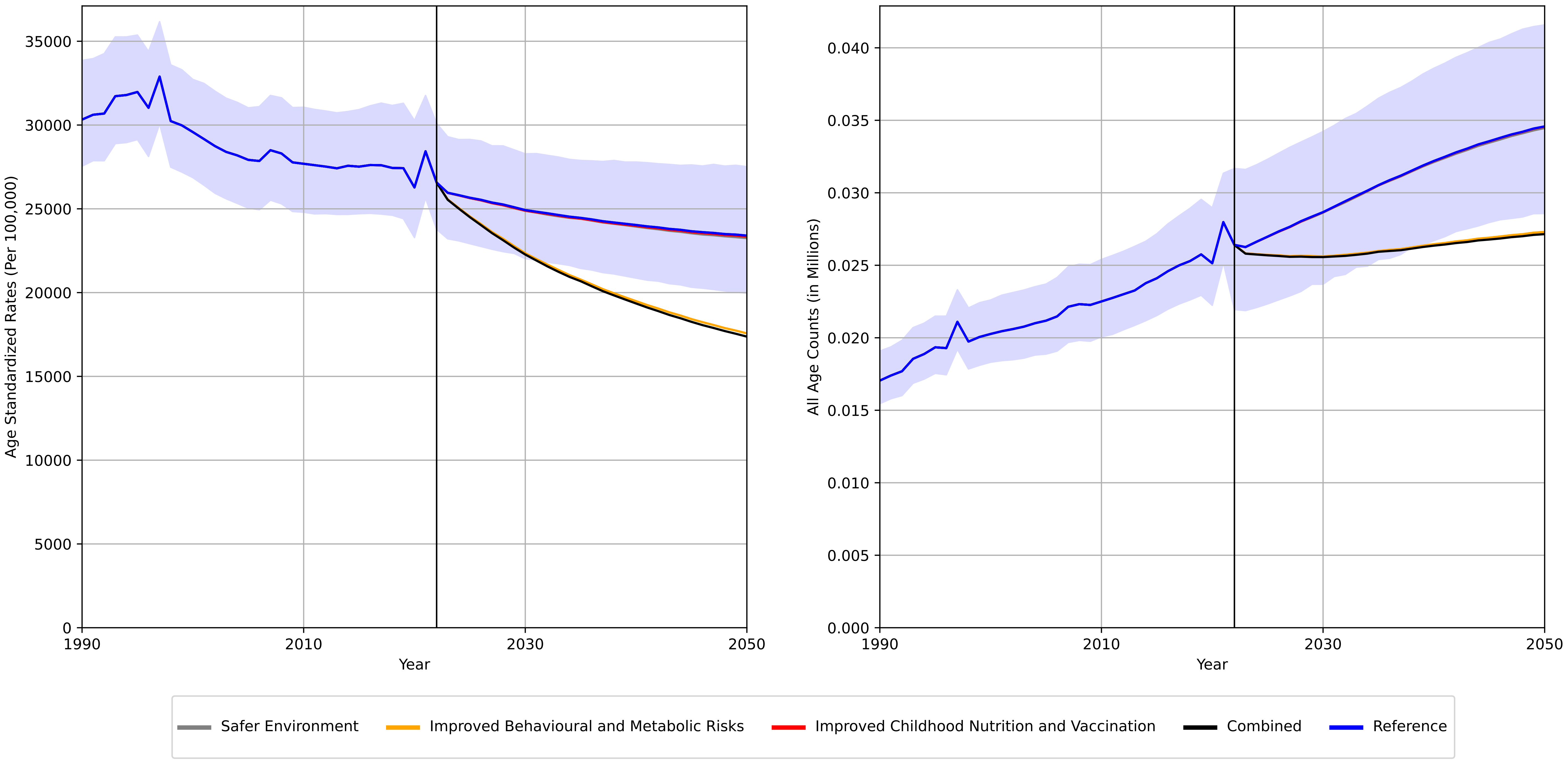

The Bahamas

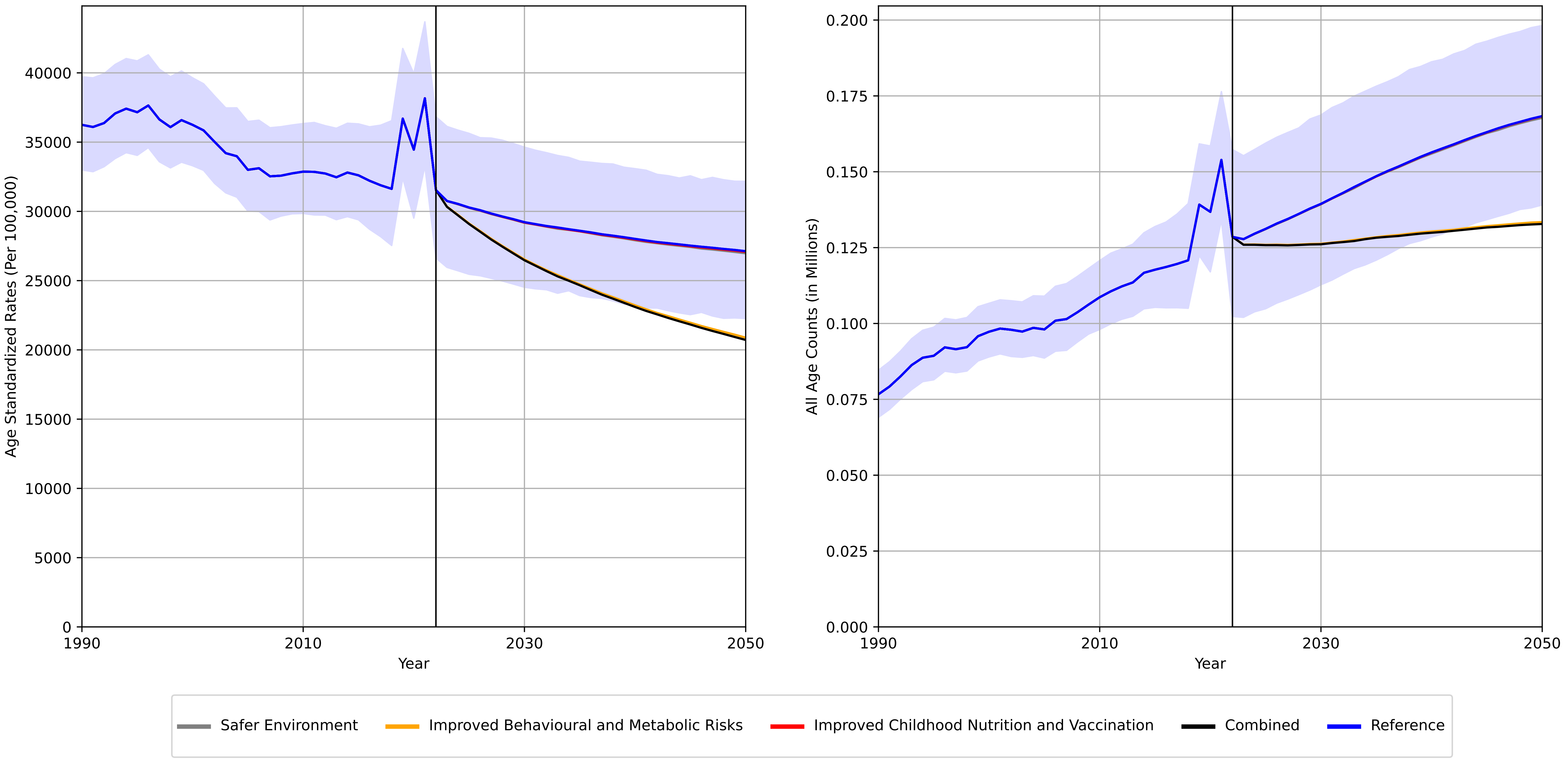

Age Standardized Rates (Per 100,000)
40000
35000
30000
25000
20000
15000
10000
5000
0
1990
2010
2030
2050
Year
All Age Counts (in Millions)
0.200
0.175
0.150
0.125
0.100
0.075
0.050
0.025
0.000
1990
2010
2030
2050
Year
Safer Environment
Improved Behavioural and Metabolic Risks
Improved Childhood Nutrition and Vaccination
Combined
Reference

Barbados

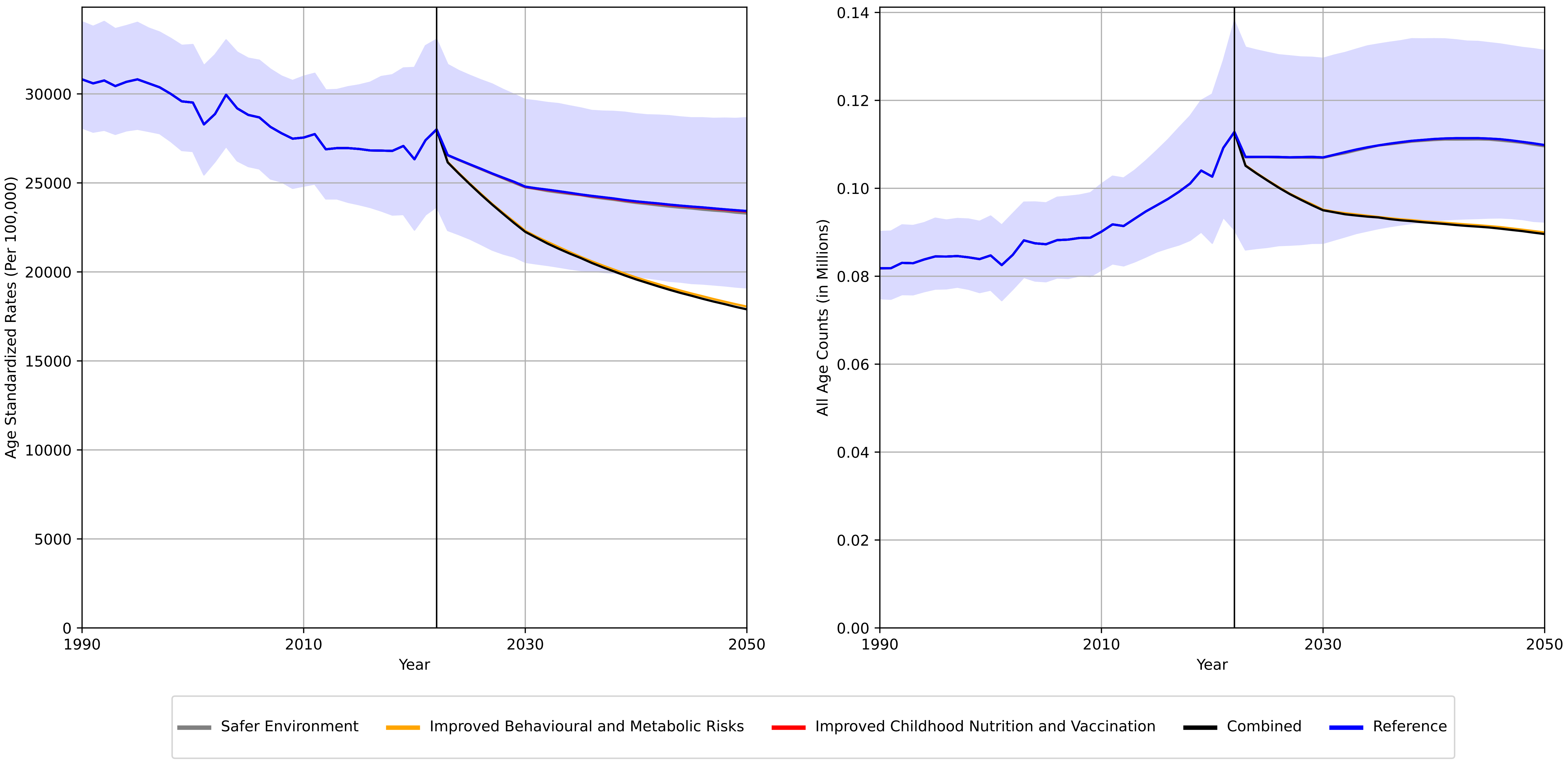

Age Standardized Rates (Per 100,000)
0
5000
10000
15000
20000
25000
30000
1990
2010
2030
2050
Year
All Age Counts (in Millions)
0.00
0.02
0.04
0.06
0.08
0.10
0.12
0.14
1990
2010
2030
2050
Year
Safer Environment
Improved Behavioural and Metabolic Risks
Improved Childhood Nutrition and Vaccination
Combined
Reference

Belize

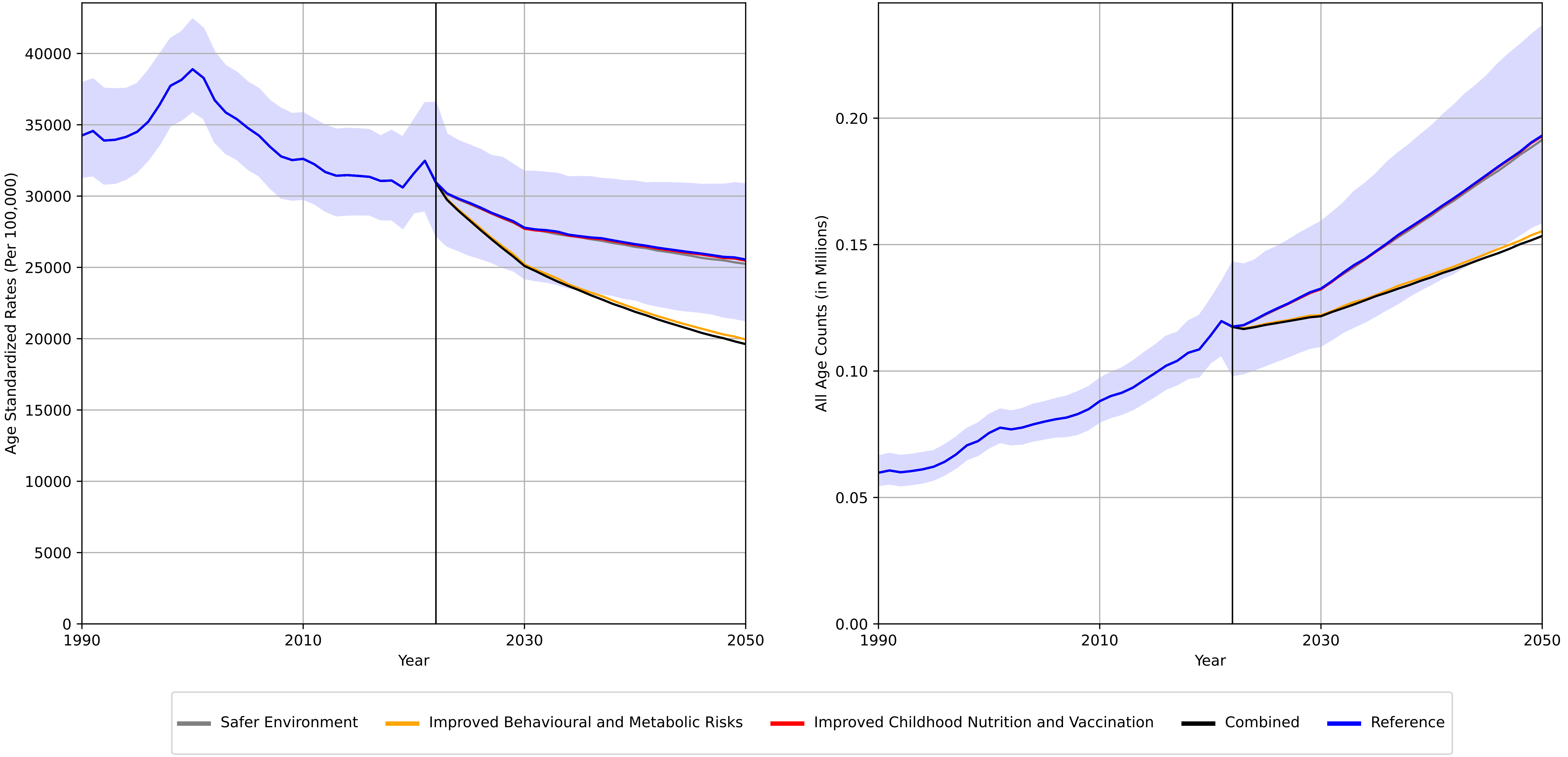
Age Standardized Rates (Per 100,000)
0
5000
10000
15000
20000
25000
30000
35000
40000
1990
2010
2030
2050
Year
All Age Counts (in Millions)
0.00
0.05
0.10
0.15
0.20
1990
2010
2030
2050
Year
Safer Environment
Improved Behavioural and Metabolic Risks
Improved Childhood Nutrition and Vaccination
Combined
Reference

Bermuda

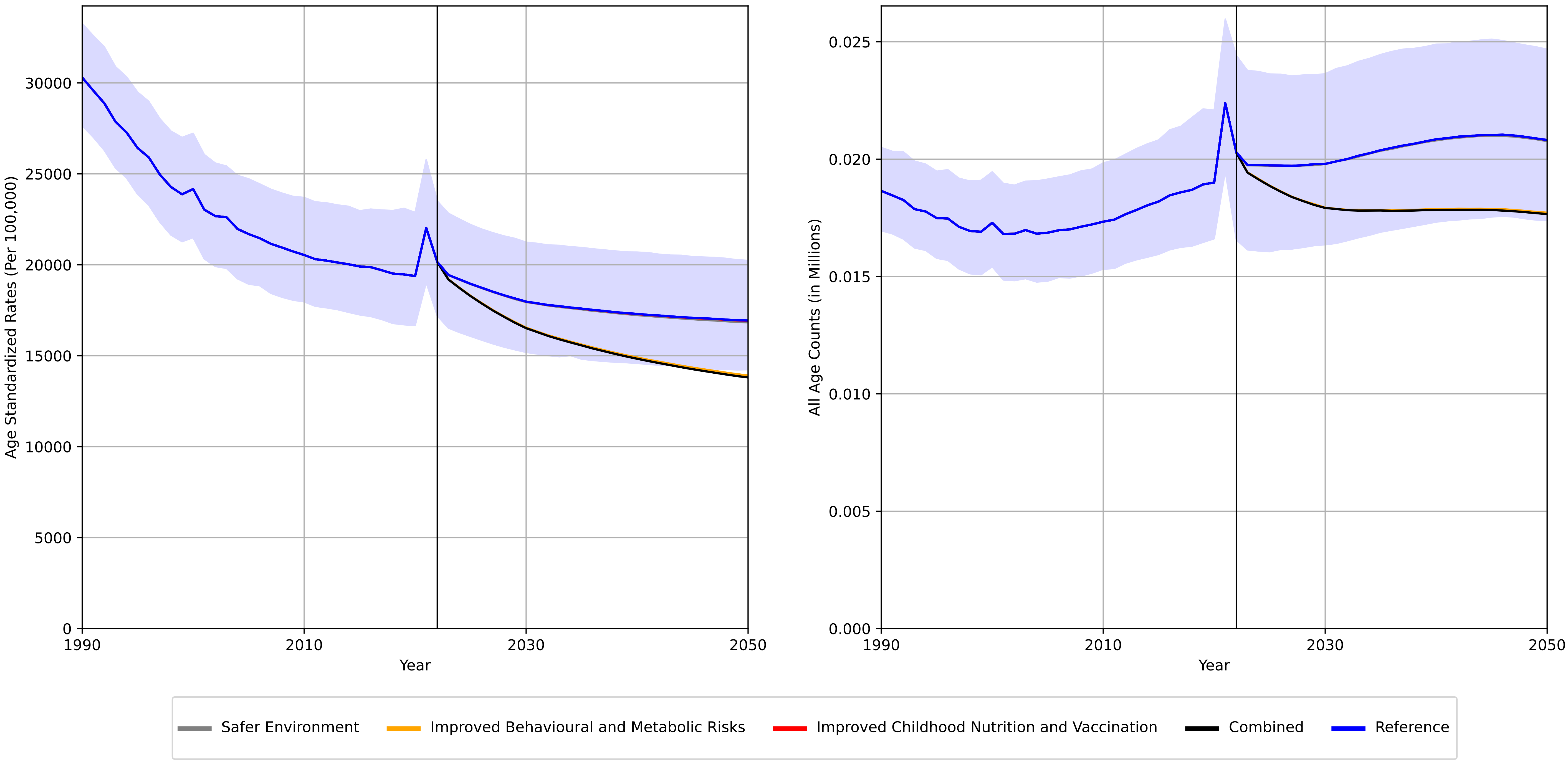
Age Standardized Rates (Per 100,000)
30000
25000
20000
15000
10000
5000
0
1990
2010
2030
2050
Year
All Age Counts (in Millions)
0.025
0.020
0.015
0.010
0.005
0.000
1990
2010
2030
2050
Year
Safer Environment
Improved Behavioural and Metabolic Risks
Improved Childhood Nutrition and Vaccination
Combined
Reference

Cuba

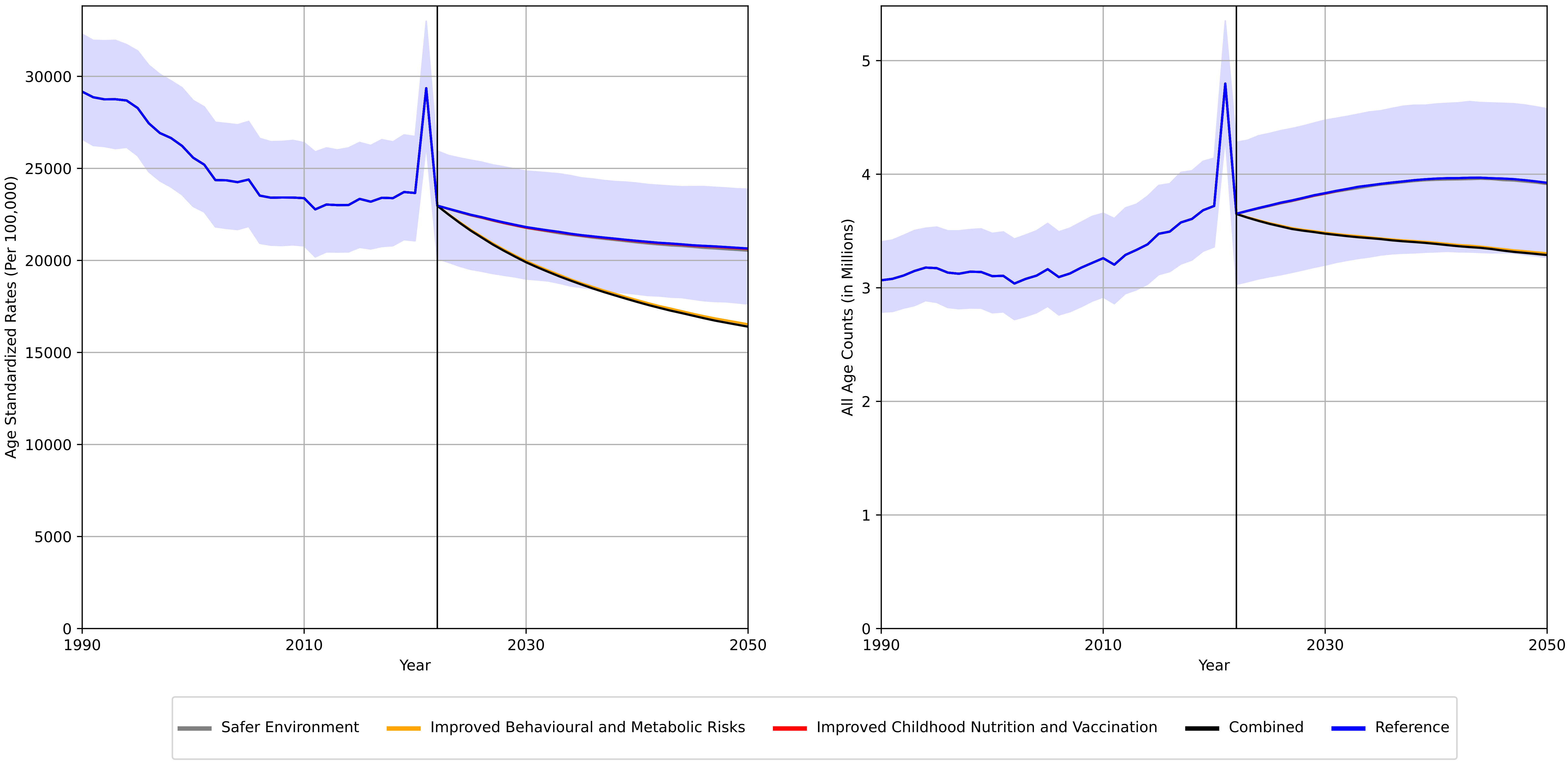
Age Standardized Rates (Per 100,000)
30000
25000
20000
15000
10000
5000
0
1990
2010
2030
2050
Year
All Age Counts (in Millions)
5
4
3
2
1
0
1990
2010
2030
2050
Year
Safer Environment
Improved Behavioural and Metabolic Risks
Improved Childhood Nutrition and Vaccination
Combined
Reference

Dominica

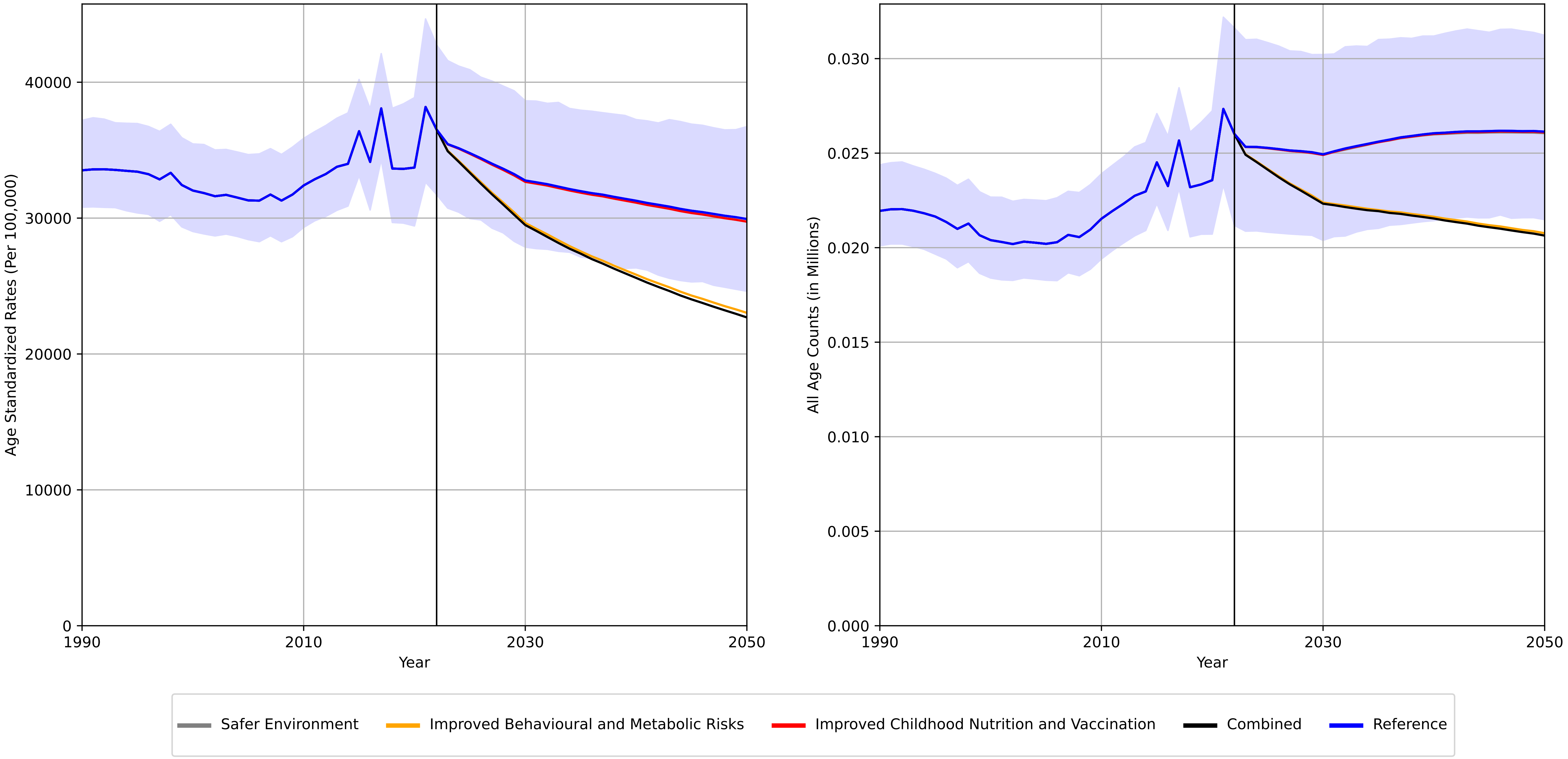
Age Standardized Rates (Per 100,000)
0
10000
20000
30000
40000
1990
2010
2030
2050
Year
All Age Counts (in Millions)
0.000
0.005
0.010
0.015
0.020
0.025
0.030
1990
2010
2030
2050
Year
Safer Environment
Improved Behavioural and Metabolic Risks
Improved Childhood Nutrition and Vaccination
Combined
Reference

Dominican Republic

Supplemental Results Figure S10. All-cause age standardised DALY rates (A) and all age DALY counts (B), for the past and for five future scenarios, 1990–2050

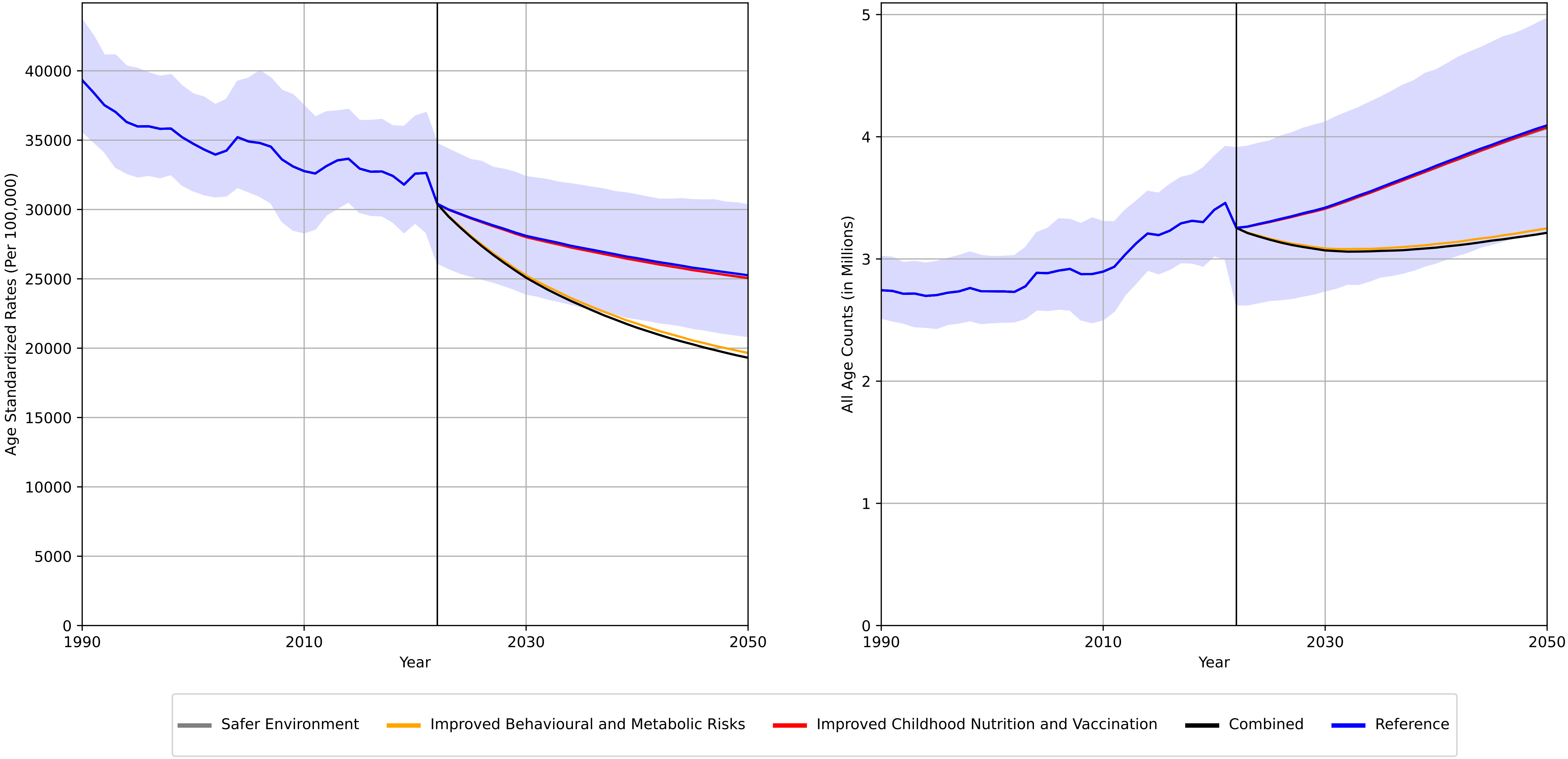

Grenada

Supplemental Results Figure S10. All-cause age standardised DALY rates (A) and all age DALY counts (B), for the past and for five future scenarios, 1990–2050

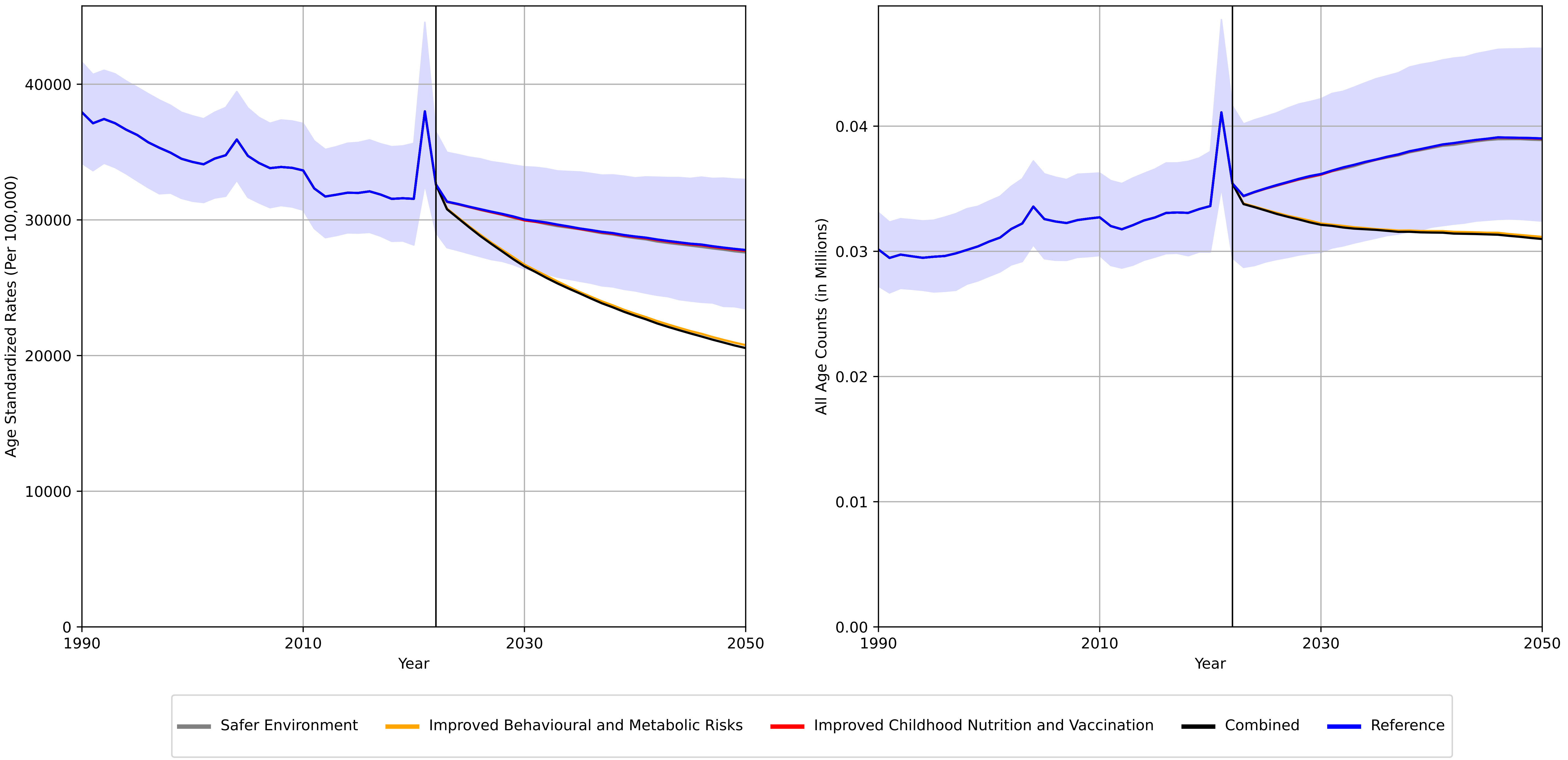

Guyana

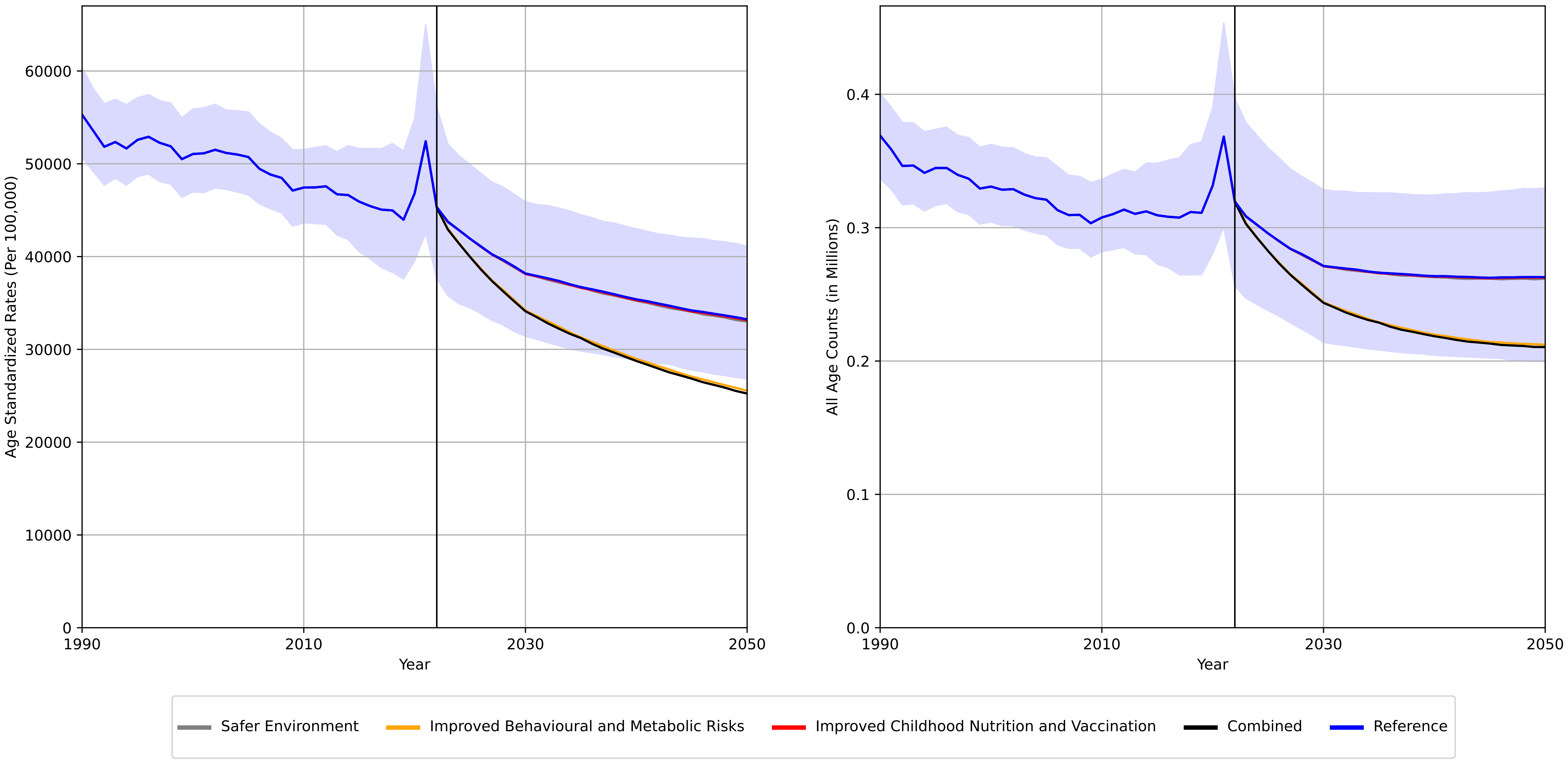
Age Standardized Rates (Per 100,000)
60000
50000
40000
30000
20000
10000
0
1990
2010
2030
2050
Year
All Age Counts (in Millions)
0.4
0.3
0.2
0.1
0.0
1990
2010
2030
2050
Year
Safer Environment
Improved Behavioural and Metabolic Risks
Improved Childhood Nutrition and Vaccination
Combined
Reference

Haiti

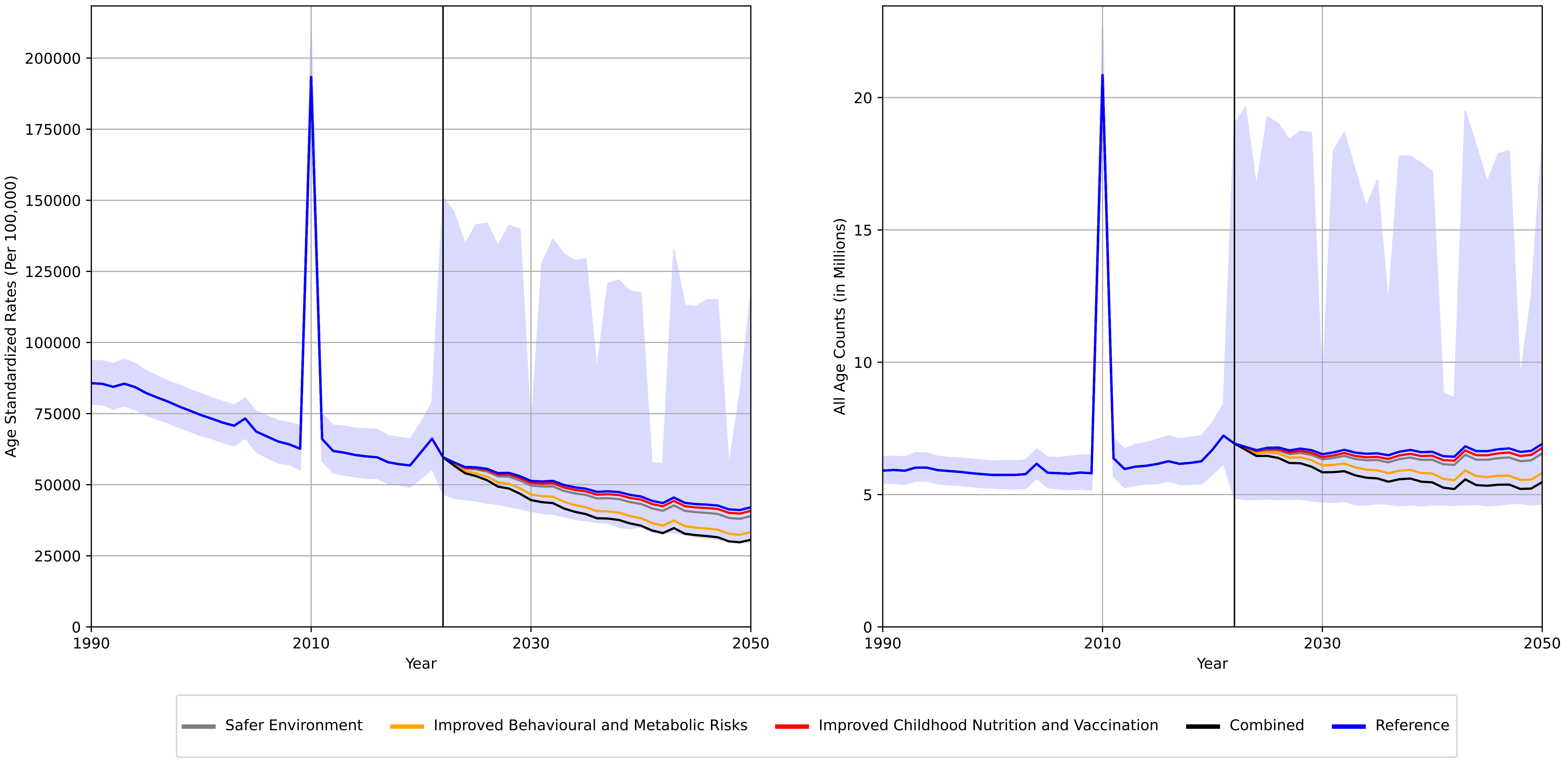
Age Standardized Rates (Per 100,000)
200000
175000
150000
125000
100000
75000
50000
25000
0
1990
2010
2030
2050
Year
All Age Counts (in Millions)
20
15
10
5
0
1990
2010
2030
2050
Year
Safer Environment
Improved Behavioural and Metabolic Risks
Improved Childhood Nutrition and Vaccination
Combined
Reference

Jamaica

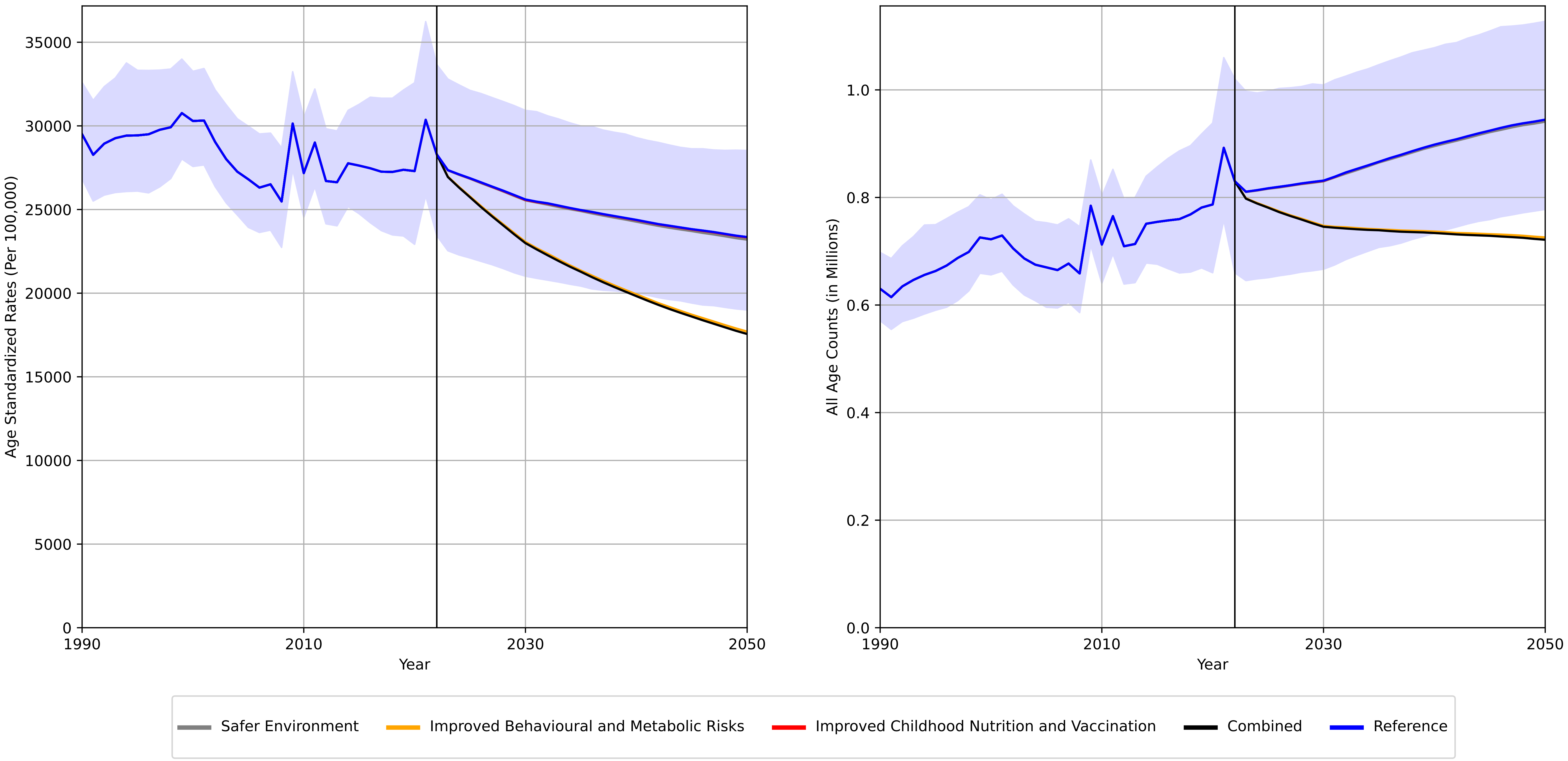
Age Standardized Rates (Per 100,000)
35000
30000
25000
20000
15000
10000
5000
0
1990
2010
2030
2050
Year
All Age Counts (in Millions)
1.0
0.8
0.6
0.4
0.2
0.0
1990
2010
2030
2050
Year
Safer Environment
Improved Behavioural and Metabolic Risks
Improved Childhood Nutrition and Vaccination
Combined
Reference

Puerto Rico

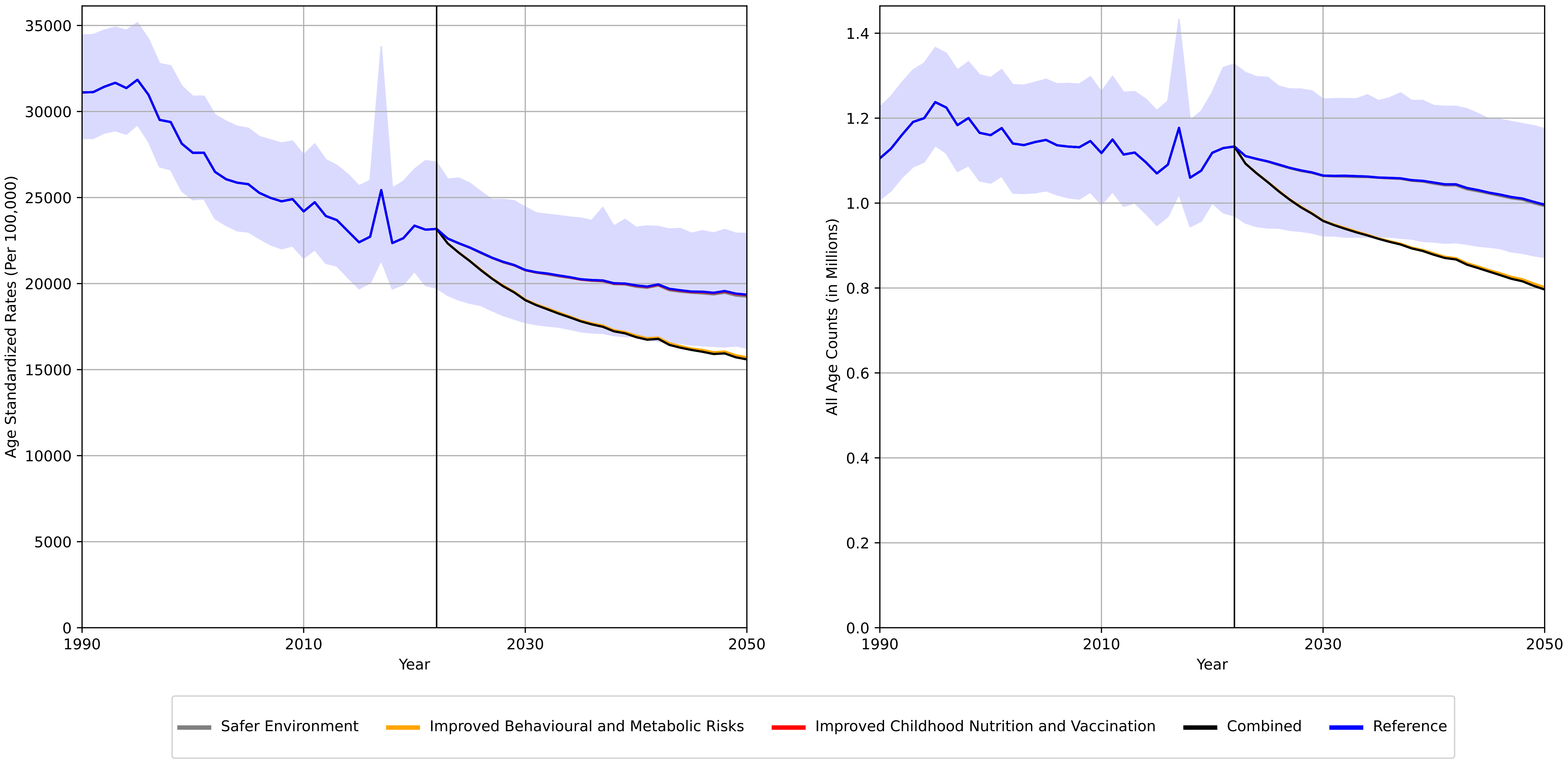

Age Standardized Rates (Per 100,000)
35000
30000
25000
20000
15000
10000
5000
0
1990
2010
2030
2050
Year
All Age Counts (in Millions)
1.4
1.2
1.0
0.8
0.6
0.4
0.2
0.0
1990
2010
2030
2050
Year
Safer Environment
Improved Behavioural and Metabolic Risks
Improved Childhood Nutrition and Vaccination
Combined
Reference

Saint Kitts And Nevis

Supplemental Results Figure S10. All-cause age standardised DALY rates (A) and all age DALY counts (B), for the past and for five future scenarios, 1990–2050

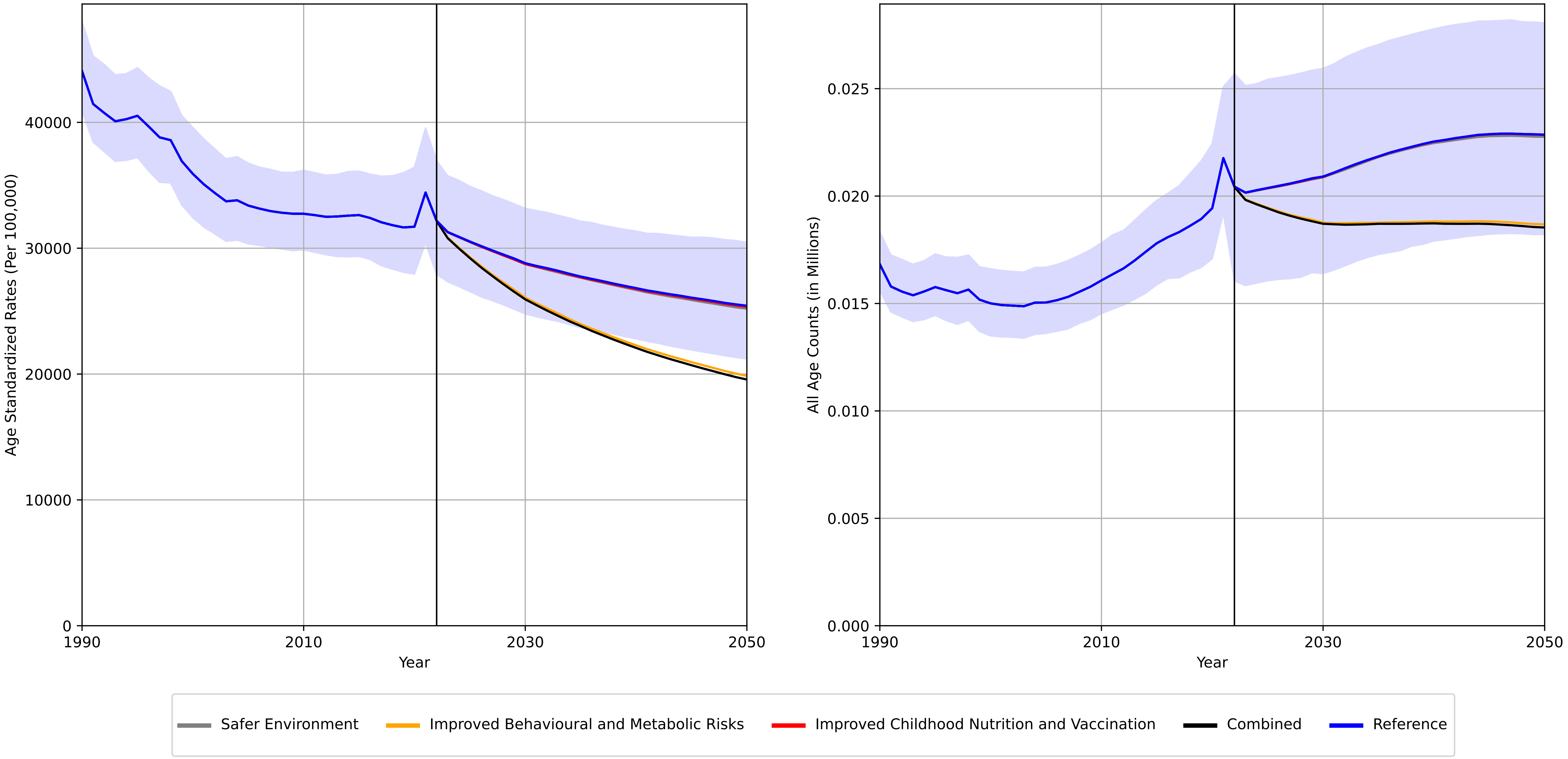

Saint Lucia

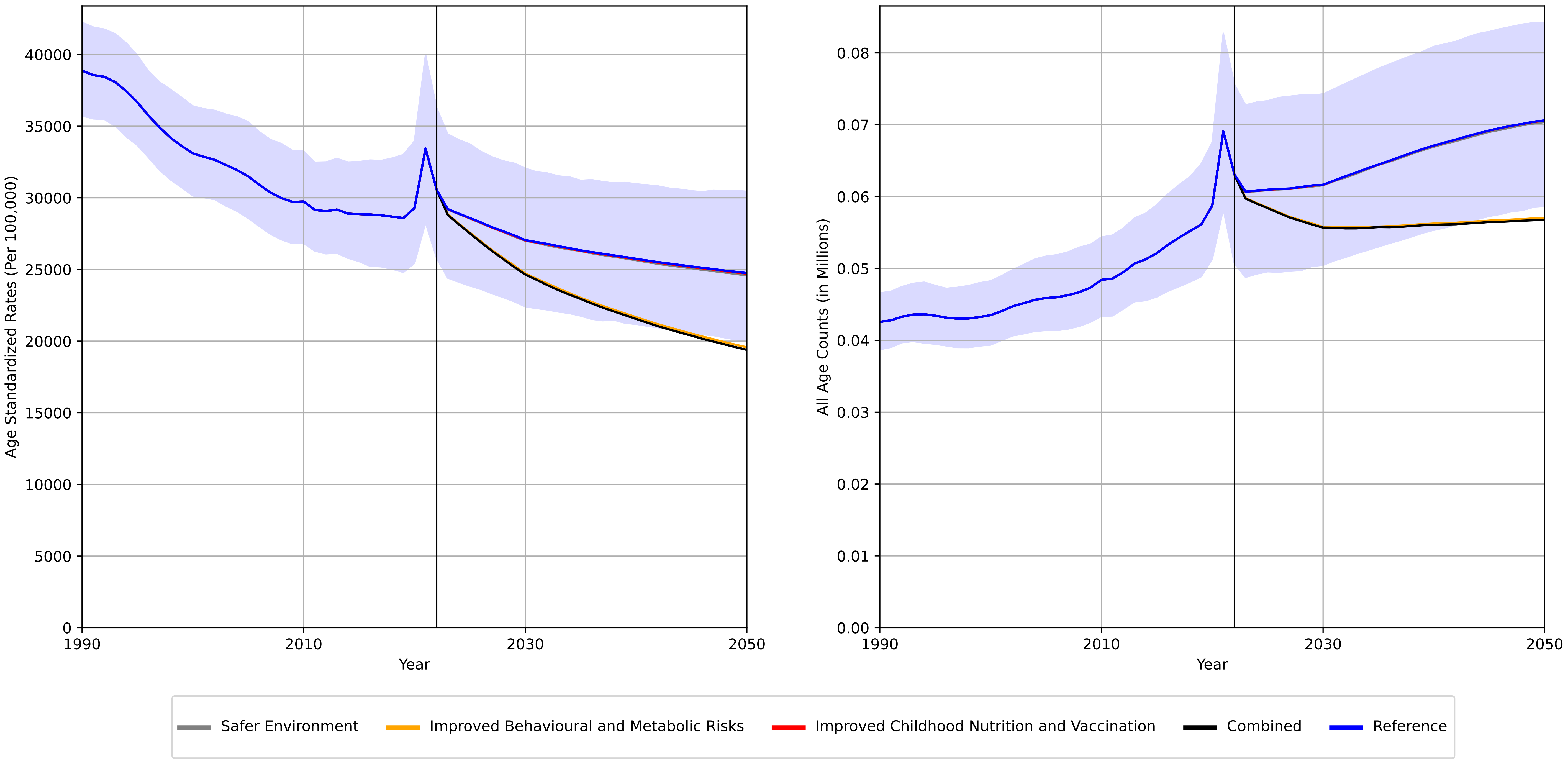

Age Standardized Rates (Per 100,000)
0
5000
10000
15000
20000
25000
30000
35000
40000
1990
2010
2030
2050
Year
All Age Counts (in Millions)
0.00
0.01
0.02
0.03
0.04
0.05
0.06
0.07
0.08
1990
2010
2030
2050
Year
Safer Environment
Improved Behavioural and Metabolic Risks
Improved Childhood Nutrition and Vaccination
Combined
Reference

Saint Vincent And The Grenadines

Supplemental Results Figure S10. All-cause age standardised DALY rates (A) and all age DALY counts (B), for the past and for five future scenarios, 1990–2050

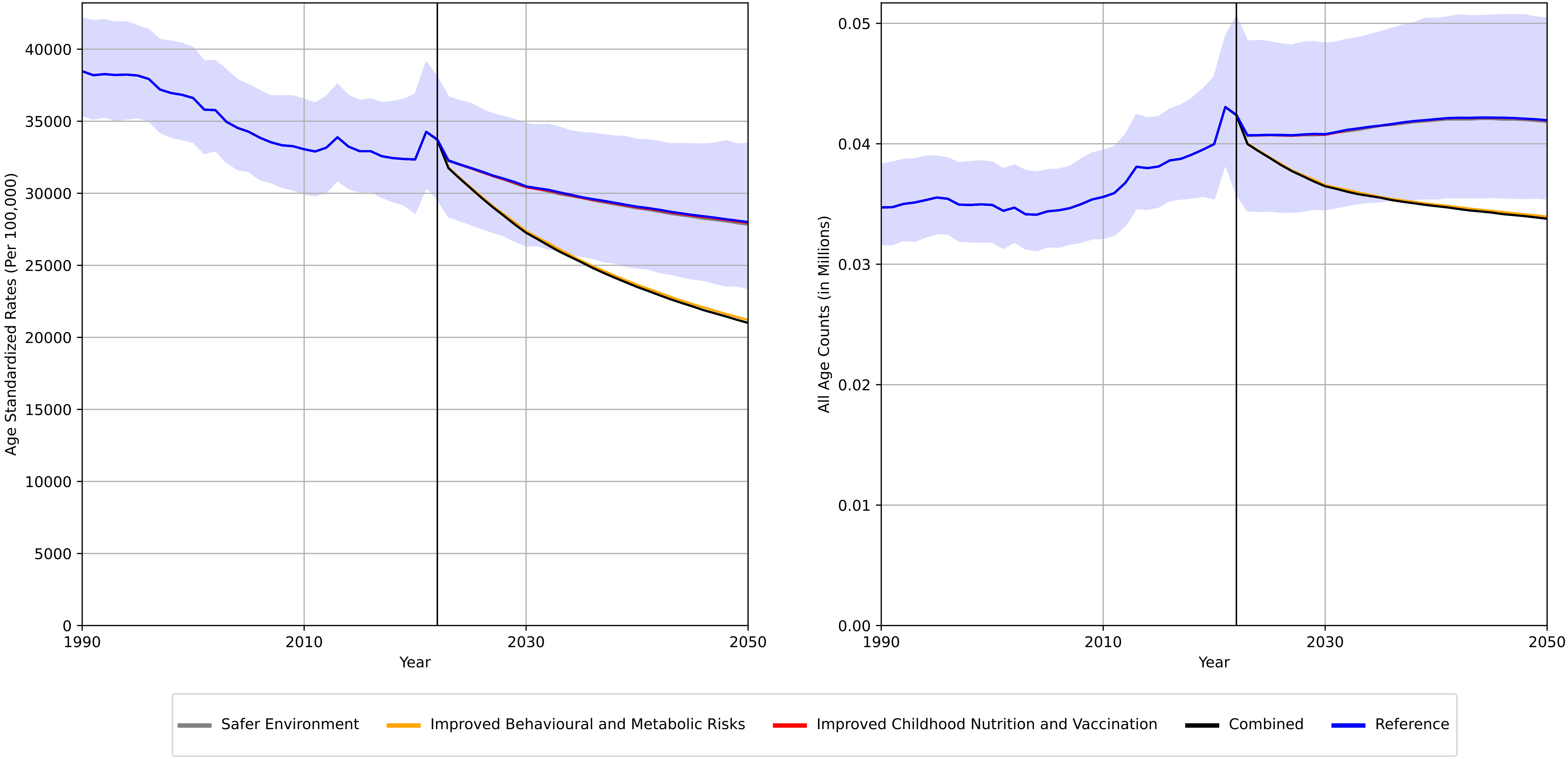

Suriname

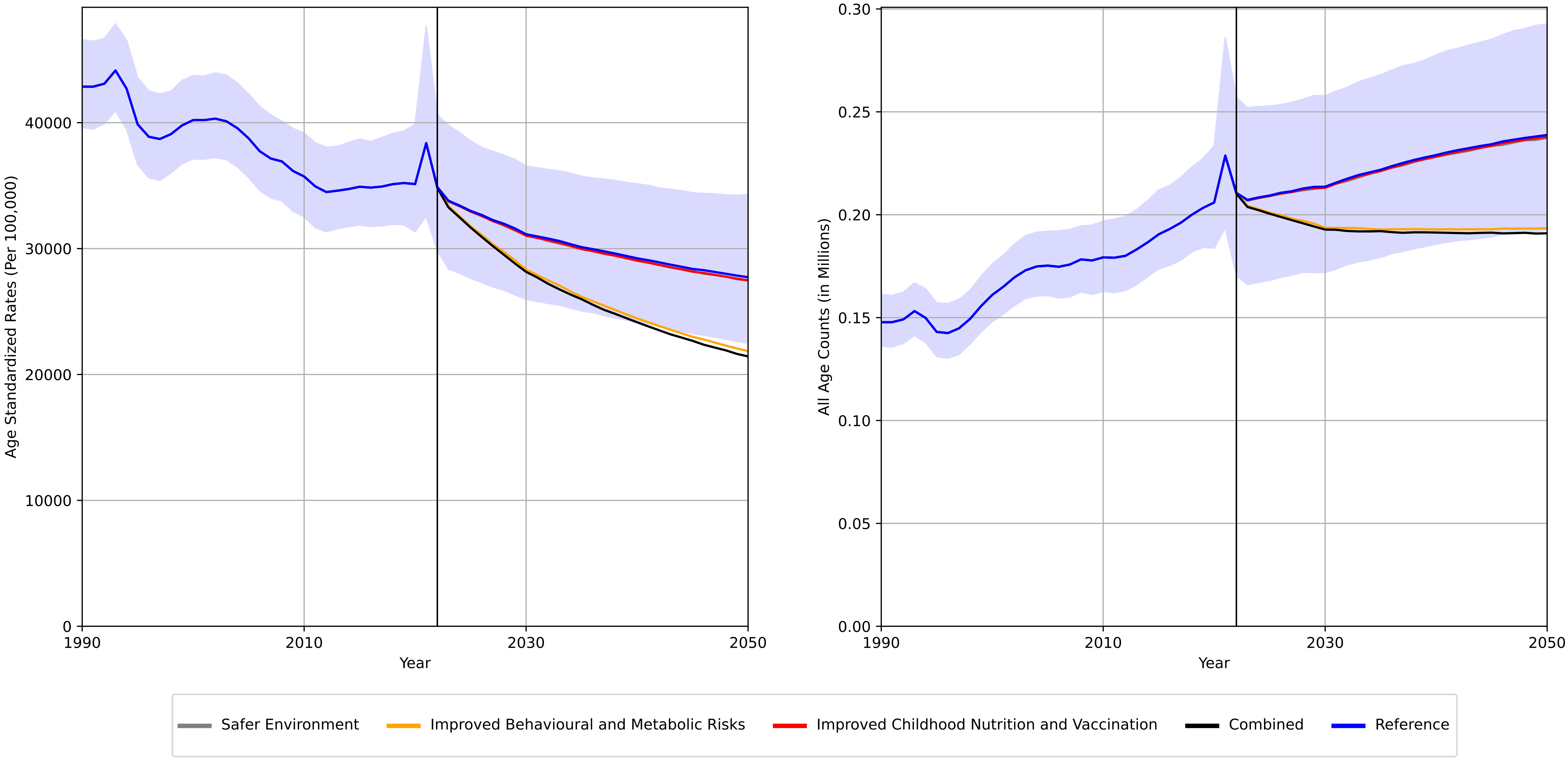
Age Standardized Rates (Per 100,000)
0
10000
20000
30000
40000
1990
2010
2030
2050
Year
All Age Counts (in Millions)
0.00
0.05
0.10
0.15
0.20
0.25
0.30
1990
2010
2030
2050
Year
Safer Environment
Improved Behavioural and Metabolic Risks
Improved Childhood Nutrition and Vaccination
Combined
Reference

Trinidad And Tobago

Supplemental Results Figure S10. All-cause age standardised DALY rates (A) and all age DALY counts (B), for the past and for five future scenarios, 1990–2050

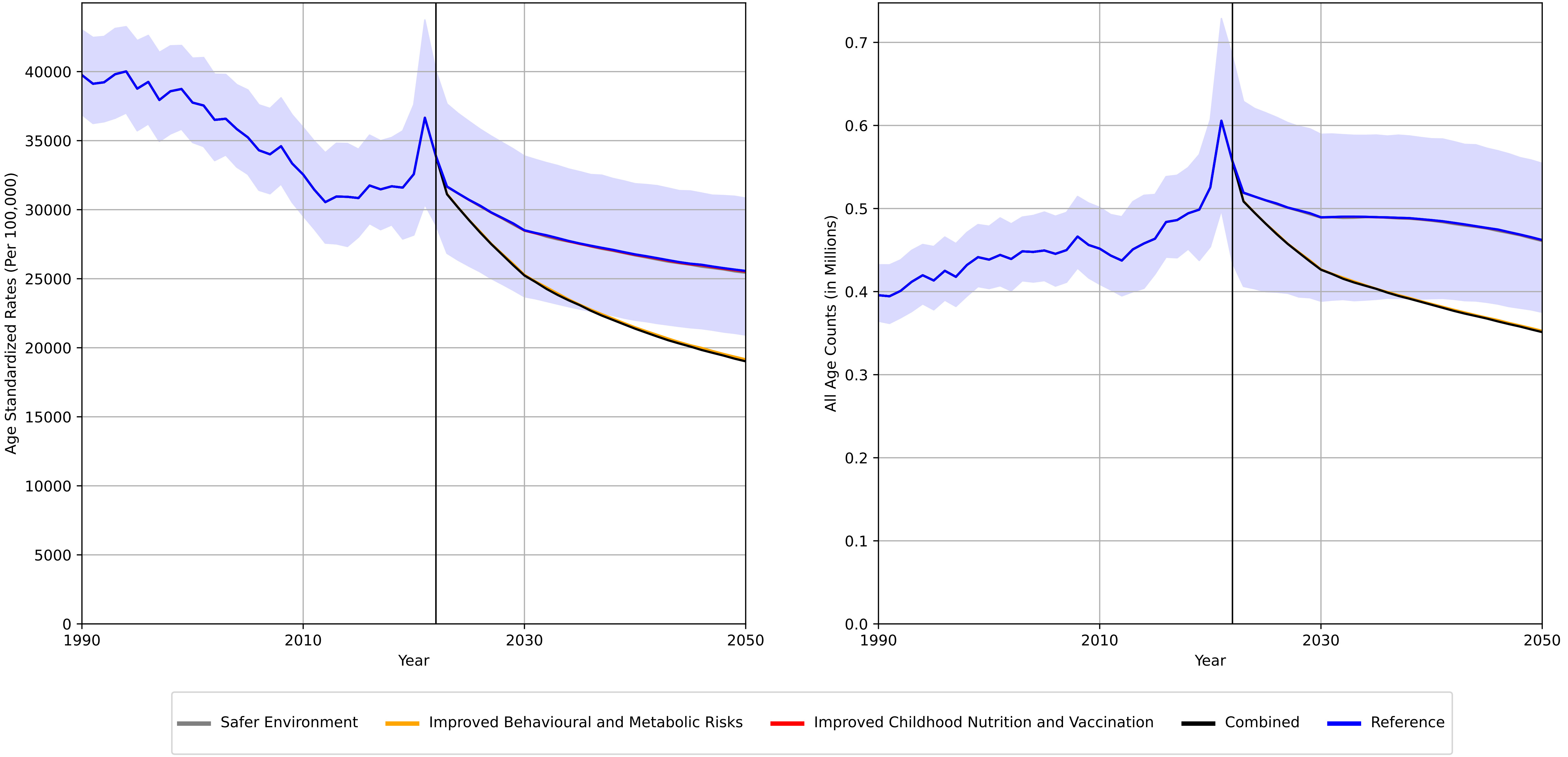

Virgin Islands

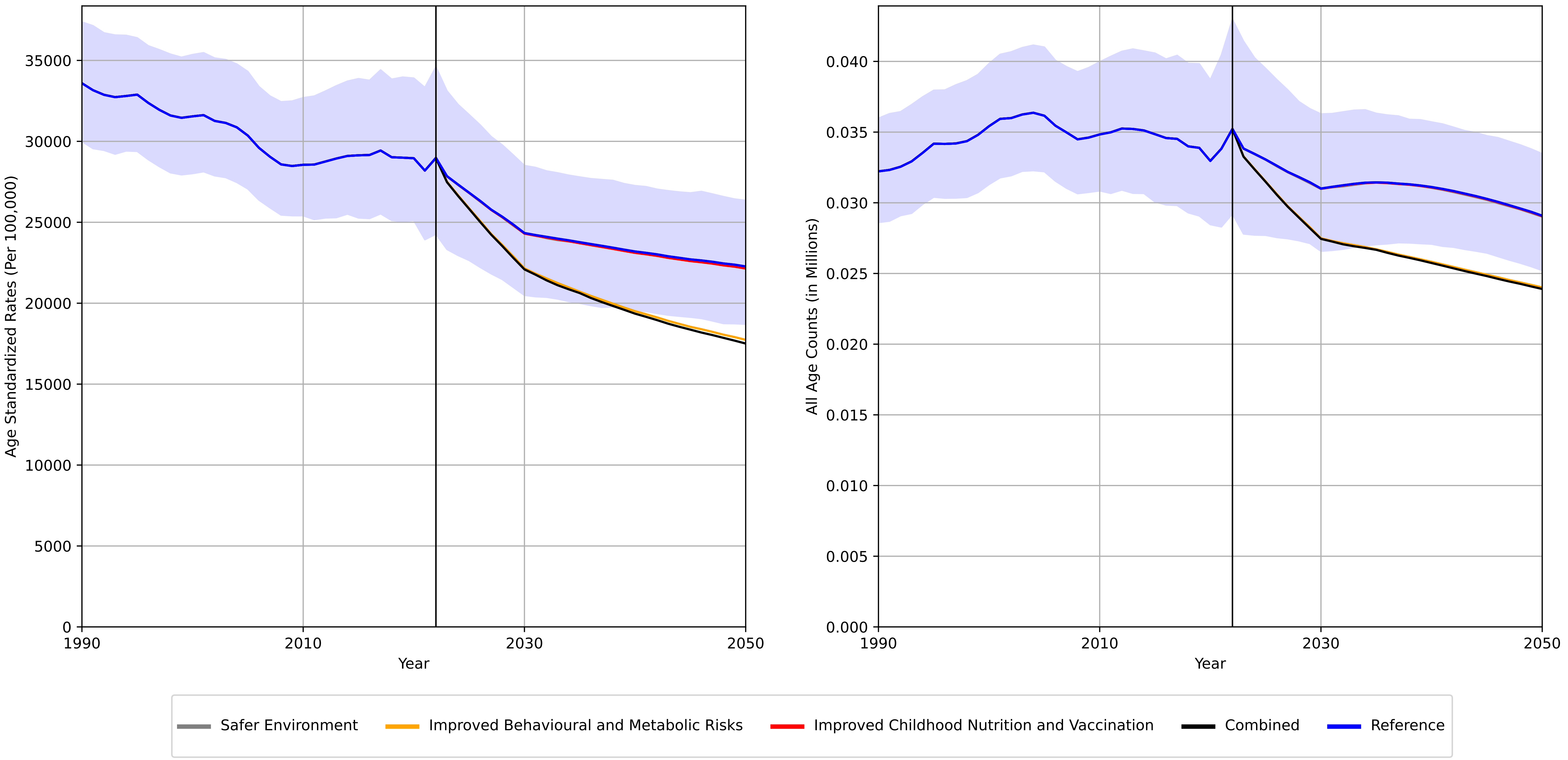

Colombia

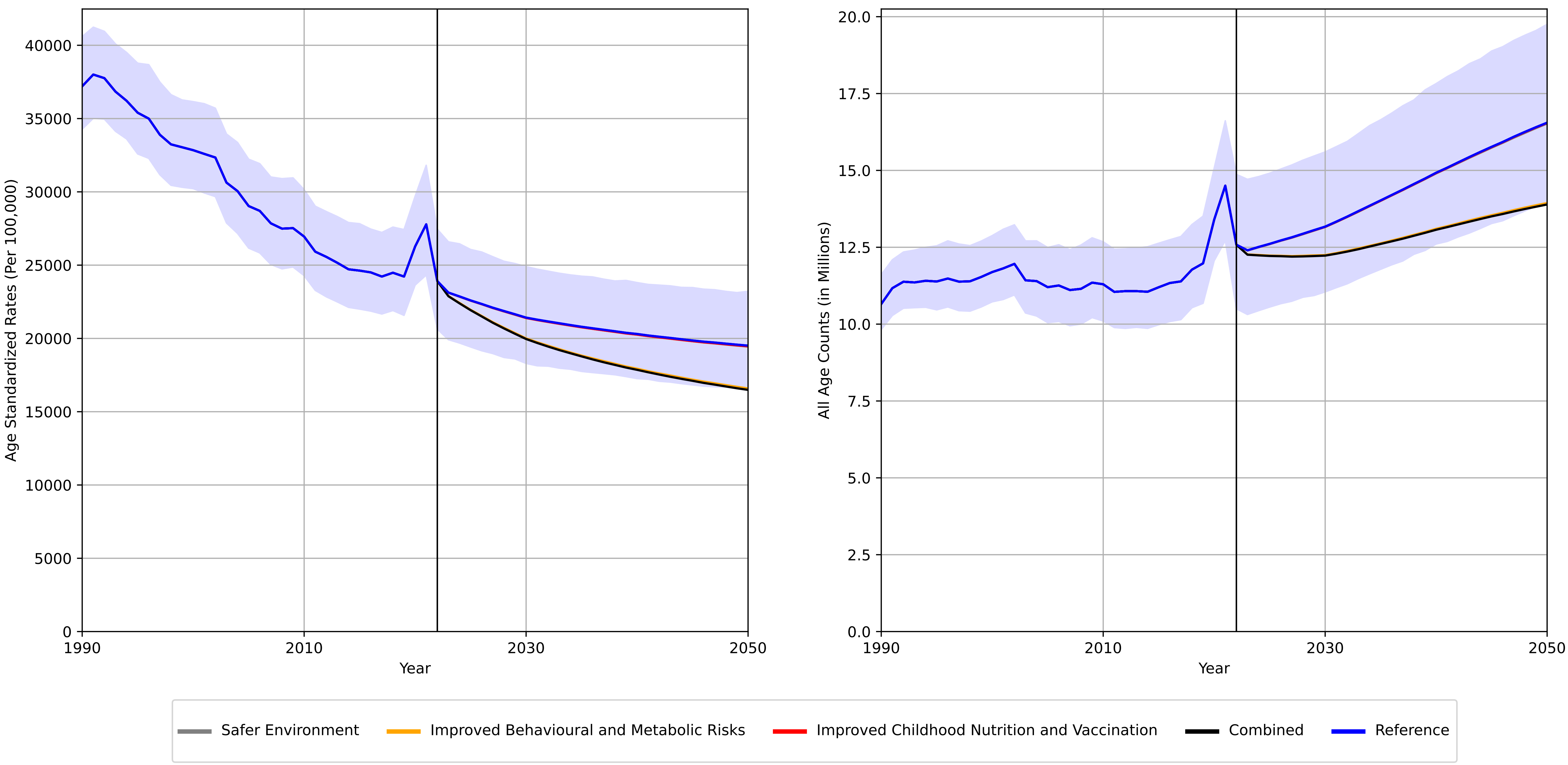
Age Standardized Rates (Per 100,000)
40000
35000
30000
25000
20000
15000
10000
5000
0
1990
2010
2030
2050
Year
All Age Counts (in Millions)
20.0
17.5
15.0
12.5
10.0
7.5
5.0
2.5
0.0
1990
2010
2030
2050
Year
Safer Environment
Improved Behavioural and Metabolic Risks
Improved Childhood Nutrition and Vaccination
Combined
Reference

Costa Rica

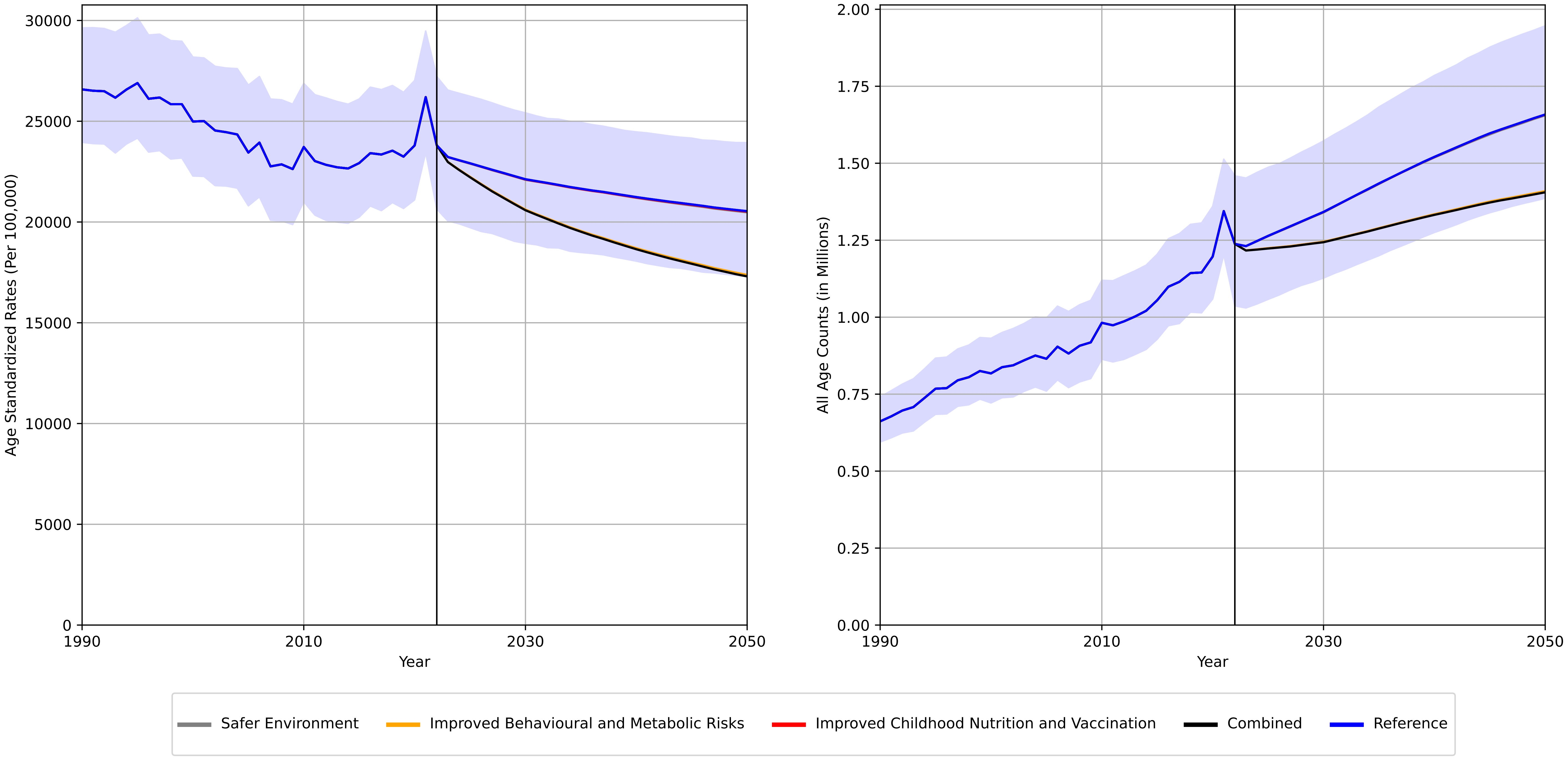
Age Standardized Rates (Per 100,000)
30000
25000
20000
15000
10000
5000
0
1990
2010
2030
2050
Year
All Age Counts (in Millions)
2.00
1.75
1.50
1.25
1.00
0.75
0.50
0.25
0.00
Safer Environment
Improved Behavioural and Metabolic Risks
Improved Childhood Nutrition and Vaccination
Combined
Reference

El Salvador

Supplemental Results Figure S10. All-cause age standardised DALY rates (A) and all age DALY counts (B), for the past and for five future scenarios, 1990–2050

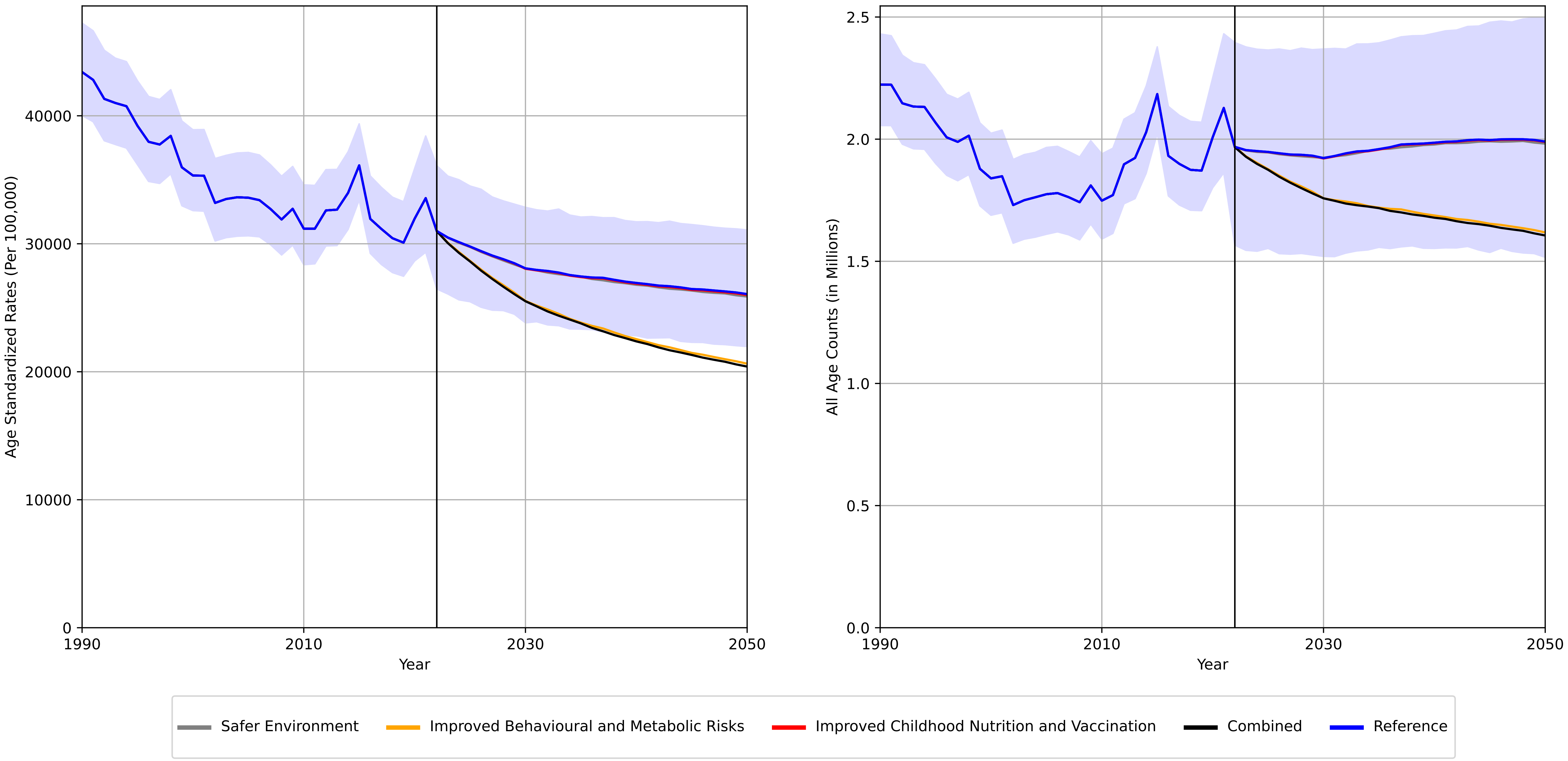

Guatemala

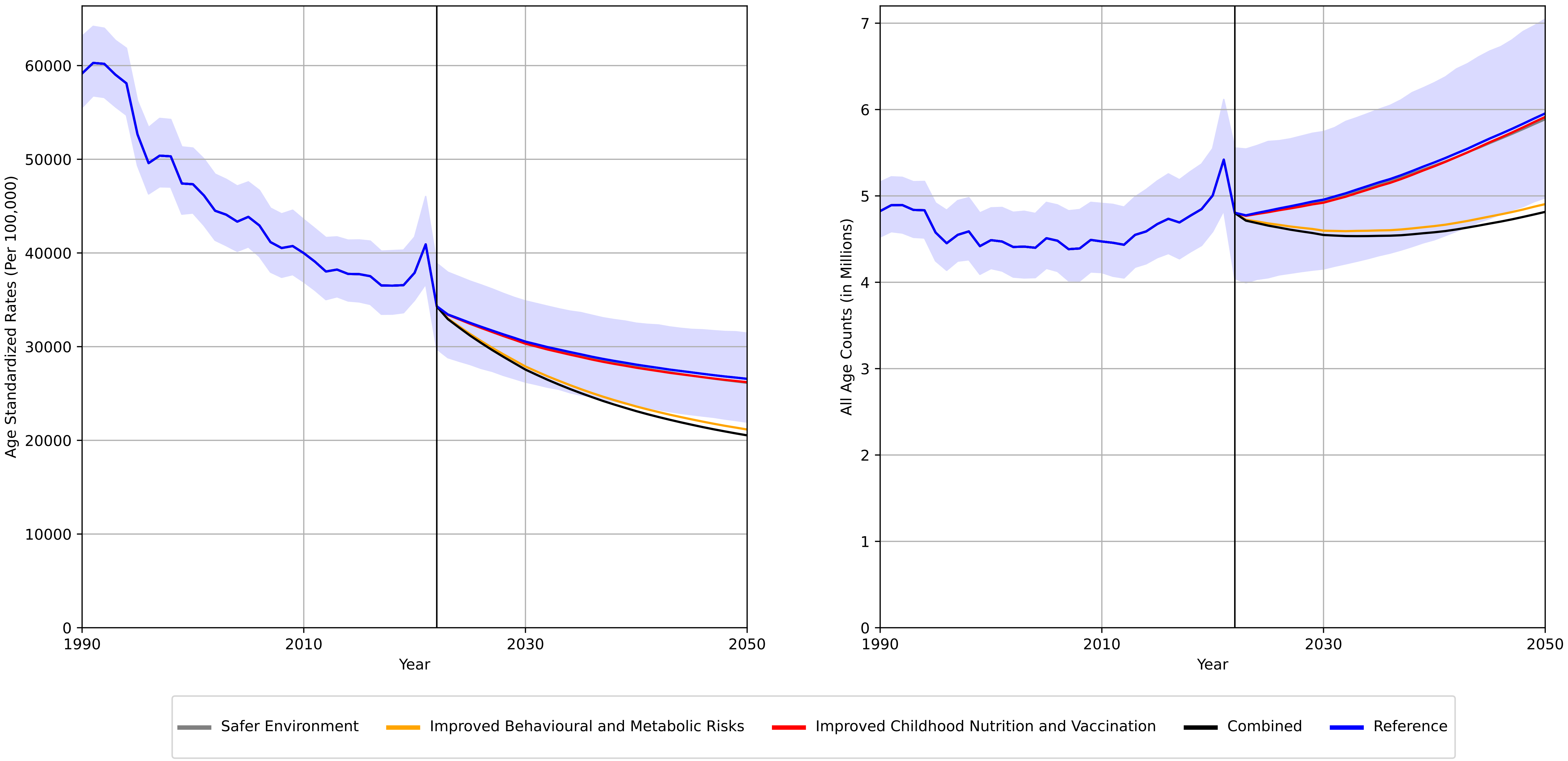
Age Standardized Rates (Per 100,000)
60000
50000
40000
30000
20000
10000
0
1990
2010
2030
2050
Year
All Age Counts (in Millions)
7
6
5
4
3
2
1
0
1990
2010
2030
2050
Year
Safer Environment
Improved Behavioural and Metabolic Risks
Improved Childhood Nutrition and Vaccination
Combined
Reference

Honduras

Supplemental Results Figure S10. All-cause age standardised DALY rates (A) and all age DALY counts (B), for the past and for five future scenarios, 1990–2050

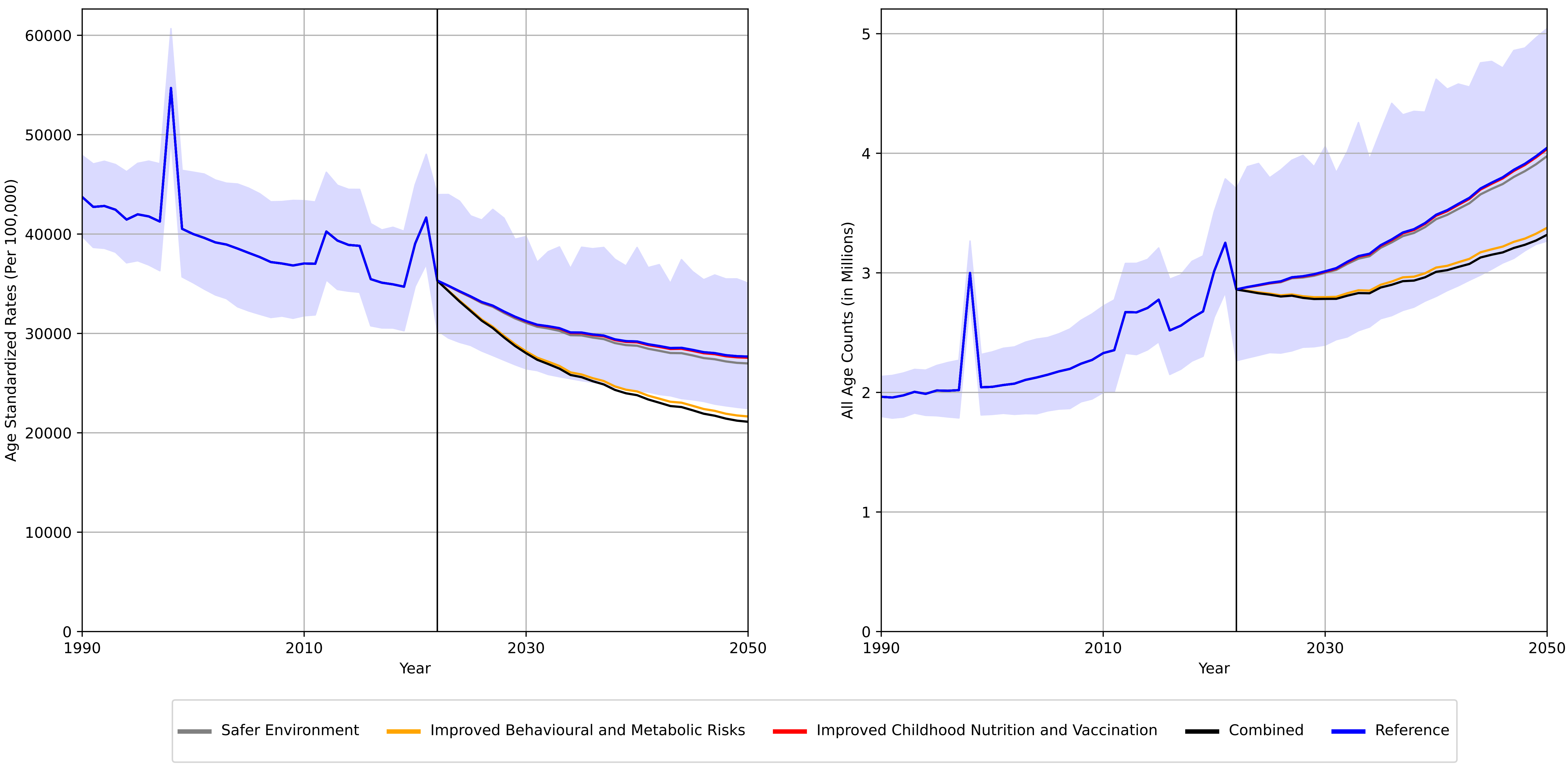

Mexico

Supplemental Results Figure S10. All-cause age standardised DALY rates (A) and all age DALY counts (B), for the past and for five future scenarios, 1990–2050

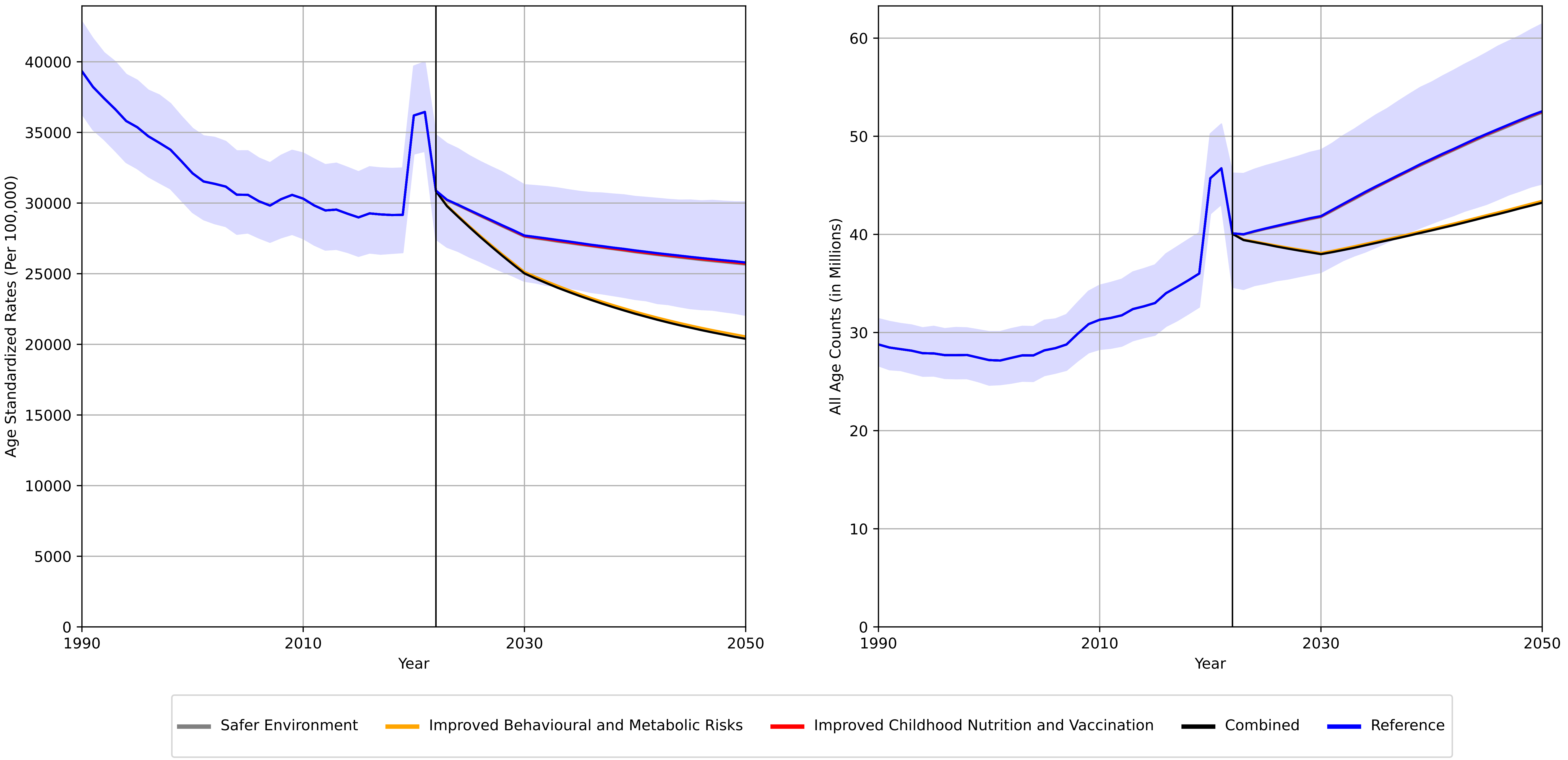

Nicaragua

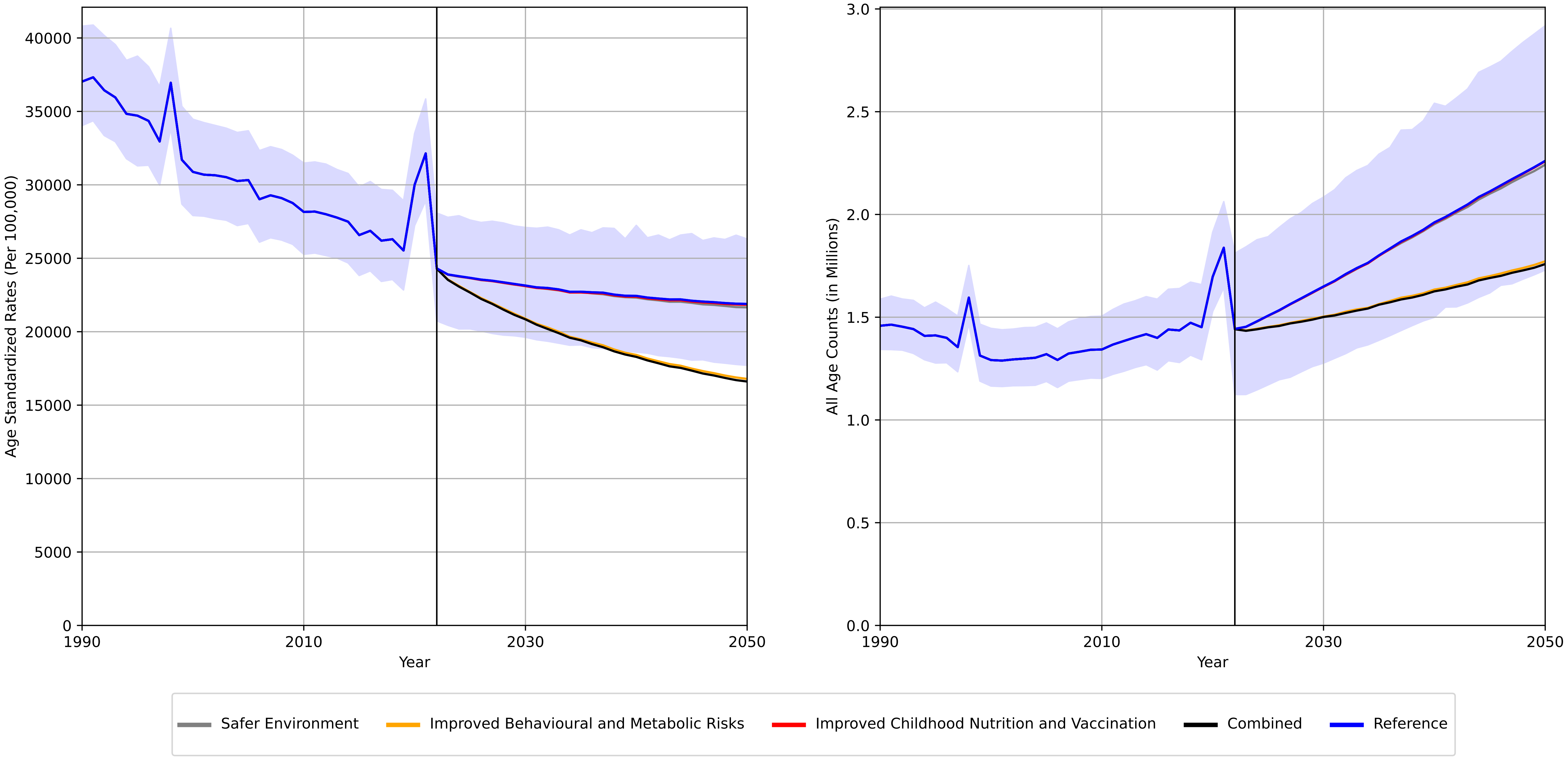
Age Standardized Rates (Per 100,000)
40000
35000
30000
25000
20000
15000
10000
5000
0
1990
2010
2030
2050
Year
All Age Counts (in Millions)
3.0
2.5
2.0
1.5
1.0
0.5
0.0
Safer Environment
Improved Behavioural and Metabolic Risks
Improved Childhood Nutrition and Vaccination
Combined
Reference

Panama

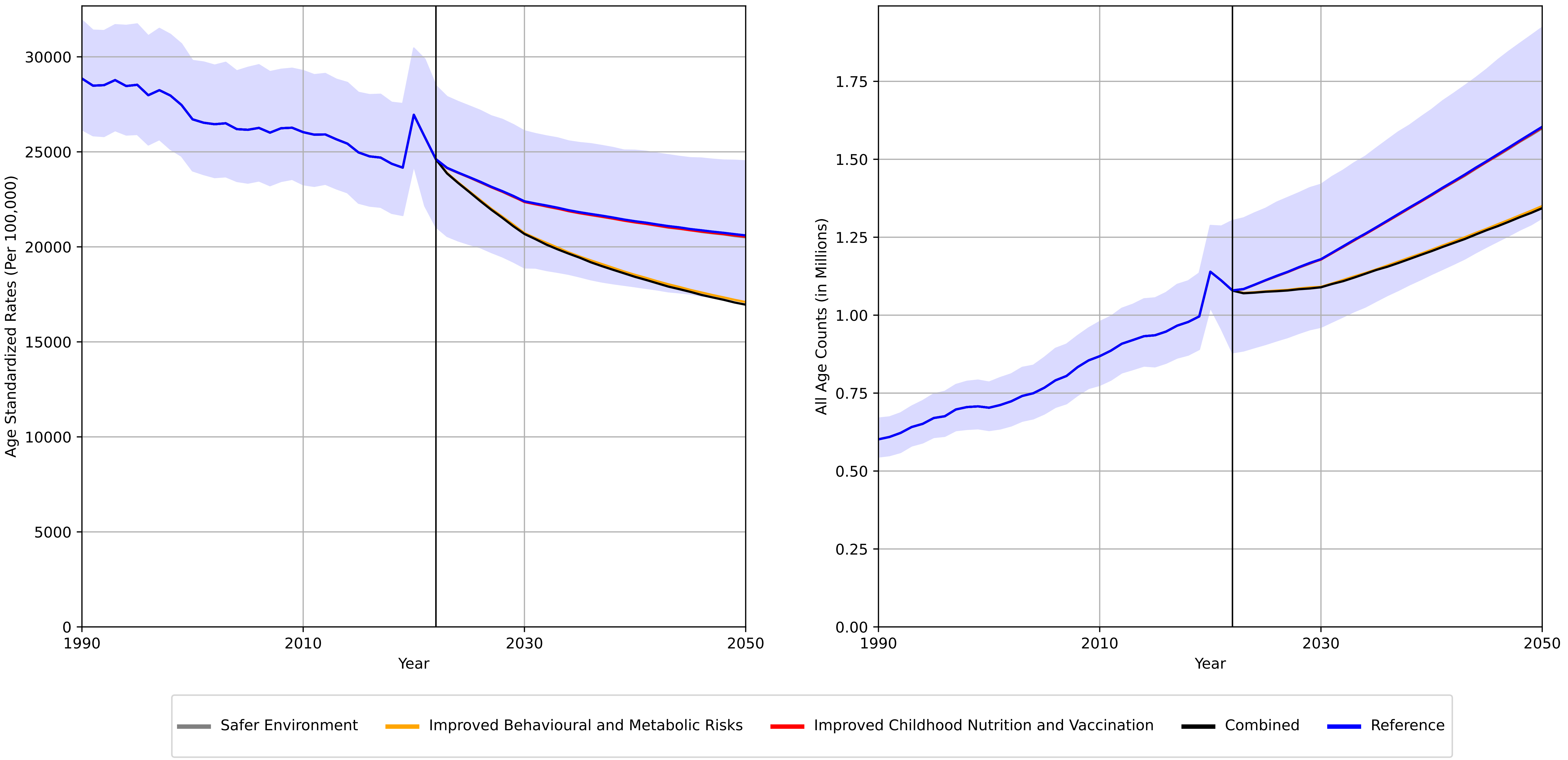
Age Standardized Rates (Per 100,000)
30000
25000
20000
15000
10000
5000
0
1990
2010
2030
2050
Year
All Age Counts (in Millions)
1.75
1.50
1.25
1.00
0.75
0.50
0.25
0.00
1990
2010
2030
2050
Year
Safer Environment
Improved Behavioural and Metabolic Risks
Improved Childhood Nutrition and Vaccination
Combined
Reference

Venezuela

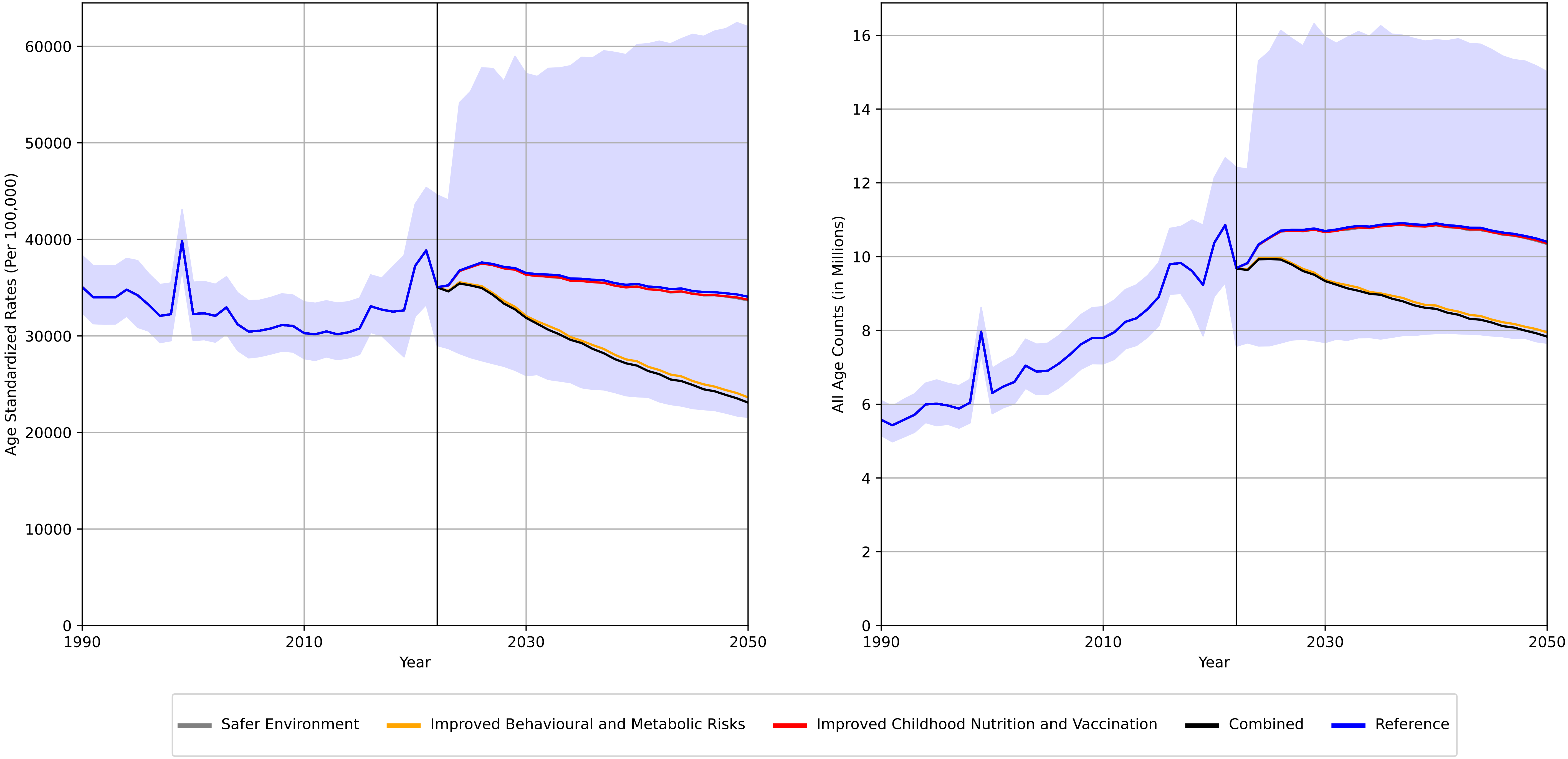

Age Standardized Rates (Per 100,000)
60000
50000
40000
30000
20000
10000
0
1990
2010
2030
2050
Year
All Age Counts (in Millions)
16
14
12
10
8
6
4
2
0
Safer Environment
Improved Behavioural and Metabolic Risks
Improved Childhood Nutrition and Vaccination
Combined
Reference

Brazil

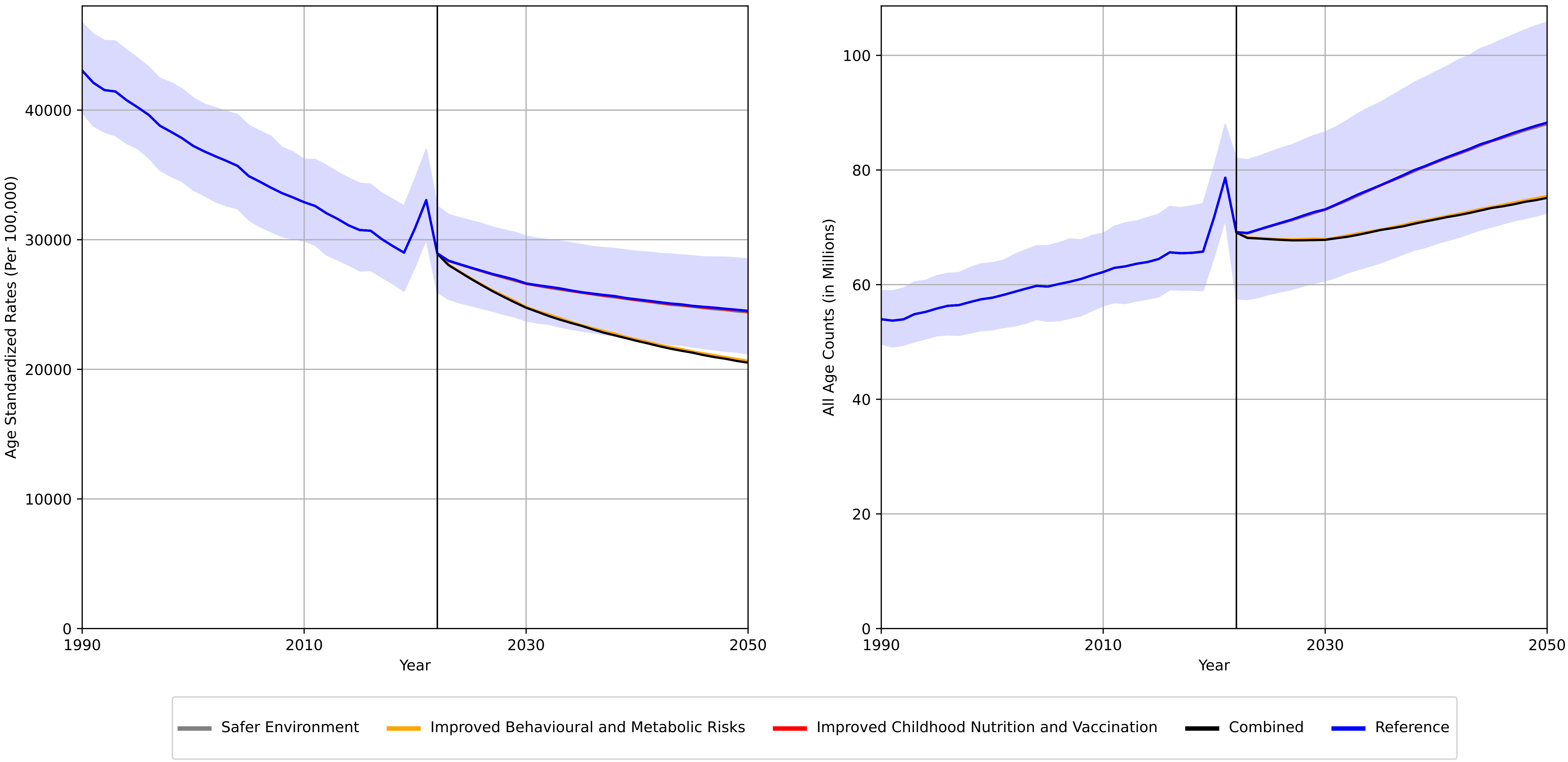
Age Standardized Rates (Per 100,000)
40000
30000
20000
10000
0
1990
2010
2030
2050
Year
All Age Counts (in Millions)
100
80
60
40
20
0
Safer Environment
Improved Behavioural and Metabolic Risks
Improved Childhood Nutrition and Vaccination
Combined
Reference

Paraguay

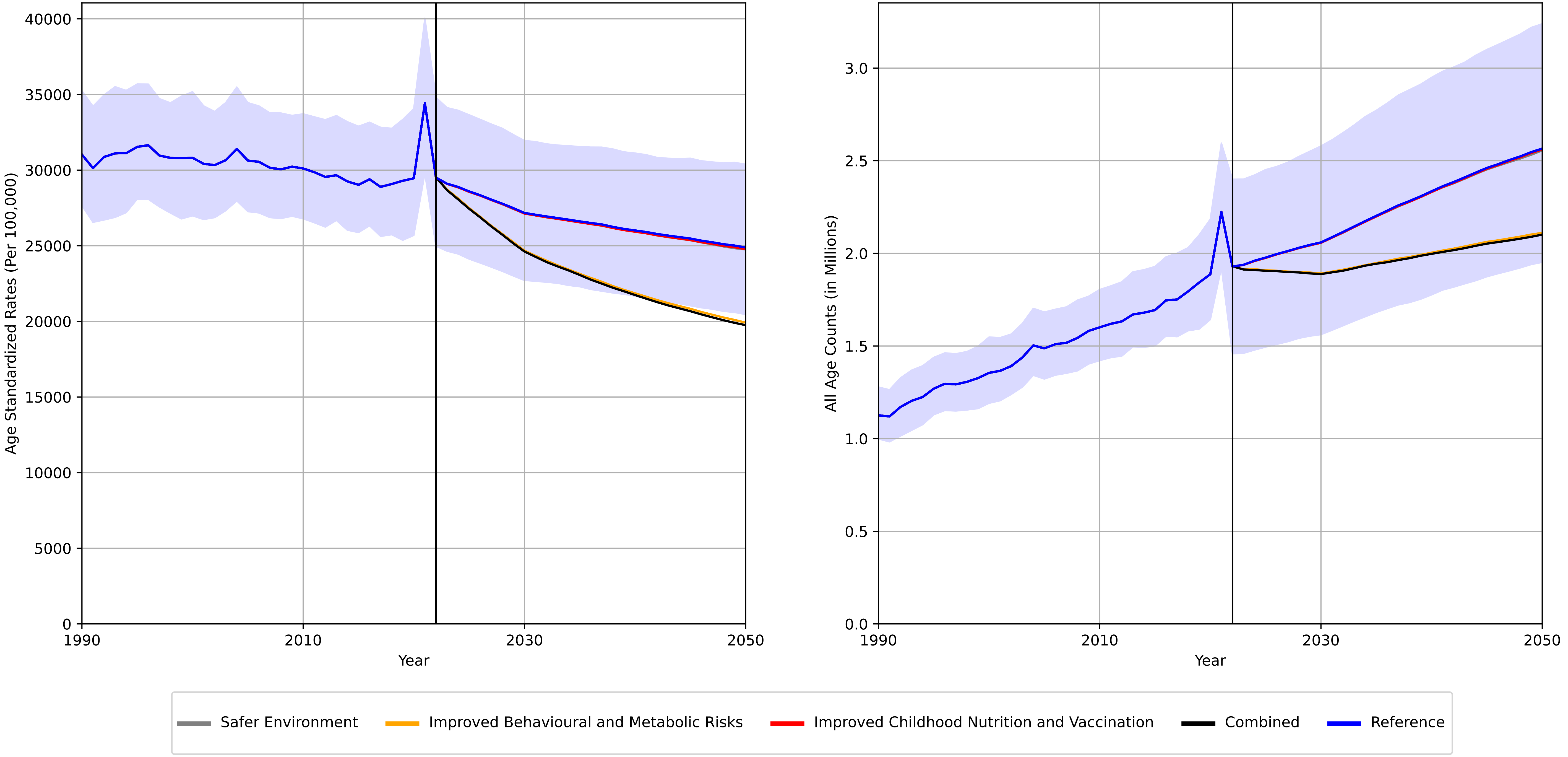

Age Standardized Rates (Per 100,000)
40000
35000
30000
25000
20000
15000
10000
5000
0
1990
2010
2030
2050
Year
All Age Counts (in Millions)
3.0
2.5
2.0
1.5
1.0
0.5
0.0
1990
2010
2030
2050
Year
Safer Environment
Improved Behavioural and Metabolic Risks
Improved Childhood Nutrition and Vaccination
Combined
Reference

Afghanistan

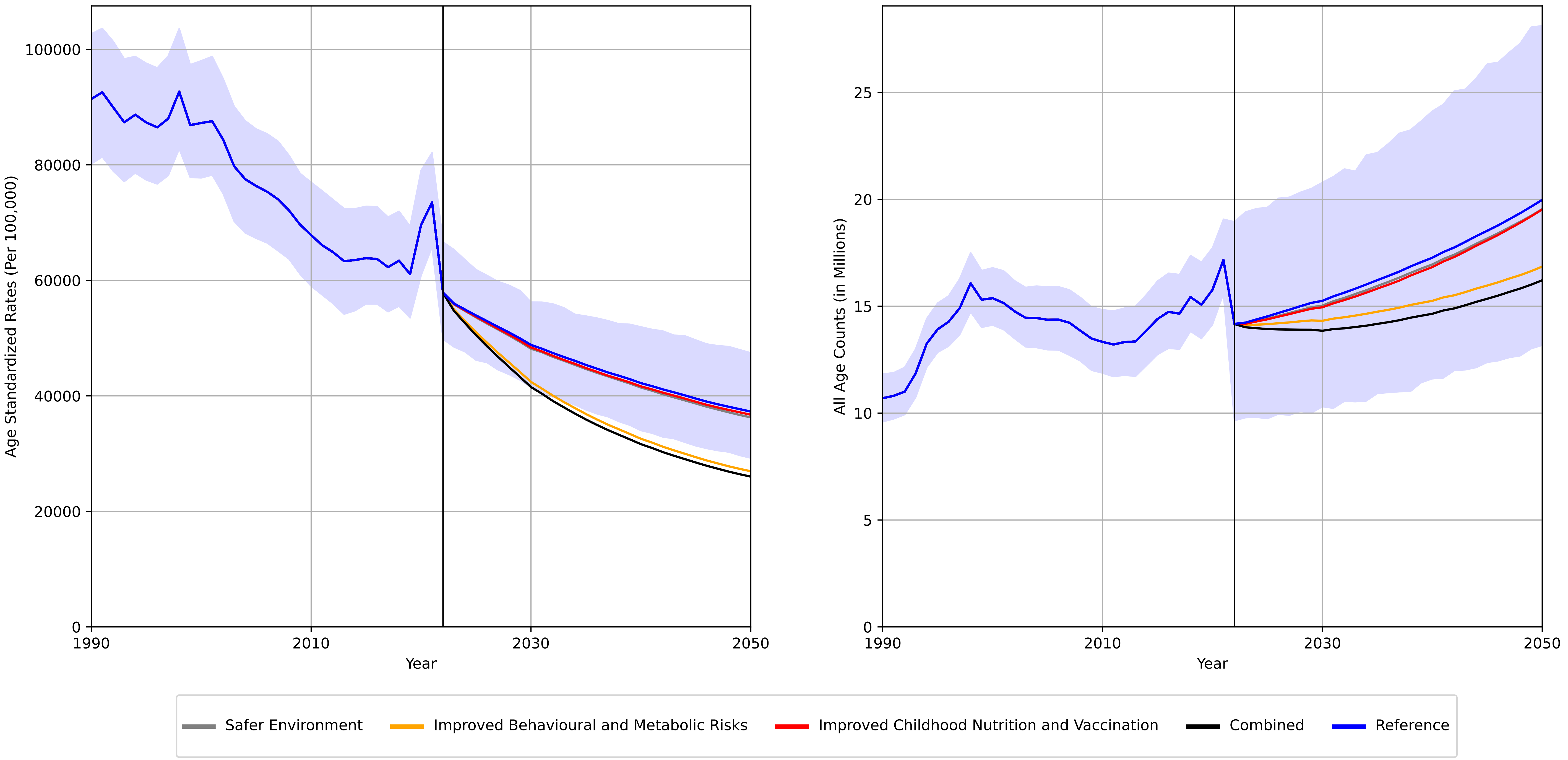

Algeria

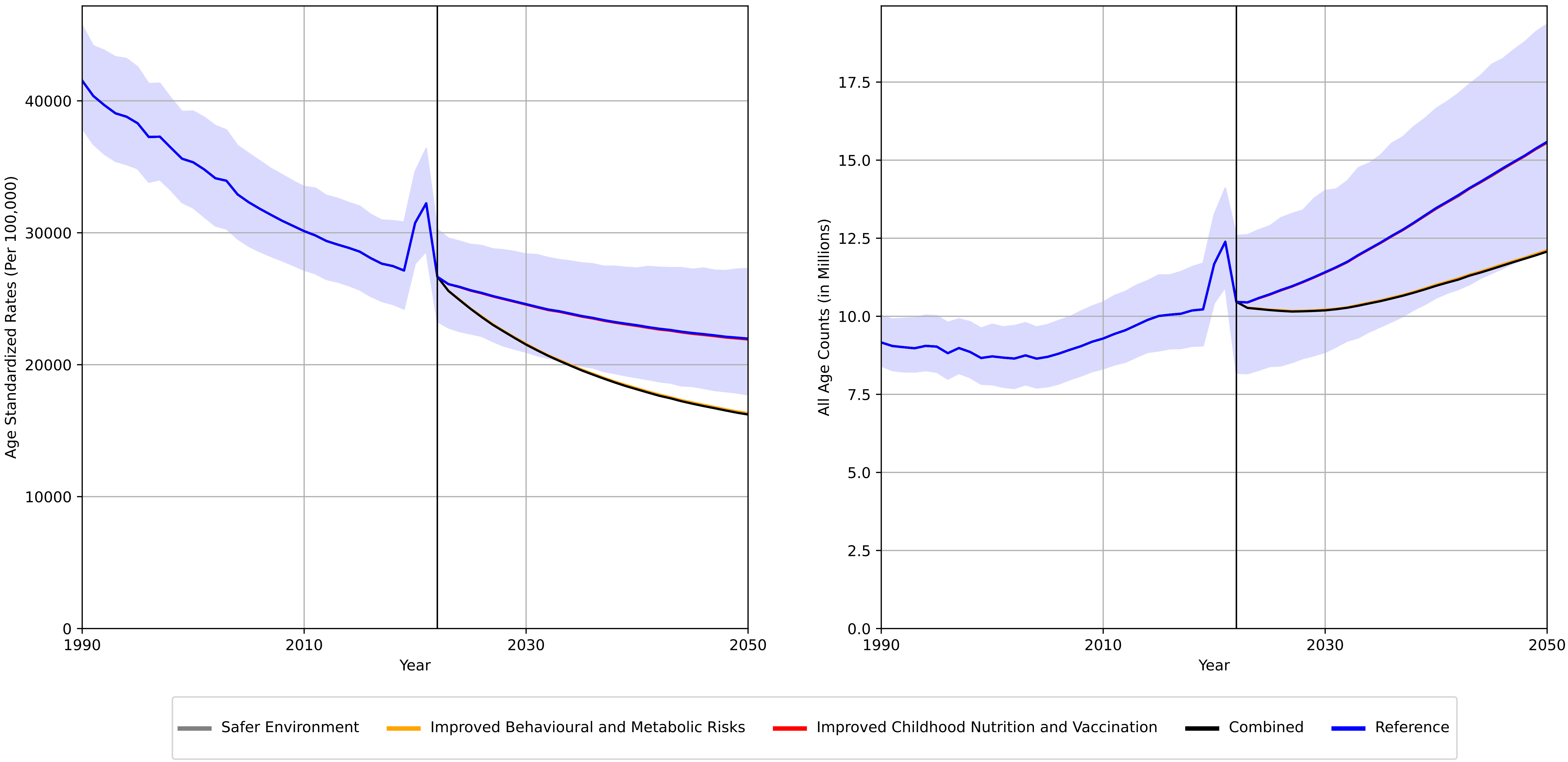
Age Standardized Rates (Per 100,000)
40000
30000
20000
10000
0
1990
2010
2030
2050
Year
All Age Counts (in Millions)
17.5
15.0
12.5
10.0
7.5
5.0
2.5
0.0
Safer Environment
Improved Behavioural and Metabolic Risks
Improved Childhood Nutrition and Vaccination
Combined
Reference

Bahrain

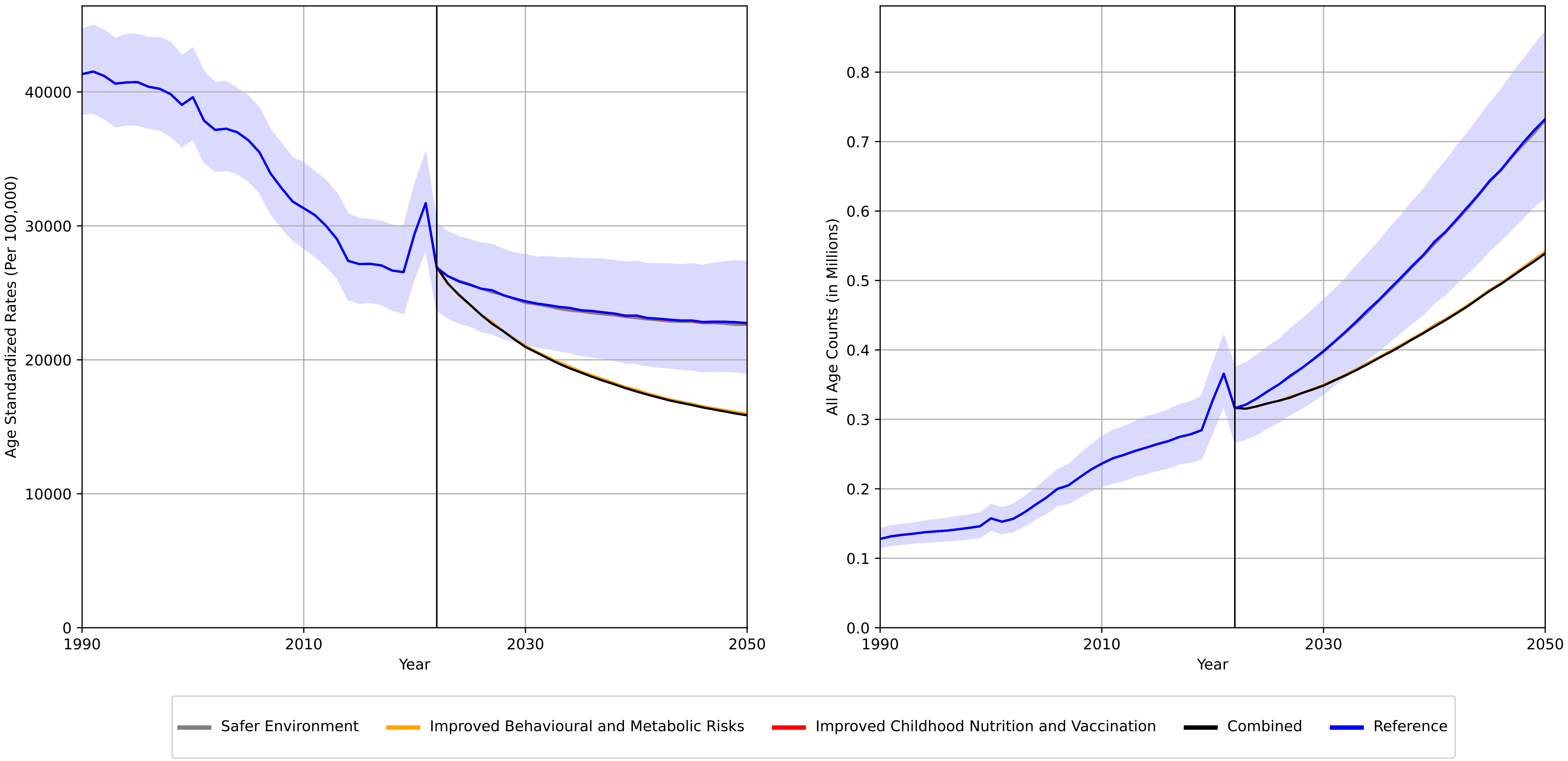

Age Standardized Rates (Per 100,000)
0
10000
20000
30000
40000
1990
2010
2030
2050
Year
All Age Counts (in Millions)
0.0
0.1
0.2
0.3
0.4
0.5
0.6
0.7
0.8
1990
2010
2030
2050
Year
Safer Environment
Improved Behavioural and Metabolic Risks
Improved Childhood Nutrition and Vaccination
Combined
Reference

Egypt

Supplemental Results Figure S10. All-cause age standardised DALY rates (A) and all age DALY counts (B), for the past and for five future scenarios, 1990–2050

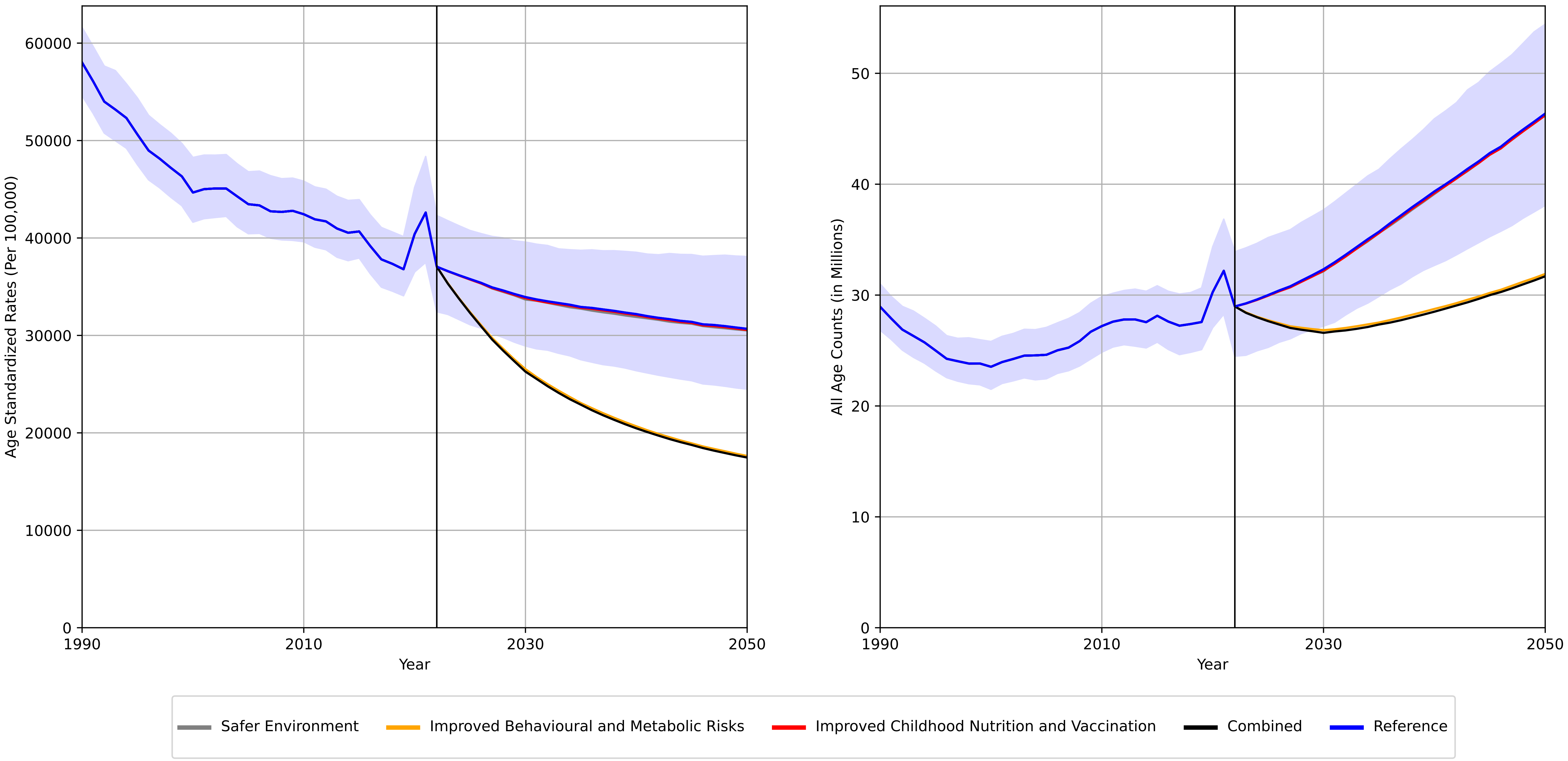

Iran

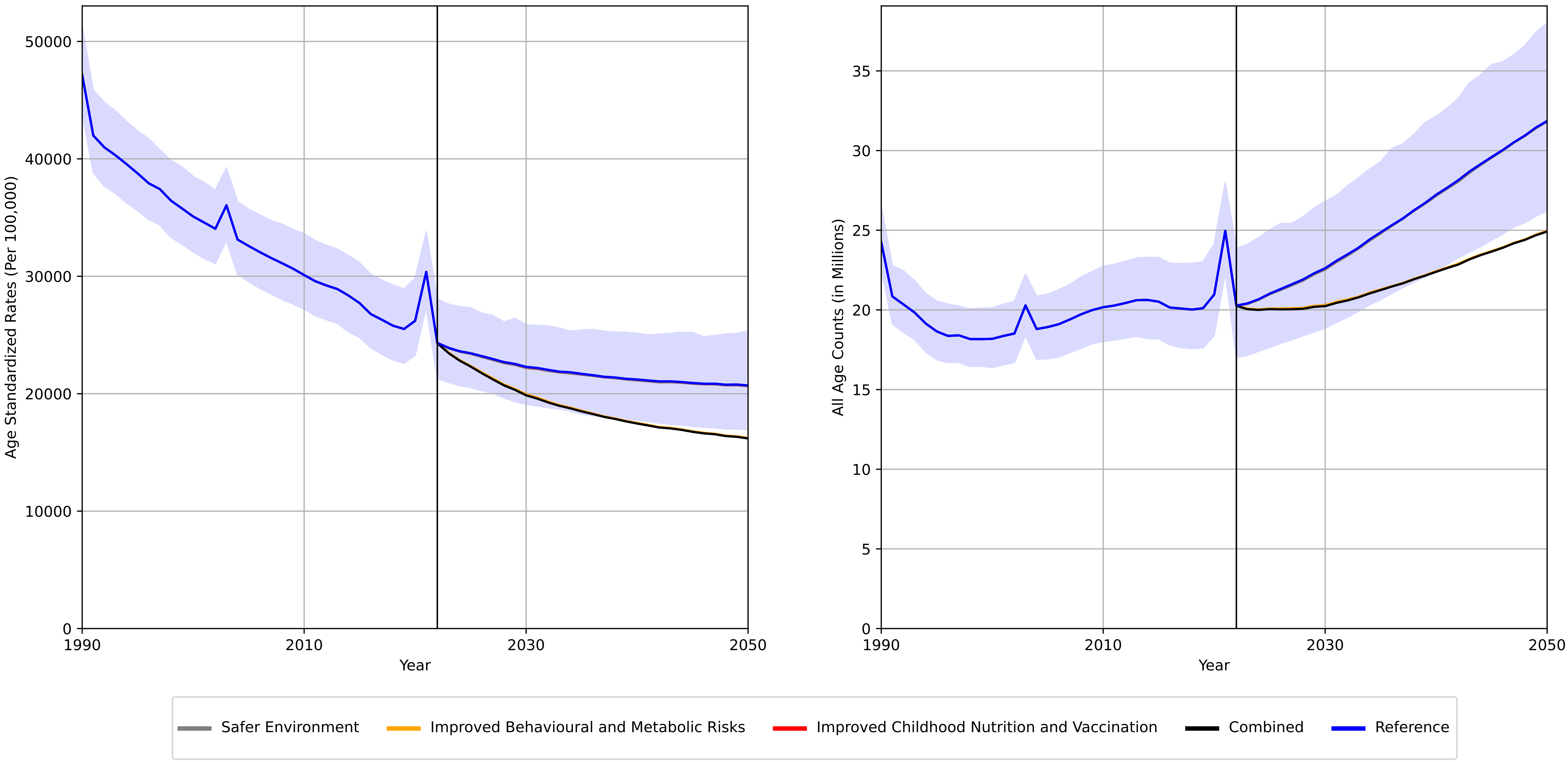

Age Standardized Rates (Per 100,000)
50000
40000
30000
20000
10000
0
1990
2010
2030
2050
Year
All Age Counts (in Millions)
35
30
25
20
15
10
5
0
1990
2010
2030
2050
Year
Safer Environment
Improved Behavioural and Metabolic Risks
Improved Childhood Nutrition and Vaccination
Combined
Reference

Iraq

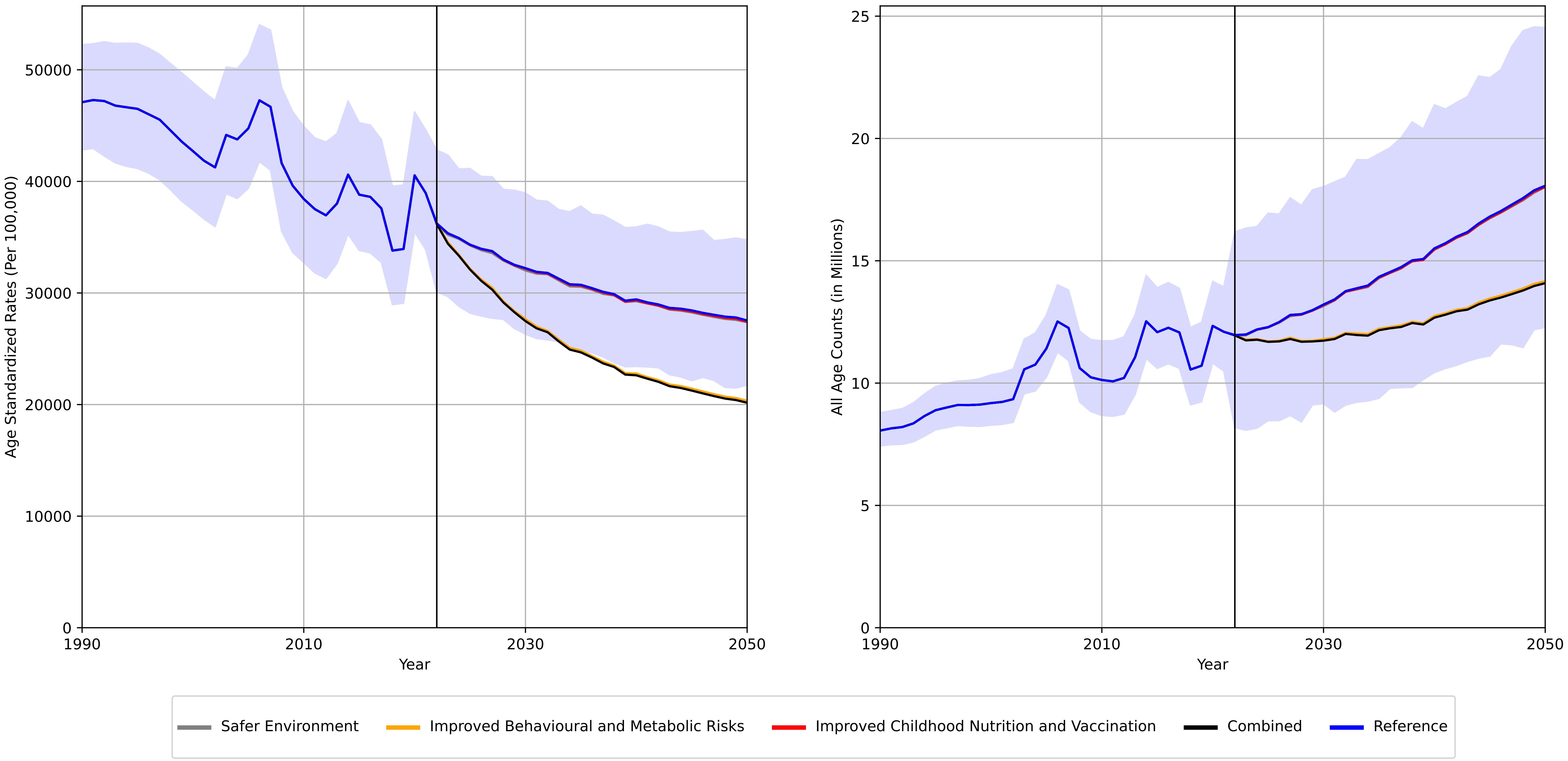

Age Standardized Rates (Per 100,000)
50000
40000
30000
20000
10000
0
1990
2010
2030
2050
Year
All Age Counts (in Millions)
25
20
15
10
5
0
1990
2010
2030
2050
Year
Safer Environment
Improved Behavioural and Metabolic Risks
Improved Childhood Nutrition and Vaccination
Combined
Reference

Jordan

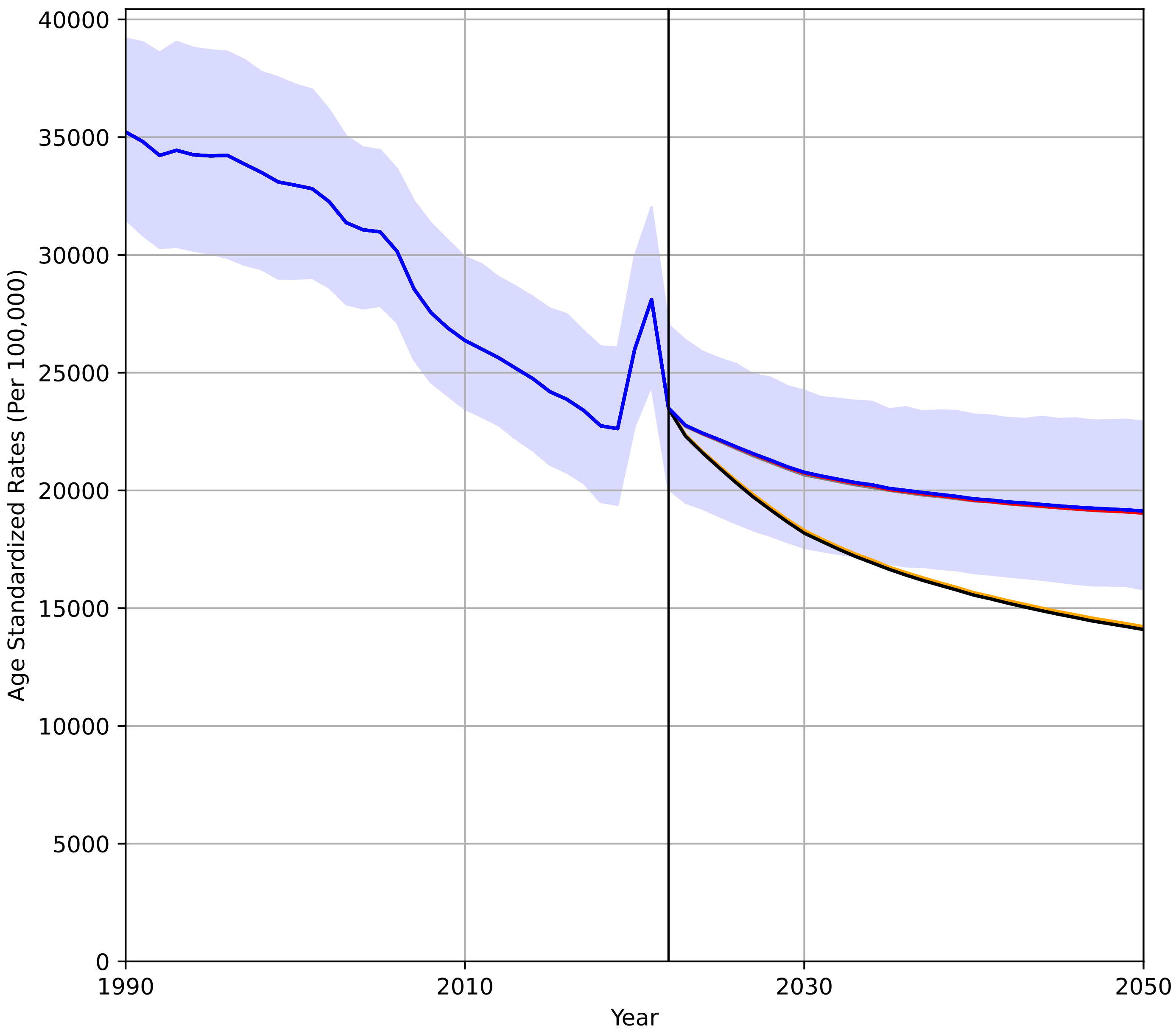

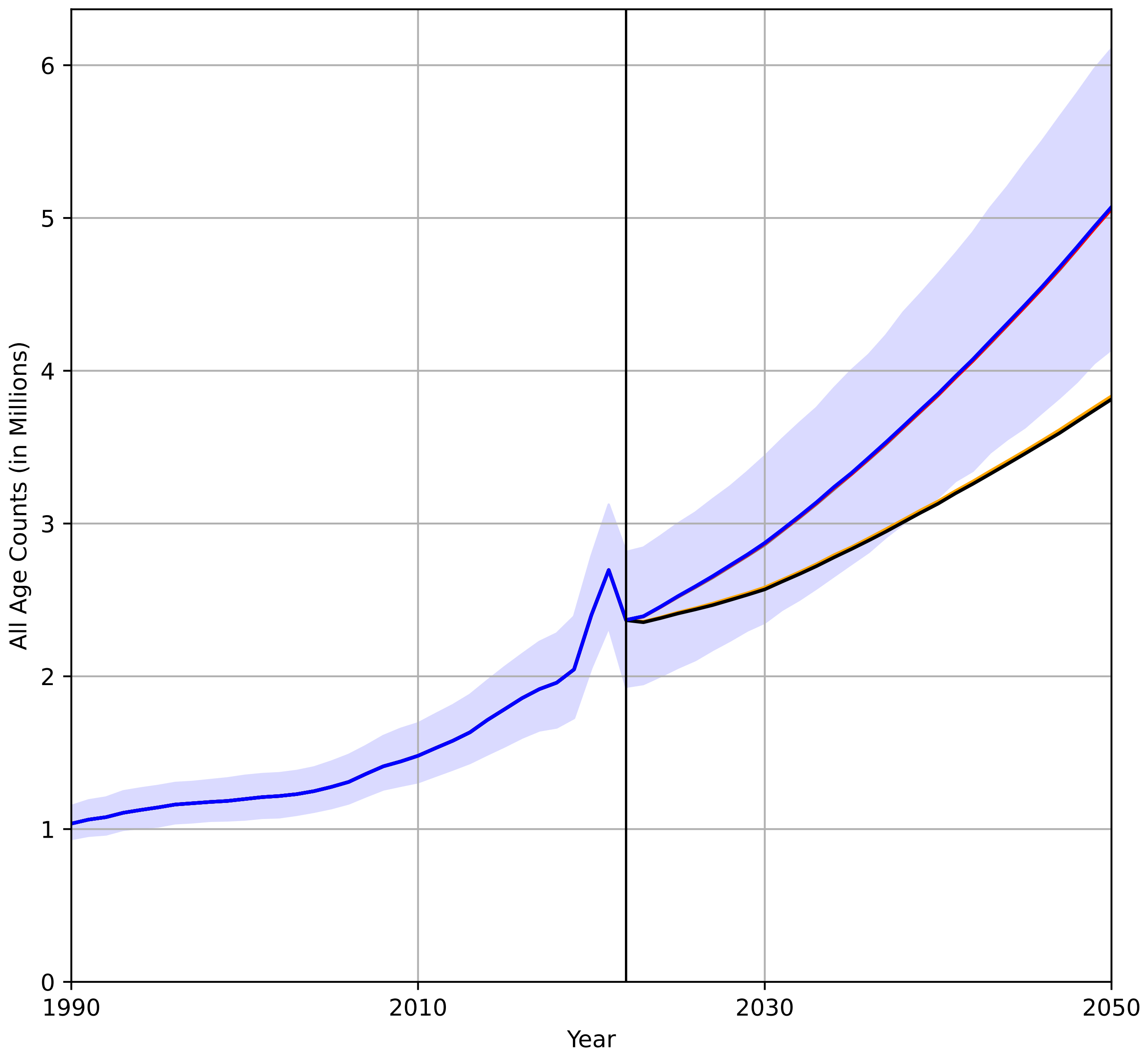

Safer Environment
Improved Behavioural and Metabolic Risks
Improved Childhood Nutrition and Vaccination
Combined
Reference

Kuwait

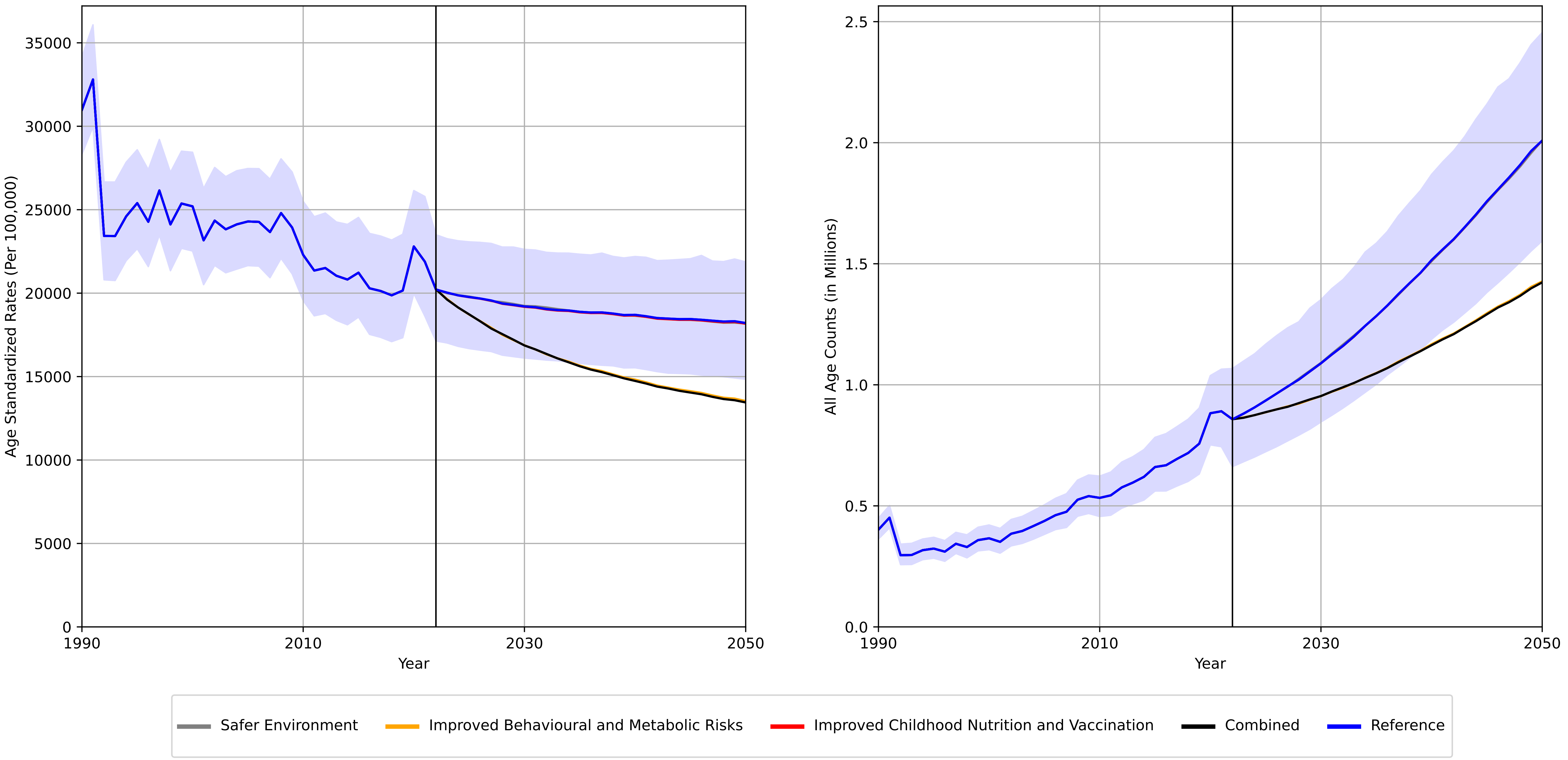
Age Standardized Rates (Per 100,000)
35000
30000
25000
20000
15000
10000
5000
0
1990
2010
2030
2050
Year
All Age Counts (in Millions)
2.5
2.0
1.5
1.0
0.5
0.0
1990
2010
2030
2050
Year
Safer Environment
Improved Behavioural and Metabolic Risks
Improved Childhood Nutrition and Vaccination
Combined
Reference

Lebanon

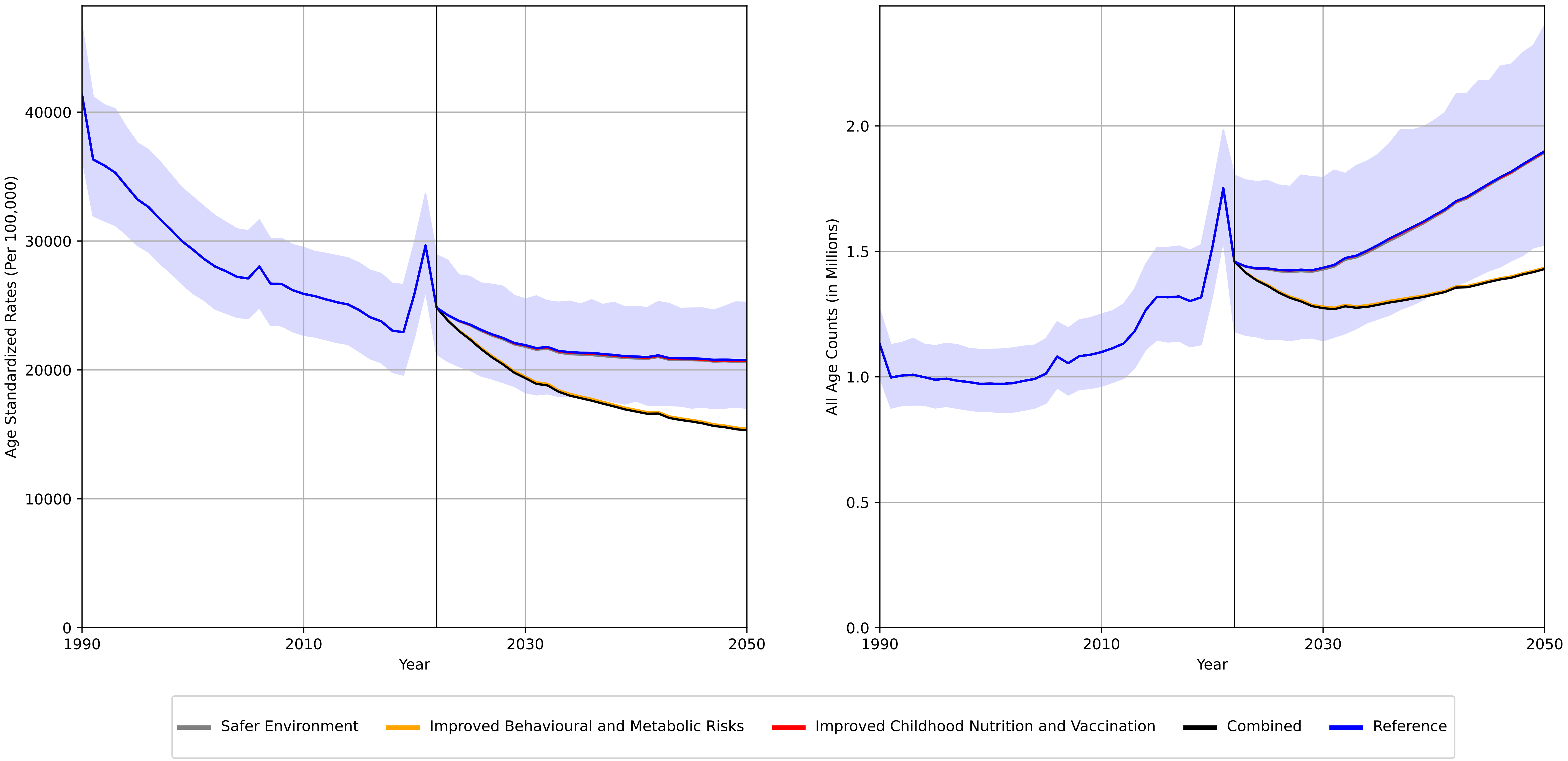
Age Standardized Rates (Per 100,000)
40000
30000
20000
10000
0
1990
2010
2030
2050
Year
All Age Counts (in Millions)
2.0
1.5
1.0
0.5
0.0
Safer Environment
Improved Behavioural and Metabolic Risks
Improved Childhood Nutrition and Vaccination
Combined
Reference

Libya

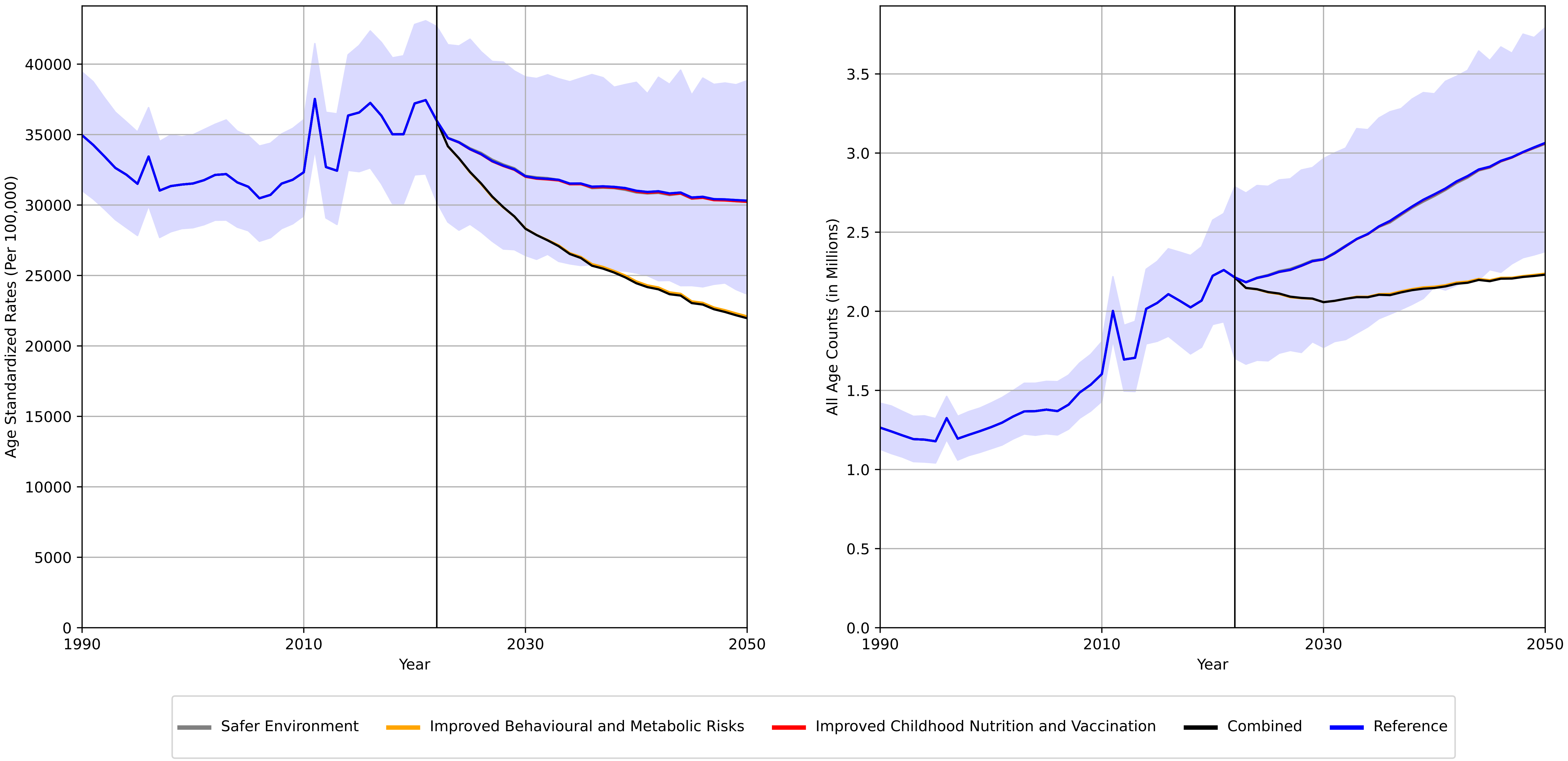
Age Standardized Rates (Per 100,000)
0
5000
10000
15000
20000
25000
30000
35000
40000
1990
2010
2030
2050
Year
All Age Counts (in Millions)
0.0
0.5
1.0
1.5
2.0
2.5
3.0
3.5
1990
2010
2030
2050
Year
Safer Environment
Improved Behavioural and Metabolic Risks
Improved Childhood Nutrition and Vaccination
Combined
Reference

Morocco

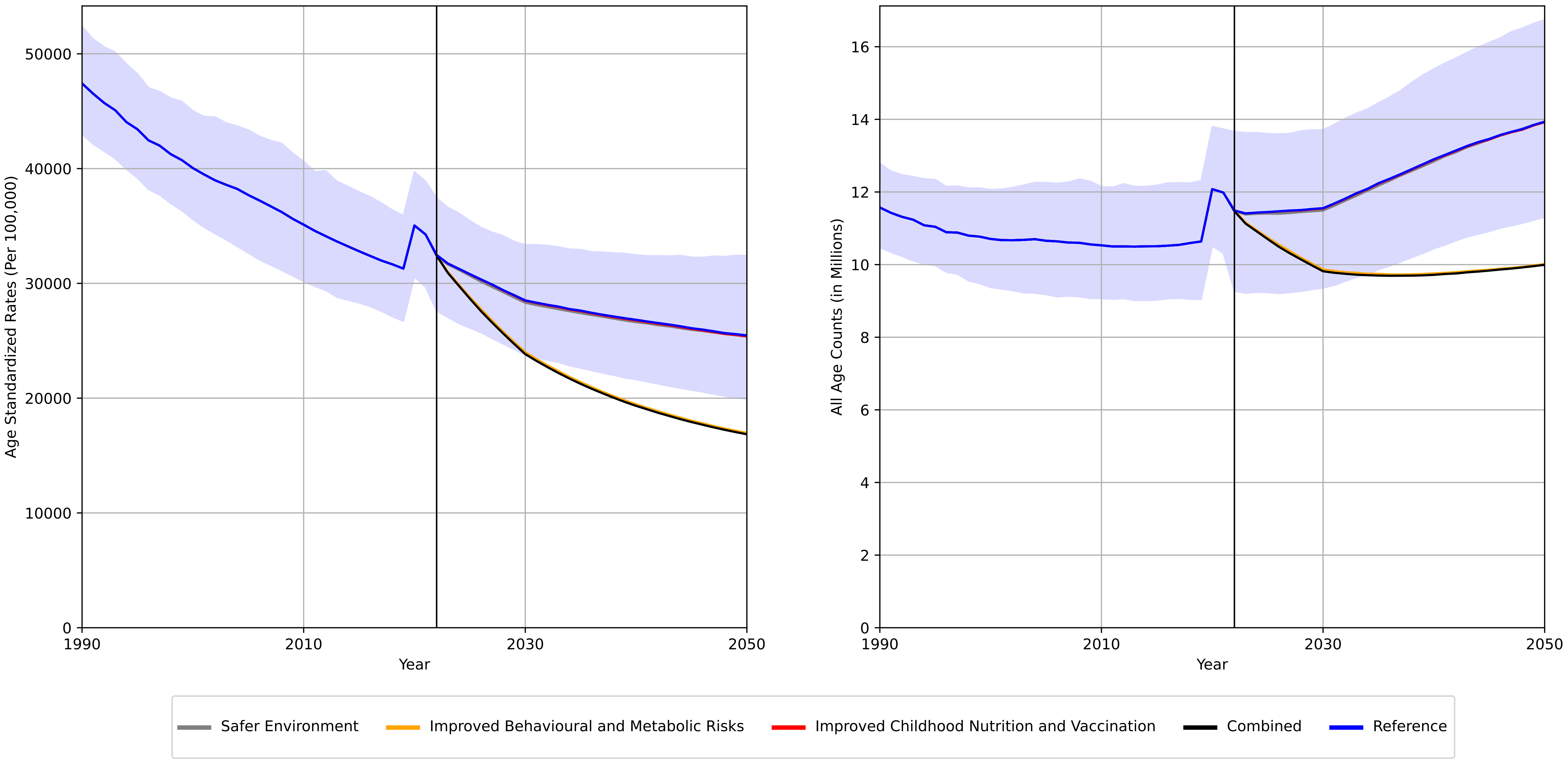

Age Standardized Rates (Per 100,000)
50000
40000
30000
20000
10000
0
1990
2010
2030
2050
Year
All Age Counts (in Millions)
16
14
12
10
8
6
4
2
0
Safer Environment
Improved Behavioural and Metabolic Risks
Improved Childhood Nutrition and Vaccination
Combined
Reference

Oman

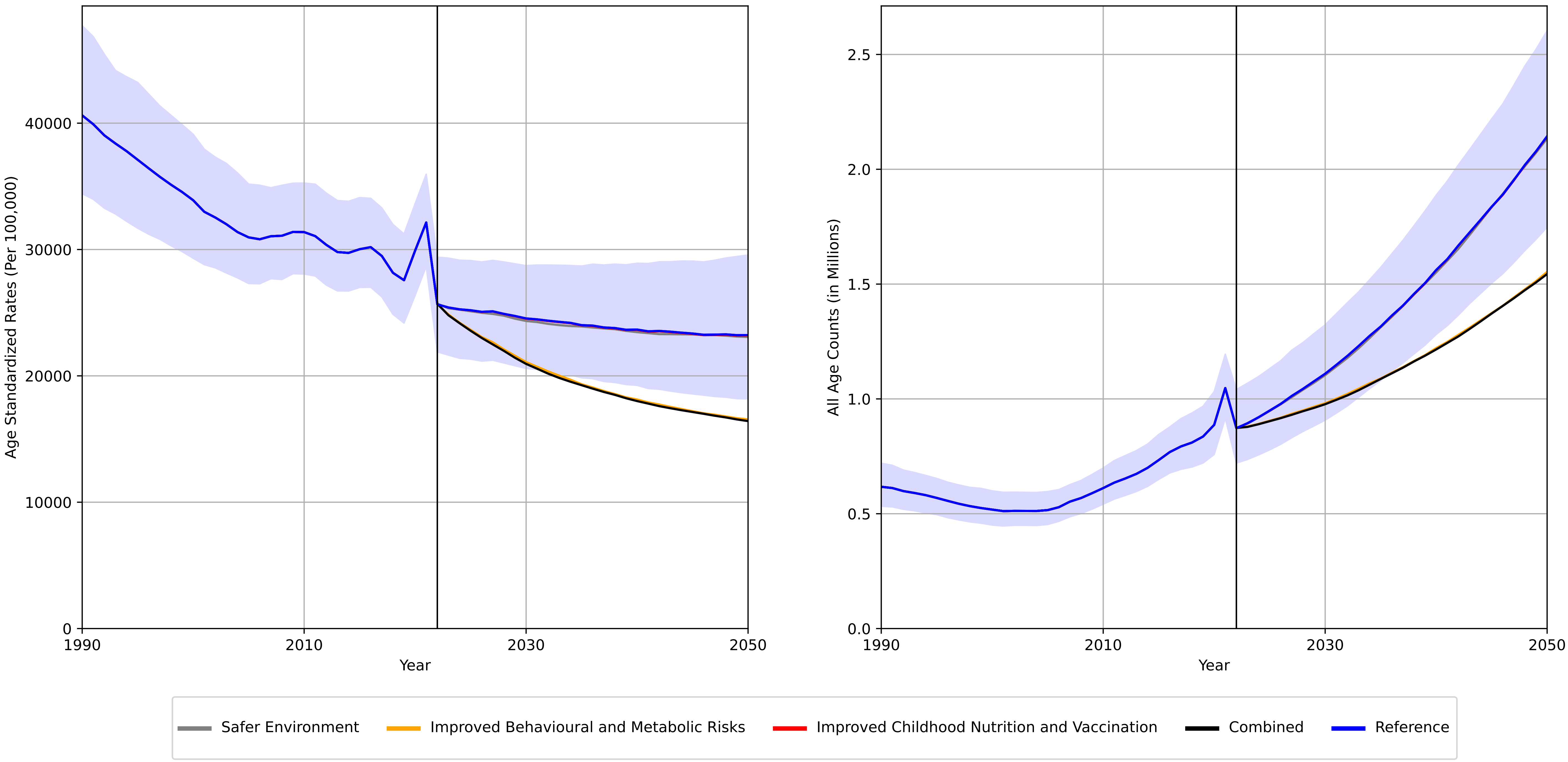
Age Standardized Rates (Per 100,000)
0
10000
20000
30000
40000
1990
2010
2030
2050
Year
All Age Counts (in Millions)
0.0
0.5
1.0
1.5
2.0
2.5
1990
2010
2030
2050
Year
Safer Environment
Improved Behavioural and Metabolic Risks
Improved Childhood Nutrition and Vaccination
Combined
Reference

Palestine

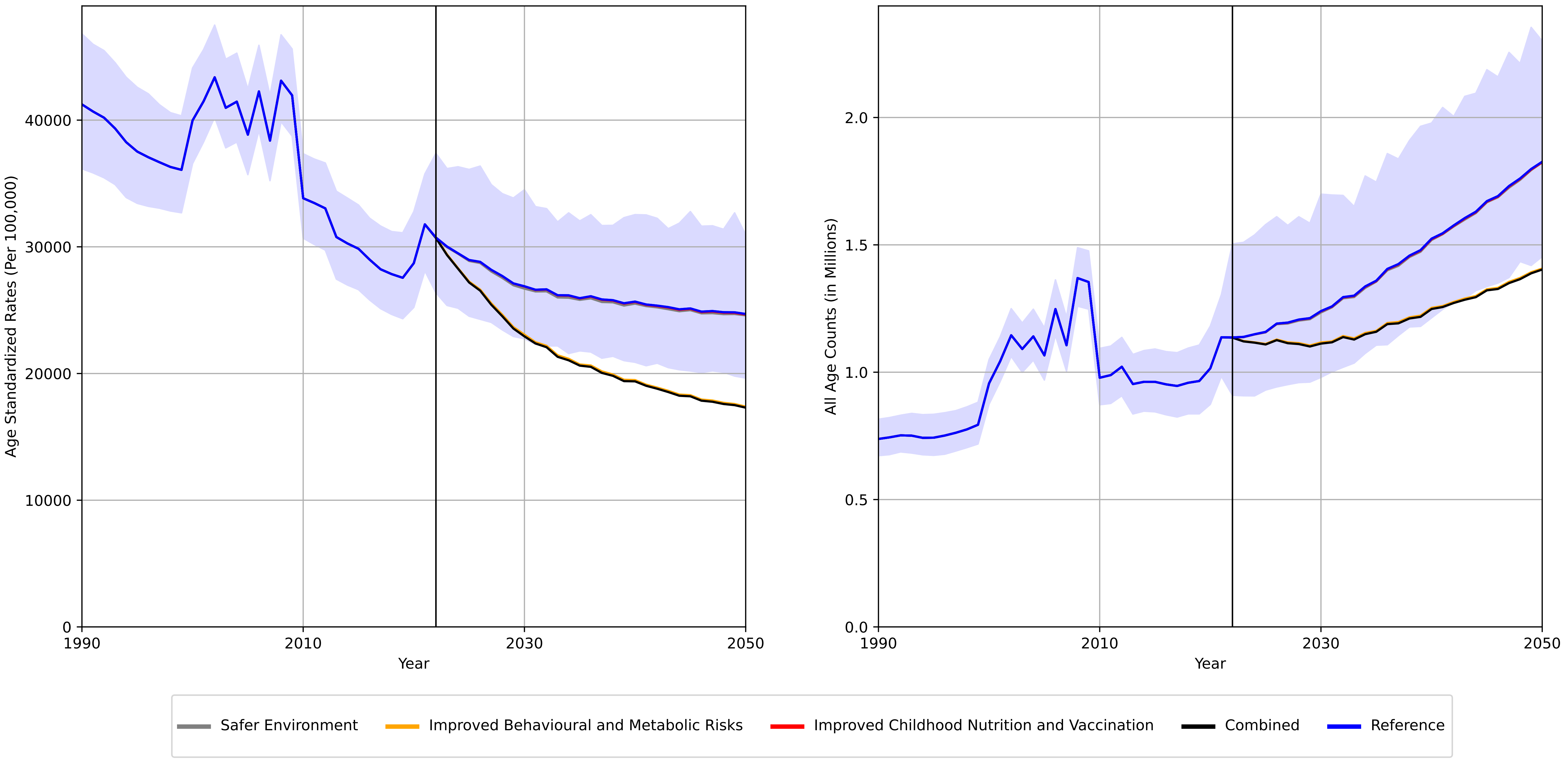

Age Standardized Rates (Per 100,000)
0
10000
20000
30000
40000
1990
2010
2030
2050
Year
All Age Counts (in Millions)
0.0
0.5
1.0
1.5
2.0
1990
2010
2030
2050
Year
Safer Environment
Improved Behavioural and Metabolic Risks
Improved Childhood Nutrition and Vaccination
Combined
Reference

Qatar

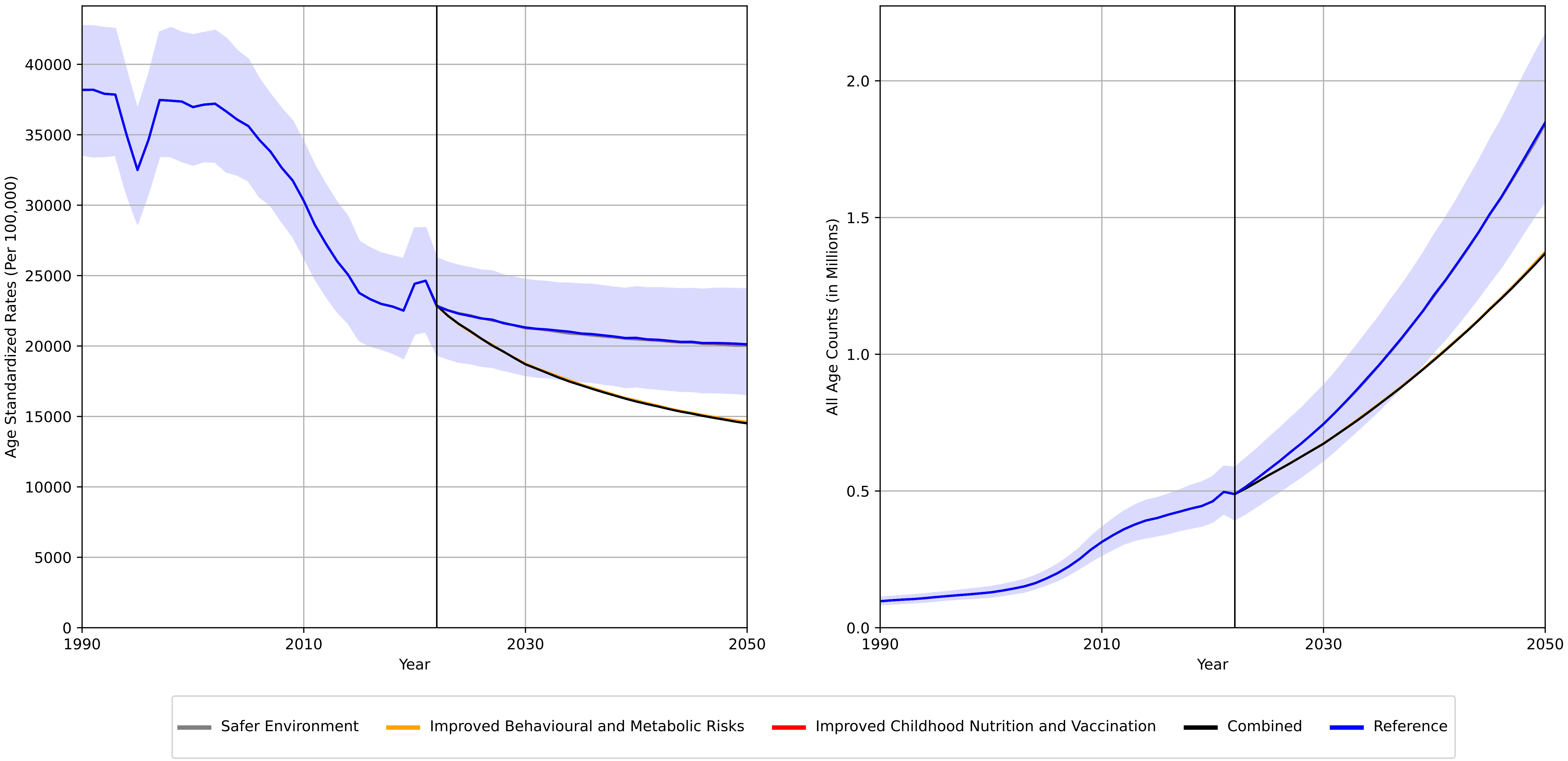

Age Standardized Rates (Per 100,000)
0
5000
10000
15000
20000
25000
30000
35000
40000
1990
2010
2030
2050
Year
All Age Counts (in Millions)
0.0
0.5
1.0
1.5
2.0
1990
2010
2030
2050
Year
Safer Environment
Improved Behavioural and Metabolic Risks
Improved Childhood Nutrition and Vaccination
Combined
Reference

Saudi Arabia

Supplemental Results Figure S10. All-cause age standardised DALY rates (A) and all age DALY counts (B), for the past and for five future scenarios, 1990–2050

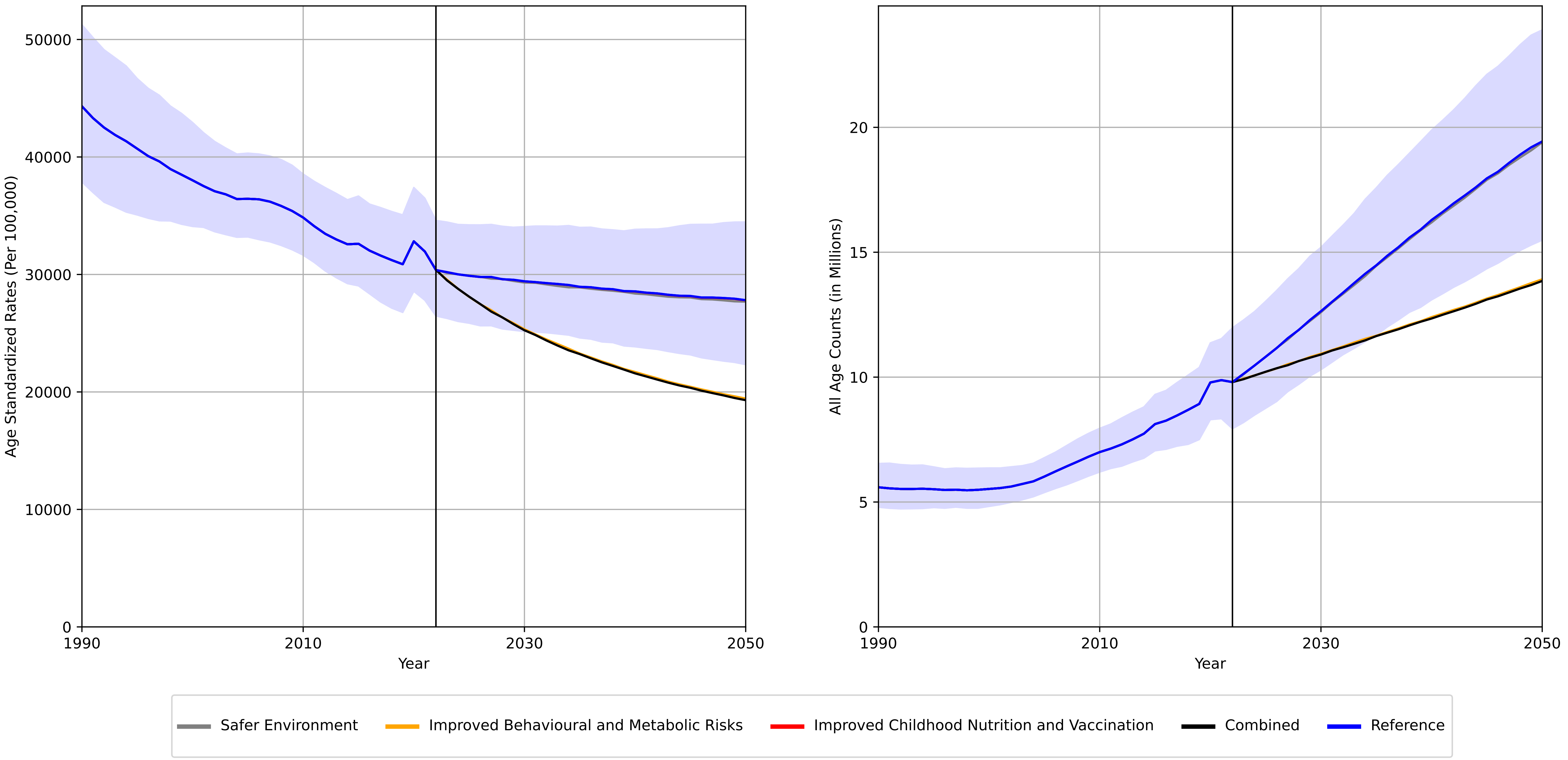

Sudan

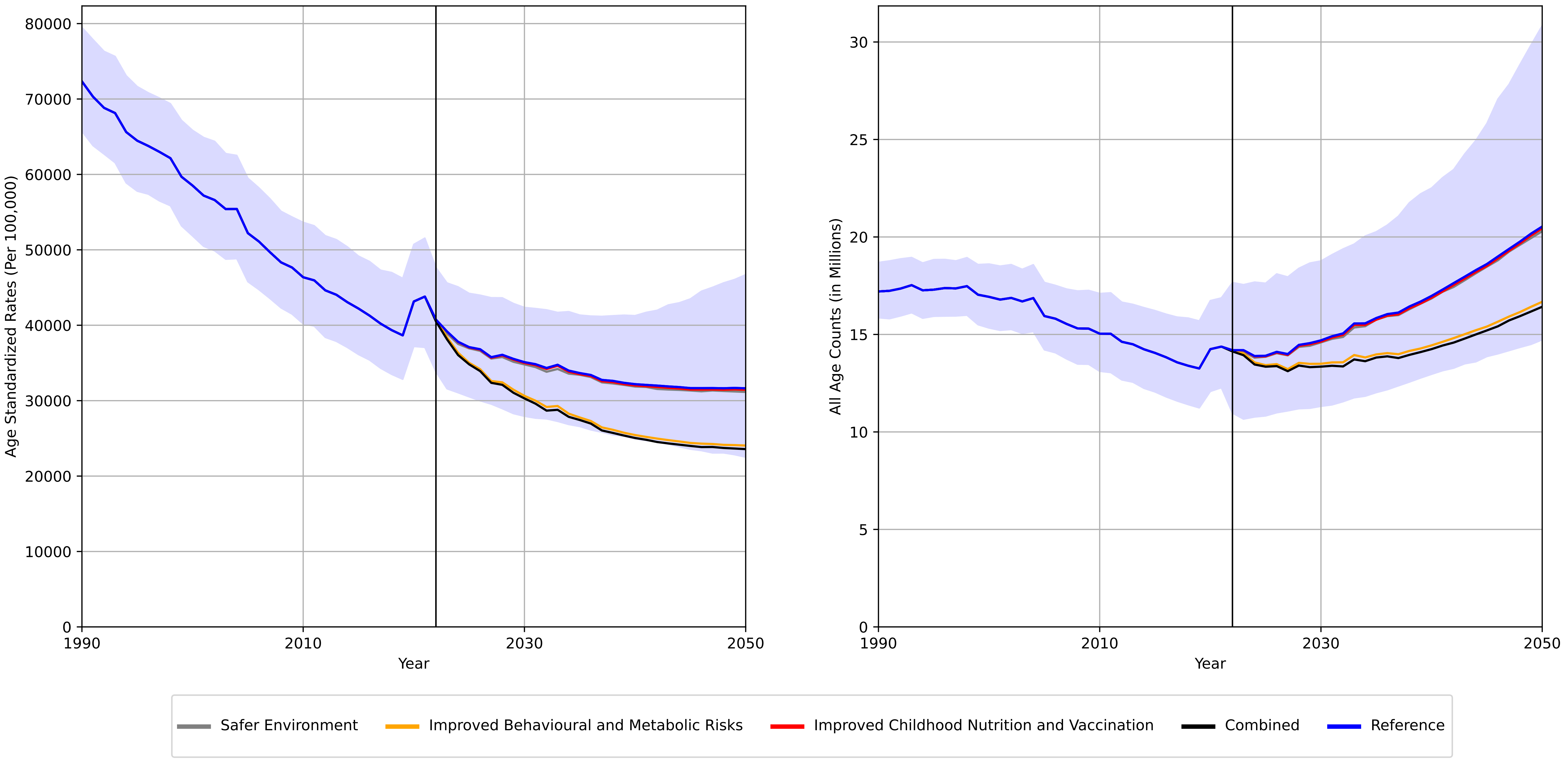

Age Standardized Rates (Per 100,000)
80000
70000
60000
50000
40000
30000
20000
10000
0
1990
2010
2030
2050
Year
All Age Counts (in Millions)
30
25
20
15
10
5
0
1990
2010
2030
2050
Year
Safer Environment
Improved Behavioural and Metabolic Risks
Improved Childhood Nutrition and Vaccination
Combined
Reference

Syria

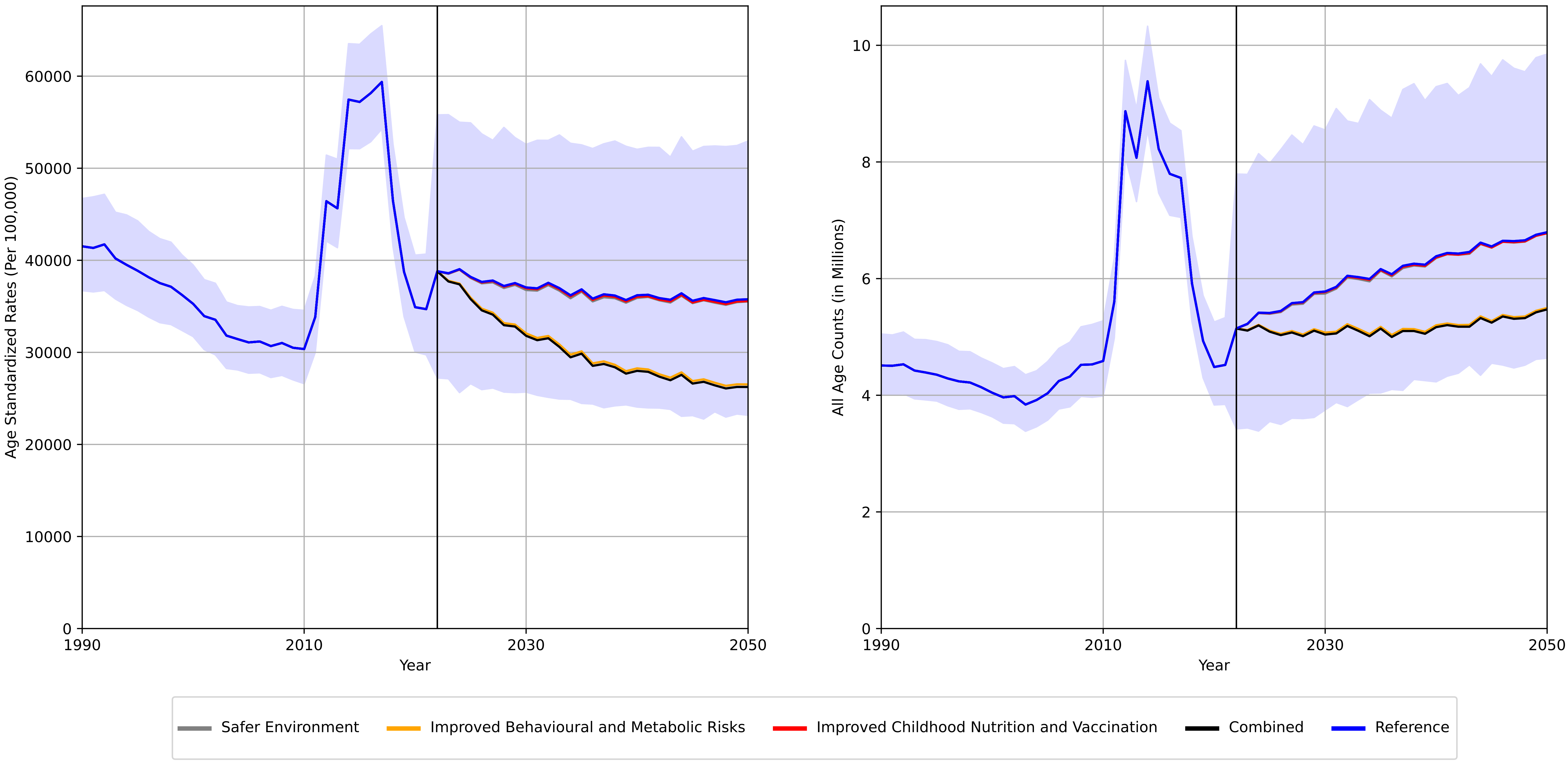
Age Standardized Rates (Per 100,000)
0
10000
20000
30000
40000
50000
60000
1990
2010
2030
2050
Year
All Age Counts (in Millions)
0
2
4
6
8
10
1990
2010
2030
2050
Year
Safer Environment
Improved Behavioural and Metabolic Risks
Improved Childhood Nutrition and Vaccination
Combined
Reference

Tunisia

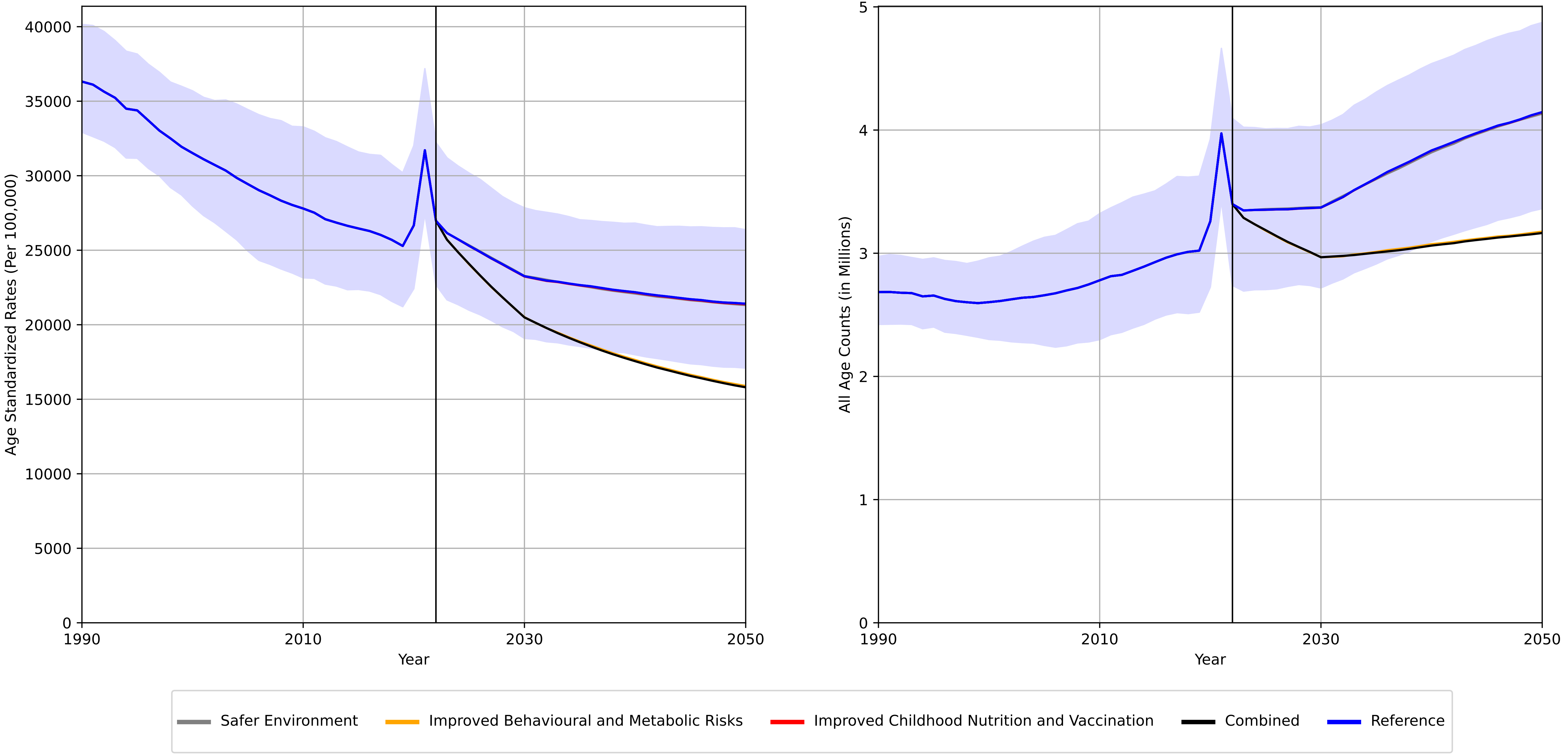

Age Standardized Rates (Per 100,000)
40000
35000
30000
25000
20000
15000
10000
5000
0
1990
2010
2030
2050
Year
All Age Counts (in Millions)
5
4
3
2
1
0
1990
2010
2030
2050
Year
Safer Environment
Improved Behavioural and Metabolic Risks
Improved Childhood Nutrition and Vaccination
Combined
Reference

Türkiye

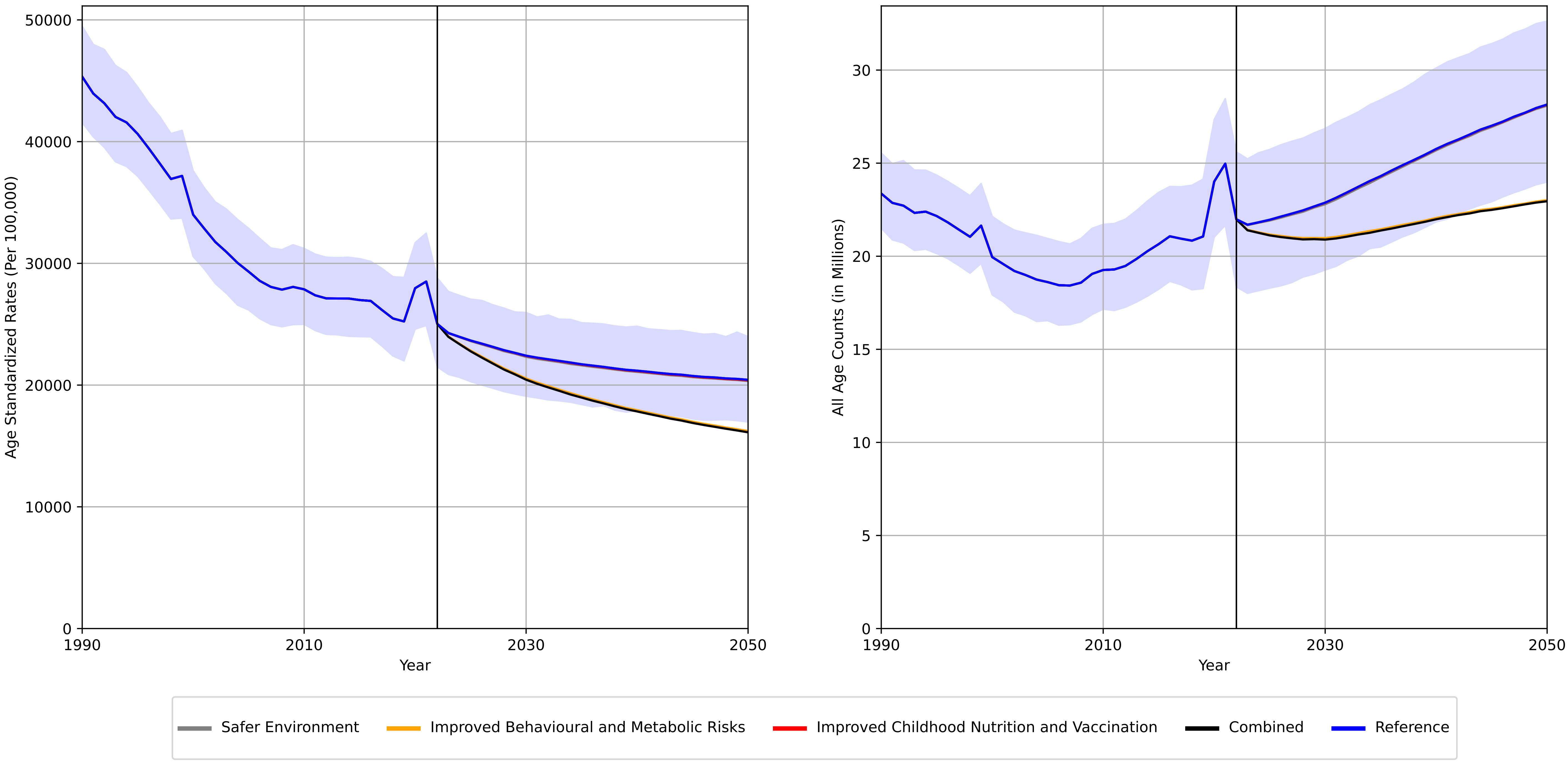
Age Standardized Rates (Per 100,000)
0
10000
20000
30000
40000
50000
1990
2010
2030
2050
Year
All Age Counts (in Millions)
0
5
10
15
20
25
30
1990
2010
2030
2050
Year
Safer Environment
Improved Behavioural and Metabolic Risks
Improved Childhood Nutrition and Vaccination
Combined
Reference

United Arab Emirates

Supplemental Results Figure S10. All-cause age standardised DALY rates (A) and all age DALY counts (B), for the past and for five future scenarios, 1990–2050

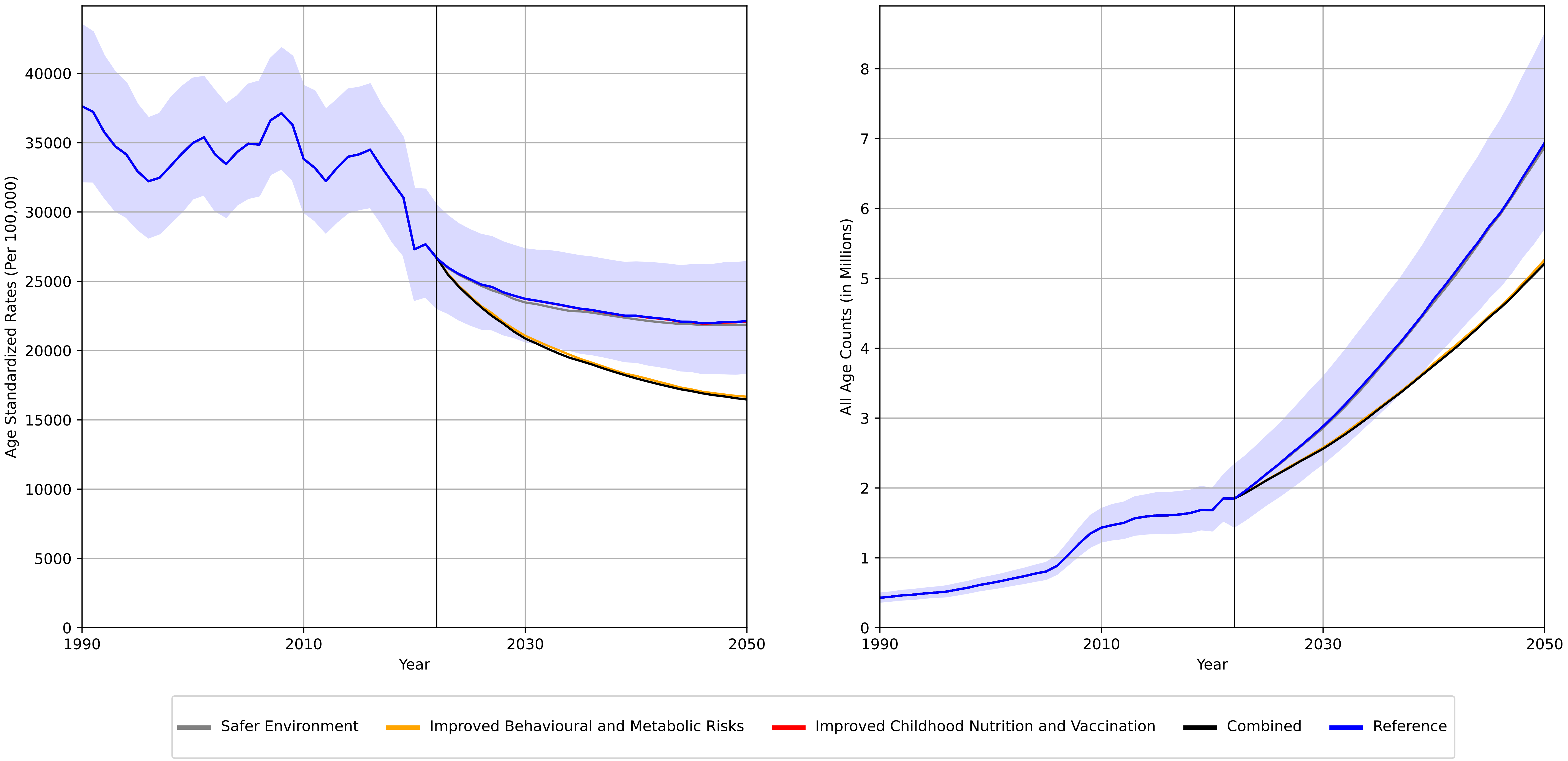

Yemen

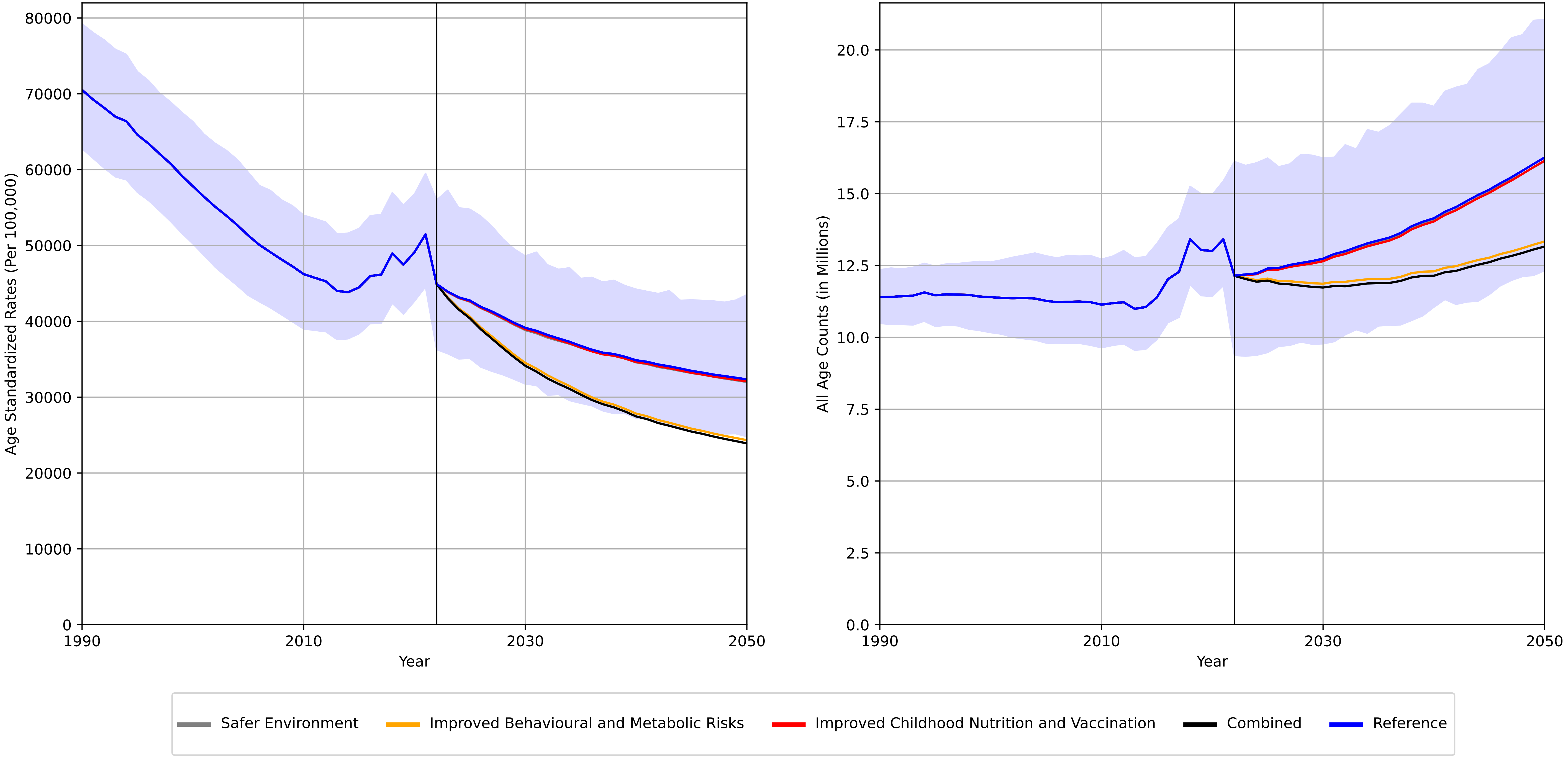

Age Standardized Rates (Per 100,000)
0
10000
20000
30000
40000
50000
60000
70000
80000
1990
2010
2030
2050
Year
All Age Counts (in Millions)
0.0
2.5
5.0
7.5
10.0
12.5
15.0
17.5
20.0
1990
2010
2030
2050
Year
Safer Environment
Improved Behavioural and Metabolic Risks
Improved Childhood Nutrition and Vaccination
Combined
Reference

Bangladesh

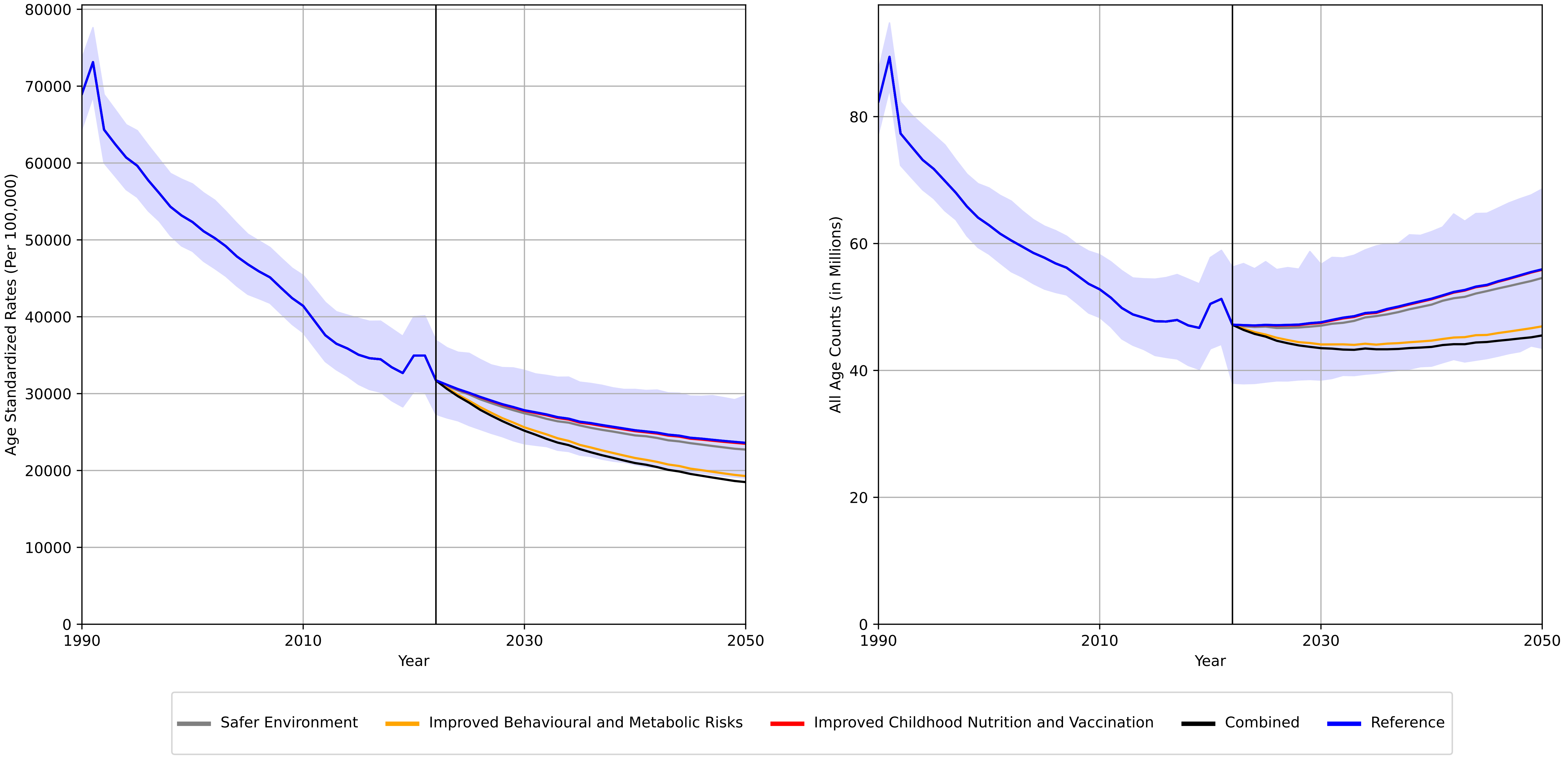

Age Standardized Rates (Per 100,000)
80000
70000
60000
50000
40000
30000
20000
10000
0
1990
2010
2030
2050
Year
All Age Counts (in Millions)
80
60
40
20
0
1990
2010
2030
2050
Year
Safer Environment
Improved Behavioural and Metabolic Risks
Improved Childhood Nutrition and Vaccination
Combined
Reference

Bhutan

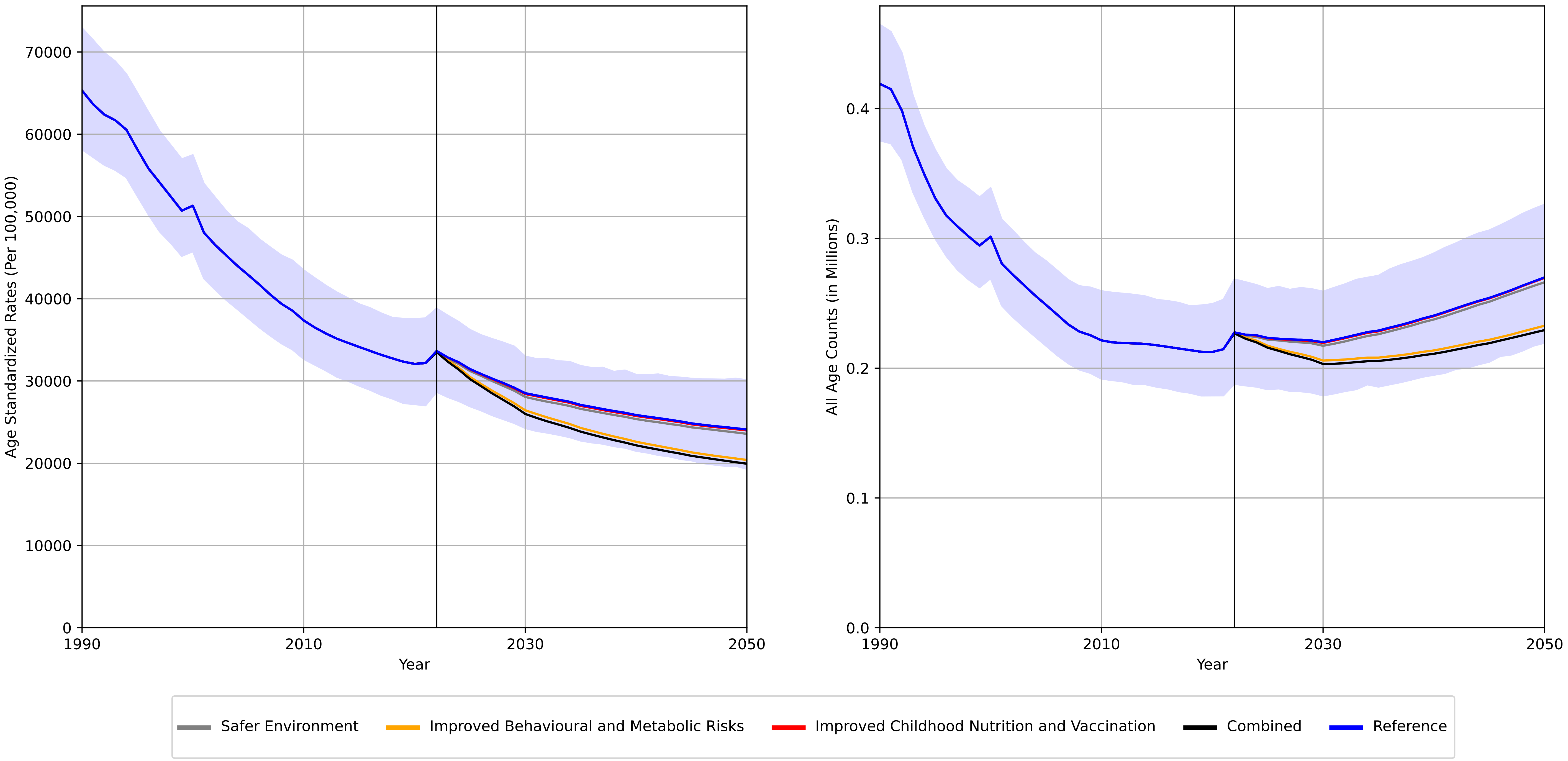

Age Standardized Rates (Per 100,000)
70000
60000
50000
40000
30000
20000
10000
0
1990
2010
2030
2050
Year
All Age Counts (in Millions)
0.4
0.3
0.2
0.1
0.0
1990
2010
2030
2050
Year
Safer Environment
Improved Behavioural and Metabolic Risks
Improved Childhood Nutrition and Vaccination
Combined
Reference

India

Supplemental Results Figure S10. All-cause age standardised DALY rates (A) and all age DALY counts (B), for the past and for five future scenarios, 1990–2050

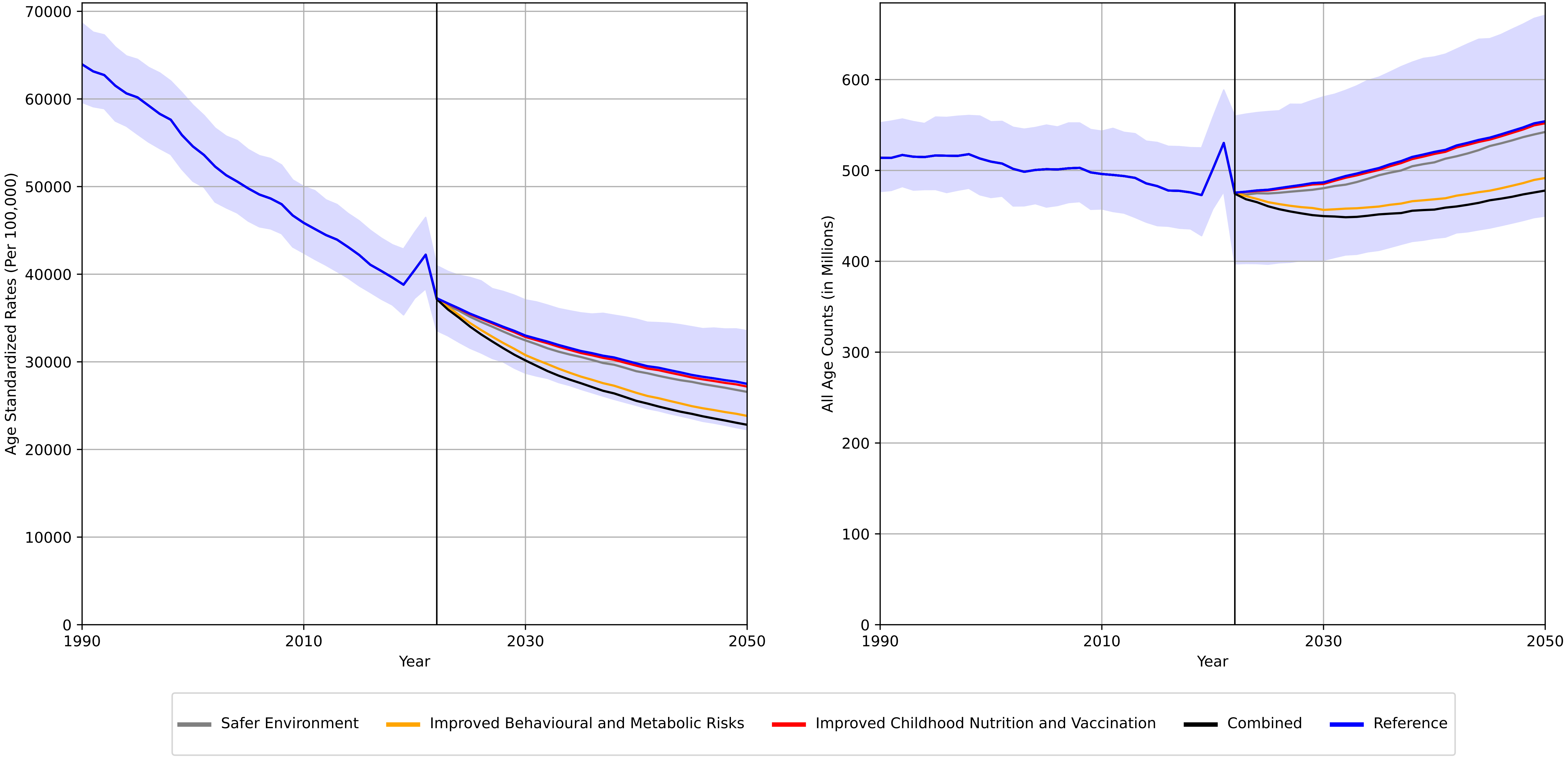

Nepal

Supplemental Results Figure S10. All-cause age standardised DALY rates (A) and all age DALY counts (B), for the past and for five future scenarios, 1990–2050

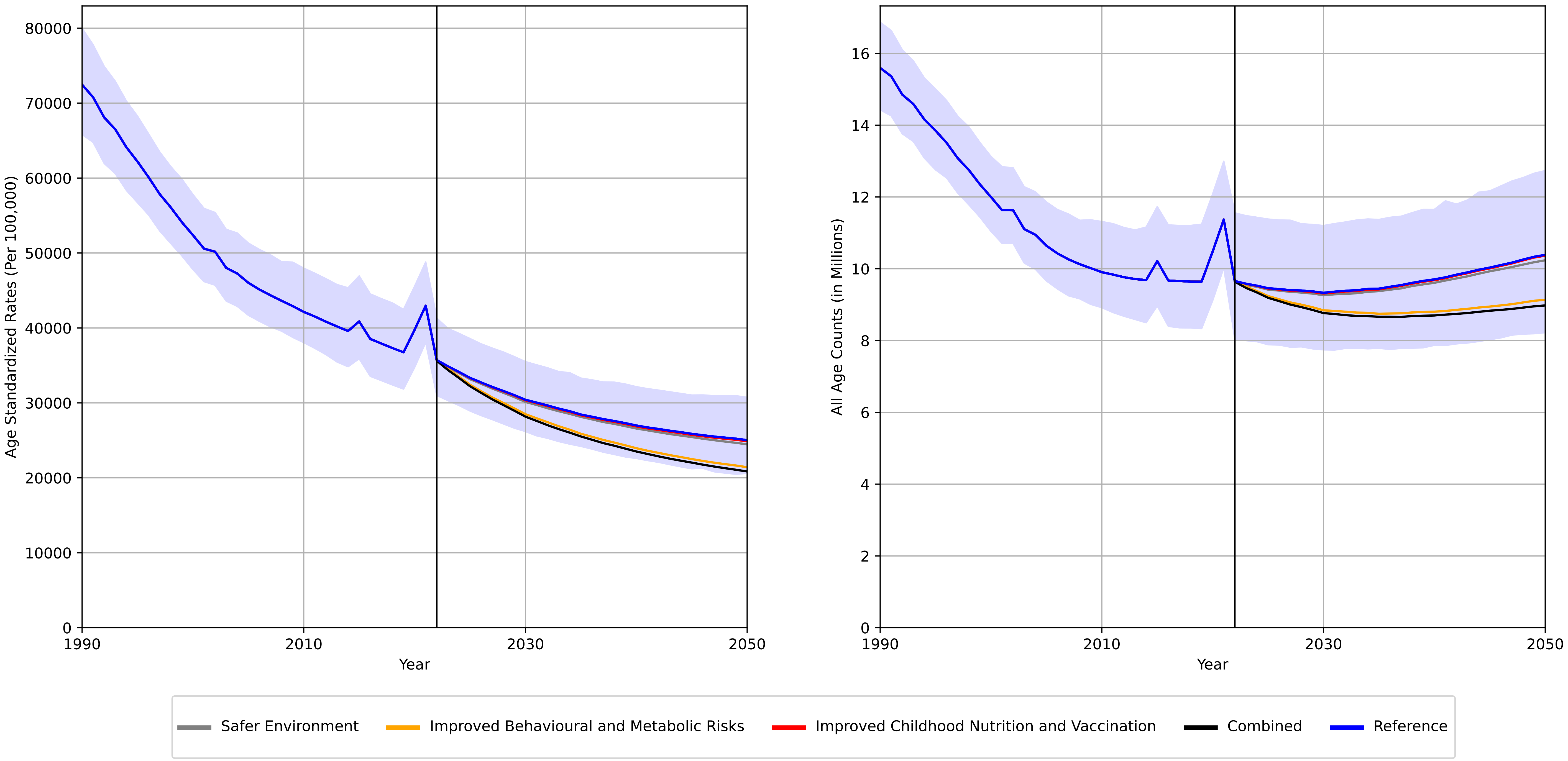

Pakistan

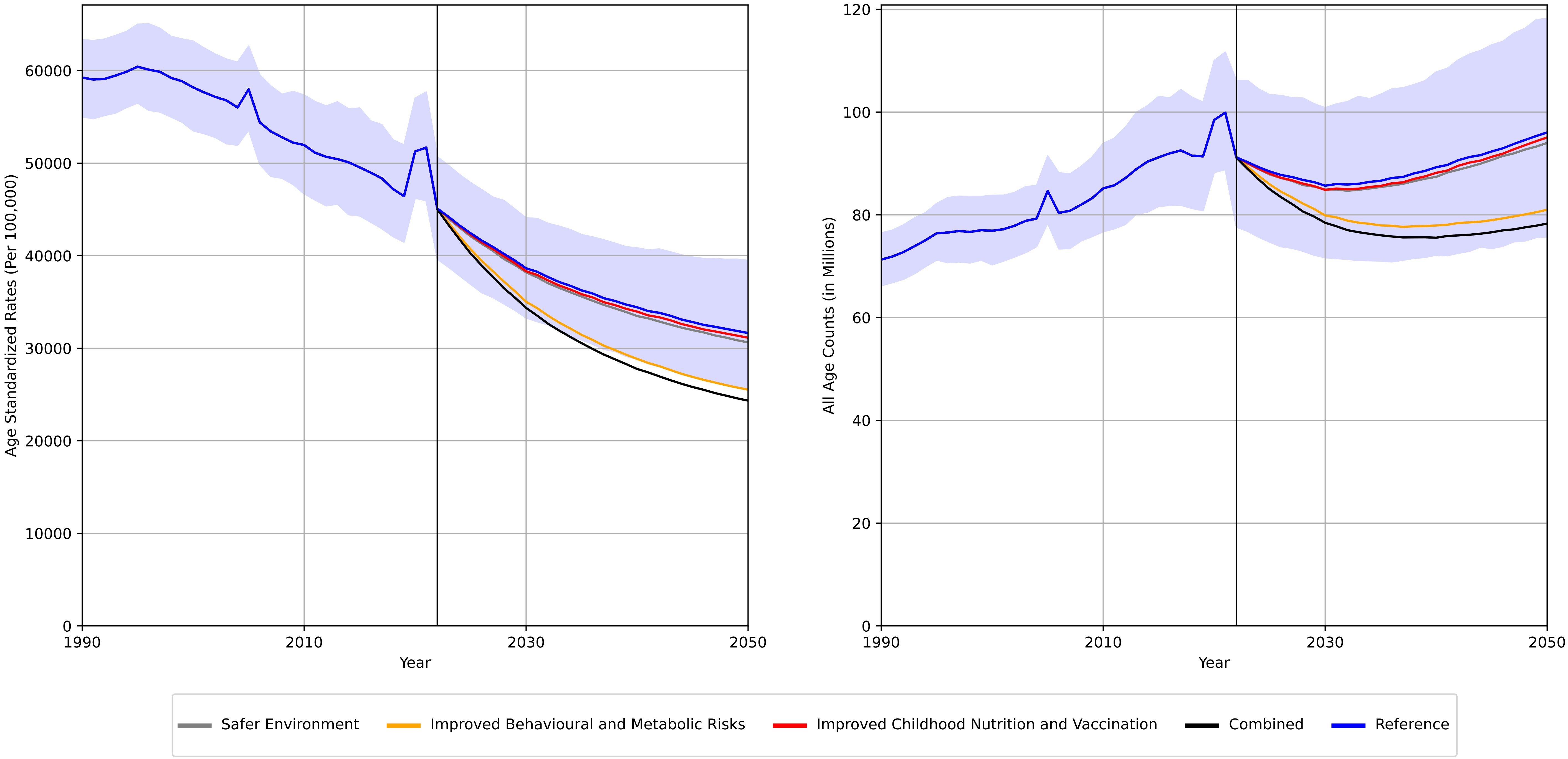

Age Standardized Rates (Per 100,000)
60000
50000
40000
30000
20000
10000
0
1990
2010
2030
2050
Year
All Age Counts (in Millions)
120
100
80
60
40
20
0
1990
2010
2030
2050
Year
Safer Environment
Improved Behavioural and Metabolic Risks
Improved Childhood Nutrition and Vaccination
Combined
Reference

China

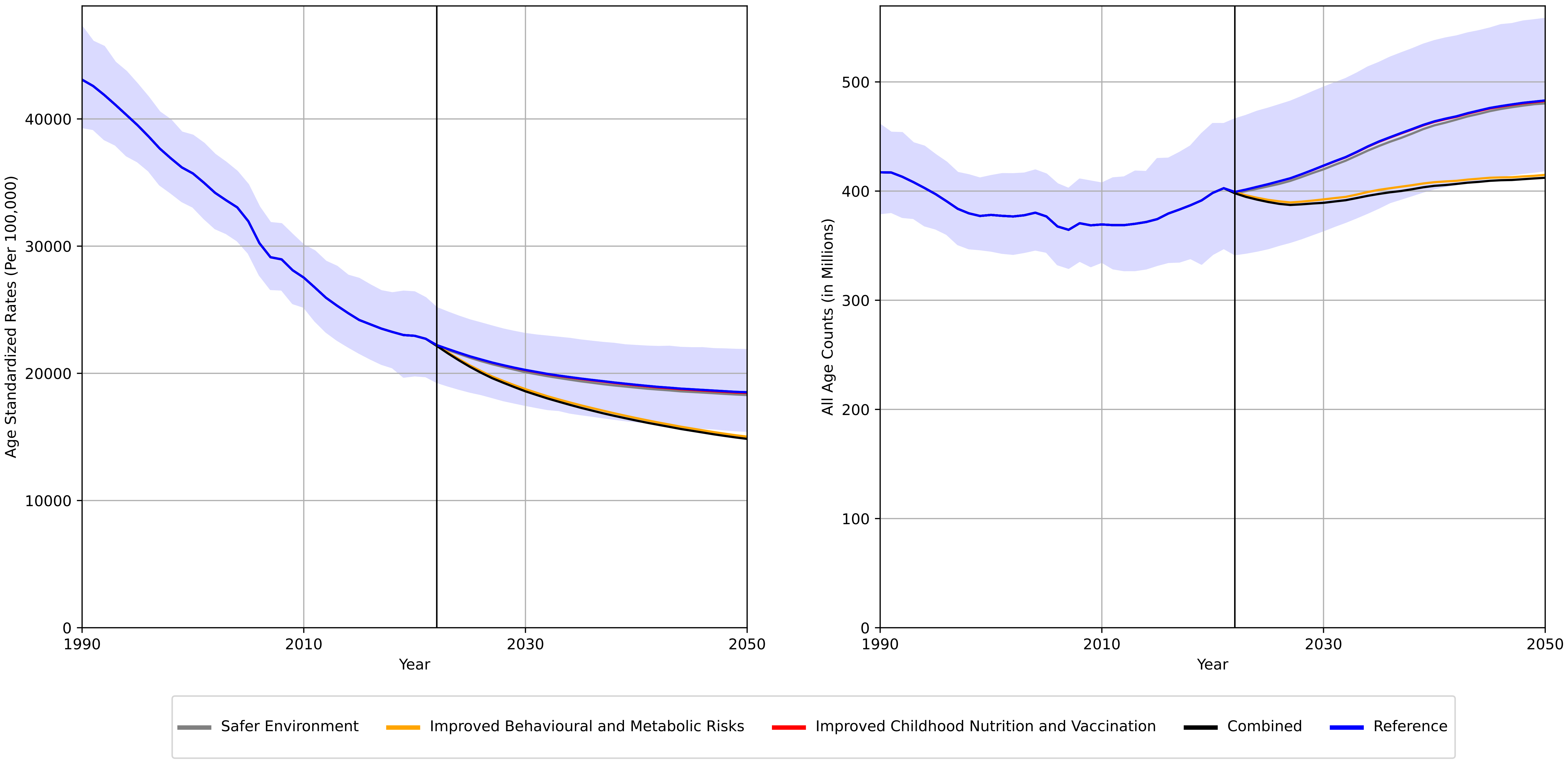
Age Standardized Rates (Per 100,000)
0
10000
20000
30000
40000
1990
2010
2030
2050
Year
All Age Counts (in Millions)
0
100
200
300
400
500
1990
2010
2030
2050
Year
Safer Environment
Improved Behavioural and Metabolic Risks
Improved Childhood Nutrition and Vaccination
Combined
Reference

North Korea

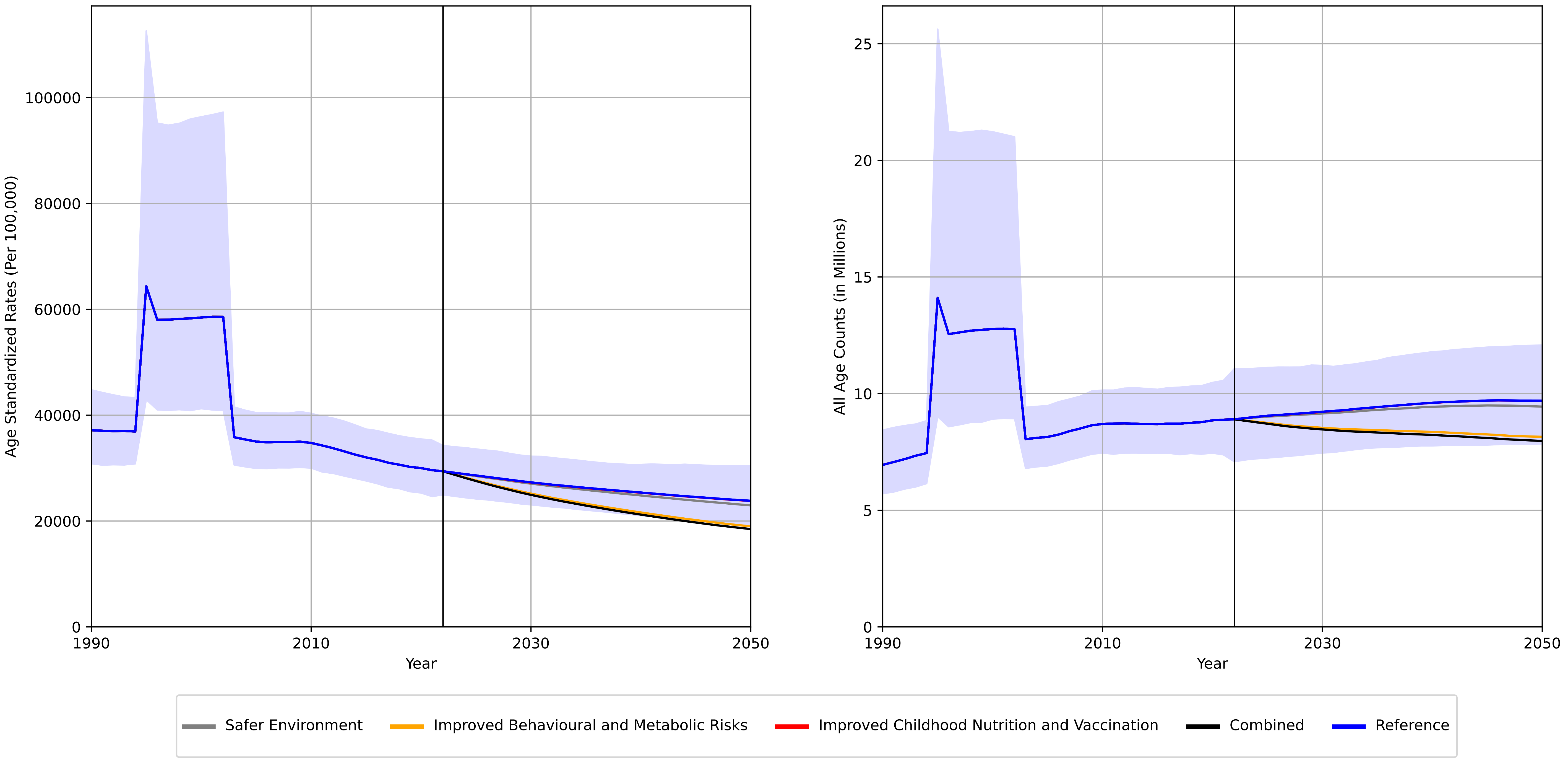

Age Standardized Rates (Per 100,000)
0
20000
40000
60000
80000
100000
1990
2010
2030
2050
Year
All Age Counts (in Millions)
0
5
10
15
20
25
1990
2010
2030
2050
Year
Safer Environment
Improved Behavioural and Metabolic Risks
Improved Childhood Nutrition and Vaccination
Combined
Reference

Taiwan (Province Of China)

Supplemental Results Figure S10. All-cause age standardised DALY rates (A) and all age DALY counts (B), for the past and for five future scenarios, 1990–2050

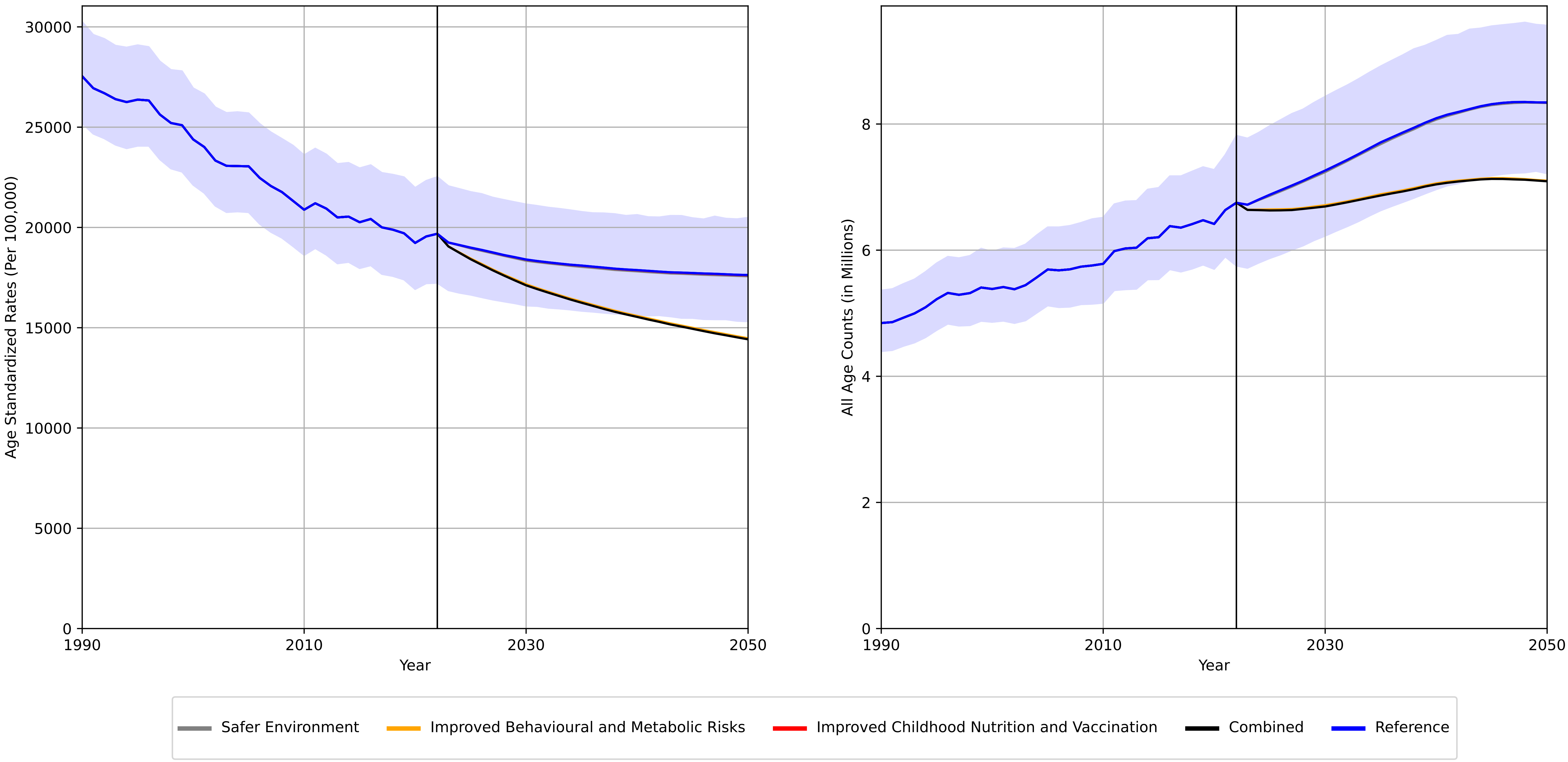

American Samoa

Supplemental Results Figure S10. All-cause age standardised DALY rates (A) and all age DALY counts (B), for the past and for five future scenarios, 1990–2050

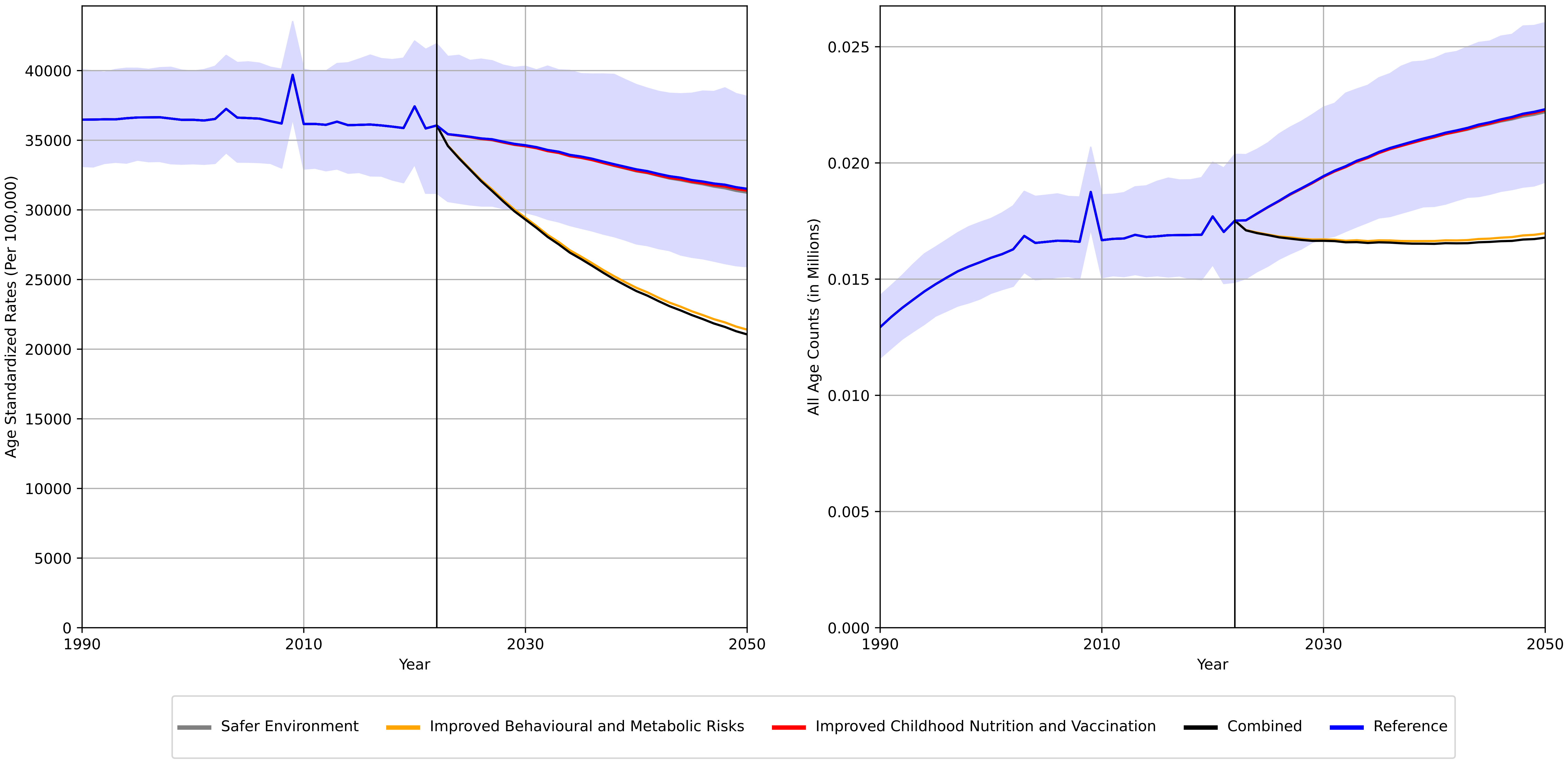

Cook Islands

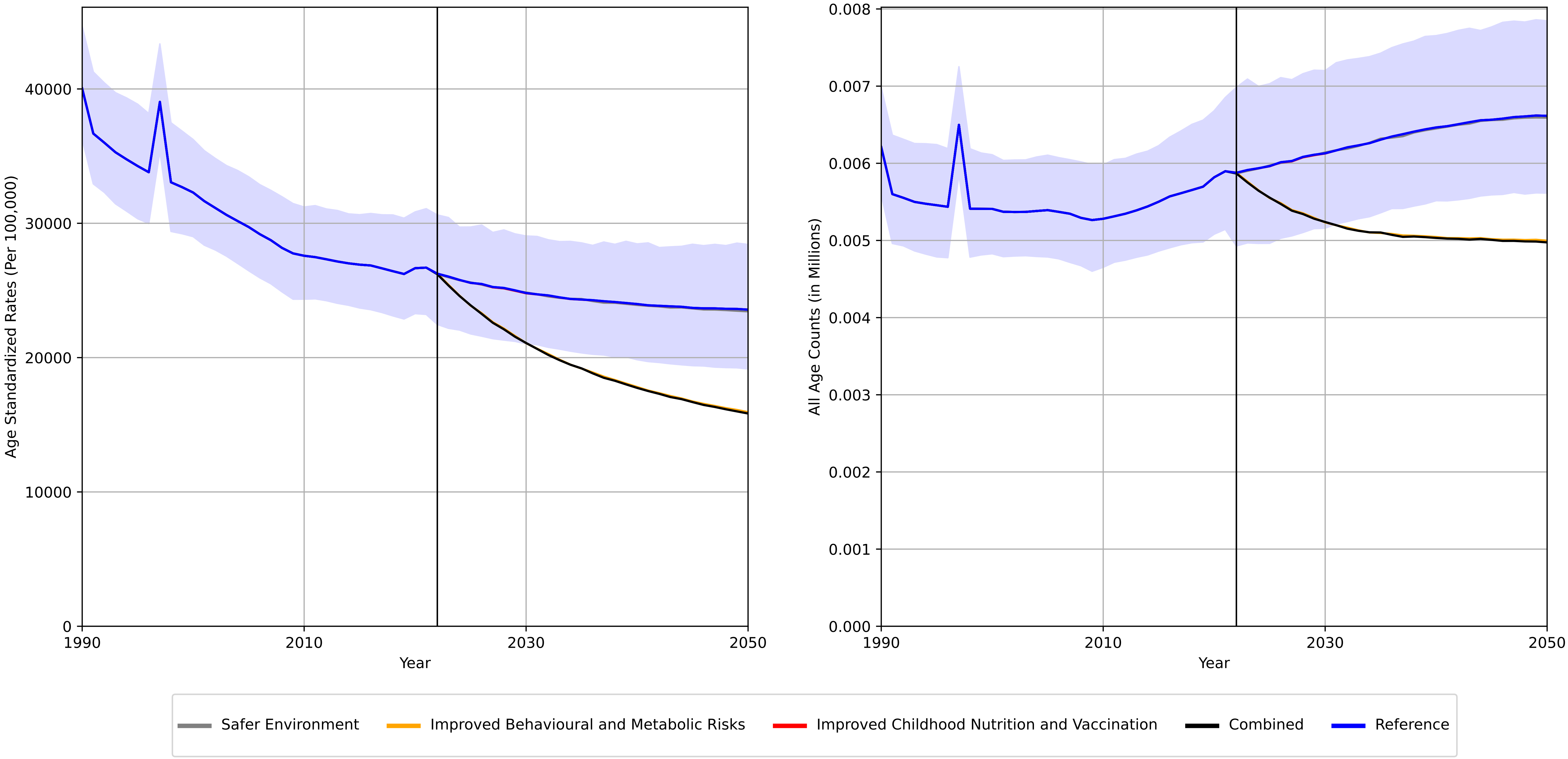

Fiji

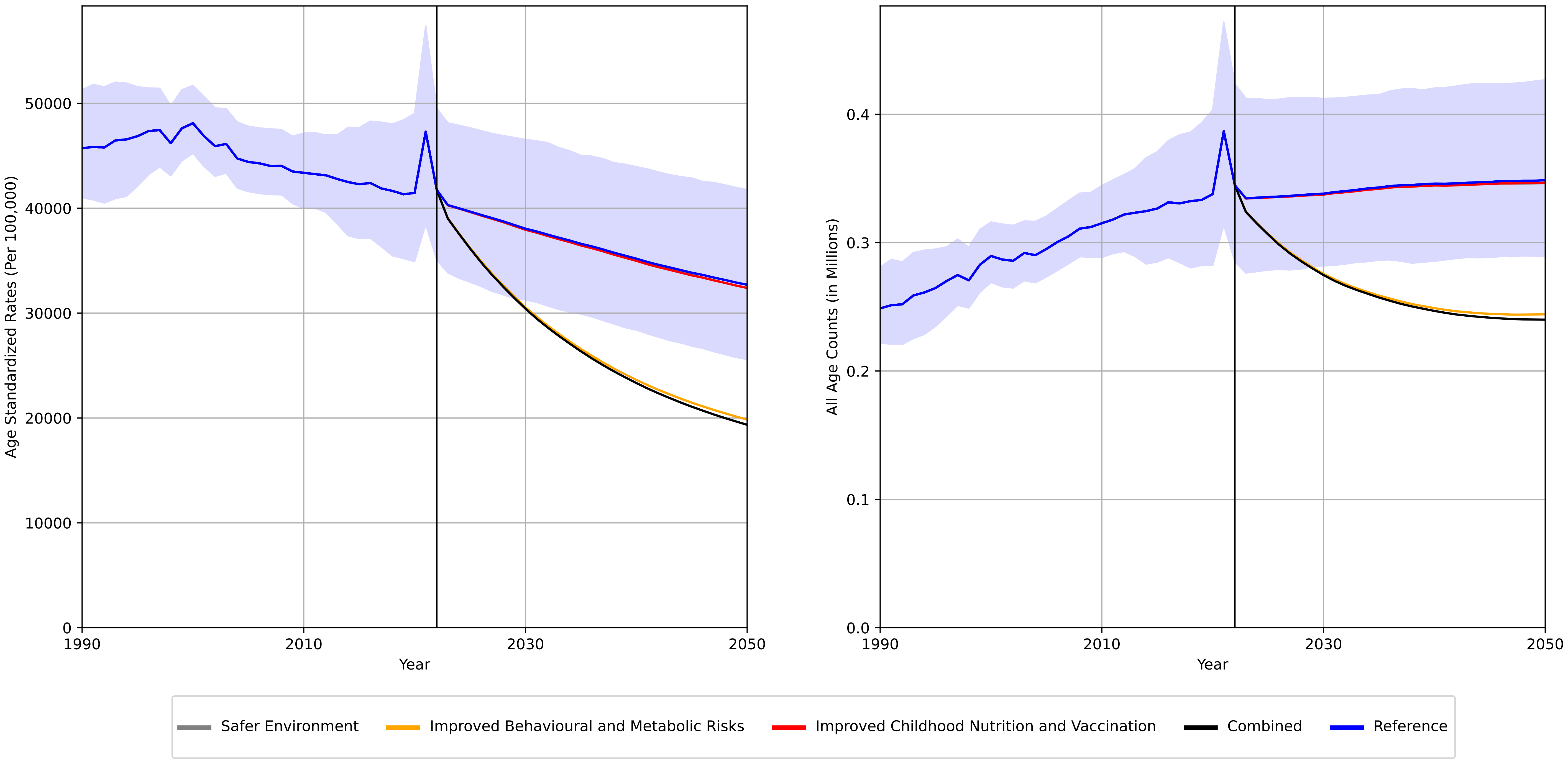

Age Standardized Rates (Per 100,000)
0
10000
20000
30000
40000
50000
1990
2010
2030
2050
Year
All Age Counts (in Millions)
0.0
0.1
0.2
0.3
0.4
1990
2010
2030
2050
Year
Safer Environment
Improved Behavioural and Metabolic Risks
Improved Childhood Nutrition and Vaccination
Combined
Reference

Guam

Supplemental Results Figure S10. All-cause age standardised DALY rates (A) and all age DALY counts (B), for the past and for five future scenarios, 1990–2050

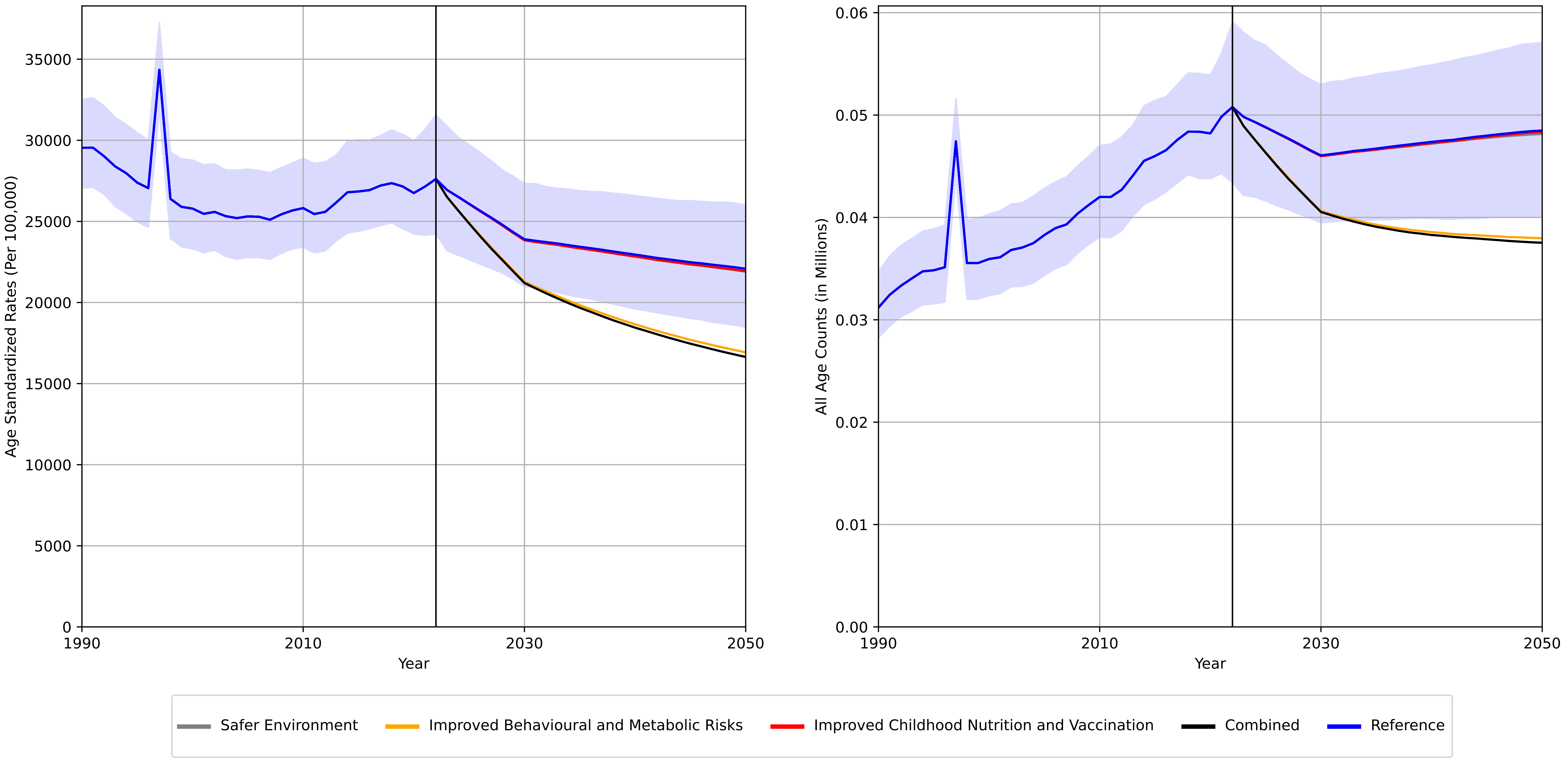

Kiribati

Supplemental Results Figure S10. All-cause age standardised DALY rates (A) and all age DALY counts (B), for the past and for five future scenarios, 1990–2050

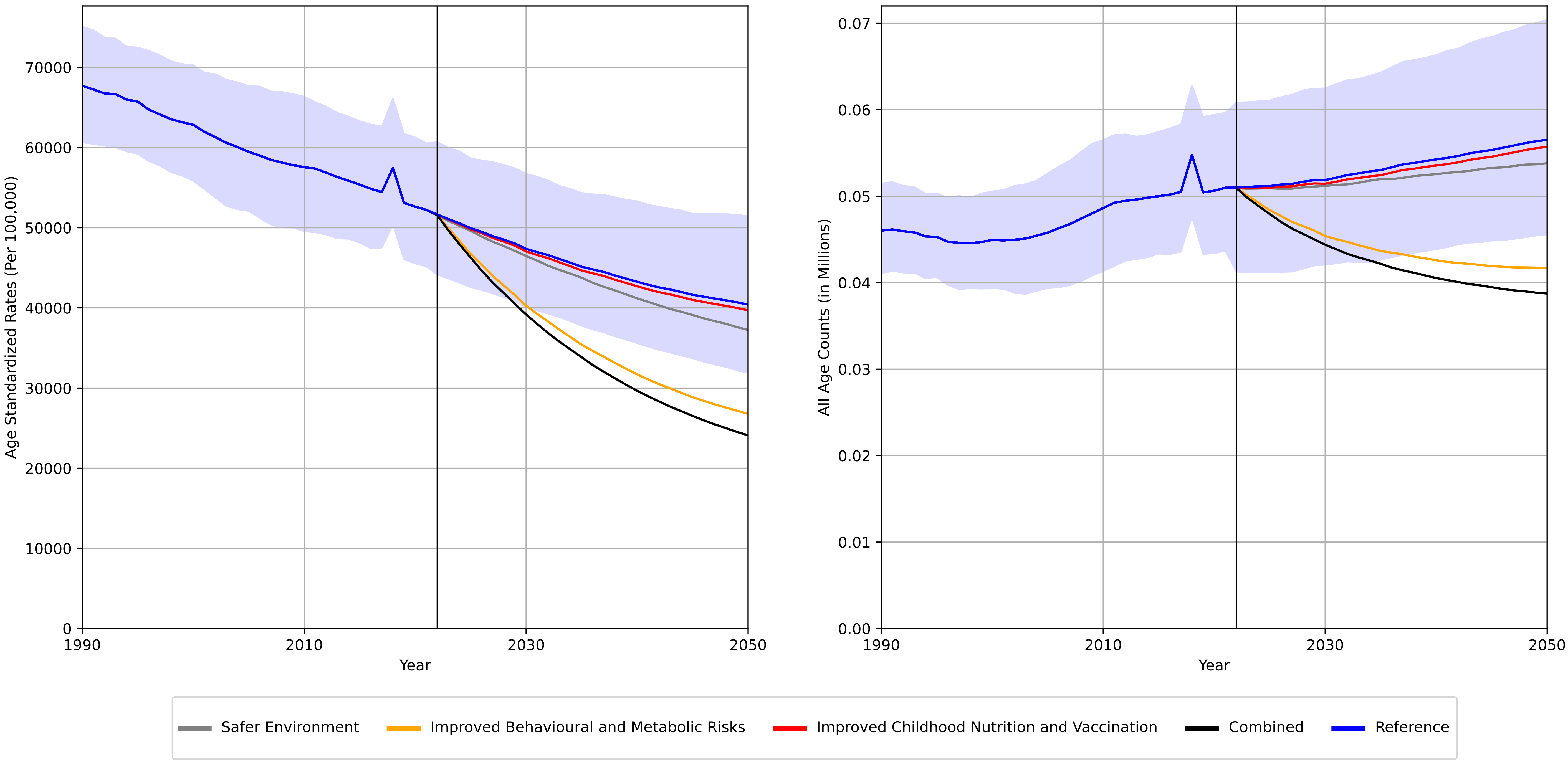

Marshall Islands

Supplemental Results Figure S10. All-cause age standardised DALY rates (A) and all age DALY counts (B), for the past and for five future scenarios, 1990–2050

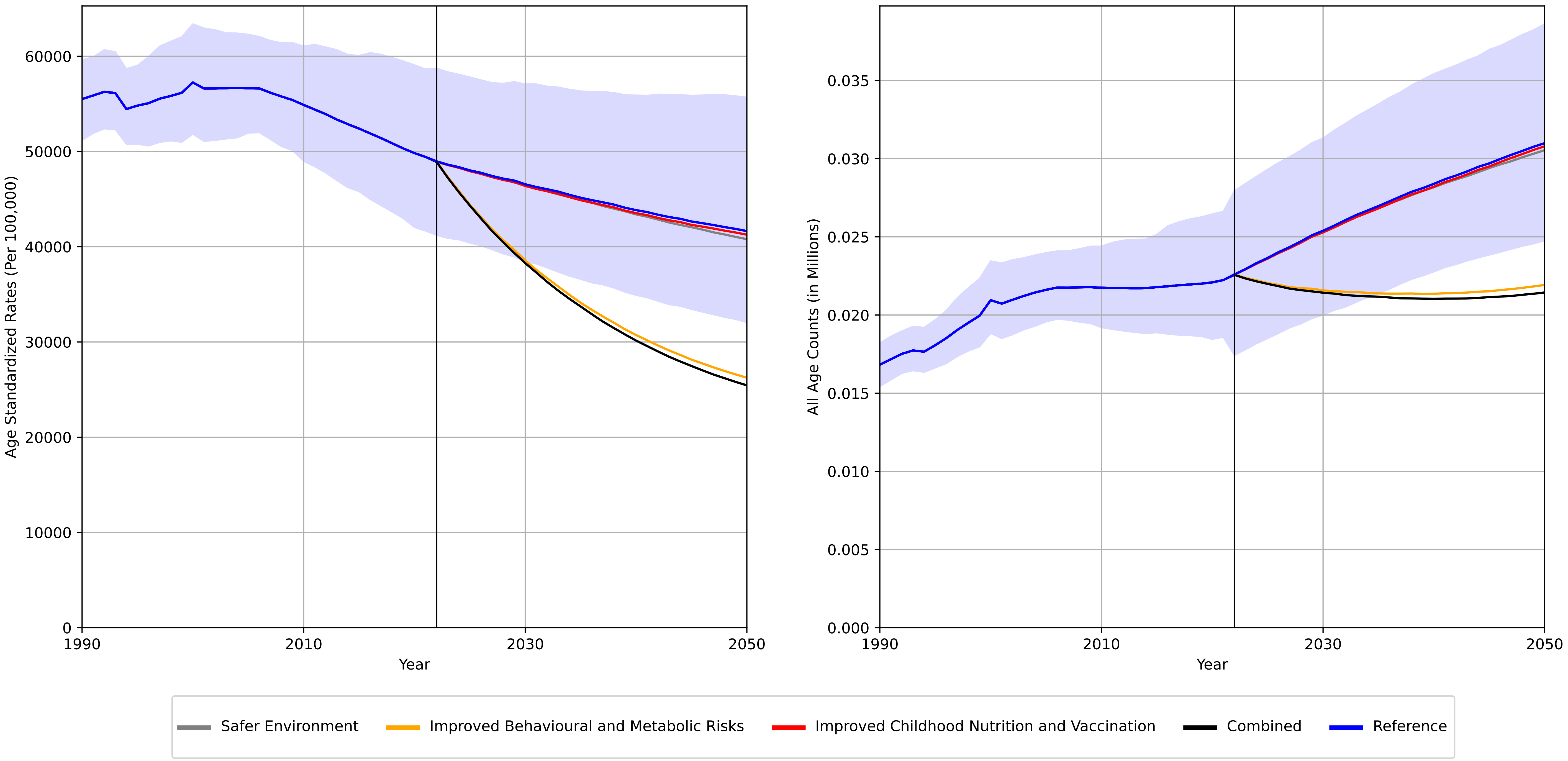

Federated States Of Micronesia

Supplemental Results Figure S10. All-cause age standardised DALY rates (A) and all age DALY counts (B), for the past and for five future scenarios, 1990–2050

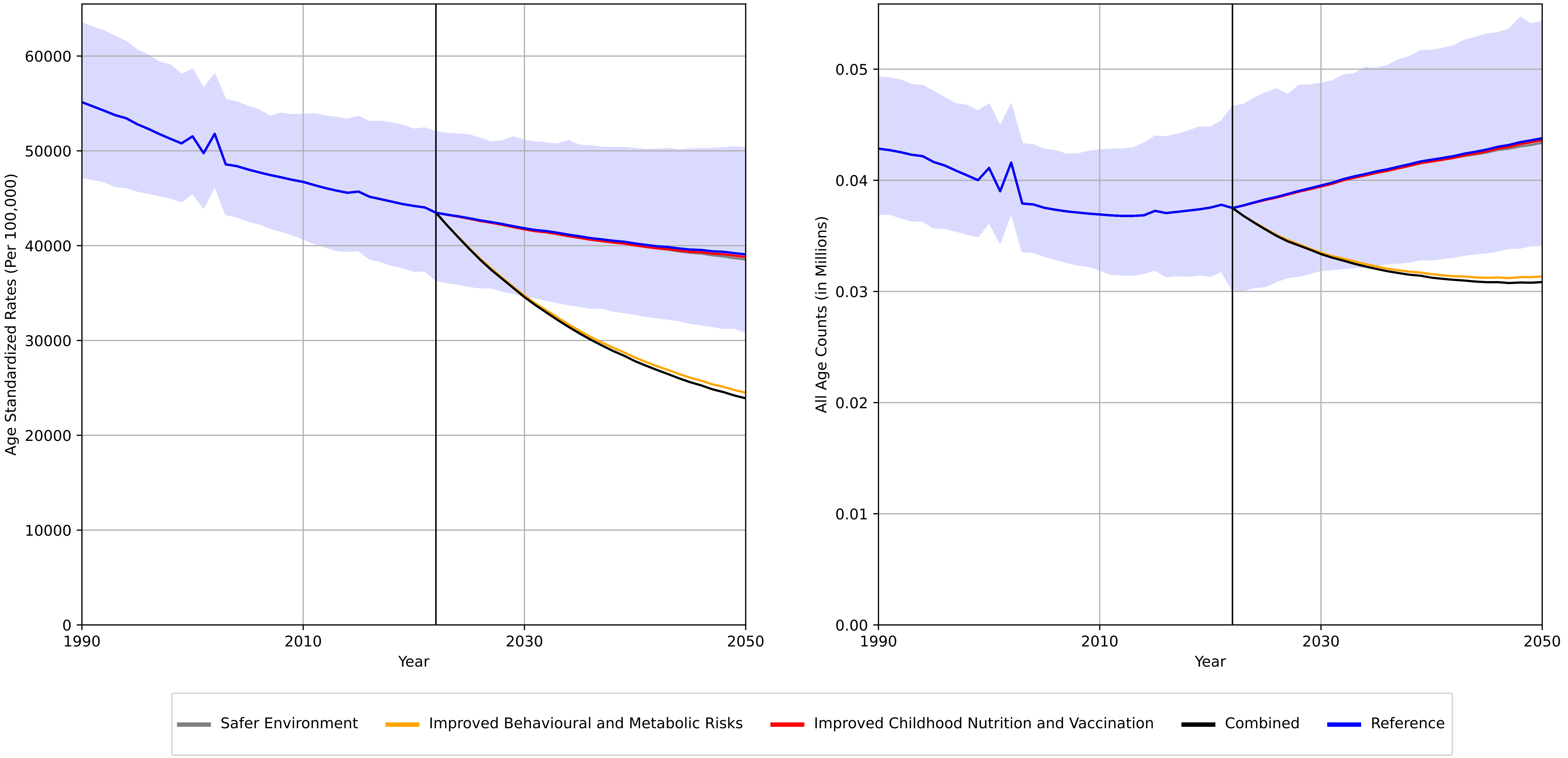

Nauru

Supplemental Results Figure S10. All-cause age standardised DALY rates (A) and all age DALY counts (B), for the past and for five future scenarios, 1990–2050

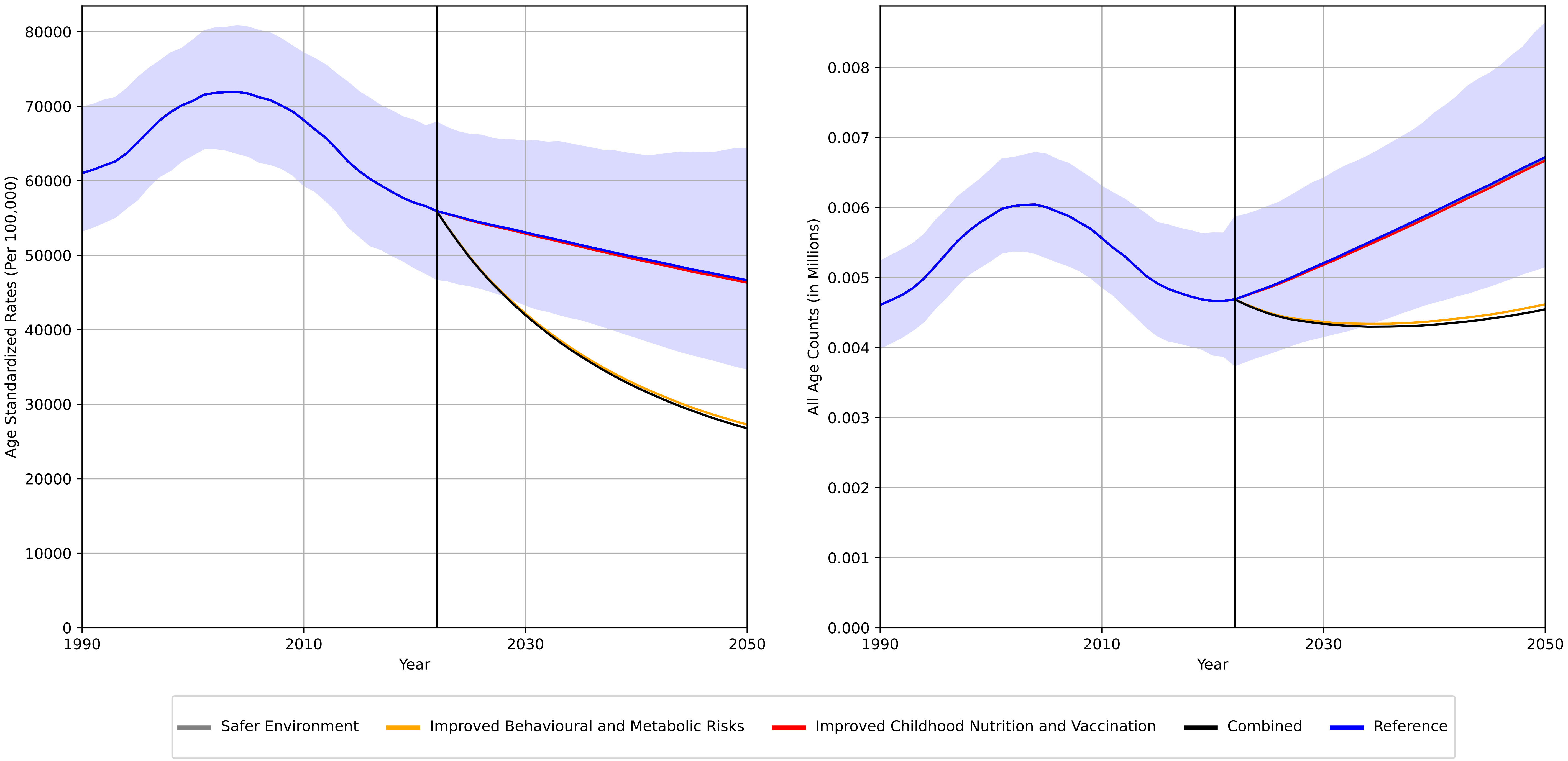

Niue

Supplemental Results Figure S10. All-cause age standardised DALY rates (A) and all age DALY counts (B), for the past and for five future scenarios, 1990–2050

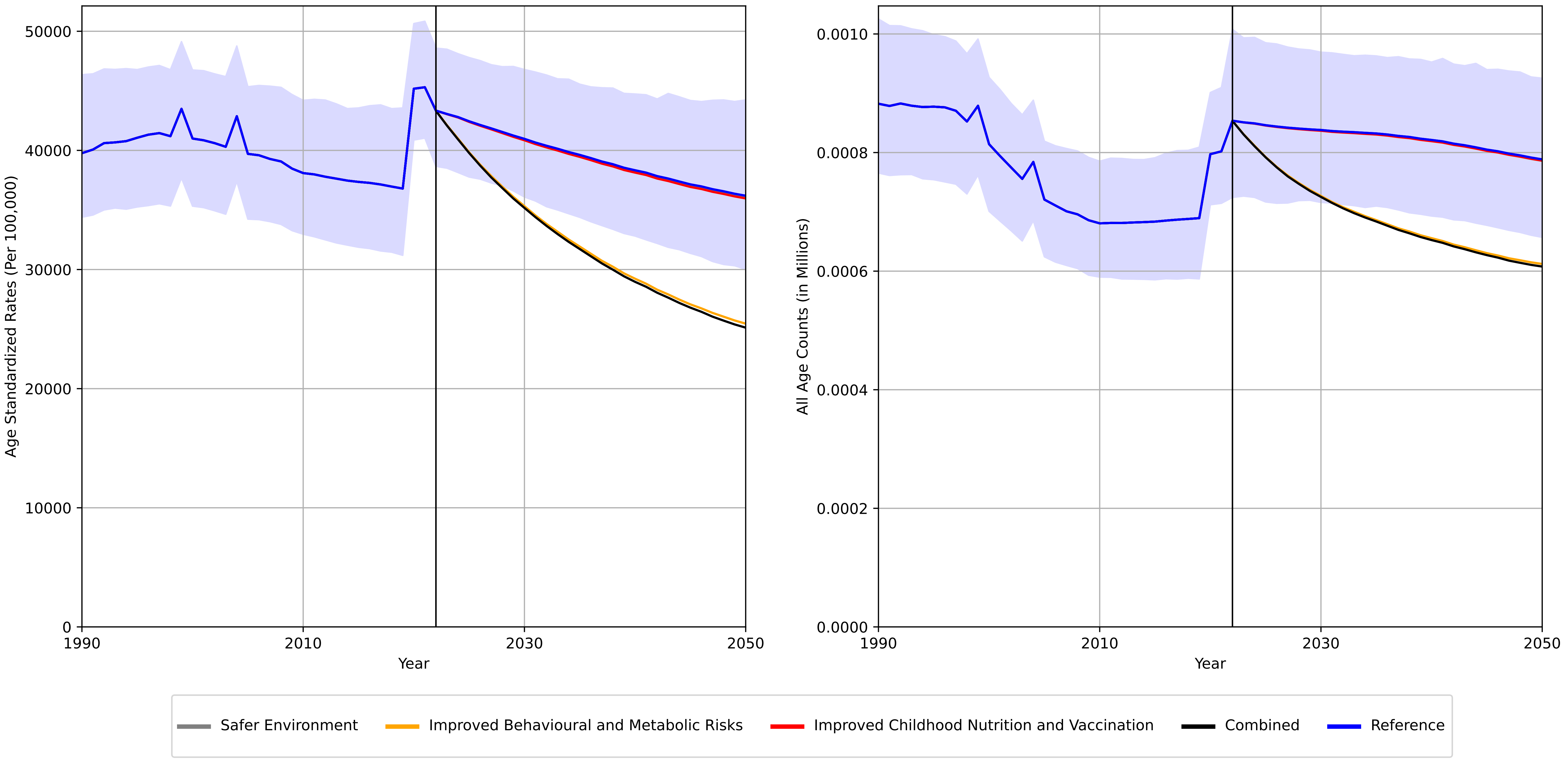

Northern Mariana Islands

Supplemental Results Figure S10. All-cause age standardised DALY rates (A) and all age DALY counts (B), for the past and for five future scenarios, 1990–2050

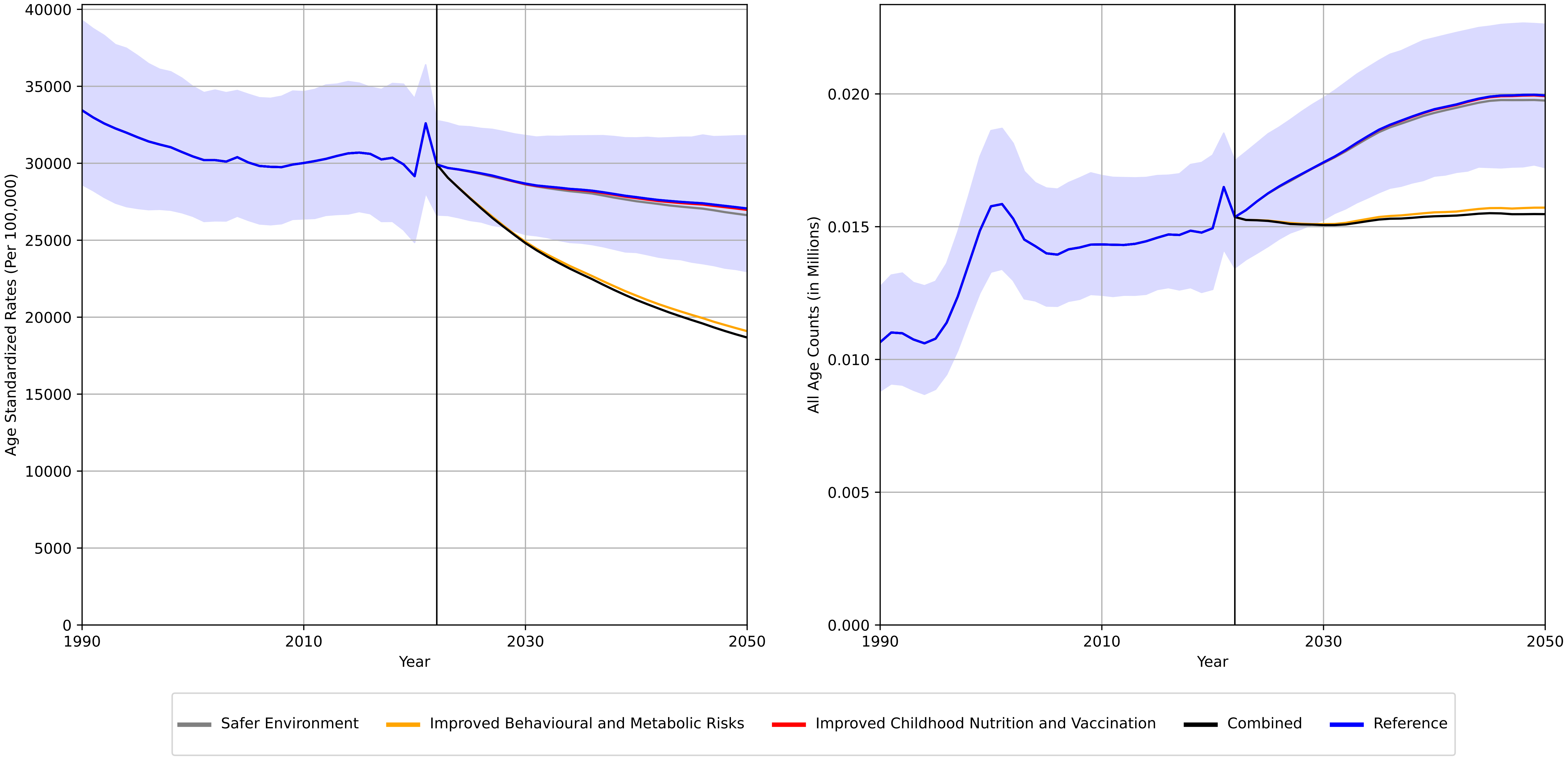

Palau

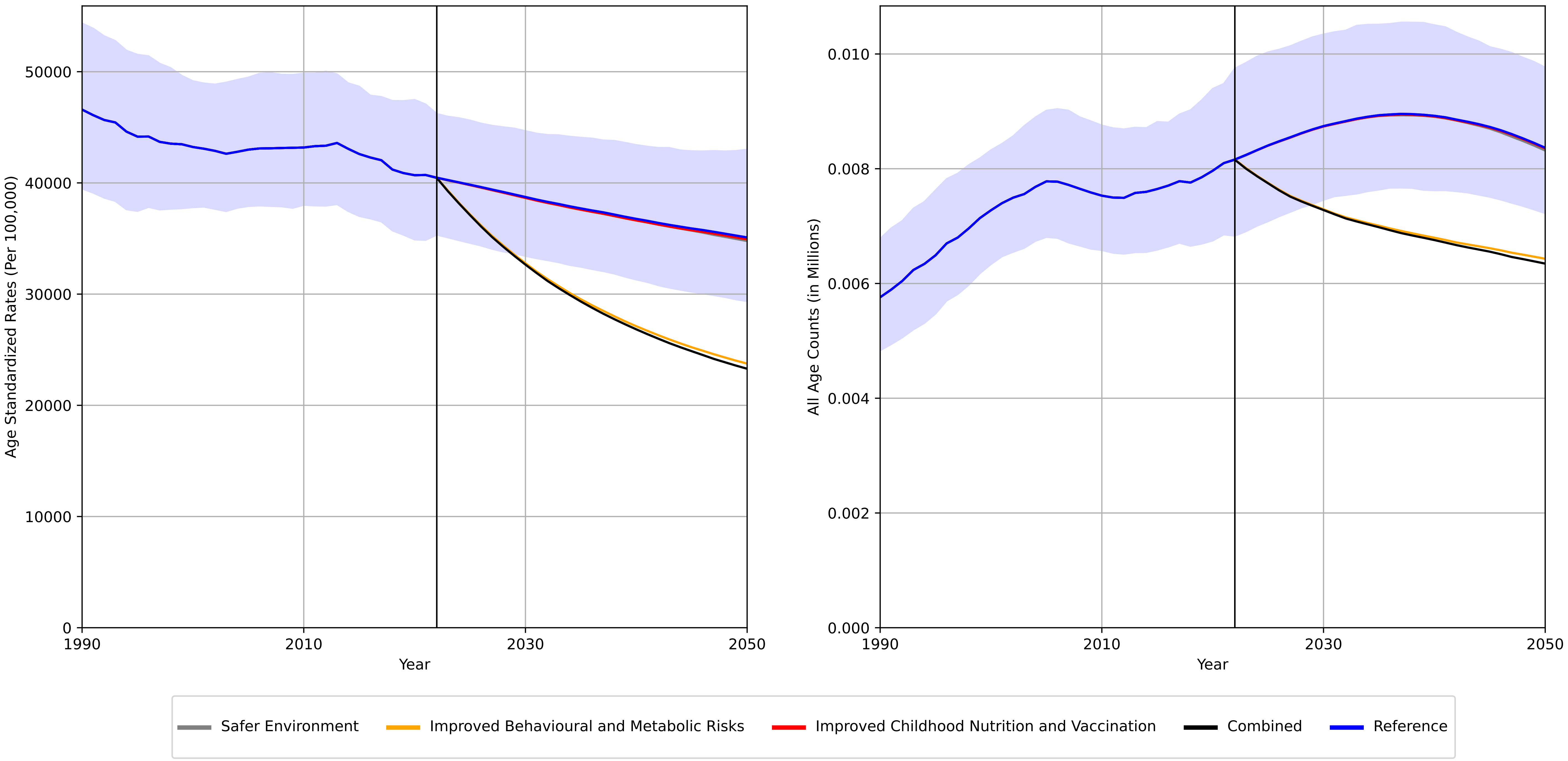
Age Standardized Rates (Per 100,000)
0
10000
20000
30000
40000
50000
1990
2010
2030
2050
Year
All Age Counts (in Millions)
0.000
0.002
0.004
0.006
0.008
0.010
1990
2010
2030
2050
Year
Safer Environment
Improved Behavioural and Metabolic Risks
Improved Childhood Nutrition and Vaccination
Combined
Reference

Papua New Guinea

Supplemental Results Figure S10. All-cause age standardised DALY rates (A) and all age DALY counts (B), for the past and for five future scenarios, 1990–2050

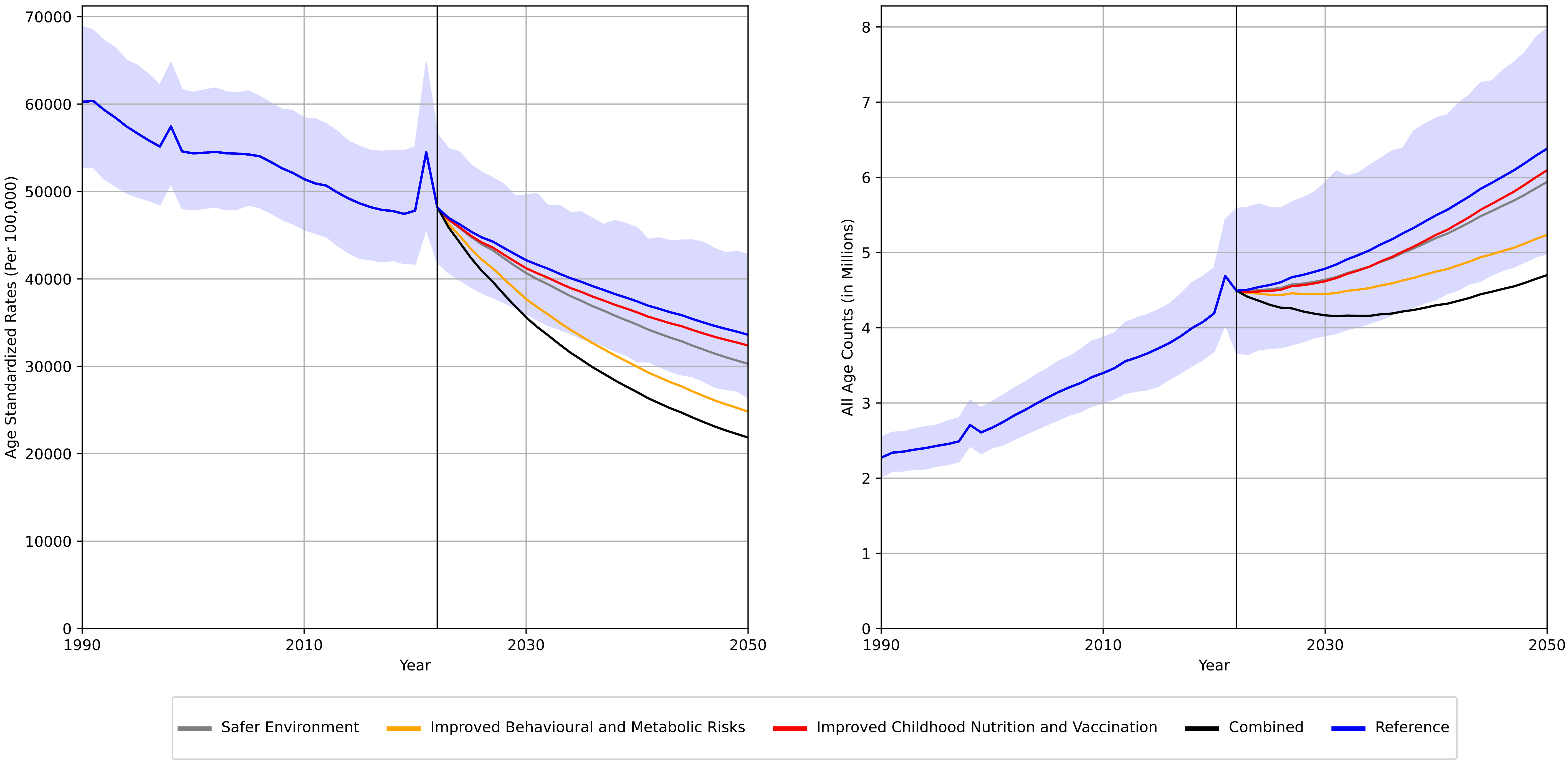

Samoa

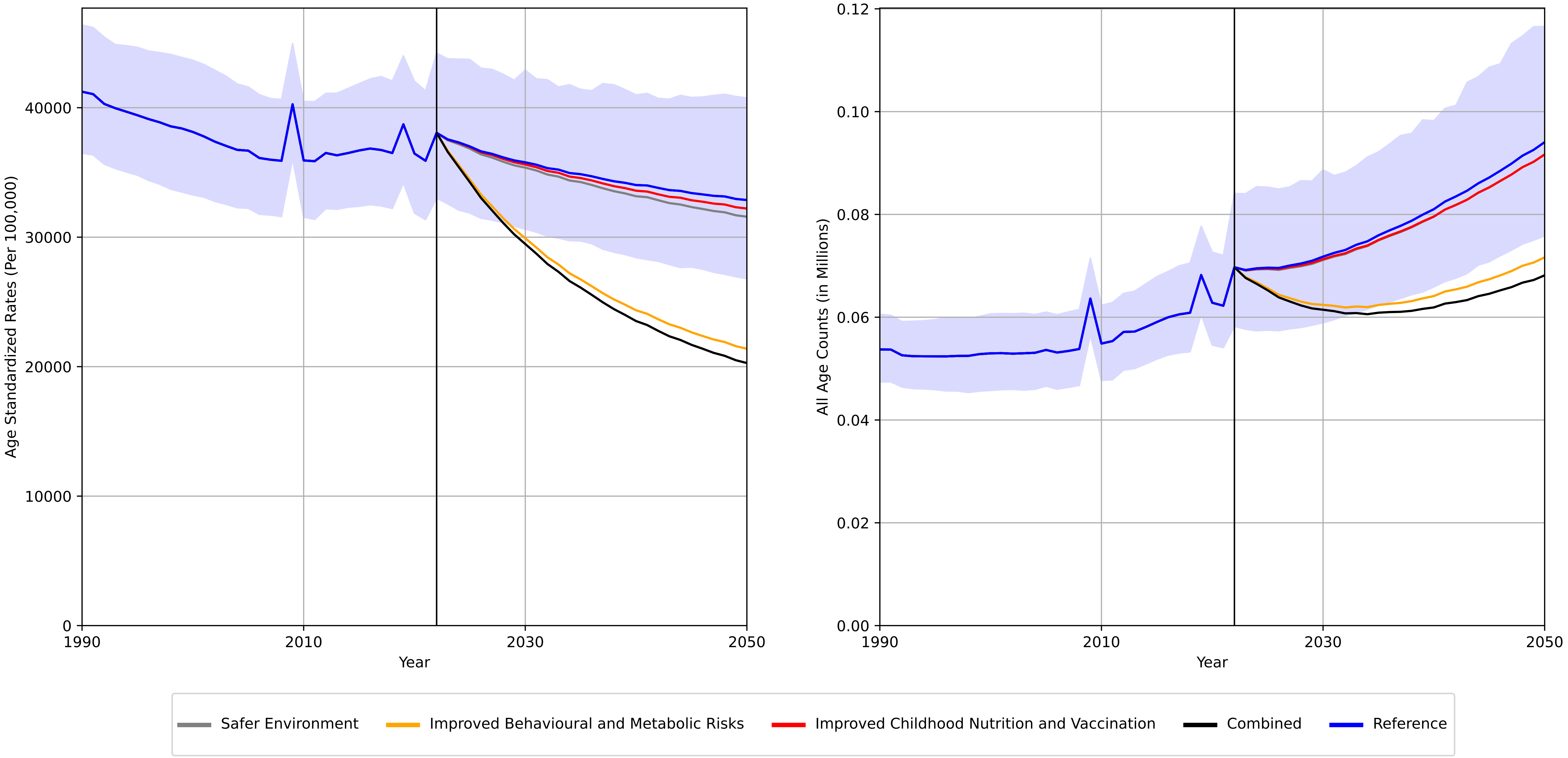
Age Standardized Rates (Per 100,000)
0
10000
20000
30000
40000
1990
2010
2030
2050
Year
All Age Counts (in Millions)
0.00
0.02
0.04
0.06
0.08
0.10
0.12
1990
2010
2030
2050
Year
Safer Environment
Improved Behavioural and Metabolic Risks
Improved Childhood Nutrition and Vaccination
Combined
Reference

Solomon Islands

Supplemental Results Figure S10. All-cause age standardised DALY rates (A) and all age DALY counts (B), for the past and for five future scenarios, 1990–2050

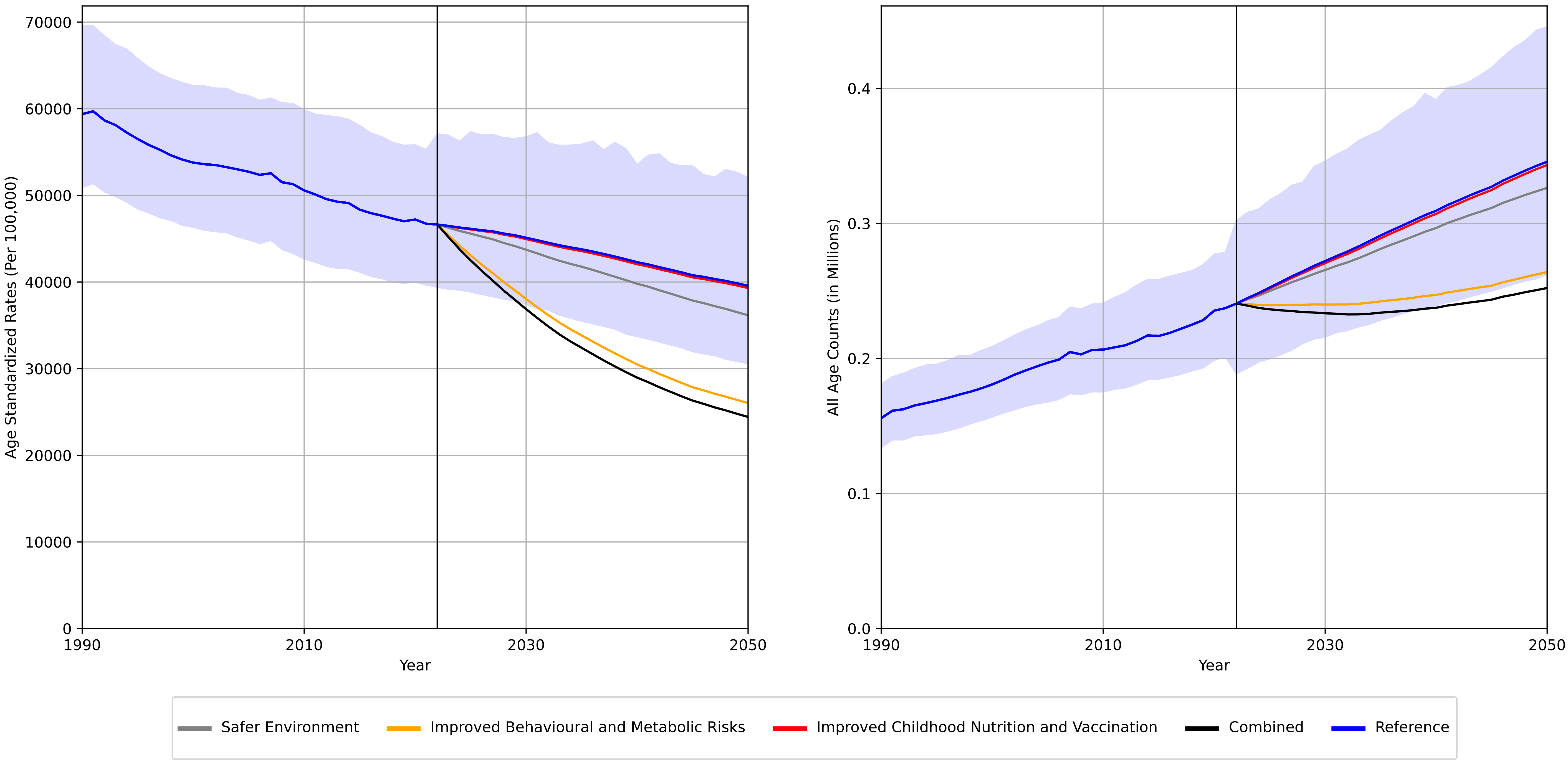

Tokelau

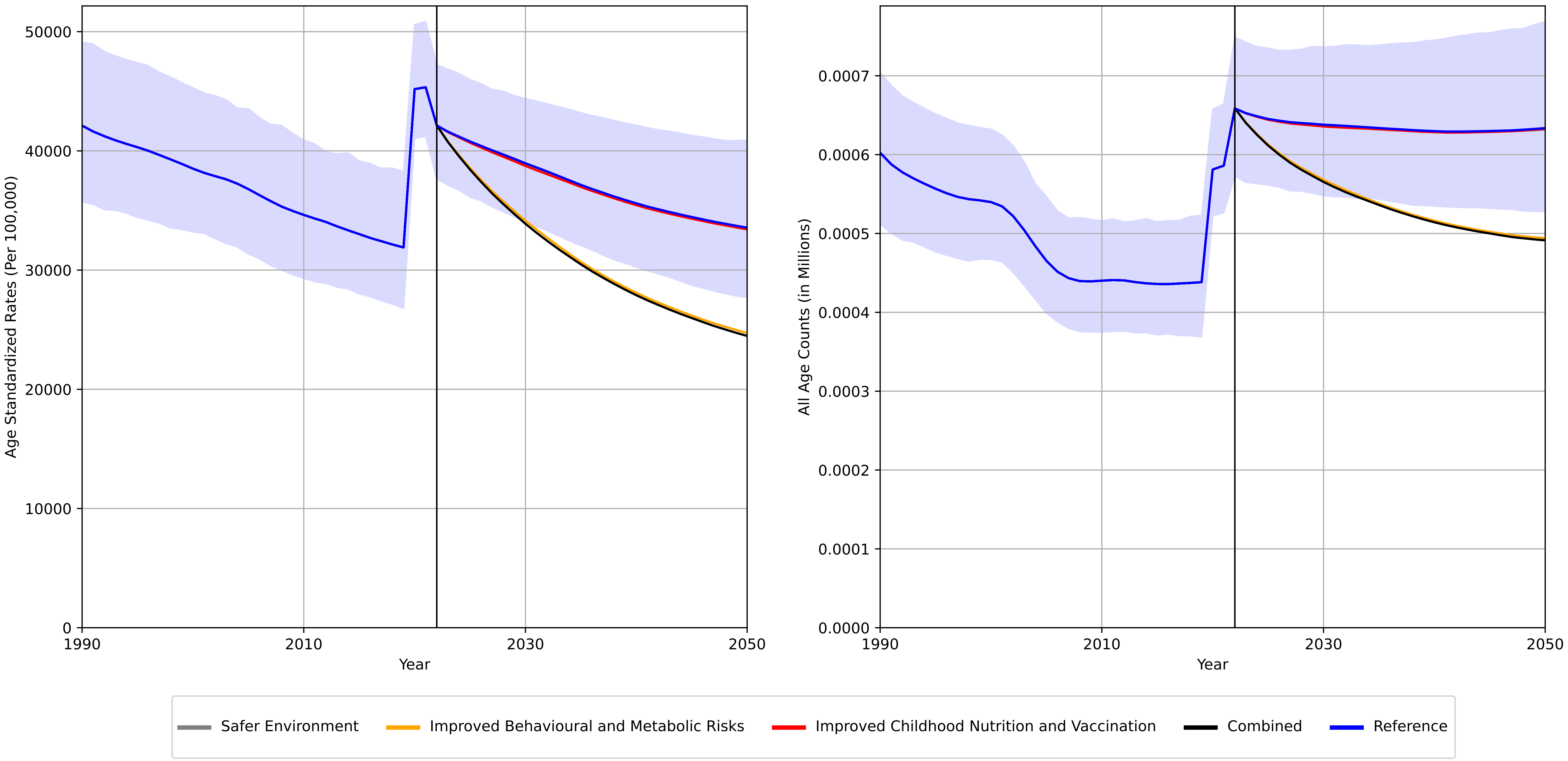

Age Standardized Rates (Per 100,000)
0
10000
20000
30000
40000
50000
1990
2010
2030
2050
Year
All Age Counts (in Millions)
0.0000
0.0001
0.0002
0.0003
0.0004
0.0005
0.0006
0.0007
1990
2010
2030
2050
Year
Safer Environment
Improved Behavioural and Metabolic Risks
Improved Childhood Nutrition and Vaccination
Combined
Reference

Tonga

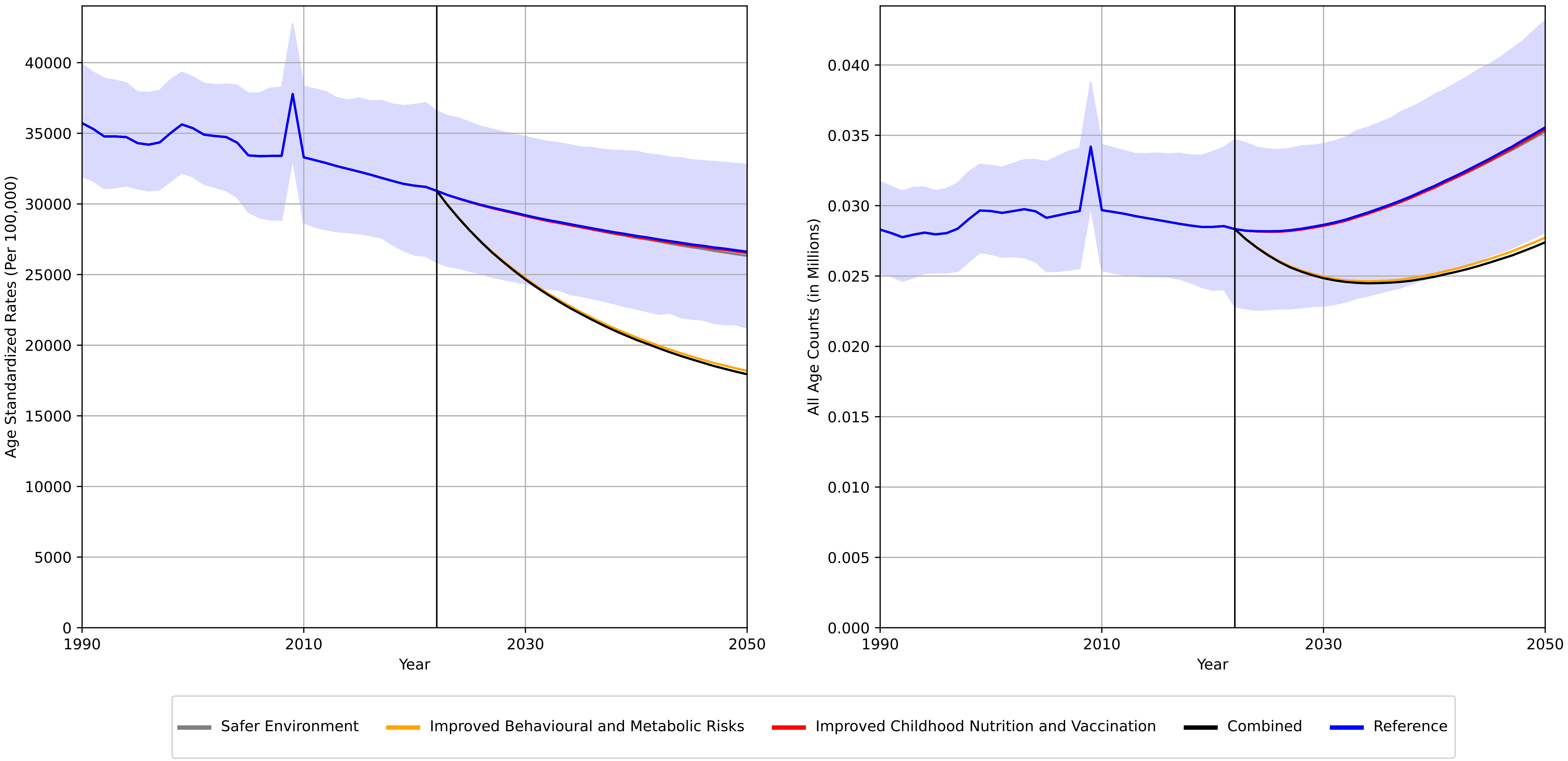
Age Standardized Rates (Per 100,000)
40000
35000
30000
25000
20000
15000
10000
5000
0
1990
2010
2030
2050
Year
All Age Counts (in Millions)
0.040
0.035
0.030
0.025
0.020
0.015
0.010
0.005
0.000
1990
2010
2030
2050
Year
Safer Environment
Improved Behavioural and Metabolic Risks
Improved Childhood Nutrition and Vaccination
Combined
Reference

Tuvalu

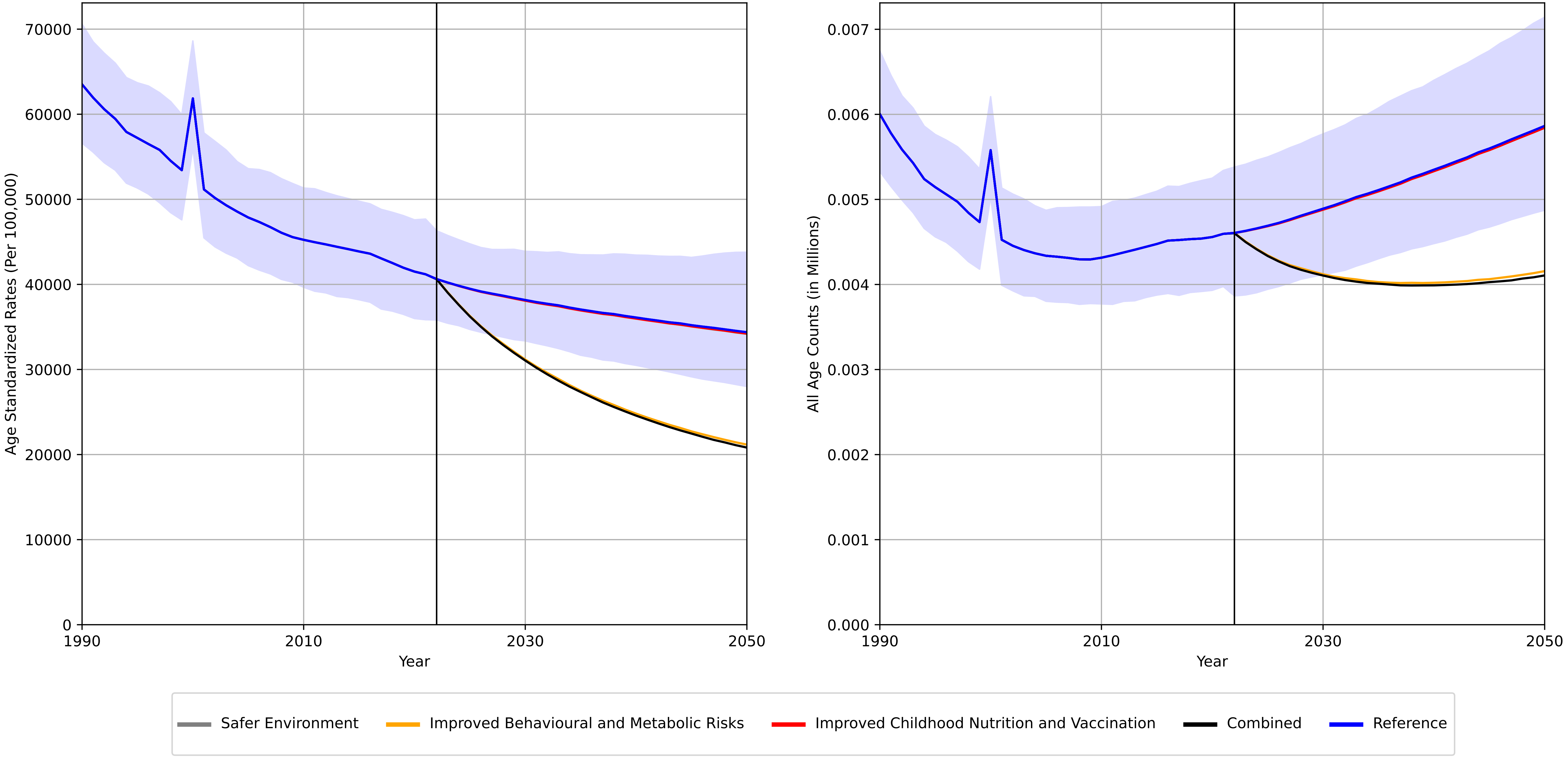

Age Standardized Rates (Per 100,000)
70000
60000
50000
40000
30000
20000
10000
0
1990
2010
2030
2050
Year
All Age Counts (in Millions)
0.007
0.006
0.005
0.004
0.003
0.002
0.001
0.000
1990
2010
2030
2050
Year
Safer Environment
Improved Behavioural and Metabolic Risks
Improved Childhood Nutrition and Vaccination
Combined
Reference

Vanuatu

Supplemental Results Figure S10. All-cause age standardised DALY rates (A) and all age DALY counts (B), for the past and for five future scenarios, 1990–2050

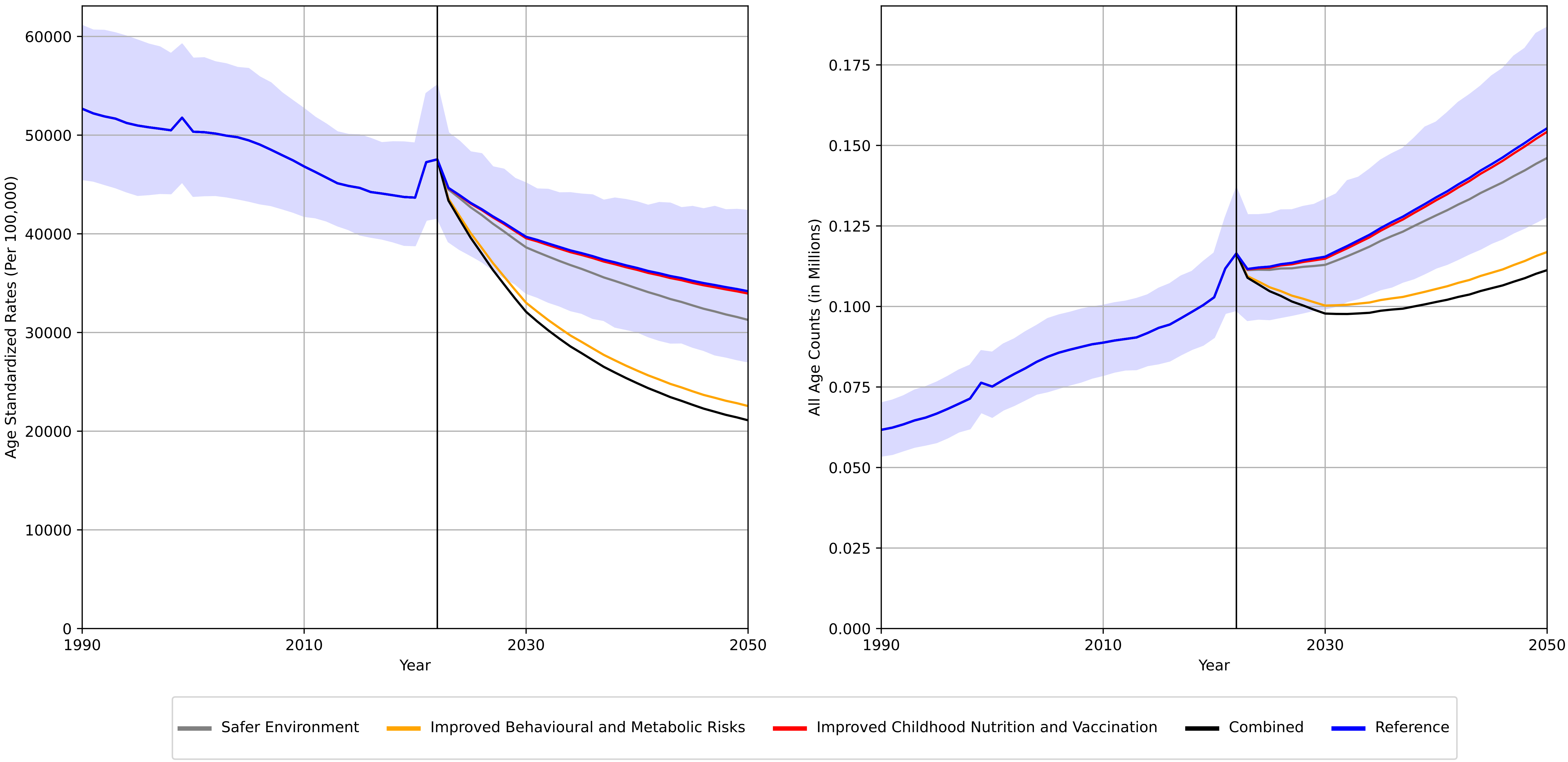

Cambodia

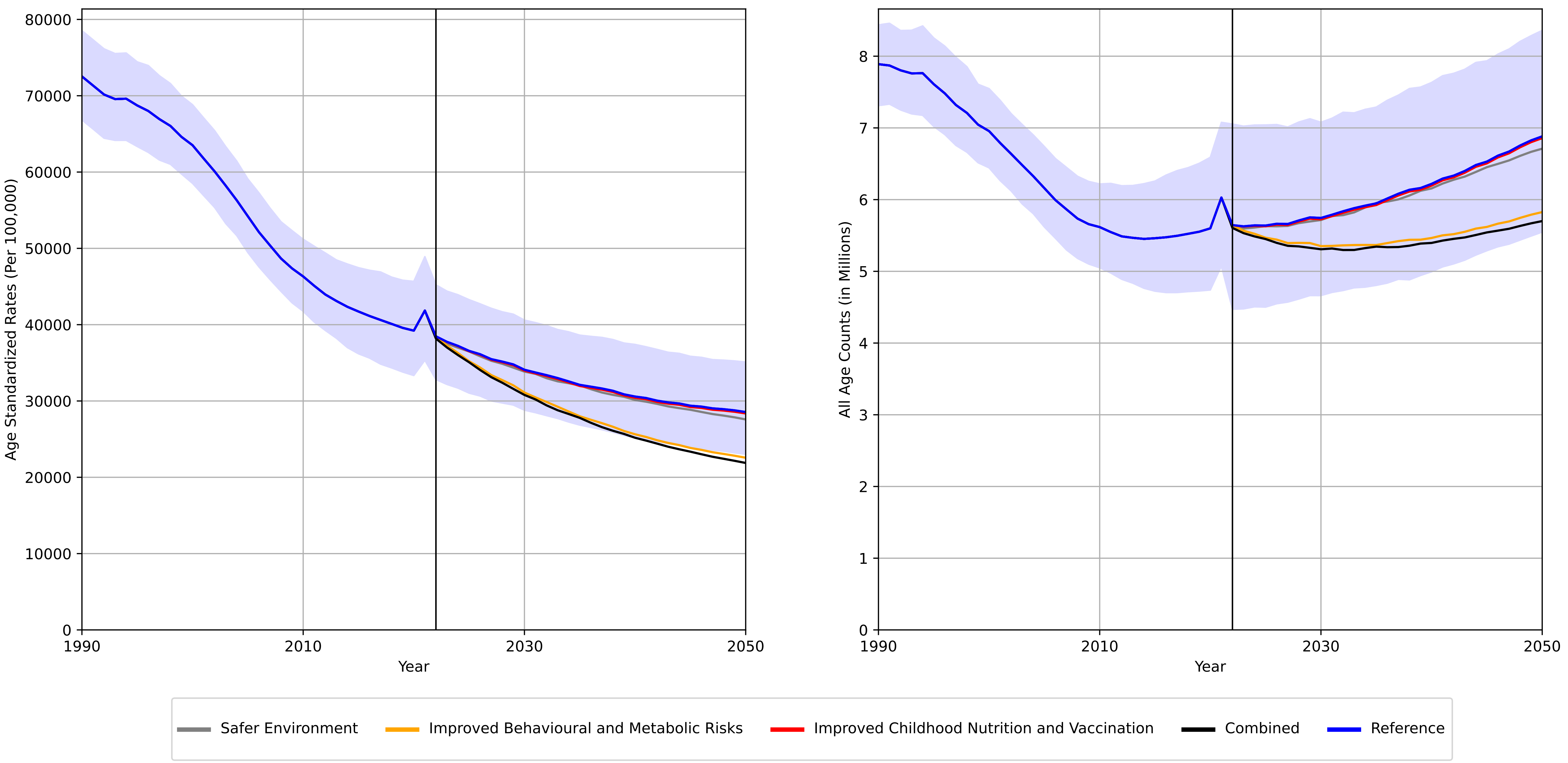
Age Standardized Rates (Per 100,000)
80000
70000
60000
50000
40000
30000
20000
10000
0
1990
2010
2030
2050
Year
All Age Counts (in Millions)
8
7
6
5
4
3
2
1
0
1990
2010
2030
2050
Year
Safer Environment
Improved Behavioural and Metabolic Risks
Improved Childhood Nutrition and Vaccination
Combined
Reference

Indonesia

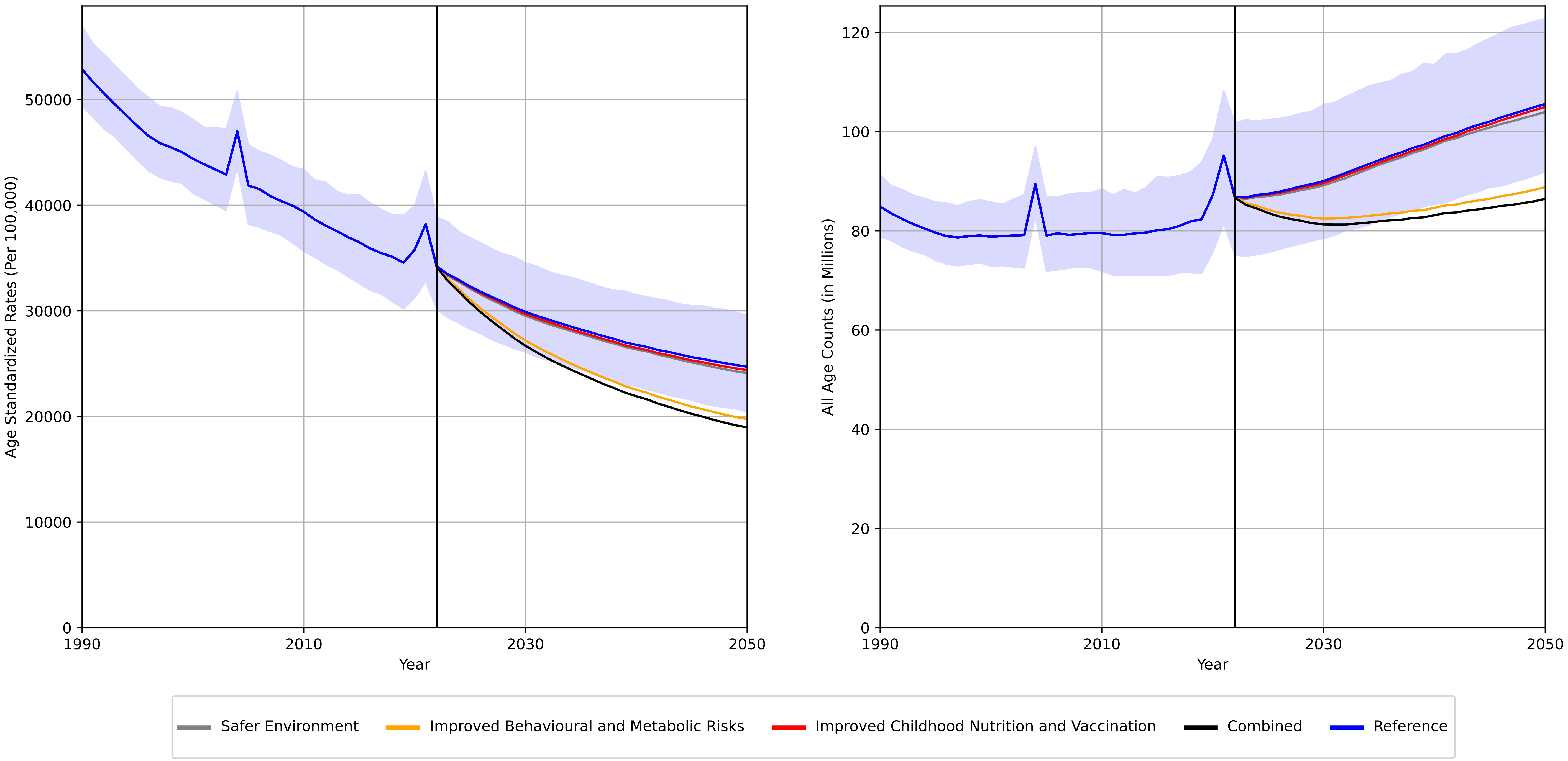
Age Standardized Rates (Per 100,000)
0
10000
20000
30000
40000
50000
1990
2010
2030
2050
Year
All Age Counts (in Millions)
0
20
40
60
80
100
120
1990
2010
2030
2050
Year
Safer Environment
Improved Behavioural and Metabolic Risks
Improved Childhood Nutrition and Vaccination
Combined
Reference

Laos

Supplemental Results Figure S10. All-cause age standardised DALY rates (A) and all age DALY counts (B), for the past and for five future scenarios, 1990–2050

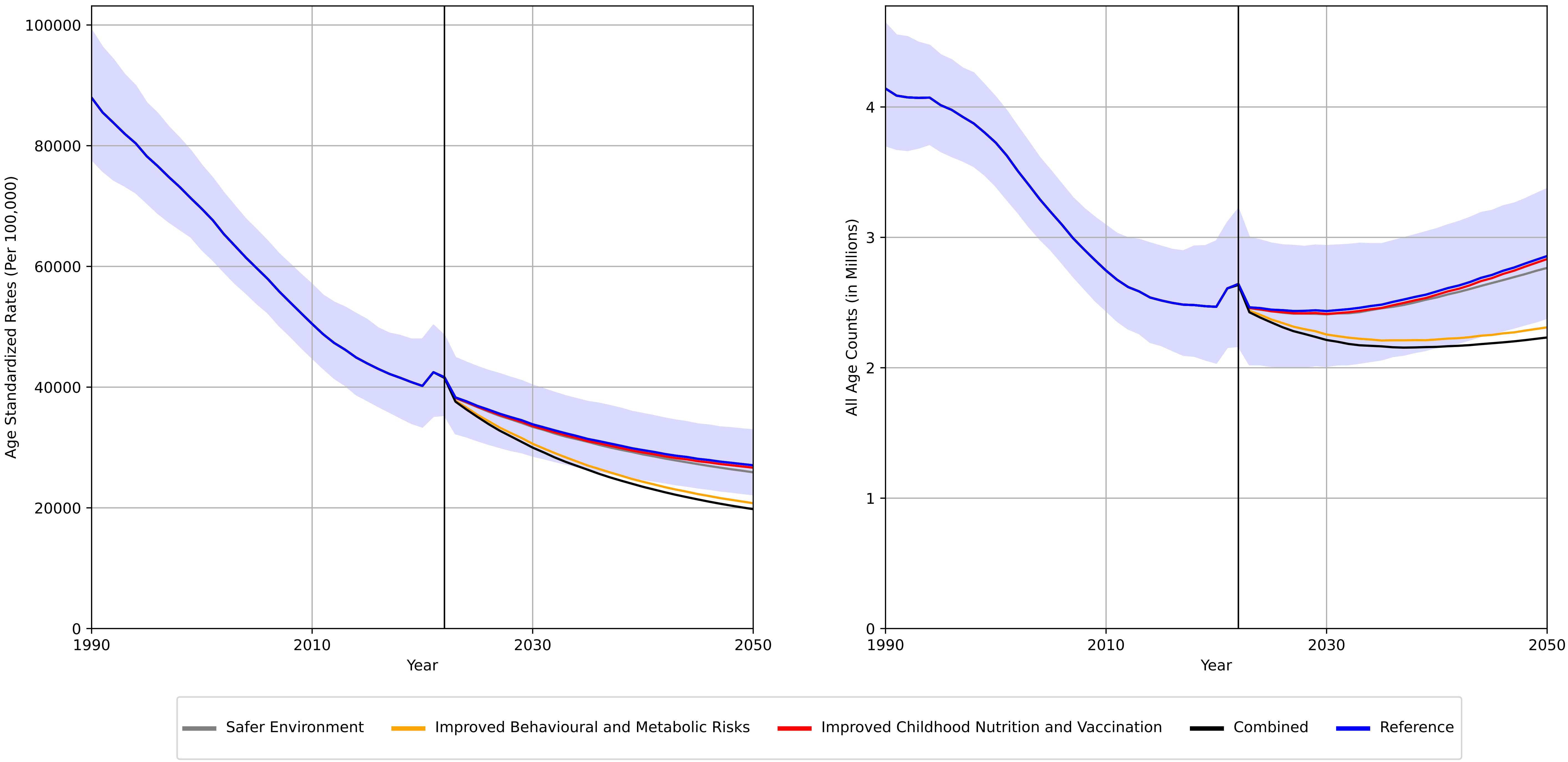

Malaysia

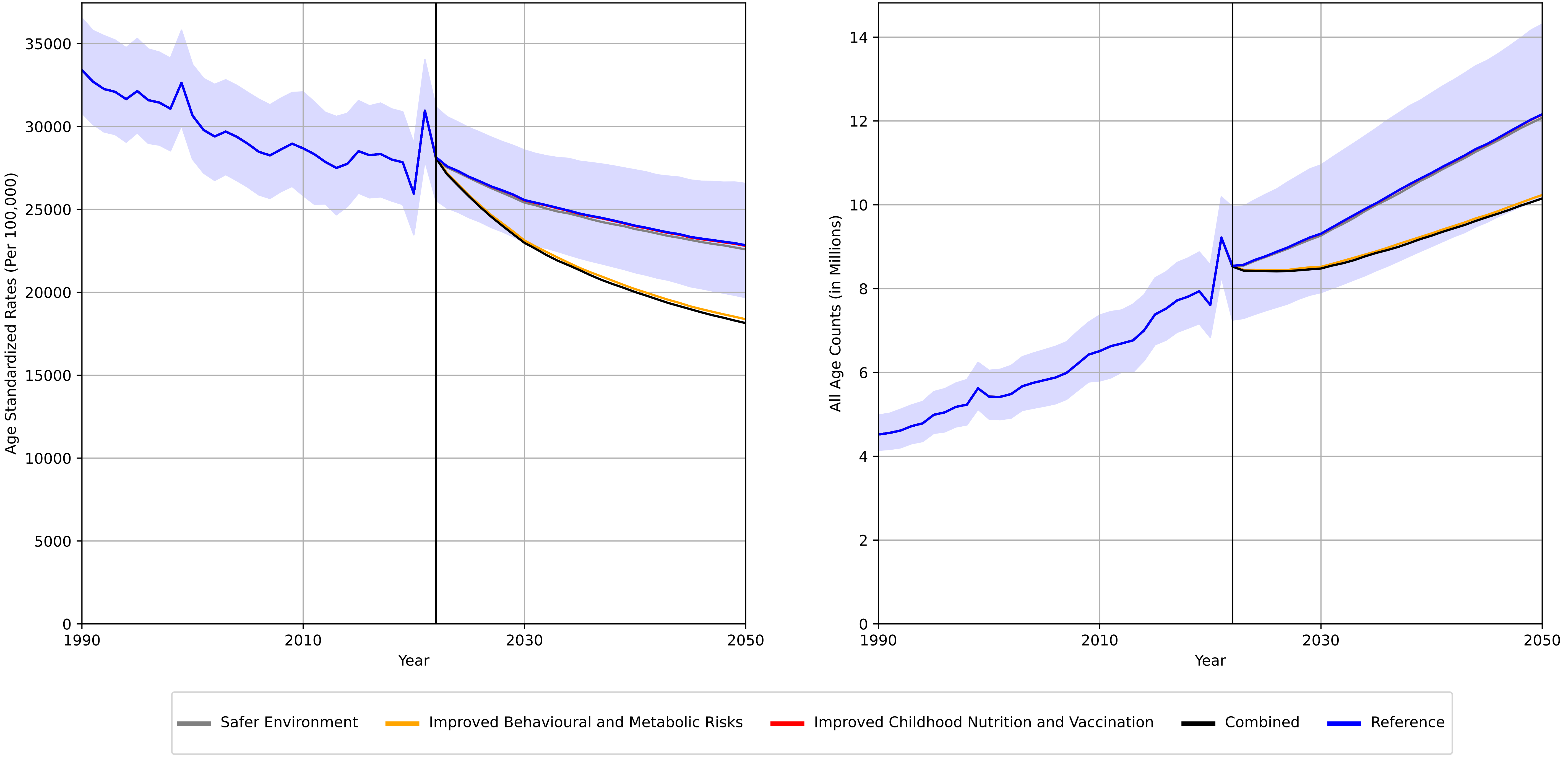

Age Standardized Rates (Per 100,000)
35000
30000
25000
20000
15000
10000
5000
0
1990
2010
2030
2050
Year
All Age Counts (in Millions)
14
12
10
8
6
4
2
0
1990
2010
2030
2050
Year
Safer Environment
Improved Behavioural and Metabolic Risks
Improved Childhood Nutrition and Vaccination
Combined
Reference

Maldives

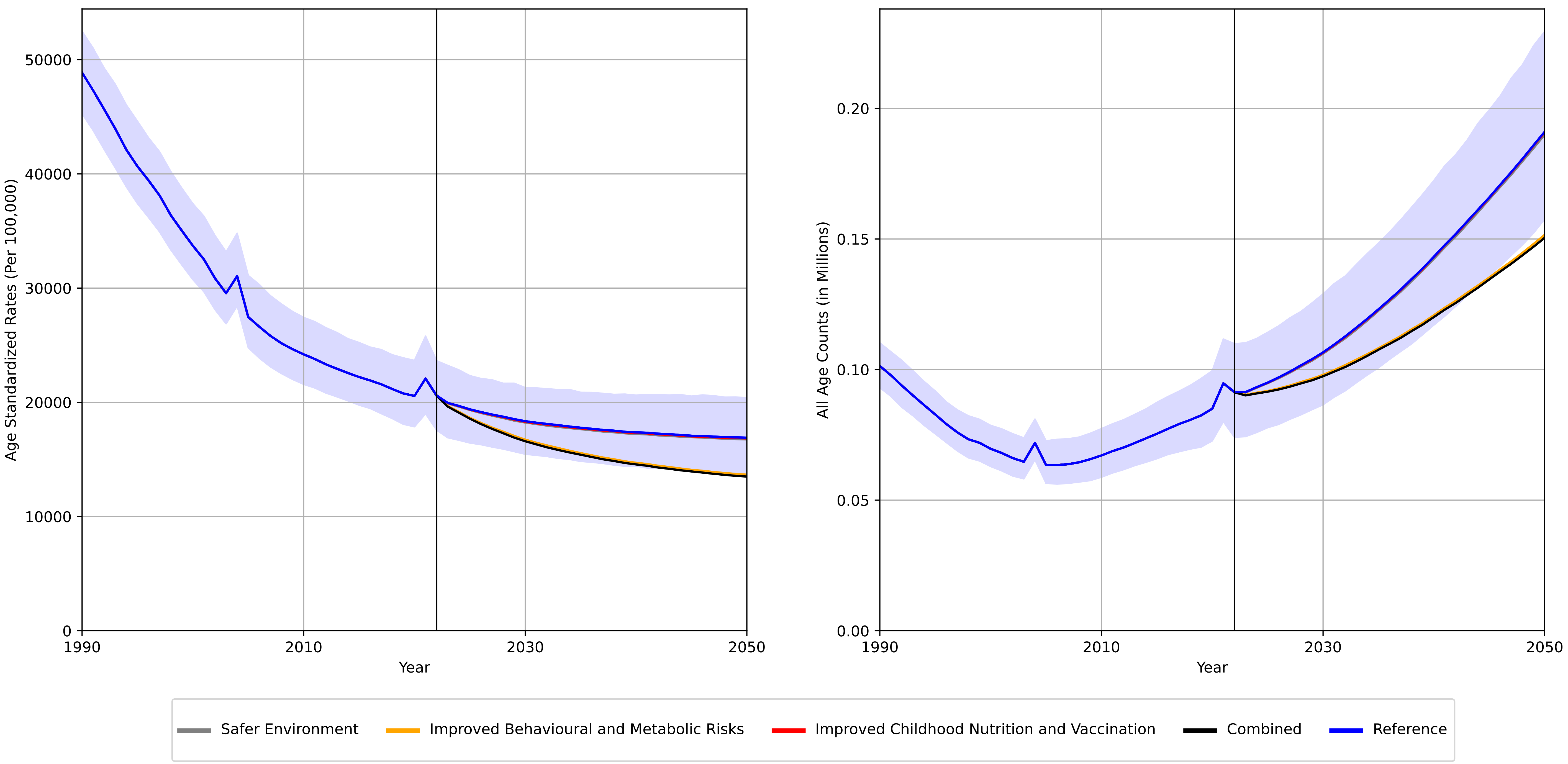

Age Standardized Rates (Per 100,000)
50000
40000
30000
20000
10000
0
1990
2010
2030
2050
Year
All Age Counts (in Millions)
0.20
0.15
0.10
0.05
0.00
1990
2010
2030
2050
Year
Safer Environment
Improved Behavioural and Metabolic Risks
Improved Childhood Nutrition and Vaccination
Combined
Reference

Mauritius

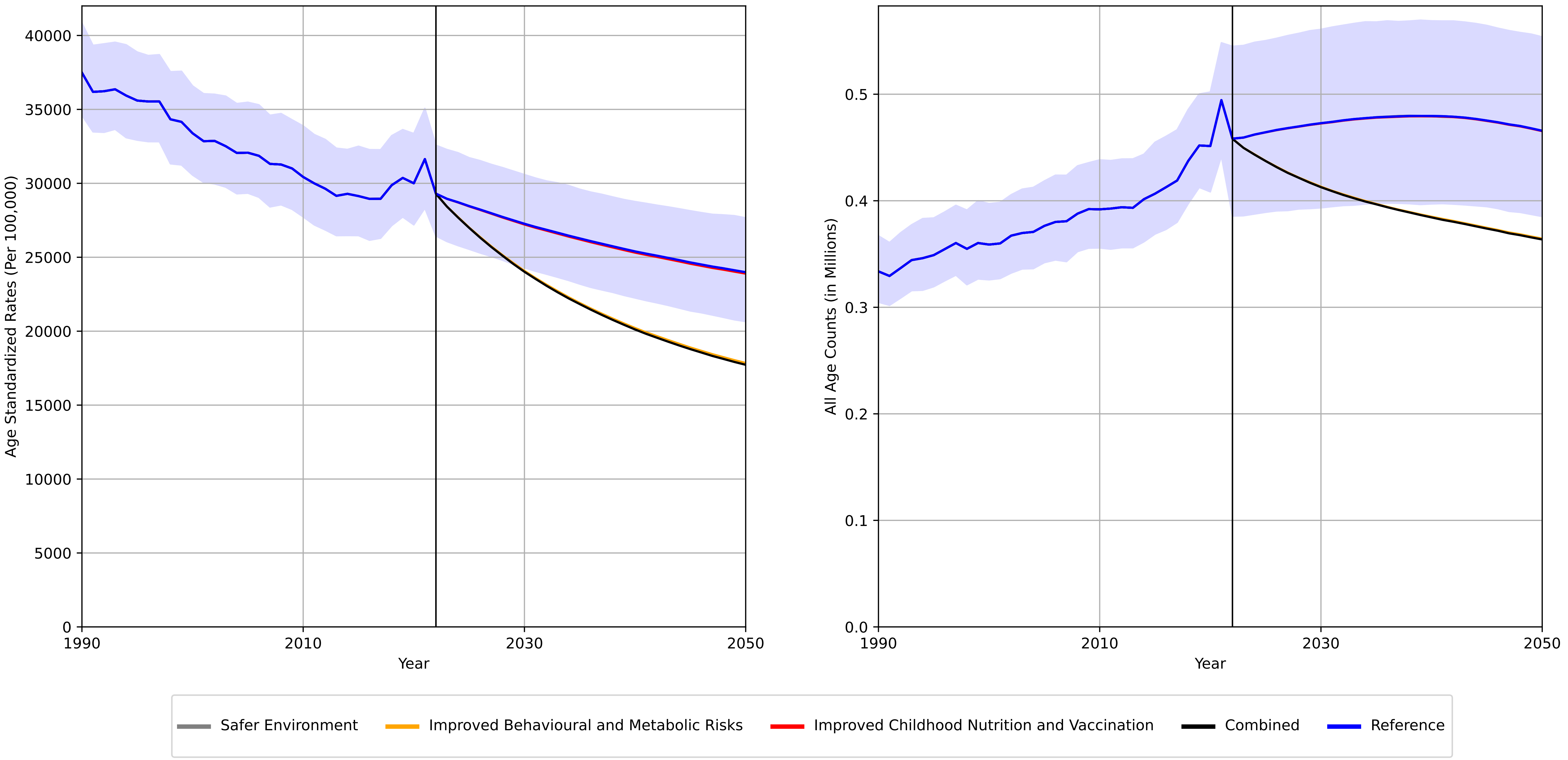
Age Standardized Rates (Per 100,000)
40000
35000
30000
25000
20000
15000
10000
5000
0
1990
2010
2030
2050
Year
All Age Counts (in Millions)
0.5
0.4
0.3
0.2
0.1
0.0
1990
2010
2030
2050
Year
Safer Environment
Improved Behavioural and Metabolic Risks
Improved Childhood Nutrition and Vaccination
Combined
Reference

Myanmar

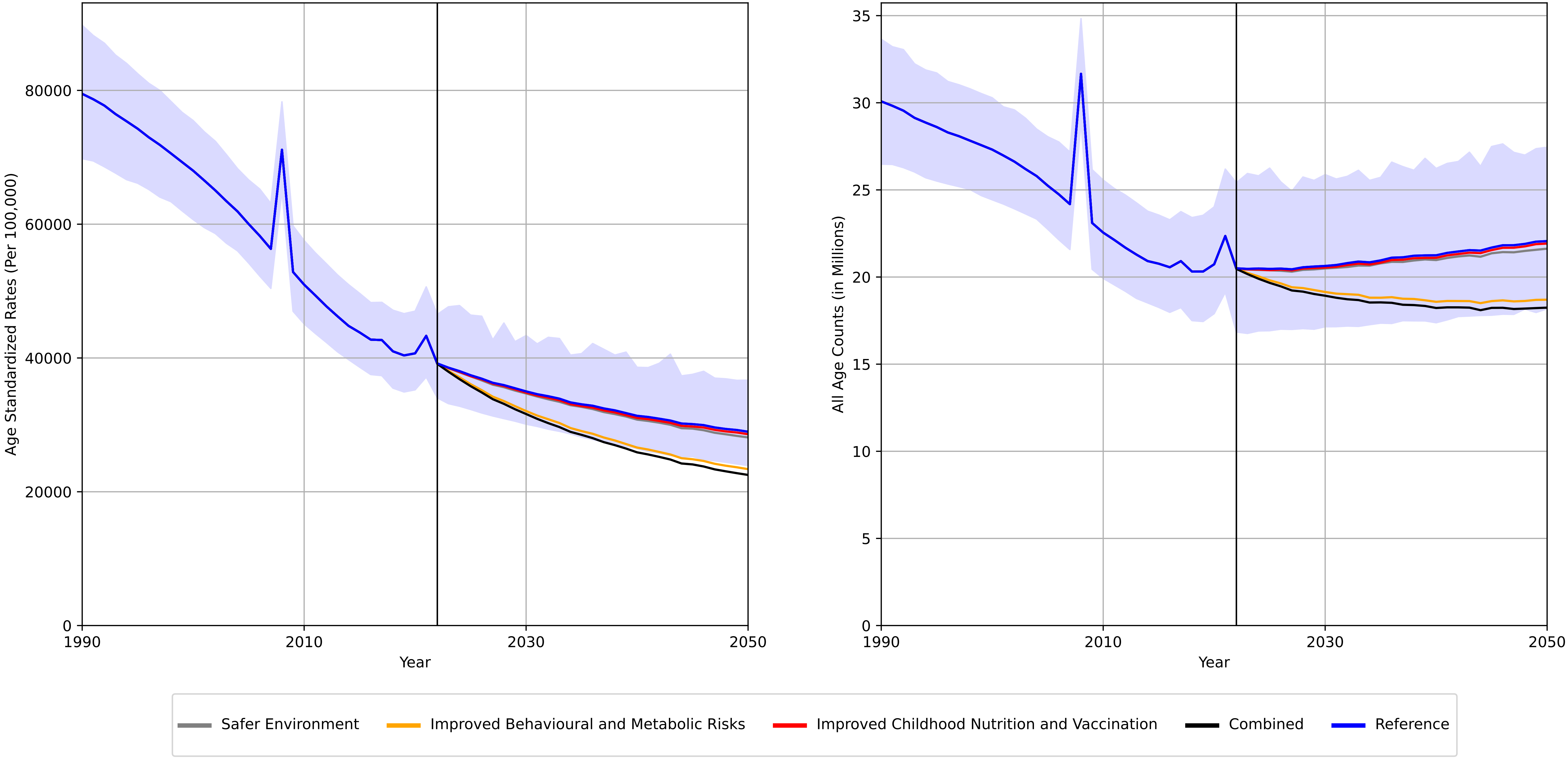

Age Standardized Rates (Per 100,000)
0
20000
40000
60000
80000
1990
2010
2030
2050
Year
All Age Counts (in Millions)
0
5
10
15
20
25
30
35
1990
2010
2030
2050
Year
Safer Environment
Improved Behavioural and Metabolic Risks
Improved Childhood Nutrition and Vaccination
Combined
Reference

Philippines

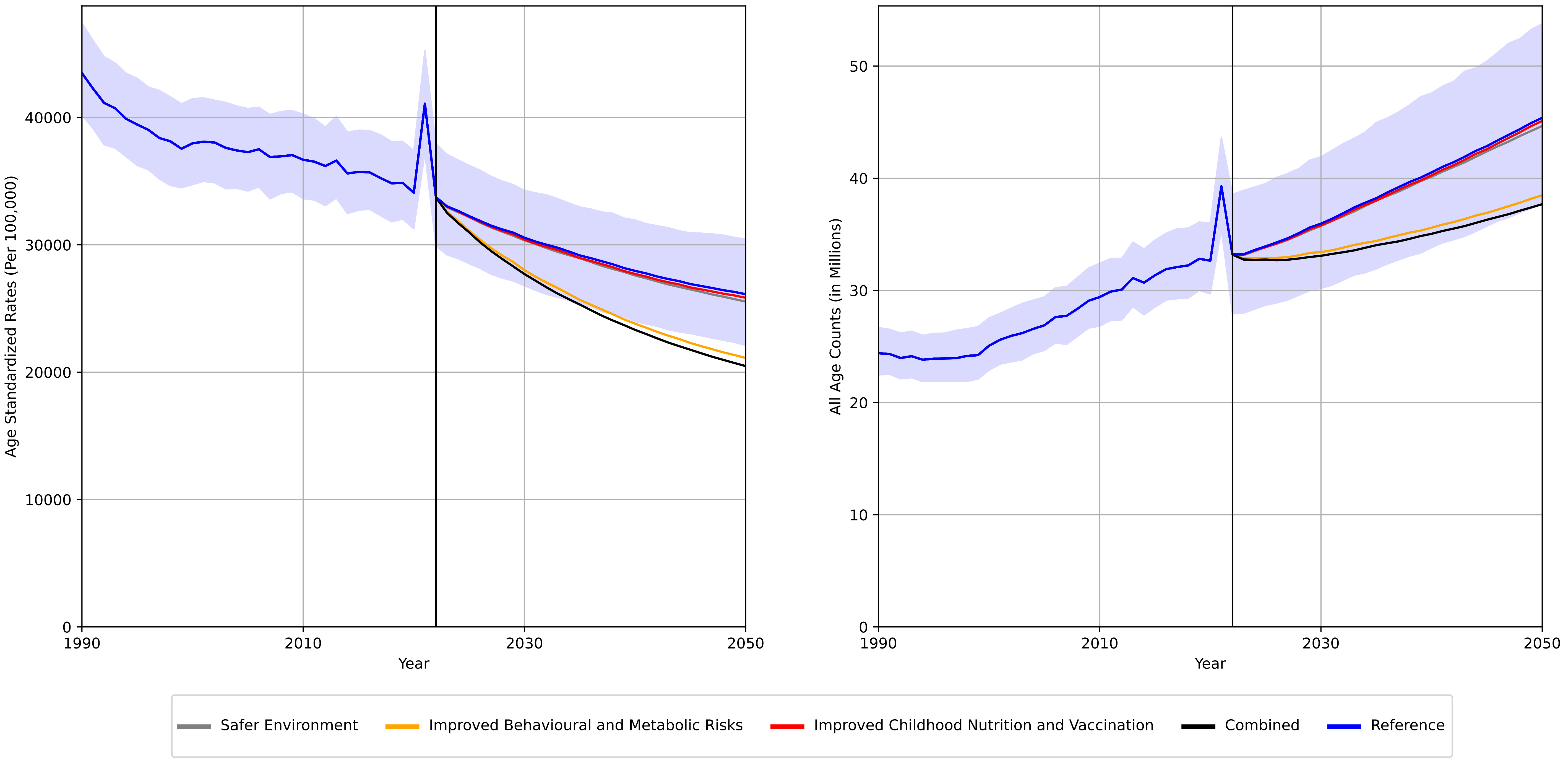

Seychelles

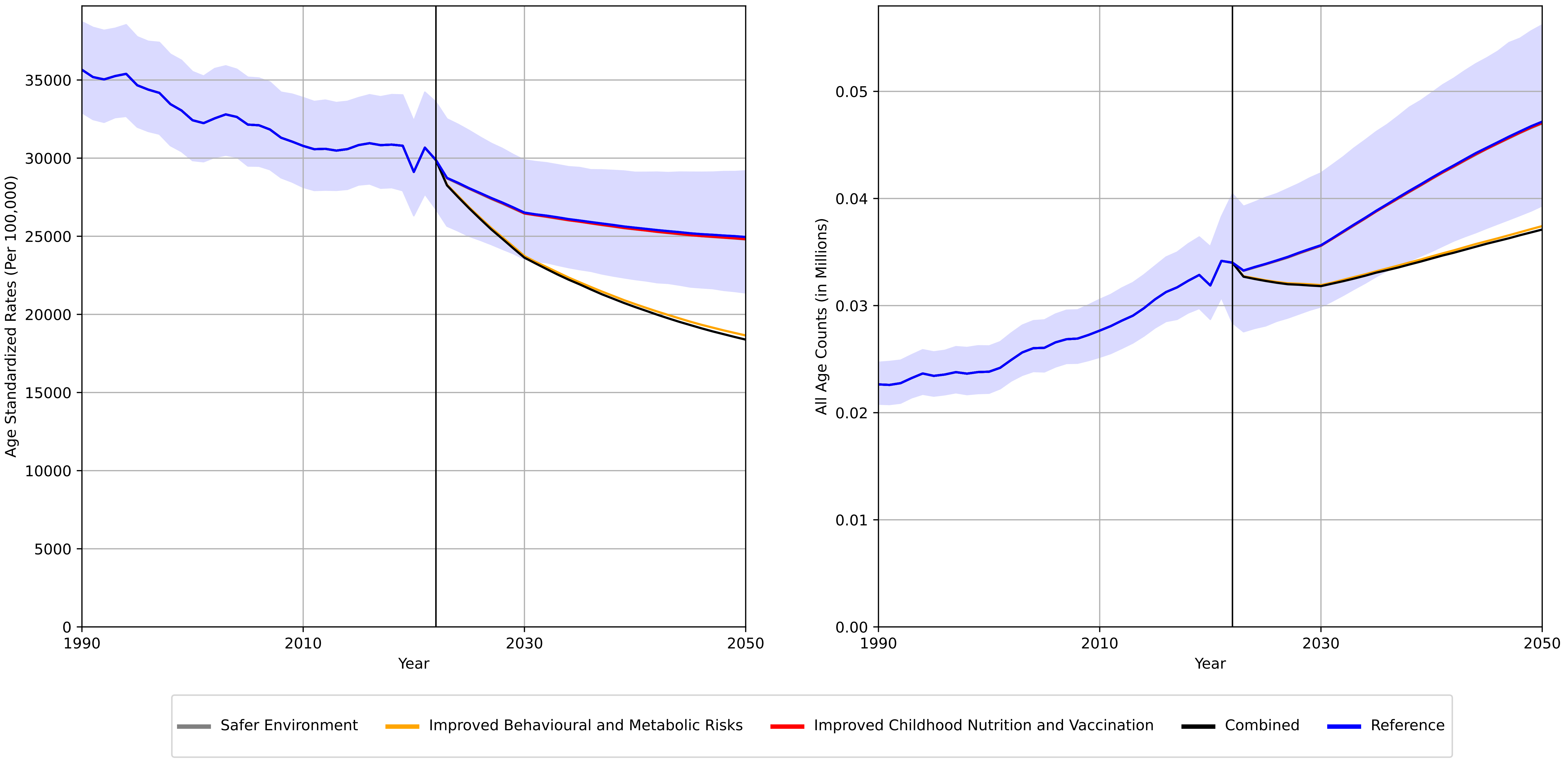

Age Standardized Rates (Per 100,000)
0
5000
10000
15000
20000
25000
30000
35000
1990
2010
2030
2050
Year
All Age Counts (in Millions)
0.00
0.01
0.02
0.03
0.04
0.05
1990
2010
2030
2050
Year
Safer Environment
Improved Behavioural and Metabolic Risks
Improved Childhood Nutrition and Vaccination
Combined
Reference

Sri Lanka

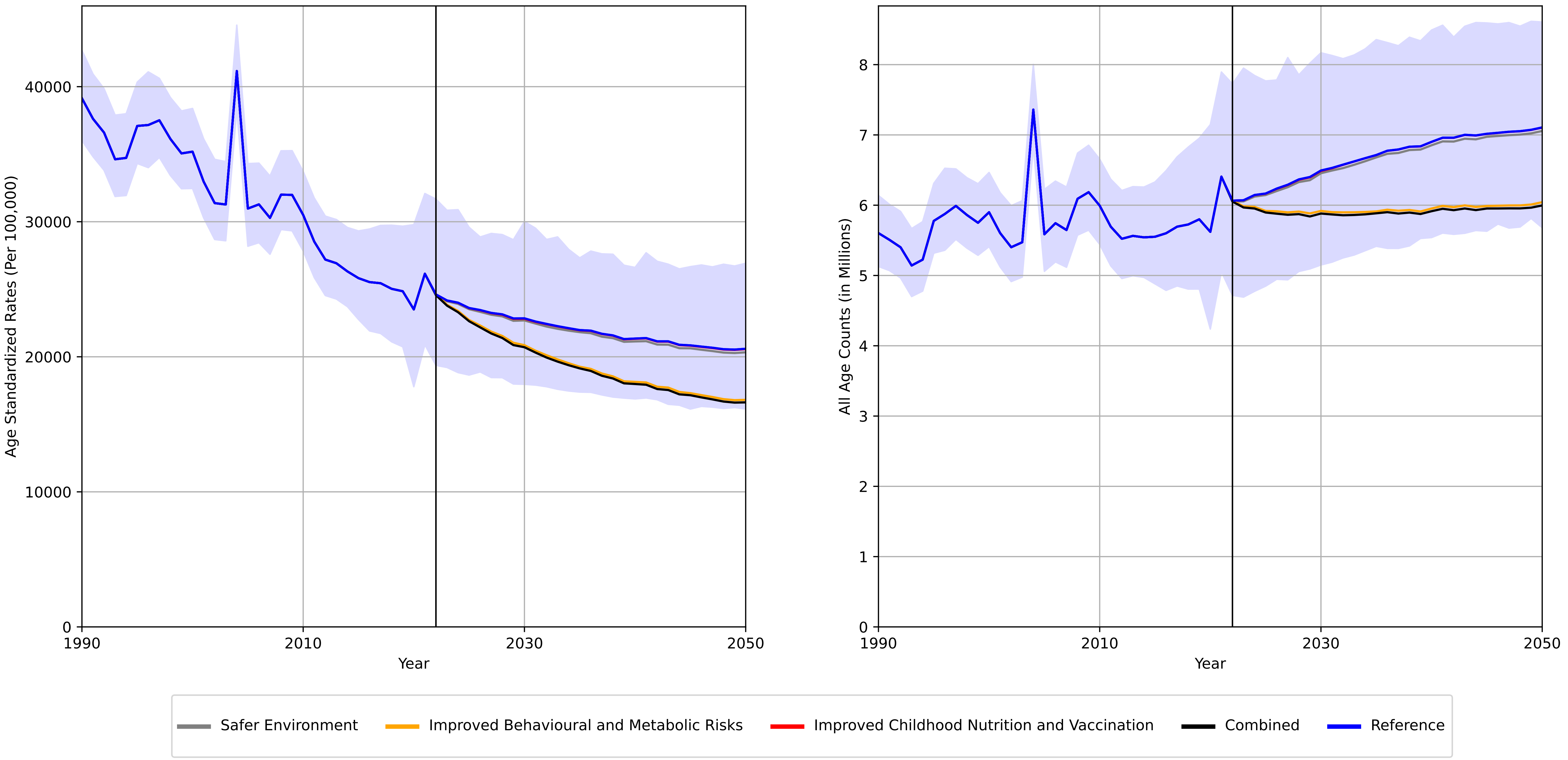
Age Standardized Rates (Per 100,000)
0
10000
20000
30000
40000
1990
2010
2030
2050
Year
All Age Counts (in Millions)
0
1
2
3
4
5
6
7
8
1990
2010
2030
2050
Year
Safer Environment
Improved Behavioural and Metabolic Risks
Improved Childhood Nutrition and Vaccination
Combined
Reference

Thailand

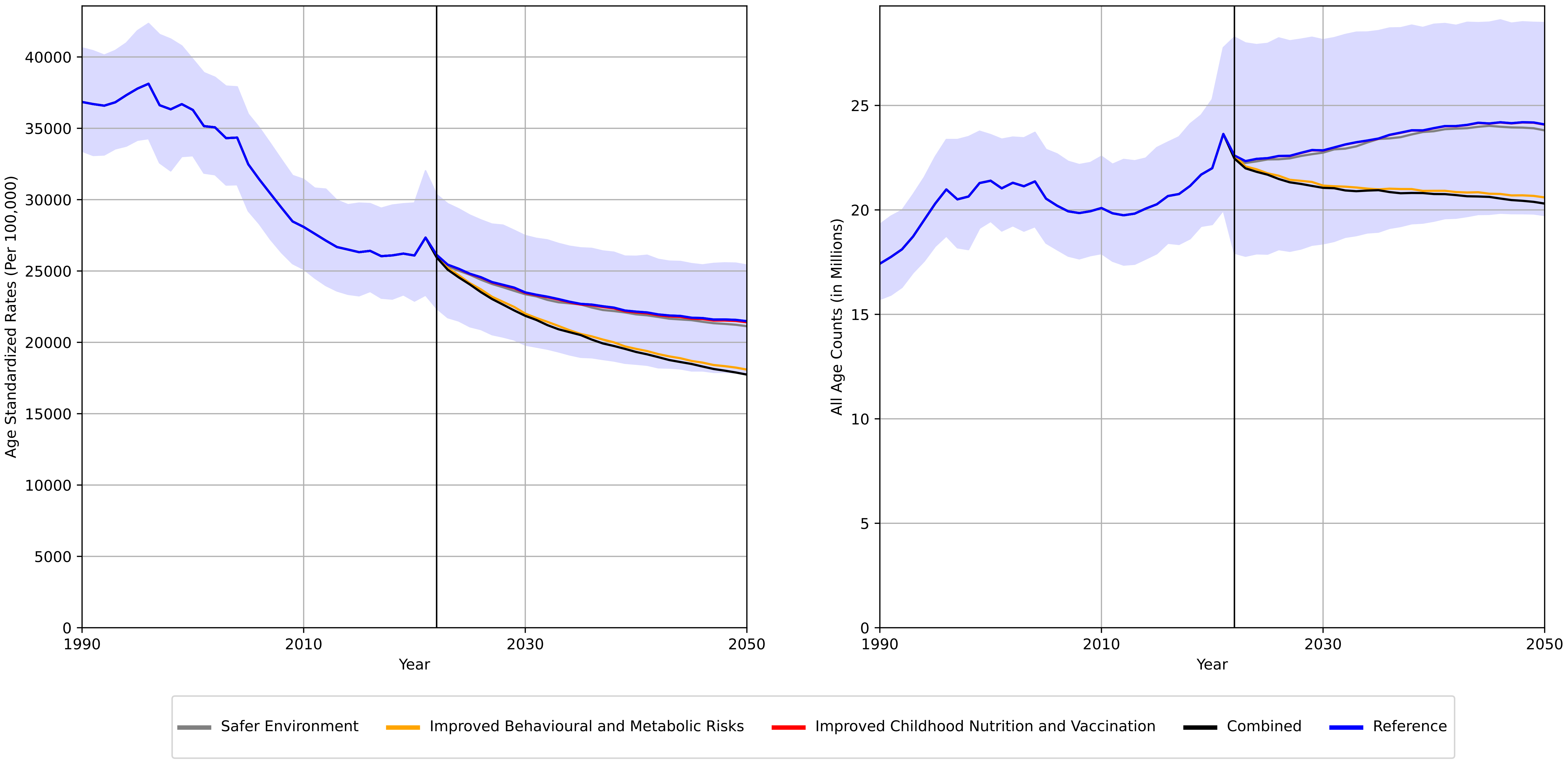
Age Standardized Rates (Per 100,000)
40000
35000
30000
25000
20000
15000
10000
5000
0
1990
2010
2030
2050
Year
All Age Counts (in Millions)
25
20
15
10
5
0
1990
2010
2030
2050
Year
Safer Environment
Improved Behavioural and Metabolic Risks
Improved Childhood Nutrition and Vaccination
Combined
Reference

Timor-Leste

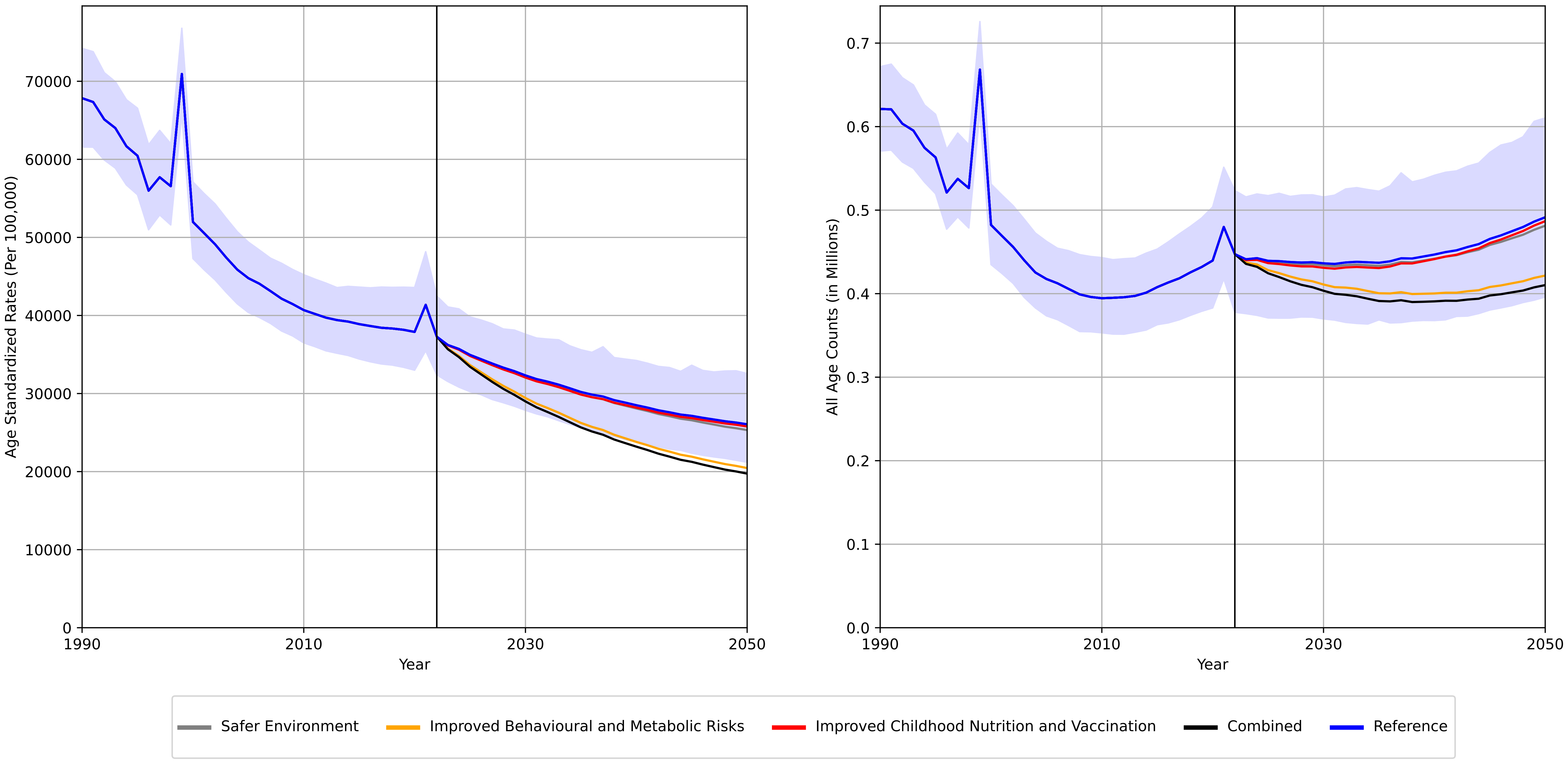
Age Standardized Rates (Per 100,000)
0
10000
20000
30000
40000
50000
60000
70000
1990
2010
2030
2050
Year
All Age Counts (in Millions)
0.0
0.1
0.2
0.3
0.4
0.5
0.6
0.7
1990
2010
2030
2050
Year
Safer Environment
Improved Behavioural and Metabolic Risks
Improved Childhood Nutrition and Vaccination
Combined
Reference

Viet Nam

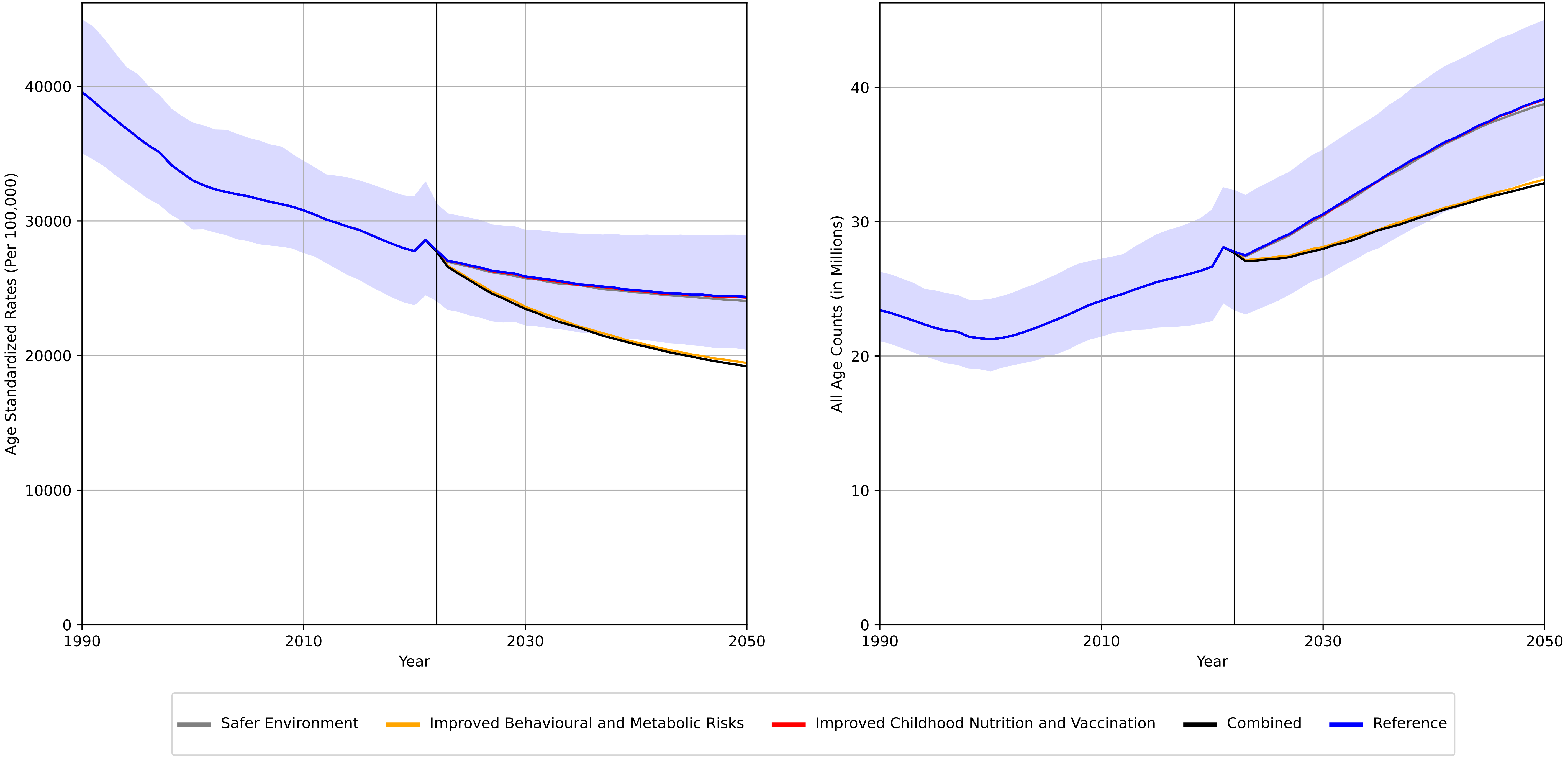
Age Standardized Rates (Per 100,000)
40000
30000
20000
10000
0
1990
2010
2030
2050
Year
All Age Counts (in Millions)
40
30
20
10
0
1990
2010
2030
2050
Year
Safer Environment
Improved Behavioural and Metabolic Risks
Improved Childhood Nutrition and Vaccination
Combined
Reference

Angola

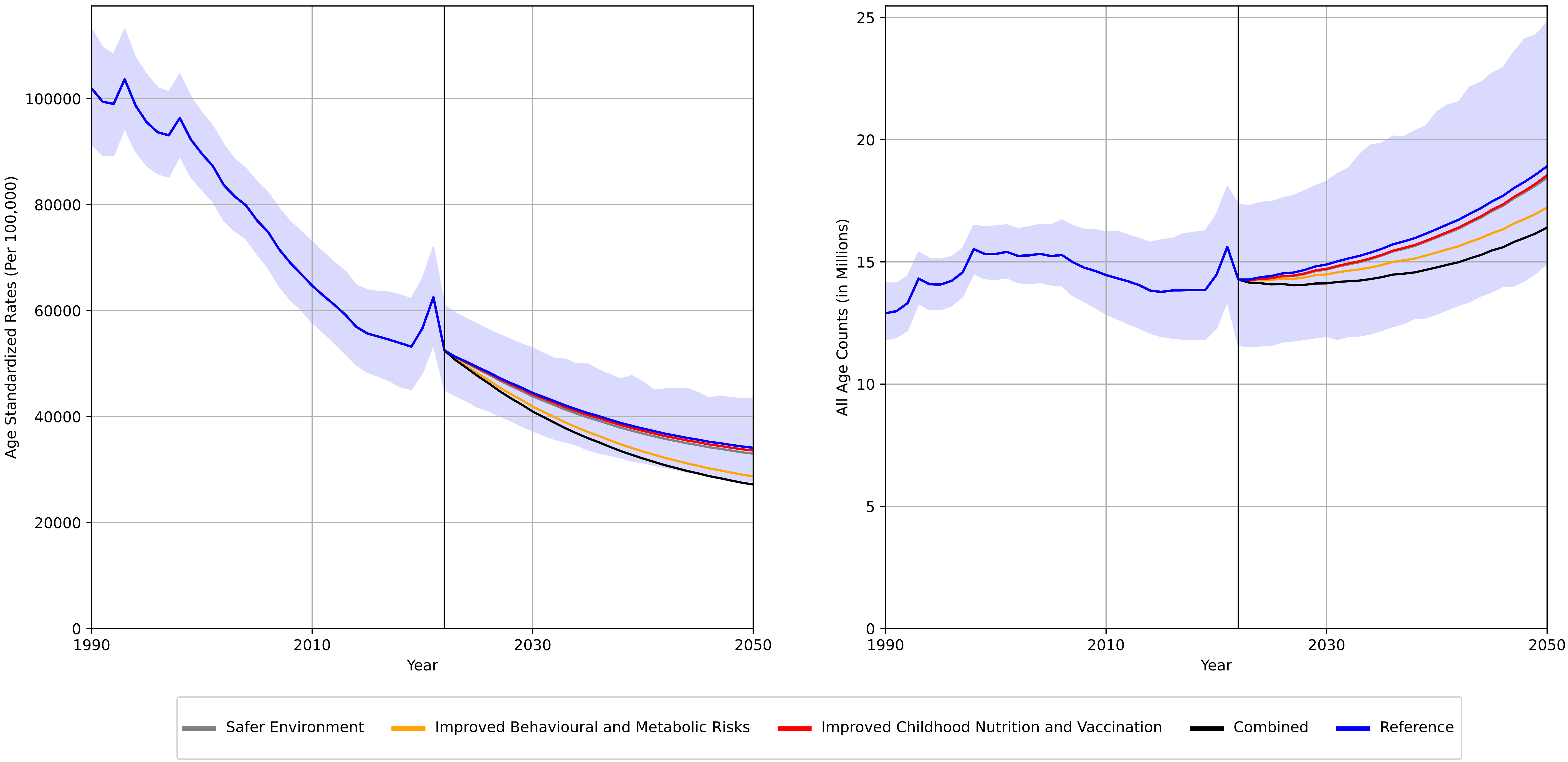

Age Standardized Rates (Per 100,000)
0
20000
40000
60000
80000
100000
1990
2010
2030
2050
Year
All Age Counts (in Millions)
0
5
10
15
20
25
1990
2010
2030
2050
Year
Safer Environment
Improved Behavioural and Metabolic Risks
Improved Childhood Nutrition and Vaccination
Combined
Reference

Central African Republic

Supplemental Results Figure S10. All-cause age standardised DALY rates (A) and all age DALY counts (B), for the past and for five future scenarios, 1990–2050

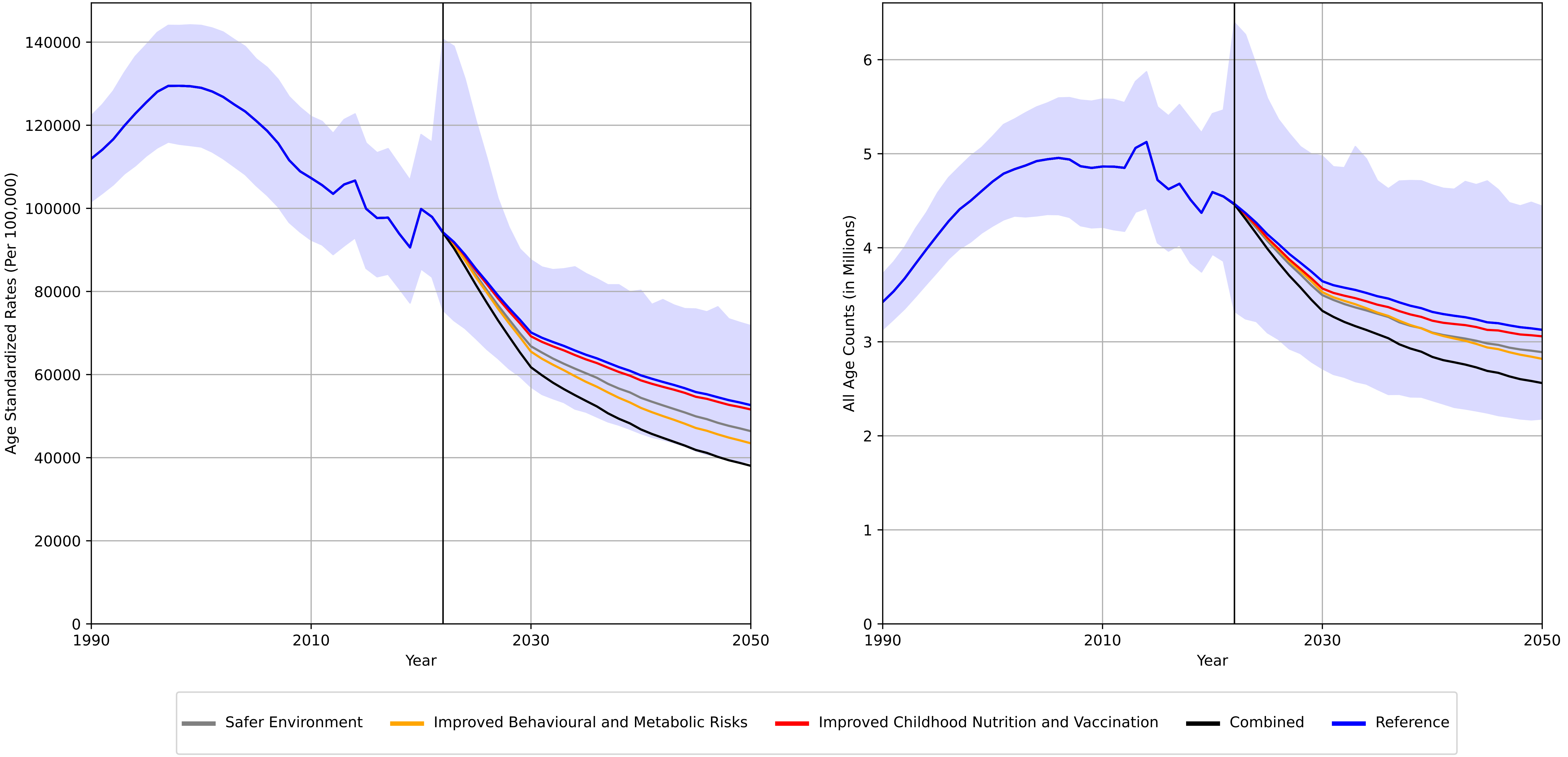

Congo (Brazzaville)

Supplemental Results Figure S10. All-cause age standardised DALY rates (A) and all age DALY counts (B), for the past and for five future scenarios, 1990–2050

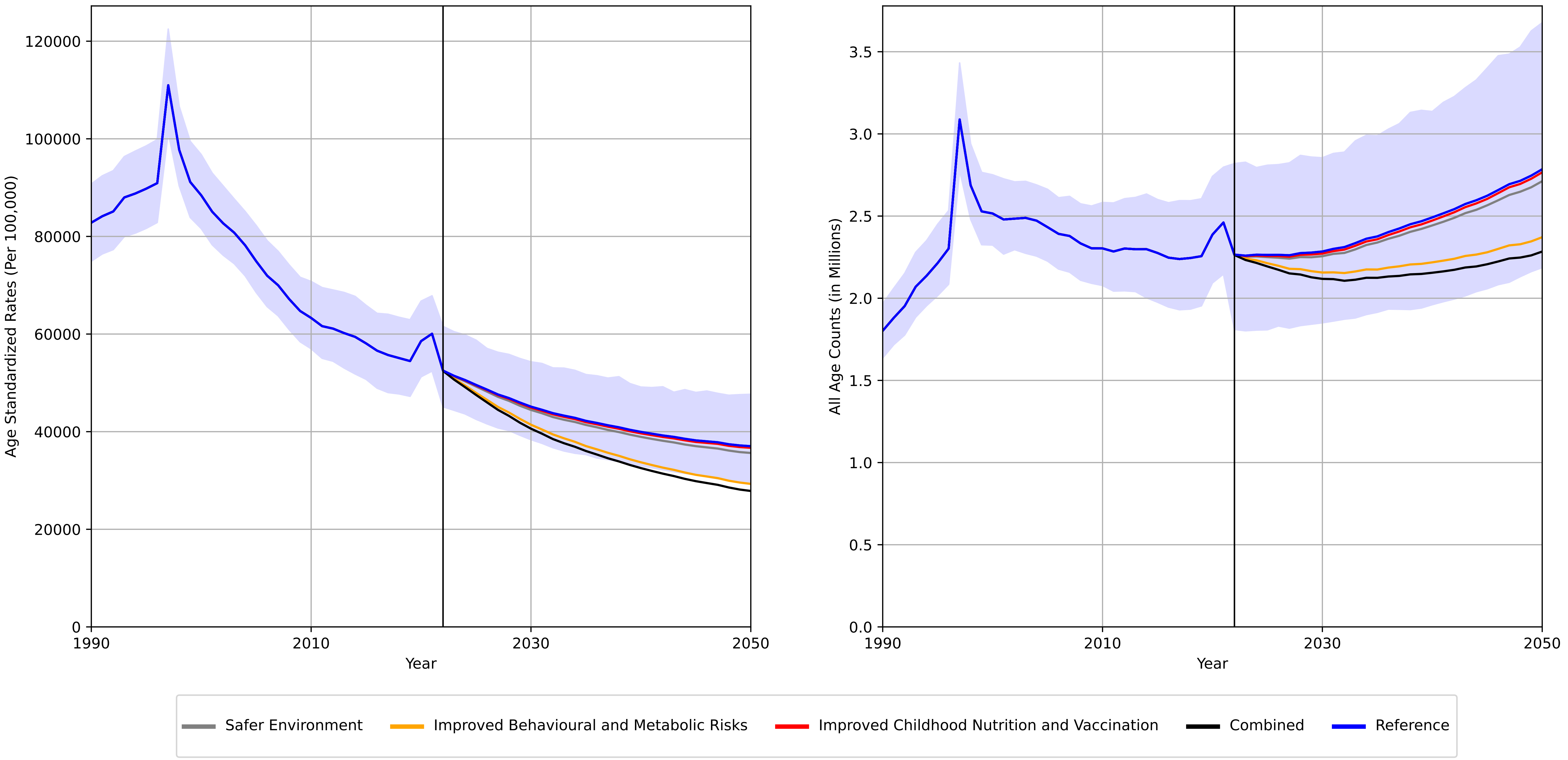

DR Congo

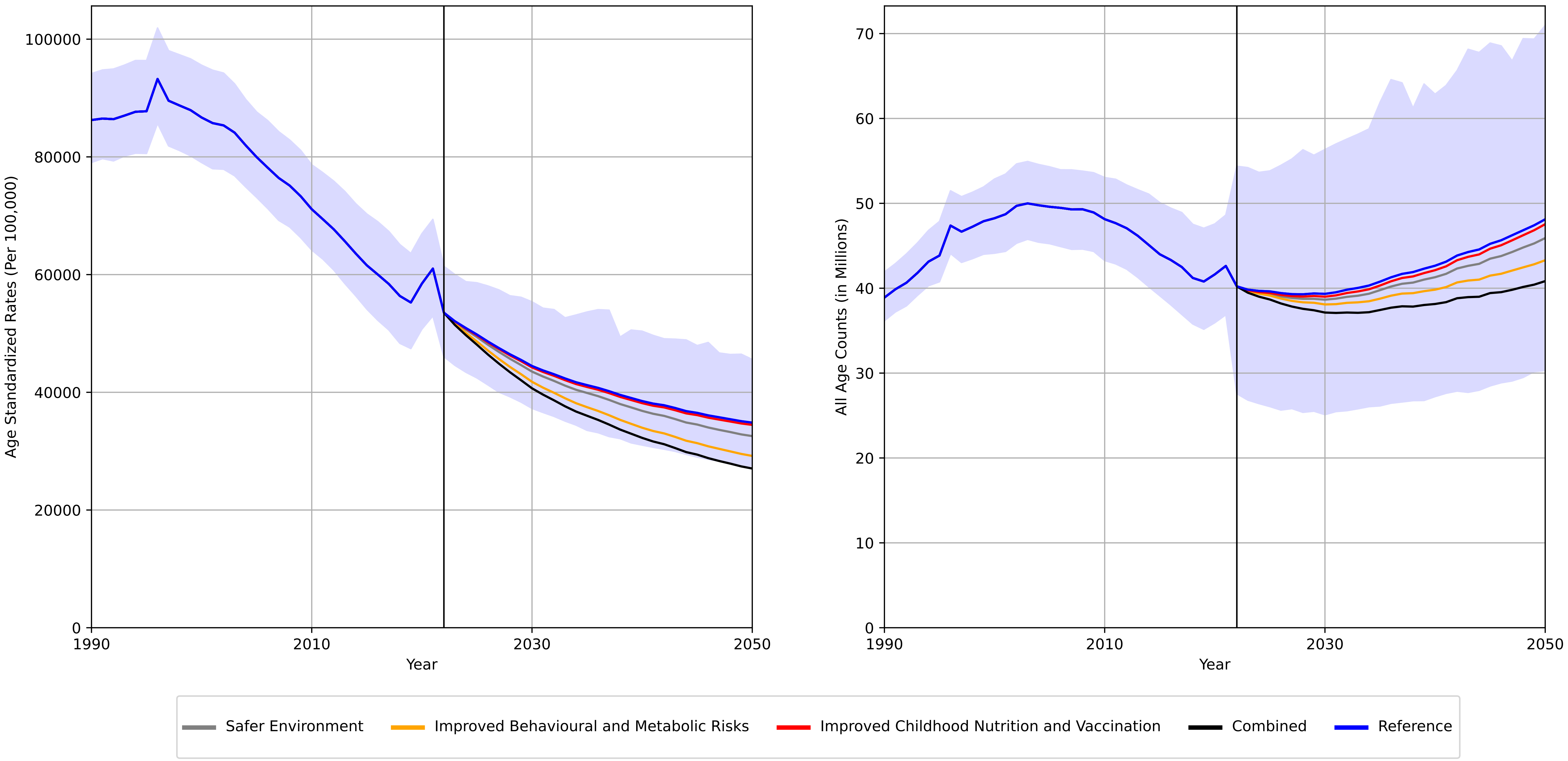
Age Standardized Rates (Per 100,000)
0
20000
40000
60000
80000
100000
1990
2010
2030
2050
Year
All Age Counts (in Millions)
0
10
20
30
40
50
60
70
1990
2010
2030
2050
Year
Safer Environment
Improved Behavioural and Metabolic Risks
Improved Childhood Nutrition and Vaccination
Combined
Reference

Equatorial Guinea

Supplemental Results Figure S10. All-cause age standardised DALY rates (A) and all age DALY counts (B), for the past and for five future scenarios, 1990–2050

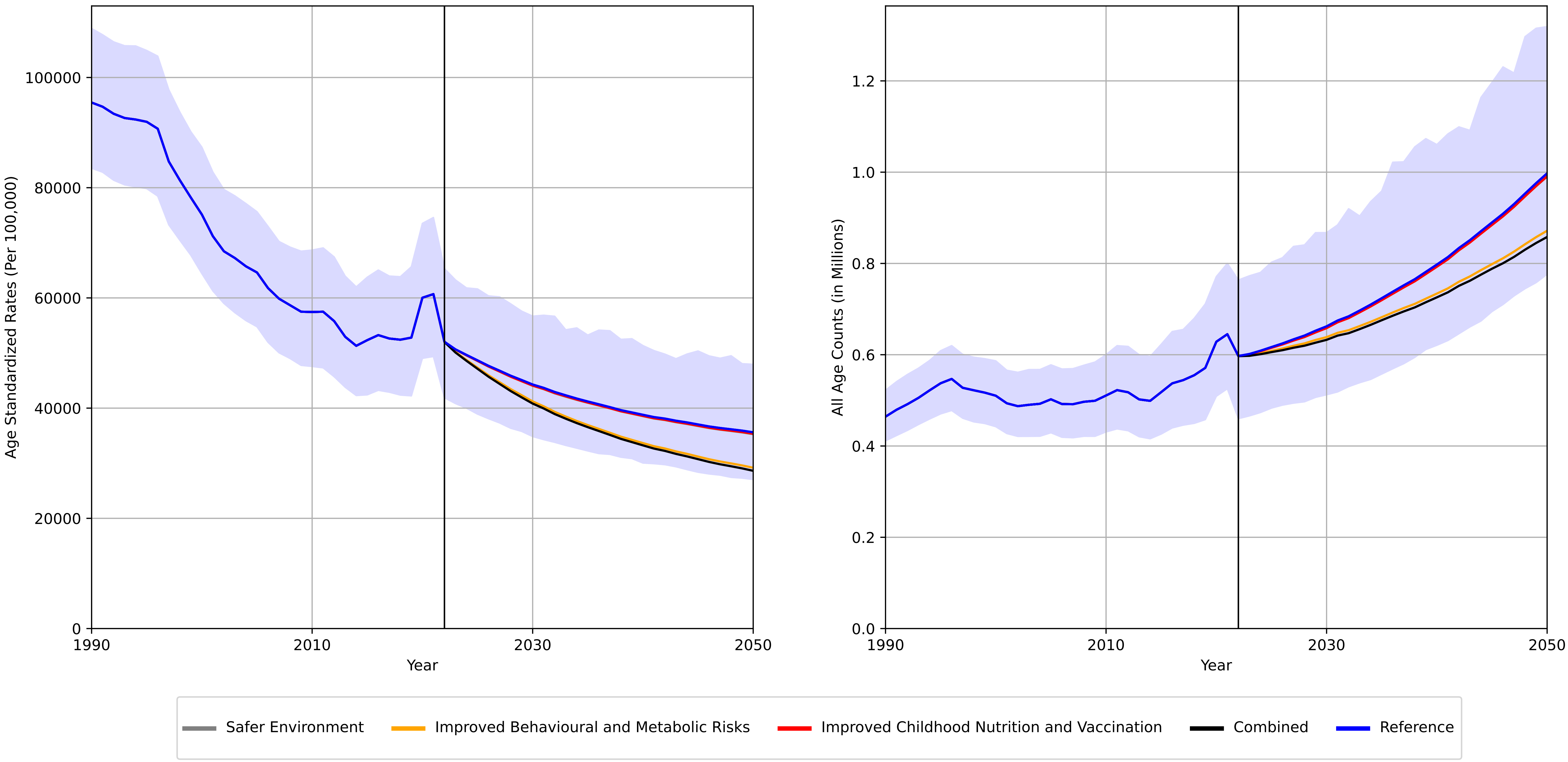

Gabon

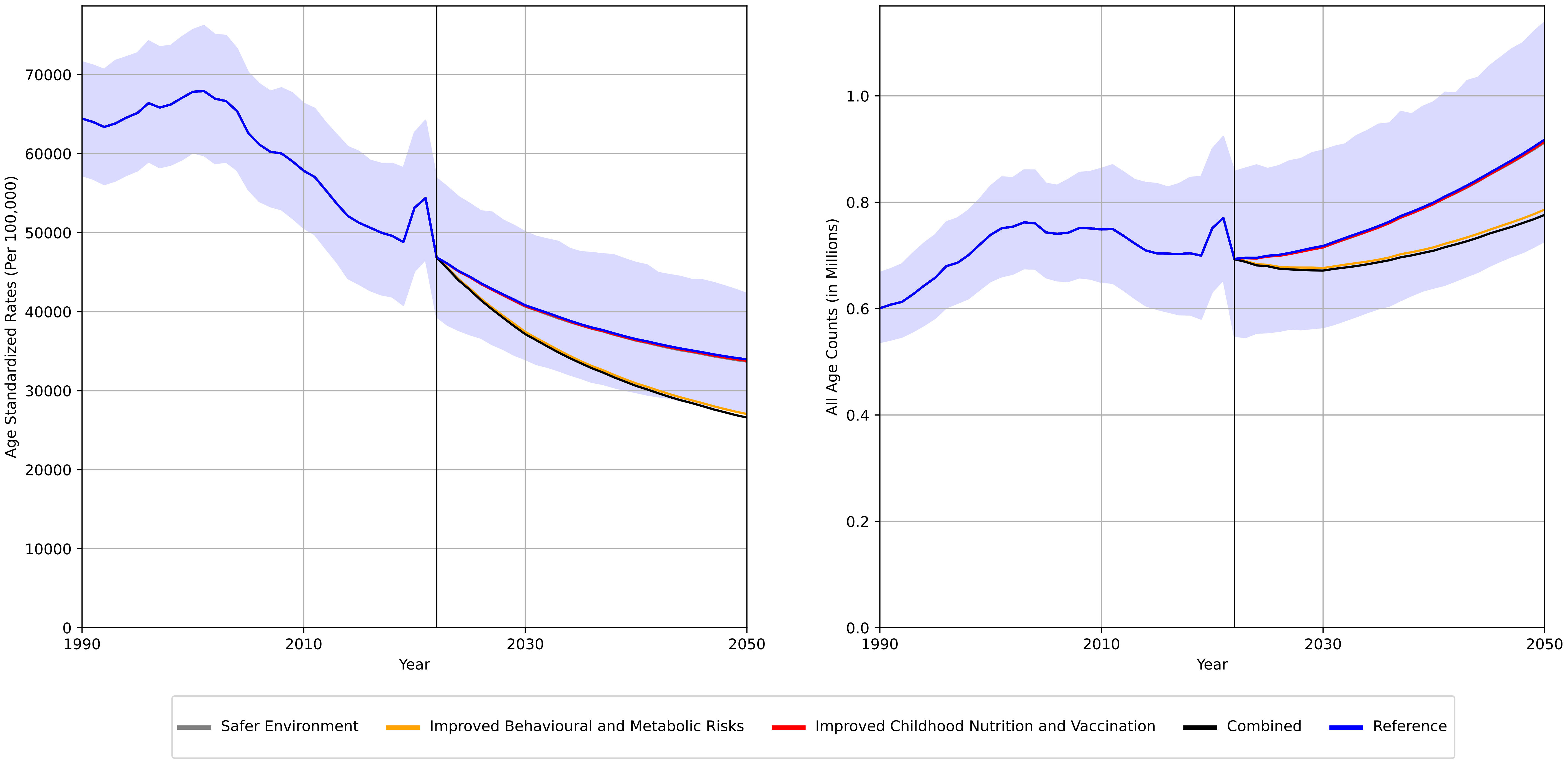

Age Standardized Rates (Per 100,000)
70000
60000
50000
40000
30000
20000
10000
0
1990
2010
2030
2050
Year
All Age Counts (in Millions)
1.0
0.8
0.6
0.4
0.2
0.0
1990
2010
2030
2050
Year
Safer Environment
Improved Behavioural and Metabolic Risks
Improved Childhood Nutrition and Vaccination
Combined
Reference

Burundi

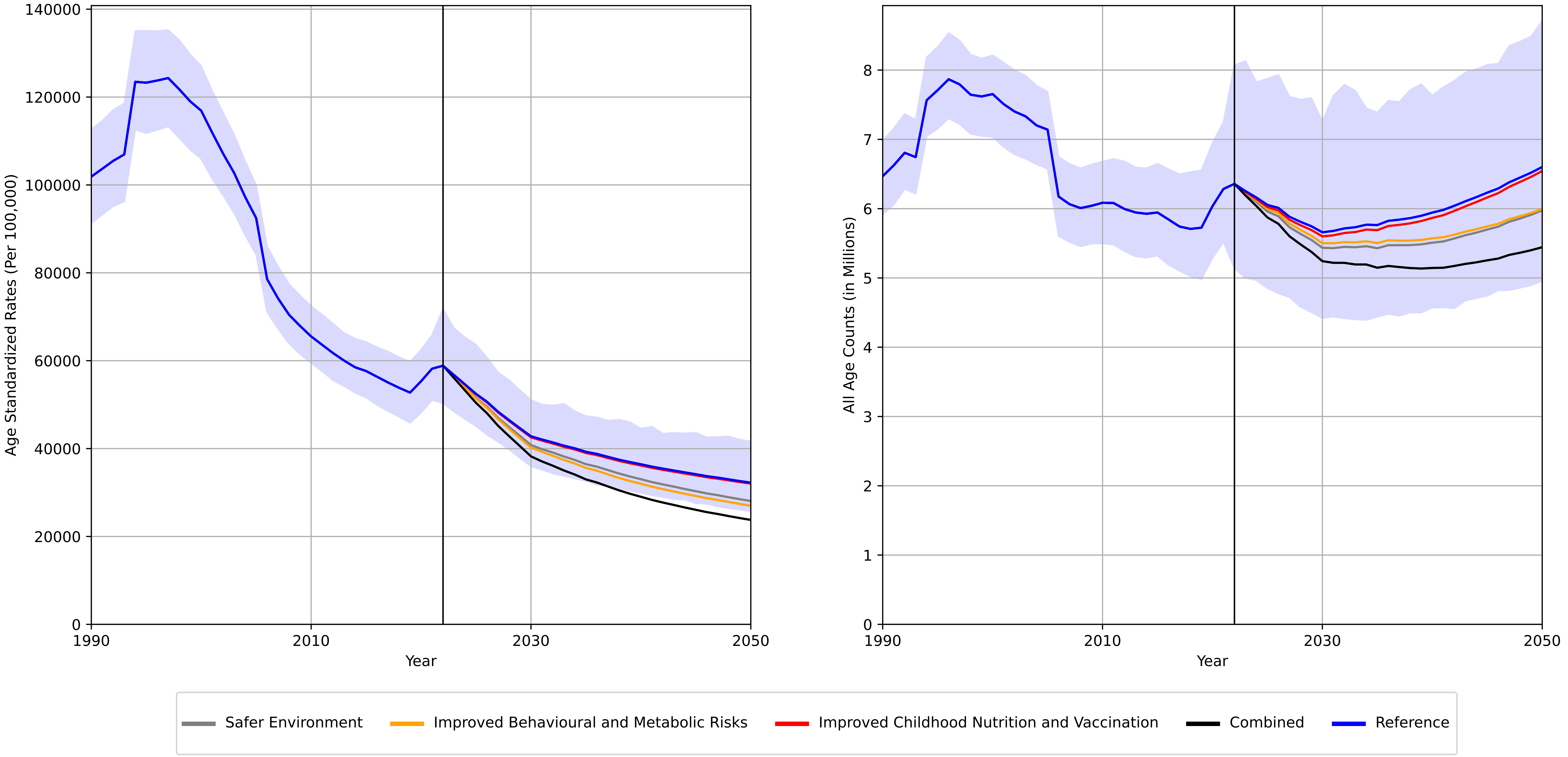
Age Standardized Rates (Per 100,000)
140000
120000
100000
80000
60000
40000
20000
0
1990
2010
2030
2050
Year
All Age Counts (in Millions)
8
7
6
5
4
3
2
1
0
1990
2010
2030
2050
Year
Safer Environment
Improved Behavioural and Metabolic Risks
Improved Childhood Nutrition and Vaccination
Combined
Reference

Comoros

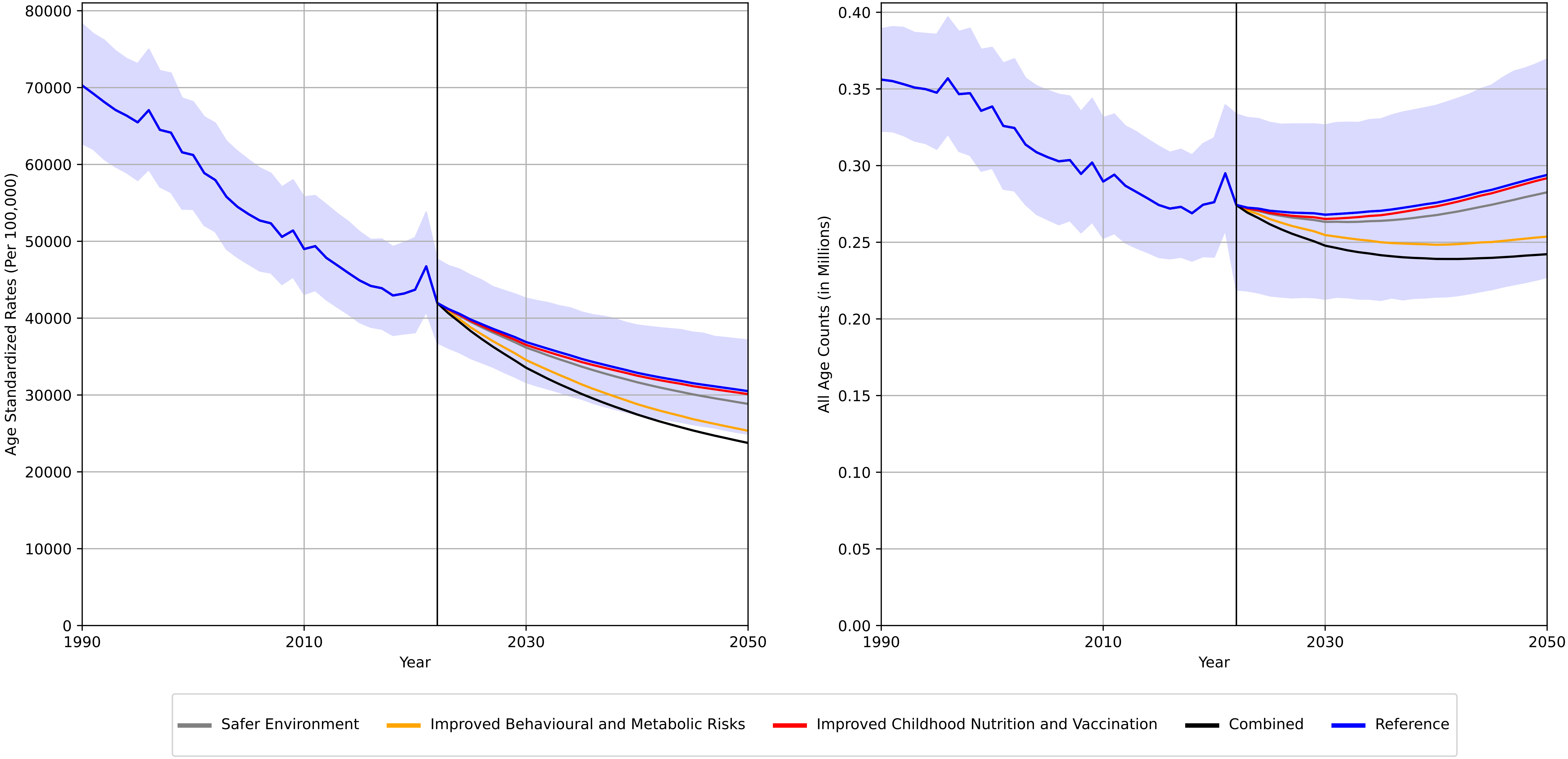

Age Standardized Rates (Per 100,000)
80000
70000
60000
50000
40000
30000
20000
10000
0
1990
2010
2030
2050
Year
All Age Counts (in Millions)
0.40
0.35
0.30
0.25
0.20
0.15
0.10
0.05
0.00
1990
2010
2030
2050
Year
Safer Environment
Improved Behavioural and Metabolic Risks
Improved Childhood Nutrition and Vaccination
Combined
Reference

Djibouti

Supplemental Results Figure S10. All-cause age standardised DALY rates (A) and all age DALY counts (B), for the past and for five future scenarios, 1990–2050

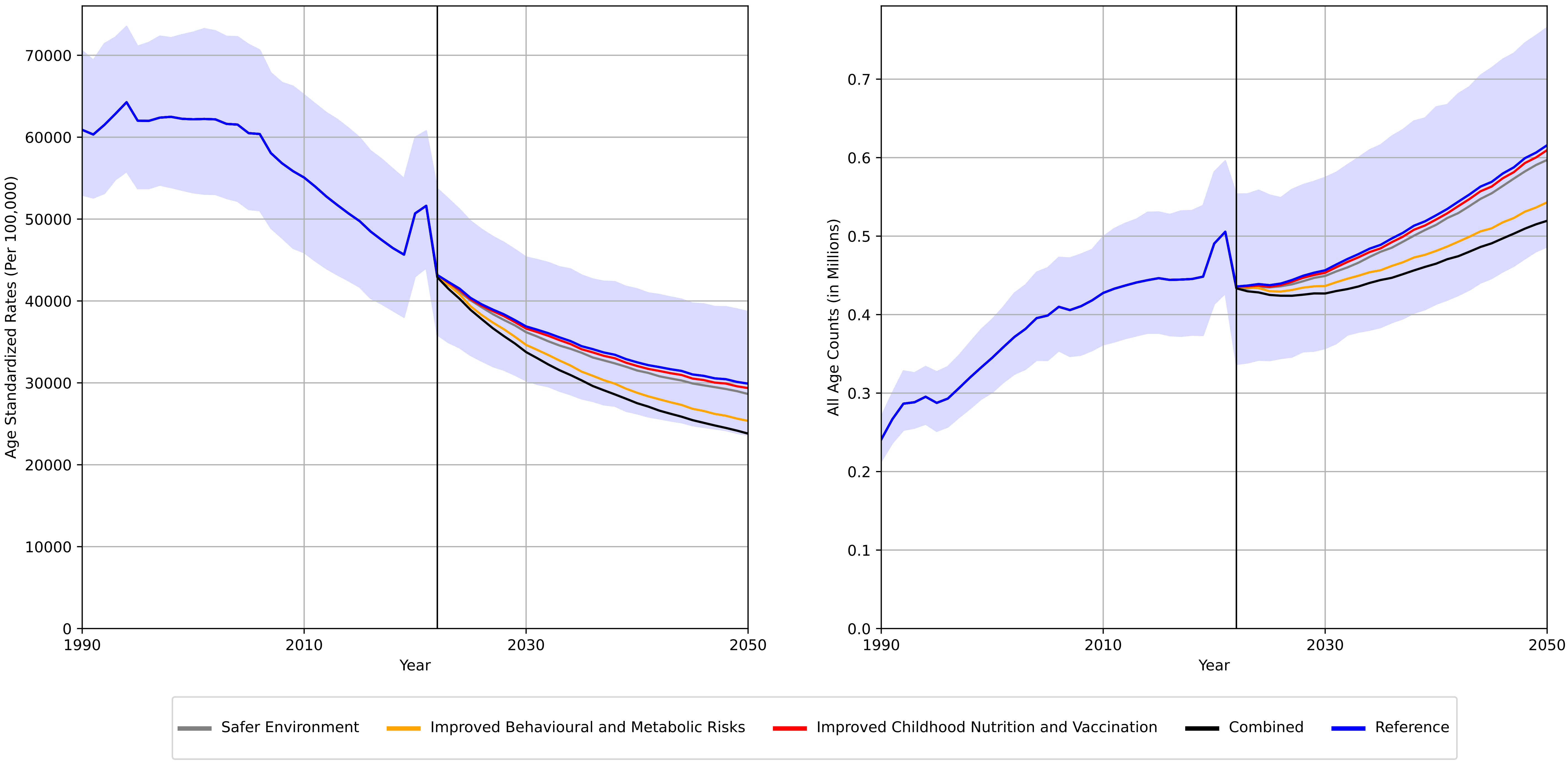

Eritrea

Supplemental Results Figure S10. All-cause age standardised DALY rates (A) and all age DALY counts (B), for the past and for five future scenarios, 1990–2050

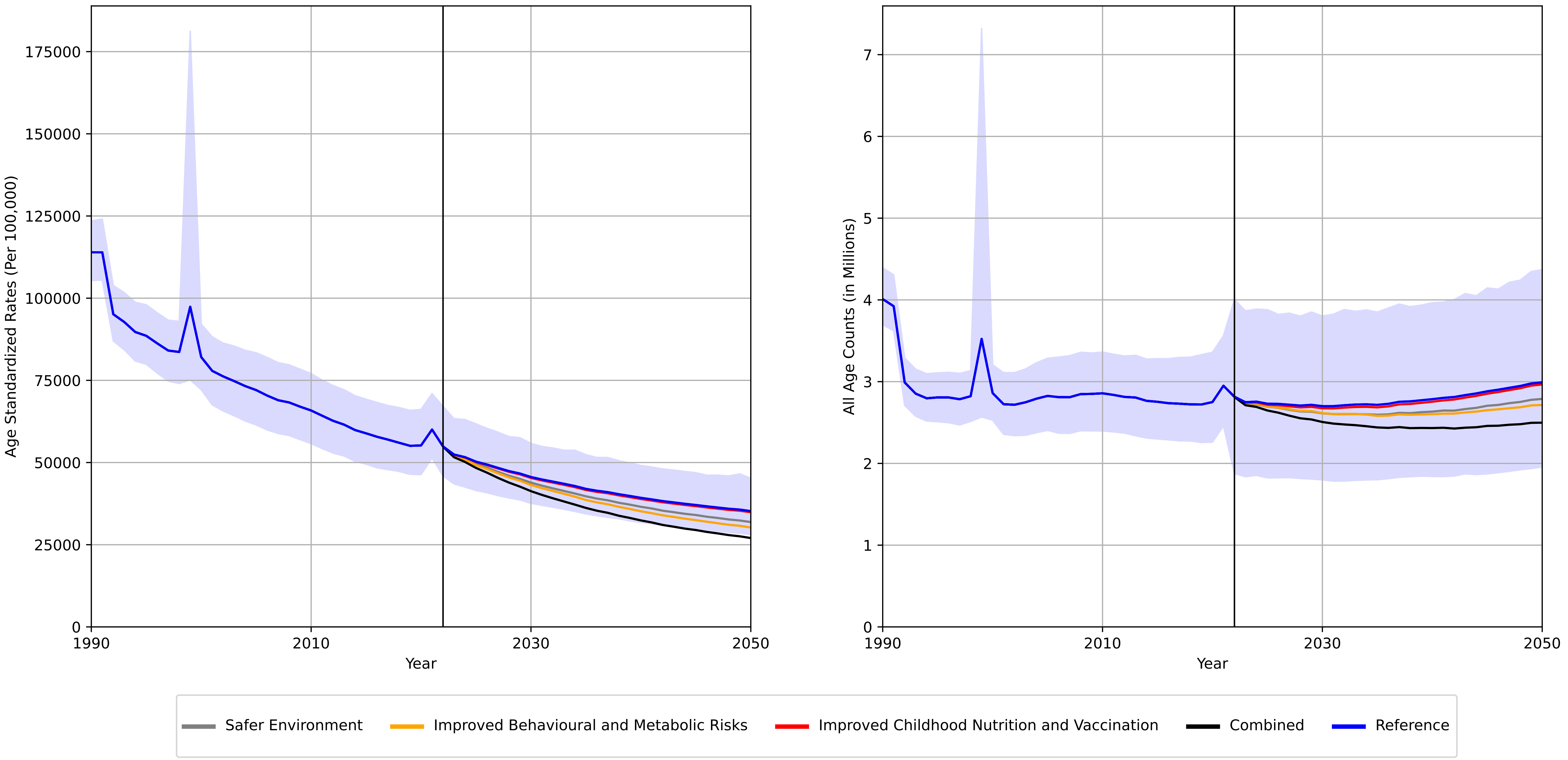

Ethiopia

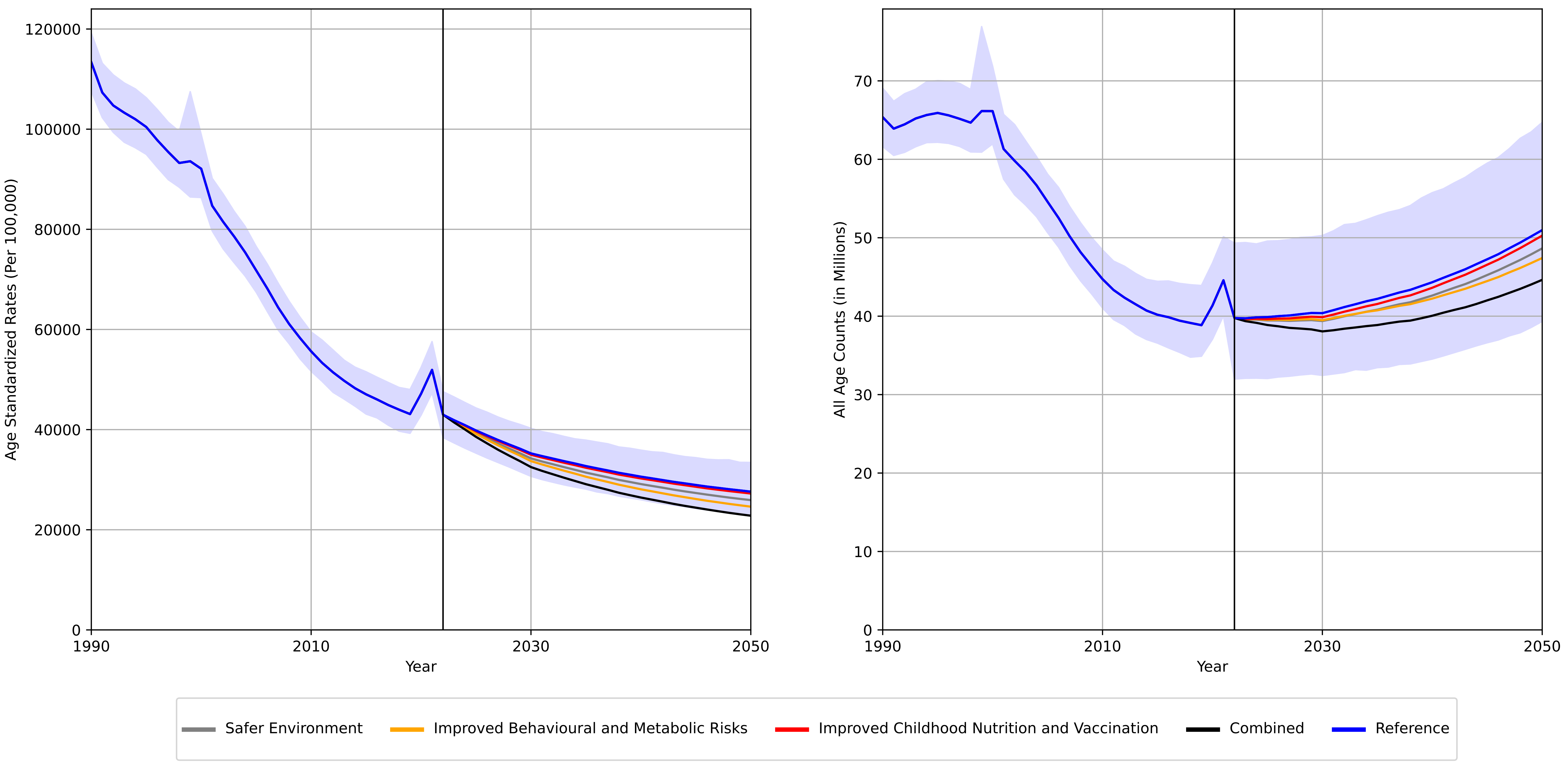
Age Standardized Rates (Per 100,000)
120000
100000
80000
60000
40000
20000
0
1990
2010
2030
2050
Year
All Age Counts (in Millions)
70
60
50
40
30
20
10
0
1990
2010
2030
2050
Year
Safer Environment
Improved Behavioural and Metabolic Risks
Improved Childhood Nutrition and Vaccination
Combined
Reference

Kenya

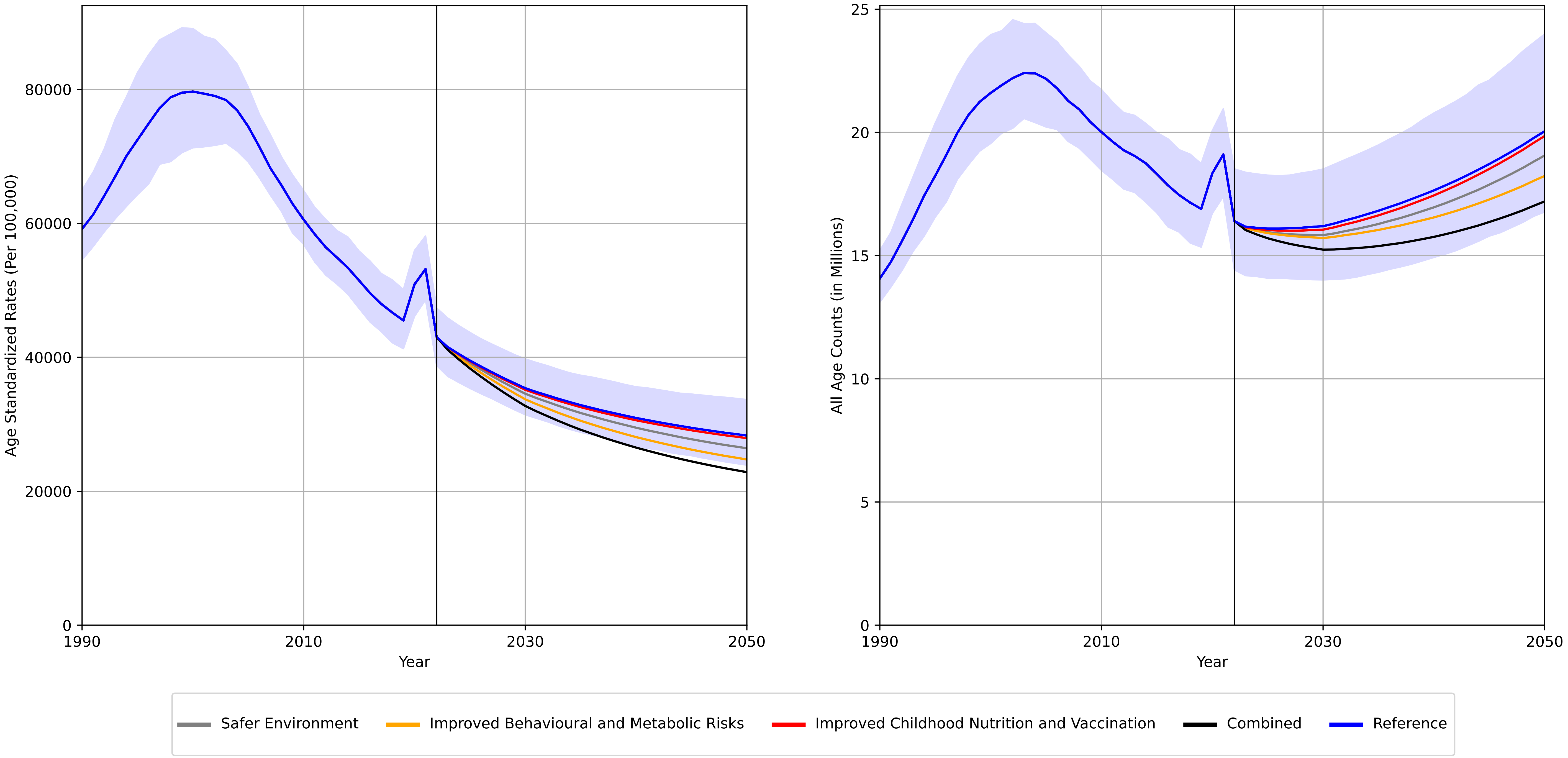
Age Standardized Rates (Per 100,000)
0
20000
40000
60000
80000
1990
2010
2030
2050
Year
All Age Counts (in Millions)
0
5
10
15
20
25
1990
2010
2030
2050
Year
Safer Environment
Improved Behavioural and Metabolic Risks
Improved Childhood Nutrition and Vaccination
Combined
Reference

Madagascar

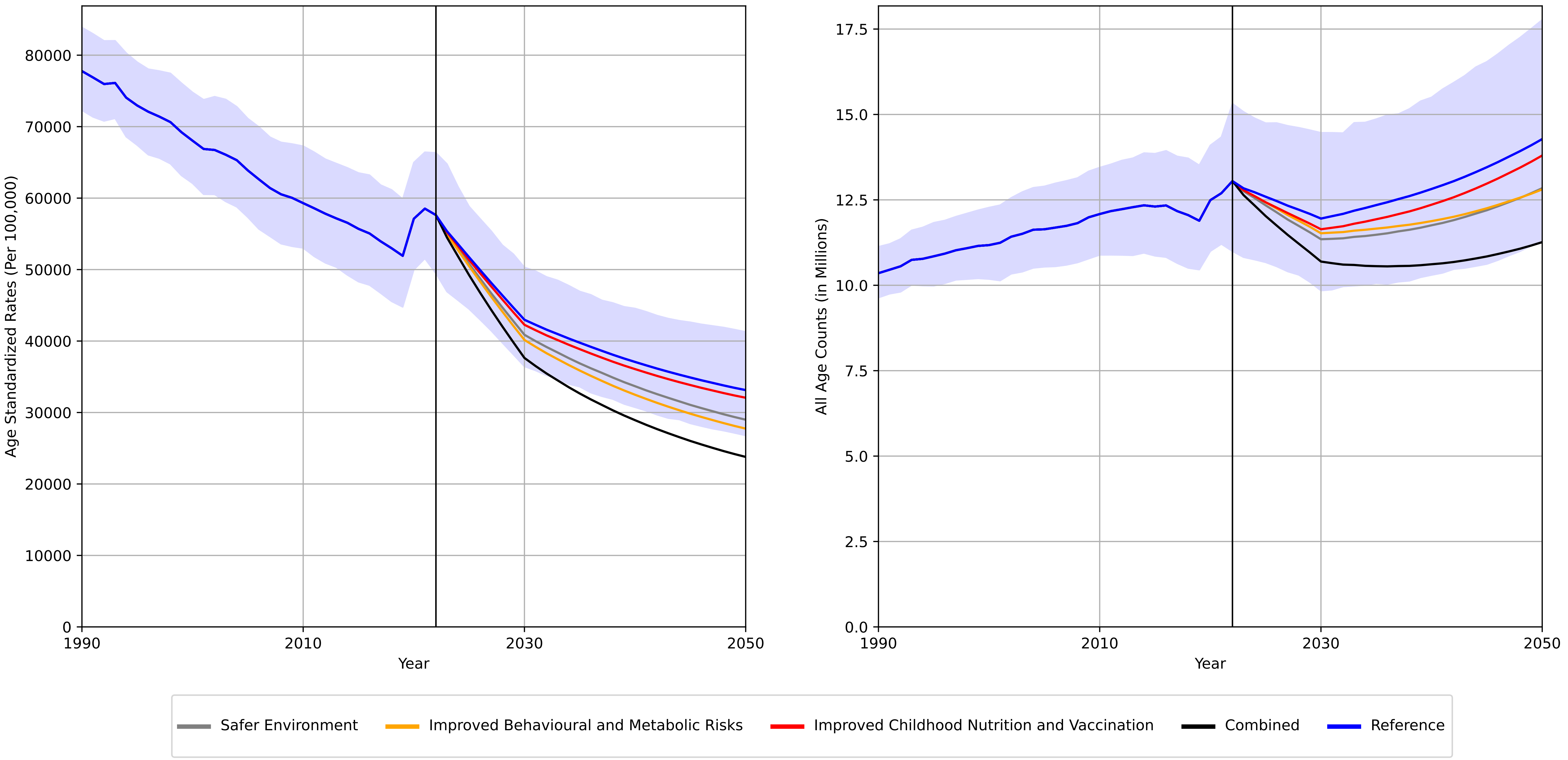
Age Standardized Rates (Per 100,000)
0
10000
20000
30000
40000
50000
60000
70000
80000
1990
2010
2030
2050
Year
All Age Counts (in Millions)
0.0
2.5
5.0
7.5
10.0
12.5
15.0
17.5
1990
2010
2030
2050
Year
Safer Environment
Improved Behavioural and Metabolic Risks
Improved Childhood Nutrition and Vaccination
Combined
Reference

Malawi

Supplemental Results Figure S10. All-cause age standardised DALY rates (A) and all age DALY counts (B), for the past and for five future scenarios, 1990–2050

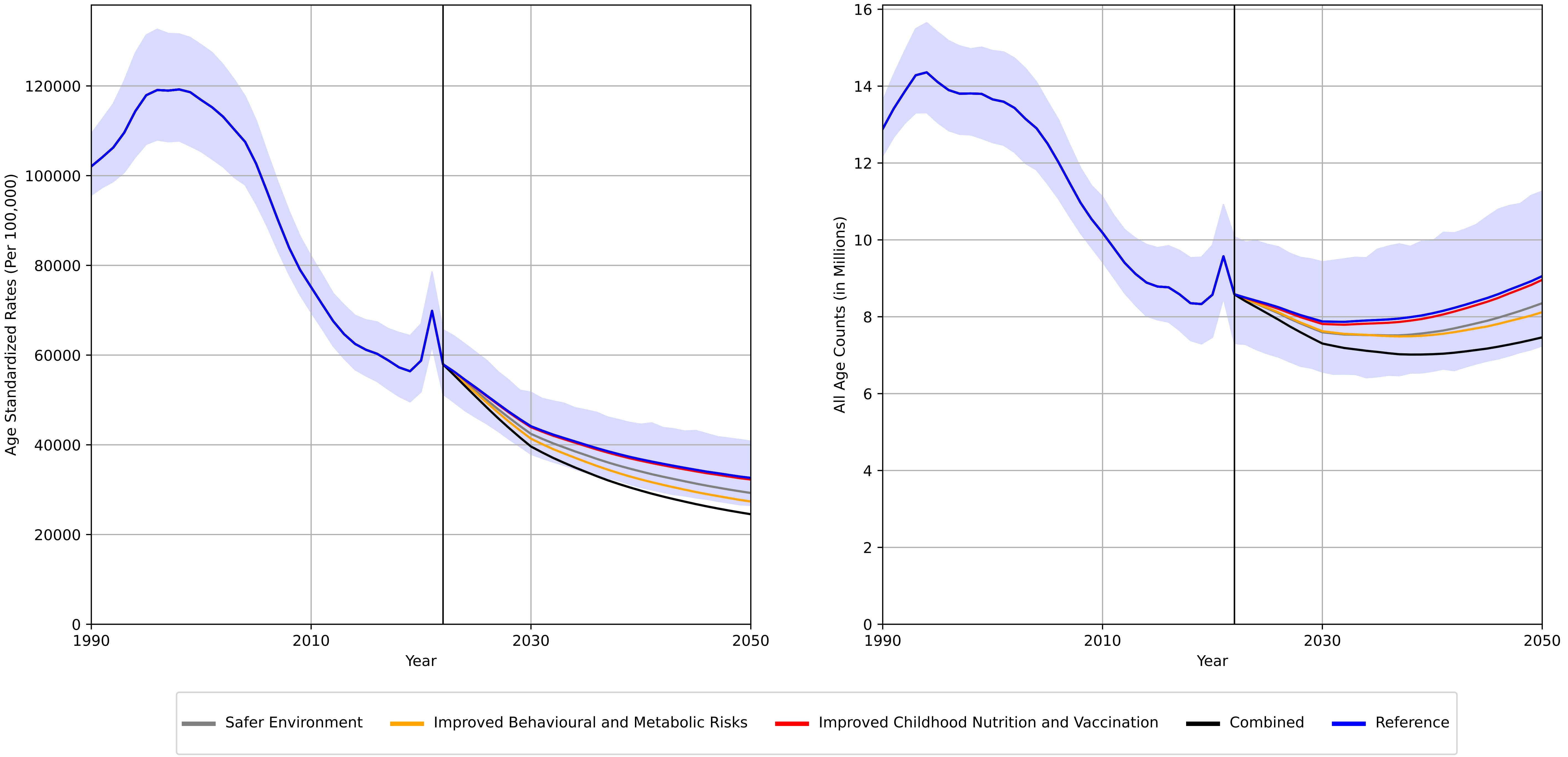

Mozambique

Supplemental Results Figure S10. All-cause age standardised DALY rates (A) and all age DALY counts (B), for the past and for five future scenarios, 1990–2050

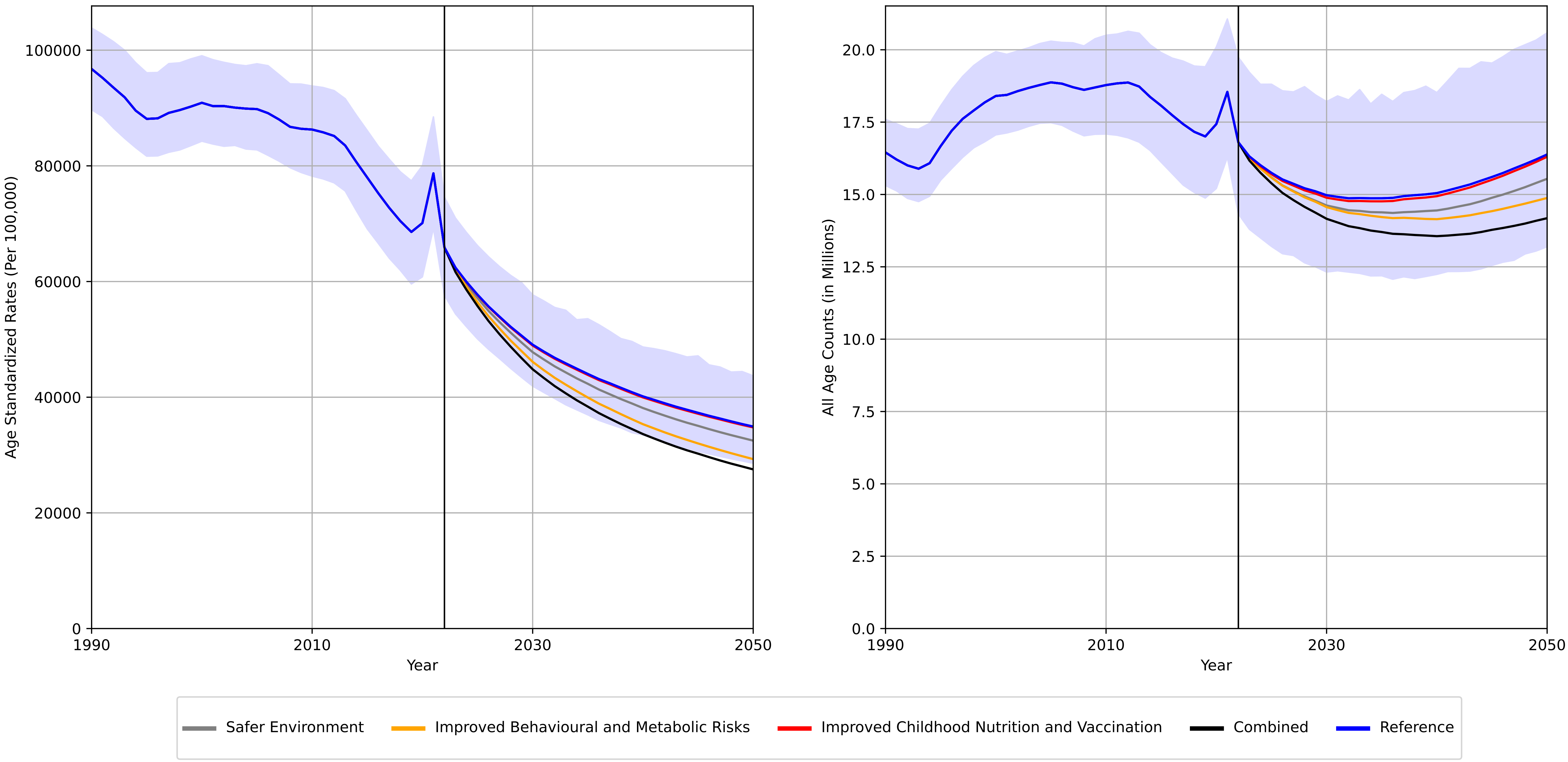

Rwanda

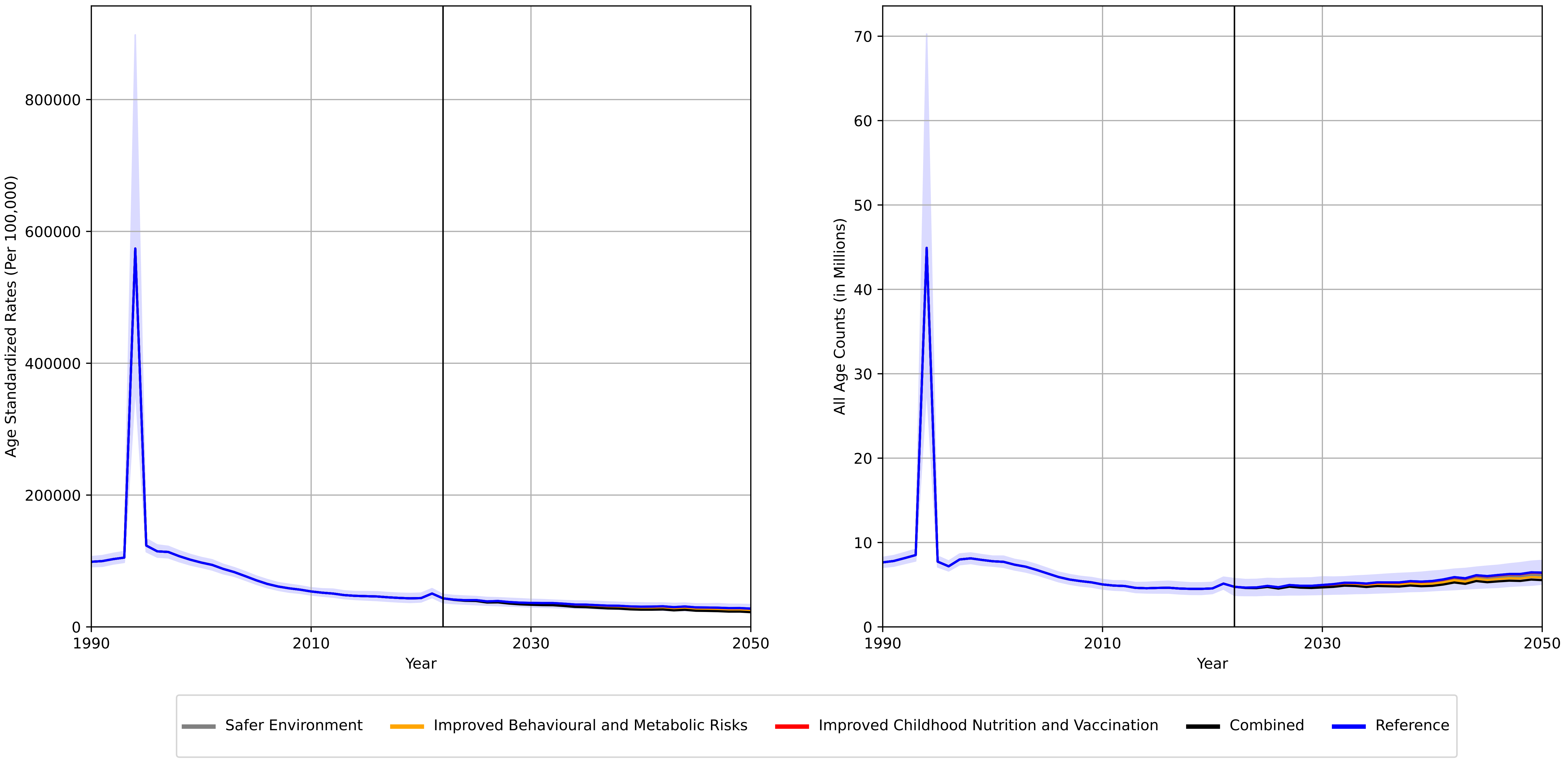
Age Standardized Rates (Per 100,000)
800000
600000
400000
200000
0
1990
2010
2030
2050
Year
All Age Counts (in Millions)
70
60
50
40
30
20
10
0
1990
2010
2030
2050
Year
Safer Environment
Improved Behavioural and Metabolic Risks
Improved Childhood Nutrition and Vaccination
Combined
Reference

Somalia

Supplemental Results Figure S10. All-cause age standardised DALY rates (A) and all age DALY counts (B), for the past and for five future scenarios, 1990–2050

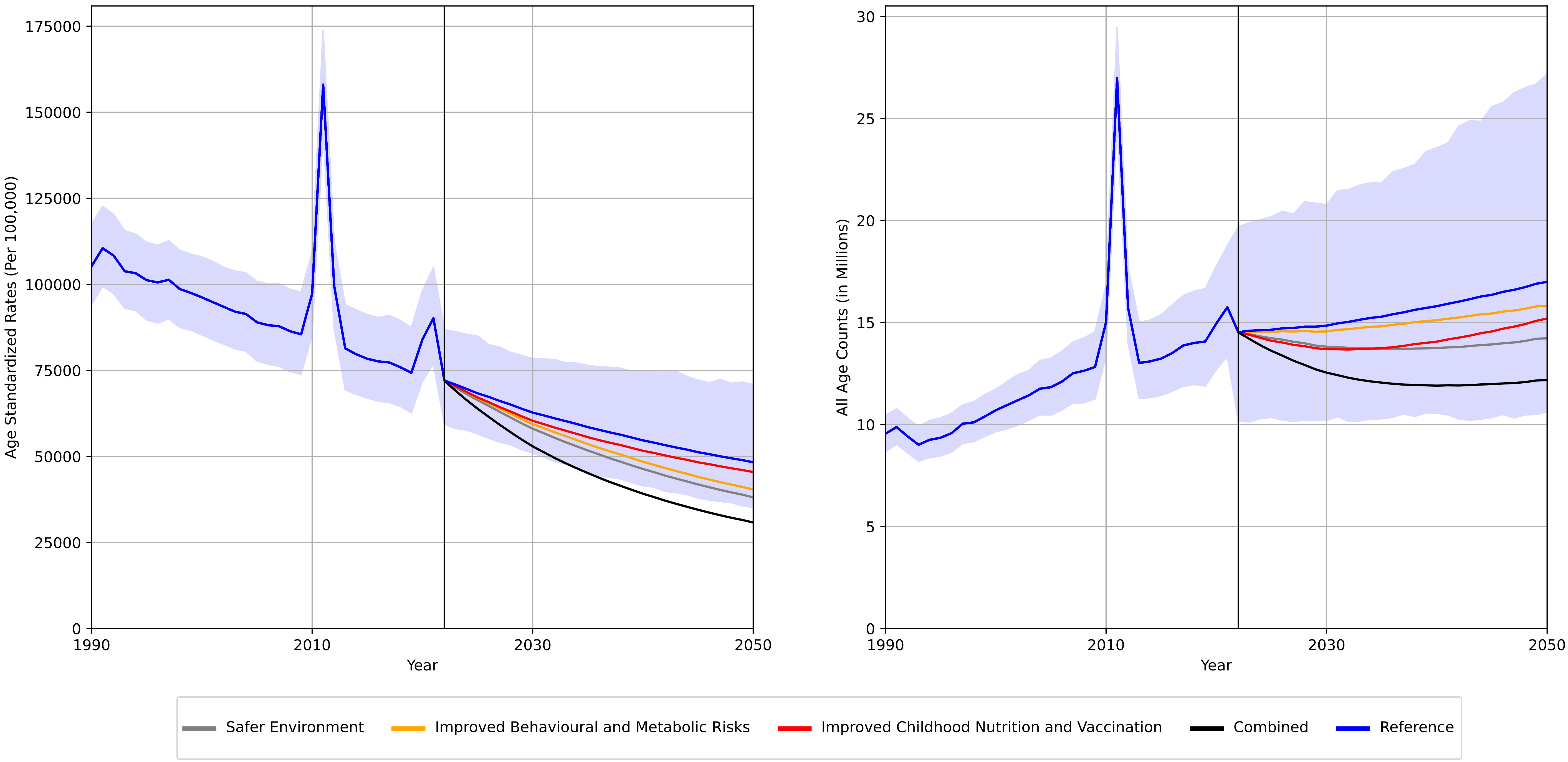

South Sudan

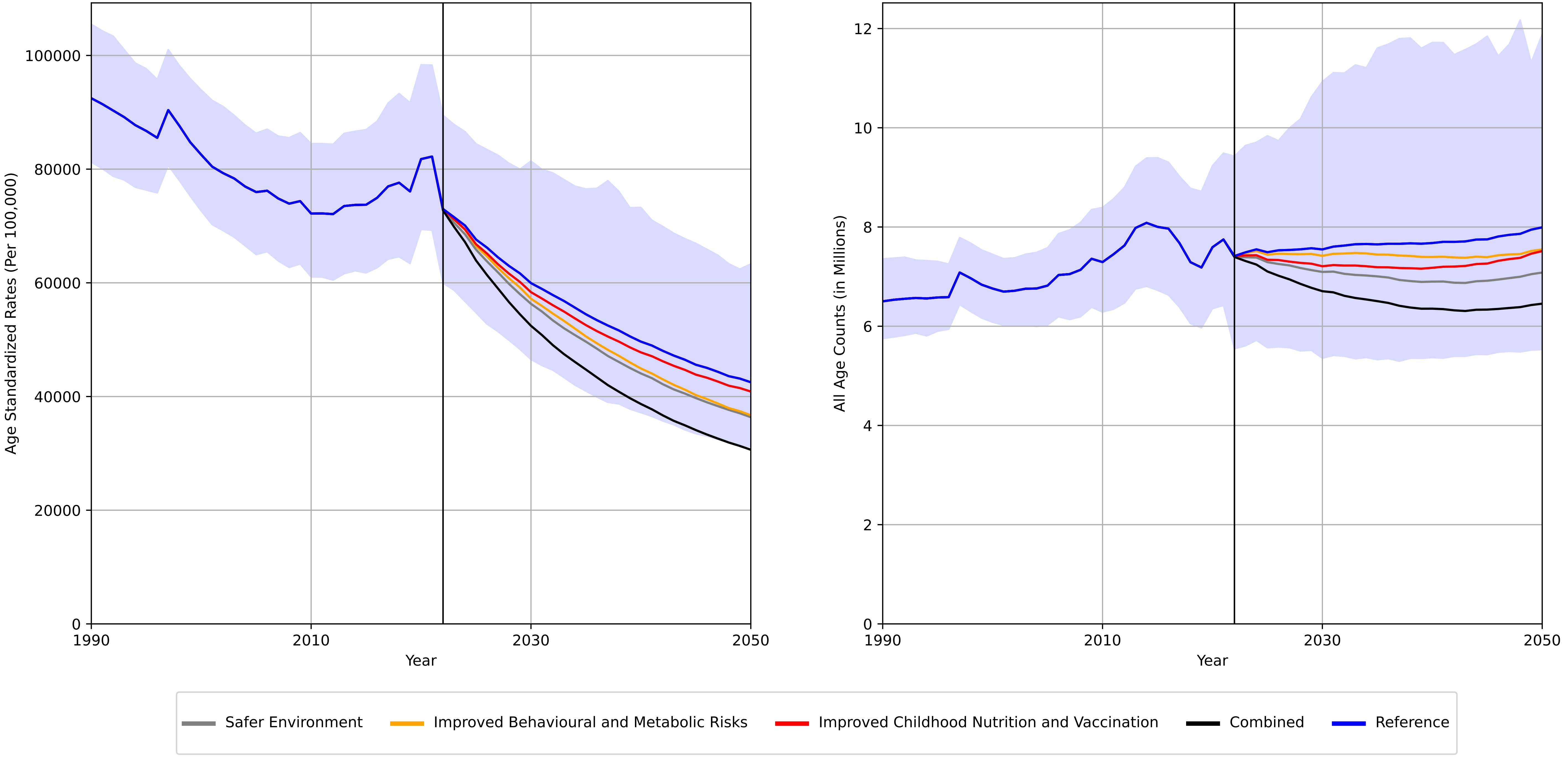

Age Standardized Rates (Per 100,000)
100000
80000
60000
40000
20000
0
1990
2010
2030
2050
Year
All Age Counts (in Millions)
12
10
8
6
4
2
0
1990
2010
2030
2050
Year
Safer Environment
Improved Behavioural and Metabolic Risks
Improved Childhood Nutrition and Vaccination
Combined
Reference

Uganda

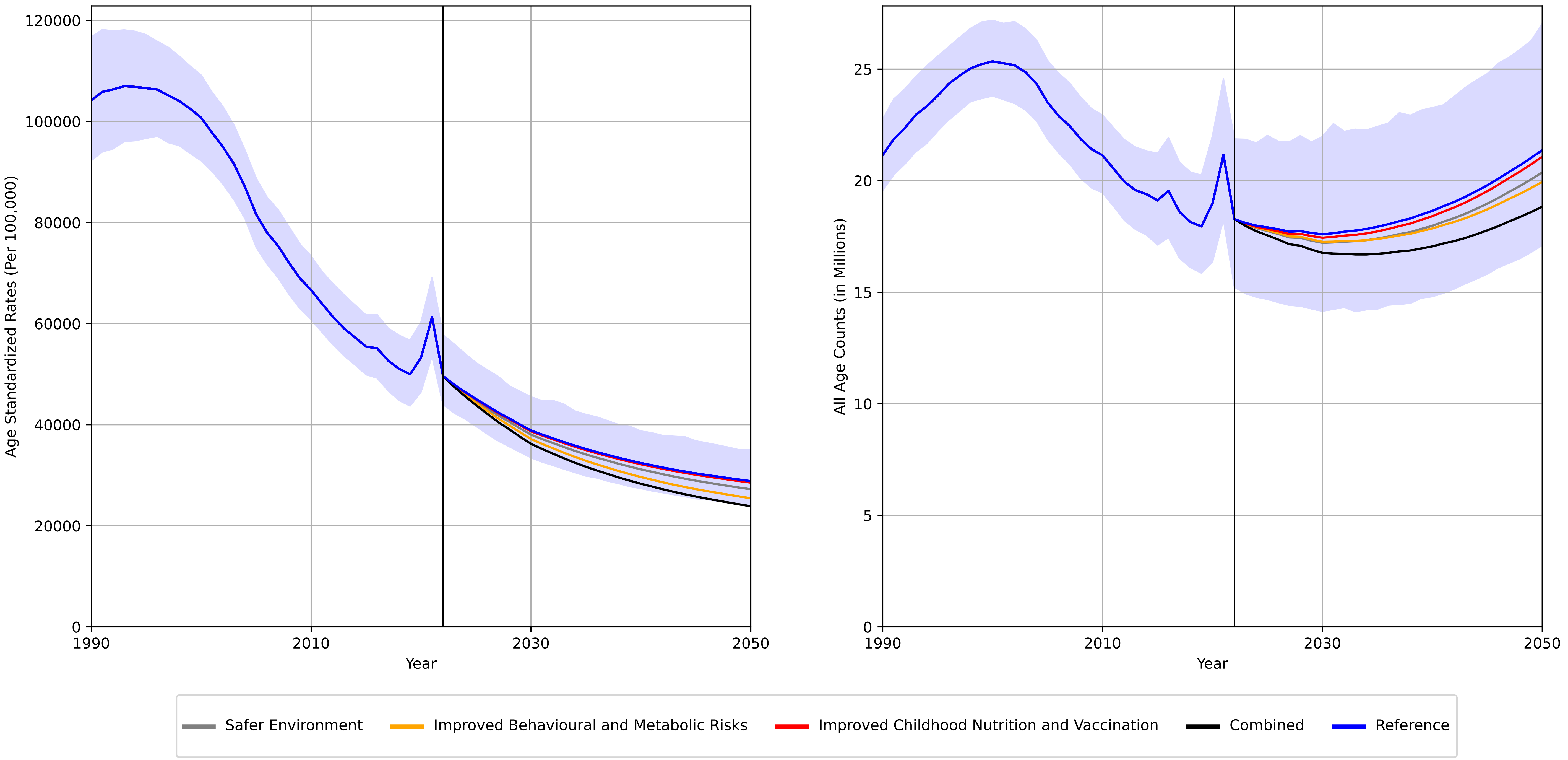

Age Standardized Rates (Per 100,000)
120000
100000
80000
60000
40000
20000
0
1990
2010
2030
2050
Year
All Age Counts (in Millions)
25
20
15
10
5
0
Safer Environment
Improved Behavioural and Metabolic Risks
Improved Childhood Nutrition and Vaccination
Combined
Reference

Tanzania

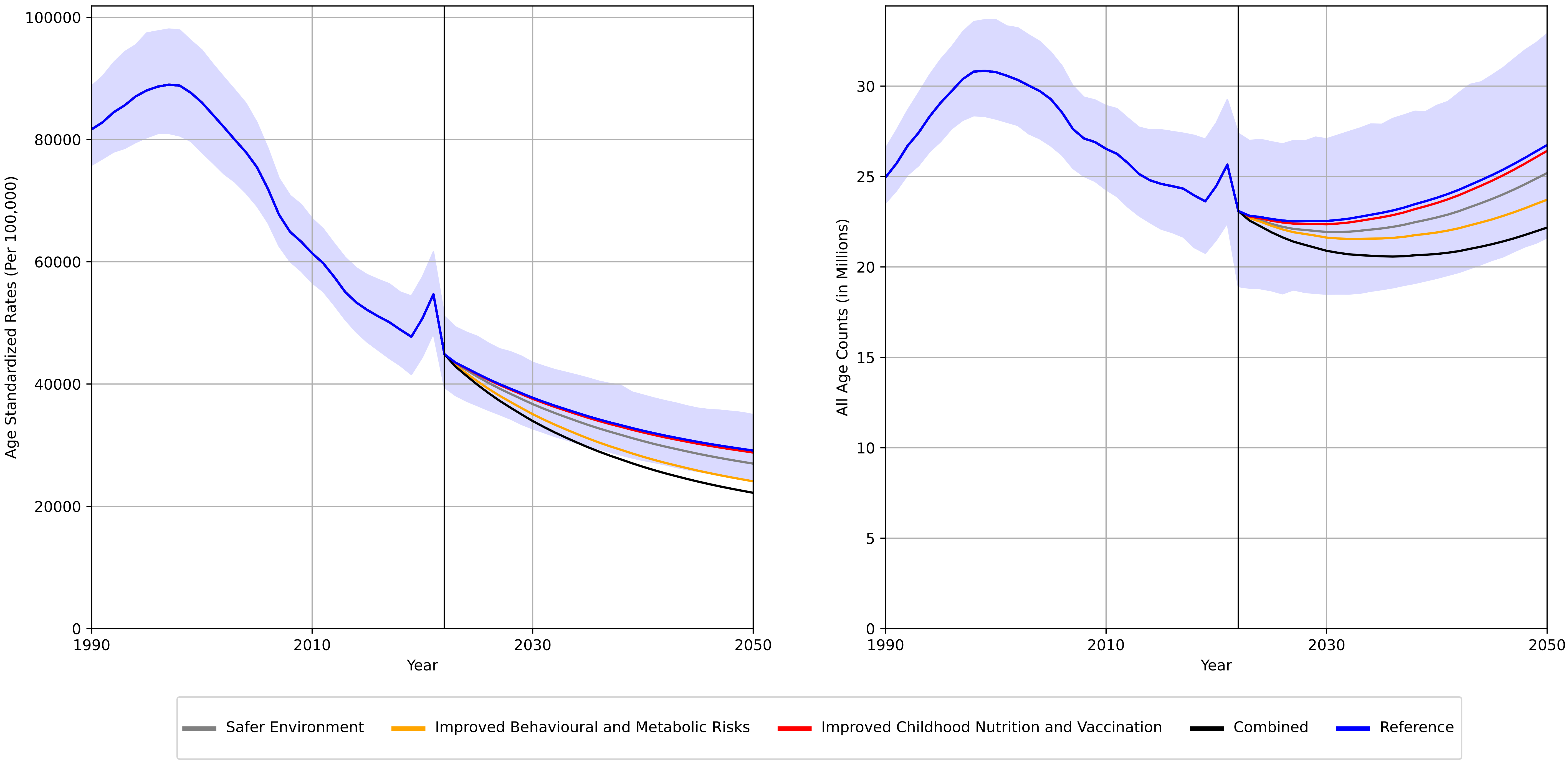

Age Standardized Rates (Per 100,000)
100000
80000
60000
40000
20000
0
1990
2010
2030
2050
Year
All Age Counts (in Millions)
30
25
20
15
10
5
0
1990
2010
2030
2050
Year
Safer Environment
Improved Behavioural and Metabolic Risks
Improved Childhood Nutrition and Vaccination
Combined
Reference

Zambia

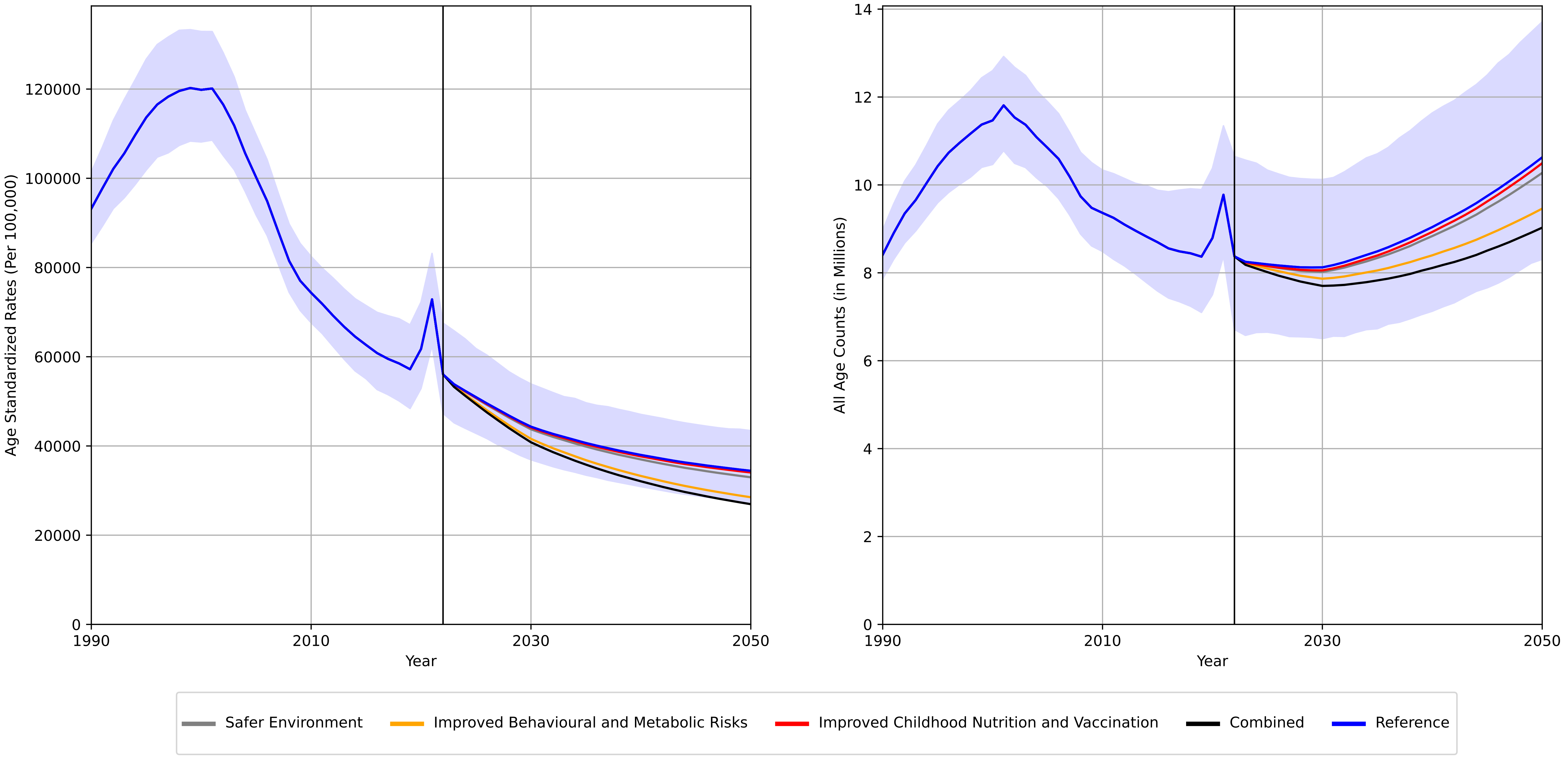
Age Standardized Rates (Per 100,000)
120000
100000
80000
60000
40000
20000
0
1990
2010
2030
2050
Year
All Age Counts (in Millions)
14
12
10
8
6
4
2
0
1990
2010
2030
2050
Year
Safer Environment
Improved Behavioural and Metabolic Risks
Improved Childhood Nutrition and Vaccination
Combined
Reference

Botswana

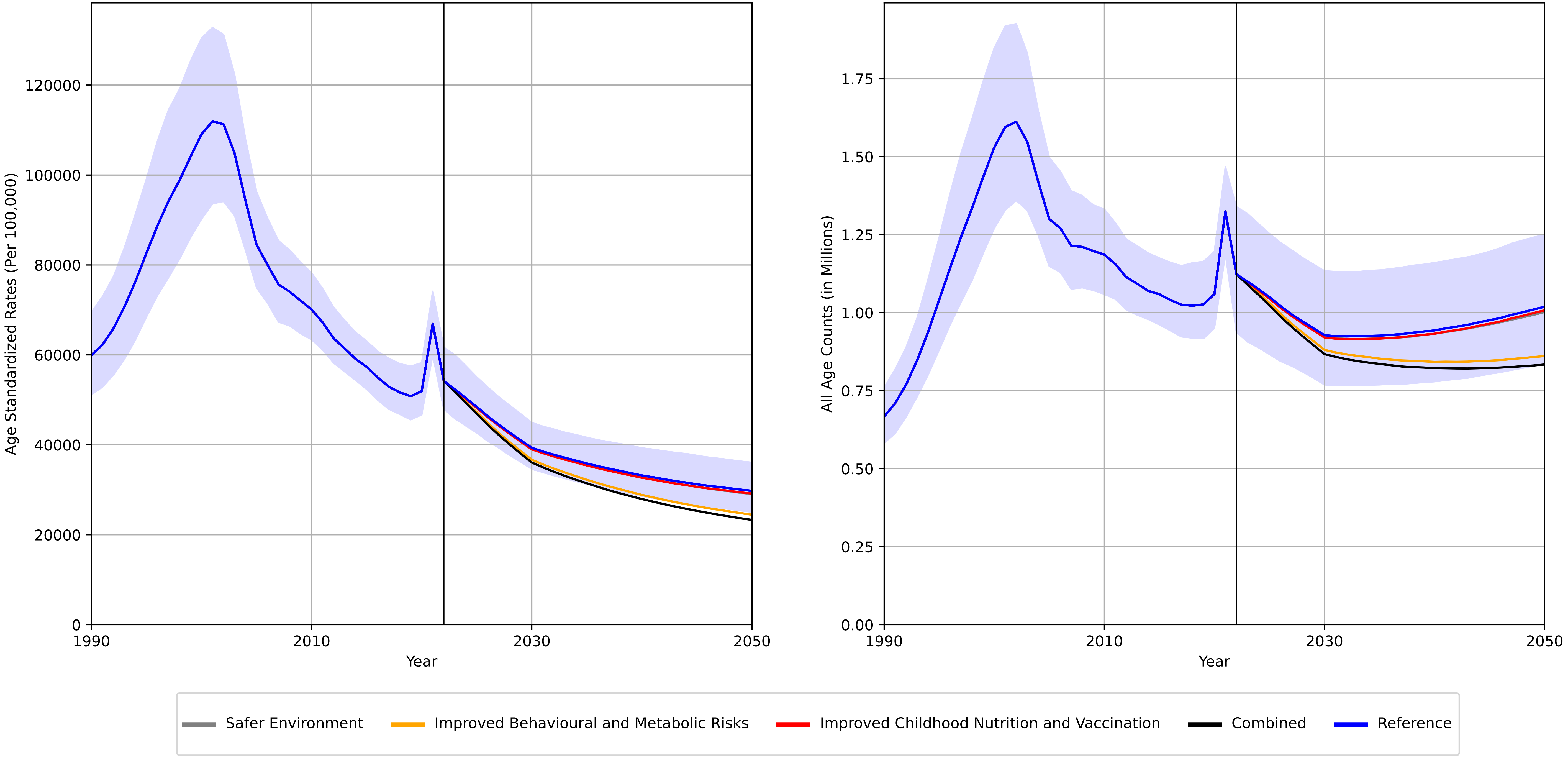
Age Standardized Rates (Per 100,000)
120000
100000
80000
60000
40000
20000
0
1990
2010
2030
2050
Year
All Age Counts (in Millions)
1.75
1.50
1.25
1.00
0.75
0.50
0.25
0.00
1990
2010
2030
2050
Year
Safer Environment
Improved Behavioural and Metabolic Risks
Improved Childhood Nutrition and Vaccination
Combined
Reference

Eswatini

Supplemental Results Figure S10. All-cause age standardised DALY rates (A) and all age DALY counts (B), for the past and for five future scenarios, 1990–2050

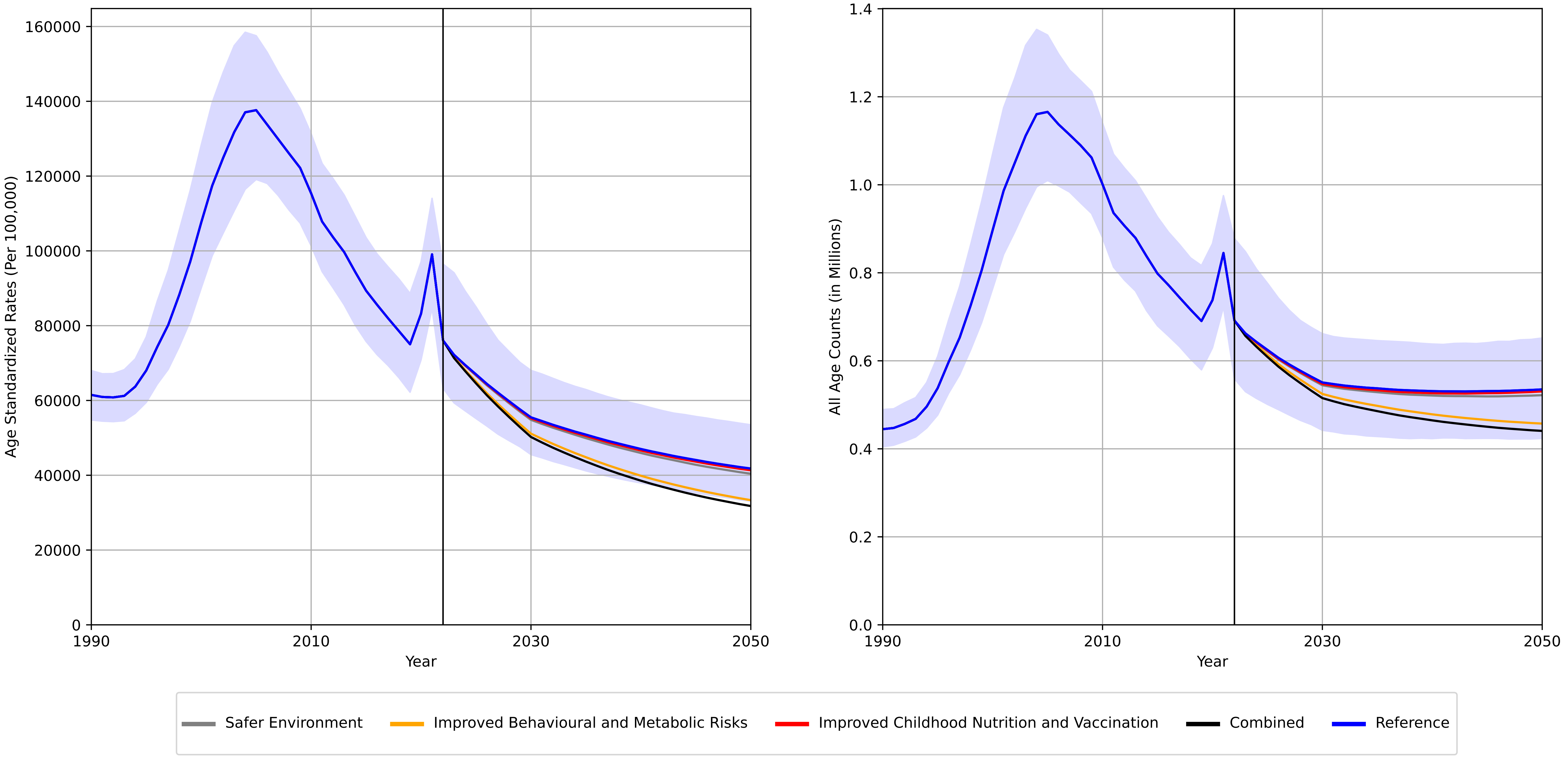

Lesotho

Supplemental Results Figure S10. All-cause age standardised DALY rates (A) and all age DALY counts (B), for the past and for five future scenarios, 1990–2050

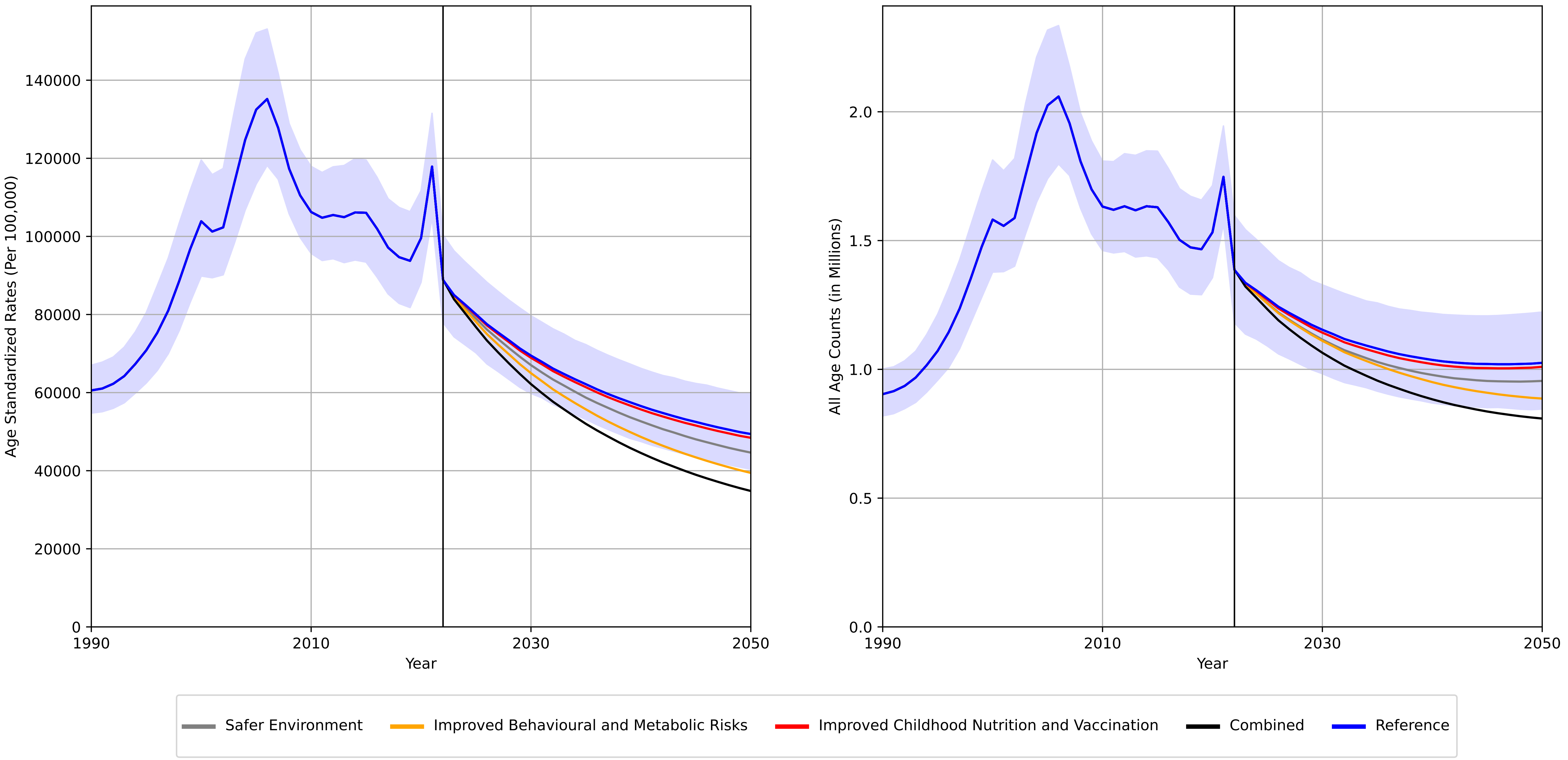

Namibia

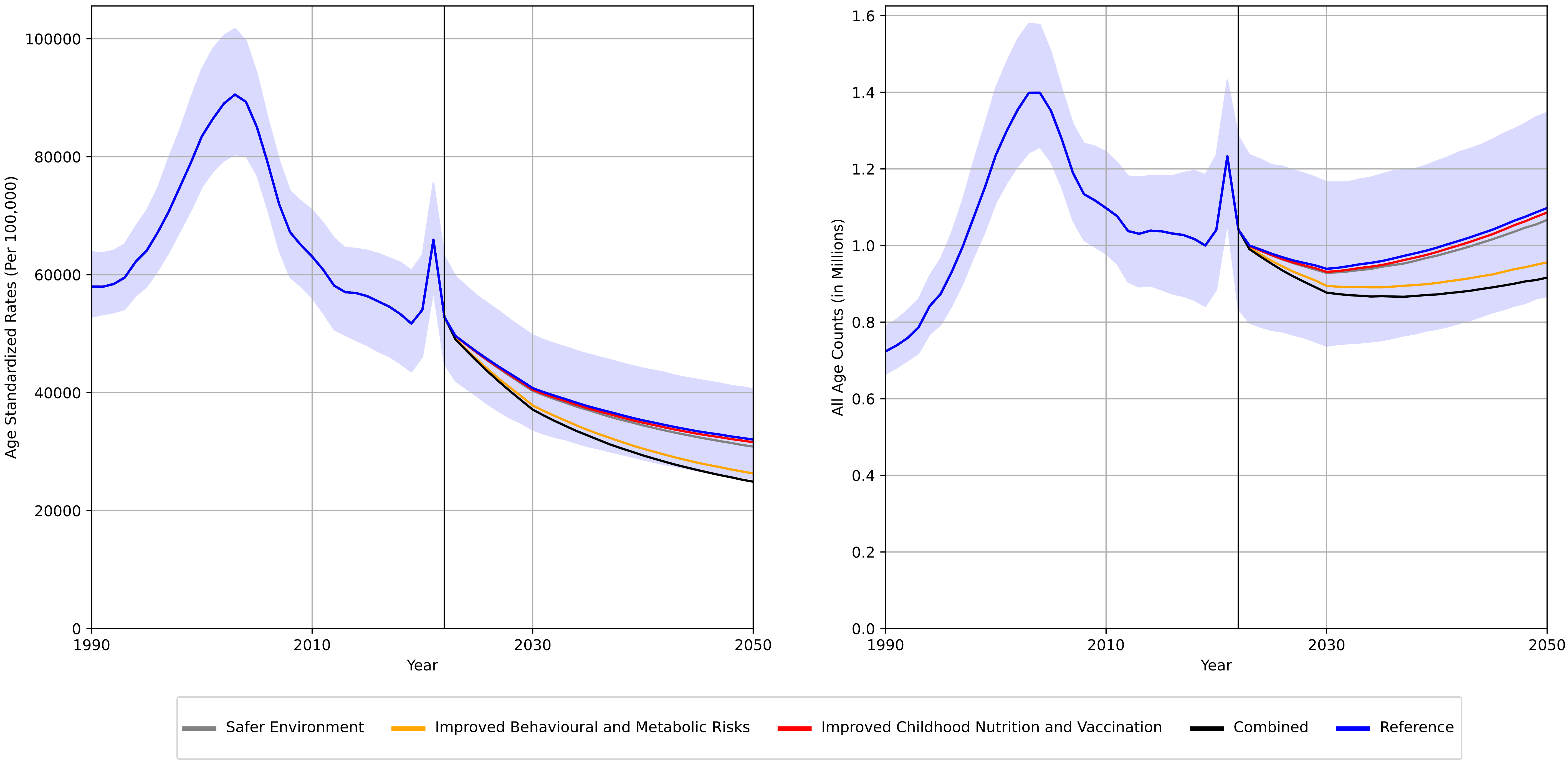
Age Standardized Rates (Per 100,000)
100000
80000
60000
40000
20000
0
1990
2010
2030
2050
Year
All Age Counts (in Millions)
1.6
1.4
1.2
1.0
0.8
0.6
0.4
0.2
0.0
Safer Environment
Improved Behavioural and Metabolic Risks
Improved Childhood Nutrition and Vaccination
Combined
Reference

South Africa

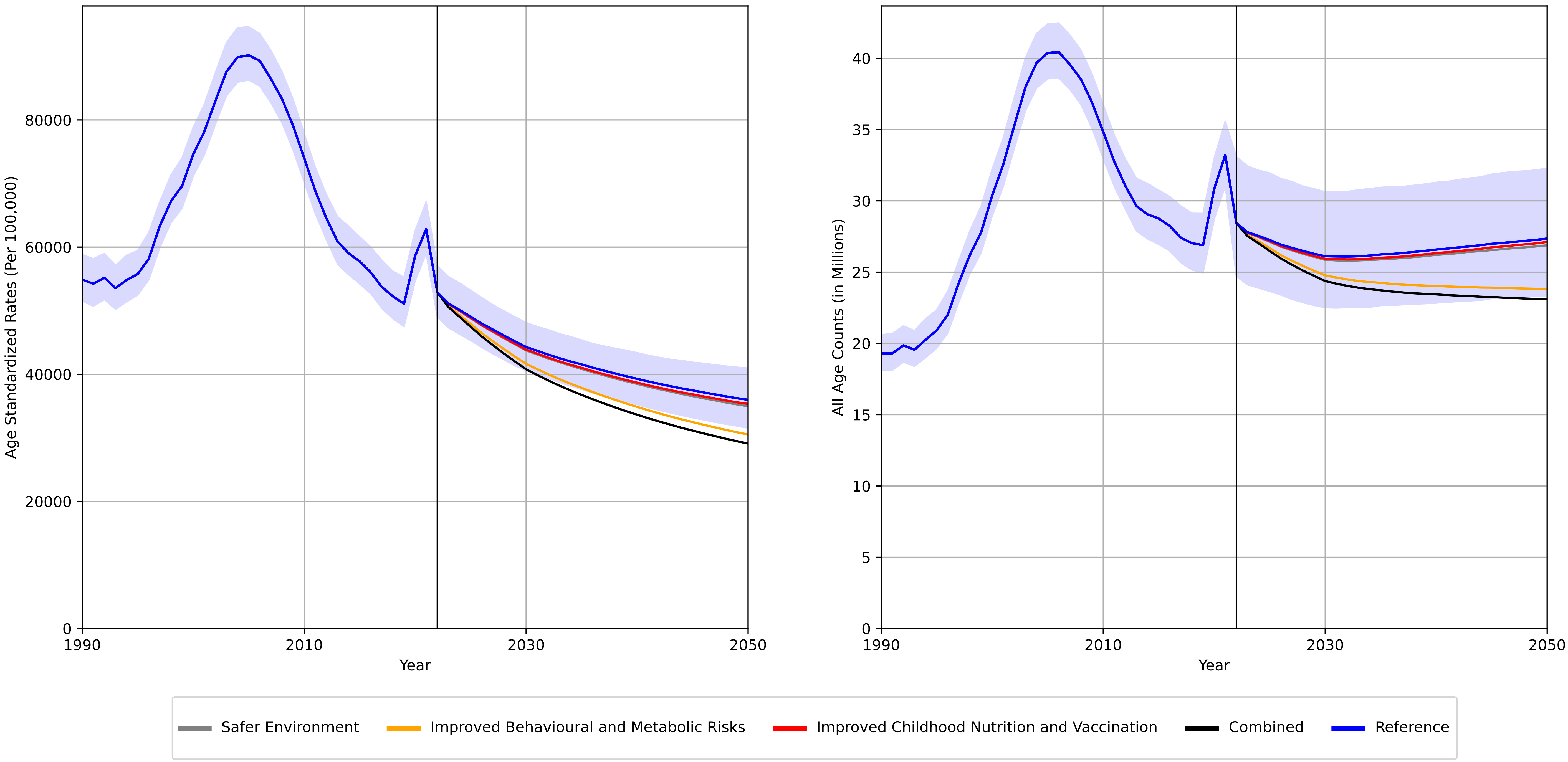

Zimbabwe

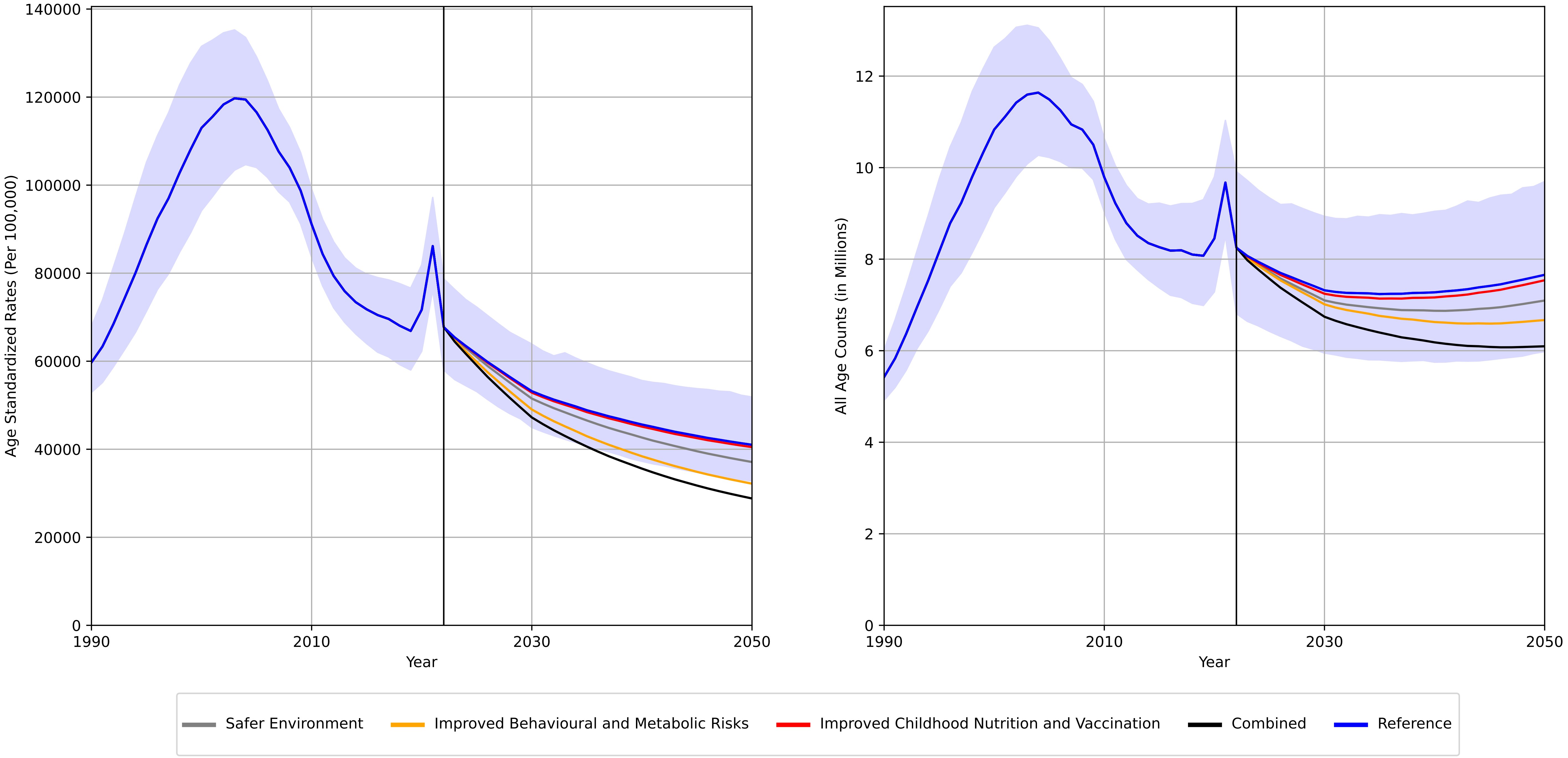
Age Standardized Rates (Per 100,000)
140000
120000
100000
80000
60000
40000
20000
0
1990
2010
2030
2050
Year
All Age Counts (in Millions)
12
10
8
6
4
2
0
1990
2010
2030
2050
Year
Safer Environment
Improved Behavioural and Metabolic Risks
Improved Childhood Nutrition and Vaccination
Combined
Reference

Benin

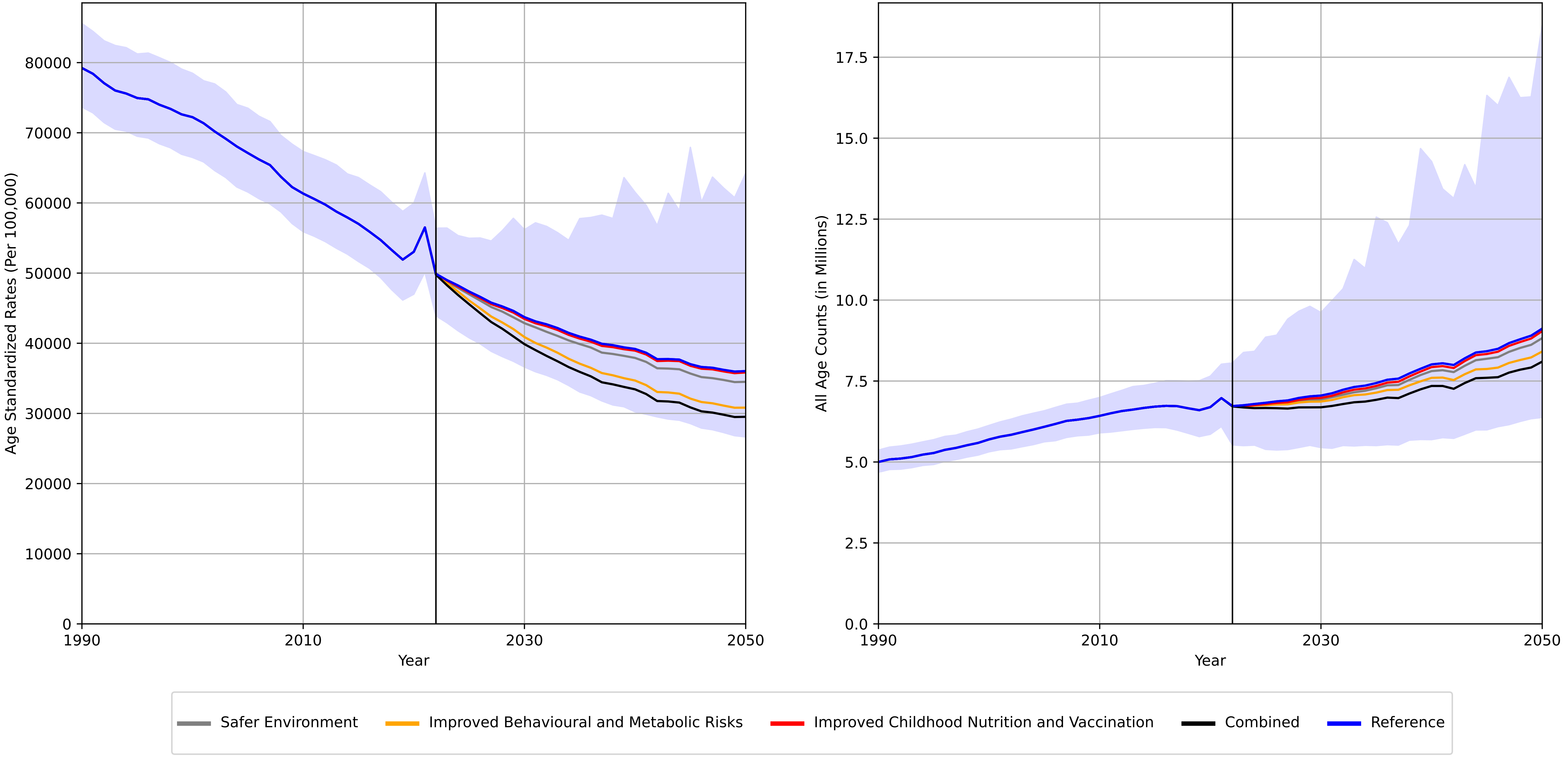

Age Standardized Rates (Per 100,000)
80000
70000
60000
50000
40000
30000
20000
10000
0
1990
2010
2030
2050
Year
All Age Counts (in Millions)
17.5
15.0
12.5
10.0
7.5
5.0
2.5
0.0
Safer Environment
Improved Behavioural and Metabolic Risks
Improved Childhood Nutrition and Vaccination
Combined
Reference

Burkina Faso

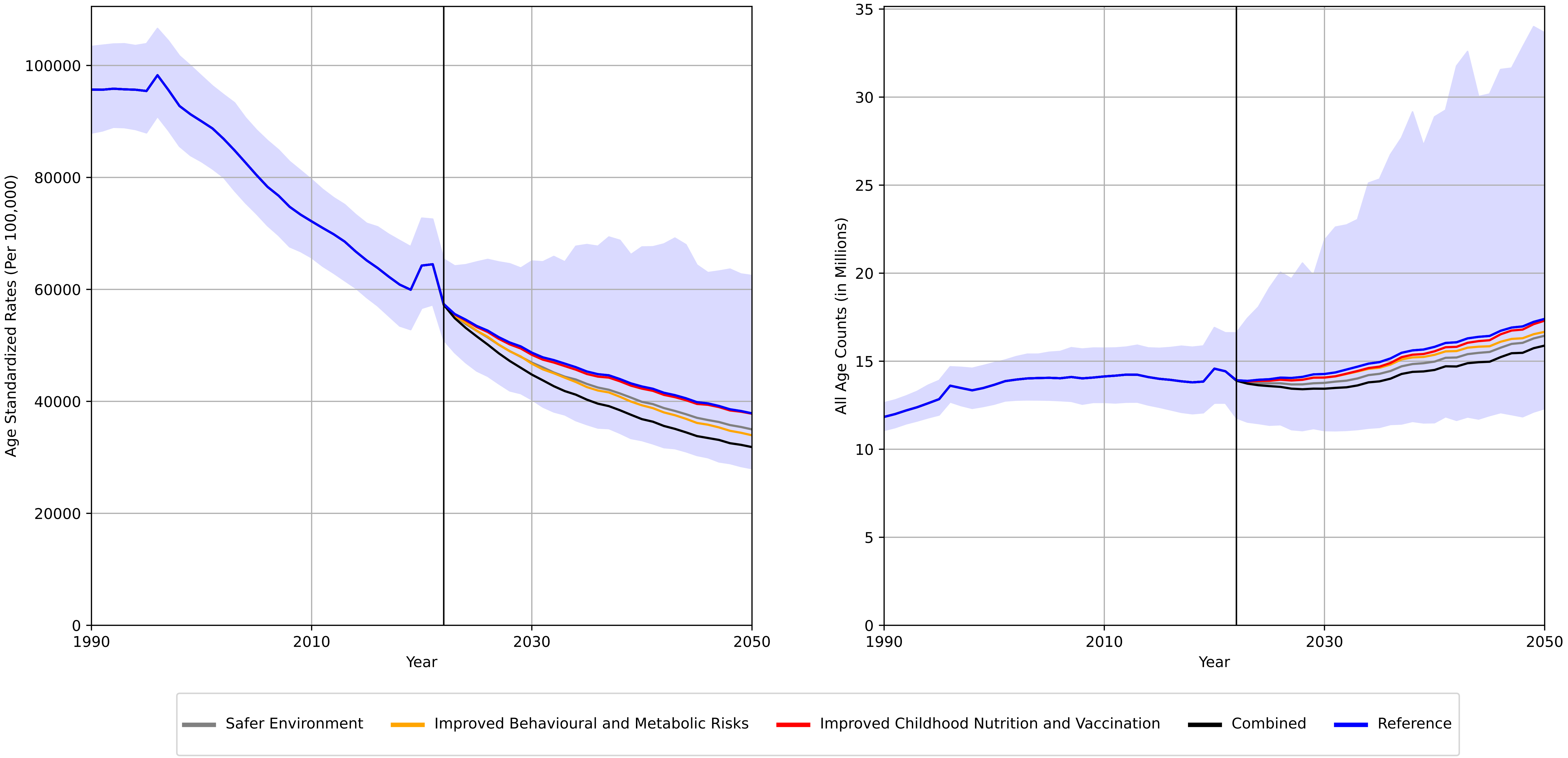

Cabo Verde

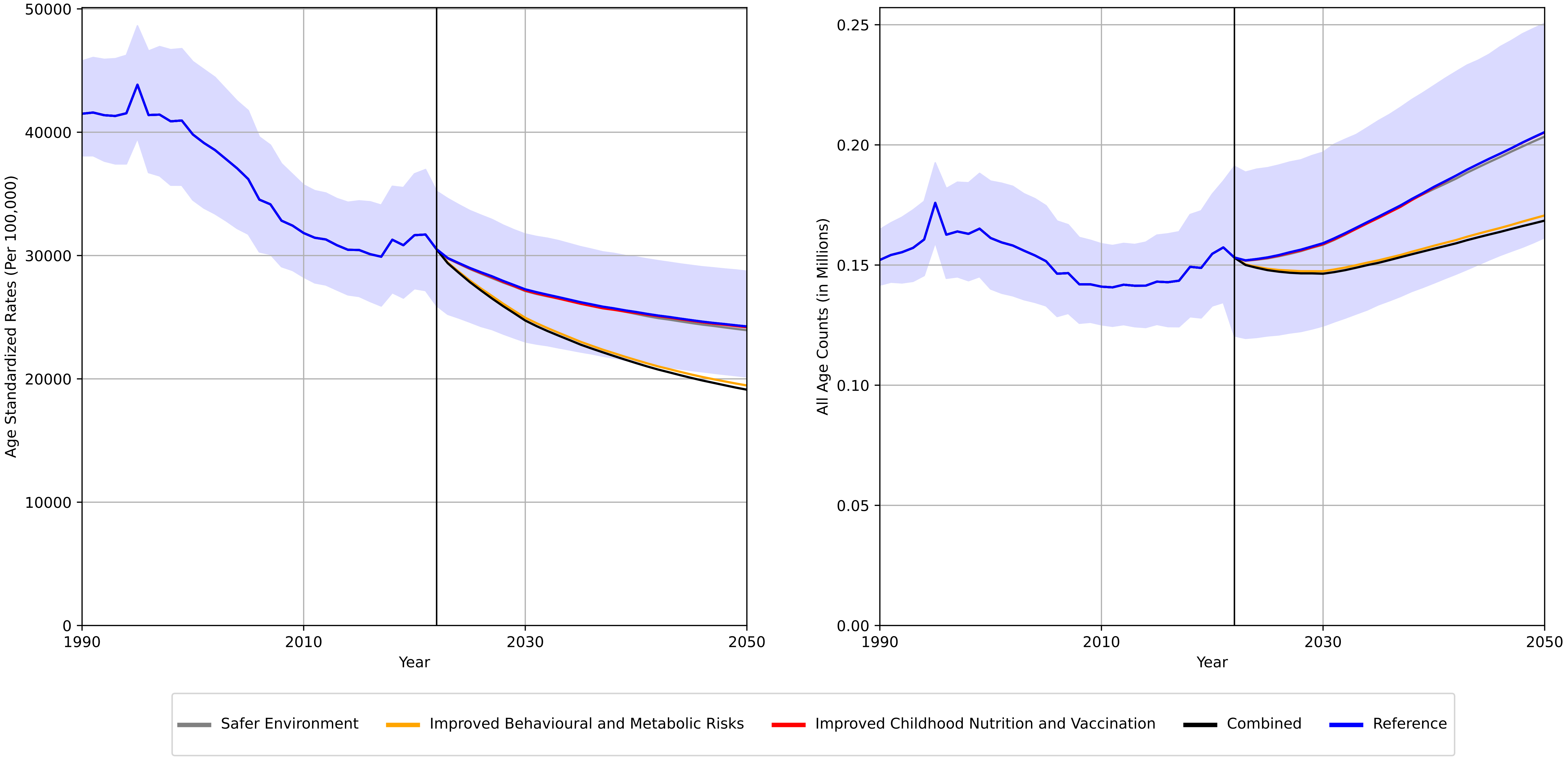
Age Standardized Rates (Per 100,000)
50000
40000
30000
20000
10000
0
1990
2010
2030
2050
Year
All Age Counts (in Millions)
0.25
0.20
0.15
0.10
0.05
0.00
1990
2010
2030
2050
Year
Safer Environment
Improved Behavioural and Metabolic Risks
Improved Childhood Nutrition and Vaccination
Combined
Reference

Cameroon

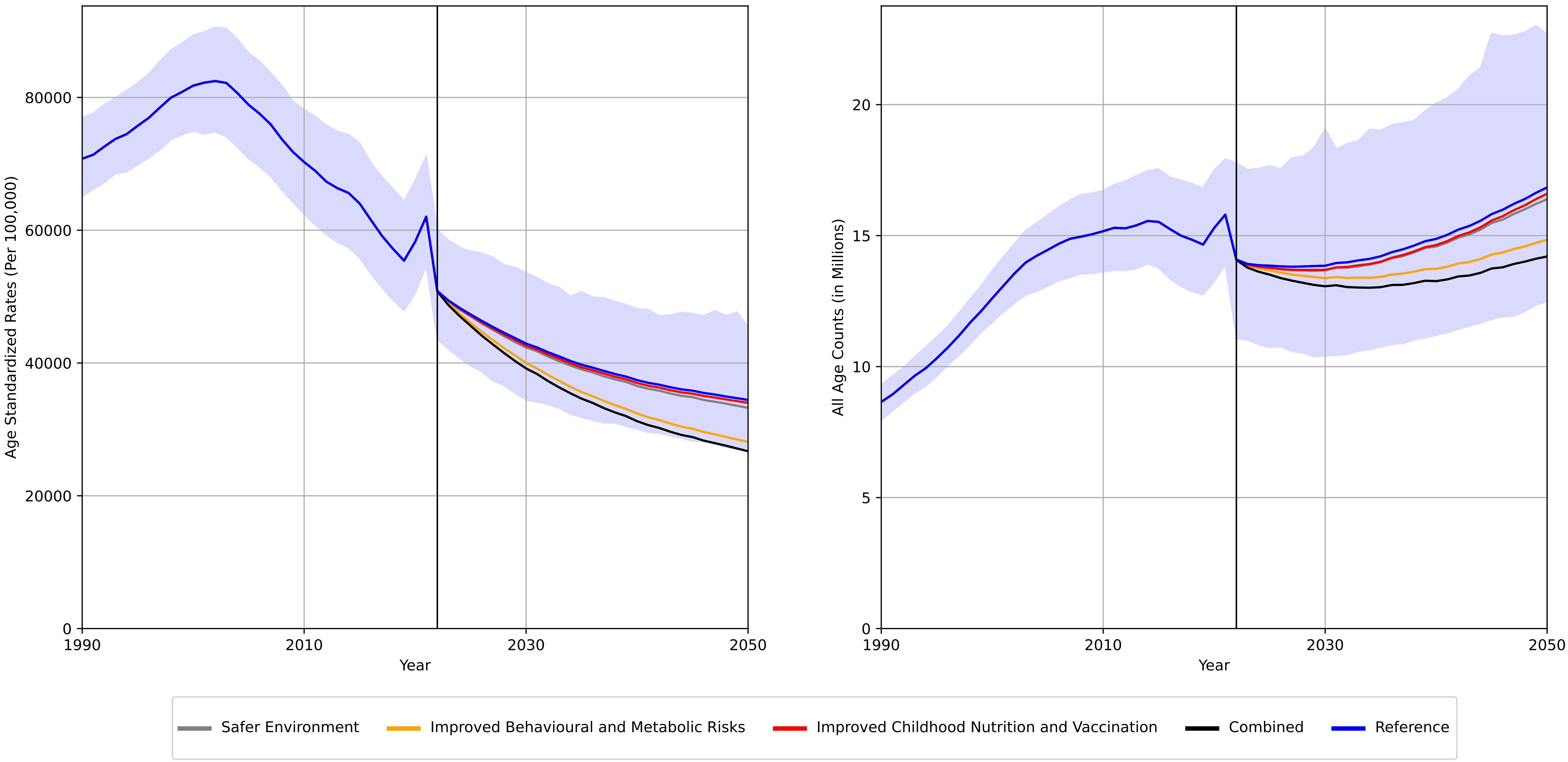
Age Standardized Rates (Per 100,000)
0
20000
40000
60000
80000
1990
2010
2030
2050
Year
All Age Counts (in Millions)
0
5
10
15
20
Safer Environment
Improved Behavioural and Metabolic Risks
Improved Childhood Nutrition and Vaccination
Combined
Reference

Chad

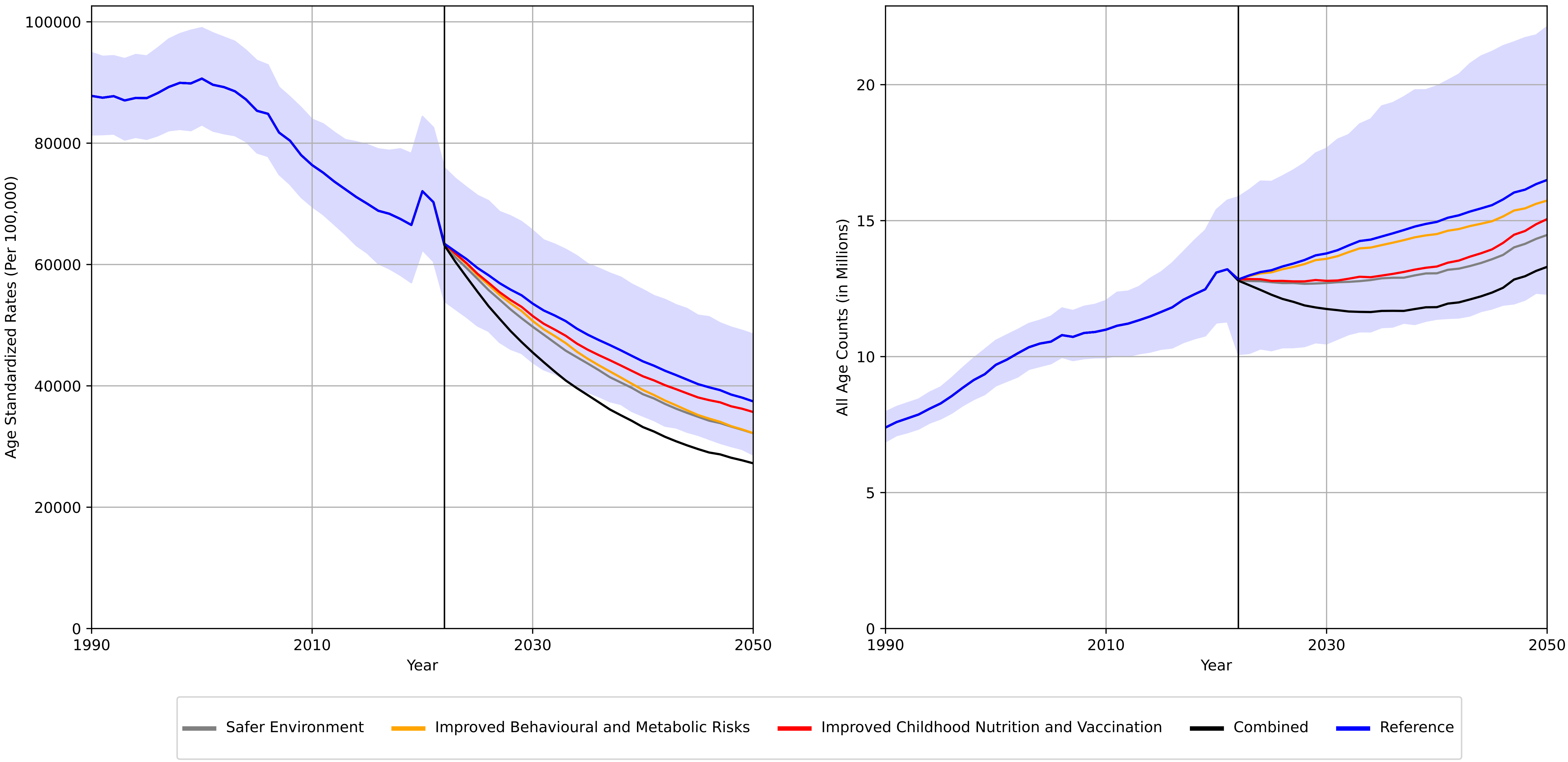

Age Standardized Rates (Per 100,000)
100000
80000
60000
40000
20000
0
1990
2010
2030
2050
Year
All Age Counts (in Millions)
20
15
10
5
0
1990
2010
2030
2050
Year
Safer Environment
Improved Behavioural and Metabolic Risks
Improved Childhood Nutrition and Vaccination
Combined
Reference

Côte D'Ivoire

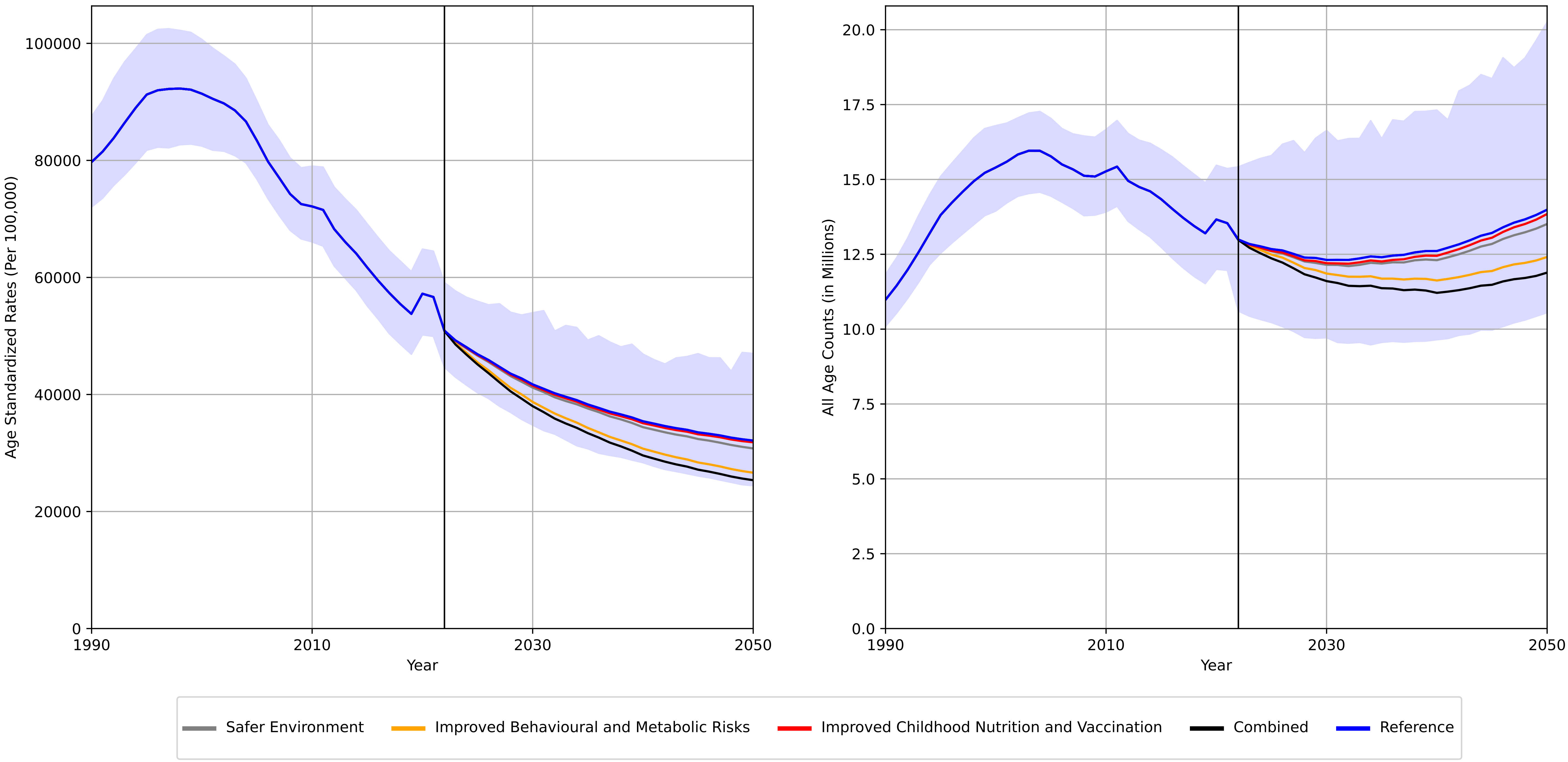

The Gambia

Supplemental Results Figure S10. All-cause age standardised DALY rates (A) and all age DALY counts (B), for the past and for five future scenarios, 1990–2050

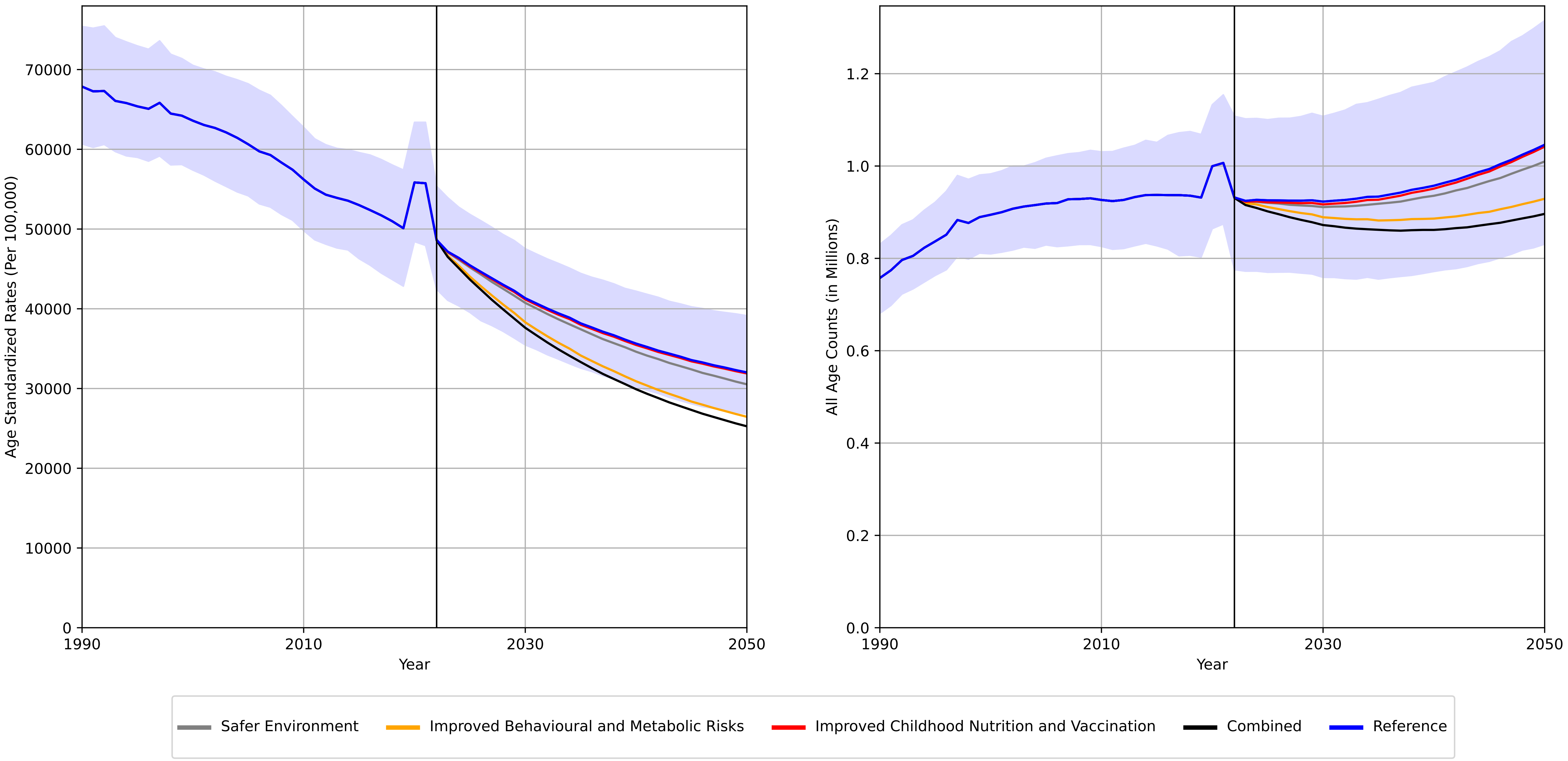

Ghana

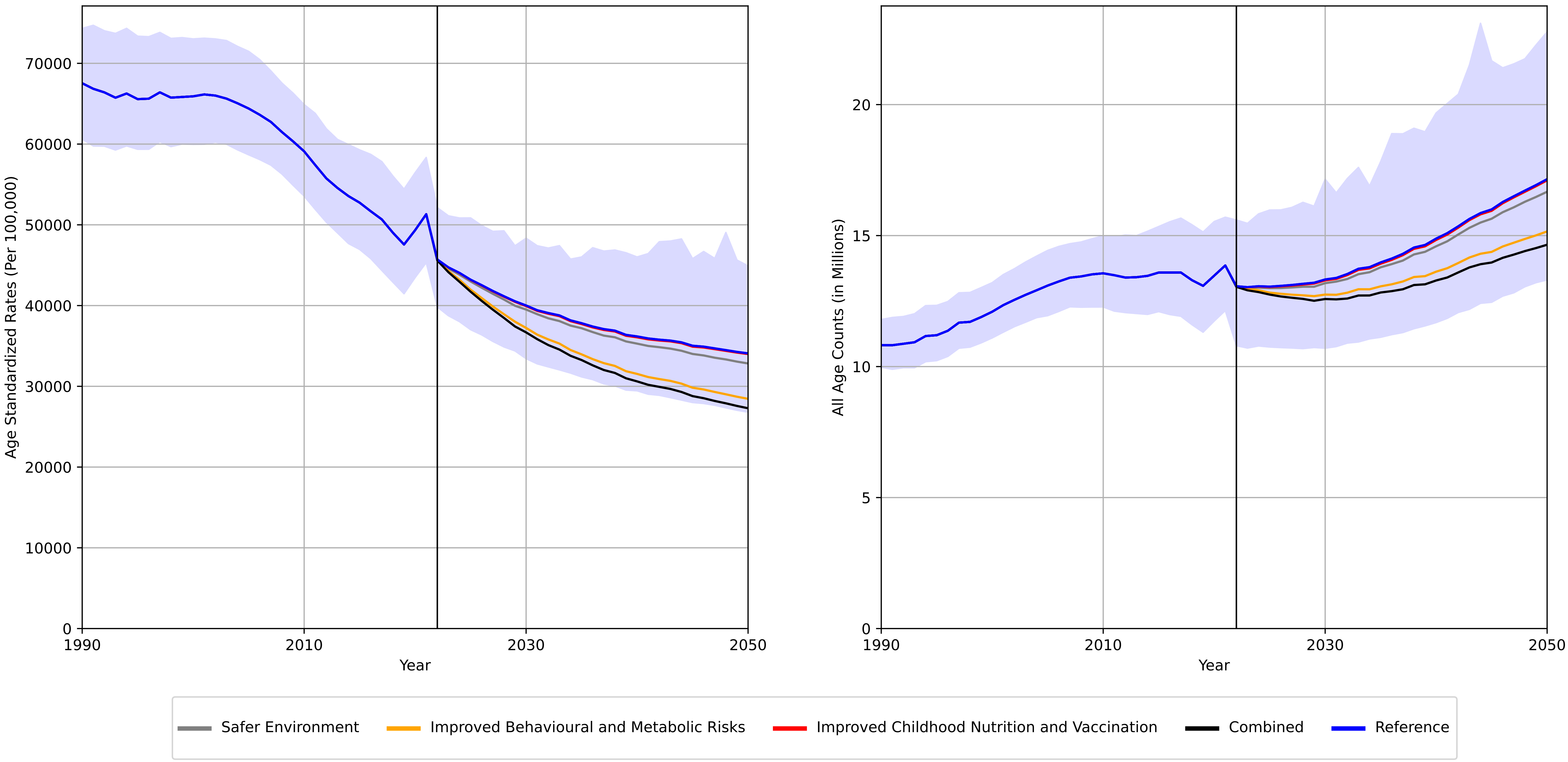

Age Standardized Rates (Per 100,000)
0
10000
20000
30000
40000
50000
60000
70000
1990
2010
2030
2050
Year
All Age Counts (in Millions)
0
5
10
15
20
1990
2010
2030
2050
Year
Safer Environment
Improved Behavioural and Metabolic Risks
Improved Childhood Nutrition and Vaccination
Combined
Reference

Guinea

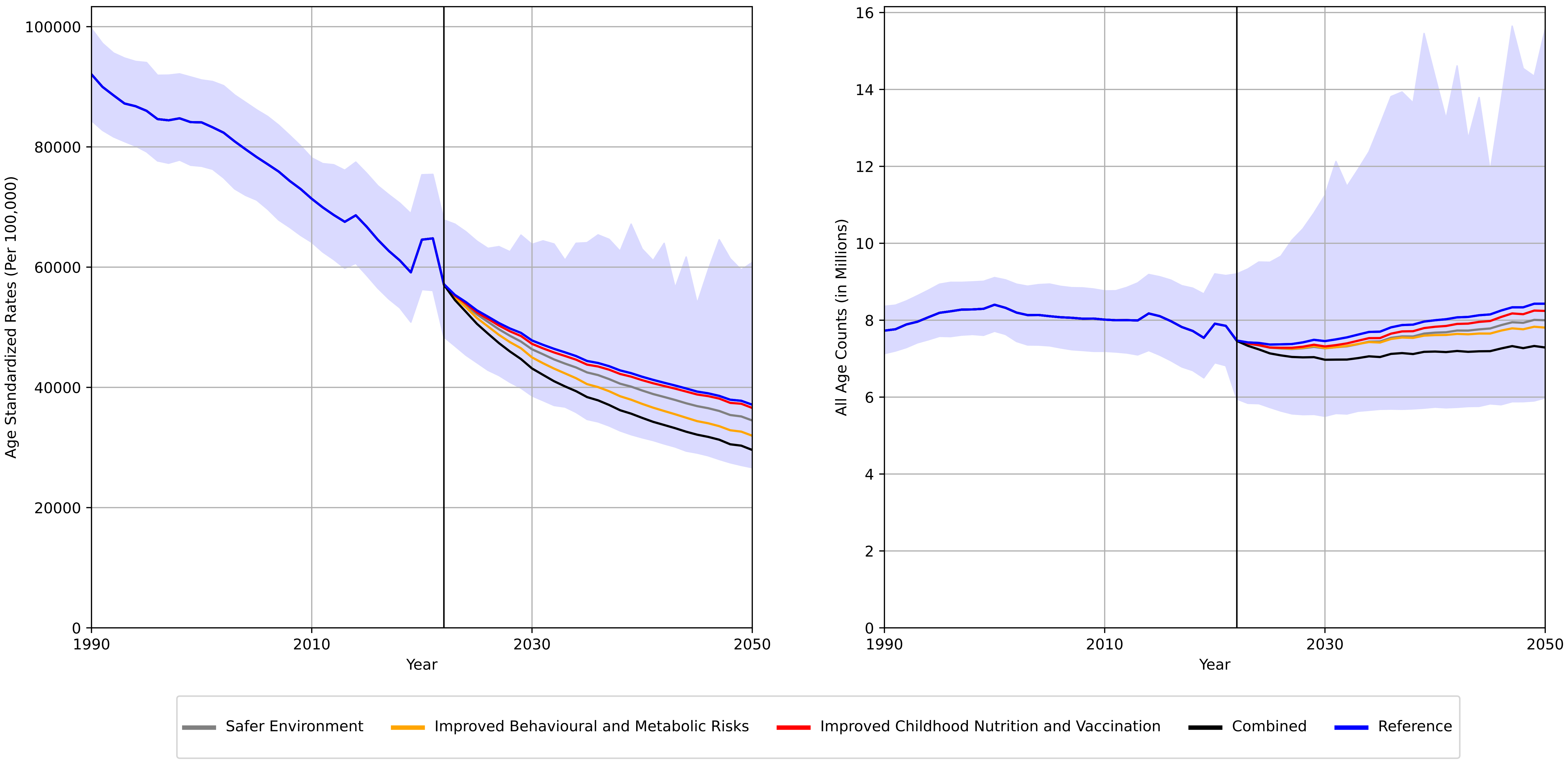
100000
80000
60000
40000
20000
0
Age Standardized Rates (Per 100,000)
1990
2010
2030
2050
Year
16
14
12
10
8
6
4
2
0
All Age Counts (in Millions)
1990
2010
2030
2050
Year
Safer Environment
Improved Behavioural and Metabolic Risks
Improved Childhood Nutrition and Vaccination
Combined
Reference

Guinea-Bissau

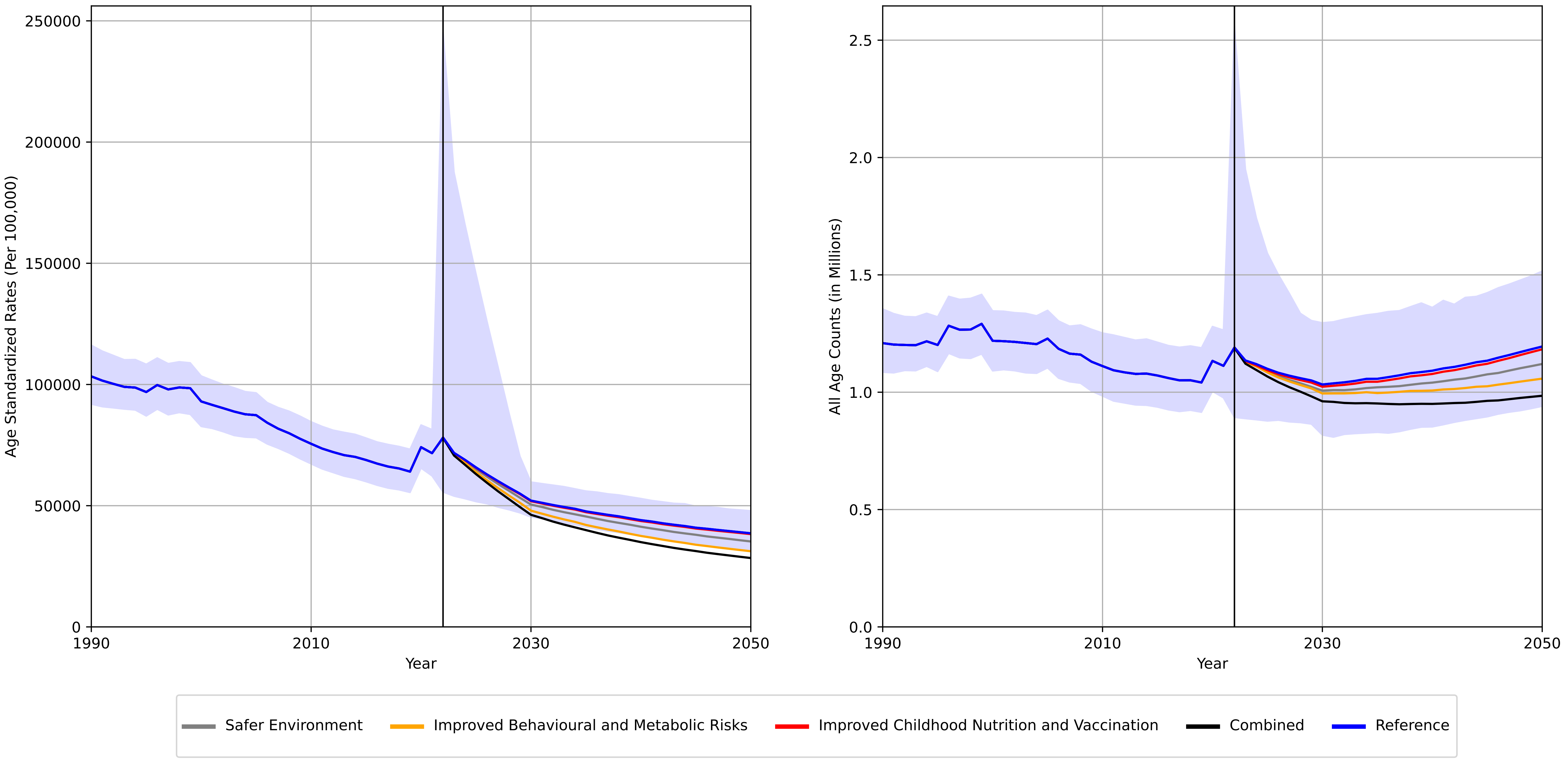

Liberia

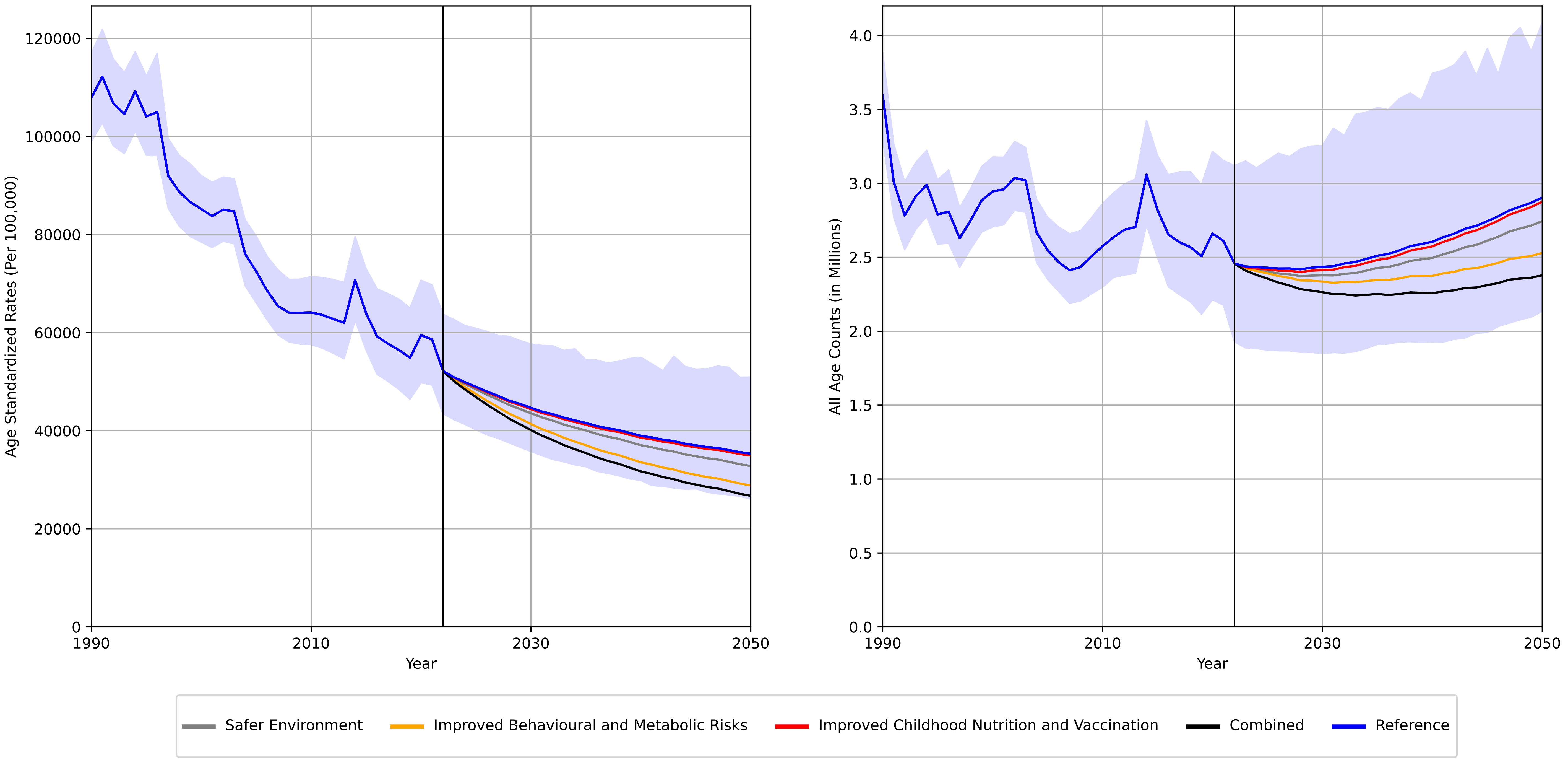
Age Standardized Rates (Per 100,000)
0
20000
40000
60000
80000
100000
120000
1990
2010
2030
2050
Year
All Age Counts (in Millions)
0.0
0.5
1.0
1.5
2.0
2.5
3.0
3.5
4.0
1990
2010
2030
2050
Year
Safer Environment
Improved Behavioural and Metabolic Risks
Improved Childhood Nutrition and Vaccination
Combined
Reference

Mali

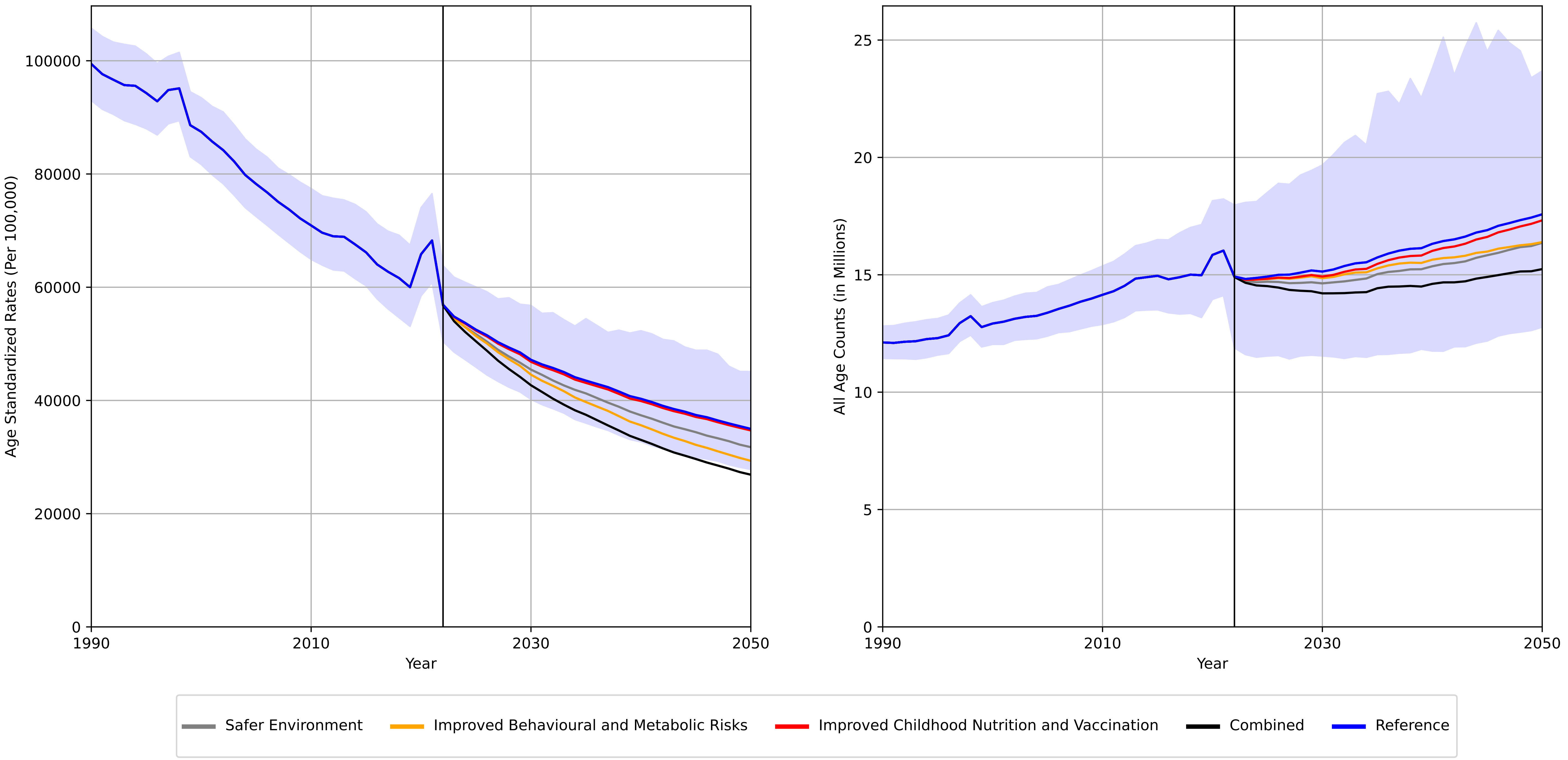

Age Standardized Rates (Per 100,000)
100000
80000
60000
40000
20000
0
1990
2010
2030
2050
Year
All Age Counts (in Millions)
25
20
15
10
5
0
1990
2010
2030
2050
Year
Safer Environment
Improved Behavioural and Metabolic Risks
Improved Childhood Nutrition and Vaccination
Combined
Reference

Mauritania

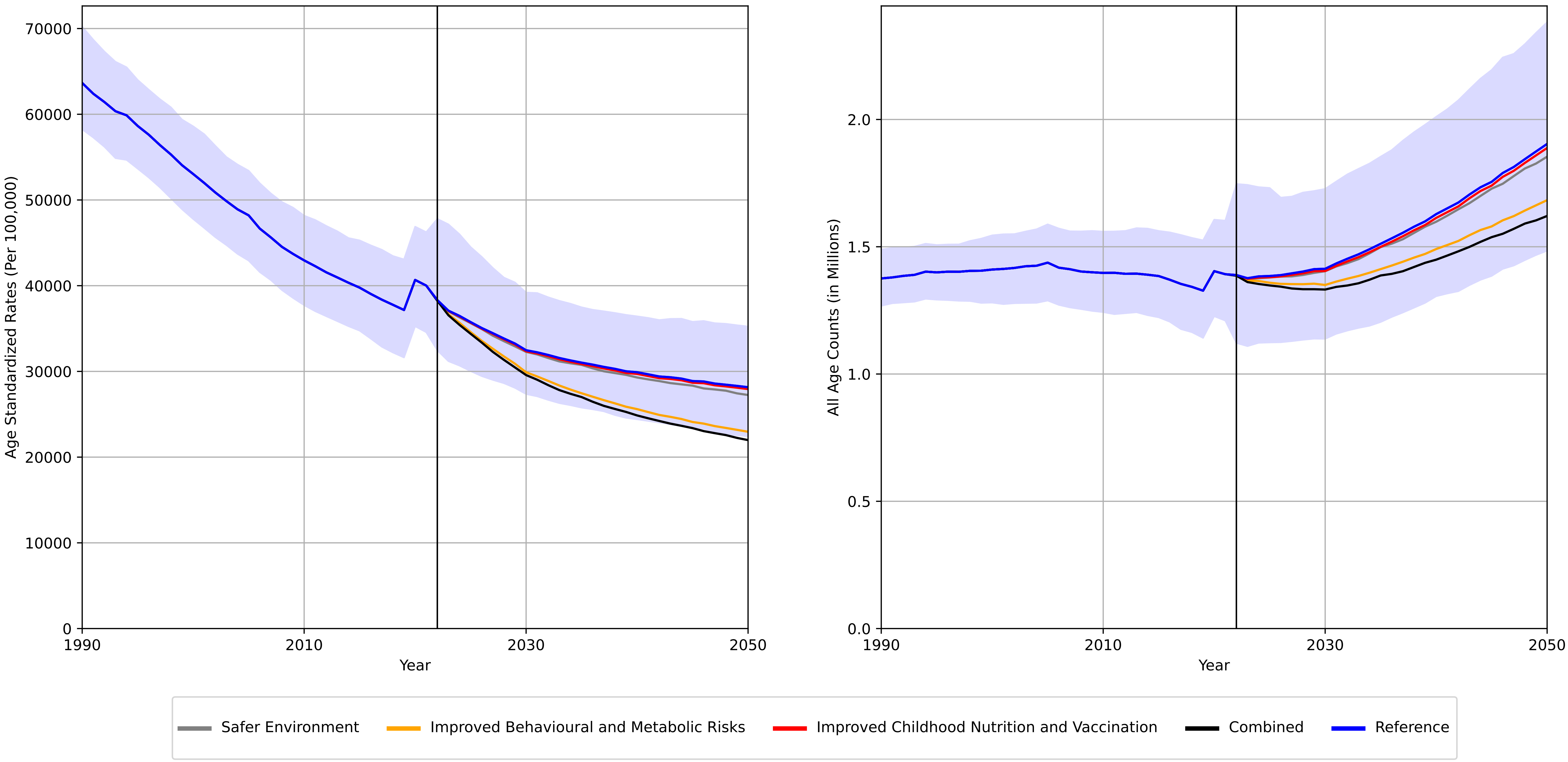

Age Standardized Rates (Per 100,000)
70000
60000
50000
40000
30000
20000
10000
0
1990
2010
2030
2050
Year
All Age Counts (in Millions)
2.0
1.5
1.0
0.5
0.0
1990
2010
2030
2050
Year
Safer Environment
Improved Behavioural and Metabolic Risks
Improved Childhood Nutrition and Vaccination
Combined
Reference

Niger

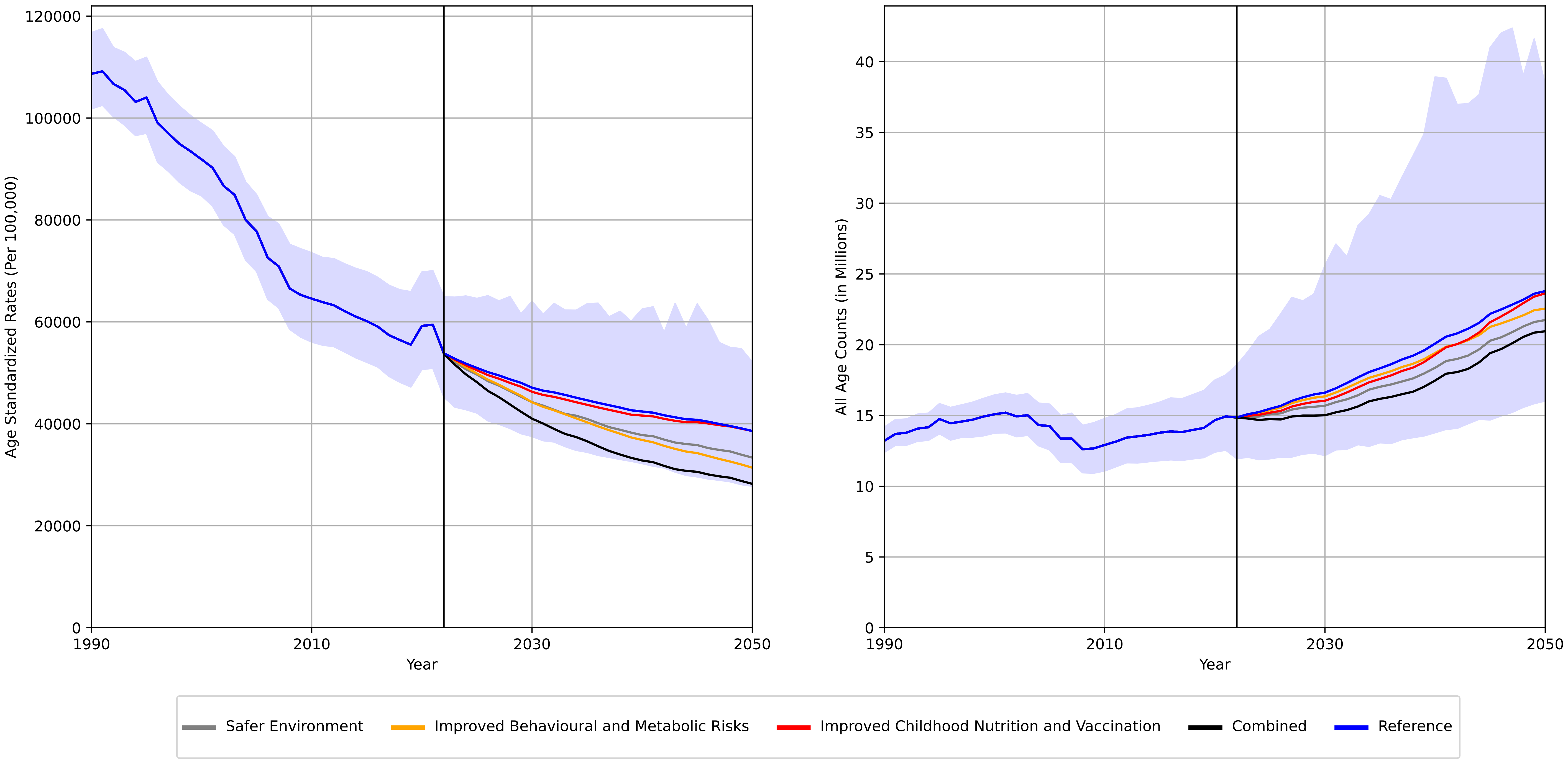

Age Standardized Rates (Per 100,000)
120000
100000
80000
60000
40000
20000
0
1990
2010
2030
2050
Year
All Age Counts (in Millions)
40
35
30
25
20
15
10
5
0
1990
2010
2030
2050
Year
Safer Environment
Improved Behavioural and Metabolic Risks
Improved Childhood Nutrition and Vaccination
Combined
Reference

Nigeria

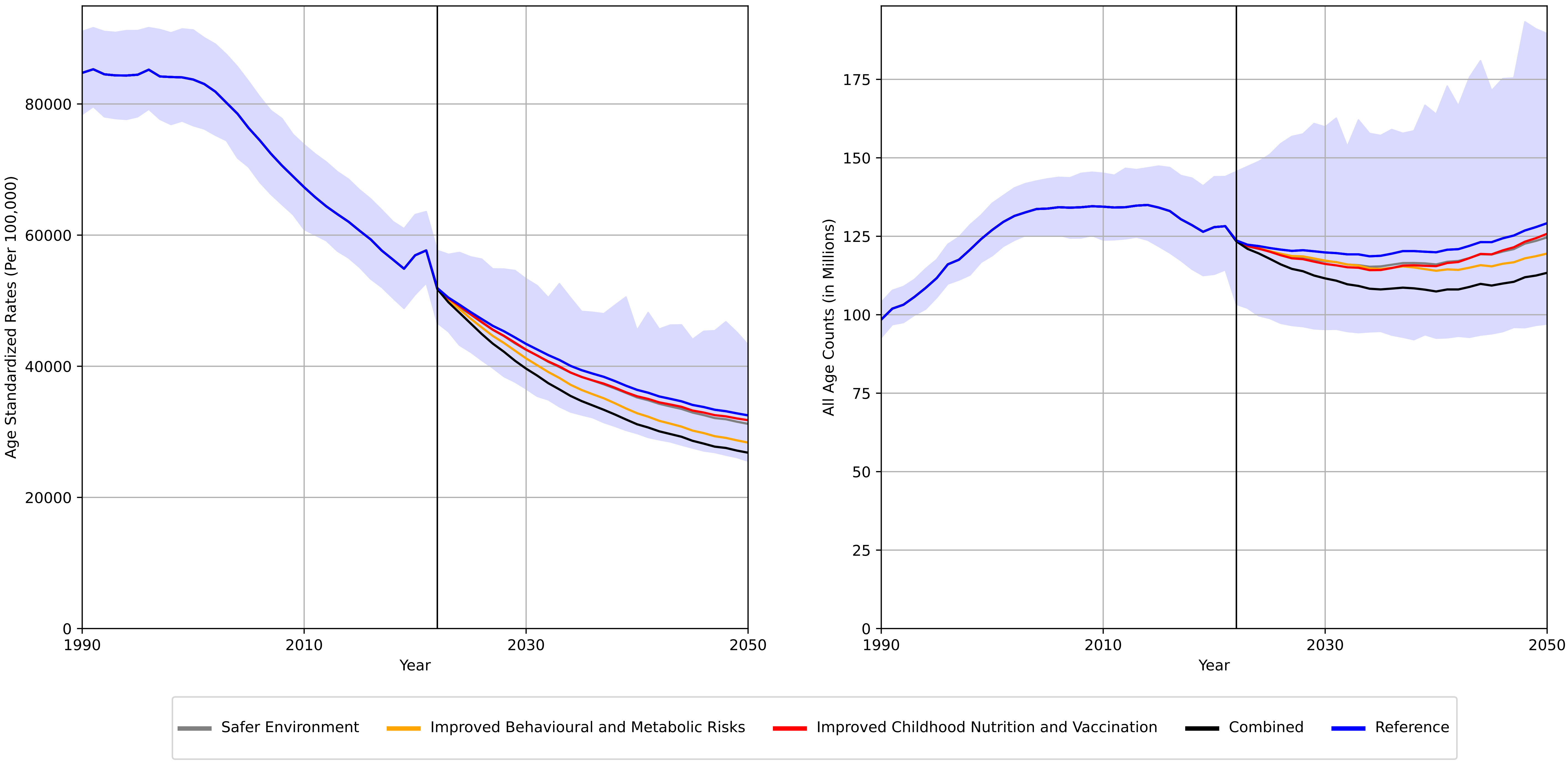

Age Standardized Rates (Per 100,000)
0
20000
40000
60000
80000
1990
2010
2030
2050
Year
All Age Counts (in Millions)
0
25
50
75
100
125
150
175
1990
2010
2030
2050
Year
Safer Environment
Improved Behavioural and Metabolic Risks
Improved Childhood Nutrition and Vaccination
Combined
Reference

São Tomé And Príncipe

Supplemental Results Figure S10. All-cause age standardised DALY rates (A) and all age DALY counts (B), for the past and for five future scenarios, 1990–2050

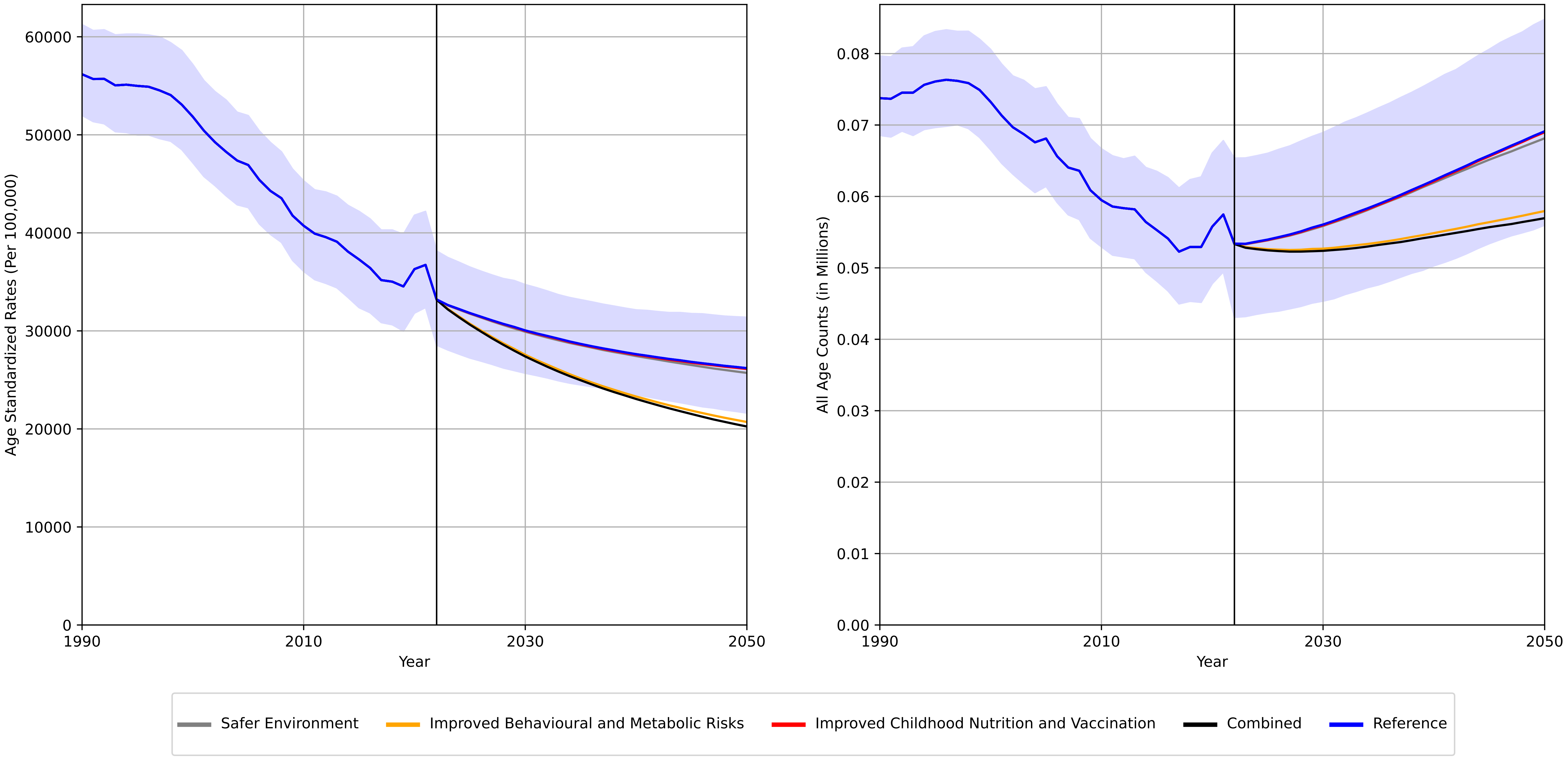

Senegal

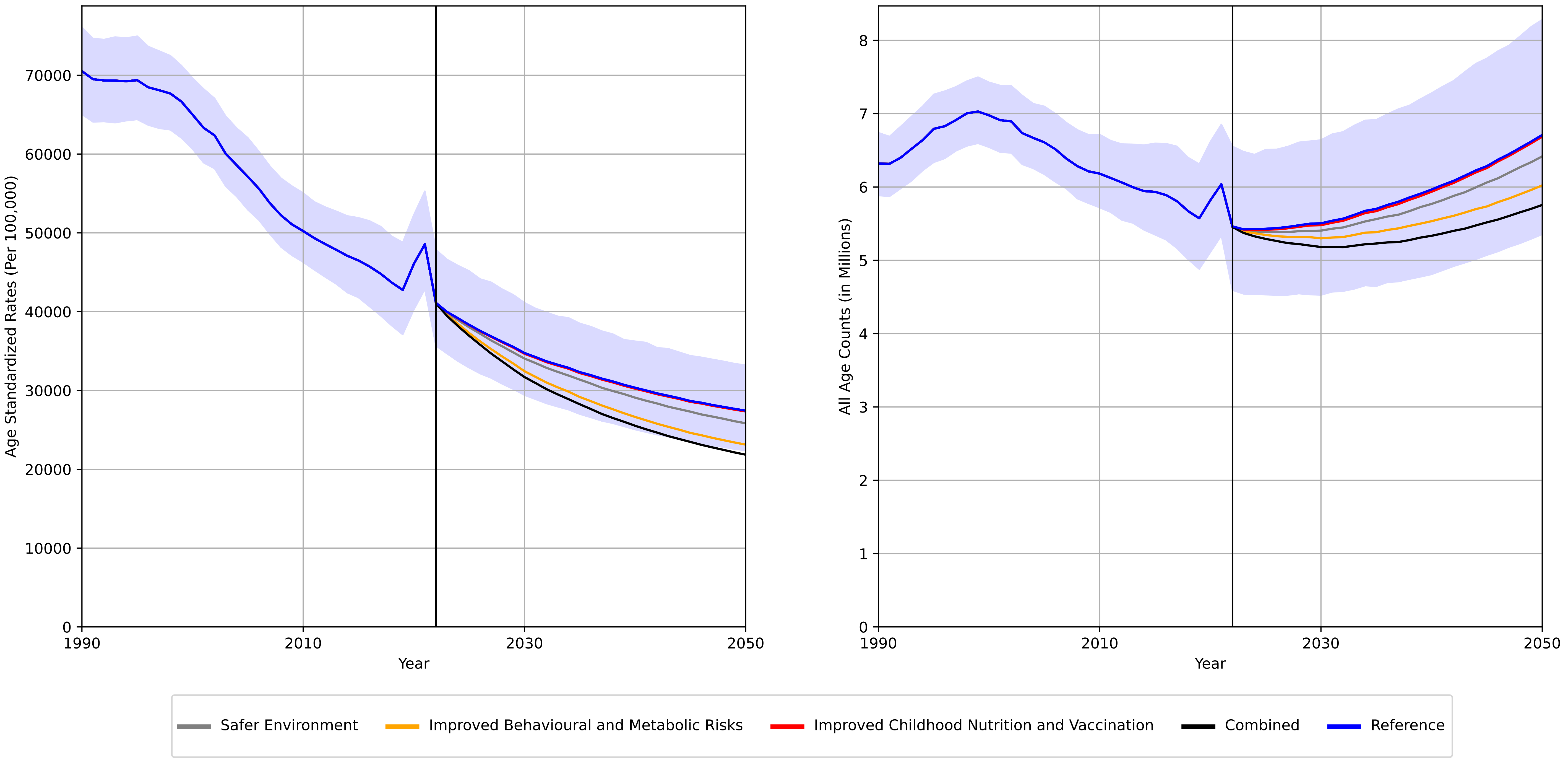
Age Standardized Rates (Per 100,000)
All Age Counts (in Millions)
Year
Year
Safer Environment
Improved Behavioural and Metabolic Risks
Improved Childhood Nutrition and Vaccination
Combined
Reference

Sierra Leone

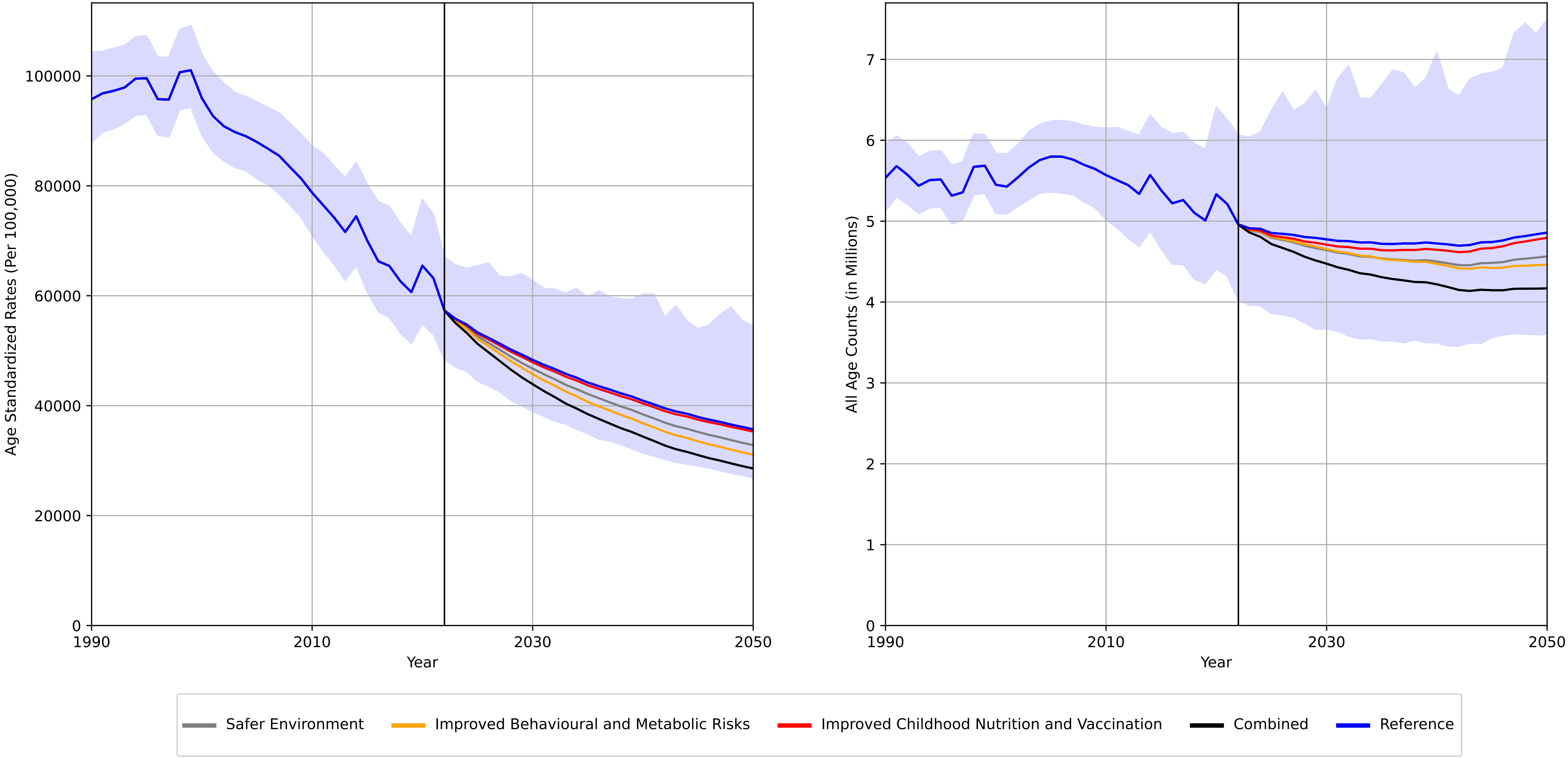

Togo

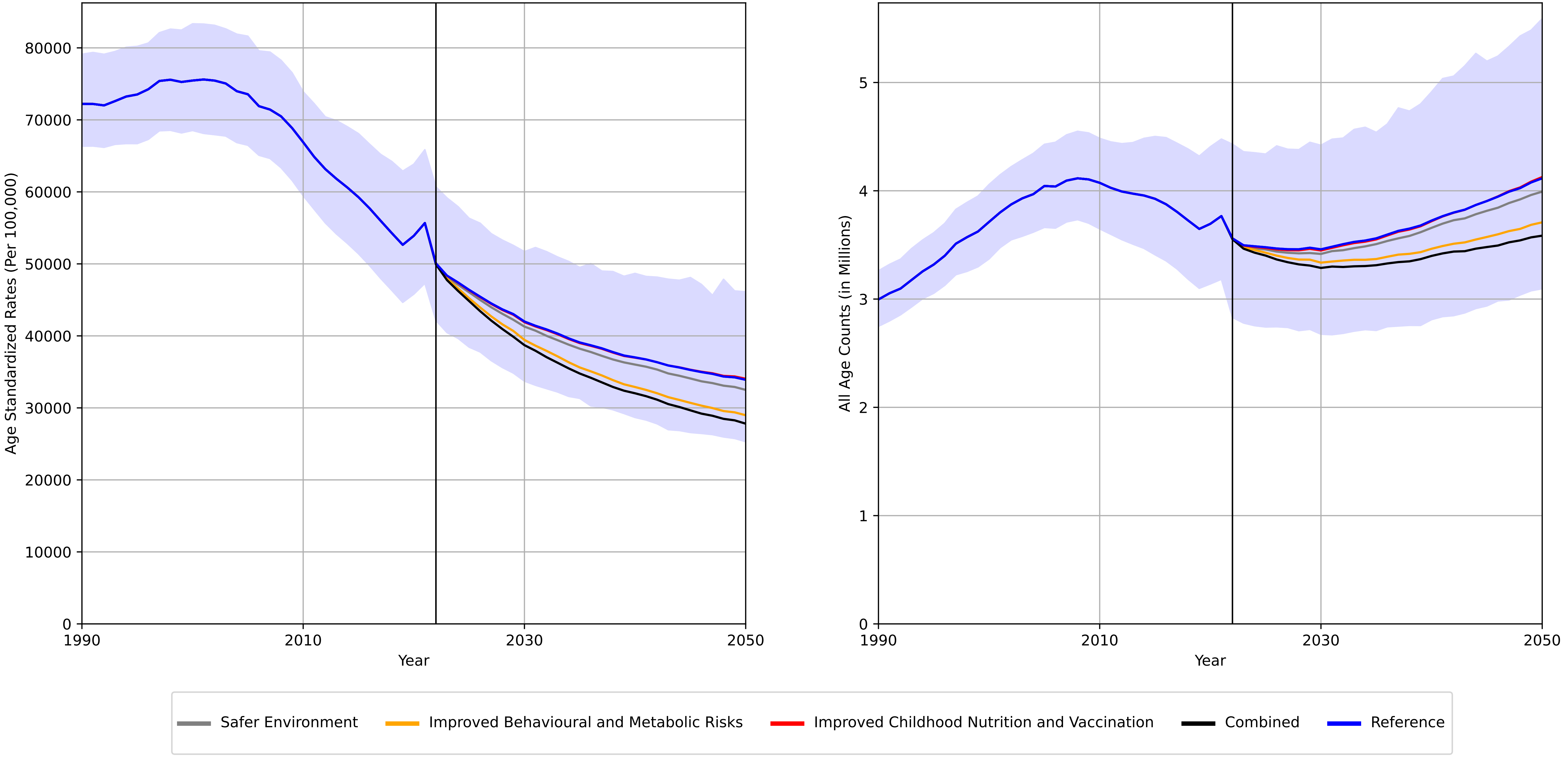
Age Standardized Rates (Per 100,000)
0
10000
20000
30000
40000
50000
60000
70000
80000
1990
2010
2030
2050
Year
All Age Counts (in Millions)
0
1
2
3
4
5
1990
2010
2030
2050
Year
Safer Environment
Improved Behavioural and Metabolic Risks
Improved Childhood Nutrition and Vaccination
Combined
Reference

Global

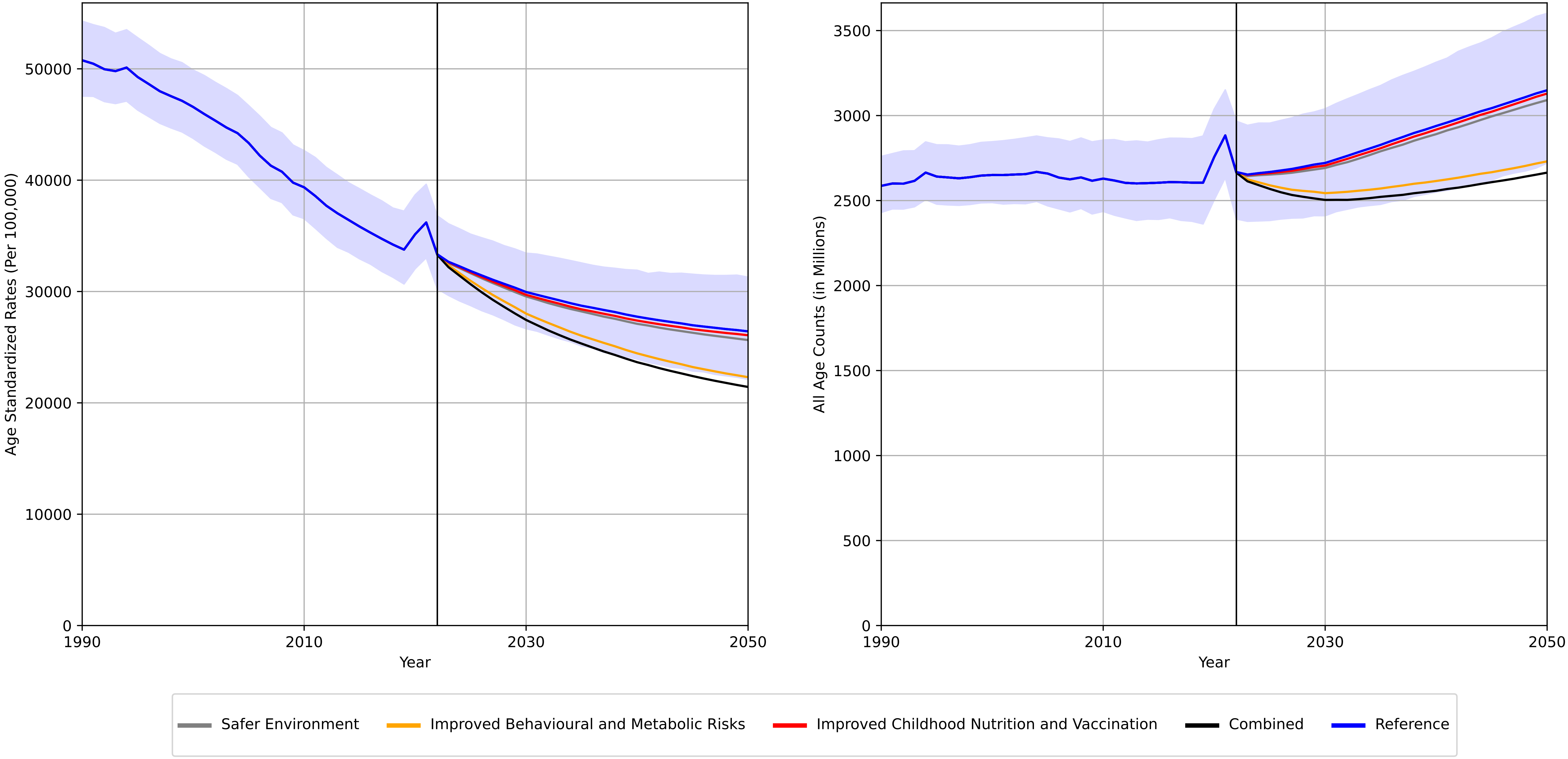
Age Standardized Rates (Per 100,000)
50000
40000
30000
20000
10000
0
1990
2010
2030
2050
Year
All Age Counts (in Millions)
3500
3000
2500
2000
1500
1000
500
0
1990
2010
2030
2050
Year
Safer Environment
Improved Behavioural and Metabolic Risks
Improved Childhood Nutrition and Vaccination
Combined
Reference

Central Europe, Eastern Europe, And Central Asia

Supplemental Results Figure S10. All-cause age standardised DALY rates (A) and all age DALY counts (B), for the past and for five future scenarios, 1990–2050

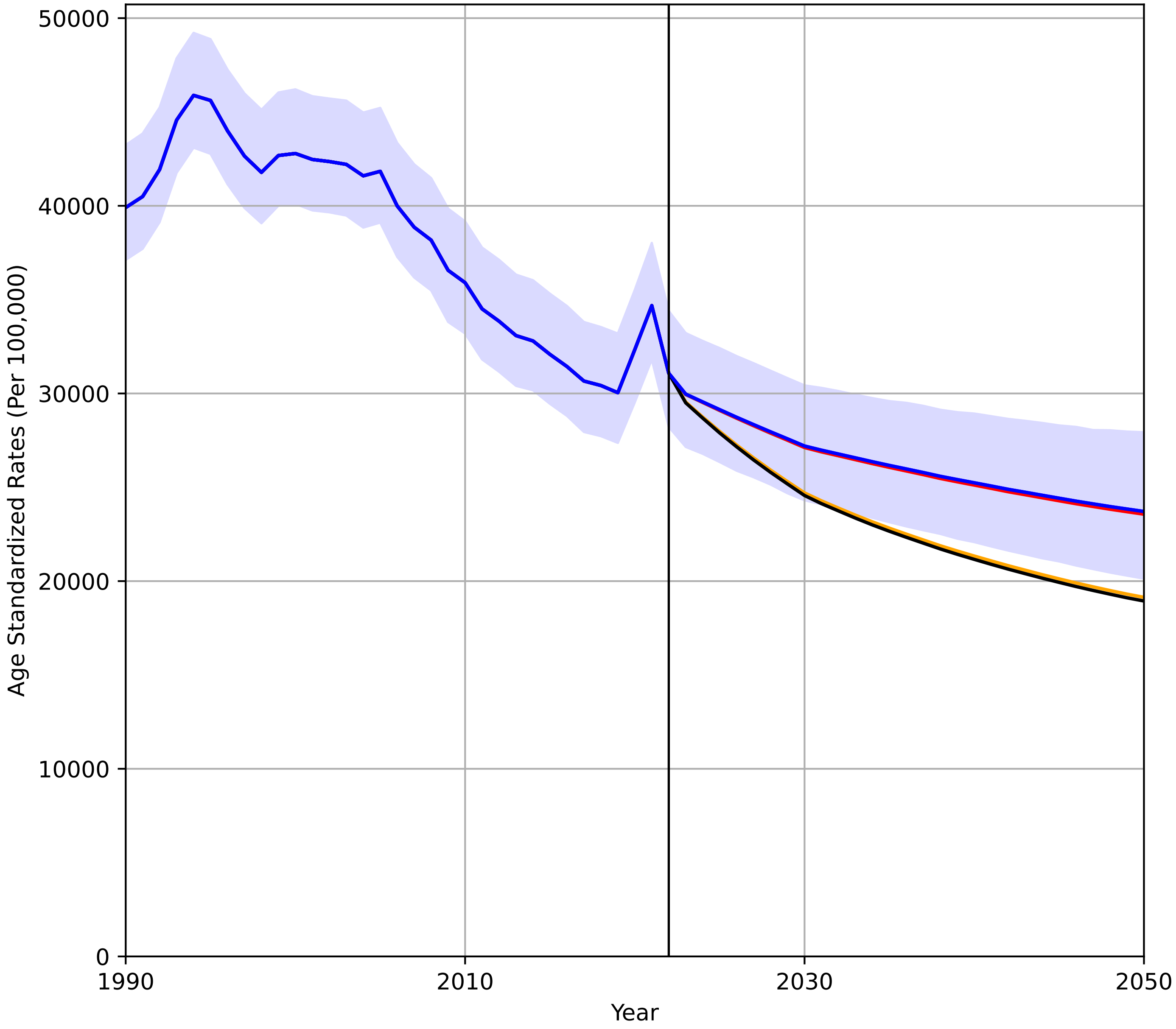

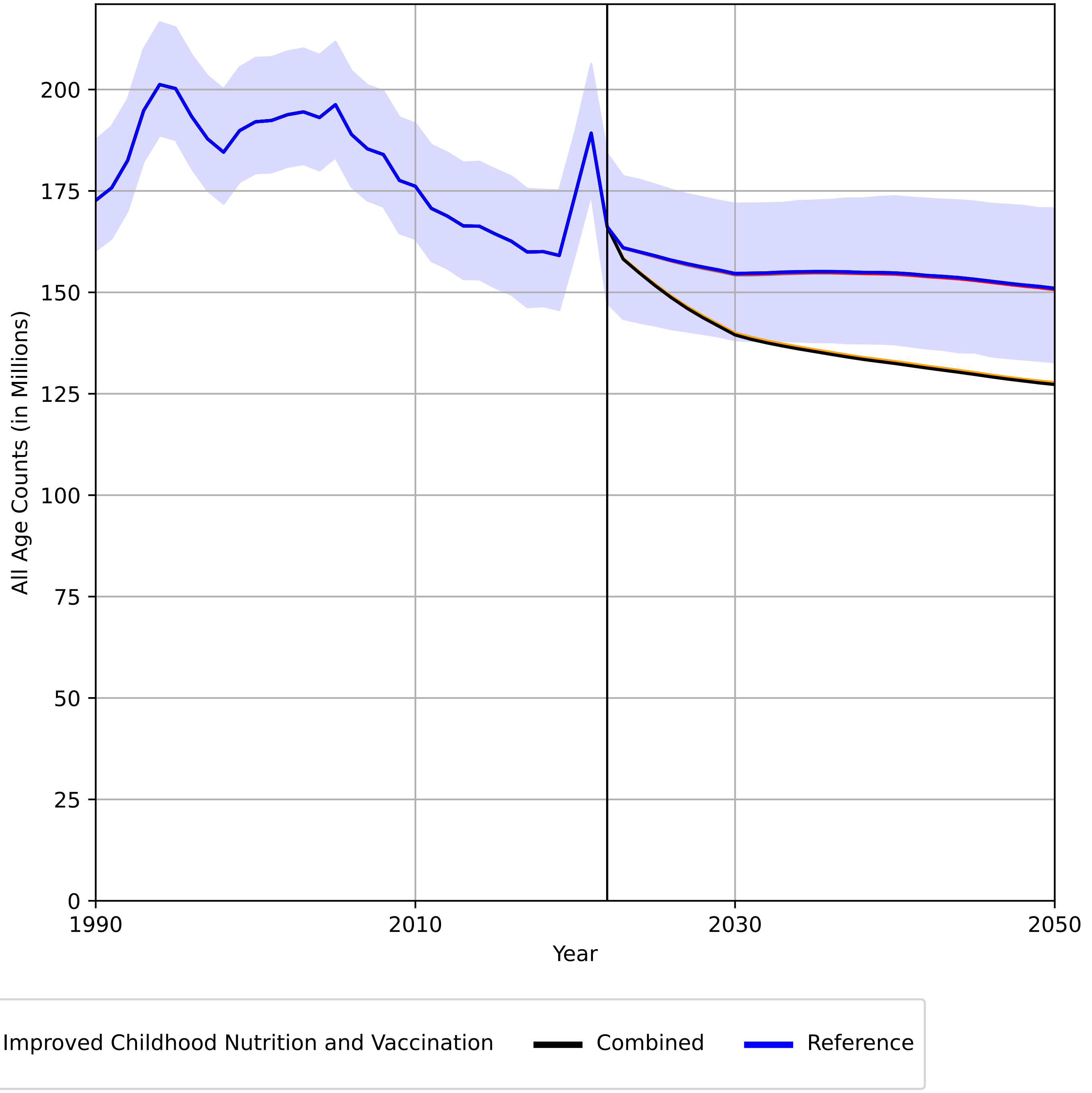

High Income

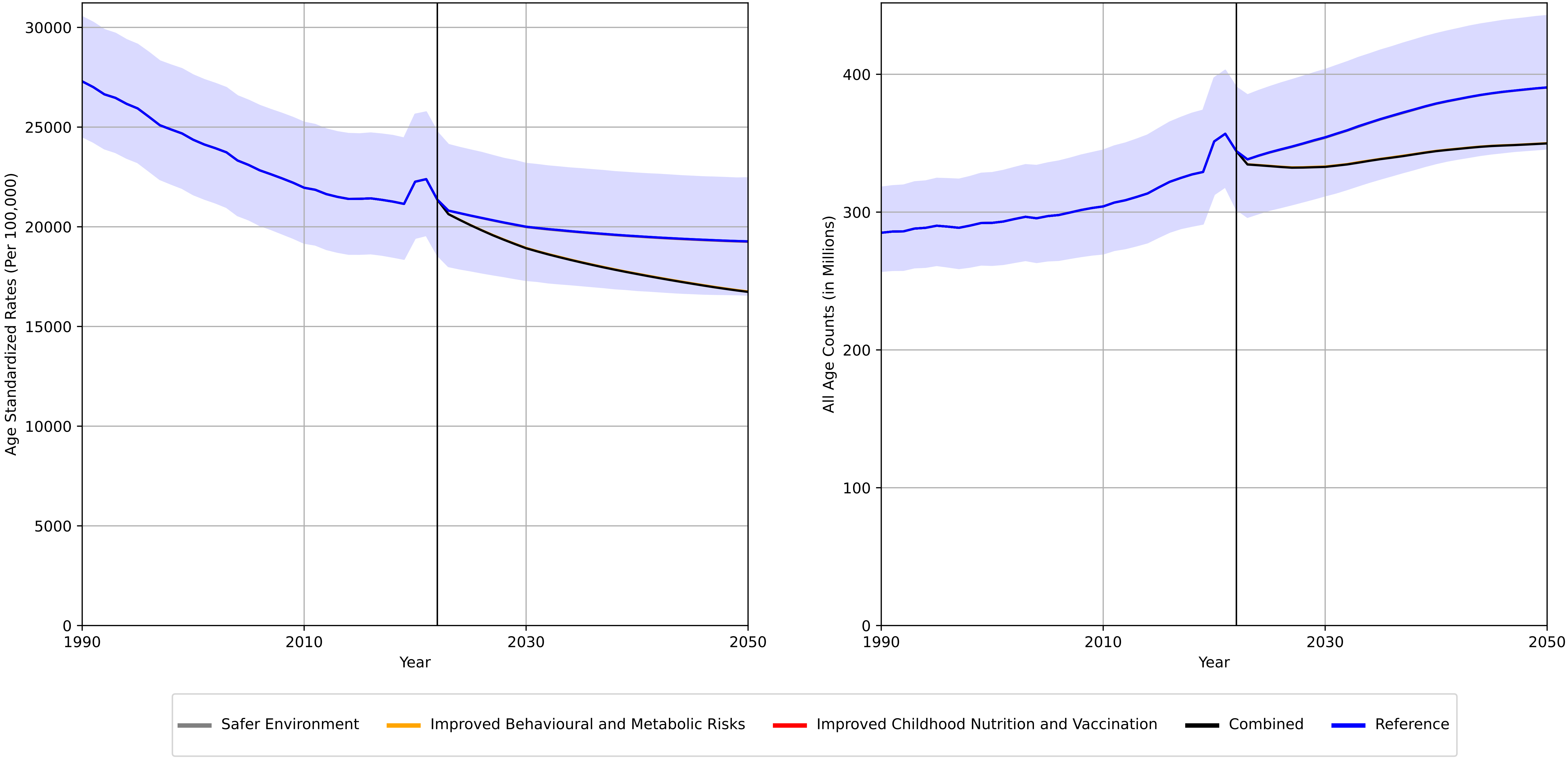
Age Standardized Rates (Per 100,000)
30000
25000
20000
15000
10000
5000
0
1990
2010
2030
2050
Year
All Age Counts (in Millions)
400
300
200
100
0
1990
2010
2030
2050
Year
Safer Environment
Improved Behavioural and Metabolic Risks
Improved Childhood Nutrition and Vaccination
Combined
Reference

Latin America And Caribbean

Supplemental Results Figure S10. All-cause age standardised DALY rates (A) and all age DALY counts (B), for the past and for five future scenarios, 1990–2050

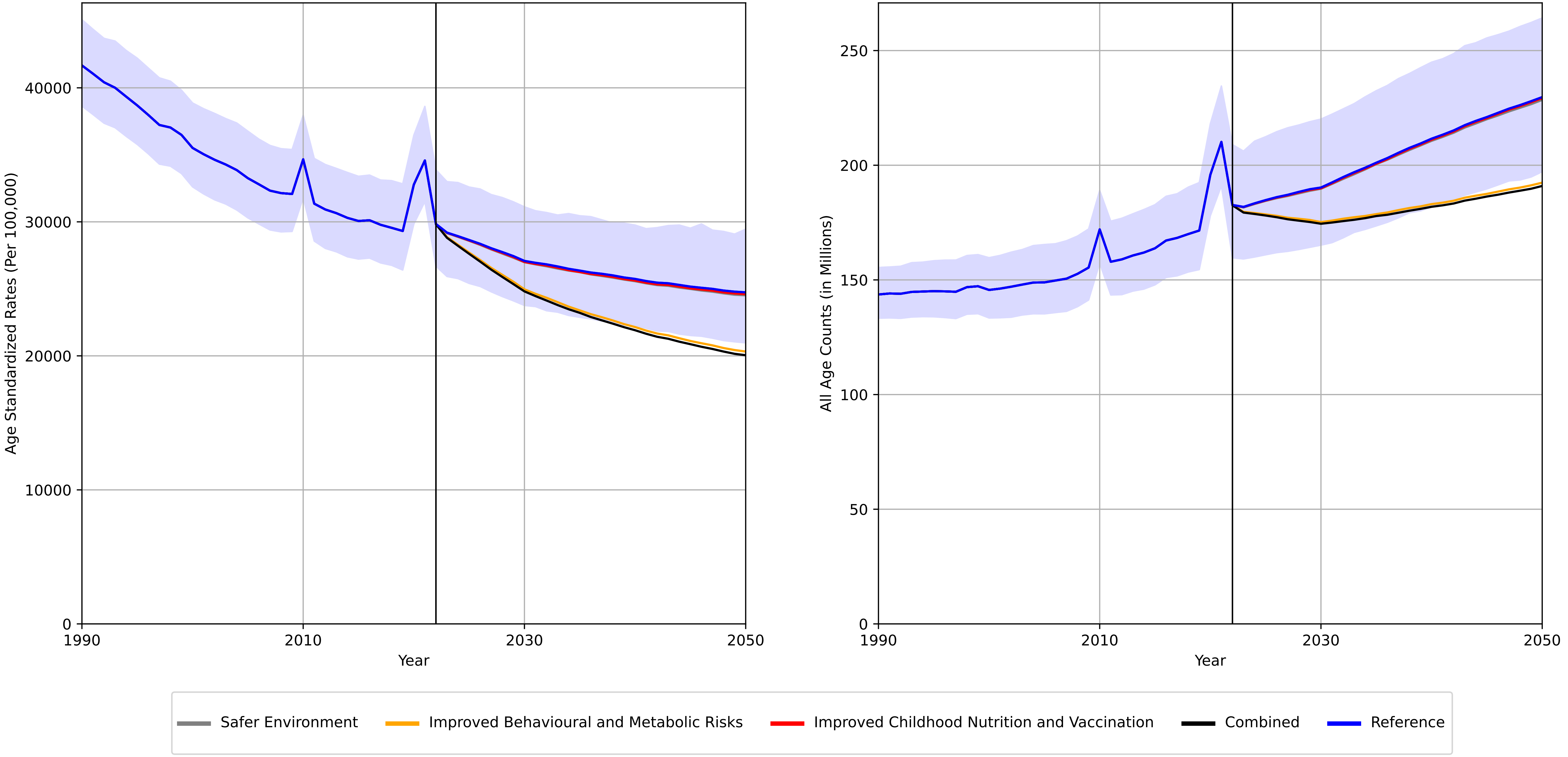

North Africa And Middle East

Supplemental Results Figure S10. All-cause age standardised DALY rates (A) and all age DALY counts (B), for the past and for five future scenarios, 1990–2050

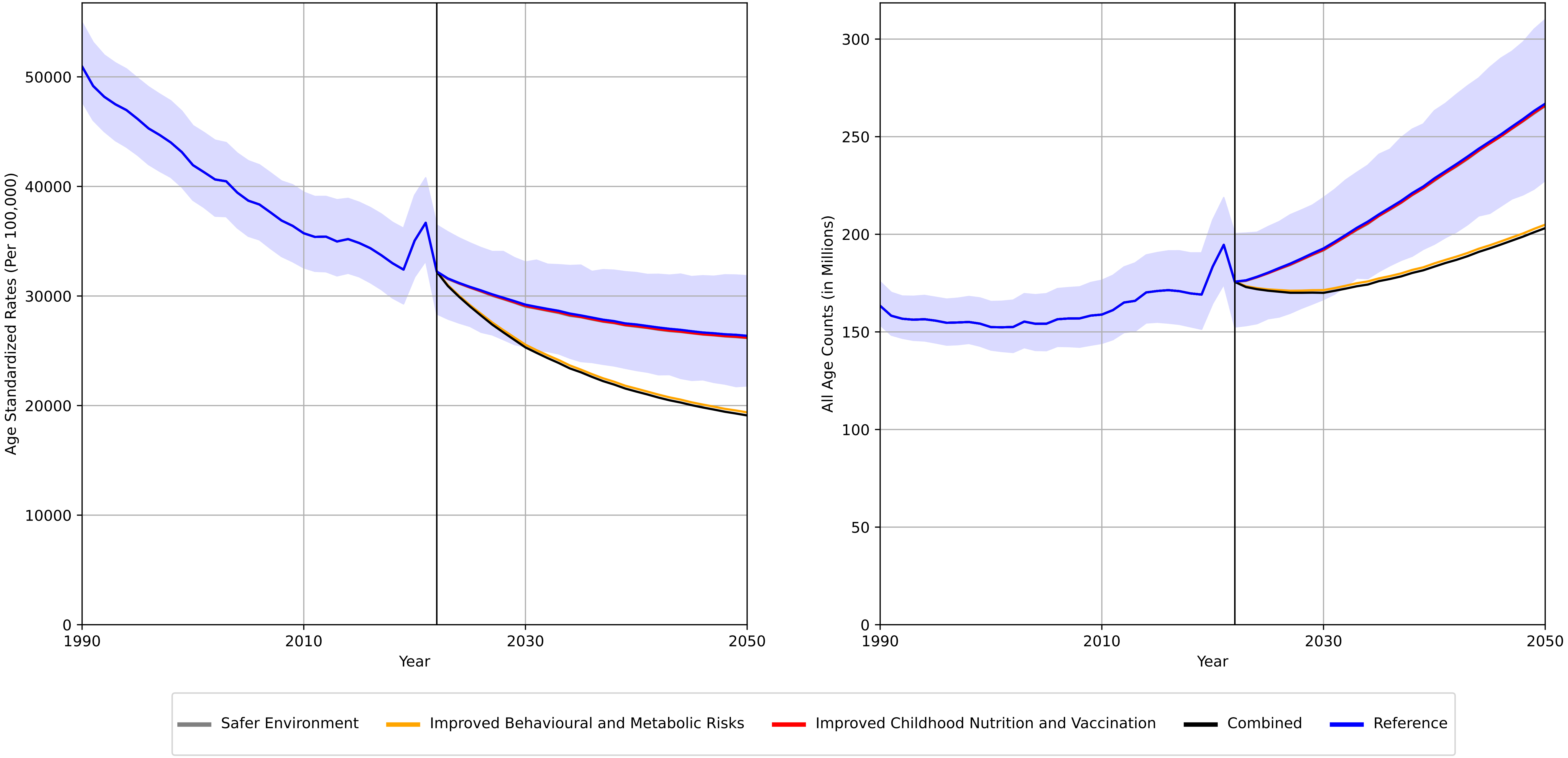

South Asia

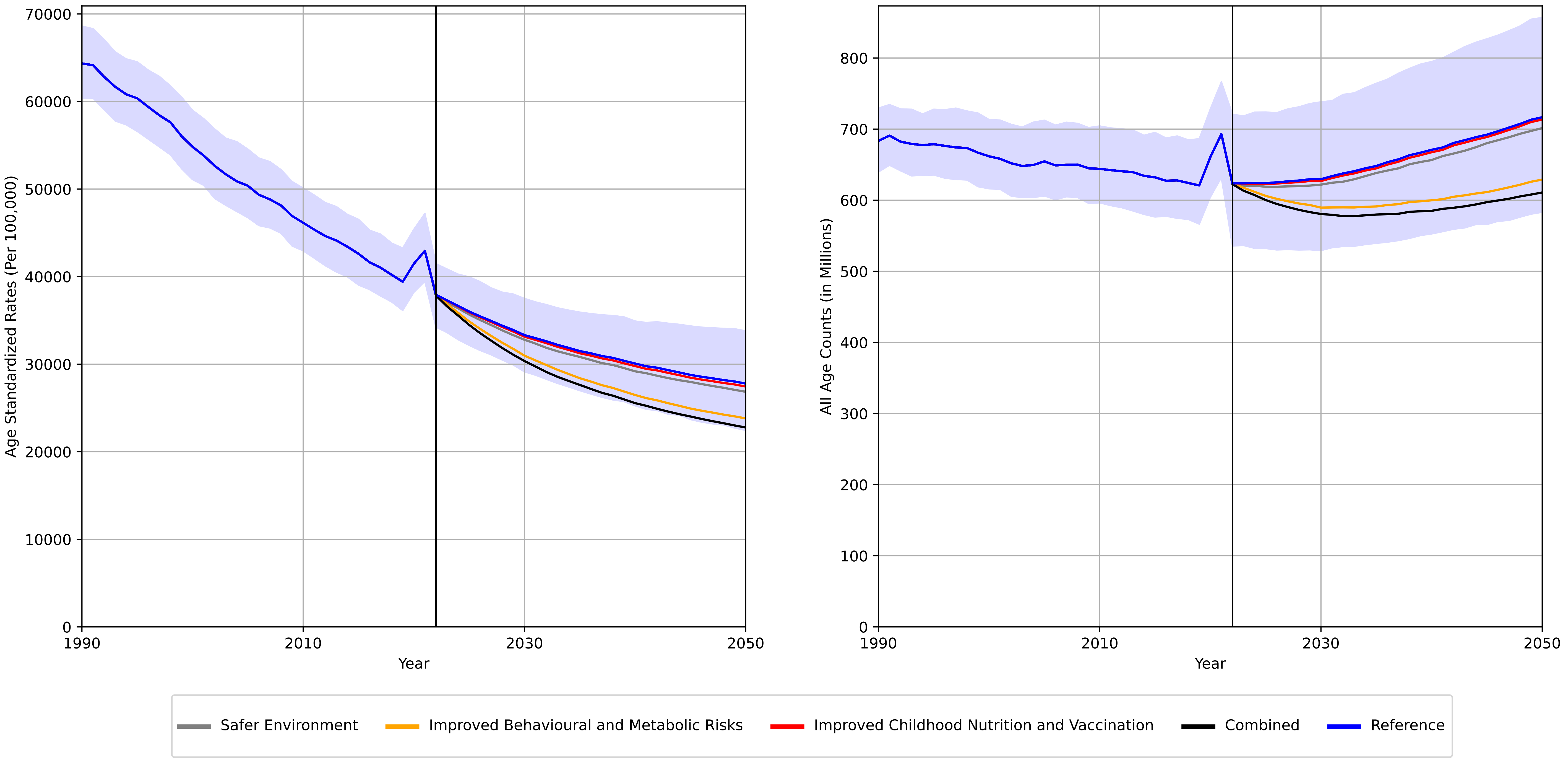

Age Standardized Rates (Per 100,000)
70000
60000
50000
40000
30000
20000
10000
0
1990
2010
2030
2050
Year
All Age Counts (in Millions)
800
700
600
500
400
300
200
100
0
1990
2010
2030
2050
Year
Safer Environment
Improved Behavioural and Metabolic Risks
Improved Childhood Nutrition and Vaccination
Combined
Reference

Southeast Asia, East Asia, And Oceania

Supplemental Results Figure S10. All-cause age standardised DALY rates (A) and all age DALY counts (B), for the past and for five future scenarios, 1990–2050

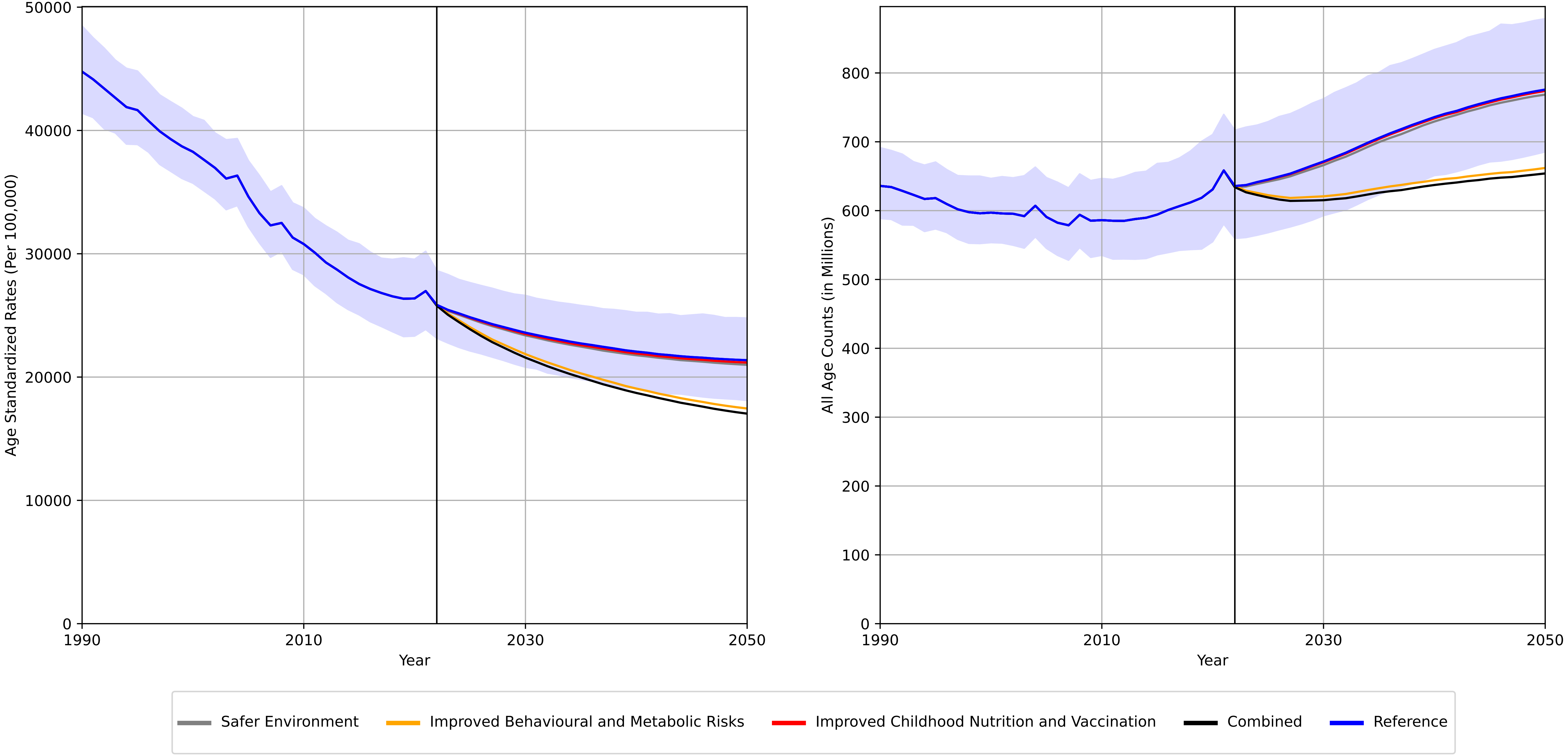

Sub-Saharan Africa

Supplemental Results Figure S10. All-cause age standardised DALY rates (A) and all age DALY counts (B), for the past and for five future scenarios, 1990–2050

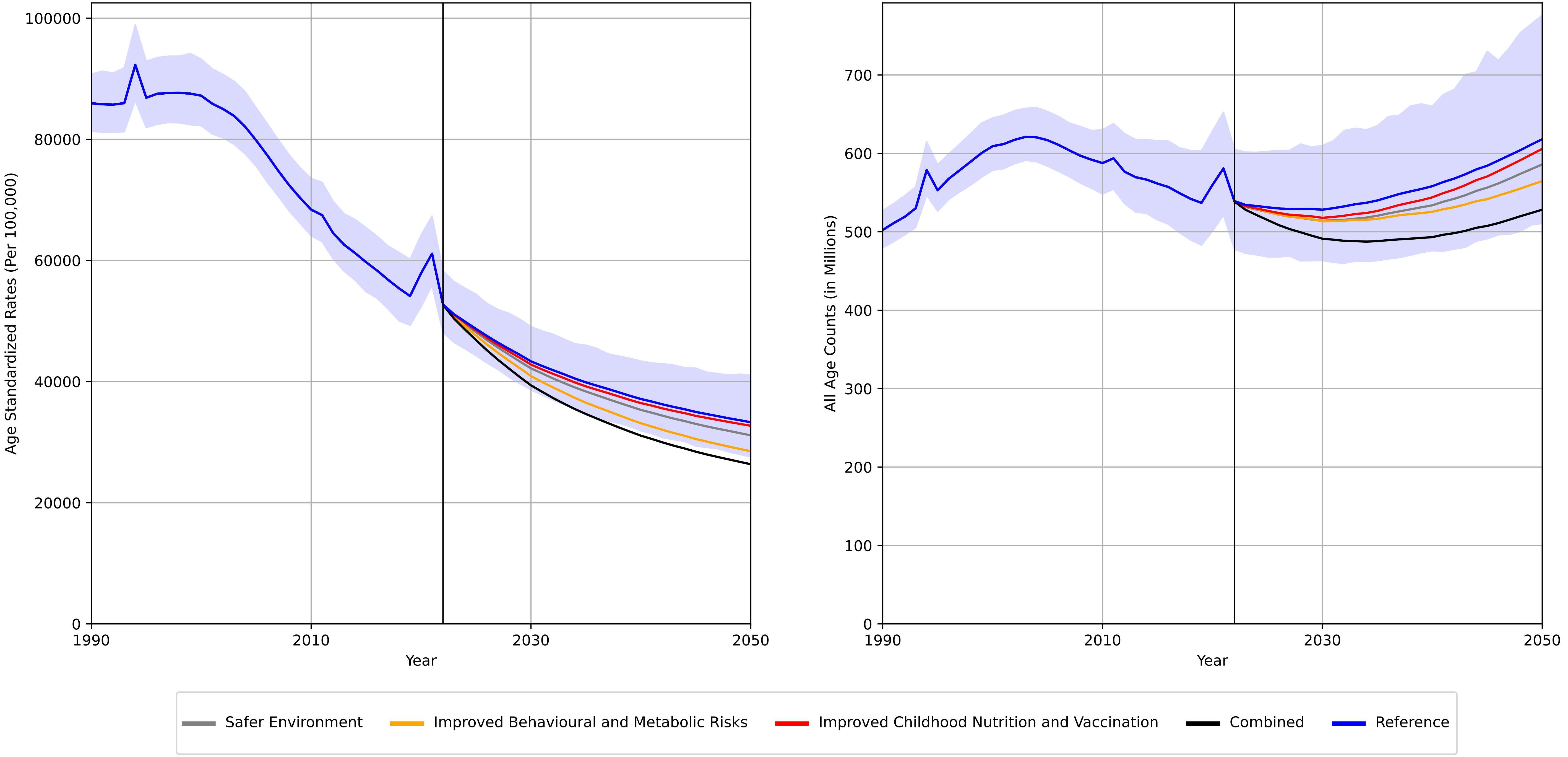

Central Asia

Supplemental Results Figure S10. All-cause age standardised DALY rates (A) and all age DALY counts (B), for the past and for five future scenarios, 1990–2050

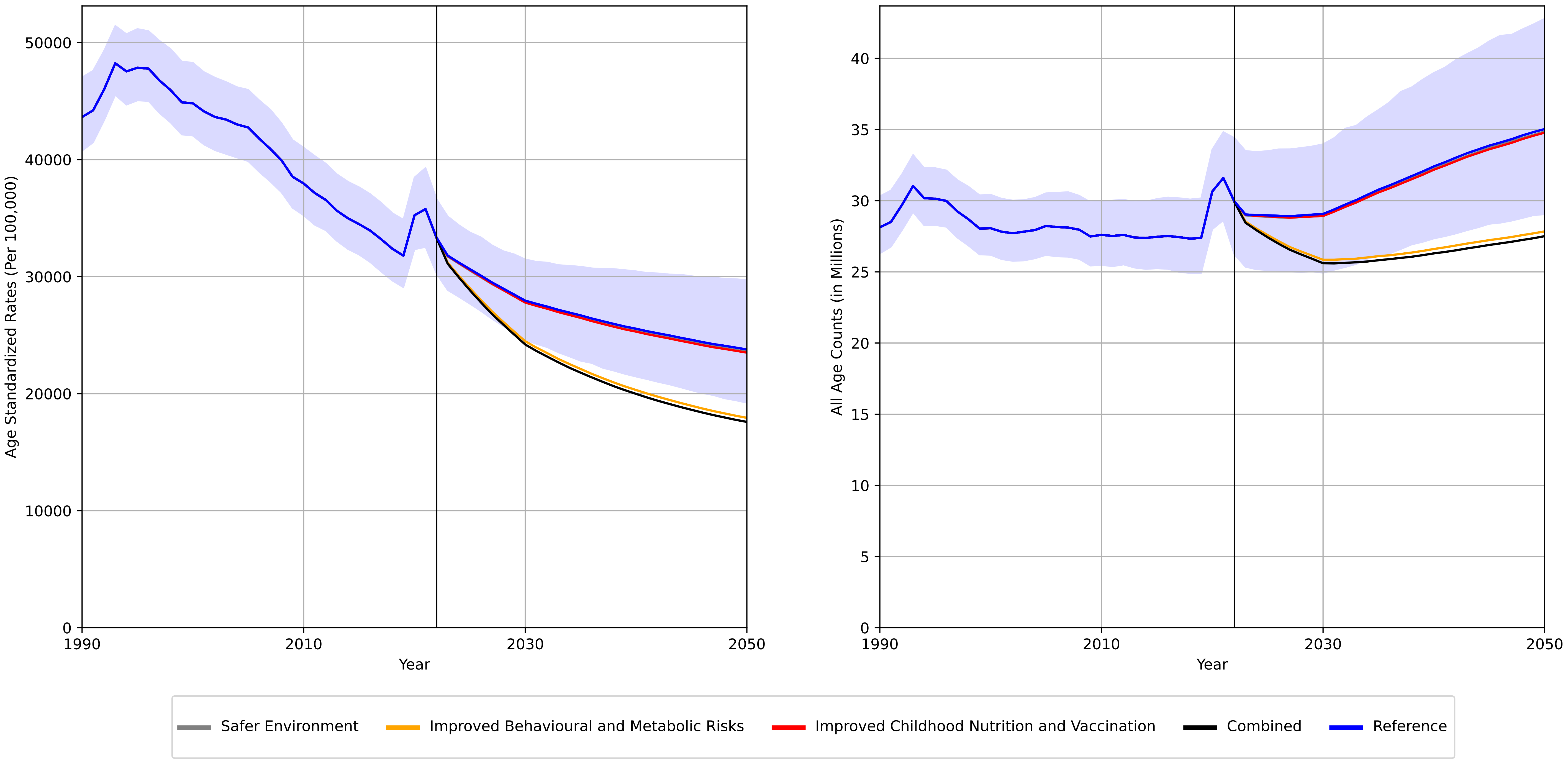

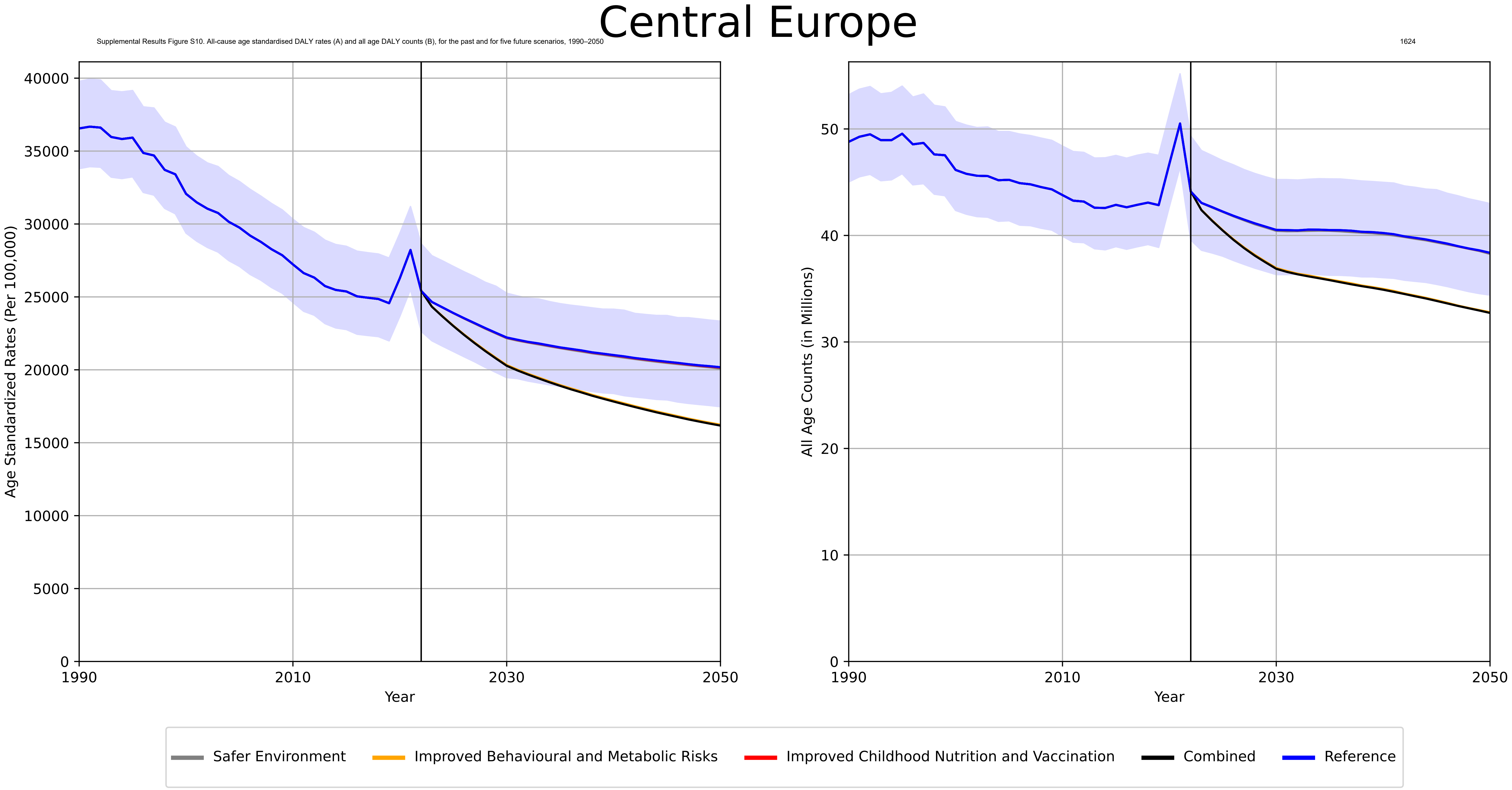
Central Europe
Age Standardized Rates (Per 100,000)
0
5000
10000
15000
20000
25000
30000
35000
40000
1990
2010
2030
2050
Year
All Age Counts (in Millions)
0
10
20
30
40
50
1990
2010
2030
2050
Year
Safer Environment
Improved Behavioural and Metabolic Risks
Improved Childhood Nutrition and Vaccination
Combined
Reference

Eastern Europe

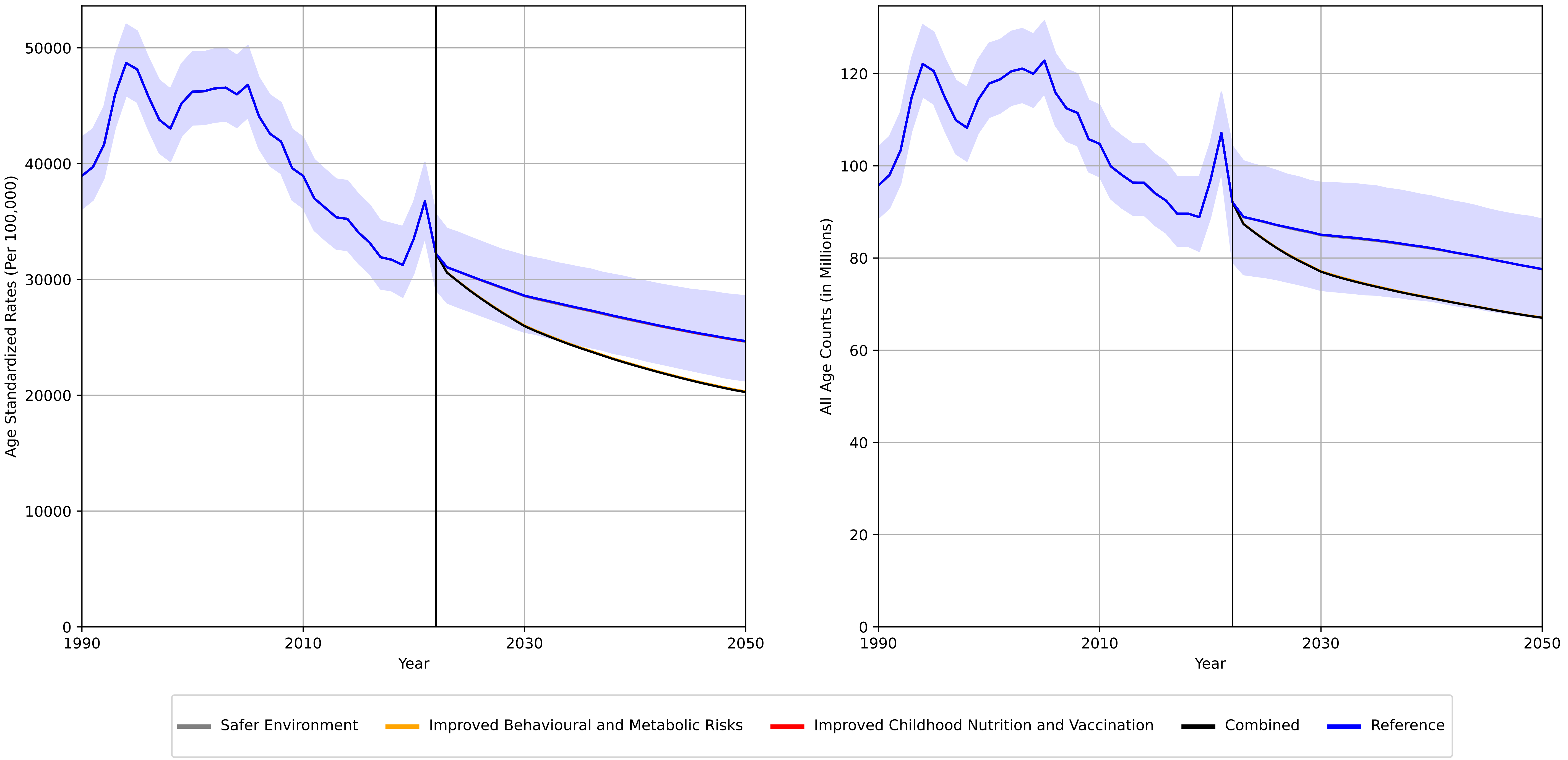

Australasia

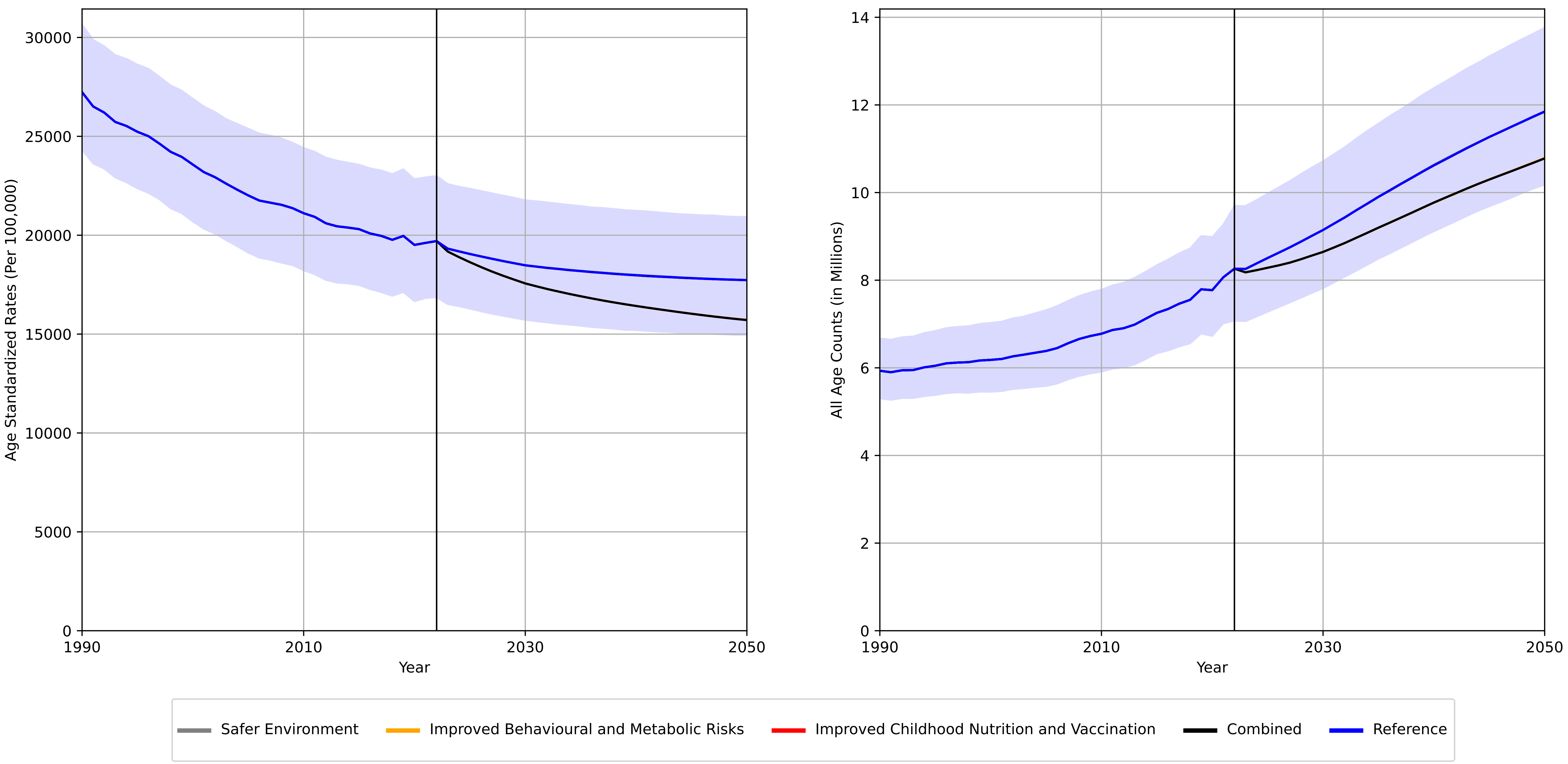

High-Income Asia Pacific

Supplemental Results Figure S10. All-cause age standardised DALY rates (A) and all age DALY counts (B), for the past and for five future scenarios, 1990–2050

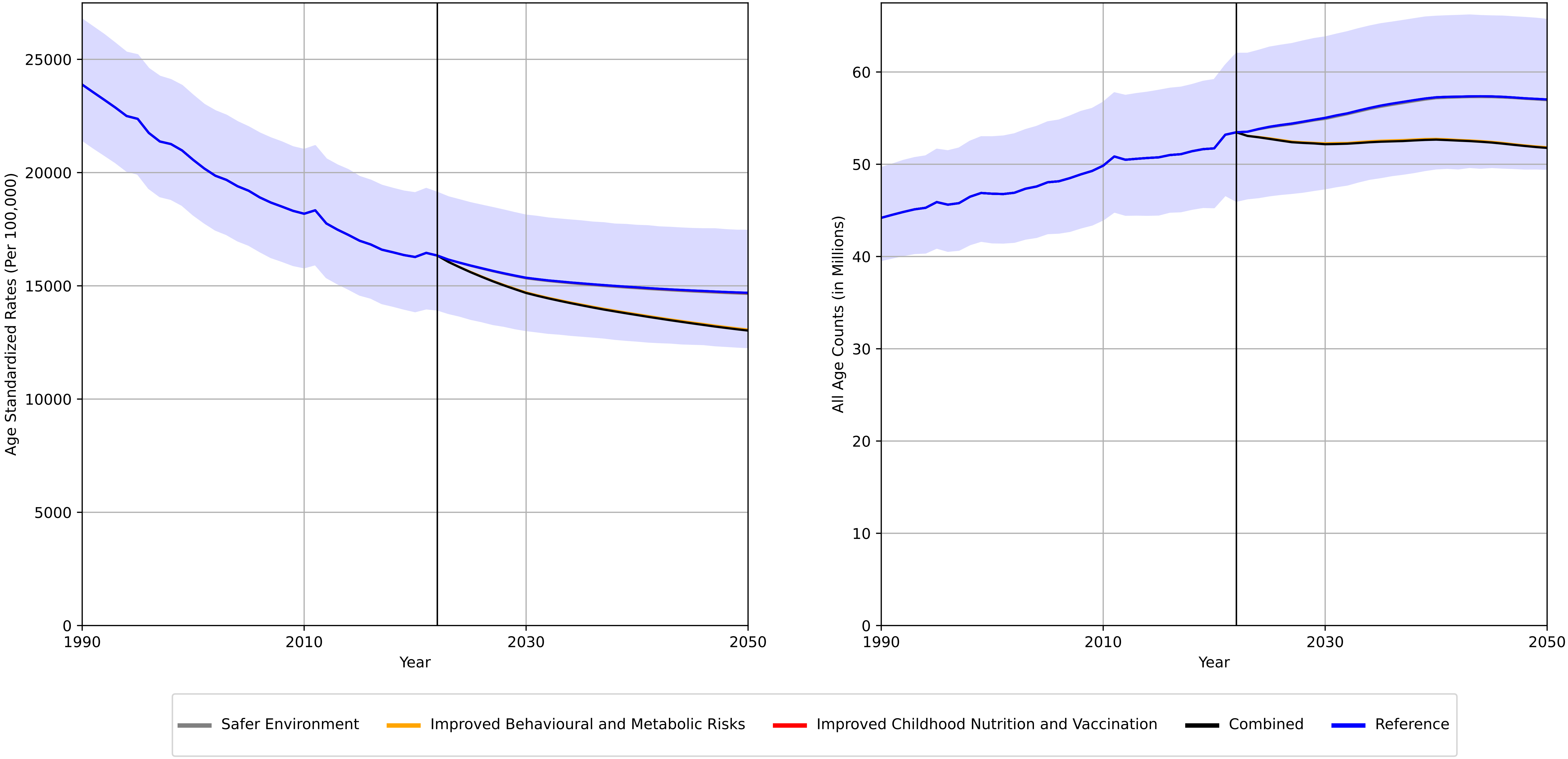

High-Income North America

Supplemental Results Figure S10. All-cause age standardised DALY rates (A) and all age DALY counts (B), for the past and for five future scenarios, 1990–2050

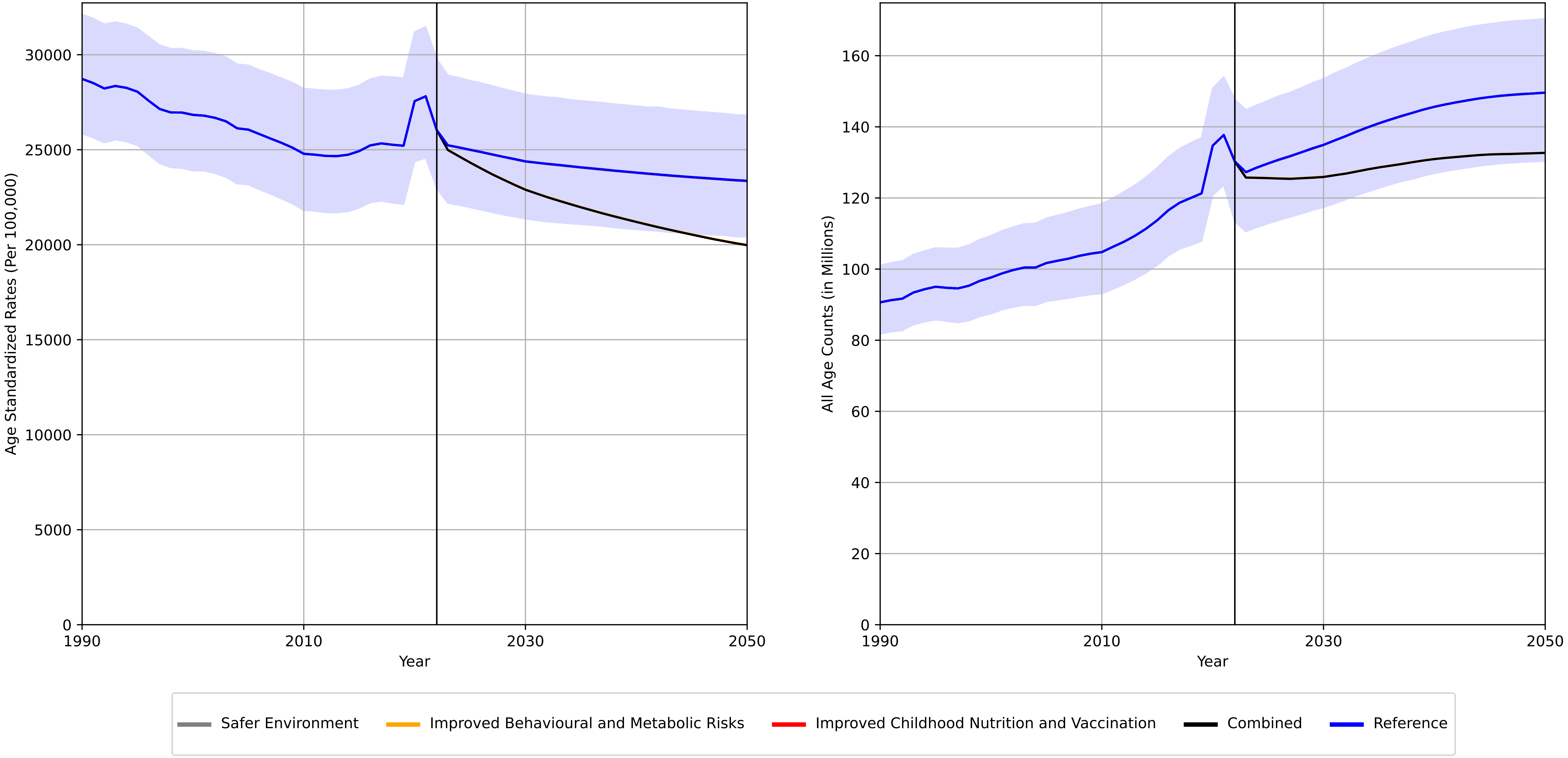

Southern Latin America

Supplemental Results Figure S10. All-cause age standardised DALY rates (A) and all age DALY counts (B), for the past and for five future scenarios, 1990–2050

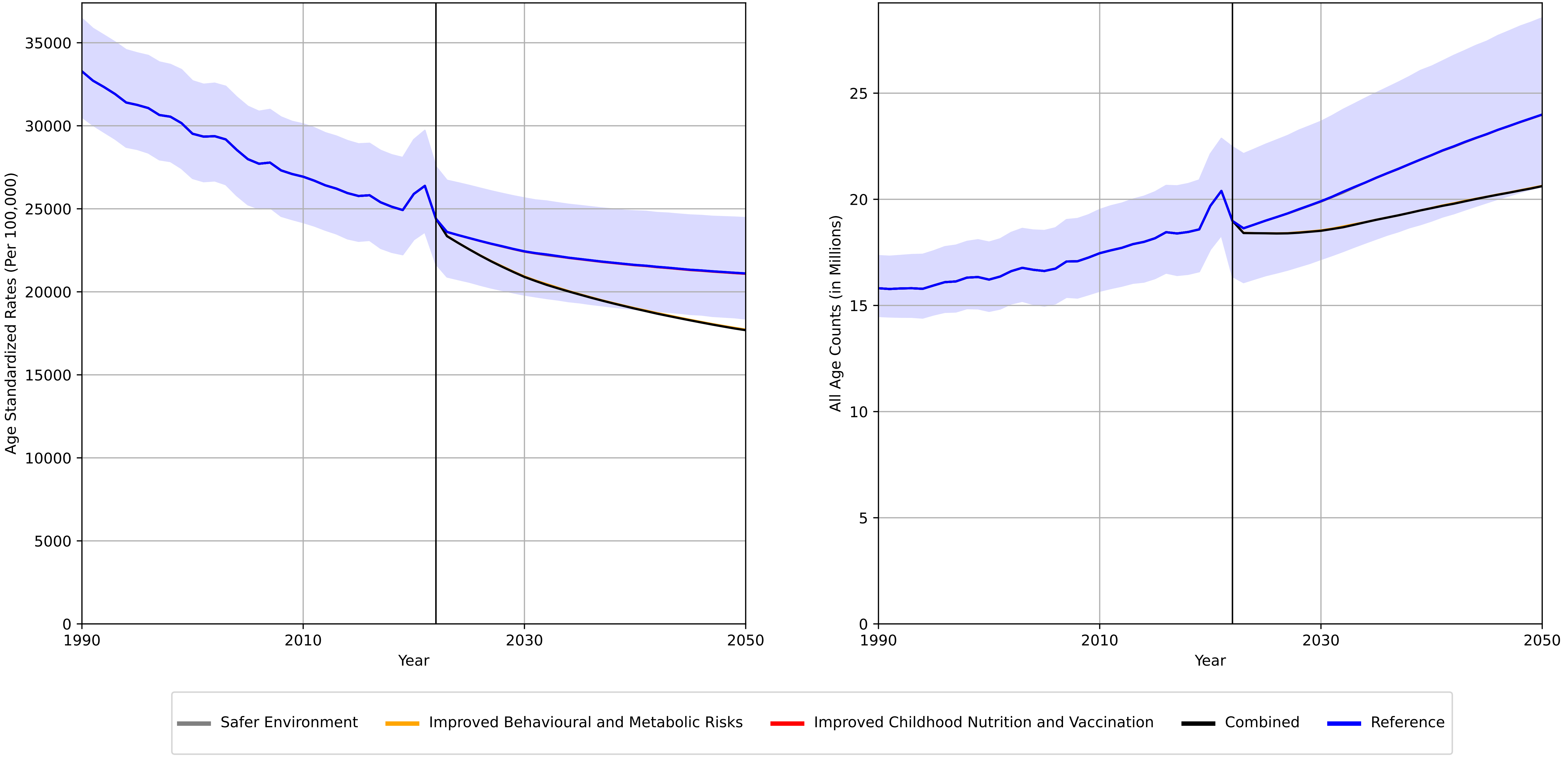

Western Europe

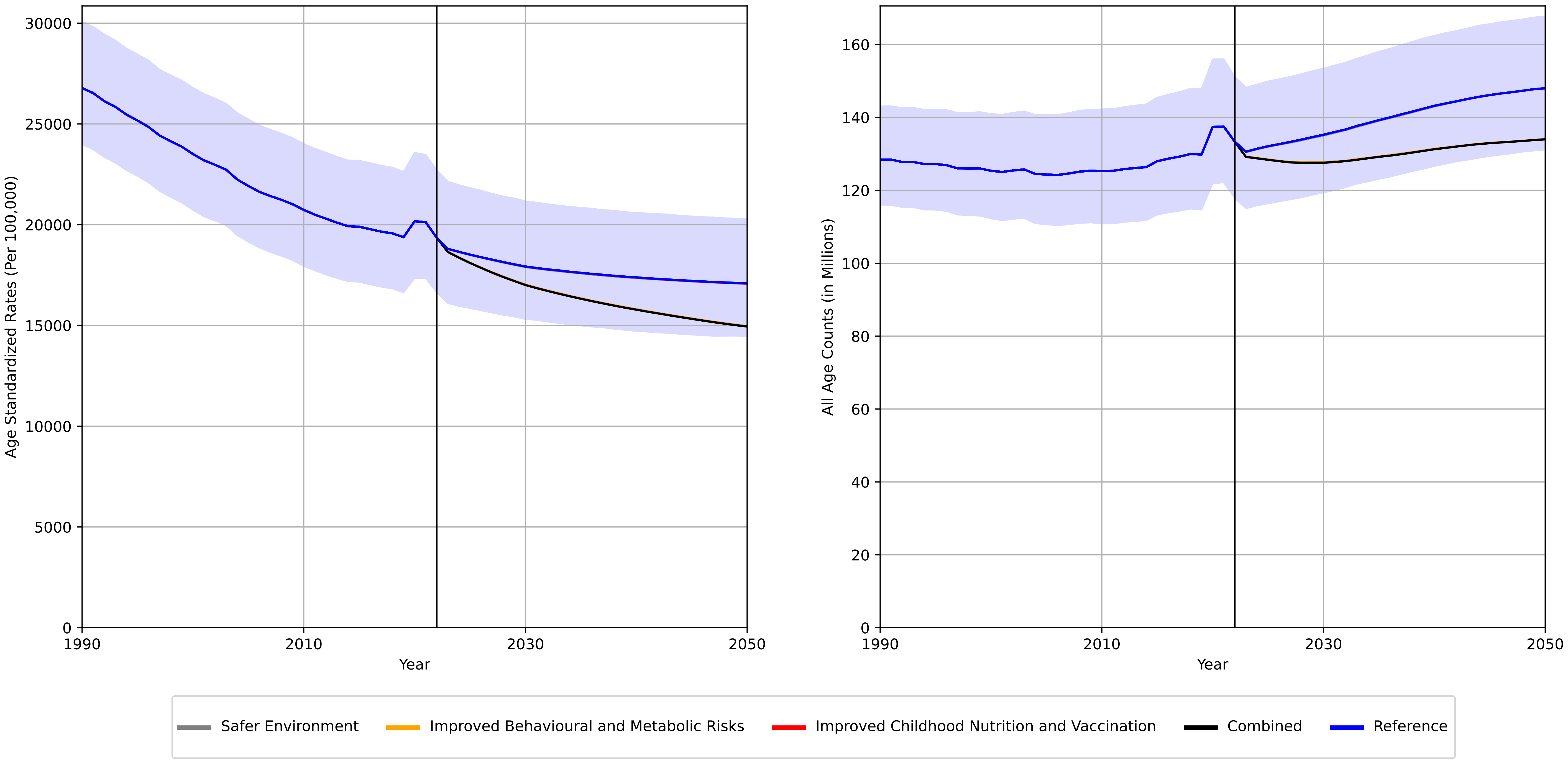

Andean Latin America

Supplemental Results Figure S10. All-cause age standardised DALY rates (A) and all age DALY counts (B), for the past and for five future scenarios, 1990–2050

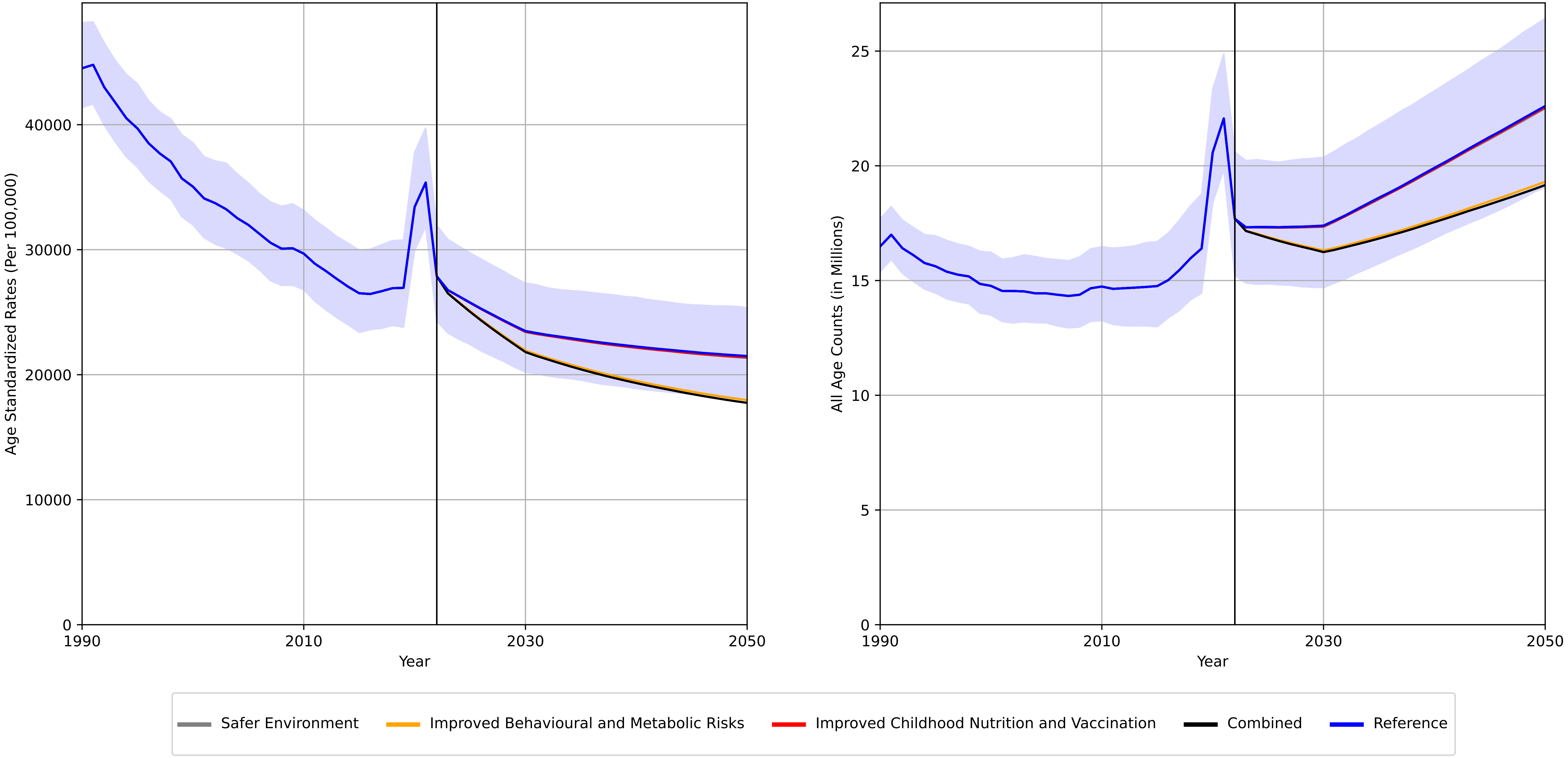

Caribbean

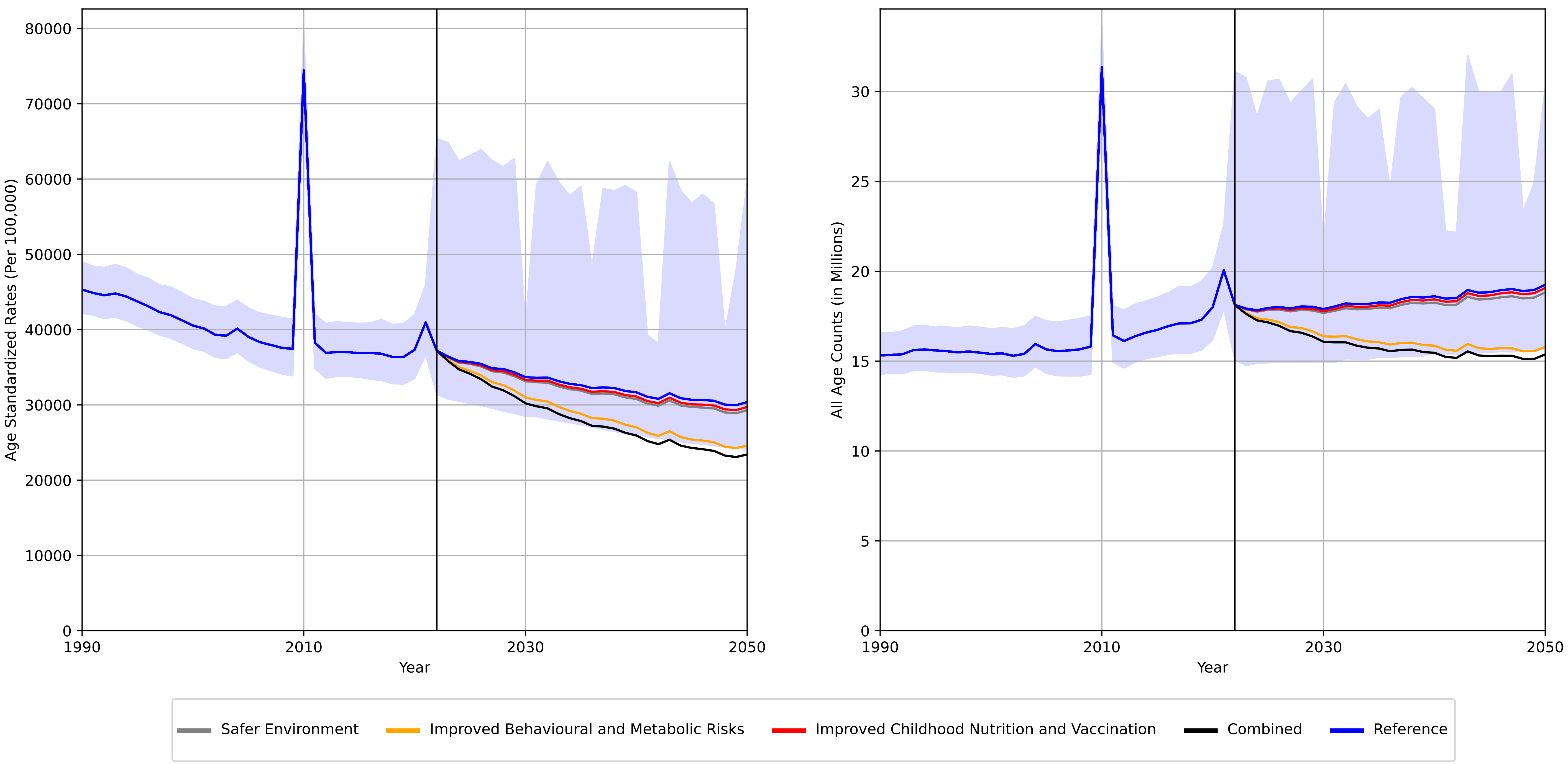
Age Standardized Rates (Per 100,000)
80000
70000
60000
50000
40000
30000
20000
10000
0
1990
2010
2030
2050
Year
All Age Counts (in Millions)
30
25
20
15
10
5
0
Safer Environment
Improved Behavioural and Metabolic Risks
Improved Childhood Nutrition and Vaccination
Combined
Reference

Supplemental Results Figure S10. All-cause age standardised DALY rates (A) and all age DALY counts (B), for the past and for five future scenarios, 1990–2050

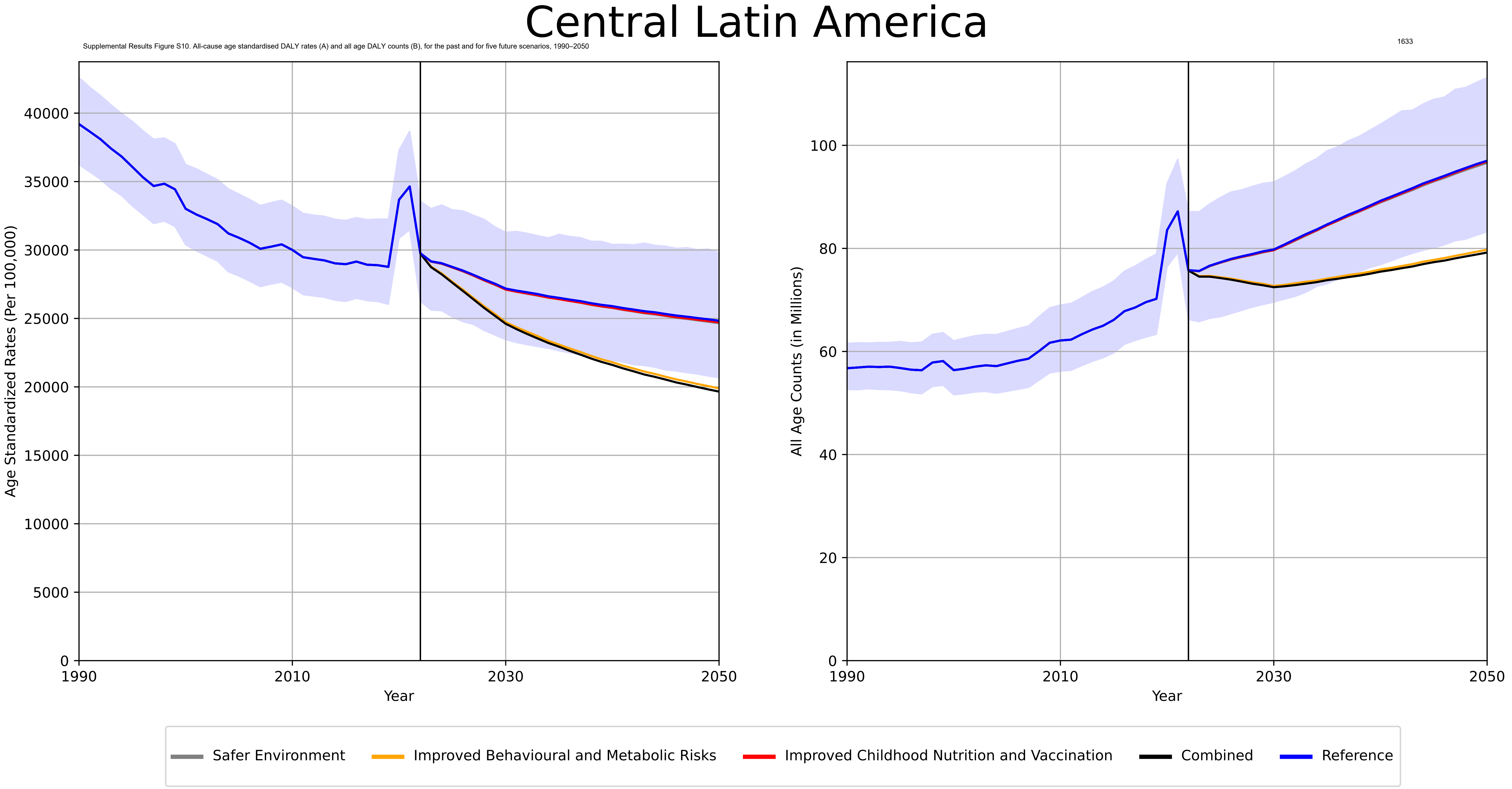

Tropical Latin America

Supplemental Results Figure S10. All-cause age standardised DALY rates (A) and all age DALY counts (B), for the past and for five future scenarios, 1990–2050

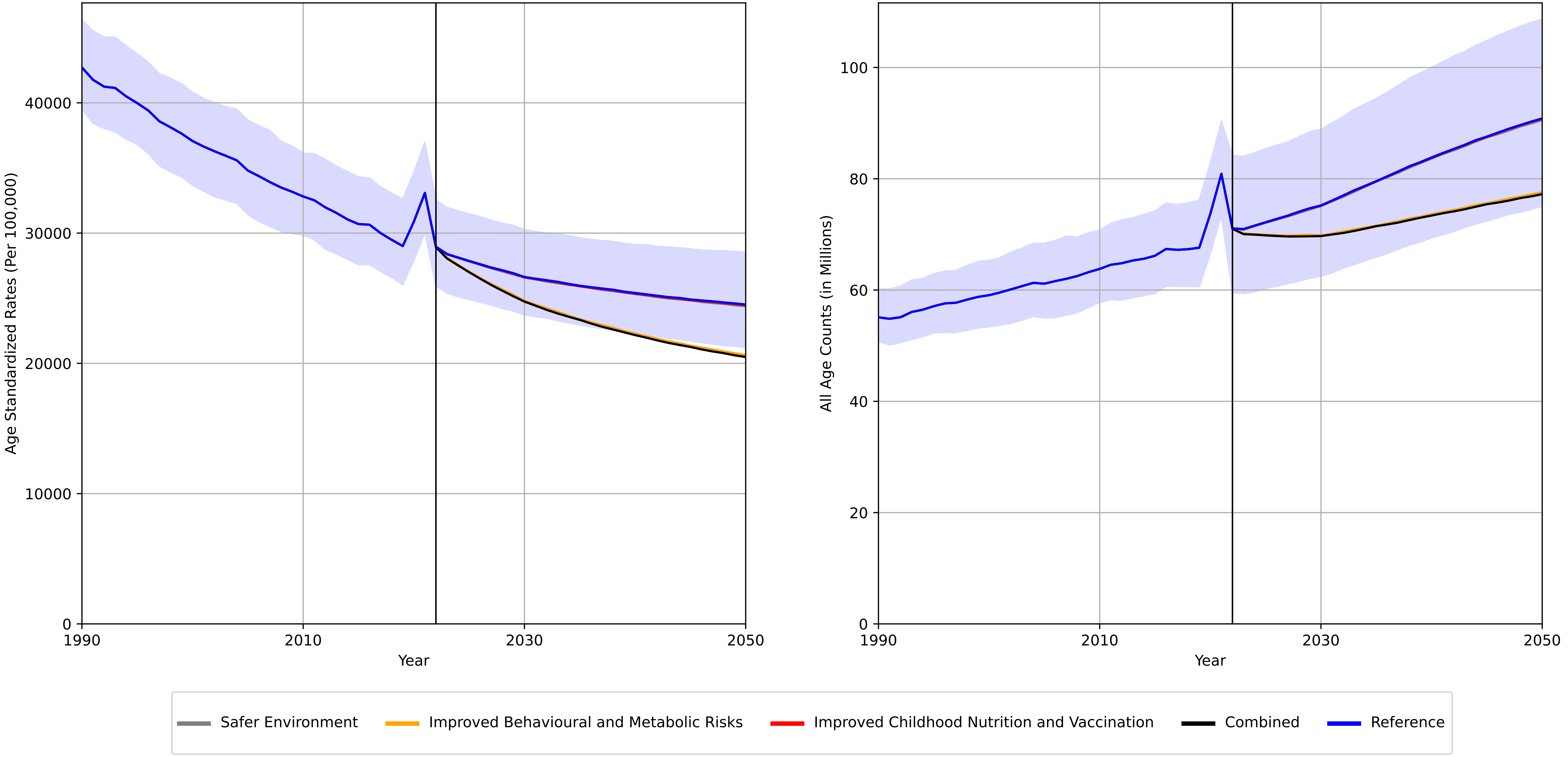

North Africa And Middle East

Supplemental Results Figure S10. All-cause age standardised DALY rates (A) and all age DALY counts (B), for the past and for five future scenarios, 1990–2050

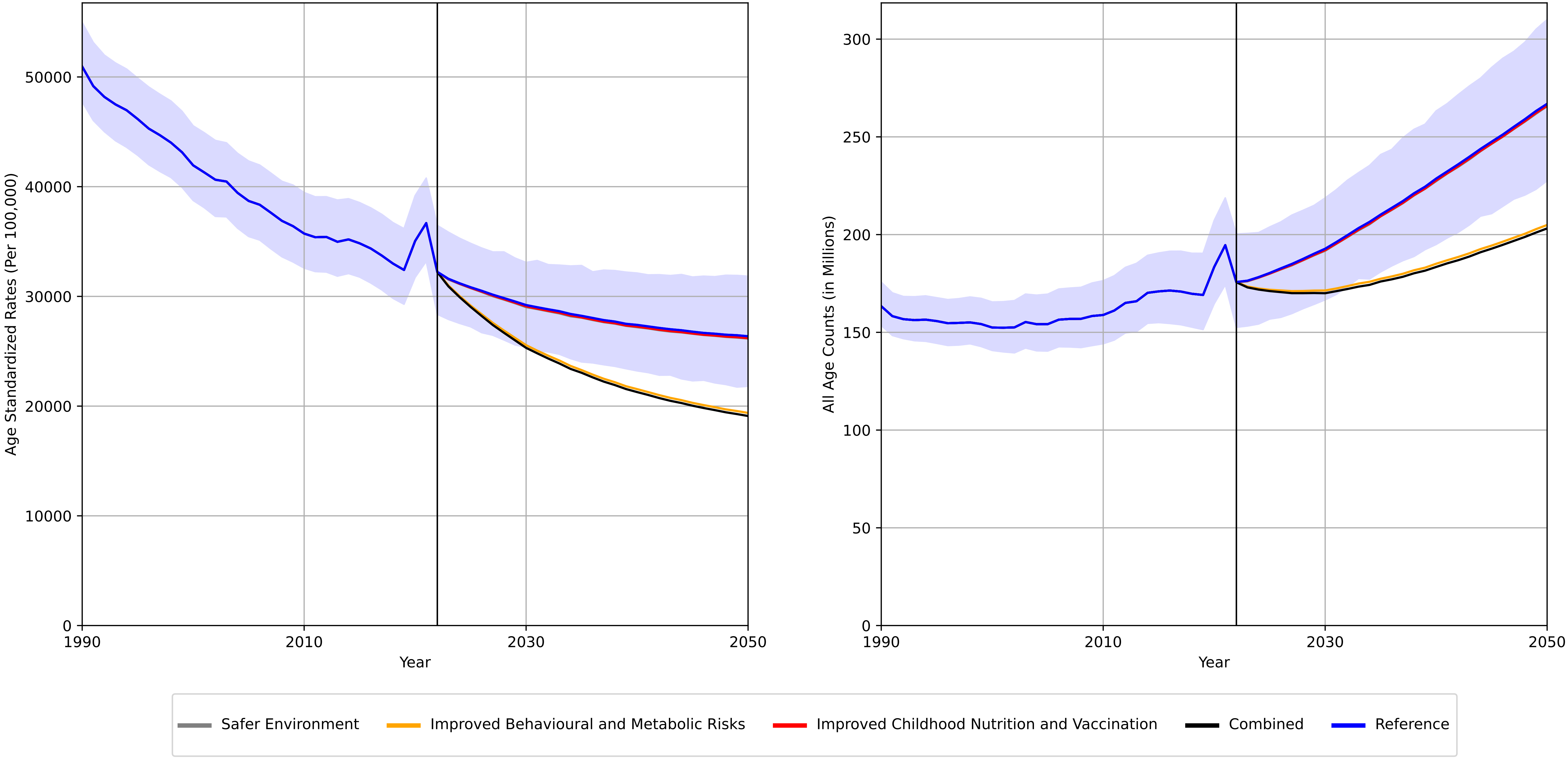

South Asia

Supplemental Results Figure S10. All-cause age standardised DALY rates (A) and all age DALY counts (B), for the past and for five future scenarios, 1990–2050

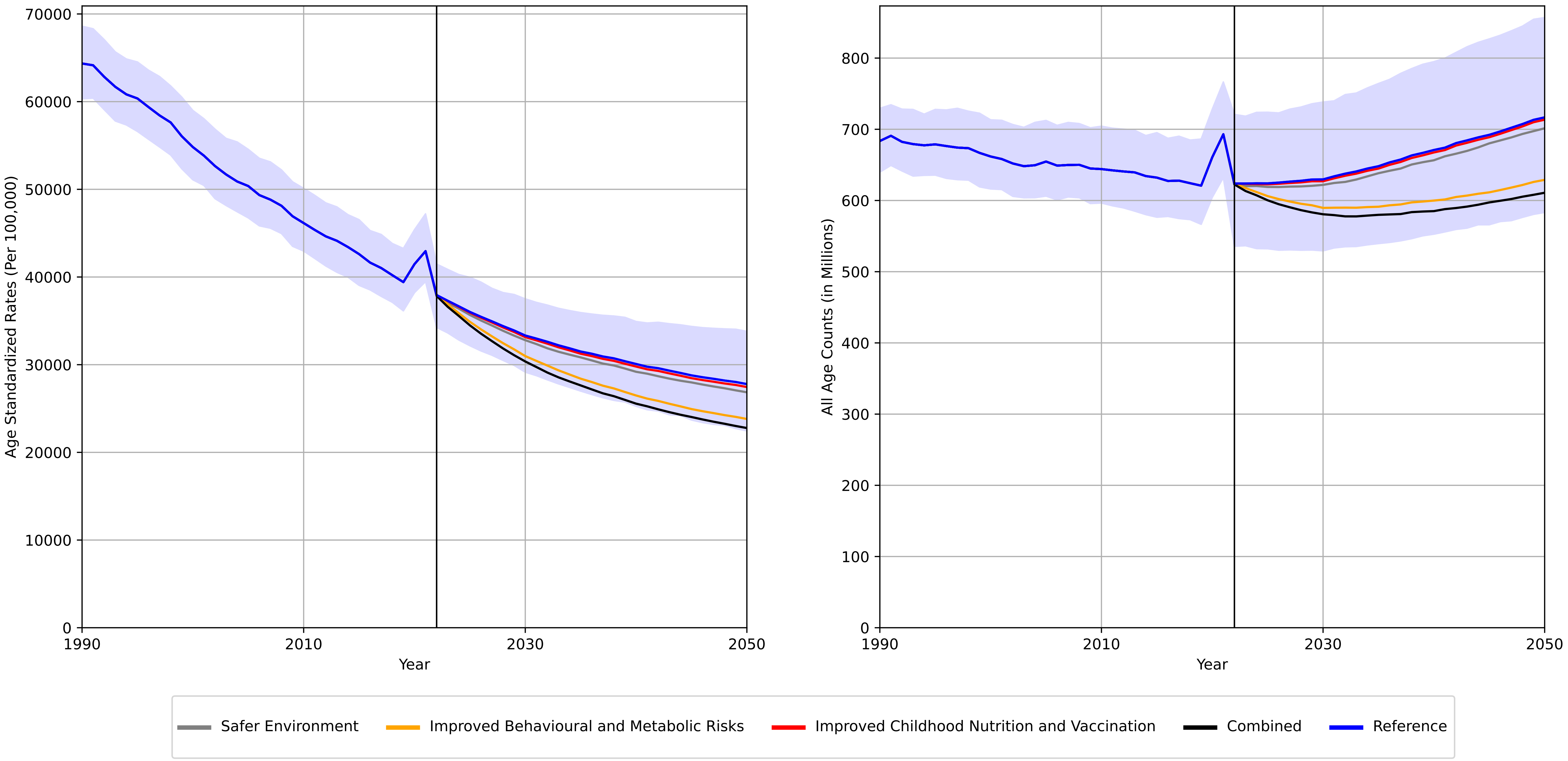

East Asia

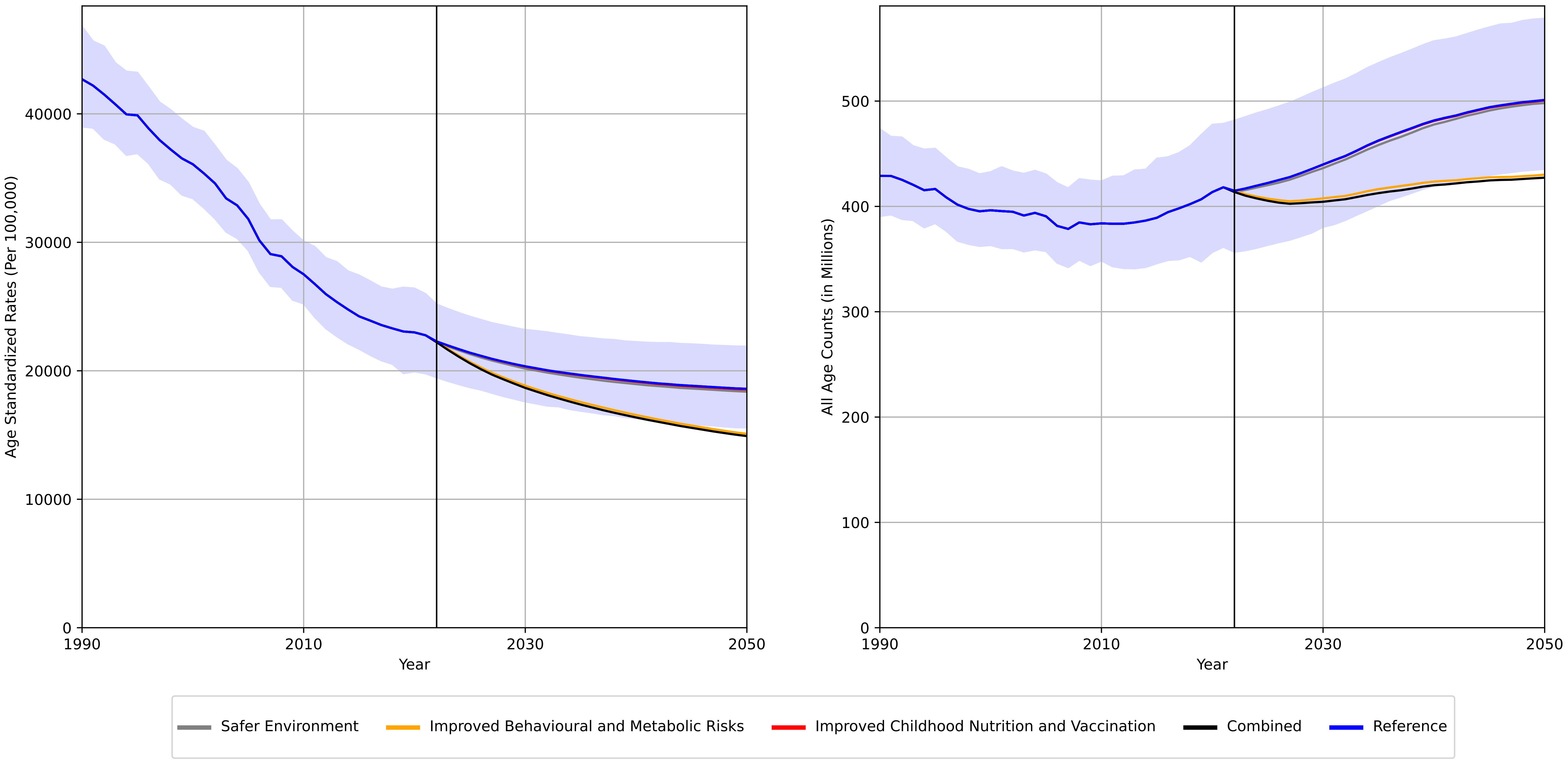
Age Standardized Rates (Per 100,000)
40000
30000
20000
10000
0
1990
2010
2030
2050
Year
All Age Counts (in Millions)
500
400
300
200
100
0
1990
2010
2030
2050
Year
Safer Environment
Improved Behavioural and Metabolic Risks
Improved Childhood Nutrition and Vaccination
Combined
Reference

Oceania

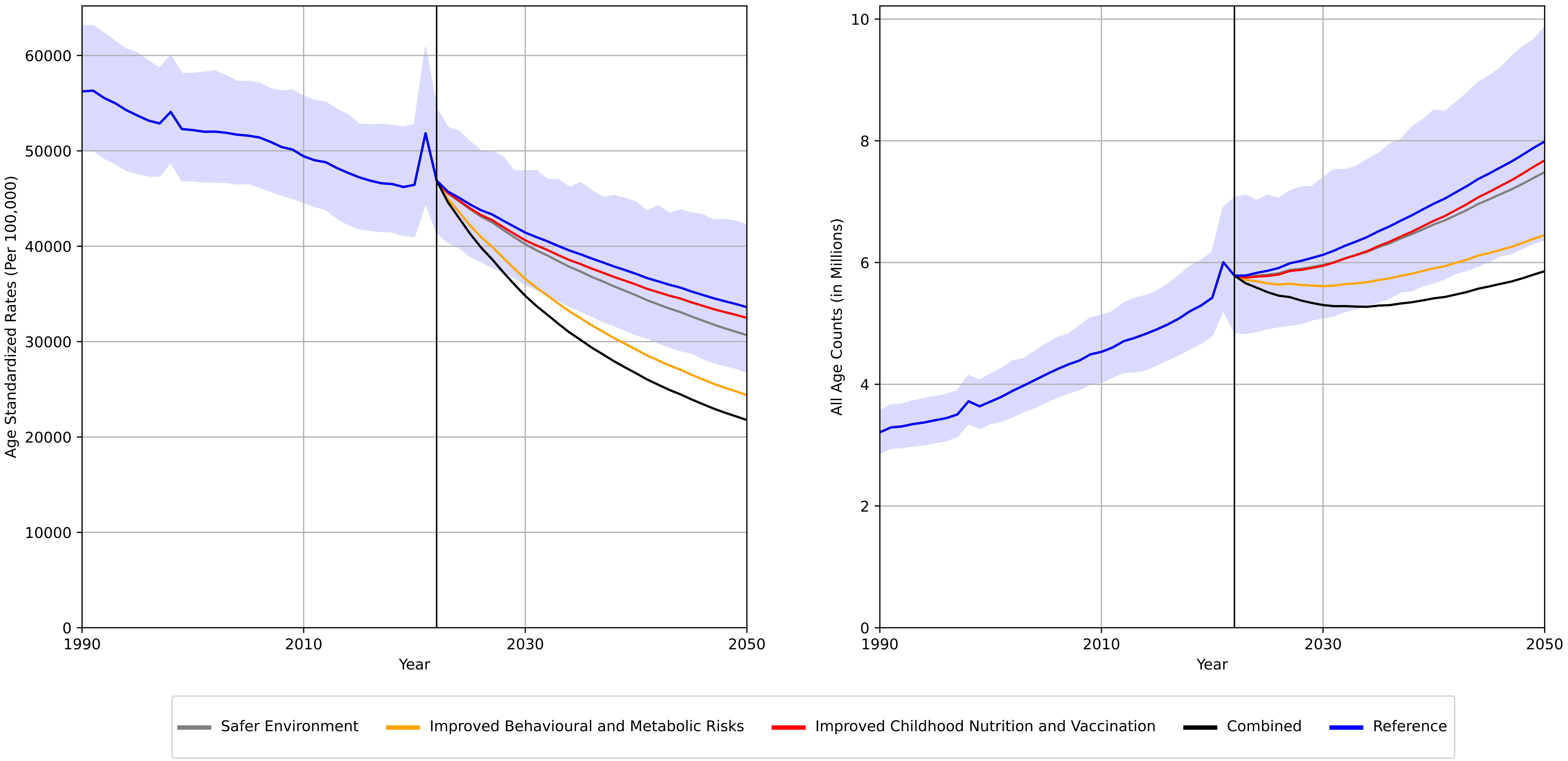
Age Standardized Rates (Per 100,000)
60000
50000
40000
30000
20000
10000
0
1990
2010
2030
2050
Year
All Age Counts (in Millions)
10
8
6
4
2
0
1990
2010
2030
2050
Year
Safer Environment
Improved Behavioural and Metabolic Risks
Improved Childhood Nutrition and Vaccination
Combined
Reference

Southeast Asia

Supplemental Results Figure S10. All-cause age standardised DALY rates (A) and all age DALY counts (B), for the past and for five future scenarios, 1990–2050

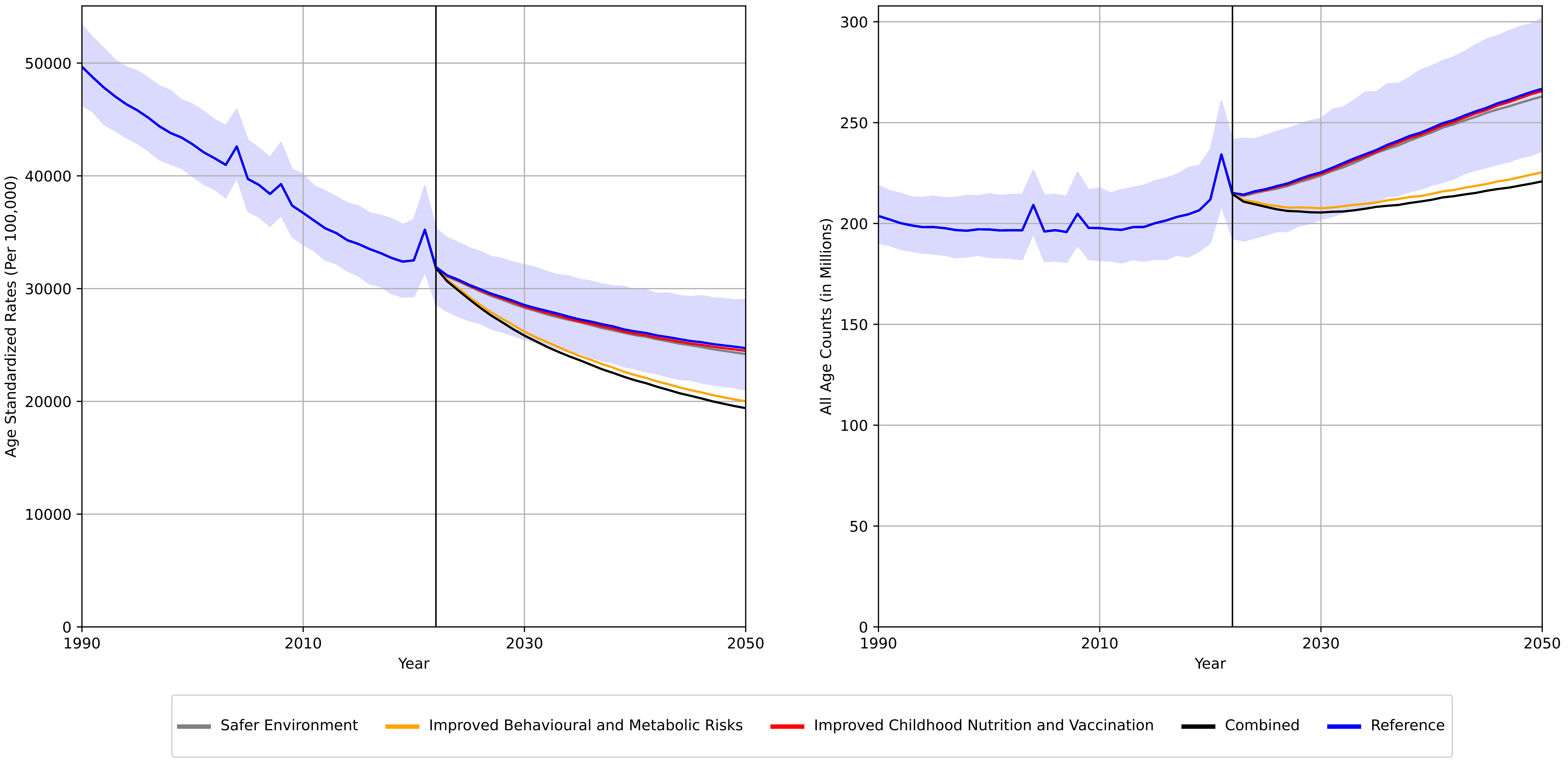

Supplemental Results Figure S10. All-cause age standardised DALY rates (A) and all age DALY counts (B), for the past and for five future scenarios, 1990–2050

Central Sub-Saharan Africa

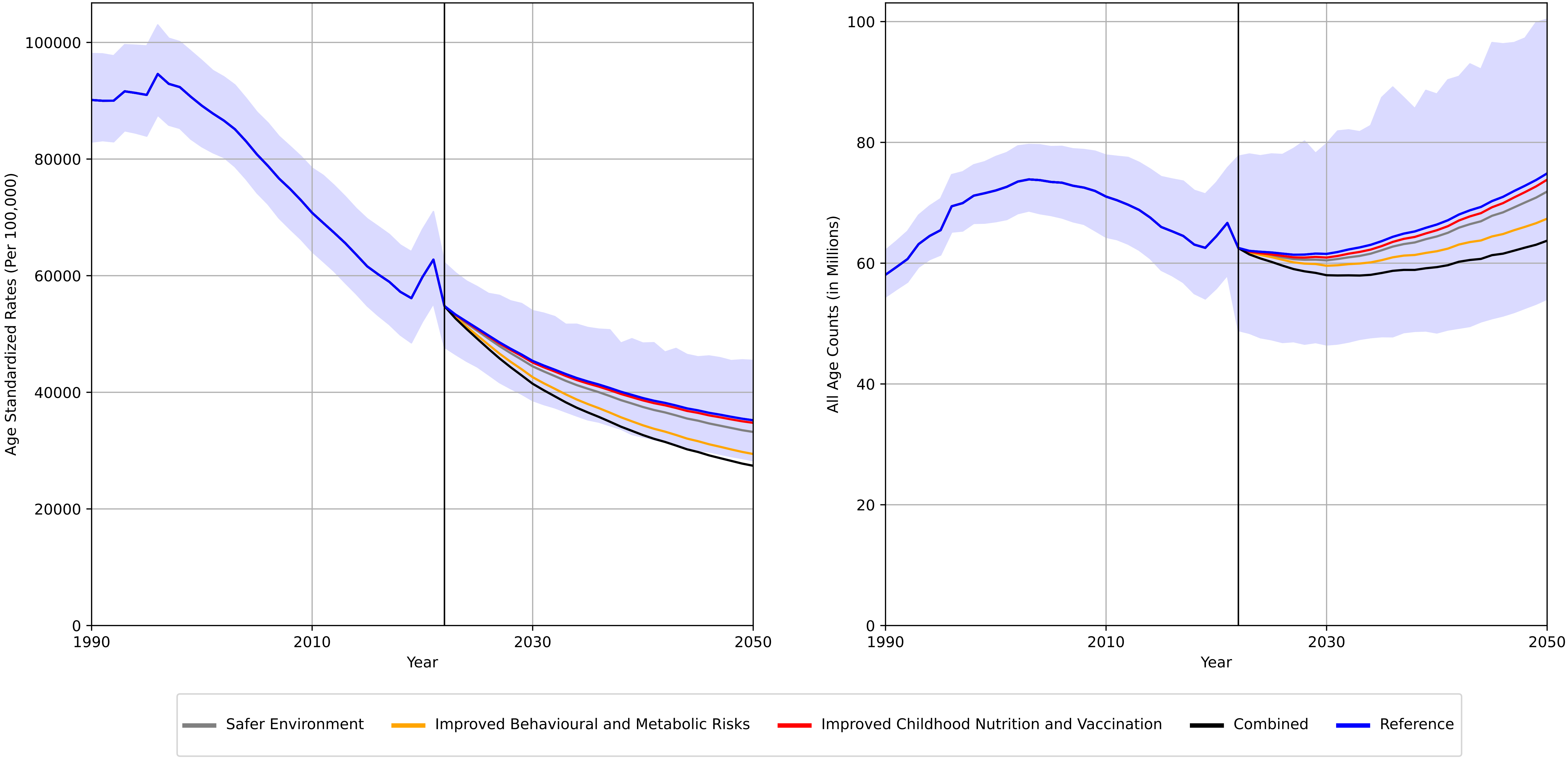

Eastern Sub-Saharan Africa

Supplemental Results Figure S10. All-cause age standardised DALY rates (A) and all age DALY counts (B), for the past and for five future scenarios, 1990–2050

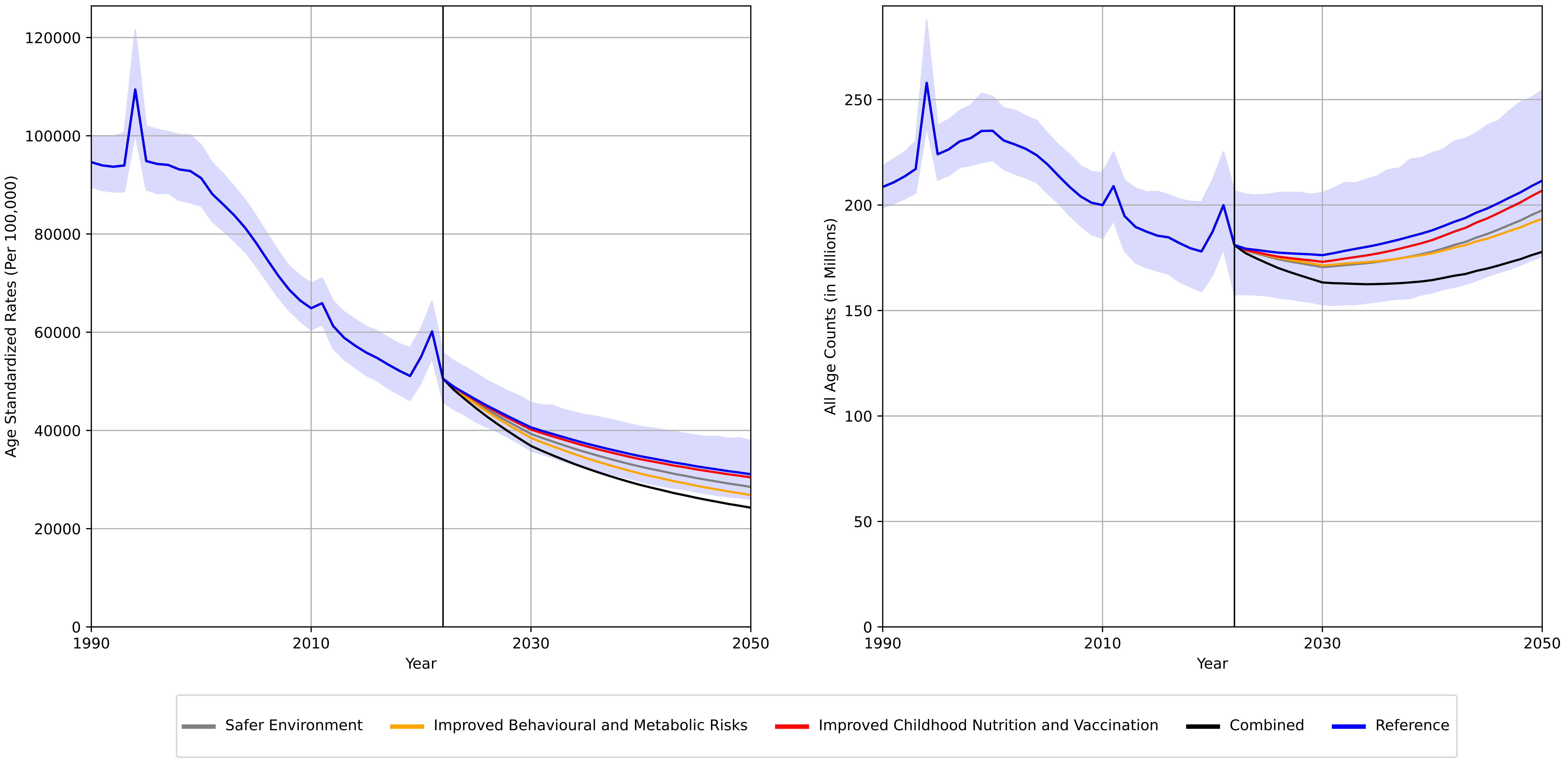

Southern Sub-Saharan Africa

Supplemental Results Figure S10. All-cause age standardised DALY rates (A) and all age DALY counts (B), for the past and for five future scenarios, 1990–2050

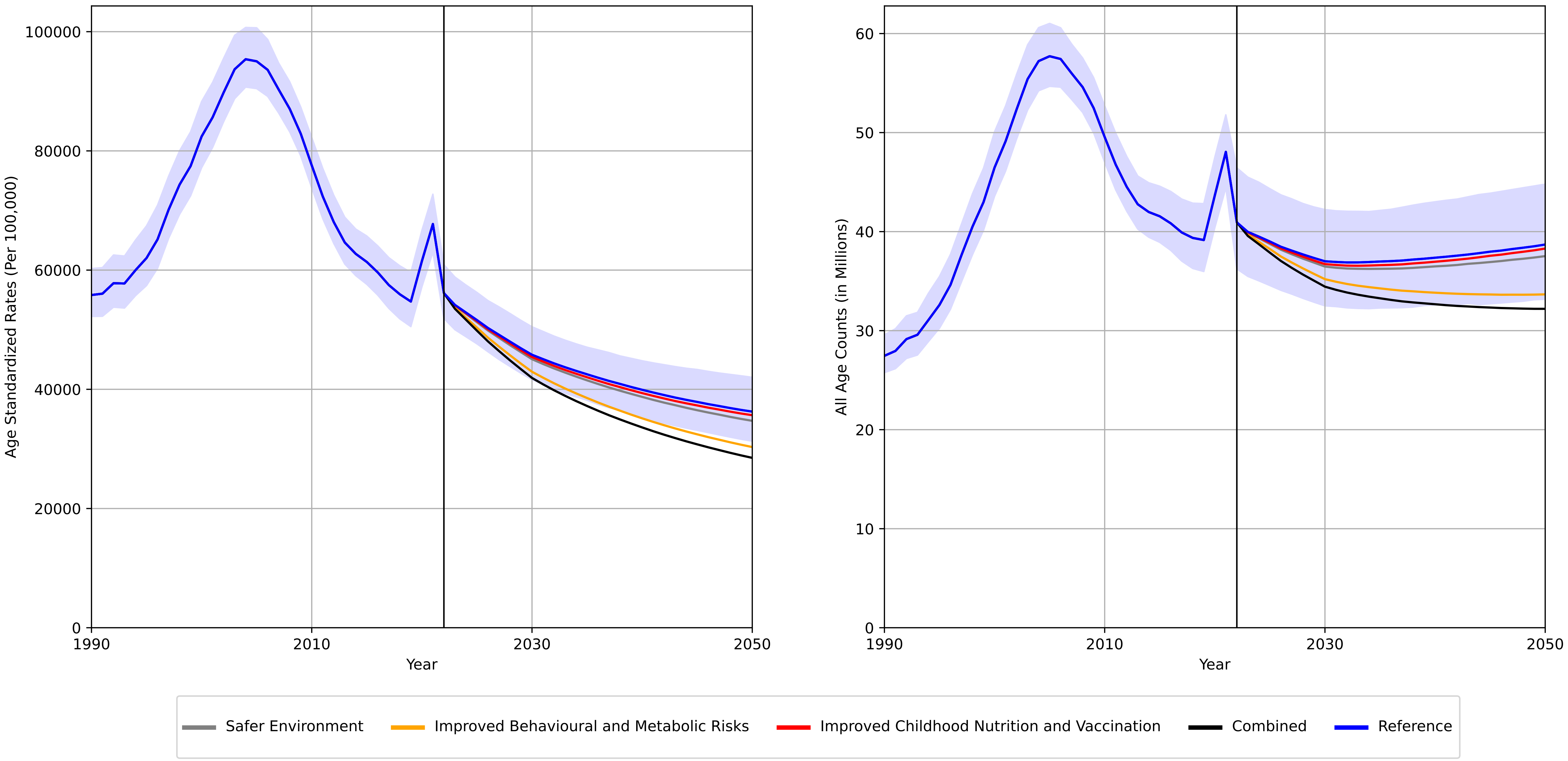

Western Sub-Saharan Africa

Supplemental Results Figure S10. All-cause age standardised DALY rates (A) and all age DALY counts (B), for the past and for five future scenarios, 1990–2050

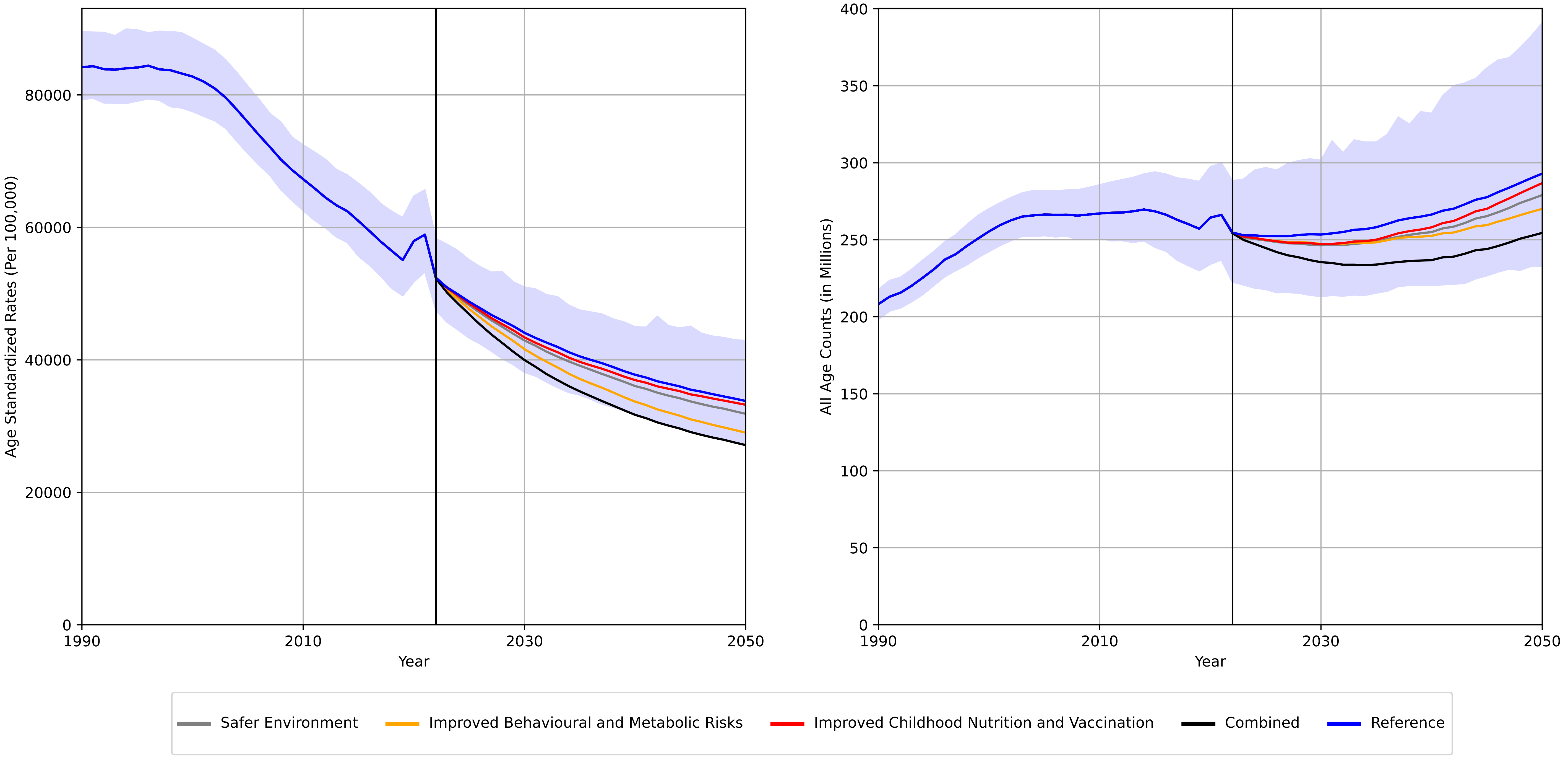

Armenia

Supplemental Results Figure S11. All-cause age standardised death rates (A) and all age death counts (B), for the past and for five future scenarios, 1990–2050

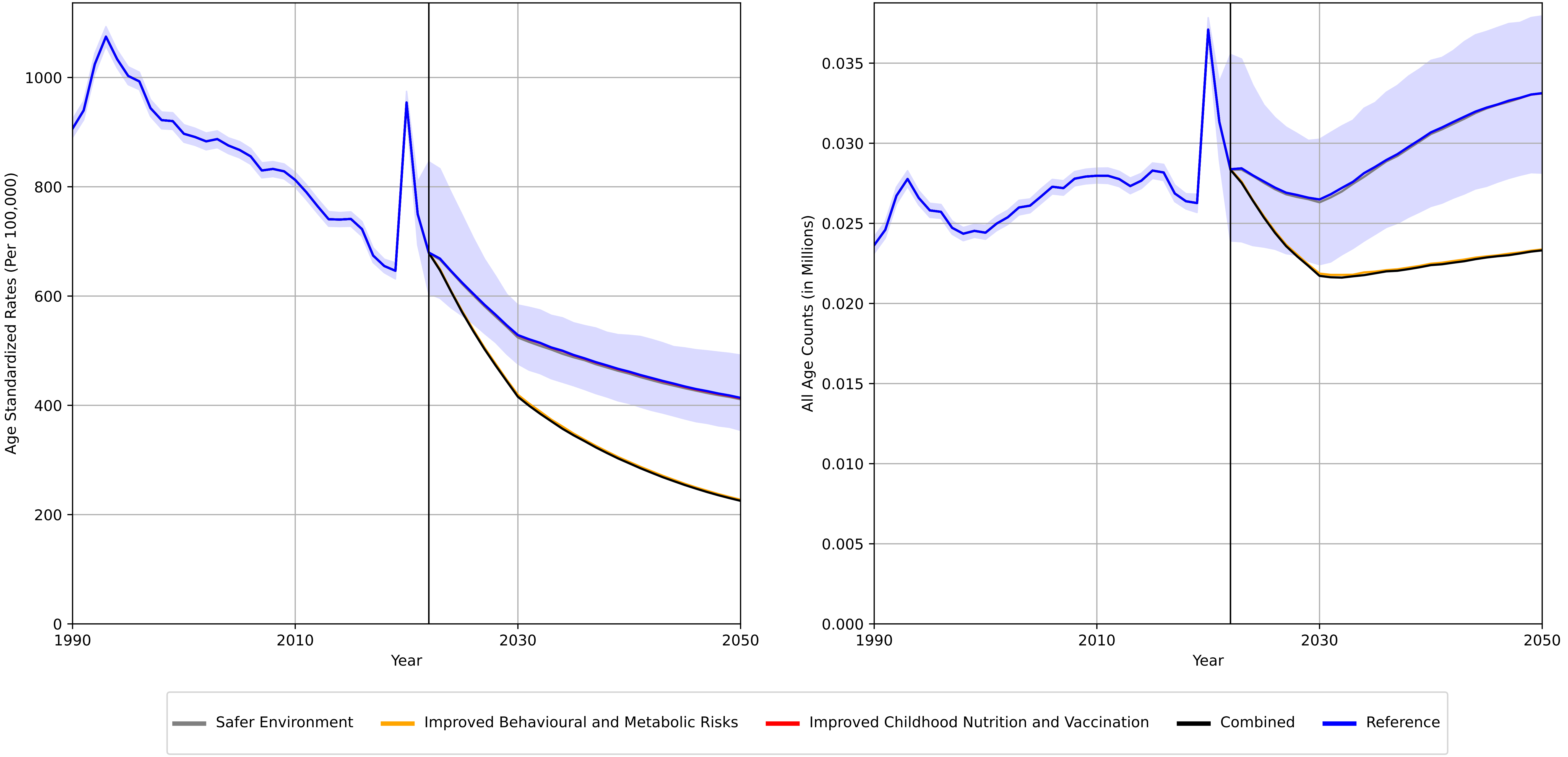

Azerbaijan

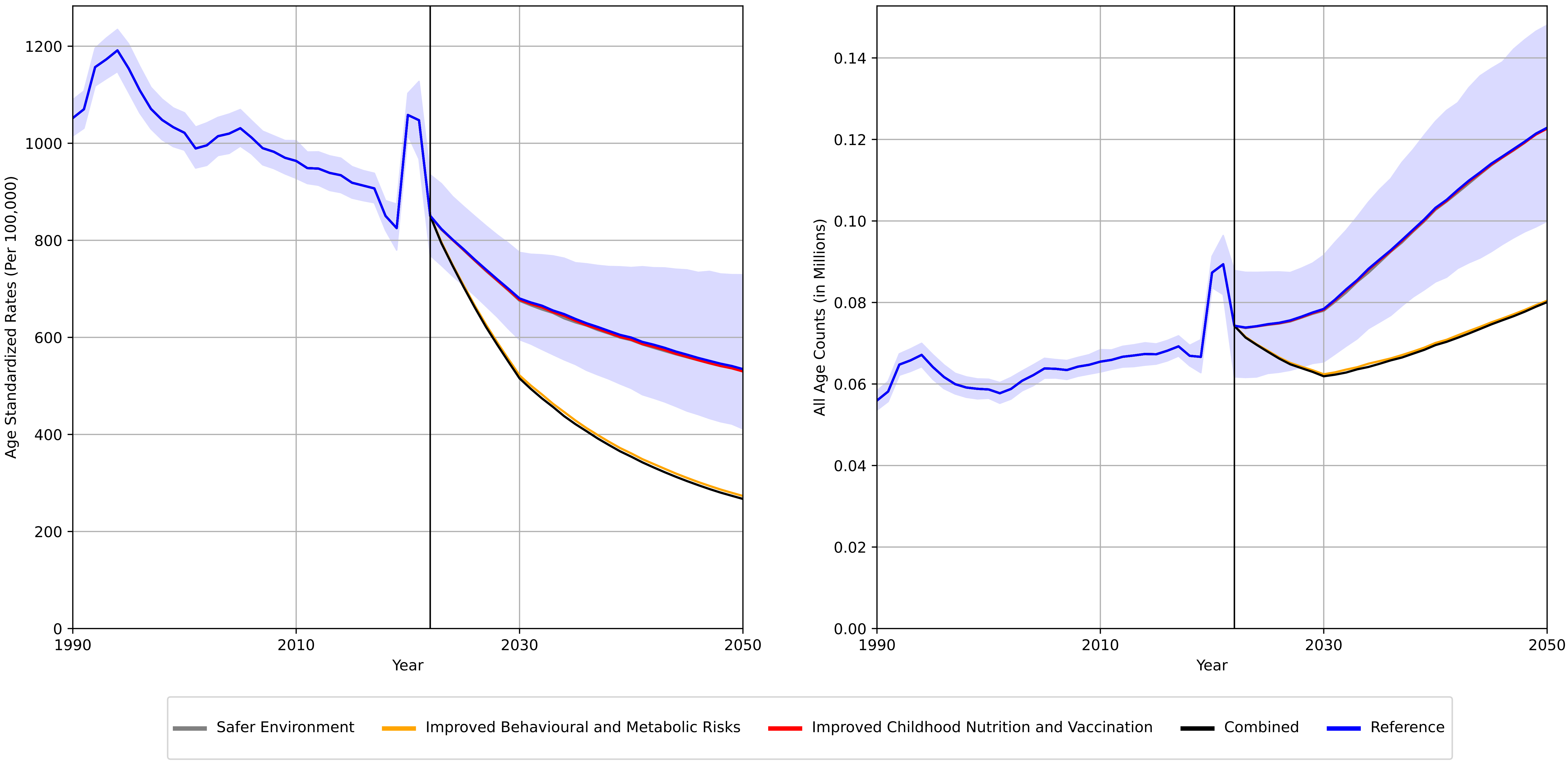

Age Standardized Rates (Per 100,000)
0
200
400
600
800
1000
1200
1990
2010
2030
2050
Year
All Age Counts (in Millions)
0.00
0.02
0.04
0.06
0.08
0.10
0.12
0.14
1990
2010
2030
2050
Year
Safer Environment
Improved Behavioural and Metabolic Risks
Improved Childhood Nutrition and Vaccination
Combined
Reference

Georgia

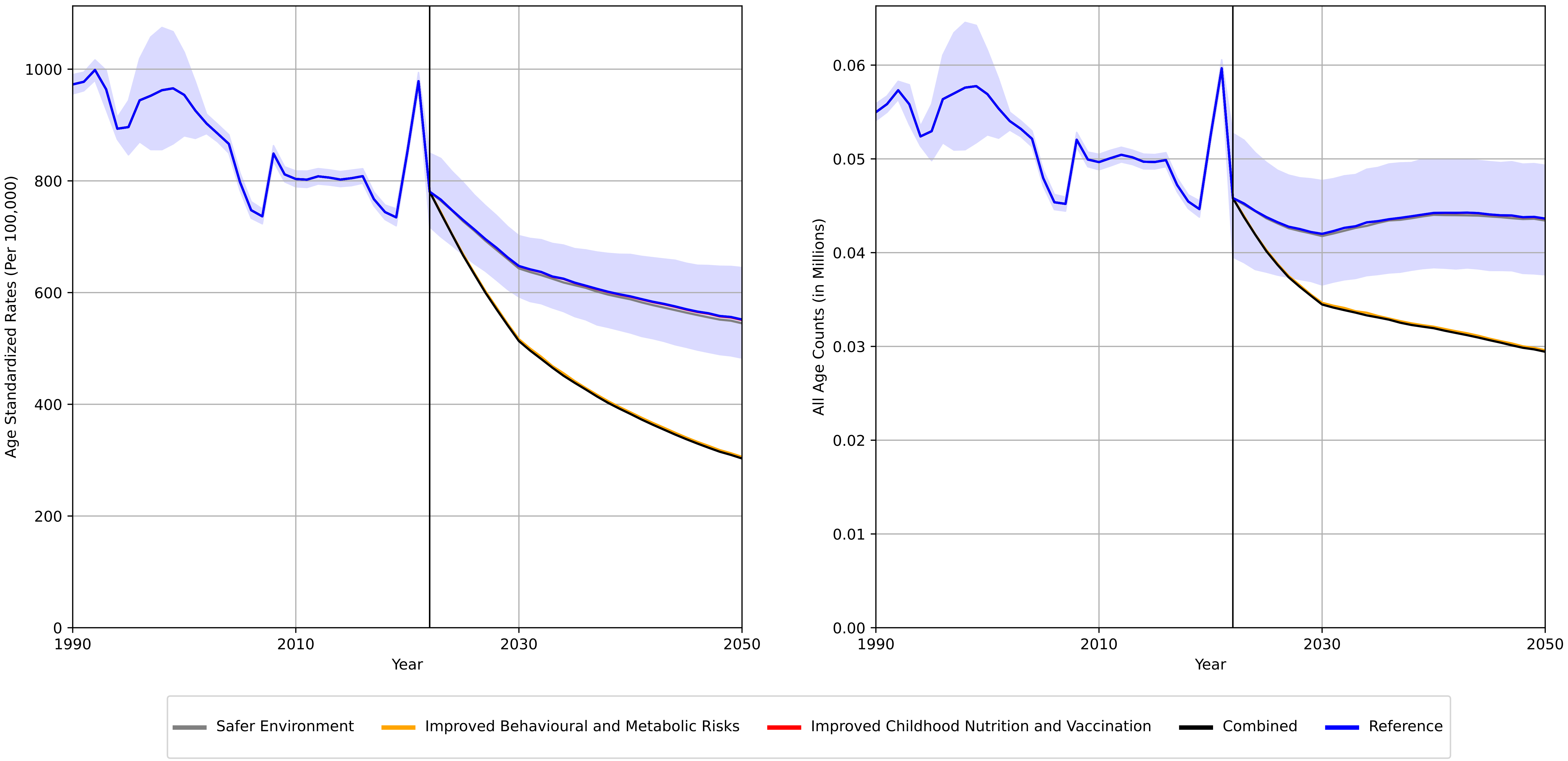
Age Standardized Rates (Per 100,000)
0
200
400
600
800
1000
1990
2010
2030
2050
Year
All Age Counts (in Millions)
0.00
0.01
0.02
0.03
0.04
0.05
0.06
1990
2010
2030
2050
Year
Safer Environment
Improved Behavioural and Metabolic Risks
Improved Childhood Nutrition and Vaccination
Combined
Reference

Kazakhstan

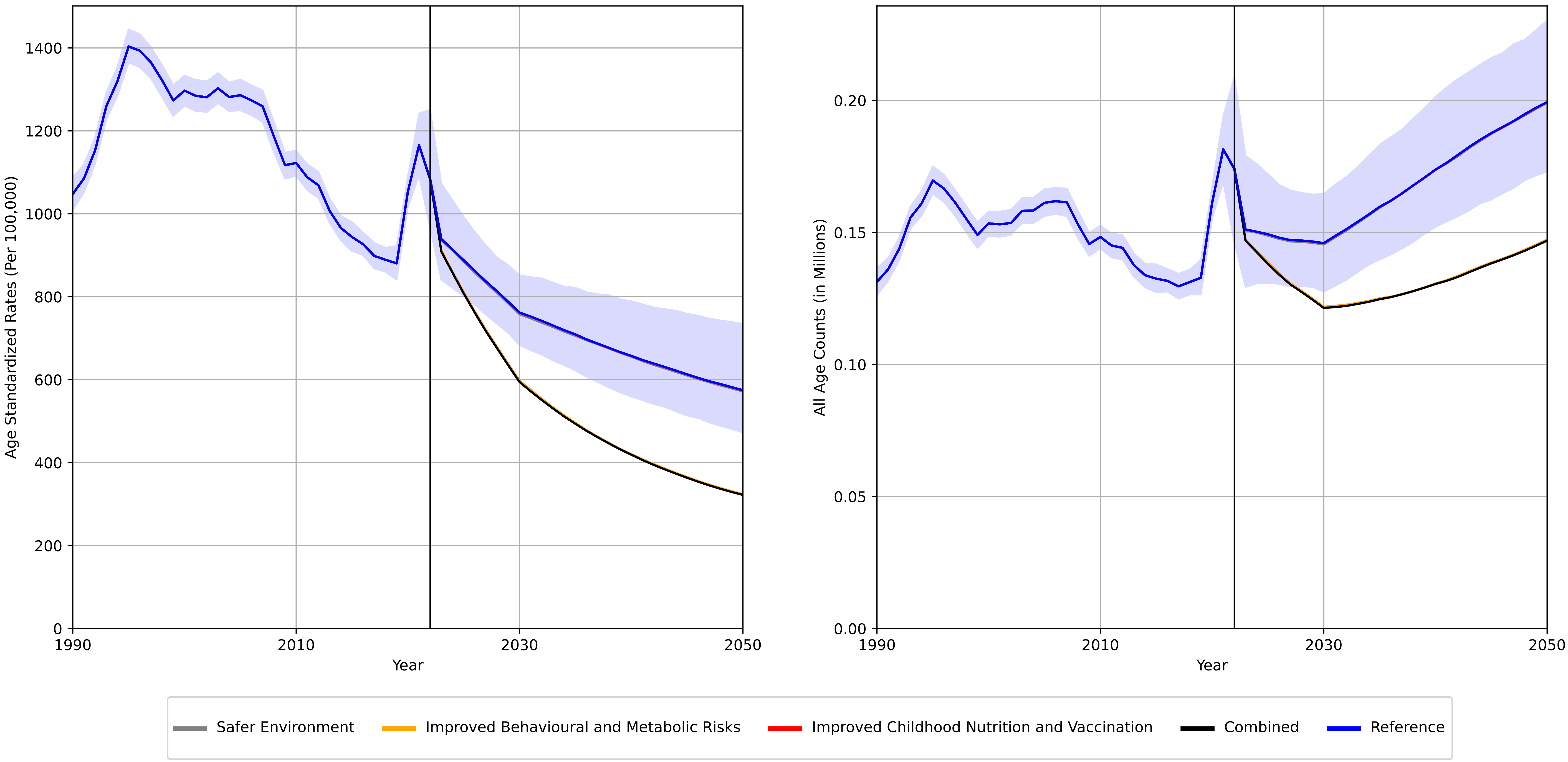
Age Standardized Rates (Per 100,000)
0
200
400
600
800
1000
1200
1400
1990
2010
2030
2050
Year
All Age Counts (in Millions)
0.00
0.05
0.10
0.15
0.20
1990
2010
2030
2050
Year
Safer Environment
Improved Behavioural and Metabolic Risks
Improved Childhood Nutrition and Vaccination
Combined
Reference

Kyrgyzstan

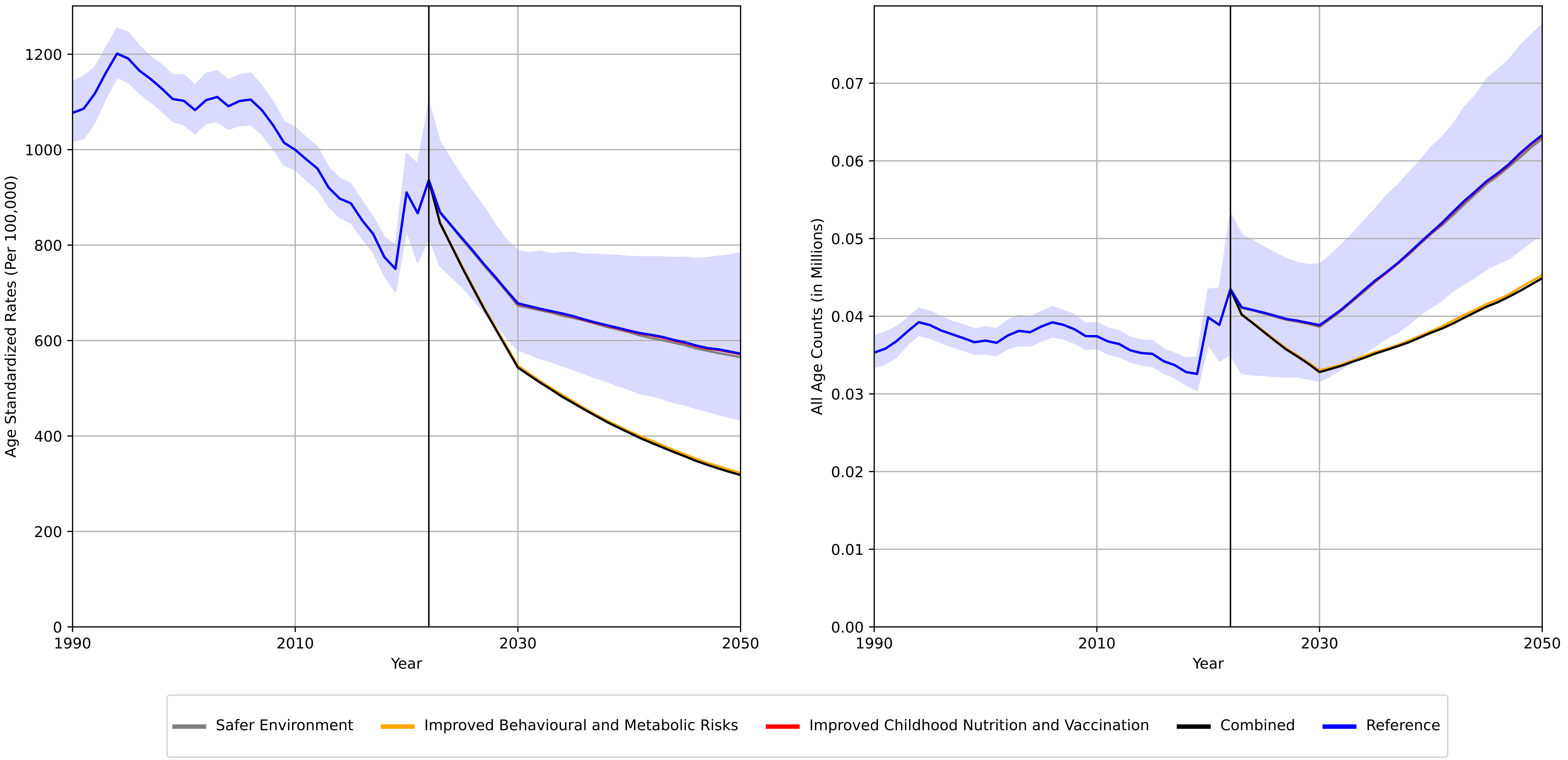
Age Standardized Rates (Per 100,000)
0
200
400
600
800
1000
1200
1990
2010
2030
2050
Year
All Age Counts (in Millions)
0.00
0.01
0.02
0.03
0.04
0.05
0.06
0.07
1990
2010
2030
2050
Year
Safer Environment
Improved Behavioural and Metabolic Risks
Improved Childhood Nutrition and Vaccination
Combined
Reference

Mongolia

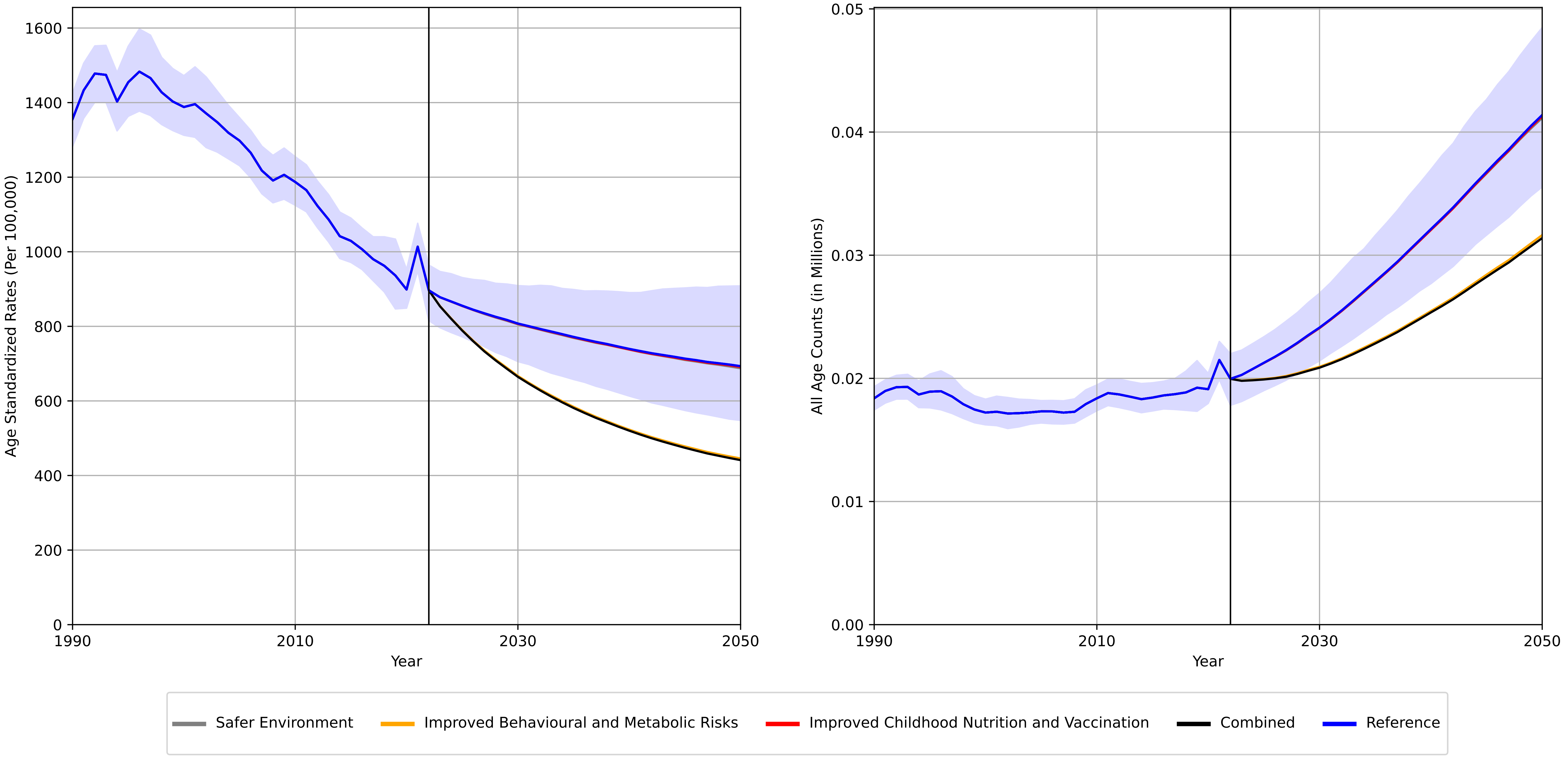

Age Standardized Rates (Per 100,000)
0
200
400
600
800
1000
1200
1400
1600
1990
2010
2030
2050
Year
All Age Counts (in Millions)
0.00
0.01
0.02
0.03
0.04
0.05
1990
2010
2030
2050
Year
Safer Environment
Improved Behavioural and Metabolic Risks
Improved Childhood Nutrition and Vaccination
Combined
Reference

Tajikistan

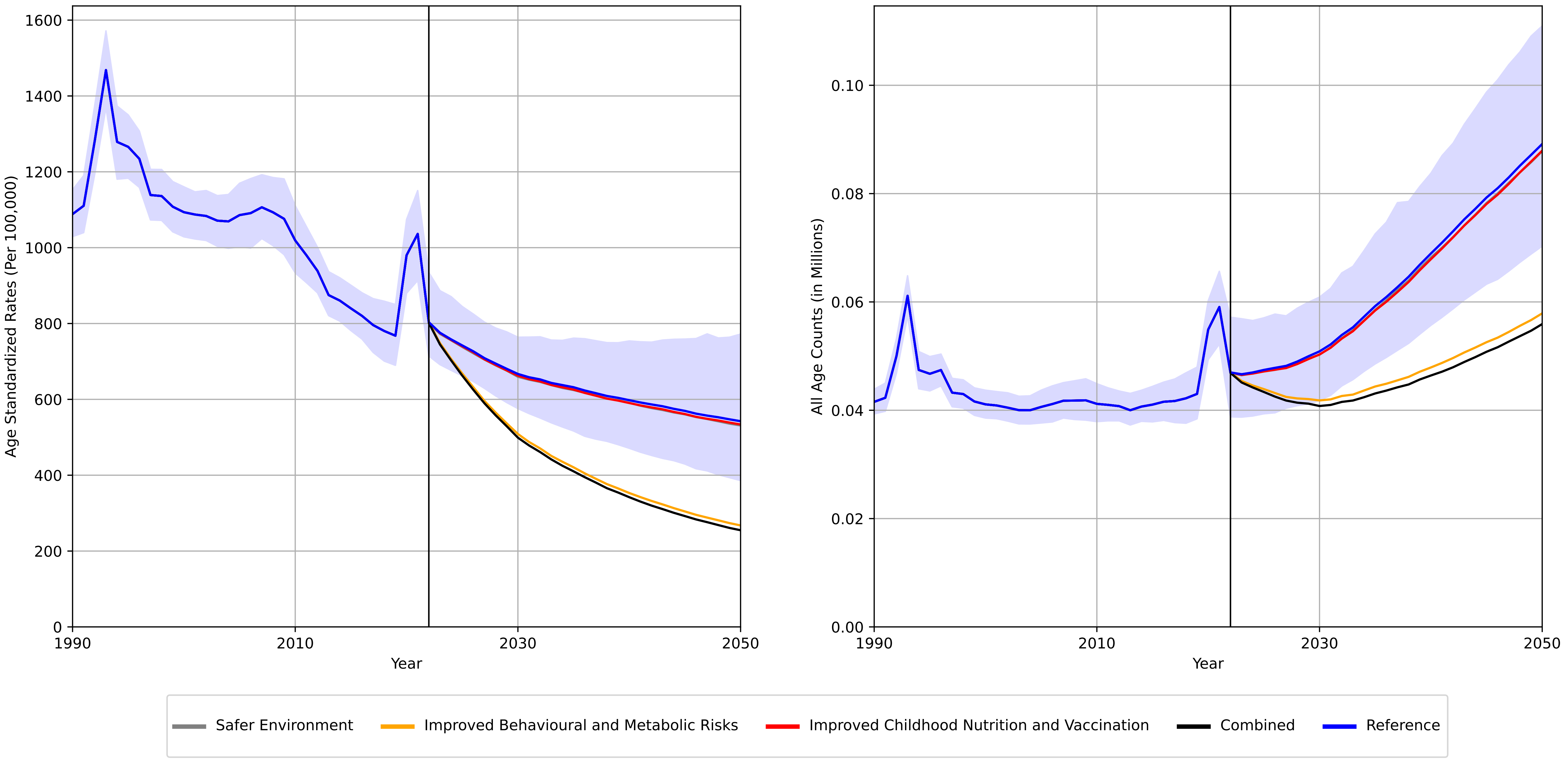

Age Standardized Rates (Per 100,000)
1600
1400
1200
1000
800
600
400
200
0
1990
2010
2030
2050
Year
All Age Counts (in Millions)
0.10
0.08
0.06
0.04
0.02
0.00
1990
2010
2030
2050
Year
Safer Environment
Improved Behavioural and Metabolic Risks
Improved Childhood Nutrition and Vaccination
Combined
Reference

Turkmenistan

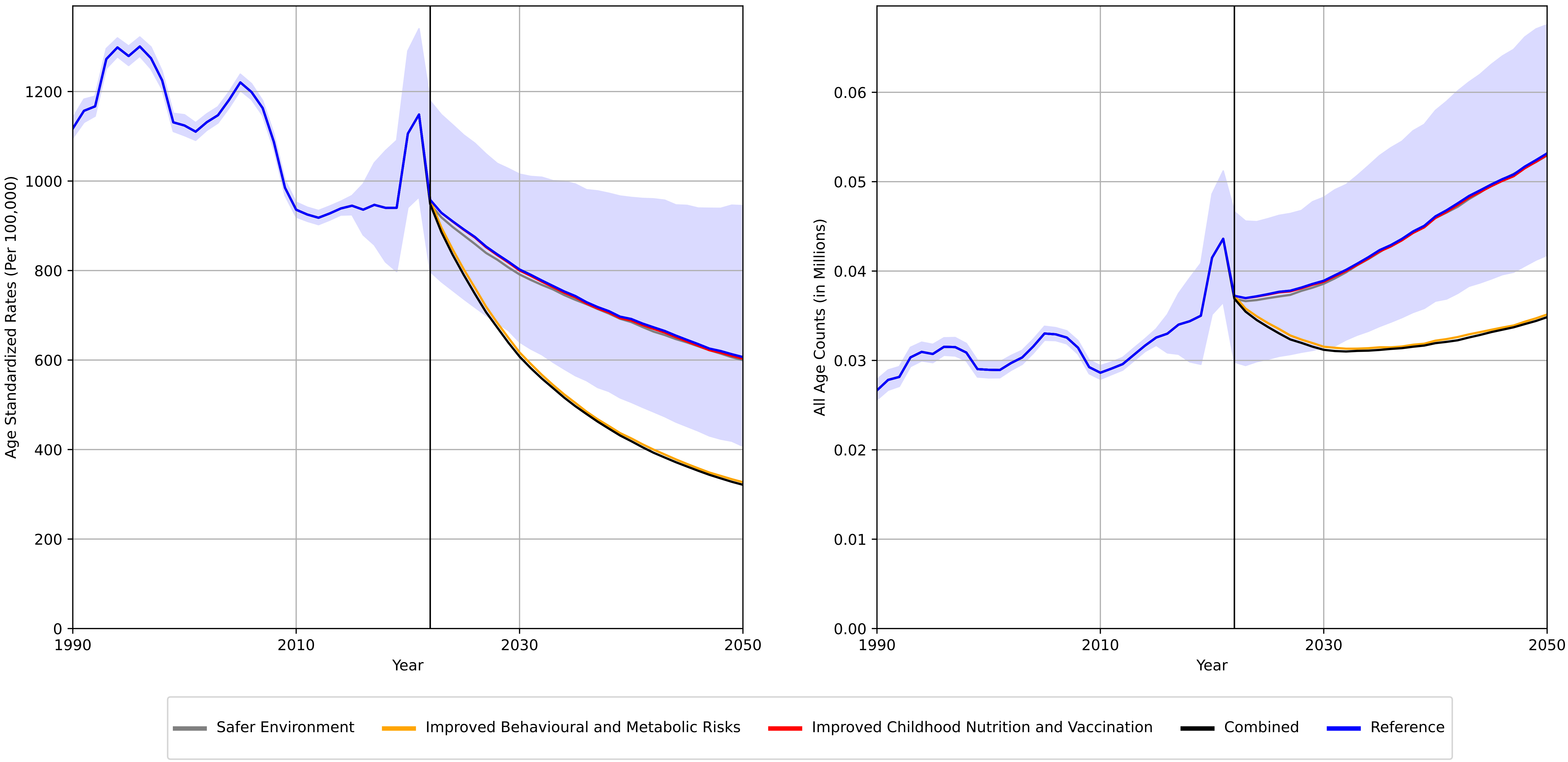

Uzbekistan

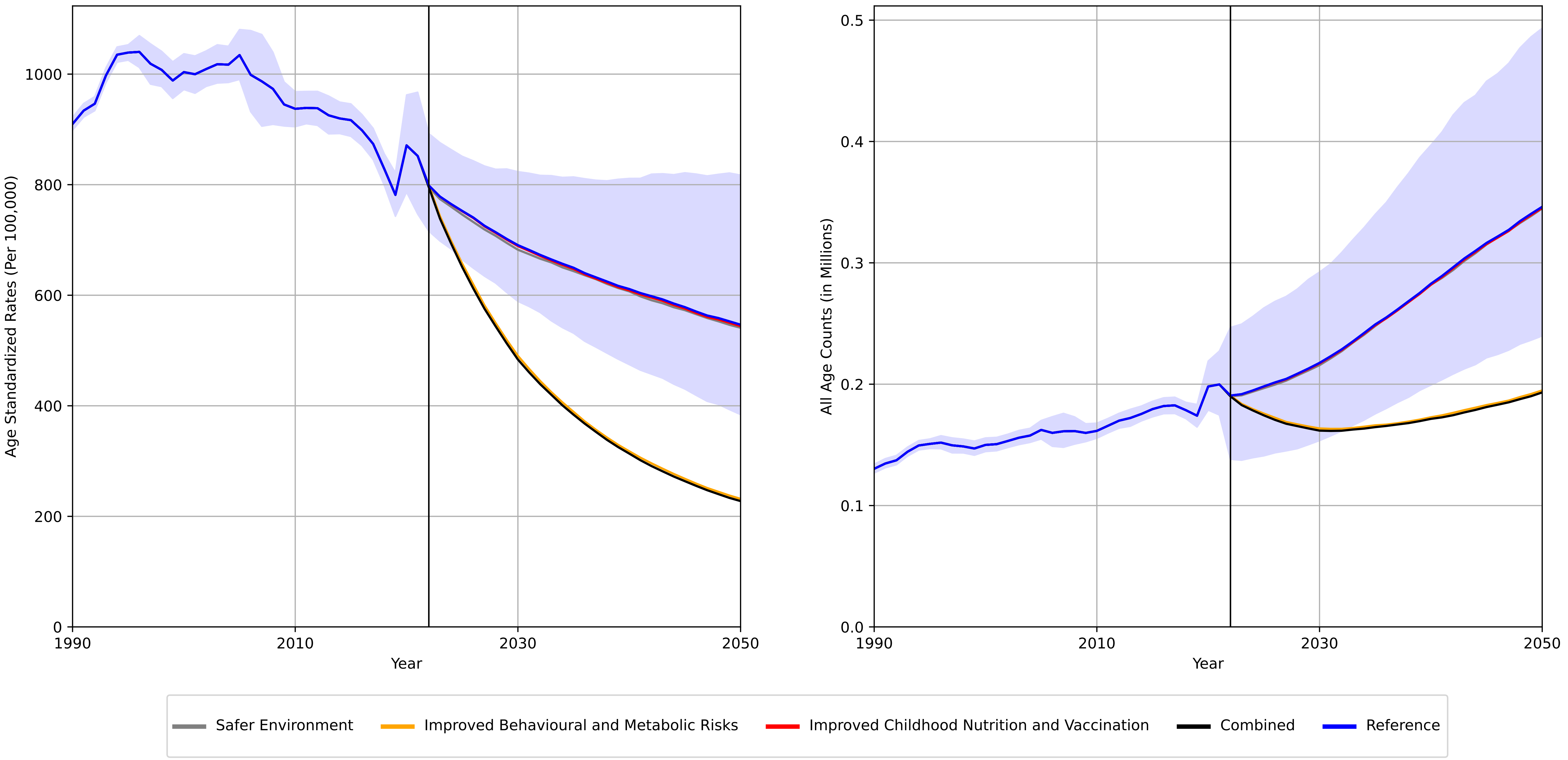

Age Standardized Rates (Per 100,000)
0
200
400
600
800
1000
1990
2010
2030
2050
Year
All Age Counts (in Millions)
0.0
0.1
0.2
0.3
0.4
0.5
1990
2010
2030
2050
Year
Safer Environment
Improved Behavioural and Metabolic Risks
Improved Childhood Nutrition and Vaccination
Combined
Reference

Albania

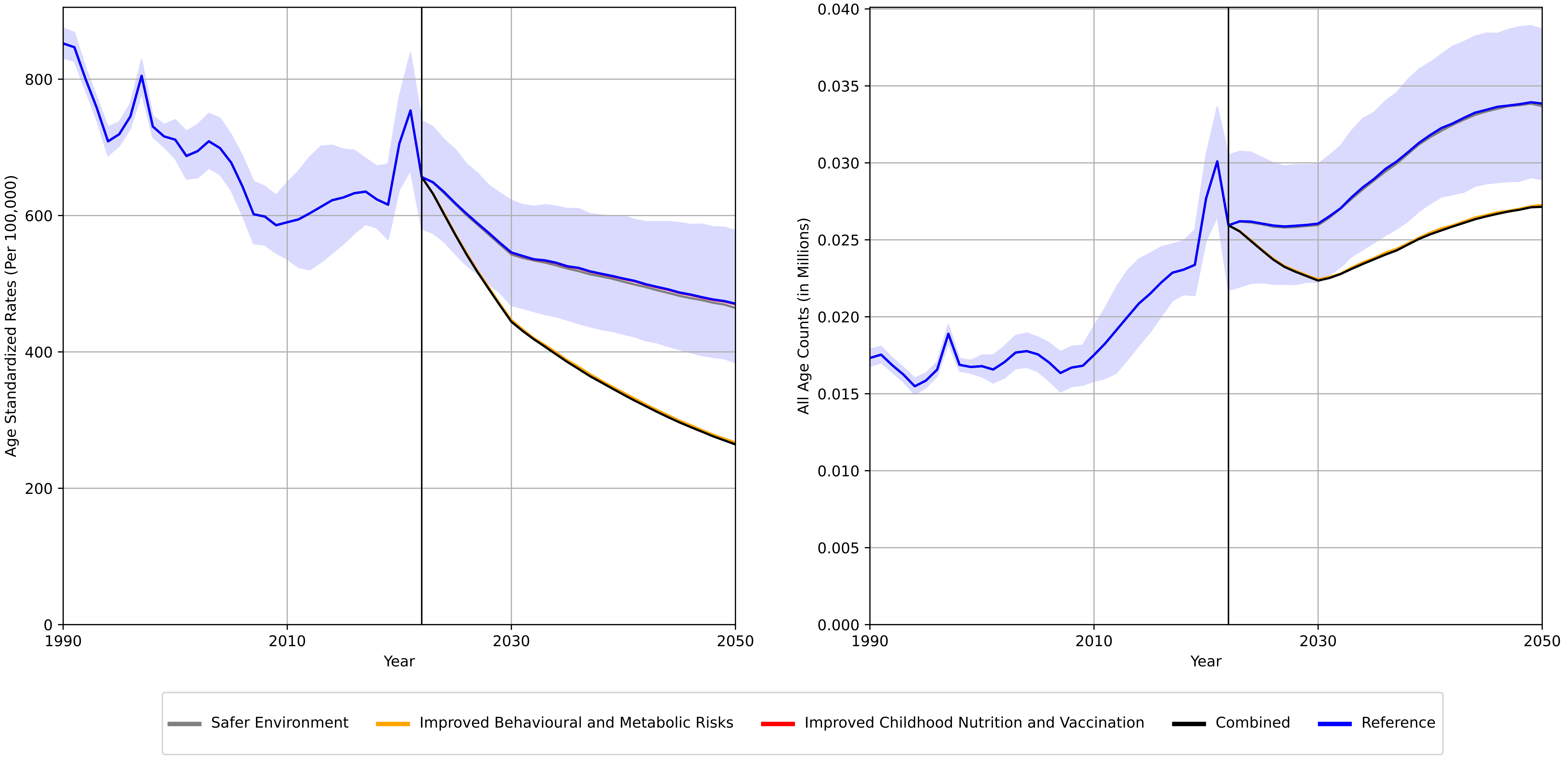
Age Standardized Rates (Per 100,000)
0
200
400
600
800
1990
2010
2030
2050
Year
All Age Counts (in Millions)
0.000
0.005
0.010
0.015
0.020
0.025
0.030
0.035
0.040
1990
2010
2030
2050
Year
Safer Environment
Improved Behavioural and Metabolic Risks
Improved Childhood Nutrition and Vaccination
Combined
Reference

Bosnia And Herzegovina

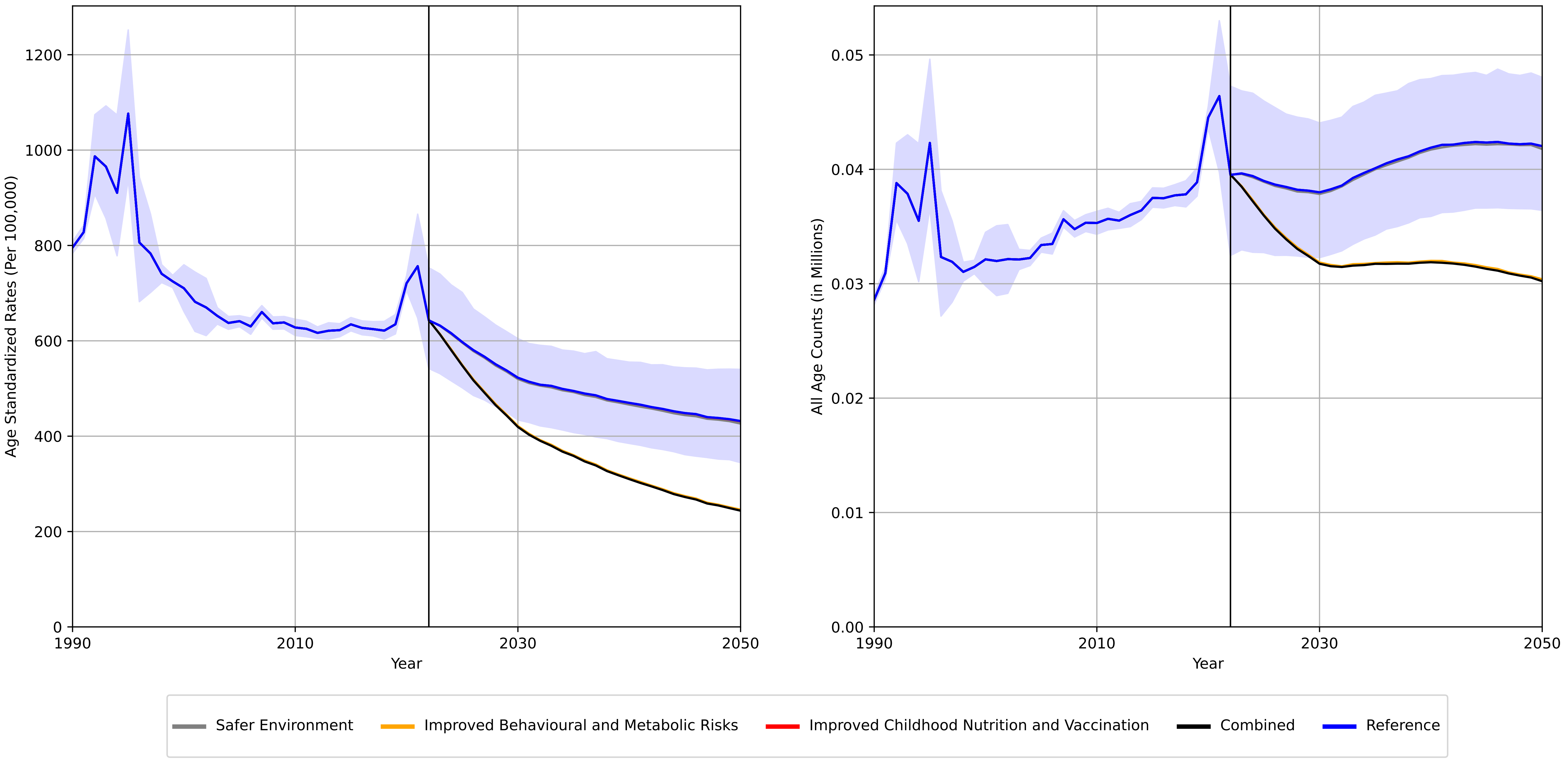

Bulgaria

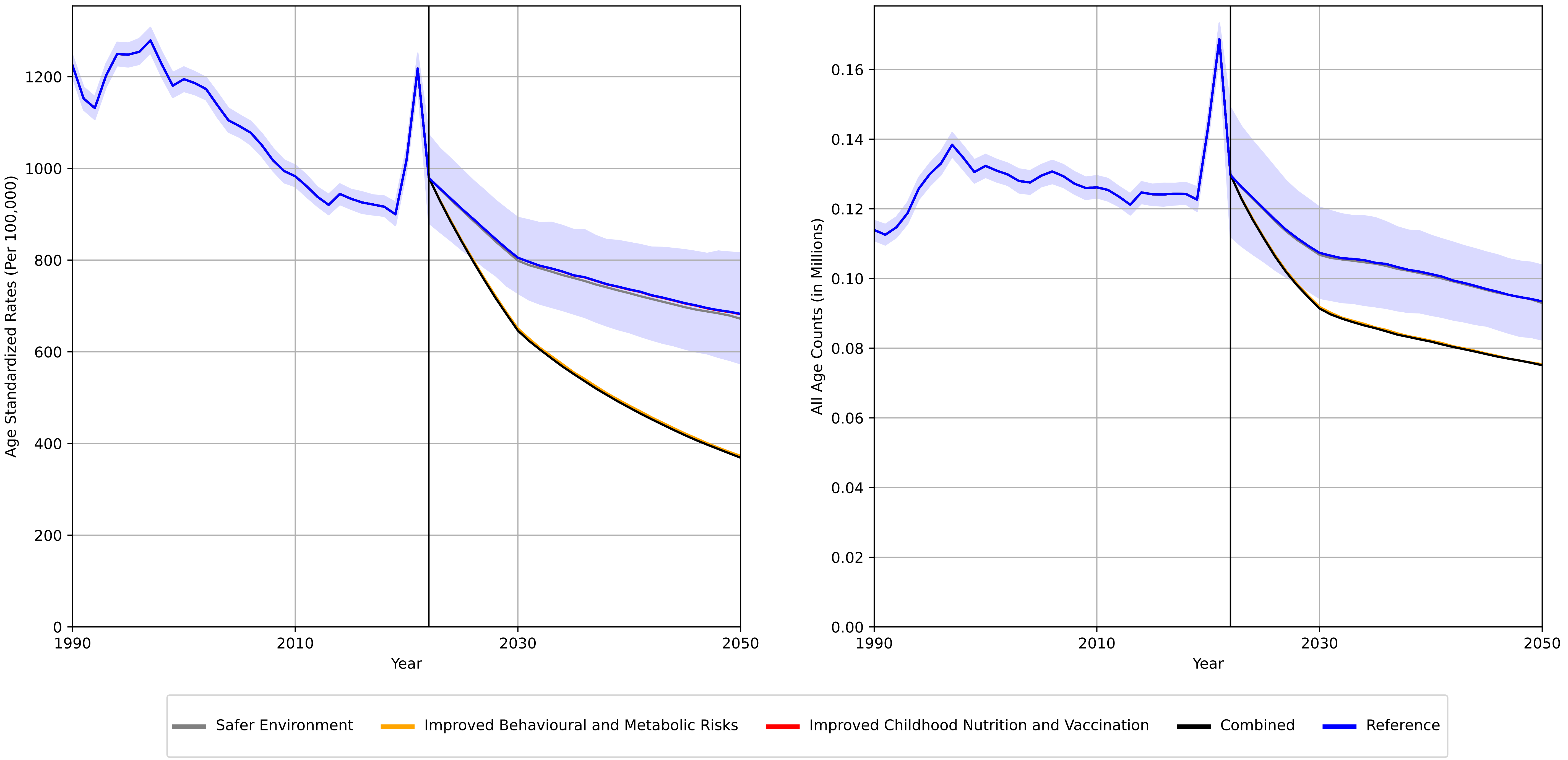
Age Standardized Rates (Per 100,000)
0
200
400
600
800
1000
1200
1990
2010
2030
2050
Year
All Age Counts (in Millions)
0.00
0.02
0.04
0.06
0.08
0.10
0.12
0.14
0.16
Safer Environment
Improved Behavioural and Metabolic Risks
Improved Childhood Nutrition and Vaccination
Combined
Reference

Croatia

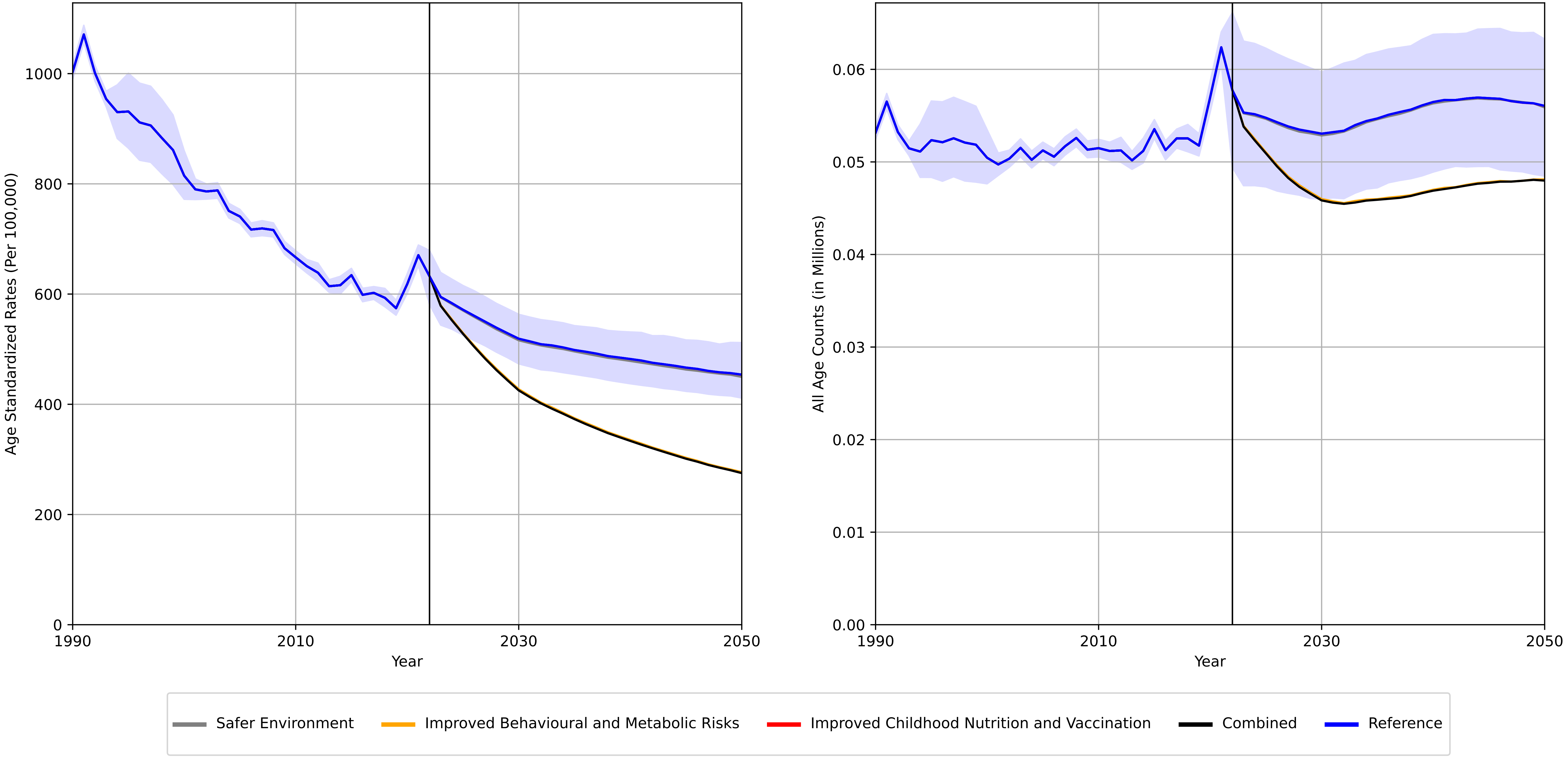

Age Standardized Rates (Per 100,000)
0
200
400
600
800
1000
1990
2010
2030
2050
Year
All Age Counts (in Millions)
0.00
0.01
0.02
0.03
0.04
0.05
0.06
1990
2010
2030
2050
Year
Safer Environment
Improved Behavioural and Metabolic Risks
Improved Childhood Nutrition and Vaccination
Combined
Reference

Czechia

Supplemental Results Figure S11. All-cause age standardised death rates (A) and all age death counts (B), for the past and for five future scenarios, 1990–2050

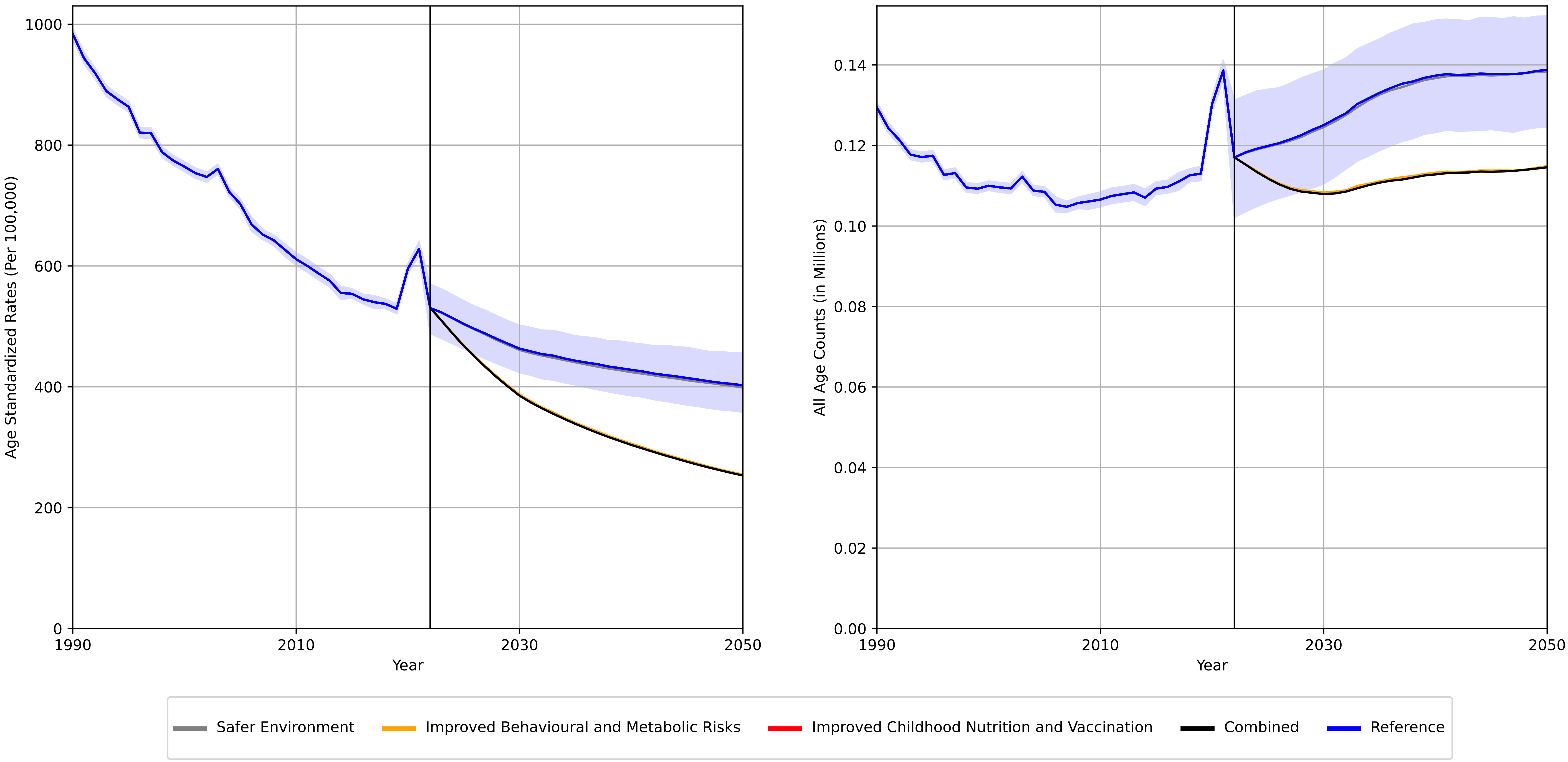

Hungary

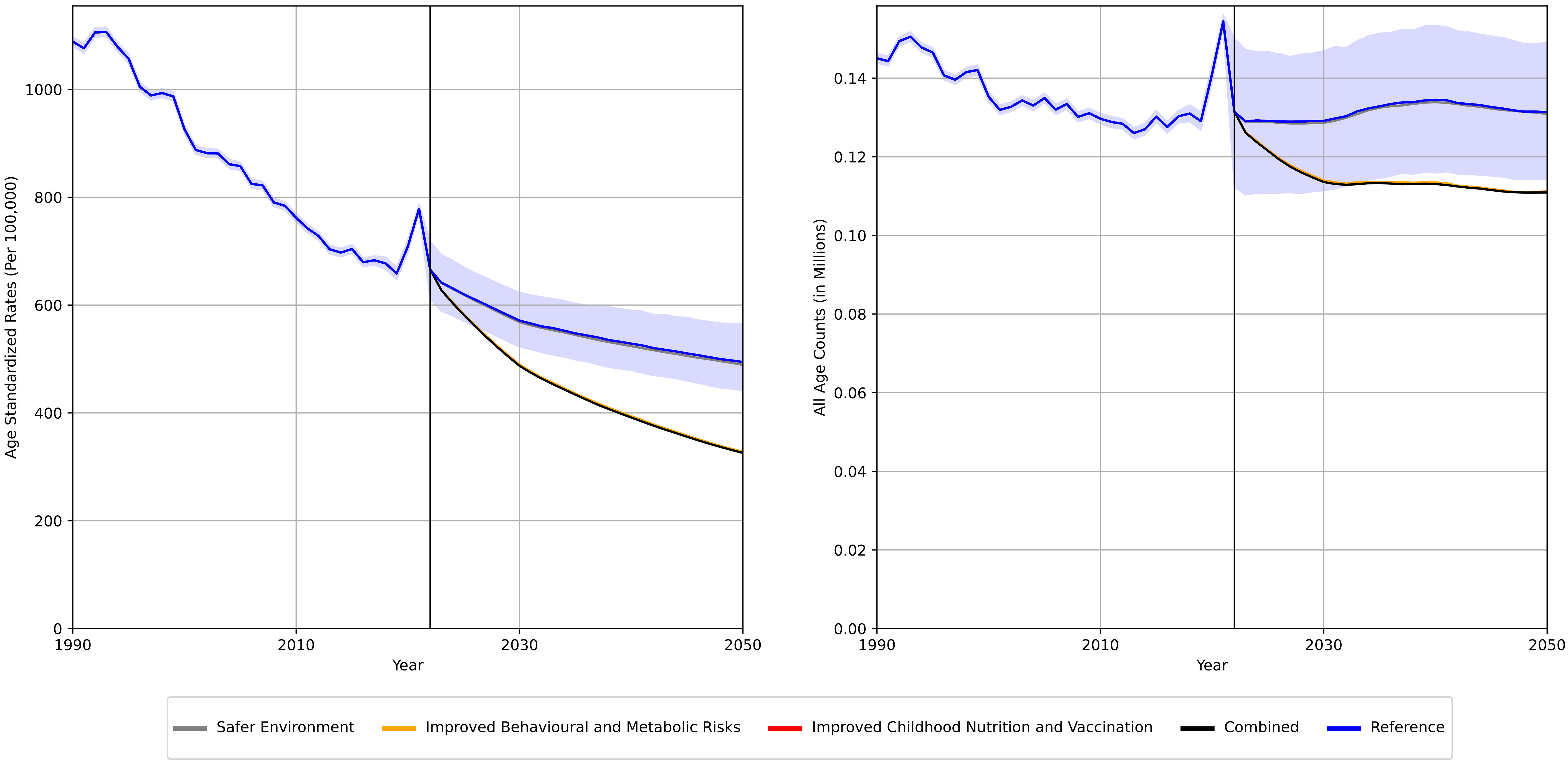
Age Standardized Rates (Per 100,000)
0
200
400
600
800
1000
1990
2010
2030
2050
Year
All Age Counts (in Millions)
0.00
0.02
0.04
0.06
0.08
0.10
0.12
0.14
1990
2010
2030
2050
Year
Safer Environment
Improved Behavioural and Metabolic Risks
Improved Childhood Nutrition and Vaccination
Combined
Reference

Montenegro

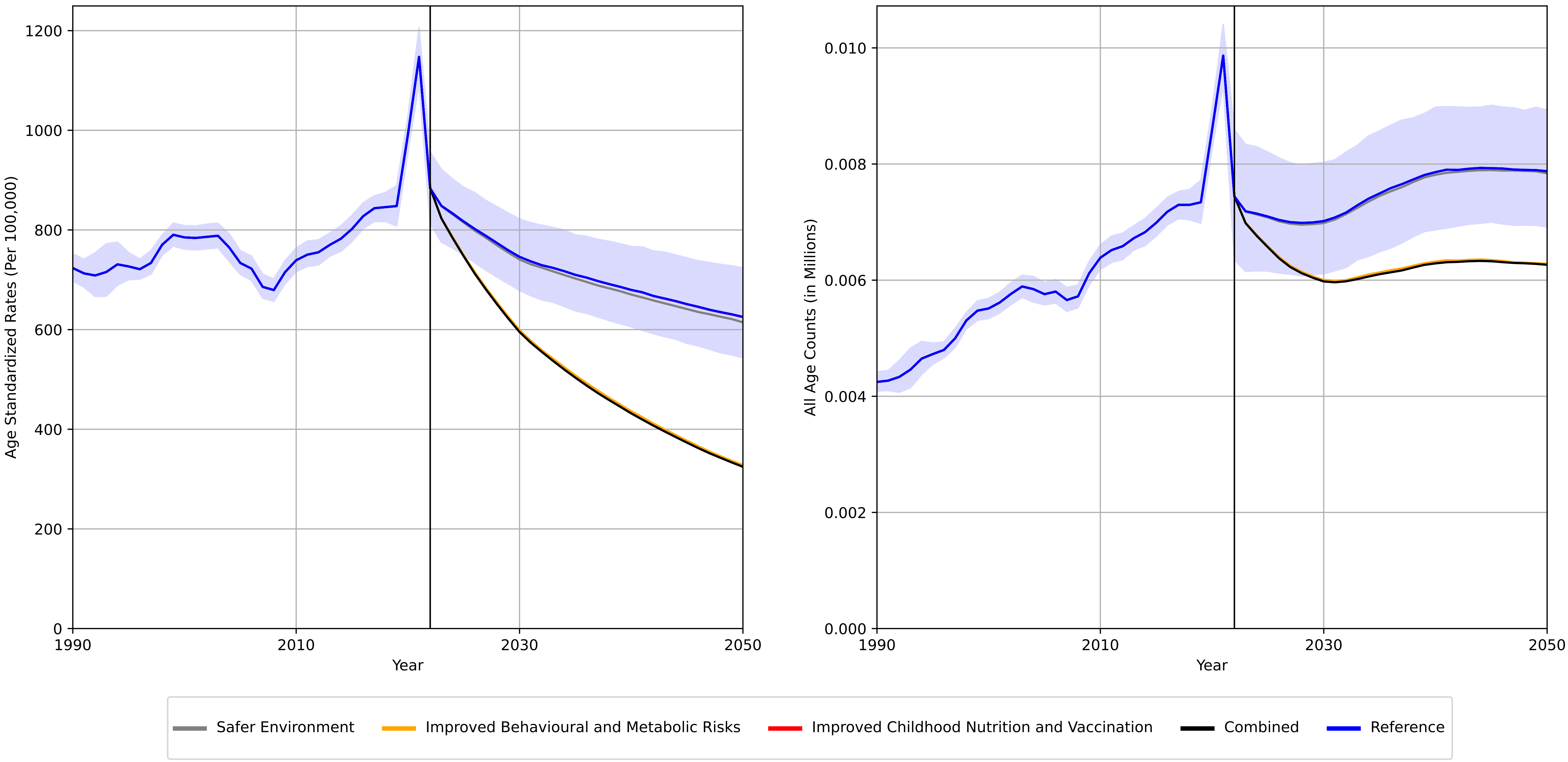

Age Standardized Rates (Per 100,000)
0
200
400
600
800
1000
1200
1990
2010
2030
2050
Year
All Age Counts (in Millions)
0.000
0.002
0.004
0.006
0.008
0.010
1990
2010
2030
2050
Year
Safer Environment
Improved Behavioural and Metabolic Risks
Improved Childhood Nutrition and Vaccination
Combined
Reference

North Macedonia

Supplemental Results Figure S11. All-cause age standardised death rates (A) and all age death counts (B), for the past and for five future scenarios, 1990–2050

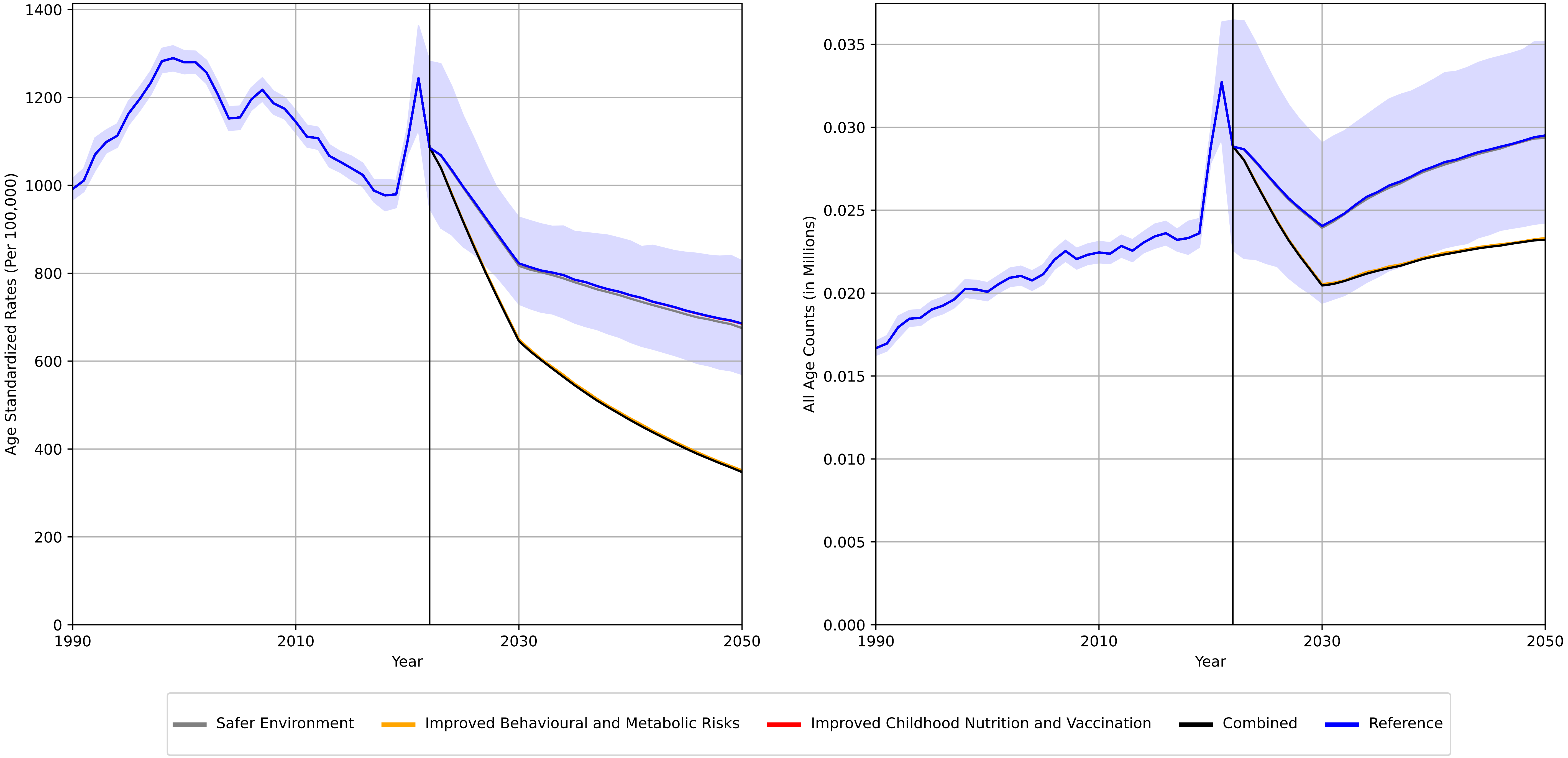

Poland

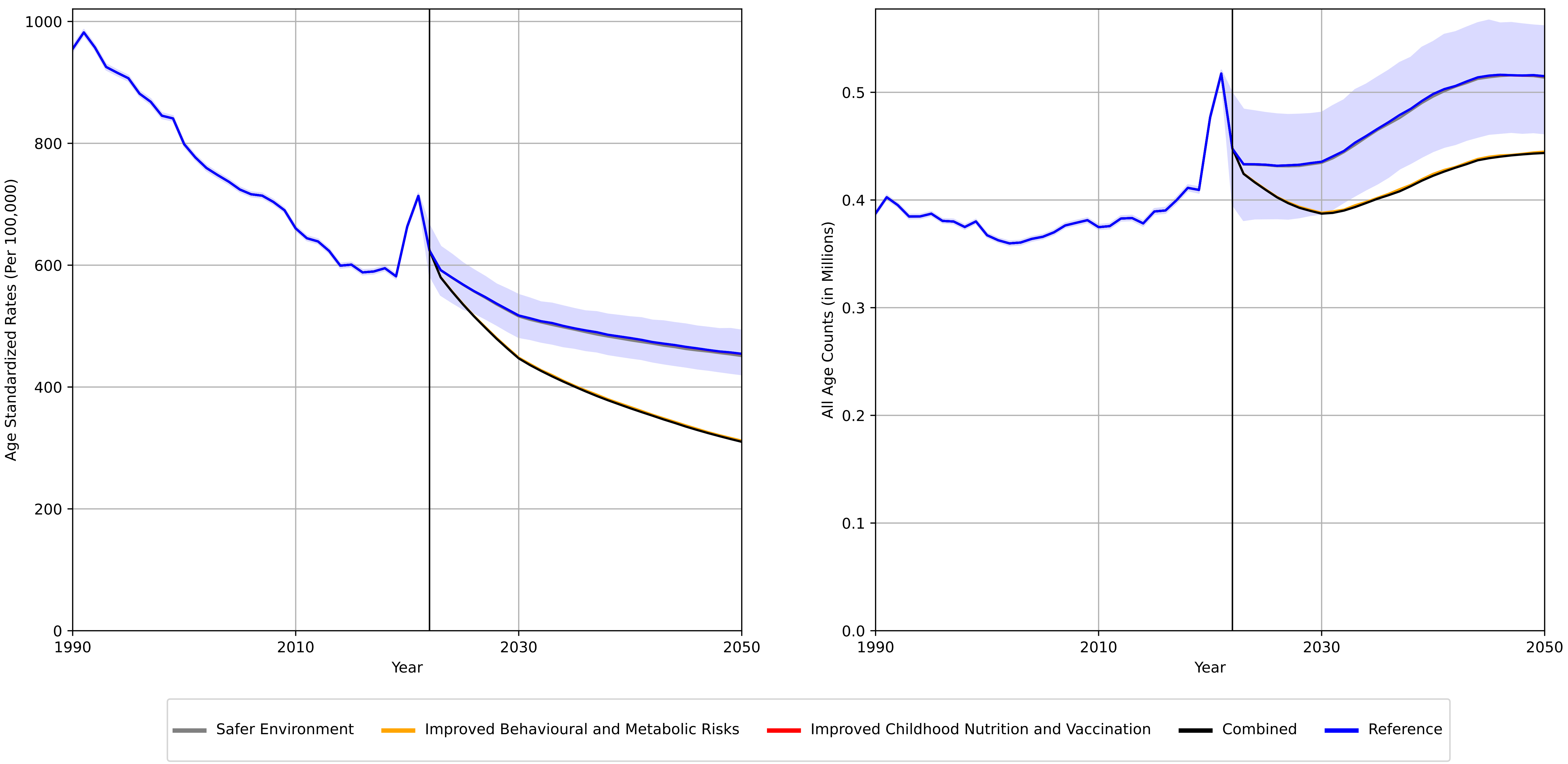

Age Standardized Rates (Per 100,000)
1000
800
600
400
200
0
1990
2010
2030
2050
Year
All Age Counts (in Millions)
0.5
0.4
0.3
0.2
0.1
0.0
Safer Environment
Improved Behavioural and Metabolic Risks
Improved Childhood Nutrition and Vaccination
Combined
Reference

Romania

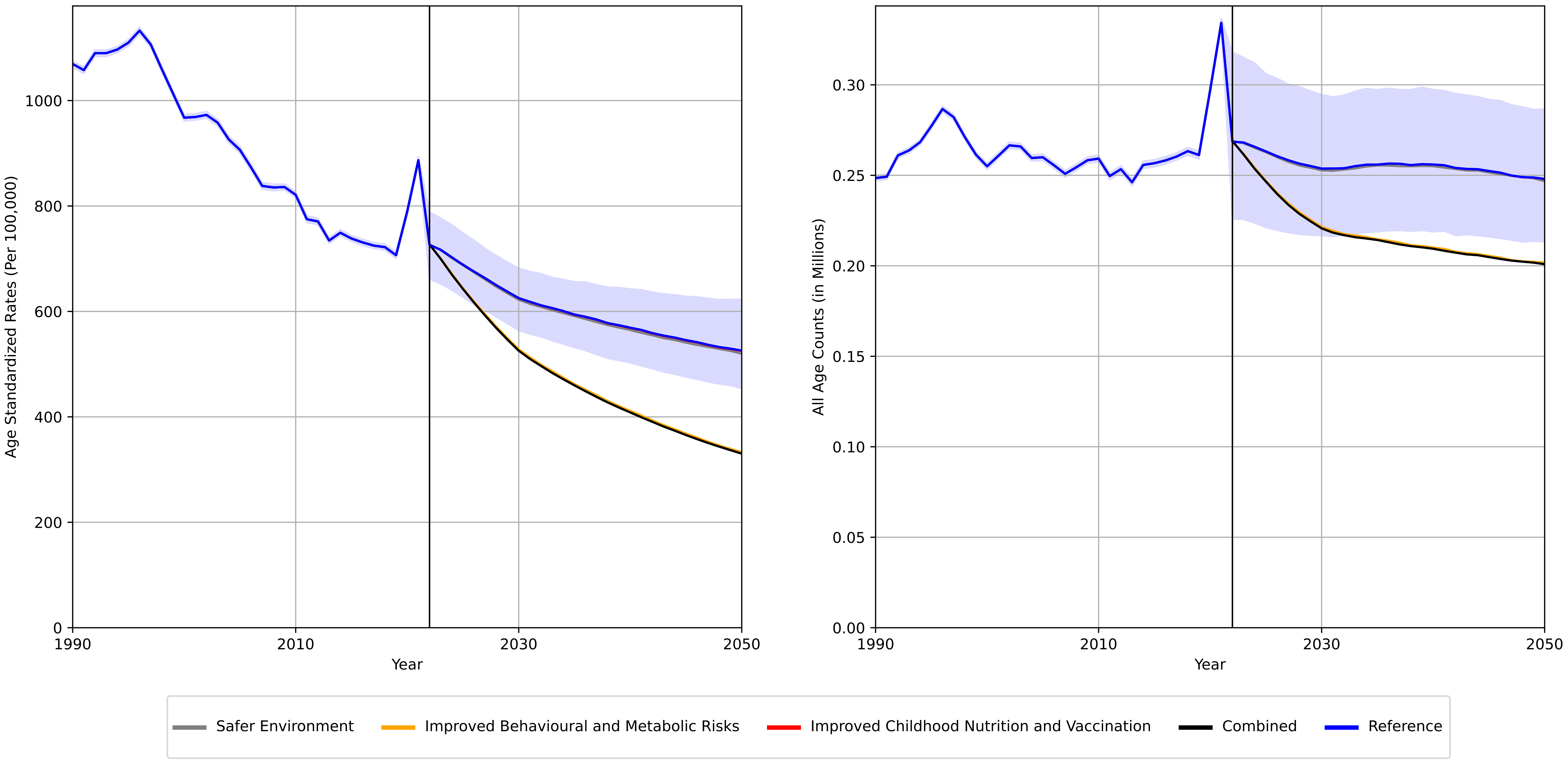

Age Standardized Rates (Per 100,000)
0
200
400
600
800
1000
1990
2010
2030
2050
Year
All Age Counts (in Millions)
0.00
0.05
0.10
0.15
0.20
0.25
0.30
1990
2010
2030
2050
Year
Safer Environment
Improved Behavioural and Metabolic Risks
Improved Childhood Nutrition and Vaccination
Combined
Reference

Serbia

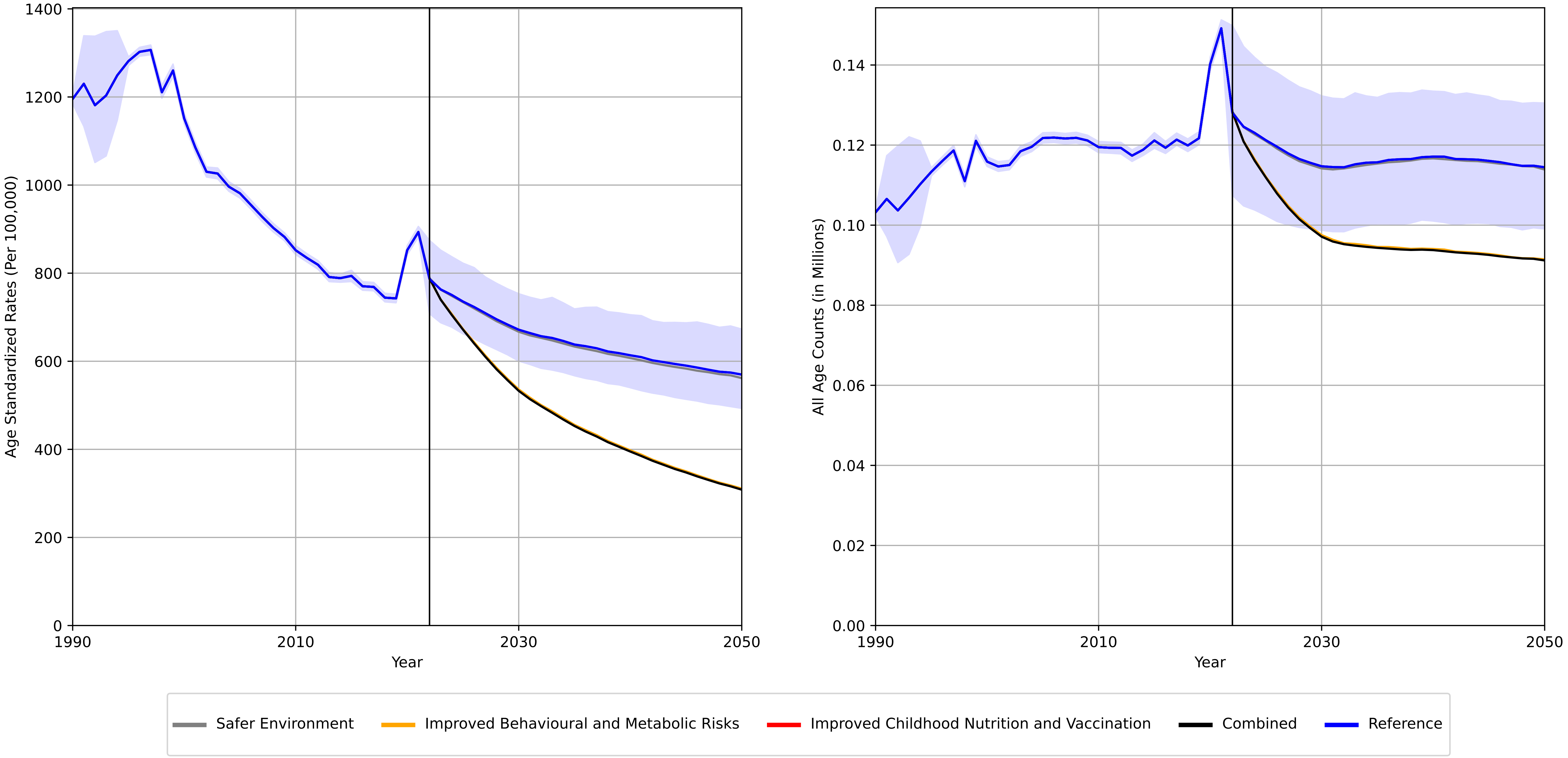
Age Standardized Rates (Per 100,000)
Year
All Age Counts (in Millions)
Year
Safer Environment
Improved Behavioural and Metabolic Risks
Improved Childhood Nutrition and Vaccination
Combined
Reference

Slovakia

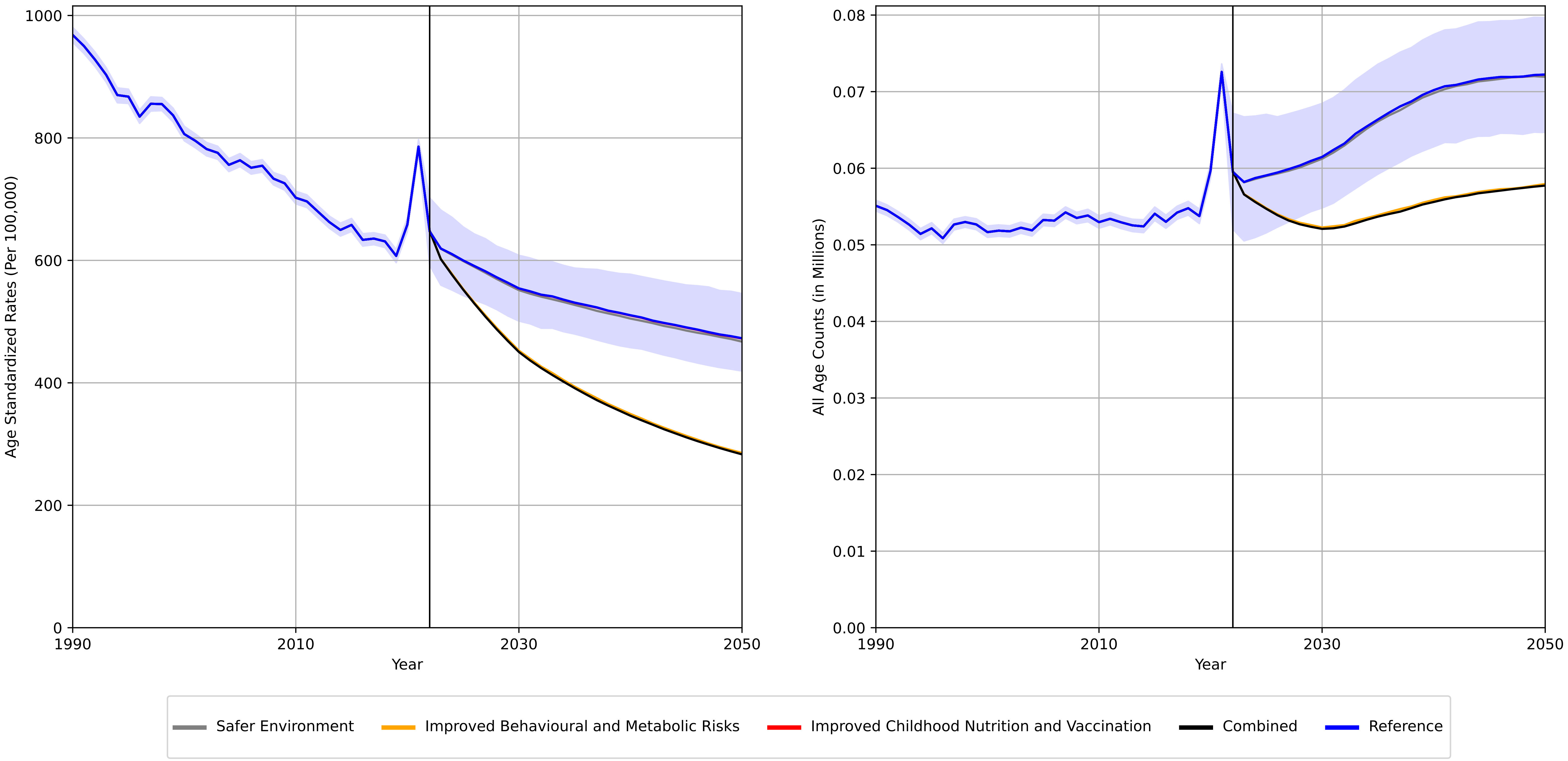
Age Standardized Rates (Per 100,000)
0
200
400
600
800
1000
1990
2010
2030
2050
Year
All Age Counts (in Millions)
0.00
0.01
0.02
0.03
0.04
0.05
0.06
0.07
0.08
1990
2010
2030
2050
Year
Safer Environment
Improved Behavioural and Metabolic Risks
Improved Childhood Nutrition and Vaccination
Combined
Reference

Slovenia

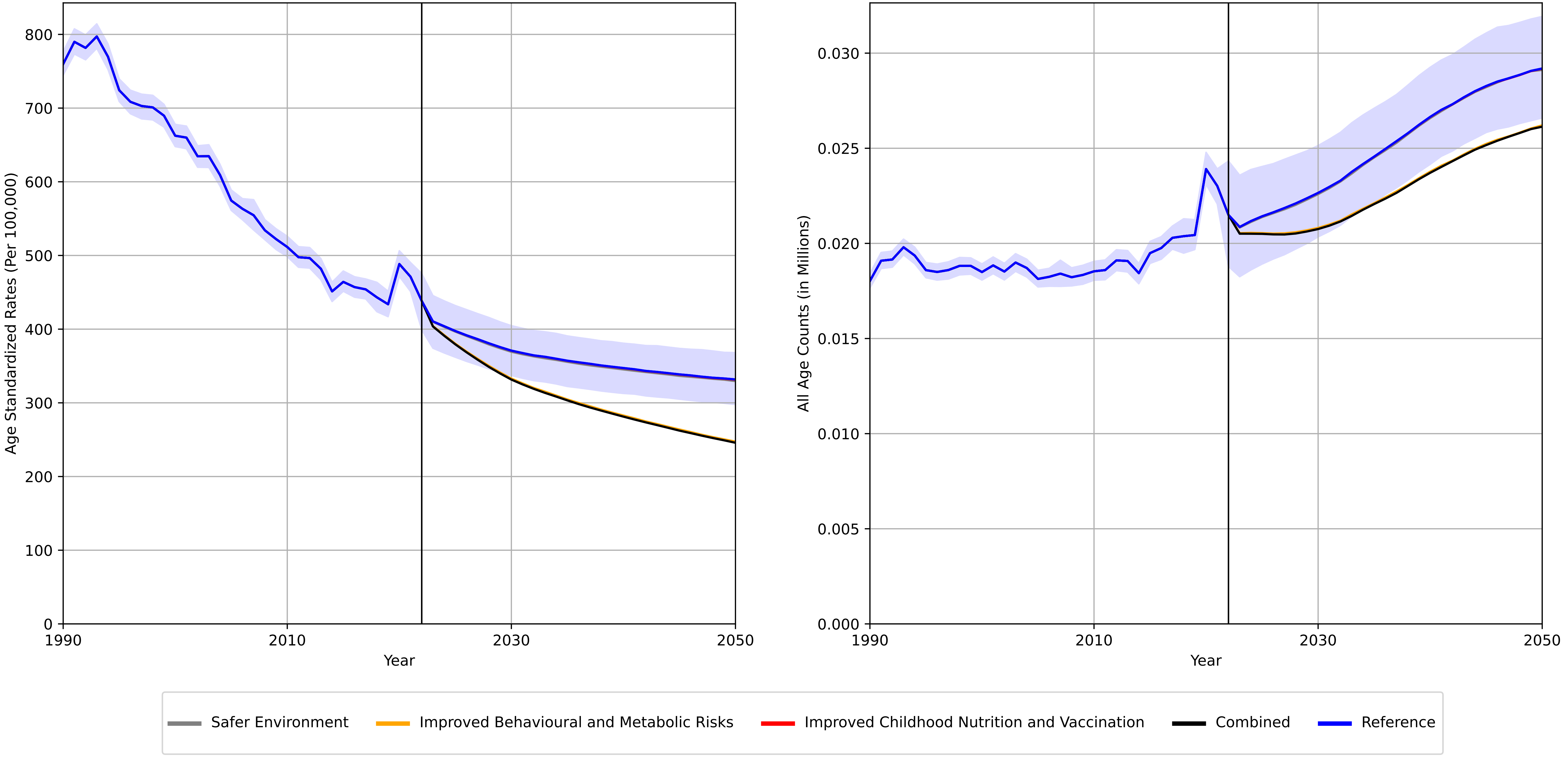

Age Standardized Rates (Per 100,000)
0
100
200
300
400
500
600
700
800
1990
2010
2030
2050
Year
All Age Counts (in Millions)
0.000
0.005
0.010
0.015
0.020
0.025
0.030
1990
2010
2030
2050
Year
Safer Environment
Improved Behavioural and Metabolic Risks
Improved Childhood Nutrition and Vaccination
Combined
Reference

Belarus

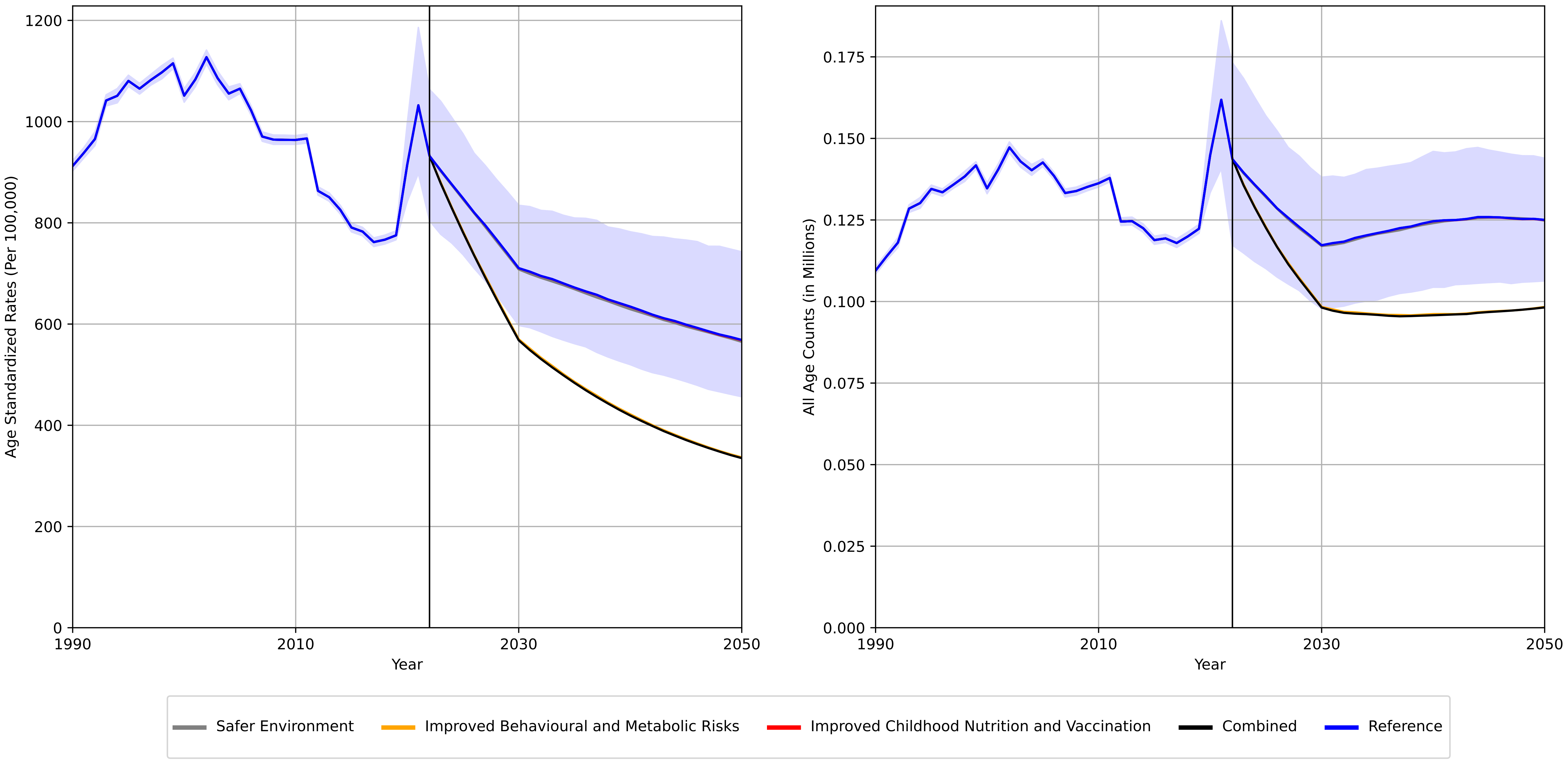
Age Standardized Rates (Per 100,000)
0
200
400
600
800
1000
1200
1990
2010
2030
2050
Year
All Age Counts (in Millions)
0.000
0.025
0.050
0.075
0.100
0.125
0.150
0.175
1990
2010
2030
2050
Year
Safer Environment
Improved Behavioural and Metabolic Risks
Improved Childhood Nutrition and Vaccination
Combined
Reference

Estonia

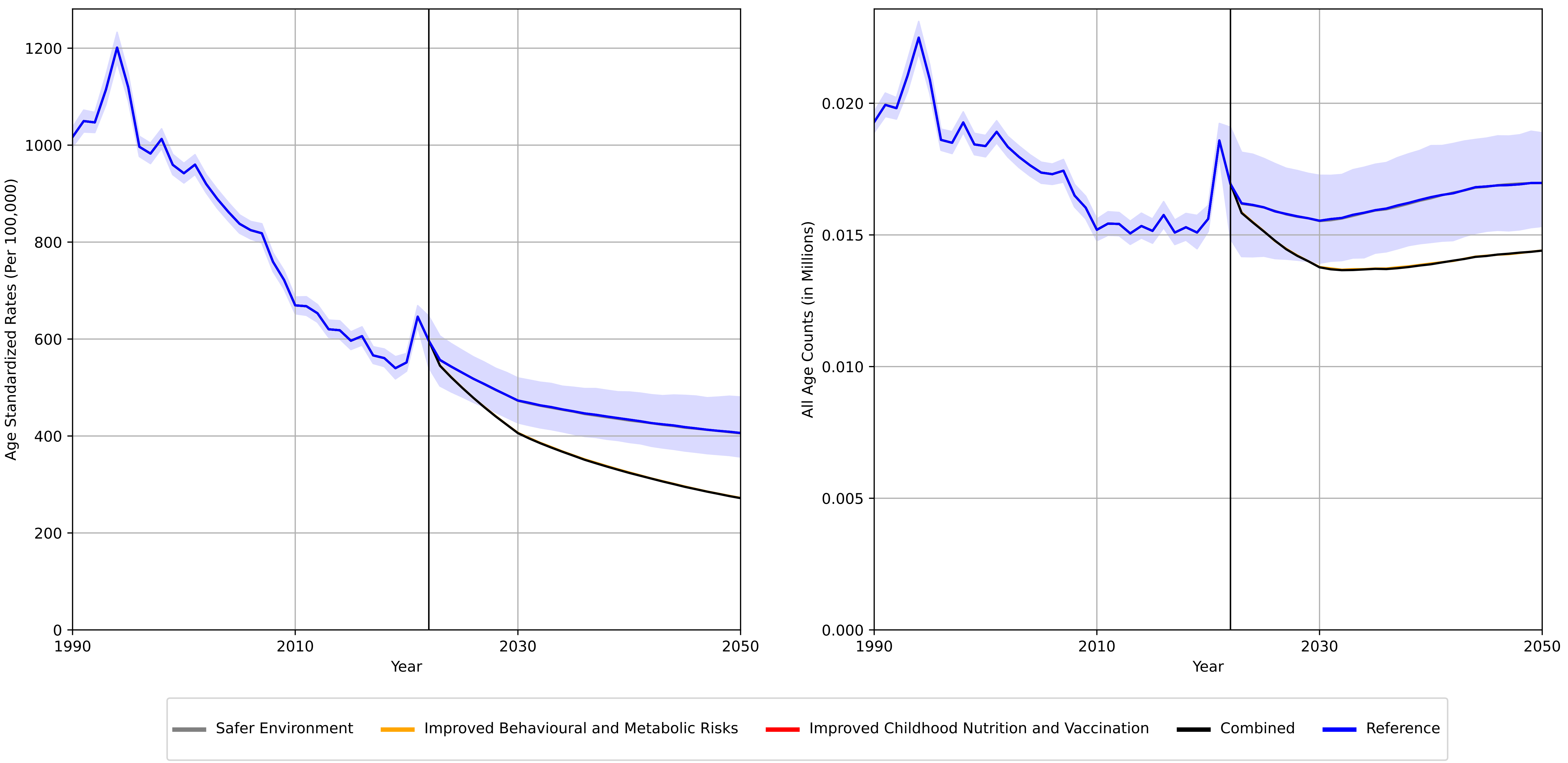
Age Standardized Rates (Per 100,000)
1200
1000
800
600
400
200
0
1990
2010
2030
2050
Year
All Age Counts (in Millions)
0.020
0.015
0.010
0.005
0.000
1990
2010
2030
2050
Year
Safer Environment
Improved Behavioural and Metabolic Risks
Improved Childhood Nutrition and Vaccination
Combined
Reference

Latvia

Supplemental Results Figure S11. All-cause age standardised death rates (A) and all age death counts (B), for the past and for five future scenarios, 1990–2050

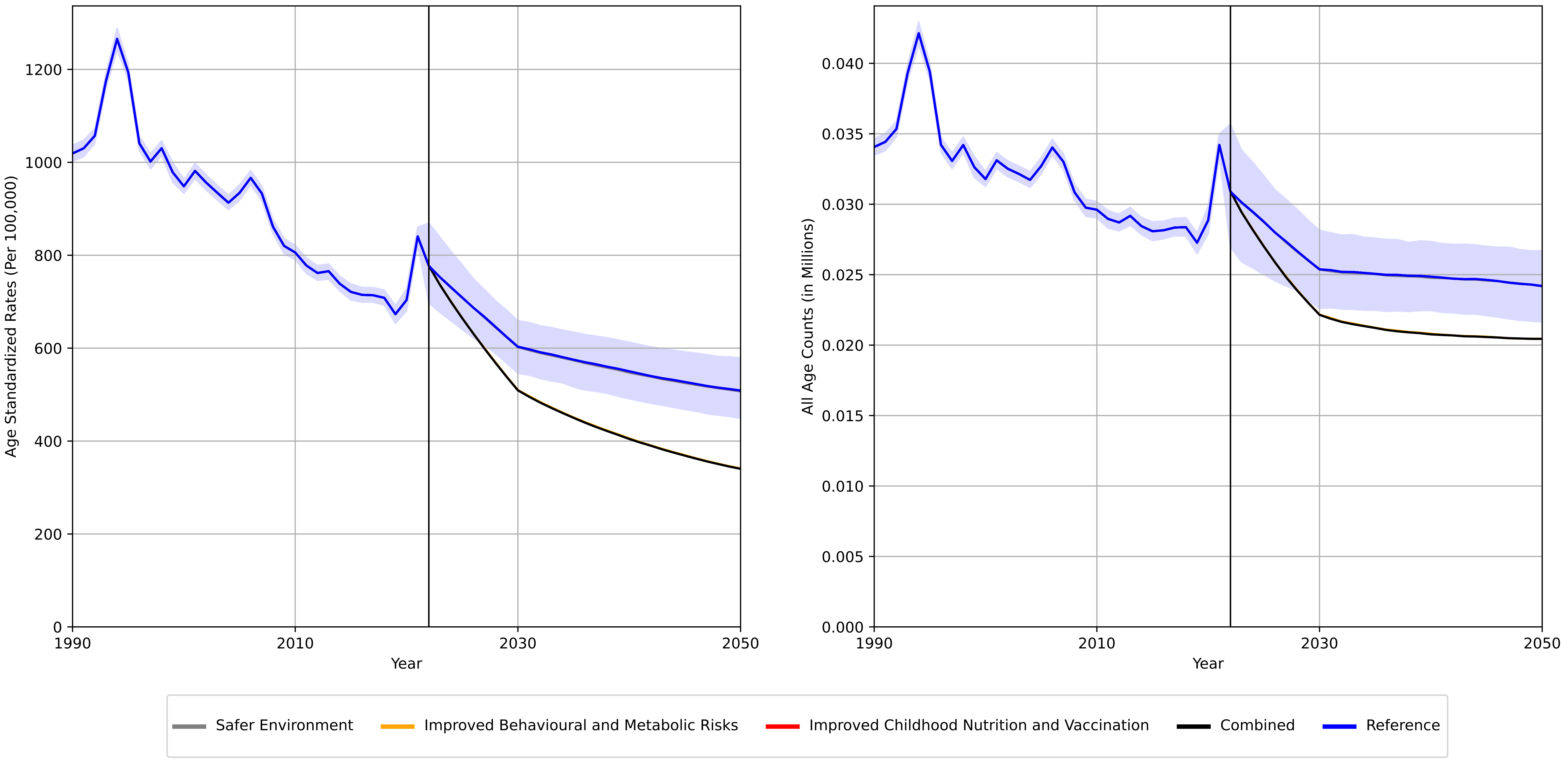

Lithuania

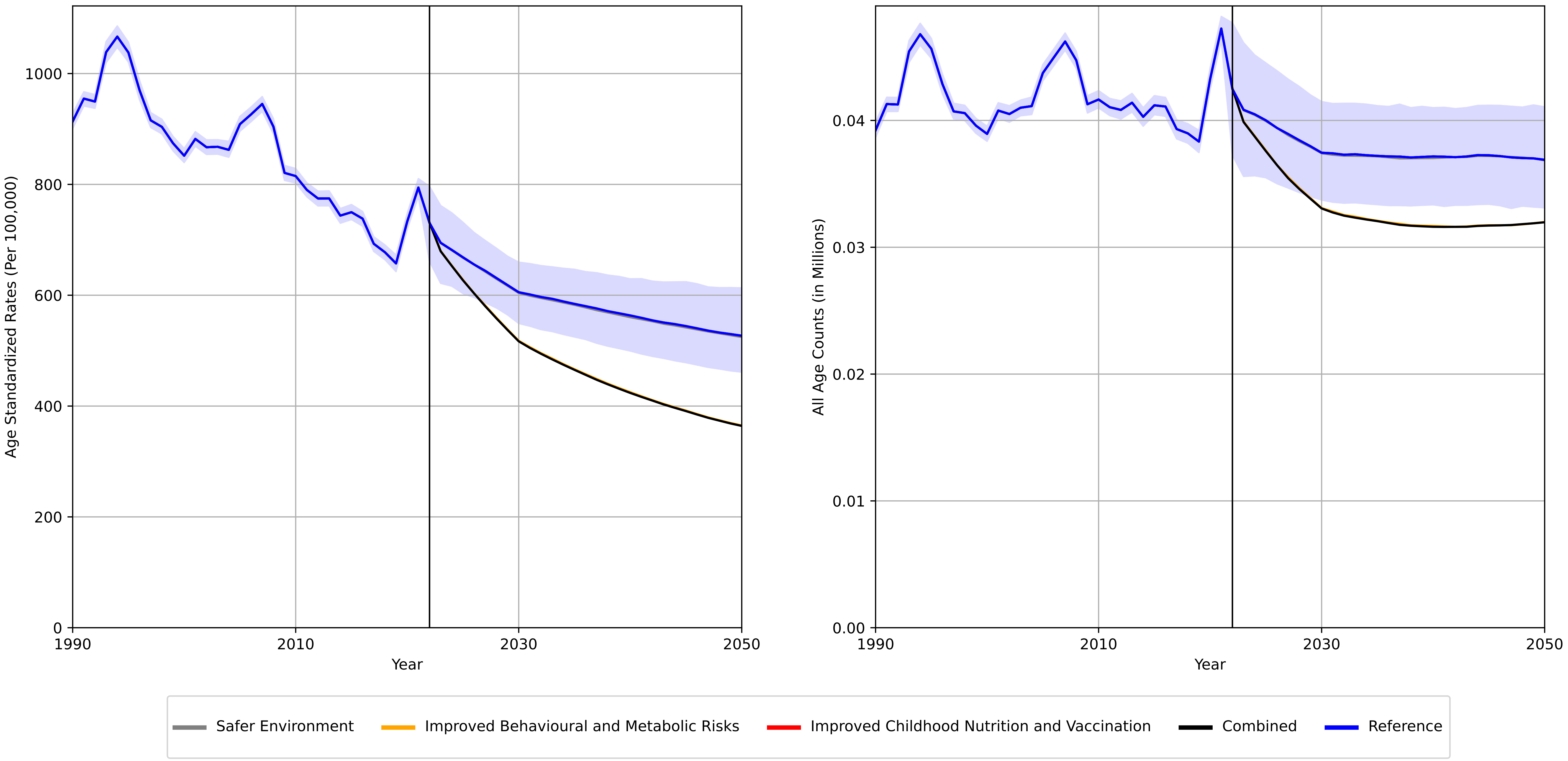

Age Standardized Rates (Per 100,000)
Year
All Age Counts (in Millions)
Year
Safer Environment
Improved Behavioural and Metabolic Risks
Improved Childhood Nutrition and Vaccination
Combined
Reference

Moldova

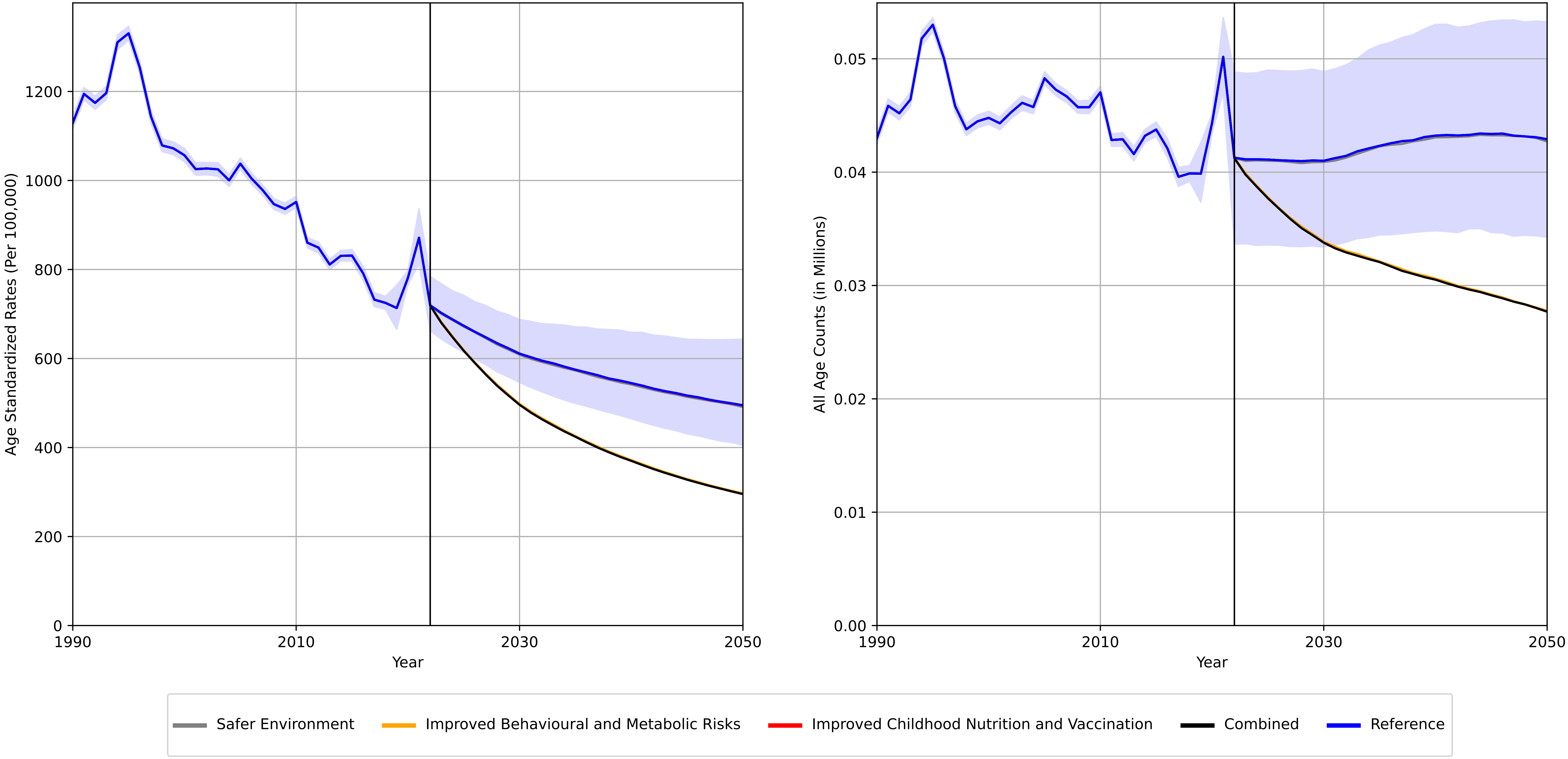

Age Standardized Rates (Per 100,000)
0
200
400
600
800
1000
1200
1990
2010
2030
2050
Year
All Age Counts (in Millions)
0.00
0.01
0.02
0.03
0.04
0.05
1990
2010
2030
2050
Year
Safer Environment
Improved Behavioural and Metabolic Risks
Improved Childhood Nutrition and Vaccination
Combined
Reference

Russia

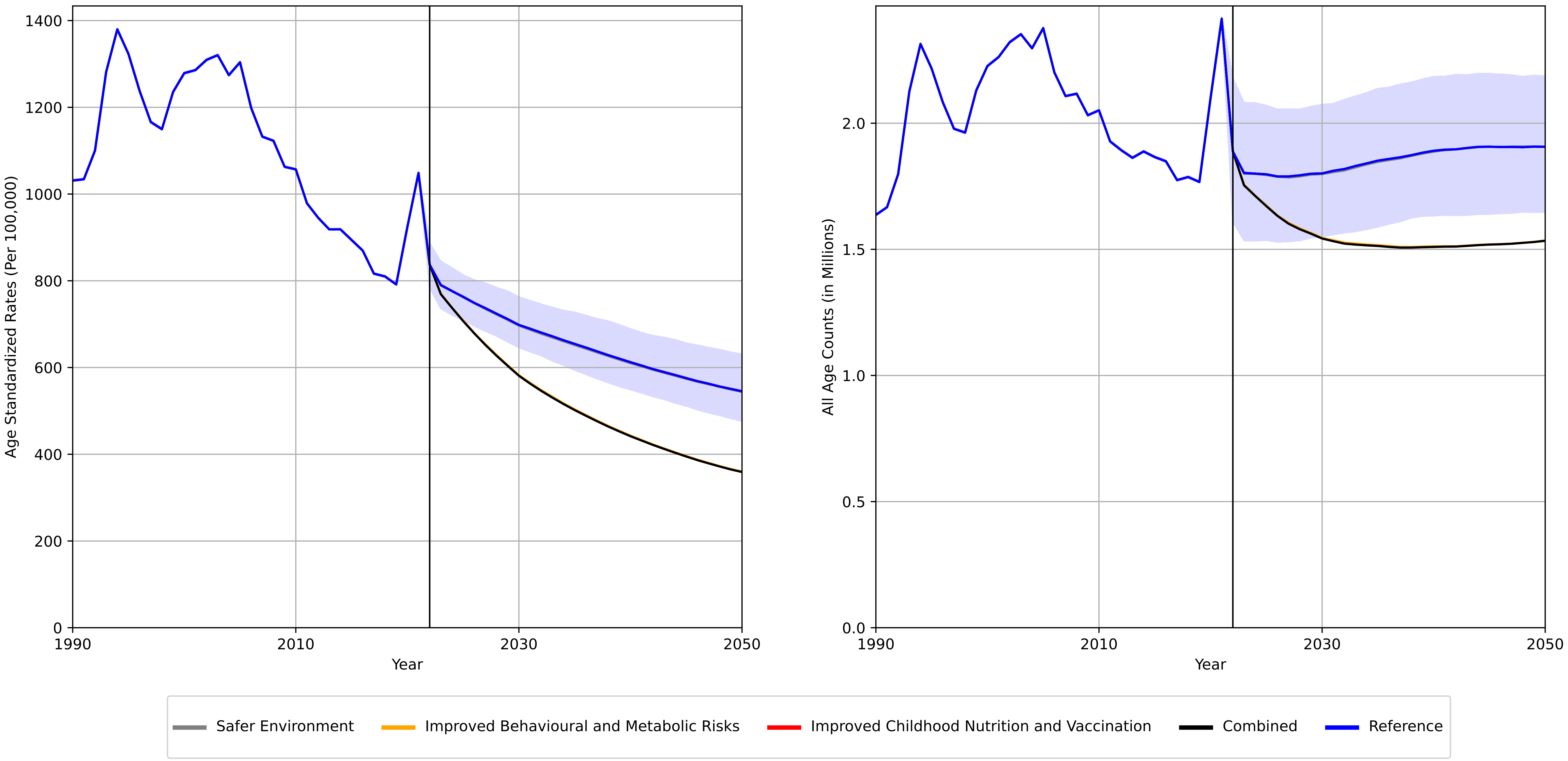
Age Standardized Rates (Per 100,000)
0
200
400
600
800
1000
1200
1400
1990
2010
2030
2050
Year
All Age Counts (in Millions)
0.0
0.5
1.0
1.5
2.0
1990
2010
2030
2050
Year
Safer Environment
Improved Behavioural and Metabolic Risks
Improved Childhood Nutrition and Vaccination
Combined
Reference

Ukraine

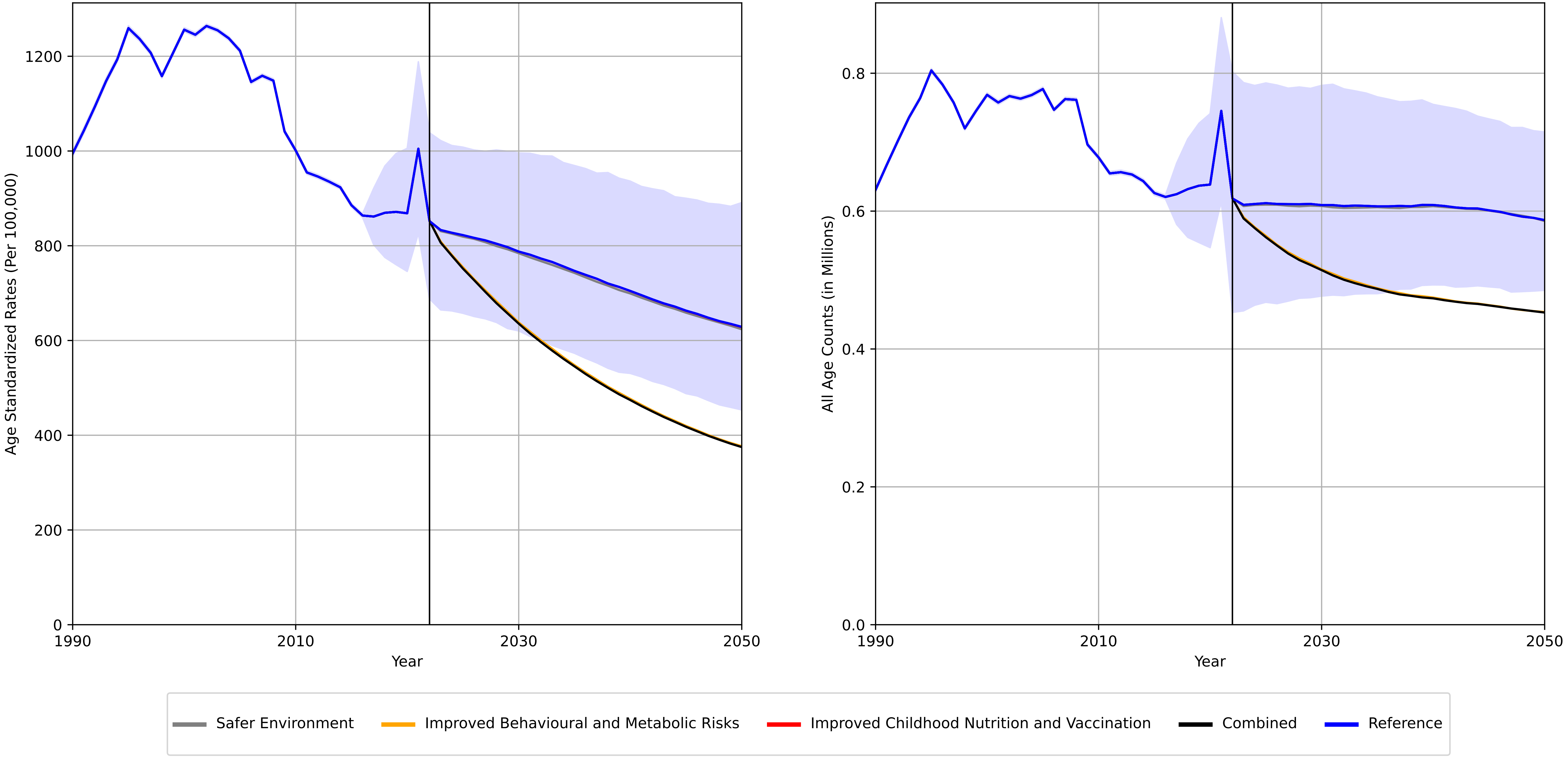
Age Standardized Rates (Per 100,000)
0
200
400
600
800
1000
1200
1990
2010
2030
2050
Year
All Age Counts (in Millions)
0.0
0.2
0.4
0.6
0.8
1990
2010
2030
2050
Year
Safer Environment
Improved Behavioural and Metabolic Risks
Improved Childhood Nutrition and Vaccination
Combined
Reference

Australia

Supplemental Results Figure S11. All-cause age standardised death rates (A) and all age death counts (B), for the past and for five future scenarios, 1990–2050

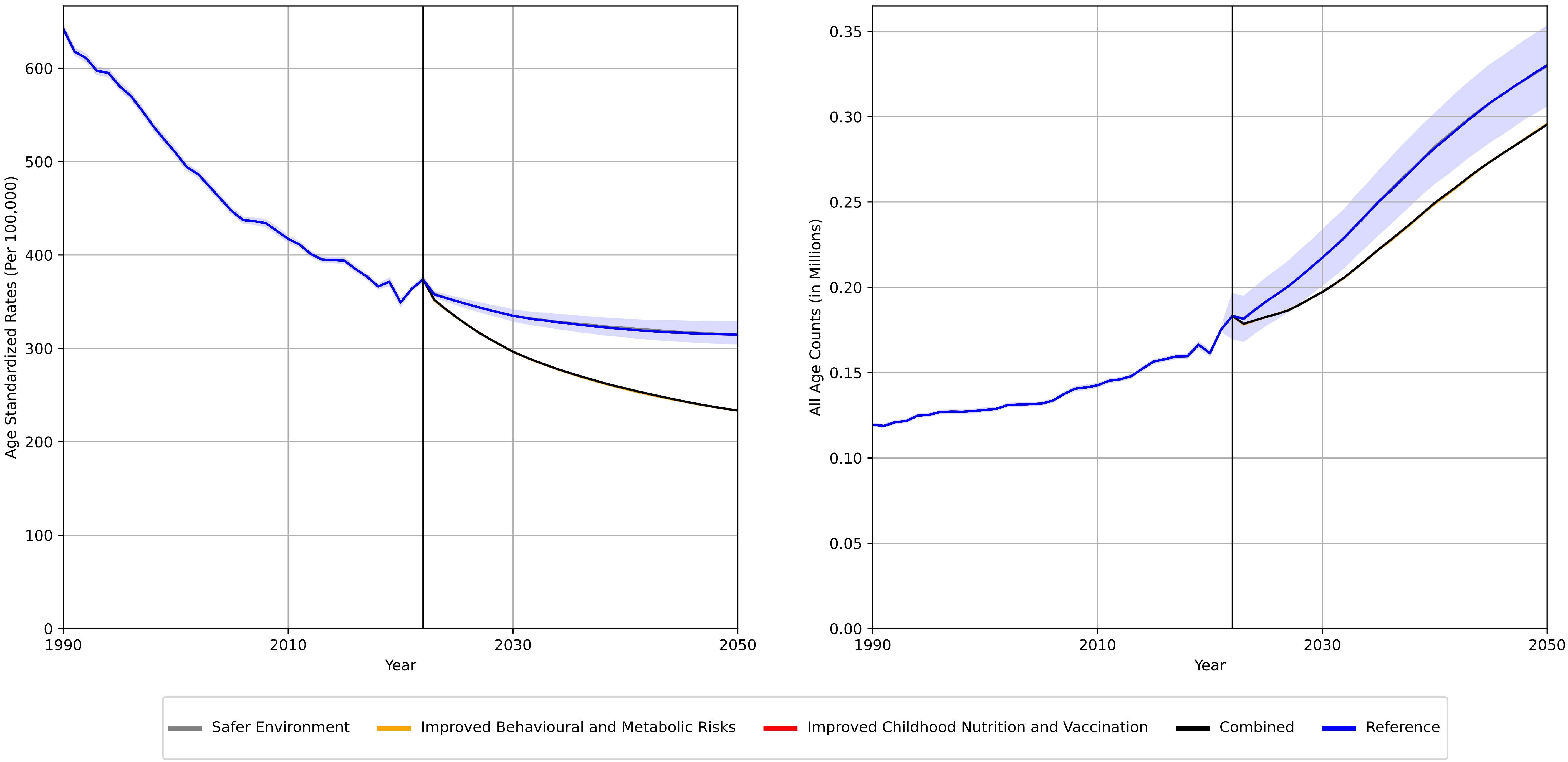

New Zealand

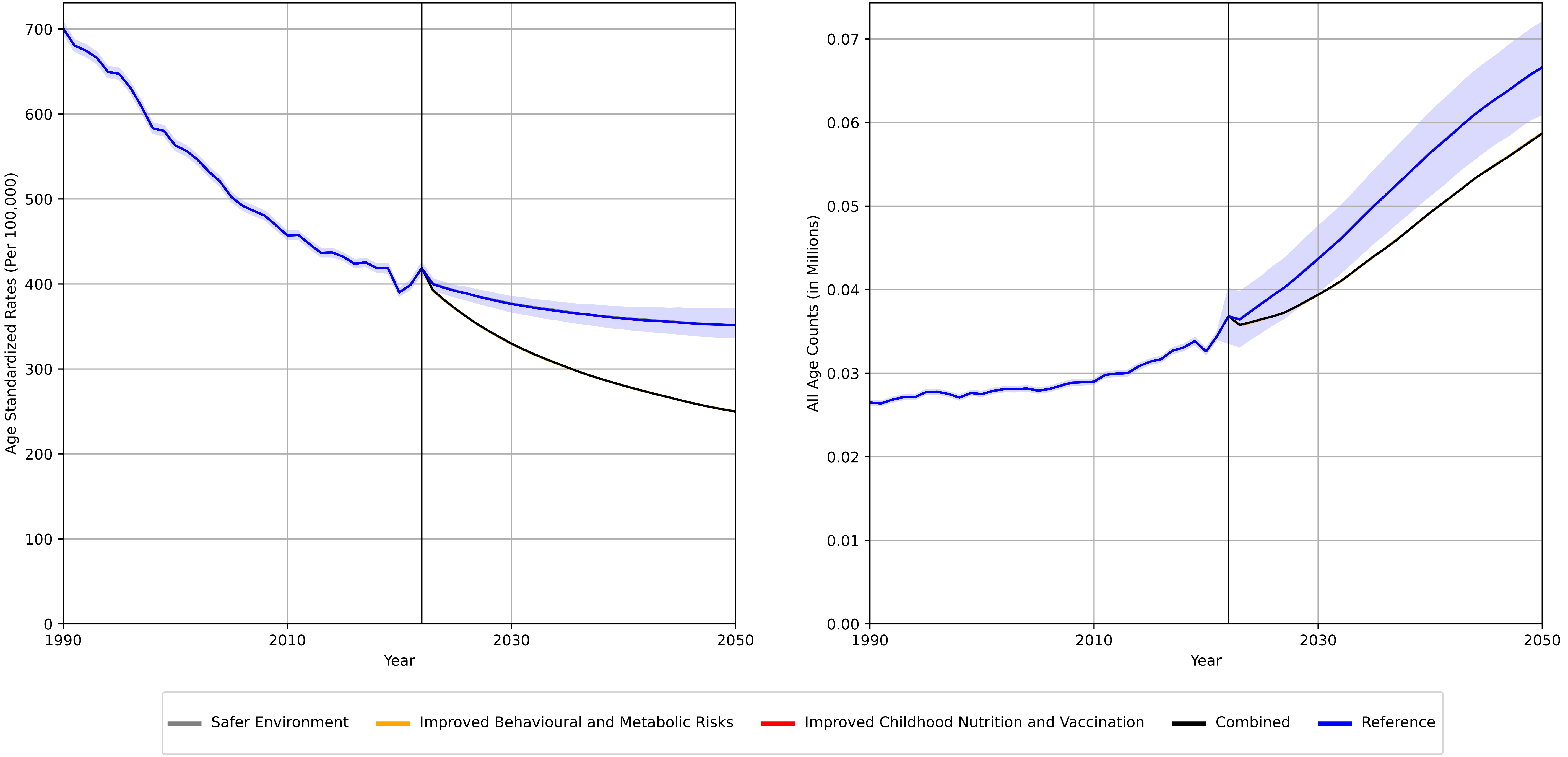
Age Standardized Rates (Per 100,000)
700
600
500
400
300
200
100
0
1990
2010
2030
2050
Year
All Age Counts (in Millions)
0.07
0.06
0.05
0.04
0.03
0.02
0.01
0.00
Safer Environment
Improved Behavioural and Metabolic Risks
Improved Childhood Nutrition and Vaccination
Combined
Reference

Brunei

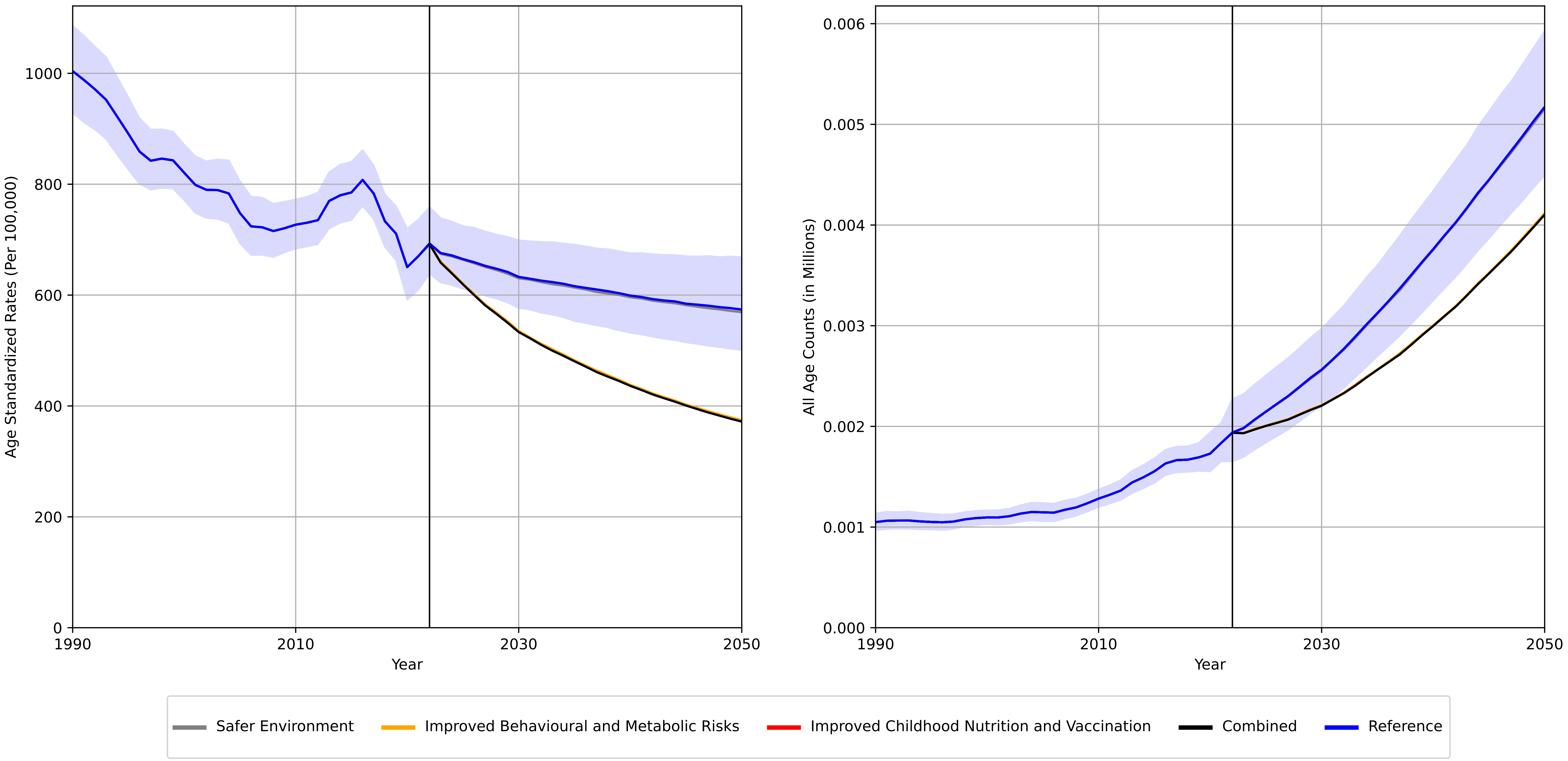
Age Standardized Rates (Per 100,000)
0
200
400
600
800
1000
1990
2010
2030
2050
Year
All Age Counts (in Millions)
0.000
0.001
0.002
0.003
0.004
0.005
0.006
1990
2010
2030
2050
Year
Safer Environment
Improved Behavioural and Metabolic Risks
Improved Childhood Nutrition and Vaccination
Combined
Reference

Japan

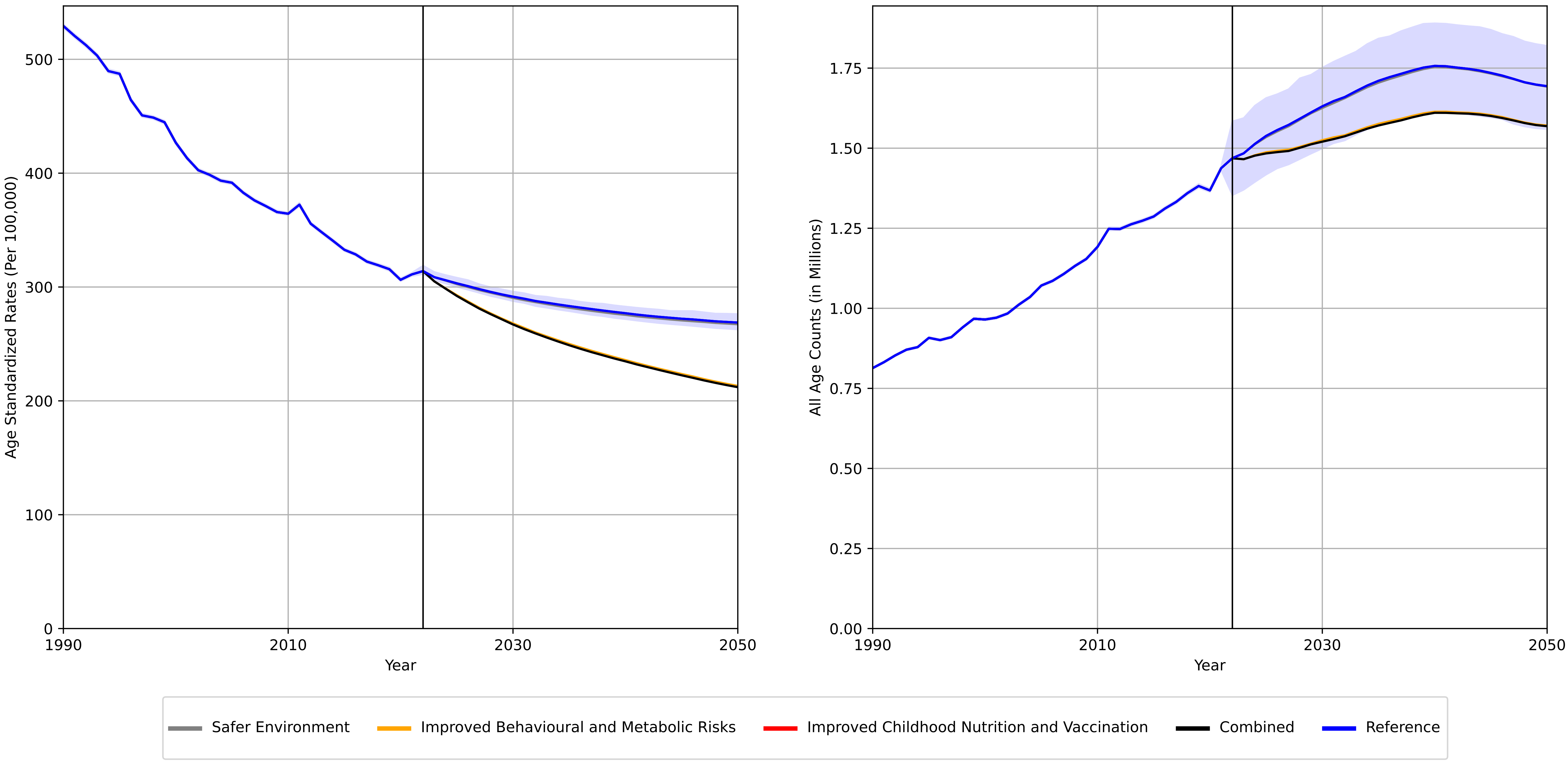

Age Standardized Rates (Per 100,000)
0
100
200
300
400
500
1990
2010
2030
2050
Year
All Age Counts (in Millions)
0.00
0.25
0.50
0.75
1.00
1.25
1.50
1.75
1990
2010
2030
2050
Year
Safer Environment
Improved Behavioural and Metabolic Risks
Improved Childhood Nutrition and Vaccination
Combined
Reference

South Korea

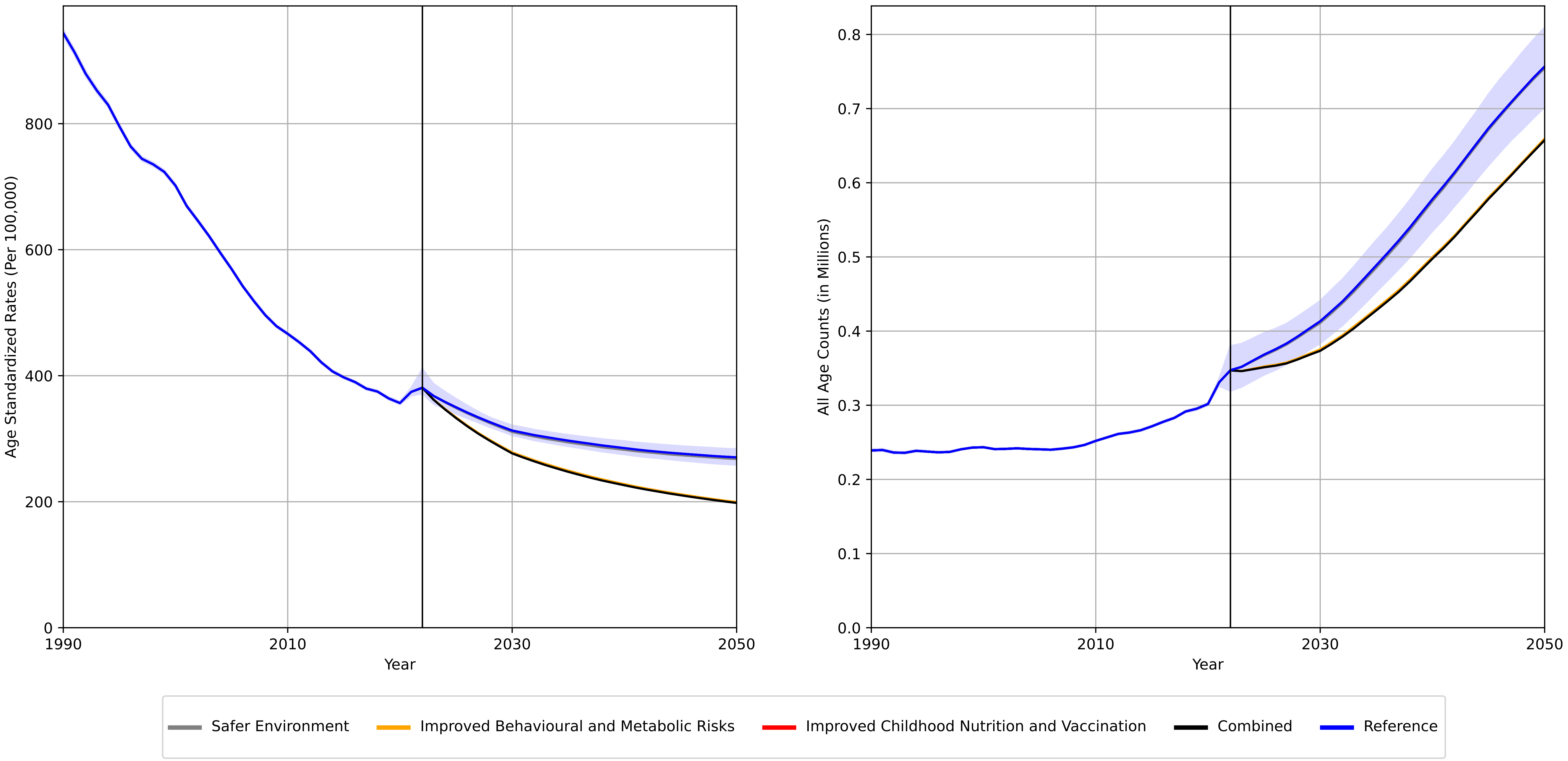
Age Standardized Rates (Per 100,000)
0
200
400
600
800
1990
2010
2030
2050
Year
All Age Counts (in Millions)
0.0
0.1
0.2
0.3
0.4
0.5
0.6
0.7
0.8
1990
2010
2030
2050
Year
Safer Environment
Improved Behavioural and Metabolic Risks
Improved Childhood Nutrition and Vaccination
Combined
Reference

Singapore

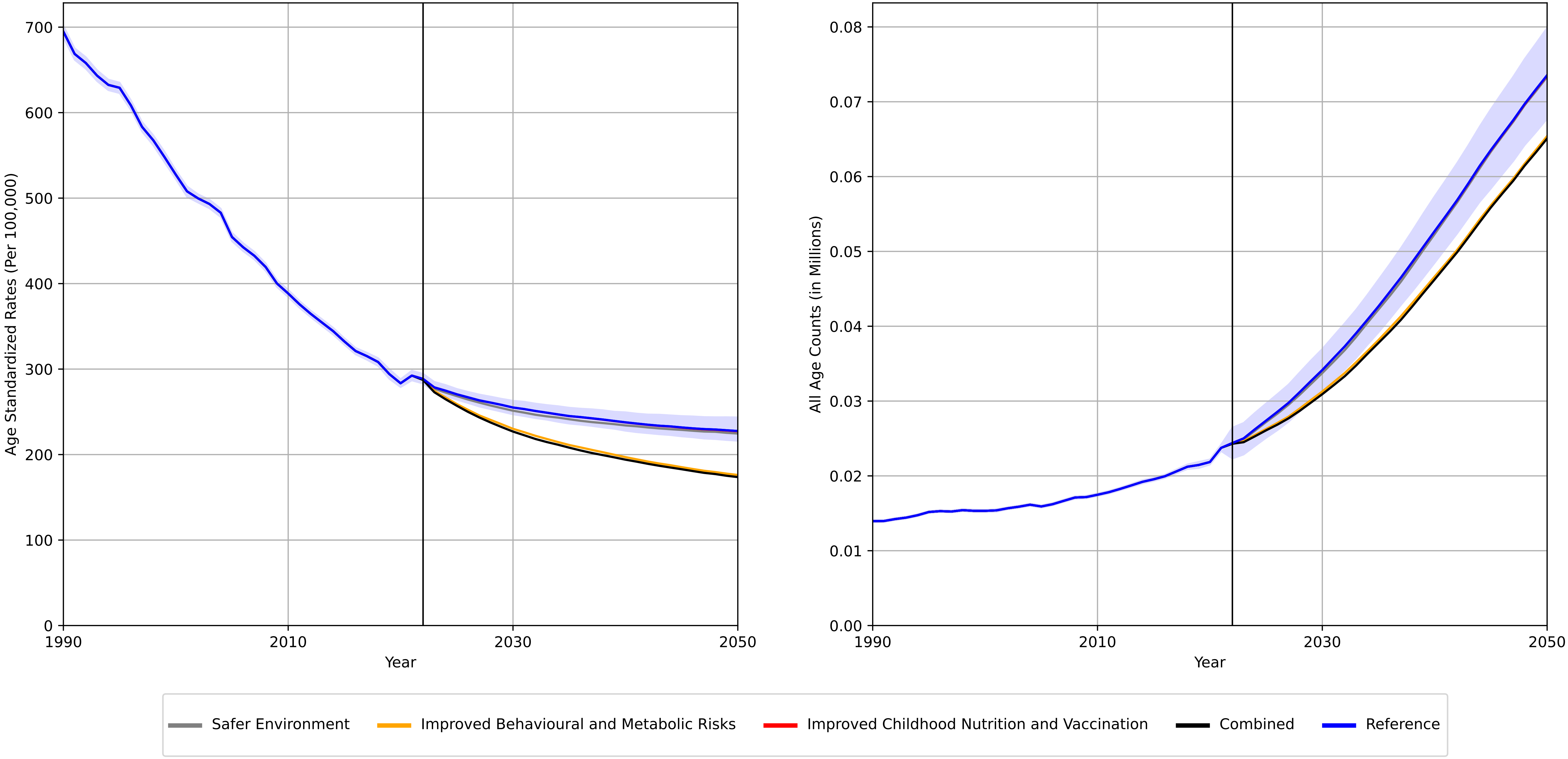
Age Standardized Rates (Per 100,000)
0
100
200
300
400
500
600
700
1990
2010
2030
2050
Year
All Age Counts (in Millions)
0.00
0.01
0.02
0.03
0.04
0.05
0.06
0.07
0.08
1990
2010
2030
2050
Year
Safer Environment
Improved Behavioural and Metabolic Risks
Improved Childhood Nutrition and Vaccination
Combined
Reference

Canada

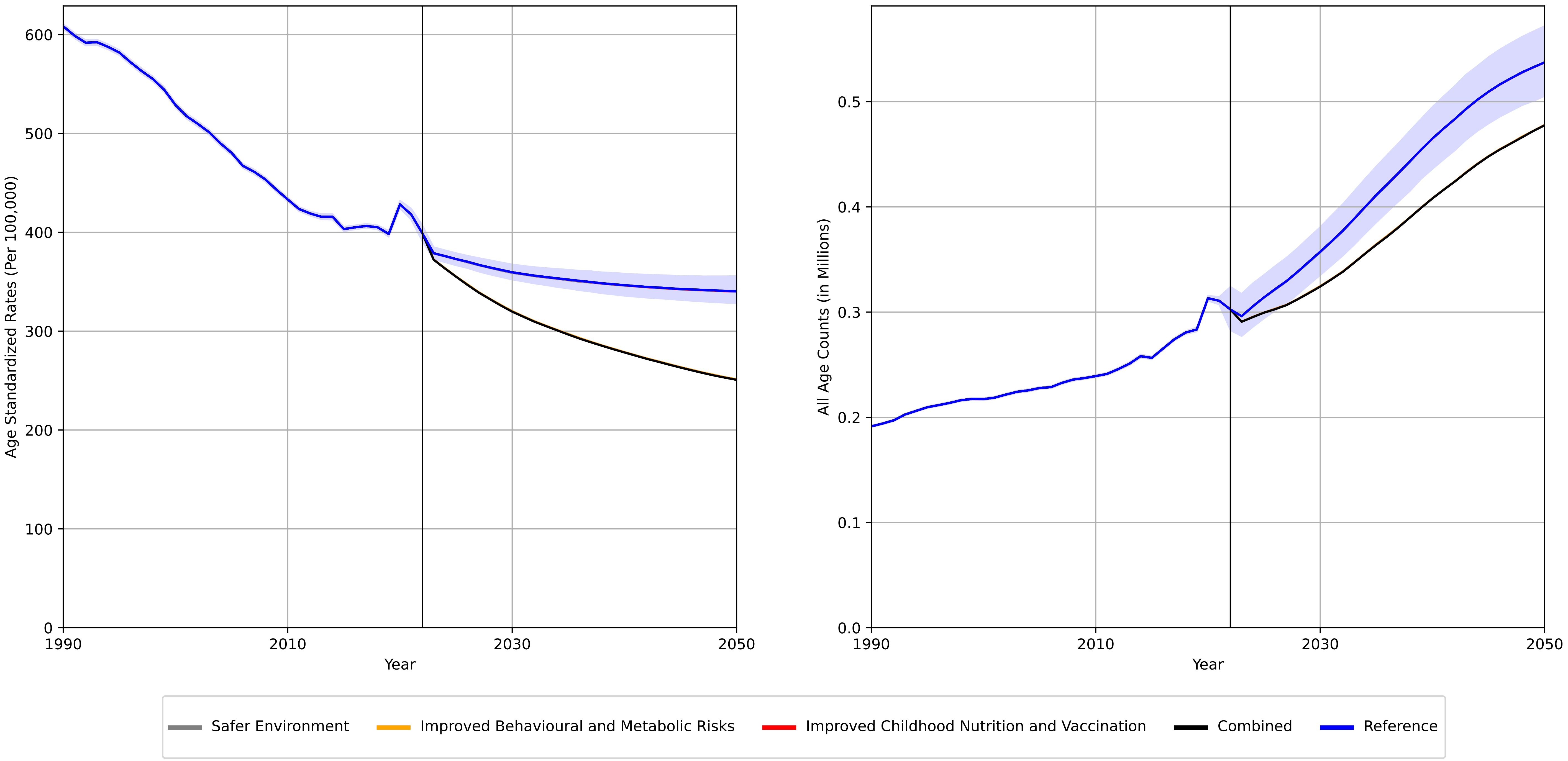

Age Standardized Rates (Per 100,000)
0
100
200
300
400
500
600
1990
2010
2030
2050
Year
All Age Counts (in Millions)
0.0
0.1
0.2
0.3
0.4
0.5
1990
2010
2030
2050
Year
Safer Environment
Improved Behavioural and Metabolic Risks
Improved Childhood Nutrition and Vaccination
Combined
Reference

Greenland

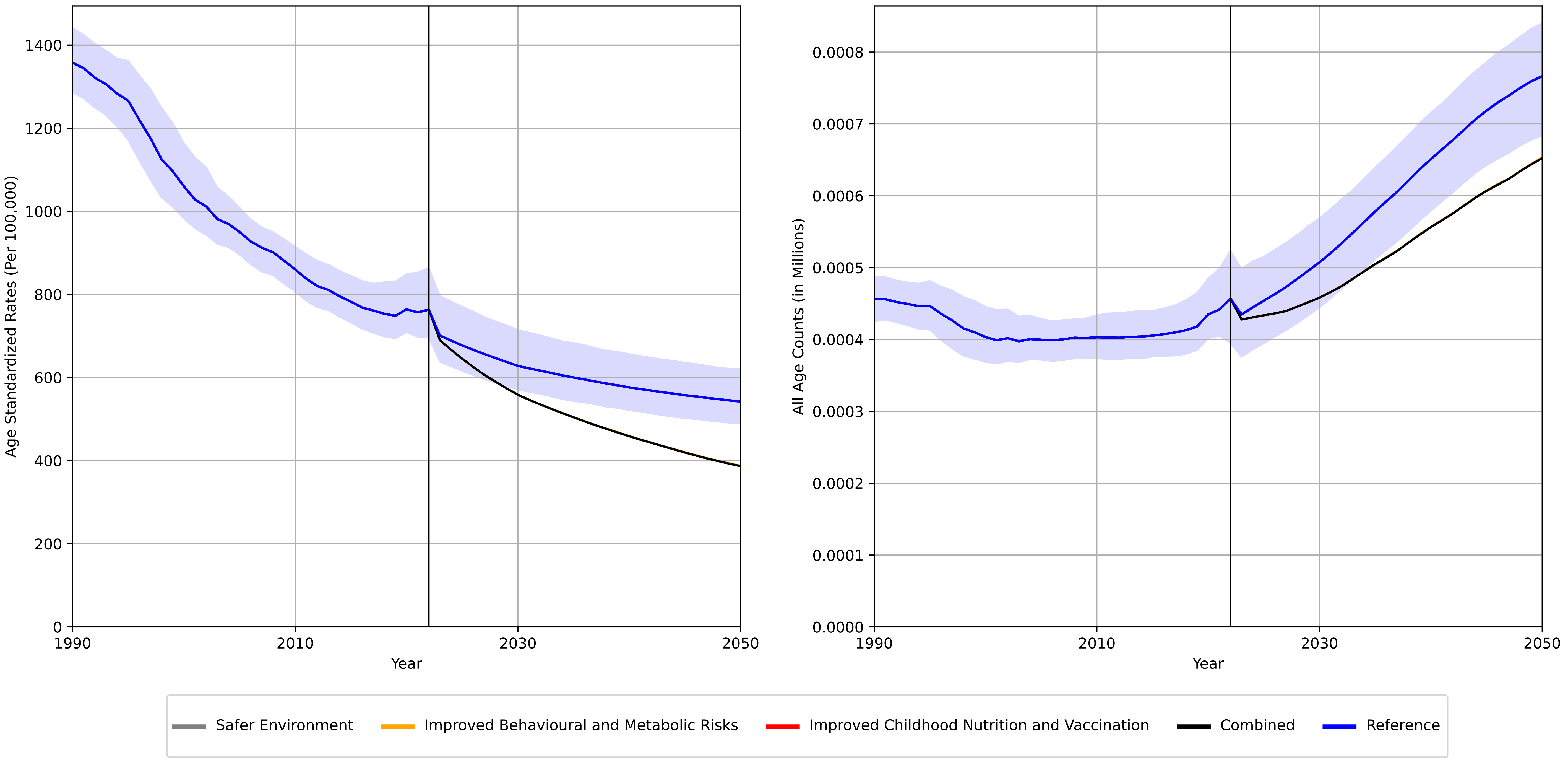

Age Standardized Rates (Per 100,000)
0
200
400
600
800
1000
1200
1400
1990
2010
2030
2050
Year
All Age Counts (in Millions)
0.0000
0.0001
0.0002
0.0003
0.0004
0.0005
0.0006
0.0007
0.0008
1990
2010
2030
2050
Year
Safer Environment
Improved Behavioural and Metabolic Risks
Improved Childhood Nutrition and Vaccination
Combined
Reference

USA

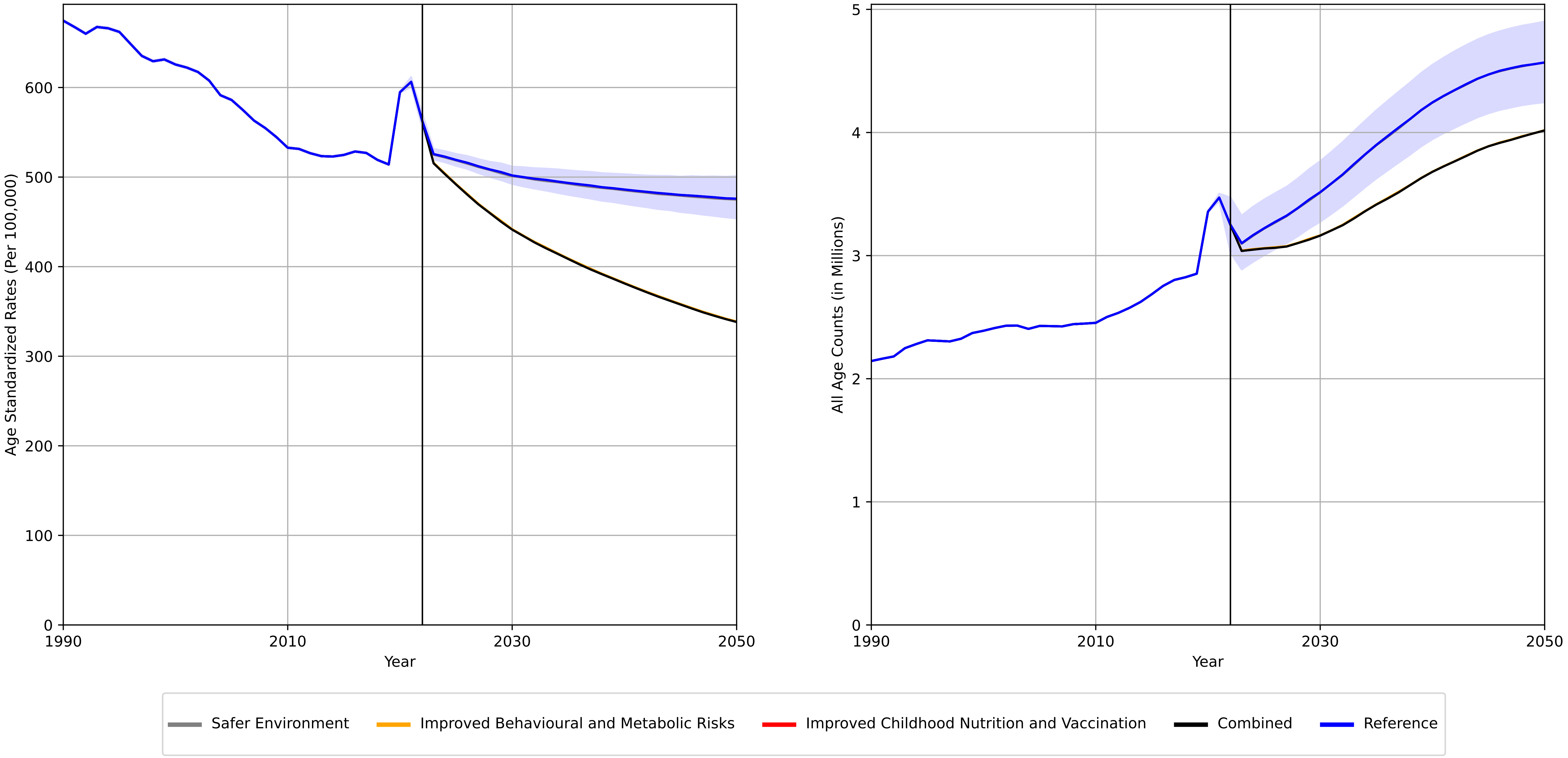

Age Standardized Rates (Per 100,000)
0
100
200
300
400
500
600
1990
2010
2030
2050
Year
All Age Counts (in Millions)
0
1
2
3
4
5
1990
2010
2030
2050
Year
Safer Environment
Improved Behavioural and Metabolic Risks
Improved Childhood Nutrition and Vaccination
Combined
Reference

Argentina

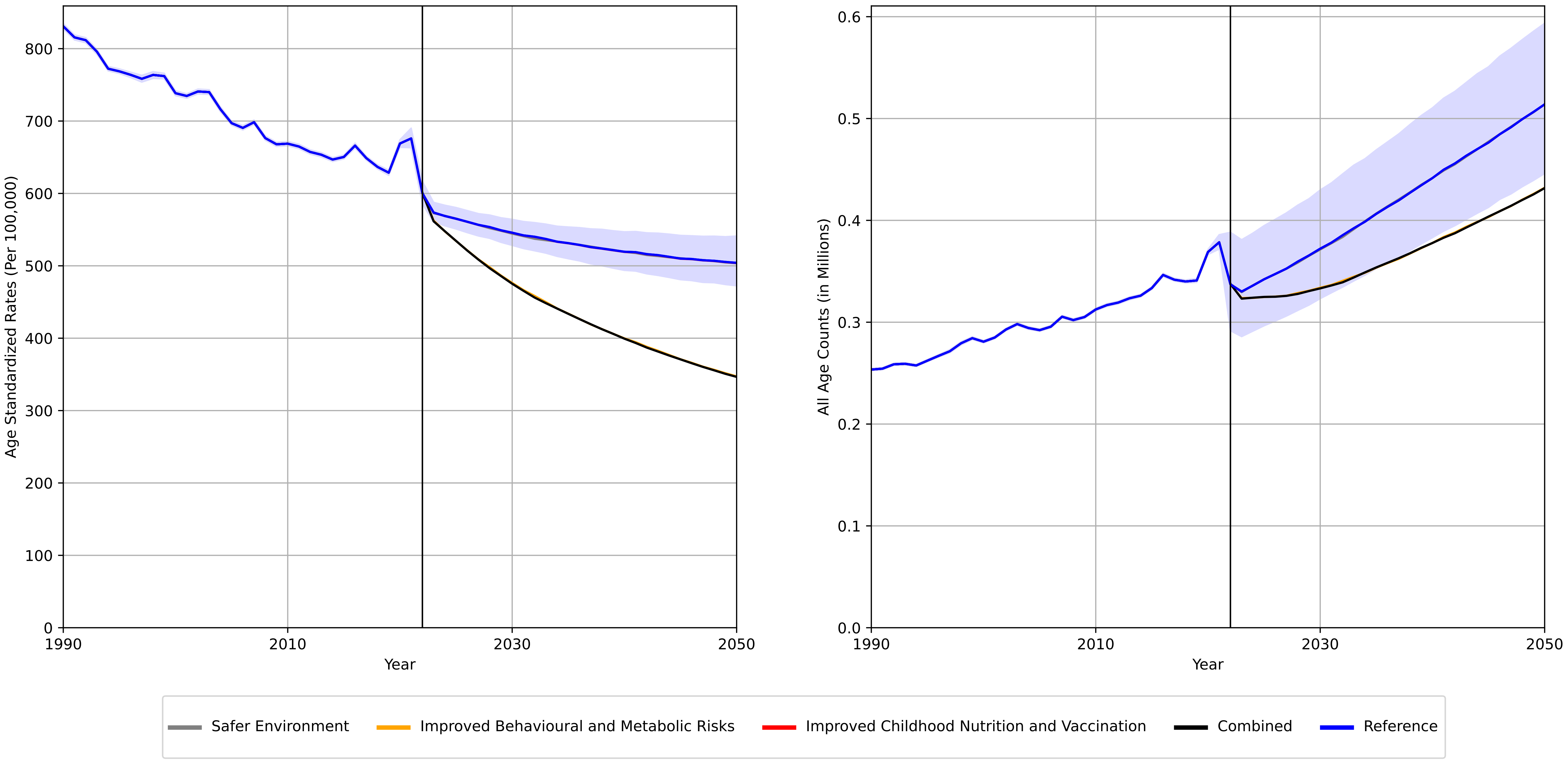

Age Standardized Rates (Per 100,000)
800
700
600
500
400
300
200
100
0
1990
2010
2030
2050
Year
All Age Counts (in Millions)
0.6
0.5
0.4
0.3
0.2
0.1
0.0
1990
2010
2030
2050
Year
Safer Environment
Improved Behavioural and Metabolic Risks
Improved Childhood Nutrition and Vaccination
Combined
Reference

Chile

Supplemental Results Figure S11. All-cause age standardised death rates (A) and all age death counts (B), for the past and for five future scenarios, 1990–2050

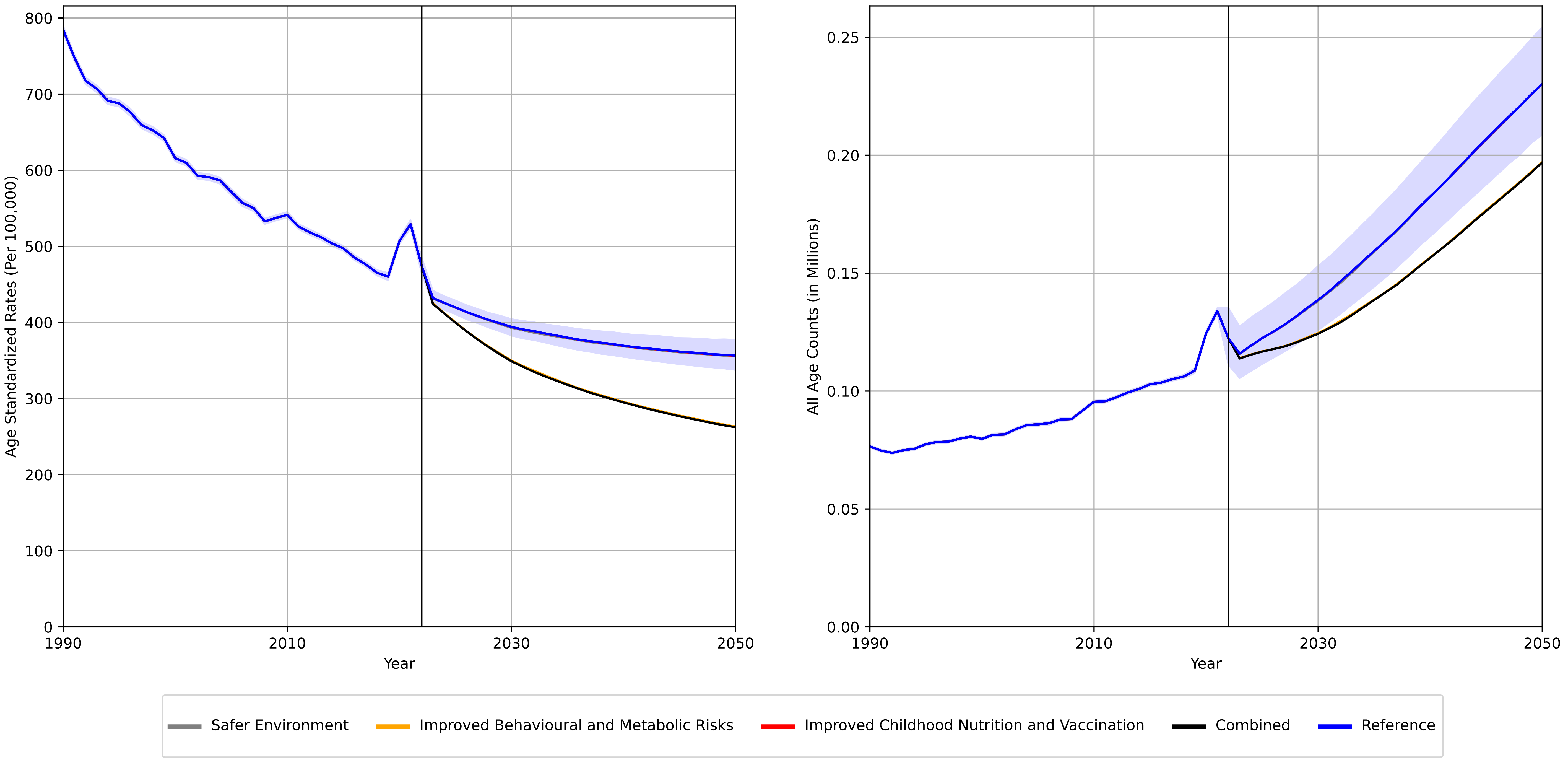

Uruguay

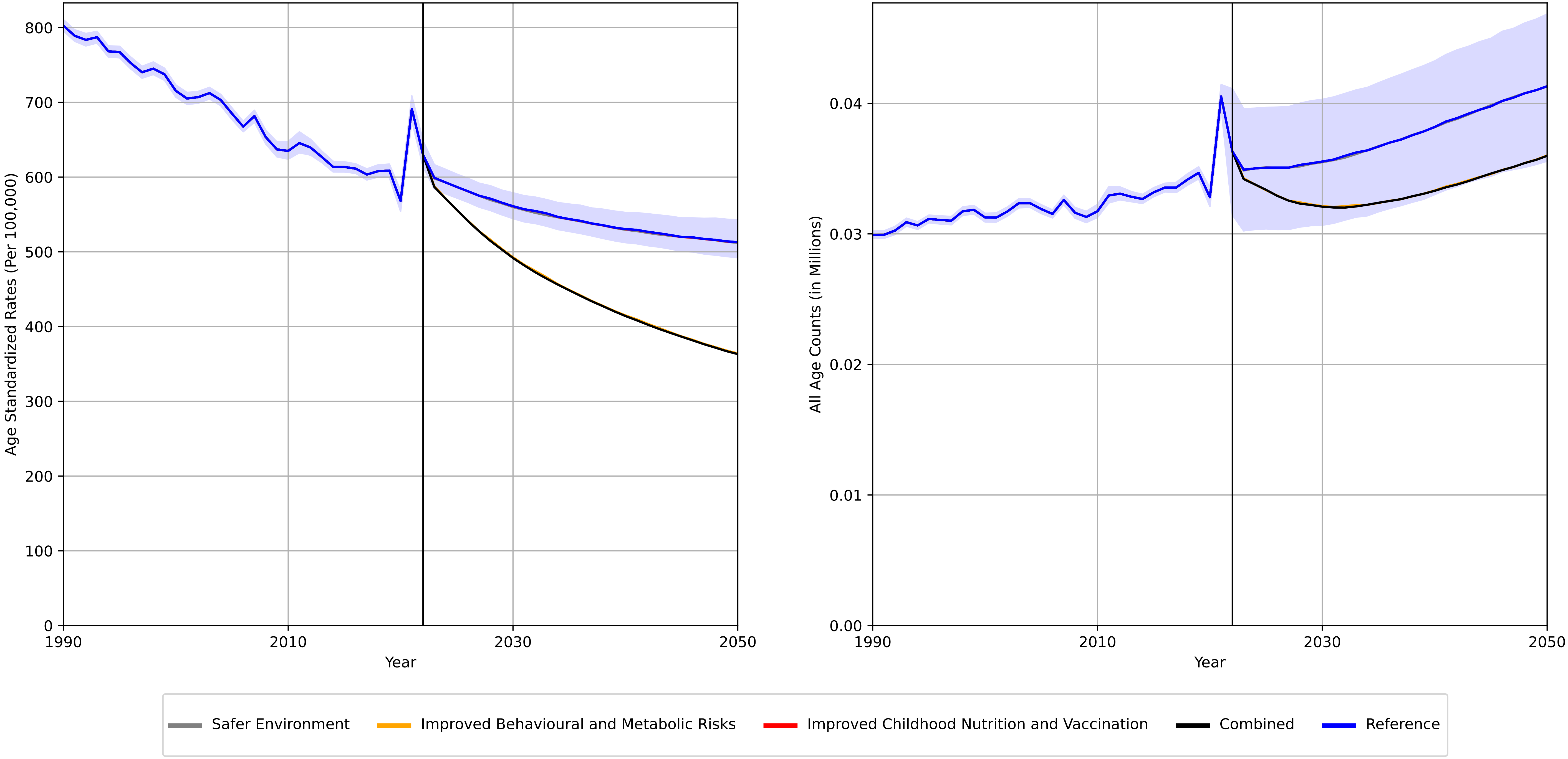

Age Standardized Rates (Per 100,000)
Year
All Age Counts (in Millions)
Year
Safer Environment
Improved Behavioural and Metabolic Risks
Improved Childhood Nutrition and Vaccination
Combined
Reference

Andorra

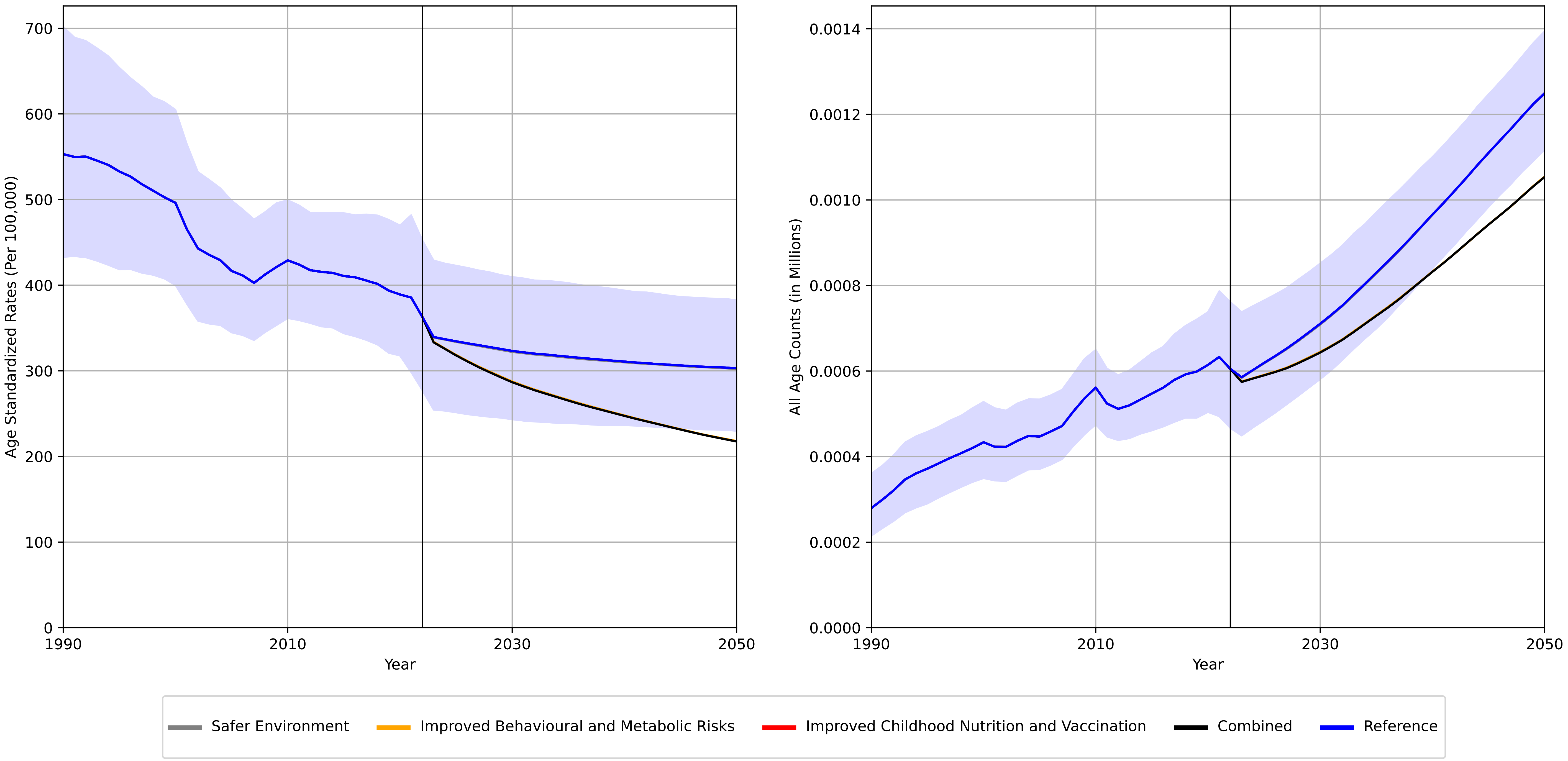
Age Standardized Rates (Per 100,000)
700
600
500
400
300
200
100
0
1990
2010
2030
2050
Year
All Age Counts (in Millions)
0.0014
0.0012
0.0010
0.0008
0.0006
0.0004
0.0002
0.0000
1990
2010
2030
2050
Year
Safer Environment
Improved Behavioural and Metabolic Risks
Improved Childhood Nutrition and Vaccination
Combined
Reference

Austria

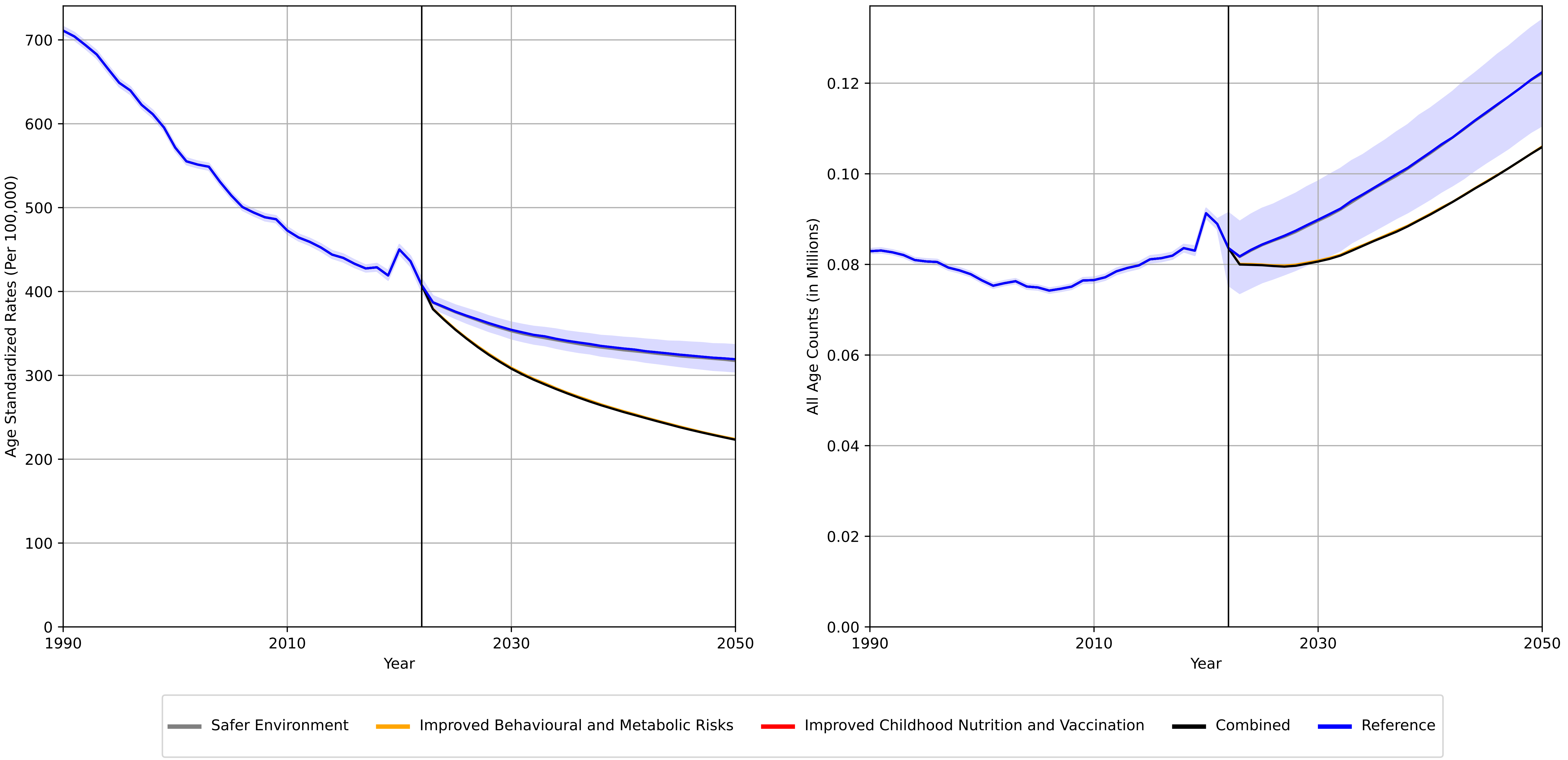
Age Standardized Rates (Per 100,000)
700
600
500
400
300
200
100
0
1990
2010
2030
2050
Year
All Age Counts (in Millions)
0.12
0.10
0.08
0.06
0.04
0.02
0.00
1990
2010
2030
2050
Year
Safer Environment
Improved Behavioural and Metabolic Risks
Improved Childhood Nutrition and Vaccination
Combined
Reference

Belgium

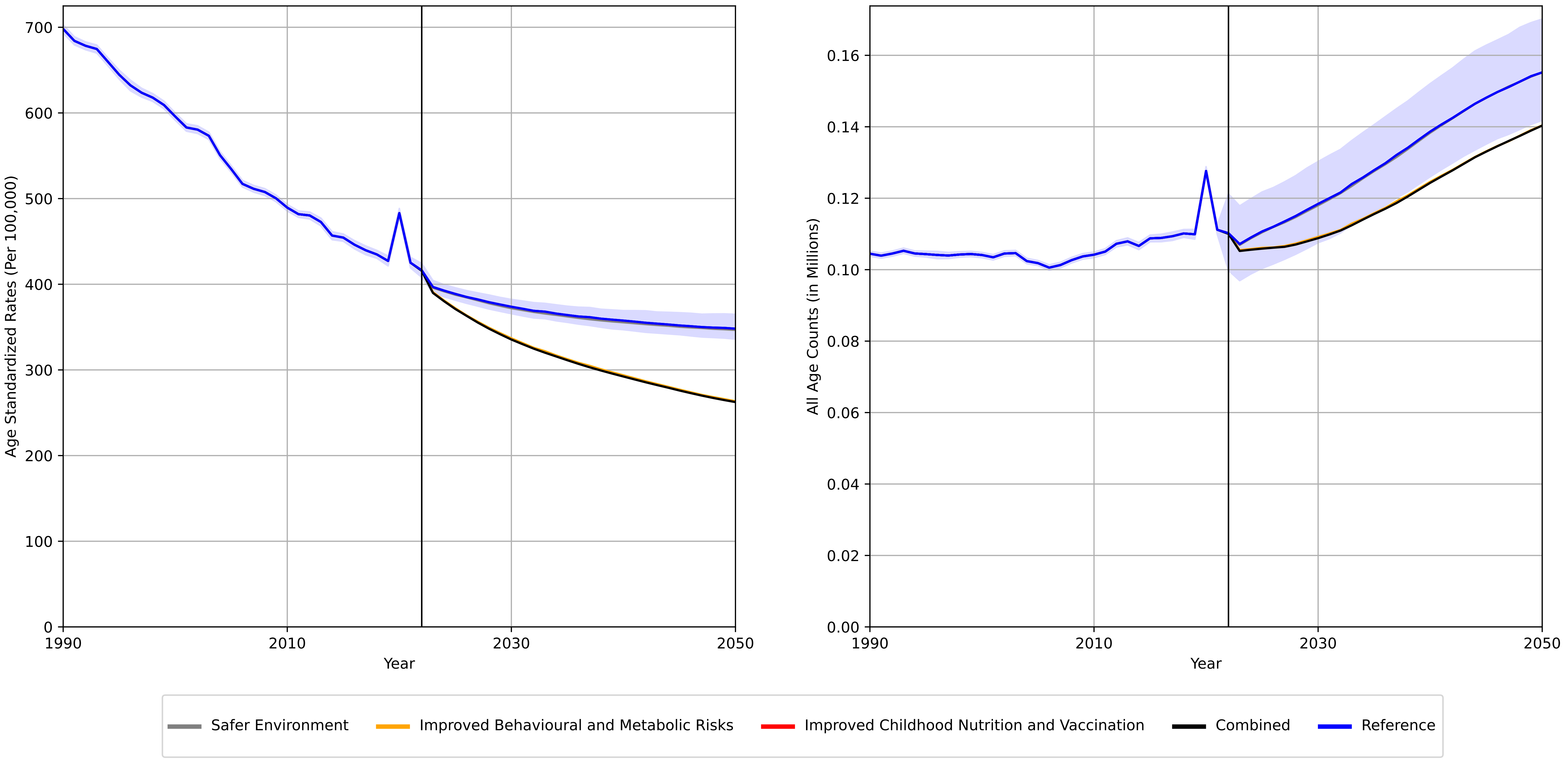

Age Standardized Rates (Per 100,000)
0
100
200
300
400
500
600
700
1990
2010
2030
2050
Year
All Age Counts (in Millions)
0.00
0.02
0.04
0.06
0.08
0.10
0.12
0.14
0.16
1990
2010
2030
2050
Year
Safer Environment
Improved Behavioural and Metabolic Risks
Improved Childhood Nutrition and Vaccination
Combined
Reference

Cyprus

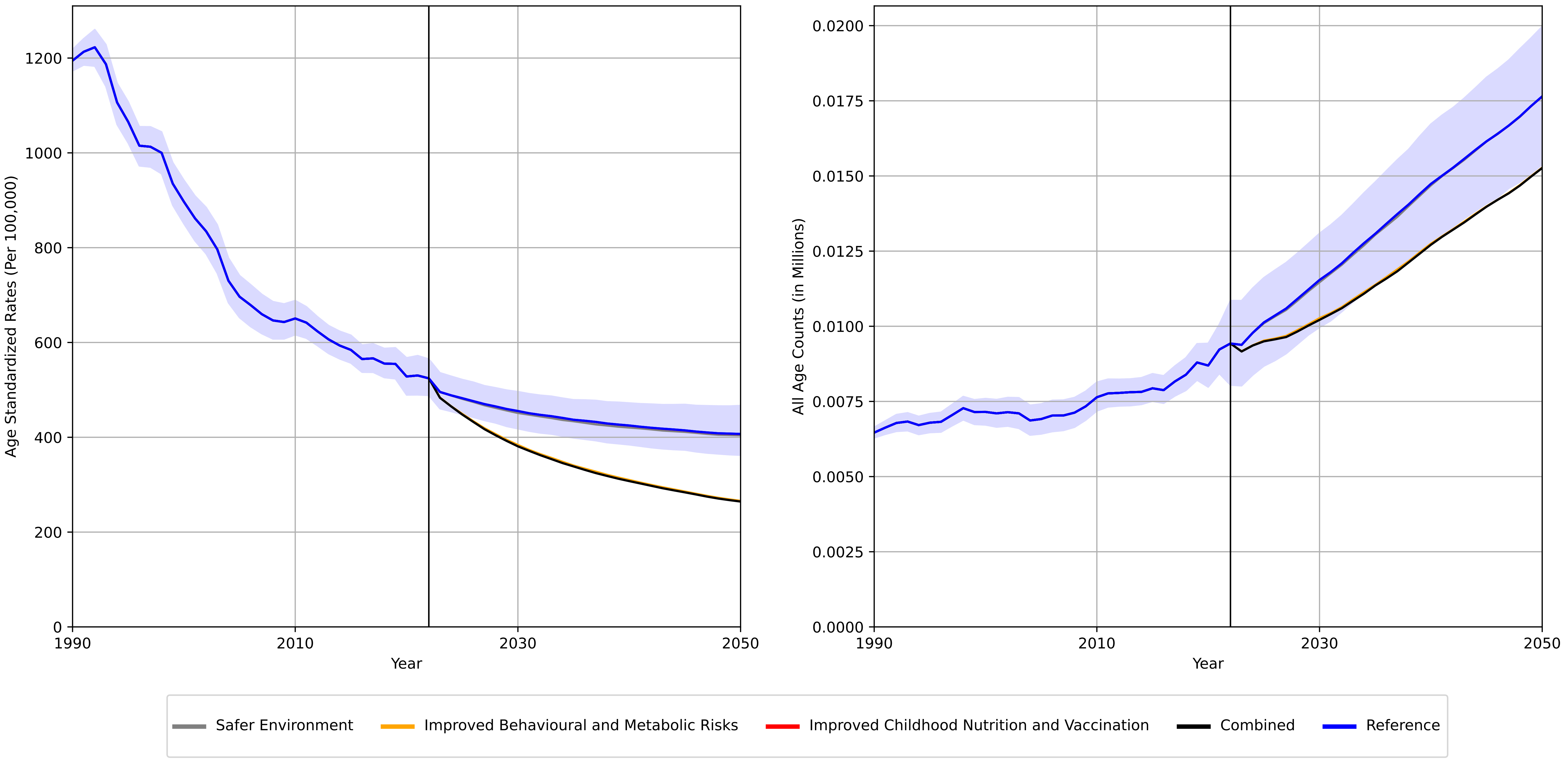

Age Standardized Rates (Per 100,000)
0
200
400
600
800
1000
1200
1990
2010
2030
2050
Year
All Age Counts (in Millions)
0.0000
0.0025
0.0050
0.0075
0.0100
0.0125
0.0150
0.0175
0.0200
1990
2010
2030
2050
Year
Safer Environment
Improved Behavioural and Metabolic Risks
Improved Childhood Nutrition and Vaccination
Combined
Reference

Denmark

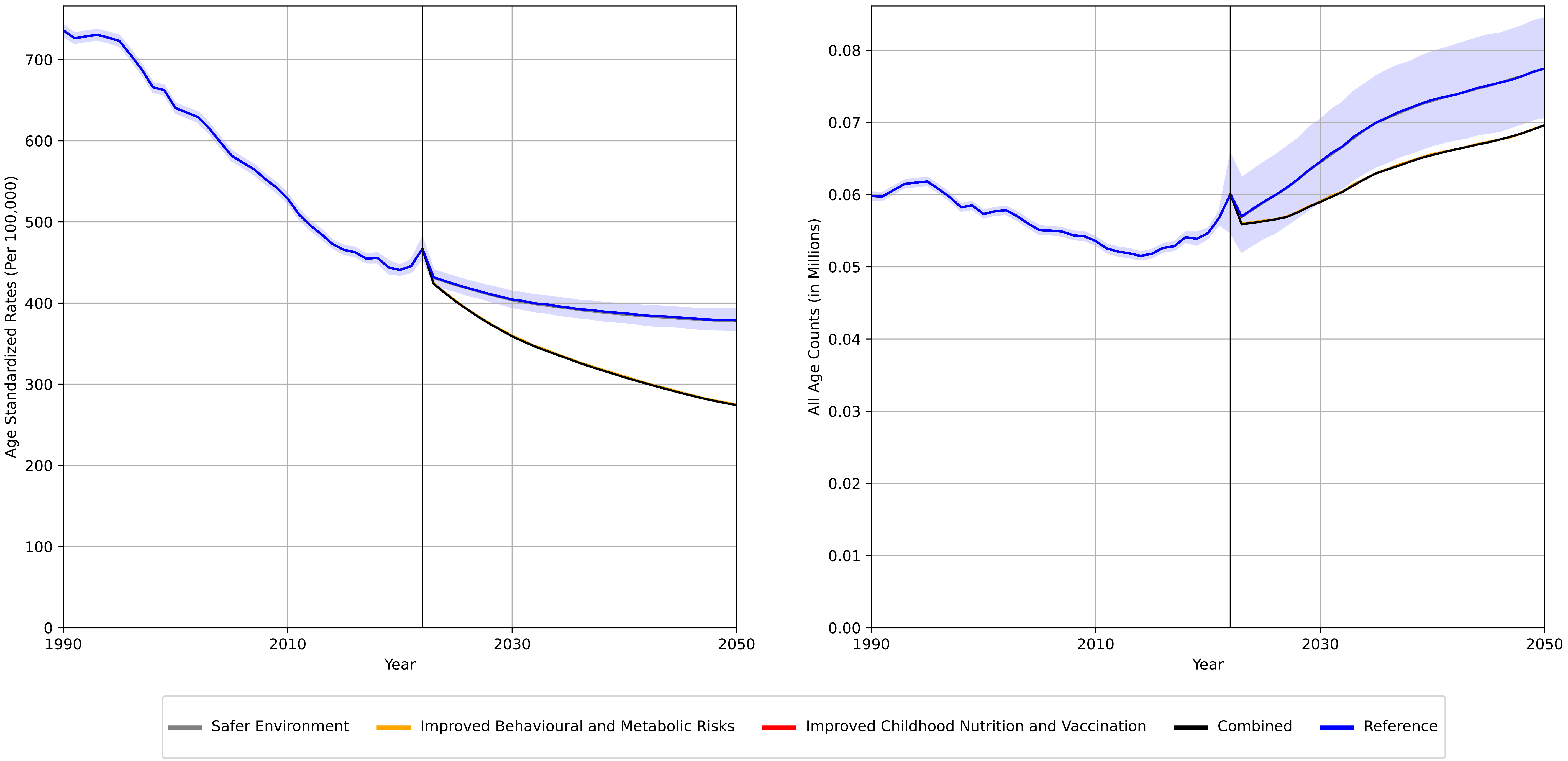

Age Standardized Rates (Per 100,000)
0
100
200
300
400
500
600
700
1990
2010
2030
2050
Year
All Age Counts (in Millions)
0.00
0.01
0.02
0.03
0.04
0.05
0.06
0.07
0.08
1990
2010
2030
2050
Year
Safer Environment
Improved Behavioural and Metabolic Risks
Improved Childhood Nutrition and Vaccination
Combined
Reference

Finland

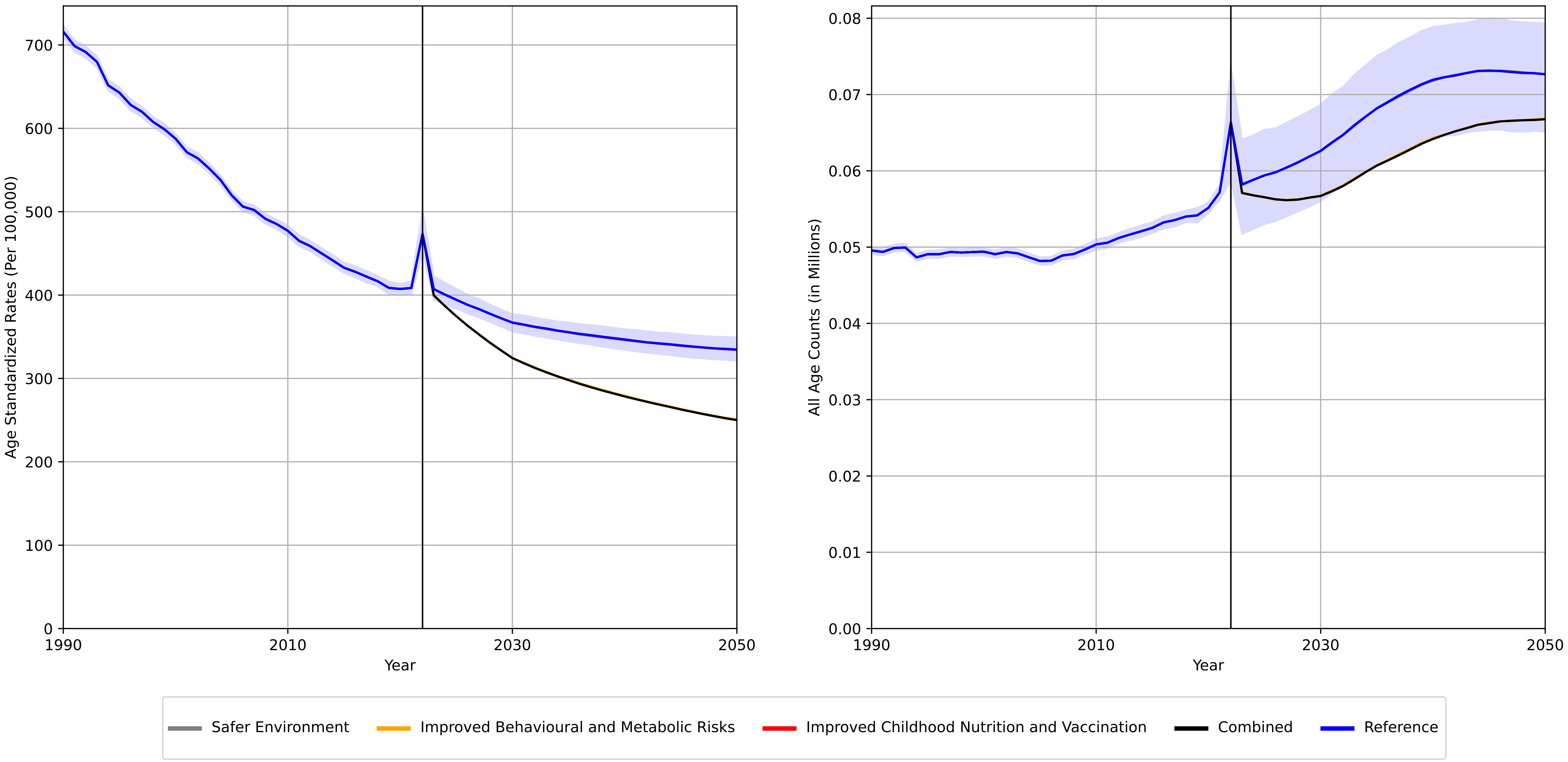
Age Standardized Rates (Per 100,000)
0
100
200
300
400
500
600
700
1990
2010
2030
2050
Year
All Age Counts (in Millions)
0.00
0.01
0.02
0.03
0.04
0.05
0.06
0.07
0.08
1990
2010
2030
2050
Year
Safer Environment
Improved Behavioural and Metabolic Risks
Improved Childhood Nutrition and Vaccination
Combined
Reference

France

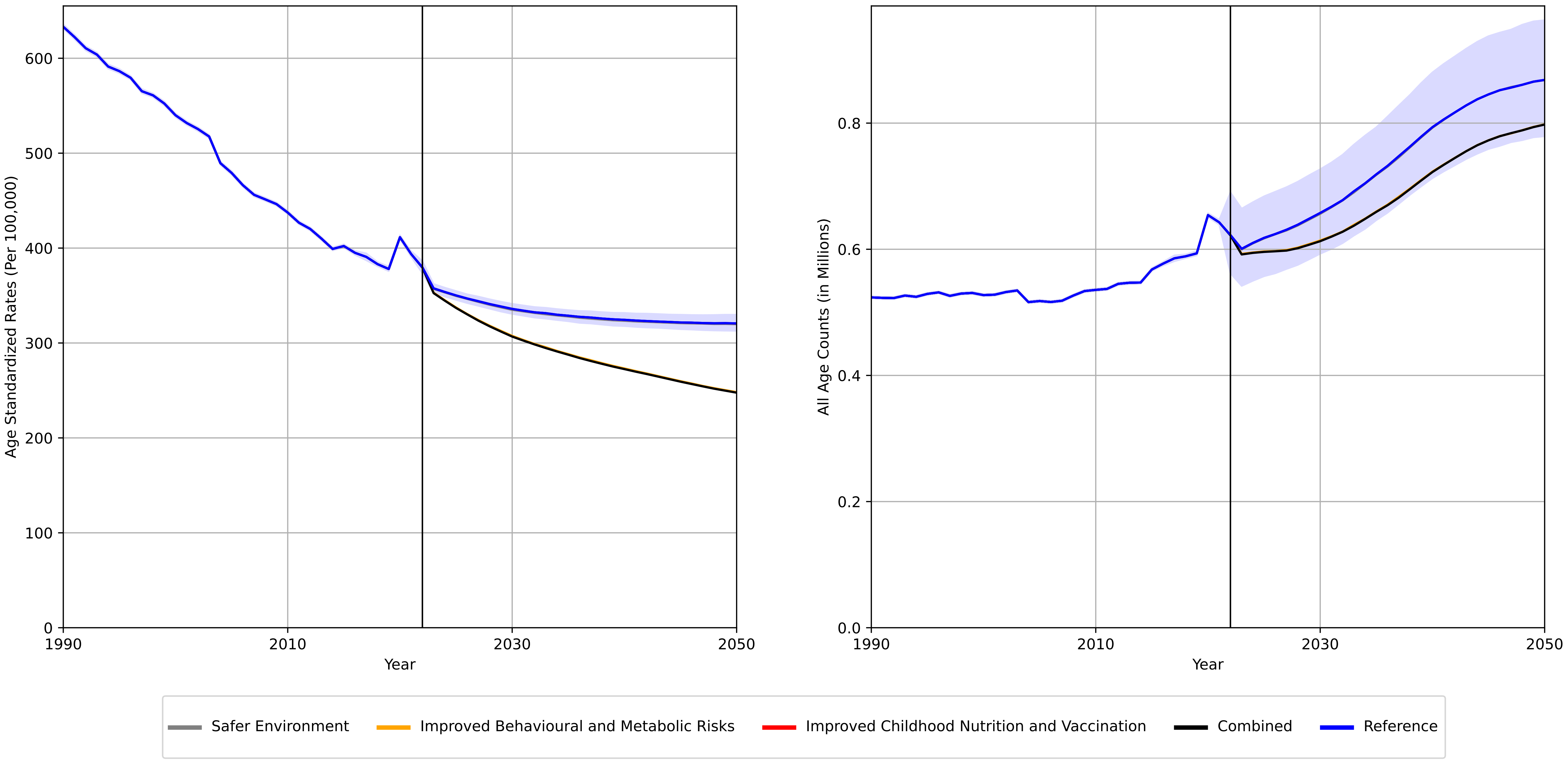

Age Standardized Rates (Per 100,000)
0
100
200
300
400
500
600
1990
2010
2030
2050
Year
All Age Counts (in Millions)
0.0
0.2
0.4
0.6
0.8
1990
2010
2030
2050
Year
Safer Environment
Improved Behavioural and Metabolic Risks
Improved Childhood Nutrition and Vaccination
Combined
Reference

Germany

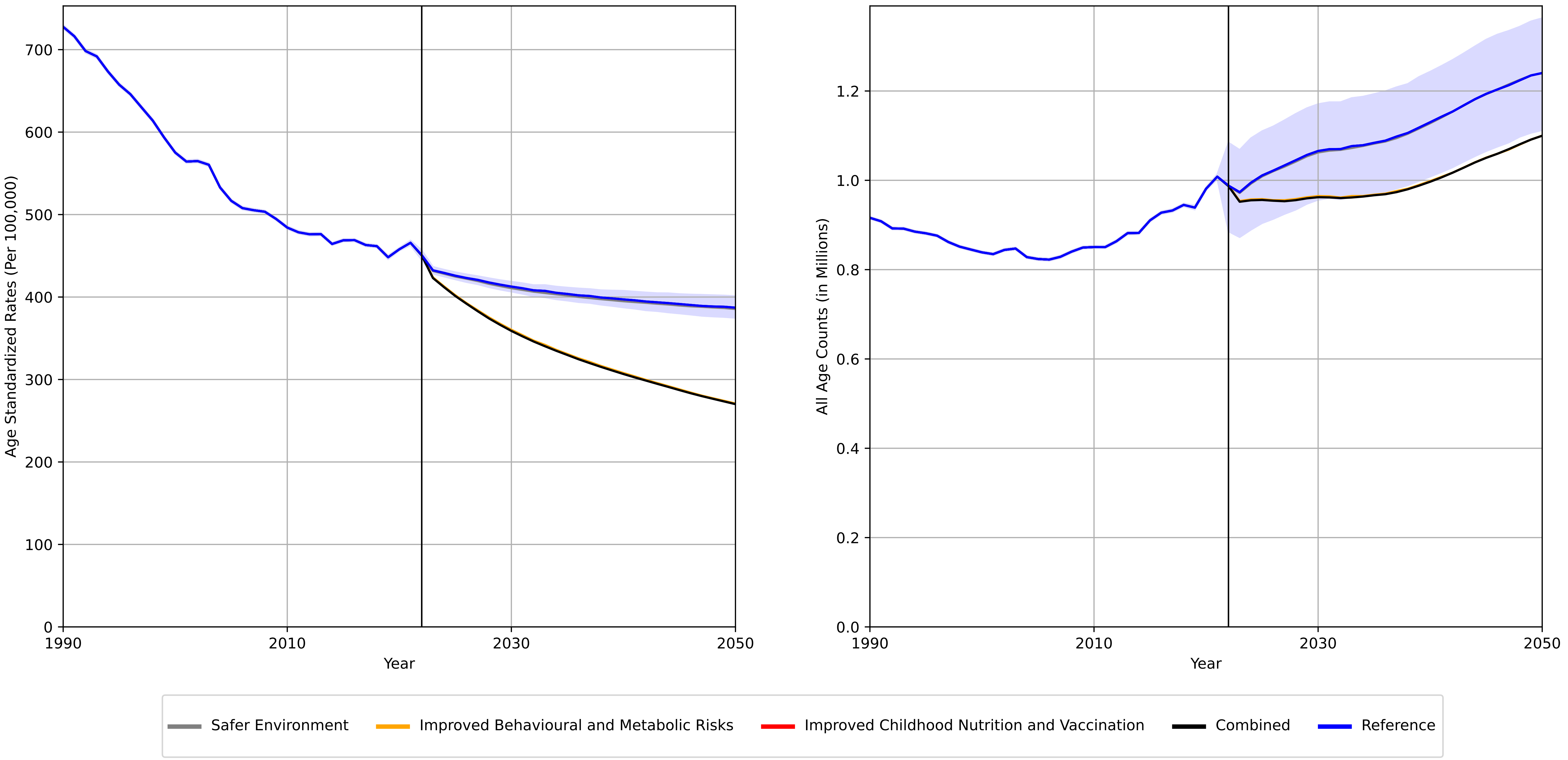
Age Standardized Rates (Per 100,000)
0
100
200
300
400
500
600
700
1990
2010
2030
2050
Year
All Age Counts (in Millions)
0.0
0.2
0.4
0.6
0.8
1.0
1.2
1990
2010
2030
2050
Year
Safer Environment
Improved Behavioural and Metabolic Risks
Improved Childhood Nutrition and Vaccination
Combined
Reference

Greece

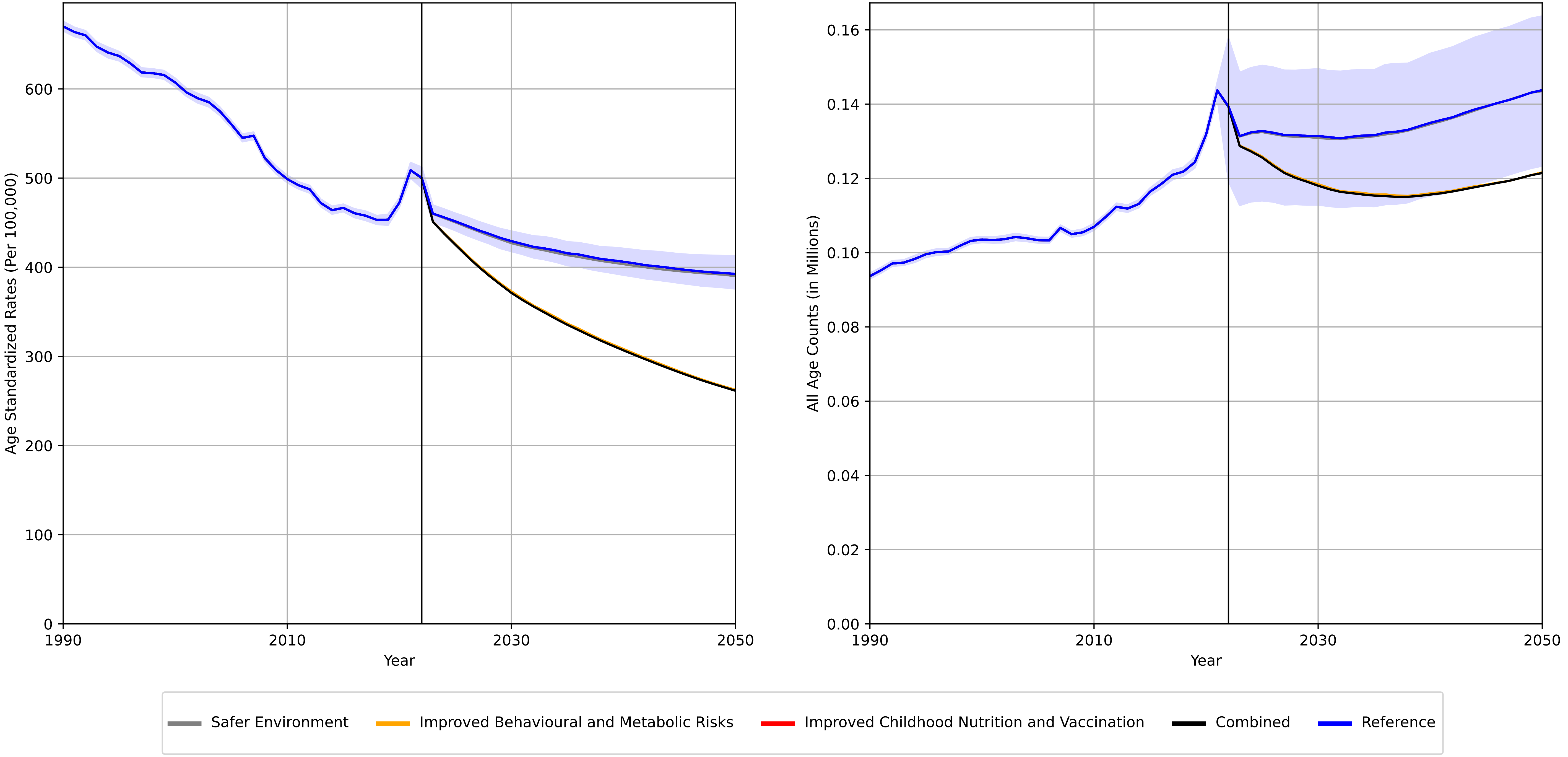

Age Standardized Rates (Per 100,000)
0
100
200
300
400
500
600
1990
2010
2030
2050
Year
All Age Counts (in Millions)
0.00
0.02
0.04
0.06
0.08
0.10
0.12
0.14
0.16
1990
2010
2030
2050
Year
Safer Environment
Improved Behavioural and Metabolic Risks
Improved Childhood Nutrition and Vaccination
Combined
Reference

Iceland

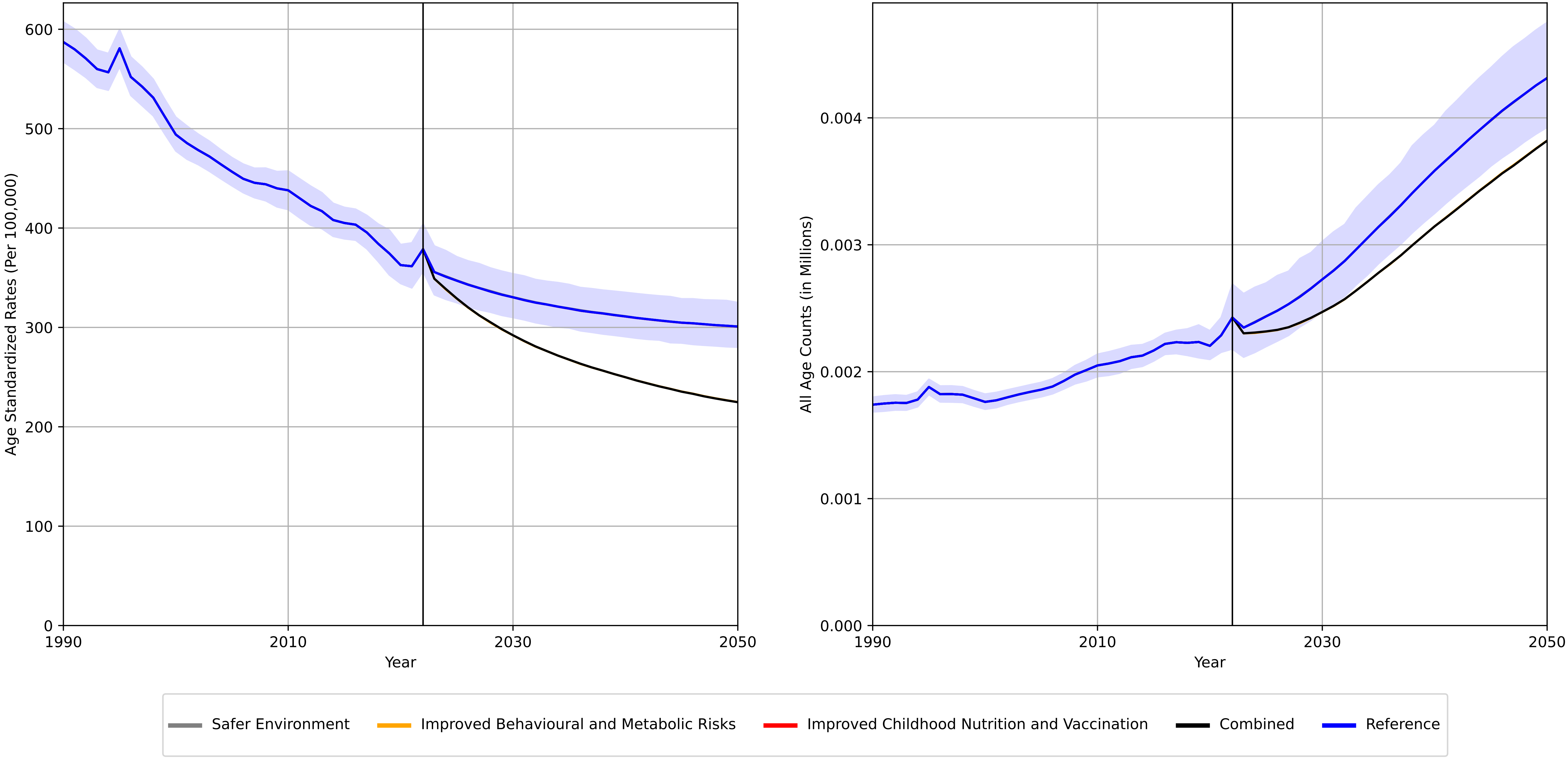

Age Standardized Rates (Per 100,000)
600
500
400
300
200
100
0
1990
2010
2030
2050
Year
All Age Counts (in Millions)
0.004
0.003
0.002
0.001
0.000
1990
2010
2030
2050
Year
Safer Environment
Improved Behavioural and Metabolic Risks
Improved Childhood Nutrition and Vaccination
Combined
Reference

Ireland

Supplemental Results Figure S11. All-cause age standardised death rates (A) and all age death counts (B), for the past and for five future scenarios, 1990–2050

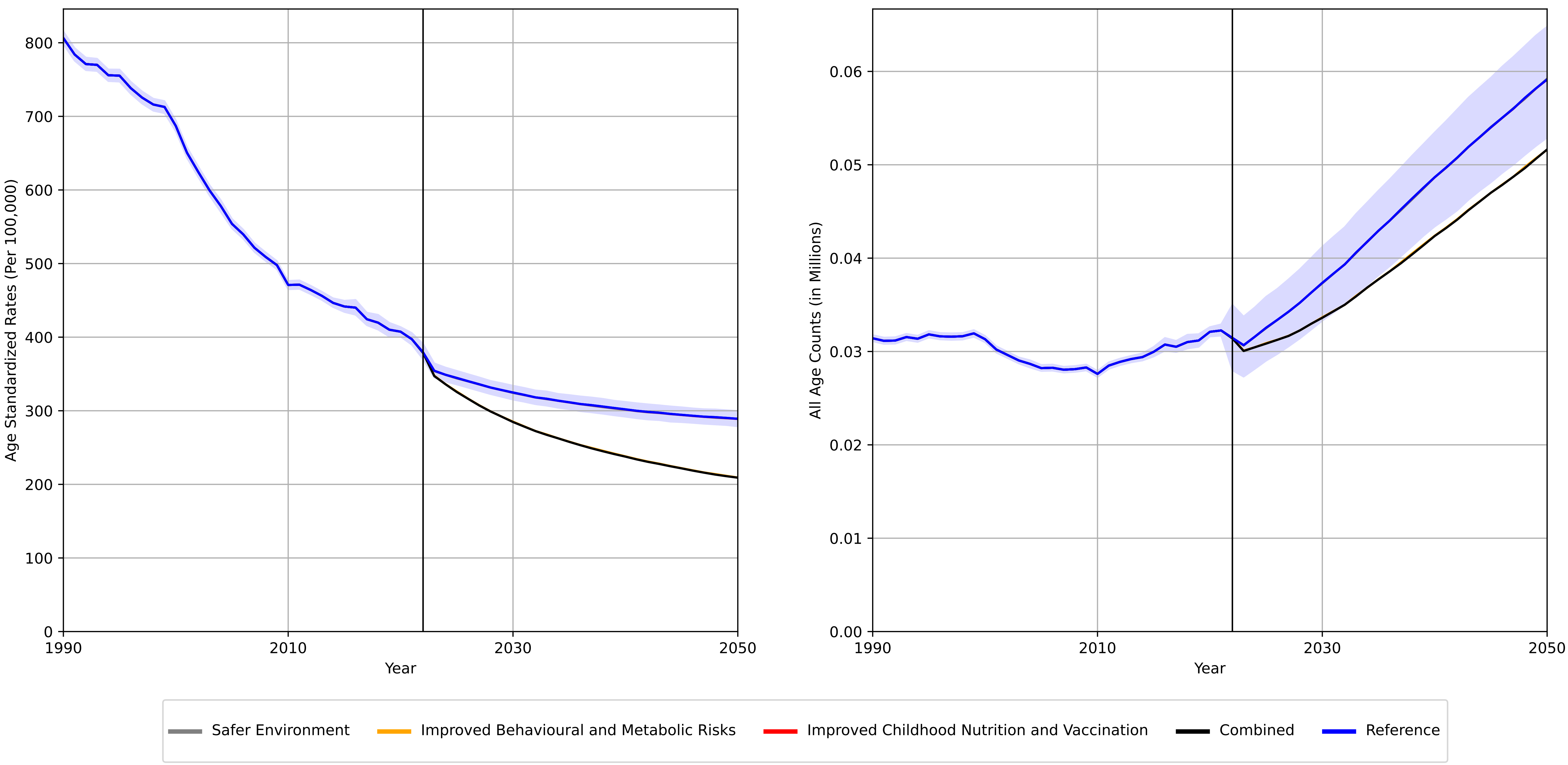

Israel

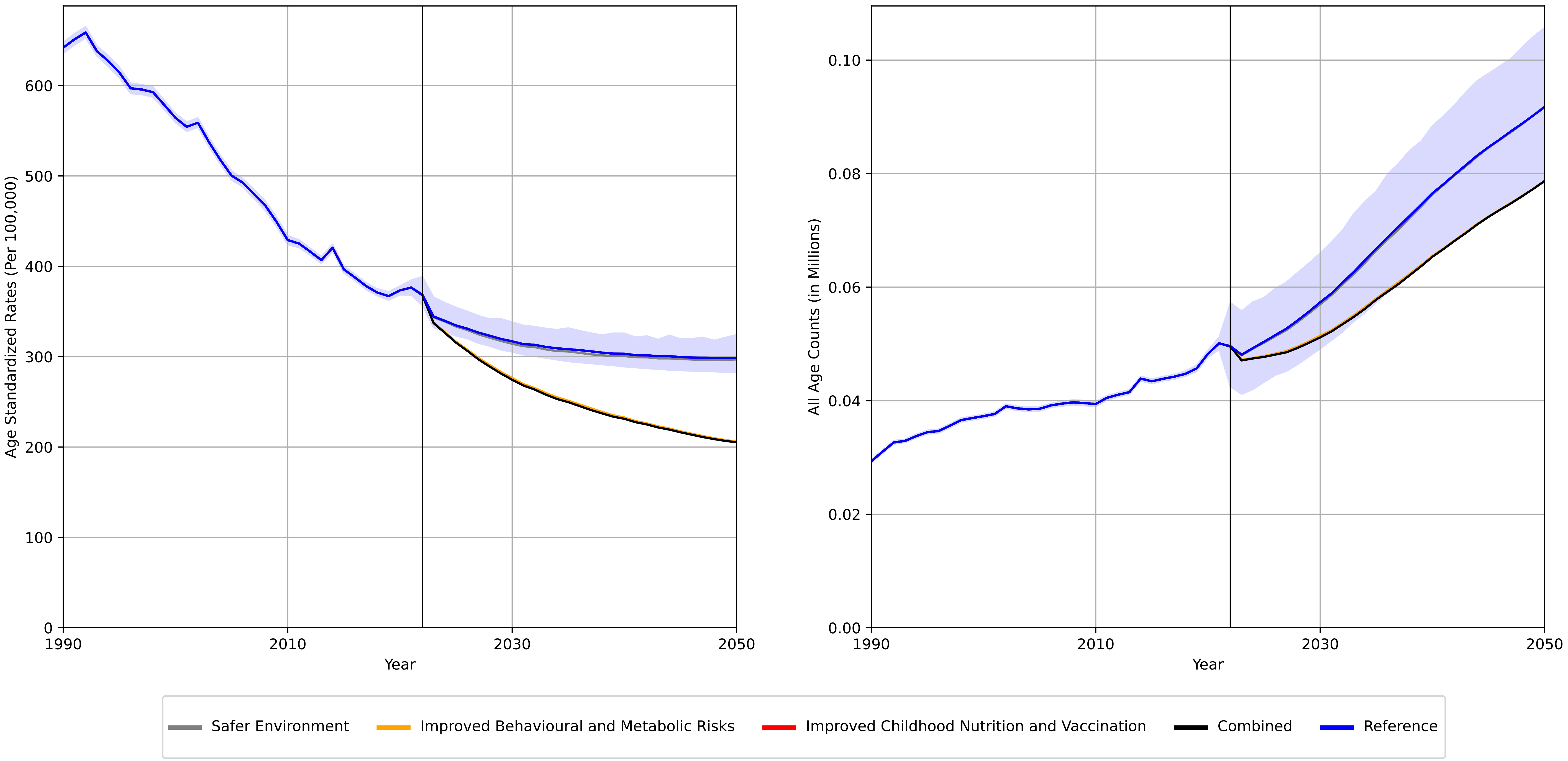

Age Standardized Rates (Per 100,000)
0
100
200
300
400
500
600
1990
2010
2030
2050
Year
All Age Counts (in Millions)
0.00
0.02
0.04
0.06
0.08
0.10
1990
2010
2030
2050
Year
Safer Environment
Improved Behavioural and Metabolic Risks
Improved Childhood Nutrition and Vaccination
Combined
Reference

Italy

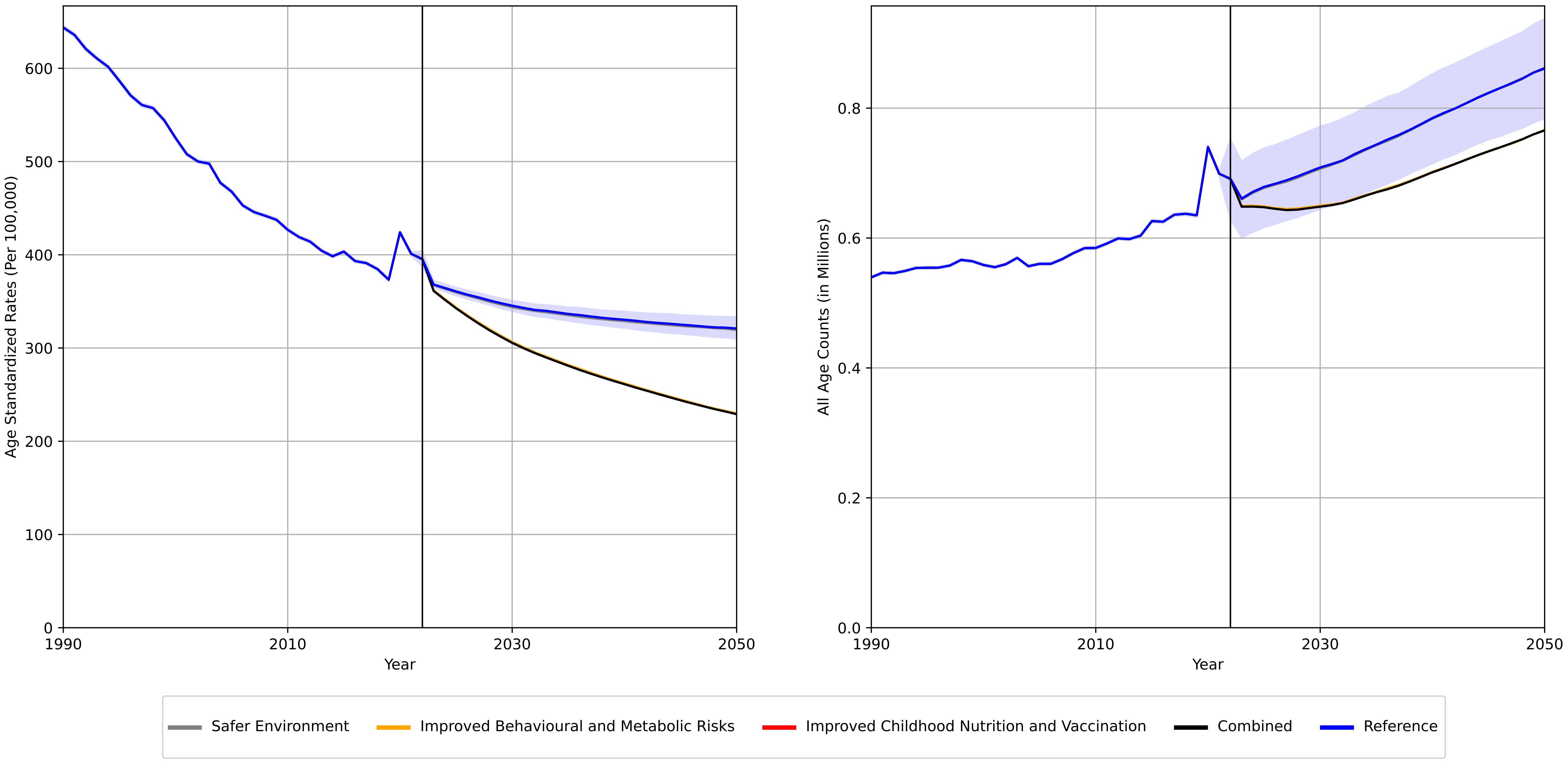

Age Standardized Rates (Per 100,000)
0
100
200
300
400
500
600
1990
2010
2030
2050
Year
All Age Counts (in Millions)
0.0
0.2
0.4
0.6
0.8
1990
2010
2030
2050
Year
Safer Environment
Improved Behavioural and Metabolic Risks
Improved Childhood Nutrition and Vaccination
Combined
Reference

Luxembourg

Supplemental Results Figure S11. All-cause age standardised death rates (A) and all age death counts (B), for the past and for five future scenarios, 1990–2050

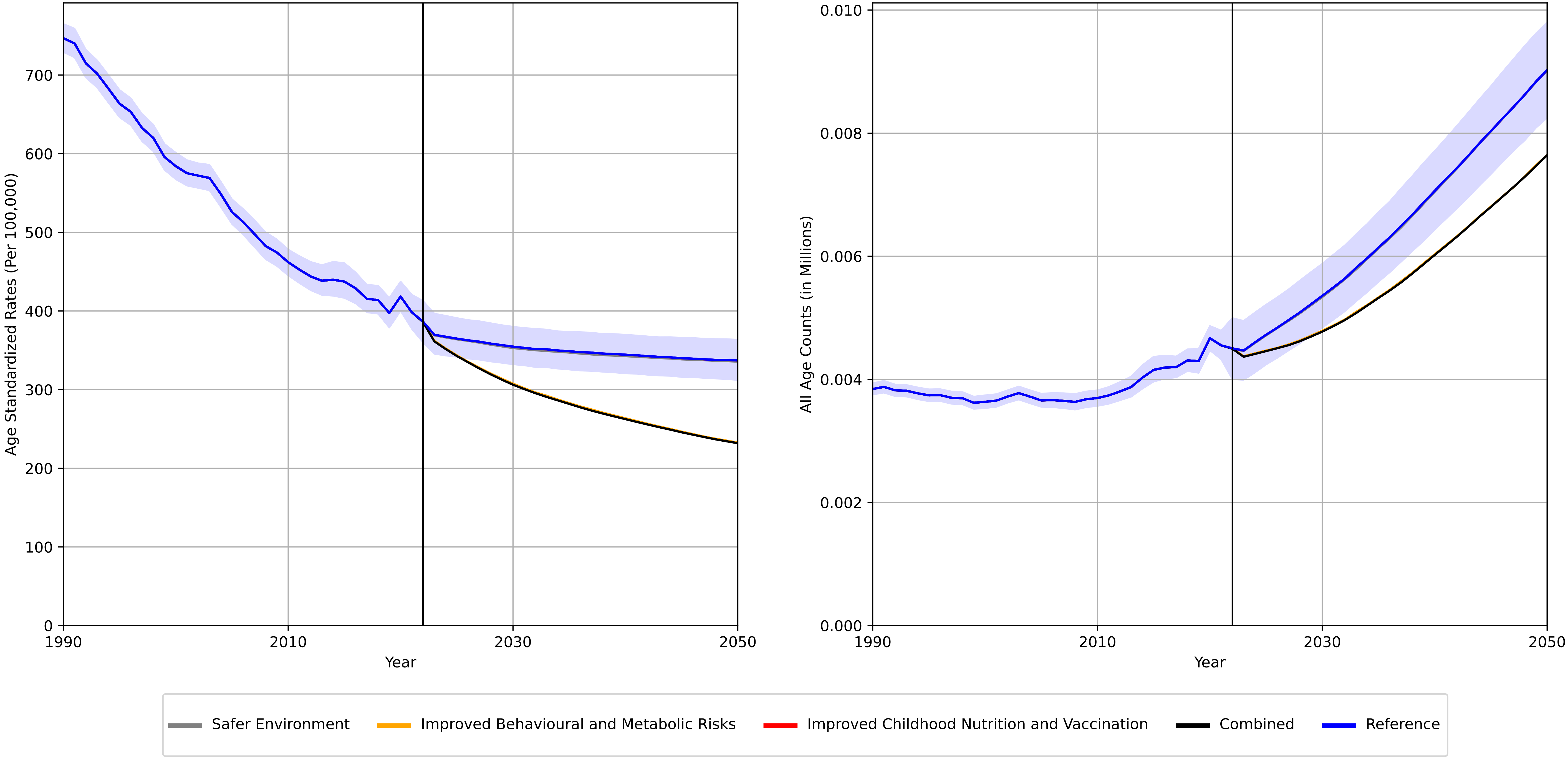

Malta

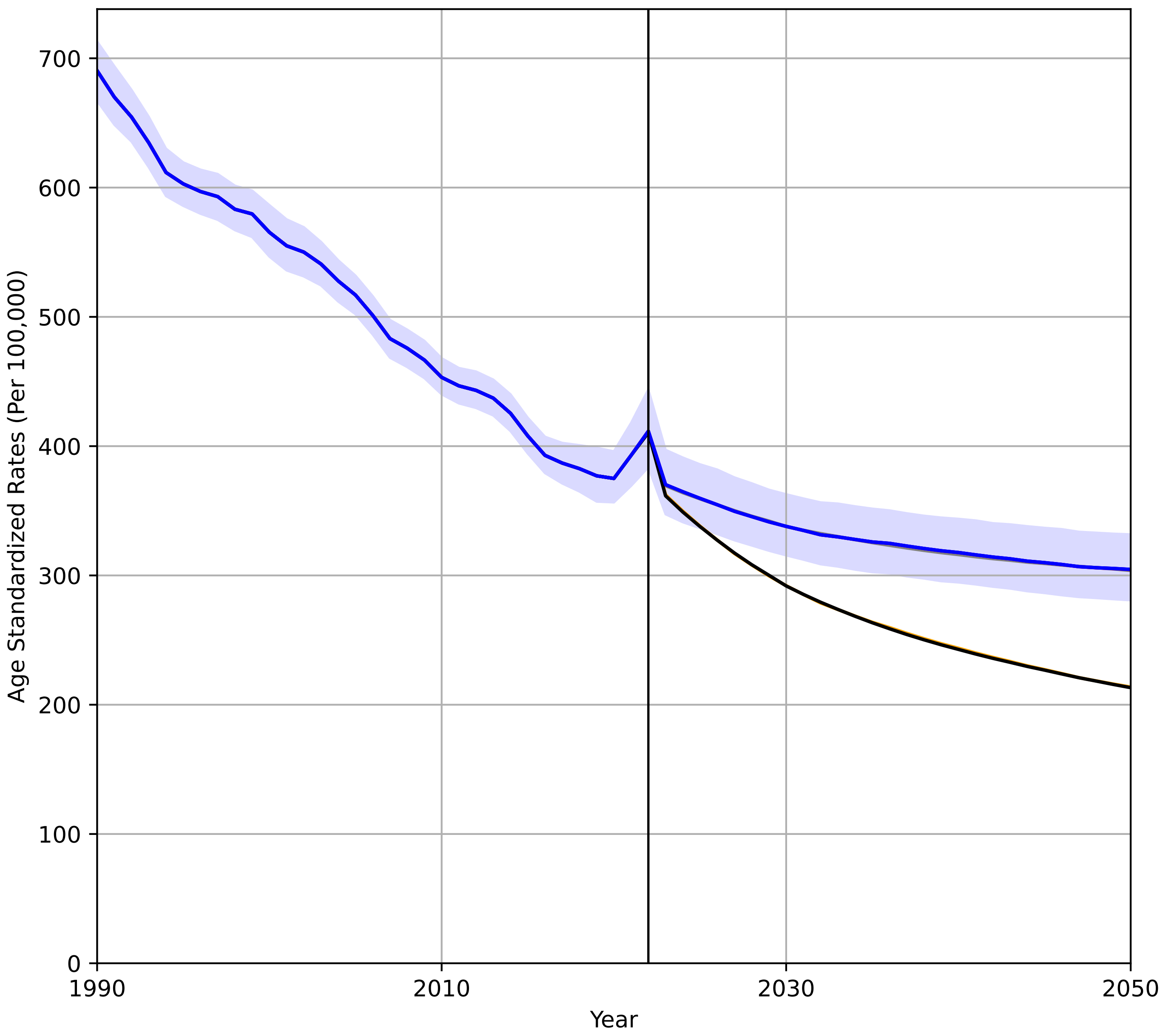
Age Standardized Rates (Per 100,000)
700
600
500
400
300
200
100
0
1990
2010
2030
2050
Year

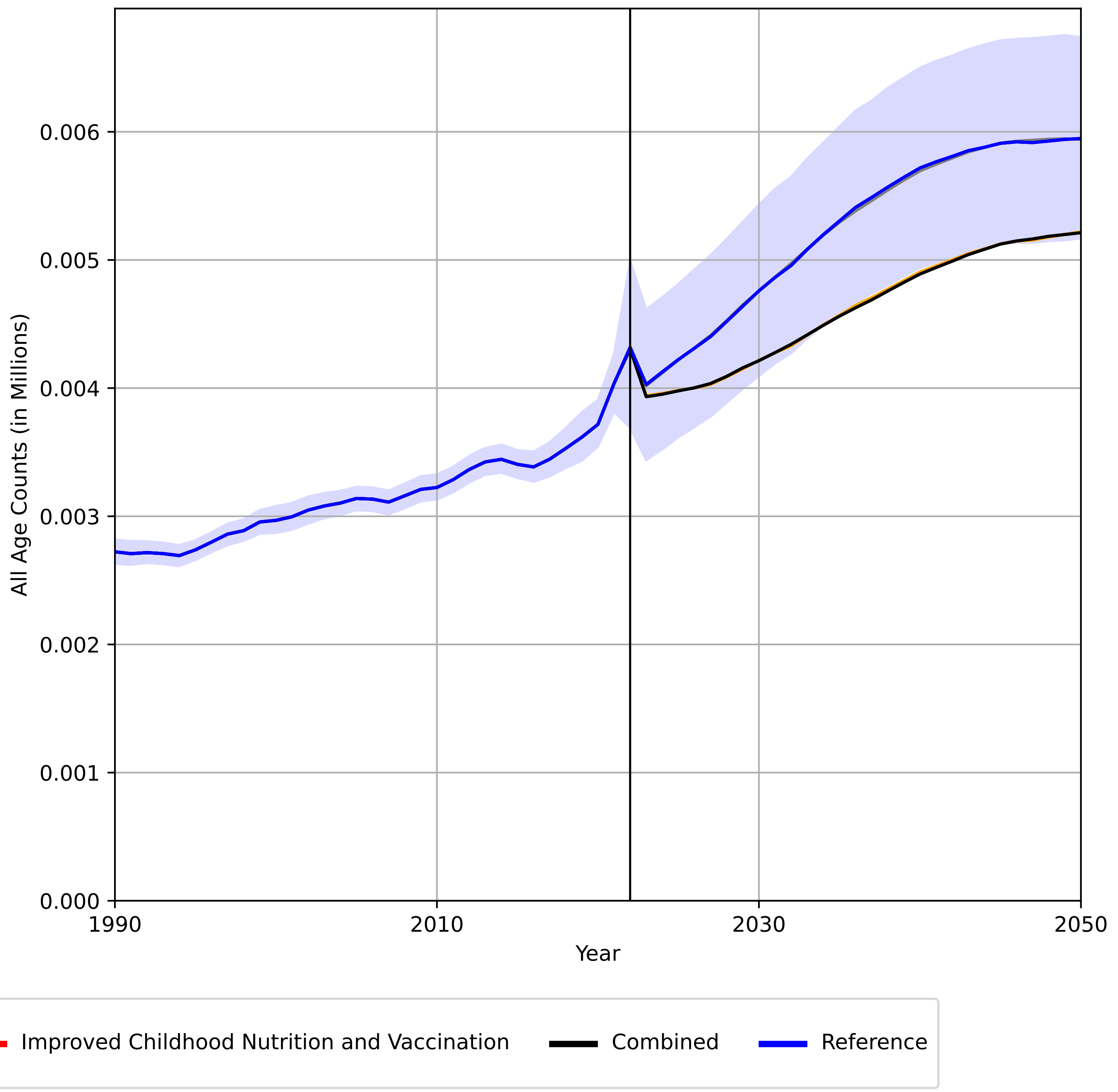
All Age Counts (in Millions)
0.006
0.005
0.004
0.003
0.002
0.001
0.000
1990
2010
2030
2050
Year
Safer Environment
Improved Behavioural and Metabolic Risks
Improved Childhood Nutrition and Vaccination
Combined
Reference

Monaco

Supplemental Results Figure S11. All-cause age standardised death rates (A) and all age death counts (B), for the past and for five future scenarios, 1990–2050

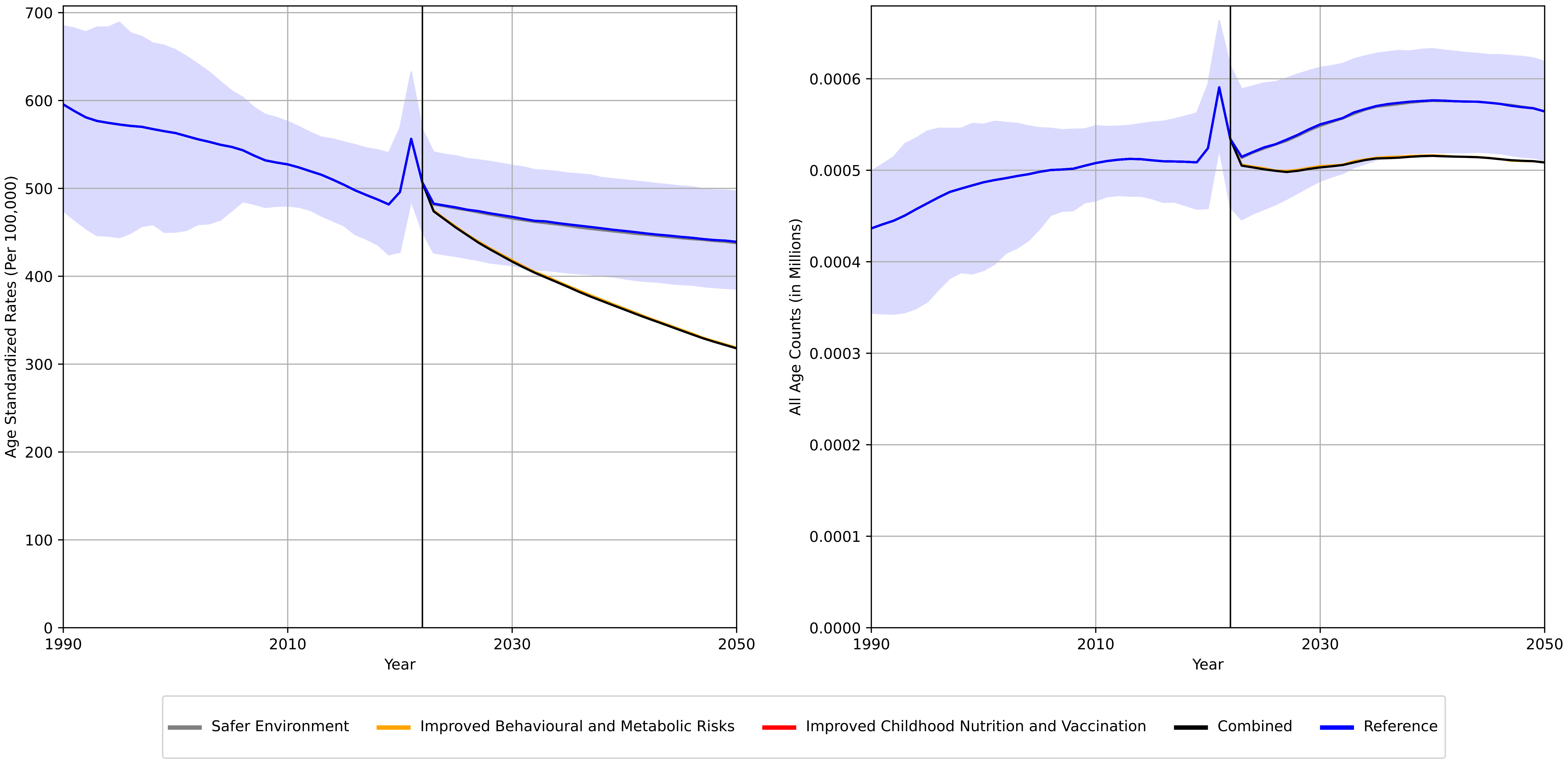

Netherlands

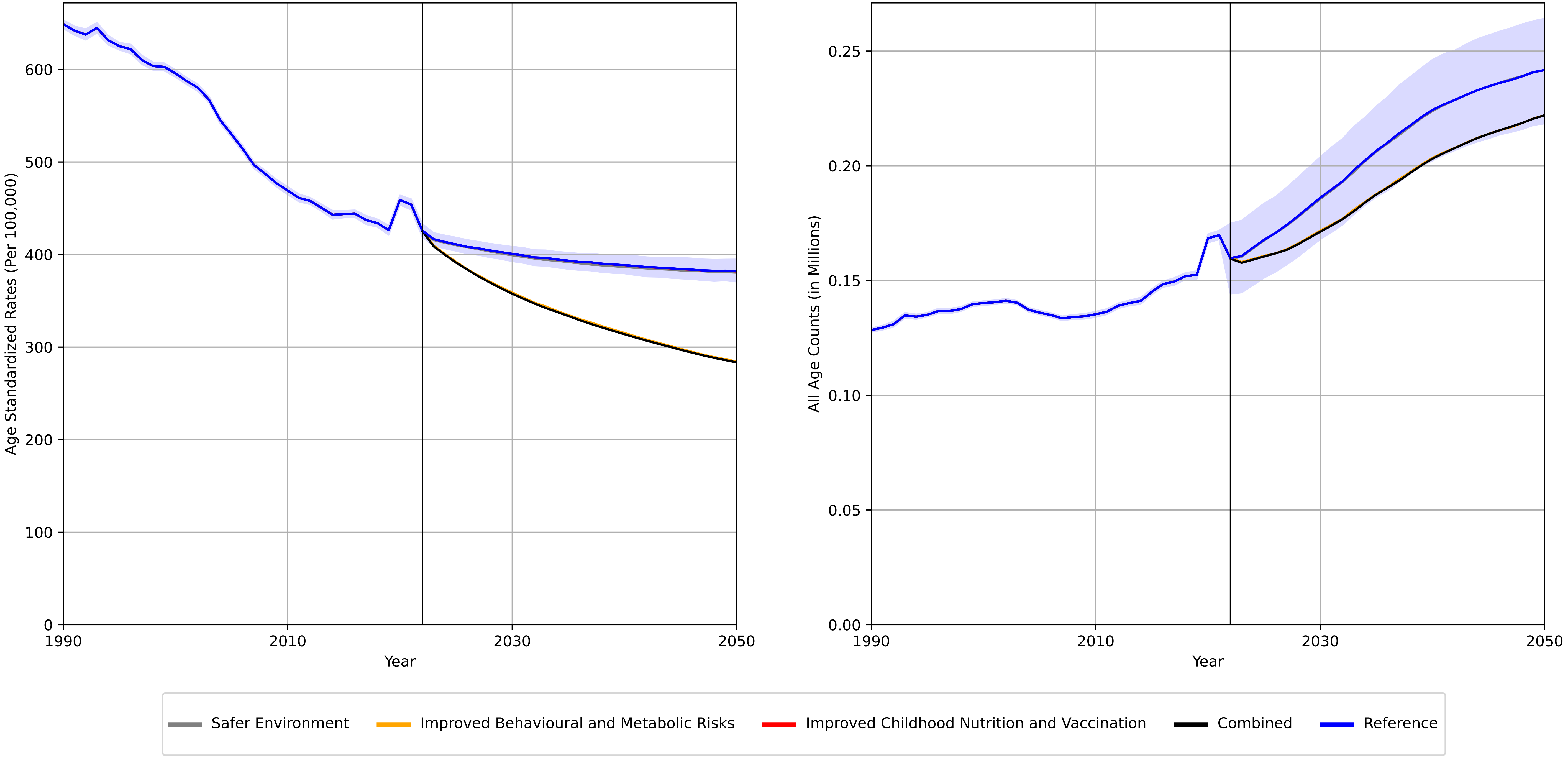

Norway

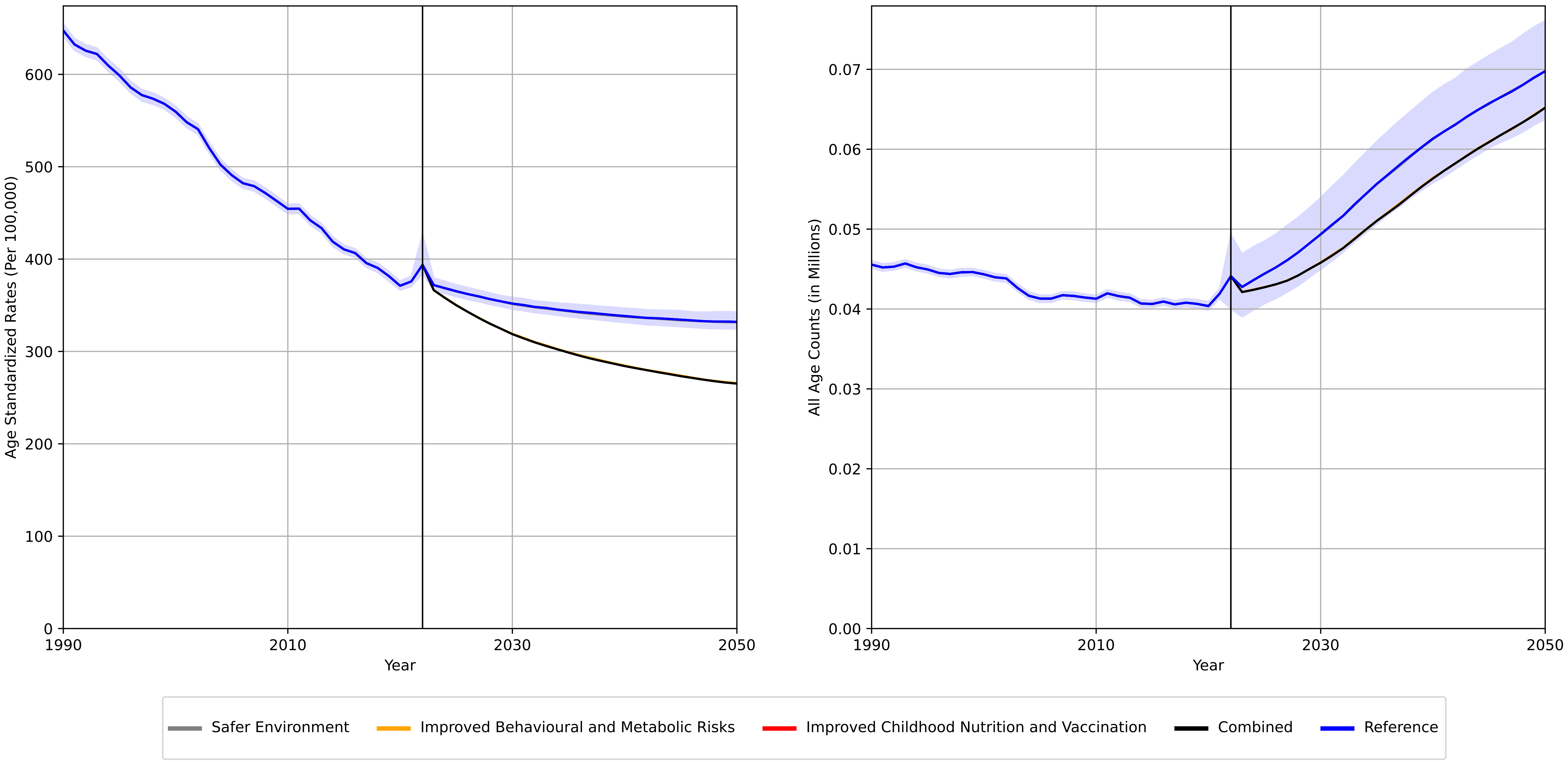
Age Standardized Rates (Per 100,000)
0
100
200
300
400
500
600
1990
2010
2030
2050
Year
All Age Counts (in Millions)
0.00
0.01
0.02
0.03
0.04
0.05
0.06
0.07
Safer Environment
Improved Behavioural and Metabolic Risks
Improved Childhood Nutrition and Vaccination
Combined
Reference

Portugal

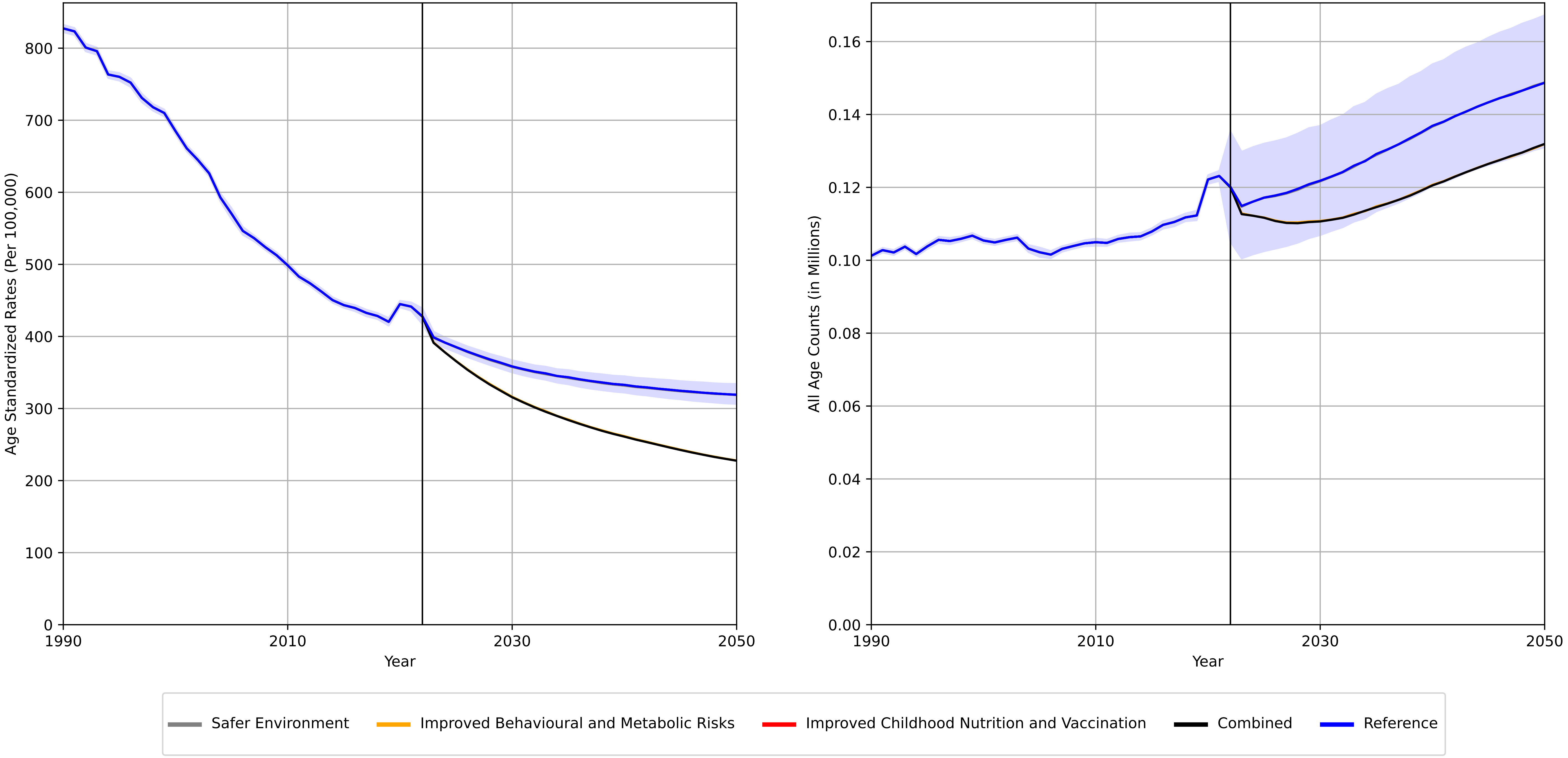
Age Standardized Rates (Per 100,000)
0
100
200
300
400
500
600
700
800
1990
2010
2030
2050
Year
All Age Counts (in Millions)
0.00
0.02
0.04
0.06
0.08
0.10
0.12
0.14
0.16
1990
2010
2030
2050
Year
Safer Environment
Improved Behavioural and Metabolic Risks
Improved Childhood Nutrition and Vaccination
Combined
Reference

San Marino

Supplemental Results Figure S11. All-cause age standardised death rates (A) and all age death counts (B), for the past and for five future scenarios, 1990–2050

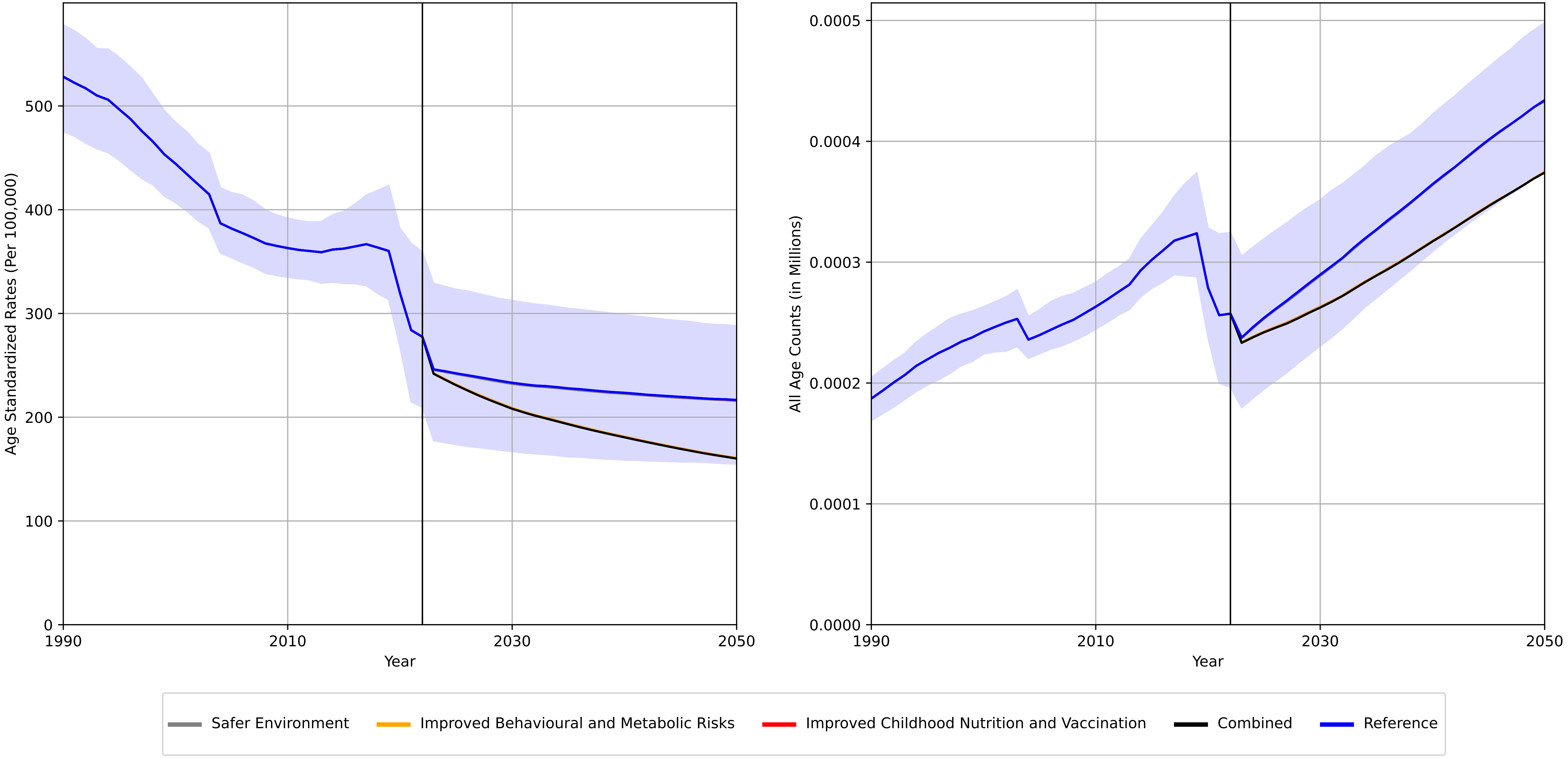

Spain

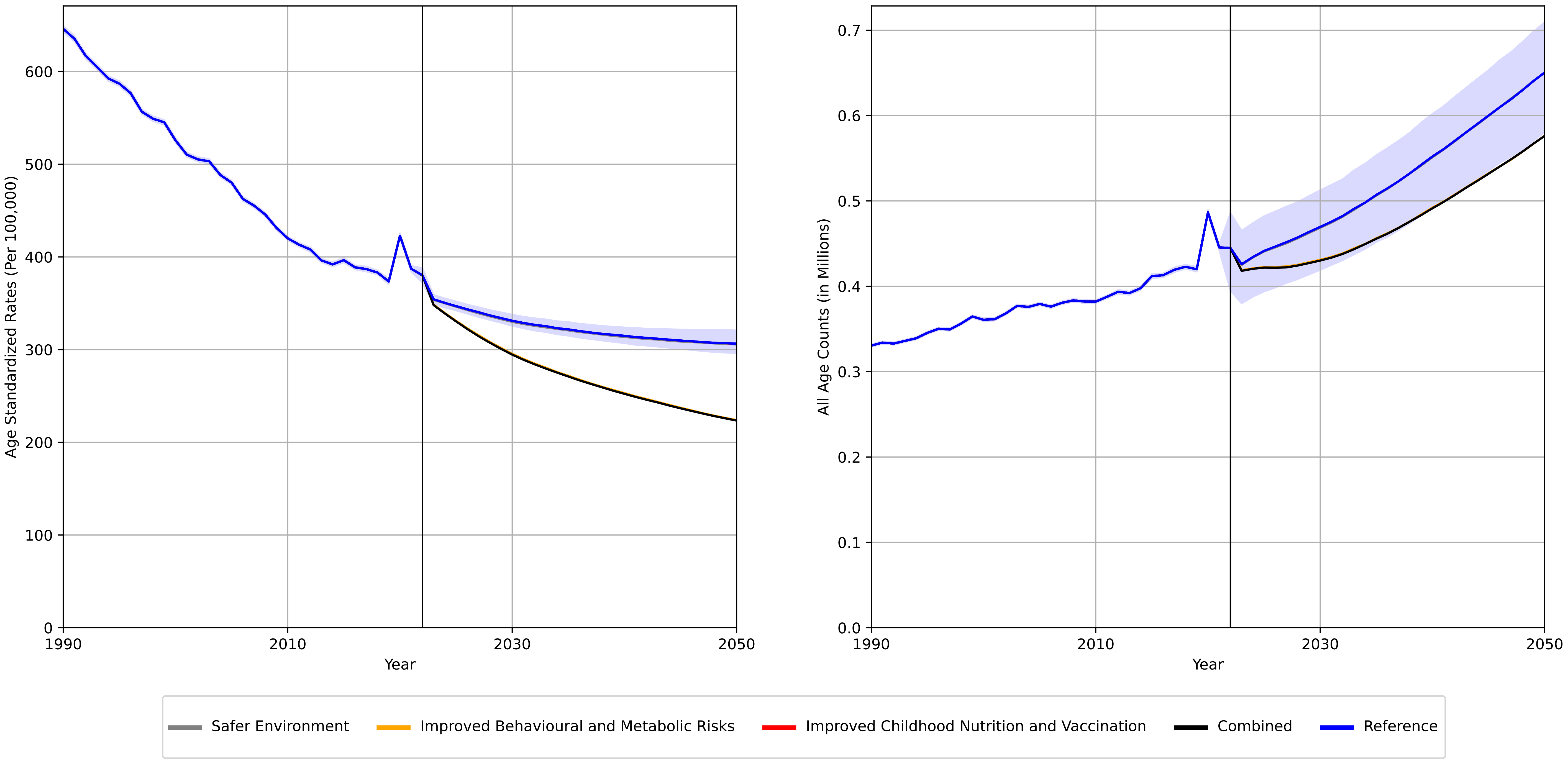

Age Standardized Rates (Per 100,000)
0
100
200
300
400
500
600
1990
2010
2030
2050
Year
All Age Counts (in Millions)
0.0
0.1
0.2
0.3
0.4
0.5
0.6
0.7
1990
2010
2030
2050
Year
Safer Environment
Improved Behavioural and Metabolic Risks
Improved Childhood Nutrition and Vaccination
Combined
Reference

Sweden

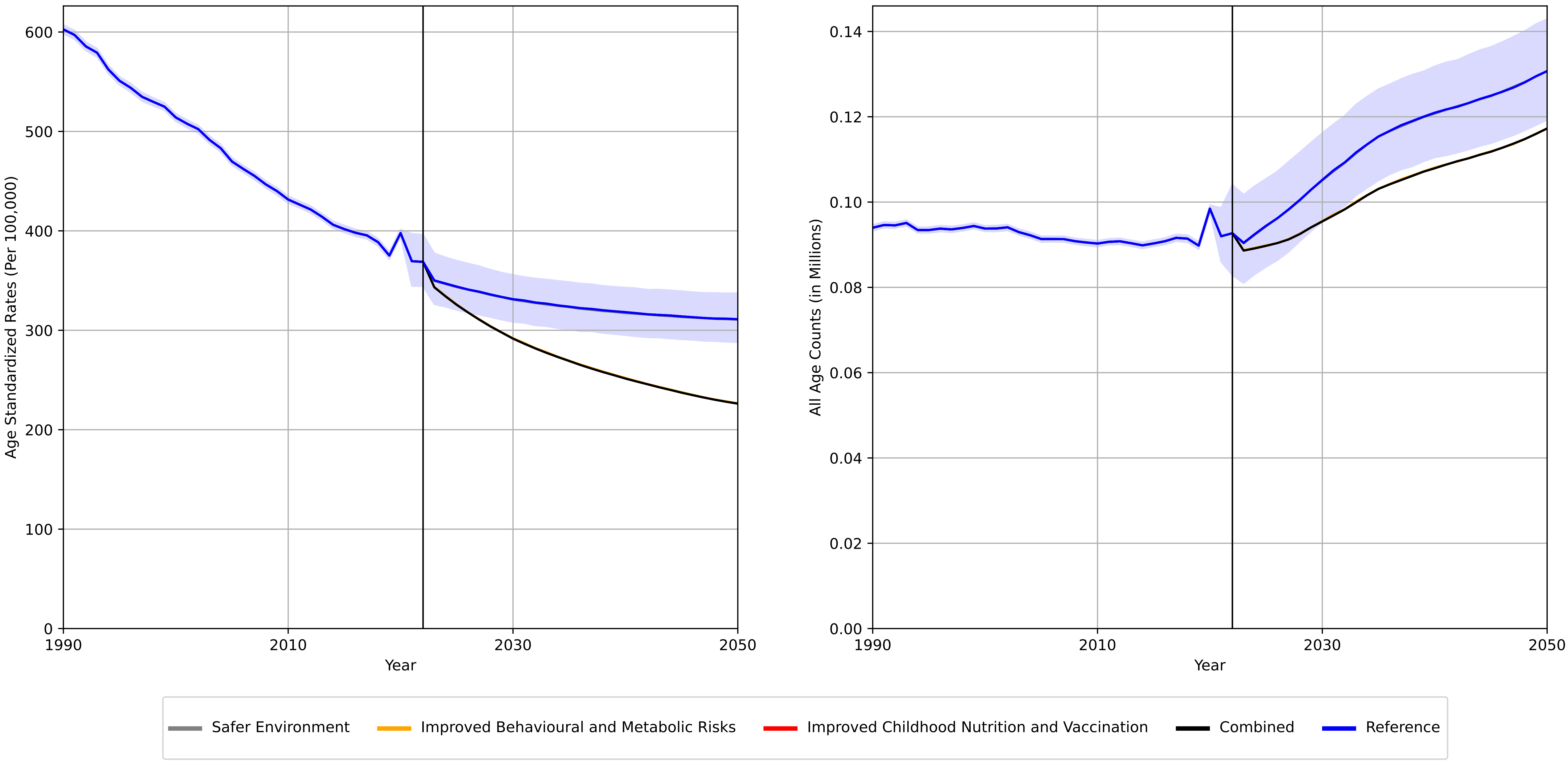

Age Standardized Rates (Per 100,000)
600
500
400
300
200
100
0
1990
2010
2030
2050
Year
All Age Counts (in Millions)
0.14
0.12
0.10
0.08
0.06
0.04
0.02
0.00
Safer Environment
Improved Behavioural and Metabolic Risks
Improved Childhood Nutrition and Vaccination
Combined
Reference

Switzerland

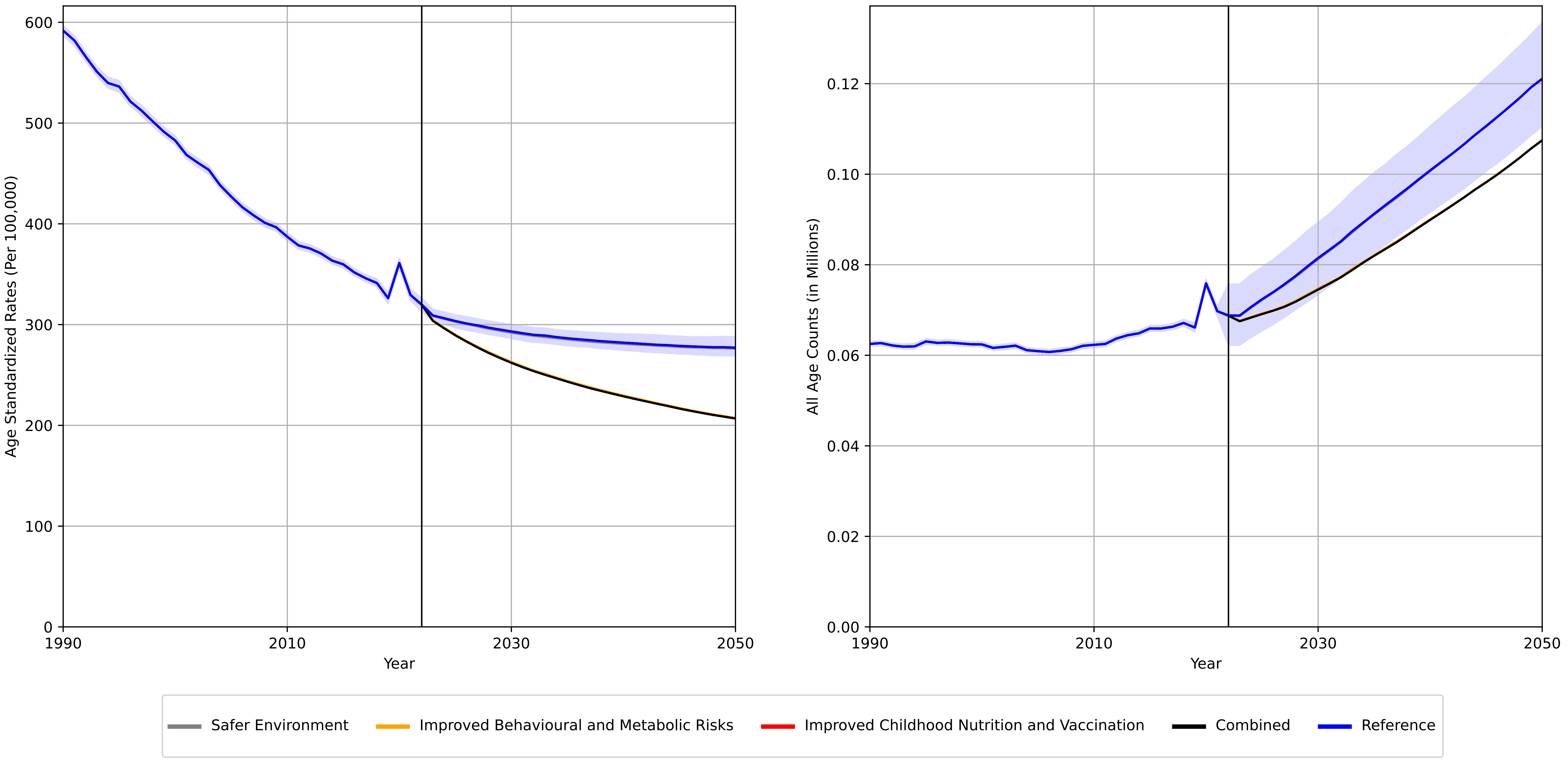

UK

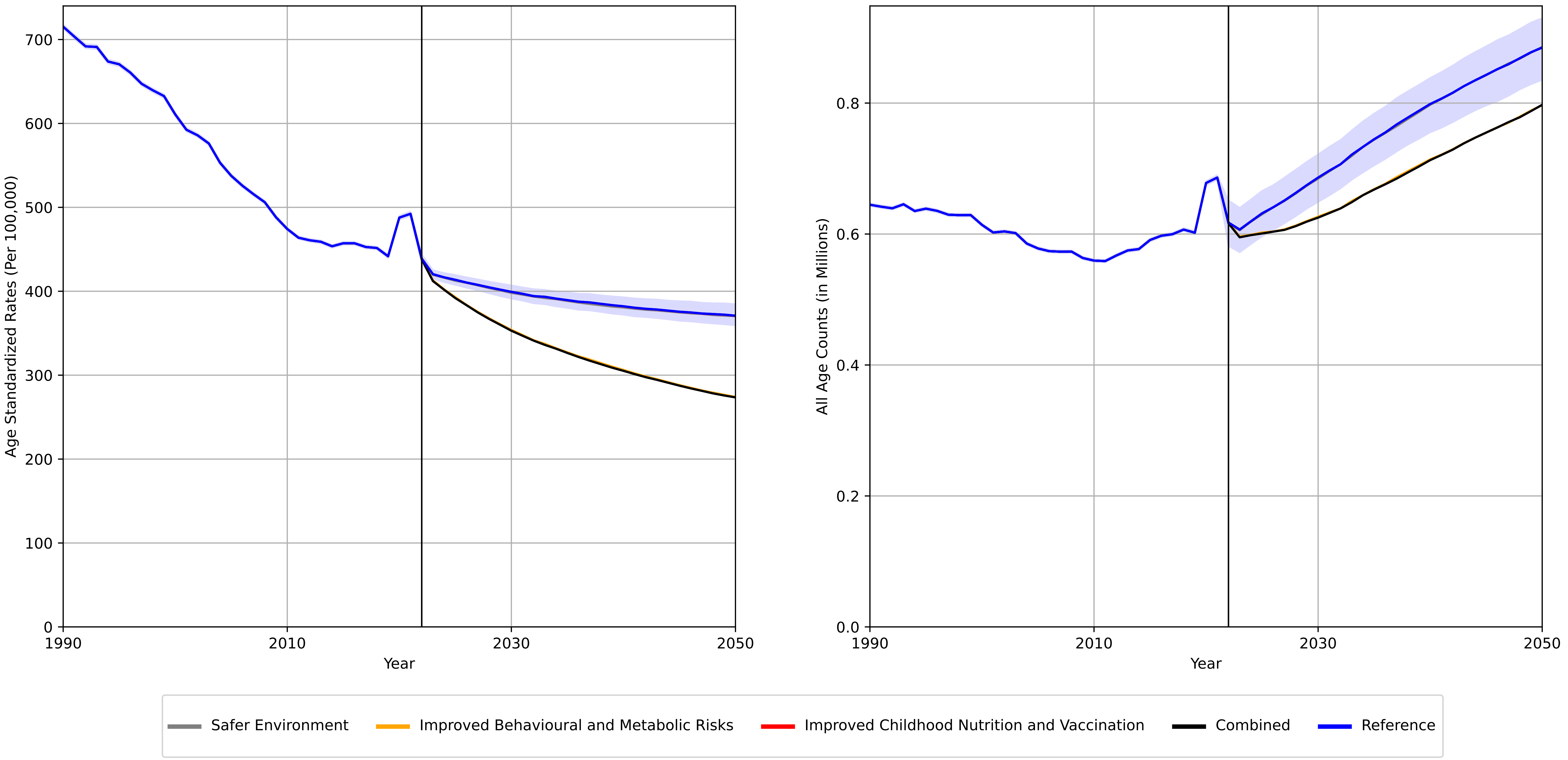

Age Standardized Rates (Per 100,000)
0
100
200
300
400
500
600
700
Year
1990
2010
2030
2050
All Age Counts (in Millions)
0.0
0.2
0.4
0.6
0.8
Safer Environment
Improved Behavioural and Metabolic Risks
Improved Childhood Nutrition and Vaccination
Combined
Reference

Bolivia

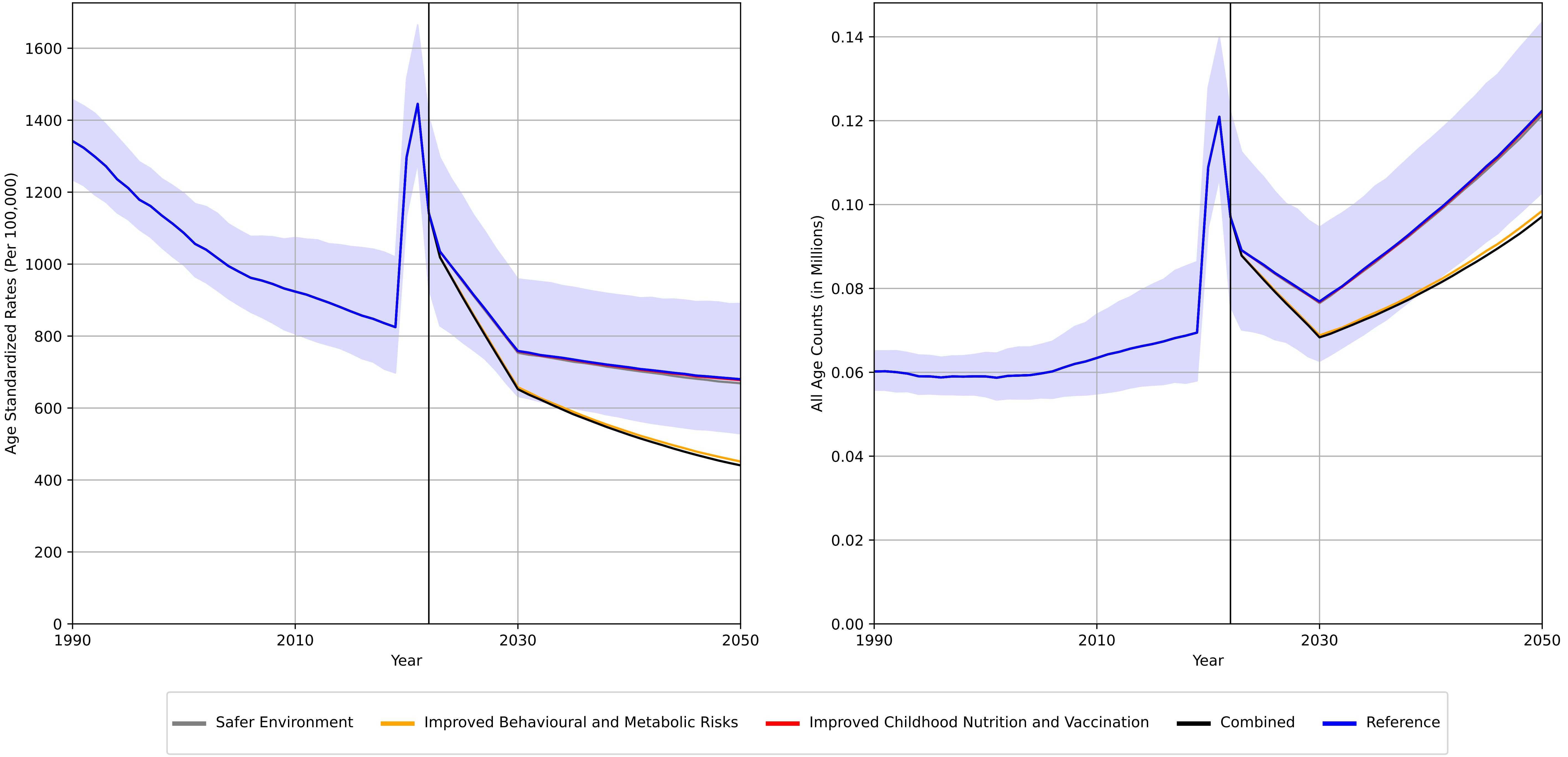
Age Standardized Rates (Per 100,000)
0
200
400
600
800
1000
1200
1400
1600
1990
2010
2030
2050
Year
All Age Counts (in Millions)
0.00
0.02
0.04
0.06
0.08
0.10
0.12
0.14
1990
2010
2030
2050
Year
Safer Environment
Improved Behavioural and Metabolic Risks
Improved Childhood Nutrition and Vaccination
Combined
Reference

Ecuador

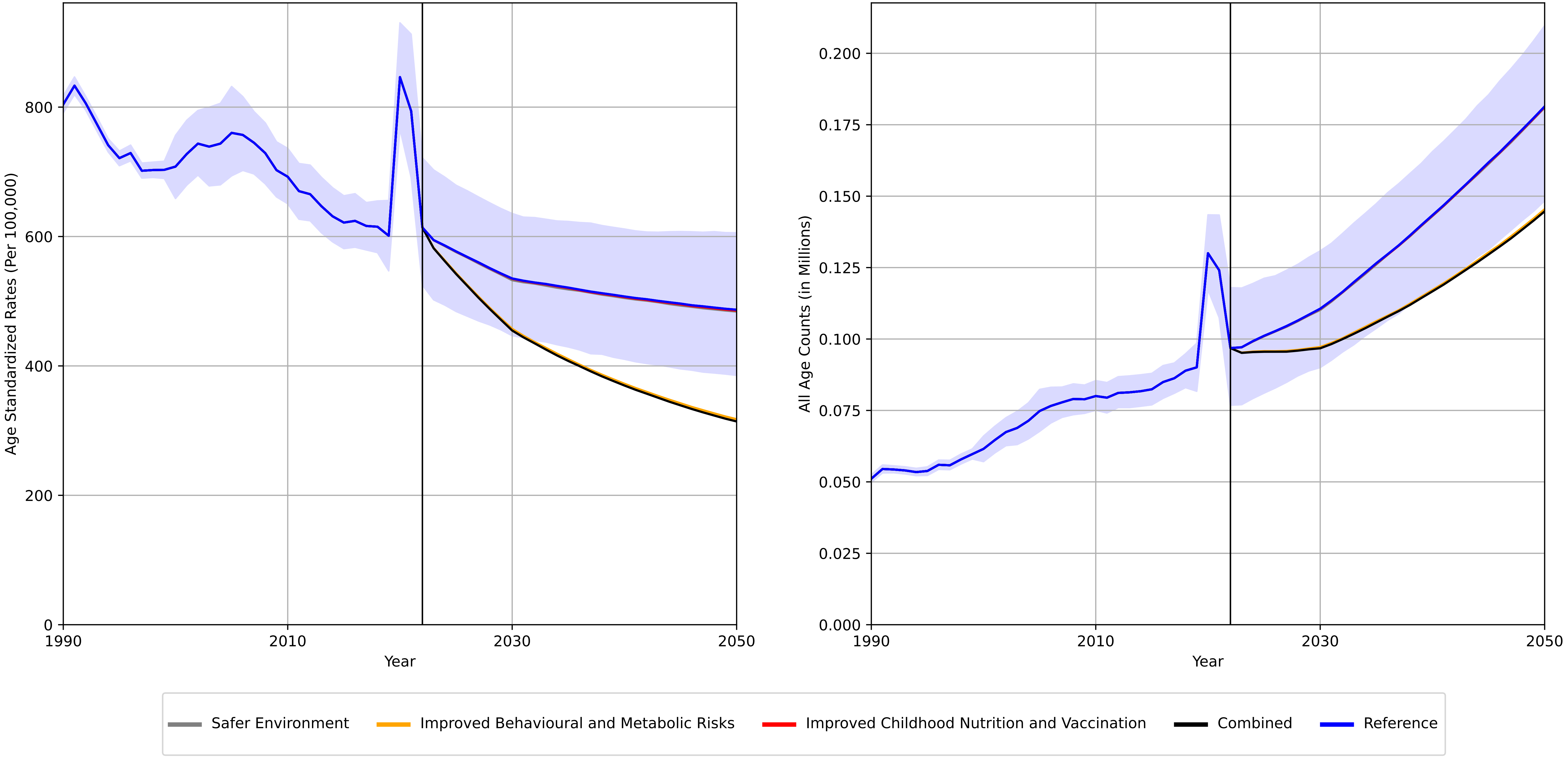

Age Standardized Rates (Per 100,000)
0
200
400
600
800
1990
2010
2030
2050
Year
All Age Counts (in Millions)
0.000
0.025
0.050
0.075
0.100
0.125
0.150
0.175
0.200
1990
2010
2030
2050
Year
Safer Environment
Improved Behavioural and Metabolic Risks
Improved Childhood Nutrition and Vaccination
Combined
Reference

Peru

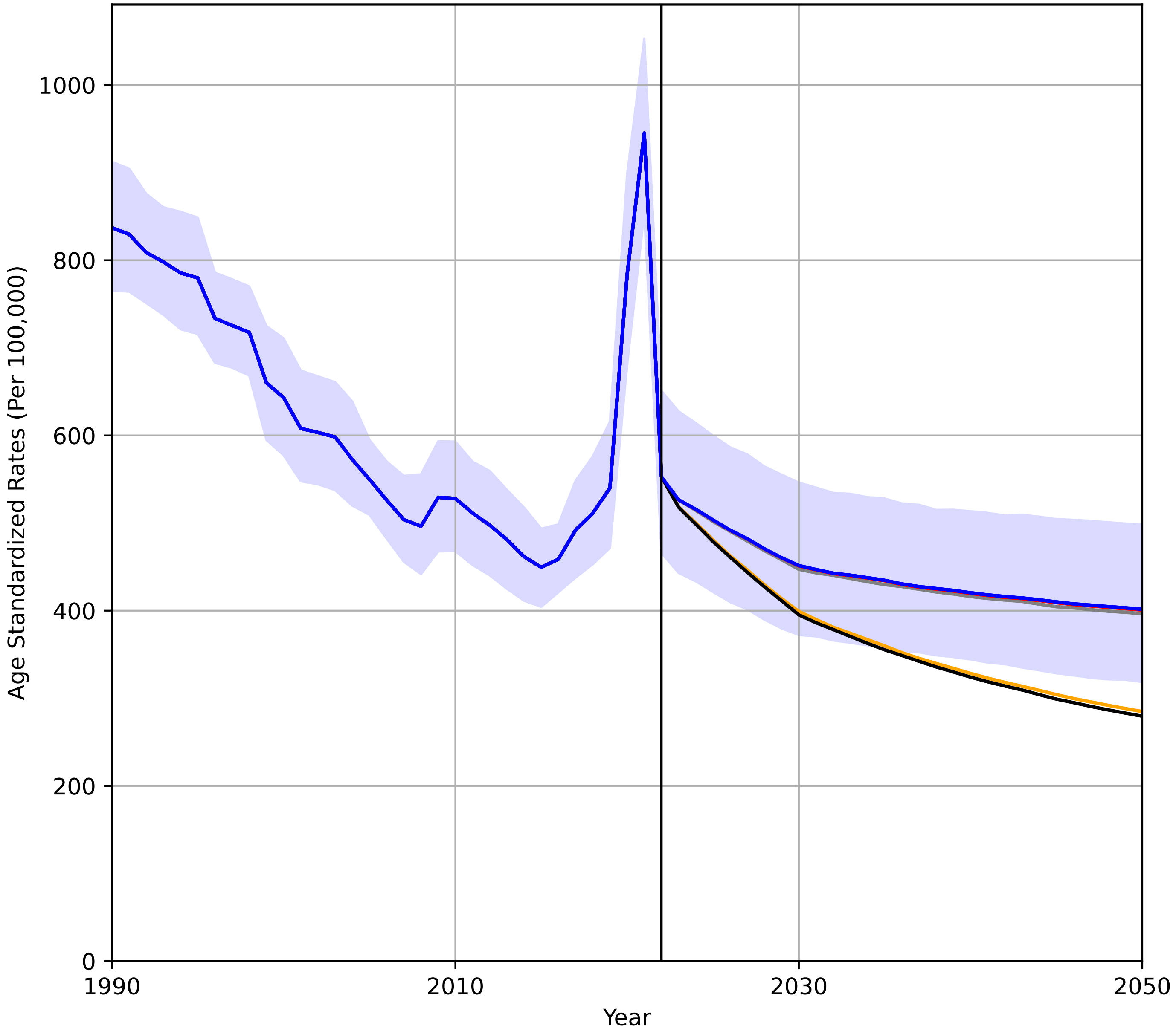

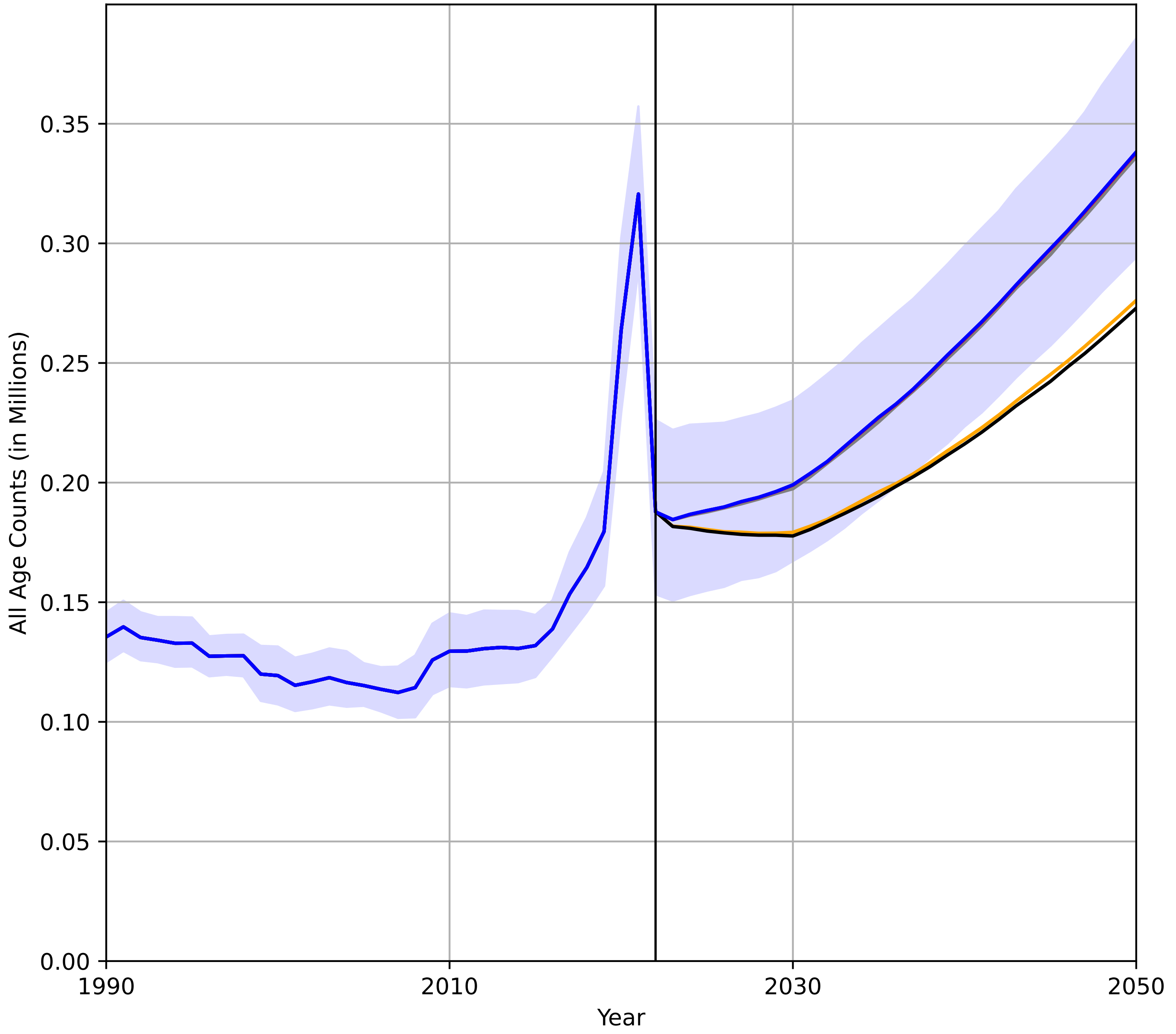

Safer Environment | Improved Behavioural and Metabolic Risks | Improved Childhood Nutrition and Vaccination | Combined | Reference

Antigua And Barbuda

Supplemental Results Figure S11. All-cause age standardised death rates (A) and all age death counts (B), for the past and for five future scenarios, 1990–2050

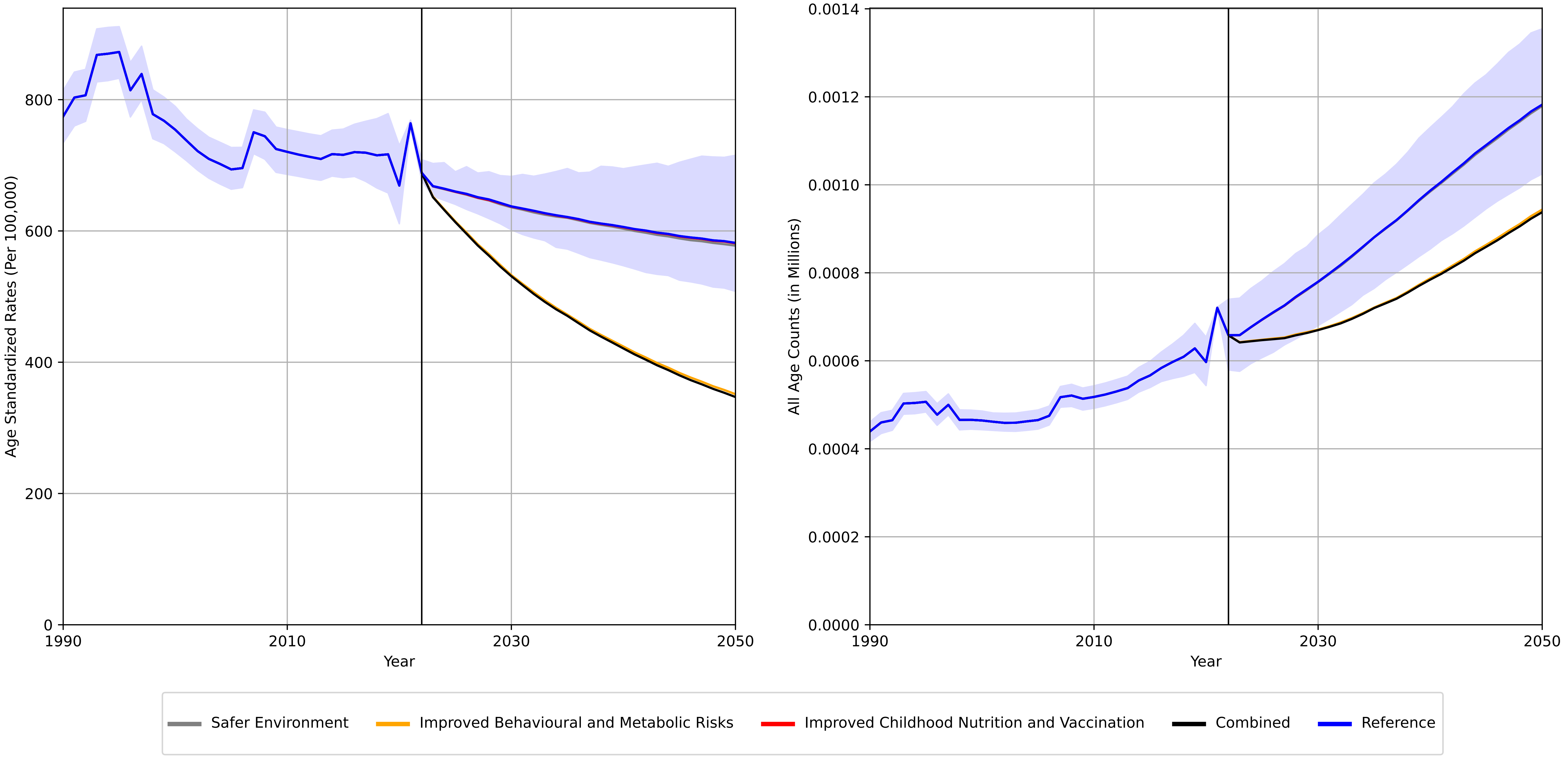

The Bahamas

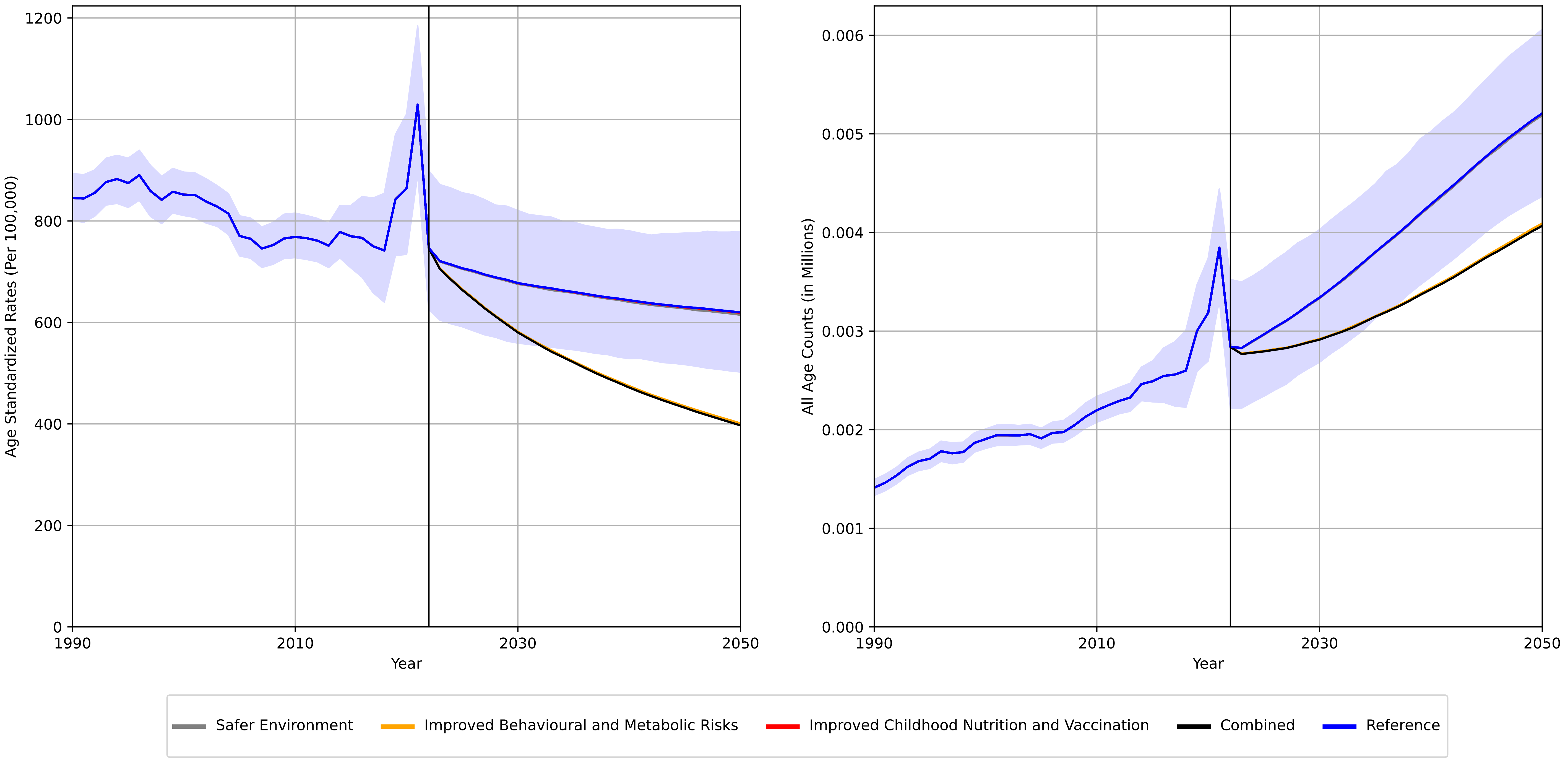
Age Standardized Rates (Per 100,000)
1200
1000
800
600
400
200
0
1990
2010
2030
2050
Year
All Age Counts (in Millions)
0.006
0.005
0.004
0.003
0.002
0.001
0.000
1990
2010
2030
2050
Year
Safer Environment
Improved Behavioural and Metabolic Risks
Improved Childhood Nutrition and Vaccination
Combined
Reference

Barbados

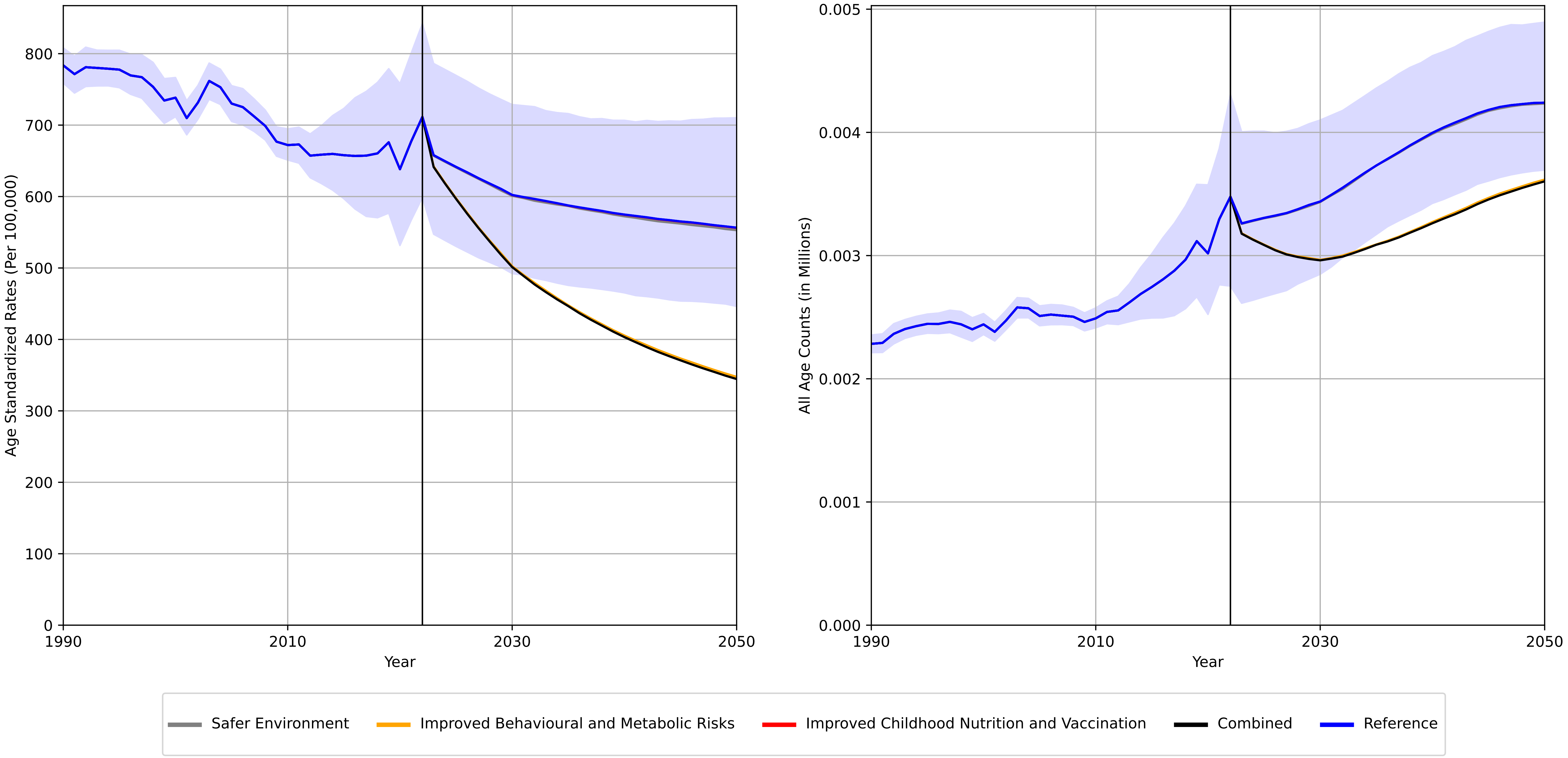
Age Standardized Rates (Per 100,000)
0
100
200
300
400
500
600
700
800
1990
2010
2030
2050
Year
All Age Counts (in Millions)
0.000
0.001
0.002
0.003
0.004
0.005
1990
2010
2030
2050
Year
Safer Environment
Improved Behavioural and Metabolic Risks
Improved Childhood Nutrition and Vaccination
Combined
Reference

Belize

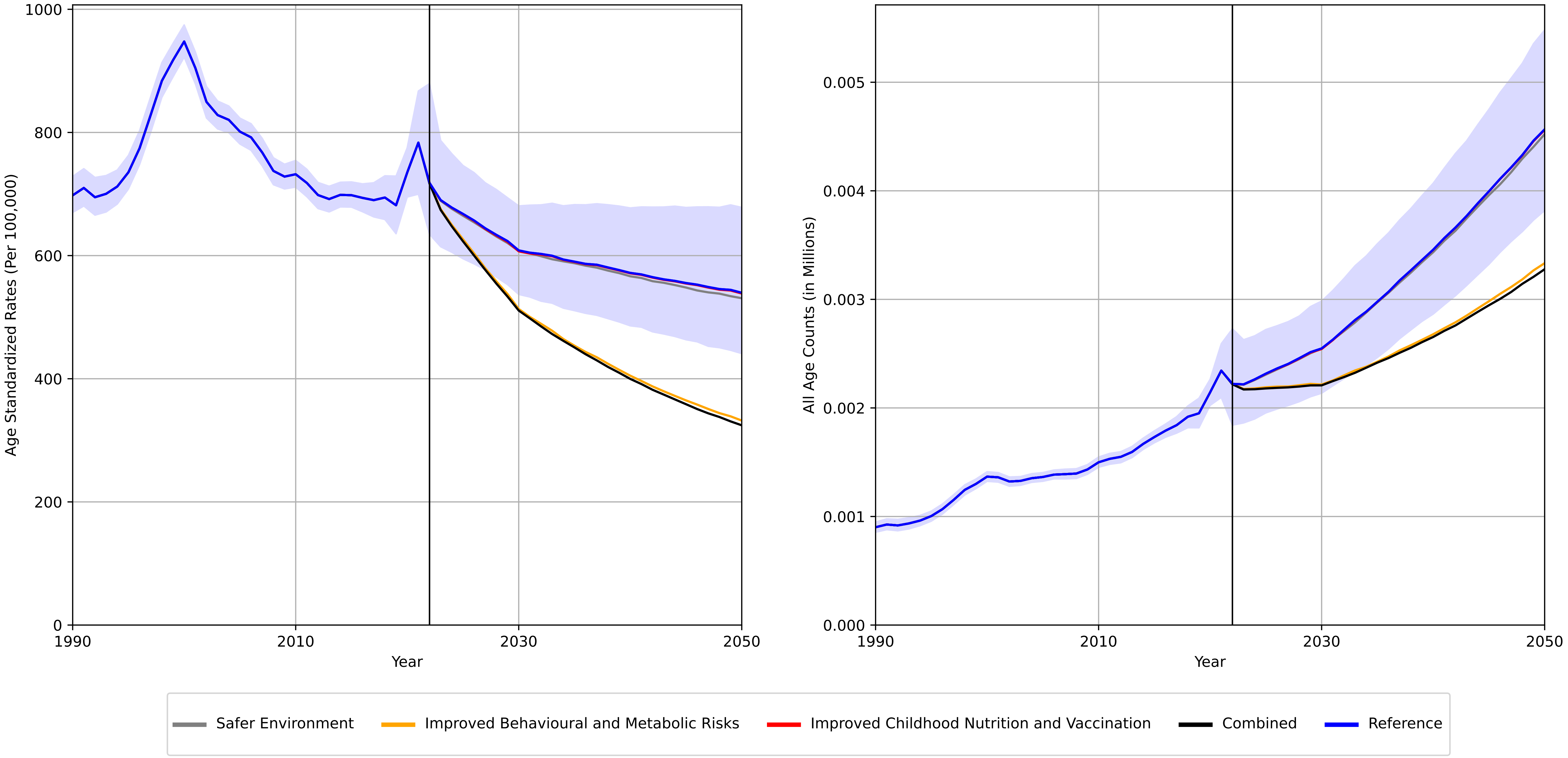

Age Standardized Rates (Per 100,000)
1000
800
600
400
200
0
1990
2010
2030
2050
Year
All Age Counts (in Millions)
0.005
0.004
0.003
0.002
0.001
0.000
1990
2010
2030
2050
Year
Safer Environment
Improved Behavioural and Metabolic Risks
Improved Childhood Nutrition and Vaccination
Combined
Reference

Bermuda

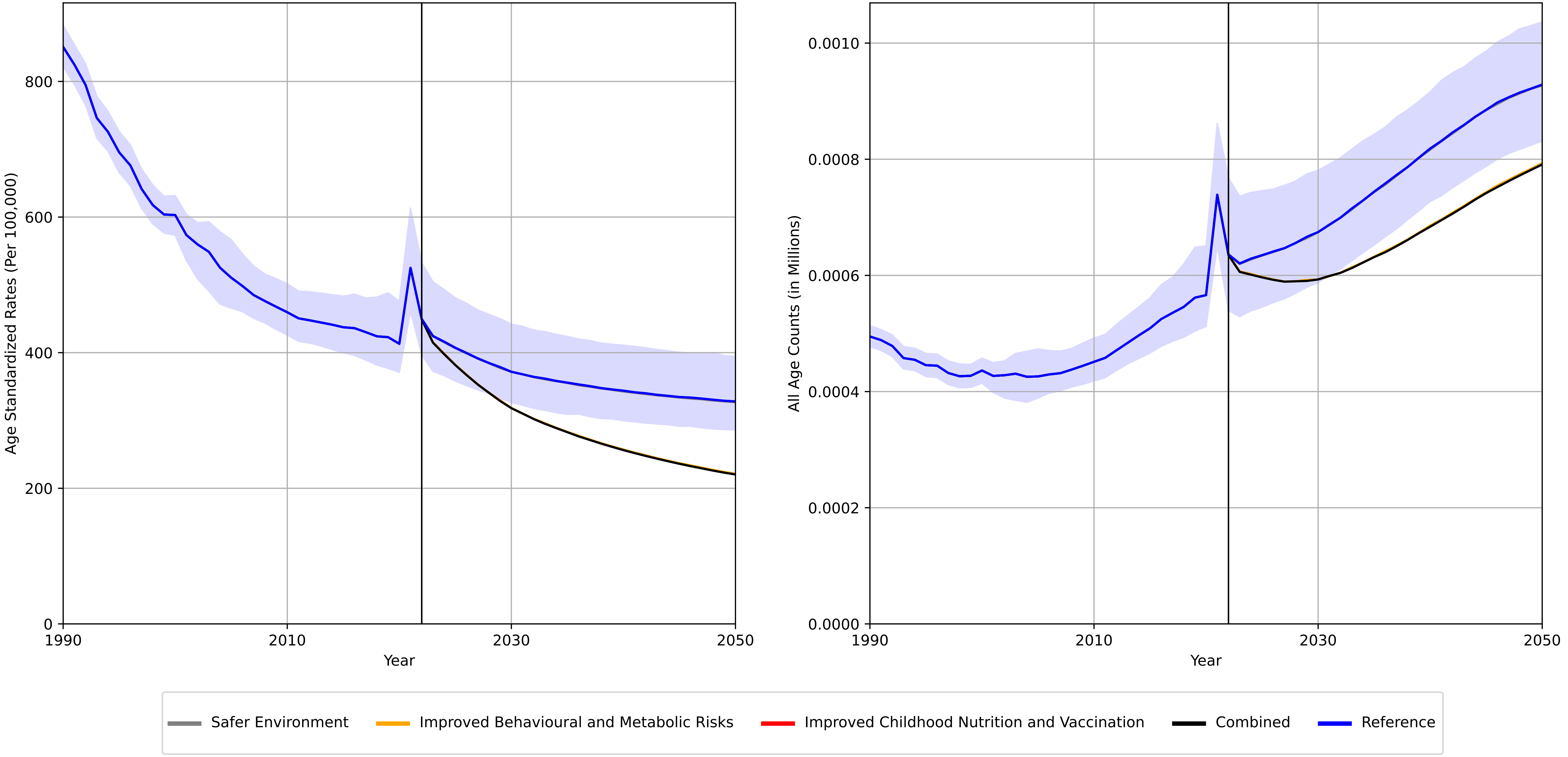

Age Standardized Rates (Per 100,000)
0
200
400
600
800
1990
2010
2030
2050
Year
All Age Counts (in Millions)
0.0000
0.0002
0.0004
0.0006
0.0008
0.0010
1990
2010
2030
2050
Year
Safer Environment
Improved Behavioural and Metabolic Risks
Improved Childhood Nutrition and Vaccination
Combined
Reference

Cuba

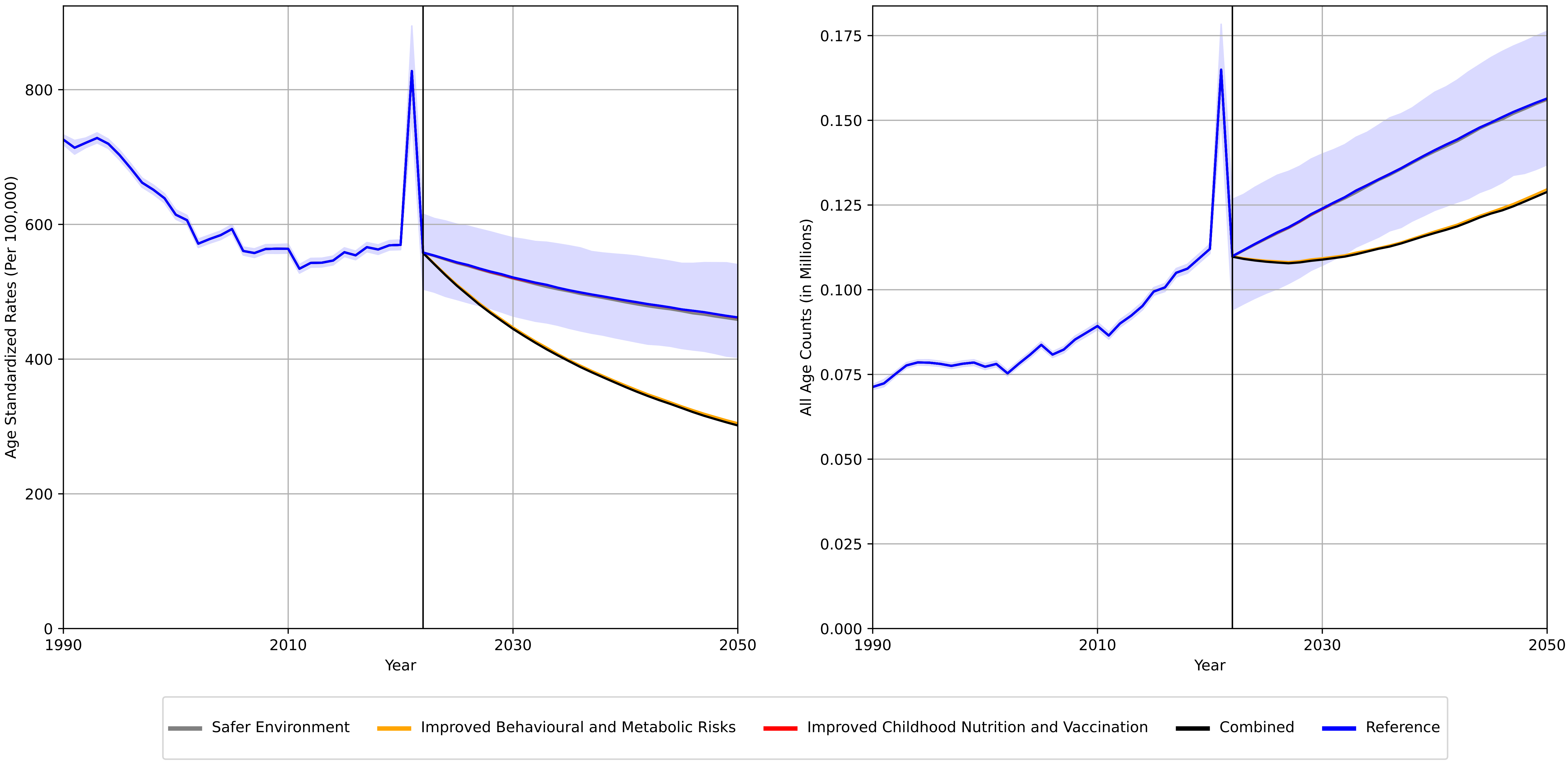

Age Standardized Rates (Per 100,000)
0
200
400
600
800
1990
2010
2030
2050
Year
All Age Counts (in Millions)
0.000
0.025
0.050
0.075
0.100
0.125
0.150
0.175
1990
2010
2030
2050
Year
Safer Environment
Improved Behavioural and Metabolic Risks
Improved Childhood Nutrition and Vaccination
Combined
Reference

Dominica

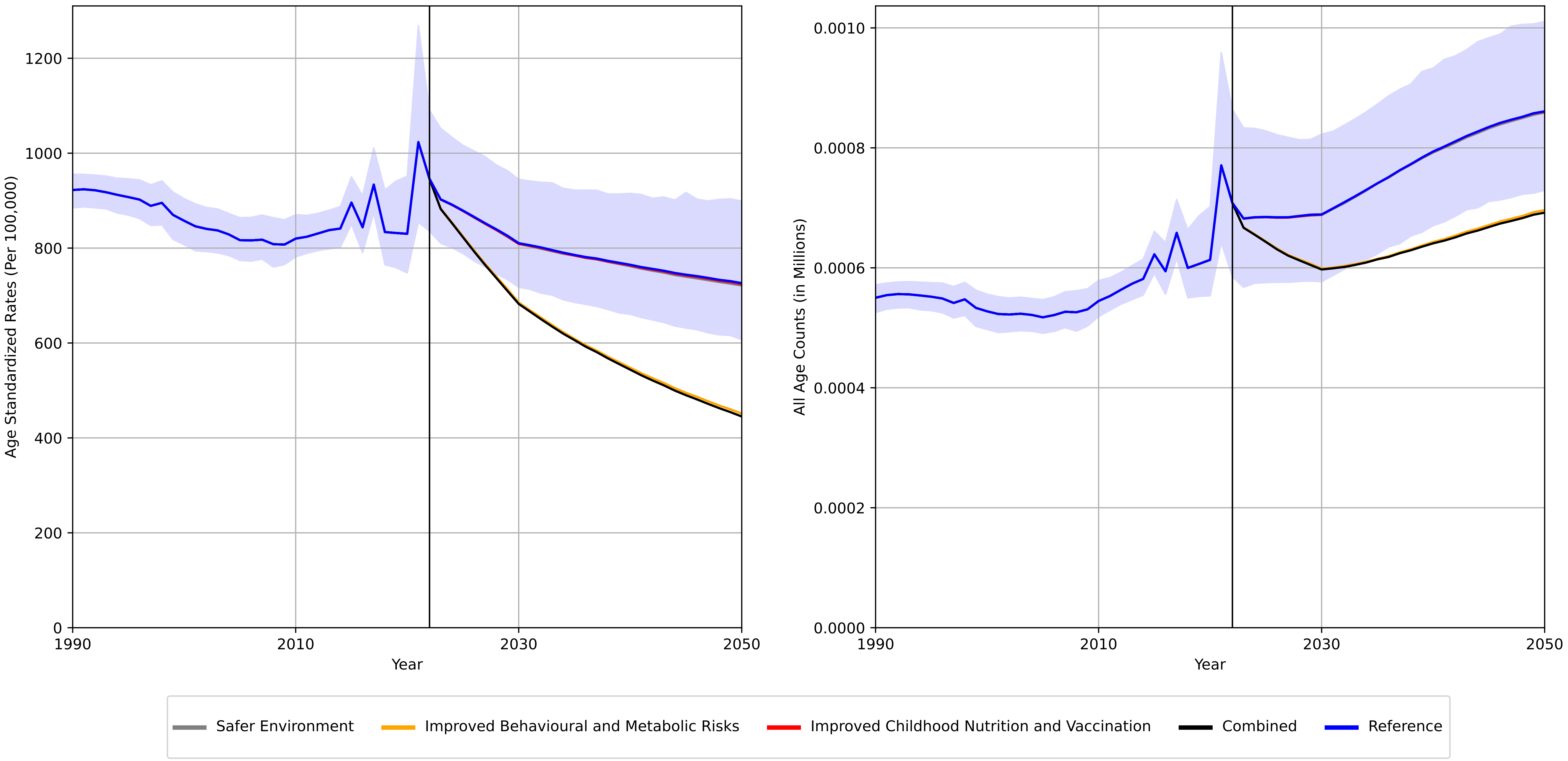

Age Standardized Rates (Per 100,000)
0
200
400
600
800
1000
1200
1990
2010
2030
2050
Year
All Age Counts (in Millions)
0.0000
0.0002
0.0004
0.0006
0.0008
0.0010
1990
2010
2030
2050
Year
Safer Environment
Improved Behavioural and Metabolic Risks
Improved Childhood Nutrition and Vaccination
Combined
Reference

Dominican Republic

Supplemental Results Figure S11. All-cause age standardised death rates (A) and all age death counts (B), for the past and for five future scenarios, 1990–2050

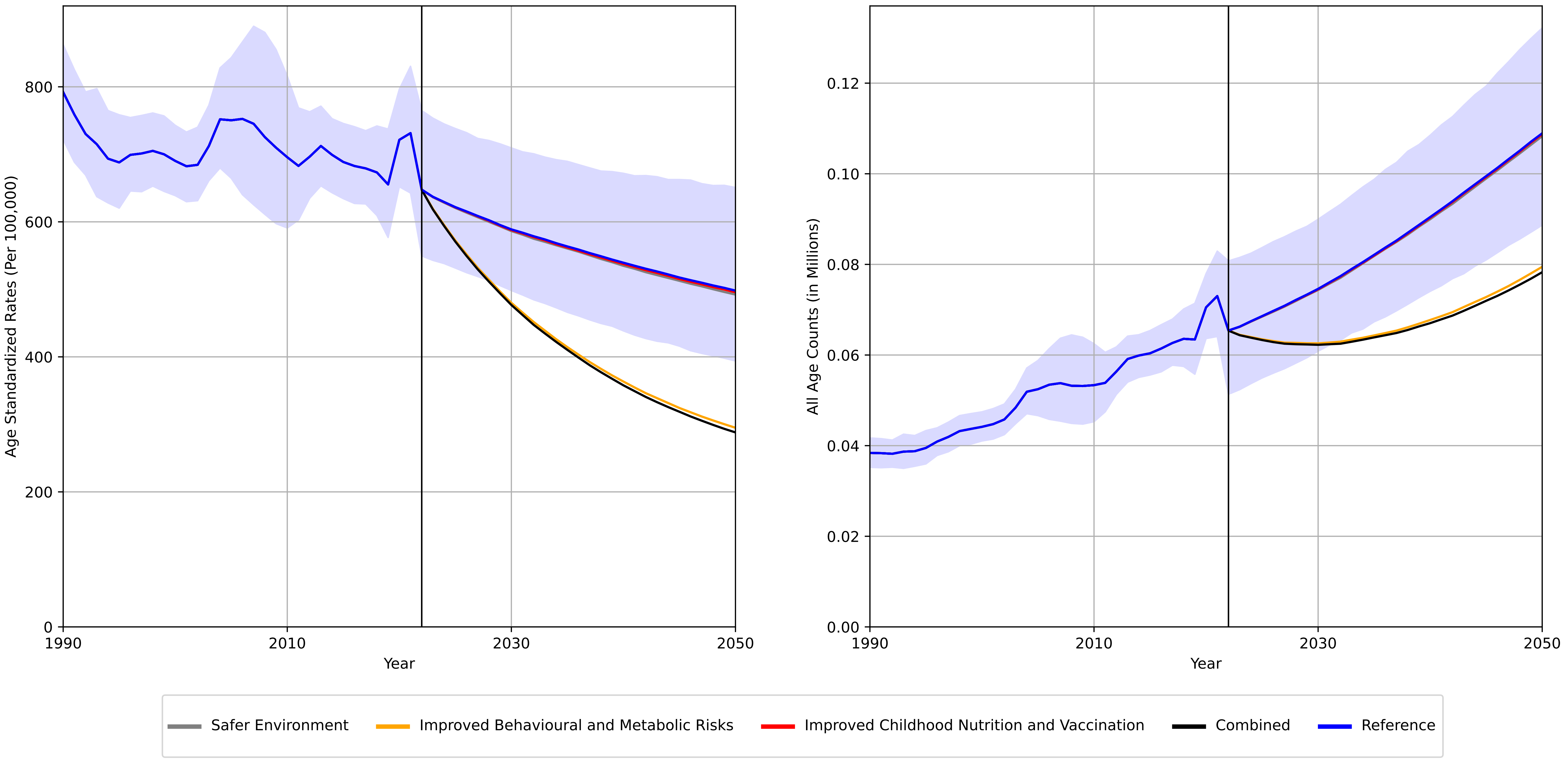

Grenada

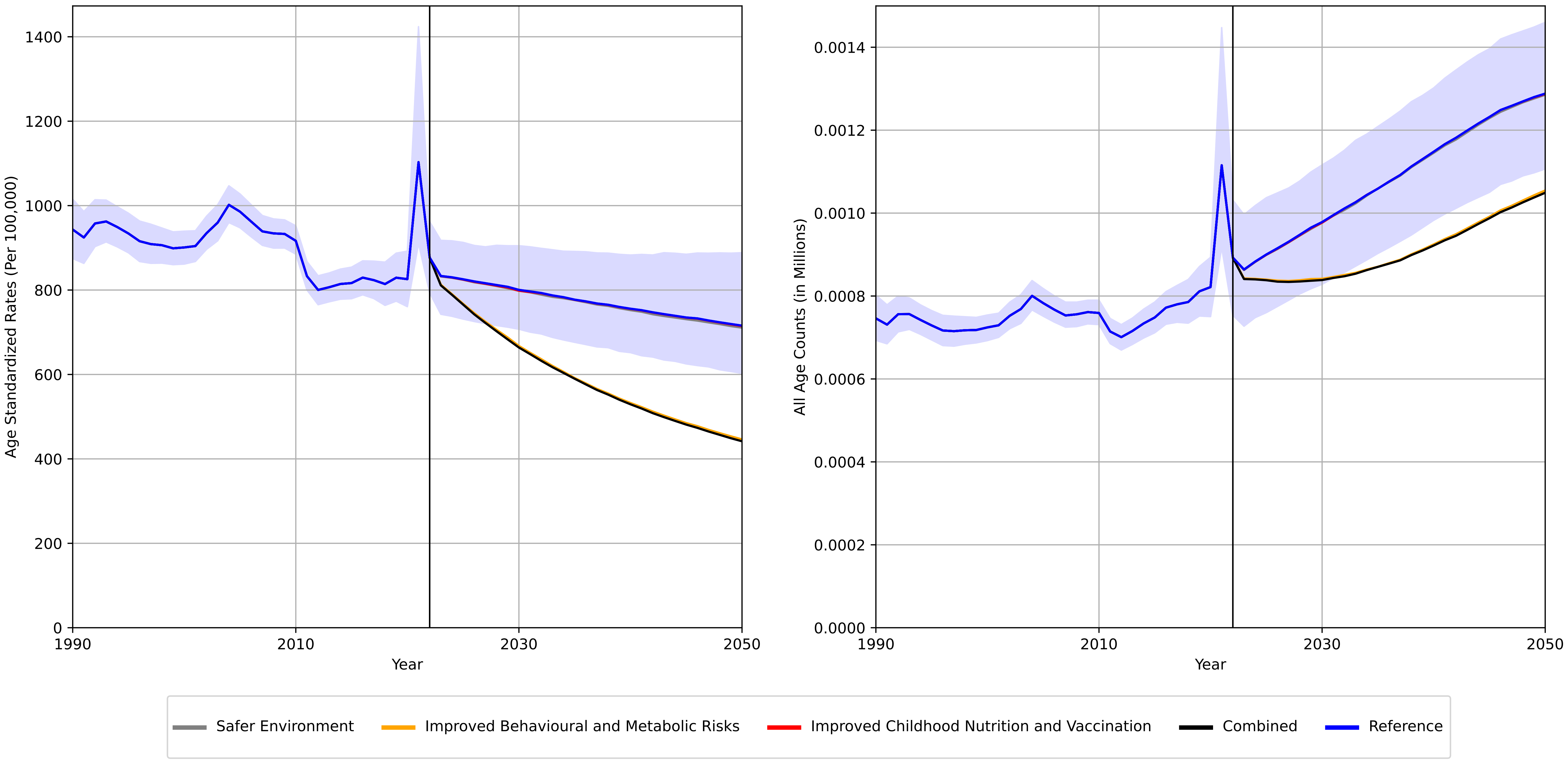
Age Standardized Rates (Per 100,000)
1400
1200
1000
800
600
400
200
0
1990
2010
2030
2050
Year
All Age Counts (in Millions)
0.0014
0.0012
0.0010
0.0008
0.0006
0.0004
0.0002
0.0000
1990
2010
2030
2050
Year
Safer Environment
Improved Behavioural and Metabolic Risks
Improved Childhood Nutrition and Vaccination
Combined
Reference

Guyana

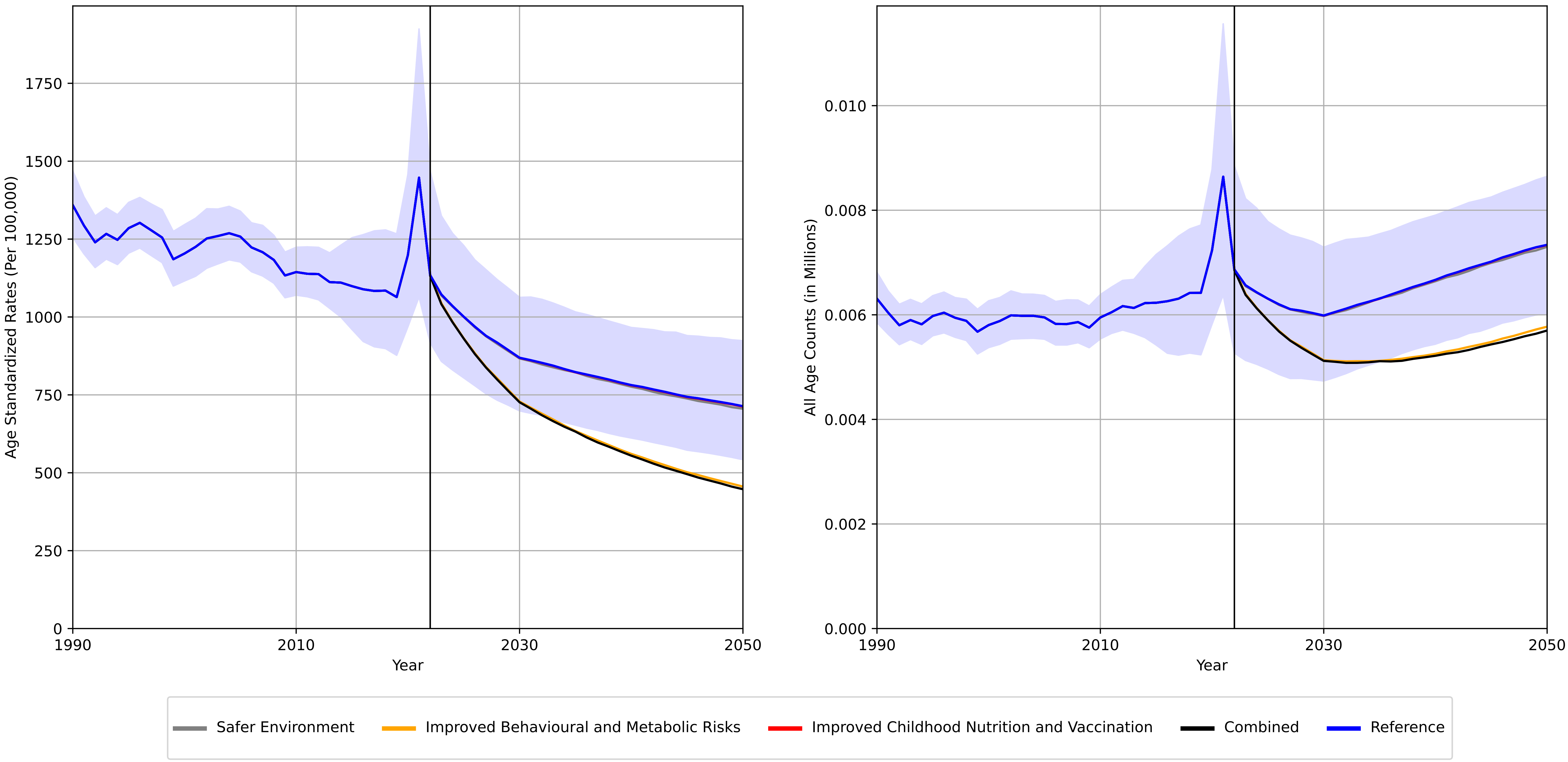
Age Standardized Rates (Per 100,000)
0
250
500
750
1000
1250
1500
1750
1990
2010
2030
2050
Year
All Age Counts (in Millions)
0.000
0.002
0.004
0.006
0.008
0.010
1990
2010
2030
2050
Year
Safer Environment
Improved Behavioural and Metabolic Risks
Improved Childhood Nutrition and Vaccination
Combined
Reference

Haiti

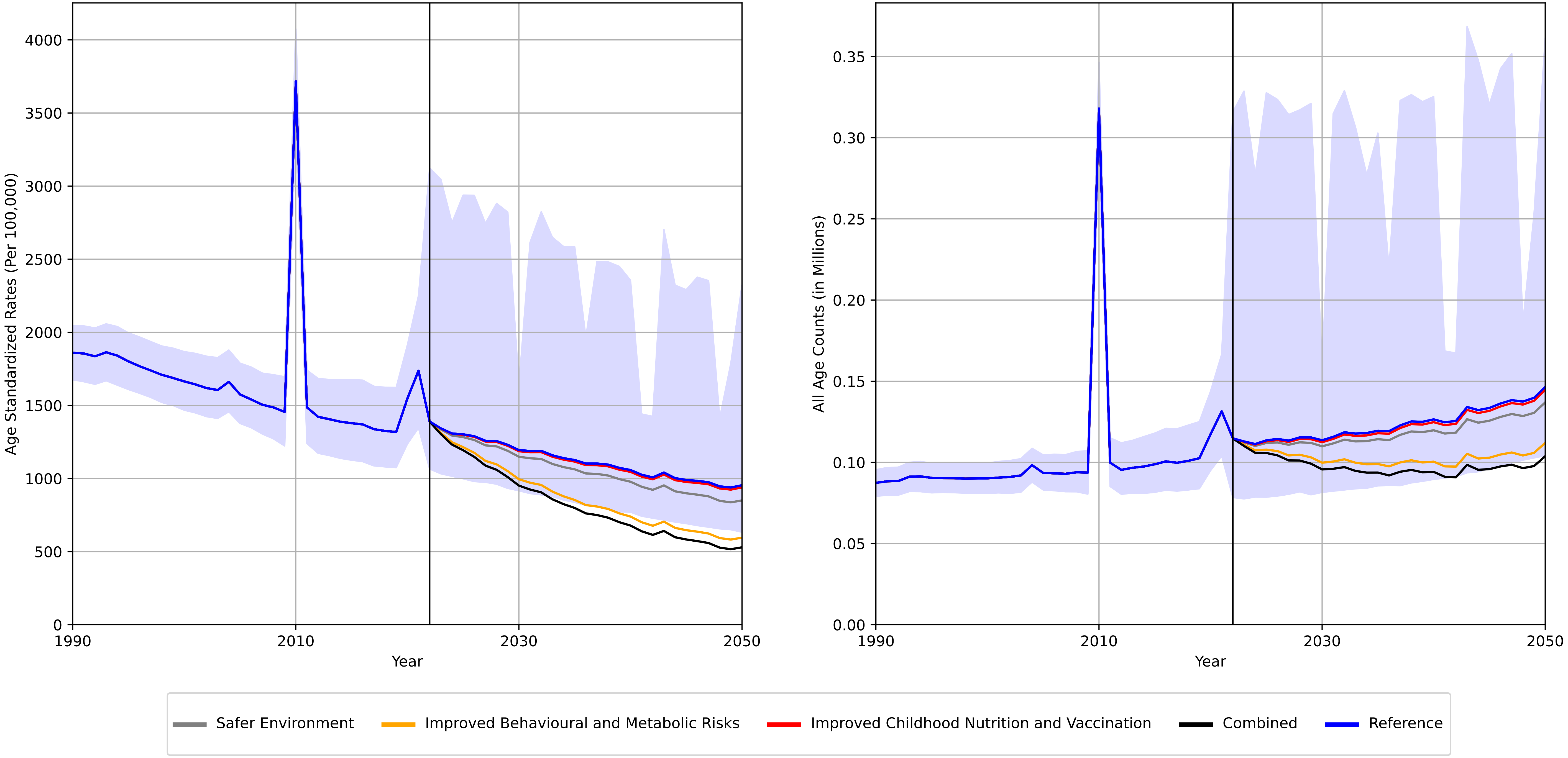
Age Standardized Rates (Per 100,000)
0
500
1000
1500
2000
2500
3000
3500
4000
1990
2010
2030
2050
Year
All Age Counts (in Millions)
0.00
0.05
0.10
0.15
0.20
0.25
0.30
0.35
1990
2010
2030
2050
Year
Safer Environment
Improved Behavioural and Metabolic Risks
Improved Childhood Nutrition and Vaccination
Combined
Reference

Jamaica

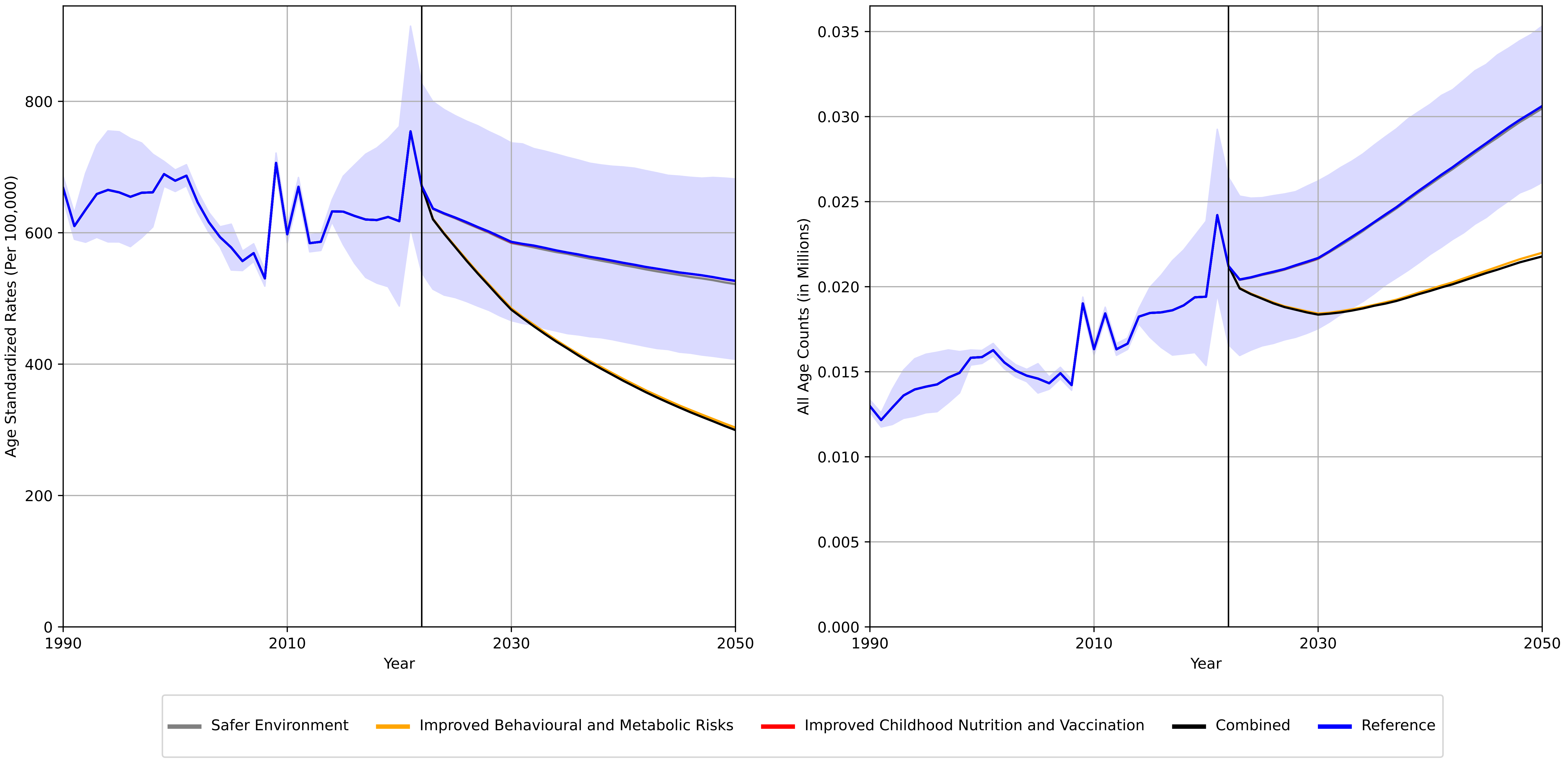

Age Standardized Rates (Per 100,000)
0
200
400
600
800
1990
2010
2030
2050
Year
All Age Counts (in Millions)
0.000
0.005
0.010
0.015
0.020
0.025
0.030
0.035
1990
2010
2030
2050
Year
Safer Environment
Improved Behavioural and Metabolic Risks
Improved Childhood Nutrition and Vaccination
Combined
Reference

Puerto Rico

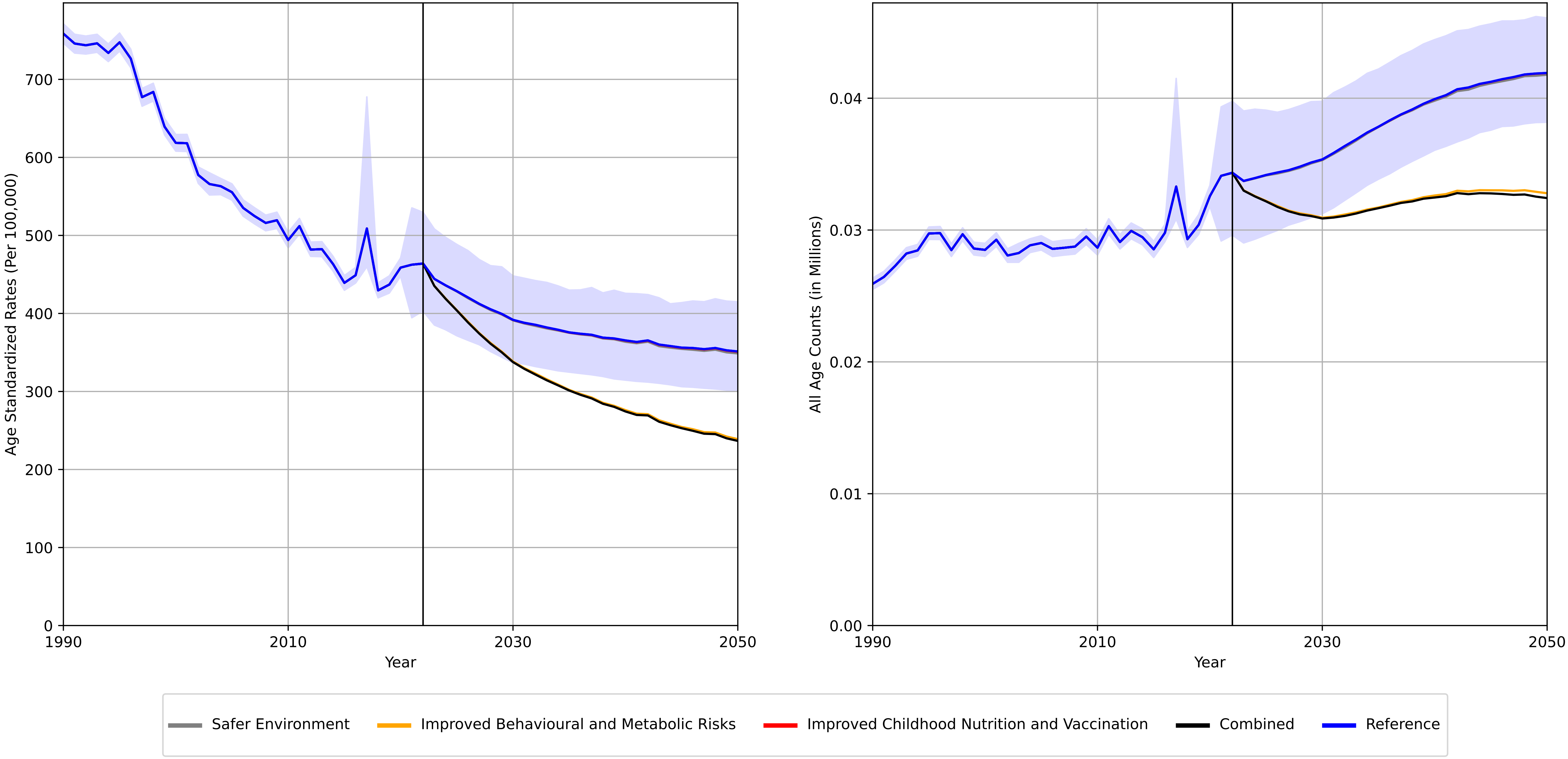
Age Standardized Rates (Per 100,000)
All Age Counts (in Millions)
Year
Year
Safer Environment
Improved Behavioural and Metabolic Risks
Improved Childhood Nutrition and Vaccination
Combined
Reference

Saint Kitts And Nevis

Supplemental Results Figure S11. All-cause age standardised death rates (A) and all age death counts (B), for the past and for five future scenarios, 1990–2050

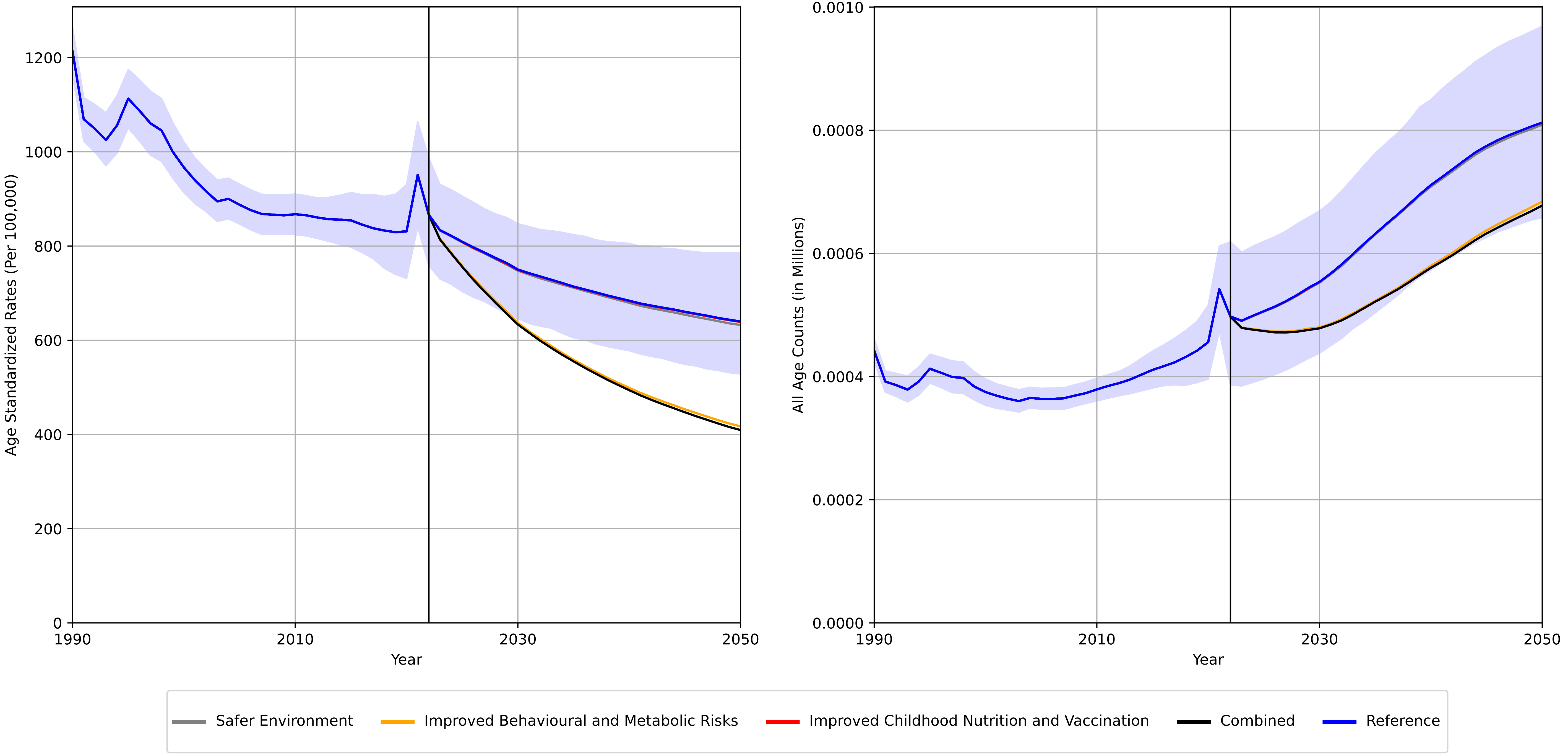

Saint Lucia

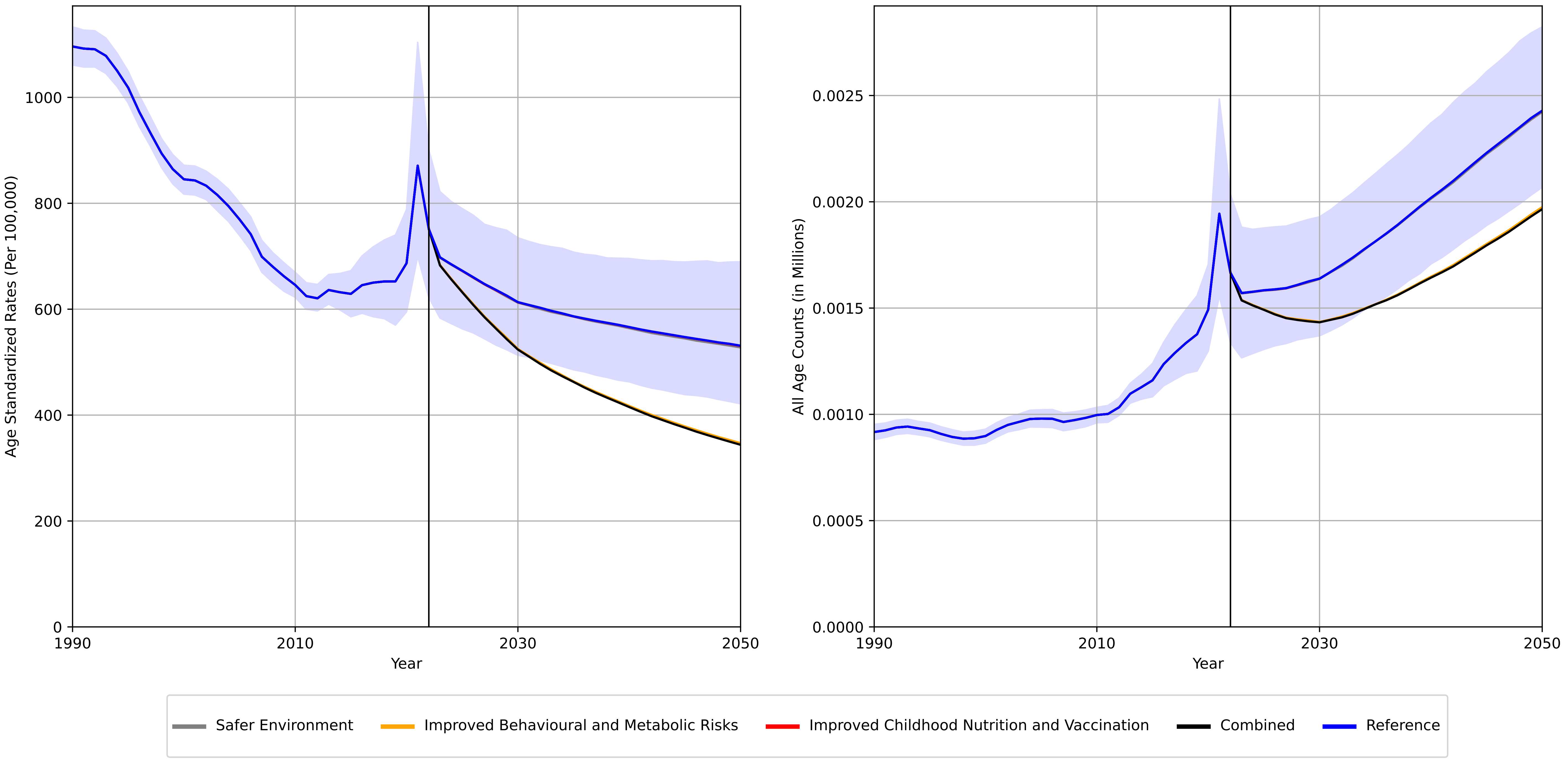

Age Standardized Rates (Per 100,000)
0
200
400
600
800
1000
1990
2010
2030
2050
Year
All Age Counts (in Millions)
0.0000
0.0005
0.0010
0.0015
0.0020
0.0025
1990
2010
2030
2050
Year
Safer Environment
Improved Behavioural and Metabolic Risks
Improved Childhood Nutrition and Vaccination
Combined
Reference

Saint Vincent And The Grenadines

Supplemental Results Figure S11. All-cause age standardised death rates (A) and all age death counts (B), for the past and for five future scenarios, 1990–2050

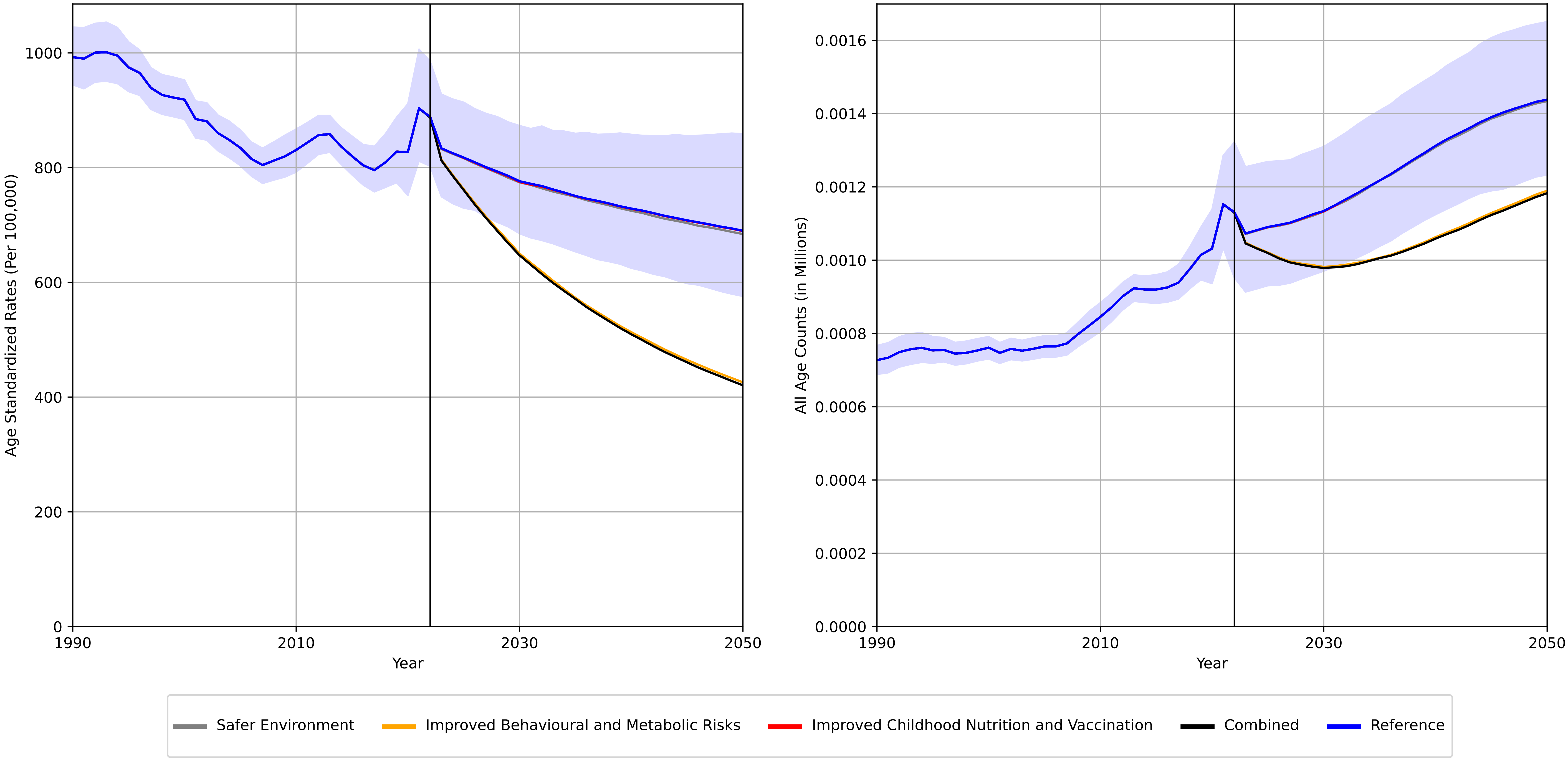

Suriname

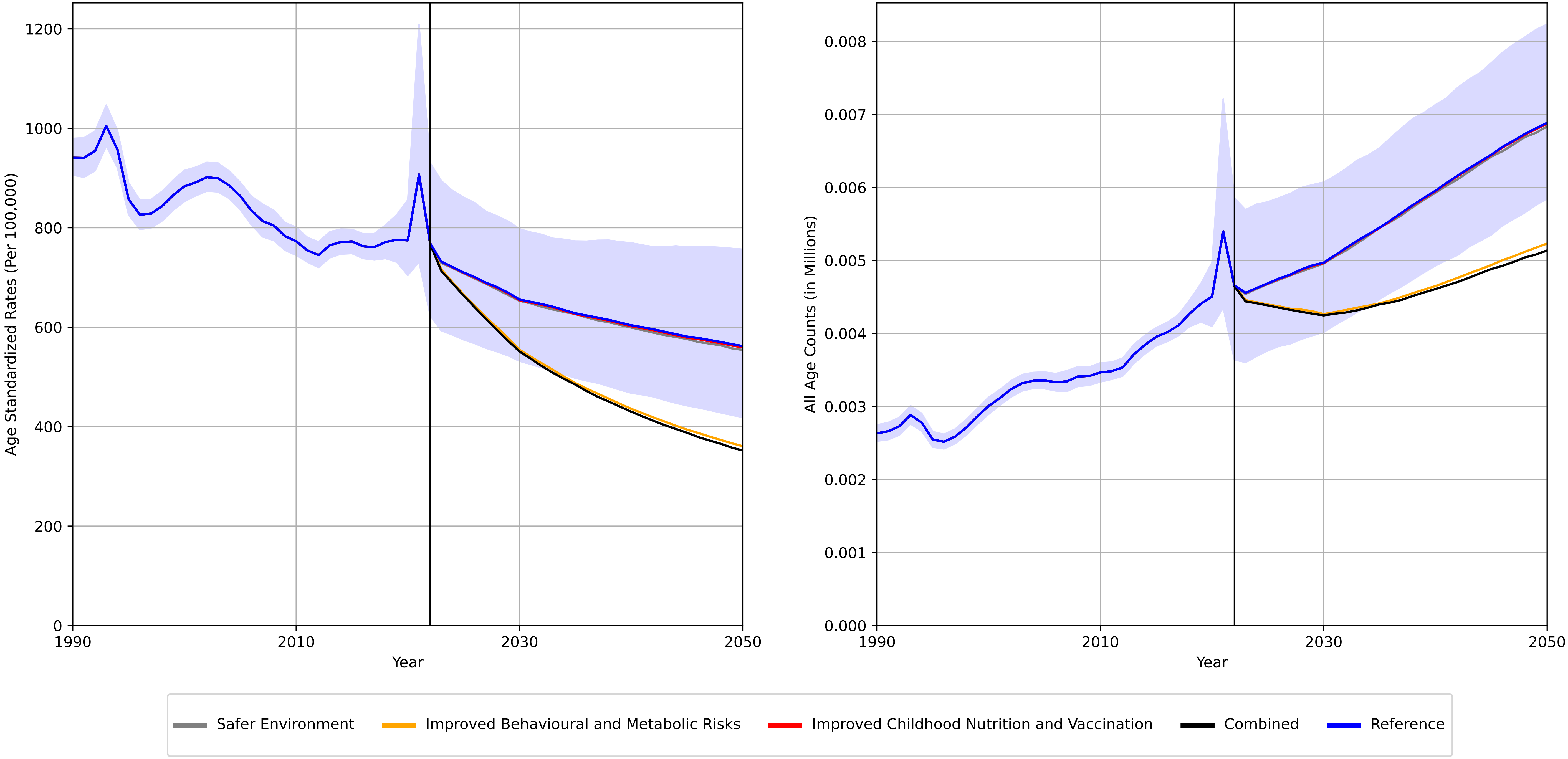

Age Standardized Rates (Per 100,000)
1200
1000
800
600
400
200
0
1990
2010
2030
2050
Year
All Age Counts (in Millions)
0.008
0.007
0.006
0.005
0.004
0.003
0.002
0.001
0.000
1990
2010
2030
2050
Year
Safer Environment
Improved Behavioural and Metabolic Risks
Improved Childhood Nutrition and Vaccination
Combined
Reference

Trinidad And Tobago

Supplemental Results Figure S11. All-cause age standardised death rates (A) and all age death counts (B), for the past and for five future scenarios, 1990–2050

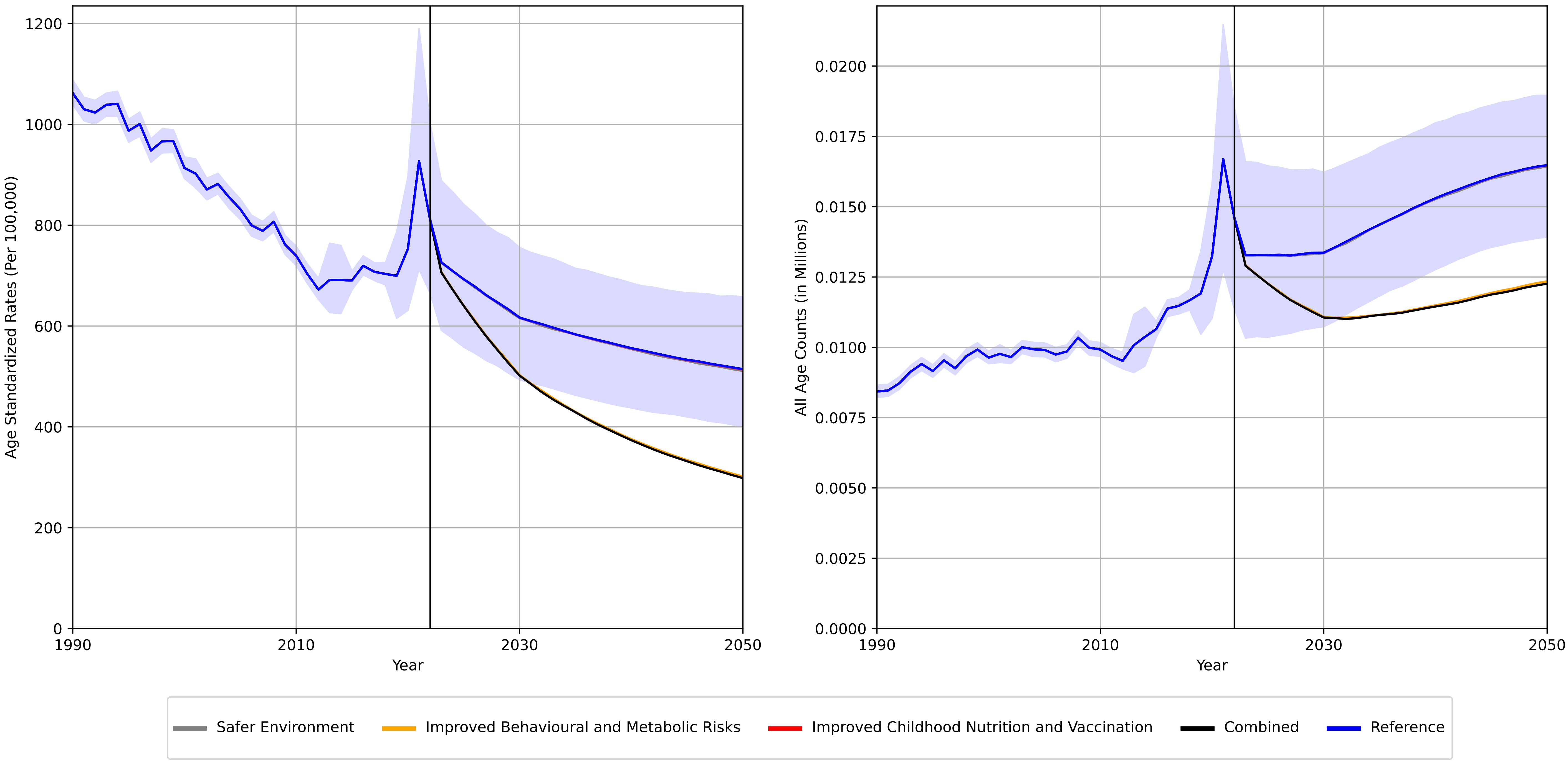

Virgin Islands

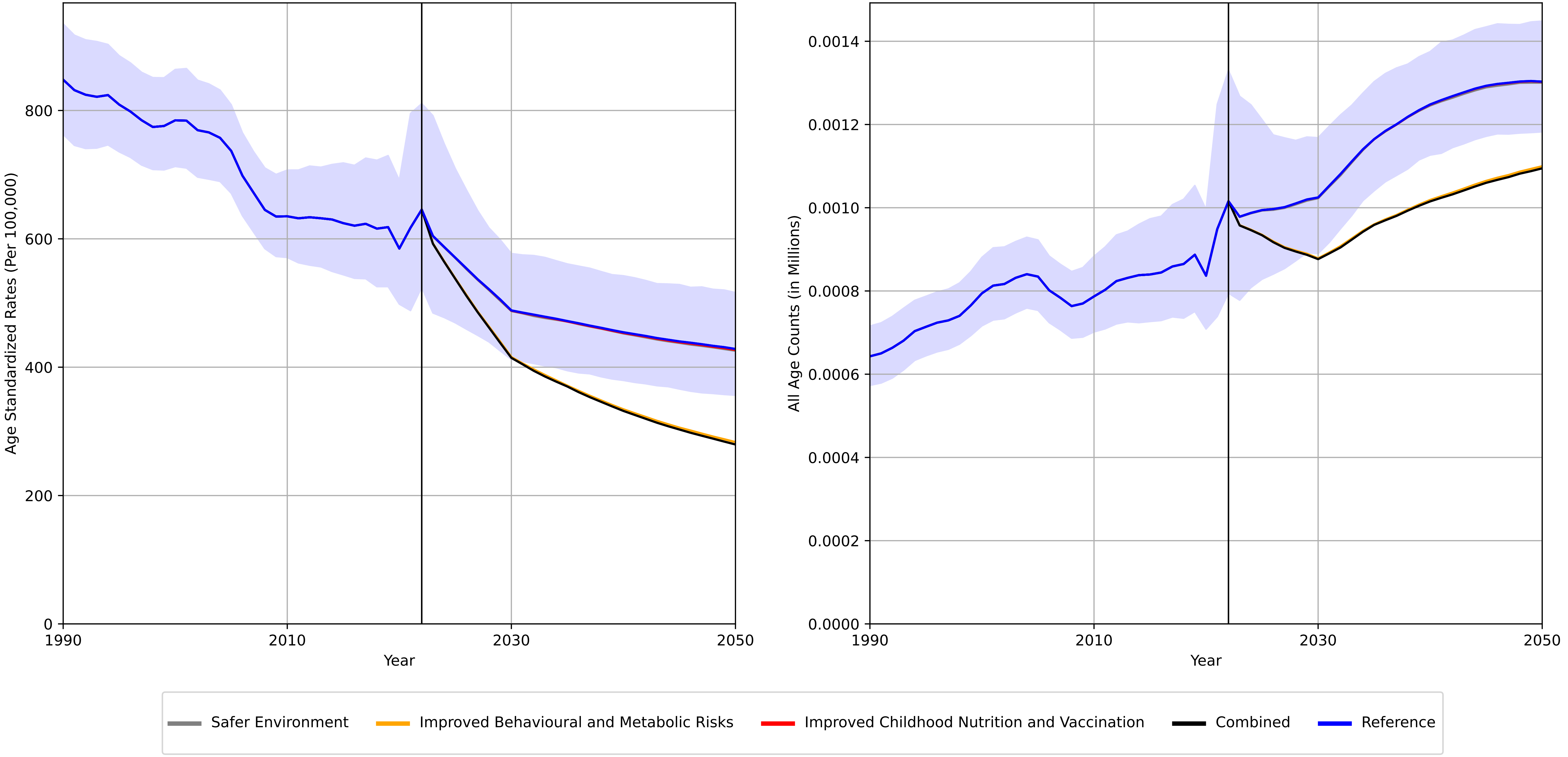

Colombia

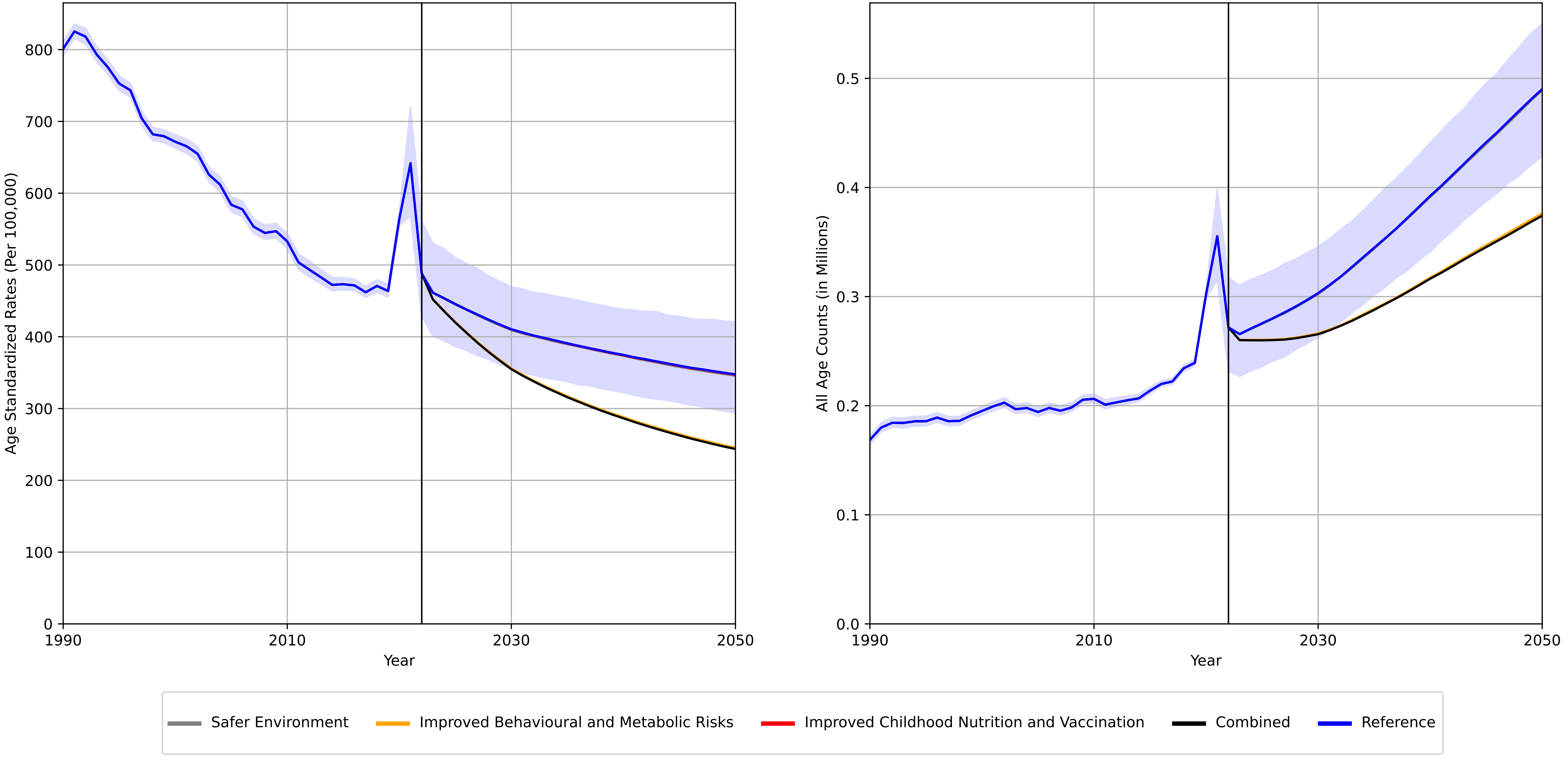

Age Standardized Rates (Per 100,000)
800
700
600
500
400
300
200
100
0
1990
2010
2030
2050
Year
All Age Counts (in Millions)
0.5
0.4
0.3
0.2
0.1
0.0
Safer Environment
Improved Behavioural and Metabolic Risks
Improved Childhood Nutrition and Vaccination
Combined
Reference

Costa Rica

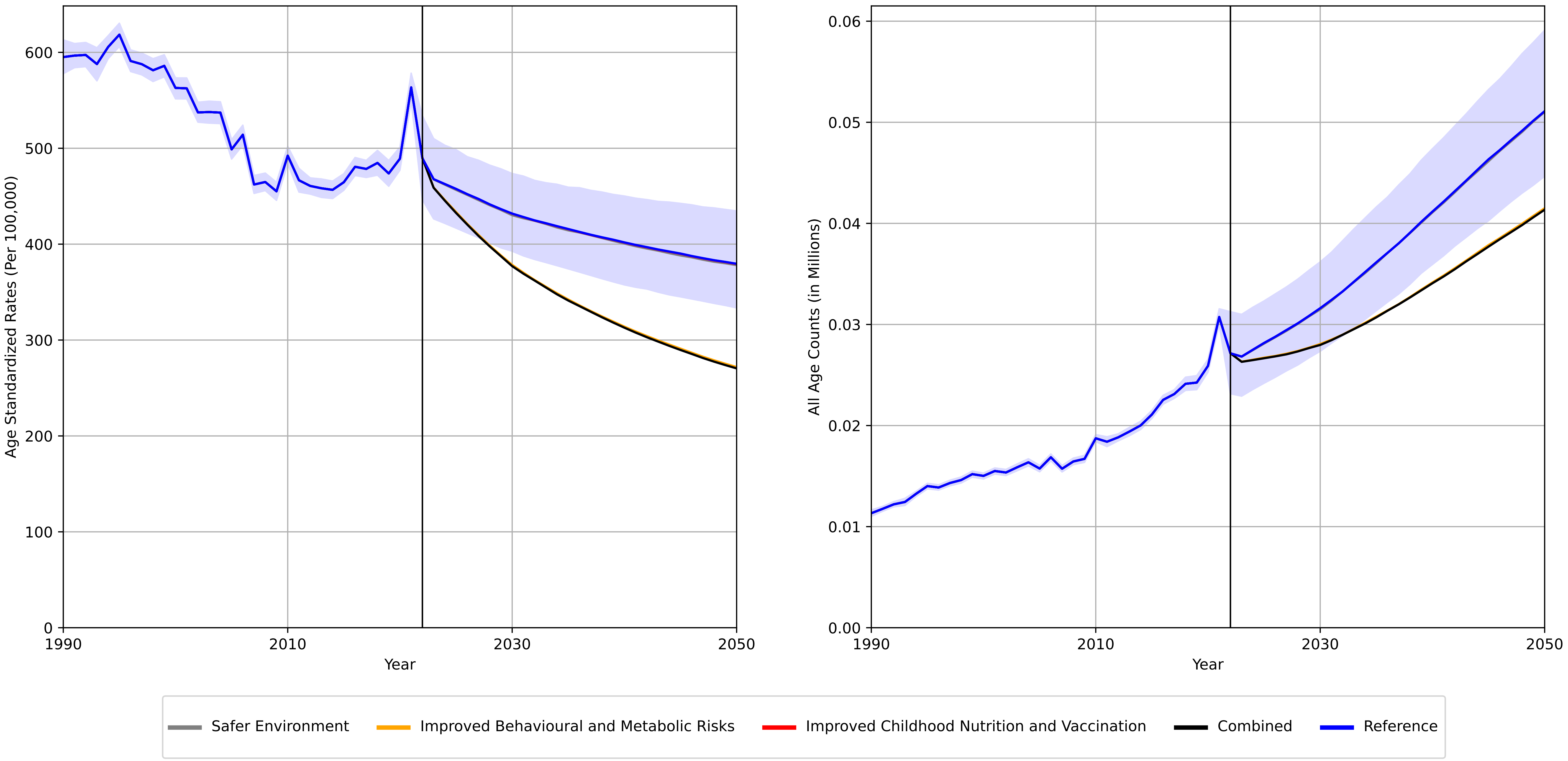

Age Standardized Rates (Per 100,000)
0
100
200
300
400
500
600
1990
2010
2030
2050
Year
All Age Counts (in Millions)
0.00
0.01
0.02
0.03
0.04
0.05
0.06
1990
2010
2030
2050
Year
Safer Environment
Improved Behavioural and Metabolic Risks
Improved Childhood Nutrition and Vaccination
Combined
Reference

El Salvador

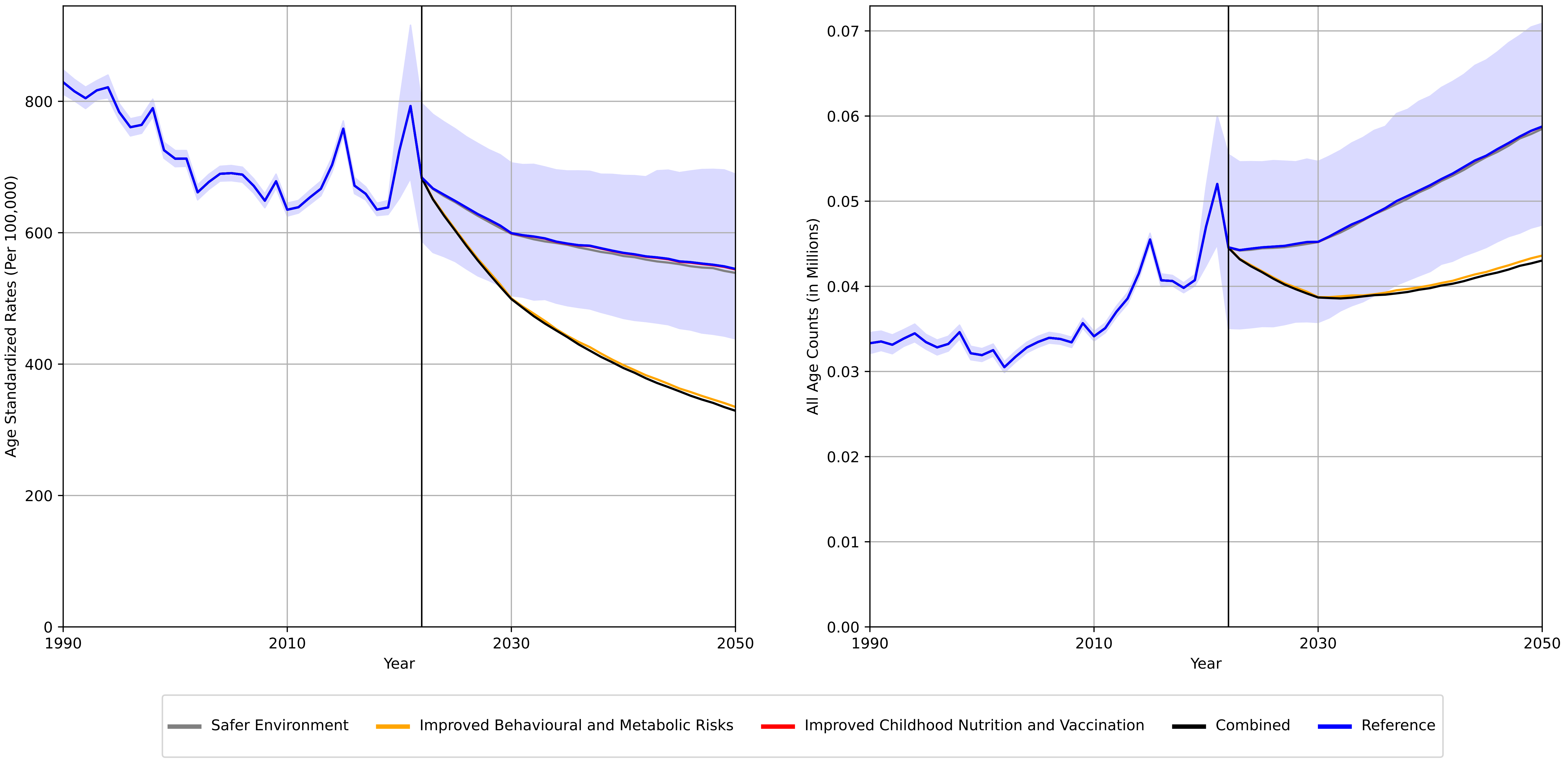

Age Standardized Rates (Per 100,000)
800
600
400
200
0
1990
2010
2030
2050
Year
All Age Counts (in Millions)
0.07
0.06
0.05
0.04
0.03
0.02
0.01
0.00
1990
2010
2030
2050
Year
Safer Environment
Improved Behavioural and Metabolic Risks
Improved Childhood Nutrition and Vaccination
Combined
Reference

Guatemala

Supplemental Results Figure S11. All-cause age standardised death rates (A) and all age death counts (B), for the past and for five future scenarios, 1990–2050

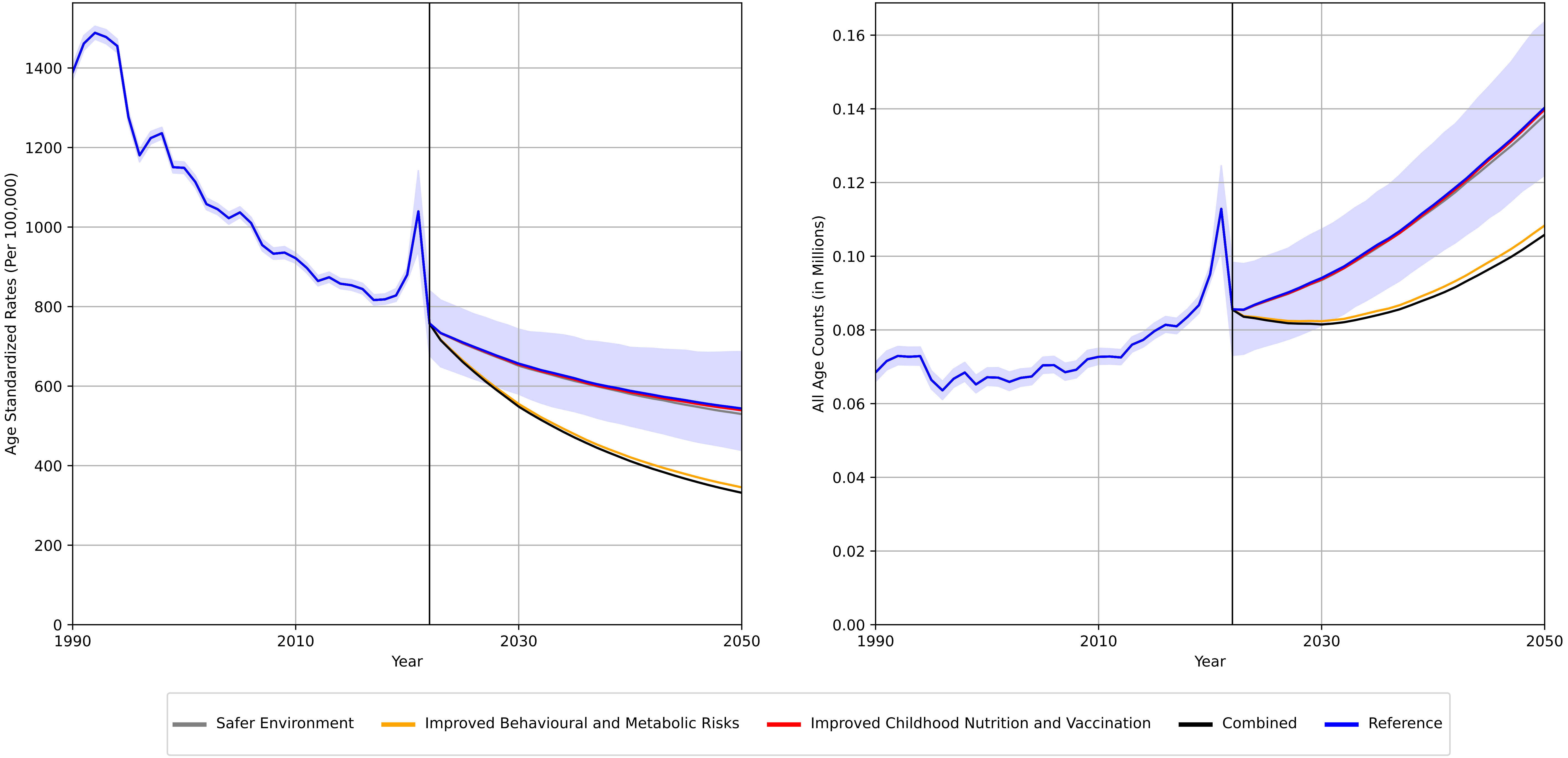

Honduras

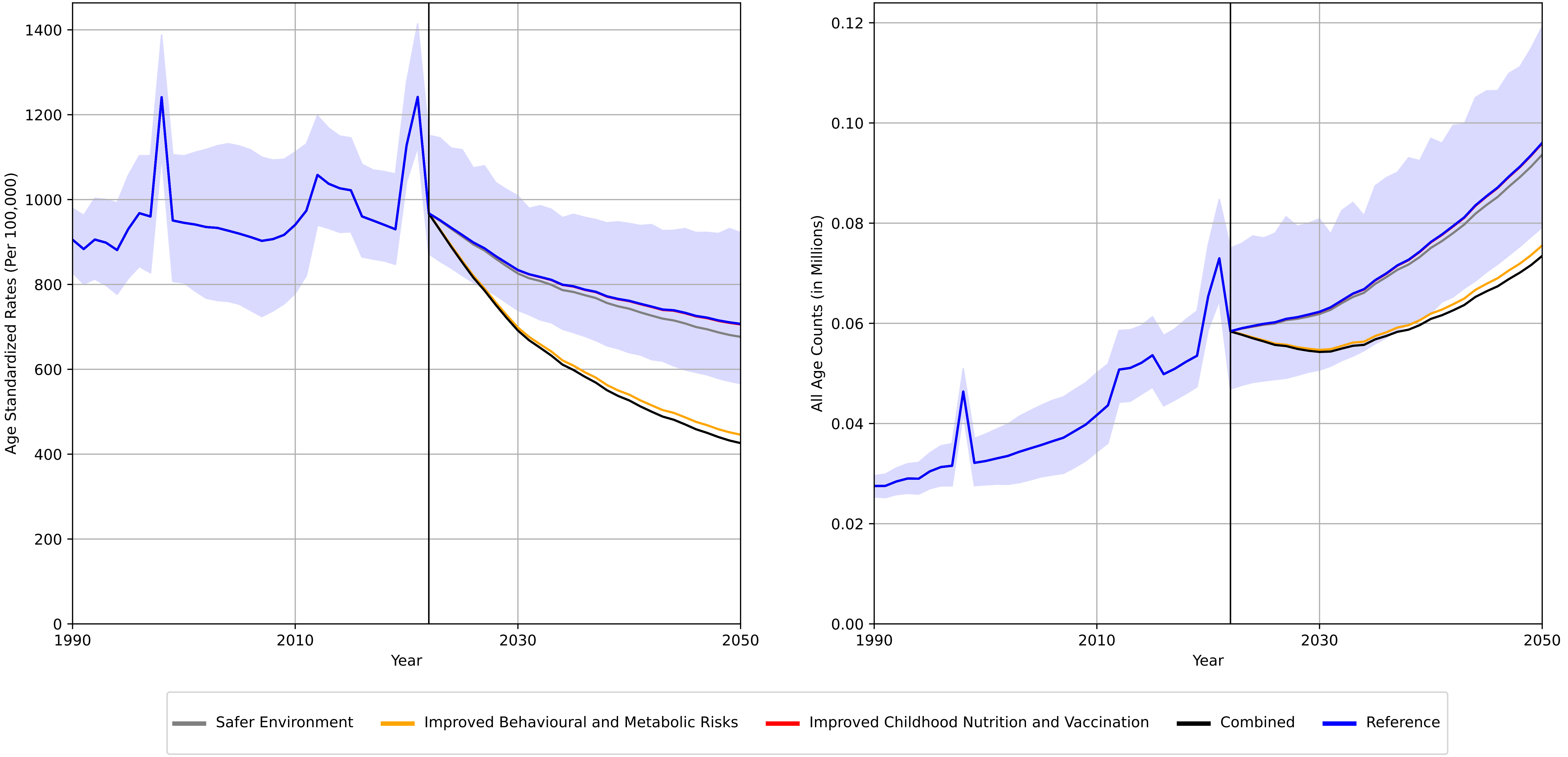
Age Standardized Rates (Per 100,000)
0
200
400
600
800
1000
1200
1400
1990
2010
2030
2050
Year
All Age Counts (in Millions)
0.00
0.02
0.04
0.06
0.08
0.10
0.12
1990
2010
2030
2050
Year
Safer Environment
Improved Behavioural and Metabolic Risks
Improved Childhood Nutrition and Vaccination
Combined
Reference

Mexico

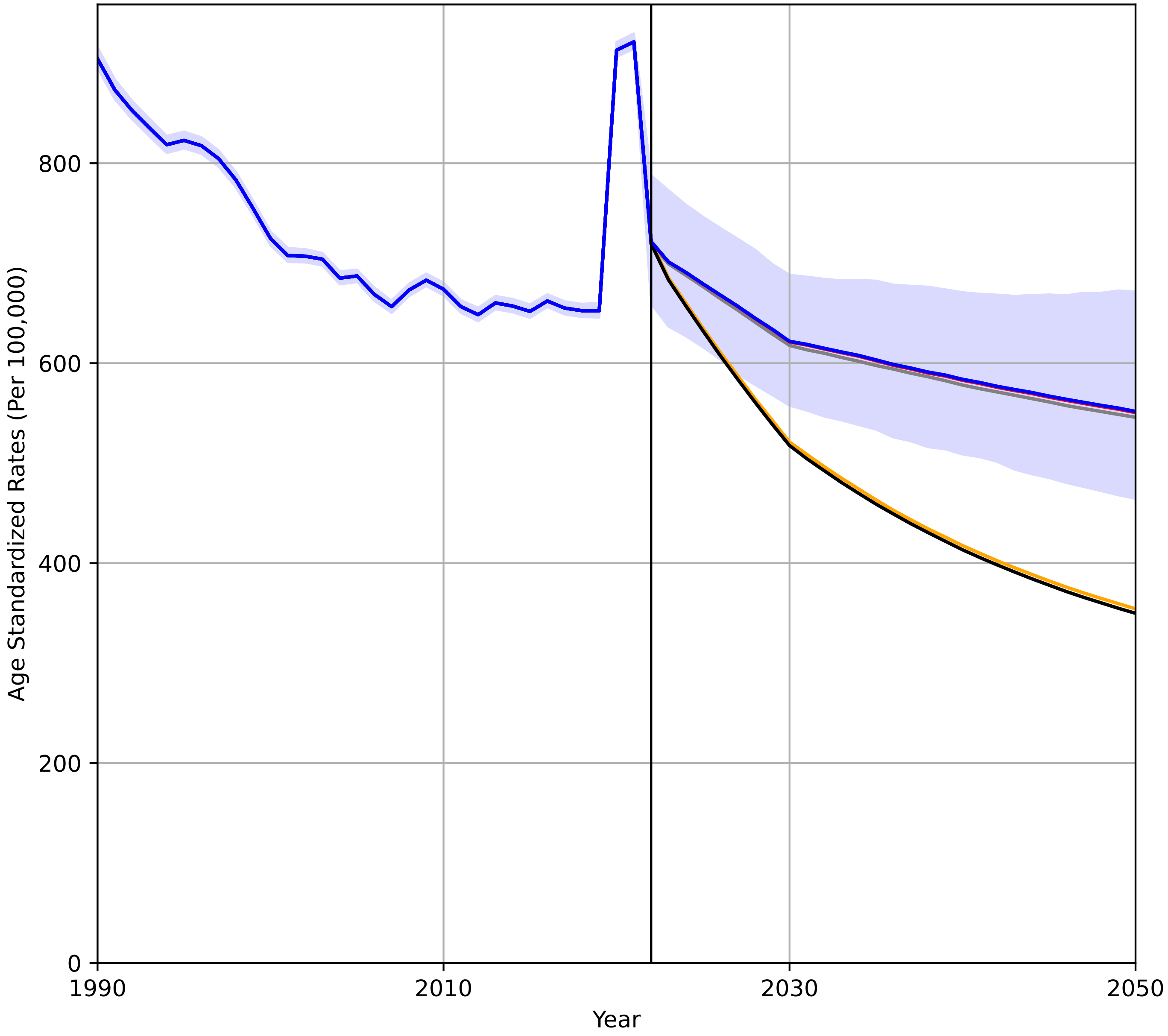

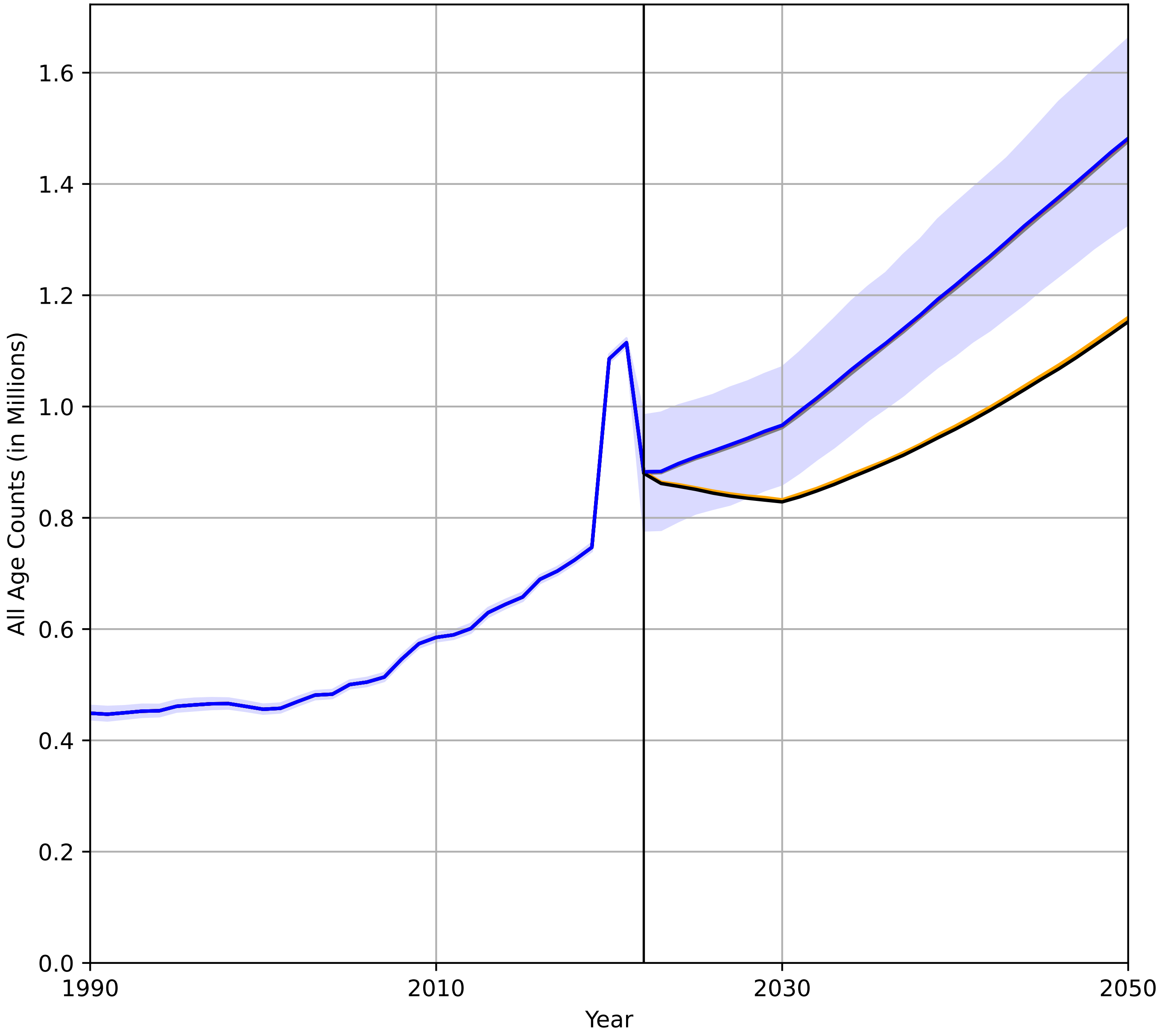

Safer Environment | Improved Behavioural and Metabolic Risks | Improved Childhood Nutrition and Vaccination | Combined | Reference

Nicaragua

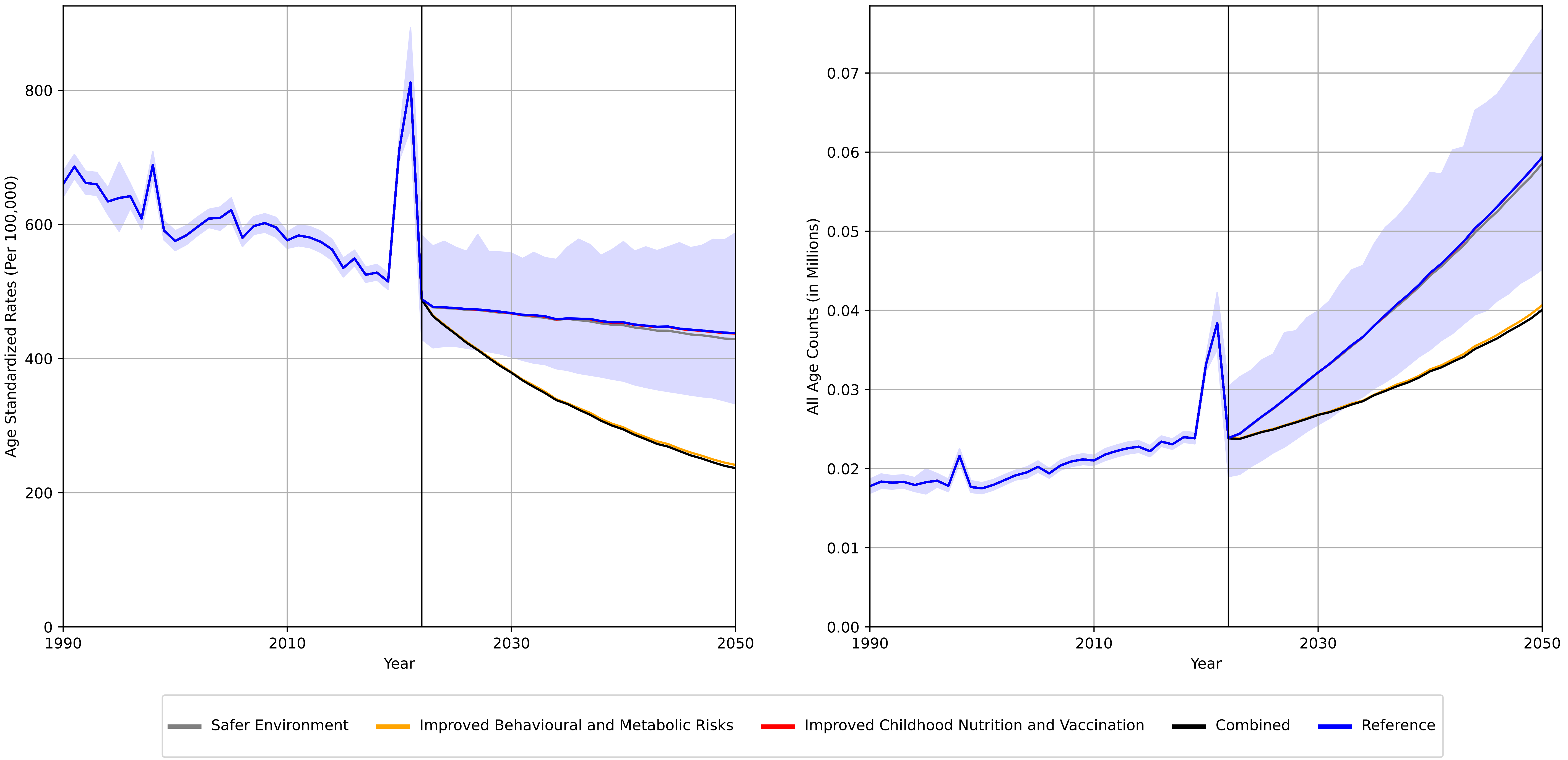
Age Standardized Rates (Per 100,000)
0
200
400
600
800
1990
2010
2030
2050
Year
All Age Counts (in Millions)
0.00
0.01
0.02
0.03
0.04
0.05
0.06
0.07
1990
2010
2030
2050
Year
Safer Environment
Improved Behavioural and Metabolic Risks
Improved Childhood Nutrition and Vaccination
Combined
Reference

Panama

Supplemental Results Figure S11. All-cause age standardised death rates (A) and all age death counts (B), for the past and for five future scenarios, 1990–2050

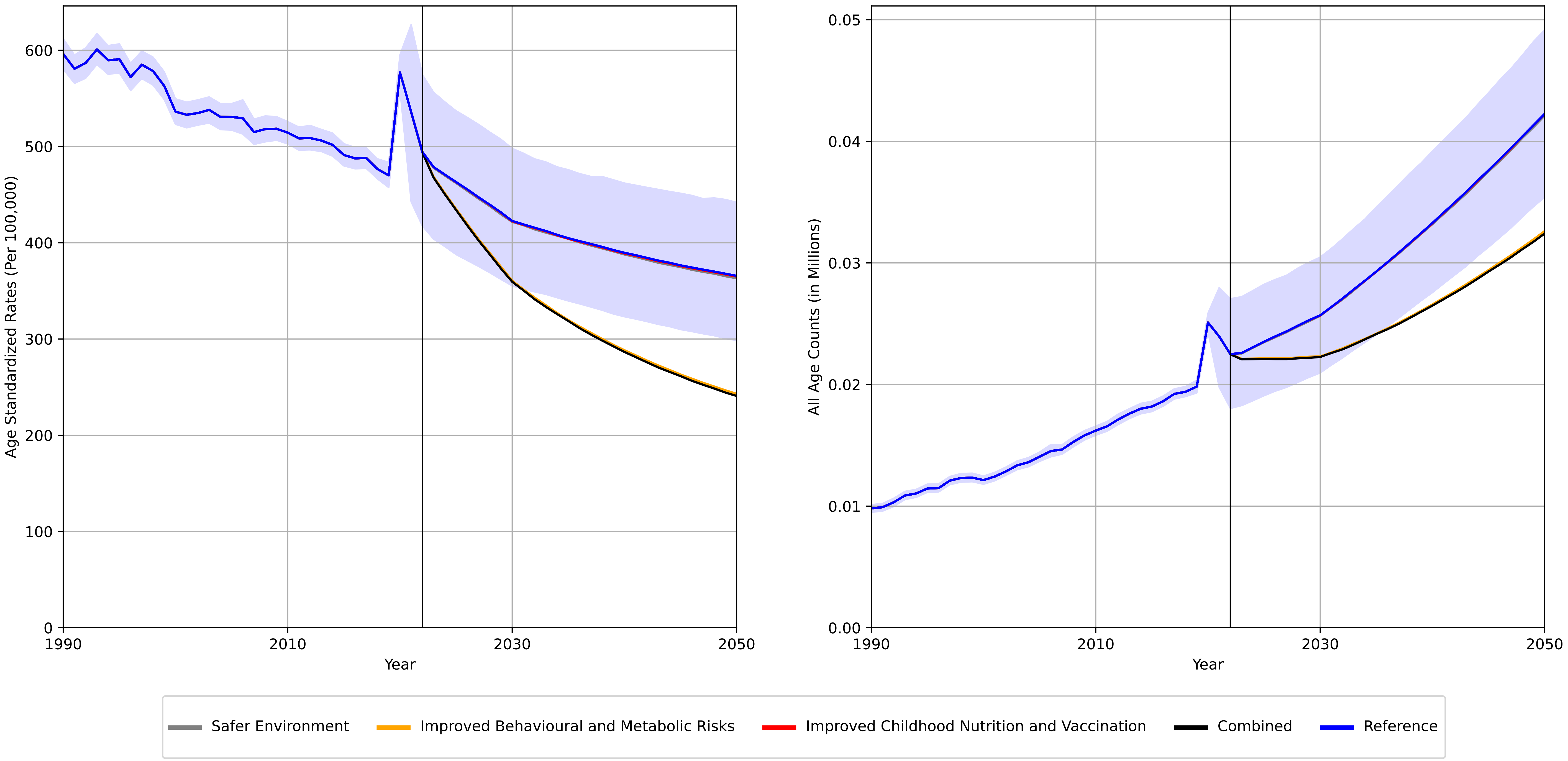

Venezuela

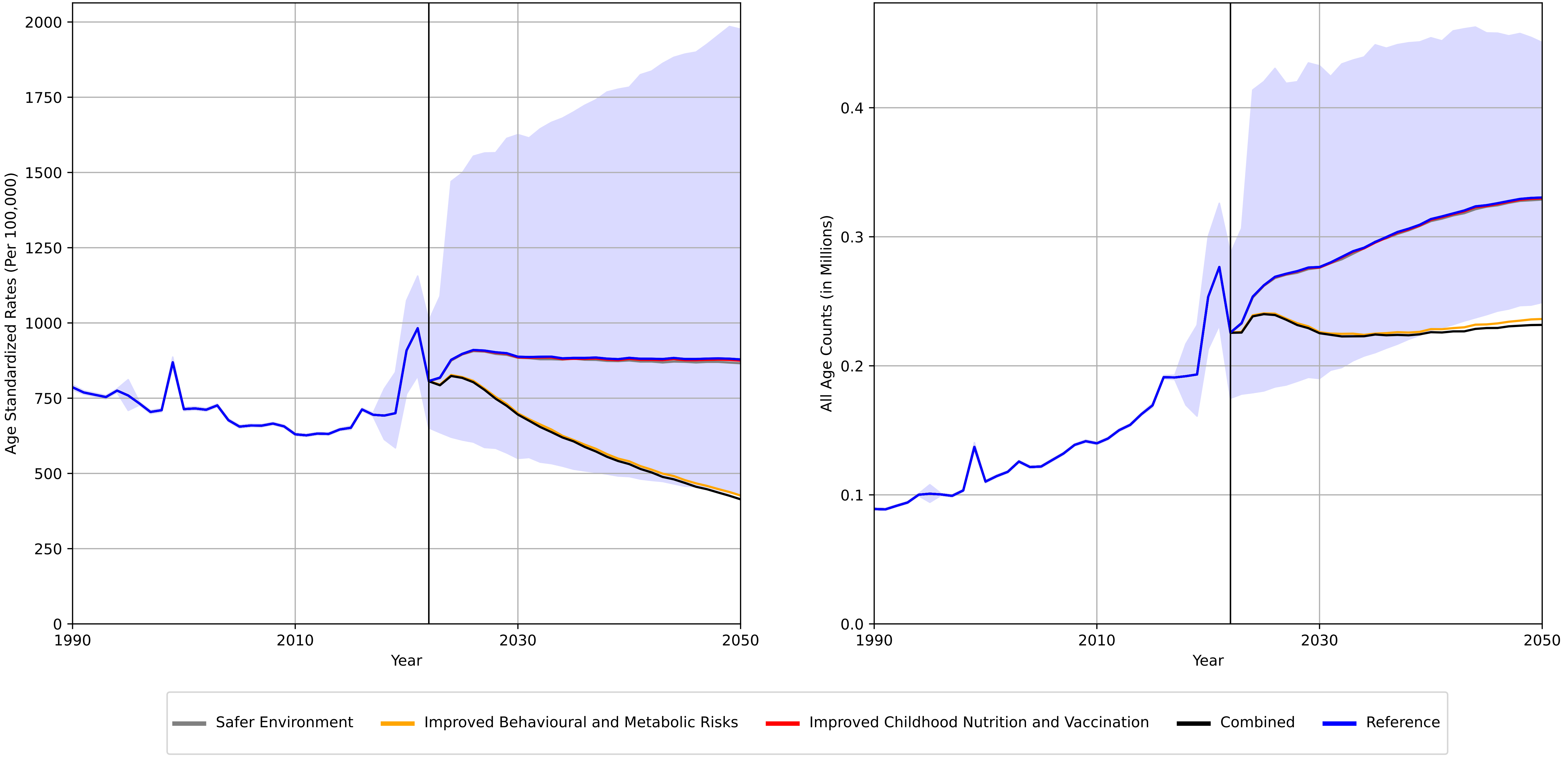

Age Standardized Rates (Per 100,000)
2000
1750
1500
1250
1000
750
500
250
0
1990
2010
2030
2050
Year
All Age Counts (in Millions)
0.4
0.3
0.2
0.1
0.0
1990
2010
2030
2050
Year
Safer Environment
Improved Behavioural and Metabolic Risks
Improved Childhood Nutrition and Vaccination
Combined
Reference

Brazil

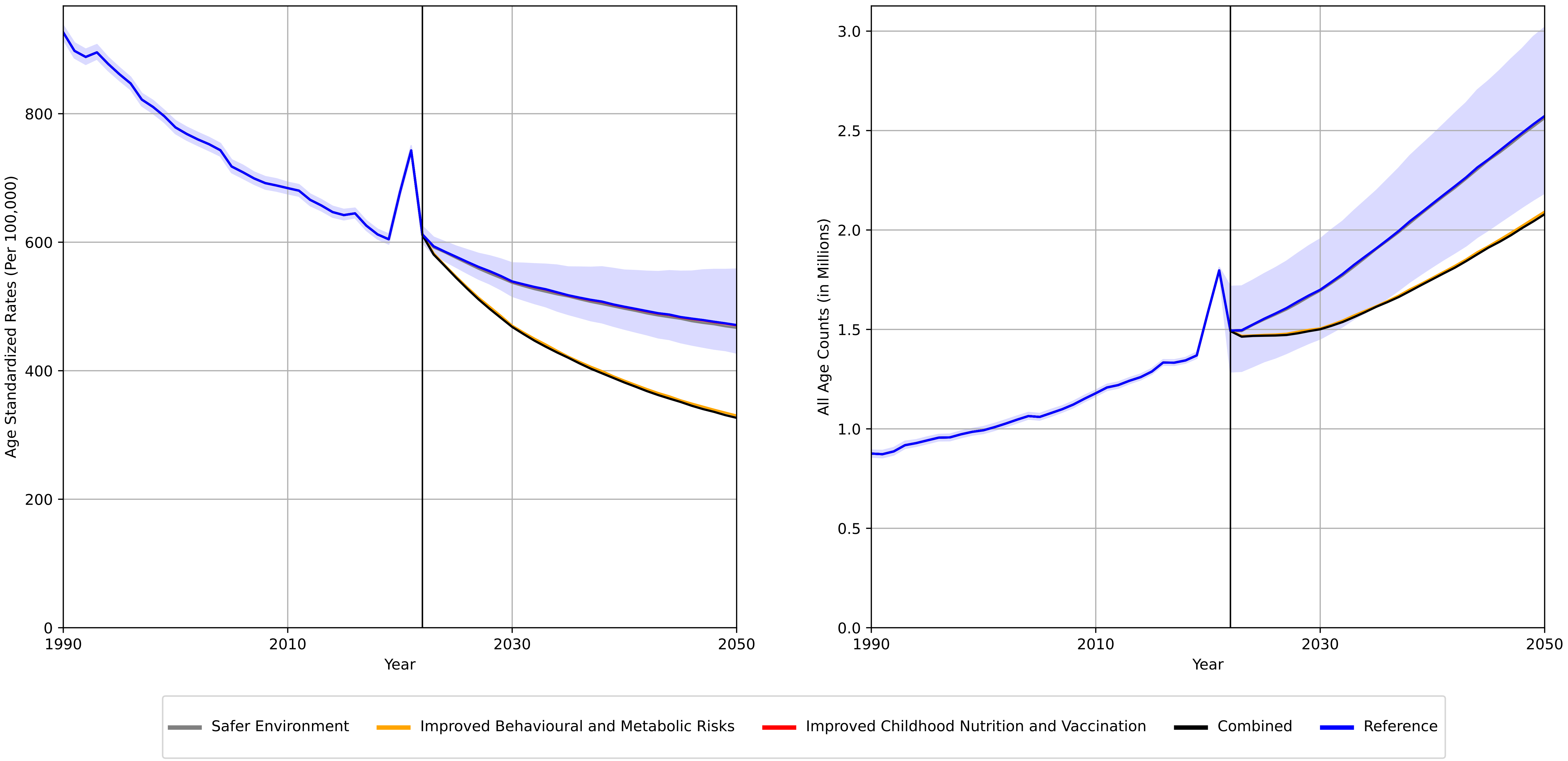
Age Standardized Rates (Per 100,000)
0
200
400
600
800
1990
2010
2030
2050
Year
All Age Counts (in Millions)
0.0
0.5
1.0
1.5
2.0
2.5
3.0
1990
2010
2030
2050
Year
Safer Environment
Improved Behavioural and Metabolic Risks
Improved Childhood Nutrition and Vaccination
Combined
Reference

Paraguay

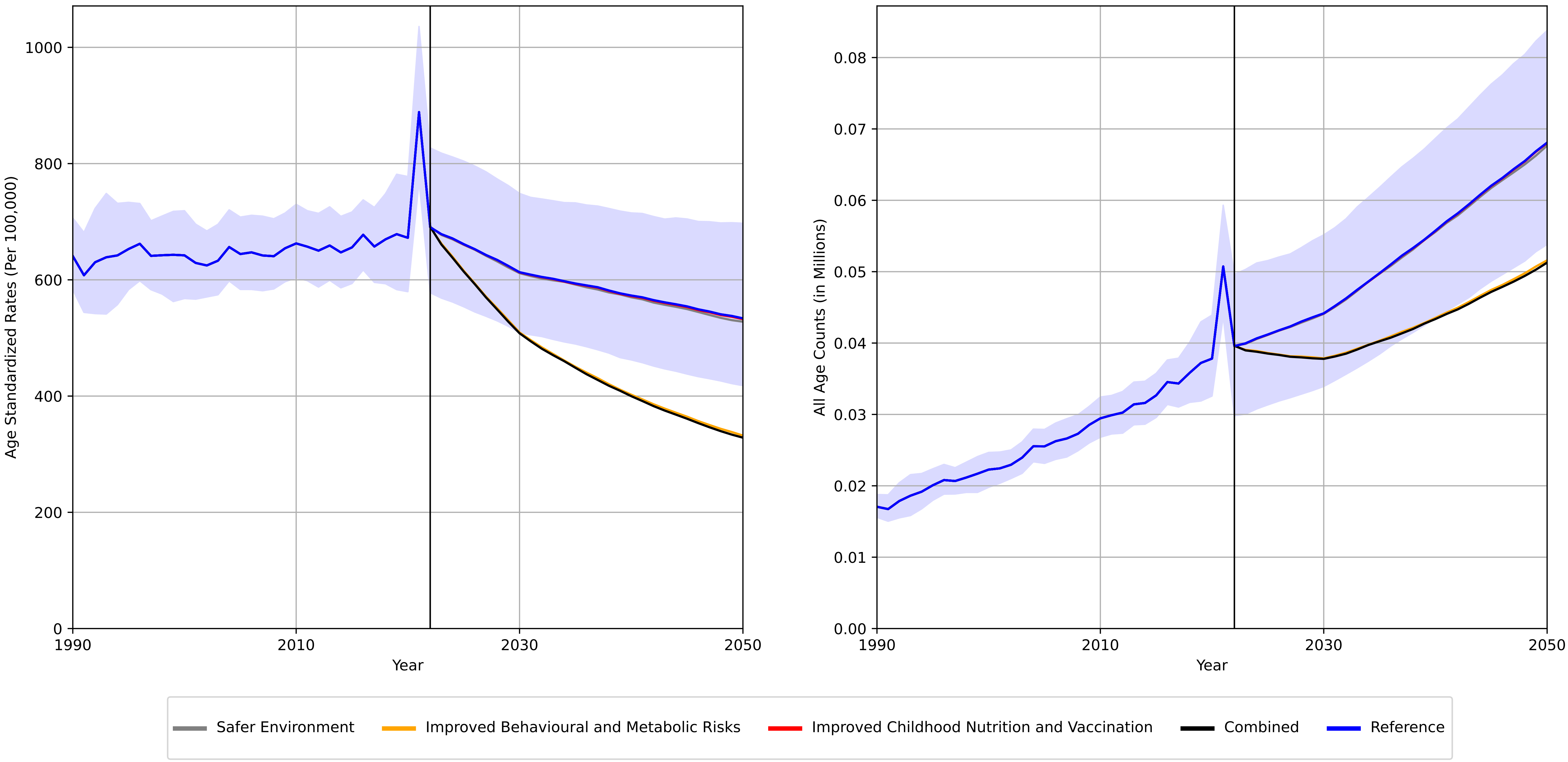

Age Standardized Rates (Per 100,000)
1000
800
600
400
200
0
1990
2010
2030
2050
Year
All Age Counts (in Millions)
0.08
0.07
0.06
0.05
0.04
0.03
0.02
0.01
0.00
1990
2010
2030
2050
Year
Safer Environment
Improved Behavioural and Metabolic Risks
Improved Childhood Nutrition and Vaccination
Combined
Reference

Afghanistan

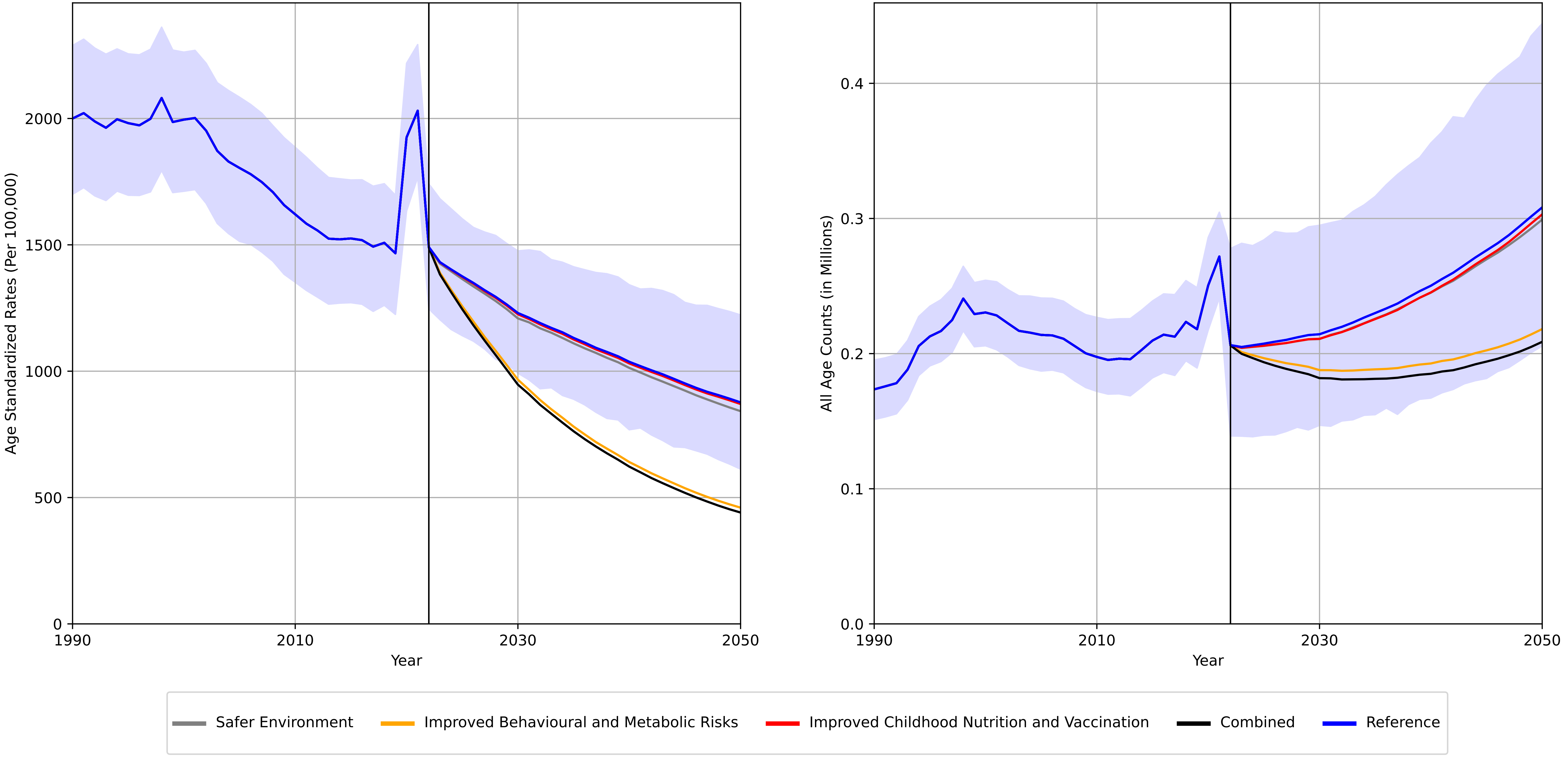

Algeria

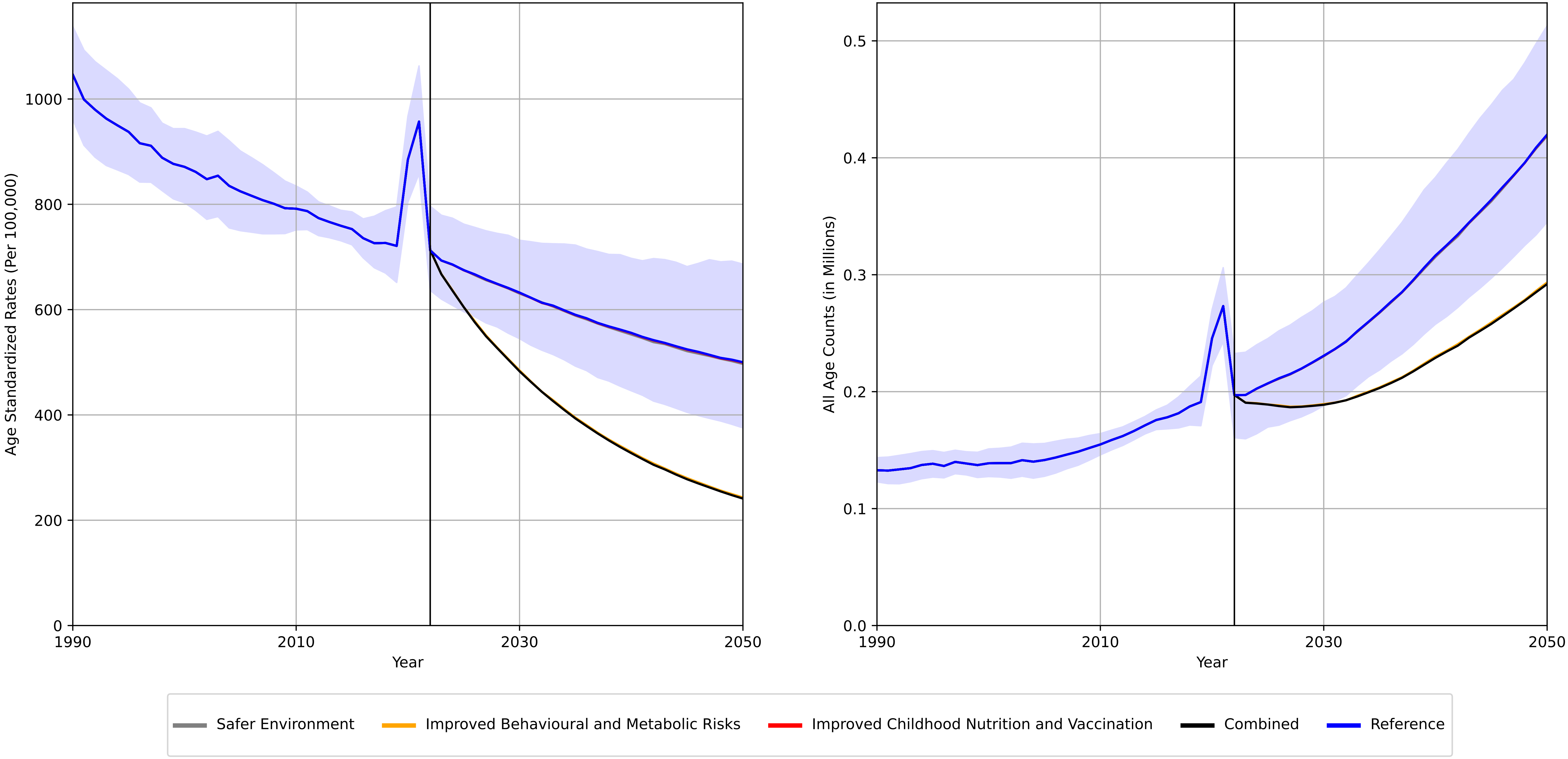

Age Standardized Rates (Per 100,000)
0
200
400
600
800
1000
1990
2010
2030
2050
Year
All Age Counts (in Millions)
0.0
0.1
0.2
0.3
0.4
0.5
1990
2010
2030
2050
Year
Safer Environment
Improved Behavioural and Metabolic Risks
Improved Childhood Nutrition and Vaccination
Combined
Reference

Bahrain

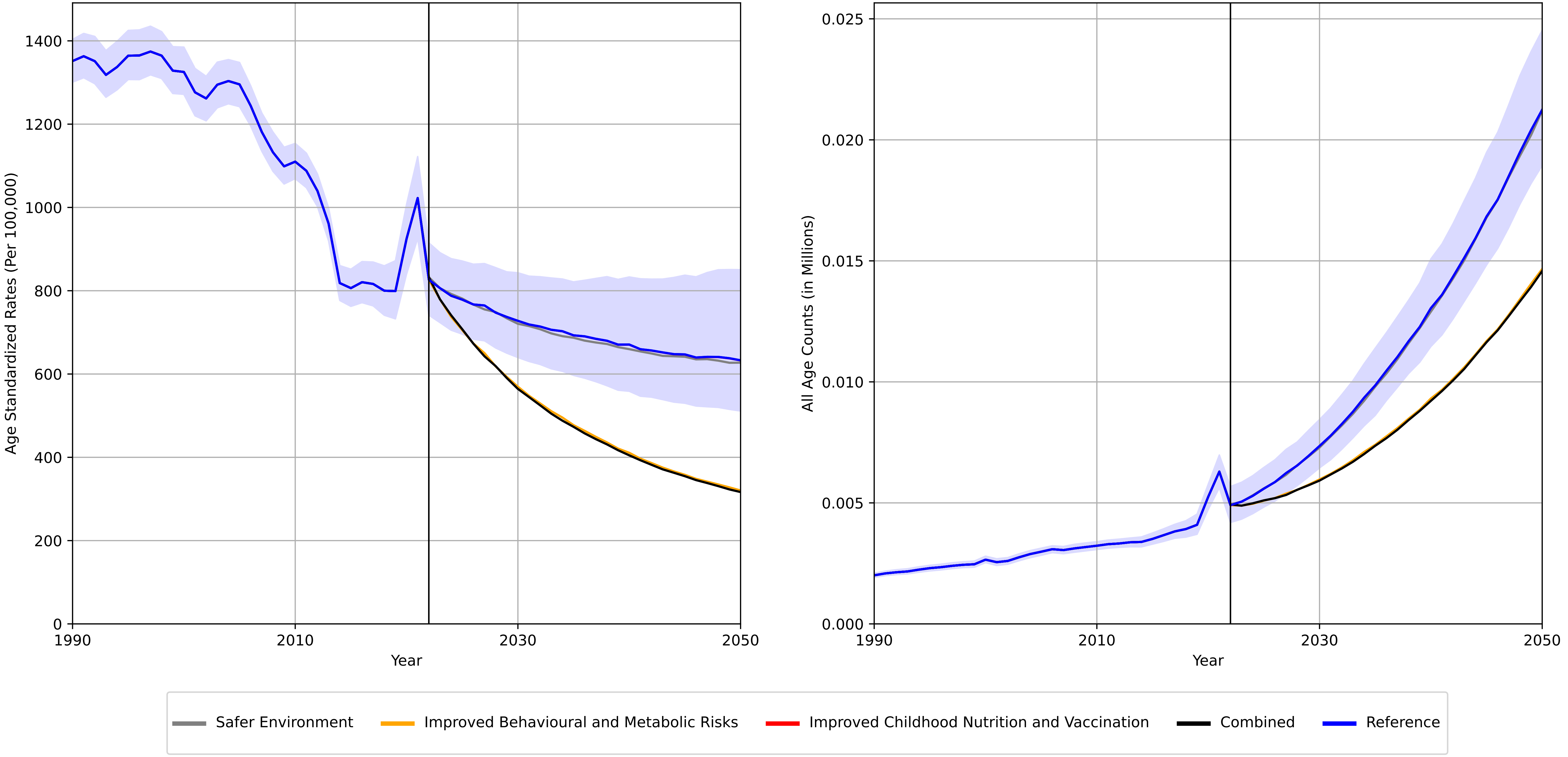

Age Standardized Rates (Per 100,000)
1400
1200
1000
800
600
400
200
0
1990
2010
2030
2050
Year
All Age Counts (in Millions)
0.025
0.020
0.015
0.010
0.005
0.000
1990
2010
2030
2050
Year
Safer Environment
Improved Behavioural and Metabolic Risks
Improved Childhood Nutrition and Vaccination
Combined
Reference

Egypt

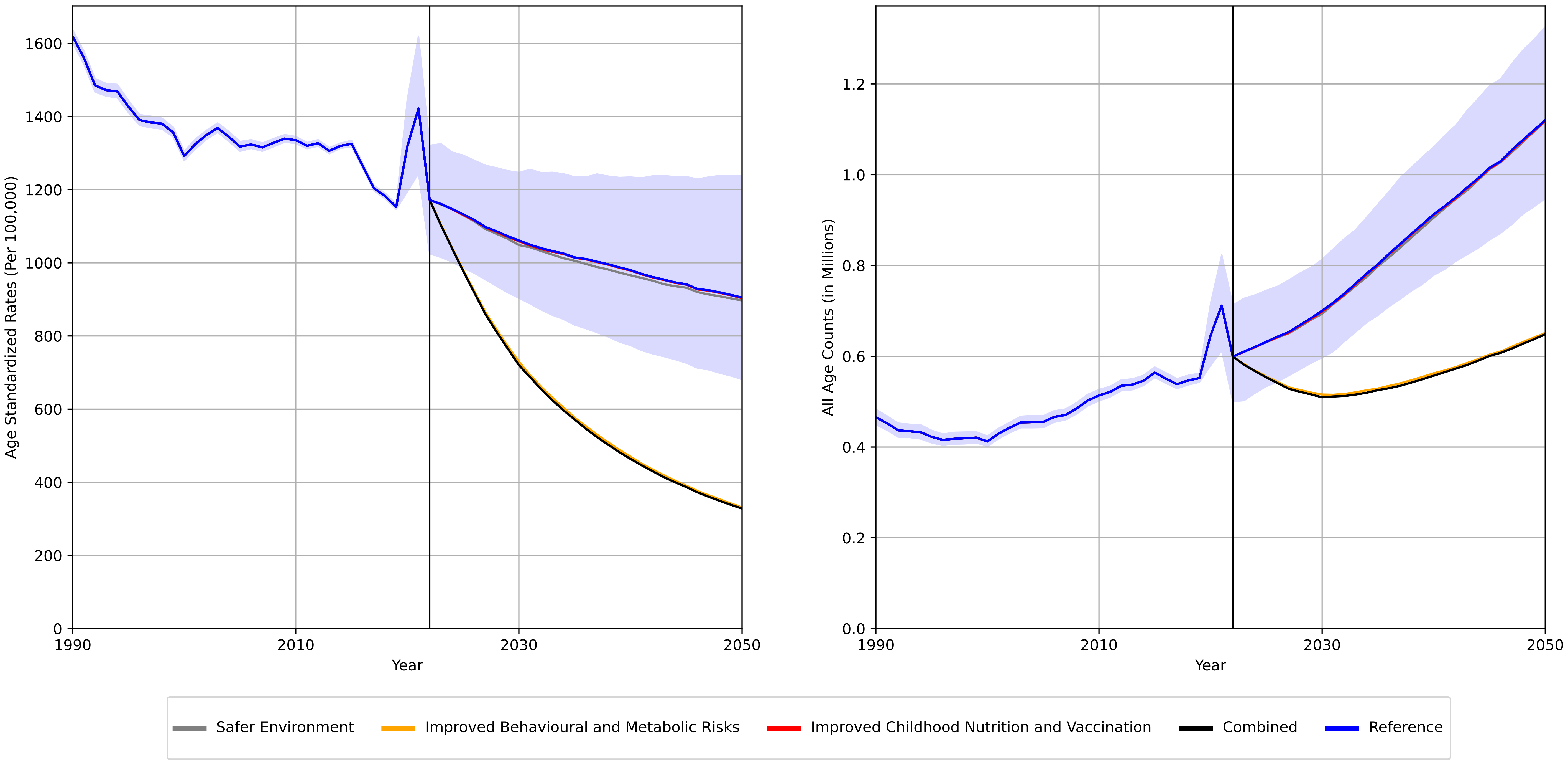
Age Standardized Rates (Per 100,000)
Year
All Age Counts (in Millions)
Year
Safer Environment
Improved Behavioural and Metabolic Risks
Improved Childhood Nutrition and Vaccination
Combined
Reference

Iran

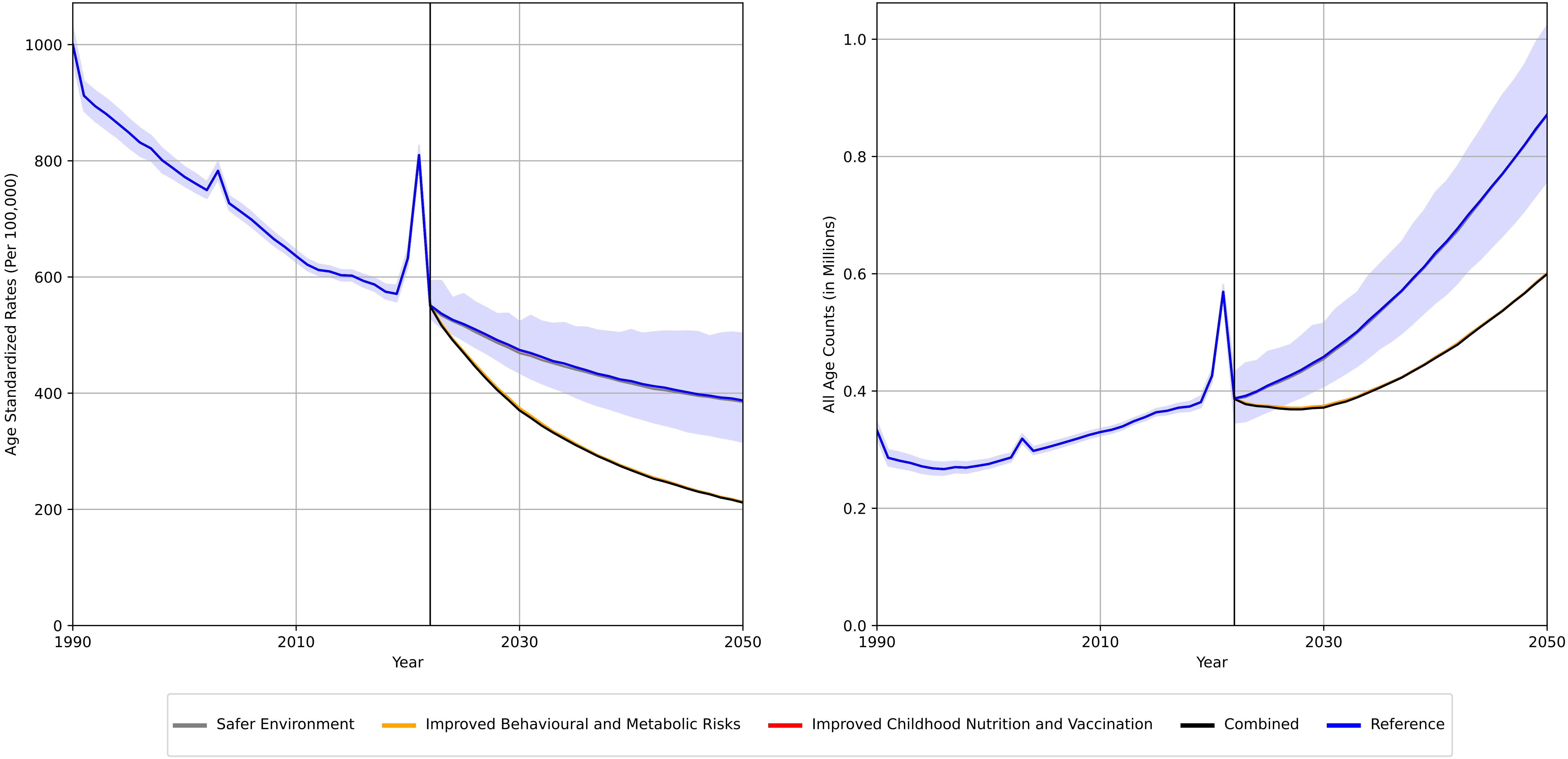

Age Standardized Rates (Per 100,000)
0
200
400
600
800
1000
1990
2010
2030
2050
Year
All Age Counts (in Millions)
0.0
0.2
0.4
0.6
0.8
1.0
1990
2010
2030
2050
Year
Safer Environment
Improved Behavioural and Metabolic Risks
Improved Childhood Nutrition and Vaccination
Combined
Reference

Iraq

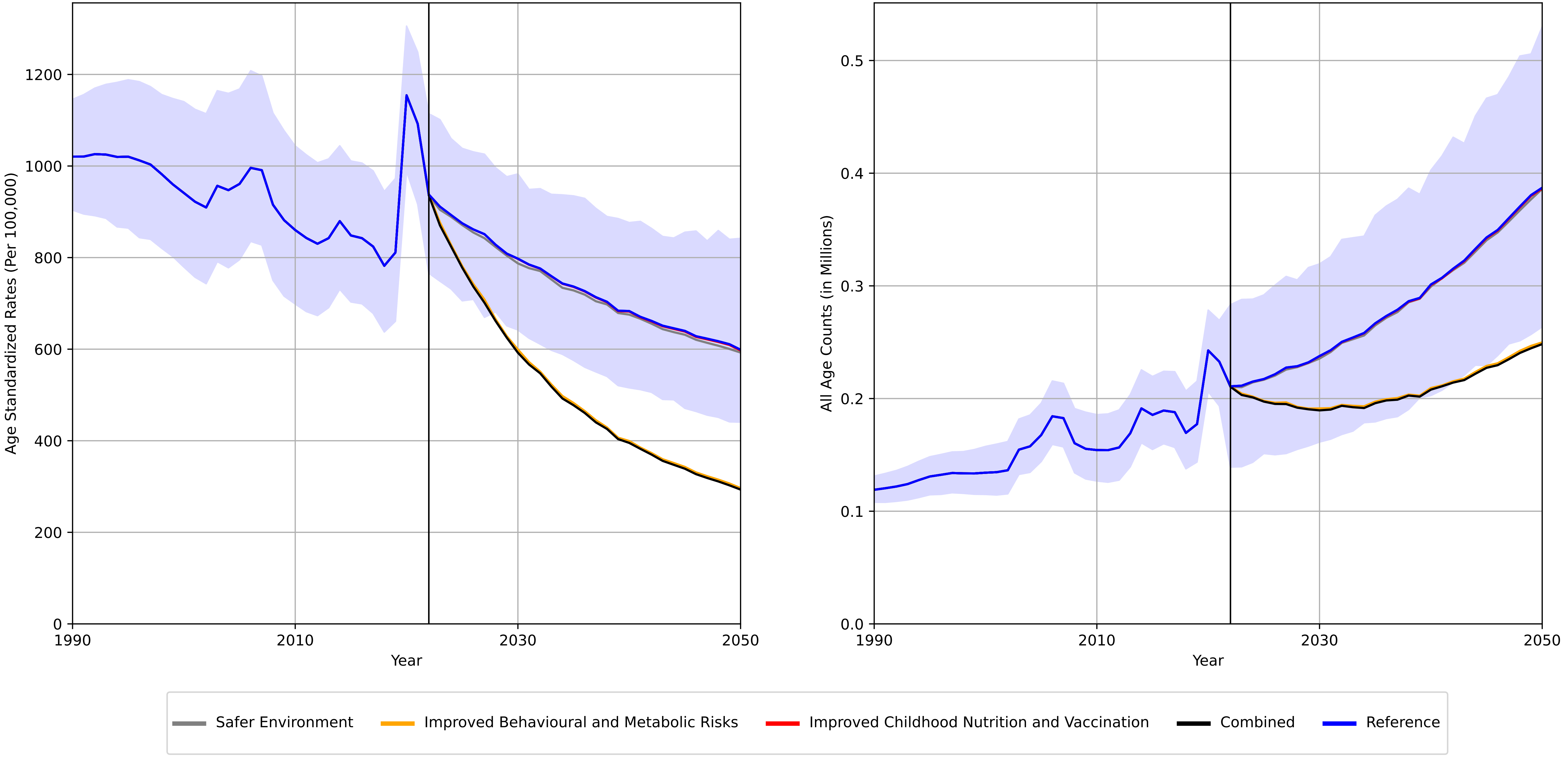

Age Standardized Rates (Per 100,000)
0
200
400
600
800
1000
1200
1990
2010
2030
2050
Year
All Age Counts (in Millions)
0.0
0.1
0.2
0.3
0.4
0.5
1990
2010
2030
2050
Year
Safer Environment
Improved Behavioural and Metabolic Risks
Improved Childhood Nutrition and Vaccination
Combined
Reference

Jordan

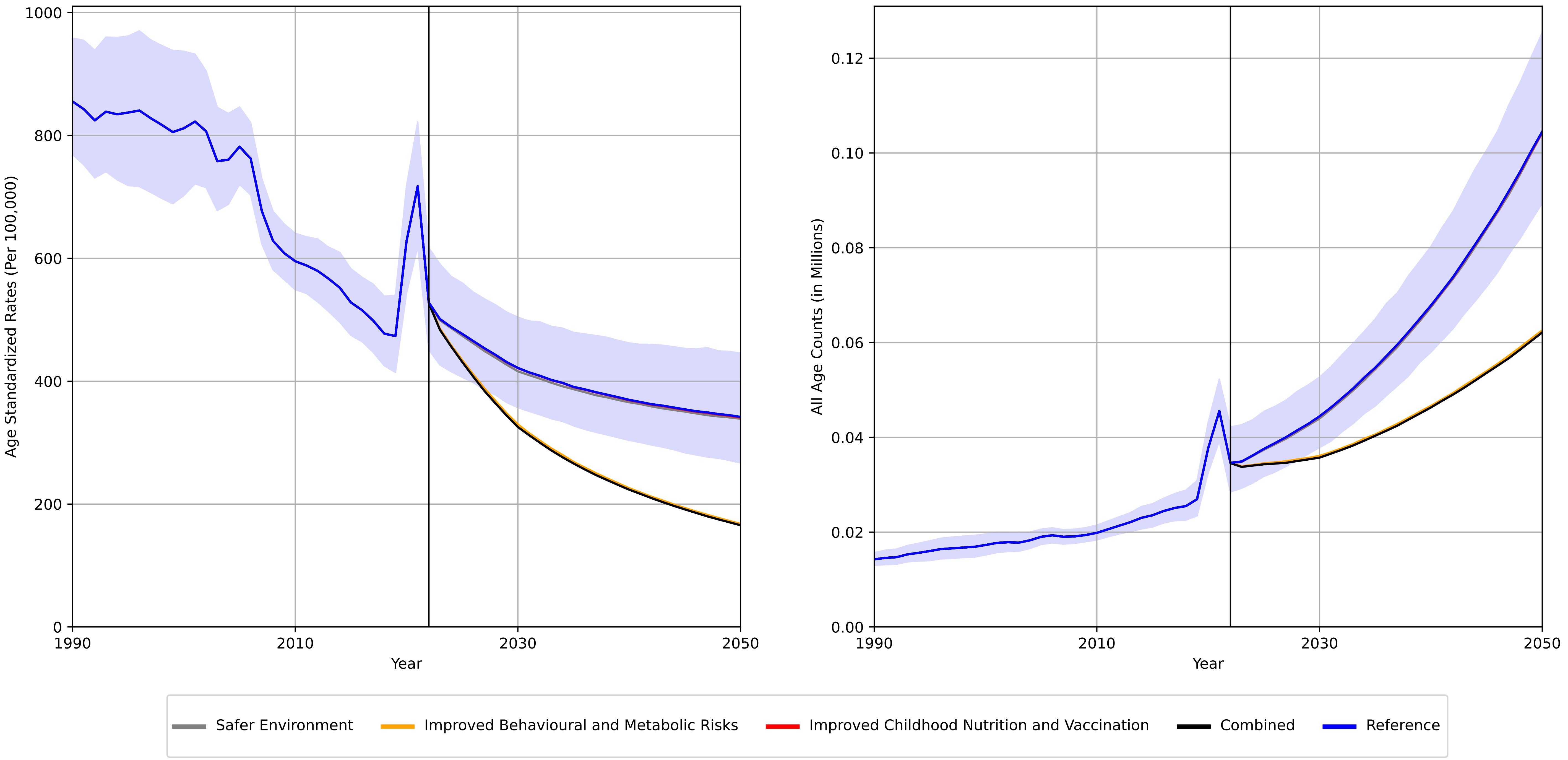

Age Standardized Rates (Per 100,000)
0
200
400
600
800
1000
1990
2010
2030
2050
Year
All Age Counts (in Millions)
0.00
0.02
0.04
0.06
0.08
0.10
0.12
1990
2010
2030
2050
Year
Safer Environment
Improved Behavioural and Metabolic Risks
Improved Childhood Nutrition and Vaccination
Combined
Reference

Kuwait

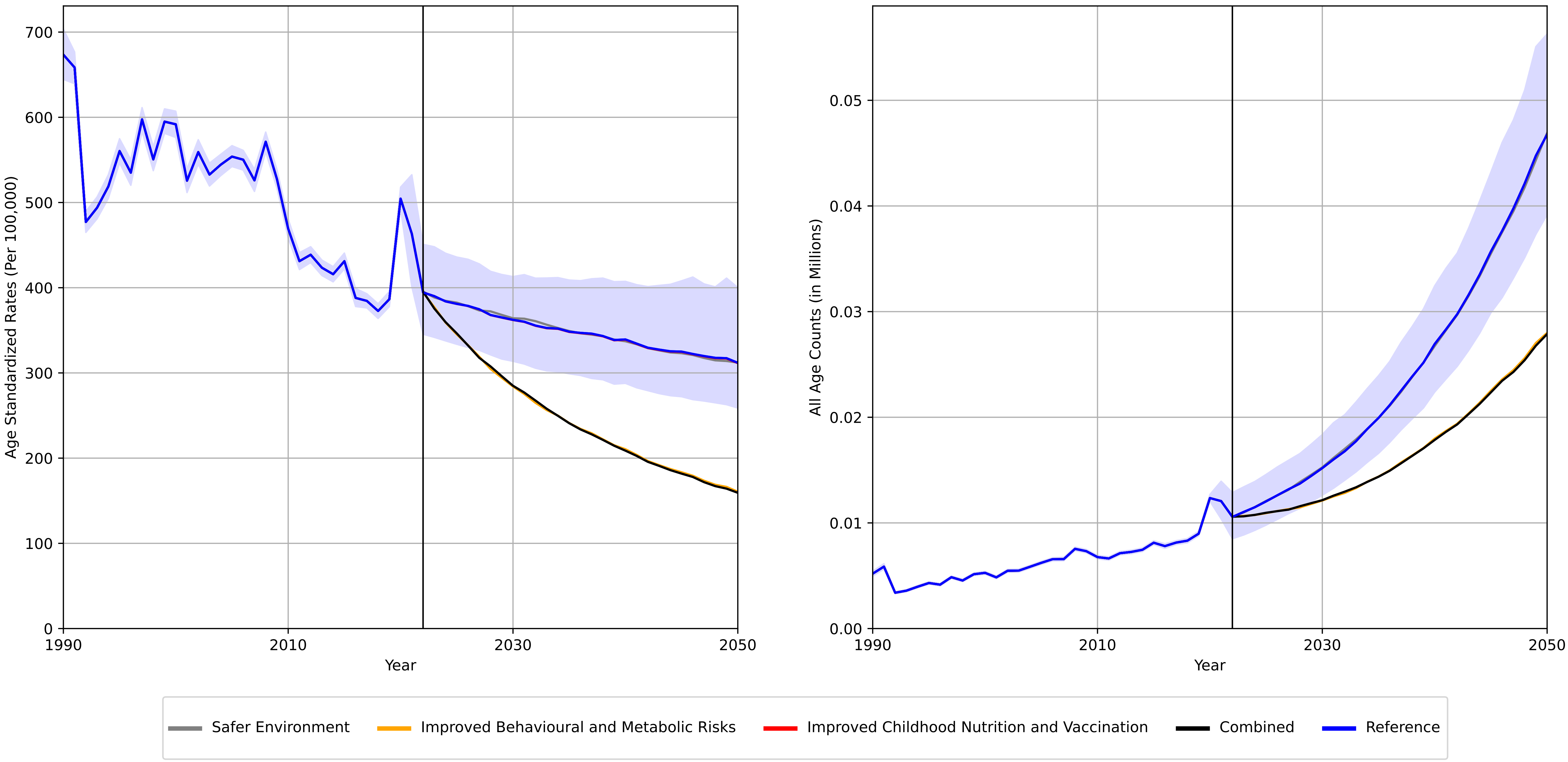

Age Standardized Rates (Per 100,000)
700
600
500
400
300
200
100
0
1990
2010
2030
2050
Year
All Age Counts (in Millions)
0.05
0.04
0.03
0.02
0.01
0.00
1990
2010
2030
2050
Year
Safer Environment
Improved Behavioural and Metabolic Risks
Improved Childhood Nutrition and Vaccination
Combined
Reference

Lebanon

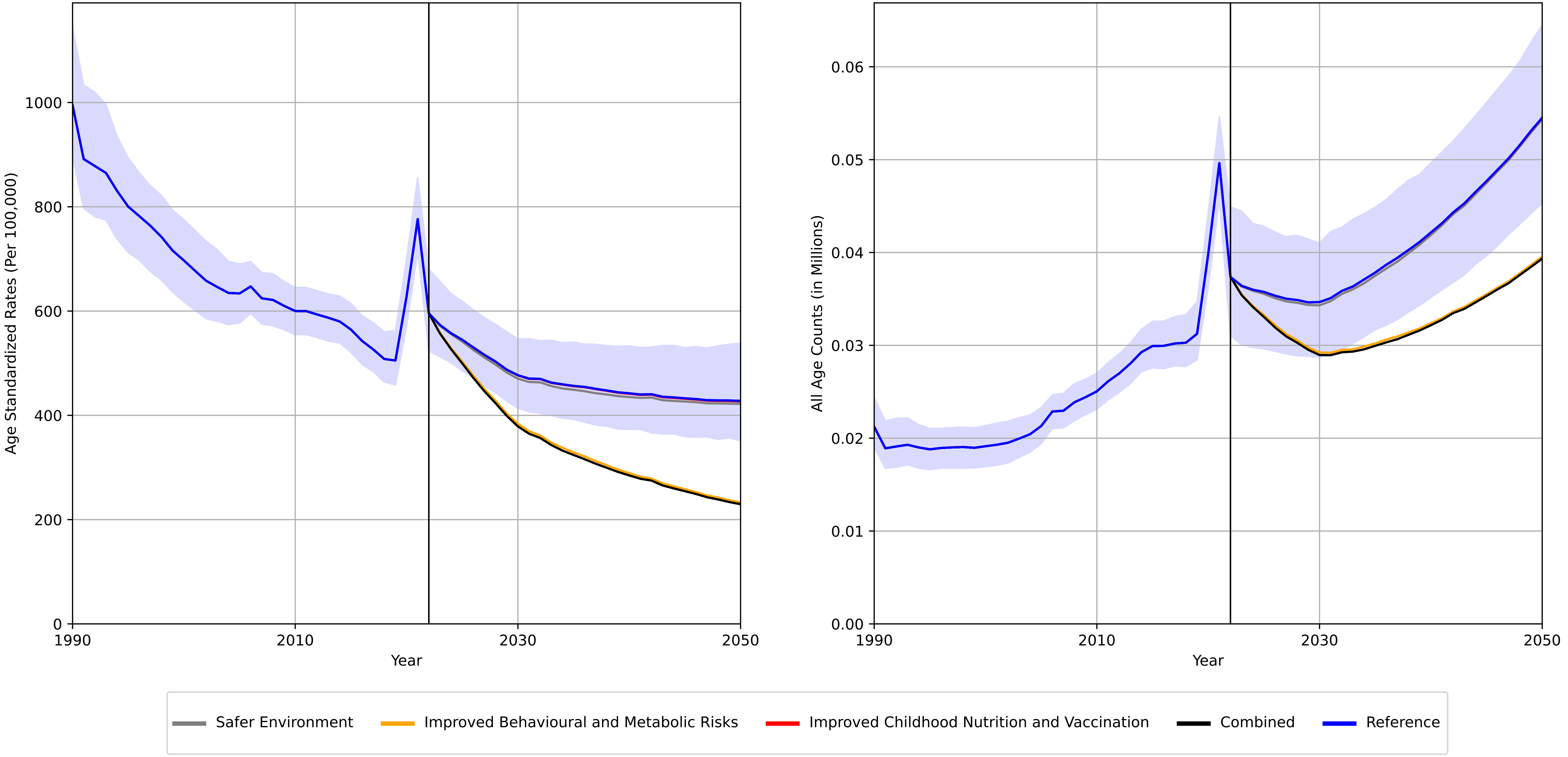
Age Standardized Rates (Per 100,000)
0
200
400
600
800
1000
1990
2010
2030
2050
Year
All Age Counts (in Millions)
0.00
0.01
0.02
0.03
0.04
0.05
0.06
1990
2010
2030
2050
Year
Safer Environment
Improved Behavioural and Metabolic Risks
Improved Childhood Nutrition and Vaccination
Combined
Reference

Libya

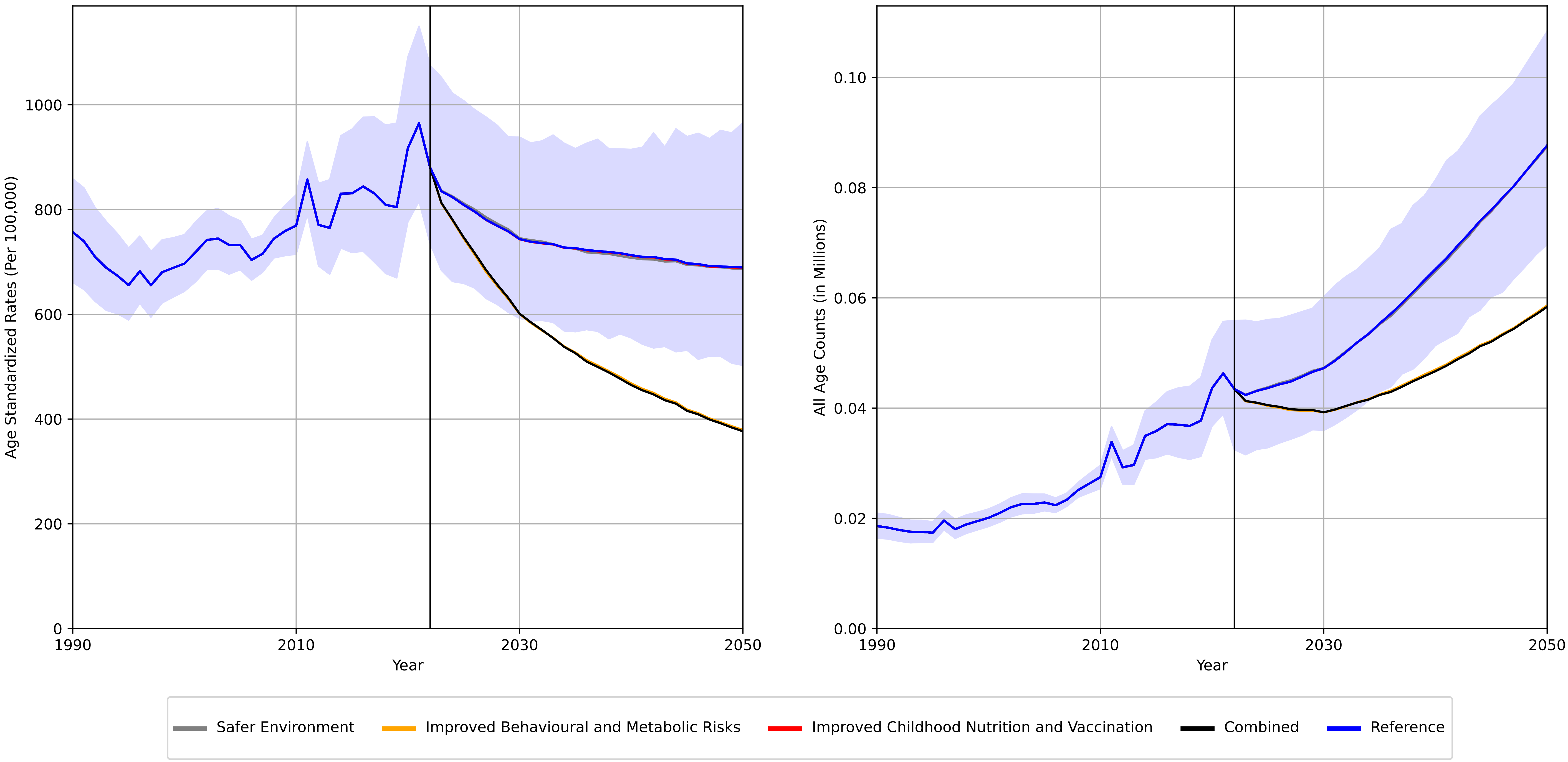
Age Standardized Rates (Per 100,000)
0
200
400
600
800
1000
1990
2010
2030
2050
Year
All Age Counts (in Millions)
0.00
0.02
0.04
0.06
0.08
0.10
1990
2010
2030
2050
Year
Safer Environment
Improved Behavioural and Metabolic Risks
Improved Childhood Nutrition and Vaccination
Combined
Reference

Morocco

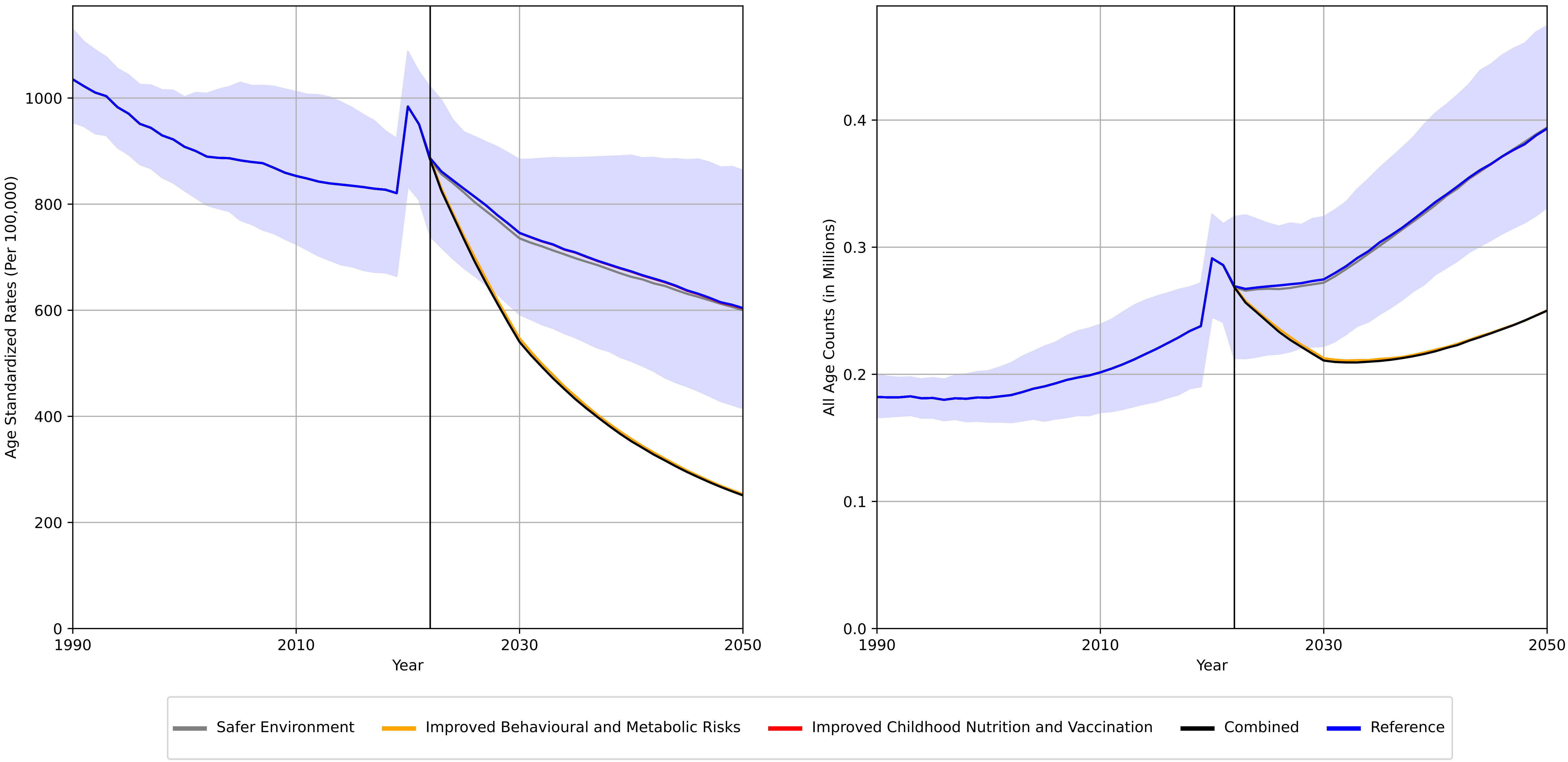

Age Standardized Rates (Per 100,000)
0
200
400
600
800
1000
1990
2010
2030
2050
Year
All Age Counts (in Millions)
0.0
0.1
0.2
0.3
0.4
1990
2010
2030
2050
Year
Safer Environment
Improved Behavioural and Metabolic Risks
Improved Childhood Nutrition and Vaccination
Combined
Reference

Oman

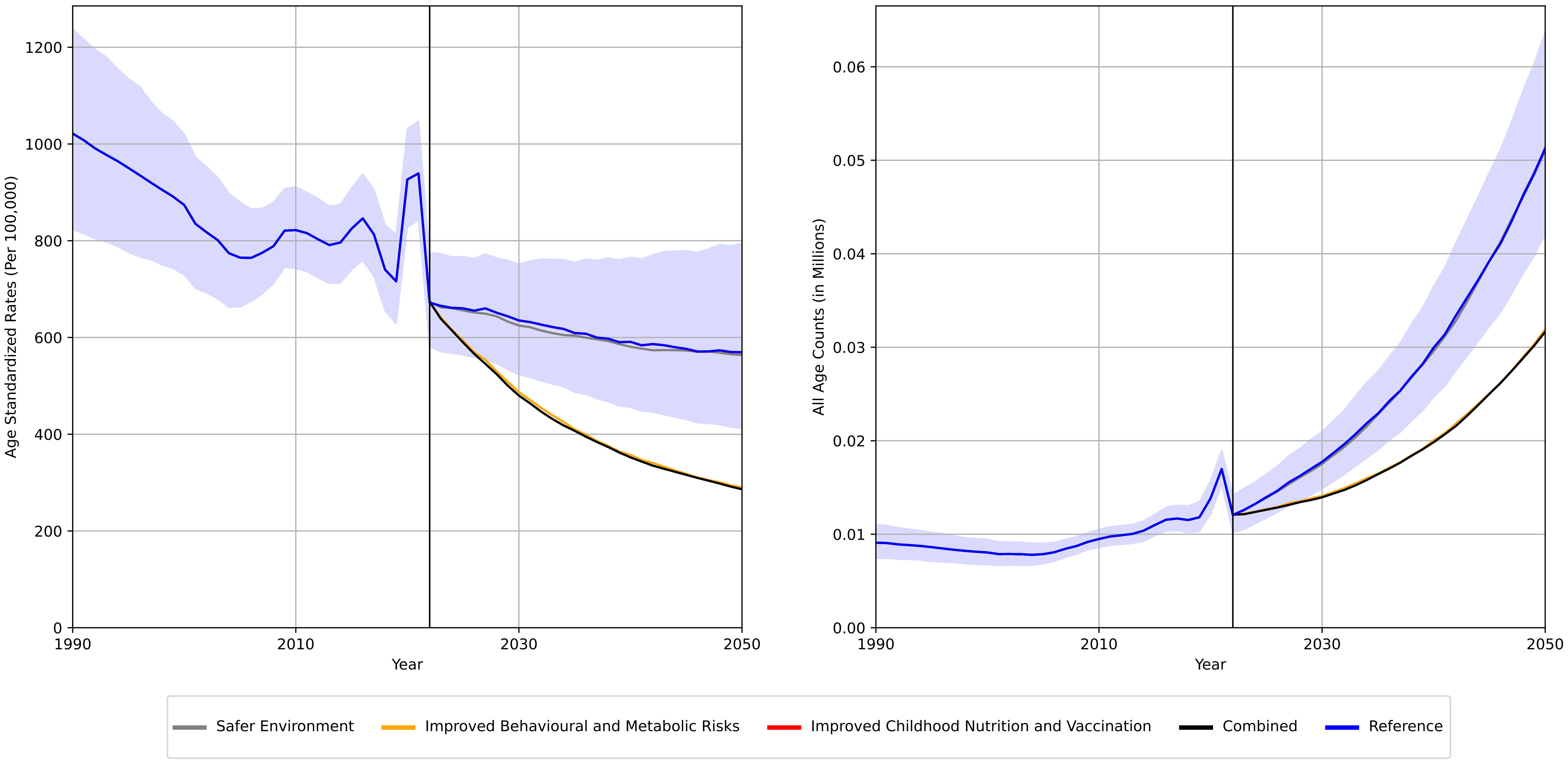
Age Standardized Rates (Per 100,000)
0
200
400
600
800
1000
1200
1990
2010
2030
2050
Year
All Age Counts (in Millions)
0.00
0.01
0.02
0.03
0.04
0.05
0.06
1990
2010
2030
2050
Year
Safer Environment
Improved Behavioural and Metabolic Risks
Improved Childhood Nutrition and Vaccination
Combined
Reference

Palestine

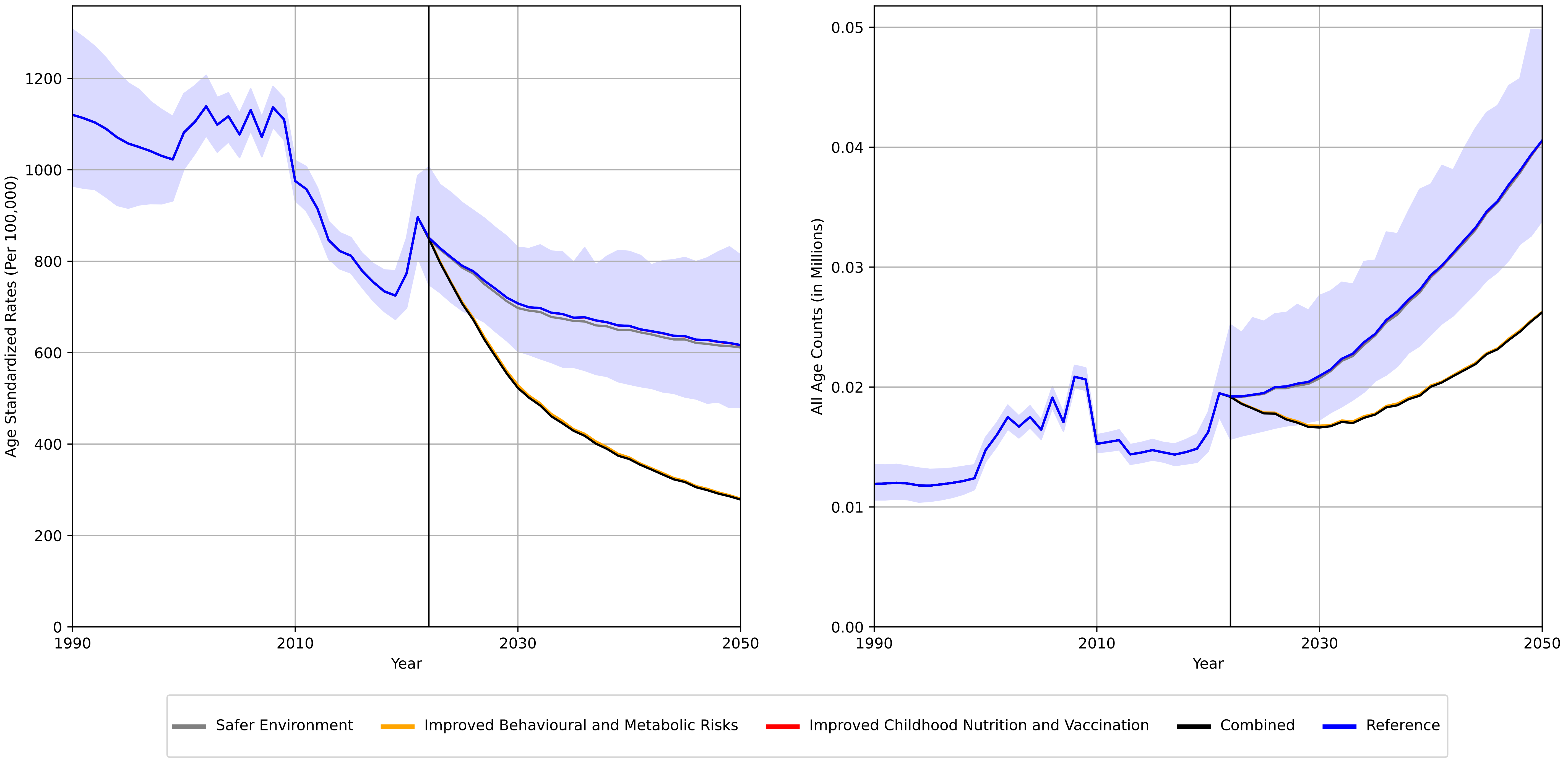
Age Standardized Rates (Per 100,000)
0
200
400
600
800
1000
1200
1990
2010
2030
2050
Year
All Age Counts (in Millions)
0.00
0.01
0.02
0.03
0.04
0.05
1990
2010
2030
2050
Year
Safer Environment
Improved Behavioural and Metabolic Risks
Improved Childhood Nutrition and Vaccination
Combined
Reference

Qatar

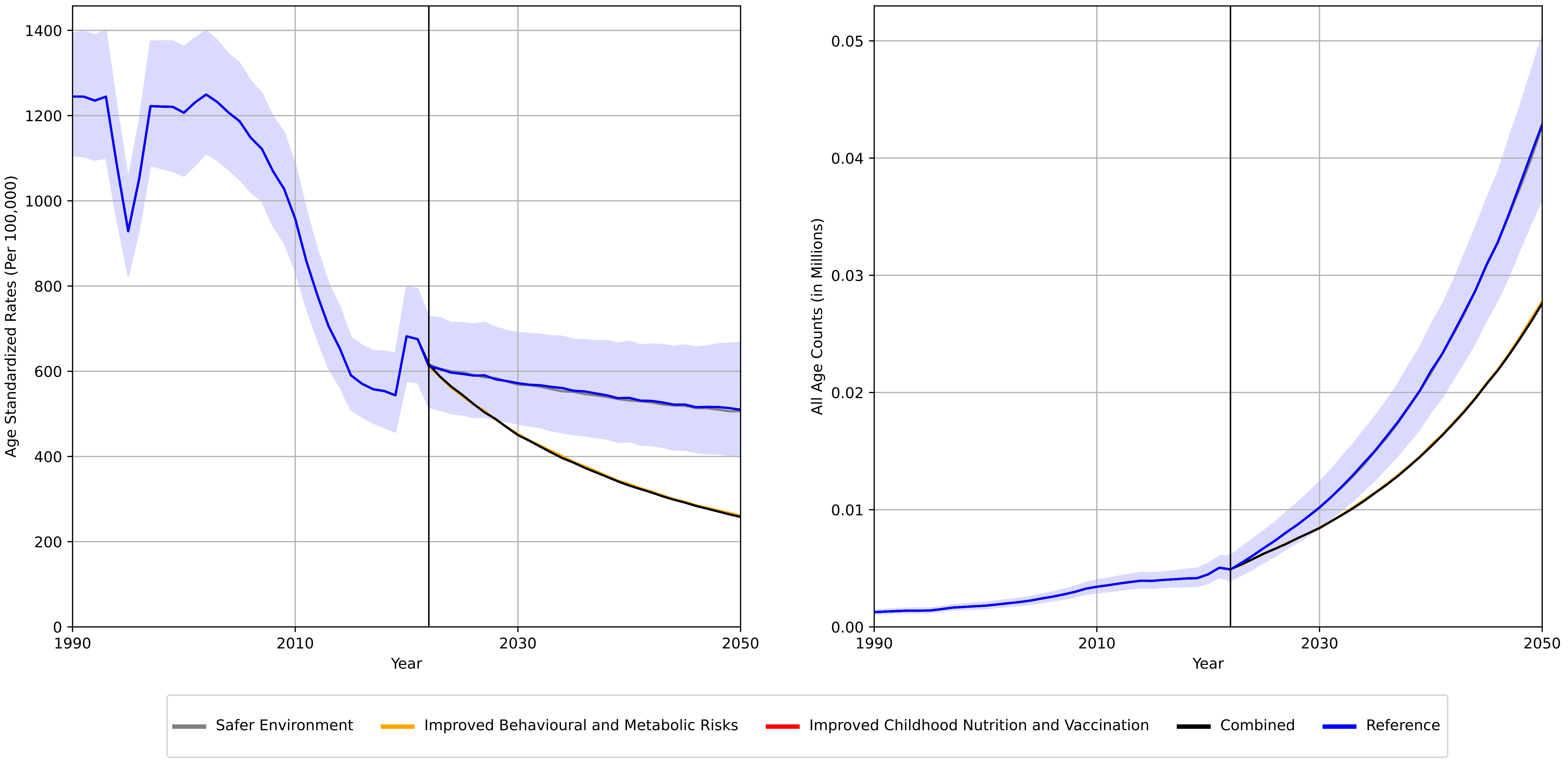

Age Standardized Rates (Per 100,000)
1400
1200
1000
800
600
400
200
0
1990
2010
2030
2050
Year
All Age Counts (in Millions)
0.05
0.04
0.03
0.02
0.01
0.00
1990
2010
2030
2050
Year
Safer Environment
Improved Behavioural and Metabolic Risks
Improved Childhood Nutrition and Vaccination
Combined
Reference

Saudi Arabia

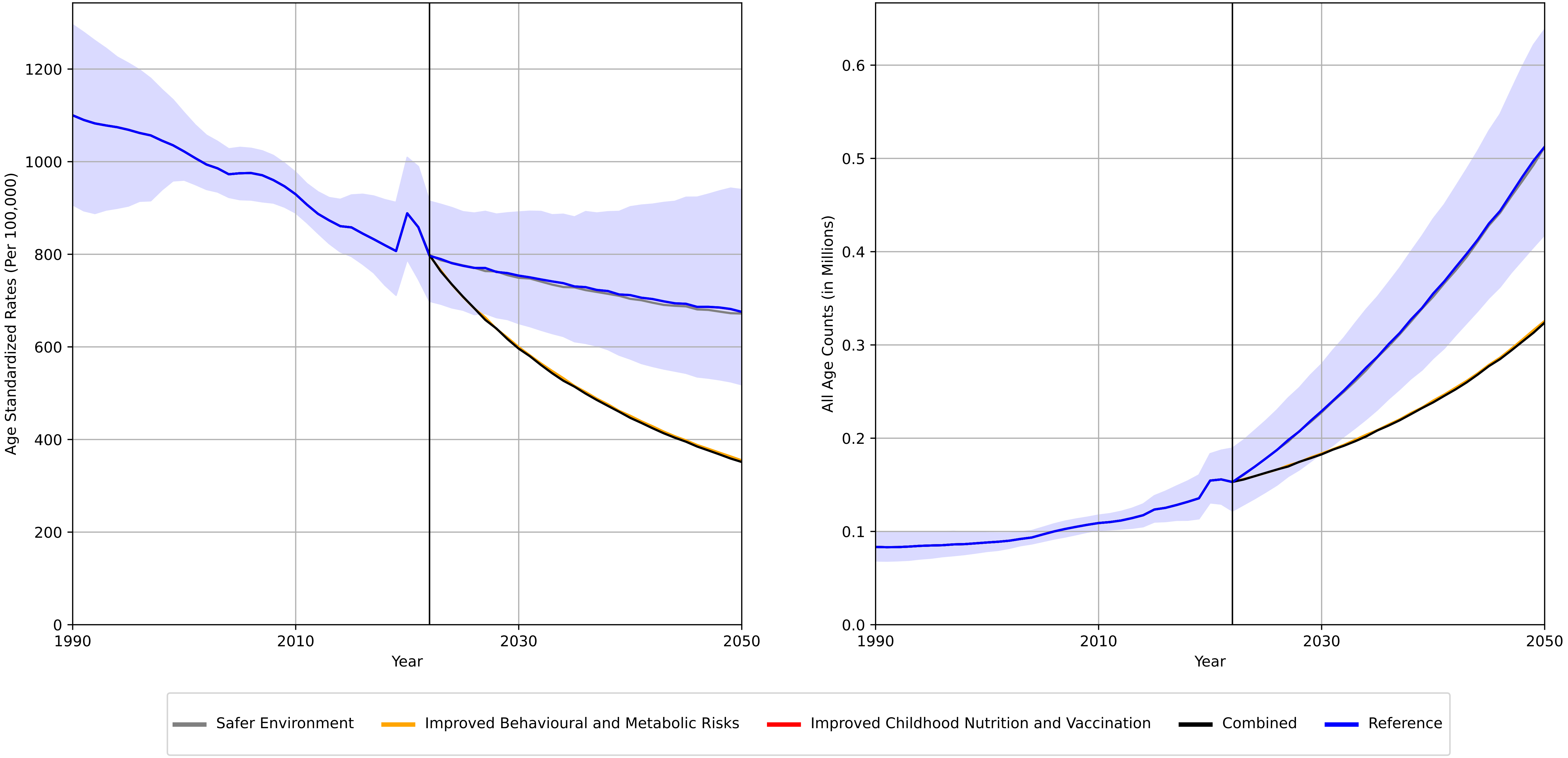

Sudan

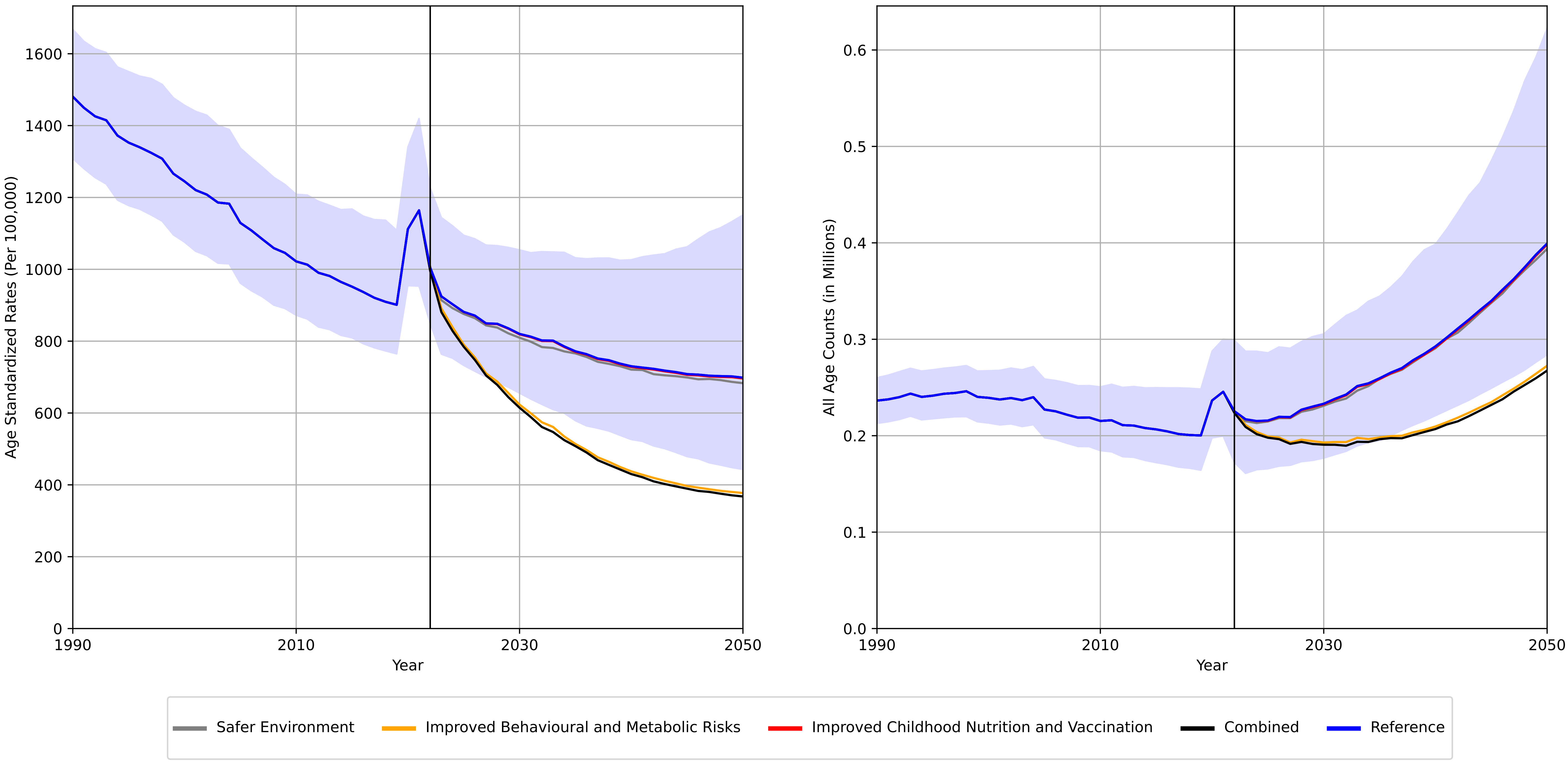
Age Standardized Rates (Per 100,000)
0
200
400
600
800
1000
1200
1400
1600
1990
2010
2030
2050
Year
All Age Counts (in Millions)
0.0
0.1
0.2
0.3
0.4
0.5
0.6
Safer Environment
Improved Behavioural and Metabolic Risks
Improved Childhood Nutrition and Vaccination
Combined
Reference

Syria

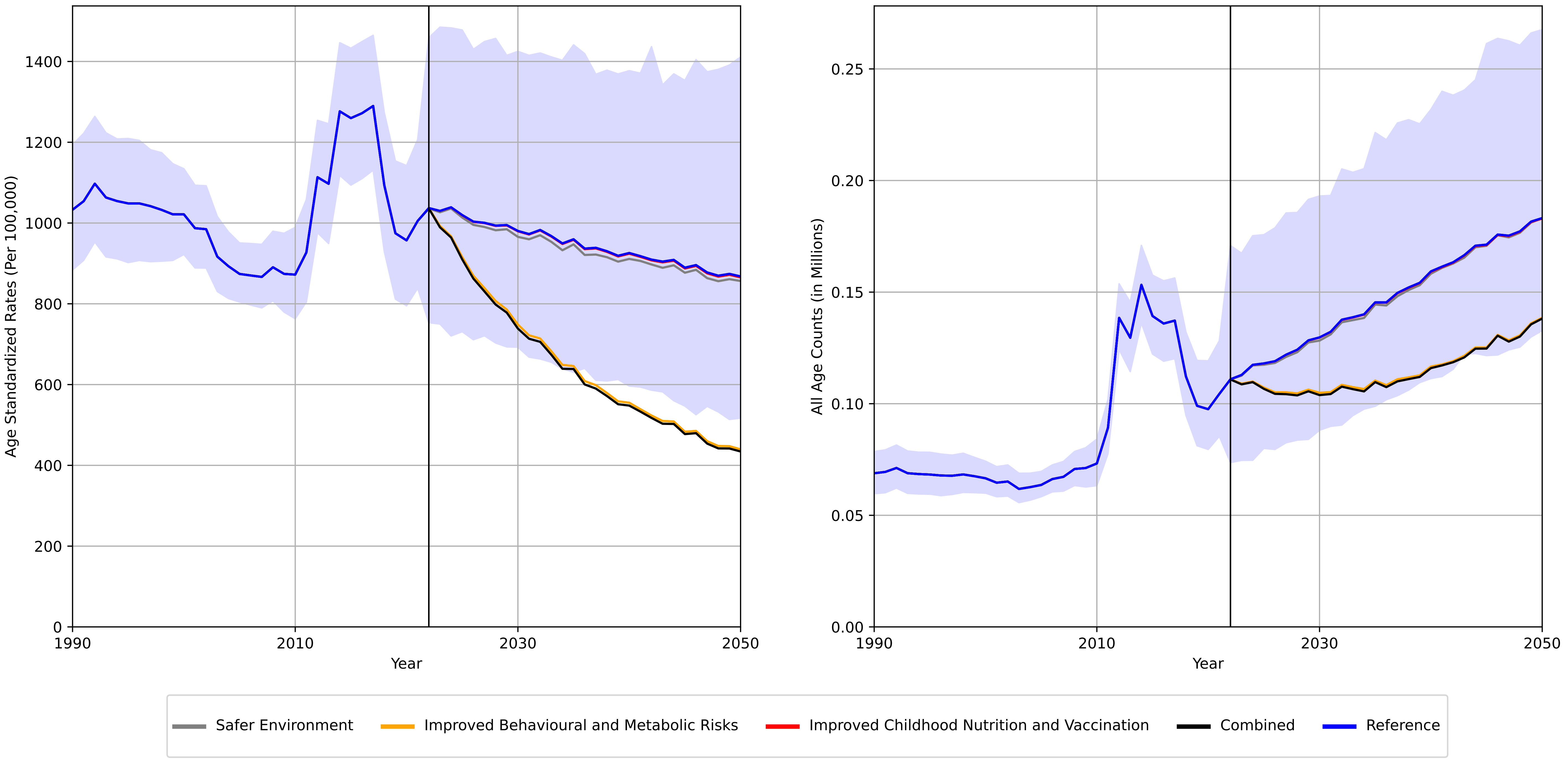

Age Standardized Rates (Per 100,000)
0
200
400
600
800
1000
1200
1400
1990
2010
2030
2050
Year
All Age Counts (in Millions)
0.00
0.05
0.10
0.15
0.20
0.25
Year
Safer Environment
Improved Behavioural and Metabolic Risks
Improved Childhood Nutrition and Vaccination
Combined
Reference

Tunisia

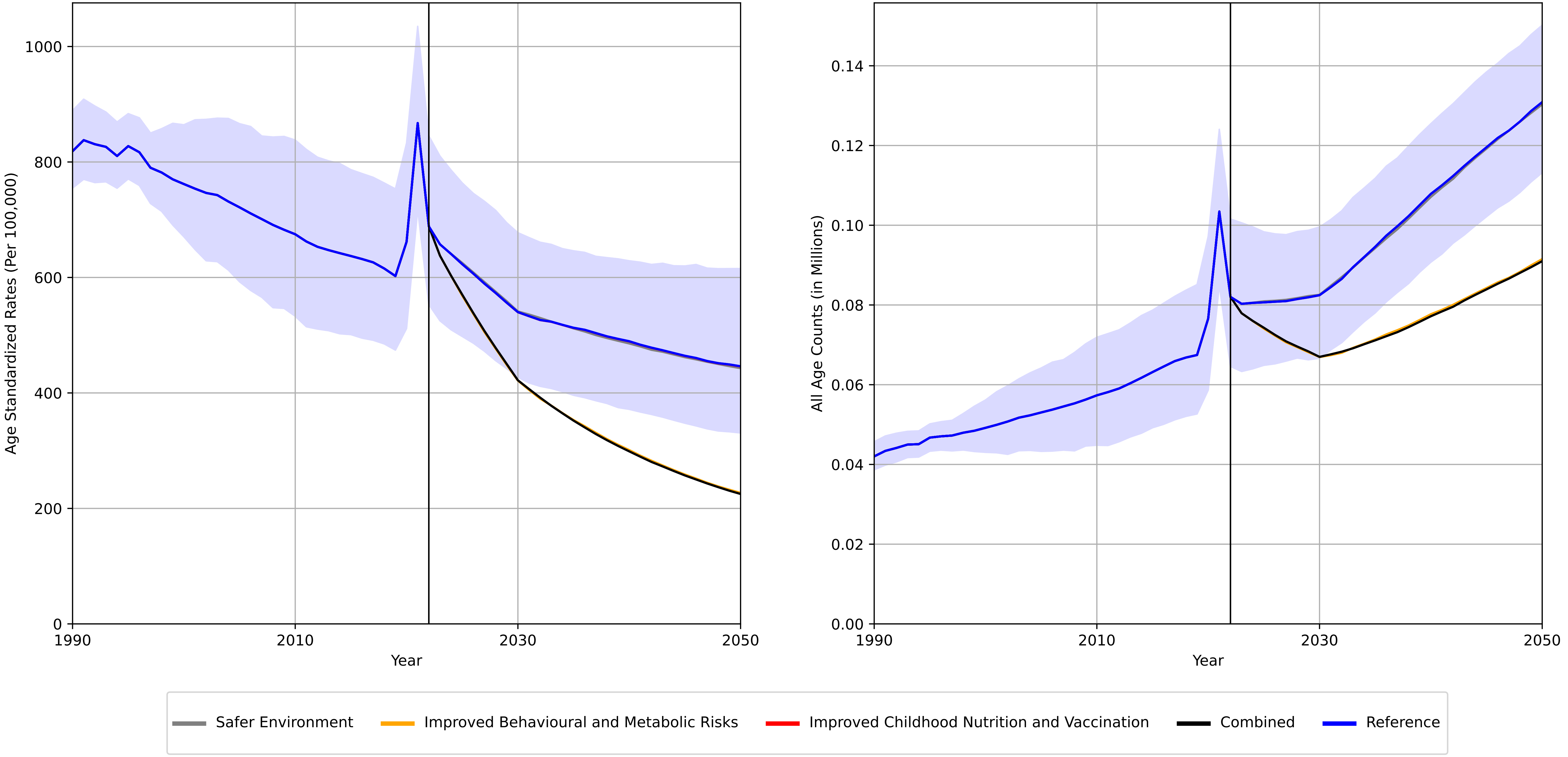
Age Standardized Rates (Per 100,000)
0
200
400
600
800
1000
1990
2010
2030
2050
Year
All Age Counts (in Millions)
0.00
0.02
0.04
0.06
0.08
0.10
0.12
0.14
1990
2010
2030
2050
Year
Safer Environment
Improved Behavioural and Metabolic Risks
Improved Childhood Nutrition and Vaccination
Combined
Reference

Türkiye

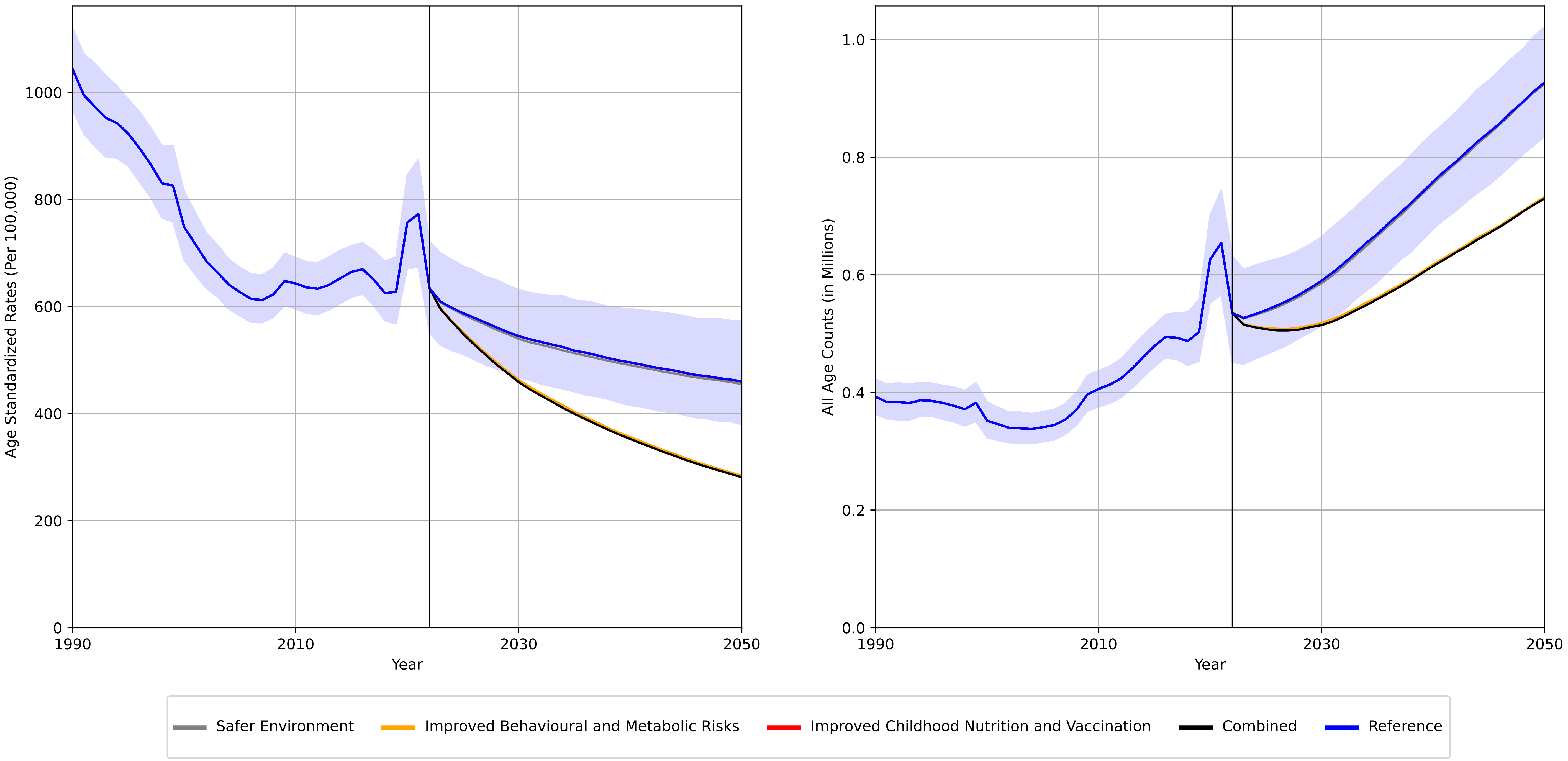

Age Standardized Rates (Per 100,000)
0
200
400
600
800
1000
1990
2010
2030
2050
Year
All Age Counts (in Millions)
0.0
0.2
0.4
0.6
0.8
1.0
1990
2010
2030
2050
Year
Safer Environment
Improved Behavioural and Metabolic Risks
Improved Childhood Nutrition and Vaccination
Combined
Reference

United Arab Emirates

Supplemental Results Figure S11. All-cause age standardised death rates (A) and all age death counts (B), for the past and for five future scenarios, 1990–2050

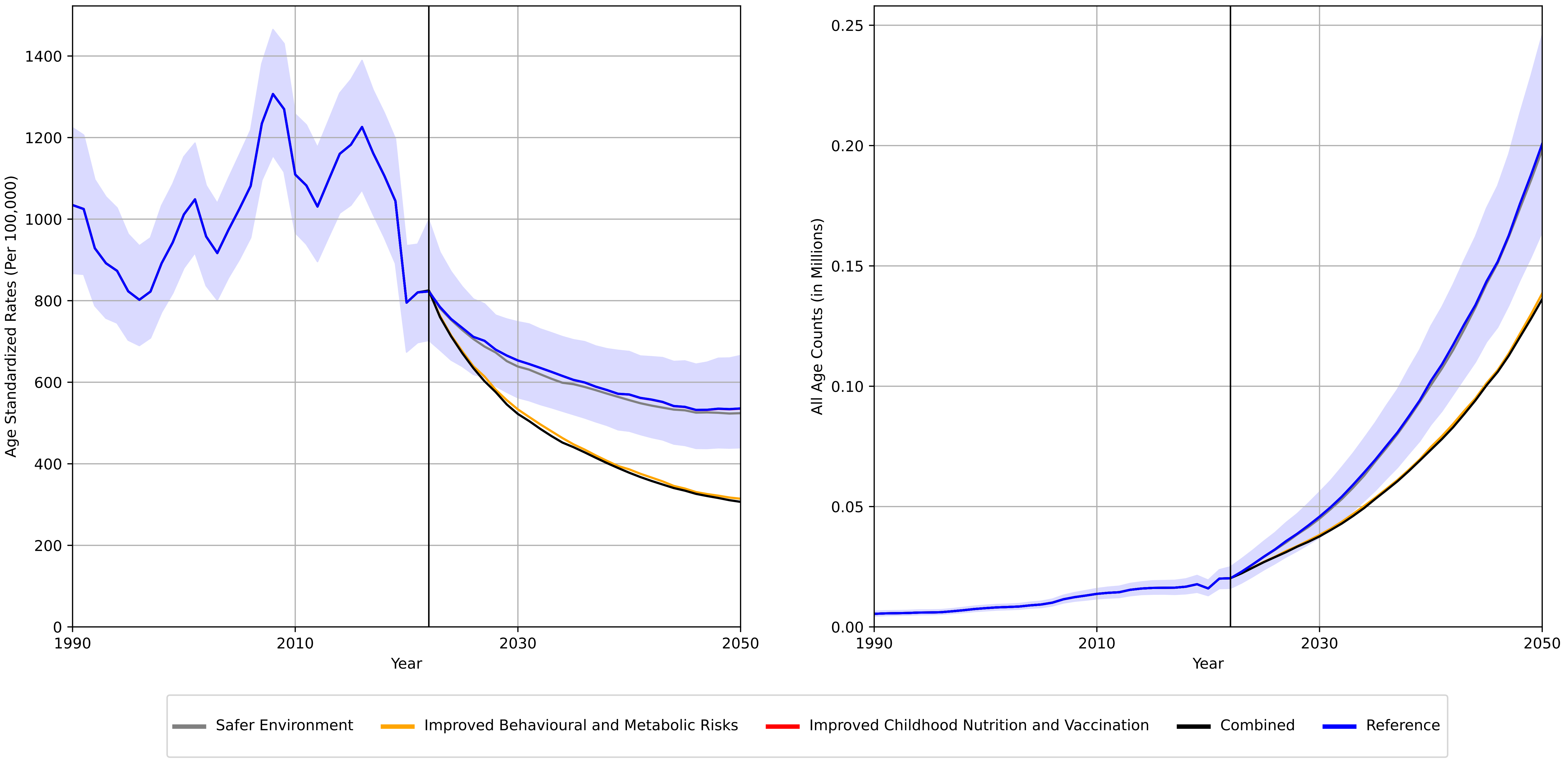

Yemen

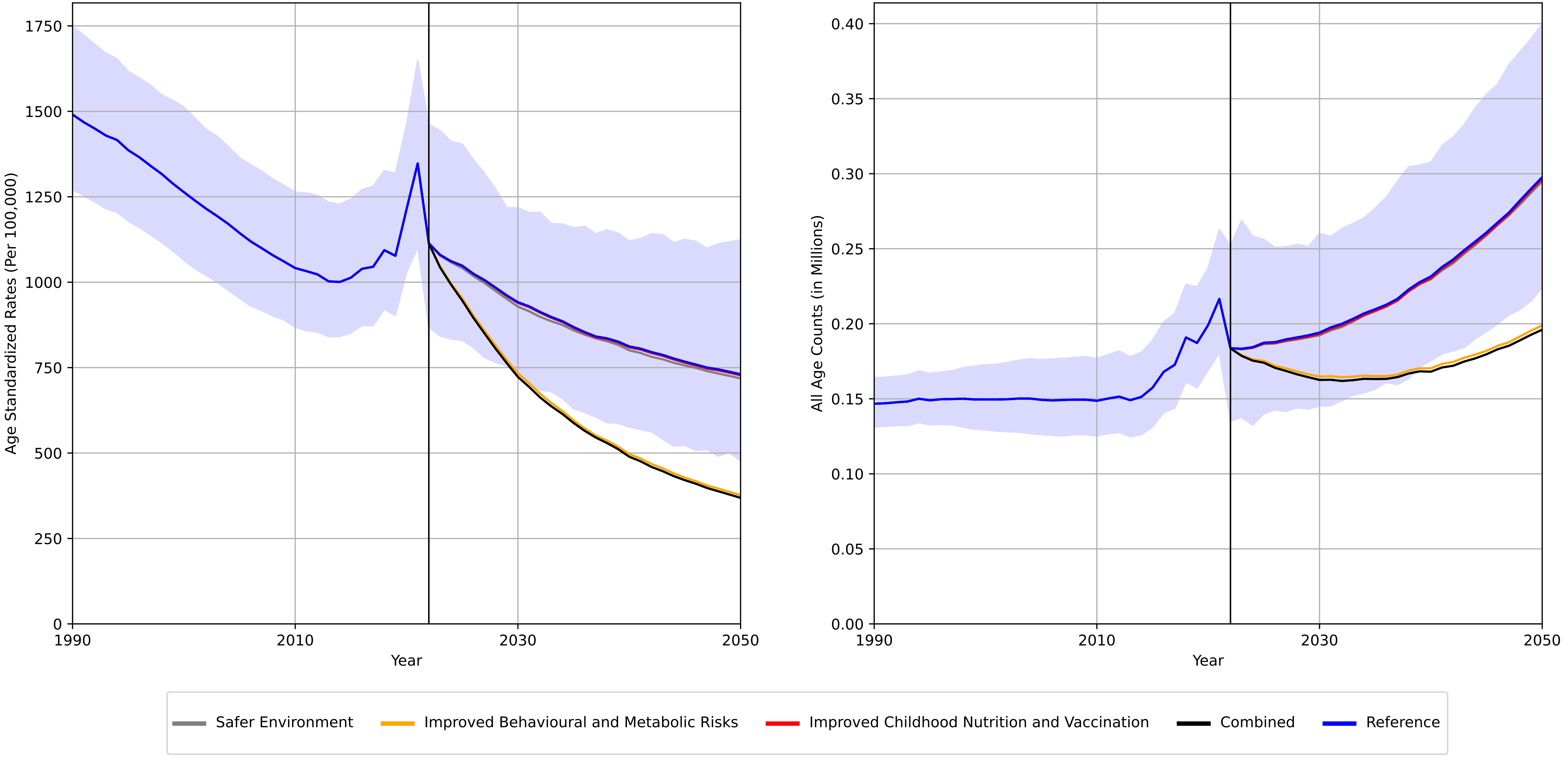
Age Standardized Rates (Per 100,000)
0
250
500
750
1000
1250
1500
1750
1990
2010
2030
2050
Year
All Age Counts (in Millions)
0.00
0.05
0.10
0.15
0.20
0.25
0.30
0.35
0.40
1990
2010
2030
2050
Year
Safer Environment
Improved Behavioural and Metabolic Risks
Improved Childhood Nutrition and Vaccination
Combined
[truncated: 409,883 more chars]
